# Supplementary material for: Single cell RNA sequencing of stem cell-derived retinal ganglion cells
Source: Sci Data. 2018 Feb 13;5:180013. doi: 10.1038/sdata.2018.13 (PMC5810423; doi:10.1038/sdata.2018.13)
Supplement: Supplementary Tables [file sdata201813-s2.zip › Supplementary tables/table s2.pdf]

id	baseMean	baseMeanA	baseMean B	foldChange	log2FoldChange	pval	padj
LGALS1	4.3548159	1.74378687	5.4865807	6.032078354	2.5926552	1.06E-106	1.06E-103
CD63	12.234922	6.48764868	14.72611	2.501273372	1.3226627	4.56E-105	4.59E-102
PMEL	5.1871522	2.04657419	6.5484528	5.30153793	2.4064109	3.08E-97	3.10E-94
PTGDS	3.572607	1.46771481	4.484984	7.451087493	2.897451	2.47E-89	2.49E-86
GSTP1	29.320636	18.1408237	34.166586	1.934947023	0.9522941	2.89E-85	2.90E-82
CTGF	2.7986266	1.19054779	3.4956571	13.0972767	3.711195	1.43E-78	1.43E-75
HSP90AA1	18.59744	11.2821169	21.768306	2.019847285	1.0142462	6.50E-77	6.54E-74
KRT18	5.2360585	2.41986063	6.4567547	3.843162204	1.9422939	1.22E-72	6.13E-70
IGFBP7	5.6012689	1.67390419	7.3036065	9.353861509	3.2255621	1.65E-72	8.31E-70
SERPINF1	4.1160212	1.86846056	5.0902386	4.709757455	2.2356528	1.74E-71	1.75E-68
PGK1	11.076016	6.38012171	13.111476	2.251152853	1.170664	7.31E-70	3.67E-67
CFL1	24.013259	16.5122893	27.264595	1.693147547	0.7597077	2.62E-68	2.63E-65
ELN	2.7127243	1.27467667	3.3360538	8.504740709	3.0882673	6.96E-68	7.01E-65
ACTB	60.322674	35.7101239	70.991118	2.016446791	1.0118153	1.27E-66	6.39E-64
FN1	3.034981	1.42962864	3.7308298	6.3562564	2.6681773	2.05E-64	6.87E-62
TUBA1A	15.992027	8.66982591	19.165875	2.368485905	1.2439651	5.02E-64	1.68E-61
ITM2B	6.5425782	3.5884045	7.8230809	2.636018003	1.3983602	1.33E-62	4.47E-60
FTH1	111.85449	81.6666832	124.93956	1.536440569	0.619592	1.85E-58	9.29E-56
TMEM98	5.5352716	3.01885526	6.6260259	2.786740584	1.4785787	9.87E-55	9.93E-52
KRT8	5.6107815	3.22776394	6.6437134	2.533353408	1.3410483	1.51E-53	1.51E-50
ENO1	32.923793	22.3073385	37.525553	1.714224106	0.7775557	3.48E-53	1.75E-50
CRYAB	4.1160924	1.43579816	5.2778805	9.816196682	3.2951642	3.14E-51	3.15E-48
STMN2	2.2562046	1.1717928	2.7262488	10.04843524	3.328899	5.89E-51	5.92E-48
S100A4	10.188261	3.5715958	13.056291	4.68825269	2.2290503	1.44E-50	7.23E-48
MYL12B	4.9564811	2.92443763	5.8372814	2.5136078	1.3297596	1.56E-50	7.83E-48
ITM2C	4.0983143	2.42488804	4.82367	2.683488027	1.4241095	8.26E-50	8.31E-47
TPM1	8.6769405	4.01690098	10.69686	3.214179031	1.6844503	3.15E-49	1.58E-46
ANXA2	12.467805	6.99681613	14.839236	2.30776386	1.2064956	8.69E-49	4.37E-46
LDHA	26.038062	16.5306078	30.15912	1.877526022	0.9088329	2.69E-48	2.70E-45
PDIA3	3.9119342	2.28320193	4.6179171	2.819444886	1.4954111	4.10E-48	1.37E-45
ANXA1	4.4205782	2.47872725	5.2622841	2.882400453	1.5272708	7.75E-48	2.60E-45
ATP5B	5.3761306	3.29064167	6.2800971	2.305073368	1.2048127	6.20E-46	2.08E-43
TPI1	28.621789	19.9482815	32.381367	1.656159025	0.7278412	8.84E-46	2.96E-43
KRT19	3.6170948	1.71701395	4.4406951	4.798644607	2.262627	1.85E-45	4.65E-43
DCT	8.9044389	1.63015914	12.057515	17.54717867	4.1331672	2.10E-45	2.11E-42
APOE	6.5746084	3.71287369	7.8150428	2.512112102	1.3289008	1.89E-43	4.74E-41
HSP90B1	7.4019544	4.38488968	8.7097175	2.277686479	1.1875692	2.08E-43	2.10E-40
PLIN2	3.5051914	2.04115204	4.1397872	3.015685602	1.592486	2.19E-42	1.10E-39
ANXA5	4.0491667	2.46512444	4.7357785	2.549802875	1.3503857	4.06E-42	4.08E-39
ALDH1A1	4.6834269	2.73230486	5.5291514	2.614523279	1.3865479	6.57E-41	3.30E-38
CALR	6.811507	4.3681674	7.8705858	2.039858756	1.0284693	9.72E-41	2.44E-38
PRDX4	5.950437	3.91228767	6.8338839	2.003196294	1.0023038	1.34E-40	6.73E-38
UBB	8.0358107	5.36012249	9.1956023	1.879672484	0.9104813	3.23E-40	1.08E-37
PKM	19.560275	14.2147867	21.877306	1.579844331	0.6597824	3.51E-40	8.82E-38
ATP5G3	9.7080278	6.9042611	10.923336	1.680707466	0.7490686	7.26E-40	3.65E-37
HLA-A	3.5500854	2.22203214	4.1257373	2.557819199	1.3549143	3.06E-39	1.54E-36
SKP1	9.1677841	5.83977288	10.610329	1.98569827	0.9896464	5.39E-39	1.81E-36
SRP14	20.428875	14.9599287	22.799419	1.561570947	0.6429981	5.92E-39	1.49E-36
MGP	1.9040902	1.02893404	2.2834313	44.35713757	5.4710944	7.41E-39	1.86E-36

MYL12A	4.0286191	2.45893248	4.7090083	2.5422755	1.3461204	1.07E-38	2.16E-36
UCHL1	4.41994	2.8581798	5.0968934	2.204788492	1.1406403	1.66E-38	4.17E-36
CLU	7.4906527	4.91107832	8.6087832	1.945443828	0.9600993	2.48E-38	5.00E-36
SDCBP	4.4368214	2.75856901	5.164269	2.367987243	1.2436613	3.86E-38	1.94E-35
MYL6	25.956456	18.955971	28.990855	1.558860541	0.6404919	4.34E-38	2.18E-35
PPIA	17.772805	13.0916459	19.801878	1.554947819	0.6368662	5.45E-38	1.83E-35
CALM2	13.081208	9.22237713	14.75384	1.672732764	0.742207	1.41E-36	3.55E-34
PRDX1	10.035159	7.02101008	11.341658	1.717595213	0.7803901	4.27E-35	8.59E-33
S100A11	10.678958	5.69840838	12.837804	2.519534948	1.3331575	7.99E-35	2.68E-32
ARPC3	5.3614423	3.66549864	6.0965583	1.912046844	0.9351179	9.84E-34	2.48E-31
ITGB1	4.581598	3.02618904	5.2557985	2.100395555	1.070661	1.00E-33	1.67E-31
LAPTM4A	4.5752015	2.98875837	5.2628538	2.143474993	1.0999516	6.03E-33	2.02E-30
PDIA6	5.4182149	3.73461212	6.1479816	1.882527171	0.9126707	8.58E-33	1.73E-30
PRDX2	14.457338	10.1644308	16.318123	1.671475621	0.7411223	1.16E-32	2.91E-30
TAGLN2	4.4162219	2.93261647	5.0592988	2.1004161	1.0706752	2.54E-32	8.51E-30
MYL9	7.4807449	4.26192037	8.8759619	2.414516909	1.2717346	5.36E-32	1.08E-29
APLP2	7.2791652	4.36747244	8.5412542	2.239440515	1.1631383	6.62E-32	2.22E-29
SFRP1	4.773819	3.09291211	5.5024172	2.151269129	1.105188	8.86E-32	1.78E-29
PGAM1	9.9426294	6.84573177	11.284997	1.759402752	0.8150858	9.54E-32	1.92E-29
HSPA5	4.1366962	2.61180304	4.7976695	2.356162238	1.2364389	1.36E-31	1.37E-28
MORF4L1	8.235658	5.78931501	9.2960386	1.732197322	0.7926033	3.74E-31	9.41E-29
PSMA7	8.0772878	5.58631765	9.1570123	1.778553713	0.8307045	2.27E-30	4.56E-28
ALDOA	31.22104	23.5293557	34.555043	1.489392027	0.5747235	2.56E-30	4.29E-28
SPARC	8.0377371	5.58813585	9.0995301	1.765320462	0.8199301	4.01E-30	6.73E-28
CPE	3.7662235	2.49378697	4.3177681	2.221044985	1.1512386	5.64E-29	9.45E-27
KRT7	1.9702628	1.19976223	2.3042404	6.528963952	2.7068541	7.62E-29	7.67E-26
ATP6V1C2	1.8047219	1.09387349	2.112843	11.85471027	3.5673885	1.21E-28	3.04E-26
TUBB2A	2.186002	1.42996742	2.5137093	3.520520815	1.8157889	1.42E-28	2.38E-26
ARPC2	6.2489853	4.37026649	7.0633262	1.799064312	0.8472468	2.46E-28	3.54E-26
PSMB1	5.028892	3.59386242	5.6509134	1.793045524	0.8424121	3.52E-28	1.18E-25
COPE	4.114494	2.81022258	4.6798376	2.032809454	1.023475	3.86E-28	6.47E-26
TUBB4B	3.1496297	2.06710376	3.6188564	2.454172203	1.2952365	3.94E-28	1.32E-25
LAPTM4B	3.2452194	2.22515486	3.6873719	2.193495671	1.1332319	4.50E-28	2.26E-25
ATP1B1	3.0033847	1.81378224	3.5190244	3.095452676	1.6301504	7.52E-28	7.55E-25
BSG	10.014028	7.26289499	11.206522	1.629681141	0.7045897	1.15E-27	2.31E-25
B2M	10.846437	7.77337353	12.178472	1.650355231	0.7227766	1.71E-27	4.29E-25
SLC16A3	3.1791075	2.200091	3.6034676	2.169391788	1.1172906	8.30E-27	2.78E-24
LDHB	14.751221	11.4020477	16.202938	1.461533158	0.5474826	1.81E-26	3.03E-24
PSMA4	4.3744798	3.05943432	4.9444934	1.9153286	0.9375919	6.91E-26	8.69E-24
PLD3	5.7842132	4.25374682	6.4476021	1.67425508	0.7435193	8.48E-26	2.13E-23
CSTB	6.6454343	4.78021872	7.4539222	1.707288034	0.7717065	1.05E-25	1.51E-23
MSI2	6.4830707	8.35000108	5.6738396	0.635896457	-0.6531362	1.24E-25	2.49E-23
MIAT	7.3840787	10.2534427	6.1403372	0.555505383	-0.8481272	1.43E-25	2.88E-23
KRT17	1.7543743	1.07819555	2.0474677	13.39548907	3.7436753	2.96E-25	4.25E-23
GJA1	3.4729376	2.15596683	4.0437858	2.633108208	1.3967668	3.58E-25	5.14E-23
HLA-C	2.5607037	1.74730827	2.9132744	2.560221092	1.3562684	4.24E-25	7.11E-23
SLC2A1	11.066841	7.63310485	12.555213	1.742051872	0.8007876	5.30E-25	8.89E-23
UQCRC1	2.6734954	1.86121892	3.0255811	2.351993257	1.2338839	1.02E-24	1.47E-22
TYRP1	1.7032166	1.05816086	1.9828196	16.8982982	4.0788061	1.34E-24	1.69E-22
NDN	3.0926896	2.17501349	3.4904613	2.119517211	1.0837357	1.76E-24	2.53E-22
CNN3	5.2064907	3.72196519	5.8499664	1.781788548	0.8333261	2.69E-24	9.01E-22

TMEM59	3.2787865	2.27225728	3.7150721	2.13405902	1.0936001	3.11E-24	6.25E-22
ACTG1	66.846338	53.6364105	72.572253	1.359747983	0.4433393	5.21E-24	6.55E-22
ARL6IP5	3.4950574	2.43727712	3.953558	2.054967651	1.0391157	8.42E-24	9.41E-22
TMEM176A	2.4299051	1.66622799	2.7609251	2.643126832	1.4022457	1.13E-23	2.84E-21
TMEFF2	1.8327646	1.19718539	2.1082598	5.62039522	2.4906716	1.29E-23	1.85E-21
ATPIF1	5.9536596	4.40212592	6.6261803	1.653724882	0.7257192	2.74E-23	1.38E-20
PSMD8	3.6069527	2.54864525	4.0656817	1.979589413	0.9852012	2.85E-23	4.77E-21
S100A9	1.7040901	1.06052655	1.9830463	16.24157205	4.0216194	3.11E-23	5.21E-21
CTSB	4.335317	2.91108483	4.9526581	2.068279801	1.0484314	4.76E-23	2.39E-20
NDUFC2	4.7619063	3.44088033	5.3345122	1.775798724	0.8284681	7.93E-23	9.97E-21
PFN1	21.550267	16.7145626	23.64633	1.441104683	0.5271751	1.21E-22	1.74E-20
ATP6AP2	2.3909146	1.7041276	2.6886061	2.398153616	1.2619241	2.40E-22	2.68E-20
SEPT7	4.9731529	3.58941527	5.5729415	1.766013187	0.8204961	3.70E-22	5.31E-20
PSMB6	3.5800227	2.54084674	4.0304592	1.966749286	0.9758131	9.08E-22	2.28E-19
HLA-B	2.69565	1.88262202	3.0480614	2.320428658	1.2143913	1.80E-21	2.01E-19
RAN	10.071957	7.8353177	11.04144	1.469052447	0.5548859	5.46E-21	1.37E-18
MFGE8	3.0204367	2.15688848	3.3947463	2.069988927	1.0496231	8.05E-21	1.16E-18
YWHAE	10.966091	8.45058998	12.056449	1.483969558	0.5694615	8.36E-21	2.80E-18
TXN	10.918587	8.56983341	11.936668	1.444769907	0.5308397	9.10E-21	1.31E-18
UBC	6.3678636	4.68033106	7.0993337	1.657278553	0.7288161	1.16E-20	1.46E-18
COL3A1	1.6023087	1.02123502	1.8541782	40.22498007	5.3300198	1.71E-20	5.71E-18
YWHAQ	7.9492105	5.9305038	8.8242299	1.58690273	0.6662137	1.95E-20	1.96E-18
RPN2	2.8168961	2.02210472	3.1614027	2.114658737	1.0804249	2.31E-20	2.32E-18
GHITM	3.2042306	2.36776956	3.5667992	1.876631345	0.9081453	2.82E-20	7.10E-18
POLR2J3	4.0798148	5.3158553	3.5440464	0.589465174	-0.7625215	3.25E-20	4.08E-18
JUNB	2.4083573	1.70847298	2.711726	2.416077969	1.272667	6.46E-20	1.20E-17
PRRT2	2.4483451	3.26828221	2.0929388	0.481835479	-1.0533875	6.55E-20	5.99E-18
SCG5	2.1619599	1.54951491	2.4274275	2.5976137	1.3771869	6.99E-20	8.79E-18
S100A10	6.7103145	3.76738817	7.985942	2.524380944	1.3359296	7.16E-20	1.20E-17
ARPC5	3.3603813	2.44523286	3.7570573	1.907690706	0.9318273	8.39E-20	1.06E-17
CCT7	3.248322	2.35363781	3.6361278	1.947439523	0.9615785	1.02E-19	1.14E-17
TRPM1	1.7158677	1.14622951	1.9627805	6.584037108	2.7189725	1.90E-19	2.12E-17
PPIB	5.7716146	4.10961499	6.4920173	1.766140618	0.8206002	2.01E-19	4.05E-17
SSB	3.7042276	2.73111131	4.1260302	1.80579389	0.8526332	2.23E-19	2.24E-17
PSMB3	4.0772233	3.00527063	4.5418671	1.766278831	0.8207131	2.34E-19	2.14E-17
PEBP1	10.103924	8.03199447	11.002014	1.422358025	0.5082847	2.78E-19	2.93E-17
HNRNPK	7.8318771	6.15432077	8.559023	1.466541057	0.5524175	2.92E-19	2.93E-17
H3F3B	18.103793	14.7177827	19.571477	1.35382499	0.4370413	3.15E-19	2.88E-17
ASAH1	2.8511794	2.08879736	3.181638	2.003713489	1.0026762	3.50E-19	4.40E-17
NDUFB4	4.9994872	3.60472135	5.604056	1.767581019	0.8217763	3.84E-19	6.43E-17
CALM1	4.2396419	3.13127692	4.7200687	1.745464716	0.8036112	3.93E-19	9.86E-17
YWHAB	4.5593033	3.46961136	5.0316363	1.632498263	0.7070815	7.68E-19	1.29E-16
ARL4A	3.5128347	2.40492261	3.9930653	2.130412912	1.0911331	8.66E-19	2.18E-16
TMEM176B	2.3314333	1.66924191	2.6184638	2.418353885	1.2740254	1.02E-18	2.06E-16
SNCA	3.0274109	2.21401899	3.3799801	1.960414211	0.9711585	1.31E-18	1.10E-16
CD164	2.9578174	2.19865787	3.2868793	1.907866548	0.9319603	1.36E-18	1.71E-16
COL2A1	2.7156042	3.66330819	2.3048167	0.489923281	-1.0293722	1.83E-18	3.67E-16
TIMP3	1.7527259	1.20670136	1.9894033	4.786631735	2.2590108	1.86E-18	3.75E-16
ERICH5	2.6114894	1.91457012	2.9135728	2.092319374	1.0651031	1.88E-18	2.10E-16
POMP	6.2020221	4.78294698	6.8171279	1.537723885	0.6207965	2.53E-18	5.09E-16
FABP5	11.864398	8.44699854	13.345688	1.65780726	0.7292763	2.73E-18	2.29E-16

STC2	2.3666865	1.68368023	2.6627393	2.432042428	1.2821684	2.79E-18	2.81E-16
CXCL14	2.4668491	1.52645435	2.8744683	3.560552454	1.8321011	3.36E-18	3.07E-16
WSB1	9.281884	11.5243292	8.309884	0.694570063	-0.5258079	4.39E-18	3.68E-16
EID1	6.4087285	4.95369973	7.0394187	1.527535997	0.6112064	6.54E-18	7.31E-16
MDH1	2.5793658	1.92203408	2.8642898	2.021931575	1.0157342	9.70E-18	1.08E-15
EIF3I	3.62181	2.70488765	4.019255	1.770940728	0.8245159	1.11E-17	1.87E-15
CTSH	1.900304	1.34040986	2.1429932	3.357697187	1.7474721	1.32E-17	1.89E-15
ATXN10	2.6571411	1.98314173	2.9492898	1.982714966	0.9874773	1.41E-17	1.41E-15
ATP5E	24.804359	21.3501279	26.301615	1.243314789	0.3141916	1.50E-17	1.89E-15
WDR1	2.4733454	1.82146027	2.7559086	2.137545337	1.095955	1.52E-17	3.06E-15
CD151	4.3442015	3.24526985	4.8205394	1.701594744	0.7668875	2.11E-17	1.63E-15
NUDC	2.6496536	1.99162962	2.9348776	1.951209987	0.964369	2.11E-17	2.13E-15
C14orf166	4.8838024	3.79150879	5.357263	1.560898892	0.6423771	2.57E-17	4.31E-15
PERP	2.6264228	1.92666832	2.9297352	2.082444373	1.058278	2.61E-17	2.02E-15
AKR1B1	2.7074967	2.02986509	3.0012198	1.943186381	0.9584243	2.90E-17	2.92E-15
PDIA4	2.7807341	2.1025415	3.0747004	1.881743556	0.91207	3.23E-17	4.65E-15
STMN1	13.959648	10.8059145	15.32665	1.461021327	0.5469772	3.38E-17	1.70E-14
ARF1	4.1747525	3.18191271	4.6051042	1.652267862	0.7244476	3.43E-17	3.14E-15
CAP1	2.4870186	1.85068728	2.7628399	2.072253743	1.0512007	5.59E-17	4.68E-15
CALU	3.4096314	2.58334769	3.7677886	1.748061165	0.8057557	5.83E-17	5.33E-15
CAPZB	4.3722543	3.37354133	4.8051518	1.603153815	0.6809129	6.01E-17	6.72E-15
ATP1B3	3.1424378	2.39596387	3.466001	1.766522096	0.8209118	6.09E-17	1.23E-14
BCAP31	2.6930108	2.02194206	2.9838893	1.941293296	0.9570181	6.15E-17	5.63E-15
MALAT1	648.82656	771.385269	595.70282	0.771955075	-0.3734112	9.80E-17	8.96E-15
CYR61	1.979583	1.4031658	2.2294342	3.049450706	1.6085494	1.06E-16	1.07E-14
HELLS	2.0564694	2.71232212	1.7721864	0.450958623	-1.148933	1.07E-16	7.71E-15
IGFBP5	13.506537	7.39824354	16.154209	2.368495229	1.2439708	1.30E-16	1.86E-14
ECH1	2.6387276	1.98499913	2.9220897	1.951361814	0.9644813	1.30E-16	1.09E-14
TUBB	23.847015	19.5069336	25.728247	1.33616123	0.4180941	1.35E-16	4.54E-14
DYNLRB1	4.0945914	3.10375875	4.5240732	1.675131817	0.7442746	1.88E-16	1.72E-14
SERPINH1	2.733437	2.04124702	3.0334705	1.952918464	0.9656317	1.98E-16	1.33E-14
PRMT1	3.4378373	2.61901474	3.7927604	1.724975258	0.7865757	2.13E-16	1.78E-14
CYP1B1	11.933555	15.149061	10.539777	0.674233931	-0.5686789	2.54E-16	1.97E-14
CST3	7.0673665	5.3822412	7.7977932	1.551213847	0.6333976	3.01E-16	5.03E-14
POLR2G	3.0630168	2.33117196	3.3802389	1.788077698	0.8384094	3.53E-16	3.55E-14
ARPC1B	2.2738228	1.72415169	2.5120807	2.08807183	1.0621713	4.08E-16	5.13E-14
ECHS1	2.9372229	2.23203123	3.242892	1.820483106	0.8643214	4.16E-16	3.49E-14
HSD17B2	2.5791297	1.81110374	2.9120348	2.35732459	1.2371504	4.20E-16	7.04E-14
RHOA	5.9994249	4.65273645	6.5831544	1.528485412	0.6121028	4.27E-16	3.22E-14
DYNLL1	8.9166285	7.06246672	9.720325	1.438412024	0.524477	4.49E-16	3.22E-14
CRABP2	6.6271252	4.83786872	7.4026881	1.668292639	0.7383724	4.69E-16	4.29E-14
PSME1	3.4701419	2.65731413	3.8224665	1.703036539	0.7681094	6.80E-16	5.26E-14
SLC7A8	1.8103477	1.3023915	2.030524	3.407913315	1.7688886	6.88E-16	1.15E-13
ATP5J2	7.3892701	5.63744398	8.1486086	1.541497563	0.6243326	7.31E-16	5.67E-14
TALDO1	2.9526595	2.2417487	3.2608076	1.820664362	0.864465	7.33E-16	5.67E-14
CD59	2.6691666	1.99925925	2.9595415	1.960994138	0.9715852	7.35E-16	1.23E-13
NQO1	1.9913287	1.467681	2.2183066	2.60499488	1.3812805	7.82E-16	5.62E-14
TIMP1	6.6651332	5.24701276	7.2798252	1.478645232	0.564276	8.70E-16	1.25E-13
PSAP	4.1834657	3.16183405	4.6262975	1.677417143	0.7462415	1.24E-15	1.39E-13
PPP2R1A	3.6119775	2.72939782	3.9945364	1.73154862	0.7920629	1.42E-15	2.03E-13
MDH2	3.5843325	2.76169499	3.9409092	1.669363462	0.7392981	1.86E-15	1.44E-13

SPATS2L	1.7284605	1.25668327	1.9329548	3.634653888	1.861818	2.07E-15	2.08E-13
DAD1	4.1972029	3.22228215	4.6197876	1.628860476	0.703863	2.20E-15	1.84E-13
RAC1	7.1502485	5.61767292	7.8145517	1.475754533	0.5614528	2.86E-15	2.05E-13
EIF1	20.146004	16.1014505	21.899138	1.383915916	0.4687563	2.97E-15	1.99E-13
ARL6IP1	4.0097888	2.84778808	4.5134643	1.901443324	0.9270949	3.42E-15	2.65E-13
TAX1BP1	2.6247563	2.01489318	2.8891048	1.861382843	0.8963748	3.70E-15	1.86E-12
LMAN2	2.6027854	1.97175104	2.8763107	1.930855372	0.9492401	3.88E-15	4.87E-13
ANP32A	2.6924498	2.06422779	2.9647561	1.846179998	0.8845432	3.96E-15	2.65E-13
TMSB4X	411.57085	327.285676	448.10472	1.370286081	0.4544771	4.43E-15	5.57E-13
ACTA2	2.4358139	1.79392157	2.7140457	2.158960939	1.1103371	4.49E-15	2.82E-13
CCT5	2.5377908	1.94182806	2.7961141	1.907050941	0.9313434	4.72E-15	3.95E-13
PRDX6	10.111444	7.99393308	11.02929	1.43399858	0.5200436	6.57E-15	7.34E-13
TMEM50A	2.6993643	2.0820169	2.9669569	1.817861494	0.8622423	7.59E-15	5.45E-13
NDUFV2	3.5160448	2.70152624	3.8691024	1.686193429	0.75377	8.65E-15	7.91E-13
ATP5A1	4.644255	3.64158851	5.0788662	1.544095984	0.6267624	8.71E-15	6.26E-13
APLP1	2.2816699	1.72876749	2.5213284	2.087536078	1.0618011	9.34E-15	5.52E-13
SDC2	2.3560223	1.78914287	2.6017393	2.029720334	1.021281	9.77E-15	1.04E-12
TMED9	4.1195546	3.15993292	4.5355078	1.636860021	0.710931	1.00E-14	5.60E-13
SAP18	4.6161093	3.65693717	5.0318677	1.517487037	0.6016842	1.01E-14	1.26E-12
ATP5J	7.4196218	5.72910519	8.1523855	1.51241835	0.5968573	1.04E-14	1.04E-12
NKAIN4	3.8488636	2.99510465	4.21893	1.613414113	0.6901168	1.21E-14	3.99E-12
GSN	2.1715976	1.64308488	2.4006843	2.178070681	1.1230508	1.31E-14	8.24E-13
UQCRB	10.5922	8.77392675	11.38034	1.33527627	0.4171383	1.59E-14	3.99E-12
FDFT1	2.3225321	1.78201977	2.5568201	1.990768157	0.9933252	1.61E-14	1.25E-12
TUBB6	1.9973931	1.50822325	2.2094264	2.379714846	1.2507887	1.76E-14	1.26E-12
P4HB	4.8235104	3.82486653	5.256378	1.506753652	0.5914436	1.94E-14	2.79E-12
GAPDH	128.85074	110.931663	136.61786	1.23365601	0.3029402	2.01E-14	1.44E-12
GPNMB	1.5184095	1.07345038	1.7112795	9.683810141	3.2755748	2.17E-14	4.36E-12
H3F3A	35.958687	30.6683959	38.251793	1.255605219	0.3283829	2.38E-14	1.59E-12
TRPM3	4.5450557	3.2236172	5.1178404	1.851865683	0.8889795	2.58E-14	1.73E-12
TBCB	3.6903753	2.9096303	4.0287934	1.586062719	0.6654498	3.93E-14	4.95E-12
SMS	5.8218492	4.55877404	6.3693361	1.508760038	0.5933634	4.51E-14	6.48E-12
RBP1	23.778062	20.0132567	25.409938	1.283837817	0.360463	4.53E-14	3.03E-12
PFDN2	2.6881257	2.07936192	2.9519977	1.808473735	0.8547726	4.64E-14	4.67E-12
SNRPB2	3.3377877	2.56600589	3.6723207	1.706456326	0.7710035	4.91E-14	2.60E-12
RAB2A	3.2871057	2.5538444	3.6049418	1.676449605	0.7454091	5.04E-14	4.22E-12
SOD1	9.447725	7.47182095	10.304191	1.437646591	0.5237091	5.14E-14	4.70E-12
PSAT1	2.2189172	1.66734726	2.4579982	2.184766906	1.1274794	5.38E-14	1.35E-11
TNFRSF12A	2.1087311	1.53229699	2.3585896	2.552315122	1.3518065	5.93E-14	6.62E-12
TPM4	6.2437643	4.91934835	6.8178396	1.484389508	0.5698697	5.96E-14	3.75E-12
RAB1A	3.0532132	2.30104638	3.3792441	1.82871579	0.8708309	6.29E-14	5.27E-12
SPCS2	2.9995165	2.33612229	3.2870684	1.711720834	0.7754474	6.58E-14	4.41E-12
MANF	3.3295701	2.61102093	3.6410292	1.63935127	0.713125	1.05E-13	1.76E-11
SOX4	10.174601	8.04748909	11.096609	1.43265344	0.5186897	1.06E-13	1.06E-11
PLS3	1.8881906	1.43875781	2.0829997	2.468331374	1.3035361	1.27E-13	1.83E-11
GGH	2.1719686	1.68445772	2.3832828	2.020990865	1.0150628	1.33E-13	8.38E-12
PLP2	2.5823703	1.99236681	2.8381105	1.852249075	0.8892781	1.43E-13	1.79E-11
PSMA5	2.2370152	1.73312807	2.4554278	1.985229932	0.9893061	1.55E-13	1.81E-11
SMARCB1	3.3438902	2.60330055	3.6649028	1.662135547	0.733038	1.56E-13	1.42E-11
VDAC2	4.978289	3.96010467	5.4196266	1.493064294	0.5782763	1.56E-13	1.42E-11
MORF4L2	10.054049	8.28306874	10.82169	1.348564753	0.4314248	1.62E-13	1.81E-11

LRRC17	3.1251076	2.29619696	3.4844034	1.916686637	0.9386145	1.67E-13	9.87E-12
PTH2	7.9749222	10.4244007	6.9131825	0.627433262	-0.6724661	1.91E-13	1.37E-11
HSPB1	8.9125595	7.0565165	9.7170714	1.439287979	0.5253553	1.92E-13	1.49E-11
BNIP3L	4.6335457	3.7078339	5.0348005	1.490047262	0.5753581	2.33E-13	2.60E-11
NDUFAB1	3.5587758	2.70258242	3.9298975	1.720855001	0.7831255	2.34E-13	1.68E-11
FOS	3.9261778	2.74264659	4.4391859	1.973541788	0.9807871	2.37E-13	1.40E-11
NECTIN2	2.280433	1.79451023	2.4910588	1.876701896	0.9081995	2.70E-13	3.02E-11
DSP	1.6972469	1.25862065	1.8873719	3.43117174	1.7787013	2.70E-13	2.27E-11
LY6E	2.6644181	2.0408798	2.9346942	1.85871044	0.894302	2.71E-13	1.70E-11
ACTR3	2.4584145	1.92994859	2.6874809	1.814595917	0.8596483	2.91E-13	2.93E-11
CDT1	1.6132189	2.04869946	1.4244575	0.404746552	-1.3049093	2.99E-13	1.67E-11
KIAA0101	3.4004873	4.53107535	2.9104278	0.541032855	-0.8862119	3.30E-13	2.55E-11
SEZ6L2	1.5756546	1.17696203	1.7484701	4.229551937	2.0805048	3.47E-13	2.69E-11
APP	3.5194654	2.73593528	3.8590908	1.647003086	0.7198433	4.02E-13	4.10E-11
TPM2	7.7653994	6.02769339	8.5186175	1.495440739	0.5805707	4.06E-13	8.17E-11
SOX2	3.6621949	4.64244685	3.2372993	0.614229774	-0.7031496	4.08E-13	4.10E-11
COX5B	9.033483	7.50151229	9.6975241	1.337769377	0.4198294	4.24E-13	2.67E-11
MUM1	3.2563483	4.04231217	2.9156679	0.629675006	-0.6673207	5.13E-13	2.58E-11
FZD5	2.0666861	2.6528472	1.8126113	0.491643341	-1.024316	5.29E-13	6.65E-11
RCN2	6.0271664	4.80218064	6.5581431	1.461830357	0.5477759	5.38E-13	2.85E-11
ILF2	3.3953067	2.70180766	3.6959076	1.58414354	0.6637031	5.40E-13	4.94E-11
RAB31	3.1408631	3.87359216	2.8232577	0.634487279	-0.6563369	5.99E-13	4.63E-11
DCTN3	2.6375603	2.07425899	2.8817264	1.751650571	0.808715	6.25E-13	6.99E-11
HNRNPA2B1	10.684777	8.60592854	11.585866	1.391791378	0.476943	7.20E-13	4.83E-11
RTN4	7.3204467	5.95635484	7.9117197	1.39451673	0.4797652	7.65E-13	4.80E-11
PRDX5	8.0462289	6.51742411	8.7088976	1.397191412	0.4825297	7.77E-13	3.91E-11
TGM2	1.7004498	1.24755491	1.8967595	3.622466914	1.8569725	9.22E-13	5.45E-11
ATP5O	7.0411993	5.68031354	7.6310827	1.416803087	0.5026393	9.25E-13	6.20E-11
TMBIM6	4.8841504	3.91931799	5.3023622	1.473755945	0.5594976	9.26E-13	5.48E-11
SLC3A2	2.3990243	1.85137885	2.6364042	1.92206353	0.942656	1.18E-12	1.19E-10
LGALS3	2.8954604	2.15949406	3.214469	1.909857978	0.9334654	1.34E-12	9.02E-11
LY6H	1.9530566	1.4772421	2.159301	2.429167605	1.280462	1.50E-12	8.37E-11
GTF2A2	3.2387442	2.53163744	3.5452434	1.661779319	0.7327288	1.65E-12	1.51E-10
TAGLN	12.320012	7.31292268	14.490362	2.136943923	1.095549	1.76E-12	1.11E-10
MAGED2	3.1361548	2.40725946	3.4520985	1.742463671	0.8011286	1.79E-12	1.12E-10
EMP3	3.7258887	2.94120731	4.0660131	1.579436202	0.6594097	2.04E-12	1.47E-10
IER2	2.4373444	1.90183715	2.6694629	1.851180017	0.8884452	2.08E-12	1.23E-10
XRCC6	3.0865878	2.44653202	3.3640235	1.634269708	0.7086461	2.20E-12	1.23E-10
ACTN1	2.0006309	1.53016281	2.2045578	2.272052575	1.1839962	2.33E-12	2.13E-10
RCN1	5.0814594	4.10331457	5.5054416	1.45181596	0.5378586	2.38E-12	1.26E-10
NME1	5.4435322	4.29129764	5.9429745	1.50183152	0.586723	2.47E-12	3.32E-10
FAM162A	7.9475251	6.30084005	8.6612897	1.445297278	0.5313663	2.64E-12	3.32E-10
GSTO1	2.7825155	2.20334959	3.0335581	1.689914667	0.7569504	2.89E-12	1.45E-10
C21orf58	1.5257318	1.91952864	1.3550384	0.386109091	-1.3729196	3.05E-12	1.81E-10
PSME2	2.4312496	1.93371606	2.6469082	1.763821257	0.8187044	3.14E-12	2.25E-10
PSMB7	3.7225488	2.94262882	4.0606093	1.575498782	0.6558086	3.31E-12	2.08E-10
SRGAP3	2.3555194	2.87796134	2.1290641	0.601217992	-0.7340399	3.46E-12	3.49E-10
WLS	1.5310492	1.13357501	1.7033365	5.265479826	2.396565	3.47E-12	1.94E-10
TMSB10	80.688025	68.4368476	85.998365	1.260414264	0.333898	3.55E-12	1.98E-10
COL1A1	4.0257368	2.7761138	4.5673926	2.008538319	1.006146	3.63E-12	2.15E-10
TXNDC17	4.6348942	3.72603399	5.0288446	1.477914286	0.5635626	4.01E-12	3.20E-10

PSMD2	2.2608832	1.77771647	2.4703145	1.890553393	0.9188086	4.10E-12	3.43E-10
FBLN1	2.6110203	2.04573841	2.8560448	1.774865303	0.8277095	4.13E-12	3.20E-10
LRPAP1	2.0098123	1.58243508	2.1950613	2.051836152	1.0369155	5.73E-12	3.84E-10
FSCN1	3.4778991	2.77689297	3.7817541	1.565515833	0.6466381	6.19E-12	3.46E-10
NDUFS5	10.821473	8.85071021	11.67571	1.359840047	0.443437	6.22E-12	8.92E-10
ERH	7.9735334	6.56022119	8.5861412	1.364359615	0.448224	6.23E-12	4.82E-10
CETN2	2.7244342	2.1789725	2.9608676	1.663200415	0.733962	6.52E-12	6.56E-10
PCMT1	2.3648108	1.87050201	2.5790717	1.813978229	0.8591571	7.03E-12	5.45E-10
SRI	4.2035275	3.31198581	4.5899711	1.552765211	0.6348397	7.05E-12	5.45E-10
CCNB1	1.7985013	1.34749795	1.9939912	2.860423183	1.5162286	7.20E-12	3.81E-10
SNU13	4.7123779	3.80150858	5.1071992	1.466066968	0.551951	7.64E-12	4.04E-10
NDUFAF3	3.476137	2.75432755	3.7890093	1.589788208	0.6688346	8.32E-12	4.93E-10
VIM	95.262411	79.2241509	102.21428	1.293900675	0.3717269	8.73E-12	4.18E-10
GRN	1.9713341	1.5516943	2.1532293	2.090341213	1.0637385	9.02E-12	1.01E-09
S100A2	2.3288208	1.54801164	2.6672668	3.042393124	1.6052066	1.08E-11	1.36E-09
GLRX3	2.4828614	1.98729733	2.6976663	1.719508649	0.7819964	1.20E-11	7.51E-10
CCT8	4.2883672	3.48322074	4.6373623	1.464776062	0.5506801	1.25E-11	1.14E-09
GABPB1-AS1	3.6793465	4.47350036	3.3351162	0.672265988	-0.5728959	1.36E-11	9.75E-10
APOC1	1.9501998	1.49159857	2.1489831	2.337238393	1.2248049	1.39E-11	7.34E-10
HERPUD1	2.0955355	1.61496596	2.303841	2.120183958	1.0841894	1.42E-11	6.49E-10
ATP5H	3.5891964	2.79477279	3.9335436	1.634493034	0.7088432	1.79E-11	9.49E-10
IGFBP3	1.7455758	1.24431064	1.962852	3.941097146	1.9785973	1.80E-11	1.83E-09
ARF4	3.0757748	2.32375301	3.4017428	1.814343585	0.8594477	1.82E-11	1.83E-09
DSTN	8.2199981	6.84486495	8.816057	1.337251943	0.4192713	2.27E-11	1.63E-09
RAB5C	2.9532233	2.37130971	3.2054569	1.60828501	0.6855231	2.27E-11	2.08E-09
PSMB2	3.3240426	2.67188221	3.6067251	1.559155947	0.6407652	2.28E-11	1.09E-09
CHPF	2.736312	2.18169089	2.9767155	1.672785596	0.7422525	2.44E-11	1.36E-09
EIF6	2.3604801	1.89246595	2.5633434	1.751712139	0.8087657	2.51E-11	2.52E-09
S100A13	6.8264614	5.46556418	7.4163497	1.436850858	0.5229103	2.52E-11	1.69E-09
ATP6V1G1	7.5660795	6.33280956	8.100647	1.331502082	0.4130547	2.67E-11	1.34E-09
EGR1	2.1811582	1.63438963	2.418158	2.235468466	1.1605772	2.89E-11	1.93E-09
HDAC2	4.1293302	3.3393054	4.4717708	1.484103275	0.5695915	3.06E-11	1.54E-09
NDUFV1	2.272662	1.81271375	2.472029	1.811251535	0.8569869	3.06E-11	2.20E-09
TFPI2	1.4831072	1.08443116	1.6559155	7.768642351	2.9576625	3.33E-11	2.09E-09
UQCRFS1	3.270928	2.60169449	3.561011	1.598938489	0.6771144	3.53E-11	2.37E-09
ATP5C1	3.8507487	2.95523887	4.2389122	1.656530202	0.7281645	3.90E-11	3.56E-09
FSTL1	2.5201875	1.99938485	2.7459322	1.747006859	0.8048853	4.13E-11	1.92E-09
PCBP4	2.0761679	1.62042879	2.2737105	2.052952007	1.0376999	4.21E-11	1.92E-09
PSMD7	2.4153825	1.92103787	2.6296589	1.769372316	0.8232377	4.33E-11	1.89E-09
FLOT1	2.5649784	2.06748205	2.7806209	1.66805697	0.7381686	4.35E-11	2.57E-09
KIF5B	3.3730437	2.74108574	3.6469694	1.520298117	0.6043543	4.35E-11	3.65E-09
SPP1	2.0664798	1.5240849	2.3015838	2.483536173	1.3123958	4.91E-11	4.11E-09
NDUFA12	3.3257721	2.65181056	3.6179044	1.584869637	0.6643642	5.04E-11	2.99E-09
CAPN2	2.8114754	2.25185882	3.0540442	1.640795395	0.7143953	5.29E-11	2.31E-09
SPINT2	4.8448699	3.95644834	5.229961	1.430757629	0.5167793	5.42E-11	2.99E-09
IL32	1.6962398	1.27481846	1.8789072	3.198137396	1.6772319	5.52E-11	2.99E-09
NDUFA13	5.6495914	4.64842052	6.0835544	1.393357591	0.4785656	5.64E-11	2.99E-09
CYB5R3	2.7467571	2.19259162	2.9869631	1.666088469	0.736465	5.77E-11	3.86E-09
HMGB1	19.632985	16.8277304	20.848937	1.254060862	0.3266074	5.80E-11	3.24E-09
VDAC1	5.7450148	4.58994551	6.2456859	1.461216024	0.5471695	6.28E-11	3.16E-09
ATP6V0B	3.0644191	2.47222438	3.3211092	1.576600145	0.6568168	7.04E-11	3.22E-09

RTFDC1	2.1841141	1.74111781	2.3761333	1.856834752	0.8928454	7.11E-11	5.11E-09
CCT2	3.4077025	2.76291869	3.6871876	1.524283333	0.6081311	7.13E-11	5.98E-09
AHSA1	1.9922423	1.58716365	2.1678258	1.988927293	0.9919905	7.37E-11	3.71E-09
CLTB	2.4947465	1.97961877	2.7180315	1.753775569	0.8104641	7.42E-11	4.66E-09
SLC25A4	1.9836079	1.58056667	2.1583083	1.995134019	0.9964857	7.69E-11	3.55E-09
CHMP5	2.6367211	2.12896079	2.8568125	1.644709514	0.7178328	7.76E-11	3.55E-09
UBXN4	3.5210146	2.85198331	3.8110098	1.517837559	0.6020174	8.54E-11	3.58E-09
DNAJB11	1.8066616	1.42477363	1.9721929	2.288731856	1.1945484	8.70E-11	5.47E-09
COX6A1	7.8578738	6.53854207	8.4297453	1.34146228	0.4238065	8.71E-11	5.15E-09
UBE2L3	3.2074973	2.56403629	3.486409	1.589738687	0.6687896	8.78E-11	4.21E-09
CYBA	3.073842	2.45731553	3.3410787	1.606432289	0.6838602	9.15E-11	4.60E-09
TUBB2B	10.956612	9.19827593	11.718772	1.307442246	0.3867472	9.65E-11	4.17E-09
LAMTOR5	6.4188013	5.29779776	6.9047063	1.373891155	0.4582677	9.95E-11	4.17E-09
NDUFA4	17.730828	15.2213396	18.81858	1.25294665	0.325325	1.00E-10	5.91E-09
AP2M1	5.998841	4.75839234	6.5365202	1.47310863	0.5588638	1.07E-10	6.30E-09
PTTG1	4.6213969	3.41364949	5.1449016	1.717275701	0.7801217	1.17E-10	6.51E-09
STRAP	4.2989812	3.44092843	4.6709088	1.503898565	0.5887073	1.20E-10	6.69E-09
SSR2	4.8210328	3.94613721	5.2002611	1.425684138	0.5116544	1.27E-10	5.10E-09
COX4I1	13.086242	10.946156	14.013875	1.308432581	0.3878396	1.28E-10	6.76E-09
MRFAP1	4.9973967	4.04004935	5.4123642	1.451412019	0.5374571	1.30E-10	5.46E-09
FKBP3	3.0382666	2.32529488	3.3473081	1.771159131	0.8246938	1.31E-10	1.01E-08
SCARB2	3.4387199	2.79224852	3.7189364	1.517053245	0.6012717	1.33E-10	5.12E-09
COX6C	11.071394	9.1918122	11.886109	1.328901208	0.4102339	1.42E-10	1.02E-08
USMG5	9.8794546	8.34887281	10.542894	1.298552018	0.3769038	1.44E-10	6.91E-09
XRCC5	3.7202349	2.99242865	4.0357064	1.523621142	0.6075042	1.52E-10	7.67E-09
C4orf48	7.3642901	6.2499329	7.8473142	1.304266999	0.3832392	1.53E-10	1.28E-08
TPM3	3.1664272	2.57356973	3.4234045	1.540068043	0.6229941	1.57E-10	1.21E-08
NDUFB2	6.8972208	5.56697295	7.473824	1.417530613	0.5033799	1.58E-10	7.59E-09
GABARAPL2	4.5954237	3.75777808	4.9585057	1.435396742	0.5214496	1.63E-10	9.09E-09
ISYNA1	3.9337471	3.17075083	4.264472	1.503844659	0.5886556	2.14E-10	1.13E-08
ESD	4.2726268	3.51941474	4.5991106	1.428550271	0.5145518	2.31E-10	1.55E-08
TSPO	2.1588387	1.7304188	2.3445396	1.840779014	0.8803164	2.45E-10	1.37E-08
NPC2	7.8618681	6.160572	8.5993042	1.472570127	0.5583363	2.75E-10	1.45E-08
ATP5L	13.169981	11.3072866	13.977376	1.259048756	0.3323342	2.85E-10	1.15E-08
COPS6	3.4316704	2.78070447	3.7138351	1.524023314	0.607885	3.06E-10	1.81E-08
NDFIP1	3.0244065	2.43556282	3.279644	1.587979286	0.6671921	3.55E-10	1.62E-08
MRPS6	5.2035062	4.14314997	5.6631234	1.483582846	0.5690855	3.59E-10	2.41E-08
XKR4	1.5893504	1.98383263	1.41836	0.425234904	-1.2336681	3.59E-10	1.57E-08
BLVRB	2.1450947	1.7322922	2.3240262	1.808057236	0.8544403	3.82E-10	2.02E-08
RAB7A	2.8923606	2.34568038	3.1293221	1.58233869	0.6620584	3.83E-10	3.50E-08
CCT6A	3.5476143	2.90514813	3.8260947	1.483398934	0.5689066	3.85E-10	2.77E-08
OTX2	1.6118744	1.24173178	1.7723147	3.19492417	1.6757817	4.10E-10	1.65E-08
EIF3K	6.2213477	5.0441675	6.7316029	1.417251607	0.5030959	4.26E-10	3.57E-08
BRCA2	1.3935822	1.73005866	1.2477346	0.339335248	-1.5592168	4.54E-10	2.28E-08
CAPZA2	2.7706917	2.23577517	3.0025542	1.620484231	0.696425	4.62E-10	2.44E-08
POLR2L	11.97873	10.2459034	12.729833	1.268651898	0.3432963	4.71E-10	1.82E-08
NDUFB10	4.3658866	3.58024714	4.7064263	1.43646176	0.5225196	4.77E-10	2.29E-08
HTN1	1.4551793	1.07682487	1.6191791	8.059617355	3.0107113	5.03E-10	3.89E-08
ETFB	3.8658221	3.13286425	4.1835267	1.49260634	0.5778337	5.39E-10	2.71E-08
DNAJC8	2.8310608	2.31556783	3.0545041	1.561686172	0.6431046	6.44E-10	3.01E-08
TGIF1	1.9938985	1.61058294	2.1600487	1.899903538	0.9259262	6.60E-10	3.01E-08

CLIC1	6.9827723	5.79023446	7.4996843	1.356861411	0.4402734	6.60E-10	3.02E-08
TXNL1	2.9391437	2.40019568	3.1727537	1.551750004	0.6338961	7.32E-10	4.60E-08
MMADHC	2.284844	1.843618	2.4760958	1.749720637	0.8071246	7.50E-10	3.28E-08
MGST1	1.6913779	1.33066994	1.8477287	2.56367025	1.3582107	8.11E-10	3.02E-08
EIF4G2	7.387281	6.31621445	7.8515406	1.288800644	0.3660291	8.13E-10	6.29E-08
RAD23B	2.3816162	1.94678888	2.5700945	1.658336463	0.7297367	8.32E-10	4.40E-08
CSRP2	3.3350011	2.71382838	3.6042518	1.51955224	0.6036463	8.77E-10	4.20E-08
ATP6V0E1	4.060226	3.32018084	4.3810027	1.457215146	0.5432139	9.44E-10	4.31E-08
CALD1	8.3162541	6.94697969	8.9097735	1.330048854	0.4114792	9.60E-10	4.83E-08
SSNA1	3.413045	2.78078886	3.6870999	1.508937958	0.5935335	9.77E-10	7.56E-08
CCL2	2.9463426	1.92205651	3.390325	2.592384477	1.3742797	1.05E-09	4.58E-08
ATAD5	1.4917771	1.83208701	1.344268	0.413740361	-1.2732024	1.07E-09	6.74E-08
C12orf75	2.1372087	1.70365389	2.3251354	1.883220512	0.9132019	1.08E-09	5.16E-08
TECR	3.6167048	2.93695207	3.9113473	1.503055939	0.5878987	1.10E-09	5.51E-08
NDUFS3	2.4011265	1.96855122	2.5886286	1.640211243	0.7138816	1.10E-09	4.10E-08
PSMC5	2.617644	2.15285914	2.8191076	1.577909634	0.6580146	1.11E-09	7.83E-08
FAM96B	3.7678851	3.12335732	4.0472592	1.435113715	0.5211651	1.17E-09	7.83E-08
C1QBP	3.5172826	2.87262566	3.7967127	1.493471308	0.5786695	1.25E-09	6.96E-08
COX5A	6.3789464	5.30383576	6.844959	1.358081335	0.4415699	1.25E-09	6.01E-08
SSR4	7.1088063	6.04853744	7.5683856	1.301047215	0.3796733	1.40E-09	8.28E-08
BEX4	6.5248625	5.48528115	6.9754748	1.332240848	0.4138549	1.50E-09	6.30E-08
RAD51AP1	1.5137233	1.86884863	1.3597923	0.414102373	-1.2719406	1.58E-09	7.20E-08
TRMT112	5.9439503	4.98150841	6.361126	1.346506258	0.4292209	1.63E-09	6.55E-08
NDUFB8	5.3076339	4.37567275	5.7115976	1.395750694	0.4810413	1.66E-09	6.95E-08
LAMP1	1.9197954	1.54599373	2.0818218	1.981381381	0.9865066	1.67E-09	7.55E-08
ARPC4	2.6943392	2.19212429	2.9120269	1.603882214	0.6815682	1.72E-09	7.50E-08
SEMA5A	1.8477232	2.24331782	1.6762506	0.543908056	-0.8785653	1.73E-09	7.55E-08
GIPC1	1.9380522	1.56973518	2.0977011	1.926686604	0.9461219	1.84E-09	8.41E-08
MAP1LC3B	2.8920781	2.37053205	3.1181451	1.545491104	0.6280654	1.84E-09	8.84E-08
PDHB	2.0876477	1.70519063	2.2534257	1.777428226	0.8297913	1.89E-09	8.64E-08
ATP5I	11.200389	9.713403	11.844931	1.244626402	0.3157128	1.93E-09	1.29E-07
GPAA1	2.2485545	1.85085316	2.4209403	1.67001823	0.7398639	2.01E-09	1.26E-07
AKAP12	5.4011812	4.22133401	5.9125923	1.52501799	0.6088263	2.05E-09	3.43E-07
PTAR1	1.8956663	2.28016611	1.7290028	0.569459557	-0.8123347	2.11E-09	1.06E-07
SQSTM1	2.3392249	1.91335014	2.5238226	1.668388244	0.7384551	2.12E-09	1.18E-07
NUCB1	2.209447	1.81739244	2.3793852	1.68754333	0.7549245	2.17E-09	1.28E-07
SAT1	6.4353496	5.00350169	7.0559918	1.51267373	0.5971008	2.32E-09	1.67E-07
SUB1	6.6060481	5.50605463	7.0828463	1.349927321	0.4328817	2.39E-09	1.09E-07
OPTN	1.8578254	1.49506807	2.0150645	2.050353431	1.0358726	2.40E-09	1.15E-07
TSPAN3	5.4425882	4.48851564	5.8561361	1.392035079	0.4771956	2.42E-09	1.28E-07
HADHB	2.0263802	1.65085044	2.1891555	1.827079585	0.8695395	2.85E-09	1.43E-07
DHRS3	1.5263049	1.18709433	1.6733376	3.598920569	1.8475643	2.95E-09	1.65E-07
CUTA	7.0024924	5.85507383	7.4998472	1.338774116	0.4209126	3.07E-09	1.34E-07
RABAC1	4.0387304	3.34551294	4.3392093	1.423658424	0.509603	3.14E-09	1.13E-07
VBP1	2.2324831	1.8358767	2.4043943	1.680145332	0.748586	3.35E-09	2.41E-07
ERP29	4.1999819	3.47485047	4.514294	1.420002554	0.5058935	3.62E-09	1.40E-07
GNG11	1.7417241	1.37630077	1.9001188	2.392019492	1.2582291	3.69E-09	1.61E-07
IGFBP6	1.7042116	1.34582192	1.8595575	2.485549546	1.3135649	3.75E-09	1.64E-07
DCTN2	3.0322643	2.51604145	3.256024	1.488101769	0.5734732	3.79E-09	2.01E-07
SCCPDH	2.2163443	1.81965501	2.3882915	1.693750959	0.7602218	3.98E-09	1.91E-07
PCSK1N	3.091968	2.50420482	3.3467371	1.560118041	0.6416552	4.09E-09	2.74E-07

FKBP4	2.173581	1.76645471	2.3500521	1.761424542	0.8167427	4.14E-09	1.67E-07
NEAT1	53.207537	63.1996177	48.876415	0.769722021	-0.3775906	4.31E-09	2.55E-07
UBL5	14.082442	12.2026838	14.897233	1.240527135	0.3109533	4.33E-09	1.61E-07
TIMP2	1.9478422	1.57932913	2.1075762	1.911825438	0.9349508	4.63E-09	1.94E-07
LGALS3BP	2.0542156	1.682479	2.2153468	1.780782735	0.8325115	4.63E-09	1.67E-07
CDCA7	1.4274455	1.75336056	1.2861758	0.379865683	-1.3964387	5.01E-09	2.80E-07
UQCR11	10.139262	8.63669293	10.790559	1.282041741	0.3584432	5.15E-09	1.78E-07
PTGES3	7.2606952	6.00821733	7.8035885	1.35848508	0.4419987	5.32E-09	2.81E-07
EIF4A1	4.0078878	3.34873554	4.2936009	1.402286825	0.4877815	5.69E-09	3.01E-07
AIMP1	2.3792501	1.97493325	2.5545034	1.594471615	0.6730784	5.78E-09	2.91E-07
SSBP1	3.3662078	2.76483294	3.6268771	1.488456503	0.5738171	5.96E-09	2.60E-07
TMSB15A	4.6944446	5.6016516	4.3012107	0.717396934	-0.4791565	6.35E-09	2.20E-07
DTD1	2.3192313	1.90000309	2.500948	1.667714309	0.7378722	6.36E-09	2.90E-07
APMAP	1.9056615	1.55339783	2.0583521	1.912461549	0.9354307	6.45E-09	4.32E-07
HMGB3	4.5787734	3.7397664	4.9424455	1.438971407	0.5250379	6.50E-09	2.72E-07
RNH1	2.4529022	2.03147715	2.6355711	1.585659118	0.6650827	7.02E-09	2.35E-07
CLTA	5.8310893	4.72158662	6.3120092	1.427350691	0.5133398	7.06E-09	2.84E-07
EMC7	1.6074743	1.28667721	1.7465256	2.604063133	1.3807644	7.79E-09	3.73E-07
REEP5	2.2369813	1.83235515	2.4123688	1.696834315	0.7628457	7.82E-09	3.28E-07
RAB11A	3.8322991	3.17680178	4.116428	1.431654449	0.5176833	8.45E-09	3.54E-07
OST4	14.106103	12.6154544	14.752232	1.183959894	0.2436202	8.83E-09	4.04E-07
TXNDC12	2.6119889	2.15794087	2.8087985	1.562081938	0.6434701	8.95E-09	3.21E-07
VPS29	2.3135648	1.91756198	2.4852143	1.618652861	0.6947936	9.09E-09	5.71E-07
PPP1CA	2.8139426	2.32549052	3.0256648	1.52823786	0.6118691	9.11E-09	3.99E-07
LINC01474	1.4088054	1.07856989	1.5519478	7.024927523	2.8124833	9.18E-09	3.99E-07
ACTR10	1.9420711	1.59039445	2.0945073	1.853857741	0.8905305	9.39E-09	3.93E-07
TUBA1C	2.8814555	2.36329201	3.1060563	1.544831423	0.6274494	9.44E-09	3.95E-07
VCP	2.4496041	2.02250766	2.6347314	1.598747337	0.676942	9.51E-09	3.99E-07
PPP4C	3.2176823	2.66443975	3.4574883	1.476465783	0.5621479	9.69E-09	3.90E-07
PRDX3	2.9186152	2.43318948	3.1290256	1.485515775	0.5709639	9.97E-09	6.27E-07
DNAJA1	3.4945364	2.90550784	3.749854	1.443108203	0.5291795	1.04E-08	3.61E-07
EIF5A	7.3930591	6.14795115	7.932758	1.346702363	0.429431	1.08E-08	5.42E-07
CTSC	1.5973877	1.2807744	1.7346255	2.616426133	1.3875975	1.15E-08	5.04E-07
PAX6	10.137849	8.63705561	10.788376	1.281694958	0.3580529	1.15E-08	4.83E-07
HN1	6.0967084	5.11270473	6.5232301	1.342967816	0.4254247	1.19E-08	4.62E-07
SEC61G	10.217898	8.52350052	10.952344	1.322834189	0.4036322	1.21E-08	7.16E-07
SLC25A3	11.529222	10.0278762	12.179989	1.238385289	0.3084602	1.28E-08	4.30E-07
RRAGA	1.9152046	1.56872988	2.065386	1.873272344	0.9055607	1.31E-08	7.30E-07
CLDN6	1.6671518	1.31933382	1.8179153	2.561317686	1.3568862	1.44E-08	5.55E-07
SNX3	5.5221239	4.67016687	5.8914093	1.332748479	0.4144045	1.46E-08	6.38E-07
CHMP4B	2.4238956	1.95667534	2.6264148	1.700069754	0.7655939	1.49E-08	2.15E-06
SEPT2	4.4230723	3.71309763	4.7308147	1.375112577	0.4595497	1.52E-08	5.92E-07
NDUFA8	2.2774267	1.88939405	2.4456215	1.62540046	0.7007952	1.53E-08	5.92E-07
IGFBP2	8.0858894	9.47195663	7.485091	0.765477362	-0.3855684	1.53E-08	6.42E-07
DDOST	2.5495216	2.1209915	2.7352703	1.547978068	0.630385	1.59E-08	5.16E-07
ATP5F1	3.5640484	2.94740497	3.8313359	1.453901944	0.53993	1.61E-08	6.35E-07
EZR	1.6472008	1.32807527	1.7855275	2.394351313	1.2596348	1.64E-08	6.35E-07
PPIC	1.8589114	1.52107933	2.0053466	1.92935422	0.948118	1.71E-08	7.46E-07
BMP4	1.591418	1.25757937	1.7361221	2.857845753	1.5149281	1.84E-08	9.26E-07
NDUFB6	2.392937	1.98144129	2.5713021	1.601014872	0.6789867	1.87E-08	6.07E-07
ILK	2.2309472	1.83738803	2.4015376	1.673701524	0.7430423	1.92E-08	7.49E-07

PHGDH	2.2411024	1.84618459	2.4122816	1.668999484	0.7389835	1.94E-08	7.49E-07
COL4A1	1.7913974	1.43732577	1.9448717	2.160567135	1.1114101	1.95E-08	9.34E-07
TUFM	3.8319564	3.1720399	4.1180007	1.435517241	0.5215707	1.97E-08	7.60E-07
ATP6V0D1	1.6156184	1.3017036	1.7516865	2.49147333	1.3169991	2.02E-08	1.07E-06
COX7C	25.057199	22.4399177	26.191674	1.174989308	0.2326476	2.17E-08	8.41E-07
HSBP1	11.054281	9.4097793	11.767099	1.280306988	0.3564898	2.18E-08	8.13E-07
RDX	3.7228548	3.09059175	3.9969127	1.433523643	0.5195657	2.22E-08	8.94E-07
METRN	2.8999011	3.40750206	2.6798787	0.697768314	-0.51918	2.26E-08	8.41E-07
CTSD	2.243967	1.84959917	2.4149078	1.66538278	0.7358538	2.39E-08	8.90E-07
HACD3	3.1770813	2.62985132	3.4142811	1.481289163	0.5668533	2.48E-08	9.60E-07
LOXL1	2.654407	2.20985386	2.847101	1.526714177	0.61043	2.51E-08	9.36E-07
NDUFS8	4.4345237	3.74089986	4.7351787	1.36275636	0.4465277	2.66E-08	1.67E-06
ADH5	2.2074901	1.84196901	2.3659271	1.62230096	0.6980415	2.66E-08	9.56E-07
CD9	2.4651789	2.01979366	2.6582335	1.626048094	0.7013699	2.72E-08	1.01E-06
PSMA2	3.9075077	3.13721333	4.2413959	1.516645951	0.6008843	2.73E-08	1.25E-06
ITGAV	2.2188184	1.83273123	2.38617	1.664606759	0.7351814	2.75E-08	8.66E-07
CAPG	1.470533	1.17535273	1.5984804	3.413008859	1.7710442	2.85E-08	1.20E-06
BRCA1	1.4321253	1.735164	1.3007716	0.409121769	-1.2893978	2.92E-08	9.79E-07
DCBLD2	8.7243115	7.04695306	9.4513716	1.397624804	0.4829771	2.92E-08	9.79E-07
COPZ1	2.5635556	2.1183092	2.7565501	1.570719491	0.6514256	2.93E-08	1.05E-06
ANXA6	1.7540797	1.4311849	1.8940402	2.073449684	1.052033	3.01E-08	1.52E-06
TRAPPC1	4.6340379	3.82735275	4.9836999	1.408985812	0.4946571	3.05E-08	1.22E-06
DDX17	5.4766545	6.41282344	5.070867	0.752078291	-0.4110452	3.22E-08	1.12E-06
TMEM14A	2.2678274	1.80650176	2.4677915	1.819948295	0.8638975	3.29E-08	1.44E-06
RAB18	1.8465069	1.51635699	1.9896121	1.916527	0.9384943	3.39E-08	1.26E-06
CCDC14	2.4574523	2.89148406	2.2693189	0.671070352	-0.5754641	3.43E-08	1.33E-06
PHB	4.2027069	3.53924032	4.4902901	1.374541063	0.45895	3.60E-08	1.10E-06
UBXN1	3.6508395	3.03938914	3.915876	1.429779133	0.5157923	3.62E-08	1.74E-06
PAIP1	2.7577283	2.30125906	2.9555873	1.502842405	0.5876937	3.72E-08	1.21E-06
TMED3	2.8068282	2.35058623	3.0045887	1.484235991	0.5697205	3.75E-08	1.34E-06
PSMC1	2.4478201	2.03654933	2.6260876	1.568750815	0.6496162	3.88E-08	1.15E-06
FKBP8	4.2400011	3.58842524	4.5224302	1.360839078	0.4444965	3.88E-08	1.85E-06
ZNF503	1.5712697	1.26465165	1.7041749	2.660761478	1.4118392	3.90E-08	1.40E-06
DHFR	1.954104	2.34407658	1.7850683	0.584094898	-0.7757253	4.01E-08	2.37E-06
TCEAL9	8.6098631	7.36934726	9.1475715	1.279184688	0.3552246	4.01E-08	1.44E-06
MIS18BP1	2.1285402	2.55962645	1.9416835	0.603787829	-0.7278864	4.05E-08	1.85E-06
PSMB5	4.335779	3.63385367	4.6400323	1.382017661	0.4667761	4.09E-08	1.29E-06
PCBP1	4.171824	3.45753491	4.4814365	1.416637646	0.5024708	4.26E-08	1.58E-06
FDCSP	1.3667746	1.04123591	1.5078811	12.31647791	3.6225178	4.33E-08	1.82E-06
NUDT5	2.1022044	1.75155433	2.2541956	1.668802285	0.738813	4.46E-08	2.04E-06
CTNNA1	2.3579694	1.951764	2.5340413	1.611787502	0.6886616	4.46E-08	1.66E-06
SERPINB6	2.785165	2.31183889	2.9903308	1.517206742	0.6014177	4.55E-08	1.83E-06
DDB2	2.0738328	2.4830861	1.8964398	0.604442153	-0.7263238	4.61E-08	2.73E-06
SH3BGRL	3.2287542	2.72154365	3.4486074	1.422332454	0.5082587	4.92E-08	2.75E-06
RBX1	6.5116404	5.57384603	6.9181325	1.293907236	0.3717342	5.19E-08	1.74E-06
EIF4E	2.2141017	1.85058145	2.3716715	1.612628026	0.6894137	5.31E-08	2.81E-06
ANKRD45	4.1632376	5.06485055	3.7724286	0.682049338	-0.552052	5.38E-08	2.24E-06
FAM84B	1.4774737	1.18068235	1.6061195	3.35461393	1.7461467	5.46E-08	1.77E-06
GNAS	6.2586929	5.32717135	6.662466	1.308583731	0.3880062	5.57E-08	2.24E-06
DEK	4.9951754	5.8467425	4.626059	0.74814353	-0.418613	5.81E-08	1.67E-06
TMEM147	4.6638798	3.94318149	4.9762704	1.351010948	0.4340394	5.98E-08	2.31E-06

TMED10	3.0876451	2.59012921	3.303296	1.448496154	0.5345559	6.24E-08	2.17E-06
RHOC	4.903801	4.09379653	5.2549018	1.375301125	0.4597475	6.25E-08	2.73E-06
CAST	2.759774	2.29246372	2.9623322	1.518288032	0.6024455	6.33E-08	2.12E-06
LUC7L3	4.4272229	5.13951513	4.118476	0.753343296	-0.4086206	6.47E-08	2.17E-06
KDELR2	3.2709988	2.72386068	3.5081588	1.454966086	0.5409855	6.50E-08	2.42E-06
H2AFY	5.1087224	4.28291944	5.4666712	1.360578979	0.4442207	6.77E-08	2.35E-06
NR2F1	5.4996085	6.53975128	5.0487529	0.730854634	-0.4523436	6.83E-08	2.22E-06
SRSF10	3.2126028	3.76591387	2.9727672	0.71324245	-0.4875355	6.93E-08	2.40E-06
SARAF	4.8307881	3.95829099	5.2089768	1.422773078	0.5087056	7.13E-08	8.97E-06
BIRC7	2.1307524	1.75810801	2.2922771	1.704608217	0.7694402	7.46E-08	2.42E-06
BASP1	3.5307415	2.95546872	3.7800966	1.421703456	0.5076206	7.57E-08	2.54E-06
SH3BGRL3	14.175337	11.7877949	15.21023	1.317250636	0.3975299	7.61E-08	3.06E-06
PSMA1	4.4702621	3.76544971	4.7757669	1.365335573	0.4492556	7.92E-08	2.84E-06
XRCC2	1.3715024	1.65828476	1.2471951	0.375513897	-1.4130618	8.21E-08	2.58E-06
TCEAL4	7.4683069	6.4025565	7.9302622	1.282774595	0.3592677	8.33E-08	2.62E-06
PGRMC1	4.5236684	3.69473606	4.8829736	1.440947638	0.5270179	8.33E-08	3.27E-06
SLC16A1-AS1	1.6259301	1.93716266	1.4910246	0.523948156	-0.932504	8.35E-08	4.20E-06
COTL1	5.2870906	4.36560474	5.6865136	1.39247297	0.4776493	8.53E-08	3.27E-06
CTSL	1.7310609	1.41998977	1.8658964	2.06170825	1.0438402	8.78E-08	3.27E-06
COX7B	7.7100757	6.60097788	8.1908202	1.283850855	0.3604776	8.79E-08	3.05E-06
MAGED1	3.0309536	2.52444936	3.2505006	1.476271112	0.5619577	8.92E-08	3.45E-06
BZW1	3.4504915	2.92351687	3.6789116	1.392715398	0.4779005	9.11E-08	3.66E-06
S100A16	4.3124591	3.3227916	4.7414358	1.610749691	0.6877323	9.32E-08	1.04E-05
DIO3OS	1.8108203	2.13765255	1.669153	0.588187521	-0.7656519	9.60E-08	2.68E-06
MTCH2	3.0607227	2.5170947	3.2963613	1.513657166	0.5980385	1.01E-07	3.64E-06
CRB1	1.9664461	2.3536929	1.7985918	0.589935758	-0.7613702	1.02E-07	3.10E-06
CDON	2.362134	2.80521888	2.1700765	0.648163236	-0.6255709	1.02E-07	4.29E-06
GNPDA1	2.1442344	1.79185057	2.2969771	1.637906375	0.7118529	1.04E-07	3.87E-06
SPCS1	4.6739072	3.93111892	4.9958728	1.363258493	0.4470591	1.09E-07	4.20E-06
CSNK2B	3.7768701	3.19038116	4.0310869	1.383817091	0.4686533	1.11E-07	3.39E-06
MINOS1	4.5158272	3.78740353	4.8315664	1.374600544	0.4590124	1.14E-07	3.81E-06
NDUFA1	5.3845337	4.49179065	5.7714981	1.366490299	0.4504752	1.21E-07	4.21E-06
NKTR	3.430805	4.01207643	3.1788497	0.72337131	-0.4671917	1.22E-07	4.37E-06
IER3	1.6395229	1.32252462	1.7769276	2.408893848	1.2683708	1.26E-07	3.42E-06
PSMC3	2.4463686	2.0411381	2.622018	1.557927834	0.6396284	1.30E-07	4.08E-06
FEZ2	1.9529148	1.62103869	2.0967683	1.766022567	0.8205038	1.44E-07	4.83E-06
LMNA	2.0867568	1.72628431	2.2430055	1.711458556	0.7752264	1.49E-07	4.85E-06
ATOX1	5.2689915	4.4763877	5.6125499	1.32682263	0.4079755	1.52E-07	5.64E-06
FRZB	1.4316482	1.14441628	1.5561504	3.851022896	1.9452417	1.56E-07	5.80E-06
HM13	2.0501257	1.71921241	2.1935618	1.659540065	0.7307835	1.59E-07	5.17E-06
GNG5	10.187541	8.73596492	10.816734	1.268973429	0.3436619	1.66E-07	5.95E-06
PLTP	1.8704756	1.5552586	2.0071081	1.813764088	0.8589868	1.72E-07	5.41E-06
COPS8	2.3540399	1.96973229	2.5206201	1.56808239	0.6490014	1.73E-07	5.63E-06
CPNE3	2.5059865	2.10781657	2.6785754	1.515210555	0.5995183	1.74E-07	7.61E-06
DYNC1I2	3.8550469	3.25622391	4.11461	1.380452537	0.4651413	1.84E-07	5.62E-06
CHRNA3	1.4170359	1.13532636	1.5391444	3.984031239	1.994229	1.88E-07	5.91E-06
SCOC	3.854059	3.27906465	4.1032934	1.361652216	0.4453583	1.89E-07	7.94E-06
PTP4A3	1.7288235	1.41296512	1.8657341	2.096385423	1.067904	1.96E-07	7.02E-06
PTGR1	2.0705534	1.73374638	2.2165443	1.657990148	0.7294354	1.98E-07	5.85E-06
COL8A1	1.3639259	1.08905874	1.4830686	5.424156883	2.4393989	2.02E-07	6.16E-06
ADRM1	1.9245043	1.6064419	2.0623702	1.751808727	0.8088453	2.09E-07	7.01E-06

CHMP2A	3.9240322	3.31430781	4.1883206	1.377656249	0.462216	2.17E-07	6.23E-06
SDHB	1.84073	1.53031959	1.9752791	1.839040289	0.8789531	2.17E-07	7.81E-06
PDLIM2	2.1863071	1.84192307	2.3355823	1.586347143	0.6657085	2.25E-07	1.08E-05
CACYBP	2.7773075	2.24590061	3.0076487	1.611403589	0.6883179	2.28E-07	7.89E-06
TNRC6B	4.2596569	4.97397765	3.9500307	0.742337003	-0.4298538	2.31E-07	6.82E-06
COLGALT2	1.8128242	1.49962109	1.9485838	1.898606478	0.9249409	2.60E-07	7.94E-06
GLO1	3.6173569	2.99725467	3.8861436	1.445055373	0.5311248	2.62E-07	8.25E-06
BNIP3	13.526323	11.7325525	14.303842	1.239578561	0.3098497	2.65E-07	7.70E-06
COX17	3.2020641	2.71172976	3.4146022	1.410621163	0.4963306	2.68E-07	7.70E-06
TWF1	1.8099515	1.50471303	1.9422588	1.866919907	0.90066	2.77E-07	2.79E-05
COX7A2	10.677624	8.7004831	11.534627	1.368047551	0.4521184	2.77E-07	8.71E-06
KDELR1	6.043914	5.0972897	6.4542334	1.331180806	0.4127065	2.93E-07	1.18E-05
CAPNS1	2.2651683	1.91110144	2.4186405	1.557060996	0.6388255	2.95E-07	1.02E-05
HIF1A	2.519207	2.13059196	2.6876542	1.492717356	0.577941	2.96E-07	1.35E-05
RHEB	5.0186434	4.2456763	5.3536901	1.341381496	0.4237196	2.98E-07	8.83E-06
PDPN	1.8349575	1.52070521	1.9711719	1.865108902	0.8992599	3.02E-07	8.93E-06
CLDN3	1.8415593	1.533093	1.9752657	1.829447512	0.871408	3.13E-07	8.74E-06
SYPL1	1.8738062	1.54016038	2.0184268	1.885415651	0.9148826	3.51E-07	1.18E-05
PSMD11	1.9146388	1.60394716	2.0493098	1.737419821	0.7969464	3.53E-07	1.97E-05
TGFBI	1.4332305	1.14489799	1.5582097	3.852432559	1.9457697	3.63E-07	1.04E-05
RBBP7	3.752143	3.18136725	3.9995488	1.375077392	0.4595128	3.69E-07	1.32E-05
TTC14	2.4242129	2.81471833	2.2549463	0.691537761	-0.5321201	3.95E-07	1.73E-05
HSD17B12	2.233341	1.89701187	2.3791247	1.537465418	0.620554	3.95E-07	1.10E-05
ANXA11	1.6745839	1.38513994	1.8000449	2.077283622	1.0546982	4.14E-07	1.48E-05
MT2A	4.5135946	3.03077948	5.1563289	2.046666793	1.0332762	4.15E-07	1.34E-05
PSMD13	1.8383071	1.5357818	1.9694383	1.809390136	0.8555035	4.15E-07	1.35E-05
MLLT11	5.6341862	4.85152125	5.9734365	1.291291464	0.3688147	4.16E-07	1.61E-05
EFEMP2	1.9981292	1.68055191	2.1357847	1.668917127	0.7389123	4.35E-07	1.22E-05
TRAPPC2L	2.2668525	1.92418053	2.4153856	1.531503334	0.6149485	4.43E-07	2.34E-05
ETFA	2.1266531	1.80166026	2.2675231	1.581122485	0.6609491	4.48E-07	1.29E-05
PTOV1	3.0690666	2.60660486	3.2695231	1.41262061	0.4983741	4.55E-07	1.39E-05
METTL5	2.1901626	1.84025317	2.3418328	1.596938633	0.6753089	4.57E-07	1.39E-05
CXCR4	2.0071235	1.5923574	2.1869061	2.003699285	1.002666	4.65E-07	1.46E-05
SLC6A15	1.5654215	1.27905648	1.6895479	2.470997676	1.3050937	4.82E-07	1.67E-05
VDAC3	2.7381029	2.30555379	2.9255937	1.474924813	0.5606414	4.85E-07	1.39E-05
DNAJC1	4.4234648	5.11405685	4.1241239	0.759377912	-0.3971101	4.88E-07	1.96E-05
SEC11C	2.3744844	2.00634873	2.5340547	1.524376888	0.6082196	4.98E-07	1.73E-05
LRP10	1.7099254	1.42023265	1.8354943	1.988170919	0.9914418	5.16E-07	1.57E-05
VPS28	3.2475669	2.75928191	3.4592167	1.397852569	0.4832122	5.29E-07	1.56E-05
ARL3	3.4115331	2.88590489	3.6393694	1.399524151	0.4849364	5.40E-07	1.76E-05
ATP6V1F	5.9679915	5.13721211	6.3280973	1.287847275	0.3649615	5.42E-07	1.76E-05
MGST3	4.8526078	4.14762927	5.1581846	1.321052854	0.4016882	5.45E-07	1.71E-05
RLBP1	1.2924693	1.03781132	1.4028522	10.65427705	3.4133608	5.47E-07	1.49E-05
SNX17	2.1356773	1.81218247	2.2758979	1.570949758	0.651637	5.50E-07	2.09E-05
SARS	2.1420226	1.79975844	2.2903788	1.613460707	0.6901584	5.60E-07	1.66E-05
COL9A3	1.5596246	1.28389861	1.6791395	2.3921905	1.2583323	5.66E-07	5.18E-05
GRSF1	2.0974861	1.77329704	2.2380076	1.600947061	0.6789256	5.69E-07	1.55E-05
CENPU	1.606087	1.89489498	1.4809016	0.537383229	-0.8959768	5.92E-07	2.09E-05
EIF4A3	1.6065655	1.3274595	1.7275456	2.221787964	1.1517211	5.94E-07	2.09E-05
NIPSNAP1	2.9315333	2.48670701	3.1243456	1.428893249	0.5148981	6.02E-07	2.09E-05
PPP2CA	2.0273179	1.71288112	2.1636123	1.632266922	0.706877	6.14E-07	1.66E-05

ROMO1	6.4501467	5.51012809	6.8576028	1.298766399	0.377142	6.15E-07	1.72E-05
TCEB2	13.625456	11.7289424	14.447511	1.253386424	0.3258313	6.26E-07	2.10E-05
GSTA4	2.12305	1.79620347	2.2647233	1.58844239	0.6676128	6.27E-07	1.66E-05
ZFP36L1	9.1618172	10.5536106	8.5585368	0.791170708	-0.3379391	6.39E-07	3.21E-05
PRPH	1.2951594	1.04061196	1.4054943	9.984604244	3.3197052	6.51E-07	2.05E-05
NDUFC1	6.1101484	5.28865502	6.4662291	1.274578882	0.3500207	6.55E-07	1.70E-05
MRPL47	2.0993783	1.78111436	2.2373315	1.584059336	0.6636264	6.60E-07	1.84E-05
ZIC2	1.6814177	1.97657175	1.5534816	0.566759795	-0.8191907	6.60E-07	1.70E-05
COL6A2	1.9179442	1.6072059	2.0526353	1.733572293	0.793748	6.70E-07	2.04E-05
CDC123	1.7565787	1.46626906	1.882415	1.892501783	0.9202947	6.92E-07	1.83E-05
DKK3	5.9007246	5.06992452	6.2608393	1.292613491	0.370291	7.18E-07	1.95E-05
C5orf24	2.6680947	2.27938082	2.8365849	1.435526332	0.5215798	7.35E-07	3.08E-05
WBP2	1.6895508	1.40498284	1.8128983	2.007241307	1.0052141	7.38E-07	2.39E-05
DSC2	1.6999236	1.41546655	1.8232231	1.98144258	0.9865512	7.51E-07	3.60E-05
SLIRP	6.2996603	5.46844613	6.6599545	1.266649385	0.3410172	7.56E-07	2.00E-05
RAB32	1.6736373	1.39168825	1.7958497	2.031844769	1.0227902	7.73E-07	2.99E-05
ERP44	1.6589983	1.3784493	1.7806038	2.06263776	1.0444905	7.93E-07	2.35E-05
TERF2IP	2.4873757	2.1078827	2.6518691	1.491014418	0.5762942	8.29E-07	2.78E-05
LGMN	1.5778336	1.30491286	1.6961325	2.283054052	1.190965	8.66E-07	2.23E-05
TUSC3	3.6338951	3.07715387	3.8752176	1.384210216	0.4690631	8.66E-07	2.81E-05
NAA20	1.8533629	1.55807499	1.981357	1.758468041	0.8143191	8.82E-07	3.06E-05
NDUFB3	3.3797898	2.86436781	3.6032022	1.396292207	0.4816009	9.16E-07	2.36E-05
YIPF3	2.4843429	2.10262962	2.6497986	1.496240008	0.5813416	9.61E-07	3.22E-05
CENPV	6.1889175	7.09577678	5.7958344	0.786747052	-0.3460282	9.64E-07	2.94E-05
CPAMD8	1.9941163	2.33286626	1.8472832	0.635685122	-0.6536158	9.93E-07	2.85E-05
CHCHD2	14.296531	12.4594553	15.092821	1.229798529	0.298422	1.01E-06	2.74E-05
HOMER2	3.2866352	3.80439602	3.0622089	0.735348677	-0.4434996	1.02E-06	2.93E-05
G6PC3	2.3402309	1.95444869	2.5074502	1.579393686	0.6593708	1.13E-06	9.50E-05
DCTN6	1.7443472	1.46100264	1.8671644	1.881040029	0.9115306	1.15E-06	3.73E-05
CDC42	5.3874215	4.5238623	5.7617359	1.351283208	0.4343301	1.15E-06	3.22E-05
GNAI3	2.1725621	1.85047496	2.3121725	1.542870212	0.6256167	1.16E-06	4.59E-05
ACTN4	2.4520788	2.0841542	2.6115578	1.486465443	0.5718859	1.18E-06	2.98E-05
CRIM1	1.6491159	1.36289664	1.7731792	2.130576784	1.091244	1.19E-06	4.59E-05
POGZ	2.2590889	2.62637945	2.0998848	0.676278076	-0.5643115	1.19E-06	3.16E-05
FIGN	2.916925	3.42289629	2.697609	0.700652761	-0.5132285	1.20E-06	3.66E-05
DDX24	2.6917055	2.30806741	2.8579955	1.420412633	0.5063101	1.26E-06	4.71E-05
SNCG	1.7310319	1.41191462	1.869355	2.110522422	1.0776002	1.27E-06	3.46E-05
BMPR1B	1.7299461	2.03520194	1.5976312	0.57730885	-0.7925848	1.29E-06	4.46E-05
NSFL1C	1.7284729	1.44732223	1.8503392	1.900954573	0.9267241	1.31E-06	0.0001013
EIF2S2	3.3887691	2.90417254	3.5988202	1.364802884	0.4486926	1.33E-06	3.27E-05
DNAJB6	3.1463155	2.67249961	3.3516936	1.406095146	0.4916942	1.33E-06	3.53E-05
BTF3	17.678478	15.8430286	18.474064	1.177257291	0.2354297	1.35E-06	3.98E-05
FUS	5.5464476	6.31539103	5.2131448	0.792631214	-0.3352783	1.35E-06	3.39E-05
RSL24D1	3.7313601	3.21033157	3.9572027	1.337900059	0.4199704	1.35E-06	5.03E-05
COX6B1	12.257297	10.7840568	12.895881	1.215843457	0.2819575	1.40E-06	4.41E-05
B4GAT1	1.6363514	1.36423713	1.7543008	2.07090589	1.050262	1.43E-06	6.53E-05
SNX2	1.8653914	1.57423016	1.9915968	1.726828101	0.7881245	1.49E-06	3.95E-05
NPM1	26.238578	23.5165972	27.418435	1.173287199	0.2305562	1.54E-06	3.79E-05
PDCD5	3.4299176	2.94301307	3.640969	1.359213182	0.4427717	1.55E-06	3.99E-05
OSBPL1A	2.4043186	2.04724949	2.5590922	1.488749523	0.574101	1.65E-06	5.17E-05
LINC00969	2.2535127	2.59312762	2.1063048	0.694423213	-0.5261129	1.65E-06	4.15E-05

PTMA	80.677227	71.8175101	84.517519	1.179334302	0.2379727	1.66E-06	5.05E-05
ECI2	2.1772277	1.86423024	2.3128982	1.519153261	0.6032674	1.71E-06	4.33E-05
PFKL	2.2602257	1.92066553	2.4074099	1.528687466	0.6122935	1.72E-06	4.94E-05
ZCCHC11	2.9182711	3.35565932	2.7286828	0.73384246	-0.4464577	1.72E-06	4.33E-05
HYAL2	2.2222672	1.90211282	2.3610399	1.508724662	0.5933295	1.80E-06	6.46E-05
PABPN1	4.1730976	4.7695531	3.9145608	0.773184693	-0.371115	1.81E-06	4.34E-05
PSMD4	2.91748	2.45512827	3.1178889	1.455465408	0.5414806	1.89E-06	5.29E-05
BAZ2B	2.8094893	3.22933541	2.6275047	0.730040315	-0.453952	1.95E-06	6.99E-05
UQCRC2	2.5586171	2.19665397	2.7155121	1.433590739	0.5196332	1.95E-06	6.76E-05
HACD1	1.6050254	1.3389892	1.7203402	2.124965145	1.0874392	1.99E-06	6.26E-05
FIS1	4.1699184	3.60349716	4.4154367	1.311864961	0.3916192	2.04E-06	8.93E-05
NRP2	1.5138125	1.24708634	1.6294264	2.547394409	1.3490223	2.09E-06	6.99E-05
CDK5	1.4871836	1.23137019	1.5980673	2.584893467	1.3701048	2.11E-06	7.07E-05
NDUFS6	4.7459467	3.98502252	5.0757734	1.365407929	0.449332	2.11E-06	0.0001518
BHLHE40	1.8360211	1.5271392	1.9699076	1.839945887	0.8796633	2.15E-06	9.00E-05
NDUFB11	6.5999537	5.69301873	6.9930696	1.277018051	0.3527789	2.16E-06	7.01E-05
ZFAND6	2.6877912	2.28821305	2.8609906	1.444629483	0.5306995	2.28E-06	5.88E-05
PSMA3	2.7174707	2.33024947	2.8853138	1.417263344	0.5031079	2.31E-06	5.67E-05
CCND1	3.6565545	3.03889489	3.9242823	1.434248696	0.5202952	2.35E-06	6.76E-05
TSPAN10	1.5431255	1.27496838	1.6593597	2.397947471	1.2618001	2.39E-06	6.50E-05
EMC4	2.6896643	2.31961387	2.8500646	1.401974207	0.4874598	2.39E-06	8.92E-05
RP11-490M8.1	1.664878	1.39158343	1.7833391	2.000439795	1.0003172	2.58E-06	8.87E-05
ELP4	1.594043	1.33226408	1.7075125	2.129367984	1.0904253	2.60E-06	5.99E-05
GNB2	3.8997689	3.36667678	4.1308406	1.322884758	0.4036874	2.62E-06	5.99E-05
MGARP	4.1483757	3.46442512	4.4448378	1.397826116	0.4831849	2.62E-06	9.43E-05
GADD45GIP1	4.8115617	4.14771767	5.0993085	1.302311375	0.3810744	2.64E-06	6.32E-05
ACTC1	1.8366423	1.29770843	2.0702461	3.5949473	1.8459706	2.65E-06	8.87E-05
MAPRE1	2.1233267	1.81512685	2.2569176	1.541990214	0.6247936	2.71E-06	6.05E-05
TSEN34	2.8003011	2.40900339	2.9699112	1.39808837	0.4834556	2.73E-06	0.0001098
RP11-71N10.1	2.1190201	2.43752401	1.9809629	0.68239758	-0.5513156	2.74E-06	8.11E-05
GDE1	1.8984628	1.61323351	2.0220969	1.666733648	0.7370236	2.84E-06	7.31E-05
HNRNPA0	6.2008838	5.42344696	6.537868	1.251934994	0.3241597	2.89E-06	8.31E-05
GPX4	13.905584	12.7304538	14.41495	1.143600295	0.1935829	2.91E-06	0.0001009
BDH2	2.117218	1.81094564	2.2499735	1.541377644	0.6242204	2.95E-06	7.42E-05
WDR61	1.888623	1.60516002	2.0114916	1.671444813	0.7410957	2.97E-06	9.97E-05
CIAPIN1	1.6447218	1.38031402	1.7593308	1.99658902	0.9975374	3.11E-06	0.0001
REXO2	2.0436044	1.74168071	2.1744749	1.583531635	0.6631457	3.14E-06	9.13E-05
BEX3	23.399359	21.2896454	24.313826	1.149050428	0.2004421	3.15E-06	8.81E-05
DUSP4	1.3948209	1.13824173	1.5060366	3.660519695	1.8720485	3.18E-06	9.13E-05
HSPA9	2.1008072	1.79030144	2.2353976	1.563197965	0.6445005	3.18E-06	0.0001
KRTCAP2	2.8318039	2.43326175	3.0045542	1.398596029	0.4839793	3.20E-06	7.48E-05
SELENOK	4.2111374	3.64673148	4.4557823	1.305679218	0.3848005	3.28E-06	7.16E-05
TMEM2	1.6972629	1.97262725	1.5779048	0.594168806	-0.7510552	3.32E-06	0.0001012
ENO2	3.1149465	2.65585532	3.313942	1.397430079	0.4827761	3.37E-06	0.0001059
DUSP1	1.7632885	1.47912975	1.8864587	1.850143356	0.8876371	3.38E-06	9.44E-05
PEPD	1.6173303	1.35642475	1.7304212	2.049299771	1.035131	3.43E-06	0.0001045
TBX2-AS1	2.5065931	2.94110268	2.3182526	0.679125616	-0.5582496	3.57E-06	0.0001199
NDUFA11	7.0662102	6.20150434	7.4410217	1.238299778	0.3083606	3.66E-06	0.0001022
P3H2	1.819361	1.54372488	1.9388369	1.726676435	0.7879978	3.79E-06	0.0001157
ALCAM	1.5891049	1.33217742	1.7004715	2.108726992	1.0763723	3.87E-06	0.0001043
PPA2	1.9192857	1.63652554	2.0418496	1.636775851	0.7108568	3.93E-06	0.0001198

NCCRP1	1.3075967	1.0585923	1.415529	7.091870261	2.8261661	3.93E-06	0.0001162
PPT1	1.9704679	1.66941529	2.1009608	1.644660441	0.7177898	3.94E-06	0.0001043
CKB	9.2493143	7.09259413	10.184156	1.507429532	0.5920906	3.98E-06	0.0001179
BTG1	10.599571	9.29493697	11.165072	1.225454965	0.2933175	4.15E-06	0.0001018
EAPP	1.7389134	1.47112304	1.8549886	1.814788442	0.8598014	4.16E-06	0.0001231
PSMC4	1.6713384	1.40820647	1.7853943	1.924012437	0.9441181	4.18E-06	0.0001202
TMED2	4.2328695	3.63798012	4.4907276	1.323257728	0.4040941	4.18E-06	0.0001079
CYCS	3.6185178	3.10664909	3.8403901	1.348297672	0.431139	4.22E-06	0.000101
CADM1	2.1532752	1.84379489	2.2874212	1.52575135	0.6095199	4.23E-06	0.0001064
SCP2	3.2771633	2.81594285	3.4770818	1.364074747	0.4479227	4.50E-06	0.0001222
SLC39A6	2.2710078	1.92050215	2.4229364	1.545826231	0.6283782	4.54E-06	0.0002802
NFU1	1.8400264	1.56461328	1.9594057	1.699226215	0.7648779	4.55E-06	0.0002802
C19orf48	2.4679309	2.83234648	2.309973	0.714915534	-0.4841553	4.58E-06	0.0001046
HNRNPM	3.4181466	2.93441376	3.6278233	1.358459766	0.4419718	4.61E-06	0.0001288
RAD9A	1.5077206	1.76429777	1.3965058	0.518784504	-0.9467927	4.67E-06	0.0001341
ANXA3	1.3260484	1.09389906	1.4266748	4.543973303	2.1839544	4.69E-06	0.0001523
CXADR	2.4016753	2.02644208	2.5643222	1.524023825	0.6078855	4.74E-06	0.0002802
DGUOK	3.921523	3.39759497	4.1486224	1.313241991	0.3931328	4.85E-06	0.0001219
MSMO1	1.8687071	1.57233409	1.9971716	1.742289392	0.8009843	4.97E-06	0.0001219
CNTN5	1.3655712	1.60939436	1.2598847	0.426463904	-1.2295045	4.97E-06	0.000139
GINS2	1.9003341	2.21585257	1.7635709	0.628012763	-0.6711342	5.00E-06	0.0001227
ARGLU1	4.9207248	5.6109248	4.6215538	0.785428953	-0.3484473	5.02E-06	0.0001122
PCCA	2.0324271	2.3494315	1.8950199	0.663256995	-0.5923601	5.04E-06	0.0001268
IVNS1ABP	2.407102	1.97927441	2.5925462	1.626251224	0.7015501	5.18E-06	0.0001446
NUDT4	5.1362917	5.96741815	4.7760355	0.760160581	-0.3956239	5.33E-06	0.0001578
UBE2D1	1.7915736	1.52213568	1.908363	1.739706708	0.7988441	5.34E-06	0.000145
ATF6B	2.2312591	2.56453974	2.0867968	0.69464314	-0.5256561	5.35E-06	0.0001312
TSPAN6	2.1835456	1.88462269	2.3131153	1.484378994	0.5698595	5.37E-06	0.0001461
TMCO1	2.7717309	2.39067574	2.9369013	1.392777082	0.4779644	5.39E-06	0.0001178
TCEB1	3.6509174	3.16227581	3.8627218	1.32393924	0.4048369	5.49E-06	0.0001304
LITAF	2.456475	2.11794981	2.6032106	1.434063137	0.5201085	5.61E-06	0.0002172
TFG	2.2695443	1.95316986	2.4066785	1.47578993	0.5614874	5.64E-06	0.0001666
CMTM6	2.0713135	1.78238541	2.196551	1.529362586	0.6129305	5.69E-06	0.0001845
HNRNPH2	1.7332439	1.4688862	1.8478312	1.808181204	0.8545393	5.70E-06	0.0001304
PTTG1IP	3.4451781	2.98106916	3.6463487	1.335818425	0.4177239	5.70E-06	0.0001304
SNRPD3	2.6025332	2.25667714	2.7524463	1.394508013	0.4797562	5.76E-06	0.0001868
TCEAL8	5.2946071	4.55604265	5.6147419	1.297718374	0.3759773	5.80E-06	0.0001535
AP1S2	5.7497701	5.0267284	6.0631765	1.257392098	0.3304346	5.92E-06	0.0001417
RFK	1.5947624	1.34200082	1.7043233	2.059420019	1.0422381	6.02E-06	0.0001591
PPP1R7	1.6961364	1.43597949	1.8089029	1.855369146	0.8917063	6.13E-06	0.000176
MAB21L2	1.5726872	1.32235439	1.6811953	2.113187649	1.0794209	6.41E-06	0.0002016
SPG21	3.098783	2.62891029	3.3024519	1.413492118	0.4992638	6.46E-06	0.0001711
NOL7	2.1676835	1.86152788	2.3003883	1.50939777	0.593973	6.98E-06	0.0001956
PNN	3.5556639	4.03990793	3.3457657	0.771656838	-0.3739687	7.00E-06	0.0001956
MAGEF1	2.4213917	2.08081846	2.5690151	1.451691585	0.537735	7.19E-06	0.000167
PDLIM3	1.5817673	1.33195179	1.6900512	2.078769346	1.0557297	7.25E-06	0.000187
ORMDL2	1.4719884	1.23146341	1.5762453	2.489574093	1.315899	7.30E-06	0.000167
DHRS7	1.6974387	1.43907473	1.809428	1.843485647	0.8824362	7.34E-06	0.000211
CD74	1.7021315	1.44423256	1.8139193	1.832191815	0.8735705	7.38E-06	0.000274
HPF1	2.0756514	1.78999661	2.1994699	1.518322902	0.6024786	7.54E-06	0.0001614
QPRT	4.2498636	3.66584942	4.5030078	1.314030625	0.3939989	7.60E-06	0.0001819

ZNF518A	2.3409512	2.68023659	2.193886	0.710546387	-0.4929993	7.61E-06	0.000163
TRAPPC3	2.4012007	2.07831517	2.5411571	1.429226995	0.5152351	7.63E-06	0.000274
VAT1	2.3680921	2.0567355	2.5030513	1.422353334	0.5082799	7.90E-06	0.0002482
OSTC	5.9812846	5.26029912	6.2937997	1.242588726	0.3133489	8.12E-06	0.0001816
IDI1	1.9918818	1.69065113	2.1224519	1.625208159	0.7006245	8.15E-06	0.0002278
RUVBL1	1.6811778	1.42575696	1.7918913	1.859960951	0.8952723	8.26E-06	0.0002245
SNX6	2.3600558	2.04527179	2.4965007	1.431685701	0.5177148	8.37E-06	0.0002159
MRPS15	2.4881375	2.16177173	2.6296026	1.402687397	0.4881935	8.47E-06	0.0002581
BTG3	2.6074829	2.23894632	2.767227	1.426395146	0.5123737	8.61E-06	0.0002277
PRSS33	1.2982611	1.06969496	1.3973343	5.70104743	2.511227	8.73E-06	0.0002043
NUCB2	2.3791474	2.04883861	2.5223215	1.451435423	0.5374804	8.87E-06	0.0002174
CCDC28B	2.3498921	2.03628688	2.485826	1.433797905	0.5198417	8.92E-06	0.0002719
COL5A1	1.4968838	1.24925661	1.6042192	2.424084783	1.2774402	8.93E-06	0.0002138
LHFP	1.408692	1.1760463	1.5095335	2.894315554	1.5332222	8.93E-06	0.0003014
RBMS1	2.2365268	1.93222452	2.3684283	1.467917058	0.5537705	8.99E-06	0.0003014
SYT7	1.6426771	1.90162909	1.530433	0.588305104	-0.7653635	9.08E-06	0.0002076
MED4	2.2460026	1.94819227	2.3750901	1.450222831	0.5362746	9.14E-06	0.0002356
MRPL21	2.1357844	1.84777222	2.2606248	1.486985255	0.5723903	9.19E-06	0.0002148
RBM8A	3.5281298	2.9569239	3.7757221	1.418410871	0.5042755	9.20E-06	0.0002436
TUBA1B	21.560511	18.5241683	22.87663	1.248369081	0.3200445	9.21E-06	0.0002436
GNB3	1.3345911	1.10068965	1.435977	4.329908503	2.1143365	9.28E-06	0.0002171
NHLRC3	2.0413397	2.32845851	1.9168864	0.69018824	-0.5349382	9.34E-06	0.0002764
TPD52L1	4.0601373	3.45171558	4.3238609	1.3557286	0.4390684	9.50E-06	0.000239
UNC50	1.6519689	1.40090381	1.7607944	1.897698161	0.9242505	9.60E-06	0.0002192
GPR143	1.3341099	1.10724052	1.4324476	4.032501961	2.0116752	9.67E-06	0.0002492
SHISA5	2.4855794	2.13798784	2.6362449	1.43784042	0.5239036	9.81E-06	0.0002192
RBM3	3.8386932	3.30718257	4.0690794	1.330228217	0.4116738	1.01E-05	0.0002819
MRPL3	2.088355	1.80109751	2.2128683	1.514008364	0.5983732	1.01E-05	0.0002265
GRB10	1.6524083	1.9111554	1.5402529	0.592931719	-0.7540621	1.02E-05	0.0002334
RFC4	1.5405167	1.78992191	1.4324107	0.547409458	-0.8693077	1.05E-05	0.0003404
PFKP	2.0119031	1.73400388	2.13236	1.542716622	0.6254731	1.07E-05	0.0003173
HTATSF1	1.8522175	1.58692374	1.9672105	1.647932127	0.7206568	1.09E-05	0.0003416
PEMT	2.1602531	1.87421317	2.2842387	1.46902233	0.5548563	1.10E-05	0.0003153
RBPMS	1.6069686	1.36146646	1.7133829	1.973579713	0.9808148	1.11E-05	0.000294
ISOC2	2.250362	1.94754652	2.3816191	1.458101556	0.5440912	1.13E-05	0.000307
ACTL6A	1.6100283	1.36459638	1.7164122	1.964945881	0.9744896	1.14E-05	0.0003591
C14orf2	11.77064	10.3741448	12.375959	1.213546331	0.2792292	1.16E-05	0.0002928
MRPL48	1.8724512	1.60626339	1.9878317	1.629377133	0.7043206	1.18E-05	0.0003031
CBR1	1.7973802	1.53195389	1.9124306	1.715243839	0.7784137	1.18E-05	0.0002582
TMEM14C	5.6774451	4.96848977	5.9847457	1.256081277	0.3289298	1.22E-05	0.0002927
COL4A2	2.2922831	1.97393327	2.4302735	1.46855393	0.5543962	1.23E-05	0.0002692
MRPL28	2.2827892	1.94586467	2.4288309	1.510608218	0.5951295	1.24E-05	0.0006937
DNAJB9	1.7105745	1.4579488	1.8200765	1.790760285	0.8405722	1.24E-05	0.0003472
MYDGF	3.016572	2.61477778	3.1907319	1.356677036	0.4400773	1.25E-05	0.0003817
RP11-96L14.7	2.4283598	2.03607001	2.5983999	1.542752828	0.6255069	1.27E-05	0.0003269
DDIT4	12.81026	10.1261867	13.973687	1.421588991	0.5075044	1.27E-05	0.0003127
SDHC	3.1525023	2.72242461	3.3389218	1.357924073	0.4414028	1.30E-05	0.0003275
TOMM7	10.735487	9.55538641	11.247009	1.197725985	0.2602979	1.33E-05	0.000284
LEPROT	2.5377861	2.18833728	2.6892566	1.421529616	0.5074442	1.37E-05	0.0002923
SERPINB9	1.4055556	1.16983823	1.5077285	2.98948316	1.5798961	1.39E-05	0.0002908
PHIP	2.708184	3.07685753	2.5483805	0.745540072	-0.4236422	1.42E-05	0.0004075

HSPA8	11.215423	9.88604816	11.791648	1.214448509	0.2803013	1.45E-05	0.0004075
SF3B6	5.2036022	4.4972596	5.5097702	1.289515423	0.366829	1.46E-05	0.0004075
MIR4435-2HG	2.1894193	1.83503955	2.3430271	1.608339565	0.685572	1.47E-05	0.0003943
ITFG1	1.7716914	1.51672093	1.8822098	1.707323535	0.7717365	1.49E-05	0.0004284
S1PR3	1.6541401	1.91578804	1.5407274	0.590450419	-0.7601122	1.49E-05	0.0003943
CITED2	2.0044059	1.69703833	2.137636	1.632099663	0.7067292	1.50E-05	0.0003677
PSMD14	2.1246353	1.84502654	2.2458332	1.474312533	0.5600424	1.51E-05	0.0003802
CYSTM1	3.0900947	2.67595467	3.269606	1.354216818	0.4374587	1.51E-05	0.0003805
ASPH	2.7345321	2.37515326	2.8903069	1.374615421	0.459028	1.54E-05	0.0003372
RAX	1.5749165	1.82149424	1.4680361	0.569737515	-0.8116307	1.60E-05	0.0003285
GBAS	2.3002978	1.99109748	2.4343223	1.447206107	0.5332704	1.60E-05	0.0003357
DBN1	2.1011829	1.80710094	2.2286543	1.52230567	0.6062581	1.61E-05	0.0003366
NMB	1.7712031	1.5116324	1.8837155	1.727246903	0.7884743	1.66E-05	0.0004076
CHCHD3	2.2733454	1.94220869	2.4168785	1.503784105	0.5885975	1.66E-05	0.000881
NDUFA6	4.2370046	3.73007313	4.4567368	1.266170026	0.3404711	1.67E-05	0.0004541
C8orf4	1.9168707	1.62964796	2.041369	1.653890811	0.725864	1.68E-05	0.0003447
NGFR	1.4297869	1.66431323	1.3281302	0.493939027	-1.0175951	1.68E-05	0.0003925
TMA7	16.027455	14.5191958	16.681219	1.159922454	0.2140284	1.69E-05	0.0004052
PKN2	2.149509	2.44993512	2.0192877	0.70298851	-0.508427	1.70E-05	0.0004079
HNRNPH3	3.8152289	3.26936804	4.0518352	1.344795199	0.4273865	1.71E-05	0.0004086
FHL2	1.4393732	1.20343826	1.5416404	2.662431516	1.4127444	1.71E-05	0.0003579
IDH3B	1.7659657	1.5124703	1.8758446	1.709064123	0.7732065	1.72E-05	0.0003459
CYC1	3.1572687	2.74337554	3.3366729	1.340315289	0.4225724	1.82E-05	0.0004251
PIGT	1.6847375	1.43873153	1.7913702	1.803768623	0.8510143	1.83E-05	0.0009205
ZYX	1.6056683	1.36535571	1.7098331	1.942854753	0.958178	1.85E-05	0.0003965
EXOSC7	1.6527463	1.40934444	1.7582502	1.852352447	0.8893586	1.87E-05	0.0003831
NDUFB7	5.1111953	4.42411279	5.4090149	1.287637175	0.3647261	1.88E-05	0.0005111
CD99	3.9254048	3.32440488	4.1859114	1.370635324	0.4548448	1.88E-05	0.0004731
HMX1	2.6270503	3.03637098	2.449628	0.71186835	-0.4903176	1.90E-05	0.0004441
MIF	15.158726	13.8722173	15.71637	1.143266108	0.1931612	1.90E-05	0.0005791
GULP1	2.8643642	2.47674862	3.0323783	1.376252033	0.4607447	1.93E-05	0.0005383
AURKAIP1	3.8756944	3.36443874	4.0973009	1.309951851	0.3895138	1.94E-05	0.0003989
PSMD3	1.8754167	1.61735765	1.9872738	1.599192559	0.6773437	2.02E-05	0.0005343
C14orf119	2.0331783	1.75544638	2.1535628	1.526994891	0.6106952	2.03E-05	0.0005512
HINT1	22.901855	20.9000372	23.769554	1.144196566	0.1943349	2.09E-05	0.0004782
MMS22L	1.4298928	1.66178665	1.3293771	0.497708903	-1.0066259	2.16E-05	0.0005724
DEF8	1.9219505	1.66120122	2.0349738	1.565293166	0.6464329	2.19E-05	0.0004885
DDX11	1.3155285	1.53764346	1.2192515	0.407800924	-1.2940631	2.19E-05	0.0004594
PDLIM1	1.9645719	1.67825922	2.0886757	1.605102674	0.6826656	2.22E-05	0.0005731
SLF1	1.8613598	2.12385942	1.7475779	0.665188085	-0.5881658	2.23E-05	0.0004492
SLITRK5	1.5829979	1.82617242	1.4775926	0.578078607	-0.7906624	2.25E-05	0.0004913
CANX	4.9248898	4.26984094	5.2088242	1.287164828	0.3641968	2.27E-05	0.0005855
UFC1	3.4529228	3.00016482	3.6491732	1.324477459	0.4054233	2.30E-05	0.0004918
SUCLG1	1.9507933	1.68635712	2.0654147	1.552274507	0.6343837	2.32E-05	0.0005679
SELENOT	2.3665507	2.04931932	2.5040564	1.433363839	0.5194049	2.32E-05	0.0004676
THUMPD3-AS1	2.4844112	2.8413577	2.3296908	0.722125215	-0.4696791	2.33E-05	0.0006173
KRT10	4.2903642	3.74765147	4.5256059	1.283134336	0.3596722	2.34E-05	0.0005468
OAZ1	12.817901	11.8256069	13.248016	1.131393002	0.1781002	2.35E-05	0.0006058
CUEDC2	2.9338913	2.54287951	3.1033775	1.363280443	0.4470824	2.38E-05	0.0005322
LAMP5	1.6322622	1.88145638	1.5242477	0.594751721	-0.7496406	2.39E-05	0.0005856
MFAP2	2.3362458	2.03368781	2.4673913	1.419569079	0.5054531	2.41E-05	0.0006221

NREP	4.645134	3.97788187	4.9343581	1.321193473	0.4018417	2.46E-05	0.0011771
DCLK1	1.5229128	1.77269288	1.4146443	0.536622342	-0.898021	2.49E-05	0.0007372
AHNAK	1.3868627	1.16698244	1.482171	2.887555167	1.5298485	2.52E-05	0.0005746
CNIH4	2.4817707	2.09444594	2.6496587	1.507300288	0.5919669	2.52E-05	0.0006022
TCP1	3.039474	2.65455279	3.2063202	1.333484301	0.4152008	2.53E-05	0.0004927
UBE2S	2.1735595	1.85635969	2.3110515	1.530958917	0.6144356	2.54E-05	0.0006084
CTD-2034I4.2	1.9589577	2.2331405	1.8401117	0.681278147	-0.5536842	2.55E-05	0.0004927
GALK1	1.6224202	1.38516562	1.7252595	1.882980751	0.9130183	2.56E-05	0.0006285
CDC20	1.4982464	1.25028929	1.6057247	2.420098345	1.2750657	2.62E-05	0.0005377
ANGPTL4	1.6146378	1.34676486	1.7307487	2.107332108	1.0754177	2.62E-05	0.0004972
MTCH1	4.4856317	3.88857114	4.7444309	1.296291732	0.3743904	2.70E-05	0.0006464
MESDC2	2.2866868	1.99148127	2.4146453	1.426799804	0.5127829	2.77E-05	0.000558
CHD9	3.0626111	3.46426937	2.8885101	0.766357023	-0.3839114	2.78E-05	0.0005592
ATP6V1D	2.1807717	1.89480636	2.3047249	1.458108671	0.5440982	2.78E-05	0.0006085
ZFAND5	2.6555814	2.31685853	2.8024026	1.36871392	0.4528209	2.82E-05	0.0007102
NDUFB9	3.9637715	3.4611354	4.1816418	1.292753654	0.3704474	2.87E-05	0.0006005
TAGLN3	1.408161	1.16609975	1.5130838	3.089010162	1.6271446	2.90E-05	0.0005691
GRHPR	2.4674829	2.14271537	2.6082551	1.407397823	0.4930302	2.92E-05	0.0006817
ADSS	1.8569291	1.6043561	1.9664082	1.599070881	0.6772339	2.93E-05	0.0006005
NDUFB5	3.2907843	2.86011309	3.4774611	1.33188733	0.413472	2.94E-05	0.0005691
TMBIM4	2.1113153	1.83971162	2.2290433	1.463649314	0.5495699	3.06E-05	0.0008316
PRCD	1.3950091	1.17678136	1.4896011	2.7695292	1.4696407	3.26E-05	0.0008199
CLRN1	1.7817113	2.05083891	1.6650565	0.632881521	-0.6599926	3.30E-05	0.0006511
TLE4	3.3721916	2.90523582	3.5745961	1.351326719	0.4343765	3.38E-05	0.0006416
CNN2	3.4236767	3.01564703	3.6005394	1.290175965	0.3675678	3.41E-05	0.0009016
UBE2V2	2.8410833	2.49021364	2.9931697	1.337506027	0.4195454	3.44E-05	0.0008038
MAGOH	1.9611011	1.70442819	2.0723574	1.522309078	0.6062613	3.61E-05	0.0008054
PNISR	7.2098272	8.16366799	6.7963797	0.809135728	-0.3055464	3.65E-05	0.0008956
ERGIC3	4.0458929	3.57739857	4.2489643	1.260559524	0.3340642	3.67E-05	0.000924
CIB1	2.4955881	2.18014157	2.6323201	1.383156151	0.467964	3.73E-05	0.0007361
MALSU1	1.6886641	1.45108753	1.7916429	1.754965169	0.8114424	3.76E-05	0.0008784
PHPT1	6.4184529	5.7111425	6.7250404	1.215212753	0.2812089	3.82E-05	0.0008732
PLIN3	1.7712703	1.52867616	1.8764241	1.657771121	0.7292448	3.84E-05	0.0008216
CCT4	4.5562296	4.00155354	4.796657	1.264897311	0.3390203	3.91E-05	0.0007572
SLC39A1	2.7914985	2.42646209	2.9497255	1.366826022	0.4508296	3.92E-05	0.0007308
RTN3	3.2227271	2.81960549	3.3974623	1.317572603	0.3978825	4.17E-05	0.0009523
MKRN1	2.3947142	2.08413011	2.5293386	1.410659649	0.4963699	4.22E-05	0.0008322
PNMA1	2.0788258	1.81686516	2.1923741	1.459695166	0.5456671	4.28E-05	0.0010258
LRRC42	1.5367176	1.31258849	1.6338676	2.027801987	1.0199168	4.40E-05	0.0008357
SPAG7	2.6116278	2.29258629	2.7499181	1.353811427	0.4370268	4.47E-05	0.0009763
CLNS1A	3.6461195	3.21114963	3.8346595	1.281984491	0.3583788	4.47E-05	0.0012581
AP2S1	5.422371	4.81750457	5.6845537	1.227124569	0.2952817	4.50E-05	0.0012581
FXR1	2.9971444	2.61687125	3.1619759	1.337135449	0.4191456	4.60E-05	0.0010281
SERPINE2	1.6096138	1.37023493	1.7133739	1.926814039	0.9462173	4.70E-05	0.0011809
SNRPN	5.4327289	4.82107411	5.6978541	1.229459024	0.2980237	4.72E-05	0.0008635
PRNP	1.6552177	1.42373207	1.7555564	1.783099359	0.8343871	4.73E-05	0.0011598
DNASE2	1.5416939	1.31821418	1.6385624	2.006706395	1.0048295	4.74E-05	0.0008828
RNF5	3.0366965	2.6604935	3.1997637	1.324765041	0.4057365	4.84E-05	0.0012661
PAPSS1	1.567383	1.34202686	1.6650649	1.944481441	0.9593855	4.91E-05	0.0012661
CFL2	2.124493	1.85989983	2.2391823	1.441077533	0.527148	4.92E-05	0.0011777
LYPD6B	1.3688719	1.15935116	1.4596898	2.88475946	1.528451	4.97E-05	0.0011343

RAB11B	2.8587868	2.50854917	3.0105992	1.332803256	0.4144638	4.97E-05	0.0012198
YWHAZ	5.6416237	5.01229727	5.9144087	1.22483664	0.2925893	4.99E-05	0.0009294
UBE2E1	2.2301961	1.95768732	2.3483165	1.40788803	0.4935326	5.02E-05	0.0013644
DRAP1	4.0779013	3.56068858	4.3020899	1.289532024	0.3668476	5.04E-05	0.0011796
RSU1	1.5406755	1.31816069	1.6371258	2.002528371	1.0018227	5.08E-05	0.0011106
ENY2	4.6556674	4.12388481	4.8861715	1.244018811	0.3150083	5.10E-05	0.0009858
GPX1	8.1463338	7.33087657	8.4997981	1.184638182	0.2444465	5.15E-05	0.0013644
UROD	1.7323169	1.49508173	1.8351478	1.686888814	0.7543649	5.17E-05	0.0009993
POLR2E	3.3628116	2.95803875	3.5382626	1.296329115	0.374432	5.24E-05	0.0009588
ANXA7	1.5676202	1.34352812	1.6647542	1.9350794	0.9523928	5.32E-05	0.0010093
SPATA33	1.7380952	1.97935579	1.6335195	0.646873694	-0.628444	5.40E-05	0.0012636
SERBP1	8.038561	7.11345596	8.4395528	1.216914429	0.2832277	5.40E-05	0.0013588
NELFE	2.3524851	2.0541291	2.4818091	1.405718821	0.491308	5.40E-05	0.0011557
STX8	1.8040228	1.56339467	1.9083244	1.612234661	0.6890617	5.46E-05	0.0009807
SNF8	3.0951029	2.73247406	3.2522863	1.300040419	0.3785565	5.65E-05	0.001457
LINC00493	3.4153745	2.99480934	3.5976707	1.302215042	0.3809677	5.70E-05	0.0012753
POLE4	2.696899	2.29003768	2.8732552	1.452093391	0.5381342	5.72E-05	0.0013058
LAMP2	1.6070705	1.38174205	1.7047403	1.846116484	0.8844936	5.86E-05	0.001436
ATP6V1E1	1.6733447	1.44347372	1.7729836	1.743020001	0.8015891	6.00E-05	0.001373
C12orf10	1.9713956	1.72170515	2.0796253	1.495936758	0.5810492	6.06E-05	0.0011084
FAM132A	1.6749545	1.44334679	1.7753461	1.748847823	0.8064048	6.06E-05	0.0014523
RHOD	1.2685622	1.06869834	1.3551943	5.170347785	2.3702613	6.09E-05	0.0012064
SMAD2	2.815741	3.18295411	2.6565704	0.758866361	-0.3980823	6.10E-05	0.0012776
TUBG1	1.8823055	1.63791316	1.9882387	1.549174364	0.6314995	6.12E-05	0.0012064
EIF1B	3.8494897	3.40601415	4.0417166	1.264213946	0.3382406	6.24E-05	0.0014593
C19orf33	1.2772303	1.06537473	1.3690603	5.645304544	2.4970514	6.37E-05	0.0016024
CMAS	1.9313416	1.68426385	2.0384389	1.517600076	0.6017917	6.45E-05	0.0013797
RAD23A	3.4494621	3.02313559	3.6342557	1.302065799	0.3808024	6.49E-05	0.0014246
SPOCK1	2.5026461	2.18446247	2.6405645	1.385070925	0.4699599	6.52E-05	0.0014246
SLC25A11	2.1996837	1.92431481	2.3190439	1.427050473	0.5130364	6.58E-05	0.0015047
ORC6	1.6352534	1.8671273	1.5347463	0.61668724	-0.6973891	6.65E-05	0.0012857
RP11-247C2.2	1.689044	1.93091497	1.5842037	0.627558627	-0.6721779	6.77E-05	0.001661
SEC13	1.7517485	1.5179465	1.8530912	1.64706435	0.7198969	6.77E-05	0.0015488
HIST1H4C	12.798276	16.6339774	11.13567	0.648310401	-0.6252434	6.90E-05	0.0016512
OXA1L	2.3566546	2.07530121	2.4786087	1.3750647	0.4594995	6.95E-05	0.0014551
ARHGDIB	1.9080099	1.64125878	2.0236347	1.596289527	0.6747223	7.04E-05	0.0013342
PRPS1	1.7595755	1.52630356	1.8606884	1.635346011	0.7095959	7.26E-05	0.0012815
TWF2	1.5418735	1.32372223	1.6364324	1.965982993	0.9752508	7.45E-05	0.0034086
PHLDA3	2.064009	1.80371086	2.1768366	1.46425375	0.5501656	7.49E-05	0.0013459
FGFBP3	1.7023444	1.93702336	1.6006215	0.640988866	-0.6416288	7.53E-05	0.0018024
DMKN	1.522175	1.30512599	1.616256	2.019677263	1.0141248	7.54E-05	0.0014319
CENPK	1.7343961	1.97706764	1.6292088	0.643976705	-0.6349196	7.56E-05	0.0015512
FAU	31.104404	28.3194774	32.311545	1.146125345	0.1967648	7.57E-05	0.0016802
NUP93	1.5908298	1.36942053	1.6868008	1.859130137	0.8946278	7.59E-05	0.0016723
HSPD1	10.567283	9.56440484	11.001985	1.167855289	0.2238615	7.61E-05	0.0019626
PLEKHA1	2.9145032	3.32316309	2.7373673	0.747845615	-0.4191876	7.65E-05	0.0016723
LAMTOR2	3.1125623	2.6978881	3.2923051	1.350091963	0.4330577	7.68E-05	0.0016802
CCDC51	1.3518967	1.1478967	1.4403215	2.977223569	1.5739676	7.74E-05	0.001588
SLC35B2	1.6440148	1.41951849	1.741324	1.767083049	0.8213698	7.75E-05	0.0016957
AK2	2.1255285	1.87020895	2.2361982	1.420576299	0.5064763	7.80E-05	0.0013658
STK17A	1.3408455	1.13862976	1.428497	3.090945347	1.6280481	7.81E-05	0.001721

TIMM17B	4.3862512	3.83795335	4.6239139	1.276946267	0.3526978	7.83E-05	0.0017482
VSX2	1.9108827	2.16232201	1.8018949	0.689907732	-0.5355247	7.87E-05	0.0017588
BCAS2	1.6946951	1.46657377	1.7935755	1.70085755	0.7662623	7.87E-05	0.0013658
GCA	1.6078949	1.3858147	1.7041568	1.825116492	0.8679886	7.88E-05	0.001721
SFT2D1	1.9571916	1.71188712	2.0635201	1.493944924	0.579127	7.99E-05	0.0013851
HSD17B10	2.3802718	2.03121407	2.5315727	1.485213176	0.57067	8.08E-05	0.0017276
KPNA2	2.4151876	2.06335268	2.5676923	1.474291975	0.5600223	8.18E-05	0.0019014
ADARB1	1.6148815	1.84265027	1.5161539	0.612536287	-0.7071328	8.21E-05	0.0018771
CCDC80	1.7955534	1.54991171	1.9020281	1.640314414	0.7139724	8.25E-05	0.0016584
CPLX3	1.231325	1.03357808	1.3170394	9.441856451	3.2390705	8.32E-05	0.0019014
SLC35B1	1.8172332	1.58224849	1.9190886	1.578516147	0.658569	8.35E-05	0.0018245
ACTR2	2.4564094	2.14610286	2.5909134	1.388106999	0.4731188	8.38E-05	0.0017927
MRPL17	2.1275749	1.87163738	2.2385125	1.420903343	0.5068084	8.44E-05	0.0018876
SAE1	1.7782659	1.5463253	1.8788018	1.608568741	0.6857776	8.53E-05	0.0017144
GOLGA4	3.174037	2.80320181	3.3347775	1.294795449	0.3727242	8.70E-05	0.0021874
KIF5C	2.2592838	1.97427926	2.3828206	1.419326708	0.5052067	8.71E-05	0.0017158
HNRNPF	2.8351934	2.51037839	2.9759862	1.308272306	0.3876629	8.85E-05	0.0019355
CDKN1A	2.2898468	1.94954039	2.4373544	1.513736984	0.5981146	8.88E-05	0.0018605
ZCRB1	3.249854	2.88141156	3.4095573	1.280717829	0.3569527	8.96E-05	0.0021996
FKBP1A	6.1726219	5.37494207	6.5183806	1.261360838	0.334981	8.99E-05	0.00393
IDH1	1.7620246	1.53152173	1.8619373	1.621640741	0.6974542	9.08E-05	0.0018648
EIF3G	3.5192106	3.09885314	3.7014168	1.287091878	0.364115	9.32E-05	0.0021805
CCT3	4.0986109	3.60779919	4.3113558	1.269789425	0.3445893	9.33E-05	0.0019962
BLVRA	1.4759818	1.26539354	1.5672625	2.137438951	1.0958832	9.34E-05	0.0020417
TSC22D3	2.0009682	1.74617519	2.1114096	1.489475424	0.5748043	9.41E-05	0.0019726
NDUFS4	3.3735586	2.98645353	3.5413514	1.27934097	0.3554008	9.55E-05	0.0023436
MFSD10	2.2219829	1.96091492	2.3351443	1.389451081	0.474515	9.80E-05	0.0020972
PXMP2	1.964738	2.21183238	1.8576336	0.707716351	-0.4987568	9.84E-05	0.0016776
HEXB	1.6293443	1.4095538	1.7246137	1.769276038	0.8231592	1.00E-04	0.0019319
ARF5	2.4982443	2.19500795	2.6296837	1.363742968	0.4475718	0.0001	0.0018628
IDH3G	1.8775461	1.64127779	1.9799579	1.528133149	0.6117703	0.0001	0.0016791
PGM1	2.1525524	1.89841621	2.2627091	1.405483479	0.4910665	0.0001	0.0020765
SYF2	2.583093	2.26539028	2.720803	1.359899023	0.4434995	0.000101	0.0020262
NAP1L3	1.4400025	1.23302915	1.5297162	2.27317573	1.1847092	0.000101	0.0020765
CDH6	4.011964	4.66591203	3.7285066	0.74429136	-0.4260606	0.000102	0.0021371
WBSCR22	1.8865938	1.65026762	1.9890307	1.52095949	0.6049817	0.000102	0.0023931
AKR7A2	2.591061	2.27395993	2.7285102	1.356801056	0.4402092	0.000102	0.0019383
FABP3	1.365883	1.16428661	1.4532661	2.758995779	1.4641432	0.000102	0.0023931
PARVA	1.613591	1.39523453	1.7082388	1.791945654	0.8415269	0.000108	0.0020033
SESN3	3.3816021	2.96501284	3.5621749	1.303897296	0.3828302	0.000108	0.0018278
MRPL52	4.0042525	3.56870119	4.1930445	1.243057985	0.3138936	0.000108	0.0023193
SERPINE1	1.3096455	1.08892211	1.4053193	4.558137883	2.1884446	0.000109	0.0019898
FOSB	1.6999676	1.47190985	1.7988205	1.692739544	0.75936	0.000109	0.0018278
CTNNB1	3.3547178	2.83034316	3.5820108	1.410670321	0.4963809	0.000114	0.0047655
TAF9	2.0102542	1.76485143	2.1166254	1.459924624	0.5458939	0.000115	0.0026197
PDHA1	1.7779553	1.54591257	1.8785354	1.609296912	0.6864305	0.000122	0.0025636
CDO1	1.5623629	1.34969532	1.6545448	1.871757376	0.9043934	0.000122	0.0022349
NDUFB1	5.0769831	4.49114081	5.3309196	1.24054566	0.3109748	0.000123	0.0027582
ATP1A1	2.1353649	1.88025734	2.2459426	1.415429963	0.5012404	0.000125	0.0022349
ACOT13	1.8612685	1.62099425	1.9654166	1.554630553	0.6365718	0.000127	0.0023654
ARPC5L	1.942629	1.70110841	2.0473174	1.493802428	0.5789893	0.000129	0.0027662

LDOC1	1.7416779	1.51785946	1.8386933	1.61953836	0.6955826	0.000132	0.0023246
MRPS23	1.7370582	1.51329258	1.8340507	1.624903037	0.7003536	0.000132	0.0028251
CERCAM	1.4190628	1.21765214	1.5063654	2.32648946	1.2181547	0.000136	0.0027969
SLC35E2B	2.158298	2.42384216	2.0431964	0.732662941	-0.4487785	0.000136	0.0026887
MEAF6	2.9937704	2.64393428	3.1454088	1.30504535	0.3840999	0.000137	0.0027568
FAM213A	3.8741192	3.34041075	4.105458	1.326885876	0.4080443	0.000137	0.0028135
FUCA2	1.5029587	1.29621052	1.5925748	2.000518981	1.0003743	0.00014	0.0031332
PIDD1	1.403832	1.60928692	1.3147765	0.516630907	-0.9527941	0.000141	0.0027215
GUCA1A	1.6206958	1.40538694	1.7140226	1.761335927	0.8166701	0.000143	0.0028189
EGLN3	1.5817478	1.35094562	1.6817902	1.942723223	0.9580804	0.000143	0.0028189
PRPF4B	2.1670426	2.42728693	2.0542383	0.738630982	-0.4370743	0.000143	0.0023638
SCAND1	3.5210357	3.10267753	3.7023753	1.285206724	0.3620004	0.000143	0.0027223
MRPL51	4.9541412	4.43456768	5.1793532	1.216849847	0.2831512	0.000144	0.0034496
NAXE	2.729985	2.34274743	2.8978352	1.413397004	0.4991668	0.000146	0.0028189
RNF114	1.7615443	1.53811088	1.8583928	1.595196814	0.6737344	0.000146	0.0023733
CHID1	2.70798	2.38261131	2.8490129	1.337333812	0.4193596	0.000147	0.0027349
SUGT1	2.1086255	1.84579647	2.2225501	1.445442436	0.5315112	0.000149	0.0059979
MRPS36	2.5354349	2.24712537	2.6604042	1.331385184	0.412928	0.00015	0.0029653
MRPL33	3.9987525	3.54271875	4.1964228	1.257088603	0.3300863	0.000152	0.0027358
DPY30	3.0078526	2.67496408	3.1521449	1.28489021	0.3616451	0.000154	0.0032365
PLK2	2.3399518	1.91359472	2.5247586	1.66896609	0.7389546	0.000156	0.0028533
TRIP6	1.6390383	1.42420675	1.7321583	1.725946758	0.787388	0.000156	0.0035695
SYAP1	2.9068775	2.58460152	3.0465698	1.291535955	0.3690878	0.000156	0.0036551
G3BP2	2.0400805	1.79956288	2.1443342	1.431199714	0.517225	0.000156	0.0030123
KRAS	1.8031116	1.57715163	1.9010552	1.561210594	0.6426652	0.000159	0.0030123
CCDC25	2.4251234	2.14138586	2.548111	1.356343269	0.4397223	0.000159	0.0029617
ZFP36L2	5.4087468	6.22678297	5.0541645	0.77565197	-0.3665186	0.000161	0.0029494
MTDH	3.5374935	3.15495578	3.7033066	1.254460355	0.3270669	0.000162	0.0033274
PSMF1	1.8834871	1.65241019	1.9836486	1.507714966	0.5923637	0.000163	0.0034881
SRSF4	2.4086017	2.12247012	2.532627	1.365405637	0.4493296	0.000165	0.0028598
PA2G4	3.5744059	3.18957616	3.7412124	1.251937468	0.3241625	0.000169	0.0038594
COL18A1	2.7319362	2.42032394	2.8670063	1.314493278	0.3945068	0.000173	0.0035667
SEC11A	5.8652328	5.27515272	6.1210063	1.197853414	0.2604514	0.000173	0.0031729
COPS4	1.6276609	1.41484285	1.7199081	1.735375378	0.7952478	0.000174	0.0035667
ZNF330	1.7158135	1.49755483	1.8104188	1.6288031	0.7038122	0.000174	0.0034337
PPID	1.7036642	1.48658514	1.7977584	1.639504166	0.7132596	0.000176	0.0029489
TINAGL1	1.2334794	1.02725694	1.3228676	11.84533708	3.5662473	0.000176	0.0029489
MAP2K1	1.7902235	1.56766364	1.8866933	1.562004707	0.6433988	0.000177	0.0037027
DYNLT3	1.7859756	1.56356762	1.8823795	1.565703064	0.6468106	0.000181	0.0028914
COMMD1	1.6680184	1.4536394	1.7609422	1.677416456	0.7462409	0.000184	0.0041115
SEPT11	2.3590575	2.08278846	2.4788078	1.36574028	0.4496832	0.000185	0.0032628
SEC62	3.506095	3.11119384	3.677267	1.268129429	0.342702	0.000189	0.0031243
ENOSF1	1.5079307	1.71655651	1.4175007	0.582648714	-0.7793018	0.00019	0.0041533
GSTM3	2.1837212	1.91139564	2.3017622	1.428317353	0.5143166	0.00019	0.0073581
SEC61B	7.0060596	6.35176348	7.2896678	1.175251448	0.2329695	0.000191	0.0038484
STXBP6	1.4384782	1.23911638	1.5248927	2.195134748	1.1343095	0.000191	0.0034391
HDDC2	4.3920073	3.90302881	4.6039577	1.241447443	0.3120232	0.000195	0.0040767
DPYSL3	1.3309571	1.14042938	1.4135424	2.944842352	1.5581904	0.000196	0.003524
RP11-798M19.6	2.3268747	2.6042551	2.2066426	0.752151323	-0.4109052	0.000197	0.0040448
UQCRQ	11.588271	9.90639241	12.317291	1.270693027	0.3456155	0.000197	0.0042137
FAM111A	1.4270881	1.62886011	1.3396289	0.540070727	-0.8887797	0.000201	0.0040455

PARK7	7.2715278	6.40495018	7.6471506	1.229826442	0.2984547	0.000204	0.0076039
EEF1E1	1.9382489	1.70612889	2.0388625	1.471208085	0.5570013	0.000204	0.0033667
CNIH1	2.4175982	2.13466252	2.5402383	1.35744174	0.4408903	0.000211	0.0034135
HOXB5	1.232585	1.02388737	1.3230461	13.52372238	3.7574204	0.000213	0.0041291
MYRF	1.311811	1.12299455	1.3936546	3.200585514	1.6783359	0.000217	0.0038867
SURF4	1.9782603	1.73451941	2.0839111	1.475673841	0.5613739	0.000217	0.0038242
TGFB1I1	1.5583258	1.35358323	1.6470726	1.830043239	0.8718777	0.000218	0.0039217
CSAD	1.5786125	1.79011199	1.4869368	0.616288386	-0.6983225	0.000219	0.0043981
VPS26A	1.7124003	1.49748864	1.8055549	1.619242796	0.6953193	0.000219	0.0038719
BRK1	8.3472902	7.59816397	8.6720031	1.162748169	0.2175387	0.00022	0.0048163
PPP1R15A	1.6630971	1.45193479	1.7546266	1.669768709	0.7396483	0.000226	0.0043924
GGCT	2.5536461	2.85847825	2.4215149	0.764881115	-0.3866926	0.000227	0.0036174
LINC00116	1.8822819	1.65774104	1.9796104	1.489355682	0.5746883	0.000227	0.0043924
INSIG1	1.5884525	1.36937564	1.6834126	1.850183197	0.8876681	0.000227	0.0039429
BRI3	2.6753251	2.37879241	2.8038588	1.308288902	0.3876812	0.00023	0.0042731
GLOD4	1.74841	1.53184282	1.8422822	1.583705123	0.6633037	0.000231	0.0047343
C5orf15	1.8046495	1.58536298	1.8997004	1.536995776	0.6201132	0.000234	0.0083989
C19orf70	3.0289018	2.68188101	3.1793198	1.295763356	0.3738023	0.000235	0.0036884
PPA1	5.0388314	4.41806267	5.307907	1.260335888	0.3338083	0.000236	0.0044765
HSBP1L1	1.4174355	1.22294076	1.5017403	2.250554349	1.1702804	0.00024	0.0047255
NAA10	2.8284871	2.50214717	2.9699408	1.31141667	0.3911261	0.00024	0.0037119
ELAVL1	2.4662037	2.18409712	2.5884844	1.34151527	0.4238635	0.000245	0.0051269
RNF181	3.0816715	2.74130568	3.2292048	1.280191562	0.3563597	0.000245	0.0050262
HEBP1	2.3375676	2.07376606	2.4519139	1.352169647	0.4352762	0.000246	0.0053726
LEPROTL1	1.7236974	1.50939096	1.8165897	1.603070561	0.6808379	0.00025	0.0045658
VKORC1	5.6698206	5.10202311	5.9159356	1.198417323	0.2611304	0.00025	0.0038052
RHBDD2	2.267251	2.00706072	2.380032	1.370356255	0.454551	0.000251	0.00443
AASDHPPT	2.2579067	1.95107212	2.3909058	1.462460873	0.548398	0.000251	0.0046761
LINC00908	1.4931959	1.69621853	1.4051947	0.581993559	-0.7809249	0.000255	0.0045781
GORASP2	1.6299951	1.42345887	1.7195193	1.699148042	0.7648116	0.000263	0.0049102
ZNF292	3.230459	3.59062357	3.0743437	0.800712133	-0.3206444	0.000264	0.0049102
LGR4	2.4623947	2.74226024	2.3410854	0.769738856	-0.377559	0.000265	0.0056827
MAT2A	2.2180066	2.52783851	2.0837082	0.709308103	-0.4955157	0.000268	0.0049024
MRPL40	1.935118	1.70970903	2.0328227	1.455276287	0.5412931	0.00027	0.0059779
CCDC151	2.0926352	2.34357658	1.9838632	0.732271797	-0.4495489	0.000271	0.0054421
DBI	4.7504199	4.26422968	4.9611617	1.213505822	0.279181	0.000273	0.0059779
MAD2L1	2.0005028	1.75397353	2.1073623	1.468701803	0.5545415	0.000274	0.0043147
DNAJC3	1.8761706	1.64177157	1.9777722	1.523551681	0.6074384	0.000276	0.0047512
CHMP1B	1.9661794	1.73901329	2.0646458	1.440631426	0.5267013	0.000278	0.0049098
ATP6AP1	1.607516	1.40278877	1.6962561	1.72858865	0.7895946	0.000279	0.0047512
SEMA6A	1.5928917	1.80106299	1.5026587	0.627489667	-0.6723364	0.000283	0.0043728
GTF3A	3.1884029	2.83445035	3.3418255	1.276581586	0.3522857	0.000286	0.0061145
HIGD1A	2.6724774	2.33524103	2.8186543	1.362041939	0.4457711	0.000287	0.0099436
CEP135	1.3684817	1.56115422	1.2849668	0.507822541	-0.9776037	0.000288	0.0059029
CCNI	12.575242	11.4355332	13.069256	1.15655381	0.2098324	0.000294	0.0044065
IGF2	5.9618965	5.05454877	6.3551913	1.320786018	0.4013968	0.000297	0.0062162
KREMEN1	1.3848852	1.19565988	1.4669059	2.386313994	1.2547839	0.000305	0.0052908
CAV1	1.6771539	1.44954022	1.7758143	1.725795115	0.7872612	0.000307	0.0049854
SLC9A3R1	1.3190565	1.13506053	1.3988106	2.952828609	1.5620976	0.000308	0.004697
TSPAN4	2.3594499	2.08897037	2.4766908	1.356043098	0.439403	0.000309	0.0059855
CHCHD10	2.5004821	2.21722266	2.6232624	1.333578874	0.4153032	0.000314	0.0055357

SPTBN1	2.001108	1.76640718	2.1028404	1.438974492	0.525041	0.000315	0.0059866
SERINC2	2.2234465	1.97860265	2.3295754	1.358646887	0.4421705	0.000318	0.0047736
CORO1B	1.768076	1.55250854	1.8615148	1.55927879	0.6408789	0.000323	0.0053951
HSPH1	1.9254742	1.69786943	2.0241308	1.467510556	0.5533709	0.000324	0.0063958
VAMP8	1.3337417	1.13659173	1.4191975	3.068981451	1.6177599	0.000326	0.0065524
DCAF13	1.7774588	1.56474538	1.8696605	1.539916136	0.6228518	0.000326	0.0056439
PDLIM4	2.4996439	2.19747219	2.6306218	1.361719975	0.4454301	0.000327	0.0053951
CISD1	2.249058	1.99201677	2.3604739	1.371422314	0.4556729	0.000327	0.0053951
G6PD	1.8229134	1.60789801	1.916113	1.50701754	0.5916962	0.00033	0.0067675
SELENOS	2.1609151	1.91716864	2.2665683	1.380954686	0.465666	0.000335	0.0064809
EIF3J	2.2639709	1.96234009	2.3947144	1.449294728	0.535351	0.000338	0.0060646
PFDN4	2.8117289	2.51122847	2.9419824	1.285035619	0.3618083	0.000345	0.0069319
NFKBIA	1.6316794	1.4172206	1.7246378	1.736821686	0.7964496	0.000346	0.0058066
PBX3	1.3142316	1.13195898	1.3932386	2.980006379	1.5753154	0.00035	0.0065134
SRSF5	6.5602369	7.27356674	6.2510403	0.837010347	-0.2566826	0.00035	0.0073432
EGFL7	1.7921629	1.57728412	1.8853032	1.533565877	0.6168901	0.000363	0.0066367
ATAD2	1.4083745	1.60043855	1.3251233	0.541476387	-0.8850297	0.000364	0.007799
COPB2	1.6890705	1.48310096	1.7783491	1.611151951	0.6880926	0.000365	0.0064344
ZMAT1	1.3994528	1.59112094	1.3163733	0.535209032	-0.9018256	0.000368	0.0072609
FAM192A	2.0123423	1.78746646	2.109816	1.409350159	0.4950301	0.000369	0.0058882
TRAPPC4	1.7361556	1.52818615	1.8263011	1.564412723	0.6456212	0.000374	0.0075339
LHX2	2.4832888	2.7903033	2.3502117	0.754180433	-0.4070184	0.000378	0.006141
FBXO32	1.3973464	1.21009078	1.4785133	2.277650228	1.1875462	0.00038	0.0072211
DMD	1.6034934	1.80945102	1.51422	0.635270067	-0.6545581	0.000382	0.007469
KLF6	1.7541602	1.54058499	1.8467356	1.566332017	0.6473901	0.000386	0.007469
YPEL5	2.3627466	2.09566003	2.4785167	1.349430136	0.4323503	0.00039	0.0076758
HIGD2A	3.8822887	3.45902728	4.0657537	1.246734327	0.3181541	0.000392	0.007312
MYH9	2.4509346	2.12730982	2.5912116	1.411512219	0.4972416	0.0004	0.0069237
ARHGDIA	2.2138468	1.97949402	2.3154283	1.342967185	0.4254241	0.000401	0.007205
ATRAID	3.7492088	3.33594678	3.9283394	1.253598529	0.3260754	0.000401	0.0064092
AP3S1	3.3237433	2.961414	3.4807969	1.264800233	0.3389095	0.000414	0.0061201
DYNLT1	4.9956189	4.42275436	5.2439302	1.239916666	0.3102432	0.000417	0.0080511
C19orf43	4.2879685	3.75344506	4.5196606	1.278275211	0.3541985	0.00042	0.0071607
KLHL7	1.5959412	1.39719059	1.6820908	1.717288413	0.7801324	0.000423	0.0081755
ALKBH7	3.4876653	3.10094328	3.6552921	1.263857124	0.3378334	0.000424	0.0066648
RAB38	1.4453366	1.25663425	1.5271306	2.054014923	1.0384467	0.000425	0.0070049
SH3BP5	1.418527	1.23174955	1.4994867	2.155286741	1.1078798	0.00043	0.007322
GAD2	2.0397814	2.35698813	1.9022865	0.664918458	-0.5887507	0.000432	0.0090504
LINC01021	3.0380407	3.47780783	2.8474213	0.745586982	-0.4235514	0.000433	0.0082208
ACBD6	2.0453052	1.81515604	2.1450646	1.404718256	0.4902808	0.000437	0.0074566
PFDN1	2.4633181	2.20776701	2.5740881	1.303304424	0.3821741	0.000439	0.0077408
ASNA1	1.8176128	1.60728758	1.9087794	1.496456388	0.5815502	0.00044	0.0088457
ARSI	1.398745	1.21452684	1.4785953	2.23093438	1.1576481	0.000446	0.0080978
MRPL22	2.1452928	1.91587833	2.2447337	1.359060085	0.4426092	0.000447	0.0081717
OCIAD1	2.9421057	2.633233	3.0759883	1.271091306	0.3460677	0.000449	0.0085149
RTCB	1.6856958	1.48334572	1.7734056	1.60010845	0.6781697	0.000451	0.0080978
TPRN	1.2493052	1.0746715	1.3250011	4.352411685	2.121815	0.000453	0.0067054
SERF2	25.022243	22.882455	25.949746	1.140171229	0.1892505	0.000456	0.0076342
NDUFA5	3.2366782	2.88552091	3.3888893	1.266965154	0.3413768	0.000457	0.0066559
DSG2	1.6107015	1.41331009	1.6962619	1.684599264	0.7524054	0.000457	0.0093852
DUSP6	1.2612764	1.08682236	1.3368944	3.880272231	1.9561579	0.000461	0.0076728

TMEM11	1.6861987	1.48413685	1.7737835	1.59827426	0.676515	0.000465	0.0080673
TBCA	9.2802458	8.37918869	9.6708139	1.175036207	0.2327052	0.000465	0.0076728
FKBP2	4.4074451	3.93334685	4.6129456	1.231680322	0.3006279	0.000484	0.0086865
OSBPL9	1.8310474	1.62094094	1.9221192	1.48503524	0.5704972	0.000484	0.0075889
TXNDC15	1.5086646	1.31767758	1.591449	1.861790181	0.8966905	0.000487	0.007539
DDAH2	5.3991233	4.84938202	5.6374116	1.204715863	0.2686929	0.00049	0.0075889
TMEM106C	3.1785773	3.53949368	3.0221362	0.796275342	-0.3286607	0.000495	0.0075407
PPP1R3E	1.5131623	1.70767153	1.4288513	0.60600329	-0.7226025	0.0005	0.0098486
LIMS1	1.9057283	1.69206033	1.9983438	1.442567578	0.5286389	0.000502	0.0081512
HILPDA	6.6564166	7.99206736	6.0774715	0.726176003	-0.4616088	0.000505	0.0101561
PHLDA2	1.5418033	1.31775587	1.6389178	2.010719189	1.0077116	0.000509	0.0170706
KHDRBS1	4.4224527	3.95426611	4.6253907	1.227171338	0.2953367	0.000521	0.0078266
UBE2B	2.5425621	2.26665229	2.6621567	1.312243875	0.3920359	0.000523	0.0101096
STIP1	1.7655048	1.5607056	1.8542761	1.523573345	0.607459	0.000524	0.0099532
PHLDA1	2.0411549	1.79487325	2.147907	1.444138415	0.530209	0.000525	0.0092628
MFF	2.4173408	2.16337718	2.5274227	1.312921299	0.3927804	0.000529	0.0108693
ZEB2	1.7198413	1.92686369	1.6301063	0.679826298	-0.5567619	0.000534	0.0089362
APEX1	4.7529636	4.29590876	4.9510765	1.198782124	0.2615695	0.000537	0.0104344
PSMD6	1.7643351	1.56041401	1.8527258	1.521599666	0.6055888	0.000538	0.0093343
RNPEP	1.6964028	1.49617072	1.7831945	1.578477839	0.658534	0.000539	0.0102118
CCNB2	1.5994697	1.38763418	1.6912909	1.783358973	0.8345971	0.000539	0.0104344
CHEK1	1.4062809	1.59298521	1.3253529	0.54866954	-0.8659906	0.000545	0.017272
NDUFA9	1.5982683	1.40419451	1.6823907	1.688273043	0.7555482	0.000546	0.0089931
TCEA1	3.7865936	3.33862894	3.9807663	1.274578551	0.3500203	0.000549	0.017272
CTDSPL2	1.4975811	1.68978977	1.4142672	0.600570287	-0.735595	0.000553	0.0111245
PBDC1	1.5391478	1.34944748	1.6213744	1.778162514	0.8303872	0.000561	0.010459
CKS2	4.9910436	4.30717236	5.2874713	1.296416032	0.3745288	0.000567	0.0092305
ERO1A	2.2009592	1.91547241	2.3247051	1.447018004	0.5330829	0.000569	0.0092305
DPM1	1.6947018	1.49540421	1.7810884	1.576668806	0.6568796	0.000574	0.0099522
RAD21	2.6331089	2.3568572	2.7528517	1.291846849	0.369435	0.000575	0.0087625
ZFR	2.0774775	1.85710715	2.1729983	1.368554979	0.4526534	0.000582	0.0099174
ATP6V1H	1.7033867	1.50347797	1.7900382	1.569161467	0.6499938	0.000586	0.0093523
ACTR1A	1.7438163	1.54233109	1.8311511	1.532552996	0.615937	0.00059	0.011202
FAM50A	2.2852543	2.05178123	2.3864544	1.318196589	0.3985655	0.000605	0.0102993
YIF1A	2.3984768	2.09141545	2.5315742	1.403291701	0.4888149	0.000609	0.0097142
DNM1	1.7410141	1.94603086	1.6521484	0.689352183	-0.5366869	0.000612	0.0114075
RAB13	4.8766005	4.29122693	5.1303339	1.254952639	0.3276329	0.000619	0.0109169
IK	2.1434598	1.91038142	2.2444889	1.366997288	0.4510104	0.000619	0.0113258
MRPL43	2.7716117	2.4153218	2.9260475	1.360854846	0.4445132	0.000625	0.0098165
PTPMT1	2.1045744	1.87925539	2.2022402	1.367338996	0.451371	0.000626	0.0098451
FH	1.4551516	1.27239013	1.5343706	1.96178403	0.9721662	0.000637	0.010672
TPD52	1.8169111	1.61183553	1.9058023	1.480466936	0.5660523	0.000639	0.0090903
TAX1BP3	2.9184481	2.61716907	3.0490392	1.267053164	0.3414771	0.000642	0.0115364
SFRP2	35.903453	31.8631605	37.65474	1.187653479	0.248114	0.000642	0.0090903
LAYN	1.4170844	1.23649778	1.4953606	2.094567689	1.0666525	0.000644	0.0099683
DBNL	1.8446133	1.63890301	1.9337795	1.461535555	0.5474849	0.00065	0.0120991
RIC3	1.8928039	2.10667337	1.800101	0.722978418	-0.4679755	0.000655	0.0091458
NEU1	1.5921083	1.40137641	1.6747821	1.681170257	0.7494658	0.000659	0.0106875
ARL1	1.9579079	1.74567653	2.0499007	1.407984171	0.4936311	0.00066	0.0120696
RSPH9	1.7987488	2.00557079	1.7091006	0.705172273	-0.5039523	0.000664	0.0109557
GALE	1.6162598	1.42427371	1.6994772	1.648646174	0.7212818	0.000668	0.0131726

SUCLA2	1.5490363	1.36085391	1.6306049	1.747535211	0.8053215	0.000682	0.010739
PYURF	3.7215409	3.33263808	3.890113	1.238989012	0.3091634	0.000684	0.010739
RIT1	1.4853059	1.30087191	1.5652498	1.878705609	0.909739	0.000686	0.0103061
COMMD6	12.530811	11.4132161	13.015239	1.153845117	0.2064496	0.000687	0.0099478
TSC22D1	11.265751	9.55521856	12.007191	1.28660548	0.3635697	0.000687	0.0111512
GYG1	2.9174737	2.6042601	3.0532379	1.279865957	0.3559927	0.000689	0.0101863
CDKN1C	1.7753853	1.55187912	1.8722653	1.580536919	0.6604147	0.000692	0.0099478
RAI14	1.9402493	1.73060941	2.0311188	1.411313304	0.4970383	0.000705	0.0118093
PLGRKT	1.6566235	1.46187283	1.7410392	1.604422502	0.6820541	0.000709	0.0122927
MARCH3	1.8384126	2.04725057	1.7478906	0.714146754	-0.4857075	0.000711	0.0134737
SLC25A37	2.1143401	2.34275074	2.0153342	0.756159859	-0.4032368	0.00072	0.0109703
GOLGA6L10	1.2740573	1.44801432	1.1986547	0.443411488	-1.1732819	0.000722	0.0131987
RPUSD3	1.8292976	1.62625937	1.9173056	1.464737496	0.5506421	0.000727	0.0100021
HEBP2	2.5386903	2.27800702	2.6516849	1.292391072	0.3700427	0.000734	0.0136815
MAML2	1.5609395	1.75240102	1.4779495	0.63523243	-0.6546435	0.000738	0.010463
RWDD4	1.6683726	1.47439225	1.7524545	1.586144084	0.6655238	0.000762	0.0123841
PSMD1	1.6756405	1.48147868	1.759801	1.578057388	0.6581497	0.000762	0.0150396
METAP2	3.5055815	3.15300182	3.6584091	1.234745385	0.3042136	0.000764	0.0123841
PSMD12	1.6893163	1.49428548	1.7738534	1.565600125	0.6467158	0.000774	0.0119622
COL9A1	1.7941759	1.99816449	1.705756	0.707053762	-0.5001082	0.000775	0.0106154
UBE2D3	5.2531318	4.70627394	5.4901704	1.211505262	0.2768007	0.000775	0.013216
KC6	2.1829	2.45326251	2.06571	0.733322409	-0.4474805	0.000776	0.0123067
ABHD2	1.369738	1.19491863	1.4455145	2.285643296	1.1926003	0.00078	0.0106154
RNF13	1.8373237	1.63459035	1.9251996	1.457947739	0.543939	0.000783	0.0123067
BANF1	6.0829359	5.52775747	6.323581	1.175765487	0.2336003	0.000784	0.0133654
DCLRE1C	1.6242787	1.81853362	1.5400778	0.659811431	-0.5998743	0.000788	0.0106154
TM9SF2	1.7377292	1.54043485	1.8232476	1.523305851	0.6072056	0.000789	0.0117571
VASP	1.7008223	1.50652124	1.7850431	1.549872042	0.6321491	0.000791	0.0106154
PRCP	1.5910532	1.40276448	1.672668	1.670127277	0.7399581	0.000795	0.0117571
HSP90AB1	24.824434	22.8478927	25.681176	1.129682235	0.175917	0.000805	0.0128433
AKIRIN1	2.3645227	2.11414146	2.4730518	1.322140746	0.4028758	0.000811	0.0110182
NIT2	1.6484173	1.45707805	1.7313544	1.60006452	0.6781301	0.000833	0.0121408
FXYD5	1.6422575	1.45195496	1.7247452	1.603578308	0.6812948	0.00084	0.0124325
ABRACL	3.0484321	2.73584848	3.1839232	1.258130061	0.3312811	0.000848	0.0142167
MACROD1	1.6040637	1.41554135	1.6857797	1.650328358	0.7227531	0.000856	0.025658
C20orf24	2.2663114	2.03119495	2.3682239	1.326833385	0.4079872	0.000862	0.0133214
ID3	10.879075	9.01373259	11.687617	1.333662832	0.415394	0.000867	0.025658
TTF2	1.5070913	1.69428214	1.4259524	0.613514857	-0.7048298	0.000869	0.0158871
WNT2B	1.5649305	1.28589712	1.685879	2.399041372	1.262458	0.000872	0.015124
PSMC3IP	1.3420903	1.5174531	1.2660783	0.514207621	-0.9595771	0.000879	0.0155211
PARD6B	1.5361358	1.35201921	1.6159421	1.749739948	0.8071405	0.000881	0.0116562
PIM3	1.4122163	1.23607583	1.4885653	2.069527123	1.0493012	0.000892	0.016013
C5orf46	1.1806562	1.02033514	1.2501482	12.30127438	3.6207359	0.000895	0.0152662
FBXO2	1.4027814	1.22749961	1.4787582	2.104435126	1.073433	0.000899	0.0167371
NSA2	3.4055162	3.05767007	3.5562921	1.24232359	0.313041	0.000913	0.0176619
TMEM55A	1.6690646	1.47836667	1.7517237	1.571438309	0.6520856	0.000919	0.0165119
MRPS35	1.7075086	1.5148698	1.791009	1.536328158	0.6194864	0.000924	0.0154957
PSMC2	1.5639507	1.37962396	1.6438481	1.696015575	0.7621494	0.000925	0.0141344
ORAI3	1.7885063	1.59155015	1.873878	1.477267834	0.5629314	0.000927	0.0141344
DAZAP2	2.3186467	2.07670228	2.4235188	1.322109955	0.4028422	0.000932	0.0156221
DNTTIP1	1.4616888	1.28362448	1.5388718	1.899948094	0.92596	0.000936	0.0142463

SNRPG	8.4864779	7.7949068	8.7862432	1.145894035	0.1964736	0.000938	0.018156
RP11-119B16.2	1.2788268	1.44985911	1.2046919	0.455013236	-1.1360196	0.000945	0.0172616
NDUFA10	1.9964232	1.78087947	2.0898519	1.395672317	0.4809603	0.000945	0.0155869
C1D	1.8341	1.63480401	1.920486	1.450031788	0.5360845	0.000948	0.0156381
CCNB1IP1	3.6938598	4.08858049	3.522766	0.816804351	-0.2919375	0.000952	0.0167824
LRRC59	1.7372458	1.54308757	1.8214048	1.512472055	0.5969085	0.000952	0.0147713
TMEM59L	1.3127852	1.1446512	1.3856639	2.666164203	1.4147656	0.000955	0.0147713
HOMER3	1.8351484	1.63623512	1.9213685	1.448157164	0.5342182	0.000968	0.0173825
ATP5D	6.2990409	5.68359484	6.5658094	1.188362689	0.2489752	0.000976	0.0146586
PSMC6	2.1492927	1.93492826	2.2422101	1.328668931	0.4099817	0.000983	0.0159565
SORCS1	1.4499225	1.63078954	1.3715247	0.588983561	-0.7637007	0.000984	0.0162254
PDCD2	2.1820367	1.96278403	2.277073	1.326437681	0.4075569	0.001	0.0176573
AKAP6	1.6771432	1.87164509	1.5928353	0.680133868	-0.5561094	0.001003	0.0162678
NDUFS7	2.9614766	2.65542946	3.0941343	1.265009701	0.3391484	0.001013	0.0175563
AKIRIN2	1.5508374	1.36877834	1.629752	1.70767068	0.7720298	0.001015	0.0164703
COPS5	1.8485873	1.64979365	1.9347555	1.438542089	0.5246074	0.001018	0.0193213
NR1H2	1.6031888	1.41804414	1.6834407	1.634852923	0.7091609	0.001029	0.0134449
RAB4A	2.9635525	2.65543388	3.0971082	1.266802784	0.3411919	0.001034	0.0150686
SUPT4H1	2.634606	2.36406436	2.7518737	1.284304268	0.360987	0.001041	0.0177395
SF3B5	4.4053331	3.98865699	4.5859436	1.199851163	0.2628555	0.001045	0.0150234
TRIB1	1.3685757	1.19762196	1.4426765	2.240016795	1.1635095	0.00105	0.0157549
FANCD2	1.2459475	1.41239728	1.1737989	0.421435565	-1.246616	0.001053	0.0153536
EFHC1	2.3837855	2.63560019	2.274635	0.77930721	-0.3597359	0.001057	0.0168712
SMYD3	1.5241115	1.34410528	1.6021362	1.749860297	0.8072397	0.001061	0.0304907
PET100	3.4315947	3.07766385	3.5850079	1.24418967	0.3152064	0.001065	0.0153044
FAH	1.4464796	1.27185104	1.5221733	1.920806754	0.9417124	0.001073	0.0186057
PFDN5	14.175557	12.9659213	14.699881	1.144908146	0.1952319	0.001081	0.0139414
SLTM	2.3474941	2.11545755	2.4480716	1.298186196	0.3764973	0.001091	0.020318
RAB14	1.8754653	1.67660294	1.9616633	1.421311157	0.5072224	0.001107	0.0210135
CAPN1	1.5924631	1.40949484	1.6717717	1.640488919	0.7141258	0.001109	0.0148554
ZNF302	1.8624148	2.06509038	1.774564	0.72722846	-0.4595194	0.00111	0.0164172
C11orf58	5.863684	5.26161073	6.1246559	1.202516185	0.2660563	0.001111	0.0177446
MPG	2.6865514	2.41737354	2.803228	1.272232008	0.3473618	0.001116	0.0158114
STXBP2	1.3782487	1.20800598	1.4520414	2.173213323	1.1198298	0.001121	0.0197805
MYH14	1.3284792	1.16125641	1.4009628	2.486492295	1.314112	0.001135	0.0184102
STON2	1.5052734	1.68743333	1.4263152	0.620155003	-0.6892992	0.001144	0.0205381
BGN	1.2841623	1.11305431	1.35833	3.169538918	1.664273	0.00116	0.0162073
DPP7	2.1432963	1.93030058	2.2356204	1.328194815	0.4094668	0.001162	0.0216182
VPS35	2.3825768	2.09413565	2.5076031	1.377894158	0.4624651	0.001162	0.0176885
ZNF451	1.6099587	1.79688726	1.5289335	0.663749492	-0.5912892	0.001175	0.0214991
HSPE1	10.259884	9.34013568	10.658554	1.158081159	0.2117364	0.001175	0.0214991
PSMD10	1.8069382	1.61280744	1.8910852	1.45410314	0.5401296	0.001176	0.0184905
CXXC5	3.2808505	2.96323742	3.4185216	1.231904826	0.3008908	0.001178	0.0179335
BTBD10	1.3834936	1.21373482	1.4570764	2.13852118	1.0966135	0.001189	0.0206182
DNAJC15	1.6976193	1.50987847	1.7789966	1.527808321	0.6114636	0.001196	0.0169451
UBA52	27.792345	25.8977575	28.613564	1.109078357	0.1493613	0.001202	0.0212
PON2	2.2018504	1.98322618	2.2966143	1.31873451	0.3991541	0.001224	0.0219835
DOC2B	1.4581726	1.63582545	1.3811679	0.599485183	-0.738204	0.001225	0.0168785
APOO	1.9211239	1.72066193	2.0080153	1.398735293	0.484123	0.001226	0.0183947
MAGI3	1.5386041	1.72025781	1.4598654	0.638473298	-0.6473018	0.001229	0.0196213
HLA-E	1.777905	1.58634613	1.8609373	1.468308947	0.5541556	0.001239	0.019789

SPINT1	1.2566225	1.0955037	1.3264603	3.418299777	1.7732789	0.001252	0.0182471
DRG1	1.6123406	1.43010873	1.69133	1.607337822	0.6846732	0.001253	0.020993
NDUFA3	4.7946602	4.37038335	4.9785653	1.180448893	0.2393356	0.001265	0.0215686
AMZ2	2.327402	2.10151177	2.4253154	1.293962945	0.3717963	0.001279	0.0183808
TMEM179B	1.9808597	1.77949713	2.0681414	1.370295518	0.4544871	0.001294	0.0191285
MARCKSL1	16.771483	15.5768661	17.289296	1.117475879	0.1602437	0.001297	0.0183834
FUNDC2	2.8529018	2.57151946	2.9748685	1.256661842	0.3295965	0.001325	0.0229875
ATP2B4	1.9393721	2.14251253	1.8513198	0.745129506	-0.4244369	0.001332	0.0209325
UBE2D2	4.8786664	4.4081631	5.0826087	1.197891223	0.2604969	0.001347	0.0188245
MRPL12	2.6458672	2.39576916	2.7542736	1.256850777	0.3298134	0.001348	0.0211938
HAUS1	2.17678	1.96422296	2.268914	1.315996449	0.3961556	0.001357	0.0247936
SAMM50	1.8256742	1.63261825	1.9093554	1.437447264	0.523509	0.001362	0.0249148
RASL11B	1.2625339	1.10213284	1.3320606	3.251261734	1.7009997	0.001366	0.0230469
CAP2	1.3662546	1.19967331	1.4384602	2.195887745	1.1348043	0.001375	0.0230469
CCDC107	2.1196333	1.90046517	2.214633	1.348894991	0.431778	0.001382	0.0217158
TFDP1	1.6728799	1.86077126	1.5914374	0.687101703	-0.5414044	0.001398	0.0192687
MITF	1.4706661	1.2978786	1.5455618	1.831490386	0.8730181	0.001413	0.0205823
CDKN2A	1.1904117	1.02534247	1.2619618	10.33686762	3.3697272	0.001426	0.0255952
ILF3-AS1	1.8613353	2.05842031	1.7759078	0.733080955	-0.4479556	0.001428	0.0256609
UXT	3.7876042	3.42316621	3.9455718	1.215588027	0.2816544	0.001429	0.0199674
NELL2	2.0411753	1.83288082	2.1314617	1.358491756	0.4420058	0.001439	0.0206797
AAGAB	1.4251532	1.25584668	1.49854	1.948588755	0.9624296	0.001444	0.0254832
EIF4H	3.7765467	3.40640038	3.9369886	1.220490434	0.287461	0.001453	0.0208677
RRAS	1.5298275	1.35494232	1.6056324	1.706283869	0.7708577	0.001454	0.020033
UBE2E2	1.7212046	1.53503454	1.801901	1.498783605	0.5837921	0.001455	0.018299
MTX1	1.7696592	1.5808819	1.8514858	1.465850052	0.5517375	0.001455	0.018299
GPN3	1.4854952	1.3130832	1.5602282	1.789390704	0.8394684	0.001465	0.0241393
MCM4	1.3691113	1.53857459	1.2956566	0.54896123	-0.8652238	0.001468	0.0410139
HNRNPA3	5.8705287	5.28519782	6.1242435	1.195800919	0.2579772	0.001479	0.0239204
CEP152	1.2301663	1.39109622	1.1604104	0.410155902	-1.2857557	0.001491	0.0256391
AHI1	2.1268546	2.35156081	2.0294545	0.761678252	-0.3927464	0.001505	0.0256391
FANCA	1.219491	1.37900615	1.1503483	0.396690954	-1.3339126	0.001511	0.0244929
MPHOSPH9	1.5000542	1.6763958	1.4236179	0.626287046	-0.6751041	0.001512	0.0262253
ANKRD1	1.3155367	1.10746252	1.4057276	3.775526506	1.9166778	0.001514	0.0198178
HTRA1	1.8696087	1.66590017	1.9579072	1.438514781	0.52458	0.001518	0.0232457
BST2	1.6781636	1.48953357	1.7599263	1.552347628	0.6344517	0.001518	0.0198178
ITIH5	1.2088495	1.05335366	1.2762501	5.177715439	2.3723157	0.00152	0.0239204
CA2	1.8264245	1.62791484	1.9124696	1.453174071	0.5392075	0.00152	0.0239204
EIF5	5.1161083	4.64372602	5.320865	1.185836963	0.2459057	0.001521	0.0214827
SBF2	3.1270274	3.57293528	2.9337463	0.751572058	-0.4120167	0.001522	0.0239204
MCTS1	1.9222483	1.7261797	2.0072354	1.387033216	0.4720023	0.001525	0.0232457
EBNA1BP2	1.9420217	1.7450433	2.027403	1.378984324	0.4636061	0.001526	0.0260279
DARS	3.214924	2.89530003	3.3534668	1.241738365	0.3123612	0.001539	0.025778
NANS	1.4654318	1.29495607	1.5393255	1.828494165	0.870656	0.001544	0.0238956
ELOVL5	1.6513299	1.46959402	1.7301043	1.554756484	0.6366886	0.001544	0.0214827
THOC1	1.5056119	1.68203116	1.429142	0.629211711	-0.6683826	0.00156	0.0214827
SSR3	3.2307784	2.91970527	3.3656147	1.232280134	0.3013303	0.001561	0.0212176
ALDH3A2	1.4019088	1.2353578	1.4741012	2.01438508	1.0103395	0.001563	0.0201332
ARL2BP	2.47997	2.24532338	2.5816789	1.270094907	0.3449363	0.001565	0.0262329
PROX1	1.3049894	1.14427744	1.3746509	2.596739393	1.3767012	0.001582	0.0224148
NEDD8	7.3703689	6.77381873	7.6289467	1.148104409	0.1992538	0.00159	0.0280456

TKT	13.532113	12.3378417	14.049777	1.150993013	0.2028791	0.001599	0.0434683
SELENOM	2.6658737	2.40712546	2.7780296	1.26358995	0.3375284	0.001611	0.021907
TSPAN13	1.5959436	1.41878895	1.6727322	1.606375214	0.6838089	0.001617	0.0280456
ID1	5.3839599	4.59630802	5.7253718	1.313950814	0.3939113	0.001628	0.020219
CYTOR	2.6282199	2.31939704	2.7620808	1.335519753	0.4174013	0.001628	0.0252
MORN2	1.6949979	1.51177473	1.774417	1.513199012	0.5976017	0.001629	0.026842
PTRF	1.4184336	1.25178307	1.4906692	1.948777509	0.9625694	0.001657	0.0255795
TIMM17A	1.92655	1.70943843	2.0206581	1.438684572	0.5247503	0.001664	0.0249587
EIF5B	3.2479333	2.93949998	3.3816255	1.227958486	0.2962618	0.001668	0.0223776
JAK1	1.8341349	1.64425623	1.9164388	1.422475687	0.508404	0.001678	0.0255795
SRPK2	1.9469019	1.74629035	2.0338581	1.38532955	0.4702292	0.001684	0.0260682
ST13	8.8863608	8.1826104	9.1914052	1.140449601	0.1896027	0.001686	0.0257014
FOXN4	1.2687483	1.42957356	1.1990377	0.463337772	-1.1098638	0.001689	0.0214854
CALCOCO1	1.6456136	1.82977444	1.5657881	0.681857665	-0.5524575	0.0017	0.0251241
FAM161A	1.6398112	1.82289306	1.5604534	0.681076835	-0.5541105	0.001712	0.0257028
SNRPC	3.255684	2.93402918	3.3951071	1.238402758	0.3084806	0.00173	0.0274339
RBM4	1.8805556	2.07594828	1.7958616	0.739683897	-0.4350192	0.001734	0.0252553
PRKCSH	2.4790285	2.2359505	2.5843919	1.281921823	0.3583083	0.001747	0.0274339
SRP19	1.6632248	1.48312394	1.7412905	1.534369274	0.6176457	0.001749	0.0231488
CTD-3184A7.4	1.4523494	1.62325579	1.3782691	0.606924399	-0.7204113	0.001762	0.0268591
ZWINT	1.424659	1.59502551	1.3508126	0.589575804	-0.7622508	0.001769	0.0261639
PCDH11X	1.2819657	1.44336578	1.212006	0.478174097	-1.0643921	0.001772	0.0231488
YWHAH	2.7688804	2.49630118	2.8870313	1.261130672	0.3347178	0.001806	0.0226924
CASC15	1.961007	2.16331216	1.8733168	0.750715773	-0.4136613	0.001824	0.0295685
CSN3	1.2039921	1.029634	1.2795685	9.434045897	3.2378766	0.001836	0.02757
GDI2	4.413321	3.93055293	4.6225795	1.236141969	0.3058444	0.001838	0.0271587
TMED1	1.9320685	1.73745816	2.0164234	1.378279396	0.4628684	0.001866	0.0229198
HMGXB4	1.9971363	2.20075907	1.9088749	0.75691699	-0.401793	0.00188	0.0229198
ISCU	2.7745983	2.50062549	2.8933533	1.261709437	0.3353797	0.001892	0.025719
STT3B	1.9228566	1.72881435	2.0069652	1.38164845	0.4663906	0.001893	0.0229198
SNRPB	5.1765962	4.72447998	5.3725684	1.174007768	0.231442	0.001914	0.0287351
EVA1A	1.3869239	1.22445748	1.4573458	2.037560991	1.0268432	0.001917	0.0323347
EPCAM	1.229632	1.06429168	1.3012996	4.686448107	2.2284949	0.001929	0.0323347
BABAM1	2.2914686	2.07472462	2.3854175	1.289090706	0.3663538	0.001956	0.0239034
NR2F1-AS1	1.7831749	1.97123394	1.7016597	0.722441478	-0.4690474	0.00196	0.0328636
CTNNBL1	1.4839919	1.31607801	1.5567751	1.761511819	0.8168142	0.001965	0.0324069
ELOVL1	1.4005307	1.23758323	1.4711612	1.983141844	0.9877879	0.001986	0.0239034
SET	7.9170511	7.24555785	8.2081135	1.154118446	0.2067913	0.001989	0.0339197
WDR45	1.9169972	1.72425999	2.0005402	1.381465528	0.4661996	0.001992	0.0317818
TMEM126A	1.7185769	1.5379905	1.796853	1.481165613	0.566733	0.001996	0.0239034
EMC8	1.6404565	1.46365843	1.7170905	1.546592252	0.6290929	0.002003	0.0530311
DYNC1H1	2.4068175	2.17105752	2.509009	1.288586561	0.3657895	0.002011	0.0293279
MTX2	1.8909874	1.70194865	1.9729272	1.386037626	0.4709664	0.002012	0.0293279
ENOPH1	1.9281563	1.73581423	2.011528	1.37470571	0.4591228	0.002012	0.0311149
UQCRH	12.47436	11.6258257	12.842162	1.114469853	0.1563576	0.002028	0.0334436
RMI2	1.4167629	1.58323228	1.3446058	0.590855156	-0.7591236	0.002054	0.0245769
SPNS1	1.5795651	1.40727955	1.6542432	1.606373841	0.6838077	0.002074	0.0315856
GOLGA8A	1.7951025	1.98230423	1.7139589	0.72682058	-0.4603288	0.002075	0.0278316
BCAS4	1.4522772	1.28722955	1.523818	1.823691194	0.8668615	0.002159	0.0361974
MRPL34	2.9375502	2.6733322	3.0520769	1.226341627	0.2943609	0.002178	0.035339
RBM42	1.4655267	1.30066223	1.5369881	1.786017831	0.8367465	0.002188	0.033343

DDX1	1.9510409	1.73519598	2.0446	1.420845685	0.5067499	0.00219	0.0318915
RNF165	1.4330529	1.59961536	1.3608555	0.6018117	-0.7326159	0.002204	0.038612
FKBP7	1.746773	1.56652356	1.8249031	1.45607903	0.5420887	0.002222	0.0366509
STARD3NL	2.048857	1.85316982	2.1336787	1.328784388	0.410107	0.002228	0.038612
PTGES	1.5478942	1.37200583	1.6241339	1.677753033	0.7465304	0.002235	0.0314938
ARHGAP11B	1.2884537	1.44631157	1.2200293	0.492994837	-1.0203556	0.002235	0.0314938
TMED7	1.7505378	1.56995572	1.8288121	1.454169234	0.5401952	0.002254	0.0314938
TMEM101	1.6714728	1.49489563	1.7480111	1.511452163	0.5959353	0.002258	0.0267218
CDK4	4.316041	3.93454843	4.481401	1.186349824	0.2465295	0.002259	0.0291366
MLF2	5.2107618	4.73261344	5.4180178	1.183625868	0.2432131	0.002275	0.0364092
VPS25	2.3306852	2.1165736	2.423493	1.274876092	0.350357	0.002277	0.0329207
CDC37	2.9633107	2.68639618	3.0833408	1.235380408	0.3049554	0.00228	0.0364092
OAT	1.5634742	1.39361823	1.6370992	1.618571424	0.694721	0.002282	0.0364399
NRCAM	1.4245144	1.59052099	1.352558	0.597028717	-0.7441278	0.002285	0.032593
MOB4	1.7777727	1.59656211	1.8563194	1.435423666	0.5214766	0.002286	0.0389364
UFD1L	1.8826775	1.6961547	1.9635268	1.384069952	0.4689169	0.002288	0.059015
RP13-942N8.1	1.5651763	1.73860287	1.4900037	0.663419659	-0.5920063	0.002297	0.0329207
PDXK	1.7228733	1.54454909	1.8001689	1.469415502	0.5552424	0.002303	0.032593
UBE2H	2.0024216	1.81006807	2.0857983	1.340379025	0.422641	0.002303	0.0329207
TIMMDC1	2.0097249	1.8169413	2.0932881	1.338270035	0.4203693	0.002307	0.0317961
VAPA	3.8003329	3.46305909	3.9465261	1.196287204	0.2585638	0.00231	0.0380357
RB1CC1	2.1766446	1.96886293	2.2667087	1.307417883	0.3867203	0.002314	0.0273568
RP4-614C10.2	2.474832	2.23862549	2.577217	1.273360708	0.3486412	0.002323	0.0329207
EIF3M	3.2767186	2.98100787	3.404896	1.213976018	0.2797399	0.002344	0.0380357
COPS9	4.7893477	4.37542229	4.968766	1.17578354	0.2336225	0.002366	0.0301241
ACO2	1.5870338	1.41640231	1.6609949	1.587394916	0.6666611	0.002367	0.0372078
P4HA1	3.2349071	2.93629718	3.3643413	1.22106321	0.2881379	0.00237	0.0396943
BRIP1	1.1654022	1.31454243	1.1007566	0.320327418	-1.6423808	0.002386	0.0320052
CBX3	5.4391295	4.96381046	5.6451591	1.17189233	0.22884	0.002396	0.0372191
CACNA2D1	1.411157	1.57573604	1.3398193	0.590234514	-0.7606398	0.002407	0.0372191
HMGN2	12.482436	13.7151491	11.948109	0.861028781	-0.2158666	0.002418	0.0595685
BRE	1.4632196	1.29957703	1.5341513	1.783018209	0.8343214	0.002437	0.0306418
PLPP3	2.2428235	2.47893693	2.1404789	0.771147751	-0.3749208	0.002437	0.0595685
NTPCR	2.3251803	2.10281819	2.4215644	1.289028801	0.3662845	0.002444	0.0285653
PRELID1	5.2123623	4.7749773	5.4019492	1.166086263	0.2216745	0.002449	0.0391068
ZNHIT1	4.1497416	3.73247292	4.3306089	1.218899151	0.2855788	0.002455	0.0368629
FRG1	1.8914678	1.70641175	1.9716813	1.375516948	0.4599739	0.002468	0.0362944
NUP107	1.5141364	1.6831816	1.4408628	0.64530843	-0.6319392	0.002479	0.0362944
LAMTOR4	4.7542273	4.31661408	4.9439132	1.189138411	0.2499166	0.002481	0.0384012
CFAP36	2.3642139	2.11736203	2.4712132	1.31668448	0.3969097	0.002487	0.0595685
UPP1	1.5728601	1.40381286	1.6461346	1.600084228	0.6781478	0.002489	0.0329462
CLDN4	1.3160826	1.14904435	1.3884863	2.606514505	1.3821219	0.002495	0.0291907
TMEM167A	2.2882793	2.08239281	2.3775219	1.27266356	0.3478511	0.002509	0.0362944
FGF14-AS2	1.4205099	1.5843535	1.349491	0.598081408	-0.7415862	0.002511	0.0340973
TMEM9	2.7371855	2.49380386	2.8426806	1.233549207	0.3028153	0.002525	0.0362944
COX8A	8.6109223	7.87015552	8.9320117	1.154560714	0.207344	0.002557	0.0389706
FBLIM1	1.4115368	1.25197041	1.4807017	1.907770415	0.9318876	0.002568	0.0416604
MSH6	1.6180067	1.7922594	1.5424759	0.684720056	-0.5464138	0.002588	0.036155
NIFK	1.7236975	1.54739815	1.8001155	1.461670059	0.5476177	0.002599	0.0378887
PRKRA	2.2628811	2.05817528	2.351612	1.277304443	0.3531024	0.002612	0.0341243
ZC3H15	2.8650827	2.59098001	2.9838941	1.246963541	0.3184193	0.002613	0.0397815

GOT1	1.2757524	1.12426251	1.3414165	2.747542353	1.4581417	0.002621	0.0393574
GPS1	1.7612544	1.58358741	1.8382652	1.436400352	0.5224579	0.002634	0.0304533
DTNA	2.0445055	2.25400554	1.9536966	0.760520208	-0.3949415	0.002648	0.0375222
WFDC2	2.3496025	2.12432654	2.4472496	1.287214646	0.3642526	0.00267	0.040047
MRPL32	1.9291495	1.74113708	2.0106445	1.363640426	0.4474633	0.002686	0.0375339
RNF167	2.0604645	1.86841324	2.1437102	1.317011444	0.3972679	0.002715	0.0379385
NEFM	1.2032436	1.03617964	1.2756583	7.61915749	2.9296315	0.002722	0.0391233
PPP1R12A	1.9928008	1.79892243	2.0768384	1.347863566	0.4306745	0.002734	0.043651
DTX4	1.4043578	1.56627913	1.3341721	0.59011907	-0.760922	0.002748	0.0378702
DOT1L	1.3350816	1.49241767	1.2668835	0.541986027	-0.8836724	0.002764	0.0380938
CALM3	4.9246425	4.41914319	5.143754	1.211927587	0.2773035	0.002768	0.0647661
SHPRH	1.8332557	2.02881632	1.7484889	0.727524371	-0.4589325	0.00277	0.0396964
NR2F2	1.8494675	1.65569866	1.9334576	1.42360763	0.5095516	0.002804	0.0396964
FAM114A1	1.5320503	1.3675993	1.6033325	1.6412777	0.7148194	0.002813	0.0416129
NAV2	1.6224786	1.79523037	1.5475984	0.688603509	-0.5382546	0.002817	0.0416748
GARS	1.5795501	1.41195474	1.6521952	1.583171993	0.662818	0.002821	0.0322476
FAM227A	1.5277516	1.69530728	1.4551237	0.654564785	-0.6113921	0.002863	0.0399658
PDCD6	2.6981094	2.43167427	2.8135971	1.266766557	0.3411507	0.002915	0.042507
AAMP	1.7863595	1.60922579	1.863139	1.416780162	0.5026159	0.002922	0.0337552
LINC01355	1.2850713	1.43890659	1.2183906	0.497578735	-1.0070033	0.002925	0.0391936
MGST2	1.3671355	1.21217656	1.4343033	2.046895561	1.0334375	0.002925	0.0330646
IGFBP4	1.7191172	1.54513839	1.7945292	1.457481679	0.5434777	0.002932	0.0460952
YTHDF2	2.3404248	2.12574365	2.4334795	1.273362245	0.3486429	0.002959	0.043135
ODC1	4.136707	3.74895176	4.3047816	1.202196996	0.2656733	0.002963	0.0419887
FAM177A1	2.0453203	1.82088998	2.1426009	1.391905018	0.4770608	0.002974	0.0409398
IKZF4	1.3313135	1.48733456	1.2636854	0.541076709	-0.886095	0.002977	0.0467956
MCM10	1.1643391	1.31058086	1.1009498	0.325035533	-1.6213307	0.002982	0.0405416
DEGS1	1.6174825	1.4495101	1.6902911	1.535652079	0.6188514	0.003016	0.0343404
PEF1	1.8912086	1.70951511	1.9699646	1.367081015	0.4510987	0.003027	0.0406065
ZNF436-AS1	2.0387198	2.24963525	1.9472974	0.758059133	-0.3996177	0.003041	0.0343404
COL6A1	2.5728559	2.33595345	2.6755425	1.254192279	0.3267585	0.003054	0.0393911
N4BP2L2	4.4791376	4.91293693	4.291105	0.841083069	-0.2496798	0.003067	0.0428482
ASCL1	1.6259299	1.81926507	1.5421277	0.661724394	-0.5956976	0.003068	0.048221
PAF1	1.4038365	1.24763875	1.4715412	1.904149619	0.9291468	0.003074	0.0454287
NDUFA2	4.6500021	4.23099423	4.8316233	1.185896054	0.2459776	0.003082	0.0442928
BAG1	2.5412221	2.31523199	2.6391787	1.246303867	0.3176559	0.003107	0.0506012
SLC52A2	1.4445724	1.28567757	1.5134463	1.797292941	0.8458256	0.003116	0.0429352
NCKAP1	1.9518591	1.76801432	2.0315477	1.343135971	0.4256054	0.003122	0.0506012
EIF4A2	11.482435	10.6367818	11.848988	1.125789523	0.1709371	0.003135	0.0412595
RER1	1.9011826	1.71966781	1.9798611	1.361546396	0.4452461	0.003138	0.0426641
SFI1	1.3670234	1.52450972	1.2987601	0.569598828	-0.8119819	0.003158	0.0429352
HMGA1	15.202491	16.41604	14.676471	0.887158521	-0.1727362	0.003161	0.0412595
MRPL9	2.0896834	1.89671244	2.1733277	1.308477136	0.3878887	0.003178	0.0430098
GNG12	1.450088	1.29160337	1.518784	1.779073963	0.8311265	0.003201	0.0459961
PARVB	1.3168626	1.16594601	1.3822783	2.303630205	1.2039091	0.003206	0.0430098
NOP10	5.4426776	4.99605266	5.6362696	1.160212344	0.2143889	0.003255	0.0363788
ODF2L	2.3056525	2.52412542	2.2109542	0.794523991	-0.3318373	0.003265	0.0449973
MIEN1	2.8124195	2.56735869	2.9186424	1.224124663	0.2917505	0.003268	0.049433
NUCKS1	7.4927537	6.72630628	7.8249745	1.191863332	0.2532188	0.003277	0.0477295
WTAP	2.0210164	1.83064461	2.1035341	1.32852739	0.409828	0.003287	0.040827
COL11A1	2.2255354	2.43948681	2.132797	0.786945006	-0.3456653	0.003288	0.0493159

SNW1	1.881871	1.70207767	1.9598035	1.367090131	0.4511084	0.003289	0.049433
VPS72	1.8072766	1.63104541	1.883665	1.400319221	0.4857557	0.003292	0.049433
ARHGAP21	1.7120965	1.88650137	1.6364998	0.717990793	-0.4779628	0.003296	0.0436251
FRMD4A	1.8340325	2.01474509	1.7557016	0.744720626	-0.4252288	0.003296	0.0442156
PPP2R5C	1.960167	1.77644704	2.0398013	1.339178712	0.4213485	0.003346	0.0442871
CFI	1.3235896	1.17321172	1.3887717	2.244488291	1.1663866	0.00338	0.0476245
ABCD4	2.7412988	3.05281636	2.6062698	0.782471245	-0.3538904	0.003383	0.0459386
RP11-1260E13.1	1.5652708	1.75996535	1.4808794	0.632764857	-0.6602586	0.003409	0.0476245
FILIP1L	1.2895752	1.14045931	1.3542103	2.521800323	1.334454	0.003416	0.0446326
TRIM36	1.2452216	1.09916227	1.3085318	3.111382994	1.637556	0.003418	0.0446505
TSG101	1.8752761	1.69696126	1.9525677	1.366744057	0.4507431	0.003444	0.0509551
IFRD1	2.5180012	2.76575194	2.4106123	0.798873427	-0.3239612	0.003471	0.038431
GTF2H5	3.4801432	3.13533738	3.6296012	1.231468733	0.30038	0.003499	0.0493168
OLFM2	1.5305468	1.36934712	1.6004196	1.625624228	0.7009938	0.003515	0.038431
VPS37A	1.5188655	1.35850687	1.5883738	1.641178688	0.7147323	0.003518	0.038431
ARID1B	2.4604614	2.68469421	2.3632664	0.809207061	-0.3054192	0.003522	0.0545054
UBE2J1	1.8220838	1.64604813	1.8983875	1.390589054	0.4756961	0.003525	0.0466611
IPO9	1.9190971	2.10613866	1.838023	0.757611131	-0.4004706	0.00353	0.0493168
NUDCD2	2.9519274	2.69258879	3.0643391	1.219634129	0.2864484	0.00354	0.0564792
SMAP1	2.4322871	2.207379	2.5297747	1.267021155	0.3414406	0.003544	0.0481811
MT1X	2.3240633	1.95096685	2.485784	1.562393094	0.6437575	0.003554	0.0435989
PRKAA2	1.7452838	1.92089782	1.6691629	0.726641847	-0.4606836	0.003558	0.0525924
NAPA	1.5029828	1.34382188	1.5719719	1.663570495	0.734283	0.003567	0.0459655
ZNF714	1.6741572	1.84462784	1.6002658	0.710686693	-0.4927144	0.003671	0.0576384
PSMA3-AS1	1.9709706	2.15562133	1.8909327	0.77095556	-0.3752804	0.003673	0.0559899
SLC25A24	1.5284206	1.36783367	1.5980279	1.625810788	0.7011594	0.003737	0.0544362
FTL	103.35319	93.4454497	107.64775	1.153629009	0.2061793	0.003747	0.0531681
USP1	1.9646214	2.16770013	1.8765958	0.750702853	-0.4136861	0.003747	0.0516423
CLPP	2.2866684	2.06055675	2.3846777	1.305613915	0.3847283	0.003753	0.0858092
MPHOSPH6	1.7171696	1.54802886	1.7904846	1.442414194	0.5284855	0.003753	0.0580294
IFT57	2.5224402	2.28686664	2.6245509	1.262408129	0.3361784	0.003756	0.0531681
SDF2L1	2.2577421	2.0530604	2.3464626	1.278618558	0.3545859	0.003769	0.0493614
GREM1	1.1797712	1.03866018	1.2409365	6.232163612	2.6397331	0.003775	0.0407967
RSRP1	2.5968516	2.84354294	2.4899219	0.80818401	-0.3072443	0.003778	0.0493614
RPRM	1.5472565	1.3874058	1.6165446	1.591469762	0.6703597	0.003857	0.0491217
PAM	1.9657551	1.7847842	2.0441979	1.330554128	0.4120272	0.003866	0.0491859
CD46	1.9312796	1.74900264	2.0102885	1.348845045	0.4317246	0.003872	0.0519341
FANCB	1.2748784	1.4233751	1.2105116	0.497222538	-1.0080364	0.003888	0.060172
CRABP1	26.449762	23.9340685	27.540202	1.15723917	0.2106871	0.003888	0.042984
RNASET2	1.7821246	1.60992036	1.8567675	1.404720244	0.4902828	0.003925	0.0563505
MRPL13	2.0997533	1.87634077	2.1965927	1.365442254	0.4493683	0.003932	0.0526879
DNAJA2	1.6101142	1.44678107	1.6809118	1.524039071	0.6078999	0.003939	0.0508047
SPSB1	1.2624855	1.11774757	1.3252229	2.762035399	1.4657318	0.003962	0.0511016
TMPO	2.0467427	2.23946667	1.9632055	0.777112848	-0.363804	0.003974	0.0476063
HK1	1.7235818	1.55471269	1.796779	1.436381472	0.5224389	0.003975	0.0476063
ILF3	3.5765496	3.87871977	3.4455723	0.849534685	-0.2352552	0.004021	0.058624
CCDC15	1.1309161	1.27002219	1.0706198	0.261533408	-1.9349328	0.004047	0.0547342
CPXM1	1.7198522	1.55124456	1.7929361	1.438446946	0.524512	0.004049	0.0529051
RTF1	3.6182338	3.26632417	3.7707709	1.222583662	0.2899332	0.004052	0.0558389
B9D1	1.6643497	1.49881836	1.7361001	1.47568767	0.5613874	0.004072	0.0620076
MRPS5	2.0606413	1.87417193	2.1414675	1.305770003	0.3849008	0.004081	0.0547342

XXbac-BPGBPG55C20.2	1.3429116	1.4940125	1.2774161	0.561556878	-0.8324959	0.004114	0.054457
PRKAR1A	2.7560557	2.51508325	2.8605065	1.227989646	0.2962984	0.004117	0.0530365
ANKRD37	1.5951727	1.41614668	1.6727725	1.61667167	0.6930267	0.004119	0.0571306
MSN	1.9880433	1.80751689	2.0662935	1.320459673	0.4010402	0.004141	0.0621207
MRPS18A	1.6189283	1.4561116	1.689502	1.511695906	0.596168	0.00415	0.0571306
LMO4	2.3814974	2.16972022	2.4732934	1.259526352	0.3328813	0.004159	0.0624504
PCP4	1.3773268	1.17516255	1.464956	2.654425548	1.4083997	0.004165	0.0530365
MRPL41	3.3516814	3.06404494	3.476359	1.199760219	0.2627461	0.004181	0.0583573
TAPBP	1.7363226	1.56685559	1.8097789	1.428545364	0.5145469	0.004184	0.0532799
ARL4D	1.4336382	1.2808267	1.4998752	1.780013054	0.8318878	0.004194	0.0458568
GADD45B	1.5131206	1.34266419	1.5870059	1.71306454	0.7765795	0.004214	0.0596496
FHL1	2.2965662	2.04968485	2.4035782	1.337142491	0.4191532	0.004234	0.0559359
ZNF519	1.3122512	1.46139438	1.2476043	0.536643567	-0.8979639	0.004269	0.0543645
MFAP4	1.3292974	1.18177431	1.393242	2.163353139	1.1132692	0.004286	0.0559359
ACAT2	1.5587313	1.39971946	1.6276558	1.570240716	0.6509857	0.004294	0.0508196
LYPD1	1.6692775	1.49498314	1.7448263	1.504750786	0.5895246	0.004295	0.0540061
HOOK2	1.9338453	2.1145172	1.8555321	0.767625775	-0.3815249	0.004312	0.0619761
THOC3	2.0752482	1.88317782	2.1585022	1.311742852	0.3914849	0.004325	0.0587936
FCGRT	1.3436564	1.19634871	1.4075077	2.075428708	1.0534094	0.004372	0.0593832
SUGP2	2.0054176	2.18799945	1.9262765	0.779694358	-0.3590194	0.004379	0.0587326
MEA1	2.7444986	2.50435242	2.8485912	1.228828581	0.2972837	0.004383	0.0551154
COMT	1.3064368	1.16114241	1.3694154	2.292477725	1.1969077	0.0044	0.0470398
CMC1	1.8743017	1.69815246	1.9506545	1.361671839	0.4453791	0.004402	0.067098
CCDC66	2.3919315	2.59988155	2.3017944	0.813681753	-0.2974635	0.004402	0.0649416
RIF1	1.8771931	2.07090892	1.7932259	0.740703442	-0.4330321	0.004416	0.0568971
KIAA0232	1.5880621	1.75119682	1.5173505	0.688701708	-0.5380488	0.004454	0.0629974
LOXL2	1.4352622	1.28374809	1.5009368	1.765427878	0.8200179	0.004457	0.0559852
UCHL3	1.4987636	1.34253158	1.5664832	1.653813155	0.7257963	0.004459	0.0649416
FDPS	2.7691038	2.52989395	2.8727906	1.224130999	0.291758	0.004462	0.0482686
ADAM28	1.1727124	1.03553699	1.2321718	6.533242611	2.7077992	0.004502	0.0595886
ARID5B	1.3702932	1.22166405	1.4347173	1.961153598	0.9717025	0.004503	0.0596006
PIAS2	2.1727053	2.38321518	2.0814586	0.781844116	-0.3550471	0.004509	0.0629974
SRP54	1.6733902	1.5093429	1.7444974	1.461682196	0.5476297	0.004514	0.0630106
HPRT1	2.3226188	2.12311098	2.4090965	1.254636909	0.3272699	0.004523	0.0669164
TDG	1.8358221	1.66465906	1.9100137	1.369143593	0.4532738	0.004532	0.0607247
UGCG	1.4906855	1.33593336	1.5577637	1.660340246	0.7314789	0.00454	0.0593167
DTL	1.2098395	1.35216974	1.1481457	0.420665591	-1.2492543	0.004545	0.056898
NOL12	1.3054314	1.45307918	1.2414327	0.532870873	-0.9081421	0.004564	0.1020291
RP11-111M22.3	1.4350275	1.589226	1.3681893	0.62486938	-0.6783734	0.004572	0.0483658
FNTA	1.8476458	1.67579083	1.9221373	1.364530656	0.4484048	0.004573	0.057504
SF3B2	3.0054212	2.73577406	3.1223013	1.222682903	0.2900503	0.004614	0.059506
CCDC74A	1.7458546	1.9159534	1.6721244	0.733797585	-0.4465459	0.004638	0.057609
BLOC1S1	3.6050733	3.28778708	3.7426028	1.198801577	0.2615929	0.004645	0.0576293
PWP1	1.687775	1.52345726	1.7589995	1.449974099	0.5360271	0.004649	0.0577413
TROVE2	2.494097	2.28309508	2.5855569	1.235728271	0.3053615	0.004658	0.0641878
HSPA4	1.8992235	1.7163964	1.9784709	1.365823245	0.4497708	0.004675	0.0675803
NUDT16L1	1.555486	1.3978601	1.6238098	1.567912442	0.648845	0.00468	0.056898
PRR13	2.9547211	2.68664365	3.0709208	1.227835405	0.2961172	0.00469	0.056898
SEC63	2.271953	2.07095269	2.3590777	1.269036155	0.3437332	0.004694	0.056898
BZW2	2.2206891	2.02929771	2.3036487	1.266541946	0.3408949	0.004702	0.0675803
RUVBL2	1.9922771	1.81181998	2.0704972	1.318638645	0.3990493	0.004731	0.0491742

WEE1	1.6604761	1.83735942	1.5838051	0.697197698	-0.5203603	0.004746	0.0491742
LRP2	1.7968663	1.97105191	1.7213647	0.742869319	-0.4288197	0.004756	0.062897
WDR18	1.6706998	1.50827921	1.7411019	1.458060702	0.5440508	0.004765	0.0684124
NAPEPLD	1.3732008	1.52373329	1.3079517	0.58799329	-0.7661284	0.004766	0.065609
PRR4	1.869992	2.04493289	1.794163	0.760013358	-0.3959033	0.004785	0.0586981
MRPL20	3.3338421	3.03879692	3.461731	1.207442967	0.271955	0.004838	0.0592918
FAM219B	2.0108757	2.19548021	1.9308579	0.778647647	-0.3609575	0.004841	0.0579822
TMEM205	2.4269575	2.20974343	2.5211102	1.257382464	0.3304235	0.004888	0.0599648
PRKD3	1.5342348	1.69233376	1.465706	0.672661063	-0.5720483	0.004895	0.0523859
CHMP2B	1.9822181	1.80478831	2.059126	1.316030562	0.396193	0.004897	0.0593008
GTF3C6	3.0725384	2.79922297	3.1910084	1.217752567	0.284221	0.004933	0.0628187
TTC3	9.1717964	9.79900436	8.8999297	0.897820859	-0.1555005	0.004934	0.0571779
GRINA	2.510134	2.29219707	2.6045999	1.241760971	0.3123875	0.004945	0.0571779
TSTD1	2.6378266	2.37871203	2.7501412	1.269403004	0.3441502	0.005006	0.1094813
WHSC1	2.0796595	2.26290716	2.0002299	0.792005867	-0.336417	0.005007	0.0700719
WDR73	1.6922657	1.85802027	1.6204185	0.723081402	-0.46777	0.005008	0.0671761
POLR2K	2.8872335	2.642132	2.9934741	1.213954846	0.2797148	0.005015	0.0700719
ZMAT5	1.6569716	1.49534753	1.7270284	1.46771386	0.5535707	0.00502	0.059408
CHD7	2.8379454	3.09517793	2.7264466	0.824009539	-0.2792671	0.005093	0.060931
POLR2C	1.9689651	1.79338311	2.0450721	1.317235175	0.3975129	0.005096	0.0596081
ARF3	1.9123061	1.73994537	1.9870167	1.33390487	0.4156558	0.005106	0.0667047
HIST2H2AC	1.3249593	1.47140572	1.2614813	0.55468415	-0.8502616	0.00511	0.0703444
RAB5A	1.815315	1.64704279	1.8882535	1.372789492	0.4571104	0.005132	0.0770495
EIF4E2	2.1700654	1.9815161	2.2517932	1.27536693	0.3509124	0.005154	0.0669684
NSMCE2	1.5369431	1.3823483	1.6039531	1.579588754	0.659549	0.005159	0.0730322
DPCD	2.7733868	2.52970767	2.8790109	1.228346375	0.2967174	0.005198	0.0669684
CTSA	1.4270594	1.27845276	1.4914737	1.765016461	0.8196816	0.005228	0.0536112
PDLIM5	2.2345333	2.04450965	2.3169001	1.260783039	0.33432	0.005234	0.0675089
GUK1	7.3137118	6.79635551	7.5379627	1.127943705	0.1736951	0.005257	0.0601011
YY1	2.6991268	2.46854249	2.7990748	1.225075095	0.2928702	0.005287	0.0782238
TAF1D	2.6544288	2.89794058	2.5488773	0.816083145	-0.2932119	0.005306	0.0613498
HSD17B14	1.4673339	1.31652959	1.5327008	1.682941549	0.7509851	0.005351	0.0746884
LTBP4	2.1913629	2.39239361	2.104225	0.793040851	-0.3345329	0.005358	0.0665404
FBXO7	2.0628643	1.88180533	2.1413453	1.294327998	0.3722033	0.005361	0.0606025
ADGRA2	1.3804701	1.52876292	1.3161918	0.597984079	-0.741821	0.005384	0.0694377
IL13RA1	1.7357291	1.57246556	1.8064966	1.40881237	0.4944795	0.005452	0.0740446
LL22NC03-2H8.5	1.395511	1.54463805	1.3308711	0.607506384	-0.7190285	0.005477	0.0754754
COX7A2L	6.7435874	6.2354938	6.9638233	1.139113814	0.1879119	0.005599	0.0587983
SHFM1	6.3217343	5.77717947	6.5577746	1.163400836	0.2183482	0.005609	0.0714259
PSMB4	1.9658836	1.79190443	2.0412959	1.314926193	0.3949818	0.005611	0.0587983
SSU72	3.7697	3.46100201	3.9035068	1.179806772	0.2385506	0.005669	0.0587983
AIP	1.6545958	1.49534195	1.7236253	1.460860141	0.5468181	0.005716	0.0760463
SRSF2	4.5572758	4.92206109	4.3991577	0.86667637	-0.2064347	0.005722	0.0777891
ALDH9A1	1.5358544	1.38308862	1.6020716	1.571624919	0.6522569	0.005751	0.0760463
CBX5	4.7086329	5.16878411	4.5091778	0.841774891	-0.2484936	0.005761	0.1233015
HPCAL1	1.3773905	1.23254904	1.4401729	1.892817381	0.9205352	0.005791	0.0587826
TBRG4	1.4277572	1.28045344	1.4916069	1.752900079	0.8097438	0.005795	0.0695258
JKAMP	1.6201353	1.46371565	1.6879362	1.48353027	0.5690344	0.005805	0.0695258
FAM111B	1.155819	1.29072518	1.0973432	0.334828887	-1.5785041	0.005821	0.0801383
JUN	2.9675487	2.58101187	3.1350952	1.350461202	0.4334522	0.005824	0.0650984
DDX21	1.912514	1.7412915	1.9867314	1.331097641	0.4126164	0.005832	0.0741874

PTPA	1.8833714	1.71248001	1.9574453	1.343820545	0.4263405	0.005845	0.0852126
DBP	1.6324793	1.47546501	1.700538	1.473374444	0.5591241	0.005887	0.0604282
CRYL1	1.7353922	1.57291507	1.8058188	1.406524061	0.4921342	0.005889	0.0696157
HES1	5.6335117	6.34349476	5.3257657	0.809538683	-0.3048281	0.005897	0.075014
PEA15	1.9319097	1.75824462	2.0071857	1.328312393	0.4095945	0.005928	0.0805028
NDRG1	2.0293758	1.83592614	2.1132276	1.331729671	0.4133013	0.00594	0.0853729
INO80C	1.5543005	1.40066891	1.620893	1.549641091	0.6319341	0.005957	0.0696157
HYI	1.5963668	1.4410859	1.6636741	1.504636922	0.5894154	0.005972	0.0682736
FAM174A	1.4076188	1.26205814	1.4707128	1.796215309	0.8449603	0.006003	0.0754148
SCAMP2	1.4068891	1.26169546	1.469824	1.795308244	0.8442316	0.00601	0.0817074
MND1	1.3634603	1.50931514	1.3002388	0.589495126	-0.7624482	0.006023	0.1262324
SH3GLB1	2.0152407	1.83962622	2.0913617	1.299818563	0.3783103	0.006023	0.0786923
FDX1	2.3685153	2.16962704	2.4547245	1.243750709	0.3146973	0.006037	0.061341
GINM1	1.9613929	1.78067942	2.0397241	1.331819519	0.4133986	0.006058	0.0717004
NDUFAF4	1.4317453	1.28500203	1.495352	1.738064865	0.7974819	0.006081	0.0672224
PSMB8	1.3547906	1.21195751	1.4167023	1.965971087	0.9752421	0.006134	0.0717497
CEP85L	2.2303579	2.43668797	2.140923	0.794134172	-0.3325453	0.00616	0.0619077
HSPG2	1.2626595	1.12528024	1.3222072	2.571891942	1.36283	0.006169	0.0827407
GLB1	1.4326202	1.28604535	1.4961539	1.734528869	0.7945439	0.006178	0.0698353
C9orf135	1.9984436	2.18697558	1.9167233	0.772318614	-0.372732	0.006182	0.0827705
AC069277.2	1.3076367	1.4500456	1.2459088	0.54640853	-0.8719481	0.006204	0.0748437
HIST1H1D	1.6017761	1.78213674	1.5235978	0.669445307	-0.5789619	0.006211	0.0718199
ILDR2	1.3238246	1.46696778	1.2617783	0.56059189	-0.8349772	0.006249	0.0748437
ALDH7A1	1.9497807	1.77688382	2.0247238	1.319018076	0.3994643	0.006259	0.0827705
RHOBTB3	3.1066348	2.81664793	3.2323312	1.228818838	0.2972722	0.006262	0.0828828
STRADB	2.0574867	1.88000458	2.1344174	1.289103929	0.3663686	0.006306	0.0813305
ALDH1A3	1.3733581	1.22792406	1.4363973	1.914660805	0.9370888	0.006324	0.0837104
IFT20	1.8882608	1.71805714	1.9620365	1.339777133	0.421993	0.006368	0.0902258
RGS16	1.425251	1.26504368	1.4946937	1.866460933	0.9003053	0.006381	0.0866552
PLOD3	1.320722	1.18104321	1.3812665	2.105941882	1.0744656	0.006385	0.0755707
WDR83OS	4.3961953	4.01364087	4.5620156	1.181964178	0.2411863	0.006453	0.078995
GOT2	1.6660964	1.50867799	1.7343302	1.443605265	0.5296763	0.006461	0.0843247
GABARAPL1	1.819864	1.65514248	1.8912634	1.36041161	0.4440432	0.006465	0.090335
UBE2A	1.7763979	1.61379885	1.8468773	1.379730968	0.464387	0.006466	0.0813046
SYNGR2	1.4838463	1.33627137	1.5478134	1.629081238	0.7040585	0.006497	0.078995
HAGH	1.9948992	1.8218591	2.0699044	1.301809998	0.3805189	0.006498	0.0848954
ZNF593	1.8857442	1.71909007	1.9579813	1.332213266	0.4138251	0.006499	0.0726495
TIMM8B	4.4800947	4.09070342	4.6488784	1.180598047	0.2395179	0.006524	0.078995
GOPC	1.5820449	1.42961621	1.6481159	1.508592853	0.5932035	0.006537	0.0657609
OCIAD2	5.0949564	4.66090881	5.2830966	1.169954466	0.2264524	0.006565	0.0825529
PPDPF	11.753439	11.0567549	12.05542	1.099302956	0.136589	0.006569	0.0905315
MRPL4	2.0889083	1.87880637	2.1799782	1.342705512	0.4251429	0.006605	0.0829729
AAMDC	2.1545137	1.97466736	2.2324691	1.26450225	0.3385696	0.00664	0.0884031
KPNA3	1.9441245	1.77524908	2.0173245	1.31225502	0.3920481	0.006679	0.0884031
GPC3	1.3593669	1.21739092	1.4209072	1.936176427	0.9532104	0.006711	0.0875852
MZT2B	9.5373295	8.89206006	9.8170251	1.117201975	0.15989	0.006728	0.0743747
HMGA2	3.6269694	3.24626727	3.7919868	1.242945059	0.3137625	0.00678	0.0775121
TAF7	4.0110381	3.69062774	4.1499217	1.170701399	0.2273731	0.006817	0.0878304
GSTK1	2.0681515	1.86190619	2.1575496	1.343011128	0.4254713	0.00687	0.0844804
PGAP1	1.5879209	1.74316365	1.5206301	0.700559143	-0.5134212	0.006873	0.1411114
DICER1-AS1	1.5022273	1.65279687	1.4369621	0.669369162	-0.579126	0.006893	0.0844804

CTSV	1.2180717	1.08479454	1.2758415	3.253056881	1.701796	0.006913	0.0848127
NNT-AS1	1.7888928	1.95241221	1.7180145	0.753890458	-0.4075732	0.006945	0.0691768
NFE2L2	1.9826866	1.81269166	2.0563718	1.299843303	0.3783377	0.00703	0.0873148
EMC3	1.4996164	1.35214631	1.5635381	1.600295328	0.6783382	0.007086	0.0818573
C18orf32	1.5365546	1.3868472	1.601446	1.554737986	0.6366715	0.007114	0.1417574
SNRNP40	1.6250098	1.47200553	1.6913303	1.464665698	0.5505714	0.007137	0.0908828
PTPRA	2.8021737	3.03337589	2.7019578	0.837010923	-0.2566816	0.007145	0.0909014
FBXO21	1.7393633	1.58094507	1.8080306	1.390889802	0.4760081	0.007182	0.0708365
DNMT1	2.0195014	2.19414164	1.9438026	0.790360672	-0.3394169	0.007185	0.0785625
SEMA3E	1.6406019	1.7972793	1.5726893	0.71830447	-0.4773326	0.007187	0.1417574
ABAT	1.8067368	1.97017083	1.7358955	0.758521567	-0.3987379	0.007256	0.0708658
ROCK1	2.0080634	1.83830359	2.0816467	1.290280421	0.3676846	0.007263	0.0948924
CREB3	1.7441513	1.58570994	1.8128286	1.387766427	0.4727648	0.00731	0.0955018
VTI1B	2.7840304	2.54378146	2.8881676	1.223079611	0.2905183	0.007332	0.0793161
RFXANK	1.8720635	1.7077891	1.9432692	1.332698076	0.41435	0.007338	0.141962
COMMD7	2.041307	1.8682353	2.1163258	1.285741087	0.3626002	0.007355	0.0936619
ZNF326	2.2459735	2.06594132	2.3240094	1.242103465	0.3127854	0.00737	0.0926715
CCPG1	1.4865146	1.34119497	1.5495042	1.610528553	0.6875342	0.007401	0.083655
LYPLAL1	1.7194984	1.56264408	1.7874878	1.399619838	0.485035	0.007404	0.0992175
H2AFY2	2.8219027	2.58335068	2.9253043	1.215968343	0.2821057	0.007426	0.0886499
MGAT2	1.4295294	1.28667307	1.4914513	1.714326677	0.7776421	0.007456	0.0741898
WDR27	1.3489582	1.49012791	1.2877674	0.587127241	-0.7682549	0.007486	0.0801117
UQCR10	8.6031953	7.76435871	8.9667936	1.177760367	0.236046	0.007498	0.0886499
PELO	1.4249185	1.28280086	1.4865202	1.720363354	0.7827133	0.007537	0.0972118
SLC35A2	1.345961	1.20753416	1.4059629	1.956125484	0.9679989	0.007546	0.0943415
SMIM14	1.8177328	1.65592432	1.8878695	1.353615827	0.4368183	0.007579	0.0918626
NARS	2.354873	2.15811329	2.4401596	1.243539484	0.3144523	0.007593	0.0867104
RALA	1.748065	1.59020174	1.8164917	1.38341115	0.46823	0.007596	0.0943415
STAP2	1.3338516	1.1959704	1.393617	2.008553411	1.0061568	0.007598	0.0932095
SLC25A5	8.3494545	7.71392219	8.6249295	1.135689281	0.1835682	0.007599	0.0932937
OPN3	1.6886453	1.53349848	1.7558945	1.416863527	0.5027008	0.007604	0.0932937
RARS	1.8485821	1.68120418	1.921133	1.352212828	0.4353222	0.007654	0.0842315
SLC25A6	15.012823	13.9746249	15.462836	1.114701687	0.1566577	0.007678	0.0924565
PRPSAP2	1.7938601	1.63477187	1.8628178	1.359256451	0.4428177	0.007688	0.0954797
UXS1	1.4103071	1.26917512	1.4714815	1.751579164	0.8086562	0.00772	0.0842315
SPAG16	2.3527733	2.15235481	2.4396458	1.249307798	0.321129	0.007722	0.0842315
RAB9A	1.7940671	1.62964472	1.8653369	1.374325612	0.4587239	0.007776	0.0924565
MRPL15	1.7078071	1.55222967	1.775243	1.403841566	0.4893801	0.007787	0.0842315
NCALD	1.4056958	1.26450529	1.4668956	1.765165632	0.8198036	0.0078	0.1006034
ADA	1.61734	1.46648023	1.682731	1.463579712	0.5495013	0.007811	0.095827
PRCC	1.5863202	1.43630913	1.6513432	1.492848068	0.5780673	0.007815	0.1033439
TTR	4.2751156	1.95916687	5.2789761	4.461138403	2.1574119	0.00782	0.0924565
ACOT7	1.6932658	1.53843868	1.7603765	1.412187752	0.4979319	0.007821	0.1484425
CHCHD6	1.8693822	1.70557385	1.9403859	1.332795781	0.4144557	0.007851	0.0951593
CWC15	2.7767273	2.54990277	2.8750456	1.20978274	0.274748	0.00786	0.0859435
NIFK-AS1	1.5698153	1.72066164	1.5044302	0.699954286	-0.5146674	0.00788	0.0843321
PPIE	1.6332914	1.48192857	1.6989005	1.450215917	0.5362677	0.007903	0.0913707
ANKRD36C	1.6066087	1.7592846	1.5404305	0.711762814	-0.4905315	0.00791	0.0913707
MEIS1	2.0837803	2.26743056	2.0041761	0.792292832	-0.3358943	0.007925	0.1022073
NINJ1	2.2281832	2.04969348	2.3055506	1.243744563	0.3146902	0.007961	0.0920807
PDGFC	1.3084513	1.17258517	1.3673432	2.1284747	1.0898199	0.007963	0.0920807

SMPD1	1.2649838	1.13170722	1.3227533	2.450536308	1.2930975	0.007995	0.084661
EIF3H	6.1720162	5.74615959	6.3566061	1.128619037	0.1745586	0.007999	0.108744
RP3-525N10.2	1.5309278	1.67998055	1.46632	0.68578433	-0.5441732	0.008001	0.0958257
INHBA	1.187713	1.05840459	1.2437624	4.173686332	2.0613222	0.008009	0.0935922
TTC9C	1.5827388	1.43336984	1.6474836	1.494067007	0.5792449	0.008041	0.1023943
MRPL36	2.0753003	1.9016239	2.1505813	1.27612114	0.3517653	0.008061	0.0871953
MARCH6	2.6218154	2.84796447	2.5237899	0.824577499	-0.278273	0.008126	0.0860482
GSPT1	1.9475258	1.78183367	2.019346	1.303788831	0.3827102	0.008131	0.0997488
PUF60	2.1706435	1.99454469	2.2469744	1.253814392	0.3263238	0.008135	0.0962813
OS9	1.6348059	1.48388554	1.7002231	1.44708417	0.5331488	0.008147	0.0929913
TET1	2.3069258	2.50471678	2.2211923	0.81157618	-0.3012016	0.00817	0.0790293
ARL2	3.9152344	3.60229973	4.0508776	1.17237747	0.2294371	0.008227	0.0929913
CDV3	1.784415	1.62342661	1.8541963	1.370163434	0.454348	0.008286	0.0868274
ZNF730	1.1927552	1.32363296	1.1360255	0.420308062	-1.250481	0.008355	0.1012681
NOSIP	2.3221491	2.13816506	2.401898	1.23171764	0.3006716	0.008424	0.1058247
EIF3D	3.4442325	3.16798941	3.5639716	1.182649518	0.2420226	0.008458	0.0945436
EMC2	1.4952214	1.35150393	1.5575165	1.586088934	0.6654737	0.008488	0.0908444
CAV2	1.2538386	1.1225234	1.3107578	2.536313952	1.3427333	0.00853	0.1072589
POLR2D	1.5598621	1.70951497	1.4949943	0.697651655	-0.5194212	0.00855	0.1088784
PLEKHB1	1.7163992	1.56189504	1.7833699	1.394156982	0.479393	0.008558	0.1089791
FZD3	2.3960443	2.59876078	2.3081758	0.818243618	-0.2893977	0.00856	0.0896995
TPBG	1.3680991	1.23067233	1.4276675	1.854004516	0.8906448	0.008564	0.1103467
EDF1	7.0864144	6.50862744	7.3368593	1.150351774	0.2020751	0.008585	0.1040537
RAP1A	1.7869074	1.63003624	1.8549041	1.356912631	0.4403278	0.008594	0.115279
CGNL1	1.2194306	1.08936681	1.2758075	3.086240653	1.6258506	0.008635	0.095456
SLC12A8	1.2016138	1.0731057	1.2573164	3.519785367	1.8154875	0.008684	0.0919579
SIVA1	3.6364088	3.94286516	3.5035736	0.850726564	-0.2332326	0.008721	0.0904504
C9orf78	1.8128507	1.65508621	1.8812346	1.345219257	0.4278413	0.008763	0.110197
COQ5	1.5895388	1.44190107	1.6535332	1.478912828	0.564537	0.008776	0.1002211
ANP32B	4.4130536	4.07156325	4.5610744	1.159368736	0.2133395	0.008784	0.0910981
DDX39B	1.434439	1.57789894	1.3722555	0.644153215	-0.6345242	0.008789	0.1052635
HNRNPC	5.7437113	5.33406798	5.9212734	1.13548598	0.1833099	0.008922	0.1108031
PLOD2	2.2260178	2.04665804	2.3037623	1.245643077	0.3168907	0.008923	0.0854875
BCL2L1	1.4382717	1.29762831	1.4992343	1.677375163	0.7462054	0.008954	0.0919135
PCDH7	3.2804849	3.56789286	3.1559063	0.8395624	-0.2522905	0.009031	0.0889854
PAX2	1.1224502	1.24837295	1.0678682	0.273251292	-1.8716998	0.009086	0.1128429
GLUD1	1.7143529	1.56196136	1.7804079	1.388721607	0.4737574	0.009118	0.1105098
PRPF40A	2.6936304	2.46064545	2.794619	1.228647947	0.2970716	0.009151	0.1150759
ITPA	1.7674659	1.61241795	1.8346723	1.362912913	0.4466934	0.009162	0.1124002
CTSZ	1.2927503	1.16059463	1.3500339	2.179611234	1.1240708	0.009241	0.0877055
AFG3L2	1.5306917	1.3866479	1.5931282	1.534026709	0.6173236	0.009254	0.1011927
JAG1	1.2844336	1.15292841	1.3414353	2.232647691	1.1587556	0.009313	0.1184773
RNF11	2.0214402	1.85450246	2.0938003	1.280043483	0.3561928	0.009388	0.112433
DRAM2	1.8570865	1.69713181	1.9264198	1.328901908	0.4102346	0.009436	0.1171935
DPY19L1	1.3988358	1.53872198	1.3382014	0.627784613	-0.6716584	0.009444	0.1066473
AHSA2	1.6965808	1.8498282	1.6301548	0.741508511	-0.4314648	0.009529	0.0895873
BCL11A	1.5540647	1.7002434	1.4907028	0.700760302	-0.513007	0.009602	0.1149928
RPE	1.3654788	1.22987028	1.4242591	1.84564577	0.8841257	0.009621	0.1138642
CSDE1	5.0578291	4.64122229	5.2384096	1.164007363	0.2191002	0.009727	0.1137829
ERRFI1	1.2965316	1.16429832	1.3538489	2.153697427	1.1068156	0.009755	0.1225492
PLAU	1.1908567	1.06499849	1.2454106	3.775635629	1.9167195	0.009784	0.1104771

EMC10	3.1937368	2.91247265	3.3156523	1.210815913	0.2759795	0.009793	0.1201471
RSBN1L	1.8553654	2.0151086	1.7861239	0.774423423	-0.3688055	0.009832	0.0959363
LINC01481	1.6119409	1.76043617	1.5475748	0.720079881	-0.4737711	0.009838	0.1164362
MED8	1.42769	1.29011433	1.4873229	1.679761606	0.7482565	0.009976	0.1209166
AIMP2	1.2982388	1.16717219	1.3550504	2.12386026	1.0866888	0.009986	0.1238999
TARBP1	1.3035761	1.43749546	1.245528	0.561212602	-0.8333807	0.010081	0.1164472
USP28	1.4906722	1.63408771	1.428508	0.675786582	-0.5653604	0.010088	0.1879313
POU2F2	1.2241186	1.35357627	1.1680045	0.475157728	-1.0735216	0.010125	0.1130629
ALDOC	1.5642341	1.42048736	1.6265419	1.490037328	0.5753485	0.010259	0.1053115
HIKESHI	2.5115034	2.2684875	2.61684	1.274620351	0.3500676	0.010297	0.1175933
IMP3	2.0340811	1.86896665	2.1056508	1.272374291	0.3475231	0.01032	0.1221398
CNDP2	1.5191463	1.37754441	1.5805244	1.537632054	0.6207103	0.010325	0.1207795
PTS	1.7623732	1.60983258	1.8284927	1.358557673	0.4420758	0.010374	0.1054172
GPX8	1.9138341	1.75259905	1.9837222	1.307100023	0.3863695	0.010448	0.1382977
RP11-486G15.2	1.5728794	1.71820439	1.5098874	0.709947534	-0.4942157	0.010488	0.1065729
STAU1	2.7038419	2.48363726	2.7992908	1.21275654	0.27829	0.01049	0.1227131
C1orf54	1.8433288	1.68757236	1.9108422	1.324721955	0.4056896	0.010535	0.1218188
SMYD4	1.3377104	1.47241078	1.2793238	0.591273128	-0.7581034	0.010542	0.1308047
TBC1D9	1.4433277	1.58365643	1.3825014	0.65535366	-0.6096544	0.01055	0.12618
SNRPF	6.082596	5.66658833	6.2629167	1.127786803	0.1734944	0.010618	0.1112222
HCG18	1.584321	1.73050247	1.5209579	0.71315005	-0.4877224	0.010648	0.1071155
MYO10	1.5040009	1.36415451	1.5646181	1.550490468	0.6327247	0.010661	0.1153245
CEBPB	1.4668535	1.32800496	1.5270382	1.606799361	0.6841898	0.010661	0.12618
RBM41	1.7582791	1.91221397	1.6915553	0.758106417	-0.3995277	0.010705	0.1223777
SNHG15	2.0332008	2.20071089	1.9605927	0.800019986	-0.3218921	0.010724	0.1112222
IFITM1	1.7234431	1.56813125	1.7907638	1.391868215	0.4770226	0.010808	0.1410602
MRPS26	3.1669032	2.91220094	3.2773053	1.190934085	0.2520936	0.010814	0.1194271
ARMCX3	2.383265	2.20297212	2.4614139	1.214836053	0.2807616	0.010817	0.1250736
P4HA2	1.5303617	1.38955987	1.5913931	1.518105709	0.6022723	0.010837	0.1090161
HADH	1.913585	1.75513881	1.9822644	1.3007733	0.3793695	0.010853	0.1114118
EPM2AIP1	2.0856211	2.26091619	2.0096385	0.800718165	-0.3206336	0.01092	0.1323544
COL8A2	1.1545477	1.03153857	1.2078667	6.590873653	2.7204697	0.010966	0.1224552
CAPZA1	2.5867683	2.37446681	2.6787915	1.221412933	0.288551	0.010985	0.1346378
ACAA2	2.7220354	2.50914565	2.8143137	1.202212447	0.2656919	0.010987	0.1285192
CSTF3	2.0106884	2.17752824	1.9383707	0.796898719	-0.3275317	0.010999	0.1062911
JPX	2.349684	2.54438171	2.2652912	0.819286544	-0.28756	0.011089	0.1104513
KCTD12	1.2168353	1.08591873	1.2735818	3.184192585	1.6709276	0.01112	0.1331755
AQP1	1.1792149	1.05578211	1.2327175	4.171902095	2.0607053	0.011141	0.1118293
RSL1D1	3.3808299	3.12225582	3.4929103	1.174651182	0.2322324	0.011145	0.1118293
ALDH2	1.5532948	1.41213912	1.6144795	1.4909516	0.5762334	0.011168	0.1040319
ARMC1	1.7554278	1.60467981	1.8207703	1.357363449	0.4408071	0.011178	0.1291065
NARS2	1.4243743	1.28862159	1.483217	1.674223312	0.743492	0.011184	0.1461212
MYBL2	1.3105891	1.44282605	1.2532702	0.571940627	-0.8060627	0.011217	0.1267896
SLC1A3	1.2838909	1.41438513	1.2273274	0.548589749	-0.8662004	0.011227	0.1118293
CDIPT	2.0106715	1.84848089	2.0809738	1.274010844	0.3493776	0.011319	0.1291065
C6orf48	5.3687177	4.96753775	5.5426113	1.144944696	0.1952779	0.011372	0.1217098
TMEM256	6.0052827	5.55049	6.202415	1.143264802	0.1931596	0.011422	0.1291065
ELAC2	1.3400003	1.20893878	1.3968097	1.899167269	0.925367	0.011653	0.1149294
MRPS33	2.5640653	2.36546059	2.6501515	1.208494437	0.2732108	0.01166	0.1363915
MRPL37	2.0775622	1.91145179	2.1495637	1.261244631	0.3348481	0.011663	0.1111201
MAP1B	7.7772961	7.05971203	8.0883369	1.169748136	0.2261979	0.011705	0.1508133

PPCS	1.95703	1.79789189	2.0260093	1.285900148	0.3627786	0.01172	0.1111201
SNRNP27	2.1857492	1.9789837	2.2753728	1.302751872	0.3815623	0.011721	0.1323542
LIPA	1.3969417	1.26333552	1.454854	1.727279426	0.7885015	0.011748	0.1297461
MITD1	1.567958	1.42699001	1.6290613	1.47324597	0.5589983	0.011868	0.2170786
GREB1	1.2389055	1.36678115	1.1834771	0.50023584	-0.9993197	0.011886	0.1328565
DHRS1	1.5276358	1.38816726	1.5880892	1.515040646	0.5993565	0.011947	0.1446651
TXLNG	1.4177358	1.28410339	1.4756596	1.674248082	0.7435133	0.012029	0.127384
GPBP1	3.9763876	4.26554951	3.8510488	0.873068632	-0.195833	0.012116	0.1486449
SNRPA1	1.9379538	1.78212193	2.0054999	1.285605065	0.3624475	0.012198	0.1551712
POLR2H	2.5453052	2.35621391	2.6272678	1.199860736	0.262867	0.0122	0.1394642
CRMP1	1.2998619	1.17156425	1.3554732	2.07195411	1.050992	0.012224	0.1217556
ANKRD36B	1.339943	1.47199788	1.282703	0.598949804	-0.739493	0.012306	0.1213667
SCAF11	3.0034895	3.24736632	2.8977798	0.844446137	-0.2439227	0.012398	0.1433563
YARS	1.3957117	1.26364506	1.4529567	1.71805502	0.7807762	0.012492	0.2244113
CERS2	1.5934982	1.45197924	1.6548404	1.448828546	0.5348869	0.012507	0.1382601
RAB10	2.2493076	2.07333355	2.3255845	1.235016406	0.3045302	0.012528	0.1368556
XXYLT1	1.7877613	1.63822382	1.8525792	1.335862334	0.4177713	0.012573	0.1621612
AKR1A1	3.3504241	3.1070487	3.4559165	1.165571778	0.2210379	0.012642	0.151734
ERV3-1	1.7586087	1.90842866	1.6936684	0.763591512	-0.389127	0.012713	0.151734
MRPS34	3.0256307	2.79790133	3.1243413	1.181567237	0.2407017	0.01279	0.162874
FAM133B	2.3582326	2.5428692	2.2782008	0.828457037	-0.2715012	0.01282	0.151734
ASF1A	1.6180706	1.47685142	1.6792829	1.424516797	0.5104726	0.01283	0.1611722
TOP1	2.2495223	2.08129808	2.32244	1.223011516	0.290438	0.012859	0.1347468
TMEM9B	1.5404246	1.40192631	1.6004574	1.493949008	0.5791309	0.012912	0.1476084
SQLE	2.1453141	1.97873926	2.2175169	1.243964545	0.3149454	0.012957	0.1498261
SPOCD1	1.2013768	1.07950462	1.254203	3.19733619	1.6768704	0.012973	0.1517502
RBBP6	1.997369	2.15728413	1.9280529	0.801923082	-0.3184642	0.013025	0.1407526
MRPS18C	2.2293171	2.05250007	2.3059594	1.240816463	0.3112897	0.01303	0.1531549
OLA1	4.0906584	3.78986285	4.2210398	1.154551323	0.2073323	0.013046	0.1598978
S100A6	1.3590397	1.18782425	1.4332539	2.306698441	1.2058294	0.013064	0.1427091
TIMM50	1.5413443	1.40351765	1.6010861	1.489615273	0.5749398	0.013091	0.143149
A2M	1.3985298	1.53153813	1.3408766	0.641302285	-0.6409235	0.013093	0.1531549
IP6K2	2.7209422	2.94248527	2.6249131	0.836512461	-0.2575411	0.013116	0.1569213
TXNL4A	2.4971226	2.28093246	2.5908314	1.241932307	0.3125865	0.013189	0.2292598
USP46	1.562878	1.42399577	1.6230773	1.469536632	0.5553613	0.013216	0.1303488
PRPF31	1.9780977	1.81973651	2.0467402	1.276922729	0.3526712	0.013218	0.2292598
PGLS	4.013166	3.69735813	4.1500547	1.167829595	0.2238298	0.013283	0.1435454
EP400	1.4755275	1.61219826	1.4162868	0.679986953	-0.556421	0.013334	0.1541805
ILVBL	1.519669	1.38280838	1.578992	1.512485133	0.596921	0.013403	0.1622924
LBH	1.4246172	1.29190342	1.4821427	1.651720024	0.7239692	0.013441	0.1312788
PCBD1	4.176733	3.86252959	4.3129262	1.157342177	0.2108155	0.013563	0.1603623
ABR	1.6387174	1.49639244	1.7004089	1.410998364	0.4967163	0.013595	0.1554139
UGP2	2.0731368	1.90568675	2.1457189	1.265027823	0.3391691	0.013681	0.2332672
ANKRD36	1.3859656	1.51801726	1.3287271	0.634587089	-0.6561099	0.01371	0.1640264
ABI2	2.9540157	3.18121137	2.8555365	0.85069081	-0.2332932	0.013729	0.153465
SRSF9	7.143102	6.62877087	7.3660417	1.130982554	0.1775767	0.013761	0.1555431
TOR1A	1.3357239	1.20845879	1.3908877	1.875131735	0.906992	0.013797	0.1720415
EIF3J-AS1	2.0151739	2.20092239	1.9346602	0.778285228	-0.3616291	0.013804	0.1541486
CUL1	1.6798032	1.5370805	1.7416671	1.380923546	0.4656334	0.013848	0.1565277
TMEM206	1.2487695	1.12561394	1.3021519	2.405401256	1.2662776	0.013852	0.1720415
SNAPIN	2.1821704	2.01716761	2.2536918	1.232532177	0.3016253	0.013934	0.1495451

FAM53C	1.6507416	1.7945145	1.5884224	0.740606275	-0.4332213	0.013973	0.1495451
P3H4	2.2346627	2.0625618	2.3092608	1.232173815	0.3012058	0.014073	0.1726462
KIF20B	1.5574834	1.69658938	1.4971872	0.713744978	-0.4865194	0.01409	0.1574987
TOP2A	3.1727998	3.65280717	2.9647381	0.740626035	-0.4331828	0.014208	0.170162
ATIC	1.6703958	1.52885551	1.7317472	1.38364298	0.4684717	0.014215	0.1591366
OBSL1	3.361607	3.60634136	3.2555255	0.865399122	-0.2085624	0.014237	0.1591366
GINS4	1.2661714	1.39167743	1.2117701	0.540674655	-0.8871674	0.014303	0.1635091
TIMM10	2.2264723	2.05644015	2.3001737	1.23071212	0.2994933	0.014363	0.1535661
TMEM261	2.9672332	2.69328644	3.0859769	1.231910238	0.3008971	0.014518	0.1599237
DHPS	1.9707031	1.81749845	2.0371105	1.268639132	0.3432817	0.014561	0.1711467
LAMTOR1	3.8250538	3.52839989	3.95364	1.168185457	0.2242693	0.014638	0.1553624
NRBP1	1.7672391	1.62055271	1.8308211	1.338840492	0.4209841	0.01464	0.1599237
MAP2	2.2171333	2.39343158	2.1407159	0.818637887	-0.2887027	0.014645	0.1711467
CREG1	1.7658935	1.62052912	1.8289024	1.335799432	0.4177034	0.01468	0.1354851
JAGN1	1.9256326	1.77385563	1.9914211	1.281144813	0.3574336	0.014692	0.155097
MAPK10	1.4432741	1.57642946	1.3855572	0.668871443	-0.5801991	0.014769	0.1553624
TCF7L2	1.5993976	1.73969352	1.5385855	0.728119834	-0.4577522	0.014801	0.155097
EMP2	1.4320079	1.30137417	1.4886317	1.621345741	0.6971918	0.014805	0.1752182
MCM8	1.2032024	1.32470904	1.1505346	0.463598537	-1.1090521	0.014864	0.1553624
ZIC3	1.2813528	1.40664338	1.2270449	0.558339215	-0.8407862	0.014902	0.1362883
RFXAP	1.3032015	1.42985105	1.2483045	0.577652523	-0.7917262	0.014916	0.1456842
VAMP3	1.9854131	1.83031881	2.0526395	1.267753462	0.3422742	0.014995	0.1553624
SYNPR	1.9627327	1.81072783	2.0286201	1.268761286	0.3434207	0.015	0.1530465
EIF2B1	1.4389118	1.30790702	1.4956966	1.609890481	0.6869625	0.015003	0.1457781
SMARCA4	2.6681311	2.4689683	2.7544593	1.194347988	0.2562232	0.01502	0.164863
ERCC1	2.6393443	2.44098067	2.725326	1.197327674	0.259818	0.015071	0.1457781
IGSF10	1.3240741	1.45153952	1.2688235	0.595348749	-0.7481931	0.015077	0.164863
TM2D1	2.0251833	1.86129842	2.0962201	1.272752963	0.3479524	0.015104	0.1461041
LINC00685	2.2374706	2.43145255	2.1533881	0.805746673	-0.3116018	0.015113	0.1764895
KITLG	1.3374269	1.46546895	1.2819263	0.605682306	-0.7233668	0.015139	0.1692251
TM7SF3	1.6727258	1.53240288	1.7335496	1.377809143	0.4623761	0.015147	0.1411038
EBPL	3.3641114	3.11078493	3.4739171	1.172036547	0.2290176	0.015211	0.1411038
UBE2N	2.7394305	2.53686247	2.8272347	1.188938321	0.2496739	0.015215	0.1530465
BOD1	2.944657	2.72651628	3.0392113	1.181113266	0.2401473	0.015271	0.1384037
LSM1	2.1425936	1.97696243	2.2143874	1.243023635	0.3138537	0.015278	0.1764895
UNC119	2.4608123	2.27287957	2.5422728	1.211640765	0.276962	0.015293	0.1559751
STON1	1.3447564	1.47287239	1.2892238	0.611631914	-0.7092644	0.015304	0.1411038
GTF2H2	1.8440633	1.99346312	1.7793051	0.784432876	-0.3502781	0.015325	0.1530465
ACTG2	1.153105	1.02659497	1.2079414	7.818825177	2.9669519	0.015346	0.1530465
PFDN6	1.9217059	1.77012316	1.9874102	1.282145882	0.3585604	0.015368	0.1559751
SSPN	1.5055443	1.37250793	1.5632097	1.511940069	0.596401	0.015393	0.1487597
MPC2	2.7469488	2.54938006	2.8325861	1.182786667	0.2421899	0.015394	0.1701749
GNG4	1.3886462	1.51829312	1.33245	0.641432424	-0.6406308	0.015403	0.1530465
SOCS3	1.8946002	1.72251592	1.9691911	1.341411447	0.4237518	0.015496	0.1607067
FGF9	1.3697268	1.49860528	1.3138637	0.629483325	-0.6677599	0.015518	0.1530465
PHB2	3.6980928	3.41523374	3.8206996	1.167878532	0.2238902	0.01552	0.1559751
APEH	1.4874471	1.35544268	1.5446652	1.532357322	0.6157528	0.015527	0.1487597
FEZ1	2.1383582	1.97629677	2.2086046	1.237948012	0.3079507	0.015606	0.1650898
MT1G	1.3968729	1.17247548	1.4941391	2.86498195	1.518526	0.015669	0.1426855
ARMCX1	1.6974687	1.55576514	1.7588909	1.365488509	0.4494172	0.015702	0.1611837
SNHG10	1.3158617	1.44181735	1.2612655	0.591342879	-0.7579332	0.015734	0.1739439

ELK3	1.4642411	1.33342198	1.5209454	1.562420555	0.6437828	0.015749	0.1703619
FAM49B	1.9708273	1.81929852	2.0365083	1.26511682	0.3392706	0.01575	0.1954177
CDC42EP1	1.298493	1.17549963	1.3518051	2.004591639	1.0033084	0.015759	0.1426855
FSTL3	1.3564671	1.23055811	1.4110431	1.782817744	0.8341592	0.015775	0.1704714
C1orf122	3.1196893	2.89273171	3.2180653	1.171885745	0.2288319	0.015784	0.1913125
IMP4	1.7779927	1.63368017	1.8405457	1.326450996	0.4075714	0.015815	0.1828756
TRIM56	1.8394886	1.98816172	1.7750454	0.784330528	-0.3504663	0.015816	0.1515351
PPP1CC	4.1941715	3.88652952	4.3275206	1.152775534	0.2051116	0.015955	0.1822105
LINC01003	1.5983188	1.737016	1.5381998	0.730241686	-0.4535541	0.015998	0.1668624
TTYH3	1.9385027	1.78859707	2.0034801	1.272487796	0.3476518	0.015999	0.1848146
TSSC1	1.4684297	1.337579	1.5251477	1.555628947	0.637498	0.016002	0.1808727
PAPPA	1.1799728	1.06089343	1.2315884	3.803175954	1.9272047	0.01604	0.1555115
TXNDC9	1.4027515	1.27494197	1.4581512	1.666355941	0.7366966	0.016077	0.1555115
MXRA8	2.1771719	2.01460202	2.2476388	1.229682905	0.2982863	0.016105	0.1668624
SAR1B	1.3640689	1.23784143	1.4187828	1.760764901	0.8162023	0.016128	0.2660851
SUMF2	2.1613545	1.98640003	2.2371895	1.254247253	0.3268218	0.016134	0.2660851
DCP2	1.6652677	1.80627856	1.6041457	0.749301508	-0.4163817	0.016158	0.1786262
RP11-242D8.1	1.4791545	1.6125033	1.4213537	0.687920661	-0.5396859	0.01617	0.1534591
UBXN2A	1.9950953	2.1547577	1.9258887	0.80180346	-0.3186795	0.01619	0.1809629
TSEN15	1.707705	1.56694099	1.7687199	1.355908075	0.4392594	0.016219	0.1634788
PPP6C	1.4432073	1.31341396	1.4994669	1.593633366	0.6723198	0.016246	0.1738641
SURF2	1.4414018	1.31130534	1.4977928	1.59904986	0.6772149	0.01625	0.1634788
SOCS6	1.3292221	1.45526831	1.2745867	0.603131664	-0.7294551	0.01637	0.155356
MCFD2	2.1174689	1.95822898	2.1864923	1.238213721	0.3082604	0.016431	0.1475857
FBN1	1.5183549	1.38613382	1.5756668	1.490847962	0.5761331	0.016478	0.1963471
ADPRHL2	1.6447277	1.50663178	1.7045861	1.390726317	0.4758385	0.016508	0.1644302
JUP	1.5030833	1.37157945	1.5600843	1.507306948	0.5919732	0.016534	0.1554529
PAQR4	1.3182058	1.44339984	1.2639398	0.595263533	-0.7483996	0.016561	0.1586664
SLC4A2	1.3206324	1.19698572	1.3742277	1.899770698	0.9258253	0.01659	0.1963471
DPY19L2	1.3066323	1.43155727	1.2524828	0.585050598	-0.7733667	0.016701	0.1768502
FAM160A2	1.3069657	1.43200671	1.2527659	0.585097242	-0.7732517	0.016716	0.1787187
MPST	2.8652348	2.64643235	2.960076	1.190498933	0.2515663	0.016759	0.1590496
TMEM70	1.4194781	1.29194937	1.4747562	1.626159275	0.7014686	0.016768	0.1653762
THOC7	2.4925888	2.30814821	2.5725356	1.202108125	0.2655667	0.016787	0.1963658
ERI3	2.3494344	2.17897916	2.4233191	1.207247065	0.271721	0.016846	0.1925788
SOX9	1.7913145	1.64244291	1.8558438	1.33217092	0.4137792	0.016887	0.2740113
RBM6	2.4384948	2.62903278	2.3559051	0.832337505	-0.2647594	0.01689	0.1907262
MMP14	1.3837819	1.258079	1.4382685	1.698195188	0.7640023	0.016927	0.1621815
RPAIN	3.321529	3.08134253	3.4256391	1.165420418	0.2208505	0.016952	0.1579082
ACBD3	1.3277871	1.20437768	1.3812796	1.865563861	0.8996117	0.01696	0.1837587
TRAPPC6A	1.8033469	1.65947375	1.8657095	1.312727736	0.3925677	0.016988	0.1837587
PDCD10	1.721087	1.58085261	1.7818724	1.346077041	0.428761	0.016999	0.1941362
SSRP1	2.7066229	2.50801144	2.7927121	1.188792138	0.2494965	0.0171	0.1977348
BMF	1.1710722	1.0555781	1.2211337	3.978791981	1.9923305	0.017145	0.1874757
AP000345.1	1.182961	1.3009849	1.1318029	0.437905418	-1.1913088	0.01719	0.1631398
DCX	1.277608	1.14539406	1.3349168	2.303511121	1.2038346	0.017197	0.1943821
WDR6	1.8814972	2.03117186	1.8166199	0.791933855	-0.3365482	0.017237	0.1884785
ACP5	1.1988333	1.08210221	1.2494311	3.03805572	1.6031483	0.017368	0.2031655
XAGE2	1.1531183	1.02342628	1.2093339	8.935857711	3.1596062	0.017394	0.1942335
LPAR6	1.4317966	1.56171585	1.3754824	0.668456094	-0.5810953	0.017442	0.1703533
HIBADH	1.8138389	1.66321334	1.8791284	1.325559022	0.4066009	0.017498	0.1851116

SHKBP1	1.6079006	1.47277539	1.6664714	1.409699761	0.4953879	0.017507	0.2754566
MLH3	1.5309542	1.66444866	1.4730903	0.712004367	-0.490042	0.017554	0.1620079
RNF126	1.7174901	1.5780621	1.7779259	1.345747912	0.4284082	0.017557	0.2007073
ZMYM2	2.4869034	2.69770159	2.3955318	0.822012397	-0.2827679	0.017584	0.2754566
TPGS2	3.0385297	2.80690461	3.1389288	1.183753053	0.2433681	0.017656	0.1660037
TMEM116	1.2241711	1.34387332	1.1722855	0.501014322	-0.9970762	0.017685	0.1574468
ZBTB38	1.9254916	2.07955247	1.8587131	0.795434378	-0.3301852	0.017732	0.2002297
SFXN5	1.3278038	1.45208283	1.2739344	0.605938437	-0.7227569	0.017839	0.1972125
EFNB1	2.0332162	2.19234743	1.9642399	0.808690409	-0.3063406	0.017884	0.2754566
FARSA	1.501197	1.37177825	1.5572943	1.498996494	0.583997	0.017973	0.1643723
R3HDM4	1.3466067	1.22334566	1.4000348	1.791101953	0.8408475	0.01801	0.15893
CEBPD	1.4313941	1.30410788	1.486567	1.599981479	0.6780552	0.018014	0.1927922
DESI2	1.790622	1.64893794	1.8520357	1.312969429	0.3928333	0.018048	0.1619471
PRIM1	1.3132088	1.43663572	1.2597087	0.594794815	-0.749536	0.01807	0.1893551
ESCO2	1.1562542	1.27198459	1.1060903	0.390059799	-1.3582328	0.018072	0.2754566
ROGDI	1.6417207	1.50604919	1.7005283	1.384308623	0.4691656	0.018141	0.1983637
MTPN	2.5043164	2.32445183	2.5822796	1.194667574	0.2566092	0.018196	0.1694926
FIGNL1	1.207832	1.3263714	1.1564505	0.479363398	-1.0608083	0.018224	0.203498
USP37	1.2587063	1.37948298	1.206355	0.543779447	-0.8789065	0.018252	0.1974409
SHARPIN	1.9771461	1.82712928	2.0421717	1.259986517	0.3334083	0.018273	0.1818278
SLF2	1.6301595	1.76708557	1.5708081	0.74412577	-0.4263816	0.018306	0.1694926
ZBTB41	1.5248483	1.65676106	1.46767	0.712085484	-0.4898777	0.018307	0.2046273
MOB3B	1.4952778	1.62597597	1.438626	0.700707366	-0.513116	0.018352	0.1985137
RP11-620J15.3	2.3531738	2.52428257	2.2790058	0.839087046	-0.2531076	0.018365	0.1694926
CASP7	1.8812319	2.03151848	1.8160893	0.791153364	-0.3379707	0.018545	0.2272912
SGCB	1.5274598	1.39752167	1.5837823	1.468554553	0.5543969	0.01866	0.1998841
MIR124-2HG	1.2962161	1.41811441	1.2433786	0.582086141	-0.7806954	0.018677	0.1998841
USP14	1.8953161	1.750569	1.9580576	1.276441685	0.3521276	0.018695	0.2064645
MRPS18B	1.8958402	1.75034903	1.9589041	1.277944098	0.3538247	0.01871	0.1742789
MAPK3	1.4633152	1.33580417	1.5185856	1.544309606	0.626962	0.018767	0.2146778
WNT5A	1.4971067	1.62760759	1.4405404	0.70193603	-0.5105885	0.018774	0.1925328
LMNB1	1.5998035	1.46639849	1.6576287	1.410014606	0.4957101	0.018779	0.2146778
UBA2	2.4010381	2.22077858	2.4791726	1.21166333	0.2769889	0.018836	0.1770932
CNOT8	1.4468237	1.31997075	1.5018089	1.568296062	0.6491979	0.018976	0.212926
LRRIQ1	1.3538795	1.47839561	1.2999073	0.62690228	-0.6736875	0.019049	0.212926
AP1M1	1.5352212	1.40477783	1.5917627	1.461944366	0.5478884	0.019066	0.1977393
SRRM1	3.7832737	4.05838545	3.664025	0.871056003	-0.1991626	0.019107	0.1747432
GMPR	1.1412142	1.02888576	1.1899036	6.574299692	2.7168372	0.019115	0.2024187
STOML2	4.2116033	3.89985783	4.346731	1.154101766	0.2067704	0.019142	0.1848293
FUNDC1	1.6019054	1.47007673	1.6590472	1.401999168	0.4874855	0.019257	0.1705225
RRAGC	1.3927687	1.26881426	1.4464974	1.66098854	0.7320421	0.019291	0.1848293
BACE2	1.2282451	1.11190537	1.2786733	2.490258155	1.3162953	0.019318	0.1689865
PAXIP1-AS1	1.765406	1.90576629	1.704566	0.777867373	-0.3624039	0.019343	0.1705225
LASP1	1.7989522	1.65822381	1.8599517	1.30647309	0.3856774	0.019361	0.2140365
FAM198B	1.5500951	1.42010154	1.6064415	1.443559404	0.5296305	0.01937	0.1755558
LOX	1.4147371	1.29041781	1.468624	1.613620083	0.6903009	0.019571	0.2094473
TP53I3	1.4419259	1.31672037	1.496197	1.566672112	0.6477033	0.019595	0.2361476
GALNT2	1.6104235	1.47747502	1.6680507	1.39913234	0.4845324	0.019605	0.1700209
LSM6	2.5669047	2.37469323	2.6502198	1.200427687	0.2635485	0.019613	0.1826938
ITGA1	1.2037745	1.08902625	1.2535128	2.847618301	1.5097558	0.019641	0.2935163
ARCN1	1.513908	1.38523188	1.5696833	1.47880623	0.564433	0.019669	0.2059074

RNF168	1.4213754	1.54770113	1.3666188	0.669377428	-0.5791082	0.019738	0.2361476
QKI	2.2539221	2.42159094	2.1812451	0.830931816	-0.267198	0.019766	0.2006518
ATP6V1B2	1.546339	1.41628135	1.6027132	1.447850621	0.5339128	0.019815	0.2098287
IRF2BPL	1.9064349	1.75966707	1.9700522	1.276943849	0.3526951	0.01984	0.2935163
BLMH	1.4708956	1.34463294	1.5256248	1.525172927	0.6089728	0.019874	0.2246432
SYT1	4.0921515	3.82292322	4.2088499	1.136711729	0.1848664	0.020033	0.2193752
BECN1	1.4217092	1.29745696	1.475567	1.598775793	0.6769676	0.020044	0.2185605
ITGB1BP1	3.641994	3.38817638	3.7520126	1.152348991	0.2045777	0.020053	0.2190543
SYCE1L	1.9490352	2.09634236	1.8851841	0.807397528	-0.3086489	0.020062	0.2193752
ARMT1	1.3648835	1.24285457	1.4177776	1.720278823	0.7826424	0.020205	0.2185605
ARPC1A	3.0953972	2.88328787	3.1873372	1.161446003	0.2159221	0.02026	0.2168229
NIN	1.4892934	1.61820456	1.4334162	0.701088625	-0.5123313	0.020388	0.1775962
PUDP	1.5322113	1.40358555	1.5879648	1.456852845	0.5428552	0.020499	0.1775962
IFNGR1	1.2618206	1.14531059	1.3123225	2.149344608	1.1038968	0.020511	0.2425183
PYCR1	2.1028598	1.95170575	2.1683784	1.227667649	0.2959201	0.020542	0.1949534
ITSN1	1.7235702	1.58696575	1.7827821	1.333607841	0.4153345	0.020546	0.2220332
TNNT1	1.509181	1.38173515	1.5644231	1.478572484	0.564205	0.020635	0.2033129
SOD3	1.3072921	1.18898961	1.358571	1.897305292	0.9239518	0.020638	0.2074079
XBP1	1.7926535	1.65435569	1.8525995	1.302960324	0.3817932	0.020748	0.2244306
POLQ	1.1420063	1.25449709	1.0932465	0.366395276	-1.4485272	0.020769	0.2176405
TTC1	1.6546497	1.52157592	1.7123313	1.365728846	0.4496711	0.020781	0.2322842
TUBB4A	1.2716349	1.15470057	1.3223207	2.083513292	1.0590183	0.020925	0.1931201
SYNGR1	1.7275312	1.59170732	1.7864048	1.329043472	0.4103883	0.020974	0.222107
NUP210	1.2109824	1.32677063	1.1607933	0.49206782	-1.0230709	0.021171	0.195395
CEP350	1.736692	1.87577281	1.6764067	0.772354085	-0.3726657	0.021197	0.1938571
KTN1	4.4839527	4.15212934	4.6277833	1.150899244	0.2027615	0.021278	0.2486612
RBM22	1.520121	1.39277958	1.5753179	1.464734734	0.5506394	0.021354	0.2360709
CCS	1.7243551	1.58913112	1.7829687	1.329022892	0.410366	0.021421	0.2244702
LRRC47	1.4735151	1.34853802	1.5276871	1.514001541	0.5983667	0.021621	0.231161
MAN1A1	1.9467865	2.097529	1.8814463	0.803118953	-0.3163144	0.021655	0.1980441
CDC73	1.4555868	1.33128598	1.5094657	1.537842657	0.6209079	0.021687	0.1936413
TTC28	1.6393878	1.77301495	1.5814664	0.752205915	-0.4108004	0.021791	0.1936413
CDKN2B	1.1374707	1.02789592	1.1849665	6.630591839	2.7291376	0.021796	0.2104906
NKAP	1.8346139	1.6955961	1.8948719	1.286482091	0.3634314	0.021943	0.1936413
NEDD4L	1.3234124	1.20475119	1.3748468	1.830742676	0.872429	0.021964	0.2104906
CNPY3	1.9626968	1.8152671	2.026601	1.259220507	0.3325309	0.021969	0.2351167
PKDCC	1.3434966	1.46398569	1.29127	0.627756446	-0.6717232	0.021974	0.232465
CCDC124	2.3561452	2.19163194	2.4274544	1.197898715	0.2605059	0.021989	0.2404408
SEPT9	2.1525902	1.99883404	2.2192366	1.220659889	0.2876613	0.021992	0.2104906
MOSPD3	1.8160713	1.67816115	1.8758492	1.291505964	0.3690543	0.022121	0.1900116
PMF1	1.9432171	1.79934207	2.0055804	1.258010172	0.3311436	0.022141	0.2344594
EPN1	2.2233589	2.05994398	2.294192	1.221000347	0.2880636	0.022189	0.2301228
CBX1	4.5569702	4.23181169	4.697912	1.144222591	0.1943677	0.022211	0.2260348
FAM127A	2.5076745	2.3312702	2.5841378	1.189944649	0.2508945	0.022244	0.2260348
CCDC8	1.6236127	1.75640429	1.5660534	0.748347696	-0.4182194	0.022261	0.2017558
TMED5	1.5205542	1.39437564	1.575247	1.458627141	0.5446111	0.022433	0.2302839
LIMA1	1.6463035	1.51523195	1.7031172	1.364661402	0.448543	0.022453	0.2034895
CLEC19A	1.2122857	1.32686169	1.1626221	0.497525864	-1.0071566	0.022464	0.1918178
CD320	2.4284228	2.2547143	2.5037177	1.198454252	0.2611748	0.022476	0.2130945
AHNAK2	1.7808638	1.64334218	1.8404734	1.306417302	0.3856158	0.0225	0.1918178
MT1E	1.3094277	1.17591477	1.3672996	2.087940478	1.0620806	0.022577	0.2355383

GAL	2.5710259	3.0760945	2.3521012	0.651271493	-0.618669	0.02258	0.1923089
FERMT2	1.4233167	1.30157461	1.4760865	1.57866895	0.6587087	0.022619	0.2440485
PDCD7	1.5804129	1.71151407	1.5235864	0.735876326	-0.4424648	0.022722	0.3312764
SYNCRIP	2.2262569	2.07415316	2.2921871	1.202982207	0.2666153	0.022793	0.2363871
CISD2	2.5106034	2.3340421	2.5871349	1.18971873	0.2506205	0.0228	0.2355383
PKIB	2.6355981	2.44385126	2.7187118	1.190366225	0.2514055	0.022806	0.2048424
RPF2	1.5536972	1.42647542	1.6088421	1.427613642	0.5136056	0.022834	0.1923803
VCL	1.7950695	1.6589239	1.8540826	1.296177838	0.3742637	0.022892	0.2277845
FAM204A	2.4409235	2.26842541	2.5156937	1.194941132	0.2569395	0.022945	0.2355383
KLF3	1.8746442	2.01569935	1.813503	0.80092898	-0.3202538	0.022948	0.1923803
GATA3	1.142384	1.03316327	1.1897263	5.720974929	2.516261	0.023025	0.2410482
MZT1	2.2271333	2.06259657	2.2984526	1.221961991	0.2891994	0.023045	0.2440485
MLEC	3.1968778	2.97899653	3.2913196	1.157818883	0.2114096	0.023046	0.2440485
CDK5RAP3	2.1716955	2.32911431	2.1034614	0.830223105	-0.268429	0.023057	0.1947293
NCAPG	1.2552192	1.37139372	1.2048627	0.551605171	-0.8582921	0.023139	0.2157149
THYN1	2.3737451	2.20055976	2.4488133	1.206781484	0.2711645	0.02314	0.235136
ATP5G2	16.907465	16.0115821	17.29579	1.085547839	0.1184233	0.023158	0.2157149
ENDOV	1.7204103	1.85581148	1.6617199	0.773207551	-0.3710724	0.023305	0.2344439
SIGMAR1	2.2154533	2.05464403	2.2851569	1.218569362	0.2851884	0.023414	0.2585799
CTBP2	1.6973301	1.56524343	1.7545838	1.334971337	0.4168088	0.023422	0.2506671
TRUB2	1.3949128	1.27504157	1.4468716	1.624742208	0.7002108	0.023621	0.1963872
HMOX2	1.5464768	1.41999754	1.6012999	1.431674716	0.5177037	0.023663	0.207003
HIF3A	1.6198622	1.49047652	1.6759451	1.37813964	0.4627221	0.023737	0.3411388
LSAMP	2.539899	2.73550376	2.4551131	0.838438465	-0.2542232	0.023776	0.245179
CNP	1.7022664	1.57021129	1.7595064	1.331973699	0.4135656	0.023807	0.2225837
C1orf21	2.0473368	1.90092036	2.1108018	1.232963376	0.3021299	0.023821	0.249625
ZDHHC12	2.174661	2.02524436	2.2394264	1.208908304	0.2737048	0.023908	0.245179
ASUN	1.3603339	1.2421851	1.4115461	1.699303843	0.7649438	0.023919	0.2225837
TEX264	1.7425228	1.60979529	1.8000542	1.312004608	0.3917728	0.02418	0.2025093
HNRNPA1	49.036519	46.5270352	50.124268	1.079013112	0.1097124	0.024357	0.2510984
VWA1	1.4028046	1.28313599	1.4546756	1.605855724	0.6833423	0.024382	0.2581899
AMFR	1.3430039	1.22580344	1.393805	1.744017086	0.8024142	0.024428	0.2821865
PDZD11	1.8923234	1.75327095	1.9525965	1.264613313	0.3386963	0.024474	0.2258831
TMBIM1	1.2907171	1.17602706	1.3404301	1.933964746	0.9515615	0.024485	0.2510984
ETHE1	2.0710222	1.92630559	2.1337504	1.223948545	0.2915429	0.024631	0.2481224
DIP2A	1.4444903	1.56744957	1.3911929	0.689388027	-0.5366119	0.024689	0.2481224
EZH2	1.7187096	1.85250825	1.6607139	0.775023464	-0.3676881	0.024692	0.2036109
STK26	1.7385815	1.60592173	1.7960835	1.313838906	0.3937884	0.024759	0.2594542
TSTA3	1.760091	1.62699217	1.8177835	1.304296142	0.3832715	0.025067	0.2271833
CADPS	1.1912321	1.08112807	1.2389573	2.945433227	1.5584798	0.025068	0.2496917
FIRRE	1.3132345	1.43017757	1.262545	0.610317635	-0.7123678	0.025155	0.3564174
CEP162	1.3776407	1.49781038	1.3255525	0.653968792	-0.6127063	0.025338	0.2767855
ZNF883	1.6410651	1.77111421	1.5846946	0.758246421	-0.3992613	0.025393	0.2899948
ANKRD26	1.8298161	1.96628736	1.7706619	0.797549356	-0.3263543	0.025406	0.2110144
PAFAH1B3	4.6051917	4.24362748	4.7619137	1.159785985	0.2138586	0.025467	0.2585259
CDKN3	1.713916	1.57157445	1.7756147	1.356979314	0.4403987	0.025511	0.2673367
NETO2	1.5813037	1.7089982	1.5259539	0.741826821	-0.4308457	0.025522	0.2567473
MICU2	1.5842766	1.45807203	1.6389806	1.394934803	0.4801977	0.025571	0.2296819
REEP6	1.6268613	1.49898989	1.6822878	1.367337891	0.4513698	0.025582	0.2358663
ETF1	1.4977367	1.37513007	1.5508812	1.468507047	0.5543502	0.025649	0.2303802
TNIK	1.3372272	1.22179811	1.3872606	1.746004894	0.8040576	0.025804	0.2676153

AP3D1	2.1221717	1.97715805	2.1850286	1.212729735	0.2782581	0.025832	0.2632997
TM7SF2	2.7145709	2.50258893	2.8064557	1.202228791	0.2657115	0.025885	0.3616756
REV3L	1.8782316	2.01804636	1.8176281	0.803134485	-0.3162865	0.025904	0.2580092
LYPLA2	1.8828046	1.74575754	1.9422085	1.263424635	0.3373396	0.025911	0.2632997
MRPL14	3.9551599	3.6786524	4.0750136	1.147970382	0.1990854	0.025977	0.2694082
RRBP1	1.8127237	1.67829725	1.8709916	1.284085404	0.3607412	0.026101	0.2706939
CRIPT	1.7704458	1.63660836	1.8284583	1.301362644	0.380023	0.026106	0.2924559
SOCS2	1.4963063	1.37404273	1.5493021	1.468554482	0.5543968	0.026261	0.2637152
CNN1	1.3197848	1.20511763	1.3694879	1.801346568	0.8490758	0.026326	0.2924559
SWI5	1.859563	1.72365704	1.9184721	1.26920911	0.3439298	0.026326	0.2702493
EIF2B4	1.4774561	1.35617607	1.5300256	1.48809995	0.5734714	0.026359	0.2678543
CLPTM1	1.46254	1.34184017	1.514858	1.506136771	0.5908528	0.026408	0.2710841
TCF25	3.3407922	3.08405304	3.4520771	1.176590565	0.2346124	0.02644	0.3643586
SKP2	1.3900427	1.50937485	1.3383175	0.664181754	-0.59035	0.026442	0.2631129
SNRNP25	2.4120375	2.58603053	2.3366193	0.842744988	-0.246832	0.026502	0.2842115
GDF15	1.1781938	1.06428663	1.2275675	3.539888064	1.8237037	0.026503	0.2637152
IFT52	1.8388217	1.70339098	1.8975249	1.275997111	0.3516251	0.026508	0.2614425
PPP2R2A	2.024789	1.8799073	2.0875888	1.236026574	0.3057098	0.026583	0.2842115
COL5A2	1.4060244	1.28802113	1.4571735	1.58729161	0.6665672	0.026622	0.2924559
GPC2	1.4776473	1.35663357	1.5301014	1.486403522	0.5718258	0.026696	0.271271
NUTF2	2.9761991	2.75141587	3.0736326	1.183974981	0.2436386	0.026764	0.2639711
COPS3	1.6583787	1.53051195	1.7138033	1.345499018	0.4281413	0.026772	0.2924559
SDF4	2.2377284	2.08287065	2.3048523	1.20499372	0.2690256	0.026879	0.2392958
HAX1	2.1956816	2.03796658	2.2640441	1.217808075	0.2842868	0.026897	0.2394558
PSMD9	1.4071204	1.28935844	1.458165	1.583382051	0.6630094	0.026905	0.2706687
PPP1R14A	1.6060996	1.47698353	1.6620656	1.388026162	0.4730348	0.026965	0.2312903
SPCS3	1.8625263	1.72713182	1.9212138	1.266914405	0.3413191	0.0271	0.2312903
SNHG18	2.4440948	2.61680684	2.3692319	0.846874127	-0.2397805	0.027129	0.2312903
LSM10	2.2998744	2.10849274	2.3828299	1.247486644	0.3190244	0.027199	0.267993
CASP8AP2	1.4819706	1.60408814	1.4290381	0.710224358	-0.4936533	0.027331	0.2499589
ZNF793	1.4388143	1.55925655	1.386608	0.691289167	-0.5326388	0.027368	0.278105
SBDS	2.1357526	1.98907494	2.1993309	1.212578381	0.278078	0.027389	0.2698579
CSNK2A1	2.0725963	1.92342297	2.1372563	1.23156591	0.3004938	0.027494	0.2685361
YIPF4	1.9272095	1.78955073	1.9868784	1.249923957	0.3218403	0.027556	0.2691424
LRRC4C	1.3562232	1.47322524	1.305508	0.645586944	-0.6313167	0.027657	0.2440596
PACSIN3	1.485613	1.36596099	1.5374768	1.46867228	0.5545125	0.027682	0.2928446
SELENOF	2.9796022	2.73168315	3.0870641	1.205222842	0.2692999	0.027738	0.2706518
S100A3	1.2383102	1.12860269	1.2858635	2.222841945	1.1524054	0.027783	0.2852033
GRPEL1	1.557878	1.4347706	1.6112396	1.405889879	0.4914836	0.027943	0.2343412
KIF21A	1.8206294	1.68737497	1.8783892	1.277889462	0.353763	0.027953	0.2343412
MRPL19	1.8415358	1.70797648	1.8994278	1.270420384	0.345306	0.027954	0.2791149
MRPL44	1.5324581	1.41036128	1.5853817	1.426503229	0.512483	0.028022	0.2791149
RBM28	1.3967211	1.51520523	1.3453634	0.670341494	-0.5770319	0.028154	0.2698947
LINC00632	2.298795	2.46423831	2.2270826	0.838034788	-0.254918	0.02817	0.2698947
CCNC	1.9324005	1.79391317	1.9924286	1.250046831	0.3219821	0.028304	0.3847865
INAFM1	1.9507671	2.09309262	1.8890753	0.813357703	-0.2980381	0.028346	0.2501398
TRIM33	1.6606808	1.78909189	1.6050204	0.766729939	-0.3832096	0.028476	0.2836349
HCCS	1.3564702	1.24203499	1.4060728	1.677744159	0.7465227	0.028648	0.238177
STX10	1.783587	1.65258654	1.8403698	1.287752327	0.3648551	0.028691	0.2554286
MAP4	1.9516867	1.81239671	2.0120627	1.24577402	0.3170424	0.028743	0.2891529
CHMP6	1.3775778	1.26253176	1.4274451	1.628165288	0.7032472	0.028894	0.3059722

UBA1	2.5904505	2.41926823	2.6646504	1.172893467	0.230072	0.028897	0.284719
BCAM	1.5502219	1.42842936	1.6030135	1.407498148	0.493133	0.029048	0.2796023
RRAS2	1.5002988	1.38022878	1.5523438	1.452661661	0.5386987	0.029111	0.2958127
ISOC1	1.4062956	1.29010567	1.4566588	1.574111795	0.654538	0.029235	0.2883365
CRK	1.6472799	1.5217195	1.7017047	1.34498469	0.4275898	0.029237	0.2671212
MAL	1.4118571	1.28678421	1.4660706	1.625161233	0.7005829	0.029361	0.2864861
ANKLE2	1.6631654	1.79132172	1.6076154	0.767848701	-0.381106	0.029447	0.2668843
TMEM18	1.7497925	1.62009517	1.8060105	1.299817446	0.378309	0.029455	0.2795423
SEPHS2	1.6125108	1.48836057	1.6663244	1.364410823	0.4482781	0.029464	0.2796023
MRPL49	1.5060424	1.3862581	1.5579636	1.444535606	0.5306058	0.029499	0.2909382
CRELD2	1.8589233	1.71536072	1.9211513	1.287673857	0.3647672	0.029507	0.2851375
CLEC2D	1.3091044	1.42259497	1.2599113	0.615036372	-0.7012564	0.029589	0.2796023
PES1	1.3994436	1.28366355	1.4496291	1.585079048	0.6645548	0.029596	0.3969804
ENO3	1.8705312	1.73253453	1.9303466	1.270037859	0.3448715	0.029712	0.259914
WRB	2.1619291	2.01117263	2.2272753	1.213714933	0.2794296	0.029715	0.3110748
GTF2F2	1.7282679	1.59952154	1.7840737	1.307832391	0.3871777	0.029739	0.2796023
ZSWIM7	2.0551669	1.91427456	2.1162374	1.220899557	0.2879445	0.029761	0.2603416
TMEM170A	1.7760909	1.90730201	1.7192167	0.792698266	-0.3351563	0.029844	0.2460911
WDR45B	2.7802638	2.58722225	2.8639387	1.174340098	0.2318503	0.029926	0.2980748
TGFB1	1.5878095	1.46558356	1.640789	1.376313747	0.4608094	0.029929	0.2864585
CDCA5	1.3157856	1.42904499	1.2666927	0.621596053	-0.6859508	0.030032	0.3975283
SYNC	1.6135463	1.73932979	1.5590248	0.756123732	-0.4033058	0.030035	0.2719341
PLAG1	1.5817148	1.70554993	1.5280377	0.748405926	-0.4181071	0.030145	0.3257586
FLNB	1.4834631	1.3652973	1.5346827	1.463692031	0.549612	0.03036	0.3151277
GABRP	1.1353252	1.01970255	1.1854425	9.412102403	3.234517	0.030395	0.3057697
GOLGA6L9	1.2293134	1.33865666	1.181918	0.537175344	-0.896535	0.030422	0.2506083
RAE1	1.3915081	1.27673819	1.4412557	1.594487811	0.6730931	0.030424	0.2488309
MRPL50	1.821295	1.69004163	1.8781875	1.272658791	0.3478457	0.030445	0.2686674
RGS12	1.5295691	1.65099101	1.4769381	0.732633856	-0.4488357	0.030652	0.2993778
GATM	1.2686495	1.15929783	1.3160485	1.984010425	0.9884196	0.03069	0.3281208
DKK1	1.3063589	1.1831907	1.3597469	1.963783454	0.9736359	0.030701	0.2662478
QTRT1	2.006263	2.14623318	1.9455922	0.824956232	-0.2776105	0.030704	0.2490997
FAM60A	3.0286189	2.79923694	3.1280457	1.182748996	0.2421439	0.030717	0.4013093
EPB41L3	1.6252989	1.50195169	1.6787644	1.352250502	0.4353624	0.030748	0.3151277
RHOT1	1.6810321	1.55485054	1.7357262	1.325989975	0.4070699	0.030763	0.2916668
ZNHIT3	2.5457645	2.36911266	2.6223352	1.184953772	0.2448308	0.031011	0.2666424
DKC1	1.6754828	1.55068657	1.7295764	1.324848771	0.4058277	0.031075	0.3005948
C15orf40	1.8521058	1.98504542	1.7944824	0.806543908	-0.310175	0.031242	0.3151277
PTP4A1	2.0599055	1.91410708	2.1231026	1.228633533	0.2970547	0.031278	0.4034049
ATG101	1.4083171	1.29369984	1.4579985	1.559410236	0.6410005	0.031292	0.3151277
KSR2	1.1051637	1.20833033	1.0604455	0.29014268	-1.7851656	0.031325	0.3151277
ZNRD1	1.5630353	1.44193553	1.6155267	1.392797489	0.4779855	0.031444	0.3326413
PQBP1	3.0370936	2.83657543	3.1240093	1.156505333	0.2097719	0.031548	0.2759774
DYNC1LI2	1.9973453	1.85844294	2.0575532	1.231943552	0.3009362	0.031671	0.3123651
PSENEN	2.5659299	2.37220176	2.6499024	1.202375922	0.265888	0.031731	0.4040656
IFI27L1	1.9477118	2.08364778	1.8887896	0.82018311	-0.2859821	0.031797	0.3180363
RP11-12G12.7	1.4792965	1.59826025	1.427731	0.71495816	-0.4840693	0.031919	0.2568449
IGBP1	2.1343195	1.99349331	2.1953614	1.203190172	0.2668647	0.031991	0.2774393
TMEM38B	1.5180959	1.39953914	1.5694849	1.425354559	0.5113208	0.032007	0.2981357
CETN3	1.8221297	1.69245282	1.8783389	1.268445819	0.3430619	0.032098	0.2625211
RP11-660L16.2	1.4282026	1.54409882	1.3779667	0.694665562	-0.5256095	0.032228	0.3180363

PAK1IP1	1.3269701	1.21614739	1.3750068	1.734958918	0.7949015	0.032243	0.2568449
TSPYL1	1.7101414	1.58415934	1.764749	1.309144534	0.3886244	0.032246	0.3180363
FAM3C	2.6764824	2.50074858	2.7526552	1.167853947	0.2238599	0.032302	0.290138
XPC	1.6489522	1.77397709	1.5947594	0.768445779	-0.3799846	0.032407	0.4074973
PTPN18	1.3995351	1.5146597	1.3496338	0.679349402	-0.5577743	0.032425	0.2568449
HCRT	1.1283729	1.23194731	1.083478	0.359900682	-1.4743293	0.032487	0.3054354
SUZ12	1.7170745	1.84437632	1.6618948	0.783886051	-0.3512841	0.032502	0.2916478
FLJ37453	1.5390968	1.65965138	1.4868417	0.738028776	-0.438251	0.032508	0.3235797
GADD45A	2.1826987	1.93437884	2.2903343	1.380954076	0.4656653	0.0328	0.3398353
HIST1H1B	1.1385249	1.24204864	1.093652	0.386914063	-1.3699149	0.032808	0.3235797
ZC3H11A	1.8289814	1.96063748	1.7719143	0.80354382	-0.3155514	0.03281	0.4074973
SYTL2	1.4052076	1.51993719	1.3554774	0.683693046	-0.5485793	0.032828	0.3057825
RTKN2	1.2816735	1.39170406	1.2339801	0.597338933	-0.7433783	0.032859	0.2801347
IWS1	1.5161527	1.39789446	1.5674124	1.426037519	0.5120119	0.032983	0.283596
ALKBH5	2.7687305	2.59312348	2.8448483	1.158007105	0.2116441	0.033059	0.3199695
TBC1D7	1.556876	1.43768467	1.6085402	1.390362124	0.4754607	0.033078	0.3199695
CHRAC1	1.6649616	1.78956581	1.6109513	0.773781327	-0.3700022	0.033104	0.3297293
PARP1	3.6140423	3.3705851	3.7195701	1.147214704	0.1981354	0.033205	0.3235791
NET1	1.3991835	1.28572935	1.4483608	1.56918014	0.650011	0.03322	0.3066013
ZGRF1	1.1581803	1.26252167	1.112953	0.430261755	-1.2167135	0.033254	0.3244676
ZNF704	1.2456588	1.35360086	1.1988708	0.562415976	-0.8302905	0.03334	0.3490327
LIG1	1.3274365	1.43870547	1.2792064	0.636432381	-0.6519209	0.033452	0.3235791
SPON2	1.1458738	1.04337477	1.1903026	4.387402173	2.133367	0.03359	0.2839664
GRB2	1.9096956	1.7764348	1.9674582	1.24602635	0.3173346	0.033595	0.3313384
MRPS10	1.7897409	1.66247593	1.8449046	1.275374046	0.3509204	0.033699	0.3256455
R3HCC1	1.5866228	1.4665773	1.6386572	1.368813206	0.4529256	0.033773	0.2868332
MIR325HG	1.3098554	1.42029589	1.2619843	0.623333087	-0.6819248	0.033861	0.2661261
POLR2J2	1.1899822	1.29555228	1.1442223	0.487975764	-1.0351186	0.03393	0.2868332
SEPT4	1.2223343	1.11039844	1.2708535	2.453417454	1.2947927	0.03404	0.3324654
RHOB	1.5277018	1.41057413	1.5784714	1.408932969	0.494603	0.034193	0.4194957
CSRP1	1.3616277	1.25070684	1.409707	1.63420731	0.708591	0.034203	0.2667328
CDC42EP3	1.8059404	1.67545623	1.8624995	1.276913921	0.3526613	0.034212	0.2853523
TRAP1	1.8883759	1.75783751	1.9449584	1.246914273	0.3183623	0.034302	0.2761052
C16orf87	1.9095632	1.77808473	1.9665532	1.242221059	0.3129219	0.034307	0.2761052
GINS1	1.1900735	1.29493659	1.14462	0.490342541	-1.0281382	0.034319	0.3273634
S100A14	1.141332	1.03197923	1.1887316	5.901690851	2.5611283	0.034322	0.2853523
YIPF5	1.623413	1.50227789	1.6759197	1.345708558	0.428366	0.034354	0.3355375
REEP3	1.7350739	1.60993071	1.7893178	1.294110678	0.371961	0.034359	0.3523529
HSDL1	1.5016577	1.38530566	1.5520912	1.432865485	0.5189032	0.034393	0.3295131
ANKRD10	2.0275255	2.16363393	1.9685286	0.832330991	-0.2647707	0.034493	0.3336549
RBM10	1.3901685	1.27805812	1.4387634	1.577955632	0.6580566	0.034494	0.3273634
PIN1	3.2740807	3.01332035	3.3871087	1.185657679	0.2456875	0.034529	0.324318
TCEAL7	4.0814974	3.80754377	4.200244	1.139873244	0.1888734	0.034582	0.3078711
ADAMTS6	1.6260699	1.7485512	1.5729798	0.765451679	-0.3856168	0.034628	0.3003118
LARP1	1.9869583	1.85123611	2.0457878	1.228551991	0.2969589	0.034687	0.2834143
EFHD1	1.2398355	1.1336822	1.2858482	2.138266938	1.096442	0.034814	0.3332218
ZMPSTE24	1.2426744	1.13704166	1.2884615	2.104918047	1.0737641	0.034917	0.3373857
C8orf33	2.6733112	2.50201309	2.7475613	1.163479407	0.2184457	0.035262	0.3343213
MTHFD2L	1.7140604	1.83937777	1.6597409	0.785988114	-0.3474206	0.035281	0.3373857
NMT1	1.9537243	1.81924927	2.0120132	1.235293412	0.3048538	0.035371	0.3373857
LSM14A	3.1064374	2.90363254	3.1943442	1.152714191	0.2050348	0.035392	0.3390857

RRM1	1.6169599	1.73860121	1.5642338	0.763922083	-0.3885026	0.035413	0.4241356
RNASEH1-AS1	1.4577761	1.57317824	1.4077545	0.711392107	-0.4912831	0.035415	0.4241356
RBFOX2	1.9222362	1.79094685	1.9791442	1.237939345	0.3079406	0.035491	0.3433061
ARL6IP4	5.987798	5.65784619	6.1308174	1.101542897	0.1395257	0.03555	0.3373857
ADI1	2.0231068	1.8873906	2.0819337	1.219230536	0.2859709	0.035665	0.317194
MYO3A	1.2481109	1.35489693	1.2018239	0.568683279	-0.8143027	0.035853	0.3446531
RP11-108M9.4	2.0781626	2.21716231	2.0179124	0.836299668	-0.2579081	0.03586	0.340329
LSM4	7.6202292	8.11527995	7.4056468	0.90026631	-0.1515763	0.035866	0.3164994
EFEMP1	1.1985784	1.09555232	1.2432356	2.545575526	1.3479919	0.035929	0.3378029
TMEM91	1.4906546	1.37518558	1.5407052	1.441167337	0.5272379	0.036009	0.3446531
SLC39A7	1.5498584	1.43258158	1.6006927	1.388622835	0.4736548	0.036207	0.3097413
ZBED6	1.4959244	1.61292507	1.4452098	0.726368994	-0.4612255	0.036267	0.3374834
FAM103A1	2.6998706	2.51765692	2.7788521	1.172104224	0.2291009	0.036331	0.3097413
NEMF	1.8872032	2.02083389	1.8292803	0.812355752	-0.2998164	0.036505	0.3432107
ARL6IP6	2.0174963	2.15267989	1.9589003	0.831887767	-0.2655392	0.036558	0.3197998
CCNL1	2.2715221	2.12837307	2.3335709	1.181852814	0.2410504	0.036567	0.3788664
G2E3	1.5049594	1.62219623	1.4541425	0.729902376	-0.4542246	0.036577	0.430755
EBP	1.6609831	1.54018423	1.7133441	1.320556996	0.4011466	0.036626	0.3389433
AC114803.3	1.3366154	1.44635077	1.28905	0.647584926	-0.6268587	0.036727	0.3452995
APOLD1	1.2323119	1.33784462	1.1865682	0.552230732	-0.8566569	0.036824	0.430755
EVA1B	1.6547262	1.53439302	1.7068853	1.322781748	0.403575	0.036832	0.3399336
TPT1	53.403826	51.5108969	54.224326	1.053719682	0.0754911	0.036889	0.3497512
ARF6	1.9774074	1.84470645	2.0349274	1.225191728	0.2930075	0.036915	0.3254373
GTF2B	1.2677689	1.16204479	1.3135956	1.9352401	0.9525126	0.036952	0.2858801
RAB40B	1.5870148	1.70662135	1.5351708	0.757365678	-0.4009381	0.037005	0.351197
QDPR	1.6170223	1.49792628	1.6686452	1.34285978	0.4253087	0.037032	0.3053647
TSR3	1.5207231	1.40512854	1.5708282	1.409005263	0.494677	0.037227	0.2858801
RPN1	1.4879056	1.37345169	1.5375163	1.439319611	0.525387	0.037264	0.3407967
CA14	1.3617821	1.25260979	1.4091034	1.619507279	0.695555	0.037283	0.3389433
FAM215B	1.5515471	1.6691673	1.500564	0.748040088	-0.4188125	0.037389	0.3389433
MED21	1.5158473	1.40049289	1.5658482	1.412879629	0.4986386	0.037398	0.3389433
DALRD3	1.7265674	1.60373619	1.7798092	1.291639038	0.369203	0.037597	0.315192
TIPRL	1.756147	1.63269762	1.8096568	1.279689895	0.3557942	0.037643	0.360331
LGI1	1.1533792	1.05278107	1.1969839	3.732094847	1.8999857	0.037659	0.3472188
ID4	1.487775	1.60688894	1.4361444	0.718656069	-0.4766266	0.037694	0.3055072
KARS	1.9918971	1.85918138	2.0494234	1.22142239	0.2885622	0.037958	0.3636752
SPA17	1.2725049	1.1672712	1.318119	1.901815512	0.9273773	0.037967	0.360331
ECI1	1.4437717	1.33192206	1.4922536	1.483039654	0.5685572	0.037971	0.3410606
ADAM10	1.7451021	1.62129515	1.7987669	1.285648056	0.3624958	0.038061	0.3477401
PEX7	1.2498184	1.14529075	1.2951264	2.03128158	1.0223902	0.038077	0.328993
TOMM20	4.54362	4.27813441	4.6586962	1.11609096	0.1584546	0.038084	0.3547457
DHRSX	1.5608654	1.44456059	1.6112784	1.375017057	0.4594495	0.038242	0.3591925
DNAJC19	2.8171149	2.99962296	2.7380058	0.869166731	-0.2022951	0.038263	0.328993
TMEM47	2.111795	1.97532479	2.1709488	1.200573202	0.2637234	0.038398	0.3610112
ELF2	1.7624217	1.88717319	1.7083475	0.798432058	-0.3247584	0.038412	0.3172963
TMEM69	1.4648971	1.35157706	1.5140163	1.462030154	0.5479731	0.038442	0.3249818
TIA1	2.20196	2.34205974	2.141233	0.850359323	-0.2338555	0.038479	0.3172963
KLC1	1.7194982	1.59784625	1.7722288	1.291684677	0.3692539	0.038513	0.3522161
CCDC47	1.5769623	1.46043398	1.6274721	1.362784053	0.446557	0.038593	0.3917752
GPRC5C	1.2478892	1.14427604	1.2928008	2.029448811	1.021088	0.03878	0.309236
SLC35A1	1.2425294	1.13881554	1.2874847	2.070983442	1.050316	0.038906	0.3502624

DNAJC2	1.5640126	1.44761382	1.6144663	1.372759898	0.4570793	0.039015	0.3595887
POLE3	1.8658801	2.00442009	1.8058292	0.802283031	-0.3178168	0.039034	0.3502624
FOSL2	1.2686334	1.16406514	1.3139591	1.913624609	0.9363078	0.039046	0.309236
PLOD1	2.1511289	2.01232259	2.2112953	1.196550689	0.2588815	0.039078	0.309236
NEK9	1.2857229	1.39203691	1.2396405	0.611270126	-0.710118	0.039086	0.3479649
SH3KBP1	1.4351336	1.32386872	1.4833619	1.492462591	0.5776948	0.039093	0.3197343
MCL1	1.9463539	1.81705418	2.0023996	1.226845987	0.2949542	0.039124	0.3595887
SPNS2	1.1195619	1.02099736	1.1622852	7.728838582	2.9502516	0.039184	0.3551236
CCDC150	1.2045227	1.30741361	1.159924	0.520224274	-0.9427944	0.039319	0.3595887
MINCR	1.8427592	1.97014825	1.7875418	0.811774661	-0.3008488	0.039324	0.3697182
XRRA1	1.7601414	1.88402296	1.7064442	0.79912429	-0.3235082	0.039397	0.3661321
PRELID2	1.4517139	1.56464812	1.402762	0.713297323	-0.4874245	0.039421	0.330479
CENPH	2.0202046	2.17558445	1.9528544	0.810536728	-0.3030505	0.039429	0.3506706
DONSON	1.3215195	1.42953342	1.2747003	0.639531874	-0.6449118	0.039526	0.3678109
TMCO3	1.8944564	1.76736716	1.9495439	1.23740492	0.3073177	0.039537	0.3207569
TMEM5	1.6057129	1.48834406	1.6565871	1.344517361	0.4270884	0.03959	0.3555991
ABCF1	1.8759603	1.7497395	1.9306713	1.241326259	0.3118823	0.03967	0.3661321
SHISA2	1.9807775	2.13860225	1.9123675	0.801304809	-0.319577	0.039861	0.3129695
ZKSCAN1	2.9538645	3.1433221	2.8717431	0.873290643	-0.1954662	0.039913	0.303255
MRPL24	1.5935755	1.47721598	1.6440122	1.34951934	0.4324457	0.040092	0.303255
ARFGAP3	1.5663702	1.45114676	1.6163144	1.366106257	0.4500697	0.040198	0.3744379
C15orf61	1.5054631	1.39217775	1.5545672	1.414071154	0.4998547	0.040215	0.33435
PMS2	1.3735769	1.48321319	1.3260544	0.674763029	-0.5675472	0.040233	0.4038165
SRRM2	3.8348792	4.06786411	3.7338907	0.891138129	-0.166279	0.040257	0.3715499
MRPS27	1.6014885	1.48434808	1.6522636	1.346683561	0.4294109	0.04026	0.465538
EID2B	1.3924957	1.50254714	1.3447933	0.686091376	-0.5435274	0.040437	0.4146822
DDX52	1.6356805	1.51808787	1.6866517	1.325357526	0.4063816	0.040437	0.3228556
YIPF6	1.5642619	1.44913774	1.6141631	1.367427044	0.4514639	0.040539	0.3674091
LINC01508	1.6154446	1.73375494	1.5641623	0.768870232	-0.379188	0.040583	0.4038165
UBE2Q2	1.57107	1.45536262	1.6212239	1.364240109	0.4480976	0.040652	0.3717831
COPS2	2.09132	1.93070318	2.1609402	1.247379697	0.3189007	0.04066	0.3584493
PRDM2	1.5251681	1.64045332	1.4751971	0.741969993	-0.4305673	0.040711	0.3757409
CDK16	3.3136349	3.11018184	3.4018227	1.13820651	0.1867623	0.040752	0.3726924
TNFRSF21	1.2556959	1.15259301	1.3003863	1.968545938	0.9771304	0.040835	0.3589909
SLC27A5	1.6645894	1.78400751	1.6128269	0.781659494	-0.3553878	0.040973	0.3069841
SCYL1	1.3620598	1.25512308	1.4084122	1.600843612	0.6788324	0.041038	0.3589909
EMC6	2.8467961	2.66160585	2.9270679	1.15976232	0.2138292	0.041059	0.3785715
DANT2	1.2679646	1.37239533	1.2226985	0.598016471	-0.7417429	0.041096	0.3201641
TFAP2A	1.1623247	1.06340944	1.2052	3.236111801	1.6942615	0.041107	0.4676899
GSK3B	1.635359	1.51775637	1.6863346	1.325593662	0.4066386	0.041125	0.4052044
CERK	1.7986656	1.92261202	1.7449403	0.807425328	-0.3085993	0.041161	0.3866093
NCOR1	2.6550379	2.48815477	2.7273743	1.160749112	0.2150562	0.041196	0.3069841
COL9A2	2.4359033	2.26682163	2.5091927	1.191322194	0.2525636	0.041293	0.3693189
COMMD9	1.469553	1.35849085	1.5176935	1.44409112	0.5301618	0.041376	0.4676899
LEMD1	1.4119144	1.52226516	1.3640822	0.697121367	-0.5205182	0.041379	0.3731308
SMOX	1.2723993	1.16895031	1.3172398	1.877710607	0.9089747	0.041484	0.3693189
CSNK1D	1.9679123	1.83787825	2.0242763	1.222464363	0.2897924	0.04151	0.3209066
IFI16	1.9973277	2.13727936	1.9366649	0.823601394	-0.2799818	0.041541	0.3731308
NUMB	1.6133937	1.73151006	1.5621956	0.768541116	-0.3798057	0.041651	0.3527182
TSN	1.7360727	1.61561876	1.7882841	1.280474473	0.3566785	0.041723	0.3527182
DMTF1	1.5152194	1.62936031	1.4657444	0.740028238	-0.4343478	0.041956	0.3837056

CDH2	3.3152889	3.10394696	3.4068963	1.143990942	0.1940756	0.042113	0.3847642
DACH1	2.496889	2.65903761	2.4266047	0.859898973	-0.2177609	0.042197	0.3687656
EIF4G1	1.8925878	1.76679643	1.9471128	1.235155446	0.3046926	0.042227	0.4286655
PIN4	2.831021	2.65466349	2.9074641	1.152780704	0.2051181	0.042398	0.3842571
NEIL1	1.352247	1.46014371	1.3054786	0.663876427	-0.5910134	0.042473	0.3849342
B4GALT3	1.4915859	1.37978805	1.5400453	1.421964828	0.5078858	0.042591	0.4155752
SSSCA1	1.5160674	1.4038285	1.564718	1.398410454	0.4837879	0.042687	0.3424059
RNASEH2C	2.907709	3.08402307	2.8312847	0.878725734	-0.1865151	0.042783	0.37103
TRIM73	1.2359231	1.33881418	1.1913244	0.564688213	-0.8244736	0.042886	0.3424059
C19orf53	5.835395	5.5088834	5.9769232	1.103803935	0.1424839	0.043123	0.3555877
RNF26	1.7796541	1.6588953	1.8319976	1.26271594	0.3365301	0.043322	0.3898096
ITGB3BP	2.1417884	2.27700417	2.0831784	0.848218375	-0.2374924	0.043366	0.3930268
NUB1	1.4260643	1.31722494	1.4732414	1.491816396	0.57707	0.043388	0.3828761
AGPAT2	2.0240837	1.87279861	2.0896591	1.248465639	0.3201561	0.043409	0.3793596
ABHD11	1.5809603	1.46629499	1.6306626	1.352497082	0.4356255	0.043441	0.3898096
TRAM1	2.2049941	2.06496509	2.2656904	1.188480673	0.2491184	0.043495	0.3906822
SMARCAD1	1.3939771	1.50243035	1.3469674	0.690578184	-0.5341233	0.043828	0.440476
CEP70	1.4454034	1.33624882	1.492717	1.465334497	0.55123	0.043903	0.3908535
FGFR1OP	1.2777919	1.38129512	1.2329279	0.61088616	-0.7110245	0.043937	0.3946472
NCL	5.5647565	5.20646204	5.7200611	1.122097637	0.1661982	0.043966	0.4091256
LINC00094	1.7825971	1.90512509	1.7294866	0.805951191	-0.3112356	0.043986	0.3484257
NDP	1.3046762	1.40956007	1.2592136	0.632907527	-0.6599334	0.04412	0.396295
RP5-1074L1.4	1.165429	1.26420477	1.122614	0.46408713	-1.1075324	0.044218	0.3936584
GPC1	1.8381856	1.71610275	1.8911031	1.24437878	0.3154257	0.044361	0.4958559
DYNC1LI1	1.4806419	1.37036799	1.5284408	1.426799247	0.5127824	0.044383	0.3916642
DPH3	1.6656019	1.54897599	1.7161541	1.304527177	0.383527	0.04442	0.3285771
CTTN	2.4937898	2.33308661	2.5634475	1.172802635	0.2299602	0.044523	0.3526784
TOP3A	1.1804743	1.28025302	1.1372246	0.489645422	-1.0301907	0.044582	0.3737484
SAMD11	2.1921769	2.37220492	2.1141427	0.811936108	-0.3005619	0.044656	0.4315313
TNFAIP1	1.5112579	1.40028175	1.5593612	1.397418579	0.4827642	0.044701	0.3638757
PPP1R1A	1.2414902	1.14006782	1.2854523	2.037957606	1.027124	0.044707	0.3656547
COPB1	1.6838099	1.56711422	1.7343922	1.294963496	0.3729114	0.044851	0.3638757
NDUFS1	1.4711885	1.36192041	1.5185514	1.432777311	0.5188144	0.044966	0.3950527
TCF19	1.1684835	1.26724738	1.1256737	0.470252434	-1.0884927	0.045022	0.3938469
RAB27A	1.2681312	1.16636	1.3122445	1.876920573	0.9083676	0.045149	0.4991245
TSPAN2	1.2239936	1.12425846	1.2672243	2.150552391	1.1047073	0.04516	0.3950527
RPA3	2.4131917	2.26680199	2.4766451	1.165647917	0.2211321	0.04529	0.4298267
CFAP20	1.7370793	1.61887318	1.7883164	1.273793144	0.349131	0.045322	0.36769
C11orf74	1.8977891	1.76118663	1.9570002	1.257247745	0.330269	0.045344	0.3883622
DBF4B	1.1584702	1.2562907	1.1160694	0.452881926	-1.1427931	0.045407	0.451826
SCN3A	1.2103113	1.31086365	1.1667263	0.53633256	-0.8988003	0.045546	0.3949932
FGFR2	1.1417488	1.04556885	1.1834385	4.025524397	2.0091767	0.045599	0.4059548
RP11-499F3.2	1.6014391	1.71669049	1.5514828	0.76948528	-0.3780344	0.045635	0.3883622
ERLEC1	2.0974562	1.96766301	2.1537157	1.19227015	0.2537112	0.045676	0.3589863
TLN2	1.2849278	1.38833027	1.2401075	0.618307466	-0.6936037	0.045753	0.3883622
AMOTL2	1.2913032	1.18916871	1.335574	1.773940371	0.8269575	0.045866	0.3604751
BDP1	2.0300471	2.16012049	1.9736661	0.839280149	-0.2527756	0.045922	0.398254
PHYH	1.4131683	1.30642037	1.4594388	1.49937405	0.5843603	0.045956	0.3951405
THAP9-AS1	1.8146639	1.9366767	1.7617767	0.813276063	-0.2981829	0.045992	0.3701469
TMEM123	3.3706016	3.11720738	3.4804367	1.171560589	0.2284316	0.046087	0.3883622
ADAM9	1.3172545	1.21424247	1.3619056	1.689233891	0.7563691	0.046166	0.3838245

SHMT1	1.2879568	1.1858927	1.332197	1.787036501	0.8375691	0.046167	0.4038595
MAB21L1	5.3453349	5.70753182	5.1883386	0.889710105	-0.1685928	0.04623	0.3360756
SNX9	1.4506093	1.34269556	1.4973851	1.45139047	0.5374357	0.046344	0.4435797
CCDC30	1.3681079	1.47465261	1.3219255	0.67823395	-0.5601451	0.046372	0.3883622
NSDHL	1.1804055	1.08299395	1.222629	2.682472962	1.4235636	0.046454	0.3708943
CLDND2	1.3483038	1.45360218	1.3026616	0.667240152	-0.583722	0.046484	0.4027313
PLEKHA3	1.7358534	1.61740074	1.7871973	1.275018398	0.3505181	0.046512	0.3360756
MAP9	1.8757494	1.75279892	1.9290429	1.234118283	0.3034807	0.046579	0.3360756
MYADM	1.5569075	1.4452894	1.605289	1.359315936	0.4428808	0.046672	0.4012989
TM2D2	1.5307956	1.42007605	1.5787876	1.3778163	0.4623836	0.04677	0.3360756
ZNF516	1.8335899	1.95745516	1.7798998	0.814554881	-0.2959162	0.046822	0.4060583
ATP2A1-AS1	1.419315	1.52701344	1.3726325	0.707064452	-0.5000864	0.046869	0.5109027
DPF2	1.9339294	2.05958697	1.8794625	0.830004997	-0.2688081	0.046891	0.3631934
C2orf40	1.2555397	1.15523986	1.2990152	1.926149704	0.9457198	0.046934	0.3631934
MIR210HG	1.9721322	2.1018996	1.9158838	0.831186286	-0.2667562	0.047005	0.4148027
INPP1	1.2869101	1.18533959	1.3309364	1.785568032	0.8363831	0.04715	0.3887908
LUM	1.1175715	1.02024793	1.1597569	7.890039036	2.9800324	0.047231	0.5109027
PRPF19	1.4641745	1.35638314	1.5108973	1.43356186	0.5196042	0.047249	0.4206417
ETV4	1.3755926	1.27096641	1.4209435	1.553489409	0.6355124	0.047276	0.4358919
DCDC1	1.6049275	1.71959203	1.5552255	0.771583779	-0.3741053	0.047478	0.4082281
PRKAA1	1.3337503	1.43815234	1.2884967	0.658439184	-0.6028779	0.047487	0.370324
SYT11	1.6788234	1.79570738	1.6281594	0.789435218	-0.3411072	0.047519	0.4211417
SNHG12	1.2647487	1.36609158	1.2208211	0.60318553	-0.7293263	0.047606	0.4093293
VGLL4	2.2806254	2.13756437	2.342636	1.180272545	0.23912	0.047719	0.4068243
RASSF8-AS1	1.5136311	1.62473016	1.4654746	0.745081012	-0.4245308	0.047737	0.378137
TADA3	2.679914	2.5158312	2.7510366	1.155165993	0.2081002	0.047773	0.3408473
WDR11	1.6637412	1.779886	1.6133976	0.786522188	-0.3464406	0.047804	0.4367528
HIC2	1.2495728	1.35043108	1.2058552	0.587434201	-0.7675008	0.047813	0.4211417
ATXN7L3B	2.3836059	2.23818219	2.4466405	1.16835839	0.2244829	0.047871	0.3704504
CDK18	1.3904011	1.49661362	1.3443627	0.6934217	-0.5281951	0.047974	0.4089964
ZNF644	2.0866827	2.21666152	2.0303427	0.846860591	-0.2398036	0.047981	0.4703621
CYP27A1	1.2638059	1.16351227	1.3072787	1.879239236	0.9101487	0.04802	0.5139117
RAP2B	1.4696755	1.36193695	1.5163753	1.426699568	0.5126816	0.048097	0.4131441
C11orf1	1.4693443	1.36133526	1.5161614	1.428483382	0.5144843	0.048142	0.4211417
ICE2	1.7177461	1.83592877	1.6665191	0.797339635	-0.3267337	0.048165	0.4250354
TMEM41A	1.4214794	1.31523016	1.4675337	1.483150266	0.5686648	0.048435	0.3719489
C9orf69	1.2697836	1.37096845	1.2259245	0.609012732	-0.7154557	0.048604	0.4569681
C14orf1	2.4382453	2.28663747	2.5039606	1.168907814	0.2251612	0.048618	0.4140803
CDC6	1.1773124	1.27471107	1.1350944	0.491769194	-1.0239467	0.048685	0.3724056
LINC01224	1.2600811	1.36089378	1.2163833	0.599576266	-0.7379848	0.048693	0.5156382
PIGM	1.3010215	1.40360419	1.2565565	0.635663708	-0.6536644	0.048745	0.4154924
DLGAP4	1.7595912	1.64186655	1.8106196	1.262909906	0.3367517	0.04879	0.4703621
WDR76	1.1912807	1.2890508	1.1489018	0.515140485	-0.9569622	0.048864	0.3724056
D2HGDH	1.3313525	1.43534891	1.2862746	0.65757516	-0.6047723	0.049108	0.4446267
PUM2	1.6503966	1.76534955	1.6005696	0.784699731	-0.3497874	0.049132	0.4703621
ZNF721	1.6367459	1.75089358	1.5872679	0.782092065	-0.3545896	0.049142	0.4703621
STAG2	2.174861	2.31472846	2.1142347	0.847501802	-0.2387117	0.049149	0.4154924
TES	1.755974	1.63905641	1.8066525	1.262255552	0.336004	0.049177	0.4153211
ZIC5	1.237804	1.33736128	1.1946504	0.576979066	-0.7934091	0.049236	0.4307048
SFT2D2	1.3737251	1.4788091	1.3281759	0.68540026	-0.5449814	0.049243	0.516022
GNL1	1.6158619	1.50317133	1.6647083	1.321037701	0.4016716	0.049286	0.4097642

YIPF2	1.5805471	1.46960942	1.6286337	1.338630947	0.4207583	0.049394	0.3736127
PCOLCE	1.6926834	1.5775267	1.7425988	1.28582584	0.3626952	0.049471	0.4097642
RPF1	1.4114461	1.30680381	1.4568039	1.488912186	0.5742587	0.049525	0.4097642
DCUN1D5	1.9367953	1.81244174	1.990697	1.219406833	0.2861795	0.049584	0.4227276
THOC2	1.9167203	2.04007357	1.8632522	0.829991483	-0.2688316	0.049693	0.4097642
RPAP3	1.3922447	1.28792258	1.4374637	1.519379672	0.6034824	0.049763	0.4718073
CNTLN	1.4739546	1.58266492	1.4268335	0.732553965	-0.4489931	0.050009	0.4414575
MIDN	2.0920256	1.95878407	2.1497798	1.199206239	0.2620798	0.050036	0.4339303
SMAD3	1.3935031	1.28912296	1.4387472	1.517510761	0.6017067	0.050076	0.4414575
LSR	1.5150632	1.4065242	1.56211	1.382722043	0.4675112	0.050151	0.382212
HCFC1R1	3.6323412	3.36638532	3.7476213	1.161104761	0.2154981	0.050234	0.417229
TMED4	2.915398	2.74485454	2.989321	1.140107078	0.1891693	0.050251	0.411
CASZ1	1.2736712	1.37438767	1.2300151	0.614376929	-0.7028041	0.050446	0.4738197
EIF4G3	1.7803795	1.89965696	1.7286781	0.809950999	-0.3040935	0.050513	0.3555082
GS1-600G8.5	1.1314041	1.02708385	1.1766222	6.521312853	2.7051624	0.050534	0.3555082
VPS4A	1.7282749	1.61262579	1.7784036	1.270602013	0.3455122	0.050545	0.406234
FADD	1.4626043	1.35653621	1.50858	1.426447056	0.5124262	0.050844	0.4447746
RASAL2	1.6112867	1.72358055	1.5626124	0.777539349	-0.3630124	0.05088	0.406234
HRASLS	1.3559608	1.45939296	1.3111275	0.677257968	-0.5622226	0.050893	0.4302407
RBM15	1.1786906	1.27528746	1.1368201	0.497008118	-1.0086587	0.050904	0.3850337
EEF1D	11.433906	10.8689895	11.678772	1.082053263	0.1137715	0.050971	0.4454389
FLNA	3.1928008	2.98525319	3.2827634	1.149860104	0.2014583	0.051156	0.4020516
COMMD3	1.6227755	1.51118356	1.6711457	1.312925007	0.3927845	0.051167	0.474202
CASP6	1.6209106	1.50979558	1.669074	1.312435928	0.392247	0.051188	0.3842905
GMPR2	1.9006436	1.77941774	1.9531896	1.2229508	0.2903664	0.051363	0.4608907
USO1	1.9364466	1.81374493	1.9896322	1.216145517	0.2823159	0.051363	0.4305952
SAP30BP	1.520784	1.41269	1.5676379	1.375458297	0.4599124	0.051378	0.4455707
ARHGAP29	1.5913487	1.47103521	1.6434993	1.36613842	0.4501037	0.05158	0.474202
ZFP14	1.5288155	1.6379657	1.4815037	0.754748611	-0.4059319	0.05172	0.4485387
CCNA2	1.6322892	1.51118044	1.6847845	1.339614053	0.4218174	0.051861	0.3978667
TONSL	1.1447792	1.23968175	1.1036432	0.432420079	-1.2094946	0.051903	0.474202
PARL	1.7602722	1.64387209	1.8107264	1.259142	0.332441	0.051913	0.4463665
STX7	1.7042375	1.59072208	1.7534414	1.275458273	0.3510157	0.052232	0.4374457
TMEM144	1.3302633	1.22908364	1.3741202	1.633116364	0.7076276	0.052284	0.5422471
PHYKPL	1.8686426	1.98911469	1.8164234	0.825408197	-0.2768203	0.05229	0.4320627
MSANTD3	1.3734439	1.27104701	1.4178284	1.541534723	0.6243674	0.052382	0.4742675
SRP68	1.4597977	1.35472377	1.5053425	1.424608596	0.5105656	0.052384	0.4653466
MSRB2	2.2654829	2.12894432	2.3246663	1.17336725	0.2306546	0.052397	0.4320627
ZMAT3	2.2777635	2.14046513	2.3372762	1.172570902	0.2296752	0.052786	0.4653466
ACYP2	1.6756886	1.56239172	1.7247978	1.288777416	0.3660031	0.05307	0.4451892
IRF3	1.6050995	1.49464296	1.6529775	1.320098719	0.4006458	0.053085	0.4310832
LARS	2.4330818	2.2866324	2.4965611	1.163161383	0.2180513	0.053101	0.3709684
ABHD4	1.1993938	1.10381993	1.2408208	2.31960111	1.2138767	0.053104	0.4451892
AC011043.1	1.58318	1.69400132	1.5351438	0.771099172	-0.3750117	0.053136	0.4310832
TTLL5	1.2249109	1.32247231	1.1826224	0.566319684	-0.8203114	0.053169	0.4952557
LNPEP	1.5072361	1.61544702	1.4603315	0.747962852	-0.4189615	0.053246	0.3967783
GPR137	1.5472723	1.43858137	1.594385	1.355244556	0.4385532	0.053337	0.4351774
SLC7A2	1.1548496	1.06110853	1.1954822	3.198935213	1.6775918	0.053443	0.456039
ADGRG6	1.1215083	1.02956761	1.1613605	5.457339727	2.4481979	0.053492	0.456039
PCMTD1	2.190113	2.33072656	2.1291633	0.848531414	-0.23696	0.053518	0.4239269
EIF3E	10.313442	9.80007907	10.535963	1.083622367	0.1158621	0.053565	0.4177231

CLDND1	1.7825372	1.66697759	1.8326272	1.248358556	0.3200324	0.0536	0.3718726
NUBP1	1.3096782	1.21019308	1.3528006	1.678459778	0.747138	0.05364	0.4351774
ELOVL4	1.2623161	1.16469147	1.304632	1.849713364	0.8873017	0.053689	0.4422741
KDM5C	1.5555163	1.66552435	1.5078328	0.763056616	-0.390138	0.053714	0.5513905
FABP7	4.3746926	4.82881113	4.1778524	0.829984123	-0.2688444	0.053961	0.4486356
EHD1	1.3174765	1.21800988	1.3605908	1.654011382	0.7259692	0.053976	0.4635926
FASTK	1.7238954	1.61032944	1.7731211	1.26672759	0.3411063	0.053995	0.4642681
IGSF9	1.3088186	1.40933623	1.2652486	0.647996942	-0.6259411	0.054016	0.4245349
SMYD2	1.5206649	1.41402391	1.566889	1.369218136	0.4533523	0.054037	0.4568198
CCDC171	1.409019	1.51243892	1.3641911	0.710701453	-0.4926844	0.054167	0.4339794
LPP	2.2135644	2.07752113	2.272533	1.180981993	0.239987	0.054171	0.4734043
GDI1	1.8279704	1.71087612	1.8787255	1.236116283	0.3058145	0.054317	0.5013068
MTF2	2.6237652	2.78025426	2.5559342	0.873995472	-0.1943023	0.054399	0.4083996
CFDP1	4.1043101	3.85146753	4.2139061	1.12710597	0.1726232	0.054419	0.4519927
CDCA4	1.6489186	1.76102087	1.6003273	0.788844711	-0.3421868	0.054521	0.4635926
ADSL	2.1337066	2.00810417	2.1881496	1.17859808	0.2370718	0.054567	0.441062
TXNRD1	1.5693732	1.46106908	1.6163182	1.336715607	0.4186926	0.054677	0.4339794
CEP290	2.1616049	2.29210981	2.1050368	0.855218976	-0.2256342	0.054741	0.4635926
SMARCE1	3.3469114	3.14521774	3.4343366	1.13477367	0.1824046	0.054787	0.4339794
C3orf14	1.6123872	1.72295991	1.5644589	0.780761028	-0.3570471	0.054804	0.441062
THAP7	1.7418179	1.85628513	1.6922015	0.808377344	-0.3068992	0.054893	0.4635926
DHCR7	1.3679651	1.26723288	1.411628	1.54033451	0.6232437	0.055026	0.4691214
WASF2	2.7999577	2.59614433	2.8883017	1.183039425	0.2424982	0.055065	0.4499238
KMT2A	1.9517171	2.07427334	1.8985945	0.836467276	-0.257619	0.055095	0.4194726
RAP1B	1.7123319	1.59919543	1.7613715	1.270656374	0.3455739	0.055106	0.4944801
DNA2	1.1621782	1.2564528	1.1213144	0.473047609	-1.0799427	0.055215	0.3804524
TRIM16	1.5468045	1.65526883	1.4997901	0.76272522	-0.3907647	0.055256	0.4632324
ENC1	1.3997999	1.29781526	1.4440056	1.490876047	0.5761603	0.055309	0.5620254
VAX2	1.372983	1.4746035	1.328935	0.693073313	-0.5289201	0.055312	0.441621
PPIL4	1.5404359	1.43375499	1.5866773	1.352554477	0.4356867	0.055515	0.4467843
ZNF215	1.2398281	1.33665348	1.1978586	0.58772174	-0.7667948	0.055541	0.5079502
RIC8A	1.6860826	1.57460741	1.7344021	1.278093634	0.3539935	0.055558	0.4330593
UBE2E3	4.8961726	4.61865409	5.0164646	1.109933266	0.1504729	0.055576	0.4649016
SLC25A1	1.9405046	1.81702553	1.9940273	1.216641653	0.2829043	0.055673	0.4667247
FIBCD1	3.3830106	3.16152044	3.4790167	1.146885605	0.1977215	0.055959	0.5305541
DTNBP1	1.391027	1.28958456	1.4349978	1.502144397	0.5870235	0.055973	0.4649016
FBN2	1.8805393	1.75941111	1.933043	1.22864016	0.2970624	0.056173	0.5091037
TATDN1	1.9184259	1.79894383	1.970216	1.214373218	0.2802119	0.05637	0.4323237
JRK	1.2216889	1.31775553	1.1800483	0.566625117	-0.8195335	0.056388	0.4330593
GLUL	2.9224797	2.74366522	2.9999878	1.147002184	0.1978681	0.056392	0.4330593
FHL3	1.3640919	1.26387377	1.407532	1.544420302	0.6270654	0.056447	0.4205862
CCDC6	1.6187114	1.50963371	1.6659917	1.306804568	0.3860434	0.05651	0.4270094
ELF3	1.1381027	1.04610288	1.1779805	3.860506628	1.9487902	0.056566	0.4702951
RRM2B	1.4109182	1.30860434	1.4552667	1.475244091	0.5609537	0.05662	0.4323237
TSPAN31	1.7373352	1.62420119	1.7863738	1.259808178	0.3332041	0.056673	0.419217
ISCA2	2.1959201	2.06528459	2.2525448	1.175784248	0.2336234	0.056726	0.4323237
UNC5D	1.1641572	1.07169972	1.2042333	2.848454001	1.5101791	0.056841	0.4682422
CDCA7L	2.29119	2.43342101	2.2295391	0.857765535	-0.2213447	0.056849	0.4683076
STAT3	1.8302927	1.71391156	1.8807387	1.233680388	0.3029687	0.056855	0.5106799
HDAC3	2.1351057	2.00971861	2.1894554	1.178006774	0.2363478	0.056859	0.4205862
NME6	1.416639	1.31462892	1.4608558	1.464759841	0.5506641	0.057078	0.4866172

SMDT1	4.2118347	3.95480237	4.3232468	1.124693414	0.1695318	0.057187	0.5086058
RND3	2.0460086	1.84804492	2.131817	1.334619128	0.4164281	0.057453	0.3931834
CHURC1	2.9227102	2.74483201	2.9998125	1.14613468	0.1967766	0.057492	0.469752
TRIP12	1.7348739	1.8488411	1.6854742	0.807541277	-0.3083921	0.057571	0.5738045
TBPL1	1.6708008	1.56012368	1.7187744	1.283242221	0.3597935	0.057586	0.466728
CLINT1	1.5447749	1.43833354	1.5909125	1.348088615	0.4309153	0.057609	0.5738045
TWISTNB	1.3397586	1.2407362	1.3826804	1.589625384	0.6686868	0.057621	0.4231147
GMCL1	1.6372314	1.74784502	1.5892854	0.787977916	-0.3437729	0.057659	0.4995482
C6orf118	1.1353054	1.22812591	1.0950719	0.416751891	-1.2627393	0.057672	0.5416826
LPCAT4	1.5308774	1.63772792	1.4845624	0.759826232	-0.3962586	0.057763	0.4802442
PTRHD1	2.40146	2.25488949	2.4649918	1.167426921	0.2233322	0.057787	0.4404077
SEPT1	1.5288773	1.63506726	1.4828487	0.760311115	-0.3953382	0.05824	0.4682511
EFNA5	3.4046679	3.17246837	3.505316	1.153211736	0.2056574	0.05825	0.4759436
CHST11	1.1942944	1.10081814	1.2348122	2.329066725	1.219752	0.058379	0.4286786
AGO3	1.7159113	1.82876889	1.6669926	0.804799315	-0.313299	0.058467	0.5766463
HIST1H1E	1.2645329	1.36161445	1.2224524	0.615164517	-0.7009558	0.058546	0.467441
FAM134B	1.2212503	1.12692731	1.2621352	2.065238363	1.0463083	0.058569	0.4829522
RDH10	2.1091252	1.97247837	2.1683556	1.20142063	0.2647413	0.0586	0.4953908
RANBP17	1.2794126	1.37669903	1.2372432	0.629795179	-0.6670454	0.058617	0.517407
PROS1	1.631948	1.52376159	1.6788421	1.296089811	0.3741657	0.058747	0.3993187
ZNF462	1.8234246	1.70816237	1.8733857	1.233312724	0.3025387	0.059012	0.517407
HAS2	1.3155633	1.41435891	1.2727398	0.658221133	-0.6033557	0.059131	0.5467477
CCDC12	1.9681841	1.8478742	2.020333	1.203401449	0.267118	0.059147	0.517407
MMP2	1.1571427	1.06545431	1.1968855	3.007983317	1.5887966	0.059269	0.5225059
LAP3	1.4396159	1.33773406	1.4837771	1.432420351	0.5184549	0.059289	0.4322073
TNNI1	1.3874798	1.28716987	1.4309597	1.500713386	0.5856485	0.05932	0.4973007
SRP72	2.2086392	2.07538755	2.2663979	1.177619972	0.235874	0.059382	0.5467477
UCP2	2.2101169	2.07427785	2.268997	1.181255882	0.2403215	0.059726	0.4517637
TMEM126B	1.9941569	1.87342581	2.0464884	1.198142297	0.2607993	0.059732	0.4841201
CCDC148	1.2688498	1.3659586	1.2267574	0.619625861	-0.6905307	0.059843	0.5467477
NSD1	2.3199468	2.4607296	2.2589238	0.86184587	-0.2144982	0.059977	0.4945614
TGIF2	1.6582074	1.76813639	1.610558	0.794856252	-0.3312341	0.060056	0.5186022
NDFIP2	1.3693934	1.27054129	1.4122413	1.523764968	0.6076404	0.060195	0.5004665
FSIP2	1.366897	1.46686565	1.323565	0.69305814	-0.5289517	0.060249	0.4845912
DIAPH3	1.2404175	1.33589615	1.1990318	0.59253959	-0.7550165	0.060269	0.4774025
RNF44	1.5053183	1.61089822	1.4595541	0.75225972	-0.4106973	0.060273	0.4845912
DTYMK	2.4113348	2.55484761	2.3491283	0.86769169	-0.2047456	0.060315	0.5186022
TAF6	1.7715335	1.65767931	1.8208842	1.248152739	0.3197945	0.060473	0.4823453
MLH1	1.347597	1.44724337	1.3044047	0.680624294	-0.5550694	0.060562	0.4580891
UBE2F	1.5070835	1.40348438	1.5519891	1.36805571	0.452127	0.060791	0.4779012
SIAH1	1.4658737	1.36373238	1.5101475	1.402535152	0.4880369	0.060807	0.4779012
ATP2B1	1.9846886	1.86380439	2.0370865	1.200603396	0.2637597	0.060921	0.489806
RP11-1094M14.11	1.5792556	1.68661753	1.532719	0.775859872	-0.366132	0.061029	0.4876395
TMEM165	2.226089	2.09949322	2.2809627	1.165048288	0.2203898	0.06106	0.4876395
BRD7	1.741518	1.85396999	1.6927751	0.811240551	-0.3017983	0.061243	0.4884889
QSOX1	1.3829641	1.2838953	1.425906	1.500222156	0.5851762	0.061339	0.5990997
NDUFV3	2.5416883	2.39460167	2.6054438	1.151184464	0.203119	0.061427	0.4876395
RCHY1	1.3993895	1.29976609	1.4425719	1.476390724	0.5620746	0.061456	0.5370739
PITHD1	1.5834363	1.47755892	1.6293294	1.317804722	0.3981366	0.061506	0.5274033
DLD	1.5083721	1.40515631	1.5531115	1.365180502	0.4490917	0.061553	0.4876395
AKTIP	1.3304709	1.2331507	1.3726548	1.598343182	0.6765772	0.061561	0.4876395

SYNE2	2.1080826	2.24248923	2.0498233	0.844935521	-0.2430868	0.061723	0.5028777
AP1S1	2.965445	2.79838305	3.0378589	1.133161735	0.1803538	0.061924	0.5274033
FAM217B	1.5414026	1.64692895	1.4956616	0.76617623	-0.3842518	0.061946	0.4650595
COMMD10	1.4398323	1.3390425	1.4835202	1.426134435	0.51211	0.061985	0.5028777
KCNG1	1.1178702	1.02887959	1.1564437	5.417102157	2.4375213	0.062106	0.5121231
UROS	2.5969844	2.44616731	2.6623569	1.149491379	0.2009956	0.062163	0.4841717
CCP110	1.5047351	1.60849764	1.4597586	0.755563542	-0.404375	0.062248	0.4925916
RPH3AL	1.4453678	1.5469726	1.4013267	0.73372358	-0.4466914	0.062285	0.5135976
TBC1D20	1.361349	1.2635391	1.4037453	1.532012815	0.6154284	0.062662	0.4958668
RP11-244M2.1	1.1591738	1.25019795	1.1197189	0.478496674	-1.0634192	0.062699	0.5001015
SIL1	1.3749937	1.27644529	1.4177101	1.511004583	0.595508	0.062743	0.4926309
VPS51	1.6540127	1.54570192	1.7009606	1.284511905	0.3612203	0.062798	0.5136181
ZFHX3	1.6171299	1.5107481	1.6632417	1.298569	0.3769227	0.063018	0.5372551
PDCL3	1.4851981	1.38346163	1.5292964	1.380311232	0.4649936	0.063366	0.4586079
ZNF385D	1.4806401	1.3789892	1.5247012	1.384475375	0.4693394	0.063519	0.4841717
DDX3X	2.1210366	1.99926655	2.1738184	1.174680014	0.2322678	0.063531	0.550421
SCMH1	1.3584846	1.45673193	1.3158988	0.691650257	-0.5318854	0.063766	0.4984287
AGPAT1	1.7390564	1.6286805	1.7868994	1.251668268	0.3238523	0.063914	0.4984287
KNOP1	2.2081314	2.33780467	2.1519238	0.861055308	-0.2158222	0.063989	0.5013468
SUMO2	16.926003	16.1143964	17.277798	1.076973082	0.1069822	0.063996	0.4985769
RAMP1	1.4826314	1.3814476	1.5264901	1.380242293	0.4649215	0.064055	0.5068946
INPP5F	1.673742	1.78280553	1.6264678	0.800285362	-0.3214136	0.064255	0.5431958
BPTF	2.6497453	2.80813295	2.5810913	0.874433112	-0.1935801	0.064352	0.5013468
GPM6A	2.1783814	2.04834325	2.2347471	1.177808049	0.2361044	0.064419	0.54435
CCK	1.395146	1.29438737	1.4388204	1.490622498	0.5759149	0.064423	0.5211908
C19orf81	2.0577946	2.17968131	2.0049622	0.851892971	-0.2312559	0.064445	0.4841717
KIF1C	1.3941013	1.29548537	1.4368469	1.478404344	0.5640409	0.064452	0.5271468
HDLBP	2.7643974	2.60531629	2.833352	1.142050327	0.1916262	0.064457	0.4841717
BIRC5	2.3634412	2.17220364	2.4463341	1.233859105	0.3031777	0.064476	0.4841717
SCG3	1.215523	1.12091437	1.2565317	2.121597989	1.0851513	0.064513	0.4702905
BRD3	2.1738947	2.04804798	2.2284436	1.172125336	0.2291268	0.064551	0.4841717
RBM33	1.4401	1.54089987	1.3964077	0.732867054	-0.4483766	0.064569	0.4639718
GLCCI1	1.3583331	1.4559951	1.3160009	0.692991978	-0.5290894	0.064719	0.4841717
MRPS7	2.3546283	2.21547278	2.414946	1.164111662	0.2192294	0.06476	0.5211908
FLRT2	1.4430922	1.54410797	1.3993064	0.733873454	-0.4463968	0.064932	0.54435
GCHFR	4.8407847	5.11893768	4.7202177	0.903198351	-0.1468852	0.064973	0.4841717
ZNF83	1.5562585	1.66071534	1.5109811	0.773375543	-0.370759	0.065235	0.5008161
GSK3A	1.3717491	1.27389962	1.4141625	1.512096036	0.5965498	0.065268	0.512967
SPC24	1.2514181	1.3449549	1.2108741	0.611309159	-0.7100259	0.065276	0.4407204
PCSK4	1.2098403	1.30222969	1.1697937	0.561803369	-0.8318628	0.065281	0.5008161
ANXA4	1.410218	1.3116646	1.4529365	1.453281912	0.5393146	0.065475	0.4844846
LRCH2	1.3012235	1.20652625	1.3422705	1.657273715	0.7288119	0.065497	0.4844846
OSTM1	1.2401071	1.14769462	1.2801638	1.896912937	0.9236535	0.065539	0.5317119
TP53RK	1.4434869	1.34392197	1.4866439	1.414983345	0.5007851	0.065625	0.4679261
BAX	3.7133762	3.49922161	3.8062026	1.122830645	0.1671403	0.06565	0.4751367
MVB12A	2.1769066	2.04652874	2.2334195	1.178581631	0.2370517	0.066053	0.4845581
FAM171B	1.5271894	1.63067711	1.4823322	0.764784623	-0.3868746	0.066133	0.5112628
RFC1	2.6779134	2.83350988	2.6104692	0.878353162	-0.187127	0.066156	0.4679261
CCDC43	1.4546963	1.35497686	1.4979202	1.402683455	0.4881895	0.06647	0.4845581
ITGB8	1.4297214	1.52990653	1.3862956	0.728988244	-0.4560325	0.066514	0.4679261
CHST12	1.4197391	1.32112782	1.4624827	1.440182518	0.5262517	0.066822	0.476996

SNRPE	8.6036663	8.17300605	8.7903383	1.086063259	0.1191081	0.066855	0.476996
CTDSP2	1.9693451	2.08729449	1.9182193	0.844499219	-0.243832	0.067046	0.5224468
KLHL5	1.3670689	1.27079615	1.4087989	1.509618513	0.594184	0.06706	0.5224468
NMRK2	1.1611599	1.07202088	1.1997977	2.774163701	1.4720529	0.067241	0.4697528
IDH2	3.3685134	3.15344348	3.4617366	1.143162846	0.1930309	0.067368	0.6468887
RCC1	1.4722329	1.57283925	1.4286245	0.748245648	-0.4184161	0.067479	0.5404676
PHF3	2.4889902	2.65163236	2.418492	0.858842491	-0.2195345	0.067518	0.6468887
CDC5L	1.7380909	1.62929145	1.7852505	1.247832852	0.3194247	0.067693	0.5404676
ZNF428	5.2206652	4.92177581	5.3502205	1.109247616	0.1495815	0.068043	0.5265511
UBE2I	4.8008409	4.5137709	4.9252729	1.117111232	0.1597728	0.068168	0.5442606
PLEKHA5	1.471609	1.37219847	1.5146991	1.382861895	0.4676571	0.068197	0.5318275
MAP3K13	1.8848697	1.99878315	1.8354932	0.836511145	-0.2575433	0.068309	0.5280797
KPNB1	4.0249237	4.25335871	3.9259073	0.89934974	-0.1530458	0.068383	0.6191473
CLDN7	1.1122845	1.02315018	1.1509202	6.51917835	2.7046901	0.068477	0.6498837
PPP1R3C	1.185109	1.09569747	1.2238649	2.339298535	1.226076	0.068736	0.5155186
SEC22B	1.6497313	1.54422717	1.6954627	1.277890359	0.3537641	0.068761	0.5431264
WDR90	1.1988157	1.28943859	1.1595347	0.551186757	-0.8593869	0.068794	0.5323596
LINC00973	1.2612064	1.16887464	1.3012281	1.783737987	0.8349037	0.068852	0.4983123
NTHL1	1.5084669	1.40768331	1.5521521	1.354365227	0.4376168	0.068904	0.5903589
PDE7A	1.4584124	1.55835776	1.4150905	0.743413205	-0.4277638	0.06905	0.4867897
DHX40	1.7757692	1.66558103	1.8235309	1.237311299	0.3072085	0.069091	0.530575
MEX3A	2.1555632	2.27762914	2.1026531	0.863046259	-0.2124902	0.069106	0.5431264
CXCL12	1.1560825	1.067935	1.1942906	2.859947873	1.5159889	0.069131	0.5391147
ELMOD3	1.3202842	1.41545603	1.2790314	0.671626793	-0.5742683	0.069196	0.4867897
PPP1R14C	1.2341989	1.1430614	1.273703	1.913185477	0.9359767	0.069212	0.5309797
NFE2L1	1.7082045	1.60103097	1.7546595	1.255608378	0.3283866	0.069315	0.6219796
CPNE1	2.1929105	2.04339379	2.2577193	1.205411906	0.2695262	0.069548	0.5295094
TTN-AS1	1.3041579	1.3984261	1.2632969	0.660842459	-0.5976217	0.069697	0.4869102
EI24	3.63997	3.44463348	3.7246397	1.114539151	0.1564473	0.069886	0.5450026
APBB1	1.5050439	1.40499275	1.5484116	1.354127007	0.4373631	0.070271	0.5903589
RP11-792A8.4	1.7067626	1.81415342	1.6602135	0.810920224	-0.3023681	0.070277	0.5191862
EXOSC8	1.9801659	1.86546727	2.0298826	1.189972938	0.2509288	0.070377	0.5404421
LRRFIP1	1.6730874	1.56798347	1.7186452	1.265257303	0.3394308	0.070482	0.5883408
GPATCH8	1.7792422	1.8892306	1.7315671	0.822696672	-0.2815675	0.070484	0.6626837
DLK1	5.8392018	6.55054346	5.5308668	0.816292471	-0.2928419	0.070671	0.5903589
EXT2	1.2851427	1.19301664	1.3250752	1.68418213	0.7520482	0.070704	0.5191862
SRA1	1.5478415	1.44649404	1.5917711	1.325372821	0.4063982	0.070836	0.5247905
TTYH1	2.9738446	3.20315951	2.8744468	0.850799386	-0.2331091	0.070864	0.4916485
TSR2	1.5696972	1.46779144	1.6138689	1.312270382	0.392065	0.070956	0.5903589
FNDC3B	1.3882821	1.29293445	1.429611	1.466577373	0.5524532	0.071016	0.5247905
PWAR6	1.795166	1.90428103	1.7478695	0.827032171	-0.2739846	0.071078	0.5903589
EGLN1	1.9953152	1.88028682	2.0451748	1.187311684	0.2476987	0.07112	0.6006346
DRG2	1.3749862	1.27944899	1.4163974	1.490065835	0.5753761	0.07115	0.5883408
NUFIP2	1.8655575	1.9772882	1.8171273	0.836117006	-0.2582232	0.071281	0.6639683
RNF19A	1.4588927	1.36112327	1.5012713	1.388089234	0.4731003	0.071328	0.5404421
FAM122B	1.3387546	1.43370436	1.2975981	0.686177269	-0.5433468	0.071349	0.5883408
RNF180	1.4365943	1.53493653	1.3939674	0.736474967	-0.4412916	0.07145	0.5404421
SEPT6	1.7278292	1.62105099	1.7741128	1.246456037	0.317832	0.072294	0.4848497
IGF2BP1	2.0284596	2.1454183	1.9777633	0.853629878	-0.2283174	0.072435	0.543801
RLF	1.5595382	1.4583141	1.6034144	1.316595743	0.3968124	0.072472	0.5316362
REEP2	1.9714503	2.08987688	1.9201176	0.844239975	-0.244275	0.072509	0.5743611

BCAP29	1.4312491	1.33491243	1.4730068	1.412329816	0.498077	0.072647	0.5621791
IMMT	1.7538403	1.64675618	1.8002565	1.237338745	0.3072405	0.072694	0.6043836
SORD	1.4689607	1.37089449	1.5114681	1.379012269	0.4636353	0.072772	0.5951939
TRAF4	1.8215156	1.71205469	1.868962	1.220358461	0.287305	0.072993	0.4862955
MET	1.1485095	1.06191498	1.1860443	3.004835078	1.5872858	0.073118	0.5615568
FGFR1	1.839264	1.72956088	1.8868153	1.21554674	0.2816054	0.073125	0.5615568
TPRA1	1.4274038	1.33064729	1.4693434	1.41946849	0.5053508	0.073147	0.5886859
HIVEP3	1.3029457	1.39611933	1.2625591	0.662828339	-0.5932928	0.073218	0.6031488
NAA40	1.25784	1.34938511	1.2181592	0.624409116	-0.6794365	0.073298	0.5541312
TOP3B	1.3410907	1.43523432	1.3002837	0.689935701	-0.5354662	0.073333	0.5541312
MUC12	1.1964204	1.28529121	1.1578988	0.553465313	-0.8534352	0.073426	0.6149463
DAB2	1.2446881	1.15484913	1.2836293	1.831649324	0.8731433	0.073487	0.5775637
TDRD3	1.412804	1.51024352	1.3705684	0.726257872	-0.4614462	0.07361	0.605958
NKIRAS2	1.76248	1.65527138	1.8089501	1.234526845	0.3039582	0.073787	0.561641
DCTN1	1.4639355	1.36648644	1.5061754	1.381157195	0.4658775	0.073875	0.5125363
EIF2S1	1.4902401	1.39171406	1.5329468	1.360550565	0.4441906	0.073919	0.5676548
A1BG	2.7687443	2.61499652	2.8353871	1.136465081	0.1845534	0.073994	0.5394076
TMEM44	1.1306551	1.21761643	1.0929613	0.427179533	-1.2270856	0.074088	0.605958
CNOT1	1.532379	1.63303114	1.4887508	0.772080114	-0.3731775	0.074253	0.561641
MAP7D1	1.5726698	1.4719334	1.6163346	1.305977815	0.3851304	0.074266	0.5791624
ZPR1	1.368116	1.27412377	1.4088575	1.491506864	0.5767706	0.074598	0.6195943
MTFR1L	1.8074681	1.69927353	1.8543656	1.221790211	0.2889966	0.074841	0.5613093
TRIM2	1.5663858	1.66768848	1.5224756	0.782514073	-0.3538114	0.07487	0.6117404
RP11-332H14.2	1.3735617	1.46919623	1.3321084	0.707824165	-0.4985371	0.074883	0.6659975
SVBP	2.6570385	2.50515744	2.7228721	1.144645802	0.1949012	0.075231	0.547876
STX4	1.4529936	1.35634227	1.4948877	1.388798785	0.4738376	0.075304	0.6012354
F2R	1.6433057	1.54115402	1.687584	1.270588299	0.3454966	0.07539	0.5194692
DAPK3	1.4555641	1.3593556	1.4972661	1.383771774	0.468606	0.075545	0.5429967
SFN	1.1060726	1.02148786	1.1427363	6.64264958	2.7317588	0.075566	0.5429967
CHD8	1.4021805	1.49793849	1.3606737	0.724333864	-0.4652733	0.07575	0.5405606
DCAKD	1.5910447	1.48961172	1.6350114	1.296969323	0.3751444	0.075764	0.5405606
CNTNAP3B	1.2916325	1.38334663	1.2518785	0.65705166	-0.6059213	0.076017	0.6167167
ZNF224	1.3896712	1.48476339	1.348453	0.718810417	-0.4763168	0.076072	0.5836083
RAPH1	1.2107181	1.29963679	1.1721758	0.57461486	-0.7993328	0.076418	0.6228909
SPAG4	2.4907542	2.64925695	2.4220503	0.862236945	-0.2138437	0.076523	0.6061594
RBP7	1.1410209	1.05537685	1.1781437	3.216934587	1.6856866	0.076603	0.5838057
CLK1	1.8772109	1.98930734	1.8286221	0.837577996	-0.2557046	0.076687	0.5471459
PROM1	1.2765523	1.36761812	1.2370794	0.644906724	-0.6328376	0.076707	0.5840163
RBM26	1.8202447	1.92892263	1.7731377	0.832295008	-0.2648331	0.076845	0.6228909
ASS1	2.0079204	2.1354547	1.95264	0.838994251	-0.2532672	0.076854	0.6228909
HMGCL	1.5105601	1.41232181	1.553142	1.341529866	0.4238792	0.076891	0.618817
CACNA1A	1.4295663	1.52577197	1.3878655	0.73770662	-0.4388809	0.077145	0.5829349
GCC2	2.0512177	2.16833955	2.0004506	0.856301241	-0.2238097	0.07718	0.5580295
TMX2	1.4731675	1.37694187	1.5148771	1.365932276	0.449886	0.077202	0.6257068
PNO1	1.1973983	1.11020949	1.2351907	2.134033304	1.0935827	0.077284	0.5475211
CD99L2	1.6718278	1.56867251	1.7165409	1.260023894	0.3334511	0.077741	0.5793144
C16orf59	1.2849111	1.37586622	1.2454861	0.653120859	-0.6145781	0.077887	0.5885434
GCK	2.1179253	2.32815701	2.0267993	0.773100828	-0.3712715	0.07804	0.5490084
RSPRY1	1.5455544	1.4464614	1.5885068	1.318158312	0.3985237	0.078093	0.5813627
KIF1A	2.1358057	2.01769297	2.1870022	1.166365761	0.2220203	0.07837	0.5972745
DDIT3	1.5494791	1.4450767	1.5947329	1.336248036	0.4181878	0.078442	0.6306702

AGO1	1.7114573	1.81595212	1.6661635	0.816424711	-0.2926082	0.078524	0.5197037
GPATCH2L	1.7900667	1.89729782	1.7435868	0.828695627	-0.2710858	0.078535	0.7208177
CLSPN	1.2465471	1.33665686	1.2074885	0.616320469	-0.6982474	0.078599	0.6929105
MPI	1.5042138	1.40671229	1.5464764	1.3436436	0.4261505	0.078674	0.5384109
SLC7A6	1.3370618	1.24535758	1.3768115	1.535764616	0.6189571	0.078744	0.6388456
TCTN3	1.6120202	1.51185394	1.6554379	1.280517415	0.3567269	0.078886	0.6104596
SDC4	1.4336904	1.33846296	1.4749673	1.403306447	0.4888301	0.078887	0.7208177
PDDC1	1.5834164	1.6841079	1.5397712	0.789014658	-0.341876	0.078888	0.5394225
MBNL1	1.4866479	1.58449826	1.4442341	0.760026449	-0.3958785	0.079022	0.5932559
LRRC14	1.268971	1.35922992	1.2298478	0.639834671	-0.6442289	0.079202	0.6224783
OCLN	1.214456	1.30305797	1.176051	0.580915198	-0.7836005	0.079249	0.521075
CELF4	1.3457695	1.25335174	1.3858284	1.522896264	0.6068177	0.079367	0.6003255
MAP1LC3A	1.7101199	1.60645281	1.755055	1.245035033	0.3161863	0.079374	0.5394225
ASPHD1	1.1348237	1.05048803	1.1713794	3.394456551	1.7631806	0.079715	0.5984589
FAM13C	1.6217974	1.72330353	1.577799	0.798833355	-0.3240335	0.080121	0.5926617
PAPD4	1.3882048	1.29480318	1.4286902	1.454157369	0.5401834	0.080173	0.5394225
SMC4	2.497638	2.67375181	2.4213005	0.849170429	-0.235874	0.080185	0.7208177
RCCD1	1.4402821	1.53643455	1.3986042	0.743062215	-0.4284451	0.08025	0.7208177
FAM89A	1.3050148	1.21488062	1.344084	1.601279828	0.6792254	0.080299	0.5394225
CLTC	2.0207285	1.90711131	2.0699764	1.179542617	0.2382275	0.080305	0.5245901
INTS6	1.5374496	1.63706034	1.4942727	0.77586483	-0.3661228	0.080381	0.5939941
FAM234A	1.8919153	1.78372434	1.9388113	1.197884539	0.2604889	0.080548	0.5394225
KDM5B	2.204978	2.08462464	2.2571459	1.159060782	0.2129562	0.08061	0.6045739
STK24	1.4596858	1.36329094	1.5014686	1.380349948	0.4650341	0.080829	0.6113836
HNRNPLL	1.6374456	1.53702677	1.6809727	1.268042383	0.342603	0.080899	0.6446395
EGFL6	1.12902	1.04463254	1.1655982	3.710256424	1.8915189	0.08095	0.6471998
NES	1.9217324	2.03420225	1.8729817	0.844111239	-0.244495	0.081048	0.5951403
NUP160	1.4317219	1.52653369	1.3906252	0.741880703	-0.4307409	0.081232	0.5754892
SHMT2	2.1094563	1.99197666	2.1603785	1.169763947	0.2262174	0.081385	0.7045634
SNX4	1.5027359	1.40664436	1.5443874	1.338730844	0.4208659	0.081492	0.5394225
KNTC1	1.1755904	1.26169275	1.1382688	0.528363261	-0.9203979	0.081642	0.6366846
H2AFJ	2.4378305	2.57006491	2.3805128	0.879271161	-0.1856199	0.081902	0.5394225
CRLF1	1.1805212	1.0952113	1.2174993	2.284385277	1.191806	0.081976	0.7045634
WDR77	1.3948151	1.30215778	1.4349779	1.43957192	0.5256399	0.082024	0.7045634
PAXBP1	1.5629221	1.6617189	1.5200981	0.785980424	-0.3474347	0.082046	0.6104541
PGM5	1.1669531	1.08206667	1.2037476	2.48270873	1.311915	0.082059	0.610884
KIF2A	1.8751009	1.76388868	1.9233065	1.208692492	0.2734473	0.082164	0.5780227
SCARA3	1.3032707	1.21400074	1.3419653	1.597963241	0.6762342	0.082167	0.6446395
UBQLN2	1.455043	1.36042144	1.4960572	1.376325365	0.4608216	0.082181	0.6471998
LIX1	1.3033538	1.39374905	1.2641715	0.670913361	-0.5758016	0.082541	0.6446395
UBE2C	2.6230513	2.39660082	2.7212075	1.232426258	0.3015013	0.082575	0.5394225
C7orf50	3.5256596	3.34159445	3.6054436	1.112679271	0.1540378	0.082576	0.5394225
SRSF1	3.0318278	3.19189126	2.9624474	0.895321533	-0.1595222	0.082621	0.6471998
FGF19	2.2139441	2.08008382	2.2719666	1.177655435	0.2359175	0.082738	0.6158117
TNKS	1.497781	1.59501162	1.4556358	0.765759518	-0.3850367	0.082741	0.6167726
MCM3	1.5822148	1.681826	1.5390378	0.790579703	-0.3390172	0.082745	0.6446395
CEP19	1.3510275	1.44325568	1.3110508	0.701741185	-0.5109891	0.08285	0.6167726
RP5-1159O4.1	1.1225519	1.20650071	1.0861638	0.417256725	-1.2609928	0.082896	0.6365894
RBM24	1.1458851	1.06190093	1.1822886	2.944843373	1.5581909	0.082922	0.6471998
RBM12B	1.5521645	1.6502857	1.5096334	0.783706878	-0.3516139	0.083005	0.6089016
DERL2	1.5422249	1.44525283	1.5842579	1.312193576	0.3919806	0.08302	0.6606093

UBE2J2	1.7328267	1.62986882	1.7774544	1.234311628	0.3037067	0.083073	0.6471998
SMARCA1	2.0711561	1.95755861	2.1203956	1.170054308	0.2265755	0.08309	0.5804733
LINC01089	1.638573	1.73932853	1.5949	0.804649039	-0.3135684	0.083251	0.6158117
PAIP2	5.8063153	5.53232187	5.9250793	1.086656998	0.1198966	0.083265	0.6606093
UBE2L6	2.1805645	2.06257815	2.2317063	1.15916773	0.2130893	0.083323	0.5407915
ZNF776	1.2188864	1.30586882	1.1811834	0.592356442	-0.7554625	0.083329	0.6255886
POP4	1.432515	1.33902202	1.47304	1.395307527	0.4805831	0.083353	0.6104541
GOLGA7	2.4022391	2.27435038	2.4576732	1.143855917	0.1939053	0.083387	0.6104541
RFX2	1.2857997	1.37513299	1.2470777	0.658640404	-0.6024371	0.083445	0.5959954
MELTF-AS1	1.2292832	1.31659386	1.1914379	0.604679684	-0.725757	0.083569	0.6606093
FASN	1.579022	1.67751671	1.536329	0.791609948	-0.3371384	0.083617	0.5959954
NAGK	1.64327	1.5435572	1.6864911	1.262960098	0.3368091	0.083689	0.6184372
FUOM	1.5877503	1.48954205	1.6303193	1.28756918	0.3646499	0.08374	0.6104541
COA6	1.6386049	1.53855728	1.681971	1.266292514	0.3406107	0.083768	0.6127032
AJUBA	1.3043828	1.21529128	1.3429999	1.593190124	0.6719184	0.083782	0.7458849
MPDU1	1.5238261	1.42796656	1.5653769	1.321077256	0.4017148	0.083834	0.5816323
NECAP2	1.5322117	1.43597027	1.573928	1.316438506	0.3966401	0.083938	0.6127032
CKLF	1.8148975	1.71042876	1.86018	1.210789998	0.2759487	0.084054	0.6606093
MAPK1	1.6083812	1.50934849	1.6513074	1.278706862	0.3546856	0.084127	0.5718363
REST	2.0579218	1.94437445	2.1071395	1.172352281	0.2294062	0.084132	0.6127032
TTLL7	1.5860398	1.68521329	1.5430526	0.792530697	-0.3354613	0.084261	0.6111776
SEMA3C	1.1664631	1.08208948	1.2030353	2.4733407	1.306461	0.084275	0.6280076
MRI1	1.2972183	1.38666882	1.2584455	0.668389833	-0.5812383	0.084323	0.6147018
HABP4	1.4001013	1.30776446	1.4401252	1.430071565	0.5160873	0.084454	0.7192906
CWC27	1.7083863	1.60673744	1.7524466	1.240151886	0.3105168	0.084471	0.5482471
SDR39U1	1.9749203	1.86503155	2.0225521	1.18209806	0.2413497	0.084652	0.653636
DNPEP	1.8184771	1.71407858	1.8637292	1.209571695	0.2744963	0.084745	0.6551413
DSN1	1.2358343	1.32300033	1.1980517	0.613162535	-0.7056585	0.084747	0.6111776
ASCC2	1.2463056	1.15950926	1.283928	1.780009223	0.8318847	0.085038	0.6631636
PPP1R11	2.0849803	1.97191615	2.1339885	1.166755532	0.2225023	0.085139	0.6111776
RDH11	1.6169968	1.51821978	1.6598122	1.273228591	0.3484915	0.0852	0.653636
RFX7	1.2665931	1.35492638	1.2283046	0.643245009	-0.6365597	0.085221	0.6494882
LACTB2	1.3154344	1.22625406	1.3540902	1.565011268	0.646173	0.085309	0.550132
CNPY4	1.5123098	1.41682154	1.5536997	1.328385439	0.4096738	0.08545	0.6415104
ADIPOR1	1.9897397	1.87830179	2.0380431	1.181875213	0.2410777	0.085912	0.6648284
ZDHHC3	1.5567116	1.45919726	1.5989797	1.304406136	0.3833931	0.08617	0.6321203
MYOF	1.257342	1.170453	1.2950045	1.730708789	0.791363	0.086198	0.6232322
CHTOP	2.1548665	2.04148309	2.2040132	1.156056379	0.2092118	0.086316	0.5827789
TMEM43	1.4359877	1.34359465	1.4760359	1.38545795	0.4703629	0.086389	0.553549
UBL7-AS1	1.3910885	1.48336152	1.3510923	0.72635546	-0.4612524	0.086452	0.730124
MT1H	1.1404887	1.0477346	1.1806935	3.785376712	1.9204369	0.086602	0.626771
EXOC4	1.511086	1.60656904	1.4696983	0.774352652	-0.3689374	0.087524	0.61145
JTB	4.2019156	3.97313626	4.3010812	1.1103027	0.150953	0.087555	0.6672693
ZNF718	1.1328995	1.21587929	1.0969314	0.449007577	-1.1551883	0.087561	0.6291886
ESYT1	1.5139074	1.60981468	1.4723359	0.774556481	-0.3685577	0.087581	0.6637166
YLPM1	1.3905319	1.48229439	1.350757	0.72726736	-0.4594423	0.087641	0.6672693
MPP6	1.3647916	1.27455212	1.4039063	1.471146331	0.5569408	0.087752	0.6637451
CCNG1	6.4564374	6.14709405	6.5905239	1.086151499	0.1192253	0.088296	0.6637166
PPP3CC	1.248166	1.16223037	1.2854153	1.759320942	0.8150187	0.088445	0.6349117
SUV39H2	1.3248731	1.41455449	1.2860002	0.689897624	-0.5355458	0.088496	0.6637166
ASMTL	1.2384223	1.15276972	1.2755489	1.803687988	0.8509498	0.088506	0.6796751

TMUB1	2.0521204	1.93766788	2.1017305	1.174968765	0.2326224	0.088533	0.6646556
MAP4K4	1.8112986	1.70748624	1.8562966	1.210336758	0.2754085	0.088606	0.6506395
TMEM151A	1.1233427	1.04148871	1.1588227	3.82809561	1.9366269	0.088794	0.5726088
MYH10	2.1709628	2.05532827	2.2210853	1.157066787	0.2104721	0.088888	0.5657292
UPF3A	2.6304729	2.48651747	2.6928711	1.13881683	0.1875357	0.08895	0.6171277
PRAF2	2.1993677	2.08557979	2.2486896	1.150251367	0.2019492	0.088966	0.6438806
GSE1	1.5155807	1.61094304	1.4742453	0.77625132	-0.3654043	0.089047	0.6135706
LINC01515	1.1673015	1.2511935	1.1309381	0.521263862	-0.9399142	0.089068	0.6716873
EIF2S3	4.9123106	5.15205592	4.8083916	0.91723033	-0.124644	0.089414	0.5657292
DHRS4-AS1	1.772153	1.87434811	1.727856	0.832455614	-0.2645547	0.089556	0.633831
ILKAP	1.4369403	1.3449873	1.4767979	1.382073648	0.4668345	0.089706	0.6532927
RAB34	3.7029931	3.51140644	3.7860374	1.109353449	0.1497191	0.089798	0.6022434
KLHDC2	2.2993174	2.18252216	2.349943	1.141579416	0.1910312	0.089931	0.6462212
TPRKB	2.0584777	1.94722035	2.1067028	1.168368898	0.2244959	0.089986	0.6413873
CALCOCO2	1.3823246	1.29265436	1.4211927	1.439215528	0.5252827	0.090021	0.638273
CHMP1A	1.6098523	1.51309052	1.6517942	1.270329888	0.3452032	0.090116	0.6429554
MSI1	2.2508788	2.39008202	2.1905404	0.856453322	-0.2235535	0.090151	0.6717933
RWDD1	3.553946	3.36649623	3.6351971	1.113543753	0.1551582	0.090516	0.6716873
ASPSCR1	1.7772109	1.8804364	1.7324672	0.831936555	-0.2654546	0.09055	0.638273
SMC5	1.7942238	1.89733009	1.7495318	0.835291008	-0.2596492	0.090588	0.6463257
METTL17	1.4979585	1.59175318	1.4573027	0.772793028	-0.371846	0.090718	0.6185162
ARG2	1.3274171	1.23978676	1.3654009	1.523857849	0.6077283	0.090719	0.6716873
MFSD14A	1.6114147	1.51439852	1.6534669	1.270351467	0.3452277	0.090791	0.6765602
TUBGCP5	1.2359685	1.32213533	1.1986191	0.616570324	-0.6976626	0.090804	0.6920373
SYNM	1.1573924	1.24048311	1.1213763	0.504718698	-0.9864486	0.090819	0.638273
DENR	2.6272429	2.49262875	2.6855921	1.129277515	0.1754001	0.090895	0.6716873
DPAGT1	1.3078231	1.22033522	1.3457452	1.569177997	0.650009	0.090962	0.6185162
RASSF6	1.1212382	1.04056532	1.1562063	3.850734014	1.9451335	0.090994	0.6185162
DUT	5.1520098	5.43273404	5.0303284	0.909219538	-0.1372994	0.091091	0.6701876
NFIB	1.2090111	1.12504472	1.2454068	1.962552337	0.9727311	0.091152	0.6770024
EGFR	1.6178145	1.71634769	1.5751048	0.802829155	-0.3168351	0.091163	0.5841422
MOAP1	1.4166747	1.32581277	1.4560592	1.399758614	0.4851781	0.091401	0.6701876
MCM2	1.2890989	1.3765981	1.2511719	0.666949463	-0.5843506	0.09153	0.6617838
RBFA	1.5340579	1.62939987	1.4927314	0.782859095	-0.3531754	0.091567	0.6487105
AK6	2.0035575	1.89210143	2.0518687	1.179090917	0.237675	0.091692	0.6782544
DPP3	1.4008189	1.31075573	1.4398572	1.415443621	0.5012543	0.091693	0.7679248
ADGRL2	1.5070803	1.60117707	1.4662936	0.775634301	-0.3665515	0.091905	0.7315339
MIR99AHG	1.5856503	1.68265661	1.5436023	0.796304197	-0.3286084	0.091934	0.6701876
CD53	1.1017576	1.02155234	1.1365229	6.334484693	2.6632273	0.091997	0.6465512
NIPSNAP3A	1.3216791	1.23422069	1.3595884	1.535254677	0.618478	0.092043	0.6770024
PIGS	1.4486368	1.35705508	1.4883334	1.367669529	0.4517197	0.092101	0.5864176
SIRT2	1.4342752	1.34313172	1.4737818	1.380757798	0.4654603	0.092288	0.6770024
ZMYND11	1.6235469	1.52657377	1.6655804	1.263983187	0.3379773	0.092351	0.7315339
NALT1	1.1680886	1.25104696	1.1321299	0.526315489	-0.9260002	0.092557	0.6789758
DHRS4L2	1.7418601	1.64190041	1.7851881	1.223224222	0.2906889	0.092616	0.8137712
DPM3	2.7010431	2.56463219	2.7601711	1.124974388	0.1698922	0.092885	0.7187836
ZBTB10	1.5240578	1.6181939	1.483254	0.781719098	-0.3552778	0.092891	0.6482996
DAPL1	13.743002	13.0730611	14.033392	1.079543282	0.1104211	0.093026	0.8137712
OSBPL8	2.4685087	2.34098172	2.523786	1.136321251	0.1843708	0.093307	0.6180554
TMPO-AS1	1.1678602	1.25063259	1.1319821	0.526596	-0.9252315	0.093336	0.6562903
CHAF1A	1.2714449	1.35749497	1.2341461	0.654963307	-0.610514	0.093384	0.6180554

SF3B1	2.3846076	2.26167536	2.4378932	1.139669732	0.1886158	0.093515	0.6810303
CENPX	3.0386993	3.20469725	2.9667466	0.89207106	-0.1647695	0.093673	0.6828613
AK4	2.22479	2.34411875	2.1730663	0.872740049	-0.1963761	0.093735	0.6496814
STAMBP	1.5826429	1.48737846	1.6239358	1.280187532	0.3563552	0.09378	0.6562903
ABCB4	1.1367163	1.21844587	1.1012901	0.46368525	-1.1087823	0.093783	0.6549006
CCNDBP1	1.8503417	1.74763104	1.8948622	1.196930234	0.2593391	0.094036	0.6562903
SIRT3	1.3423507	1.25454291	1.3804115	1.494488593	0.5796519	0.094123	0.6911479
PPP3CB	1.4252635	1.33554817	1.4641511	1.383262295	0.4680747	0.094141	0.6549006
FIBP	2.4286166	2.30499961	2.482199	1.135785044	0.1836898	0.094173	0.6192007
TSFM	1.3151454	1.2286303	1.3526458	1.542428	0.6252031	0.094228	0.7371854
PLAGL1	1.4269724	1.51853871	1.3872825	0.746872807	-0.4210655	0.094291	0.7831618
TCTEX1D2	2.2852036	2.16548846	2.3370948	1.147239818	0.198167	0.094388	0.5971994
ZC3H6	1.828893	1.93170034	1.7843306	0.841827133	-0.2484041	0.094417	0.6549006
NHLH1	1.1175306	1.034501	1.1535202	4.449731271	2.1537182	0.094483	0.6503799
POLR1D	3.7317194	3.51410738	3.8260446	1.124074727	0.1687379	0.094549	0.8199654
FUCA1	1.2791433	1.19393876	1.3160757	1.629770717	0.704669	0.094665	0.6894075
CCDC91	1.4766379	1.38452432	1.5165651	1.343387319	0.4258753	0.094727	0.6482699
POR	1.3278143	1.24106488	1.3654162	1.515841878	0.6001193	0.0949	0.6199327
SOX11	3.3385991	3.09164673	3.4456419	1.169242354	0.225574	0.095045	0.6549006
C1GALT1C1	1.1895922	1.1075376	1.2251592	2.093771861	1.0661043	0.095155	0.6795141
CLN8	1.4156585	1.32593792	1.4545484	1.394585843	0.4798367	0.095194	0.7371854
SLC41A3	1.5667752	1.47186937	1.6079127	1.288307122	0.3654766	0.095211	0.6890803
SNRPD2	10.595072	10.1918198	10.769864	1.062886832	0.087988	0.095263	0.7371854
MRPL58	1.3290589	1.24181496	1.3668753	1.517173707	0.6013863	0.095335	0.6795141
PILRB	1.2740403	1.35990493	1.2368218	0.658012182	-0.6038138	0.095424	0.5998659
ZMYM4	1.6912757	1.79004946	1.6484616	0.820786125	-0.2849218	0.096025	0.6017613
EEF1A1	200.32308	206.030772	197.84905	0.960095167	-0.0587507	0.096067	0.5998659
CCDC58	1.3365362	1.24903821	1.3744626	1.503635312	0.5884547	0.096077	0.692384
WDR60	2.0592187	2.16779019	2.0121578	0.866729179	-0.2063468	0.096193	0.6912127
MAP1A	1.5634167	1.46902256	1.6043323	1.288493084	0.3656848	0.096306	0.6017613
TMX1	1.6764612	1.57933791	1.7185598	1.240312047	0.3107031	0.096308	0.72802
GALNT11	1.716136	1.61776671	1.7587747	1.228254383	0.2966094	0.096465	0.6601606
ASCC1	1.3940391	1.30532499	1.4324927	1.416499583	0.5023302	0.096599	0.5998659
LINC01473	1.3686862	1.45720748	1.3303162	0.72246461	-0.4690012	0.096626	0.742029
SNAPC1	1.7523647	1.65218388	1.7957886	1.220190611	0.2871065	0.096888	0.6541553
ZNF43	1.4388496	1.52933548	1.3996279	0.754961572	-0.4055249	0.096923	0.7007716
GOLGA5	1.2540708	1.16996431	1.2905272	1.70934228	0.7734413	0.096973	0.72802
BEX1	4.8017045	5.10018209	4.6723278	0.895649935	-0.1589931	0.097169	0.6604882
PPP3CA	1.7553014	1.85542362	1.7119028	0.832222567	-0.2649587	0.097349	0.7096566
KIAA1147	1.2474336	1.33207259	1.2107463	0.634639343	-0.6559911	0.097352	0.7476039
ST20	1.7198428	1.6217303	1.7623702	1.226207206	0.2942028	0.09737	0.6685269
NSMCE3	1.4172345	1.32829744	1.4557848	1.388328824	0.4733493	0.097405	0.6893771
NAGA	1.2765319	1.19206435	1.3131449	1.630416421	0.7052405	0.097756	0.6070518
NHP2	5.0486997	4.80915129	5.1525333	1.090146581	0.1245221	0.097825	0.692384
DECR1	2.1490564	2.03581952	2.1981395	1.156706794	0.2100232	0.097832	0.8059147
PRSS8	1.1313304	1.05158307	1.1658973	3.216118455	1.6853205	0.097882	0.692384
RP11-112J3.16	1.1705613	1.25230912	1.1351272	0.535562135	-0.9008741	0.097981	0.692384
RNF7	5.4490552	5.15364643	5.5771018	1.101947861	0.140056	0.097985	0.6685269
DNAAF2	1.6310163	1.5354045	1.6724598	1.255984568	0.3288187	0.098047	0.7022706
SIMC1	1.4154185	1.50482485	1.3766648	0.746129703	-0.4225017	0.098094	0.6667765
ANKRD50	1.2365571	1.15295102	1.2727967	1.783555771	0.8347563	0.098103	0.689466

TOMM34	1.6134249	1.51872715	1.6544721	1.261688589	0.3353559	0.098321	0.6685269
CH507-254M2.3	1.1553824	1.23647865	1.1202308	0.508421428	-0.9759033	0.098329	0.6068648
PLPP1	1.2495136	1.16598425	1.2857198	1.721367181	0.7835549	0.09842	0.692384
AUP1	1.9506755	1.84520499	1.9963923	1.178876448	0.2374125	0.09852	0.8471056
PHYHIPL	1.8077199	1.70583766	1.8518814	1.206908371	0.2713162	0.098528	0.7022706
PMS1	1.4178947	1.50731607	1.3791345	0.747333944	-0.420175	0.098593	0.6685269
CEP164	1.466938	1.55823918	1.4273629	0.765555262	-0.3854216	0.098619	0.6882807
RAB25	1.0924036	1.01400962	1.1263839	9.02121874	3.1733224	0.098642	0.7517707
ANKRD17	1.742845	1.84262265	1.6995958	0.830259877	-0.2683651	0.098724	0.8066491
NME3	2.3713391	2.24876244	2.4244706	1.140705857	0.1899268	0.098831	0.6672751
AP006621.5	1.3104172	1.39646336	1.27312	0.688890944	-0.5376525	0.099115	0.6685269
FAT3	1.5307908	1.62413975	1.4903281	0.785606312	-0.3481216	0.09918	0.7558736
NT5DC1	1.2944915	1.20957675	1.3312982	1.580796673	0.6606518	0.099223	0.6693493
TTF1	1.5262782	1.61938503	1.4859205	0.784520898	-0.3501162	0.099266	0.6786542
CCND2	2.6076336	2.47217472	2.666349	1.131896254	0.1787417	0.099441	0.7044885
TACO1	1.3296101	1.24388136	1.3667696	1.503885537	0.5886948	0.099612	0.6465163
FKBP1B	1.728003	1.63018583	1.7704024	1.222500386	0.2898349	0.099675	0.7026716
ATPAF2	1.3005192	1.21566686	1.3372989	1.563981119	0.6452231	0.099702	0.7413553
NMD3	1.4775044	1.3869651	1.5167492	1.335389602	0.4172607	0.09973	0.6961262
PDE4C	1.2196413	1.30245515	1.1837451	0.607511984	-0.7190152	0.099789	0.6964418
CENPA	1.5303361	1.42834438	1.574545	1.341315579	0.4236487	0.0999	0.7211262
STXBP3	1.4706448	1.38062773	1.5096633	1.339007169	0.4211637	0.099982	0.7026716
WASF1	1.4507661	1.36101498	1.4896693	1.356368253	0.4397489	0.100018	0.6921059
PPFIA4	1.3840379	1.4723876	1.3457422	0.73190359	-0.4502745	0.100196	0.7578704
SENP7	1.3884659	1.47682389	1.3501667	0.734373256	-0.4454146	0.100223	0.7413553
EHMT1	1.7554303	1.85463922	1.7124276	0.833600402	-0.2625721	0.100275	0.6693493
FBXW2	1.3951377	1.30727992	1.4332201	1.409854986	0.4955468	0.100295	0.6726435
HDGF	2.4923903	2.35989601	2.5498206	1.139661122	0.1886049	0.100336	0.6961262
TMEM67	1.5142446	1.60688245	1.4740902	0.781189474	-0.3562556	0.100356	0.7211262
MATN2	1.1292458	1.04987682	1.1636487	3.281056134	1.7141603	0.100405	0.6159012
PAPLN	1.0997059	1.17839739	1.0655966	0.367699284	-1.4434017	0.100469	0.6693493
HOXB2	1.1536492	1.06730727	1.1910745	2.838839377	1.5053012	0.100545	0.6921059
ST8SIA2	1.1937996	1.11250187	1.2290385	2.03586426	1.0256414	0.100581	0.8142543
BCL2L13	1.6086329	1.51431166	1.6495169	1.262885886	0.3367243	0.100602	0.7400308
FKBP9	1.3456297	1.25964178	1.3829016	1.474730219	0.5604511	0.100651	0.761315
C8orf59	4.0059121	3.80869	4.0913991	1.10065513	0.1383625	0.100696	0.7027712
COLEC12	1.3833743	1.29624589	1.4211406	1.42159147	0.5075069	0.100954	0.7102098
FAM13A	1.6972008	1.79536485	1.654651	0.82308262	-0.2808908	0.100971	0.7414369
PYGL	1.2698139	1.18613999	1.3060827	1.64436856	0.7175337	0.100987	0.6857597
TRA2B	2.7494072	2.89126642	2.6879175	0.892480039	-0.1641082	0.101082	0.7400308
OXCT1	1.3025969	1.2180802	1.3392312	1.555533985	0.6374099	0.101198	0.8627543
H2AFX	2.0875416	2.22040624	2.0299507	0.843940829	-0.2447862	0.101275	0.8142543
CLIP3	1.5319009	1.43990484	1.5717771	1.299774491	0.3782613	0.101384	0.6979539
SRSF7	3.9702731	4.17619567	3.8810149	0.907064672	-0.1407227	0.101515	0.7400308
ETNK2	1.1471269	1.06790249	1.1814671	2.67246646	1.4181718	0.101824	0.6822225
GOLIM4	2.2524914	2.1316777	2.3048587	1.153030302	0.2054304	0.101853	0.7622505
FAM213B	1.4192118	1.33099683	1.457449	1.382034524	0.4667937	0.102057	0.6979539
MDC1	1.2114331	1.29384612	1.1757107	0.597968337	-0.741859	0.102089	0.6979539
C12orf73	1.83942	1.739335	1.8828024	1.194049311	0.2558624	0.102277	0.8157808
MYEF2	2.6867773	2.8196035	2.6292031	0.895361617	-0.1594576	0.10229	0.7622505
ZNF300	1.2579657	1.34135141	1.2218218	0.649834018	-0.6218568	0.102377	0.7152195

SPOP	1.6913244	1.59480394	1.7331617	1.232610739	0.3017173	0.102405	0.6243632
IREB2	1.8801318	1.98181524	1.8360566	0.851541707	-0.2318509	0.102507	0.6610411
RP13-1032I1.7	1.1462509	1.22621109	1.1115917	0.493307831	-1.0194399	0.10307	0.7737963
ME2	1.4798272	1.38993236	1.5187926	1.330468302	0.4119341	0.103204	0.7160193
MRPL54	2.102645	1.99230153	2.150474	1.159399644	0.2133779	0.103246	0.7526482
GFER	1.5853388	1.49266293	1.6255097	1.269650327	0.3444312	0.103256	0.6616258
ACP2	1.3297567	1.24528302	1.3663723	1.4936716	0.578863	0.103513	0.7029067
PRR14	1.4956722	1.58669344	1.4562185	0.777609639	-0.362882	0.103812	0.8215077
ANKFY1	1.3170776	1.40228494	1.280144	0.696381932	-0.5220493	0.103913	0.7513116
SMIM8	1.7423478	1.84090158	1.6996292	0.831998891	-0.2653465	0.104171	0.7432338
NUDT12	1.2496179	1.33269423	1.213608	0.642055024	-0.6392312	0.104517	0.6945062
STOM	1.2068459	1.12628387	1.2417659	1.914463792	0.9369404	0.104679	0.6945062
EIF2B3	1.2454038	1.16336516	1.2809639	1.719852125	0.7822845	0.10473	0.8816983
TGDS	1.2339583	1.15247077	1.2692795	1.766105548	0.8205716	0.104763	0.6933627
DHCR24	1.3177806	1.23370349	1.3542243	1.515699477	0.5999837	0.104906	0.7759967
NSMCE1	1.4162379	1.32867804	1.4541911	1.38187246	0.4666245	0.104938	0.7273315
PTGES2	1.7914654	1.69385126	1.8337768	1.201665032	0.2650348	0.105173	0.8816983
HAUS5	1.2679734	1.35089658	1.2320299	0.661248632	-0.5967353	0.105274	0.7100669
CHD6	1.7317216	1.82918342	1.6894763	0.831512414	-0.2661903	0.105348	0.6945062
ZZZ3	1.4583684	1.54765565	1.4196664	0.766296095	-0.3840261	0.105564	0.7025944
ZNF430	1.2983991	1.38245029	1.2619667	0.684969276	-0.5458888	0.105625	0.7582346
IKBIP	1.9881911	2.09824041	1.9404896	0.856360366	-0.2237101	0.105626	0.6945062
CXCL16	1.1059255	1.02886071	1.1393296	4.827658059	2.2713235	0.106372	0.7544628
KIF11	1.2300509	1.31152656	1.1947349	0.62509873	-0.677844	0.106495	0.7544628
DCAF16	1.4117169	1.49944596	1.3736903	0.748209735	-0.4184854	0.106536	0.7097713
PTDSS1	1.3934517	1.30719434	1.4308404	1.402501125	0.4880019	0.106634	0.7347553
LSM12	1.7016261	1.60572929	1.7431931	1.226939279	0.2950639	0.106703	0.7015865
THY1	1.1478028	1.06972935	1.1816441	2.604988167	1.3812768	0.107451	0.7018194
LLPH	1.680806	1.58645253	1.721704	1.230626494	0.299393	0.10762	0.7357667
VSIG10	1.3194437	1.40371165	1.2829174	0.700790702	-0.5129445	0.107751	0.7580249
EMG1	1.5032205	1.41418057	1.5418153	1.308162114	0.3875413	0.108013	0.8397445
LLNLF-187D8.1	1.1932595	1.27363758	1.1584192	0.578938007	-0.7885192	0.108054	0.8397445
USP49	1.1840071	1.26368373	1.1494708	0.56685626	-0.8189451	0.108133	0.7018194
ESRRG	1.3018945	1.38556789	1.2656258	0.688920965	-0.5375896	0.108241	0.7948199
TBX2	1.4525466	1.54023846	1.4145361	0.767320615	-0.3820986	0.108303	0.6563447
R3HDM2	1.7169249	1.81274713	1.6753903	0.830996841	-0.2670851	0.108309	0.7826045
ITGA3	1.3579552	1.27373685	1.39446	1.441019183	0.5270895	0.108649	0.7454731
NOVA2	1.30288	1.38664366	1.2665722	0.689451962	-0.5364781	0.108739	0.7703641
GLT8D1	1.8752095	1.77633992	1.9180651	1.182555537	0.2419079	0.109036	0.8397445
NACA	32.774899	31.8040002	33.19574	1.045180477	0.0637521	0.109132	0.7019423
CTB-50L17.10	1.6099493	1.51800188	1.6498045	1.25444425	0.3270484	0.109242	0.7454731
CHCHD5	2.9553496	2.78783258	3.0279607	1.13431242	0.1818181	0.109382	0.909406
MOXD1	1.4042395	1.3190506	1.4411651	1.382743522	0.4675336	0.10944	0.7233745
PRELP	1.2686379	1.35140045	1.2327641	0.662389817	-0.5942476	0.109459	0.8397445
ZC3H14	1.6244606	1.71772536	1.5840345	0.813729721	-0.2973784	0.109564	0.7826045
PPM1G	2.8719091	2.72978132	2.9335151	1.117780081	0.1606364	0.109581	0.7930801
SEC23B	1.2021825	1.12272781	1.2366226	1.928027214	0.9471254	0.109608	0.8167806
ZFP36	1.1734511	1.09494526	1.2074799	2.185258274	1.1278038	0.109742	0.7019423
PIGH	1.3352618	1.25167736	1.371492	1.476064367	0.5617556	0.10982	0.7672157
ATAD1	1.6505102	1.55822975	1.6905096	1.236963079	0.3068024	0.109904	0.7019423
RABL2A	1.4077136	1.49407893	1.3702781	0.749431046	-0.4161324	0.10993	0.7826045

TBCC	1.2991193	1.21679251	1.3348043	1.5443538	0.6270033	0.109941	0.7836198
SLC30A9	1.727421	1.63169242	1.7689151	1.217230231	0.2836021	0.110001	0.7019423
PLEKHA2	1.1801547	1.10150475	1.214246	2.110699297	1.0777211	0.110016	0.7233745
SLC6A6	1.3229949	1.40690011	1.2866258	0.704413096	-0.5055064	0.110122	0.7477908
CBX6	1.9702641	2.08824523	1.9191246	0.844593259	-0.2436714	0.110245	0.7019423
SEPT10	1.657239	1.56398136	1.697662	1.237030263	0.3068808	0.110379	0.7027913
AAK1	1.7431867	1.64764368	1.7846004	1.21146926	0.2767578	0.110386	0.7600863
FANCM	1.1557611	1.23443646	1.1216588	0.518941435	-0.9463564	0.110394	0.7454731
MTFP1	1.7339346	1.8288721	1.6927834	0.835814642	-0.2587451	0.110467	0.7826045
HSDL2	1.4969992	1.40882369	1.5352193	1.309169043	0.3886514	0.110523	0.7454731
ZMAT2	2.0670891	1.96239658	2.1124687	1.155935833	0.2090613	0.110703	0.8070071
TNC	1.5303436	1.43446716	1.5719018	1.316329245	0.3965204	0.110855	0.7792935
ATP6V1B1	2.6079745	2.43733733	2.6819382	1.170176366	0.226726	0.110867	0.7428065
UQCC2	4.7978532	4.5321689	4.9130155	1.107822299	0.1477265	0.11103	0.9155417
PTBP2	1.3920007	1.47744412	1.3549647	0.743468617	-0.4276562	0.111403	0.7994055
ASPM	1.9598574	2.11003464	1.8947622	0.806066952	-0.3110284	0.111492	0.7520093
BRAT1	1.2732028	1.35549323	1.2375336	0.66818028	-0.5816907	0.111504	0.7600863
CHST15	1.3200612	1.40353767	1.2838779	0.703473129	-0.5074328	0.111549	0.7792935
KIF5A	1.2454443	1.32646399	1.2103259	0.644254519	-0.6342973	0.111696	0.7483611
LRRC41	1.767858	1.67247222	1.8092034	1.20332621	0.2670278	0.11175	0.6877684
CERS4	1.5825306	1.49168781	1.6219069	1.264841028	0.3389561	0.111771	0.8509852
LUC7L	1.6965453	1.79132545	1.6554623	0.828309368	-0.2717584	0.111928	0.750465
RP11-54O7.1	1.1818985	1.26090051	1.1476546	0.565942186	-0.8212734	0.11201	0.750465
KANSL1	1.486741	1.5748281	1.4485592	0.780336216	-0.3578322	0.112087	0.7776538
NDUFA7	2.2816147	2.16938406	2.3302616	1.137574613	0.1859612	0.112123	0.6877684
C2orf68	1.542399	1.6323141	1.5034249	0.796162657	-0.3288649	0.112219	0.7994055
SRPX	1.3790104	1.29489646	1.4154701	1.40886774	0.4945362	0.11239	0.7600863
MCUB	1.6971573	1.60396164	1.7375535	1.22119256	0.2882907	0.112586	0.7123356
MPV17	2.1641946	2.0582886	2.2101001	1.143450008	0.1933933	0.112668	0.7710487
MAL2	1.1136426	1.03772032	1.1465515	3.885213974	1.9579941	0.112805	0.6877684
COX14	3.0377228	2.8866243	3.1032173	1.114804535	0.1567908	0.112839	0.7994055
RNF216	1.4391341	1.35293466	1.4764977	1.350101816	0.4330682	0.112866	0.7372967
TIAL1	2.1783062	2.28767719	2.1308988	0.878247135	-0.1873011	0.112965	0.7469081
ATG16L2	1.2093759	1.28940094	1.1746886	0.603621516	-0.7282839	0.112997	0.8358422
BUB3	2.1924422	2.08389568	2.2394922	1.14355305	0.1935233	0.11309	0.7977557
HIST1H1C	1.3967422	1.48219128	1.3597038	0.745977444	-0.4227961	0.113229	0.6819221
SURF1	1.9880214	1.88661143	2.0319781	1.163957595	0.2190385	0.113552	0.6881538
ZNF688	1.481219	1.56940207	1.4429956	0.778001318	-0.3621555	0.113681	0.7793325
IAH1	2.0592241	1.95484018	2.1044699	1.156706511	0.2100229	0.113779	0.7623165
TMEM243	1.3811405	1.46608427	1.3443211	0.738752998	-0.436836	0.113783	0.7526883
MED10	2.2915702	2.18237921	2.3388996	1.132377475	0.179355	0.113839	0.7526883
EHD4	1.208269	1.12955327	1.2423887	1.870957993	0.9037772	0.113992	0.8512018
ZNF706	4.6080569	4.3958852	4.7000238	1.089560932	0.1237469	0.113997	0.6819221
SPPL2B	1.4872861	1.57538752	1.449098	0.780514039	-0.3575035	0.114052	0.8512018
CTC-260E6.6	1.135018	1.21141466	1.1019035	0.482007851	-1.0528715	0.114135	0.7552603
TOMM22	2.8884587	2.74186629	2.9519999	1.120637075	0.1643191	0.114228	0.7552603
PXDN	2.1980975	2.08723157	2.2461529	1.146170669	0.1968219	0.114341	0.8512018
GLIPR2	1.432192	1.34656118	1.4693092	1.354188586	0.4374287	0.114393	0.935608
CASP3	1.5491261	1.46045991	1.587559	1.276026371	0.3516581	0.114406	0.737771
GLYR1	1.3774562	1.29372038	1.4137519	1.408659124	0.4943225	0.114454	0.7977557
PDLIM7	4.6146321	4.38055379	4.7160946	1.099256174	0.1365276	0.114513	0.7521963

CENPT	1.3998795	1.48516188	1.3629134	0.748025317	-0.418841	0.114546	0.7977557
CYB5A	1.8468071	1.74980646	1.8888526	1.185442692	0.2454259	0.114557	0.6819221
EPHX2	1.6086607	1.69975857	1.5691738	0.813386013	-0.2979879	0.114906	0.7600863
BMPR2	1.5587634	1.46989825	1.5972825	1.271088892	0.3460649	0.114965	0.7749957
HOXB6	1.1053932	1.02609754	1.1397643	5.355459454	2.4210104	0.115026	0.7793325
FAM189B	1.6669603	1.75971096	1.6267571	0.824994123	-0.2775443	0.115042	0.7981564
TMEM33	1.6431761	1.55148313	1.6829209	1.238335138	0.3084018	0.115076	0.809553
MSRA	1.3715486	1.28804804	1.4077424	1.415536208	0.5013487	0.115099	0.7977557
TMEM134	2.7752028	2.64063909	2.8335302	1.117570693	0.1603661	0.115137	0.8454572
PNRC1	4.3137986	4.11395528	4.4004217	1.091994403	0.1269655	0.115142	0.7749957
RP11-146F11.1	1.3350085	1.41783356	1.2991075	0.715853255	-0.4822642	0.115218	0.7793325
TIMM9	2.1539522	2.04305406	2.2020216	1.152405883	0.2046489	0.115428	0.7793325
CREBZF	1.5271897	1.61565679	1.4888432	0.794018971	-0.3327546	0.115556	0.7749957
SSFA2	1.5642426	1.475113	1.6028763	1.268911327	0.3435913	0.115595	0.7546959
DDA1	1.6435562	1.55199978	1.6832419	1.23775747	0.3077287	0.115645	0.7546959
NEUROD1	1.1957611	1.10911516	1.2333183	2.138275672	1.0964479	0.115773	0.6974115
YEATS2	1.3810689	1.46549799	1.3444727	0.740009012	-0.4343853	0.116104	0.8402892
CDKN2D	1.2824881	1.20174581	1.3174863	1.573694466	0.6541555	0.11626	0.7556868
RRP1B	1.5844928	1.49493908	1.6233104	1.259367896	0.3326998	0.116275	0.8476251
TOPBP1	1.449187	1.53523724	1.411888	0.76954292	-0.3779263	0.116738	0.7569114
FLNC	1.358905	1.2761055	1.3947949	1.429869846	0.5158838	0.117075	0.8178987
IER3IP1	2.6746962	2.54391646	2.7313834	1.121423007	0.1653306	0.117079	0.6890943
MTMR11	1.3193571	1.4013744	1.2838063	0.707086154	-0.5000421	0.117113	0.9439396
TM2D3	1.7002928	1.60800635	1.7402948	1.217577453	0.2840135	0.117229	0.757991
CREM	1.9907128	1.89060804	2.0341038	1.161121138	0.2155185	0.117249	0.7600863
GUSB	1.3027807	1.22183863	1.3378655	1.523023719	0.6069384	0.117333	0.6890943
GEM	1.3438404	1.25639544	1.3817439	1.488887399	0.5742346	0.117357	0.7556868
FGD4	1.5254795	1.43814922	1.5633332	1.285710928	0.3625663	0.117438	0.7808217
TRIP10	1.3906859	1.30756349	1.4267158	1.38740714	0.4723912	0.117514	0.7829065
MRPS22	1.3612596	1.27880585	1.3969996	1.423928472	0.5098767	0.117541	0.757991
CNTFR	1.6316331	1.72239248	1.592293	0.819904725	-0.2864718	0.117602	0.8011067
UNG	1.4720228	1.55876616	1.4344234	0.777469035	-0.3631429	0.11771	0.9439396
SLC2A1-AS1	1.4604039	1.54688973	1.4229162	0.773311597	-0.3708782	0.117817	0.6890943
PHACTR2	1.4851705	1.39908916	1.5224829	1.30918842	0.3886727	0.117818	0.7556868
NARF	2.8418462	2.69955887	2.9035215	1.120009134	0.1635105	0.117819	0.7739093
DAAM2	1.1211489	1.04597511	1.1537334	3.343839509	1.7415056	0.117947	0.7600863
ARID3A	2.1000747	2.20785224	2.0533579	0.872091669	-0.1974483	0.118053	0.7556868
BCAR1	1.627779	1.53787661	1.6667476	1.23959214	0.3098655	0.118094	0.7808217
TMEM63A	1.361175	1.44416181	1.3252039	0.732174351	-0.4497409	0.118107	0.7425947
IDH3A	1.194272	1.11685199	1.2278301	1.949732211	0.963276	0.118145	0.8550632
ZNF711	1.3851585	1.46955634	1.3485757	0.74235128	-0.4298261	0.118176	0.873284
DKK2	1.0960592	1.01740877	1.1301506	7.476154759	2.9022964	0.118227	0.9439396
PPM1B	1.5256745	1.43829461	1.5635498	1.285778644	0.3626423	0.118266	0.7600863
SMIM1	1.8625965	1.76473415	1.9050155	1.183438082	0.2429842	0.118631	0.7600863
RP11-83A24.2	1.6341239	1.72497524	1.5947438	0.820364323	-0.2856633	0.118643	0.6899138
PLXNC1	1.2262878	1.30500332	1.1921681	0.630052597	-0.6664558	0.118684	0.7899175
SLC4A8	1.3020361	1.38338726	1.2667741	0.695834445	-0.523184	0.118734	0.7649197
TOM1L1	1.5397936	1.45179676	1.5779363	1.279195388	0.3552366	0.118808	0.8186388
HINT2	2.1426426	2.03897548	2.1875777	1.143027665	0.1928603	0.118909	0.8011067
MAP4K5	1.5941477	1.50495297	1.6328096	1.253205131	0.3256226	0.118999	0.8011067
CHN1	1.6086803	1.51919373	1.6474688	1.247065839	0.3185376	0.119077	0.8011067

UBXN7	1.7393106	1.83335817	1.6985452	0.838229224	-0.2545833	0.119147	0.8561587
NBPF19	1.2357234	1.31475835	1.2014652	0.640063234	-0.6437137	0.11949	0.7600863
RHOF	1.3416401	1.26007247	1.376996	1.449580627	0.5356356	0.119547	0.7652516
MKLN1	1.662816	1.75362616	1.6234539	0.827272049	-0.2735663	0.119568	0.8011067
CNPPD1	1.3276771	1.24647165	1.362876	1.472282921	0.5580549	0.119704	0.7600863
FRRS1L	1.2415476	1.32042604	1.2073573	0.647129926	-0.6278727	0.119807	0.7580248
CHCHD1	2.4834092	2.36380994	2.5352501	1.125706813	0.1708311	0.119863	0.7150734
C6orf203	1.40187	1.31891261	1.4378283	1.372878518	0.457204	0.119953	0.7939007
NEMP1	1.2081147	1.28617224	1.1742802	0.609004502	-0.7154752	0.119976	0.7600863
SORBS2	1.6113537	1.70097426	1.5725071	0.816730591	-0.2920678	0.120017	0.7883486
ERGIC2	2.1041104	2.00263933	2.1480935	1.145071329	0.1954375	0.120095	0.7695237
WHRN	1.2507589	1.17195595	1.2849165	1.656915463	0.7285	0.120237	0.7150734
UBE2Z	1.5181731	1.43194698	1.5555483	1.286149339	0.3630582	0.120296	0.8824619
NDRG2	1.1619763	1.08602787	1.1948965	2.265504105	1.1798321	0.120503	0.8081706
MFSD4B	1.2753423	1.35525094	1.2407054	0.677564425	-0.56157	0.120538	0.7866298
MID1	2.3219821	2.44170856	2.270086	0.88095893	-0.1828533	0.120837	0.7150734
KRCC1	1.938755	1.8390365	1.9819785	1.170364428	0.2269578	0.120888	0.8625088
FABP4	1.0978634	1.02438265	1.1297141	5.319934302	2.4114084	0.120892	0.7889368
TRMT13	1.3949344	1.47872936	1.3586131	0.749093549	-0.4167822	0.121072	0.8285627
CCDC189	1.1919709	1.26927209	1.1584643	0.588491342	-0.7649069	0.121116	0.7600863
RRP9	1.2167638	1.13964565	1.2501911	1.79161376	0.8412597	0.121283	0.771455
UFSP2	1.685678	1.59444495	1.7252235	1.220001129	0.2868825	0.121349	0.772638
RPP25	1.2029428	1.12575708	1.2363993	1.879809126	0.9105862	0.121484	0.7600863
MRPS12	2.0875613	1.98493207	2.1320465	1.149365084	0.2008371	0.121657	0.7600863
KHNYN	1.2947144	1.37537346	1.2597523	0.691983746	-0.5311899	0.121667	0.7600863
TMEM208	2.1408591	2.03769826	2.1855747	1.142504322	0.1921996	0.121742	0.8004757
SRSF11	3.680924	3.48688482	3.7650314	1.111845384	0.1529562	0.121874	0.8119554
RDH14	1.5194148	1.43378372	1.5565321	1.282971434	0.359489	0.121882	0.7809762
HK2	1.7724593	1.67036478	1.8167127	1.218310824	0.2848823	0.121885	0.7600863
IQGAP1	1.6239731	1.53488547	1.6625887	1.238748659	0.3088835	0.122212	0.8645365
PLA2G16	2.0684086	1.96524913	2.1131237	1.153198328	0.2056407	0.122264	0.7927438
SMC3	2.5865927	2.45771237	2.6424565	1.126735669	0.1721491	0.1224	0.7600863
PIP5K1A	1.4142176	1.49780394	1.3779867	0.759308326	-0.3972423	0.122891	0.8645365
HERC4	1.6132205	1.70210355	1.5746937	0.818531213	-0.2888907	0.122894	0.7824792
NAA50	1.8153133	1.72101989	1.8561853	1.187464251	0.2478841	0.122935	0.780953
CRISPLD1	1.3970357	1.31452035	1.4328024	1.37607116	0.4605551	0.122952	0.7771474
GOSR2	1.4835344	1.39836783	1.5204503	1.306456553	0.3856591	0.123197	0.9756442
NOC4L	1.2010571	1.12468692	1.2341602	1.877985324	0.9091858	0.12342	0.8050754
PTPRO	1.1482226	1.22345252	1.1156138	0.517397706	-0.9506544	0.123617	0.7932864
C3orf67	1.2324725	1.31031072	1.1987331	0.640432573	-0.6428814	0.123659	0.780953
MYO19	1.2426733	1.32105903	1.2086966	0.650025775	-0.6214312	0.123666	0.8018331
CHCHD7	1.8893507	1.79383354	1.9307532	1.172479045	0.2295621	0.123926	0.7932864
RP5-882C2.2	1.1355476	1.21069012	1.1029767	0.488759151	-1.0328044	0.123937	0.7841521
GEN1	1.1380588	1.21281197	1.1056567	0.496478903	-1.0101957	0.124042	0.8050754
GRID2	1.1142375	1.04040825	1.1462391	3.619040978	1.8556074	0.124138	0.9756442
OFD1	1.763799	1.85707398	1.7233685	0.843997691	-0.244689	0.124149	0.7643133
RP11-796E2.4	1.1969469	1.27413836	1.1634878	0.596369615	-0.7457213	0.124408	0.815222
PHF19	1.3716377	1.29030782	1.4068906	1.401583299	0.4870575	0.124663	0.815222
CELSR2	1.3076464	1.38787555	1.2728706	0.703500414	-0.5073768	0.124791	0.780953
DENND4B	1.1814471	1.2579031	1.1483068	0.575048596	-0.7982442	0.124824	0.7643133
GNAI1	1.3286333	1.24862349	1.363314	1.461301825	0.5472542	0.124862	0.7811172

MINPP1	1.2178846	1.1411437	1.2511484	1.779380571	0.8313751	0.124977	0.9101621
DCTPP1	2.2989287	2.19227069	2.3451602	1.128233857	0.1740661	0.125134	0.7811172
AKT3	1.5652273	1.65244158	1.5274238	0.808384682	-0.3068861	0.125145	0.815222
SLC16A1	2.2890679	2.18173707	2.335591	1.130193062	0.1765692	0.125196	0.8509936
C9orf142	2.5358589	2.65547969	2.4840086	0.896422088	-0.1577499	0.125241	0.7825601
EFNA4	1.73466	1.64337978	1.7742259	1.203373146	0.2670841	0.125253	0.7368687
PABPC1L	1.1927396	1.2692749	1.159565	0.592572832	-0.7549356	0.125511	0.7643133
PLCD3	1.3050121	1.22560977	1.3394294	1.504497989	0.5892822	0.125702	0.878171
AIFM1	1.3154598	1.23601	1.3498978	1.482554744	0.5680854	0.125703	0.8721185
RBM7	1.8290061	1.72985806	1.8719824	1.194728684	0.256683	0.125909	0.815222
MYCN	1.3300421	1.41046093	1.2951841	0.719152671	-0.47563	0.125991	0.780953
TMEM181	1.320977	1.40172439	1.2859766	0.711872742	-0.4903087	0.126119	0.7643133
C7orf73	4.4696874	4.24838418	4.5656125	1.097657265	0.1344277	0.126279	0.780953
RP11-214N9.1	1.1247877	1.19898736	1.0926254	0.465483966	-1.1031966	0.126462	0.8896585
BCLAF1	2.9246451	3.06261733	2.8648403	0.904113574	-0.1454241	0.126475	0.7846122
PSMG4	1.5227214	1.60917309	1.4852484	0.796569069	-0.3281286	0.126542	0.815222
RNF24	1.5943133	1.68201233	1.5562997	0.815673913	-0.2939356	0.126662	0.780953
CDH7	1.3772048	1.29610617	1.4123575	1.392600089	0.477781	0.126696	0.8124006
STAU2	1.6091411	1.52127417	1.6472275	1.241625852	0.3122305	0.126786	0.8124006
ZNF660	1.2961101	1.37578445	1.2615748	0.696076568	-0.5226821	0.126897	0.8035835
RBM23	2.1570945	2.25916995	2.1128494	0.883795999	-0.1782147	0.126978	0.7885192
MCC	1.1628544	1.08821652	1.1952066	2.21281254	1.1458812	0.127107	0.9071208
SPICE1	1.3210458	1.40112837	1.2863335	0.713820145	-0.4863675	0.127134	0.8035835
FADS1	3.0892227	2.95044435	3.149377	1.101993476	0.1401157	0.127141	0.9071208
EIF3F	8.0565689	7.71534119	8.2044759	1.072838405	0.1014328	0.127219	0.7895496
CADM2	1.6061678	1.69427353	1.5679779	0.818089509	-0.2896694	0.127286	0.7848012
RP11-1000B6.3	1.2054669	1.28203776	1.1722768	0.610828838	-0.7111599	0.127329	0.8005813
MKKS	2.3747825	2.25775019	2.4255108	1.133381525	0.1806336	0.12741	0.7895496
RP11-195F19.5	1.4934074	1.4091571	1.5299261	1.295165306	0.3731362	0.127444	0.7895496
TMEM30A	1.6521477	1.56378781	1.6904478	1.224658925	0.29238	0.127513	0.8214797
YES1	1.7554811	1.66418179	1.7950552	1.197044623	0.2594769	0.12786	0.7891246
TRIM24	1.4098685	1.49229403	1.3741407	0.759994339	-0.3959394	0.128667	0.8140835
MTA1	1.6451224	1.73423977	1.606494	0.826016261	-0.2757579	0.128701	0.8007487
EIF2B2	1.3845559	1.30384855	1.419539	1.380750429	0.4654526	0.128748	0.7755714
RRP1	1.2855241	1.20738826	1.3193925	1.540070316	0.6229962	0.128836	0.9000653
EIF5AL1	1.3050428	1.22640419	1.3391291	1.497892487	0.5829341	0.128876	1
LINC01503	1.2165558	1.14045926	1.2495402	1.776602307	0.8291208	0.129119	0.7895496
ACAT1	1.5253684	1.44032597	1.5622305	1.276850563	0.3525897	0.129211	0.7895496
ZBTB20	2.2698739	2.38481011	2.2200542	0.881026335	-0.182743	0.129236	0.7878522
CDC42SE1	1.5209802	1.43495964	1.5582663	1.283489879	0.3600719	0.129358	0.8733818
PINK1	1.309002	1.23021992	1.3431505	1.490533415	0.5758287	0.129449	0.8007487
NEFL	1.1120565	1.03757514	1.1443408	3.841392009	1.9416292	0.129519	0.8526886
TRIAP1	2.0869752	1.98671749	2.1304324	1.145649505	0.1961657	0.129616	0.7581002
MTX3	1.2913545	1.36982921	1.2573392	0.695832498	-0.523188	0.129635	0.82983
LECT1	1.1113308	1.03859143	1.14286	3.701858831	1.8882499	0.129802	0.8007487
GPR107	1.2279195	1.30476962	1.1946084	0.638542504	-0.6471454	0.12985	0.8267658
BOC	1.4662125	1.54968015	1.4300331	0.782333235	-0.3541448	0.129915	0.7969146
ECE2	1.1970582	1.12192136	1.2296266	1.883399529	0.9133391	0.130143	0.7878522
CDC16	1.5780909	1.49209346	1.6153669	1.250508274	0.3225146	0.130217	0.7895496
GRIA4	1.9037217	1.99922453	1.8623255	0.862994737	-0.2125763	0.130218	0.8666855
SEPHS1	1.4985632	1.41447019	1.5350137	1.290837665	0.3683076	0.130266	0.8526886

CENPJ	1.2380406	1.31482134	1.2047596	0.650399336	-0.6206023	0.130358	0.7895496
FBXW5	1.7973796	1.70587406	1.8370431	1.185825035	0.2458912	0.130381	0.8197706
NUDT9	1.297028	1.21905991	1.3308236	1.510197088	0.5947368	0.130399	0.7878522
PARP2	1.3415951	1.42149486	1.3069621	0.728270106	-0.4574545	0.130753	0.8272829
MARCH5	1.8872602	1.79321885	1.928023	1.169945693	0.2264416	0.130917	0.7878522
NUSAP1	1.9154438	1.80786817	1.9620731	1.190878779	0.2520266	0.130937	0.8526886
GPI	4.0851132	4.27263657	4.0038302	0.917862453	-0.1236501	0.13105	0.8335801
DLG4	1.3093959	1.38852483	1.275097	0.70805516	-0.4980663	0.131057	0.9475671
TSGA10	1.311637	1.39065799	1.2773849	0.710045427	-0.4940168	0.131135	0.8007487
NBPF15	1.1801007	1.2550392	1.1476182	0.578806094	-0.788848	0.131192	0.7628864
CEP57	2.221574	2.35021983	2.1658118	0.863423722	-0.2118594	0.131234	0.8400669
KMT2E	3.5280856	3.7006389	3.4532915	0.908411509	-0.1385821	0.131319	0.7895496
POLD3	1.336262	1.41585956	1.30176	0.72562967	-0.4626946	0.131336	0.8007487
CROT	1.3328421	1.25385429	1.3670798	1.446025565	0.5320931	0.131593	0.9322694
PCDH19	1.2304812	1.15486801	1.2632562	1.699874519	0.7654283	0.131736	1
LRRC39	1.1344196	1.06126145	1.1661304	2.711825812	1.4392645	0.131848	0.9147538
ZFYVE21	1.6394446	1.55249325	1.6771341	1.225597066	0.2934847	0.131853	0.7895496
SGTA	1.5411957	1.45674491	1.5778013	1.265041553	0.3391848	0.131966	0.9036005
ALAS1	1.2965249	1.21849545	1.3303472	1.511917804	0.5963797	0.132037	0.9036005
ALKBH3	1.1993503	1.12487215	1.2316333	1.854963798	0.891391	0.132071	0.8299637
TCEAL1	2.1562332	2.25754126	2.1123207	0.884520214	-0.177033	0.13214	0.8049025
INTU	1.5172479	1.60191941	1.4805466	0.798357051	-0.324894	0.132148	0.8049025
ELAVL3	1.1512962	1.07758314	1.1832475	2.361950269	1.2399786	0.132494	0.8526886
APPL2	1.2298757	1.15440921	1.262587	1.70059164	0.7660368	0.132709	0.911232
PLEKHB2	1.5751891	1.48957057	1.612301	1.250689868	0.3227241	0.132827	0.8299637
FBXO5	1.4681333	1.55135494	1.4320605	0.783634	-0.3517481	0.132872	0.9538279
FIP1L1	1.6122345	1.52626115	1.6495001	1.234178397	0.3035509	0.133002	0.8526886
CEP95	1.3854967	1.46617867	1.3505247	0.751910545	-0.4113671	0.133074	0.8526886
RP11-410L14.2	1.218788	1.29486634	1.1858114	0.630154717	-0.666222	0.133078	0.8925098
MRPL46	1.2946218	1.21721036	1.3281762	1.510868282	0.5953779	0.133116	0.911232
ACTR3B	1.684223	1.7731146	1.6456924	0.835183344	-0.2598352	0.133152	0.911232
VAV3	1.1282764	1.05597934	1.159614	2.85130212	1.5116209	0.133483	0.8081356
SMIM10L1	2.0487122	1.95046264	2.091299	1.148176682	0.1993447	0.13387	0.9379441
TMCC1-AS1	1.2567721	1.3334201	1.2235486	0.670471407	-0.5767523	0.133871	0.8008341
HTT	1.3017276	1.37988455	1.2678501	0.705082773	-0.5041355	0.133963	0.9544583
BNIP2	1.4736727	1.39139437	1.5093367	1.301338957	0.3799968	0.134075	0.8528217
TCAF1	2.5184992	2.63427931	2.4683137	0.89844719	-0.1544944	0.134183	0.8871961
KIN	1.3214345	1.24361323	1.3551665	1.457911377	0.543903	0.134217	0.7759922
PXN	1.4747393	1.39217703	1.5105264	1.301775264	0.3804804	0.134301	0.839171
PSIP1	4.4257189	4.19530243	4.5255942	1.103367921	0.1419139	0.134411	1
UBXN6	1.5025054	1.41942078	1.5385188	1.283958361	0.3605984	0.13442	0.9262097
CYB5B	2.0033802	1.90490659	2.0460642	1.155991315	0.2091306	0.134592	0.9148615
SRP9	10.653592	10.2084036	10.846561	1.06930168	0.0966689	0.134678	0.8161813
ARHGEF9	1.3061486	1.22864148	1.3397444	1.485926537	0.5713628	0.134929	0.8989281
PLXNB1	1.2804883	1.20361681	1.3138087	1.541172585	0.6240284	0.135042	0.8233457
MBIP	1.4410276	1.35971627	1.4762725	1.32402258	0.4049277	0.1351	0.9544583
NCOA1	1.2880578	1.36553571	1.2544746	0.69616883	-0.5224909	0.135228	0.9379441
NCOA4	1.5913724	1.50592439	1.6284104	1.242103327	0.3127852	0.135322	0.7823815
FDX2	1.4885366	1.40585709	1.5243745	1.292017666	0.3696258	0.135357	0.8145764
TRNT1	1.3401348	1.26169411	1.3741354	1.429666854	0.515679	0.13539	0.8407554
C21orf59	1.9659825	1.87085729	2.007215	1.156578728	0.2098635	0.135711	0.8070412

PDE4D	1.2694051	1.19290138	1.3025661	1.56850149	0.6493869	0.135808	0.9544583
MXD3	1.3443834	1.4236967	1.3100046	0.73166622	-0.4507424	0.135922	0.813911
B3GAT3	2.0888565	1.99045777	2.131508	1.142409083	0.1920794	0.136434	0.9379441
HPS1	1.8437793	1.75218584	1.883481	1.174551452	0.2321099	0.136475	0.7845389
STX3	1.4492184	1.36801131	1.4844181	1.316313015	0.3965026	0.136605	0.8973088
NOP16	1.2974319	1.22086004	1.3306224	1.496977076	0.5820521	0.136794	0.8494755
ETV5	1.3782137	1.29896679	1.4125637	1.379964935	0.4646316	0.136848	0.9550864
RASGEF1B	1.3761284	1.45484878	1.3420066	0.751912672	-0.411363	0.136908	0.8717031
FOXG1	1.2578219	1.33404034	1.2247846	0.672926532	-0.5714791	0.136958	0.9379441
MAD2L1BP	1.2738816	1.19793101	1.3068028	1.55004903	0.6323139	0.137003	1
PDCD11	1.4514599	1.53322313	1.4160191	0.780197066	-0.3580895	0.137028	0.8457072
ATP6V1A	1.4491497	1.36781404	1.4844052	1.316983876	0.3972377	0.137055	0.9379441
RP11-120I21.2	1.2415026	1.16604498	1.2742101	1.651420743	0.7237077	0.137138	0.8482341
SLC25A39	3.8336461	3.66148928	3.9082684	1.092722181	0.1279266	0.137638	0.791222
KLF5	1.1496409	1.07753107	1.1808972	2.333222298	1.2223238	0.13764	0.8482341
SDHD	1.6696876	1.58292363	1.707296	1.213359584	0.2790072	0.137664	0.8496338
ATP5EP2	1.3824412	1.3028255	1.4169511	1.376869125	0.4613914	0.137745	0.9116564
BRD4	1.7741945	1.68436016	1.8131337	1.188166317	0.2487368	0.137988	0.8482341
SPG7	1.737969	1.82720156	1.6992907	0.845369139	-0.2423466	0.138019	0.7883363
FAM107A	1.1166152	1.04536814	1.1474976	3.251126559	1.7009397	0.138073	0.932223
EZH1	1.3040323	1.38128995	1.2705446	0.709550716	-0.4950223	0.13831	0.8482341
TYMS	3.1772321	3.0205349	3.2451534	1.111167839	0.1520767	0.138328	0.9027259
BPHL	1.6626475	1.57662699	1.6999335	1.213841028	0.2795795	0.13848	1
C1RL	1.3305362	1.40873629	1.29664	0.725749012	-0.4624574	0.138567	0.9418833
COX20	3.4363041	3.26068701	3.5124263	1.111355192	0.15232	0.138634	0.8771419
COX11	1.8878126	1.79197524	1.9293539	1.17346329	0.2307727	0.13867	0.8222711
CAT	1.4867791	1.40500711	1.5222236	1.289418461	0.3667205	0.138858	0.9438622
SLC19A1	1.1733871	1.10062374	1.2049268	2.036565246	1.026138	0.138952	0.8222711
EPPK1	1.1284162	1.05723103	1.1592718	2.782962844	1.4766216	0.138973	0.8235444
DIMT1	1.3295278	1.25239596	1.362961	1.438061851	0.5241257	0.13898	0.8525227
SLU7	1.4145527	1.33467509	1.449176	1.342125682	0.4245198	0.139168	0.8235444
GGT7	1.258764	1.33525592	1.2256081	0.672942916	-0.571444	0.13926	0.8276018
DCPS	1.2449714	1.17018644	1.2773874	1.62990308	0.7047862	0.139308	0.8545386
EIF1AX	3.489951	3.32841081	3.5599715	1.099450104	0.1367821	0.139405	0.9452831
GTF2F1	1.8341422	1.74372829	1.8733326	1.174263003	0.2317556	0.139519	0.7883363
RAB8A	1.49704	1.41552333	1.5323738	1.281212829	0.3575101	0.139535	0.8482341
CUBN	1.1400206	1.06835021	1.1710865	2.503086894	1.3237084	0.139702	0.9351831
B4GALT4	1.4230624	1.34369745	1.4574636	1.331006742	0.4125179	0.139728	0.7883363
ANKRD49	1.4724978	1.55446053	1.4369707	0.788100574	-0.3435483	0.139987	0.9452831
CADM4	1.4898875	1.40874296	1.52506	1.284572685	0.3612885	0.139996	0.8798424
SEMA4C	1.2302089	1.15563826	1.2625319	1.686808469	0.7542962	0.140007	0.9452831
NEDD9	1.2971296	1.37399315	1.2638126	0.705394298	-0.5034982	0.140074	0.8798424
LSM7	5.654382	5.89725719	5.5491065	0.928909038	-0.1063908	0.140148	0.8482341
CSRNP2	1.1731532	1.10059699	1.2046031	2.033889064	1.024241	0.140271	0.7883363
WBP11	2.3080458	2.20294634	2.3536018	1.125238705	0.1702311	0.1404	0.9231522
LTV1	1.1972525	1.12414167	1.2289428	1.844206229	0.883	0.140536	0.9698966
SDHA	1.4343652	1.35477194	1.4688654	1.321596501	0.4022818	0.140552	0.8807959
C4orf3	10.701702	10.2964767	10.877349	1.062483026	0.0874398	0.140791	0.8482341
SRR	1.2119145	1.1384645	1.2437518	1.760392288	0.815897	0.1409	0.9698966
KLHL20	1.9505331	2.04586297	1.9092119	0.869341343	-0.2020053	0.14095	0.8482341
SASS6	1.2368534	1.31134311	1.2045654	0.657041828	-0.6059429	0.140962	0.8807959

HARS	1.4919921	1.41107326	1.5270668	1.282172509	0.3585904	0.141006	0.9142653
THUMPD3	1.5854515	1.5016168	1.6217901	1.239571865	0.3098419	0.141007	1
DENND1A	1.2632649	1.33890958	1.2304762	0.680052294	-0.5562824	0.141056	0.8586401
RP11-722G7.1	1.3430249	1.42101304	1.3092205	0.734467813	-0.4452288	0.141127	0.8276018
C6orf106	1.2809173	1.20536067	1.3136677	1.527399115	0.6110771	0.141137	0.8276018
FAM208A	1.864148	1.95621417	1.8242414	0.861984041	-0.2142669	0.141184	0.9351831
SETX	1.4350172	1.51559619	1.4000898	0.775975134	-0.3659177	0.141319	0.8540337
CREB3L1	1.1551097	1.08336744	1.1862067	2.233566506	1.1593492	0.141357	0.8316065
DYNC2LI1	1.6017166	1.51742176	1.6382546	1.23352878	0.3027914	0.141372	0.9481378
KAT2A	1.411164	1.49073864	1.376672	0.767561329	-0.3816461	0.141412	0.8518577
SGO1	1.4286879	1.50871304	1.3940005	0.774504462	-0.3686545	0.141516	0.8276018
FURIN	1.2601419	1.18527013	1.2925954	1.579290948	0.659277	0.141639	0.8276018
IFNGR2	2.3317027	2.22645931	2.377321	1.123005868	0.1673655	0.141684	0.8586401
MAGOHB	1.5434757	1.46125785	1.5791135	1.255509154	0.3282725	0.141773	0.8540337
ECHDC2	1.3019819	1.22577471	1.3350143	1.483843374	0.5693388	0.141974	0.9274421
FAHD2B	1.3726452	1.29463964	1.4064571	1.379505834	0.4641516	0.142054	0.9351831
SFXN1	2.3491557	2.45753717	2.3021771	0.893409181	-0.162607	0.14223	0.9351831
CNOT9	1.4945584	1.57607699	1.4592237	0.797156899	-0.3270644	0.14232	0.8513774
SNHG25	1.7200235	1.80734207	1.6821748	0.844963773	-0.2430386	0.14269	0.8435483
MSX2	1.0996349	1.02990769	1.1298586	4.341978768	2.1183527	0.142911	0.8579255
STMN3	1.8461392	1.93655805	1.8069467	0.86160883	-0.2148951	0.143043	0.856557
SLC25A36	2.8545228	2.99234432	2.7947833	0.900839931	-0.1506573	0.143076	0.8417238
NRL	1.1344091	1.06348642	1.1651509	2.601357394	1.3792646	0.143105	0.7998004
NDUFAF2	2.0776516	1.977287	2.1211552	1.147211856	0.1981318	0.143114	0.8887174
POLR2J	3.7431533	3.53828771	3.8319534	1.115694399	0.1579419	0.143135	0.8889978
GPRC5A	1.1200851	1.04954002	1.1506632	3.04124296	1.6046611	0.143224	0.967002
DENND4A	1.3399477	1.41761126	1.3062841	0.733419065	-0.4472903	0.143301	0.8889978
GPR82	1.1436293	1.21552828	1.1124643	0.521807783	-0.9384096	0.143323	1
RAB30-AS1	1.6548728	1.74022728	1.6178754	0.83471039	-0.2606524	0.143419	0.8579255
LINC00954	1.2688257	1.34380242	1.2363266	0.6873907	-0.5407978	0.143523	0.8579255
ADGRV1	1.3574632	1.43480573	1.3239387	0.745019341	-0.4246502	0.143528	0.8579255
TMEM8A	1.2568439	1.1824632	1.2890846	1.584344892	0.6638864	0.143699	0.9702106
DOLK	1.168215	1.09640113	1.1993432	2.067850684	1.048132	0.144049	0.9349262
CIAO1	1.4823978	1.40197994	1.5172554	1.286769216	0.3637533	0.144051	0.8574899
LENG8	2.0014662	2.09590142	1.9605327	0.876477263	-0.1902114	0.144204	0.8592203
CPVL	1.1161222	1.0462327	1.1464162	3.16694087	1.6630899	0.144241	0.8436438
EMC9	1.7689981	1.68136934	1.8069812	1.184352138	0.2440981	0.144342	0.8592203
TRIM9	1.2243603	1.29846068	1.192241	0.644108403	-0.6346246	0.144562	0.8596759
SERP1	3.5972303	3.43271283	3.6685412	1.096940509	0.1334853	0.14458	0.9283228
DYNC2H1	1.5296061	1.61173505	1.4940069	0.807550443	-0.3083757	0.144611	0.8925081
MYL6B	6.5774763	6.33257579	6.6836297	1.065831964	0.09198	0.144749	0.8579255
FARP1	2.0402479	1.94392506	2.0819996	1.146277028	0.1969558	0.144753	0.9896401
YIF1B	1.7161521	1.6296296	1.7536557	1.196982675	0.2594023	0.144942	0.847744
TMEM131	1.252086	1.32654679	1.2198105	0.673136418	-0.5710292	0.144977	0.8579255
LCMT1	2.1123831	2.01426274	2.1549138	1.138673243	0.1873538	0.145228	0.9124713
RNF41	1.4264908	1.34726654	1.460831	1.327023866	0.4081943	0.14551	0.8610787
C18orf54	1.1580628	1.22968589	1.1270174	0.553004761	-0.8546362	0.145539	0.8612479
STOX2	1.4191345	1.49861291	1.3846842	0.77150875	-0.3742456	0.145575	0.845679
FAM183A	1.2203534	1.29596893	1.1875774	0.633773832	-0.65796	0.145748	0.8565913
SETD3	1.3531565	1.27668284	1.3863044	1.396199483	0.4815051	0.145847	0.978144
COL4A3BP	1.5145225	1.43351616	1.5496352	1.26785394	0.3423886	0.146144	0.9283228

ANAPC10	1.4195844	1.34102023	1.4536384	1.330238961	0.4116854	0.146346	0.9124713
AURKB	1.5957834	1.68222162	1.5583162	0.818379581	-0.2891579	0.146432	0.9028483
CCDC106	1.6045362	1.52095118	1.6407666	1.229993627	0.2986508	0.146522	0.8826391
CAMK2G	1.2456512	1.17194816	1.2775981	1.614429305	0.6910243	0.146525	0.811883
HDAC10	1.283158	1.35844573	1.2505241	0.69891776	-0.5168054	0.14657	0.8631989
TMOD3	1.6548575	1.57022554	1.6915416	1.212751094	0.2782835	0.146649	0.9283228
GPER1	1.0920692	1.16142294	1.0620075	0.384130639	-1.3803311	0.146672	0.9124713
ZNF618	1.3743615	1.45234995	1.3405569	0.752861622	-0.4095434	0.146862	0.9555977
AMIGO2	1.1412748	1.21263803	1.110342	0.518919516	-0.9464173	0.146942	0.8631989
MBP	1.1972306	1.1251483	1.228475	1.825634205	0.8683977	0.146955	0.9283228
ISY1	1.3508949	1.27458334	1.3839726	1.398382685	0.4837592	0.147234	0.9555977
CYB5D2	1.5163701	1.42636228	1.5553845	1.302611655	0.381407	0.14731	0.8566111
GSTZ1	1.3043047	1.2292842	1.3368228	1.469018696	0.5548528	0.147404	0.811883
PCGF6	1.3443019	1.42102315	1.3110467	0.738787536	-0.4367686	0.147446	0.8674336
DDX10	1.3280168	1.25207675	1.3609334	1.431839166	0.5178694	0.147732	0.8631989
CRYZL1	1.9976182	2.09196569	1.9567228	0.876147289	-0.1907547	0.147931	0.9124713
PLCXD1	1.4946114	1.57535301	1.4596135	0.798837377	-0.3240263	0.147993	0.9124713
PHF14	3.5254026	3.68196055	3.4575417	0.916322829	-0.1260721	0.14801	0.8460124
TMTC4	1.6670426	1.75186493	1.6302759	0.838283473	-0.2544899	0.148211	0.9056929
SKA1	1.1247516	1.19479089	1.0943926	0.484584388	-1.0451802	0.148241	0.811883
RFWD3	1.2429768	1.31624069	1.2112201	0.667909432	-0.5822756	0.14842	0.9283228
HDAC1	1.4435721	1.36505829	1.4776043	1.308296017	0.387689	0.14843	0.9888803
EML1	1.8175569	1.90544821	1.7794599	0.860855285	-0.2161574	0.148442	1
SVIL	1.5120329	1.43187665	1.5467771	1.266049236	0.3403335	0.148548	0.9056929
NME7	1.6873694	1.6025527	1.7241337	1.20177647	0.2651686	0.148619	0.9863613
NTMT1	1.7982076	1.71035938	1.8362859	1.177271599	0.2354472	0.148687	1
KLF10	1.45028	1.37183459	1.4842825	1.302413909	0.381188	0.148705	0.858899
SCPEP1	1.4824662	1.40248733	1.5171335	1.28484422	0.3615935	0.148716	0.9283228
RP11-37B2.1	1.1749753	1.24657633	1.1439394	0.583752047	-0.7765724	0.148929	0.811883
DPYSL5	1.4201	1.49821187	1.386242	0.775256563	-0.3672543	0.148967	0.8920298
PCM1	3.1631943	3.306135	3.1012359	0.911150422	-0.1342388	0.149005	0.9608907
SHC1	2.2055224	2.30455079	2.1625979	0.891186419	-0.1662008	0.149033	0.9863613
SCYL2	1.4158246	1.33812093	1.4495056	1.329422738	0.4107999	0.149097	0.8720429
HEXIM1	1.3973685	1.31996071	1.4309213	1.346794323	0.4295295	0.149443	1
BSDC1	1.7779911	1.86529557	1.7401485	0.855370677	-0.2253783	0.149635	1
ZMYM5	1.6099448	1.69304577	1.5739242	0.828118792	-0.2720904	0.149972	0.8720924
PICALM	1.4465554	1.36816973	1.4805321	1.305191874	0.3842619	0.150013	0.9863629
PGGHG	1.2851598	1.35962388	1.2528829	0.703187188	-0.5080193	0.150031	0.9929657
AC069513.4	1.1727034	1.24384858	1.1418651	0.581775551	-0.7814654	0.150037	0.811883
PPP4R2	2.3388707	2.23126423	2.3855133	1.125276982	0.1702802	0.150096	0.9193938
MTFR1	1.8824628	1.79336087	1.9210846	1.160990649	0.2153564	0.150105	0.8488445
ISLR2	1.1335903	1.20348578	1.1032937	0.507621305	-0.9781755	0.15011	0.811883
SLC5A3	1.9562649	2.05371479	1.9140247	0.867430831	-0.2051794	0.150158	0.8759823
GLRX2	1.2141542	1.14220359	1.2453416	1.725283945	0.7868338	0.150181	0.8590583
MAN1B1	1.3876245	1.31064907	1.4209899	1.355194329	0.4384997	0.150193	0.8488445
WIPI1	1.2792115	1.20518302	1.3112995	1.517179826	0.6013921	0.150442	0.8590583
PLEKHM3	1.1740166	1.24509352	1.143208	0.584299215	-0.7752207	0.150521	0.9337854
DDT	3.8059831	3.63499269	3.8800999	1.093020069	0.1283199	0.150659	0.8752183
TENM4	1.2422972	1.31559644	1.2105253	0.667071261	-0.5840872	0.150705	1
RP11-496I9.1	1.1808673	1.25221087	1.149943	0.594514455	-0.7502162	0.150895	0.8774583
PTPN11	1.6762922	1.59190878	1.7128687	1.20435568	0.2682615	0.151022	0.9193938

FLJ46066	1.0713464	1.13931905	1.0418832	0.30062823	-1.7339476	0.151052	0.8759823
FTX	1.3331803	1.40911589	1.3002656	0.73393765	-0.4462706	0.151225	0.9214093
TIMELESS	1.4438887	1.52291823	1.4096329	0.783359442	-0.3522537	0.151276	0.9214093
TMEM107	2.0831843	2.1835751	2.0396694	0.878414374	-0.1870264	0.15134	0.8749875
NIPA2	1.5432667	1.46228051	1.5783706	1.251124864	0.3232258	0.15143	0.9956779
DENND6B	1.2044454	1.27656092	1.1731865	0.626214594	-0.675271	0.151555	0.8517574
RP11-488L18.10	1.1740056	1.24507084	1.143202	0.584329105	-0.7751469	0.151663	0.8759823
SLC7A5	1.1974165	1.12597172	1.2283846	1.812983166	0.8583655	0.151681	0.9192214
TMEM141	2.5584972	2.44696737	2.6068404	1.110488373	0.1511943	0.152481	0.9934024
ARMCX2	1.5238477	1.44416087	1.5583884	1.257176126	0.3301868	0.152496	0.8807977
PSMG2	2.5062537	2.39088895	2.5562592	1.118895383	0.1620752	0.152596	0.9193938
RGP1	1.5317462	1.61227831	1.4968392	0.811459725	-0.3014086	0.152775	0.9193938
RP11-527L4.2	1.1302607	1.19957466	1.1002161	0.502148544	-0.9938139	0.152818	0.935796
MS4A6A	1.1117971	1.04201182	1.1420459	3.381093539	1.7574899	0.153017	0.9760282
EFHD2	2.1301927	2.22939478	2.087193	0.884331881	-0.1773402	0.153084	0.8687954
BMP1	1.2186325	1.14704389	1.249663	1.697880719	0.7637351	0.153189	1
PIR	1.627874	1.54538178	1.6636307	1.216818625	0.2831141	0.153192	0.9934024
TMEM245	1.7504973	1.83696273	1.7130183	0.851911688	-0.2312242	0.153293	0.9760282
LDLRAD4	1.2129475	1.28511414	1.1816665	0.63717112	-0.6502472	0.153315	0.8558902
TMEM183A	2.50008	2.38801168	2.5486567	1.115737491	0.1579976	0.153469	0.896401
GNPDA2	1.3429595	1.26823412	1.3753496	1.39933559	0.484742	0.153631	0.935796
ADAR	1.9377081	2.02817949	1.8984928	0.873867663	-0.1945133	0.153638	0.935796
TSNAX	1.4982497	1.41917759	1.532524	1.270401907	0.345285	0.153645	0.8799474
CDS1	1.1936521	1.1227021	1.2244058	1.828866529	0.8709498	0.153703	0.9911847
CNOT10	1.4566369	1.53514074	1.4226091	0.789715713	-0.3405947	0.153712	0.8836217
CYHR1	2.425136	2.53259315	2.3785581	0.899493831	-0.1528147	0.153779	0.819107
ZNF331	1.2146013	1.28643224	1.1834658	0.640520925	-0.6426824	0.153851	0.819107
SPHK2	1.3117755	1.38633796	1.279456	0.723345859	-0.4672425	0.153876	0.8687954
TMEM161A	1.2911268	1.21778683	1.3229164	1.482717518	0.5682438	0.153888	0.819107
IMPDH2	6.1919818	6.43516407	6.0865732	0.935863779	-0.0956295	0.153992	0.8558902
MRPS16	2.6703108	2.55131892	2.7218885	1.109951332	0.1504964	0.154026	0.896401
GPC6	2.6984486	2.82255989	2.6446519	0.902385671	-0.1481839	0.154047	0.9934024
GPN1	1.4146601	1.33780269	1.4479743	1.32614193	0.4072352	0.154312	0.892174
SLC22A18	1.2437613	1.1716991	1.274997	1.601621713	0.6795334	0.154383	0.9299965
RAB9B	1.2520744	1.32498456	1.2204711	0.678404794	-0.5597817	0.154928	0.896401
APOA1	1.1763618	1.24688716	1.1457922	0.590521778	-0.7599378	0.154997	0.923592
SPTSSA	2.3531823	2.24869092	2.3984746	1.1199526	0.1634377	0.155044	0.896401
SLC24A5	1.1254097	1.05633759	1.1553494	2.757473975	1.4633473	0.155057	0.9800793
PPM1L	1.2218859	1.29393036	1.1906578	0.648649555	-0.6244888	0.155073	0.8571642
CRY1	1.1858619	1.11563318	1.216303	1.870595895	0.9034979	0.15531	0.923592
UHRF1	1.2799003	1.35328772	1.2480901	0.702232438	-0.5099795	0.155388	0.8799474
TOPORS	1.4402131	1.51851643	1.4062722	0.783528043	-0.3519432	0.155588	0.9957295
CKS1B	4.3735489	4.59471551	4.277683	0.911805944	-0.1332013	0.155645	0.8240993
CLASP2	1.4767475	1.55524138	1.4427239	0.797353999	-0.3267077	0.15567	0.8897984
MLXIP	1.2430264	1.17143016	1.2740602	1.598669502	0.6768717	0.155681	0.8740741
TLE3	1.4157718	1.49252944	1.3825008	0.776604913	-0.3647473	0.155877	0.9800793
VPS41	1.3828025	1.30668765	1.4157949	1.355760129	0.4391019	0.156302	0.903679
SH3RF1	1.3774288	1.30176515	1.4102256	1.359420043	0.4429913	0.156352	0.8799474
MARCH7	1.8435923	1.75628462	1.8814363	1.165482294	0.2209271	0.156387	0.9957295
MRS2	1.6858203	1.60206323	1.7221253	1.199417679	0.2623341	0.156493	0.9473588
KIAA1551	1.2773302	1.35061325	1.2455652	0.700387674	-0.5137744	0.156504	0.8799474

PGM2	1.2297063	1.15831665	1.2606505	1.6463871	0.7193036	0.156847	1
TRADD	1.2096964	1.13858743	1.240519	1.735503729	0.7953545	0.156934	0.9501126
TRIM45	1.1733198	1.24338395	1.1429502	0.587344228	-0.7677218	0.157065	0.9405228
LPAR4	1.288835	1.36180976	1.2572037	0.710881055	-0.4923199	0.157106	0.8799474
SUPT6H	1.3158015	1.39001071	1.2836352	0.727249672	-0.4594774	0.157149	1
GGA1	1.8005673	1.88749681	1.7628872	0.859594349	-0.2182721	0.157224	0.9359039
NPDC1	2.6257466	2.51342973	2.6744309	1.106381686	0.1458492	0.157422	0.9473588
DNAH14	1.6414546	1.7240166	1.6056677	0.836538357	-0.2574964	0.157566	0.9271984
AP3M2	1.3998115	1.47621448	1.3666942	0.770019027	-0.377034	0.157603	0.8799474
SNAP29	1.5754437	1.49542018	1.6101304	1.231541213	0.3004649	0.157762	0.9271984
KLHL13	1.1953285	1.1247905	1.2259036	1.810262444	0.8561989	0.15781	0.8811037
THAP4	1.3213284	1.24727355	1.3534279	1.429299257	0.515308	0.158128	1
RP11-128M1.1	1.1402898	1.20899829	1.1105077	0.52874915	-0.9193447	0.158487	0.8307389
ONECUT2	1.1283435	1.05983012	1.158041	2.641495965	1.4013552	0.158556	1
BAG2	1.3862041	1.31079126	1.4188922	1.347824964	0.4306332	0.158593	0.9487285
ELFN1	1.3761965	1.45179672	1.3434272	0.76013654	-0.3956695	0.158732	0.8725928
ST6GALNAC3	1.28173	1.20922526	1.3131576	1.496748695	0.581832	0.158801	1
RAB4B	1.2066026	1.13630635	1.2370729	1.739265457	0.7984781	0.158921	0.8824058
ECHDC1	1.9912292	1.90121118	2.030248	1.143181577	0.1930546	0.159198	0.8307389
MTERF3	1.248895	1.17767102	1.2797674	1.574637434	0.6550197	0.159286	0.9027314
C19orf24	2.0776205	1.98624324	2.1172285	1.132812306	0.1799088	0.159291	0.884462
GLIPR1	1.1494662	1.08088526	1.179193	2.215397372	1.1475655	0.159297	1
CENPO	1.1127186	1.18072634	1.0832403	0.460587572	-1.1184526	0.159376	0.8307389
TMEM167B	1.4254842	1.34898146	1.4586447	1.314237927	0.3942265	0.159413	0.9489334
RP11-51J9.5	1.2754361	1.34822659	1.2438847	0.700362038	-0.5138272	0.159556	1
RALB	1.4024805	1.32684	1.4352673	1.331744347	0.4133172	0.159608	0.9027314
MAP7D3	1.5164312	1.43766557	1.5505726	1.257975498	0.3311038	0.159725	0.9980359
KRBA2	1.13778	1.20670599	1.1079037	0.522015102	-0.9378366	0.159866	0.9027314
RTKN	1.3503209	1.27636934	1.3823756	1.383567348	0.4683929	0.15987	1
DET1	1.2534405	1.32507096	1.222392	0.684133551	-0.5476501	0.159871	1
AASS	1.2522033	1.32447296	1.2208777	0.680727484	-0.5548507	0.159887	0.9027314
ZNF682	1.1645568	1.23381149	1.1345379	0.575411879	-0.7973331	0.159935	1
EPHB2	1.5747776	1.65468943	1.5401393	0.825031409	-0.2774791	0.159998	0.9322354
TBL1X	1.5724931	1.65316178	1.5375268	0.822961264	-0.2811036	0.160005	0.9197976
GPR162	1.5899308	1.67033687	1.5550783	0.828058797	-0.2721949	0.160091	0.9452583
JAM3	1.5718835	1.49228474	1.606386	1.231778911	0.3007433	0.160474	0.9322354
LXN	1.1767967	1.10801911	1.2066087	1.912705504	0.9356148	0.160542	0.8865085
RP11-841O20.2	1.4635222	1.38633881	1.4969778	1.28637809	0.3633147	0.160732	0.9135399
INA	1.1402241	1.07173647	1.1699104	2.368536043	1.2439956	0.160903	1
FAM172A	1.7549017	1.84002936	1.7180027	0.854735223	-0.2264505	0.160979	0.9996593
PCDH9	4.1999582	3.96303481	4.3026539	1.114618648	0.1565502	0.161016	1
RAP1GDS1	1.3963188	1.3213274	1.4288242	1.334539897	0.4163424	0.161124	0.9209694
SMNDC1	1.5201285	1.44151823	1.5542026	1.255220131	0.3279404	0.16115	1
DNAJB14	1.6922978	1.60961347	1.7281378	1.194425323	0.2563167	0.161246	0.9703754
RNF121	1.2153426	1.14516614	1.245761	1.692963835	0.7595512	0.161263	0.9589909
CDH11	1.587815	1.6689935	1.5526277	0.826058358	-0.2756844	0.161404	0.9513119
MMP24-AS1	3.2019969	3.33724764	3.1433717	0.91704947	-0.1249285	0.161661	0.9452583
COQ4	2.0200513	2.1114643	1.9804278	0.882104664	-0.1809782	0.161751	0.9298355
ABCC5	1.272199	1.20031948	1.3033555	1.514358548	0.5987068	0.161776	0.9299783
NFATC2IP	1.4691392	1.54639786	1.435651	0.797314619	-0.326779	0.162076	0.8404554
UTP23	1.3486221	1.27508949	1.3804953	1.383169068	0.4679775	0.162156	0.9513119

THTPA	1.4551416	1.37837266	1.4884176	1.29083742	0.3683073	0.1623	1
APPL1	2.2212146	2.12212156	2.2641671	1.126586559	0.1719582	0.16247	1
IGF2BP3	1.7934802	1.70907054	1.830068	1.170642352	0.2273004	0.16265	0.9513119
CAND1	2.3373935	2.43969221	2.2930516	0.898144478	-0.1549806	0.162874	1
PLK4	1.1242186	1.1920435	1.0948195	0.493739486	-1.0181781	0.163092	1
FBXO18	1.3068495	1.23454345	1.3381909	1.441911717	0.5279828	0.163174	1
PDE4DIP	1.3459208	1.27245266	1.377766	1.386538176	0.4714873	0.163187	1
AGBL5	1.3471833	1.27345701	1.3791403	1.386471479	0.4714179	0.163201	0.8922821
GNB4	1.500769	1.4233862	1.534311	1.261994261	0.3357053	0.163234	0.8421207
NOG	1.1020332	1.03521621	1.1309954	3.719745772	1.895204	0.16352	1
TMEM218	1.5202208	1.59843951	1.4863165	0.812641031	-0.2993099	0.163564	0.9452583
NAE1	2.5168697	2.40937277	2.5634648	1.10933378	0.1496935	0.16363	1
AARS	1.2995752	1.22765778	1.3307482	1.452830734	0.5388666	0.163853	0.901699
ZFAND1	1.5625259	1.64197088	1.52809	0.822607374	-0.2817241	0.163943	1
AC007246.3	1.4790315	1.55638752	1.4455012	0.800703034	-0.3206608	0.16396	0.9371811
REPS1	1.2862014	1.21425689	1.3173862	1.481334888	0.5668978	0.16403	1
LARP7	1.9918703	1.8984794	2.0323511	1.148998015	0.2003763	0.164206	0.928041
MT1F	1.2102024	1.13794766	1.2415216	1.750820713	0.8080314	0.164438	1
AMMECR1	1.309402	1.38178882	1.2780255	0.72821806	-0.4575576	0.164557	0.901699
REEP4	1.3057956	1.23401194	1.3369106	1.43971554	0.5257838	0.164635	1
FAM32A	3.1649507	3.02894061	3.223905	1.096091697	0.1323685	0.165071	0.8976283
POLR2I	4.8794165	5.10034506	4.7836539	0.922764747	-0.1159652	0.165087	0.901699
CDC25B	1.5866395	1.50689114	1.6212068	1.225523102	0.2933977	0.165183	0.9388351
TBRG1	1.5213051	1.44368381	1.5549505	1.250779147	0.3228271	0.165198	0.9284308
ADGRB2	1.1919583	1.26141665	1.1618512	0.619131083	-0.6916832	0.165265	0.9533165
CMPK1	2.567274	2.45753985	2.6148389	1.107920954	0.147855	0.165334	0.9452583
NAA60	1.4712224	1.39470026	1.5043913	1.277909721	0.3537859	0.165414	1
NFIA	1.1987434	1.1297837	1.2286344	1.761656881	0.816933	0.165432	0.9732447
ZCCHC7	1.8038326	1.88796521	1.7673649	0.864183479	-0.2105904	0.165612	0.9027933
RTN1	1.2027844	1.12807134	1.2351692	1.836235904	0.8767514	0.165717	0.9027933
NTRK2	1.580653	1.50071876	1.615301	1.228835432	0.2972917	0.16623	0.8532012
NRBF2	1.5567206	1.4786224	1.5905728	1.233901197	0.3032269	0.166488	1
TOLLIP	1.4989114	1.42212511	1.5321948	1.260751418	0.3342838	0.16657	1
UBE3A	2.2967082	2.19961728	2.3387927	1.116016539	0.1583584	0.16661	1
RP11-544I20.2	1.0869568	1.15282551	1.0584056	0.382171788	-1.3877068	0.16674	0.9533165
CDK10	1.4237646	1.49878748	1.3912456	0.784393341	-0.3503508	0.166778	0.9425794
CHPT1	1.47193	1.39576956	1.5049421	1.275848724	0.3514573	0.166851	0.9452583
RRNAD1	1.3191852	1.39149877	1.2878405	0.735227035	-0.4437383	0.16691	0.9027933
SFXN3	1.4114687	1.33704676	1.4437273	1.316515682	0.3967247	0.166921	0.9973805
AGL	1.2941884	1.36603769	1.263045	0.718628188	-0.4766826	0.167084	0.9027933
MAFB	1.4313096	1.35234243	1.4655384	1.321266871	0.4019219	0.167411	0.949257
KIF15	1.1616099	1.22956654	1.1321538	0.575666611	-0.7966946	0.167533	0.9533165
NUP37	1.4950017	1.41856726	1.5281327	1.261762959	0.3354409	0.167667	0.9452583
SLC2A4	1.1177169	1.18477344	1.0886508	0.479781123	-1.0595517	0.167772	1
AIG1	1.7918419	1.70837314	1.8280219	1.168906436	0.2251595	0.167781	1
ERC1	1.3833281	1.30967218	1.4152547	1.340949238	0.4232546	0.167898	0.9533165
TAF15	1.7474508	1.66489282	1.783236	1.177988394	0.2363253	0.167904	0.9926108
NCSTN	1.3832699	1.30985849	1.4150904	1.339612959	0.4218162	0.16796	0.949257
MRPL45	1.8552254	1.77016302	1.8920962	1.158321263	0.2120354	0.168121	0.9452583
ANAPC7	1.2661144	1.19548276	1.2967301	1.517934673	0.6021097	0.168177	1
RP5-827C21.4	1.0974364	1.16354008	1.0687835	0.420590876	-1.2495105	0.168183	0.9399542

PRMT2	3.7312834	3.54104098	3.813745	1.10731981	0.147072	0.168377	0.9973805
UTP18	1.4323012	1.35740874	1.4647638	1.300370478	0.3789227	0.16844	1
ACSL3	1.4710925	1.39524205	1.5039703	1.27509293	0.3506024	0.168651	0.9587635
SLC25A38	1.427084	1.35253304	1.4593985	1.303136091	0.3819878	0.168689	0.9587635
FXYD6	3.0805108	3.2423705	3.0103519	0.896529754	-0.1575766	0.168711	0.9973805
ZNF536	1.2100125	1.14123729	1.2398235	1.698018184	0.7638519	0.168749	0.9869875
TH	1.2413244	1.17171829	1.2714955	1.581051566	0.6608844	0.168825	1
ARMC10	2.2728748	2.17416632	2.3156605	1.120506071	0.1641505	0.169106	0.914626
TLE1	1.5396187	1.46245023	1.5730678	1.239198844	0.3094077	0.169147	1
PPP1R13L	1.2991407	1.37115722	1.2679247	0.721863212	-0.4702026	0.169272	0.9195598
CCDC159	1.2099178	1.27893635	1.1800014	0.645313488	-0.6319279	0.169732	0.9452583
GPR137B	1.356815	1.28423957	1.3882732	1.366006952	0.4499648	0.169919	0.9593718
SLC38A10	1.3031853	1.23219961	1.3339544	1.438221076	0.5242855	0.17013	1
MRPS28	1.6585493	1.57836417	1.693306	1.198736075	0.2615141	0.170153	0.914461
TAF10	1.8937759	1.80821367	1.9308633	1.1517539	0.2038325	0.170189	0.9896564
RCC1L	1.5212197	1.44450135	1.5544736	1.247405947	0.318931	0.170337	0.9452583
SENP6	1.9797456	1.89243459	2.017591	1.140241509	0.1893394	0.170547	0.9452583
YKT6	1.5032679	1.42702872	1.5363142	1.255920685	0.3287454	0.170707	1
CTD-2547G23.4	1.2815636	1.35237783	1.2508688	0.711931147	-0.4901904	0.170797	0.949289
GTPBP4	1.4734659	1.3974639	1.5064094	1.274101754	0.3494805	0.171271	1
IGLON5	1.1972126	1.12876323	1.2268823	1.762011627	0.8172234	0.171283	0.9680387
NIT1	1.6164819	1.53784851	1.6505659	1.209570872	0.2744953	0.171527	0.9223623
RAC3	2.3024272	2.39904342	2.2605483	0.901007298	-0.1503893	0.171533	0.9452583
RHOBTB1	1.5425531	1.46560534	1.5759065	1.236898462	0.3067271	0.171607	1
NMT2	1.5121886	1.43603081	1.5451997	1.250369551	0.3223546	0.17161	1
YTHDF3-AS1	1.150797	1.21801718	1.12166	0.558029407	-0.8415869	0.171815	0.9646601
BOLA2B	1.1620672	1.09481003	1.1912202	2.016876938	1.0121231	0.171839	0.877514
CARHSP1	3.2246467	3.09009989	3.2829668	1.092276386	0.127338	0.171866	0.9452583
NUTM2B-AS1	2.4141216	2.51338533	2.3710952	0.905978898	-0.1424506	0.171964	0.9942262
NUMA1	1.6474278	1.56742643	1.6821049	1.202102804	0.2655603	0.172038	1
C1orf56	1.4772219	1.40211635	1.5097768	1.267734474	0.3422526	0.172199	0.9173958
KIAA1328	1.2107484	1.27905863	1.1811389	0.649107104	-0.6234716	0.172349	1
PBXIP1	1.3664235	1.29428576	1.3976919	1.351380105	0.4344335	0.17237	0.9223623
TP53TG1	2.615933	2.5074686	2.6629475	1.103139075	0.1416147	0.172505	1
HN1L	1.9496434	2.03688223	1.9118292	0.879395191	-0.1854165	0.17251	0.9644007
GNB1	2.4490823	2.34627698	2.4936438	1.109462519	0.1498609	0.172525	0.9173958
C20orf27	2.5875686	2.69671983	2.5402564	0.907784764	-0.1395778	0.172531	1
ERCC4	1.265598	1.19585224	1.2958297	1.510473671	0.595001	0.172608	0.9644007
ADK	1.3990816	1.3264434	1.430567	1.318963677	0.3994048	0.173157	1
ALKBH4	1.4130192	1.33969042	1.4448039	1.309439108	0.388949	0.173305	1
CACNA2D3	1.2629227	1.33260966	1.2327165	0.699668408	-0.5152567	0.173325	0.9731359
MRPL10	2.1050846	2.01394906	2.1445877	1.128841458	0.1748429	0.173368	0.9227924
CCHCR1	1.2583087	1.32825783	1.2279888	0.694541871	-0.5258664	0.17341	1
TMEM35A	1.2659537	1.19588851	1.2963238	1.512716766	0.5971419	0.173457	1
LCNL1	1.0862807	1.02143083	1.1143902	5.337647334	2.416204	0.173515	0.9644007
ATN1	1.5456389	1.46824607	1.5791853	1.236925002	0.306758	0.173518	1
MBOAT7	1.7088369	1.62871108	1.7435679	1.182686215	0.2420674	0.173738	1
GID8	1.8634899	1.77887866	1.9001652	1.155719385	0.2087911	0.173883	1
RFC2	1.4347482	1.50923846	1.40246	0.79031737	-0.339496	0.173931	1
IFT81	1.9231861	2.00932925	1.8858469	0.877659013	-0.1882676	0.1742	1
FJX1	1.3485641	1.42071367	1.3172905	0.75417209	-0.4070343	0.174306	1

DCN	1.0810211	1.01646731	1.1090023	6.619312881	2.7266815	0.174334	0.9345519
MFSD1	1.1878145	1.12053305	1.216978	1.800153801	0.8481202	0.174339	0.9698952
KAT6A	1.49807	1.5735981	1.4653319	0.811250735	-0.3017802	0.174397	1
COMTD1	1.159916	1.09351366	1.1886984	2.017870193	1.0128334	0.174407	0.9345519
TMSB15B	1.493351	1.56886179	1.4606204	0.809722953	-0.3044997	0.174656	1
STAG1	1.5138293	1.59067103	1.4805218	0.813518517	-0.2977529	0.174678	0.9698952
EIF3A	3.2978217	3.16201118	3.3566895	1.090045004	0.1243877	0.174679	0.9248818
FOXM1	1.2490301	1.31804282	1.2191161	0.688951671	-0.5375253	0.174717	0.9452583
PPP1CB	3.5276142	3.35621861	3.6019065	1.10427211	0.1430957	0.174754	1
EXOSC1	1.4792371	1.4044984	1.511633	1.264857844	0.3389753	0.174815	1
SAC3D1	1.6865114	1.76647153	1.6518522	0.850458476	-0.2336873	0.174822	0.9345519
KAT8	1.3996101	1.32646655	1.4313145	1.32115994	0.4018051	0.175015	0.9836031
CCM2	1.4569926	1.38300548	1.4890628	1.276908084	0.3526547	0.175225	0.9268493
RNF113A	1.3225172	1.25166424	1.3532289	1.403571898	0.489103	0.175606	1
FAM171A1	1.4100297	1.33683118	1.441758	1.311511491	0.3912304	0.175615	1
KEAP1	1.5937892	1.51663068	1.6272339	1.214085685	0.2798702	0.175647	1
BICD1	1.6458391	1.7253635	1.6113688	0.842844782	-0.2466611	0.17582	1
ANKRD39	1.4740154	1.39939478	1.5063602	1.267818689	0.3423484	0.175887	1
NAT14	2.2958565	2.20033032	2.3372628	1.114079003	0.1558515	0.175993	0.9452583
DLC1	1.1035539	1.03912324	1.1314818	3.360707975	1.7487652	0.176015	0.9452583
RANBP1	7.1211749	6.8176536	7.2527379	1.074786908	0.1040507	0.176035	1
FAM63B	1.9427409	1.85538223	1.9806071	1.146396346	0.1971059	0.176335	1
TCIRG1	1.1534884	1.08722152	1.1822121	2.089072351	1.0628625	0.176352	1
FBF1	1.1581251	1.22465068	1.1292892	0.575512344	-0.7970812	0.17649	0.9452583
FBL	3.6583734	3.50928806	3.7229953	1.08516648	0.1179164	0.176525	0.9452583
AIDA	1.9034855	1.81868495	1.9402428	1.148479365	0.1997249	0.176601	0.9751871
AK3	2.1933416	2.10037096	2.2336403	1.121113087	0.1649318	0.177	0.9947599
NIP7	1.2622261	1.19311334	1.2921835	1.513015368	0.5974266	0.177087	0.9283691
GRAMD1A	2.0546198	1.96601324	2.0930268	1.131482209	0.1782139	0.177184	0.9283691
KMO	1.1197299	1.05468966	1.1479219	2.70475161	1.4354961	0.17719	1
CRYBB2	1.0977618	1.02408062	1.1296993	5.386045972	2.4292265	0.177246	1
GSKIP	1.3748506	1.30282806	1.4060691	1.340923089	0.4232265	0.17735	1
PCGF2	2.0135375	1.92672485	2.051167	1.134281615	0.1817789	0.177361	0.9452583
SCRN3	1.3383761	1.26759123	1.3690581	1.379186188	0.4638172	0.177456	0.9452583
FAM45A	1.7813587	1.69868937	1.8171922	1.169607287	0.2260242	0.17752	0.9812368
SYMPK	2.0519241	2.13973567	2.0138617	0.88955866	-0.1688384	0.177524	1
ZDHHC24	1.5145366	1.43977101	1.5469442	1.24370225	0.3146411	0.177562	1
FGFR3	1.1427387	1.07706473	1.1712054	2.22157906	1.1515855	0.177578	1
TSHZ2	1.2665678	1.19753873	1.2964889	1.500915253	0.5858425	0.177587	1
DAAM1	2.5128129	2.4031479	2.5603477	1.112033698	0.1532005	0.177737	1
ABHD14A	1.8652855	1.7822387	1.9012825	1.152183505	0.2043705	0.178032	0.9949988
ARL8A	1.3146801	1.24483108	1.3449566	1.408957428	0.494628	0.178047	1
CYB5RL	1.1129646	1.1779083	1.0848144	0.476731269	-1.0687518	0.178181	0.9920015
IARS2	1.9081452	1.82401175	1.9446133	1.146358987	0.1970589	0.178296	0.9452583
FAM118B	1.1790459	1.112188	1.2080259	1.854261287	0.8908446	0.178471	1
ZNF678	1.1916356	1.25860275	1.1626083	0.628795626	-0.6693369	0.178528	0.9452583
RABEPK	1.3568121	1.28551868	1.3877146	1.357930654	0.4414098	0.178724	1
MRPL16	1.5591789	1.48332464	1.5920583	1.2249702	0.2927467	0.17876	0.9317748
ADM	2.3463037	2.19430853	2.4121868	1.182430475	0.2417554	0.1791	0.9999766
FAM228B	1.4922355	1.56734406	1.4596792	0.810230112	-0.3035964	0.179409	0.9920015
RIMKLB	1.4558454	1.52987083	1.4237587	0.799739653	-0.3223977	0.179467	0.9920015

TUG1	2.0230896	2.11543144	1.9830635	0.881330261	-0.1822454	0.17956	0.9870897
AUH	1.2667486	1.19815164	1.2964823	1.496239287	0.5813409	0.179908	1
SIPA1	1.2910009	1.22206365	1.3208821	1.445000516	0.53107	0.180621	0.9604429
TRPM7	1.3270601	1.39744017	1.2965535	0.746158836	-0.4224453	0.180674	0.9506646
ESF1	2.5236921	2.63020682	2.4775227	0.90634062	-0.1418748	0.180761	1
ALOX5AP	1.093316	1.02988503	1.1208105	4.042508308	2.0152507	0.180795	1
STUB1	2.9791479	2.852868	3.0338846	1.097695372	0.1344777	0.180801	1
DPF3	1.1073355	1.17189207	1.0793531	0.461644658	-1.1151453	0.180857	0.990687
PIGU	1.3215068	1.25202285	1.351625	1.395210786	0.4804831	0.180943	1
PPP2R5B	1.2397318	1.17204961	1.2690691	1.56390395	0.6451519	0.181524	0.990687
ADAMTS9	1.1887532	1.12253018	1.217458	1.774730068	0.8275996	0.181612	1
SLC2A4RG	1.363214	1.29183173	1.3941551	1.350624495	0.4336266	0.181649	0.9229222
NAALAD2	1.2483535	1.31637227	1.2188704	0.691812868	-0.5315462	0.181651	0.956758
SGIP1	1.3331565	1.40366912	1.3025924	0.74960495	-0.4157976	0.181765	1
HECTD1	1.9955115	1.90736723	2.0337181	1.139250036	0.1880844	0.181831	1
OXTR	1.1248159	1.06035012	1.1527589	2.531210945	1.3398277	0.181922	1
FBXO9	1.6252487	1.54773898	1.6588456	1.202845993	0.2664519	0.181957	1
PKIG	2.2857685	2.18820011	2.32806	1.117707363	0.1605425	0.182365	0.990687
MAF1	3.0252308	2.87053357	3.0922851	1.118549879	0.1616296	0.182441	1
EHD2	1.1531266	1.08820527	1.1812671	2.055059453	1.0391801	0.182949	1
MYO9B	1.4309559	1.50383475	1.3993662	0.792653099	-0.3352385	0.183166	0.955764
SIPA1L2	1.1747757	1.1089453	1.2033103	1.866168251	0.9000791	0.183319	0.9267262
ATP2C1	1.5818882	1.50608218	1.6147468	1.214717259	0.2806205	0.183526	1
SLC38A1	2.2578505	2.16734333	2.2970813	1.111139501	0.15204	0.183545	0.955764
WDR43	1.4537888	1.38117949	1.4852617	1.273053031	0.3482925	0.183807	1
PRRC2B	2.4648454	2.56485919	2.4214939	0.908384564	-0.1386249	0.183891	1
MTR	1.4818363	1.55605104	1.4496675	0.80868031	-0.3063586	0.183993	0.9941531
HES4	2.7829225	2.65356103	2.838995	1.112142172	0.1533412	0.184051	0.9643496
LIM2	1.1505037	1.08215455	1.18013	2.192574632	1.132626	0.184228	1
ARFIP1	1.2641581	1.1961073	1.293655	1.49742021	0.5824791	0.184288	1
LIPE	1.1915145	1.25780751	1.1627795	0.631399265	-0.6633755	0.184303	1
SMCO4	1.4669083	1.54015891	1.4351574	0.805609989	-0.3118465	0.184459	1
TACC1	1.2784718	1.21021326	1.3080589	1.465458906	0.5513525	0.18446	1
ABI1	1.3672255	1.296882	1.3977163	1.339644467	0.4218502	0.184476	1
CCZ1	1.4637478	1.39085493	1.4953436	1.267333534	0.3417963	0.184758	1
PLEKHG1	1.3742389	1.44561119	1.3433022	0.770407399	-0.3763065	0.184863	1
BOP1	1.307993	1.23903353	1.3378838	1.413541409	0.4993141	0.184989	0.9592724
NTM	1.1485202	1.08417564	1.1764107	2.095745448	1.0674635	0.185002	1
GAS1	2.1125913	2.01129376	2.1564993	1.1435839	0.1935622	0.185181	1
MLF1	1.5036467	1.43005632	1.5355448	1.245289961	0.3164817	0.185308	0.9801793
AC116614.1	1.1331439	1.19764499	1.1051855	0.532194214	-0.9099753	0.18577	1
TCF7	1.2387558	1.30620032	1.2095216	0.68426305	-0.547377	0.185834	1
LSM8	2.7379854	2.85201914	2.6885568	0.911738324	-0.1333083	0.186142	0.9603031
MED28	2.8880644	2.77031184	2.9391049	1.095346485	0.1313873	0.186173	0.9339127
ZDHHC2	1.2260211	1.15942878	1.2548859	1.598744789	0.6769397	0.186187	1
DDX54	1.5911677	1.51551387	1.6239603	1.210365663	0.275443	0.186477	1
SLBP	1.9500617	1.86519003	1.9868498	1.140616288	0.1898135	0.186485	1
NR2F2-AS1	1.2179865	1.28483736	1.1890096	0.663570315	-0.5916787	0.18649	1
ABHD17A	1.5400732	1.46497199	1.5726263	1.231528527	0.30045	0.186499	1
MELK	1.2028367	1.26913784	1.1740982	0.646873615	-0.6284442	0.186614	1
WASF3	1.718269	1.79737122	1.6839817	0.857795783	-0.2212939	0.187166	1

FOXP2	1.6731791	1.75118861	1.6393655	0.851138437	-0.2325343	0.187458	1
NFYB	1.5965342	1.52044701	1.6295146	1.209565133	0.2744885	0.187488	1
KLHL8	1.2976358	1.36646021	1.2678035	0.730784614	-0.4524818	0.187495	1
PRR3	1.4442344	1.37189392	1.4755908	1.27883448	0.3548295	0.18751	0.9339127
NDUFA4L2	3.2456629	3.53586847	3.1198717	0.835954913	-0.258503	0.187517	0.9866758
CDH20	1.3536925	1.4236974	1.3233485	0.763159073	-0.3899443	0.187525	0.9339127
KIAA0907	1.5887711	1.66452958	1.5559332	0.836581499	-0.257422	0.187595	0.9718198
MKI67	2.2879695	2.46100035	2.2129683	0.830231389	-0.2684146	0.187785	1
CCDC50	1.8671413	1.78581689	1.9023918	1.148348747	0.1995608	0.187814	1
CLCN3	2.3615753	2.45701697	2.3202056	0.906101753	-0.142255	0.18782	1
PSME3	1.408635	1.33775125	1.4393599	1.30083873	0.3794421	0.187931	1
CCDC115	1.8524117	1.77131402	1.887564	1.150716779	0.2025328	0.188206	1
S100PBP	1.4339307	1.50614648	1.4026283	0.795477907	-0.3301062	0.188213	0.9660344
ASH1L	2.1487364	2.23884177	2.1096797	0.895739657	-0.1588486	0.188342	1
NR2E1	1.1669571	1.23181533	1.138844	0.598942064	-0.7395116	0.188753	0.9344826
KAT6B	1.4788454	1.40565454	1.5105704	1.258633518	0.3318583	0.188811	1
PITPNA	1.4029188	1.33214328	1.4335969	1.305451372	0.3845487	0.189069	1
AMH	1.1132595	1.17679375	1.0857202	0.484859886	-1.0443602	0.189139	0.9719668
SNAI2	1.1184867	1.0549906	1.1460094	2.655170367	1.4088044	0.189305	1
SDC1	1.2625468	1.19541549	1.2916453	1.492436723	0.5776698	0.189357	1
ATF7IP	1.9903015	2.07476096	1.9536921	0.887352797	-0.1724203	0.189376	1
SH3BGR	1.2618983	1.19471211	1.2910206	1.494619662	0.5797784	0.189497	0.9344826
MPC1	1.6407791	1.56439762	1.6738871	1.193993568	0.2557951	0.189527	1
DMAP1	1.4761334	1.40334233	1.5076851	1.258695339	0.3319291	0.189558	0.9719668
KCTD16	1.3175198	1.38626053	1.2877237	0.744895461	-0.4248901	0.189627	1
VTA1	1.3514761	1.28220418	1.3815024	1.351866705	0.4349529	0.190019	1
TXNIP	4.0714952	3.87082184	4.1584782	1.1002	0.1377658	0.190342	1
C8orf76	1.4485443	1.37646488	1.4797876	1.274455072	0.3498805	0.190437	1
POLR2B	1.8467104	1.76518497	1.8820481	1.152725327	0.2050488	0.190548	0.9720824
SOX6	1.1644537	1.09985641	1.1924537	1.927304698	0.9465847	0.190694	1
ETFDH	1.2284846	1.29499873	1.1996536	0.676794906	-0.5632094	0.190765	1
DNAJC9-AS1.1	1.141486	1.20542803	1.1137699	0.553818915	-0.8525138	0.191377	1
AC009403.2	1.2949371	1.36281297	1.265516	0.731826057	-0.4504273	0.191529	0.9709213
RP11-395A13.2	1.2332671	1.29971193	1.2044662	0.682209215	-0.5517139	0.191745	0.9709213
TEX2	1.1646652	1.10046896	1.1924914	1.915929136	0.9380442	0.191784	1
SLC30A5	1.3908487	1.32084126	1.4211938	1.312779565	0.3926247	0.191914	1
ANKRD54	1.3938358	1.46420723	1.3633329	0.782695374	-0.3534772	0.191926	1
FLYWCH1	1.2351454	1.16891347	1.2638539	1.562065674	0.6434551	0.191946	1
NSG1	1.1058996	1.04290481	1.133205	3.104662735	1.6344366	0.191955	1
CDC37L1	1.2745695	1.2075362	1.3036254	1.462999702	0.5489295	0.191959	1
SLC45A2	1.097295	1.03492462	1.1243297	3.559944546	1.8318548	0.191999	0.9421984
HMMR	1.2312861	1.16551383	1.2597954	1.569629456	0.650424	0.192154	1
PIEZO2	1.2870249	1.35459288	1.2577372	0.726853796	-0.4602629	0.192161	1
CCDC85C	1.4805796	1.4083343	1.5118947	1.253616726	0.3260963	0.192263	1
KDELC1	1.2232437	1.15759331	1.2517003	1.597150714	0.6755005	0.192383	1
ARRDC4	1.2301837	1.16449431	1.2586571	1.572437924	0.6530031	0.192576	1
CNTN2	1.1083097	1.04542654	1.1355668	2.984307732	1.5773963	0.192603	1
CMTM8	1.3406543	1.27177078	1.3705123	1.363326345	0.4471309	0.192796	1
UNC45A	1.4693733	1.39760164	1.5004831	1.258755115	0.3319976	0.192802	1
COL27A1	1.3101012	1.24228705	1.3394956	1.401212394	0.4866757	0.192832	0.9709213
MIR503HG	1.1037992	1.16647158	1.0766336	0.460340215	-1.1192276	0.192918	1

RAD54L	1.1005409	1.16297911	1.0734768	0.450835566	-1.1493268	0.192986	1
TIMM22	1.4664256	1.39463182	1.497545	1.260782719	0.3343197	0.193026	0.9709213
MIR1302-9	1.1506407	1.21452367	1.1229503	0.573131765	-0.8030612	0.193029	1
GTF2I	4.4637637	4.63463313	4.3896995	0.93261117	-0.1006524	0.193036	0.9426915
PSPC1	1.3918768	1.32210782	1.4221185	1.310488261	0.3901044	0.193159	1
MTBP	1.0882852	1.15034742	1.061384	0.40828136	-1.2923644	0.193252	1
AL132709.1	1.121106	1.18441227	1.0936655	0.507913672	-0.9773448	0.193297	1
PHF23	1.5418042	1.46847184	1.5735905	1.224386291	0.2920588	0.193733	1
ARPP21	1.1550119	1.21891938	1.1273109	0.581542448	-0.7820436	0.193816	1
ANKRD46	1.3875867	1.31780441	1.4178342	1.314752578	0.3947913	0.193821	1
C7orf26	1.2233073	1.15788329	1.2516657	1.593998529	0.6726503	0.193831	1
URI1	1.7152373	1.79217709	1.6818873	0.860776358	-0.2162896	0.193886	1
HS3ST3B1	1.0858024	1.02419099	1.1125081	4.650829331	2.217488	0.194098	1
TMEM199	1.2771071	1.21004438	1.3061759	1.457672232	0.5436664	0.194112	1
ECE1	1.4358851	1.36533801	1.4664641	1.276801337	0.3525341	0.194184	0.9856331
FZD6	1.2228332	1.15750784	1.2511489	1.594516716	0.6731192	0.19425	1
HNRNPR	4.0132329	3.8316318	4.0919489	1.091931848	0.1268828	0.194528	1
SCO1	1.3208602	1.2532062	1.3501852	1.383004076	0.4678054	0.194679	1
ATF3	1.1336283	1.07055301	1.1609686	2.281526929	1.1899997	0.195054	1
TMEM169	1.2620416	1.19560986	1.2908369	1.486821029	0.572231	0.195124	1
ZC3H13	2.0986845	2.00941898	2.1373771	1.126764099	0.1721855	0.195211	1
C9orf47	1.1089941	1.17151647	1.0818935	0.477467012	-1.066527	0.195243	1
CELF5	1.3175007	1.38520348	1.2881545	0.748057917	-0.4187781	0.195251	1
GALNT18	1.1346533	1.07181885	1.1618893	2.254133625	1.172573	0.195303	1
KIAA0100	1.3740479	1.44367688	1.3438668	0.775038761	-0.3676596	0.195433	0.942965
DIXDC1	1.7850841	1.7065534	1.8191236	1.159323022	0.2132826	0.195527	0.942965
CLK3	1.4105929	1.34083526	1.4408297	1.293380462	0.3711467	0.195527	1
ANKS1B	1.1938379	1.25841361	1.1658473	0.641789939	-0.6398269	0.195788	1
NUDT14	1.589107	1.51498085	1.6212374	1.206331026	0.2706258	0.195904	0.942965
PUM1	1.9793946	2.06284378	1.9432231	0.887452272	-0.1722586	0.195952	1
ATP1A2	1.1570763	1.22076243	1.1294712	0.586472981	-0.7698635	0.196018	1
ZCCHC10	1.6144381	1.53961855	1.6468691	1.198752535	0.2615339	0.196237	1
C5orf34	1.113579	1.17607141	1.0864913	0.491228749	-1.0255331	0.196293	0.9814211
LIN54	1.1547434	1.218145	1.1272616	0.583380682	-0.7774905	0.197221	1
PDE6D	1.3714091	1.30233201	1.4013509	1.327517021	0.4087304	0.197234	1
LMBR1L	1.2936793	1.22694573	1.3226053	1.421508479	0.5074227	0.197279	1
BPNT1	1.2713183	1.20478115	1.3001592	1.465756208	0.5516452	0.197485	0.9814211
C6orf62	2.2141161	2.30468123	2.1748601	0.900495929	-0.1512083	0.197558	0.946396
ZNF324	1.2134765	1.27843892	1.1853181	0.665561093	-0.587357	0.197598	1
RP11-793J2.1	1.1424052	1.20558608	1.1150191	0.559469469	-0.8378687	0.197716	1
CFAP97	2.1327377	2.04690364	2.1699429	1.117526866	0.1603095	0.197763	1
TSPAN5	1.4337671	1.36389699	1.4640527	1.275230947	0.3507585	0.197936	1
ZNF785	1.3913155	1.46039292	1.3613736	0.78492426	-0.3493746	0.19804	0.9814211
ACAA1	1.36663	1.29843875	1.3961879	1.327535022	0.4087499	0.19818	1
ZBTB4	1.2434425	1.17809501	1.2717678	1.525970739	0.6097273	0.198202	1
TOMM70	1.3483061	1.28050273	1.3776958	1.346496132	0.4292101	0.19826	1
RELA	1.3894935	1.32059743	1.4193568	1.308048007	0.3874155	0.198375	1
KIAA1191	1.9821665	1.90022454	2.0176846	1.130478644	0.1769337	0.198379	1
NPRL3	1.6472947	1.57178322	1.6800256	1.189306656	0.2501208	0.198415	1
PDHX	1.3997389	1.33066176	1.4296808	1.299457268	0.3779092	0.198508	1
SORT1	1.2579561	1.19206941	1.2865151	1.491726882	0.5769834	0.198528	1

PPIL1	1.7669562	1.69008764	1.8002753	1.159672012	0.2137168	0.198631	1
ISG15	1.4743366	1.38958189	1.511074	1.311852638	0.3916057	0.19864	1
C15orf57	1.3936831	1.32487112	1.42351	1.303624544	0.3825284	0.19868	1
ADD2	1.2357738	1.30123968	1.2073972	0.688479123	-0.5385152	0.198707	1
KIFAP3	1.3484638	1.28054955	1.3779017	1.347005051	0.4297553	0.198719	1
IL10RB	1.2723392	1.20638403	1.3009278	1.458096451	0.5440862	0.198759	0.9476382
RP11-398K22.12	1.3162532	1.38335341	1.2871682	0.749095192	-0.416779	0.198879	1
NAT6	1.2835681	1.21720327	1.3123342	1.437981232	0.5240448	0.199078	1
ADGRB3	1.1952016	1.25950949	1.167327	0.64478173	-0.6331172	0.199085	0.9817609
CIZ1	1.5935846	1.5197995	1.6255672	1.203477833	0.2672096	0.199206	1
CTHRC1	1.2735303	1.20774401	1.3020458	1.45393253	0.5399603	0.199259	1
MAGI1	1.5418893	1.61471209	1.5103238	0.830183505	-0.2684978	0.199397	1
MLLT6	1.3509538	1.41930909	1.3213249	0.766319812	-0.3839815	0.199522	1
RARB	1.1703452	1.1069785	1.1978118	1.849079882	0.8868076	0.199682	1
ANK3	1.4586258	1.38791666	1.4892751	1.261289216	0.3348991	0.199737	1
ZCCHC18	1.2849049	1.35097508	1.2562665	0.730155772	-0.4537238	0.199751	1
SORCS2	1.1362368	1.19897926	1.1090407	0.54800027	-0.8677515	0.199755	1
GRAMD1B	1.1205459	1.05853655	1.1474242	2.518498425	1.3325638	0.199867	1
DDX41	1.6203056	1.54591994	1.6525485	1.1953191	0.2573958	0.200044	1
GEMIN7	1.7259422	1.64943085	1.7591064	1.168879504	0.2251262	0.200114	1
RAP2C-AS1	1.2533202	1.31935762	1.2246959	0.703586982	-0.5071993	0.200173	1
ZNF521	1.3559718	1.42430788	1.3263511	0.769137594	-0.3786864	0.200335	1
SMKR1	1.222351	1.28712781	1.1942731	0.676608444	-0.5636069	0.200376	1
BCKDK	2.6711583	2.56467813	2.7173127	1.097550169	0.1342869	0.200386	0.9833589
TGFB2	2.0014509	1.91543491	2.038735	1.134690154	0.1822984	0.200393	1
UBN2	1.3729724	1.44146405	1.3432843	0.777604133	-0.3628922	0.200399	1
IPO5	2.4485383	2.54591851	2.4063284	0.909704079	-0.1365308	0.200465	1
TMEM97	2.4048398	2.50083101	2.3632318	0.908318007	-0.1387306	0.200656	1
ATP1A3	1.1000881	1.0383675	1.1268412	3.305953842	1.7250666	0.200904	1
ATXN2L	1.5713077	1.64457132	1.5395512	0.837069858	-0.2565801	0.200972	1
NOVA1	2.7305478	2.84975652	2.6788762	0.907620082	-0.1398396	0.201007	1
MTIF3	2.386354	2.28770982	2.4291118	1.109808895	0.1503113	0.201023	1
MFAP3L	1.1146494	1.05289381	1.1414176	2.673614147	1.4187913	0.201071	1
SGTB	1.2377915	1.17274271	1.2659872	1.539788353	0.6227321	0.201141	1
CPQ	1.1659846	1.10308598	1.1932484	1.874632939	0.9066081	0.20117	1
CPT2	1.2016689	1.13781607	1.2293463	1.664147531	0.7347833	0.201298	0.9524555
ENDOG	1.24097	1.17629098	1.2690055	1.525917394	0.6096769	0.201392	1
ANKRD11	2.2589056	2.34912781	2.2197983	0.904138433	-0.1453844	0.201398	1
HIRIP3	1.2659054	1.33184911	1.2373217	0.715149527	-0.4836832	0.201565	1
SPEF2	1.1717643	1.2354234	1.1441709	0.612389748	-0.707478	0.201583	1
RSPH1	1.1188737	1.18075191	1.0920522	0.509273938	-0.9734862	0.201663	0.9524555
CREBRF	1.3763177	1.44490476	1.3465882	0.779016704	-0.3602738	0.201669	1
UBAP1	1.4785407	1.40779689	1.509205	1.248673026	0.3203957	0.201823	1
SCAMP1	1.5619059	1.48921422	1.5934146	1.212995305	0.278574	0.201877	1
APRT	3.4558982	3.31781539	3.515751	1.085397485	0.1182235	0.201888	1
RALBP1	2.1307044	2.02756836	2.1754092	1.143874499	0.1939288	0.201906	1
GNS	1.4636021	1.39357052	1.4939576	1.255067654	0.3277651	0.202068	1
ARHGEF3	1.2178428	1.1538481	1.2455816	1.596260364	0.674696	0.202271	1
PGP	1.9508853	2.03309788	1.9152498	0.885927503	-0.1747394	0.202432	1
HAGHL	1.3793019	1.31129095	1.4087816	1.31318183	0.3930667	0.202502	1
RGS17	1.2368741	1.30158816	1.2088235	0.69241279	-0.5302957	0.202539	1

RP11-448A19.1	1.2477799	1.31313365	1.219452	0.70082545	-0.5128729	0.20255	1
DDR1	2.2877043	2.18701381	2.3313492	1.121595382	0.1655523	0.202725	1
EFS	1.3578317	1.42562904	1.3284445	0.771668518	-0.3739468	0.202787	0.9857169
MADCAM1	1.1146897	1.17657761	1.087864	0.497594113	-1.0069587	0.202826	0.9857169
DIO3	3.1544975	3.39592413	3.0498499	0.85555708	-0.225064	0.202841	1
HEPH	1.327879	1.26092098	1.3569023	1.367856025	0.4519164	0.203221	1
SRGAP2B	1.312927	1.37942153	1.2841045	0.748783338	-0.4173798	0.203425	1
NEK2	1.2644199	1.19813115	1.2931531	1.479591337	0.5651988	0.203557	1
CXXC4	1.5981076	1.67155147	1.566273	0.843230956	-0.2460003	0.203717	1
FANCG	1.2863003	1.35237315	1.2576607	0.731215329	-0.4516318	0.203944	1
EPDR1	1.1773989	1.11451219	1.2046574	1.787210948	0.8377099	0.204054	1
ITPKB	1.1513038	1.08904304	1.178291	2.002301385	1.0016591	0.204116	1
IFT122	1.3183936	1.38496492	1.2895379	0.752114948	-0.4109749	0.204305	1
UBE2R2	2.4960725	2.39449304	2.5401027	1.104417628	0.1432858	0.204438	1
NCLN	1.2051361	1.14182045	1.2325806	1.639965071	0.7136651	0.204683	0.9899592
DCTD	2.0857317	1.99917074	2.1232521	1.124184322	0.1688786	0.204705	1
DYRK2	1.3535167	1.28652322	1.3825554	1.33516363	0.4170166	0.204854	1
TLN1	1.9467832	1.86538034	1.9820677	1.13483939	0.1824881	0.204886	1
CPSF1	1.3375823	1.40440484	1.3086177	0.763140418	-0.3899796	0.205046	1
CEP170	1.840931	1.76048892	1.875799	1.151626261	0.2036726	0.205065	1
CAHM	1.2259389	1.29011942	1.1981195	0.682889443	-0.5502761	0.205233	1
APIP	1.5270795	1.45597271	1.5579012	1.223540694	0.2910621	0.205303	1
LIN7C	1.3708729	1.30350231	1.400075	1.318194321	0.3985631	0.205361	1
SLC35D3	1.0958106	1.0352064	1.1220798	3.467545165	1.7939147	0.205367	1
WFDC1	1.2135111	1.14992304	1.2410737	1.607983273	0.6852524	0.205466	1
CTA-392E5.1	1.0753025	1.01566441	1.101153	6.457502953	2.6909764	0.205548	1
MEIS3	1.7395905	1.81555052	1.7066653	0.86648867	-0.2067472	0.205748	0.990349
PCCB	1.2222508	1.15862735	1.2498287	1.574940696	0.6552975	0.205805	1
ZNF507	1.2133451	1.27711122	1.1857054	0.670147418	-0.5774496	0.206254	1
MICA	1.2961461	1.23038349	1.3246513	1.409177912	0.4948538	0.206317	1
LARP1B	1.3733202	1.30604355	1.4024816	1.31511227	0.395186	0.206401	1
KLHL14	1.2092213	1.14618024	1.2365468	1.61818578	0.6943772	0.206458	1
TIMM23	1.2808518	1.21580593	1.3090463	1.432056772	0.5180887	0.206519	1
CYP46A1	1.147511	1.20946558	1.1206564	0.576020219	-0.7958086	0.206555	0.9670483
NPAT	1.2046668	1.26862524	1.1769436	0.658700693	-0.602305	0.206601	1
VPS16	1.1699626	1.10764819	1.1969731	1.829785142	0.8716743	0.206668	1
FBXO42	1.2888759	1.35465509	1.2603635	0.734131631	-0.4458893	0.20697	1
CYB5R2	1.1425914	1.08119732	1.169203	2.083848897	1.0592507	0.207066	1
THRAP3	2.3899379	2.29203973	2.4323724	1.108613295	0.1487562	0.207149	1
TTC26	1.2145317	1.27843759	1.1868313	0.670998912	-0.5756177	0.207229	1
DLEU1	1.3396366	1.40645978	1.3106717	0.764335549	-0.387722	0.20744	0.9670483
L3MBTL1	1.2293771	1.29350988	1.2015784	0.686785739	-0.542068	0.207471	1
CHKA	1.1710476	1.10876891	1.1980427	1.820765721	0.8645453	0.207567	1
RP11-294J22.6	1.2303708	1.16615737	1.2582045	1.553975615	0.6359639	0.207568	1
TNFRSF10B	1.7929971	1.71591487	1.8264088	1.154339456	0.2070675	0.207624	1
PLEC	1.4234317	1.35498204	1.4531016	1.276407005	0.3520884	0.207637	0.9670483
RABGGTB	2.0892475	2.00138416	2.1273323	1.125774025	0.1709173	0.207859	1
GABRB3	1.1145002	1.05382439	1.1408005	2.615922972	1.3873201	0.208022	1
SERTAD3	1.3188702	1.2532523	1.3473127	1.371409877	0.4556598	0.208052	1
HADHA	2.6475414	2.5433299	2.6927125	1.096792405	0.1332905	0.208101	1
MRPL27	2.1149596	2.03196109	2.1509357	1.115289801	0.1574186	0.208342	1

POLDIP2	1.8889794	1.81100824	1.9227765	1.137813953	0.1862647	0.208706	1
NBPF1	1.2871182	1.35242469	1.2588108	0.734371815	-0.4454174	0.208716	1
CTDSP1	1.597009	1.66972278	1.5654908	0.844365432	-0.2440606	0.208908	0.9684863
LINC01170	1.1093982	1.16988492	1.0831798	0.489624629	-1.030252	0.209105	1
TMEM223	1.6102717	1.53838564	1.6414312	1.191397257	0.2526545	0.209471	1
ZNF385A	1.5481002	1.47637391	1.5791904	1.215831396	0.2819432	0.209547	1
TYRO3	1.1944483	1.25715639	1.1672672	0.650449353	-0.6204914	0.209733	1
BAIAP2	1.4211995	1.35316184	1.4506908	1.276159531	0.3518087	0.209756	1
ABCF2	1.2645114	1.20016578	1.2924024	1.460800984	0.5467596	0.209764	1
SNN	1.4667885	1.39732351	1.4968985	1.250614381	0.322637	0.210067	1
MPDZ	1.7034697	1.7784944	1.6709499	0.861855793	-0.2144816	0.210274	1
JOSD2	1.7071891	1.63278142	1.7394415	1.168557566	0.2247288	0.210364	1
SIX3	3.2972776	3.42179847	3.2433034	0.926296462	-0.1104541	0.210544	1
SPSB3	1.7607436	1.68543264	1.7933876	1.157498952	0.2110109	0.210611	1
GM2A	1.2062699	1.14349553	1.2334799	1.627087986	0.7022923	0.210682	1
ELL2	1.3298283	1.39576999	1.3012455	0.761163111	-0.3937225	0.210765	1
PGAM2	1.3198137	1.24935842	1.3503529	1.405017531	0.4905881	0.211112	0.9727054
C20orf194	1.2298911	1.29332149	1.2023969	0.690017198	-0.5352958	0.211148	1
SNX14	1.4592483	1.39030971	1.48913	1.2531844	0.3255987	0.211185	1
NDC1	1.2860392	1.3508356	1.2579529	0.735252879	-0.4436876	0.211567	1
ZKSCAN2	1.1184527	1.17904188	1.09219	0.514907546	-0.9576147	0.211592	1
BLOC1S2	2.0589116	1.97522427	2.0951864	1.12300977	0.1673705	0.211633	1
CDC7	1.1916056	1.25443324	1.1643726	0.646034201	-0.6303176	0.211655	1
LRRC49	1.3320538	1.39778137	1.3035638	0.763142436	-0.3899757	0.211752	0.9727054
ADGRL1	1.4602084	1.52937072	1.4302297	0.812719041	-0.2991714	0.211772	1
BIN1	2.3848428	2.29252778	2.4248572	1.102380329	0.1406221	0.211794	1
WDR74	1.3542995	1.28833931	1.3828904	1.327915946	0.4091638	0.21183	1
MAP3K11	1.4274234	1.49620155	1.3976112	0.801309834	-0.3195679	0.211916	1
SYNJ2BP	1.7193435	1.64417802	1.7519244	1.167261747	0.2231281	0.211963	1
TMEM92	1.118373	1.05848408	1.1443322	2.467888622	1.3032773	0.212263	1
KDM1A	1.85205	1.77524156	1.8853431	1.142022213	0.1915907	0.212312	1
RNF2	1.4436352	1.37534528	1.4732358	1.260801232	0.3343408	0.21254	1
PMAIP1	1.5804137	1.50932985	1.6112253	1.200057963	0.2631041	0.212596	1
CAPN7	1.4780622	1.54739703	1.4480086	0.81843446	-0.2890612	0.212744	1
IL6ST	1.7800085	1.85621275	1.7469773	0.872420223	-0.1969049	0.212844	1
FGF11	1.6388373	1.71153393	1.6073265	0.853545399	-0.2284602	0.212929	1
NPB	1.4123601	1.47988254	1.3830921	0.798303876	-0.3249901	0.21328	1
GOLGA2	1.4589775	1.39067093	1.4885854	1.250631621	0.3226569	0.213301	1
FAM160B2	1.2331113	1.29650194	1.2056344	0.693534597	-0.5279602	0.213313	1
CUTC	1.3726934	1.30654829	1.4013644	1.309302197	0.3887981	0.213576	1
EPHA4	1.2215604	1.2846864	1.1941981	0.682147313	-0.5518448	0.213903	1
KHSRP	1.8088445	1.88541101	1.7756563	0.876040946	-0.1909298	0.21399	1
CSNK1G2	1.3354558	1.40129516	1.3069173	0.764816915	-0.3868137	0.214102	1
BEX5	1.4046189	1.33774337	1.4336064	1.283834035	0.3604587	0.214115	1
PATL1	1.2447919	1.18128649	1.2723187	1.502145681	0.5870247	0.214116	1
DIABLO	1.2153416	1.15287875	1.2424165	1.585678203	0.6651	0.214254	1
GFY	1.1240714	1.18446673	1.0978927	0.530679616	-0.914087	0.214509	1
CNIH2	1.157361	1.21877918	1.130739	0.597584348	-0.7427857	0.214823	1
ERI1	1.2524739	1.31626326	1.2248241	0.710876302	-0.4923296	0.214986	1
SPATA20	1.454887	1.38659245	1.4844896	1.253230978	0.3256523	0.21515	0.9805263
LRRC23	1.5747764	1.50328055	1.6057667	1.203636151	0.2673993	0.215269	1

LMAN1	4.0462161	3.89799875	4.1104617	1.073313682	0.1020718	0.215404	0.9805263
TAP1	1.1599324	1.09938299	1.1861779	1.873338069	0.9056113	0.21566	1
PRKAG1	1.7653155	1.69048951	1.7977493	1.155338693	0.2083158	0.215666	1
TMEM25	1.4035172	1.33692205	1.4323832	1.283333073	0.3598957	0.215712	1
CCSER2	1.5897201	1.51812471	1.6207536	1.198077512	0.2607212	0.215766	1
ZNF419	1.1231787	1.18340653	1.0970726	0.529275513	-0.9179092	0.21578	1
GKAP1	1.5317402	1.60158989	1.5014635	0.833563641	-0.2626357	0.215809	1
LLNLR-245B6.1	2.5695122	2.6715196	2.5252966	0.912520919	-0.1320705	0.216047	1
TSPAN15	1.1889208	1.12735252	1.2156079	1.693000434	0.7595823	0.216064	1
MARVELD1	1.46669	1.53508592	1.4370435	0.816772538	-0.2919937	0.216185	1
ZNF724	1.1037595	1.16345509	1.0778842	0.476486697	-1.0694922	0.216561	1
FAM127B	2.8586717	2.7526822	2.9046134	1.086684964	0.1199338	0.216609	1
ORC4	1.5573161	1.48722499	1.5876975	1.206213751	0.2704856	0.216699	1
PINLYP	1.3135171	1.24892062	1.3415168	1.371990832	0.4562708	0.216856	1
SGSM1	1.0786462	1.13761923	1.053084	0.385730614	-1.3743344	0.216875	1
GXYLT1	1.4364178	1.36896147	1.465657	1.262074985	0.3357976	0.217035	1
HLA-DPB1	1.5741785	1.5033426	1.6048826	1.20173147	0.2651146	0.217084	1
HP1BP3	3.0703191	3.19293887	3.0171689	0.919847299	-0.1205337	0.217111	1
HOXB4	1.1237042	1.06164534	1.150604	2.443071204	1.2886959	0.217249	1
STK4	1.6370862	1.70946905	1.6057115	0.853753164	-0.2281091	0.217292	1
CD24	9.0891126	8.66013786	9.275054	1.080274813	0.1113984	0.217357	1
THOC6	1.4377127	1.37057405	1.4668143	1.259705713	0.3330867	0.217432	1
PEX19	1.5333676	1.46406755	1.563406	1.214060392	0.2798402	0.21759	1
SUDS3	1.8693893	1.79272438	1.9026201	1.138630479	0.1872996	0.217807	1
CDCP1	1.0789242	1.02059986	1.1042053	5.058542296	2.3387217	0.217877	0.9873166
YWHAG	2.2365599	2.14823152	2.2748463	1.110269404	0.1509098	0.218088	1
FAM118A	1.7719795	1.84691923	1.7394965	0.873160552	-0.1956811	0.218152	1
PIM1	1.2201711	1.15830111	1.246989	1.560247751	0.6417751	0.218157	1
RASA4	1.4288434	1.49629358	1.3996067	0.805182131	-0.3126129	0.21826	1
SLC48A1	1.3313749	1.26671721	1.3594011	1.34749877	0.430284	0.218327	1
SMIM3	1.3657787	1.30008674	1.3942533	1.313797785	0.3937432	0.218445	1
RPP38	1.39074	1.3244298	1.4194825	1.292983941	0.3707044	0.218588	1
PEG10	2.5316789	2.63308951	2.487722	0.910986183	-0.1344989	0.218714	1
TMEM88	1.1621341	1.10153908	1.1883994	1.855437247	0.8917592	0.218812	1
CATSPER2	1.271925	1.3353246	1.2444442	0.728977699	-0.4560534	0.21894	0.9876852
WDR48	1.4055694	1.33920922	1.4343336	1.280429783	0.3566281	0.218959	1
ADAMTS7	1.2568422	1.19355422	1.2842747	1.468708445	0.554548	0.219223	1
CRACR2B	2.5291832	2.62937361	2.4857552	0.911856669	-0.133121	0.219304	1
RASD1	1.0887784	1.03018146	1.1141775	3.783034578	1.919544	0.219509	1
ZNF891	1.2995035	1.36363193	1.2717067	0.747202424	-0.420429	0.219543	1
CKAP4	2.4072875	2.31685106	2.4464877	1.098444437	0.1354619	0.21962	1
LINC00467	1.8767575	1.96012123	1.8406231	0.87553849	-0.1917575	0.220132	1
EHMT2	2.0153062	2.09451073	1.9809746	0.896267712	-0.1579984	0.220342	1
WDR12	1.3557676	1.29086304	1.3839008	1.319868101	0.4003938	0.220382	1
WDFY1	1.3940414	1.32797625	1.4226777	1.288744742	0.3659665	0.220712	1
SLC22A17	1.5297604	1.46115683	1.5594971	1.213246812	0.2788731	0.220731	1
SGK1	1.17993	1.11926453	1.2062257	1.729145598	0.7900594	0.22076	1
NFS1	1.1704637	1.11015939	1.1966029	1.784713271	0.8356923	0.220784	1
DDX27	1.3546513	1.28958513	1.3828547	1.322079825	0.4028093	0.220851	1
CKAP2L	1.2452967	1.3078102	1.2181998	0.708877817	-0.4963911	0.221127	1
LAMA4	1.1217686	1.06263028	1.1474024	2.35353274	1.2348279	0.221182	1

COL4A5	1.652577	1.72527815	1.6210643	0.856311924	-0.2237917	0.221216	1
POLH	1.1481666	1.20798903	1.1222363	0.587705472	-0.7668348	0.221553	1
MARCH2	1.2271416	1.1650989	1.2540343	1.53867979	0.621693	0.221635	1
STC1	1.557092	1.48680983	1.5875562	1.206952204	0.2713685	0.221656	0.9954716
UBE2G1	1.6022535	1.53213167	1.6326482	1.188894107	0.2496202	0.221916	1
COMMD2	1.6534997	1.58193488	1.6845199	1.176282611	0.2342347	0.221929	1
TMEM242	1.4859602	1.41825337	1.5153081	1.23204762	0.301058	0.221974	1
NFX1	1.228583	1.29054816	1.2017239	0.694287338	-0.5263952	0.222091	1
PHYHD1	1.238708	1.17660918	1.2656251	1.504027655	0.5888311	0.222204	1
FAM126A	1.381577	1.31592633	1.4100336	1.297877358	0.3761541	0.222258	1
GANAB	1.7873539	1.86206273	1.754971	0.87577271	-0.1913716	0.22252	1
CUX1	1.9187884	1.99669727	1.8850184	0.887951043	-0.171448	0.22255	1
OC90	1.0787493	1.02056357	1.1039702	5.056036787	2.338007	0.222754	1
SCHIP1.1	2.2963322	2.18856695	2.3430437	1.129968878	0.176283	0.222822	1
WDPCP	1.1366874	1.19615465	1.1109109	0.565425932	-0.82259	0.222894	1
GALNT7	1.7199826	1.64784413	1.7512515	1.15961761	0.2136491	0.222897	1
ERCC6L2	1.353124	1.41817841	1.3249258	0.777002862	-0.3640082	0.223022	1
DERL3	1.1725632	1.23302783	1.1463545	0.628055876	-0.6710352	0.223091	1
ZNF827	1.5287637	1.59727909	1.4990653	0.835564613	-0.2591767	0.223129	1
GOLM1	2.6191395	2.51939736	2.6623733	1.094100438	0.1297452	0.223465	1
ZNF136	1.1960389	1.25710154	1.1695709	0.659548412	-0.6004495	0.223541	1
NBL1	1.8873587	1.804388	1.9233228	1.147857502	0.1989436	0.223623	1
RAB22A	1.3902335	1.45616786	1.3616539	0.792808752	-0.3349552	0.223832	1
EXTL2	1.7696705	1.69568654	1.8017392	1.152443156	0.2046956	0.223836	1
CCZ1B	1.3606443	1.29631376	1.3885288	1.311207301	0.3908958	0.223916	1
XPO7	1.3036259	1.36702048	1.2761472	0.752402659	-0.4104231	0.22393	1
C8orf44	1.2679019	1.33035942	1.2408293	0.728991737	-0.4560256	0.224114	1
SNHG8	3.1959537	3.3195901	3.1423629	0.923595457	-0.114667	0.224223	1
DDAH1	1.5142632	1.44640332	1.5436774	1.217906281	0.2844031	0.224513	1
CH507-154B10.1	1.1068299	1.16534204	1.0814675	0.492720746	-1.0211579	0.224534	1
LAMB2	1.340444	1.27623536	1.3682755	1.333194709	0.4148875	0.22456	1
RGS3	1.4968223	1.42944503	1.5260274	1.224900429	0.2926645	0.224566	1
ZNF76	1.278908	1.34165928	1.2517081	0.736722697	-0.4408064	0.224575	1
TNFRSF19	1.1413292	1.08220349	1.1669576	2.031027599	1.0222098	0.224927	1
GLYATL2	1.1207828	1.17975713	1.09522	0.529714887	-0.916712	0.225007	1
SLFN13	1.1321232	1.19135102	1.1064506	0.556310533	-0.8460377	0.225085	1
GALNT10	1.2047097	1.14406214	1.2309978	1.603459188	0.6811876	0.225196	1
MFSD8	1.1792946	1.2393967	1.153243	0.640121521	-0.6435823	0.225428	1
CMTM3	1.8234908	1.74918624	1.8556985	1.142170616	0.1917782	0.225626	1
COPS7B	1.5043404	1.43677601	1.5336266	1.221739675	0.2889369	0.225769	1
ANAPC13	3.2680374	3.14092237	3.3231362	1.085109948	0.1178412	0.225783	1
PDE10A	1.0892163	1.14723748	1.0640667	0.435125112	-1.2004978	0.225912	1
CCNH	1.4371177	1.37130321	1.4656454	1.25408401	0.326634	0.226042	1
ATG3	1.5755141	1.50655024	1.6054069	1.195156653	0.2571997	0.226075	1
ZNF423	1.322515	1.38613634	1.2949379	0.763818156	-0.3886989	0.22659	1
EIF2A	1.7987658	1.72467635	1.8308803	1.146553678	0.1973039	0.226714	1
AP001347.6	1.2488458	1.31085826	1.2219662	0.714042992	-0.4859172	0.226872	1
C1orf174	1.624591	1.69502282	1.594062	0.854737402	-0.2264468	0.226892	1
NFE2L3	1.1618857	1.10252901	1.1876141	1.829863609	0.8717361	0.227038	1
FBXW8	1.1228068	1.18159298	1.0973256	0.53595458	-0.8998174	0.227091	1
WDR20	1.2517582	1.19023832	1.2784244	1.463555694	0.5494776	0.227128	1

FRMD4B	1.8564195	1.93201749	1.8236512	0.88372935	-0.1783235	0.227261	1
SNX27	1.5166923	1.4487471	1.5461436	1.217040875	0.2833776	0.227446	1
ACOT9	1.2604808	1.19862986	1.2872904	1.446360519	0.5324272	0.227462	1
MAATS1	1.1701475	1.2300261	1.1441927	0.626853833	-0.673799	0.227562	1
ING5	1.4354277	1.50138187	1.4068396	0.811436505	-0.3014499	0.227743	1
USP11	2.5775508	2.48132323	2.6192612	1.093118075	0.1284492	0.22788	1
EFR3A	1.3094567	1.24665147	1.3366799	1.365002704	0.4489038	0.228048	1
HBP1	1.5784703	1.50954122	1.6083479	1.193913109	0.2556978	0.22813	1
LAMC2	1.0960011	1.03865422	1.1208584	3.126655712	1.6446204	0.228287	1
EBLN3P	1.9956686	2.07276539	1.9622506	0.896981397	-0.15685	0.228301	1
CCDC53	1.55346	1.48519486	1.5830499	1.201681879	0.265055	0.228306	1
ZC3H7A	1.5611936	1.63010177	1.531325	0.843236834	-0.2459902	0.228925	1
XPO4	1.1852977	1.24548374	1.1592098	0.648555339	-0.6246984	0.22917	1
VEGFA	1.9812287	1.89087379	2.0203935	1.145385071	0.1958327	0.229223	1
ZBTB1	1.2863967	1.34914396	1.2591986	0.742383057	-0.4297643	0.22941	1
SELENOP	2.4563264	2.55977892	2.4114844	0.904925944	-0.1441284	0.229465	1
RP3-467N11.1	1.1728914	1.23250147	1.147053	0.63248221	-0.6609032	0.229738	1
ACLY	1.540097	1.47228378	1.569491	1.205823756	0.2700191	0.229777	1
LRWD1	1.2168298	1.15650796	1.2429766	1.552487049	0.6345812	0.229793	1
MIR1302-10	1.1063508	1.16442785	1.0811769	0.493693396	-1.0183127	0.229843	1
TBP	1.2460142	1.30720572	1.2194903	0.714473476	-0.4850476	0.229903	1
EPHB6	1.1609561	1.22021816	1.1352686	0.614248164	-0.7031065	0.229936	1
MED13L	1.434923	1.50076841	1.4063819	0.811516655	-0.3013074	0.230062	1
DES	1.1428454	1.084189	1.1682703	1.998720907	0.999077	0.230213	1
SMARCC1	2.8519337	2.75031824	2.8959795	1.083219861	0.1153261	0.23029	1
TMEM63B	1.2917773	1.22981621	1.3186347	1.386476118	0.4714228	0.230446	1
ZNF614	1.1486043	1.207599	1.1230327	0.592645749	-0.7547581	0.230547	1
MRPS9	1.5856914	1.51725533	1.6153554	1.189655096	0.2505434	0.23058	1
RAB29	1.243499	1.18260176	1.2698952	1.478053558	0.5636985	0.230626	1
RHNO1	1.86661	1.940783	1.8344593	0.886983812	-0.1730203	0.230701	1
HMBS	1.4701119	1.40306086	1.4991755	1.238461855	0.3085494	0.230714	1
RP11-455F5.3	2.4027611	2.31386689	2.4412928	1.096985381	0.1335443	0.230876	1
DPY19L3	1.2839997	1.34562628	1.2572873	0.744408975	-0.4258326	0.231001	1
MSL1	1.8172408	1.89052557	1.7854752	0.882035484	-0.1810914	0.231079	1
ARPIN	1.2238382	1.28477142	1.1974264	0.693280072	-0.5284898	0.231492	1
CDCA8	1.3333113	1.27049087	1.3605412	1.332914576	0.4145843	0.231566	1
TINF2	1.5445087	1.61274394	1.5149317	0.84037017	-0.2509031	0.231676	1
TK1	1.3663567	1.43050256	1.3385524	0.786411999	-0.3466428	0.231794	1
NBAS	1.4120824	1.34714061	1.4402317	1.268165398	0.3427429	0.231897	1
CD2BP2	1.47396	1.40792059	1.5025851	1.232066101	0.3010797	0.231994	1
RP11-158I9.8	1.1087175	1.16644065	1.0836972	0.502864838	-0.9917574	0.232075	1
C5orf42	1.5290614	1.59649474	1.4998321	0.837948831	-0.2550659	0.232081	1
HIST2H2BE	1.1324639	1.07421827	1.1577107	2.124958113	1.0874344	0.232144	1
ZNF107	1.1404605	1.19877302	1.1151847	0.579478384	-0.7871732	0.232191	1
RMI1	1.2567631	1.31811341	1.2301705	0.723548517	-0.4668383	0.232238	1
RMDN3	1.2912155	1.22939702	1.3180111	1.386291218	0.4712304	0.232315	1
HIP1	1.5154522	1.58267908	1.4863124	0.834614456	-0.2608182	0.232581	1
USB1	1.5720208	1.50377717	1.6016014	1.194181616	0.2560223	0.232876	1
TANK	1.4138446	1.34936933	1.4417917	1.264540707	0.3386135	0.233033	1
ATP13A2	1.4155688	1.35123371	1.4434552	1.262564451	0.336357	0.233065	1
FN3KRP	1.3699933	1.30635465	1.3975778	1.297769821	0.3760345	0.233095	1

DNAJC27-AS1	1.1683867	1.22735499	1.1428266	0.628209706	-0.6706819	0.233311	1
NUP155	1.2048869	1.26471542	1.178954	0.676023922	-0.5648538	0.233398	1
CTC-524C5.2	1.2260958	1.16576459	1.2522466	1.521716083	0.6056992	0.23344	1
TMEM189	1.430211	1.36519696	1.4583916	1.255190201	0.327906	0.233601	1
CXorf57	1.1338224	1.19191113	1.1086435	0.566113471	-0.8208368	0.233662	1
C11orf57	1.5840329	1.65267493	1.5542796	0.849243017	-0.2357506	0.233675	1
AC090498.1	4.2322236	4.37711523	4.1694195	0.938499062	-0.0915728	0.23372	1
NELFB	1.371016	1.30751308	1.3985416	1.296015214	0.3740827	0.23374	1
FAM57B	1.2074956	1.14741116	1.2335395	1.584272831	0.6638208	0.233903	1
RBFOX1	1.1025403	1.04538375	1.1273151	2.805301261	1.4881557	0.233907	1
MPV17L2	1.1821178	1.12280512	1.2078273	1.692333894	0.7590142	0.233958	1
CTNNBIP1	2.2591316	2.34496199	2.221928	0.90852234	-0.1384061	0.234658	1
CLSTN2	1.4486746	1.38368749	1.4768436	1.242791668	0.3135845	0.234887	1
TATDN3	1.2255186	1.16539399	1.2515799	1.521094781	0.6051101	0.234932	1
RP11-111M22.2	1.2258097	1.2860286	1.1997075	0.698208275	-0.5182706	0.235126	1
SEC23A	2.0490111	1.97060362	2.0829972	1.115797627	0.1580754	0.235174	1
GNL3	2.022093	1.94124917	2.0571352	1.123119397	0.1675113	0.235175	1
PXN-AS1	1.3908218	1.45501113	1.3629986	0.797779587	-0.3259379	0.235287	1
LRRCC1	1.6126028	1.68113375	1.5828977	0.855775723	-0.2246953	0.23536	1
SGCE	1.2748323	1.2138573	1.3012623	1.408706999	0.4943716	0.235368	1
RNF219	1.3235051	1.38628407	1.2962933	0.76703461	-0.3826364	0.235551	1
CIT	1.1326741	1.19076677	1.1074935	0.563481101	-0.8275609	0.235705	1
CTD-3131K8.2	1.1627477	1.22148687	1.1372869	0.619842141	-0.6900273	0.23572	1
SUMO3	3.1683212	3.04682741	3.2209833	1.085085767	0.1178091	0.235784	1
KDM4B	1.5528569	1.62053549	1.5235213	0.843660543	-0.2452655	0.235818	1
PPP2R5E	1.8850626	1.81100722	1.9171623	1.130892834	0.1774622	0.236137	1
ATRX	3.5877669	3.71224765	3.53381	0.934210406	-0.0981806	0.236243	1
LINC00342	1.124887	1.18254116	1.0998966	0.547255004	-0.8697149	0.236269	1
GTF2E2	1.7288676	1.65816653	1.7595134	1.153983608	0.2066227	0.236338	1
ATF4	2.7576212	2.65670676	2.8013631	1.087315624	0.1207708	0.236642	1
TNIP1	2.050092	1.97387375	2.0831292	1.112186451	0.1533987	0.236818	1
TMEM45A	2.1601779	2.0621901	2.2026513	1.132237325	0.1791764	0.23683	1
CCDC18	1.2067171	1.26629232	1.1808939	0.679305729	-0.5578671	0.236883	1
NCBP2	3.0962372	2.97052329	3.1507285	1.091450451	0.1262466	0.236943	1
GORASP1	1.1495678	1.09184868	1.1745864	1.900804896	0.9266105	0.236964	1
SCAMP3	1.3630992	1.30027856	1.3903292	1.299890223	0.3783898	0.237342	1
TRIM38	1.1566841	1.09857372	1.1818724	1.845039325	0.8836516	0.237379	1
FAM69C	1.1475572	1.2055827	1.1224058	0.595408977	-0.7480471	0.237381	1
VEPH1	1.1126038	1.05574733	1.1372485	2.461975214	1.2998162	0.23756	1
PTK2B	1.1145535	1.05753984	1.1392664	2.420346557	1.2752136	0.237857	1
RRP36	1.1933059	1.13440884	1.2188352	1.628131009	0.7032168	0.237968	1
SNAP23	1.5135718	1.44782067	1.5420719	1.2104665	0.2755632	0.238004	1
PRSS23	3.0127431	2.87581476	3.0720955	1.104637575	0.1435731	0.238046	1
OGFOD3	1.3521522	1.28992248	1.379126	1.307680619	0.3870102	0.238174	1
TMEM115	1.4717791	1.40672949	1.4999752	1.229257294	0.2977869	0.238366	1
CRADD	1.2415808	1.18175712	1.2675117	1.471808843	0.5575903	0.238639	1
PRPF38A	1.8267065	1.75199606	1.8590902	1.14241316	0.1920845	0.238648	1
NPRL2	1.4915786	1.42572988	1.5201211	1.221716295	0.2889093	0.239094	1
PRRC1	1.4443453	1.37968836	1.4723711	1.244102259	0.3151051	0.239144	1
STX18	1.2019693	1.14277366	1.227628	1.59432756	0.6729481	0.239236	1
CD44	1.218522	1.15965625	1.2440376	1.528519067	0.6121345	0.239244	1

MAP2K3	1.2648061	1.20453781	1.2909297	1.422376202	0.5083031	0.239446	1
PDE9A	1.1976198	1.1386746	1.2231699	1.609306225	0.6864389	0.23957	1
MGEA5	2.2993738	2.38346675	2.2629233	0.912868552	-0.131521	0.239585	1
ZNF22	2.3278752	2.24100899	2.3655278	1.100336782	0.1379452	0.239599	1
EAF1	1.2140438	1.27368515	1.1881919	0.687621918	-0.5403126	0.239709	1
SERINC1	1.8356756	1.76344235	1.8669854	1.135626612	0.1834886	0.239754	1
MLANA	1.079617	1.02344045	1.1039671	4.435370793	2.1490547	0.23992	1
CHTF18	1.1814048	1.23977607	1.1561035	0.651038704	-0.6191848	0.240396	1
ANKH	1.1998333	1.25877002	1.1742868	0.67352007	-0.5702072	0.240979	1
MFAP1	1.6684128	1.59955554	1.6982593	1.164628279	0.2198696	0.240994	1
SRGAP1	1.6130418	1.68115228	1.5835189	0.85666439	-0.223198	0.241052	1
ZCCHC3	1.395146	1.33182969	1.4225908	1.273517158	0.3488184	0.241081	1
TSC2	1.4326777	1.49675442	1.4049033	0.815097464	-0.2949555	0.241355	1
ALDH16A1	1.1531543	1.09552707	1.1781331	1.864739882	0.8989744	0.241659	1
GALNT1	1.8539206	1.78127639	1.8854087	1.133284826	0.1805105	0.242174	1
SNHG21	1.3444065	1.40647672	1.3175018	0.781106988	-0.3564079	0.242182	1
NFKBIE	1.175635	1.1177111	1.2007424	1.705381792	0.7700948	0.242296	1
ACP6	1.5986979	1.66635853	1.56937	0.85444988	-0.2269322	0.242301	1
CTBS	1.457595	1.39317171	1.4855197	1.234879442	0.3043702	0.242532	1
CCDC191	1.1712047	1.22932008	1.1460143	0.636726974	-0.6512532	0.242868	1
SECISBP2	2.0794987	2.15647533	2.0461328	0.904587199	-0.1446685	0.243004	1
PTK2	1.9543421	1.87936385	1.9868419	1.122222507	0.1663588	0.243106	1
DCP1B	1.2931345	1.35407654	1.2667189	0.753280375	-0.4087412	0.243218	1
POC1A	1.1962394	1.25484517	1.1708364	0.670353682	-0.5770056	0.243294	1
SETD6	1.1996406	1.25824441	1.1742384	0.674703483	-0.5676745	0.243364	1
ZNF605	1.4596414	1.52378737	1.431837	0.824451045	-0.2784943	0.24351	1
SGCD	1.0950556	1.03897958	1.1193621	3.062169412	1.6145541	0.243558	1
TBL2	1.3657618	1.30402184	1.3925234	1.291102507	0.3686035	0.243583	1
CES2	1.6617401	1.7302242	1.6320553	0.865563366	-0.2082887	0.243978	1
TSPAN9	1.1917627	1.13350482	1.2170149	1.625520897	0.7009021	0.244059	1
PPP1R2	1.8570181	1.78476045	1.8883386	1.131986978	0.1788574	0.244202	1
TRMT1L	1.3420725	1.28083829	1.3686148	1.312551735	0.3923743	0.244544	1
SNX7	1.533392	1.46810963	1.561689	1.199909059	0.2629251	0.244721	1
ATG2A	1.1814364	1.2398288	1.156126	0.650989174	-0.6192945	0.244765	1
ZNF320	1.1714279	1.11372587	1.1964391	1.727303885	0.7885219	0.244979	1
A2M-AS1	1.2433008	1.3026869	1.2175595	0.718761003	-0.476416	0.244999	1
GOLGA8B	1.6590536	1.72700565	1.6295994	0.866017147	-0.2075325	0.245114	1
STARD10	1.7658323	1.83615209	1.7353518	0.879447444	-0.1853307	0.245386	1
HINT3	1.4965818	1.43132877	1.5248661	1.216858567	0.2831615	0.245487	1
RP5-1172A22.1	1.087223	1.03176167	1.1112631	3.503060423	1.8086159	0.24557	1
RP11-572O17.1	1.0795221	1.13499473	1.0554772	0.410958263	-1.2829362	0.245608	1
RAD51B	1.1614904	1.21877969	1.136658	0.62463756	-0.6789088	0.245614	1
MORN4	1.3291996	1.26798243	1.3557345	1.327454653	0.4086626	0.245889	1
SLFN5	1.2405476	1.1817487	1.2660343	1.463748116	0.5496673	0.246217	1
STK25	2.3451996	2.25997613	2.3821402	1.096957416	0.1335075	0.246289	1
FAM126B	1.2680516	1.32787779	1.2421196	0.738444594	-0.4374384	0.246468	1
LINC01560	1.2791832	1.3391618	1.2531852	0.746502621	-0.4217808	0.246708	1
PSMG1	1.5263785	1.46128958	1.5545916	1.20226339	0.265753	0.246799	1
NRXN2	1.242022	1.30089977	1.216501	0.719512155	-0.474909	0.246841	1
DIRC3	1.1350067	1.19180161	1.1103886	0.57553543	-0.7970234	0.246963	1
NDRG4	1.4656673	1.5301046	1.4377367	0.82575525	-0.2762139	0.246977	1

AP2A2	1.6570594	1.72521497	1.6275171	0.865284195	-0.208754	0.247141	1
ZFYVE16	1.5551579	1.62080978	1.5267008	0.848409277	-0.2371677	0.247265	1
NR1D1	1.1633765	1.10616398	1.1881755	1.772498942	0.8257848	0.247296	1
HOOK3	1.8286141	1.90006038	1.7976453	0.886213092	-0.1742745	0.247309	1
RP11-178L8.7	1.1990332	1.25682964	1.1739811	0.677418267	-0.5618812	0.247428	1
BROX	1.7429462	1.81289955	1.7126245	0.876645184	-0.1899351	0.247616	1
GMPPA	1.2687235	1.20933932	1.2944638	1.406634084	0.4922471	0.247648	1
INPP5K	1.15888	1.10167256	1.1836768	1.806552775	0.8532394	0.247853	1
SPATA7	1.4857373	1.42157827	1.5135474	1.218154384	0.284697	0.247967	1
PNP	1.1803569	1.12310726	1.2051721	1.666612372	0.7369186	0.24802	1
NOP56	2.2061175	2.28717361	2.1709832	0.909732173	-0.1364862	0.248032	1
PHKG2	1.3168862	1.2561151	1.3432278	1.340131005	0.422374	0.248341	1
LMCD1	1.1971115	1.13952923	1.2220708	1.591572063	0.6704525	0.248721	1
C16orf62	1.289012	1.22919182	1.3149414	1.374138972	0.4585279	0.248811	1
PTPRN2	1.3767077	1.31486271	1.4035147	1.281557747	0.3578985	0.248906	1
ZNF346	1.2503799	1.30962146	1.2247014	0.725729341	-0.4624965	0.249163	1
PCNX4	1.7998462	1.87001903	1.7694294	0.884382259	-0.177258	0.249258	1
PLK1	1.3464012	1.28435938	1.3732936	1.312752876	0.3925954	0.249355	1
ZFYVE19	1.2726738	1.21330542	1.2984074	1.398967771	0.4843627	0.249421	1
MTMR4	1.7047726	1.77337963	1.6750345	0.872837144	-0.1962156	0.249532	1
BMS1	1.3959567	1.33349739	1.42303	1.268465568	0.3430844	0.249629	1
PCYOX1	1.7931625	1.72323917	1.8234711	1.138587568	0.1872453	0.249757	1
TMEM54	1.1031388	1.04785416	1.1271022	2.656031864	1.4092725	0.249808	1
EPB41L4A-AS1	2.955241	3.07286359	2.9042568	0.918659986	-0.1223971	0.249827	1
SMU1	1.4468968	1.3836064	1.4743303	1.236502662	0.3062653	0.249857	1
PFN2	7.2604809	7.02360405	7.3631564	1.056370305	0.0791157	0.249947	1
CLVS2	1.1257645	1.18173345	1.1015045	0.558534887	-0.8402807	0.249948	1
GRK6	1.2847801	1.22522574	1.3105942	1.379035062	0.4636591	0.250083	1
PRPF4	1.3587391	1.29734043	1.3853527	1.29599821	0.3740637	0.250155	1
NUPL2	1.3040727	1.24405175	1.3300891	1.352537206	0.4356683	0.250172	1
SHTN1	1.3511671	1.29003623	1.3776646	1.302128961	0.3808723	0.250363	1
GABPA	1.3624498	1.42452088	1.3355447	0.790408011	-0.3393305	0.250447	1
WDR25	1.667119	1.59722506	1.697415	1.167759035	0.2237426	0.25054	1
LBR	1.7093383	1.64142083	1.7387775	1.151782773	0.2038686	0.250888	1
BRIX1	1.4485107	1.38528919	1.4759144	1.235213392	0.3047603	0.250932	1
HSPBP1	1.539937	1.47532764	1.5679423	1.194843876	0.2568221	0.251052	1
CDKL2	1.2293096	1.28781541	1.2039499	0.708613519	-0.4969291	0.251162	1
OGFOD1	1.2409177	1.18229425	1.2663284	1.460980587	0.546937	0.25122	1
ATM	1.8823925	1.95387651	1.8514074	0.892576112	-0.1639529	0.251288	1
PRPF8	1.677968	1.74583509	1.6485506	0.869562984	-0.2016376	0.251299	1
TTLL4	1.2164608	1.15842845	1.2416153	1.525075041	0.6088802	0.25138	1
SRM	1.9182765	1.84634734	1.9494545	1.121826082	0.165849	0.251811	1
NPAS3	1.2757793	1.33504153	1.2500917	0.746449965	-0.4218825	0.251841	1
SLC35G1	1.4974108	1.43316939	1.5252566	1.212589326	0.278091	0.252281	1
MRPL39	1.4061416	1.34434162	1.4329292	1.257266578	0.3302906	0.252359	1
AP000344.3	1.0907959	1.14558522	1.0670472	0.460535811	-1.1186147	0.252465	1
LRRC75B	1.1016506	1.15680116	1.0777452	0.495820492	-1.0121102	0.252563	1
STK33	1.4220437	1.48477884	1.3948509	0.814496941	-0.2960188	0.252616	1
GALM	1.284426	1.225343	1.3100359	1.375839792	0.4603125	0.252778	1
TCF3	1.710218	1.77830799	1.6807041	0.874594743	-0.1933134	0.253053	1
TOB1	1.377242	1.31622721	1.4036891	1.276579333	0.3522832	0.253306	1

SPRED1	1.4980088	1.56199686	1.4702728	0.836788969	-0.2570643	0.253348	1
GGA2	1.7798183	1.70990038	1.8101247	1.141180776	0.1905273	0.253596	1
SUGCT	1.104159	1.04912261	1.1280148	2.606025181	1.381851	0.253636	1
OSGEP	1.5668486	1.50170083	1.5950873	1.186139758	0.246274	0.253643	1
ATP6V0E2	2.1633728	2.08568735	2.197046	1.102569741	0.1408699	0.253772	1
ACADVL	3.4593737	3.33295048	3.5141725	1.077679308	0.1079279	0.25382	1
INTS3	1.3773213	1.43840386	1.3508447	0.800277316	-0.3214281	0.254231	1
TMTC2	1.2496862	1.30834674	1.2242595	0.727296544	-0.4593844	0.254378	1
PPP2CB	1.5536683	1.48897725	1.5817089	1.1896442	0.2505302	0.254824	1
C2CD5	1.2046438	1.26193754	1.1798095	0.686459484	-0.5427535	0.254967	1
ZNF790	1.2700074	1.32918151	1.2443581	0.742320192	-0.4298865	0.255037	1
RRAD	1.0700311	1.01622129	1.0933552	5.755105505	2.5248424	0.255037	1
SLC50A1	1.6371232	1.57079019	1.6658757	1.166585675	0.2222923	0.255191	1
VWA9	1.3610299	1.30048824	1.387272	1.28880923	0.3660387	0.255519	1
FAAH	1.1581504	1.21448401	1.1337323	0.623506852	-0.6815227	0.255522	1
EXT1	1.2237058	1.16626828	1.2486024	1.49518859	0.5803275	0.255935	1
ACACB	1.1269023	1.18215931	1.1029508	0.565168897	-0.823246	0.256022	1
ZNF467	1.2562104	1.19791615	1.2814783	1.422210074	0.5081346	0.256063	1
EXOSC4	1.6054302	1.54010011	1.6337479	1.1733897	0.2306822	0.25609	1
CRX	1.1408719	1.08475779	1.1651949	1.949023362	0.9627514	0.256347	1
FNIP2	1.3876817	1.32650168	1.4142004	1.268601251	0.3432387	0.25642	1
ABCC4	1.1995353	1.256485	1.1748501	0.681716792	-0.5527556	0.256506	1
RB1	1.5043358	1.56774549	1.4768505	0.83990196	-0.2517072	0.256567	1
BAIAP2-AS1	1.2522874	1.31073432	1.2269533	0.730377153	-0.4532865	0.25671	1
FAM71E1	1.264984	1.20648237	1.2903418	1.406133643	0.4917337	0.256891	1
H1FX-AS1	1.1626263	1.21868446	1.1383275	0.63254399	-0.6607623	0.256992	1
DUSP11	1.3241912	1.26465638	1.3499969	1.322457725	0.4032216	0.257031	1
TSPAN17	1.4549472	1.39230383	1.4821004	1.228895366	0.2973621	0.257062	1
SIX6	4.6775117	4.84378542	4.6054395	0.93799188	-0.0923527	0.257094	1
AFAP1	1.3633843	1.30273241	1.3896742	1.287190351	0.3642254	0.257133	1
RP11-128P17.4	1.2259983	1.28368711	1.2009928	0.708501605	-0.497157	0.257436	1
BNC2	1.1384174	1.08317064	1.1623644	1.952184063	0.9650891	0.257459	1
AC074289.1	1.1541801	1.20989957	1.1300282	0.619478297	-0.6908744	0.257486	1
RBMS3	1.1547656	1.09934504	1.1787879	1.799666483	0.8477296	0.257621	1
PPP1R3G	1.1340415	1.18964153	1.1099414	0.579732904	-0.7865397	0.257912	1
KATNA1	1.3209835	1.26180154	1.3466363	1.324042222	0.4049491	0.258276	1
CTXN1	1.9778961	2.05122863	1.9461097	0.900003777	-0.151997	0.258286	1
SMC1A	1.8187533	1.88839605	1.7885662	0.887629123	-0.1719711	0.258531	1
ERAP1	1.1058987	1.16061248	1.0821828	0.511683657	-0.9666759	0.258793	1
RP11-1017G21.5	1.1150453	1.16974744	1.0913343	0.53805993	-0.8941612	0.258916	1
RBM14	1.4930131	1.55612236	1.465658	0.837330086	-0.2561316	0.259038	1
FAHD2A	1.7556286	1.68743713	1.7851865	1.142193909	0.1918076	0.259135	1
MIIP	1.4254856	1.36398583	1.4521431	1.242199675	0.3128971	0.259333	1
BOLA3	2.2843914	2.20139622	2.3203662	1.099026399	0.136226	0.259375	1
POLDIP3	1.5580672	1.62243307	1.5301675	0.85176628	-0.2314705	0.259647	1
CPNE8	1.3812114	1.32073407	1.4074256	1.270291041	0.3451591	0.259902	1
SDK1	1.1482439	1.2036926	1.1242094	0.609788258	-0.7136197	0.260001	1
TRIM65	1.1755437	1.23172744	1.1511905	0.652449907	-0.616061	0.260009	1
TRAF2	1.2300751	1.17308072	1.2547797	1.472027966	0.5578051	0.260255	1
DHX29	1.5811501	1.51645675	1.6091918	1.179560087	0.2382489	0.260331	1
COA1	3.6789256	3.79861602	3.6270451	0.938694357	-0.0912726	0.260445	1

LINC00941	1.1275656	1.1827832	1.1036312	0.56696249	-0.8186748	0.260633	1
RP11-348J24.2	1.1853301	1.24132265	1.1610599	0.667404701	-0.5833662	0.260637	1
NEK1	1.4735335	1.41077931	1.5007346	1.218986886	0.2856826	0.260693	1
TRMT10C	1.5508763	1.48740508	1.5783883	1.186668551	0.246917	0.260828	1
PDPK1	1.2762823	1.21820339	1.301457	1.381541211	0.4662786	0.260851	1
ADAL	1.513106	1.57668626	1.4855468	0.841960039	-0.2481763	0.261023	1
MAIP1	1.2825538	1.22463977	1.307657	1.369557183	0.4537095	0.261056	1
CENPL	1.1687225	1.22447335	1.144557	0.643983127	-0.6349052	0.261163	1
TK2	1.2436192	1.30083153	1.2188202	0.727384506	-0.4592099	0.261475	1
GDF11	1.5495344	1.61349685	1.5218096	0.850549718	-0.2335325	0.261571	1
RAB1B	1.4822988	1.41980509	1.5093871	1.213389406	0.2790426	0.261679	1
PLEKHJ1	2.7541106	2.66138189	2.7943044	1.080007208	0.1110409	0.262652	1
SERTAD1	1.276632	1.21881021	1.3016951	1.378798172	0.4634113	0.262661	1
GGPS1	2.0245619	1.95066325	2.0565936	1.111427862	0.1524143	0.262661	1
ST3GAL1	1.1123332	1.05825393	1.1357741	2.330729393	1.2207815	0.26297	1
AGPAT4	1.3676564	1.42792284	1.3415336	0.798119622	-0.3253231	0.263129	1
MGLL	1.1654741	1.11018616	1.189439	1.719262911	0.7817902	0.263297	1
IER5L	1.3621545	1.30236105	1.3880724	1.283473369	0.3600534	0.26333	1
TIMM21	1.2993712	1.3580807	1.2739233	0.764976471	-0.3865127	0.263352	1
DEAF1	2.099681	2.17838411	2.0655668	0.904260968	-0.1451889	0.263373	1
SH2B1	1.4311149	1.36999795	1.4576064	1.23678077	0.3065898	0.263394	1
GPD1	1.0829786	1.02955721	1.1061344	3.590811737	1.84431	0.263454	1
PCGF5	1.3175123	1.2585279	1.3430794	1.327049981	0.4082227	0.263564	1
ALG3	1.6366237	1.5715889	1.6648134	1.163097134	0.2179716	0.263565	1
ANAPC1	1.2403998	1.297482	1.2156572	0.724941909	-0.4640627	0.264111	1
RBPJ	2.9693068	2.85124332	3.0204821	1.091418967	0.126205	0.264206	1
PROSER3	1.2989652	1.35729964	1.2736798	0.765967119	-0.3846456	0.264347	1
GBE1	1.8019708	1.73402458	1.8314225	1.132690237	0.1797534	0.264465	1
SLITRK1	1.0745373	1.12777544	1.0514609	0.40274508	-1.3120611	0.264533	1
KIAA1524	1.2128449	1.26941739	1.1883233	0.699002024	-0.5166315	0.264571	1
CCNL2	1.7189096	1.78580558	1.6899132	0.877969343	-0.1877575	0.264721	1
NEK6	1.5845763	1.52071476	1.6122574	1.175801845	0.2336449	0.264774	1
CLOCK	1.405958	1.46658711	1.3796779	0.813734302	-0.2973703	0.264808	1
EML6	1.1089114	1.16280045	1.0855528	0.525507448	-0.9282169	0.265094	1
RP11-85O21.2	1.3444097	1.40348637	1.3188025	0.790119735	-0.3398568	0.265366	1
MVD	1.283398	1.22584009	1.3083468	1.365332554	0.4492524	0.265712	1
NENF	3.549336	3.43068783	3.6007648	1.0699707	0.0975713	0.266082	1
APH1A	2.6768536	2.58311877	2.7174834	1.084873389	0.1175267	0.26609	1
TOLLIP-AS1	1.1300552	1.18442742	1.1064873	0.577393888	-0.7923723	0.266166	1
CHD2	1.7386728	1.80561966	1.7096543	0.880880119	-0.1829824	0.266347	1
TBC1D14	1.4055604	1.34539137	1.431641	1.249715743	0.3216	0.266908	1
FNDC10	1.1945063	1.25016415	1.1703811	0.68107726	-0.5541096	0.26697	1
DYM	1.6404317	1.57529462	1.6686658	1.162301441	0.2169843	0.267149	1
ICAM1	1.1275878	1.07359369	1.1509918	2.051694882	1.0368162	0.267264	1
SCUBE3	1.3071475	1.36483987	1.2821404	0.773326743	-0.37085	0.267355	1
AC159540.1	1.1983293	1.25390932	1.1742378	0.686220444	-0.543256	0.267458	1
AAAS	1.4355548	1.37531243	1.4616672	1.23008782	0.2987613	0.2676	1
FANCI	1.3050162	1.36322231	1.2797865	0.770289993	-0.3765264	0.267741	1
ENAH	2.8792991	2.77614715	2.9240108	1.083249688	0.1153658	0.267799	1
OCEL1	1.5513571	1.61500053	1.5237705	0.851658637	-0.2316528	0.267828	1
PRPS2	1.5315886	1.4693031	1.5585867	1.190247104	0.2512611	0.26789	1

MKRN2	1.4764256	1.41499729	1.503052	1.212181386	0.2776056	0.268211	1
SLC25A13	1.6690439	1.6038911	1.6972847	1.154653018	0.2074594	0.268274	1
ZWILCH	1.3159916	1.37386298	1.290907	0.778111159	-0.3619518	0.26843	1
MANEA-AS1	1.2104068	1.26590732	1.1863498	0.700807301	-0.5129103	0.268872	1
HMGN3	9.3094261	9.63106789	9.1700087	0.946581443	-0.0792015	0.268963	1
MYC	1.0746016	1.02196551	1.097417	4.434996393	2.1489329	0.268985	1
CDS2	1.4808865	1.41960711	1.5074483	1.209341599	0.2742218	0.269147	1
SDC3	1.322671	1.38112579	1.2973335	0.78014537	-0.3581851	0.26921	1
GOLGA3	1.2073494	1.15182719	1.2314158	1.524204901	0.6080569	0.269414	1
METTL10	1.3304326	1.27195553	1.3557798	1.308227902	0.3876139	0.269422	1
PPAN	1.3615481	1.30246098	1.3871598	1.280032107	0.35618	0.269552	1
CREBBP	1.4483653	1.51022033	1.4215539	0.826219296	-0.2754033	0.269737	1
MBD5	1.4189232	1.47947035	1.3926788	0.818984467	-0.288092	0.269761	1
DGKH	1.2638324	1.32081354	1.2391336	0.745397571	-0.423918	0.269765	1
ARSG	1.2012073	1.25688524	1.1770733	0.689309139	-0.536777	0.270088	1
MRPL42	2.9516322	2.8496136	2.9958528	1.079064718	0.1097814	0.270108	1
CAD	1.2654831	1.32218951	1.2409034	0.747707025	-0.419455	0.27019	1
CPSF6	1.7870631	1.85428333	1.7579261	0.887206946	-0.1726574	0.270256	1
BCOR	1.2404357	1.29673205	1.2160337	0.728043173	-0.4579041	0.27069	1
PGRMC2	1.8154073	1.74842964	1.8444392	1.12828129	0.1741268	0.271046	1
OLFML3	1.1643289	1.10986824	1.1879351	1.71055003	0.7744603	0.271052	1
CD70	1.1129631	1.05961848	1.1360856	2.282606878	1.1906824	0.271092	1
MTAP	1.4773259	1.41658662	1.5036537	1.209001078	0.2738155	0.271121	1
ACTR6	1.2948322	1.23783461	1.3195381	1.343530702	0.4260293	0.271208	1
NEO1	1.4080215	1.34788864	1.4340865	1.247774155	0.3193568	0.271337	1
UBTF	1.5176112	1.45575963	1.5444211	1.194535615	0.2564499	0.271357	1
TBCD	1.3719494	1.31278735	1.3975935	1.271130457	0.3461121	0.271592	1
HMCES	1.3075193	1.25020626	1.3323619	1.328351641	0.4096371	0.271746	1
CHMP7	1.3033924	1.2462738	1.3281507	1.332463088	0.4140956	0.271747	1
MAPK6	2.0361088	1.96382177	2.0674421	1.107509805	0.1473195	0.271752	1
STAM	1.2492822	1.19311024	1.2736303	1.416964045	0.5028032	0.271812	1
ITGB6	1.1720433	1.11726176	1.1957886	1.669671774	0.7395645	0.271931	1
TIMM29	1.2582437	1.20193853	1.2826495	1.399680782	0.4850978	0.271986	1
C9orf116	1.6318673	1.69581726	1.6041479	0.868256569	-0.2038067	0.272211	1
RP11-115D19.1	3.2443759	3.09863877	3.3075465	1.099544388	0.1369058	0.272262	1
KIAA1586	1.3449585	1.40345412	1.3196032	0.792167351	-0.3361229	0.27233	1
MUT	1.2784257	1.22130445	1.3031852	1.369991597	0.454167	0.272771	1
LPCAT3	1.200331	1.14511005	1.2242668	1.545494476	0.6280685	0.272876	1
RP11-395G23.3	1.9082331	1.97935037	1.8774069	0.895907036	-0.1585791	0.272971	1
LINC00115	1.1781312	1.23286931	1.1544046	0.663052816	-0.5928043	0.273091	1
SST	1.0722114	1.01685718	1.096205	5.707065023	2.512749	0.273491	1
CCNT2	1.476674	1.53763448	1.4502504	0.837465651	-0.2558981	0.2735	1
BCHE	1.654297	1.58999192	1.6821705	1.156236939	0.2094371	0.273562	1
CHORDC1	1.5658417	1.5032198	1.5929855	1.178382728	0.2368082	0.273584	1
NPHP3	1.3198187	1.37768463	1.2947364	0.780377048	-0.3577567	0.273649	1
SPG20	1.706699	1.64139072	1.7350073	1.145958722	0.1965551	0.273809	1
CEP192	1.1959114	1.25086198	1.1720928	0.686006056	-0.5437068	0.273848	1
ASB13	1.1962291	1.14119024	1.220086	1.5587908	0.6404273	0.273891	1
DNMT3A	1.8148253	1.88180525	1.7857925	0.891117911	-0.1663118	0.273949	1
UHRF1BP1	1.2112014	1.26657075	1.1872013	0.702257326	-0.5099283	0.27414	1
MCM6	1.2846928	1.34152518	1.2600585	0.761462105	-0.3931559	0.274261	1

MASTL	1.153226	1.20717555	1.1298413	0.626721002	-0.6741048	0.274339	1
ADSSL1	1.2206084	1.16543057	1.2445255	1.478115625	0.5637591	0.274427	1
SOCS7	1.2023087	1.25734214	1.1784541	0.693450708	-0.5281348	0.274451	1
FAM136A	2.5623207	2.47475806	2.6002752	1.085110354	0.1178418	0.274592	1
ZNF32	2.2060912	2.13094023	2.2386658	1.095253075	0.1312643	0.275041	1
BTAF1	1.2995368	1.3564828	1.2748533	0.771014211	-0.3751706	0.275143	1
SLC35C2	1.635739	1.57207177	1.6633359	1.159532687	0.2135435	0.275327	1
KIZ	1.3180182	1.37495395	1.2933391	0.782333771	-0.3541439	0.275461	1
LRP6	1.4476086	1.5076144	1.4215987	0.830549224	-0.2678624	0.275677	1
SEMA5B	1.0781627	1.13012846	1.0556378	0.427560851	-1.2257983	0.27575	1
ZCCHC17	2.4749297	2.39302441	2.510432	1.084282499	0.1167407	0.275838	1
GTPBP8	1.2831226	1.22671409	1.3075732	1.356656515	0.4400555	0.275866	1
ACSL4	1.5758377	1.638817	1.548539	0.858679334	-0.2198086	0.275895	1
TMEM164	1.3072377	1.25009444	1.3320067	1.327525471	0.4087395	0.275948	1
ANP32E	3.1729589	3.06124633	3.2213813	1.077688416	0.1079401	0.276192	1
LINC00339	1.2001378	1.25517971	1.1762796	0.690805866	-0.5336478	0.276208	1
TOM1L2	1.2522659	1.30850439	1.227889	0.738689813	-0.4369594	0.276237	1
IRF9	1.2296604	1.28488641	1.2057224	0.722120748	-0.469688	0.276316	1
DNM2	1.3033975	1.24655653	1.3280356	1.330468041	0.4119339	0.276365	1
WBSCR17	1.1812905	1.23589912	1.1576201	0.668167391	-0.5817185	0.27638	1
COL16A1	1.093257	1.04100637	1.1159054	2.826521407	1.4990276	0.276566	1
PLAC9	1.0838712	1.03175589	1.1064608	3.352475116	1.7452266	0.276605	1
B4GALT1	1.3827638	1.32442783	1.4080499	1.257752338	0.3308479	0.276613	1
COLGALT1	1.2922122	1.34851287	1.2678084	0.768431796	-0.3800109	0.276785	1
DZIP3	1.7313313	1.79639886	1.7031275	0.882883583	-0.1797049	0.276832	1
RABL2B	1.6852612	1.74967643	1.6573401	0.876831742	-0.1896281	0.27727	1
PPP1R8	1.405061	1.34648972	1.430449	1.242313948	0.3130298	0.277335	1
CCNA1	1.1253865	1.07258533	1.1482734	2.042745849	1.0305097	0.277339	1
FAM129A	1.1123568	1.05970394	1.1351794	2.264162691	1.1789776	0.277355	1
TMEM14B	4.1124967	3.98324446	4.1685218	1.062105987	0.0869277	0.277452	1
RFX3	1.5520356	1.61402688	1.5251651	0.855280365	-0.2255307	0.277465	1
TBK1	1.1933204	1.24799976	1.1696194	0.683949783	-0.5480377	0.277508	1
LMAN2L	1.2903939	1.23396615	1.3148528	1.345719427	0.4283776	0.277762	1
DUOX1	1.2007082	1.25561659	1.1769079	0.692083036	-0.530983	0.278006	1
DCLRE1A	1.1257052	1.17871632	1.1027272	0.5748061	-0.7988527	0.278019	1
FGD1	1.1455752	1.19889193	1.1224648	0.615735554	-0.6996172	0.278135	1
PCNX2	1.1832047	1.12905401	1.2066766	1.601473555	0.6794	0.278145	1
PUS7L	1.4470614	1.50693025	1.421111	0.830707943	-0.2675867	0.278162	1
RIDA	1.778698	1.71305054	1.8071533	1.131972017	0.1788383	0.278215	1
PRKAG2	1.2244425	1.16970661	1.2481681	1.462336301	0.5482751	0.278319	1
SC5D	1.3691439	1.31150791	1.3941266	1.265221678	0.3393902	0.278637	1
VSX1	1.050277	1.10942342	1.0246397	0.225177662	-2.1508644	0.2787	1
SUCO	2.0241271	1.95365696	2.0546728	1.105924705	0.1452532	0.278743	1
HDHD3	1.430556	1.3711399	1.4563102	1.229483034	0.2980518	0.278783	1
LMO1	1.3130636	1.25325126	1.3389896	1.338550669	0.4206718	0.278853	1
BRMS1	1.4545014	1.39507997	1.480258	1.215596913	0.2816649	0.279087	1
RP11-440L14.1	1.181359	1.23551475	1.157885	0.670382451	-0.5769437	0.279147	1
TWSG1	1.7122076	1.77739631	1.6839512	0.879797341	-0.1847569	0.279181	1
AAED1	1.2710669	1.21533979	1.2952221	1.370959589	0.455186	0.279361	1
KNSTRN	1.3865024	1.32856095	1.4116174	1.252788552	0.3251429	0.279495	1
EXOSC9	1.3155976	1.25902674	1.3401185	1.313063316	0.3929365	0.279514	1

COL13A1	1.2403951	1.29559775	1.2164672	0.732303122	-0.4494871	0.279743	1
HYOU1	1.3719445	1.4300627	1.3467529	0.806284547	-0.310639	0.280061	1
LINC00511	1.1100818	1.16225277	1.087468	0.539084538	-0.8914166	0.280221	1
COL26A1	1.1050254	1.05294908	1.1275981	2.409826645	1.2689294	0.280245	1
DOK5	1.1949665	1.14033581	1.2186465	1.55802382	0.6397173	0.280493	1
MAFG-AS1	1.40194	1.46074267	1.3764517	0.817054172	-0.2914964	0.280669	1
RRAGB	1.3102022	1.25320206	1.3349092	1.322695227	0.4034807	0.281087	1
KCTD17	1.2936426	1.23763539	1.3179192	1.337844685	0.4199106	0.281374	1
UCHL5	1.3800793	1.32217798	1.4051769	1.257618315	0.3306941	0.281513	1
PAWR	2.0010974	1.93258892	2.0307928	1.105302482	0.1444412	0.281562	1
AP3B1	1.4395029	1.38060769	1.4650314	1.221812937	0.2890234	0.281734	1
KCMF1	1.426532	1.36801554	1.4518963	1.227927135	0.296225	0.282013	1
ATP6V1C1	1.4416596	1.38236135	1.4673628	1.222306488	0.2896061	0.282013	1
YBX1	37.128534	36.4181386	37.436458	1.028751363	0.0408943	0.282133	1
RP11-77P6.2	1.0974098	1.14911864	1.0749963	0.502930546	-0.9915689	0.282144	1
SPTY2D1	1.2706634	1.21500924	1.294787	1.371043545	0.4552744	0.282262	1
CNRIP1	1.2196448	1.27403681	1.1960683	0.715481761	-0.4830131	0.28229	1
TBC1D23	1.2220566	1.16777916	1.2455835	1.463730455	0.5496499	0.282525	1
GAP43	2.8235293	2.71514978	2.870507	1.090579394	0.1250948	0.282622	1
SLC22A23	1.1315099	1.1839388	1.1087843	0.591415821	-0.7577553	0.282987	1
NEXN	1.1749206	1.12162332	1.1980227	1.628163508	0.7032456	0.283038	1
SLC4A3	1.1323678	1.07984896	1.1551324	1.942823113	0.9581546	0.283123	1
WDR54	4.2401113	4.37407267	4.1820451	0.943087294	-0.0845368	0.283154	1
RP11-159D12.2	1.1668982	1.22033661	1.143735	0.652342853	-0.6162977	0.283329	1
VARS2	1.1391609	1.08669837	1.161901	1.867405749	0.9010354	0.283508	1
RNF214	1.3555969	1.41290643	1.3307558	0.801043015	-0.3200484	0.283538	1
RP11-352M15.2	1.7968129	1.86200264	1.7685561	0.891593681	-0.1655417	0.283733	1
RP11-545I5.3	1.1537877	1.20661296	1.1308903	0.633504903	-0.6585723	0.284179	1
PSMA6	1.450735	1.39178619	1.4762867	1.215680219	0.2817638	0.284299	1
CDK9	1.3521003	1.29498511	1.3768573	1.277546717	0.353376	0.284457	1
HIST1H1A	1.2316181	1.28607339	1.2080141	0.727135552	-0.4597038	0.284494	1
ZDHHC4	1.8036059	1.73560989	1.8330791	1.13250121	0.1795126	0.28458	1
CEP83	1.3884062	1.44618697	1.3633609	0.81436904	-0.2962454	0.284638	1
SPATA13	1.1604464	1.21318703	1.1375857	0.64537535	-0.6317896	0.284668	1
MEF2A	1.4809649	1.42161867	1.5066889	1.201770496	0.2651614	0.284842	1
GRAMD3	1.1581097	1.21124267	1.1350789	0.639449056	-0.6450987	0.284874	1
LRP3	1.2001073	1.25401623	1.1767402	0.695782946	-0.5232908	0.284913	1
STIM2	1.3606849	1.41748506	1.3360646	0.804973838	-0.3129862	0.285061	1
EEFSEC	1.1830735	1.12975678	1.206184	1.589003354	0.6681222	0.285151	1
PDPR	1.2387839	1.29324296	1.2151783	0.73378856	-0.4465637	0.28564	1
RAB36	1.1966939	1.25025354	1.1734782	0.693209958	-0.5286357	0.285696	1
BMP7	1.353556	1.29646823	1.378301	1.276025276	0.3516569	0.285763	1
MRNIP	1.6604443	1.59729098	1.6878185	1.151563502	0.203594	0.285765	1
MDFIC	1.2993051	1.35525587	1.2750529	0.774238936	-0.3691492	0.285786	1
ZNF770	1.7973061	1.86445556	1.7681998	0.888651546	-0.1703103	0.285788	1
CA8	1.2451757	1.19045624	1.2688942	1.41184256	0.4975792	0.285919	1
DERA	1.6240705	1.56202491	1.6509646	1.158248607	0.2119449	0.285938	1
MDP1	1.2480297	1.19343119	1.2716957	1.404611779	0.4901714	0.285974	1
ANGPTL2	1.1015364	1.05015224	1.1238091	2.468664806	1.303731	0.285994	1
ARSD	1.2758796	1.220616	1.2998339	1.359075951	0.4426261	0.28618	1
ANOS1	1.332669	1.27634211	1.3570842	1.29218144	0.3698087	0.286372	1

ALDH6A1	1.3331872	1.38977132	1.3086605	0.791901604	-0.3366069	0.286428	1
SORL1	1.110137	1.05854913	1.132498	2.263022211	1.1782507	0.286541	1
SLC38A2	2.0185286	1.94797605	2.04911	1.106684048	0.1462434	0.286626	1
SKA3	1.1375413	1.1895507	1.1149976	0.606684973	-0.7209805	0.286669	1
SMIM4	1.7712778	1.70629728	1.799444	1.131880262	0.1787213	0.286708	1
RBM38	1.1834963	1.23655099	1.1604994	0.67849823	-0.559583	0.286765	1
ZSWIM6	1.2445604	1.29923665	1.2208607	0.738080453	-0.43815	0.286946	1
PAK2	2.6783706	2.58689115	2.7180229	1.082634372	0.1145461	0.286953	1
PDCL	1.3984331	1.3410017	1.423327	1.241422039	0.3119937	0.287039	1
SULF1	1.2612621	1.20646427	1.2850145	1.38045433	0.4651432	0.287248	1
ZNF92	1.2141199	1.26794213	1.1907904	0.712058197	-0.4899329	0.287252	1
GTF3C2	1.3128422	1.36956825	1.2882539	0.779974838	-0.3585005	0.287261	1
RP11-390P2.4	1.5975363	1.65825134	1.571219	0.867782577	-0.2045945	0.287307	1
DCAF8	1.4852396	1.42582121	1.5109949	1.200022112	0.263061	0.287427	1
H1FX	3.0198381	3.12552472	2.9740276	0.928724853	-0.1066769	0.287485	1
TMEM187	1.3219686	1.26608728	1.3461907	1.301041758	0.3796673	0.287634	1
MYD88	1.2142004	1.16057575	1.2374443	1.478706178	0.5643354	0.287798	1
ARL10	1.3162662	1.37198373	1.2921152	0.785290249	-0.3487021	0.288072	1
EPN2	1.4869099	1.54621973	1.4612017	0.844352011	-0.2440835	0.288082	1
PRKCZ	1.3779565	1.32099413	1.4026472	1.25437562	0.3269694	0.288167	1
STN1	1.482435	1.42299303	1.5082005	1.201439413	0.2647639	0.288205	1
KIF1BP	1.2046526	1.15145029	1.2277135	1.503552467	0.5883752	0.288306	1
PRKACB	1.4266105	1.36810963	1.451968	1.2278082	0.2960852	0.288646	1
CCSAP	1.194745	1.14152611	1.217813	1.539030504	0.6220218	0.288647	1
TOMM40	1.9760457	1.90798535	2.0055468	1.107448264	0.1472393	0.288843	1
ZNF470	1.1530039	1.2057548	1.1301387	0.632494214	-0.6608758	0.28886	1
MED1	1.2697594	1.32493382	1.2458438	0.756596377	-0.4024042	0.288878	1
LAMC1	1.6047194	1.5437345	1.6311536	1.160775322	0.2150888	0.289105	1
CDK11B	1.3469066	1.29081204	1.371221	1.27649821	0.3521915	0.289157	1
NRIP1	2.3203077	2.39956301	2.285954	0.918825358	-0.1221374	0.289232	1
USF3	1.2620206	1.31658502	1.2383693	0.752939405	-0.4093943	0.289424	1
NPIPA1	1.2226381	1.27651549	1.1992847	0.720699982	-0.4725293	0.289437	1
RP11-834C11.4	1.0849795	1.03398548	1.1070832	3.150851347	1.6557417	0.289614	1
UPF3B	1.7368667	1.67314146	1.7644888	1.13570307	0.1835857	0.289634	1
ALDH1B1	1.1701035	1.11769343	1.1928209	1.638332306	0.712228	0.289672	1
CDC26	2.3483424	2.26787274	2.3832225	1.090978945	0.1256233	0.289727	1
SLC35A5	1.3549397	1.29853816	1.3793873	1.270816741	0.345756	0.290196	1
RAB11B-AS1	1.4452035	1.50335097	1.4199992	0.834406266	-0.2611781	0.290221	1
SOX12	1.522651	1.58212689	1.4968709	0.853543984	-0.2284626	0.290527	1
PREP	1.2030253	1.1498953	1.2260548	1.5080847	0.5927175	0.290748	1
ENTPD1-AS1	1.1666424	1.21927319	1.1438293	0.655936528	-0.6083719	0.290997	1
PTPRJ	1.1897849	1.2426527	1.1668691	0.687686787	-0.5401765	0.291006	1
MXRA5	1.1894945	1.13663707	1.2124058	1.554525328	0.6364741	0.291156	1
ZC4H2	1.2889286	1.23396597	1.3127525	1.336743602	0.4187228	0.291229	1
CRLS1	1.7481836	1.68435988	1.7758483	1.133684641	0.1810194	0.291518	1
ZNF783	1.1881444	1.24132793	1.1650917	0.684097148	-0.5477269	0.291538	1
JMJD1C	2.4318342	2.51600001	2.395352	0.920416907	-0.1196406	0.291546	1
PSRC1	1.4184492	1.4761153	1.3934535	0.826382839	-0.2751178	0.291736	1
LAMA5	1.4052834	1.34840932	1.4299359	1.233996418	0.3033382	0.291824	1
TBXAS1	1.0794677	1.12990287	1.0576063	0.443456678	-1.1731349	0.29193	1
NUS1	1.4284788	1.37091697	1.4534293	1.222454854	0.2897812	0.291975	1

GSX2	1.0707995	1.02056357	1.0925746	4.50187089	2.1705247	0.292052	1
ASAP2	1.4437534	1.50214901	1.4184415	0.833301474	-0.2630896	0.292113	1
LINC01572	1.0933403	1.14432401	1.0712412	0.493619864	-1.0185276	0.292241	1
MROH1	1.1286802	1.07721728	1.1509871	1.955353221	0.9674292	0.292295	1
GTF2IRD2	1.2870967	1.34215486	1.2632315	0.769334373	-0.3783173	0.292344	1
AMD1	2.2059423	2.13253274	2.237762	1.092915011	0.1281812	0.292394	1
LENG1	1.2522947	1.19832714	1.2756872	1.390062847	0.4751501	0.29244	1
WNT6	1.0701985	1.02009375	1.0919166	4.574388148	2.1935788	0.292543	1
CACNA1D	1.255248	1.3093624	1.2317919	0.749256793	-0.4164678	0.292578	1
PJA1	1.6924656	1.62981255	1.7196229	1.142598492	0.1923185	0.292579	1
BCL7B	1.5321642	1.59193507	1.5062562	0.855256313	-0.2255712	0.292669	1
PPY	1.0799148	1.13021855	1.0581103	0.44625219	-1.1640688	0.292744	1
CCDC90B	2.5238044	2.43808073	2.5609617	1.085447923	0.1182905	0.292848	1
TMEM158	1.0791488	1.02895341	1.1009062	3.485124286	1.8012101	0.293028	1
MICU1	1.5170198	1.45787998	1.5426542	1.185145131	0.2450637	0.293088	1
RBMX2	1.3978472	1.341089	1.4224493	1.238531007	0.30863	0.293232	1
RNF150	1.2593772	1.31351782	1.2359097	0.752460306	-0.4103126	0.293255	1
OTX2-AS1	1.072683	1.02252858	1.0944228	4.191243395	2.0673783	0.293371	1
SSC4D	1.3221961	1.26694422	1.3461453	1.296695406	0.3748396	0.293427	1
RP11-88I18.2	1.119561	1.17064502	1.0974183	0.570882785	-0.8087335	0.293475	1
SNHG7	5.0146552	5.16812202	4.9481342	0.94722136	-0.0782265	0.293628	1
SLC39A14	1.2307725	1.17777128	1.2537462	1.427374611	0.513364	0.293644	1
YAP1	1.8378764	1.7722156	1.8663374	1.121885342	0.1659252	0.293734	1
TSKU	1.299405	1.24429442	1.3232929	1.323374142	0.404221	0.293777	1
CCDC34	2.8833146	2.97741294	2.8425271	0.931786733	-0.1019283	0.293942	1
FIG4	1.2543459	1.20059206	1.2776458	1.384131572	0.4689811	0.294086	1
CTC-338M12.5	1.4117972	1.46884371	1.3870701	0.825584438	-0.2765123	0.294274	1
OGT	1.4567498	1.51495212	1.4315216	0.837983984	-0.2550054	0.294398	1
MKNK2	1.4010109	1.45808574	1.3762715	0.821399802	-0.2838435	0.294421	1
FAM98A	1.2934977	1.23906232	1.3170931	1.326403316	0.4075195	0.294437	1
ZNF606	1.2967501	1.35146164	1.273035	0.776855823	-0.3642812	0.294545	1
NBPF9	1.210282	1.26363955	1.1871539	0.709885527	-0.4943417	0.294685	1
RP11-801F7.1	1.0847034	1.13496549	1.062917	0.466170992	-1.1010689	0.294738	1
PTK7	1.5697888	1.50982614	1.5957799	1.168594321	0.2247742	0.294754	1
PRSS35	1.3082898	1.25328472	1.332132	1.311299112	0.3909968	0.294873	1
NCAPG2	1.1715312	1.22355251	1.1489823	0.666430818	-0.585473	0.29519	1
RRP8	1.2753461	1.32986304	1.2517155	0.76309089	-0.3900732	0.295243	1
CHML	1.6225491	1.68361404	1.5960801	0.871954185	-0.1976758	0.29552	1
WNK1	2.0910496	2.02247528	2.1207735	1.096137486	0.1324288	0.295673	1
LRRC58	1.6069404	1.66751939	1.5806821	0.86991049	-0.2010611	0.296138	1
SLC7A1	1.1848489	1.23716968	1.1621702	0.683773001	-0.5484106	0.296153	1
TXLNB	1.074672	1.12458642	1.0530363	0.425698746	-1.2320953	0.296206	1
UBTD1	1.3837354	1.32765202	1.408045	1.245360984	0.316564	0.296423	1
NEPRO	1.3880935	1.44474395	1.3635381	0.817409816	-0.2908685	0.296423	1
COG1	1.2241351	1.27742346	1.201037	0.724657671	-0.4646285	0.296473	1
ICA1L	1.2484944	1.30226937	1.2251853	0.744982253	-0.424722	0.296545	1
USF1	1.3110609	1.25618955	1.3348451	1.307021057	0.3862824	0.29657	1
IFI6	1.8303045	1.73751574	1.8705243	1.180346742	0.2392107	0.296667	1
GFPT1	1.4091666	1.35258009	1.4336943	1.230059057	0.2987276	0.29673	1
CPTP	1.2706629	1.21681745	1.2940025	1.355991059	0.4393477	0.296766	1
USP48	1.5892549	1.52938147	1.6152073	1.162124807	0.216765	0.296899	1

UPF2	1.5559804	1.61565494	1.5301141	0.861057228	-0.215819	0.297065	1
FAXDC2	1.2693096	1.32307766	1.2460035	0.761437769	-0.393202	0.297085	1
UBAC2	2.0773276	2.00948063	2.1067362	1.096342225	0.1326982	0.297172	1
FAM65A	1.1858769	1.13339779	1.2086242	1.563925883	0.6451721	0.297223	1
QARS	3.1129385	3.01048414	3.157348	1.073048987	0.1017159	0.297294	1
BBC3	1.5359582	1.59477905	1.5104619	0.858237896	-0.2205505	0.297481	1
RAD51D	1.21861	1.27146655	1.1956991	0.720895812	-0.4721373	0.297538	1
TMEM184B	1.4468696	1.38928414	1.4718303	1.212045937	0.2774444	0.297663	1
COL6A3	1.0669355	1.01724954	1.0884722	5.128960004	2.3586663	0.297745	1
ATF1	1.4183526	1.36195998	1.4427964	1.223329596	0.2908132	0.297902	1
GLMN	1.3729035	1.42895923	1.3486058	0.812678103	-0.2992441	0.297925	1
ARV1	1.2486763	1.19523869	1.2718391	1.392342278	0.4775139	0.29793	1
PRPF18	1.2627772	1.20913285	1.2860296	1.367693169	0.4517446	0.298029	1
TNIP2	1.378489	1.322523	1.4027478	1.248741362	0.3204747	0.29811	1
MAPK1IP1L	2.2922977	2.21862056	2.3242335	1.086665947	0.1199085	0.298213	1
FMC1	2.3299223	2.25065428	2.3642815	1.090854189	0.1254583	0.29858	1
TMEM39A	1.1876384	1.13571166	1.2101463	1.548476241	0.6308492	0.298675	1
MAP3K4	1.3177986	1.37267012	1.2940143	0.78893984	-0.3420128	0.29899	1
GAR1	1.6243772	1.56397929	1.6505569	1.1535121	0.2060331	0.299039	1
TMEM230	4.5628801	4.41756391	4.6258682	1.060951096	0.0853582	0.299461	1
ZDHHC6	1.2925069	1.34651524	1.2690968	0.776579855	-0.3647938	0.299478	1
TMX4	1.8098862	1.74665332	1.8372948	1.12139702	0.1652971	0.299514	1
FBXO30	1.1296672	1.07891507	1.151666	1.921889295	0.9425252	0.299564	1
COQ10B	1.27255	1.21888625	1.2958109	1.351436637	0.4344939	0.29966	1
MRTO4	1.4870595	1.42943474	1.5120372	1.192351627	0.2538098	0.299737	1
FLRT3	2.0654628	2.15846815	2.0251492	0.884917889	-0.1763845	0.299951	1
UBA6	1.7483129	1.8110841	1.7211044	0.889062418	-0.1696434	0.299981	1
BRINP2	1.0747496	1.12426696	1.053286	0.428802724	-1.221614	0.299997	1
KCNQ2	1.1058907	1.15587309	1.0842256	0.540347362	-0.888041	0.30002	1
ZNF529	1.2226119	1.27519095	1.1998212	0.726118488	-0.4617231	0.300086	1
ECEL1	1.1538843	1.10273811	1.1760538	1.713617614	0.7770452	0.300167	1
FAM83D	1.1481763	1.09734932	1.1702075	1.748419687	0.8060515	0.30038	1
NUF2	1.4340897	1.37758214	1.4585832	1.214525727	0.2803931	0.300384	1
LYPLA1	3.0619829	2.95261369	3.1093896	1.080290301	0.1114191	0.300387	1
METTL6	1.2082951	1.26079379	1.1855393	0.711440635	-0.4911847	0.300491	1
PLPPR1	1.1281814	1.07770837	1.1500593	1.931056466	0.9493904	0.300549	1
UBLCP1	1.3580138	1.30270926	1.3819858	1.261890046	0.3355862	0.300699	1
FRMD6	1.1706726	1.11935135	1.1929181	1.616387776	0.6927733	0.300865	1
CAMK1	1.3550066	1.30046777	1.3786468	1.260191064	0.3336425	0.301063	1
RAB33A	1.2051825	1.15278903	1.2278927	1.491551735	0.576814	0.30117	1
WDR19	1.2788833	1.33257983	1.2556083	0.768562236	-0.379766	0.301322	1
UNC5B	1.2669656	1.21322013	1.2902618	1.361324724	0.4450112	0.301371	1
DPP10	1.1378081	1.0871124	1.1597824	1.834209162	0.8751582	0.301373	1
PLEKHA8	1.4746827	1.53220253	1.4497505	0.845073868	-0.2428506	0.301568	1
VPS36	1.5474835	1.60678098	1.5217807	0.85991605	-0.2177323	0.301612	1
SUMO1	6.2159318	6.03957457	6.2923747	1.050163	0.0706133	0.301695	1
ARHGAP1	1.22855	1.17556016	1.2515187	1.432663725	0.5187	0.301727	1
SF3A3	1.6034807	1.54373531	1.6293777	1.157507472	0.2110215	0.301787	1
INTS6-AS1	1.1305877	1.18097208	1.1087483	0.60091178	-0.7347749	0.301877	1
KLHL24	1.7506705	1.68874954	1.7775104	1.128872515	0.1748826	0.301968	1
EPHX1	1.2583103	1.20502141	1.2814086	1.372581623	0.4568919	0.302103	1

UBN1	1.5887664	1.64873815	1.5627714	0.867486178	-0.2050873	0.302136	1
MAP3K12	1.1940665	1.24585651	1.1716178	0.69804042	-0.5186175	0.302209	1
PARD3	1.3883891	1.4445299	1.3640546	0.818965277	-0.2881258	0.302216	1
TARSL2	1.5392433	1.48080747	1.5645726	1.174217549	0.2316997	0.302328	1
DACT2	1.0833772	1.13270124	1.0619975	0.467196038	-1.0979001	0.302505	1
FAM96A	2.4862492	2.40344498	2.5221411	1.084574837	0.1171296	0.30255	1
GTPBP1	1.2337912	1.2863768	1.2109977	0.736783538	-0.4406873	0.302673	1
IMPA2	1.6859284	1.74707228	1.6594252	0.882679293	-0.1800387	0.302692	1
FANCL	1.6522718	1.71289614	1.6259938	0.878099572	-0.1875436	0.302824	1
CLSTN3	1.2327119	1.18005514	1.2555363	1.419211416	0.5050895	0.30291	1
SENP2	1.3923828	1.44828403	1.3681522	0.821247541	-0.284111	0.302983	1
IQCH	1.06966	1.11867716	1.0484133	0.407940855	-1.2935681	0.303017	1
WASH1	1.5653117	1.62444354	1.5396808	0.864258683	-0.2104649	0.303036	1
SMARCA2	1.2447722	1.19220313	1.2675585	1.392061088	0.4772225	0.30324	1
SPAG9	2.1531997	2.22658001	2.1213925	0.91424329	-0.12935	0.303264	1
PCBP2	7.1468651	6.94563447	7.2340895	1.04851544	0.0683481	0.303987	1
CACHD1	1.1381621	1.08784136	1.159974	1.82116912	0.8648649	0.304046	1
CENPC	1.434551	1.49097185	1.4100951	0.835272056	-0.2596819	0.30433	1
TMEM60	1.7778146	1.7154993	1.8048255	1.124844625	0.1697257	0.304537	1
C11orf54	1.4264046	1.37049267	1.45064	1.216326318	0.2825303	0.304784	1
B3GALNT1	1.4582448	1.51517801	1.4335668	0.84158639	-0.2488167	0.304796	1
GMDS	1.2544171	1.20184429	1.277205	1.373360791	0.4577107	0.305142	1
SRFBP1	1.3554515	1.30073224	1.3791699	1.260822174	0.3343648	0.305218	1
FAIM	1.2763931	1.22345822	1.299338	1.339570224	0.4217702	0.305241	1
RP11-315A16.1	1.0717986	1.12085973	1.0505328	0.418111049	-1.2580419	0.305245	1
CEP89	1.4469303	1.50318996	1.4225442	0.839730971	-0.2520009	0.305327	1
RP11-386G11.10	1.2456795	1.29815814	1.2229323	0.747698222	-0.419472	0.305393	1
RAD51	1.1380297	1.18846565	1.116168	0.616387936	-0.6980895	0.305574	1
VWA5A	1.2680251	1.21509293	1.2909688	1.352758751	0.4359046	0.305656	1
RP3-512B11.3	1.1557617	1.10539395	1.1775938	1.685047465	0.7527892	0.305724	1
KIF23	1.2486432	1.30188793	1.2255639	0.747177754	-0.4204766	0.305973	1
LONP2	1.6134503	1.5542954	1.6390912	1.152979524	0.2053669	0.306052	1
LRPPRC	1.6239076	1.56366197	1.6500214	1.153211395	0.205657	0.306078	1
ZNF488	1.1340752	1.18448652	1.1122241	0.60830519	-0.7171328	0.306268	1
MLLT10	1.5508705	1.60966776	1.5253845	0.861755401	-0.2146497	0.306272	1
CHIC2	1.3312465	1.2770507	1.354738	1.280408081	0.3566037	0.306354	1
POM121	1.3657405	1.42044908	1.3420267	0.813479534	-0.297822	0.306375	1
PPIH	1.5175954	1.46024609	1.5424538	1.178616898	0.2370949	0.306508	1
NBR1	1.5188133	1.46139674	1.5437008	1.178380237	0.2368051	0.306512	1
UVSSA	1.1493575	1.2001117	1.1273578	0.636433325	-0.6519187	0.306577	1
SYNGAP1	1.4608433	1.51745766	1.4363035	0.843167606	-0.2461087	0.306676	1
GMFB	1.5043533	1.44704139	1.5291955	1.183772853	0.2433923	0.306927	1
KIAA2026	1.4371425	1.49353814	1.4126975	0.836201917	-0.2580767	0.307257	1
FBXL7	1.1804459	1.12951372	1.2025227	1.563715968	0.6449785	0.307329	1
GMPS	1.8852548	1.82078713	1.9131986	1.112588893	0.1539206	0.307332	1
GPR180	1.279679	1.22638128	1.3027812	1.337483515	0.4195211	0.307384	1
TMEM161B	1.2282772	1.28060139	1.2055971	0.732701499	-0.4487025	0.307461	1
PRMT9	1.3348779	1.38940692	1.311242	0.799271974	-0.3232416	0.307618	1
SPATA6	1.4335856	1.48945945	1.4093667	0.836364991	-0.2577954	0.307626	1
LHX1	1.0808237	1.03198219	1.1019944	3.189098953	1.6731489	0.30765	1
CENPM	1.5031671	1.56073008	1.4782161	0.852845485	-0.2296437	0.307804	1

PODNL1	1.1090198	1.15840426	1.0876139	0.553103046	-0.8543798	0.307897	1
IRF2	1.2420506	1.18991803	1.2646478	1.393484399	0.4786969	0.307947	1
METTL3	1.402645	1.45845604	1.3784534	0.825495568	-0.2766676	0.308097	1
AC005076.5	1.1174564	1.1670588	1.095956	0.574384774	-0.7999106	0.308202	1
IKBKAP	1.1911637	1.13992649	1.2133727	1.524891692	0.6087068	0.308294	1
MRPS31	1.5589849	1.50094857	1.5841411	1.16606993	0.2216543	0.308336	1
KIAA1522	1.2209512	1.16916503	1.2433983	1.438821383	0.5248875	0.308591	1
TMEM219	3.3784079	3.27300783	3.4240942	1.066469778	0.0928431	0.308592	1
SLC35E3	1.6581264	1.71830183	1.632043	0.879912871	-0.1845674	0.308631	1
SHROOM3	1.4643827	1.52107273	1.4398101	0.844047442	-0.244604	0.308696	1
PREX2	1.1406857	1.19059554	1.119052	0.624631637	-0.6789225	0.308785	1
SLC30A7	1.3725109	1.31765785	1.3962872	1.247528511	0.3190728	0.308801	1
UFL1	1.4887153	1.43219701	1.5132134	1.187452421	0.2478697	0.308837	1
CYB5R1	1.403303	1.34786343	1.4273335	1.228451959	0.2968414	0.30897	1
CAPS2	1.1629849	1.21356065	1.1410626	0.660527003	-0.5983106	0.3092	1
KCNIP4	1.1139139	1.06476901	1.135216	2.087665361	1.0618905	0.30921	1
TSPAN19	1.1464647	1.1966248	1.1247225	0.634317477	-0.656723	0.309307	1
TNFAIP2	1.1002385	1.05125059	1.1214725	2.37016846	1.2449896	0.309323	1
KIF20A	1.2038197	1.15247565	1.2260751	1.48269661	0.5682234	0.309378	1
C9orf72	1.4284302	1.48427067	1.4042258	0.834710468	-0.2606522	0.309704	1
IGSF8	1.3038755	1.25096267	1.3268108	1.302228741	0.3809829	0.309753	1
GPR85	1.3024469	1.35555812	1.2794255	0.785878628	-0.3476216	0.309765	1
PRRX1	1.0757177	1.02694115	1.0968602	3.595251012	1.8460925	0.309831	1
NUDT15	1.6228301	1.56345994	1.6485645	1.151039162	0.2029369	0.309977	1
SOSTDC1	1.2044433	1.15339314	1.2265713	1.477062567	0.5627309	0.309995	1
EME1	1.0746816	1.12310439	1.0536925	0.436154347	-1.1970893	0.310016	1
PTPRD	1.7282213	1.78891425	1.7019136	0.889720999	-0.1685751	0.310059	1
PHF20L1	1.7016284	1.64088302	1.7279589	1.135868536	0.1837959	0.310068	1
NUP153	1.2667791	1.31919078	1.2440609	0.764623981	-0.3871776	0.310282	1
DNPH1	3.3128849	3.21289312	3.3562269	1.064772115	0.0905447	0.310546	1
MTRF1L	1.5677229	1.62593735	1.5424896	0.866683538	-0.2064228	0.310547	1
VTCN1	1.0753399	1.02661987	1.0964579	3.623529578	1.8573957	0.31067	1
PPP6R3	1.532464	1.58994841	1.5075471	0.860324624	-0.217047	0.310784	1
PIH1D1	2.80989	2.71787437	2.8497746	1.07678109	0.106725	0.31096	1
SNRNP200	1.8758643	1.93924993	1.8483894	0.903262673	-0.1467825	0.310968	1
LHX5-AS1	1.0738381	1.02457518	1.0951913	3.873475196	1.9536285	0.310969	1
TCEB3-AS1	1.2416952	1.29370615	1.2191508	0.746156635	-0.4224496	0.311046	1
TTC8	1.2231147	1.17170444	1.2453988	1.42919295	0.5152007	0.31112	1
PATJ	1.1931125	1.14224126	1.215163	1.512662048	0.5970897	0.311134	1
ST14	1.0697505	1.02144353	1.0906895	4.229222837	2.0803926	0.311184	1
AFDN	1.5917985	1.65088885	1.5661855	0.86986516	-0.2011363	0.311287	1
FAM66C	1.0812777	1.12965481	1.0603084	0.46514566	-1.1042455	0.311687	1
NMNAT3	1.1544624	1.10454379	1.1760999	1.68446035	0.7522865	0.311725	1
NIF3L1	1.2298501	1.1783829	1.2521588	1.413581816	0.4993554	0.311888	1
HMGCR	1.3803326	1.32596098	1.4039003	1.239106238	0.3092999	0.31189	1
PDRG1	1.5971196	1.53894515	1.6223357	1.1547292	0.2075546	0.312056	1
FBXO34	1.3077819	1.25490195	1.330703	1.297373183	0.3755935	0.31276	1
CDH24	1.1374783	1.18705577	1.1159887	0.620075241	-0.6894848	0.3129	1
C1orf53	1.3548966	1.30088071	1.3783101	1.25734249	0.3303777	0.313014	1
ERAL1	1.4639826	1.40823817	1.4881454	1.195736742	0.2578998	0.313124	1
KLHL9	1.5105405	1.45387365	1.5351031	1.178969209	0.237526	0.313185	1

AP001372.2	1.2127125	1.2638361	1.1905527	0.722239065	-0.4694516	0.313223	1
NAA16	1.3243522	1.37768185	1.3012361	0.797592214	-0.3262768	0.313235	1
POLE	1.1465418	1.19634115	1.124956	0.636422927	-0.6519423	0.313267	1
RP11-672L10.6	1.2209868	1.2721123	1.1988262	0.730677094	-0.4526941	0.313512	1
WDR46	1.3276013	1.27443511	1.3506465	1.277702774	0.3535523	0.313544	1
TNPO1	2.0389697	1.97288604	2.0676141	1.097368063	0.1340475	0.313576	1
PQLC1	1.2933451	1.34583266	1.270594	0.782442123	-0.3539441	0.313594	1
ADCY6	1.1910969	1.24196602	1.1690474	0.698640928	-0.5173769	0.313821	1
F2RL1	1.078991	1.03108021	1.0997582	3.209701654	1.6824392	0.313853	1
BAD	3.094223	2.99369001	3.1377996	1.072282851	0.1006855	0.313986	1
MIR34A	1.128029	1.17720887	1.1067117	0.602180169	-0.7317329	0.31407	1
DDX6	2.0213313	2.08857313	1.9921849	0.911454551	-0.1337574	0.314112	1
API5	1.5056891	1.44917016	1.5301876	1.180371361	0.2392408	0.314113	1
HSD17B11	1.4245883	1.36958533	1.4484296	1.213331783	0.2789741	0.314143	1
TPMT	1.3309834	1.2773701	1.3542224	1.277074887	0.3528431	0.314256	1
EPHA3	1.0881263	1.13655393	1.0671351	0.491637731	-1.0243325	0.314311	1
KLHL18	1.1248733	1.07572232	1.1461781	1.930449352	0.9489367	0.314675	1
U2SURP	2.3906814	2.47623476	2.3535979	0.916925898	-0.1251229	0.314756	1
CCDC102B	1.1731286	1.22335619	1.1513571	0.677649164	-0.5613895	0.314794	1
HSF4	1.1943271	1.24519597	1.1722778	0.702612482	-0.5091989	0.31482	1
KCTD11	1.2385347	1.18720707	1.260783	1.39301883	0.4782148	0.314952	1
RP11-18H7.1	1.0565859	1.10405245	1.0360113	0.346088281	-1.530788	0.315035	1
RP5-991G20.1	1.0804232	1.12857156	1.059553	0.463189863	-1.1103244	0.315075	1
ACOT11	1.2296957	1.28092189	1.2074915	0.738609188	-0.4371169	0.31516	1
KCTD6	1.5826233	1.52499856	1.6076011	1.157338619	0.210811	0.315173	1
C5orf49	1.1325147	1.18145357	1.1113019	0.613390244	-0.7051229	0.315229	1
C12orf45	1.6129789	1.55469338	1.6382431	1.150623317	0.2024156	0.315581	1
PPP1R9A	1.5306128	1.5877776	1.5058344	0.860588079	-0.2166052	0.315791	1
HOOK1	1.2898137	1.23762213	1.3124365	1.314845845	0.3948937	0.315808	1
LINC01122	1.1326552	1.18181532	1.1113465	0.612415483	-0.7074173	0.315839	1
EPC1	2.02098	1.95607254	2.0491144	1.097316764	0.1339801	0.315881	1
PMPCB	2.1728199	2.10238076	2.2033522	1.091593965	0.1264363	0.316387	1
GTPBP6	1.3863132	1.44025226	1.362933	0.82437505	-0.2786273	0.316434	1
BYSL	1.1966214	1.14633976	1.2184162	1.492528061	0.5777581	0.31656	1
MCRIP2	1.8526453	1.78986284	1.8798586	1.113938523	0.1556696	0.316613	1
TEX9	1.4256774	1.48062399	1.4018606	0.836122544	-0.2582137	0.31671	1
ADHFE1	1.0982537	1.146684	1.0772613	0.526719044	-0.9248945	0.31681	1
TMEM186	1.2627786	1.3147019	1.2402721	0.763491238	-0.3893165	0.31692	1
DAPK1	1.3033523	1.2508913	1.3260918	1.299733294	0.3782156	0.317045	1
SMIM15	1.9748819	2.03939	1.9469205	0.911034805	-0.1344219	0.317121	1
CLPTM1L	1.2820935	1.23025037	1.3045652	1.322756783	0.4035478	0.317361	1
EFNB2	1.5164195	1.57260318	1.4920664	0.859349686	-0.2186828	0.317444	1
UBTD2	1.3289742	1.27623666	1.3518336	1.273667463	0.3489887	0.31752	1
IQSEC2	1.3125816	1.36519648	1.2897754	0.793478168	-0.3337376	0.317567	1
FKBP5	1.2369097	1.28807135	1.2147334	0.74541755	-0.4238793	0.317725	1
N6AMT1	1.1501575	1.10091556	1.1715017	1.699457478	0.7650743	0.317762	1
LRRC37A2	1.1002734	1.14848523	1.0793758	0.534570058	-0.9035491	0.317868	1
PAK4	1.4899224	1.43401862	1.5141542	1.184636355	0.2444443	0.317951	1
PCNA	1.8871077	1.82397289	1.9144739	1.109834894	0.1503451	0.317963	1
HIPK2	1.9359268	1.99965949	1.9083015	0.908610856	-0.1382656	0.318026	1
ADORA2B	1.1053064	1.15390692	1.0842402	0.54734542	-0.8694765	0.318087	1

ABHD8	1.5057388	1.44961337	1.5300667	1.178938974	0.237489	0.318145	1
MRPL55	2.6358822	2.71718471	2.6006411	0.932131004	-0.1013954	0.318246	1
MED19	1.5145207	1.45811565	1.5389697	1.176492764	0.2344924	0.318605	1
BARD1	1.3051055	1.3577187	1.2823001	0.789167746	-0.3415961	0.318714	1
BAP1	1.327911	1.2752051	1.3507566	1.27452804	0.3499631	0.318838	1
ENDOD1	1.1757053	1.12596208	1.1972668	1.566080848	0.6471587	0.318845	1
U2AF1L4	1.1906768	1.24055506	1.1690568	0.702778075	-0.5088589	0.318855	1
SYT4	1.0890481	1.04089894	1.1099187	2.68756753	1.426301	0.31889	1
TNRC6A	1.7888075	1.72776826	1.8152654	1.120226604	0.1637906	0.319249	1
CCDC3	1.1829428	1.13286675	1.2046486	1.540254405	0.6231687	0.319297	1
NGEF	1.0958531	1.14370315	1.0751123	0.522690507	-0.9359711	0.319376	1
KIAA1429	1.2737646	1.22210598	1.2961563	1.333400775	0.4151105	0.319486	1
CLVS1	1.2332184	1.28397346	1.2112183	0.743795969	-0.4270212	0.319525	1
RAB28	1.4969995	1.44094294	1.5212976	1.18223366	0.2415152	0.31954	1
RALGPS2	1.5370047	1.59377414	1.5123977	0.862950534	-0.2126502	0.319711	1
SLC38A5	1.1278008	1.07912208	1.1489009	1.881913744	0.9122005	0.319881	1
HMGN5	1.8767347	1.93947327	1.8495404	0.904273073	-0.1451696	0.319924	1
TGOLN2	1.4479737	1.39301653	1.4717952	1.200446274	0.2635708	0.319962	1
AC062017.1	1.2242346	1.27479524	1.2023188	0.736253076	-0.4417263	0.320129	1
AHDC1	1.3679229	1.4214875	1.344705	0.817829798	-0.2901275	0.320269	1
MAF	1.804616	1.86620331	1.7779207	0.89808094	-0.1550826	0.320478	1
MBD4	1.6996707	1.75954938	1.6737159	0.886994199	-0.1730034	0.320719	1
GPLD1	1.0733188	1.12069384	1.0527839	0.437336861	-1.1931831	0.320749	1
NPLOC4	1.2311736	1.18072897	1.2530391	1.400102366	0.4855323	0.321186	1
RP11-655M14.13	1.1824886	1.23210889	1.1609805	0.693555807	-0.5279161	0.321474	1
NRDC	2.1575817	2.09082194	2.1865192	1.087729453	0.1213198	0.321506	1
BCL2L11	1.2088025	1.25894497	1.1870679	0.722423467	-0.4690833	0.321515	1
ARHGEF26	1.2557391	1.30665505	1.2336693	0.761993931	-0.3921486	0.321518	1
DPYSL4	1.6216182	1.56355784	1.6467848	1.147681255	0.198722	0.321661	1
LSM5	4.9392969	4.79766909	5.0006862	1.053458355	0.0751333	0.321684	1
POLR2M	1.4662457	1.52164617	1.442232	0.847762388	-0.2382681	0.321723	1
ZNF106	1.9454723	1.8822444	1.9728788	1.102731673	0.1410818	0.32175	1
SMC6	1.437235	1.38277382	1.4608414	1.203952316	0.2677783	0.321832	1
ZNF620	1.2455684	1.29614036	1.2236477	0.755208385	-0.4050533	0.321971	1
TBC1D10A	1.1188138	1.07052429	1.1397452	1.981518244	0.9866063	0.322068	1
ADAM19	1.0999503	1.05229021	1.1206088	2.306527402	1.2057224	0.322146	1
TIGAR	1.2144393	1.16437211	1.2361413	1.436626143	0.5226847	0.322172	1
ZEB1-AS1	1.3090907	1.3610199	1.2865817	0.793811483	-0.3331317	0.322195	1
LIN9	1.1267204	1.17491138	1.1058318	0.605059282	-0.7248516	0.322315	1
RP11-382A20.3	2.3164046	2.24299234	2.3482255	1.084661155	0.1172444	0.322394	1
LPGAT1	1.6586279	1.60038888	1.6838719	1.1390482	0.1878288	0.322435	1
IPMK	1.1380403	1.18683456	1.1168901	0.625634318	-0.6766084	0.322528	1
ANKS3	1.4001459	1.45383738	1.3768731	0.830414373	-0.2680967	0.322833	1
CLMP	1.1153837	1.06756257	1.136112	2.014607332	1.0104987	0.322856	1
ADGRG1	1.2161888	1.16594299	1.2379681	1.434035224	0.5200805	0.323013	1
CCDC102A	1.170276	1.12114004	1.1915742	1.581427491	0.6612274	0.32308	1
RAD18	1.1515989	1.20044465	1.1304264	0.650685383	-0.6199679	0.323171	1
MMAB	1.8586704	1.9214046	1.8314779	0.902402546	-0.148157	0.323343	1
HGSNAT	1.4175782	1.47145361	1.3942257	0.836191866	-0.2580941	0.323443	1
TRRAP	1.203833	1.25375757	1.1821929	0.717979967	-0.4779845	0.323539	1
GLRX5	2.48861	2.41256226	2.5215733	1.077172575	0.1072494	0.323785	1

ATG12	2.2063487	2.13716728	2.2363357	1.087206577	0.1206261	0.32388	1
APBA2	1.4426329	1.49695282	1.4190876	0.843314679	-0.245857	0.323911	1
KIAA0430	1.3477045	1.40081829	1.3246821	0.810048091	-0.3039205	0.323926	1
BAMBI	1.4652058	1.41084572	1.4887685	1.189664407	0.2505547	0.323933	1
CCNF	1.2245313	1.2748072	1.2027389	0.73774954	-0.438797	0.324039	1
SRSF6	2.0171261	2.08118101	1.9893612	0.915074508	-0.1280389	0.324247	1
TP53BP2	1.3547544	1.30196625	1.3776358	1.250589292	0.3226081	0.324412	1
DHRS4	1.2636843	1.21266991	1.2857968	1.343851615	0.4263738	0.32461	1
TAF13	1.1181005	1.07020674	1.1388604	1.977878078	0.9839535	0.324634	1
CEP295	1.3300926	1.38225469	1.3074827	0.804392194	-0.314029	0.324788	1
EML4	1.7329368	1.67339859	1.758744	1.126738325	0.1721525	0.324821	1
POU4F2	1.0714696	1.02424935	1.0919375	3.791338087	1.9227071	0.324965	1
GNAQ	1.5598436	1.50368133	1.5841875	1.159835543	0.2139203	0.325027	1
DGKZ	1.3129441	1.36503207	1.2903662	0.795454041	-0.3301495	0.325115	1
UBE2V1	1.1689635	1.21784783	1.1477744	0.678337551	-0.5599247	0.325403	1
SEC23IP	1.3047202	1.25294537	1.3271623	1.293411006	0.3711808	0.325617	1
HJURP	1.2005262	1.24993128	1.1791112	0.716641917	-0.4806757	0.32571	1
CRTAC1	1.9016949	1.840169	1.9283636	1.104972417	0.1440104	0.325757	1
RNF6	1.3358822	1.28334079	1.3586566	1.265813499	0.3400649	0.3258	1
HIBCH	2.0380048	1.97368591	2.0658842	1.094689979	0.1305224	0.325909	1
WDR82	1.5646162	1.62088874	1.5402246	0.870082759	-0.2007755	0.325985	1
NKILA	1.1586148	1.10957339	1.1798721	1.64156739	0.715074	0.326195	1
PFKM	1.8276226	1.76662992	1.8540602	1.114044953	0.1558074	0.326238	1
BBS12	1.1694173	1.1206564	1.190553	1.57930264	0.6592877	0.326371	1
ARHGEF1	1.2670754	1.21632518	1.2890734	1.336290835	0.418234	0.326412	1
ADAT2	1.1417858	1.19015396	1.1208204	0.635381813	-0.6543043	0.326436	1
UQCC1	1.4118255	1.46559813	1.3885174	0.834447956	-0.261106	0.326702	1
B4GALT7	1.7253022	1.66663441	1.750732	1.126152559	0.1714023	0.326853	1
AHCYL1	2.055888	1.98900257	2.0848798	1.096943361	0.133489	0.327097	1
TRIM52	1.1533802	1.20206618	1.1322771	0.654622469	-0.611265	0.327317	1
PRKAB2	1.3153457	1.36646205	1.2931891	0.800053135	-0.3218323	0.327544	1
XAB2	1.2520463	1.20180322	1.2738244	1.356888027	0.4403017	0.327717	1
RP11-430G17.3	1.0783088	1.12512071	1.058018	0.463696111	-1.1087485	0.327721	1
TTC31	1.1689282	1.21751532	1.1478678	0.679804021	-0.5568092	0.327934	1
ROBO1	1.3000033	1.2487193	1.3222326	1.295567215	0.3735839	0.328182	1
GLYATL1	1.0765423	1.12328195	1.0562827	0.456536509	-1.1311979	0.328363	1
EDA2R	1.2138096	1.26339601	1.192316	0.730140321	-0.4537543	0.328532	1
SPATA6L	1.076121	1.12301946	1.0557926	0.453526967	-1.1407398	0.328762	1
AP006222.2	1.6960649	1.63772722	1.7213518	1.131129004	0.1777635	0.328825	1
FAM221A	1.3564276	1.40871409	1.3337638	0.816619265	-0.2922645	0.329107	1
LAS1L	1.2507875	1.20039284	1.2726313	1.360484246	0.4441203	0.329115	1
PP7080	1.3413086	1.39308851	1.3188644	0.811177058	-0.3019112	0.32918	1
CACNA1H	1.1562935	1.20444141	1.1354236	0.662407745	-0.5942086	0.329255	1
ARHGAP18	1.1995585	1.15043284	1.2208523	1.468112125	0.5539622	0.329274	1
FKBP14	1.3309783	1.27963093	1.3532351	1.263218879	0.3371046	0.329425	1
FEZF1-AS1	1.1675924	1.21598147	1.1466179	0.678845041	-0.5588458	0.329443	1
ZNF793-AS1	1.229707	1.27948881	1.2081288	0.744676731	-0.4253138	0.32945	1
PLEKHG4B	1.2973415	1.34827543	1.2752639	0.790362567	-0.3394135	0.3295	1
MCM5	1.3033976	1.35452943	1.2812342	0.793260497	-0.3341334	0.329842	1
SLC2A6	1.1279109	1.0802151	1.1485849	1.852330356	0.8893414	0.329897	1
MIR7-3HG	1.0845455	1.03763687	1.1048784	2.786585511	1.4784984	0.329968	1

DCAF6	1.2817618	1.23074165	1.3038767	1.316956614	0.3972078	0.330126	1
R3HDM1	1.547746	1.60316825	1.523723	0.868286732	-0.2037566	0.330248	1
B4GALT2	1.3012506	1.35229198	1.2791265	0.792315679	-0.3358527	0.330378	1
NRN1	1.3801852	1.43214137	1.3576646	0.82765636	-0.2728962	0.330505	1
ARMC6	1.3014468	1.25055096	1.3235079	1.2911862	0.3686971	0.330602	1
PTX3	1.1130301	1.06588881	1.1334637	2.025589983	1.0183422	0.330699	1
USP18	1.137703	1.09001022	1.1583756	1.759529543	0.8151897	0.331052	1
TFDP2	3.0345771	3.12624887	2.9948415	0.938197556	-0.0920364	0.331053	1
ZNF213-AS1	1.2822223	1.33284283	1.2602805	0.781992274	-0.3547737	0.331118	1
BCAT2	1.6194707	1.56278415	1.6440418	1.144385151	0.1945727	0.331171	1
MPRIP	1.352818	1.30078379	1.3753726	1.247981388	0.3195964	0.331211	1
DEXI	1.5654897	1.5094034	1.5898006	1.15782617	0.2114187	0.331336	1
STX6	1.3208561	1.26955584	1.3430925	1.272806751	0.3480134	0.331432	1
SCFD1	1.7862895	1.72679099	1.8120795	1.117349385	0.1600804	0.331553	1
WDHD1	1.2380792	1.28783336	1.216513	0.752216538	-0.4107801	0.331663	1
SDF2	1.7626133	1.70364849	1.788172	1.120121735	0.1636555	0.331758	1
MGRN1	1.5118837	1.56674487	1.4881039	0.861240875	-0.2155113	0.331857	1
PKN1	1.409457	1.35697792	1.4322044	1.210731469	0.2758789	0.332566	1
INTS2	1.1619272	1.20999707	1.1410911	0.671871676	-0.5737424	0.332735	1
SMIM12	1.5884201	1.53296424	1.6124577	1.149153421	0.2005714	0.332838	1
ATXN3	1.5640434	1.61954246	1.539987	0.871590006	-0.1982784	0.332965	1
ZDHHC17	1.344409	1.39580975	1.3221291	0.813848412	-0.297168	0.333024	1
RDM1	1.122967	1.17023245	1.1024795	0.601997445	-0.7321707	0.333176	1
TBC1D1	1.4474078	1.50043861	1.4244213	0.848098653	-0.237696	0.333314	1
KIAA0753	1.1819184	1.23030647	1.1609443	0.698826553	-0.5169937	0.333484	1
N4BP1	1.2126032	1.26163219	1.1913513	0.731375296	-0.4513162	0.333685	1
RPA2	1.7334815	1.67578129	1.758492	1.122392768	0.1665776	0.333846	1
FAM122A	1.4217489	1.36893167	1.4446428	1.205217128	0.2692931	0.333995	1
PPP1R14B	2.1121201	2.17868851	2.0832656	0.919043169	-0.1217955	0.334106	1
DMC1	1.0703721	1.11654878	1.0503566	0.432064823	-1.2106803	0.334247	1
BEST1	1.2820888	1.2315081	1.3040133	1.313186506	0.3930718	0.334423	1
YAF2	1.9150648	1.85352752	1.9417385	1.103348753	0.1418889	0.334501	1
STX17	1.3821889	1.32995595	1.4048295	1.226919858	0.295041	0.334505	1
AC007040.11	1.136936	1.18424292	1.1164305	0.63194025	-0.6621399	0.334631	1
FAM234B	1.1940791	1.24259078	1.1730515	0.71334732	-0.4873234	0.334684	1
AC013461.1	1.4750005	1.42159882	1.4981478	1.181568264	0.240703	0.334714	1
ZNF124	1.3275736	1.37881973	1.3053607	0.806084356	-0.3109973	0.334768	1
B3GNT5	1.1883753	1.23706789	1.1672693	0.705575379	-0.5031279	0.334774	1
PTGFRN	1.3795774	1.32730227	1.4022363	1.228944533	0.2974198	0.334807	1
TMEM132B	1.0924472	1.13887733	1.0723218	0.520760342	-0.9413085	0.334899	1
PARM1	1.1720394	1.12405949	1.1928366	1.554388046	0.6363467	0.335118	1
TMEM214	1.225044	1.17584942	1.2463677	1.401015237	0.4864726	0.335179	1
CREB5	1.3057413	1.35665675	1.2836717	0.79536325	-0.3303142	0.335299	1
MGAT1	1.6167736	1.56090444	1.6409904	1.142780022	0.1925477	0.335371	1
KCNAB3	1.1314716	1.1785905	1.1110476	0.621800273	-0.6854768	0.335453	1
ARHGEF2	1.2586399	1.2089746	1.2801676	1.340677845	0.4229626	0.335538	1
ACADS	1.2514786	1.20192256	1.2729589	1.351799893	0.4348816	0.335914	1
GAMT	2.6362638	2.71510541	2.6020895	0.934105547	-0.0983425	0.335929	1
RNF115	1.7313943	1.67322434	1.7566083	1.12385769	0.1684594	0.33593	1
PANK3	1.4992345	1.44501989	1.5227342	1.17463103	0.2322077	0.336115	1
HNRNPU	5.1667225	5.32503691	5.0981002	0.947529544	-0.0777572	0.336241	1

RNF220	1.311546	1.26090107	1.3334983	1.278255869	0.3541767	0.336261	1
KIF26A	1.1896587	1.23801035	1.1687004	0.70879439	-0.4965609	0.336289	1
RMND1	1.2804958	1.23057263	1.3021352	1.310368951	0.3899731	0.336517	1
BRD2	2.356839	2.42930051	2.3254302	0.92732788	-0.1088486	0.337064	1
TRO	1.2357041	1.28487636	1.2143901	0.752572579	-0.4100974	0.337081	1
C18orf25	1.1581187	1.20571646	1.1374873	0.668333754	-0.5813594	0.337105	1
CNBP	5.5537072	5.38007707	5.6289681	1.056823425	0.0797344	0.337522	1
RICTOR	1.6470165	1.59040113	1.6715567	1.137458349	0.1858137	0.337699	1
TMEM159	1.4625952	1.40938409	1.4856599	1.186318427	0.2464913	0.338069	1
XRCC3	1.1101918	1.15661739	1.0900684	0.575085609	-0.7981514	0.338136	1
LIPG	1.0600864	1.01466374	1.0797751	5.440299	2.4436859	0.33814	1
PEX1	1.3136115	1.36432175	1.2916309	0.800476124	-0.3210697	0.338175	1
ZNF254	1.4246049	1.47689755	1.4019383	0.842819037	-0.2467052	0.338236	1
CHPF2	1.1357586	1.08889586	1.1560716	1.755667451	0.8120196	0.338289	1
RNF207	1.2762505	1.22663154	1.2977581	1.313842245	0.3937921	0.33832	1
CPEB4	1.4621548	1.40873693	1.4853091	1.187338431	0.2477312	0.338332	1
RCAN3	1.1745043	1.12691326	1.195133	1.53753011	0.6206147	0.33846	1
FUBP1	2.1154721	2.05120812	2.1433277	1.087632087	0.1211906	0.338471	1
PAAF1	1.5292897	1.47583301	1.5524609	1.161039356	0.2154169	0.338501	1
ATP5SL	1.747931	1.68970007	1.7731716	1.12102582	0.1648195	0.338516	1
C6orf52	1.151497	1.1041979	1.1719991	1.650696411	0.7230748	0.339031	1
UBA6-AS1	1.1888029	1.23667182	1.1680538	0.710071198	-0.4939644	0.339139	1
ZNF839	1.149639	1.19705351	1.1290869	0.655085586	-0.6102447	0.339155	1
CPSF2	1.4697311	1.41673498	1.4927025	1.182292263	0.2415867	0.339443	1
PHKB	1.5840764	1.63912007	1.5602174	0.876544913	-0.1901001	0.339513	1
OSER1-AS1	1.3547169	1.40550898	1.3327008	0.820452249	-0.2855087	0.339661	1
PSEN2	1.179765	1.13206051	1.2004427	1.51780968	0.6019909	0.339696	1
TMEM41B	1.5695673	1.62465186	1.5456906	0.873591582	-0.1949691	0.339821	1
ECT2	1.3467194	1.29581998	1.368782	1.246643232	0.3180486	0.339831	1
PLXNA4	1.0987988	1.14486058	1.078833	0.544199327	-0.8777929	0.339896	1
CMSS1	2.3133522	2.24357712	2.3435966	1.080428837	0.1116041	0.340174	1
COL23A1	1.2275278	1.27622818	1.2064183	0.747274627	-0.4202896	0.34039	1
CNOT7	3.0813883	2.98575006	3.1228433	1.069038507	0.0963138	0.340507	1
RP3-510D11.2	1.2228326	1.27120631	1.2018648	0.744321773	-0.4260017	0.340592	1
TSC22D4	1.6803516	1.62373663	1.7048916	1.13011098	0.1764645	0.340733	1
RERE	2.0791232	2.01388858	2.1073995	1.092229973	0.1272767	0.340734	1
TMEM177	1.2447961	1.19607005	1.2659167	1.356233304	0.4396054	0.340772	1
DNAJB1	1.7577211	1.69860579	1.7833449	1.121297517	0.1651691	0.340807	1
SERPING1	1.7005519	1.64335028	1.7253462	1.127451441	0.1730653	0.340913	1
WAC-AS1	1.6817545	1.73859037	1.6571187	0.889693035	-0.1686204	0.340926	1
KBTBD2	1.2118061	1.16333298	1.232817	1.425413513	0.5113805	0.340957	1
CD81	1.8503621	1.79124818	1.8759853	1.107093009	0.1467764	0.341293	1
IPO7	2.4393074	2.36711698	2.4705987	1.075693399	0.1052669	0.341383	1
BTBD8	1.1291543	1.17560389	1.1090205	0.620831738	-0.6877258	0.341451	1
ATL1	1.2004341	1.15267753	1.2211344	1.448375476	0.5344357	0.341892	1
TSHZ3	1.105087	1.15102737	1.0851739	0.563963319	-0.8263268	0.341988	1
GLMP	1.783377	1.72416661	1.8090421	1.117204384	0.1598931	0.341999	1
SPON1	1.0790673	1.12467064	1.0593003	0.475655702	-1.0720104	0.342053	1
HTATIP2	1.0888459	1.04312185	1.1086652	2.519956875	1.333399	0.342176	1
STIL	1.1010643	1.14687216	1.0812086	0.552920249	-0.8548567	0.342179	1
TMEM161B-AS1	1.8320161	1.89007166	1.8068516	0.906501856	-0.1416181	0.342256	1

PJA2	2.2898019	2.21936458	2.3203333	1.082804383	0.1147726	0.342551	1
FALEC	1.1687986	1.21583135	1.1484121	0.68762975	-0.5402961	0.342602	1
SEMA4D	1.1686376	1.21607137	1.1480772	0.685316036	-0.5451586	0.342602	1
FBXW9	1.2703794	1.22117518	1.2917072	1.318896641	0.3993315	0.342656	1
AZIN1-AS1	1.2029274	1.2508141	1.1821706	0.726317227	-0.4613283	0.342781	1
SAMD1	1.4929679	1.54600856	1.4699772	0.860750567	-0.2163329	0.342803	1
AGA	1.3415776	1.29102234	1.363491	1.249014079	0.3207897	0.342989	1
DCAF5	1.4298041	1.37762491	1.4524214	1.198070808	0.2607132	0.34301	1
OTOS	1.2056782	1.15115414	1.2293119	1.517073484	0.601291	0.343012	1
SMIM20	2.6901806	2.77213405	2.6546575	0.933708973	-0.0989551	0.343029	1
ZDHHC15	1.1392852	1.0922002	1.1596944	1.732039758	0.792472	0.3431	1
PFKFB4	1.6096974	1.6646829	1.5858637	0.881418296	-0.1821013	0.34312	1
POM121C	1.4543862	1.5067332	1.4316961	0.851919915	-0.2312103	0.343163	1
CHD3	1.4813838	1.42808894	1.5044848	1.178457893	0.2369002	0.343337	1
GLIS1	1.1228909	1.07614746	1.1431521	1.879932851	0.9106811	0.343355	1
RGL3	1.2163204	1.26413698	1.195594	0.74050213	-0.4334242	0.343594	1
XKR6	1.2026353	1.25054925	1.1818668	0.725872346	-0.4622122	0.34361	1
MALL	1.0718121	1.02627467	1.0915505	3.484362983	1.8008949	0.343614	1
ACBD5	1.3898561	1.33867165	1.4120423	1.216642412	0.2829052	0.343638	1
TNFAIP8	1.1949915	1.14736074	1.2156373	1.463329065	0.5492542	0.34369	1
CXCL2	1.0446311	1.08917729	1.0253224	0.28395546	-1.8162634	0.343749	1
ZDHHC8	1.176345	1.22375269	1.1557959	0.69628612	-0.5222478	0.343752	1
RP3-402G11.26	1.176449	1.22360474	1.156009	0.697699972	-0.5193213	0.343752	1
FSD1L	1.2729285	1.32210417	1.2516131	0.78115429	-0.3563206	0.343763	1
FO538757.1	1.4253359	1.47707718	1.4029084	0.844534984	-0.2437709	0.343898	1
COL25A1	1.2011691	1.24911235	1.1803879	0.724122539	-0.4656942	0.343949	1
ZNRF1	1.2066999	1.25443175	1.1860103	0.731081268	-0.4518963	0.344099	1
DUSP12	1.6098146	1.554446	1.6338144	1.143148948	0.1930134	0.344135	1
DCAF10	1.5391394	1.48492586	1.5626385	1.160256732	0.2144441	0.344153	1
DERL1	1.4804964	1.42818538	1.5031709	1.175124069	0.2328131	0.344209	1
SH3D19	1.3153751	1.36505155	1.2938426	0.804934414	-0.3130569	0.344327	1
RBPMS2	1.2317642	1.18286843	1.2529584	1.383280669	0.4680939	0.344563	1
PDE4B	1.1157946	1.16175412	1.0958732	0.592709458	-0.754603	0.344732	1
AKAP8L	1.5966074	1.54190406	1.6203188	1.144702234	0.1949724	0.344742	1
SLC35F1	1.2365371	1.28502918	1.2155179	0.756125825	-0.4033018	0.344809	1
TFB1M	1.2711295	1.22235985	1.292269	1.31439652	0.3944006	0.344829	1
DZIP1L	1.1343539	1.18063215	1.1142943	0.632746085	-0.6603014	0.345014	1
PKIA	1.4519131	1.50398126	1.4293439	0.851904464	-0.2312364	0.345034	1
MMP16	1.2744854	1.32372253	1.2531433	0.781976289	-0.3548032	0.345069	1
MAPK15	1.1017392	1.14736864	1.0819609	0.556162692	-0.8464211	0.34516	1
FCF1	1.2228831	1.17471916	1.2437601	1.395153779	0.4804241	0.34539	1
BCO2	1.4169438	1.46883391	1.3944518	0.841346472	-0.2492281	0.345419	1
IDS	2.3515701	2.28143275	2.3819715	1.078458085	0.1089701	0.345428	1
CECR5-AS1	1.1289357	1.17505876	1.1089434	0.622324576	-0.6842609	0.34558	1
RSAD1	1.4691709	1.52220442	1.4461832	0.85442257	-0.2269783	0.345942	1
ADNP2	1.2251459	1.27311763	1.2043523	0.748220997	-0.4184636	0.346004	1
GSS	1.3975208	1.34678651	1.4195119	1.20971221	0.2746639	0.346191	1
RING1	1.5576587	1.5033025	1.5812198	1.15481197	0.207658	0.346402	1
TUBGCP3	1.2154471	1.26345316	1.1946386	0.738797628	-0.4367489	0.346411	1
UBE2T	1.8315008	1.7718809	1.8573434	1.110719763	0.1514949	0.346514	1
POGLUT1	1.2309298	1.18258275	1.2518861	1.379572174	0.4642209	0.346652	1

EMD	1.6035492	1.54896585	1.6272086	1.142527572	0.192229	0.347076	1
HNRNPAB	2.6126069	2.53529113	2.6461198	1.072187411	0.1005571	0.347091	1
VPS26B	1.4837311	1.4309467	1.5066107	1.175576284	0.2333682	0.347194	1
PM20D2	1.4609276	1.51305513	1.4383327	0.854357861	-0.2270876	0.347221	1
PAPOLA	4.0350403	3.91965826	4.0850532	1.056648744	0.0794959	0.347274	1
ACVR1B	1.1798949	1.22673387	1.1595924	0.703875224	-0.5066084	0.347386	1
TRMU	1.5221611	1.57529873	1.4991283	0.86759851	-0.2049005	0.347468	1
LTBP3	1.5702416	1.62485218	1.5465704	0.874719531	-0.1931076	0.347516	1
MYO6	1.8118193	1.75399688	1.8368827	1.109928608	0.1504669	0.347541	1
MED12L	1.2134558	1.26129828	1.1927182	0.737541114	-0.4392046	0.347587	1
IQCB1	1.4538914	1.50593888	1.4313311	0.852535934	-0.2301674	0.347797	1
CDC42SE2	1.4576386	1.50957686	1.4351258	0.85389622	-0.2278674	0.347817	1
RP5-1120P11.1	1.1316874	1.17773618	1.1117274	0.628613581	-0.6697547	0.34797	1
ZBED5	1.5608289	1.50678661	1.5842538	1.152859576	0.2052168	0.347991	1
TTK	1.1410979	1.18747311	1.1209962	0.64540587	-0.6317214	0.3481	1
MED7	1.2828052	1.23408719	1.3039223	1.298329268	0.3766563	0.348446	1
POP7	2.0919424	2.15721987	2.0636476	0.919140423	-0.1216428	0.348759	1
METTL26	3.3984049	3.50226555	3.3533859	0.940502047	-0.088497	0.348863	1
MSX1	1.1361302	1.09019412	1.1560414	1.730062251	0.7908239	0.349162	1
TOR1AIP1	1.2381272	1.19023897	1.2588846	1.36083881	0.4444962	0.349176	1
PQLC2	1.2821806	1.23360188	1.3032373	1.298094556	0.3763955	0.349193	1
TIMM13	5.5442338	5.36484615	5.6219903	1.058912539	0.0825834	0.349194	1
SLC25A22	1.4013189	1.45217602	1.3792746	0.838776383	-0.2536419	0.34926	1
ZNF7	1.3298081	1.37947271	1.3082807	0.812392397	-0.2997514	0.349414	1
NTS	1.15631	1.10690958	1.1777229	1.662366416	0.7332384	0.349708	1
PIGP	2.0412542	1.97961107	2.0679737	1.090201762	0.1245952	0.349863	1
PIEZO1	1.1592989	1.11287752	1.1794205	1.589515291	0.6685869	0.349936	1
PGM5-AS1.1	1.1146567	1.06912894	1.1343909	1.944061577	0.9590739	0.350005	1
MDM4	2.0075526	2.07081734	1.9801302	0.915310327	-0.1276671	0.350035	1
AGBL3	1.1047475	1.15021717	1.0850384	0.566103246	-0.8208629	0.350125	1
C9orf3	1.2872823	1.33612575	1.2661108	0.791700253	-0.3369738	0.350366	1
COQ9	1.6998527	1.64395934	1.72408	1.124418847	0.1691795	0.350848	1
H2AFZ	18.064586	17.6013094	18.265396	1.040002035	0.0565864	0.350887	1
SNAP47	1.559799	1.50648286	1.5829092	1.150896259	0.2027578	0.351001	1
WDR83	1.3965088	1.44768937	1.3743243	0.836124962	-0.2582095	0.351239	1
SLC35B3	1.2552049	1.20725739	1.275988	1.331619539	0.4131819	0.35129	1
CNOT11	1.3031834	1.2545266	1.3242739	1.274027557	0.3493965	0.351483	1
AGTPBP1	1.5246494	1.57720889	1.5018672	0.869472443	-0.2017878	0.351489	1
RP5-1068E13.7	1.1182396	1.16362967	1.098565	0.602366306	-0.731287	0.352094	1
IL17RE	1.1417332	1.18762458	1.1218413	0.649388903	-0.6228454	0.352139	1
RP11-128P17.2	1.0731159	1.1175619	1.0538505	0.458060864	-1.1263888	0.352147	1
PPIP5K2	1.6219262	1.67609219	1.5984477	0.885156873	-0.1759949	0.352574	1
VKORC1L1	1.3067581	1.25785756	1.3279543	1.271843013	0.3469206	0.352686	1
MRPL2	1.6325345	1.57829982	1.6560428	1.134433656	0.1819722	0.352791	1
KCTD5	1.2475112	1.1995702	1.2682915	1.344346305	0.4269048	0.352987	1
ABHD12	1.5792689	1.52590585	1.6023995	1.145451176	0.195916	0.353033	1
DOHH	1.1933835	1.14675958	1.2135929	1.45539313	0.5414089	0.353079	1
CXorf23	1.1631109	1.20908953	1.1431812	0.684784209	-0.5462787	0.353081	1
PPP2R5A	1.1898924	1.14322606	1.2101201	1.467052502	0.5529205	0.35323	1
SERINC3	1.5779774	1.52413128	1.6013173	1.147264601	0.1981982	0.353374	1
PIGBOS1	1.6407587	1.58660771	1.6642307	1.132325239	0.1792884	0.353464	1

ABCE1	1.7537428	1.69777553	1.7780022	1.114974847	0.1570112	0.353488	1
GNAI2	2.5034133	2.43200282	2.5343666	1.071482925	0.0996089	0.353514	1
ZMYND8	1.9755291	2.03537079	1.9495904	0.917150073	-0.1247703	0.35355	1
LGALS8	1.3651122	1.31555798	1.3865917	1.225105159	0.2929056	0.353569	1
NAP1L1	18.438164	18.8777262	18.247634	0.964755461	-0.0517648	0.353583	1
SETD5	2.5372381	2.61372793	2.5040832	0.932055004	-0.101513	0.353657	1
GOSR1	1.4212009	1.37055773	1.4431524	1.195906503	0.2581046	0.354068	1
BICD2	1.2637051	1.31203234	1.2427575	0.777988164	-0.3621799	0.35407	1
MRPL57	4.8837509	4.74316705	4.9446877	1.05383694	0.0756517	0.354222	1
MOB2	1.3600844	1.3107645	1.3814624	1.22749666	0.2957191	0.354309	1
RP13-766D20.4	1.1110545	1.15602356	1.0915624	0.58684956	-0.7689374	0.354322	1
PCDHB16	1.1945106	1.24111084	1.1743115	0.722951691	-0.4680288	0.354409	1
MAP1S	1.1925465	1.14582789	1.2127969	1.459233157	0.5452104	0.354422	1
C17orf58	1.5175139	1.56977498	1.4948611	0.868520205	-0.2033687	0.35449	1
ATF2	1.7394364	1.79542809	1.7151666	0.899096424	-0.1534522	0.354494	1
VRK2	1.2726139	1.32069754	1.2517717	0.785075321	-0.349097	0.354793	1
ATP5G1	4.2386532	4.11967086	4.2902268	1.054671119	0.0767932	0.354926	1
FZD8	1.1014078	1.14639621	1.0819074	0.559491126	-0.8378128	0.35494	1
FAM175B	1.2171044	1.16999915	1.2375224	1.39719758	0.482536	0.355027	1
SP1	1.2827874	1.33086175	1.2619493	0.791718168	-0.3369411	0.355094	1
ZNF585A	1.1576287	1.20357593	1.1377126	0.676467957	-0.5639065	0.355171	1
KHDRBS2	1.1561996	1.20220426	1.1362586	0.673866134	-0.5694661	0.355213	1
KLHL28	1.3901224	1.44028755	1.3683781	0.836676152	-0.2572588	0.355273	1
PTRH1	1.3958228	1.34575072	1.4175268	1.207594877	0.2721365	0.355402	1
N4BP2L1	1.1971315	1.24356624	1.1770041	0.726718555	-0.4605314	0.355436	1
TAF8	1.249923	1.29741748	1.2293362	0.771091897	-0.3750253	0.355534	1
PIAS1	1.8810543	1.93914229	1.8558757	0.911337593	-0.1339425	0.355623	1
NPTN	1.1640319	1.1181156	1.1839346	1.557242289	0.6389934	0.355887	1
CGN	1.2367523	1.28408405	1.2162362	0.761169656	-0.39371	0.355971	1
HMGB2	6.1819812	5.89358013	6.3069902	1.084480078	0.1170036	0.356169	1
TIAM1	1.3546089	1.4041245	1.3331462	0.824365163	-0.2786446	0.356185	1
NECAB2	1.231946	1.27906832	1.2115206	0.757952702	-0.3998203	0.356253	1
CTA-445C9.14	1.1786711	1.22474934	1.1586983	0.706112243	-0.5020306	0.356519	1
GTF2H2C	1.3037252	1.35213612	1.2827413	0.802931705	-0.3166508	0.356858	1
KCTD10	1.2734151	1.22511377	1.2943516	1.307568145	0.3868861	0.356871	1
MBTPS1	1.6113615	1.55785157	1.6345556	1.137499041	0.1858653	0.356992	1
TMEM145	1.1113811	1.15608175	1.0920054	0.589469162	-0.7625118	0.357261	1
DPYSL2	3.1831218	3.27142033	3.1448484	0.94427629	-0.082719	0.357346	1
LONRF2	1.1420839	1.18742539	1.1224304	0.653222227	-0.6143542	0.357434	1
NOTCH3	1.4731683	1.52445451	1.4509381	0.859823014	-0.2178884	0.357582	1
RBL2	1.4479348	1.49922589	1.4257024	0.852725102	-0.2298474	0.357674	1
C14orf28	1.1961346	1.24275124	1.1759284	0.724726934	-0.4644906	0.35772	1
TOM1	1.2421341	1.19463753	1.2627218	1.349800325	0.432746	0.357913	1
SSBP4	3.0786132	2.99049166	3.11681	1.063460891	0.088767	0.358218	1
PDS5A	1.6613999	1.7153659	1.638008	0.891862454	-0.1651069	0.358371	1
C8orf46	1.1588664	1.10888556	1.1805309	1.657987381	0.729433	0.35844	1
SOCS1	1.0815055	1.0372293	1.1006973	2.704786092	1.4355145	0.358546	1
UHMK1	1.3988717	1.3488313	1.420562	1.205631431	0.2697889	0.358546	1
PGM3	1.4863572	1.43514743	1.5085543	1.168694322	0.2248976	0.358707	1
AC004381.6	1.0891609	1.13311394	1.0701093	0.526686225	-0.9249844	0.358801	1
ANKRD52	1.166773	1.12108456	1.1865769	1.54088116	0.6237556	0.358852	1

PEX6	1.2072341	1.25362014	1.1871277	0.737826859	-0.4386458	0.358887	1
C2orf81	1.178147	1.22384626	1.1583385	0.707353607	-0.4994965	0.359037	1
RABEP1	1.7824783	1.72657295	1.8067107	1.110295548	0.1509438	0.359122	1
CGRRF1	1.2990772	1.25115833	1.3198479	1.273490989	0.3487888	0.359222	1
CYB561D2	1.2925648	1.24484643	1.3132486	1.279367601	0.3554309	0.359229	1
GPR37	1.4967822	1.44527574	1.519108	1.165812431	0.2213357	0.359272	1
MEST	9.0433044	8.73222976	9.1781415	1.057669225	0.0808885	0.359308	1
MAPK12	1.2616775	1.30915313	1.2410989	0.779868876	-0.3586965	0.359613	1
FAM214A	1.1972891	1.24324491	1.1773693	0.729179929	-0.4556532	0.359833	1
PTPN1	1.2928541	1.24509728	1.3135545	1.279306271	0.3553617	0.360638	1
PAG1	1.1902479	1.14402013	1.2102855	1.460111989	0.546079	0.360641	1
M6PR	1.7824592	1.72669969	1.8066285	1.109988779	0.1505451	0.360881	1
AQP3	1.0822876	1.03818824	1.1014027	2.655337358	1.4088952	0.360956	1
PRKCI	1.5392389	1.48737002	1.5617218	1.152557129	0.2048383	0.361147	1
MRPL53	1.2266252	1.17942578	1.2470841	1.377082472	0.461615	0.361221	1
HSD17B8	1.2859739	1.23822982	1.3066689	1.287281661	0.3643278	0.361361	1
CLGN	1.318493	1.27031574	1.3393757	1.25547892	0.3282378	0.361387	1
VIPR2	1.0825954	1.126436	1.0635924	0.50296156	-0.99148	0.361433	1
RGS2	1.1993871	1.15326081	1.2193808	1.43142155	0.5174486	0.361644	1
BORCS8	1.2696505	1.22208279	1.290269	1.307030554	0.3862929	0.361723	1
C3orf38	1.3814244	1.33221967	1.4027525	1.21230766	0.2777559	0.361937	1
TBX6	1.0835986	1.127411	1.0646079	0.507082874	-0.9797065	0.361958	1
GLRB	1.1469417	1.10184198	1.1664904	1.634790991	0.7091062	0.361974	1
ANAPC11	6.974481	6.77003888	7.0630975	1.050789714	0.071474	0.362189	1
PRMT5	1.3419909	1.29350997	1.3630052	1.236773113	0.3065809	0.362229	1
FAM179B	1.4650762	1.51546839	1.4432335	0.859865529	-0.217817	0.362348	1
ZNF738	1.4648018	1.51522926	1.4429437	0.859702163	-0.2180912	0.362348	1
RP11-115C21.2	1.479322	1.53037601	1.4571923	0.862015504	-0.2142143	0.362353	1
PHF11	1.2718873	1.31945576	1.2512684	0.786551617	-0.3463867	0.362689	1
NUDT22	1.8965055	1.839014	1.9214255	1.098224204	0.1351726	0.362807	1
CD83	1.1061224	1.06174369	1.1253586	2.030306032	1.0216972	0.363084	1
PEX10	1.7137998	1.65905236	1.7375304	1.119077069	0.1623094	0.363236	1
VGF	1.4898315	1.43901635	1.5118576	1.165919281	0.2214679	0.36354	1
DGCR8	1.1899961	1.23567068	1.1701981	0.722186264	-0.4695571	0.363581	1
ALG2	1.1969473	1.15105969	1.2168376	1.435443178	0.5214962	0.363591	1
MTMR6	1.1917069	1.14594008	1.2115448	1.449531838	0.535587	0.363593	1
UBXN11	1.4367801	1.48667474	1.415153	0.853039942	-0.2293148	0.363656	1
TRIB3	1.1513675	1.1063456	1.1708824	1.606859403	0.6842437	0.363662	1
ZDHHC1	1.2275655	1.27407309	1.2074066	0.756756528	-0.4020989	0.363674	1
MLST8	2.1936172	2.25545953	2.1668112	0.929389764	-0.1056443	0.363902	1
PPP2R3A	1.3331605	1.3817205	1.3121119	0.817645084	-0.2904533	0.363907	1
LIAS	1.3373049	1.38584003	1.3162671	0.819684637	-0.2868591	0.363938	1
MESP1	1.3349919	1.28662014	1.355959	1.241918853	0.3125709	0.363943	1
LAMB1	1.2013584	1.15571004	1.221145	1.420235907	0.5061306	0.364093	1
CIRBP	11.704044	11.92349	11.608923	0.97120273	-0.0421556	0.364099	1
GAS2L3	1.3563998	1.40520798	1.3352436	0.8273371	-0.2734528	0.364452	1
GPC4	1.4624323	1.51316189	1.4404433	0.858293098	-0.2204577	0.364542	1
ZNF791	1.573923	1.62624208	1.551245	0.880242711	-0.1840267	0.3646	1
PDCD2L	1.1720674	1.12682545	1.1916778	1.511351365	0.5958391	0.364914	1
KLHDC10	1.491739	1.54263234	1.469679	0.865556663	-0.2082998	0.365378	1
GLG1	1.9762878	2.03538383	1.9506723	0.918183482	-0.1231456	0.365409	1

EDIL3	1.107903	1.06387504	1.1269871	1.988055334	0.9913579	0.365646	1
CCDC59	1.8753104	1.8182055	1.9000628	1.100044891	0.1375624	0.365864	1
FTO	1.2905676	1.24316098	1.3111162	1.279465909	0.3555417	0.365897	1
RP11-149I23.3	1.0933461	1.04976161	1.112238	2.255513701	1.173456	0.36593	1
HOXB8	1.0834878	1.03854515	1.1029684	2.671370899	1.4175803	0.365953	1
SEMA4F	1.2043863	1.15852025	1.2242672	1.414754014	0.5005512	0.366146	1
FAM219A	1.3035293	1.3507973	1.2830407	0.806849621	-0.3096283	0.366224	1
RCL1	1.14022	1.0954421	1.1596293	1.672524746	0.7420276	0.366284	1
AGTRAP	2.0137613	1.95391769	2.0397009	1.089927263	0.1242319	0.366509	1
HSPA1B	1.1015895	1.0578656	1.1205418	2.083134332	1.0587559	0.36667	1
TFEC	1.0791067	1.03579628	1.0978799	2.734359739	1.4512031	0.366677	1
SND1	2.2816775	2.34675747	2.2534683	0.930730502	-0.1035646	0.366942	1
SZRD1	1.8036334	1.85892844	1.7796655	0.907718849	-0.1396826	0.366947	1
GNB5	1.3978672	1.34825433	1.4193721	1.204212189	0.2680896	0.367083	1
KIAA1033	1.3962147	1.4451073	1.375022	0.842542887	-0.247178	0.367171	1
CDC45	1.1089263	1.15284852	1.089888	0.588085791	-0.7659015	0.367185	1
ZNF117	1.2800988	1.3268516	1.2598335	0.794958766	-0.3310481	0.367205	1
BTN2A1	1.3593028	1.31102998	1.380227	1.222476869	0.2898072	0.36728	1
EMP1	1.2993638	1.25190839	1.3199336	1.270039423	0.3448733	0.367576	1
MMP11	1.2196315	1.17393012	1.239441	1.376650822	0.4611627	0.367823	1
RELB	1.1022815	1.05848632	1.1212648	2.073386963	1.0519894	0.367881	1
ANGPT2	1.1096309	1.0659316	1.1285727	1.950091645	0.9635419	0.367997	1
TTC9	1.0805967	1.03726119	1.0993807	2.667137805	1.4152924	0.368248	1
FBXO17	1.756545	1.70167237	1.7803298	1.112100017	0.1532865	0.368349	1
LINC00877	1.0563542	1.09901139	1.0378642	0.382422861	-1.3867593	0.368381	1
TMEM231	1.3318224	1.3795384	1.3111397	0.819784524	-0.2866833	0.368539	1
UBL3	1.6959815	1.64217026	1.7193063	1.120117787	0.1636504	0.368645	1
SPOCK2	1.2381951	1.19179227	1.2583087	1.346815055	0.4295518	0.368794	1
E2F1	1.117971	1.16183884	1.0989562	0.611448844	-0.7096963	0.368965	1
BPGM	1.5192975	1.4682563	1.5414216	1.156250447	0.2094539	0.368993	1
MAST4	1.4338746	1.48334995	1.4124293	0.853272589	-0.2289214	0.36905	1
CUL2	1.2972835	1.25035968	1.3176229	1.268666386	0.3433127	0.369122	1
MAP6	1.4850071	1.53470443	1.4634655	0.866769602	-0.2062795	0.36931	1
SACS	1.583667	1.53231655	1.6059252	1.138279749	0.1868552	0.36932	1
GMNN	1.7819236	1.72695951	1.8057481	1.108380981	0.1484539	0.369437	1
C12orf43	1.3088221	1.26156447	1.3293062	1.258986804	0.3322632	0.369456	1
RWDD2A	1.1725965	1.12786063	1.1919875	1.501536954	0.58644	0.369496	1
RBM25	2.8012605	2.71900293	2.8369154	1.068593543	0.0957132	0.369581	1
ZNF263	1.264238	1.31075289	1.2440759	0.785434113	-0.3484378	0.36973	1
TMEM106B	2.144306	2.08350766	2.1706594	1.080434844	0.1116121	0.370419	1
RNF25	1.0986305	1.05499351	1.1175452	2.137436873	1.0958818	0.370441	1
MFSD11	1.2656337	1.31207777	1.2455023	0.786670158	-0.3461692	0.370865	1
RP11-644F5.11	1.1710936	1.21617694	1.151552	0.70105545	-0.5123995	0.370926	1
CSRNP1	1.1294553	1.08539243	1.1485546	1.739669165	0.798813	0.371028	1
RP11-271F18.4	1.0540358	1.09642694	1.0356612	0.36982565	-1.4350828	0.371072	1
CREB1	1.8421638	1.78646424	1.8663071	1.101521253	0.1394973	0.371197	1
C12orf57	4.2927186	4.1782121	4.342352	1.051645361	0.0726483	0.371331	1
AKT1S1	1.6246886	1.57212805	1.6474713	1.131689453	0.1784781	0.37151	1
ABCA3	1.1958585	1.24092698	1.1763233	0.731853646	-0.4503729	0.371545	1
PRADC1	1.3613345	1.40932588	1.3405324	0.831934638	-0.2654579	0.371678	1
POSTN	1.0595715	1.01686089	1.0780847	4.631112021	2.2113587	0.371753	1

IGF1R	2.0305607	1.97203078	2.0559307	1.086314105	0.1194413	0.371755	1
VPS33B	1.1434634	1.09920523	1.1626473	1.639503287	0.7132588	0.371772	1
RGS20	1.4253111	1.47392424	1.4042395	0.85296234	-0.229446	0.371781	1
ULK3	1.3030133	1.25597216	1.3234036	1.263432642	0.3373488	0.371793	1
CASC4	1.9091019	1.96574683	1.8845489	0.915922102	-0.1267032	0.371872	1
GART	1.3388416	1.29116259	1.3595083	1.23473376	0.3042	0.37214	1
NORAD	2.9907772	2.90849278	3.0264438	1.061803223	0.0865164	0.372179	1
SPOCK3	1.0912746	1.04814866	1.1099678	2.283923023	1.191514	0.372282	1
KIAA0586	1.2926404	1.34001219	1.2721068	0.800285468	-0.3214134	0.372436	1
LTBP1	1.2993287	1.25239353	1.3196731	1.266565993	0.3409222	0.372647	1
PAPSS2	1.1222619	1.07863871	1.1411707	1.795180083	0.8441286	0.372745	1
SBSPON	1.0615235	1.01907602	1.0799225	4.189686347	2.0668422	0.372861	1
PPP2R3C	1.8984133	1.8417441	1.9229768	1.096505228	0.1329127	0.372892	1
XPO5	1.29825	1.34479114	1.2780765	0.806507099	-0.3102409	0.373103	1
SP8	1.0729231	1.11545889	1.0544857	0.471905545	-1.08343	0.37319	1
NCOA3	1.234369	1.18862925	1.2541952	1.347591571	0.4303833	0.373305	1
PIGZ	1.0602014	1.10253468	1.0418517	0.40817166	-1.2927521	0.37339	1
DST	2.5625071	2.63716146	2.5301477	0.934634573	-0.0975257	0.373578	1
FBXL15	1.7554583	1.70161395	1.7787975	1.110008564	0.1505708	0.373666	1
MTHFSD	1.1467456	1.19090075	1.1276063	0.668442962	-0.5811236	0.373717	1
SSX2IP	1.6336247	1.58078531	1.6565282	1.130414572	0.176852	0.37384	1
PDGFD	1.1061988	1.06282127	1.125001	1.989787546	0.9926144	0.374049	1
RAB3A	1.4235932	1.37404238	1.4450713	1.189895488	0.2508349	0.374194	1
SLC25A14	1.2609873	1.30707646	1.2410096	0.784852223	-0.3495071	0.374343	1
TACC3	1.6124767	1.56076698	1.6348906	1.132182486	0.1791065	0.374474	1
APAF1	1.3125206	1.35933081	1.2922305	0.813263148	-0.2982059	0.37454	1
COQ8A	1.177238	1.13272289	1.1965334	1.480779625	0.5663569	0.374653	1
ZNF506	1.2602664	1.21393693	1.2803481	1.310424046	0.3900337	0.374661	1
CGGBP1	2.0025107	2.05982991	1.9776654	0.922473909	-0.11642	0.374708	1
EDRF1	1.2447875	1.29008367	1.2251537	0.776168015	-0.3655591	0.374744	1
LINC00958	1.0903016	1.13312161	1.0717411	0.538913941	-0.8918732	0.374882	1
TRIM8	1.2896889	1.24325347	1.3098166	1.273637079	0.3489542	0.374888	1
KCTD13	1.1945051	1.14976516	1.2138978	1.42822136	0.5142196	0.374985	1
METAP1	1.3929704	1.34510406	1.4137183	1.198821951	0.2616174	0.375169	1
CTD-2008L17.2	1.2792966	1.32544079	1.2592952	0.796750678	-0.3277998	0.375189	1
EXOSC10	1.2789949	1.32514258	1.258992	0.796548901	-0.3281652	0.375189	1
CYB5R4	1.3496943	1.30198062	1.3703761	1.226489684	0.2945351	0.375229	1
TEAD3	1.1847611	1.1401142	1.2041135	1.456765309	0.5427685	0.375291	1
SH3YL1	1.6258866	1.67780063	1.6033842	0.890208999	-0.167784	0.375312	1
DDX5	6.1181159	6.30936026	6.0352199	0.948366601	-0.0764832	0.37536	1
LRRC1	1.2173819	1.17230297	1.2369216	1.375028845	0.4594619	0.375417	1
RBM5	1.7855544	1.83954683	1.7621511	0.907812542	-0.1395337	0.375459	1
PRKAB1	1.2033763	1.15848395	1.2228351	1.406041883	0.4916396	0.375476	1
MARCH1	1.1083856	1.06531735	1.1270538	1.945176079	0.9599008	0.375515	1
PI4KA	1.3241266	1.37122489	1.3037116	0.818133844	-0.2895912	0.375539	1
APEX2	1.1562279	1.11233029	1.1752556	1.560181062	0.6417135	0.375541	1
UBE3C	1.2462599	1.20040388	1.2661365	1.328000791	0.409256	0.375731	1
TICRR	1.0575308	1.09948198	1.0393468	0.395517251	-1.3381875	0.37593	1
GYPC	2.3531884	2.28886785	2.3810686	1.071536201	0.0996806	0.375977	1
GFRA1	1.2828114	1.32904532	1.262771	0.798586058	-0.3244802	0.376009	1
SAMD4B	1.5555235	1.60592517	1.5336766	0.880763235	-0.1831738	0.376142	1

SNRNP35	1.3439493	1.29679074	1.3643905	1.227769025	0.2960392	0.37619	1
TPP2	1.3834558	1.33516351	1.4043883	1.206540407	0.2708762	0.376203	1
HLCS	1.1717897	1.21606775	1.1525972	0.706247023	-0.5017552	0.376237	1
SMARCC2	1.763241	1.81682846	1.7400132	0.905959185	-0.142482	0.376288	1
GSDMD	1.1798175	1.13550696	1.1990242	1.468737768	0.5545768	0.376328	1
STARD3	1.630645	1.68226788	1.6082687	0.891539432	-0.1656295	0.376442	1
BLOC1S4	1.8329302	1.77852573	1.8565121	1.100171918	0.137729	0.376494	1
CBLL1	1.2188028	1.26383751	1.1992823	0.75532207	-0.4048362	0.376623	1
ZDBF2	1.2100007	1.25487223	1.1905509	0.747633025	-0.4195978	0.376643	1
SLC25A40	1.1929881	1.23726763	1.1737949	0.73248468	-0.4491295	0.376907	1
AP4M1	1.5157932	1.56587457	1.4940851	0.873135353	-0.1957228	0.376932	1
NT5DC2	1.920665	1.86437856	1.9450627	1.093343525	0.1287468	0.37721	1
AADAT	1.2662909	1.31200615	1.2464753	0.789969514	-0.3401311	0.377427	1
SLC25A29	1.3621934	1.31481386	1.3827303	1.215735288	0.2818291	0.377522	1
NEK5	1.1367176	1.18005738	1.1179317	0.654967312	-0.6105052	0.377549	1
CPS1	1.2359025	1.28092073	1.2163891	0.770285189	-0.3765354	0.377778	1
MAGI2	1.2233494	1.26847969	1.2037874	0.75904225	-0.3977479	0.377869	1
KPNA1	1.4005517	1.35268487	1.4212998	1.194550191	0.2564675	0.378013	1
CCNT1	1.2681301	1.31395495	1.248267	0.79077279	-0.3386649	0.378197	1
CBWD5	1.464701	1.51382741	1.4434069	0.862949145	-0.2126526	0.378406	1
MAD1L1	1.4167516	1.36825105	1.4377744	1.188793408	0.249498	0.378515	1
ZNF511	1.8906873	1.82985037	1.9170574	1.105087596	0.1441607	0.378649	1
CCDC142	1.1301962	1.17337012	1.1114822	0.643029903	-0.6370423	0.378814	1
MANBAL	2.028021	1.97021304	2.0530781	1.085409149	0.118239	0.378975	1
GNPNAT1	1.378628	1.4262607	1.3579813	0.839817811	-0.2518517	0.379	1
CNOT3	1.3785088	1.4261716	1.3578492	0.839683281	-0.2520828	0.379	1
MDGA1	1.0896789	1.13196533	1.0713497	0.540669795	-0.8871803	0.379041	1
LINC00893	1.1104259	1.1532133	1.0918794	0.599683032	-0.7377279	0.379488	1
FBLN7	1.1314767	1.17476103	1.1127149	0.644965913	-0.6327052	0.379547	1
BLM	1.129883	1.17291625	1.11123	0.643259376	-0.6365275	0.379668	1
SRPRB	1.3034007	1.25717027	1.3234396	1.257686492	0.3307723	0.379755	1
ETV1	1.3178708	1.27174109	1.3378659	1.243337615	0.3142181	0.380039	1
MYF6	1.0969544	1.0544326	1.1153857	2.119790142	1.0839214	0.38064	1
BBS7	1.3887757	1.34124791	1.4093769	1.199646652	0.2626095	0.380936	1
TMEM57	1.2359963	1.1909842	1.255507	1.337843625	0.4199095	0.381067	1
GS1-124K5.11	1.0861626	1.12829588	1.0678997	0.529242773	-0.9179984	0.381113	1
KLF7	1.2928129	1.24689238	1.3127173	1.26661397	0.3409769	0.381166	1
RP11-141B14.1	1.1388904	1.18230594	1.1200717	0.658627238	-0.6024659	0.381222	1
RP11-128I7.1	1.1186291	1.16125034	1.1001548	0.621113451	-0.6870713	0.381262	1
RARS2	1.7121628	1.65895712	1.7352251	1.115740475	0.1580015	0.381287	1
ARL17B	1.1179448	1.16061802	1.0994478	0.619157348	-0.691622	0.381419	1
CCDC180	1.0955792	1.13776813	1.0772922	0.561031188	-0.8338471	0.381512	1
ZBTB24	1.1837991	1.22795129	1.1646611	0.722352009	-0.469226	0.381522	1
RBM45	1.1268711	1.08399242	1.1454571	1.731788159	0.7922625	0.38153	1
C17orf82	1.0734722	1.11529762	1.0553427	0.479998506	-1.0588982	0.381588	1
KIDINS220	1.853214	1.90774135	1.8295788	0.913893359	-0.1299023	0.381683	1
AC142528.1	1.0825985	1.12468954	1.0643539	0.516112691	-0.954242	0.381708	1
TMEM51	1.1522094	1.10878163	1.1710334	1.572263474	0.652843	0.381742	1
C4orf47	1.3502878	1.39711525	1.3299901	0.830968138	-0.2671349	0.381808	1
ASTN1	1.1821527	1.22622958	1.1630473	0.720716094	-0.472497	0.381881	1
ASB7	1.1573036	1.20097824	1.1383726	0.688495363	-0.5384812	0.381935	1

E2F8	1.0620515	1.10366348	1.0440145	0.424590227	-1.2358569	0.381967	1
ZFPL1	1.4137785	1.365933	1.4345174	1.187423489	0.2478346	0.382111	1
NOP2	1.0975077	1.05521517	1.1158396	2.097967632	1.0689924	0.382177	1
RPP14	1.2533065	1.29826794	1.2338177	0.783918385	-0.3512246	0.38234	1
SNRNP70	3.5108916	3.60748717	3.4690217	0.946896985	-0.0787206	0.382526	1
TNFAIP3	1.0841442	1.04209802	1.1023694	2.431691115	1.28196	0.382598	1
TPD52L2	1.6150462	1.56391755	1.6372082	1.129966899	0.1762805	0.382648	1
EPS8L1	1.0847435	1.04252919	1.1030415	2.422841096	1.2766998	0.382684	1
APBB2	1.2222414	1.17785097	1.2414827	1.357781296	0.4412511	0.382816	1
CEP104	1.3736975	1.42072193	1.3533145	0.839781585	-0.2519139	0.382914	1
PAPOLG	1.1698272	1.12613321	1.1887666	1.496565309	0.5816552	0.38312	1
CTD-2095E4.5	1.1698441	1.21307203	1.1511067	0.709181212	-0.4957738	0.383171	1
C2orf74	1.4119137	1.45984851	1.3911361	0.850576022	-0.2334879	0.383254	1
CTB-31O20.2	1.2279681	1.27284734	1.208515	0.764218394	-0.3879431	0.38332	1
LPIN3	1.1587378	1.20222321	1.1398888	0.691754347	-0.5316683	0.383464	1
SHC2	1.2163425	1.26079186	1.1970757	0.755682125	-0.4041486	0.383499	1
ZNF665	1.119934	1.16250186	1.1014828	0.624502353	-0.6792211	0.383594	1
DNAH7	1.0723635	1.11400719	1.0543128	0.47639834	-1.0697597	0.383676	1
NCS1	1.1752762	1.13147502	1.194262	1.47755865	0.5632154	0.383684	1
RP11-563J2.3	1.0635594	1.10508297	1.0455608	0.433570178	-1.2056626	0.383795	1
ZDHHC7	1.3527732	1.30600453	1.3730453	1.219084313	0.2857979	0.383815	1
RP11-192H23.6	1.194118	1.23778463	1.1751905	0.736761402	-0.4407306	0.383831	1
NSRP1	1.8849801	1.93991125	1.8611699	0.916224721	-0.1262266	0.383902	1
EVL	1.8639993	1.91886074	1.8402193	0.914414141	-0.1290804	0.383994	1
RWDD3	1.090654	1.13271899	1.0724207	0.545669811	-0.8738999	0.384079	1
TTC39B	1.1068252	1.14916605	1.0884724	0.593113282	-0.7536204	0.384101	1
TSC1	1.2526743	1.29733046	1.2333178	0.784708786	-0.3497707	0.384466	1
PKMYT1	1.094568	1.13656123	1.0763658	0.559205468	-0.8385496	0.384471	1
FASTKD2	1.2399903	1.1954453	1.2592985	1.32670645	0.4078492	0.384484	1
RP11-11N9.4	2.2094547	2.1462344	2.2368579	1.079061958	0.1097777	0.384839	1
UTP14C	1.2117258	1.25600776	1.1925315	0.752053393	-0.411093	0.384914	1
SLC39A13	1.4371182	1.38940085	1.4578015	1.175656084	0.2334661	0.385189	1
ZSCAN16	1.0956163	1.05349888	1.1138723	2.128499561	1.0898368	0.385452	1
GUF1	1.383263	1.33625256	1.40364	1.200407216	0.2635239	0.385453	1
NID1	1.5196107	1.56951015	1.4979815	0.874403125	-0.1936295	0.385494	1
STAT5B	1.1832436	1.22698442	1.1642839	0.723767103	-0.4664026	0.385516	1
SURF6	1.4448887	1.39686948	1.4657029	1.173441033	0.2307453	0.385536	1
ASF1B	1.3479785	1.39433754	1.3278839	0.831480419	-0.2662458	0.385748	1
LIPE-AS1	1.2027443	1.246648	1.183714	0.744842935	-0.4249919	0.38615	1
CPNE2	1.2914338	1.24573604	1.3112418	1.266569612	0.3409264	0.386266	1
DHRS2	1.0678965	1.02656865	1.0858103	3.229758243	1.6914262	0.386385	1
CACNG7	1.1492327	1.19215138	1.1306293	0.679825188	-0.5567643	0.386391	1
C14orf132	1.5819929	1.63162825	1.5604782	0.887354553	-0.1724174	0.386516	1
SEPT8	1.3588077	1.31191718	1.3791327	1.215491531	0.2815398	0.386988	1
PNPLA4	1.5529175	1.50327585	1.5744349	1.141391767	0.1907941	0.387108	1
GCAT	1.3466305	1.30046168	1.3666426	1.22026424	0.2871936	0.387223	1
FLII	1.37281	1.32626132	1.3929868	1.204515462	0.2684529	0.387374	1
MAVS	1.4821718	1.53100051	1.4610067	0.868185015	-0.2039256	0.387632	1
PCLO	1.2425453	1.19819268	1.2617701	1.320786115	0.4013969	0.387965	1
ARID4A	1.5516514	1.60149498	1.5300464	0.881214966	-0.1824341	0.388158	1
RP11-9G1.3	1.0688013	1.10990944	1.0509827	0.46386109	-1.1082353	0.388564	1

TOR3A	1.2901927	1.24529505	1.3096539	1.262373034	0.3361383	0.388638	1
MCU	1.1324522	1.09020533	1.1507644	1.67134704	0.7410113	0.388776	1
UNC5B-AS1	1.1046341	1.06262539	1.122843	1.961552173	0.9719957	0.388856	1
CTSK	1.0713827	1.03009406	1.0892794	2.966679353	1.568849	0.38908	1
CALHM2	1.1456215	1.1882619	1.1271388	0.675329554	-0.5663364	0.389273	1
NRGN	1.0764825	1.11767026	1.0586295	0.498252532	-1.005051	0.389291	1
ACAP2	1.7417149	1.79451246	1.7188295	0.904742901	-0.1444202	0.38932	1
AC009948.5	1.1448068	1.1873336	1.1263733	0.674589739	-0.5679177	0.389325	1
KCTD1	1.1722643	1.12919751	1.1909319	1.477829381	0.5634797	0.389418	1
RP11-1114A5.4	1.2766019	1.32108891	1.2573188	0.80139408	-0.3194162	0.389543	1
MEIS2	3.2239185	3.12787279	3.2655501	1.064701854	0.0904495	0.389656	1
GFM1	1.2739707	1.22907406	1.2934314	1.280945489	0.3572091	0.389678	1
ZNF814	1.3489632	1.39581531	1.3286549	0.830323894	-0.2682539	0.389863	1
POLR3K	1.5176704	1.46910141	1.5387228	1.148414477	0.1996434	0.389866	1
LRR1	1.2653985	1.31011466	1.246016	0.793306637	-0.3340495	0.389915	1
C19orf25	1.9743344	2.03265024	1.9490571	0.919049865	-0.121785	0.390009	1
ITGA2	1.2536146	1.20974955	1.2726282	1.299779503	0.3782669	0.3901	1
PCDHB14	1.1501696	1.10723867	1.1687783	1.573856437	0.6543039	0.39029	1
NUP214	1.2422117	1.2863489	1.2230802	0.779050164	-0.3602119	0.390303	1
WDR13	1.8977727	1.84339704	1.9213422	1.092418075	0.1275251	0.390694	1
EIF5A2	1.1576268	1.11498412	1.1761105	1.531607494	0.6150466	0.390808	1
PTMS	12.339099	12.1027534	12.441545	1.030514201	0.0433644	0.390846	1
CRELD1	1.8615461	1.80788212	1.884807	1.095217948	0.131218	0.391054	1
MYO5B	1.2543377	1.21016401	1.273485	1.30129342	0.3799463	0.391138	1
UACA	1.7603511	1.81307647	1.737497	0.907045021	-0.1407539	0.391211	1
ATAD3A	1.2668715	1.22238058	1.2861563	1.28678625	0.3637724	0.39127	1
FER	1.3706304	1.41720433	1.3504427	0.839978493	-0.2515757	0.391338	1
SARS2	1.1921675	1.14901695	1.2108714	1.4150831	0.5008868	0.391769	1
TMTC3	1.3183874	1.27302115	1.3380516	1.2381883	0.3082307	0.391843	1
SFRP5	1.0687195	1.02760803	1.0865394	3.134574001	1.6482694	0.391849	1
LIN7B	1.2218161	1.178056	1.2407842	1.352294808	0.4354097	0.391875	1
STIM1	1.1662771	1.12334405	1.1848866	1.498950247	0.5839525	0.391928	1
UTP11	1.6402542	1.58984906	1.6621026	1.122494937	0.1667089	0.391955	1
NGLY1	1.5474564	1.49884041	1.5685293	1.139701831	0.1886564	0.392058	1
PLPP2	1.0662924	1.02535514	1.0840368	3.314389556	1.7287432	0.392379	1
TRAF7	1.8436373	1.89723431	1.8204053	0.914371357	-0.1291479	0.39273	1
KIF3C	1.1755678	1.1327033	1.1941477	1.463020727	0.5489502	0.392808	1
ARHGAP11A	1.308727	1.35367226	1.2892453	0.817834183	-0.2901197	0.392812	1
C1orf109	1.3170834	1.36268006	1.2973193	0.819784047	-0.2866842	0.392896	1
NME4	6.1596844	6.34109483	6.0810511	0.951312651	-0.0720085	0.393214	1
SELENON	1.5151022	1.56324187	1.4942358	0.877484149	-0.188555	0.393262	1
LATS2	1.1795442	1.13663576	1.1981432	1.450155987	0.5362081	0.39341	1
HBEGF	1.082526	1.04148641	1.1003149	2.418018157	1.2738251	0.393419	1
FCHO1	1.1936356	1.15066398	1.2122619	1.408843113	0.494511	0.393455	1
PAPD5	1.2077256	1.25125226	1.1888588	0.751669923	-0.4118288	0.393504	1
ZNF692	1.3895015	1.43593329	1.3693754	0.847320846	-0.2390197	0.393612	1
LIMK2	1.438327	1.39080571	1.4589253	1.174305497	0.2318078	0.393786	1
STXBP1	1.4048257	1.35796784	1.4251365	1.187638725	0.248096	0.393818	1
RP1-315G1.3	1.1204003	1.16196149	1.1023854	0.632159097	-0.6616404	0.394177	1
TMEM185B	1.1143036	1.07273174	1.1323232	1.819331545	0.8634085	0.394178	1
TNRC18	1.1971972	1.24029772	1.178515	0.742891102	-0.4287773	0.394314	1

DDI2	1.226744	1.18280892	1.2457879	1.344507049	0.4270773	0.394587	1
TMEM17	1.1179866	1.15980093	1.0998619	0.624914292	-0.6782698	0.394592	1
CTA-243E7.1	1.105948	1.14732559	1.0880126	0.597402063	-0.7432259	0.394819	1
TIGD1	1.2356599	1.27921707	1.2167798	0.776384488	-0.3651568	0.395045	1
MDFI	1.9489913	1.89349896	1.9730448	1.089027349	0.1230402	0.395178	1
ZNF664	1.4875632	1.53531328	1.4668657	0.872135499	-0.1973758	0.395207	1
SS18	1.5694798	1.61893696	1.5480423	0.88545746	-0.1755051	0.395288	1
LOXL4	1.0776567	1.03681652	1.0953591	2.590116981	1.3730173	0.395367	1
HIPK3	1.2656732	1.2214632	1.2848363	1.286156369	0.3630661	0.395572	1
LINC01139	1.2303667	1.18676745	1.249265	1.334627629	0.4164373	0.396019	1
PCYT1A	1.188582	1.14563334	1.2071984	1.422739913	0.508672	0.396491	1
SEMA3D	1.1947701	1.23769866	1.1761625	0.741116806	-0.4322272	0.396527	1
BCS1L	1.2481903	1.20425918	1.2672324	1.308300777	0.3876943	0.396569	1
IBTK	1.8949505	1.94876734	1.8716233	0.918690272	-0.1223495	0.396655	1
FOXP1	2.8109232	2.88515823	2.7787456	0.943552433	-0.0838254	0.396845	1
C21orf91	1.2354293	1.19156884	1.2544409	1.328195506	0.4094675	0.396907	1
ZNF672	1.4660965	1.41864051	1.4866665	1.162492584	0.2172215	0.396931	1
SLC2A11	1.4203181	1.4668782	1.4001363	0.857046545	-0.2225545	0.396936	1
RORB	1.5180594	1.56734831	1.4966949	0.875467222	-0.1918749	0.396956	1
CYP2U1	1.1770809	1.13454817	1.1955169	1.45313697	0.5391707	0.397035	1
KPNA4	1.9251113	1.8649708	1.9511795	1.099666574	0.1370662	0.397165	1
WDR41	1.5680486	1.5193943	1.5891381	1.134279105	0.1817757	0.397634	1
CXCL3	1.0465957	1.08642418	1.0293318	0.339393231	-1.5589703	0.397753	1
ZNHIT2	1.1546561	1.11275942	1.1728165	1.53261252	0.615993	0.397835	1
NBEA	2.1204355	2.06324938	2.1452232	1.077097441	0.1071488	0.397897	1
COPA	1.8194491	1.87205947	1.7966449	0.913521321	-0.1304897	0.397925	1
NOP58	1.5398587	1.5876856	1.5191278	0.883342782	-0.1789547	0.397999	1
FNBP1L	1.9463574	1.89235952	1.9697631	1.086740332	0.1200073	0.398085	1
CHSY3	1.1096133	1.15083249	1.0917466	0.608268192	-0.7172205	0.398197	1
METTL13	1.1933722	1.15064192	1.2118939	1.406606169	0.4922184	0.398204	1
IQGAP2	1.1082495	1.06695953	1.1261468	1.883926548	0.9137427	0.398215	1
EXOG	1.3143573	1.35908979	1.2949677	0.821431656	-0.2837875	0.398384	1
LDAH	1.3708522	1.32545977	1.3905278	1.199926362	0.2629459	0.398562	1
FAM46A	1.3314183	1.28689008	1.3507193	1.22248669	0.2898188	0.398651	1
PANX1	1.1468264	1.1049442	1.1649804	1.572077539	0.6526724	0.398665	1
CEBPZ	1.8289788	1.77646021	1.8517433	1.096956811	0.1335067	0.398877	1
PNPLA2	1.2980025	1.25371374	1.3171997	1.250226798	0.3221898	0.398994	1
HAUS8	1.1560716	1.19789526	1.1379429	0.697049927	-0.5206661	0.39915	1
KANK1	1.2021689	1.15927396	1.220762	1.38605192	0.4709813	0.399319	1
SLC25A21-AS1	1.266077	1.30973874	1.2471516	0.79793562	-0.3256557	0.399412	1
BHMT	1.0617608	1.02064644	1.079582	3.854513987	1.946549	0.399782	1
PIK3R3	1.2143984	1.25773607	1.1956135	0.75896831	-0.3978884	0.399842	1
CFD	1.1250455	1.08355503	1.1430298	1.711804008	0.7755175	0.399898	1
PFKFB3	1.567637	1.51830795	1.5890189	1.136426471	0.1845043	0.399944	1
SLC20A2	1.2355515	1.19274106	1.2541079	1.318389961	0.3987772	0.399991	1
TCEA3	1.0896058	1.13024413	1.0719909	0.552737905	-0.8553325	0.400194	1
SMAD4	1.4473724	1.49380956	1.4272439	0.86519974	-0.2088949	0.400194	1
NCAM1	1.6689065	1.71813276	1.6475691	0.901740113	-0.1492164	0.400303	1
PCDH18	1.119357	1.07801802	1.1372756	1.759537095	0.8151959	0.400313	1
HERC1	1.3208166	1.36542038	1.3014828	0.82503002	-0.2774815	0.400846	1
RFTN2	1.1627647	1.20455227	1.1446517	0.707162297	-0.4998867	0.400858	1

FOXRED1	1.1966051	1.2391759	1.1781526	0.744860018	-0.4249588	0.401121	1
DENND4C	1.1999397	1.15702711	1.2185404	1.391736481	0.4768861	0.401167	1
OPA3	1.197903	1.15552453	1.2162721	1.390598316	0.4757057	0.401287	1
CCDC144NL-AS1	2.8337267	2.90953589	2.8008668	0.943091357	-0.0845306	0.401461	1
ZNF503-AS2	1.1391747	1.0976792	1.1571611	1.608951911	0.6861212	0.401484	1
C10orf10	1.0566753	1.01677497	1.0739703	4.409564675	2.1406362	0.401491	1
CEBPA-AS1	1.0518734	1.09175904	1.0345848	0.376908416	-1.4077141	0.401497	1
ABCA1	1.0862856	1.04581651	1.1038271	2.266151277	1.1802442	0.401649	1
CADM3	1.210173	1.25282749	1.1916842	0.758161986	-0.399422	0.401658	1
SIX3-AS1	1.8261666	1.87883448	1.8033374	0.914094036	-0.1295855	0.401899	1
E2F7	1.1588361	1.20048	1.1407853	0.702240925	-0.509962	0.402048	1
MAP4K3	1.254452	1.29800004	1.2355758	0.790522808	-0.339121	0.402101	1
RAMP2-AS1	1.1314088	1.17265537	1.1135303	0.657554231	-0.6048182	0.402108	1
NUP62	1.4931364	1.54030887	1.4726892	0.874850009	-0.1928924	0.40211	1
ZNF576	1.3097912	1.26569165	1.3289063	1.237924986	0.3079239	0.402364	1
CLIC4	1.7308028	1.68025184	1.7527144	1.106523098	0.1460336	0.402602	1
DYNLL2	1.3248978	1.28040373	1.344184	1.227458553	0.2956743	0.402681	1
MME	1.0653202	1.02531946	1.0826588	3.264635094	1.7069217	0.4027	1
NCBP2-AS2	2.3187085	2.38070275	2.2918368	0.935637136	-0.095979	0.4031	1
P2RX4	1.1836415	1.14169065	1.2018253	1.424408359	0.5103628	0.403373	1
UBE4B	1.3589604	1.4038698	1.3394942	0.840602973	-0.2505035	0.403532	1
RNPEPL1	1.1580589	1.11636926	1.1761296	1.513540181	0.597927	0.403553	1
CRTAP	2.77695	2.70101089	2.8098662	1.063994474	0.0894907	0.40364	1
FBXO43	1.0828214	1.12312116	1.0653533	0.530804899	-0.9137464	0.403736	1
UBAC1	1.7431334	1.7939115	1.7211234	0.908317121	-0.138732	0.403933	1
SLITRK6	1.1534783	1.11199455	1.1714597	1.530964738	0.6144411	0.404242	1
CCDC138	1.2298775	1.27232543	1.2114783	0.776564466	-0.3648224	0.404608	1
OTULIN	1.2851702	1.3287517	1.2662795	0.809971606	-0.3040568	0.404703	1
PGPEP1	1.292618	1.3361704	1.27374	0.814289503	-0.2963863	0.404755	1
MNAT1	1.7746158	1.724285	1.796432	1.099611321	0.1369937	0.40484	1
ZNF33B	1.3887477	1.43414748	1.369069	0.850100464	-0.2342947	0.404898	1
NT5C3B	2.7538195	2.66411187	2.7927038	1.077273589	0.1073847	0.404986	1
ATP8B2	1.0964341	1.1367018	1.0789798	0.577752525	-0.7914764	0.405211	1
COASY	1.6678356	1.61848672	1.6892262	1.114375074	0.1562349	0.405375	1
BUD31	2.9269353	2.85084549	2.9599168	1.058930562	0.082608	0.405377	1
STRIP2	1.1371125	1.09563673	1.1550904	1.621661069	0.6974723	0.40538	1
ZC3H7B	1.3149174	1.35910171	1.2957655	0.82362594	-0.2799388	0.405528	1
NSMCE4A	1.5284832	1.48135126	1.5489129	1.140358194	0.1894871	0.405565	1
EMSY	1.2592026	1.30211229	1.2406032	0.796403283	-0.3284289	0.405824	1
FECH	1.1755353	1.1337179	1.1936613	1.448282189	0.5343427	0.405834	1
RAF1	1.6818332	1.63164206	1.7035888	1.113904236	0.1556252	0.405915	1
ZNF397	1.9295926	1.98535881	1.9054205	0.918873875	-0.1220612	0.405919	1
HAUS6	1.4730829	1.51936449	1.4530219	0.872261933	-0.1971667	0.406017	1
PARPBP	1.2910862	1.3347887	1.2721431	0.812880059	-0.2988856	0.406062	1
AARS2	1.1020087	1.14241566	1.084494	0.593291775	-0.7531863	0.406198	1
AGO2	1.5676397	1.61587952	1.5467299	0.887722223	-0.1718198	0.406339	1
PIGK	1.1764215	1.13475297	1.1944829	1.44325526	0.5293265	0.406555	1
VASN	1.0850747	1.04504877	1.1024242	2.27362985	1.1849974	0.406757	1
DOK4	1.282544	1.23886987	1.3014748	1.262088102	0.3358126	0.406946	1
IKBKB	1.2829515	1.3264964	1.2640768	0.808819834	-0.3061097	0.407031	1
KDSR	1.5983151	1.55038047	1.6190927	1.124844896	0.1697261	0.407056	1

JPH4	1.4643182	1.51049249	1.4443038	0.870343395	-0.2003434	0.407123	1
LINC00294	1.0963598	1.13664478	1.078898	0.577394838	-0.7923699	0.407127	1
CPD	1.4875316	1.53471203	1.4670809	0.873518669	-0.1950896	0.407228	1
DNAJB2	2.1861883	2.12637562	2.2121144	1.076119175	0.1058379	0.407359	1
MXRA7	1.6815783	1.63206291	1.703041	1.112295857	0.1535406	0.407557	1
EDN2	1.0987865	1.05868913	1.1161669	1.979359087	0.9850334	0.407945	1
SELENBP1	1.2644206	1.30770819	1.2456574	0.79834522	-0.3249154	0.408083	1
ZC2HC1A	1.5701062	1.52225836	1.5908461	1.131329216	0.1780188	0.408186	1
MOSPD1	1.2601658	1.21709133	1.2788367	1.284421241	0.3611184	0.408217	1
FNDC3A	2.3736985	2.43387599	2.3476143	0.939840182	-0.0895126	0.408236	1
ALS2	1.2301041	1.27256235	1.2117003	0.776704102	-0.364563	0.408286	1
DIS3L2	1.2812976	1.32449828	1.262572	0.809162927	-0.3054979	0.408839	1
PRRC2A	1.9991162	2.05451538	1.9751031	0.924693078	-0.1129535	0.40896	1
RP11-486O12.2	1.0778363	1.11751491	1.0606374	0.515997717	-0.9545634	0.409148	1
SLC30A6	1.1482398	1.18905853	1.1305467	0.690509694	-0.5342664	0.409319	1
RP11-161M6.2	1.2539708	1.29634775	1.2356023	0.795019656	-0.3309376	0.40953	1
CEP128	1.1230277	1.1632758	1.1055819	0.646647701	-0.6289482	0.409611	1
SEC14L2	1.2920647	1.24875621	1.310837	1.249564947	0.3214259	0.409663	1
MGME1	1.5423011	1.58963291	1.5217849	0.884931708	-0.176362	0.409732	1
EFR3B	1.4202115	1.37458587	1.4399881	1.174598851	0.2321681	0.409775	1
AC142472.6	1.1049769	1.14504158	1.0876107	0.604038213	-0.7272883	0.40994	1
ATG9B	1.1012437	1.14151537	1.0837877	0.592075158	-0.7561478	0.409993	1
SMAGP	1.1520518	1.11074895	1.1699547	1.534594593	0.6178576	0.410019	1
CNTN1	1.0691977	1.02952487	1.0863941	2.926144812	1.5490012	0.410104	1
LRCH4	1.2602983	1.21750144	1.2788488	1.282054822	0.358458	0.410182	1
USP31	1.1122319	1.15237397	1.0948321	0.622364439	-0.6841685	0.410251	1
NBEAL1	3.9763971	4.07890982	3.9319624	0.952272918	-0.070553	0.410508	1
NAA35	1.3083472	1.2648656	1.3271945	1.235322834	0.3048881	0.410595	1
IFT27	2.0461553	1.99021233	2.0704041	1.080984414	0.1123457	0.410659	1
TTC21A	1.1367382	1.17729136	1.1191602	0.672115274	-0.5732194	0.41066	1
LSM3	3.5109737	3.41947521	3.5506343	1.054209713	0.0761619	0.410709	1
RRP7A	1.4597444	1.41418913	1.4794906	1.157661115	0.211213	0.410717	1
RP1-39G22.7	1.4605926	1.41424512	1.4806821	1.160380902	0.2145985	0.410717	1
HIVEP2	1.0883689	1.04834478	1.1057176	2.186742545	1.1287834	0.410744	1
CREB3L2	1.372329	1.32739294	1.3918067	1.196747607	0.2591189	0.411181	1
HACD2	1.2489488	1.20644604	1.2673719	1.295117586	0.3730831	0.411229	1
ELF1	1.3968535	1.35215064	1.4162302	1.181966422	0.2411891	0.411432	1
LINC00665	1.9432434	1.99825294	1.9193992	0.921008221	-0.1187141	0.411484	1
ZNF114	1.0804201	1.11996248	1.0632802	0.527499794	-0.9227576	0.411601	1
C22orf15	1.0567374	1.09575754	1.0398239	0.415882855	-1.2657509	0.411716	1
FRMD6-AS1	1.1469009	1.18785102	1.1291509	0.687517465	-0.5405317	0.411732	1
CLIP2	1.1953863	1.15328165	1.2136368	1.393752955	0.4789749	0.411749	1
MLX	1.3871908	1.34238072	1.406614	1.187607636	0.2480583	0.41177	1
METTL14	1.2964762	1.33973197	1.2777267	0.817487735	-0.290731	0.41197	1
MYO1E	1.221296	1.17933623	1.2394836	1.33538911	0.4172602	0.412132	1
MED31	1.674625	1.62587669	1.6957552	1.111649071	0.1527014	0.412152	1
EHBP1	1.4874738	1.53344862	1.4675457	0.876458807	-0.1902418	0.412227	1
SSR1	2.4324762	2.37107515	2.4590908	1.064194609	0.089762	0.412239	1
UNC119B	1.3626376	1.406808	1.3434916	0.844358115	-0.2440731	0.412347	1
DHX36	2.4938028	2.42829291	2.5221985	1.065746697	0.0918646	0.41236	1
HAT1	1.5999518	1.55288625	1.6203526	1.122025785	0.1661058	0.412617	1

VPS4B	1.4341143	1.38903922	1.4536523	1.166083654	0.2216713	0.412737	1
RP11-345P4.9	1.4161693	1.46104931	1.3967158	0.860462937	-0.216815	0.412904	1
DDX56	1.4063242	1.36199826	1.4255376	1.175523806	0.2333038	0.412925	1
CECR5	2.2470214	2.1909911	2.2713081	1.067437118	0.0941511	0.413095	1
MGAT4A	1.1184288	1.15851069	1.101055	0.63752807	-0.6494392	0.413103	1
CHP1	1.7392458	1.6873379	1.7617456	1.108254924	0.1482898	0.413109	1
VPS37D	1.2533625	1.21073254	1.2718407	1.289979571	0.3673482	0.413163	1
MTMR14	1.2357709	1.1937966	1.2539648	1.310471079	0.3900855	0.413423	1
C8orf88	1.2694474	1.22684172	1.287915	1.269233014	0.343957	0.413477	1
ZNF813	1.1227874	1.16285646	1.1054193	0.647314096	-0.6274622	0.413548	1
SWAP70	1.1982501	1.15681343	1.2162111	1.37877906	0.4633913	0.41367	1
SLC16A9	1.1781518	1.2190933	1.1604055	0.732133407	-0.4498215	0.413734	1
DFNA5	1.1554772	1.11490781	1.1730622	1.506096133	0.5908139	0.413771	1
ANKRD40	1.3197457	1.27611165	1.3386591	1.226529381	0.2945818	0.413988	1
PCOLCE2	1.1508583	1.11002651	1.1685571	1.531968363	0.6153865	0.414061	1
LRP12	1.2420469	1.19972103	1.2603932	1.303784827	0.3827058	0.414148	1
ATXN2	1.8338724	1.88512092	1.8116584	0.917002863	-0.1250019	0.414236	1
SERPINB1	1.2459039	1.20407686	1.264034	1.293796935	0.3716112	0.414272	1
PLXDC2	1.6536522	1.70169961	1.6328257	0.901847064	-0.1490453	0.414293	1
PDCD6IP	1.7855608	1.83566298	1.7638438	0.914057212	-0.1296436	0.414329	1
DHRS7B	1.3588258	1.40336702	1.3395191	0.841712648	-0.2486003	0.414465	1
SERTAD2	1.3720376	1.32741351	1.3913802	1.195369604	0.2574568	0.414528	1
DGCR6	1.4386838	1.39310337	1.4584409	1.166209678	0.2218272	0.414605	1
TFPI	1.4984719	1.45218435	1.5185355	1.146734736	0.1975317	0.41462	1
STK32C	1.458375	1.50337372	1.4388701	0.871857436	-0.1978358	0.414643	1
LETM1	1.335996	1.29221632	1.3549726	1.214759635	0.2806709	0.414862	1
TNFRSF1B	1.1701623	1.21110575	1.1524151	0.721984659	-0.4699599	0.41491	1
PRICKLE2	1.1706485	1.21154719	1.1529208	0.722868468	-0.4681949	0.41491	1
DYRK4	1.8764697	1.92763135	1.8542934	0.920940596	-0.11882	0.415417	1
RP11-351D16.3	1.0985037	1.13807735	1.0813502	0.58916415	-0.7632584	0.415456	1
MICAL1	1.1268905	1.08681434	1.1442617	1.661726917	0.7326833	0.415476	1
ADAM17	1.7169158	1.76604138	1.695622	0.908073639	-0.1391188	0.415528	1
MED15	1.4160971	1.37123293	1.4355437	1.173235648	0.2304928	0.415604	1
PMM1	1.5195248	1.47327055	1.5395739	1.140096082	0.1891554	0.415768	1
PLEKHH2	1.086187	1.12523845	1.0692599	0.553024046	-0.8545859	0.415998	1
AC093375.1	1.1316606	1.1718294	1.1142492	0.664898759	-0.5887934	0.416014	1
DGCR6L	2.9538142	2.87442305	2.9882268	1.060714006	0.0850357	0.416103	1
TMEM99	1.4623685	1.41618231	1.4823881	1.159078916	0.2129788	0.416269	1
CDC25A	1.0952244	1.13466543	1.0781285	0.580167469	-0.7854587	0.416281	1
TRIM41	1.1742345	1.21510608	1.1565185	0.727633905	-0.4587153	0.416552	1
SAMD5	1.0694688	1.03050792	1.0863566	2.830628518	1.5011224	0.416599	1
IDE	1.2697352	1.31209205	1.2513754	0.805452763	-0.3121281	0.416608	1
FAM161B	1.0865791	1.1260007	1.0694916	0.551517392	-0.8585217	0.416882	1
EFTUD2	1.2926896	1.33517895	1.2742723	0.818286264	-0.2893225	0.416977	1
WDR37	1.1532352	1.19371436	1.1356893	0.700460915	-0.5136235	0.417037	1
UAP1	1.6024698	1.55469011	1.6231802	1.12347452	0.1679674	0.41705	1
POLA1	1.2883463	1.33084727	1.2699241	0.815856972	-0.2936118	0.417307	1
LRRC28	1.4378697	1.39238104	1.457587	1.166180087	0.2217906	0.417324	1
HMGCS1	1.7575262	1.70449865	1.7805113	1.107896053	0.1478225	0.417335	1
SLC2A3	2.4404136	2.36282863	2.4740432	1.081605658	0.1131746	0.417404	1
TIPARP	1.2319729	1.19004616	1.2501463	1.316239952	0.3964225	0.417542	1

RAB6A	2.1803599	2.12524548	2.2042496	1.070210533	0.0978946	0.417553	1
ATRN	1.2139136	1.2551198	1.1960526	0.768472745	-0.379934	0.417772	1
HIST1H2AC	1.09922	1.05974121	1.1163324	1.947271647	0.9614542	0.417782	1
P3H1	1.5008845	1.45581256	1.5204212	1.141743868	0.191239	0.417868	1
IRF7	1.2089897	1.16757068	1.226943	1.354311826	0.43756	0.417919	1
INPP4A	1.2175113	1.2587714	1.1996269	0.771441039	-0.3743722	0.417947	1
SKAP2	1.0765229	1.03745191	1.0934585	2.495426752	1.3192866	0.417948	1
REV1	1.3302658	1.37367444	1.3114501	0.833479724	-0.262781	0.417965	1
CSNK1A1	3.7953425	3.69813392	3.837478	1.051644618	0.0726473	0.418097	1
HNRNPDL	11.764659	12.0497235	11.641097	0.963019288	-0.0543634	0.418265	1
NUP98	1.2213286	1.26258574	1.2034455	0.774777365	-0.3681463	0.418379	1
MZF1-AS1	1.2127081	1.25383126	1.194883	0.767766053	-0.3812613	0.418432	1
RP11-15A1.3	1.1400766	1.18009244	1.1227315	0.681491403	-0.5532326	0.418462	1
ROPN1L	1.0794677	1.11825258	1.0626562	0.529850588	-0.9163425	0.418531	1
TYW5	1.2611187	1.3033642	1.2428072	0.800381853	-0.3212396	0.418596	1
OXSM	1.165761	1.12533961	1.1832819	1.462282284	0.5482218	0.418609	1
RGS14	1.3837227	1.33965001	1.4028262	1.186003884	0.2461087	0.418684	1
FANCF	1.3761394	1.3324112	1.3950936	1.188568769	0.2492254	0.418759	1
PYGO1	1.2659689	1.30781984	1.2478283	0.80510839	-0.3127451	0.418914	1
PDE3B	1.1726474	1.21270568	1.155284	0.730041518	-0.4539496	0.419017	1
PHAX	1.5287963	1.48331453	1.5485106	1.134893754	0.1825572	0.419106	1
KIAA1217	1.4765201	1.52215458	1.4567396	0.874720963	-0.1931052	0.419133	1
IQCE	1.2479789	1.28965815	1.2299128	0.793738256	-0.3332648	0.41918	1
PAOX	1.1308429	1.09099106	1.1481168	1.627817371	0.7029388	0.419204	1
S100B	1.2341307	1.19280387	1.252044	1.307256107	0.3865418	0.419538	1
ARFGEF2	1.1573463	1.11701414	1.1748285	1.494080307	0.5792577	0.419642	1
PPP4R3A	1.6283341	1.58076938	1.6489513	1.117399269	0.1601448	0.419679	1
DMWD	1.243193	1.28486147	1.2251316	0.790319672	-0.3394918	0.419725	1
STX5	1.5589604	1.51238262	1.5791499	1.130307378	0.1767152	0.419798	1
KIAA1841	1.3551641	1.39829954	1.3364668	0.84475829	-0.2433895	0.419823	1
C1orf27	1.3798267	1.33621036	1.3987325	1.185961344	0.246057	0.419826	1
KCNMB4	1.3585557	1.3147085	1.3775615	1.199718259	0.2626956	0.420044	1
CHMP4C	1.3075707	1.26497335	1.3260347	1.230443384	0.2991783	0.420197	1
RP11-701H24.9	1.2586808	1.30080195	1.2404232	0.799273957	-0.323238	0.420347	1
MBNL2	1.4841518	1.52947961	1.4645042	0.877284455	-0.1888834	0.420589	1
PLAT	1.402914	1.35915581	1.4218812	1.174646713	0.2322269	0.420603	1
FAM168A	1.1248765	1.16432036	1.1077794	0.655909918	-0.6084304	0.420743	1
ZNF112	1.0834649	1.12248454	1.0665516	0.543347295	-0.8800535	0.420769	1
UBR4	1.3665306	1.40999127	1.3476923	0.848048036	-0.2377821	0.420845	1
ARFGEF1	1.549263	1.59559518	1.5291801	0.88848956	-0.1705733	0.420851	1
ZNF19	1.1472721	1.18711986	1.1299999	0.694741524	-0.5254518	0.42089	1
TNRC6C	1.3805104	1.42423144	1.3615593	0.852269072	-0.2306191	0.420912	1
PDE8B	1.0721478	1.11076361	1.0554096	0.500251007	-0.9992759	0.420994	1
CDK11A	1.2255457	1.18454985	1.2433156	1.318427373	0.3988181	0.421072	1
FAM104A	1.5405709	1.49419873	1.5606711	1.134505387	0.1820635	0.42115	1
DDB1	1.8158571	1.76596396	1.8374835	1.09337194	0.1287843	0.421165	1
NECTIN3	1.1961741	1.15541475	1.2138415	1.375940696	0.4604183	0.421173	1
SCLY	1.1321773	1.17178865	1.1150075	0.669470867	-0.5789068	0.421223	1
STOX1	1.1070957	1.14636588	1.0900738	0.615401789	-0.7003995	0.421282	1
CDKAL1	1.3379215	1.29473411	1.3566413	1.210044034	0.2750595	0.421351	1
PDS5B	1.5687149	1.61494158	1.5486777	0.892243577	-0.1644905	0.421592	1

ARHGAP23	1.1311099	1.0916471	1.1482152	1.617238462	0.6935324	0.421738	1
NHSL2	1.237384	1.19572415	1.2554416	1.305110476	0.3841719	0.421818	1
MINK1	1.1874396	1.22765287	1.170009	0.746790323	-0.4212249	0.421835	1
GATAD2B	1.406922	1.45083375	1.3878882	0.860379743	-0.2169545	0.421888	1
RP11-1338A24.1	1.3325827	1.3753467	1.3140464	0.8366834	-0.2572463	0.421901	1
ENTPD1	1.1485904	1.18826115	1.1313949	0.697939639	-0.5188258	0.421924	1
RNASEH1	1.4330486	1.38875685	1.4522471	1.163316034	0.2182431	0.421929	1
ETNK1	1.7439021	1.69482342	1.7651755	1.101251744	0.1391443	0.422096	1
NCOA2	1.2706693	1.31242161	1.2525716	0.808431747	-0.3068021	0.422228	1
STK39	1.2143354	1.17337599	1.2320894	1.338648018	0.4207767	0.422428	1
LINC00271	1.0660569	1.10453624	1.0493778	0.472350594	-1.08207	0.422608	1
FPGS	1.3201839	1.27712515	1.338848	1.22272556	0.2901006	0.422612	1
CEP85	1.1366178	1.17607049	1.1195168	0.678800832	-0.5589398	0.42264	1
TMEM209	1.1797609	1.22007803	1.1622852	0.73739848	-0.4394837	0.4227	1
SBNO1	1.843517	1.7927639	1.8655162	1.091770402	0.1266695	0.422795	1
MAP2K2	1.4618273	1.41725101	1.4811491	1.153140729	0.2055686	0.423138	1
ARHGAP10	1.1651955	1.12523341	1.1825173	1.457416858	0.5434136	0.423419	1
PTRH2	1.1907842	1.15018301	1.2083831	1.387527626	0.4725165	0.423432	1
PPP1R32	1.1119542	1.15090004	1.095073	0.630039484	-0.6664859	0.423577	1
ZNF532	1.4750927	1.43017965	1.4945605	1.149660306	0.2012076	0.423665	1
NIPAL2	1.0882219	1.12724512	1.0713071	0.560391924	-0.8354919	0.423754	1
PEX16	1.3419012	1.29863704	1.3606543	1.207667771	0.2722236	0.423914	1
SNX16	1.1729334	1.13276815	1.1903432	1.433651258	0.5196941	0.42399	1
ZNF266	1.1556264	1.19547465	1.1383539	0.707784417	-0.4986181	0.424173	1
ZNF587	1.2928551	1.25119619	1.3109124	1.237727501	0.3076937	0.424231	1
TCTN1	1.7588511	1.80775349	1.737654	0.913216766	-0.1309707	0.424247	1
CDC34	1.6922856	1.64500382	1.7127801	1.105078876	0.1441493	0.42427	1
KLF9	1.1027352	1.06382354	1.1196016	1.873941819	0.9060762	0.424344	1
ELOF1	2.2842989	2.22504287	2.3099837	1.069337027	0.0967166	0.424524	1
TMEM87B	1.1844215	1.22500652	1.1668297	0.741443867	-0.4315906	0.424526	1
GATS	1.1664822	1.20641718	1.1491722	0.722673243	-0.4685846	0.424572	1
MYO1C	1.2056994	1.1652762	1.2232211	1.35059445	0.4335945	0.424707	1
EPB41L2	1.5156121	1.56117333	1.4958633	0.883618846	-0.1785039	0.424711	1
LRRC10B	1.0859419	1.12444522	1.0692524	0.556489165	-0.8455745	0.424847	1
C21orf62-AS1	1.0859904	1.12442708	1.0693298	0.557191958	-0.8437537	0.424847	1
LCORL	1.54235	1.49649595	1.5622257	1.132387356	0.1793675	0.424861	1
CLCN5	1.4622858	1.5064383	1.4431477	0.875028028	-0.1925989	0.425104	1
LRRC4B	1.296532	1.25467176	1.3146765	1.235615911	0.3052304	0.425213	1
PIF1	1.2857223	1.32806844	1.2673671	0.814973563	-0.2951748	0.425228	1
ZNF581	2.2507753	2.19243252	2.2760644	1.070135483	0.0977935	0.425373	1
PLEKHG3	1.1368565	1.09746808	1.1539296	1.579282126	0.6592689	0.425631	1
TCTE3	1.1510248	1.19054405	1.133895	0.702698361	-0.5090226	0.425637	1
MTMR2	1.3916164	1.34782289	1.4105989	1.180482821	0.239377	0.425759	1
RP13-131K19.7	1.0449939	1.0827456	1.0286302	0.346002616	-1.5311451	0.425838	1
DXO	1.2424011	1.28350429	1.2245847	0.792174006	-0.3361107	0.425955	1
BCL10	1.2884856	1.24653207	1.3066705	1.243937671	0.3149142	0.426241	1
DNAH11	1.1132935	1.15224854	1.0964082	0.633229026	-0.6592007	0.426543	1
GPALPP1	1.4111337	1.45441401	1.3923736	0.863471638	-0.2117793	0.426621	1
NXPE3	1.2845069	1.3264136	1.2663422	0.81596536	-0.2934202	0.426806	1
MYPOP	1.1197001	1.15852127	1.1028728	0.64895281	-0.6238145	0.426843	1
LPCAT1	1.4016345	1.44513773	1.3827778	0.859908694	-0.2177446	0.426883	1

TMEM234	1.2781407	1.23646797	1.296204	1.252617954	0.3249465	0.426886	1
SMAD7	1.1516021	1.19106631	1.1344961	0.703923627	-0.5065092	0.427064	1
C1orf123	1.7497805	1.70148943	1.7707126	1.098680232	0.1357716	0.427261	1
ENSA	3.2631765	3.17966105	3.2993767	1.054923963	0.077139	0.427341	1
HIST1H2AH	1.0517668	1.08942444	1.0354439	0.396355924	-1.3351316	0.427642	1
ACSS3	1.3347056	1.37740598	1.316197	0.837816504	-0.2552938	0.427696	1
TMCC1	1.3021458	1.34394801	1.2840263	0.825782784	-0.2761658	0.427877	1
CDKL5	1.0924527	1.13116809	1.0756713	0.576903091	-0.7935991	0.427921	1
LEMD2	1.3591802	1.31661921	1.3776286	1.192689981	0.2542191	0.427979	1
MYLK-AS1	1.3080792	1.3497897	1.2899995	0.82906827	-0.2704372	0.42848	1
DAZAP1	1.6835178	1.63602525	1.7041038	1.107037427	0.146704	0.428492	1
ISCA1	2.2277441	2.17295549	2.2514925	1.066956546	0.0935014	0.428496	1
TMEM160	3.4683773	3.38708722	3.503613	1.048815034	0.0687603	0.428508	1
RNF40	1.3452288	1.38752404	1.3268958	0.843549638	-0.2454551	0.428834	1
CHD4	3.5687588	3.66056988	3.5289628	0.95053425	-0.0731895	0.429247	1
ORAI1	1.1780076	1.13825263	1.1952397	1.412195005	0.4979393	0.429404	1
TSPYL4	1.3203766	1.36236473	1.3021766	0.8339017	-0.2620508	0.429408	1
SNX12	1.4747007	1.43025222	1.4939672	1.148087411	0.1992325	0.430186	1
NEK4	1.2594647	1.30028638	1.2417704	0.805132623	-0.3127016	0.430202	1
ERCC5	1.1604197	1.19969501	1.1433956	0.718072992	-0.4777976	0.430374	1
PRKAR1B	1.1552332	1.11577153	1.1723381	1.488605019	0.573961	0.430673	1
TBC1D24	1.2019131	1.24176951	1.1846371	0.763690779	-0.3889395	0.430688	1
KCNJ3	1.0847733	1.04627692	1.1014598	2.192449412	1.1325436	0.430766	1
ZNF429	1.1704899	1.20978503	1.1534572	0.731497248	-0.4510757	0.430846	1
PYGM	1.0922671	1.05391854	1.1088895	2.019519382	1.014012	0.431167	1
ABT1	1.8127817	1.76174176	1.8349052	1.096047617	0.1323105	0.431257	1
ALG9	1.1251281	1.16375373	1.1083856	0.661881584	-0.595355	0.431302	1
ADNP	1.9665503	2.0175838	1.9444296	0.928109904	-0.1076324	0.431311	1
TTLL12	1.3078408	1.26631925	1.3258385	1.223488263	0.2910003	0.431548	1
MIGA2	1.1139027	1.15218965	1.0973071	0.639380224	-0.645254	0.43162	1
STK16	1.7665848	1.71809506	1.7876029	1.096794825	0.1332937	0.431657	1
EWSR1	2.0703603	2.01545354	2.0941599	1.077508576	0.1076994	0.431766	1
RNF175	1.5770502	1.62265977	1.5572804	0.894999864	-0.1600406	0.431934	1
C16orf72	1.533459	1.57849838	1.5139365	0.888397398	-0.1707229	0.431961	1
LINC00662	1.5898945	1.63549349	1.5701294	0.897144358	-0.1565879	0.431986	1
ITPKC	1.075143	1.03715875	1.0916075	2.465299683	1.301763	0.432115	1
SETBP1	1.1217351	1.16026298	1.105035	0.65539138	-0.6095714	0.432275	1
MYNN	1.1921212	1.23184848	1.1749012	0.754377072	-0.4066423	0.432428	1
KRIT1	1.6745727	1.72105669	1.654424	0.907590247	-0.139887	0.432811	1
GFOD2	1.412576	1.45593275	1.3937828	0.863686112	-0.211421	0.432827	1
CA13	1.0809468	1.11850223	1.0646681	0.545712396	-0.8737873	0.433185	1
DTX3	1.6211477	1.66717151	1.6011984	0.90111522	-0.1502165	0.433213	1
MTUS1	1.0852671	1.04726525	1.1017392	2.152515572	1.1060237	0.433253	1
PISD	1.2938742	1.33511488	1.2759982	0.823592774	-0.2799969	0.433429	1
CTCF	1.6240905	1.57828264	1.6439462	1.113549189	0.1551653	0.433574	1
SRP14-AS1	1.1900375	1.15029323	1.2072648	1.379069535	0.4636952	0.433655	1
TRIM21	1.0867546	1.04882765	1.1031943	2.113439456	1.0795928	0.433688	1
TEAD2	2.1283167	2.07209815	2.1526849	1.075167305	0.1045612	0.433864	1
AC009005.2	1.3416125	1.38326237	1.3235591	0.84422362	-0.2443029	0.43387	1
PAQR3	1.2505539	1.29129595	1.232894	0.799509981	-0.3228121	0.434006	1
RP11-390F4.6	1.2179028	1.25789267	1.200569	0.777722637	-0.3626724	0.434075	1

TIAM2	1.1726127	1.13306119	1.1897565	1.426084803	0.5120598	0.434127	1
SEC31A	1.8844942	1.83054479	1.9078789	1.093112552	0.128442	0.434298	1
GLTSCR2	5.3382541	5.22151646	5.3888546	1.039639348	0.0560831	0.434307	1
TRPT1	2.3301067	2.39004875	2.3041246	0.938186202	-0.0920538	0.434318	1
ARL13B	1.3315741	1.3736389	1.3133409	0.838619642	-0.2539115	0.434419	1
PTBP3	1.3822583	1.33964922	1.4007275	1.179827392	0.2385758	0.434423	1
PLEKHF2	1.157687	1.1185844	1.1746362	1.472674631	0.5584387	0.434496	1
MPND	1.2748639	1.23377057	1.292676	1.251979747	0.3242112	0.434603	1
C1orf50	1.1849358	1.14506421	1.2022183	1.393991741	0.479222	0.434739	1
CCAR2	1.2394637	1.19929311	1.2568759	1.28893526	0.3661798	0.435275	1
CLEC18B	1.0782924	1.11583846	1.0620178	0.535382112	-0.9013592	0.4355	1
KANK4	1.0713935	1.03386546	1.0876602	2.588483542	1.3721071	0.435788	1
FBXL20	1.3604433	1.40263298	1.342156	0.849796224	-0.2348112	0.435925	1
ITGAE	2.3904174	2.32918573	2.4169586	1.066035095	0.0922549	0.436178	1
CTBP1	1.6072802	1.65283554	1.5875341	0.899972526	-0.1520471	0.436209	1
KMT2E-AS1	1.1617116	1.2003829	1.1449493	0.723361788	-0.4672107	0.436262	1
ARSA	1.1210628	1.08260193	1.1377339	1.667441468	0.7376361	0.436305	1
JMJD4	1.2319955	1.19164098	1.2494875	1.301848185	0.3805612	0.436419	1
PPOX	1.3597512	1.31775461	1.3779549	1.189455257	0.250301	0.436529	1
LINC00998	4.1886718	4.08961118	4.2316101	1.045960141	0.0648279	0.436576	1
TULP1	1.1100535	1.07144883	1.1267869	1.77451263	0.8274228	0.436707	1
ZNF337-AS1	1.1013566	1.13893002	1.0850702	0.612323948	-0.707633	0.436753	1
ZSCAN32	1.2051805	1.16522885	1.2224978	1.346603954	0.4293256	0.436814	1
SLC35E4	1.1127754	1.07467858	1.1292887	1.73126876	0.7918297	0.436907	1
CCDC97	1.2613585	1.30175922	1.2438465	0.808083164	-0.3074243	0.437272	1
IKZF2	1.1095313	1.14744007	1.0930996	0.63144004	-0.6632823	0.437373	1
ITSN2	1.3107636	1.26969171	1.3285664	1.218303804	0.2848739	0.437425	1
BLOC1S5	1.3216566	1.28030915	1.3395788	1.211443892	0.2767276	0.437632	1
SDHAF1	1.4492657	1.49206901	1.4307124	0.875309012	-0.1921357	0.437683	1
MFSD13A	1.0747191	1.11194044	1.0585853	0.52336144	-0.9341205	0.437764	1
ZNF544	1.4779648	1.52151262	1.4590888	0.880302402	-0.1839289	0.437839	1
IMPACT	1.4965158	1.4530192	1.5153696	1.137633021	0.1860352	0.437897	1
TTC5	1.3200542	1.36140121	1.3021321	0.83600204	-0.2584216	0.438049	1
RGL2	1.4641263	1.42085485	1.4828825	1.14738494	0.1983495	0.438198	1
GPX3	1.6964747	1.74502295	1.6754312	0.906591134	-0.141476	0.43823	1
TRIM22	1.1563734	1.11773142	1.173123	1.470491027	0.556298	0.438273	1
SLC5A6	1.3510914	1.3096133	1.3690704	1.192036518	0.2534284	0.438351	1
RP11-1055B8.4	1.140443	1.17897495	1.1237412	0.691388247	-0.532432	0.43838	1
TLE6	1.2312089	1.2708014	1.2140474	0.790421921	-0.3393051	0.438383	1
NUDT3	2.0278556	2.07920962	2.0055959	0.931789191	-0.1019245	0.438419	1
TMEM168	1.219711	1.259271	1.2025635	0.781281116	-0.3560864	0.438772	1
SPR	1.0886746	1.05108708	1.1049671	2.054670616	1.0389071	0.438858	1
TFR2	1.0933848	1.13069243	1.0772135	0.590803471	-0.7592498	0.438902	1
RFX5	1.3879414	1.4306535	1.3694276	0.857830313	-0.2212358	0.438969	1
METRNL	1.131856	1.09340919	1.148521	1.590004272	0.6690306	0.439053	1
SHCBP1	1.0818471	1.11922656	1.0656448	0.550588926	-0.8609525	0.439304	1
PARN	1.2082914	1.16877959	1.2254179	1.335575866	0.4174619	0.439319	1
COX16	1.3867323	1.3447869	1.4049138	1.174388605	0.2319099	0.439388	1
VPS33A	1.1841772	1.1452433	1.2010533	1.38425176	0.4691064	0.439524	1
ZNF558	1.1173955	1.15532702	1.1009539	0.649943949	-0.6216128	0.439699	1
RANGRF	2.0322871	2.08288087	2.010357	0.933026881	-0.1000094	0.439712	1

FAM20B	1.2464367	1.2863195	1.2291493	0.800327199	-0.3213382	0.439783	1
IFI27L2	3.721267	3.8113247	3.682231	0.954080824	-0.0678166	0.439842	1
PDK1	2.2255251	2.280885	2.201529	0.938045964	-0.0922695	0.439953	1
PSPH	1.4983461	1.45455028	1.5173297	1.138113161	0.186644	0.439989	1
SOD2	1.4655745	1.42218161	1.4843834	1.147334246	0.1982857	0.440111	1
PPP1R21	1.2182417	1.17881332	1.2353321	1.316076959	0.3962439	0.440182	1
MVK	1.1919152	1.15297293	1.2087949	1.364913893	0.4488099	0.440192	1
MYO1B	1.5155602	1.47185678	1.5345036	1.132766683	0.1798507	0.440341	1
NSMF	1.4685417	1.51176064	1.4498082	0.878942475	-0.1861593	0.440536	1
BACH1	1.3914822	1.3493436	1.4097474	1.172906524	0.230088	0.440556	1
SMAD5	1.9979549	2.04858843	1.9760075	0.930782255	-0.1034844	0.440889	1
RAB6B	1.3291464	1.28811659	1.346931	1.204134104	0.2679961	0.441263	1
FGF8	1.081342	1.11846549	1.0652506	0.550798593	-0.8604032	0.44137	1
WDFY2	1.3070828	1.26649415	1.3246762	1.218323736	0.2848975	0.441418	1
IST1	1.8737254	1.82520477	1.8947569	1.084284697	0.1167436	0.441563	1
GRIPAP1	1.5902864	1.6351625	1.5708346	0.898722167	-0.1540529	0.441954	1
CARS	1.3116795	1.27093077	1.3293423	1.21559567	0.2816634	0.441956	1
RHBDF2	1.1134326	1.07570862	1.1297843	1.714260799	0.7775866	0.441957	1
MEF2C	1.1945715	1.23346166	1.1777143	0.761214103	-0.3936258	0.442273	1
CDC40	1.4814318	1.43855297	1.5000178	1.140153781	0.1892284	0.442314	1
DPH2	1.1622099	1.12398879	1.1787771	1.441880858	0.527952	0.44232	1
NUP50	1.4406289	1.48291591	1.4222994	0.874478113	-0.1935058	0.442469	1
SLC16A2	1.5140652	1.55767243	1.4951634	0.887910835	-0.1715133	0.442617	1
MRPL18	2.3117143	2.2551595	2.3362283	1.064588466	0.0902958	0.442646	1
TUBGCP2	1.6954002	1.64922796	1.7154138	1.101945478	0.1400528	0.442965	1
ACSF3	1.1964352	1.15750486	1.2133098	1.354305897	0.4375536	0.442994	1
EFCAB2	1.2125998	1.17297147	1.2297768	1.328408965	0.4096994	0.44301	1
RP1-267D11.6	1.1723631	1.21093	1.155646	0.737903658	-0.4384956	0.443033	1
UIMC1	1.3271025	1.28541677	1.3451715	1.209359443	0.2742431	0.443039	1
APTR	1.4024595	1.44470793	1.3841467	0.863817894	-0.2112009	0.443118	1
RNF169	1.1544835	1.19273732	1.1379022	0.715492962	-0.4829905	0.443179	1
GGCX	1.3363398	1.29537548	1.354096	1.198799502	0.2615904	0.443238	1
CHTF8	1.6302169	1.5849872	1.649822	1.110831137	0.1516395	0.443488	1
PMPCA	1.4520777	1.40935659	1.4705954	1.149597761	0.2011292	0.443551	1
DHRS13	1.3293379	1.28861223	1.3469906	1.202272677	0.2657641	0.443747	1
FAM120AOS	1.5664781	1.52178649	1.58585	1.122777175	0.1670716	0.443875	1
INTS10	1.68534	1.63946774	1.7052235	1.102828921	0.141209	0.44412	1
PTPN12	1.5999349	1.55468771	1.6195476	1.116930423	0.1595393	0.444157	1
FGF18	1.0471444	1.08331245	1.0314671	0.377700395	-1.4046858	0.444187	1
MCHR1	1.1464219	1.18433535	1.1299882	0.705172308	-0.5039523	0.444333	1
COPG1	1.2848305	1.24468559	1.3022315	1.235183228	0.3047251	0.444348	1
ZNF367	1.0693068	1.10573578	1.0535165	0.506133808	-0.9824093	0.444489	1
CBX7	1.1346494	1.17274995	1.1181345	0.683847034	-0.5482544	0.44459	1
RBM43	1.2089636	1.24796687	1.1920574	0.774528298	-0.3686101	0.444649	1
SLC39A8	1.272343	1.31226727	1.2550377	0.816728827	-0.2920709	0.444659	1
P2RX6	1.0879992	1.12513206	1.0719037	0.574622723	-0.7993131	0.444668	1
CBLN1	1.0776804	1.1144371	1.061748	0.539579967	-0.8900913	0.444822	1
KPTN	1.1497216	1.11176665	1.1661734	1.486788443	0.5721994	0.444876	1
CERS5	1.3906572	1.34920632	1.4086243	1.170151507	0.2266953	0.445048	1
HCN3	1.0767225	1.11364894	1.0607166	0.534246925	-0.9044214	0.445098	1
C20orf196	1.1158465	1.07827371	1.1321326	1.688083787	0.7553865	0.445181	1

RP11-437B10.1	1.0801008	1.11696433	1.0641221	0.5482193	-0.867175	0.445493	1
TYR	1.0537337	1.01724954	1.0695479	4.031871798	2.0114498	0.445523	1
ABCA5	1.1502074	1.11201299	1.166763	1.488782545	0.574133	0.445602	1
ZNF638	2.6412082	2.70326933	2.6143075	0.947769972	-0.0773911	0.445706	1
HID1	1.1264992	1.08886656	1.1428112	1.607029742	0.6843966	0.445778	1
FAM21A	1.163297	1.12491398	1.1799344	1.44046618	0.5265358	0.445893	1
FBXO10	1.0941089	1.05680544	1.1102783	1.941332588	0.9570473	0.44594	1
ZSCAN18	1.8858671	1.83774707	1.906725	1.082337424	0.1141503	0.445994	1
WAPL	1.7372493	1.78398681	1.7169906	0.914544231	-0.1288751	0.445997	1
ACADM	1.7244414	1.67862188	1.7443021	1.096784731	0.1332804	0.446068	1
NADSYN1	1.2441626	1.204657	1.2612865	1.276704371	0.3524245	0.446076	1
TDP2	1.333264	1.29253816	1.3509169	1.199559414	0.2625046	0.446178	1
SPIN3	1.2190179	1.2580409	1.2021032	0.783221382	-0.3525079	0.44618	1
MVP	1.1434448	1.10552762	1.1598802	1.515055644	0.5993708	0.446187	1
SIRT7	1.1886924	1.2271586	1.172019	0.757263665	-0.4011324	0.446211	1
RRM2	1.3402666	1.38084871	1.3226761	0.847255319	-0.2391313	0.446561	1
PTCH1	1.1326197	1.17005097	1.1163949	0.684470621	-0.5469395	0.446896	1
NUP133	1.2532072	1.21381734	1.270281	1.264074179	0.3380811	0.446901	1
RPUSD4	1.2653229	1.22546573	1.2825992	1.253402085	0.3258493	0.447009	1
PEX5	1.2331091	1.19395531	1.2500805	1.289371767	0.3666683	0.447097	1
RRAGD	1.325887	1.28515158	1.343544	1.204776622	0.2687657	0.447217	1
IPO4	1.2141622	1.17519311	1.2310536	1.318851056	0.3992816	0.447243	1
DNAJC30	1.3450197	1.38613418	1.3271984	0.847369598	-0.2389367	0.447294	1
SAMD12	1.1265052	1.08879883	1.1428493	1.608684	0.685881	0.44742	1
IQCH-AS1	1.11831	1.15540685	1.1022302	0.657823007	-0.6042286	0.447426	1
TJP2	1.1981462	1.23625097	1.1816294	0.768798696	-0.3793222	0.447735	1
PARD6G	1.174817	1.21292351	1.1582995	0.743457038	-0.4276787	0.447785	1
VAMP7	1.372981	1.33115001	1.3911129	1.181074757	0.2401003	0.448057	1
EDN1	1.0800406	1.04342451	1.0959121	2.208707931	1.1432027	0.448195	1
TIMM8A	1.2839124	1.32359008	1.2667138	0.82423365	-0.2788747	0.448517	1
SLC12A2	1.4321088	1.47393449	1.4139792	0.87349458	-0.1951293	0.448759	1
FAN1	1.252869	1.29178	1.2360028	0.808838149	-0.3060771	0.448849	1
FRYL	1.5652284	1.6087811	1.5463502	0.897449397	-0.1560975	0.448915	1
ABCB1	1.0851093	1.12182301	1.0691956	0.568000814	-0.8160351	0.448926	1
NF1	1.9488636	1.99695679	1.9280174	0.930850163	-0.1033791	0.448955	1
YAE1D1	1.2889165	1.24919551	1.3061338	1.228488323	0.2968841	0.448998	1
ELP2	1.6142248	1.65840854	1.5950731	0.903805194	-0.1459162	0.449041	1
ZNF385C	1.2032043	1.24171091	1.1865134	0.771638395	-0.3740032	0.449101	1
RTCA	1.5837413	1.53986199	1.602761	1.116509458	0.1589955	0.449236	1
SGO2	1.4747271	1.51715151	1.456338	0.882406712	-0.1804843	0.449574	1
CEP78	1.7673012	1.81387449	1.7471138	0.917971731	-0.1234784	0.449761	1
LSG1	1.1359589	1.09854685	1.1521754	1.544193572	0.6268536	0.449803	1
TAF1C	1.1636404	1.2013616	1.14729	0.731470007	-0.4511294	0.449842	1
SEL1L	1.3581659	1.31681004	1.3760918	1.187120732	0.2474667	0.449928	1
RNASEH2A	1.8229684	1.776438	1.8431373	1.085904202	0.1188968	0.449969	1
FAM196A	1.0534747	1.08949584	1.0378612	0.423049837	-1.2411005	0.450018	1
PLPP4	1.1777027	1.14003337	1.1940306	1.385602724	0.4705137	0.450129	1
RP11-140K17.3	1.3545567	1.39518039	1.3369481	0.852643798	-0.2299849	0.450143	1
SLC15A4	1.262616	1.30190687	1.2455851	0.813446584	-0.2978805	0.450197	1
ENHO	1.1106338	1.14736407	1.0947129	0.64271347	-0.6377524	0.45031	1
LINC01001	1.1301652	1.16725682	1.1140876	0.682110601	-0.5519224	0.450431	1

PMP22	1.15899	1.12140323	1.1752822	1.443801475	0.5298724	0.450491	1
UNC5C	1.1937401	1.23178052	1.1772513	0.7647377	-0.3869631	0.450662	1
AKT2	1.5538386	1.59706389	1.5351023	0.896222856	-0.1580706	0.450668	1
C2orf48	1.0528397	1.08885411	1.0372291	0.418991562	-1.2550069	0.450727	1
ANKRD28	1.2836351	1.32326884	1.2664557	0.824254148	-0.2788389	0.450924	1
CTD-2017D11.1	1.2267909	1.26544002	1.2100382	0.791283094	-0.3377342	0.450945	1
GBP1	1.057523	1.02146071	1.0731543	3.408756701	1.7692456	0.451214	1
OAZ2	4.9978908	4.89216604	5.0437178	1.038937634	0.0551091	0.451221	1
FAM89B	1.5864486	1.54291047	1.6053205	1.114954526	0.1569849	0.451223	1
ANGEL2	1.2500837	1.28928818	1.2330903	0.805737392	-0.3116184	0.45129	1
OVOS2	1.0398833	1.07570165	1.0243577	0.32175914	-1.635947	0.45136	1
USP39	1.4263352	1.38484563	1.4443191	1.154538461	0.2073162	0.45139	1
MPP5	1.3126357	1.35271599	1.2952627	0.837111767	-0.2565078	0.451457	1
GPRC5B	1.3571039	1.39787265	1.3394325	0.853118362	-0.2291822	0.451489	1
ZFP30	1.2278401	1.26635556	1.2111454	0.792720092	-0.3351166	0.451497	1
STAT6	1.2765447	1.23726328	1.2935714	1.237323479	0.3072227	0.451583	1
TNS3	1.3369514	1.2967017	1.3543978	1.194458216	0.2563564	0.451599	1
PAN3	1.3732691	1.41372196	1.3557346	0.859839718	-0.2178603	0.451601	1
POLG	1.2867836	1.24727171	1.3039102	1.229053835	0.2975481	0.451632	1
RPGRIP1L	1.3224069	1.36224393	1.3051394	0.842358894	-0.2474931	0.451755	1
ZNF431	1.4287542	1.47006047	1.4108498	0.874035967	-0.1942354	0.451775	1
HDAC11	1.1832149	1.14507106	1.1997485	1.3769011	0.4614249	0.451837	1
HSD11B1L	1.9152679	1.96391746	1.8941805	0.927652548	-0.1083435	0.451952	1
GNRH1	1.0524882	1.08824147	1.0369907	0.41919878	-1.2542936	0.452019	1
RP11-554J4.1	1.0852455	1.12164559	1.0694677	0.571066493	-0.8082694	0.452087	1
RNF146	1.6364453	1.59204117	1.6556924	1.107511567	0.1473218	0.452103	1
RARA-AS1	1.2308348	1.26955308	1.2140522	0.794100425	-0.3326066	0.452131	1
GDF7	1.0659967	1.03002247	1.08159	2.717630723	1.4423494	0.452168	1
IFT22	2.4602784	2.40090679	2.4860133	1.060751027	0.0850861	0.45218	1
RP11-25K19.1	1.4986668	1.5414551	1.4801199	0.886721618	-0.1734468	0.452221	1
ATXN7	1.2846739	1.3237036	1.2677563	0.827165073	-0.2737528	0.45239	1
RILPL1	1.1898358	1.22786732	1.1733508	0.760753116	-0.3944998	0.452603	1
C6orf1	1.6366082	1.59240608	1.6557679	1.10695672	0.1465988	0.452727	1
MRPS2	1.802732	1.75532722	1.8232799	1.089964568	0.1242812	0.452975	1
ETS1	1.0573283	1.0213688	1.0729152	3.412227678	1.7707139	0.45308	1
HPCA	1.1160307	1.15276337	1.1001088	0.655319088	-0.6097305	0.453151	1
C8orf37	1.1082029	1.14477115	1.0923522	0.637918188	-0.6485567	0.453187	1
THAP11	1.4376621	1.39617498	1.455645	1.150110381	0.2017723	0.45333	1
VEGFB	1.5347487	1.49172702	1.5533967	1.12541437	0.1704563	0.453425	1
APCDD1L	1.2024323	1.16444376	1.2188987	1.331145988	0.4126688	0.453696	1
BBIP1	1.9539315	2.00210749	1.9330494	0.931087096	-0.103012	0.453718	1
WWTR1	1.5469494	1.50355255	1.5657599	1.123537013	0.1680477	0.453845	1
LINC01011	1.1339831	1.17106044	1.1179117	0.689298399	-0.5367994	0.453864	1
CYB561A3	1.2377437	1.27659568	1.2209032	0.798650043	-0.3243646	0.453885	1
RP11-219G17.4	1.0957358	1.13209113	1.0799774	0.605471607	-0.7238688	0.453967	1
GCFC2	1.3612447	1.3206689	1.3788326	1.181382299	0.2404759	0.454107	1
PIP5K1C	1.2581833	1.21903572	1.275152	1.256196786	0.3290625	0.454282	1
RP11-354P11.3	1.0933127	1.12959834	1.0775845	0.598653815	-0.7402061	0.454449	1
CAMK1D	1.3320605	1.37204284	1.3147299	0.84595067	-0.2413546	0.454466	1
SARNP	1.1880417	1.14973869	1.2046444	1.366676613	0.4506719	0.454509	1
EIF4E3	1.1369307	1.1737112	1.1209879	0.696488889	-0.5218278	0.454652	1

MIGA1	1.3268917	1.3668044	1.3095913	0.844022763	-0.2446462	0.454733	1
LINC00526	1.2587901	1.29724627	1.242121	0.814546887	-0.2959303	0.454756	1
MFSD2A	1.0676062	1.03151855	1.0832486	2.641258501	1.4012255	0.454849	1
SF3B4	1.434832	1.39348706	1.4527532	1.150617715	0.2024086	0.455802	1
PCNP	3.8174129	3.73252142	3.8542095	1.044533262	0.0628584	0.455848	1
EML3	1.2533679	1.21459737	1.2701731	1.258976891	0.3322518	0.455857	1
ZFP1	1.2341593	1.27253819	1.2175238	0.798140681	-0.325285	0.455935	1
CTC-378H22.2	1.0688262	1.0327302	1.0844723	2.580866602	1.3678556	0.456238	1
ADAMTS12	1.1582036	1.19537482	1.1420915	0.727276581	-0.459424	0.456393	1
CCDC130	1.4352102	1.47651388	1.4173069	0.875749814	-0.1914093	0.456546	1
PGBD2	1.1021526	1.1386895	1.0863155	0.622365317	-0.6841664	0.456573	1
OSGIN2	1.1319933	1.09527206	1.1479103	1.55250433	0.6345973	0.456834	1
RP11-579D7.4	1.0903761	1.12632573	1.0747935	0.592068981	-0.7561628	0.45692	1
FBXL12	1.296324	1.25743434	1.3131809	1.216546773	0.2827918	0.456978	1
EFNA1	1.2450181	1.20636441	1.2617727	1.26849728	0.3431204	0.457186	1
CYB5D1	1.1136668	1.15007135	1.097887	0.652269779	-0.6164593	0.457235	1
CD276	1.8934049	1.84612538	1.9138984	1.080098128	0.1111624	0.457342	1
BCAS1	1.0595532	1.02372068	1.075085	3.165380896	1.6623791	0.457351	1
RAPGEFL1	1.0905646	1.12654005	1.0749709	0.592467668	-0.7551917	0.457379	1
IPPK	1.0721268	1.03613054	1.0877295	2.428126728	1.2798437	0.457393	1
OTX1	1.1023936	1.06616741	1.1180961	1.784807644	0.8357686	0.457609	1
KLHDC3	2.5382338	2.59751234	2.5125392	0.946809115	-0.0788545	0.45761	1
LRRFIP2	1.4240043	1.38293213	1.4418073	1.153748375	0.2063286	0.457661	1
ROM1	1.1363511	1.09927012	1.1524241	1.535448208	0.6186598	0.4577	1
CAB39L	1.1088773	1.07229374	1.1247346	1.725385508	0.7869187	0.457778	1
WARS	1.2770264	1.23833322	1.2937982	1.232720364	0.3018456	0.45787	1
PHF10	1.5347696	1.57725924	1.5163523	0.894489381	-0.1608637	0.457951	1
BISPR	1.0683176	1.1041526	1.0527848	0.50680208	-0.9805056	0.458044	1
TMEM150A	1.2927288	1.25329225	1.3098228	1.223183268	0.2906406	0.458048	1
RP11-567M16.6	1.1261071	1.16274926	1.1102244	0.677265189	-0.5622073	0.458074	1
INTS4	1.1558043	1.19279968	1.1397685	0.724941455	-0.4640636	0.458359	1
SSBP3	1.7743507	1.72897383	1.7940196	1.089229198	0.1233076	0.458432	1
CLEC2A	1.5170563	1.55857621	1.4990593	0.893448941	-0.1625428	0.458617	1
NCEH1	1.1006789	1.06442846	1.1163919	1.806529522	0.8532208	0.458698	1
KIFC1	1.30547	1.34473149	1.2884519	0.836743824	-0.2571421	0.458706	1
CABP7	1.0699679	1.03405088	1.0855363	2.512013477	1.3288442	0.458761	1
DDIAS	1.0656633	1.10115617	1.0502788	0.497041002	-1.0085632	0.458992	1
GSTM2	1.0761216	1.11206158	1.0605432	0.540267311	-0.8882547	0.45908	1
PRTG	1.5187231	1.56082603	1.5004734	0.892386143	-0.16426	0.459088	1
CROCC	1.1937585	1.23105138	1.1775937	0.768633007	-0.3796332	0.459143	1
ST7L	1.2397788	1.27796166	1.2232282	0.803090051	-0.3163663	0.459192	1
HEXA	1.6890731	1.73418591	1.6695188	0.911919935	-0.1330209	0.459206	1
SRRM2-AS1	1.0918265	1.12775173	1.0762545	0.596896124	-0.7444482	0.459393	1
ZNF217	1.2217978	1.1837112	1.2383066	1.297180549	0.3753793	0.459484	1
ARRDC1	1.5355719	1.5782854	1.5170574	0.894121533	-0.1614572	0.459582	1
NCKAP5	1.0768016	1.04094953	1.0923419	2.255016363	1.1731379	0.459615	1
ZNF273	1.2579149	1.29655022	1.2411682	0.813245745	-0.2982367	0.459621	1
UBL4A	1.518714	1.47638064	1.5370636	1.127383373	0.1729782	0.459677	1
DCP1A	1.5369436	1.49425259	1.5554482	1.123814444	0.1684038	0.459778	1
RP11-563K23.1	1.0904615	1.1261176	1.0750062	0.594732371	-0.7496875	0.45982	1
RP11-1275H24.2	1.0639028	1.09934521	1.0485401	0.488599857	-1.0332747	0.459888	1

ITGA5	1.1497864	1.11306999	1.1657014	1.465476108	0.5513694	0.460046	1
PLBD1	1.1894439	1.1520534	1.205651	1.352491648	0.4356197	0.460149	1
SPAG17	1.1085179	1.14448313	1.0929286	0.643179539	-0.6367066	0.460161	1
CEP76	1.1349448	1.09826875	1.1508423	1.534997405	0.6182362	0.460205	1
CSTF2T	1.1756429	1.21272306	1.1595703	0.750131734	-0.4147841	0.460275	1
ZFX	1.5085285	1.55079521	1.4902078	0.890000049	-0.1681227	0.460275	1
ALKBH2	1.6351651	1.6787966	1.6162528	0.907860763	-0.139457	0.460348	1
ST6GALNAC6	1.5049744	1.46301965	1.52316	1.129887202	0.1761788	0.460402	1
LOH12CR2	1.0969746	1.13297593	1.0813696	0.611912156	-0.7086035	0.460487	1
C16orf45	2.8113322	2.74395241	2.8405383	1.055383338	0.0777671	0.460512	1
PBX1	3.4106343	3.48893472	3.3766946	0.954904347	-0.0665719	0.460606	1
ZDHHC21	1.5079492	1.54985121	1.4897866	0.890762088	-0.1668879	0.46061	1
YARS2	1.2989472	1.26000041	1.3158289	1.214724526	0.2806292	0.460661	1
KAZN	1.3980392	1.43820259	1.3806302	0.868616931	-0.203208	0.460827	1
CALY	1.2548321	1.21632276	1.2715242	1.255180931	0.3278953	0.460884	1
AIFM2	1.1200271	1.08380546	1.1357275	1.619554893	0.6955974	0.461052	1
CCDC82	1.5110565	1.55268806	1.493011	0.892024031	-0.1648455	0.461136	1
NONO	5.267737	5.39828961	5.2111482	0.957451328	-0.0627289	0.461419	1
MOB3A	1.2309862	1.26925777	1.2143971	0.796252392	-0.3287023	0.461451	1
HIF1A-AS2	1.0745247	1.11013289	1.0590902	0.536535204	-0.8982553	0.461495	1
TMEM50B	2.0605622	2.10984504	2.0392003	0.936347172	-0.0948846	0.461499	1
FAM114A2	1.2075181	1.1699753	1.2237912	1.316610373	0.3968285	0.461535	1
CRYBB3	1.106644	1.06881786	1.12304	1.787907612	0.8382722	0.461557	1
THAP2	1.5755099	1.61804685	1.557072	0.901342612	-0.1498525	0.461886	1
AEBP2	1.4324628	1.47277039	1.4149912	0.87778598	-0.1880589	0.462053	1
TSEN2	1.2878965	1.32668076	1.2710852	0.82981687	-0.2691351	0.462106	1
TPST2	1.556798	1.59922747	1.5384067	0.898501406	-0.1544073	0.462134	1
RFC5	1.4440597	1.40314151	1.4617959	1.145493387	0.1959691	0.462507	1
USP15	1.3584333	1.39833104	1.3411395	0.856422026	-0.2236062	0.462516	1
ST3GAL2	1.2973817	1.33604302	1.2806238	0.835082887	-0.2600087	0.46263	1
DLST	1.3682082	1.32833441	1.3854918	1.174082715	0.2315341	0.462749	1
VARS	1.4174263	1.37678377	1.435043	1.154622482	0.2074212	0.462796	1
PDGFA	1.0721905	1.03690879	1.0874835	2.37026197	1.2450465	0.462857	1
RP11-268J15.5	1.1637483	1.20025551	1.1479241	0.738676871	-0.4369847	0.463158	1
HOMEZ	1.2280863	1.26595365	1.2116726	0.795900178	-0.3293406	0.46328	1
DUSP26	1.2555143	1.21726709	1.2720927	1.25234216	0.3246288	0.463357	1
LCLAT1	1.1315771	1.16767466	1.1159305	0.691401258	-0.5324049	0.463742	1
CTC-459F4.3	1.1507673	1.18717099	1.1349879	0.721201024	-0.4715266	0.464117	1
INSR	1.5432458	1.58458657	1.5253264	0.898628964	-0.1542025	0.464215	1
RAMP2	2.159287	2.20911544	2.1376886	0.940926326	-0.0878463	0.464278	1
HMP19	1.056625	1.0213674	1.0719075	3.365292004	1.7507317	0.464378	1
C19orf73	1.1409941	1.17720476	1.1252984	0.707082522	-0.5000495	0.464675	1
FOXRED2	1.1820831	1.21867368	1.1662228	0.760140769	-0.3956615	0.46473	1
RAP2A	1.325077	1.28598158	1.3420231	1.195961887	0.2581714	0.464865	1
RP11-412D9.4	1.1564958	1.19309705	1.1406308	0.728290539	-0.457414	0.465088	1
SULT1A1	1.5221876	1.56393671	1.5040912	0.893879046	-0.1618485	0.46511	1
ARHGEF37	1.1034159	1.13922437	1.0878946	0.631316052	-0.6635657	0.465138	1
RP11-89K21.1	2.2317766	2.28557515	2.2084574	0.940013019	-0.0892474	0.465242	1
CCNK	1.4435312	1.40240551	1.4613573	1.146498507	0.1972345	0.465242	1
ZNF550	1.1520348	1.18837581	1.1362826	0.723461505	-0.4670118	0.4653	1
VAPB	1.4703219	1.42939763	1.4880608	1.136617289	0.1847466	0.465343	1

MECR	1.3793087	1.33976598	1.3964487	1.16682862	0.2225927	0.465351	1
MYBL1	1.1141243	1.14982539	1.0986495	0.658429693	-0.6028987	0.465352	1
SH3GL1	1.4763222	1.43537304	1.4940719	1.134824179	0.1824688	0.465414	1
PIK3IP1	1.1885349	1.15166409	1.2045167	1.348484959	0.4313394	0.465602	1
CTD-2325M2.1	1.1472582	1.18341682	1.131585	0.717409606	-0.479131	0.465711	1
ARID5A	1.2266002	1.18912293	1.242845	1.284058913	0.3607114	0.465883	1
GMPPB	1.2808429	1.2423142	1.2975434	1.227923744	0.296221	0.466043	1
CAPRIN1	2.433974	2.37721561	2.4585762	1.059076167	0.0828063	0.466097	1
NPEPL1	1.296571	1.33516729	1.2798413	0.83493007	-0.2602727	0.466108	1
FOXO1	1.1209161	1.08479643	1.1365723	1.610590214	0.6875895	0.466267	1
PSME4	1.3513008	1.390618	1.3342585	0.855717115	-0.2247941	0.466362	1
SLC29A4	1.3403734	1.30090004	1.3574834	1.188046878	0.2485918	0.466447	1
PTPRZ1	1.1940606	1.23078831	1.1781407	0.771879245	-0.3735529	0.466606	1
C2orf82	1.1555884	1.19199948	1.1398058	0.728157096	-0.4576784	0.466612	1
TCF12	1.962612	2.01054239	1.9418364	0.932010738	-0.1015815	0.46662	1
C10orf11	1.0760719	1.04068047	1.0914126	2.247087013	1.168056	0.466816	1
RMND5A	1.4236765	1.46389978	1.4062415	0.875709584	-0.1914756	0.466955	1
CEBPG	1.3821426	1.42168028	1.3650047	0.865595879	-0.2082345	0.467119	1
EIF2AK4	2.3857165	2.44742461	2.3589688	0.938887453	-0.0909759	0.467369	1
LTA4H	2.829402	2.76437531	2.8575882	1.052830538	0.0742732	0.467528	1
PPM1K	1.3462502	1.30700186	1.3632626	1.183258739	0.2427656	0.467605	1
RABEP2	1.2131132	1.17590443	1.2292415	1.303216156	0.3820764	0.467785	1
PABPC4	2.7716943	2.84003881	2.74207	0.946757211	-0.0789336	0.4678	1
RP11-966I7.4	1.0498917	1.08465783	1.0348221	0.411327885	-1.2816392	0.467825	1
VMA21	1.9232986	1.9701796	1.9029778	0.93073257	-0.1035614	0.467876	1
LATS1	1.2584409	1.22051009	1.2748822	1.246574331	0.3179689	0.468023	1
ZNF90	1.590043	1.63205556	1.5718324	0.90471853	-0.1444591	0.468075	1
ZNF454	1.043153	1.07759807	1.0282225	0.363701362	-1.4591738	0.468094	1
CTIF	1.1801267	1.14316114	1.1961497	1.370132052	0.4543149	0.468131	1
TAF1	1.213387	1.25040035	1.1973434	0.788111469	-0.3435284	0.468187	1
NUFIP1	1.1814102	1.14459249	1.197369	1.365001926	0.448903	0.468209	1
PDZRN4	1.0679279	1.03299639	1.0830692	2.517523664	1.3320053	0.468361	1
FNIP1	1.8011061	1.84583271	1.7817191	0.924200658	-0.113722	0.468502	1
SCAI	1.409605	1.44935059	1.3923771	0.87320921	-0.1956007	0.468805	1
PRKCDBP	2.4225311	2.36194577	2.4487922	1.063766423	0.0891814	0.46887	1
PDZRN3	1.274936	1.23689377	1.2914256	1.230195303	0.2988874	0.468922	1
LINC00920	1.0559336	1.09060555	1.0409049	0.45146121	-1.1473261	0.468973	1
SEC22A	1.1866922	1.14991649	1.2026328	1.351637948	0.4347088	0.469049	1
EPAS1	1.1083347	1.07292621	1.1236826	1.695997144	0.7621337	0.469239	1
STT3A	1.2871685	1.2491537	1.3036462	1.218710426	0.2853554	0.469476	1
PPM1A	1.7996365	1.75497669	1.8189946	1.08479453	0.1174218	0.469719	1
TRIM13	1.5816491	1.62383151	1.5633649	0.903072221	-0.1470867	0.469789	1
GNAL	1.2734675	1.31087881	1.2572514	0.82749747	-0.2731732	0.469949	1
GLI2	1.1785272	1.2149021	1.1627603	0.757369286	-0.4009312	0.470028	1
SV2A	1.3424646	1.3041897	1.359055	1.180365568	0.2392337	0.470093	1
NASP	3.1714567	3.10175168	3.2016708	1.047540857	0.0670065	0.470096	1
RNF139	1.3415728	1.3029698	1.3583055	1.182644193	0.2420161	0.470427	1
ERVMER34-1	1.0618044	1.02720434	1.0768021	2.823154316	1.497308	0.470778	1
GPRASP1	1.0999672	1.13508229	1.0847464	0.627368575	-0.6726148	0.47078	1
NUDT8	1.4100141	1.37019293	1.4272747	1.154194763	0.2068867	0.470845	1
CELF1	2.2612533	2.20408632	2.2860327	1.068056878	0.0949885	0.470917	1

PI4KB	1.3046511	1.26639267	1.3212344	1.205867935	0.2700719	0.471025	1
KIF18A	1.2175952	1.25447941	1.2016076	0.792235257	-0.3359992	0.471153	1
MCPH1	1.196483	1.23337255	1.180493	0.773411399	-0.3706921	0.471294	1
SORBS1	1.1006792	1.13584872	1.0854348	0.628896586	-0.6691053	0.471399	1
TRMT11	1.4794576	1.52002283	1.4618743	0.888180838	-0.1710746	0.471405	1
NOTCH1	1.2815625	1.3195368	1.2651023	0.829645732	-0.2694327	0.471647	1
ZNF318	1.207949	1.24457913	1.1920714	0.785314003	-0.3486585	0.471656	1
HYDIN	1.0868298	1.1216853	1.0717214	0.589400926	-0.7626788	0.471708	1
ZNF800	1.4371038	1.47756294	1.4195666	0.878557653	-0.1867911	0.471796	1
GSPT2	1.2563389	1.21908292	1.2724877	1.24376492	0.3147138	0.47187	1
PHF8	1.1514442	1.18726269	1.1359184	0.725816923	-0.4623224	0.471898	1
CDC14B	1.1606107	1.19677072	1.144937	0.736577858	-0.4410901	0.472003	1
CTD-2287O16.5	1.107191	1.14214852	1.0920384	0.647480698	-0.6270909	0.47215	1
CAMK4	1.5485613	1.58984626	1.530666	0.899668399	-0.1525347	0.472634	1
FAM135A	1.3212778	1.35940895	1.3047496	0.84791891	-0.2380018	0.472704	1
PPWD1	1.3354839	1.37397702	1.3187989	0.852455768	-0.2303031	0.472755	1
RNF10	1.9754	1.92851022	1.9957246	1.072389491	0.100829	0.472832	1
THAP12	1.4497448	1.48954731	1.4324922	0.883453362	-0.1787741	0.47288	1
NDE1	1.2152561	1.17854639	1.2311681	1.294722866	0.3726433	0.472893	1
ZNF681	1.1902823	1.2267658	1.1744684	0.769377014	-0.3782374	0.473009	1
VTN	1.0628057	1.02829132	1.0777661	2.748762924	1.4587825	0.473175	1
CNTNAP3	1.0992109	1.1340404	1.0841139	0.627526324	-0.6722521	0.473204	1
SP100	1.0852918	1.05035037	1.1004374	1.994769349	0.9962219	0.473216	1
C12orf29	1.4153156	1.37576604	1.4324586	1.150871923	0.2027273	0.473276	1
ZNF277	1.5456151	1.58648401	1.5279003	0.900110333	-0.1518262	0.473374	1
EDEM2	1.2868856	1.24936006	1.3031513	1.215717138	0.2818076	0.473451	1
JMJD6	1.1670685	1.13089395	1.1827485	1.396156912	0.4814611	0.473528	1
RP5-1092A3.4	1.1627098	1.19851644	1.1471892	0.741445732	-0.431587	0.473859	1
KYAT1	1.1828323	1.14623537	1.1986954	1.358736824	0.442266	0.473892	1
FAM212B	1.0902589	1.12520508	1.0751113	0.59990579	-0.7371921	0.473914	1
LCOR	1.6608083	1.70319014	1.6424377	0.913604516	-0.1303583	0.474077	1
TTC21B	1.1940294	1.23011026	1.1783899	0.775236797	-0.367291	0.474214	1
APBA3	1.1195879	1.08426136	1.1349004	1.60097542	0.6789512	0.474229	1
CA11	1.8238636	1.77857268	1.8434952	1.083386534	0.1155481	0.474237	1
SACM1L	1.3005546	1.33836755	1.2841644	0.839809945	-0.2518652	0.474344	1
AP2B1	2.9039532	2.83782671	2.9326161	1.051576881	0.0725543	0.474462	1
CINP	1.3868155	1.34794323	1.4036649	1.160145928	0.2143063	0.474579	1
DLEU2	1.1327168	1.16814134	1.1173618	0.697994925	-0.5187115	0.474697	1
MYCBPAP	1.0518789	1.08599474	1.0370912	0.431318888	-1.2131732	0.474731	1
TTBK2	1.3863756	1.4254034	1.3694587	0.868490309	-0.2034183	0.474936	1
LPIN2	1.5957771	1.55379948	1.6139726	1.108655045	0.1488105	0.475021	1
LINC00205	1.1491625	1.18478775	1.1337205	0.723643711	-0.4666485	0.475058	1
RIC8B	1.1803453	1.21667565	1.1645977	0.759650293	-0.3965927	0.475094	1
SH3BP2	1.1946868	1.23073878	1.1790598	0.776028317	-0.3658188	0.475101	1
SNX5	1.8777224	1.92301214	1.8580913	0.929664164	-0.1052184	0.475168	1
ELP6	1.8094691	1.85355125	1.7903614	0.925968287	-0.1109653	0.475177	1
POLR2F	3.6960097	3.61820244	3.7297357	1.042599164	0.0601846	0.475359	1
SIN3A	1.401296	1.44023344	1.3844183	0.873214691	-0.1955917	0.475398	1
RSRC1	1.7222712	1.67881548	1.7411074	1.091765537	0.1266631	0.475698	1
DGKE	1.1177576	1.15296156	1.1024982	0.670091377	-0.5775703	0.475746	1
CLEC4F	1.0455336	1.07948776	1.0308159	0.387681554	-1.367056	0.475863	1

VAMP2	3.7448184	3.66083581	3.7812211	1.045243424	0.063839	0.47589	1
E2F2	1.0644792	1.09864598	1.0496694	0.503511255	-0.9899041	0.475986	1
TNFRSF1A	2.1528791	2.10042572	2.1756153	1.068327689	0.0953542	0.476013	1
BBOX1-AS1	1.2755305	1.23778484	1.2918915	1.227544811	0.2957757	0.476181	1
ZC3H8	1.6744603	1.71673686	1.6561353	0.915447956	-0.1274502	0.476555	1
CD58	1.2006542	1.16495609	1.2161277	1.310213698	0.3898021	0.476678	1
LRP11	1.3462459	1.30776886	1.362924	1.179209678	0.2378203	0.476709	1
VPS18	1.1227586	1.08744566	1.1380651	1.578867881	0.6588905	0.476895	1
NEK3	1.2100065	1.1735816	1.2257951	1.300801134	0.3794004	0.476917	1
PIH1D2	1.1084855	1.14351121	1.0933034	0.65014702	-0.6211621	0.476963	1
CISD3	1.4243752	1.46377217	1.4072984	0.878229453	-0.1873302	0.477015	1
LGR5	1.1414896	1.17694113	1.1261229	0.712796118	-0.4884386	0.477096	1
C5orf38	1.0727357	1.0382106	1.0877008	2.295196017	1.1986174	0.477251	1
RP11-1055B8.9	1.1671263	1.20302965	1.1515637	0.746510346	-0.4217658	0.477267	1
NFYC	1.5008079	1.46030647	1.5183635	1.126126793	0.1713693	0.477296	1
EMILIN2	1.0878996	1.05338558	1.1028599	1.926736435	0.9461592	0.477364	1
ZNF616	1.1499964	1.18560929	1.1345598	0.724962717	-0.4640213	0.477397	1
PPTC7	1.1126112	1.0776462	1.127767	1.645502554	0.7185283	0.477461	1
TMEM53	1.3231195	1.28505322	1.3396196	1.191425191	0.2526884	0.477612	1
ARHGEF12	1.7294671	1.68635776	1.7481531	1.09003376	0.1243728	0.477783	1
ZNF460	1.0890889	1.12359463	1.0741323	0.599801767	-0.7374423	0.477887	1
CFAP74	1.0890928	1.12351723	1.0741714	0.600493978	-0.7357783	0.477887	1
ATP6V0A2	1.1371571	1.17232505	1.1219133	0.70746124	-0.499277	0.477991	1
RANBP3	1.3072025	1.2697314	1.3234445	1.199135515	0.2619947	0.478243	1
RP11-182L21.6	1.4596843	1.49897654	1.4426528	0.887121539	-0.1727963	0.478249	1
CSNK1E	2.2639476	2.31588612	2.2414346	0.943420987	-0.0840264	0.478274	1
CRB2	2.7868989	2.86509278	2.7530053	0.939902448	-0.0894171	0.478406	1
ZNF391	1.0844067	1.11863102	1.069572	0.586457329	-0.769902	0.478472	1
CPT1C	1.3164526	1.35379277	1.3002673	0.848709546	-0.2366572	0.478533	1
ESCO1	1.6368069	1.67898417	1.618525	0.910956421	-0.1345461	0.47869	1
BET1L	1.2189052	1.18267643	1.2346088	1.284286086	0.3609666	0.478725	1
JADE1	1.3731168	1.41155036	1.3564576	0.866133674	-0.2073384	0.478753	1
FHAD1	1.1708385	1.20638393	1.1554312	0.75311679	-0.4090545	0.478793	1
CMBL	1.8428657	1.79869387	1.8620123	1.079277418	0.1100657	0.478957	1
SYNE4	1.0991012	1.06446376	1.114115	1.770219898	0.8239286	0.478977	1
SREBF1	1.0920509	1.05743297	1.1070562	1.864019266	0.8984168	0.479038	1
MRE11	1.3705026	1.40866046	1.3539628	0.866153855	-0.2073048	0.479064	1
AC084219.4	1.134103	1.16919482	1.1188923	0.70269471	-0.5090301	0.479084	1
BSN	1.0803676	1.11446606	1.0655875	0.572986117	-0.8034279	0.479149	1
PPP1R1C	1.1130454	1.07815812	1.1281675	1.639848927	0.7135629	0.479229	1
HRAS	1.4544882	1.41510508	1.471559	1.135999068	0.1839617	0.479385	1
PRDM16	1.0768493	1.04255945	1.0917124	2.154923734	1.1076368	0.479411	1
RHPN1	1.345933	1.38402656	1.3294211	0.857808173	-0.221273	0.479544	1
METTL9	4.1692234	4.07700189	4.2091974	1.042962424	0.0606872	0.479653	1
CBX2	1.2220905	1.25826618	1.20641	0.799214039	-0.3233462	0.479955	1
CENPP	1.0850531	1.11931179	1.0702035	0.588403671	-0.7651218	0.480048	1
IP6K1	1.3131528	1.35074826	1.2968569	0.846353112	-0.2406684	0.480146	1
EMX2	1.1126863	1.07600572	1.1285858	1.691790665	0.7585511	0.480306	1
LRRN1	1.4145633	1.37569555	1.4314108	1.148298852	0.1994982	0.480352	1
PAPPA2	1.3167947	1.27967815	1.3328831	1.190236175	0.2512479	0.48057	1
NEK8	1.1103958	1.14528933	1.095271	0.655732667	-0.6088203	0.480606	1

CRYZ	1.1628822	1.12739424	1.1782646	1.399314672	0.4847204	0.480929	1
DNAJC12	1.4486548	1.40918495	1.4657633	1.138270717	0.1868437	0.481247	1
CAMK2A	1.0664811	1.03232734	1.0812852	2.51444161	1.3302381	0.481279	1
PAK3	1.3477233	1.38561028	1.331301	0.85916014	-0.219001	0.481483	1
DENND3	1.1801656	1.14447535	1.1956358	1.354111804	0.4373469	0.481811	1
STEAP3	1.0926816	1.12683808	1.0778763	0.613982429	-0.7037307	0.48187	1
GALNT6	1.0508547	1.0171592	1.0654602	3.814877008	1.9316365	0.48188	1
PCAT7	1.4747542	1.51415105	1.4576774	0.890161349	-0.1678612	0.48207	1
NUP54	1.3767281	1.41489905	1.3601827	0.868121196	-0.2040316	0.482154	1
FNDC4	1.6289524	1.66989389	1.611206	0.912392334	-0.1322738	0.48226	1
HAS1	1.1027736	1.0684929	1.1176327	1.717444163	0.7802632	0.482359	1
POLR3E	1.2405805	1.27709098	1.2247548	0.811122699	-0.3020079	0.48261	1
RAB23	1.1962868	1.16047743	1.2118085	1.319864791	0.4003901	0.482628	1
YPEL1	1.5493665	1.58901524	1.5321806	0.903509013	-0.1463891	0.482887	1
B3GNT7	1.0601565	1.02627663	1.0748419	2.848231897	1.5100666	0.4829	1
TYMP	1.2685612	1.30548688	1.2525556	0.826731319	-0.2745096	0.48332	1
REC8	1.9783295	1.93191122	1.9984498	1.071400087	0.0994973	0.483322	1
LAMA1	1.1399874	1.10479494	1.1552418	1.481386391	0.566948	0.483501	1
NUAK2	1.1757407	1.21094742	1.1604802	0.760759035	-0.3944885	0.483534	1
CWF19L2	1.5138113	1.55361263	1.4965592	0.896943367	-0.1569112	0.483829	1
CP	1.9397139	1.99458032	1.9159318	0.920922898	-0.1188477	0.483841	1
ZNF180	1.1205246	1.1550367	1.1055652	0.680904594	-0.5544754	0.484127	1
LRCH3	1.3855577	1.42363978	1.3690508	0.871142967	-0.1990186	0.484182	1
SLC26A6	1.1809644	1.14516727	1.1964809	1.353478999	0.4366725	0.484269	1
COL12A1	1.0927684	1.05866276	1.1075517	1.833390335	0.874514	0.484278	1
TOMM5	1.6568761	1.61487786	1.6750804	1.097909773	0.1347595	0.484329	1
LRP5	1.3161746	1.35295508	1.3002319	0.850623672	-0.2334071	0.484425	1
HOTAIRM1	1.0604497	1.02656728	1.0751362	2.828148211	1.4998577	0.484429	1
KLC4	1.2722305	1.23557769	1.2881179	1.223026975	0.2904562	0.484487	1
SH3PXD2B	1.2190607	1.2549152	1.2035194	0.798380759	-0.3248511	0.484491	1
GPAT4	1.3668502	1.3287646	1.3833587	1.166058174	0.2216398	0.484553	1
UBE2O	1.2492791	1.28563734	1.2335194	0.817538212	-0.2906419	0.484838	1
PRKACA	1.3045911	1.34152657	1.2885812	0.844974486	-0.2430203	0.484859	1
FXYD3	1.0555867	1.02191321	1.0701827	3.202758285	1.6793149	0.484859	1
C6orf141	1.0783348	1.04433906	1.0930704	2.099061881	1.0697447	0.484929	1
SMIM7	2.2807616	2.22933263	2.3030538	1.059968443	0.0840213	0.484972	1
HAPLN3	1.0768231	1.04294547	1.0915076	2.130784986	1.091385	0.485043	1
UHRF1BP1L	1.1986084	1.23421714	1.1831736	0.782067443	-0.3546351	0.485177	1
CARS2	1.3894404	1.42761793	1.3728922	0.872021813	-0.1975639	0.485179	1
THUMPD2	1.3683996	1.4062923	1.3519748	0.866309182	-0.2070461	0.485226	1
KDELR3	1.4708766	1.43214358	1.4876656	1.128480402	0.1743814	0.485314	1
RNF14	1.3124822	1.27532607	1.3285877	1.193449355	0.2551373	0.485361	1
RARRES2	3.4947023	3.57470992	3.4600226	0.955456218	-0.0657383	0.485591	1
C21orf2	1.4095714	1.37032893	1.4265813	1.151898334	0.2040134	0.485779	1
ZBTB37	1.2109504	1.24654252	1.1955228	0.793058976	-0.3344999	0.485794	1
CBY1	1.8555666	1.89918122	1.8366616	0.930470457	-0.1039678	0.485837	1
TMC6	1.1173775	1.08307335	1.1322468	1.591927904	0.670775	0.48592	1
TBC1D9B	1.5271962	1.48708227	1.5445838	1.118053061	0.1609887	0.485984	1
TNFRSF13C	1.0536654	1.08701186	1.0392112	0.45064162	-1.1499475	0.485995	1
HSPB11	2.4390272	2.38561207	2.4621802	1.055259455	0.0775978	0.485997	1
MRPS21	5.1725198	5.0487939	5.2261495	1.043804543	0.0618516	0.486053	1

C3orf62	1.1515102	1.18600929	1.1365564	0.734137548	-0.4458777	0.48616	1
ZRANB2	2.648332	2.7089718	2.6220474	0.949136439	-0.0753126	0.486266	1
VCAN	2.0053643	2.05527863	1.9837287	0.932198105	-0.1012915	0.486275	1
C18orf21	1.4586025	1.41947317	1.4755634	1.133715811	0.181059	0.486362	1
SMURF2	1.3603793	1.39777299	1.3441708	0.865244324	-0.2088205	0.486422	1
RUFY2	1.4283505	1.38954451	1.4451712	1.142799308	0.1925721	0.486447	1
PNPLA3	1.0869457	1.05286383	1.1017187	1.924163807	0.9442316	0.486462	1
EIF2AK2	1.7654977	1.80847238	1.7468701	0.923804081	-0.1143412	0.486718	1
TMEM19	1.2669004	1.23048507	1.2826849	1.226478087	0.2945215	0.486786	1
KIF3A	1.5577613	1.51760233	1.5751684	1.111216815	0.1521403	0.486884	1
ADGRA3	1.3521475	1.38980078	1.3358265	0.861533701	-0.2150209	0.486963	1
TPCN1	1.1310403	1.09647927	1.146021	1.513496102	0.597885	0.486978	1
ZDHHC20	1.3122289	1.34917826	1.2962129	0.848314402	-0.237329	0.487012	1
PYCARD	1.0620246	1.0285951	1.0765148	2.675801868	1.4199713	0.487177	1
NSUN6	1.3408367	1.37813643	1.3246689	0.858602442	-0.2199378	0.487185	1
ASGR1	1.3984086	1.43622718	1.3820159	0.875726998	-0.1914469	0.487228	1
MCM3AP-AS1	1.4364898	1.47513386	1.4197394	0.88341288	-0.1788402	0.487299	1
RILPL2	1.2949819	1.25835783	1.3108568	1.20320267	0.2668797	0.487571	1
VANGL2	1.2923584	1.3287151	1.2765995	0.841456472	-0.2490395	0.487591	1
PTHLH	1.0653052	1.03189505	1.0797871	2.501550153	1.3228224	0.48764	1
EBAG9	1.5524214	1.51212868	1.5698865	1.112779857	0.1541682	0.487673	1
BRWD1	2.1829334	2.2318414	2.1617339	0.943087268	-0.0845368	0.487726	1
ZNRF3	1.161055	1.12595367	1.1762698	1.399481624	0.4848925	0.48827	1
HAUS7	1.1273604	1.09303212	1.1422402	1.528936596	0.6125286	0.488276	1
TRIM47	1.0754394	1.04190292	1.089976	2.14724819	1.102489	0.488517	1
ISPD	1.0802326	1.04654289	1.0948357	2.037596763	1.0268686	0.488627	1
PMVK	2.1272761	2.07980007	2.1478549	1.063025365	0.088176	0.488778	1
HDHD2	1.6325619	1.59156545	1.6503321	1.099340862	0.1366388	0.489029	1
OPTC	1.1711819	1.13631584	1.1862947	1.366640369	0.4506336	0.48905	1
PEX3	1.204247	1.16872912	1.2196424	1.301745925	0.3804479	0.489071	1
HINFP	1.2652539	1.22852214	1.2811754	1.230407845	0.2991366	0.489206	1
RNF8	1.5958094	1.63631252	1.5782531	0.908756379	-0.1380345	0.489357	1
PNPLA6	1.2180549	1.18247032	1.2334792	1.279546446	0.3556325	0.489562	1
F3	1.1034779	1.06969912	1.1181195	1.694705517	0.7610346	0.489597	1
SDK2	1.9736349	2.01869653	1.9541027	0.93659172	-0.0945078	0.489691	1
GDPD1	1.1878619	1.22312097	1.1725786	0.773475546	-0.3705724	0.490053	1
FILIP1	1.1250257	1.09066596	1.1399191	1.543237349	0.62596	0.490138	1
LINC00472	1.0427963	1.07561436	1.0285712	0.377853715	-1.4041003	0.490188	1
BBS4	1.4417695	1.48062328	1.4249281	0.884118805	-0.1776878	0.490362	1
GPM6B	3.0702923	3.14311741	3.0387258	0.95128984	-0.0720431	0.490381	1
HEXIM2	1.1939619	1.15865548	1.2092656	1.318993822	0.3994378	0.490666	1
SLC10A3	1.1697226	1.13501916	1.1847649	1.368434924	0.4525268	0.49106	1
SKIL	2.4989356	2.56010702	2.4724205	0.943794525	-0.0834553	0.491374	1
LYST	1.1165339	1.08262276	1.1312329	1.588338001	0.667518	0.491527	1
AFTPH	1.3062537	1.34273862	1.2904391	0.847407047	-0.238873	0.491534	1
TRMT2A	1.2708147	1.23463227	1.2864982	1.221052114	0.2881248	0.491561	1
PLAUR	1.2980828	1.33449986	1.2822976	0.843939419	-0.2447887	0.491669	1
TMEM200A	1.0572903	1.02425204	1.0716109	2.952779937	1.5620738	0.491721	1
JAZF1	1.1433425	1.10917981	1.1581505	1.448532059	0.5345916	0.491918	1
ZNF577	1.0911619	1.12465827	1.0766427	0.614822638	-0.7017578	0.491954	1
JOSD1	1.3142432	1.3512396	1.2982069	0.849012801	-0.2361418	0.492028	1

MTHFD1L	1.2866695	1.25078171	1.3022253	1.205132832	0.2691922	0.492032	1
MAEA	1.4903818	1.4520888	1.5069801	1.121416986	0.1653228	0.49222	1
SDSL	1.2688604	1.23308457	1.2843676	1.220019183	0.2869038	0.492355	1
AATF	1.3358377	1.2991734	1.3517301	1.175673021	0.2334869	0.49243	1
EXO1	1.0818619	1.11524287	1.0673928	0.584789395	-0.7740109	0.492436	1
ANKMY2	1.2278339	1.19214392	1.2433039	1.266258821	0.3405723	0.492462	1
PLAA	1.2263625	1.19085458	1.2417536	1.266690066	0.3410636	0.492727	1
UTRN	2.0775761	2.12670085	2.0562826	0.937500525	-0.0931086	0.492835	1
CTD-2089N3.2	1.0634939	1.09639934	1.0492309	0.510697034	-0.9694604	0.492998	1
C22orf46	1.1331505	1.16714833	1.1184139	0.708435939	-0.4972907	0.492998	1
FAM229B	2.7647869	2.82393679	2.7391481	0.953513335	-0.068675	0.493097	1
HS6ST1	1.2123493	1.24741098	1.1971517	0.796859073	-0.3276035	0.493112	1
IGF2R	1.279995	1.31604441	1.2643692	0.836493782	-0.2575733	0.493226	1
ZNF677	1.7647654	1.80732228	1.7463188	0.924437295	-0.1133526	0.493393	1
TBX3	1.1627393	1.1278955	1.1778425	1.390529954	0.4756348	0.493531	1
HNRNPA1L2	1.7704846	1.72717149	1.7892588	1.085381994	0.1182029	0.493664	1
BIRC2	1.5891334	1.54894076	1.6065551	1.104955551	0.1439883	0.493685	1
ZNF436	1.2172809	1.2525162	1.202008	0.799980495	-0.3219633	0.493693	1
DHX8	1.1880269	1.15316812	1.2031366	1.326232654	0.4073339	0.493769	1
DIS3	1.3727612	1.33556401	1.3888846	1.158898363	0.212754	0.493837	1
IMMP2L	1.5005145	1.46182921	1.5172829	1.120073954	0.163594	0.493855	1
MAPKAPK2	1.2781363	1.24219982	1.2937132	1.212689513	0.2782102	0.493935	1
DPP10-AS1	1.0601292	1.02714881	1.0744247	2.741359774	1.4548917	0.493957	1
ASIP	1.1019192	1.13518691	1.0874991	0.647245645	-0.6276147	0.494151	1
MTO1	1.1225762	1.08853409	1.1373319	1.551175025	0.6333615	0.494153	1
MARK3	1.6911086	1.64980587	1.7090114	1.091112693	0.1258001	0.49428	1
CHL1	1.1127976	1.1464067	1.0982295	0.670935798	-0.5757534	0.4943	1
S1PR2	1.1440225	1.17783746	1.1293653	0.72743541	-0.4591089	0.494334	1
RP11-351I21.11	1.0602624	1.09305828	1.0460469	0.494817684	-1.015031	0.49445	1
ATAD2B	1.1860635	1.22057124	1.1711059	0.775740051	-0.3663548	0.494553	1
KAZALD1	1.0833819	1.05000059	1.0978512	1.957000771	0.9686443	0.49481	1
B3GNT8	1.0917704	1.0584618	1.1062082	1.816710823	0.8613288	0.494837	1
DLAT	1.2497565	1.21384037	1.2653246	1.240759954	0.311224	0.494998	1
AC007325.4	1.3141982	1.3503534	1.2985266	0.852072683	-0.2309516	0.495107	1
LENG8-AS1	1.0719111	1.10487533	1.0576226	0.549438668	-0.8639696	0.495234	1
WFS1	1.158347	1.12413072	1.1731783	1.395128455	0.480398	0.495421	1
CWF19L1	1.1255944	1.15920511	1.1110256	0.6973747	-0.5199941	0.49551	1
RABGGTA	1.3101929	1.27409117	1.3258414	1.188806661	0.2495141	0.495511	1
NOL8	1.3376393	1.3009643	1.3535363	1.174678633	0.2322661	0.495546	1
AARSD1	1.2201712	1.25504779	1.2050538	0.803981705	-0.3147654	0.495592	1
SLC9A3R2	1.1653316	1.13095503	1.1802324	1.376292177	0.4607868	0.495687	1
URM1	1.9866013	1.94018458	2.0067209	1.070769426	0.0986479	0.495709	1
MAP10	1.0817039	1.11463523	1.0674297	0.588210786	-0.7655949	0.495905	1
TMEM106A	1.0925664	1.12567538	1.0782151	0.622357894	-0.6841836	0.495991	1
RP11-111F5.4	1.1022833	1.13564464	1.0878226	0.647446408	-0.6271673	0.496042	1
TMCO6	1.175034	1.20936931	1.1601511	0.764921734	-0.386616	0.496147	1
LARP6	1.5489273	1.50971678	1.5659234	1.110270212	0.1509108	0.496181	1
C9orf85	1.2655731	1.30106784	1.2501877	0.831001244	-0.2670775	0.496335	1
HMGXB3	1.3204303	1.28413356	1.3361634	1.18311751	0.2425934	0.496568	1
AC104655.3	1.1411046	1.10705041	1.1558655	1.456001248	0.5420116	0.496634	1
PHF7	1.1084176	1.14180302	1.0939465	0.662514091	-0.593977	0.49676	1

XPNPEP1	1.7997164	1.75774867	1.8179075	1.079391548	0.1102183	0.497082	1
PHF6	3.1168271	3.05341578	3.1443131	1.044266419	0.0624898	0.497399	1
VEZF1	1.8897898	1.93262181	1.871224	0.934166448	-0.0982485	0.49748	1
TOR1AIP2	1.8690984	1.8246773	1.8883529	1.077212794	0.1073033	0.497558	1
CCDC57	1.2007503	1.23543911	1.1857143	0.788799585	-0.3422693	0.497666	1
FAM102B	1.1176903	1.1508071	1.1033357	0.685217532	-0.545366	0.497683	1
RASSF5	1.078344	1.11119303	1.0641054	0.576523141	-0.7945496	0.497735	1
WDCP	1.1094909	1.07595442	1.1240274	1.632919369	0.7074536	0.497773	1
YTHDC2	1.3284043	1.36508747	1.3125038	0.855969633	-0.2243685	0.497795	1
INTS8	1.4067242	1.44431362	1.3904308	0.878728021	-0.1865114	0.497804	1
IL17D	1.0686535	1.10124137	1.0545281	0.538594925	-0.8927275	0.497883	1
OTUB1	2.2426885	2.18988193	2.2655777	1.06361623	0.0889777	0.497981	1
ZDHHC18	1.1391541	1.17288864	1.1245317	0.720299843	-0.4733305	0.49842	1
RP1-179N16.6	1.0535068	1.08595044	1.0394439	0.458914895	-1.1237015	0.498567	1
PXDC1	1.1265284	1.09283745	1.1411319	1.520204725	0.6042656	0.498689	1
NT5C	2.3956092	2.34575234	2.41722	1.05310607	0.0746508	0.498702	1
EDEM1	1.1414903	1.1077131	1.1561312	1.449509836	0.5355651	0.498892	1
ZNF689	1.2205427	1.25549475	1.2053925	0.803901149	-0.31491	0.499235	1
ZNF200	1.1374898	1.17105017	1.1229428	0.71875299	-0.476432	0.499246	1
TOP2B	2.364573	2.31500325	2.3860592	1.05403482	0.0759225	0.49928	1
MAN1B1-AS1	1.1968038	1.23145038	1.181786	0.785421104	-0.3484617	0.499415	1
LINC01311	1.0708355	1.10346173	1.0566934	0.547965394	-0.8678433	0.499541	1
CHRNB1	1.4305111	1.39339496	1.4465992	1.135243946	0.1830023	0.499576	1
ARAP1	1.2492063	1.21367922	1.2646058	1.238331775	0.3083979	0.499676	1
ZNF500	1.190799	1.22503084	1.1759611	0.781942025	-0.3548664	0.499836	1
ZNF765	1.2018528	1.16700367	1.2169584	1.299123331	0.3775384	0.499884	1
PLA2G12A	2.1547964	2.20382487	2.1335448	0.941619364	-0.0867841	0.499886	1
EMC1	1.148394	1.18238819	1.133659	0.732827305	-0.4484548	0.499935	1
AP1AR	1.1432973	1.17673005	1.1288057	0.728827214	-0.4563513	0.500044	1
FAM210A	1.4958925	1.45779093	1.5124079	1.119305441	0.1626038	0.500094	1
SRD5A3	1.2012385	1.16666737	1.2162235	1.297335487	0.3755516	0.500119	1
OGG1	1.4371625	1.47455222	1.4209557	0.887058739	-0.1728985	0.500446	1
RNF145	2.2378208	2.18841781	2.2592347	1.059589262	0.0835051	0.500476	1
ANKRD18A	1.0894558	1.12227907	1.0752284	0.615218662	-0.7008288	0.500486	1
MIEF1	1.3497245	1.31330363	1.3655114	1.166636211	0.2223548	0.500503	1
ERICH1	1.7394262	1.69809507	1.7573414	1.084868525	0.1175202	0.500592	1
LA16c-431H6.6	1.0946392	1.12755158	1.0803731	0.630122484	-0.6662958	0.500641	1
B3GNT2	1.1040896	1.0710623	1.1184055	1.666221271	0.73658	0.501024	1
ANKRD6	1.1385663	1.1049398	1.153142	1.45933162	0.5453078	0.501035	1
MDM2	1.5660194	1.60517035	1.5490493	0.907263986	-0.1404057	0.501258	1
RBBP8	1.2444163	1.27933474	1.2292807	0.820809731	-0.2848803	0.50171	1
CRAT	1.356121	1.31960447	1.3719493	1.163779996	0.2188184	0.501901	1
LRP4-AS1	1.1088609	1.14177343	1.0945948	0.667225158	-0.5837544	0.5021	1
RBL1	1.1086977	1.14148145	1.0944874	0.667843282	-0.5824185	0.5021	1
SDAD1	1.5712097	1.53254029	1.5879712	1.104087714	0.1428548	0.502348	1
RP11-545E17.3	1.1476757	1.11410214	1.1622283	1.421781815	0.5077001	0.502385	1
CACNG8	1.1518104	1.18558495	1.1371707	0.739126032	-0.4361077	0.502396	1
STK36	1.1603899	1.19372569	1.1459403	0.753334731	-0.4086371	0.502573	1
DNASE1	1.3842611	1.42097658	1.3683466	0.87498119	-0.1926761	0.502614	1
MMACHC	1.0961115	1.06296144	1.1104805	1.754733226	0.8112517	0.502842	1
SLC35F6	1.3090713	1.27321438	1.3246137	1.188127983	0.2486902	0.502922	1

GOLT1B	1.3351976	1.29923121	1.3507874	1.172295468	0.2293362	0.502944	1
PPP2R5D	1.2089419	1.17449209	1.2238744	1.283005977	0.3595279	0.503045	1
RHOQ	1.4038313	1.36717929	1.4197184	1.143088379	0.192937	0.503061	1
ZMYM3	1.4646966	1.50223314	1.4484262	0.89286459	-0.1634867	0.503114	1
PAN3-AS1	1.0811569	1.11375762	1.0670259	0.589199574	-0.7631717	0.503135	1
HCFC1	1.5699558	1.60867484	1.5531728	0.90881497	-0.1379415	0.503246	1
RBM12	1.2361137	1.20122091	1.2512381	1.248568622	0.3202751	0.503254	1
GPBP1L1	1.7257773	1.76640056	1.708169	0.924019365	-0.114005	0.503314	1
PLK3	1.1862933	1.15228679	1.2010336	1.320098556	0.4006456	0.503433	1
TMEM251	1.4981792	1.53597465	1.4817966	0.8989168	-0.1537405	0.503484	1
ANAPC15	2.0149825	1.97077233	2.0341456	1.065281328	0.0912345	0.503596	1
GDF10	1.0648022	1.03246632	1.0788183	2.427695059	1.2795872	0.503775	1
PRRT3	1.2234319	1.25811192	1.2083996	0.807400156	-0.3086442	0.503813	1
AAR2	1.2443245	1.2094821	1.2594272	1.238421788	0.3085028	0.503847	1
PITPNC1	1.2041403	1.16995616	1.2189576	1.288318038	0.3654888	0.503887	1
GEMIN2	1.2688707	1.2334595	1.2842199	1.217426836	0.2838351	0.50399	1
EPHA2	1.2687172	1.23370193	1.2838948	1.214772864	0.2806866	0.50399	1
TPGS1	1.7205257	1.67962559	1.7382541	1.086265935	0.1193773	0.504087	1
MORC4	1.3660919	1.32964642	1.3818893	1.158481693	0.2122352	0.504107	1
EFNB3	1.2515847	1.28611258	1.2366184	0.827011686	-0.2740204	0.504207	1
RP11-586K2.1	1.0536382	1.08569068	1.0397449	0.463817928	-1.1083695	0.504287	1
MBD3	1.385027	1.42155117	1.3691953	0.875801979	-0.1913234	0.504376	1
THAP6	1.2945419	1.32953703	1.2793731	0.847774519	-0.2382475	0.5046	1
RP11-108L7.15	1.0640112	1.09602972	1.0501326	0.522053002	-0.9377318	0.504685	1
TSPOAP1	1.1567782	1.12306309	1.1713922	1.392718036	0.4779032	0.504817	1
MAN2B2	1.1191726	1.08626375	1.1334371	1.546850478	0.6293337	0.504866	1
SYT14	1.0662883	1.03408358	1.0802476	2.354437258	1.2353823	0.504892	1
IMMP1L	2.8643142	2.8007873	2.8918503	1.050568446	0.0711702	0.504904	1
UBE2W	1.4746874	1.43621498	1.4913635	1.126425194	0.1717515	0.505012	1
OSTF1	1.2038964	1.16986703	1.2186466	1.287163057	0.3641948	0.505033	1
EEF2K	1.2443295	1.27879894	1.2293886	0.822774225	-0.2814315	0.505118	1
KIF21B	1.0668777	1.03437251	1.0809673	2.355582427	1.2360838	0.505141	1
DUS1L	1.1537371	1.18696044	1.1393362	0.745270989	-0.424163	0.505177	1
FKBP10	2.8687981	2.9290475	2.8426827	0.955229288	-0.066081	0.505259	1
KLHL42	1.3565616	1.39270778	1.3408938	0.868059818	-0.2041336	0.505308	1
SPRY1	1.3992996	1.36239466	1.4152963	1.145978006	0.1965794	0.505357	1
DRC3	1.1155025	1.14834577	1.1012664	0.682637295	-0.5508089	0.505397	1
LINC00649	1.2411058	1.2754011	1.2262404	0.82149411	-0.2836779	0.505523	1
PDK4	1.1270026	1.15989474	1.1127454	0.705122451	-0.5040543	0.50571	1
TCEB3	1.289634	1.2544147	1.3049	1.198436943	0.261154	0.505747	1
TMEM178A	1.3433596	1.37962171	1.3276415	0.863073759	-0.2124442	0.505804	1
ZNF788	1.0929798	1.12543899	1.0789102	0.629072167	-0.6687026	0.505834	1
RNF182	1.2604006	1.29527304	1.245285	0.830705743	-0.2675906	0.506379	1
PORCN	1.1505634	1.11705116	1.1650894	1.4104041	0.4961086	0.506475	1
IRAK4	1.1254468	1.15832122	1.1111973	0.702352307	-0.5097332	0.506475	1
CH507-154B10.2	1.0543584	1.08616135	1.0405733	0.470898782	-1.0865111	0.506548	1
NPAS2	1.0818679	1.04956892	1.095868	1.934034526	0.9516135	0.506679	1
TRMT6	1.157449	1.12421718	1.1718535	1.383492114	0.4683144	0.506774	1
TTC13	1.0842871	1.11672521	1.0702266	0.601640214	-0.7330271	0.506793	1
LMO3	1.0590251	1.02694501	1.0729303	2.706635753	1.4365007	0.506882	1
EFCAB3	1.0395252	1.07104224	1.0258639	0.364063741	-1.457737	0.506974	1

ALG13	1.4018482	1.36531552	1.4176835	1.143350007	0.1932671	0.506979	1
AP4E1	1.0967867	1.12914625	1.0827602	0.640825544	-0.6419964	0.507026	1
RP11-452L6.1	1.1049025	1.13715962	1.0909204	0.662880431	-0.5931794	0.50716	1
COQ8B	1.452093	1.48931252	1.43596	0.890964386	-0.1665603	0.507163	1
LRTOMT	1.2047259	1.23849238	1.1900896	0.797046917	-0.3272634	0.507174	1
ST6GAL2	1.1184676	1.08550517	1.1327553	1.552599943	0.6346861	0.507184	1
CANT1	1.4439887	1.40666471	1.4601669	1.131563455	0.1783175	0.50733	1
LYRM2	2.1547327	2.20049851	2.1348953	0.945353371	-0.0810744	0.507515	1
SNRPD1	4.7945503	4.70124559	4.8349937	1.036135964	0.0512133	0.507927	1
FRMD8	1.1577038	1.12426638	1.1721975	1.385712638	0.4706281	0.508078	1
SEMA6C	1.1943082	1.22792151	1.1797383	0.788597459	-0.342639	0.508183	1
MRPS17	1.3696909	1.33376556	1.3852629	1.15429197	0.2070082	0.508209	1
ZNF24	1.7738158	1.81592158	1.7555649	0.926026351	-0.1108748	0.50821	1
MURC	1.1628913	1.19608603	1.1485029	0.757335348	-0.4009958	0.508437	1
CDC27	1.4592355	1.42204453	1.4753561	1.126317353	0.1716134	0.50855	1
COL7A1	1.11141	1.14384853	1.0973493	0.676748439	-0.5633084	0.508663	1
FMR1	1.4437224	1.40682506	1.4597157	1.130008345	0.1763334	0.509061	1
OLMALINC	1.1194007	1.15188639	1.1053196	0.693410628	-0.5282181	0.509185	1
VPS8	1.3681394	1.40407832	1.3525615	0.872507884	-0.1967599	0.50922	1
HSD3B7	1.15846	1.12514879	1.1728989	1.381546557	0.4662842	0.509259	1
STX12	1.4058551	1.36955059	1.4215915	1.140822187	0.1900739	0.509318	1
TEAD4	1.1037194	1.07124083	1.1177974	1.653509959	0.7255317	0.509613	1
METTL23	1.7942825	1.75222182	1.8125139	1.080152012	0.1112344	0.509622	1
LEFTY1	1.0885925	1.05626865	1.1026035	1.823457579	0.8666766	0.509627	1
FN3K	1.1917729	1.22550632	1.177151	0.785570077	-0.3481881	0.509748	1
TSPAN1	1.0522094	1.02059969	1.0659107	3.199598676	1.677891	0.509758	1
PCED1A	1.4332393	1.46951788	1.4175142	0.889240263	-0.1693548	0.5098	1
SNHG9	1.764889	1.80540422	1.7473275	0.92789121	-0.1079724	0.509877	1
LPAR1	1.1717842	1.20493368	1.1574154	0.768128547	-0.3805803	0.509882	1
PCDHB2	1.3223504	1.2867651	1.337775	1.177880442	0.2361931	0.509964	1
SUOX	1.1213933	1.15393558	1.1072877	0.696964619	-0.5208427	0.509972	1
ZNF586	1.1181248	1.08543256	1.1322954	1.548536213	0.6309051	0.510221	1
B4GALT6	1.131256	1.16385649	1.1171251	0.714803239	-0.4843819	0.510293	1
FBXO8	1.2206997	1.18672231	1.2354274	1.260842623	0.3343882	0.510388	1
PCDHA12	1.1165291	1.08397957	1.1306378	1.55559038	0.6374622	0.510473	1
ERI2	1.2071554	1.24047605	1.1927123	0.801378497	-0.3194443	0.510628	1
CAMK2N1	1.295566	1.26054102	1.3107478	1.192701878	0.2542335	0.510858	1
CAPN10-AS1	1.1941502	1.22730043	1.179781	0.79093995	-0.3383599	0.511037	1
LYRM9	1.3815299	1.41766501	1.365867	0.875981895	-0.191027	0.511108	1
RAB11FIP5	1.0699113	1.03797185	1.0837557	2.205731127	1.1412569	0.511153	1
LINC01116	1.1428593	1.17541463	1.128748	0.733963838	-0.4462191	0.51127	1
ASNS	1.3693677	1.33339256	1.3849614	1.154679013	0.2074919	0.511351	1
ALPK3	1.1042535	1.07181839	1.1183127	1.647387992	0.7201804	0.511367	1
MAOB	1.1070901	1.07471979	1.1211212	1.621005171	0.6968887	0.511495	1
CTC-425F1.4	1.1752921	1.14180751	1.1898062	1.338477875	0.4205933	0.511563	1
ALAD	1.2592027	1.22472007	1.2741494	1.219959659	0.2868334	0.511568	1
RSPO3	1.0789986	1.04707903	1.0928343	1.971881796	0.9795731	0.511613	1
ZNF311	1.0577393	1.08928759	1.0440646	0.493512659	-1.018841	0.511673	1
PRH1	1.1270998	1.15982677	1.1129142	0.70647841	-0.5012826	0.51179	1
ASAH2	1.0743404	1.10592531	1.0606497	0.572570612	-0.8044745	0.512379	1
PRR11	1.2105711	1.24392511	1.1961136	0.803990938	-0.3147489	0.512438	1

ANG	1.237845	1.27181134	1.2231222	0.820871401	-0.2847719	0.512515	1
CCDC74B	1.2223057	1.25613494	1.2076422	0.810675223	-0.302804	0.512561	1
TERF1	1.9704904	2.01332942	1.9519216	0.939399988	-0.0901885	0.512603	1
IARS	1.616011	1.6547937	1.5992005	0.915098097	-0.1280017	0.512834	1
CCDC149	1.17056	1.20358386	1.1562457	0.767475727	-0.381807	0.512878	1
ZCCHC2	1.1516459	1.11850249	1.1660121	1.400916547	0.486371	0.51309	1
FXR2	1.2014268	1.16796583	1.2159306	1.285562929	0.3624002	0.513133	1
PPM1E	1.1393452	1.17204763	1.1251702	0.727532361	-0.4589167	0.513143	1
GEMIN5	1.1053164	1.13754235	1.0913479	0.664144127	-0.5904317	0.513171	1
C9orf40	1.243716	1.27775801	1.2289602	0.824315559	-0.2787314	0.513213	1
SLC43A3	1.1375968	1.17041951	1.1233696	0.723917331	-0.4661031	0.51324	1
GREB1L	1.1217235	1.15387242	1.1077884	0.700505163	-0.5135324	0.513298	1
ZRANB3	1.1710556	1.203738	1.1568893	0.770054142	-0.3769682	0.513383	1
OPA1	1.4397988	1.40305501	1.4557256	1.130678357	0.1771886	0.513414	1
UBAP2	1.4158669	1.45199832	1.4002056	0.885413866	-0.1755761	0.513418	1
PREB	1.5923503	1.55413908	1.6089131	1.098845341	0.1359883	0.513426	1
PPP1R12B	1.1842196	1.21723343	1.1699096	0.782151962	-0.3544792	0.513528	1
ZNF552	1.1292287	1.1616964	1.1151554	0.712170733	-0.4897049	0.513597	1
PAFAH1B2	2.094348	2.04998103	2.1135791	1.0605707	0.0848408	0.513605	1
CAMSAP1	1.3468905	1.38245647	1.3314742	0.866697819	-0.206399	0.513672	1
DAPK2	1.0328117	1.06372713	1.0194112	0.304598504	-1.7150192	0.513807	1
ESPN	1.5022049	1.53909088	1.4862164	0.901919257	-0.1489298	0.513832	1
ZNF138	1.2688972	1.30310285	1.2540706	0.838232239	-0.2545781	0.514071	1
AC004556.1	1.6485323	1.60904032	1.6656503	1.09294956	0.1282268	0.51421	1
RGMA	1.1292036	1.09685197	1.1432266	1.478820207	0.5644467	0.514241	1
LETMD1	1.7545878	1.71405521	1.7721569	1.081368625	0.1128584	0.514243	1
ZNF667-AS1	1.5321399	1.56945971	1.5159634	0.906057853	-0.1423249	0.514334	1
MAPKAP1	1.6853558	1.64656409	1.7021703	1.086002707	0.1190277	0.514511	1
COG8	1.2002033	1.23342551	1.1858029	0.795983782	-0.3291891	0.514528	1
SPTAN1	1.6675194	1.62900197	1.684215	1.087778716	0.1213851	0.5147	1
PLEKHO1	1.8994672	1.94065999	1.8816119	0.937226969	-0.0935296	0.514762	1
CTPS2	1.3959259	1.35972682	1.4116167	1.144247892	0.1943996	0.514843	1
GPR155	1.2780917	1.31236839	1.2632343	0.842704746	-0.2469008	0.514843	1
IL4R	1.1008948	1.06890745	1.1147598	1.66541965	0.7358858	0.514849	1
NDUFAF1	1.1811948	1.14833331	1.1954387	1.317564604	0.3978737	0.514877	1
TOP1MT	1.2925434	1.25804168	1.3074984	1.191661574	0.2529746	0.515006	1
ZCCHC9	1.3474002	1.31203738	1.3627284	1.162451621	0.2171707	0.515099	1
KHDC1	1.3970909	1.4321761	1.3818831	0.883628373	-0.1784884	0.515491	1
MED20	1.1929454	1.15949796	1.2074434	1.300601942	0.3791795	0.515586	1
SMARCD1	1.8135361	1.85398499	1.7960033	0.932104529	-0.1014363	0.515598	1
FAM76B	1.2951973	1.32957002	1.2802983	0.850496873	-0.2336222	0.515607	1
ZNF74	1.2427226	1.2760967	1.2282564	0.826726307	-0.2745183	0.515802	1
RP11-212P7.2	1.1489213	1.18135661	1.134862	0.743628666	-0.4273457	0.515816	1
SCGB3A1	1.1129608	1.14479143	1.0991637	0.684872424	-0.5460928	0.515835	1
RP11-55K13.1	1.0411642	1.07208372	1.027762	0.385135769	-1.376561	0.515869	1
RFWD2	1.3821748	1.3468414	1.3974902	1.146028753	0.1966432	0.515888	1
FLYWCH2	1.6512456	1.68963669	1.6346048	0.920201648	-0.1199781	0.516337	1
LINC00672	1.2739756	1.30809558	1.259186	0.841252061	-0.24939	0.5164	1
RPIA	1.5903458	1.55201703	1.6069596	1.099530582	0.1368877	0.516425	1
MFNG	1.098295	1.06606929	1.1122634	1.699176905	0.7648361	0.51645	1
IGFBPL1	1.0811312	1.11263589	1.0674753	0.599056866	-0.7392351	0.516482	1

MEG3	1.0545101	1.08571308	1.040985	0.478165091	-1.0644193	0.516501	1
BHLHE41	1.2187564	1.18492672	1.23342	1.262229778	0.3359746	0.516511	1
BTBD19	1.0837904	1.11540439	1.0700871	0.607317299	-0.7194776	0.516554	1
ST8SIA4	1.0890333	1.12069297	1.0753102	0.623981913	-0.6804239	0.516576	1
CENPBD1	1.1800684	1.14702894	1.1943895	1.32211748	0.4028504	0.5167	1
SLC25A25	1.0902059	1.05840914	1.1039883	1.780343592	0.8321557	0.516703	1
C4orf36	1.0842719	1.11572801	1.070637	0.610371091	-0.7122415	0.516712	1
KDM4C	1.1266286	1.15869665	1.1127285	0.710339816	-0.4934187	0.516758	1
WDR70	1.3920696	1.35635867	1.4075487	1.143647565	0.1936425	0.516822	1
LYRM1	1.7854777	1.74455543	1.8032156	1.078785523	0.1094081	0.516841	1
ARFRP1	1.3859344	1.35034857	1.4013592	1.145599728	0.1961031	0.517143	1
HERC2	1.862218	1.90355603	1.8442998	0.934418896	-0.0978586	0.517364	1
TAF9B	1.1615235	1.12897122	1.1756335	1.36180377	0.4455188	0.517445	1
PDZD4	1.0731034	1.10431043	1.0595765	0.571146218	-0.808068	0.517484	1
RP11-798L4.1	1.0516444	1.0825346	1.0382549	0.463501224	-1.1093549	0.517608	1
HS1BP3	1.2203192	1.18694211	1.2347866	1.255932194	0.3287586	0.517693	1
DPM2	2.2084083	2.16014605	2.2293278	1.059631953	0.0835633	0.517935	1
CH17-189H20.1	1.5144592	1.4772323	1.5305954	1.111817813	0.1529204	0.51819	1
B3GAT2	1.4108955	1.4464098	1.3955016	0.885960751	-0.1746853	0.518288	1
SLC12A6	1.1514333	1.1836631	1.1374631	0.748452491	-0.4180174	0.518324	1
ETS2	1.1074599	1.07558287	1.1212772	1.604559622	0.6821774	0.518356	1
SHISA4	1.4889898	1.45222643	1.504925	1.11653147	0.1590239	0.518389	1
SF1	2.4738628	2.52389628	2.4521755	0.952935927	-0.0695489	0.518398	1
HIPK1	1.3571784	1.39195856	1.3421027	0.872803256	-0.1962716	0.518466	1
UNC13D	1.0902044	1.05832893	1.104021	1.783352399	0.8345918	0.518534	1
PLBD2	1.116569	1.08475164	1.1303604	1.538146221	0.6211927	0.518662	1
RAB35	1.1850412	1.15214823	1.1992988	1.309899003	0.3894556	0.518699	1
USP9X	1.7613978	1.72148528	1.7786981	1.07929864	0.1100941	0.518764	1
NPHP4	1.093833	1.12525274	1.080214	0.640416904	-0.6429167	0.518794	1
ADAM23	1.0729647	1.10435324	1.0593592	0.568829426	-0.813932	0.518841	1
CEP57L1	1.3533146	1.38840382	1.338105	0.870498582	-0.2000861	0.518844	1
ZZEF1	1.1438798	1.17628171	1.1298351	0.736520345	-0.4412027	0.518916	1
MAFF	1.1476882	1.11554442	1.1616212	1.398779377	0.4841684	0.518925	1
ALG1L2	1.1274123	1.15930936	1.1135864	0.712992483	-0.4880412	0.519028	1
GOLGA6L4	1.1015903	1.13324184	1.0878707	0.65948285	-0.600593	0.519179	1
RP11-343J3.2	1.0680253	1.09902056	1.0545902	0.551301712	-0.859086	0.51929	1
MIPOL1	1.2389966	1.2722554	1.2245804	0.824888558	-0.2777289	0.519293	1
HMGN3-AS1	1.0508028	1.08161131	1.0374486	0.458865916	-1.1238554	0.519392	1
NAP1L4	2.3565245	2.30844312	2.3773656	1.052675194	0.0740604	0.519419	1
EDNRB	1.0656046	1.03428852	1.0791787	2.309189988	1.2073869	0.519435	1
ANKZF1	1.5901136	1.62762141	1.5738556	0.914334001	-0.1292068	0.519464	1
RAVER2	1.1210216	1.15283182	1.1072333	0.701642831	-0.5111913	0.519494	1
MIF4GD	1.6966372	1.65738936	1.7136493	1.085580913	0.1184673	0.519522	1
RBM27	1.1945034	1.22734653	1.1802673	0.792918618	-0.3347553	0.519533	1
FGF13-AS1	1.0519	1.08265409	1.0385694	0.466636686	-1.0996284	0.519768	1
PRKCB	1.0716718	1.04024509	1.0852938	2.119359219	1.0836281	0.519926	1
ZNF253	1.2506916	1.28416281	1.2361833	0.831154744	-0.266811	0.520048	1
CMTR2	1.5062719	1.54272863	1.4904695	0.903710422	-0.1460675	0.520079	1
ATP6V0A1	1.5067516	1.54315824	1.490971	0.903918844	-0.1457348	0.520079	1
RIOK2	1.2399629	1.20639703	1.2545121	1.233119222	0.3023123	0.520428	1
RP11-498P14.5	1.1239179	1.15560852	1.1101815	0.708068396	-0.4980394	0.520523	1

FAM65C	1.0611847	1.09211724	1.0477769	0.518652697	-0.9471593	0.520673	1
PACRGL	1.2171731	1.25017961	1.2028663	0.810882445	-0.3024353	0.520729	1
RP11-637O19.2	1.0678737	1.09906396	1.0543541	0.548676508	-0.8659723	0.520741	1
ASIC4	1.0929618	1.06138134	1.1066506	1.737508252	0.7970198	0.520939	1
EPS8L2	1.5759287	1.5385159	1.5921454	1.099587641	0.1369626	0.520966	1
ARAP3	1.1199912	1.15165003	1.1062686	0.700748678	-0.513031	0.521085	1
PTPRK	1.3557001	1.32106975	1.3707109	1.154611623	0.2074077	0.521774	1
IQSEC1	1.3685706	1.333459	1.3837899	1.150935733	0.2028073	0.521959	1
PCSK5	1.0776466	1.04633665	1.091218	1.968593627	0.9771653	0.521989	1
GSTCD	1.2343852	1.26748488	1.220038	0.822618415	-0.2817047	0.52202	1
SGK3	1.0899944	1.12129036	1.0764289	0.630131975	-0.6662741	0.52206	1
CTNND2	1.3182137	1.35234139	1.3034208	0.861155843	-0.2156538	0.522177	1
METAP1D	1.2349601	1.2679646	1.2206542	0.823445205	-0.2802554	0.522183	1
RP11-1042B17.3	1.1607118	1.19289245	1.1467629	0.760853396	-0.3943096	0.52228	1
BRI3BP	1.1974217	1.22967007	1.1834435	0.798726048	-0.3242273	0.522651	1
SRPRA	1.3069904	1.27306764	1.3216944	1.178075758	0.2364323	0.522779	1
INTS5	1.1334911	1.10170674	1.1472682	1.447969232	0.5340309	0.522781	1
MYO1D	1.0924861	1.06100821	1.1061303	1.739607412	0.7987618	0.522812	1
LRRC6	1.0892946	1.12040692	1.0758088	0.629605077	-0.6674809	0.522847	1
PKNOX1	1.257996	1.22446968	1.2725281	1.214097627	0.2798844	0.522859	1
ZNF75A	1.4941163	1.53007523	1.4785298	0.902758202	-0.1475885	0.52293	1
NAPRT	1.1740497	1.14160797	1.1881117	1.328397724	0.4096872	0.523017	1
RP11-631N16.2	1.087351	1.11861996	1.0737973	0.622132411	-0.6847064	0.523086	1
MFSD14B	1.2654651	1.29917436	1.2508537	0.838486658	-0.2541403	0.523271	1
LRRC32	1.0521592	1.02135183	1.0655129	3.068255467	1.6174186	0.523274	1
CRYGC	1.0515501	1.02089516	1.0648377	3.102999621	1.6336635	0.523425	1
SMURF1	1.1101345	1.07855694	1.123822	1.576206756	0.6564568	0.523491	1
CCDC86	1.1993803	1.1667475	1.2135252	1.280530039	0.3567411	0.523582	1
ALMS1	1.2985511	1.33201707	1.2840452	0.855513698	-0.2251371	0.523704	1
SNRPA	2.1429529	2.18765605	2.123576	0.946044959	-0.0800193	0.523713	1
FAM129B	1.1898283	1.15734098	1.2039101	1.295975522	0.3740385	0.523991	1
SON	4.027011	3.94619114	4.0620429	1.039322544	0.0556435	0.523996	1
DPYD	1.0981834	1.12920883	1.0847353	0.655800925	-0.6086702	0.524141	1
SYS1	1.4763654	1.44031279	1.4919927	1.117370792	0.160108	0.524178	1
ARL14EP	1.6966786	1.65796215	1.7134605	1.08434889	0.116829	0.524356	1
CHAF1B	1.1334855	1.16513585	1.1197664	0.725260101	-0.4634296	0.524392	1
PCDH17	1.1010117	1.06959413	1.1146298	1.647118706	0.7199445	0.524673	1
BAG4	1.3134079	1.27927855	1.3282015	1.175176108	0.232877	0.524699	1
SCOC-AS1	1.1121948	1.08051814	1.1259252	1.563935199	0.6451807	0.524759	1
GCLM	1.2839726	1.25025355	1.2985883	1.193143294	0.2547673	0.524779	1
CATIP	1.0374004	1.06766065	1.0242839	0.358907409	-1.4783164	0.524807	1
PPFIBP1	1.6459589	1.60814168	1.6623509	1.089139198	0.1231883	0.524843	1
SLC35F5	1.3078026	1.27382587	1.3225299	1.177865137	0.2361744	0.525269	1
ARHGAP33	1.1642924	1.19622809	1.1504497	0.766708413	-0.3832501	0.52534	1
RFTN1	1.0648243	1.03403126	1.0781717	2.297056105	1.1997861	0.525673	1
SUPT7L	1.3086019	1.27486826	1.323224	1.175923275	0.2337939	0.525714	1
SRGAP2C	1.3406373	1.37496105	1.3257595	0.86878219	-0.2029336	0.525763	1
PER1	1.1289239	1.16023818	1.1153506	0.719869317	-0.4741931	0.525866	1
EPS15	1.4060388	1.37112186	1.4211738	1.13486651	0.1825226	0.525889	1
TRPM4	1.1538079	1.12169936	1.1677255	1.37819516	0.4627802	0.525894	1
BRCC3	1.2488486	1.21572844	1.2632047	1.220074007	0.2869687	0.525955	1

NOC3L	1.3800843	1.34543802	1.395102	1.143770919	0.1937981	0.52621	1
KIF9	1.4296183	1.39417526	1.4449813	1.128892071	0.1749076	0.526216	1
SIDT2	1.2048574	1.23729817	1.1907958	0.80403405	-0.3146715	0.52652	1
LARS2	1.0973032	1.12824924	1.0838895	0.654113224	-0.6123877	0.526704	1
SSBP2	2.5494745	2.59925714	2.527896	0.955378567	-0.0658556	0.526944	1
PSMB10	1.2434213	1.21031181	1.2577728	1.225669491	0.29357	0.527009	1
BTG2	1.9336609	1.97462345	1.9159054	0.939753107	-0.0896463	0.527141	1
CHST1	1.0983312	1.12916643	1.0849655	0.657798677	-0.604282	0.527272	1
NBPF12	1.1488259	1.18034566	1.1351635	0.749468839	-0.4160596	0.527291	1
LINC01420	5.1591704	5.0525547	5.2053836	1.037711747	0.0534058	0.527357	1
SSH3	1.1957069	1.16338855	1.2097154	1.283537942	0.3601259	0.52741	1
POMT2	1.1801588	1.21217782	1.16628	0.783682351	-0.3516591	0.527747	1
ZP1	1.0640848	1.094445	1.050925	0.539202482	-0.891101	0.527821	1
RP11-297D21.4	1.137738	1.16940766	1.1240107	0.732025223	-0.4500347	0.52819	1
RAB20	1.120932	1.08936504	1.1346149	1.506348684	0.5910558	0.528214	1
CH507-9B2.5	1.0750099	1.10549959	1.061794	0.585727192	-0.7716992	0.528346	1
KIFC3	1.2605231	1.22729934	1.2749242	1.209524717	0.2744403	0.52838	1
BBS2	1.2695643	1.23591055	1.2841517	1.204489112	0.2684214	0.528519	1
CLASRP	1.2921542	1.3253498	1.2777654	0.853743989	-0.2281246	0.528635	1
SP3	1.5555369	1.59211356	1.5396825	0.911451062	-0.1337629	0.528689	1
TIGD5	1.0934375	1.1242089	1.0800995	0.644877362	-0.6329033	0.528737	1
C11orf68	1.1881394	1.15576481	1.2021724	1.29793383	0.3762168	0.528766	1
RP11-513M16.7	1.063657	1.09393052	1.0505348	0.538002108	-0.8943163	0.529163	1
RP11-505K9.1	1.1274045	1.15847284	1.1139377	0.718972968	-0.4759906	0.529294	1
EDNRA	1.0808393	1.04994273	1.0942316	1.886792606	0.9159359	0.529419	1
UPF1	1.1650561	1.19702156	1.1512004	0.767430918	-0.3818912	0.529533	1
KBTBD4	1.1941491	1.16215199	1.2080185	1.282860946	0.3593648	0.529561	1
EMB	1.080364	1.04957584	1.0937093	1.890220301	0.9185544	0.529618	1
ZNF554	1.0742447	1.04350017	1.0875711	2.01312103	1.0094339	0.529733	1
SULT1C4	1.4741684	1.50994969	1.4586588	0.899419721	-0.1529336	0.529739	1
MMD	1.8002039	1.83910126	1.7833437	0.933550809	-0.0991996	0.529839	1
RAB30	1.2207763	1.18813091	1.2349266	1.24874033	0.3204735	0.529887	1
RNF212	1.1843373	1.15195329	1.1983744	1.305495829	0.3845978	0.530191	1
TFEB	1.0893492	1.05839314	1.1027672	1.759919105	0.8155091	0.530309	1
MDH1B	1.0752158	1.1056091	1.0620417	0.587465702	-0.7674235	0.5305	1
CTD-3222D19.8	1.0710518	1.10140327	1.0578958	0.570946549	-0.8085724	0.530554	1
ADD1	1.7665199	1.72774771	1.7833259	1.076370087	0.1061742	0.530595	1
DUSP14	1.4012335	1.36641324	1.4163265	1.136221231	0.1842438	0.530691	1
VLDLR-AS1	1.112509	1.1433551	1.0991386	0.691559893	-0.5320739	0.530792	1
LYAR	1.3115307	1.27772518	1.3261839	1.174484463	0.2320276	0.531023	1
RP11-326C3.11	1.1409942	1.17224135	1.1274499	0.739949665	-0.434501	0.531026	1
FLVCR1-AS1	1.0906731	1.12125785	1.077416	0.638441232	-0.6473743	0.531057	1
CCDC127	1.3208677	1.28706185	1.335521	1.168810714	0.2250413	0.531058	1
SATB1	1.2401026	1.27270102	1.2259727	0.82864629	-0.2711717	0.53123	1
CHST14	1.2285617	1.19601668	1.2426686	1.237999626	0.3080109	0.531542	1
DCLK2	1.1551935	1.18631264	1.1417048	0.760575325	-0.394837	0.531786	1
TNNI3	1.5225907	1.55823728	1.5071395	0.908465828	-0.1384958	0.531804	1
LINC00618	1.0448277	1.07465542	1.0318987	0.427278816	-1.2267503	0.531985	1
TCEANC2	1.1983688	1.23036859	1.1844983	0.800882912	-0.3203368	0.531986	1
RP11-596C23.2	1.0794223	1.10981551	1.0662482	0.60326824	-0.7291285	0.531988	1
RP11-432J24.5	1.082791	1.11321717	1.0696027	0.614771191	-0.7018785	0.532172	1

OGFR	1.3940806	1.35938905	1.4091178	1.138370231	0.1869698	0.53222	1
PIBF1	1.3838069	1.41779426	1.3690749	0.883389083	-0.1788791	0.532342	1
WDR7	1.1133273	1.14399094	1.100036	0.694738286	-0.5254585	0.53239	1
RIBC2	1.0632587	1.09333197	1.0502233	0.538114236	-0.8940156	0.532401	1
RP11-469H8.6	1.0899537	1.12036386	1.0767722	0.637834304	-0.6487464	0.532644	1
FAM188B	1.0898469	1.12026638	1.0766615	0.637430596	-0.6496598	0.532644	1
RBCK1	1.7897549	1.75089079	1.8066007	1.074191808	0.1032516	0.53273	1
FAM117B	1.1864705	1.21816641	1.1727318	0.791743196	-0.3368955	0.53274	1
HMG20A	1.2821689	1.31520026	1.2678513	0.849781342	-0.2348364	0.53275	1
RPRD1A	1.7173911	1.75538058	1.7009243	0.927908836	-0.107945	0.53283	1
GAS2L1	1.1302448	1.099006	1.1437855	1.452290373	0.5383299	0.53288	1
AASDH	1.4599934	1.49513566	1.4447608	0.89826041	-0.1547943	0.532885	1
OAS1	1.0520879	1.08207606	1.0390894	0.476257707	-1.0701857	0.533202	1
ETV3	1.1442335	1.17536035	1.1307414	0.745558745	-0.4236061	0.533203	1
NOL9	1.1777695	1.20910992	1.1641848	0.785160069	-0.3489413	0.533231	1
ACTR8	1.1486703	1.11727896	1.162277	1.38368381	0.4685143	0.533287	1
USP5	1.3853411	1.41948504	1.3705412	0.883324002	-0.1789854	0.533384	1
ZNF687	1.1106863	1.1413471	1.0973963	0.689057489	-0.5373037	0.533438	1
CTD-3193K9.4	1.1465432	1.17774497	1.1330187	0.74836816	-0.4181799	0.533503	1
CSK	1.2153833	1.18309294	1.2293798	1.25280514	0.325162	0.533665	1
WNT10A	1.0473797	1.01740877	1.0603708	3.467837644	1.7940364	0.533866	1
MBTPS2	1.2082838	1.17630222	1.2221464	1.260032072	0.3334605	0.533944	1
TBC1D31	1.1420961	1.17336215	1.1285436	0.741474608	-0.4315308	0.534038	1
RP11-93H24.3	1.155629	1.18673566	1.1421456	0.761213132	-0.3936276	0.534098	1
CCDC157	1.2177184	1.24949796	1.2039434	0.817415265	-0.2908589	0.534115	1
CUL4B	1.3630852	1.39675289	1.3484917	0.878359567	-0.1871165	0.534215	1
NOL10	1.2453235	1.21309726	1.2592921	1.216778229	0.2830662	0.534247	1
MAP2K6	1.214638	1.18256588	1.2285398	1.25182101	0.3240283	0.534263	1
NLK	1.1866752	1.15481631	1.2004847	1.294984163	0.3729345	0.534287	1
VIPAS39	1.2393238	1.20688726	1.2533836	1.224742642	0.2924786	0.534308	1
HDAC4	1.1087295	1.13908349	1.0955724	0.687158296	-0.5412856	0.534401	1
SIX5	1.3206158	1.35398891	1.3061501	0.864857803	-0.2094651	0.534494	1
STK35	1.1729832	1.14139503	1.1866753	1.320239569	0.4007997	0.534609	1
RALGAPB	1.2975639	1.33061436	1.2832381	0.856702237	-0.2231342	0.534635	1
PYM1	1.3964565	1.36215022	1.4113268	1.135790427	0.1836967	0.534855	1
CLEC18C	1.0489915	1.07863294	1.0361433	0.45964522	-1.1214074	0.535112	1
POLG2	1.1956587	1.22752562	1.1818459	0.799232561	-0.3233127	0.535145	1
RP11-708J19.1	1.1241622	1.15486667	1.1108532	0.71579793	-0.4823757	0.53525	1
LINC01023	1.1430537	1.17393064	1.1296699	0.745526471	-0.4236685	0.535332	1
CREB3L4	1.4054882	1.37061424	1.4206045	1.134885014	0.1825461	0.535388	1
CLUAP1	1.5426995	1.5070027	1.5581725	1.10092602	0.1387175	0.535398	1
PIP4K2C	1.1015226	1.07101992	1.1147441	1.615661311	0.6921248	0.535488	1
ZNF334	1.278537	1.31130865	1.264332	0.849099357	-0.2359947	0.535565	1
ZNF568	1.1081864	1.13857312	1.0950151	0.685667424	-0.5444191	0.535744	1
SLC41A1	1.2568997	1.28945402	1.2427889	0.838782233	-0.2536318	0.535792	1
TSPAN7	1.1026807	1.07194005	1.1160054	1.612528378	0.6893245	0.5359	1
CTD-3001H11.2	1.1085931	1.13893655	1.0954406	0.686936768	-0.5417508	0.535901	1
GPR19	1.046098	1.07570712	1.0332637	0.439373867	-1.186479	0.53593	1
C12orf4	1.1505889	1.18169409	1.1371062	0.754599089	-0.4062177	0.536099	1
CYP24A1	1.0465818	1.01683833	1.0594742	3.532072077	1.8205148	0.536173	1
ZNF771	1.3516955	1.38556713	1.3370136	0.874072557	-0.1941751	0.53628	1

POLA2	1.1505358	1.18145296	1.1371347	0.755758679	-0.4040025	0.536307	1
ATP11B	1.2014784	1.23315287	1.187749	0.805261262	-0.3124712	0.536524	1
PTPDC1	1.1821436	1.21346216	1.1685684	0.789687693	-0.3406459	0.536567	1
RP11-421L21.3	1.3105482	1.34376602	1.2961497	0.861486334	-0.2151002	0.536664	1
TRIM25	1.2173671	1.24925895	1.2035435	0.816594389	-0.2923084	0.536729	1
ORC1	1.0584625	1.08821674	1.0455654	0.516516196	-0.9531145	0.536759	1
RPP30	1.430868	1.39625806	1.4458699	1.125200755	0.1701824	0.536945	1
B3GNT9	1.1186953	1.08804108	1.1319826	1.49910219	0.5840987	0.537028	1
SENP5	1.5636209	1.59952235	1.5480593	0.914159851	-0.1294816	0.53707	1
MTA2	1.1349487	1.10370668	1.1484907	1.431833899	0.5178641	0.537432	1
GMDS-AS1	1.1326589	1.16328008	1.119386	0.73117338	-0.4517145	0.537611	1
PLEKHA4	1.7776424	1.73941138	1.7942138	1.074116281	0.1031502	0.537634	1
SUN1	1.7741391	1.81231955	1.7575896	0.932625042	-0.1006309	0.537652	1
DNAL4	1.4661721	1.50111819	1.4510245	0.900036134	-0.1519452	0.537684	1
GNG3	1.4561142	1.42082567	1.4714102	1.12020302	0.1637602	0.53769	1
FAM65B	1.036991	1.06629054	1.024291	0.366432435	-1.4483809	0.538052	1
CAMSAP2	1.5044286	1.4690193	1.519777	1.108220852	0.1482454	0.538087	1
SERINC5	1.3245378	1.35753956	1.310233	0.867688485	-0.2047509	0.538162	1
ACTR1B	2.2168647	2.2600784	2.1981336	0.950840481	-0.0727248	0.538197	1
FBXW11	1.6682671	1.70512797	1.6522895	0.925065463	-0.1123726	0.538206	1
ZFYVE27	1.258072	1.29038194	1.244067	0.840503452	-0.2506744	0.538211	1
FAM175A	1.8201942	1.78059667	1.837358	1.072715298	0.1012672	0.538344	1
FAM220A	1.189335	1.15789238	1.2029639	1.285457282	0.3622817	0.538388	1
SLK	1.449514	1.41512489	1.4644202	1.11874811	0.1618852	0.538424	1
MMRN1	1.2374573	1.269282	1.2236627	0.83058929	-0.2677928	0.53846	1
FAM160B1	1.2080792	1.17624453	1.2218781	1.258921685	0.3321885	0.538515	1
HECTD3	1.1193609	1.08891427	1.1325582	1.490853961	0.5761389	0.538552	1
PIGX	2.1500708	2.19455686	2.130788	0.946617167	-0.079147	0.538622	1
C14orf37	1.2482021	1.28091964	1.2340205	0.833051563	-0.2635223	0.538626	1
KIF2C	1.219488	1.18744929	1.2333754	1.245005238	0.3161518	0.538634	1
LRP1B	1.0743751	1.10424566	1.0614276	0.589257848	-0.763029	0.538652	1
CTD-2371O3.3	1.1503331	1.18109395	1.1369996	0.756511273	-0.4025665	0.538734	1
UBA5	1.4065938	1.37272893	1.4212727	1.130238875	0.1766277	0.538854	1
EMX1	1.0860892	1.11613633	1.0730651	0.629131929	-0.6685655	0.538857	1
PCF11	1.6702413	1.70677656	1.6544049	0.925900696	-0.1110706	0.538913	1
RALGDS	1.4171735	1.4509645	1.4025266	0.892590422	-0.1639298	0.539003	1
GABRA2	1.1054502	1.13561122	1.0923768	0.681188495	-0.553874	0.539046	1
THBS1	1.1023259	1.07201088	1.1154661	1.603453462	0.6811825	0.539057	1
RBM4B	1.4924825	1.52734563	1.4773709	0.905233418	-0.1436383	0.539092	1
RP11-480A16.1	1.1006719	1.13060856	1.0876957	0.671439296	-0.5746711	0.539175	1
TFPT	1.2459921	1.21381694	1.2599386	1.215706275	0.2817947	0.539472	1
NBPF14	1.2769578	1.30916403	1.2629978	0.850673999	-0.2333217	0.539559	1
LINC00937	1.0629972	1.09264466	1.0501463	0.541275832	-0.8855641	0.539621	1
MYB	1.0422664	1.0715249	1.0295842	0.413621136	-1.2736182	0.539722	1
TBC1D17	1.2684144	1.23592862	1.2824955	1.19737715	0.2598776	0.53974	1
ARHGEF11	1.0981082	1.12817123	1.0850773	0.66377816	-0.5912269	0.539906	1
SYDE2	1.1470164	1.17796868	1.1336	0.750693901	-0.4137033	0.539986	1
CPSF3L	1.5046315	1.46974485	1.5197533	1.106458741	0.1459497	0.540025	1
AC074117.10	1.1831775	1.21411378	1.169768	0.792886964	-0.3348129	0.540082	1
G0S2	1.0799662	1.04992307	1.0929886	1.862638243	0.8973475	0.540291	1
CCDC184	1.080052	1.0500131	1.0930725	1.860962463	0.896049	0.540291	1

TARS	1.7073698	1.67065802	1.7232827	1.078467187	0.1089823	0.540587	1
CLN6	1.120411	1.15067687	1.1072921	0.712067754	-0.4899136	0.54061	1
ZHX3	1.1294296	1.09860212	1.142792	1.448163154	0.5342241	0.540729	1
MAPK14	1.3163965	1.2835122	1.3306504	1.16626523	0.2218959	0.54076	1
DENND5B	1.3278352	1.3611099	1.3134121	0.867913241	-0.2043773	0.5408	1
TRIP13	1.1934807	1.22466677	1.1799629	0.801021579	-0.320087	0.540851	1
RASGRF2	1.1138525	1.08332406	1.1270852	1.52519253	0.6089914	0.540935	1
PYCR2	1.6604809	1.62411492	1.6762439	1.083524651	0.115732	0.540955	1
NBPF3	1.1053983	1.07505507	1.1185507	1.579516737	0.6594832	0.541038	1
FUT11	1.5710372	1.60589783	1.5559266	0.917525322	-0.1241801	0.541142	1
SPDL1	1.4663726	1.43202512	1.4812607	1.113964722	0.1557035	0.54158	1
RNF157	1.1953117	1.22634564	1.1818599	0.803461092	-0.3156999	0.541621	1
B3GAT1	1.0669854	1.09643087	1.0542221	0.562290243	-0.8306131	0.541756	1
SUN2	1.2283949	1.19681289	1.2420842	1.230022235	0.2986844	0.541962	1
CDC25C	1.1241446	1.15437366	1.1110417	0.719304506	-0.4753255	0.54198	1
STRA6	1.9777998	1.93731855	1.9953466	1.061908524	0.0866595	0.542064	1
KIF27	1.2193583	1.25067869	1.2057824	0.820900961	-0.2847199	0.542081	1
RC3H2	1.3151389	1.34778666	1.3009876	0.865437492	-0.2084985	0.542102	1
ORAI2	1.862883	1.90134982	1.8462094	0.938824623	-0.0910724	0.542277	1
MOCS3	1.0894816	1.0596165	1.1024267	1.718093693	0.7808087	0.542516	1
PEAK1	1.3066402	1.3387168	1.2927364	0.864251305	-0.2104772	0.542711	1
SLC38A7	1.1455482	1.11492043	1.1588239	1.382033786	0.4667929	0.542905	1
PEX14	1.21442	1.1830989	1.2279963	1.245208694	0.3163876	0.542908	1
FAM49A	1.1007273	1.07063257	1.113772	1.610758693	0.6877404	0.542992	1
ZNF93	1.2120131	1.24318705	1.1985006	0.816246521	-0.2929232	0.543041	1
CCDC71	1.2623559	1.23008909	1.2763421	1.201022163	0.2642628	0.543051	1
DKKL1	1.1483356	1.11749583	1.1617033	1.376247086	0.4607395	0.543061	1
PLA2G15	1.1720792	1.14154489	1.1853145	1.309227706	0.388716	0.543238	1
DIP2C	1.2770815	1.24474326	1.2910987	1.189404422	0.2502393	0.543281	1
SLC16A14	1.1123078	1.14240621	1.0992615	0.697030966	-0.5207053	0.543322	1
DESI1	1.384388	1.35096813	1.398874	1.136496323	0.184593	0.543395	1
RECQL	1.4470168	1.48125282	1.432177	0.898024891	-0.1551727	0.543492	1
SDHAF4	1.3110598	1.27825177	1.3252807	1.169015707	0.2252943	0.543509	1
MCF2L	1.1916515	1.22286363	1.1781224	0.799243867	-0.3232923	0.543571	1
SPTBN2	1.1737395	1.14282667	1.1871389	1.310251536	0.3898438	0.543592	1
ST18	1.0475596	1.01804205	1.0603541	3.345190052	1.7420882	0.543624	1
FAM200B	2.7007886	2.64801284	2.7236645	1.045904765	0.0647515	0.543695	1
SNTA1	1.1321596	1.10175124	1.1453402	1.428387672	0.5143876	0.543869	1
ING4	1.9442703	1.90493428	1.9613207	1.062309955	0.0872048	0.54391	1
STRN	1.1541527	1.18478958	1.140873	0.762342811	-0.3914882	0.544014	1
TMEM191C	1.1462477	1.11567542	1.1594994	1.378852752	0.4634684	0.544042	1
TMEM175	1.155318	1.12460966	1.1686287	1.353255558	0.4364343	0.544609	1
DOCK10	1.0574956	1.02826308	1.0701666	2.482623486	1.3118655	0.544685	1
UTP6	1.2658856	1.2340171	1.2796992	1.19520849	0.2572623	0.544779	1
KLHL15	1.1467053	1.11587761	1.1600678	1.381352301	0.4660813	0.544882	1
SOGA3	1.0405356	1.06960003	1.0279375	0.401400043	-1.3168873	0.544909	1
P2RX2	1.0757868	1.04602639	1.0886866	1.92686395	0.9462547	0.544927	1
WWC1	1.1481077	1.11761412	1.1613253	1.371649048	0.4559114	0.545119	1
POLR1A	1.1374274	1.16778455	1.1242689	0.740645505	-0.4331449	0.545199	1
ASB1	1.2469573	1.21529386	1.2606819	1.210819232	0.2759835	0.545295	1
CTC-367J11.1	1.0717762	1.10115754	1.0590406	0.583650276	-0.7768239	0.545318	1

RFT1	1.173015	1.14241916	1.1862769	1.307948471	0.3873057	0.545527	1
TBC1D22B	1.0998014	1.06985761	1.1127806	1.614435884	0.6910301	0.545761	1
SYNJ2	1.0832789	1.11252587	1.0706017	0.627425924	-0.672483	0.545933	1
ZNF283	1.0794541	1.10871249	1.066772	0.614206875	-0.7032034	0.545978	1
BCDIN3D	1.1337762	1.16397841	1.1206848	0.735980022	-0.4422615	0.546212	1
SLC35D1	1.0912344	1.12084119	1.0784011	0.64879474	-0.624166	0.546267	1
KDM7A	1.139149	1.1693596	1.126054	0.744297838	-0.4260481	0.546337	1
IFT46	1.2377777	1.20613106	1.2514951	1.220073959	0.2869686	0.546406	1
APBB3	1.1976302	1.2283405	1.1843186	0.807209502	-0.3089849	0.546799	1
ZNF700	1.1006007	1.13012699	1.0878023	0.67474328	-0.5675894	0.546894	1
HNRNPH1	3.9421964	3.82299291	3.9938658	1.060528995	0.0847841	0.546952	1
MRPS24	1.0598369	1.08885791	1.0472576	0.531833165	-0.9109543	0.547006	1
RBMS2	1.1808793	1.14985878	1.1943253	1.296723068	0.3748704	0.54708	1
TEFM	1.1555691	1.12524165	1.1687147	1.347113679	0.4298716	0.547201	1
SKIV2L2	1.5295681	1.5640009	1.5146431	0.91248625	-0.1321253	0.54729	1
KIF14	1.2189376	1.2499719	1.2054856	0.822034617	-0.2827289	0.547318	1
NGDN	1.4967529	1.46236947	1.5116566	1.106596767	0.1461296	0.547391	1
CDH1	1.0430023	1.01400962	1.0555693	3.966512047	1.9878709	0.547475	1
NUP43	1.1535852	1.18381365	1.1404825	0.764265976	-0.3878533	0.54752	1
RP11-32K4.1	1.1307658	1.16082593	1.1177361	0.732071834	-0.4499429	0.547569	1
FOXO3	1.8085685	1.77003986	1.825269	1.071722447	0.0999313	0.547581	1
FBXL13	1.0777141	1.10692347	1.0650531	0.60840826	-0.7168884	0.547755	1
UQCC3	2.3856631	2.42932126	2.3667392	0.956215524	-0.0645923	0.547803	1
ARMCX4	1.2095379	1.2402369	1.1962312	0.816823681	-0.2919034	0.547978	1
DLX1	1.0502238	1.02087759	1.0629441	3.014914199	1.5921169	0.548074	1
RP11-1391J7.1	1.187162	1.21763766	1.1739521	0.799274005	-0.3232379	0.548168	1
HOXB3	1.0665946	1.03723232	1.0793219	2.130457361	1.0911632	0.548225	1
FAAP24	1.1823363	1.21297646	1.1690552	0.793774083	-0.3331996	0.54824	1
RAB3D	1.2480312	1.21618157	1.2618366	1.211188452	0.2764234	0.548404	1
TMEM238	1.0561063	1.08484599	1.043649	0.514449426	-0.9588988	0.548483	1
LINC00894	1.0560745	1.08509768	1.0434942	0.511109688	-0.9682952	0.548483	1
C10orf54	1.0518949	1.02296211	1.064436	2.806189359	1.4886124	0.548536	1
CTD-2623N2.11	1.0358742	1.06465409	1.0233994	0.361916553	-1.466271	0.548566	1
MON1A	1.0741752	1.10319569	1.061596	0.59688588	-0.744473	0.548629	1
KRT4	1.0451681	1.01623264	1.0577103	3.555202104	1.8299316	0.548663	1
KIF18B	1.0820322	1.11116657	1.0694037	0.624322133	-0.6796375	0.548671	1
BTN2A2	1.2408721	1.20948005	1.2544792	1.214813347	0.2807347	0.548709	1
CEP63	1.5058776	1.47178617	1.5206547	1.103581893	0.1421937	0.548745	1
PASK	1.1106948	1.14033002	1.0978492	0.697279138	-0.5201918	0.548787	1
MAMDC4	1.1230189	1.15262753	1.1101849	0.721920056	-0.470089	0.548884	1
FAM184B	1.244205	1.2750533	1.2308336	0.83923213	-0.2528582	0.548898	1
GATAD1	1.6704912	1.70630662	1.6549668	0.92731228	-0.1088728	0.548902	1
PHF21A	1.5064945	1.54080533	1.4916223	0.9090559	-0.1375591	0.549036	1
MTERF4	1.4013133	1.43445311	1.3869487	0.890657053	-0.1670581	0.549039	1
DAP3	1.7588075	1.7220874	1.774724	1.072895015	0.1015089	0.54907	1
RP11-192H23.4	1.0593494	1.08819382	1.0468466	0.531177348	-0.9127345	0.549313	1
ZNF354A	1.2516097	1.22004972	1.2652896	1.205589268	0.2697385	0.549529	1
SNX21	1.2158094	1.18469434	1.2292964	1.241491474	0.3120744	0.549737	1
NCOR2	1.4868368	1.52115579	1.471961	0.905604449	-0.143047	0.549879	1
PRRG3	1.1196921	1.1490731	1.1069567	0.717478466	-0.4789926	0.549989	1
ANO6	1.4202005	1.38682304	1.4346681	1.123687158	0.1682404	0.550064	1

SNRNP48	1.3495784	1.38219098	1.3354423	0.877682336	-0.1882292	0.550202	1
DNAJB12	1.2731354	1.30481568	1.2594035	0.851017465	-0.2327394	0.550218	1
ULK4	1.1506791	1.180731	1.137653	0.76164579	-0.3928079	0.550276	1
HSD17B4	1.4668614	1.43303454	1.4815239	1.111975642	0.1531252	0.550426	1
KLC2	1.1912589	1.16058039	1.2045567	1.273858768	0.3492053	0.550614	1
PHC1	1.2924291	1.26052029	1.3062602	1.175571359	0.2333621	0.550625	1
PPP1R26	1.1802658	1.21044559	1.1671842	0.794429444	-0.332009	0.550639	1
CCDC61	1.0617586	1.03273429	1.0743394	2.270994038	1.1833239	0.550708	1
WRN	1.2787677	1.3100709	1.2651991	0.855285407	-0.2255222	0.550867	1
ACAP3	1.3629387	1.39505624	1.3490172	0.883462127	-0.1787598	0.550959	1
BNIP1	1.1986434	1.16763535	1.2120841	1.265151211	0.3393098	0.550972	1
SNX18	1.2151825	1.18436029	1.2285426	1.239651951	0.3099351	0.550988	1
RP11-258C19.7	1.1821097	1.21249971	1.168937	0.794998501	-0.330976	0.55101	1
EXPH5	1.0937686	1.06424063	1.1065677	1.658883386	0.7302125	0.551135	1
VPS13B	1.2065873	1.23747504	1.1931988	0.813554228	-0.2976896	0.551197	1
POMGNT2	1.2892611	1.25761322	1.302979	1.176100459	0.2340113	0.551204	1
CTD-2308L22.1	1.0405433	1.06911768	1.0281576	0.407386758	-1.295529	0.551404	1
PVRL3-AS1	1.5206202	1.5551933	1.5056344	0.910735716	-0.1348956	0.551435	1
CLCC1	1.2499839	1.28111539	1.2364898	0.841255197	-0.2493846	0.551637	1
KBTBD7	1.2423286	1.21121471	1.2558151	1.211161487	0.2763912	0.551708	1
FAM120A	1.492407	1.45829441	1.5071933	1.106697553	0.146261	0.551713	1
TACSTD2	1.0455428	1.01685236	1.0579788	3.440397642	1.7825753	0.551915	1
SLAIN2	1.4651913	1.43190147	1.4796209	1.110486897	0.1511924	0.552089	1
SMAP2	1.1950588	1.2255561	1.1818395	0.806183077	-0.3108206	0.552238	1
RP11-736K20.5	1.1042987	1.07493018	1.1170286	1.561835392	0.6432424	0.552307	1
SLC19A2	1.1157639	1.08609588	1.1286237	1.493958262	0.5791398	0.552362	1
STYX	1.232402	1.20145611	1.2458157	1.220194883	0.2871116	0.552374	1
TRMT12	1.0957587	1.06647096	1.1084536	1.631594239	0.7062823	0.552392	1
TOR4A	1.0628728	1.03391755	1.0754236	2.22373365	1.152984	0.552506	1
RBM26-AS1	1.0999601	1.12912549	1.0873182	0.676227516	-0.5644194	0.552642	1
QRSL1	1.2502068	1.21903893	1.2637167	1.203972041	0.2678019	0.55267	1
MSL3	1.9221641	1.96085191	1.9053947	0.942283265	-0.0857673	0.552675	1
PCBD2	1.1931275	1.22317947	1.1801013	0.806979493	-0.3093961	0.552801	1
GTF3C5	1.4859984	1.45240501	1.5005597	1.106441582	0.1459273	0.552864	1
SOX5	1.2193086	1.18800846	1.2328758	1.238645522	0.3087634	0.552875	1
YTHDF3	1.3130512	1.34498713	1.2992083	0.867302918	-0.2053921	0.5531	1
WDR24	1.1526154	1.18250956	1.1396577	0.765207389	-0.3860773	0.553117	1
NAV1	1.7665144	1.72958486	1.7825217	1.072557504	0.101055	0.553254	1
UTP14A	1.120584	1.09109943	1.1333642	1.463940968	0.5498574	0.553319	1
ANKRD9	1.1522973	1.18198353	1.1394297	0.766166368	-0.3842704	0.553358	1
PCDH15	1.043751	1.07204135	1.0314884	0.437088353	-1.1940032	0.55354	1
SPIRE2	1.0669686	1.09547177	1.0546137	0.572040032	-0.805812	0.553575	1
SERF1A	1.0790147	1.0498705	1.0916474	1.837708467	0.8779079	0.55388	1
TCERG1	1.8844953	1.9220905	1.8681995	0.941555628	-0.0868818	0.554001	1
ZBBX	1.0463446	1.0746469	1.0340769	0.456507589	-1.1312893	0.554025	1
TRAM2	1.1877365	1.15745117	1.2008638	1.275721539	0.3513135	0.554083	1
COA3	2.944399	2.89005462	2.9679549	1.041215867	0.0582692	0.554121	1
GNB1L	1.1432511	1.11324027	1.1562595	1.379893162	0.4645566	0.554139	1
COL1A2	4.2096654	4.11453249	4.2509013	1.04378469	0.0618241	0.554312	1
DCC	1.1031021	1.07327832	1.1160294	1.583407055	0.6630322	0.554491	1
USP47	1.915922	1.95441763	1.8992358	0.942182756	-0.0859212	0.554598	1

MORC2	1.1818558	1.21190868	1.1688293	0.796707585	-0.3278778	0.554643	1
VCPKMT	1.1378509	1.10810493	1.1507444	1.394426856	0.4796723	0.554707	1
KNL1	1.2409283	1.27174936	1.2275688	0.837421636	-0.2559739	0.55473	1
MAGT1	1.4908415	1.45729378	1.5053829	1.105160341	0.1442557	0.554808	1
SEC24B	1.2129733	1.24328299	1.1998354	0.821411438	-0.2838231	0.554833	1
MTERF2	1.5273624	1.5608236	1.5128585	0.914473785	-0.1289863	0.555154	1
STAT2	1.2385619	1.26924184	1.2252634	0.836658394	-0.2572894	0.555293	1
DCUN1D4	1.3207392	1.2888107	1.3345788	1.158470913	0.2122218	0.555331	1
TMF1	1.8392682	1.80192793	1.8554535	1.066746152	0.0932169	0.555367	1
FAM76A	1.2512419	1.28217921	1.2378319	0.842839976	-0.2466694	0.555574	1
CDC42BPA	1.6155584	1.65054062	1.6003952	0.922917337	-0.1157267	0.555625	1
STPG1	1.131623	1.10206537	1.1444349	1.415121681	0.5009261	0.555656	1
VAC14	1.2444819	1.21396956	1.2577077	1.204412776	0.2683299	0.555747	1
MACROD2	1.0924829	1.12148525	1.0799117	0.657789138	-0.6043029	0.55582	1
ZNF501	1.0924047	1.12141056	1.079832	0.657537398	-0.6048551	0.55582	1
SNTB1	1.0603996	1.03175396	1.0728162	2.293137818	1.1973231	0.555911	1
RGS5	1.2471828	1.21636066	1.2605428	1.204206114	0.2680823	0.555966	1
PDE6B	1.1554282	1.18502557	1.1425991	0.770699505	-0.3757596	0.556124	1
NRAV	1.168817	1.19836877	1.1560076	0.786452341	-0.3465688	0.556171	1
SYNRG	1.3708548	1.33848486	1.3848857	1.137083902	0.1853387	0.556172	1
ACAD9	1.2368079	1.20636554	1.2500033	1.211458331	0.2767448	0.556191	1
CACNB4	1.1785116	1.20835276	1.1655768	0.79469437	-0.331528	0.556468	1
TSNAXIP1	1.0740548	1.10249185	1.0617286	0.602277859	-0.7314989	0.556591	1
CTSS	1.0641387	1.03569195	1.0764691	2.142475673	1.0992788	0.556652	1
CXCL10	1.0416506	1.01338003	1.0539046	4.028733783	2.0103265	0.556773	1
ANLN	1.2111311	1.24119283	1.1981007	0.821337485	-0.283953	0.556913	1
RP11-210M15.2	1.1548838	1.18434788	1.1421125	0.770892907	-0.3753976	0.55698	1
SEC14L1	1.9158678	1.95370132	1.8994687	0.943134614	-0.0844644	0.557025	1
MEGF10	1.0816184	1.11032379	1.0691759	0.627025797	-0.6734033	0.557062	1
COPS7A	1.6846993	1.64919206	1.70009	1.078402043	0.1088951	0.557206	1
U2AF2	1.5998922	1.5659687	1.6145965	1.085919521	0.1189172	0.557454	1
SLCO4A1	1.0502939	1.02191748	1.0625938	2.855884026	1.5139374	0.557525	1
ZNF275	1.1060907	1.13508695	1.0935222	0.692310989	-0.5305078	0.557565	1
ARMC7	1.1301442	1.10045952	1.1430112	1.423570588	0.509514	0.557694	1
GLB1L	1.1065405	1.07748597	1.1191344	1.537496328	0.620583	0.557712	1
FAM98C	1.2702375	1.23918809	1.283696	1.18607917	0.2462003	0.557778	1
BORCS7	1.998337	2.03674433	1.9816891	0.946896015	-0.0787221	0.55795	1
NABP2	1.7929072	1.75595124	1.808926	1.070076926	0.0977145	0.558005	1
KAT5	1.296325	1.26527557	1.3097835	1.167779872	0.2237683	0.558072	1
FAM200A	1.2387672	1.20831867	1.2519653	1.209518339	0.2744326	0.558142	1
ANKRD18B	1.1361736	1.1652969	1.1235499	0.747442575	-0.4199654	0.558303	1
NRDE2	1.34234	1.37398913	1.3286215	0.878692591	-0.1865696	0.558529	1
CLDN15	1.0985846	1.06944207	1.1112165	1.601572666	0.6794893	0.558603	1
CCDC85B	3.0978547	3.03819602	3.1237141	1.041957709	0.0592967	0.558686	1
SLC38A6	1.2705656	1.30135937	1.2572179	0.853525429	-0.228494	0.55871	1
LRRN3	1.0918918	1.06321933	1.10432	1.650128084	0.722578	0.558729	1
CDR2L	1.1306763	1.10141989	1.1433576	1.413505826	0.4992778	0.558795	1
WRNIP1	1.2568948	1.22640385	1.2701112	1.193050464	0.2546551	0.558887	1
SNHG23	1.0850991	1.11354369	1.0727696	0.640895195	-0.6418396	0.558952	1
YBEY	1.7033315	1.73853098	1.6880742	0.931679477	-0.1020944	0.559007	1
FAM227B	1.2786413	1.30980875	1.2651317	0.855791406	-0.2246689	0.55908	1

CEP126	1.411189	1.44316705	1.3973279	0.896564608	-0.1575205	0.559151	1
RNGTT	1.1830449	1.21313523	1.1700021	0.797625482	-0.3262166	0.55921	1
GAS6	1.2384278	1.20739253	1.2518803	1.214509825	0.2803742	0.559278	1
SAFB	1.6078878	1.6424018	1.5929275	0.922985359	-0.1156203	0.559353	1
RGN	1.1027119	1.07346825	1.1153877	1.570578368	0.6512959	0.559552	1
ZNF445	1.3988999	1.43091976	1.3850207	0.89348588	-0.1624832	0.560156	1
CLEC16A	1.1122891	1.08342712	1.1247994	1.495909508	0.5810229	0.560268	1
MNS1	1.1097238	1.13836014	1.0973112	0.703318458	-0.50775	0.560413	1
DOC2A	1.0902925	1.11884383	1.0779167	0.655623001	-0.6090616	0.560428	1
SLC11A2	1.3832181	1.41503931	1.369425	0.890096449	-0.1679664	0.560448	1
OPHN1	1.1214182	1.15026685	1.1089136	0.72480112	-0.4643429	0.560514	1
CHAC2	1.0592697	1.03107619	1.0714904	2.300488259	1.2019401	0.56058	1
GANC	1.1049225	1.13355812	1.0925103	0.692659378	-0.529782	0.560598	1
TAMM41	1.1739357	1.14428215	1.1867892	1.294610588	0.3725182	0.560625	1
FDXR	1.2315648	1.20127291	1.244695	1.215737521	0.2818318	0.56069	1
PAGR1	1.237703	1.26801578	1.2245637	0.837875007	-0.2551931	0.560944	1
ADCY7	1.1200702	1.14913407	1.1074722	0.720641783	-0.4726458	0.560947	1
BEX2	5.1198618	5.0336658	5.1572239	1.030631717	0.0435289	0.560968	1
ARID2	1.5658743	1.59972502	1.5512015	0.919090355	-0.1217214	0.560991	1
GTF3C3	1.3037155	1.27250613	1.3172434	1.16416963	0.2193013	0.561759	1
MLXIPL	1.0466428	1.07455696	1.0345433	0.463313683	-1.1099388	0.561762	1
SDCCAG8	1.7829222	1.81872142	1.7674048	0.937321036	-0.0933848	0.561788	1
AC009506.1	1.1586527	1.12898936	1.1715104	1.32964798	0.4110443	0.562034	1
WDR62	1.0691722	1.0972551	1.0569995	0.586082334	-0.7708247	0.562068	1
TBL1XR1	2.2026908	2.16217512	2.2202526	1.049973072	0.0703523	0.562189	1
SERP2	1.3884996	1.35621453	1.4024937	1.12991937	0.1762198	0.562191	1
PRR5	1.2413568	1.21128877	1.2543899	1.203991461	0.2678252	0.5622	1
CYB561D2.1	1.1393434	1.16831565	1.1267852	0.75325828	-0.4087835	0.562385	1
TUSC2	2.0937903	2.05198392	2.1119116	1.056966305	0.0799294	0.562403	1
ZNF595	1.161304	1.19039517	1.1486943	0.780977083	-0.3566479	0.56273	1
EPB41L5	1.2175256	1.18701881	1.2307489	1.233827279	0.3031404	0.562758	1
SALL2	1.3299991	1.36105729	1.3165367	0.876693904	-0.1898549	0.562767	1
GOLPH3L	1.2660505	1.23567761	1.2792158	1.184736363	0.2445661	0.562922	1
SKI	1.3164352	1.28494204	1.3300861	1.158432362	0.2121738	0.563069	1
RAB12	1.0610047	1.03280121	1.0732296	2.232528962	1.1586789	0.563196	1
ZIC1	1.4003181	1.43225355	1.3864754	0.894094326	-0.1615011	0.563279	1
CRB3	1.0482785	1.02026985	1.060419	2.980734293	1.5756678	0.563293	1
NRXN3	1.0482693	1.02028119	1.060401	2.978175387	1.5744287	0.563293	1
CTA-212A2.4	1.0342855	1.06184245	1.0223408	0.361253201	-1.4689177	0.563434	1
PLEKHM2	1.2169389	1.18696363	1.2299318	1.229821181	0.2984486	0.563626	1
ZNF561	1.4937873	1.52643796	1.4796347	0.91109445	-0.1343275	0.563735	1
CCAR1	1.8738777	1.83769986	1.8895592	1.061906822	0.0866572	0.56376	1
LRRC75A	1.3503083	1.31823775	1.3642094	1.144457064	0.1946633	0.563773	1
SLX4	1.0808736	1.10913814	1.0686222	0.628764634	-0.669408	0.563782	1
TRMT10B	1.2896984	1.32047664	1.2763574	0.862332317	-0.2136841	0.563838	1
DYNC1I1	1.0521245	1.02407559	1.0642825	2.670026638	1.4168541	0.563934	1
ZNF384	1.1538847	1.18294279	1.1412893	0.772313927	-0.3727407	0.563965	1
CACUL1	1.9425276	1.9815074	1.9256316	0.943071477	-0.084561	0.564075	1
ULK2	1.2658404	1.29598637	1.2527734	0.854003518	-0.2276861	0.564105	1
CARF	1.3021138	1.33265378	1.288876	0.868398435	-0.203571	0.564278	1
METTL2B	1.3496509	1.38090676	1.3361029	0.882375865	-0.1805348	0.56428	1

DIRC2	1.1118984	1.08354073	1.1241902	1.486583175	0.5720002	0.564454	1
CBLB	1.4187151	1.386049	1.4328744	1.121293959	0.1651645	0.564506	1
ATG16L1	1.1188087	1.08998961	1.1313005	1.459063198	0.5450424	0.564614	1
PSMD5	1.2400134	1.20995817	1.253041	1.205197106	0.2692691	0.564648	1
RP3-414A15.10	1.072598	1.10060766	1.060457	0.600918636	-0.7347584	0.564671	1
ZFP62	1.2620224	1.29238925	1.2488598	0.851124951	-0.2325571	0.564735	1
LINC00545	1.0695258	1.04152479	1.081663	1.966608421	0.9757097	0.564858	1
MIR217HG	1.0987733	1.12704258	1.0865198	0.681030014	-0.5542097	0.564886	1
RECQL4	1.1306575	1.15925138	1.1182634	0.742620828	-0.4293023	0.564984	1
FGFR1OP2	1.6788038	1.64406442	1.6938617	1.077317284	0.1074432	0.565031	1
ANKHD1	1.1427046	1.17155827	1.1301978	0.758913182	-0.3979932	0.565043	1
RP1-198K11.5	1.0912608	1.11958438	1.0789839	0.660486429	-0.5983992	0.565113	1
ACAD10	1.2130747	1.24262751	1.2002649	0.82540073	-0.2768334	0.565272	1
SLC9A6	1.1174503	1.08866988	1.1299253	1.465269481	0.551166	0.565302	1
LARP4	1.5133981	1.54664857	1.4989855	0.912808585	-0.1316157	0.565316	1
MAP7D2	1.0486416	1.07624693	1.036676	0.481015573	-1.0558445	0.56533	1
C6orf226	1.2118893	1.18204542	1.2248254	1.234995947	0.3045063	0.565361	1
CCDC186	1.5563305	1.52292226	1.5708114	1.091579876	0.1264177	0.565577	1
NOL4L	1.4932864	1.46014371	1.5076523	1.103247303	0.1417562	0.565675	1
ZNF697	1.0706026	1.04248354	1.082791	1.948777941	0.9625697	0.565734	1
ADAMTS18	1.5191838	1.4865402	1.5333334	1.096175385	0.1324786	0.565755	1
SH3BP1	1.1806568	1.15113539	1.193453	1.279998273	0.3561419	0.565771	1
RAB27B	1.0429816	1.07067146	1.0309792	0.438355561	-1.1898265	0.565822	1
LRRC27	1.3742886	1.40586451	1.3606019	0.888478536	-0.1705912	0.565826	1
CTD-2186M15.3	1.1410306	1.16990305	1.1285157	0.756406053	-0.4027672	0.565826	1
ENOX2	1.2602404	1.29015657	1.2472731	0.852205795	-0.2307262	0.566205	1
KAT7	1.3973533	1.42923537	1.3835338	0.893528024	-0.1624151	0.566242	1
SAFB2	1.4362238	1.40409445	1.4501505	1.113973465	0.1557149	0.5663	1
FAM58A	1.3528116	1.32164054	1.3663229	1.138920209	0.1876667	0.566311	1
LNX1	1.1376946	1.10896801	1.1501463	1.377893598	0.4624645	0.566429	1
GLI4	1.3767571	1.40813829	1.3631547	0.889783446	-0.1684738	0.56645	1
DNAJC24	1.4386631	1.47059413	1.4248224	0.902736247	-0.1476236	0.566506	1
ANKLE1	1.0578844	1.08549288	1.0459174	0.537090548	-0.8967628	0.56676	1
PPIG	3.0918581	3.1479785	3.0675323	0.962547968	-0.0550697	0.56694	1
PTP4A2	2.7511356	2.70122374	2.7727701	1.042055825	0.0594326	0.566965	1
CPLX2	1.0450567	1.0171592	1.0571491	3.330519611	1.7357473	0.567201	1
LYPD6	1.1377985	1.10927193	1.1501635	1.374218695	0.4586116	0.56725	1
NR3C1	1.1746738	1.20378287	1.1620563	0.795240111	-0.3305376	0.567646	1
MCEE	1.1998294	1.22906342	1.1871577	0.817056395	-0.2914924	0.56784	1
ZMYM1	1.2348157	1.20484438	1.2478069	1.209732412	0.274688	0.567949	1
SLC40A1	1.0952195	1.06673133	1.1075678	1.611953554	0.6888102	0.56807	1
C14orf169	1.1057965	1.07750734	1.1180586	1.523192425	0.6070982	0.568117	1
NKD2	1.0891131	1.06074987	1.1014073	1.669258642	0.7392075	0.568371	1
ZSCAN5A	1.1394484	1.11072399	1.1518992	1.371872154	0.456146	0.568434	1
ZNF446	1.1970531	1.22661779	1.1842381	0.812990629	-0.2986894	0.568518	1
PDZD8	1.315596	1.346494	1.3022031	0.872173979	-0.1973121	0.568524	1
RP1-40E16.12	1.0764163	1.10413576	1.0644011	0.618434504	-0.6933073	0.568673	1
PIK3CB	1.1275119	1.15607073	1.115133	0.737697341	-0.4388991	0.568692	1
JRKL	1.1945462	1.22378528	1.1818723	0.812709155	-0.2991889	0.56878	1
EYA2	1.1116224	1.0830516	1.1240066	1.493126676	0.5783366	0.568918	1
RBM47	1.1448236	1.11589777	1.1573617	1.35776313	0.4412318	0.568971	1

MAPK9	1.196161	1.16693592	1.2088288	1.250951592	0.323026	0.569153	1
NLGN2	1.4488409	1.48054269	1.4350995	0.905433666	-0.1433191	0.569292	1
C10orf95	1.0716599	1.09917128	1.059735	0.602341417	-0.7313466	0.569362	1
NAT10	1.2613522	1.29084062	1.2485703	0.854661473	-0.226575	0.569442	1
LINC00667	1.7042876	1.73891421	1.6892784	0.932826073	-0.10032	0.569448	1
RP11-467L13.7	1.0898602	1.11769587	1.0777947	0.66098072	-0.5973199	0.569462	1
PIANP	1.2906942	1.32098159	1.277566	0.864741203	-0.2096597	0.569552	1
GADD45G	1.2356479	1.20486314	1.2489917	1.215404916	0.281437	0.569573	1
AC093673.5	1.3449671	1.31394691	1.358413	1.141635671	0.1911023	0.569591	1
CAMKK1	1.051307	1.0786005	1.0394765	0.502242245	-0.9935447	0.569694	1
ZNF772	1.103591	1.07535211	1.1158314	1.537201414	0.6203062	0.569824	1
SLC25A20	1.0767647	1.04887262	1.0888547	1.818087079	0.8624213	0.569906	1
AVEN	1.1380444	1.10937293	1.1504722	1.375771494	0.4602409	0.570025	1
SCRG1	1.1536874	1.18210565	1.1413694	0.776304317	-0.3653058	0.570154	1
MCM3AP	1.2323272	1.26201013	1.2194609	0.837604675	-0.2556586	0.570179	1
NAT9	1.6405583	1.67432422	1.6259223	0.928221578	-0.1074589	0.570317	1
LHPP	1.246478	1.21675795	1.2593603	1.196543216	0.2588725	0.570332	1
ZNF197	1.2580633	1.28791033	1.2451259	0.851396691	-0.2320966	0.570525	1
PIK3CD-AS2	1.4345231	1.46603199	1.4208654	0.903082659	-0.1470701	0.570574	1
RP3-395M20.12	1.0971278	1.12492272	1.0850799	0.681060631	-0.5541449	0.570777	1
ACOT2	1.0649344	1.03717559	1.0769666	2.070353361	1.049877	0.570792	1
PYROXD1	1.1300066	1.10148737	1.1423685	1.40281967	0.4883296	0.570794	1
UBQLN1	1.7248715	1.75959519	1.7098204	0.934471938	-0.0977768	0.570831	1
DYRK1B	1.3615311	1.392344	1.348175	0.887422802	-0.1723065	0.570847	1
CCDC40	1.1286277	1.1567548	1.1164359	0.742790096	-0.4289735	0.570944	1
ITGA6	1.3361591	1.30576314	1.3493344	1.14250015	0.1921944	0.571016	1
PDGFB	1.0741498	1.04644066	1.0861605	1.855282449	0.8916388	0.57126	1
SART1	1.2066862	1.17748693	1.2193429	1.23582543	0.305475	0.571263	1
RNF170	1.5986216	1.63189721	1.584198	0.924514336	-0.1132324	0.571465	1
TMEM55B	1.4210068	1.38955053	1.4346417	1.115751694	0.158016	0.571497	1
NDC80	1.2303627	1.25976451	1.2176184	0.837752473	-0.2554041	0.571585	1
FYTTD1	1.9868394	1.94976365	2.0029101	1.055957534	0.0785518	0.571713	1
MICALL1	1.0806113	1.05271881	1.0927015	1.758414577	0.8142753	0.571804	1
ZNF865	1.0931435	1.12081247	1.0811502	0.671703995	-0.5741025	0.571847	1
FOXI3	1.0547904	1.08225813	1.0428843	0.521338214	-0.9397085	0.571851	1
SCAMP1-AS1	1.3058114	1.27530231	1.3190358	1.15885609	0.2127014	0.571899	1
CH17-264L24.1	1.1174151	1.14533674	1.1053123	0.724608609	-0.4647261	0.572072	1
NSUN7	1.2098631	1.23892127	1.1972677	0.825659834	-0.2763806	0.572117	1
COL4A6	1.1886956	1.21745549	1.1762295	0.810416402	-0.3032647	0.572227	1
ADGRL3	1.2721871	1.30199146	1.2592683	0.858528422	-0.2200622	0.572236	1
NLGN4X	1.2368525	1.20716557	1.2497205	1.205415003	0.2695299	0.57224	1
SLC35B4	1.3632391	1.33251741	1.3765555	1.132438599	0.1794328	0.572297	1
ACSF2	1.1358046	1.16397552	1.1235937	0.753732656	-0.4078752	0.572544	1
PIKFYVE	1.2698733	1.2996752	1.2569555	0.857446706	-0.2218811	0.572622	1
TRAPPC8	1.1693828	1.14038452	1.1819523	1.296099616	0.3741766	0.572679	1
SDCCAG3	1.1655526	1.19423913	1.1531182	0.788297546	-0.3431878	0.573005	1
FBXL5	1.5364356	1.50412845	1.5504393	1.091863139	0.126792	0.573173	1
UBE2G2	1.5341963	1.56697733	1.5199872	0.917121634	-0.124815	0.5732	1
RP11-156K23.3	1.1137393	1.14178392	1.1015833	0.716465298	-0.4810313	0.573211	1
CLSTN1	1.7635303	1.79822771	1.7484905	0.937690397	-0.0928164	0.573351	1
ELP5	2.0394748	2.000359	2.0564297	1.056050565	0.0786789	0.573355	1

MECOM	1.2211426	1.1918472	1.2338408	1.218891017	0.2855691	0.573399	1
TNNC1	1.1408539	1.1125283	1.1531318	1.360829074	0.4444859	0.573477	1
ZNF440	1.0898988	1.11748308	1.0779422	0.663433205	-0.5919769	0.573502	1
WDR17	1.0826525	1.11016457	1.0707273	0.642014807	-0.6393215	0.573546	1
PRELID3A	1.1201556	1.14810351	1.1080414	0.729499083	-0.4550219	0.573784	1
FCHSD1	1.1107886	1.13872836	1.098678	0.711303686	-0.4914625	0.573954	1
PLPPR2	1.2093877	1.17997626	1.2221363	1.234253079	0.3036382	0.57397	1
EIF3C	1.0795564	1.10700875	1.0676571	0.632257381	-0.6614161	0.574039	1
MEX3B	1.2854265	1.31538265	1.2724418	0.863845369	-0.211155	0.574046	1
MORN3	1.0731175	1.10050872	1.0612446	0.609345957	-0.7146665	0.574119	1
RFNG	1.2981962	1.32819098	1.2851948	0.868990306	-0.202588	0.574171	1
MCM9	1.1072022	1.13515101	1.0950876	0.703565458	-0.5072434	0.574172	1
PHKG1	1.2167528	1.18723149	1.229549	1.226016971	0.2939789	0.574403	1
COL22A1	1.1082403	1.08039637	1.1203095	1.496453826	0.5815478	0.574414	1
IFT140	1.1709785	1.19935871	1.158677	0.795937144	-0.3292736	0.574474	1
PCDHGB6	1.1837338	1.15505959	1.1961628	1.265079815	0.3392284	0.574558	1
CEP44	1.2752408	1.3048493	1.2624068	0.860775377	-0.2162913	0.574582	1
ANKIB1	1.4785393	1.51076445	1.4645711	0.909560362	-0.1367587	0.574667	1
MRPL11	2.8102368	2.86111086	2.7881852	0.960816064	-0.0576678	0.574674	1
TDP1	1.2575518	1.28689292	1.2448337	0.853397483	-0.2287102	0.574722	1
PLP1	1.2654237	1.23540729	1.2784345	1.182777702	0.242179	0.574793	1
RITA1	1.5838164	1.61638164	1.5697009	0.924266414	-0.1136193	0.574998	1
DDR2	1.0921515	1.06444328	1.1041618	1.616333812	0.6927252	0.57502	1
PRSS36	1.0619068	1.0890349	1.050148	0.563239936	-0.8281785	0.5752	1
TMEM121	1.2976183	1.26771261	1.3105811	1.160128671	0.2142848	0.575287	1
ZCCHC14	1.2204132	1.24942798	1.2078366	0.833253162	-0.2631732	0.57532	1
PFAS	1.1215568	1.14934852	1.1095103	0.733253172	-0.4476167	0.57533	1
LDLRAD3	1.3330653	1.30249222	1.3463173	1.144880064	0.1951965	0.575362	1
TUBGCP4	1.5459392	1.57825057	1.5319337	0.919901638	-0.1204485	0.575431	1
ESRRA	1.2067643	1.17783738	1.2193029	1.233165274	0.3023662	0.575523	1
AZIN2	1.1563708	1.12780975	1.1687507	1.320327136	0.4008954	0.575538	1
RP11-495P10.6	1.0712514	1.09839925	1.059484	0.604516466	-0.7261465	0.575587	1
EYA3	1.3764389	1.406796	1.3632805	0.893028643	-0.1632216	0.575645	1
EPOR	1.2871368	1.3169824	1.2742001	0.865032635	-0.2091735	0.575655	1
TCTN2	1.1839072	1.21229367	1.1716029	0.808327843	-0.3069876	0.575663	1
LURAP1	1.0769794	1.04935688	1.0889525	1.802231542	0.8497844	0.575724	1
RGMB	1.2309644	1.20136132	1.2437961	1.210739411	0.2758884	0.575754	1
SPATA18	1.1523655	1.18042064	1.1402049	0.777100164	-0.3638275	0.57585	1
MROH8	1.150823	1.17890897	1.138649	0.774969644	-0.3677883	0.57621	1
TMEM184C	1.2762592	1.24639584	1.2892037	1.173735993	0.2311079	0.576411	1
SFXN2	1.1157675	1.14347569	1.1037573	0.723169631	-0.467594	0.576498	1
TRMT44	1.0818152	1.10915265	1.0699656	0.640988437	-0.6416298	0.576501	1
COQ6	1.1721288	1.14361245	1.1844894	1.284633447	0.3613568	0.576911	1
MED11	1.5110583	1.47895006	1.5249758	1.096097213	0.1323758	0.577028	1
ENKUR	1.1410346	1.16918687	1.1288318	0.761476614	-0.3931284	0.577034	1
KLHDC1	1.0704878	1.09752901	1.0587666	0.602555323	-0.7308344	0.5776	1
HDAC9	1.2930175	1.32271126	1.2801466	0.868102813	-0.2040622	0.577658	1
RPUSD1	1.2671904	1.23759256	1.2800198	1.178571419	0.2370392	0.57774	1
JADE2	1.0857816	1.05837114	1.0976628	1.673135377	0.7425542	0.577801	1
TAF1B	1.3112165	1.34100859	1.2983029	0.874766526	-0.1930301	0.577911	1
GHRL	1.0798914	1.10700695	1.0681381	0.636763014	-0.6511716	0.577977	1

WWC2	1.3026129	1.33235279	1.2897219	0.87173009	-0.1980466	0.578027	1
BUB1B	1.1589208	1.18712707	1.1466947	0.78393068	-0.351202	0.578174	1
AC062029.1	1.0761141	1.10332905	1.0643176	0.622454295	-0.6839602	0.578177	1
ANAPC2	1.1274987	1.09962225	1.1395819	1.401112091	0.4865724	0.578454	1
AIF1	1.1611685	1.13263402	1.1735369	1.308388956	0.3877915	0.578485	1
RALY	3.2284078	3.17178962	3.2529493	1.037369962	0.0529305	0.578533	1
ZNF747	1.1516699	1.17946442	1.1396222	0.777993999	-0.3621691	0.578551	1
FAM149A	1.0783639	1.10524887	1.0667104	0.633834749	-0.6578213	0.578579	1
KIAA0141	1.2809627	1.31061028	1.2681117	0.863177299	-0.2122712	0.578747	1
ATXN7L1	1.088179	1.11559583	1.076295	0.660014794	-0.5994297	0.578758	1
SREBF2	1.5129636	1.54429579	1.4993825	0.917483679	-0.1242456	0.578764	1
AP5M1	1.4250382	1.39409518	1.4384506	1.112549966	0.1538701	0.578799	1
CYTH3	1.1430236	1.17086654	1.1309549	0.766416573	-0.3837993	0.57886	1
ARHGEF25	1.1835673	1.2117934	1.1713325	0.8089606	-0.3058587	0.578887	1
EXOC6	1.2453892	1.27465859	1.2327022	0.847241605	-0.2391547	0.579083	1
RABL3	1.1179136	1.0902676	1.1298968	1.439019582	0.5250862	0.579152	1
AEN	1.2235307	1.25225127	1.2110816	0.836791024	-0.2570607	0.579184	1
CHRNA5	1.1793712	1.2076565	1.1671108	0.804746411	-0.3133939	0.579313	1
SLC26A11	1.2932127	1.26359666	1.3060499	1.161053913	0.215435	0.579354	1
HCG11	1.2340859	1.20488962	1.2467413	1.204264323	0.2681521	0.579728	1
USF2	1.7078074	1.74097125	1.6934324	0.935842462	-0.0956624	0.579751	1
PINX1	1.1515595	1.1234842	1.1637289	1.3259094	0.4069822	0.579765	1
GUCD1	1.3422126	1.31186115	1.3553685	1.139508892	0.1884122	0.579907	1
MYH7B	1.0769251	1.10401687	1.065182	0.626648783	-0.674271	0.579923	1
TMTC1	1.1903196	1.21860111	1.1780609	0.814546985	-0.2959302	0.580096	1
PRRT1	1.2439909	1.27266595	1.2315615	0.849249854	-0.235739	0.580168	1
SCN1B	1.0723502	1.04502112	1.0841961	1.870146516	0.9031513	0.580185	1
NUDCD1	1.2903886	1.26106091	1.3031009	1.161035222	0.2154117	0.5802	1
PALLD	3.3274937	3.26615831	3.3540799	1.038797651	0.0549147	0.580291	1
IGHMBP2	1.1158975	1.14327703	1.1040297	0.726073881	-0.4618117	0.580307	1
AP1B1	1.3174204	1.28749592	1.3303913	1.14920344	0.2006342	0.580386	1
ZNF141	1.3860509	1.41633149	1.3729256	0.895741868	-0.1588451	0.580535	1
ST6GALNAC5	1.1393845	1.16714862	1.1273499	0.761896414	-0.3923332	0.580611	1
AC245100.1	1.1279327	1.1555503	1.1159618	0.745493599	-0.4237321	0.580782	1
PPP1R3B	1.2111762	1.1825228	1.2235961	1.225031165	0.2928185	0.581045	1
NNMT	1.1596854	1.18746976	1.1476421	0.787551688	-0.3445535	0.581063	1
LTN1	1.4459539	1.4145627	1.4595606	1.108542941	0.1486647	0.581201	1
TMEM248	1.714329	1.68048218	1.7290002	1.071299405	0.0993617	0.581222	1
PTPN6	1.0627195	1.03578071	1.0743963	2.079229443	1.056049	0.581249	1
ZNF417	1.0629788	1.08977032	1.0513658	0.572191777	-0.8054293	0.581279	1
GLA	1.2531838	1.2240729	1.2658021	1.186230611	0.2463845	0.581604	1
NHS	1.1272451	1.15508952	1.1151758	0.742640451	-0.4292642	0.581661	1
N4BP2	2.0547182	2.09092811	2.0390228	0.952420949	-0.0703287	0.581673	1
CNOT2	1.8763133	1.84155419	1.8913798	1.05920664	0.0829841	0.581822	1
RP11-379H18.1	1.1256248	1.15297591	1.1137693	0.743707227	-0.4271933	0.581881	1
WAC	1.962293	1.9253386	1.9783111	1.057246622	0.080312	0.58194	1
CCDC110	1.0800529	1.05293252	1.0918084	1.734441785	0.7944714	0.581995	1
GPS2	1.2380764	1.20921597	1.2505861	1.197738808	0.2603133	0.582007	1
MAMDC2	1.1518386	1.1241217	1.1638527	1.320097093	0.400644	0.582047	1
SNAPC3	1.9778193	2.01356219	1.9623264	0.949449789	-0.0748364	0.582102	1
LINC01125	1.0566086	1.08305542	1.0451451	0.543554498	-0.8795034	0.582221	1

TRIM16L	1.2682873	1.2386945	1.2811144	1.177716335	0.2359921	0.582235	1
SMCHD1	1.9308094	1.8945206	1.9465389	1.058152188	0.0815471	0.582236	1
RASA2	1.2377115	1.26627064	1.2253324	0.846253426	-0.2408383	0.582302	1
AGAP5	1.0550383	1.08159982	1.0435251	0.533396913	-0.9067186	0.582402	1
CYTL1	1.0561658	1.02916583	1.067869	2.327004504	1.218474	0.582439	1
ZDHHC14	1.0790379	1.05205809	1.0907325	1.742909067	0.8014973	0.582675	1
RP11-426L16.3	1.0550752	1.08160158	1.0435772	0.534023479	-0.9050249	0.582725	1
CRLF3	1.123463	1.15072958	1.1116442	0.740692228	-0.4330539	0.58291	1
PRUNE1	1.1458716	1.11793824	1.1579795	1.339510538	0.4217059	0.582916	1
KLHL23	1.839893	1.87426322	1.8249951	0.943646081	-0.0836822	0.58296	1
MPV17L	1.0539106	1.08050564	1.0423828	0.526457575	-0.9256108	0.583071	1
COPRS	2.1436606	2.1042663	2.1607363	1.051138008	0.0719521	0.583143	1
VSTM2B	1.0645701	1.0911785	1.0530366	0.581678797	-0.7817054	0.58322	1
SLC29A2	1.2031677	1.17501921	1.2153688	1.230544011	0.2992963	0.583306	1
CCDC113	1.1586656	1.18639304	1.1466469	0.786761798	-0.3460012	0.583735	1
KCTD3	1.430266	1.39955057	1.4435798	1.110196987	0.1508157	0.583903	1
CMTM4	1.2402729	1.21167816	1.2526674	1.193639376	0.255367	0.583905	1
NFIC	1.4211654	1.39088719	1.4342896	1.111035605	0.1519051	0.584679	1
SORBS3	1.7673928	1.73353941	1.7820667	1.066154958	0.0924171	0.584746	1
MUC20	1.0630119	1.08943081	1.0515605	0.576540438	-0.7945063	0.584763	1
OGFOD2	1.2254918	1.19708676	1.2378041	1.206596155	0.2709429	0.584889	1
ALDH5A1	1.1747331	1.20242229	1.162731	0.803918501	-0.3148788	0.585001	1
CCDC94	1.1472374	1.11973767	1.1591573	1.329216507	0.4105761	0.585002	1
ATP11A	1.155231	1.12736487	1.1673098	1.313625786	0.3935544	0.585104	1
ZNF211	1.0834116	1.05645946	1.0950942	1.684291336	0.7521417	0.585246	1
ZSCAN1	1.2439562	1.21487199	1.2565628	1.194026457	0.2558348	0.585399	1
FEM1B	1.4385875	1.40781722	1.451925	1.108155797	0.1481607	0.585458	1
ARHGAP26	1.0752518	1.10181766	1.0637367	0.625988275	-0.6757925	0.585483	1
DBF4	1.5973234	1.62914034	1.5835321	0.927507084	-0.1085698	0.585538	1
SPRYD7	1.2556036	1.22705864	1.2679766	1.180208711	0.239042	0.585686	1
PCGF1	1.1461517	1.11846129	1.1581542	1.335070957	0.4169164	0.58571	1
ZNF444	1.5792823	1.54733571	1.5931297	1.083667082	0.1159216	0.585762	1
LRP8	1.1490554	1.12120414	1.1611277	1.329390758	0.4107652	0.585782	1
EEF1AKMT1	1.3389325	1.30925857	1.3517948	1.137542552	0.1859205	0.585859	1
MGAT4B	1.5967915	1.62905056	1.5828086	0.926489359	-0.1101537	0.586042	1
VPS37B	1.1654184	1.1931565	1.1533951	0.794149361	-0.3325177	0.586043	1
ZNF774	1.0635308	1.08986948	1.0521141	0.579886855	-0.7861567	0.586091	1
MED17	1.397031	1.42707319	1.3840091	0.899164672	-0.1533427	0.586147	1
KATNB1	1.3981681	1.36773967	1.4113575	1.118610662	0.161708	0.586168	1
TCF4	2.2625959	2.30050036	2.2461661	0.958220458	-0.0615705	0.586218	1
DEDD	1.1975477	1.16963993	1.2096444	1.235820055	0.3054687	0.586403	1
TSPAN14	1.6920751	1.72538848	1.6776352	0.934168684	-0.098245	0.586419	1
IFT43	1.6837194	1.65068789	1.6980372	1.07276801	0.1013381	0.58656	1
DMXL1	1.2777943	1.30678576	1.2652278	0.864537464	-0.2099996	0.586562	1
RP11-284M14.1	1.0333602	1.05933545	1.0221011	0.372476979	-1.4247768	0.586673	1
BCL2L2	1.1335314	1.10608386	1.1454287	1.370884737	0.4551073	0.586712	1
ITPR2	1.1590922	1.13141166	1.1710905	1.301942945	0.3806662	0.586808	1
RMDN1	1.4461561	1.41519642	1.4595758	1.106887618	0.1465088	0.586836	1
SLC44A1	1.4385067	1.46903344	1.4252747	0.906704355	-0.1412959	0.586876	1
PAFAH1B1	2.3310374	2.37098208	2.3137232	0.958235102	-0.0615484	0.586891	1
SGMS1	1.2032509	1.1749602	1.2155136	1.231786621	0.3007524	0.586999	1

CIB2	1.4579931	1.48841855	1.444805	0.91070462	-0.1349449	0.587341	1
ANO10	1.305576	1.27620997	1.3183048	1.152401563	0.2046435	0.58737	1
RCAN2	1.1675233	1.1399797	1.1794623	1.282059322	0.358463	0.587382	1
RMND5B	1.4211819	1.39069059	1.4343986	1.111873647	0.1529928	0.587411	1
LINC00599	1.0483701	1.02171652	1.0599233	2.759341307	1.4643239	0.587514	1
GATA3-AS1	1.0421126	1.01574718	1.0535408	3.400028731	1.7655469	0.587567	1
NCK1	1.5001046	1.46920052	1.5135001	1.09441509	0.13016	0.587598	1
RP11-400F19.6	1.229957	1.20140807	1.2423317	1.203187603	0.2668616	0.587627	1
PLXNB2	1.6804713	1.71332146	1.6662323	0.93398601	-0.0985272	0.58766	1
PENK	1.0417566	1.01516282	1.0532839	3.514113827	1.8131609	0.58773	1
SPSB2	1.1556652	1.12779196	1.167747	1.312657092	0.3924901	0.587788	1
ZFHX4	1.984313	1.94840553	1.9998774	1.054271961	0.0762471	0.5878	1
LFNG	1.0840245	1.11065211	1.0724826	0.65504932	-0.6103246	0.587851	1
UBAC2-AS1	1.1545346	1.18172737	1.1427478	0.785505199	-0.3483073	0.587884	1
SPIN2B	1.2234275	1.19501217	1.2357443	1.208869599	0.2736586	0.588027	1
RP11-356K23.1	1.0316415	1.05742211	1.0204667	0.356425187	-1.4883288	0.588053	1
RP11-795F19.5	1.069224	1.0955614	1.0578079	0.60492959	-0.7251609	0.588144	1
CD27	1.0293958	1.05531562	1.0181607	0.328310003	-1.6068694	0.588156	1
ZNF426	1.3562384	1.32649279	1.3691318	1.13059703	0.1770848	0.588239	1
SMO	1.4022102	1.43219305	1.389214	0.900555925	-0.1511122	0.588389	1
CLDN19	1.0686714	1.04211405	1.0801829	1.903946195	0.9289927	0.588466	1
TIPIN	1.2803375	1.3092867	1.2677894	0.865828948	-0.2078461	0.588509	1
RP11-295G20.2	1.151774	1.17880315	1.1400581	0.783308637	-0.3523472	0.588668	1
PDIK1L	1.1517718	1.17892976	1.1400001	0.782430356	-0.3539658	0.588668	1
NBPF10	1.0921087	1.11851698	1.0806619	0.68059363	-0.5551344	0.588684	1
NANOS3	1.1313676	1.10413983	1.1431697	1.37478294	0.4592039	0.588745	1
WNK2	1.3268674	1.29730047	1.3396833	1.142558841	0.1922685	0.588759	1
XPR1	1.4778293	1.50846973	1.464548	0.91361976	-0.1303342	0.588778	1
EID2	1.2247649	1.19665277	1.2369502	1.204916663	0.2689334	0.588802	1
FAM109A	1.1612989	1.13366216	1.1732782	1.296389705	0.3744995	0.588807	1
SIAH2	1.3143812	1.28538198	1.3269511	1.145661471	0.1961808	0.588819	1
SLC35G2	1.1971343	1.22475644	1.1851614	0.823831326	-0.2795791	0.588975	1
CA12	1.0653371	1.03897521	1.0767638	1.969554073	0.977869	0.589009	1
MUTYH	1.3045973	1.33351939	1.2920608	0.875693745	-0.1915017	0.58903	1
PHKA1	1.1088696	1.13567332	1.0972514	0.716805738	-0.4803459	0.589092	1
CELSR1	1.1710383	1.1432541	1.1830815	1.278019373	0.3539097	0.589095	1
TRPV1	1.085722	1.11218644	1.0742508	0.661852115	-0.5954192	0.589208	1
CTC-471J1.11	1.080353	1.10673838	1.0689162	0.645655042	-0.6311645	0.589289	1
ZNF680	1.2068417	1.23444236	1.194878	0.831240504	-0.2666621	0.589365	1
TMEM263	1.8617309	1.82722062	1.8766896	1.059801472	0.083794	0.58939	1
SAR1A	2.1986775	2.1521231	2.2188567	1.057922282	0.0812336	0.589405	1
ACACA	1.2734781	1.30180266	1.2612006	0.865468276	-0.2084472	0.589692	1
OSGIN1	1.0578798	1.03146675	1.0693287	2.203236201	1.1396242	0.589802	1
TANC1	1.3260239	1.35499104	1.313468	0.88303075	-0.1794644	0.589934	1
MICAL3	1.1442539	1.17141117	1.1324825	0.772892985	-0.3716594	0.589939	1
LINC01164	1.1256793	1.0986531	1.137394	1.392697774	0.4778822	0.589954	1
SCN5A	1.1789078	1.15109092	1.1909652	1.263908887	0.3378925	0.589964	1
CDKN2C	1.101547	1.12793253	1.0901101	0.704356508	-0.5056223	0.590093	1
PTPRU	1.4083447	1.37837535	1.421335	1.113537237	0.1551498	0.590187	1
GPRIN3	1.0639231	1.03751211	1.075371	2.009245636	1.0066539	0.590204	1
TAF3	1.2603107	1.2317669	1.2726831	1.176540377	0.2345508	0.590324	1

LMLN	1.1333847	1.16029373	1.1217208	0.759361064	-0.3971421	0.590377	1
ZBTB8OS	1.6603863	1.69246433	1.6464819	0.93359591	-0.0991299	0.590468	1
MEN1	1.3425475	1.31298276	1.3553624	1.135405775	0.183208	0.59054	1
TMEM62	1.076571	1.05013013	1.0880319	1.756067201	0.8123481	0.590811	1
CDCA3	1.3370063	1.36610077	1.3243951	0.886081421	-0.1744888	0.590929	1
ZNF35	1.108503	1.0816377	1.1201479	1.471720376	0.5575036	0.590979	1
SP4	1.1526108	1.18001469	1.1407324	0.781782785	-0.3551603	0.591009	1
RILP	1.169265	1.14180638	1.1811671	1.277566513	0.3533984	0.591122	1
LRRC37B	1.3154273	1.28636843	1.3280231	1.14545815	0.1959247	0.591196	1
MAPK8IP3	1.2057539	1.17780835	1.2178671	1.225291607	0.2931251	0.591197	1
PRKDC	3.214442	3.26964057	3.1905159	0.965137808	-0.0511931	0.591288	1
OSBPL11	1.3101256	1.28114019	1.3226895	1.147788707	0.1988571	0.591365	1
GCH1	1.0585393	1.03234894	1.0698917	2.160555917	1.1114026	0.591439	1
RP11-97C16.1	1.0863444	1.11270173	1.0749196	0.664760231	-0.589094	0.591684	1
ZNF236	1.2092742	1.23664992	1.1974081	0.83417776	-0.2615732	0.591739	1
FOXN2	1.1422439	1.1691759	1.1305701	0.771801097	-0.373699	0.591781	1
TMEM254	1.4078743	1.37745673	1.421059	1.115515859	0.157711	0.59195	1
SRBD1	1.1439531	1.11671488	1.1557596	1.334530929	0.4163327	0.591999	1
LINC00562	1.0491645	1.07505538	1.037942	0.505519842	-0.9841604	0.592082	1
WHSC1L1	2.9136199	2.96240359	2.8924744	0.964365529	-0.052348	0.59215	1
RP11-254F7.2	1.1531125	1.18030411	1.1413262	0.783821025	-0.3514038	0.592153	1
UCKL1-AS1	1.0358073	1.061653	1.0246044	0.399077977	-1.3252574	0.592159	1
WSCD1	1.0583625	1.08422362	1.0471528	0.559852635	-0.836881	0.592213	1
CFAP65	1.0626874	1.03642367	1.0740715	2.033609707	1.0240428	0.592333	1
TXNDC16	1.2799811	1.30834965	1.2676846	0.868120366	-0.204033	0.592362	1
TRIM71	1.0990047	1.12546306	1.0875361	0.697704361	-0.5193122	0.592562	1
MST1	1.2755187	1.30383983	1.2632427	0.866386401	-0.2069175	0.592566	1
RP11-677M14.7	1.0596854	1.08557199	1.0484647	0.566361333	-0.8202053	0.592687	1
BTBD1	1.2498259	1.22139559	1.2621492	1.18407594	0.2437616	0.592914	1
NT5M	1.1392939	1.16605269	1.1276951	0.76900359	-0.3789378	0.593017	1
LZTS2	1.5704684	1.53933064	1.5839652	1.082759172	0.1147124	0.593035	1
NPEPPS	2.5547238	2.59974843	2.5352076	0.959655619	-0.0594113	0.593268	1
RP11-247A12.2	1.0850127	1.05852188	1.0964952	1.648874418	0.7214815	0.593392	1
ZNF571	1.0992882	1.12547966	1.0879353	0.700793556	-0.5129386	0.593598	1
RP11-87C12.5	1.0369978	1.06265507	1.0258765	0.412999183	-1.2757892	0.593696	1
RP11-79P5.9	1.071681	1.09765703	1.0604215	0.61871161	-0.692661	0.593937	1
AIF1L	1.4622396	1.49239646	1.4491679	0.91220784	-0.1325655	0.593975	1
MFSD3	1.5636703	1.53236505	1.5772398	1.084293129	0.1167548	0.594042	1
CYP27C1	1.1277856	1.10097838	1.1394053	1.380546472	0.4652395	0.594125	1
RP11-154H23.3	1.059177	1.08487802	1.0480367	0.565949973	-0.8212536	0.594344	1
GPR137C	1.1850324	1.21223448	1.1732415	0.816274308	-0.292874	0.594439	1
RP11-110G21.1	1.1817678	1.20880985	1.1700463	0.814359408	-0.2962624	0.594582	1
TMEM44-AS1	1.1190255	1.14539716	1.1075946	0.74000481	-0.4343934	0.594663	1
MFN2	1.2352982	1.20699833	1.2475649	1.195975214	0.2581875	0.594668	1
RDH13	1.1407664	1.16732372	1.129255	0.772484668	-0.3724218	0.594705	1
SELENOW	6.770529	6.64661758	6.8242391	1.031456263	0.0446826	0.59496	1
AHCYL2	1.1688373	1.19592907	1.1570942	0.801791238	-0.3187014	0.595062	1
MAST3	1.0863786	1.11240268	1.0750983	0.66811853	-0.581824	0.595176	1
UBE2D4	1.4055926	1.43511908	1.3927942	0.902728149	-0.1476365	0.595472	1
TRIB2	1.5977462	1.62879594	1.5842876	0.929216538	-0.1059133	0.595506	1
AFF1	1.2517999	1.2796224	1.2397401	0.857370831	-0.2220088	0.595538	1

COL24A1	1.0421391	1.06769256	1.0310629	0.458881596	-1.1238061	0.595556	1
ASB6	1.1283837	1.1015371	1.1400204	1.37900775	0.4636306	0.59561	1
CCND3	1.3519449	1.38093194	1.3393803	0.890921094	-0.1666304	0.595712	1
TAPT1	1.1125536	1.08599847	1.1240641	1.442631317	0.5287026	0.595731	1
RP11-61K9.3	1.4221956	1.4516171	1.4094426	0.906614554	-0.1414388	0.595733	1
SECISBP2L	1.6761343	1.64400778	1.6900596	1.071508237	0.0996429	0.595795	1
TBCK	1.3025308	1.33078458	1.290284	0.877562123	-0.1884268	0.595823	1
NUP205	1.1877984	1.21479996	1.1760945	0.819806943	-0.2866439	0.595832	1
TGFBR3L	1.2070461	1.17959328	1.2189456	1.219119374	0.2858394	0.595849	1
EXOSC2	1.2524074	1.28037855	1.2402831	0.856995458	-0.2226405	0.595959	1
SLC25A46	1.3985758	1.36906675	1.4113667	1.114613246	0.1565432	0.596033	1
TUBE1	1.2381948	1.21031151	1.250281	1.190048752	0.2510207	0.596053	1
SBF1	1.2198713	1.24729877	1.2079827	0.841018105	-0.2497912	0.596101	1
FASTKD1	1.1597342	1.18664681	1.1480688	0.793310309	-0.3340428	0.59621	1
CHST2	1.3210539	1.34989884	1.3085508	0.881828699	-0.1814297	0.596249	1
NFRKB	1.1960301	1.22359695	1.1840811	0.823272133	-0.2805587	0.596305	1
LYN	1.228656	1.20094907	1.2406657	1.197645254	0.2602006	0.596359	1
P3H2-AS1	1.0752228	1.1010457	1.0640298	0.633671487	-0.658193	0.59639	1
RP11-484K9.4	1.0463881	1.0719218	1.0353203	0.491093611	-1.02593	0.596482	1
C15orf41	1.1980605	1.17075039	1.2098982	1.229269101	0.2978008	0.596598	1
FZR1	1.2377149	1.21004374	1.2497091	1.188843503	0.2495588	0.596613	1
RNF128	1.0532487	1.02739839	1.0644537	2.352463709	1.2341725	0.596643	1
WSCD2	1.0974696	1.12337719	1.0862398	0.698993043	-0.51665	0.596686	1
BANK1	1.0389711	1.01301898	1.0502201	3.857454668	1.9476492	0.596723	1
EAF2	1.0946844	1.12087333	1.0833327	0.68942197	-0.5365408	0.596898	1
CLIP4	1.169385	1.14221071	1.1811638	1.273911138	0.3492646	0.597055	1
WDFY3	1.2615849	1.28927918	1.2495806	0.862767248	-0.2129567	0.597133	1
MECP2	1.7663754	1.73234599	1.7811256	1.066607317	0.0930291	0.5972	1
HEXDC	1.2414867	1.26920271	1.229473	0.852417299	-0.2303682	0.597217	1
CYP39A1	1.097343	1.07105025	1.1087397	1.530461723	0.613967	0.597348	1
MRPS25	1.7912705	1.75848946	1.8054796	1.061952293	0.086719	0.59741	1
ORMDL3	1.2616421	1.233651	1.273775	1.171726061	0.2286353	0.597415	1
CES4A	1.1786857	1.20549807	1.1670638	0.812970155	-0.2987257	0.597589	1
CALB1	3.6106914	3.50372855	3.657055	1.061239252	0.0857499	0.597606	1
PHLPP1	1.1144651	1.14066067	1.1031106	0.733044682	-0.448027	0.597879	1
BAHD1	1.1049744	1.07848166	1.1164578	1.483885963	0.5693802	0.598117	1
RAB11FIP1	1.4045454	1.3754959	1.4171371	1.110896482	0.1517244	0.598123	1
GHR	1.0417434	1.0670321	1.0307819	0.459211526	-1.1227692	0.598137	1
IRAK1	1.1239641	1.15021283	1.1125864	0.749512703	-0.4159752	0.598268	1
MAP3K9	1.1287223	1.10192923	1.1403358	1.376796756	0.4613156	0.598284	1
PLD1	1.1067444	1.08060666	1.1180739	1.464816135	0.5507196	0.598357	1
RP11-521B24.3	1.1968542	1.22380579	1.1851719	0.827377754	-0.2733819	0.598504	1
CEBPZOS	1.9528366	1.91727869	1.9682494	1.055567278	0.0780185	0.598794	1
GIGYF1	1.3695859	1.39843564	1.3570808	0.896206862	-0.1580963	0.59882	1
HPS4	1.8277228	1.86051276	1.8135097	0.945377893	-0.081037	0.598996	1
PRIMPOL	1.2282225	1.25548296	1.2164063	0.847048045	-0.2394843	0.599031	1
C2orf49	1.5116342	1.48124402	1.524807	1.090521522	0.1250182	0.599167	1
BACE1	1.3958587	1.36679343	1.4084572	1.113589246	0.1552172	0.599286	1
DNAJC13	1.1880259	1.21499888	1.1763343	0.820163912	-0.2860158	0.599295	1
TTC12	1.1124687	1.13882421	1.1010447	0.72786113	-0.4582649	0.599393	1
CTD-2366F13.1	1.1167109	1.1429028	1.1053579	0.737269754	-0.4397355	0.599407	1

ATOH7	1.0884391	1.06182357	1.0999758	1.617114751	0.6934221	0.599438	1
FBXO25	1.2079376	1.18041323	1.2198682	1.218692371	0.285334	0.599461	1
GMEB2	1.1427224	1.16920045	1.1312453	0.775679211	-0.366468	0.599548	1
CNST	1.145901	1.11931589	1.1574245	1.319392469	0.3998738	0.599548	1
CABP1	1.0604601	1.08603824	1.0493732	0.57385132	-0.8012511	0.599559	1
MPZL2	1.0557294	1.02993243	1.0669113	2.235410813	1.16054	0.599674	1
SIK3	1.1936008	1.2204737	1.1819527	0.825280543	-0.2770435	0.599733	1
ZNF14	1.0777835	1.10346722	1.0666507	0.64417215	-0.6344818	0.599849	1
DNAAF1	1.0465799	1.07188003	1.0356135	0.495457017	-1.0131682	0.599959	1
IRGQ	1.2226113	1.19534893	1.2344284	1.200049318	0.2630937	0.600043	1
BRSK2	1.0730882	1.09863969	1.0620127	0.628679251	-0.6696039	0.600135	1
KCNK1	1.2486059	1.22096646	1.2605864	1.179302815	0.2379342	0.600139	1
NAGLU	1.1380922	1.11183421	1.1494739	1.336566846	0.418532	0.60016	1
SPRY2	1.173619	1.14676984	1.1852569	1.262227555	0.335972	0.600198	1
DNTTIP2	1.3665589	1.33812825	1.3788823	1.120528269	0.164179	0.600295	1
SLC37A3	1.3397157	1.36811316	1.3274066	0.889418363	-0.1690659	0.600472	1
INO80D	1.2742055	1.30193208	1.2621873	0.86836516	-0.2036263	0.60054	1
SNX13	1.3617833	1.39055038	1.3493141	0.894414857	-0.1609839	0.600619	1
C6orf47	1.2915245	1.26338202	1.3037231	1.153165551	0.2055996	0.600698	1
AC004076.5	1.101226	1.12711934	1.0900024	0.708015005	-0.4981482	0.60074	1
PVR	1.1052928	1.07899164	1.1166932	1.477286119	0.5629493	0.600769	1
PRKCD	1.0919586	1.06609735	1.1031683	1.560853584	0.6423352	0.600822	1
ST6GAL1	1.1577036	1.13104744	1.1692579	1.291577275	0.369134	0.60116	1
PLD5	1.0498713	1.02437926	1.0609209	2.498883938	1.3212839	0.601227	1
COA5	1.8296141	1.79658382	1.8439313	1.059438167	0.0832994	0.601232	1
FBLN5	1.0413278	1.01585048	1.052371	3.304065361	1.7242422	0.601285	1
ADCK5	1.1409626	1.16730124	1.129546	0.774327836	-0.3689836	0.601336	1
KIAA1549	1.3131508	1.3410663	1.3010506	0.882674745	-0.1800462	0.601373	1
SYNGR3	1.1764884	1.20309849	1.1649541	0.812187946	-0.3001145	0.60143	1
IFRD2	1.5082577	1.47804628	1.521353	1.090590964	0.1251101	0.601756	1
SYDE1	1.1730952	1.14645615	1.184642	1.260732357	0.334262	0.601776	1
GNG8	1.1002016	1.07377622	1.1116558	1.513438356	0.5978299	0.60178	1
LPIN1	1.3100253	1.33810887	1.2978524	0.880936333	-0.1828903	0.601916	1
MAK16	1.1419593	1.11535256	1.1534921	1.330634789	0.4121147	0.601946	1
C1orf43	3.7000124	3.63033892	3.7302128	1.037969952	0.0537647	0.601964	1
RCN3	1.1152545	1.08915966	1.1265654	1.419536666	0.5054201	0.602271	1
ZNF337	1.1412829	1.16759495	1.1298778	0.774950521	-0.3678239	0.602606	1
IRF2BP2	1.6844191	1.71555281	1.670924	0.937630326	-0.0929089	0.602689	1
RAB3IP	1.6031325	1.5722471	1.6165199	1.077366628	0.1075093	0.602753	1
PAX5	1.0427105	1.06766197	1.0318952	0.471390614	-1.0850051	0.602856	1
CHDH	1.1373748	1.16348382	1.1260576	0.771071038	-0.3750643	0.602913	1
IRAK1BP1	1.20839	1.18133061	1.220119	1.213909982	0.2796614	0.603124	1
ITGB4	1.0583242	1.03263304	1.0694602	2.128523354	1.0898529	0.603183	1
RP1-168L15.5	1.0623895	1.08764589	1.0514419	0.586929397	-0.7687411	0.603249	1
STEAP2	1.1437515	1.16972493	1.1324932	0.780634825	-0.3572803	0.603332	1
CSTF2	1.1003425	1.07450941	1.1115399	1.496991029	0.5820656	0.603378	1
SLC35F3	1.2673891	1.29475123	1.2555288	0.866930381	-0.206012	0.603399	1
ATP6V1E2	1.1390571	1.1649538	1.127832	0.774956408	-0.3678129	0.603419	1
AGPAT5	1.3182299	1.34634134	1.3060448	0.88365084	-0.1784517	0.603506	1
PUM3	1.3577974	1.32926571	1.3701647	1.124212646	0.1689149	0.603614	1
NMRAL1	1.7351976	1.76746269	1.7212121	0.939735718	-0.089673	0.603907	1

TMEM232	1.1225315	1.14827838	1.1113713	0.751096145	-0.4129305	0.60393	1
DUSP22	1.3557177	1.38388393	1.3435089	0.894825028	-0.1603225	0.603975	1
SHISA9	1.15416	1.18029898	1.1428299	0.79218353	-0.3360934	0.603987	1
DGKD	1.2255729	1.19842206	1.2373415	1.196144812	0.2583921	0.604028	1
LINC01551	1.4448524	1.47400284	1.4322171	0.911844866	-0.1331397	0.604035	1
RBM39	4.9283967	5.00901699	4.8934514	0.971173588	-0.0421989	0.604079	1
CAB39	1.3507282	1.32234609	1.3630306	1.126213651	0.1714805	0.60416	1
SIX1	1.0841916	1.05857769	1.0952941	1.626797962	0.7020351	0.604201	1
C17orf100	1.2881765	1.25972309	1.3005098	1.157039299	0.2104379	0.604363	1
IQCK	1.4783939	1.50794554	1.4655845	0.916603244	-0.1256307	0.604411	1
CA9	1.4909297	1.46088904	1.5039511	1.09343249	0.1288641	0.604456	1
AP001258.4	1.0949751	1.12056384	1.0838835	0.695760395	-0.5233375	0.604475	1
AC004540.4	1.3276802	1.3551249	1.3157842	0.889220073	-0.1693876	0.604557	1
C14orf39	1.2774795	1.30502473	1.2655398	0.870551813	-0.1999979	0.604583	1
NHSL1	1.5376115	1.56789714	1.5244841	0.92355473	-0.1147306	0.604707	1
VPS53	1.4023621	1.3730962	1.4150476	1.112441237	0.1537291	0.604736	1
TRAPPC2	1.2848682	1.25683404	1.2970198	1.156465929	0.2097228	0.605001	1
KLK6	1.2823586	1.25486399	1.2942763	1.154640472	0.2074437	0.605024	1
EEF2	28.755271	29.1342893	28.590984	0.980688862	-0.0281326	0.605197	1
PLPP6	1.1333598	1.15936916	1.1220859	0.766057314	-0.3844758	0.605392	1
ELMOD2	1.4834943	1.51269173	1.4708385	0.918365787	-0.1228592	0.605502	1
MAPK8IP2	1.0581481	1.08311858	1.0473245	0.569361047	-0.8125843	0.605729	1
OCRL	1.1613836	1.13476268	1.1729226	1.283163463	0.359705	0.605919	1
RASSF7	1.5134265	1.48334629	1.526465	1.089208706	0.1232804	0.605937	1
PIFO	1.2569581	1.22950752	1.2688567	1.171450606	0.2282961	0.606031	1
ACAP1	1.3678757	1.39604888	1.3556639	0.89803028	-0.155164	0.606045	1
NOM1	1.1800627	1.20622785	1.1687213	0.818130455	-0.2895972	0.606148	1
ZBTB26	1.1536959	1.17984177	1.1423628	0.791600347	-0.3371559	0.606218	1
KATNAL2	1.2295673	1.25607921	1.2180756	0.851594392	-0.2317616	0.60631	1
BIN3	1.2999376	1.32757508	1.287958	0.879059556	-0.1859672	0.606358	1
NPPB	1.0456148	1.0204288	1.0565318	2.767261156	1.4684588	0.606358	1
ZNF414	1.3871843	1.41569172	1.3748276	0.90169622	-0.1492866	0.606386	1
SETD1A	1.111402	1.13711663	1.1002559	0.731172548	-0.4517162	0.606484	1
GALNT3	1.0605318	1.03519914	1.0715124	2.031652283	1.0226535	0.606567	1
MTSS1L	1.1110107	1.08517581	1.1222089	1.43478456	0.5208341	0.606752	1
TRABD	1.6541255	1.62291388	1.6676543	1.071824477	0.1000687	0.60688	1
ENKD1	1.1856616	1.21203851	1.1742284	0.821682774	-0.2833466	0.606889	1
PCGF3	1.4356585	1.40648916	1.4483021	1.102863553	0.1412543	0.606952	1
TRIM27	1.6387745	1.60797115	1.6521264	1.072627249	0.1011488	0.606971	1
ERP27	1.0468902	1.07186544	1.0360645	0.501834134	-0.9947175	0.607068	1
PALMD	1.3757024	1.40380025	1.3635233	0.900255164	-0.1515941	0.60709	1
UBQLNL	1.0493128	1.0741701	1.0385383	0.519593639	-0.9445443	0.607139	1
DPPA4	1.0457222	1.02057042	1.0566244	2.752708437	1.4608518	0.60717	1
MRVI1	1.1064013	1.1318764	1.095359	0.723093492	-0.4677459	0.607182	1
TTI1	1.1728816	1.19901719	1.161553	0.811753857	-0.3008858	0.607259	1
SREK1	2.1510757	2.18996043	2.134221	0.953158541	-0.0692119	0.607266	1
MAPK8	1.3695174	1.34146907	1.3816752	1.117744448	0.1605904	0.607369	1
ANKAR	1.0979158	1.12334138	1.0868949	0.70450752	-0.505313	0.607567	1
ITPRIPL1	1.0868018	1.11203351	1.0758649	0.677162657	-0.5624257	0.607607	1
RP11-206L10.9	1.1697738	1.19601663	1.1583987	0.808087892	-0.3074159	0.607614	1
PIGQ	1.3923145	1.42070964	1.3800065	0.903251147	-0.1468009	0.607663	1

RP11-732A21.3	1.1086803	1.13403891	1.0976884	0.728806333	-0.4563926	0.607711	1
TCEAL5	2.0500558	2.08605475	2.0344519	0.952485923	-0.0702303	0.607766	1
TNK2	1.1471125	1.1730613	1.1358649	0.785068	-0.3491105	0.608051	1
MAPK11	1.0871798	1.11250555	1.0762023	0.677319951	-0.5620906	0.608202	1
KHDRBS3	1.147308	1.17317303	1.1360967	0.785899954	-0.3475824	0.608271	1
RP5-984P4.6	1.0247443	1.04917514	1.0141546	0.287839759	-1.7966622	0.608279	1
TMEM94	1.1914779	1.21776012	1.1800858	0.826991616	-0.2740554	0.6083	1
RP11-497H16.9	1.0464692	1.07110725	1.0357897	0.503319746	-0.9904529	0.608332	1
SYT6	1.1186229	1.14433045	1.1074798	0.74467862	-0.4253102	0.60839	1
ZIC4	1.135695	1.16144858	1.1245319	0.771341099	-0.3745591	0.608403	1
ZNF329	1.106812	1.13227747	1.0957739	0.72403776	-0.4658632	0.608515	1
TPX2	1.7900148	1.82645857	1.774218	0.936789907	-0.0942026	0.608707	1
FST	1.1972137	1.1705081	1.2087894	1.224512863	0.2922079	0.608819	1
RP11-445F12.1	1.0948589	1.06957977	1.1058163	1.52079176	0.6048226	0.608911	1
ERICH6-AS1	1.0950275	1.06945652	1.1061114	1.527738832	0.6113979	0.608911	1
NMI	1.0714672	1.04627014	1.082389	1.780608776	0.8323706	0.608912	1
SLC26A2	1.2630872	1.29010507	1.2513762	0.866500427	-0.2067276	0.608956	1
EPSTI1	1.0603403	1.03524674	1.0712173	2.020536123	1.0147381	0.608974	1
TRMT61B	1.2916815	1.31885957	1.279901	0.877818903	-0.1880048	0.608991	1
CTNS	1.1140658	1.08832376	1.1252239	1.417782253	0.503636	0.609024	1
MIR92B	1.0638826	1.08874598	1.0531055	0.598398896	-0.7408206	0.609107	1
WRAP73	1.2385864	1.21161712	1.2502764	1.182685038	0.2420659	0.609187	1
POMGNT1	1.5231059	1.49319269	1.5360719	1.086942134	0.1202751	0.609336	1
RP11-393I2.4	1.0662972	1.09117006	1.0555159	0.608926674	-0.7156596	0.609362	1
NCK2	1.2322578	1.25869165	1.2207999	0.853525474	-0.2284939	0.609507	1
CCDC120	1.0867426	1.06144715	1.097707	1.590098582	0.6691162	0.609656	1
ADAMTS8	1.0644061	1.03926553	1.0753034	1.917800402	0.9394526	0.609788	1
SGK494	1.1138734	1.13936993	1.1028218	0.737761787	-0.438773	0.60981	1
ERCC8	1.1723352	1.14607053	1.1837197	1.257746711	0.3308414	0.60985	1
CELF2	2.0651535	2.10262786	2.04891	0.951282	-0.072055	0.609909	1
IFIT5	1.2273621	1.20053318	1.2389913	1.191779395	0.2531172	0.609937	1
SCML1	1.2654556	1.29237183	1.2537886	0.868033674	-0.2041771	0.609952	1
RP11-560J1.2	1.0454769	1.07014467	1.0347845	0.495897171	-1.0118871	0.609993	1
KCNMB2	1.0881114	1.11314234	1.0772616	0.682870591	-0.5503159	0.610246	1
AC005253.2	1.0777443	1.05241348	1.0887241	1.692771827	0.7593875	0.610267	1
AC007405.6	1.1873401	1.16090475	1.1987987	1.23550557	0.3051015	0.610301	1
MAPRE2	1.420271	1.44850456	1.408033	0.909763268	-0.1364369	0.610308	1
CTH	1.080398	1.05502489	1.0913962	1.66099722	0.7320497	0.610339	1
PCYOX1L	1.1441136	1.11829919	1.155303	1.312798894	0.3926459	0.610642	1
C1orf226	1.0671682	1.09191759	1.0564405	0.614033633	-0.7036104	0.610787	1
ID2	4.157657	4.06405102	4.198231	1.043791696	0.0618338	0.610821	1
UNK	1.3648828	1.33624885	1.3772943	1.122068759	0.1661611	0.610993	1
MRPS14	2.012846	1.97861379	2.0276842	1.05014275	0.0705855	0.611011	1
NIPSNAP3B	1.0359932	1.06044261	1.0253954	0.42015769	-1.2509972	0.611037	1
MAPKAPK3	1.8806731	1.91607817	1.8653266	0.944599065	-0.082226	0.611274	1
CDK5RAP2	1.3714631	1.39909024	1.3594879	0.900768462	-0.1507718	0.61141	1
C1orf220	1.0426456	1.06707581	1.0320562	0.477909576	-1.0651904	0.611458	1
TRIO	1.4731999	1.44400711	1.4858536	1.094247431	0.129939	0.611485	1
RSF1	2.5746293	2.53399872	2.5922409	1.037967541	0.0537613	0.611522	1
SERPINI1	1.0594364	1.03458225	1.0702095	2.030218534	1.021635	0.611633	1
CHM	1.2187954	1.24531194	1.2073016	0.845053098	-0.2428861	0.611635	1

SLC18B1	1.2793162	1.25226572	1.2910413	1.153709443	0.2062799	0.611636	1
SIAE	1.1124073	1.08699118	1.1234241	1.418811423	0.5046829	0.611687	1
ZSCAN16-AS1	2.1265268	2.16171206	2.1112755	0.9565843	-0.064036	0.6117	1
CDR1-AS	1.2685098	1.29518766	1.2569461	0.870450075	-0.2001665	0.611881	1
FKBP11	1.2678946	1.29487849	1.2561983	0.868826582	-0.2028599	0.611881	1
SPPL2A	1.3034383	1.27629205	1.315205	1.140840066	0.1900966	0.611886	1
YY2	1.0819546	1.10677319	1.0711968	0.666804004	-0.5846653	0.611905	1
ZNF280B	1.2411551	1.21439918	1.2527526	1.178887975	0.2374266	0.612011	1
MAT2B	1.3891395	1.36109467	1.4012956	1.111330789	0.1522883	0.612037	1
FOXJ1	1.0824768	1.10748921	1.071635	0.666438629	-0.5854561	0.612071	1
PTPN7	1.0558202	1.08033372	1.0451946	0.562586118	-0.8298541	0.612094	1
BAG5	1.3742806	1.34631778	1.3864013	1.11574197	0.1580034	0.612118	1
RNF103	1.1232355	1.09788414	1.1342242	1.371255761	0.4554977	0.612125	1
TSSC4	1.5861408	1.61647261	1.5729933	0.929470881	-0.1055184	0.612256	1
SLC7A11	1.157966	1.1837432	1.1467928	0.798901977	-0.3239096	0.612296	1
SNX32	1.1135448	1.13866008	1.1026584	0.74036009	-0.433701	0.612507	1
ZSCAN22	1.0461783	1.07070405	1.0355475	0.502765262	-0.9920431	0.612532	1
CYTIP	1.08944	1.06442484	1.100283	1.556588527	0.6383876	0.612659	1
DIRAS3	1.0410876	1.01647485	1.0517561	3.141520626	1.6514631	0.612698	1
NKAIN3	1.1974259	1.17130121	1.2087498	1.218612263	0.2852392	0.612729	1
BACE1-AS	1.1556028	1.18125297	1.1444845	0.797142903	-0.3270897	0.612901	1
AMER2	1.0559119	1.03110616	1.0666641	2.143116595	1.0997103	0.612912	1
HTRA2	1.4983956	1.46944219	1.5109456	1.088409997	0.1222221	0.61302	1
CSE1L	1.4604292	1.43186055	1.4728125	1.094826747	0.1307026	0.613534	1
ZNF853	1.0807038	1.05562062	1.0915763	1.646444501	0.7193539	0.613561	1
NLGN1	1.2378012	1.21093631	1.2494459	1.18256501	0.2419195	0.613736	1
POLM	1.2576789	1.28409092	1.2462304	0.866731158	-0.2063435	0.614037	1
ARID4B	2.6574357	2.61332026	2.6765577	1.039197092	0.0554693	0.614081	1
NBPF26	1.0752181	1.09988845	1.0645245	0.645965967	-0.6304699	0.614084	1
AC053503.4	1.040157	1.06454002	1.029588	0.458444568	-1.1251808	0.614101	1
ZNF343	1.0907603	1.11568637	1.0799559	0.691143634	-0.5329425	0.61411	1
DDX18	2.2213842	2.17780785	2.2402726	1.053034762	0.0745531	0.614158	1
FBXO36	1.1801014	1.15384821	1.191481	1.244609878	0.3156936	0.61434	1
PTBP1	2.5023583	2.54379696	2.4843965	0.961523148	-0.0566065	0.614422	1
TXLNA	1.4289974	1.40042687	1.4413814	1.102277188	0.1404871	0.614529	1
TFRC	1.3578316	1.32977856	1.3699914	1.121938836	0.165994	0.614566	1
ZDHHC19	1.0361609	1.0603168	1.0256903	0.425923467	-1.2313339	0.614587	1
LZTS3	1.073782	1.09832492	1.0631437	0.642194079	-0.6389187	0.614615	1
TMEM132D	1.0754658	1.05076201	1.0861738	1.697604193	0.7635001	0.614831	1
CCT6B	1.0816976	1.10641296	1.0709846	0.66706767	-0.584095	0.614834	1
NAB2	1.3899697	1.3620783	1.4020594	1.110421008	0.1511068	0.614939	1
NPHP1	1.2642548	1.29098287	1.2526693	0.868330496	-0.2036838	0.615161	1
CASD1	1.2594808	1.28596686	1.2480003	0.867234351	-0.2055062	0.61526	1
MTOR	1.2077962	1.18141538	1.2192311	1.208448257	0.2731557	0.615322	1
CDK19	1.6087789	1.63830135	1.5959822	0.933700348	-0.0989685	0.615374	1
RP11-464F9.20	1.0747188	1.09921558	1.0641006	0.646073444	-0.6302299	0.615393	1
ABLIM1	1.5938483	1.56439084	1.6066168	1.074816888	0.1040909	0.615412	1
DRAM1	1.0839403	1.05918974	1.0946686	1.599408538	0.6775385	0.615582	1
RP11-384P7.7	1.0388957	1.063066	1.0284189	0.450621614	-1.1500116	0.615598	1
CXXC1	1.3328704	1.36045611	1.3209132	0.89029761	-0.1676404	0.615679	1
ZNF48	1.2834685	1.25654056	1.2951406	1.150463605	0.2022153	0.615745	1

WDR26	1.5275561	1.49854892	1.5401294	1.08340303	0.11557	0.615798	1
L1CAM	1.1997226	1.17374038	1.2109848	1.214368027	0.2802057	0.615889	1
PLA2G6	1.1109021	1.13578954	1.1001145	0.737277299	-0.4397208	0.615891	1
METTL7A	1.0499579	1.02534715	1.0606256	2.391812767	1.2581045	0.615893	1
OAF	1.642771	1.67382111	1.6293122	0.933945488	-0.0985897	0.616024	1
SEC22C	1.7934622	1.76162842	1.8072607	1.059914132	0.0839474	0.616026	1
CD82	1.1315701	1.10621416	1.1425608	1.342201256	0.424601	0.616037	1
BICDL1	1.0609777	1.03626864	1.0716879	1.976582114	0.9830079	0.616061	1
ISG20L2	1.5390426	1.5093836	1.5518985	1.083463442	0.1156505	0.616103	1
ATG5	1.4724842	1.44355546	1.4850235	1.093490193	0.1289403	0.616136	1
SSC5D	1.1397508	1.16509327	1.1287659	0.779958811	-0.3585302	0.616201	1
TRIM69	1.3389877	1.36612088	1.3272267	0.893766789	-0.1620297	0.616282	1
RP11-166O4.6	1.0889619	1.11350958	1.0783216	0.6899999	-0.5353319	0.616332	1
FAT1	1.6766738	1.70740622	1.6633526	0.937725194	-0.0927629	0.616352	1
FXYD1	1.3800597	1.35273745	1.3919026	1.111032042	0.1519004	0.616389	1
TRAM1L1	1.1478626	1.12235983	1.1589169	1.29876696	0.3771426	0.616486	1
TMEM139	1.136192	1.11069249	1.1472449	1.330215882	0.4116604	0.616505	1
EFCAB11	1.2230141	1.2491784	1.2116731	0.849484014	-0.2353413	0.616545	1
HLA-DQB1	1.0735212	1.04874888	1.0842589	1.728427007	0.7894597	0.617056	1
PRKX	1.5776376	1.60714396	1.5648479	0.930336006	-0.1041762	0.617106	1
ADM5	1.0397195	1.06387287	1.02925	0.45794117	-1.1267658	0.617196	1
EEF2KMT	1.1840769	1.15826502	1.1952652	1.23378642	0.3030927	0.617267	1
ALKBH6	1.2646833	1.29108287	1.2532402	0.869993578	-0.2009233	0.61732	1
BORCS5	1.1575682	1.13210749	1.1686043	1.27626609	0.3519291	0.617536	1
RP11-923I11.8	1.0921163	1.06721399	1.1029104	1.531086035	0.6145554	0.617622	1
EP300	1.3688934	1.39645386	1.3569471	0.900349673	-0.1514427	0.617702	1
TRIT1	1.4501429	1.42152284	1.4625484	1.097326972	0.1339935	0.617708	1
PDIA5	1.1926995	1.16695473	1.2038587	1.221041612	0.2881124	0.617714	1
RP11-147L13.11	1.1210672	1.14615687	1.110192	0.753929627	-0.4074982	0.617761	1
SERTAD4-AS1	1.0866376	1.06181127	1.0973987	1.575743822	0.656033	0.617764	1
ATP6AP1L	1.1927135	1.21818353	1.1816734	0.83266307	-0.2641953	0.617857	1
FUBP3	1.309305	1.28229645	1.321012	1.137144857	0.185416	0.617993	1
ZNF264	1.2015458	1.17543474	1.2128638	1.213350464	0.2789963	0.618125	1
ZNF808	1.112086	1.13671041	1.1014124	0.741804423	-0.4308892	0.618152	1
ZCWPW1	1.1574431	1.13159582	1.1686468	1.281551127	0.357891	0.618212	1
LINC00524	1.0602329	1.08451052	1.0497096	0.588206186	-0.7656061	0.618293	1
SERGEF	1.699429	1.72970311	1.6863065	0.940528367	-0.0884566	0.618386	1
COG5	1.2096268	1.18328828	1.2210433	1.205987264	0.2702147	0.618472	1
KMT2B	1.1411122	1.166364	1.1301666	0.782420585	-0.3539838	0.618487	1
C19orf12	1.5729651	1.54347888	1.5857461	1.077771588	0.1080515	0.618617	1
GNAS-AS1	1.0574325	1.0816462	1.0469369	0.574881257	-0.7986641	0.618628	1
OPRK1	1.1086499	1.13334519	1.0979456	0.734526971	-0.4451126	0.61863	1
TMEM222	1.4017273	1.4295211	1.3896799	0.907242842	-0.1404393	0.618746	1
RP11-480C16.1	1.0719011	1.09621035	1.0613641	0.637811732	-0.6487975	0.618787	1
CD7	1.0720549	1.0961449	1.061613	0.640834971	-0.6419752	0.618787	1
ALG10B	1.1636644	1.1892262	1.1525846	0.806360592	-0.310503	0.618832	1
SPOUT1	1.2393525	1.26532828	1.2280932	0.859664031	-0.2181552	0.618907	1
RPP21	1.3377737	1.31034295	1.3496637	1.126701071	0.1721048	0.61891	1
CTA-373H7.7	1.0480654	1.07216706	1.0376185	0.521269069	-0.9398998	0.61899	1
LYNX1	1.0762094	1.0515955	1.0868784	1.683837137	0.7517526	0.619027	1
TRAM2-AS1	1.1798023	1.20491555	1.1689168	0.824324099	-0.2787164	0.61904	1

THAP10	1.1278984	1.15291892	1.1170531	0.76545845	-0.385604	0.61913	1
CDK5RAP1	1.1672122	1.14181258	1.1782218	1.25674186	0.3296883	0.619193	1
NFAT5	1.5726693	1.60144739	1.5601953	0.931411939	-0.1025087	0.619239	1
CRYM	1.1004146	1.07572688	1.1111156	1.467320493	0.553184	0.619261	1
TACC2	1.2255303	1.25156916	1.2142436	0.851629142	-0.2317028	0.619509	1
ZBED3-AS1	1.1418868	1.16687253	1.1310566	0.785369437	-0.3485566	0.619601	1
RP11-392O17.2	1.1233859	1.14796593	1.1127316	0.761875122	-0.3923735	0.619627	1
BCL7C	2.3001158	2.26371264	2.315895	1.04129291	0.0583759	0.61966	1
TSC22D2	1.3710588	1.34348049	1.3830128	1.115093242	0.1571644	0.619699	1
MID1IP1	1.8215551	1.78977617	1.8353299	1.057679234	0.0809022	0.619843	1
FRS2	1.2434303	1.26950218	1.2321293	0.861326054	-0.2153686	0.619871	1
FARS2	1.2067421	1.18090346	1.217942	1.204741871	0.2687241	0.619872	1
RP11-154J22.1	1.0456243	1.06964013	1.0352145	0.505663211	-0.9837513	0.619929	1
DDX50	1.7551621	1.72437582	1.7685065	1.060922403	0.0853191	0.619932	1
PUS3	1.1764332	1.15101724	1.1874498	1.241247928	0.3117913	0.619952	1
TRMT61A	1.1187482	1.09382241	1.1295524	1.380825472	0.465531	0.620022	1
ZNF487	1.2502143	1.27659465	1.2387796	0.863283419	-0.2120938	0.620054	1
IKBKE	1.0933689	1.06865909	1.1040795	1.515887633	0.6001628	0.620149	1
FAM229A	1.1795254	1.20480878	1.1685662	0.823041919	-0.2809622	0.620174	1
NR4A1	1.0560555	1.03174475	1.0665932	2.097770167	1.0688566	0.620217	1
TIMP4	1.0367727	1.06045125	1.026509	0.438519307	-1.1892877	0.620387	1
PSTK	1.3100909	1.33679595	1.2985155	0.886339326	-0.174069	0.620406	1
ZNF44	1.1616463	1.18676775	1.1507572	0.807190995	-0.309018	0.620414	1
GUCY1A3	1.2111183	1.18520127	1.2223521	1.200597209	0.2637522	0.620556	1
NCAPD2	1.3361543	1.30897224	1.3479365	1.12610939	0.171347	0.620607	1
ZNF799	1.0682744	1.09244366	1.0577981	0.625225596	-0.6775513	0.620623	1
TYW3	1.3265272	1.2995009	1.3382419	1.129352008	0.1754952	0.620727	1
FAM168B	1.5437487	1.57219949	1.5314166	0.928726129	-0.1066749	0.620759	1
ZNF563	1.1197944	1.14456627	1.1090569	0.754373309	-0.4066495	0.620811	1
AC144652.1	1.0609465	1.08503662	1.0505045	0.593914924	-0.7516718	0.620828	1
PITRM1	1.2483135	1.27445311	1.2369832	0.863474292	-0.2117749	0.620859	1
P3H3	1.348297	1.37548103	1.3365139	0.89622071	-0.158074	0.621005	1
DHX30	2.0209696	2.05538052	2.0060539	0.953261803	-0.0690556	0.621107	1
CCNJ	1.2604403	1.2862035	1.2492731	0.870964568	-0.1993141	0.621155	1
EIF2D	1.5970727	1.62596569	1.5845488	0.933835284	-0.09876	0.621222	1
KDM4A	1.2687428	1.29497051	1.2573742	0.872542203	-0.1967032	0.621235	1
CYTH1	1.1364513	1.16123806	1.1257073	0.779638082	-0.3591235	0.621245	1
SPRR2F	1.0513591	1.02710934	1.0618704	2.282252496	1.1904584	0.621321	1
ANGPTL7	1.0375699	1.01362911	1.0479471	3.517991155	1.8147519	0.621354	1
ANO1	1.0585254	1.08250098	1.048133	0.583423392	-0.7773849	0.621356	1
BMP2	1.0620087	1.03767051	1.0725582	1.926127217	0.945703	0.621436	1
CD40	1.1350067	1.11003021	1.1458329	1.325389703	0.4064166	0.621478	1
FMN2	1.1293178	1.1539567	1.118638	0.770593021	-0.375959	0.621496	1
NID2	1.0534682	1.02913636	1.0640149	2.19708033	1.1355876	0.621556	1
KCTD15	1.1591825	1.18408723	1.1483875	0.806071537	-0.3110202	0.621768	1
MESTIT1	1.0365202	1.0602826	1.0262202	0.434954982	-1.201062	0.621867	1
TARBP2	1.4129109	1.38527691	1.424889	1.102814645	0.1411903	0.621923	1
CUL4A	1.4127055	1.38533665	1.4245687	1.101812319	0.1398785	0.621923	1
DSCR3	1.4796911	1.45141252	1.4919487	1.089798435	0.1240613	0.622123	1
HBD	1.046657	1.02253204	1.0571141	2.534795037	1.3418691	0.622154	1
GRIN2B	1.0949759	1.11949719	1.0843471	0.705849808	-0.5025669	0.622162	1

MCOLN3	1.0429361	1.01886772	1.0533686	2.828567494	1.5000716	0.622249	1
ZNF439	1.3628603	1.3896068	1.3512668	0.901593142	-0.1494516	0.622251	1
RP1-313I6.12	1.176392	1.20151478	1.1655024	0.821291576	-0.2840336	0.622405	1
COG8.1	1.1007277	1.12544336	1.0900146	0.71757154	-0.4788054	0.622474	1
LZTR1	1.1523269	1.12731982	1.1631663	1.281546768	0.3578861	0.622754	1
C16orf95	1.0856954	1.06108728	1.0963619	1.577446887	0.6575914	0.622848	1
VAT1L	1.1832033	1.15782943	1.1942017	1.230453199	0.2991898	0.622901	1
PLCG2	1.196092	1.17049112	1.2071889	1.215247273	0.2812499	0.622968	1
SYVN1	1.1283554	1.15300627	1.1176704	0.769055788	-0.3788398	0.622988	1
C11orf63	1.0845726	1.10882339	1.0740609	0.680560571	-0.5552045	0.623122	1
ZNF627	1.1608518	1.18617418	1.1498756	0.805029015	-0.3128873	0.623137	1
RP11-372K14.2	1.0540435	1.02993952	1.0644916	2.154061289	1.1070593	0.623155	1
RBBP4	3.5180927	3.57182822	3.4948007	0.970049525	-0.0438697	0.623216	1
ZSCAN2	1.1432607	1.11838496	1.1540433	1.301206653	0.3798501	0.623294	1
CLUL1	1.1375419	1.11276181	1.1482829	1.315009926	0.3950737	0.623385	1
RCBTB2	1.2162523	1.24186089	1.2051521	0.848223754	-0.2374832	0.62343	1
ACVR2B	1.5922884	1.62055375	1.5800367	0.934708161	-0.0974121	0.623441	1
PAFAH2	1.1191844	1.14353013	1.1086316	0.756855826	-0.4019096	0.623516	1
ZNF573	1.1530523	1.17783705	1.1423093	0.800222859	-0.3215263	0.623612	1
LACE1	1.0671146	1.09105452	1.0567377	0.623118387	-0.6824218	0.623655	1
ARSK	1.2478144	1.22180812	1.259087	1.168068041	0.2241243	0.623666	1
RP11-318E3.9	1.0384367	1.06218929	1.028141	0.452505962	-1.1439913	0.623667	1
GRIA2	1.0536832	1.02948736	1.064171	2.176220564	1.1218248	0.623686	1
CTC-543D15.8	1.108231	1.13252743	1.0976996	0.737202536	-0.4398671	0.623702	1
SHROOM4	1.1546407	1.17946418	1.1438808	0.801724578	-0.3188214	0.62395	1
RP4-816N1.6	1.1180291	1.14251929	1.1074137	0.753678263	-0.4079793	0.623964	1
RNASE1	1.0712275	1.04697126	1.0817415	1.740246116	0.7992914	0.624151	1
FUT8	1.2406571	1.26661386	1.2294059	0.860442639	-0.2168491	0.624195	1
HUS1	1.1627448	1.13738804	1.1737358	1.264562861	0.3386388	0.624265	1
LNX2	1.0961138	1.12031983	1.0856215	0.711615906	-0.4908293	0.624269	1
SUSD1	1.0499177	1.02599853	1.0602856	2.318808356	1.2133836	0.624277	1
ZNF274	1.271474	1.29735969	1.2602538	0.875215356	-0.19229	0.62428	1
MTMR1	1.1033747	1.12765123	1.0928519	0.727387613	-0.4592037	0.624312	1
FAM98B	1.3459378	1.37271902	1.3343294	0.897001139	-0.1568183	0.624321	1
PURG	1.1729814	1.19794677	1.16216	0.819210391	-0.2876941	0.624354	1
AL049794.1	1.0396408	1.06319092	1.0294329	0.465777539	-1.102287	0.624366	1
AJ011932.1	1.0526998	1.02862006	1.0631373	2.20605064	1.1414659	0.62443	1
GPT2	1.3474317	1.37453992	1.3356815	0.896250309	-0.1580264	0.624471	1
ABTB1	1.1041119	1.07962806	1.1147246	1.440755495	0.5268255	0.624569	1
SCAF8	1.2827897	1.30835004	1.2717104	0.881175195	-0.1824992	0.624588	1
ABHD14B	1.4293712	1.40170867	1.4413616	1.098710798	0.1358117	0.62461	1
C9orf43	1.0345628	1.05803654	1.024388	0.420217254	-1.2507927	0.62464	1
APOL2	1.2793285	1.30541249	1.2680223	0.877574622	-0.1884063	0.624659	1
PLEKHA7	1.0649845	1.08870432	1.054703	0.616689418	-0.697384	0.624968	1
LINC00240	1.0830298	1.10705345	1.0726166	0.678321393	-0.5599591	0.625098	1
LINC00535	1.0323512	1.0559311	1.0221304	0.395671887	-1.3376235	0.625324	1
CNIH3	1.1568374	1.18164998	1.1460823	0.804196456	-0.3143801	0.625347	1
COG4	1.2909172	1.26444079	1.3023936	1.14352093	0.1934828	0.625363	1
MCAM	1.1061917	1.0819106	1.1167165	1.424924964	0.5108859	0.625485	1
AMDHD2	1.2307177	1.25603181	1.2197452	0.85827315	-0.2204912	0.625527	1
PURB	1.3650718	1.33788828	1.3768547	1.115323365	0.1574621	0.625712	1

SLC2A12	1.0956269	1.11984261	1.0851304	0.710351934	-0.4933941	0.625716	1
GLDC	1.0979741	1.12217815	1.0874827	0.716025331	-0.4819175	0.625875	1
CLYBL	1.1034077	1.12773444	1.0928632	0.727001733	-0.4599693	0.62592	1
SLC39A9	1.2370593	1.21104946	1.2483334	1.17665991	0.2346974	0.625932	1
AC000068.5	1.0759246	1.09974338	1.0656003	0.657690325	-0.6045196	0.625933	1
SEMA3B	1.2811119	1.25482174	1.2925075	1.147890844	0.1989855	0.626095	1
ALG12	1.3386413	1.31210392	1.350144	1.121882735	0.1659219	0.626133	1
SPC25	1.2501417	1.27564856	1.2390856	0.867356776	-0.2053025	0.626175	1
ZFP41	1.0742877	1.09806632	1.0639807	0.652423124	-0.6161202	0.626341	1
MAP2K7	1.5150755	1.48655933	1.527436	1.084011785	0.1163804	0.626536	1
AC012354.6	1.1324869	1.1077858	1.1431937	1.328502509	0.409801	0.626548	1
HS3ST1	1.0904004	1.06612289	1.1009236	1.526304182	0.6100425	0.626641	1
RP5-1198O20.4	1.1031061	1.12722807	1.0926502	0.728221652	-0.4575505	0.626804	1
CTB-129O4.1	1.0749281	1.09860401	1.0646656	0.655811329	-0.6086473	0.626857	1
TRPC4	1.0574804	1.03365289	1.0678085	2.014939027	1.0107362	0.626954	1
TRMT1	1.5215659	1.54948887	1.5094625	0.92715704	-0.1091144	0.626987	1
RIMBP2	1.052192	1.07573267	1.0419882	0.554426639	-0.8509315	0.627149	1
NAA38	3.9462368	4.005939	3.9203586	0.971529562	-0.0416702	0.62718	1
ZHX1	1.6603904	1.68937823	1.6478254	0.939724204	-0.0896907	0.62727	1
PIGL	1.258167	1.28375313	1.2470766	0.870744955	-0.1996779	0.627348	1
ARHGAP12	1.2965088	1.27020064	1.3079122	1.139568717	0.1884879	0.627458	1
VPS52	1.1955069	1.2205858	1.1846363	0.837027303	-0.2566534	0.627468	1
ATXN1	1.2566985	1.28235233	1.2455786	0.869759596	-0.2013114	0.627557	1
EXOC7	1.8832341	1.91478237	1.8695593	0.950564143	-0.0731441	0.627589	1
FAAP20	3.5453347	3.48906728	3.5697241	1.032404452	0.0460083	0.627763	1
DNAJC4	2.313195	2.34793499	2.2981368	0.963055953	-0.0543085	0.627784	1
AC092835.2	1.0637369	1.08749	1.053441	0.610824597	-0.7111699	0.627956	1
VPS37C	1.1580336	1.13356175	1.1686411	1.262644984	0.3364491	0.628035	1
CYFIP1	1.3906287	1.36395409	1.402191	1.105059653	0.1441243	0.628079	1
INTS7	1.1217331	1.1458597	1.1112752	0.762892247	-0.3904488	0.628105	1
ZNF181	1.1765402	1.20107603	1.165905	0.825086053	-0.2773835	0.628218	1
NISCH	1.3787345	1.40532469	1.3672088	0.905962157	-0.1424773	0.628251	1
NEK10	1.0636696	1.08733135	1.0534133	0.61161706	-0.7092994	0.62826	1
PKD1	1.2997469	1.32583981	1.2884367	0.885210231	-0.175908	0.62846	1
DIRAS2	1.1562844	1.18065284	1.1457217	0.806639474	-0.3100041	0.628598	1
LMNB2	1.4238261	1.45056487	1.4122361	0.91493165	-0.1282641	0.628644	1
PDE3A	1.0625304	1.08605963	1.0523315	0.60808456	-0.7176561	0.628682	1
RP11-385D13.3	1.1261958	1.15040835	1.1157008	0.769244414	-0.378486	0.628686	1
HSPA12A	1.0716318	1.04759683	1.0820499	1.723851247	0.7856353	0.628705	1
CCDC24	1.1397761	1.11513141	1.1504585	1.306841613	0.3860843	0.628713	1
SLC1A4	1.0828973	1.10694295	1.0724745	0.677693468	-0.5612952	0.628723	1
LACC1	1.049779	1.0261008	1.0600424	2.300403817	1.2018871	0.628742	1
HIF1AN	1.349859	1.37600639	1.3385253	0.900317893	-0.1514936	0.628971	1
AC009133.12	1.1251347	1.14932402	1.1146497	0.767791118	-0.3812142	0.629314	1
THAP5	1.582065	1.61009074	1.5699171	0.934151366	-0.0982718	0.629353	1
TUBA4A	1.0498951	1.02623844	1.0601492	2.292407925	1.1968638	0.629568	1
C15orf52	1.0576379	1.03400163	1.0678831	1.99646651	0.9974489	0.629636	1
RORA-AS1	1.0445095	1.06792822	1.0343585	0.505805811	-0.9833445	0.629714	1
AP5B1	1.0669848	1.04322137	1.0772851	1.788123263	0.8384462	0.629732	1
MSS51	1.0386144	1.06183461	1.0285495	0.46170758	-1.1149487	0.629791	1
C1orf35	1.6642309	1.63518062	1.6768229	1.065559692	0.0916114	0.629802	1

MEIG1	1.0595481	1.08311078	1.0493348	0.593602511	-0.7524309	0.629821	1
SESN1	1.3270228	1.30038799	1.3385678	1.127101708	0.1726177	0.629833	1
TNPO2	1.3269733	1.30047418	1.3384595	1.126417794	0.171742	0.629833	1
SRPK1	2.0120395	1.9797164	2.0260501	1.047292999	0.0666651	0.629849	1
NCBP3	1.5548178	1.52652302	1.5670823	1.077032289	0.1070615	0.629923	1
LINC01006	1.267543	1.29298926	1.2565132	0.875503769	-0.1918147	0.629941	1
GAPVD1	1.335852	1.30960514	1.3472288	1.121521387	0.1654571	0.630142	1
SOBP	1.1422375	1.11772368	1.1528631	1.298491032	0.376836	0.630206	1
GNGT1	1.0648737	1.04104894	1.0752007	1.831977495	0.8734018	0.630274	1
SP140L	1.062902	1.08630433	1.0527581	0.611302715	-0.7100411	0.630605	1
HIST3H2A	1.6304682	1.65928722	1.6179764	0.937340188	-0.0933554	0.63068	1
RP11-727F15.12	1.0492976	1.07254745	1.0392199	0.540609961	-0.88734	0.630686	1
SMIM10L2A	1.1405627	1.16471324	1.1300946	0.789824554	-0.3403959	0.630733	1
OPCML	1.1492648	1.12446979	1.1600123	1.285551604	0.3623875	0.630833	1
CHGB	1.0432568	1.01968058	1.053476	2.717196315	1.4421188	0.630857	1
WHAMM	1.3584758	1.38484826	1.3470445	0.901769651	-0.1491691	0.630872	1
FTSJ1	1.233437	1.20817967	1.2443849	1.173913597	0.2313262	0.630906	1
AC010883.5	1.0601439	1.08354556	1.0500004	0.598480149	-0.7406247	0.630971	1
MIF-AS1	1.1704252	1.14542931	1.1812598	1.246377229	0.3177408	0.631085	1
CCDC22	1.409443	1.38207939	1.4213039	1.10266058	0.1409888	0.631105	1
MFSD9	1.127189	1.10288163	1.1377252	1.338675799	0.4208066	0.631205	1
MOSPD2	1.6431481	1.67223535	1.6305401	0.937975235	-0.0923783	0.631209	1
COG2	1.21696	1.19193609	1.2278068	1.186888753	0.2471847	0.631268	1
FAM43A	1.1631497	1.1875587	1.1525694	0.813448872	-0.2978764	0.631518	1
MED29	1.5484574	1.52058148	1.5605404	1.076758203	0.1066943	0.63156	1
LA16c-325D7.2	1.0423705	1.06553194	1.0323311	0.493364072	-1.0192754	0.631678	1
DOCK5	1.0505525	1.02701672	1.0607542	2.248761583	1.1691307	0.631834	1
UNC93B1	1.0758256	1.05218346	1.0860734	1.649438989	0.7219754	0.631946	1
RDH5	1.1828298	1.20737944	1.1721886	0.830306973	-0.2682833	0.632119	1
ADAM12	1.1829391	1.2076547	1.172226	0.829386253	-0.269884	0.632119	1
DPP8	1.2910965	1.31703509	1.2798533	0.882720323	-0.1799717	0.632143	1
C6orf89	1.4090319	1.38179668	1.4208372	1.102254777	0.1404577	0.632294	1
B9D2	1.2181421	1.19295065	1.2290615	1.18715069	0.2475031	0.632338	1
CTB-58E17.1	1.0918556	1.11537711	1.0816601	0.707766648	-0.4986543	0.632378	1
CTC-499B15.5	1.0507325	1.07391794	1.0406826	0.55037582	-0.861511	0.63248	1
RP11-481J2.3	1.0507149	1.07387025	1.040678	0.550668433	-0.8607442	0.63248	1
TAOK3	1.5316394	1.55898144	1.5197878	0.929883902	-0.1048775	0.632691	1
PECR	1.2085145	1.18364537	1.2192942	1.194117696	0.255945	0.632693	1
PTPRB	1.0592604	1.03583143	1.0694158	1.93728919	0.9540393	0.632709	1
ZNF260	1.3539467	1.38011254	1.342605	0.901325058	-0.1498806	0.632738	1
ZDHHC13	1.2041669	1.22908173	1.1933674	0.844097734	-0.244518	0.632878	1
CTC-444N24.8	1.0776827	1.05400836	1.0879445	1.628350055	0.7034109	0.632884	1
MIER2	1.2413706	1.21589527	1.2524131	1.169145933	0.225455	0.632885	1
LRP4	1.1225774	1.14662237	1.1121549	0.764923653	-0.3866123	0.632938	1
NIPAL3	1.2627093	1.28792068	1.2517813	0.874481464	-0.1935003	0.633106	1
CLIC3	1.049161	1.0257863	1.0592929	2.29939732	1.2012558	0.633107	1
RP11-420A23.1	1.0582816	1.08149213	1.0482209	0.591724591	-0.7570022	0.633124	1
AGK	1.213421	1.18830525	1.2243076	1.19119128	0.2524051	0.633181	1
KLF4	1.0763395	1.05274953	1.0865647	1.64105095	0.71462	0.633226	1
ATE1	1.3356058	1.30949431	1.346924	1.120938294	0.1647069	0.633298	1
ITPR3	1.0591673	1.03581055	1.0692914	1.934944679	0.9522923	0.633304	1

ZNF79	1.0404973	1.06368925	1.0304446	0.478017945	-1.0648633	0.633489	1
ARL5A	1.7352757	1.70569964	1.7480956	1.060076559	0.0841685	0.63351	1
F2RL2	1.1006388	1.12419967	1.0904262	0.728070816	-0.4578493	0.633588	1
PDE1C	1.0849796	1.06127003	1.0952567	1.554702723	0.6366387	0.633672	1
FIBIN	1.0439645	1.02055602	1.054111	2.632368694	1.3963616	0.633682	1
RNF20	1.3700589	1.34331728	1.3816501	1.11165434	0.1527083	0.633722	1
GPX7	2.4668559	2.43034888	2.48268	1.036586284	0.0518402	0.633728	1
RNF34	1.3773652	1.35064968	1.3889451	1.109212823	0.1495362	0.63374	1
U2AF1L5	1.0690421	1.0922421	1.058986	0.639468988	-0.6450537	0.633748	1
FBXO48	1.0780853	1.10154828	1.0679151	0.66879608	-0.5803617	0.633775	1
RXRB	1.3553841	1.32937762	1.3666568	1.11318057	0.1546876	0.633817	1
RGS4	1.0669723	1.04346105	1.0771634	1.775461902	0.8281944	0.633917	1
RP11-367N14.2	1.0376878	1.06056135	1.0277731	0.458594249	-1.1247098	0.633931	1
DNAJB4	1.3953844	1.36864717	1.4069737	1.103965438	0.142695	0.634163	1
CELF3	1.0666777	1.04294998	1.0769627	1.791913764	0.8415012	0.634216	1
NFYA	1.1690168	1.14444934	1.1796657	1.243797479	0.3147516	0.634232	1
ARHGEF5	1.0724266	1.04886799	1.0826382	1.69104974	0.7579191	0.634435	1
PIK3R1	1.9432343	1.97431951	1.9297602	0.954266201	-0.0675363	0.634472	1
MSRB1	1.4832881	1.51018727	1.4716285	0.924422256	-0.1133761	0.634584	1
VTI1A	1.1515486	1.17562573	1.1411122	0.803482599	-0.3156613	0.634636	1
CEP55	1.2020513	1.17717663	1.2128334	1.20124998	0.2645364	0.634722	1
BRICD5	1.0708723	1.09418594	1.0607669	0.64518027	-0.6322258	0.634748	1
PIK3CA	1.2535727	1.27842025	1.2428024	0.872071742	-0.1974813	0.634814	1
FAM193A	1.205132	1.18007323	1.2159938	1.199477634	0.2624063	0.634963	1
RP11-849H4.2	1.0894444	1.11284794	1.0793	0.702715969	-0.5089864	0.635036	1
CCDC181	1.3933389	1.36711534	1.4047057	1.102393816	0.1406397	0.635052	1
TRAPPC11	1.222082	1.24672594	1.2114	0.856821195	-0.2229339	0.635107	1
KDM4A-AS1	1.1012135	1.12462048	1.0910676	0.730759435	-0.4525315	0.635159	1
RNF135	1.2296083	1.20488662	1.240324	1.172960836	0.2301548	0.635236	1
ZNF222	1.0695232	1.09278009	1.0594424	0.640680787	-0.6423224	0.635245	1
LARGE1	1.1418811	1.16588317	1.1314773	0.792589713	-0.3353539	0.635349	1
GATB	1.1838574	1.15948688	1.1944209	1.219040292	0.2857458	0.63555	1
FASTKD3	1.0954597	1.07183811	1.1056986	1.471344441	0.557135	0.635654	1
SRRM4	1.0852713	1.06138721	1.095624	1.557718838	0.6394349	0.635735	1
TBKBP1	1.1339831	1.15774921	1.1236816	0.784039454	-0.3510018	0.635824	1
EVI5	1.5877225	1.61610435	1.5754202	0.933965434	-0.0985589	0.635884	1
UPRT	1.1550079	1.13056528	1.1656028	1.268352212	0.3429554	0.636122	1
LSS	1.2619329	1.2367711	1.2728395	1.15233433	0.2045594	0.636153	1
C16orf58	1.3136824	1.28784383	1.3248823	1.128675584	0.1746309	0.636183	1
CCDC183	1.0597406	1.08305709	1.0496339	0.597587928	-0.7427771	0.636233	1
COMMD5	1.3922409	1.3658537	1.4036786	1.103388116	0.1419403	0.636237	1
ZNF239	1.0646661	1.08788975	1.0545998	0.62123006	-0.6868005	0.636401	1
AC009501.4	1.1279235	1.10372486	1.1384125	1.334419936	0.4162127	0.636674	1
MORF4L2-AS1	1.0955	1.11897923	1.0853228	0.717123315	-0.4797069	0.636986	1
TMEM147-AS1	1.2393745	1.26393081	1.2287304	0.866630066	-0.2065118	0.637027	1
WNT7B	1.0865933	1.06301745	1.0968124	1.536279825	0.619441	0.637222	1
ANKRD65	1.1399083	1.1637149	1.1295892	0.791554164	-0.33724	0.637249	1
ADAMTSL4	1.0717411	1.09492681	1.0616911	0.649880351	-0.621754	0.637289	1
ARHGAP5-AS1	1.1643083	1.13988709	1.1748938	1.250249396	0.3222159	0.637327	1
MEGF8	1.2718876	1.24659526	1.2828507	1.14702413	0.1978957	0.637393	1
ZNF461	1.0687523	1.09189795	1.0587197	0.638966563	-0.6461877	0.637429	1

DPH7	1.3310619	1.30524274	1.3422533	1.121249562	0.1651074	0.637455	1
EBF1	1.0481282	1.02473755	1.058267	2.355405284	1.2359753	0.637459	1
DTWD1	2.049724	2.01737875	2.0637442	1.045573394	0.0642943	0.637496	1
HUWE1	1.7643121	1.79324429	1.7517712	0.947717172	-0.0774715	0.637807	1
FANK1	1.2584554	1.28323452	1.2477148	0.874592516	-0.1933171	0.637869	1
MYL5	1.1596045	1.18364787	1.1491827	0.812330212	-0.2998618	0.637884	1
TMEM87A	1.5226568	1.49514074	1.5345838	1.079660333	0.1105775	0.637944	1
XXbac-BPG32J3.19	1.0405488	1.06331424	1.030681	0.484582694	-1.0451852	0.637948	1
NAA25	1.3372456	1.36255908	1.3262733	0.899917703	-0.152135	0.637964	1
LINC01301	1.0533931	1.07615558	1.0435266	0.571548761	-0.8070515	0.637998	1
TOX4	1.7161455	1.68721732	1.7286846	1.060340874	0.0845281	0.638156	1
POLD2	3.6515052	3.71788332	3.6227332	0.964991084	-0.0514125	0.638315	1
H6PD	1.2572189	1.23213937	1.2680897	1.154865361	0.2077247	0.638438	1
SLC2A10	1.2010572	1.22562281	1.1904091	0.843926814	-0.2448102	0.638534	1
RP11-651L5.3	1.1153145	1.13888612	1.1050973	0.756715704	-0.4021767	0.638638	1
ENPP2	1.0872643	1.06378384	1.0974421	1.527692219	0.6113539	0.638912	1
AC002117.1	1.1257264	1.14923484	1.1155366	0.77419306	-0.3692347	0.638915	1
URGCP	1.1752201	1.19966238	1.1646254	0.824519005	-0.2783753	0.638917	1
WWP1	1.2879165	1.26264083	1.2988724	1.137950945	0.1864384	0.638927	1
TMEM138	1.3774773	1.40375669	1.3660864	0.906700529	-0.141302	0.63903	1
HSPA13	1.4985069	1.47195282	1.5100169	1.080652327	0.1119024	0.639042	1
APH1B	1.1455847	1.12139743	1.1560688	1.285602342	0.3624445	0.639206	1
TRIL	1.0703433	1.04699867	1.0804621	1.712008551	0.7756899	0.639264	1
GK	1.099035	1.12239314	1.0889103	0.726431809	-0.4611007	0.639355	1
EFCAB10	1.0526351	1.07542508	1.0427566	0.566875618	-0.8188959	0.639418	1
CAMKMT	1.2246984	1.24934766	1.214014	0.858295469	-0.2204537	0.639747	1
BANP	1.2409285	1.21615762	1.2516655	1.164268585	0.2194239	0.639772	1
KIF16B	1.1868767	1.16250983	1.1974387	1.214933781	0.2808777	0.639909	1
MYO1F	1.0751503	1.09812156	1.0651933	0.664413652	-0.5898464	0.639925	1
APPBP2	1.592178	1.56444672	1.6041982	1.070425631	0.0981846	0.639941	1
IFT172	1.2666155	1.29104381	1.2560269	0.879685108	-0.1849409	0.639945	1
VMAC	1.0811074	1.05765837	1.0912715	1.58297112	0.6626349	0.64015	1
NABP1	1.1465423	1.17034792	1.1362237	0.799679094	-0.3225069	0.640231	1
TMCC2	1.0632344	1.04017201	1.0732309	1.822933831	0.8662622	0.640306	1
SNX10	1.4053234	1.43126212	1.3940801	0.913783361	-0.1300759	0.640311	1
CD2AP	1.5165454	1.54343596	1.5048896	0.929069088	-0.1061422	0.640552	1
CARNS1	1.047156	1.02429327	1.057066	2.349046991	1.2320756	0.640592	1
SNX29	1.157239	1.13317039	1.1676717	1.259076334	0.3323658	0.640751	1
PDAP1	3.9433612	3.88327013	3.9694079	1.029875039	0.0424693	0.640942	1
HNRNPUL2	1.4084344	1.38201145	1.4198876	1.099149136	0.1363871	0.641004	1
AC009404.2	1.0413683	1.06398503	1.031565	0.493318275	-1.0194094	0.641017	1
NEIL2	1.9885134	1.95743821	2.0019831	1.046525058	0.0656069	0.64118	1
MAPK13	1.1144492	1.09109647	1.1245715	1.367468416	0.4515075	0.641397	1
KIAA1958	1.3871349	1.41255978	1.3761144	0.911660311	-0.1334317	0.641448	1
RP11-389G6.3	1.0325602	1.0551115	1.0227852	0.413437518	-1.2742588	0.641513	1
TLE2	1.1322725	1.1556911	1.1221216	0.784384056	-0.3503679	0.641516	1
DLGAP4-AS1	1.0638627	1.08670291	1.0539625	0.622383419	-0.6841245	0.64153	1
WASIR2	1.0644692	1.08717926	1.0546254	0.626587198	-0.6744128	0.641692	1
GNPAT	1.8441186	1.81419407	1.8570895	1.052684523	0.0740731	0.6417	1
FGF13	1.4449286	1.41811961	1.4565491	1.091910405	0.1268545	0.64172	1
LGALSL	1.3755236	1.34957851	1.3867696	1.106388444	0.145858	0.641742	1

STAC	1.0889351	1.1119235	1.0789707	0.705577395	-0.5031238	0.64176	1
B4GALT5	1.3058291	1.28048602	1.3168142	1.129518772	0.1757082	0.642108	1
HOXB-AS3	1.0551281	1.03208519	1.0651161	2.029476193	1.0211074	0.642136	1
GLIS2	1.2582413	1.23346924	1.2689789	1.152095789	0.2042607	0.642241	1
THEM4	1.4666954	1.49323665	1.455191	0.922865341	-0.1158079	0.642287	1
EMG1.1	1.0766463	1.09944728	1.0667632	0.671342208	-0.5748797	0.642439	1
PTPRD-AS1	1.2005605	1.17606592	1.2111778	1.199424757	0.2623427	0.642451	1
MEF2D	1.1009963	1.12440983	1.0908475	0.730227779	-0.4535815	0.642659	1
LYG2	1.0430822	1.06557087	1.0333344	0.508371674	-0.9760444	0.642723	1
KIAA1456	1.1751705	1.19895878	1.1648593	0.828610335	-0.2712343	0.642848	1
IFT74	1.3693873	1.34359445	1.3805673	1.107606155	0.147445	0.64305	1
MYLIP	1.1131788	1.08980513	1.1233102	1.37308665	0.4574227	0.64307	1
CALB2	1.039592	1.01685068	1.0494494	2.93456244	1.5531454	0.643084	1
ARHGAP42	1.1136851	1.13667581	1.1037196	0.758873043	-0.3980695	0.643282	1
LZTS1	1.060395	1.08293616	1.0506243	0.610401416	-0.7121698	0.643311	1
TAF2	1.2348286	1.21045091	1.2453952	1.16604489	0.2216233	0.643428	1
AMPH	1.1160356	1.13898486	1.1060882	0.763307445	-0.3896638	0.643479	1
ZBTB8B	1.1161832	1.13922192	1.1061969	0.762788666	-0.3906447	0.643479	1
PSMG3	1.420664	1.39441991	1.4320396	1.095379793	0.1314312	0.643497	1
IGF2-AS	1.0482703	1.02543753	1.0581673	2.286673163	1.1932502	0.643549	1
NEIL3	1.0730517	1.09576788	1.0632053	0.659984525	-0.5994959	0.643615	1
RHOG	1.4519946	1.42561018	1.4634311	1.088862792	0.1228222	0.643855	1
ISG20	1.07294	1.04990338	1.0829254	1.661718487	0.732676	0.64387	1
CTDSPL	1.184323	1.16013089	1.1948092	1.216562135	0.28281	0.643978	1
MAML1	1.1924132	1.21640032	1.1820159	0.841107187	-0.2496384	0.644067	1
NPC1	1.1137083	1.13690164	1.1036551	0.757149906	-0.4013491	0.644362	1
B3GALNT2	1.3669936	1.39261881	1.3558862	0.906442032	-0.1417133	0.644394	1
ZBTB7A	1.1575277	1.13397829	1.1677353	1.251958956	0.3241873	0.644427	1
ADORA1	1.0628073	1.08541719	1.053007	0.620565764	-0.688344	0.644516	1
CBWD2	1.413596	1.38759319	1.4248671	1.09616762	0.1324684	0.644539	1
SULT1C2	1.1332398	1.10985334	1.1433768	1.305165492	0.3842327	0.644637	1
IFI35	1.1452197	1.16887117	1.1349678	0.799235232	-0.3233079	0.644732	1
GPCPD1	1.1993015	1.17486473	1.2098937	1.200320306	0.2634194	0.644822	1
WARS2	1.1990678	1.17486484	1.2095587	1.198404003	0.2611143	0.644822	1
DYRK3	1.1094699	1.08604062	1.1196254	1.390336397	0.475434	0.645074	1
RASSF2	1.1321389	1.15515482	1.1221625	0.787358666	-0.3449071	0.645248	1
LYRM4	2.5069188	2.47022563	2.5228237	1.035775501	0.0507113	0.64529	1
ZDHHC11	1.061309	1.08369114	1.0516073	0.61663979	-0.6975001	0.645324	1
ADAMTS9-AS1	1.2598744	1.23499666	1.2706578	1.151751815	0.2038299	0.645515	1
PER3	1.0912377	1.06820226	1.1012225	1.484151679	0.5696385	0.64574	1
EPHA5-AS1	1.0492545	1.02657069	1.059087	2.223764543	1.153004	0.645762	1
PITX1	1.0413505	1.01869874	1.051169	2.736497386	1.4523305	0.645779	1
EHD3	1.1076438	1.084257	1.1177809	1.397876632	0.483237	0.645828	1
CTA-246H3.12	1.0302727	1.05244505	1.020662	0.393974066	-1.3438274	0.64586	1
PDE4A	1.0607286	1.08315165	1.0510092	0.613448292	-0.7049864	0.645927	1
BMI1	1.1634047	1.18681099	1.1532592	0.820396891	-0.2856061	0.646076	1
ARRB2	1.5143398	1.54069373	1.5029165	0.930131892	-0.1044928	0.646228	1
IQCG	1.1717697	1.1950446	1.161681	0.828943962	-0.2706535	0.646275	1
SEMA3A	1.6741465	1.64665854	1.6860612	1.060932765	0.0853332	0.646284	1
RP5-1042K10.10	1.1126293	1.13561012	1.1026682	0.757083318	-0.401476	0.646347	1
AVL9	1.4040612	1.37818499	1.4152773	1.098079853	0.134983	0.646396	1

FLCN	1.3402575	1.36493871	1.3295593	0.903053887	-0.147116	0.646408	1
KIRREL	1.1997678	1.17564168	1.2102253	1.196898916	0.2593013	0.646425	1
DR1	1.6909725	1.66267333	1.7032389	1.061215088	0.0857171	0.646652	1
ZNF354C	1.0507103	1.07281776	1.0411276	0.564802267	-0.8241822	0.646724	1
NUDT16	1.3791213	1.35393655	1.3900378	1.101999082	0.140123	0.64679	1
GAB1	1.1539141	1.17702532	1.1438965	0.81285816	-0.2989245	0.646824	1
XKR8	1.2940723	1.2695459	1.3047034	1.130432515	0.1768749	0.646843	1
BCL3	1.2197049	1.24348834	1.2093958	0.859983056	-0.2176199	0.646855	1
GRPEL2	1.1189031	1.14201844	1.1088836	0.76668652	-0.3832913	0.647012	1
RIOK1	1.1658382	1.14216175	1.1761008	1.238735711	0.3088684	0.647338	1
ZNF792	1.065701	1.04282544	1.0756165	1.765691286	0.8202331	0.647536	1
ADAM22	1.1310833	1.15398754	1.1211554	0.786786905	-0.3459551	0.647558	1
VGLL3	1.0704571	1.04762685	1.0803531	1.687137938	0.7545779	0.647684	1
PTPN13	1.4149121	1.44028893	1.4039123	0.917380098	-0.1244085	0.647745	1
ZBED3	1.1729154	1.14917475	1.183206	1.228129782	0.296463	0.647791	1
KRR1	1.5415998	1.51459924	1.5533033	1.075211991	0.1046211	0.647986	1
APLF	1.0772692	1.05457416	1.0871065	1.596112727	0.6745625	0.648002	1
RNLS	1.079037	1.05635097	1.0888704	1.577088122	0.6572633	0.648136	1
RP1-223E5.4	1.058464	1.0807162	1.0488187	0.604819165	-0.7254242	0.648241	1
FAM167A	1.0902715	1.11279096	1.0805103	0.713801007	-0.4864062	0.648364	1
RP11-390E23.6	1.401735	1.42692493	1.3908163	0.915421493	-0.1274919	0.648366	1
DDX39A	1.5010105	1.47506616	1.5122563	1.078284075	0.1087373	0.648493	1
GPD2	1.1517542	1.17479421	1.1417673	0.811052867	-0.3021321	0.648749	1
RP11-21L23.3	1.0783099	1.10071926	1.0685965	0.681066251	-0.554133	0.648852	1
NAP1L5	1.1755636	1.15166509	1.1859225	1.225875514	0.2938125	0.648869	1
ZNF287	1.1348778	1.15798972	1.1248598	0.790303215	-0.3395218	0.648919	1
ZNF333	1.1374699	1.16041872	1.1275227	0.794936329	-0.3310888	0.649027	1
CHD1	1.8936292	1.92316937	1.8808248	0.954131298	-0.0677403	0.649227	1
LPL	1.0662712	1.04370503	1.0760526	1.740132978	0.7991976	0.649248	1
LACTB	1.3077276	1.28300041	1.3184458	1.125248378	0.1702435	0.649253	1
SPATA12	1.0664652	1.08874687	1.0568071	0.640102783	-0.6436245	0.649276	1
SAPCD1-AS1	1.0565488	1.07878022	1.0469125	0.595485301	-0.7478622	0.649376	1
KALRN	1.1533819	1.12987792	1.1635698	1.259411785	0.3327501	0.649472	1
CLASP1	1.3504965	1.375552	1.339636	0.904364764	-0.1450233	0.649484	1
ABCD3	1.2980418	1.27332654	1.3087548	1.129618616	0.1758358	0.649487	1
CC2D2A	1.4729833	1.44639068	1.48451	1.085394549	0.1182196	0.649708	1
TRDMT1	1.218193	1.24191934	1.2079087	0.85941343	-0.2185758	0.649717	1
ZBED5-AS1	1.2179691	1.19383307	1.228431	1.178493453	0.2369437	0.649867	1
CTC-304I17.6	1.028268	1.05012829	1.0187925	0.374888718	-1.4154657	0.649868	1
MFN1	1.2301061	1.20622596	1.2404571	1.165988326	0.2215533	0.649874	1
FBXL19	1.1897309	1.21279702	1.1797327	0.844620368	-0.2436251	0.64988	1
ZKSCAN7	1.1016024	1.07856689	1.1115872	1.420283194	0.5061786	0.649902	1
RP1-193H18.2	1.1273552	1.15021113	1.1174482	0.781887772	-0.3549665	0.649983	1
KXD1	2.5752325	2.61359648	2.5586034	0.965918933	-0.050026	0.650058	1
AP1G2	1.3191002	1.34369699	1.3084386	0.897414426	-0.1561537	0.650209	1
MIB1	1.8796281	1.90836425	1.8671723	0.954652601	-0.0669523	0.650295	1
RBFOX3	1.043955	1.02167	1.0536146	2.474138307	1.3069262	0.650427	1
GTPBP3	1.3616208	1.38673466	1.350735	0.906913891	-0.1409625	0.650466	1
WDR66	1.1159029	1.13859804	1.1060656	0.765274601	-0.3859506	0.650565	1
RANGAP1	1.3299049	1.30524001	1.3405961	1.115830449	0.1581178	0.650576	1
PI4K2B	1.1097726	1.08680421	1.1197284	1.379292549	0.4639285	0.650582	1

BID	1.8188748	1.78952177	1.831598	1.053293355	0.0749073	0.650639	1
TEAD1	1.4915471	1.46521012	1.5029631	1.081152461	0.11257	0.650678	1
ATP9A	1.1791954	1.20258116	1.1690587	0.834523048	-0.2609762	0.650679	1
MAD2L2	2.7776744	2.73741722	2.7951242	1.033214203	0.0471394	0.650726	1
RP9	1.5193315	1.49292804	1.5307762	1.076782288	0.1067266	0.650768	1
MTRNR2L8	1.0524735	1.03007039	1.0621843	2.067957156	1.0482063	0.650844	1
RP4-639F20.1	1.271957	1.24746911	1.2825714	1.141845306	0.1913672	0.650949	1
BET1	1.8556128	1.82426446	1.8692009	1.054516996	0.0765823	0.651009	1
ZCCHC6	1.2389767	1.21510353	1.2493247	1.159091427	0.2129944	0.651078	1
HKDC1	1.0481687	1.02595869	1.0577958	2.226451398	1.1547461	0.65109	1
TNFRSF10A	1.0446532	1.02248618	1.0542617	2.413111344	1.2708945	0.651349	1
SLC39A3	1.5929831	1.56599659	1.6046806	1.068346704	0.0953799	0.651436	1
SPAG5	1.1576603	1.18085183	1.1476078	0.816180759	-0.2930394	0.651493	1
STOML1	1.1377839	1.11465665	1.1478085	1.289140496	0.3664095	0.651534	1
COPZ2	1.1974143	1.22061762	1.1873567	0.849237185	-0.2357606	0.651775	1
RP11-188D8.1	1.0386106	1.06042244	1.0291561	0.482537888	-1.0512859	0.651783	1
RP11-346D14.1	1.029012	1.05071531	1.0196046	0.386561339	-1.3712307	0.651801	1
SEL1L3	1.3462649	1.37106736	1.3355141	0.904186522	-0.1453077	0.651812	1
ATG13	1.3033646	1.27859415	1.3141015	1.127452022	0.173066	0.651953	1
PRICKLE4	1.1124134	1.13503076	1.1026097	0.759898977	-0.3961205	0.652029	1
AL592528.1	1.0452199	1.06704312	1.0357605	0.533395661	-0.906722	0.652129	1
CD52	1.0619228	1.03979342	1.0715149	1.797153445	0.8457136	0.652181	1
NFIX	1.0542	1.03200224	1.0638217	1.994288024	0.9958738	0.65221	1
PRR14L	1.5550232	1.58115556	1.543696	0.935543055	-0.096124	0.652424	1
C18orf65	1.0464745	1.06826853	1.0370278	0.54238397	-0.8826136	0.652589	1
RACGAP1	1.2351809	1.25888728	1.2249052	0.868737911	-0.2030071	0.652608	1
PDSS2	1.1705099	1.19347209	1.1605568	0.8298706	-0.2690417	0.652613	1
PLRG1	1.484476	1.45851647	1.4957283	1.081157088	0.1125762	0.652651	1
CTD-2256P15.2	1.136152	1.11315864	1.1461186	1.291272139	0.3687931	0.652816	1
FRRS1	1.0380807	1.05993284	1.0286088	0.477347464	-1.0668883	0.652949	1
SMIM10	1.2594726	1.28327837	1.2491538	0.879537008	-0.1851838	0.65301	1
ACKR3	1.0639588	1.08616787	1.0543321	0.630538373	-0.6653439	0.653084	1
CUX2	1.0473441	1.06909889	1.0379144	0.548697434	-0.8659173	0.653201	1
PROCR	1.0618416	1.03964775	1.0714616	1.802412518	0.8499292	0.653238	1
NCAPH	1.1043293	1.12669823	1.0946334	0.746919815	-0.4209747	0.653307	1
RBAK-RBAKDN	1.2472899	1.22337676	1.2576552	1.153455661	0.2059625	0.65331	1
L3MBTL2	1.1679157	1.1910117	1.1579047	0.826675381	-0.2746072	0.653366	1
WNK3	1.4075597	1.43239804	1.3967934	0.917657673	-0.123972	0.65354	1
MYT1	1.0376948	1.0156563	1.0472475	3.017798805	1.5934966	0.653673	1
RABGAP1L	1.1791906	1.15555147	1.1894371	1.217842165	0.2843272	0.653961	1
MAMDC2-AS1	1.0754219	1.09753315	1.0658377	0.675028752	-0.5669791	0.654073	1
CTB-12O2.1	1.0462931	1.02421417	1.0558633	2.307048779	1.2060485	0.654073	1
RAD17	1.3584614	1.33357158	1.36925	1.106958731	0.1466014	0.65415	1
ALKBH8	1.1160634	1.13837088	1.1063941	0.768905415	-0.379122	0.654231	1
PBX4	1.1367931	1.15946646	1.1269652	0.796187201	-0.3288204	0.654442	1
AES	2.9020922	2.85473572	2.9226192	1.036600076	0.0518594	0.654472	1
COLCA1	1.0572328	1.07894316	1.0478223	0.605781141	-0.7231314	0.654565	1
DDX28	1.0866395	1.06426039	1.0963398	1.49920977	0.5842023	0.654661	1
RP11-324I22.4	1.0647016	1.08655135	1.0552307	0.638125905	-0.648087	0.654778	1
DZANK1	1.1224736	1.14483546	1.1127807	0.778681711	-0.3608944	0.654877	1
CASC3	1.5744889	1.60082131	1.563075	0.937175491	-0.0936089	0.655046	1

UBFD1	1.3827712	1.35804824	1.3934875	1.098978892	0.1361637	0.65516	1
STK3	1.4192113	1.44451673	1.4082426	0.918396454	-0.122811	0.655269	1
SYTL1	1.2415349	1.21755404	1.2519296	1.158009303	0.2116468	0.655323	1
DEPDC1B	1.2573763	1.23345622	1.2677446	1.146873041	0.1977057	0.655384	1
PARD3-AS1	1.0516389	1.07342465	1.0421957	0.574680584	-0.7991678	0.655453	1
RASSF1	1.2521381	1.22837528	1.2624382	1.149153036	0.2005709	0.655547	1
CEP41	1.5173034	1.54315586	1.5060975	0.931772073	-0.101951	0.655573	1
FAM131A	1.2844653	1.26020667	1.2949803	1.133638589	0.1809608	0.655712	1
CTC-444N24.13	1.033693	1.05524163	1.0243527	0.440838998	-1.1816762	0.655803	1
ZYG11B	1.4360446	1.41088957	1.4469482	1.087757416	0.1213569	0.655936	1
SCO2	1.3001963	1.27610128	1.3106404	1.125095711	0.1700477	0.655961	1
SLC25A32	1.1127479	1.09023973	1.1225041	1.357540744	0.4409955	0.656017	1
TMEM120B	1.1821826	1.15905745	1.1922063	1.208408069	0.2731077	0.656056	1
MCF2L2	1.0972004	1.11928911	1.087626	0.734568152	-0.4450317	0.656121	1
RP11-118F19.1	1.0460555	1.0676143	1.0367108	0.542943664	-0.8811256	0.656123	1
RP11-20D14.6	1.0343664	1.05586479	1.0250478	0.448364381	-1.1572564	0.656144	1
BDH1	1.073073	1.0949492	1.0635907	0.669733707	-0.5783405	0.656414	1
ZNF780B	1.136001	1.15836542	1.1263071	0.797567211	-0.326322	0.65646	1
SELENOI	1.1945799	1.21776296	1.184531	0.847394092	-0.238895	0.656537	1
TYSND1	1.2578295	1.23395271	1.268179	1.14629561	0.1969791	0.656537	1
ZNF582-AS1	1.1900434	1.16682425	1.2001079	1.199513289	0.2624491	0.656569	1
CPA5	1.0354004	1.05682606	1.0261134	0.45953184	-1.1217633	0.6568	1
ZNF140	1.1810465	1.15785789	1.1910978	1.210568421	0.2756846	0.656806	1
OTUD7A	1.0535727	1.07538482	1.0441182	0.585239539	-0.7729009	0.656815	1
METTL12	1.5178054	1.54329057	1.5067587	0.932758222	-0.1004249	0.656958	1
ZNF441	1.124798	1.14691906	1.1152094	0.784169462	-0.3507626	0.656992	1
STARD4-AS1	1.4324604	1.45699023	1.4218278	0.923056505	-0.1155091	0.656997	1
CHAMP1	1.3621332	1.38669101	1.3514884	0.908964574	-0.137704	0.657104	1
SBK1	1.4122913	1.43709406	1.4015404	0.918659036	-0.1223986	0.657175	1
SNAPC5	1.5262289	1.55197914	1.5150673	0.933128157	-0.0998529	0.657256	1
GLRX	1.5135935	1.48758024	1.5248691	1.076477312	0.1063179	0.657319	1
SELENOH	7.7335398	7.83299195	7.6904317	0.979136483	-0.0304181	0.657348	1
NUP88	1.2033557	1.22677629	1.1932039	0.851958285	-0.2311453	0.657368	1
BCL9	1.3178222	1.3418725	1.3073974	0.899158032	-0.1533534	0.657552	1
TANGO2	1.0906292	1.11257369	1.0811173	0.720570632	-0.4727882	0.657707	1
MAP3K7	1.6779611	1.704767	1.6663419	0.945478242	-0.0808838	0.657712	1
EFCAB12	1.047457	1.06898611	1.0381251	0.552649016	-0.8555646	0.657733	1
ZFYVE26	1.1012355	1.1232711	1.091684	0.743759182	-0.4270925	0.657757	1
STK38L	1.4308943	1.45610153	1.419968	0.920777533	-0.1190755	0.657782	1
LINC01252	1.0997985	1.07760413	1.1094188	1.409960849	0.4956551	0.65787	1
CERS1	1.0727897	1.09472058	1.0632836	0.668107962	-0.5818468	0.657878	1
FAM124A	1.0496974	1.02785441	1.0591653	2.124090911	1.0868455	0.657931	1
SYT5	1.2429256	1.26618783	1.2328424	0.874729843	-0.1930906	0.657995	1
SNAPC4	1.1027928	1.08038211	1.1125068	1.399649503	0.4850656	0.658014	1
RP11-54H7.4	1.0629236	1.04103363	1.072412	1.764697519	0.8194209	0.658048	1
TRIM26	1.1327816	1.11029288	1.1425295	1.292281754	0.3699207	0.658411	1
NOL3	1.8218485	1.79239828	1.8346139	1.053275705	0.0748831	0.658473	1
RP11-635N19.1	1.2259619	1.20235488	1.2361945	1.167228879	0.2230875	0.658555	1
BOLA1	1.5002291	1.52525681	1.4893807	0.931698006	-0.1020657	0.65858	1
CTD-2017F17.2	1.0621704	1.08366692	1.0528526	0.631702181	-0.6626835	0.658592	1
RP5-1180E21.5	1.0549755	1.07648952	1.0456501	0.596815575	-0.7446429	0.65862	1

TRAFD1	1.1998743	1.17673637	1.2099036	1.187664958	0.2481279	0.658839	1
GLYCTK	1.0620193	1.08362674	1.0526534	0.62962368	-0.6674383	0.658956	1
RP11-499E18.1	1.2163412	1.23907995	1.206485	0.863664978	-0.2114563	0.659216	1
PTPRF	2.0364414	2.07074403	2.0215728	0.95407751	-0.0678216	0.659314	1
ERCC6L	1.042768	1.06422442	1.0334675	0.521102883	-0.9403599	0.65941	1
CTDNEP1	1.9710632	2.00010228	1.958476	0.958377962	-0.0613334	0.659477	1
TRAPPC10	1.1017701	1.12364403	1.0922887	0.74640615	-0.4219672	0.659604	1
NSMAF	1.3262682	1.30223615	1.336685	1.113980067	0.1557234	0.659694	1
FAM19A5	1.2309427	1.25364219	1.2211035	0.871714187	-0.1980729	0.659785	1
POLR1E	1.2163162	1.19300871	1.2264189	1.173102021	0.2303285	0.6598	1
DENND2A	1.1547785	1.13205429	1.1646284	1.246672255	0.3180822	0.660008	1
RP11-522I20.3	1.0474232	1.0257028	1.056838	2.211355027	1.1449307	0.660069	1
CC2D1A	1.3096793	1.3337886	1.299229	0.896462623	-0.1576847	0.660136	1
METTL21B	1.1578328	1.13478936	1.1678211	1.245061941	0.3162175	0.660194	1
TMEM127	1.1846818	1.16151299	1.1947244	1.205626791	0.2697834	0.660427	1
MLLT1	1.3883307	1.36346835	1.3991075	1.098052907	0.1349476	0.660554	1
NACA2	1.1623169	1.13938802	1.1722556	1.235798895	0.305444	0.660681	1
AP3M1	1.4230646	1.44777928	1.412352	0.920882171	-0.1189115	0.660819	1
MMP23B	1.3020307	1.27788787	1.3124956	1.124538331	0.1693328	0.6609	1
PLCB4	1.2543334	1.27783701	1.2441456	0.878736711	-0.1864971	0.660997	1
AP001462.6	1.0999441	1.12157134	1.0905697	0.744991864	-0.4247034	0.66108	1
C22orf39	2.0694322	2.03764008	2.0832126	1.043919412	0.0620103	0.661107	1
NUP58	1.4716238	1.44622524	1.4826329	1.081590365	0.1131542	0.661153	1
PPARGC1A	1.0831042	1.10470589	1.0737408	0.704265605	-0.5058085	0.661213	1
SLC1A6	1.2217661	1.24469729	1.2118264	0.865667148	-0.2081157	0.661242	1
RPTOR	1.1119947	1.13418975	1.1023741	0.762905416	-0.3904239	0.661362	1
OXNAD1	1.1300781	1.15233934	1.1204288	0.790529981	-0.3391079	0.661576	1
PML	1.2542243	1.27740329	1.2441772	0.880224523	-0.1840565	0.6616	1
HDAC6	1.3800457	1.3554213	1.3907193	1.099313241	0.1366025	0.661603	1
LINC00471	1.0514139	1.0728007	1.0421436	0.578890275	-0.7886382	0.661652	1
ROR1	1.1775632	1.15496811	1.1873572	1.209004591	0.2738197	0.661657	1
CSNK2A3	1.0498265	1.02829643	1.0591589	2.090683463	1.0639746	0.661714	1
ACOXL	1.0420265	1.06328939	1.0328099	0.518411296	-0.9478309	0.661722	1
TTC34	1.0775385	1.09900452	1.0682339	0.689200048	-0.5370053	0.661795	1
TRIM23	1.1527852	1.17503822	1.1431395	0.817761629	-0.2902477	0.661828	1
TOR1B	1.0819418	1.06007024	1.0914221	1.521920529	0.605893	0.661851	1
TNS1	1.0879553	1.06602856	1.0974595	1.476020658	0.5617129	0.661864	1
GSAP	1.0593408	1.08064973	1.0501043	0.621257681	-0.6867363	0.661884	1
RGS22	1.0835216	1.10509647	1.0741698	0.705730477	-0.5028108	0.6619	1
NMRK1	1.3227616	1.34645108	1.3124933	0.901984	-0.1488263	0.661993	1
MUL1	1.2152745	1.19233742	1.2252167	1.170945947	0.2276745	0.662028	1
C2orf15	1.1817979	1.20402082	1.1721653	0.843861458	-0.2449219	0.662079	1
TARS2	1.2511509	1.22785829	1.2612472	1.146533856	0.197279	0.662102	1
WDTC1	1.3329343	1.30867259	1.3434506	1.112669657	0.1540253	0.662159	1
KPNA5	1.4585689	1.48377452	1.4476434	0.925314042	-0.111985	0.662192	1
DTWD2	1.1025265	1.08043645	1.1121016	1.393666232	0.4788851	0.662407	1
DEPDC5	1.0685919	1.09023859	1.059209	0.656137892	-0.6079291	0.66243	1
COMMD4	1.7687944	1.74116179	1.7807719	1.053443265	0.0751126	0.662548	1
NDEL1	1.1790903	1.15622758	1.1890003	1.209775566	0.2747394	0.662667	1
RHBDD3	1.4303772	1.40555549	1.4411364	1.087733669	0.1213254	0.662794	1
RPP40	1.1353772	1.11317885	1.1449992	1.28115116	0.3574407	0.662887	1

RP11-239L20.6	1.0282006	1.04917346	1.0191098	0.388619913	-1.3635683	0.663026	1
KLHL3	1.2017592	1.17876559	1.2117259	1.184377433	0.2441289	0.663451	1
LUZP1	1.5656771	1.54018311	1.5767275	1.067651923	0.0944414	0.663483	1
EFHB	1.0721615	1.09370205	1.0628246	0.670471876	-0.5767513	0.663577	1
KLHL29	1.0918101	1.11339013	1.0824561	0.727189682	-0.4595964	0.663641	1
DIP2B	1.2828091	1.305989	1.2727617	0.891410124	-0.1658387	0.663751	1
EPG5	1.1896754	1.21231402	1.1798626	0.847153725	-0.2393043	0.663827	1
GNPTAB	1.1840411	1.16129235	1.1939017	1.202175221	0.2656472	0.66386	1
DUSP9	1.1196811	1.14144309	1.1102483	0.779453262	-0.3594656	0.663981	1
NME2	1.2033975	1.18028949	1.2134138	1.183728607	0.2433384	0.664284	1
PYY	1.1371327	1.15915791	1.1275857	0.80162987	-0.3189918	0.664325	1
POLRMT	1.2056633	1.18292357	1.21552	1.178196908	0.2365807	0.664491	1
DPF1	1.0681517	1.08934439	1.0589657	0.659981698	-0.5995021	0.664591	1
CDC42BPB	1.4600788	1.43530999	1.4708149	1.081562441	0.113117	0.66461	1
DDX42	1.5406132	1.51495641	1.5517343	1.0714194	0.0995233	0.664651	1
FLOT2	1.465047	1.43993446	1.4759322	1.081825293	0.1134675	0.664724	1
PARP8	1.1393831	1.16130987	1.1298788	0.8051508	-0.3126691	0.664901	1
ALDH4A1	1.1108293	1.08882082	1.1203689	1.355188361	0.4384934	0.664949	1
MRPL23	2.4706606	2.43451841	2.4863266	1.036115412	0.0511847	0.665047	1
ARF4-AS1	1.0687893	1.09006253	1.0595683	0.661410827	-0.5963814	0.665049	1
DHX38	1.190192	1.21261718	1.1804717	0.848810336	-0.2364859	0.66518	1
RP11-95D17.1	1.0783357	1.09960234	1.0691176	0.693935095	-0.5271274	0.665199	1
LYPD2	1.0512103	1.0298718	1.0604596	2.023968875	1.0171871	0.665209	1
ALPK1	1.1689872	1.19121129	1.159354	0.833392149	-0.2629326	0.665263	1
TTC38	1.1704131	1.14811571	1.1800781	1.215793116	0.2818978	0.665286	1
PCDHA7	1.0262348	1.04711804	1.0171829	0.364676736	-1.4553099	0.665496	1
EPHA6	1.0861838	1.10771206	1.0768523	0.713497805	-0.4870191	0.665507	1
SLFN12	1.1714022	1.19367457	1.1617481	0.835154213	-0.2598855	0.665612	1
GGNBP2	1.8875523	1.9158618	1.8752813	0.955691508	-0.0653831	0.665615	1
RPUSD2	1.1471942	1.16919387	1.1376583	0.813612787	-0.2975857	0.665673	1
FCHO2	1.1974356	1.17467474	1.2073015	1.186785564	0.2470593	0.665675	1
ARIH1	1.5692698	1.54356012	1.5804138	1.067800538	0.0946422	0.665718	1
TTC4	1.0733093	1.05174238	1.0826576	1.597482894	0.6758005	0.665762	1
ENPP4	1.135226	1.11301045	1.1448555	1.281788369	0.3581581	0.665886	1
PAN2	1.1203174	1.14206775	1.1108896	0.780540453	-0.3574547	0.666056	1
PPP6R1	1.3208329	1.3444066	1.3106147	0.901883809	-0.1489865	0.666074	1
EFHC2	1.0686517	1.04696884	1.0780503	1.661745566	0.7326995	0.666082	1
CYTH2	1.9134735	1.88522994	1.9257159	1.04573493	0.0645172	0.666139	1
ZNF146	2.1675959	2.19841657	2.1542365	0.963134639	-0.0541906	0.666402	1
SGK2	1.0297235	1.0505673	1.0206887	0.409131249	-1.2893644	0.66647	1
SETD2	1.5906496	1.61620471	1.5795726	0.940551964	-0.0884204	0.66654	1
DUSP15	1.2204316	1.24277483	1.2107469	0.868075415	-0.2041077	0.666567	1
KCTD20	1.6168075	1.64228408	1.6057645	0.943141071	-0.0844545	0.666612	1
SLC20A1	1.4250145	1.40064361	1.4355783	1.087196338	0.1206125	0.666681	1
GTF2A1	1.3949378	1.41937392	1.3843458	0.916475322	-0.1258321	0.66669	1
ANKRD13B	1.1402869	1.16225322	1.1307655	0.805934672	-0.3112652	0.666789	1
CTD-2270P14.5	1.0364709	1.05739463	1.0274015	0.47742197	-1.0666631	0.666821	1
CTD-2116N20.1	1.0255758	1.04623085	1.0166228	0.359560144	-1.475695	0.666932	1
ARIH2	1.6763556	1.6499049	1.6878208	1.058340752	0.0818042	0.666938	1
RTTN	1.1057877	1.12727002	1.096476	0.758041865	-0.3996506	0.66694	1
ING2	1.7998648	1.82696833	1.7881167	0.953019164	-0.0694229	0.667057	1

GNA12	1.2146191	1.1918486	1.2244891	1.170136905	0.2266773	0.667097	1
ADAMTS17	1.0282553	1.04900628	1.0192607	0.393024725	-1.347308	0.667238	1
ADGRA1	1.034649	1.05546497	1.0256262	0.462025089	-1.1139569	0.667447	1
OARD1	2.2556809	2.28677434	2.2422033	0.965362174	-0.0508578	0.667659	1
SVEP1	1.0349151	1.05575852	1.0258804	0.464151342	-1.1073328	0.667665	1
CTD-3138B18.5	1.2068091	1.22913382	1.1971323	0.86033688	-0.2170264	0.667723	1
ADAMTS3	1.106787	1.12814062	1.0975312	0.761125987	-0.3937928	0.667793	1
GALNT16	1.1366465	1.15865192	1.1271081	0.801175703	-0.3198094	0.667831	1
RP11-324E6.10	1.0385223	1.05925006	1.0295377	0.498526202	-1.0042588	0.667921	1
RP11-727F15.11	1.1037299	1.12493139	1.09454	0.756735199	-0.4021395	0.667965	1
PLCD1	1.121461	1.09975937	1.1308677	1.311833695	0.3915848	0.667979	1
EPS15L1	1.3797547	1.40377162	1.3693445	0.914736171	-0.1285724	0.668059	1
YIPF1	1.3159459	1.29252218	1.326099	1.114783769	0.1567639	0.668211	1
MFSD7	1.0449229	1.02376223	1.0540951	2.276514268	1.1868265	0.668241	1
RP11-727F15.9	1.0881086	1.06655872	1.0974495	1.464113056	0.550027	0.668285	1
DOCK4	1.1291247	1.15067496	1.1197836	0.794980158	-0.3310092	0.66842	1
C19orf57	1.0656402	1.08662284	1.0565452	0.652774258	-0.6153439	0.668435	1
SLC33A1	1.3044641	1.2807601	1.3147388	1.12102392	0.1648171	0.668576	1
CD55	1.0711433	1.04984705	1.0803743	1.612418564	0.6892263	0.668673	1
CORO2A	1.0445719	1.02341681	1.0537416	2.295002001	1.1984954	0.668778	1
ARL8B	1.3502793	1.32632824	1.360661	1.105209167	0.1443194	0.668821	1
MED14	1.2082906	1.18569968	1.2180827	1.174383887	0.2319041	0.668828	1
USP33	1.5122312	1.48737859	1.5230037	1.073095435	0.1017784	0.668874	1
SERAC1	1.2418087	1.21907129	1.2516644	1.148778498	0.2001007	0.668896	1
SAYSD1	1.2836438	1.26055573	1.2936514	1.127019586	0.1725126	0.668951	1
CD200	1.4886153	1.46305954	1.4996926	1.079110823	0.109843	0.669339	1
POLR2A	1.4114277	1.43559242	1.4009534	0.920478348	-0.1195443	0.66948	1
LDLR	1.1672746	1.14495887	1.1769474	1.220673174	0.287677	0.669537	1
FBXO22	1.7332901	1.70689403	1.7447316	1.053526567	0.0752267	0.669606	1
REXO4	1.7111444	1.68502264	1.7224671	1.054661573	0.0767801	0.669754	1
RHPN2	1.1222193	1.10057389	1.1316017	1.308507422	0.3879221	0.669845	1
HS3ST3A1	1.0884926	1.06707149	1.0977777	1.457812649	0.5438053	0.669897	1
PGF	1.2063758	1.18398301	1.216082	1.174467355	0.2320066	0.66991	1
RAD54B	1.1504726	1.17208402	1.141105	0.819977154	-0.2863444	0.669981	1
DNAJC27	1.1431753	1.16476378	1.1338176	0.812178565	-0.3001311	0.670071	1
RABGEF1	1.114246	1.09258529	1.123635	1.335362745	0.4172317	0.670182	1
ZNF708	1.2044761	1.22680974	1.1947955	0.85884985	-0.2195222	0.670224	1
ZSWIM4	1.0973671	1.07591747	1.1066645	1.405005896	0.4905762	0.670239	1
CXorf56	1.0965871	1.07503577	1.1059287	1.411709071	0.4974428	0.670416	1
ZCCHC4	1.0937297	1.11502314	1.0845	0.734634545	-0.4449014	0.670422	1
CNTRL	1.3550059	1.37841834	1.3448577	0.911313283	-0.133981	0.670448	1
PARP6	1.2819971	1.305108	1.2719795	0.891420421	-0.1658221	0.670685	1
ARHGAP44	1.1454744	1.16696933	1.1361574	0.815463321	-0.2943081	0.670687	1
PLCB1	1.3018454	1.32509998	1.2917656	0.897464277	-0.1560736	0.670713	1
BTBD2	1.3521077	1.3753399	1.3420376	0.911274389	-0.1340426	0.670923	1
PITPNM1	1.3541054	1.33025724	1.3644425	1.103511058	0.1421011	0.670935	1
ZNF468	1.0708197	1.049618	1.0800097	1.612513217	0.689311	0.670955	1
RP11-521O16.2	1.0717765	1.09264267	1.0627319	0.677138539	-0.5624771	0.670966	1
PDXDC1	1.5920077	1.56643234	1.6030934	1.064722791	0.0904779	0.67098	1
POU6F2	1.1771414	1.19864275	1.1678216	0.844841222	-0.2432479	0.671058	1
RP11-839D17.3	1.0720111	1.09291432	1.0629504	0.677510706	-0.5616844	0.671136	1

ST3GAL3	1.2877803	1.3103479	1.2779982	0.895763118	-0.1588108	0.671226	1
ZNF555	1.1145786	1.09282028	1.1240099	1.336021436	0.4179432	0.671228	1
LEFTY2	3.8512514	3.682312	3.9244792	1.090282999	0.1247027	0.671247	1
YTHDC1	2.0916085	2.06070215	2.105005	1.041767446	0.0590333	0.671263	1
STK17B	1.1049581	1.08342709	1.1142908	1.369948169	0.4541213	0.671306	1
OTUD6B	1.1896523	1.16728396	1.199348	1.191674307	0.25299	0.671307	1
PREX1	1.1138291	1.13513683	1.1045932	0.77398008	-0.3696317	0.671341	1
ERGIC1	1.9875736	1.95857255	2.0001442	1.043368296	0.0612485	0.671356	1
PPRC1	1.0796008	1.05834372	1.0888148	1.522268896	0.6062232	0.67136	1
NOA1	1.463229	1.4382556	1.4740538	1.08168336	0.1132782	0.671364	1
CDIP1	1.6519573	1.62616181	1.6631385	1.059052859	0.0827746	0.671414	1
ANKRD13C	1.2451444	1.26767511	1.2353784	0.879343681	-0.185501	0.671426	1
ICAM3	1.165152	1.14327716	1.1746337	1.218852506	0.2855236	0.671497	1
NXPH4	1.1231403	1.10147495	1.1325312	1.306048638	0.3852086	0.671513	1
NKAIN1	1.0946151	1.11561622	1.0855121	0.739619986	-0.4351439	0.671519	1
P2RY1	1.1146809	1.13601867	1.1054319	0.775128015	-0.3674935	0.671521	1
RGCC	1.0406171	1.01968849	1.0496887	2.523743436	1.3355653	0.671555	1
TMEM105	1.040583	1.01968023	1.0496434	2.522500944	1.3348548	0.671555	1
PPP1R12C	1.1119387	1.13322021	1.1027141	0.771009941	-0.3751786	0.671647	1
NUDT1	1.7871691	1.81412686	1.7754841	0.952534687	-0.0701565	0.671656	1
C9orf172	1.1316433	1.15294666	1.1224092	0.800339255	-0.3213164	0.671683	1
ZNF17	1.062723	1.08353357	1.0537026	0.642886444	-0.6373642	0.671752	1
HLTF	2.0477204	2.07817632	2.0345192	0.959508325	-0.0596328	0.671799	1
CTD-2336O2.1	1.7613967	1.78735202	1.7501462	0.952745623	-0.069837	0.672032	1
POT1	1.1825327	1.16030281	1.1921683	1.198783395	0.261571	0.672111	1
ADPGK	1.4134996	1.43814624	1.4028163	0.919365058	-0.1212903	0.672185	1
RELT	1.06533	1.08606817	1.056341	0.654608887	-0.6112949	0.67219	1
UBR5	1.846043	1.87256975	1.8345448	0.956421922	-0.0642809	0.672299	1
CFLAR	1.2568196	1.23371409	1.2668348	1.14171457	0.191202	0.672402	1
MARC1	1.1932508	1.21527323	1.1837051	0.853357691	-0.2287775	0.672515	1
EFCAB7	1.1571688	1.1351912	1.1666951	1.233032486	0.3022108	0.672618	1
RP11-527D7.1	1.0543407	1.03315104	1.0635254	1.916241745	0.9382796	0.672619	1
XXbac-BPG299F13.17	1.0732093	1.09403535	1.0641821	0.682531403	-0.5510327	0.672861	1
CLCN7	1.2444463	1.22197228	1.2541878	1.145133049	0.1955152	0.672883	1
AZI2	1.9192343	1.89078709	1.9315649	1.045777291	0.0645756	0.672929	1
KIAA1257	1.1050311	1.12597166	1.0959542	0.761712866	-0.3926808	0.673089	1
CPA4	1.0837712	1.06246798	1.0930051	1.488845064	0.5741936	0.673306	1
CDK2AP2	2.1766132	2.14738739	2.1892813	1.036512392	0.0517374	0.673526	1
FASTKD5	1.0925066	1.07126475	1.101714	1.427269192	0.5132575	0.673633	1
DCBLD1	1.1075974	1.08635706	1.1168042	1.352572209	0.4357056	0.673698	1
GRIK1	1.0440183	1.02312404	1.0530751	2.295234997	1.1986419	0.673702	1
MARCH4	1.0568191	1.07743107	1.0478847	0.618416753	-0.6933487	0.67374	1
ZNF267	1.1096629	1.13110429	1.100369	0.76556616	-0.385401	0.673804	1
RP11-634H22.1	1.0605816	1.08117327	1.051656	0.636367387	-0.6520682	0.673829	1
RASA3	1.1490941	1.12744449	1.1584783	1.243508303	0.3144161	0.674088	1
GPHN	1.2179984	1.19544876	1.2277727	1.165383221	0.2208044	0.674235	1
CYP4V2	1.091246	1.1120908	1.0822107	0.733429919	-0.447269	0.674246	1
EEF1B2	19.900311	20.0599938	19.831096	0.987990648	-0.0174307	0.674279	1
KIF9-AS1	1.0740242	1.09492949	1.0649628	0.68432634	-0.5472436	0.67435	1
SGCG	1.0412877	1.02055942	1.0502725	2.445231054	1.2899708	0.674408	1
FBXL3	1.2269877	1.2045421	1.2367169	1.157301594	0.2107649	0.67456	1

XXbac-B135H6.18	1.1006189	1.12166554	1.0914961	0.752029477	-0.4111389	0.674581	1
ZNF567	1.1722823	1.19398308	1.162876	0.839640311	-0.2521567	0.674653	1
CCDC153	1.0905836	1.06953911	1.0997054	1.43380361	0.5198474	0.674664	1
WWP2	1.1402924	1.16175067	1.1309912	0.809834246	-0.3043014	0.674765	1
RP11-419I17.1	1.0715541	1.09227236	1.0625736	0.67814028	-0.5603444	0.674774	1
U73166.2	1.093889	1.11469561	1.0848702	0.739960663	-0.4344795	0.674785	1
RP11-712L6.5	1.072274	1.09291001	1.0633292	0.681618389	-0.5529638	0.674891	1
LINC00982	1.0773566	1.05641099	1.0864355	1.532245916	0.6156479	0.675013	1
PLSCR1	1.16094	1.13919527	1.1703653	1.223930427	0.2915216	0.675106	1
DCAF12	1.6608721	1.63506729	1.6720573	1.058245812	0.0816748	0.675126	1
CTNNA2	1.200171	1.22179997	1.1907959	0.860215961	-0.2172292	0.675185	1
CTD-3222D19.12	1.0586234	1.07925438	1.0496808	0.626852556	-0.673802	0.675218	1
MMP7	1.0502305	1.02918849	1.0593513	2.033379134	1.0238792	0.675337	1
CTD-3025N20.3	1.0457531	1.06617101	1.0369028	0.557688771	-0.8424679	0.675443	1
CHRNA7	1.0843959	1.10510451	1.0754197	0.717568212	-0.4788121	0.675518	1
MSANTD2	1.1779638	1.1562276	1.1873855	1.199438896	0.2623597	0.675819	1
IRF2BP1	1.1625382	1.18424407	1.1531296	0.831123842	-0.2668646	0.675839	1
TBC1D2B	1.1658552	1.14388971	1.1753763	1.218824262	0.2854901	0.676097	1
TMEM74B	1.0652623	1.04443584	1.0742896	1.671840797	0.7414375	0.676108	1
SCLT1	1.2777941	1.2552662	1.2875589	1.126506172	0.1718552	0.676113	1
NOS1AP	1.1753955	1.15314452	1.1850403	1.208272458	0.2729458	0.676247	1
MMS19	1.3021443	1.27901977	1.3121677	1.118801546	0.1619542	0.676248	1
CTPS1	1.2771452	1.29968579	1.2673748	0.892183762	-0.1645872	0.676256	1
NVL	1.2122683	1.23367609	1.2029889	0.868676423	-0.2031092	0.676322	1
MATN1-AS1	1.0404869	1.0608101	1.0316777	0.520927847	-0.9408445	0.676332	1
TRIM14	1.0379051	1.05825839	1.0290829	0.499204789	-1.0022963	0.676406	1
AGT	1.039539	1.01886772	1.048499	2.570477873	1.3620366	0.676474	1
AP1S3	1.1248	1.10347872	1.1340418	1.295356044	0.3733487	0.676477	1
EXOC2	1.0737178	1.05285392	1.0827614	1.565850986	0.6469469	0.676505	1
DDX49	1.3490642	1.32613261	1.359004	1.100791631	0.1385414	0.67651	1
TTYH2	1.066771	1.0458953	1.0758196	1.652013092	0.7242251	0.676544	1
RP11-436D23.1	1.0734114	1.05245879	1.0824934	1.572537361	0.6530943	0.676676	1
ST3GAL6	1.1424292	1.16355186	1.1332734	0.814869519	-0.295359	0.676716	1
C1orf159	1.1529229	1.1741652	1.1437153	0.825166357	-0.2772431	0.676786	1
DHX16	1.1858209	1.16405072	1.1952573	1.190225389	0.2512348	0.676825	1
DRD2	1.0583334	1.07854714	1.0495716	0.63110657	-0.6640445	0.676845	1
TRAPPC5	1.1395516	1.16059889	1.1304286	0.812138789	-0.3002018	0.676901	1
MICAL2	1.0480317	1.02743132	1.0569611	2.076498685	1.054153	0.676903	1
CSNK1G1	1.2509692	1.27328242	1.2412974	0.882959956	-0.1795801	0.67692	1
ARHGAP32	1.2041383	1.22582828	1.1947367	0.862322064	-0.2137013	0.677058	1
MFSD2B	1.0928371	1.11361623	1.0838303	0.737837264	-0.4386254	0.67719	1
CD68	1.117154	1.0961129	1.1262743	1.313812581	0.3937595	0.67723	1
IQCD	1.1006464	1.07960305	1.1097678	1.378939098	0.4635587	0.677245	1
MIER1	1.7908733	1.81677412	1.7796464	0.95454349	-0.0671172	0.677431	1
KLK8	1.0507509	1.02999765	1.0597465	1.991705075	0.994004	0.677447	1
RP11-705O1.8	1.0300013	1.05019547	1.0212481	0.423306245	-1.2402263	0.677702	1
SPG11	1.3463171	1.36978475	1.3361449	0.909028483	-0.1376026	0.677819	1
USP10	1.7017121	1.67608926	1.7128184	1.05432594	0.0763209	0.677846	1
CRIPAK	1.1021004	1.12279483	1.0931302	0.758421289	-0.3989286	0.677924	1
NATD1	1.1020294	1.12293307	1.0929686	0.756253767	-0.4030577	0.677924	1
SEC61A1	1.8374426	1.8108788	1.8489568	1.046958958	0.0662049	0.678108	1

AP000253.1	1.0371072	1.05731895	1.0283464	0.494537694	-1.0158476	0.678131		1
KMT5A	1.2861145	1.26340979	1.295956	1.12355718	0.1680735	0.678227		1
ZNF70	1.0618471	1.08231394	1.0529756	0.643579908	-0.6358088	0.678428		1
WDR4	1.0582545	1.037708	1.0671605	1.781066754	0.8327416	0.678508		1
ITGB5	1.1191351	1.09781249	1.1283774	1.312485157	0.3923011	0.678622		1
WIPF3	1.0553391	1.0345937	1.0643313	1.85962622	0.8950127	0.678713		1
IKBKG	1.1374973	1.11625106	1.1467066	1.261980906	0.3356901	0.678861		1
TIPARP-AS1	1.0609763	1.04034245	1.0699202	1.733167027	0.7934107	0.678877		1
STYXL1	1.3308184	1.30802168	1.3406997	1.106089999	0.1454688	0.67888		1
RGS10	1.1972837	1.21877306	1.1879691	0.859196545	-0.2189399	0.678886		1
PRC1	1.5268969	1.50257332	1.5374401	1.069376564	0.09677	0.678889		1
FRAT2	1.0504063	1.02981717	1.0593307	1.98981826	0.9926367	0.6789		1
ERBIN	1.7694763	1.74355963	1.78071	1.049962885	0.0703383	0.678979		1
FKBP15	1.1919557	1.21342137	1.1826512	0.855824506	-0.2246131	0.679012		1
KLRG1	1.1063407	1.12708642	1.0973484	0.766001215	-0.3845814	0.679132		1
TPK1	1.0895181	1.11010474	1.0805948	0.731982589	-0.4501188	0.67916		1
ST3GAL5	1.1697809	1.14810899	1.1791747	1.209749236	0.274708	0.679369		1
DMTN	1.0588516	1.03826208	1.0677763	1.771369921	0.8248655	0.67951		1
CHRDL1	1.1763789	1.197441	1.1672494	0.847085239	-0.2394209	0.679514		1
ADCK1	1.1093512	1.13013037	1.1003443	0.771105912	-0.3749991	0.679537		1
LPAR2	1.2254545	1.24732271	1.2159756	0.87325424	-0.1955264	0.679665		1
NYAP1	1.1082638	1.12897259	1.0992874	0.769833332	-0.377382	0.679804		1
MED24	1.393319	1.37035335	1.4032736	1.088888636	0.1228564	0.679806		1
SPACA6	1.2063961	1.2276162	1.1971981	0.866362277	-0.2069577	0.679815		1
CEP97	1.265931	1.2880024	1.256364	0.890145255	-0.1678873	0.679856		1
RP11-290O12.2	1.066792	1.08716024	1.0579633	0.665020258	-0.5885298	0.680041		1
TRH	3.442689	3.50844518	3.4141866	0.962423508	-0.0552562	0.680116		1
NEBL-AS1	1.0829309	1.10328786	1.0741071	0.717480983	-0.4789875	0.680199		1
STAR	1.0481252	1.06822671	1.0394121	0.577664044	-0.7916974	0.680201		1
TYK2	1.2901458	1.26765403	1.299895	1.120457622	0.1640881	0.680225		1
AP001505.10	1.0800316	1.10042839	1.0711905	0.708868662	-0.4964097	0.680344		1
RP11-774O3.3	1.0352569	1.05529866	1.0265697	0.480477095	-1.0574604	0.680365		1
ACTA1	1.0589897	1.03854346	1.0678523	1.760410071	0.8159115	0.680401		1
ERCC2	1.2128324	1.19094922	1.2223178	1.164277328	0.2194347	0.680502		1
DGCR2	1.4472528	1.42330167	1.4576346	1.081107386	0.1125098	0.680534		1
DNAJC18	1.2915946	1.26885835	1.3014497	1.121221232	0.165071	0.680577		1
ZBTB7B	1.0871111	1.06631141	1.0961268	1.449627343	0.5356821	0.680618		1
NECTIN4	1.0371883	1.01683833	1.0460091	2.732400581	1.450169	0.680636		1
LINC01356	1.0397703	1.05977298	1.0311	0.520301305	-0.9425808	0.680715		1
CFAP70	1.0899915	1.11034248	1.0811703	0.735621429	-0.4429646	0.680767		1
C1orf228	1.0557103	1.07584204	1.0469841	0.61949955	-0.6908249	0.680771		1
CHMP3	1.7588797	1.73300702	1.7700944	1.050596144	0.0712082	0.680772		1
RP11-736N17.11	1.0283812	1.04837953	1.0197128	0.407460724	-1.2952671	0.680835		1
RP11-368I23.4	1.0283647	1.04833487	1.0197086	0.407751012	-1.2942396	0.680835		1
MRRF	1.4675464	1.49095601	1.4573994	0.931650459	-0.1021393	0.681149		1
PCTP	1.1951989	1.17331622	1.204684	1.180986062	0.2399919	0.681284		1
ZNF654	1.2741581	1.29650089	1.2644735	0.891982174	-0.1649132	0.681464		1
PRKAG2-AS1	1.1044776	1.12501192	1.0955769	0.764541915	-0.3873325	0.681495		1
TMEM258	9.3495731	9.26235893	9.3873765	1.015130981	0.0216659	0.681558		1
CHSY1	1.2034805	1.18192769	1.2128227	1.169820087	0.2262867	0.681576		1
FUT10	1.1750972	1.19611275	1.1659879	0.84639024	-0.2406051	0.681599		1

PIK3R4	1.0971165	1.11767141	1.0882068	0.749602658	-0.415802	0.68161	1
LCMT2	1.0709793	1.05039644	1.0799011	1.585450539	0.6648929	0.68168	1
PPIL2	1.2547609	1.27662492	1.2452838	0.886701762	-0.1734792	0.68168	1
SLC13A3	1.0847689	1.06404582	1.0937514	1.463818222	0.5497364	0.681704	1
EXTL3-AS1	1.0274728	1.04726676	1.0188929	0.39970868	-1.3229792	0.681769	1
RP4-798A10.2	1.0447507	1.02432168	1.0536058	2.204035288	1.1401473	0.681809	1
MAZ	2.1331368	2.16054415	2.1212569	0.966147569	-0.0496845	0.681823	1
REXO1	1.1085165	1.12917409	1.0995623	0.770760901	-0.3756447	0.68192	1
ABCB10	1.0780637	1.09834403	1.0692731	0.704395857	-0.5055417	0.681927	1
TESK1	1.1492557	1.12786007	1.1585298	1.239869534	0.3101883	0.682039	1
RP11-966I7.2	1.0568225	1.07685232	1.0481405	0.626402811	-0.6748374	0.682353	1
MAPK8IP1	1.1973991	1.21845833	1.1882708	0.861815649	-0.2145488	0.68272	1
GRTP1	1.1105661	1.08962488	1.1196432	1.334932607	0.4167669	0.682768	1
ARHGAP19	1.1060555	1.12654923	1.0971724	0.767862164	-0.3810807	0.682776	1
ELAVL4	1.0432959	1.02292863	1.0521242	2.273325472	1.1848042	0.682785	1
BIK	1.0463959	1.02607209	1.0552053	2.117410726	1.0823011	0.682955	1
NFKBIZ	1.0791857	1.05867954	1.0880742	1.500935054	0.5858616	0.683078	1
SPATA24	1.119115	1.09833164	1.1281237	1.302975049	0.3818095	0.683108	1
CARMN	1.0517836	1.03144125	1.0606011	1.927439076	0.9466853	0.683159	1
CABIN1	1.5650386	1.54068768	1.5755936	1.064558364	0.090255	0.683239	1
C5	1.0791203	1.0990855	1.0704662	0.71116581	-0.4917421	0.683266	1
TIGD3	1.0404955	1.0604198	1.0318593	0.52729858	-0.923308	0.68329	1
AC005537.2	1.0279067	1.04758419	1.0193774	0.407224514	-1.2961037	0.683333	1
PPP1R37	1.3793804	1.35647093	1.3893106	1.092124413	0.1271372	0.68334	1
UCKL1	1.2489159	1.22699222	1.2584189	1.138448081	0.1870685	0.683385	1
C16orf91	1.4062967	1.42896199	1.3964723	0.924259661	-0.1136299	0.683416	1
HDAC8	1.2635415	1.28548562	1.2540297	0.889816216	-0.1684207	0.683484	1
PPCDC	1.1711244	1.192063	1.1620484	0.843725253	-0.2451548	0.683504	1
PCSK7	1.5113731	1.4874063	1.5217617	1.070486113	0.0982661	0.683619	1
RASGRF2-AS1	1.0354	1.05509958	1.0268612	0.487501949	-1.0365201	0.683649	1
PCDHGA10	1.1465068	1.16720113	1.1375367	0.82258237	-0.2817679	0.683959	1
SFMBT2	1.0479678	1.06771948	1.0394063	0.581904469	-0.7811458	0.684043	1
CTD-2298J14.2	1.0964312	1.11671721	1.0876382	0.750859084	-0.4133859	0.684138	1
GABARAP	1.8449189	1.8183841	1.8564205	1.046477475	0.0655413	0.684839	1
CDK6	1.5338149	1.51031257	1.5440021	1.066017538	0.0922312	0.684925	1
NPIPB2	1.0552467	1.03494142	1.0640481	1.833014342	0.8742181	0.684971	1
RFX3-AS1	1.0571483	1.07710834	1.0484965	0.628939427	-0.669007	0.685012	1
RP11-60L3.6	1.0928604	1.1130321	1.0841169	0.744186153	-0.4262645	0.685034	1
HEATR1	1.234917	1.25639976	1.2256051	0.879895942	-0.1845952	0.6851	1
IER5	1.1127297	1.13309167	1.1039036	0.780692347	-0.357174	0.685152	1
LINC00869	1.412342	1.43517555	1.4024446	0.924786849	-0.1128072	0.685195	1
SOAT1	1.175782	1.196642	1.1667401	0.847937255	-0.2379706	0.685204	1
ELOVL7	1.0483787	1.02833081	1.0570686	2.014366541	1.0103262	0.685677	1
CCER2	1.0249047	1.04452886	1.0163985	0.36826782	-1.4411728	0.685728	1
TAF6L	1.1208416	1.10008925	1.1298368	1.297209848	0.3754119	0.685874	1
TNFRSF25	1.092631	1.11287972	1.0838541	0.742862236	-0.4288334	0.685946	1
EXOSC3	1.3699618	1.34734069	1.379767	1.093355981	0.1287632	0.68595	1
RP11-498C9.15	1.0662009	1.08599899	1.0576194	0.670000495	-0.5777659	0.685979	1
RP11-48G14.2	1.0446947	1.0644348	1.0361383	0.560850525	-0.8343118	0.686142	1
RP11-319G6.1	1.0954926	1.11594426	1.0866277	0.74714948	-0.4205312	0.686155	1
ERLIN2	1.3344422	1.35639622	1.3249261	0.911699123	-0.1333703	0.686214	1

ZNF154	1.1128837	1.13329153	1.1040378	0.780528494	-0.3574768	0.6863	1
ACCS	1.0762233	1.0963537	1.0674977	0.700520113	-0.5135016	0.68636	1
FZD2	1.4146796	1.39205965	1.4244844	1.082703575	0.1146383	0.686454	1
ZNF469	1.0471673	1.02717387	1.0558336	2.05468027	1.0389139	0.686519	1
PPP4R1	1.3294662	1.35167971	1.3198377	0.909457274	-0.1369222	0.686736	1
BLACAT1	1.1037412	1.12419675	1.0948746	0.763905533	-0.3885339	0.686784	1
HSF2	1.3842435	1.40680945	1.3744622	0.920485541	-0.119533	0.68682	1
ANAPC5	2.2133888	2.2436889	2.2002551	0.965076635	-0.0512846	0.686837	1
ZNF420	1.2224973	1.24386098	1.2132371	0.874420833	-0.1936003	0.686962	1
LINC00106	1.0325482	1.05213278	1.0240592	0.461497842	-1.1156042	0.687073	1
RP11-7F17.8	1.0325007	1.05204461	1.0240293	0.461706238	-1.1149529	0.687073	1
ENOX1	1.0472795	1.02725828	1.0559578	2.052872369	1.0376439	0.687115	1
ATF6	1.4609435	1.48458253	1.450697	0.930072654	-0.1045847	0.687295	1
RP11-15A1.2	1.035118	1.05467449	1.0266411	0.487268045	-1.0372125	0.687383	1
MGAT3	1.103059	1.12330171	1.0942847	0.764666784	-0.3870969	0.687507	1
ATRIP	1.0231874	1.04268602	1.0147356	0.345208854	-1.5344586	0.687706	1
FMN1	1.0516953	1.03170653	1.0603596	1.903695757	0.9288029	0.687793	1
GPR27	1.1176059	1.09685556	1.1266002	1.307103354	0.3863732	0.687914	1
NCK1-AS1	1.1563156	1.17713005	1.1472934	0.831555264	-0.2661159	0.68794	1
PRDM10	1.0904989	1.07014106	1.0993231	1.416047596	0.5018698	0.687987	1
CTD-2319I12.2	1.0405801	1.06025279	1.0320528	0.531972259	-0.9105771	0.688172	1
GID4	1.1807708	1.20160318	1.1717408	0.851875702	-0.2312852	0.688199	1
RP11-814H16.2	1.0326614	1.05221644	1.0241852	0.463171262	-1.1103824	0.688264	1
RP11-1057B6.1	1.0301666	1.04970616	1.021697	0.436505954	-1.1959268	0.688338	1
CTB-13F3.1	1.0301524	1.0497562	1.021655	0.435222063	-1.2001764	0.688338	1
PIP5KL1	1.0604207	1.08026282	1.05182	0.645628561	-0.6312237	0.688359	1
TRIP4	1.1828131	1.16189795	1.1918788	1.185183786	0.2451108	0.688374	1
MIR4500HG	1.0904335	1.07014739	1.0992267	1.414545622	0.5003387	0.688396	1
FOXP4-AS1	1.0948145	1.11486102	1.0861252	0.749820944	-0.415382	0.688411	1
RNF139-AS1	1.117756	1.13808136	1.1089459	0.788998058	-0.3419063	0.688546	1
TRAPPC6B	1.2955093	1.27340848	1.305089	1.115872636	0.1581724	0.688683	1
CEP112	1.262756	1.28402898	1.2535351	0.892638197	-0.1638526	0.688779	1
IFI27	1.1075565	1.08708882	1.1164283	1.336891371	0.4188822	0.688862	1
PKP3	1.0378577	1.01804205	1.0464468	2.57436592	1.3642171	0.688885	1
AGBL2	1.0521435	1.07172782	1.0436545	0.608613941	-0.7164007	0.688962	1
LINC01063	1.0395188	1.05914538	1.0310116	0.524328428	-0.9314573	0.688968	1
GEMIN8	1.4753758	1.45215539	1.4854408	1.073614912	0.1024766	0.689095	1
ATAT1	1.6116405	1.63573983	1.6011945	0.945661203	-0.0806047	0.689101	1
ECSIT	1.6739611	1.6492536	1.6846707	1.05455055	0.0766283	0.689107	1
TERC	1.0239324	1.04335155	1.015515	0.357888857	-1.4824165	0.689173	1
RASAL2-AS1	1.0532035	1.07274314	1.0447339	0.614956972	-0.7014426	0.689329	1
STMN4	1.3186663	1.34094847	1.3090079	0.906318603	-0.1419098	0.689449	1
STX16	1.6249597	1.64858485	1.6147192	0.947785299	-0.0773678	0.689569	1
PROSER2	1.0577179	1.03786039	1.0663252	1.751836035	0.8088678	0.68969	1
RCOR2	1.309121	1.33067821	1.299777	0.906551907	-0.1415385	0.689698	1
NRM	1.3712764	1.39370154	1.3615561	0.918350722	-0.1228829	0.689763	1
C17orf49	1.1853842	1.16449107	1.1944404	1.182072823	0.2413189	0.689816	1
ZKSCAN8	1.1714271	1.19175845	1.1626143	0.848016522	-0.2378357	0.68984	1
PRDM1	1.0639311	1.04366182	1.0727169	1.665457545	0.7359186	0.689968	1
GABRA5	1.041641	1.02191834	1.0501899	2.289859418	1.195259	0.689977	1
FAM159A	1.0714604	1.09115898	1.062922	0.690244145	-0.5348213	0.690501	1

SETDB2	1.2334779	1.2545939	1.224325	0.881109045	-0.1826075	0.690537	1
THBS4	1.0811102	1.060971	1.0898396	1.473481265	0.5592287	0.690651	1
ZNF133	1.0785867	1.09838303	1.0700059	0.711564455	-0.4909337	0.690668	1
C1GALT1	1.5638823	1.58796655	1.5534429	0.941282957	-0.0872996	0.690686	1
FOXC2	1.0472006	1.02750394	1.0557383	2.026554939	1.0190293	0.690709	1
GJC1	1.2582271	1.27964457	1.2489436	0.890214295	-0.1677754	0.690784	1
RP11-680F8.1	1.0937373	1.11361314	1.085122	0.749226753	-0.4165257	0.690835	1
LINC00668	1.0283006	1.04767494	1.0199027	0.417467718	-1.2602635	0.690905	1
KIAA0895	1.1744255	1.15365601	1.1834281	1.193757898	0.2555103	0.691002	1
SLC25A27	1.1414683	1.16189689	1.1326134	0.819122688	-0.2878485	0.691016	1
OLFML2A	1.0582401	1.03853664	1.0667806	1.732912333	0.7931987	0.691027	1
XRN2	2.9930681	2.95337145	3.0102748	1.02913085	0.0414264	0.691139	1
ATPAF1	1.5411643	1.51811839	1.5511536	1.063760041	0.0891727	0.691199	1
SHC3	1.0806995	1.10044823	1.0721393	0.718174065	-0.4775945	0.691228	1
IRF6	1.0325967	1.01301898	1.0410828	3.155605495	1.6579169	0.691288	1
ROCK2	1.4173616	1.39462807	1.4272155	1.082577683	0.1144706	0.691449	1
MIR24-2	1.106996	1.0867331	1.1157791	1.334889307	0.4167201	0.691622	1
FREM2	1.0402982	1.02060533	1.0488342	2.369977265	1.2448732	0.691652	1
SLC30A1	1.1096426	1.08937252	1.1184288	1.325114287	0.4061168	0.691698	1
RP11-513M16.8	1.0392066	1.05858624	1.0308064	0.525829452	-0.9273331	0.691705	1
ZNF226	1.607304	1.63118775	1.5969515	0.945758965	-0.0804555	0.691991	1
MACF1	2.193697	2.22274366	2.1811066	0.965947873	-0.0499828	0.69209	1
RP11-17M16.2	1.0379292	1.05724584	1.0295563	0.51630445	-0.9537061	0.692161	1
GJB2	1.0334632	1.01394208	1.0419247	3.007060704	1.588354	0.692227	1
COMMD8	1.5568155	1.53352985	1.5669088	1.062562458	0.0875476	0.692303	1
PRDM4	1.3055377	1.28350744	1.3150868	1.111387987	0.1523626	0.692323	1
DHRS11	1.1957083	1.17478106	1.2047794	1.171633547	0.2285214	0.69233	1
MAP4K2	1.1308617	1.15118985	1.1220504	0.807265649	-0.3088846	0.692334	1
AHCY	3.5774826	3.62282871	3.557827	0.975216954	-0.0362049	0.692438	1
PPT2	1.1349728	1.11458087	1.1438118	1.255111519	0.3278156	0.692481	1
C2orf27B	1.069923	1.08950221	1.0614363	0.686421608	-0.5428331	0.692517	1
FBXL17	1.2265735	1.2473058	1.217587	0.879829549	-0.184704	0.692577	1
GLT8D2	1.093027	1.07302999	1.1016948	1.392507201	0.4776848	0.692598	1
MPLKIP	2.5810393	2.5477335	2.5954758	1.03084662	0.0438297	0.692606	1
STX1A	1.1296826	1.14968896	1.1210107	0.808414218	-0.3068334	0.692669	1
PVT1	1.1296982	1.14984822	1.1209641	0.80724393	-0.3089234	0.692676	1
ZNF41	1.0426835	1.0620526	1.0342878	0.552560549	-0.8557955	0.692713	1
EPB41L4B	1.0617718	1.0418671	1.0703997	1.681503159	0.7497515	0.692715	1
C20orf85	1.0295502	1.0486192	1.0212846	0.437781698	-1.1917165	0.692717	1
GPN2	1.2350383	1.21377997	1.2442528	1.142542923	0.1922484	0.692857	1
RP11-285F7.2	1.0626275	1.08210843	1.0541834	0.659900374	-0.5996799	0.692944	1
CAPN5	1.077349	1.05741176	1.0859909	1.497792958	0.5828382	0.693083	1
ZNF732	1.0403722	1.05958385	1.0320448	0.537810455	-0.8948303	0.693102	1
TLK2	1.3478013	1.36950137	1.3383953	0.915816039	-0.1268703	0.693242	1
MSH3	1.3690539	1.39100402	1.3595395	0.919528877	-0.1210332	0.693259	1
RP11-589C21.6	1.0439561	1.06331437	1.0355651	0.561723111	-0.8320689	0.693367	1
APCDD1	1.1793203	1.19948608	1.1705793	0.855093742	-0.2258455	0.693503	1
ARHGAP31	1.041184	1.06038491	1.0328613	0.544196612	-0.8778001	0.69351	1
TSTD2	1.2506953	1.27161978	1.2416254	0.889572348	-0.1688162	0.693596	1
METTL25	1.0800703	1.09959373	1.0716078	0.718998772	-0.4759388	0.693683	1
TUB	1.1316759	1.15157588	1.1230502	0.811805874	-0.3007933	0.693734	1

PRR5L	1.1313322	1.15143046	1.1226206	0.809748346	-0.3044545	0.693734	1
EIF4ENIF1	1.1821239	1.20265321	1.1732254	0.854787404	-0.2263624	0.693758	1
DHX32	1.5578429	1.58176082	1.5474756	0.941066453	-0.0876315	0.693829	1
KIAA0408	1.0394345	1.05860489	1.031125	0.531098325	-0.9129491	0.693905	1
MYBPHL	1.042436	1.0229374	1.0508878	2.218550794	1.1496176	0.694	1
FBXL2	1.3044452	1.32598936	1.2951068	0.905265038	-0.1435879	0.694087	1
DKFZP434L187	1.191151	1.21135547	1.1823933	0.862969445	-0.2126186	0.694092	1
USP32	1.257674	1.23628039	1.2669471	1.129789647	0.1760542	0.694172	1
PI4K2A	1.1235292	1.10343198	1.1322404	1.278525698	0.3544812	0.694328	1
LDLRAP1	1.2367519	1.21579996	1.2458336	1.139173715	0.1879878	0.694456	1
ABCC10	1.1619562	1.14157011	1.1707926	1.20641739	0.2707291	0.694563	1
SFMBT1	1.0758276	1.09531302	1.0673816	0.706950634	-0.5003186	0.694607	1
ABHD5	1.1434095	1.16352356	1.134691	0.823679262	-0.2798454	0.694616	1
NTF4	1.0664742	1.04677608	1.0750124	1.603648727	0.6813582	0.694666	1
TMEM109	1.4321588	1.41002829	1.4417514	1.07736821	0.1075114	0.694683	1
ZNF12	1.3800029	1.40188316	1.3705188	0.921956451	-0.1172295	0.694695	1
RP11-806H10.4	1.179356	1.15868748	1.1883149	1.1867032	0.2469592	0.694857	1
FHIT	1.2547672	1.23331221	1.2640669	1.131817889	0.1786418	0.694886	1
TBCE	1.1597312	1.13958266	1.1684647	1.206917187	0.2713267	0.694915	1
LINC01144	1.0292214	1.04825789	1.0209699	0.43453826	-1.2024449	0.694933	1
RECQL5	1.1110231	1.13065438	1.1025138	0.784617902	-0.3499378	0.694984	1
PPL	1.1525746	1.13207451	1.1614605	1.222495681	0.2898294	0.694998	1
PTGR2	1.2829518	1.30419796	1.2737426	0.899882941	-0.1521908	0.695338	1
PAIP2B	1.2685886	1.28927828	1.2596205	0.897476619	-0.1560537	0.695391	1
TIMM44	1.6663084	1.64201106	1.6768403	1.054250156	0.0762172	0.695521	1
RCOR3	1.3387973	1.36064535	1.3293272	0.913160854	-0.1310591	0.695522	1
DDX23	1.3478309	1.36936306	1.3384976	0.916435989	-0.125894	0.695529	1
DAB2IP	1.2461116	1.22488233	1.2553136	1.135320805	0.1831	0.695649	1
NUTM2A-AS1	1.3035202	1.32460419	1.2943812	0.906892876	-0.1409959	0.695721	1
TCAIM	1.4299234	1.40763689	1.4395837	1.078370659	0.1088531	0.695728	1
THAP3	1.3933303	1.37132848	1.4028672	1.084934719	0.1176082	0.69594	1
GABPB2	1.1359042	1.11555593	1.1447243	1.252417359	0.3247154	0.695965	1
MTG2	1.2101156	1.18939535	1.2190969	1.156822944	0.2101681	0.696101	1
PRPF40B	1.1889454	1.20918539	1.1801722	0.861304022	-0.2154055	0.696101	1
LIMS2	1.0780108	1.05842009	1.0865025	1.480697906	0.5662773	0.696127	1
SMG9	1.3837242	1.40525896	1.3743898	0.923828675	-0.1143028	0.6964	1
SLC43A1	1.0474061	1.06666727	1.0390572	0.585853446	-0.7713883	0.69641	1
TTPA	1.0448004	1.064004	1.0364765	0.569909228	-0.8111959	0.696485	1
LIPT1	1.1218356	1.1019472	1.1304564	1.279646431	0.3557452	0.696521	1
PROSER1	1.1581974	1.17829343	1.1494866	0.838430274	-0.2542373	0.69659	1
ZBTB7C	1.0401708	1.05940893	1.031832	0.535811079	-0.9002037	0.696707	1
USP4	1.1780216	1.19816343	1.1692911	0.854300335	-0.2271847	0.696751	1
RUFY3	2.127355	2.154699	2.1155025	0.966054816	-0.049823	0.696872	1
SAMD15	1.1298075	1.10960584	1.1385641	1.264203482	0.3382287	0.696874	1
TUFT1	1.1298631	1.10980664	1.1385567	1.26182441	0.3355112	0.696874	1
PLA2G3	1.0584843	1.0775571	1.0502171	0.647485245	-0.6270808	0.696899	1
RP11-722E23.2	1.0601055	1.07942318	1.0517322	0.651348317	-0.6184988	0.696904	1
FAM201A	1.0798771	1.09928451	1.0714648	0.719798067	-0.4743359	0.697072	1
CACNG4	1.1261253	1.14577073	1.1176099	0.806814288	-0.3096915	0.697344	1
ANTXR2	1.2436676	1.22298457	1.2526328	1.132960787	0.1800979	0.697415	1
CHST10	1.1537983	1.13323748	1.1627105	1.221206414	0.2883071	0.697432	1

DIAPH2-AS1	1.0681473	1.04865057	1.0765982	1.574457089	0.6548544	0.697599	1
SYCE2	1.061181	1.08028734	1.0528992	0.658873338	-0.6019269	0.697622	1
MAEL	1.064984	1.08409902	1.0566984	0.674186433	-0.5687805	0.697635	1
SLIT3	1.1128046	1.09293865	1.1214156	1.306405427	0.3856027	0.69767	1
EIF4EBP1	3.2334563	3.187728	3.2532775	1.029962347	0.0425916	0.697702	1
RP3-399L15.3	1.0362723	1.05518315	1.0280752	0.508764411	-0.9749303	0.697724	1
YME1L1	1.8446161	1.86942354	1.8338632	0.959098932	-0.0602485	0.697811	1
F8A1	1.1050113	1.12464015	1.0965031	0.774253931	-0.3691213	0.697951	1
CHRNB4	1.069429	1.04981767	1.0779296	1.564296154	0.6455137	0.698062	1
UBR7	1.1733045	1.19316763	1.1646948	0.852600317	-0.2300585	0.698604	1
NADK	1.37019	1.39201514	1.3607297	0.920193406	-0.119991	0.698664	1
PIK3C3	1.4642919	1.48643668	1.4546931	0.934742564	-0.097359	0.698679	1
UPK2	1.0451636	1.02579129	1.0535606	2.076694861	1.0542892	0.698742	1
GXYLT2	1.0867346	1.06696344	1.0953046	1.423233012	0.5091719	0.698881	1
KDM2B	1.2922924	1.2709474	1.3015445	1.112926521	0.1543583	0.698887	1
FUT9	1.075489	1.09455558	1.0672245	0.710951986	-0.492176	0.699097	1
BCL6	1.0951056	1.07546896	1.1036171	1.37297695	0.4573074	0.699168	1
NOP14	1.2280029	1.24841583	1.2191548	0.882209556	-0.1808067	0.699413	1
ZNF395	1.3807824	1.35851282	1.3904353	1.089041313	0.1230587	0.699461	1
DUSP3	1.3937526	1.41549011	1.3843304	0.925004823	-0.1124672	0.699513	1
CRNDE	1.6025644	1.57892909	1.6128093	1.058522184	0.0820515	0.699734	1
RP1-90J20.8	1.0562465	1.03687623	1.0646426	1.7529621	0.8097948	0.699796	1
FAM63A	1.1240092	1.1434805	1.1155693	0.805470461	-0.3120964	0.6998	1
CARMIL3	1.0352622	1.05400031	1.02714	0.502590547	-0.9925446	0.700002	1
HMGN1	15.275961	15.417406	15.214651	0.985936813	-0.0204329	0.700051	1
SLC25A23	1.6330115	1.6560899	1.6230081	0.949577366	-0.0746425	0.700058	1
BCL9L	1.1136812	1.09385856	1.1222735	1.302742087	0.3815515	0.700066	1
ALG5	1.6796996	1.65652448	1.689745	1.05060054	0.0712142	0.700121	1
GS1-124K5.3	1.0575301	1.07647212	1.0493195	0.64493457	-0.6327753	0.700162	1
LMNTD2	1.0653676	1.08434875	1.05714	0.677426167	-0.5618644	0.700411	1
MYCBP2	1.6453989	1.66896143	1.6351856	0.949509991	-0.0747449	0.70045	1
CLP1	1.1075019	1.08791602	1.1159914	1.319343634	0.3998204	0.700467	1
PAQR5	1.1634393	1.14301718	1.1722914	1.204690183	0.2686622	0.700516	1
FBXL4	1.2638418	1.28454114	1.2548695	0.89572124	-0.1588783	0.700639	1
TESK2	1.0550104	1.03575796	1.0633554	1.771784641	0.8252033	0.700648	1
OSCP1	1.1719396	1.15181314	1.1806635	1.190038684	0.2510085	0.700745	1
TMEM40	1.0378054	1.01870983	1.0460825	2.463011362	1.3004233	0.700758	1
GALC	1.249232	1.26993962	1.2402561	0.890036572	-0.1680635	0.700773	1
LINC00629	1.0256401	1.04424325	1.0175765	0.397269487	-1.3318101	0.700904	1
TSPEAR-AS2	1.0972235	1.11646423	1.0888835	0.763182777	-0.3898995	0.701126	1
LRRTM2	1.0454115	1.06413145	1.0372972	0.581574992	-0.7819629	0.701195	1
VRK3	1.5198981	1.54216505	1.5102464	0.941127486	-0.0875379	0.701327	1
SMAD1	1.1826003	1.1625405	1.1912954	1.176909159	0.235003	0.701469	1
DACT3	1.0929991	1.11227764	1.0846427	0.753870001	-0.4076123	0.701612	1
XIAP	1.4978254	1.52073144	1.4878966	0.936944735	-0.0939641	0.70171	1
TINCR	1.0568032	1.03752108	1.0651612	1.736656188	0.7963122	0.701738	1
AC009228.1	1.0426804	1.06151977	1.0345144	0.561028911	-0.833853	0.701793	1
ZNF443	1.0624941	1.08136789	1.0543131	0.667500846	-0.5831584	0.701843	1
IL7R	1.0535561	1.07236253	1.0454043	0.627456071	-0.6724136	0.70191	1
PREPL	1.7357589	1.75972598	1.7253702	0.954778679	-0.0667617	0.70197	1
ZNF584	1.2099895	1.23006069	1.2012895	0.874940908	-0.1927425	0.701981	1

FYCO1	1.1044699	1.08489399	1.1129551	1.330543487	0.4120157	0.702047	1
C10orf107	1.0458201	1.02670975	1.0541036	2.025612498	1.0183582	0.702095	1
CDH12	1.0395852	1.02056217	1.0478308	2.326156257	1.217948	0.702155	1
RGMB-AS1	1.070892	1.05154074	1.0792798	1.538197871	0.6212411	0.70229	1
FAM169A	1.3770964	1.35557109	1.3864266	1.086777467	0.1200566	0.702297	1
YPEL3	1.581124	1.6041247	1.5711542	0.945424358	-0.0809661	0.702439	1
ZNF383	1.1138542	1.1330455	1.1055356	0.793229616	-0.3341896	0.702449	1
LAMA2	1.053429	1.07224892	1.0452714	0.626602786	-0.6743769	0.702455	1
TCERG1L	1.0971548	1.0778232	1.1055342	1.356076536	0.4394386	0.702559	1
TRA2A	2.1011169	2.06956937	2.1147914	1.04228061	0.0597437	0.702641	1
SPRR3	1.0377495	1.01850742	1.04609	2.490353551	1.3163506	0.702679	1
ESRP1	1.0375149	1.01850742	1.0457538	2.472186449	1.3057876	0.702679	1
SEMA6B	1.1057333	1.12498184	1.0973899	0.779232165	-0.3598749	0.702722	1
FPGT-TNNI3K	1.0447641	1.06347484	1.0366538	0.577454561	-0.7922207	0.702745	1
RP11-95O2.5	1.0447594	1.06349689	1.0366375	0.576996431	-0.7933657	0.702745	1
SCGB1B2P	1.0354374	1.05398563	1.0273975	0.507496901	-0.9785291	0.70276	1
ARL17A	1.1058748	1.08631769	1.1143519	1.324779464	0.4057522	0.702777	1
B3GALT5	1.0394893	1.02056357	1.0476928	2.319286135	1.2136808	0.702924	1
THBS3	1.2885637	1.26758416	1.2976573	1.112387783	0.1536598	0.702926	1
RP5-1112D6.8	1.0646744	1.08369607	1.0564294	0.674217848	-0.5687133	0.702948	1
DUSP10	1.1001797	1.11947367	1.0918167	0.768509583	-0.3798648	0.702967	1
RP11-368I23.2	1.0530079	1.07165228	1.0449263	0.627004867	-0.6734515	0.702974	1
PCNX1	1.3951259	1.37370111	1.4044126	1.082181867	0.113943	0.702979	1
FBXO33	1.1500517	1.13012592	1.1586887	1.219500908	0.2862908	0.70302	1
ASAH2B	1.0926728	1.11150854	1.0845084	0.757864541	-0.3999881	0.703116	1
MKS1	1.1719909	1.19183928	1.1633875	0.851689728	-0.2316001	0.703119	1
TNFSF10	1.0455811	1.02661383	1.0538026	2.021604148	1.0155005	0.703163	1
WSB2	1.5187096	1.49596343	1.528569	1.065741954	0.0918582	0.703229	1
ORC3	1.3492356	1.32760013	1.3586136	1.094668697	0.1304943	0.703349	1
ARFIP2	1.5965859	1.57376715	1.6064768	1.057008646	0.0799872	0.703481	1
TPP1	1.4005146	1.37904081	1.4098225	1.081209339	0.1126459	0.703585	1
TOX2	1.1213901	1.14075499	1.1129962	0.802786523	-0.3169117	0.703674	1
LANCL2	1.1462804	1.12632524	1.1549301	1.226438417	0.2944748	0.703732	1
ADCY8	1.0314126	1.04991176	1.0233941	0.468709078	-1.0932354	0.703782	1
CTC-559E9.1	1.1232807	1.14270497	1.1148612	0.804885715	-0.3131441	0.703867	1
RFX4	1.0621848	1.08106824	1.0539997	0.666102148	-0.5861847	0.703973	1
TFIP11	1.1310486	1.11150688	1.1395191	1.251214802	0.3233295	0.704099	1
POU5F2	1.0628098	1.0816077	1.0546617	0.669811014	-0.578174	0.704152	1
CSPG5	1.1470243	1.16626088	1.1386861	0.834147598	-0.2616254	0.704251	1
CDK17	1.2096402	1.18945882	1.2183879	1.152693236	0.2050086	0.704371	1
IL17RD	1.2032997	1.22313732	1.194701	0.872561281	-0.1966716	0.704592	1
CHST9	1.1024242	1.12128485	1.094249	0.777087728	-0.3638506	0.704719	1
TAF11	2.3163405	2.288263	2.3285109	1.031241977	0.0443829	0.70475	1
CCKBR	1.2238806	1.20341182	1.2327529	1.144244917	0.1943959	0.704947	1
ZFP28	1.0997089	1.08013915	1.1081915	1.350045293	0.4330078	0.704984	1
RP11-434H6.7	1.121377	1.14027956	1.1131836	0.806843104	-0.3096399	0.705105	1
FANCC	1.1061698	1.08675531	1.1145852	1.320785727	0.4013964	0.705163	1
FAM206A	1.1327435	1.15221759	1.1243024	0.816610083	-0.2922807	0.705378	1
MAP3K14	1.0704832	1.08924884	1.0623492	0.698599398	-0.5174627	0.705461	1
SPIRE1	1.214046	1.19386133	1.2227951	1.149249996	0.2006927	0.705471	1
KDM6B	1.5600114	1.58229003	1.5503546	0.945155419	-0.0813765	0.705646	1

RBM19	1.2542854	1.23396275	1.2630943	1.124513803	0.1693014	0.705662	1
CD22	1.0311215	1.04950135	1.0231546	0.467757593	-1.096167	0.705767	1
SNHG19	1.5660961	1.58815783	1.5565333	0.946231249	-0.0797353	0.705776	1
NEDD1	1.374306	1.39532528	1.365195	0.923783698	-0.114373	0.705848	1
AXIN1	1.1413349	1.12174919	1.1498244	1.230599107	0.2993609	0.705984	1
CTD-2006H14.2	1.0708929	1.08949573	1.0628294	0.702038288	-0.5103784	0.706018	1
APOPT1	2.0305055	2.00413022	2.0419379	1.037652205	0.053323	0.706021	1
CBX8	1.1356617	1.15503058	1.1272661	0.820909979	-0.2847041	0.70613	1
HEY1	1.4169214	1.3962135	1.4258974	1.07491898	0.1042279	0.706167	1
LINC00159	1.0326213	1.01394208	1.040718	2.920508866	1.5462198	0.706229	1
CNFN	1.201834	1.18155448	1.2106242	1.160115815	0.2142688	0.706306	1
HIRA	1.1106446	1.12964447	1.102409	0.789921774	-0.3402183	0.706331	1
RP11-334C17.5	1.0909353	1.10997868	1.0826808	0.751789486	-0.4115994	0.706403	1
ENTPD3-AS1	1.1586688	1.13904977	1.1671727	1.202251024	0.2657382	0.706447	1
CRTC2	1.152304	1.13249333	1.160891	1.214332812	0.2801639	0.706476	1
RP11-446N19.1	1.0682513	1.08706483	1.0600964	0.690248955	-0.5348113	0.70653	1
RND1	1.0621554	1.04332679	1.0703168	1.622939393	0.6986091	0.706577	1
RP11-350J20.5	1.0488129	1.06731082	1.0407949	0.606066834	-0.7224512	0.706729	1
PPP1R35	1.4035783	1.42493221	1.3943224	0.927965351	-0.1078572	0.706841	1
ZNF214	1.142994	1.16220571	1.1346666	0.830221217	-0.2684323	0.7069	1
TASP1	1.1427995	1.16203658	1.1344611	0.82981966	-0.2691303	0.7069	1
AK9	1.1948709	1.17484019	1.2035533	1.164225112	0.21937	0.707179	1
ADAMTS1	1.1215848	1.14071826	1.1132913	0.805093126	-0.3127724	0.707273	1
CYP2S1	1.0325752	1.01400962	1.0406226	2.899620013	1.5358639	0.707304	1
CASK	1.4439434	1.46555768	1.4345745	0.933449421	-0.0993562	0.707433	1
SPINK5	1.0792152	1.0977255	1.0711918	0.728487182	-0.4570245	0.707484	1
GGA3	1.1480449	1.16750801	1.1396085	0.833443474	-0.2628437	0.707561	1
RP11-430B1.2	1.0420449	1.06048439	1.0340522	0.562991628	-0.8288146	0.707617	1
AURKA	1.3775967	1.35685455	1.3865875	1.083319574	0.1154589	0.707637	1
SERPINA5	1.0498246	1.0311112	1.0579361	1.862226101	0.8970282	0.707731	1
NAPG	1.2641682	1.24394761	1.272933	1.118817982	0.1619753	0.707842	1
CMIP	1.385541	1.36432784	1.394736	1.083463778	0.1156509	0.707865	1
HELB	1.2102147	1.18998993	1.2189813	1.152594127	0.2048846	0.70789	1
DAGLA	1.0602182	1.07868566	1.0522133	0.663568532	-0.5916826	0.70814	1
KBTBD6	1.1991268	1.21876615	1.190614	0.871314149	-0.1987351	0.708247	1
C2CD2	1.1954397	1.17575785	1.2039709	1.16052221	0.2147741	0.708249	1
AF127936.9	1.0871873	1.10594614	1.0790562	0.746192153	-0.4223809	0.708275	1
UG0898H09	1.1793192	1.19892142	1.1708225	0.858743855	-0.2197002	0.708294	1
CRYBG3	1.2011765	1.22039638	1.1928455	0.874993877	-0.1926552	0.708351	1
OXSR1	1.5369508	1.51475594	1.5465714	1.061806792	0.0865213	0.708354	1
NDUFAF8	3.6852313	3.7272785	3.6670057	0.977900033	-0.0322411	0.708687	1
GABPB1	2.3312875	2.35906446	2.3192475	0.970702659	-0.0428987	0.708688	1
CRCP	1.2700824	1.29031051	1.2613144	0.900120458	-0.15181	0.708781	1
C19orf68	1.1716481	1.19109897	1.1632171	0.854097102	-0.227528	0.70895	1
PRPF39	1.2666796	1.28653908	1.2580714	0.900649943	-0.1509616	0.708984	1
IBA57	1.1121819	1.13111673	1.1039745	0.792991954	-0.3346219	0.709021	1
LINC01137	1.0987686	1.1174484	1.0906717	0.772013159	-0.3733027	0.709035	1
ELK4	1.2653568	1.28543189	1.2566551	0.899181676	-0.1533155	0.709051	1
RP11-53I6.3	1.030052	1.04824483	1.0221662	0.459451444	-1.1220157	0.709127	1
EEF1G	1.5534891	1.57518229	1.544086	0.945936724	-0.0801844	0.709128	1
UMPS	1.2094395	1.22928947	1.2008354	0.875903497	-0.1911562	0.709295	1

UHRF2	1.2265188	1.20634069	1.2352651	1.140177716	0.1892587	0.709295	1
PODXL	1.1001196	1.08089854	1.1084511	1.34058173	0.4228592	0.709535	1
CFAP43	1.0534695	1.07179253	1.0455273	0.634150555	-0.6571027	0.709567	1
ZBTB49	1.0571538	1.07549276	1.0492047	0.651779944	-0.6175431	0.709638	1
LIMCH1	1.4982905	1.47591658	1.5079887	1.067390157	0.0940876	0.709849	1
KLHL21	1.1124687	1.09334087	1.1207597	1.293749675	0.3715585	0.709861	1
HSPA6	1.0334095	1.01490147	1.0414319	2.780386705	1.4752856	0.71013	1
GALNT14	1.0333981	1.01490147	1.0414156	2.77929676	1.4747199	0.71013	1
MAN2A1	1.3300895	1.30948017	1.3390227	1.095458499	0.1315348	0.710193	1
CDK8	1.6087	1.63118872	1.5989521	0.948927087	-0.0756309	0.710224	1
C14orf93	1.128361	1.14728455	1.1201584	0.815825054	-0.2936683	0.710271	1
GNG10	1.1031291	1.0841154	1.1113707	1.324023078	0.4049283	0.710301	1
RP11-615I2.6	1.0561458	1.07445071	1.0482114	0.647561536	-0.6269108	0.710328	1
ANKRD33	1.0362737	1.05445522	1.0283929	0.521398557	-0.9395415	0.710343	1
RBM17	2.5593459	2.58932482	2.5463513	0.972961161	-0.0395459	0.710683	1
GPATCH2	1.4022096	1.38105832	1.4113777	1.079566293	0.1104518	0.710727	1
NR2C2AP	1.2975608	1.31751233	1.2889128	0.909926121	-0.1361787	0.710822	1
PPM1N	1.0750066	1.09340873	1.0670301	0.717599552	-0.4787491	0.711008	1
RP13-582O9.7	1.0874987	1.10605324	1.0794561	0.749209271	-0.4165593	0.711023	1
AC021188.4	1.0290241	1.04708071	1.0211974	0.450235735	-1.1512475	0.711107	1
KIF4A	1.1981125	1.21749685	1.1897103	0.87224389	-0.1971965	0.711195	1
MRFAP1L1	1.5301412	1.55244529	1.5204734	0.942126583	-0.0860072	0.711204	1
EXOC1	1.4636698	1.48486745	1.4544816	0.937331604	-0.0933686	0.71131	1
SH3BGRL2	1.3150491	1.33499075	1.3064053	0.914668048	-0.1286798	0.711379	1
COL14A1	1.0361085	1.01771531	1.0440812	2.488309841	1.3151661	0.711438	1
PHKA2	1.1457207	1.16464457	1.1375181	0.835242292	-0.2597333	0.711441	1
GAL3ST3	1.2139194	1.23333056	1.2055055	0.880748372	-0.1831982	0.711515	1
DPH6	1.3238278	1.30345404	1.3326589	1.096241494	0.1325656	0.711693	1
TTI2	1.1641786	1.18319101	1.1559376	0.851229566	-0.2323798	0.711777	1
AKT1	1.5053329	1.52663564	1.4960992	0.942015953	-0.0861766	0.711779	1
KIAA1024L	1.0593037	1.07751476	1.05141	0.663228399	-0.5924223	0.711786	1
ZNF669	1.1567413	1.17579478	1.1484825	0.844635432	-0.2435993	0.711859	1
XYLB	1.0461228	1.06432521	1.0382328	0.594367428	-0.750573	0.711901	1
SRRD	1.2552337	1.27533456	1.2465209	0.89535042	-0.1594757	0.712005	1
PCBP3	1.1356232	1.11618222	1.14405	1.239862621	0.3101803	0.712016	1
SENP3	1.0891093	1.10753067	1.0811245	0.754430885	-0.4065394	0.712169	1
GATAD2A	1.3175094	1.33765602	1.3087767	0.914471265	-0.1289903	0.712193	1
SEC31B	1.1344868	1.15346155	1.1262621	0.822760315	-0.2814559	0.712288	1
SLC12A4	1.1281573	1.1091307	1.1364045	1.249918338	0.3218338	0.712305	1
MYOZ3	1.0670611	1.08529408	1.0591579	0.693575429	-0.5278753	0.712325	1
GNA11	1.2158319	1.19581768	1.2245071	1.14651105	0.1972503	0.712375	1
SPEF1	1.0743041	1.09260318	1.0663723	0.716738908	-0.4804804	0.712503	1
ARSJ	1.0512754	1.03280015	1.0592836	1.807419695	0.8539315	0.712506	1
TEX26	1.056868	1.0751323	1.0489512	0.651533919	-0.6180878	0.712517	1
ZNF525	1.1091764	1.12792424	1.1010501	0.789921413	-0.340219	0.712545	1
MCMBP	1.4823491	1.50385153	1.4730288	0.938825796	-0.0910706	0.712573	1
ZNF66	1.0543061	1.07256629	1.0463911	0.639292336	-0.6454523	0.712593	1
BHLHB9	1.1558963	1.17492914	1.1476464	0.844035358	-0.2446247	0.712754	1
RP11-89F3.2	1.0496732	1.06778528	1.0418225	0.616984283	-0.6966944	0.712922	1
RP11-74J13.8	1.0588225	1.07701627	1.0509363	0.661369998	-0.5964705	0.713008	1
MIR181A2HG	1.1829259	1.16309549	1.1915215	1.174290347	0.2317892	0.71303	1

TRAK2	1.1077564	1.12651586	1.099625	0.787450527	-0.3447388	0.7131	1
CD47	2.4833915	2.45338912	2.4963962	1.029590906	0.0420712	0.713109	1
FGD6	1.1185444	1.13739834	1.1103721	0.80330009	-0.3159891	0.71311	1
ZNF189	1.1474864	1.16627606	1.139342	0.838015714	-0.2549508	0.713145	1
RP11-74E22.3	1.0274751	1.04541858	1.0196974	0.433685588	-1.2052786	0.713178	1
FAM208B	1.3020695	1.32225029	1.2933221	0.910230553	-0.1356961	0.713205	1
GATSL3	1.2590321	1.23893673	1.2677426	1.120558366	0.1642178	0.71321	1
SETD7	1.1643854	1.14509062	1.1727489	1.190627558	0.2517222	0.713253	1
ZNF165	1.0459432	1.02760984	1.0538899	1.951835417	0.9648314	0.713357	1
XXbac-B135H6.15	1.0455026	1.0635725	1.0376701	0.592553791	-0.754982	0.7134	1
C1orf112	1.1248318	1.14346884	1.1167535	0.813790057	-0.2972714	0.713412	1
OXER1	1.0309942	1.04895418	1.0232094	0.474103838	-1.076725	0.713444	1
CLDN1	2.1273974	2.0994704	2.1395025	1.036410356	0.0515953	0.713494	1
AURKC	1.0807091	1.06205269	1.0887958	1.430973968	0.5169974	0.71368	1
CHRM3	1.1654203	1.18458796	1.157112	0.851149837	-0.232515	0.713701	1
CTU1	1.0382592	1.05614154	1.030508	0.543412418	-0.8798806	0.713781	1
PPP6R2	1.4133401	1.39227092	1.4224727	1.076992091	0.1070077	0.713801	1
PDK2	1.4879538	1.46650976	1.4972488	1.065891499	0.0920606	0.713884	1
METTL15	1.3263357	1.3462858	1.3176882	0.917416085	-0.1243519	0.713917	1
DENND1B	1.3262979	1.34653649	1.3175254	0.916282726	-0.1261353	0.713917	1
KIAA0391	1.0457006	1.06371447	1.0378924	0.594721935	-0.7497128	0.713952	1
RP11-66N11.8	1.1854938	1.20447961	1.1772642	0.866904163	-0.2060556	0.713979	1
QSOX2	1.1431659	1.16168308	1.1351396	0.835830011	-0.2587185	0.714155	1
DOCK9	1.1429856	1.16197609	1.134754	0.831937915	-0.2654522	0.714155	1
GCDH	1.2280845	1.20849613	1.2365751	1.134673958	0.1822778	0.714352	1
PARP11	1.21673	1.2361566	1.2083094	0.882081826	-0.1810156	0.714406	1
AKAP9	3.0165126	3.0585448	2.9982935	0.970731127	-0.0428563	0.714413	1
LRIG1	1.1205282	1.10170391	1.1286877	1.265317276	0.3394992	0.714441	1
CCDC112	1.4416077	1.42072535	1.4506592	1.071148268	0.0991582	0.714451	1
RPAP2	1.4656092	1.44463347	1.4747013	1.067623814	0.0944034	0.71449	1
RP11-136K7.2	1.1181793	1.13671266	1.110146	0.805675088	-0.3117299	0.714546	1
MARCH9	1.1775745	1.19658224	1.1693355	0.861397745	-0.2152485	0.714659	1
RP11-396C23.4	1.0257607	1.04367127	1.0179973	0.412109156	-1.2789016	0.714661	1
BOD1L1	1.4489294	1.42794094	1.4580269	1.070304093	0.0980208	0.714733	1
ARMCX5	1.2512808	1.27113809	1.2426735	0.895018191	-0.1600111	0.714774	1
APC	1.4225099	1.4013853	1.4316665	1.075441632	0.1049292	0.714852	1
ARL6	1.0965377	1.1150298	1.0885222	0.769558845	-0.3778964	0.715048	1
FDXACB1	1.0523991	1.07037997	1.0446052	0.633777152	-0.6579524	0.715163	1
ATCAY	1.1316384	1.11264569	1.1398709	1.241689236	0.3123041	0.715227	1
ERMP1	1.0886229	1.07006564	1.0966667	1.379659388	0.4643121	0.715311	1
AC093818.1	1.0885916	1.0700294	1.0966376	1.379956976	0.4646233	0.715311	1
CCNE2	1.0462145	1.06409239	1.0384652	0.600152179	-0.7365997	0.715315	1
TXN2	2.9752951	2.94094932	2.9901824	1.025365486	0.0361382	0.715325	1
AP1M2	1.0401526	1.02188353	1.0480714	2.196691495	1.1353323	0.715375	1
ANKS1A	1.1948245	1.21385337	1.1865763	0.872449699	-0.1968561	0.715471	1
UTP15	1.0972026	1.07870815	1.1052191	1.336826088	0.4188118	0.715479	1
VWCE	1.0830911	1.10153569	1.0750961	0.739603309	-0.4351764	0.715546	1
CFAP221	1.0859565	1.10409751	1.0780932	0.750193201	-0.4146659	0.715547	1
EPHB4	1.2440031	1.22421814	1.2525791	1.126488058	0.171832	0.715617	1
MED27	1.096648	1.07795209	1.1047519	1.343798613	0.4263169	0.715661	1
TMEM120A	1.2529493	1.23344331	1.2614043	1.119776318	0.1632106	0.715664	1

MBOAT2	1.6998023	1.72220673	1.690091	0.95553111	-0.0656253	0.715668	1
SLC25A28	1.0885787	1.07001534	1.0966251	1.380056009	0.4647268	0.715698	1
RP11-456O19.2	1.1804296	1.19946352	1.1721793	0.863211788	-0.2122135	0.715847	1
MBTD1	1.2998379	1.31967055	1.2912414	0.911067352	-0.1343704	0.715873	1
RP11-406A9.2	1.063457	1.08156064	1.0556099	0.681822682	-0.5525315	0.71588	1
ATP11C	1.491576	1.51292275	1.4823231	0.940342507	-0.0887418	0.715903	1
SFXN4	1.3481704	1.32731835	1.3572089	1.091319414	0.1260734	0.715966	1
SLC17A5	1.1998418	1.18061007	1.208178	1.152637659	0.2049391	0.716022	1
CNOT4	1.7273373	1.75003794	1.7174976	0.956615011	-0.0639897	0.716085	1
HRH1	1.0449173	1.02671971	1.0528052	1.976263465	0.9827753	0.716107	1
RP11-342K6.4	1.0352219	1.05311918	1.0274642	0.51703029	-0.9516793	0.71613	1
GLB1L2	1.1141667	1.13254253	1.1062016	0.801264049	-0.3196503	0.716133	1
PIPOX	1.1319754	1.11291529	1.1402371	1.241966994	0.3126268	0.716308	1
LRRC73	1.2073067	1.18753729	1.2158758	1.15110871	0.2030241	0.716349	1
UGGT1	1.2424715	1.26211166	1.2339583	0.892590159	-0.1639302	0.716524	1
KRI1	1.1466532	1.16555738	1.1384591	0.836321021	-0.2578713	0.716745	1
ZRSR2	1.350851	1.37099409	1.3421198	0.922170555	-0.1168945	0.716861	1
RNMT	3.3497256	3.3095241	3.3671512	1.024951948	0.0355563	0.716883	1
ITPR1-AS1	1.0323864	1.05019553	1.024667	0.491417392	-1.0249792	0.716921	1
NLE1	1.1645605	1.18341191	1.1563892	0.85266658	-0.2299464	0.716976	1
YDJC	1.7202514	1.69803924	1.7298794	1.045613761	0.06435	0.717102	1
FBXO4	1.1223076	1.14071473	1.1143289	0.812486783	-0.2995838	0.717328	1
SLC41A2	1.0722945	1.05411291	1.0801754	1.481632456	0.5671876	0.717487	1
KAT14	1.1795999	1.16045057	1.1879002	1.171078527	0.2278378	0.717527	1
REPIN1	2.1319529	2.10673692	2.1428829	1.032659955	0.0463653	0.717579	1
TROAP	1.4006234	1.37983917	1.4096325	1.078436599	0.1089414	0.717608	1
AP001412.1	1.0483903	1.06622965	1.0406577	0.61388993	-0.7039481	0.717618	1
RNF166	1.2347351	1.21527517	1.2431701	1.129578195	0.1757841	0.717694	1
DNAJC10	2.0166421	1.99142838	2.0275711	1.036455189	0.0516577	0.717706	1
PAQR6	1.1026968	1.12103838	1.0947465	0.782780419	-0.3533204	0.717959	1
C6orf132	1.1227549	1.14132175	1.114707	0.811672357	-0.3010306	0.718016	1
TMEM179	1.0335488	1.05123437	1.0258829	0.505185396	-0.9851152	0.718249	1
RFLNB	1.2582941	1.27736381	1.2500282	0.901445022	-0.1496886	0.71825	1
SYP	1.2282268	1.20840747	1.2368176	1.136320262	0.1843695	0.718281	1
ZNF624	1.100426	1.11850192	1.0925908	0.78134471	-0.3559689	0.718286	1
SCD5	1.4397619	1.46067048	1.430699	0.934939349	-0.0970553	0.718301	1
IFIT1	1.0438058	1.0615153	1.0361295	0.587325281	-0.7677684	0.718309	1
CYFIP2	1.4713521	1.49214147	1.4623409	0.939447109	-0.0901162	0.718386	1
FAM91A1	1.3227757	1.30207699	1.3317476	1.098222094	0.1351698	0.718417	1
ANKRA2	1.6967551	1.71914012	1.6870522	0.955380127	-0.0658532	0.718426	1
LRRC29	1.0918797	1.07350876	1.0998426	1.358240934	0.4417394	0.718432	1
MCRIP1	4.1326333	4.17848735	4.1127577	0.979320457	-0.0301471	0.71846	1
TRIM32	1.1005207	1.08217031	1.1084748	1.320122087	0.4006714	0.718537	1
ARRDC3	2.0255528	2.0577558	2.0115943	0.95635901	-0.0643758	0.718626	1
ZFHX2	1.1841634	1.20312142	1.175946	0.866210975	-0.2072096	0.718716	1
LYSMD1	1.1143345	1.09550603	1.1224957	1.28259692	0.3590678	0.719015	1
RP11-96H19.1	1.074009	1.05566752	1.0819592	1.472298338	0.55807	0.719032	1
SLC35C1	1.184219	1.16530966	1.1924154	1.163969416	0.2190532	0.719059	1
PLEKHG5	1.1262294	1.10735168	1.134412	1.252071639	0.3243171	0.719086	1
AMHR2	1.0388916	1.02086411	1.0467058	2.238570319	1.1625776	0.71912	1
RP11-456H18.2	1.2091712	1.22803243	1.2009956	0.881434454	-0.1820748	0.719121	1

EEPD1	1.1225886	1.10387469	1.1307002	1.2582486	0.331417	0.719217	1
POLR1C	1.2968248	1.31645484	1.288316	0.911081033	-0.1343487	0.719371	1
FSIP2-AS1	1.1004322	1.11858172	1.0925652	0.780602706	-0.3573396	0.719394	1
INTS6L	1.100301	1.11849189	1.0924161	0.77993601	-0.3585723	0.719394	1
HELZ2	1.1205255	1.13888155	1.112569	0.810539656	-0.3030453	0.719445	1
FHOD1	1.1590839	1.17757715	1.1510679	0.850716808	-0.2332491	0.719522	1
C7orf49	1.5886063	1.61000033	1.5793329	0.949725625	-0.0744173	0.719569	1
LANCL1	1.4487626	1.4278716	1.457818	1.06998918	0.0975962	0.719646	1
OGDH	1.2006783	1.18132661	1.2090664	1.152982231	0.2053703	0.719647	1
INSIG2	2.5111753	2.54167517	2.497955	0.971641105	-0.0415046	0.71967	1
MTIF2	1.4965944	1.47564853	1.5056736	1.063124438	0.0883105	0.719679	1
ASRGL1	1.489405	1.50978457	1.4805714	0.942695054	-0.0851369	0.719812	1
C2orf76	1.2963128	1.27665919	1.3048317	1.101831179	0.1399032	0.719909	1
TMLHE	1.1514264	1.13260948	1.1595827	1.20340381	0.2671208	0.719923	1
RP11-53O19.3	1.1192712	1.13752414	1.1113594	0.809744248	-0.3044618	0.719925	1
AK1	2.2704255	2.2440709	2.2818491	1.030366591	0.0431577	0.720062	1
RAPGEF2	1.2029718	1.22219709	1.1946385	0.875972185	-0.191043	0.720086	1
GLTSCR1	1.0839564	1.10191444	1.0761724	0.747414968	-0.4200186	0.720089	1
WRAP53	1.1902049	1.20896961	1.1820713	0.871281167	-0.1987897	0.720146	1
MALT1	1.4289196	1.44925361	1.4201058	0.935119376	-0.0967775	0.720185	1
UNC5A	1.0325271	1.05005967	1.0249275	0.497956292	-1.005909	0.720206	1
DOCK7	1.5743858	1.59541671	1.5652698	0.949368453	-0.07496	0.720277	1
MED23	1.2370653	1.25575423	1.2289645	0.895252012	-0.1596342	0.720381	1
PHLDB3	1.1640167	1.18247569	1.1560155	0.854993337	-0.2260149	0.720508	1
KCNJ4	1.0690402	1.08689927	1.061299	0.705403297	-0.5034798	0.720557	1
SLC39A10	2.7130678	2.74529216	2.6991	0.973533254	-0.0386978	0.7206	1
RP11-465L10.10	1.0517542	1.06945199	1.0440829	0.634725168	-0.655796	0.720641	1
PBK	1.5133008	1.49188213	1.5225848	1.062418821	0.0873526	0.720653	1
DGKI	1.0772814	1.0589829	1.085213	1.444706682	0.5307766	0.720866	1
IFNAR1	1.4915663	1.51248788	1.4824977	0.941481278	-0.0869957	0.720972	1
NAXD	1.2811579	1.30034418	1.2728415	0.908429532	-0.1385535	0.721027	1
CTB-178M22.2	1.0934289	1.1113711	1.0856518	0.769066728	-0.3788193	0.72103	1
RP11-129M6.1	1.0711481	1.08891446	1.0634471	0.713574835	-0.4868634	0.721051	1
CIR1	1.9266562	1.90264056	1.9370659	1.038138501	0.0539989	0.721068	1
MYCL	1.1101486	1.09170352	1.1181438	1.288323062	0.3654944	0.721109	1
MMGT1	1.1690416	1.15023059	1.1771953	1.17948865	0.2381615	0.721118	1
C1S	1.1045645	1.08604598	1.1125914	1.308503019	0.3879173	0.721148	1
SCAF4	1.4009084	1.42070975	1.3923254	0.932532291	-0.1007744	0.721305	1
SCAMP5	1.1611594	1.17955311	1.1531866	0.853154645	-0.2291208	0.721315	1
SMIM19	2.1136323	2.13922104	2.1025407	0.96780228	-0.0472158	0.72134	1
RP11-418J17.1	1.0735951	1.05537374	1.0814932	1.471693748	0.5574775	0.721536	1
RNF187	1.3738869	1.35358808	1.3826855	1.082291976	0.1140898	0.721582	1
PRRG1	1.3780862	1.35748695	1.387015	1.082598941	0.1144989	0.721622	1
MATR3	1.0456908	1.06332536	1.0380471	0.600818987	-0.7349977	0.72167	1
SDHAF3	2.1033619	2.07832579	2.1142139	1.033281314	0.0472331	0.721711	1
TSPOAP1-AS1	1.082174	1.0640527	1.0900288	1.405542694	0.4911273	0.721743	1
MGA	1.4265351	1.44671952	1.4177861	0.935231263	-0.0966049	0.721843	1
RP11-932O9.8	1.0294513	1.04689725	1.0218893	0.466749575	-1.0992794	0.721929	1
LINC01579	1.0294081	1.04675094	1.0218908	0.468243463	-1.0946692	0.721929	1
FAM184A	1.1324148	1.1504673	1.1245898	0.828019012	-0.2722642	0.722127	1
RP11-171I2.4	1.0297597	1.04733854	1.02214	0.467694687	-1.0963611	0.722153	1

FUT4	1.0559592	1.03810587	1.0636979	1.671602206	0.7412316	0.722187	1
GMIP	1.065236	1.04722491	1.073043	1.546704395	0.6291975	0.722237	1
RP11-504A18.1	1.0411792	1.05866013	1.033602	0.572825533	-0.8038323	0.722248	1
COQ3	1.1168564	1.09864289	1.1247511	1.264674173	0.3387657	0.722283	1
CPSF4	1.4151845	1.43531765	1.4064576	0.933703568	-0.0989635	0.722293	1
MUC16	1.0342477	1.01646702	1.0419548	2.547809238	1.3492573	0.722311	1
MARK1	1.1257807	1.10735628	1.1337669	1.246009143	0.3173147	0.722461	1
CTBP1-AS2	1.2104358	1.22895761	1.2024074	0.884038602	-0.1778187	0.722476	1
STRBP	1.5902139	1.56845914	1.5996436	1.054857884	0.0770486	0.72257	1
RP11-35G9.5	1.0284771	1.04591934	1.0209167	0.455510248	-1.1344446	0.722614	1
TCTEX1D1	1.0584195	1.07586229	1.0508588	0.670408965	-0.5768867	0.722847	1
ZBTB6	1.0910517	1.07293964	1.0989024	1.355948889	0.4393028	0.72287	1
SNAI3-AS1	1.0870548	1.10480239	1.079362	0.757253494	-0.4011518	0.722883	1
ZNF280C	1.1111479	1.12905384	1.1033865	0.801111041	-0.3199259	0.722944	1
GAN	1.385833	1.36575755	1.3945348	1.078678424	0.1092648	0.723008	1
ZSCAN29	1.1482362	1.16642972	1.14035	0.843299183	-0.2458835	0.723029	1
NUP85	1.2773746	1.29645559	1.2691039	0.907737518	-0.1396529	0.723058	1
TMEM150C	1.1853831	1.16643007	1.1935985	1.163242065	0.2181513	0.723072	1
ZNF790-AS1	1.0332298	1.05061938	1.0256921	0.507555526	-0.9783624	0.723096	1
TWNK	1.0672839	1.08500409	1.059603	0.701177538	-0.5121483	0.723112	1
SPRED3	1.0925989	1.07444493	1.1004679	1.349559692	0.4324888	0.723142	1
RASAL3	1.0396187	1.0217216	1.0473763	2.181067422	1.1250344	0.723194	1
IFNLR1	1.0419813	1.02424391	1.0496696	2.048747537	1.0347422	0.723248	1
ING1	1.2575379	1.23784581	1.2660735	1.118680841	0.1617985	0.723265	1
IL21R-AS1	1.021293	1.03857775	1.0138008	0.357740512	-1.4830146	0.723319	1
CNTNAP2	1.3972731	1.41713918	1.3886621	0.931732377	-0.1020125	0.723361	1
KIAA1549L	1.0309467	1.0481838	1.0234752	0.487200208	-1.0374133	0.723413	1
SARM1	1.160053	1.17838217	1.1521082	0.852709499	-0.2298738	0.723429	1
TTC17	1.8544018	1.87732034	1.8444677	0.962553433	-0.0550615	0.723431	1
FBXO15	1.0778944	1.09563415	1.070205	0.734099687	-0.4459521	0.723584	1
ZNF354B	1.1287501	1.11045646	1.1366796	1.23740682	0.3073199	0.723632	1
THRB	1.2402167	1.2588894	1.2321229	0.896610179	-0.1574472	0.723678	1
PRIM2	1.1982252	1.21658923	1.1902652	0.87846123	-0.1869495	0.72377	1
NAT8L	1.2037782	1.18489368	1.2119638	1.146408858	0.1971217	0.723838	1
PRICKLE3	1.1036672	1.0854423	1.1115669	1.305756827	0.3848862	0.723848	1
RP4-798A10.7	1.0714715	1.08909996	1.0638304	0.716390641	-0.4811816	0.72388	1
HARS2	1.1718357	1.15331688	1.1798628	1.173144444	0.2303807	0.723932	1
C1orf52	2.0037359	1.9794408	2.0142667	1.035556965	0.0504069	0.723936	1
RP5-940J5.9	1.2588003	1.27748566	1.250701	0.903473788	-0.1464453	0.723953	1
NTF3	1.0334422	1.01586192	1.0410625	2.588747368	1.3722542	0.724159	1
STK40	1.2321443	1.21298417	1.2404494	1.128954247	0.174987	0.724189	1
CYP20A1	1.2295042	1.21053022	1.2377285	1.129189476	0.1752876	0.724215	1
COG3	1.1688084	1.18714735	1.1608593	0.859532922	-0.2183752	0.724219	1
ADAMTS10	1.1863452	1.20482538	1.1783348	0.870667484	-0.1998062	0.724223	1
HES7	1.1066804	1.12483383	1.0988116	0.791545397	-0.337256	0.724341	1
ITGA7	1.2244662	1.24321822	1.2163381	0.889481373	-0.1689637	0.724358	1
PBLD	1.0498277	1.06726618	1.0422689	0.628382442	-0.6702852	0.724414	1
HES2	1.0410947	1.0234733	1.0487328	2.076093645	1.0538715	0.724439	1
LMBR1	1.2760242	1.29496943	1.2678122	0.907932199	-0.1393435	0.72467	1
ALOX12-AS1	1.1024365	1.08444833	1.1102336	1.305338076	0.3844235	0.724729	1
PLXDC1	1.0764358	1.05857602	1.0841773	1.43706012	0.5231204	0.724754	1

KLK1	1.0379127	1.02027264	1.0455589	2.247308398	1.1681981	0.724846	1
ZNF646	1.0884267	1.10595046	1.080831	0.762913129	-0.3904093	0.724952	1
ANAPC4	1.2041691	1.22215199	1.1963744	0.883963957	-0.1779405	0.725008	1
NUP62CL	1.0907594	1.10841043	1.0831085	0.766609898	-0.3834355	0.72502	1
AC105760.2	1.0618081	1.07937327	1.0541944	0.682779223	-0.5505089	0.72505	1
ZNF562	1.0685858	1.08613126	1.0609806	0.707996425	-0.498186	0.72509	1
ZNF416	1.0625142	1.04487144	1.0701615	1.563611334	0.6448819	0.725246	1
SUPT16H	2.196914	2.17180143	2.2077991	1.030719954	0.0436524	0.7253	1
IQCC	1.0973289	1.11492858	1.0897003	0.780486997	-0.3575535	0.725336	1
CDC37L1-AS1	1.1289479	1.14682777	1.1211978	0.825441773	-0.2767616	0.725452	1
RP11-490B18.5	1.039495	1.05675435	1.0320138	0.564076194	-0.826038	0.725471	1
SAXO2	1.1091357	1.12681833	1.1014711	0.800129548	-0.3216945	0.725486	1
CTD-2541J13.1	1.0866109	1.10421666	1.0789796	0.757840143	-0.4000345	0.7255	1
MTFR2	1.08628	1.10398568	1.0786053	0.755924495	-0.403686	0.7255	1
RGS19	1.2067	1.22525479	1.1986573	0.881922596	-0.1812761	0.725597	1
RRP12	1.1550979	1.17307711	1.1473047	0.851092947	-0.2326114	0.725647	1
EIF4EBP2	2.0386924	2.06177423	2.0286874	0.968838208	-0.0456723	0.725655	1
PPFIA3	1.4466019	1.46628272	1.4380711	0.939496838	-0.0900398	0.725737	1
PXK	1.1887966	1.17011406	1.1968946	1.157426981	0.2109212	0.725767	1
SFPQ	2.9325109	2.89878291	2.9471305	1.025462417	0.0362746	0.725782	1
BRD8	1.5310414	1.50974327	1.5402732	1.0598927	0.0839182	0.725815	1
LZTFL1	1.3358614	1.35519747	1.3274801	0.921966274	-0.1172141	0.725895	1
NCDN	1.0946732	1.0767311	1.1024503	1.335186271	0.417041	0.725938	1
BBS9	1.2554061	1.2361437	1.2637555	1.116927809	0.1595359	0.725985	1
MED13	1.4384665	1.4585702	1.4297524	0.937157246	-0.093637	0.726164	1
VIM-AS1	1.2260824	1.24470951	1.2180084	0.890886463	-0.1666865	0.726168	1
ELMO2	1.1533677	1.13477597	1.1614264	1.197738448	0.2603129	0.726176	1
VN1R1	1.0307622	1.04797127	1.0233028	0.485765448	-1.0416682	0.726221	1
CCDC170	1.0505853	1.06782033	1.0431147	0.63571976	-0.6535372	0.726283	1
RP11-444D3.1	1.0386106	1.05577889	1.0311689	0.558793954	-0.8396117	0.726333	1
BLNK	1.0419084	1.02421992	1.0495756	2.046892296	1.0334352	0.726341	1
RP11-481J2.4	1.0680135	1.08537027	1.0604901	0.708561606	-0.4970348	0.726379	1
GABRE	1.0482048	1.06549889	1.0407086	0.621516631	-0.6861351	0.726412	1
IFT74-AS1	1.0283031	1.04537194	1.0209046	0.460737702	-1.1179824	0.726423	1
MAST4-AS1	1.0457395	1.06297485	1.0382687	0.607682063	-0.7186114	0.726582	1
ANXA8L1	1.0457074	1.06295488	1.0382314	0.607281954	-0.7195616	0.726582	1
TBC1D22A	1.2509409	1.231848	1.2592169	1.118046669	0.1609804	0.726584	1
WDR55	1.2769822	1.25769047	1.2853443	1.107314049	0.1470644	0.726646	1
SH3BP4	1.1720134	1.15364007	1.1799774	1.171422544	0.2282616	0.72665	1
ZSCAN21	1.1215956	1.10355166	1.1294169	1.249780678	0.3216749	0.72669	1
NANOS1	1.0590993	1.04146352	1.0667437	1.609696453	0.6867887	0.726765	1
AGRN	1.4821886	1.50245133	1.4734056	0.942191851	-0.0859072	0.726792	1
GPKOW	1.2038331	1.18533314	1.211852	1.143087548	0.1929359	0.726827	1
NCOA7	1.3581458	1.33828121	1.3667562	1.084175628	0.1165985	0.726835	1
TADA1	1.1349446	1.15262517	1.1272809	0.833944577	-0.2619766	0.726884	1
IVD	1.4442681	1.46438252	1.4355494	0.937910854	-0.0924773	0.726969	1
FAM84A	1.4142026	1.39399108	1.4229634	1.073535495	0.1023699	0.727023	1
FNBP1	1.5848942	1.56441939	1.5937691	1.051999765	0.0731344	0.727179	1
MFSD5	1.1368978	1.11881955	1.1447339	1.218098614	0.2846309	0.727205	1
RP11-1191J2.5	1.0781757	1.09543372	1.0706951	0.740776613	-0.4328895	0.727502	1
KIF12	1.0481885	1.06530964	1.0407673	0.624215059	-0.6798849	0.727509	1

PCSK6	1.0662701	1.0834825	1.0588094	0.704451303	-0.5054281	0.727515	1
PEX2	2.1800318	2.15429075	2.1911894	1.031966502	0.0453961	0.727517	1
EEA1	1.6400561	1.66152329	1.630751	0.953482671	-0.0687214	0.72755	1
GIPR	1.0463784	1.06360639	1.0389108	0.611743263	-0.7090018	0.72761	1
TSC22D1-AS1	1.0518121	1.06901551	1.0443552	0.642684322	-0.6378178	0.72778	1
RUNX2	1.0554906	1.07290164	1.0479436	0.657648118	-0.6046122	0.727842	1
CLPX	1.4417616	1.42155703	1.4505194	1.068703358	0.0958615	0.727918	1
STRC	1.0390811	1.05608944	1.0317088	0.565325878	-0.8228454	0.728039	1
VEZT	1.9346209	1.95732122	1.9247814	0.966009494	-0.0498907	0.72805	1
GRK5	1.1240917	1.14173027	1.1164462	0.821604262	-0.2834844	0.72806	1
BEGAIN	1.0664184	1.08367235	1.0589396	0.704409734	-0.5055132	0.728076	1
LRRC8B	1.1662491	1.18436553	1.1583965	0.85914368	-0.2190287	0.728084	1
MGAT5	1.1582266	1.17635464	1.1503688	0.852650369	-0.2299738	0.728165	1
PHLDB1	1.2412138	1.22241111	1.2493639	1.121184708	0.165024	0.728199	1
SLCO6A1	1.0357579	1.0529161	1.0283206	0.535197806	-0.9018559	0.728261	1
HLA-DRB1	1.1042326	1.12208252	1.0964955	0.79041193	-0.3393234	0.728342	1
CLHC1	1.2354738	1.25398197	1.2274513	0.895541091	-0.1591685	0.728401	1
IL17RB	1.0273524	1.04432758	1.0199945	0.451061581	-1.1486037	0.728413	1
RP11-165E7.1	1.0274008	1.04439396	1.020035	0.451299905	-1.1478416	0.728413	1
RP11-932O9.10	1.0274199	1.04438177	1.0200677	0.452161215	-1.1450908	0.728466	1
THAP7-AS1	1.1390373	1.15703865	1.1312345	0.835682893	-0.2589725	0.728491	1
PPP1R18	1.4993933	1.51963394	1.4906199	0.944164485	-0.0828899	0.728589	1
RP11-156E8.1	1.0525877	1.03511167	1.0601628	1.713469992	0.7769209	0.728793	1
C11orf49	1.6419589	1.62089942	1.6510872	1.048619446	0.0684912	0.728915	1
ZDHHC9	1.3174382	1.297962	1.3258803	1.093697415	0.1292137	0.72892	1
RP4-773N10.4	1.0483512	1.06545306	1.0409384	0.62546156	-0.6770069	0.72921	1
ZNF282	1.1049628	1.08723731	1.1126459	1.29125884	0.3687782	0.729352	1
XPO1	2.1693802	2.19429193	2.1585821	0.97009956	-0.0437953	0.729403	1
VCX3B	1.0334507	1.05044897	1.0260828	0.517012867	-0.9517279	0.729612	1
NOV	1.0479093	1.03061659	1.055405	1.809638876	0.8557018	0.729684	1
RP5-1125N11.2	1.0351191	1.05206321	1.0277745	0.533476771	-0.9065026	0.729703	1
GRIN3B	1.0351104	1.05215491	1.0277224	0.531538787	-0.9117531	0.729703	1
ARAP2	1.0803573	1.09776705	1.072811	0.744739594	-0.425192	0.729752	1
LIX1L	1.6403568	1.66116589	1.631337	0.95488443	-0.066602	0.729777	1
ACTN3	1.0405641	1.05756808	1.0331936	0.576598061	-0.7943621	0.729828	1
RP11-375N15.2	1.0279407	1.04495793	1.0205644	0.457415307	-1.1284234	0.730066	1
RP11-387M24.5	1.027937	1.04485644	1.0206032	0.459314718	-1.1224451	0.730066	1
ZFHX4-AS1	1.0541712	1.03677581	1.0617113	1.678041068	0.746778	0.730106	1
RP5-1112D6.4	1.1129532	1.13046335	1.1053634	0.80760898	-0.3082711	0.730201	1
EIF2B5	1.3463066	1.32700814	1.3546717	1.084595891	0.1171576	0.730206	1
NOP9	1.1127347	1.09497409	1.1204332	1.268063571	0.3426271	0.730215	1
NEURL2	1.1050791	1.12264646	1.0974645	0.794678139	-0.3315574	0.730223	1
CRYBA1	1.0440782	1.02678883	1.0515723	1.925143134	0.9449657	0.730224	1
SOX7	1.0441561	1.0267181	1.0517147	1.935568381	0.9527573	0.730224	1
BRF1	1.1763493	1.19417672	1.1686219	0.86839407	-0.2035782	0.73028	1
H2AFV	3.7189629	3.75841414	3.7018626	0.979498537	-0.0298848	0.730308	1
FNDC5	1.0878717	1.10504993	1.0804257	0.76559515	-0.3853464	0.730331	1
NOMO1	1.1238846	1.10602848	1.1316245	1.241406846	0.311976	0.730357	1
PNMA6A	1.0800556	1.06267992	1.0875872	1.397372808	0.482717	0.730398	1
UBAP2L	1.4741391	1.45385	1.4829335	1.064081842	0.0896091	0.730446	1
CDKN1B	1.4124971	1.43218314	1.4039641	0.934705922	-0.0974156	0.730491	1

CYP1B1-AS1	1.0928997	1.11025223	1.0853781	0.774389138	-0.3688694	0.730492	1
STAM2	1.1271072	1.10897783	1.1349654	1.238466887	0.3085553	0.730508	1
PNKP	1.732191	1.7539416	1.7227631	0.958646034	-0.0609299	0.730651	1
TMEM135	1.1760245	1.15749528	1.184056	1.168644663	0.2248363	0.730674	1
E2F5	1.2865701	1.26740548	1.2948772	1.102734213	0.1410851	0.73068	1
LOXL3	1.2281606	1.24661837	1.2201599	0.892714978	-0.1637285	0.730687	1
RP11-22A3.2	1.0431769	1.06016826	1.0358119	0.595196596	-0.7485618	0.730689	1
FAM72B	1.0430672	1.05999605	1.0357293	0.595527345	-0.7477603	0.730689	1
THAP8	1.4160157	1.3963102	1.4245572	1.071274874	0.0993287	0.730781	1
DYX1C1	1.2190237	1.20026111	1.2271564	1.134301302	0.1818039	0.730832	1
ZNF674-AS1	1.1324229	1.14998467	1.1248107	0.832156213	-0.2650737	0.730844	1
C20orf96	1.7271506	1.74880996	1.7177622	0.958537184	-0.0610937	0.730964	1
ITGA2B	1.0530857	1.07025017	1.0456457	0.649759629	-0.622022	0.731025	1
PIM2	1.3105916	1.32982257	1.3022559	0.916419693	-0.1259196	0.731034	1
ZNF737	1.0663077	1.08355175	1.0588332	0.704153019	-0.5060391	0.731065	1
NXT2	1.3529986	1.3332157	1.3615736	1.08510363	0.1178328	0.731173	1
CARM1	1.3965473	1.37642172	1.4052708	1.076640361	0.1065364	0.731233	1
ABCB8	1.1512088	1.16902422	1.1434865	0.84891115	-0.2363145	0.731258	1
CTD-2555O16.4	1.0332411	1.0501162	1.0259265	0.517328582	-0.9508472	0.731264	1
STOML3	1.0279629	1.04477574	1.0206753	0.461752971	-1.1148069	0.731337	1
CDK2AP1	1.2023166	1.22058227	1.1943992	0.881300178	-0.1822946	0.731424	1
RP11-111M22.4	1.0347951	1.05174571	1.0274477	0.530434655	-0.9147531	0.731431	1
RNF38	1.4064262	1.42608951	1.3979031	0.933848576	-0.0987395	0.731558	1
SLC22A15	1.0359132	1.05270354	1.0286353	0.54332817	-0.8801042	0.731564	1
BCL7A	1.7033442	1.68130407	1.7128976	1.046372172	0.0653961	0.73171	1
KANSL2	1.2105138	1.19199861	1.2185394	1.138234191	0.1867974	0.731748	1
YY1AP1	1.2362283	1.21772079	1.2442505	1.121851887	0.1658822	0.731822	1
SEH1L	1.2447629	1.2262165	1.2528019	1.117522133	0.1603034	0.73184	1
CDC42EP4	1.3182376	1.3372139	1.3100122	0.919333918	-0.1213391	0.731906	1
PARG	1.2931766	1.31184563	1.2850843	0.914184201	-0.1294432	0.731912	1
RGS8	1.0528741	1.06976602	1.0455522	0.652928304	-0.6150035	0.73194	1
NAPB	1.1631058	1.1809237	1.1553825	0.858829088	-0.219557	0.732012	1
ZNF559	1.1628618	1.18058927	1.1551777	0.859285513	-0.2187905	0.732012	1
PARP10	1.0652813	1.04790953	1.0728112	1.519763591	0.6038469	0.732058	1
BMPR1B-AS1	1.0366437	1.05351828	1.0293294	0.548025428	-0.8676853	0.732128	1
LINGO2	1.0396907	1.02255938	1.0471164	2.088548507	1.0625007	0.732248	1
ZNF607	1.0937717	1.11098562	1.0863102	0.777670158	-0.3627697	0.732282	1
AKAP13	1.7895094	1.8109741	1.7802053	0.9620595	-0.055802	0.732324	1
ANO8	1.0716523	1.08874686	1.0642426	0.723885556	-0.4661665	0.732393	1
AC019172.2	1.0331843	1.05002506	1.0258846	0.517433015	-0.950556	0.732396	1
NELFCD	2.2183186	2.19396217	2.228876	1.029242014	0.0415823	0.732402	1
SAMD4A	1.1255254	1.10783799	1.1331921	1.235113388	0.3046435	0.732421	1
SLC8A1	1.2356461	1.21694278	1.2437531	1.123582395	0.1681059	0.732513	1
ATP10D	1.1930452	1.21114583	1.1851993	0.877115741	-0.1891609	0.732609	1
IGSF3	1.2028582	1.18464036	1.2107548	1.141433935	0.1908474	0.732871	1
USP36	1.2028157	1.1845559	1.2107306	1.141825092	0.1913417	0.732871	1
COA4	2.584357	2.61243299	2.5721873	0.975040385	-0.0364661	0.732879	1
MAP3K1	1.1409971	1.12327347	1.1486795	1.206094973	0.2703435	0.733018	1
ASB16-AS1	1.2578483	1.27598852	1.2499853	0.905781476	-0.1427651	0.73306	1
RUNX1	1.1333416	1.15098363	1.1256945	0.832504371	-0.2644702	0.733137	1
RARRES3	1.0471629	1.03008137	1.054567	1.813979984	0.8591585	0.733233	1

TECPR1	1.1523641	1.17000752	1.1447165	0.851236015	-0.2323689	0.733254	1
RAPGEF1	1.1542032	1.17182489	1.146565	0.852990314	-0.2293987	0.733414	1
CYP7B1	1.0507369	1.06772474	1.0433735	0.640437302	-0.6428708	0.733471	1
NANP	1.1016297	1.11911735	1.0940496	0.789554567	-0.3408891	0.733529	1
LRRC8D	1.1174452	1.13485474	1.1098989	0.814942903	-0.2952291	0.73356	1
ZNF502	1.064325	1.08120032	1.0570103	0.702094803	-0.5102622	0.733564	1
PRX	1.0445217	1.06129223	1.0372524	0.607783875	-0.7183697	0.733604	1
RP5-943J3.2	1.0344046	1.05113678	1.0271519	0.530966117	-0.9133083	0.733663	1
TMEM257	1.0523822	1.0692972	1.0450503	0.650102971	-0.6212598	0.733685	1
INCENP	1.0723476	1.05508836	1.0798288	1.449104238	0.5351614	0.733762	1
MIS18A	1.5145144	1.4944239	1.5232227	1.058247193	0.0816767	0.733921	1
ZNF746	1.1224356	1.13977999	1.1149176	0.822132088	-0.2825579	0.734053	1
FAM86B1	1.1737961	1.19156595	1.1660936	0.86703091	-0.2058447	0.734165	1
FEZF1	1.1102035	1.12752268	1.1026964	0.80531861	-0.3123684	0.73421	1
MAPRE3	1.2579104	1.27655114	1.2498304	0.903378747	-0.1465971	0.734253	1
TRPV2	1.0343939	1.01740877	1.0417561	2.39856953	1.2621743	0.734289	1
ITGA8	1.0345521	1.01740877	1.041983	2.411599988	1.2699906	0.734289	1
ZNF781	1.1981308	1.21612289	1.190332	0.880665751	-0.1833335	0.734328	1
RASSF9	1.0852155	1.10218694	1.0778591	0.761928432	-0.3922726	0.734342	1
OSBPL3	1.2314277	1.24974301	1.2234888	0.894875198	-0.1602416	0.734373	1
MBNL3	1.0327831	1.04944214	1.0255621	0.517010697	-0.951734	0.734378	1
ZNF121	1.2423145	1.26081063	1.2342973	0.898342528	-0.1546625	0.734413	1
BTRC	1.2015325	1.21944641	1.1937676	0.882983772	-0.1795412	0.734426	1
RP11-707G18.1	1.0725627	1.08962712	1.0651661	0.727079926	-0.4598141	0.734593	1
RP11-435I10.4	1.0231639	1.03969701	1.0159976	0.402991369	-1.3111792	0.734668	1
DLX2	1.0380729	1.02102198	1.0454638	2.162676941	1.1128182	0.734835	1
VLDLR	1.2804942	1.26189559	1.2885558	1.101797136	0.1398586	0.734897	1
WBP1L	1.3033274	1.28446593	1.311503	1.095044922	0.1309901	0.735019	1
POLL	1.3679378	1.38647292	1.3599037	0.931252058	-0.1027564	0.735031	1
TXNRD3	1.0636243	1.08052067	1.0563005	0.699205633	-0.5162113	0.735125	1
ZNF45	1.0911634	1.07381747	1.0986821	1.336839683	0.4188265	0.735132	1
HOPX	1.0326597	1.04934991	1.0254252	0.515203438	-0.9567859	0.735167	1
KMT5B	1.667225	1.68847095	1.6580158	0.955764007	-0.0652737	0.735169	1
EARS2	1.1485642	1.16627349	1.1408881	0.847327242	-0.2390088	0.735202	1
RP11-332H18.5	1.0240749	1.04065359	1.0168887	0.415429943	-1.2673229	0.7353	1
AP5S1	1.1673974	1.18505898	1.1597419	0.86319454	-0.2122424	0.735315	1
NSUN4	1.1258255	1.1081453	1.1334891	1.234349162	0.3037505	0.735359	1
RINT1	1.1062822	1.08877761	1.1138696	1.282639087	0.3591153	0.735369	1
ROBO3	1.1408802	1.15818994	1.1333773	0.843146247	-0.2461452	0.735383	1
LYRM7	1.4063025	1.42531079	1.3980632	0.935934895	-0.0955199	0.735548	1
TAB1	1.2363151	1.21810971	1.2442063	1.119649105	0.1630467	0.735642	1
NEGR1	1.0609911	1.07794678	1.0536416	0.688182329	-0.5391372	0.735647	1
SRSF3	5.4685865	5.40767988	5.4949869	1.01980792	0.0282974	0.735825	1
ZNF862	1.0664416	1.08314373	1.059202	0.712043692	-0.4899623	0.735863	1
CDC23	1.2783491	1.29670172	1.270394	0.911332775	-0.1339501	0.735957	1
EXTL3	1.2542354	1.27243303	1.2463475	0.904249793	-0.1452067	0.736016	1
ASXL2	1.2398884	1.25787321	1.2320927	0.900026477	-0.1519607	0.736157	1
PACSIN2	1.3345858	1.35401055	1.3261661	0.921345594	-0.1181857	0.736158	1
FARP2	1.1404478	1.15765645	1.1329886	0.843534328	-0.2454813	0.736238	1
AMOTL1	1.3694379	1.35050737	1.3776434	1.077419316	0.1075798	0.736249	1
IL17RA	1.0729831	1.05569703	1.0804759	1.444886324	0.530956	0.736425	1

ANO5	1.1333625	1.11567414	1.1410296	1.219197007	0.2859313	0.736463	1
GPR161	1.304335	1.28566662	1.3124269	1.093676552	0.1291861	0.736681	1
COL19A1	1.0317822	1.04826284	1.0246386	0.510509294	-0.9699909	0.736693	1
NT5C3A	1.7309452	1.70994967	1.7400459	1.042392015	0.0598979	0.736731	1
ZNF227	1.1805713	1.19826497	1.1729019	0.872074815	-0.1974762	0.736786	1
NARFL	1.2610262	1.27924536	1.253129	0.906475323	-0.1416603	0.736825	1
UBE2Q1	1.2462892	1.22807951	1.2541823	1.11444609	0.1563268	0.736866	1
RP1-239B22.5	1.058619	1.07531828	1.0513807	0.682180542	-0.5517745	0.736874	1
RP11-66N24.3	1.0939653	1.11087621	1.0866351	0.781367903	-0.3559261	0.736886	1
TMEM130	1.0675713	1.05051529	1.0749643	1.483992038	0.5694834	0.73701	1
NMNAT2	1.0509474	1.0339855	1.0582996	1.715426167	0.778567	0.737127	1
BRAF	1.2308476	1.24869987	1.2231095	0.897103398	-0.1566538	0.737205	1
CTA-292E10.6	1.1516279	1.16896942	1.1441112	0.852883277	-0.2295798	0.737333	1
ATR	1.147294	1.16454749	1.1398153	0.849695956	-0.2349814	0.737474	1
TPR	3.0998197	3.0667832	3.1141396	1.022913072	0.0326835	0.737542	1
RP11-160E2.6	1.114901	1.13201402	1.1074833	0.81418121	-0.2965782	0.737543	1
CYB561D1	1.0695851	1.08638929	1.0623012	0.721168157	-0.4715924	0.737592	1
KCNIP3	1.0481479	1.06488585	1.0408928	0.630226679	-0.6660573	0.737603	1
PMM2	1.0755621	1.05854064	1.0829401	1.416795999	0.502632	0.737631	1
VPS9D1	1.102827	1.08553832	1.1103209	1.289725346	0.3670639	0.737634	1
PPIL3	1.707065	1.72768203	1.6981284	0.959386616	-0.0598158	0.73767	1
HOGA1	1.0515715	1.06820014	1.0443638	0.650493934	-0.6203925	0.737685	1
ZNF611	1.1201961	1.10279648	1.1277381	1.242631048	0.313398	0.737767	1
BCKDHB	1.2753245	1.29349366	1.267449	0.911259769	-0.1340657	0.737838	1
EFNA3	1.5676108	1.54756017	1.5763018	1.052490436	0.0738071	0.737926	1
PCDHB12	1.0353303	1.05181582	1.0281846	0.543938347	-0.878485	0.738056	1
DHX15	1.7610176	1.78215013	1.7518576	0.961270158	-0.0569861	0.73813	1
CTB-32O4.2	1.0325337	1.0489606	1.0254133	0.519056648	-0.9460361	0.738201	1
OTUB2	1.0843717	1.06720115	1.0918143	1.366261143	0.4502333	0.738364	1
RP11-260M2.1	1.0933355	1.0757705	1.1009492	1.33230211	0.4139213	0.738445	1
DISC1	1.0602265	1.07681454	1.0530363	0.690446267	-0.534399	0.738558	1
NDUFAF5	1.2138837	1.1957527	1.2217426	1.132769298	0.1798541	0.738622	1
ELL	1.0670197	1.05002095	1.0743879	1.487135806	0.5725364	0.738643	1
ZNF256	1.0506627	1.06716785	1.0435084	0.647756248	-0.6264771	0.738656	1
FBLN2	1.0842095	1.06721742	1.0915748	1.362367057	0.4461155	0.738745	1
RP11-44F21.5	1.1273459	1.14436955	1.1199669	0.830971134	-0.2671297	0.738812	1
RNF130	2.1146349	2.09109073	2.1248403	1.03093192	0.0439491	0.738907	1
PINK1-AS	1.0429068	1.02608139	1.0501998	1.924736248	0.9446608	0.739005	1
LINC00311	1.0474312	1.06398595	1.0402555	0.629129701	-0.6685706	0.739098	1
KCNS3	1.0482929	1.03145441	1.0555916	1.767370392	0.8216044	0.7391	1
RP11-110I1.12	1.0413453	1.05776153	1.0342296	0.592601875	-0.7548649	0.739336	1
AP001469.9	1.0719135	1.05470158	1.0793741	1.451037991	0.5370853	0.739346	1
RP3-508I15.21	1.0410156	1.02406289	1.0483638	2.009892388	1.0071183	0.739414	1
RAB3B	1.2760197	1.25747348	1.2840587	1.103254295	0.1417654	0.739471	1
DLGAP1-AS2	1.1291689	1.11182737	1.1366857	1.222292003	0.289589	0.739495	1
SPPL3	1.4629033	1.44371269	1.4712216	1.061997127	0.0867799	0.739529	1
LL22NC03-86G7.1	1.0874761	1.10428923	1.0801883	0.768903141	-0.3791262	0.73953	1
ESR2	1.0279719	1.0443179	1.0208866	0.471290788	-1.0853106	0.739586	1
SLITRK4	1.1895741	1.17186752	1.1972491	1.147680972	0.1987217	0.739599	1
RP11-706O15.1	1.5785954	1.55801131	1.5875177	1.05287773	0.0743379	0.739649	1
KIAA2022	1.1524872	1.13494748	1.16009	1.186313099	0.2464848	0.739663	1

LLGL2	1.0963378	1.0792518	1.1037438	1.309040338	0.3885096	0.739691	1
AMN1	1.3178357	1.33630065	1.3098319	0.921294465	-0.1182657	0.73975	1
TMOD2	1.1138416	1.09646681	1.1213728	1.258181822	0.3313404	0.739849	1
FBN3	1.0675322	1.08398256	1.0604017	0.719217142	-0.4755007	0.739867	1
CRTC1	1.1887825	1.17109841	1.1964478	1.148156542	0.1993194	0.740044	1
KLHDC8B	2.5501708	2.57910723	2.5376281	0.973732542	-0.0384025	0.740068	1
KIAA0368	1.3624761	1.34332764	1.3707761	1.079948437	0.1109624	0.740074	1
ZNF480	1.3442157	1.3627393	1.3361865	0.926799214	-0.1096713	0.740123	1
XDH	1.0863874	1.10314028	1.0791257	0.76716625	-0.3823888	0.740239	1
PURA	1.3441181	1.36259789	1.3361079	0.926943804	-0.1094462	0.740303	1
AC078883.3	1.0989931	1.11571353	1.0917455	0.792867945	-0.3348475	0.740345	1
ANKRD12	2.6090383	2.6375261	2.5966902	0.975062416	-0.0364335	0.740346	1
ADRB1	1.1400784	1.15695906	1.1327613	0.845834083	-0.2415534	0.740431	1
LAMB3	1.0420362	1.02537771	1.0492569	1.940952283	0.9567647	0.740519	1
RALGAPA1	1.5417889	1.56155552	1.533221	0.949542783	-0.0746951	0.740651	1
UBP1	1.2620725	1.2799848	1.2543084	0.908293461	-0.1387696	0.740677	1
MEIS1-AS2	1.1097593	1.12657297	1.1024713	0.809582786	-0.3047495	0.740786	1
LINC00924	1.0292468	1.04558374	1.0221654	0.486257061	-1.0402089	0.740832	1
KCNA1	1.0292699	1.04553161	1.0222212	0.488038123	-1.0349342	0.740832	1
PARP14	1.2528653	1.27086146	1.2450647	0.904760486	-0.1443922	0.740856	1
TOB2	1.641716	1.66189239	1.6329704	0.956304153	-0.0644586	0.741203	1
ASTN2	1.0987491	1.11541618	1.0915246	0.792996304	-0.334614	0.741426	1
EIF4B	4.6959639	4.64669592	4.7173194	1.019366426	0.0276727	0.7415	1
PELP1	1.2423275	1.22454694	1.2500346	1.113506974	0.1551106	0.741534	1
RP11-259K5.2	1.0356264	1.01904442	1.0428139	2.248108789	1.1687119	0.741556	1
FAIM2	1.0787124	1.06185102	1.086021	1.390777455	0.4758916	0.741731	1
C7orf33	1.05359	1.03685576	1.0608436	1.65085632	0.7232146	0.741881	1
ZMIZ1	1.3895009	1.40814226	1.3814207	0.934528825	-0.0976889	0.741918	1
RP11-1379J22.5	1.0291686	1.04533175	1.0221626	0.488897977	-1.0323947	0.741926	1
PUS1	1.1394052	1.15649279	1.1319985	0.843479416	-0.2455752	0.741938	1
GNA13	1.2589873	1.2407956	1.2668725	1.108294874	0.1483418	0.742071	1
RP11-473A10.2	1.02571	1.04198619	1.018655	0.44431337	-1.1703505	0.74212	1
RP11-571M6.17	1.0412514	1.02457435	1.0484802	1.972796089	0.9802418	0.742213	1
SPECC1	1.3969853	1.37800973	1.4052103	1.071957462	0.1002477	0.742289	1
B3GALT1	1.1139341	1.1306314	1.1066966	0.816776104	-0.2919874	0.742368	1
TMEM80	1.4648626	1.44546485	1.4732707	1.062419903	0.0873541	0.742431	1
UBIAD1	1.1399629	1.12296876	1.147329	1.198101463	0.2607501	0.742441	1
MAGEE1	1.0751717	1.09172105	1.0679983	0.74135961	-0.4317546	0.742452	1
HSF1	1.8826062	1.85843255	1.8930844	1.040366386	0.0570917	0.742502	1
SLC25A26	1.7287976	1.70738511	1.738079	1.043390632	0.0612794	0.74269	1
ZNF629	1.3129494	1.33127511	1.305006	0.920702933	-0.1191924	0.742718	1
FAM66B	1.0283159	1.04443445	1.0213292	0.48001435	-1.0588506	0.742889	1
CTD-2517O10.6	1.1471182	1.16388741	1.1398495	0.853326493	-0.2288303	0.742949	1
GOLPH3	1.4953994	1.47612487	1.5037541	1.058029327	0.0813796	0.743044	1
RP11-91G21.1	1.1250063	1.14199658	1.1176418	0.828483515	-0.2714551	0.743056	1
SLC39A11	1.0783705	1.09493266	1.0711915	0.749915442	-0.4152002	0.743107	1
NANOG	1.0353562	1.05157524	1.028326	0.549217011	-0.8645518	0.743119	1
ZNF843	1.0600405	1.07629846	1.0529934	0.694554003	-0.5258412	0.743362	1
IGSF5	1.0453508	1.06158983	1.0383118	0.622048197	-0.6849017	0.743423	1
MTF1	1.1748679	1.191997	1.1674432	0.872113519	-0.1974122	0.743439	1
LRSAM1	1.1385095	1.12141351	1.1459198	1.201841576	0.2652467	0.743493	1

SPINK1	1.0335359	1.0171592	1.0406344	2.368083941	1.2437202	0.743613	1
YEATS4	2.0738146	2.05189377	2.0833163	1.029872316	0.0424655	0.743762	1
CHST7	1.0947185	1.07761965	1.1021302	1.315777163	0.3959152	0.743857	1
RP11-458D21.1	1.0270151	1.04316669	1.0200141	0.463646422	-1.1089031	0.743895	1
KIF22	1.4967825	1.47767801	1.5050634	1.057330281	0.0804261	0.743908	1
C2CD4C	1.279226	1.29681582	1.2716016	0.915050939	-0.128076	0.743932	1
ZNF543	1.1079698	1.12469278	1.1007211	0.807753869	-0.3080123	0.743933	1
ZNF829	1.0281802	1.04429961	1.0211932	0.478405518	-1.0636941	0.743936	1
MED22	1.2559523	1.23780467	1.2638184	1.109391267	0.1497683	0.744026	1
MUM1L1	1.1491774	1.13180044	1.1567095	1.18899105	0.2497379	0.744072	1
ATG10	1.5807374	1.56076345	1.5893952	1.051058536	0.071843	0.744157	1
MCCC2	1.3449663	1.32633958	1.3530401	1.081818241	0.1134581	0.744166	1
SCG2	1.1807625	1.16345638	1.1882639	1.151768682	0.203851	0.744276	1
KLHL26	1.0704131	1.05386279	1.0775869	1.440455329	0.5265249	0.744323	1
MYO15B	1.1375611	1.15418715	1.1303544	0.845429836	-0.2422431	0.744403	1
BTF3L4	3.3629714	3.32829896	3.3780004	1.021346667	0.0304726	0.744412	1
DOPEY2	1.102725	1.119311	1.0955356	0.800727846	-0.3206161	0.744618	1
RP11-1007O24.2	1.0489184	1.06516127	1.0418779	0.642680283	-0.6378269	0.744644	1
LINC00621	1.0362843	1.05237205	1.029311	0.559667846	-0.8373572	0.744647	1
RP11-458F8.4	1.0363208	1.05247476	1.0293187	0.558720425	-0.8398015	0.744647	1
ZC3H12B	1.1115844	1.09451185	1.1189847	1.258939111	0.3322085	0.744649	1
ZNF615	1.1116333	1.09468955	1.1189776	1.256501888	0.3294128	0.744649	1
AHRR	1.0251033	1.04109398	1.0181721	0.442207661	-1.1772041	0.744683	1
PNMAL1	1.3586102	1.34007943	1.3666425	1.078108396	0.1085022	0.744684	1
C17orf53	1.0639031	1.0800698	1.0568955	0.710573648	-0.4929439	0.744684	1
SATB2	1.0734427	1.08974209	1.0663777	0.739649416	-0.4350865	0.744743	1
PPME1	1.5196291	1.53896059	1.5112497	0.948584582	-0.0761517	0.744827	1
NFKBIL1	1.7108736	1.73080182	1.7022355	0.960911048	-0.0575252	0.744871	1
MDM1	1.2267656	1.20920607	1.2343768	1.120315546	0.1639051	0.744911	1
PIGC	1.6306099	1.61031427	1.6394071	1.047668675	0.0671825	0.745037	1
LY96	1.034876	1.01850742	1.041971	2.267794542	1.1812899	0.745215	1
SELENOO	1.0686564	1.08491892	1.0616074	0.725484695	-0.4629829	0.745285	1
SMPDL3B	1.0446842	1.02826036	1.0518032	1.833068421	0.8742606	0.745287	1
SLC9A9	1.0539688	1.0701496	1.0469551	0.66935696	-0.5791523	0.745313	1
SPIN4	1.1449627	1.16164135	1.1377332	0.852091448	-0.2309198	0.745328	1
SPIN1	1.9983679	1.97613061	2.0080067	1.032655611	0.0463592	0.745377	1
SIN3B	1.2105575	1.22795091	1.2030183	0.890622695	-0.1671137	0.745713	1
RP11-276E15.4	1.0289062	1.04483766	1.0220006	0.490673486	-1.0271648	0.745812	1
CDK12	1.6909609	1.66977263	1.7001451	1.045347475	0.0639826	0.745856	1
ZIK1	1.1247719	1.14123618	1.1176354	0.832898603	-0.2637872	0.746044	1
RP11-1398P2.1	1.0298595	1.01362911	1.0368946	2.707044158	1.4367184	0.746049	1
PHLDB2	1.1073449	1.09055647	1.1146219	1.265751134	0.3399938	0.746135	1
KIAA0355	1.5511874	1.57076934	1.5426994	0.950820948	-0.0727544	0.746195	1
MEX3D	1.1924334	1.20935714	1.1850977	0.884124207	-0.177679	0.746228	1
DMXL2	1.5079083	1.52718152	1.4995542	0.947594256	-0.0776586	0.746279	1
RP11-666A20.4	1.0539323	1.07012468	1.0469136	0.669002939	-0.5799155	0.746463	1
NOL4	1.1154761	1.098174	1.1229758	1.252630983	0.3249615	0.746569	1
MCPH1-AS1	1.0819188	1.09813165	1.0748912	0.763171084	-0.3899216	0.746589	1
PCDH1	1.045556	1.06153326	1.0386305	0.627798893	-0.6716256	0.746882	1
CH507-9B2.3	1.0527334	1.0686779	1.0458221	0.667203478	-0.5838013	0.746935	1
ZP3	1.1252848	1.10833288	1.1326327	1.224307112	0.2919655	0.746956	1

EPB41	1.4826225	1.50141278	1.4744777	0.946281644	-0.0796585	0.746964	1
UBALD2	1.229046	1.21138662	1.2367005	1.119751707	0.1631789	0.746996	1
MSH5	1.0620859	1.07832786	1.0550458	0.702761441	-0.5088931	0.747009	1
PLCB3	1.1596608	1.17628651	1.1524543	0.864809939	-0.209545	0.747182	1
HFM1	1.0626038	1.07886519	1.0555552	0.704432115	-0.5054674	0.747186	1
RP11-501C14.5	1.0772784	1.09361599	1.0701968	0.749838083	-0.415349	0.74719	1
MAPKAPK5	1.720269	1.74035582	1.7115623	0.961108581	-0.0572287	0.747194	1
MKNK1	1.2631394	1.2806303	1.2555578	0.910656485	-0.1350211	0.747209	1
SPACA9	1.2521501	1.26940275	1.2446719	0.908201171	-0.1389162	0.74725	1
CTD-2369P2.10	1.1183775	1.13498949	1.1111769	0.823596598	-0.2799902	0.747445	1
CHKB	1.2421146	1.22414054	1.2499055	1.114950216	0.1569793	0.747547	1
AKAP17A	1.5132702	1.49389758	1.5216674	1.056225791	0.0789183	0.747565	1
AEBP1	2.0769301	2.05450115	2.086652	1.030489132	0.0433293	0.747566	1
LIMK1	1.3651009	1.34679617	1.3730351	1.075661084	0.1052236	0.747606	1
RP11-98D18.9	1.0750307	1.09113002	1.0680523	0.746760457	-0.4212826	0.747653	1
UQCRHL	1.2680881	1.25002032	1.2759196	1.103588713	0.1422026	0.747661	1
RP11-499P20.2	1.082253	1.09839711	1.0752552	0.764811373	-0.3868241	0.747702	1
CHMP4A	2.0331804	2.05637759	2.0231254	0.968522424	-0.0461426	0.747759	1
RP11-158M9.1	1.0375521	1.05355682	1.0306147	0.571630502	-0.8068452	0.747771	1
INTS1	1.2082638	1.22563046	1.2007362	0.889667878	-0.1686612	0.747811	1
GRAMD1C	1.0533214	1.06933811	1.0463788	0.668879486	-0.5801818	0.747975	1
LINC01621	1.0352327	1.05103794	1.0283819	0.556093734	-0.8466	0.748076	1
AC093495.4	1.0316604	1.04748155	1.0248026	0.522363403	-0.9368743	0.748223	1
CD3EAP	1.1301333	1.11343228	1.1373724	1.211052064	0.2762609	0.748238	1
RAP2C	1.7235457	1.74394088	1.7147052	0.960701667	-0.0578396	0.748298	1
SEC24C	1.256967	1.27423029	1.2494841	0.909761163	-0.1364402	0.74832	1
ZBTB43	1.2873341	1.30502445	1.2796661	0.916864544	-0.1252195	0.748418	1
LINC00327	1.0410486	1.02475549	1.0481109	1.943445627	0.9586167	0.748591	1
SWT1	1.0998253	1.08320973	1.1070274	1.286236049	0.3631554	0.748632	1
CEND1	1.1086236	1.09188867	1.1158774	1.261062926	0.3346403	0.748636	1
CD302	1.0621761	1.04578527	1.0692807	1.513166516	0.5975708	0.74876	1
ZNF709	1.0462994	1.06219607	1.0394088	0.633622795	-0.6583039	0.748767	1
OSBPL6	1.1637602	1.18057362	1.1564723	0.866528982	-0.2066801	0.748881	1
ANKRD42	1.1249146	1.10810711	1.1321999	1.222860775	0.2902602	0.749066	1
PXMP4	1.1415535	1.1579847	1.1344313	0.850913511	-0.2329156	0.749117	1
RP11-329B9.3	1.0240349	1.03977608	1.0172118	0.432718275	-1.2085	0.749165	1
LINC00630	1.0508051	1.06666492	1.0439305	0.658975168	-0.601704	0.749295	1
CCNJL	1.0718992	1.0556069	1.0789612	1.419988507	0.5058793	0.749333	1
TMEM259	1.639855	1.65983758	1.6311935	0.956589164	-0.0640286	0.749339	1
ZNF823	1.0646358	1.08076952	1.0576425	0.713667009	-0.486677	0.74935	1
NMU	1.0630297	1.04672966	1.070095	1.500010972	0.5849731	0.749462	1
DTNB	1.2355575	1.25244993	1.2282354	0.904082002	-0.1454745	0.749497	1
UBAP1L	1.0835267	1.09955785	1.0765779	0.769180024	-0.3786068	0.749572	1
AC012358.8	1.0449017	1.06095393	1.0379437	0.622498792	-0.6838571	0.749584	1
CASKIN1	1.0552712	1.07119122	1.0483706	0.679445537	-0.5575702	0.749595	1
TMEM42	1.7552846	1.77576009	1.7464094	0.96216521	-0.0556435	0.749672	1
TFAP4	1.1097077	1.12592204	1.1026795	0.815421536	-0.294382	0.749809	1
PTCD2	1.1197338	1.13627552	1.1125637	0.826000542	-0.2757854	0.749932	1
ATMIN	1.2780399	1.2603903	1.2856902	1.09716133	0.1337757	0.75003	1
INPP5A	1.0817143	1.06537968	1.0887946	1.358137947	0.44163	0.750162	1
RP11-37C7.3	1.0745163	1.09044931	1.0676101	0.747491043	-0.4198718	0.75029	1

TRMT10A	1.2228864	1.24000441	1.2154665	0.897760468	-0.1555975	0.75032	1
ZDHHC16	1.4983111	1.47963617	1.5064059	1.05581249	0.0783536	0.750386	1
WDR44	1.166965	1.15007507	1.1742861	1.161326032	0.2157731	0.750395	1
FAXC	1.1705129	1.18737341	1.1632047	0.871012871	-0.1992341	0.75041	1
RP11-124N14.3	1.0445752	1.06047237	1.0376845	0.623169234	-0.6823041	0.750468	1
AC079922.3	1.0824981	1.09854173	1.0755439	0.766618741	-0.3834188	0.750563	1
DCUN1D3	1.0722891	1.08827914	1.0653582	0.740357834	-0.4337054	0.750624	1
GLT1D1	1.0563062	1.04004475	1.0633548	1.582100152	0.6618409	0.750676	1
FAM117A	1.0794715	1.09543522	1.0725519	0.760221303	-0.3955086	0.7507	1
RADIL	1.0422866	1.05803409	1.0354607	0.611033007	-0.7106778	0.750756	1
PCDH10	1.0843931	1.06781995	1.0915768	1.35029229	0.4332717	0.750811	1
TNFRSF14	1.075743	1.05940459	1.082825	1.394252698	0.4794921	0.750825	1
SIKE1	1.7104319	1.68994674	1.7193113	1.042560668	0.0601313	0.75099	1
ODF3B	1.1479799	1.16444743	1.140842	0.856456199	-0.2235486	0.751012	1
TPST1	1.2210103	1.20370485	1.2285114	1.121777011	0.1657859	0.751021	1
FAM102A	1.1239741	1.10727222	1.1312136	1.223183334	0.2906407	0.751031	1
DSC3	1.101043	1.08479172	1.1080872	1.274737978	0.3502007	0.751033	1
JAK2	1.0666744	1.05039942	1.0737289	1.462890848	0.5488221	0.751095	1
NDST1	1.2161289	1.23288055	1.2088679	0.896888401	-0.1569996	0.751099	1
PCIF1	1.3240557	1.30616166	1.331812	1.083780314	0.1160723	0.751187	1
RP5-1112D6.7	1.0841112	1.10019071	1.0771414	0.769945971	-0.3771709	0.751213	1
XXbac-BPG181B23.7	1.0231313	1.03867085	1.0163956	0.423978531	-1.2379369	0.751224	1
NDRG3	1.4282622	1.40975238	1.4362854	1.064753921	0.09052	0.751243	1
ETV3L	1.0328325	1.01683833	1.0397652	2.361587503	1.239757	0.75125	1
AC006942.4	1.1688966	1.15199081	1.1762245	1.15944194	0.2134306	0.751286	1
PDK3	1.2724983	1.25498381	1.28009	1.09846208	0.1354851	0.751321	1
ARL5B	1.4882459	1.46930516	1.4964558	1.057852838	0.0811389	0.751322	1
POPDC2	1.0500779	1.0340476	1.0570264	1.674901014	0.7440758	0.751342	1
PTPRR	1.0902064	1.10645225	1.0831645	0.781237476	-0.3561669	0.751444	1
CDK5R1	1.0847317	1.06828209	1.0918619	1.345329502	0.4279596	0.751473	1
PAICS	2.8472083	2.81714144	2.860241	1.02371833	0.0338188	0.751483	1
RP11-468E2.5	1.0302303	1.04594612	1.0234182	0.509688302	-0.9723129	0.751495	1
LRRIQ3	1.0615931	1.07741313	1.0547358	0.707061152	-0.5000931	0.751581	1
ZNF319	1.0548669	1.07066501	1.0480192	0.67953224	-0.5573861	0.751632	1
MUC1	1.0832948	1.06695644	1.0903768	1.349784875	0.4327295	0.751652	1
ARPP19	3.1736624	3.14134872	3.187669	1.021631362	0.0308747	0.751716	1
ALYREF	1.0957284	1.11172908	1.0887928	0.794715224	-0.3314901	0.751752	1
AMY2B	1.0400746	1.05569044	1.0333059	0.598053811	-0.7416528	0.751776	1
USP16	1.8553409	1.8760999	1.8463428	0.966034614	-0.0498532	0.751865	1
MCCC1	1.2390967	1.22166178	1.246654	1.112749389	0.1541287	0.751981	1
SLC24A1	1.0759899	1.09196692	1.0690646	0.750972301	-0.4131684	0.752022	1
ZNF322	1.8498463	1.86983973	1.8411801	0.967051785	-0.0483349	0.752049	1
C1orf106	1.0987064	1.11475213	1.0917513	0.799560451	-0.322721	0.752071	1
KCNRG	1.0241893	1.03968463	1.0174728	0.440290464	-1.1834725	0.75208	1
PITX2	1.0317391	1.01574718	1.0386709	2.455736812	1.296156	0.752114	1
CTA-228A9.3	1.02297	1.03844074	1.0162642	0.423096707	-1.2409406	0.752272	1
C22orf29	1.2304703	1.21305204	1.2380204	1.11719347	0.159879	0.752321	1
RTCA-AS1	1.0307652	1.04629182	1.0240351	0.519207895	-0.9456158	0.752434	1
SEMA3B-AS1	1.0362114	1.05174182	1.0294797	0.569746453	-0.8116081	0.752513	1
RP11-214K3.24	1.0509997	1.06673501	1.0441791	0.662007568	-0.5950804	0.752683	1
UGDH-AS1	1.0976133	1.11374061	1.0906228	0.796749909	-0.3278011	0.752782	1

RP11-317P15.4	1.0400237	1.05564764	1.0332514	0.597533967	-0.7429074	0.752922	1
PIP4K2B	1.2988942	1.31599476	1.2914818	0.922426104	-0.1164948	0.75293	1
PRDM15	1.0479956	1.03197858	1.0549382	1.717970049	0.7807049	0.752955	1
ARMC2	1.0515754	1.0672558	1.0447786	0.665795625	-0.5868487	0.75298	1
PLCXD2	1.056629	1.04053268	1.0636061	1.569254788	0.6500796	0.753001	1
NUDT18	1.2068708	1.22382876	1.1995203	0.891396989	-0.16586	0.753009	1
C17orf67	1.0833537	1.09920865	1.0764813	0.770913227	-0.3753596	0.753175	1
TTC30A	1.1396979	1.15606819	1.1326021	0.84964206	-0.2350729	0.7533	1
P4HTM	1.7791995	1.75942237	1.7877719	1.03733044	0.0528755	0.753338	1
TNPO3	1.4733354	1.49138286	1.4655126	0.947352067	-0.0780274	0.753349	1
RP11-363E6.4	1.0214401	1.03689453	1.0147413	0.399553208	-1.3235405	0.753358	1
DHX34	1.1314383	1.11485602	1.138626	1.20695467	0.2713715	0.753364	1
IRF1	1.0967483	1.08038353	1.1038417	1.291828214	0.3694142	0.753432	1
FGF2	1.1370173	1.15322604	1.1299915	0.848364619	-0.2372436	0.753465	1
LINC00882	1.0909673	1.10688624	1.0840672	0.786511105	-0.346461	0.753551	1
NEK7	1.2885657	1.27080109	1.2962659	1.094035068	0.129659	0.753687	1
KLC3	1.040061	1.02410956	1.0469753	1.948410248	0.9622975	0.753717	1
SLC5A12	1.2402364	1.2572134	1.2328776	0.905386696	-0.143394	0.753722	1
LOXL1-AS1	1.3144326	1.29670008	1.3221189	1.085671774	0.118588	0.753729	1
RP11-410E4.1	1.0421941	1.05789564	1.0353882	0.611241513	-0.7101856	0.75374	1
C10orf88	1.1649469	1.14807518	1.1722601	1.163328615	0.2182587	0.753751	1
BRPF1	1.1015674	1.11743889	1.0946879	0.80627339	-0.310659	0.753789	1
LRIG2	1.2269419	1.24340675	1.2198051	0.903035995	-0.1471446	0.753797	1
ZMYND10	1.088349	1.10428648	1.0814409	0.780934126	-0.3567272	0.753871	1
NPR3	1.0513651	1.06693245	1.0446174	0.666603517	-0.5850992	0.754042	1
KB-226F1.2	1.0288579	1.04426736	1.0221786	0.501014197	-0.9970766	0.754101	1
ERMAP	1.1117719	1.1278351	1.1048092	0.819878119	-0.2865186	0.754138	1
ZBTB22	1.1880289	1.20441305	1.1809271	0.885105637	-0.1760784	0.754247	1
BAALC	1.7100497	1.68949098	1.718961	1.042741772	0.0603819	0.754252	1
GOLGA8N	1.0315747	1.0470061	1.0248858	0.529416656	-0.9175245	0.754263	1
LINC01426	1.0315756	1.04695348	1.02491	0.530525542	-0.9145059	0.754263	1
CAMKV	1.0377551	1.05335175	1.0309946	0.580948596	-0.7835176	0.754306	1
ZNF26	1.1728755	1.18946096	1.1656864	0.874514863	-0.1934452	0.754474	1
NSF	1.1963132	1.17945045	1.2036224	1.134699789	0.1823106	0.754549	1
BAZ1B	2.0450915	2.02330426	2.0545352	1.030519738	0.0433721	0.754554	1
TRIM35	1.060197	1.07576299	1.0534499	0.705488118	-0.5033063	0.754774	1
ATP1B2	1.433112	1.41449019	1.4411837	1.064400812	0.0900415	0.754808	1
OLFM1	1.0671217	1.05112685	1.0740548	1.448451558	0.5345114	0.754837	1
SLCO3A1	1.0842867	1.06825424	1.091236	1.336707972	0.4186843	0.754844	1
RP11-147L13.15	1.1395037	1.15550942	1.1325659	0.852462434	-0.2302918	0.754847	1
OTUD5	1.5577795	1.57663867	1.5496049	0.953118417	-0.0692726	0.755144	1
USHBP1	1.0288069	1.04418719	1.0221403	0.501056149	-0.9969558	0.755242	1
GPRIN1	1.0300485	1.04551612	1.023344	0.512874007	-0.9633236	0.755324	1
CH17-373J23.1	1.0829867	1.06679351	1.0900057	1.347521433	0.4303082	0.75549	1
ZNF10	1.0895889	1.10537322	1.0827472	0.785277004	-0.3487264	0.755546	1
PITX3	1.0847095	1.06873264	1.0916348	1.333206871	0.4149007	0.755579	1
RP11-474G23.3	1.0663899	1.08200816	1.05962	0.727001246	-0.4599703	0.755636	1
WDYHV1	1.515612	1.53364282	1.5077964	0.951566096	-0.0716242	0.75568	1
FAM222B	1.3675441	1.34955151	1.3753431	1.073784676	0.1027047	0.75587	1
PARD3B	1.2896512	1.30684645	1.2821978	0.919671139	-0.12081	0.755903	1
RWDD2B	1.1282601	1.14428975	1.1213119	0.840752087	-0.2502476	0.755904	1

PIGV	1.1289872	1.1123819	1.1361849	1.211804584	0.2771571	0.755914	1
ATF5	1.1997365	1.18287382	1.2070458	1.132178211	0.1791011	0.75593	1
MTHFS	1.41747	1.43588583	1.4094875	0.939437553	-0.0901308	0.756055	1
ZMYM6	1.206191	1.22245996	1.1991392	0.895168496	-0.1597688	0.756135	1
FAM19A3	1.03236	1.04775854	1.0256854	0.537817039	-0.8948126	0.756151	1
RP11-317L10.1	1.0323447	1.0477684	1.0256593	0.537159594	-0.8965773	0.756151	1
CIART	1.204125	1.18764974	1.2112663	1.125854655	0.1710206	0.756211	1
CSF1	1.0887432	1.1044527	1.0819339	0.784411132	-0.3503181	0.756291	1
CXorf40B	1.2070243	1.19014381	1.2143412	1.127258606	0.1728185	0.756294	1
LINC00324	1.0805048	1.09614808	1.0737241	0.76677679	-0.3831214	0.756306	1
ANK2	1.2679291	1.25011695	1.2756499	1.102084167	0.1402344	0.756404	1
RC3H1	1.4227065	1.44077493	1.4148746	0.941239169	-0.0873667	0.756407	1
THRA1/BTR	1.0342212	1.0185237	1.0410254	2.214750659	1.1471443	0.756453	1
SUCLG2	1.6182581	1.59909654	1.6265638	1.045847776	0.0646729	0.756591	1
PROSC	1.5867227	1.5682313	1.594738	1.046647663	0.0657759	0.756628	1
CLUH	1.0687285	1.08433682	1.061963	0.734708925	-0.4447553	0.756665	1
ST3GAL6-AS1	1.0265133	1.04179364	1.0198899	0.475907337	-1.0712474	0.756667	1
DLG2	1.163416	1.17965587	1.1563768	0.870423883	-0.20021	0.756875	1
COX18	1.2707249	1.2874798	1.2634624	0.916455472	-0.1258633	0.757031	1
LRRN2	1.1549408	1.1386411	1.1620059	1.168527579	0.2246918	0.757065	1
DYNLRB2	1.0561065	1.07151984	1.0494255	0.691073258	-0.5330894	0.757129	1
NT5E	1.0421872	1.02654844	1.0489659	1.844399752	0.8831514	0.757137	1
ZKSCAN5	1.137591	1.12130277	1.1446513	1.192481384	0.2539667	0.757167	1
AFDN-AS1	1.26678	1.28338845	1.259581	0.915990126	-0.126596	0.757213	1
ZNF135	1.1687526	1.18481002	1.1617924	0.87545229	-0.1918995	0.757215	1
LMF1	1.1205416	1.1042435	1.127606	1.224115084	0.2917392	0.757257	1
DLGAP5	1.4914211	1.50990369	1.4834098	0.948041347	-0.0769781	0.757336	1
FUT2	1.029144	1.04451681	1.0224806	0.504991454	-0.9856691	0.757372	1
ATP2A1	1.0291059	1.04433719	1.0225038	0.507559659	-0.9783507	0.757372	1
ARID1A	2.179683	2.20245353	2.169813	0.972855094	-0.0397032	0.757424	1
LINC00638	1.0483462	1.06375105	1.0416689	0.653619169	-0.6134778	0.757585	1
ENG	1.0333085	1.01775155	1.0400518	2.256241484	1.1739215	0.757664	1
ZNF619	1.0610578	1.07652389	1.0543539	0.710286988	-0.493526	0.757686	1
FTSJ3	1.1787704	1.16239968	1.1858663	1.144499416	0.1947167	0.757711	1
KCTD7	1.1875651	1.17092059	1.1947798	1.139592338	0.1885178	0.757752	1
C8orf48	1.0962486	1.08026747	1.1031757	1.285398847	0.3622161	0.757936	1
ZADH2	1.4314756	1.44885861	1.4239408	0.944486242	-0.0823983	0.75795	1
SLC12A1	1.0328519	1.0171592	1.0396539	2.310942786	1.2084815	0.757984	1
PRPH2	1.0409944	1.05631082	1.0343555	0.610103951	-0.712873	0.75807	1
ANKEF1	1.0585158	1.07405657	1.0517796	0.699190006	-0.5162435	0.758073	1
RP11-11M20.4	1.028326	1.04357218	1.0217175	0.498425271	-1.0045509	0.758082	1
CTD-2021H9.3	1.0283259	1.04360779	1.0217019	0.497660462	-1.0067663	0.758082	1
ZDHHC11B	1.0375308	1.0218318	1.0443356	2.030779033	1.0220333	0.758147	1
CTD-2231E14.8	1.0257828	1.04101088	1.0191821	0.467731326	-1.096248	0.758158	1
SCNN1D	1.0568066	1.07211995	1.050169	0.695632685	-0.5236024	0.758176	1
PIAS3	1.2937198	1.27669499	1.3010994	1.088199495	0.1219431	0.758188	1
SERPINA1	1.0387106	1.02314871	1.0454561	1.963654076	0.9735408	0.758203	1
RP11-480I12.10	1.0197621	1.03491805	1.0131926	0.377816651	-1.4042418	0.758206	1
PCK2	1.1673934	1.15125248	1.1743898	1.152971435	0.2053568	0.758273	1
TRAPPC12	1.264185	1.28076551	1.2569981	0.915347736	-0.1276082	0.758284	1
IRS1	1.2406767	1.25754691	1.2333643	0.906103976	-0.1422515	0.758284	1

IFFO1	1.1242378	1.10800477	1.1312742	1.215447764	0.2814879	0.758322	1
TXNL4B	1.1651481	1.18143203	1.1580897	0.871344071	-0.1986856	0.758382	1
MIR181A1HG	1.1650303	1.18103211	1.1580942	0.873293933	-0.1954608	0.758452	1
CCR10	1.1625618	1.1786359	1.1555944	0.871014009	-0.1992322	0.758463	1
PWWP2B	1.1019628	1.08591794	1.1089175	1.267692201	0.3422045	0.758472	1
PID1	1.0352127	1.01962419	1.0419696	2.138664571	1.0967102	0.758475	1
FLAD1	1.4859801	1.46764101	1.4939292	1.056214561	0.0789029	0.758536	1
PYGB	1.3092279	1.2920304	1.3166823	1.084415392	0.1169175	0.758568	1
E2F6	1.4577883	1.43959969	1.4656722	1.059309613	0.0831243	0.758583	1
ARL15	1.1398607	1.12356713	1.1469233	1.189015952	0.2497681	0.758669	1
SLC16A12	1.0336422	1.01815945	1.0403533	2.222164028	1.1519653	0.758679	1
LINC01410	1.0696227	1.08501623	1.0629503	0.740450032	-0.4335257	0.758755	1
AFMID	1.3998301	1.41743142	1.3922008	0.939557336	-0.0899469	0.758778	1
CCDC136	1.280215	1.29653643	1.2731404	0.921102248	-0.1185668	0.758817	1
GRB14	1.06717	1.08251714	1.0605177	0.733395199	-0.4473373	0.758858	1
MRPS11	1.7888261	1.76879057	1.7975106	1.03735747	0.0529131	0.758886	1
EPHA5	1.0362643	1.02062141	1.0430448	2.087384434	1.0616963	0.758937	1
TTC25	1.0647281	1.08013892	1.0580482	0.724344994	-0.4652511	0.758972	1
ARFGAP2	1.5450138	1.52697327	1.5528336	1.049073322	0.0691155	0.759009	1
TPRG1	1.0296054	1.04475244	1.0230398	0.514828483	-0.9578362	0.759016	1
KIAA0930	1.1904707	1.17401721	1.1976025	1.135534288	0.1833713	0.759048	1
PPARA	1.1466441	1.13013735	1.1537991	1.181821249	0.2410118	0.759138	1
AL356053.1	1.0473454	1.06258798	1.0407385	0.650899659	-0.6194929	0.759198	1
TNKS1BP1	1.2182301	1.20166868	1.2254088	1.117718381	0.1605567	0.759257	1
RAI2	1.0438836	1.02824817	1.0506609	1.793422783	0.8427156	0.759578	1
IFITM3	9.2411494	9.34536564	9.1959764	0.982099134	-0.0260594	0.759661	1
LHCGR	1.0548437	1.07011177	1.0482257	0.68784046	-0.5398541	0.75968	1
GIN1	1.1447257	1.16058158	1.1378529	0.858460399	-0.2201765	0.759786	1
LINC01534	1.0482208	1.06347732	1.0416078	0.655474645	-0.6093881	0.759844	1
SF3A2	1.7429323	1.76165087	1.7348187	0.964771054	-0.0517415	0.760109	1
HLA-DPA1	1.0768203	1.06095591	1.0836968	1.373070842	0.4574061	0.760144	1
RP11-424N24.2	1.0389158	1.05404818	1.0323566	0.598661679	-0.7401872	0.760169	1
CFAP126	1.0388443	1.05394996	1.0322967	0.598642542	-0.7402333	0.760169	1
GAL3ST4	1.0939448	1.07818205	1.1007772	1.289007004	0.3662601	0.760263	1
APC2	1.0955952	1.11112662	1.0888631	0.799656146	-0.3225483	0.760364	1
RP11-576D8.4	1.0297017	1.04477927	1.0231663	0.517343509	-0.9508056	0.760367	1
SLC30A4	1.1122462	1.12793191	1.1054471	0.824244278	-0.2788561	0.760412	1
RUNX1T1	1.037702	1.0222656	1.044393	1.993791789	0.9955148	0.760415	1
OTUD7B	1.3236917	1.30627077	1.3312428	1.081535923	0.1130816	0.760421	1
B3GLCT	1.1269607	1.14271176	1.1201334	0.841790358	-0.2484671	0.760448	1
FEM1A	1.1333432	1.14912313	1.1265032	0.848314078	-0.2373296	0.760558	1
RP5-933K21.3	1.0274709	1.04246852	1.0209702	0.493781395	-1.0180556	0.760626	1
TRIM3	1.1159521	1.0998089	1.1229495	1.231849324	0.3008258	0.760781	1
EIF3B	1.5164547	1.53457488	1.5086004	0.951411014	-0.0718594	0.760808	1
UBL7	1.6922181	1.67356423	1.7003037	1.03969844	0.0561651	0.760837	1
L1TD1	1.0467573	1.03111005	1.0535397	1.720978105	0.7832287	0.760871	1
C9orf16	6.606089	6.54225248	6.6337593	1.016510756	0.0236255	0.760912	1
TAF5L	1.0662968	1.08153102	1.0596935	0.732156902	-0.4497752	0.760968	1
SRSF12	1.2608032	1.24409295	1.2680463	1.098132108	0.1350516	0.761088	1
ODF2	1.4718526	1.4897353	1.4641013	0.947657421	-0.0775625	0.761209	1
APTX	1.5594689	1.57762855	1.5515975	0.954934591	-0.0665262	0.76123	1

RP11-118K6.3	1.0385399	1.05367442	1.0319798	0.595810957	-0.7470734	0.761238	1
AKAP8	1.2010133	1.18470882	1.2080806	1.126533248	0.1718899	0.76131	1
GRK2	1.1344521	1.15012022	1.1276606	0.850389407	-0.2338045	0.761335	1
PRKAR2A-AS1	1.0349158	1.05000834	1.0283739	0.567383725	-0.8176033	0.761346	1
LCA5L	1.0766802	1.09190219	1.0700821	0.762572819	-0.391053	0.761397	1
RP11-153I24.5	1.0259056	1.04091467	1.0193999	0.474154762	-1.0765701	0.761409	1
SPAG1	1.1033705	1.11889829	1.0966399	0.81279469	-0.2990371	0.761571	1
CBX4	1.0800359	1.06442365	1.0868031	1.34738005	0.4301568	0.761742	1
CNTN3	1.1556978	1.17133088	1.1489216	0.86920471	-0.2022321	0.761789	1
MEIOC	1.0457228	1.06089179	1.0391477	0.642905896	-0.6373205	0.761795	1
GLCE	1.2328692	1.2492845	1.2257539	0.905607417	-0.1430423	0.761982	1
TMA16	1.9939131	2.01554359	1.9845372	0.9694682	-0.0447345	0.762364	1
TMEM178B	1.0632206	1.07848222	1.0566054	0.721251438	-0.4714258	0.762436	1
DDX25	1.0619526	1.04629314	1.0687403	1.48489202	0.570358	0.7625	1
RP11-352G9.1	1.0361374	1.05102176	1.0296857	0.581823699	-0.781346	0.762567	1
KRBOX4	1.2113683	1.19503733	1.218447	1.120026788	0.1635332	0.762657	1
CDKN2AIPNL	1.8858974	1.90753971	1.8765165	0.965816117	-0.0501796	0.762671	1
KCNQ1OT1	7.2559096	7.17580367	7.2906319	1.018593249	0.0265781	0.762791	1
RASGRP1	1.1325895	1.11664399	1.1395011	1.195956407	0.2581648	0.762803	1
NECAB1	1.0441682	1.02859123	1.0509201	1.780970947	0.832664	0.76287	1
SLC6A17	1.0298915	1.01466374	1.036492	2.488590343	1.3153288	0.762873	1
ELAVL2	1.2954502	1.27809418	1.3029733	1.089462766	0.1236169	0.762914	1
RP11-218F10.3	1.0469066	1.06192981	1.0403948	0.652266968	-0.6164655	0.762927	1
SZT2	1.1241065	1.13976796	1.117318	0.839376844	-0.2526094	0.762946	1
KCNE5	1.0368163	1.05181903	1.0303133	0.5849837	-0.7735317	0.762987	1
PIWIL1	1.0327263	1.01740877	1.0393658	2.261263757	1.1771293	0.763019	1
NNT	1.4667018	1.48453462	1.458972	0.947243045	-0.0781935	0.763048	1
CYBRD1	1.2274522	1.21078071	1.2346785	1.113377461	0.1549428	0.763187	1
KB-1125A3.12	1.0270764	1.04195461	1.0206274	0.491660084	-1.0242669	0.763218	1
PODXL2	1.4873935	1.46949217	1.495153	1.054656452	0.0767731	0.763331	1
CFAP46	1.134721	1.15020509	1.1280093	0.852230066	-0.2306851	0.763454	1
SCD	2.3150876	2.29116681	2.3254561	1.026556861	0.0378135	0.763469	1
CLEC11A	1.9941003	2.01676132	1.9842778	0.96805198	-0.0468436	0.76347	1
RP11-16P6.1	1.0233866	1.0382977	1.0169233	0.441887732	-1.1782482	0.763497	1
PNCK	1.1232876	1.13866922	1.1166204	0.840996763	-0.2498278	0.763591	1
ATP8A2	1.064528	1.04919933	1.0711724	1.446612187	0.5326782	0.763652	1
RP11-105N14.1	1.0646814	1.04919725	1.0713931	1.451160688	0.5372073	0.763652	1
CCDC69	1.0622671	1.04685276	1.0689486	1.471601538	0.5573871	0.763674	1
FAM53A	1.0236389	1.03854127	1.0171794	0.445739663	-1.1657268	0.763723	1
KIAA1644	1.0455582	1.06045805	1.0390997	0.646724796	-0.6287762	0.764024	1
CHUK	1.103323	1.11879465	1.0966168	0.813309051	-0.2981244	0.764059	1
ABCA2	1.1709062	1.18668782	1.1640656	0.878822992	-0.1863555	0.764176	1
CHD1L	1.3590286	1.34160279	1.3665819	1.073123124	0.1018156	0.764178	1
CCL28	1.0292146	1.01400962	1.0358053	2.555766339	1.3537559	0.764392	1
GTF3C1	1.2489578	1.26514072	1.2419433	0.912508988	-0.1320893	0.764644	1
OSBPL7	1.0630263	1.07802045	1.0565269	0.724514411	-0.4649137	0.764781	1
LMCD1-AS1	1.083144	1.09844306	1.0765125	0.777225627	-0.3635946	0.764843	1
LMTK3	1.1091487	1.09336036	1.1159923	1.242414311	0.3131464	0.764908	1
PEX13	1.5086487	1.52603493	1.5011125	0.952622188	-0.0700239	0.764916	1
HMBOX1	1.4108886	1.42797675	1.4034817	0.942765369	-0.0850293	0.764919	1
HEMK1	1.4078364	1.42487258	1.4004519	0.942522439	-0.0854011	0.764947	1

C15orf65	1.1704229	1.18622174	1.1635748	0.878387025	-0.1870714	0.764986	1
CYGB	1.1140574	1.09834793	1.1208668	1.228971226	0.2974511	0.76502	1
PPIP5K1	1.0574418	1.04215257	1.064069	1.519931121	0.6040059	0.765077	1
BCDIN3D-AS1	1.0557925	1.07063162	1.0493605	0.698843763	-0.5169581	0.765114	1
AC138969.4	1.0284094	1.04326776	1.021969	0.507744495	-0.9778254	0.765116	1
CDH4	1.0939872	1.10915408	1.087413	0.800822411	-0.3204457	0.76512	1
ZNF174	1.1919726	1.17573822	1.1990095	1.132419901	0.179409	0.765135	1
PPP1R13B	1.0831418	1.06764631	1.0898584	1.328356701	0.4096426	0.765136	1
RP11-250B2.5	1.0431189	1.05802636	1.0366571	0.631732718	-0.6626138	0.765218	1
FCHSD2	1.2327326	1.24869428	1.2258139	0.907997889	-0.1392392	0.765356	1
ACTRT3	1.0485193	1.03337103	1.0550854	1.650696214	0.7230746	0.765397	1
USP20	1.1183611	1.10253548	1.1252208	1.221243898	0.2883514	0.765402	1
KIAA1024	1.0359623	1.05071787	1.0295663	0.582957293	-0.7785379	0.765429	1
RGS5.1	1.0508702	1.06565436	1.044462	0.677213047	-0.5623183	0.765512	1
AQR	1.3493405	1.36653019	1.3418896	0.932773299	-0.1004016	0.76556	1
DDX31	1.0911139	1.10622821	1.0845626	0.796046457	-0.3290755	0.765583	1
RP11-315I20.1	1.0322447	1.04697905	1.025858	0.550414629	-0.8614093	0.765585	1
NOTCH2	1.4323176	1.44958197	1.4248342	0.944953844	-0.0816842	0.765753	1
WIPF1	1.3157288	1.3322318	1.3085755	0.928795747	-0.1065667	0.765792	1
SH3GLB2	1.2807469	1.29711599	1.2736516	0.921026321	-0.1186857	0.765819	1
ESYT2	1.2810579	1.29742033	1.2739656	0.921139353	-0.1185087	0.765819	1
DNAAF3	1.1214277	1.13679365	1.1147673	0.838980867	-0.2532902	0.765844	1
ZNF131	1.6038048	1.62184978	1.5959831	0.958403587	-0.0612948	0.765862	1
SND1-IT1	1.0938127	1.10909939	1.0871866	0.799147939	-0.3234655	0.76603	1
RP11-513G11.4	1.0805224	1.09566403	1.0739592	0.773113877	-0.3712472	0.766042	1
CCDC175	1.0310645	1.04574831	1.0246997	0.539903973	-0.8892253	0.766055	1
POPDC3	1.0307932	1.01564979	1.0373572	2.387070697	1.2552413	0.766077	1
RP11-326K13.4	1.0405202	1.05536427	1.034086	0.615667744	-0.6997761	0.7661	1
KMT5C	1.5574375	1.57507867	1.5497909	0.956027267	-0.0648763	0.766137	1
RAD52	1.0994423	1.11466941	1.092842	0.809649026	-0.3046314	0.766171	1
ARFGAP1	1.2658686	1.28219651	1.2587912	0.91706007	-0.1249119	0.766192	1
GPATCH3	1.1252086	1.1094603	1.1320348	1.20623458	0.2705105	0.766452	1
INSM1	1.0562967	1.04087612	1.0629808	1.540772575	0.6236539	0.766694	1
RP11-620J15.4	1.0334885	1.04817488	1.0271226	0.56300229	-0.8287873	0.766775	1
NIPBL	2.1969615	2.17583348	2.2061196	1.025757118	0.0366892	0.766947	1
RSPO2	1.0300612	1.04487281	1.0236411	0.526846547	-0.9245453	0.767018	1
SLC8A3	1.029971	1.0447063	1.0235839	0.527529027	-0.9226776	0.767018	1
CERS6	1.4028977	1.42005863	1.3954593	0.941438268	-0.0870616	0.767049	1
JMJD1C-AS1	1.0394912	1.02437693	1.0460425	1.888773274	0.9174495	0.767085	1
PNPT1	1.3579167	1.37487735	1.350565	0.935145754	-0.0967369	0.767109	1
RP11-490O6.2	1.0342349	1.04890442	1.0278763	0.570015406	-0.8109272	0.767195	1
RP11-422P24.12	1.0717713	1.08674795	1.0652796	0.752520929	-0.4101964	0.767232	1
TWIST1	1.0299668	1.01490147	1.0364969	2.449215225	1.2923196	0.767287	1
GCSH	4.4905996	4.44165983	4.5118128	1.020383462	0.0291114	0.767315	1
FICD	1.059668	1.07450095	1.0532386	0.714603312	-0.4847855	0.767333	1
LL22NC03-32F9.1	1.0445671	1.05933613	1.0381654	0.643206208	-0.6366468	0.767366	1
CFAP44	1.044582	1.0593419	1.0381843	0.643462416	-0.6360722	0.767366	1
ZNF285	1.0524173	1.06730099	1.0459659	0.682990464	-0.5500627	0.767379	1
RP11-16N11.2	1.0409094	1.05569658	1.0344998	0.619424459	-0.6909997	0.767428	1
RP11-307C12.11	1.0669469	1.05170484	1.0735537	1.422568427	0.508498	0.767486	1
EMILIN1	1.0785622	1.06324063	1.0852035	1.347290293	0.4300607	0.767577	1

PABPC4L	1.0699325	1.05461579	1.0765716	1.402005113	0.4874916	0.767635	1
CARD8	1.3730435	1.38962154	1.3658576	0.939007716	-0.0907911	0.767673	1
PRKCA	1.0638586	1.04860066	1.0704722	1.450025943	0.5360787	0.767732	1
SLC18A2	1.0341356	1.01899597	1.040698	2.142452476	1.0992632	0.767771	1
CNKSR2	1.1157931	1.10020598	1.1225494	1.222974415	0.2903942	0.767808	1
MAP3K8	1.1157221	1.10006793	1.1225075	1.224243464	0.2918905	0.767808	1
DNHD1	1.0628619	1.07766808	1.0564441	0.726734351	-0.4605	0.767811	1
ZNF608	1.8053462	1.82493242	1.7968564	0.965965649	-0.0499562	0.767823	1
RP5-1050D4.3	1.0643943	1.07939796	1.0578908	0.729122637	-0.4557666	0.767893	1
ZNF566	1.2815889	1.26483622	1.2888505	1.090675954	0.1252225	0.768085	1
HMOX1	1.0392079	1.02409636	1.0457581	1.898964136	0.9252127	0.768114	1
RIMKLA	1.2552423	1.23858461	1.2624627	1.100082127	0.1376112	0.76822	1
G3BP1	2.0384626	2.01778075	2.0474272	1.029128538	0.0414232	0.768398	1
SGSM2	1.1885702	1.20405981	1.1818562	0.891190638	-0.166194	0.768409	1
PTPRM	1.1803727	1.19582724	1.1736738	0.886872517	-0.1732014	0.76841	1
KIAA0196	1.1601548	1.14432721	1.1670153	1.157198982	0.210637	0.768516	1
GPBAR1	1.0292667	1.04391508	1.0229174	0.521856099	-0.9382761	0.768518	1
CAPN12	1.0413443	1.02626019	1.0478826	1.823389389	0.8666227	0.768526	1
EDEM3	1.2279914	1.24399189	1.2210559	0.905997009	-0.1424218	0.768586	1
BTD	1.118136	1.1025846	1.1248769	1.217306045	0.2836919	0.768678	1
RP11-936I5.1	1.0297824	1.04447066	1.0234156	0.526540995	-0.9253822	0.768693	1
ADAM15	1.3334133	1.3171907	1.3404451	1.073313725	0.1020718	0.768801	1
RP11-345J4.3	1.0245724	1.03922945	1.0182192	0.46442681	-1.1064768	0.768853	1
RORA	1.3609698	1.37703973	1.3540043	0.938904426	-0.0909498	0.768869	1
PSEN1	1.3500192	1.33338339	1.3572301	1.071529332	0.0996713	0.768882	1
DDRGK1	2.0463845	2.02626523	2.0551053	1.028101933	0.0399833	0.768962	1
C18orf8	1.0748339	1.05973062	1.0813805	1.362458579	0.4462124	0.768983	1
LINC00158	1.0222943	1.03681449	1.0160005	0.434623884	-1.2021606	0.76908	1
ZFAND2A	1.2370005	1.25297494	1.2300763	0.909482515	-0.1368822	0.769121	1
NUAK1	1.3012675	1.28479954	1.3084056	1.082886422	0.1148819	0.769135	1
ADCYAP1	1.0318275	1.01687956	1.0383067	2.269413773	1.1823197	0.769208	1
MARK4	1.3105044	1.29395889	1.3176761	1.080682116	0.1119422	0.769248	1
PLEKHG2	1.1615098	1.17660916	1.154965	0.877445807	-0.1886181	0.769334	1
KCNMA1	1.3005684	1.28363593	1.3079079	1.085574543	0.1184588	0.769337	1
PKNOX2	1.1489838	1.16425427	1.1423648	0.866734234	-0.2063384	0.769357	1
SLC4A1AP	1.1710643	1.15530702	1.1778944	1.145437023	0.1958981	0.769379	1
CTC-498J12.3	1.0230775	1.0376067	1.0167797	0.446189122	-1.1642728	0.769498	1
TRIM6	1.1115213	1.09619098	1.1181662	1.228454472	0.2968444	0.769527	1
RP11-533E19.7	1.0797427	1.09458025	1.0733113	0.775123141	-0.3675026	0.769565	1
TNFRSF4	1.0733953	1.05817636	1.079992	1.374990953	0.4594221	0.769581	1
MAPKBP1	1.1251312	1.10968887	1.1318248	1.201806367	0.2652045	0.769678	1
CAPN10	1.2091668	1.22502243	1.2022941	0.898995411	-0.1536143	0.769967	1
RP11-333E1.1	1.0599125	1.0745577	1.0535644	0.718428914	-0.4770827	0.770117	1
SAMD13	1.1869857	1.20238892	1.180309	0.890903598	-0.1666588	0.770132	1
CMTM7	1.4939701	1.47624242	1.5016543	1.053359032	0.0749973	0.770414	1
APOL6	1.0742296	1.08890301	1.0678693	0.763408806	-0.3894723	0.77043	1
ARL9	1.0895701	1.10428508	1.0831919	0.797735056	-0.3260184	0.770474	1
LIMD2	2.7475309	2.72095768	2.7590492	1.022133888	0.0315842	0.770518	1
TMEM255A	1.0506586	1.06525725	1.0443307	0.679322517	-0.5578314	0.770581	1
C12orf66	1.0890444	1.10414111	1.0825007	0.792200891	-0.3360618	0.770772	1
RP11-159D12.5	1.0816437	1.09635952	1.075265	0.781085137	-0.3564483	0.770795	1

FAM167B	1.0581352	1.07254867	1.0518877	0.71521172	-0.4835577	0.770831	1
EPHA7	1.0863163	1.10110455	1.0799063	0.790333544	-0.3394665	0.770851	1
RGL1	1.1112402	1.09589198	1.1178929	1.229434318	0.2979947	0.770985	1
SGSM3	1.5626525	1.58079613	1.554788	0.955219922	-0.0660952	0.771	1
ZNF404	1.1000438	1.11499579	1.0935627	0.81361881	-0.2975751	0.771059	1
WNT5B	1.0852761	1.07009175	1.0918578	1.310536181	0.3901572	0.771143	1
LIFR	1.1432516	1.12760574	1.1500334	1.17575727	0.2335903	0.771262	1
RP11-115D19.2	1.0506867	1.03575229	1.05716	1.598779973	0.6769714	0.771307	1
RP11-401F2.3	1.0908799	1.10566587	1.0844709	0.79941502	-0.3229834	0.771371	1
STX18-AS1	1.0587606	1.04375953	1.0652629	1.49139767	0.576665	0.771454	1
CDC14A	1.0971847	1.1120769	1.0907296	0.809530323	-0.304843	0.77151	1
TRIM28	1.7705699	1.75138735	1.7788847	1.036595419	0.0518529	0.771607	1
ZNF775	1.1102182	1.09484955	1.1168798	1.232264883	0.3013124	0.771755	1
ASL	1.1955386	1.21097158	1.1888491	0.895140089	-0.1598146	0.77184	1
SLMAP	1.4984384	1.48060669	1.5061676	1.053184627	0.0747584	0.771857	1
MREG	1.1043399	1.08887579	1.111043	1.249417423	0.3212556	0.77188	1
SLC26A1	1.0299542	1.04439929	1.0236929	0.533631262	-0.9060849	0.771969	1
HYKK	1.0865374	1.10134206	1.0801203	0.790592552	-0.3389937	0.771977	1
CILP2	1.0411462	1.02630218	1.0475804	1.808988605	0.8551833	0.772086	1
LBX2-AS1	1.0782257	1.06322121	1.0847294	1.340205815	0.4224546	0.772132	1
HNF1A	1.0465809	1.06110633	1.0402847	0.659256073	-0.6010891	0.772152	1
LRP2BP	1.0694956	1.08410793	1.0631617	0.750960591	-0.4131909	0.772224	1
PTPN2	1.5967134	1.57812378	1.6047711	1.04609276	0.0650108	0.772269	1
MYO18A	1.0829658	1.09772537	1.0765682	0.783503752	-0.3519879	0.772283	1
POTEF	1.0510051	1.06551911	1.0447139	0.682455773	-0.5511925	0.772361	1
EME2	1.0900218	1.10464411	1.0836837	0.799697839	-0.3224731	0.77239	1
SIGLEC15	1.0318449	1.0171592	1.0382106	2.226825969	1.1549888	0.772449	1
TGFB2-AS1	1.048599	1.06300266	1.0423556	0.672282961	-0.5728595	0.7725	1
TMEM163	1.0452925	1.0303686	1.0517613	1.704436082	0.7692945	0.772574	1
ZNF514	1.356664	1.37281543	1.3496631	0.93789878	-0.0924959	0.772577	1
FITM2	1.0666576	1.08144581	1.0602476	0.739726707	-0.4349357	0.772621	1
CHN2	1.0602717	1.04548153	1.0666825	1.466145112	0.5520279	0.772711	1
CTNND1	1.6346065	1.61658381	1.6424185	1.041899727	0.0592164	0.772712	1
FRA10AC1	2.1131447	2.09267877	2.1220158	1.026848738	0.0382237	0.772718	1
TEX10	1.1722659	1.15674398	1.178994	1.141951407	0.1915013	0.772722	1
MAPKAPK5-AS1	1.7347988	1.71623461	1.7428455	1.037153865	0.0526299	0.772759	1
STARD13	1.0955104	1.11032588	1.0890885	0.807503006	-0.3084605	0.772778	1
SIRT5	1.2844089	1.30023762	1.2775479	0.924427337	-0.1133682	0.772921	1
ZNF691	1.1769511	1.19253098	1.1701979	0.884002733	-0.1778773	0.773076	1
SETD9	1.3790708	1.39533471	1.3720211	0.941028093	-0.0876903	0.773157	1
AP000695.4	1.0416151	1.02673224	1.0480661	1.798058602	0.84644	0.773168	1
RP11-104N10.1	1.0432012	1.05774056	1.036899	0.639048636	-0.6460024	0.773176	1
SH2D4A	1.0610618	1.04622565	1.0674926	1.460068079	0.5460356	0.773181	1
LCA5	1.6618445	1.64358548	1.6697589	1.040668193	0.0575102	0.773238	1
CACFD1	1.2887949	1.30472533	1.2818898	0.925061805	-0.1123783	0.773262	1
DUSP16	1.1738808	1.18907651	1.1672942	0.884796352	-0.1765827	0.773275	1
CTB-58E17.3	1.0583996	1.07287593	1.0521248	0.71525406	-0.4834723	0.773447	1
WDR75	1.2687188	1.25262221	1.2756959	1.091336699	0.1260963	0.773486	1
KBTBD3	1.3460766	1.32945628	1.3532807	1.072314414	0.100728	0.773619	1
SLC25A51	1.1747891	1.19003328	1.1681814	0.885010041	-0.1762343	0.773777	1
RALGPS1	1.11826	1.13298317	1.1118781	0.841295498	-0.2493155	0.773781	1

CEBPB-AS1	1.0307332	1.04498126	1.0245573	0.54594428	-0.8731744	0.773864	1
CATSPERG	1.0578717	1.07234091	1.0516	0.713288789	-0.4874418	0.773865	1
RP11-131L12.4	1.0268197	1.04108784	1.0206351	0.502219438	-0.9936102	0.773887	1
PNMA3	1.0553087	1.0697381	1.0490542	0.703406442	-0.5075695	0.773943	1
DNM3	1.0456528	1.06000364	1.0394324	0.657166877	-0.6056683	0.773963	1
TCEAL2	1.5292173	1.54657065	1.5216954	0.954488533	-0.0672002	0.774097	1
GATC	1.3499276	1.3329246	1.3572977	1.073208958	0.101931	0.774125	1
LPCAT2	1.1110259	1.09591594	1.1175754	1.225817082	0.2937437	0.774145	1
NKRF	1.1024826	1.08728344	1.1090708	1.249616051	0.3214849	0.774151	1
DIEXF	1.1256614	1.14063207	1.1191723	0.847405009	-0.2388764	0.774273	1
EIF2AK3	1.1036245	1.11816873	1.0973202	0.823570094	-0.2800367	0.774326	1
RP11-378J18.8	1.0846229	1.06953802	1.0911616	1.310959982	0.3906236	0.774332	1
RP11-597D13.9	1.0785216	1.06374054	1.0849285	1.332408814	0.4140368	0.774405	1
AC114271.2	1.066107	1.08058239	1.0598326	0.742502219	-0.4295328	0.774406	1
MRPS30	1.5728144	1.55528685	1.5804117	1.045246705	0.0638435	0.774408	1
SH2B3	1.0605492	1.04593928	1.0668819	1.455876183	0.5418877	0.77448	1
GBF1	1.2336581	1.21809264	1.240405	1.102306659	0.1405256	0.77452	1
ZFR2	1.1666115	1.18160613	1.1601119	0.881643863	-0.1817321	0.774614	1
ITM2A	1.0708739	1.05613715	1.0772616	1.376301441	0.4607965	0.774704	1
MAP2K4	1.5067809	1.489357	1.5143334	1.051039245	0.0718165	0.774745	1
ZNF579	1.2357013	1.25108431	1.2290334	0.912177308	-0.1326138	0.77482	1
SNTB2	1.2008338	1.21635401	1.1941065	0.897170822	-0.1565454	0.774938	1
USP43	1.0961514	1.08120839	1.1026285	1.263767173	0.3377307	0.774964	1
REL	1.4149373	1.43122685	1.4078766	0.945851481	-0.0803144	0.775008	1
R3HCC1L	1.190908	1.17557091	1.197556	1.125220614	0.1702079	0.775032	1
NSUN5	1.6245539	1.64217045	1.6169179	0.960676276	-0.0578777	0.775045	1
FNDC11	1.0671088	1.08148599	1.0608769	0.747084066	-0.4206575	0.775163	1
MSTO1	1.1569388	1.1417801	1.1635094	1.153260955	0.205719	0.77521	1
LINC00574	1.0283484	1.0426534	1.0221478	0.519250872	-0.9454964	0.775242	1
ERMARD	1.1353363	1.15023025	1.1288804	0.857885667	-0.2211427	0.775253	1
ZBTB3	1.0556319	1.07010018	1.0493606	0.704143924	-0.5060578	0.775285	1
TERF2	1.1479358	1.13265526	1.1545592	1.165119488	0.2204779	0.775371	1
CYP3A5	1.1038072	1.11854461	1.0974192	0.821793421	-0.2831523	0.775463	1
CCDC88A	1.8790624	1.86016492	1.8872536	1.031492371	0.0447332	0.775472	1
CD164L2	1.0391294	1.05339497	1.0329459	0.617022997	-0.6966038	0.775521	1
CHCHD4	1.2412006	1.22527688	1.2481029	1.101324197	0.1392392	0.775544	1
AC068057.1	1.0248011	1.03898056	1.0186549	0.478569647	-1.0631992	0.775581	1
SHF	1.156466	1.17134391	1.1500171	0.87553198	-0.1917682	0.775589	1
NR2C2	1.278455	1.26240222	1.2854132	1.087693632	0.1212723	0.775656	1
TBC1D25	1.1039532	1.11873168	1.0975474	0.821578302	-0.28353	0.775699	1
LRFN5	1.0681584	1.05334505	1.0745794	1.398056026	0.4834222	0.775846	1
RP11-299L17.3	1.0357038	1.04988701	1.029556	0.592459601	-0.7552113	0.775936	1
HLA-F	1.0369497	1.05126398	1.0307452	0.59974192	-0.7375863	0.776019	1
PTPN4	1.4462746	1.42925253	1.4536529	1.056843896	0.0797623	0.776019	1
PCID2	1.5941124	1.57682308	1.6016066	1.042965513	0.0606915	0.776118	1
ZNF408	1.1127779	1.12739095	1.1064437	0.835567494	-0.2591717	0.776223	1
GNMT	1.0273889	1.04157936	1.0212379	0.510780836	-0.9692237	0.776227	1
ATP2A2	1.7353986	1.75359666	1.7275105	0.965384515	-0.0508244	0.77631	1
KPNA6	1.5719133	1.58923203	1.5644063	0.95786772	-0.0621017	0.77633	1
ZNF407	1.0841803	1.09861805	1.0779221	0.790140727	-0.3398185	0.776385	1
PPP1R3F	1.0842753	1.09890267	1.077935	0.787997225	-0.3437375	0.776385	1

HES5	1.0439645	1.05820308	1.0377927	0.64932447	-0.6229885	0.776403	1
XRN1	1.4280833	1.44439617	1.4210124	0.947380773	-0.0779837	0.776522	1
C7orf31	1.095609	1.08062961	1.102102	1.266308342	0.3406287	0.776535	1
GEMIN4	1.1141983	1.12921719	1.1076883	0.83338952	-0.2629371	0.776645	1
RP11-538P18.2	1.1993432	1.18343138	1.2062402	1.124345284	0.1690852	0.776654	1
SPRY3	1.0534894	1.06778155	1.0472943	0.697746248	-0.5192256	0.77666	1
ZNF551	1.1230951	1.137842	1.1167029	0.846642764	-0.2401747	0.776812	1
ATP23	1.2005559	1.18530548	1.2071663	1.11797179	0.1608838	0.776854	1
CPNE5	1.092445	1.0774604	1.0989401	1.277299249	0.3530966	0.776882	1
ZNF16	1.1435505	1.15842719	1.1371021	0.865394732	-0.2085698	0.776915	1
DTX2	1.1084672	1.09353746	1.1149386	1.228798033	0.2972478	0.77697	1
FBXO28	1.2261839	1.21043985	1.2330083	1.107244192	0.1469734	0.776976	1
RP11-819C21.1	1.0637765	1.04890153	1.0702241	1.436030339	0.5220862	0.777026	1
TCEAL3	2.7615972	2.78959929	2.7494596	0.97757055	-0.0327273	0.777038	1
SUCLG2-AS1	1.0575406	1.07178573	1.051366	0.715545901	-0.4828838	0.777077	1
B3GALT4	1.06649	1.05179958	1.0728577	1.406530054	0.4921404	0.777175	1
RAP1GAP	1.0603164	1.04579369	1.0666114	1.454596877	0.5406194	0.777266	1
ZNF512B	1.165643	1.15029015	1.1722977	1.146433919	0.1971532	0.777375	1
SRPX2	1.0290755	1.01466374	1.0353224	2.408823613	1.2683288	0.777379	1
RP11-506H21.5	1.0441445	1.0582975	1.0380098	0.651997027	-0.6170627	0.777509	1
DMBX1	1.0318763	1.01740877	1.0381474	2.191274446	1.1317702	0.777525	1
FBXL18	1.055847	1.07000246	1.0497112	0.710134752	-0.4938353	0.777539	1
CCDC160	1.395175	1.3785255	1.4023918	1.063050673	0.0882104	0.777611	1
RP11-46F15.2	1.0445161	1.02982598	1.0508836	1.70601711	0.7706321	0.77776	1
MLIP	1.0282708	1.01394208	1.0344816	2.473203273	1.3063808	0.777799	1
SOX10	1.0283421	1.01394208	1.0345838	2.480536578	1.3106522	0.777799	1
SENP1	1.1218986	1.10691342	1.128394	1.200915762	0.264135	0.77782	1
RP11-225H22.4	1.0279613	1.04194267	1.0219011	0.522166499	-0.9374182	0.777849	1
FUZ	1.4893004	1.50587538	1.4821159	0.953032996	-0.0694019	0.777862	1
LRFN4	1.1229702	1.10814774	1.129395	1.196465311	0.2587786	0.777898	1
KCNK2	1.0354176	1.04945158	1.0293345	0.593197117	-0.7534165	0.777908	1
NYNRIN	1.2983337	1.28240483	1.3052382	1.080853412	0.1121709	0.777958	1
ONECUT1	1.052822	1.03813508	1.0591882	1.552066647	0.6341905	0.777984	1
NIPA1	1.1740691	1.18922901	1.167498	0.885160236	-0.1759895	0.778018	1
IGSF1	1.1742458	1.18920638	1.1677611	0.886656558	-0.1735527	0.778018	1
ST5	1.6990671	1.6810915	1.7068587	1.037832176	0.0535732	0.778076	1
RP11-318A15.2	1.0360068	1.05006026	1.0299153	0.597585231	-0.7427836	0.77809	1
PHF5A	1.8628355	1.88119692	1.8548766	0.97013121	-0.0437482	0.778146	1
RP11-14N7.2	1.5333768	1.55042468	1.5259873	0.955602741	-0.0655171	0.778362	1
HS6ST3	1.0464688	1.06042271	1.0404204	0.668959807	-0.5800086	0.778535	1
FBXO45	1.1523085	1.16708141	1.1459051	0.873257323	-0.1955213	0.778579	1
PLCG1	1.248439	1.26405047	1.2416722	0.915249936	-0.1277623	0.778596	1
RNF215	1.0557678	1.06988449	1.0496488	0.710441065	-0.4932131	0.778714	1
ZBTB25	1.3171173	1.33236965	1.310506	0.93421895	-0.0981674	0.778726	1
ROR2	1.0842886	1.09862085	1.0780762	0.791680189	-0.3370103	0.778777	1
LRCH1	1.1222377	1.1074342	1.1286543	1.197517181	0.2600464	0.778781	1
DNASE1L1	1.3434578	1.32716563	1.3505197	1.071383119	0.0994745	0.778782	1
SLC30A10	1.0327195	1.04677409	1.0266275	0.569278973	-0.8127923	0.778885	1
NGF	1.0443361	1.02998803	1.0505553	1.685848805	0.7534752	0.778917	1
RP11-617F23.1	1.1420348	1.12686535	1.14861	1.171399761	0.2282335	0.778937	1
SLC16A10	1.0825449	1.06791036	1.0888883	1.308906973	0.3883626	0.779024	1

IL17RC	1.2368847	1.25218962	1.2302508	0.913006495	-0.131303	0.779137	1
CCDC146	1.0812252	1.09537619	1.0750913	0.787317249	-0.344983	0.779214	1
ECM1	1.2483479	1.26401295	1.2415578	0.914946716	-0.1282404	0.779216	1
IMPA1	1.351003	1.3350384	1.3579229	1.068304191	0.0953225	0.779244	1
RP11-488C13.5	1.0491157	1.06318572	1.043017	0.680802444	-0.5546919	0.779281	1
RP11-430H10.1	1.1811076	1.19598204	1.1746602	0.89120533	-0.1661702	0.779443	1
SAMD10	1.1088012	1.09416541	1.1151451	1.222796292	0.2901841	0.779445	1
KMT2C	1.9529512	1.97251593	1.9444708	0.971162318	-0.0422157	0.779585	1
AGFG1	1.269597	1.25437162	1.2761966	1.085799564	0.1187578	0.779762	1
FEM1C	1.1592758	1.14426538	1.1657822	1.149147366	0.2005638	0.779775	1
USP54	1.1497168	1.16447862	1.1433182	0.871348651	-0.198678	0.779821	1
CEP120	1.1508883	1.1359156	1.1573782	1.157911595	0.2115251	0.779857	1
CKAP2	1.8726164	1.89169528	1.8643465	0.969329448	-0.044941	0.78001	1
SLC27A3	1.1502757	1.13538712	1.1567293	1.157638198	0.2111844	0.780049	1
RNF149	1.3514726	1.36722233	1.3446457	0.93852065	-0.0915396	0.780066	1
KLHDC8A	1.2095629	1.22436445	1.2031471	0.905433507	-0.1433194	0.780257	1
ATP6V1B1-AS1	1.0241851	1.03802955	1.0181842	0.478158819	-1.0644382	0.780321	1
ZNF569	1.1678639	1.18246953	1.161533	0.885260233	-0.1758265	0.78034	1
SPACA3	1.0551736	1.06930257	1.0490494	0.707757004	-0.498674	0.780388	1
C16orf70	1.1048742	1.11932579	1.0986101	0.82639359	-0.275099	0.780432	1
EML2	1.386826	1.37020555	1.3940302	1.064355133	0.0899796	0.780508	1
NFATC3	1.4326981	1.41581372	1.4400167	1.058206336	0.081621	0.780557	1
YJEFN3	1.1298001	1.14439167	1.1234753	0.855141403	-0.2257651	0.780574	1
TRIM66	1.079554	1.06466779	1.0860065	1.329974843	0.411399	0.780608	1
RSRC2	3.3634276	3.39249951	3.3508262	0.982581701	-0.0253507	0.780739	1
GTF2H1	1.3191218	1.303277	1.3259898	1.074891407	0.1041909	0.780785	1
PRKD2	1.142963	1.15771317	1.1365695	0.865935901	-0.2076679	0.780887	1
TRPC4AP	1.5858836	1.56833481	1.5934902	1.044261637	0.0624832	0.780888	1
RP11-166P13.3	1.0816511	1.06722972	1.0879021	1.307488785	0.3867986	0.78096	1
ZFC3H1	1.4125274	1.42871791	1.4055095	0.945865551	-0.080293	0.78103	1
TLCD1	1.1294454	1.11469222	1.1358402	1.184389336	0.2441434	0.781057	1
RP11-462G2.2	1.0366516	1.05055832	1.0306236	0.605708274	-0.723305	0.781119	1
MORC3	1.2548507	1.23911936	1.2616695	1.094304995	0.1300149	0.781148	1
CDKN2AIP	1.246078	1.23118003	1.2525356	1.092376422	0.1274701	0.781235	1
CECR2	1.2708871	1.28554705	1.2645326	0.926406505	-0.1102827	0.781247	1
TATDN2	1.1006461	1.08609304	1.1069543	1.242310284	0.3130256	0.781285	1
C19orf47	1.0548898	1.06883796	1.0488438	0.709548113	-0.4950276	0.781286	1
RP11-248G5.9	1.0259206	1.0397287	1.0199353	0.501787059	-0.9948528	0.781375	1
AC009120.11	1.0259966	1.03989853	1.0199707	0.500536266	-0.9984535	0.781375	1
FES	1.0472707	1.03292	1.0534911	1.62488214	0.7003351	0.781426	1
PHOSPHO2	1.1497144	1.13452895	1.1562966	1.161806714	0.2163701	0.781549	1
ZNF134	1.3799812	1.36368977	1.3870427	1.064211273	0.0897846	0.781559	1
KDR	1.0549203	1.04062055	1.0611185	1.504621157	0.5894003	0.78161	1
RP11-580I16.2	1.0235395	1.03736514	1.0175467	0.469600718	-1.0904935	0.781624	1
LINC00960	1.2556801	1.2706697	1.2491828	0.920615797	-0.1193289	0.781752	1
WDR63	1.0415357	1.05540007	1.0355261	0.641263676	-0.6410104	0.781777	1
RP11-139K4.2	1.035124	1.04899149	1.029113	0.594246448	-0.7508667	0.781803	1
RP13-577H12.2	1.0292452	1.01490147	1.0354626	2.379803582	1.2508425	0.781815	1
ZSCAN20	1.0532729	1.06721504	1.0472297	0.702665083	-0.5090909	0.781849	1
ZNF621	1.2248916	1.23990768	1.2183829	0.91027874	-0.1356197	0.781856	1
ENTPD6	1.2825564	1.26701713	1.2892919	1.083420853	0.1155938	0.781925	1

APLN	1.0490132	1.03464326	1.0552419	1.59459335	0.6731886	0.781986	1
ETNPPL	1.0489649	1.03475551	1.055124	1.586050662	0.6654389	0.781986	1
TIFA	1.2494707	1.26461201	1.2429077	0.917976741	-0.1234705	0.782013	1
FGF7	1.1717946	1.18646966	1.1654335	0.887187444	-0.1726891	0.782028	1
ADGRE5	1.0742149	1.05973419	1.0804916	1.347496979	0.430282	0.782077	1
CPPED1	1.142009	1.12722375	1.1484177	1.166588066	0.2222952	0.782107	1
RP13-401N8.1	1.0430191	1.05687992	1.0370111	0.650687515	-0.6199632	0.782122	1
SEPT5	1.0328166	1.04658041	1.0268506	0.576434844	-0.7947705	0.782173	1
CAAP1	1.5077012	1.49147359	1.5147351	1.047330223	0.0667164	0.782189	1
STKLD1	1.0653794	1.05097651	1.0716224	1.405007867	0.4905782	0.782191	1
TMEM100	1.1007618	1.08617012	1.1070866	1.242734611	0.3135182	0.782201	1
LHX5	1.0333718	1.01899597	1.0396031	2.084817408	1.059921	0.782302	1
FLJ37035	1.0933167	1.10738672	1.0872179	0.812185152	-0.3001194	0.782486	1
MACC1	1.0557293	1.04132128	1.0619746	1.499822903	0.5847922	0.78255	1
NIPAL4	1.0308527	1.01680903	1.03694	2.19762531	1.1359454	0.782621	1
OTUD6B-AS1	2.5073091	2.48438169	2.5172471	1.022140782	0.0315939	0.782623	1
PTGIS	1.0726131	1.05829205	1.0788206	1.352166932	0.4352733	0.782719	1
HGH1	1.1537608	1.13900603	1.1601563	1.152153776	0.2043333	0.782726	1
DAG1	1.4114493	1.42718919	1.4046267	0.947183945	-0.0782835	0.782777	1
VPS13D	1.1803133	1.19491912	1.1739824	0.892587528	-0.1639344	0.78278	1
CCDC173	1.0609607	1.07487529	1.0549293	0.733610488	-0.4469138	0.782817	1
AC016549.1	1.0988907	1.11317951	1.0926972	0.819027817	-0.2880156	0.782829	1
AVPI1	1.2444297	1.25943615	1.2379251	0.917085231	-0.1248723	0.782857	1
SALL1	1.1975195	1.21207783	1.1912092	0.901599045	-0.1494421	0.782897	1
NPIPB5	1.1476352	1.16193176	1.1414383	0.873444058	-0.1952128	0.782931	1
VPS54	1.1853831	1.17042326	1.1918676	1.125829771	0.1709887	0.783018	1
ARHGAP28	1.1858072	1.20046175	1.1794551	0.895208884	-0.1597037	0.783078	1
SCARF1	1.0318318	1.01772642	1.0379458	2.140635662	1.0980393	0.783086	1
RP11-219B4.7	1.0327208	1.0465246	1.0267375	0.574696281	-0.7991284	0.783105	1
CPOX	1.196459	1.1812935	1.2030325	1.119910658	0.1633836	0.783223	1
ADARB2	1.0346043	1.04843518	1.0286092	0.590669573	-0.7595768	0.783231	1
SFTPD-AS1	1.0241115	1.03788476	1.0181414	0.478856635	-1.0623343	0.783313	1
KCNE2	1.0305707	1.0443077	1.0246163	0.555575196	-0.8479459	0.783358	1
RBM34	1.1177089	1.10328385	1.1239614	1.200201659	0.2632768	0.783562	1
RBM48	1.151371	1.16577257	1.1451286	0.875468216	-0.1918733	0.783689	1
RP11-274B21.10	1.0620483	1.07579799	1.0560884	0.739972155	-0.4344571	0.783691	1
GRWD1	1.2129283	1.19822761	1.2193004	1.106306073	0.1457506	0.783707	1
WDR31	1.1005585	1.08601222	1.1068637	1.242424256	0.3131579	0.783721	1
CTA-384D8.36	1.0504594	1.0643293	1.0444475	0.690936502	-0.533375	0.783782	1
SCAF1	1.2087148	1.22350917	1.2023021	0.905117548	-0.1438229	0.783818	1
GLTP	1.6713138	1.68887191	1.6637031	0.963463725	-0.0536977	0.783869	1
RP3-508I15.9	1.0750973	1.06075206	1.0813153	1.33847785	0.4205933	0.783918	1
ASNSD1	1.5458702	1.5290014	1.553182	1.045709915	0.0644827	0.783922	1
BRWD3	1.3750817	1.39057558	1.3683658	0.943135679	-0.0844628	0.783937	1
RP11-181E10.3	1.0413523	1.05505245	1.0354139	0.643276262	-0.6364896	0.784006	1
FAM127C	1.3277601	1.31240912	1.334414	1.07043611	0.0981987	0.784026	1
PPP1R10	1.5992299	1.58207716	1.6066649	1.042241377	0.0596894	0.784028	1
ARHGEF10	1.3882174	1.40356783	1.3815636	0.945475765	-0.0808876	0.784085	1
CCDC117	1.364808	1.34901872	1.371652	1.064848284	0.0906479	0.784158	1
CTC-529I10.2	1.0342864	1.04805996	1.0283162	0.589185027	-0.7632073	0.78426	1
HLA-DMA	1.0675222	1.05320886	1.0737263	1.385602886	0.4705138	0.784265	1

RFC3	1.3324787	1.34791176	1.3257892	0.936413175	-0.0947829	0.784486	1
POLD1	1.2254736	1.24037574	1.2190141	0.9111324	-0.1342674	0.784511	1
IMPDH1	1.2900367	1.27483468	1.296626	1.079288985	0.1100812	0.784566	1
MYCBP	1.2723227	1.28730522	1.2658284	0.925247541	-0.1120887	0.78458	1
ZNF830	1.3429249	1.35861748	1.3361229	0.93727409	-0.0934571	0.784633	1
TM9SF4	1.3624285	1.34649713	1.3693341	1.065907982	0.0920829	0.784642	1
LUC7L2	1.8594585	1.84095224	1.8674802	1.031545095	0.0448069	0.784654	1
AGPS	1.290419	1.30557813	1.2838482	0.928889007	-0.1064219	0.78469	1
QTRT2	1.1840092	1.16916955	1.1904416	1.125743748	0.1708785	0.784729	1
CH17-340M24.3	1.2647687	1.24933693	1.2714577	1.088718198	0.1226306	0.784895	1
DDX19A	1.3261343	1.31060312	1.3328664	1.07167752	0.0998708	0.7849	1
ARSB	1.1879905	1.20239282	1.1817477	0.897994659	-0.1552212	0.784965	1
RP11-46H11.3	1.0293043	1.0429599	1.0233852	0.54434917	-0.8773957	0.784985	1
RP4-671G15.2	1.0293177	1.04293143	1.0234167	0.545444736	-0.8744951	0.784985	1
RP11-692D12.1	1.0293252	1.0430409	1.02338	0.543204514	-0.8804326	0.784985	1
CCDC60	1.0392645	1.05299961	1.033311	0.628514167	-0.6699828	0.785133	1
LRRC40	1.2057585	1.22052454	1.1993581	0.904017706	-0.1455771	0.785276	1
CCDC121	1.0820535	1.06760583	1.0883159	1.306335281	0.3855252	0.785352	1
GRIP1	1.1360181	1.15040175	1.1297834	0.862911327	-0.2127158	0.785387	1
CHERP	1.3130277	1.32805365	1.3065146	0.934343011	-0.0979758	0.785411	1
ANO4	1.0386333	1.02464663	1.0446959	1.81346833	0.8587516	0.785445	1
PRNCR1	1.0705036	1.08431533	1.0645169	0.765185575	-0.3861184	0.785516	1
C16orf74	1.06714	1.05290387	1.0733108	1.385735399	0.4706518	0.785527	1
RAB24	1.1455993	1.13101023	1.151923	1.159627162	0.213661	0.785642	1
RP11-266L9.5	1.0317851	1.04537553	1.0258942	0.570665119	-0.8092837	0.785645	1
RP11-1072C15.4	1.0233257	1.03686467	1.0174572	0.473548082	-1.0784172	0.785668	1
RP5-991G20.6	1.0233754	1.03699572	1.0174716	0.472259297	-1.0823489	0.785668	1
IBA57-AS1	1.0233238	1.03686009	1.0174564	0.473585321	-1.0783037	0.785668	1
OGFRL1	1.4553496	1.47129694	1.4484372	0.95149607	-0.0717304	0.785737	1
RIT2	1.0221382	1.03566635	1.0162743	0.456293301	-1.1319666	0.785814	1
LINC01134	1.0326438	1.01860175	1.0387304	2.082086465	1.05803	0.785903	1
RP11-603J24.21	1.0352985	1.04886133	1.0294196	0.602103664	-0.7319162	0.785921	1
LRP1	1.569099	1.58573729	1.5618871	0.959281722	-0.0599735	0.786105	1
RP11-315O6.1	1.042834	1.05649283	1.0369136	0.653420204	-0.613917	0.786135	1
SPRYD4	1.2513445	1.26619012	1.2449097	0.920055383	-0.1202074	0.786198	1
DND1	1.0227268	1.03618751	1.0168921	0.46679479	-1.0991396	0.786387	1
KIAA2012	1.0227531	1.03626618	1.0168958	0.465882836	-1.1019609	0.786387	1
CLCN4	1.2497941	1.23489027	1.2562542	1.09095285	0.1255888	0.786454	1
SMN2	1.0803217	1.0661102	1.0864817	1.308144461	0.3875219	0.786511	1
C11orf70	1.1909671	1.176182	1.1973758	1.120294906	0.1638786	0.786521	1
MZF1	1.2914835	1.27605852	1.2981695	1.080095251	0.1111585	0.786582	1
RAPGEF5	1.0499274	1.06359585	1.0440027	0.69191121	-0.5313412	0.786634	1
RP6-24A23.3	1.0499936	1.0636144	1.0440897	0.693076695	-0.5289131	0.786634	1
HHAT	1.1455716	1.13102743	1.1518758	1.159114423	0.213023	0.786814	1
ARRDC1-AS1	1.4194428	1.43487391	1.4127542	0.949135247	-0.0753144	0.786819	1
AREL1	1.1592297	1.17328039	1.1531393	0.883766048	-0.1782636	0.786949	1
FLJ35934	1.0786863	1.09245879	1.0727165	0.786475045	-0.3465271	0.786959	1
RP11-276H19.2	1.1101211	1.09579944	1.1163289	1.214296663	0.2801209	0.786991	1
LAD1	1.0310436	1.0171592	1.0370618	2.159881416	1.1109521	0.787004	1
SMIM5	1.0342673	1.02039227	1.0402815	1.975330486	0.982094	0.787015	1
HTRA3	1.1450041	1.15925594	1.1388266	0.871720233	-0.1980629	0.787031	1

RNF213	1.482688	1.49853003	1.4758213	0.954448538	-0.0672607	0.787053	1
C2orf42	1.0927661	1.07868988	1.0988675	1.256419612	0.3293184	0.78712	1
MMP24	1.0357643	1.02186796	1.0417877	1.910909957	0.9342598	0.787137	1
TMEM38A	1.1185368	1.10414871	1.1247735	1.198031631	0.260666	0.787162	1
TAF1A	1.1158431	1.12990468	1.1097481	0.844835354	-0.2432579	0.787164	1
RAB15	1.0841291	1.06992629	1.0902854	1.291151641	0.3686584	0.787179	1
RP11-179B2.2	1.1371118	1.15102414	1.1310814	0.867949654	-0.2043167	0.787424	1
DLX3	1.0410718	1.02696857	1.0471849	1.749625197	0.8070459	0.787435	1
NUDT7	1.1475779	1.1332588	1.1537846	1.154029571	0.2066802	0.787439	1
SNX11	1.1809143	1.16623756	1.187276	1.126556728	0.17192	0.78744	1
RP11-173D3.1	1.0275249	1.04096671	1.0216985	0.529662166	-0.9168556	0.787496	1
DCAF12L2	1.0579862	1.04399821	1.0640494	1.45572698	0.5417398	0.787574	1
RP13-753N3.1	1.0214932	1.03496699	1.0156529	0.447648862	-1.1595606	0.787606	1
GVQW2	1.0439456	1.05751898	1.0380622	0.66173228	-0.5956804	0.787772	1
NLN	1.3965021	1.41203166	1.3897707	0.945972662	-0.0801296	0.787853	1
PRRG2	1.0411519	1.05466143	1.0352961	0.645721497	-0.631016	0.787921	1
GRK4	1.1883817	1.2025905	1.1822229	0.899464146	-0.1528623	0.788132	1
VEGFD	1.1271189	1.11267912	1.1333779	1.183696906	0.2432997	0.788243	1
RP11-337C18.8	1.0698327	1.08366763	1.0638358	0.762969008	-0.3903036	0.788245	1
CCL27	1.0388338	1.05235336	1.0329736	0.629828526	-0.666969	0.788276	1
OBSCN	1.19039	1.17560258	1.1967997	1.12071053	0.1644137	0.78828	1
HPS5	1.1529609	1.16709874	1.1468328	0.878719006	-0.1865262	0.788475	1
LINC01088	1.0496113	1.03571361	1.0556353	1.557818969	0.6395276	0.788554	1
FBXO44	1.4493962	1.46489962	1.4426761	0.952197275	-0.0706676	0.788575	1
CLDN5	1.0410346	1.02713465	1.0470596	1.73430089	0.7943542	0.788594	1
MRM1	1.0490449	1.03512712	1.0550776	1.567950555	0.6488801	0.788731	1
POU2F1	1.5666091	1.54977705	1.573905	1.04388679	0.0619653	0.788761	1
CRYGS	1.0318027	1.01804205	1.0377674	2.093297264	1.0657772	0.788807	1
ZSWIM3	1.0518755	1.03781574	1.0579699	1.53295578	0.6163161	0.788809	1
RP11-258F22.1	1.0280003	1.04142638	1.0221807	0.535423789	-0.9012469	0.788827	1
LMO7	1.0598187	1.04585882	1.0658696	1.436357191	0.5224146	0.78885	1
WDR33	1.8363593	1.85371312	1.8288372	0.970861463	-0.0426626	0.788863	1
NR2C1	1.3627753	1.37805281	1.3561532	0.942072632	-0.0860898	0.7889	1
KB-431C1.5	1.0190197	1.03235898	1.0132377	0.409088171	-1.2895163	0.788925	1
GPAM	1.1558808	1.16989291	1.1498072	0.881774514	-0.1815183	0.788986	1
NAALADL1	1.0338707	1.02010384	1.039838	1.98161038	0.9866733	0.789088	1
IRX6	1.0196713	1.03313693	1.0138345	0.417494653	-1.2601704	0.789106	1
GAK	1.2642569	1.27863183	1.2580261	0.926046603	-0.1108433	0.789161	1
ZNF212	1.1472015	1.13282798	1.1534318	1.155116836	0.2080388	0.789166	1
TMEM151B	1.0406508	1.05408561	1.0348274	0.643930875	-0.6350223	0.78934	1
SYTL3	1.0359617	1.02218088	1.041935	1.890592328	0.9188383	0.789367	1
B3GALT5-AS1	1.0359221	1.02220411	1.0418683	1.885610202	0.9150315	0.789367	1
ZNF382	1.1662462	1.15189014	1.1724689	1.135484427	0.1833079	0.789408	1
PHF13	1.195944	1.21023786	1.1897482	0.902540542	-0.1479364	0.789462	1
PTEN	1.5082316	1.49216595	1.5151953	1.04679192	0.0659747	0.789673	1
PDE1A	1.0272528	1.01338003	1.033266	2.486244853	1.3139684	0.78978	1
ABCC6	1.0975185	1.11104521	1.0916553	0.825387394	-0.2768567	0.789879	1
PTPN23	1.1463955	1.13199543	1.1526373	1.15638339	0.2096198	0.789894	1
COX15	1.2973809	1.31229898	1.2909146	0.931525942	-0.1023321	0.789902	1
MCAT	1.2427542	1.22778494	1.2492427	1.094201607	0.1298786	0.79013	1
MFSD14C	1.2533086	1.23811005	1.2598964	1.091497064	0.1263083	0.790158	1

TEX261	1.2809859	1.26594085	1.2875073	1.081094766	0.112493	0.790182	1
GABRB2	1.0350297	1.02140077	1.0409372	1.912885023	0.9357502	0.790202	1
RP11-47I22.2	1.0350075	1.021355	1.0409252	1.916423869	0.9384167	0.790202	1
STAG3	1.0482032	1.06154344	1.0424208	0.689281978	-0.5368338	0.790264	1
ITGA4	1.0695761	1.05580257	1.0755463	1.35381329	0.4370288	0.790271	1
DTD2	1.1921699	1.20627582	1.1860557	0.901975085	-0.1488405	0.790292	1
RP11-503P10.1	1.0256579	1.03892831	1.0199057	0.511342448	-0.9676383	0.790318	1
WNK4	1.0256127	1.03885064	1.0198746	0.511564518	-0.9670119	0.790318	1
MFAP5	1.0264327	1.01281342	1.032336	2.523605752	1.3354865	0.790353	1
ANKRD34A	1.061012	1.04721263	1.0669935	1.418973781	0.5048479	0.79039	1
ZNF8	1.1145729	1.10050493	1.1206708	1.200645296	0.26381	0.790426	1
SGMS2	1.058464	1.07190403	1.0526384	0.732064158	-0.449958	0.790669	1
ACBD4	1.1492424	1.1350203	1.155407	1.150990143	0.2028755	0.790693	1
LCN12	1.0852264	1.09876364	1.0793586	0.803520917	-0.3155925	0.790713	1
KLHL2	1.0972262	1.08321725	1.1032985	1.241310862	0.3118645	0.790751	1
FAM181B	1.0276848	1.04108061	1.0218783	0.532570624	-0.9089552	0.790752	1
RHBDL1	1.0600228	1.07353695	1.054165	0.736568262	-0.4411089	0.790798	1
RP11-468E2.4	1.0678261	1.08131163	1.0619807	0.762261785	-0.3916415	0.790806	1
GRK3	1.1751622	1.18939134	1.1689945	0.892303444	-0.1643937	0.790824	1
CTA-293F17.1	1.1406174	1.12628546	1.1468297	1.16268069	0.2174549	0.79086	1
MNT	1.2585344	1.27317904	1.2521865	0.923154779	-0.1153555	0.790889	1
TBL3	1.2462264	1.2316308	1.2525529	1.090325215	0.1247585	0.790895	1
ZNF432	1.144867	1.15873474	1.1388559	0.874766981	-0.1930293	0.790924	1
RUSC1	1.4696843	1.45378355	1.4765766	1.050228843	0.0707037	0.790946	1
ATOH8	1.1566642	1.17046353	1.1506829	0.883959487	-0.1779478	0.790999	1
TSEN54	1.2686181	1.25351234	1.2751658	1.085413886	0.1182453	0.791055	1
PPP2R2D	1.3692175	1.38442982	1.3626236	0.943276554	-0.0842473	0.791108	1
BSCL2	1.0576092	1.07112841	1.0517493	0.727547092	-0.4588875	0.791143	1
CCDC33	1.4167814	1.43179605	1.4102732	0.950154937	-0.0737653	0.791241	1
YRDC	1.1028518	1.08898056	1.1088644	1.223462717	0.2909701	0.791255	1
NFASC	1.0516231	1.0378844	1.0575782	1.519838536	0.6039181	0.791261	1
ZC3H12C	1.1619916	1.17636813	1.15576	0.883152989	-0.1792647	0.791262	1
ANO9	1.035148	1.02156801	1.0410344	1.902557363	0.92794	0.791268	1
CDK7	1.2932011	1.27848261	1.2995809	1.07576175	0.1053586	0.79129	1
GK5	1.295219	1.30982489	1.288888	0.932423391	-0.1009429	0.791316	1
RP11-483F11.7	1.0468707	1.06011739	1.0411288	0.684141553	-0.5476332	0.791321	1
ZBTB2	1.0742012	1.06043502	1.0801682	1.326518947	0.4076453	0.791375	1
GAB2	1.0626878	1.04888526	1.0686706	1.404730518	0.4902934	0.791378	1
LSP1	1.034178	1.02062385	1.0400531	1.9420753	0.9575991	0.791479	1
CCNO	1.0768309	1.06313401	1.0827679	1.310987398	0.3906538	0.791499	1
RP11-98I9.4	1.0401728	1.05349141	1.0343998	0.643090937	-0.6369053	0.791503	1
RP11-290F5.2	1.0401222	1.05339544	1.0343688	0.643666029	-0.6356158	0.791503	1
USP6	1.0730554	1.0590569	1.0791232	1.33977858	0.4219946	0.79153	1
ZNF565	1.065191	1.07881368	1.0592861	0.752231473	-0.4107514	0.79153	1
RASGRF1	1.0952243	1.10880624	1.0893371	0.821066282	-0.2844294	0.791545	1
RP11-138P22.1	1.0585472	1.07187189	1.0527715	0.734243979	-0.4456686	0.791572	1
RP11-15A1.7	1.0584598	1.07187319	1.0526457	0.732481005	-0.4491367	0.791572	1
HCG23	1.0455182	1.03179572	1.0514662	1.618652281	0.6947931	0.79168	1
WDR89	1.1926705	1.20686239	1.1865189	0.901657033	-0.1493493	0.79169	1
SHOX2	1.0274908	1.0407369	1.0217492	0.53389416	-0.9053743	0.79175	1
CTD-2007H13.3	1.0274169	1.04060893	1.0216987	0.534334186	-0.9041858	0.79175	1

LINC01574	1.037807	1.05108895	1.0320498	0.627333592	-0.6726953	0.791766	1
AC007383.3	1.024937	1.03819901	1.0191885	0.502331105	-0.9932895	0.791827	1
OAS3	1.0249298	1.03817572	1.0191882	0.502629389	-0.9924331	0.791827	1
RP4-561L24.3	1.0249221	1.03812955	1.0191973	0.503474783	-0.9900086	0.791827	1
LINC01099	1.0561563	1.06953031	1.0503592	0.724277129	-0.4653863	0.791867	1
ZC3HC1	1.2073926	1.19310716	1.2135847	1.106042374	0.1454067	0.792022	1
RP6-65G23.5	1.0738375	1.08726189	1.0680187	0.77947758	-0.3594206	0.792061	1
ALOX12B	1.0269429	1.04009859	1.0212405	0.529706569	-0.9167347	0.792062	1
RSAD2	1.0269664	1.04019078	1.0212342	0.528334921	-0.9204753	0.792062	1
RP11-624L4.1	1.0449937	1.05833663	1.0392101	0.67213582	-0.5731753	0.792114	1
BCL2L12	1.5973782	1.58085604	1.6045399	1.040774002	0.0576568	0.792239	1
SMC2	1.7922713	1.80962455	1.7847495	0.969275812	-0.0450208	0.792322	1
FAM166B	1.0452948	1.05844856	1.0395933	0.677404156	-0.5619113	0.792346	1
AC016700.5	1.1047442	1.11820684	1.0989087	0.836742429	-0.2571445	0.792622	1
LTBP2	1.1300613	1.11605132	1.136134	1.173049841	0.2302643	0.792693	1
HECA	1.1298957	1.11583931	1.1359885	1.173941031	0.2313599	0.792693	1
CYB561	1.1584719	1.14412631	1.1646901	1.142679224	0.1924205	0.792693	1
SERTAD4	1.0370334	1.02339344	1.0429457	1.835799399	0.8764084	0.792719	1
CCDC92	1.1926624	1.17844609	1.1988245	1.114199332	0.1560074	0.792744	1
FAM122C	1.1114684	1.12538859	1.1054346	0.840862879	-0.2500575	0.792785	1
NACC1	1.282333	1.26754024	1.288745	1.079258323	0.1100402	0.792891	1
CACNA2D2	1.0846175	1.09810331	1.078772	0.802949557	-0.3166187	0.792916	1
SCYL3	1.1324916	1.1184359	1.1385841	1.170119154	0.2266554	0.792958	1
BBS10	1.1322277	1.11835279	1.1382419	1.168049495	0.2241014	0.792958	1
SHROOM1	1.1209662	1.10685906	1.127081	1.189239612	0.2500394	0.792962	1
ZNF77	1.085236	1.09884295	1.079338	0.802667114	-0.3171263	0.793103	1
AC002398.11	1.0266803	1.03988608	1.0209562	0.5254024	-0.9285053	0.793112	1
ALDH18A1	1.3588741	1.34346592	1.3655528	1.064305991	0.089913	0.793178	1
CASC2	1.0770761	1.09037587	1.0713112	0.789051373	-0.3418089	0.793197	1
RP1-187B23.1	1.0738086	1.08717988	1.0680128	0.78014321	-0.3581891	0.793243	1
ERLIN1	1.2960253	1.31070823	1.289661	0.932260337	-0.1011952	0.793249	1
ARMC3	1.0487574	1.06207495	1.0429848	0.692466345	-0.5301841	0.793291	1
RND2	1.5140339	1.49804849	1.5209629	1.04600834	0.0648944	0.793299	1
TPPP3	2.6575069	2.62046615	2.6735624	1.032766005	0.0465134	0.793348	1
AGAP4	1.0890834	1.07500214	1.095187	1.269123627	0.3438326	0.793353	1
LINC01607	1.0710674	1.05747916	1.0769573	1.33887231	0.4210184	0.793519	1
RP11-817O13.8	1.0518387	1.03813377	1.0577792	1.515172348	0.5994819	0.793544	1
ZBTB14	1.1694577	1.1834966	1.1633724	0.890329383	-0.1675889	0.793619	1
ATF7IP2	1.3143575	1.29959931	1.3207545	1.070611503	0.0984351	0.793686	1
RP4-613B23.1	1.0572256	1.07052597	1.0514605	0.7296672	-0.4546895	0.793802	1
ARNT	1.2815833	1.26694859	1.2879268	1.078585318	0.1091403	0.793821	1
KLHL22	1.1909515	1.17663708	1.1971562	1.116165461	0.1585509	0.793851	1
SYT17	1.4168357	1.43234813	1.4101118	0.948568342	-0.0761764	0.793889	1
AC006262.5	1.0366322	1.02298153	1.0425491	1.851447783	0.8886539	0.793925	1
GGACT	1.1407377	1.12665683	1.1468411	1.159361748	0.2133308	0.794021	1
NOTUM	1.0265655	1.03959192	1.0209192	0.528369579	-0.9203807	0.794227	1
EED	1.2391953	1.22465838	1.2454964	1.092754468	0.1279693	0.794319	1
IQGAP3	1.1109211	1.12435085	1.1050999	0.845188342	-0.2426552	0.794358	1
SMG7	1.3521034	1.33674514	1.3587605	1.065377076	0.0913641	0.794457	1
PNPLA8	1.5227578	1.53932801	1.5155753	0.955958752	-0.0649797	0.794581	1
FEN1	1.3426943	1.32782341	1.3491402	1.065025319	0.0908877	0.794599	1

UGDH	1.6437822	1.62733102	1.6509131	1.037591118	0.053238	0.794678	1
IGFLR1	1.0337142	1.04677169	1.0280543	0.599813649	-0.7374137	0.794684	1
RP11-686F15.2	1.0340389	1.04715061	1.0283556	0.601382921	-0.7336442	0.794914	1
LINC00235	1.0340199	1.04716452	1.0283222	0.600498988	-0.7357663	0.794914	1
PCDHB4	1.0611035	1.04756461	1.066972	1.408022094	0.49367	0.795014	1
GPSM3	1.1602821	1.14629256	1.166346	1.137077732	0.1853309	0.795062	1
TTC37	2.0922184	2.07309673	2.1005068	1.025542915	0.0363879	0.795064	1
GABBR2	1.0289878	1.01564798	1.03477	2.222015344	1.1518688	0.795177	1
CFAP157	1.02571	1.03868103	1.0200877	0.519316512	-0.945314	0.795208	1
KB-1125A3.11	1.1001214	1.11365356	1.0942558	0.829325509	-0.2699896	0.795341	1
BRF2	1.2023129	1.2166432	1.1961014	0.905181496	-0.143721	0.7954	1
PFN4	1.0516221	1.0647652	1.0459252	0.70910246	-0.495934	0.795494	1
ANKMY1	1.0623547	1.07555929	1.0566311	0.749492607	-0.4160138	0.795563	1
MIR940	1.0442792	1.0573251	1.0386243	0.673777185	-0.5696565	0.795612	1
SUFU	1.1317327	1.14541268	1.1258031	0.865145136	-0.2089859	0.795636	1
PGM5P4-AS1	1.0291213	1.01574718	1.0349184	2.217440166	1.1488952	0.795682	1
NFXL1	1.2183952	1.23243511	1.2123096	0.913414438	-0.1306585	0.795734	1
FAM92B	1.0950588	1.10849287	1.0892357	0.822502572	-0.2819079	0.795743	1
ZNF18	1.0824951	1.06890941	1.0883838	1.282609311	0.3590818	0.795773	1
RP11-930P14.2	1.0375039	1.05056927	1.0318407	0.629645098	-0.6673892	0.795806	1
HMGCLL1	1.0557634	1.06895768	1.0500442	0.72572356	-0.462508	0.79582	1
RIMS3	1.0739803	1.06031764	1.0799025	1.324695499	0.4056608	0.795949	1
KLHL25	1.0636296	1.07682465	1.0579101	0.753796374	-0.4077532	0.796042	1
RP11-600F24.7	1.0352134	1.04811272	1.0296221	0.615681802	-0.6997432	0.796053	1
ACOX1	1.2640517	1.24964322	1.2702971	1.082733489	0.1146782	0.796058	1
ZNF358	1.4316427	1.41613514	1.4383646	1.053418762	0.0750791	0.79615	1
RP11-531A24.3	1.0803811	1.09368609	1.0746139	0.796425029	-0.3283895	0.796263	1
RP11-804H8.6	1.0757802	1.06216082	1.0816836	1.314068952	0.394041	0.796273	1
CH507-39O4.2	1.0334232	1.04634274	1.0278232	0.600378768	-0.7360551	0.796298	1
AOC2	1.0317423	1.04471695	1.0261184	0.58408183	-0.7757576	0.796396	1
CDCA2	1.1966041	1.18253549	1.2027022	1.110481107	0.1511848	0.796401	1
ARC	1.0283187	1.01490147	1.0341344	2.290675767	1.1957733	0.79643	1
RP1-43E13.2	1.0834315	1.09664256	1.0777051	0.8040462	-0.3146497	0.796488	1
SEC16A	1.1983115	1.21240128	1.1922043	0.904911028	-0.1441521	0.796539	1
CPB1	1.0410343	1.02758104	1.0468657	1.699201492	0.7648569	0.796795	1
RP11-950C14.3	1.026181	1.03906793	1.0205951	0.527160735	-0.9236852	0.796849	1
AC018816.3	1.0944658	1.10773725	1.0887133	0.823422553	-0.2802951	0.797026	1
MON2	1.2384675	1.25236536	1.2324433	0.921058785	-0.1186349	0.797049	1
ENO1-AS1	1.0413854	1.05439634	1.0357457	0.657134713	-0.6057389	0.797131	1
RP11-1038A11.3	1.051336	1.06442277	1.0456635	0.708809969	-0.4965292	0.797204	1
HIP1R	1.1958533	1.18161083	1.2020268	1.112416235	0.1536967	0.797346	1
NRBP2	1.209523	1.22341977	1.2034994	0.910838785	-0.1347324	0.797403	1
IL10RB-AS1	1.0240589	1.03691763	1.0184852	0.500713854	-0.9979417	0.797428	1
KCTD21-AS1	1.0672587	1.08031092	1.0616011	0.767033335	-0.3826388	0.797458	1
KDM6A	1.1452985	1.1315071	1.1512765	1.150329377	0.202047	0.797486	1
PGS1	1.2368551	1.25093109	1.2307539	0.919590503	-0.1209365	0.797523	1
DBT	1.2976767	1.31209289	1.2914279	0.933785807	-0.0988364	0.797578	1
SYCP2L	1.064557	1.07757577	1.058914	0.75943838	-0.3969952	0.797609	1
FBXO27	1.127743	1.14142564	1.1218122	0.861315912	-0.2153856	0.797625	1
ZNF184	1.1601435	1.17358977	1.1543151	0.888964215	-0.1698027	0.797764	1
SPATS2	2.3907938	2.37037409	2.3996448	1.021359664	0.030491	0.79777	1

TCFL5	1.101241	1.08768109	1.1071186	1.22168452	0.2888718	0.797802	1
CCDC78	1.0439235	1.0568008	1.0383418	0.675022833	-0.5669918	0.797863	1
HAUS2	1.2078547	1.22168827	1.2018585	0.910551251	-0.1351879	0.797878	1
RP6-65G23.3	1.2085177	1.22241466	1.202494	0.910434552	-0.1353728	0.79791	1
RP11-715J22.3	1.0354842	1.04838777	1.0298911	0.617741274	-0.6949254	0.79793	1
ARFGEF3	1.2567608	1.24228038	1.2630374	1.085673751	0.1185906	0.797993	1
GCGR	1.0890136	1.07541302	1.0949089	1.25852165	0.33173	0.798	1
KB-1460A1.5	1.0995113	1.11271966	1.093786	0.83202869	-0.2652948	0.79805	1
CTC-523E23.11	1.0314534	1.04428535	1.0258914	0.584649115	-0.7743571	0.79809	1
CYYR1	1.3974837	1.41234903	1.3910402	0.948323412	-0.0765489	0.798166	1
ADAT1	1.1590728	1.17275307	1.153143	0.886484797	-0.1738322	0.798211	1
C12orf76	2.5121868	2.48971101	2.521929	1.021627009	0.0308686	0.798258	1
RP11-421M1.8	1.0374772	1.02421789	1.0432245	1.784815087	0.8357746	0.798334	1
NR1D2	1.4929111	1.4774462	1.4996145	1.046430934	0.0654771	0.798356	1
ANGPTL1	1.0482683	1.03490059	1.0540626	1.549045321	0.6313794	0.798511	1
LINC00461	1.0484345	1.03493439	1.0542861	1.55394557	0.635936	0.798511	1
ALX3	1.0370443	1.02379012	1.0427894	1.798621099	0.8468913	0.798512	1
FOSL1	1.0476896	1.03433705	1.0534773	1.557424019	0.6391618	0.798638	1
XXbac-BPG154L12.4	1.0423883	1.0552696	1.0368049	0.665915659	-0.5865886	0.798647	1
ADAMTS4	1.0595567	1.07260589	1.0539004	0.742369762	-0.4297901	0.798724	1
AC144450.2	1.0275469	1.04035766	1.021994	0.544976543	-0.875734	0.798818	1
C16orf82	1.0429384	1.05581769	1.0373559	0.669247657	-0.5793879	0.798831	1
PELI1	1.1879281	1.20175638	1.1819341	0.901751372	-0.1491984	0.798834	1
HSPB9	1.0221652	1.03491443	1.016639	0.476565268	-1.0692543	0.798835	1
PRMT3	1.1180098	1.10448052	1.1238742	1.185619852	0.2456415	0.798925	1
THSD4	1.0586591	1.04539641	1.0644079	1.418787819	0.5046588	0.799172	1
POLR1B	1.0533338	1.06623341	1.0477424	0.720821242	-0.4722866	0.799181	1
MIR3911	1.0314707	1.04438836	1.0258715	0.582844384	-0.7788174	0.799251	1
TPRG1L	1.1086921	1.09512739	1.1145718	1.204404118	0.2683195	0.799282	1
LMOD1	1.1342306	1.12065743	1.1401139	1.161254033	0.2156836	0.79937	1
SSH2	1.3404664	1.35486386	1.3342257	0.941842096	-0.0864429	0.799377	1
CTC-444N24.11	1.3745832	1.38923023	1.3682343	0.946057847	-0.0799997	0.799389	1
METTL4	1.2664355	1.25173171	1.2728089	1.083728832	0.1160038	0.799394	1
FAM13B	1.2166113	1.23036345	1.2106504	0.914426413	-0.129061	0.799413	1
SCRN2	1.7664044	1.74964565	1.7736686	1.032045706	0.0455069	0.799534	1
AC240274.1	1.0974753	1.11068798	1.0917482	0.828890206	-0.2707471	0.799561	1
FZD1	1.3023926	1.28783695	1.3087018	1.072488328	0.1009619	0.799601	1
RAB21	1.4603505	1.44521946	1.4669091	1.048716689	0.068625	0.799622	1
TAOK1	2.0856114	2.06711427	2.0936291	1.024847201	0.0354088	0.799688	1
VCAM1	1.0301827	1.04298085	1.0246353	0.573169086	-0.8029673	0.799726	1
RNFT1	1.27378	1.25959118	1.2799303	1.078350514	0.1088262	0.799765	1
TMEM237	1.5431286	1.55811922	1.5366308	0.961498512	-0.0566435	0.799791	1
CTC-479C5.12	1.0402377	1.02705414	1.0459522	1.69852708	0.7642842	0.799801	1
FABP6	1.0400859	1.05287309	1.0345432	0.65332337	-0.6141308	0.799811	1
DCDC2	1.0428618	1.05573791	1.0372805	0.66885378	-0.5802372	0.799929	1
CASC1	1.0561305	1.06902489	1.0505414	0.732220099	-0.4496507	0.799934	1
WNT9A	1.0315607	1.01856629	1.0371932	2.003262583	1.0023515	0.799996	1
ARHGDIG	1.0315737	1.01850257	1.0372394	2.012662297	1.0091051	0.799996	1
ANKRD27	1.1457453	1.13223361	1.1516021	1.146471387	0.1972003	0.800004	1
BTN3A3	1.0577165	1.04432561	1.0635208	1.433049946	0.5190889	0.800062	1
FAM83G	1.0656136	1.05234727	1.0713639	1.363278155	0.44708	0.800091	1

RP11-96L14.8	1.0349841	1.04778262	1.0294365	0.616051012	-0.6988783	0.800127	1
RP11-20I23.6	1.0490049	1.03575611	1.0547476	1.53114006	0.6146063	0.800217	1
FAM174B	1.105346	1.09188643	1.1111801	1.209972793	0.2749746	0.800231	1
TMEM133	1.0569255	1.04373072	1.0626448	1.432511773	0.518547	0.800247	1
HBS1L	1.430658	1.41554618	1.4372083	1.052129161	0.0733118	0.800322	1
RAB42	1.0707965	1.08374848	1.0651823	0.77831058	-0.3615821	0.800328	1
AK8	1.0403155	1.02712976	1.046031	1.696697083	0.762729	0.80037	1
WFIKKN2	1.0403912	1.02716929	1.0461223	1.697590929	0.7634889	0.80037	1
RP11-442H21.2	1.0681163	1.0809874	1.0625373	0.772185105	-0.3729814	0.800407	1
MORN1	1.1015757	1.11471001	1.0958826	0.83586979	-0.2586499	0.80041	1
KIF13B	1.085154	1.07178251	1.0909499	1.267020117	0.3414394	0.800474	1
SLC38A9	1.168008	1.18118122	1.1622979	0.895776801	-0.1587888	0.800474	1
RNU12	1.0317197	1.01860763	1.0374032	2.010098877	1.0072665	0.800521	1
FIZ1	1.1094139	1.12270766	1.1036517	0.844704332	-0.2434816	0.800527	1
XXbac-BPGBPG55C20.3	1.0229704	1.03559606	1.0174978	0.491564487	-1.0245474	0.80059	1
IRS2	1.3482331	1.36255221	1.3420265	0.943385365	-0.0840809	0.800636	1
NUDT2	1.7404841	1.75707628	1.7332922	0.968584288	-0.0460505	0.800681	1
PRMT7	1.3063826	1.2919083	1.3126566	1.071078211	0.0990638	0.800759	1
CNPY2	5.614358	5.5728302	5.6323584	1.013017803	0.0186595	0.800849	1
TMEM268	1.0678391	1.05458261	1.0735852	1.348142865	0.4309734	0.800863	1
RNF185	1.2317379	1.24572039	1.2256771	0.918430592	-0.1227574	0.80087	1
GMEB1	1.2567004	1.27058797	1.2506807	0.926429524	-0.1102469	0.800879	1
NEK11	1.0846089	1.09763679	1.0789618	0.808730367	-0.3062693	0.800985	1
CBARP	1.0635768	1.07665701	1.0579071	0.75540479	-0.4046782	0.801083	1
BAZ2A	1.6281691	1.61184159	1.6352463	1.03825288	0.0541579	0.80109	1
FXN	1.3963114	1.41099022	1.3899487	0.948802909	-0.0758197	0.801107	1
HIST1H4J	1.039478	1.02639276	1.0451498	1.710689508	0.7745779	0.801123	1
SOX15	1.0695721	1.05618909	1.075373	1.341417524	0.4237584	0.801166	1
ENGASE	1.1103229	1.12359602	1.1045696	0.846059291	-0.2411693	0.801202	1
C16orf86	1.1825895	1.16864555	1.1886336	1.118520801	0.1615921	0.801272	1
GIP	1.0672077	1.05398878	1.0729374	1.350974012	0.4339999	0.801295	1
AC073321.4	1.0482851	1.06096154	1.0427905	0.701926025	-0.5106091	0.801337	1
SLC25A25-AS1	1.0317285	1.04435742	1.0262544	0.591883733	-0.7566143	0.801344	1
DUS4L	1.371485	1.38605246	1.3651706	0.945909362	-0.0802261	0.801344	1
LINC00863	1.0794986	1.0925751	1.0738305	0.797519994	-0.3264074	0.801358	1
FLJ27354	1.0316863	1.04437546	1.0261861	0.590103629	-0.7609598	0.80137	1
CCDC7	1.0611147	1.07381831	1.0556082	0.753311543	-0.4086815	0.80143	1
UBASH3B	1.0474326	1.03437936	1.0530906	1.544256963	0.6269128	0.80143	1
GNL3L	1.1379097	1.15123279	1.1321347	0.873717063	-0.1947619	0.801443	1
GGN	1.0388014	1.02570223	1.0444793	1.730561348	0.7912401	0.801538	1
HSCB	1.7272516	1.74310595	1.7203794	0.969416786	-0.044811	0.801607	1
PIAS4	1.2069981	1.19306235	1.2130387	1.10347087	0.1420485	0.80162	1
C10orf35	1.4972784	1.51234852	1.4907462	0.957836584	-0.0621486	0.801644	1
TIMM23B	1.1010554	1.11410451	1.0953992	0.836068558	-0.2583068	0.801656	1
RP11-128A17.1	1.0514361	1.06417081	1.0459161	0.715529801	-0.4829162	0.801731	1
BARX2	1.0351144	1.02183822	1.040869	1.871444533	0.9041523	0.801762	1
GFM2	1.2172055	1.20344228	1.2231712	1.096975742	0.1335316	0.801804	1
ARHGAP5	2.3249717	2.34496793	2.3163043	0.978688237	-0.0310787	0.801901	1
RBMXL1	1.4851827	1.4691536	1.4921306	1.048975431	0.0689809	0.801964	1
SPTLC1	1.128293	1.1415078	1.1225649	0.866135505	-0.2073353	0.801967	1
RP11-356J5.12	1.1454593	1.15852611	1.1397954	0.881844676	-0.1814035	0.802002	1

SPAG8	1.0770091	1.08994794	1.0714008	0.793800934	-0.3331508	0.802075	1
HIST1H2AE	1.0484274	1.03539707	1.0540755	1.527683722	0.6113459	0.802141	1
RP11-211N8.2	1.0412179	1.05389237	1.0357241	0.662879453	-0.5931816	0.802155	1
VASH2	1.1377127	1.12398904	1.1436612	1.158660809	0.2124583	0.802215	1
ZNF844	1.1125012	1.0989638	1.1183691	1.196084473	0.2583193	0.802224	1
PPARD	1.1457939	1.15902125	1.1400604	0.88076507	-0.1831708	0.802245	1
APELA	1.0284214	1.04109484	1.022928	0.557929404	-0.8418455	0.802266	1
DUSP5	1.0519626	1.06463688	1.0464688	0.718921568	-0.4760937	0.802315	1
FAM83B	1.0311152	1.01815945	1.036731	2.022691992	1.0162766	0.802348	1
WWOX	1.3766169	1.39127315	1.3702641	0.946305918	-0.0796214	0.802375	1
SLC45A4	1.0394179	1.05203321	1.0339497	0.652461966	-0.6160343	0.802384	1
ARID3B	1.2031692	1.18911169	1.2092625	1.106555087	0.1460753	0.802425	1
ZNF784	1.1085313	1.12149429	1.1029125	0.847055957	-0.2394708	0.802438	1
RP11-383I23.2	1.0288665	1.04157427	1.0233583	0.561845459	-0.8317547	0.802443	1
GSTO2	1.4087104	1.39360209	1.4152592	1.055022836	0.0772742	0.802464	1
SEMA4G	1.0878204	1.10064253	1.0822626	0.817374122	-0.2909315	0.802484	1
RP11-303E16.5	1.0304693	1.04320204	1.0249503	0.577525401	-0.7920437	0.802502	1
LRTM1	1.042	1.05469776	1.0364961	0.667231679	-0.5837403	0.802582	1
STXBP5	1.1634569	1.14974794	1.1693992	1.131228798	0.1778908	0.802582	1
MANBA	1.3123934	1.29789006	1.31868	1.06979065	0.0973285	0.802762	1
SMPD4	1.2526744	1.23865726	1.2587502	1.084191603	0.1166197	0.802798	1
SREK1IP1	2.6570616	2.63123317	2.6682571	1.022696884	0.0323786	0.802863	1
SCN3B	1.078199	1.06497127	1.0839326	1.291842435	0.3694301	0.802864	1
TIRAP	1.0780921	1.06490883	1.0838065	1.291141401	0.368647	0.802864	1
LRIG3	1.0740725	1.06099924	1.0797392	1.307217052	0.3864987	0.802943	1
AKIP1	1.5825066	1.597615	1.5759578	0.963760537	-0.0532534	0.802954	1
ZNF549	1.0959956	1.10901136	1.0903538	0.828847708	-0.270821	0.802999	1
KCTD18	1.2388268	1.2527459	1.2327935	0.921057502	-0.1186369	0.803014	1
NRF1	1.0768294	1.0635715	1.0825761	1.298948639	0.3773444	0.803022	1
CMYA5	1.0769815	1.06366709	1.0827526	1.299771018	0.3782575	0.803022	1
IFI44L	1.0768622	1.06370418	1.0825656	1.296077643	0.3741521	0.803022	1
CTTNBP2NL	1.479987	1.46492064	1.4865177	1.046453141	0.0655077	0.803062	1
MAP1LC3B2	1.069542	1.05650621	1.0751924	1.330692728	0.4121775	0.803088	1
LRRC8A	1.2314146	1.21754852	1.2374249	1.091365343	0.1261341	0.80313	1
GS1-166A23.2	1.0249641	1.03757628	1.0194973	0.51887212	-0.9465491	0.803152	1
C5orf47	1.0249409	1.03752359	1.0194869	0.519324461	-0.9452919	0.803152	1
SAPCD2	1.1556925	1.16895026	1.1499458	0.887514678	-0.1721571	0.803155	1
REEP1	1.0771146	1.08972376	1.0716492	0.798552776	-0.3245403	0.803208	1
PITPNA-AS1	1.6461713	1.66160383	1.639482	0.966563375	-0.0490638	0.803301	1
FGF5	1.033576	1.02056357	1.0392163	1.907077106	0.9313632	0.803532	1
RP11-305K5.1	1.0300631	1.04255682	1.0246476	0.579169956	-0.7879413	0.803558	1
FAM47E	1.1541054	1.16735133	1.1483639	0.886541407	-0.1737401	0.803591	1
RP1-90G24.11	1.0288155	1.04139992	1.0233608	0.564271318	-0.8255391	0.803604	1
RNPS1	4.0429438	4.07397484	4.0294932	0.985529593	-0.0210289	0.803643	1
SMOC1	1.088244	1.10120677	1.0826252	0.816399849	-0.2926522	0.803647	1
NT5DC3	1.0807421	1.09345258	1.0752326	0.805035016	-0.3128766	0.803665	1
PPP5D1	1.1007243	1.11371457	1.0950936	0.836248192	-0.2579969	0.803669	1
XPA	1.445962	1.46047086	1.439673	0.954833552	-0.0666788	0.803671	1
BCCIP	1.5863139	1.57079044	1.5930427	1.038984977	0.0551748	0.803726	1
EVC	1.0198601	1.03230823	1.0144645	0.447701947	-1.1593895	0.80375	1
IFT88	1.3879025	1.40218976	1.3817096	0.949078347	-0.0754009	0.80378	1

EXOSC5	1.3587214	1.34439926	1.3649294	1.059611463	0.0835354	0.803839	1
CFHR3	1.020576	1.03307594	1.0151579	0.458274469	-1.1257162	0.803858	1
RP11-1299A16.3	1.0205713	1.03297064	1.0151968	0.46091769	-1.117419	0.803858	1
KCNMB2-AS1	1.0433936	1.03049354	1.0489852	1.60641389	0.6838436	0.804013	1
DENND2C	1.0414878	1.05402092	1.0360552	0.667429996	-0.5833116	0.804025	1
RP11-89C3.4	1.041464	1.05417551	1.0359541	0.66365884	-0.5914863	0.804025	1
RP11-46H11.12	1.0465769	1.05912594	1.0411375	0.695760959	-0.5233364	0.804133	1
NFATC4	1.4172861	1.40266557	1.4236234	1.052047852	0.0732003	0.804135	1
RP11-77K12.9	1.0375246	1.05003573	1.0321015	0.641572292	-0.6403163	0.804152	1
RIPK1	1.1032364	1.08986691	1.1090315	1.213254968	0.2788828	0.804155	1
MAP3K10	1.0600218	1.04691169	1.0657044	1.400597659	0.4860426	0.804159	1
APOBEC3G	1.0309328	1.04341734	1.0255212	0.587812272	-0.7665726	0.804209	1
MYH3	1.0403298	1.05279225	1.0349278	0.661608961	-0.5959493	0.804222	1
ZCCHC12	1.1709007	1.15736643	1.1767672	1.123283881	0.1677226	0.804245	1
SLX1A	1.044213	1.03113771	1.0498805	1.601931994	0.6798129	0.804298	1
HAPLN4	1.0506166	1.06315101	1.0451835	0.715483111	-0.4830104	0.804364	1
RP11-872J21.3	1.0947313	1.08148932	1.1004712	1.232936743	0.3020988	0.804376	1
IL13RA2	1.0261948	1.01338003	1.0317495	2.372900478	1.2466516	0.804447	1
SMG1	1.7099635	1.69342801	1.717131	1.034182273	0.0484905	0.804472	1
BAZ1A	1.9195728	1.93669367	1.9121517	0.973799315	-0.0383036	0.804494	1
GDAP1L1	1.0768717	1.06368753	1.0825865	1.296745155	0.374895	0.804664	1
IDNK	1.1473667	1.16037854	1.1417266	0.88370058	-0.1783705	0.804737	1
NTRK1	1.0603101	1.04728346	1.0659566	1.394919141	0.4801815	0.804834	1
POGK	1.286062	1.27203386	1.2921426	1.073919951	0.1028865	0.804931	1
TUBG2	1.2608465	1.24677275	1.2669468	1.081751492	0.1133691	0.804961	1
SPIDR	1.3779689	1.3633891	1.3842887	1.057512875	0.0806752	0.804974	1
KCNH2	1.1721786	1.15878643	1.1779835	1.120898512	0.1646557	0.804998	1
AF003626.1	1.0256012	1.01281342	1.0311441	2.43058852	1.2813057	0.805023	1
MIR4453	1.118543	1.1314895	1.1129312	0.858861211	-0.2195031	0.805033	1
CCNG2	1.6550993	1.63932664	1.6619361	1.03536446	0.0501387	0.805194	1
ABHD17B	1.3182502	1.33275515	1.3119629	0.937515011	-0.0930863	0.805202	1
SUPT5H	1.5975659	1.612884	1.5909261	0.964172901	-0.0526362	0.805273	1
ZSCAN9	1.1379465	1.12459119	1.1437355	1.153656711	0.206214	0.805351	1
BMPR1A	1.3790617	1.39296504	1.3730352	0.949283393	-0.0750893	0.80537	1
HKR1	1.2154266	1.20159472	1.221422	1.09835234	0.1353409	0.805424	1
AC093609.1	1.0285346	1.04100069	1.0231311	0.564163311	-0.8258152	0.805524	1
NCOA5	1.135319	1.14820474	1.1297336	0.875367624	-0.1920391	0.805576	1
CYP2R1	1.2821402	1.29603982	1.2761153	0.93269642	-0.1005205	0.805584	1
TBC1D5	1.5969477	1.58112286	1.6038071	1.039035207	0.0552445	0.805766	1
RP11-196G18.24	1.050651	1.03777553	1.056232	1.488582652	0.5739393	0.805767	1
RP11-452L6.5	1.076024	1.06296382	1.081685	1.297332626	0.3755484	0.805846	1
SEPSECS	1.1214736	1.10831115	1.1271789	1.174199324	0.2316773	0.805905	1
GCC1	1.0720966	1.0590504	1.0777515	1.316697876	0.3969243	0.80592	1
ASXL1	1.3043301	1.31791463	1.2984418	0.938748335	-0.0911897	0.80601	1
CYSRT1	1.0488005	1.06123199	1.043412	0.708975323	-0.4961927	0.806012	1
GPR108	1.5738389	1.55854499	1.5804682	1.039250557	0.0555435	0.806016	1
FUT8-AS1	1.0333762	1.02061195	1.038909	1.887690644	0.9166224	0.806107	1
SCUBE2	1.0188488	1.03116399	1.0135107	0.433537043	-1.2057728	0.806111	1
SKIDA1	1.2661627	1.25208536	1.2722646	1.080049086	0.1110969	0.806132	1
MTMR10	1.0878697	1.10062953	1.0823388	0.818237201	-0.289409	0.806175	1
CTD-2561B21.11	1.0743037	1.08691027	1.0688393	0.792073454	-0.3362939	0.806351	1

KRTCAP3	1.0414544	1.02863087	1.0470129	1.642034746	0.7154847	0.806372	1
KBTBD11-OT1	1.0414531	1.02862692	1.0470126	1.642252213	0.7156757	0.806372	1
MTFMT	1.286252	1.27203933	1.2924126	1.074890919	0.1041903	0.806454	1
CARMIL1	1.1547002	1.14148117	1.16043	1.133932107	0.1813343	0.806471	1
CGA	1.0328502	1.02000327	1.0384188	1.920624406	0.9415754	0.806499	1
CTD-2139B15.2	1.0282924	1.04067848	1.0229236	0.563530689	-0.8274339	0.806559	1
PCDH11Y	1.0303386	1.04271376	1.0249745	0.584695016	-0.7742438	0.806589	1
KIF17	1.0387416	1.02596565	1.0442794	1.705305581	0.7700303	0.80659	1
RNF208	1.2147631	1.22840784	1.2088487	0.914367644	-0.1291537	0.806608	1
RP11-65L3.4	1.0258216	1.03815856	1.0204741	0.536554186	-0.8982042	0.806636	1
GAA	1.277316	1.26325166	1.2834123	1.07658299	0.1064595	0.806639	1
ANKRD13D	1.3173198	1.30314236	1.3234651	1.067040354	0.0936147	0.806685	1
WDR81	1.0683575	1.05557081	1.0738999	1.329833881	0.411246	0.8067	1
LINC00844	1.0460177	1.05831713	1.0406864	0.697675155	-0.5193726	0.806783	1
ZNF764	1.1418422	1.12852866	1.147613	1.148482858	0.1997293	0.806846	1
IKZF5	1.1207226	1.10748171	1.1264619	1.176590162	0.2346119	0.80685	1
C10orf111	1.0497592	1.06236672	1.0442944	0.710225727	-0.4936505	0.806858	1
SDPR	1.0351449	1.02260882	1.0405787	1.794815818	0.8438358	0.806867	1
KIF1B	1.9564646	1.97282186	1.9493744	0.975897482	-0.0351985	0.806882	1
ORMDL1	2.7260388	2.70448122	2.7353831	1.018129773	0.0259215	0.806923	1
CAPN3	1.0783919	1.06540008	1.0840233	1.284758625	0.3614973	0.806964	1
UBXN8	1.2019508	1.21525054	1.1961859	0.911430391	-0.1337956	0.806968	1
WIPI2	2.1636531	2.1463752	2.1711423	1.021604712	0.0308371	0.806976	1
FGGY	1.1315203	1.1182963	1.1372523	1.160241399	0.214425	0.806978	1
SAV1	1.491948	1.50636379	1.4856993	0.959190492	-0.0601107	0.807043	1
DOPEY1	1.1460895	1.158928	1.1405245	0.884202574	-0.1775512	0.807069	1
KIAA0825	1.0542815	1.06670036	1.0488985	0.733106515	-0.4479053	0.80734	1
ACAD11	1.0637513	1.07611597	1.0583918	0.767142501	-0.3824335	0.807453	1
HIST1H2AG	1.037783	1.05009592	1.0324459	0.647676051	-0.6266557	0.807455	1
PAH	1.0223418	1.03460932	1.0170244	0.491901773	-1.0235578	0.807502	1
PROM2	1.0275086	1.01486911	1.0329873	2.218509654	1.1495908	0.807541	1
MARCKS	6.8509324	6.79027934	6.8772228	1.015015425	0.0215017	0.807607	1
CCDC28A	1.2700553	1.25610804	1.2761009	1.078064104	0.108443	0.807608	1
GLS	1.376613	1.36246408	1.382746	1.055955599	0.0785492	0.807619	1
EHBP1L1	1.0778267	1.09036407	1.0723923	0.801118377	-0.3199127	0.807628	1
USH2A	1.0195871	1.03180935	1.0142893	0.449218525	-1.1545107	0.80765	1
CDR1	1.074698	1.08724542	1.0692593	0.793844215	-0.3330722	0.80772	1
RP11-285A1.1	1.0259349	1.03816822	1.0206323	0.540561754	-0.8874687	0.807744	1
UNC5CL	1.0482475	1.06058864	1.0428982	0.708023163	-0.4981315	0.807758	1
GLIDR	1.0795634	1.06671233	1.0851337	1.276131865	0.3517774	0.80778	1
CD96	1.0262293	1.03850429	1.0209087	0.543022447	-0.8809163	0.807974	1
ARHGEF28	1.124166	1.13679371	1.1186924	0.867674264	-0.2047746	0.808066	1
FRAS1	1.235586	1.22206899	1.241445	1.087252397	0.1206869	0.808074	1
RP11-320G10.1	1.0340604	1.02136411	1.0395637	1.851879172	0.88899	0.808138	1
RP11-171I2.1	1.0340187	1.02135881	1.0395062	1.849644586	0.8872481	0.808138	1
ST20-AS1	1.1671492	1.15373757	1.1729625	1.125050198	0.1699894	0.808153	1
ST8SIA1	1.1233063	1.11007085	1.1290433	1.172365436	0.2294223	0.808187	1
DDC	1.017949	1.03008175	1.0126899	0.421848779	-1.2452022	0.808208	1
TTC32	1.2569652	1.27034972	1.2511636	0.929032168	-0.1061995	0.808214	1
ELOVL6	1.4324198	1.41813471	1.4386118	1.048972403	0.0689767	0.808282	1
UBOX5	1.063223	1.05042609	1.0687698	1.363774752	0.4476054	0.808319	1

SH2B2	1.1230846	1.11002908	1.1287436	1.170087505	0.2266164	0.808324	1
LAIR1	1.0344357	1.0468093	1.0290723	0.621079706	-0.6871497	0.808331	1
DDX46	2.7972834	2.81830011	2.7881737	0.983431529	-0.0241035	0.808423	1
RSPH4A	1.0455534	1.05784706	1.0402247	0.695362349	-0.5241631	0.808446	1
ZNF594	1.150402	1.16317102	1.1448672	0.887824159	-0.1716541	0.808478	1
TGFBR3	1.0726729	1.059791	1.0782567	1.308836912	0.3882853	0.808503	1
RP11-284F21.10	1.0348539	1.04710484	1.0295437	0.627190919	-0.6730234	0.80851	1
TRPS1	1.2274222	1.24081423	1.2216174	0.920283661	-0.1198495	0.808529	1
TMEM185A	1.3147033	1.30072866	1.3207607	1.066611787	0.0930352	0.808529	1
DRC1	1.0356036	1.02298878	1.0410716	1.786591347	0.8372097	0.808564	1
DNAJC5	1.3574046	1.34300541	1.3636461	1.060175913	0.0843037	0.808763	1
GPR87	1.0377529	1.02506626	1.0432521	1.725509423	0.7870224	0.808777	1
DUSP2	1.0459204	1.03331934	1.0513824	1.542119283	0.6249144	0.808799	1
KCNK5	1.0296876	1.04190245	1.024393	0.582138861	-0.7805648	0.808818	1
MUS81	1.4298675	1.41544498	1.436119	1.04976354	0.0700644	0.808826	1
CBFA2T2	1.4248	1.43902592	1.4186337	0.953551306	-0.0686175	0.808891	1
ZC3HAV1	1.2086048	1.22165742	1.2029471	0.915589123	-0.1272278	0.809031	1
ZNF169	1.1223059	1.13497878	1.1168128	0.865415809	-0.2085346	0.809163	1
KANSL1L	1.0879981	1.07513389	1.0935742	1.245432353	0.3166467	0.809191	1
ENO4	1.0463784	1.05874488	1.0410181	0.698241425	-0.5182021	0.809204	1
LDOC1L	1.2897992	1.30343546	1.2838885	0.935581012	-0.0960655	0.809262	1
SYT9	1.0739917	1.08636007	1.0686305	0.794701934	-0.3315142	0.809282	1
TMEM216	1.3784027	1.36402921	1.3846329	1.056599019	0.079428	0.809331	1
KLHL11	1.0282671	1.0404945	1.0229671	0.567164759	-0.8181602	0.809442	1
ERG	1.0294206	1.01683833	1.0348745	2.071135932	1.0504222	0.809495	1
RP11-758H9.2	1.220226	1.23327208	1.214571	0.919831639	-0.1205583	0.809514	1
TAS2R4	1.1714776	1.18410731	1.1660031	0.901665187	-0.1493363	0.809579	1
DNMBP	1.1338097	1.12059533	1.1395375	1.157072535	0.2104793	0.809608	1
SCN8A	1.0360583	1.04826459	1.0307674	0.637474238	-0.6495611	0.809663	1
RP11-307B6.3	1.036116	1.04836458	1.0308067	0.636968811	-0.6507054	0.809663	1
PKP4	1.6189191	1.60343464	1.625631	1.036783335	0.0521144	0.809742	1
ZFP91	1.3017471	1.3154446	1.2958099	0.937755329	-0.0927165	0.809896	1
TADA2A	1.1232498	1.13580527	1.1178075	0.867473723	-0.205108	0.809949	1
PEX11A	1.2558815	1.24205605	1.2618742	1.081874302	0.1135329	0.810053	1
RP11-167P20.1	1.0187223	1.03085103	1.0134651	0.4364556	-1.1960932	0.810109	1
RELL1	1.0631239	1.07546477	1.0577747	0.765585343	-0.3853649	0.810127	1
DNAI1	1.0437169	1.05584201	1.0384612	0.688750891	-0.5379458	0.810189	1
DICER1	2.0537942	2.03644449	2.0613146	1.023995585	0.0342095	0.810275	1
MAMLD1	1.0450559	1.05728105	1.0397568	0.694066049	-0.5268551	0.810298	1
RHEBL1	1.0362261	1.02370427	1.0416538	1.75722777	0.8133012	0.810368	1
THSD7A	1.2310145	1.21748801	1.2368777	1.089152702	0.1232062	0.810379	1
TTC19	2.3643006	2.34182583	2.3740424	1.024009524	0.0342291	0.810556	1
MFAP3	1.0716434	1.08392898	1.0663181	0.790169746	-0.3397655	0.810642	1
GPR146	1.1350064	1.12205641	1.1406197	1.152087472	0.2042503	0.810668	1
EXD2	1.1745685	1.16149673	1.1802346	1.11602609	0.1583708	0.810725	1
PLA2R1	1.1154263	1.10245687	1.1210479	1.181452522	0.2405617	0.810757	1
LINC00926	1.0612607	1.04867416	1.0667163	1.370672741	0.4548842	0.810902	1
TBC1D32	1.2030544	1.18958072	1.2088946	1.101876813	0.1399629	0.810951	1
TLK1	1.9508896	1.93405699	1.9581858	1.025832322	0.0367949	0.810991	1
FBXL6	1.0636709	1.05111991	1.0691112	1.351942916	0.4350342	0.810995	1
RP11-215P8.3	1.0342336	1.04631547	1.0289966	0.6260668	-0.6756115	0.811051	1

THEM6	1.2286442	1.24176879	1.2229553	0.922183861	-0.1168737	0.811059	1
KLHL32	1.0471597	1.0593281	1.0418852	0.705992998	-0.5022742	0.811155	1
EPB41L4A	1.1178489	1.10499982	1.1234184	1.175415323	0.2331706	0.811203	1
RP11-190A12.8	1.0252425	1.03731518	1.0200096	0.536231937	-0.8990709	0.811227	1
MYO9A	1.3495605	1.33517436	1.3557962	1.061525778	0.0861394	0.811281	1
NDUFAF7	1.2627458	1.24908315	1.268668	1.078627679	0.109197	0.811378	1
SCNM1	1.8299664	1.84586203	1.8230763	0.973062145	-0.0393961	0.811491	1
ZNF280D	1.1313466	1.11843154	1.1369446	1.156319039	0.2095395	0.811533	1
NRK	1.0622563	1.04958568	1.0677485	1.366290839	0.4502646	0.81162	1
STON1-GTF2A1L	1.0345344	1.04672096	1.029252	0.626100472	-0.6755339	0.811622	1
SLN	1.0559777	1.04343662	1.0614137	1.413868859	0.4996483	0.8117	1
HSD17B1	1.1158583	1.10296578	1.1214466	1.179485365	0.2381575	0.811728	1
ICE1	1.3652965	1.37903849	1.35934	0.948030435	-0.0769947	0.811762	1
CLN3	1.0611079	1.07327081	1.0558359	0.762048151	-0.3920459	0.811773	1
SLC4A4	1.2084364	1.22122152	1.2028947	0.917156188	-0.1247607	0.811794	1
ZC3H12A	1.0449087	1.03247947	1.0502962	1.548554505	0.6309222	0.811914	1
RHBDD1	1.1165904	1.12916444	1.1111402	0.860454842	-0.2168286	0.812033	1
SEMA3F-AS1	1.0233774	1.03539691	1.0181674	0.513248924	-0.9622694	0.812131	1
PPP1R9B	1.2054631	1.19214757	1.2112347	1.099336045	0.1366325	0.812139	1
RP11-736N17.10	1.0653338	1.07760022	1.0600168	0.773410752	-0.3706933	0.812152	1
SCML2	1.0652671	1.07756032	1.0599385	0.77279893	-0.371835	0.812152	1
DCUN1D2	1.1030418	1.11552958	1.0976289	0.845055644	-0.2428818	0.812198	1
LINC00441	1.0248824	1.03687174	1.0196855	0.533890997	-0.9053829	0.81225	1
PLEKHA6	1.1098107	1.09688043	1.1154154	1.191318009	0.2525586	0.812345	1
KCNJ2	1.0300782	1.01771135	1.0354387	2.000904748	1.0006525	0.812378	1
KRT23	1.030049	1.01772858	1.0353893	1.996171707	0.9972358	0.812378	1
TRAK1	1.298892	1.28523146	1.3048132	1.06865222	0.0957924	0.812417	1
TMEM254-AS1	1.0807468	1.0929568	1.0754543	0.811713957	-0.3009567	0.812453	1
UNKL	1.2084237	1.22136759	1.2028131	0.916182521	-0.1262931	0.812621	1
UCK2	1.2152362	1.20215152	1.2209079	1.092783579	0.1280077	0.812653	1
AC008746.12	1.0363966	1.04835842	1.0312116	0.645422733	-0.6316837	0.812692	1
NUDT10	1.153264	1.14022059	1.1589177	1.13334061	0.1805815	0.812742	1
KCNK6	1.0661342	1.05374628	1.0715038	1.330394947	0.4118546	0.812793	1
C15orf48	1.0377538	1.02550378	1.0430636	1.688520011	0.7557593	0.812807	1
RP11-1260E13.2	1.0223867	1.03432141	1.0172136	0.501540936	-0.9955606	0.812838	1
RP11-399K21.13	1.0534967	1.04102075	1.0589045	1.435969238	0.5220248	0.81289	1
RP11-559M23.1	1.025809	1.0377875	1.0206169	0.54560116	-0.8740814	0.812901	1
CS	1.6876661	1.70243657	1.6812638	0.969858039	-0.0441545	0.812933	1
GCC2-AS1	1.0271116	1.03905375	1.0219352	0.561666998	-0.8322131	0.813189	1
RP11-384K6.8	1.0271251	1.03908327	1.0219418	0.561411339	-0.8328699	0.813189	1
METTL8	1.3118696	1.32521657	1.3060842	0.941170409	-0.0874721	0.813195	1
HCG17	1.0468436	1.03442088	1.0522283	1.517343199	0.6015474	0.813222	1
HIPK1-AS1	1.0248635	1.03680183	1.0196888	0.534995299	-0.9024019	0.813371	1
RP11-977G19.12	1.0805115	1.09276096	1.0752019	0.81070588	-0.3027495	0.813409	1
NBPF11	1.1083394	1.09568471	1.1138247	1.189580996	0.2504535	0.813452	1
CCSER1	1.0666471	1.05404684	1.0721088	1.334191101	0.4159653	0.813463	1
RP11-121C2.2	1.0608838	1.07299938	1.0556322	0.762091208	-0.3919644	0.8135	1
PRSS22	1.0296421	1.01724954	1.0350137	2.029834912	1.0213624	0.81358	1
MED30	1.5386541	1.52422175	1.5449099	1.039464505	0.0558405	0.813596	1
GSTT2B	1.0992035	1.08669433	1.1046257	1.20683483	0.2712282	0.813672	1
C10orf126	1.02304	1.03499024	1.0178602	0.510432848	-0.9702069	0.81368	1

TMEM220-AS1	1.0230239	1.03492432	1.0178655	0.511550104	-0.9670525	0.81368	1
ZNF142	1.0767424	1.0888169	1.0715086	0.805124331	-0.3127165	0.813721	1
RP11-35G9.3	1.0768417	1.0889992	1.071572	0.804187082	-0.3143969	0.813721	1
SAMD8	1.246471	1.23328554	1.2521863	1.081019855	0.112393	0.813773	1
CLDN11	1.166051	1.17887246	1.1604934	0.897250556	-0.1564172	0.813794	1
AC016907.3	1.0256431	1.03749946	1.0205039	0.54677974	-0.8709683	0.813796	1
OLFM3	1.1807883	1.16780992	1.1864139	1.110863427	0.1516815	0.81381	1
CWC25	1.3990194	1.41264953	1.3931113	0.952651812	-0.0699791	0.813856	1
MIR193BHG	1.0492402	1.03684769	1.0546118	1.482095367	0.5676383	0.813903	1
RP11-126K1.2	1.0491851	1.03687113	1.0545227	1.478735744	0.5643643	0.813903	1
DPH5	1.7457277	1.73044043	1.7523541	1.030000613	0.0426452	0.813913	1
ANKRD35	1.1691871	1.15593898	1.1749295	1.121781853	0.1657921	0.81402	1
TKFC	1.2407734	1.2276092	1.2464795	1.082906502	0.1149087	0.814049	1
FAM19A4	1.0322923	1.04413964	1.0271571	0.615253059	-0.7007482	0.81405	1
LINC00174	1.032248	1.04422762	1.0270553	0.611729114	-0.7090352	0.81405	1
TULP3	1.2962231	1.28252271	1.3021616	1.069512484	0.0969533	0.814093	1
GPR63	1.1092347	1.12160603	1.1038723	0.854170951	-0.2274033	0.814215	1
SUMF1	1.2049428	1.19189699	1.2105976	1.097451039	0.1341566	0.814221	1
TTC30B	1.0990467	1.08635578	1.1045477	1.210662036	0.2757962	0.81426	1
BMP6	1.031955	1.01968943	1.0372715	1.892971958	0.920653	0.814337	1
RP11-649A18.12	1.0240068	1.03587245	1.0188636	0.525853173	-0.9272681	0.814357	1
ZNF345	1.1520326	1.13920255	1.1575939	1.132119498	0.1790262	0.814391	1
TRAF1	1.0449644	1.05697475	1.0397584	0.697824757	-0.5190633	0.814424	1
RP11-314N13.3	1.0310553	1.04298267	1.0258853	0.602227359	-0.7316198	0.814528	1
SLC29A1	1.2574978	1.27062742	1.2518067	0.930455403	-0.1039911	0.814555	1
PSD3	1.5530961	1.5380875	1.5596017	1.039982663	0.0565595	0.814617	1
LINC01588	1.0436482	1.05563228	1.0384537	0.6912118	-0.5328002	0.814622	1
DNAH6	1.0425501	1.05449157	1.037374	0.685868082	-0.543997	0.814644	1
DIAPH1	1.3486475	1.36205542	1.3428357	0.946915084	-0.078693	0.814645	1
ALG11	1.0826464	1.09489351	1.0773378	0.814996134	-0.2951349	0.814658	1
THAP9	1.0921237	1.10401168	1.0869709	0.836164349	-0.2581416	0.814716	1
PIGN	1.1609836	1.17353506	1.1555431	0.89632114	-0.1579124	0.814767	1
WDR92	1.1944427	1.18130708	1.2001365	1.103853504	0.1425487	0.814899	1
PTOV1-AS1	1.0691793	1.08116169	1.0639855	0.788371007	-0.3430534	0.81495	1
DLGAP1	1.1661683	1.17855878	1.1607976	0.900530284	-0.1511533	0.814977	1
CST6	1.0307774	1.01858676	1.0360615	1.940172709	0.9561851	0.81522	1
KCTD21	1.0308028	1.01860021	1.0360921	1.940413443	0.9563641	0.81522	1
SNIP1	1.0834817	1.07104062	1.0888743	1.251035156	0.3231223	0.815238	1
FKBP1C	1.0238174	1.03556199	1.0187266	0.526590824	-0.9252457	0.815428	1
DDX58	1.0825412	1.09465873	1.0772888	0.816499576	-0.292476	0.815578	1
RP4-666F24.3	1.0210393	1.03275716	1.0159601	0.487224533	-1.0373413	0.815605	1
HBZ	1.0210552	1.03280984	1.0159601	0.486442245	-1.0396596	0.815605	1
RP11-464F9.22	1.0226677	1.03450868	1.0175352	0.508138342	-0.9767068	0.815615	1
FAR1	1.3757314	1.36188201	1.3817345	1.054858907	0.07705	0.815713	1
LINC01456	1.0881386	1.10024731	1.08289	0.82685487	-0.274294	0.815756	1
MTSS1	1.1587904	1.17105848	1.1534727	0.897194478	-0.1565074	0.815801	1
IP6K3	1.0497581	1.03754223	1.0550532	1.466433051	0.5523112	0.815812	1
PNKD	2.853542	2.83153528	2.863081	1.017223635	0.0246369	0.815865	1
RALYL	1.344665	1.33041314	1.3508425	1.061829841	0.0865526	0.815868	1
SLC25A35	1.128888	1.14113881	1.1235778	0.875575944	-0.1916958	0.815887	1
FAS	1.0919233	1.07956139	1.0972817	1.2227248	0.2900997	0.81614	1

KCNQ4	1.037992	1.02572772	1.0433081	1.683322744	0.7513118	0.816195	1
TEPP	1.0496004	1.06163483	1.044384	0.720112918	-0.4737049	0.816201	1
RBPMS-AS1	1.0247393	1.03654115	1.0196237	0.537029814	-0.8969259	0.816267	1
CMTR1	1.2167079	1.20355498	1.2224091	1.092624093	0.1277971	0.816312	1
SESN2	1.2187059	1.20571419	1.2243372	1.09052868	0.1250277	0.816317	1
IMPAD1	1.4191868	1.43314547	1.4131364	0.95380517	-0.0682335	0.816344	1
KIRREL2	1.0929724	1.10525392	1.0876489	0.832737886	-0.2640656	0.816381	1
RP1-102K2.8	1.0648876	1.05264345	1.0701949	1.333401389	0.4151111	0.816404	1
IPO13	1.1193436	1.10677009	1.1247936	1.168806777	0.2250364	0.816697	1
FMO1	1.031817	1.01963698	1.0370965	1.889116557	0.9177117	0.816738	1
GRHL1	1.0318075	1.01963624	1.0370832	1.888509131	0.9172478	0.816738	1
RP11-166P13.4	1.0605232	1.07250044	1.0553317	0.763190845	-0.3898842	0.816754	1
ZNF789	1.1451758	1.15765457	1.1397668	0.88653795	-0.1737457	0.8168	1
DNM1L	1.6839889	1.66912404	1.6904322	1.031844792	0.045226	0.817002	1
AC005592.1	1.0292324	1.04101996	1.024123	0.588079005	-0.7659181	0.817097	1
PLCB2	1.0402784	1.05205286	1.0351747	0.675748944	-0.5654407	0.81712	1
ZBTB46	1.0576307	1.04541254	1.0629267	1.385668115	0.4705818	0.817129	1
NAT1	1.0576728	1.04543158	1.0629788	1.386233374	0.4711702	0.817129	1
NUP188	1.1473208	1.15969518	1.1419571	0.888925275	-0.1698659	0.817135	1
JAM2	1.4472045	1.43305924	1.4533359	1.046821893	0.066016	0.817209	1
SBNO2	1.0856251	1.07332152	1.0909582	1.240538567	0.3109666	0.817228	1
RP11-103J8.1	1.0198238	1.03153158	1.014749	0.467753501	-1.0961796	0.817242	1
BAHCC1	1.1368991	1.12430693	1.1423572	1.145207304	0.1956088	0.817258	1
TECTA	1.0232198	1.03493302	1.0181426	0.519354857	-0.9452075	0.817272	1
DKFZp779M0652	1.0231814	1.03485208	1.0181227	0.519988469	-0.9434485	0.817272	1
RLN1	1.0429831	1.05466474	1.0379196	0.693676489	-0.5276651	0.817304	1
TSPYL5	1.0495711	1.06135652	1.0444627	0.72466144	-0.464621	0.817378	1
GBGT1	1.1284628	1.11595539	1.1338842	1.154618229	0.2074159	0.817432	1
KIAA0895L	1.3416329	1.35501368	1.3358329	0.945971643	-0.0801312	0.817469	1
HERC3	1.088905	1.10084635	1.0837289	0.830262123	-0.2683612	0.817475	1
PLCH1	1.2748977	1.28785763	1.2692801	0.93546274	-0.0962479	0.817627	1
ARHGEF19	1.1105757	1.1226694	1.1053337	0.858679344	-0.2198086	0.81763	1
SLC25A43	1.1352838	1.14761919	1.129937	0.880217221	-0.1840685	0.817709	1
AC008079.10	1.0588945	1.04648708	1.0642726	1.382589779	0.4673732	0.817777	1
RRP15	1.5331276	1.51858193	1.5394326	1.040207001	0.0568707	0.817814	1
AP4S1	1.0894668	1.10147583	1.0842614	0.830359119	-0.2681927	0.817847	1
NCOA6	1.2826677	1.26914982	1.288527	1.071994181	0.1002971	0.817852	1
ZNF25	1.2004664	1.18751407	1.2060807	1.099014367	0.1362102	0.817967	1
MESP2	1.0257886	1.03745552	1.0207315	0.553495735	-0.8533559	0.818019	1
LINC00276	1.0258227	1.03751433	1.020755	0.553254476	-0.8539849	0.818019	1
AC079354.3	1.0204289	1.03205455	1.0153897	0.480109886	-1.0585635	0.818068	1
DNAH9	1.0763184	1.06413768	1.0815982	1.272235595	0.3473659	0.818103	1
CLDN10	1.0815933	1.0934742	1.0764434	0.817802419	-0.2901758	0.818118	1
TNNT2	1.0300996	1.01804205	1.035326	1.957983481	0.9693686	0.818181	1
ITIH3	1.0300891	1.01804205	1.035311	1.957150187	0.9687545	0.818181	1
TCTA	1.3130653	1.29964116	1.318884	1.064219738	0.0897961	0.818246	1
TNFRSF10D	1.183607	1.19609807	1.1781927	0.908691763	-0.1381371	0.818266	1
RP11-147L13.14	1.030881	1.04253758	1.0258284	0.607189509	-0.7197812	0.818363	1
RP1-257A7.4	1.0309223	1.04262885	1.025848	0.606349175	-0.7217793	0.818363	1
FAM86C1	1.1001174	1.08758566	1.1055494	1.205099062	0.2691517	0.818434	1
HS3ST4	1.0296487	1.04132175	1.0245889	0.595059329	-0.7488946	0.818442	1

RBSN	1.2811479	1.29369832	1.2757079	0.93874519	-0.0911945	0.818579	1
OMA1	1.1444056	1.13169127	1.1499167	1.138395306	0.1870016	0.818677	1
RP11-989E6.10	1.0214222	1.03298437	1.0164106	0.497525056	-1.0071589	0.818728	1
JUND	2.0842793	2.06651118	2.091981	1.023881463	0.0340487	0.818729	1
C9orf135-AS1	1.0423414	1.05397063	1.0373007	0.691129689	-0.5329716	0.818769	1
TNFAIP8L3	1.0284438	1.04006	1.0234087	0.584341145	-0.7751172	0.81892	1
CCDC154	1.0284213	1.04001655	1.0233953	0.584640223	-0.774379	0.81892	1
ZFAT	1.0618176	1.07359628	1.0567121	0.770584197	-0.3759755	0.818958	1
PRR12	1.1464523	1.15856713	1.1412011	0.890481431	-0.1673426	0.81897	1
RACK1	58.992068	58.7101203	59.11428	1.007003278	0.0100684	0.819025	1
UBQLN4	1.2347919	1.24746797	1.2292974	0.926574008	-0.1100219	0.819123	1
PCNX3	1.0345484	1.02253893	1.039754	1.763793451	0.8186816	0.819173	1
ZBTB39	1.1455824	1.13302659	1.1510248	1.135297828	0.1830708	0.81918	1
ESRP2	1.0252967	1.01338003	1.0304621	2.276683739	1.1869339	0.819193	1
CTC1	1.0691398	1.08098677	1.0640047	0.790310281	-0.3395089	0.819194	1
RP11-629G13.1	1.095511	1.08318679	1.100853	1.21236829	0.277828	0.819228	1
POLR3D	1.4659421	1.45194857	1.4720076	1.044383511	0.0626516	0.819502	1
MAP2K5	1.2805707	1.2676109	1.2861882	1.069418988	0.0968272	0.819527	1
PKP2	1.1803504	1.16768875	1.1858386	1.108235493	0.1482645	0.819613	1
CTC-548K16.2	1.0224852	1.03405686	1.0174694	0.512949138	-0.9631123	0.819702	1
NUPR1	1.0309275	1.01886772	1.0361549	1.9162311	0.9382716	0.819704	1
CLDN2	1.0345092	1.04607648	1.0294953	0.640137494	-0.6435463	0.819767	1
ZER1	1.1196283	1.10720146	1.1250148	1.166166545	0.2217738	0.81978	1
FLJ20021	1.2311389	1.24367036	1.2257071	0.926280466	-0.110479	0.81979	1
AF131216.1	1.0212601	1.03278348	1.0162652	0.496139583	-1.011182	0.819799	1
RP11-734K2.4	1.0902741	1.10219523	1.0851068	0.832786154	-0.263982	0.81982	1
FOXD2-AS1	1.0316641	1.04325869	1.0266383	0.615791235	-0.6994868	0.819917	1
ZNF667	1.0811108	1.06887053	1.0864163	1.254765169	0.3274174	0.819928	1
FGD5-AS1	1.9790571	1.96248312	1.9862411	1.024684062	0.0351792	0.819972	1
TRUB1	1.2472643	1.23417926	1.2529361	1.080095882	0.1111594	0.819974	1
RP11-327J17.9	1.0334901	1.02145228	1.038708	1.804377447	0.8515012	0.820059	1
FAM149B1	1.1317194	1.11924914	1.1371247	1.149901379	0.2015101	0.820129	1
TCEA2	2.4843304	2.50355527	2.4759973	0.981671462	-0.0266878	0.820153	1
GABBR1	1.1597991	1.17219647	1.1544254	0.896797586	-0.1571457	0.820225	1
CSF1R	1.0327706	1.02086466	1.0379313	1.817967223	0.8623262	0.820243	1
ACSL1	1.0750661	1.0630259	1.080285	1.273841918	0.3491863	0.820268	1
KCP	1.0491811	1.06082866	1.0441324	0.725519361	-0.462914	0.820308	1
JARID2	1.4365013	1.42292378	1.4423865	1.046019555	0.0649098	0.820324	1
MISP3	1.164553	1.17652008	1.1593658	0.902819726	-0.1474902	0.820329	1
OSBPL10	1.049753	1.03780594	1.0549316	1.452987939	0.5390227	0.820351	1
RASA4B	1.047115	1.05863149	1.0421231	0.718437313	-0.4770658	0.820566	1
RP11-451G4.2	1.0335701	1.02157086	1.0387712	1.797389366	0.845903	0.820571	1
ATP13A1	1.2138333	1.22618068	1.2084813	0.921746843	-0.1175575	0.820675	1
ZUFSP	1.1884716	1.20092497	1.1830736	0.911153996	-0.1342332	0.820877	1
EBLN2	1.1278631	1.13989654	1.1226471	0.876698803	-0.1898468	0.82088	1
CBFA2T3	1.0677588	1.0556836	1.0729929	1.310851262	0.390504	0.820948	1
THUMPD1	1.6016396	1.58754796	1.6077477	1.034379787	0.048766	0.820951	1
HSF2BP	1.076228	1.06402512	1.0815173	1.273208875	0.3484691	0.820993	1
SMARCD2	1.28189	1.29478299	1.2763015	0.937304851	-0.0934097	0.82106	1
RP11-1348G14.4	1.0452589	1.03334877	1.0504214	1.511940855	0.5964017	0.821079	1
SLC25A17	1.2265687	1.2138287	1.232091	1.085406129	0.118235	0.82116	1

IDH1-AS1	1.0675424	1.05523187	1.0728785	1.319500275	0.3999917	0.821184	1
RP11-771K4.1	1.0395556	1.05121106	1.0345035	0.673750433	-0.5697138	0.82122	1
HECW1	1.0318965	1.02000151	1.0370525	1.85248399	0.8894611	0.821222	1
PPP1R3D	1.1219237	1.10953147	1.1272952	1.16217952	0.2168329	0.821261	1
PLEKHH1	1.2095096	1.22172834	1.2042134	0.92100701	-0.118716	0.821276	1
DNER	1.4985431	1.51224286	1.4926049	0.96166285	-0.0563969	0.821278	1
ZNF529-AS1	1.1787497	1.1662313	1.1841758	1.107949257	0.1478918	0.821407	1
SOCS5	1.1790071	1.1665761	1.1843953	1.106973481	0.1466207	0.821407	1
RP1-317E23.3	1.037697	1.04930356	1.0326661	0.662550489	-0.5938977	0.821482	1
PMFBP1	1.0298711	1.04134408	1.0248981	0.602216437	-0.731646	0.821557	1
C21orf33	1.0344339	1.02252057	1.0395979	1.758296699	0.8141785	0.821562	1
LONP1	1.3711678	1.38418539	1.3655252	0.951429208	-0.0718318	0.821613	1
KB-1208A12.3	1.0361526	1.04745508	1.0312534	0.658590105	-0.6025473	0.821613	1
MOCS1	1.0953313	1.08317919	1.1005987	1.209421715	0.2743174	0.821714	1
IFIT2	1.0414389	1.02954545	1.0465942	1.577033708	0.6572135	0.821748	1
TMEM200B	1.0414668	1.02962037	1.0466017	1.573297812	0.6537918	0.821748	1
STARD9	1.2119553	1.19906351	1.2175433	1.092833864	0.1280741	0.821812	1
SMIM22	1.0258228	1.01394208	1.0309726	2.221517635	1.1515456	0.821829	1
FCRLB	1.0603669	1.04826933	1.0656106	1.359261213	0.4428227	0.821894	1
APBA1	1.057798	1.06947367	1.0527371	0.75909528	-0.3976471	0.821929	1
RP5-940J5.8	1.021958	1.03343366	1.0169839	0.507987492	-0.9771351	0.821981	1
FAM216B	1.0219091	1.0332717	1.0169839	0.51046033	-0.9701292	0.821981	1
RP5-894A10.2	1.0680279	1.0798107	1.0629205	0.788372134	-0.3430513	0.822007	1
GLTPD2	1.0197148	1.03106021	1.014797	0.476397306	-1.0697628	0.82213	1
STX17-AS1	1.0597257	1.07140736	1.0546622	0.765497851	-0.3855298	0.822159	1
FAM157C	1.0596894	1.07125477	1.0546763	0.767334926	-0.3820717	0.822159	1
C3orf58	1.3180295	1.30487444	1.3237316	1.06185225	0.086583	0.822195	1
ZNF517	1.0386278	1.05012792	1.033643	0.671142604	-0.5753088	0.822241	1
EGF	1.075509	1.08721744	1.070434	0.807567488	-0.3083453	0.822253	1
NFKB2	1.1428981	1.15489445	1.1376982	0.888981069	-0.1697754	0.822342	1
B4GALNT1	1.0606508	1.04867555	1.0658415	1.352661369	0.4358007	0.822616	1
KHDC1L	1.0268084	1.0382188	1.0218625	0.572036014	-0.8058221	0.822618	1
AC010761.8	1.0343817	1.02256458	1.0395039	1.750705647	0.8079365	0.822688	1
ZBTB21	1.1136565	1.10135066	1.1189905	1.17404722	0.2314904	0.82273	1
GUCY1B3	1.2346626	1.22201155	1.2401463	1.081683836	0.1132789	0.822826	1
RP11-11N5.3	1.1154532	1.12717705	1.1103714	0.867855965	-0.2044725	0.822834	1
C11orf84	1.1322136	1.14411468	1.127055	0.8816242	-0.1817643	0.8229	1
SSPO	1.0860922	1.09748907	1.0811521	0.832422617	-0.2646119	0.822907	1
FAM134C	1.3189694	1.33181807	1.3134	0.944493511	-0.0823872	0.822949	1
SPHK1	2.9851124	2.96223872	2.9950271	1.016709662	0.0239078	0.822991	1
TDRD9	1.0181931	1.02962552	1.0132377	0.446833536	-1.1621906	0.823041	1
RP11-1E4.1	1.0327953	1.02089966	1.0379516	1.815893508	0.8606796	0.823047	1
ZNF91	1.5112644	1.52517084	1.5052365	0.962042214	-0.0558279	0.823057	1
NPAP1	1.0188234	1.03013341	1.0139211	0.461981811	-1.114092	0.823116	1
SKIV2L	1.1663752	1.15418542	1.171659	1.113328113	0.1548788	0.823194	1
SLC9B2	1.2469056	1.25912253	1.2416102	0.932416701	-0.1009532	0.823203	1
NKAIN2	1.0517598	1.06331255	1.0467523	0.738435758	-0.4374557	0.823215	1
CTD-2311M21.3	1.0446848	1.05603818	1.0397636	0.709579954	-0.4949628	0.823229	1
C2orf88	1.0347456	1.02295519	1.0398562	1.736258878	0.7959821	0.823281	1
LRRC20	1.2668505	1.27945152	1.2613885	0.935362513	-0.0964025	0.823331	1
PTCD3	1.5191243	1.50504913	1.5252253	1.039948899	0.0565126	0.823417	1

KCNA4	1.1184846	1.10624276	1.123791	1.165170713	0.2205413	0.823471	1
SS18L1	1.2389433	1.22617525	1.2444777	1.080921521	0.1122618	0.823562	1
GIT1	1.264837	1.25180585	1.2704855	1.074182605	0.1032393	0.823618	1
HYLS1	1.0877019	1.07572183	1.0928948	1.226790188	0.2948885	0.823619	1
LINC01504	1.0365785	1.024759	1.0417017	1.684305305	0.7521537	0.823725	1
RPRML	1.028111	1.01645797	1.033162	2.014953111	1.0107463	0.823764	1
BEND6	1.0542676	1.06565476	1.0493318	0.751381763	-0.412382	0.823856	1
ATP6V1G2	1.2655385	1.27775444	1.2602435	0.936955157	-0.0939481	0.823862	1
RP5-1157M23.2	1.02835	1.03969559	1.0234322	0.59029757	-0.7604857	0.823864	1
PDP2	1.095427	1.08346767	1.1006108	1.205386712	0.2694961	0.82387	1
MOB1A	2.0093846	2.02428207	2.0029272	0.979151367	-0.0303962	0.823913	1
MSANTD4	1.9088524	1.89328807	1.9155989	1.024976037	0.0355902	0.823923	1
EXD3	1.3890497	1.3756912	1.39484	1.050969565	0.0717209	0.823956	1
MAP3K3	1.0950143	1.10667323	1.0899607	0.843330014	-0.2458308	0.823959	1
BTBD11	1.0875228	1.09923275	1.0824471	0.830845929	-0.2673471	0.824073	1
TFAP2E	1.0199198	1.03127365	1.0149985	0.479587652	-1.0601336	0.824106	1
AC007880.1	1.0198791	1.03113906	1.0149985	0.48166049	-1.0539115	0.824106	1
RGAG4	1.1393535	1.12710886	1.1446611	1.138087899	0.186612	0.824244	1
CXCL8	1.0285369	1.01683833	1.0336077	1.995905458	0.9970434	0.824261	1
OSMR-AS1	1.0286008	1.01683833	1.0336993	2.001343219	1.0009686	0.824261	1
PATZ1	1.4188691	1.43141319	1.4134319	0.958319927	-0.0614207	0.824297	1
CRYBB1	1.0358668	1.02429886	1.040881	1.682426211	0.7505432	0.824307	1
SCARB1	1.2932206	1.30584515	1.2877484	0.940830371	-0.0879935	0.824325	1
SGF29	1.2275855	1.21488731	1.2330896	1.084706108	0.1173042	0.824383	1
VPS50	1.1033017	1.11516707	1.0981585	0.852314198	-0.2305427	0.824403	1
KB-1183D5.13	1.024762	1.03601971	1.0198823	0.55198463	-0.8573	0.824405	1
TFB2M	1.1360882	1.14825563	1.1308141	0.882355215	-0.1805685	0.824436	1
RIN1	1.1213553	1.13320719	1.116218	0.87246069	-0.196838	0.824611	1
SYCE3	1.0447797	1.03285266	1.0499495	1.520409874	0.6044603	0.824637	1
IDUA	1.0836623	1.07175302	1.0888244	1.237918808	0.3079167	0.824644	1
BRMS1L	1.218859	1.20641917	1.2242512	1.086387293	0.1195385	0.824663	1
RP11-111F5.3	1.0466031	1.05814222	1.0416014	0.715510375	-0.4829554	0.82469	1
UBR2	1.3055989	1.31825797	1.3001117	0.942982564	-0.084697	0.824723	1
TM6SF2	1.0441482	1.03235132	1.0492616	1.522709101	0.6066404	0.824822	1
SYNPO2	1.0534112	1.06467012	1.0485309	0.750438121	-0.414195	0.82483	1
LMTK2	1.0549808	1.06642449	1.0500205	0.753042548	-0.4091967	0.824832	1
EXOC6B	1.1606911	1.14840825	1.1660152	1.118638665	0.1617441	0.824876	1
PAK1	1.5668619	1.55322783	1.5727716	1.035326813	0.0500862	0.824921	1
MAPT	1.1340621	1.12189813	1.1393347	1.143042197	0.1928787	0.824992	1
RP11-109A6.5	1.0195576	1.03078186	1.0146924	0.477306045	-1.0670135	0.825026	1
SYT12	1.0195721	1.03075993	1.0147227	0.478632747	-1.063009	0.825026	1
SEC16B	1.0574932	1.06892318	1.0525389	0.762281369	-0.3916045	0.825066	1
MT3	1.0378656	1.04913486	1.0329809	0.671231679	-0.5751173	0.825108	1
ARHGEF40	1.4650055	1.47838388	1.4592066	0.95991244	-0.0590253	0.825184	1
SF3B3	1.6502292	1.63591548	1.6564335	1.032265399	0.0458139	0.825191	1
URB1	1.0959224	1.08398066	1.1010986	1.203832437	0.2676346	0.825322	1
COL17A1	1.0267461	1.01516282	1.0317669	2.09505439	1.0669877	0.825329	1
MAFA-AS1	1.104	1.11545714	1.0990339	0.857754495	-0.2213633	0.825518	1
RASL10B	1.1331983	1.12113852	1.1384257	1.142705997	0.1924543	0.825602	1
TTC23	1.2209514	1.23285879	1.21579	0.926699107	-0.1098271	0.825612	1
RIPK4	1.0351424	1.02345047	1.0402103	1.714691694	0.7779492	0.825649	1

AQP11	1.0886235	1.07672668	1.0937802	1.222263782	0.2895557	0.825793	1
CTD-2024P10.2	1.0285679	1.03985052	1.0236774	0.594155227	-0.7510882	0.825816	1
C1orf162	1.0265732	1.03781285	1.0217013	0.573913143	-0.8010957	0.825852	1
ANGPTL6	1.0265851	1.0378729	1.0216924	0.572767993	-0.8039772	0.825852	1
ZNF703	1.3009605	1.28784459	1.3066456	1.065316698	0.0912824	0.825865	1
TNFRSF10C	1.0479348	1.05920535	1.0430494	0.727120961	-0.4597327	0.825872	1
LAMA3	1.0264363	1.01490147	1.0314362	2.109600738	1.07697	0.8259	1
SEMA4B	1.2955386	1.30822302	1.2900405	0.941008447	-0.0877204	0.82595	1
ZNF223	1.0678677	1.07940469	1.0628669	0.791727914	-0.3369234	0.82598	1
ZEB1	1.4143108	1.40113406	1.4200223	1.047087179	0.0663816	0.826039	1
TIGD2	1.1131249	1.1011632	1.1183098	1.169494019	0.2258845	0.826051	1
HIST2H2AB	1.0245744	1.0358495	1.0196871	0.549159145	-0.8647038	0.826108	1
ANKRD33B	1.0529586	1.04130247	1.058011	1.404541053	0.4900988	0.826141	1
CTAGE5	1.1500822	1.16173626	1.1450306	0.896710724	-0.1572854	0.826198	1
FBXO16	1.1730184	1.18503783	1.1678085	0.906887639	-0.1410043	0.826216	1
HAS2-AS1	1.0844175	1.09586533	1.0794553	0.828822134	-0.2708656	0.826216	1
ZNF484	1.0526147	1.06379941	1.0477666	0.748699799	-0.4175407	0.826377	1
AL133245.2	1.030591	1.01899597	1.035617	1.874974605	0.9068711	0.826393	1
FZD9	1.0354142	1.04661056	1.0305611	0.655669651	-0.608959	0.826435	1
DHDDS	1.2199688	1.20799999	1.2251567	1.082484407	0.1143462	0.826458	1
AKAP5	1.0571458	1.06845374	1.0522443	0.763205327	-0.3898569	0.826496	1
GALNT12	1.0789027	1.06703748	1.0840458	1.253712957	0.3262071	0.826704	1
CNNM2	1.1014048	1.11275062	1.0964868	0.855754405	-0.2247313	0.826753	1
ALG1	1.1128728	1.12461781	1.1077819	0.864899747	-0.2093952	0.82679	1
BHMT2	1.0206338	1.03181042	1.0157893	0.496356491	-1.0105514	0.826811	1
ELMSAN1	1.2377876	1.25003373	1.2324795	0.929792622	-0.1050191	0.826855	1
GLIS3	1.2571786	1.26953872	1.251821	0.934266562	-0.0980939	0.827058	1
CFAP69	1.0716523	1.08297401	1.0667449	0.804407488	-0.3140016	0.827069	1
RP11-710C12.1	1.0855621	1.09699676	1.0806057	0.831013959	-0.2670554	0.827127	1
RP11-425D10.10	1.0257279	1.03689827	1.020886	0.566043099	-0.8210162	0.82713	1
FBXO6	1.1469863	1.1348938	1.1522278	1.128501398	0.1744082	0.827166	1
ZNF527	1.0726173	1.06072997	1.0777699	1.280585335	0.3568034	0.827173	1
TRIM59	1.3830403	1.39546912	1.3776529	0.954949151	-0.0665042	0.827188	1
PPP2R1B	1.1967241	1.18458428	1.2019861	1.094275879	0.1299765	0.827201	1
AGGF1	1.9729353	1.98827173	1.9662876	0.977754959	-0.0324551	0.82723	1
CEP68	1.3429091	1.35541153	1.3374898	0.949574749	-0.0746465	0.827266	1
LYSMD4	1.1479113	1.15938196	1.1429393	0.896834752	-0.1570859	0.827359	1
VPS39	1.1774266	1.165289	1.1826877	1.105262033	0.1443884	0.827415	1
RHOBTB2	1.1544416	1.14236826	1.1596749	1.121562154	0.1655096	0.827417	1
ZNF524	1.1974089	1.20935201	1.1922321	0.918224366	-0.1230814	0.827455	1
CTD-2240J17.4	1.0162592	1.02732787	1.0114615	0.419405925	-1.2535809	0.82752	1
POLR3H	1.5280381	1.54094342	1.5224442	0.965801959	-0.0502007	0.827521	1
HAUS4	1.2128793	1.2007025	1.2181574	1.086969158	0.120311	0.827617	1
KIAA1143	1.9490383	1.93365531	1.9557061	1.023617724	0.033677	0.827682	1
RP11-575H3.1	1.0331957	1.04436454	1.0283544	0.639123839	-0.6458326	0.827735	1
RP11-962G15.1	1.0266638	1.03773919	1.0218631	0.579321695	-0.7875634	0.827783	1
ITGA9	1.0283339	1.01686603	1.0333048	1.974666333	0.9816089	0.827794	1
ZNF610	1.0929999	1.10443781	1.0880421	0.843009746	-0.2463788	0.827838	1
AC007228.11	1.0729313	1.06125053	1.0779944	1.273367551	0.3486489	0.827839	1
ANKS6	1.1286442	1.14021119	1.1236305	0.881744632	-0.1815672	0.827869	1
PSTPIP2	1.1495817	1.13735883	1.1548798	1.127556041	0.1731991	0.82787	1

EDA	1.0460091	1.03438143	1.0510492	1.484789636	0.5702585	0.827881	1
KIAA1109	1.6193457	1.63284057	1.6134963	0.969432642	-0.0447874	0.827919	1
NGRN	1.2815551	1.26836944	1.2872705	1.070429315	0.0981895	0.827959	1
ABHD6	1.0847133	1.09616103	1.0797512	0.82935013	-0.2699468	0.828072	1
PEX11B	1.3021559	1.28958	1.307607	1.06225226	0.0871264	0.828082	1
CTC-492K19.7	1.0280246	1.03914108	1.0232062	0.592884945	-0.7541759	0.828089	1
RP4-605O3.4	1.195962	1.20778839	1.1908358	0.918413957	-0.1227835	0.828217	1
MTHFD1	1.3475682	1.33455869	1.3532073	1.055740828	0.0782557	0.828224	1
PET117	1.615822	1.62932921	1.6099672	0.969233871	-0.0450833	0.828241	1
HRC	1.0881943	1.0763295	1.0933371	1.222818366	0.2902101	0.828249	1
MIER3	1.1426135	1.15415675	1.13761	0.892662736	-0.1638129	0.828257	1
HELZ	1.5592684	1.57236805	1.5535902	0.967192779	-0.0481246	0.828274	1
KIAA1683	1.0447114	1.05577316	1.0399166	0.715696387	-0.4825804	0.828293	1
TSPEAR-AS1	1.0446785	1.05594114	1.0397966	0.711402021	-0.491263	0.828293	1
VPS11	1.1122108	1.10038675	1.1173359	1.168838718	0.2250759	0.828417	1
POFUT2	1.2222291	1.20989023	1.2275774	1.08426873	0.1167224	0.828474	1
ST6GALNAC2	1.0391367	1.02763237	1.0441234	1.596800405	0.675184	0.82853	1
U47924.31	1.0292129	1.04024862	1.0244294	0.606961511	-0.7203231	0.828533	1
UTP20	1.0980639	1.08628963	1.1031675	1.195595237	0.2577291	0.828558	1
RCOR1	1.1936529	1.18133107	1.1989938	1.097405942	0.1340973	0.82863	1
INPP5E	1.1618991	1.17360161	1.1568266	0.903370822	-0.1466098	0.828754	1
TRIM44	2.1823338	2.16641591	2.1892335	1.019562117	0.0279497	0.82877	1
ZBED9	1.0895253	1.07779363	1.0946105	1.21617253	0.2823479	0.828833	1
RP11-583F2.5	1.0222502	1.03341044	1.0174127	0.521176849	-0.9401551	0.828847	1
LYPLAL1-AS1	1.0221825	1.03318817	1.017412	0.524645419	-0.9305854	0.828847	1
ZNF100	1.1981985	1.2099365	1.1931106	0.919852427	-0.1205257	0.828847	1
RIC1	1.1497975	1.16127496	1.1448226	0.897985648	-0.1552357	0.828891	1
MBD1	1.3300395	1.31749603	1.3354765	1.056632111	0.0794732	0.828904	1
TMEM27	1.0638755	1.05231343	1.0688871	1.316815142	0.3970528	0.829077	1
NCAPH2	1.2550554	1.2672945	1.2497503	0.934363634	-0.097944	0.829083	1
TGM1	1.0384698	1.02703458	1.0434264	1.60632828	0.6837668	0.829114	1
PLXNA2	1.0507348	1.06182791	1.0459264	0.74280967	-0.4289355	0.829215	1
POLN	1.0508585	1.06190619	1.0460698	0.744187691	-0.4262616	0.829215	1
KSR1	1.0913307	1.07955961	1.0964329	1.212083991	0.2774897	0.829328	1
SLC36A1	1.0434006	1.05468226	1.0385105	0.704259955	-0.50582	0.829424	1
LINP1	1.0298517	1.01849616	1.0347738	1.880057888	0.9107771	0.829432	1
MSLN	1.0298766	1.01851935	1.0347995	1.879086463	0.9100315	0.829432	1
CPB2-AS1	1.0472336	1.03586011	1.0521634	1.454637056	0.5406592	0.829443	1
ZNF69	1.0850928	1.07337813	1.0901706	1.228848476	0.297307	0.829615	1
MYO5A	1.2404781	1.22813137	1.2458298	1.077580061	0.1077951	0.829653	1
IRAK2	1.0366251	1.04763042	1.0318547	0.668789584	-0.5803757	0.829759	1
AC008088.4	1.0366693	1.04770384	1.0318863	0.668422557	-0.5811677	0.829759	1
PLXNA1	1.2928915	1.30529797	1.2875139	0.941748386	-0.0865864	0.829925	1
USP6NL	1.2530448	1.26500034	1.2478626	0.935329436	-0.0964535	0.829945	1
PPFIA1	1.524214	1.51059821	1.5301159	1.038225156	0.0541193	0.83004	1
COQ10A	1.2746329	1.28659068	1.2694497	0.940190016	-0.0889757	0.830042	1
THG1L	1.3003177	1.28756586	1.3058451	1.0635655	0.0889089	0.830092	1
SMG8	1.02594	1.03690241	1.0211883	0.574170211	-0.8004496	0.830224	1
SNCB	1.0632397	1.05144806	1.0683509	1.328541581	0.4098434	0.830233	1
SLC29A3	1.0898795	1.07843853	1.0948386	1.209081758	0.2739118	0.830263	1
RP11-147L13.2	1.0239722	1.03491871	1.0192274	0.550634238	-0.8608338	0.830293	1

PCDHGA12	1.0240085	1.03496895	1.0192576	0.550704613	-0.8606494	0.830293	1
CKMT1B	1.0247443	1.03568983	1.0199998	0.560379118	-0.8355249	0.830322	1
TNKS2	1.493647	1.50649892	1.4880762	0.963627413	-0.0534527	0.830382	1
RHCE	1.0376871	1.02629766	1.0426239	1.620824843	0.6967282	0.830478	1
NDUFS2	2.4220751	2.40460011	2.4296497	1.017833977	0.0255023	0.830489	1
RP11-38L15.3	1.0300192	1.01870446	1.0349236	1.867126676	0.9008198	0.83051	1
DNAJC9	1.7068067	1.72090312	1.7006966	0.971970486	-0.0410156	0.830511	1
LONRF1	1.1722105	1.16044786	1.1773091	1.105088895	0.1441624	0.830587	1
ACVR1	1.115594	1.10413713	1.12056	1.157704164	0.2112666	0.830724	1
POU3F4	1.029113	1.01776634	1.0340313	1.915491475	0.9377146	0.83075	1
PTPRE	1.188377	1.20004111	1.1833212	0.916417451	-0.1259232	0.830794	1
THPO	1.0327886	1.04376886	1.0280292	0.640390853	-0.6429754	0.830829	1
WIPF2	1.3971639	1.38419896	1.4027836	1.048372448	0.0681513	0.830838	1
DCK	1.5041227	1.49101855	1.5098027	1.038255487	0.0541615	0.830868	1
PRSS56	1.0316391	1.02036551	1.0365257	1.79350586	0.8427825	0.831074	1
MON1B	1.2715908	1.28385893	1.2662731	0.938047135	-0.0922677	0.83114	1
CPED1	1.0285034	1.0171592	1.0334206	1.947677063	0.9617545	0.831155	1
POT1-AS1	1.1443688	1.13245243	1.149534	1.128964201	0.1749997	0.831159	1
TST	1.2224226	1.21012177	1.2277545	1.083916758	0.116254	0.831164	1
ARMC4	1.0438676	1.0547894	1.0391335	0.714253043	-0.4854928	0.831172	1
RUBCN	1.1362376	1.12428509	1.1414184	1.137855287	0.1863171	0.831264	1
NME8	1.1405412	1.15178204	1.1356688	0.893839516	-0.1619123	0.83128	1
TIGD4	1.1206148	1.13188096	1.1157314	0.877544684	-0.1884555	0.831365	1
NMNAT1	1.1743738	1.16273691	1.1794179	1.102502689	0.1407822	0.831402	1
SUPV3L1	1.174324	1.16240453	1.1794905	1.105206233	0.1443156	0.831402	1
CA3	2.0786193	2.09697944	2.070661	0.976008259	-0.0350347	0.831461	1
CPNE4	1.0308539	1.01962546	1.0357209	1.820131128	0.8640424	0.831502	1
RP11-266K4.14	1.0231669	1.03407728	1.0184377	0.541054477	-0.8861542	0.831537	1
VGLL1	1.0296964	1.01839445	1.0345953	1.880744373	0.9113038	0.831634	1
CAMK2D	1.5746795	1.56135823	1.5804536	1.034016395	0.0482591	0.831725	1
ZNF740	1.1494378	1.13768745	1.154531	1.122332033	0.1664995	0.831739	1
RTN2	1.126742	1.11524029	1.1317275	1.14306811	0.1929114	0.831755	1
CLRN1-AS1	1.0501994	1.06110129	1.0454739	0.744238412	-0.4261632	0.831889	1
NKIRAS1	1.2314087	1.21909165	1.2367475	1.080586836	0.111815	0.831931	1
RP11-817G13.3	1.0301456	1.04102112	1.0254315	0.61996215	-0.689748	0.831984	1
TRIM58	1.0301153	1.04103717	1.0253812	0.618492976	-0.6931709	0.831984	1
CA5B	1.1826308	1.17049816	1.1878898	1.102004883	0.1401306	0.832091	1
RNASEK	1.1649252	1.17629	1.1599991	0.9075902	-0.1398871	0.832131	1
CTC-523E23.1	1.0306024	1.04143537	1.0259068	0.62523279	-0.6775347	0.832163	1
WDR5B	1.0664605	1.05506868	1.0713983	1.296531801	0.3746576	0.832169	1
RP11-277B15.3	1.0321469	1.04300463	1.0274405	0.638083483	-0.6481829	0.83219	1
LGALS7	1.0206959	1.03157127	1.0159819	0.50621651	-0.9821735	0.832194	1
MIR762HG	1.2810378	1.26864525	1.2864095	1.066125243	0.0923769	0.832206	1
TMEM203	1.7001585	1.68609266	1.7062554	1.029387776	0.0417866	0.832208	1
CENPS	1.3607266	1.37286765	1.3554639	0.953324663	-0.0689605	0.832314	1
PGM2L1	1.4453972	1.45786987	1.4399909	0.960951834	-0.057464	0.832406	1
BCR	1.1708123	1.15912955	1.1758763	1.105239806	0.1443594	0.832455	1
SPATA41	1.0253996	1.03619782	1.0207191	0.57238393	-0.8049449	0.832513	1
P2RY6	1.0464108	1.03513408	1.0512987	1.460084761	0.5460521	0.832538	1
ICOSLG	1.0465052	1.03520457	1.0514035	1.460136941	0.5461037	0.832538	1
SLC16A5	1.0966035	1.10764098	1.0918192	0.853013597	-0.2293594	0.832613	1

CTA-228A9.4	1.0339215	1.04470211	1.0292486	0.654300516	-0.6119747	0.832621	1
AIFM3	1.0420795	1.05292584	1.0373781	0.706234531	-0.5017807	0.832649	1
DOLPP1	1.1015372	1.09011862	1.1064867	1.181627623	0.2407755	0.832656	1
MIR296	1.0231375	1.03393843	1.0184558	0.543801787	-0.8788472	0.832663	1
ATP9B	1.1898346	1.17794182	1.1949896	1.095805649	0.1319919	0.832935	1
ZNF175	1.1158084	1.12709824	1.1109148	0.872669875	-0.1964921	0.832941	1
PRKCQ	1.0292446	1.01804205	1.0341004	1.890049321	0.9184239	0.832982	1
CACNB2	1.0317841	1.02056357	1.0366476	1.782162578	0.833629	0.83306	1
GPRASP2	1.2151536	1.22682127	1.2100961	0.92626295	-0.1105063	0.833077	1
KCNC4	1.0302639	1.04113744	1.0255507	0.62110516	-0.6870905	0.833102	1
CSTF3-AS1	1.0302551	1.04105422	1.0255742	0.62293619	-0.6828437	0.833102	1
U2AF1	1.0894141	1.10027508	1.0847064	0.844740422	-0.24342	0.833103	1
ASIC2	1.0318112	1.04260818	1.0271312	0.636761278	-0.6511755	0.833224	1
FAM83H	1.0623965	1.07336629	1.0576417	0.785669559	-0.3480054	0.833337	1
DNAJC16	1.2052256	1.21677231	1.2002207	0.923644944	-0.1145897	0.833386	1
RFFL	1.2239954	1.23537926	1.219061	0.93067259	-0.1036544	0.833414	1
PRMT6	1.2940179	1.30611329	1.2887751	0.943360303	-0.0841192	0.833434	1
SIRT6	1.2972342	1.30929343	1.292007	0.944110108	-0.082973	0.833436	1
ZFAND3	1.8661476	1.88042094	1.8599607	0.976760837	-0.0339227	0.833492	1
CHADL	1.0423794	1.03120423	1.0472233	1.513361583	0.5977567	0.833647	1
RP11-91I8.3	1.0671653	1.05572806	1.0721229	1.294193249	0.3720531	0.833649	1
PAPOLB	1.0222706	1.032969	1.0176334	0.534847269	-0.9028011	0.833654	1
POLR3F	1.2092259	1.22091265	1.2041602	0.924167223	-0.1137742	0.833687	1
RP11-861E21.2	1.0591007	1.07007966	1.0543418	0.775428686	-0.366934	0.83371	1
ACTL6B	1.0247794	1.01362911	1.0296125	2.17273885	1.1195148	0.833751	1
TRIM55	1.0247094	1.01362911	1.0295122	2.165380232	1.1146204	0.833751	1
RP11-338N10.1	1.0221961	1.03289757	1.0175575	0.533701888	-0.905894	0.833762	1
RP11-158H5.7	1.0221832	1.03291226	1.0175327	0.532709863	-0.9085781	0.833762	1
PRRC2C	3.6744039	3.64970313	3.6851106	1.013362793	0.0191508	0.833768	1
SMCR8	1.0767423	1.08809489	1.0718215	0.815273973	-0.2946431	0.833889	1
ABLIM3	1.0336281	1.02253201	1.0384377	1.705917513	0.7705479	0.833947	1
LEAP2	1.1111794	1.122544	1.1062533	0.867062765	-0.2057917	0.834	1
KLK7	1.0244725	1.01338003	1.0292807	2.188385719	1.129867	0.834013	1
WIF1	1.0245351	1.01338003	1.0293703	2.195083695	1.1342759	0.834013	1
SPTLC2	1.311234	1.32294559	1.3061575	0.948015784	-0.077017	0.834021	1
DARS-AS1	1.0878919	1.09880973	1.0831596	0.841613101	-0.2487709	0.834047	1
CENPQ	1.2539743	1.26574277	1.2488732	0.936519277	-0.0946194	0.834078	1
DNAJC9-AS1	1.0341109	1.04475157	1.0294986	0.659163166	-0.6012925	0.83417	1
ZNF766	1.3645814	1.37680625	1.3592824	0.953493847	-0.0687045	0.834182	1
RP11-90D4.3	1.0386724	1.02745806	1.0435334	1.585449239	0.6648917	0.834296	1
AC093388.3	1.0506516	1.0614415	1.0459747	0.748267145	-0.4183747	0.834318	1
HAP1	1.1446546	1.15572816	1.1398547	0.898069155	-0.1551016	0.834367	1
TMEM241	1.1448229	1.13337891	1.1497833	1.122990818	0.1673461	0.834456	1
NOC2L	1.6451087	1.65864068	1.6392432	0.970549227	-0.0431267	0.834459	1
HVCN1	1.0687403	1.05752635	1.073601	1.27943172	0.3555032	0.834569	1
DEGS2	1.0299106	1.04059337	1.0252802	0.622765668	-0.6832387	0.834666	1
LEKR1	1.0406098	1.02952886	1.0454129	1.537915868	0.6209766	0.834725	1
ZFYVE1	1.1470893	1.13557481	1.1520803	1.121744521	0.1657441	0.834727	1
EPO	1.0221386	1.03279279	1.0175204	0.534276934	-0.9043404	0.83473	1
DISP1	1.1124243	1.12342244	1.1076572	0.87226571	-0.1971604	0.83479	1
TTPAL	1.1838558	1.19520413	1.1789369	0.916665321	-0.125533	0.834797	1

AP001596.6	1.0325548	1.02144834	1.037369	1.742276843	0.8009739	0.834836	1
GLIPR1L2	1.0209499	1.03167272	1.016302	0.514702593	-0.958189	0.834983	1
TMEM39B	1.3382218	1.32644536	1.3433264	1.051711585	0.0727391	0.835068	1
RP1	1.0911262	1.10225755	1.0863013	0.843959678	-0.244754	0.835138	1
ZNF592	1.1188254	1.10726887	1.1238347	1.154432626	0.207184	0.835206	1
RP11-803D5.4	1.0518933	1.06280304	1.0471644	0.750988362	-0.4131375	0.835216	1
ADD3	1.6890498	1.67558077	1.6948881	1.028578841	0.0406524	0.835279	1
PGBD5	1.0402863	1.02920186	1.0450909	1.544109055	0.6267746	0.8353	1
MRC2	1.3271282	1.33960203	1.3217213	0.947347987	-0.0780336	0.835337	1
AC099850.1	1.0307822	1.04146406	1.0261521	0.630716505	-0.6649364	0.835425	1
SMTNL2	1.1481543	1.15944653	1.1432597	0.898481059	-0.15444	0.835487	1
POU3F1	1.0230076	1.03363066	1.0184029	0.547206705	-0.8698422	0.835573	1
TLR6	1.0229949	1.03363112	1.0183846	0.546655622	-0.8712958	0.835573	1
TMEM119	1.0240501	1.01301898	1.0288316	2.214583165	1.1470352	0.835669	1
AC025335.1	1.0803898	1.09126641	1.0756753	0.829169632	-0.2702608	0.83574	1
MBLAC2	1.1469687	1.13530586	1.152024	1.123558176	0.1680748	0.835742	1
AFF2	1.0583498	1.04700184	1.0632686	1.34608827	0.428773	0.835752	1
CORO1C	1.5835962	1.57039218	1.5893196	1.03318317	0.047096	0.835826	1
PDGFRB	1.5695473	1.55566734	1.5755636	1.035806105	0.050754	0.835835	1
FRY	1.0342417	1.02321937	1.0390194	1.680465729	0.7488611	0.835899	1
UPK1B	1.0343302	1.02323906	1.0391377	1.684132387	0.7520056	0.835899	1
MN1	1.1892032	1.17748179	1.1942839	1.094669699	0.1304956	0.835941	1
BBOF1	1.1483047	1.1594739	1.1434633	0.89960353	-0.1526388	0.835947	1
CAMK2B	1.09296	1.08157885	1.0978932	1.19998312	0.2630141	0.835952	1
CASP4	1.0310912	1.02002985	1.0358859	1.791619684	0.8412644	0.836019	1
CTD-2269F5.1	1.0310832	1.02000206	1.0358864	1.794136041	0.8432893	0.836019	1
CCDC134	1.035704	1.02470719	1.0404706	1.638009738	0.7119439	0.836089	1
CCDC174	1.4908621	1.47794503	1.4964611	1.038741038	0.054836	0.836268	1
RP1-78B3.1	1.0476643	1.05840851	1.0430071	0.736316224	-0.4416026	0.83629	1
C6orf136	1.255179	1.24317209	1.2603835	1.070778594	0.0986602	0.836347	1
LIN28B	1.303737	1.31553275	1.2986241	0.946412501	-0.079459	0.836358	1
PCYT2	1.2099292	1.19805283	1.215077	1.085957757	0.118968	0.836373	1
NLRP3	1.0275127	1.03805285	1.022944	0.602951855	-0.7298853	0.836428	1
HIST1H4H	1.0369276	1.04759941	1.0323019	0.678619907	-0.5593243	0.836472	1
INPP4B	1.0593131	1.04823968	1.0641129	1.329049019	0.4103943	0.836481	1
RP11-458J1.1	1.0760464	1.08683245	1.0713712	0.821941408	-0.2828925	0.836599	1
LRRC25	1.0280147	1.03867468	1.023394	0.604892307	-0.7252498	0.836607	1
AC104135.2	1.0279902	1.03857236	1.0234034	0.606739271	-0.7208514	0.836607	1
NAA30	1.3244964	1.33640083	1.3193363	0.949273314	-0.0751046	0.836695	1
TOPORS-AS1	1.6691724	1.65520031	1.6752287	1.030568347	0.0434402	0.836893	1
PIK3R2	1.0940752	1.10513127	1.0892829	0.849251998	-0.2357354	0.836933	1
LINC01258	1.022805	1.03338363	1.0182196	0.545763697	-0.8736517	0.836954	1
ARRB1	1.0617008	1.05063556	1.0664971	1.313249508	0.393141	0.837132	1
RHOJ	1.1105326	1.0994022	1.1153572	1.160509327	0.2147581	0.83723	1
PANK1	1.176065	1.16448654	1.1810837	1.10090311	0.1386875	0.837232	1
LRRC24	1.1206143	1.10945165	1.1254527	1.146193158	0.1968502	0.837289	1
ACSM3	1.049084	1.03805072	1.0538664	1.415646528	0.5014611	0.837364	1
CDK1	1.7374492	1.72227646	1.7440259	1.030112351	0.0428017	0.83739	1
KIFC2	1.1418094	1.15298062	1.1369672	0.895323694	-0.1595187	0.837418	1
RP11-209D14.2	1.0514561	1.06213438	1.0468275	0.753649415	-0.4080345	0.837436	1
C11orf24	1.261744	1.27313121	1.2568082	0.940237378	-0.0889031	0.837465	1

PRKRIP1	1.4199054	1.4323774	1.4144994	0.958651834	-0.0609211	0.837527	1
RP11-109G23.3	1.0323006	1.02135442	1.0370453	1.734782735	0.794755	0.837672	1
PLEKHM1	1.0932016	1.08207732	1.0980235	1.19428292	0.2561446	0.837713	1
RASD2	1.0279098	1.03843515	1.0233476	0.607453083	-0.7191551	0.837779	1
CCDC168	1.0279311	1.03848851	1.023355	0.606803914	-0.7206977	0.837779	1
SYNE3	1.0279241	1.0384461	1.0233633	0.607689188	-0.7185945	0.837779	1
AC012360.4	1.0280088	1.03852168	1.0234519	0.608798526	-0.7159632	0.837779	1
CKMT2-AS1	1.0958792	1.10662241	1.0912225	0.855566336	-0.2250484	0.837818	1
UGT2B7	1.0318827	1.02091354	1.0366373	1.751845859	0.8088758	0.837851	1
TMEM65	1.2015679	1.18993044	1.2066122	1.087830652	0.121454	0.837908	1
SLC27A1	1.2266148	1.21477926	1.231745	1.078991618	0.1096837	0.837952	1
ZRANB1	1.3541822	1.34191336	1.3595002	1.051436678	0.072362	0.837956	1
ASPRV1	1.0361394	1.04670601	1.0315592	0.675699451	-0.5655464	0.838011	1
RP11-151A6.6	1.0190272	1.02955365	1.0144645	0.489432503	-1.0308182	0.838019	1
OXR1	1.819342	1.83236145	1.8136987	0.977578568	-0.0327154	0.838058	1
MOGS	1.2135712	1.22505569	1.2085932	0.926851591	-0.1095897	0.838064	1
C1orf115	1.0366446	1.04723081	1.032056	0.678708817	-0.5591353	0.838191	1
CTB-193M12.1	1.0195985	1.03006157	1.0150633	0.501080544	-0.9968856	0.838203	1
APOBEC3A	1.0196128	1.03008785	1.0150724	0.50094554	-0.9972743	0.838203	1
FAM133A	1.1460748	1.1570888	1.1413008	0.899496253	-0.1528108	0.838208	1
SNAPC2	1.49939	1.48705406	1.5047371	1.036306131	0.0514502	0.838301	1
CNTNAP1	1.1050701	1.09394375	1.1098928	1.169772644	0.2262282	0.838344	1
ZNF641	1.1049616	1.09373845	1.1098263	1.17162471	0.2285105	0.838344	1
NLRX1	1.102655	1.11367614	1.0978778	0.861023124	-0.2158761	0.838434	1
MSMP	1.0473988	1.05793897	1.0428301	0.739228159	-0.4359084	0.838516	1
FAM13A-AS1	1.0272801	1.01646229	1.0319692	1.941964795	0.957517	0.838586	1
SUPT3H	1.3058793	1.29363182	1.3111881	1.059790037	0.0837785	0.838711	1
LHFPL2	1.1640988	1.15277226	1.1690084	1.106276568	0.1457121	0.838715	1
KDM2A	1.3033938	1.31519177	1.2982799	0.946344329	-0.0795629	0.83877	1
HOXA2	1.027179	1.01622108	1.0319288	1.968352742	0.9769888	0.838847	1
ZNF821	1.4265795	1.43880657	1.4212796	0.960057533	-0.0588072	0.83888	1
CNEP1R1	1.1839524	1.17243711	1.1889438	1.095725942	0.131887	0.838986	1
ABCB7	1.2885245	1.27648402	1.2937435	1.06242472	0.0873606	0.83908	1
CD226	1.0273494	1.03774908	1.0228416	0.605089152	-0.7247804	0.839093	1
ASCC3	1.7919604	1.80573199	1.785991	0.975499312	-0.0357872	0.839097	1
RBP4	1.0276532	1.01683833	1.0323409	1.920673122	0.941612	0.839101	1
RP11-1152H14.1	1.0364182	1.04695603	1.0318506	0.678305818	-0.5599922	0.839134	1
IL11RA	1.3295637	1.31754365	1.3347739	1.054260978	0.076232	0.839174	1
C1orf204	1.0299081	1.04039677	1.0253618	0.627816887	-0.6715843	0.839194	1
MGAM	1.0299771	1.04051006	1.0254116	0.627290487	-0.6727944	0.839194	1
KCNIP2-AS1	1.0299412	1.04048305	1.0253718	0.626726381	-0.6740924	0.839194	1
NSUN2	1.1403421	1.12909866	1.1452157	1.124842495	0.169723	0.839262	1
CNTN4	1.2082872	1.2194497	1.2034487	0.92708594	-0.109225	0.839336	1
MTERF1	1.1408502	1.15185997	1.136078	0.896075234	-0.1583082	0.839348	1
CCNE1	1.0817335	1.07061741	1.0865518	1.225644387	0.2935405	0.839367	1
RP3-466P17.1	1.0365999	1.04711563	1.0320417	0.6800659	-0.5562535	0.839368	1
NDST3	1.0264658	1.01564826	1.0311547	1.990935298	0.9934463	0.83943	1
EFCAB5	1.0448918	1.05540375	1.0403354	0.728025675	-0.4579388	0.839579	1
PAX8	1.0651516	1.07583493	1.0605208	0.798060261	-0.3254304	0.839655	1
NEU3	1.0730157	1.06201912	1.0777822	1.254165496	0.3267277	0.839679	1
BTBD17	1.086739	1.07561886	1.0915591	1.210796839	0.2759568	0.839735	1

FAM173A	1.9636262	1.94720905	1.9707424	1.024844885	0.0354056	0.839829	1
SIPA1L1	1.2109573	1.19929346	1.2160131	1.083894558	0.1162244	0.839845	1
RP5-994D16.11	1.035382	1.04582722	1.0308545	0.673278997	-0.5707236	0.83985	1
FAT4	1.0965724	1.08531778	1.1014507	1.189092371	0.2498608	0.839859	1
WFIKKN1	1.1115251	1.12226987	1.1068677	0.874031478	-0.1942429	0.839921	1
UTP3	1.2466783	1.2347856	1.2518333	1.072609598	0.1011251	0.840016	1
MIR22HG	1.0595424	1.04867948	1.0642509	1.319876839	0.4004033	0.840061	1
ANTXR1	1.4894091	1.50215175	1.4838857	0.963624372	-0.0534572	0.840075	1
CTD-2371O3.2	1.0401357	1.0506733	1.0355681	0.701910761	-0.5106405	0.840225	1
DYRK1A	1.3436422	1.35541876	1.3385376	0.95250354	-0.0702036	0.840305	1
KIAA1614	1.0454148	1.03457754	1.0501123	1.449271827	0.5353282	0.840365	1
H3F3C	1.0485848	1.05901375	1.0440643	0.746678216	-0.4214415	0.840375	1
RNFT2	1.2663386	1.27747963	1.2615094	0.942445481	-0.0855189	0.840413	1
CTD-2047H16.4	1.0378223	1.04818226	1.0333317	0.69178401	-0.5316064	0.840427	1
TNFAIP8L1	1.1742186	1.18532716	1.1694035	0.914078149	-0.1296106	0.840465	1
PIK3C2A	1.5467591	1.55915119	1.5413877	0.96823136	-0.0465763	0.840514	1
PPP4R4	1.0692062	1.05822251	1.0739671	1.270420835	0.3453065	0.840515	1
C1QTNF3	1.1039436	1.11479985	1.0992378	0.8644423	-0.2101584	0.840517	1
RP11-408B11.2	1.0256743	1.01490147	1.0303439	2.036301834	1.0259514	0.840745	1
RP5-827C21.6	1.0274639	1.03784308	1.0229649	0.606846007	-0.7205976	0.84081	1
CD247	1.0249182	1.03526581	1.0204329	0.579397106	-0.7873756	0.840889	1
PICK1	1.1851419	1.17381998	1.1900495	1.093369566	0.1287811	0.840919	1
CDH13	1.0382929	1.02760037	1.0429276	1.555326802	0.6372177	0.840928	1
TM9SF3	2.1370634	2.12250233	2.143375	1.018594797	0.0265803	0.840954	1
C14orf159	1.438699	1.45038302	1.4336345	0.962812699	-0.0546729	0.841031	1
RP11-167N5.5	1.0188673	1.02912238	1.0144222	0.495226946	-1.0138383	0.841055	1
CCDC88B	1.1139595	1.12442613	1.1094226	0.879418552	-0.1853781	0.841132	1
SLC4A5	1.0773838	1.08808115	1.0727469	0.825908207	-0.2759466	0.841237	1
SCEL	1.0298357	1.01899597	1.0345343	1.817979533	0.862336	0.84124	1
RP4-594I10.3	1.0297292	1.01899597	1.0343815	1.809936753	0.8559393	0.84124	1
GOLGB1	2.3533975	2.37022587	2.3461032	0.982395113	-0.0256247	0.841263	1
GRIN2D	1.053918	1.06434144	1.0493999	0.767777546	-0.3812397	0.841313	1
NPTX2	1.0432172	1.03244855	1.047885	1.475719497	0.5614185	0.841323	1
SLC16A13	1.036907	1.04716395	1.032461	0.688258794	-0.538977	0.841485	1
KCNIP4-IT1	1.0274257	1.0167906	1.0320355	1.907944236	0.932019	0.841501	1
ZBTB5	1.176937	1.18784735	1.1722079	0.91674371	-0.1254096	0.841567	1
CTA-392C11.1	1.0345099	1.02386623	1.0391234	1.639279105	0.7130615	0.841591	1
AC002456.2	1.0290113	1.03933156	1.0245379	0.62387196	-0.6806781	0.841688	1
LRRC34	1.0709987	1.06006695	1.0757371	1.260878621	0.3344294	0.841697	1
DTX3L	1.1212522	1.1318859	1.116643	0.884423598	-0.1771906	0.841879	1
CDH3	1.0604372	1.04958447	1.0651414	1.313745407	0.3936857	0.841919	1
ZBTB16	1.380866	1.39226924	1.3759232	0.958329542	-0.0614063	0.841944	1
PCDHGA5	1.0187327	1.02897849	1.0142916	0.493179391	-1.0198156	0.841955	1
CTD-2630F21.1	1.0187385	1.02899408	1.0142931	0.492966583	-1.0204382	0.841955	1
FAM90A1	1.0187273	1.02896827	1.0142883	0.493238243	-1.0196434	0.841955	1
RCAN1	1.1061055	1.09517669	1.1108427	1.164599042	0.2198333	0.841993	1
RP11-83J21.3	1.0629666	1.0521444	1.0676575	1.297502528	0.3757373	0.841999	1
CHIC1	1.1150258	1.12568971	1.1104035	0.878381238	-0.1870809	0.842058	1
ZGLP1	1.0465125	1.05690739	1.0420068	0.738160342	-0.4379939	0.842073	1
JMJD8	1.3860396	1.37446588	1.3910562	1.044304067	0.0625418	0.84211	1
RP11-227G15.11	1.0253527	1.03567249	1.0208795	0.585310992	-0.7727247	0.842239	1

USP45	1.2041977	1.19285164	1.2091157	1.084334427	0.1168098	0.842361	1
PLPP7	1.0456366	1.03492726	1.0502787	1.43952575	0.5255936	0.842528	1
PLCL2	1.0471258	1.03640998	1.0517706	1.421880063	0.5077998	0.842644	1
RP11-522B15.3	1.0356049	1.04589662	1.0311439	0.678565409	-0.5594402	0.842665	1
ADAMTS2	1.1109479	1.12135464	1.106437	0.877073928	-0.1892296	0.842679	1
REPS2	1.0933257	1.10401563	1.0886922	0.852681007	-0.229922	0.842768	1
FAM64A	1.5985564	1.61136026	1.5930065	0.969978738	-0.043975	0.842826	1
RP11-890B15.3	1.0641974	1.07476857	1.0596153	0.79733049	-0.3267503	0.842998	1
SLC25A21	1.0913091	1.10189567	1.0867202	0.851068761	-0.2326524	0.843033	1
NRTN	1.0454915	1.05575621	1.0410423	0.736102225	-0.442022	0.843078	1
TIGD6	1.1140748	1.10299823	1.1188761	1.154156404	0.2068387	0.843091	1
RP13-516M14.2	1.0265732	1.03679158	1.0221439	0.601875099	-0.732464	0.843192	1
TRIM5	1.4117373	1.42328697	1.406731	0.960887039	-0.0575613	0.843259	1
USP3	1.5690723	1.58166478	1.563614	0.968966965	-0.0454806	0.84344	1
GHET1	1.0301516	1.04031601	1.0257458	0.638600659	-0.6470141	0.843471	1
ALG14	1.2112319	1.19952571	1.216306	1.084100725	0.1164988	0.843513	1
PMEPA1	1.4865504	1.47440207	1.4918162	1.036707506	0.0520089	0.843523	1
ACYP1	1.9505701	1.93612843	1.95683	1.022113989	0.0315561	0.843556	1
STAT5A	1.0388574	1.02825642	1.0434525	1.537792896	0.6208612	0.843607	1
TENM2	1.0825108	1.07189076	1.0871142	1.211757541	0.2771011	0.843666	1
PCDH9-AS1	1.0631389	1.0523328	1.0678229	1.295992683	0.3740576	0.843674	1
RP11-517P14.2	1.0295984	1.01906734	1.0341631	1.791709369	0.8413366	0.843675	1
HS2ST1	1.5711492	1.5834039	1.5658374	0.96988962	-0.0441075	0.843728	1
CASP2	1.2848595	1.27327372	1.2898814	1.060773154	0.0851162	0.843788	1
RP11-253M7.1	1.0340217	1.04419003	1.0296141	0.670153842	-0.5774358	0.843814	1
TAZ	1.281745	1.27030804	1.2867025	1.060650931	0.0849499	0.843914	1
USP2	1.0448192	1.05515967	1.040337	0.73127753	-0.4515091	0.843933	1
SERPINB8	1.0467325	1.0570499	1.0422603	0.740760261	-0.4329214	0.844	1
RP4-621F18.2	1.090814	1.10148874	1.0861869	0.849226372	-0.2357789	0.844002	1
ZBED8	1.1302668	1.1409693	1.1256278	0.891171266	-0.1662254	0.844005	1
NPIPA5	1.0543821	1.04366572	1.0590271	1.351796084	0.4348775	0.844011	1
CITED4	1.0544142	1.04373548	1.059043	1.350001803	0.4329613	0.844011	1
CDYL	1.3023757	1.31371839	1.2974592	0.948172716	-0.0767782	0.844094	1
RP11-66B24.4	1.0250182	1.03516403	1.0206204	0.586406842	-0.7700262	0.84411	1
CTD-2012K14.8	1.0801851	1.09067418	1.0756385	0.834179512	-0.2615702	0.84413	1
TAOK2	1.2074415	1.21818418	1.2027851	0.929421595	-0.1055949	0.84415	1
KCNK10	1.0453395	1.05556707	1.0409062	0.736159943	-0.4419088	0.844169	1
C1R	1.0980307	1.08734261	1.1026635	1.175411983	0.2331665	0.844174	1
CMTM1	1.0175514	1.02761899	1.0131875	0.477479165	-1.0664903	0.844175	1
PFKFB2	1.0767752	1.06614074	1.0813848	1.230478592	0.2992196	0.844192	1
TMEM117	1.1809146	1.19158402	1.1762899	0.920170223	-0.1200273	0.844245	1
RP11-1191J2.2	1.0442917	1.054695	1.0397823	0.727348487	-0.4592813	0.844254	1
VSTM4	1.0289634	1.01851066	1.0334942	1.809455331	0.8555555	0.844259	1
NTNG1	1.0256394	1.03572432	1.0212681	0.595338904	-0.7482169	0.844295	1
RP4-548D19.3	1.0457292	1.03514302	1.0503178	1.431799877	0.5178299	0.844348	1
ABHD18	1.1198281	1.13025557	1.1153083	0.885246286	-0.1758492	0.844409	1
LINC01431	1.0977972	1.08696556	1.1024922	1.178538021	0.2369983	0.844414	1
TRNP1	1.0755449	1.06487338	1.0801706	1.235801024	0.3054465	0.84443	1
POLK	1.5185307	1.53053102	1.5133292	0.967576122	-0.0475529	0.844443	1
ZNF30	1.0731328	1.06251265	1.0777361	1.243526695	0.3144375	0.844477	1
WDR59	1.380539	1.36879809	1.3856282	1.04563507	0.0643794	0.844606	1

SPAST	1.3732164	1.36114393	1.3784492	1.047917975	0.0675258	0.844614	1
MMAA	1.084499	1.0946292	1.080108	0.846546709	-0.2403384	0.844617	1
RAB40C	1.203219	1.19179947	1.2081689	1.085346641	0.1181559	0.844646	1
AUNIP	1.0540887	1.06433671	1.0496466	0.771668637	-0.3739466	0.844713	1
KDM3A	1.5428916	1.55494839	1.5376655	0.968856754	-0.0456447	0.844743	1
TTLL3	1.1387148	1.14911995	1.1342046	0.899977684	-0.1520389	0.844913	1
PSORS1C1	1.045372	1.03487171	1.0499235	1.431632477	0.5176612	0.844927	1
CBL	1.3578574	1.34589073	1.3630444	1.04959288	0.0698298	0.844999	1
ZBTB42	1.0462723	1.05644544	1.0418627	0.741648447	-0.4311926	0.845023	1
DIDO1	1.4730207	1.4609049	1.4782724	1.037681235	0.0533633	0.84504	1
KATNAL1	1.2580322	1.24674911	1.2629229	1.065547539	0.091595	0.845074	1
TRAPPC2B	1.2679479	1.25653631	1.2728943	1.063764807	0.0891792	0.845144	1
RBM5-AS1	1.0268109	1.03690533	1.0224355	0.607919513	-0.7180478	0.845185	1
CNBD2	1.2641267	1.25232402	1.2692426	1.067050891	0.093629	0.845224	1
RP11-820I16.4	1.0255671	1.03564771	1.0211976	0.59464109	-0.7499089	0.845268	1
PRDM8	1.0359875	1.04615866	1.0315788	0.684136104	-0.5476447	0.845271	1
ZNF749	1.0291754	1.01872619	1.0337046	1.799866308	0.8478897	0.84534	1
PHTF2	1.3014353	1.31277904	1.2965183	0.948011972	-0.0770228	0.845364	1
CES3	1.0497189	1.03899799	1.0543659	1.394068631	0.4793016	0.845427	1
WBSCR27	1.0534036	1.06366935	1.0489539	0.768877112	-0.3791751	0.845471	1
UPB1	1.0243856	1.0344975	1.0200025	0.579825448	-0.7863094	0.845521	1
RP5-892K4.1	1.0243924	1.03449084	1.0200151	0.580302323	-0.7851234	0.845521	1
VMP1	2.4743163	2.49491405	2.465388	0.980249018	-0.0287798	0.845682	1
CEP170B	1.1028954	1.11322537	1.0984178	0.869220375	-0.2022061	0.845797	1
FAM43B	1.0539353	1.06407485	1.0495403	0.773163051	-0.3711554	0.845809	1
RP11-535A19.2	1.0261617	1.03619362	1.0218133	0.602683614	-0.7305273	0.845852	1
HGF	1.0414389	1.05169643	1.0369927	0.715575103	-0.4828249	0.845859	1
RP11-295I5.3	1.0414675	1.05173112	1.0370187	0.715599014	-0.4827767	0.845859	1
AGAP3	1.2536501	1.2645953	1.2489058	0.940703726	-0.0881877	0.845966	1
PQLC3	1.2542703	1.26543085	1.2494327	0.939727505	-0.0896856	0.845966	1
POFUT1	1.2251743	1.2366225	1.220212	0.930646793	-0.1036944	0.845967	1
ZSCAN30	1.2181195	1.22923686	1.2133006	0.930481279	-0.103951	0.845973	1
CAMLG	4.0475226	4.07305249	4.0364565	0.988091336	-0.0172837	0.845996	1
DOCK8	1.0275529	1.0171592	1.0320581	1.868276001	0.9017076	0.84602	1
LGALS8-AS1	1.0335968	1.02313192	1.0381329	1.648496796	0.7211511	0.846056	1
AARD	1.0336206	1.02314912	1.0381596	1.648425324	0.7210885	0.846056	1
ZNF491	1.0627596	1.07293996	1.0583468	0.79992948	-0.3220553	0.846292	1
PTDSS2	1.366542	1.37811681	1.3615248	0.956119371	-0.0647373	0.846294	1
RP13-467H17.1	1.0225065	1.03254454	1.0181555	0.557865132	-0.8420117	0.846483	1
ODAM	1.0288092	1.01839445	1.0333235	1.811607576	0.8572705	0.8465	1
CRISPLD2	1.0287663	1.01839445	1.033262	1.808263336	0.8546048	0.8465	1
PPP1R16B	1.0240298	1.03403322	1.0196938	0.578664577	-0.7892008	0.846504	1
GPR176	1.0913704	1.10161131	1.0869315	0.855529324	-0.2251108	0.846633	1
AMOT	1.2334293	1.22246962	1.2381798	1.070617271	0.0984428	0.846706	1
RP11-744I24.2	1.0227436	1.03268805	1.0184332	0.563912015	-0.826458	0.846716	1
MIPEP	1.1644649	1.15367336	1.1691426	1.100663135	0.138373	0.846764	1
MYLPF	1.0650717	1.05443721	1.0696813	1.280029687	0.3561773	0.846777	1
ZNF20	1.0549573	1.04439265	1.0595366	1.341136622	0.4234562	0.846817	1
FAM69B	1.3406626	1.32929804	1.3455886	1.049470548	0.0696617	0.846822	1
SLC45A3	1.0341751	1.04423578	1.0298143	0.673985911	-0.5692097	0.846926	1
TET2	1.195521	1.18446287	1.2003143	1.085932656	0.1189346	0.846931	1

F12	1.3518479	1.34021068	1.3568921	1.049032732	0.0690597	0.846939	1
KISS1R	1.2971695	1.30848166	1.2922662	0.947434726	-0.0779015	0.846955	1
DFNB59	1.0622351	1.07217224	1.0579278	0.802633298	-0.3171871	0.846996	1
ITCH	1.2249523	1.23561544	1.2203304	0.935126958	-0.0967658	0.847095	1
ERICH2	1.0460632	1.03559917	1.0505989	1.421350293	0.5072622	0.847102	1
RP11-452F19.3	1.1195497	1.12973952	1.1151329	0.887416031	-0.1723175	0.847212	1
SETDB1	1.3101896	1.32110503	1.3054582	0.951271954	-0.0720703	0.847254	1
RP11-396F22.1	1.0884975	1.09868818	1.0840803	0.851979547	-0.2311093	0.847384	1
ARHGAP45	1.0374723	1.02712096	1.0419591	1.547111446	0.6295771	0.847388	1
PYCRL	1.1736626	1.18441617	1.1690015	0.916413555	-0.1259293	0.847405	1
RP11-455F5.6	1.0500069	1.06015256	1.0456092	0.758225439	-0.3993012	0.847407	1
LIN52	1.2043872	1.21513776	1.1997273	0.928369287	-0.1072293	0.847476	1
SART3	1.2165544	1.20539012	1.2213937	1.077917705	0.108247	0.847493	1
NEXN-AS1	1.0429067	1.0529996	1.0385318	0.727021207	-0.4599306	0.847518	1
SLC9B1	1.0533921	1.06346132	1.0490275	0.77255714	-0.3722865	0.84761	1
TFAP2B	1.02887	1.01850742	1.0333617	1.802614398	0.8500908	0.847673	1
VSTM2L	1.1209763	1.13131498	1.1164949	0.887141216	-0.1727643	0.84787	1
SLC2A8	1.2058838	1.19491226	1.2106394	1.080688412	0.1119506	0.847875	1
HSPBAP1	1.071321	1.08154132	1.066891	0.820332101	-0.28572	0.847939	1
SPATA17	1.0937099	1.08323653	1.0982496	1.180366632	0.239235	0.847981	1
CDC42EP2	1.0740395	1.06365473	1.0785409	1.233857849	0.3031762	0.848079	1
TBC1D2	1.040813	1.03062517	1.0452289	1.476854636	0.5625278	0.848092	1
FAF2	1.4115044	1.40026203	1.4163775	1.04026226	0.0569473	0.848117	1
PVALB	1.1264267	1.1157018	1.1310755	1.132873221	0.1799864	0.848191	1
STRN4	1.2840529	1.27278352	1.2889377	1.059219751	0.0830019	0.848257	1
ZNF252P-AS1	1.0314101	1.0413337	1.0271087	0.655849937	-0.6085623	0.848277	1
POTEE	1.0422451	1.05208987	1.0379778	0.729082318	-0.4558464	0.848382	1
VPS13A	1.4935543	1.48158077	1.4987443	1.035639987	0.0505226	0.848455	1
USP51	1.0956795	1.08506188	1.1002818	1.178927793	0.2374754	0.84847	1
INHA	1.091631	1.10189478	1.0871822	0.855609675	-0.2249753	0.848505	1
SH2D3C	1.0428107	1.05279212	1.0384841	0.728975268	-0.4560582	0.848568	1
RP11-85A1.3	1.0224511	1.0323149	1.0181756	0.562452721	-0.8301963	0.84857	1
BGLAP	1.0224647	1.03236137	1.0181749	0.561623746	-0.8323242	0.84857	1
RAI1	1.1825528	1.17159704	1.1873017	1.0915203	0.126339	0.848616	1
ST3GAL4-AS1	1.1405761	1.15091796	1.1360934	0.901770402	-0.1491679	0.848633	1
ANKRD16	1.1279127	1.13830739	1.123407	0.892266309	-0.1644537	0.848667	1
SGPP1	1.0907464	1.08021017	1.0953134	1.188296164	0.2488945	0.848693	1
UBE2M	1.3136629	1.30228671	1.318594	1.053946286	0.0758013	0.848751	1
BCL2	1.0725595	1.08263405	1.0681926	0.825235716	-0.2771218	0.848775	1
ZNF497	1.0726605	1.08274043	1.0682913	0.825368117	-0.2768904	0.848775	1
AC006994.2	1.0335915	1.04359344	1.0292561	0.67111192	-0.5753747	0.848793	1
IGSF11	1.0336153	1.04355567	1.0293066	0.672853956	-0.5716347	0.848793	1
ARHGEF4	1.2031417	1.21372733	1.1985532	0.929002608	-0.1062454	0.84889	1
ASTE1	1.0702507	1.08027301	1.0659065	0.821029577	-0.2844939	0.848932	1
C2orf69	1.3871743	1.39872259	1.3821686	0.958482521	-0.061176	0.849075	1
LHX9	1.034897	1.04475374	1.0306245	0.684288635	-0.5473231	0.8491	1
FBXW4	1.2703613	1.28106406	1.2657221	0.945414658	-0.0809809	0.849222	1
USE1	1.5558893	1.54343132	1.5612892	1.032861414	0.0466467	0.849369	1
ZNF155	1.0635197	1.07336007	1.0592544	0.807719977	-0.3080729	0.849404	1
TSIX	1.0229904	1.01281342	1.0274016	2.138511651	1.0966071	0.849485	1
ZNF251	1.1078199	1.11799995	1.1034073	0.876333538	-0.190448	0.849513	1

FAM105A	1.0361446	1.0460282	1.0318605	0.692195824	-0.5307479	0.849549	1
TNFRSF11B	1.0361528	1.04616444	1.0318132	0.689127253	-0.5371577	0.849549	1
LRRK1	1.0362682	1.04622726	1.0319514	0.691180743	-0.5328651	0.849549	1
LIN7A	1.5128683	1.52457234	1.5077951	0.968017227	-0.0468954	0.849621	1
MBD2	1.1465919	1.13598428	1.1511898	1.111818437	0.1529212	0.849633	1
RP11-415F23.2	1.0241983	1.03402956	1.0199369	0.585868776	-0.7713505	0.849644	1
PDCD4	1.7203115	1.70766684	1.7257924	1.025613097	0.0364866	0.849664	1
GS1-393G12.14	1.0333966	1.04327587	1.0291144	0.672762527	-0.5718307	0.8497	1
WDR47	1.1201813	1.10958357	1.1247749	1.138627906	0.1872964	0.84975	1
GNG2	1.1886076	1.1774168	1.1934584	1.090417361	0.1248804	0.849782	1
CH507-338C24.1	1.0197179	1.02948819	1.0154829	0.525053356	-0.9294641	0.849859	1
MARK2	1.2330358	1.22213272	1.2377618	1.070359258	0.0980951	0.849963	1
CTD-2005H7.1	1.0217729	1.03156713	1.0175275	0.555244847	-0.848804	0.849997	1
PHF1	1.3805986	1.36869766	1.3857571	1.046269526	0.0652545	0.850022	1
CTB-113P19.4	1.0202311	1.03008455	1.0159601	0.53050796	-0.9145537	0.850038	1
SSTR2	1.0744863	1.06403782	1.0790152	1.233883742	0.3032065	0.850049	1
FBXO31	1.0970668	1.10725926	1.0926489	0.863784261	-0.2112571	0.850081	1
CSDC2	1.179514	1.1686909	1.1842054	1.091969986	0.1269332	0.85011	1
SLC31A2	1.0659595	1.05563254	1.0704358	1.2660898	0.3403797	0.850121	1
HFE	1.0487732	1.0587114	1.0444655	0.757357137	-0.4009543	0.850167	1
RP11-42I10.1	1.0420468	1.0318023	1.0464873	1.46175948	0.5477059	0.850338	1
SCAP	1.3345099	1.32307067	1.3394683	1.050755583	0.0714271	0.850414	1
SSH1	1.1843135	1.1735224	1.188991	1.089144834	0.1231958	0.850432	1
BCORL1	1.1291299	1.11857964	1.133703	1.127537166	0.173175	0.850438	1
VAMP5	1.997756	2.01204815	1.991561	0.979756774	-0.0295045	0.850494	1
SHROOM2	1.128952	1.13901957	1.1245881	0.89619107	-0.1581217	0.850498	1
DMGDH	1.0230866	1.01301898	1.0274504	2.108491135	1.076211	0.850564	1
C1orf131	1.4244243	1.43591194	1.4194449	0.962223868	-0.0555555	0.850597	1
AC091729.9	1.1466135	1.15671604	1.1422345	0.907594056	-0.1398809	0.85075	1
GSTM4	1.4488376	1.43749397	1.4537546	1.037167572	0.052649	0.850919	1
GK-AS1	1.0199158	1.029661	1.0156917	0.529033528	-0.9185689	0.850976	1
RP11-443O13.3	1.0199479	1.02976716	1.0156917	0.527146681	-0.9237236	0.850976	1
LRRC3-AS1	1.0199015	1.02961373	1.0156917	0.529877841	-0.9162683	0.850976	1
RP13-20L14.6	1.0248664	1.01466374	1.0292888	1.997360679	0.9980949	0.851129	1
CRH	1.0248611	1.01466374	1.0292813	1.996848337	0.9977248	0.851129	1
ZNF317	1.1486437	1.13828125	1.1531353	1.107419079	0.1472013	0.851178	1
RP11-350N15.5	1.039448	1.049213	1.0352153	0.715568607	-0.482838	0.851185	1
RP11-410N8.4	1.0201494	1.02984911	1.015945	0.534185568	-0.9045871	0.851209	1
RP11-247A12.7	1.031595	1.04126172	1.0274048	0.664171191	-0.5903729	0.851354	1
YBX2	1.0377343	1.0474825	1.0335088	0.70570884	-0.502855	0.85138	1
RP11-479O16.1	1.0377232	1.04752775	1.0334733	0.704289933	-0.5057586	0.85138	1
FBRS	1.2557696	1.24483608	1.2605087	1.064012837	0.0895156	0.851415	1
MYOM2	1.1198218	1.10931105	1.1243777	1.137833092	0.1862889	0.851542	1
CYP2W1	1.0240095	1.01394208	1.0283732	2.035079093	1.0250849	0.851558	1
KIAA0513	1.0733988	1.08326279	1.0691232	0.830180621	-0.2685028	0.851566	1
RP3-329A5.8	1.0304198	1.04011655	1.0262167	0.65351388	-0.6137102	0.851573	1
KB-1507C5.4	1.0304748	1.04018025	1.0262679	0.6537521	-0.6131844	0.851573	1
FLJ21408	1.0288771	1.03854796	1.0246851	0.64037479	-0.6430116	0.851579	1
ACOT1	1.0334847	1.02340066	1.0378557	1.617720183	0.6939621	0.851639	1
RP11-306O13.1	1.0189243	1.02855688	1.014749	0.516478275	-0.9532204	0.851689	1
XXbac-BPG154L12.5	1.0189446	1.02862406	1.014749	0.515266076	-0.9566105	0.851689	1

FAM120C	1.0964613	1.10637434	1.0921644	0.866416148	-0.206868	0.851711	1
BBX	3.2142869	3.19658993	3.2219577	1.0115487	0.0165658	0.85172	1
ZSWIM8	1.1560462	1.16626709	1.1516159	0.911881786	-0.1330813	0.851748	1
CDK5R2	1.0403764	1.05013541	1.0361462	0.720972323	-0.4719842	0.851892	1
C6orf163	1.0321999	1.04187542	1.028006	0.668793306	-0.5803677	0.851941	1
C2orf72	1.0322681	1.04206194	1.0280229	0.666228165	-0.5859117	0.851941	1
E2F4	1.4319786	1.44334698	1.4270509	0.963243028	-0.0540283	0.852	1
SLX4IP	1.2725256	1.28280859	1.2680684	0.947879307	-0.0772247	0.852064	1
KYAT3	1.2965117	1.2857455	1.3011784	1.054009329	0.0758876	0.852065	1
MIR155HG	1.0989286	1.10906816	1.0945336	0.866738607	-0.2063311	0.852082	1
RNF4	1.4818271	1.47032591	1.4868123	1.03505318	0.0497049	0.852161	1
THBD	1.0507961	1.04064172	1.0551976	1.358150202	0.441643	0.85219	1
NXNL2	1.0401077	1.02992927	1.0445195	1.487492094	0.572882	0.852236	1
ACP1	4.4896008	4.4612611	4.5018848	1.011736665	0.0168338	0.8523	1
RP13-554M15.8	1.0249453	1.03463799	1.020744	0.598879287	-0.7396629	0.852372	1
DROSHA	1.2414923	1.25219842	1.2368516	0.939147846	-0.0905758	0.85244	1
CTC-487M23.8	1.0196005	1.02931613	1.0153892	0.524939217	-0.9297777	0.852472	1
PIWIL4	1.0286011	1.03838208	1.0243615	0.634709158	-0.6558324	0.852522	1
RP11-102G14.1	1.022179	1.03183974	1.0179915	0.565065022	-0.8235112	0.852522	1
ZNF37A	1.0221648	1.03178035	1.017997	0.566291813	-0.8203824	0.852522	1
RP1-122P22.2	1.0221715	1.03179187	1.0180016	0.566231243	-0.8205367	0.852522	1
GNAO1	1.0314482	1.02135805	1.0358218	1.677202564	0.7460569	0.852543	1
AC003075.4	1.0416692	1.0315648	1.046049	1.458872425	0.5448537	0.852581	1
FAM199X	1.5415149	1.52942751	1.5467542	1.032727288	0.0464593	0.852602	1
DNAH5	1.0571056	1.06688883	1.052865	0.790341405	-0.3394521	0.852634	1
C3orf33	1.1837802	1.1732262	1.1883549	1.087335071	0.1207966	0.852663	1
USP21	1.130108	1.11965125	1.1346405	1.125274841	0.1702774	0.852669	1
TET3	1.1040955	1.09394778	1.1084941	1.154833929	0.2076854	0.852698	1
KIAA1211L	1.0309579	1.02091297	1.035312	1.688520186	0.7557594	0.852722	1
ZNF347	1.1258484	1.13583815	1.1215183	0.894581449	-0.1607153	0.852723	1
GJD2	1.0335217	1.02344895	1.0378878	1.615755745	0.6922091	0.852801	1
RRS1	1.1660761	1.15556239	1.1706333	1.096880055	0.1334058	0.852802	1
SIK1	1.0395334	1.04916324	1.0353593	0.719222823	-0.4754893	0.852846	1
CPXM2	1.2111514	1.20050143	1.2157678	1.076140754	0.1058668	0.852848	1
ZC3HAV1L	1.0433486	1.0333373	1.0476881	1.430471387	0.5164906	0.852886	1
SAMHD1	1.0750145	1.08493139	1.070716	0.832624913	-0.2642614	0.852903	1
RHOV	1.033016	1.022963	1.0373735	1.627551728	0.7027034	0.852926	1
ZNF599	1.2405722	1.25103876	1.2360354	0.94023502	-0.0889067	0.852962	1
MLYCD	1.2566359	1.24564782	1.2613987	1.064119751	0.0896605	0.852998	1
S100A1	1.068776	1.05875573	1.0731194	1.244463679	0.3155241	0.853002	1
ZNF202	1.0765066	1.08632986	1.0722487	0.836890605	-0.256889	0.853006	1
ZNF207	2.8553621	2.87432441	2.8471428	0.985497933	-0.0210752	0.853047	1
VANGL1	1.1213423	1.11103222	1.1258112	1.13310529	0.1802819	0.853069	1
RP11-616M22.12	1.0205543	1.03010815	1.0164131	0.545138252	-0.8753059	0.853088	1
CCDC126	1.2354732	1.22491071	1.2400516	1.067319441	0.093992	0.853363	1
RP11-1008C21.2	1.0686114	1.07842591	1.0643572	0.820611437	-0.2852288	0.853404	1
SAMD14	1.3529683	1.36424025	1.3480824	0.955639622	-0.0654614	0.853435	1
TMEM8B	1.3025191	1.29120271	1.3074242	1.055705264	0.0782071	0.853439	1
SF3A1	1.4084782	1.41958883	1.4036623	0.962042527	-0.0558274	0.853452	1
ZNF837	1.1105099	1.12044156	1.1062049	0.881796361	-0.1814826	0.853472	1
ADAMTS20	1.0489593	1.05869396	1.0447397	0.762254212	-0.3916559	0.853525	1

GNG7	1.1417626	1.15175884	1.1374296	0.905579207	-0.1430873	0.853559	1
PLEKHH3	1.1703503	1.18028405	1.1660445	0.921015992	-0.1187019	0.853576	1
PABPC1	13.709687	13.7722902	13.682551	0.992973891	-0.0101723	0.853626	1
CCDC137	1.4704708	1.48167211	1.4656155	0.966664894	-0.0489122	0.853661	1
ABCB11	1.0261922	1.01622158	1.030514	1.881074268	0.9115568	0.853738	1
FOXJ3	1.3343692	1.34512777	1.3297059	0.955315508	-0.0659508	0.853767	1
ZNF286B	1.1307524	1.12007521	1.1353805	1.127464356	0.1730818	0.853773	1
WASL	1.8028577	1.79033791	1.8082845	1.022707526	0.0323936	0.853851	1
RP11-799D4.4	1.0403336	1.03030033	1.0446826	1.474657585	0.56038	0.853961	1
NINL	1.3694273	1.35804574	1.3743608	1.045566855	0.0642853	0.854006	1
CDKL3	1.2041954	1.19327689	1.2089281	1.080978102	0.1123373	0.854032	1
ELOVL2	1.1677652	1.17781746	1.163408	0.918964791	-0.1219185	0.85404	1
FAM181A	1.0371823	1.04685227	1.0329909	0.704146258	-0.506053	0.854053	1
COLQ	1.0371529	1.04678904	1.032976	0.704780544	-0.504754	0.854053	1
ADAP1	1.0307913	1.04043273	1.0266121	0.65818237	-0.6034407	0.854145	1
SPDYA	1.0308476	1.04045779	1.0266819	0.659500831	-0.6005536	0.854145	1
CCDC122	1.2084894	1.21881068	1.2040156	0.932384002	-0.1010038	0.854174	1
ZNF580	3.3815585	3.36084747	3.3905358	1.012575299	0.0180292	0.854184	1
PACRG	1.094331	1.10418359	1.0900604	0.864439522	-0.2101631	0.854191	1
UPK3A	1.0316832	1.02170759	1.0360072	1.658736971	0.7300851	0.854261	1
CTTNBP2	1.0618406	1.05176095	1.0662097	1.279143343	0.3551779	0.854308	1
PLCH2	1.0255984	1.01564798	1.0299114	1.911520401	0.9347206	0.854322	1
AGAP2-AS1	1.065916	1.07562525	1.0617074	0.815963215	-0.293424	0.854374	1
TUBGCP6	1.1056196	1.11548498	1.1013434	0.877545986	-0.1884534	0.854407	1
DRP2	1.0457629	1.05525854	1.0416469	0.753673582	-0.4079883	0.854436	1
TMEM170B	1.1624932	1.15205432	1.1670179	1.098409605	0.1354161	0.854525	1
SRC	1.162445	1.15197662	1.1669826	1.098738876	0.1358486	0.854525	1
RECK	1.2200392	1.23019576	1.2156367	0.936753622	-0.0942584	0.854653	1
GPATCH1	1.0979791	1.10788722	1.0936843	0.86835401	-0.2036448	0.854666	1
RP11-138A9.2	1.0337276	1.0237356	1.0380587	1.603441685	0.6811719	0.854703	1
RP11-544A12.8	1.1824953	1.19247573	1.1781692	0.925671063	-0.1114285	0.854749	1
STK10	1.0980113	1.08787483	1.102405	1.165351335	0.220765	0.854754	1
SMIM13	1.0978777	1.08783879	1.1022291	1.163825992	0.2188754	0.854754	1
TMEM64	1.251654	1.24097661	1.2562821	1.063514529	0.0888397	0.854763	1
PPT2-EGFL8	1.0290973	1.03861608	1.0249713	0.646654961	-0.628932	0.854821	1
NDUFV2-AS1	1.064823	1.07439994	1.0606719	0.815483103	-0.2942731	0.854865	1
RIOK3	1.597984	1.60930185	1.5930782	0.973373372	-0.0389348	0.854881	1
NDUFB2-AS1	1.0256328	1.01574718	1.0299177	1.89988006	0.9259083	0.854898	1
IGSF21	1.0419865	1.03204434	1.046296	1.444748966	0.5308188	0.854903	1
FKTN	1.1920914	1.18113704	1.1968396	1.086688854	0.1199389	0.855063	1
ABCC1	1.1906009	1.20083641	1.1861643	0.926944793	-0.1094447	0.855209	1
PITPNM3	1.0599098	1.04998049	1.0642137	1.284775828	0.3615167	0.85523	1
PRPSAP1	2.3988049	2.4137302	2.3923355	0.984866499	-0.0219999	0.855233	1
GTDC1	1.2203921	1.20982046	1.2249745	1.072223766	0.100606	0.855258	1
DCAF7	2.5330549	2.54823275	2.5264759	0.985947317	-0.0204175	0.855346	1
OIP5	1.2896607	1.27894966	1.2943034	1.055041313	0.0772995	0.855413	1
FAM46B	1.035919	1.02608348	1.0401822	1.540522812	0.62342	0.855553	1
AP3B2	1.0438137	1.03395318	1.0480878	1.416298834	0.5021257	0.85557	1
USP42	1.2668857	1.25583137	1.2716773	1.061938786	0.0867006	0.855608	1
TM4SF18	1.0246893	1.01490147	1.0289319	1.941543134	0.9572038	0.855657	1
KLRD1	1.0387469	1.02887269	1.0430269	1.490226558	0.5755317	0.855704	1

RP11-365P13.5	1.0302922	1.0398709	1.0261403	0.655622674	-0.6090623	0.855715	1
KIT	1.0394759	1.0490128	1.0353421	0.72107862	-0.4717715	0.855752	1
RP11-629O1.2	1.047373	1.05705038	1.0431783	0.756844278	-0.4019316	0.855763	1
CTA-14H9.5	1.077203	1.06710505	1.08158	1.215706114	0.2817945	0.85577	1
RP11-256I23.2	1.077415	1.06734089	1.0817817	1.214443312	0.2802951	0.85577	1
CNOT6	1.4234735	1.41214772	1.4283827	1.039391272	0.0557388	0.855772	1
CH17-360D5.2	1.0289912	1.03840824	1.0249093	0.648540618	-0.6247312	0.855798	1
PRR29-AS1	1.0283488	1.03781889	1.024244	0.641054749	-0.6414805	0.855816	1
HMGA1P4	1.0282958	1.03780442	1.0241743	0.639456862	-0.6450811	0.855816	1
CTB-58E17.5	1.0427204	1.03274756	1.0470432	1.436540174	0.5225983	0.855893	1
TCTEX1D4	1.025772	1.03528997	1.0216464	0.613386703	-0.7051312	0.855895	1
MRGBP	1.4378253	1.42696204	1.442534	1.036471532	0.0516805	0.855961	1
SOST	1.0274041	1.01751621	1.03169	1.809181379	0.8553371	0.85597	1
TXNDC5	1.0351643	1.02534637	1.03942	1.555251809	0.6371482	0.855985	1
SCAPER	1.4553621	1.46634098	1.4506033	0.966252879	-0.0495273	0.855995	1
AP001059.6	1.0278577	1.03727441	1.023776	0.637863172	-0.6486811	0.856052	1
SMC5-AS1	1.0758429	1.08541392	1.0716942	0.83937426	-0.2526139	0.856054	1
RP11-816J6.3	1.0262485	1.03571425	1.0221455	0.620074149	-0.6894873	0.856074	1
CNTN6	1.0548268	1.06455549	1.0506098	0.783973949	-0.3511224	0.856115	1
RERG	1.1222055	1.13204649	1.1179398	0.893168829	-0.1629952	0.856211	1
FAR2	1.3009236	1.31137759	1.2963922	0.951873919	-0.0711576	0.856279	1
FZD7	1.4135255	1.40234803	1.4183705	1.039822407	0.0563371	0.856296	1
ZW10	1.1353115	1.12509819	1.1397385	1.117030683	0.1596688	0.85631	1
RP11-615I2.2	1.0720146	1.06191568	1.076392	1.233807135	0.3031169	0.856343	1
PLXND1	1.2723728	1.26215842	1.2768003	1.055851202	0.0784065	0.856376	1
PTPRQ	1.0296875	1.03908925	1.0256122	0.655223693	-0.6099406	0.856385	1
MANEAL	1.0265605	1.01678166	1.0307993	1.835291924	0.8760096	0.8564	1
DDX20	1.0964577	1.10614679	1.0922579	0.869154262	-0.2023158	0.856403	1
CKAP5	1.5035718	1.51492589	1.4986503	0.968392312	-0.0463365	0.856407	1
ATG2B	1.1577764	1.14754206	1.1622125	1.099432336	0.1367588	0.856435	1
FRAT1	1.0361088	1.02624531	1.0403842	1.538719222	0.62173	0.856461	1
SMG5	1.2194407	1.22965556	1.2150131	0.936241582	-0.0950473	0.856462	1
DSCC1	1.1323915	1.14222658	1.1281285	0.900875669	-0.1506001	0.856541	1
TMCC3	1.097414	1.08738652	1.1017604	1.164486265	0.2196936	0.856561	1
ORC5	1.133561	1.12333968	1.1379915	1.118792478	0.1619425	0.856582	1
NTN5	1.02986	1.03927385	1.0257795	0.656404546	-0.6073429	0.856648	1
SLC14A2	1.0298899	1.03929363	1.0258138	0.656946793	-0.6061516	0.856648	1
MX1	1.0662288	1.07580275	1.0620789	0.818953009	-0.2881474	0.856673	1
RP11-1336O20.2	1.0438139	1.03400178	1.048067	1.413662638	0.4994379	0.856712	1
TMX3	1.4105268	1.42157796	1.4057366	0.962423537	-0.0552562	0.856904	1
PHACTR4	1.683942	1.67215037	1.6890531	1.025147227	0.0358311	0.856916	1
ZNF418	1.0712335	1.0612742	1.0755504	1.232988747	0.3021596	0.856941	1
C8orf89	1.0169936	1.02637833	1.0129257	0.490011098	-1.0291137	0.856964	1
LA16c-306E5.2	1.0196147	1.02904499	1.0155271	0.534588796	-0.9034985	0.856997	1
ZBTB12	1.0259821	1.03537479	1.0219108	0.619389341	-0.6910815	0.857017	1
ZNF649	1.1704155	1.16024624	1.1748234	1.090967022	0.1256075	0.857021	1
TRAF3IP1	1.2545568	1.26508042	1.2499952	0.943092109	-0.0845294	0.857052	1
CCDC68	1.0352223	1.02545096	1.0394577	1.550343271	0.6325877	0.857122	1
RPRD1B	1.2869344	1.29755749	1.2823298	0.948824339	-0.0757871	0.857193	1
WIZ	1.3155691	1.32595441	1.3110675	0.954328154	-0.0674427	0.857244	1
NUP35	1.1659761	1.15555572	1.1704929	1.09602483	0.1322805	0.857286	1

RP11-342K6.1	1.0655612	1.07528123	1.061348	0.814917063	-0.2952749	0.857336	1
RP11-46J23.1	1.0438633	1.05343846	1.0397129	0.743152708	-0.4282694	0.857351	1
HCFC2	1.0914714	1.1012312	1.0872409	0.861798577	-0.2145774	0.8574	1
TDO2	1.0376046	1.04696342	1.0335479	0.714341141	-0.4853149	0.857413	1
DNAJC25	1.2659944	1.25541907	1.2705784	1.059350635	0.0831802	0.85746	1
NRSN2-AS1	1.0674498	1.07695774	1.0633285	0.822899513	-0.2812118	0.85754	1
UBA3	1.460933	1.4500288	1.4656595	1.034732624	0.049258	0.857544	1
RAB40A	1.0350218	1.04452076	1.0309044	0.694156342	-0.5266675	0.857586	1
HELQ	1.1878174	1.17742297	1.1923229	1.083979837	0.1163379	0.857599	1
IL1RAPL1	1.0361839	1.02630374	1.0404665	1.538431964	0.6214606	0.857663	1
HIST1H2BM	1.0178605	1.0271487	1.0138345	0.509582107	-0.9726135	0.857722	1
DRD1	1.0178847	1.02722886	1.0138345	0.508081872	-0.9768671	0.857722	1
FRMPD2	1.0178644	1.02716156	1.0138345	0.50934071	-0.9732971	0.857722	1
PNMA5	1.0178828	1.0272224	1.0138345	0.508202456	-0.9765247	0.857722	1
AC006116.17	1.0249717	1.03433003	1.0209153	0.609240763	-0.7149156	0.857733	1
SDCBP2-AS1	1.1391479	1.14864066	1.1350332	0.908454168	-0.1385144	0.857832	1
RUNDC3B	1.0689511	1.05889996	1.0733078	1.244615921	0.3157006	0.857847	1
CTC-534A2.2	1.0690678	1.05915134	1.0733661	1.240311708	0.3107027	0.857847	1
IQUB	1.0568496	1.06635997	1.0527273	0.79456476	-0.3317633	0.858002	1
LEF1	1.1869776	1.17665388	1.1914524	1.083771463	0.1160606	0.858062	1
SLCO5A1	1.1436743	1.15331783	1.1394942	0.90983686	-0.1363202	0.858073	1
THNSL2	1.3018232	1.29095507	1.306534	1.053544179	0.0752508	0.858112	1
GJB7	1.0373453	1.02765038	1.0415476	1.502605835	0.5874666	0.858119	1
PRRG4	1.0269918	1.01724954	1.0312146	1.80958971	0.8556626	0.858138	1
RP11-347P5.1	1.0290284	1.01933168	1.0332315	1.719019182	0.7815856	0.858302	1
DOK1	1.2179838	1.22825834	1.2135303	0.935476407	-0.0962268	0.858304	1
AC137932.4	1.0847512	1.07477221	1.0890766	1.191306003	0.252544	0.858349	1
EPS8	1.2021384	1.19155045	1.2067278	1.079234006	0.1100077	0.858438	1
ADCY4	1.0309831	1.04034293	1.026926	0.667429101	-0.5833135	0.858455	1
TSGA10IP	1.0310037	1.04032731	1.0269623	0.668587063	-0.5808127	0.858455	1
TRHDE	1.0345674	1.04391735	1.0305147	0.694820373	-0.525288	0.858533	1
TRABD2A	1.025587	1.03487385	1.0215616	0.618272869	-0.6936844	0.858537	1
APLNR	1.0256711	1.03496132	1.0216442	0.619091329	-0.6917758	0.858537	1
MYL4	1.0916096	1.10153119	1.0873091	0.859923508	-0.2177198	0.858558	1
SPRTN	1.1903697	1.20038502	1.1860285	0.92835521	-0.1072512	0.858602	1
GYG2	1.2450541	1.2552156	1.2406495	0.942926464	-0.0847828	0.858628	1
SMIM24	1.0809787	1.07107898	1.0852698	1.199649126	0.2626125	0.858636	1
RP11-459F6.3	1.0178399	1.02712282	1.0138162	0.509392934	-0.9731491	0.858643	1
ZFP82	1.290623	1.28004912	1.2952063	1.054123254	0.0760436	0.858654	1
CIPC	1.1572985	1.1670138	1.1530874	0.916615043	-0.1256121	0.858693	1
ZNF675	1.1314125	1.14106278	1.1272295	0.901935039	-0.1489046	0.858695	1
EIF3L	7.6260057	7.66395233	7.6095575	0.991837446	-0.0118244	0.858756	1
BCKDHA	1.04568	1.05495481	1.0416597	0.758072542	-0.3995922	0.858823	1
EEF1A2	1.0785583	1.06879495	1.0827903	1.203435199	0.2671585	0.858823	1
BTBD3	1.6189371	1.63040552	1.613966	0.973922359	-0.0381213	0.858861	1
CADPS2	1.2843566	1.27358489	1.2890256	1.05643843	0.0792087	0.858967	1
TMEM246	1.6154462	1.626784	1.6105318	0.974070487	-0.0379019	0.859001	1
FBXL22	1.0462786	1.05574168	1.0421768	0.756647106	-0.4023075	0.859013	1
LINC00909	1.2896867	1.3001414	1.2851551	0.950069266	-0.0738954	0.859037	1
COCH	1.1272091	1.11727069	1.1315169	1.121481335	0.1654056	0.859075	1
CPT1B	1.0435866	1.05286693	1.0395639	0.748368077	-0.4181801	0.859084	1

ADRA2C	1.0259383	1.03510974	1.0219628	0.625548321	-0.6768068	0.859113	1
DUSP7	1.0818656	1.07201821	1.086134	1.196003732	0.2582219	0.859124	1
ACTR5	1.0699141	1.06003636	1.0741956	1.235845039	0.3054979	0.859176	1
WDR53	1.0881989	1.07842956	1.0924335	1.178554672	0.2370187	0.859208	1
VSTM2A	1.0184273	1.02766584	1.0144228	0.521321019	-0.9397561	0.859226	1
TMIE	1.0264761	1.03574265	1.0224595	0.628367901	-0.6703186	0.859298	1
JAKMIP3	1.0265197	1.03583961	1.02248	0.627237743	-0.6729157	0.859298	1
RP11-410N8.3	1.0265143	1.03576641	1.0225039	0.629191003	-0.6684301	0.859298	1
RP11-756H6.1	1.0265669	1.03582286	1.0225549	0.629623273	-0.6674392	0.859298	1
PRDM6	1.0531039	1.04342347	1.0572999	1.31956029	0.4000573	0.859316	1
TEF	1.1138686	1.10398244	1.1181538	1.136285906	0.1843259	0.859332	1
OSBPL5	1.1164362	1.10655105	1.120721	1.132987075	0.1801314	0.859413	1
AC005618.6	1.0186774	1.02788039	1.0146883	0.526833233	-0.9245817	0.859489	1
CTA-984G1.5	1.0186906	1.02788531	1.0147051	0.52734299	-0.9231865	0.859489	1
RP11-793H13.3	1.018683	1.02791414	1.0146817	0.5259582	-0.9269799	0.859489	1
INIP	1.4278506	1.43873365	1.4231333	0.964442407	-0.052233	0.859513	1
TRANK1	1.1452659	1.15494436	1.1410707	0.910460696	-0.1353314	0.859519	1
KCNIP1	1.0383152	1.04754309	1.0343153	0.721771982	-0.470385	0.859595	1
RP11-359E3.4	1.0383393	1.04767087	1.0342945	0.719400562	-0.4751328	0.859595	1
CUL7	1.2041744	1.21374223	1.2000272	0.935833769	-0.0956758	0.859629	1
COX6B2	1.0586052	1.0488218	1.0628459	1.287250122	0.3642924	0.859646	1
HECTD4	1.2720382	1.28236927	1.2675601	0.947553994	-0.0777199	0.859688	1
PKI55	1.1337205	1.123755	1.13804	1.115430003	0.1576	0.859794	1
TMEM125	1.0369615	1.02728855	1.0411543	1.508115667	0.5927471	0.859795	1
CHAC1	1.0368819	1.02731731	1.0410278	1.501896932	0.5867858	0.859795	1
ROBO2	1.1083303	1.09838878	1.1126394	1.144840207	0.1951462	0.85984	1
GNPTG	1.1973891	1.20748715	1.1930121	0.930236294	-0.1043309	0.859863	1
LLNLR-284B4.2	1.0162462	1.02540272	1.0122773	0.483306592	-1.0489894	0.859929	1
GS1-590J6.3	1.0162909	1.02555029	1.0122773	0.48051514	-1.0573462	0.859929	1
RP3-323A16.1	1.0162485	1.02541018	1.0122773	0.483164776	-1.0494128	0.859929	1
TYW1	1.1420591	1.13205217	1.1463967	1.108627856	0.1487752	0.859993	1
ADCK2	1.2234295	1.23342597	1.2190965	0.938612522	-0.0913984	0.860002	1
CTDP1	1.1002683	1.10988746	1.0960988	0.874520088	-0.1934366	0.860005	1
RLIM	1.296576	1.2859055	1.3012012	1.053499272	0.0751893	0.860024	1
RNASEL	1.0645665	1.07397171	1.0604897	0.817741503	-0.2902832	0.860045	1
LINC01547	1.0397099	1.0490368	1.0356671	0.727353647	-0.4592711	0.860047	1
IRX2	1.0500476	1.04024589	1.0542962	1.349112526	0.4320107	0.860081	1
ITPK1	1.1553578	1.1456385	1.1595707	1.095662763	0.1318038	0.860116	1
MPP2	1.1803715	1.17018153	1.1847884	1.085831215	0.1187999	0.860136	1
SMPDL3A	1.2544428	1.26487308	1.2499218	0.943553067	-0.0838244	0.860204	1
RP11-250B2.3	1.0283209	1.0187158	1.0324843	1.735660587	0.7954849	0.860236	1
ENPEP	1.0626901	1.07212345	1.0586012	0.812512801	-0.2995376	0.860261	1
SPDYE3	1.0435144	1.05280213	1.0394885	0.747858464	-0.4191628	0.860271	1
TRMT2B	1.0719764	1.08131146	1.0679301	0.835430386	-0.2594085	0.860271	1
CALCRL	1.1090015	1.11861203	1.1048357	0.883854049	-0.1781199	0.860272	1
RP11-796E10.1	1.0231968	1.03242345	1.0191975	0.592085724	-0.756122	0.860406	1
RP11-894P9.1	1.0653454	1.07453603	1.0613617	0.823248637	-0.2805999	0.860484	1
TMEM143	1.1322463	1.14176257	1.1281215	0.903775109	-0.1459643	0.860536	1
RP5-1116H23.5	1.0171436	1.0262586	1.0131926	0.502411462	-0.9930587	0.860585	1
DSCAML1	1.0236665	1.03289312	1.0196671	0.597910169	-0.7419993	0.860586	1
RP11-575L7.8	1.0236596	1.03285035	1.0196758	0.598951467	-0.739489	0.860586	1

RP11-977B10.2	1.0354267	1.02571441	1.0396366	1.541415648	0.6242559	0.860593	1
MYSM1	1.2650864	1.27496268	1.2608055	0.94851242	-0.0762614	0.8607	1
AC002310.12	1.23393	1.24359574	1.2297403	0.94312116	-0.084485	0.860718	1
RP11-306G20.1	1.0422799	1.0515609	1.038257	0.741977664	-0.4305523	0.860757	1
MTG1	1.196368	1.20633853	1.1920463	0.930734039	-0.1035591	0.860785	1
METTL18	1.1606882	1.1704104	1.156474	0.918218543	-0.1230905	0.860787	1
RSG1	1.0747259	1.06487282	1.0789967	1.217717246	0.2841792	0.860794	1
PTPN9	1.3086728	1.29813982	1.3132383	1.050642343	0.0712716	0.860797	1
ZNF564	1.083342	1.09282883	1.0792299	0.853505275	-0.228528	0.860902	1
MXD1	1.1083861	1.09849645	1.1126728	1.143927849	0.1939961	0.860966	1
TBCEL	1.1078839	1.09800617	1.1121654	1.144472757	0.1946831	0.861024	1
RP11-707M3.3	1.0273694	1.03653912	1.0233948	0.64026727	-0.6432538	0.861108	1
GALR2	1.0291719	1.01968762	1.0332828	1.690546786	0.7574899	0.861234	1
MSRB3	1.2999817	1.28937553	1.3045791	1.052539122	0.0738739	0.861243	1
RP11-114H21.2	1.0197779	1.02897982	1.0157893	0.54483799	-0.8761008	0.861338	1
C10orf76	1.1270248	1.13668536	1.1228374	0.898687555	-0.1541085	0.861479	1
THBS2	1.0805561	1.08987557	1.0765165	0.851360217	-0.2321584	0.861492	1
ERN1	1.0804334	1.08980793	1.0763699	0.850369466	-0.2338383	0.861492	1
MIEF2	1.2180608	1.22793707	1.2137799	0.937889845	-0.0925096	0.861498	1
GRM7	1.0248624	1.03402323	1.0208916	0.614038015	-0.7036001	0.861516	1
SLFN11	1.0233492	1.0324927	1.0193858	0.596621366	-0.7451125	0.861528	1
CTD-2033A16.3	1.0233921	1.03257911	1.01941	0.595780863	-0.7471463	0.861528	1
ARL4C	1.9549506	1.94107124	1.9609667	1.021141331	0.0301826	0.861539	1
ZNF622	1.2833399	1.27317114	1.2877476	1.053360063	0.0749987	0.861622	1
CCBE1	1.042904	1.05206396	1.0389335	0.747801524	-0.4192727	0.861639	1
RTN4R	1.0911829	1.08142793	1.0954112	1.171726182	0.2286355	0.861658	1
A4GALT	1.0276861	1.01815945	1.0318155	1.752007117	0.8090086	0.861665	1
DLG1	1.6951674	1.70637579	1.690309	0.97725463	-0.0331936	0.861698	1
DDX60L	1.0915758	1.10110765	1.0874442	0.864862034	-0.2094581	0.861701	1
RCC2	1.6401837	1.62836304	1.6453074	1.026965827	0.0383882	0.86174	1
KCNJ8	1.0533439	1.06255286	1.0493522	0.788967896	-0.3419615	0.861752	1
COG7	1.1880524	1.19793444	1.1837689	0.928433388	-0.1071297	0.861798	1
CTD-3185P2.1	1.0555013	1.04577867	1.0597156	1.304440544	0.3834312	0.861833	1
TRIM17	1.0603294	1.06972753	1.0562557	0.806792894	-0.3097297	0.861854	1
PRELID3B	1.27929	1.26897631	1.2837605	1.054964825	0.0771949	0.861973	1
PUS7	1.1407676	1.15040378	1.1365908	0.908160617	-0.1389806	0.862039	1
BCL2L14	1.0153849	1.02443645	1.0114615	0.469031788	-1.0922424	0.862053	1
RP11-480D4.6	1.0153744	1.02440158	1.0114615	0.46970208	-1.0901821	0.862053	1
USP12	1.2037952	1.19400663	1.2080381	1.072324709	0.1007418	0.862092	1
SCX	1.1054082	1.11489614	1.1012956	0.881627339	-0.1817591	0.862102	1
TEKT2	1.3022676	1.31210964	1.2980015	0.954797517	-0.0667333	0.862126	1
AJ006998.2	1.0257931	1.0349423	1.0218273	0.62466819	-0.678838	0.862175	1
MCUR1	1.8924346	1.90437817	1.8872576	0.981069243	-0.0275731	0.862236	1
MPPE1	1.2318994	1.2417541	1.2276278	0.941567651	-0.0868633	0.862363	1
BTBD9	1.0717294	1.08093013	1.0677413	0.837034344	-0.2566413	0.862364	1
BARHL2	1.0362512	1.02658168	1.0404425	1.521444401	0.6054416	0.86244	1
MARC2	1.0902502	1.08067088	1.0944025	1.1702173	0.2267765	0.862443	1
PCDHA4	1.0741082	1.06438804	1.0783215	1.216398337	0.2826157	0.86249	1
AKNA	1.1515276	1.14135666	1.1559363	1.103140942	0.1416171	0.862562	1
AC016723.4	1.0279637	1.01850742	1.0320625	1.732414807	0.7927844	0.862608	1
ALX1	1.0280084	1.01850742	1.0321266	1.735877724	0.7956653	0.862608	1

COL4A4	1.0279628	1.01850742	1.0320612	1.732344323	0.7927257	0.862608	1
RASGEF1A	1.0716137	1.06194044	1.0758067	1.223863837	0.2914431	0.862624	1
PCDHA3	1.0249375	1.03396371	1.0210251	0.61904605	-0.6918814	0.862651	1
CTB-113D17.1	1.0249164	1.03412035	1.0209269	0.613325211	-0.7052758	0.862651	1
PPP1R16A	1.5254366	1.51456759	1.5301478	1.030278273	0.0430341	0.862737	1
SLC25A42	1.313613	1.32366417	1.3092563	0.955485064	-0.0656948	0.862787	1
ERCC3	1.1833503	1.19296582	1.1791824	0.928570835	-0.1069161	0.862802	1
TSR1	1.2976599	1.28751712	1.3020563	1.050568197	0.0711698	0.862843	1
CDK20	1.1629065	1.1529455	1.1672241	1.093357577	0.1287653	0.862927	1
RENBP	1.2895982	1.27921153	1.2941004	1.053324575	0.0749501	0.863019	1
XCL1	1.023031	1.03204704	1.019123	0.596716483	-0.7448825	0.86303	1
RP11-883G14.1	1.0230713	1.03206276	1.0191739	0.598011263	-0.7417554	0.86303	1
RP4-671O14.7	1.0230281	1.03211374	1.0190899	0.594445977	-0.7503824	0.86303	1
SLC25A18	1.0256212	1.03470777	1.0216826	0.624719435	-0.6787197	0.863082	1
TJP1	1.9821269	1.99417489	1.9769047	0.982628619	-0.0252818	0.863086	1
UBE3B	1.3262082	1.33641627	1.3217835	0.956504076	-0.064157	0.863095	1
CH17-353B19.1	1.0316196	1.0221641	1.0357182	1.611533223	0.6884339	0.863123	1
RP13-39P12.3	1.0152803	1.02436032	1.0113445	0.465696484	-1.1025381	0.863133	1
RP11-54A4.2	1.0152803	1.02436032	1.0113445	0.465696484	-1.1025381	0.863133	1
CDHR2	1.0152454	1.02424487	1.0113445	0.467914182	-1.0956841	0.863133	1
RP11-495P10.1	1.0786925	1.08763742	1.0748153	0.853691392	-0.2282135	0.863171	1
LL0XNC01-237H1.2	1.0785853	1.08772831	1.0746222	0.850605264	-0.2334383	0.863171	1
GALNT13	1.0569555	1.06615359	1.0529685	0.800689313	-0.3206855	0.863187	1
LINC00623	1.0569117	1.0660967	1.0529304	0.800801959	-0.3204826	0.863187	1
VHL	1.2995238	1.30915597	1.2953487	0.955338817	-0.0659156	0.863249	1
HRK	1.0322606	1.04123818	1.0283692	0.68793468	-0.5396565	0.863264	1
AC129492.6	1.0407077	1.0311875	1.0448343	1.437571886	0.5236341	0.86327	1
ZNF213	1.1267953	1.1361327	1.122748	0.90167901	-0.1493142	0.863281	1
L3HYPDH	1.1266145	1.13594991	1.1225681	0.901567733	-0.1494922	0.863281	1
IZUMO4	1.061974	1.07114	1.058001	0.815307881	-0.2945831	0.863294	1
ZBTB11-AS1	1.0567624	1.04727361	1.0608754	1.287724217	0.3648237	0.863326	1
RP11-35O15.1	1.0404502	1.04943279	1.0365567	0.739523025	-0.435333	0.863385	1
ADAMTSL5	1.031279	1.02193758	1.035328	1.610389453	0.6874096	0.863387	1
TTC7B	1.0980115	1.10731237	1.0939799	0.875760295	-0.1913921	0.863397	1
INMT	1.0437578	1.05275019	1.03986	0.755637659	-0.4042335	0.863445	1
CCND2-AS1	1.0786076	1.06897444	1.0827831	1.200199476	0.2632742	0.863506	1
COA7	1.1819794	1.17218332	1.1862255	1.08155367	0.1131053	0.863506	1
KLHL31	1.039435	1.02992766	1.043556	1.455376035	0.541392	0.863508	1
ESPNL	1.040993	1.05007167	1.0370578	0.740095062	-0.4342175	0.863566	1
CSRNP3	1.4145732	1.40367865	1.4192954	1.038686201	0.0547599	0.863581	1
NTAN1	1.6752078	1.66399702	1.6800672	1.024202171	0.0345005	0.863648	1
TNFSF12	1.0644017	1.05493199	1.0685064	1.247112639	0.3185918	0.863683	1
MPP1	1.2803243	1.27015654	1.2847316	1.053950344	0.0758069	0.863787	1
RP11-482D24.2	1.023899	1.03289082	1.0200015	0.608116962	-0.7175793	0.863792	1
GAD1	1.1187947	1.10929589	1.122912	1.124580084	0.1693864	0.863805	1
ARHGEF17	1.2121064	1.22176324	1.2079206	0.937579391	-0.0929872	0.863854	1
ST6GALNAC1	1.0227741	1.01338003	1.026846	2.006420423	1.0046239	0.863856	1
EXOC3-AS1	1.3307459	1.34044101	1.3265434	0.959177743	-0.0601299	0.863866	1
NAAA	1.0824224	1.07296662	1.0865211	1.185762731	0.2458154	0.864061	1
CDK15	1.0394663	1.03001567	1.0435627	1.451333656	0.5373792	0.864168	1
TADA2B	1.066305	1.05668545	1.0704746	1.243258054	0.3141258	0.864243	1

LINC01569	1.0662589	1.05677844	1.0703682	1.239347537	0.3095808	0.864243	1
AC100830.3	1.0584323	1.04903717	1.0625046	1.274637685	0.3500872	0.864258	1
CHGA	1.0283193	1.01886772	1.0324162	1.718076097	0.7807939	0.864327	1
TECPR2	1.2075009	1.19757115	1.2118049	1.072043913	0.100364	0.864347	1
MARVELD3	1.0389059	1.02951587	1.0429761	1.456031891	0.542042	0.864354	1
COIL	1.5158576	1.52685216	1.5110919	0.970085993	-0.0438155	0.864498	1
RP11-16E18.3	1.0308193	1.0214725	1.0348707	1.623968665	0.6995238	0.86459	1
CTB-193M12.5	1.0600297	1.05054875	1.0641392	1.268858941	0.3435317	0.864604	1
LETM2	1.1012064	1.09176272	1.1052998	1.147522424	0.1985223	0.864604	1
RP11-159H10.3	1.0804253	1.08943388	1.0765205	0.855609872	-0.224975	0.864669	1
RP11-245J9.6	1.0250809	1.03402529	1.0212039	0.623180412	-0.6822782	0.864685	1
RP13-753N3.3	1.0238523	1.03279081	1.0199778	0.609249548	-0.7148948	0.864734	1
NRG1	1.0517838	1.06076604	1.0478904	0.788110736	-0.3435297	0.864773	1
TMLHE-AS1	1.0231202	1.03209241	1.0192312	0.599244353	-0.7387837	0.864855	1
RP11-536C5.7	1.0231206	1.0320778	1.019238	0.599730108	-0.7376147	0.864855	1
GHRLOS	1.0300056	1.03904367	1.026088	0.668175956	-0.5817	0.864871	1
TMEM217	1.0385058	1.02919718	1.0425407	1.457013045	0.5430138	0.864934	1
TAB2	1.4259055	1.41534639	1.4304824	1.036441797	0.0516391	0.864965	1
GOLGA8H	1.0226424	1.03160191	1.0187589	0.593599147	-0.7524391	0.865055	1
GALNTL6	1.0226313	1.03155007	1.0187653	0.594779798	-0.7495724	0.865055	1
AC093382.1	1.0226602	1.03156749	1.0187992	0.595525438	-0.747765	0.865055	1
AC026202.3	1.0226259	1.03147909	1.0187884	0.596853164	-0.744552	0.865055	1
DDX51	1.136989	1.14632039	1.1329443	0.908583504	-0.138309	0.865084	1
OVGP1	1.0210846	1.03005591	1.017196	0.572132793	-0.8055781	0.865113	1
LRRTM3	1.021075	1.0300242	1.017196	0.572737009	-0.8040553	0.865113	1
RP11-230G5.2	1.0210581	1.02997434	1.0171933	0.573602156	-0.8018776	0.865113	1
SLC35G5	1.0210647	1.02999358	1.0171945	0.573271831	-0.8027087	0.865113	1
SLC22A18AS	1.0210612	1.0299782	1.017196	0.573618314	-0.801837	0.865113	1
CMC4	1.0564262	1.04694971	1.0605339	1.289334118	0.3666262	0.865175	1
NPR2	1.06814	1.07726427	1.064185	0.830720339	-0.2675652	0.865188	1
GTF2IRD2B	1.1846712	1.19416242	1.1805572	0.929928602	-0.1048081	0.865273	1
DOK6	1.0950696	1.08560583	1.0991717	1.158469162	0.2122196	0.865431	1
ZNF613	1.0610942	1.07017473	1.0571582	0.814513072	-0.2959902	0.865457	1
FAM155A	1.0610894	1.06991128	1.0572656	0.819117631	-0.2878574	0.865457	1
TOX	1.6992743	1.70967894	1.6947643	0.978983947	-0.0306429	0.865498	1
PGGT1B	1.2718483	1.28172039	1.2675692	0.949768821	-0.0743517	0.865577	1
RP11-312B8.1	1.0247326	1.03357906	1.020898	0.622353555	-0.6841937	0.865584	1
LINC01460	1.024743	1.03364982	1.0208823	0.620575498	-0.6883214	0.865584	1
F11R	1.1528017	1.16228705	1.1486903	0.916217711	-0.1262376	0.865599	1
USP46-AS1	1.0325329	1.02326597	1.0365497	1.570951875	0.651639	0.86563	1
EFCAB14	1.5475158	1.5579402	1.5429973	0.973217687	-0.0391656	0.865666	1
ZBTB34	1.0769131	1.06758337	1.0809571	1.197885539	0.2604901	0.865776	1
RP11-395B7.4	1.0752961	1.06596293	1.0793416	1.202820392	0.2664212	0.865811	1
TP53	1.5357327	1.52487071	1.5404409	1.029664847	0.0421748	0.865848	1
LRRC8C	1.0531946	1.06225375	1.0492679	0.791404928	-0.337512	0.865877	1
MARCH10	1.0531137	1.062085	1.0492251	0.792865916	-0.3348512	0.865877	1
RP11-723D22.3	1.0207707	1.02963919	1.0169265	0.571086119	-0.8082198	0.866055	1
TAS2R14	1.0507468	1.05971788	1.0468582	0.784659243	-0.3498618	0.866187	1
UTP4	1.2153091	1.20566484	1.2194894	1.067219028	0.0938563	0.86622	1
ZC2HC1C	1.0673315	1.05787785	1.0714293	1.234138069	0.3035038	0.866265	1
AC007743.1	1.0210399	1.02992993	1.0171864	0.574222617	-0.8003179	0.866288	1

RP11-737O24.2	1.0210271	1.0299136	1.0171752	0.574160202	-0.8004748	0.866288	1
C22orf31	1.0305003	1.0212367	1.0345156	1.625280513	0.7006887	0.866297	1
LCTL	1.0565573	1.04712316	1.0606465	1.286979769	0.3639894	0.866369	1
AP000487.5	1.0764507	1.06710717	1.0805007	1.199584137	0.2625343	0.866376	1
ZNF295-AS1	1.0292231	1.03803937	1.0254017	0.66777346	-0.5825693	0.866415	1
STAC2	1.029245	1.03810135	1.0254062	0.666806053	-0.5846609	0.866415	1
TMEM128	1.6019788	1.59134043	1.60659	1.025788189	0.0367329	0.866423	1
DNAJA3	1.4217319	1.4320803	1.4172464	0.965668612	-0.0503999	0.866462	1
LIG3	1.3891801	1.39924902	1.3848156	0.963848575	-0.0531216	0.866463	1
DSCAM	1.0231339	1.01394208	1.0271181	1.945054023	0.9598102	0.86652	1
SV2B	1.0231423	1.01394208	1.0271302	1.945924252	0.9604556	0.86652	1
ADCY2	1.0302499	1.02104603	1.0342393	1.62687713	0.7021053	0.866531	1
DLG5	1.2670706	1.27673549	1.2628813	0.949937005	-0.0740963	0.866534	1
RP11-10L12.4	1.1628372	1.15324385	1.1669955	1.089736919	0.1239799	0.866586	1
RNF31	1.0232104	1.03209885	1.0193576	0.603062266	-0.7296211	0.866667	1
RP6-24A23.6	1.0231997	1.03202412	1.0193747	0.605003519	-0.7249846	0.866667	1
ZFP69	1.047798	1.03862498	1.0517742	1.340431744	0.4226978	0.866737	1
BDNF	1.1181564	1.10874574	1.1222355	1.124048429	0.1687042	0.866743	1
RGS11	1.0404433	1.03115301	1.0444702	1.427476176	0.5134667	0.866747	1
JPH3	1.040321	1.03109701	1.0443192	1.425192566	0.5111569	0.866747	1
RP11-415F23.4	1.0198215	1.02867328	1.0159847	0.557477148	-0.8430154	0.86677	1
ABCA7	1.0690328	1.07792494	1.0651785	0.836426256	-0.2576897	0.866771	1
FAM66A	1.0331638	1.04200244	1.0293327	0.698356785	-0.5179638	0.866909	1
ZBTB44	1.7317638	1.72078003	1.7365247	1.021843977	0.0311749	0.866945	1
TAF4	1.0845236	1.0751011	1.0886078	1.179846651	0.2385994	0.866979	1
CLDN16	1.0499918	1.04068173	1.0540273	1.328047433	0.4093067	0.866998	1
RP11-553A21.3	1.0245196	1.03332092	1.0207045	0.621367826	-0.6864806	0.867042	1
RP11-449H3.3	1.0245573	1.03350167	1.0206803	0.617292352	-0.6959742	0.867042	1
LA16c-358B7.3	1.0245195	1.03333213	1.0206995	0.621008962	-0.687314	0.867042	1
PDE6H	1.0393015	1.03000898	1.0433295	1.443883078	0.5299539	0.867106	1
BOLA3-AS1	1.172502	1.18149451	1.1686041	0.928976558	-0.1062859	0.867134	1
AC092301.3	1.0223238	1.03120179	1.0184756	0.592131236	-0.7560111	0.867143	1
RP11-218M22.1	1.0614799	1.0704439	1.0575945	0.817593199	-0.2905449	0.867168	1
TXNRD2	1.0983602	1.08907629	1.1023844	1.149401026	0.2008822	0.867286	1
CTC-360G5.9	1.0294148	1.03823651	1.025591	0.669281028	-0.579316	0.867541	1
PTGES2-AS1	1.0294588	1.03830866	1.0256228	0.668850509	-0.5802443	0.867541	1
RP1-257A7.5	1.0230358	1.03178996	1.0192413	0.605262316	-0.7243676	0.867574	1
AC079117.1	1.0230262	1.03180682	1.0192201	0.604277209	-0.7267176	0.867574	1
RP5-837J1.6	1.0230421	1.03183853	1.0192292	0.603959758	-0.7274757	0.867574	1
RP11-20I23.13	1.0543646	1.06326554	1.0505064	0.798323811	-0.3249541	0.867621	1
RP5-1098D14.1	1.0231587	1.01400962	1.0271244	1.936123691	0.9531711	0.867653	1
CNNM3	1.0774651	1.08633108	1.0736221	0.852788469	-0.2297402	0.86767	1
SLC31A1	1.4180029	1.42868984	1.4133706	0.96426509	-0.0524983	0.867753	1
NFYC-AS1	1.0709075	1.07994862	1.0669885	0.837894778	-0.255159	0.867874	1
TAL2	1.0342952	1.02508022	1.0382894	1.526678877	0.6103966	0.867935	1
CASC10	1.3437808	1.33334934	1.3483024	1.044857134	0.0633057	0.868058	1
AIM2	1.0383144	1.04709717	1.0345075	0.732686763	-0.4487315	0.868063	1
MFSD6	1.0431516	1.03400417	1.0471166	1.385612136	0.4705235	0.868082	1
TUNAR	1.0207125	1.02941849	1.0169389	0.575789873	-0.7963857	0.868115	1
Z83844.1	1.0207293	1.0294654	1.0169425	0.574997243	-0.7983731	0.868115	1
RP11-96K19.5	1.0214104	1.03017463	1.0176115	0.583652147	-0.7768193	0.868141	1

FAM21C	1.2553257	1.24513594	1.2597426	1.059585858	0.0835005	0.868169	1
RP11-38M8.1	1.0340309	1.0247967	1.0380335	1.533813564	0.6171231	0.8682	1
TTC7A	1.0811812	1.07192388	1.0851939	1.184500342	0.2442786	0.868224	1
DNAJC22	1.0602186	1.06904288	1.0563937	0.816792367	-0.2919587	0.868385	1
SLC44A5	1.1263873	1.13544323	1.122462	0.904157374	-0.1453542	0.868474	1
KIF6	1.0481226	1.03884353	1.0521447	1.342429424	0.4248462	0.868475	1
BRD1	1.1351622	1.14417278	1.1312565	0.91041125	-0.1354097	0.868506	1
TULP4	1.7247615	1.71355972	1.729617	1.022503098	0.0321052	0.868668	1
PPP1R1B	1.0253484	1.01624157	1.0292958	1.803753643	0.8510023	0.86869	1
ZNHIT6	1.2822928	1.27244043	1.2865634	1.051838745	0.0729135	0.868747	1
SPEN	1.4954007	1.50526902	1.4911232	0.972003472	-0.0409666	0.868754	1
CCDC163	1.0507437	1.04158254	1.0547147	1.315808328	0.3959493	0.868916	1
CIC	1.3585594	1.34825079	1.3630277	1.042431682	0.0599528	0.868973	1
ABHD17C	1.0687718	1.05957434	1.0727585	1.221306816	0.2884257	0.868982	1
SH2D3A	1.0420625	1.03289345	1.0460369	1.399575439	0.4849893	0.868982	1
ZNF493	1.4393758	1.44933499	1.4350589	0.968228443	-0.0465806	0.868996	1
RP11-77P16.4	1.0845965	1.09332719	1.0808122	0.86590175	-0.2077248	0.869004	1
C17orf50	1.0380797	1.04692025	1.0342476	0.729911771	-0.454206	0.869014	1
NOLC1	1.419287	1.42965458	1.4147931	0.965410671	-0.0507853	0.869019	1
BORA	1.1103687	1.10095448	1.1144494	1.133672971	0.1810045	0.869045	1
ITPR1	1.0665517	1.05731705	1.0705545	1.230951831	0.2997743	0.869086	1
ZNF2	1.0776783	1.0866249	1.0738003	0.851953013	-0.2311542	0.869094	1
RP11-629B11.5	1.0395261	1.04824989	1.0357447	0.740823987	-0.4327973	0.869108	1
KIAA1211	1.1146281	1.12338055	1.1108343	0.898312241	-0.1547111	0.869145	1
GIT2	1.1914283	1.20041735	1.187532	0.935707436	-0.0958706	0.869171	1
DAGLB	1.1041559	1.11316587	1.1002505	0.885872138	-0.1748296	0.869183	1
RP11-147L13.13	1.0764064	1.06707164	1.0804526	1.199502486	0.2624361	0.869187	1
RP11-355F16.1	1.022479	1.03115937	1.0187164	0.600666854	-0.735363	0.869193	1
BSN-AS2	1.0224483	1.03107774	1.0187079	0.601970668	-0.7322349	0.869193	1
CTD-3076O17.2	1.0224741	1.03113361	1.0187205	0.601296545	-0.7338514	0.869193	1
CTC-338M12.9	1.0225064	1.0312168	1.0187308	0.600023427	-0.7369093	0.869193	1
TMEM129	1.7968787	1.80791822	1.7920936	0.980413123	-0.0285383	0.869215	1
TTC33	1.2014859	1.19177404	1.2056956	1.072593678	0.1011037	0.869234	1
DUS3L	1.1271786	1.13639259	1.1231847	0.903162567	-0.1469424	0.869365	1
CHEK2	1.1488843	1.13939421	1.1529979	1.097591458	0.1343412	0.869373	1
RAPGEF6	1.1341496	1.14324181	1.1302086	0.909012397	-0.1376281	0.869385	1
DEC1	1.0420049	1.03301556	1.0459014	1.390295607	0.4753917	0.869396	1
PARS2	1.1199723	1.12921594	1.1159655	0.897455255	-0.1560881	0.869411	1
PLEKHG6	1.0334176	1.02434056	1.0373521	1.534560111	0.6178252	0.869425	1
AANAT	1.0524417	1.04315592	1.0564667	1.308433588	0.3878407	0.869491	1
TESC	1.0487164	1.05729762	1.0449969	0.785318258	-0.3486507	0.869565	1
MIR548XHG	1.0200672	1.02875346	1.016302	0.566958997	-0.8186837	0.869581	1
CTC-428H11.2	1.0300149	1.03862313	1.0262836	0.680514407	-0.5553024	0.869708	1
ABLIM2	1.1148462	1.12377757	1.1109748	0.896566445	-0.1575176	0.869747	1
DGKB	1.0411738	1.03205775	1.0451253	1.407624166	0.4932622	0.86975	1
ELK1	1.2417915	1.25145781	1.2376016	0.944896556	-0.0817717	0.869758	1
CTB-43P18.1	1.0218893	1.03056911	1.0181271	0.592985984	-0.7539301	0.869811	1
RP11-282O18.3	1.0218763	1.03049997	1.0181383	0.59469999	-0.749766	0.869811	1
LTBR	1.0674966	1.05826949	1.0714962	1.226991793	0.2951256	0.869817	1
PSTPIP1	1.0247671	1.01574718	1.0286768	1.821075531	0.8647908	0.86987	1
RP11-96D1.11	1.0247553	1.01574718	1.0286599	1.820002307	0.8639403	0.86987	1

GREM2	1.0248236	1.01574718	1.0287579	1.826225706	0.8688651	0.86987	1
SLC25A16	1.1326988	1.12318268	1.1368236	1.110737331	0.1515177	0.869872	1
OSBPL2	1.3332556	1.32318084	1.3376225	1.044686052	0.0630695	0.870109	1
PRR15	1.0324937	1.0234792	1.0364011	1.550355766	0.6325993	0.87019	1
RSPH14	1.078703	1.08737944	1.0749421	0.85766298	-0.2215172	0.870244	1
GOLGA1	1.141326	1.13203161	1.1453547	1.100908267	0.1386943	0.87035	1
ZNF696	1.0789527	1.08781178	1.0751127	0.855382543	-0.2253583	0.870514	1
AC034243.1	1.0196931	1.0282704	1.0159752	0.565085132	-0.8234599	0.870517	1
PLB1	1.0196872	1.02825506	1.0159735	0.565330998	-0.8228323	0.870517	1
GDAP1	1.2220497	1.21231499	1.2262692	1.065724322	0.0918343	0.87058	1
RP1-178F15.4	1.0376749	1.04630746	1.033933	0.732776902	-0.4485541	0.870583	1
ZXDB	1.0661801	1.07501275	1.0623516	0.831213051	-0.2667098	0.870605	1
FAHD1	1.3791819	1.36902515	1.3835844	1.039453235	0.0558249	0.870687	1
ADTRP	1.0469409	1.05556779	1.0432015	0.77745632	-0.3631665	0.870709	1
RP1-197B17.3	1.0677067	1.05858555	1.0716603	1.223174364	0.2906301	0.87078	1
ERVK3-1	1.2760152	1.28560943	1.2718565	0.951847048	-0.0711983	0.870782	1
SHB	1.1850642	1.1755364	1.1891941	1.077805272	0.1080965	0.870905	1
PRLR	1.0573985	1.06618169	1.0535913	0.809760719	-0.3044324	0.87104	1
ZFP69B	1.0789395	1.06982879	1.0828887	1.187026958	0.2473527	0.871084	1
MARCH11	1.0270999	1.03567192	1.0233843	0.655538078	-0.6092485	0.871123	1
RP11-61A14.1	1.0279808	1.01899597	1.0318753	1.678004476	0.7467466	0.87113	1
DNM3OS	1.0280635	1.01899597	1.0319938	1.684243081	0.7521004	0.87113	1
CTD-2132N18.2	1.0868321	1.0955935	1.0830345	0.8686206	-0.2032019	0.871156	1
RP11-161H23.5	1.020619	1.0292122	1.0168943	0.578329551	-0.7900363	0.871175	1
RIPK2	1.2734726	1.26397611	1.2775889	1.05156823	0.0725425	0.871177	1
TLCD2	1.123469	1.13247158	1.1195668	0.90258459	-0.1478659	0.871185	1
RP11-795F19.1	1.0664685	1.07513297	1.0627128	0.834690739	-0.2606863	0.871193	1
MLPH	1.0304701	1.03905827	1.0267475	0.684811117	-0.546222	0.871386	1
RP11-301O19.1	1.0305104	1.03912314	1.0267772	0.684433241	-0.5470183	0.871386	1
RETSAT	1.1097366	1.1005873	1.1137024	1.130385612	0.176815	0.871474	1
C1RL-AS1	1.0716999	1.06265398	1.0756209	1.206960301	0.2713782	0.871494	1
SMTNL1	1.0219912	1.03065963	1.0182338	0.594715742	-0.7497278	0.871567	1
GPR35	1.0219294	1.03049857	1.018215	0.597240714	-0.7436156	0.871567	1
SPTB	1.0219176	1.03046267	1.0182137	0.597901835	-0.7420195	0.871567	1
TDRP	1.2652661	1.25536806	1.2695565	1.055560727	0.0780096	0.871631	1
DHX58	1.0307841	1.03941671	1.0270423	0.68606106	-0.5435911	0.87165	1
RP11-344N10.5	1.0266271	1.01773008	1.0304836	1.719313054	0.7818323	0.871837	1
C1QTNF6	1.2246715	1.23389354	1.2206741	0.943481018	-0.0839346	0.871907	1
KIAA1324	1.0553451	1.06400754	1.0515903	0.806003505	-0.311142	0.871917	1
RP11-490B18.6	1.0232306	1.03184586	1.0194963	0.612207577	-0.7079072	0.871931	1
AC010729.2	1.0632197	1.0540891	1.0671774	1.241975815	0.3126371	0.871957	1
CCDC77	1.2679639	1.27715588	1.2639796	0.952458951	-0.0702712	0.87204	1
NEDD4	1.1032642	1.11195574	1.0994968	0.888715649	-0.1702062	0.872051	1
AC007403.2	1.0178394	1.02633612	1.0141565	0.537531972	-0.8955775	0.872064	1
CTD-2002J20.1	1.0268086	1.03536148	1.0231013	0.653289936	-0.6142047	0.872069	1
MGC45922	1.0204759	1.02904424	1.0167618	0.577113954	-0.7930719	0.872081	1
RP11-244H3.4	1.0204666	1.02904418	1.0167486	0.576659563	-0.7942082	0.872081	1
CTD-2031P19.5	1.0204586	1.0289841	1.0167632	0.578357625	-0.7899662	0.872081	1
WDR38	1.0204413	1.0288933	1.0167777	0.580677582	-0.7841908	0.872081	1
TMEM173	1.0412782	1.03241716	1.045119	1.391825155	0.476978	0.872127	1
MIS12	1.3414484	1.35134409	1.337159	0.959626203	-0.0594555	0.872183	1

ZNF396	1.0488799	1.05753196	1.0451296	0.78442636	-0.3502901	0.872222	1
RP4-622L5.7	1.0398227	1.04839673	1.0361062	0.746045961	-0.4226636	0.872251	1
MARCH8	1.1990276	1.20799257	1.1951417	0.938214551	-0.0920102	0.872262	1
CCDC114	1.0378408	1.04648652	1.0340932	0.733400317	-0.4473272	0.872316	1
CELSR3	1.077485	1.08622341	1.0736973	0.854724889	-0.226468	0.872336	1
FAM19A2	1.0829829	1.0916063	1.079245	0.865060699	-0.2091267	0.872484	1
NLRC5	1.0257938	1.01686321	1.0296649	1.759146619	0.8148757	0.872496	1
PBRM1	1.8249467	1.81366928	1.829835	1.019867637	0.0283819	0.872503	1
RD3	1.0327365	1.02374774	1.0366327	1.542576672	0.6253422	0.872517	1
MAP7	1.3129078	1.3223636	1.3088092	0.957952954	-0.0619733	0.872526	1
C1orf132	1.0357765	1.04433532	1.0320666	0.723273549	-0.4673867	0.872576	1
CTD-2366F13.2	1.018128	1.02656568	1.0144707	0.544712758	-0.8764324	0.87264	1
GPATCH11	1.1898003	1.19884276	1.1858808	0.934812944	-0.0972504	0.872676	1
CLPB	1.0705934	1.0615377	1.0745186	1.210942472	0.2761303	0.872691	1
XXbac-B444P24.14	1.0292641	1.03776501	1.0255794	0.677329818	-0.5620696	0.87272	1
NALCN-AS1	1.0292512	1.03772012	1.0255803	0.678159409	-0.5603037	0.87272	1
ASIC3	1.036818	1.02790151	1.0406829	1.458088116	0.5440779	0.87274	1
SGCA	1.0368206	1.02794677	1.040667	1.455160567	0.5411784	0.87274	1
HSD17B7	1.3139051	1.32359717	1.3097041	0.957066621	-0.0633087	0.872779	1
S100A5	1.0457023	1.03688488	1.0495243	1.342671336	0.4251062	0.872886	1
TMEM190	1.1331077	1.12414937	1.1369907	1.103434837	0.1420014	0.873006	1
ACRBP	1.0592745	1.0678328	1.0555649	0.819144548	-0.28781	0.873062	1
DCST2	1.0305499	1.03898205	1.026895	0.689932528	-0.5354728	0.873079	1
RUNDC1	1.2553952	1.26470966	1.2513578	0.94956051	-0.0746682	0.873189	1
CTD-2162K18.4	1.0283623	1.036819	1.0246967	0.670758839	-0.5761339	0.873198	1
RCVRN	1.0281684	1.01933938	1.0319954	1.654417518	0.7263234	0.873252	1
SLC9A8	1.0751483	1.06611538	1.0790637	1.19584353	0.2580286	0.873427	1
SIGIRR	2.0611998	2.07260306	2.056257	0.984760383	-0.0221554	0.873488	1
ZNF232	1.240607	1.23107592	1.2447383	1.059125237	0.0828732	0.873497	1
B3GNT4	1.0279319	1.01908283	1.0317676	1.664722154	0.7352814	0.873517	1
AF003625.3	1.027885	1.01910532	1.0316906	1.658732901	0.7300816	0.873517	1
MILR1	1.0354807	1.04401161	1.0317829	0.722149072	-0.4696314	0.873527	1
LRRC37A3	1.0962343	1.10502729	1.0924229	0.879989861	-0.1844412	0.873537	1
ZNF639	1.2962867	1.30574549	1.2921868	0.955653682	-0.0654402	0.873554	1
RP4-635E18.7	1.0291471	1.03750174	1.0255257	0.680655079	-0.5550042	0.873631	1
SLC27A6	1.0291033	1.03759036	1.0254246	0.676358942	-0.564139	0.873631	1
IL18BP	1.0290886	1.03748232	1.0254503	0.678994051	-0.5585292	0.873631	1
RP11-441O15.3	1.0654975	1.07413344	1.0617542	0.833013679	-0.2635879	0.873649	1
CTD-3074O7.5	1.1247004	1.13350496	1.120884	0.905464737	-0.1432696	0.873662	1
IRX3	1.0533352	1.04448147	1.0571729	1.285319952	0.3621275	0.873685	1
SLC47A1	1.0533963	1.06188426	1.0497171	0.803389017	-0.3158294	0.873695	1
CTD-2245F17.6	1.0198778	1.02830586	1.0162246	0.57318796	-0.8029198	0.873717	1
AKAP14	1.0198808	1.02830863	1.0162277	0.573241462	-0.8027851	0.873717	1
RP11-131K5.1	1.0198879	1.02831091	1.016237	0.573523443	-0.8020756	0.873717	1
KLHDC7A	1.0198828	1.02830131	1.0162338	0.57360633	-0.8018672	0.873717	1
BCL11B	1.0575182	1.06597954	1.0538506	0.816170489	-0.2930575	0.873743	1
RP11-666O2.2	1.0772122	1.08552699	1.0736082	0.860642425	-0.2165141	0.873776	1
SLC10A7	1.1031837	1.11183449	1.099434	0.889117645	-0.1695538	0.873833	1
NACC2	1.1333807	1.12447971	1.1372389	1.102500427	0.1407792	0.873851	1
LIF	1.0790057	1.06999753	1.0829104	1.184475817	0.2442487	0.873936	1
RP11-379F4.9	1.0247699	1.03319628	1.0211174	0.636138719	-0.6525867	0.873944	1

NXN	1.6309792	1.62039183	1.6355683	1.024462744	0.0348675	0.874014	1
DCTN4	1.4453797	1.45498991	1.4412141	0.969722801	-0.0443557	0.874059	1
ARAF	1.2708571	1.27993936	1.2669203	0.953493321	-0.0687053	0.874074	1
ADAM33	1.0267727	1.03514858	1.0231422	0.658409742	-0.6029424	0.874156	1
CLPSL2	1.026803	1.03520197	1.0231624	0.65798609	-0.603871	0.874156	1
ZNF81	1.0446617	1.03580649	1.0485	1.354502857	0.4377634	0.874185	1
GPSM2	1.2655657	1.27493584	1.2615041	0.951145882	-0.0722615	0.874248	1
TTLL11	1.0379174	1.0464923	1.0342006	0.735618541	-0.4429703	0.874251	1
AC062028.1	1.0193024	1.02768536	1.0156688	0.565958502	-0.8212318	0.874318	1
CTD-2081C10.7	1.019263	1.02761454	1.0156429	0.566474586	-0.8199169	0.874318	1
PRDM5	1.0947889	1.10348058	1.0910215	0.879599418	-0.1850814	0.874371	1
RNASEH2B	1.7346422	1.72381032	1.7393374	1.021451872	0.0306212	0.874475	1
HGS	1.4701065	1.47973388	1.4659335	0.971233318	-0.0421102	0.874483	1
STAC3	1.0477862	1.05636844	1.0440662	0.781753674	-0.355214	0.87449	1
CTD-2036P10.3	1.070044	1.06112598	1.0739095	1.209134695	0.273975	0.874586	1
INTS12	1.1601554	1.1510357	1.1641084	1.086553419	0.1197591	0.874649	1
CLCF1	1.0359865	1.02722957	1.0397822	1.460994116	0.5469504	0.874687	1
PROCA1	1.213907	1.22284935	1.2100309	0.942479317	-0.0854671	0.874813	1
STK11	1.207596	1.21657015	1.2037061	0.940600881	-0.0883454	0.874876	1
ARNT2	1.161497	1.17014706	1.1577475	0.927124652	-0.1091648	0.874925	1
GNAZ	1.1438511	1.13478668	1.1477801	1.096400257	0.1327746	0.874927	1
LSM2	4.5319446	4.50609558	4.543149	1.01056829	0.0151668	0.874959	1
TTC39A	1.115588	1.10648334	1.1195344	1.122564387	0.1667982	0.874977	1
RP11-387H17.4	1.0171163	1.02543483	1.0135106	0.531184782	-0.9127143	0.87504	1
PPEF1	1.0171139	1.02543977	1.013505	0.53086126	-0.9135932	0.87504	1
RP11-466F5.10	1.0171205	1.02545336	1.0135086	0.530718192	-0.9139821	0.87504	1
FYN	1.6015455	1.61131568	1.5973106	0.977090296	-0.0334362	0.875089	1
RP11-195F19.9	1.0376799	1.04610693	1.0340271	0.738004215	-0.438299	0.875165	1
IL15	1.0273967	1.01871147	1.0311613	1.66535857	0.7358328	0.875191	1
MYBBP1A	1.1002339	1.10892491	1.0964668	0.885626327	-0.17523	0.87529	1
PANK2	1.4374115	1.42747285	1.4417195	1.033327627	0.0472977	0.875295	1
CDK14	1.1777969	1.18659797	1.1739821	0.932390027	-0.1009945	0.87532	1
RP11-803B1.8	1.0832348	1.09173841	1.0795489	0.867127703	-0.2056836	0.875384	1
SOWAHC	1.1256521	1.11656118	1.1295926	1.111799129	0.1528962	0.875415	1
SLAIN1	1.1153664	1.10647466	1.1192205	1.119707839	0.1631223	0.875579	1
LACTB2-AS1	1.0260933	1.03444554	1.0224729	0.652419551	-0.6161281	0.87561	1
LINC01549	1.0261085	1.03444998	1.0224929	0.652913363	-0.6150365	0.87561	1
CTD-2541J13.2	1.02452	1.03285031	1.0209092	0.636498704	-0.6517705	0.875667	1
ENPP1	1.04316	1.05150907	1.0395411	0.767652758	-0.3814742	0.875695	1
ARHGAP6	1.0430581	1.05142642	1.0394308	0.766741712	-0.3831874	0.875695	1
RP11-407N17.5	1.0360677	1.04434215	1.0324811	0.732511325	-0.449077	0.875738	1
STXBP4	1.1978489	1.20665981	1.1940297	0.938884671	-0.0909801	0.875751	1
URB2	1.0644692	1.07285875	1.0608327	0.834940825	-0.2602541	0.875819	1
RP11-413N10.3	1.0278895	1.03617414	1.0242985	0.671709441	-0.5740908	0.875878	1
CTD-2012K14.7	1.0278615	1.0361771	1.0242571	0.670510304	-0.5766686	0.875878	1
DIS3L	1.2779376	1.26840695	1.2820688	1.050899632	0.0716249	0.875878	1
ARNTL	1.1266809	1.11782441	1.1305198	1.107748332	0.1476302	0.875914	1
MRGPRG-AS1	1.0306795	1.0218366	1.0345125	1.580489503	0.6603715	0.87598	1
GALNT5	1.0305666	1.02184923	1.0343453	1.571920877	0.6525286	0.87598	1
CTD-3064M3.7	1.0306104	1.02188668	1.0343917	1.57135359	0.6520079	0.87598	1
ZCCHC24	1.121146	1.12961439	1.1174754	0.906345203	-0.1418675	0.876038	1

ZNF582	1.1183318	1.12703621	1.1145589	0.901781374	-0.1491504	0.87612	1
PCDHGA6	1.0171145	1.02537716	1.013533	0.533273787	-0.9070517	0.876174	1
C9orf129	1.0171058	1.02535508	1.0135301	0.533623804	-0.9061051	0.876174	1
RBM11	1.0993246	1.10805768	1.0955392	0.884149831	-0.1776372	0.876179	1
DNAH10OS	1.0283031	1.01963469	1.0320605	1.632850777	0.707393	0.876193	1
ZKSCAN4	1.0904227	1.08167034	1.0942165	1.15361891	0.2061667	0.876196	1
ANAPC16	5.9843982	5.95655039	5.996469	1.008053716	0.0115725	0.876201	1
CCNYL1	1.1589743	1.14990684	1.1629046	1.086705497	0.119961	0.876315	1
AC002467.7	1.0924118	1.10111478	1.0886394	0.876621765	-0.1899736	0.876321	1
SYNDIG1	1.0455337	1.03680353	1.0493178	1.340029581	0.4222648	0.876343	1
PLD2	1.1523211	1.14298981	1.1563658	1.093545301	0.129013	0.876371	1
DZIP1	1.8462485	1.85688337	1.8416388	0.982209282	-0.0258976	0.876396	1
RAD1	1.3826096	1.39187781	1.3785923	0.966097707	-0.049759	0.87645	1
SPDYE16	1.0206275	1.02893453	1.0170268	0.588458183	-0.7649882	0.876454	1
LINC01571	1.0206008	1.02886337	1.0170193	0.589651301	-0.762066	0.876454	1
RARA	1.508444	1.49795549	1.5129903	1.030192989	0.0429146	0.876534	1
GDPD5	1.0526364	1.04394411	1.0564042	1.28354338	0.3601321	0.87655	1
RP11-382D12.2	1.0542297	1.0625956	1.0506034	0.808417844	-0.3068269	0.8766	1
RP5-903G2.2	1.0178499	1.02605909	1.0142915	0.548427913	-0.8666261	0.876604	1
MCTP1	1.0826185	1.09122771	1.0788868	0.86472415	-0.2096881	0.876608	1
EID3	1.0545658	1.04575527	1.0583847	1.27602186	0.351653	0.876623	1
RGR	1.0267733	1.01815945	1.0305071	1.679954821	0.7484224	0.876656	1
RP11-345J4.5	1.0641843	1.05554478	1.0679292	1.222962755	0.2903805	0.876821	1
TBX19	1.0617631	1.07006847	1.058163	0.830088395	-0.2686631	0.876854	1
USP8	1.7841339	1.77299132	1.7889637	1.020663107	0.0295068	0.87706	1
TMEM201	1.1301076	1.13885131	1.1263176	0.90973284	-0.1364852	0.877119	1
NME5	1.1779896	1.18655363	1.1742775	0.934195299	-0.0982039	0.877141	1
CORO6	1.044016	1.05228795	1.0404304	0.773226057	-0.3710378	0.877147	1
ATG7	1.1977054	1.20620397	1.1940217	0.940921015	-0.0878545	0.877162	1
TSSK6	1.0162287	1.02439734	1.012688	0.520054672	-0.9432648	0.877172	1
RP11-713P17.5	1.0162387	1.02441994	1.0126924	0.519756714	-0.9440916	0.877172	1
RBMS3-AS2	1.0162458	1.02443868	1.0126946	0.51944745	-0.9449503	0.877172	1
SNTG1	1.0266369	1.03487912	1.0230643	0.661263267	-0.5967033	0.877229	1
KCTD2	1.1406416	1.14949004	1.1368063	0.915153016	-0.1279151	0.877261	1
PCDHA9	1.0732869	1.08158948	1.0696881	0.854130818	-0.227471	0.877282	1
GJA5	1.0232895	1.03149178	1.0197341	0.626643211	-0.6742838	0.877331	1
RP11-65J3.3	1.0232889	1.03159721	1.0196876	0.623081708	-0.6825067	0.877331	1
ANGPTL8	1.0232604	1.03150418	1.0196871	0.624905292	-0.6782905	0.877331	1
NUBP2	2.1938756	2.20598169	2.1886282	0.985610446	-0.0209105	0.877367	1
PIGB	1.101421	1.10977088	1.0978017	0.890962286	-0.1665637	0.877368	1
RGPD5	1.0914606	1.08275172	1.0952355	1.150858623	0.2027106	0.877421	1
PARP3	1.1195275	1.11060251	1.123396	1.115671256	0.157912	0.877465	1
PLEKHO2	1.0900391	1.09836224	1.0864314	0.878704611	-0.1865498	0.877564	1
SEZ6L	1.0271867	1.01850742	1.0309488	1.672236805	0.7417792	0.877602	1
RP11-66H6.3	1.0280228	1.03627198	1.0244471	0.673995414	-0.5691893	0.877604	1
KCNQ5	1.0279861	1.03617744	1.0244355	0.675434372	-0.5661125	0.877604	1
MED12	1.1863641	1.19533496	1.1824756	0.934167698	-0.0982465	0.877609	1
BRPF3	1.1071875	1.09832086	1.1110308	1.129269697	0.1753901	0.877639	1
TBC1D19	1.2214072	1.23052423	1.2174553	0.943307991	-0.0841992	0.877772	1
SETD1B	1.0826325	1.09098695	1.0790112	0.868379121	-0.2036031	0.877778	1
ATP6V0D2	1.0265915	1.01804205	1.0302973	1.679260964	0.7478264	0.877782	1

AKR1C3	1.0614742	1.06975632	1.0578843	0.829807809	-0.2691509	0.877817	1
PRTFDC1	1.2526069	1.24327671	1.2566512	1.05497636	0.0772107	0.877902	1
CHST3	1.0988927	1.10737756	1.0952148	0.886729303	-0.1734343	0.877964	1
SDE2	1.1109105	1.1021409	1.1147117	1.123073036	0.1674518	0.877995	1
C11orf88	1.0343187	1.04251271	1.030767	0.723713296	-0.4665098	0.878081	1
GS1-24F4.2	1.0239068	1.03206655	1.02037	0.635240356	-0.6546255	0.878104	1
ATG4A	1.1734254	1.18213082	1.169652	0.931484576	-0.1023962	0.878131	1
ZNF143	1.2139978	1.20474133	1.2180101	1.064807375	0.0905925	0.878147	1
PHRF1	1.1439733	1.13501404	1.1478567	1.095120762	0.13109	0.878167	1
CREBL2	1.3055056	1.29582557	1.3097015	1.046905798	0.0661316	0.878187	1
SLCO1A2	1.0902971	1.08141328	1.0941479	1.156418991	0.2096642	0.878249	1
TSHZ1	1.2626796	1.25302911	1.2668627	1.054671879	0.0767942	0.878249	1
CWC22	1.3463074	1.35551493	1.3423164	0.962874858	-0.0545798	0.878377	1
DFFA	1.9067548	1.89567766	1.9115562	1.017727995	0.025352	0.878425	1
CTA-212A2.2	1.0221851	1.03034694	1.0186473	0.614471788	-0.7025813	0.878504	1
RP11-16C1.1	1.0221709	1.03029199	1.0186508	0.615700668	-0.699699	0.878504	1
ERAP2	1.0446244	1.03611825	1.0483114	1.337590187	0.4196362	0.878516	1
KRT5	1.0221314	1.01362911	1.0258167	1.894234307	0.9216148	0.878605	1
HENMT1	1.1074894	1.09875618	1.1112748	1.126762861	0.1721839	0.878621	1
B3GALT2	1.0311611	1.02253389	1.0349006	1.548805563	0.631156	0.878661	1
GAS6-AS2	1.0241665	1.0323549	1.0206173	0.637221999	-0.650132	0.878682	1
SUSD6	1.1384642	1.14688532	1.134814	0.917818102	-0.1237198	0.87873	1
UST	1.0532475	1.06147627	1.0496806	0.808126808	-0.3073464	0.87877	1
CTD-2537I9.18	1.0167124	1.02482703	1.013195	0.531478713	-0.9119162	0.878841	1
CTD-2619J13.19	1.0167042	1.02481838	1.013187	0.531340566	-0.9122912	0.878841	1
RP11-352G18.2	1.0167232	1.02488209	1.0131866	0.529965002	-0.916031	0.878841	1
AKR7A3	1.0247918	1.03289497	1.0212795	0.646892477	-0.6284022	0.878868	1
AP001059.7	1.021885	1.01338003	1.0255715	1.911172626	0.9344581	0.878869	1
NPM3	2.9981301	3.01463132	2.9909776	0.98825903	-0.0170389	0.878927	1
ATP5L2	1.13618	1.14467073	1.1324997	0.915870833	-0.1267839	0.879003	1
LINC01219	1.0169718	1.02507181	1.0134608	0.536890173	-0.8973011	0.879104	1
DHX57	1.2138032	1.22243972	1.2100597	0.944344445	-0.0826149	0.879172	1
ZNF587B	1.0455141	1.05361749	1.0420017	0.783357656	-0.3522569	0.87922	1
AC002451.3	1.0454864	1.05360056	1.0419693	0.783001543	-0.3529129	0.87922	1
NUDT13	1.0627582	1.07096349	1.0592016	0.834254953	-0.2614397	0.879245	1
AC011747.4	1.0380128	1.02942591	1.0417348	1.418302511	0.5041653	0.879259	1
PDZD2	1.0273553	1.01886772	1.0310343	1.644833532	0.7179416	0.879326	1
SCRT1	1.0274896	1.01886772	1.0312268	1.65504032	0.7268664	0.879326	1
TWIST2	1.0274582	1.01886772	1.0311818	1.652651719	0.7247827	0.879326	1
EPHX4	1.0931955	1.08458147	1.0969293	1.145987125	0.1965908	0.879336	1
ENPP5	1.1023834	1.11090748	1.0986886	0.889828086	-0.1684015	0.879379	1
CLIC6	1.2330183	1.24175208	1.2292326	0.94821341	-0.0767163	0.879412	1
AGR2	1.0213269	1.01281342	1.0250171	1.952414328	0.9652592	0.879455	1
PLSCR4	1.0387117	1.03013064	1.0424313	1.408242475	0.4938958	0.879489	1
GPANK1	1.679323	1.66878332	1.6838915	1.022590585	0.0322286	0.87955	1
RET	1.0301143	1.02151195	1.033843	1.573220652	0.653721	0.879558	1
UGT3A2	1.0642445	1.05549944	1.068035	1.225868766	0.2938045	0.879779	1
ZBTB40	1.1395344	1.13086265	1.1432932	1.094989346	0.1309168	0.87978	1
GATA2	1.0377811	1.02913278	1.0415298	1.425535698	0.5115042	0.87984	1
STPG2-AS1	1.0308958	1.03908321	1.027347	0.699710947	-0.515169	0.879919	1
METTL2A	1.3061627	1.29680727	1.3102179	1.045182794	0.0637553	0.879954	1

LENG9	1.0942185	1.0855311	1.0979841	1.145595748	0.196098	0.879965	1
FAAP100	1.2318411	1.24085047	1.2279359	0.946379485	-0.0795093	0.879993	1
APOL4	1.0300419	1.02156397	1.0337167	1.563567697	0.6448417	0.880013	1
RP11-977P2.1	1.0214454	1.0294421	1.0179792	0.610663941	-0.7115494	0.880046	1
RHD	1.0214308	1.02945686	1.0179519	0.60943025	-0.714467	0.880046	1
RP11-420L9.5	1.0653276	1.05678938	1.0690285	1.21551823	0.2815715	0.880109	1
RP13-1032I1.11	1.0235282	1.03156003	1.0200468	0.6351947	-0.6547292	0.880152	1
CAPN6	1.0431357	1.03464906	1.0468143	1.351100061	0.4341345	0.880214	1
LINC00475	1.1237149	1.11479328	1.127582	1.111406427	0.1523865	0.880242	1
MYOCD	1.1311103	1.12225197	1.13495	1.103867701	0.1425673	0.880259	1
XXbac-BPG252P9.9	1.1073736	1.11576087	1.1037381	0.89614157	-0.1582014	0.880311	1
VSIG1	1.0252944	1.03335818	1.021799	0.65348439	-0.6137753	0.880385	1
PTCD1	1.1184663	1.12672247	1.1148877	0.90660849	-0.1414484	0.880408	1
ZNF655	1.4303119	1.43960894	1.4262821	0.969684748	-0.0444123	0.880508	1
GTSE1	1.6724591	1.6830937	1.6678494	0.977683509	-0.0325606	0.880519	1
ZNFX1	1.1197225	1.11101723	1.1234959	1.112402868	0.1536794	0.880536	1
VPREB3	1.0214176	1.01301898	1.025058	1.924728006	0.9446546	0.88054	1
LIPC	1.0214335	1.01301898	1.0250808	1.926476608	0.9459647	0.88054	1
RP11-150C16.1	1.0256361	1.03369068	1.0221448	0.657297302	-0.605382	0.88065	1
RP11-553A10.1	1.7664768	1.75565928	1.7711657	1.020520324	0.0293049	0.880727	1
RALGAPA2	1.2618448	1.2525513	1.2658731	1.05274872	0.0741611	0.880807	1
EMX2OS	1.0331697	1.02471961	1.0368325	1.490010619	0.5753226	0.880823	1
ZNF350	1.1177444	1.1259915	1.1141697	0.90616984	-0.1421466	0.880996	1
BEST3	1.0465167	1.05457617	1.0430233	0.788315676	-0.3431546	0.881012	1
RP11-390P24.1	1.0515566	1.05963544	1.0480547	0.805808332	-0.3114914	0.881064	1
RP11-425A6.5	1.0231359	1.01466374	1.0268083	1.828202396	0.8704258	0.881109	1
ANO3	1.0230272	1.01466374	1.0266523	1.817568288	0.8620096	0.881109	1
LINC01562	1.021618	1.02959101	1.0181621	0.613770691	-0.7042283	0.881172	1
MMRN2	1.0216324	1.0296413	1.0181609	0.612690403	-0.7067698	0.881172	1
RP11-453A12.1	1.0216269	1.02963439	1.018156	0.61266749	-0.7068238	0.881172	1
OVOL1-AS1	1.0345858	1.04258662	1.0311178	0.730694783	-0.4526592	0.881256	1
SIRT1	1.1682356	1.17681684	1.164516	0.930431778	-0.1040277	0.881304	1
BCAR3	1.1529553	1.14410893	1.1567898	1.087995238	0.1216722	0.881316	1
TFE3	1.457667	1.46711794	1.4535704	0.970997561	-0.0424604	0.881391	1
GNG12-AS1	1.0218584	1.02981404	1.0184099	0.617491655	-0.6955085	0.881406	1
RFPL3S	1.021878	1.02982948	1.0184314	0.617892808	-0.6945715	0.881406	1
CCDC167	3.0778197	3.09327637	3.0711199	0.989415406	-0.0153517	0.881413	1
CITED1	1.0577482	1.04933818	1.0613935	1.244341399	0.3153824	0.881434	1
SOCS4	1.3413824	1.35048306	1.3374376	0.962778724	-0.0547238	0.881473	1
NR2F6	1.8789324	1.86745468	1.8839075	1.018966738	0.027107	0.881481	1
PDE6G	1.0293801	1.02101614	1.0330056	1.570486385	0.6512114	0.88152	1
SMIM18	1.0223515	1.01394208	1.0259966	1.864616238	0.8988787	0.88154	1
ELMO3	1.0471301	1.03856968	1.0508407	1.318152582	0.3985174	0.881602	1
CC2D2B	1.032146	1.04021283	1.0286494	0.712443266	-0.489153	0.88161	1
THEMIS2	1.0492488	1.05725104	1.0457801	0.799638219	-0.3225807	0.881626	1
SLC12A5	1.1921833	1.20057432	1.1885462	0.940031579	-0.0892189	0.881632	1
NHLRC4	1.0413962	1.04945846	1.0379016	0.766331358	-0.3839598	0.881642	1
VSNL1	1.0394836	1.03115687	1.0430929	1.383093951	0.4678992	0.881643	1
AGAP6	1.0704816	1.07859167	1.0669663	0.852078786	-0.2309413	0.881747	1
TXNDC11	1.1640186	1.15511779	1.1678768	1.082253332	0.1140382	0.881753	1
FAM155B	1.024026	1.03197212	1.0205818	0.643741243	-0.6354472	0.881753	1

METTL22	1.3162033	1.32490066	1.3124333	0.961627324	-0.0564502	0.881827	1
RP4-728D4.2	1.0206861	1.0286784	1.0172218	0.600515937	-0.7357256	0.881889	1
HIVEP1	1.2102559	1.21897743	1.2064755	0.942907817	-0.0848114	0.88192	1
RP11-279O9.4	1.0750728	1.06658368	1.0787524	1.182758346	0.2421553	0.882069	1
PRPF38B	2.1006131	2.11229882	2.0955479	0.984940297	-0.0218918	0.882091	1
DHX37	1.0859152	1.07735192	1.0896269	1.158690501	0.2124953	0.882181	1
RP11-17A19.2	1.0404911	1.03204407	1.0441524	1.377866183	0.4624358	0.882295	1
SLC4A7	1.8822655	1.87153577	1.8869163	1.017647611	0.0252381	0.882465	1
SPNS3	1.0296912	1.02135499	1.0333046	1.559568239	0.6411467	0.882468	1
ATAD3B	1.060234	1.06833024	1.0567247	0.830155278	-0.2685469	0.882536	1
B3GALT6	1.1092139	1.11760018	1.1055789	0.897778182	-0.1555691	0.882583	1
CTD-2501E16.2	1.0317773	1.03968378	1.0283503	0.714404169	-0.4851876	0.88259	1
LL09NC01-251B2.3	1.0303647	1.03838669	1.0268875	0.700437274	-0.5136722	0.882598	1
DPP9	1.1354045	1.14365521	1.1318282	0.917670643	-0.1239516	0.882637	1
LINC01235	1.029335	1.02090619	1.0329885	1.5779286	0.6580319	0.882648	1
RP11-344B5.3	1.0239147	1.03186827	1.0204672	0.642244872	-0.6388046	0.882663	1
LINC-PINT	1.0670959	1.05861296	1.0707728	1.207460125	0.2719755	0.882663	1
AC092652.1	1.0223284	1.01400962	1.0259343	1.851176057	0.8884421	0.882677	1
SLC43A2	1.102408	1.09392868	1.1060834	1.12940353	0.175561	0.882757	1
UBA7	1.0305396	1.03852212	1.0270796	0.702961597	-0.5084822	0.882834	1
USP44	1.1076122	1.11580405	1.1040615	0.89859946	-0.1542499	0.883001	1
CTD-2020K17.3	1.0392486	1.0471869	1.0358077	0.758847753	-0.3981176	0.883058	1
GON7	1.402533	1.41191568	1.398466	0.967348425	-0.0478925	0.883068	1
LILRB5	1.0196015	1.0274867	1.0161837	0.588781683	-0.7641953	0.88308	1
ELOVL3	1.0330939	1.02484237	1.0366706	1.476132266	0.561822	0.883146	1
RAB3IL1	1.0611045	1.0692067	1.0575926	0.83218245	-0.2650282	0.883209	1
LINC00525	1.0215817	1.02947475	1.0181605	0.616136132	-0.698679	0.883222	1
PLD6	1.022344	1.03021249	1.0189333	0.626672598	-0.6742162	0.883232	1
PCAT6	1.7785616	1.78852664	1.7742422	0.981884578	-0.0263747	0.883301	1
LAGE3	3.5212717	3.5026836	3.5293288	1.010646636	0.0152787	0.883307	1
MEF2C-AS1	1.0292984	1.03711863	1.0259087	0.697997267	-0.5187067	0.883319	1
SNX24	1.4220493	1.41227347	1.4262866	1.033989917	0.0482221	0.883383	1
RP11-126K1.6	1.1828649	1.19126015	1.1792259	0.937079414	-0.0937568	0.883504	1
CD36	1.0339774	1.04185373	1.0305633	0.730240708	-0.453556	0.883613	1
USP7	1.38056	1.37109088	1.3846645	1.036577502	0.051828	0.883689	1
STX1B	1.0602502	1.06821383	1.0567984	0.832651909	-0.2642146	0.883695	1
WDR86	1.3241857	1.31448748	1.3283894	1.044204936	0.0624049	0.883723	1
ARMC12	1.0324754	1.02419786	1.0360634	1.490355094	0.5756561	0.883735	1
COBLL1	1.049808	1.04149698	1.0534105	1.287093977	0.3641174	0.88374	1
PRKG1	1.1030107	1.0943624	1.1067594	1.131376455	0.1780791	0.883774	1
TP53I13	1.8465438	1.83545517	1.8513502	1.019025604	0.0271903	0.883816	1
GBA	1.1644226	1.15590569	1.1681143	1.078307459	0.1087686	0.884001	1
LONRF3	1.0251027	1.01683833	1.028685	1.703552968	0.7685468	0.884004	1
HLA-DOA	1.0352952	1.04316877	1.0318823	0.73855	-0.4372325	0.884016	1
IPO8	1.2561812	1.26494485	1.2523825	0.952585069	-0.0700802	0.884039	1
LINC00504	1.0299214	1.03780302	1.0265051	0.701137253	-0.5122312	0.884114	1
FER1L5	1.0490083	1.04065493	1.0526291	1.29453135	0.3724299	0.884151	1
SMC2-AS1	1.0500733	1.05796255	1.0466537	0.804893666	-0.3131299	0.884191	1
WAS	1.0391798	1.04709517	1.0357488	0.759075696	-0.3976843	0.884248	1
BTBD16	1.0238904	1.01566013	1.0274579	1.753361977	0.8101239	0.884287	1
XKR5	1.023883	1.0156614	1.0274466	1.752501958	0.8094161	0.884287	1

RP11-102N12.3	1.0505276	1.05838972	1.0471197	0.806986976	-0.3093827	0.884337	1
C1orf198	1.409899	1.40089283	1.4138028	1.032203083	0.0457268	0.884356	1
SLC22A5	1.1421859	1.1336881	1.1458694	1.091116986	0.1258058	0.884377	1
RP11-637A17.2	1.0871662	1.09509295	1.0837303	0.880510308	-0.1835882	0.884384	1
AHCTF1	1.3218307	1.3304836	1.3180801	0.962468477	-0.0551888	0.884395	1
WTIP	1.1267346	1.1347253	1.123271	0.914980665	-0.1281868	0.884473	1
RP5-858L17.1	1.0282585	1.03609625	1.0248612	0.688746332	-0.5379554	0.884479	1
RP11-491F9.8	1.0214036	1.02926194	1.0179973	0.615042149	-0.7012428	0.884542	1
PTGS1	1.0213782	1.02917789	1.0179973	0.616813907	-0.6970928	0.884542	1
RP11-40E6.2	1.0213762	1.02917128	1.0179973	0.616953781	-0.6967657	0.884542	1
CLEC12A	1.0213917	1.02922262	1.0179973	0.615869841	-0.6993026	0.884542	1
KRTAP5-10	1.021406	1.02927009	1.0179973	0.61487103	-0.7016443	0.884542	1
RP11-160N1.10	1.0668295	1.05836582	1.0704981	1.207866704	0.2724613	0.884553	1
DNAAF5	1.2066433	1.19788254	1.2104407	1.063462817	0.0887696	0.884575	1
MTPAP	1.2673747	1.27607693	1.2636027	0.954816065	-0.0667053	0.884614	1
RP11-386G21.2	1.0188641	1.02666459	1.0154829	0.580652974	-0.7842519	0.884621	1
AC013275.2	1.0188589	1.02664763	1.0154829	0.581022396	-0.7833343	0.884621	1
SIT1	1.0209422	1.02873182	1.0175658	0.611369246	-0.7098841	0.88471	1
CTD-2574D22.2	1.0583088	1.04989945	1.0619538	1.241573637	0.3121698	0.884767	1
PCDHGA3	1.0193243	1.02708564	1.0159601	0.589245628	-0.7630589	0.884801	1
HIGD2B	1.019328	1.02709785	1.0159601	0.588980071	-0.7637093	0.884801	1
MX2	1.1373889	1.12890878	1.1410646	1.094297913	0.1300056	0.884853	1
SERPINF2	1.023921	1.01574718	1.027464	1.744057082	0.8024473	0.884899	1
TAT-AS1	1.0227069	1.03048191	1.0193368	0.634370125	-0.6566033	0.884908	1
CD81-AS1	1.0227212	1.03048885	1.0193543	0.634798562	-0.6556292	0.884908	1
FAM212A	1.0580164	1.04979702	1.0615792	1.236603446	0.3063829	0.885006	1
ABHD13	1.266266	1.25749208	1.2700691	1.048844486	0.0688008	0.885077	1
LINC01030	1.0233923	1.01516282	1.0269594	1.777994542	0.8302509	0.885085	1
RP11-197K6.1	1.1474378	1.13852807	1.1512998	1.092195841	0.1272316	0.885155	1
INCA1	1.0444722	1.03621433	1.0480516	1.326865755	0.4080224	0.885156	1
RP11-344P13.6	1.0868105	1.07840326	1.0904547	1.153710779	0.2062816	0.885163	1
FAM104B	1.5584008	1.54923401	1.5623743	1.023924694	0.0341096	0.885314	1
MYO3B	1.0342687	1.02619217	1.0377695	1.442015716	0.5280869	0.8854	1
EOGT	1.049351	1.04106178	1.052944	1.289373231	0.3666699	0.88546	1
RAB11FIP3	1.0493296	1.04104164	1.0529221	1.289472992	0.3667816	0.88546	1
RP13-270P17.3	1.0489903	1.05681568	1.0455983	0.802565651	-0.3173087	0.885544	1
CTD-3065J16.9	1.0586434	1.06646696	1.0552522	0.831273235	-0.2666053	0.885573	1
C10orf67	1.0477462	1.05555343	1.0443621	0.798548492	-0.3245481	0.885649	1
FOXN3	1.5032876	1.49426122	1.5072001	1.026178262	0.0372814	0.885662	1
UVRAG	1.3731339	1.38172541	1.3694098	0.967737052	-0.047313	0.885664	1
MSH5-SAPCD1	1.0190279	1.02672486	1.0156917	0.587155884	-0.7681845	0.885746	1
RP11-11N5.1	1.0190501	1.02679824	1.0156917	0.58554822	-0.7721401	0.885746	1
CTD-2336O2.3	1.0190336	1.02674357	1.0156917	0.586745226	-0.7691939	0.885746	1
CTD-3051D23.4	1.0190655	1.02684906	1.0156917	0.584439942	-0.7748733	0.885746	1
AC007395.3	1.0190655	1.02684906	1.0156917	0.584439942	-0.7748733	0.885746	1
MBNL1-AS1	1.0429754	1.03480566	1.0465167	1.336468757	0.4184261	0.885774	1
DDX11-AS1	1.030713	1.03844895	1.0273598	0.711587756	-0.4908864	0.885833	1
SNRK	1.1163356	1.10778631	1.1200414	1.113697939	0.155358	0.885873	1
HIST1H4E	1.0508511	1.0426317	1.0544139	1.276371862	0.3520487	0.885907	1
SCUBE1	1.0507371	1.04253035	1.0542943	1.27660193	0.3523087	0.885907	1
RP11-264B17.4	1.0256194	1.01751783	1.0291311	1.662939737	0.7337359	0.885945	1

RP13-192B19.2	1.0192862	1.02699443	1.015945	0.590676238	-0.7595605	0.88598	1
CTD-2162K18.3	1.0192841	1.02698778	1.015945	0.590821614	-0.7592055	0.88598	1
GLIS2-AS1	1.0192738	1.02695343	1.015945	0.591574719	-0.7573677	0.88598	1
AUTS2	1.3100026	1.30095347	1.313925	1.043101335	0.0608793	0.886061	1
HLA-DMB	1.0388791	1.04663799	1.035516	0.761524841	-0.393037	0.886152	1
CTD-2587M2.1	1.0295476	1.03725999	1.0262046	0.703291578	-0.5078052	0.886153	1
PRKAR2A	1.8033835	1.79305139	1.8078621	1.018675562	0.0266946	0.886175	1
CENPE	1.4056026	1.41472941	1.4016465	0.968454457	-0.0462439	0.886223	1
BEST4	1.0869143	1.07862198	1.0905087	1.15118773	0.2031231	0.886284	1
C14orf80	1.1451159	1.15305094	1.1416765	0.925681806	-0.1114117	0.886301	1
RP11-783K16.14	1.0395003	1.04712698	1.0361945	0.768021504	-0.3807814	0.88634	1
TMEM37	1.1076309	1.09933333	1.1112275	1.119740175	0.163164	0.886376	1
SLC24A4	1.0248968	1.01677692	1.0284164	1.693781036	0.7602474	0.886377	1
ZC3H3	1.1408051	1.14891686	1.137289	0.921916951	-0.1172913	0.886422	1
PRPF6	1.7282311	1.7379905	1.7240008	0.981043525	-0.027611	0.886439	1
KIAA0319L	1.4734286	1.48274102	1.4693921	0.972347611	-0.0404559	0.886459	1
CTD-2199O4.6	1.0180763	1.0257524	1.014749	0.572723607	-0.804089	0.886464	1
SLCO1B1	1.0180819	1.02577117	1.014749	0.57230653	-0.80514	0.886464	1
SCN4B	1.0180734	1.0257429	1.014749	0.572935069	-0.8035564	0.886464	1
SLC6A9	1.1040895	1.1119868	1.1006664	0.898912681	-0.1537471	0.886588	1
FNTB	1.1873177	1.19563299	1.1837134	0.939071634	-0.0906929	0.88662	1
JADE3	1.0884514	1.09634116	1.0850316	0.882609287	-0.1801532	0.886672	1
RP11-1109F11.3	1.0317317	1.03946552	1.0283794	0.719093196	-0.4757493	0.886688	1
XX-C2158C12.2	1.0490408	1.0568421	1.0456593	0.803264957	-0.3160522	0.886697	1
KLF13	1.168424	1.17677363	1.1648048	0.932292856	-0.1011449	0.886701	1
RP11-1250I15.3	1.0228122	1.03047982	1.0194886	0.63939236	-0.6452266	0.886702	1
CBWD7	1.1060689	1.09771645	1.1096893	1.122526738	0.1667498	0.886725	1
ATG14	1.2384057	1.2466057	1.2348513	0.952335323	-0.0704585	0.886731	1
RP11-122G18.12	1.0205644	1.02828835	1.0172165	0.608606432	-0.7164185	0.886769	1
STARD7	2.2645153	2.27775155	2.258778	0.98515082	-0.0215835	0.886784	1
RP11-67L2.2	1.0740623	1.08200176	1.0706209	0.861212487	-0.2155589	0.886796	1
MAFG	1.1879661	1.19603669	1.1844678	0.940986128	-0.0877546	0.886815	1
UAP1L1	1.0757236	1.06739775	1.0793325	1.177079138	0.2352113	0.886838	1
RP11-778D9.13	1.0257577	1.01772173	1.029241	1.650007895	0.7224729	0.886855	1
RP1-120G22.11	1.0526205	1.06041613	1.0492415	0.815038502	-0.2950599	0.886982	1
RP11-656D10.3	1.047248	1.05498312	1.0438952	0.798340163	-0.3249245	0.887012	1
ZNF221	1.0471106	1.05485869	1.0437521	0.797542226	-0.3263672	0.887012	1
SLC25A45	1.0646972	1.07253393	1.0613003	0.845126193	-0.2427613	0.887023	1
RP11-452L6.7	1.0565933	1.06454493	1.0531466	0.82340419	-0.2803273	0.887037	1
ARHGEF7-AS2	1.0240577	1.03173034	1.020732	0.653381385	-0.6140027	0.88705	1
CTB-5E10.3	1.0241081	1.03182975	1.0207611	0.652255869	-0.6164901	0.88705	1
CSMD3	1.0240598	1.03169709	1.0207493	0.654613009	-0.6112858	0.88705	1
RP4-694B14.8	1.0240969	1.03181017	1.0207536	0.652420352	-0.6161263	0.88705	1
RP11-473E2.4	1.0240687	1.03173098	1.0207474	0.653854013	-0.6129595	0.88705	1
RGPD2	1.0291659	1.03685626	1.0258325	0.70089746	-0.5127247	0.887114	1
RP11-47A8.5	1.0430969	1.03497353	1.046618	1.332950483	0.4146232	0.887132	1
RP5-1159O4.2	1.0276647	1.0353279	1.0243431	0.689061379	-0.5372956	0.887157	1
AGAP2	1.0277046	1.03539299	1.024372	0.688611756	-0.5382373	0.887157	1
CPEB1	1.0837425	1.09169781	1.0802942	0.875638894	-0.1915921	0.88717	1
COL28A1	1.0187097	1.02636973	1.0153895	0.583602937	-0.776941	0.887201	1
NOSTRIN	1.0186969	1.02634546	1.0153817	0.583844678	-0.7763435	0.887201	1

RP11-1017G21.6	1.0213081	1.02894873	1.0179962	0.621658553	-0.6858057	0.887201	1
PCDHA13	1.021319	1.02894838	1.018012	0.622211543	-0.6845229	0.887201	1
ACOT8	1.3111986	1.30229257	1.315059	1.042232068	0.0596765	0.887223	1
RP11-477D19.2	1.0455131	1.05325109	1.042159	0.791702233	-0.3369702	0.887238	1
RP11-1029J19.2	1.0279405	1.03559787	1.0246214	0.691652634	-0.5318804	0.887393	1
PALM2	1.0279489	1.03574787	1.0245684	0.687268257	-0.5410548	0.887393	1
PGD	2.7931846	2.80668252	2.7873338	0.989290479	-0.0155339	0.887415	1
MPP7	1.1171581	1.12501495	1.1137526	0.909911785	-0.1362014	0.887447	1
TTC27	1.1214142	1.11310186	1.1250173	1.105351087	0.1445047	0.887516	1
MYBPC2	1.0248504	1.01685675	1.0283153	1.67976326	0.7482579	0.887516	1
AP000769.1	1.1563334	1.14779068	1.1600362	1.082857405	0.1148433	0.887539	1
MAP6D1	1.0869432	1.09485269	1.0835148	0.880468711	-0.1836564	0.887551	1
DVL3	1.4755741	1.48442767	1.4717365	0.973801697	-0.0383001	0.887559	1
PPARGC1B	1.038215	1.045965	1.0348557	0.758309299	-0.3991417	0.887582	1
RP11-290L1.2	1.0475156	1.05517162	1.0441971	0.801083904	-0.3199747	0.887597	1
SLC28A2	1.0301085	1.0377414	1.0267999	0.7100937	-0.4939187	0.887778	1
RP11-425D17.1	1.0190086	1.02664179	1.0157	0.58929809	-0.7629305	0.887778	1
RP3-428L16.2	1.1237741	1.11558425	1.127324	1.101569119	0.13956	0.887787	1
CDHR3	1.0605323	1.05226699	1.0641149	1.226681044	0.2947602	0.887829	1
RP11-20B24.4	1.0196007	1.02721379	1.0163008	0.598988584	-0.7393996	0.887964	1
RP11-642C5.1	1.0196008	1.02720026	1.0163067	0.599506767	-0.7381521	0.887964	1
MARVELD2	1.0860973	1.09396538	1.0826868	0.87997128	-0.1844717	0.888044	1
NCKIPSD	1.1970302	1.18863029	1.2006711	1.063833032	0.0892717	0.888076	1
TP53I11	1.4296276	1.42022697	1.4337024	1.032066918	0.0455365	0.888099	1
FNBP4	1.8978967	1.8878423	1.9022548	1.016233185	0.0232315	0.888109	1
ASCL2	1.0314337	1.03907218	1.0281228	0.719764462	-0.4744032	0.888154	1
SH3BP5-AS1	1.0524838	1.04433054	1.0560178	1.263639883	0.3375854	0.888162	1
TCF7L1	1.4091852	1.41751784	1.4055734	0.971391793	-0.0418748	0.88817	1
ATAD3C	1.0402548	1.04792759	1.0369289	0.77051532	-0.3761045	0.88819	1
MZT2A	6.1286388	6.09754116	6.1421183	1.008744829	0.0125613	0.888239	1
MBD6	1.2095958	1.21757888	1.2061355	0.947405629	-0.0779459	0.888253	1
DIAPH2	1.578502	1.56929031	1.5824948	1.023194686	0.0330807	0.88833	1
DDHD2	1.4843569	1.47502992	1.4883997	1.028145178	0.040044	0.888436	1
SCN9A	1.1536211	1.14534243	1.1572095	1.081649202	0.1132327	0.888455	1
SLC37A4	1.777814	1.76817132	1.7819936	1.017993807	0.0257288	0.888463	1
RP1-93H18.1	1.0272193	1.01908646	1.0307446	1.61080595	0.6877827	0.888523	1
WBP1	1.073814	1.0815276	1.0704704	0.864375355	-0.2102702	0.888564	1
LY6G5C	1.0583258	1.06600356	1.0549978	0.833254702	-0.2631705	0.888599	1
TNXB	1.0783131	1.06991078	1.0819551	1.172281687	0.2293193	0.888608	1
PLAC8	1.0413057	1.03323644	1.0448033	1.348017806	0.4308396	0.888609	1
GEMIN6	1.3874089	1.39624732	1.3835779	0.968026394	-0.0468817	0.88865	1
GSG2	1.0272954	1.03484334	1.0240237	0.689477503	-0.5364246	0.888655	1
RP11-70D24.2	1.0299295	1.03754566	1.0266283	0.709223223	-0.4956883	0.888691	1
TMEM86B	1.0299902	1.03762082	1.0266826	0.709250913	-0.495632	0.888691	1
NLGN3	1.0299323	1.03760396	1.0266069	0.707556868	-0.499082	0.888691	1
USP53	1.3667581	1.35759737	1.3707288	1.036721376	0.0520282	0.888761	1
ADCY9	1.0365796	1.044212	1.0332713	0.752540831	-0.4101582	0.888792	1
TNNC2	1.0365719	1.04408553	1.0333151	0.755693268	-0.4041273	0.888792	1
RP11-83N9.5	1.0195468	1.02705878	1.0162906	0.602045863	-0.7320547	0.888858	1
AC000068.9	1.0195597	1.02707065	1.016304	0.602276753	-0.7315015	0.888858	1
ACSBG1	1.02076	1.02834163	1.0174738	0.616540522	-0.6977324	0.88886	1

PCAT29	1.0207405	1.0282916	1.0174674	0.617404669	-0.6957117	0.88886	1
AQP7	1.0207275	1.02825801	1.0174634	0.617998113	-0.6943257	0.88886	1
AC005932.1	1.0431809	1.03520087	1.0466399	1.324964275	0.4059535	0.888874	1
SNX33	1.0969428	1.08875719	1.1004909	1.132199865	0.1791287	0.888945	1
SLC35E2	1.1309045	1.13885765	1.1274572	0.917898008	-0.1235942	0.888945	1
RP11-359I18.5	1.0468747	1.05452687	1.0435578	0.798832073	-0.3240358	0.888997	1
RP13-49I15.6	1.0256716	1.03321892	1.0224002	0.674319656	-0.5684954	0.889055	1
NOMO2	1.025656	1.03319772	1.022387	0.674354972	-0.5684199	0.889055	1
RP11-468E2.10	1.0256808	1.03324543	1.0224018	0.673831426	-0.5695404	0.889055	1
FTCD	1.0438607	1.03581428	1.0473485	1.322055952	0.4027832	0.889068	1
SLC25A53	1.1887922	1.19691611	1.1852709	0.940861871	-0.0879452	0.889082	1
HIC1	1.0277047	1.03540329	1.0243677	0.688288374	-0.538915	0.889235	1
USP12-AS2	1.0283843	1.03585447	1.0251464	0.701345047	-0.5118037	0.889263	1
SLC16A4	1.0979559	1.08987699	1.1014578	1.128851607	0.1748558	0.889269	1
LINC01482	1.0183695	1.02597538	1.0150726	0.580266761	-0.7852118	0.889283	1
RP11-386D6.1	1.0183437	1.02589015	1.0150726	0.582177078	-0.7804701	0.889283	1
SNTN	1.0183666	1.02596586	1.0150726	0.580479406	-0.7846832	0.889283	1
RP11-341N2.1	1.0183263	1.02583254	1.0150726	0.583475337	-0.7772564	0.889283	1
UCK1	1.5546546	1.56347103	1.550833	0.977571116	-0.0327264	0.88929	1
ERCC6	1.1150153	1.10651401	1.1187003	1.114410037	0.1562802	0.889392	1
TAPT1-AS1	1.020128	1.02762705	1.0168775	0.61090501	-0.71098	0.889446	1
NEFH	1.0705327	1.06252306	1.0740046	1.183636568	0.2432262	0.889578	1
RFX1	1.2749972	1.2665653	1.278652	1.045342541	0.0639758	0.889634	1
ATP13A3	1.302037	1.31040581	1.2984095	0.961352852	-0.056862	0.889659	1
RAB39A	1.0204159	1.0279411	1.017154	0.613934179	-0.7038441	0.88971	1
AC007386.2	1.0204256	1.02795925	1.01716	0.61375216	-0.7042719	0.88971	1
MEI4	1.020403	1.02788239	1.017161	0.615476699	-0.7002239	0.88971	1
SEMA6A-AS1	1.0203938	1.02785443	1.01716	0.616059791	-0.6988577	0.88971	1
TMEM108	1.2499154	1.24151433	1.2535568	1.049862531	0.0702004	0.889878	1
SMCR5	1.3803598	1.38892294	1.376648	0.968438691	-0.0462674	0.889929	1
RP4-742C19.13	1.0450076	1.05251898	1.0417518	0.794984544	-0.3310013	0.88994	1
RP13-977J11.2	1.0449832	1.05264483	1.0416623	0.791384371	-0.3375495	0.88994	1
SLC45A1	1.0342496	1.02633829	1.0376788	1.430571292	0.5165914	0.889982	1
SPRYD3	1.1276495	1.11933127	1.1312551	1.099922343	0.1374017	0.889998	1
RP11-713P17.3	1.0464136	1.0539045	1.0431666	0.800797002	-0.3204915	0.890038	1
PDE12	1.1869618	1.19497025	1.1834904	0.941120169	-0.0875491	0.890094	1
SLC15A2	1.0359763	1.04344845	1.0327374	0.75347787	-0.408363	0.890095	1
DDX59	1.1919228	1.18361132	1.1955255	1.064887971	0.0907017	0.890125	1
CCDC155	1.0386739	1.04611991	1.0354463	0.768569311	-0.3797527	0.890167	1
LEPR	1.1344781	1.14231839	1.1310797	0.9210314	-0.1186778	0.89017	1
RP11-370I10.12	1.0179871	1.02545588	1.0147498	0.579425042	-0.7873061	0.890187	1
RP11-491F9.1	1.0179698	1.02543321	1.0147348	0.579352862	-0.7874858	0.890187	1
RP11-685M7.3	1.0179819	1.02542234	1.0147568	0.580464912	-0.7847192	0.890187	1
RP11-305L7.1	1.0179975	1.025488	1.0147508	0.578733186	-0.7890297	0.890187	1
DNAJC7	2.2706371	2.28193727	2.265739	0.987364227	-0.0183457	0.890264	1
RP11-15H20.8	1.0293722	1.03693249	1.0260952	0.706565001	-0.5011058	0.890321	1
ZNF570	1.0975294	1.089509	1.1010058	1.12844319	0.1743338	0.890446	1
DARS2	1.0709822	1.06311612	1.0743918	1.178649202	0.2371344	0.890451	1
RP4-555D20.2	1.0255587	1.01774337	1.0289463	1.631388011	0.7061	0.890508	1
SLC1A7	1.0255765	1.01775367	1.0289674	1.631626609	0.7063109	0.890508	1
RP11-347C18.5	1.0256904	1.01775578	1.0291297	1.640575283	0.7142018	0.890508	1

ZNF670-ZNF695	1.0274962	1.03495995	1.0242609	0.693963834	-0.5270676	0.890523	1
RP6-191P20.4	1.0274322	1.03486487	1.0242105	0.694410152	-0.5261401	0.890523	1
ITPKA	1.0274707	1.03497624	1.0242174	0.692394644	-0.5303335	0.890523	1
DNAJB13	1.0274378	1.03489027	1.0242075	0.69381889	-0.527369	0.890523	1
CD4	1.0654235	1.07297346	1.0621509	0.851692396	-0.2315956	0.890636	1
PARK2	1.0440172	1.05161144	1.0407254	0.789076981	-0.341762	0.890667	1
DACT1	1.0533111	1.04538712	1.0567458	1.25026255	0.3222311	0.890678	1
DEPTOR	1.0362368	1.04375621	1.0329775	0.753665017	-0.4080047	0.890678	1
SRCAP	1.0432408	1.03524752	1.0467055	1.325072513	0.4060713	0.890743	1
TMEM56	1.2728592	1.28122582	1.2692327	0.957354119	-0.0628754	0.890766	1
CRTC3-AS1	1.0282825	1.02036941	1.0317124	1.556866209	0.638645	0.890773	1
TUBD1	1.1896721	1.19731136	1.1863609	0.944501437	-0.0823751	0.890777	1
IL17B	1.0253682	1.03283099	1.0221334	0.674161367	-0.5688341	0.890783	1
PNRC2	2.7341806	2.74717009	2.7285502	0.989342813	-0.0154576	0.890825	1
NAPSA	1.0573039	1.06465924	1.0541158	0.836937695	-0.2568079	0.890884	1
RP11-138I1.4	1.0309766	1.02311287	1.0343852	1.487708955	0.5730923	0.890924	1
MYEOV	1.0309068	1.02312511	1.0342798	1.482361876	0.5678977	0.890924	1
SRSF8	2.0665905	2.05579226	2.0712711	1.014660838	0.0209976	0.89097	1
FMO5	1.0249947	1.0171592	1.028391	1.654563761	0.7264509	0.890983	1
RP11-867G23.8	1.1357741	1.14334413	1.1324928	0.924298774	-0.1135688	0.890998	1
NPHS1	1.0297626	1.0218327	1.0331999	1.520650787	0.6046889	0.891009	1
FGF14	1.0488733	1.05650123	1.0455669	0.80647707	-0.3102946	0.891017	1
NUDCD3	1.4048311	1.41328229	1.401168	0.970687497	-0.0429212	0.891025	1
NACAD	1.0907097	1.09842425	1.0873658	0.887645206	-0.171945	0.891056	1
BBS5	1.1071559	1.11484837	1.1038216	0.903988422	-0.1456238	0.891171	1
RP11-449J10.1	1.0464536	1.05398945	1.0431871	0.799917248	-0.3220773	0.891191	1
SLC26A4	1.0274238	1.01960382	1.0308134	1.571807068	0.6524241	0.891206	1
RP11-10C24.3	1.0345738	1.02675346	1.0379635	1.419013991	0.5048888	0.891222	1
MMP15	1.1273396	1.11946824	1.1307514	1.094445086	0.1301996	0.891272	1
AC026188.1	1.0202009	1.0276228	1.0169839	0.614850186	-0.7016932	0.891274	1
RP11-417F21.1	1.020188	1.02757999	1.0169839	0.615804474	-0.6994557	0.891274	1
EXD1	1.0179671	1.02534722	1.0147681	0.582632279	-0.7793425	0.891324	1
PTGIR	1.0262128	1.01839445	1.0296017	1.609272326	0.6864085	0.891468	1
TC2N	1.0261797	1.01839445	1.0295543	1.606695688	0.6840967	0.891468	1
MPP3	1.0757033	1.08323409	1.0724391	0.870305423	-0.2004063	0.891486	1
MPZ	1.0543934	1.04642419	1.0578476	1.246066594	0.3173812	0.891489	1
INVS	1.1017384	1.09376931	1.1051926	1.121823632	0.1658459	0.891562	1
GPR141	1.026677	1.03402636	1.0234914	0.690389393	-0.5345178	0.891654	1
SLC37A2	1.0265745	1.03396489	1.0233711	0.688096494	-0.5393172	0.891654	1
RP5-1050D4.2	1.0259771	1.01815945	1.0293657	1.617102828	0.6934114	0.891703	1
TBC1D8	1.0625871	1.07003312	1.0593596	0.847593799	-0.2385551	0.891705	1
LINC00284	1.0250919	1.03250888	1.0218769	0.672952942	-0.5714225	0.891732	1
LINC01513	1.0250594	1.03245133	1.0218554	0.673482194	-0.5702883	0.891732	1
RP11-50D9.3	1.0187297	1.02612886	1.0155225	0.594074467	-0.7512843	0.891756	1
TMPRSS5	1.0380216	1.04548394	1.0347871	0.764820766	-0.3868064	0.891769	1
C1orf143	1.0380306	1.04549811	1.0347938	0.764730475	-0.3869767	0.891769	1
RP11-467P9.1	1.0380331	1.04548889	1.0348014	0.765051619	-0.386371	0.891769	1
CTC-529L17.1	1.0161232	1.0234999	1.0129257	0.550030914	-0.8624154	0.891773	1
RP11-161M6.3	1.0161229	1.02349898	1.0129257	0.550052456	-0.8623589	0.891773	1
RP11-115C10.1	1.1024497	1.11030068	1.0990467	0.897969742	-0.1552613	0.891777	1
LAMTOR3	1.761727	1.77121494	1.7576144	0.98236481	-0.0256692	0.891935	1

RP11-45P15.4	1.0335149	1.04100541	1.0302681	0.738149888	-0.4380143	0.89198	1
CCDC96	1.0335259	1.04089929	1.0303299	0.741575829	-0.4313339	0.89198	1
RUFY1	1.281596	1.28988155	1.2780046	0.959028147	-0.0603549	0.892049	1
BDNF-AS	1.1404185	1.14818271	1.1370531	0.924892615	-0.1126422	0.892079	1
ZBTB8A	1.2532454	1.24488381	1.2568698	1.048945643	0.0689399	0.892085	1
NT5C2	1.3496686	1.35769603	1.346189	0.967830151	-0.0471742	0.892139	1
ZNF777	1.0455383	1.05303006	1.042291	0.797491421	-0.3264591	0.892206	1
GAS6-AS1	1.0455213	1.05293335	1.0423085	0.79927849	-0.3232298	0.892206	1
PACSIN1	1.0943941	1.10183872	1.0911673	0.895212102	-0.1596986	0.892241	1
TBC1D8B	1.1513752	1.1592125	1.1479781	0.929437562	-0.1055701	0.89227	1
RP11-755F10.1	1.0171108	1.02445328	1.0139282	0.569585544	-0.8120156	0.892325	1
CTD-2035E11.3	1.0171082	1.02444688	1.0139272	0.569691299	-0.8117477	0.892325	1
LINC00323	1.027482	1.01971687	1.0308479	1.564540756	0.6457392	0.892347	1
RP11-166B2.8	1.0164253	1.02377932	1.0132377	0.556688462	-0.8450579	0.89235	1
RP11-95O2.1	1.016417	1.02375196	1.0132377	0.55732985	-0.8433967	0.89235	1
IGSF23	1.0164168	1.02375113	1.0132377	0.557349311	-0.8433463	0.89235	1
RYK	1.5178655	1.52670023	1.5140361	0.975955701	-0.0351124	0.892401	1
MTRNR2L10	1.0241233	1.03147976	1.0209346	0.665017349	-0.5885361	0.892452	1
LINC01481.1	1.0241479	1.03152058	1.0209522	0.664714353	-0.5891936	0.892452	1
RP1-150O5.3	1.0241355	1.03151337	1.0209375	0.66440169	-0.5898724	0.892452	1
KIF25-AS1	1.0366294	1.02868725	1.0400719	1.396853879	0.4821811	0.892492	1
CLK4	1.3309621	1.33930857	1.3273442	0.964739019	-0.0517894	0.892519	1
HDC	1.0170421	1.02444205	1.0138345	0.566011825	-0.8210959	0.892536	1
TAS2R46	1.0170194	1.02436711	1.0138345	0.567752535	-0.8166659	0.892536	1
RP5-864K19.6	1.017013	1.02434586	1.0138345	0.568248126	-0.8154071	0.892536	1
RP11-692C24.2	1.0170067	1.02432519	1.0138345	0.568731153	-0.8141813	0.892536	1
RP11-900F13.2	1.017006	1.02432295	1.0138345	0.568783327	-0.8140489	0.892536	1
ZBTB17	1.224843	1.21666047	1.2283897	1.054136653	0.0760619	0.892648	1
EDN3	1.0262725	1.01850742	1.0296384	1.601431942	0.6793625	0.892651	1
NBEAL2	1.0623749	1.06981439	1.0591502	0.847249617	-0.239141	0.892671	1
RP11-147L13.8	1.0288913	1.03626617	1.0256946	0.708499415	-0.4971614	0.89271	1
CTD-2377O17.1	1.0288439	1.03622881	1.0256429	0.707803126	-0.49858	0.89271	1
DCHS1	1.259091	1.25070209	1.2627272	1.047965719	0.0675915	0.892733	1
RP11-29B2.6	1.02663	1.0339452	1.0234591	0.691088656	-0.5330573	0.892796	1
KCNB2	1.1460623	1.15374234	1.1427333	0.928393226	-0.1071921	0.892813	1
KCNN4	1.0257429	1.01804205	1.0290808	1.611836524	0.6887054	0.892831	1
CTB-55O6.12	1.0707391	1.07827801	1.0674713	0.861944298	-0.2143335	0.892863	1
RAP1GAP2	1.0624711	1.06984429	1.0592751	0.848675314	-0.2367154	0.89291	1
RP11-457M11.5	1.058756	1.06622185	1.0555199	0.838391847	-0.2543034	0.892924	1
RP4-625H18.2	1.0752119	1.08267712	1.0719761	0.870568205	-0.1999708	0.892962	1
TBC1D4	1.0897761	1.09730839	1.0865112	0.889041197	-0.1696778	0.892964	1
VCX	1.0301486	1.03746836	1.0269757	0.719960701	-0.4740099	0.893078	1
RP11-386G11.5	1.1231491	1.13067044	1.119889	0.917491398	-0.1242335	0.893183	1
KRT86	1.0273472	1.03471214	1.0241548	0.695860367	-0.5231303	0.89323	1
PLXNA3	1.204838	1.21274186	1.201412	0.946743657	-0.0789542	0.893324	1
LRRC43	1.0296792	1.02192978	1.0330382	1.506543474	0.5912423	0.893337	1
RP11-770J1.4	1.0169562	1.02422668	1.0138047	0.569814307	-0.8114362	0.893412	1
XXyac-YX155B6.7	1.0169555	1.02422273	1.0138055	0.569938756	-0.8111212	0.893412	1
RP11-276M12.1	1.0169815	1.02431506	1.0138028	0.567664198	-0.8168903	0.893412	1
PIP5K1B	1.3087652	1.31671587	1.3053189	0.964015255	-0.0528721	0.893418	1
AC113167.1	1.0181535	1.02543219	1.0149985	0.589743011	-0.7618417	0.893431	1

RP11-49K24.8	1.0181503	1.02542184	1.0149985	0.58998322	-0.7612542	0.893431	1
LINC00950	1.043908	1.03599258	1.047339	1.315243329	0.3953297	0.893441	1
SLC19A3	1.0230424	1.03025835	1.0199147	0.658154589	-0.6035016	0.893646	1
DSEL	1.6552191	1.66392675	1.6514448	0.981199742	-0.0273812	0.893656	1
AP001062.7	1.0457226	1.03800071	1.0490697	1.291283181	0.3688054	0.89367	1
TEX19	1.0303081	1.02260365	1.0336477	1.488596432	0.5739527	0.89368	1
ADIRF	1.030296	1.02255758	1.0336503	1.491752812	0.5770085	0.89368	1
HYAL3	1.0540558	1.04631555	1.0574109	1.239560247	0.3098284	0.893713	1
FGF22	1.0250363	1.03236107	1.0218613	0.675542908	-0.5658807	0.893791	1
CLCNKA	1.0250293	1.03228175	1.0218857	0.677959343	-0.5607293	0.893791	1
BEND3	1.066127	1.07353985	1.0629138	0.855506172	-0.2251498	0.893826	1
TJP3	1.0622063	1.05429023	1.0656376	1.209013015	0.2738298	0.893836	1
SGSH	1.1085481	1.11607948	1.1052836	0.906995695	-0.1408324	0.893872	1
IL1RAP	1.0921497	1.09955587	1.0889395	0.893362373	-0.1626826	0.893875	1
PCDHB13	1.0351723	1.02743467	1.0385262	1.40428746	0.4898383	0.893909	1
APOBEC3D	1.0256497	1.03289369	1.0225098	0.684318553	-0.54726	0.893977	1
CTB-171A8.1	1.0175416	1.02474369	1.0144199	0.582769774	-0.779002	0.893999	1
CTB-46B19.2	1.0175605	1.02480687	1.0144195	0.581271311	-0.7827164	0.893999	1
TCTE1	1.017614	1.02496724	1.0144267	0.577823784	-0.7912985	0.893999	1
HNMT	1.1557261	1.14778301	1.159169	1.077045685	0.1070794	0.894071	1
VWA8	1.1124904	1.11970919	1.1093613	0.913558227	-0.1304314	0.894083	1
PACS2	1.3397218	1.33101736	1.3434948	1.037694269	0.0533815	0.894085	1
MFSD12	1.2707673	1.27895623	1.2672177	0.957919871	-0.0620231	0.894091	1
LINC00957	1.0462848	1.05362558	1.0431029	0.803774231	-0.3151378	0.894171	1
THOC5	1.2331022	1.22477602	1.2367112	1.053097886	0.0746395	0.894184	1
SLC36A4	2.0102729	2.02048469	2.0058465	0.985655675	-0.0208443	0.894191	1
CTC-503J8.4	1.0353088	1.04265028	1.0321266	0.753255464	-0.4087889	0.894255	1
TBC1D13	1.1889683	1.18083844	1.1924923	1.06444339	0.0900992	0.894255	1
FAM20A	1.0560277	1.04822823	1.0594085	1.231819582	0.300791	0.894282	1
RP11-356C4.3	1.0536035	1.04585203	1.0569635	1.242332396	0.3130512	0.894334	1
ADAMTS13	1.0672867	1.07469884	1.0640739	0.857762641	-0.2213496	0.894359	1
MYH15	1.0378148	1.03009745	1.0411599	1.36755584	0.4515997	0.894431	1
SPEG	1.2328377	1.2246827	1.2363725	1.052028047	0.0731732	0.894442	1
LARP4B	1.2980216	1.28972627	1.3016173	1.04104237	0.0580288	0.894469	1
ZNF850	1.042395	1.04966008	1.039246	0.790291968	-0.3395423	0.894483	1
SDCBP2	1.1197718	1.12727402	1.1165199	0.915504264	-0.1273615	0.894507	1
C11orf96	1.0203995	1.01281342	1.0236877	1.84866275	0.8864821	0.894523	1
RP11-884K10.7	1.0631147	1.05535491	1.0664782	1.200945064	0.2641702	0.894569	1
RP13-516M14.4	1.0535259	1.06084464	1.0503535	0.827574773	-0.2730384	0.894615	1
SCFD2	1.1170115	1.10925784	1.1203724	1.101727852	0.1397679	0.894631	1
ELP3	1.1756005	1.18352915	1.1721637	0.938072916	-0.092228	0.894637	1
PDCD4-AS1	1.119614	1.12713568	1.1163536	0.915192549	-0.1278528	0.894716	1
SPATA2	1.0802801	1.07247402	1.0836638	1.154396425	0.2071387	0.894746	1
FAM157B	1.0153871	1.02256139	1.0122773	0.544173094	-0.8778625	0.894757	1
FGR	1.0153797	1.02253707	1.0122773	0.544760401	-0.8763063	0.894757	1
ZNF841	1.0540096	1.0613974	1.0508073	0.827515647	-0.2731415	0.894764	1
SLC35A3	1.2528307	1.26050988	1.2495022	0.957745505	-0.0622857	0.894774	1
EXOC3	1.1578125	1.16520882	1.1546066	0.935825165	-0.0956891	0.894799	1
FOXK2	1.1960569	1.18795461	1.1995688	1.061792657	0.0865021	0.894801	1
C17orf97	1.1033041	1.11088619	1.1000176	0.901984417	-0.1488256	0.894873	1
C1orf186	1.0255606	1.03280198	1.0224218	0.683550845	-0.5488794	0.894893	1

TAS2R31	1.0255567	1.03273452	1.0224454	0.685680582	-0.5443914	0.894893	1
MRGPRF	1.0620669	1.06940709	1.0588853	0.848404828	-0.2371753	0.894926	1
BCAS3	1.2103591	1.20195888	1.2140002	1.059622452	0.0835503	0.895002	1
ZNF71	1.3319766	1.3237256	1.335553	1.036535162	0.0517691	0.895071	1
FPGT	1.0846318	1.07693615	1.0879676	1.143384326	0.1933104	0.895104	1
RP11-572M11.3	1.0248839	1.03222489	1.021702	0.673453763	-0.5703492	0.895113	1
RP11-380B4.3	1.024836	1.03207594	1.0216978	0.676452164	-0.5639402	0.895113	1
SYT15	1.0336483	1.04095361	1.0304818	0.74430102	-0.4260419	0.895218	1
ZNF471	1.0831005	1.09062425	1.0798394	0.88099327	-0.1827971	0.895225	1
VCPIP1	1.2047497	1.21229736	1.2014781	0.949037143	-0.0754635	0.895244	1
PPM1H	1.1585559	1.16617345	1.1552541	0.934289413	-0.0980586	0.895259	1
KCTD9	1.1873572	1.17908891	1.1909411	1.066180737	0.092452	0.89527	1
VSIG8	1.0244472	1.03172916	1.0212908	0.671017655	-0.5755774	0.895282	1
POLE2	1.1116614	1.11922349	1.1083836	0.909079085	-0.1375223	0.89535	1
AL450992.2	1.0227702	1.02996088	1.0196534	0.65596888	-0.6083007	0.895373	1
ULBP1	1.0227675	1.02991589	1.019669	0.657475138	-0.6049918	0.895373	1
RP11-599B13.7	1.0227905	1.0299944	1.019668	0.655722491	-0.6088427	0.895373	1
RP11-310E22.4	1.0227885	1.03002041	1.0196538	0.654679877	-0.6111385	0.895373	1
RELL2	1.1741843	1.18183484	1.1708682	0.939688966	-0.0897448	0.895376	1
RP11-526F3.1	1.0163344	1.02358271	1.0131926	0.559419316	-0.837998	0.895417	1
PCDHGA11	1.016287	1.0234257	1.0131926	0.563168792	-0.8283607	0.895417	1
CH507-254M2.1	1.0162952	1.02345305	1.0131926	0.562512073	-0.830044	0.895417	1
RP4-535B20.4	1.0162961	1.02345586	1.0131926	0.56244454	-0.8302172	0.895417	1
RNF123	1.1071137	1.11458105	1.1038769	0.906580426	-0.1414931	0.895608	1
ZFAND4	1.1130353	1.10500977	1.116514	1.109553413	0.1499791	0.895693	1
DUSP23	2.079318	2.06846485	2.0840224	1.01456064	0.0208551	0.895738	1
ARHGAP17	1.2355462	1.22771138	1.2389422	1.04932034	0.0694552	0.895788	1
AMT	1.105547	1.09777855	1.1089142	1.113886714	0.1556025	0.895799	1
RP11-262H14.3	1.0275674	1.02000979	1.0308433	1.541412386	0.6242529	0.89584	1
TRPC1	1.1946551	1.20222404	1.1913743	0.946347832	-0.0795575	0.89587	1
CTD-2537I9.19	1.0154168	1.02259522	1.0123053	0.544599423	-0.8767326	0.895894	1
RP11-1070A24.2	1.0153888	1.02250232	1.0123053	0.546847852	-0.8707886	0.895894	1
HIST1H2BG	1.0396477	1.03201216	1.0429573	1.341907283	0.424285	0.895923	1
EDARADD	1.0525305	1.05970748	1.0494195	0.827694295	-0.2728301	0.895974	1
RNPC3	1.4042879	1.41194139	1.4009704	0.973367573	-0.0389434	0.895993	1
GDPGP1	1.0639981	1.07119826	1.0608772	0.855038091	-0.2259394	0.896032	1
GSR	1.1281738	1.13572896	1.124899	0.920208624	-0.1199671	0.896096	1
RAB3GAP1	1.3865408	1.37792543	1.3902752	1.032677768	0.0463902	0.896096	1
AP000265.1	1.0188816	1.02601559	1.0157893	0.606917196	-0.7204284	0.896174	1
GAS1RR	1.0188686	1.02597281	1.0157893	0.607916816	-0.7180542	0.896174	1
ZNF846	1.0999295	1.10735135	1.0967125	0.900897146	-0.1505657	0.896185	1
AC046143.3	1.0543806	1.04671284	1.0577043	1.235298272	0.3048594	0.896209	1
KCNK15-AS1	1.0674824	1.05971473	1.0708493	1.186463239	0.2466674	0.896226	1
RPPH1	1.0674533	1.05969843	1.0708148	1.186208009	0.246357	0.896226	1
ALDH8A1	1.0224884	1.02961366	1.0194	0.655102136	-0.6102082	0.896321	1
RP11-80H18.3	1.0161369	1.02324071	1.0130577	0.561847049	-0.8317507	0.896325	1
RP11-73M7.9	1.0161584	1.02331177	1.0130577	0.56013454	-0.8361547	0.896325	1
CTB-147C22.9	1.0161347	1.02323341	1.0130577	0.56202367	-0.8312972	0.896325	1
GATA1	1.0161443	1.02326512	1.0130577	0.561257645	-0.8332649	0.896325	1
GAPLINC	1.0249219	1.01740877	1.0281786	1.618642843	0.6947847	0.896332	1
HPSE2	1.0309316	1.02342596	1.0341849	1.459275938	0.5452527	0.896505	1

HIST1H2AB	1.0227087	1.02976161	1.0196515	0.660297433	-0.5988121	0.896556	1
RP11-923I11.6	1.022729	1.02986474	1.019636	0.657496632	-0.6049446	0.896556	1
MT1M	1.0214179	1.01394208	1.0246584	1.76862874	0.8226312	0.896613	1
NTRK3	1.0422749	1.04942584	1.0391753	0.792606986	-0.3353224	0.896632	1
DNAJC21	1.5233351	1.51443242	1.5271941	1.02480724	0.0353526	0.896645	1
TMEM253	1.04977	1.04213447	1.0530796	1.259766442	0.3331563	0.896647	1
DUSP19	1.0882959	1.09557715	1.0851397	0.890795916	-0.1668332	0.896674	1
PHF2	1.1417725	1.14913539	1.1385809	0.929229119	-0.1058937	0.896702	1
C6orf120	1.4225554	1.41421538	1.4261705	1.028862013	0.0410495	0.896751	1
BEND7	1.1289902	1.1212739	1.1323349	1.091206487	0.1259241	0.896757	1
NAP1L2	1.0851568	1.07747848	1.0884849	1.14205816	0.1916361	0.896813	1
TNFSF13B	1.042893	1.05013557	1.0397537	0.792923795	-0.3347459	0.89682	1
PCDHAC1	1.0248814	1.03193436	1.0218243	0.683410653	-0.5491754	0.896872	1
RP11-323F24.3	1.0248904	1.0319696	1.0218219	0.682583365	-0.5509228	0.896872	1
ASPG	1.0145264	1.02159719	1.0114615	0.530692609	-0.9140516	0.896894	1
AC025171.1	1.0145198	1.02157545	1.0114615	0.531227496	-0.9125983	0.896894	1
C7orf55-LUC7L2	1.0145174	1.02156745	1.0114615	0.531424404	-0.9120636	0.896894	1
ECT2L	1.014518	1.02156943	1.0114615	0.53137582	-0.9121955	0.896894	1
AC078883.4	1.0145468	1.02166489	1.0114615	0.52903428	-0.9185669	0.896894	1
ERN2	1.0548926	1.06211516	1.051762	0.833322693	-0.2630528	0.896938	1
RP11-138A9.1	1.0374722	1.02997341	1.0407226	1.358623984	0.4421462	0.897014	1
NR4A2	1.0215319	1.02864985	1.0184466	0.643862991	-0.6351744	0.897041	1
LMNTD1	1.0215217	1.02862603	1.0184423	0.644250886	-0.6343055	0.897041	1
PRSS53	1.0215186	1.02859583	1.0184509	0.645230823	-0.6321127	0.897041	1
CEP131	1.2274101	1.21935737	1.2309006	1.052622822	0.0739886	0.897052	1
C2orf54	1.0223529	1.01486911	1.0255967	1.721470862	0.7836418	0.897091	1
KCNJ13	1.0791329	1.08636353	1.0759987	0.879985793	-0.1844479	0.8971	1
ZNF304	1.0596521	1.06692824	1.0564982	0.844160492	-0.2444108	0.897183	1
AC013271.5	1.0370198	1.02949825	1.04028	1.365504509	0.4494341	0.897195	1
LSMEM1	1.0410432	1.03363573	1.044254	1.315683073	0.395812	0.89726	1
RASSF4	1.8350251	1.84452076	1.8309092	0.983882451	-0.0234421	0.89727	1
NFATC1	1.0500244	1.04251912	1.0532776	1.253027375	0.3254179	0.897359	1
PTGER4	1.0500615	1.04250609	1.0533365	1.254796461	0.3274534	0.897359	1
SDHAF2	1.4390699	1.43072957	1.442685	1.027756323	0.0394982	0.897402	1
ZNF653	1.0560412	1.06324535	1.0529185	0.836717193	-0.257188	0.897417	1
RNF122	1.0727345	1.07989212	1.0696319	0.871574553	-0.198304	0.897427	1
TBCCD1	1.1367579	1.14430471	1.1334867	0.925033388	-0.1124227	0.897459	1
ZNF219	1.5434956	1.55191886	1.5398445	0.978123027	-0.0319122	0.897526	1
ARHGEF35	1.0793682	1.0718668	1.0826197	1.149622108	0.2011597	0.897558	1
JAK3	1.0921989	1.08438177	1.0955873	1.132795918	0.179888	0.897568	1
XKR9	1.0310996	1.0382417	1.0280038	0.732284076	-0.4495247	0.897688	1
ADGRL4	1.044053	1.05124606	1.0409351	0.798794804	-0.3241031	0.897727	1
SMIM11A	1.0214544	1.01400962	1.0246814	1.761745182	0.8170053	0.897753	1
CARD14	1.0214283	1.01400962	1.024644	1.759075813	0.8148177	0.897753	1
HAUS3	1.1464196	1.15375879	1.1432383	0.931578138	-0.1022513	0.897757	1
RP11-461M2.2	1.0309342	1.02343705	1.0341838	1.458538393	0.5445234	0.897772	1
SNHG22	1.0221574	1.02919728	1.0191059	0.654373477	-0.6118138	0.897783	1
RP11-400N9.1	1.0247746	1.0317965	1.0217309	0.683436725	-0.5491203	0.897784	1
UBE2E1-AS1	1.0324981	1.02502405	1.0357377	1.42813462	0.514132	0.897877	1
LRRC37A	1.0143793	1.02138065	1.0113445	0.530597369	-0.9143106	0.89798	1
RP11-697M17.2	1.0143841	1.02139654	1.0113445	0.530203348	-0.9153823	0.89798	1

THCAT158	1.0143697	1.02134907	1.0113445	0.531382405	-0.9121776	0.89798	1
RP11-48B3.5	1.0416066	1.04871326	1.0385262	0.790877283	-0.3384742	0.898065	1
LINC00575	1.0401439	1.04716772	1.0370993	0.786540489	-0.3464071	0.898085	1
ME1	1.0734694	1.06593244	1.0767363	1.163862562	0.2189207	0.898089	1
PHC3	1.4022125	1.41034398	1.3986879	0.971594398	-0.0415739	0.898112	1
EDC3	1.1850026	1.17725898	1.1883591	1.062620724	0.0876268	0.898128	1
STRIP1	1.1624397	1.16997455	1.1591737	0.936456206	-0.0947166	0.898177	1
SPIN2A	1.0515963	1.05874526	1.0484976	0.82555785	-0.2765588	0.898222	1
BCL2L2-PABPN1	1.0204541	1.02746963	1.0174132	0.633906304	-0.6576585	0.898252	1
AC006004.1	1.020463	1.02747852	1.0174221	0.63402787	-0.6573818	0.898252	1
RP11-574F21.2	1.0204668	1.02747588	1.0174286	0.634324927	-0.6567061	0.898252	1
ELF4	1.0488345	1.04139858	1.0520576	1.257472801	0.3305272	0.898268	1
GUCY1A2	1.0489164	1.04141371	1.0521685	1.259690928	0.3330698	0.898268	1
RP11-893F2.13	1.0301926	1.03725592	1.0271309	0.728231235	-0.4575315	0.898411	1
USP27X-AS1	1.0484178	1.04087521	1.0516871	1.264510729	0.3385793	0.898457	1
RP11-46C24.7	1.0876486	1.0947853	1.0845552	0.892071103	-0.1647694	0.898463	1
ZMYND15	1.0238547	1.01647837	1.027052	1.641665342	0.7151601	0.898497	1
SIGLEC7	1.0230705	1.03008674	1.0200292	0.665716277	-0.5870207	0.898548	1
DMRT1	1.014986	1.02197195	1.0119579	0.544236403	-0.8776946	0.898567	1
SLC18A3	1.0150132	1.0220619	1.0119579	0.542017279	-0.8835893	0.898567	1
ACOT4	1.0150238	1.02209683	1.0119579	0.541160617	-0.8858712	0.898567	1
OLFML2B	1.0437072	1.05077309	1.0406444	0.800510233	-0.3210083	0.898678	1
SH3PXD2A	1.2672476	1.27472172	1.2640079	0.961001094	-0.05739	0.898703	1
CDK13	1.3451805	1.3529818	1.341799	0.968318969	-0.0464457	0.898706	1
COBL	1.0377098	1.03027173	1.0409339	1.352214728	0.4353243	0.898753	1
HACL1	1.1425564	1.14981974	1.139408	0.930504894	-0.1039144	0.898806	1
ACER2	1.0152454	1.0221992	1.0122313	0.550979387	-0.8599297	0.898831	1
UXT-AS1	1.0152496	1.02221307	1.0122313	0.550635437	-0.8608306	0.898831	1
RP11-95I16.4	1.0152524	1.0222222	1.0122313	0.550409295	-0.8614233	0.898831	1
RP11-121C2.3	1.0152637	1.02225973	1.0122313	0.549481272	-0.8638578	0.898831	1
RP11-98O2.1	1.0152729	1.02229004	1.0122313	0.548734141	-0.8658208	0.898831	1
RP1-296L11.1	1.0152478	1.02220682	1.0122313	0.550790366	-0.8604248	0.898831	1
LINC00605	1.0403555	1.03287053	1.0435999	1.326411784	0.4075287	0.898836	1
ZNF281	1.4430975	1.45099013	1.4396764	0.97491349	-0.0366539	0.899016	1
ATP6V0E2-AS1	1.0397687	1.03233706	1.04299	1.329433778	0.4108119	0.899023	1
PLA2G4A	1.0412601	1.04832114	1.0381994	0.790532465	-0.3391034	0.899071	1
TRAPPC13	1.1464882	1.13863258	1.1498932	1.081226407	0.1126687	0.899166	1
WI2-85898F10.1	1.0308022	1.03777551	1.0277796	0.735386719	-0.443425	0.899192	1
PNPLA7	1.0308235	1.03778672	1.0278053	0.735847352	-0.4425216	0.899192	1
UEVLD	1.1327077	1.12546855	1.1358456	1.082706077	0.1146416	0.899227	1
RP11-23F23.2	1.0334218	1.0403249	1.0304297	0.754612235	-0.4061926	0.899229	1
THAP1	1.2818638	1.27418081	1.2851941	1.040167923	0.0568165	0.899242	1
RP11-171I2.5	1.0401113	1.04706927	1.0370953	0.788100604	-0.3435483	0.899278	1
SNAP91	1.0534934	1.0461422	1.0566798	1.228372048	0.2967476	0.899346	1
GFRA3	1.0229569	1.01565391	1.0261224	1.668745711	0.7387641	0.899351	1
KIF13A	1.1930824	1.18514401	1.1965233	1.061461955	0.0860527	0.89937	1
LSM14B	1.2126429	1.2047666	1.2160569	1.055137369	0.0774308	0.899425	1
RP11-502I4.3	1.0503566	1.05741585	1.0472967	0.823757508	-0.2797084	0.899437	1
CEACAM1	1.0706147	1.07773088	1.0675301	0.868767882	-0.2029573	0.899467	1
EIF2S3L	1.0830115	1.07542029	1.0863019	1.144280196	0.1944404	0.899478	1
POMC	1.0780866	1.07083245	1.081231	1.146804458	0.1976194	0.899526	1

ADM2	1.0291308	1.0360968	1.0261113	0.723369402	-0.4671955	0.89959	1
TBC1D10B	1.2182366	1.21047031	1.221603	1.052894189	0.0743605	0.899614	1
AATK	1.09501	1.10217495	1.0919043	0.899480206	-0.1528366	0.899668	1
RP11-420N3.2	1.0222286	1.02915233	1.0192274	0.659550613	-0.6004447	0.899719	1
HIST2H4B	1.0222501	1.02920012	1.0192376	0.658818916	-0.6020461	0.899719	1
ANXA8	1.0222342	1.02915045	1.0192363	0.659898822	-0.5996833	0.899719	1
AOC3	1.0310884	1.03807862	1.0280585	0.73685725	-0.4405429	0.899773	1
RP11-305L7.7	1.0197484	1.02672297	1.0167252	0.625872561	-0.6760592	0.899798	1
CSPP1	1.2601839	1.26776247	1.2568989	0.95942825	-0.0597532	0.899852	1
SLC9A1	1.1067051	1.11389426	1.1035889	0.909518094	-0.1368258	0.899863	1
GSG1	1.0218099	1.02875144	1.0188011	0.653917657	-0.6128191	0.89987	1
GDPD3	1.0218057	1.02874849	1.0187963	0.653817241	-0.6130407	0.89987	1
AC105760.3	1.0217908	1.0286874	1.0188014	0.655387866	-0.6095791	0.89987	1
CTD-2296D1.2	1.0217873	1.02870872	1.0187872	0.654408751	-0.6117361	0.89987	1
C9orf50	1.0317123	1.03865966	1.0287009	0.74239825	-0.4297348	0.899961	1
PERM1	1.0201917	1.0271029	1.017196	0.63447247	-0.6563705	0.899979	1
RP11-541G9.2	1.0202077	1.02714818	1.0171992	0.63353209	-0.6585104	0.899979	1
NPAS4	1.0201975	1.02712224	1.017196	0.634017339	-0.6574058	0.899979	1
ULBP3	1.0202115	1.02717901	1.0171914	0.632523221	-0.6608096	0.899979	1
HPGD	1.0230105	1.01574718	1.0261589	1.661180276	0.7322086	0.89998	1
CTD-2396E7.11	1.0230657	1.01574718	1.0262379	1.666198625	0.7365604	0.89998	1
MPP4	1.0230358	1.01574718	1.0261951	1.663479933	0.7342045	0.89998	1
USP22	2.5289953	2.54012098	2.5241728	0.989644882	-0.0150172	0.900028	1
RP4-593H12.1	1.0236095	1.0305374	1.0206066	0.674798023	-0.5674723	0.900036	1
ZNF362	1.224774	1.23219297	1.2215582	0.954198599	-0.0676385	0.900084	1
ERC2	1.2318684	1.23926224	1.2286635	0.95570256	-0.0653664	0.90012	1
LGI4	1.0523045	1.05918483	1.0493221	0.833357942	-0.2629918	0.900123	1
CREG2	1.030839	1.02346726	1.0340343	1.450287033	0.5363385	0.900174	1
LRRC46	1.0307713	1.0234583	1.0339411	1.446869797	0.5329351	0.900174	1
GPR157	1.0585136	1.05111026	1.0617226	1.20763623	0.2721859	0.900193	1
PARP4	1.2216196	1.22919626	1.2183354	0.952613229	-0.0700375	0.90025	1
PNMT	1.048588	1.0410924	1.051837	1.2614732	0.3351096	0.90037	1
CCDC17	1.0231577	1.01586655	1.0263181	1.658717397	0.7300681	0.900441	1
RNF217	1.3860522	1.39364684	1.3827603	0.972344379	-0.0404607	0.900529	1
SGMS1-AS1	1.0761361	1.08320497	1.073072	0.878216988	-0.1873507	0.900574	1
RP13-436F16.1	1.0394474	1.04634474	1.0364577	0.786662674	-0.346183	0.900588	1
DBNDD1	1.191496	1.19870814	1.1883698	0.947972409	-0.077083	0.900714	1
LIMD1	1.1321609	1.12467212	1.135407	1.086104521	0.1191629	0.90072	1
MVB12B	1.2295817	1.22197454	1.232879	1.049125049	0.0691866	0.900743	1
ELMO1	1.0498538	1.04250569	1.0530388	1.247805252	0.3193928	0.900787	1
ZBTB33	1.1889206	1.19627367	1.1857334	0.946298221	-0.0796332	0.900874	1
AC009961.3	1.0377814	1.04466876	1.0347961	0.77898082	-0.3603403	0.900916	1
RP4-781K5.2	1.0198922	1.02675026	1.0169196	0.632502025	-0.660858	0.900926	1
RP11-8L8.2	1.0199039	1.02678001	1.0169234	0.631941733	-0.6621366	0.900926	1
RP11-180C16.1	1.0199	1.02676806	1.0169231	0.632211241	-0.6615214	0.900926	1
CIDEA	1.0199122	1.02678038	1.0169352	0.632373184	-0.6611519	0.900926	1
LINC01010	1.0198827	1.02670201	1.0169269	0.633918211	-0.6576314	0.900926	1
RP11-80H5.9	1.0199359	1.02685752	1.0169356	0.630572567	-0.6652657	0.900926	1
CDHR4	1.0198984	1.02676575	1.0169218	0.632217248	-0.6615077	0.900926	1
EPB42	1.0689677	1.07584571	1.0659864	0.870008838	-0.200898	0.900984	1
RP3-473L9.4	1.0689872	1.07594734	1.0659703	0.8686324	-0.2031823	0.900988	1

UBXN10-AS1	1.0568531	1.04948306	1.0600477	1.213500758	0.279175	0.901	1
LRAT	1.0247954	1.01752676	1.0279461	1.594480449	0.6730864	0.901012	1
PALD1	1.0808842	1.08788551	1.0778495	0.88580554	-0.1749381	0.901051	1
PHC2	1.4471969	1.43852934	1.4509539	1.028332326	0.0403066	0.901096	1
RP11-569D9.5	1.0201408	1.0269774	1.0171774	0.636732245	-0.6512413	0.901161	1
RP1-140A9.1	1.0201461	1.02700328	1.0171739	0.635991808	-0.6529199	0.901161	1
HNF1B	1.0201397	1.02698004	1.0171748	0.636573143	-0.6516018	0.901161	1
PCSK9	1.0275294	1.02025339	1.0306832	1.514965146	0.5992846	0.901164	1
STK19	1.3915965	1.38369165	1.3950228	1.02953205	0.0419887	0.901234	1
OSER1	1.3919463	1.38390801	1.3954305	1.030013653	0.0426635	0.901234	1
RUNDC3A-AS1	1.0262654	1.01899597	1.0294164	1.548560872	0.6309281	0.901246	1
CDNF	1.0404454	1.04729186	1.0374777	0.792476622	-0.3355597	0.90136	1
AKAP2	1.0311116	1.02388943	1.0342421	1.433358209	0.5193992	0.901412	1
ZSCAN26	1.2405137	1.24783957	1.2373382	0.957628463	-0.0624621	0.901416	1
LA16c-60D12.2	1.0223002	1.02913257	1.0193387	0.663816576	-0.5911434	0.901441	1
TAC1	1.0240175	1.01677692	1.0271559	1.618648206	0.6947895	0.901445	1
RBKS	1.0946617	1.101529	1.0916851	0.903043427	-0.1471327	0.901456	1
MDGA2	1.0321983	1.03905887	1.0292246	0.748219304	-0.4184669	0.901491	1
ARHGAP8	1.0482899	1.04103465	1.0514347	1.253445347	0.3258991	0.901512	1
FERMT1	1.0502666	1.04292055	1.0534507	1.245341482	0.3165414	0.901573	1
FAM110D	1.0498567	1.05679218	1.0468505	0.824945892	-0.2776286	0.90164	1
RP11-876N24.4	1.0189529	1.02576969	1.0159982	0.620812714	-0.68777	0.901646	1
TRPM8	1.0189304	1.02574095	1.0159783	0.620735348	-0.6879498	0.901646	1
JAKMIP2	1.4530578	1.46105695	1.4495905	0.975130142	-0.0363333	0.901794	1
CD160	1.0236572	1.03048345	1.0206984	0.679003948	-0.5585081	0.901868	1
SPTBN4	1.0655372	1.07245084	1.0625405	0.863212732	-0.212212	0.901873	1
RP11-476H16.1	1.0214474	1.02833779	1.0184607	0.651453026	-0.6182669	0.901919	1
RP11-485G7.5	1.0214156	1.02822418	1.0184644	0.654204751	-0.6121859	0.901919	1
PGAP2	1.3690442	1.37700716	1.3655926	0.969723353	-0.0443549	0.901998	1
ACHE	1.1539195	1.14641393	1.1571729	1.073483044	0.1022994	0.902036	1
ZNF490	1.0248956	1.03166878	1.0219597	0.693417463	-0.5282039	0.902201	1
ATP8A1	1.126155	1.13332284	1.1230481	0.922933047	-0.1157021	0.90221	1
DNAJC28	1.0513749	1.04412652	1.0545168	1.235465425	0.3050546	0.902269	1
SNUPN	1.2931188	1.30088752	1.2897513	0.96298894	-0.0544089	0.902298	1
OTUD4	1.2487124	1.24090895	1.2520949	1.046432082	0.0654787	0.902321	1
RANBP3L	1.0222201	1.02908755	1.0192434	0.661566961	-0.5960409	0.902353	1
CAMK2N2	1.0195555	1.02629784	1.016633	0.632484111	-0.6608989	0.902369	1
CHST6	1.0395725	1.04634764	1.0366357	0.790454679	-0.3392453	0.902396	1
SRRT	1.3320545	1.33924942	1.3289359	0.969598981	-0.0445399	0.902426	1
NOTCH2NL	1.1054392	1.11240835	1.1024184	0.911128271	-0.1342739	0.902467	1
RP11-219B17.3	1.0240051	1.01686603	1.0270996	1.606758262	0.6841529	0.902587	1
BNIPL	1.0239956	1.01684949	1.0270932	1.607952134	0.6852245	0.902587	1
PODN	1.0334908	1.02628097	1.036616	1.393249779	0.4784539	0.902624	1
C16orf52	1.2247896	1.21681018	1.2282483	1.052756512	0.0741718	0.902688	1
SEMA7A	1.0440665	1.03683608	1.0472006	1.281369219	0.3576862	0.902727	1
RANBP6	1.1771542	1.16981634	1.1803349	1.061940839	0.0867034	0.902792	1
MOB3C	1.0610035	1.06793437	1.0579993	0.853754552	-0.2281067	0.902831	1
ACVR2A	1.1430928	1.15027482	1.1399797	0.931491403	-0.1023856	0.902854	1
ZNF556	1.0198426	1.02658503	1.01692	0.636449119	-0.6518829	0.902948	1
FAM231D	1.0275695	1.03438103	1.024617	0.716004205	-0.48196	0.902999	1
CTC-575N7.1	1.0276229	1.03439452	1.0246877	0.717780909	-0.4783845	0.902999	1

ERBB2	1.3660272	1.35812588	1.3694521	1.031626418	0.0449206	0.903009	1
FAM193B	1.114038	1.12080872	1.1111032	0.919662262	-0.120824	0.903086	1
FXYD2	1.0770167	1.08394143	1.0740151	0.88174743	-0.1815626	0.903092	1
LDHD	1.020453	1.02715724	1.0175471	0.646128942	-0.630106	0.903134	1
RP11-297P16.3	1.0204827	1.02718739	1.0175765	0.646495065	-0.6292887	0.903134	1
ATG4C	1.2741288	1.26623512	1.2775503	1.042500733	0.0600484	0.903154	1
SAAL1	1.3289595	1.33623876	1.3258042	0.968966814	-0.0454808	0.903189	1
SEPT7-AS1	1.0411128	1.04783444	1.0381993	0.79857337	-0.3245031	0.9032	1
SMAD9	1.7855547	1.79371318	1.7820183	0.985265592	-0.0214154	0.903228	1
JMY	1.2041642	1.19649038	1.2074904	1.055982499	0.0785859	0.903231	1
C1QL2	1.0244599	1.01724954	1.0275853	1.599189807	0.6773412	0.903245	1
PPP5C	1.3385947	1.33099064	1.3418908	1.032931843	0.0467451	0.903357	1
RP11-262H14.4	1.1729253	1.18000244	1.1698576	0.94364072	-0.0836904	0.903385	1
RP11-398C13.6	1.0592465	1.06612471	1.0562651	0.85089425	-0.2329483	0.903484	1
MAX	1.5621801	1.57008601	1.5587533	0.980121016	-0.0289682	0.903515	1
SOS2	1.1995455	1.19205689	1.2027915	1.055892886	0.0784635	0.903526	1
MMP28	1.0342436	1.02709222	1.0373434	1.378379316	0.462973	0.903532	1
TLX2	1.026184	1.01906529	1.0292697	1.535232579	0.6184572	0.903579	1
UBXN10	1.0261542	1.01910733	1.0292086	1.528661564	0.612269	0.903579	1
HERPUD2	1.4028994	1.39496327	1.4063393	1.028802766	0.0409664	0.903659	1
IFITM2	5.6070674	5.63015575	5.5970596	0.992852039	-0.0103494	0.903762	1
RBM18	1.3268582	1.31885982	1.3303252	1.03595743	0.0509647	0.903769	1
KDM1B	1.1664473	1.1588147	1.1697558	1.068891955	0.096116	0.903966	1
AC079354.5	1.0204457	1.02714812	1.0175404	0.646101407	-0.6301675	0.904014	1
CEBPA	1.0203901	1.02699882	1.0175255	0.649119883	-0.6234431	0.904014	1
STBD1	1.0216129	1.02831274	1.0187088	0.660790107	-0.597736	0.904032	1
RP11-260M19.2	1.0216172	1.0282931	1.0187235	0.661768408	-0.5956017	0.904032	1
NPW	3.9306237	3.95106548	3.921763	0.990070551	-0.0143968	0.904095	1
PTTG2	1.0317736	1.02456233	1.0348994	1.420852267	0.5067566	0.904099	1
RANBP9	1.2777863	1.26981816	1.2812402	1.04233226	0.0598152	0.904162	1
PTPRG	1.593322	1.60169258	1.5896937	0.980058131	-0.0290608	0.904168	1
CACNA1C	1.0696601	1.07654578	1.0666754	0.871053032	-0.1991675	0.904187	1
RP11-639B1.1	1.026555	1.03332308	1.0236214	0.708858664	-0.4964301	0.904196	1
RP2	1.1132986	1.12018313	1.1103144	0.91788596	-0.1236132	0.904279	1
NEMP2	1.0547871	1.04760758	1.0578991	1.216174896	0.2823507	0.90433	1
RP11-26J3.1	1.0292164	1.03588402	1.0263263	0.733649636	-0.4468368	0.904355	1
TCP10L	1.0600195	1.05289625	1.0631072	1.193036546	0.2546382	0.904394	1
C11orf98	1.0671179	1.07401125	1.06413	0.866489665	-0.2067456	0.904434	1
RP5-1070A16.1	1.0191958	1.02586048	1.0163069	0.630573981	-0.6652625	0.904473	1
FOXH1	1.0291498	1.03584543	1.0262475	0.732241647	-0.4496083	0.904531	1
GPATCH4	1.282074	1.28990671	1.2786788	0.961270683	-0.0569854	0.90456	1
RP11-84G21.1	1.0446086	1.0374769	1.0476999	1.272781885	0.3479852	0.904577	1
PRKAR2B	1.1141952	1.12092729	1.1112771	0.920198327	-0.1199833	0.904599	1
AC002310.7	1.0210931	1.02772805	1.0182171	0.656991654	-0.6060531	0.904603	1
ABCD1	1.1512936	1.14391606	1.1544914	1.073482517	0.1022987	0.904655	1
SLITRK2	1.1571845	1.14968524	1.1604351	1.071816628	0.1000581	0.904677	1
EFCAB1	1.0706677	1.0772901	1.0677972	0.877178756	-0.1890572	0.904722	1
ZNF707	1.0387359	1.04539884	1.0358478	0.789618773	-0.3407718	0.904761	1
LTC4S	1.0848225	1.09153766	1.0819118	0.894842769	-0.1602939	0.904802	1
CLK2	1.1506477	1.143037	1.1539466	1.07627124	0.1060417	0.904813	1
CRIP3	1.0255894	1.01859059	1.0286231	1.539654822	0.6226069	0.904819	1

LMX1A	1.0256138	1.01859376	1.0286566	1.541196153	0.6240505	0.904819	1
MDK	26.789327	26.7128114	26.822493	1.004265639	0.0061409	0.904826	1
CCDC144A	1.051386	1.04442907	1.0544015	1.224457488	0.2921427	0.904932	1
KIF19	1.0459924	1.05246223	1.043188	0.823221287	-0.2806478	0.904939	1
TMEM155	1.1903603	1.18286785	1.1936079	1.058731045	0.0823361	0.904948	1
RP11-843A23.1	1.0249751	1.0180094	1.0279945	1.55443556	0.6363908	0.905005	1
MAN2B1	1.06862	1.06146856	1.0717198	1.166772611	0.2225234	0.905069	1
PTH1R	1.0813118	1.07409959	1.084438	1.139520964	0.1884275	0.905091	1
FARSB	1.3030494	1.29524092	1.3064341	1.03791191	0.053684	0.905112	1
C19orf45	1.0575061	1.06421189	1.0545995	0.850301898	-0.2339529	0.905176	1
CTD-2147F2.1	1.148524	1.14114163	1.1517239	1.074976468	0.1043051	0.905198	1
C2	1.0394023	1.04602863	1.0365301	0.793637884	-0.3334472	0.905198	1
RP11-402D21.2	1.0360004	1.02891712	1.0390707	1.351125399	0.4341616	0.905204	1
EPC2	1.2373962	1.24460511	1.2342714	0.957753711	-0.0622734	0.905285	1
C17orf62	1.7546295	1.74655794	1.7581282	1.015498167	0.0221876	0.905312	1
TPRG1-AS1	1.0188363	1.02547205	1.0159599	0.626566949	-0.6744594	0.905363	1
ZFYVE9	1.2699873	1.27730074	1.2668173	0.962194522	-0.0555995	0.905386	1
MIA3	1.5035784	1.49568192	1.5070011	1.022835588	0.0325743	0.905423	1
RAB26	1.028974	1.03556573	1.0261167	0.734322355	-0.4455146	0.905451	1
LINC01567	1.0290554	1.03562037	1.0262098	0.735808211	-0.4425983	0.905451	1
RNF152	1.042634	1.03554551	1.0457066	1.285861717	0.3627355	0.905473	1
ALOX5	1.0425732	1.03559218	1.0455991	1.281155034	0.3574451	0.905473	1
ZNF225	1.0635384	1.05651656	1.0665821	1.178099163	0.236461	0.905503	1
THSD1	1.0351368	1.04177346	1.0322601	0.772262134	-0.3728375	0.905505	1
TNK1	1.035237	1.04191441	1.0323427	0.771635866	-0.3740079	0.905505	1
DNAH1	1.0352604	1.04173628	1.0324535	0.777583698	-0.3629301	0.905505	1
NRARP	1.0351873	1.04180239	1.03232	0.773161689	-0.3711579	0.905505	1
KTI12	1.140394	1.13328901	1.1434737	1.076410197	0.106228	0.90553	1
ECHDC3	1.0524482	1.04545349	1.0554801	1.220591098	0.28758	0.905552	1
RP11-10A14.5	1.0247528	1.01776147	1.0277832	1.564239503	0.6454614	0.905585	1
GCNT2	1.1105796	1.11738445	1.10763	0.916901263	-0.1251617	0.905748	1
HESX1	1.107972	1.1147438	1.1050367	0.915402068	-0.1275225	0.90575	1
MALRD1	1.0273905	1.02037851	1.0304299	1.493236043	0.5784422	0.905834	1
AC226118.1	1.0274019	1.02036252	1.0304531	1.4955466	0.5806729	0.905834	1
NCF2	1.0272902	1.02036586	1.0302916	1.487372858	0.5727664	0.905834	1
SLC6A16	1.1052757	1.11185677	1.1024231	0.91566291	-0.1271115	0.905908	1
XX-FW83563B9.5	1.037765	1.0443491	1.0349111	0.787189074	-0.3452179	0.905914	1
ARL16	2.5303027	2.54223912	2.5251288	0.988905514	-0.0160954	0.905969	1
ZBED1	1.3227769	1.31519345	1.326064	1.034488588	0.0489177	0.906026	1
SPN	1.0296267	1.03626126	1.0267509	0.737725385	-0.4388442	0.906042	1
RP11-314B1.2	1.0241244	1.0171592	1.0271435	1.581860177	0.6616221	0.906077	1
EAF1-AS1	1.0474175	1.05404262	1.0445458	0.824271144	-0.2788091	0.906162	1
RAB11FIP4	1.176288	1.16886057	1.1795075	1.063051743	0.0882118	0.906188	1
RP11-465N4.4	1.0337332	1.02678608	1.0367444	1.371772975	0.4560417	0.906253	1
WDR5	1.2558914	1.26298914	1.2528149	0.961313015	-0.0569218	0.906255	1
RNF183	1.0265902	1.01964674	1.0295999	1.506607503	0.5913036	0.906268	1
PRKCH	1.0508586	1.04382593	1.053907	1.230025671	0.2986884	0.906315	1
RP11-373I8.1	1.065549	1.07233906	1.0626058	0.865449588	-0.2084783	0.906319	1
SEC24D	1.2939039	1.28656079	1.2970868	1.036732115	0.0520432	0.906333	1
MDN1	1.2037507	1.19622364	1.2070134	1.054987086	0.0772253	0.906396	1
C9orf163	1.0509103	1.05747301	1.0480656	0.836315921	-0.2578801	0.906447	1

RP11-324I22.2	1.0210583	1.02762243	1.018213	0.659356258	-0.6008699	0.90647	1
RP5-1116H23.4	1.0210598	1.02760175	1.0182241	0.660253155	-0.5989088	0.90647	1
KB-1592A4.15	1.0210536	1.02760549	1.0182137	0.659784956	-0.5999322	0.90647	1
SAXO1	1.0188407	1.02536357	1.0160133	0.631350713	-0.6634865	0.906503	1
LLNLR-268E12.1	1.0188597	1.0254292	1.0160122	0.62967631	-0.6673177	0.906503	1
ARHGAP35	1.4032556	1.41091459	1.3999358	0.973281981	-0.0390702	0.906565	1
ACOX3	1.1096628	1.11675856	1.106587	0.912884105	-0.1314964	0.906642	1
AFAP1L2	1.0426874	1.03564453	1.0457401	1.283229342	0.359779	0.906668	1
ZFP64	1.2303649	1.22305303	1.2335343	1.046990141	0.0662479	0.906731	1
TCERG1L-AS1	1.0274213	1.03392131	1.0246039	0.725322144	-0.4633062	0.906769	1
ODF3L2	1.0223203	1.02883421	1.0194969	0.676171221	-0.5645395	0.906785	1
VWA3A	1.0223416	1.02883844	1.0195255	0.677065932	-0.5626318	0.906785	1
SYTL5	1.0352011	1.02823239	1.0382217	1.353823566	0.4370397	0.906788	1
DRC7	1.0251273	1.01815945	1.0281476	1.550024626	0.6322911	0.906798	1
BIRC3	1.0462867	1.03936774	1.0492857	1.251931185	0.3241553	0.906805	1
ATP5S	1.494045	1.50153602	1.490798	0.978589669	-0.031224	0.906849	1
AP000251.3	1.0868874	1.09347209	1.0840333	0.899020153	-0.1535746	0.906862	1
AC090616.2	1.0259528	1.03251136	1.0231099	0.710825513	-0.4924326	0.90688	1
RP11-1148L6.9	1.0259945	1.03252846	1.0231623	0.712063443	-0.4899223	0.90688	1
ZNF473	1.0952469	1.10178189	1.0924143	0.907964315	-0.1392925	0.90688	1
RP11-817O13.9	1.0952553	1.10172802	1.0924496	0.90879211	-0.1379778	0.90688	1
DAXX	1.3934835	1.40081232	1.3903068	0.973789523	-0.0383181	0.906889	1
TRIP11	1.8168496	1.82559829	1.8130575	0.984810033	-0.0220826	0.906898	1
RP1-59D14.5	1.0170099	1.02357649	1.0141635	0.60074857	-0.7351668	0.90697	1
AC005009.1	1.0169682	1.02345614	1.014156	0.603507979	-0.7285553	0.90697	1
CTD-3193K9.11	1.0370276	1.0435609	1.0341957	0.785010099	-0.3492169	0.906984	1
AQP4	1.0261839	1.03263849	1.0233861	0.716520586	-0.4809199	0.907116	1
PLAC8L1	1.0261962	1.03268314	1.0233843	0.715486149	-0.4830043	0.907116	1
MAST2	1.1550547	1.14790923	1.158152	1.06925013	0.0965994	0.907255	1
RP11-582E3.6	1.1136621	1.12036607	1.1107562	0.920161505	-0.120041	0.907255	1
GARNL3	1.0583555	1.06495849	1.0554934	0.85429074	-0.2272009	0.907355	1
VRK1	1.4289994	1.43640124	1.4257911	0.975687174	-0.0355094	0.907373	1
RAB17	1.0335862	1.02666449	1.0365865	1.372104104	0.4563899	0.907388	1
PART1	1.026597	1.01971668	1.0295793	1.50021818	0.5851723	0.907412	1
RP11-44N21.1	1.0670946	1.07353127	1.0643047	0.874521261	-0.1934346	0.90745	1
EXTL1	1.0360992	1.02917925	1.0390987	1.339947412	0.4221764	0.907468	1
NAMA	1.0179641	1.02445742	1.0151495	0.619423135	-0.6910028	0.907507	1
TNNT3	1.0356307	1.02873718	1.0386187	1.343857378	0.42638	0.907509	1
RP11-196G18.23	1.0884382	1.08134201	1.0915141	1.125053006	0.169993	0.907511	1
DDX55	1.2254418	1.2324554	1.2224017	0.956750123	-0.0637859	0.907541	1
PRSS45	1.0172633	1.0237146	1.0144669	0.610042082	-0.7130193	0.907549	1
CH507-145C22.1	1.0172793	1.02373788	1.0144799	0.609989459	-0.7131438	0.907549	1
RP11-413H22.2	1.0559799	1.06253084	1.0531403	0.849825848	-0.2347609	0.90755	1
RP11-193M21.1	1.0250075	1.03155154	1.022171	0.70269027	-0.5090392	0.907602	1
RP11-3D4.3	1.0249767	1.03149449	1.0221515	0.703346301	-0.5076929	0.907602	1
AXL	1.9608783	1.97143848	1.956301	0.984417447	-0.0226579	0.907729	1
TMEM45B	1.0178707	1.02433431	1.015069	0.619250155	-0.6914058	0.907735	1
AHSG	1.0178877	1.02438228	1.0150726	0.618180181	-0.6939007	0.907735	1
MIR137HG	1.017883	1.02435981	1.0150755	0.618869501	-0.6922929	0.907735	1
RP11-151A10.3	1.0178727	1.02434942	1.0150653	0.618713807	-0.6926559	0.907735	1
SLC44A3	1.1053181	1.09834718	1.1083396	1.101603847	0.1396055	0.907842	1

PCDHB5	1.0540679	1.06062597	1.0512252	0.844938679	-0.2430815	0.907865	1
B4GALNT4	1.3244949	1.33110975	1.3216276	0.971362527	-0.0419183	0.908007	1
THOP1	1.3435011	1.33589346	1.3467986	1.032466153	0.0460945	0.908026	1
RP4-563E14.1	1.0269586	1.0201059	1.0299289	1.488563991	0.5739212	0.90809	1
ORAOV1	1.1880414	1.19487146	1.1850809	0.94975905	-0.0743665	0.908096	1
ZNF596	1.0290344	1.02217617	1.0320072	1.443314564	0.5293858	0.908122	1
ZNF546	1.0956876	1.10216265	1.092881	0.909147825	-0.1374132	0.908182	1
RP5-884G6.2	1.0347071	1.04106378	1.0319517	0.778100006	-0.3619725	0.908201	1
CSNK1G3	1.5434012	1.53582089	1.5466869	1.020279126	0.0289639	0.908258	1
PLIN5	1.0282209	1.03474544	1.0253928	0.730825019	-0.4524021	0.90835	1
C10orf82	1.0281952	1.03461886	1.0254109	0.734018005	-0.4461126	0.90835	1
SYT13	1.073351	1.06623844	1.0764339	1.153920839	0.2065443	0.908368	1
MYOZ1	1.1007322	1.10734578	1.0978655	0.911684874	-0.1333929	0.908419	1
ENTPD5	1.1252633	1.13194697	1.1223662	0.927389063	-0.1087534	0.908432	1
FMNL3	1.0992657	1.09252097	1.1021893	1.104498609	0.1433916	0.908461	1
RAPGEF4	1.0550391	1.06149287	1.0522417	0.849557399	-0.2352167	0.908572	1
SLC35E1	1.310398	1.31752419	1.3073091	0.967828952	-0.047176	0.908589	1
RP4-591C20.9	1.0896052	1.09625463	1.0867229	0.900974104	-0.1504425	0.908618	1
LINC00854	1.0190096	1.02541555	1.016233	0.638701918	-0.6467853	0.908632	1
AC008067.2	1.0190119	1.02542402	1.0162326	0.638473678	-0.647301	0.908632	1
RP11-763F8.1	1.0190216	1.0254561	1.0162326	0.637669089	-0.6491201	0.908632	1
BMT2	1.3242323	1.31670571	1.3274948	1.034066463	0.0483289	0.908635	1
NWD1	1.0239164	1.03036913	1.0211194	0.69542334	-0.5240366	0.908816	1
C1QL1	1.036998	1.04347697	1.0341897	0.786385945	-0.3466906	0.908831	1
PRKD1	1.0828727	1.089504	1.0799983	0.893795872	-0.1619827	0.908875	1
P2RY11	1.0829663	1.08948134	1.0801423	0.895631203	-0.1590233	0.908875	1
PRR16	1.0343493	1.02755842	1.0372928	1.35322631	0.4364031	0.908945	1
SULT1A2	1.0645575	1.05761324	1.0675675	1.172777849	0.2299298	0.908999	1
DNM1P35	1.0201741	1.01338003	1.023119	1.727874081	0.7889981	0.909051	1
RP3-465N24.6	1.0264796	1.03282122	1.0237308	0.72303111	-0.4678704	0.909115	1
HECW2	1.0808301	1.08741014	1.077978	0.892093006	-0.164734	0.909143	1
SASH1	1.2969331	1.2891048	1.3003263	1.038814523	0.0549381	0.909148	1
SYNE1	1.3509581	1.35839676	1.3477338	0.970248104	-0.0435744	0.909158	1
ANKRD34B	1.0184209	1.02481821	1.015648	0.630504426	-0.6654216	0.909185	1
RP3-461P17.10	1.0184428	1.02484222	1.0156689	0.630738043	-0.6648871	0.909185	1
TTBK1	1.0184137	1.02474627	1.0156688	0.633179706	-0.6593131	0.909185	1
MED25	1.3870837	1.37936678	1.3904286	1.029158659	0.0414654	0.909222	1
RFESD	1.1276764	1.12054078	1.1307694	1.084856273	0.1175039	0.909251	1
KCNJ12	1.0360762	1.04250102	1.0332913	0.78330542	-0.3523532	0.909313	1
SMARCAL1	1.1351581	1.12824308	1.1381555	1.077294222	0.1074123	0.909325	1
SLC37A1	1.1624223	1.16924074	1.1594668	0.942248359	-0.0858207	0.909338	1
HCST	1.0623021	1.05537483	1.0653048	1.179322875	0.2379588	0.909405	1
OSGEPL1	1.2240388	1.23092394	1.2210544	0.957260626	-0.0630163	0.909442	1
RP11-475O6.1	1.0186889	1.02504847	1.0159324	0.636061252	-0.6527624	0.90945	1
RP11-744I24.3	1.0186787	1.0250031	1.0159374	0.637417373	-0.6496898	0.90945	1
PWRN1	1.0186902	1.02503867	1.0159384	0.636553197	-0.651647	0.90945	1
HEY2	1.0256842	1.01886772	1.0286388	1.517874535	0.6020525	0.90948	1
RP11-165J3.6	1.025663	1.01886772	1.0286085	1.516267663	0.6005245	0.90948	1
CTB-47B11.3	1.0257034	1.01886772	1.0286664	1.51933339	0.6034385	0.90948	1
ENTPD8	1.0257862	1.01886772	1.0287851	1.525625968	0.6094013	0.90948	1
ZNF652	1.4425989	1.43466354	1.4460385	1.026169578	0.0372692	0.909486	1

AKR1C2	1.1176492	1.12441608	1.114716	0.922035391	-0.117106	0.909499	1
CNNM4	1.0562139	1.06266949	1.0534157	0.852339108	-0.2305006	0.909557	1
DDIT4L	1.0748272	1.06829003	1.0776607	1.137218761	0.1855098	0.909558	1
RP1-138B7.7	1.0397141	1.04615789	1.036921	0.799885943	-0.3221338	0.909597	1
C1orf194	1.0396363	1.04605429	1.0368543	0.800236543	-0.3215016	0.909597	1
PPARG	1.019522	1.01281342	1.0224299	1.750500683	0.8077676	0.90964	1
RP11-295P9.13	1.0195463	1.01281342	1.0224648	1.753221667	0.8100084	0.90964	1
GCLC	1.1626425	1.15552072	1.1657295	1.065642419	0.0917234	0.909711	1
RP11-69E11.4	1.0464316	1.03957538	1.0494035	1.248339719	0.3200106	0.909789	1
EVA1C	1.0439306	1.03721154	1.0468431	1.258832013	0.3320858	0.909793	1
SH3TC2	1.0360032	1.02916943	1.0389654	1.335828797	0.4177351	0.909805	1
BTN3A1	1.1017019	1.1082873	1.0988475	0.912826295	-0.1315877	0.909876	1
KIAA0556	1.121992	1.12854225	1.1191528	0.926954256	-0.1094299	0.909935	1
CTD-2256P15.4	1.0162534	1.02258347	1.0135097	0.598209913	-0.7412763	0.909962	1
C6orf99	1.0162535	1.02259623	1.0135042	0.597629624	-0.7426764	0.909962	1
RP11-228B15.4	1.0162667	1.0226255	1.0135104	0.597130816	-0.7438811	0.909962	1
AC005042.4	1.0162544	1.0225916	1.0135075	0.597900864	-0.7420218	0.909962	1
ADGRD1	1.0276865	1.03412631	1.0248951	0.72949938	-0.4550213	0.910017	1
FAM66D	1.0265151	1.03277571	1.0238014	0.726191063	-0.4615789	0.910018	1
RP11-110I1.13	1.0326473	1.03890197	1.0299362	0.769528124	-0.377954	0.910109	1
HMCN2	1.0325886	1.03887998	1.0298616	0.76804472	-0.3807378	0.910109	1
SQRDL	1.0451639	1.03848393	1.0480594	1.248818033	0.3205633	0.910284	1
RP11-176H8.1	1.0452147	1.03843081	1.0481552	1.253036747	0.3254287	0.910284	1
VILL	1.0733238	1.07974726	1.0705395	0.884538725	-0.1770028	0.91029	1
VEGFC	1.0549422	1.04816699	1.057879	1.201631635	0.2649947	0.910308	1
SUSD2	1.0231888	1.02955233	1.0204305	0.691332717	-0.5325479	0.910367	1
MYLK4	1.0231801	1.02954669	1.0204205	0.691125805	-0.5329797	0.910367	1
AC087294.2	1.0273619	1.02066285	1.0302656	1.464737483	0.5506421	0.910417	1
ZNF233	1.0273327	1.02062976	1.0302381	1.465750332	0.5516394	0.910417	1
CTC-366B18.4	1.0252359	1.03157829	1.0224867	0.712093406	-0.4898616	0.91044	1
HIST1H2BE	1.0252198	1.03159183	1.0224579	0.710876037	-0.4923301	0.91044	1
GRB7	1.0449546	1.03829925	1.0478394	1.24909633	0.3208847	0.910522	1
SLC51A	1.0236473	1.02996633	1.0209082	0.697723977	-0.5192717	0.910548	1
RP11-514P8.6	1.0171595	1.02346257	1.0144274	0.614912727	-0.7015464	0.910624	1
DLEC1	1.0171444	1.02341519	1.0144263	0.616109619	-0.698741	0.910624	1
FLT1	1.0171389	1.02341108	1.0144201	0.615953821	-0.6991059	0.910624	1
ARMCX3-AS1	1.0171548	1.0234203	1.014439	0.616516472	-0.6977887	0.910624	1
FGL2	1.0171641	1.02348848	1.0144227	0.614034255	-0.703609	0.910624	1
CCDC182	1.0171458	1.02343643	1.0144191	0.615243252	-0.7007712	0.910624	1
AC005616.2	1.0171913	1.02358271	1.0144209	0.6115038	-0.7095666	0.910624	1
RP11-320M2.1	1.0447761	1.05109596	1.0420368	0.822702234	-0.2815577	0.910699	1
ALDH1L2	1.0447205	1.05101551	1.0419918	0.823119163	-0.2808268	0.910699	1
SUPT20H	1.9205142	1.91193716	1.9242319	1.013482016	0.0193205	0.910711	1
NF2	1.3371589	1.34444943	1.3339987	0.96965973	-0.0444495	0.910722	1
NPY	1.0196758	1.01301898	1.0225612	1.732946	0.7932267	0.91073	1
SMN1	1.1168042	1.11013245	1.1196962	1.086838276	0.1201373	0.910779	1
PTENP1-AS	1.0327055	1.02597092	1.0356246	1.371713242	0.4559789	0.910799	1
DOCK3	1.0796522	1.0858678	1.076958	0.896238366	-0.1580456	0.910861	1
IFFO2	1.0539	1.04707535	1.0568582	1.207812278	0.2723962	0.910926	1
PKN3	1.1178558	1.12414079	1.1151315	0.927426851	-0.1086946	0.910928	1
CTD-3193O13.12	1.048315	1.05468027	1.0455559	0.833132347	-0.2633824	0.910984	1

ABHD15	1.0957706	1.0888082	1.0987885	1.112380308	0.1536501	0.910999	1
LNPK	1.9435183	1.95177931	1.9399375	0.987558211	-0.0180623	0.911091	1
CIITA	1.0162617	1.02254322	1.0135389	0.600575663	-0.7355821	0.911102	1
CTC-420A11.2	1.01629	1.02265247	1.0135321	0.597379573	-0.7432802	0.911102	1
BLZF1	1.1822215	1.18914241	1.1792216	0.947548269	-0.0777287	0.911263	1
RP11-71L14.4	1.0212907	1.01466374	1.0241632	1.647816863	0.7205559	0.911301	1
YIPF7	1.0213601	1.01466374	1.0242627	1.654604009	0.726486	0.911301	1
PHF12	1.2511871	1.2582831	1.2481113	0.960617544	-0.0579659	0.911318	1
LY6G5B	1.0248773	1.03118554	1.022143	0.710039201	-0.4940294	0.911353	1
ARHGEF6	1.1001311	1.10652219	1.0973609	0.913996161	-0.12974	0.911386	1
CNTNAP5	1.027927	1.02123502	1.0308276	1.451734615	0.5377777	0.911415	1
AC113189.5	1.1519771	1.14493298	1.1550304	1.069669734	0.0971654	0.911423	1
CRIP2	1.6974227	1.68868905	1.7012083	1.018178368	0.0259903	0.911453	1
CTD-3110H11.1	1.0233525	1.0296412	1.0206266	0.695877296	-0.5230952	0.911499	1
RHOU	1.4484381	1.44047318	1.4518906	1.025920793	0.0369194	0.911508	1
AC004067.5	1.0170014	1.02326082	1.0142883	0.614262974	-0.7030717	0.911535	1
KLHDC9	1.4769584	1.46934633	1.4802578	1.023248327	0.0331563	0.911548	1
SRRM3	1.0740416	1.06717036	1.07702	1.146637304	0.1974091	0.911553	1
EIF1AD	1.2156728	1.22242825	1.2127446	0.956463789	-0.0642177	0.911582	1
GDPD2	1.0894333	1.09590774	1.0866269	0.903231651	-0.1468321	0.911629	1
AC005523.2	1.0277293	1.0210172	1.0306387	1.457789547	0.5437825	0.911651	1
RHBDL3	1.0277495	1.02102545	1.030664	1.4584245	0.5444107	0.911651	1
AP000223.42	1.0318834	1.03812795	1.0291767	0.765231724	-0.3860314	0.911667	1
SMYD5	1.1185177	1.11172034	1.121464	1.087214894	0.1206371	0.911703	1
RP11-59H7.4	1.0205536	1.01394208	1.0234194	1.679767375	0.7482615	0.911734	1
ZDHHC5	1.1743881	1.16719718	1.177505	1.061650564	0.086309	0.911769	1
RP11-849F2.9	1.0338314	1.0400595	1.0311317	0.777137646	-0.3637579	0.911778	1
HEATR5B	1.1008202	1.09391946	1.1038113	1.105322966	0.144468	0.911835	1
GRIK5	1.1008582	1.09415594	1.1037634	1.102037726	0.1401736	0.911835	1
PIGW	1.0799003	1.08636479	1.0770982	0.892703913	-0.1637463	0.911856	1
SLC27A4	1.0652788	1.05864033	1.0681563	1.162277017	0.216954	0.911952	1
NEUROD4	1.0366505	1.02991338	1.0395708	1.322845604	0.4036447	0.91204	1
GRAMD4	1.1464279	1.13934495	1.1494981	1.07286314	0.1014661	0.912053	1
UNC80	1.0606129	1.06687551	1.0578983	0.865762547	-0.2079567	0.912093	1
AC009264.1	1.0154003	1.02163643	1.0126972	0.586844653	-0.7689494	0.912105	1
AC137932.6	1.0153776	1.02157322	1.012692	0.588322225	-0.7653216	0.912105	1
GRIN2C	1.0215029	1.01486911	1.0243784	1.63953332	0.7132852	0.912213	1
RP11-3P17.4	1.0214676	1.01486911	1.0243277	1.636122449	0.7102807	0.912213	1
TFAP2D	1.0215269	1.01486911	1.0244128	1.641848361	0.7153209	0.912213	1
RP11-60A24.3	1.0215163	1.01486911	1.0243975	1.640818583	0.7144157	0.912213	1
VWA5B2	1.0214968	1.01486911	1.0243696	1.638939065	0.7127622	0.912213	1
CTD-2283N19.1	1.0224099	1.0286275	1.0197148	0.688667855	-0.5381198	0.91222	1
MRAS	1.3104901	1.30318535	1.3136564	1.034536901	0.0489851	0.912422	1
RP11-78F17.1	1.0271015	1.03328248	1.0244223	0.733787818	-0.4465651	0.912445	1
HCG27	1.0248402	1.03100102	1.0221698	0.715130457	-0.4837216	0.912497	1
SMIM2-AS1	1.024832	1.03101544	1.0221518	0.714217841	-0.4855639	0.912497	1
ZNF598	1.1367208	1.14319151	1.133916	0.935223252	-0.0966173	0.9125	1
RP11-809O17.1	1.0541804	1.06033714	1.0515117	0.853731816	-0.2281452	0.912512	1
FAM171A2	1.1475185	1.14077534	1.1504414	1.068662846	0.0958068	0.91256	1
ZHX1-C8orf76	1.0566864	1.05011846	1.0595333	1.187851379	0.2483543	0.912576	1
RP11-68L18.1	1.0280475	1.02138256	1.0309364	1.446807274	0.5328728	0.912603	1

RHPN1-AS1	1.0644238	1.05772647	1.0673269	1.166308134	0.221949	0.912735	1
PCYT1B	1.1568479	1.16327348	1.1540627	0.943586928	-0.0837727	0.912851	1
DPH1	1.0300056	1.0234297	1.0328559	1.402319872	0.4878155	0.912863	1
OGDHL	1.043001	1.04921048	1.0403095	0.819124596	-0.2878452	0.912875	1
SCRT2	1.0205689	1.01400962	1.023412	1.671139237	0.7408319	0.912876	1
EFNA2	1.0206171	1.01400962	1.0234812	1.676077338	0.7450887	0.912876	1
RP11-686D22.4	1.0205328	1.01400962	1.0233604	1.667450992	0.7376444	0.912876	1
C4orf19	1.0205384	1.01400962	1.0233684	1.668024039	0.7381401	0.912876	1
SECTM1	1.0205442	1.01400962	1.0233767	1.668614773	0.7386509	0.912876	1
ZNF530	1.0978933	1.10430442	1.0951143	0.911891441	-0.133066	0.912884	1
HIST1H2AJ	1.0294879	1.02297438	1.0323113	1.40640507	0.4920122	0.912926	1
RP11-546K22.1	1.0256088	1.03176285	1.0229414	0.72226994	-0.46939	0.912932	1
RP11-135A1.3	1.0230256	1.02918946	1.0203539	0.697303453	-0.5201415	0.912947	1
SRXN1	1.0230237	1.0292192	1.0203382	0.696057186	-0.5227223	0.912947	1
CD72	1.0822845	1.07550525	1.0852231	1.12870376	0.1746669	0.913026	1
TVP23A	1.1462002	1.15257592	1.1434365	0.940099518	-0.0891146	0.913081	1
KIF3B	1.2465712	1.25304025	1.2437672	0.963353558	-0.0538627	0.913187	1
RP4-539M6.22	1.0152369	1.0213835	1.0125726	0.587956733	-0.7662181	0.913194	1
AXIN2	1.0638369	1.05729315	1.0666733	1.163721609	0.218746	0.913433	1
DNASE2B	1.0213247	1.02747584	1.0186584	0.679083731	-0.5583386	0.913451	1
TMC8	1.0213186	1.02747602	1.0186496	0.678760804	-0.5590248	0.913451	1
KHDC3L	1.0239731	1.030102	1.0213165	0.708141401	-0.4978906	0.913505	1
COG6	1.2455367	1.23857899	1.2485525	1.041803995	0.0590839	0.913534	1
ADCY5	1.0230144	1.01648539	1.0258445	1.56772036	0.6486682	0.913606	1
LEMD1-AS1	1.0231152	1.01647906	1.0259916	1.577251401	0.6574126	0.913606	1
KCNMB3	1.0906834	1.08391143	1.0936187	1.115685282	0.1579301	0.913627	1
MRM2	1.4367077	1.44368889	1.4336817	0.977445383	-0.032912	0.913655	1
KLHL12	1.2751187	1.2817593	1.2722402	0.966215648	-0.0495829	0.913674	1
WDR91	1.0601349	1.06633772	1.0574463	0.865966964	-0.2076161	0.91373	1
GPHA2	1.0158279	1.02192214	1.0131863	0.601508419	-0.7333432	0.913783	1
RP11-180M15.6	1.0158797	1.02209683	1.0131848	0.596684655	-0.7449594	0.913783	1
FUT1	1.022794	1.01622207	1.0256426	1.580722634	0.6605842	0.913871	1
ZNF33A	1.6801943	1.68753287	1.6770134	0.984699669	-0.0222443	0.913919	1
RIN3	1.0650612	1.05849846	1.0679059	1.160815576	0.2151388	0.913928	1
IFT80	1.2752293	1.28190852	1.2723342	0.966037479	-0.0498489	0.913938	1
FOXK1	1.4426197	1.44971867	1.4395427	0.977372516	-0.0330196	0.913993	1
AC007879.2	1.0160955	1.02218093	1.0134578	0.60672772	-0.7208789	0.914047	1
C9orf153	1.040631	1.04684194	1.0379388	0.809931807	-0.3041277	0.914097	1
MGAT5B	1.0405987	1.046818	1.0379029	0.809579959	-0.3047545	0.914097	1
KCNAB1	1.0407385	1.04679998	1.038111	0.814339	-0.2962986	0.914097	1
TNKS2-AS1	1.0525683	1.04606405	1.0553876	1.202404853	0.2659227	0.914268	1
COX8C	1.0342304	1.04037573	1.0315667	0.78182284	-0.3550864	0.914316	1
TLR5	1.034335	1.04042924	1.0316934	0.783921579	-0.3512188	0.914316	1
RP13-516M14.1	1.0410179	1.04702036	1.0384161	0.817009044	-0.291576	0.914334	1
RP3-403A15.5	1.0409905	1.04704604	1.0383657	0.815493477	-0.2942548	0.914334	1
RP11-927P21.1	1.0282244	1.02172523	1.0310415	1.428821572	0.5148258	0.914338	1
RP11-800A3.7	1.0282535	1.02172243	1.0310844	1.430982578	0.5170061	0.914338	1
GCNT1	1.0282556	1.02171963	1.0310886	1.431361486	0.5173881	0.914338	1
AC007773.2	1.1169765	1.1233262	1.1142242	0.926195765	-0.1106109	0.914399	1
FADS2	1.6905606	1.69799193	1.6873394	0.984738367	-0.0221876	0.914456	1
RP11-188P20.3	1.0221669	1.01566168	1.0249867	1.595402123	0.6739201	0.914461	1

P2RY2	1.0221809	1.01564865	1.0250124	1.598373779	0.6766048	0.914461	1
RPGR	1.1752805	1.18173332	1.1724835	0.949102013	-0.0753649	0.914496	1
NAPA-AS1	1.0770229	1.07034236	1.0799187	1.136138537	0.1841388	0.914523	1
FAM207A	1.5166323	1.52405862	1.5134133	0.979686692	-0.0296077	0.914547	1
C5orf30	1.2361586	1.24259406	1.2333692	0.961973967	-0.0559302	0.91456	1
LINC01545	1.023892	1.02989316	1.0212908	0.712229087	-0.4895867	0.914599	1
SEL1L2	1.025049	1.03111629	1.0224191	0.720494184	-0.4729413	0.914615	1
SPAG6	1.0250438	1.03111339	1.022413	0.720363508	-0.473203	0.914615	1
RP11-546J1.1	1.0303067	1.02380039	1.0331268	1.39186042	0.4770145	0.914656	1
CYP2C8	1.0299851	1.03608425	1.0273413	0.75770834	-0.4002855	0.914728	1
RP5-855D21.2	1.0300713	1.03608781	1.0274634	0.761015444	-0.3940024	0.914728	1
RP11-121M22.1	1.0300424	1.03630963	1.0273259	0.752579441	-0.4100842	0.914728	1
NOMO3	1.0759934	1.06951212	1.0788027	1.133654708	0.1809813	0.914836	1
KDELC2	1.2729655	1.28022903	1.2698171	0.962844832	-0.0546248	0.914844	1
MTRNR2L3	1.0308764	1.0244481	1.0336627	1.376905297	0.4614293	0.914876	1
MYCNOS	1.0871323	1.09325891	1.0844767	0.905830032	-0.1426877	0.914899	1
LRRC45	1.1719456	1.16511746	1.1749053	1.059277915	0.0830811	0.914904	1
GRIK1-AS1	1.0231152	1.02918525	1.0204841	0.701863991	-0.5107366	0.914922	1
AC009133.21	1.0231264	1.02926194	1.020467	0.699439481	-0.5157289	0.914922	1
FRMD7	1.0231385	1.02925518	1.0204872	0.700291527	-0.5139725	0.914922	1
RP11-307E17.8	1.0230909	1.02914857	1.0204651	0.702097312	-0.5102571	0.914922	1
LIPT2	1.1070022	1.10046814	1.1098344	1.093226183	0.1285919	0.914999	1
RP11-1081M5.2	1.0206108	1.02668796	1.0179766	0.673585508	-0.570067	0.915001	1
SULF2	1.3249657	1.33159405	1.3220926	0.971346212	-0.0419425	0.915034	1
SOGA1	1.2683578	1.27512299	1.2654254	0.964751634	-0.0517705	0.915053	1
CTD-2588E21.1	1.0226322	1.0286771	1.020012	0.697837431	-0.5190371	0.915057	1
RARG	1.0974633	1.10376743	1.0947308	0.912914303	-0.1314487	0.915061	1
RP11-414H17.5	1.0326421	1.03866866	1.0300298	0.776592667	-0.36477	0.915066	1
RP11-115D19.3	1.0478785	1.04136072	1.0507037	1.225889691	0.2938292	0.915086	1
TRPV6	1.0223178	1.01574718	1.0251659	1.598121629	0.6763772	0.915107	1
TRABD2B	1.0221801	1.01574718	1.0249685	1.585586625	0.6650167	0.915107	1
FGFBP2	1.0221715	1.01574718	1.0249561	1.584797671	0.6642987	0.915107	1
SLC38A3	1.0427991	1.03624516	1.04564	1.259202749	0.3325106	0.915112	1
FAM26F	1.1362067	1.14239283	1.1335253	0.93772514	-0.092763	0.915133	1
MCOLN1	1.1999103	1.20661484	1.1970042	0.953485085	-0.0687177	0.915163	1
SRF	1.1204659	1.11387064	1.1233246	1.083023912	0.1150651	0.915182	1
NLRP12	1.0210518	1.02711652	1.018423	0.679399566	-0.5576678	0.915182	1
AC073957.15	1.0210454	1.02707733	1.0184308	0.680671195	-0.55497	0.915182	1
HIST1H4F	1.0210442	1.02710174	1.0184186	0.679608797	-0.5572236	0.915182	1
PMP2	1.021086	1.02716133	1.0184526	0.679369487	-0.5577317	0.915182	1
AC108938.5	1.0244744	1.03063263	1.0218051	0.711824742	-0.490406	0.91519	1
SLC25A34	1.0244427	1.03050592	1.0218145	0.715090858	-0.4838015	0.91519	1
MTRNR2L1	1.0244412	1.03046584	1.0218298	0.71653385	-0.4808932	0.91519	1
F7	1.0403984	1.03396182	1.0431884	1.271676177	0.3467313	0.915218	1
CTD-2515O10.5	1.0216511	1.01516282	1.0244635	1.613385739	0.6900914	0.915294	1
RP11-110I1.11	1.0216024	1.01516282	1.0243937	1.608782136	0.685969	0.915294	1
CDH18	1.0475354	1.04103079	1.0503549	1.22724651	0.2954251	0.915324	1
TGFB3	1.0476314	1.04114012	1.050445	1.226175736	0.2941658	0.915324	1
APOBEC3F	1.0456552	1.05172684	1.0430235	0.831743764	-0.265789	0.915535	1
CFAP52	1.0222417	1.01586347	1.0250064	1.576350348	0.6565882	0.915553	1
SPATA2L	1.1392999	1.13276798	1.1421311	1.070522681	0.0983154	0.915556	1

ARRDC3-AS1	1.0352871	1.02882007	1.0380903	1.321659564	0.4023506	0.915603	1
FBXL19-AS1	1.0352476	1.02883539	1.0380269	1.318759498	0.3991815	0.915603	1
CASC9	1.3073479	1.3000143	1.3105266	1.035039452	0.0496858	0.915635	1
ANK1	1.0690758	1.07517511	1.066432	0.883696412	-0.1783773	0.915638	1
ETFBKMT	1.0690035	1.07519316	1.0663206	0.882002885	-0.1811447	0.915638	1
IPO11	1.1253615	1.13166604	1.1226288	0.931362201	-0.1025858	0.915651	1
CFAP77	1.0391255	1.03271121	1.0419059	1.281085441	0.3573667	0.91567	1
CFAP53	1.1307928	1.12411735	1.1336863	1.077096133	0.107147	0.915711	1
SKAP1	1.0341796	1.02776144	1.0369615	1.33139858	0.4129425	0.915742	1
TTC9B	1.0341317	1.02763182	1.0369491	1.337193551	0.4192083	0.915742	1
TBC1D15	1.1110223	1.10448676	1.1138551	1.089660831	0.1238792	0.915751	1
HOXB7	1.0293281	1.02284558	1.0321379	1.406746577	0.4923625	0.91577	1
RP11-329B9.5	1.029335	1.02287457	1.0321353	1.40484659	0.4904126	0.91577	1
RRN3	1.1673364	1.16078958	1.1701742	1.058365974	0.0818386	0.915807	1
SULT1A3	1.03178	1.02534076	1.0345712	1.364251823	0.44811	0.915886	1
CALCB	1.0318048	1.02535158	1.0346019	1.364883188	0.4487775	0.915886	1
MID2	1.0317425	1.02537605	1.034502	1.359629509	0.4432136	0.915886	1
TSLP	1.0222693	1.02826297	1.0196714	0.696011811	-0.5228163	0.915951	1
BMP2K	1.2285293	1.23547977	1.2255165	0.957689547	-0.06237	0.916026	1
BRAP	1.1841653	1.19061941	1.1813678	0.951465437	-0.0717768	0.916064	1
FBXL16	1.0238612	1.01751383	1.0266125	1.519516351	0.6036122	0.916125	1
TM9SF1	1.135132	1.14147494	1.1323827	0.935732399	-0.0958321	0.916194	1
MADD	1.1791152	1.18547546	1.1763583	0.950844224	-0.0727191	0.916245	1
MKL1	1.0764902	1.07020986	1.0792125	1.128224603	0.1740543	0.91625	1
CAMK1G	1.0766624	1.07013863	1.0794902	1.133330101	0.1805681	0.91625	1
RP11-307C19.1	1.0267252	1.0202778	1.0295198	1.455770921	0.5417834	0.916277	1
RP11-392E22.9	1.0292321	1.03522327	1.0266352	0.756180655	-0.4031972	0.916285	1
TRIM68	1.0969402	1.10313254	1.0942561	0.913932067	-0.1298412	0.916326	1
STAM-AS1	1.1063068	1.11240919	1.1036616	0.922181276	-0.1168777	0.916348	1
VWA3B	1.0210129	1.02700962	1.0184136	0.681740805	-0.5527048	0.916367	1
RP11-589M4.1	1.021038	1.0270967	1.0184118	0.679486788	-0.5574826	0.916367	1
OPRD1	1.0210551	1.02707276	1.0184467	0.681376483	-0.5534759	0.916367	1
NHEJ1	1.0210035	1.02697492	1.0184152	0.682678616	-0.5507215	0.916367	1
EFCC1	1.0210127	1.02700274	1.0184164	0.682017992	-0.5521183	0.916367	1
RP11-370I10.11	1.0209914	1.02695389	1.0184069	0.682902744	-0.550248	0.916367	1
AC015849.19	1.0210257	1.02699173	1.0184397	0.683162649	-0.549699	0.916367	1
RP11-526I2.5	1.0209982	1.02693806	1.0184236	0.683924826	-0.5480903	0.916367	1
SCIN	1.0254076	1.01899597	1.0281868	1.483828357	0.5693242	0.916375	1
TMEM61	1.0254383	1.01899597	1.0282307	1.486141571	0.5715716	0.916375	1
H1F0	4.0406416	4.05442931	4.0346652	0.993529363	-0.0093655	0.916441	1
C2orf91	1.0297666	1.03579056	1.0271554	0.758731527	-0.3983386	0.916466	1
DAP	2.0635061	2.07175238	2.0599316	0.988970647	-0.0160004	0.916477	1
RP11-91J19.3	1.0302735	1.02385081	1.0330574	1.386009266	0.4709369	0.916494	1
TNIP3	1.0494789	1.04313126	1.0522304	1.210963589	0.2761555	0.916514	1
TANGO6	1.0500799	1.04363832	1.0528721	1.211597747	0.2769108	0.91655	1
SLC9A5	1.0580666	1.06408383	1.0554584	0.865403298	-0.2085555	0.916647	1
MANEA	1.2705859	1.26361619	1.273607	1.037899132	0.0536662	0.916722	1
RSBN1	1.6061204	1.61326193	1.6030249	0.983307252	-0.0242858	0.916782	1
PLEKHG4	1.0782684	1.07177029	1.0810851	1.12978635	0.17605	0.916837	1
CTD-2012K14.6	1.0333683	1.03933553	1.0307818	0.782543483	-0.3537572	0.916851	1
MIA	1.0197859	1.02571513	1.0172159	0.669483807	-0.5788789	0.916853	1

SPINK6	1.0198151	1.02576783	1.0172348	0.668849113	-0.5802473	0.916853	1
FAM78A	1.0197945	1.02576233	1.0172077	0.667939733	-0.5822102	0.916853	1
RP5-958B11.1	1.0197871	1.02570468	1.0172221	0.670000397	-0.5777661	0.916853	1
ATP4A	1.0198071	1.02578945	1.017214	0.667482362	-0.5831984	0.916853	1
GUSBP1	1.0673086	1.06084668	1.0701096	1.152233423	0.204433	0.916876	1
XPOT	1.6744417	1.66695331	1.6776876	1.01609451	0.0230346	0.916907	1
DDX60	1.0669671	1.0730174	1.0643446	0.881222286	-0.1824221	0.916934	1
HIST1H2BD	1.1519782	1.14510555	1.1549572	1.06789323	0.0947674	0.916972	1
GNGT2	1.0240916	1.01771982	1.0268535	1.515449282	0.5997456	0.917039	1
TISP43	1.024567	1.03053976	1.0219781	0.719653809	-0.474625	0.91706	1
CNOT6L	1.3260804	1.31883601	1.3292205	1.032569953	0.0462395	0.917067	1
ZKSCAN3	1.0947746	1.08830111	1.0975805	1.105088266	0.1441616	0.917134	1
RP11-53O19.1	1.1653527	1.15841185	1.1683613	1.062807219	0.0878799	0.917139	1
RP11-493L12.4	1.0424096	1.04831345	1.0398505	0.824832592	-0.2778268	0.917314	1
RP11-646I6.5	1.0257809	1.03174889	1.0231941	0.730547609	-0.4529498	0.917377	1
CARD6	1.0258052	1.03177947	1.0232156	0.730520778	-0.4530028	0.917377	1
TBC1D29	1.0258067	1.03177253	1.0232207	0.730843343	-0.4523659	0.917377	1
CARD9	1.0257837	1.03172844	1.0232069	0.731422919	-0.4512223	0.917377	1
LRBA	1.6215494	1.62824649	1.6186464	0.984719299	-0.0222156	0.917397	1
DECR2	1.1712719	1.17780935	1.1684382	0.947296751	-0.0781117	0.91742	1
HIST1H2AM	1.0294111	1.03535599	1.0268343	0.758974735	-0.3978762	0.91742	1
RTN4RL1	1.0306111	1.02426895	1.0333601	1.374599461	0.4590113	0.917448	1
RP11-255M2.3	1.0603617	1.06633251	1.0577736	0.870969249	-0.1993063	0.917464	1
GSG1L	1.0404116	1.04636709	1.0378302	0.81588481	-0.2935626	0.917476	1
CTD-2015H6.3	1.1641392	1.17024819	1.1614913	0.948563766	-0.0761833	0.917511	1
MORC2-AS1	1.1644204	1.17060415	1.16174	0.948042818	-0.0769759	0.917511	1
RP11-728F11.4	1.0230253	1.02893655	1.020463	0.707168889	-0.4998733	0.917529	1
CXCL17	1.0230371	1.02893989	1.0204786	0.707624132	-0.4989448	0.917529	1
FAM163A	1.0230531	1.02894217	1.0205004	0.708322894	-0.4975209	0.917529	1
ASMTL-AS1	1.023081	1.02897208	1.0205275	0.708525638	-0.497108	0.917529	1
AP001059.5	1.0204198	1.02630518	1.0178688	0.679286691	-0.5579075	0.917562	1
SEPT3	1.1535158	1.15942826	1.150953	0.946839445	-0.0788083	0.917696	1
KIAA1755	1.0231411	1.01685271	1.0258669	1.534880184	0.618126	0.917703	1
FZD10	1.0231543	1.01687251	1.0258772	1.533690061	0.617007	0.917703	1
BSPRY	1.0231145	1.01684635	1.0258315	1.533356137	0.6166928	0.917703	1
HEYL	1.0231767	1.01687616	1.0259077	1.535163396	0.6183922	0.917703	1
OXGR1	1.0231469	1.01687449	1.0258657	1.532829419	0.6161972	0.917703	1
DGKQ	1.0616063	1.06768922	1.0589696	0.871181795	-0.1989543	0.917755	1
ASB3	1.1763563	1.16982241	1.1791885	1.055152354	0.0774513	0.917862	1
CRTC3	1.2694603	1.2628906	1.272308	1.035822547	0.0507769	0.917875	1
UMAD1	1.2661469	1.27241211	1.2634312	0.967032048	-0.0483644	0.917925	1
RP11-49I11.1	1.0318747	1.03774683	1.0293294	0.777003255	-0.3640075	0.917979	1
ASAP1	1.7112791	1.71862784	1.7080938	0.98534145	-0.0213043	0.918003	1
RP11-539L10.3	1.2969339	1.30343062	1.2941178	0.969308383	-0.0449724	0.918051	1
RAD51C	1.5793981	1.57184121	1.5826737	1.018943119	0.0270735	0.918053	1
CDC42EP5	1.0794538	1.08547007	1.0768461	0.899099342	-0.1534476	0.918061	1
NKX2-1	1.0187298	1.02460392	1.0161837	0.657767768	-0.6043498	0.918101	1
RP4-584D14.6	1.0187591	1.02470071	1.0161837	0.655190372	-0.6100139	0.918101	1
RP11-207C16.4	1.0187301	1.02460488	1.0161837	0.657742091	-0.6044061	0.918101	1
RP11-344E13.4	1.0187325	1.02461288	1.0161837	0.657528289	-0.6048751	0.918101	1
AP000255.6	1.0187276	1.02459658	1.0161837	0.657964171	-0.6039191	0.918101	1

GABRD	1.0187196	1.02457016	1.0161837	0.658671758	-0.6023684	0.918101	1
RP11-235D19.4	1.0187297	1.02460335	1.0161837	0.657783153	-0.604316	0.918101	1
RP11-440L14.4	1.0214054	1.02728742	1.0188559	0.691009007	-0.5332236	0.918101	1
CTD-2090I13.1	1.0214411	1.02727211	1.0189137	0.693516521	-0.5279978	0.918101	1
ANKRD53	1.058318	1.05190939	1.0610958	1.17696993	0.2350775	0.918108	1
AC092198.1	1.0284366	1.03437426	1.0258629	0.752390582	-0.4104463	0.918145	1
CBR4	2.0334671	2.04094385	2.0302262	0.989703932	-0.0149311	0.918251	1
SPTY2D1-AS1	1.097421	1.10362005	1.094734	0.914243795	-0.1293492	0.918258	1
DGCR14	1.1649218	1.17112186	1.1622344	0.948063305	-0.0769447	0.918341	1
YOD1	1.1650774	1.15849591	1.1679301	1.059523494	0.0834156	0.918347	1
AATK-AS1	1.0235125	1.01724954	1.0262272	1.520456563	0.6045046	0.918378	1
ZNF804A	1.0235218	1.01724954	1.0262405	1.521229097	0.6052374	0.918378	1
PRICKLE1	1.1121168	1.11805852	1.1095414	0.927856385	-0.1080266	0.918386	1
CTC-308K20.1	1.0309445	1.03675931	1.028424	0.773246769	-0.3709992	0.918461	1
PSKH1	1.1761455	1.16966901	1.1789528	1.054717093	0.0768561	0.91847	1
ZNF415	1.158377	1.1518689	1.161198	1.061428602	0.0860073	0.918484	1
GBA2	1.2112716	1.20476913	1.2140902	1.045519876	0.0642205	0.918503	1
CUL9	1.1128152	1.11874985	1.1102428	0.928361637	-0.1072412	0.918575	1
AC093627.9	1.0395261	1.04537518	1.0369908	0.815220312	-0.2947381	0.918661	1
KRT13	1.0253218	1.01910226	1.0280177	1.466724226	0.5525976	0.918681	1
LINC01004	1.038069	1.04410816	1.0354512	0.803734224	-0.3152096	0.918697	1
RP11-48B3.4	1.0344359	1.04025658	1.0319129	0.792736347	-0.335087	0.918744	1
CTB-186G2.1	1.0344325	1.04036068	1.0318629	0.789454127	-0.3410727	0.918744	1
C2CD4D	1.0497409	1.04348161	1.052454	1.206348969	0.2706473	0.918752	1
PDE8A	1.1409507	1.14707238	1.1382972	0.940334061	-0.0887547	0.918754	1
LSINCT5	1.0290597	1.03491288	1.0265226	0.759678836	-0.3965385	0.918893	1
PSMB8-AS1	1.0316395	1.03749655	1.0291008	0.776091335	-0.3657016	0.918897	1
FKRP	1.1426817	1.13619496	1.1454934	1.068273376	0.0952809	0.918969	1
CUEDC1	1.267721	1.26109298	1.270594	1.036389274	0.051566	0.919016	1
FAM134A	1.7981863	1.80567242	1.7949414	0.986680713	-0.0193448	0.919131	1
RP1-101K10.6	1.0973656	1.10324069	1.094819	0.918427083	-0.1227629	0.919205	1
RP11-328K4.1	1.0247177	1.01848226	1.0274205	1.483611372	0.5691132	0.919272	1
RP11-81A22.5	1.0680582	1.07396951	1.0654959	0.885445121	-0.1755252	0.919285	1
RP4-549L20.3	1.0503626	1.05624867	1.0478112	0.849996849	-0.2344706	0.919334	1
PADI2	1.0273801	1.03320867	1.0248536	0.748407043	-0.418105	0.919361	1
CAMTA2	1.1340729	1.1276793	1.1368442	1.071780753	0.1000098	0.919379	1
DNAJC6	1.0779096	1.07152742	1.080676	1.127903499	0.1736436	0.919403	1
MAFK	1.0949624	1.10110597	1.0922994	0.912897572	-0.1314751	0.919522	1
AC096670.3	1.0205085	1.02630176	1.0179973	0.684263256	-0.5473766	0.91957	1
RP5-944M2.3	1.0205155	1.02632495	1.0179973	0.683660573	-0.5486479	0.91957	1
RP1-60O19.2	1.0205261	1.02636023	1.0179973	0.682745585	-0.55058	0.91957	1
AC079154.1	1.0205197	1.02633889	1.0179973	0.683298599	-0.5494119	0.91957	1
RP11-326E22.1	1.020526	1.02635959	1.0179973	0.682762091	-0.5505451	0.91957	1
PTOV1-AS2	1.0205198	1.02633915	1.0179973	0.683291947	-0.549426	0.91957	1
HIST1H2AI	1.0204979	1.02626684	1.0179973	0.685172886	-0.54546	0.91957	1
RP11-519G16.2	1.0205142	1.02632071	1.0179973	0.683770677	-0.5484155	0.91957	1
KIZ-AS1	1.0205294	1.02637103	1.0179973	0.682466036	-0.5511708	0.91957	1
TCF23	1.0205068	1.02629624	1.0179973	0.68440689	-0.5470738	0.91957	1
AC009480.3	1.0179956	1.02379265	1.0154829	0.650741748	-0.619843	0.91965	1
RP11-335L23.5	1.0179961	1.02379419	1.0154829	0.650699638	-0.6199363	0.91965	1
RP11-268P4.5	1.0179951	1.02379095	1.0154829	0.650788228	-0.6197399	0.91965	1

AC074363.1	1.0179785	1.02373595	1.0154829	0.652296389	-0.6164005	0.91965	1
AF011889.5	1.0179985	1.02380201	1.0154829	0.650485817	-0.6204105	0.91965	1
LINC01352	1.0180034	1.02381846	1.0154829	0.650036582	-0.6214072	0.91965	1
RP11-455B3.1	1.017994	1.02378743	1.0154829	0.650884498	-0.6195265	0.91965	1
RP11-1376P16.1	1.0179862	1.0237614	1.0154829	0.651597543	-0.6179469	0.91965	1
RP11-875O11.1	1.0179935	1.02378574	1.0154829	0.650930904	-0.6194237	0.91965	1
NXF3	1.0179966	1.02379598	1.0154829	0.650650684	-0.6200449	0.91965	1
LINC00484.1	1.0180028	1.02381634	1.0154829	0.650094521	-0.6212786	0.91965	1
ARID3C	1.017992	1.02378069	1.0154829	0.651069044	-0.6191175	0.91965	1
KANK2	1.198866	1.20529655	1.1960786	0.955099477	-0.0662771	0.919655	1
DIO2	1.0334973	1.02726508	1.0361987	1.327658633	0.4088842	0.919669	1
SYT10	1.0334802	1.02726492	1.0361742	1.326767799	0.4079159	0.919669	1
RP5-1116H23.6	1.0200629	1.02584483	1.0175567	0.679312338	-0.557853	0.919688	1
CTB-49A3.4	1.0200399	1.02579101	1.0175471	0.680355615	-0.5556391	0.919688	1
RP11-439M11.1	1.0200375	1.02582546	1.0175286	0.67873479	-0.5590801	0.919688	1
RP11-119J18.1	1.0200884	1.02596935	1.0175392	0.675382347	-0.5662236	0.919688	1
PCAT4	1.0200481	1.02578214	1.0175626	0.681191927	-0.5538668	0.919688	1
SLC12A7	1.1410971	1.13458519	1.1439198	1.069358058	0.096745	0.91971	1
ICA1	1.2302141	1.22366426	1.2330531	1.041977479	0.0593241	0.919784	1
NRG3	1.0218493	1.02763946	1.0193395	0.69970524	-0.5151808	0.919785	1
SRCIN1	1.0602512	1.05398861	1.0629657	1.16627751	0.2219111	0.919794	1
SIRT4	1.0525055	1.04635077	1.0551733	1.190342663	0.2513769	0.919795	1
WDR93	1.0395566	1.04536554	1.0370387	0.816449003	-0.2925653	0.919815	1
CTD-3195I5.4	1.0184749	1.0242767	1.0159601	0.65742444	-0.605103	0.919831	1
RP11-362F19.1	1.0184749	1.0242767	1.0159601	0.65742444	-0.605103	0.919831	1
KCTD4	1.0184809	1.02429652	1.0159601	0.656887987	-0.6062807	0.919831	1
LA16c-316G12.2	1.0184809	1.02429652	1.0159601	0.656887987	-0.6062807	0.919831	1
LINC01305	1.0184548	1.02421019	1.0159601	0.659230361	-0.6011454	0.919831	1
RP11-256L11.3	1.0184721	1.02426751	1.0159601	0.65767326	-0.6045571	0.919831	1
RP11-173P15.10	1.0184721	1.02426751	1.0159601	0.65767326	-0.6045571	0.919831	1
RP11-442O1.4	1.0184855	1.02431176	1.0159601	0.656476239	-0.6071853	0.919831	1
RP11-134L10.1	1.0184637	1.02423954	1.0159601	0.658432284	-0.602893	0.919831	1
ARHGAP19-SLIT1	1.0248141	1.01861175	1.0275025	1.477695393	0.5633489	0.919939	1
CTB-147N14.6	1.0247963	1.01860105	1.0274816	1.477424392	0.5630843	0.919939	1
FOXL1	1.0247424	1.0185845	1.0274116	1.474972471	0.560688	0.919939	1
ANGPT1	1.024791	1.01859376	1.0274772	1.477762936	0.5634148	0.919939	1
EXOC8	1.0577532	1.06371702	1.0551682	0.865831427	-0.2078419	0.919984	1
PLEKHN1	1.0405412	1.03431091	1.0432417	1.260289912	0.3337556	0.919993	1
ZNF695	1.069225	1.06292113	1.0719574	1.143613222	0.1935992	0.920029	1
MTTP	1.022135	1.0278971	1.0196374	0.703923211	-0.50651	0.920051	1
PAQR7	1.1816111	1.18760638	1.1790125	0.954191894	-0.0676487	0.920061	1
PRL	1.024177	1.01802041	1.0268456	1.489732901	0.5750537	0.920126	1
ASAP3	1.131955	1.13788362	1.1293852	0.938364945	-0.091779	0.920143	1
DRAXIN	1.070019	1.06365322	1.0727782	1.143355179	0.1932736	0.92016	1
SPSB4	1.1032637	1.10920816	1.100687	0.92197356	-0.1172027	0.920191	1
KAT2B	1.0877985	1.08146075	1.0905457	1.111525141	0.1525406	0.920247	1
GATM-AS1	1.0557288	1.06157123	1.0531963	0.863980071	-0.2109301	0.920249	1
RELN	1.9950633	2.00274371	1.9917341	0.989020546	-0.0159276	0.92026	1
COQ2	1.2228539	1.21647361	1.2256195	1.042249275	0.0597004	0.920281	1
SEMA6D	1.1254323	1.11921141	1.1281288	1.074803157	0.1040725	0.920352	1
MYOM1	1.0248526	1.01874306	1.0275009	1.467257002	0.5531216	0.920367	1

VPS13C	1.5378269	1.54484402	1.5347853	0.981538312	-0.0268835	0.920478	1
ZNF316	1.0852435	1.07895015	1.0879714	1.114265337	0.1560928	0.920505	1
RP3-522D1.1	1.0703409	1.064098	1.073047	1.13961393	0.1885452	0.920513	1
HNRNPL	1.6254062	1.63230521	1.6224157	0.984359651	-0.0227426	0.92052	1
KCNC3	1.2290842	1.22250058	1.231938	1.042415165	0.05993	0.920554	1
DGCR9	1.0197031	1.02542876	1.0172213	0.677238379	-0.5622644	0.920563	1
TTLL10	1.0196923	1.02544191	1.0172001	0.676053385	-0.5647909	0.920563	1
POMK	1.0311286	1.03694794	1.0286061	0.774228461	-0.3691688	0.920568	1
NPIPA2	1.0319283	1.02574655	1.0346078	1.344172841	0.4267187	0.920602	1
RP11-157P1.4	1.0360146	1.04175295	1.0335273	0.802992076	-0.3165423	0.920609	1
MIOS	1.205927	1.2122066	1.2032051	0.957581554	-0.0625327	0.920634	1
LRRC3B	1.0608121	1.05464986	1.0634832	1.161634504	0.2161562	0.920658	1
DUSP8	1.0414723	1.03533265	1.0441336	1.249088131	0.3208753	0.920721	1
STRN3	1.4797405	1.47301431	1.482656	1.02038348	0.0291114	0.920749	1
GON4L	1.4472607	1.44058789	1.4501531	1.021710166	0.030986	0.920761	1
SHOC2	1.769659	1.76177046	1.7730783	1.014844113	0.0212581	0.920773	1
DUS2	1.1210249	1.12713546	1.1183763	0.931103387	-0.1029867	0.920775	1
AY269186.1	1.0181761	1.02390781	1.0156917	0.656340339	-0.607484	0.92078	1
CTD-2154I11.2	1.0181702	1.02388823	1.0156917	0.656878232	-0.6063021	0.92078	1
AC026167.1	1.0181828	1.02392983	1.0156917	0.655736385	-0.6088121	0.92078	1
WI2-85898F10.2	1.0181723	1.02389518	1.0156917	0.656687252	-0.6067216	0.92078	1
PKHD1L1	1.0181724	1.02389573	1.0156917	0.656672271	-0.6067546	0.92078	1
RP11-80H18.4	1.0181816	1.02392606	1.0156917	0.655839729	-0.6085848	0.92078	1
RP11-46E17.6	1.0181766	1.02390935	1.0156917	0.656298087	-0.6075769	0.92078	1
CTD-2297D10.1	1.0181784	1.02391531	1.0156917	0.656134588	-0.6079363	0.92078	1
RP5-1024G6.2	1.0380166	1.04379286	1.0355129	0.810927982	-0.3023543	0.920798	1
TRIM37	1.4653265	1.47211874	1.4623824	0.979377275	-0.0300634	0.920822	1
TRERF1	1.1421336	1.13594471	1.1448161	1.0652577	0.0912025	0.920825	1
MYRIP	1.0291849	1.03489113	1.0267114	0.765565498	-0.3854023	0.920837	1
RP11-25H12.1	1.0292453	1.03490879	1.0267904	0.767439634	-0.3818748	0.920837	1
ZNF684	1.0731364	1.07910932	1.0705474	0.891770771	-0.1652552	0.920937	1
C1orf74	1.073175	1.07925509	1.0705396	0.89003234	-0.1680703	0.920937	1
PRAP1	1.0264896	1.02040835	1.0291256	1.427139303	0.5131262	0.92094	1
SPATA5	1.1236479	1.11736813	1.1263699	1.076697101	0.1066124	0.920949	1
NAGS	1.1612866	1.15495029	1.1640331	1.05861772	0.0821817	0.920978	1
CD248	1.0385588	1.04431316	1.0360645	0.813855292	-0.2971558	0.920987	1
RP11-700H6.1	1.0184316	1.02416836	1.015945	0.659745555	-0.6000184	0.921015	1
RP11-1143G9.5	1.0184235	1.02414166	1.015945	0.660475146	-0.5984238	0.921015	1
RP5-1120P11.3	1.0184245	1.02414505	1.015945	0.660382298	-0.5986266	0.921015	1
RGSL1	1.0184305	1.02416463	1.015945	0.659847413	-0.5997956	0.921015	1
AC004051.2	1.0184194	1.02412798	1.015945	0.660849601	-0.5976061	0.921015	1
CASC21	1.0184209	1.02413299	1.015945	0.660712398	-0.5979057	0.921015	1
CTC-454I21.3	1.0184175	1.02412174	1.015945	0.661020407	-0.5972333	0.921015	1
FGF17	1.0184134	1.02410805	1.015945	0.661395958	-0.5964139	0.921015	1
CTD-2154B17.4	1.0184159	1.02411643	1.015945	0.661166199	-0.5969151	0.921015	1
TM4SF19-AS1	1.027134	1.03286793	1.0246485	0.749926863	-0.4151782	0.921098	1
LCAT	1.0429308	1.03682303	1.0455783	1.237765281	0.3077378	0.921148	1
PRR7	2.1329204	2.14117533	2.1293423	0.989630804	-0.0150377	0.921156	1
MICU3	1.1870656	1.18045517	1.1899309	1.052510462	0.0738346	0.921164	1
RP11-234B24.2	1.0279797	1.0218795	1.0306239	1.399663049	0.4850796	0.921209	1
SLC8A2	1.0233032	1.0171592	1.0259663	1.513258087	0.5976581	0.921216	1

RP11-253E3.3	1.0205872	1.02628568	1.0181172	0.689241681	-0.5369181	0.921227	1
RP11-115H18.1	1.0205925	1.02626675	1.018133	0.690339994	-0.534621	0.921227	1
RP11-693J15.5	1.0536863	1.04756331	1.0563403	1.184532497	0.2443178	0.921324	1
AFAP1L1	1.0328176	1.02668598	1.0354753	1.329362393	0.4107344	0.921328	1
C2CD2L	1.064318	1.07024626	1.0617484	0.879027368	-0.18602	0.921487	1
RP11-977G19.10	1.0172096	1.02288614	1.014749	0.644451575	-0.6338561	0.921501	1
AC005330.2	1.0172162	1.02290827	1.014749	0.643829044	-0.6352504	0.921501	1
KB-1517D11.4	1.0172082	1.02288159	1.014749	0.644579655	-0.6335694	0.921501	1
CTD-2006K23.1	1.0172082	1.02288159	1.014749	0.644579655	-0.6335694	0.921501	1
AF196970.3	1.0171964	1.02284268	1.014749	0.64567772	-0.6311138	0.921501	1
RP11-485F13.1	1.0172113	1.02289202	1.014749	0.644285908	-0.6342271	0.921501	1
HBA1	1.0171974	1.0228459	1.014749	0.645586699	-0.6313172	0.921501	1
SAP30L	1.2176066	1.21132677	1.2203286	1.042596863	0.0601814	0.921585	1
MED18	1.0924998	1.09853566	1.0898835	0.912192362	-0.13259	0.921612	1
CAMSAP3	1.0876913	1.09359666	1.0851316	0.909557799	-0.1367628	0.921613	1
ACOX2	1.1135721	1.1074564	1.116223	1.081583067	0.1131445	0.92164	1
CNTROB	1.2121502	1.20523911	1.2151458	1.048269204	0.0680093	0.921679	1
RP1-45I4.3	1.0219211	1.02762411	1.0194491	0.704061597	-0.5062264	0.921689	1
AC079145.4	1.0219437	1.02759234	1.0194952	0.706544542	-0.5011476	0.921689	1
UBALD1	1.2330441	1.22651498	1.2358743	1.041318531	0.0584114	0.921696	1
BCAN	1.0244496	1.01839445	1.0270743	1.471871576	0.5576518	0.921702	1
RP11-505K9.4	1.024506	1.01839445	1.0271551	1.476263329	0.5619501	0.921702	1
CTD-3076O17.1	1.0244744	1.01839445	1.0271098	1.473804263	0.5595449	0.921702	1
KCNB1	1.02445	1.01839445	1.0270748	1.471901687	0.5576813	0.921702	1
LCN15	1.0197572	1.02534033	1.0173372	0.684173743	-0.5475654	0.921706	1
WNT2	1.0197209	1.02540095	1.0172588	0.679456493	-0.5575469	0.921706	1
TMEM191B	1.1432159	1.13694671	1.1459333	1.065620894	0.0916943	0.921812	1
ZNF34	1.1132223	1.10689412	1.1159653	1.084861132	0.1175104	0.921829	1
RP11-326G21.1	1.028337	1.03406597	1.0258537	0.758929961	-0.3979613	0.921908	1
ARRDC2	1.1159159	1.10955801	1.1186718	1.083187141	0.1152825	0.921912	1
NRG2	1.0544241	1.06016666	1.0519349	0.863184885	-0.2122585	0.921949	1
LINC01521	1.0800837	1.07380992	1.082803	1.121841745	0.1658692	0.921972	1
PABPC1L2B	1.0232034	1.02886003	1.0207515	0.719040638	-0.4758548	0.921988	1
B4GALT1-AS1	1.0268134	1.0324825	1.0243561	0.74982158	-0.4153807	0.922052	1
RP3-467K16.2	1.0379098	1.04356075	1.0354603	0.81404362	-0.296822	0.922092	1
AP001437.1	1.0204447	1.02607573	1.0180039	0.69044683	-0.5343978	0.92214	1
FAM218A	1.0472425	1.0529644	1.0447623	0.845138717	-0.2427399	0.922176	1
RP11-446H18.6	1.017833	1.02347677	1.0153867	0.655399379	-0.6095538	0.922191	1
RP11-268G13.1	1.0178329	1.02347778	1.0153862	0.655349485	-0.6096636	0.922191	1
CTC-457L16.2	1.0178323	1.02348898	1.0153804	0.654790045	-0.6108957	0.922191	1
ANKRD24	1.017841	1.02349436	1.0153905	0.6550709	-0.610277	0.922191	1
RP4-616B8.5	1.0178246	1.02344318	1.0153892	0.656447515	-0.6072484	0.922191	1
CISTR	1.0178274	1.02344006	1.0153946	0.656763024	-0.6065552	0.922191	1
AKAP3	1.0270455	1.03266063	1.0246117	0.753557103	-0.4082113	0.922288	1
FAM222A	1.0916566	1.097256	1.0892295	0.917469949	-0.1242672	0.922358	1
RP11-22P6.2	1.0752508	1.06899707	1.0779615	1.129924528	0.1762264	0.922373	1
SH3TC1	1.0368214	1.03082942	1.0394187	1.278607402	0.3545734	0.92249	1
NIPAL1	1.0257389	1.01971836	1.0283485	1.437671399	0.523734	0.92252	1
SLC38A8	1.035199	1.02918529	1.0378057	1.295368742	0.3733628	0.922545	1
RP1-78O14.1	1.029225	1.03493901	1.0267483	0.765570876	-0.3853921	0.922575	1
C1QTNF9B-AS1	1.0654238	1.07117249	1.062932	0.884218099	-0.1775258	0.922634	1

TSPAN33	1.0445374	1.03849317	1.0471573	1.225082251	0.2928786	0.922635	1
ANKRD31	1.0445152	1.03843487	1.0471507	1.226769752	0.2948645	0.922635	1
C12orf65	1.504372	1.49774125	1.5072462	1.019096132	0.0272901	0.922697	1
PCAT14	1.0188672	1.02442986	1.0164561	0.673605872	-0.5700234	0.922712	1
BEAN1	1.0188287	1.02440199	1.0164129	0.672606706	-0.5721649	0.922712	1
LINC00176	1.0188282	1.02441489	1.0164066	0.671992341	-0.5734833	0.922712	1
RP11-435O5.4	1.018827	1.02444154	1.0163933	0.670715794	-0.5762265	0.922712	1
RP11-73K9.2	1.0950701	1.10083857	1.0925697	0.917999417	-0.1234349	0.922718	1
GPR1	1.0181541	1.02378392	1.0157138	0.660689179	-0.5979564	0.922771	1
TMEM140	1.0181434	1.02372961	1.015722	0.662549502	-0.5938998	0.922771	1
RP11-126O1.5	1.0604157	1.06602531	1.0579841	0.87821086	-0.1873607	0.922773	1
PRR35	1.0393478	1.04504356	1.036879	0.818739994	-0.2885227	0.922797	1
FBXO3	1.3782894	1.37144899	1.3812545	1.026397921	0.0375902	0.922814	1
RUSC2	1.0963533	1.09020843	1.0990169	1.097645559	0.1344123	0.92283	1
ATP8B1	1.0244965	1.01850742	1.0270925	1.46387502	0.5497924	0.922889	1
OSTN	1.0245044	1.01850742	1.0271039	1.464487147	0.5503955	0.922889	1
DLX6	1.0245176	1.01850742	1.0271228	1.465508515	0.5514014	0.922889	1
CCDC9	1.0634827	1.05739566	1.0661212	1.152024468	0.2041714	0.922914	1
SLC16A6	1.057645	1.06323782	1.0552208	0.873224598	-0.1955753	0.922927	1
LINC00652	1.0187254	1.02430364	1.0163074	0.670987613	-0.575642	0.922958	1
HSPA12B	1.0187223	1.02431185	1.0162995	0.670433973	-0.5768328	0.922958	1
KBTBD8	1.0187348	1.02434276	1.016304	0.669769304	-0.5782638	0.922958	1
ZNF878	1.0187227	1.02432038	1.0162963	0.670069572	-0.5776172	0.922958	1
SLC9C1	1.0187542	1.02436711	1.0163213	0.669808368	-0.5781797	0.922958	1
SLC52A3	1.0187333	1.0243611	1.0162939	0.668848656	-0.5802483	0.922958	1
TP53BP1	1.6346685	1.64086245	1.6319837	0.98614558	-0.0201275	0.922969	1
MKL2	1.264349	1.27049831	1.2616836	0.967412928	-0.0477963	0.923025	1
ZNF280A	1.0240349	1.01804205	1.0266325	1.476135664	0.5618253	0.923071	1
MCEMP1	1.0266141	1.02056357	1.0292367	1.42177252	0.5076907	0.92315	1
RP11-217B1.2	1.0260825	1.02011257	1.0286702	1.425487322	0.5114552	0.923216	1
RP11-1348G14.8	1.0354789	1.0410717	1.0330547	0.804804916	-0.313289	0.923312	1
ZHX2	1.1869273	1.19300501	1.1842929	0.954860491	-0.0666381	0.923312	1
RP11-54O7.2	1.0465552	1.05223244	1.0440943	0.844194024	-0.2443535	0.923335	1
ST3GAL4	1.2429447	1.23683431	1.2455933	1.036983468	0.0523929	0.923335	1
RP11-679C8.2	1.0318614	1.03749706	1.0294187	0.784559066	-0.350046	0.92334	1
KCTD8	1.0318336	1.03750268	1.0293762	0.783310494	-0.3523438	0.92334	1
CYP26A1	1.1073855	1.10131437	1.1100171	1.085897946	0.1188885	0.923342	1
PAPD7	1.1842621	1.18990918	1.1818143	0.957375147	-0.0628437	0.923398	1
FMO4	1.0552667	1.06092634	1.0528134	0.866840804	-0.206161	0.923406	1
MEGF9	1.3297998	1.32334228	1.3325988	1.028627695	0.0407209	0.923449	1
ALG10	1.0558953	1.06148848	1.0534709	0.869608715	-0.2015617	0.92351	1
MMP9	1.0459187	1.05157651	1.0434664	0.842754808	-0.2468151	0.923564	1
AKR1C1	1.08995	1.08380853	1.092612	1.105042795	0.1441022	0.923699	1
CENPF	3.6280553	3.60551797	3.6378243	1.012399192	0.0177783	0.923702	1
NSL1	1.6543003	1.64749804	1.6572488	1.015059085	0.0215637	0.923722	1
ZMIZ2	1.2073222	1.20107683	1.2100293	1.044522597	0.0628437	0.923746	1
CTD-2129N1.1	1.0187121	1.02419568	1.0163352	0.675129979	-0.5667628	0.923805	1
GLDN	1.0186891	1.02424495	1.0162809	0.671516391	-0.5745055	0.923805	1
ANXA2R	1.0186682	1.02420639	1.0162677	0.672041069	-0.5733787	0.923805	1
RP11-62F24.2	1.028524	1.02256109	1.0311086	1.378861231	0.4634773	0.923858	1
RYR2	1.0284987	1.02253769	1.0310825	1.3791331	0.4637617	0.923858	1

MOV10	1.3091268	1.31539155	1.3064113	0.971526647	-0.0416745	0.923887	1
RP1-159A19.4	1.0247713	1.03032828	1.0223626	0.737351802	-0.439575	0.924009	1
PLCD4	1.0248089	1.03046379	1.0223577	0.733911053	-0.4463229	0.924009	1
SCT	1.0334684	1.02743525	1.0360834	1.315221828	0.3953061	0.924025	1
RARRES1	1.1184115	1.12418839	1.1159075	0.933320052	-0.0995562	0.924037	1
ZSWIM5	1.0807772	1.07449561	1.0835	1.120871822	0.1646213	0.924114	1
CNR1	1.0438087	1.03781857	1.0464052	1.22704846	0.2951922	0.924183	1
AK7	1.0437002	1.03775189	1.0462786	1.225861317	0.2937958	0.924183	1
AC067968.3	1.0174747	1.02301631	1.0150726	0.654868186	-0.6107236	0.924333	1
KLRC1	1.0174677	1.02299304	1.0150726	0.65553091	-0.6092643	0.924333	1
RP11-452H21.1	1.0174754	1.02301874	1.0150726	0.654798986	-0.610876	0.924333	1
AL035610.2	1.0174638	1.02298038	1.0150726	0.655891996	-0.6084698	0.924333	1
RP11-285E23.2	1.0174736	1.02301259	1.0150726	0.654974063	-0.6104903	0.924333	1
RP11-888D10.3	1.0174701	1.02300098	1.0150726	0.655304619	-0.6097624	0.924333	1
RP11-731D1.4	1.0174517	1.02294034	1.0150726	0.657036857	-0.6059538	0.924333	1
RP11-1090M7.1	1.017481	1.02303704	1.0150726	0.654278972	-0.6120222	0.924333	1
CTD-2531D15.5	1.0174674	1.02299213	1.0150726	0.655556961	-0.609207	0.924333	1
HEPACAM2	1.0174644	1.02298236	1.0150726	0.6558355	-0.6085941	0.924333	1
OLFM4	1.0174665	1.02298914	1.0150726	0.655642266	-0.6090192	0.924333	1
CTD-2616J11.2	1.0192822	1.02479339	1.0168933	0.681364057	-0.5535023	0.924395	1
AP000350.5	1.0193008	1.02483792	1.0169007	0.680438374	-0.5554636	0.924395	1
CTD-2189E23.1	1.0193388	1.02498364	1.0168921	0.676125379	-0.5646373	0.924395	1
CTD-3157E16.2	1.0816017	1.07563013	1.0841901	1.113181544	0.1546889	0.924491	1
TMEM182	1.0627953	1.05681592	1.0653871	1.150859214	0.2027114	0.924523	1
RAB11FIP2	1.4246937	1.41810091	1.4275513	1.022603243	0.0322465	0.924525	1
RP11-500C11.3	1.045578	1.05117961	1.04315	0.84310851	-0.2462098	0.924561	1
CDHR1	1.0248153	1.01886772	1.0273933	1.451861203	0.5379035	0.924624	1
LINC00551	1.0195478	1.02504327	1.0171657	0.68544266	-0.5448921	0.92466	1
AC108488.4	1.0478821	1.04192496	1.0504643	1.20368141	0.2674536	0.92469	1
AC005609.20	1.0274621	1.02147005	1.0300594	1.400061055	0.4854897	0.92476	1
DLX5	1.0186616	1.01281342	1.0211965	1.65424316	0.7261713	0.9248	1
TMEM52B	1.0186927	1.01281342	1.021241	1.657719179	0.7291996	0.9248	1
ZFAS1	16.458379	16.4846642	16.446985	0.997566685	-0.0035148	0.92485	1
AC005754.8	1.0646708	1.07023135	1.0622605	0.886506146	-0.1737975	0.924884	1
ZNF486	1.0981741	1.09208613	1.1008129	1.094767479	0.1306245	0.924901	1
TSPYL2	1.2306387	1.23665861	1.2280293	0.963536844	-0.0535883	0.924948	1
MPPED2	1.3393517	1.33263832	1.3422616	1.028930246	0.0411452	0.924955	1
KLHL35	1.0898401	1.08374013	1.0924841	1.104418018	0.1432863	0.924967	1
VWDE	1.0376868	1.03184695	1.0402181	1.262856208	0.3366904	0.92503	1
LINC00852	1.0171089	1.0225626	1.0147449	0.65351151	-0.6137154	0.92519	1
SOWAHD	1.0171102	1.02255921	1.0147483	0.653759819	-0.6131674	0.92519	1
LRRC63	1.0171184	1.02258427	1.0147492	0.653074699	-0.6146801	0.92519	1
CTC-428G20.6	1.0171053	1.02256171	1.0147402	0.653330176	-0.6141158	0.92519	1
RP1-92C4.2	1.0171274	1.02262324	1.0147453	0.651774707	-0.6175547	0.92519	1
RP11-130F10.1	1.0744557	1.06846233	1.0770535	1.125488117	0.1705508	0.9252	1
SP2-AS1	1.033441	1.03904358	1.0310125	0.794304989	-0.332235	0.92524	1
ZFP42	1.0334766	1.03893133	1.0311123	0.799157551	-0.3234481	0.92524	1
RP11-539G18.3	1.0617447	1.05576759	1.0643356	1.15363739	0.2061898	0.925252	1
NOTCH4	1.100917	1.10652272	1.0984872	0.924565	-0.1131533	0.925254	1
BFAR	1.937145	1.94413999	1.934113	0.989379721	-0.0154038	0.925278	1
EGR2	1.0443208	1.03842421	1.0468766	1.219976636	0.2868535	0.925287	1

DGAT2	1.0392545	1.033374	1.0418034	1.252573601	0.3248954	0.925359	1
FGF12	1.0392836	1.03329113	1.0418811	1.258025698	0.3311614	0.925359	1
GAS2	1.0392457	1.03338955	1.041784	1.251409935	0.3235545	0.925359	1
MTMR3	1.1581827	1.15208095	1.1608275	1.05751269	0.080675	0.925368	1
ZSCAN23	1.036085	1.04153145	1.0337243	0.812017395	-0.3004175	0.925409	1
TMEM198	1.1557448	1.16144443	1.1532743	0.949393526	-0.0749219	0.925506	1
SH3BP5L	1.1262738	1.12026673	1.1288776	1.071598198	0.0997641	0.92556	1
RP3-453C12.15	1.0240672	1.02961988	1.0216604	0.731278451	-0.4515072	0.925563	1
EIF3CL	1.0240344	1.02948778	1.0216706	0.73490174	-0.4443767	0.925563	1
RP11-80H5.2	1.0240421	1.02952162	1.021667	0.733936801	-0.4462723	0.925563	1
ACVR2B-AS1	1.024027	1.02952081	1.0216457	0.733235457	-0.4476515	0.925563	1
ATP2A3	1.0240589	1.02954739	1.0216799	0.733732014	-0.4466749	0.925563	1
CPEB3	1.0360544	1.0416217	1.0336413	0.808263043	-0.3071032	0.925583	1
RP11-475I24.3	1.0260981	1.03155483	1.0237329	0.752115077	-0.4109747	0.925621	1
ACAD8	1.2126	1.2064823	1.2152517	1.04247035	0.0600064	0.925722	1
DLG3	1.1851933	1.19091514	1.1827131	0.957038372	-0.0633513	0.92576	1
MAU2	1.2624874	1.25604489	1.2652799	1.036067829	0.0511185	0.925765	1
COPG2	1.3360708	1.3293411	1.3389879	1.029291125	0.0416511	0.925826	1
PLPPR3	1.3979146	1.39107107	1.4008809	1.025084635	0.035743	0.925843	1
RP11-703H8.7	1.0179991	1.02341841	1.0156501	0.668280122	-0.5814751	0.925853	1
ZNF324B	1.0542428	1.05975604	1.0518531	0.867745763	-0.2046557	0.92587	1
ZNF438	1.1092718	1.11501466	1.1067826	0.92842582	-0.1071415	0.925895	1
OXLD1	1.9948486	2.0023852	1.9915817	0.989222259	-0.0156334	0.925942	1
CRY2	1.1270729	1.12104217	1.129687	1.071419579	0.0995236	0.925952	1
PITPNB	1.4972588	1.49054976	1.5001669	1.019604893	0.0280102	0.926001	1
ZMYND12	1.0282134	1.03357271	1.0258904	0.771174906	-0.37487	0.926023	1
ARVCF	1.2759663	1.28178994	1.2734421	0.970375584	-0.0433848	0.926025	1
MCM7	1.9037369	1.91110044	1.9005451	0.988414713	-0.0168116	0.926043	1
GORAB	1.1147755	1.12034308	1.1123622	0.933682217	-0.0989965	0.926073	1
HSPA2	1.0282969	1.022481	1.0308179	1.370841712	0.455062	0.92618	1
TMEM200C	1.0807563	1.07487325	1.0833064	1.112632253	0.1539768	0.926236	1
FAM107B	1.2821926	1.27596351	1.2848927	1.032356289	0.045941	0.926275	1
RP13-465B17.5	1.0171058	1.02248553	1.0147739	0.657041785	-0.605943	0.926332	1
KCNG3	1.0170992	1.02248553	1.0147645	0.656623726	-0.6068612	0.926332	1
RP11-351A20.1	1.019323	1.02471961	1.0169839	0.687061119	-0.5414897	0.926334	1
RP11-574K11.29	1.0193175	1.02470116	1.0169839	0.687574329	-0.5404124	0.926334	1
FAM95C	1.0193439	1.0247886	1.0169839	0.685148936	-0.5455105	0.926334	1
AC130469.2	1.019345	1.02479231	1.0169839	0.685046435	-0.5457263	0.926334	1
MKRN3-AS1	1.019346	1.02479547	1.0169839	0.684959048	-0.5459104	0.926334	1
GRID2IP	1.0193352	1.02475992	1.0169839	0.685942581	-0.5438403	0.926334	1
LINC00384	1.0193387	1.02477147	1.0169839	0.685622586	-0.5445135	0.926334	1
RP11-755B10.3	1.0193232	1.02472002	1.0169839	0.687049785	-0.5415135	0.926334	1
AE000658.22	1.0193402	1.02477622	1.0169839	0.685491255	-0.5447898	0.926334	1
C10orf62	1.0193428	1.02478508	1.0169839	0.685246233	-0.5453056	0.926334	1
ALPL	1.0901461	1.09568588	1.0877448	0.917008781	-0.1249925	0.926361	1
LINC00936	1.0998339	1.09380667	1.1024465	1.092101986	0.1271076	0.926435	1
SHISA3	1.0204266	1.01466374	1.0229245	1.563345899	0.644637	0.926464	1
MAK	1.0444613	1.0385199	1.0470367	1.221101012	0.2881825	0.926486	1
RP4-738P15.1	1.0257405	1.03111922	1.023409	0.752237234	-0.4107404	0.926518	1
FTCDNL1	1.0257457	1.03116652	1.023396	0.75067897	-0.413732	0.926518	1
UPK1A-AS1	1.1207074	1.12636632	1.1182545	0.935807389	-0.0957165	0.926541	1

JAG2	1.0570256	1.05118059	1.0595592	1.163706334	0.218727	0.926574	1
RP11-186B7.7	1.0206123	1.02601651	1.0182698	0.70223854	-0.5099669	0.926614	1
RP4-616B8.4	1.0205972	1.02601116	1.0182504	0.701638676	-0.5111998	0.926614	1
MIR2052HG	1.0205936	1.02600288	1.018249	0.701806056	-0.5108557	0.926614	1
RP4-790G17.7	1.0206296	1.02606895	1.018272	0.700908732	-0.5127015	0.926614	1
NPPA	1.0232322	1.01740877	1.0257564	1.479506202	0.5651157	0.926616	1
ELSPBP1	1.0231844	1.01740877	1.0256879	1.475572296	0.5612746	0.926616	1
MYL10	1.0231912	1.01740877	1.0256977	1.476133299	0.561823	0.926616	1
RP11-77H9.2	1.0232342	1.01740877	1.0257593	1.479674097	0.5652795	0.926616	1
ANO7	1.1275523	1.13331012	1.1250565	0.938086975	-0.0922064	0.926653	1
RP11-107N15.1	1.0796313	1.08492102	1.0773385	0.910710579	-0.1349355	0.926682	1
RP11-636O21.2	1.079269	1.08465628	1.0769339	0.908779619	-0.1379976	0.926682	1
SPACA6P-AS	1.0242209	1.02963683	1.0218733	0.738046297	-0.4382168	0.926698	1
RP11-536G4.2	1.0242307	1.02960772	1.0219	0.739671079	-0.4350442	0.926698	1
GDF9	1.0242277	1.029581	1.0219073	0.740586393	-0.4332601	0.926698	1
BVES	1.046511	1.04068318	1.0490372	1.205342403	0.269443	0.92675	1
FGFRL1	1.1407232	1.13477759	1.1433003	1.06323523	0.0884608	0.926758	1
AC006273.4	1.017867	1.0232171	1.015548	0.669680021	-0.5784562	0.926766	1
RP11-738E22.3	1.0178552	1.02324766	1.0155178	0.667500234	-0.5831598	0.926766	1
SLC17A7	1.047645	1.04192929	1.0501225	1.195405846	0.2575005	0.92679	1
TLR4	1.0326683	1.03812385	1.0303035	0.7948711	-0.3312072	0.926802	1
ABC12-49244600F4.4	1.0152625	1.02065365	1.0129257	0.625829959	-0.6761574	0.926835	1
CTD-2376I4.1	1.0152431	1.02058944	1.0129257	0.62778171	-0.6716651	0.926835	1
CTC-482H14.5	1.0152501	1.02061269	1.0129257	0.627073654	-0.6732932	0.926835	1
RP11-511B23.2	1.0152599	1.02064516	1.0129257	0.626087229	-0.6755644	0.926835	1
KB-1836B5.4	1.015256	1.02063232	1.0129257	0.626477056	-0.6746664	0.926835	1
RP11-575L7.4	1.0152603	1.02064644	1.0129257	0.62604841	-0.6756539	0.926835	1
RP11-317G6.1	1.0152636	1.02065733	1.0129257	0.625718376	-0.6764146	0.926835	1
GCM2	1.0152527	1.02062116	1.0129257	0.62681601	-0.6738861	0.926835	1
RP11-124N2.1	1.0152517	1.0206181	1.0129257	0.626909091	-0.6736718	0.926835	1
RP11-65M17.3	1.0152601	1.02064587	1.0129257	0.626065887	-0.6756136	0.926835	1
RP11-703G6.1	1.0152555	1.02063059	1.0129257	0.626529589	-0.6745455	0.926835	1
CEMP1	1.0152555	1.02063059	1.0129257	0.626529589	-0.6745455	0.926835	1
RP11-624L4.2	1.0152518	1.02061822	1.0129257	0.626905233	-0.6736807	0.926835	1
HIST1H3C	1.0152453	1.02059669	1.0129257	0.627560561	-0.6721734	0.926835	1
PATL2	1.0347857	1.04021732	1.0324313	0.806400543	-0.3104315	0.926896	1
TP63	1.0196698	1.01394208	1.0221525	1.588897581	0.6680261	0.926898	1
NPAS1	1.1338419	1.12776651	1.1364754	1.068162409	0.095131	0.9269	1
DUBR	1.1402165	1.14553173	1.1379126	0.94764613	-0.0775797	0.926925	1
DNALI1	1.5674788	1.56066057	1.5704342	1.017432424	0.024933	0.926931	1
HYAL1	1.0245195	1.029846	1.0222107	0.744178079	-0.4262802	0.926934	1
RP11-452H21.4	1.024462	1.02979234	1.0221515	0.743529316	-0.4275385	0.926934	1
RP11-20I23.8	1.024465	1.02986721	1.0221234	0.740725455	-0.4329892	0.926934	1
ARMC8	1.418903	1.42501747	1.4162527	0.97937788	-0.0300625	0.926962	1
RREB1	1.238858	1.24491709	1.2362317	0.964537433	-0.0520909	0.927105	1
SEMA4A	1.0365418	1.04192001	1.0342106	0.81609161	-0.293197	0.927106	1
HIST1H3H	1.0357413	1.02994723	1.0382528	1.27734078	0.3531435	0.927109	1
ZNF84	1.5312571	1.5246268	1.5341311	1.018116328	0.0259024	0.927117	1
MTM1	1.1098158	1.10375412	1.1124433	1.083747384	0.1160285	0.927168	1
ACIN1	1.6537093	1.64641108	1.6568728	1.016184312	0.0231621	0.927172	1
CARD8-AS1	1.0266464	1.03206294	1.0242986	0.757841394	-0.4000322	0.927185	1

AKAP10	1.3439127	1.34988655	1.3413233	0.975525652	-0.0357483	0.92725	1
HARBI1	1.0617275	1.06715378	1.0593755	0.884172346	-0.1776005	0.927264	1
AC137934.1	1.0283473	1.02260394	1.0308369	1.364224971	0.4480816	0.927328	1
C12orf56	1.0283143	1.02259455	1.0307935	1.362873956	0.4466521	0.927328	1
NPIPA3	1.0162401	1.02160166	1.0139161	0.644215724	-0.6343842	0.927338	1
SCN7A	1.0368715	1.04216644	1.0345763	0.819996278	-0.2863107	0.927374	1
RP11-219A15.1	1.020586	1.01486911	1.023064	1.551136429	0.6333256	0.927377	1
RASL12	1.0206341	1.01486911	1.0231329	1.555770246	0.637629	0.927377	1
RP11-79N23.1	1.0205909	1.01486911	1.023071	1.551604677	0.633761	0.927377	1
HIST1H4D	1.0155451	1.02086854	1.0132377	0.634336273	-0.6566803	0.927414	1
CTC-242N15.1	1.0155493	1.02088237	1.0132377	0.633916289	-0.6576358	0.927414	1
RP11-611O2.5	1.0155501	1.02088492	1.0132377	0.633838981	-0.6578117	0.927414	1
RP11-66N11.7	1.0155501	1.02088492	1.0132377	0.633838981	-0.6578117	0.927414	1
RP11-47I22.1	1.015548	1.02087801	1.0132377	0.63404876	-0.6573343	0.927414	1
WASF3-AS1	1.0155563	1.02090532	1.0132377	0.633220273	-0.6592207	0.927414	1
AGXT2	1.0155438	1.02086411	1.0132377	0.634471074	-0.6563737	0.927414	1
RP13-228J13.1	1.0155513	1.02088908	1.0132377	0.633712648	-0.6580993	0.927414	1
CD93	1.0155359	1.02083808	1.0132377	0.635263496	-0.654573	0.927414	1
AC010504.2	1.0155572	1.02090855	1.0132377	0.633122674	-0.659443	0.927414	1
AC124944.3	1.0155497	1.0208838	1.0132377	0.633872822	-0.6577347	0.927414	1
RP11-725P16.2	1.0232497	1.02865316	1.0209076	0.729678793	-0.4546666	0.927421	1
CDH8	1.1731572	1.16703263	1.1758119	1.052560065	0.0739026	0.927506	1
AC005220.3	1.0161369	1.0214486	1.0138345	0.645006706	-0.6326139	0.927601	1
APOBEC4	1.0161373	1.02145004	1.0138345	0.644963482	-0.6327106	0.927601	1
XIRP2	1.0161522	1.02149935	1.0138345	0.64348421	-0.6360233	0.927601	1
RP11-21C4.1	1.0161485	1.02148706	1.0138345	0.643852256	-0.6351984	0.927601	1
LDHAL6A	1.0161422	1.02146619	1.0138345	0.644478265	-0.6337964	0.927601	1
RP4-668E10.4	1.0161344	1.02144035	1.0138345	0.645254982	-0.6320587	0.927601	1
MEG8	1.016147	1.0214821	1.0138345	0.644000729	-0.6348658	0.927601	1
CTC-321K16.1	1.0279864	1.03337129	1.0256523	0.768692438	-0.3795216	0.92763	1
ICMT	1.4513165	1.45841978	1.4482375	0.977788399	-0.0324058	0.92766	1
ANKRD29	1.0257731	1.03107297	1.0234758	0.755504713	-0.4044873	0.927666	1
SLC6A8	1.2793531	1.27298857	1.2821119	1.033419993	0.0474267	0.92781	1
RGS9	1.2792952	1.27306294	1.2819966	1.032716525	0.0464443	0.92781	1
TRIQK	1.5392433	1.54570672	1.5364417	0.983022042	-0.0247043	0.927831	1
DSE	1.2378501	1.23165669	1.2405346	1.038323634	0.0542562	0.927897	1
PNOC	1.0266264	1.02088765	1.0291139	1.393833029	0.4790577	0.927917	1
MAP3K7CL	1.0291934	1.02341718	1.0316972	1.353587652	0.4367883	0.927997	1
RP11-127B20.3	1.1757475	1.18120861	1.1733803	0.956799378	-0.0637116	0.928026	1
ADAMTS9-AS2	1.1253505	1.13094618	1.1229251	0.938745123	-0.0911946	0.928032	1
RP11-318M2.3	1.0197062	1.01400962	1.0221754	1.58286967	0.6625425	0.928041	1
DSCR8	1.0197172	1.01400962	1.0221912	1.583995877	0.6635686	0.928041	1
ANKRD22	1.0197395	1.01400962	1.0222231	1.586275221	0.6656431	0.928041	1
RP11-363J20.1	1.0286809	1.02292371	1.0311764	1.360007851	0.443615	0.928044	1
WDR3	1.1842346	1.18982611	1.1818109	0.957776096	-0.0622397	0.928078	1
RP11-314C16.1	1.0729277	1.06723432	1.0753955	1.121384713	0.1652813	0.928163	1
IGFL2	1.0446659	1.03898216	1.0471296	1.209003642	0.2738186	0.928238	1
ZNF268	1.3345711	1.32842271	1.3372361	1.026835532	0.0382051	0.928241	1
EMID1	1.109688	1.10369481	1.1122858	1.082848467	0.1148314	0.928268	1
MAPK4	1.0549428	1.04918251	1.0574397	1.167888006	0.2239019	0.928269	1
PCSK2	1.0305554	1.02477662	1.0330602	1.334332152	0.4161178	0.928273	1

ZNF75D	1.0851741	1.07915337	1.0877839	1.109035259	0.1493052	0.928276	1
ZNF658	1.078731	1.08409583	1.0764056	0.908554484	-0.1383551	0.928278	1
GOLGA6L7P	1.0160951	1.02136669	1.0138101	0.646338066	-0.6296391	0.928429	1
RP11-188P17.2	1.0161023	1.02140425	1.0138042	0.64492763	-0.6327908	0.928429	1
SZT2-AS1	1.0160865	1.02136263	1.0137995	0.645963388	-0.6304757	0.928429	1
SCIMP	1.0160807	1.02132751	1.0138064	0.647351236	-0.6273794	0.928429	1
FAM47E-STBD1	1.0172741	1.02252394	1.0149985	0.665889455	-0.5866454	0.928499	1
AJ003147.9	1.0172836	1.02255565	1.0149985	0.664953333	-0.588675	0.928499	1
RP11-403I13.7	1.0172838	1.02255621	1.0149985	0.664936908	-0.5887106	0.928499	1
NUP210L	1.0173005	1.02261134	1.0149985	0.66331582	-0.5922322	0.928499	1
SEC62-AS1	1.0173019	1.02261603	1.0149985	0.663178144	-0.5925316	0.928499	1
RP11-10A14.4	1.0172946	1.02259191	1.0149985	0.663886121	-0.5909923	0.928499	1
AC079767.4	1.0172854	1.02256135	1.0149985	0.664785391	-0.5890394	0.928499	1
RP11-31I22.2	1.0172835	1.02255508	1.0149985	0.664970235	-0.5886383	0.928499	1
SMARCA5-AS1	1.0172854	1.02256156	1.0149985	0.664779136	-0.589053	0.928499	1
PHLPP2	1.0815314	1.08691361	1.0791985	0.911232691	-0.1341086	0.928567	1
INPPL1	1.3858548	1.39169397	1.3833239	0.978630969	-0.0311632	0.928618	1
PTCH2	1.0610138	1.06633557	1.058707	0.885001072	-0.1762489	0.928648	1
RP13-631K18.2	1.0222259	1.02749093	1.0199437	0.72546478	-0.4630225	0.928671	1
TAF12	1.912478	1.90562895	1.9154468	1.010840897	0.0155559	0.928739	1
SSUH2	1.0221178	1.016462	1.0245693	1.492483979	0.5777154	0.928756	1
PSMB9	1.0220996	1.01647431	1.0245379	1.489464439	0.5747937	0.928756	1
RP11-573D15.2	1.0299004	1.02419556	1.0323733	1.337983301	0.4200601	0.928866	1
AMIGO1	1.0298741	1.0242009	1.0323331	1.336030145	0.4179526	0.928866	1
ZBTB20-AS2	1.0247943	1.03007714	1.0225045	0.748225077	-0.4184558	0.928903	1
RP11-309M23.1	1.0247861	1.03001758	1.0225185	0.750177532	-0.414696	0.928903	1
CACNG6	1.0247502	1.03001109	1.0224698	0.748716009	-0.4175095	0.928903	1
NRROS	1.0247777	1.03009632	1.0224724	0.746681681	-0.4214348	0.928903	1
TCOF1	1.3973954	1.40336281	1.3948088	0.978793241	-0.030924	0.928932	1
DCAF4	1.081066	1.07532768	1.0835533	1.109198267	0.1495173	0.928939	1
POC1B	1.2426748	1.24834466	1.2402172	0.967273605	-0.0480041	0.929005	1
IL36G	1.0166998	1.02197012	1.0144154	0.656135047	-0.6079353	0.929019	1
DNAJC25-GNG10	1.0166858	1.02191996	1.014417	0.657709143	-0.6044784	0.929019	1
AC005534.8	1.0166798	1.02190273	1.0144159	0.658178716	-0.6034487	0.929019	1
RP11-547D24.3	1.0167202	1.02197119	1.0144441	0.657409653	-0.6051355	0.929019	1
RP11-46A10.5	1.0166875	1.0219238	1.0144178	0.657630551	-0.6046508	0.929019	1
KCNA5	1.0218668	1.0162132	1.0243174	1.499853202	0.5848213	0.929021	1
VOPP1	1.6963102	1.68962946	1.6992059	1.013886426	0.0198961	0.929119	1
LBX2	1.0980638	1.10338121	1.0957589	0.926269645	-0.1104959	0.929124	1
INO80	1.2188301	1.22483732	1.2162262	0.961700579	-0.0563403	0.92914	1
TTL	1.2922544	1.2859958	1.2949672	1.03136883	0.0445604	0.929152	1
GUCY2D	1.0386287	1.03297029	1.0410814	1.246012696	0.3173188	0.929227	1
SLC46A1	1.1063637	1.11191856	1.1039559	0.928853421	-0.1064771	0.929257	1
DNMT3B	1.1478471	1.15297427	1.1456247	0.951955816	-0.0710335	0.929259	1
RP11-961A15.3	1.0379567	1.04323044	1.0356707	0.825129357	-0.2773078	0.929271	1
AC005037.3	1.016965	1.02220078	1.0146955	0.66193788	-0.5952323	0.929284	1
C10orf113	1.0169645	1.0221781	1.0147047	0.663027623	-0.5928591	0.929284	1
RP11-326I11.4	1.0169797	1.02225973	1.014691	0.659983291	-0.5994986	0.929284	1
RP11-830F9.6	1.0169549	1.02218262	1.0146889	0.662178873	-0.5947071	0.929284	1
RP11-122L9.1	1.0169675	1.02222466	1.0146887	0.660920669	-0.597451	0.929284	1
LRGUK	1.0169614	1.02217399	1.014702	0.663029698	-0.5928546	0.929284	1

NCKAP5L	1.2562589	1.2619675	1.2537844	0.968762992	-0.0457843	0.929306	1
CDK2	1.3578941	1.35161472	1.3606159	1.025599559	0.0364675	0.929319	1
BMP3	1.022453	1.01683833	1.0248867	1.477977088	0.5636239	0.929375	1
GPAT3	1.0224554	1.01683833	1.0248901	1.478180632	0.5638226	0.929375	1
SMCO3	1.0224345	1.01683833	1.0248602	1.476407152	0.5620906	0.929375	1
PPP1R36	1.0224375	1.01683833	1.0248645	1.476663535	0.5623411	0.929375	1
RP11-977G19.11	1.0737897	1.07925302	1.0714215	0.901183791	-0.1501067	0.92939	1
MTMR7	1.0519887	1.05724816	1.049709	0.868306751	-0.2037233	0.929413	1
ZNF628	1.061838	1.06719024	1.059518	0.885813346	-0.1749254	0.929431	1
C19orf66	1.5067232	1.51290602	1.5040432	0.982720357	-0.0251472	0.9296	1
OSMR	1.0376556	1.03203926	1.04009	1.251276619	0.3234008	0.929647	1
DHTKD1	1.2784294	1.27254834	1.2809785	1.03093104	0.0439478	0.929657	1
PSD	1.1401262	1.13430656	1.1426488	1.062113527	0.086938	0.929672	1
LINGO1	1.0679535	1.06218524	1.0704538	1.132966392	0.1801051	0.929759	1
CTD-2126E3.4	1.0259025	1.03113573	1.0236341	0.759067965	-0.397699	0.929805	1
RP11-1020A11.2	1.0258869	1.03107938	1.0236362	0.760509735	-0.3949614	0.929805	1
POLB	1.2839123	1.27799668	1.2864765	1.03050328	0.0433491	0.929828	1
AC006449.2	1.0145382	1.01975418	1.0122773	0.621504207	-0.6861639	0.929832	1
RP11-218E20.5	1.0145278	1.01971963	1.0122773	0.622593101	-0.6836385	0.929832	1
RP11-196H14.3	1.0145341	1.01974059	1.0122773	0.621931785	-0.6851717	0.929832	1
FETUB	1.0145325	1.01973539	1.0122773	0.622095752	-0.6847914	0.929832	1
LATS2-AS1	1.0300011	1.02441951	1.0324205	1.327649069	0.4088739	0.929964	1
U91319.1	1.0334688	1.03864108	1.0312268	0.808124002	-0.3073514	0.930001	1
RP11-120D5.1	1.0328119	1.03805858	1.0305377	0.802385811	-0.317632	0.930004	1
CENPI	1.0468989	1.04122577	1.0493579	1.197257911	0.259734	0.930081	1
FLJ16779	1.2358387	1.24149882	1.2333853	0.966403273	-0.0493028	0.930103	1
EFL1	1.1299082	1.12417676	1.1323926	1.066162385	0.0924272	0.930119	1
RP11-114O18.1	1.0239854	1.02919538	1.021727	0.744194733	-0.4262479	0.930145	1
CTD-2537I9.12	1.0239726	1.02915239	1.0217274	0.745305021	-0.4240971	0.930145	1
STK32A	1.0240009	1.0291887	1.0217523	0.745229232	-0.4242438	0.930145	1
SUCLA2-AS1	1.0239693	1.02918307	1.0217093	0.743902051	-0.4268154	0.930145	1
C19orf60	3.1007174	3.11115685	3.0961923	0.992911704	-0.0102627	0.930149	1
AC068831.6	1.0214589	1.02664047	1.019213	0.721194839	-0.471539	0.930224	1
LINC00907	1.0214118	1.02656464	1.0191782	0.721946371	-0.4700364	0.930224	1
PCAT1	1.0370585	1.03141918	1.0395029	1.257285004	0.3303117	0.930238	1
MPPED1	1.0235206	1.0286796	1.0212844	0.742143826	-0.4302293	0.930264	1
RP5-1102E8.3	1.0235018	1.02874125	1.0212307	0.738685194	-0.4369684	0.930264	1
PSMG3-AS1	1.0664155	1.06071969	1.0688844	1.134464825	0.1820119	0.930275	1
RP11-386G11.8	1.0213811	1.01574718	1.0238232	1.512854006	0.5972728	0.930276	1
SLC9A7	1.2206024	1.21514523	1.2229679	1.036359782	0.0515249	0.930277	1
GALNT8	1.0431143	1.04840244	1.0408222	0.843391169	-0.2457262	0.930296	1
PSD2	1.0467456	1.04110851	1.049189	1.196564113	0.2588977	0.930319	1
AC007392.3	1.0253227	1.03048783	1.0230838	0.757147992	-0.4013528	0.930364	1
MYH7	1.0253236	1.03055238	1.0230572	0.754677616	-0.4060676	0.930364	1
RP11-245J9.4	1.0219035	1.02707343	1.0196626	0.726268859	-0.4614244	0.930406	1
RP11-230C9.2	1.021938	1.02713007	1.0196875	0.725669651	-0.4626152	0.930406	1
DNAJB5	1.1489401	1.14320183	1.1514274	1.057440169	0.080576	0.930439	1
ALG8	1.5891004	1.59523802	1.58644	0.985219257	-0.0214833	0.93046	1
AP006216.10	1.0207691	1.01516282	1.0231992	1.53000349	0.6135349	0.930463	1
BICDL2	1.0207247	1.01516282	1.0231355	1.525805692	0.6095713	0.930463	1
RP11-410K21.2	1.0207317	1.01516282	1.0231456	1.526473152	0.6102022	0.930463	1

DMPK	1.1637127	1.16916583	1.161349	0.953791898	-0.0682536	0.930467	1
RP5-837J1.4	1.0154199	1.02055822	1.0131926	0.641719891	-0.6399844	0.930494	1
C20orf144	1.0154223	1.02056612	1.0131926	0.641473531	-0.6405384	0.930494	1
HHIPL1	1.0154269	1.02058152	1.0131926	0.640993666	-0.641618	0.930494	1
CTD-2540B15.6	1.0154274	1.02058303	1.0131926	0.640946625	-0.6417239	0.930494	1
RP11-676J12.7	1.0154486	1.02065338	1.0131926	0.638763378	-0.6466465	0.930494	1
RP11-384F7.1	1.0154347	1.02060723	1.0131926	0.640193898	-0.6434192	0.930494	1
ANKRD66	1.0154226	1.02056732	1.0131926	0.641435969	-0.6406228	0.930494	1
GPR132	1.0154247	1.02057407	1.0131926	0.641225681	-0.6410959	0.930494	1
RP5-1039K5.18	1.0154538	1.02067032	1.0131926	0.638239935	-0.6478292	0.930494	1
SCGB3A2	1.0154191	1.02055557	1.0131926	0.641802652	-0.6397983	0.930494	1
TMPRSS11D	1.0154494	1.0206558	1.0131926	0.638688426	-0.6468158	0.930494	1
RP4-621N11.2	1.0154217	1.02056411	1.0131926	0.641536119	-0.6403976	0.930494	1
RP11-103J8.2	1.0154382	1.02061901	1.0131926	0.639828121	-0.6442437	0.930494	1
BCAT1	2.8023878	2.7906284	2.807485	1.009413764	0.0135177	0.9305	1
CTD-2349P21.9	1.0465285	1.05171575	1.04428	0.856219071	-0.2239481	0.930582	1
RP5-821D11.7	1.0465672	1.05176227	1.0443153	0.856131879	-0.224095	0.930582	1
ZNF512	1.5090215	1.50242914	1.511879	1.018808265	0.0268826	0.930588	1
ARHGEF39	1.1520126	1.14631579	1.1544819	1.055811495	0.0783523	0.9306	1
DSTYK	1.3790638	1.38506623	1.376462	0.977655079	-0.0326025	0.930626	1
TIMM10B	1.4035781	1.4095886	1.4009728	0.978964669	-0.0306713	0.930628	1
HPD	1.0256155	1.03076289	1.0233844	0.76014937	-0.3956452	0.930631	1
SALL4	1.0481664	1.0426616	1.0505524	1.18496342	0.2448425	0.930675	1
NRAP	1.0706279	1.07577271	1.0683979	0.902671317	-0.1477273	0.930691	1
GABRB1	1.0213909	1.01586389	1.0237866	1.499421194	0.5844057	0.930706	1
SNTG2	1.0404701	1.034859	1.0429022	1.230735678	0.299521	0.930727	1
SLC23A2	1.1002916	1.10566038	1.0979645	0.927164222	-0.1091032	0.930856	1
AC010547.9	1.0145475	1.01972023	1.0123053	0.623995946	-0.6803914	0.930973	1
CACNA1C-AS1	1.0145328	1.01967151	1.0123053	0.625541192	-0.6768232	0.930973	1
GS1-304P7.3	1.0145253	1.01964674	1.0123053	0.626330018	-0.6750051	0.930973	1
RP11-12M5.3	1.0145369	1.01968506	1.0123053	0.625110704	-0.6778164	0.930973	1
AC009060.2	1.0145369	1.01968506	1.0123053	0.625110704	-0.6778164	0.930973	1
DENND6A-AS1	1.0145269	1.01965208	1.0123053	0.626159801	-0.6753972	0.930973	1
CTC-806A22.1	1.0145188	1.0196252	1.0123053	0.627017435	-0.6734225	0.930973	1
SLC35G6	1.0145299	1.01966195	1.0123053	0.625845515	-0.6761215	0.930973	1
RP11-83M16.5	1.0145323	1.01967011	1.0123053	0.625585835	-0.6767202	0.930973	1
RP3-467K16.7	1.0145285	1.01965743	1.0123053	0.625989517	-0.6757896	0.930973	1
SULT1B1	1.0145432	1.01970602	1.0123053	0.624445853	-0.6793516	0.930973	1
RP11-147L13.12	1.0758589	1.07004525	1.0783789	1.118975575	0.1621785	0.931017	1
TMEM229B	1.0204739	1.01490147	1.0228893	1.536040964	0.6192167	0.931044	1
RP11-1069G10.2	1.0203989	1.01490147	1.0227818	1.528827151	0.6124253	0.931044	1
FAM160A1	1.0395228	1.04465339	1.037299	0.835299849	-0.2596339	0.931089	1
NFKBIB	1.1819761	1.17610432	1.1845213	1.047795225	0.0673568	0.931132	1
ZFAND2B	1.4829294	1.48901248	1.4802926	0.982168476	-0.0259576	0.93114	1
KLHDC4	1.0987267	1.1040037	1.0964393	0.927268364	-0.1089412	0.93114	1
RASL10A	1.02314	1.02830553	1.020901	0.73840517	-0.4375154	0.931143	1
PANO1	1.0731792	1.06755219	1.0756183	1.119405509	0.1627328	0.931156	1
SEC24B-AS1	1.1211837	1.11556963	1.1236172	1.069634003	0.0971172	0.931195	1
SMARCA5	2.69942	2.70759593	2.6958761	0.993136668	-0.0099358	0.931252	1
RP11-387H17.6	1.0180179	1.02315934	1.0157893	0.681768325	-0.5526465	0.931255	1
CTD-2008A1.3	1.0180041	1.02311359	1.0157893	0.68311798	-0.5497933	0.931255	1

RP3-380B4.1	1.0180075	1.02312511	1.0157893	0.682777538	-0.5505125	0.931255	1
CTD-3234P18.6	1.0180264	1.0231875	1.0157893	0.680940449	-0.5543995	0.931255	1
RSPH10B2	1.018015	1.02314977	1.0157893	0.682050256	-0.55205	0.931255	1
AC016722.3	1.0180151	1.02315018	1.0157893	0.682038017	-0.5520759	0.931255	1
CXorf36	1.0180085	1.02312821	1.0157893	0.682686007	-0.5507059	0.931255	1
RP11-624G17.3	1.0180148	1.02314919	1.0157893	0.682067226	-0.5520142	0.931255	1
LINC00534	1.0180148	1.02314919	1.0157893	0.682067226	-0.5520142	0.931255	1
LINC00403	1.0180209	1.02316928	1.0157893	0.681475947	-0.5532654	0.931255	1
ADAM2	1.0180037	1.02311232	1.0157893	0.683155426	-0.5497142	0.931255	1
TAS2R5	1.0180236	1.02317824	1.0157893	0.681212598	-0.553823	0.931255	1
TCEANC	1.0505725	1.04499698	1.0529893	1.177618407	0.2358721	0.931263	1
AC004540.5	1.2755169	1.28089687	1.2731849	0.972545273	-0.0401627	0.931271	1
RABL6	1.6922169	1.68556448	1.6951004	1.013909609	0.019929	0.931278	1
RP11-932O9.7	1.0230091	1.0174976	1.0253981	1.451516292	0.5375608	0.931279	1
YTHDF1	1.2450707	1.2392937	1.2475748	1.034606537	0.0490822	0.931283	1
DAB1	1.2080894	1.21345246	1.2057648	0.963984152	-0.0529187	0.931283	1
AC007228.9	1.0420423	1.0472076	1.0398034	0.843155692	-0.246129	0.931315	1
VPS45	1.2378497	1.23194417	1.2404095	1.036497399	0.0517165	0.931322	1
MAGIX	1.0851976	1.07965022	1.0876021	1.099835506	0.1372878	0.931324	1
LTB4R2	1.0216503	1.02681868	1.01941	0.723748305	-0.46644	0.931358	1
GDF6	1.0326484	1.03795874	1.0303467	0.799464193	-0.3228947	0.931366	1
ANKRD20A3	1.0327183	1.03783242	1.0305016	0.806229348	-0.3107378	0.931366	1
POU6F2-AS2	1.0152698	1.02037329	1.0130577	0.640923917	-0.641775	0.931406	1
FOXB1	1.0152833	1.02041783	1.0130577	0.639525836	-0.6449255	0.931406	1
DNMBP-AS1	1.0152804	1.02040835	1.0130577	0.639822877	-0.6442555	0.931406	1
TERT	1.0152628	1.02035008	1.0130577	0.641654943	-0.6401304	0.931406	1
NMUR2	1.0152819	1.02041331	1.0130577	0.639667431	-0.6446061	0.931406	1
ACOT12	1.0152698	1.020373	1.0130577	0.640932927	-0.6417547	0.931406	1
MIXL1	1.0152765	1.02039515	1.0130577	0.640236807	-0.6433225	0.931406	1
DAB1-AS1	1.0152756	1.02039227	1.0130577	0.640327218	-0.6431188	0.931406	1
AC108051.3	1.0152726	1.02038246	1.0130577	0.640635482	-0.6424244	0.931406	1
RP11-417L19.4	1.0258996	1.02025087	1.0283481	1.399845976	0.4852681	0.931431	1
CSMD2	1.0258301	1.02027518	1.028238	1.392735767	0.4779216	0.931431	1
KBTBD11	1.1376706	1.13208161	1.1400932	1.060656147	0.084957	0.931434	1
SWSAP1	1.1772183	1.17146365	1.1797127	1.048109887	0.06779	0.931446	1
FAM178B	1.0465979	1.04105592	1.0490001	1.193495835	0.2551935	0.931466	1
TMC4	1.046444	1.04085665	1.0488659	1.196032065	0.2582561	0.931466	1
KCNJ14	1.0420451	1.04718499	1.0398172	0.843853922	-0.2449348	0.931471	1
MRVI1-AS1	1.0321521	1.03727047	1.0299335	0.803141866	-0.3162732	0.931523	1
EPYC	1.0245278	1.01899597	1.0269256	1.417438863	0.5032865	0.931546	1
C6orf223	1.0245314	1.01899597	1.0269307	1.417706042	0.5035584	0.931546	1
AGBL1	1.021916	1.02712798	1.0196569	0.724597872	-0.4647475	0.931594	1
RP11-1C8.7	1.0218627	1.0269948	1.0196382	0.727480786	-0.459019	0.931594	1
AP001469.7	1.0218803	1.02697377	1.0196725	0.729318949	-0.4553782	0.931594	1
HBB	1.0218712	1.02697885	1.0196573	0.728617858	-0.4567657	0.931594	1
RNF212B	1.0218743	1.02700412	1.0196507	0.727691968	-0.4586002	0.931594	1
COL6A6	1.0219004	1.02702755	1.019678	0.728070683	-0.4578496	0.931594	1
FBLL1	1.0218935	1.02704563	1.0196603	0.726931532	-0.4601086	0.931594	1
AKR1E2	1.0294017	1.02388418	1.0317933	1.33114623	0.4126691	0.931617	1
BRWD1-AS2	1.0673334	1.0725241	1.0650834	0.897404379	-0.1561699	0.931643	1
PLBD1-AS1	1.022387	1.01677525	1.0248195	1.479528956	0.5651379	0.931714	1

LINC01229	1.0222743	1.0167867	1.024653	1.468601269	0.5544428	0.931714	1
ITPRIP	1.1735057	1.16787072	1.1759482	1.048117511	0.0678005	0.931752	1
AC137932.5	1.024103	1.02925399	1.0218703	0.747601019	-0.4196596	0.931809	1
CKMT1A	1.0341836	1.03929869	1.0319665	0.813423449	-0.2979215	0.931965	1
FAM71F2	1.0136676	1.01875717	1.0114615	0.611044892	-0.7106497	0.931977	1
LINC01419	1.0136597	1.01873096	1.0114615	0.611900012	-0.7086322	0.931977	1
RP11-1038A11.1	1.0136582	1.01872619	1.0114615	0.612055882	-0.7082647	0.931977	1
AC006946.15	1.0136582	1.01872619	1.0114615	0.612055882	-0.7082647	0.931977	1
ACER1	1.013657	1.01872224	1.0114615	0.612184713	-0.7079611	0.931977	1
RP11-303E16.7	1.0136668	1.01875461	1.0114615	0.6111282	-0.710453	0.931977	1
RP11-1099M24.7	1.0136584	1.01872676	1.0114615	0.612037167	-0.7083088	0.931977	1
RP4-697K14.15	1.0136494	1.01869706	1.0114615	0.613009385	-0.7060189	0.931977	1
RP11-77E14.2	1.0136566	1.01872094	1.0114615	0.612227494	-0.7078603	0.931977	1
SRPK3	1.0136533	1.01870983	1.0114615	0.612590942	-0.7070041	0.931977	1
C6	1.0136603	1.01873298	1.0114615	0.611833774	-0.7087883	0.931977	1
LINC00896	1.0136661	1.01875238	1.0114615	0.61120104	-0.7102811	0.931977	1
C1QTNF4	1.3927671	1.39832532	1.3903579	0.979997752	-0.0291497	0.93198	1
NR6A1	1.162966	1.16806871	1.1607543	0.956479391	-0.0641942	0.931993	1
RABGAP1	1.4894285	1.48313952	1.4921545	1.018659186	0.0266714	0.932019	1
CHRNB2	1.0206536	1.02575572	1.018442	0.716035992	-0.481896	0.932081	1
RASGEF1C	1.0206705	1.02575074	1.0184684	0.717198354	-0.4795559	0.932081	1
RP11-93K22.6	1.0206637	1.02574934	1.0184592	0.716882334	-0.4801918	0.932081	1
RP11-1212A22.4	1.0206483	1.02573587	1.018443	0.716627906	-0.4807039	0.932081	1
CRKL	1.2652537	1.2708877	1.2628116	0.970186532	-0.0436659	0.932124	1
FOXO6	1.0398807	1.0343529	1.0422768	1.230661184	0.2994336	0.932176	1
RP11-1000B6.8	1.039954	1.03445186	1.0423389	1.228928541	0.297401	0.932176	1
RP11-33H15.1	1.0232034	1.01773008	1.0255759	1.442515149	0.5285865	0.932194	1
HSPB2	1.0231454	1.02823632	1.0209387	0.741553565	-0.4313772	0.93229	1
CTD-2342J14.6	1.0231152	1.02814881	1.0209333	0.743665178	-0.4272749	0.93229	1
GPR61	1.0231464	1.02820838	1.0209523	0.742766962	-0.4290184	0.93229	1
PTPRH	1.0578061	1.06296165	1.0555715	0.882623976	-0.1801292	0.932339	1
RP11-473M20.16	1.0432979	1.04840461	1.0410844	0.848770528	-0.2365535	0.932381	1
AC023590.1	1.0710418	1.07621959	1.0687975	0.902622384	-0.1478055	0.932383	1
BRD9	1.4653785	1.45902599	1.468132	1.019837758	0.0283397	0.932495	1
NUDT19	1.1839151	1.17790366	1.1865208	1.048437352	0.0682407	0.932557	1
DOCK1	1.2637134	1.25755926	1.2663809	1.034250995	0.0485863	0.932562	1
CTD-2600O9.1	1.0266822	1.03176416	1.0244794	0.770662058	-0.3758297	0.932573	1
UBE3D	1.2668925	1.2608946	1.2694923	1.032954699	0.046777	0.932629	1
PSMD6-AS2	1.023869	1.02892166	1.0216789	0.749571765	-0.4158615	0.932725	1
CRHR1	1.0238866	1.02896278	1.0216863	0.748764997	-0.4174151	0.932725	1
STRADA	1.0507612	1.05581024	1.0485726	0.870317015	-0.2003871	0.932737	1
ERBB3	1.0423333	1.03683399	1.044717	1.214013889	0.2797849	0.932746	1
CPLX1	1.0424178	1.03690617	1.0448068	1.214073157	0.2798554	0.932746	1
RP11-700J17.2	1.0304886	1.03552652	1.0283049	0.796726041	-0.3278444	0.932821	1
OTUD3	1.1670739	1.16135232	1.169554	1.050830829	0.0715304	0.932907	1
ANXA13	1.0135111	1.01850938	1.0113445	0.612906426	-0.7062613	0.933068	1
AC114765.1	1.0135147	1.01852145	1.0113445	0.612507045	-0.7072017	0.933068	1
HPDL	1.0135126	1.01851442	1.0113445	0.612739405	-0.7066545	0.933068	1
RP3-428L16.1	1.0135136	1.01851767	1.0113445	0.612632138	-0.706907	0.933068	1
RP11-676J12.6	1.0135355	1.01859025	1.0113445	0.610240176	-0.7125509	0.933068	1
RP11-856F16.2	1.0135175	1.01853074	1.0113445	0.612199917	-0.7079252	0.933068	1

GPC6-AS1	1.0135117	1.01851135	1.0113445	0.612841281	-0.7064146	0.933068	1
DAW1	1.0135074	1.01849712	1.0113445	0.613312519	-0.7053057	0.933068	1
MID1IP1-AS1	1.0135109	1.01850891	1.0113445	0.612921845	-0.706225	0.933068	1
RP11-653J6.1	1.0135162	1.01852622	1.0113445	0.612349223	-0.7075734	0.933068	1
RP11-15E1.5	1.0135097	1.01850492	1.0113445	0.613053981	-0.705914	0.933068	1
EXOC5	1.6842897	1.67791786	1.6870516	1.013473283	0.0193081	0.93308	1
ABL2	1.2848692	1.2791018	1.2873691	1.029620949	0.0421133	0.93308	1
EPRS	1.8352104	1.82834663	1.8381855	1.011877777	0.017035	0.93313	1
TUBB3	1.1419719	1.14750318	1.1395743	0.946246129	-0.0797126	0.933158	1
MTURN	1.105014	1.11026546	1.1027377	0.931730855	-0.1020148	0.933238	1
LINC01317	1.022252	1.02728996	1.0200682	0.735371242	-0.4434553	0.933298	1
CTD-2249K22.1	1.0223012	1.02737257	1.0201029	0.734418588	-0.4453255	0.933298	1
TUSC5	1.0196063	1.02464663	1.0174216	0.706855807	-0.5005121	0.933348	1
RP3-483K16.4	1.0195909	1.02461933	1.0174113	0.70722048	-0.499768	0.933348	1
RP11-464F9.21	1.0195838	1.02459152	1.0174132	0.708096509	-0.4979821	0.933348	1
FITM1	1.0195832	1.02458814	1.0174138	0.70821849	-0.4977336	0.933348	1
RP1-278C19.8	1.0216152	1.02663282	1.0194402	0.729934804	-0.4541605	0.933356	1
CEL	1.0215899	1.02654112	1.0194437	0.732588645	-0.4489248	0.933356	1
RIMS4	1.0215884	1.02663418	1.0194013	0.72843731	-0.4571233	0.933356	1
TSGA13	1.02162	1.02664651	1.0194413	0.729600023	-0.4548223	0.933356	1
TLDC1	1.2950876	1.2891133	1.2976772	1.029621363	0.0421139	0.933422	1
PDZD7	1.0842637	1.07868238	1.086683	1.101682638	0.1397087	0.933462	1
LRRTM4	1.1175787	1.11196681	1.1200111	1.071845739	0.1000973	0.933483	1
JAKMIP1	1.0221609	1.02710725	1.0200169	0.73843276	-0.4374615	0.933543	1
CECR6	1.0317899	1.03684394	1.0295992	0.803367218	-0.3158685	0.933594	1
CES1	1.0141154	1.01909287	1.0119579	0.626303586	-0.675066	0.933658	1
C19orf38	1.014108	1.0190683	1.0119579	0.627110569	-0.6732083	0.933658	1
CTB-55O6.10	1.0141125	1.01908329	1.0119579	0.626617972	-0.6743419	0.933658	1
C16orf54	1.0141269	1.01913089	1.0119579	0.625058926	-0.6779359	0.933658	1
CTD-2550O8.5	1.0141295	1.01913941	1.0119579	0.624780507	-0.6785787	0.933658	1
AP001172.2	1.0141179	1.01910101	1.0119579	0.626036586	-0.6756811	0.933658	1
RP11-91P24.6	1.0141183	1.01910226	1.0119579	0.625995791	-0.6757751	0.933658	1
MIR7515HG	1.0141183	1.01910226	1.0119579	0.625995791	-0.6757751	0.933658	1
RP11-264B17.2	1.0141167	1.01909706	1.0119579	0.626166086	-0.6753827	0.933658	1
RP11-153K11.3	1.014106	1.0190616	1.0119579	0.627330888	-0.6727015	0.933658	1
CD109	1.056918	1.05152822	1.0592542	1.149936728	0.2015545	0.933707	1
MPRIP-AS1	1.058877	1.06378558	1.0567494	0.889690203	-0.168625	0.933802	1
ARHGAP20	1.0353086	1.04031522	1.0331385	0.821984052	-0.2828177	0.933878	1
KLHL10	1.0143938	1.01938286	1.0122313	0.631037198	-0.664203	0.933923	1
RP5-1177M21.1	1.0143938	1.01938286	1.0122313	0.631037198	-0.664203	0.933923	1
SERINC4	1.014367	1.0192942	1.0122313	0.63393673	-0.6575892	0.933923	1
RP11-1008C21.1	1.0143864	1.01935824	1.0122313	0.631839771	-0.6623693	0.933923	1
RP11-264L1.4	1.0143885	1.01936524	1.0122313	0.631611133	-0.6628915	0.933923	1
GS1-259H13.13	1.0143817	1.01934269	1.0122313	0.632347668	-0.6612101	0.933923	1
ART4	1.0143832	1.01934784	1.0122313	0.632179339	-0.6615942	0.933923	1
NCKAP1L	1.0143893	1.01936788	1.0122313	0.631525231	-0.6630877	0.933923	1
RP11-728E14.3	1.0143883	1.01936449	1.0122313	0.631635698	-0.6628354	0.933923	1
ALDH1L1-AS1	1.0143811	1.01934078	1.0122313	0.632410198	-0.6610675	0.933923	1
RP11-304F15.6	1.0143765	1.01932565	1.0122313	0.632905231	-0.6599386	0.933923	1
CYP27B1	1.0143765	1.01932565	1.0122313	0.632905231	-0.6599386	0.933923	1
FOXP4	1.5025548	1.49620887	1.5053055	1.018332302	0.0262084	0.933941	1

PARP16	1.2101106	1.20398765	1.2127646	1.043026853	0.0607763	0.933944	1
FLVCR1	1.3284168	1.33417108	1.3259226	0.975316636	-0.0360574	0.933949	1
SAP130	1.1930756	1.18735117	1.1955569	1.043798538	0.0618433	0.933972	1
LA16c-312E8.2	1.0299027	1.0348992	1.027737	0.794774939	-0.3313817	0.934044	1
ATG9A	1.2724473	1.2780156	1.2700336	0.971289534	-0.0420267	0.934074	1
LINC01250	1.121521	1.11615146	1.1238484	1.066266243	0.0925677	0.934306	1
CAMKK2	1.1991798	1.20427847	1.1969697	0.964221761	-0.0525631	0.934312	1
NEU4	1.0302122	1.02463249	1.0326307	1.324700952	0.4056667	0.934313	1
CPEB1-AS1	1.0221299	1.02704349	1.02	0.739550409	-0.4352796	0.934394	1
LYPD3	1.1352821	1.14038674	1.1330695	0.9478779	-0.0772269	0.9344	1
KCNK15	1.0412419	1.04623371	1.0390782	0.84523164	-0.2425813	0.934402	1
GTF2IRD1	1.2707672	1.2762334	1.2683978	0.971634079	-0.041515	0.934404	1
TGFBR1	1.4066836	1.40093146	1.4091769	1.020565722	0.0293691	0.934417	1
CLIC2	1.0233073	1.02825106	1.0211645	0.749156565	-0.4166608	0.934445	1
RP11-253M7.4	1.023357	1.02828999	1.0212188	0.750047616	-0.4149459	0.934445	1
AC004878.2	1.0233297	1.02830623	1.0211726	0.747985297	-0.4189182	0.934445	1
CCDC93	1.2876223	1.28177825	1.2901555	1.029729913	0.042266	0.934529	1
NLRP14	1.0282397	1.03318789	1.0260949	0.786276653	-0.3468911	0.934545	1
AP000254.8	1.031893	1.02656225	1.0342036	1.287678887	0.3647729	0.934562	1
HMCN1	1.0429424	1.03751023	1.045297	1.207590101	0.2721308	0.934571	1
GAREM2	1.0781631	1.07268364	1.0805382	1.108065434	0.1480431	0.934571	1
CFAP206	1.0517461	1.04634844	1.0540858	1.166939168	0.2227294	0.934749	1
ETV2	1.1336013	1.13885528	1.131324	0.945761333	-0.0804519	0.934788	1
PCDH8	1.0308153	1.03576223	1.028671	0.801712351	-0.3188434	0.934816	1
RP11-206L10.4	1.0213777	1.02633915	1.0192271	0.72998313	-0.454065	0.93482	1
RP11-350G8.5	1.0213814	1.02632065	1.0192405	0.731004802	-0.4520472	0.93482	1
CAPN9	1.0213583	1.02625595	1.0192355	0.732612991	-0.4488768	0.93482	1
RP11-265N6.2	1.0213711	1.02629978	1.0192347	0.731362227	-0.451342	0.93482	1
RP11-129K12.1	1.0213725	1.02629075	1.0192407	0.731843234	-0.4503934	0.93482	1
MKRN2OS	1.0213705	1.02631519	1.0192271	0.730647743	-0.4527521	0.93482	1
RP11-7F17.3	1.0213602	1.02627201	1.0192312	0.732003144	-0.4500783	0.93482	1
RP11-4B16.3	1.0213687	1.026317	1.0192238	0.730471201	-0.4531007	0.93482	1
CCNY	1.3210802	1.31535755	1.3235607	1.026012107	0.0370478	0.934887	1
RP3-382I10.7	1.0188584	1.02379265	1.0167196	0.702719811	-0.5089785	0.9349	1
IZUMO2	1.0188501	1.02377414	1.0167157	0.703104746	-0.5081885	0.9349	1
RP11-506O24.1	1.0188572	1.02380201	1.0167138	0.702202346	-0.5100413	0.9349	1
MED4-AS1	1.0188483	1.02377826	1.0167113	0.70279935	-0.5088152	0.9349	1
TIE1	1.0188629	1.02378069	1.0167312	0.703563043	-0.5072484	0.9349	1
RP11-529E10.7	1.0188481	1.02378388	1.0167087	0.702521604	-0.5093855	0.9349	1
CTD-2105E13.16	1.0188559	1.02378388	1.0167198	0.702990696	-0.5084225	0.9349	1
RP11-22C11.2	1.0188472	1.02377413	1.0167115	0.702929206	-0.5085487	0.9349	1
ARHGEF15	1.0188643	1.02379652	1.0167264	0.702894229	-0.5086205	0.9349	1
AC004637.1	1.0188627	1.02381634	1.0167155	0.701850746	-0.5107638	0.9349	1
RP1-267L14.6	1.0209102	1.02583507	1.0187756	0.726746592	-0.4604757	0.934921	1
RP11-186N15.3	1.0209432	1.02586489	1.0188099	0.727235171	-0.4595061	0.934921	1
RP13-131K19.6	1.0209303	1.02586645	1.0187907	0.726450412	-0.4610638	0.934921	1
GTSE1-AS1	1.0209139	1.02586016	1.0187699	0.725822799	-0.4623107	0.934921	1
RP11-820I16.1	1.0227617	1.02772094	1.0206121	0.74355647	-0.4274858	0.934986	1
KCNN3	1.0477435	1.05257986	1.0456472	0.868149679	-0.2039843	0.935003	1
ZC3H4	1.3201844	1.31419704	1.3227797	1.027316144	0.0388802	0.935045	1
AC010524.2	1.0193621	1.02433976	1.0172045	0.70684879	-0.5005265	0.935081	1

IGFL4	1.0193315	1.02427037	1.0171907	0.708299219	-0.4975691	0.935081	1
BPIFB1	1.0193295	1.02426427	1.0171905	0.708468672	-0.497224	0.935081	1
CTB-113P19.1	1.0193352	1.02426328	1.0171991	0.70885165	-0.4964444	0.935081	1
ERMN	1.0193267	1.02424558	1.0171946	0.70918622	-0.4957636	0.935081	1
CTC-250I14.6	1.0193127	1.0241959	1.017196	0.710698527	-0.4926904	0.935081	1
RP1-286D6.5	1.0193293	1.02425063	1.0171961	0.709099652	-0.4959397	0.935081	1
AMER1	1.0439689	1.04883661	1.041859	0.857123642	-0.2224248	0.935172	1
ARMC5	1.1124812	1.10694102	1.1148826	1.074261327	0.103345	0.935204	1
SAMD9L	1.0230168	1.02791276	1.0208946	0.748566741	-0.4177971	0.935252	1
RP11-413H22.3	1.0229813	1.02786571	1.0208642	0.748740306	-0.4174627	0.935252	1
TMEM267	1.1957023	1.19007309	1.1981423	1.042453211	0.0599826	0.935282	1
RP11-395D3.1	1.0233005	1.01801438	1.0255918	1.420629005	0.5065298	0.935285	1
RP11-190C22.8	1.0233128	1.01801944	1.0256072	1.421085836	0.5069937	0.935285	1
AC007405.4	1.0233901	1.01801276	1.0257209	1.427928251	0.5139235	0.935285	1
RP11-673E11.2	1.023358	1.01801305	1.0256748	1.42534283	0.511309	0.935285	1
PPAT	1.3480598	1.3537835	1.3455789	0.976808877	-0.0338518	0.935297	1
GAREM1	1.0385054	1.0434761	1.0363509	0.836111948	-0.258232	0.935302	1
KIAA1161	1.0341952	1.02898751	1.0364524	1.257522306	0.330584	0.935325	1
NAF1	1.1675316	1.1725832	1.1653419	0.958041618	-0.0618398	0.935335	1
RIMS2	1.1677152	1.17266211	1.1655709	0.958929966	-0.0605026	0.935335	1
BTBD7	1.5420242	1.53631989	1.5444968	1.015246249	0.0218297	0.935366	1
MAN1C1	1.1779523	1.17241066	1.1803543	1.046073971	0.0649849	0.935483	1
RP11-226E21.4	1.0239863	1.01873612	1.026262	1.401676266	0.4871532	0.93551	1
GPD1L	1.0578239	1.0525374	1.0601154	1.144239382	0.1943889	0.935595	1
SIRPA	1.0757138	1.08058882	1.0736006	0.91328573	-0.1308618	0.935681	1
RP13-638C3.4	1.0307477	1.03562913	1.0286318	0.803607175	-0.3154377	0.935708	1
AGBL5-AS1	1.0310639	1.02574286	1.0333703	1.296292468	0.3743913	0.935715	1
LINC01376	1.0319826	1.03682426	1.029884	0.811530599	-0.3012826	0.935722	1
SEZ6	1.0320132	1.0368904	1.0298991	0.810484425	-0.3031436	0.935722	1
REM2	1.0974688	1.09203517	1.099824	1.084629151	0.1172019	0.935743	1
NAA15	1.6013679	1.5953862	1.6039607	1.014401576	0.0206289	0.935779	1
RP11-7K24.3	1.0205431	1.02540428	1.0184359	0.725702293	-0.4625503	0.935782	1
RP11-371A19.2	1.0205801	1.0254764	1.0184578	0.724506461	-0.4649295	0.935782	1
FSBP	1.0229982	1.01775345	1.0252716	1.423475191	0.5094173	0.935867	1
ABCA4	1.0230347	1.01775323	1.0253239	1.426441372	0.5124205	0.935867	1
SMPD5	1.0230178	1.01775058	1.025301	1.425359421	0.5113258	0.935867	1
AC010524.4	1.0838131	1.08878334	1.0816588	0.919753244	-0.1206812	0.935913	1
MEGF6	1.1232185	1.12833836	1.1209992	0.942814018	-0.0849549	0.935935	1
CLN5	1.4212665	1.41581718	1.4236286	1.018785601	0.0268505	0.93594	1
FLVCR2	1.0300458	1.0348987	1.0279423	0.800669723	-0.3207208	0.936024	1
RP11-128P10.1	1.0190466	1.02392983	1.0169299	0.707481284	-0.4992361	0.936032	1
AC079610.2	1.0190459	1.02394408	1.0169227	0.706759562	-0.5007086	0.936032	1
TBX22	1.0190314	1.0238852	1.0169274	0.708699855	-0.4967533	0.936032	1
RP11-166B2.5	1.0190323	1.02389157	1.016926	0.708451467	-0.4972591	0.936032	1
RP11-665J16.1	1.019045	1.02394855	1.0169196	0.706497405	-0.5012438	0.936032	1
BCL2A1	1.0190196	1.02385855	1.0169221	0.709269192	-0.4955948	0.936032	1
RP11-85G18.6	1.0190374	1.02389467	1.016932	0.708611713	-0.4969328	0.936032	1
RP11-103H7.3	1.0190395	1.02390781	1.0169292	0.708104924	-0.4979649	0.936032	1
ZNRF3-AS1	1.0513886	1.05627158	1.049272	0.875610471	-0.1916389	0.93604	1
KIAA0040	1.0256452	1.02036036	1.0279359	1.372074626	0.456359	0.936084	1
RP11-219D15.3	1.025601	1.02037669	1.0278656	1.367521181	0.4515632	0.936084	1

RP11-348N5.9	1.0275695	1.03237899	1.0254848	0.787078672	-0.3454202	0.936104	1
PNPO	1.0672817	1.07245527	1.0650392	0.897646885	-0.1557801	0.93614	1
L3MBTL4	1.0296036	1.03455675	1.0274567	0.794539178	-0.3318097	0.936163	1
CTD-2020K17.4	1.0646284	1.06954384	1.0624978	0.898682672	-0.1541163	0.936221	1
ARHGAP9	1.0283897	1.02309979	1.0306826	1.328265309	0.4095433	0.936236	1
LINC01280	1.0285218	1.0231481	1.030851	1.332765295	0.4144227	0.936236	1
NAV3	1.0528932	1.04763019	1.0551744	1.158392171	0.2121238	0.936265	1
RP11-439C15.5	1.0192758	1.02412051	1.0171759	0.712085183	-0.4898783	0.936268	1
RTP1	1.0193003	1.02416836	1.0171902	0.711270034	-0.4915307	0.936268	1
RP1-167A14.2	1.0192732	1.02410429	1.0171791	0.712700264	-0.4886326	0.936268	1
RP1-28O10.1	1.0192849	1.02415363	1.0171745	0.711051209	-0.4919746	0.936268	1
SMIM17	1.0192778	1.02413545	1.0171722	0.711491595	-0.4910814	0.936268	1
ZNF32-AS3	1.0192652	1.02408189	1.0171774	0.713290372	-0.4874386	0.936268	1
LINC00884	1.019291	1.02414704	1.0171862	0.71173045	-0.4905971	0.936268	1
RP11-85O21.5	1.0192724	1.0240991	1.0171803	0.712901714	-0.4882249	0.936268	1
C12orf50	1.0192666	1.02406986	1.0171846	0.713947015	-0.4861111	0.936268	1
CTC-336P14.1	1.0192833	1.02412919	1.0171829	0.71211892	-0.4898099	0.936268	1
LRRC56	1.0576103	1.06254832	1.0554698	0.886831616	-0.1732679	0.936272	1
B4GALNT3	1.0313303	1.03617929	1.0292284	0.807877592	-0.3077914	0.936302	1
ZNF394	1.1792263	1.18462872	1.1768846	0.958055608	-0.0618187	0.936357	1
GOLGA7B	1.0271447	1.0218357	1.0294459	1.348519003	0.4313759	0.93637	1
HOXA3	1.0271126	1.02183822	1.0293988	1.346210605	0.4289041	0.93637	1
CTC-270D5.1	1.0224921	1.0171592	1.0248037	1.445505874	0.5315745	0.936393	1
SFTA2	1.0224054	1.0171592	1.0246794	1.438261081	0.5243256	0.936393	1
ADAMTS5	1.0223798	1.0171592	1.0246427	1.436121992	0.5221783	0.936393	1
DRD5	1.0223989	1.0171592	1.02467	1.437713878	0.5237766	0.936393	1
RP11-557C18.4	1.022367	1.0171592	1.0246243	1.435048797	0.5210998	0.936393	1
AC007383.4	1.021494	1.02630972	1.0194067	0.737623062	-0.4390443	0.936447	1
RP1-92O14.3	1.0248841	1.01967286	1.027143	1.379717182	0.4643726	0.93652	1
SNX22	1.0670749	1.061771	1.0693739	1.123081373	0.1674625	0.93653	1
RP11-277P12.20	1.0471479	1.05204256	1.0450262	0.865181174	-0.2089258	0.936549	1
CNNM1	1.0497782	1.05462569	1.0476771	0.872795659	-0.1962842	0.936609	1
MAP3K2	1.6048756	1.61045862	1.6024555	0.986890072	-0.0190387	0.936681	1
ZNF574	1.131002	1.13592413	1.1288685	0.948091724	-0.0769015	0.936701	1
AP000230.1	1.0411199	1.0357618	1.0434424	1.214772377	0.280686	0.936731	1
CDC42BPG	1.0410473	1.03567876	1.0433743	1.215690248	0.2817757	0.936731	1
DCAF1	1.1304174	1.12497385	1.1327769	1.06243773	0.0873783	0.936732	1
CD1D	1.0180641	1.02287087	1.0159806	0.69873294	-0.5171869	0.936755	1
RP3-406P24.4	1.0180829	1.02293407	1.0159802	0.696787388	-0.5212096	0.936755	1
RP11-69C13.1	1.0180754	1.02291022	1.0159798	0.69749443	-0.5197464	0.936755	1
OR52H1	1.0180649	1.02287552	1.0159797	0.698548659	-0.5175675	0.936755	1
RP11-234K24.6	1.0180687	1.02288666	1.0159803	0.698235908	-0.5182135	0.936755	1
CFAP58-AS1	1.0180812	1.02292863	1.0159801	0.696950304	-0.5208723	0.936755	1
SCN4A	1.0180753	1.02291891	1.0159759	0.697059848	-0.5206456	0.936755	1
TTC23L	1.0404043	1.03521343	1.0426544	1.211309808	0.2765679	0.936914	1
AC004854.4	1.0227708	1.02759498	1.0206797	0.749402371	-0.4161876	0.936927	1
IGSF6	1.022794	1.02764171	1.0206928	0.748606263	-0.417721	0.936927	1
RP11-252I13.2	1.0228336	1.02762854	1.0207552	0.751223263	-0.4126864	0.936927	1
RP11-112L6.3	1.0205542	1.02534034	1.0184796	0.729254449	-0.4555058	0.936927	1
CAND2	1.0551433	1.06003922	1.0530211	0.883107805	-0.1793385	0.936932	1
RP11-177G23.2	1.0425545	1.03726735	1.0448463	1.203367526	0.2670773	0.936964	1

IL34	1.0257995	1.02058599	1.0280594	1.36303352	0.446821	0.937001	1
CTD-3105H18.14	1.0257549	1.02057441	1.0280003	1.360930148	0.444593	0.937001	1
LINC01239	1.0257616	1.02059468	1.0280012	1.359634706	0.4432191	0.937001	1
RP13-463N16.6	1.0233475	1.01815945	1.0255963	1.409530349	0.4952145	0.937116	1
CHST8	1.0234173	1.01815945	1.0256963	1.415038061	0.5008409	0.937116	1
BNC1	1.0233772	1.01815945	1.0256389	1.411877261	0.4976147	0.937116	1
AF127936.7	1.0233349	1.01815945	1.0255783	1.408536659	0.4941971	0.937116	1
CTD-2325P2.4	1.0687479	1.07361994	1.0666361	0.905136072	-0.1437934	0.937182	1
RAB2B	1.2540661	1.24857594	1.2564459	1.031660073	0.0449677	0.937187	1
LINC01251	1.0240581	1.02887337	1.0219708	0.76093813	-0.3941489	0.93721	1
ETAA1	1.3950457	1.40059411	1.3926408	0.980146111	-0.0289313	0.937227	1
RP11-486A14.2	1.0276657	1.03245537	1.0255896	0.788455691	-0.3428984	0.937242	1
HOXB-AS1	1.0213224	1.02617191	1.0192203	0.734387109	-0.4453874	0.937362	1
CHRNA10	1.021321	1.02605346	1.0192696	0.739619257	-0.4351453	0.937362	1
SPDYE1	1.0213332	1.02608179	1.0192748	0.739015727	-0.436323	0.937362	1
TMED8	1.2983649	1.30350813	1.2961355	0.975708774	-0.0354775	0.937374	1
SCAMP4	1.5012396	1.49563115	1.5036706	1.016220568	0.0232136	0.937387	1
C5orf56	1.0777943	1.08266646	1.0756824	0.915515258	-0.1273442	0.937396	1
PPP4R3B	1.6905204	1.69629121	1.688019	0.988119571	-0.0172425	0.937407	1
CTC-203F4.2	1.018697	1.02347581	1.0166256	0.708201955	-0.4977673	0.93743	1
FSTL5	1.0279794	1.03286537	1.0258616	0.786894987	-0.345757	0.937479	1
RP11-532F12.5	1.0971347	1.09162283	1.0995239	1.086234528	0.1193356	0.937495	1
LRRC69	1.2629698	1.26787437	1.2608439	0.973754541	-0.0383699	0.937577	1
ZNF816	1.0763779	1.08113719	1.0743149	0.915916775	-0.1267116	0.937578	1
RAPGEF3	1.031836	1.02664187	1.0340874	1.279467694	0.3555437	0.93758	1
AIM1L	1.0317707	1.02656626	1.0340266	1.280819363	0.357067	0.93758	1
BRINP1	1.0318046	1.02663126	1.0340469	1.278457583	0.3544043	0.93758	1
KLLN	1.0399721	1.04470754	1.0379195	0.848167883	-0.2375782	0.937606	1
MXD4	1.5882102	1.5939278	1.5857319	0.986200564	-0.020047	0.937638	1
FBRSL1	1.1521341	1.14673497	1.1544743	1.05274377	0.0741543	0.937719	1
POLR3B	1.0612834	1.06623548	1.0591369	0.892828572	-0.1635449	0.937726	1
IGF1	1.0682688	1.06290637	1.0705932	1.122194333	0.1663225	0.937781	1
OR2A1-AS1	1.0681651	1.06295839	1.0704219	1.118547512	0.1616265	0.937781	1
LYSMD3	1.1877499	1.19269356	1.1856071	0.963224225	-0.0540564	0.937889	1
SPANXB1	1.0267873	1.03165221	1.0246786	0.779680102	-0.3590458	0.937967	1
PALM	1.5593945	1.55323178	1.5620658	1.015968033	0.022855	0.937971	1
RP11-483I13.5	1.0524923	1.04725726	1.0547615	1.158795852	0.2126264	0.937976	1
SNX31	1.0190111	1.02376797	1.0169492	0.71311294	-0.4877975	0.938011	1
TUSC1	1.2258606	1.22069944	1.2280978	1.033522139	0.0475693	0.938058	1
LANCL3	1.0236432	1.01850742	1.0258693	1.397779793	0.4831371	0.938069	1
XKR7	1.0236428	1.01850742	1.0258687	1.397748861	0.4831052	0.938069	1
RNF111	1.2805793	1.27493824	1.2830244	1.029410824	0.0418189	0.938075	1
MTRF1	1.1144369	1.10920207	1.116706	1.068716117	0.0958787	0.938114	1
RP11-875O11.2	1.0196064	1.02435375	1.0175486	0.720570808	-0.4727879	0.938198	1
PARD6G-AS1	1.0196127	1.02442073	1.0175286	0.717774284	-0.4783979	0.938198	1
RP11-713D19.1	1.0195843	1.02431693	1.0175329	0.721015664	-0.4718975	0.938198	1
ASPHD2	1.0671302	1.07184142	1.065088	0.905996136	-0.1424232	0.938205	1
ADIPOR2	1.4054572	1.39984441	1.4078901	1.020122135	0.0287419	0.938248	1
TRIM29	1.0231669	1.01804205	1.0253884	1.40717697	0.4928038	0.938251	1
RP5-1158E12.3	1.0231927	1.01804205	1.0254252	1.409221753	0.4948986	0.938251	1
RP11-670E13.6	1.0643557	1.05910425	1.066632	1.127363598	0.1729529	0.938262	1

FLJ31104	1.0479982	1.05272767	1.0459483	0.871425921	-0.1985501	0.9383	1
ADAM11	1.0377764	1.04253314	1.0357145	0.839687404	-0.2520757	0.938336	1
ZMIZ1-AS1	1.0272814	1.02216225	1.0295003	1.331105267	0.4126247	0.938347	1
LRFN1	1.0815981	1.07639182	1.0838548	1.097693566	0.1344754	0.938357	1
LAT	1.0473632	1.05220024	1.0452666	0.867171656	-0.2056105	0.938399	1
TEX30	1.3764872	1.37119733	1.3787802	1.02042812	0.0291746	0.9384	1
CAPN15	1.1300269	1.13505246	1.1278485	0.946658292	-0.0790843	0.938404	1
KRTDAP	1.0350848	1.02995303	1.0373091	1.245588029	0.316827	0.93842	1
AQP5	1.0889274	1.08368475	1.0911998	1.089801719	0.1240657	0.938432	1
GHRH	1.036388	1.04121606	1.0342952	0.832083523	-0.2651997	0.93844	1
ZNF880	1.0847079	1.08956212	1.0826038	0.922307394	-0.1166804	0.938464	1
KDM8	1.0411897	1.03600706	1.0434361	1.206322966	0.2706162	0.938482	1
ABHD11-AS1	1.0327597	1.03751768	1.0306973	0.818208422	-0.2894597	0.938499	1
RP11-355B11.2	1.0735873	1.0783382	1.0715281	0.913067499	-0.1312066	0.938551	1
LA16c-390H2.4	1.0299022	1.03465212	1.0278433	0.803508058	-0.3156156	0.938652	1
SYT3	1.0273473	1.03210404	1.0252855	0.787610834	-0.3444451	0.938682	1
NOL6	1.0781004	1.07292397	1.0803442	1.101752838	0.1398006	0.938827	1
TNS2	1.0485617	1.05328133	1.046516	0.873025758	-0.1959039	0.938899	1
GDF5	1.176741	1.17166776	1.17894	1.042362149	0.0598566	0.938978	1
RP11-932O9.9	1.0276425	1.02254694	1.0298511	1.323955516	0.4048546	0.939006	1
PROC	1.0276436	1.02256707	1.0298441	1.322461297	0.4032255	0.939006	1
CDH15	1.0277919	1.0225355	1.0300703	1.334351398	0.4161386	0.939006	1
CTNNA3	1.0276853	1.02256771	1.0299035	1.325057788	0.4060553	0.939006	1
LINC00864	1.0388924	1.04360574	1.0368494	0.845058751	-0.2428764	0.939007	1
USP40	1.1814822	1.17632519	1.1837175	1.041924318	0.0592505	0.939023	1
RP11-105C19.1	1.0195632	1.02431236	1.0175046	0.719987822	-0.4739556	0.93903	1
AC008074.3	1.0195509	1.02420967	1.0175316	0.724156134	-0.4656273	0.93903	1
SLC44A4	1.0195475	1.02422173	1.0175214	0.723376095	-0.4671822	0.93903	1
RP11-497H17.1	1.0195528	1.0242744	1.0175062	0.721179195	-0.4715703	0.93903	1
ZNF623	1.1140561	1.10878475	1.116341	1.069460144	0.0968827	0.939084	1
DCAF11	1.5596025	1.55389667	1.5620757	1.014766286	0.0211475	0.93909	1
MIR378D2	1.0207468	1.0254396	1.0187127	0.735572743	-0.4430601	0.939099	1
RP11-247I13.11	1.020733	1.02543401	1.0186953	0.735049375	-0.4440869	0.939099	1
IGLL1	1.0207381	1.02540041	1.0187172	0.736885273	-0.4404881	0.939099	1
RP11-1069G10.1	1.0207799	1.0255173	1.0187265	0.733875166	-0.4463934	0.939099	1
RBM15B	1.6121148	1.61754632	1.6097605	0.98739233	-0.0183047	0.939135	1
C15orf59	1.0186744	1.01362911	1.0208613	1.530645006	0.6141397	0.939142	1
RP11-739B23.1	1.018737	1.01362911	1.0209511	1.537227684	0.6203309	0.939142	1
CCL20	1.0186588	1.01362911	1.020839	1.52900567	0.6125938	0.939142	1
MUC3A	1.0187087	1.01362911	1.0209105	1.534251669	0.6175352	0.939142	1
LUZP2	1.0351566	1.0299864	1.0373977	1.247155257	0.3186411	0.939166	1
IQSEC3	1.0264171	1.02132017	1.0286264	1.342690956	0.4251273	0.939206	1
BMS1P14	1.0265017	1.02136652	1.0287275	1.344509881	0.4270804	0.939206	1
ANKHD1-EIF4EBP3	1.0256509	1.03031631	1.0236287	0.779405763	-0.3595535	0.939219	1
CNDP1	1.0256533	1.03039658	1.0235974	0.776316647	-0.3652829	0.939219	1
FRY-AS1	1.0256708	1.03041124	1.0236161	0.776557305	-0.3648357	0.939219	1
AC023115.2	1.0257008	1.03043876	1.0236472	0.776876818	-0.3642422	0.939219	1
RP5-1085F17.3	1.1518898	1.14652824	1.1542138	1.052451145	0.0737533	0.939221	1
BUB1	1.1802284	1.18569676	1.1778582	0.957788175	-0.0622215	0.939285	1
RP11-319E16.2	1.0345598	1.02946678	1.0367673	1.247755615	0.3193354	0.939355	1
RBMS3-AS3	1.0345092	1.02945437	1.0367003	1.24600565	0.3173106	0.939355	1

SS18L2	1.8862069	1.8802354	1.8887953	1.009724574	0.0139618	0.939384	1
DNASE1L3	1.0184243	1.01338003	1.0206108	1.540414471	0.6233186	0.939408	1
ANXA9	1.0184172	1.01338003	1.0206006	1.539651202	0.6226036	0.939408	1
PLCL1	1.018411	1.01338003	1.0205917	1.538987431	0.6219814	0.939408	1
ST8SIA6	1.0183943	1.01338003	1.0205678	1.537198902	0.6203039	0.939408	1
RYR1	1.0184486	1.01338003	1.0206456	1.543014709	0.6257518	0.939408	1
FAAH2	1.0482953	1.04326295	1.0504765	1.166738236	0.2224809	0.93948	1
ATXN7L3	1.2328062	1.23774121	1.230667	0.970244289	-0.0435801	0.939523	1
S1PR1	1.0972437	1.09215755	1.0994484	1.079112452	0.1098452	0.939548	1
CTB-127C13.1	1.018338	1.02302653	1.0163057	0.708125053	-0.4979239	0.939593	1
MYLK-AS2	1.0183258	1.02299411	1.0163023	0.708978728	-0.4961858	0.939593	1
TAS2R10	1.0183304	1.02299393	1.016309	0.709274374	-0.4955843	0.939593	1
TRIM62	1.0647967	1.06962212	1.0627051	0.900649406	-0.1509625	0.939604	1
RP11-1263C18.2	1.0202109	1.02494954	1.0181569	0.727743219	-0.4584986	0.939622	1
RP11-963H4.3	1.0201409	1.02482497	1.0181106	0.729531829	-0.4549572	0.939622	1
ABCC3	1.0201333	1.02479223	1.0181139	0.730627598	-0.4527918	0.939622	1
RP3-388M5.9	1.0201349	1.0247854	1.0181191	0.731038636	-0.4519804	0.939622	1
KATNBL1	2.2188851	2.2252096	2.2161437	0.992600527	-0.0107149	0.939732	1
CEP83-AS1	1.0717792	1.06659895	1.0740246	1.111498228	0.1525056	0.939762	1
CTD-2168K21.2	1.0239453	1.01886772	1.0261463	1.385766747	0.4706844	0.939806	1
RP11-22A3.1	1.0239046	1.01886772	1.0260878	1.382669126	0.467456	0.939806	1
TTN	1.0239856	1.01886772	1.0262039	1.388823107	0.4738629	0.939806	1
LRFN3	1.1444535	1.14931313	1.142347	0.95334575	-0.0689286	0.939857	1
RP11-799B12.4	1.0204046	1.02500149	1.0184121	0.736438534	-0.441363	0.939887	1
CACNA1F	1.0265336	1.02141756	1.0287511	1.342408156	0.4248234	0.93991	1
GALNT9	1.026543	1.0214624	1.0287453	1.339331184	0.4215127	0.93991	1
DCUN1D2-AS	1.0360568	1.04067898	1.0340532	0.837120707	-0.2564924	0.939924	1
TRNAU1AP	1.6065312	1.6120451	1.6041411	0.987085934	-0.0187524	0.939955	1
MCOLN2	1.0178437	1.01281342	1.0200241	1.562748359	0.6440855	0.939998	1
ATP6V1G3	1.0178203	1.01281342	1.0199905	1.560125272	0.6416619	0.939998	1
AMPD3	1.1024993	1.10727113	1.100431	0.936235118	-0.0950572	0.940106	1
ZBED4	1.2528391	1.24747529	1.255164	1.031068711	0.0441405	0.940129	1
MANSC1	1.2202847	1.22540594	1.2180649	0.967431762	-0.0477682	0.94015	1
L2HGDH	1.1518662	1.15658832	1.1498194	0.956772264	-0.0637525	0.940209	1
NFIL3	1.0785479	1.08333237	1.0764741	0.917699565	-0.1239062	0.940234	1
INO80E	1.4937204	1.48818736	1.4961187	1.016246527	0.0232504	0.940237	1
AMN	1.0435769	1.03838897	1.0458257	1.193719839	0.2554643	0.940326	1
DYDC2	1.0434739	1.03849374	1.0456326	1.185455125	0.2454411	0.940326	1
CLEC4A	1.0281406	1.03275369	1.026141	0.79810881	-0.3253426	0.940327	1
BHLHE22	1.0605366	1.05530599	1.0628039	1.135570686	0.1834175	0.940353	1
ARHGAP4	1.180148	1.17495681	1.1823982	1.042532787	0.0600928	0.940421	1
NAMPT	1.7928194	1.7870925	1.7953018	1.010429915	0.0149693	0.940425	1
MAP3K19	1.0179734	1.02259239	1.0159713	0.706933657	-0.5003533	0.940435	1
VAX1	1.0179695	1.02256413	1.015978	0.708115377	-0.4979437	0.940435	1
COX10	1.154359	1.1490413	1.156664	1.051144734	0.0719613	0.940463	1
NBPF20	1.2041492	1.20916884	1.2019734	0.965599942	-0.0505025	0.940497	1
CTD-2054N24.2	1.0363304	1.04092044	1.0343408	0.839209338	-0.2528974	0.94051	1
ZNF85	1.1556659	1.16039299	1.1536169	0.957753507	-0.0622737	0.940596	1
RP11-494H4.3	1.0256604	1.02058877	1.0278588	1.353106616	0.4362755	0.94068	1
AC061992.2	1.0274282	1.03198829	1.0254516	0.795652617	-0.3297894	0.940697	1
AC009120.10	1.0368687	1.04153865	1.0348445	0.838846229	-0.2535217	0.940699	1

ZFP37	1.1019408	1.10663755	1.0999049	0.936864215	-0.0940881	0.940747	1
RP11-626G11.5	1.0248964	1.02951819	1.022893	0.775555497	-0.3666981	0.940777	1
RP11-351I24.1	1.0249744	1.02952389	1.0230024	0.779113158	-0.3600952	0.940777	1
AC005944.2	1.0249269	1.02957896	1.0229104	0.774551507	-0.3685669	0.940777	1
TGFBR2	1.0635818	1.0584415	1.0658099	1.126080987	0.1713106	0.9408	1
RALY-AS1	1.0521345	1.04717904	1.0542824	1.150562791	0.2023397	0.940845	1
ICAM5	1.0281891	1.02321421	1.0303455	1.307194659	0.386474	0.940879	1
RP11-521O16.1	1.025368	1.0299162	1.0233965	0.782068173	-0.3546337	0.940958	1
RP11-775C24.5	1.0253669	1.0299411	1.0233842	0.78100819	-0.3565904	0.940958	1
RP11-802D6.1	1.0254007	1.02998919	1.0234118	0.780675566	-0.357205	0.940958	1
RP11-775D22.2	1.0254255	1.0301273	1.0233875	0.77629081	-0.3653309	0.940958	1
MATN3	1.0309071	1.02599636	1.0330358	1.270784163	0.345719	0.941032	1
PEX11G	1.0990919	1.0939593	1.1013166	1.078303219	0.1087629	0.941069	1
SAMMSON	1.0179746	1.01301898	1.0201227	1.545642575	0.6282067	0.941091	1
PRDM13	1.0188956	1.02344355	1.0169242	0.721914463	-0.4701002	0.9411	1
RP11-78O7.2	1.0188706	1.02342827	1.0168951	0.721141835	-0.4716451	0.9411	1
RP11-61E11.2	1.0188755	1.02345031	1.0168926	0.720355761	-0.4732185	0.9411	1
RP11-382A20.7	1.0188898	1.0234488	1.0169137	0.721303916	-0.4713208	0.9411	1
C1orf234	1.0188733	1.02346345	1.0168837	0.719573765	-0.4747855	0.9411	1
ADCY3	1.0833665	1.08811938	1.0813063	0.922683696	-0.1160919	0.941181	1
RP11-250B2.6	1.0249899	1.02000075	1.0271525	1.357573198	0.44103	0.941226	1
GVQW1	1.0345677	1.02959408	1.0367235	1.240907923	0.3113961	0.941255	1
DHX9	2.1508695	2.1451879	2.1533323	1.007111817	0.0102239	0.94141	1
PLPPR5	1.0818664	1.08643623	1.0798856	0.924214141	-0.1137009	0.941427	1
TCHP	1.2054956	1.20046202	1.2076774	1.035993943	0.0510156	0.941463	1
ETV6	1.163334	1.15823281	1.1655452	1.046212655	0.0651761	0.941463	1
SNURF	1.0559349	1.06052568	1.053945	0.891273777	-0.1660594	0.941521	1
RP11-449J21.5	1.0430385	1.04759622	1.041063	0.862735966	-0.213009	0.941547	1
ADAMTSL2	1.0179621	1.02249816	1.0159959	0.710988568	-0.4921017	0.941579	1
CTD-2033A16.1	1.0180347	1.02262554	1.0160447	0.709142688	-0.4958522	0.941579	1
GLIPR1L1	1.0179661	1.02251376	1.0159949	0.710449618	-0.4931958	0.941579	1
SLC25A30	1.0548798	1.04989595	1.0570401	1.143180236	0.1930529	0.941589	1
MRLN	1.0201718	1.02468046	1.0182176	0.738136786	-0.4380399	0.941597	1
RP13-890H12.2	1.0201857	1.02473508	1.0182137	0.736350328	-0.4415358	0.941597	1
AC092684.1	1.0201713	1.02468513	1.0182148	0.737884515	-0.4385331	0.941597	1
RP11-286E11.2	1.0202012	1.02473454	1.0182362	0.737277662	-0.43972	0.941597	1
RP11-239A17.1	1.0201953	1.02476303	1.0182154	0.735590172	-0.4430259	0.941597	1
SLFNL1	1.0201868	1.02472687	1.0182189	0.736805758	-0.4406438	0.941597	1
KB-1410C5.3	1.0368409	1.04139562	1.0348666	0.84227825	-0.2476312	0.941612	1
KDF1	1.019608	1.01466374	1.0217511	1.483324455	0.5688342	0.941664	1
TMEM196	1.0195744	1.01466374	1.0217029	1.480038243	0.5656345	0.941664	1
RP11-42O15.3	1.0196264	1.01466374	1.0217776	1.485130765	0.57059	0.941664	1
ZSWIM2	1.0195788	1.01466374	1.0217092	1.480469757	0.566055	0.941664	1
AC005614.3	1.0195808	1.01466374	1.0217121	1.480668375	0.5662486	0.941664	1
RP11-33O4.1	1.0266055	1.03115631	1.024633	0.790624862	-0.3389348	0.941701	1
PPFIBP2	1.0450572	1.04008882	1.0472108	1.177655466	0.2359175	0.941742	1
TEP1	1.0450967	1.04025253	1.0471964	1.172506996	0.2295965	0.941742	1
RP11-108M9.6	1.0449393	1.04941696	1.0429985	0.87011538	-0.2007214	0.941743	1
C11orf80	1.250243	1.25507222	1.2481498	0.972860739	-0.0396948	0.941755	1
MAG	1.0456328	1.04056325	1.0478302	1.179150793	0.2377482	0.941791	1
KIF26B	1.0794803	1.07452302	1.081629	1.095353149	0.1313961	0.941813	1

MMP1	1.0223427	1.01740877	1.0244814	1.406266451	0.49187	0.941816	1
LA16c-444G7.2	1.0223327	1.01740877	1.0244671	1.405445565	0.4910276	0.941816	1
RP11-680F8.3	1.0223115	1.01740877	1.0244367	1.403699733	0.4892344	0.941816	1
PIK3C2G	1.0223595	1.01740877	1.0245054	1.407649683	0.4932883	0.941816	1
ZBTB20-AS4	1.0214802	1.02600576	1.0195186	0.75054902	-0.4139818	0.941862	1
AC005592.2	1.057351	1.06195833	1.0553539	0.893405076	-0.1626136	0.941862	1
ACY1	1.0828282	1.07770963	1.0850469	1.094418736	0.1301648	0.941898	1
HIST1H3A	1.0250815	1.02957382	1.0231343	0.782256838	-0.3542857	0.941913	1
RP11-378J18.9	1.0250925	1.02959	1.0231431	0.782125237	-0.3545285	0.941913	1
GS1-279B7.2	1.0187292	1.02325423	1.0167678	0.721066335	-0.4717961	0.942014	1
TTC36	1.0187172	1.02320736	1.0167709	0.722653152	-0.4686247	0.942014	1
AC016738.3	1.0187069	1.0232212	1.0167501	0.721328178	-0.4712723	0.942014	1
HIST1H2AL	1.0187023	1.02321623	1.0167457	0.721294126	-0.4713404	0.942014	1
NDUFAF6	1.2970552	1.30227887	1.2947909	0.975228388	-0.036188	0.942059	1
FBXW7	1.4607215	1.45517475	1.4631257	1.017468005	0.0249834	0.942097	1
CLEC7A	1.0188333	1.01394208	1.0209534	1.502889749	0.5877392	0.942098	1
FSCN3	1.0188604	1.01394208	1.0209923	1.505679437	0.5904146	0.942098	1
KIAA0319	1.0188131	1.01394208	1.0209245	1.500813028	0.5857443	0.942098	1
EWSAT1	1.0161162	1.02061641	1.0141656	0.687101634	-0.5414046	0.942099	1
RP11-283I3.2	1.0161128	1.02063059	1.0141546	0.686097607	-0.5435143	0.942099	1
LINC00636	1.016103	1.02059482	1.0141561	0.687360079	-0.540862	0.942099	1
AC005540.3	1.0161135	1.02062246	1.014159	0.686582708	-0.5424946	0.942099	1
OGFRP1	1.0161069	1.02061711	1.0141519	0.686413056	-0.5428511	0.942099	1
RP11-573J24.1	1.0161165	1.02062043	1.0141643	0.686904144	-0.5418193	0.942099	1
RP5-999L4.2	1.0161112	1.02061193	1.0141603	0.686996749	-0.5416248	0.942099	1
RP11-553P9.2	1.0161137	1.02062849	1.0141567	0.686268975	-0.543154	0.942099	1
RP11-168L7.1	1.0161027	1.02059858	1.0141539	0.687131807	-0.5413412	0.942099	1
LINGO3	1.0340537	1.03854579	1.0321066	0.832947614	-0.2637023	0.942135	1
HRH2	1.0341234	1.03862142	1.0321737	0.833054148	-0.2635178	0.942135	1
CFHR1	1.0253395	1.02981911	1.0233978	0.784656664	-0.3498666	0.94215	1
AR	1.1947875	1.19932266	1.1928217	0.96738495	-0.047838	0.942279	1
RAB3C	1.0588122	1.06342118	1.0568144	0.895826598	-0.1587086	0.942297	1
OCA2	1.0462627	1.04130944	1.0484097	1.171880392	0.2288253	0.942383	1
RP11-302B13.5	1.0343999	1.02953039	1.0365107	1.236376271	0.3061179	0.942396	1
AC021224.1	1.0344614	1.02953075	1.0365986	1.23933721	0.3095688	0.942396	1
SPATC1L	2.0267	2.02092211	2.0292044	1.008112569	0.0116567	0.9424	1
PPM1D	1.163196	1.16787069	1.1611698	0.960082838	-0.0587692	0.942459	1
NAALADL2-AS2	1.0197053	1.01486911	1.0218016	1.466234931	0.5521163	0.942578	1
S100A7	1.0197559	1.01486911	1.0218741	1.47110786	0.556903	0.942578	1
CHRNB3	1.0197465	1.01486911	1.0218606	1.470201163	0.5560136	0.942578	1
AC007620.3	1.0197537	1.01486911	1.021871	1.470899425	0.5566986	0.942578	1
MAT1A	1.024118	1.02859696	1.0221766	0.775486507	-0.3668264	0.942638	1
CTC-548K16.1	1.0241527	1.02863198	1.0222112	0.775746602	-0.3663426	0.942638	1
PLS1	1.074065	1.06928696	1.0761361	1.098852078	0.1359972	0.942665	1
ZMAT4	1.2062368	1.21122492	1.2040747	0.966148564	-0.049683	0.942677	1
LINC01241	1.0164075	1.02087727	1.0144701	0.693102277	-0.5288598	0.94268	1
LINC00167	1.0164116	1.02087429	1.0144773	0.693545113	-0.5279384	0.94268	1
SMCR2	1.0164081	1.02089506	1.0144631	0.692179437	-0.530782	0.94268	1
RP11-242G20.1	1.0163981	1.02082972	1.0144773	0.695029157	-0.5248546	0.94268	1
RP11-768F21.1	1.0164153	1.02090072	1.014471	0.692369935	-0.530385	0.94268	1
CTD-2619J13.27	1.0164082	1.02088492	1.0144678	0.692738303	-0.5296176	0.94268	1

CTD-3096M3.2	1.0164046	1.02087801	1.0144656	0.69286331	-0.5293573	0.94268	1
SLC1A5	1.3709943	1.37594534	1.3688483	0.981122075	-0.0274954	0.942691	1
FSIP1	1.0549794	1.05004006	1.0571204	1.141494027	0.1909233	0.942702	1
CTB-113I20.2	1.1868012	1.19148823	1.1847696	0.964913468	-0.0515285	0.942767	1
DOCK9-AS2	1.0466856	1.05114928	1.0447508	0.87490625	-0.1927997	0.942845	1
RP11-21B23.3	1.0170113	1.02148041	1.0150741	0.701760804	-0.5109487	0.942867	1
RP11-84C10.4	1.0170138	1.02147448	1.0150803	0.70224477	-0.5099541	0.942867	1
AC016629.3	1.0170024	1.02146619	1.0150675	0.701916908	-0.5106278	0.942867	1
AC005775.2	1.0169982	1.02145756	1.0150653	0.702098548	-0.5102545	0.942867	1
ARSE	1.026263	1.02136197	1.0283874	1.328876916	0.4102075	0.942905	1
ZNF836	1.2186668	1.2134943	1.2209089	1.03472959	0.0492538	0.942912	1
PLEKHF1	1.1192741	1.12387464	1.11728	0.94676324	-0.0789244	0.942992	1
ATXN7L2	1.0541467	1.05866245	1.0521893	0.889654931	-0.1686822	0.943035	1
ZNF23	1.0448121	1.04937501	1.0428342	0.86752898	-0.2050161	0.943038	1
FAM85B	1.0447862	1.0493402	1.0428122	0.86769412	-0.2047415	0.943038	1
FAM173B	1.228783	1.23335653	1.2268006	0.971905982	-0.0411113	0.943104	1
RP13-131K19.2	1.0301299	1.03458721	1.0281979	0.81526962	-0.2946508	0.943134	1
DVL1	1.1454259	1.15016526	1.1433716	0.954758778	-0.0667918	0.943137	1
MPHOSPH8	2.1639836	2.17012978	2.1613196	0.992470745	-0.0109035	0.943153	1
RP11-315D16.4	1.0376881	1.0327615	1.0398235	1.215557698	0.2816184	0.943183	1
RASSF1-AS1	1.0933714	1.08841711	1.0955188	1.080320511	0.1114594	0.943205	1
UPK3B	1.0188447	1.01400962	1.0209405	1.494719267	0.5798745	0.943243	1
HPR	1.0188186	1.01400962	1.0209031	1.492049501	0.5772954	0.943243	1
CGB7	1.0188209	1.01400962	1.0209063	1.492284255	0.5775224	0.943243	1
AP001439.2	1.0188409	1.01400962	1.020935	1.494328348	0.5794972	0.943243	1
GBP3	1.0188202	1.01400962	1.0209054	1.492213982	0.5774544	0.943243	1
NCBP1	1.2141027	1.21880756	1.2120634	0.969177519	-0.0451672	0.943243	1
IL11	1.0717161	1.06680413	1.0738453	1.105399795	0.1445682	0.943248	1
TAF4B	1.0637469	1.06821638	1.0618096	0.906081008	-0.1422881	0.943252	1
CX3CL1	1.0636552	1.06819927	1.0616855	0.904488833	-0.1448254	0.943252	1
TRPV3	1.027391	1.03180567	1.0254774	0.801032113	-0.320068	0.943287	1
PIGA	1.1479963	1.14291118	1.1502005	1.051005872	0.0717707	0.943329	1
RP11-181G12.2	1.0458522	1.04100389	1.0479538	1.169493153	0.2258834	0.94338	1
NTNG2	1.0816496	1.08624559	1.0796575	0.923612357	-0.1146406	0.943384	1
RUNDC3A	1.059632	1.05467715	1.0617798	1.129900841	0.1761962	0.94344	1
RP11-265E18.1	1.0169602	1.02136541	1.0150507	0.70444382	-0.5054434	0.94368	1
HSD17B3	1.0169515	1.02136285	1.0150394	0.703995993	-0.5063609	0.94368	1
CTD-2203K17.1	1.0169558	1.02136203	1.0150458	0.704325756	-0.5056853	0.94368	1
OTUD1	1.0634603	1.05855659	1.0655859	1.120043254	0.1635544	0.943702	1
CT55	1.018152	1.02256678	1.0162383	0.719567451	-0.4747982	0.943767	1
RP11-1136J12.1	1.0181525	1.02259191	1.0162282	0.718318704	-0.477304	0.943767	1
RP11-495P10.3	1.0181506	1.02256135	1.0162387	0.719759119	-0.4744139	0.943767	1
CTB-35F21.5	1.0181403	1.02255066	1.0162285	0.719648044	-0.4746366	0.943767	1
CTD-3224I3.3	1.0181481	1.02256464	1.0162337	0.719431397	-0.475071	0.943767	1
NECTIN1	1.1375579	1.13266219	1.1396799	1.052899152	0.0743673	0.943777	1
CTD-2033C11.1	1.0302429	1.02543105	1.0323287	1.271228695	0.3462236	0.943806	1
EIF4EBP3	1.0257137	1.03015255	1.0237896	0.788974225	-0.3419499	0.943862	1
CTD-2145A24.5	1.0256957	1.03008664	1.0237924	0.79079447	-0.3386253	0.943862	1
FRMD3	1.0256993	1.03015492	1.023768	0.788197622	-0.3433707	0.943862	1
TBC1D16	1.4173104	1.42208647	1.4152402	0.983779995	-0.0235924	0.943869	1
RP11-2C24.7	1.0231037	1.027513	1.0211925	0.770271145	-0.3765617	0.943907	1

CDR2	1.1365326	1.1317045	1.1386254	1.052548714	0.073887	0.943918	1
MCF2	1.0213083	1.01648173	1.0234004	1.419775288	0.5056626	0.943942	1
MAFTRR	1.0376858	1.03297129	1.0397293	1.204967746	0.2689945	0.943948	1
RP5-884C9.2	1.0290911	1.02421755	1.0312036	1.288468876	0.3656577	0.943988	1
MKRN3	1.0734831	1.07788113	1.0715767	0.919050856	-0.1217834	0.943991	1
SCN2A	1.0375155	1.04196762	1.0355857	0.847931907	-0.2379797	0.944053	1
RCE1	1.2128932	1.21784493	1.2107468	0.967416492	-0.047791	0.944054	1
RP11-486I11.2	1.0256259	1.03002222	1.0237203	0.790090293	-0.3399106	0.944106	1
C5AR1	1.0256389	1.030061	1.0237221	0.789130896	-0.3416635	0.944106	1
PIK3CD	1.0645197	1.05965405	1.0666288	1.116919939	0.1595258	0.944117	1
ZNF852	1.0371105	1.03227469	1.0392066	1.214779198	0.2806941	0.944137	1
MFHAS1	1.183491	1.18821379	1.1814438	0.964030566	-0.0528492	0.944208	1
CCM2L	1.0210134	1.01622582	1.0230886	1.422951601	0.5088866	0.944208	1
SLC16A8	1.0509033	1.04612692	1.0529736	1.14843122	0.1996645	0.944226	1
ATP7B	1.0833229	1.08787491	1.0813498	0.925746012	-0.1113117	0.944232	1
HIST1H2BH	1.0747248	1.07931463	1.0727352	0.917047092	-0.1249323	0.944264	1
RP11-178C3.2	1.0175606	1.0219528	1.0156568	0.713204584	-0.4876121	0.944271	1
LA16c-380H5.5	1.0175509	1.02194033	1.0156483	0.713219652	-0.4875816	0.944271	1
MISP	1.0175437	1.02194054	1.0156379	0.712741264	-0.4885496	0.944271	1
RP11-73M18.10	1.0175346	1.02189545	1.0156444	0.714502588	-0.4849889	0.944271	1
ZNF557	1.1467247	1.14173302	1.1488884	1.050484843	0.0710553	0.944284	1
TSNARE1	1.0378148	1.03303696	1.0398858	1.207307939	0.2717937	0.944287	1
PIGF	1.9366368	1.94246953	1.9341086	0.991128735	-0.0128556	0.944413	1
RP11-293A21.1	1.0529754	1.05737239	1.0510696	0.890142033	-0.1678925	0.944426	1
MAST1	1.1119441	1.11655975	1.1099434	0.943236494	-0.0843086	0.944452	1
DDHD1	1.6081792	1.61337279	1.605928	0.987862614	-0.0176177	0.944454	1
CCDC81	1.0513531	1.05570666	1.049466	0.887973373	-0.1714117	0.944457	1
LYG1	1.0514412	1.05583852	1.0495351	0.887113209	-0.1728099	0.944457	1
RP11-524O1.4	1.0178174	1.02218079	1.015926	0.718008745	-0.4779267	0.944537	1
CD8A	1.0178323	1.02219985	1.0159391	0.71798259	-0.4779792	0.944537	1
SLC7A3	1.0178183	1.02219018	1.0159233	0.717584908	-0.4787785	0.944537	1
RP11-474P2.6	1.0333639	1.03778699	1.0314466	0.832208137	-0.2649837	0.944559	1
PRRX2	1.0216215	1.01683833	1.0236948	1.407193673	0.4928209	0.944579	1
PLD4	1.0215899	1.01683833	1.0236495	1.404504921	0.4900617	0.944579	1
RP13-349O20.2	1.0216023	1.01683833	1.0236673	1.405559307	0.4911443	0.944579	1
RP13-616I3.1	1.0217074	1.01683833	1.023818	1.41451017	0.5003025	0.944579	1
RP11-172H24.4	1.0669647	1.06211177	1.0690683	1.11199956	0.1531562	0.944619	1
RP11-700H13.1	1.0265546	1.02172243	1.0286491	1.318872729	0.3993054	0.944645	1
TENM3	1.697623	1.69218675	1.6999793	1.011257933	0.016151	0.944703	1
SLC27A2	1.0290347	1.02429589	1.0310888	1.279590062	0.3556817	0.944712	1
PCP4L1	1.0546804	1.05889784	1.0528523	0.897356	-0.1562476	0.94475	1
NXF2B	1.0204325	1.01567847	1.0224932	1.434655908	0.5207048	0.9448	1
XYLT2	1.173615	1.17816516	1.1716427	0.96339079	-0.053807	0.944803	1
MED16	1.3015769	1.30653977	1.2994258	0.976792629	-0.0338758	0.944834	1
ZFYVE28	1.0285213	1.02372983	1.0305983	1.289442686	0.3667476	0.9449	1
C4orf33	1.1828434	1.17798913	1.1849476	1.039094667	0.0553271	0.944906	1
ZNF286A	1.1723569	1.16715811	1.1746104	1.044582095	0.0629259	0.94493	1
SHD	1.0913038	1.08629382	1.0934754	1.083222788	0.11533	0.944933	1
AC022007.5	1.0482192	1.04344421	1.050289	1.157553505	0.2110789	0.944999	1
DENND1C	1.083452	1.0785725	1.0855671	1.089020794	0.1230315	0.945026	1
SPATA5L1	1.0833602	1.07849156	1.0854705	1.088913711	0.1228896	0.945026	1

ZNF773	1.0648161	1.06003799	1.0668872	1.114081074	0.1558542	0.945037	1
AP000569.9	1.0291525	1.02443925	1.0311955	1.276452379	0.3521397	0.945088	1
ZNF483	1.0993814	1.09452695	1.1014856	1.073615077	0.1024768	0.9451	1
AP001604.3	1.0153875	1.01970552	1.0135158	0.685887082	-0.543957	0.945102	1
CA10	1.0153751	1.01968307	1.0135078	0.686266604	-0.5431589	0.945102	1
CTD-2037L6.2	1.0153918	1.01973103	1.0135109	0.684753677	-0.546343	0.945102	1
CTD-2568A17.8	1.0153835	1.01970073	1.0135122	0.685874496	-0.5439835	0.945102	1
NPL	1.046032	1.04127369	1.0480945	1.165258826	0.2206504	0.94511	1
SPOPL	1.1710375	1.1756535	1.1690367	0.962330597	-0.0553955	0.945197	1
STX2	1.2919035	1.28702354	1.2940187	1.02437135	0.0347388	0.94525	1
ATP6V0C	1.0362614	1.03148357	1.0383323	1.217534662	0.2839628	0.94533	1
CTD-2541M15.3	1.0362177	1.03144053	1.0382883	1.217802347	0.28428	0.94533	1
SHANK2	1.0594958	1.06390121	1.0575863	0.901177107	-0.1501174	0.945418	1
SMAD6	1.0832944	1.08789969	1.0812983	0.92489827	-0.1126334	0.94545	1
C7orf57	1.0223321	1.0267029	1.0204375	0.765366965	-0.3857765	0.945463	1
PRSS57	1.0223122	1.0265906	1.0204578	0.769360666	-0.378268	0.945463	1
RP3-508I15.14	1.0223036	1.02664043	1.0204237	0.766643976	-0.3833713	0.945463	1
LINC00457	1.0204564	1.01574718	1.0224976	1.428676742	0.5146795	0.94548	1
S100A8	1.0204578	1.01574718	1.0224996	1.428801702	0.5148057	0.94548	1
ABHD12B	1.0205492	1.01574718	1.0226307	1.437124423	0.523185	0.94548	1
C1orf116	1.020438	1.01574718	1.0224713	1.427004647	0.51299	0.94548	1
RP11-449D8.1	1.0204463	1.01574718	1.0224831	1.427756126	0.5137496	0.94548	1
AP000221.1	1.0243678	1.02874537	1.0224703	0.781701919	-0.3553095	0.945486	1
EPB41L1	1.3462628	1.3405577	1.3487356	1.024013371	0.0342346	0.945505	1
LINC01184	1.6009991	1.59561041	1.6033348	1.012968928	0.0185899	0.945511	1
ZFP2	1.0714277	1.07580648	1.0695297	0.917200582	-0.1246908	0.945518	1
FBXO38	1.1473119	1.15182998	1.1453535	0.957343545	-0.0628914	0.945609	1
ADAM8	1.0198812	1.01516282	1.0219264	1.446065268	0.5321327	0.945668	1
NCAN	1.0198931	1.01516282	1.0219434	1.447185648	0.53325	0.945668	1
GSC	1.0199148	1.01516282	1.0219746	1.449243246	0.5352998	0.945668	1
AF131215.8	1.019846	1.01516282	1.0218759	1.442735009	0.5288063	0.945668	1
FAM209B	1.0198564	1.01516282	1.0218908	1.443719187	0.5297902	0.945668	1
MGAT4C	1.0473055	1.0426142	1.0493389	1.157804936	0.2113922	0.945673	1
IPO9-AS1	1.0580873	1.05329439	1.0601648	1.12891455	0.1749363	0.945701	1
AC005786.5	1.0162821	1.02056548	1.0144255	0.701441808	-0.5116047	0.945766	1
C5orf63	1.0163047	1.02063177	1.0144292	0.699366457	-0.5158795	0.945766	1
ADGRG3	1.0162794	1.02056171	1.0144232	0.701459827	-0.5115676	0.945766	1
COMP	1.0162884	1.0205796	1.0144284	0.70110084	-0.5123061	0.945766	1
NAGPA	1.1084629	1.11304167	1.1064782	0.941937939	-0.0862961	0.945778	1
CRBN	1.6209904	1.61614065	1.6230925	1.011282956	0.0161867	0.945801	1
MBLAC1	1.0747615	1.07003387	1.0768107	1.09676565	0.1332553	0.945818	1
VAMP4	1.1584238	1.15337734	1.1606112	1.047163539	0.0664868	0.945818	1
PDE6A	1.0264761	1.03077977	1.0246106	0.799570485	-0.3227029	0.94582	1
LINC01248	1.0205703	1.01586094	1.0226116	1.425614206	0.5115836	0.945895	1
RP11-273G15.2	1.0205714	1.01586389	1.022612	1.425373344	0.5113398	0.945895	1
XRCC1	1.5918911	1.58636497	1.5942864	1.01350938	0.0193594	0.945966	1
SMOC2	1.0324323	1.0275904	1.034531	1.251558015	0.3237252	0.945988	1
RHBDF1	1.1387645	1.13408335	1.1407936	1.050045536	0.0704519	0.945998	1
CHRNA1	1.0275699	1.02284382	1.0296184	1.296561457	0.3746906	0.946048	1
COQ7	1.3907522	1.38528017	1.3931241	1.020358952	0.0290768	0.946065	1
ATL2	1.2202474	1.22479812	1.2182749	0.970981678	-0.042484	0.946065	1

SOX8	1.0872596	1.09162859	1.0853658	0.931650542	-0.1021392	0.946154	1
NKG7	1.015383	1.0196435	1.0135363	0.689098099	-0.5372187	0.946245	1
SBK2	1.0153938	1.01967011	1.0135402	0.688366329	-0.5387516	0.946245	1
RP11-143I21.1	1.0153824	1.01963652	1.0135384	0.689447952	-0.5364865	0.946245	1
ERICH6B	1.015386	1.01965348	1.0135363	0.68874827	-0.5379513	0.946245	1
TP53AIP1	1.0153804	1.01963484	1.0135363	0.689402218	-0.5365822	0.946245	1
XIST	1.0153805	1.01963189	1.0135378	0.68957983	-0.5362105	0.946245	1
RIMS1	1.0497273	1.04506195	1.0517495	1.14840876	0.1996362	0.946246	1
AMTN	1.019534	1.01490147	1.021542	1.445628757	0.5316971	0.946249	1
RP11-325N19.3	1.01956	1.01490147	1.0215793	1.448130624	0.5341917	0.946249	1
CARD19	1.6428786	1.6371376	1.6453671	1.012916351	0.018515	0.946253	1
PC	1.058699	1.05398161	1.0607438	1.12526765	0.1702682	0.946253	1
LMF2	1.3832165	1.37826175	1.3853641	1.018776421	0.0268375	0.946296	1
ACVR1C	1.0240098	1.02831381	1.0221442	0.782099036	-0.3545768	0.94635	1
NLRP1	2.8291131	2.82110884	2.8325826	1.006300439	0.0090611	0.946371	1
LINC00836	1.018865	1.02310543	1.0170269	0.736922527	-0.4404151	0.946528	1
RP11-47L3.1	1.0188772	1.02310704	1.0170437	0.737599981	-0.4390895	0.946528	1
HIST1H4B	1.01888	1.02316561	1.0170223	0.734811197	-0.4445545	0.946528	1
ST8SIA3	1.0188663	1.0230969	1.0170325	0.737438728	-0.4394049	0.946528	1
RP11-541P9.3	1.0188717	1.02313342	1.0170245	0.735925834	-0.4423677	0.946528	1
ST8SIA5	1.0289019	1.02416913	1.0309534	1.280698959	0.3569314	0.946537	1
WISP2	1.0335184	1.03785177	1.03164	0.835893077	-0.2586097	0.946542	1
KCNIP2	1.048306	1.043591	1.0503497	1.155047718	0.2079525	0.946577	1
RP11-90M2.5	1.0224837	1.02673069	1.0206429	0.772253993	-0.3728527	0.946599	1
RP1-251M9.2	1.0225024	1.02676375	1.0206553	0.771762317	-0.3737715	0.946599	1
RP11-296O14.3	1.0662819	1.06148343	1.0683618	1.111873154	0.1529922	0.946615	1
DRD4	1.0610844	1.06526657	1.0592717	0.908147672	-0.1390012	0.946652	1
RP11-307C12.12	1.061078	1.06541668	1.0591974	0.904927583	-0.1441258	0.946652	1
RAET1E	1.0161295	1.02037867	1.0142877	0.701109293	-0.5122887	0.946679	1
RP11-281O15.4	1.0161396	1.02041331	1.0142871	0.699891946	-0.5147959	0.946679	1
MSANTD3-TMEFF1	1.0161209	1.02035285	1.0142866	0.701943976	-0.5105722	0.946679	1
PDSS1	1.100581	1.10500897	1.0986616	0.939554423	-0.0899514	0.946699	1
SMUG1	1.5506701	1.55557481	1.5485441	0.987345189	-0.0183735	0.946699	1
UBR5-AS1	1.1099989	1.11412223	1.1082117	0.948208348	-0.076724	0.946718	1
EVC2	1.0685393	1.0728272	1.0666807	0.915601835	-0.1272077	0.946738	1
RIPK3	1.0236311	1.01899597	1.0256403	1.349773048	0.4327169	0.946752	1
CDA	1.0237194	1.01899597	1.0257668	1.356434066	0.4398189	0.946752	1
TLR3	1.0236879	1.01899597	1.0257217	1.354058988	0.4372906	0.946752	1
Z69720.2	1.0227337	1.0269752	1.0208952	0.774608062	-0.3684616	0.946835	1
RP4-784A16.5	1.0227526	1.02700299	1.0209103	0.774369373	-0.3689062	0.946835	1
LINC00900	1.0561241	1.06048525	1.0542338	0.896644315	-0.1573923	0.946882	1
UGT8	1.0214221	1.01676898	1.023439	1.397761167	0.4831179	0.946903	1
FOXE3	1.0214054	1.01676898	1.0234151	1.396335683	0.4816458	0.946903	1
CASC18	1.0214878	1.016786	1.0235259	1.401516753	0.486989	0.946903	1
TENM1	1.1029989	1.09834014	1.1050183	1.067908789	0.0947884	0.946935	1
AC012123.1	1.1175644	1.12171745	1.1157642	0.951089502	-0.072347	0.946944	1
CTD-2545M3.8	1.0249394	1.02920805	1.0230891	0.790504878	-0.3391537	0.947017	1
MTRR	1.1329469	1.12816639	1.1350191	1.053467237	0.0751454	0.947103	1
RP11-538I12.3	1.0370233	1.0323433	1.0390519	1.20741767	0.2719248	0.947202	1
SNX1	1.3488738	1.35377539	1.3467491	0.980139165	-0.0289415	0.947229	1
TMX2-CTNND1	1.0145247	1.01875717	1.0126901	0.676548159	-0.5637355	0.947252	1

RP11-428O18.6	1.0145159	1.01872094	1.0126932	0.678019314	-0.5606017	0.947252	1
RP11-881M11.1	1.0145153	1.01872956	1.0126887	0.677468154	-0.561775	0.947252	1
CTB-40H15.4	1.0145175	1.01871888	1.0126964	0.678265601	-0.5600778	0.947252	1
SLC34A3	1.0145204	1.01874306	1.0126901	0.677057472	-0.5626498	0.947252	1
RP11-326N17.2	1.0145165	1.0187196	1.0126946	0.67814561	-0.560333	0.947252	1
RP11-379L18.3	1.0145209	1.01872758	1.0126975	0.678012032	-0.5606172	0.947252	1
AHSP	1.0145094	1.01870544	1.0126907	0.678448492	-0.5596888	0.947252	1
GFI1B	1.0145214	1.01873433	1.0126952	0.677644562	-0.5613993	0.947252	1
AC007255.8	1.0145248	1.01874051	1.0126975	0.677544468	-0.5616125	0.947252	1
SFRP4	1.0145208	1.01874496	1.0126898	0.676969166	-0.562838	0.947252	1
RP11-585P4.5	1.0145162	1.01872956	1.0126899	0.677536078	-0.5616303	0.947252	1
RP11-295K3.1	1.014527	1.01876113	1.0126917	0.676489134	-0.5638613	0.947252	1
TMC3	1.0145164	1.01873236	1.0126889	0.677379366	-0.5619641	0.947252	1
RNU6ATAC35P	1.0145211	1.01872094	1.0127007	0.678421488	-0.5597462	0.947252	1
RP11-403P17.6	1.0145301	1.01875238	1.0126999	0.677241168	-0.5622584	0.947252	1
RP11-651P23.5	1.0145047	1.01869104	1.0126901	0.678941672	-0.5586405	0.947252	1
RP11-680F20.4	1.0300597	1.0254048	1.0320774	1.262650308	0.3364551	0.947252	1
RP11-54O7.3	1.1121366	1.10731534	1.1142265	1.064400092	0.0900405	0.947266	1
DOCK2	1.0394869	1.03489311	1.0414781	1.188718185	0.2494067	0.947282	1
NALCN	1.0215328	1.02574727	1.019706	0.765362217	-0.3857854	0.947323	1
UCN	1.0621127	1.06638367	1.0602615	0.907775931	-0.1395919	0.94733	1
RXRA	1.2207786	1.2161262	1.2227951	1.030856703	0.0438438	0.947341	1
TMEM260	1.338714	1.33372098	1.3408782	1.02144669	0.0306139	0.947433	1
MOK	1.266245	1.26136598	1.2683598	1.026758656	0.0380971	0.94749	1
ARMC9	1.2660161	1.26110025	1.268147	1.026988499	0.03842	0.94749	1
RP11-706O15.5	1.0240106	1.0282284	1.0221824	0.785819061	-0.3477309	0.947499	1
KIF24	1.0325917	1.03674384	1.030792	0.838018173	-0.2549466	0.947588	1
ZNF235	1.0326021	1.03684387	1.0307635	0.834969871	-0.260204	0.947588	1
CARNMT1	1.3144412	1.31918175	1.3123863	0.978709896	-0.0310468	0.947613	1
PAXBP1-AS1	1.0516375	1.05589742	1.0497911	0.890757975	-0.1668946	0.947619	1
RP11-286N22.8	1.0422967	1.04659525	1.0404334	0.867758237	-0.2046349	0.94769	1
HEATR3	1.068289	1.07252488	1.0664529	0.916277313	-0.1261438	0.947713	1
PAX7	1.031212	1.03537261	1.0294085	0.831392677	-0.2663981	0.947762	1
RP11-976B16.1	1.0275018	1.03170869	1.0256782	0.809817007	-0.3043322	0.947782	1
SIPA1L3	1.0958556	1.0912721	1.0978423	1.071984756	0.1002844	0.947853	1
ABALON	1.0284167	1.02373512	1.0304459	1.282736684	0.359225	0.947913	1
RP11-356J5.13	1.0283656	1.02373468	1.0303729	1.279685695	0.3557895	0.947913	1
CLTCL1	1.0401494	1.03558819	1.0421265	1.183722489	0.2433309	0.947935	1
DLX4	1.0248481	1.02893506	1.0230765	0.79752821	-0.3263925	0.947935	1
RP3-439F8.1	1.0247841	1.02893474	1.022985	0.794375488	-0.332107	0.947935	1
RTN4IP1	1.0743045	1.06974461	1.076281	1.09371921	0.1292424	0.947965	1
ZEB2-AS1	1.0308943	1.02629257	1.0328889	1.250883572	0.3229475	0.947993	1
MLKL	1.0221439	1.02631469	1.020336	0.772801119	-0.3718309	0.948001	1
CHRNA9	1.0221437	1.02632565	1.020331	0.772290357	-0.3727847	0.948001	1
RNF223	1.0222173	1.02637077	1.0204169	0.774224859	-0.3691755	0.948001	1
RP11-236L14.2	1.0304104	1.02581373	1.0324028	1.255254956	0.3279804	0.948004	1
MTA3	1.6089209	1.61365715	1.606868	0.98893657	-0.0160501	0.94801	1
DGAT1	1.2193902	1.22374336	1.2175033	0.972110831	-0.0408073	0.948016	1
PUSL1	1.252979	1.2576117	1.2509709	0.974221631	-0.0376781	0.948086	1
LURAP1L	1.125812	1.13022259	1.1239002	0.951449729	-0.0718007	0.948101	1
RP11-347C12.10	1.064697	1.06896075	1.0628489	0.911372172	-0.1338878	0.948109	1

CRYGD	1.1292044	1.12465472	1.1311765	1.052318432	0.0735713	0.94813	1
ASIC1	1.0593315	1.06359952	1.0574816	0.903804919	-0.1459167	0.948167	1
KANSL1-AS1	1.1169153	1.12112006	1.1150927	0.950236277	-0.0736418	0.948227	1
LINC01353	1.0143737	1.01852145	1.0125759	0.67899119	-0.5585352	0.948345	1
TGM4	1.0143708	1.01850897	1.0125771	0.679512815	-0.5574273	0.948345	1
RP11-276H7.3	1.0143745	1.01853529	1.012571	0.678219763	-0.5601753	0.948345	1
DENND5B-AS1	1.0143741	1.01853128	1.0125721	0.678428306	-0.5597317	0.948345	1
TUBA8	1.0143742	1.01851767	1.0125782	0.679256496	-0.5579716	0.948345	1
ZNF32-AS2	1.0143778	1.01854113	1.0125732	0.678123253	-0.5603806	0.948345	1
CTD-2013N17.4	1.0143749	1.01852075	1.0125778	0.679121365	-0.5582587	0.948345	1
TLR2	1.0231329	1.0272385	1.0213534	0.783940679	-0.3511836	0.94851	1
RP11-533E19.5	1.023125	1.02726707	1.0213296	0.782249219	-0.3542998	0.94851	1
SENP8	1.0858921	1.08119344	1.0879287	1.082953405	0.1149712	0.948547	1
FOXJ2	1.0858296	1.08118098	1.0878446	1.08208307	0.1138113	0.948547	1
RP11-2B6.2	1.0224256	1.02655733	1.0206347	0.776987283	-0.3640371	0.948584	1
RP11-643M14.1	1.0224319	1.02659665	1.0206267	0.77553728	-0.366732	0.948584	1
RP11-343L5.2	1.0204519	1.02462287	1.018644	0.757182592	-0.4012869	0.948608	1
SYNDIG1L	1.0204842	1.02469663	1.0186583	0.75549873	-0.4044988	0.948608	1
RP11-38L15.2	1.0873346	1.08266343	1.0893593	1.081001798	0.1123689	0.948625	1
FAM57A	1.3113617	1.3158939	1.3093973	0.979434112	-0.0299797	0.948656	1
GTPBP10	1.1703304	1.16551455	1.1724178	1.041708002	0.0589509	0.948685	1
AREG	1.0238417	1.01933632	1.0257946	1.33399767	0.4157561	0.948732	1
LINC00664	1.0326822	1.03675901	1.0309151	0.841021185	-0.249786	0.948741	1
KCTD19	1.0316235	1.02703787	1.0336112	1.243116352	0.3139613	0.94876	1
SYTL4	1.0217635	1.01724954	1.0237201	1.3751124	0.4595495	0.94876	1
RP11-10C24.2	1.0217905	1.01724954	1.0237588	1.377356487	0.461902	0.94876	1
TMEM56-RWDD3	1.021752	1.01724954	1.0237037	1.374162505	0.4585526	0.94876	1
ACTR3C	1.0218235	1.01724954	1.0238062	1.380103535	0.4647765	0.94876	1
RP11-307L14.2	1.0218164	1.01724954	1.023796	1.379514788	0.4641609	0.94876	1
FOXS1	1.0230494	1.02721631	1.0212432	0.780533485	-0.3574676	0.948771	1
TBC1D12	1.1889287	1.19338479	1.1869972	0.966969417	-0.0484578	0.948809	1
CECR1	1.3309558	1.3349405	1.3292285	0.982946361	-0.0248154	0.948819	1
ABCB9	1.1079054	1.10331081	1.1098969	1.063750656	0.08916	0.948829	1
RP3-394A18.1	1.0770624	1.08125473	1.0752452	0.926040313	-0.1108531	0.948842	1
SPTLC3	1.0397315	1.03521025	1.0416913	1.184066099	0.2437496	0.948909	1
TMEM86A	1.0396658	1.03515276	1.041622	1.184032763	0.243709	0.948909	1
STARD5	1.0398324	1.04397208	1.038038	0.865049317	-0.2091457	0.948915	1
RP11-24N18.1	1.0149673	1.01907878	1.0131851	0.691089065	-0.5330564	0.948935	1
TMEM51-AS1	1.0149672	1.01907079	1.0131885	0.691553172	-0.5320879	0.948935	1
CCDC36	1.0149778	1.01909706	1.0131922	0.690798758	-0.5336626	0.948935	1
RP11-45A12.1	1.0149719	1.01907948	1.0131915	0.691394989	-0.5324179	0.948935	1
RP13-476E20.1	1.0149766	1.01910101	1.0131888	0.690477968	-0.5343327	0.948935	1
C11orf52	1.0149669	1.01907565	1.0131859	0.691244005	-0.532733	0.948935	1
RP11-78A19.3	1.0149844	1.01912599	1.0131892	0.689593616	-0.5361817	0.948935	1
RP11-114H23.2	1.0149806	1.01912129	1.0131859	0.689590865	-0.5361874	0.948935	1
EYS	1.0149685	1.01906519	1.0131928	0.691982115	-0.5311933	0.948935	1
VWA8-AS1	1.014963	1.01905761	1.0131882	0.692018686	-0.5311171	0.948935	1
AC008522.1	1.014989	1.01914905	1.0131859	0.688591153	-0.5382804	0.948935	1
PAQR8	1.1777736	1.17313666	1.1797836	1.038391177	0.05435	0.948997	1
C4orf46	1.3460377	1.35059729	1.3440614	0.981357766	-0.0271489	0.949033	1
TOE1	1.1447345	1.14901318	1.1428799	0.958840801	-0.0606368	0.949044	1

GYS1	1.2582148	1.25366689	1.2601861	1.025699798	0.0366085	0.949059	1
CABLES2	1.0771231	1.08138462	1.0752759	0.924940004	-0.1125683	0.949085	1
SFSWAP	1.5723779	1.56752696	1.5744805	1.012252371	0.017569	0.949153	1
SHQ1	1.1407931	1.14535779	1.1388145	0.954984985	-0.06645	0.94918	1
USPL1	1.1752026	1.17016986	1.1773841	1.042394499	0.0599014	0.949183	1
SSBP3-AS1	1.0152362	1.01933632	1.0134589	0.696044222	-0.5227491	0.9492	1
RP11-1C1.6	1.015238	1.01933632	1.0134615	0.696178745	-0.5224703	0.9492	1
RP11-104O19.4	1.0152592	1.01936788	1.0134783	0.695909954	-0.5230275	0.9492	1
ZNF648	1.0152351	1.01932762	1.0134611	0.696470271	-0.5218663	0.9492	1
UBE2Q2L	1.0152467	1.01933518	1.0134745	0.696889446	-0.5209983	0.9492	1
DKFZP434A062	1.0421496	1.04632573	1.0403395	0.870779599	-0.1996205	0.949487	1
DNAL1	1.2705186	1.26586098	1.2725374	1.025112594	0.0357824	0.949494	1
ARHGAP39	1.0937143	1.08911757	1.0957068	1.073938429	0.1029113	0.949584	1
DCHS2	1.0230151	1.0185068	1.0249693	1.349193399	0.4320972	0.949591	1
XPNPEP3	1.3580215	1.36229773	1.3561679	0.98308075	-0.0246182	0.949603	1
LMO2	1.0993717	1.09468549	1.1014029	1.070944369	0.0988835	0.949624	1
KDM3B	1.4678664	1.47240369	1.4658997	0.986232085	-0.0200009	0.949626	1
BAIAP2L1	1.1790063	1.18335018	1.1771234	0.966038673	-0.0498471	0.949659	1
HEG1	1.1398064	1.13536184	1.1417329	1.047066864	0.0663536	0.949773	1
NAB1	1.3215331	1.32600896	1.319593	0.980319743	-0.0286757	0.949937	1
PRKCG	1.0683163	1.07235658	1.0665651	0.919959103	-0.1203584	0.949944	1
RP13-131K19.1	1.0596081	1.06370329	1.0578331	0.907850883	-0.1394727	0.949955	1
POLD4	1.2082918	1.21253859	1.206451	0.971357614	-0.0419256	0.949962	1
ZNF671	1.0600001	1.06413536	1.0582076	0.907575015	-0.1399112	0.949967	1
CTA-276F8.1	1.0600496	1.06420805	1.0582471	0.907162376	-0.1405673	0.949967	1
TMUB2	1.5362974	1.54136631	1.5341003	0.986578393	-0.0194944	0.95002	1
RBMXL2	1.0222369	1.02633433	1.0204609	0.776966375	-0.3640759	0.950084	1
SLC6A11	1.0222328	1.02627341	1.0204814	0.779547817	-0.3592906	0.950084	1
RP11-567J20.2	1.0222361	1.02632405	1.0204642	0.777395842	-0.3632787	0.950084	1
CCDC144NL	1.0222487	1.02628845	1.0204977	0.779722274	-0.3589677	0.950084	1
AC007038.7	1.0222384	1.02630614	1.0204752	0.778341187	-0.3615254	0.950084	1
RP11-770J1.3	1.0689132	1.07315617	1.0670741	0.916862286	-0.125223	0.950136	1
ZNF782	1.0688759	1.07276939	1.0671882	0.923303477	-0.1151232	0.950136	1
LINC01502	1.0197237	1.02378574	1.017963	0.75519857	-0.4050721	0.950163	1
CTC-301O7.4	1.0196982	1.02374186	1.0179455	0.755857101	-0.4038146	0.950163	1
CMP21-97G8.1	1.0197206	1.02378968	1.0179568	0.754813484	-0.4058079	0.950163	1
S1PR5	1.0197235	1.02379012	1.0179608	0.754969676	-0.4055094	0.950163	1
ABCG1	1.0197266	1.02379012	1.0179652	0.755154413	-0.4051564	0.950163	1
RP11-362K14.5	1.0218022	1.02581399	1.0200632	0.77722281	-0.3635999	0.950168	1
C1orf101	1.0217542	1.0258146	1.0199941	0.774528462	-0.3686098	0.950168	1
RP11-80A15.1	1.0235445	1.02760139	1.021786	0.789308596	-0.3413386	0.9502	1
PANK4	1.1318799	1.1272627	1.1338812	1.05200645	0.0731436	0.950265	1
CACNA1E	1.0230621	1.01860316	1.0249948	1.343578803	0.4260809	0.950291	1
ISLR	1.0230859	1.01861598	1.0250234	1.344186742	0.4267336	0.950291	1
NAIF1	1.0774416	1.07288439	1.079417	1.089629875	0.1238382	0.95033	1
AQP4-AS1	1.0202134	1.02426895	1.0184554	0.760454381	-0.3950664	0.950345	1
NUTM2E	1.0201932	1.02426622	1.0184277	0.759396991	-0.3970738	0.950345	1
RP11-770G2.5	1.0201927	1.02426622	1.018427	0.759369712	-0.3971256	0.950345	1
RP11-83B20.1	1.0202009	1.02427207	1.0184363	0.759567916	-0.3967491	0.950345	1
AC006994.3	1.0202124	1.02430267	1.0184394	0.758741472	-0.3983197	0.950345	1
TMEM9B-AS1	1.0394887	1.043535	1.0377349	0.866770508	-0.206278	0.950405	1

GTF3C4	1.2926545	1.28746361	1.2949045	1.025884611	0.0368685	0.950422	1
LUCAT1	1.0238657	1.02784642	1.0221402	0.795082093	-0.3308243	0.950467	1
GPR135	1.0765379	1.08072868	1.0747214	0.925587108	-0.1115593	0.95052	1
INTS9	1.0767748	1.07219875	1.0787584	1.090854968	0.1254593	0.950522	1
DGKA	1.0819622	1.08620556	1.080123	0.92944076	-0.1055652	0.950532	1
TYW1B	1.0421982	1.03767832	1.0441574	1.171956677	0.2289192	0.950545	1
LRRC8E	1.0400221	1.03565869	1.0419135	1.175406412	0.2331597	0.950614	1
WNT16	1.0231492	1.01871323	1.025072	1.339800412	0.4220181	0.950688	1
SULT2B1	1.0231143	1.01870789	1.0250243	1.337631681	0.4196809	0.950688	1
PLS3-AS1	1.0231948	1.01871777	1.0251354	1.342864273	0.4253135	0.950688	1
RANBP2	1.4913546	1.4860626	1.4936485	1.015606803	0.022342	0.950717	1
KIAA1324L	1.2432619	1.24768075	1.2413465	0.974425777	-0.0373758	0.95077	1
MARS	1.340857	1.33616008	1.3428929	1.020028652	0.0286097	0.950823	1
CCDC85A	1.03288	1.03692129	1.0311283	0.84309836	-0.2462271	0.950889	1
KHK	1.2140414	1.20950788	1.2160065	1.031018495	0.0440702	0.9509	1
TVP23B	1.2143253	1.20990594	1.2162409	1.030179764	0.0428961	0.9509	1
ZDHHC23	1.0213988	1.02539713	1.0196657	0.77432787	-0.3689835	0.951014	1
EFCAB13	1.0214227	1.0254116	1.0196936	0.77498617	-0.3677575	0.951014	1
MORN5	1.0702817	1.0741234	1.0686165	0.925706839	-0.1113727	0.95106	1
CLDN9	1.022143	1.01774131	1.024051	1.355648531	0.4389832	0.951061	1
KCNAB2	1.095514	1.09094254	1.0974956	1.072057095	0.1003817	0.951105	1
NCAM2	1.0687226	1.06426239	1.070656	1.09949143	0.1368364	0.951109	1
TFCP2	1.1309337	1.12644382	1.1328799	1.050900711	0.0716264	0.951145	1
DCTN5	1.6715044	1.67608346	1.6695195	0.990291223	-0.0140752	0.951147	1
GRIA3	1.0403133	1.04434229	1.038567	0.869755884	-0.2013176	0.951181	1
RP11-346C20.3	1.0308656	1.03487819	1.0291264	0.835088519	-0.259999	0.951225	1
RP1-234P15.4	1.024758	1.02036772	1.026661	1.308983159	0.3884465	0.951262	1
CTD-2587H24.5	1.0247171	1.02040164	1.0265876	1.303209937	0.3820695	0.951262	1
MSANTD1	1.0199078	1.02391531	1.0181708	0.759796881	-0.3963143	0.951298	1
RP1-122O8.7	1.0198964	1.02389518	1.0181631	0.760114754	-0.3957109	0.951298	1
RP11-563N4.1	1.0682351	1.0637756	1.0701681	1.10023455	0.1378111	0.951301	1
LL09NC01-139C3.1	1.0284326	1.03251811	1.0266617	0.819902593	-0.2864756	0.951305	1
SMIM11B	1.031392	1.02700061	1.0332955	1.233139043	0.3023355	0.95135	1
RP11-342M1.3	1.0336939	1.02911621	1.0356781	1.225370158	0.2932176	0.951359	1
RP11-348P10.2	1.0274951	1.02312753	1.0293882	1.270701453	0.3456251	0.951415	1
JPH1	1.0288068	1.03284065	1.0270583	0.823925563	-0.2794141	0.951487	1
PTCHD1	1.0201469	1.02413066	1.0184201	0.763350224	-0.389583	0.951534	1
RP11-506H20.1	1.0201185	1.02407391	1.018404	0.764480349	-0.3874487	0.951534	1
RP11-297A16.2	1.0201585	1.02413821	1.0184334	0.763661954	-0.3889939	0.951534	1
LINC01358	1.0201685	1.02416547	1.018436	0.762907413	-0.3904201	0.951534	1
GBP7	1.0201486	1.02416913	1.0184059	0.761544179	-0.3930004	0.951534	1
XXbac-BPG170G13.32	1.0201525	1.02411893	1.0184332	0.764262176	-0.3878605	0.951534	1
MLN	1.0201509	1.02414278	1.0184206	0.762985962	-0.3902716	0.951534	1
JSRP1	1.0262381	1.02186264	1.0281347	1.28688409	0.3638821	0.951565	1
KCNA7	1.0262463	1.0218318	1.0281598	1.289851467	0.3672049	0.951565	1
PLA2G4C	1.0311386	1.02674397	1.0330434	1.235546746	0.3051496	0.951588	1
TCAF2	1.0480573	1.0520531	1.0463252	0.889961241	-0.1681856	0.95159	1
RP11-434P11.2	1.0215244	1.0171592	1.0234165	1.364660277	0.4485418	0.951604	1
SLC6A20	1.0215205	1.0171592	1.0234109	1.364333436	0.4481963	0.951604	1
RP5-858B6.3	1.0507131	1.05481198	1.0489363	0.892803758	-0.163585	0.951634	1
FAM188A	1.1899025	1.1854931	1.1918137	1.034074858	0.0483406	0.951684	1

SLC7A4	1.0239684	1.01965348	1.0258387	1.314716156	0.3947514	0.951698	1
ZNF626	1.3398913	1.3354258	1.3418269	1.01908347	0.0272722	0.951705	1
PRRT3-AS1	1.0325187	1.0363997	1.0308364	0.847160483	-0.2392928	0.951722	1
JDP2	1.0436518	1.03925925	1.0455558	1.160383665	0.2146019	0.951809	1
LTB4R	1.0593243	1.06337012	1.0575707	0.90848306	-0.1384685	0.951913	1
PHTF1	1.8329833	1.82758286	1.8353242	1.009354106	0.0134324	0.95201	1
PRC1-AS1	1.0189388	1.02290897	1.0172178	0.751576187	-0.4120087	0.952021	1
RP3-508I15.10	1.0189156	1.0228554	1.0172078	0.752900095	-0.4094697	0.952021	1
RP4-569D19.8	1.0189585	1.02296885	1.0172202	0.749717776	-0.4155805	0.952021	1
BBOX1	1.0189342	1.02287455	1.0172262	0.753074473	-0.4091356	0.952021	1
RP11-17J14.2	1.0189261	1.02289202	1.0172071	0.751662556	-0.411843	0.952021	1
RP11-646I6.6	1.0189332	1.02284902	1.0172358	0.754335478	-0.4067218	0.952021	1
CSE1L-AS1	1.0189412	1.02291403	1.0172192	0.751468801	-0.4122149	0.952021	1
C1orf158	1.0189294	1.022894	1.017211	0.751766747	-0.411643	0.952021	1
RP11-10K16.1	1.0326339	1.02825862	1.0345305	1.221944021	0.2891782	0.952029	1
RP11-165A20.3	1.0416409	1.03718292	1.0435732	1.171861459	0.228802	0.952032	1
RP11-339B21.13	1.0416448	1.03726588	1.0435428	1.168436163	0.2245789	0.952032	1
FAM27C	1.1114325	1.11538092	1.109721	0.95094618	-0.0725644	0.95208	1
RP11-34P13.7	1.0227678	1.01839445	1.0246635	1.340809983	0.4231048	0.952092	1
LINC01615	1.022716	1.01839445	1.0245892	1.33677345	0.418755	0.952092	1
DEDD2	1.4505026	1.44570079	1.452584	1.015443478	0.0221099	0.952103	1
RP5-916L7.2	1.0236849	1.02757787	1.0219975	0.797651471	-0.3261696	0.952178	1
CTD-3220F14.3	1.0236551	1.02763129	1.0219316	0.793722879	-0.3332927	0.952178	1
RP11-416N2.4	1.0249636	1.02060005	1.026855	1.30363986	0.3825454	0.95218	1
RP1-67K17.4	1.0225175	1.01815945	1.0244066	1.344015681	0.42655	0.952328	1
RP11-436K8.1	1.0225106	1.01815945	1.0243967	1.343469358	0.4259634	0.952328	1
RP5-1068H6.6	1.022519	1.01815945	1.0244087	1.344133915	0.4266769	0.952328	1
RAC2	1.0224683	1.01815945	1.024336	1.340130761	0.4223738	0.952328	1
GCN1	1.1879428	1.19174713	1.1862937	0.971559448	-0.0416258	0.952403	1
RP11-763B22.4	1.072427	1.06779991	1.0744326	1.097827485	0.1346514	0.952411	1
CCDC65	1.0285106	1.03241949	1.0268163	0.82716717	-0.2737492	0.952444	1
CTA-929C8.6	1.0249294	1.02889174	1.0232118	0.803407701	-0.3157958	0.952445	1
RP11-611O2.1	1.0249127	1.02884122	1.0232098	0.804744184	-0.3133979	0.952445	1
RP11-697E2.6	1.0249185	1.02884786	1.0232153	0.804749082	-0.3133891	0.952445	1
SNAP25	1.1510441	1.15461906	1.1494945	0.966856808	-0.0486259	0.952449	1
PHF21B	1.0984901	1.09399327	1.1004393	1.068579932	0.0956948	0.952492	1
FAP	1.0314017	1.02708044	1.0332748	1.228739379	0.2971789	0.952545	1
GPR89B	1.1059771	1.10156903	1.1078878	1.062211842	0.0870715	0.952646	1
RP11-28H5.2	1.0195678	1.02347922	1.0178724	0.761201681	-0.3936493	0.952681	1
RP5-907D15.4	1.0287384	1.03265085	1.0270425	0.828233003	-0.2718914	0.952681	1
C9orf106	1.0288581	1.03283714	1.0271333	0.826298931	-0.2752643	0.952681	1
RP5-1065J22.8	1.0621579	1.06612627	1.0604378	0.913975974	-0.1297719	0.952691	1
AVIL	1.0621899	1.0661878	1.060457	0.913415264	-0.1306572	0.952691	1
MICB	1.0407615	1.04472315	1.0390442	0.873020727	-0.1959122	0.952697	1
DNAJC17	1.2301899	1.22575545	1.232112	1.028156758	0.0400602	0.9527	1
SLC30A2	1.0311045	1.02662199	1.0330474	1.241358608	0.3119199	0.952728	1
ADNP-AS1	1.0330164	1.02862252	1.034921	1.220052263	0.2869429	0.952801	1
FBXO41	1.0647169	1.0604348	1.066573	1.101567436	0.1395578	0.952864	1
XRCC4	1.2819192	1.28619066	1.2800677	0.978605459	-0.0312008	0.952898	1
NPPC	1.0347925	1.03050835	1.0366495	1.201294795	0.2645902	0.952925	1
TAF1A-AS1	1.0860499	1.08160976	1.0879745	1.077989533	0.1083432	0.952927	1

CSNK2A2	1.4024672	1.39745684	1.4046389	1.018070155	0.025837	0.952971	1
NHLRC2	1.408457	1.4034171	1.4106416	1.01790829	0.0256076	0.953037	1
TCN2	1.5626968	1.55802813	1.5647204	1.011992761	0.017199	0.95305	1
MOCS2	1.9105533	1.90567287	1.9126688	1.00772457	0.0111014	0.953214	1
CTD-2240J17.1	1.0198691	1.02372104	1.0181995	0.767230952	-0.3822672	0.953263	1
OTOGL	1.019843	1.0236949	1.0181734	0.766973599	-0.3827512	0.953263	1
SCHLAP1	1.0198553	1.02374558	1.018169	0.765152182	-0.3861814	0.953263	1
AF196972.9	1.0198858	1.02376931	1.0182025	0.765796373	-0.3849673	0.953263	1
ARPC4-TTLL3	1.0198771	1.02375627	1.0181957	0.765932708	-0.3847104	0.953263	1
ACTL10	1.0227713	1.01850742	1.0246195	1.330252166	0.4116998	0.953282	1
AC002076.10	1.0227846	1.01850742	1.0246386	1.331280509	0.4128146	0.953282	1
LINC00862	1.0227559	1.01850742	1.0245975	1.329061244	0.4104076	0.953282	1
LINC01012	1.0501976	1.05411235	1.0485008	0.896297556	-0.1579503	0.953285	1
CTD-2396E7.9	1.0178641	1.0217409	1.0161837	0.744387965	-0.4258734	0.953324	1
SLCO1C1	1.0178629	1.02173707	1.0161837	0.744519342	-0.4256188	0.953324	1
RP11-193H5.1	1.0178552	1.02171164	1.0161837	0.745391314	-0.4239301	0.953324	1
CTD-2192J16.22	1.0178528	1.02170354	1.0161837	0.745669455	-0.4233919	0.953324	1
RP11-65B7.2	1.0178609	1.02173046	1.0161837	0.744745848	-0.4251799	0.953324	1
RP11-667K14.4	1.0178569	1.02171697	1.0161837	0.745208456	-0.4242841	0.953324	1
AC107057.1	1.0178572	1.02171824	1.0161837	0.745164872	-0.4243684	0.953324	1
CTD-2245E15.3	1.0178529	1.02170395	1.0161837	0.745655354	-0.4234191	0.953324	1
PLVAP	1.0178529	1.02170395	1.0161837	0.745655354	-0.4234191	0.953324	1
KB-1568E2.1	1.0178577	1.02171963	1.0161837	0.745116932	-0.4244612	0.953324	1
AF127577.12	1.0178577	1.02171963	1.0161837	0.745116932	-0.4244612	0.953324	1
RP11-45M22.3	1.0178534	1.02170563	1.0161837	0.745597811	-0.4235305	0.953324	1
AC135178.7	1.0178675	1.0217521	1.0161837	0.744004948	-0.4266159	0.953324	1
RP11-158M2.5	1.0178579	1.02172033	1.0161837	0.745092955	-0.4245077	0.953324	1
HIST1H2BB	1.0178585	1.02172243	1.0161837	0.745020998	-0.424647	0.953324	1
NOS1	1.0178585	1.02172243	1.0161837	0.745020998	-0.424647	0.953324	1
ZNF485	1.0587009	1.05433766	1.0605921	1.115103754	0.157178	0.953406	1
KCNF1	1.0204566	1.02432274	1.0187808	0.772149724	-0.3730475	0.95345	1
MIOX	1.0204627	1.02430968	1.0187953	0.77315976	-0.3711615	0.95345	1
NYAP2	1.0204813	1.02433171	1.0188123	0.773159344	-0.3711623	0.95345	1
CDKN2B-AS1	1.02234	1.01804205	1.024203	1.341478971	0.4238244	0.953464	1
CIB4	1.0223431	1.01804205	1.0242074	1.341719983	0.4240836	0.953464	1
DMRTA2	1.022291	1.01804205	1.0241327	1.337579442	0.4196246	0.953464	1
AC104532.4	1.0341923	1.02998197	1.0360173	1.201297403	0.2645934	0.953521	1
NXPH1	1.0248466	1.02056357	1.0267031	1.298564919	0.3769181	0.953543	1
AC012507.3	1.0248495	1.02056357	1.0267072	1.298764466	0.3771398	0.953543	1
TFAP2C	1.0248524	1.02056357	1.0267114	1.298968946	0.3773669	0.953543	1
EFCAB6	1.0455956	1.04949372	1.043906	0.88710255	-0.1728272	0.953556	1
LINC01127	1.0243536	1.02009416	1.0261998	1.303851789	0.3827799	0.953576	1
SKA2	2.4066217	2.39937412	2.4097632	1.007424065	0.0106711	0.953611	1
ITFG2	1.2626398	1.26685926	1.2608109	0.977334951	-0.033075	0.953613	1
GINS3	1.0945524	1.09852984	1.0928283	0.942133904	-0.085996	0.95363	1
RP11-380L11.4	1.0620713	1.05774115	1.0639482	1.107497825	0.1473039	0.953642	1
ZNF518B	1.3644975	1.36885129	1.3626103	0.983079932	-0.0246194	0.953671	1
EVI5L	1.1229218	1.1183712	1.1248943	1.055106764	0.077389	0.95369	1
AGO4	1.1127556	1.11666683	1.1110603	0.951943687	-0.0710519	0.953763	1
RP11-318A15.8	1.0262777	1.02196563	1.0281468	1.2814039	0.3577253	0.953778	1
VASH1	1.3174954	1.32166496	1.3156881	0.981418905	-0.027059	0.95383	1

AGAP1	1.389465	1.39352682	1.3877044	0.985204571	-0.0215048	0.95388	1
UBE2K	2.0310456	2.03594775	2.0289208	0.99321686	-0.0098193	0.953954	1
PEX12	1.0949819	1.09061287	1.0968757	1.069116081	0.0964185	0.954006	1
RP11-540A21.2	1.0267528	1.02255792	1.0285711	1.266566255	0.3409225	0.954187	1
DDN	1.0203922	1.02422881	1.0187292	0.773015267	-0.3714312	0.954268	1
CTD-2291D10.4	1.020416	1.02417106	1.0187884	0.777309157	-0.3634396	0.954268	1
ABHD16A	1.0361824	1.04006534	1.0344993	0.861076829	-0.2157861	0.954309	1
RP4-758J18.13	1.0216102	1.02544509	1.019948	0.783961231	-0.3511458	0.954353	1
HIST1H3B	1.0215767	1.02539706	1.0199208	0.78437396	-0.3503865	0.954353	1
POU3F3	1.0216002	1.02544365	1.0199342	0.783465635	-0.3520581	0.954353	1
CCR6	1.0255857	1.02135716	1.0274185	1.283809853	0.3604315	0.954371	1
RP11-205M3.3	1.0178406	1.01362911	1.019666	1.442943311	0.5290146	0.954371	1
PRMT8	1.01788	1.01362911	1.0197225	1.447088712	0.5331534	0.954371	1
POU3F2	1.0178164	1.01362911	1.0196314	1.440398236	0.5264677	0.954371	1
RP5-890E16.5	1.0292187	1.03300413	1.0275779	0.835588973	-0.2591346	0.9544	1
TMIGD2	1.0264732	1.03027651	1.0248246	0.819930167	-0.2864271	0.954441	1
BANCR	1.0265248	1.03030528	1.0248862	0.821182578	-0.2842251	0.954441	1
MERTK	1.0656031	1.06945467	1.0639337	0.92050942	-0.1194956	0.954526	1
APOOL	1.3267834	1.33103642	1.3249399	0.981583686	-0.0268168	0.954609	1
MAFA	1.0343478	1.03016276	1.0361618	1.198889833	0.2616991	0.954624	1
ZC3H11B	1.0343436	1.03017222	1.0361517	1.198176847	0.2608409	0.954624	1
SMPD3	1.0175425	1.01338003	1.0193468	1.445943602	0.5320113	0.954637	1
CAPNS2	1.0176277	1.01338003	1.0194689	1.455068476	0.541087	0.954637	1
TLL2	1.0175647	1.01338003	1.0193786	1.448324725	0.5343851	0.954637	1
RP1-40E16.11	1.0175684	1.01338003	1.0193839	1.448719944	0.5347787	0.954637	1
MED26	1.1007794	1.09632766	1.102709	1.066246353	0.0925408	0.954746	1
RCBTB1	1.1887461	1.18436903	1.1906434	1.034031732	0.0482805	0.954767	1
MOG	1.0196406	1.02343168	1.0179973	0.768076866	-0.3806774	0.954798	1
AP000350.10	1.0196422	1.02343705	1.0179973	0.767900787	-0.3810082	0.954798	1
LINC01618	1.0196396	1.02342829	1.0179973	0.768187832	-0.380469	0.954798	1
CDH9	1.0196397	1.02342871	1.0179973	0.768174238	-0.3804945	0.954798	1
SLC3A1	1.0196313	1.02340079	1.0179973	0.769090715	-0.3787743	0.954798	1
CTD-2026G6.3	1.0196313	1.02340079	1.0179973	0.769090715	-0.3787743	0.954798	1
RP11-156K13.1	1.0196313	1.02340107	1.0179973	0.769081359	-0.3787919	0.954798	1
FGD2	1.0196312	1.0234005	1.0179973	0.769100069	-0.3787568	0.954798	1
OTOF	1.0196439	1.0234425	1.0179973	0.767722177	-0.3813438	0.954798	1
ZIC4-AS1	1.019642	1.02343647	1.0179973	0.767919676	-0.3809727	0.954798	1
RP1-266L20.4	1.0196385	1.02342491	1.0179973	0.768298699	-0.3802608	0.954798	1
DNAH2	1.0196333	1.0234075	1.0179973	0.768870298	-0.3791878	0.954798	1
AC016745.3	1.0196333	1.0234075	1.0179973	0.768870298	-0.3791878	0.954798	1
GNAT1	1.0196435	1.02344128	1.0179973	0.767762288	-0.3812684	0.954798	1
CTD-2308B18.3	1.0196405	1.02343139	1.0179973	0.768086298	-0.3806597	0.954798	1
GNLY	1.0196316	1.02340177	1.0179973	0.769058505	-0.3788347	0.954798	1
RP11-354M20.3	1.0196388	1.0234259	1.0179973	0.768266283	-0.3803217	0.954798	1
RP11-545D19.1	1.0196365	1.02341816	1.0179973	0.768520137	-0.379845	0.954798	1
SAMD12-AS1	1.01963	1.02339675	1.0179973	0.769223603	-0.3785251	0.954798	1
RP11-14N9.2	1.0196416	1.02343507	1.0179973	0.767965801	-0.380886	0.954798	1
QPCT	1.0196416	1.02343507	1.0179973	0.767965801	-0.380886	0.954798	1
NXF1	1.1776952	1.18177944	1.1759249	0.967793048	-0.0472295	0.954849	1
CCDC152	1.0191756	1.0229784	1.0175272	0.762767688	-0.3906844	0.954864	1
CTD-2555O16.1	1.0191765	1.02294973	1.017541	0.764322536	-0.3877465	0.954864	1

RP11-136L23.2	1.0191923	1.02299336	1.0175448	0.763036544	-0.3901759	0.954864	1
KCNJ11	1.0192018	1.02298824	1.0175605	0.763890752	-0.3885618	0.954864	1
SERPINI2	1.0191732	1.02293571	1.0175423	0.764846555	-0.3867578	0.954864	1
RP11-675F6.3	1.0191859	1.02299213	1.017536	0.762697302	-0.3908175	0.954864	1
TRDN	1.0191862	1.02299054	1.0175372	0.76280173	-0.39062	0.954864	1
RP11-290M5.4	1.0192175	1.0230072	1.0175749	0.763884863	-0.3885729	0.954864	1
PRDM12	1.0191771	1.0229756	1.0175307	0.763013379	-0.3902197	0.954864	1
RP4-604G5.1	1.0191906	1.0229756	1.01755	0.763853845	-0.3886315	0.954864	1
SHANK3	1.0191761	1.02297719	1.0175285	0.76286491	-0.3905005	0.954864	1
TRG-AS1	1.0171211	1.02090042	1.0154829	0.740792486	-0.4328586	0.954878	1
CTD-2270F17.1	1.0171211	1.02090042	1.0154829	0.740792486	-0.4328586	0.954878	1
AL513523.2	1.0171169	1.02088661	1.0154829	0.741282286	-0.4319051	0.954878	1
RP11-495K9.5	1.0171172	1.02088765	1.0154829	0.741245174	-0.4319773	0.954878	1
TRIM61	1.0171165	1.02088546	1.0154829	0.741322889	-0.431826	0.954878	1
RP11-429B14.4	1.0171284	1.02092466	1.0154829	0.739934077	-0.4345314	0.954878	1
RP11-315E17.1	1.0171264	1.02091812	1.0154829	0.740165522	-0.4340802	0.954878	1
C1orf140	1.0171143	1.02087821	1.0154829	0.741580528	-0.4313247	0.954878	1
PDCD1LG2	1.0171231	1.02090725	1.0154829	0.740550438	-0.4333301	0.954878	1
NPIPB7	1.0171185	1.02089192	1.0154829	0.741093747	-0.432272	0.954878	1
SYNE1-AS1	1.0171246	1.02091205	1.0154829	0.74038022	-0.4336617	0.954878	1
RP11-334A14.8	1.0171142	1.02087759	1.0154829	0.741602443	-0.4312821	0.954878	1
MIRLET7DHG	1.0171142	1.02087759	1.0154829	0.741602443	-0.4312821	0.954878	1
ANKRD30BL	1.0171243	1.020911	1.0154829	0.740417564	-0.433589	0.954878	1
RP11-503E24.2	1.0171224	1.02090479	1.0154829	0.740637501	-0.4331605	0.954878	1
GCSAM	1.0171169	1.02088681	1.0154829	0.741274981	-0.4319193	0.954878	1
CTC-564N23.2	1.0171259	1.02091656	1.0154829	0.74022062	-0.4339728	0.954878	1
AP001055.6	1.0171204	1.02089837	1.0154829	0.740865098	-0.4327172	0.954878	1
RTL1	1.0171211	1.02090062	1.0154829	0.740785155	-0.4328729	0.954878	1
CTB-180A7.3	1.0171188	1.02089284	1.0154829	0.741061121	-0.4323356	0.954878	1
AC147651.4	1.0171151	1.02088073	1.0154829	0.74149075	-0.4314994	0.954878	1
OBP2B	1.0171151	1.02088073	1.0154829	0.74149075	-0.4314994	0.954878	1
RP11-665I14.1	1.0171226	1.02090556	1.0154829	0.740610345	-0.4332134	0.954878	1
CFAP47	1.0423072	1.046075	1.040674	0.88277857	-0.1798765	0.954886	1
BACH2	1.0902499	1.08605959	1.0920661	1.069795317	0.0973348	0.954911	1
CITF22-92A6.1	1.0904081	1.0860184	1.0923108	1.073151561	0.1018538	0.954911	1
TRIM46	1.1128204	1.11676742	1.1111095	0.951545676	-0.0716552	0.954961	1
CTU2	1.1254403	1.1213032	1.1272336	1.048888847	0.0688618	0.954963	1
RP1-102K2.6	1.023055	1.01886772	1.02487	1.318127122	0.3984895	0.95502	1
FAM26D	1.0230825	1.01886772	1.0249095	1.320216784	0.4007748	0.95502	1
KISS1	1.0230929	1.01886772	1.0249243	1.321001154	0.4016317	0.95502	1
MPZL3	1.0230382	1.01886772	1.0248459	1.316848398	0.3970893	0.95502	1
HSD17B6	1.0394131	1.04323385	1.0377569	0.873318413	-0.1954203	0.95504	1
PTPN3	1.0333442	1.02911127	1.035179	1.208433969	0.2731386	0.955058	1
RP11-688G15.3	1.0175934	1.0213615	1.0159601	0.747142975	-0.4205437	0.955059	1
ZGPAT	1.0175934	1.0213615	1.0159601	0.747142975	-0.4205437	0.955059	1
NR1I2	1.0175995	1.02138173	1.0159601	0.746436108	-0.4219093	0.955059	1
RP11-19O2.2	1.0175942	1.02136411	1.0159601	0.747051794	-0.4207198	0.955059	1
LY6G6C	1.0176026	1.02139206	1.0159601	0.746075672	-0.4226061	0.955059	1
XXyac-YX155B6.5	1.0175921	1.02135737	1.0159601	0.747287499	-0.4202647	0.955059	1
SLC32A1	1.0175921	1.02135737	1.0159601	0.747287499	-0.4202647	0.955059	1
CTC-542B22.2	1.0176002	1.02138396	1.0159601	0.746358103	-0.4220601	0.955059	1

RP11-769O8.1	1.0175928	1.02135955	1.0159601	0.747211007	-0.4204124	0.955059	1
RP11-1105G2.3	1.0175939	1.02136306	1.0159601	0.747088505	-0.4206489	0.955059	1
AC092625.1	1.0175924	1.02135805	1.0159601	0.747263731	-0.4203106	0.955059	1
SHANK1	1.0175935	1.02136197	1.0159601	0.747126405	-0.4205757	0.955059	1
FAM3B	1.0175964	1.02137142	1.0159601	0.746796129	-0.4212136	0.955059	1
RP1-292B18.3	1.0175948	1.02136612	1.0159601	0.746981229	-0.4208561	0.955059	1
AC009487.4	1.0175985	1.02137838	1.0159601	0.746552801	-0.4216838	0.955059	1
MYBPC3	1.0176011	1.02138711	1.0159601	0.74624823	-0.4222725	0.955059	1
RP11-42O4.2	1.0175949	1.02136649	1.0159601	0.746968313	-0.4208811	0.955059	1
LHX6	1.0175981	1.02137701	1.0159601	0.746600915	-0.4215908	0.955059	1
KLF11	1.1407597	1.13648203	1.1426138	1.044927468	0.0634028	0.955096	1
PLGLB1	1.0212741	1.02503917	1.0196421	0.78445481	-0.3502378	0.955126	1
GTF2H4	1.0212874	1.02503702	1.0196621	0.785321083	-0.3486455	0.955126	1
GNL2	1.4484927	1.45268585	1.4466751	0.986722085	-0.0192843	0.955127	1
DHX35	1.0959112	1.09171549	1.0977299	1.065576521	0.0916342	0.955132	1
RP1-76B20.11	1.0169808	1.01281342	1.0187871	1.466206507	0.5520883	0.955228	1
RP11-196E1.3	1.0169659	1.01281342	1.0187659	1.464549335	0.5504568	0.955228	1
MSTN	1.0169841	1.01281342	1.0187919	1.466578457	0.5524543	0.955228	1
DLGAP1-AS3	1.0170033	1.01281342	1.0188194	1.468722696	0.554562	0.955228	1
CDADC1	1.2767295	1.28043869	1.2751218	0.981040703	-0.0276151	0.95537	1
RP11-373N22.3	1.0290529	1.03279426	1.0274312	0.836462775	-0.2576268	0.955502	1
RP11-420A6.2	1.029104	1.03293715	1.0274425	0.83317817	-0.2633031	0.955502	1
TMEM81	1.0301849	1.03391569	1.0285678	0.842318222	-0.2475627	0.95555	1
CSTF1	1.2981791	1.30243898	1.2963326	0.979809635	-0.0294266	0.955573	1
FKBPL	1.1391123	1.1348511	1.1409593	1.045296073	0.0639116	0.955575	1
C8orf82	1.508187	1.50364089	1.5101575	1.012938949	0.0185472	0.95559	1
RP11-48G14.3	1.0352261	1.0310369	1.0370419	1.19347957	0.2551739	0.95564	1
GIGYF2	1.4900989	1.48506519	1.4922808	1.01487554	0.0213028	0.955657	1
ZNF425	1.063939	1.05972826	1.0657641	1.101055255	0.1388869	0.955686	1
RP4-799P18.2	1.0188401	1.02259237	1.0172137	0.761926123	-0.392277	0.955691	1
LINC01285	1.0188356	1.02257041	1.0172168	0.762803856	-0.390616	0.955691	1
OCM	1.0188248	1.02258282	1.0171958	0.761455526	-0.3931683	0.955691	1
RP11-644F5.10	1.0188381	1.02257311	1.0172192	0.762817692	-0.3905898	0.955691	1
CTB-31O20.3	1.0188256	1.02257885	1.0171988	0.761721289	-0.3926649	0.955691	1
PGAM5	1.0952692	1.09911746	1.0936011	0.944344997	-0.0826141	0.955692	1
DHX33	1.2597844	1.26392062	1.2579915	0.977534554	-0.0327804	0.955722	1
CHODL	1.0714303	1.06718088	1.0732722	1.090670779	0.1252157	0.955804	1
ACKR2	1.0247888	1.02059369	1.0266071	1.292005064	0.3696117	0.955863	1
FANCE	1.0748465	1.07858325	1.0732267	0.931836546	-0.1018512	0.955876	1
PILRA	1.0283427	1.03206048	1.0267311	0.833772404	-0.2622745	0.955921	1
RP11-981G7.6	1.0283577	1.0321237	1.0267253	0.831948824	-0.2654333	0.955921	1
POC5	1.164931	1.16874047	1.1632797	0.967638146	-0.0474605	0.955986	1
KIRREL3	1.025737	1.02951535	1.0240992	0.816498629	-0.2924776	0.956001	1
NKX1-2	1.0172971	1.02100086	1.0156917	0.747191489	-0.4204501	0.956011	1
AC013480.2	1.0173072	1.0210344	1.0156917	0.745999913	-0.4227526	0.956011	1
GOLGA8J	1.0173072	1.0210344	1.0156917	0.745999913	-0.4227526	0.956011	1
CCDC105	1.0172997	1.02100954	1.0156917	0.746882564	-0.4210467	0.956011	1
RP11-433A10.3	1.0172968	1.02100002	1.0156917	0.74722133	-0.4203925	0.956011	1
IL12RB2	1.0173018	1.02101647	1.0156917	0.746636411	-0.4215222	0.956011	1
TFDP3	1.0173058	1.02102966	1.0156917	0.746167969	-0.4224277	0.956011	1
ZCCHC16	1.0172975	1.02100233	1.0156917	0.747139106	-0.4205512	0.956011	1

RP13-188A5.1	1.0172975	1.02100233	1.0156917	0.747139106	-0.4205512	0.956011	1
LSMEM2	1.0173105	1.02104531	1.0156917	0.745613196	-0.4235007	0.956011	1
RGPD6	1.0173029	1.02102022	1.0156917	0.746503329	-0.4217794	0.956011	1
RP11-20I23.7	1.0173037	1.02102273	1.0156917	0.746414055	-0.4219519	0.956011	1
AC026904.1	1.0173009	1.02101361	1.0156917	0.746738019	-0.4213259	0.956011	1
RP11-142M10.2	1.0173044	1.02102506	1.0156917	0.746331453	-0.4221116	0.956011	1
RP11-454C18.1	1.0172972	1.02100114	1.0156917	0.747181346	-0.4204697	0.956011	1
RP11-275I14.4	1.0173017	1.02101628	1.0156917	0.746643075	-0.4215094	0.956011	1
RP11-632L2.2	1.0529253	1.04883998	1.0546961	1.119904905	0.1633762	0.956016	1
RHOXF1	1.0278674	1.03170247	1.026205	0.826592948	-0.274751	0.956026	1
RP3-323P24.3	1.0340251	1.02983332	1.0358421	1.201410116	0.2647287	0.956152	1
SIK2	1.1695806	1.1734124	1.1679197	0.968325713	-0.0464357	0.956171	1
AACS	1.3193713	1.31473344	1.3213816	1.021123245	0.030157	0.956192	1
GATA2-AS1	1.0301146	1.02600433	1.0318962	1.22657265	0.2946327	0.956198	1
AC114765.2	1.0175544	1.02126748	1.015945	0.749734553	-0.4155482	0.956247	1
FA2H	1.0175492	1.02125015	1.015945	0.750345939	-0.4143722	0.956247	1
RP4-591N18.2	1.0175535	1.02126454	1.015945	0.749838381	-0.4153484	0.956247	1
RP11-195C7.1	1.0175501	1.02125308	1.015945	0.750242491	-0.4145711	0.956247	1
RP11-80H5.6	1.0175501	1.02125308	1.015945	0.750242491	-0.4145711	0.956247	1
RP11-100M12.3	1.0175505	1.02125448	1.015945	0.750193312	-0.4146657	0.956247	1
RP11-352D3.2	1.0175446	1.02123502	1.015945	0.75088071	-0.4133444	0.956247	1
SLA	1.0175468	1.02124228	1.015945	0.750624165	-0.4138374	0.956247	1
RP11-118G23.3	1.0175506	1.0212548	1.015945	0.75018183	-0.4146878	0.956247	1
ZNF366	1.01755	1.02125283	1.015945	0.750251547	-0.4145537	0.956247	1
RNF148	1.0175509	1.02125579	1.015945	0.750146959	-0.4147548	0.956247	1
RP11-510M2.1	1.0175441	1.02123334	1.015945	0.750940001	-0.4132305	0.956247	1
AC005339.2	1.0175482	1.02124707	1.015945	0.750454849	-0.4141628	0.956247	1
RP11-160E2.11	1.017545	1.02123628	1.015945	0.750835936	-0.4134304	0.956247	1
ANKRD20A1	1.017545	1.02123628	1.015945	0.750835936	-0.4134304	0.956247	1
RP11-118B22.4	1.0175463	1.02124075	1.015945	0.750678037	-0.4137338	0.956247	1
DLG3-AS1	1.0171149	1.01301898	1.0188903	1.450982963	0.5370306	0.956322	1
ADAD2	1.0170995	1.01301898	1.0188683	1.449289784	0.5353461	0.956322	1
AGR3	1.0171048	1.01301898	1.0188759	1.449873181	0.5359267	0.956322	1
RP11-545M17.1	1.0171184	1.01301898	1.0188953	1.451367128	0.5374125	0.956322	1
RP11-767I20.1	1.0171173	1.01301898	1.0188937	1.451243134	0.5372892	0.956322	1
NFAM1	1.0171159	1.01301898	1.0188918	1.451096717	0.5371437	0.956322	1
AMDHD1	1.0171001	1.01301898	1.0188691	1.449352651	0.5354087	0.956322	1
IL20RB	1.0170886	1.01301898	1.0188526	1.448088252	0.5341495	0.956322	1
METTL24	1.0170963	1.01301898	1.0188637	1.448935806	0.5349937	0.956322	1
RP11-57A19.7	1.017087	1.01301898	1.0188503	1.447909103	0.533971	0.956322	1
RP11-108K3.2	1.0197376	1.02346535	1.0181218	0.772278792	-0.3728063	0.956357	1
TYROBP	1.0298808	1.03372512	1.0282145	0.836600909	-0.2573885	0.956364	1
SLC44A2	1.3872089	1.38306739	1.389004	1.015497556	0.0221868	0.95638	1
GS1-124K5.4	1.3527522	1.35681658	1.3509905	0.983672154	-0.0237505	0.956408	1
EBF4	1.1079949	1.10384819	1.1097924	1.057239031	0.0803016	0.95641	1
AC097724.3	1.0241231	1.02001906	1.0259021	1.293871096	0.3716939	0.956425	1
VWF	1.0240897	1.01999725	1.0258636	1.293358709	0.3711225	0.956425	1
RASA1	1.2646651	1.26025917	1.2665748	1.024266709	0.0345914	0.95644	1
BMP5	1.0266329	1.02256581	1.0283957	1.258352003	0.3315355	0.956482	1
NDST2	1.0553362	1.05136882	1.0570559	1.110709878	0.151482	0.956499	1
ZNF230	1.1234311	1.11932199	1.1252122	1.049363934	0.0695151	0.956511	1

ZSCAN31	1.0463304	1.04223134	1.0481071	1.139133777	0.1879372	0.956539	1
HS6ST2	1.207466	1.21118449	1.2058542	0.974760109	-0.0368809	0.956722	1
RP1-8B1.4	1.0375756	1.04128992	1.0359656	0.871051062	-0.1991708	0.956725	1
RP11-599B13.3	1.0163421	1.02001758	1.014749	0.736802858	-0.4406494	0.956734	1
NCR3	1.0163421	1.02001758	1.014749	0.736802858	-0.4406494	0.956734	1
RP11-407G23.7	1.0163447	1.02002611	1.014749	0.736488993	-0.4412641	0.956734	1
GRAPL	1.0163371	1.02000075	1.014749	0.737422728	-0.4394362	0.956734	1
RP11-375O18.2	1.0163371	1.02000075	1.014749	0.737422728	-0.4394362	0.956734	1
HAMP	1.0163371	1.02000075	1.014749	0.737422728	-0.4394362	0.956734	1
LL22NC03-N95F10.1	1.016336	1.01999725	1.014749	0.737551638	-0.439184	0.956734	1
XAF1	1.0163462	1.02003089	1.014749	0.736313223	-0.4416085	0.956734	1
TEN1-CDK3	1.0163462	1.02003089	1.014749	0.736313223	-0.4416085	0.956734	1
AC006000.5	1.0163389	1.02000682	1.014749	0.737198983	-0.439874	0.956734	1
RP11-101E5.1	1.0163404	1.02001179	1.014749	0.737015901	-0.4402323	0.956734	1
WFDC10A	1.0163398	1.02000972	1.014749	0.737092052	-0.4400833	0.956734	1
RP11-24F11.5	1.0163407	1.02001287	1.014749	0.736976219	-0.44031	0.956734	1
SLC28A1	1.0163444	1.02002512	1.014749	0.736525402	-0.4411928	0.956734	1
WNT8B	1.0163399	1.02000999	1.014749	0.737082123	-0.4401027	0.956734	1
AC005624.2	1.0163422	1.02001772	1.014749	0.73679758	-0.4406598	0.956734	1
RP11-1070N10.4	1.0163398	1.02000979	1.014749	0.737089722	-0.4400879	0.956734	1
RP5-937E21.8	1.0163418	1.0200163	1.014749	0.736849692	-0.4405577	0.956734	1
THRA	1.7249465	1.71971428	1.7272144	1.010421013	0.0149565	0.956741	1
WDR11-AS1	1.0422598	1.03815094	1.0440409	1.154384764	0.2071242	0.956782	1
PLAGL2	1.2578456	1.26167043	1.2561877	0.979047284	-0.0305496	0.956795	1
TEX22	1.0627917	1.05868109	1.0645735	1.100413803	0.1380461	0.956819	1
SLC26A10	1.0463932	1.05007445	1.0447975	0.894617713	-0.1606568	0.956827	1
ZNF510	1.098766	1.0944848	1.1006216	1.064950623	0.0907865	0.95683	1
HAAO	1.0828699	1.08650388	1.0812947	0.939781219	-0.0896032	0.956831	1
SPATA21	1.0188261	1.02247499	1.0172444	0.767272827	-0.3821884	0.956837	1
ADCYAP1R1	1.0210603	1.02473995	1.0194654	0.786800513	-0.3459302	0.956872	1
RP11-588G21.2	1.0210524	1.0247355	1.019456	0.786562136	-0.3463674	0.956872	1
CTC-487M23.5	1.021038	1.02471358	1.0194449	0.786808565	-0.3459154	0.956872	1
ECM2	1.0210494	1.02475039	1.0194452	0.78565172	-0.3480382	0.956872	1
NAP1L6	1.0274902	1.03121243	1.0258767	0.829051792	-0.2704659	0.956894	1
GPR83	1.0187004	1.01466374	1.0204502	1.394607786	0.4798594	0.956895	1
SPHKAP	1.0187003	1.01466374	1.02045	1.394597703	0.479849	0.956895	1
HSPA1L	1.0186998	1.01466374	1.0204492	1.394542349	0.4797917	0.956895	1
BOLA2	1.0252948	1.02122762	1.0270578	1.274648746	0.3500997	0.956914	1
COL11A2	1.025315	1.02125015	1.0270769	1.274196552	0.3495878	0.956914	1
FAM216A	1.811081	1.80624898	1.8131755	1.008591024	0.0123413	0.957001	1
KCNH8	1.0214863	1.01740877	1.0232538	1.33575132	0.4176514	0.957047	1
HPSE	1.0215116	1.01740877	1.02329	1.337831416	0.4198963	0.957047	1
OSBPL10-AS1	1.0214582	1.01740877	1.0232134	1.333430773	0.4151429	0.957047	1
NYX	1.0214769	1.01740877	1.0232402	1.334970973	0.4168084	0.957047	1
CLIP1	1.4724252	1.46781646	1.4744229	1.014121763	0.0202309	0.957114	1
RP11-108E14.1	1.0223381	1.02599472	1.0207532	0.798360658	-0.3248875	0.95712	1
RP11-126L15.4	1.0223705	1.02606085	1.020771	0.797017758	-0.3273162	0.95712	1
PMF1-BGLAP	1.022332	1.02599453	1.0207444	0.798029859	-0.3254854	0.95712	1
RP11-798G7.6	1.0259377	1.02960844	1.0243466	0.822285702	-0.2822884	0.957139	1
RP13-714J12.1	1.0259476	1.02964004	1.0243472	0.821427954	-0.2837941	0.957139	1
RP11-399K21.11	1.0366344	1.04021789	1.0350811	0.872276986	-0.1971418	0.957177	1

STK32B	1.0363927	1.04006624	1.0348004	0.868570372	-0.2032854	0.957177	1
SFTA3	1.0195935	1.02324174	1.0180121	0.774989023	-0.3677522	0.957273	1
MATK	1.0195766	1.02323465	1.017991	0.774318343	-0.3690013	0.957273	1
OSCAR	1.0195748	1.02322201	1.017994	0.774866497	-0.3679803	0.957273	1
GALNS	1.1378542	1.13361012	1.1396938	1.045533077	0.0642387	0.957282	1
RP11-528I4.2	1.0349352	1.03870959	1.0332991	0.860229817	-0.217206	0.957297	1
TMEM31	1.046667	1.04252808	1.0484611	1.139507689	0.1884107	0.957318	1
C15orf62	1.0179755	1.01394208	1.0197238	1.414692601	0.5004886	0.95733	1
RP11-290L1.3	1.0180603	1.01394208	1.0198454	1.423419801	0.5093612	0.95733	1
DOK3	1.0179657	1.01394208	1.0197097	1.413685598	0.4994613	0.95733	1
ADORA2A-AS1	1.0179561	1.01394208	1.0196959	1.412697746	0.4984528	0.95733	1
RP4-758J18.10	1.0179835	1.01394208	1.0197352	1.415514096	0.5013261	0.95733	1
PTK6	1.0179971	1.01394208	1.0197548	1.416922121	0.5027605	0.95733	1
GS1-72M22.1	1.0340687	1.02993617	1.0358599	1.197879477	0.2604828	0.95735	1
RCSD1	1.0169666	1.02061472	1.0153853	0.746325078	-0.4221239	0.957375	1
CTD-2616J11.14	1.0169822	1.02059386	1.0154167	0.748604142	-0.4177251	0.957375	1
RP11-797D24.4	1.0169612	1.02059499	1.0153861	0.747080556	-0.4206643	0.957375	1
PCDHGA1	1.0169919	1.02064516	1.0154084	0.746344097	-0.4220872	0.957375	1
ACADL	1.0262194	1.02997262	1.0245925	0.82050053	-0.2854238	0.957376	1
RP11-746M1.1	1.0262348	1.02987672	1.0246562	0.825264548	-0.2770714	0.957376	1
PCDHB15	1.0540556	1.05778203	1.0524404	0.907554899	-0.1399432	0.957377	1
KCND1	1.0376902	1.03360166	1.0394624	1.174417454	0.2319453	0.957406	1
FAM110C	1.0542088	1.05008112	1.0559979	1.118144575	0.1611067	0.957416	1
FBXO11	1.4749263	1.47054055	1.4768274	1.013360802	0.0191479	0.95753	1
QRICH1	1.2908912	1.28647483	1.2928055	1.022098524	0.0315343	0.957541	1
AKAP1	1.1461236	1.14999341	1.1444462	0.963017335	-0.0543663	0.957541	1
SMPD2	1.1341356	1.12981154	1.1360098	1.047748252	0.0672921	0.95755	1
GOLGA8Q	1.028353	1.03198442	1.0267789	0.83724871	-0.2562718	0.957562	1
RP11-48B3.3	1.0283577	1.03201749	1.0267713	0.836145757	-0.2581736	0.957562	1
C8orf58	1.2179878	1.21415747	1.219648	1.025637939	0.0365215	0.957582	1
RP11-507K2.2	1.0188679	1.01486911	1.0206012	1.385500813	0.4704076	0.957811	1
C3	1.0188939	1.01486911	1.0206385	1.388013713	0.4730218	0.957811	1
CAPN14	1.0188728	1.01486911	1.0206082	1.385977236	0.4709036	0.957811	1
FLJ13224	1.0188624	1.01486911	1.0205934	1.384976403	0.4698614	0.957811	1
CTB-58E17.9	1.0179611	1.02154868	1.016406	0.761346211	-0.3933754	0.957846	1
DLEU7-AS1	1.0179401	1.02154002	1.0163797	0.760429861	-0.3951129	0.957846	1
KCNN2	1.0179706	1.02157487	1.0164083	0.760529189	-0.3949245	0.957846	1
CAPSL	1.0249488	1.0285904	1.0233703	0.817417105	-0.2908557	0.957865	1
LDB3	1.025006	1.02861552	1.0234415	0.819188111	-0.2877333	0.957865	1
RP11-677I18.3	1.0172704	1.02088123	1.0157053	0.752125309	-0.4109551	0.957956	1
CTD-2091N23.1	1.0172632	1.02088422	1.0156936	0.751457335	-0.4122369	0.957956	1
KIF25	1.0172657	1.02087718	1.0157003	0.75203224	-0.4111336	0.957956	1
SVOPL	1.0172705	1.02090027	1.0156972	0.751051355	-0.4130165	0.957956	1
SPESP1	1.0172664	1.02085823	1.0157095	0.753156991	-0.4089775	0.957956	1
IGFALS	1.0172917	1.02092775	1.0157156	0.750946556	-0.4132179	0.957956	1
KLK10	1.0274442	1.03104276	1.0258844	0.83382952	-0.2621756	0.958045	1
PPP2R2B	1.1821846	1.18594166	1.1805562	0.971036598	-0.0424024	0.958075	1
APOM	1.0709351	1.06683137	1.0727139	1.088020466	0.1217057	0.95812	1
BLOC1S6	1.8786886	1.87421332	1.8806284	1.007338135	0.010548	0.958133	1
RP11-404F10.2	1.0178594	1.02144545	1.016305	0.760299258	-0.3953607	0.958143	1
RP11-169E6.1	1.0178608	1.02148041	1.0162919	0.758455013	-0.3988645	0.958143	1

FAM19A1	1.0178523	1.02142887	1.0163021	0.760752733	-0.3945005	0.958143	1
AC006262.4	1.0178472	1.02142969	1.0162944	0.760366022	-0.395234	0.958143	1
LVRN	1.0178699	1.02149935	1.0162967	0.758010695	-0.3997099	0.958143	1
SLC16A11	1.0455289	1.04143641	1.0473028	1.141575593	0.1910264	0.958232	1
RP11-848P1.5	1.0558832	1.0517916	1.0576567	1.113244056	0.1547699	0.958249	1
LINC00449	1.0451431	1.04867518	1.0436121	0.895983068	-0.1584566	0.958317	1
LINC01550	1.0310046	1.03461118	1.0294413	0.850630945	-0.2333948	0.958329	1
RIPPLY1	1.0274901	1.0234266	1.0292514	1.248640776	0.3203585	0.958368	1
CHI3L1	1.0274113	1.02343421	1.0291353	1.243278985	0.3141501	0.958368	1
RP4-597N16.4	1.026952	1.02293868	1.0286915	1.250793047	0.3228431	0.958382	1
ADPRH	1.0269493	1.02296089	1.028678	1.248994916	0.3207676	0.958382	1
KIAA1614-AS1	1.02699	1.02299106	1.0287234	1.249327109	0.3211513	0.958382	1
RNASE4	1.0585623	1.05452528	1.0603121	1.106131585	0.145523	0.958404	1
GDAP2	1.1747632	1.17066592	1.1765392	1.034413752	0.0488134	0.958434	1
HPS3	1.1665224	1.16259729	1.1682237	1.034603361	0.0490778	0.958442	1
SH3GL2	1.0179884	1.01400962	1.019713	1.407104476	0.4927295	0.958476	1
ANO2	1.0179778	1.01400962	1.0196978	1.406016681	0.4916137	0.958476	1
LINC01483	1.0180203	1.01400962	1.0197587	1.410368657	0.4960723	0.958476	1
CNGA1	1.0179746	1.01400962	1.0196933	1.405696532	0.4912852	0.958476	1
GPT	1.0179482	1.01400962	1.0196554	1.402991634	0.4885064	0.958476	1
AC007319.1	1.0179812	1.01400962	1.0197027	1.406367193	0.4919733	0.958476	1
CSTA	1.0179736	1.01400962	1.0196919	1.405594786	0.4911807	0.958476	1
MOCOS	1.0255993	1.02912972	1.024069	0.826269281	-0.2753161	0.958547	1
MR1	1.0256069	1.02921675	1.0240422	0.822892397	-0.2812243	0.958547	1
CFB	1.0256044	1.02921817	1.024038	0.822705827	-0.2815514	0.958547	1
LINC01561	1.0255535	1.02909767	1.0240172	0.825400125	-0.2768344	0.958547	1
TOMM40L	1.0824241	1.07834759	1.0841911	1.07458493	0.1037795	0.958666	1
ZNF589	1.126318	1.12999565	1.1247239	0.959446365	-0.0597259	0.958716	1
AC004069.2	1.0471022	1.05068858	1.0455476	0.898578086	-0.1542842	0.958807	1
DRAIC	1.1451682	1.14089278	1.1470215	1.043499013	0.0614292	0.958853	1
AC004951.6	1.1267935	1.13031923	1.1252653	0.961218902	-0.0570631	0.958907	1
RP11-707A18.1	1.0178274	1.02138517	1.0162853	0.761521682	-0.393043	0.958942	1
CFAP61	1.0178114	1.02136479	1.0162712	0.761589491	-0.3929145	0.958942	1
LGI2	1.0178066	1.02135995	1.0162664	0.761535393	-0.393017	0.958942	1
AL358852.1	1.017821	1.02139595	1.0162714	0.760491721	-0.3949956	0.958942	1
C14orf178	1.017819	1.0213615	1.0162834	0.762278876	-0.3916092	0.958942	1
ZBTB47	1.0446316	1.04068845	1.0463408	1.138916804	0.1876624	0.959011	1
RAET1E-AS1	1.0189998	1.02254049	1.0174651	0.774831956	-0.3680446	0.959045	1
TAS1R3	1.019003	1.0225506	1.0174652	0.774490524	-0.3686805	0.959045	1
KRTAP11-1	1.0190235	1.02258848	1.0174782	0.773765355	-0.370032	0.959045	1
AMBRA1	1.0906895	1.08657735	1.0924719	1.068084512	0.0950258	0.959056	1
AC005498.3	1.0258919	1.02944537	1.0243516	0.827008091	-0.2740267	0.959131	1
PRR22	1.0281297	1.02422196	1.0298235	1.231258951	0.3001342	0.959141	1
TMC3-AS1	1.0239095	1.02746785	1.0223671	0.814300815	-0.2963662	0.959152	1
LRRC7	1.0446978	1.04817228	1.0431918	0.896610158	-0.1574473	0.959267	1
DCLRE1B	1.1257106	1.12159635	1.1274939	1.048500928	0.0683281	0.959289	1
BST1	1.0264861	1.03005841	1.0249377	0.82963983	-0.2694429	0.959319	1
AC108142.1	1.17367	1.16980939	1.1753434	1.032589577	0.0462669	0.959355	1
RP11-128A17.2	1.0433461	1.04705401	1.0417389	0.88704242	-0.172925	0.959366	1
AC091878.1	1.0201353	1.01622143	1.0218318	1.345861157	0.4285296	0.959425	1
LGALS7B	1.0201838	1.01622422	1.0219002	1.349843122	0.4327917	0.959425	1

RP5-875O13.1	1.0201233	1.01624157	1.0218059	1.342598107	0.4250275	0.959425	1
FMOD	1.0201376	1.01621508	1.0218379	1.346763406	0.4294964	0.959425	1
SMTN	1.1367984	1.1327435	1.1385561	1.043788095	0.0618289	0.959448	1
SERHL2	1.0522593	1.05592994	1.0506683	0.905924459	-0.1425373	0.959496	1
ABCA13	1.048766	1.04472922	1.0505157	1.129367513	0.175515	0.95952	1
PTCHD4	1.1473329	1.15084934	1.1458086	0.966584596	-0.0490321	0.959527	1
RP11-728K20.3	1.0184125	1.02191405	1.0168948	0.770955663	-0.3752802	0.959533	1
ZNF528	1.0184258	1.02193211	1.016906	0.770833333	-0.3755091	0.959533	1
RP11-134K13.4	1.0184377	1.02198354	1.0169007	0.768788122	-0.379342	0.959533	1
CTB-50L17.14	1.018401	1.02191664	1.0168771	0.770057558	-0.3769618	0.959533	1
RP11-466P24.6	1.0165925	1.02009879	1.0150726	0.749928101	-0.4151758	0.959574	1
CLPS	1.0165944	1.02010524	1.0150726	0.7496875	-0.4156387	0.959574	1
RP11-903H12.3	1.0165902	1.02009109	1.0150726	0.75021561	-0.4146228	0.959574	1
SSTR1	1.0165902	1.02009109	1.0150726	0.75021561	-0.4146228	0.959574	1
RP11-298A10.1	1.0165991	1.02012083	1.0150726	0.749106561	-0.4167571	0.959574	1
RP3-400B16.1	1.0165932	1.02010117	1.0150726	0.749839255	-0.4153467	0.959574	1
NAV2-AS5	1.0165974	1.02011496	1.0150726	0.749325252	-0.416336	0.959574	1
RP11-462G2.1	1.0165974	1.02011496	1.0150726	0.749325252	-0.416336	0.959574	1
IL37	1.0165877	1.02008312	1.0150726	0.750513187	-0.4140507	0.959574	1
CLCN1	1.0165931	1.02010089	1.0150726	0.749849927	-0.4153262	0.959574	1
UST-AS1	1.0165907	1.02009277	1.0150726	0.750152951	-0.4147433	0.959574	1
GLIS3-AS1	1.0165911	1.02009416	1.0150726	0.750100919	-0.4148434	0.959574	1
RP1-225E12.2	1.0165961	1.02011088	1.0150726	0.749477377	-0.4160432	0.959574	1
KRTAP10-4	1.0166004	1.02012513	1.0150726	0.748946637	-0.4170652	0.959574	1
RIMBP3C	1.0165929	1.02010019	1.0150726	0.74987599	-0.4152761	0.959574	1
RP3-395M20.9	1.0165918	1.02009638	1.0150726	0.750018018	-0.4150028	0.959574	1
RP11-475C16.2	1.0165965	1.02011199	1.0150726	0.749435809	-0.4161232	0.959574	1
LINC01082	1.0165881	1.02008439	1.0150726	0.750465983	-0.4141414	0.959574	1
CPO	1.0165966	1.02011235	1.0150726	0.749422425	-0.4161489	0.959574	1
SAT2	5.5221361	5.51331892	5.5259579	1.002800372	0.0040344	0.959585	1
DOCK6	1.1018258	1.09781393	1.1035648	1.058794258	0.0824223	0.959657	1
A1BG-AS1	1.0825468	1.07858105	1.0842658	1.072342882	0.1007663	0.959672	1
EPHX3	1.0439897	1.04006098	1.0456926	1.140575968	0.1897625	0.959676	1
AC144831.1	1.034229	1.03776081	1.0326981	0.865927192	-0.2076824	0.959709	1
CPSF7	1.4429817	1.44709186	1.4412001	0.986822016	-0.0191382	0.95976	1
FSD1	1.4897306	1.48566956	1.4914909	1.011986233	0.0171897	0.959797	1
IRF4	1.0186805	1.02221713	1.0171475	0.771815046	-0.3736729	0.959799	1
CTD-2027I19.3	1.0186843	1.02221566	1.0171536	0.77213826	-0.3730689	0.959799	1
NEURL3	1.020731	1.01683833	1.0224183	1.331383829	0.4129266	0.959813	1
CLDN8	1.0207411	1.01683833	1.0224328	1.332247826	0.4138625	0.959813	1
AC004156.3	1.0207346	1.01683833	1.0224235	1.331692154	0.4132606	0.959813	1
ERBB4	1.1514339	1.15498879	1.149893	0.967121437	-0.048231	0.959828	1
GNE	1.0698592	1.0734405	1.0683069	0.930098273	-0.1045449	0.959877	1
ADPRM	1.2076783	1.20347764	1.209499	1.029592448	0.0420734	0.959909	1
RP11-388C12.1	1.0386137	1.03473554	1.0402948	1.160044747	0.2141805	0.959955	1
RP11-477N3.1	1.0195229	1.01564421	1.0212042	1.355400049	0.4387187	0.960017	1
STXBP5L	1.0276394	1.02370377	1.0293453	1.238002194	0.3080139	0.960069	1
SNAI1	1.0276247	1.02374577	1.029306	1.234155735	0.3035245	0.960069	1
ZNF442	1.0422872	1.04583058	1.0407512	0.889171402	-0.1694665	0.960098	1
ZSCAN12	1.1266948	1.13036101	1.1251057	0.959686475	-0.0593649	0.960157	1
LAMC3	1.0264192	1.02993336	1.024896	0.831714707	-0.2658394	0.960158	1

ZNF768	1.2578301	1.26149117	1.2562432	0.979930537	-0.0292486	0.96016	1
CORO7	1.1459298	1.14958605	1.1443449	0.964962652	-0.051455	0.960162	1
FBXL8	1.1086387	1.11219205	1.1070985	0.954599531	-0.0670325	0.960216	1
DCUN1D1	1.4887438	1.49310373	1.486854	0.987325663	-0.0184021	0.960319	1
DIRAS1	1.1007858	1.09683054	1.1025003	1.058553124	0.0820937	0.960349	1
RP5-902P8.10	1.0162466	1.01971455	1.0147433	0.747840859	-0.4191968	0.960382	1
LHX4	1.0162548	1.01968937	1.0147661	0.749951861	-0.4151301	0.960382	1
AC003092.1	1.0162443	1.01973307	1.0147321	0.746571136	-0.4216484	0.960382	1
RP11-343H5.6	1.0162537	1.0197084	1.0147562	0.748728509	-0.4174854	0.960382	1
RP3-333H23.8	1.0162434	1.01971589	1.0147382	0.747530163	-0.4197963	0.960382	1
AC012593.1	1.0162553	1.01972399	1.0147518	0.747911033	-0.4190614	0.960382	1
AC011298.2	1.0162539	1.01975418	1.0147367	0.74600621	-0.4227405	0.960382	1
RP11-325L7.1	1.0162478	1.01973568	1.014736	0.746666858	-0.4214634	0.960382	1
LINC01140	1.0162486	1.01971548	1.0147459	0.747933279	-0.4190185	0.960382	1
PCAT5	1.016253	1.0197175	1.0147513	0.748130295	-0.4186385	0.960382	1
TAS2R19	1.0162458	1.01968699	1.0147542	0.749440287	-0.4161146	0.960382	1
RP11-849F2.7	1.0162414	1.01970235	1.0147413	0.748198041	-0.4185079	0.960382	1
CORO2B	1.0994964	1.10293493	1.098006	0.952115819	-0.070791	0.960414	1
CXorf38	1.2007489	1.19684589	1.2024406	1.028421875	0.0404322	0.960423	1
CNKSR1	1.0447379	1.04820961	1.0432331	0.896774215	-0.1571833	0.960428	1
TRAPPC9	1.1092548	1.11288922	1.1076794	0.953850022	-0.0681657	0.9605	1
NR1H3	1.1156521	1.11909002	1.114162	0.958619088	-0.0609704	0.960552	1
DNASE1L2	1.0256888	1.02918128	1.024175	0.82844125	-0.2715287	0.960631	1
CYP4A22-AS1	1.0256836	1.02915206	1.0241801	0.829448205	-0.2697762	0.960631	1
TIAF1	1.0231957	1.02662812	1.0217078	0.815222291	-0.2947346	0.960711	1
RP11-399K21.14	1.0232005	1.02665795	1.0217018	0.814084869	-0.2967489	0.960711	1
TNMD	1.019627	1.01574718	1.0213087	1.353177563	0.4363512	0.960715	1
KRTAP10-2	1.0196803	1.01574718	1.0213852	1.358033859	0.4415195	0.960715	1
HSPA1A	1.0195916	1.01574718	1.021258	1.349958612	0.4329152	0.960715	1
RP11-488L18.8	1.0252586	1.02868729	1.0237725	0.828676211	-0.2711196	0.960718	1
C8orf34	1.0252362	1.02869157	1.0237384	0.827366073	-0.2734023	0.960718	1
FAM95B1	1.0252548	1.02870424	1.0237596	0.827738953	-0.2727522	0.960718	1
CPLX4	1.0271091	1.0305803	1.0256045	0.837286081	-0.2562075	0.960752	1
ACO1	1.3172772	1.31338743	1.3189633	1.017792237	0.0254431	0.960769	1
BAK1	1.3363943	1.33241365	1.3381198	1.017165787	0.0245548	0.960815	1
RP11-231E19.1	1.0300074	1.02609211	1.0317045	1.215099459	0.2810744	0.960818	1
RNF144B	1.0561833	1.0521821	1.0579177	1.109914882	0.150449	0.960874	1
RP11-61A14.4	1.0237311	1.02721801	1.0222197	0.816358433	-0.2927254	0.960893	1
SLC25A48	1.0189914	1.01516282	1.0206509	1.361943348	0.4456667	0.960903	1
RFX8	1.0190465	1.01516282	1.02073	1.367157261	0.4511792	0.960903	1
FLNB-AS1	1.0190014	1.01516282	1.0206652	1.362886695	0.4466656	0.960903	1
CBLC	1.018964	1.01516282	1.0206116	1.359350483	0.4429175	0.960903	1
SIX4	1.1123314	1.10852669	1.1139805	1.050253586	0.0707377	0.96092	1
SLPI	1.0326968	1.02884161	1.0343678	1.191606299	0.2529077	0.960971	1
PALM3	1.0326846	1.0288142	1.0343623	1.192546789	0.2540459	0.960971	1
AC073283.4	1.21792	1.21371333	1.2197434	1.028215561	0.0401428	0.961011	1
GET4	1.0171501	1.02059587	1.0156565	0.760175471	-0.3955956	0.961047	1
GRK1	1.0171616	1.02064552	1.0156514	0.758103759	-0.3995328	0.961047	1
LINC01270	1.0171495	1.02060005	1.0156539	0.759894895	-0.3961282	0.961047	1
SPINK9	1.0171501	1.02060899	1.0156508	0.759415245	-0.3970391	0.961047	1
RP11-757F18.5	1.0171429	1.0205667	1.0156588	0.76136749	-0.3933351	0.961047	1

RP11-560I19.1	1.0171417	1.02056426	1.0156582	0.761427189	-0.393222	0.961047	1
CFAP100	1.0171523	1.02057897	1.015667	0.761312354	-0.3934396	0.961047	1
CNTD1	1.0196973	1.01586794	1.0213572	1.345933072	0.4286067	0.961113	1
RDH16	1.0196745	1.01585885	1.0213284	1.344887815	0.4274858	0.961113	1
RP11-397A16.3	1.019699	1.01586528	1.0213608	1.34638555	0.4290916	0.961113	1
LAPTM5	1.0314137	1.02764366	1.0330478	1.195492266	0.2576048	0.961159	1
ADAP2	1.0362847	1.03248026	1.0379338	1.167902157	0.2239194	0.961182	1
LINC00910	1.0363213	1.03250305	1.0379763	1.168392499	0.224525	0.961182	1
CYLD	1.1850804	1.18882462	1.1834574	0.971575723	-0.0416017	0.961274	1
SLC25A10	1.178661	1.17484237	1.1803162	1.031307085	0.044474	0.96131	1
RAB8B	1.3536065	1.34948593	1.3553926	1.01690109	0.0241794	0.961337	1
NDST4	1.0734779	1.07708204	1.0719156	0.932974974	-0.1000897	0.961379	1
WDSUB1	1.1893218	1.19300118	1.187727	0.97267274	-0.0399736	0.961472	1
LMO7-AS1	1.0187449	1.01490147	1.0204108	1.369719829	0.4538808	0.961484	1
RP1-97J1.2	1.0187149	1.01490147	1.0203678	1.366830053	0.4508339	0.961484	1
RP5-1021I20.8	1.0187168	1.01490147	1.0203706	1.367018295	0.4510326	0.961484	1
RP1-124C6.1	1.0187367	1.01490147	1.0203991	1.368928973	0.4530476	0.961484	1
RP11-266O8.1	1.0187167	1.01490147	1.0203704	1.367003947	0.4510174	0.961484	1
RP11-429J17.2	1.0556732	1.05899417	1.0542337	0.919306615	-0.121382	0.96149	1
LINC00242	1.0162408	1.01963624	1.014769	0.752130477	-0.4109451	0.961527	1
RP11-422J15.1	1.0162416	1.01963887	1.014769	0.752027886	-0.4111419	0.961527	1
CPEB2-AS1	1.0162561	1.01962643	1.0147951	0.753837894	-0.4076738	0.961527	1
SLC23A3	1.0162526	1.01962825	1.0147894	0.75347576	-0.408367	0.961527	1
GAL3ST1	1.0162476	1.01966195	1.0147676	0.751073737	-0.4129735	0.961527	1
RAD51AP2	1.0162371	1.01963383	1.0147648	0.752009967	-0.4111763	0.961527	1
RP11-316M1.3	1.01624	1.01965743	1.0147587	0.750796806	-0.4135056	0.961527	1
BFSP2	1.0162353	1.01962224	1.0147673	0.752577657	-0.4100876	0.961527	1
CPNE7	1.0248503	1.02825006	1.0233767	0.827491853	-0.273183	0.961567	1
RP11-392P7.7	1.0184558	1.02185161	1.0169839	0.777237002	-0.3635735	0.96158	1
CTD-2527I21.15	1.0184579	1.02185836	1.0169839	0.776996915	-0.3640192	0.96158	1
CTA-503F6.2	1.0184655	1.02188353	1.0169839	0.776103224	-0.3656795	0.96158	1
RP11-295I5.4	1.0184518	1.02183822	1.0169839	0.777713675	-0.362689	0.96158	1
UBXN7-AS1	1.0184597	1.02186441	1.0169839	0.776782067	-0.3644182	0.96158	1
C17orf77	1.0184594	1.0218635	1.0169839	0.776814264	-0.3643584	0.96158	1
RP11-542B15.1	1.0184557	1.02185112	1.0169839	0.777254541	-0.363541	0.96158	1
NICN1	1.018462	1.02187206	1.0169839	0.776510361	-0.3649229	0.96158	1
RP11-378A12.1	1.0184508	1.02183518	1.0169839	0.777821776	-0.3624885	0.96158	1
AC011738.4	1.0184552	1.02184971	1.0169839	0.777304842	-0.3634476	0.96158	1
AF121898.3	1.0184591	1.02186264	1.0169839	0.776844877	-0.3643016	0.96158	1
RP5-940J5.3	1.0184551	1.02184929	1.0169839	0.777319582	-0.3634202	0.96158	1
LINC01098	1.0184518	1.02183836	1.0169839	0.777708598	-0.3626984	0.96158	1
TMEM240	1.0438035	1.04720469	1.0423292	0.89671695	-0.1572754	0.961639	1
TRAF3IP2	1.1899206	1.19357073	1.1883384	0.972969384	-0.0395337	0.961668	1
PTGS2	1.0212842	1.01751018	1.0229201	1.308959366	0.3884203	0.961687	1
LRRC74B	1.0213129	1.01750514	1.0229634	1.311809336	0.391558	0.961687	1
GOLT1A	1.0213043	1.01749871	1.0229539	1.311748854	0.3914915	0.961687	1
ODF3L1	1.0213035	1.0175148	1.0229457	1.310077247	0.3896519	0.961687	1
TANC2	1.3010755	1.30458992	1.2995521	0.983460426	-0.0240611	0.961705	1
PYROXD2	1.1550366	1.15862812	1.1534798	0.96754466	-0.0475998	0.961721	1
PTGER1	1.0343881	1.03783147	1.0328956	0.869530467	-0.2016915	0.961727	1
GPR39	1.0317701	1.0352273	1.0302716	0.859322114	-0.2187291	0.961807	1

TRIM7	1.0318024	1.03518352	1.0303368	0.86224436	-0.2138313	0.961807	1
METTL7B	1.0318172	1.03521257	1.0303454	0.861778645	-0.2146107	0.961807	1
RP11-231I16.1	1.0197632	1.0231694	1.0182867	0.789262204	-0.3414234	0.96181	1
REP15	1.0197289	1.02313041	1.0182544	0.789196226	-0.341544	0.96181	1
TRPV4	1.0197558	1.02317383	1.0182742	0.788571057	-0.3426873	0.96181	1
RP11-491F9.5	1.0197373	1.0231694	1.0182497	0.787664226	-0.3443473	0.96181	1
SCGB1D4	1.0197516	1.02317599	1.0182673	0.788197796	-0.3433704	0.96181	1
RP11-159F24.5	1.0197562	1.02316731	1.0182777	0.788941771	-0.3420093	0.96181	1
TM6SF1	1.0197388	1.02314683	1.0182616	0.788947067	-0.3419996	0.96181	1
ASB16	1.0197329	1.02314159	1.0182554	0.78885475	-0.3421684	0.96181	1
LPXN	1.0824286	1.07841046	1.0841703	1.073457829	0.1022655	0.961812	1
RP5-1142A6.2	1.0241021	1.02026679	1.0257646	1.271270203	0.3462707	0.96184	1
COL5A3	1.0233687	1.02685982	1.0218555	0.813686368	-0.2974553	0.961848	1
RP11-540E16.2	1.0233306	1.0266884	1.0218752	0.81965045	-0.2869193	0.961848	1
ZBTB9	1.0233484	1.02677851	1.0218615	0.816383876	-0.2926804	0.961848	1
IAPP	1.0338637	1.03725047	1.0323956	0.869670359	-0.2014594	0.961851	1
RNF43	1.0569344	1.06039645	1.0554337	0.917830317	-0.1237006	0.961892	1
RP11-61J19.5	1.0569293	1.06035588	1.0554441	0.918618955	-0.1224615	0.961892	1
QRICH2	1.0569483	1.06018517	1.0555452	0.922904954	-0.115746	0.961892	1
ACKR1	1.0169891	1.02038275	1.0155181	0.761336444	-0.393394	0.961962	1
RP11-471B22.3	1.017	1.02040599	1.0155237	0.760740246	-0.3945242	0.961962	1
TUSC8	1.0169845	1.02037457	1.015515	0.76148852	-0.3931058	0.961962	1
AC017002.2	1.0169777	1.02034096	1.0155199	0.762990184	-0.3902636	0.961962	1
GP1BA	1.027714	1.02388772	1.0293725	1.229606247	0.2981964	0.961962	1
HOXA5	1.0227697	1.01899597	1.0244055	1.284770078	0.3615102	0.961987	1
FGF10	1.0227692	1.01899597	1.0244047	1.284730545	0.3614658	0.961987	1
RP11-74C3.1	1.0227751	1.01899597	1.0244131	1.285173721	0.3619634	0.961987	1
GRID1	1.0227816	1.01899597	1.0244225	1.285668903	0.3625192	0.961987	1
SNAP25-AS1	1.0227724	1.01899597	1.0244092	1.284969399	0.361734	0.961987	1
CEP250	1.4347177	1.43874237	1.4329732	0.986850732	-0.0190962	0.961996	1
TESMIN	1.1526757	1.15621039	1.1511436	0.967564049	-0.0475709	0.962018	1
RP13-726E6.1	1.0143864	1.01775627	1.0129257	0.727949781	-0.4580892	0.962082	1
OPN1SW	1.0143821	1.01774218	1.0129257	0.728527737	-0.4569442	0.962082	1
RP11-82O19.1	1.0143824	1.01774302	1.0129257	0.728493259	-0.4570125	0.962082	1
RP11-227D13.4	1.0143843	1.01774939	1.0129257	0.728231824	-0.4575303	0.962082	1
TARID	1.0143818	1.01774131	1.0129257	0.728563527	-0.4568733	0.962082	1
ADGRG5	1.0143855	1.01775345	1.0129257	0.728065331	-0.4578602	0.962082	1
AC004014.3	1.0143849	1.01775155	1.0129257	0.72814342	-0.4577055	0.962082	1
CTC-508F8.1	1.0143813	1.01773938	1.0129257	0.728642665	-0.4567166	0.962082	1
RP11-325K19.1	1.0143815	1.01774002	1.0129257	0.728616686	-0.4567681	0.962082	1
CTC-756D1.2	1.0143851	1.01775192	1.0129257	0.728128311	-0.4577354	0.962082	1
CD86	1.0143881	1.01776197	1.0129257	0.72771624	-0.4585521	0.962082	1
FAM86B2	1.0143856	1.01775367	1.0129257	0.728056249	-0.4578782	0.962082	1
RP11-256I23.1	1.0143879	1.01776132	1.0129257	0.727742919	-0.4584992	0.962082	1
RP11-22L13.1	1.0143836	1.01774722	1.0129257	0.728320966	-0.4573537	0.962082	1
RP11-446H18.5	1.014382	1.01774168	1.0129257	0.7285484	-0.4569033	0.962082	1
STEAP3-AS1	1.0143847	1.01775073	1.0129257	0.728177144	-0.4576386	0.962082	1
RP1-261G23.5	1.0143847	1.01775073	1.0129257	0.728177144	-0.4576386	0.962082	1
XXbac-BPG308J9.3	1.0143839	1.01774806	1.0129257	0.728286487	-0.457422	0.962082	1
SLC13A5	1.0236265	1.02702805	1.022152	0.819593917	-0.2870188	0.962085	1
SLC6A4	1.0236628	1.02701245	1.0222108	0.822244754	-0.2823602	0.962085	1

FMNL1	1.0366703	1.03283877	1.0383311	1.167250035	0.2231136	0.962143	1
LLGL1	1.300128	1.29625121	1.3018084	1.01875843	0.026812	0.962185	1
AC005363.11	1.0258613	1.02933284	1.0243566	0.830352861	-0.2682036	0.962235	1
SUMO4	1.0699125	1.06605463	1.0715847	1.083719587	0.1159915	0.962247	1
PSPN	1.0560579	1.05234525	1.0576671	1.101668877	0.1396907	0.962329	1
ACSS2	1.1938588	1.19751643	1.1922734	0.973454982	-0.0388138	0.962349	1
DCXR	2.6764635	2.68085958	2.674558	0.996250959	-0.0054189	0.962358	1
SMIM10L2B	1.1233654	1.11946778	1.1250549	1.046766615	0.0659398	0.962385	1
KMT2D	1.1537661	1.15728767	1.1522396	0.967905687	-0.0470616	0.962386	1
ZNF583	1.2010779	1.20452269	1.1995847	0.975855934	-0.0352599	0.962388	1
ENTPD7	1.0498597	1.05326075	1.0483855	0.908464912	-0.1384973	0.96239	1
ADAMTS19	1.0499301	1.05340214	1.0484251	0.906801098	-0.141142	0.96239	1
RP11-225B17.2	1.0399839	1.03619436	1.0416264	1.150081163	0.2017357	0.962413	1
C2orf80	1.0153736	1.01870684	1.0139287	0.744579829	-0.4255016	0.962535	1
C10orf105	1.0153651	1.01870236	1.0139186	0.74421637	-0.426206	0.962535	1
SLC4A1	1.015372	1.01869581	1.0139313	0.745154761	-0.424388	0.962535	1
RP11-70D24.3	1.0153744	1.01870278	1.0139317	0.744899992	-0.4248813	0.962535	1
RP11-159D12.10	1.0153737	1.01872758	1.0139199	0.743285194	-0.4280122	0.962535	1
HIST1H3F	1.0153726	1.01873311	1.013916	0.742856426	-0.4288447	0.962535	1
RP11-927P21.2	1.0153916	1.01875461	1.0139338	0.742954919	-0.4286534	0.962535	1
LINC01389	1.0153752	1.01870642	1.0139313	0.744732042	-0.4252067	0.962535	1
RP11-274H2.3	1.0223931	1.02581309	1.0209107	0.810080033	-0.3038636	0.962574	1
PAK5	1.064441	1.06760254	1.0630706	0.932962442	-0.1001091	0.96262	1
RP1-224A6.9	1.0146837	1.01801971	1.0132377	0.734621901	-0.4449262	0.962663	1
RP3-406P24.3	1.0146837	1.01801971	1.0132377	0.734621901	-0.4449262	0.962663	1
CLDN34	1.0146765	1.01799599	1.0132377	0.735590332	-0.4430256	0.962663	1
WEE2	1.0146775	1.01799924	1.0132377	0.735457548	-0.443286	0.962663	1
AC003956.1	1.0146775	1.01799924	1.0132377	0.735457548	-0.443286	0.962663	1
MAGEA8-AS1	1.014677	1.01799766	1.0132377	0.735521944	-0.4431597	0.962663	1
RP11-758N13.1	1.0146816	1.01801276	1.0132377	0.734905209	-0.4443699	0.962663	1
RP11-507K2.3	1.0146895	1.01803881	1.0132377	0.733844223	-0.4464542	0.962663	1
RP5-983L19.2	1.0146767	1.01799675	1.0132377	0.735559285	-0.4430865	0.962663	1
DAND5	1.0146856	1.01802605	1.0132377	0.734363556	-0.4454336	0.962663	1
SLC5A5	1.0146806	1.0180094	1.0132377	0.735042455	-0.4441005	0.962663	1
AC073342.12	1.0146829	1.01801712	1.0132377	0.734727657	-0.4447185	0.962663	1
CHRM4	1.0146827	1.01801628	1.0132377	0.734762014	-0.4446511	0.962663	1
CTB-30L5.1	1.0146818	1.01801346	1.0132377	0.734876714	-0.4444259	0.962663	1
RP11-154H23.5	1.0146818	1.01801346	1.0132377	0.734876714	-0.4444259	0.962663	1
KLK3	1.0146766	1.01799619	1.0132377	0.735581952	-0.443042	0.962663	1
AC024132.1	1.0146766	1.01799619	1.0132377	0.735581952	-0.443042	0.962663	1
RP11-169K17.3	1.0146777	1.01800003	1.0132377	0.735425126	-0.4433496	0.962663	1
RP11-473O4.5	1.0146849	1.01802387	1.0132377	0.734452545	-0.4452588	0.962663	1
LINC00838	1.0146802	1.01800821	1.0132377	0.735090981	-0.4440053	0.962663	1
INSRR	1.0146778	1.01800032	1.0132377	0.735413496	-0.4433724	0.962663	1
RP11-244N9.4	1.0146819	1.01801375	1.0132377	0.734865046	-0.4444488	0.962663	1
IYD	1.0146819	1.01801375	1.0132377	0.734865046	-0.4444488	0.962663	1
FCRL2	1.0146854	1.01802521	1.0132377	0.734397987	-0.445366	0.962663	1
RP11-227G15.10	1.0248716	1.02819352	1.0234317	0.831103218	-0.2669004	0.962717	1
CASKIN2	1.0781825	1.08181708	1.0766071	0.93632172	-0.0949238	0.962722	1
CTD-2302E22.4	1.0271101	1.03050495	1.0256386	0.840472301	-0.2507278	0.962732	1
SELENOV	1.0270961	1.03046923	1.025634	0.841306599	-0.2492964	0.962732	1

NKD1	1.0797148	1.08308992	1.0782518	0.941772783	-0.0865491	0.962782	1
IPP	1.163511	1.16714125	1.1619375	0.96886603	-0.0456309	0.962835	1
RP11-383M4.6	1.0152782	1.01860899	1.0138345	0.743430397	-0.4277304	0.96285	1
STK24-AS1	1.0152743	1.01859593	1.0138345	0.743952435	-0.4267177	0.96285	1
DLGAP2-AS1	1.0152743	1.01859593	1.0138345	0.743952435	-0.4267177	0.96285	1
HIST1H3I	1.0152729	1.01859124	1.0138345	0.744140229	-0.4263536	0.96285	1
ABCC11	1.0152754	1.01859973	1.0138345	0.743800637	-0.4270121	0.96285	1
RP11-705C15.4	1.0152739	1.01859459	1.0138345	0.744006354	-0.4266132	0.96285	1
AC092415.1	1.0152759	1.01860127	1.0138345	0.743738998	-0.4271317	0.96285	1
AC009502.4	1.0152777	1.01860724	1.0138345	0.743500477	-0.4275944	0.96285	1
CTD-3203P2.3	1.0152741	1.01859544	1.0138345	0.743972115	-0.4266795	0.96285	1
RP5-1056H1.2	1.0152705	1.01858349	1.0138345	0.744450624	-0.4257519	0.96285	1
RP11-417B4.2	1.0152705	1.01858349	1.0138345	0.744450624	-0.4257519	0.96285	1
RP1-302D9.1	1.0152749	1.01859789	1.0138345	0.743874297	-0.4268692	0.96285	1
LINC00216	1.0152768	1.01860429	1.0138345	0.743618184	-0.427366	0.96285	1
AC140542.2	1.0152758	1.01860103	1.0138345	0.743748462	-0.4271133	0.96285	1
SRD5A2	1.015278	1.01860828	1.0138345	0.743459015	-0.4276749	0.96285	1
LINC01337	1.0152714	1.01858627	1.0138345	0.744339162	-0.425968	0.96285	1
AF064858.8	1.0152721	1.01858872	1.0138345	0.744241016	-0.4261582	0.96285	1
RP1-228P16.5	1.0152728	1.01859094	1.0138345	0.744152413	-0.42633	0.96285	1
GRPEL2-AS1	1.0152753	1.01859924	1.0138345	0.743820335	-0.4269739	0.96285	1
RP11-319E12.2	1.015271	1.01858502	1.0138345	0.744389228	-0.4258709	0.96285	1
LRRC9	1.0695893	1.07289028	1.0681585	0.935083927	-0.0968322	0.962874	1
RP11-1072A3.4	1.0404797	1.04379701	1.0390419	0.891427538	-0.1658106	0.962892	1
SPECC1L	1.2214046	1.22460022	1.2200194	0.979604736	-0.0297283	0.962894	1
CD101	1.0279814	1.02423536	1.0296051	1.221565078	0.2887307	0.962919	1
CHRFAM7A	1.0481539	1.04438007	1.0497898	1.121894438	0.1659369	0.962972	1
DLGAP3	1.0283986	1.03171116	1.0269627	0.850258629	-0.2340264	0.963001	1
RP11-316O14.1	1.0284008	1.03175566	1.0269466	0.848559943	-0.2369115	0.963001	1
C17orf75	1.4473909	1.45135748	1.4456716	0.987402725	-0.0182895	0.963021	1
DBR1	1.1520946	1.15581212	1.1504832	0.965798966	-0.0502052	0.963032	1
RP11-677M14.3	1.0316899	1.02794591	1.0333127	1.192042929	0.2534362	0.963037	1
TNFSF4	1.0316208	1.02789793	1.0332344	1.191286909	0.2525209	0.963037	1
GPR89A	1.2019479	1.19828923	1.2035338	1.026449191	0.0376622	0.963059	1
C15orf39	1.0656172	1.06909473	1.0641099	0.927855255	-0.1080283	0.96306	1
ISL1	1.0275199	1.02372877	1.0291632	1.229022412	0.2975112	0.963101	1
IFI44	1.0275519	1.02372753	1.0292096	1.231044187	0.2998825	0.963101	1
RP11-482M8.3	1.0256215	1.02892498	1.0241896	0.836287301	-0.2579294	0.963154	1
RP11-517B11.4	1.0299875	1.0262833	1.0315931	1.202021925	0.2654632	0.963181	1
RP11-92G12.3	1.0300084	1.02627323	1.0316274	1.203787346	0.2675806	0.963181	1
CHRNE	1.0300736	1.02629001	1.0317136	1.206299357	0.270588	0.963181	1
RP11-541G9.1	1.0300554	1.02638423	1.0316467	1.199454053	0.2623779	0.963181	1
AC002116.7	1.0322925	1.03560259	1.0308577	0.866727265	-0.20635	0.963186	1
MINA	1.1997635	1.20335929	1.1982048	0.974653443	-0.0370388	0.963218	1
NOX4	1.0819145	1.07814981	1.0835464	1.069054075	0.0963348	0.963225	1
RP11-50I19.2	1.0229934	1.02630192	1.0215593	0.819685605	-0.2868574	0.963236	1
AC004221.2	1.023026	1.02633332	1.0215925	0.819968346	-0.2863599	0.963236	1
RP11-998D10.4	1.0206043	1.01686463	1.0222253	1.317863513	0.398201	0.96327	1
RP5-1057I20.4	1.0205633	1.01685488	1.0221707	1.315390193	0.3954908	0.96327	1
NPIPB15	1.0205669	1.01684433	1.0221805	1.316794391	0.3970301	0.96327	1
RP11-618L22.1	1.0205602	1.01686399	1.0221624	1.314184554	0.3941679	0.96327	1

CTB-78F1.1	1.0205557	1.01686463	1.0221557	1.313735165	0.3936745	0.96327	1
STS	1.0847274	1.08097069	1.0863557	1.066506206	0.0928924	0.963307	1
MAGEL2	1.0652055	1.06138347	1.0668621	1.089253117	0.1233392	0.96336	1
LINC01138	1.1829349	1.18635883	1.1814507	0.973663072	-0.0385055	0.963433	1
EXO5	1.071474	1.07485101	1.0700103	0.935328605	-0.0964548	0.963434	1
HAND1	1.0152401	1.01853074	1.0138137	0.74544842	-0.4238196	0.963629	1
FERMT3	1.015228	1.018505	1.0138076	0.746156298	-0.4224502	0.963629	1
HRASLS2	1.0152367	1.01852539	1.0138112	0.745527146	-0.4236672	0.963629	1
TMPRSS2	1.0152352	1.01849921	1.0138204	0.747080931	-0.4206636	0.963629	1
RP11-211G23.2	1.0152346	1.01850938	1.0138152	0.746387491	-0.4220033	0.963629	1
CTD-2340E1.2	1.015231	1.01850557	1.0138117	0.746352823	-0.4220703	0.963629	1
AC007285.6	1.0152285	1.01850767	1.0138072	0.746025493	-0.4227032	0.963629	1
APOBEC2	1.0152372	1.01850048	1.0138227	0.747153924	-0.4205226	0.963629	1
GLE1	1.1663684	1.16247701	1.1680552	1.034331953	0.0486993	0.963663	1
RP11-2E11.6	1.0310098	1.03431641	1.0295765	0.8618763	-0.2144473	0.963677	1
AP000442.1	1.0310041	1.03439295	1.0295352	0.85875719	-0.2196778	0.963677	1
TGFA	1.0240154	1.02739649	1.0225498	0.823092343	-0.2808738	0.963729	1
RP11-697E22.1	1.0164182	1.01969366	1.0149985	0.761587988	-0.3929174	0.963751	1
RP5-867C24.5	1.0164188	1.01969545	1.0149985	0.761518949	-0.3930482	0.963751	1
AC092431.3	1.0164122	1.01967371	1.0149985	0.762360399	-0.3914549	0.963751	1
RP11-930P14.1	1.0164167	1.01968849	1.0149985	0.761788186	-0.3925382	0.963751	1
RP11-649A16.1	1.0164167	1.01968849	1.0149985	0.761788186	-0.3925382	0.963751	1
HCG16	1.0164188	1.0196957	1.0149985	0.761509245	-0.3930665	0.963751	1
DCST1	1.0164155	1.01968477	1.0149985	0.761931904	-0.392266	0.963751	1
RP11-13P5.2	1.0164212	1.01970367	1.0149985	0.761201089	-0.3936505	0.963751	1
CLYBL-AS2	1.016422	1.01970621	1.0149985	0.761103024	-0.3938363	0.963751	1
C8A	1.0164166	1.01968834	1.0149985	0.761793718	-0.3925277	0.963751	1
CTD-2013N24.2	1.0164198	1.01969875	1.0149985	0.761391488	-0.3932897	0.963751	1
RP11-756G20.1	1.0164129	1.01967618	1.0149985	0.762264767	-0.3916359	0.963751	1
C3orf49	1.0164179	1.01969263	1.0149985	0.761627829	-0.3928419	0.963751	1
ACTL8	1.0164124	1.01967457	1.0149985	0.762327224	-0.3915177	0.963751	1
CTD-2562J15.6	1.0164124	1.01967457	1.0149985	0.762327224	-0.3915177	0.963751	1
RP11-478K15.6	1.0164215	1.01970466	1.0149985	0.761162853	-0.3937229	0.963751	1
ERICD	1.0164203	1.0197005	1.0149985	0.761323743	-0.393418	0.963751	1
GAS5-AS1	1.0164194	1.01969755	1.0149985	0.761437718	-0.3932021	0.963751	1
LINC00244	1.0164222	1.01970677	1.0149985	0.761081436	-0.3938773	0.963751	1
RP11-280H21.1	1.0164202	1.01970037	1.0149985	0.761328706	-0.3934086	0.963751	1
NAV2-AS3	1.0319819	1.02833369	1.0335632	1.184570399	0.2443639	0.963752	1
ZNF398	1.1032699	1.09955788	1.1048788	1.053445808	0.0751161	0.963763	1
NQO2	1.9621748	1.95752064	1.9641921	1.006967466	0.0100171	0.963786	1
RP11-381K20.2	1.0394408	1.03574666	1.0410421	1.14813835	0.1992965	0.963813	1
CTD-2034I21.2	1.0233183	1.02653637	1.0219234	0.82616272	-0.2755021	0.96382	1
SLC34A2	1.0232917	1.0265828	1.0218652	0.822532735	-0.281855	0.96382	1
TUT1	1.1534202	1.15691395	1.1519058	0.96808345	-0.0467967	0.963837	1
LINC00847	1.1395558	1.13573126	1.1412136	1.040390813	0.0571256	0.963872	1
RP11-536K7.3	1.0213423	1.02466095	1.0199038	0.807096973	-0.3091861	0.963878	1
RP11-171I2.3	1.0368977	1.03320858	1.0384968	1.15924228	0.2131821	0.963894	1
RP1-191J18.66	1.0308001	1.02713139	1.0323904	1.193833401	0.2556015	0.9639	1
IFIH1	1.0307863	1.02705546	1.0324035	1.197668009	0.2602281	0.9639	1
CTD-2376I4.2	1.0229903	1.01930328	1.0245884	1.273796513	0.3491348	0.963936	1
EPHB1	1.0230219	1.01936449	1.0246072	1.27073687	0.3456653	0.963936	1

SLIT2	1.4577749	1.45425653	1.4593	1.011102717	0.0159296	0.96396	1
CXCL1	1.0491635	1.0524402	1.0477432	0.910430934	-0.1353785	0.963969	1
CTD-3035K23.7	1.0491583	1.05247831	1.0477192	0.909313762	-0.1371499	0.963969	1
RP11-326C3.7	1.0209582	1.01724954	1.0225657	1.308192494	0.3875748	0.963997	1
RP11-370I10.6	1.0210179	1.01724954	1.0226513	1.313156244	0.3930386	0.963997	1
ALDH1L1	1.0209227	1.01724954	1.0225149	1.305243805	0.3843193	0.963997	1
HORMAD2	1.0209466	1.01724954	1.022549	1.307225474	0.386508	0.963997	1
RHBDL2	1.0239499	1.0271707	1.0225539	0.830081353	-0.2686754	0.964008	1
TIGD7	1.1057971	1.10914862	1.1043444	0.955984667	-0.0649406	0.964123	1
DHDH	1.0496453	1.05304615	1.0481712	0.908099732	-0.1390773	0.964153	1
CTBP1-AS	1.0227563	1.0190532	1.0243614	1.278598183	0.3545629	0.964203	1
LINC01024	1.0227342	1.01907037	1.0243223	1.27539856	0.3509482	0.964203	1
TMEM74	1.0227445	1.01907806	1.0243337	1.275482386	0.351043	0.964203	1
SCARNA2	1.0158466	1.01913743	1.0144202	0.753505279	-0.4083105	0.96422	1
RP4-798A10.4	1.0158359	1.01906956	1.0144342	0.756926102	-0.4017756	0.96422	1
RP11-235C23.5	1.0158372	1.01910733	1.0144198	0.754674211	-0.4060741	0.96422	1
CTD-2235C13.3	1.0158306	1.01908933	1.0144181	0.755298502	-0.4048812	0.96422	1
RP13-20L14.1	1.0158286	1.01908178	1.0144185	0.755615853	-0.4042751	0.96422	1
RP1-137D17.1	1.0158375	1.01910946	1.0144193	0.754564027	-0.4062848	0.96422	1
AF131217.1	1.0409176	1.04415408	1.0395147	0.894928463	-0.1601557	0.964278	1
TRAF3	1.1757232	1.17175448	1.1774435	1.03312288	0.0470119	0.964305	1
LZIC	1.6780106	1.6739165	1.6797853	1.008708435	0.0125092	0.964309	1
BAG3	1.2871406	1.28343607	1.2887463	1.01873514	0.026779	0.964467	1
RP4-569M23.5	1.0161096	1.01936615	1.0146981	0.758956434	-0.397911	0.964486	1
TPSB2	1.0161017	1.01933435	1.0147004	0.760327119	-0.3953078	0.964486	1
C10orf128	1.0161021	1.01931893	1.0147077	0.761309178	-0.3934456	0.964486	1
HEPN1	1.0160959	1.01933435	1.0146922	0.759901012	-0.3961166	0.964486	1
LINC01133	1.0160933	1.019325	1.0146925	0.760284377	-0.3953889	0.964486	1
SPATA22	1.0161011	1.01933938	1.0146974	0.759973673	-0.3959787	0.964486	1
RP11-110I1.14	1.0161071	1.01936615	1.0146944	0.758766466	-0.3982722	0.964486	1
ZBTB48	1.0846143	1.08088934	1.0862289	1.066010314	0.0922214	0.964521	1
HNRNPUL1	2.6650954	2.67041743	2.6627885	0.995432927	-0.006604	0.964534	1
OTUD6A	1.0233653	1.01968203	1.0249618	1.268253316	0.3428429	0.96461	1
AC006160.5	1.0233191	1.01969235	1.0248912	1.264002497	0.3379993	0.96461	1
SERF1B	1.0233173	1.01969235	1.0248886	1.263870023	0.3378481	0.96461	1
RP11-201E8.1	1.0233615	1.01969895	1.024949	1.266516381	0.3408657	0.96461	1
LINC01405	1.0233023	1.01970559	1.0248614	1.261639851	0.3353001	0.96461	1
LINC01119	1.023345	1.01970037	1.0249248	1.265196139	0.3393611	0.96461	1
RP5-1186N24.3	1.0308333	1.02719157	1.0324119	1.191982712	0.2533633	0.96466	1
LINC00886	1.0282887	1.02461128	1.0298827	1.214186361	0.2799899	0.964663	1
ULK1	1.2013218	1.20466422	1.1998729	0.97658959	-0.0341757	0.964713	1
B3GNTL1	1.1340786	1.13763829	1.1325357	0.962927517	-0.0545009	0.964722	1
RP11-98G7.1	1.0499104	1.04625467	1.051495	1.113294131	0.1548348	0.964752	1
HDAC5	1.4703009	1.46637646	1.4720019	1.012062098	0.0172978	0.964819	1
TMEM102	1.0827772	1.07914753	1.0843505	1.065737235	0.0918518	0.964828	1
RNF217-AS1	1.0238439	1.02702434	1.0224653	0.831296824	-0.2665644	0.964828	1
CDRT4	1.0302422	1.02661352	1.031815	1.195445352	0.2575482	0.964848	1
SCN2B	1.030205	1.02659989	1.0317676	1.194276821	0.2561373	0.964848	1
NCR3LG1	1.0477357	1.0441663	1.0492829	1.115849473	0.1581424	0.96493	1
CTD-2378E12.1	1.0299643	1.03319665	1.0285633	0.860426137	-0.2168767	0.964948	1
DISP3	1.0308897	1.02734377	1.0324267	1.185889661	0.2459698	0.965002	1

JPH2	1.030879	1.0273157	1.0324235	1.186992754	0.2473111	0.965002	1
TDRD7	1.0308544	1.02722478	1.0324276	1.191107262	0.2523033	0.965002	1
RP11-357K6.1	1.044536	1.04766672	1.043179	0.905853139	-0.1426509	0.965006	1
RP11-138C9.1	1.01366	1.01684984	1.0122773	0.728630152	-0.4567414	0.965086	1
CTB-73N10.1	1.013662	1.01685654	1.0122773	0.728340695	-0.4573146	0.965086	1
CSNK1A1L	1.0136629	1.01685957	1.0122773	0.728209641	-0.4575743	0.965086	1
RP11-410D17.2	1.0136662	1.01687033	1.0122773	0.7277454	-0.4584943	0.965086	1
FOXL2	1.0136662	1.01687033	1.0122773	0.7277454	-0.4584943	0.965086	1
DNAI2	1.0136616	1.01685523	1.0122773	0.728397417	-0.4572023	0.965086	1
RP11-872D17.4	1.0136609	1.01685285	1.0122773	0.728500127	-0.4569989	0.965086	1
ZFP3	1.0136591	1.01684711	1.0122773	0.728748422	-0.4565072	0.965086	1
UGT2B28	1.0136626	1.01685853	1.0122773	0.728254882	-0.4574846	0.965086	1
DUX4	1.0136593	1.0168476	1.0122773	0.728727225	-0.4565492	0.965086	1
AIRE	1.0136638	1.0168625	1.0122773	0.728083359	-0.4578245	0.965086	1
RP11-337L12.1	1.0136612	1.01685404	1.0122773	0.728448779	-0.4571006	0.965086	1
RP11-402L1.4	1.013657	1.01684015	1.0122773	0.729049711	-0.4559109	0.965086	1
TMIGD3	1.0136581	1.01684363	1.0122773	0.728898803	-0.4562096	0.965086	1
SARDH	1.0426796	1.03914175	1.0442131	1.12956394	0.1757659	0.965098	1
PITPNM2	1.042675	1.03910096	1.0442243	1.131027341	0.1776338	0.965098	1
MYO16	1.0231013	1.02631192	1.0217096	0.825085858	-0.2773838	0.965355	1
RP11-306I1.2	1.0231232	1.02630416	1.0217444	0.826654322	-0.2746439	0.965355	1
RP11-394I13.1	1.0231164	1.02628412	1.0217434	0.827244675	-0.273614	0.965355	1
RP11-18B16.2	1.023108	1.02633202	1.0217105	0.824489956	-0.2784262	0.965355	1
ANTXRL	1.0230983	1.02632405	1.0217001	0.824344657	-0.2786804	0.965355	1
PPP1R15B	1.2638705	1.26747304	1.2623089	0.980692828	-0.0281268	0.965423	1
KRT80	1.0298887	1.02635784	1.0314191	1.192022511	0.2534115	0.965433	1
C11orf91	1.0205629	1.02375079	1.0191811	0.807596821	-0.3082929	0.965435	1
RP11-152N13.16	1.0205681	1.02372514	1.0191997	0.8092567	-0.3053307	0.965435	1
RP11-863K10.2	1.0205965	1.02380442	1.019206	0.806825186	-0.309672	0.965435	1
ACTN2	1.0221591	1.01858613	1.0237079	1.275568902	0.3511408	0.965513	1
RP11-51J9.6	1.0222151	1.01858188	1.02379	1.280277458	0.3564565	0.965513	1
RP11-1275H24.3	1.0802687	1.08325732	1.0789733	0.948544429	-0.0762127	0.965521	1
CPEB2	1.0799514	1.08322218	1.0785337	0.943662816	-0.0836566	0.965521	1
PEBP4	1.0325413	1.02892465	1.0341089	1.179233548	0.2378495	0.96558	1
RP4-761J14.10	1.0210588	1.02423294	1.019683	0.812242096	-0.3000183	0.965617	1
AC009065.1	1.0210554	1.02425081	1.0196704	0.811121838	-0.3020095	0.965617	1
CTC-325H20.4	1.0210975	1.02426539	1.0197243	0.812859167	-0.2989227	0.965617	1
PDIA2	1.0216732	1.01802859	1.023253	1.289782581	0.3671279	0.965701	1
ZNF341	1.0391138	1.03549274	1.0406834	1.146246267	0.196917	0.965724	1
FLJ22447	1.0352488	1.03173609	1.0367714	1.158663097	0.2124611	0.965733	1
AC005256.1	1.0145563	1.01770242	1.0131926	0.745243834	-0.4242156	0.96575	1
STYK1	1.0145594	1.01771261	1.0131926	0.744815418	-0.4250452	0.96575	1
EPN2-AS1	1.0145569	1.01770451	1.0131926	0.745156127	-0.4243854	0.96575	1
LINC00345	1.01456	1.01771463	1.0131926	0.744730152	-0.4252103	0.96575	1
TMEM92-AS1	1.0145598	1.01771394	1.0131926	0.744759464	-0.4251535	0.96575	1
RP11-618K13.2	1.014565	1.01773105	1.0131926	0.744040457	-0.426547	0.96575	1
SLC5A10	1.0145564	1.01770283	1.0131926	0.745226571	-0.424249	0.96575	1
AC009505.2	1.0145571	1.017705	1.0131926	0.745135517	-0.4244253	0.96575	1
CEACAM8	1.0145642	1.01772858	1.0131926	0.74414429	-0.4263457	0.96575	1
UROC1	1.0145616	1.01772004	1.0131926	0.744502827	-0.4256508	0.96575	1
ZC3H12D	1.0145601	1.01771485	1.0131926	0.744720863	-0.4252283	0.96575	1

RP11-622A1.2	1.0145611	1.01771822	1.0131926	0.74457954	-0.4255021	0.96575	1
LINC01392	1.0145611	1.01771822	1.0131926	0.74457954	-0.4255021	0.96575	1
NKX6-1	1.0145611	1.01771822	1.0131926	0.74457954	-0.4255021	0.96575	1
RP11-435O5.7	1.0145609	1.01771746	1.0131926	0.744611533	-0.4254401	0.96575	1
CTD-2288F12.1	1.0145601	1.01771504	1.0131926	0.744713009	-0.4252435	0.96575	1
RP11-573G6.8	1.014559	1.01771135	1.0131926	0.744868069	-0.4249432	0.96575	1
BLOC1S3	1.095195	1.09839955	1.093806	0.953317218	-0.0689717	0.965816	1
MAP3K14-AS1	1.0222782	1.01870348	1.0238277	1.273971904	0.3493335	0.965893	1
DBH-AS1	1.0223274	1.01874306	1.023881	1.274124176	0.3495059	0.965893	1
BLCAP	1.5321779	1.52792936	1.5340194	1.011535723	0.0165473	0.965915	1
DACH2	1.0605351	1.05683735	1.0621379	1.093257778	0.1286336	0.965929	1
TMEM255B	1.0678152	1.06424712	1.0693617	1.079608548	0.1105083	0.966002	1
PCDHB3	1.0385885	1.04186183	1.0371696	0.887912123	-0.1715112	0.96601	1
RPA4	1.0317737	1.0283145	1.0332731	1.175126523	0.2328161	0.966018	1
RP11-778D9.12	1.0317957	1.02825269	1.0333315	1.179762175	0.2384961	0.966018	1
IPCEF1	1.0293575	1.02575061	1.0309209	1.20078264	0.263975	0.966035	1
SCRN1	1.7686085	1.77215388	1.7670717	0.993418137	-0.009527	0.96604	1
COLEC11	1.0337947	1.03684928	1.0324707	0.881175788	-0.1824982	0.966064	1
CTD-3113P16.11	1.0405627	1.04384118	1.0391416	0.892805522	-0.1635821	0.966103	1
SLIT1	1.0533108	1.05632614	1.0520037	0.923260833	-0.1151898	0.966128	1
NCAPD3	1.2046494	1.20816948	1.2031237	0.975761072	-0.0354002	0.966135	1
RP11-783K16.5	1.1023309	1.0986011	1.1039476	1.054223606	0.0761809	0.966165	1
FAM8A1	1.2720302	1.27537783	1.2705791	0.982574134	-0.0253618	0.966229	1
RP11-166N6.3	1.0136634	1.01679638	1.0123053	0.73261876	-0.4488655	0.96623	1
RP11-336A10.2	1.0136634	1.01679638	1.0123053	0.73261876	-0.4488655	0.96623	1
RP11-286N22.10	1.0136623	1.01679273	1.0123053	0.732778194	-0.4485515	0.96623	1
ATOH1	1.0136623	1.01679273	1.0123053	0.732778194	-0.4485515	0.96623	1
LINC01247	1.0136649	1.01680144	1.0123053	0.732398114	-0.4493	0.96623	1
PDYN	1.0136597	1.01678432	1.0123053	0.733145052	-0.4478294	0.96623	1
RP11-166B2.7	1.0136597	1.01678432	1.0123053	0.733145052	-0.4478294	0.96623	1
PTCHD1-AS	1.0136617	1.01679089	1.0123053	0.732858458	-0.4483935	0.96623	1
TNFAIP8L2	1.0136589	1.01678166	1.0123053	0.733261194	-0.4476009	0.96623	1
CTC-232P5.3	1.0136618	1.01679104	1.0123053	0.732851623	-0.448407	0.96623	1
DCAF4L2	1.0136618	1.01679133	1.0123053	0.732839147	-0.4484315	0.96623	1
C7orf65	1.0136593	1.01678293	1.0123053	0.733205887	-0.4477097	0.96623	1
RP5-978I12.1	1.0136611	1.01678895	1.0123053	0.73294301	-0.4482271	0.96623	1
CTD-2193G5.1	1.0136663	1.01680609	1.0123053	0.73219527	-0.4496996	0.96623	1
RP11-353K11.1	1.0136628	1.01679441	1.0123053	0.732704736	-0.4486962	0.96623	1
NPFFR1	1.0136604	1.01678657	1.0123053	0.733046815	-0.4480228	0.96623	1
RP11-281O15.8	1.0136627	1.01679425	1.0123053	0.732711577	-0.4486827	0.96623	1
TOMM20L	1.0136627	1.01679425	1.0123053	0.732711577	-0.4486827	0.96623	1
AC093702.1	1.0136648	1.01680115	1.0123053	0.732410619	-0.4492754	0.96623	1
AC145110.1	1.022283	1.02537299	1.0209436	0.825430902	-0.2767806	0.966253	1
RP11-455O6.8	1.0213002	1.01775508	1.0228368	1.286214011	0.3631307	0.966284	1
ERFE	1.0212943	1.01775999	1.0228262	1.285260424	0.3620607	0.966284	1
DUOXA1	1.0213001	1.01775323	1.0228375	1.286384411	0.3633218	0.966284	1
PPIL6	1.1942903	1.19072695	1.1958349	1.026781371	0.038129	0.966363	1
CTA-363E6.2	1.0238734	1.02036293	1.025395	1.247120483	0.3186008	0.966468	1
LCMT1-AS1	1.0239274	1.02036138	1.0254731	1.251049494	0.3231389	0.966468	1
RP11-285J16.1	1.0327573	1.02918838	1.0343043	1.175271779	0.2329944	0.966501	1
HIST1H3G	1.0326846	1.02912593	1.0342271	1.175143133	0.2328365	0.966501	1

CYP26B1	1.032747	1.02924479	1.034265	1.17166226	0.2285568	0.966501	1
RN7SL832P	1.0326433	1.02918321	1.0341431	1.169957624	0.2264563	0.966501	1
RP1-151B14.6	1.0171359	1.02024263	1.0157893	0.780002518	-0.3584493	0.966512	1
CTD-3179P9.1	1.0171433	1.02026708	1.0157893	0.779061729	-0.3601905	0.966512	1
RP1-221C16.8	1.0171412	1.02026017	1.0157893	0.779327229	-0.3596989	0.966512	1
AC073657.1	1.0171417	1.02026188	1.0157893	0.779261721	-0.3598201	0.966512	1
IL1RAPL2	1.0171458	1.02027518	1.0157893	0.778750522	-0.3607669	0.966512	1
RP11-117L5.4	1.0171458	1.02027518	1.0157893	0.778750522	-0.3607669	0.966512	1
RP1-269M15.3	1.0171414	1.02026075	1.0157893	0.779305117	-0.3597398	0.966512	1
RP11-552D8.1	1.0171464	1.0202774	1.0157893	0.778665361	-0.3609246	0.966512	1
RP11-404E16.1	1.0171464	1.0202774	1.0157893	0.778665361	-0.3609246	0.966512	1
LINC00971	1.0171382	1.02025032	1.0157893	0.779706632	-0.3589967	0.966512	1
CTD-2240J17.2	1.0171432	1.02026664	1.0157893	0.779078791	-0.3601589	0.966512	1
BARHL1	1.0171462	1.02027646	1.0157893	0.778701445	-0.3608578	0.966512	1
RP11-617D20.2	1.017139	1.02025283	1.0157893	0.779609706	-0.359176	0.966512	1
PCDHA6	1.0171392	1.0202536	1.0157893	0.779580393	-0.3592303	0.966512	1
ZNF705A	1.0171404	1.02025732	1.0157893	0.779437029	-0.3594956	0.966512	1
RP11-650J17.1	1.0171419	1.02026242	1.0157893	0.779241072	-0.3598584	0.966512	1
AS3MT	1.0171407	1.02025837	1.0157893	0.779396738	-0.3595702	0.966512	1
C1QTNF3-AMACR	1.0171409	1.02025907	1.0157893	0.77936987	-0.3596199	0.966512	1
PRH1-PRR4	1.0171465	1.02027765	1.0157893	0.778655433	-0.360943	0.966512	1
AC105052.1	1.0171433	1.02026694	1.0157893	0.77906724	-0.3601802	0.966512	1
LINC00887	1.0313036	1.03450952	1.029914	0.866833157	-0.2061738	0.966539	1
RP11-16K12.1	1.0313062	1.03440096	1.0299648	0.871045227	-0.1991805	0.966539	1
ASXL3	1.0941006	1.09720737	1.0927539	0.954185691	-0.067658	0.966566	1
LINC00346	1.0207684	1.0238643	1.0194264	0.814037733	-0.2968324	0.966571	1
TMEM72-AS1	1.020771	1.02387856	1.019424	0.813449001	-0.2978762	0.966571	1
RP11-431N15.2	1.0208093	1.02394198	1.0194514	0.81243756	-0.2996712	0.966571	1
C19orf18	1.0266792	1.02312406	1.0282202	1.220383736	0.2873349	0.966621	1
LINC00473	1.0266489	1.023105	1.028185	1.219867354	0.2867243	0.966621	1
COL15A1	1.0266779	1.02309953	1.0282289	1.222056521	0.289311	0.966621	1
AC098973.2	1.2524409	1.24870426	1.2540606	1.021536955	0.0307414	0.966634	1
RP5-908M14.9	1.1257811	1.12875896	1.1244903	0.966847516	-0.0486397	0.966643	1
LINC00923	1.0144022	1.01750394	1.0130577	0.745988062	-0.4227756	0.966664	1
RP11-138J23.1	1.0144041	1.01751032	1.0130577	0.745716008	-0.4233018	0.966664	1
RP11-429P3.3	1.0144041	1.01751018	1.0130577	0.745722089	-0.42329	0.966664	1
LINC00620	1.0144034	1.0175078	1.0130577	0.745823339	-0.4230942	0.966664	1
CTB-176F20.3	1.0144034	1.0175078	1.0130577	0.745823339	-0.4230942	0.966664	1
TEX13C	1.0144058	1.01751599	1.0130577	0.745474747	-0.4237686	0.966664	1
ZYG11A	1.0144029	1.01750627	1.0130577	0.745888781	-0.4229676	0.966664	1
LINC00578	1.0144023	1.01750428	1.0130577	0.74597323	-0.4228042	0.966664	1
RP11-284F21.11	1.0144026	1.01750514	1.0130577	0.745936679	-0.4228749	0.966664	1
PNLDC1	1.0144081	1.01752345	1.0130577	0.745157396	-0.4243829	0.966664	1
KLHL41	1.014401	1.01749997	1.0130577	0.746157078	-0.4224487	0.966664	1
RP5-965G21.5	1.0144099	1.01752944	1.0130577	0.744902524	-0.4248764	0.966664	1
RP11-9J18.1	1.0144068	1.01751915	1.0130577	0.745340139	-0.4240291	0.966664	1
RP11-240G22.4	1.0144049	1.01751305	1.0130577	0.745599836	-0.4235266	0.966664	1
GAPDHS	1.0144059	1.01751621	1.0130577	0.745465321	-0.4237869	0.966664	1
RP11-610P16.1	1.0144009	1.01749977	1.0130577	0.746165828	-0.4224318	0.966664	1
CTD-2544H17.1	1.0144035	1.01750815	1.0130577	0.745808494	-0.4231229	0.966664	1
LL22NC03-2H8.4	1.0144003	1.0174976	1.0130577	0.746258081	-0.4222534	0.966664	1

LAMP5-AS1	1.0144042	1.01751059	1.0130577	0.745704487	-0.4233241	0.966664	1
STPG3-AS1	1.0296928	1.03285166	1.0283235	0.862165015	-0.2139641	0.966695	1
RP11-873E20.1	1.0297843	1.03286994	1.0284469	0.865437506	-0.2084984	0.966695	1
GPR153	1.16057	1.16377966	1.1591788	0.971908348	-0.0411078	0.966743	1
CACNB3	1.4747645	1.47106159	1.4763696	1.011268149	0.0161656	0.966794	1
ACSL6	1.021008	1.02415363	1.0196444	0.813312046	-0.2981191	0.966807	1
RP11-347C12.3	1.0210084	1.02415441	1.0196448	0.813300252	-0.29814	0.966807	1
RP11-324O2.3	1.0210024	1.02410853	1.019656	0.815313351	-0.2945735	0.966807	1
VIT	1.0206543	1.0171592	1.0221693	1.291974578	0.3695777	0.966843	1
AC144831.3	1.0206946	1.0171592	1.022227	1.295338248	0.3733289	0.966843	1
RHOXF1-AS1	1.0206893	1.0171592	1.0222195	1.294902596	0.3728436	0.966843	1
CARD11	1.020652	1.0171592	1.022166	1.291783075	0.3693638	0.966843	1
RP5-875H18.9	1.0206353	1.0171592	1.0221421	1.2903909	0.3678082	0.966843	1
BBS1	1.0593233	1.05583296	1.0608362	1.089611556	0.1238139	0.966849	1
TLDC2	1.03331	1.03644317	1.0319519	0.87675918	-0.1897475	0.966881	1
RBM14-RBM4	1.0231495	1.01963357	1.0246735	1.256701984	0.3296426	0.966905	1
ESYT3	1.0231652	1.02628199	1.0218142	0.830007394	-0.2688039	0.96692	1
RP11-227G15.8	1.0393578	1.03575384	1.0409199	1.14448971	0.1947045	0.966925	1
RBBP9	1.2059238	1.20914797	1.2045263	0.977902443	-0.0322375	0.966981	1
CDH23	1.2165862	1.21968791	1.2152418	0.979761562	-0.0294974	0.966988	1
USP24	1.2718892	1.27494004	1.2705667	0.984093574	-0.0231326	0.966997	1
CTC-273B12.10	1.0424356	1.04556453	1.0410793	0.901563935	-0.1494983	0.967205	1
ZNF625	1.0960035	1.0923121	1.0976035	1.057321201	0.0804137	0.967208	1
LINC01180	1.0127927	1.01586389	1.0114615	0.722488302	-0.4689539	0.967237	1
CTB-35F21.1	1.0127909	1.01585787	1.0114615	0.722762356	-0.4684067	0.967237	1
CTC-367F4.1	1.0127909	1.01585787	1.0114615	0.722762356	-0.4684067	0.967237	1
RP5-1069C8.2	1.0127909	1.01585787	1.0114615	0.722762356	-0.4684067	0.967237	1
RP11-388M20.6	1.0127886	1.0158502	1.0114615	0.723112313	-0.4677084	0.967237	1
NRN1L	1.0127894	1.01585313	1.0114615	0.722978439	-0.4679755	0.967237	1
CXCR5	1.0127894	1.01585313	1.0114615	0.722978439	-0.4679755	0.967237	1
RP11-706C16.7	1.0127937	1.01586721	1.0114615	0.722336989	-0.469256	0.967237	1
RP4-613A2.1	1.0127939	1.01586779	1.0114615	0.722310602	-0.4693087	0.967237	1
CXCL11	1.0127916	1.01586024	1.0114615	0.722654227	-0.4686226	0.967237	1
Z83001.1	1.0127916	1.01586024	1.0114615	0.722654227	-0.4686226	0.967237	1
UBE2L5P	1.0127952	1.01587201	1.0114615	0.722118476	-0.4696925	0.967237	1
CTD-2555A7.3	1.0127932	1.01586557	1.0114615	0.72241179	-0.4691067	0.967237	1
AC007228.5	1.0127932	1.01586557	1.0114615	0.72241179	-0.4691067	0.967237	1
CRACR2A	1.0127932	1.01586557	1.0114615	0.72241179	-0.4691067	0.967237	1
CTC-498M16.4	1.0127948	1.0158709	1.0114615	0.722169062	-0.4695915	0.967237	1
SLC11A1	1.0498628	1.05296762	1.048517	0.915975262	-0.1266195	0.96725	1
RP11-479G22.8	1.0664739	1.06974504	1.0650561	0.932769836	-0.100407	0.967254	1
AP3S2	1.2053844	1.20175797	1.2069563	1.025765045	0.0367003	0.967268	1
CATSPER3	1.0197899	1.02287989	1.0184506	0.806409531	-0.3104154	0.967295	1
RP11-114N19.3	1.0197991	1.02290257	1.0184539	0.805757456	-0.3115825	0.967295	1
GUCA1B	1.0197733	1.02284459	1.018442	0.807280822	-0.3088575	0.967295	1
IRF8	1.0198449	1.02289301	1.0185237	0.809143595	-0.3055323	0.967295	1
RP11-102M11.2	1.01982	1.02286948	1.0184982	0.808859447	-0.3060391	0.967295	1
RP11-546O6.4	1.0198037	1.02290049	1.0184614	0.806156479	-0.3108682	0.967295	1
RP11-644K8.1	1.0197939	1.0229067	1.0184446	0.805205697	-0.3125707	0.967295	1
ITCH-AS1	1.0198185	1.0229067	1.01848	0.806749258	-0.3098077	0.967295	1
RP11-626G11.1	1.0197997	1.02287422	1.018467	0.807330213	-0.3087692	0.967295	1

HCAR2	1.0218934	1.01839445	1.0234101	1.272671491	0.3478601	0.967331	1
AC022182.1	1.0219467	1.01839445	1.0234865	1.276823789	0.3525594	0.967331	1
NEUROG1	1.0218895	1.01839445	1.0234044	1.272362827	0.3475101	0.967331	1
KIF7	1.0592929	1.06236815	1.0579599	0.929318264	-0.1057553	0.967374	1
MGC16275	1.0240955	1.02056915	1.0256241	1.245752299	0.3170172	0.967386	1
RP11-510J16.5	1.0222679	1.0253841	1.0209172	0.824028176	-0.2792344	0.967402	1
RP4-635E18.6	1.0309232	1.0339822	1.0295973	0.870964403	-0.1993143	0.96741	1
RP11-109D9.4	1.0245189	1.02761071	1.0231788	0.839485549	-0.2524226	0.967435	1
HSD17B13	1.0245609	1.02766145	1.0232169	0.839322613	-0.2527026	0.967435	1
SNAI3	1.021648	1.01815945	1.0231601	1.275376757	0.3509235	0.967568	1
RP11-546K22.3	1.0378541	1.04102977	1.0364775	0.889050476	-0.1696628	0.96758	1
SLC22A1	1.0258696	1.028907	1.024553	0.849379753	-0.2355184	0.967686	1
MCF2L-AS1	1.0258033	1.02884623	1.0244843	0.84878645	-0.2365265	0.967686	1
GFOD1	1.0306081	1.02710407	1.032127	1.185318535	0.2452748	0.96772	1
RASIP1	1.0341508	1.03074101	1.0356287	1.158997079	0.2128769	0.967784	1
RFLNA	1.0416773	1.0447285	1.0403547	0.902215117	-0.1484566	0.967793	1
SLC25A15	1.0638419	1.06045712	1.0653091	1.080255115	0.1113721	0.967803	1
PRR19	1.0500714	1.04662473	1.0515654	1.10596774	0.1453093	0.967892	1
KLF15	1.0204366	1.02355451	1.0190851	0.810253104	-0.3035555	0.967939	1
DNLZ	1.0204354	1.02349744	1.0191081	0.81320012	-0.2983177	0.967939	1
ZNF609	1.41604	1.4122872	1.4176666	1.013047717	0.0187021	0.967972	1
RP11-16E12.1	1.1321288	1.13521342	1.1307918	0.96729874	-0.0479666	0.967986	1
RP3-475N16.1	1.0231921	1.01975642	1.0246813	1.249279626	0.3210964	0.968054	1
RP11-57A19.2	1.041971	1.04507741	1.0406245	0.901215553	-0.1500559	0.968063	1
FOXN3-AS1	1.0419936	1.0450557	1.0406662	0.902577199	-0.1478778	0.968063	1
ABHD14A-ACY1	1.1042422	1.10060085	1.1058206	1.051886036	0.0729784	0.96809	1
ETFRF1	1.8809841	1.87685751	1.8827728	1.006745996	0.0096997	0.968117	1
CENPN	1.48177	1.48473349	1.4804854	0.991236232	-0.0126992	0.968216	1
LDB1	1.1902426	1.19358288	1.1887947	0.975265634	-0.0361329	0.968239	1
PWWP2A	1.360291	1.35668655	1.3618534	1.014485629	0.0207484	0.968315	1
RP11-817J15.3	1.012652	1.01566841	1.0113445	0.724037806	-0.4658631	0.96833	1
BTN1A1	1.012652	1.01566841	1.0113445	0.724037806	-0.4658631	0.96833	1
UPK3BL	1.0126523	1.01566955	1.0113445	0.723984982	-0.4659683	0.96833	1
RP11-67L3.4	1.0126502	1.01566238	1.0113445	0.724316228	-0.4653084	0.96833	1
DPP4	1.0126511	1.01566545	1.0113445	0.724174422	-0.4655909	0.96833	1
AC009227.2	1.0126511	1.01566545	1.0113445	0.724174422	-0.4655909	0.96833	1
CTD-3126B10.1	1.0126469	1.0156516	1.0113445	0.724815338	-0.4643146	0.96833	1
RP5-1007H16.1	1.0126494	1.01565972	1.0113445	0.724439483	-0.4650629	0.96833	1
WNT4	1.0126473	1.015653	1.0113445	0.724750217	-0.4644442	0.96833	1
IL26	1.0126454	1.01564645	1.0113445	0.7250539	-0.4638398	0.96833	1
U47924.32	1.0126457	1.01564765	1.0113445	0.724998026	-0.463951	0.96833	1
RP11-745O10.4	1.0126464	1.01564979	1.0113445	0.724898819	-0.4641485	0.96833	1
RP11-542G1.1	1.0126468	1.01565123	1.0113445	0.724832322	-0.4642808	0.96833	1
RP11-135J2.3	1.0126508	1.01566441	1.0113445	0.724222276	-0.4654955	0.96833	1
LINC01538	1.0126508	1.01566441	1.0113445	0.724222276	-0.4654955	0.96833	1
AC012370.3	1.0126438	1.01564129	1.0113445	0.725292879	-0.4633644	0.96833	1
C15orf32	1.0126485	1.01565699	1.0113445	0.724565585	-0.4648118	0.96833	1
RP1-100J12.1	1.0284637	1.03146631	1.0271621	0.863213159	-0.2122112	0.968381	1
PCDHA5	1.0284548	1.03146549	1.0271499	0.86284549	-0.2128259	0.968381	1
IRS4	1.0213832	1.02442638	1.0200641	0.821412009	-0.2838221	0.968413	1
ZNF845	1.0497543	1.04631688	1.0512443	1.106384371	0.1458527	0.968483	1

MROH6	1.1585278	1.16162613	1.1571849	0.972521349	-0.0401982	0.968495	1
FCGR2A	1.0345942	1.03114846	1.0360878	1.158574835	0.2123512	0.968518	1
SNCA-AS1	1.034643	1.03120175	1.0361346	1.15809546	0.2117542	0.968518	1
C4orf26	1.0207368	1.02376798	1.0194229	0.817186458	-0.2912628	0.968522	1
CTD-2555O16.2	1.021984	1.01850742	1.023491	1.269273455	0.3440029	0.968522	1
RP11-394I13.2	1.021959	1.01850742	1.0234552	1.267338672	0.3418021	0.968522	1
KCNC2	1.0219614	1.01850742	1.0234586	1.267523775	0.3420128	0.968522	1
RP11-459I19.1	1.021963	1.01850742	1.0234608	1.267642808	0.3421483	0.968522	1
POU5F1B	1.0420466	1.0385841	1.0435475	1.128637812	0.1745826	0.968561	1
SFT2D3	1.1849165	1.18147161	1.1864097	1.027211499	0.0387333	0.968599	1
RP11-411H5.1	1.018723	1.0217477	1.017412	0.800634389	-0.3207845	0.968616	1
RP11-411K7.1	1.0187226	1.0217403	1.0174146	0.801029646	-0.3200725	0.968616	1
ACSM6	1.0187264	1.02173215	1.0174235	0.801738604	-0.3187962	0.968616	1
C1orf141	1.0187168	1.02171963	1.0174153	0.801821549	-0.3186469	0.968616	1
CTD-2058B24.3	1.018715	1.02171833	1.0174132	0.801773241	-0.3187338	0.968616	1
RP11-794P6.6	1.018716	1.02172018	1.0174138	0.801732657	-0.3188069	0.968616	1
RP11-90L1.8	1.0187219	1.02172272	1.0174212	0.801979915	-0.318362	0.968616	1
PPM1F	1.1879916	1.18450162	1.1895043	1.027114629	0.0385972	0.968685	1
ZNF57	1.055249	1.05192592	1.0566894	1.091736236	0.1266243	0.96869	1
MELTF	1.0255581	1.02219067	1.0270178	1.21752947	0.2839567	0.968702	1
IL1R1	1.0214336	1.01804205	1.0229037	1.269463779	0.3442192	0.968704	1
VIP	1.0214154	1.01804205	1.0228776	1.268016883	0.342574	0.968704	1
RP11-108M9.3	1.0214482	1.01804205	1.0229246	1.270621718	0.3455346	0.968704	1
LINC01616	1.0213281	1.02432862	1.0200276	0.823209641	-0.2806682	0.968709	1
ZNF816-ZNF321P	1.038041	1.04105493	1.0367346	0.89476614	-0.1604174	0.968726	1
MFSD4A	1.0380476	1.04105466	1.0367442	0.895006061	-0.1600306	0.968726	1
SFTA1P	1.023982	1.02056357	1.0254637	1.238291101	0.3083505	0.968783	1
CTD-2571L23.8	1.023942	1.02056357	1.0254064	1.235502818	0.3050983	0.968783	1
LIPH	1.023982	1.02056357	1.0254638	1.238295526	0.3083557	0.968783	1
RP11-679B19.2	1.0239948	1.02056357	1.0254821	1.239186646	0.3093935	0.968783	1
KCNJ15	1.0239428	1.02056357	1.0254075	1.235560904	0.3051661	0.968783	1
NTN1	1.0239557	1.02056357	1.0254261	1.236462357	0.3062183	0.968783	1
HOXC5	1.0240449	1.02056357	1.0255539	1.242675972	0.3134502	0.968783	1
ZNF276	1.178095	1.18128415	1.1767126	0.974782435	-0.0368478	0.968851	1
MTHFR	1.1889104	1.19218805	1.1874897	0.975553195	-0.0357076	0.968869	1
CPSF3	1.468996	1.47213886	1.4676338	0.990458099	-0.0138322	0.968904	1
BORCS6	1.1056778	1.10890363	1.1042796	0.957540195	-0.062595	0.968908	1
SLC9C2	1.0132484	1.01622563	1.0119579	0.736978068	-0.4403064	0.96892	1
C10orf91	1.0132465	1.0162192	1.0119579	0.737270129	-0.4397348	0.96892	1
AC107081.5	1.0132486	1.0162263	1.0119579	0.736947592	-0.4403661	0.96892	1
ALOX15B	1.0132454	1.01621571	1.0119579	0.737428936	-0.4394241	0.96892	1
TSPYL6	1.0132477	1.01622324	1.0119579	0.737086705	-0.4400938	0.96892	1
LYZ	1.0132513	1.01623531	1.0119579	0.736538425	-0.4411673	0.96892	1
OVOL2	1.0132516	1.01623616	1.0119579	0.736500182	-0.4412422	0.96892	1
CTA-992D9.8	1.013249	1.0162276	1.0119579	0.73688877	-0.4404812	0.96892	1
RP11-25O10.2	1.013249	1.0162276	1.0119579	0.73688877	-0.4404812	0.96892	1
AC011893.3	1.0132462	1.01621835	1.0119579	0.737308907	-0.4396589	0.96892	1
DTHD1	1.0132472	1.01622158	1.0119579	0.737162276	-0.4399459	0.96892	1
LRRC61	1.0132472	1.01622158	1.0119579	0.737162276	-0.4399459	0.96892	1
RP11-646J21.4	1.0132497	1.01622975	1.0119579	0.736790756	-0.4406731	0.96892	1
SLC24A2	1.013247	1.01622088	1.0119579	0.737193916	-0.4398839	0.96892	1

RP11-773H22.4	1.0132466	1.01621953	1.0119579	0.737254998	-0.4397644	0.96892	1
JAKMIP2-AS1	1.0132481	1.01622458	1.0119579	0.73702557	-0.4402134	0.96892	1
RP5-1077I2.3	1.0132471	1.01622129	1.0119579	0.73717523	-0.4399205	0.96892	1
AC110615.1	1.0132451	1.01621459	1.0119579	0.737479936	-0.4393243	0.96892	1
BOK-AS1	1.0132439	1.01621055	1.0119579	0.737663679	-0.4389649	0.96892	1
CCDC70	1.0132464	1.01621892	1.0119579	0.737283076	-0.4397095	0.96892	1
CACNA2D3-AS1	1.01325	1.01623082	1.0119579	0.736742514	-0.4407676	0.96892	1
LINC01354	1.0132517	1.01623651	1.0119579	0.736484307	-0.4412733	0.96892	1
SEC14L5	1.0383255	1.04133659	1.0370203	0.895582255	-0.1591022	0.968965	1
KCNK12	1.0383232	1.04126298	1.0370489	0.89787264	-0.1554173	0.968965	1
RP11-334G22.1	1.0253762	1.0219097	1.0268788	1.226797608	0.2948973	0.968969	1
CRIP1	1.0539227	1.05040149	1.055449	1.10014602	0.137695	0.968994	1
ADO	1.2131315	1.21651092	1.2116666	0.977625642	-0.032646	0.968998	1
AC097662.2	1.0316736	1.03465583	1.030381	0.876648782	-0.1899291	0.969002	1
MTHFD2	1.7189202	1.71545857	1.7204206	1.006935452	0.0099712	0.969022	1
XXbac-BPG157A10.21	1.0290568	1.03205099	1.027759	0.866089048	-0.2074127	0.969065	1
RP11-624D11.2	1.0290605	1.03203607	1.0277708	0.866859186	-0.2061304	0.969065	1
RP1-40E16.9	1.1528819	1.1490662	1.1545359	1.036692989	0.0519887	0.969113	1
MYO15A	1.0710831	1.0675779	1.0726024	1.074351023	0.1034654	0.969114	1
SBF2-AS1	1.1339582	1.13707665	1.1326065	0.967389352	-0.0478314	0.969135	1
RP11-497G19.1	1.0135158	1.01647906	1.0122313	0.742232942	-0.4300561	0.969186	1
RP11-253I19.3	1.0135161	1.01648033	1.0122313	0.742175722	-0.4301673	0.969186	1
AP000688.29	1.013516	1.01647976	1.0122313	0.742201485	-0.4301172	0.969186	1
ALK	1.0135107	1.01646229	1.0122313	0.742989362	-0.4285865	0.969186	1
AC004953.1	1.0135124	1.016468	1.0122313	0.742731371	-0.4290876	0.969186	1
RP11-568A7.2	1.0135178	1.0164859	1.0122313	0.741925085	-0.4306546	0.969186	1
HNF4G	1.0135178	1.0164859	1.0122313	0.741925085	-0.4306546	0.969186	1
AC010761.6	1.0135112	1.01646396	1.0122313	0.742913852	-0.4287332	0.969186	1
RP11-693J15.6	1.0135155	1.01647808	1.0122313	0.742277271	-0.4299699	0.969186	1
RP11-760H22.2	1.0135094	1.01645797	1.0122313	0.743184419	-0.4282078	0.969186	1
RP11-717K11.2	1.0135166	1.01648173	1.0122313	0.742112784	-0.4302896	0.969186	1
AC011995.1	1.0135133	1.01647107	1.0122313	0.742592976	-0.4293564	0.969186	1
RP11-561O23.8	1.0135159	1.01647948	1.0122313	0.742214365	-0.4300922	0.969186	1
LRRC19	1.0135177	1.01648539	1.0122313	0.741948159	-0.4306097	0.969186	1
RP11-260O18.1	1.0135177	1.01648539	1.0122313	0.741948159	-0.4306097	0.969186	1
AC034187.2	1.0135147	1.0164757	1.0122313	0.74238446	-0.4297616	0.969186	1
H2BFWT	1.0135153	1.01647767	1.0122313	0.742295842	-0.4299338	0.969186	1
RP11-460B17.3	1.0135177	1.01648548	1.0122313	0.741944177	-0.4306175	0.969186	1
RP1-125I3.2	1.0135173	1.01648425	1.0122313	0.741999617	-0.4305097	0.969186	1
TTLL2	1.0135167	1.01648202	1.0122313	0.742099897	-0.4303147	0.969186	1
DHFR2	1.133406	1.12999184	1.1348859	1.037648983	0.0533185	0.969255	1
ZNF234	1.1572798	1.16046806	1.1558979	0.971519684	-0.0416849	0.969267	1
RP11-338K17.8	1.0260221	1.0225724	1.0275174	1.219072567	0.285784	0.969395	1
RP3-467K16.4	1.0259476	1.02256487	1.0274139	1.214891569	0.2808276	0.969395	1
RP11-996F15.2	1.0259619	1.02256289	1.0274352	1.215944451	0.2820773	0.969395	1
IFNAR2	1.3863492	1.3830285	1.3877886	1.012427549	0.0178187	0.969468	1
RP11-352D13.6	1.0212827	1.02420712	1.0200152	0.826829253	-0.2743387	0.969511	1
SYT16	1.0578952	1.06082317	1.056626	0.930994061	-0.1031561	0.969607	1
RP11-74E22.8	1.0224729	1.02544062	1.0211865	0.832782614	-0.2639881	0.969613	1
GABRQ	1.0224618	1.02543819	1.0211717	0.832279662	-0.2648597	0.969613	1
ACTA2-AS1	1.0224824	1.02546263	1.0211906	0.832224855	-0.2649547	0.969613	1

FRGCA	1.0224575	1.02541777	1.0211744	0.833054293	-0.2635176	0.969613	1
WBSCR28	1.0224751	1.02541615	1.0212002	0.834124223	-0.2616658	0.969613	1
RP11-341G23.4	1.0169539	1.01362911	1.0183951	1.349690191	0.4326283	0.969628	1
SLC7A10	1.016998	1.01362911	1.0184583	1.354326807	0.4375759	0.969628	1
LINC01185	1.0169819	1.01362911	1.0184353	1.352637749	0.4357755	0.969628	1
WFDC5	1.0169887	1.01362911	1.018445	1.35335172	0.4365368	0.969628	1
PRLH	1.0169608	1.01362911	1.0184049	1.350410131	0.4333976	0.969628	1
MEOX1	1.0169586	1.01362911	1.0184018	1.350185709	0.4331579	0.969628	1
MYL7	1.0670026	1.07006488	1.0656752	0.937348966	-0.0933418	0.969646	1
HERC6	1.0293423	1.03228536	1.0280667	0.869331099	-0.2020223	0.969651	1
TMEM256-PLSCR3	1.0293427	1.03234054	1.0280433	0.867123836	-0.2056901	0.969651	1
USP25	1.1494624	1.14588687	1.1510122	1.03513198	0.0498147	0.969685	1
RP11-127B20.2	1.0869616	1.08338194	1.0885133	1.061539926	0.0861586	0.969769	1
ING3	1.3314067	1.32793403	1.3329119	1.015179437	0.0217348	0.969803	1
HEATR6	1.151627	1.1482137	1.1531065	1.033011498	0.0468563	0.969821	1
UFM1	2.2178506	2.21293551	2.2199811	1.005808711	0.008356	0.96983	1
LNP1	1.1885606	1.19167599	1.1872102	0.976701589	-0.0340103	0.969859	1
ATRNL1	1.1151351	1.11185448	1.1165571	1.04204229	0.0594138	0.969886	1
RP11-400N13.1	1.0167106	1.01338003	1.0181543	1.356821347	0.4402308	0.969894	1
LINC01291	1.0167042	1.01338003	1.0181451	1.356135052	0.4395009	0.969894	1
CYBB	1.0166987	1.01338003	1.0181372	1.355544262	0.4388722	0.969894	1
MAMSTR	1.1603215	1.16342362	1.1589768	0.972789814	-0.0398	0.970046	1
KLHL6-AS1	1.0205023	1.0234075	1.019243	0.822088464	-0.2826344	0.970092	1
RP4-734G22.3	1.0205071	1.02343168	1.0192395	0.821088723	-0.28439	0.970092	1
RP11-177F15.1	1.0205085	1.0234383	1.0192386	0.820820096	-0.284862	0.970092	1
GSN-AS1	1.0204983	1.02343267	1.0192264	0.82049596	-0.2854319	0.970092	1
ST8SIA6-AS1	1.0204976	1.02341816	1.0192316	0.821226843	-0.2841473	0.970092	1
RP1-197B17.4	1.0204996	1.02342928	1.0192297	0.820756736	-0.2849734	0.970092	1
LINC00951	1.0205012	1.02343366	1.0192302	0.8206216	-0.285211	0.970092	1
UTS2B	1.0205015	1.02343564	1.0192297	0.820533993	-0.285365	0.970092	1
ITGB7	1.0204908	1.02339744	1.0192309	0.821923024	-0.2829248	0.970092	1
RP1-153G14.4	1.0218564	1.02475415	1.0206003	0.832195643	-0.2650054	0.970104	1
LINC00052	1.0219028	1.02481282	1.0206415	0.831887554	-0.2655396	0.970104	1
FSCN2	1.0218582	1.02475821	1.0206012	0.832094202	-0.2651812	0.970104	1
UPK1A	1.0590384	1.06188675	1.0578038	0.934024823	-0.0984672	0.970124	1
SLC35A4	1.4286236	1.43220006	1.4270734	0.988138181	-0.0172153	0.970129	1
PDE5A	1.1080799	1.10460413	1.1095864	1.04763016	0.0671295	0.970132	1
RP11-15L13.4	1.0200456	1.02301259	1.0187595	0.815183704	-0.2948029	0.970142	1
TGM3	1.0200521	1.02294776	1.0187969	0.819118499	-0.2878559	0.970142	1
FOXF1	1.0200443	1.02294753	1.0187858	0.818642297	-0.2886949	0.970142	1
SNED1	1.0200416	1.02296003	1.0187765	0.817792664	-0.290193	0.970142	1
SEC14L4	1.020042	1.0229756	1.0187705	0.816973466	-0.2916389	0.970142	1
RP11-38G5.4	1.0200461	1.02298357	1.0187729	0.816794288	-0.2919553	0.970142	1
MIR194-2HG	1.0179806	1.02090062	1.0167149	0.799733066	-0.3224096	0.970172	1
RP11-362K14.7	1.0179761	1.020888	1.0167139	0.800166764	-0.3216274	0.970172	1
RP5-855F16.1	1.0179778	1.02089138	1.0167149	0.800086836	-0.3217715	0.970172	1
RP5-1021I20.4	1.0179832	1.02089649	1.0167204	0.800152686	-0.3216528	0.970172	1
RP11-480D4.1	1.0179845	1.02091389	1.0167147	0.799216787	-0.3233412	0.970172	1
LINC01173	1.0179834	1.02091656	1.016712	0.798981717	-0.3237656	0.970172	1
SIGLEC11	1.0179788	1.02088422	1.0167195	0.800579315	-0.3208838	0.970172	1
RP11-434D9.1	1.017978	1.02088765	1.0167167	0.800316818	-0.3213569	0.970172	1

WDR34	1.8384534	1.84196006	1.8369335	0.994029872	-0.0086389	0.970177	1
ZNF670	1.1301406	1.13313611	1.1288422	0.967747661	-0.0472972	0.970188	1
HES6	2.4919114	2.50350338	2.4868867	0.988948051	-0.0160334	0.9702	1
ZNF761	1.1131831	1.10974534	1.1146733	1.044903124	0.0633692	0.970202	1
RP11-1055B8.3	1.0222063	1.01886772	1.0236534	1.253642554	0.3261261	0.970261	1
RP11-1H15.2	1.0222622	1.01886772	1.0237335	1.25789105	0.331007	0.970261	1
GLI3	1.3703597	1.36667522	1.3719568	1.014403912	0.0206322	0.970349	1
PASD1	1.0184513	1.02136341	1.0171891	0.804603838	-0.3136495	0.970353	1
RP11-667M19.2	1.0184498	1.02135099	1.0171922	0.805218987	-0.3125469	0.970353	1
RP11-346C20.4	1.0184499	1.02135955	1.0171887	0.804732272	-0.3134192	0.970353	1
NKX3-2	1.0184664	1.02139206	1.0171982	0.803952062	-0.3148186	0.970353	1
AP001058.3	1.0184598	1.02138173	1.0171932	0.804108704	-0.3145375	0.970353	1
CTD-2542L18.1	1.0184509	1.02135835	1.0171907	0.804869282	-0.3131736	0.970353	1
ZNF726	1.0184524	1.02136577	1.0171896	0.804539004	-0.3137657	0.970353	1
AF064858.7	1.0184503	1.02135868	1.0171896	0.804806141	-0.3132868	0.970353	1
CYP17A1	1.0184553	1.02137959	1.0171877	0.803931202	-0.3148561	0.970353	1
OFCC1	1.0184674	1.02137838	1.0172056	0.804814681	-0.3132715	0.970353	1
RP11-252E2.1	1.0184622	1.02138711	1.0171943	0.803957018	-0.3148097	0.970353	1
CTC-490G23.6	1.0184683	1.02138711	1.0172031	0.804367437	-0.3140734	0.970353	1
CASC16	1.0184529	1.02135259	1.017196	0.805334901	-0.3123392	0.970353	1
FAM3A	1.5863876	1.58994851	1.5848441	0.991347768	-0.0125368	0.970439	1
SLC17A8	1.016083	1.01281342	1.0175002	1.365769545	0.4497141	0.970485	1
RP4-761J14.9	1.0161007	1.01281342	1.0175256	1.367750976	0.4518056	0.970485	1
LINC01535	1.0160742	1.01281342	1.0174876	1.364785309	0.448674	0.970485	1
GRIN2A	1.0160997	1.01281342	1.0175241	1.367637203	0.4516856	0.970485	1
RP11-54C4.3	1.0161135	1.01281342	1.0175439	1.369184524	0.4533169	0.970485	1
PTGER2	1.0160918	1.01281342	1.0175128	1.366753144	0.4507527	0.970485	1
LINC01393	1.0160965	1.01281342	1.0175195	1.367278259	0.4513069	0.970485	1
RP11-503C24.4	1.0161243	1.01281342	1.0175594	1.370388331	0.4545848	0.970485	1
AC072062.1	1.0457286	1.04866406	1.0444562	0.913533224	-0.1304709	0.970523	1
FAM69A	1.0664096	1.06944077	1.0650958	0.937428693	-0.0932191	0.970647	1
HSPA14	1.2700006	1.2732933	1.2685733	0.982729207	-0.0251342	0.970662	1
RP11-140I16.3	1.0248237	1.02156553	1.026236	1.216570333	0.2828197	0.970662	1
CDH10	1.0248441	1.02152924	1.0262809	1.220707431	0.2877175	0.970662	1
TMEM52	1.0540049	1.05066831	1.0554512	1.094395481	0.1301342	0.970666	1
GS1-259H13.2	1.0756221	1.07860694	1.0743283	0.945569666	-0.0807443	0.970681	1
ZNF835	1.0298926	1.03283982	1.0286151	0.871354663	-0.198668	0.970682	1
PRUNE2	1.0939169	1.09683316	1.0926529	0.95682984	-0.0636657	0.970686	1
DDX47	1.0360284	1.03902824	1.0347281	0.889819986	-0.1684146	0.970712	1
RP11-514O12.4	1.0360151	1.03892134	1.0347554	0.892964028	-0.163326	0.970712	1
ABTB2	1.0319424	1.02866174	1.0333644	1.164074235	0.2191831	0.970823	1
RP11-736K20.6	1.0318853	1.02855826	1.0333275	1.166998722	0.222803	0.970823	1
CACTIN	1.0698609	1.06657485	1.0712852	1.070753057	0.0986258	0.970876	1
PER2	1.064247	1.06720432	1.0629652	0.936921419	-0.094	0.970914	1
STK31	1.0196927	1.02259405	1.0184351	0.815925439	-0.2934908	0.970953	1
LINC00856	1.0196919	1.02258025	1.01844	0.816640812	-0.2922264	0.970953	1
AF146191.4	1.0196993	1.02258033	1.0184506	0.817107729	-0.2914018	0.970953	1
AC078941.1	1.0197097	1.02259915	1.0184572	0.816720941	-0.2920849	0.970953	1
DENND5A	1.3698012	1.36614657	1.3713854	1.01430793	0.0204957	0.971024	1
MED9	1.2156864	1.21225004	1.217176	1.02320815	0.0330997	0.971059	1
FAM212B-AS1	1.0238894	1.02061037	1.0253107	1.228055588	0.2963759	0.971072	1

C7orf61	1.0238506	1.02058845	1.0252646	1.227124647	0.2952818	0.971072	1
OSTM1-AS1	1.02911	1.03201503	1.0278509	0.869930928	-0.2010272	0.971151	1
FAM3D	1.0292194	1.03208294	1.0279782	0.872059345	-0.1975018	0.971151	1
LINC00237	1.029281	1.03201351	1.0280966	0.877648016	-0.1882856	0.971151	1
TJAP1	1.1910318	1.18764882	1.1924982	1.025842622	0.0368094	0.97117	1
RP11-83M16.6	1.0266449	1.02950556	1.025405	0.861023821	-0.2158749	0.971231	1
RP11-38C17.1	1.0353104	1.03196045	1.0367625	1.150250066	0.2019475	0.971253	1
RP11-626G11.3	1.1324668	1.12875599	1.1340752	1.0413125	0.0584031	0.971262	1
RP11-793H13.11	1.0674541	1.07029042	1.0662247	0.942157898	-0.0859592	0.971275	1
RP11-269C23.5	1.0304877	1.03343202	1.0292114	0.87375659	-0.1946967	0.971278	1
RGS6	1.0304284	1.0332703	1.0291966	0.877557587	-0.1884343	0.971278	1
MEIOB	1.0489437	1.05178185	1.0477134	0.921431982	-0.1180504	0.9713	1
LINC01290	1.0181718	1.02104603	1.016926	0.804237899	-0.3143058	0.971307	1
RP1-156L9.1	1.0181758	1.02104276	1.0169332	0.804702967	-0.3134717	0.971307	1
CTD-2544H17.2	1.0181612	1.02101628	1.0169236	0.8052607	-0.3124722	0.971307	1
TEKT4	1.018159	1.02101628	1.0169204	0.805111033	-0.3127403	0.971307	1
RP11-589B3.6	1.0181673	1.02101994	1.0169308	0.805464829	-0.3121065	0.971307	1
RP5-837I24.4	1.0181613	1.02101826	1.0169229	0.805153414	-0.3126644	0.971307	1
RP4-665J23.2	1.0181586	1.02100086	1.0169266	0.805996769	-0.311154	0.971307	1
LINC01032	1.0181604	1.0210172	1.0169221	0.805156147	-0.3126595	0.971307	1
LINC00890	1.0331317	1.02986028	1.0345497	1.15704496	0.2104449	0.971313	1
OPRL1	1.0544875	1.05115427	1.0559322	1.093403103	0.1288254	0.971503	1
GAS8	1.0530745	1.04991466	1.0544441	1.090743302	0.1253116	0.971539	1
PCDHB9	1.1049877	1.10787558	1.1037359	0.961625167	-0.0564534	0.971541	1
C3orf80	1.018409	1.02125031	1.0171774	0.808335299	-0.3069742	0.971543	1
RP5-851M4.1	1.0184079	1.02125509	1.0171737	0.807982756	-0.3076036	0.971543	1
LINC00540	1.0184049	1.02124469	1.017174	0.808392535	-0.3068721	0.971543	1
RP11-254F7.4	1.0184119	1.02125719	1.0171786	0.808133178	-0.307335	0.971543	1
SAPCD1	1.018402	1.02122999	1.0171762	0.80905472	-0.3056908	0.971543	1
FOXD1	1.0184119	1.02125719	1.0171787	0.808133792	-0.3073339	0.971543	1
RP11-583F2.2	1.0184135	1.02126156	1.017179	0.80798182	-0.3076053	0.971543	1
AC008753.6	1.0162433	1.01301898	1.0176409	1.35501031	0.4383038	0.97158	1
GCKR	1.0162489	1.01301898	1.0176489	1.355630276	0.4389638	0.97158	1
TFAP2A-AS1	1.0162502	1.01301898	1.0176508	1.355773067	0.4391157	0.97158	1
CTD-2021J15.1	1.016223	1.01301898	1.0176119	1.352783187	0.4359306	0.97158	1
TBR1	1.016271	1.01301898	1.0176806	1.358060579	0.4415478	0.97158	1
IRF5	1.0162173	1.01301898	1.0176036	1.352146097	0.435251	0.97158	1
HLF	1.0454266	1.04219864	1.0468257	1.10965065	0.1501055	0.971589	1
UBR3	1.2236664	1.22669123	1.2223553	0.980872849	-0.027862	0.971602	1
CLCN2	1.1279984	1.13101205	1.1266921	0.96702654	-0.0483726	0.971616	1
RP11-640N11.2	1.0206433	1.02357699	1.0193717	0.821636035	-0.2834286	0.971619	1
FOXG1-AS1	1.0205835	1.02340032	1.0193626	0.827449498	-0.2732568	0.971619	1
SRGAP2	1.1679377	1.17076745	1.1667112	0.976246863	-0.0346821	0.971632	1
RP11-573D15.9	1.0232356	1.01999955	1.0246382	1.231939168	0.300931	0.971651	1
CTD-2383M3.1	1.0232727	1.02000206	1.0246903	1.234389436	0.3037976	0.971651	1
RP11-10C24.1	1.0232261	1.02002609	1.0246132	1.229058435	0.2975535	0.971651	1
CTC-340A15.2	1.0232873	1.02001123	1.0247074	1.234676424	0.304133	0.971651	1
PCDHA10	1.0769487	1.07980514	1.0757105	0.948692539	-0.0759875	0.971672	1
TM4SF1	1.025706	1.02246967	1.0271088	1.206460151	0.2707803	0.971675	1
GRPR	1.0257093	1.02250932	1.0270963	1.203782856	0.2675752	0.971675	1
POLR3C	1.1781296	1.17483839	1.1795562	1.026983726	0.0384133	0.971837	1

CH507-9B2.9	1.0385071	1.04134368	1.0372775	0.901650149	-0.1493603	0.971843	1
RP11-54O7.16	1.0171984	1.0200086	1.0159803	0.798670089	-0.3243284	0.97203	1
RP11-282A11.4	1.0171967	1.01999982	1.0159816	0.799088881	-0.3235721	0.97203	1
ARTN	1.0172017	1.02001906	1.0159806	0.798267766	-0.3250553	0.97203	1
RP11-252A24.5	1.0172004	1.02001801	1.0159791	0.798235383	-0.3251139	0.97203	1
RP5-1028K7.3	1.0171966	1.02000151	1.0159808	0.798980099	-0.3237685	0.97203	1
PYHIN1	1.0171986	1.02000845	1.0159806	0.798693963	-0.3242853	0.97203	1
ANKUB1	1.0172023	1.02001179	1.0159845	0.798751997	-0.3241805	0.97203	1
RP1-136B1.1	1.0172031	1.02002611	1.0159794	0.797927874	-0.3256697	0.97203	1
MNX1-AS1	1.0171962	1.01998936	1.0159855	0.79969923	-0.3224706	0.97203	1
RP11-421F16.3	1.0171961	1.02000173	1.01598	0.798929084	-0.3238606	0.97203	1
RP4-753F5.1	1.0283205	1.03113397	1.0271009	0.870461866	-0.200147	0.972092	1
RP11-479O9.4	1.0197351	1.02253005	1.0185236	0.822173017	-0.2824861	0.972101	1
PRAM1	1.0197035	1.02248549	1.0184976	0.822646219	-0.281656	0.972101	1
RP11-93K22.13	1.0196762	1.02247778	1.0184618	0.821334536	-0.2839581	0.972101	1
AC007193.10	1.0197196	1.02260718	1.0184679	0.816903806	-0.2917619	0.972101	1
LINC01516	1.017891	1.01466374	1.0192898	1.315478962	0.3955882	0.972153	1
FLJ42969	1.017891	1.01466374	1.0192898	1.315478962	0.3955882	0.972153	1
RP11-680F20.9	1.0178575	1.01466374	1.0192419	1.312208637	0.3919971	0.972153	1
RP4-555D20.4	1.0266669	1.02342901	1.0280704	1.198102858	0.2607518	0.972157	1
EGFR-AS1	1.0265898	1.02339566	1.0279743	1.195705069	0.2578616	0.972157	1
C9orf24	1.0975294	1.1003584	1.0963031	0.959591845	-0.0595072	0.972168	1
HSPB8	1.2204158	1.21701599	1.2218895	1.022456736	0.0320398	0.972245	1
ATXN1L	1.1872845	1.19037641	1.1859443	0.976719071	-0.0339844	0.972301	1
LA16c-358B7.4	1.0206021	1.01740877	1.0219862	1.262940494	0.3367867	0.972305	1
RP11-268G12.1	1.0206041	1.01740877	1.0219891	1.26310438	0.3369739	0.972305	1
RP11-1094H24.4	1.0205713	1.01740877	1.0219421	1.2604065	0.3338891	0.972305	1
AC098973.1	1.0206262	1.01740877	1.0220209	1.264929769	0.3390573	0.972305	1
GPR3	1.020583	1.01740877	1.0219589	1.261371019	0.3349927	0.972305	1
RP11-498C9.2	1.0205971	1.01740877	1.0219792	1.262533677	0.3363219	0.972305	1
PELI2	1.1504048	1.15320107	1.1491927	0.973836092	-0.0382491	0.972346	1
FCER1G	1.0268507	1.02964604	1.025639	0.864838054	-0.2094981	0.972371	1
CARMIL2	1.0268006	1.02959314	1.0255902	0.864734914	-0.2096702	0.972371	1
DEPDC7	1.0241972	1.02100002	1.025583	1.218237263	0.2847951	0.972376	1
LAT2	1.0231956	1.02601228	1.0219746	0.844779195	-0.2433538	0.972384	1
RP11-334J6.7	1.0232411	1.02601366	1.0220394	0.847223471	-0.2391855	0.972384	1
CASC6	1.023196	1.02601705	1.0219733	0.844571837	-0.243708	0.972384	1
RP11-686G8.5	1.0231925	1.02593029	1.0220057	0.848649077	-0.23676	0.972384	1
ME3	1.3589103	1.36214269	1.3575091	0.987205142	-0.0185782	0.972402	1
ZNF597	1.0391096	1.04199029	1.037861	0.901660649	-0.1493435	0.972441	1
LINC01556	1.0357527	1.03854821	1.034541	0.896045455	-0.1583562	0.972464	1
WWC3	1.2066972	1.20338503	1.2081329	1.02334415	0.0332914	0.97251	1
C4orf22	1.0204181	1.02319615	1.0192139	0.828321835	-0.2717367	0.972536	1
RP11-365O16.6	1.0204564	1.02322737	1.0192554	0.828994329	-0.2705659	0.972536	1
RP11-45A17.4	1.0204646	1.02321637	1.0192718	0.830095826	-0.2686502	0.972536	1
RP11-498C9.3	1.0204431	1.02322851	1.0192358	0.828109845	-0.2721059	0.972536	1
HOXB9	1.017107	1.01394208	1.0184788	1.325399749	0.4064276	0.972588	1
AC008697.1	1.0171696	1.01394208	1.0185686	1.331836877	0.4134174	0.972588	1
AB015752.3	1.017166	1.01394208	1.0185634	1.331467454	0.4130172	0.972588	1
MAGED4	1.0170978	1.01394208	1.0184657	1.324459629	0.4054039	0.972588	1
SCML4	1.0171392	1.01394208	1.018525	1.328714015	0.4100306	0.972588	1

RP11-54O7.17	1.0270594	1.0298573	1.0258466	0.865671484	-0.2081085	0.972608	1
GPR173	1.1791111	1.17568195	1.1805976	1.027980126	0.0398124	0.972616	1
C10orf25	1.0663938	1.06930546	1.0651317	0.939777601	-0.0896087	0.97262	1
AC005592.3	1.0178393	1.02062878	1.0166302	0.806166295	-0.3108506	0.972655	1
RP11-230B22.1	1.0178356	1.02060959	1.0166332	0.807063113	-0.3092466	0.972655	1
QPCTL	1.0326612	1.02952741	1.0340196	1.152135721	0.2043107	0.972694	1
RP11-1109F11.5	1.0292279	1.03204097	1.0280086	0.874149117	-0.1940487	0.972761	1
USP34	2.2868486	2.2822851	2.2888266	1.005101466	0.0073411	0.972764	1
ADCY1	1.0533565	1.0563318	1.0520669	0.924289599	-0.1135831	0.972772	1
ZFPM1	1.0627256	1.05947014	1.0641367	1.078469102	0.1089848	0.972777	1
BAIAP3	1.0365194	1.03333951	1.0378977	1.13672027	0.1848773	0.972788	1
AC005224.2	1.0257741	1.02259406	1.0271525	1.201754635	0.2651424	0.972826	1
RP5-1148A21.3	1.0257395	1.02259023	1.0271046	1.199839158	0.262841	0.972826	1
ACER3	1.2428931	1.24608433	1.2415098	0.981410694	-0.0270711	0.972903	1
CTD-2538C1.2	1.0552126	1.05809671	1.0539625	0.928839319	-0.1064991	0.972979	1
RP11-505E24.2	1.0180199	1.01486911	1.0193857	1.303755584	0.3826734	0.973069	1
SELPLG	1.0180043	1.01486911	1.0193632	1.302244873	0.3810008	0.973069	1
FOXC2-AS1	1.0179807	1.01486911	1.0193294	1.299973558	0.3784823	0.973069	1
LMX1B	1.0180035	1.01486911	1.0193621	1.302167278	0.3809148	0.973069	1
DDR1-AS1	1.0180293	1.01486911	1.0193991	1.304661033	0.383675	0.973069	1
LPAR5	1.0179904	1.01486911	1.0193433	1.300903932	0.3795144	0.973069	1
ITGA10	1.0180323	1.01486911	1.0194034	1.304948784	0.3839932	0.973069	1
RASSF8	1.3428069	1.33960968	1.3441927	1.013494966	0.0193389	0.973071	1
TP53INP1	1.3117106	1.31465669	1.3104336	0.986578871	-0.0194937	0.973095	1
CTD-2083E4.6	1.0188083	1.02157655	1.0176083	0.816086291	-0.2932064	0.973111	1
AKAP11	1.3816359	1.37799141	1.3832156	1.013820878	0.0198028	0.973182	1
RP11-368I7.6	1.0181231	1.02087111	1.016932	0.811266172	-0.3017528	0.973237	1
AP000304.12	1.0181518	1.02088006	1.0169693	0.812702359	-0.299201	0.973237	1
GALNT15	1.0181283	1.0208942	1.0169294	0.810242878	-0.3035737	0.973237	1
RP11-287D1.2	1.0181496	1.02090532	1.0169551	0.811041417	-0.3021525	0.973237	1
ZDHHC22	1.018142	1.02084633	1.0169697	0.814039046	-0.2968301	0.973237	1
HMGN4	1.0548839	1.05169261	1.0562672	1.088495592	0.1223356	0.973237	1
RP11-299G20.2	1.0466159	1.04936369	1.0454248	0.920207363	-0.1199691	0.973241	1
RP11-130L8.1	1.028333	1.0310829	1.027141	0.873181619	-0.1956463	0.973245	1
LL21NC02-21A1.1	1.0637391	1.06061091	1.065095	1.073981551	0.1029692	0.973268	1
ZNF674	1.0371899	1.03409423	1.0385317	1.130154162	0.1765196	0.973273	1
COX19	1.5207738	1.51711268	1.5223607	1.01014865	0.0145676	0.973295	1
BUD13	1.166089	1.16925178	1.1647181	0.973213399	-0.0391719	0.973315	1
ST7-AS1	1.0244888	1.02137206	1.0258397	1.209042506	0.273865	0.973332	1
LCP1	1.0244842	1.02134662	1.0258442	1.210693665	0.2758339	0.973332	1
LRRC66	1.0187152	1.021459	1.0175259	0.816714102	-0.292097	0.973425	1
RP11-532M24.1	1.0187529	1.02150913	1.0175581	0.816311535	-0.2928083	0.973425	1
RP11-286N3.2	1.0187225	1.02148111	1.0175268	0.815916553	-0.2935065	0.973425	1
RP11-386I8.6	1.0187411	1.02149422	1.0175477	0.816390577	-0.2926686	0.973425	1
CTC-325J23.2	1.0187248	1.02146381	1.0175376	0.817077067	-0.2914559	0.973425	1
MYLK	1.1184025	1.12136897	1.1171167	0.964964278	-0.0514526	0.973444	1
C1orf145	1.0420087	1.03879682	1.0434009	1.118672631	0.1617879	0.973453	1
RP11-568A7.4	1.0318424	1.03458182	1.0306549	0.886446933	-0.1738938	0.973529	1
RP5-1096D14.6	1.0318401	1.03458804	1.030649	0.886116611	-0.1744315	0.973529	1
AP4B1	1.0830316	1.07981595	1.0844254	1.057750604	0.0809995	0.973549	1
RP11-190C22.9	1.0266234	1.02343747	1.0280044	1.194855394	0.256836	0.973594	1

SV2C	1.0265831	1.02343168	1.0279491	1.192791863	0.2543423	0.973594	1
RP11-158H5.8	1.0477593	1.05048653	1.0465772	0.922566416	-0.1162753	0.973594	1
CTSO	1.1757289	1.17871444	1.1744348	0.976053051	-0.0349685	0.973607	1
BCO1	1.0290937	1.03185563	1.0278965	0.875717866	-0.191462	0.973682	1
C1orf168	1.0290755	1.03185525	1.0278707	0.874915496	-0.1927844	0.973682	1
RP11-216B9.6	1.0290181	1.03172626	1.0278443	0.877640523	-0.188298	0.973682	1
EP400NL	1.1388255	1.14170731	1.1375763	0.970848383	-0.0426821	0.973715	1
C1orf216	1.1591459	1.16211612	1.1578584	0.973736786	-0.0383962	0.973721	1
APOL1	1.027886	1.02480637	1.0292208	1.177956196	0.2362859	0.973724	1
LINC01198	1.0171126	1.01400962	1.0184575	1.317489979	0.397792	0.973734	1
FLT3	1.0171061	1.01400962	1.0184483	1.316828827	0.3970678	0.973734	1
CA4	1.0171093	1.01400962	1.0184528	1.317152641	0.3974225	0.973734	1
KCNJ6	1.017122	1.01400962	1.0184711	1.318454586	0.3988479	0.973734	1
PDE7B	1.1221815	1.12496331	1.1209757	0.968089536	-0.0467876	0.973774	1
FAM72D	1.0613077	1.05817352	1.0626662	1.077228496	0.1073243	0.973836	1
DMRTA1	1.1669135	1.16972661	1.1656942	0.976241649	-0.0346898	0.973886	1
ZC3H18	1.3711576	1.36765577	1.3726754	1.013653148	0.0195641	0.973941	1
KTN1-AS1	1.090313	1.09308352	1.0891121	0.957334756	-0.0629046	0.973986	1
DEPDC1	1.2918008	1.28845026	1.2932531	1.016650555	0.0238239	0.973996	1
TRIM39	1.12442	1.12137802	1.1257386	1.03592547	0.0509202	0.974012	1
GHDC	1.0826437	1.08536008	1.0814662	0.954383494	-0.067359	0.974146	1
LINC01424	1.0345026	1.03730764	1.0332867	0.892221313	-0.1645265	0.974156	1
FXYD7	1.0344669	1.03718333	1.0332894	0.895277688	-0.1595929	0.974156	1
CTC-246B18.10	1.036029	1.03298553	1.0373482	1.132260983	0.1792065	0.974184	1
PROB1	1.0359876	1.03286656	1.0373404	1.136122432	0.1841183	0.974184	1
AGMAT	1.0359894	1.03290808	1.037325	1.134220547	0.1817012	0.974184	1
RP11-173M1.4	1.0186708	1.02133475	1.0175162	0.82101516	-0.2845192	0.974207	1
ANKRD55	1.0186665	1.02136304	1.0174976	0.819059615	-0.2879596	0.974207	1
TSACC	1.0186537	1.02131233	1.0175013	0.821182084	-0.2842259	0.974207	1
RBP5	1.0186996	1.0213647	1.0175444	0.821188546	-0.2842146	0.974207	1
CTD-2036P10.6	1.0186828	1.0213707	1.0175177	0.819708753	-0.2868167	0.974207	1
ADGRA1-AS1	1.0186698	1.02134214	1.0175115	0.820514064	-0.2854	0.974207	1
AC091729.8	1.0186892	1.0214204	1.0175054	0.817229369	-0.291187	0.974207	1
RP11-111H13.1	1.0186948	1.02136109	1.017539	0.821073234	-0.2844172	0.974207	1
RPP25L	1.7090601	1.71274824	1.7074615	0.992582534	-0.010741	0.974211	1
EPHA10	1.0274902	1.03016872	1.0263291	0.872729475	-0.1963936	0.974259	1
SLC2A13	1.1949817	1.19779731	1.1937612	0.979594822	-0.0297429	0.974279	1
NCMAP	1.0198625	1.02257699	1.0186859	0.827653969	-0.2729004	0.974327	1
AC015849.2	1.019859	1.02253044	1.018701	0.830034125	-0.2687574	0.974327	1
CTB-55O6.13	1.0198928	1.02255916	1.0187371	0.830576394	-0.2678152	0.974327	1
NEURL1	1.0525839	1.05544734	1.0513428	0.925973675	-0.1109569	0.974354	1
RGS7	1.0195695	1.0164985	1.0209006	1.266819773	0.3412113	0.974402	1
CTD-2049O4.1	1.0195527	1.01648548	1.0208822	1.266703125	0.3410784	0.974402	1
CMTM2	1.0248305	1.02761108	1.0236252	0.855641564	-0.2249215	0.974403	1
TNFRSF9	1.1565301	1.15366294	1.1577728	1.026746049	0.0380794	0.97442	1
RP11-266J6.2	1.027375	1.03001407	1.0262311	0.873959261	-0.1943621	0.974536	1
DEUP1	1.0273758	1.03003122	1.0262247	0.87324935	-0.1955344	0.974536	1
RP11-850A17.1	1.0405162	1.0432498	1.0393314	0.909399762	-0.1370135	0.974665	1
GRIP2	1.0192722	1.01624192	1.0205856	1.267438892	0.3419162	0.974668	1
CFAP54	1.0563332	1.05903133	1.0551636	0.934480574	-0.0977634	0.974737	1
TACR3	1.0192814	1.02198899	1.0181077	0.823490671	-0.2801758	0.9748	1

MTMR8	1.0192991	1.02204103	1.0181106	0.821677991	-0.283355	0.9748	1
RP1-35C21.2	1.0192702	1.02192689	1.0181187	0.826321412	-0.275225	0.9748	1
ZNF805	1.0350517	1.03774044	1.0338863	0.897876875	-0.1554105	0.974862	1
ADGRE2	1.0174493	1.02010187	1.0162996	0.810847557	-0.3024974	0.974874	1
RP11-118G23.1	1.0174532	1.02011129	1.016301	0.810539804	-0.3030451	0.974874	1
MRGPRF-AS1	1.01745	1.02008508	1.0163078	0.811935121	-0.3005636	0.974874	1
GP6	1.0174576	1.02011554	1.0163055	0.810591661	-0.3029528	0.974874	1
RP3-510O8.4	1.0174463	1.02009179	1.0162996	0.811254618	-0.3017733	0.974874	1
RP11-871F6.3	1.0174561	1.02010524	1.0163078	0.81112093	-0.3020111	0.974874	1
P2RY14	1.017453	1.02010314	1.0163043	0.811034009	-0.3021657	0.974874	1
RP11-395B7.2	1.0174584	1.02011088	1.0163087	0.810939404	-0.302334	0.974874	1
GALR1	1.0174597	1.02011496	1.0163087	0.810774805	-0.3026268	0.974874	1
MED6	1.6364003	1.63951746	1.6350491	0.993012976	-0.0101155	0.974931	1
CTB-179K24.3	1.0351051	1.03200788	1.0364476	1.138705677	0.1873949	0.97496	1
RHOT2	1.6126949	1.60955903	1.6140541	1.007374348	0.0105999	0.97506	1
NPPA-AS1	1.0195284	1.02218401	1.0183773	0.828401616	-0.2715977	0.975066	1
POLI	1.3111025	1.31370422	1.3099748	0.988111741	-0.0172539	0.975066	1
RP11-278C7.4	1.0377088	1.03469218	1.0390164	1.124645169	0.1694699	0.97507	1
RP11-111J6.2	1.0198829	1.01683833	1.0212026	1.259186867	0.3324924	0.975072	1
KRT16	1.0198521	1.01683833	1.0211585	1.256567756	0.3294885	0.975072	1
KCNK7	1.0198838	1.01683833	1.0212039	1.259261264	0.3325776	0.975072	1
RP11-923I11.4	1.0198925	1.01683833	1.0212164	1.260003292	0.3334275	0.975072	1
RP4-575N6.4	1.019871	1.01683833	1.0211856	1.258175366	0.331333	0.975072	1
KB-68A7.2	1.0198893	1.01683833	1.0212118	1.259731745	0.3331165	0.975072	1
TNN	1.0198809	1.01683833	1.0211997	1.259013665	0.3322939	0.975072	1
RP11-37N22.1	1.0248223	1.02172272	1.0261658	1.204534769	0.268476	0.975074	1
CCNB3	1.0247577	1.02172495	1.0260723	1.200109266	0.2631658	0.975074	1
HMG20B	2.6131258	2.61771057	2.6111386	0.995937469	-0.0058729	0.975169	1
XYLT1	1.0405288	1.03745961	1.0418592	1.11744803	0.1602077	0.975223	1
CTD-2331H12.7	1.0187044	1.01566	1.020024	1.278672406	0.3546467	0.97526	1
RP11-359E10.1	1.0186662	1.01567558	1.0199625	1.273478758	0.3487749	0.97526	1
AGFG2	1.0657085	1.06266688	1.0670269	1.069574098	0.0970364	0.975316	1
KLF12	1.1747998	1.17168712	1.176149	1.025988339	0.0370143	0.975353	1
ACADSB	1.1378483	1.13473758	1.1391967	1.033094785	0.0469726	0.975354	1
RP11-525K10.3	1.036011	1.03867172	1.0348577	0.901373977	-0.1498023	0.975398	1
RP4-569M23.2	1.0360284	1.03868601	1.0348764	0.901526107	-0.1495588	0.975398	1
NPM2	1.0274223	1.02440169	1.0287316	1.177443269	0.2356576	0.97542	1
F8	1.0391493	1.03609497	1.0404732	1.121297619	0.1651693	0.975467	1
AC004893.11	1.0455939	1.04822601	1.044453	0.921764302	-0.1175302	0.975503	1
SNX19	1.1665512	1.16932737	1.1653479	0.976498325	-0.0343105	0.97552	1
SPATA4	1.03452	1.03146164	1.0358457	1.139345565	0.1882054	0.975599	1
PIGG	1.1477804	1.14469419	1.1491182	1.030574657	0.043449	0.975603	1
LMBRD2	1.1205776	1.12319906	1.1194414	0.969499187	-0.0446884	0.97561	1
METTL1	1.1644586	1.16124405	1.1658519	1.02857706	0.0406499	0.975617	1
RP3-486I3.7	1.03601	1.03865818	1.0348621	0.901805072	-0.1491125	0.975638	1
CACNA2D4	1.035977	1.03859824	1.0348408	0.902652636	-0.1477572	0.975638	1
AC004878.8	1.0171436	1.01972863	1.0160231	0.812177287	-0.3001334	0.975666	1
RP11-245J9.5	1.017104	1.0196979	1.0159797	0.811239961	-0.3017994	0.975666	1
CTD-2653M23.3	1.0171234	1.01975062	1.0159846	0.809320748	-0.3052165	0.975666	1
ANKFN1	1.0171112	1.01974059	1.0159715	0.809068266	-0.3056667	0.975666	1
IRX5	1.0171373	1.01973817	1.01601	0.811118991	-0.3020145	0.975666	1

SUGP1	1.1572732	1.15423861	1.1585885	1.028202293	0.0401241	0.975741	1
ZNF778	1.0836135	1.08602614	1.0825678	0.959798561	-0.0591964	0.975778	1
RP11-473I1.5	1.0492457	1.05195436	1.0480716	0.925266691	-0.1120588	0.97579	1
ARMCX6	1.7561505	1.7527541	1.7576227	1.006467757	0.009301	0.975792	1
RAG1	1.026474	1.02348259	1.0277707	1.182605887	0.2419694	0.975848	1
ADAMTSL1	1.0265574	1.02916885	1.0254254	0.871662229	-0.1981589	0.975883	1
RP11-80H5.7	1.0265223	1.02916716	1.0253759	0.87001752	-0.2008836	0.975883	1
LINC00637	1.0261418	1.02878179	1.0249975	0.868518992	-0.2033707	0.975953	1
AZIN1	1.9917014	1.99472799	1.9903895	0.995638532	-0.006306	0.975958	1
RP11-439E19.10	1.0240519	1.0266535	1.0229242	0.860080863	-0.2174558	0.975963	1
RP11-318K12.2	1.0240287	1.02666939	1.0228841	0.858064466	-0.2208421	0.975963	1
CTD-2199O4.1	1.0240648	1.02665566	1.0229418	0.860674398	-0.2164605	0.975963	1
EOMES	1.0187819	1.01574718	1.0200974	1.276251185	0.3519123	0.975975	1
ASB2	1.0187483	1.01574718	1.0200492	1.273193055	0.3484512	0.975975	1
LINC00092	1.0187753	1.01574718	1.0200878	1.275646309	0.3512284	0.975975	1
GOLGA6L2	1.0187841	1.01574718	1.0201005	1.276450451	0.3521375	0.975975	1
ARHGEF33	1.0187112	1.01574718	1.019996	1.269814096	0.3446173	0.975975	1
CFH	1.0187422	1.01574718	1.0200404	1.272635218	0.347819	0.975975	1
CTD-2035E11.5	1.0380414	1.03489757	1.0394041	1.129134971	0.1752179	0.975994	1
YBX3	3.1002597	3.09614792	3.102042	1.00281188	0.004051	0.976033	1
C12orf60	1.0803417	1.07723855	1.0816867	1.057590308	0.0807809	0.976133	1
RP5-968P14.2	1.0181543	1.01516282	1.0194509	1.282804624	0.3593015	0.976162	1
DSC1	1.0181751	1.01516282	1.0194808	1.28477386	0.3615144	0.976162	1
SLCO2A1	1.0181209	1.01516282	1.019403	1.279646242	0.355745	0.976162	1
P2RX5	1.0181337	1.01516282	1.0194214	1.280856558	0.3571089	0.976162	1
RP3-326I13.1	1.0181323	1.01516282	1.0194195	1.280729426	0.3569657	0.976162	1
DMRT2	1.0181676	1.01516282	1.01947	1.284061625	0.3607144	0.976162	1
TNF	1.0181265	1.01516282	1.0194111	1.280178697	0.3563452	0.976162	1
LINC01094	1.0181381	1.01516282	1.0194277	1.281271578	0.3575763	0.976162	1
RP3-496C20.1	1.0181401	1.01516282	1.0194307	1.2814683	0.3577978	0.976162	1
RP11-59C5.3	1.0181448	1.01516282	1.0194374	1.281911578	0.3582968	0.976162	1
FRK	1.0181397	1.01516282	1.01943	1.28142602	0.3577502	0.976162	1
METTL21A	1.7685957	1.76513467	1.7700959	1.006484086	0.0093244	0.97619	1
NAIP	1.253936	1.2511401	1.2551478	1.015958215	0.0228411	0.976286	1
OPLAH	1.0726314	1.0752016	1.0715173	0.951007479	-0.0724714	0.97631	1
RP1-79C4.4	1.0180298	1.02058583	1.0169219	0.82201449	-0.2827643	0.976331	1
DCAF4L1	1.0180244	1.02058599	1.016914	0.821628336	-0.2834422	0.976331	1
MMP19	1.0180106	1.02058303	1.0168956	0.820849136	-0.284811	0.976331	1
RP11-284F21.7	1.0180438	1.02058567	1.016942	0.822999252	-0.281037	0.976331	1
AJAP1	1.0307385	1.02760871	1.0320952	1.162500923	0.2172319	0.976354	1
RP11-506K6.4	1.0188496	1.01585689	1.0201468	1.270539187	0.3454409	0.976357	1
MIB2	1.4724721	1.47540361	1.4712014	0.991160723	-0.0128091	0.976369	1
ERF	1.2207683	1.21745823	1.2222031	1.021819574	0.0311405	0.976423	1
FAM35A	1.1995572	1.19621508	1.2010058	1.024415696	0.0348013	0.976538	1
NBN	1.3037282	1.30646618	1.3025414	0.987193336	-0.0185954	0.976649	1
WDR72	1.0369893	1.03402476	1.0382743	1.124895876	0.1697915	0.976666	1
KIAA1468	1.1293405	1.13204239	1.1281693	0.970667732	-0.0429506	0.976688	1
PCDHB11	1.046532	1.04360514	1.0478007	1.096217436	0.132534	0.976721	1
RP11-958F21.1	1.017837	1.01490147	1.0191094	1.282384262	0.3588286	0.976744	1
LINC00607	1.0178194	1.01490147	1.0190842	1.280690933	0.3569224	0.976744	1
CLEC14A	1.0178206	1.01490147	1.0190859	1.28080739	0.3570535	0.976744	1

RP11-503G7.1	1.0178489	1.01490147	1.0191264	1.283524784	0.3601112	0.976744	1
LAMP3	1.0178267	1.01490147	1.0190947	1.281396553	0.357717	0.976744	1
NUMBL	1.1573288	1.16007241	1.1561396	0.975431126	-0.0358881	0.976759	1
BTC	1.0257441	1.02826158	1.0246529	0.872311464	-0.1970847	0.976787	1
RYR3	1.0257196	1.02832746	1.0245891	0.868031974	-0.2041799	0.976787	1
RP11-46D6.1	1.0171182	1.01965743	1.0160176	0.814835628	-0.295419	0.976812	1
ABCD2	1.0171012	1.01962979	1.0160051	0.815348872	-0.2945106	0.976812	1
CTD-2587H24.10	1.0171137	1.01964802	1.0160152	0.815106682	-0.2949392	0.976812	1
IGFBP7-AS1	1.0171041	1.01963484	1.0160072	0.815243924	-0.2946963	0.976812	1
KIAA2013	1.3136056	1.31050323	1.3149504	1.014322299	0.0205161	0.976856	1
ADPGK-AS1	1.019315	1.02185063	1.0182159	0.833657102	-0.262474	0.976881	1
KB-1471A8.1	1.019312	1.02182886	1.018221	0.834722025	-0.2606323	0.976881	1
FLJ40288	1.0193191	1.02185316	1.0182207	0.833780234	-0.2622609	0.976881	1
FP325317.1	1.0193274	1.02184971	1.018234	0.834520862	-0.26098	0.976881	1
RP11-588H23.3	1.0193219	1.02188013	1.0182131	0.832402936	-0.264646	0.976881	1
OCLM	1.0193156	1.02183781	1.0182223	0.834440084	-0.2611196	0.976881	1
PDE1B	1.0204426	1.01751515	1.0217116	1.239588088	0.3098608	0.976931	1
KCNK9	1.0204483	1.01753604	1.0217106	1.238057574	0.3080784	0.976931	1
CYP11A1	1.1007431	1.10331559	1.099628	0.964307591	-0.0524347	0.97701	1
GRASP	1.0549059	1.05752972	1.0537686	0.934622779	-0.0975439	0.977061	1
CORIN	1.0232076	1.02027912	1.0244769	1.207001142	0.271427	0.977083	1
CTA-212A2.3	1.0232029	1.02026426	1.0244767	1.207873338	0.2724692	0.977083	1
RP11-680G24.6	1.0205967	1.02312942	1.0194989	0.843035758	-0.2463343	0.977095	1
ESRRB	1.0205994	1.02315742	1.0194906	0.841655077	-0.248699	0.977095	1
CTD-2588C8.8	1.0206046	1.02316731	1.0194937	0.8414332	-0.2490794	0.977095	1
RP11-867G23.4	1.0206067	1.0231357	1.0195105	0.843307077	-0.24587	0.977095	1
CYP19A1	1.0242523	1.02676245	1.0231642	0.865550022	-0.2083109	0.977102	1
AC072062.3	1.0242254	1.02674115	1.023135	0.865145753	-0.2089849	0.977102	1
RP11-745L13.2	1.0242346	1.02674153	1.023148	0.865617943	-0.2081977	0.977102	1
RP11-307C19.2	1.0242215	1.02677978	1.0231127	0.863063625	-0.2124612	0.977102	1
RP11-140M13.1	1.0242645	1.02688271	1.0231297	0.860392777	-0.2169327	0.977102	1
WNT3A	1.0242371	1.02685343	1.023103	0.860338163	-0.2170243	0.977102	1
NOS2	1.0242474	1.02673834	1.0231677	0.866460294	-0.2067945	0.977102	1
AP000704.5	1.0484583	1.04541081	1.0497792	1.096197247	0.1325074	0.97711	1
ADAM20	1.0267997	1.02390483	1.0280545	1.173590354	0.2309289	0.977173	1
RP11-1079K10.3	1.0178538	1.020373	1.0167618	0.822746266	-0.2814805	0.977247	1
RP3-500L14.2	1.0178439	1.02035759	1.0167543	0.822998751	-0.2810379	0.977247	1
KLHL6	1.0178561	1.02037457	1.0167644	0.822812348	-0.2813646	0.977247	1
CXCL5	1.0178539	1.02037769	1.01676	0.822467531	-0.2819694	0.977247	1
CYSLTR1	1.017847	1.02037867	1.0167496	0.821918649	-0.2829325	0.977247	1
RP1-261G23.7	1.0178579	1.02038714	1.0167616	0.822167695	-0.2824954	0.977247	1
AF011889.2	1.0178492	1.02037207	1.0167556	0.82248077	-0.2819461	0.977247	1
RP11-9N20.3	1.0178518	1.02039008	1.0167516	0.821555081	-0.2835708	0.977247	1
SNX15	1.0219608	1.01899597	1.0232459	1.223725375	0.2912798	0.977248	1
RP5-968D22.3	1.0219388	1.01899597	1.0232144	1.222069517	0.2893264	0.977248	1
RP11-804N13.1	1.0219418	1.01899597	1.0232187	1.222297811	0.2895958	0.977248	1
RP11-503C24.2	1.0219159	1.01899597	1.0231816	1.220341793	0.2872853	0.977248	1
C7orf43	1.0552651	1.05224194	1.0565755	1.082952392	0.1149698	0.977319	1
IGDCC3	1.3730721	1.3755378	1.3720033	0.990588171	-0.0136427	0.977332	1
FAM150A	1.0245183	1.02697291	1.0234543	0.869548922	-0.2016609	0.977339	1
LINC01554	1.0244667	1.02699835	1.0233694	0.865585808	-0.2082512	0.977339	1

RP3-412A9.16	1.024486	1.02698046	1.0234047	0.86747042	-0.2051135	0.977339	1
TAC3	1.019688	1.0168068	1.0209368	1.245735971	0.3169983	0.977367	1
BAG6	2.0281872	2.02473412	2.029684	1.004830422	0.006952	0.977376	1
RP11-556E13.1	1.0152409	1.01774939	1.0141536	0.79741338	-0.3266003	0.977384	1
RP11-474D1.2	1.015244	1.01774149	1.0141615	0.798211123	-0.3251577	0.977384	1
RP11-90E5.1	1.0152462	1.01774939	1.0141611	0.797838089	-0.3258321	0.977384	1
BMP8B	1.0152381	1.01774131	1.014153	0.797742683	-0.3260046	0.977384	1
SLC52A1	1.0152396	1.01774806	1.0141523	0.797401621	-0.3266216	0.977384	1
RP11-1348G14.5	1.0152406	1.01774806	1.0141537	0.797480177	-0.3264794	0.977384	1
ATP1A4	1.0152397	1.01774687	1.014153	0.797492756	-0.3264567	0.977384	1
RERGL	1.015242	1.01775367	1.0141534	0.797207647	-0.3269725	0.977384	1
GOLGA8M	1.0152435	1.01774218	1.0141605	0.798125143	-0.3253131	0.977384	1
CTB-43E15.1	1.0152393	1.01773514	1.0141575	0.79827456	-0.3250431	0.977384	1
RP11-425A23.1	1.0152374	1.01773649	1.0141541	0.798023913	-0.3254961	0.977384	1
RP11-557N21.1	1.0152481	1.01775023	1.0141635	0.797935419	-0.3256561	0.977384	1
HTR7	1.0152433	1.01775985	1.0141524	0.796877302	-0.3275705	0.977384	1
IL7	1.0152448	1.01776513	1.0141524	0.796640643	-0.327999	0.977384	1
GTF2H3	1.2840118	1.28662692	1.2828783	0.986921459	-0.0189928	0.977385	1
ZMYND19	1.0904741	1.09291991	1.0894139	0.962268701	-0.0554883	0.977469	1
CTR9	1.3395176	1.34221275	1.3383494	0.98871069	-0.0163797	0.977469	1
RP11-176N18.2	1.0354032	1.03234263	1.0367298	1.13564683	0.1835142	0.977474	1
FAM81A	1.0373625	1.03439102	1.0386506	1.123856443	0.1684578	0.977494	1
NUP50-AS1	1.0814814	1.08409448	1.0803488	0.955458311	-0.0657352	0.9775	1
C19orf44	1.0790613	1.08162804	1.0779487	0.954925831	-0.0665394	0.977584	1
KLHL17	1.0525913	1.05507596	1.0515143	0.935331091	-0.0964509	0.977623	1
NUBPL	1.3282283	1.32517204	1.3295531	1.013473024	0.0193077	0.977669	1
LEMD3	1.199006	1.20155811	1.1978997	0.981849573	-0.0264261	0.977802	1
SLC25A12	1.1036771	1.10616562	1.1025984	0.96639967	-0.0493081	0.977804	1
RINL	1.1136033	1.11064995	1.1148835	1.038260635	0.0541686	0.977809	1
PRCAT47	1.016235	1.01873682	1.0151506	0.808600103	-0.3065017	0.977821	1
RP11-111A22.1	1.0162399	1.01875602	1.0151493	0.807701837	-0.3081053	0.977821	1
CH507-42P11.8	1.016256	1.01874051	1.0151791	0.809961969	-0.3040739	0.977821	1
CTC-332L22.1	1.0162416	1.01871691	1.0151687	0.81042547	-0.3032486	0.977821	1
RP11-847H18.2	1.0162655	1.01871888	1.0152021	0.81212575	-0.300225	0.977821	1
ECSCR	1.0162157	1.0186955	1.0151409	0.809866122	-0.3042447	0.977821	1
RP11-1081M5.1	1.0162439	1.01875717	1.0151545	0.8079334	-0.3076917	0.977821	1
AC129492.1	1.0162546	1.01874217	1.0151764	0.809745135	-0.3044602	0.977821	1
GABRG2	1.0162255	1.01870879	1.0151491	0.809733765	-0.3044805	0.977821	1
P2RX5-TAX1BP3	1.0162408	1.0187383	1.0151583	0.808944864	-0.3058867	0.977821	1
FRS3	1.0834024	1.08037901	1.0847129	1.053917922	0.0757625	0.977862	1
IL27RA	1.1654085	1.16261999	1.1666172	1.024580237	0.035033	0.977923	1
RNF19B	1.0575443	1.05468772	1.0587825	1.074875332	0.1041693	0.977929	1
SLC7A7	1.0257049	1.02818905	1.0246281	0.873677098	-0.1948279	0.977938	1
RP11-489D6.2	1.0155413	1.01799924	1.0144759	0.804253508	-0.3142778	0.977966	1
AP4B1-AS1	1.0155404	1.01802331	1.0144642	0.802525003	-0.3173818	0.977966	1
ALKBH3-AS1	1.015541	1.01802041	1.0144663	0.802771334	-0.316939	0.977966	1
CTD-2527I21.7	1.0155408	1.01801944	1.0144665	0.802824826	-0.3168429	0.977966	1
LRRD1	1.0155431	1.01801305	1.0144724	0.803440721	-0.3157365	0.977966	1
AC006116.20	1.0155387	1.01801845	1.0144639	0.802724586	-0.317023	0.977966	1
CTD-2530H12.4	1.0155397	1.01800052	1.014473	0.804032337	-0.3146746	0.977966	1
RP11-20G13.3	1.0155352	1.01799924	1.0144671	0.803763895	-0.3151563	0.977966	1

RP11-640L9.1	1.0155499	1.01802521	1.014477	0.803152253	-0.3162546	0.977966		1
MSC-AS1	1.0155405	1.01802	1.0144658	0.802764704	-0.3169509	0.977966		1
KB-1732A1.1	1.0279641	1.03043583	1.0268927	0.883585567	-0.1785582	0.977969		1
CDAN1	1.0841973	1.081215	1.08549	1.052637998	0.0740094	0.978082		1
CTD-2291D10.1	1.0161406	1.01859924	1.0150749	0.810513496	-0.3030919	0.978153		1
AC079779.6	1.0161364	1.01858439	1.0150754	0.811184052	-0.3018988	0.978153		1
AC009495.3	1.0161342	1.01860021	1.0150653	0.809954674	-0.3040869	0.978153		1
SOX1	1.0161332	1.01859747	1.015065	0.810055676	-0.303907	0.978153		1
RP11-114B7.6	1.0161407	1.01862213	1.0150651	0.808986646	-0.3058122	0.978153		1
RP3-324O17.8	1.0161314	1.01859094	1.0150653	0.810358627	-0.3033676	0.978153		1
FAM9C	1.016132	1.01859593	1.015064	0.810069294	-0.3038828	0.978153		1
CTD-3064H18.4	1.0161341	1.01860394	1.0150636	0.80969775	-0.3045446	0.978153		1
HAVCR2	1.0161444	1.01861712	1.0150726	0.809612036	-0.3046974	0.978153		1
C11orf97	1.0161359	1.01859327	1.0150707	0.810547702	-0.303031	0.978153		1
CTD-2525I3.5	1.0161344	1.01860175	1.0150649	0.809864694	-0.3042472	0.978153		1
RASGRP3	1.0307136	1.02791691	1.0319259	1.143602906	0.1935862	0.9782		1
NDNF	1.0307605	1.02793483	1.0319854	1.144999856	0.1953474	0.9782		1
PCF11-AS1	1.0418516	1.04440182	1.0407462	0.91766928	-0.1239538	0.978202		1
AL022341.3	1.0292205	1.03169755	1.0281468	0.887978903	-0.1714027	0.978223		1
SDR42E2	1.0292578	1.0317328	1.028185	0.888197618	-0.1710474	0.978223		1
ASH1L-AS1	1.1089606	1.1060985	1.1102011	1.038668164	0.0547348	0.978281		1
LINC01106	1.0265935	1.02379907	1.0278048	1.16831271	0.2244265	0.978313		1
UBR1	1.218935	1.22151091	1.2178184	0.983330479	-0.0242517	0.978367		1
USP19	1.1276084	1.12468067	1.1288774	1.033659714	0.0477613	0.978399		1
HCG9	1.0196964	1.01687616	1.0209189	1.239553013	0.30982	0.978514		1
DLL4	1.0197131	1.0168739	1.0209438	1.241193682	0.3117283	0.978514		1
ZNF3	1.5413216	1.53810524	1.5427157	1.008568003	0.0123084	0.978566		1
HBG2	1.0352837	1.03767785	1.034246	0.908914807	-0.137783	0.97857		1
SH3D21	1.087739	1.09015697	1.0866909	0.961555419	-0.0565581	0.978582		1
CCDC71L	1.1381777	1.13512044	1.1395028	1.032433279	0.0460485	0.978625		1
SPRY4	1.0611824	1.06369787	1.060092	0.94339175	-0.0840711	0.978697		1
RP5-1021I20.1	1.0544872	1.05696108	1.0534149	0.937744131	-0.0927338	0.978732		1
ST7	1.1103157	1.10746196	1.1115526	1.038065892	0.053898	0.978789		1
RP11-422P24.11	1.079036	1.08151125	1.077963	0.956469544	-0.0642091	0.978809		1
MSL2	1.1819893	1.18454907	1.1808798	0.980117548	-0.0289733	0.97886		1
XX-FW83128A1.2	1.0161015	1.01853346	1.0150473	0.811899612	-0.3006267	0.978917		1
CTD-2516F10.2	1.0161088	1.01849485	1.0150746	0.815070964	-0.2950024	0.978917		1
RP11-374M1.5	1.0386229	1.03580691	1.0398435	1.112732924	0.1541074	0.978929		1
C3orf35	1.0248511	1.02729316	1.0237926	0.871743182	-0.1980249	0.978951		1
ERVW-1	1.0248341	1.02724863	1.0237875	0.872980628	-0.1959785	0.978951		1
RP11-148L24.1	1.0752544	1.07222306	1.0765683	1.060164536	0.0842882	0.978956		1
PARD6A	1.31096	1.31337587	1.3099129	0.988949406	-0.0160314	0.978965		1
EGLN2	1.857449	1.86034803	1.8561924	0.995169792	-0.0069854	0.978985		1
C2orf73	1.0429756	1.04536089	1.0419416	0.924621092	-0.1130658	0.979		1
APITD1-CORT	1.0429752	1.04541675	1.0419169	0.922939726	-0.1156917	0.979		1
CACNA1C-AS2	1.0518078	1.04884638	1.0530914	1.086906389	0.1202277	0.979014		1
CACNB1	1.0754257	1.07258356	1.0766576	1.056129351	0.0787865	0.979016		1
CTNNAL1	2.0536858	2.05636021	2.0525266	0.996370946	-0.0052451	0.979046		1
FAM81B	1.0365914	1.03910123	1.0355035	0.907990114	-0.1392515	0.979048		1
AC022182.3	1.0172772	1.01969216	1.0162304	0.824208295	-0.2789191	0.979055		1
CCR7	1.0172755	1.01968877	1.0162294	0.824297959	-0.2787622	0.979055		1

RP11-39E3.3	1.0172819	1.01968058	1.0162422	0.825289464	-0.2770279	0.979055	1
CH17-140K24.5	1.0172715	1.01968376	1.0162259	0.824327812	-0.2787099	0.979055	1
SOX21	1.0172817	1.01968849	1.0162384	0.824766714	-0.277942	0.979055	1
RP11-385F5.5	1.0172692	1.01967052	1.0162283	0.825007227	-0.2775213	0.979055	1
MOGAT1	1.0172791	1.01969586	1.0162316	0.824112039	-0.2790876	0.979055	1
LINC01314	1.0172806	1.0197005	1.0162317	0.823923435	-0.2794178	0.979055	1
RP11-301G7.1	1.0172746	1.01968877	1.0162282	0.824235928	-0.2788707	0.979055	1
TRPM5	1.0172785	1.01970037	1.0162287	0.823775803	-0.2796763	0.979055	1
C11orf16	1.0172726	1.01968849	1.0162254	0.824104052	-0.2791016	0.979055	1
RP11-66D17.3	1.0172728	1.01968203	1.0162285	0.824535784	-0.278346	0.979055	1
AC092667.2	1.0172716	1.01968547	1.0162254	0.82423026	-0.2788807	0.979055	1
RP5-1180C18.1	1.0172778	1.01969263	1.0162311	0.8242206	-0.2788976	0.979055	1
RP11-400N13.3	1.0172728	1.01967988	1.0162294	0.824670447	-0.2781104	0.979055	1
LINC01273	1.1003128	1.0972954	1.1016207	1.044455421	0.0627509	0.979113	1
TEKT1	1.0379089	1.03508069	1.0391348	1.115563927	0.1577732	0.979118	1
RP11-227H15.5	1.0222065	1.02464663	1.0211487	0.858078847	-0.2208179	0.97915	1
GDA	1.0222172	1.02462146	1.0211751	0.86002614	-0.2175476	0.97915	1
RP11-393I23.4	1.0221601	1.02457374	1.0211138	0.859203687	-0.2189279	0.97915	1
AC141928.1	1.0221878	1.02461916	1.021134	0.858435518	-0.2202183	0.97915	1
NPIPB13	1.0221974	1.02464304	1.0211374	0.857741538	-0.2213851	0.97915	1
RP11-290D2.6	1.0416384	1.04392282	1.0406483	0.925447457	-0.111777	0.979166	1
PUS10	1.1298752	1.12708798	1.1310834	1.03143806	0.0446572	0.979199	1
DGKG	1.0504368	1.05288568	1.0493753	0.933623384	-0.0990874	0.979217	1
MIR497HG	1.0247754	1.02721864	1.0237164	0.871330575	-0.1987079	0.979247	1
ID2-AS1	1.0247949	1.02719461	1.0237547	0.873507014	-0.1951088	0.979247	1
SCARF2	1.0247903	1.02723783	1.0237294	0.871191481	-0.1989382	0.979247	1
RP11-318C24.2	1.0200454	1.01724954	1.0212573	1.232340036	0.3014004	0.979258	1
RP11-20I20.4	1.0200413	1.01724954	1.0212514	1.231997338	0.3009991	0.979258	1
RP11-351J23.1	1.0200425	1.01724954	1.0212532	1.232099991	0.3011193	0.979258	1
PAXIP1-AS2	1.0200313	1.01724954	1.0212371	1.23116619	0.3000255	0.979258	1
AP000708.1	1.0378969	1.040294	1.0368579	0.914723362	-0.1285926	0.97934	1
CMC2	1.8646999	1.86706372	1.8636753	0.996092081	-0.005649	0.979417	1
C1orf167	1.0166832	1.01906514	1.0156508	0.820911383	-0.2847016	0.979508	1
LINC01143	1.0166974	1.01910312	1.0156546	0.819480257	-0.2872189	0.979508	1
RP11-213G2.2	1.016688	1.01907185	1.0156547	0.820825226	-0.284853	0.979508	1
RAB5B	1.7174936	1.72030004	1.7162771	0.994414962	-0.0080801	0.979543	1
TRIM11	1.2561359	1.25332212	1.2573556	1.01592234	0.0227901	0.979559	1
NUDT6	1.2215635	1.21890248	1.222717	1.017425476	0.0249231	0.979606	1
ATP7A	1.2355623	1.23820278	1.2344178	0.984110189	-0.0231082	0.979634	1
RP11-158M2.3	1.0169474	1.01932692	1.0159159	0.823511752	-0.2801389	0.979774	1
PKD2L2	1.0169666	1.01930985	1.015951	0.826052722	-0.2756942	0.979774	1
PALM2-AKAP2	1.0169561	1.01932901	1.0159275	0.824021226	-0.2792466	0.979774	1
KANSL3	1.3208197	1.32346359	1.3196737	0.988283523	-0.0170031	0.979842	1
PDE2A	1.0225019	1.01969984	1.0237165	1.203892639	0.2677067	0.979854	1
AC003090.1	1.0224484	1.01967387	1.0236511	1.202155637	0.2656237	0.979854	1
RP11-284F21.8	1.0225215	1.01969756	1.0237456	1.20550767	0.2696408	0.979854	1
EPB41L4A-AS2	1.0273935	1.02457702	1.0286144	1.164272982	0.2194294	0.979875	1
CD37	1.0274385	1.0246983	1.0286263	1.159038783	0.2129288	0.979875	1
ABHD3	1.120887	1.12336246	1.119814	0.971235888	-0.0421064	0.979952	1
MYO5C	1.1917398	1.18891456	1.1929643	1.021437125	0.0306004	0.979952	1
PANX2	1.0612285	1.06356796	1.0602145	0.947245538	-0.0781897	0.979956	1

RTN4RL2	1.046972	1.04406063	1.0482339	1.094717276	0.1305583	0.98	1
DLGAP2	1.0212431	1.01850667	1.0224292	1.211950743	0.2773311	0.980024	1
AIM1	1.0212408	1.01850713	1.0224258	1.211735844	0.2770752	0.980024	1
DISP2	1.0293796	1.02662266	1.0305746	1.148442426	0.1996785	0.980028	1
LA16c-313D11.9	1.0293436	1.0266172	1.0305254	1.146828093	0.1976492	0.980028	1
RP11-497E19.2	1.024709	1.02704933	1.0236946	0.875978655	-0.1910324	0.980052	1
RP11-797H7.5	1.0328251	1.03519427	1.0317981	0.903502674	-0.1463992	0.980142	1
NIM1K	1.0259364	1.02826629	1.0249265	0.881843883	-0.1814048	0.980152	1
KCNH3	1.0259394	1.02831492	1.0249097	0.87973638	-0.1848568	0.980152	1
SAP25	1.0418541	1.03910375	1.0430462	1.100820862	0.1385797	0.980214	1
RAD50	2.2417608	2.2387504	2.2430657	1.003483592	0.005017	0.98039	1
RP11-77H9.5	1.0145255	1.01686526	1.0135113	0.80113281	-0.3198867	0.980391	1
RP11-353N14.1	1.0145202	1.01685607	1.0135078	0.801359794	-0.319478	0.980391	1
CYP17A1-AS1	1.0145252	1.01687616	1.0135062	0.800310757	-0.3213678	0.980391	1
SBSN	1.0145298	1.01686089	1.0135194	0.80181804	-0.3186532	0.980391	1
RP11-15H20.7	1.014517	1.01684335	1.0135086	0.802011724	-0.3183048	0.980391	1
ZNF492	1.0145202	1.01685621	1.0135076	0.801342442	-0.3195092	0.980391	1
RP11-532L16.3	1.0145205	1.01686294	1.0135052	0.800878522	-0.3203447	0.980391	1
RP5-1186P10.2	1.0145255	1.01685607	1.0135153	0.801806799	-0.3186734	0.980391	1
RP11-244F12.2	1.0145204	1.01685271	1.0135094	0.801616794	-0.3190154	0.980391	1
RP11-335K5.2	1.0145157	1.01684949	1.0135042	0.801458742	-0.3192998	0.980391	1
CPNE6	1.0145209	1.01684949	1.0135115	0.801895216	-0.3185144	0.980391	1
RP11-79O8.1	1.0145256	1.01686913	1.0135097	0.800854335	-0.3203882	0.980391	1
KCNT2	1.1935755	1.19056183	1.1948817	1.022669284	0.0323397	0.98048	1
PPP2R3B	1.1791188	1.1817606	1.1779738	0.979165836	-0.0303749	0.980552	1
C3orf18	1.1877104	1.18484898	1.1889506	1.022189219	0.0316623	0.980595	1
SERPINB7	1.0290436	1.02635375	1.0302096	1.146309804	0.196997	0.980614	1
CTC-529I10.1	1.0290689	1.02630962	1.030265	1.150337816	0.2020576	0.980614	1
KB-1466C5.1	1.0239903	1.02627491	1.0230001	0.875361916	-0.1920485	0.980628	1
TMEFF1	1.0240156	1.02635927	1.0229997	0.872546694	-0.1966958	0.980628	1
MAS1	1.0239708	1.02633774	1.0229448	0.871177505	-0.1989614	0.980628	1
LINC01018	1.0253058	1.02761274	1.0243059	0.880240833	-0.1840298	0.980646	1
TSPAN32	1.0235288	1.0258228	1.0225345	0.872658375	-0.1965111	0.98068	1
ACE2	1.0214957	1.02379525	1.0204989	0.861471766	-0.2151246	0.980708	1
RP11-21A7A.3	1.0214348	1.02377053	1.0204223	0.859143829	-0.2190284	0.980708	1
LINC01091	1.0214367	1.02378388	1.0204193	0.858533382	-0.2200539	0.980708	1
AP001468.58	1.0214566	1.0237514	1.0204619	0.861502952	-0.2150724	0.980708	1
RP11-584P21.2	1.0214605	1.02373432	1.0204749	0.862671459	-0.2131169	0.980708	1
DPY19L4	1.3411682	1.33824115	1.3424369	1.012404754	0.0177862	0.98071	1
CGREF1	1.0404014	1.03768767	1.0415777	1.103217637	0.1417174	0.980724	1
WWC2-AS2	1.0405767	1.03782033	1.0417715	1.104471171	0.1433558	0.980724	1
RP1-266L20.2	1.0213064	1.01860175	1.0224788	1.208422582	0.273125	0.980758	1
RP11-21B23.2	1.0213329	1.01861576	1.0225107	1.209228423	0.2740868	0.980758	1
AC093627.10	1.0213	1.01859264	1.0224735	1.208730457	0.2734926	0.980758	1
ARHGEF10L	1.1074173	1.109826	1.1063733	0.968562088	-0.0460836	0.980784	1
ANGEL1	1.1074674	1.10985122	1.1064342	0.968893752	-0.0455896	0.980784	1
NECAP1	1.2801075	1.28276607	1.2789551	0.986522466	-0.0195762	0.980784	1
MEX3C	1.2729589	1.27550772	1.2718541	0.98673856	-0.0192602	0.980798	1
TMEM110	1.0383058	1.03553168	1.0395082	1.111914567	0.1530459	0.980857	1
PEAR1	1.021912	1.02424759	1.0208996	0.861924272	-0.214367	0.98089	1
KCNC1	1.0219634	1.02425204	1.0209713	0.864724676	-0.2096872	0.98089	1

RP11-363E6.3	1.0219109	1.02425278	1.0208958	0.861582648	-0.2149389	0.98089	1
ASB9	1.0596395	1.05692617	1.0608155	1.068322961	0.0953478	0.980905	1
KCNJ2-AS1	1.0491782	1.05147523	1.0481826	0.936034851	-0.0953658	0.98092	1
RP11-539L10.2	1.0207314	1.01800877	1.0219115	1.216715531	0.2829919	0.980946	1
RP11-109E12.1	1.0207157	1.0180245	1.0218823	1.214028161	0.2798019	0.980946	1
DDX19B	1.4037739	1.40088783	1.4050248	1.010319632	0.0148118	0.980985	1
LMOD3	1.0506013	1.04800158	1.0517282	1.077635716	0.1078696	0.98099	1
ACSS1	1.0672683	1.06962451	1.066247	0.951490177	-0.0717393	0.981035	1
LINCR-0001	1.0154229	1.01771598	1.0144289	0.814456234	-0.2960909	0.981056	1
FAM159B	1.01542	1.01772004	1.014423	0.813936824	-0.2970113	0.981056	1
NKX6-3	1.0154192	1.01771746	1.014423	0.814055669	-0.2968006	0.981056	1
MYLK2	1.01542	1.01772173	1.0144223	0.81382246	-0.297214	0.981056	1
TBX20	1.0154167	1.01771787	1.0144192	0.813824013	-0.2972112	0.981056	1
LINC01126	1.0154205	1.01771135	1.0144276	0.814595163	-0.2958448	0.981056	1
SP5	1.0154204	1.01771531	1.0144257	0.814308419	-0.2963528	0.981056	1
RFPL3	1.0154165	1.01771164	1.0144217	0.814250005	-0.2964563	0.981056	1
ESM1	1.0154329	1.01771822	1.0144423	0.815110848	-0.2949318	0.981056	1
RP11-122K13.15	1.0154189	1.01771219	1.0144248	0.814400622	-0.2961894	0.981056	1
RD3L	1.0214068	1.01872015	1.0225714	1.205727918	0.2699044	0.981122	1
RP5-1024C24.1	1.0214648	1.0188028	1.0226186	1.202937387	0.2665616	0.981122	1
HIST1H2BJ	1.0213837	1.01870502	1.0225448	1.205281642	0.2693703	0.981122	1
SH3RF2	1.0420951	1.04438334	1.0411033	0.926097867	-0.1107634	0.981183	1
XK	1.0470506	1.04424842	1.0482652	1.090777626	0.125357	0.981206	1
PRDM11	1.0693282	1.07175647	1.0682757	0.951491432	-0.0717374	0.981213	1
CTB-131K11.1	1.0284644	1.02579828	1.0296201	1.148141356	0.1993003	0.981231	1
TCAP	1.0346115	1.03690382	1.0336178	0.910957376	-0.1345445	0.98124	1
RP11-73E17.2	1.0645573	1.06179611	1.0657542	1.064050357	0.0895664	0.981297	1
PCMTD2	1.5465532	1.5492714	1.545375	0.992906187	-0.0102707	0.981335	1
SVIP	2.4008603	2.39629916	2.4028373	1.004682475	0.0067396	0.981421	1
NUDT21	1.7420341	1.74508243	1.7407128	0.994135364	-0.0084858	0.981457	1
TMEM136	1.5753975	1.57203252	1.5768561	1.008432285	0.0121142	0.98149	1
SSTR3	1.0231801	1.02541255	1.0222125	0.874074386	-0.194172	0.981495	1
EXOC3L1	1.0231262	1.02540289	1.0221393	0.871528536	-0.1983802	0.981495	1
RP4-635E18.8	1.023135	1.02542098	1.0221441	0.87109488	-0.1990982	0.981495	1
HIST1H3E	1.0231166	1.02538073	1.0221352	0.872127886	-0.1973884	0.981495	1
PLIN1	1.0204883	1.01776549	1.0216685	1.219695575	0.2865211	0.981529	1
ARHGAP27	1.0204116	1.01773381	1.0215723	1.216449152	0.282676	0.981529	1
AC023481.1	1.0204088	1.01774595	1.0215631	1.215098054	0.2810727	0.981529	1
RAB6C	1.0145159	1.01678293	1.0135333	0.806371336	-0.3104837	0.981537	1
ABCA10	1.0145206	1.01679133	1.0135363	0.806148264	-0.3108829	0.981537	1
CCL18	1.0145277	1.01679675	1.0135441	0.806354679	-0.3105135	0.981537	1
CHRNA6	1.0145221	1.01680144	1.0135341	0.805533457	-0.3119836	0.981537	1
RP11-727A23.10	1.0145203	1.01679454	1.0135345	0.805889764	-0.3113456	0.981537	1
AC084809.3	1.014514	1.01678166	1.0135311	0.806302674	-0.3106066	0.981537	1
ADAMTS19-AS1	1.0145227	1.0168044	1.0135337	0.805366118	-0.3122833	0.981537	1
RP11-7F17.5	1.014523	1.01679675	1.0135375	0.805956459	-0.3112262	0.981537	1
RP11-304L19.12	1.0145224	1.01679791	1.013536	0.805814533	-0.3114803	0.981537	1
TPSG1	1.0145187	1.01678236	1.0135375	0.806647753	-0.3099893	0.981537	1
RP11-1103G16.1	1.0145149	1.0167853	1.0135308	0.806109916	-0.3109515	0.981537	1
PKD1L1	1.0145226	1.01679791	1.0135363	0.805832488	-0.3114481	0.981537	1
PAXIP1	1.143057	1.14518272	1.1421355	0.979011351	-0.0306025	0.981594	1

FOXC1	1.0326354	1.03489282	1.0316569	0.907260869	-0.1404107	0.981642	1
RP11-758M4.4	1.0318303	1.02909343	1.0330166	1.134848858	0.1825002	0.981666	1
CSGALNACT2	1.1691651	1.16652504	1.1703095	1.022725827	0.0324194	0.98167	1
TFAM	1.6431372	1.64074154	1.6441756	1.005359564	0.0077116	0.98167	1
RP11-173C1.1	1.0180144	1.02026694	1.017038	0.840680507	-0.2503705	0.981819	1
RP11-659E9.4	1.0180035	1.02026694	1.0170224	0.839912141	-0.2516897	0.981819	1
RP11-120K19.4	1.0180071	1.02027646	1.0170234	0.839565555	-0.2522851	0.981819	1
RP11-296K13.4	1.0180025	1.02026714	1.0170209	0.839827988	-0.2518342	0.981819	1
CTA-150C2.13	1.01801	1.02027481	1.0170283	0.839872459	-0.2517578	0.981819	1
CALN1	1.0180041	1.02025802	1.0170272	0.840515908	-0.250653	0.981819	1
FLJ12825	1.0180018	1.02026124	1.0170225	0.840149114	-0.2512827	0.981819	1
IL20RA	1.0180008	1.02024793	1.0170268	0.8409137	-0.2499703	0.981819	1
RP11-661A12.7	1.0179995	1.02026124	1.0170191	0.839983639	-0.2515669	0.981819	1
RP1-309F20.3	1.0216278	1.02392983	1.02063	0.862105179	-0.2140642	0.981845	1
AC010287.1	1.021626	1.02388382	1.0206474	0.864491707	-0.210076	0.981845	1
DUSP13	1.0216136	1.02387772	1.0206323	0.864080261	-0.2107628	0.981845	1
SLC6A12	1.0216567	1.02387976	1.0206931	0.866555203	-0.2066364	0.981845	1
RP11-230C9.4	1.0216205	1.02383695	1.0206597	0.866710877	-0.2063773	0.981845	1
RP1-151F17.2	1.0257925	1.02313041	1.0269463	1.16497471	0.2202986	0.98185	1
C5orf22	1.1160494	1.1182088	1.1151134	0.973814296	-0.0382814	0.981861	1
HIST1H4I	1.030628	1.03284288	1.029668	0.903330147	-0.1466747	0.981905	1
TRAF3IP2-AS1	1.3349964	1.33700442	1.3341261	0.991459029	-0.0123749	0.981926	1
RP1-23E21.2	1.0152608	1.01751032	1.0142857	0.81584563	-0.2936319	0.981971	1
HIPK4	1.0152639	1.01751599	1.0142878	0.81569845	-0.2938922	0.981971	1
CSF2RB	1.0152672	1.01751783	1.0142916	0.815831304	-0.2936572	0.981971	1
TXK	1.0152594	1.01750123	1.0142877	0.816379897	-0.2926874	0.981971	1
C16orf96	1.0152628	1.01751515	1.0142865	0.815665807	-0.2939499	0.981971	1
CTD-2561B21.5	1.0152635	1.01751383	1.0142881	0.815816798	-0.2936829	0.981971	1
CDX1	1.0152585	1.01750722	1.0142838	0.815879803	-0.2935715	0.981971	1
CFAP73	1.0152654	1.01751851	1.0142888	0.81564162	-0.2939927	0.981971	1
GFRA2	1.0584578	1.05570173	1.0596525	1.070927151	0.0988603	0.982024	1
RP11-298I3.4	1.0584464	1.05578116	1.0596016	1.068489955	0.0955733	0.982024	1
GCNT4	1.0245071	1.02185836	1.0256552	1.173701409	0.2310654	0.982033	1
XPO6	1.2533506	1.25596359	1.2522179	0.985366423	-0.0212678	0.98205	1
PGBD1	1.1499315	1.14732137	1.1510629	1.025397171	0.0361828	0.982066	1
MTCP1	1.0218622	1.02409951	1.0208924	0.866921613	-0.2060265	0.982082	1
PIWIL2	1.0218952	1.02416913	1.0209096	0.865135456	-0.2090021	0.982082	1
RP11-129M16.4	1.0218515	1.02412898	1.0208642	0.864696485	-0.2097343	0.982082	1
FGD3	1.0198227	1.0171592	1.0209772	1.222504987	0.2898404	0.982105	1
RP11-408A13.3	1.0197866	1.0171592	1.0209255	1.219491879	0.2862802	0.982105	1
C11orf53	1.019845	1.0171592	1.0210091	1.224364136	0.2920327	0.982105	1
RP11-388M20.1	1.019787	1.0171592	1.020926	1.219521148	0.2863148	0.982105	1
WNT9B	1.0198021	1.0171592	1.0209476	1.220780536	0.2878039	0.982105	1
RP5-1184F4.7	1.0198442	1.0171592	1.021008	1.224301638	0.291959	0.982105	1
CTC-350I8.1	1.0198191	1.0171592	1.020972	1.222202714	0.2894836	0.982105	1
PTH2R	1.0197983	1.0171592	1.0209422	1.220465974	0.2874321	0.982105	1
SLC22A8	1.0197911	1.0171592	1.0209319	1.219862849	0.286719	0.982105	1
KCND3	1.0222826	1.01963218	1.0234315	1.193524272	0.2552279	0.982133	1
FAM135B	1.0222778	1.01967063	1.0234079	1.189994346	0.2509547	0.982133	1
PAMR1	1.0240508	1.026341	1.0230582	0.875371387	-0.1920329	0.982162	1
NEBL	1.3739759	1.37643542	1.3729098	0.990634105	-0.0135758	0.982166	1

DOCK11	1.0779727	1.08023544	1.0769919	0.959574691	-0.059533	0.982238	1
RAVER1	1.0500886	1.05236074	1.0491037	0.93779583	-0.0926542	0.98228	1
NRAS	1.6227139	1.62033244	1.6237461	1.005502975	0.0079174	0.982295	1
AC009120.4	1.0136522	1.01587201	1.0126899	0.799517494	-0.3227985	0.982544	1
RP11-881M11.4	1.0136539	1.01587061	1.012693	0.799781127	-0.3223229	0.982544	1
SYNJ2BP-COX16	1.0136511	1.01585269	1.0126968	0.800923822	-0.3202631	0.982544	1
GRTP1-AS1	1.0136556	1.01587258	1.0126946	0.799782352	-0.3223206	0.982544	1
KLK13	1.0136505	1.01586528	1.0126904	0.799886829	-0.3221322	0.982544	1
CTC-518P12.6	1.0136513	1.01586458	1.012692	0.8000213	-0.3218897	0.982544	1
CHI3L2	1.0136455	1.01585048	1.0126898	0.80059147	-0.3208618	0.982544	1
RP11-234G16.5	1.013656	1.0158758	1.0126939	0.799574564	-0.3226955	0.982544	1
RP4-710M16.2	1.013651	1.01587061	1.0126889	0.799522455	-0.3227895	0.982544	1
HIST1H4A	1.0136582	1.01587258	1.0126984	0.800019855	-0.3218923	0.982544	1
C6orf47-AS1	1.0136549	1.01586417	1.0126972	0.80037112	-0.321259	0.982544	1
RP5-940J5.6	1.0136559	1.01586458	1.0126985	0.800431094	-0.3211509	0.982544	1
LINC01372	1.0206666	1.02290453	1.0196966	0.859942751	-0.2176875	0.98257	1
PRKG2	1.0206486	1.02286431	1.0196882	0.861089206	-0.2157654	0.98257	1
SLC8A1-AS1	1.0206365	1.02284766	1.019678	0.861269766	-0.2154629	0.98257	1
TMEM110-MUSTN1	1.0207087	1.02290257	1.0197577	0.862685608	-0.2130932	0.98257	1
ACSL5	1.0206328	1.02285336	1.0196703	0.86071601	-0.2163908	0.98257	1
PGAM4	1.0210224	1.01839445	1.0221615	1.204791329	0.2687833	0.982593	1
ADGRF3	1.0210662	1.01839445	1.0222243	1.208209183	0.2728703	0.982593	1
RP11-434B12.1	1.0209989	1.01839445	1.0221279	1.202963651	0.2665931	0.982593	1
RP11-218L14.4	1.0209917	1.01839445	1.0221174	1.202397724	0.2659142	0.982593	1
C11orf87	1.0210222	1.01839445	1.0221612	1.204773706	0.2687622	0.982593	1
SP110	1.0575935	1.05502757	1.0587058	1.066842989	0.0933479	0.98261	1
SEMA3G	1.0254178	1.02759561	1.0244738	0.886872586	-0.1732012	0.982694	1
PKD2	1.216346	1.2189226	1.2152292	0.983129237	-0.024547	0.982725	1
RNF144A	1.2132839	1.21542904	1.2123541	0.985726238	-0.0207411	0.982812	1
CTD-2396E7.10	1.0207494	1.01815945	1.021872	1.204444339	0.2683677	0.982829	1
RP11-115D19.4	1.0207929	1.01815945	1.0219344	1.207875714	0.272472	0.982829	1
BATF	1.0207668	1.01815945	1.021897	1.205817389	0.2700114	0.982829	1
CD300E	1.0207745	1.01815945	1.021908	1.206422612	0.2707354	0.982829	1
JMJD7-PLA2G4B	1.0207618	1.01815945	1.0218898	1.205424187	0.2695409	0.982829	1
LINC01598	1.0207924	1.01815945	1.0219337	1.207840235	0.2724296	0.982829	1
SLCO4C1	1.0207652	1.01815945	1.0218947	1.205693002	0.2698626	0.982829	1
TSSK3	1.0303159	1.03243431	1.0293977	0.906375698	-0.1418189	0.982864	1
ALDH1A2	1.0303365	1.03258021	1.029364	0.901283714	-0.1499468	0.982864	1
RP11-314A20.2	1.0955536	1.09286765	1.0967179	1.04145917	0.0586063	0.982889	1
CACNA1G	1.026684	1.02886776	1.0257375	0.891564416	-0.1655891	0.982929	1
ARHGEF16	1.1951842	1.19744914	1.1942024	0.983556652	-0.0239199	0.982973	1
LMBRD1	1.4068577	1.40435993	1.4079403	1.008854448	0.012718	0.983036	1
AC007292.6	1.023912	1.02612254	1.0229538	0.878699108	-0.1865589	0.983081	1
LINC01186	1.0292076	1.02667444	1.0303056	1.136127407	0.1841246	0.9831	1
STEAP1B	1.0313152	1.02873022	1.0324357	1.128974107	0.1750124	0.983141	1
NECAB3	1.3610513	1.35808198	1.3623384	1.011886623	0.0170477	0.983153	1
RP11-31F15.2	1.0428805	1.04519328	1.041878	0.926643168	-0.1099142	0.983161	1
AC092669.3	1.0428281	1.04503974	1.0418694	0.929610622	-0.1053015	0.983161	1
ADAT3	1.0213019	1.02346861	1.0203627	0.867658305	-0.2048011	0.983198	1
SMAD5-AS1	1.0212645	1.02345162	1.0203165	0.866316989	-0.2070331	0.983198	1
NPIPB4	1.0331012	1.03050547	1.0342264	1.121974583	0.16604	0.9832	1

KCNMB1	1.0330527	1.03046908	1.0341726	1.121551545	0.1654959	0.9832	1
ARNTL2	1.0664671	1.06390972	1.0675755	1.057359383	0.0804658	0.983245	1
PNMAL2	1.0866687	1.08875257	1.0857655	0.966343668	-0.0493917	0.983255	1
BATF2	1.0326432	1.03487671	1.0316751	0.908200978	-0.1389165	0.983259	1
RGS7BP	1.0327453	1.03493977	1.0317941	0.909969298	-0.1361102	0.983259	1
AGMO	1.0223164	1.01971493	1.023444	1.189151585	0.2499326	0.983283	1
CYYR1-AS1	1.0222969	1.01971905	1.0234143	1.187393407	0.247798	0.983283	1
ZACN	1.0223189	1.01971905	1.0234458	1.188990705	0.2497374	0.983283	1
ARIH2OS	1.1041668	1.10660832	1.1031085	0.967171138	-0.0481569	0.983324	1
CTD-2651B20.3	1.0593469	1.06162533	1.0583593	0.947002174	-0.0785604	0.983377	1
RPAP1	1.2052742	1.20748864	1.2043143	0.984701138	-0.0222422	0.983381	1
LYSMD2	1.1127947	1.11009242	1.113966	1.035184981	0.0498886	0.983389	1
IFIT3	1.0765204	1.0788633	1.0755048	0.957414102	-0.062785	0.983433	1
RP11-428J1.5	1.0750775	1.07256069	1.0761684	1.049719732	0.0700042	0.983557	1
EGFL8	1.0293406	1.03153494	1.0283895	0.900254112	-0.1515958	0.983592	1
AP000473.5	1.0135053	1.0156558	1.0125732	0.803099797	-0.3163488	0.983639	1
LINC01304	1.013509	1.01566841	1.012573	0.802444948	-0.3175257	0.983639	1
PYDC1	1.0135068	1.01566077	1.0125731	0.802842497	-0.3168111	0.983639	1
RP11-472N13.2	1.0135121	1.01565413	1.0125836	0.803852671	-0.314997	0.983639	1
RP11-89N17.4	1.0135125	1.01565986	1.0125818	0.803441943	-0.3157343	0.983639	1
CTD-3224K15.3	1.0135008	1.01564547	1.0125712	0.803504892	-0.3156213	0.983639	1
ABCB6	1.0135079	1.01566298	1.0125738	0.802772376	-0.3169371	0.983639	1
PABPC5-AS1	1.0135106	1.01566042	1.0125787	0.803217212	-0.3161379	0.983639	1
RP11-342K6.3	1.0135038	1.01565117	1.012573	0.803328529	-0.315938	0.983639	1
SLC7A9	1.0135069	1.01566168	1.0125729	0.802782595	-0.3169188	0.983639	1
SLC35F4	1.0135118	1.01566357	1.0125791	0.803080795	-0.316383	0.983639	1
EDRF1-AS1	1.0135037	1.01565117	1.0125729	0.803321821	-0.31595	0.983639	1
RP11-214N1.1	1.0135093	1.01565812	1.0125778	0.803278988	-0.316027	0.983639	1
RP11-278H7.1	1.0135041	1.01564994	1.012574	0.803451209	-0.3157177	0.983639	1
RP11-227D13.1	1.0500057	1.05222968	1.0490417	0.938962725	-0.0908602	0.983642	1
AC005477.1	1.022277	1.02444548	1.021337	0.872840874	-0.1962094	0.983657	1
MAN1A2	1.9746588	1.97190328	1.9758532	1.004064129	0.0058514	0.983662	1
FAM71D	1.0411956	1.03861399	1.0423147	1.095837646	0.1320341	0.983662	1
RP11-356I2.4	1.0473457	1.04949012	1.0464162	0.937888727	-0.0925113	0.983723	1
INSL3	1.0474464	1.04950616	1.0465536	0.940359972	-0.088715	0.983723	1
CTA-223H9.9	1.021572	1.0237341	1.0206349	0.869419334	-0.2018759	0.983782	1
NPNT	1.0210406	1.01850742	1.0221387	1.196206009	0.2584659	0.983784	1
RP11-41O4.1	1.0210543	1.01850742	1.0221583	1.197266211	0.259744	0.983784	1
SYT8	1.0210434	1.01850742	1.0221426	1.19641763	0.2587211	0.983784	1
EVPL	1.021048	1.01850742	1.0221492	1.196776833	0.2591542	0.983784	1
RP11-982M15.2	1.0210582	1.01850742	1.0221639	1.197568323	0.260108	0.983784	1
RP11-705O24.1	1.0210391	1.01850742	1.0221365	1.196089951	0.2583259	0.983784	1
RP5-1024G6.5	1.0388585	1.04096553	1.0379452	0.926271776	-0.1104925	0.983873	1
INPP5D	1.2449589	1.24196744	1.2462556	1.017721841	0.0253433	0.983877	1
TBX5-AS1	1.0196041	1.02171824	1.0186877	0.860462834	-0.2168152	0.983909	1
GLI1	1.0196019	1.02173707	1.0186764	0.859193689	-0.2189447	0.983909	1
ISL2	1.0195932	1.02171697	1.0186726	0.85981659	-0.2178991	0.983909	1
HMX2	1.0195756	1.02170089	1.0186543	0.85961161	-0.2182431	0.983909	1
DHRS9	1.0195866	1.02174273	1.018652	0.857847835	-0.2212063	0.983909	1
CTD-2410N18.3	1.0195851	1.02172243	1.0186587	0.858960001	-0.2193371	0.983909	1
LINC00871	1.0195938	1.02170465	1.0186789	0.860593658	-0.2165959	0.983909	1

KLK2	1.019593	1.02172243	1.0186701	0.859482858	-0.2184592	0.983909	1
FAM189A2	1.0195765	1.02169824	1.0186569	0.85983334	-0.217871	0.983909	1
FOXR1	1.0195805	1.02173116	1.0186483	0.858137478	-0.2207193	0.983909	1
TBPL2	1.0195738	1.02170354	1.0186506	0.859335752	-0.2187062	0.983909	1
RP11-276H7.2	1.0195847	1.02172609	1.0186565	0.858714533	-0.2197495	0.983909	1
RP11-111M22.5	1.0247219	1.02214038	1.0258409	1.167137717	0.2229748	0.983915	1
LIMS3	1.0247222	1.02218164	1.0258235	1.164182407	0.2193171	0.983915	1
CABS1	1.0247432	1.02217392	1.0258569	1.166093971	0.2216841	0.983915	1
HYPK	1.0753215	1.0774542	1.0743971	0.960529831	-0.0580977	0.983935	1
RP11-13K12.1	1.0205628	1.01804205	1.0216554	1.200276085	0.2633663	0.983966	1
CTD-2562J15.4	1.0205952	1.01804205	1.0217018	1.202847244	0.2664534	0.983966	1
PRSS12	1.0205991	1.01804205	1.0217075	1.203161832	0.2668307	0.983966	1
RP11-1055B8.10	1.0205703	1.01804205	1.0216662	1.200872805	0.2640834	0.983966	1
WNT5A-AS1	1.0353465	1.03753498	1.0343979	0.916421503	-0.1259168	0.984028	1
RP11-497E19.1	1.0531342	1.05053722	1.0542599	1.073663063	0.1025413	0.984033	1
SULT1E1	1.0231358	1.02056357	1.0242507	1.179305682	0.2379377	0.984046	1
RP11-2H3.6	1.0231189	1.02056357	1.0242266	1.178130384	0.2364992	0.984046	1
RSPH6A	1.0231451	1.02056357	1.0242641	1.179953823	0.2387304	0.984046	1
FKBP6	1.0231048	1.02056357	1.0242064	1.177147707	0.2352954	0.984046	1
KLRC2	1.0231436	1.02056357	1.024262	1.179852465	0.2386065	0.984046	1
C5orf66-AS1	1.0231307	1.02056357	1.0242434	1.178947648	0.2374997	0.984046	1
RP11-81H3.2	1.023125	1.02056357	1.0242353	1.178555658	0.2370199	0.984046	1
NRIP2	1.0231169	1.02056357	1.0242236	1.177985841	0.2363222	0.984046	1
TMEM235	1.0244437	1.0219225	1.0255365	1.164852641	0.2201475	0.984182	1
RP11-288L9.1	1.0141061	1.01621667	1.0131913	0.813439277	-0.2978934	0.98423	1
RP11-231N3.1	1.0141081	1.0162299	1.0131885	0.81260313	-0.2993772	0.98423	1
CTD-3051D23.1	1.0141034	1.01621753	1.013187	0.81313256	-0.2984375	0.98423	1
CHKB-CPT1B	1.0141103	1.01624242	1.0131861	0.811829675	-0.300751	0.98423	1
ADAM21	1.0141013	1.01621389	1.0131856	0.813226205	-0.2982714	0.98423	1
KLK4	1.0413208	1.04336585	1.0404344	0.932401342	-0.100977	0.984346	1
TMEM68	1.5420593	1.53901707	1.5433779	1.008090345	0.0116249	0.984373	1
FRMD5	1.0861298	1.08831274	1.0851836	0.964567584	-0.0520458	0.984373	1
MED14OS	1.0402553	1.03773144	1.0413492	1.095881961	0.1320924	0.984441	1
THNSL1	1.1698637	1.16733779	1.1709585	1.021637206	0.030883	0.984479	1
AP000355.2	1.0143723	1.01647341	1.0134615	0.817167412	-0.2912964	0.984495	1
HIST1H2BI	1.0143724	1.01647808	1.0134597	0.816822048	-0.2919063	0.984495	1
RP11-417L19.2	1.0143775	1.01647906	1.0134666	0.817196295	-0.2912454	0.984495	1
AC092580.4	1.0143673	1.01645797	1.0134611	0.817908482	-0.2899887	0.984495	1
SLC30A3	1.0143765	1.01646604	1.0134707	0.818090207	-0.2896682	0.984495	1
RP11-27K13.3	1.0143769	1.01648507	1.0134632	0.816688258	-0.2921426	0.984495	1
NPSR1	1.0143714	1.016468	1.0134627	0.817505619	-0.2906994	0.984495	1
RP11-506F3.1	1.0143716	1.01647554	1.0134597	0.816947761	-0.2916843	0.984495	1
CTD-2236F14.1	1.0143728	1.01647596	1.0134611	0.817015423	-0.2915648	0.984495	1
NAT2	1.0143778	1.0164757	1.0134685	0.817477636	-0.2907488	0.984495	1
C12orf80	1.0143712	1.01646731	1.0134627	0.81754015	-0.2906385	0.984495	1
GABRA3	1.0428601	1.04038618	1.0439325	1.087809762	0.1214263	0.984515	1
TCF20	1.1974082	1.19952555	1.1964904	0.984787954	-0.022115	0.98454	1
CCDC169	1.0680079	1.07005116	1.0671222	0.958188963	-0.0616179	0.984575	1
RP11-467J12.4	1.0325038	1.03006983	1.0335589	1.116031072	0.1583772	0.984592	1
PRTN3	1.0697125	1.06710779	1.0708415	1.055637353	0.0781143	0.984606	1
FAM50B	1.1127432	1.11015335	1.1138658	1.033702453	0.047821	0.984633	1

AMMECR1L	1.1317235	1.12919535	1.1328194	1.028050665	0.0399114	0.984647	1
C1orf105	1.0299355	1.02742998	1.0310215	1.130935209	0.1775163	0.984665	1
DLEU7	1.0495312	1.04699906	1.0506288	1.077230086	0.1073264	0.984735	1
SHISA7	1.0221378	1.02420378	1.0212423	0.877645419	-0.1882899	0.984756	1
RP11-162A12.2	1.0221166	1.02420451	1.0212116	0.876349594	-0.1904216	0.984756	1
FMNL2	1.4520114	1.4542428	1.4510442	0.992958429	-0.0101948	0.984889	1
SRD5A1	1.4521129	1.4545333	1.4510637	0.992366755	-0.0110547	0.984889	1
PTGER3	1.0282604	1.03030911	1.0273724	0.903108867	-0.1470282	0.984898	1
ZNF385B	1.0281793	1.03026216	1.0272764	0.901337538	-0.1498606	0.984898	1
LIMD1-AS1	1.0282364	1.03033468	1.0273269	0.900848168	-0.1506441	0.984898	1
ZNF157	1.028265	1.03035319	1.0273599	0.901383065	-0.1497877	0.984898	1
RP11-342I1.2	1.0160987	1.01362911	1.0171691	1.259740479	0.3331266	0.984906	1
TMEM30B	1.0161036	1.01362911	1.0171762	1.260258985	0.3337202	0.984906	1
ALG1L	1.0161073	1.01362911	1.0171815	1.260647254	0.3341646	0.984906	1
CD300C	1.016087	1.01362911	1.0171524	1.258513035	0.3317202	0.984906	1
LINC00310	1.016084	1.01362911	1.0171481	1.258193103	0.3313534	0.984906	1
CYSLTR2	1.0161004	1.01362911	1.0171716	1.259919953	0.3333321	0.984906	1
RP11-329J18.3	1.0161043	1.01362911	1.0171771	1.260325471	0.3337963	0.984906	1
CHST4	1.0161134	1.01362911	1.0171902	1.261282942	0.334892	0.984906	1
GCG	1.0160869	1.01362911	1.0171522	1.258499026	0.3317041	0.984906	1
NUDT11	1.6065539	1.60371362	1.6077851	1.006743993	0.0096969	0.984907	1
C12orf49	1.5437164	1.54583993	1.542796	0.994423403	-0.0080678	0.984981	1
WDR78	1.1589825	1.15660726	1.160012	1.021740676	0.0310291	0.985022	1
CBLN3	1.0396296	1.03715764	1.040701	1.095361366	0.1314069	0.985119	1
GNG13	1.0158408	1.01338003	1.0169075	1.263637002	0.3375821	0.985172	1
RP11-78L16.1	1.0158453	1.01338003	1.0169138	1.264111702	0.338124	0.985172	1
LINC01135	1.0158261	1.01338003	1.0168863	1.262053685	0.3357733	0.985172	1
RP3-395M20.8	1.0158279	1.01338003	1.016889	1.262253075	0.3360012	0.985172	1
RP11-282A11.3	1.0158361	1.01338003	1.0169007	1.263127494	0.3370003	0.985172	1
RP1-137D17.2	1.0158302	1.01338003	1.0168923	1.262499094	0.3362824	0.985172	1
CTC-431G16.2	1.0158403	1.01338003	1.0169068	1.263581183	0.3375184	0.985172	1
CTD-2287O16.4	1.0158224	1.01338003	1.0168811	1.261666023	0.3353301	0.985172	1
RP11-131L12.2	1.0158782	1.01338003	1.0169611	1.267642647	0.3421481	0.985172	1
TEX29	1.0158262	1.01338003	1.0168865	1.26206667	0.3357881	0.985172	1
ZG16B	1.0158274	1.01338003	1.0168882	1.262191506	0.3359308	0.985172	1
RDH10-AS1	1.0158468	1.01338003	1.016916	1.264276226	0.3383117	0.985172	1
CCL7	1.0158337	1.01338003	1.0168973	1.262873751	0.3367104	0.985172	1
RP11-469A15.2	1.13618	1.13371355	1.1372491	1.026440921	0.0376506	0.985178	1
FAM92A	2.4864425	2.48247882	2.4881606	1.003832609	0.0055187	0.985179	1
ATP5J2-PTCD1	1.0213736	1.0234075	1.020492	0.875447913	-0.1919067	0.985387	1
RP11-432J22.2	1.0213456	1.0234068	1.0204522	0.873770208	-0.1946742	0.985387	1
SLC47A2	1.0213605	1.02340946	1.0204723	0.874532048	-0.1934168	0.985387	1
CTD-2349P21.11	1.0214001	1.02344725	1.0205128	0.874847994	-0.1928957	0.985387	1
GPR50-AS1	1.0213707	1.02342829	1.0204789	0.874109396	-0.1941142	0.985387	1
RP11-305F18.1	1.0213832	1.02343747	1.0204928	0.874360342	-0.1937001	0.985387	1
GALP	1.0209005	1.02297097	1.0200031	0.87079901	-0.1995883	0.98542	1
RP11-689P11.2	1.0209878	1.02298856	1.0201205	0.875241529	-0.1922469	0.98542	1
ICAM4	1.0209117	1.02298038	1.0200151	0.870962507	-0.1993175	0.98542	1
CNKSR3	1.1610111	1.15861132	1.1620512	1.02168772	0.0309543	0.985438	1
NRSN2	1.4876704	1.48984685	1.486727	0.993630888	-0.0092181	0.985442	1
CYP21A2	1.0188602	1.02091015	1.0179717	0.859472414	-0.2184768	0.985466	1

CTD-2310F14.1	1.0188664	1.020878	1.0179944	0.861883263	-0.2144356	0.985466	1
RP11-322E11.6	1.01885	1.02091474	1.017955	0.858484431	-0.2201361	0.985466	1
CTA-363E19.2	1.0188412	1.02090042	1.0179486	0.858768588	-0.2196587	0.985466	1
SIM2	1.0188402	1.0209061	1.0179447	0.858346255	-0.2203683	0.985466	1
VAC14-AS1	1.0188386	1.0209097	1.0179409	0.858019881	-0.220917	0.985466	1
EXOC3L2	1.0188397	1.02089284	1.0179498	0.859134295	-0.2190444	0.985466	1
CENPB	1.1752709	1.17761281	1.1742558	0.981099408	-0.0275288	0.985502	1
CASP9	1.1444728	1.14177578	1.1456418	1.027268761	0.0388137	0.985503	1
LINC00304	1.0213144	1.01886772	1.0223749	1.185884353	0.2459633	0.985524	1
CTD-2568A17.1	1.0213281	1.01886772	1.0223945	1.18692153	0.2472246	0.985524	1
RP11-184I16.4	1.0213284	1.01886772	1.022395	1.186949548	0.2472586	0.985524	1
FGFR4	1.0238997	1.02144615	1.0249632	1.163994748	0.2190845	0.98553	1
RP11-696N14.1	1.0767146	1.07874443	1.0758348	0.963049355	-0.0543184	0.985535	1
AZU1	1.0674505	1.06946962	1.0665753	0.958336792	-0.0613953	0.985609	1
RP11-277A4.4	1.0370289	1.034569	1.0380952	1.102005089	0.1401309	0.985633	1
SLC9A2	1.0193123	1.02135873	1.0184253	0.862659506	-0.2131369	0.985648	1
KCNJ10	1.0193206	1.02135099	1.0184405	0.863683621	-0.2114252	0.985648	1
FRMD6-AS2	1.0193162	1.02136245	1.0184293	0.86269632	-0.2130753	0.985648	1
RP11-278A23.1	1.0193285	1.02135258	1.0184512	0.864119662	-0.210697	0.985648	1
LRRC71	1.0193149	1.02135737	1.0184295	0.862912906	-0.2127131	0.985648	1
RP4-533D7.5	1.0193183	1.02135258	1.0184365	0.863433707	-0.2118427	0.985648	1
GPRC6A	1.0193118	1.02135483	1.0184263	0.862863186	-0.2127963	0.985648	1
MYOG	1.019338	1.02136577	1.018459	0.863951527	-0.2109777	0.985648	1
SEC24A	1.1611505	1.15855743	1.1622745	1.023443285	0.0334312	0.985669	1
AMPD2	1.3225046	1.32002282	1.3235803	1.011116233	0.0159489	0.985713	1
RP11-540K16.1	1.0233005	1.02086099	1.0243579	1.167629304	0.2235823	0.985718	1
CTD-2526A2.2	1.0152371	1.01281342	1.0162877	1.271145037	0.3461287	0.985763	1
RP11-439K3.3	1.0152324	1.01281342	1.016281	1.270617465	0.3455298	0.985763	1
RP4-742J24.2	1.0152659	1.01281342	1.0163289	1.274362958	0.3497762	0.985763	1
RP11-18I14.11	1.0152306	1.01281342	1.0162783	1.270410984	0.3452953	0.985763	1
CTC-527H23.3	1.0152227	1.01281342	1.016267	1.269532399	0.3442972	0.985763	1
RP11-384P7.5	1.0152434	1.01281342	1.0162966	1.271841373	0.3469187	0.985763	1
SPARCL1	1.0792449	1.07706149	1.0801914	1.040615366	0.0574369	0.985815	1
CC2D1B	1.0791734	1.07681368	1.0801962	1.044035411	0.0621706	0.985815	1
RP5-1136G13.2	1.1499909	1.14747376	1.151082	1.024467227	0.0348738	0.98585	1
STARD4	1.5755679	1.5727512	1.5767888	1.007049544	0.0101347	0.98587	1
AC007879.4	1.02401	1.02154814	1.0250771	1.1637733	0.2188101	0.985876	1
SGPP2	1.0388806	1.04102529	1.0379509	0.925062161	-0.1123778	0.985903	1
ZSWIM1	1.0335099	1.03121295	1.0345055	1.105485508	0.1446801	0.985918	1
LINC00987	1.0320087	1.03408217	1.03111	0.912794144	-0.1316386	0.985944	1
KLHL7-AS1	1.082903	1.0847706	1.0820934	0.968418869	-0.0462969	0.98602	1
MESDC1	1.4791832	1.47631544	1.4804263	1.008630484	0.0123977	0.986101	1
TGFBRAP1	1.1223084	1.12453595	1.1213429	0.974360605	-0.0374723	0.986163	1
NUDT17	1.1224851	1.12468257	1.1215325	0.974735558	-0.0369172	0.986163	1
PRR7-AS1	1.0865034	1.08404973	1.0875669	1.041846621	0.0591429	0.986195	1
NPIPB11	1.0205682	1.02254032	1.0197134	0.87458576	-0.1933282	0.986215	1
ELMOD1	1.0205787	1.02254854	1.0197248	0.874772691	-0.1930199	0.986215	1
PBX2	1.8273115	1.82468397	1.8284505	1.004567216	0.0065741	0.986246	1
SHPK	1.0801026	1.07769545	1.081146	1.044410659	0.0626891	0.98631	1
PTPRN	1.0955236	1.09299335	1.0966203	1.039002596	0.0551993	0.986324	1
NXT1	1.7387493	1.73612052	1.7398887	1.00511898	0.0073663	0.986416	1

ORC2	1.1881241	1.1902849	1.1871875	0.983722216	-0.0236771	0.986444	1
CISH	1.1131334	1.11091288	1.1140959	1.028698483	0.0408202	0.986449	1
SPI1	1.0295843	1.03157835	1.02872	0.909483303	-0.1368809	0.986453	1
RP5-967N21.11	1.0294746	1.03151465	1.0285904	0.907208533	-0.1404939	0.986453	1
ANKRD23	1.0275288	1.02953068	1.0266611	0.902826141	-0.1474799	0.986461	1
C19orf84	1.0275124	1.02950432	1.026649	0.903222292	-0.146847	0.986461	1
SETSIP	1.0275497	1.02949511	1.0267064	0.905451729	-0.1432904	0.986461	1
RUSC1-AS1	1.0585938	1.06061508	1.0577177	0.952200833	-0.0706622	0.986492	1
RP11-506M13.3	1.0190156	1.02101676	1.0181482	0.863511983	-0.2117119	0.986602	1
SLC10A5	1.0190174	1.02102273	1.0181482	0.86326365	-0.2121269	0.986602	1
RP5-1125A11.7	1.0190318	1.02102525	1.0181678	0.864093677	-0.2107404	0.986602	1
AC005753.1	1.0190398	1.02102525	1.0181792	0.864637138	-0.2098333	0.986602	1
CERS3	1.0190191	1.0209998	1.0181605	0.864793641	-0.2095722	0.986602	1
LRFN2	1.0190207	1.02101628	1.0181557	0.863887584	-0.2110845	0.986602	1
RP11-119D9.1	1.0190281	1.02101628	1.0181663	0.864389693	-0.2102462	0.986602	1
CCDC192	1.0283713	1.02597124	1.0294117	1.132470971	0.1794741	0.986604	1
UBE2QL1	1.0279972	1.02997826	1.0271386	0.90527428	-0.1435731	0.986644	1
PAQR9	1.0279873	1.02998218	1.0271226	0.90462328	-0.144611	0.986644	1
ICK	1.2458096	1.24360677	1.2467645	1.012962392	0.0185806	0.986672	1
TEC	1.0315976	1.03355569	1.0307488	0.916352052	-0.1260261	0.986727	1
RP11-135L13.4	1.0316499	1.03366231	1.0307776	0.914304123	-0.129254	0.986727	1
ACD	1.4726447	1.47477275	1.4717223	0.993574964	-0.0092993	0.986733	1
DMRT3	1.0271589	1.02471637	1.0282176	1.141655763	0.1911277	0.986806	1
NPTX1	1.0271055	1.02470199	1.0281473	1.13947569	0.1883701	0.986806	1
PCOLCE-AS1	1.0410347	1.03863353	1.0420755	1.089092406	0.1231264	0.986815	1
TRIOBP	2.0807229	2.07879903	2.0815568	1.002556367	0.0036834	0.986829	1
YPEL2	1.1706188	1.16829207	1.1716273	1.019817968	0.0283117	0.986833	1
RP11-798K3.4	1.0192897	1.02124075	1.018444	0.868330399	-0.203684	0.986839	1
ZAR1L	1.0192663	1.02123319	1.0184137	0.867215595	-0.2055374	0.986839	1
RP11-63L7.5	1.0192693	1.0212586	1.0184071	0.86586452	-0.2077868	0.986839	1
TNS4	1.0193016	1.02126495	1.0184506	0.867653786	-0.2048086	0.986839	1
MMEL1	1.0192813	1.02127713	1.0184162	0.865537514	-0.2083317	0.986839	1
KB-1562D12.1	1.0192847	1.02128694	1.0184168	0.865168177	-0.2089475	0.986839	1
RP1-265C24.8	1.0154097	1.01301898	1.016446	1.26323331	0.3371211	0.986858	1
SLC14A2-AS1	1.0153648	1.01301898	1.0163817	1.258290867	0.3314655	0.986858	1
RP11-84A19.4	1.0153795	1.01301898	1.0164027	1.259905882	0.333316	0.986858	1
RP13-270P17.1	1.0153676	1.01301898	1.0163856	1.258593759	0.3318127	0.986858	1
OGFR-AS1	1.0153829	1.01301898	1.0164076	1.26028309	0.3337478	0.986858	1
DNAJB7	1.015385	1.01301898	1.0164106	1.26051078	0.3340085	0.986858	1
RP11-764K9.1	1.0153879	1.01301898	1.0164147	1.260827326	0.3343707	0.986858	1
ACR	1.0153958	1.01301898	1.0164261	1.2617017	0.3353709	0.986858	1
CALML4	1.0214703	1.0234635	1.0206064	0.87823206	-0.1873259	0.986882	1
LINC01118	1.0215165	1.02344427	1.0206809	0.882129649	-0.1809374	0.986882	1
FAM162B	1.0215156	1.02345871	1.0206734	0.881265379	-0.1823516	0.986882	1
AIPL1	1.021452	1.02343555	1.0205922	0.878675369	-0.1865978	0.986882	1
HOXA4	1.0224087	1.02000327	1.0234513	1.17237428	0.2294332	0.986897	1
CBS	1.0425204	1.04025388	1.0435029	1.080712031	0.1119822	0.986982	1
ZNF571-AS1	1.0406669	1.04261345	1.0398232	0.934520904	-0.0977012	0.987004	1
SOS1	1.3703809	1.37287981	1.3692978	0.990393682	-0.013926	0.987273	1
ZNF496	1.1897701	1.1872189	1.1908759	1.019533154	0.0279087	0.987306	1
CTC-523E23.14	1.018058	1.02000327	1.0172149	0.860603338	-0.2165797	0.987326	1

CNR2	1.0180717	1.02000845	1.0172322	0.861246715	-0.2155015	0.987326	1
AC109642.1	1.0180634	1.02002611	1.0172127	0.859511739	-0.2184108	0.987326	1
CTRC	1.0180911	1.02003202	1.0172498	0.861111136	-0.2157286	0.987326	1
CTD-2292P10.4	1.0180683	1.02001517	1.0172245	0.860569845	-0.2166358	0.987326	1
NLRC3	1.0180629	1.01999996	1.0172232	0.861162456	-0.2156427	0.987326	1
RP11-71E19.1	1.0180676	1.01999982	1.01723	0.861508625	-0.2150629	0.987326	1
RP11-174G6.1	1.0180869	1.02002935	1.0172449	0.860980942	-0.2159468	0.987326	1
PSD4	1.1236218	1.12126095	1.1246452	1.027908691	0.0397121	0.987369	1
C17orf64	1.0258472	1.02342925	1.0268953	1.147935076	0.1990411	0.987371	1
IMPG2	1.0235937	1.02123502	1.0246161	1.159223251	0.2131584	0.987386	1
MMP3	1.0236461	1.02125719	1.0246816	1.161095469	0.2154866	0.987386	1
RP11-706O15.3	1.0584089	1.0603558	1.057565	0.95376128	-0.0682999	0.987427	1
RP11-278A23.4	1.0227976	1.0247123	1.0219676	0.888935706	-0.169849	0.987431	1
SLFNL1-AS1	1.0228165	1.02478508	1.0219632	0.886145688	-0.1743842	0.987431	1
CTC-304I17.5	1.0227784	1.02475039	1.0219236	0.885787769	-0.174967	0.987431	1
RP11-1084I9.1	1.0170194	1.01466374	1.0180405	1.230281974	0.298989	0.987432	1
PLK5	1.0169951	1.01466374	1.0180056	1.227900065	0.2961931	0.987432	1
OVOL1	1.0169737	1.01466374	1.0179749	1.225806213	0.2937309	0.987432	1
CAPS	1.0933156	1.09523326	1.0924844	0.971135261	-0.0422558	0.987473	1
RP11-367J11.3	1.0382169	1.04023009	1.0373443	0.928269025	-0.1073851	0.987478	1
RBAK	1.2578217	1.25945291	1.2571147	0.990987907	-0.0130606	0.987534	1
ZNF548	1.0773137	1.07923926	1.0764791	0.965166093	-0.0511509	0.987546	1
RP11-563J2.2	1.0400894	1.04206041	1.0392351	0.932827561	-0.1003177	0.987559	1
CATSPER1	1.0197458	1.01740877	1.0207587	1.192429539	0.253904	0.987584	1
LINC00639	1.0197666	1.01740877	1.0207886	1.194144289	0.2559772	0.987584	1
RP11-354P11.8	1.0197547	1.01740877	1.0207715	1.19316268	0.2547908	0.987584	1
ADGRF4	1.0197262	1.01740877	1.0207307	1.190820776	0.2519563	0.987584	1
AC005152.3	1.0197311	1.01740877	1.0207378	1.191226448	0.2524477	0.987584	1
WDR97	1.0197334	1.01740877	1.020741	1.191413181	0.2526738	0.987584	1
RP11-676J12.4	1.0197753	1.01740877	1.020801	1.194858488	0.2568398	0.987584	1
RP11-168E17.1	1.0233482	1.02102541	1.0243551	1.158364089	0.2120888	0.987623	1
MYO7A	1.0233323	1.02102316	1.0243332	1.157445909	0.2109448	0.987623	1
CTD-2619J13.3	1.0234338	1.02102444	1.0244781	1.164270739	0.2194266	0.987623	1
RP6-74O6.2	1.0233607	1.02100918	1.02438	1.160445968	0.2146794	0.987623	1
RP11-893F2.6	1.0240615	1.02601898	1.023213	0.892155653	-0.1646327	0.987647	1
RP11-266K4.9	1.0240466	1.0259808	1.0232081	0.893280834	-0.1628143	0.987647	1
USP13	1.1947048	1.19674428	1.1938208	0.985140606	-0.0215984	0.987663	1
RBBP5	1.2514436	1.24888301	1.2525536	1.014748046	0.0211216	0.987664	1
ZNF365	1.0323291	1.02997673	1.0333487	1.112487818	0.1537895	0.987693	1
QSER1	1.6318523	1.62861541	1.6332554	1.007381254	0.0106098	0.98771	1
TSPAN11	1.0851015	1.08696676	1.084293	0.96925523	-0.0450515	0.987733	1
LCN2	1.0212906	1.02323714	1.0204469	0.879921359	-0.1845535	0.9878	1
RP13-650J16.1	1.0213205	1.02324696	1.0204855	0.881211895	-0.1824391	0.9878	1
RP11-143K11.7	1.0213609	1.02323341	1.0205492	0.884468462	-0.1771174	0.9878	1
RP11-944C7.1	1.0212946	1.02324616	1.0204486	0.879657195	-0.1849867	0.9878	1
RP11-548H3.1	1.0212929	1.02321836	1.0204582	0.881123605	-0.1825837	0.9878	1
FOPNL	1.5081993	1.50601524	1.509146	1.006187076	0.0088986	0.987827	1
MKX	1.0162644	1.01394208	1.017271	1.238770669	0.3089091	0.987867	1
RP11-534C12.1	1.0162439	1.01394208	1.0172416	1.236661512	0.3064507	0.987867	1
LINC00355	1.0162634	1.01394208	1.0172695	1.238663685	0.3087845	0.987867	1
LINC01564	1.0163131	1.01394208	1.0173409	1.243781323	0.3147329	0.987867	1

RP11-403A3.3	1.0162407	1.01394208	1.0172371	1.236337544	0.3060727	0.987867	1
U95743.1	1.0162714	1.01394208	1.017281	1.239488229	0.3097446	0.987867	1
RP11-265N7.1	1.016243	1.01394208	1.0172404	1.236570991	0.3063451	0.987867	1
RP11-638I2.4	1.0162708	1.01394208	1.0172802	1.239425001	0.309671	0.987867	1
NODAL	1.0162511	1.01394208	1.0172519	1.237397727	0.3073093	0.987867	1
RP11-90J7.2	1.0162513	1.01394208	1.0172522	1.237419853	0.3073351	0.987867	1
GFAP	1.0162418	1.01394208	1.0172386	1.236447055	0.3062005	0.987867	1
LGR6	1.0186828	1.02059668	1.0178533	0.866803316	-0.2062234	0.987935	1
FOCAD-AS1	1.0186974	1.02062189	1.0178632	0.866226198	-0.2071843	0.987935	1
RP1-56K13.5	1.0186956	1.02058789	1.0178753	0.868243872	-0.2038278	0.987935	1
RP11-64C12.8	1.0186838	1.02061641	1.0178461	0.865624439	-0.2081869	0.987935	1
RP11-472I20.3	1.0186987	1.02061934	1.0178661	0.866475188	-0.2067697	0.987935	1
CTB-50L17.16	1.0343996	1.03205619	1.0354154	1.104790577	0.1437729	0.987956	1
DBX2	1.1152129	1.11281231	1.1162535	1.030503235	0.043349	0.987979	1
CMTM5	1.0595624	1.05723302	1.0605721	1.058342409	0.0818065	0.988075	1
METTL16	1.3789937	1.3812377	1.3780211	0.991562613	-0.0122242	0.988207	1
ZNF513	1.1299498	1.13177	1.1291609	0.980199233	-0.0288531	0.988219	1
RP11-385F7.1	1.0804481	1.07820424	1.0814207	1.041129068	0.0581489	0.988311	1
NPTXR	1.0803483	1.07796254	1.0813824	1.043865001	0.0619351	0.988311	1
GLUD2	1.0267233	1.02860808	1.0259064	0.905562139	-0.1431145	0.988329	1
ESAM	1.0171349	1.01486911	1.0181171	1.218436165	0.2850307	0.988347	1
SLC10A4	1.0171398	1.01486911	1.018124	1.218903397	0.2855838	0.988347	1
RP1-137H15.2	1.0171372	1.01486911	1.0181203	1.218655792	0.2852907	0.988347	1
RP11-603J24.17	1.0171705	1.01486911	1.018168	1.221864708	0.2890845	0.988347	1
GADL1	1.0171454	1.01486911	1.0181321	1.219447442	0.2862276	0.988347	1
HHLA2	1.0171339	1.01486911	1.0181156	1.21833968	0.2849164	0.988347	1
MUC15	1.0171555	1.01486911	1.0181466	1.220423502	0.2873819	0.988347	1
DAPP1	1.0171268	1.01486911	1.0181054	1.217651626	0.2841014	0.988347	1
RP11-269M20.3	1.0171532	1.01486911	1.0181433	1.220199462	0.287117	0.988347	1
FAM150B	1.0196723	1.02155993	1.0188542	0.874500288	-0.1934692	0.988374	1
SCN11A	1.0363954	1.03405672	1.0374091	1.098433953	0.1354481	0.988423	1
AC009473.1	1.0190248	1.02089506	1.0182142	0.871696981	-0.1981014	0.988518	1
TCP11	1.0190023	1.02089263	1.0181829	0.870300203	-0.200415	0.988518	1
RP11-83N9.6	1.019002	1.02090118	1.0181788	0.869748376	-0.20133	0.988518	1
PCED1B	1.0350688	1.03277897	1.0360613	1.100136175	0.1376821	0.988518	1
WDR36	1.1802028	1.17785941	1.1812186	1.018886917	0.0269939	0.988546	1
AC068831.10	1.0412043	1.03893948	1.0421861	1.083374799	0.1155324	0.988571	1
RTBDN	1.0236745	1.02138094	1.0246687	1.153770745	0.2063566	0.988578	1
RP11-718O11.1	1.023642	1.02138173	1.0246218	1.151533105	0.2035559	0.988578	1
HAS3	1.0236763	1.02137396	1.0246742	1.15440612	0.2071509	0.988578	1
PCDHGC3	1.0363406	1.03819372	1.0355373	0.930449221	-0.1040007	0.988593	1
RP11-631N16.4	1.0438277	1.04158689	1.044799	1.077238104	0.1073372	0.988639	1
RP11-44N11.2	1.0485299	1.05043568	1.0477038	0.945834165	-0.0803408	0.98865	1
ITK	1.0169953	1.01886772	1.0161837	0.85774384	-0.2213812	0.988682	1
RP11-547D23.1	1.0169953	1.01886772	1.0161837	0.85774384	-0.2213812	0.988682	1
SERPINA3.1	1.0169953	1.01886772	1.0161837	0.85774384	-0.2213812	0.988682	1
ACRV1	1.0169953	1.01886772	1.0161837	0.85774384	-0.2213812	0.988682	1
RP11-141O15.1	1.0169953	1.01886772	1.0161837	0.85774384	-0.2213812	0.988682	1
RP11-465B22.8	1.0169953	1.01886772	1.0161837	0.85774384	-0.2213812	0.988682	1
RP3-523C21.2	1.0169953	1.01886772	1.0161837	0.85774384	-0.2213812	0.988682	1
AP000679.2	1.0169953	1.01886772	1.0161837	0.85774384	-0.2213812	0.988682	1

RP11-463J10.3	1.0169953	1.01886772	1.0161837	0.85774384	-0.2213812	0.988682	1
RP5-965G21.4	1.0169953	1.01886772	1.0161837	0.85774384	-0.2213812	0.988682	1
LHB	1.0169953	1.01886772	1.0161837	0.85774384	-0.2213812	0.988682	1
SCHIP1	1.0169953	1.01886772	1.0161837	0.85774384	-0.2213812	0.988682	1
NPHP3-AS1	1.0169953	1.01886772	1.0161837	0.85774384	-0.2213812	0.988682	1
RS1	1.0169953	1.01886772	1.0161837	0.85774384	-0.2213812	0.988682	1
TRPM2	1.0169953	1.01886772	1.0161837	0.85774384	-0.2213812	0.988682	1
RP11-524H19.2	1.0169953	1.01886772	1.0161837	0.85774384	-0.2213812	0.988682	1
RP11-96B5.4	1.0169953	1.01886772	1.0161837	0.85774384	-0.2213812	0.988682	1
GSTA3	1.0169953	1.01886772	1.0161837	0.85774384	-0.2213812	0.988682	1
CTD-2516F10.4	1.0169953	1.01886772	1.0161837	0.85774384	-0.2213812	0.988682	1
MS4A7	1.0169953	1.01886772	1.0161837	0.85774384	-0.2213812	0.988682	1
AC008753.4	1.0169953	1.01886772	1.0161837	0.85774384	-0.2213812	0.988682	1
RP11-320M16.2	1.0169953	1.01886772	1.0161837	0.85774384	-0.2213812	0.988682	1
LINC00968	1.0169953	1.01886772	1.0161837	0.85774384	-0.2213812	0.988682	1
RP5-823G15.5	1.0169953	1.01886772	1.0161837	0.85774384	-0.2213812	0.988682	1
SCGN	1.0195937	1.02147448	1.0187785	0.874456783	-0.193541	0.988705	1
SELL	1.0195915	1.02148108	1.0187725	0.87390666	-0.1944489	0.988705	1
CD79A	1.0195823	1.0214302	1.0187814	0.876397207	-0.1903432	0.988705	1
SFTPD	1.0326829	1.03455817	1.0318701	0.922216257	-0.116823	0.988729	1
GPR150	1.0326736	1.03457493	1.0318495	0.92117207	-0.1184574	0.988729	1
FGF1	1.0252407	1.02293195	1.0262414	1.144316786	0.1944865	0.988822	1
CHRND	1.0252151	1.02299848	1.0261758	1.138155135	0.1866972	0.988822	1
RP11-403P17.3	1.0365702	1.03845693	1.0357523	0.929672406	-0.1052057	0.988832	1
RP11-367N14.3	1.0256879	1.02344128	1.0266617	1.137383555	0.1857189	0.98884	1
EHHADH	1.0256915	1.02339675	1.0266861	1.140591641	0.1897824	0.98884	1
RP5-864K19.4	1.048842	1.05062898	1.0480674	0.949405304	-0.074904	0.988921	1
LINC00337	1.0270942	1.02476593	1.0281035	1.13476272	0.1823907	0.988922	1
RP11-214O1.3	1.0270235	1.02476339	1.0280032	1.130830205	0.1773823	0.988922	1
ATF7	1.2276123	1.22497769	1.2287543	1.016786694	0.0240171	0.988945	1
PIP4K2A	1.1722575	1.16994334	1.1732606	1.019519859	0.0278899	0.988959	1
UNC13B	1.1722419	1.17011022	1.1731659	1.017962747	0.0256848	0.988962	1
RP4-673M15.1	1.0162583	1.01400962	1.017233	1.230085956	0.2987591	0.989013	1
STT3A-AS1	1.0162605	1.01400962	1.0172362	1.230308456	0.2990201	0.989013	1
AC005481.5	1.0162864	1.01400962	1.0172733	1.232957101	0.3021226	0.989013	1
CTD-2184C24.2	1.0162297	1.01400962	1.017192	1.227155491	0.2953181	0.989013	1
PAX3	1.0162713	1.01400962	1.0172517	1.231417162	0.3003196	0.989013	1
LINC01013	1.0162386	1.01400962	1.0172048	1.228070231	0.2963931	0.989013	1
RP11-473C18.3	1.0162633	1.01400962	1.0172402	1.230597436	0.2993589	0.989013	1
CFLAR-AS1	1.0162676	1.01400962	1.0172464	1.231036378	0.2998734	0.989013	1
ENTPD4	1.5385383	1.53632646	1.539497	1.005911627	0.0085036	0.989032	1
NOB1	1.9367393	1.93876541	1.9358611	0.996906269	-0.0044702	0.989194	1
DCAF15	1.1987501	1.20063105	1.1979348	0.9865614	-0.0195193	0.989213	1
RP11-245D16.4	1.1724791	1.17021253	1.1734615	1.019087874	0.0272785	0.989238	1
TOX3	1.1098896	1.10747129	1.1109378	1.032255564	0.0458002	0.989324	1
ZNF713	1.0786832	1.080389	1.0779438	0.969583342	-0.0445632	0.989389	1
PCDHB10	1.0942332	1.09597201	1.0934795	0.974028677	-0.0379638	0.989408	1
ZNF699	1.0731774	1.0751295	1.0723312	0.962754124	-0.0547607	0.989441	1
NR3C2	1.0326715	1.03043315	1.0336417	1.105429161	0.1446066	0.989443	1
SETMAR	1.1904264	1.18827196	1.1913602	1.016403191	0.0234728	0.989445	1
CSPG4	1.0282837	1.03014537	1.0274768	0.911475956	-0.1337235	0.989459	1

RP11-764E7.1	1.0282972	1.03005814	1.0275339	0.916022156	-0.1265456	0.989459	1
TMEM154	1.0282838	1.03012966	1.0274837	0.912180647	-0.1326085	0.989459	1
RP11-415J8.5	1.0195335	1.02135911	1.0187422	0.877478148	-0.1885649	0.989472	1
RP11-360I2.1	1.0195448	1.0213741	1.0187519	0.877320495	-0.1888241	0.989472	1
RP11-360L9.4	1.0195818	1.02137102	1.0188063	0.879989457	-0.1844419	0.989472	1
HBE1	1.0195303	1.02136061	1.018737	0.87717522	-0.189063	0.989472	1
STXBP5-AS1	1.0556844	1.0575869	1.0548598	0.952643015	-0.0699924	0.989501	1
C15orf59-AS1	1.026477	1.02429872	1.0274212	1.128503905	0.1744114	0.989516	1
AC010136.2	1.0345773	1.03237768	1.0355307	1.097382583	0.1340666	0.989522	1
INPP5B	1.0756318	1.07743983	1.0748482	0.966532946	-0.0491092	0.989545	1
MARS2	1.0351962	1.03299083	1.0361521	1.095823454	0.1320154	0.989624	1
IGF2BP2	3.4718283	3.46889168	3.4731012	1.001705034	0.0024577	0.989643	1
HIST1H2BN	1.0447522	1.04252528	1.0457175	1.075066917	0.1044265	0.989662	1
RP11-783K16.13	1.0186816	1.0164859	1.0196333	1.190915073	0.2520705	0.989664	1
TMEM132E	1.0186693	1.01647808	1.0196191	1.190616609	0.2517089	0.989664	1
ZNF195	1.4386242	1.43655129	1.4395227	1.006806453	0.0097864	0.989794	1
POLR3A	1.1635109	1.16126952	1.1644824	1.019922267	0.0284592	0.989892	1
SP2	1.1313599	1.12914513	1.1323199	1.024583215	0.0350372	0.98994	1
RP1-17K7.2	1.0386327	1.04035394	1.0378866	0.93885767	-0.0910216	0.989955	1
RP11-379F4.4	1.0360059	1.03787166	1.0351971	0.929378772	-0.1056614	0.990015	1
TMPRSS9	1.0201283	1.02191698	1.019353	0.883014974	-0.1794902	0.990065	1
TBC1D26	1.0201839	1.02195973	1.0194141	0.88407863	-0.1777534	0.990065	1
LINC00354	1.0187733	1.02056357	1.0179973	0.875204339	-0.1923082	0.990158	1
RP11-519M16.1	1.0187733	1.02056357	1.0179973	0.875204339	-0.1923082	0.990158	1
RP11-338I21.1	1.0187733	1.02056357	1.0179973	0.875204339	-0.1923082	0.990158	1
RP11-344F13.1	1.0187733	1.02056357	1.0179973	0.875204339	-0.1923082	0.990158	1
ZFP92	1.0187733	1.02056357	1.0179973	0.875204339	-0.1923082	0.990158	1
RP11-456N14.4	1.0187733	1.02056357	1.0179973	0.875204339	-0.1923082	0.990158	1
NADK2-AS1	1.0187733	1.02056357	1.0179973	0.875204339	-0.1923082	0.990158	1
DDX39B-AS1	1.0187733	1.02056357	1.0179973	0.875204339	-0.1923082	0.990158	1
EHF	1.0187733	1.02056357	1.0179973	0.875204339	-0.1923082	0.990158	1
CFTR	1.0187733	1.02056357	1.0179973	0.875204339	-0.1923082	0.990158	1
RP5-1039K5.17	1.0187733	1.02056357	1.0179973	0.875204339	-0.1923082	0.990158	1
RP11-758P17.2	1.0187733	1.02056357	1.0179973	0.875204339	-0.1923082	0.990158	1
TXNDC2	1.0187733	1.02056357	1.0179973	0.875204339	-0.1923082	0.990158	1
RP11-767C1.1	1.0187733	1.02056357	1.0179973	0.875204339	-0.1923082	0.990158	1
TBC1D3I	1.0187733	1.02056357	1.0179973	0.875204339	-0.1923082	0.990158	1
CXorf21	1.0187733	1.02056357	1.0179973	0.875204339	-0.1923082	0.990158	1
RP11-390F4.10	1.0183151	1.02009277	1.0175445	0.873175501	-0.1956564	0.990172	1
CCDC62	1.0183261	1.02012513	1.0175464	0.871863044	-0.1978266	0.990172	1
ITGAX	1.0183172	1.02012837	1.0175321	0.871016401	-0.1992282	0.990172	1
RP11-95P2.3	1.0183134	1.02011551	1.0175323	0.871579394	-0.198296	0.990172	1
FAM198A	1.0183206	1.02011554	1.0175425	0.872089415	-0.197452	0.990172	1
ANKK1	1.0183038	1.02009613	1.0175269	0.87215229	-0.197348	0.990172	1
AC009299.2	1.0183114	1.02009908	1.0175366	0.87250543	-0.196764	0.990172	1
AC092295.4	1.0183277	1.02009375	1.0175622	0.87401307	-0.1942732	0.990172	1
SH3GL3	1.0652644	1.06703166	1.0644984	0.962208053	-0.0555792	0.9902	1
AC016912.3	1.0162567	1.01804205	1.0154829	0.858154795	-0.2206902	0.990237	1
GS1-594A7.3	1.0162567	1.01804205	1.0154829	0.858154795	-0.2206902	0.990237	1
RP11-291L22.4	1.0162567	1.01804205	1.0154829	0.858154795	-0.2206902	0.990237	1
RP11-593F23.1	1.0162567	1.01804205	1.0154829	0.858154795	-0.2206902	0.990237	1

LA16c-359F1.1	1.0162567	1.01804205	1.0154829	0.858154795	-0.2206902	0.990237	1
POC1B-GALNT4	1.0162567	1.01804205	1.0154829	0.858154795	-0.2206902	0.990237	1
RP11-328C8.4	1.0162567	1.01804205	1.0154829	0.858154795	-0.2206902	0.990237	1
RP11-552F3.9	1.0162567	1.01804205	1.0154829	0.858154795	-0.2206902	0.990237	1
RP11-830F9.5	1.0162567	1.01804205	1.0154829	0.858154795	-0.2206902	0.990237	1
RP4-669P10.20	1.0162567	1.01804205	1.0154829	0.858154795	-0.2206902	0.990237	1
CTD-3037G24.5	1.0162567	1.01804205	1.0154829	0.858154795	-0.2206902	0.990237	1
SLC6A2	1.0162567	1.01804205	1.0154829	0.858154795	-0.2206902	0.990237	1
RP11-44N12.5	1.0162567	1.01804205	1.0154829	0.858154795	-0.2206902	0.990237	1
RP11-616M22.1	1.0162567	1.01804205	1.0154829	0.858154795	-0.2206902	0.990237	1
RP11-739L10.1	1.0162567	1.01804205	1.0154829	0.858154795	-0.2206902	0.990237	1
RP11-143J12.3	1.0162567	1.01804205	1.0154829	0.858154795	-0.2206902	0.990237	1
AC011525.2	1.0162567	1.01804205	1.0154829	0.858154795	-0.2206902	0.990237	1
MIRLET7BHG	1.0265103	1.0243341	1.0274536	1.128194266	0.1740155	0.990289	1
RP11-489E7.4	1.1009845	1.09878511	1.1019378	1.031914502	0.0453234	0.990297	1
SUSD4	1.0238755	1.02172609	1.0248071	1.141813228	0.1913267	0.990321	1
HTN3	1.0238806	1.02170354	1.0248243	1.143791392	0.193824	0.990321	1
RP3-466P17.2	1.0239335	1.02173553	1.0248862	1.144953641	0.1952892	0.990321	1
CMP21-97G8.2	1.020415	1.02216148	1.019658	0.887036821	-0.1729341	0.990331	1
AF129408.17	1.0204009	1.0221735	1.0196326	0.885407999	-0.1755857	0.990331	1
SLC38A4	1.0557663	1.05356295	1.0567214	1.058967322	0.0826581	0.990346	1
RP1-121G13.2	1.0190299	1.01683833	1.0199798	1.18656951	0.2467966	0.990351	1
RP11-158I9.5	1.0190139	1.01683833	1.019957	1.185210179	0.2451429	0.990351	1
GRHL2	1.0190057	1.01683833	1.0199451	1.18450782	0.2442877	0.990351	1
TAF7L	1.0190363	1.01683833	1.019989	1.187114559	0.2474592	0.990351	1
AP000769.7	1.0190154	1.01683833	1.019959	1.185333836	0.2452934	0.990351	1
LINC00645	1.0190242	1.01683833	1.0199717	1.18608328	0.2462053	0.990351	1
COL4A3	1.0190176	1.01683833	1.0199623	1.185525058	0.2455262	0.990351	1
RP11-261N11.8	1.0190207	1.01683833	1.0199666	1.185782824	0.2458398	0.990351	1
MARCO	1.0190007	1.01683833	1.019938	1.184082744	0.2437699	0.990351	1
HCAR3	1.01905	1.01683833	1.0200087	1.188280962	0.248876	0.990351	1
C9orf139	1.0189999	1.01683833	1.0199368	1.184012177	0.2436839	0.990351	1
RP11-731C17.2	1.0435895	1.04127071	1.0445946	1.080539071	0.1117512	0.990355	1
FAM110B	1.4232306	1.42064131	1.4243529	1.008823599	0.0126739	0.990367	1
MAP3K6	1.1106887	1.11274259	1.1097985	0.973886392	-0.0381746	0.990385	1
RP11-576I22.2	1.0167304	1.01850742	1.0159601	0.862361964	-0.2136345	0.990419	1
RP11-864G5.3	1.0167304	1.01850742	1.0159601	0.862361964	-0.2136345	0.990419	1
RNF224	1.0167304	1.01850742	1.0159601	0.862361964	-0.2136345	0.990419	1
CTC-327F10.5	1.0167304	1.01850742	1.0159601	0.862361964	-0.2136345	0.990419	1
LINC01031	1.0167304	1.01850742	1.0159601	0.862361964	-0.2136345	0.990419	1
THSD4-AS1	1.0167304	1.01850742	1.0159601	0.862361964	-0.2136345	0.990419	1
AP000654.4	1.0167304	1.01850742	1.0159601	0.862361964	-0.2136345	0.990419	1
TMEM229A	1.0167304	1.01850742	1.0159601	0.862361964	-0.2136345	0.990419	1
MMP13	1.0167304	1.01850742	1.0159601	0.862361964	-0.2136345	0.990419	1
RP11-109J4.1	1.0167304	1.01850742	1.0159601	0.862361964	-0.2136345	0.990419	1
RP11-175K6.1	1.0167304	1.01850742	1.0159601	0.862361964	-0.2136345	0.990419	1
AC006129.2	1.0167304	1.01850742	1.0159601	0.862361964	-0.2136345	0.990419	1
PLCE1-AS1	1.0167304	1.01850742	1.0159601	0.862361964	-0.2136345	0.990419	1
RP11-160H22.5	1.0167304	1.01850742	1.0159601	0.862361964	-0.2136345	0.990419	1
AC010976.2	1.0167304	1.01850742	1.0159601	0.862361964	-0.2136345	0.990419	1
RP11-292D4.1	1.0167304	1.01850742	1.0159601	0.862361964	-0.2136345	0.990419	1

TCP11L2	1.081721	1.07965499	1.0826166	1.037179918	0.0526662	0.990438	1
AC007040.8	1.0258957	1.02370712	1.0268444	1.132333087	0.1792984	0.990478	1
LINS1	1.1567977	1.15861451	1.1560103	0.983581243	-0.0238839	0.990486	1
IGSF9B	1.0178148	1.01565117	1.0187526	1.198161719	0.2608226	0.990523	1
MTRNR2L4	1.0178132	1.01565812	1.0187474	1.19729284	0.2597761	0.990523	1
FBXO24	1.0178021	1.01566267	1.0187294	1.195797813	0.2579735	0.990523	1
HERC5	1.0487094	1.05052773	1.0479213	0.94841546	-0.0764089	0.990531	1
GRM3	1.0369396	1.03865237	1.0361972	0.93648144	-0.0946777	0.990534	1
GDF5OS	1.0265685	1.02449194	1.0274686	1.121535412	0.1654752	0.990618	1
PCDHGA8	1.0266034	1.02446614	1.0275297	1.125218428	0.1702051	0.990618	1
XXbac-BPG294E21.9	1.0361546	1.03390144	1.0371312	1.095269112	0.1312854	0.990643	1
AC079807.2	1.0293748	1.03114037	1.0286095	0.918727146	-0.1222916	0.99066	1
SLC8B1	1.0903354	1.08818803	1.0912662	1.03490445	0.0494976	0.990665	1
MICALL2	1.1356554	1.13730071	1.1349422	0.982822658	-0.024997	0.990688	1
HDX	1.1261978	1.12799937	1.1254169	0.979824387	-0.0294049	0.99069	1
LKAAEAR1	1.0336486	1.03143246	1.0346092	1.101065549	0.1389004	0.990765	1
AP1G1	1.3459431	1.34372572	1.3469043	1.009247312	0.0132797	0.990798	1
BIVM	1.3847437	1.38246849	1.3857299	1.008527278	0.0122501	0.990819	1
PPP1R26-AS1	1.0447525	1.04256518	1.0457007	1.073663291	0.1025416	0.990824	1
ARHGAP25	1.0517915	1.04954738	1.0527642	1.064924202	0.0907507	0.990854	1
TMC7	1.0516595	1.04949685	1.052597	1.06263252	0.0876428	0.990854	1
LINC01277	1.0179825	1.01975418	1.0172145	0.871435972	-0.1985334	0.990948	1
DTX1	1.0179614	1.01971358	1.0172019	0.872591399	-0.1966218	0.990948	1
LINC00702	1.0179616	1.01972313	1.017198	0.871973198	-0.1976443	0.990948	1
FADS6	1.0179776	1.01972009	1.0172222	0.873335049	-0.1953929	0.990948	1
RP11-65J3.2	1.0179871	1.0197175	1.0172371	0.87420085	-0.1939633	0.990948	1
AC195454.1	1.0179674	1.01973103	1.017203	0.871874831	-0.1978071	0.990948	1
KNDC1	1.0796108	1.07739694	1.0805704	1.041002612	0.0579737	0.990974	1
SAMD9	1.0255882	1.02350566	1.0264908	1.126998734	0.1724859	0.991062	1
TEPSIN	1.1021383	1.09985561	1.1031277	1.032768219	0.0465165	0.991109	1
RP11-307C12.13	1.0274628	1.02918429	1.0267166	0.915443877	-0.1274567	0.991134	1
AF213884.2	1.0274525	1.02918801	1.0267003	0.914769728	-0.1285195	0.991134	1
RMRP	1.027484	1.02927009	1.0267099	0.912531564	-0.1320536	0.991134	1
CCDC39	1.028768	1.03045247	1.0280378	0.92070869	-0.1191833	0.991156	1
RP11-327P2.5	1.0287342	1.03050041	1.0279686	0.916992244	-0.1250186	0.991156	1
AP000866.1	1.0269284	1.02861642	1.0261968	0.915445516	-0.1274541	0.991187	1
LINC01546	1.0269363	1.02865826	1.0261899	0.913869181	-0.1299404	0.991187	1
RP11-73K9.3	1.0269528	1.02864019	1.0262213	0.915543172	-0.1273002	0.991187	1
DPH6-AS1	1.0282222	1.02611157	1.0291371	1.115868796	0.1581674	0.991194	1
RAB3GAP2	1.5052644	1.50700355	1.5045105	0.995082857	-0.0071114	0.991218	1
AC007163.3	1.0549599	1.0527336	1.0559248	1.060516218	0.0847667	0.99122	1
RP11-1228E12.1	1.0178938	1.01574718	1.0188243	1.195404859	0.2574993	0.991254	1
GAS2L2	1.0178738	1.01574718	1.0187955	1.193580838	0.2552963	0.991254	1
HCG20	1.0178518	1.01574718	1.0187641	1.191585436	0.2528824	0.991254	1
KLHL33	1.0178717	1.01574718	1.0187926	1.193397854	0.2550751	0.991254	1
CCDC177	1.017894	1.01574718	1.0188246	1.195427534	0.2575267	0.991254	1
ACKR4	1.0178507	1.01574718	1.0187625	1.191482943	0.2527583	0.991254	1
C1orf229	1.0527666	1.05452724	1.0520034	0.953713863	-0.0683716	0.991287	1
RIBC1	1.0525479	1.05428822	1.0517936	0.954048795	-0.067865	0.991287	1
BAALC-AS2	1.0309769	1.02884318	1.0319017	1.106040012	0.1454036	0.991347	1
MYH11	1.03102	1.0288797	1.0319477	1.106234988	0.1456579	0.991347	1

RP1-127D3.4	1.0164379	1.01815945	1.0156917	0.864104389	-0.2107225	0.991372	1
AC005082.12	1.0164379	1.01815945	1.0156917	0.864104389	-0.2107225	0.991372	1
FUT3	1.0164379	1.01815945	1.0156917	0.864104389	-0.2107225	0.991372	1
BVES-AS1	1.0164379	1.01815945	1.0156917	0.864104389	-0.2107225	0.991372	1
RP11-181G12.4	1.0164379	1.01815945	1.0156917	0.864104389	-0.2107225	0.991372	1
MFSD6L	1.0164379	1.01815945	1.0156917	0.864104389	-0.2107225	0.991372	1
RP11-426C22.8	1.0164379	1.01815945	1.0156917	0.864104389	-0.2107225	0.991372	1
AC084809.2	1.0164379	1.01815945	1.0156917	0.864104389	-0.2107225	0.991372	1
RP5-1057J7.7	1.0164379	1.01815945	1.0156917	0.864104389	-0.2107225	0.991372	1
FAM26E	1.0164379	1.01815945	1.0156917	0.864104389	-0.2107225	0.991372	1
RP1-142L7.5	1.0164379	1.01815945	1.0156917	0.864104389	-0.2107225	0.991372	1
REC114	1.0164379	1.01815945	1.0156917	0.864104389	-0.2107225	0.991372	1
RP11-9M16.2	1.0164379	1.01815945	1.0156917	0.864104389	-0.2107225	0.991372	1
SLC35G4	1.0164379	1.01815945	1.0156917	0.864104389	-0.2107225	0.991372	1
CTB-133G6.2	1.0253969	1.02715105	1.0246365	0.907387405	-0.1402095	0.991396	1
SH2D5	1.0253705	1.02711417	1.0246147	0.907815396	-0.1395291	0.991396	1
POLR3G	1.1166987	1.11833389	1.11599	0.980192349	-0.0288632	0.991401	1
RP11-1007O24.3	1.0290197	1.03074724	1.0282709	0.919459859	-0.1211415	0.991424	1
PQLC2L	1.0172521	1.01516282	1.0181578	1.197519051	0.2600486	0.991441	1
AC011997.1	1.0172814	1.01516282	1.0181997	1.200287574	0.2633801	0.991441	1
GATA5	1.0173316	1.01516282	1.0182716	1.205028641	0.2690674	0.991441	1
RP11-338N10.3	1.0172851	1.01516282	1.0182049	1.200630947	0.2637928	0.991441	1
CASC8	1.0172696	1.01516282	1.0181828	1.199168627	0.2620345	0.991441	1
CTA-212D2.2	1.0173113	1.01516282	1.0182426	1.203114717	0.2667742	0.991441	1
AC015987.1	1.0172837	1.01516282	1.018203	1.200500105	0.2636355	0.991441	1
ATP10B	1.0172667	1.01516282	1.0181786	1.198895154	0.2617055	0.991441	1
RP11-138I18.1	1.0172711	1.01516282	1.018185	1.199312543	0.2622077	0.991441	1
TNFSF9	1.0172527	1.01516282	1.0181585	1.197568654	0.2601084	0.991441	1
CTD-2337A12.1	1.0173289	1.01516282	1.0182678	1.204775393	0.2687642	0.991441	1
RP11-351J23.2	1.017275	1.01516282	1.0181905	1.199677782	0.262647	0.991441	1
RP11-675F6.4	1.0172738	1.01516282	1.0181888	1.199567925	0.2625149	0.991441	1
LPO	1.0172719	1.01516282	1.0181862	1.19939199	0.2623032	0.991441	1
FBP1	1.0173133	1.01516282	1.0182455	1.203305283	0.2670027	0.991441	1
NPY5R	1.0172808	1.01516282	1.0181988	1.200226665	0.2633069	0.991441	1
RP11-318C2.1	1.0172794	1.01516282	1.0181968	1.200094614	0.2631482	0.991441	1
CKLF-CMTM1	1.0172594	1.01516282	1.0181681	1.198202831	0.2608721	0.991441	1
DNAJC11	1.1622556	1.16412816	1.161444	0.983645741	-0.0237893	0.991484	1
ABO	1.0346001	1.03249232	1.0355137	1.092987463	0.1282769	0.991495	1
TVP23C	1.0711982	1.07307747	1.0703836	0.963137276	-0.0541867	0.991498	1
VPS9D1-AS1	1.0636872	1.06532138	1.0629788	0.964138046	-0.0526884	0.991502	1
RP11-467D6.1	1.0296901	1.0275875	1.0306014	1.109250007	0.1495846	0.991568	1
DEF6	1.0296676	1.02761646	1.0305567	1.106467155	0.1459606	0.991568	1
TMEM192	1.3830544	1.38099348	1.3839477	1.007754082	0.0111436	0.991582	1
RSPH3	1.3033437	1.30497276	1.3026375	0.99234282	-0.0110895	0.991605	1
RP11-89H19.2	1.0166857	1.01839445	1.015945	0.866835614	-0.2061697	0.991608	1
RP11-254F7.3	1.0166857	1.01839445	1.015945	0.866835614	-0.2061697	0.991608	1
RP11-262K1.1	1.0166857	1.01839445	1.015945	0.866835614	-0.2061697	0.991608	1
PRR27	1.0166857	1.01839445	1.015945	0.866835614	-0.2061697	0.991608	1
CTC-265N9.1	1.0166857	1.01839445	1.015945	0.866835614	-0.2061697	0.991608	1
RP11-75C23.1	1.0166857	1.01839445	1.015945	0.866835614	-0.2061697	0.991608	1
PLA2G1B	1.0166857	1.01839445	1.015945	0.866835614	-0.2061697	0.991608	1

AC241585.1	1.0166857	1.01839445	1.015945	0.866835614	-0.2061697	0.991608	1
PRIMA1	1.0166857	1.01839445	1.015945	0.866835614	-0.2061697	0.991608	1
ADH1C	1.0166857	1.01839445	1.015945	0.866835614	-0.2061697	0.991608	1
CTB-70G10.1	1.0166857	1.01839445	1.015945	0.866835614	-0.2061697	0.991608	1
GDNF-AS1	1.0166857	1.01839445	1.015945	0.866835614	-0.2061697	0.991608	1
HNF4A	1.0166857	1.01839445	1.015945	0.866835614	-0.2061697	0.991608	1
ACTN1-AS1	1.0166857	1.01839445	1.015945	0.866835614	-0.2061697	0.991608	1
RP11-810M2.2	1.0166857	1.01839445	1.015945	0.866835614	-0.2061697	0.991608	1
HNRNPCL2	1.0166857	1.01839445	1.015945	0.866835614	-0.2061697	0.991608	1
GLYAT	1.0166857	1.01839445	1.015945	0.866835614	-0.2061697	0.991608	1
PLIN4	1.0166857	1.01839445	1.015945	0.866835614	-0.2061697	0.991608	1
VAV1	1.0166857	1.01839445	1.015945	0.866835614	-0.2061697	0.991608	1
ANKDD1B	1.0166857	1.01839445	1.015945	0.866835614	-0.2061697	0.991608	1
SLC6A14	1.0166857	1.01839445	1.015945	0.866835614	-0.2061697	0.991608	1
SRL	1.0166857	1.01839445	1.015945	0.866835614	-0.2061697	0.991608	1
C1QTNF2	1.0188662	1.02057389	1.0181261	0.88102238	-0.1827494	0.991614	1
F2RL3	1.0188899	1.02061011	1.0181443	0.880356924	-0.1838395	0.991614	1
RP11-182J1.5	1.0188864	1.02059587	1.0181454	0.88102135	-0.1827511	0.991614	1
CDX4	1.0188655	1.02058158	1.0181216	0.880476661	-0.1836433	0.991614	1
TNNI2	1.0179837	1.01586053	1.018904	1.19189274	0.2532544	0.991619	1
DGUOK-AS1	1.0179508	1.01584852	1.0188621	1.190149339	0.2511426	0.991619	1
RP5-991G20.2	1.017946	1.0158709	1.0188454	1.187418478	0.2478285	0.991619	1
PHEX	1.0179373	1.01586053	1.0188375	1.187696336	0.248166	0.991619	1
RP11-481J13.1	1.0179602	1.01585521	1.0188726	1.190311233	0.2513388	0.991619	1
ARHGAP15	1.0274608	1.02538686	1.0283597	1.117101066	0.1597597	0.991632	1
MXI1	1.5913669	1.59344361	1.5904668	0.99498383	-0.007255	0.991642	1
C16orf71	1.0249679	1.02287609	1.0258745	1.131073236	0.1776923	0.991677	1
MAP3K15	1.0380147	1.03963158	1.0373139	0.941518197	-0.0869391	0.991703	1
CTB-25B13.12	1.0430549	1.04089627	1.0439906	1.075663743	0.1052272	0.991743	1
NEURL4	1.0624548	1.06422606	1.061687	0.960467247	-0.0581917	0.991759	1
LRRTM1	1.036122	1.03414181	1.0369804	1.08314037	0.1152202	0.9918	1
RP11-267M23.4	1.0361998	1.03406103	1.0371268	1.090008302	0.1243391	0.9918	1
TAB3	1.1283292	1.12996544	1.12762	0.981953308	-0.0262737	0.991803	1
GOLGA8R	1.0456038	1.04727834	1.0448779	0.949228384	-0.0751729	0.991821	1
LINC00491	1.0709061	1.06876062	1.0718361	1.044727105	0.0631261	0.991868	1
PRSS27	1.0842848	1.08223106	1.0851751	1.035801434	0.0507475	0.991992	1
RP11-366L20.2	1.0266404	1.02833985	1.0259038	0.914041158	-0.129669	0.992005	1
GZMA	1.0169862	1.01490147	1.0178898	1.20053889	0.2636821	0.992023	1
RP3-470B24.5	1.0169759	1.01490147	1.0178751	1.199553552	0.2624976	0.992023	1
AC108004.2	1.0169822	1.01490147	1.0178841	1.200159596	0.2632263	0.992023	1
RP5-958B11.2	1.0169825	1.01490147	1.0178846	1.200186818	0.263259	0.992023	1
TAPBPL	1.0169652	1.01490147	1.0178598	1.198524573	0.2612595	0.992023	1
GTPBP2	1.1416586	1.14347641	1.1408707	0.981838436	-0.0264424	0.992051	1
SLC4A9	1.0154778	1.0171592	1.014749	0.859539253	-0.2183646	0.992095	1
AC006547.15	1.0154778	1.0171592	1.014749	0.859539253	-0.2183646	0.992095	1
LINC01510	1.0154778	1.0171592	1.014749	0.859539253	-0.2183646	0.992095	1
RP11-638I2.8	1.0154778	1.0171592	1.014749	0.859539253	-0.2183646	0.992095	1
RP11-1101H11.1	1.0154778	1.0171592	1.014749	0.859539253	-0.2183646	0.992095	1
AP001257.1	1.0154778	1.0171592	1.014749	0.859539253	-0.2183646	0.992095	1
TBC1D3D	1.0154778	1.0171592	1.014749	0.859539253	-0.2183646	0.992095	1
LINC00613	1.0154778	1.0171592	1.014749	0.859539253	-0.2183646	0.992095	1

RP11-531F16.4	1.0154778	1.0171592	1.014749	0.859539253	-0.2183646	0.992095	1
RP11-99J16__A.2	1.0154778	1.0171592	1.014749	0.859539253	-0.2183646	0.992095	1
CTC-591M7.1	1.0154778	1.0171592	1.014749	0.859539253	-0.2183646	0.992095	1
RP11-540O11.1	1.0154778	1.0171592	1.014749	0.859539253	-0.2183646	0.992095	1
ABCA12	1.0154778	1.0171592	1.014749	0.859539253	-0.2183646	0.992095	1
RP11-46O21.2	1.0154778	1.0171592	1.014749	0.859539253	-0.2183646	0.992095	1
NME1-NME2	1.0154778	1.0171592	1.014749	0.859539253	-0.2183646	0.992095	1
AP003068.23	1.0179676	1.019649	1.0172388	0.877334945	-0.1888004	0.992095	1
RP11-902B17.1	1.0179975	1.01962895	1.0172904	0.880861459	-0.183013	0.992095	1
MAP3K5	1.0179769	1.01963313	1.017259	0.879074868	-0.1859421	0.992095	1
RBM20	1.0361081	1.03788823	1.0353366	0.932652543	-0.1005884	0.992101	1
CTD-2547L16.1	1.036115	1.03787014	1.0353542	0.93356518	-0.0991773	0.992101	1
SPRY4-IT1	1.028337	1.02631648	1.0292129	1.110059879	0.1506375	0.992115	1
UFSP1	1.0284005	1.0263453	1.0292913	1.11182342	0.1529277	0.992115	1
UNC13A	1.0283503	1.02623197	1.0292685	1.11575729	0.1580232	0.992115	1
TMCO4	1.0283793	1.02630801	1.0292772	1.112861522	0.1542741	0.992115	1
RP11-767C1.2	1.0262201	1.02412237	1.0271294	1.124659005	0.1694876	0.992166	1
HIST1H2BL	1.0201802	1.02184734	1.0194576	0.890615477	-0.1671254	0.992181	1
CSGALNACT1	1.0202006	1.0218616	1.0194807	0.891090255	-0.1663565	0.992181	1
MYO1G	1.0201798	1.02184804	1.0194567	0.890545383	-0.167239	0.992181	1
RP5-1142A6.8	1.0201812	1.02185163	1.0194572	0.890423033	-0.1674372	0.992181	1
TFF1	1.0201917	1.02186676	1.0194656	0.890191556	-0.1678123	0.992181	1
RP11-91K8.5	1.020166	1.02183221	1.0194438	0.890601441	-0.1671481	0.992181	1
BMPER	1.019586	1.0174976	1.0204912	1.171087479	0.2278488	0.992193	1
C5orf66	1.0196059	1.01751368	1.0205129	1.17124701	0.2280454	0.992193	1
APOBEC3B	1.0195589	1.01751032	1.0204468	1.167699981	0.2236696	0.992193	1
ADGRB1	1.0462522	1.04418592	1.0471479	1.067033555	0.0936055	0.99222	1
C11orf65	1.0462647	1.04417641	1.0471699	1.067761696	0.0945897	0.99222	1
KCND2	1.0373901	1.03908542	1.0366553	0.937824582	-0.09261	0.992239	1
ZXDC	1.0999699	1.09788218	1.1008748	1.030573786	0.0434478	0.992242	1
DHODH	1.1253128	1.12707646	1.1245483	0.98010535	-0.0289913	0.992327	1
RMDN2	1.0549461	1.05293005	1.0558199	1.054598039	0.0766932	0.992337	1
RPE65	1.0223322	1.02026673	1.0232275	1.146091487	0.1967222	0.992346	1
TRAPPC12-AS1	1.0223418	1.02025345	1.023247	1.147803067	0.1988751	0.992346	1
GOLGA8S	1.025129	1.02675032	1.0244263	0.913120884	-0.1311222	0.992353	1
ADGB	1.0340177	1.03567489	1.0332994	0.933411473	-0.0994149	0.992365	1
RP11-328P23.4	1.0214642	1.02310803	1.0207516	0.898026249	-0.1551705	0.992379	1
ARHGAP31-AS1	1.021479	1.02317383	1.0207443	0.895162176	-0.159779	0.992379	1
ZNF578	1.0259634	1.02388943	1.0268623	1.124443057	0.1692106	0.992404	1
CXCL6	1.0259346	1.02394624	1.0267964	1.119024887	0.1622421	0.992404	1
CRAMP1	1.107184	1.10905404	1.1063735	0.975419746	-0.0359049	0.992408	1
RP11-57G10.8	1.0349606	1.03285799	1.035872	1.091727775	0.1266132	0.992456	1
GFPT2	1.1044941	1.10612514	1.1037872	0.977969747	-0.0321383	0.992501	1
RP11-310N16.1	1.0210533	1.01899597	1.021945	1.1552452	0.2081991	0.992527	1
RP11-527J8.1	1.0211053	1.01899597	1.0220196	1.15917033	0.2130926	0.992527	1
RP11-265N6.1	1.0210796	1.01899597	1.0219828	1.157234802	0.2106816	0.992527	1
SLC51B	1.0210335	1.01899597	1.0219166	1.153751652	0.2063327	0.992527	1
PTPN5	1.0210711	1.01899597	1.0219706	1.156590223	0.2098778	0.992527	1
RP11-161H23.9	1.0210511	1.01899597	1.0219419	1.15508101	0.207994	0.992527	1
GPA33	1.0210447	1.01899597	1.0219327	1.154595692	0.2073877	0.992527	1
LINC01445	1.0211105	1.01899597	1.022027	1.15956206	0.21358	0.992527	1

SNX30	1.1550446	1.15662702	1.1543587	0.985517745	-0.0210462	0.99253	1
AC003005.2	1.0187207	1.02039924	1.0179931	0.882046266	-0.1810738	0.992531	1
C11orf71	1.3676424	1.36534417	1.3686386	1.009017371	0.012951	0.992544	1
STAT1	1.3724586	1.37030628	1.3733916	1.008331687	0.0119703	0.992567	1
RP5-1116H23.3	1.0188412	1.01678895	1.0197308	1.175226861	0.2329393	0.992629	1
CTD-2012J19.3	1.0382393	1.03620364	1.0391217	1.080601976	0.1118352	0.992631	1
FAM83H-AS1	1.0364277	1.03447128	1.0372758	1.081356791	0.1128426	0.992644	1
RP11-366L5.1	1.0160987	1.01774002	1.0153873	0.867377595	-0.2052679	0.992684	1
FAM138B	1.0161149	1.01775627	1.0154035	0.867494687	-0.2050732	0.992684	1
RP11-21M24.2	1.0161124	1.01775345	1.0154011	0.86749761	-0.2050683	0.992684	1
CYP3A43	1.0160985	1.01774106	1.0153865	0.86728269	-0.2054258	0.992684	1
COX4I2	1.08235	1.08026603	1.0832534	1.037218106	0.0527193	0.992688	1
TMEM104	1.0807652	1.0788163	1.0816099	1.035444838	0.0502507	0.992702	1
RP4-612B15.3	1.0369623	1.03490416	1.0378544	1.084525384	0.1170638	0.99272	1
RPRD2	1.3226768	1.32423796	1.3220001	0.99309803	-0.009992	0.992724	1
GRIA1	1.0983727	1.09633203	1.0992572	1.030365273	0.0431559	0.992755	1
RP11-649E7.5	1.027451	1.02541817	1.0283321	1.11463837	0.1565757	0.992784	1
UGGT2	1.3947143	1.39241834	1.3957095	1.008386763	0.0120491	0.992847	1
CTXN2	1.0540868	1.0520263	1.0549799	1.05677158	0.0796636	0.99292	1
CENPW	1.8182573	1.81956054	1.8176924	0.9977206	-0.0032922	0.992938	1
TMEM79	1.0848027	1.08280583	1.0856682	1.034567243	0.0490274	0.993001	1
CUL3	1.4332472	1.43463507	1.4326457	0.995422825	-0.0066186	0.993009	1
SPRED2	1.1503544	1.15210588	1.1495953	0.983494303	-0.0240114	0.993056	1
NOS3	1.0447146	1.04632003	1.0440187	0.950316395	-0.0735202	0.993061	1
LIFR-AS1	1.0618552	1.06351114	1.0611374	0.962625186	-0.0549539	0.993069	1
AC018890.6	1.0241391	1.02570865	1.0234588	0.912485031	-0.1321272	0.993079	1
SGPL1	1.2907183	1.28841194	1.291718	1.011463095	0.0164437	0.99308	1
RP11-503L19.1	1.0171476	1.0187379	1.0164583	0.878342491	-0.1871445	0.993105	1
KB-1043D8.6	1.0170925	1.01870879	1.0163919	0.876161151	-0.1907318	0.993105	1
RP11-21A7A.4	1.0171285	1.01874496	1.0164279	0.876388975	-0.1903568	0.993105	1
ARL11	1.0170985	1.01872956	1.0163916	0.875171351	-0.1923626	0.993105	1
CA5A	1.0171017	1.01874306	1.0163903	0.874471716	-0.1935164	0.993105	1
LINC00976	1.0171238	1.01875973	1.0164147	0.874997621	-0.192649	0.993105	1
AC007966.1	1.0170832	1.01871216	1.0163771	0.875213383	-0.1922933	0.993105	1
RP11-981P6.1	1.0197509	1.01771598	1.0206329	1.164650101	0.2198966	0.99311	1
DEPDC4	1.0907924	1.0922573	1.0901574	0.977238925	-0.0332168	0.993113	1
RAD54L2	1.2547716	1.2563618	1.2540823	0.991108192	-0.0128855	0.993153	1
RP11-408A13.4	1.0288334	1.03049179	1.0281146	0.922037441	-0.1171028	0.993205	1
ANKRD44	1.0288709	1.03048738	1.0281702	0.923994956	-0.1140431	0.993205	1
RP4-794H19.1	1.0288522	1.03045269	1.0281584	0.924659989	-0.1130051	0.993205	1
PCDHGA7	1.0164121	1.01800471	1.0157218	0.873206615	-0.195605	0.993266	1
SLC2A9	1.0164089	1.01799675	1.0157207	0.873530149	-0.1950706	0.993266	1
SPATA45	1.0163983	1.01800471	1.015702	0.872105773	-0.197425	0.993266	1
SPINK2	1.0163989	1.01801845	1.0156968	0.871153308	-0.1990015	0.993266	1
RP11-507M3.1	1.0163899	1.01800032	1.0156918	0.871752253	-0.1980099	0.993266	1
CTC-498J12.1	1.0164043	1.01802	1.015704	0.871477417	-0.1984648	0.993266	1
RP11-757G1.6	1.0163956	1.01799924	1.0157005	0.872286258	-0.1971264	0.993266	1
AC100830.4	1.0164072	1.01801276	1.0157112	0.872228428	-0.1972221	0.993266	1
TREM1	1.0164007	1.01801276	1.0157019	0.87170941	-0.1980808	0.993266	1
LINC00683	1.0457509	1.04376624	1.0466111	1.065001685	0.0908557	0.993286	1
WDR49	1.0428224	1.04444533	1.042119	0.947657964	-0.0775617	0.993325	1

RSPO1	1.0427056	1.04449747	1.0419289	0.942275349	-0.0857794	0.993325	1
RP11-299H21.1	1.0337877	1.03529535	1.0331342	0.938768202	-0.0911591	0.993325	1
SLC7A6OS	1.3464829	1.34802347	1.3458151	0.993654445	-0.0091839	0.993333	1
AC093901.1	1.0262711	1.0242767	1.0271355	1.117760602	0.1606112	0.993361	1
HCG25	1.0263496	1.02423334	1.027267	1.125183856	0.1701608	0.993361	1
RP11-510M2.2	1.0262407	1.02424935	1.0271038	1.11771255	0.1605492	0.993361	1
NOXA1	1.1043824	1.10248122	1.1052064	1.026592379	0.0378635	0.993371	1
HBQ1	1.1091447	1.10713	1.110018	1.026957656	0.0383767	0.993439	1
RP11-181C3.1	1.0604022	1.0618305	1.0597831	0.96688649	-0.0485816	0.993449	1
RP11-347C12.11	1.0169972	1.01860735	1.0162992	0.875956435	-0.191069	0.993454	1
TEX41	1.0170017	1.01860302	1.0163077	0.87661327	-0.1899876	0.993454	1
LINC00336	1.0169872	1.01859173	1.0162917	0.876286371	-0.1905257	0.993454	1
LY86-AS1	1.0170123	1.01861999	1.0163154	0.876228851	-0.1906204	0.993454	1
KCNT1	1.0169915	1.01858439	1.016301	0.877135177	-0.1891289	0.993454	1
LINC01471	1.0170214	1.01860394	1.0163355	0.878066569	-0.1875978	0.993454	1
DDTL	1.0169924	1.01860668	1.0162926	0.87563271	-0.1916022	0.993454	1
RP11-402J6.1	1.017001	1.0185845	1.0163146	0.877859307	-0.1879384	0.993454	1
RP11-23E10.3	1.0169945	1.01859059	1.0163027	0.876933044	-0.1894614	0.993454	1
C17orf80	1.163637	1.16522675	1.1629479	0.986207672	-0.0200366	0.993515	1
EMILIN3	1.1131642	1.11474501	1.1124789	0.980251355	-0.0287764	0.993522	1
ZNF879	1.0809632	1.0825485	1.0802761	0.972471425	-0.0402722	0.993556	1
RP11-1275H24.1	1.2042779	1.20598777	1.2035367	0.988101082	-0.0172695	0.993566	1
PTER	1.1288306	1.13073512	1.1280051	0.979117699	-0.0304458	0.993581	1
ADPRHL1	1.0278443	1.02579818	1.0287312	1.113689137	0.1553466	0.993586	1
LINC00290	1.0274127	1.02889124	1.0267718	0.926641131	-0.1099174	0.993595	1
EYA4	1.0283007	1.02630484	1.0291658	1.108761995	0.1489497	0.993623	1
LRP5L	1.0622169	1.06377377	1.0615421	0.965006014	-0.0513902	0.993664	1
AC009518.4	1.0247285	1.02632939	1.0240346	0.912841403	-0.1315639	0.99371	1
LA16c-OS12.2	1.0188113	1.01684335	1.0196643	1.16748125	0.2233994	0.993777	1
ERO1B	1.1532978	1.15481395	1.1526405	0.985961213	-0.0203972	0.993781	1
RP11-589P10.5	1.0569626	1.05496785	1.0578272	1.052018582	0.0731602	0.993855	1
PCNT	1.2280376	1.22960044	1.2273601	0.99024251	-0.0141462	0.993932	1
AC079305.10	1.0534803	1.05517682	1.0527449	0.955924818	-0.0650309	0.994012	1
CDPF1	1.2945388	1.29641815	1.2937242	0.990911532	-0.0131718	0.99404	1
USP3-AS1	1.0438874	1.04536486	1.043247	0.953314786	-0.0689754	0.994092	1
C17orf96	1.0786295	1.08014457	1.0779728	0.972902149	-0.0396334	0.994172	1
MCRS1	1.652815	1.6506506	1.6537531	1.004768304	0.0068629	0.994173	1
RP11-723O4.9	1.0169614	1.01849675	1.0162959	0.881012961	-0.1827649	0.994201	1
GACAT3	1.0169422	1.01850267	1.0162659	0.879109968	-0.1858845	0.994201	1
LENEP	1.0169408	1.01848801	1.0162702	0.880041348	-0.1843568	0.994201	1
ITPRIPL2	1.2523992	1.25393011	1.2517356	0.991357736	-0.0125223	0.994212	1
ATP13A4	1.0375343	1.03904373	1.03688	0.944583077	-0.0822504	0.99422	1
TNFRSF18	1.1412698	1.13951661	1.1420298	1.018013456	0.0257566	0.994225	1
MRPL35	1.3257587	1.32742898	1.3250347	0.992687496	-0.0105885	0.994243	1
ETV7	1.0290478	1.02707493	1.0299029	1.104449086	0.1433269	0.994245	1
TICAM1	1.0919509	1.09350231	1.0912785	0.976216086	-0.0347276	0.994249	1
RP11-647K16.1	1.0250501	1.02659759	1.0243794	0.916600778	-0.1256346	0.994295	1
CTB-25B13.9	1.0250168	1.02654355	1.0243551	0.917550991	-0.1241398	0.994295	1
PAM16	1.0250006	1.02658092	1.0243156	0.914775122	-0.128511	0.994295	1
C11orf95	1.2759786	1.27394892	1.2768584	1.010620557	0.0152414	0.99431	1
PRLHR	1.0181667	1.01969327	1.017505	0.888882175	-0.1699359	0.994356	1

RP11-443B20.1	1.0181417	1.01970037	1.0174661	0.886584963	-0.1736692	0.994356	1
SLAMF7	1.0181601	1.01967569	1.0175031	0.88958055	-0.1688028	0.994356	1
KB-1107E3.1	1.0181682	1.0197005	1.0175039	0.888502847	-0.1705517	0.994356	1
RP11-384F7.2	1.018131	1.01968506	1.0174574	0.886837066	-0.173259	0.994356	1
RP11-319F12.2	1.0181605	1.01968912	1.017498	0.888713018	-0.1702105	0.994356	1
RAB7B	1.0181421	1.01970037	1.0174666	0.886614089	-0.1736218	0.994356	1
EPN3	1.0212767	1.01936449	1.0221056	1.141551322	0.1909957	0.99441	1
KB-68A7.1	1.0230617	1.02467038	1.0223644	0.906529003	-0.1415749	0.994419	1
RP1-292L20.3	1.0230278	1.02459183	1.0223498	0.908832304	-0.137914	0.994419	1
RP11-572O6.1	1.0230492	1.02460151	1.0223764	0.909552181	-0.1367717	0.994419	1
RP11-334C17.6	1.0230908	1.02456762	1.0224506	0.913830189	-0.130002	0.994419	1
STPG2	1.0256331	1.02721182	1.0249488	0.916835626	-0.125265	0.994483	1
SALRNA2	1.0191871	1.01724954	1.020027	1.161015918	0.2153878	0.994537	1
AC005863.1	1.0191801	1.01724954	1.0200169	1.160429876	0.2146593	0.994537	1
RP11-473M20.5	1.0192046	1.01724954	1.0200521	1.162471326	0.2171951	0.994537	1
LINC00482	1.0191847	1.01724954	1.0200235	1.160810836	0.2151329	0.994537	1
KLK11	1.019167	1.01724954	1.0199981	1.159341459	0.2133055	0.994537	1
IL18R1	1.0191785	1.01724954	1.0200147	1.160301779	0.2145001	0.994537	1
ENTPD3	1.019218	1.01724954	1.0200712	1.163580353	0.2185708	0.994537	1
HAPLN1	1.0191818	1.01724954	1.0200193	1.160568971	0.2148323	0.994537	1
ONECUT3	1.0210372	1.01912909	1.0218643	1.142984369	0.1928057	0.994677	1
LINC00867	1.02099	1.01906944	1.0218225	1.144371592	0.1945556	0.994677	1
CKM	1.0210881	1.01908428	1.0219566	1.150507775	0.2022707	0.994677	1
SLC25A33	1.3584748	1.35639441	1.3593765	1.008367436	0.0120214	0.99474	1
DUSP28	1.1843146	1.18234783	1.1851672	1.015461301	0.0221353	0.994772	1
RP11-315D16.2	1.0175468	1.01906933	1.0168869	0.88555141	-0.175352	0.994793	1
TRIM67	1.0175414	1.01906832	1.0168796	0.885215653	-0.1758991	0.994793	1
RP11-629N8.3	1.0157309	1.01724954	1.0150726	0.87379998	-0.194625	0.994937	1
RP11-192H23.7	1.0157309	1.01724954	1.0150726	0.87379998	-0.194625	0.994937	1
AC084149.1	1.0157309	1.01724954	1.0150726	0.87379998	-0.194625	0.994937	1
MLIP-AS1	1.0157309	1.01724954	1.0150726	0.87379998	-0.194625	0.994937	1
MOGAT2	1.0157309	1.01724954	1.0150726	0.87379998	-0.194625	0.994937	1
RP11-392O17.1	1.0157309	1.01724954	1.0150726	0.87379998	-0.194625	0.994937	1
CYP26C1	1.0157309	1.01724954	1.0150726	0.87379998	-0.194625	0.994937	1
AC109333.10	1.0157309	1.01724954	1.0150726	0.87379998	-0.194625	0.994937	1
AC105393.1	1.0157309	1.01724954	1.0150726	0.87379998	-0.194625	0.994937	1
CPA3	1.0157309	1.01724954	1.0150726	0.87379998	-0.194625	0.994937	1
SLC25A47	1.0157309	1.01724954	1.0150726	0.87379998	-0.194625	0.994937	1
RP11-470P21.2	1.0157309	1.01724954	1.0150726	0.87379998	-0.194625	0.994937	1
RP11-334J6.6	1.0157309	1.01724954	1.0150726	0.87379998	-0.194625	0.994937	1
AP001628.7	1.0157309	1.01724954	1.0150726	0.87379998	-0.194625	0.994937	1
RP11-587P21.3	1.0157309	1.01724954	1.0150726	0.87379998	-0.194625	0.994937	1
RP11-403A3.1	1.0157309	1.01724954	1.0150726	0.87379998	-0.194625	0.994937	1
LINC00589	1.0157309	1.01724954	1.0150726	0.87379998	-0.194625	0.994937	1
RP11-431K24.3	1.0157309	1.01724954	1.0150726	0.87379998	-0.194625	0.994937	1
AC074366.3	1.0157309	1.01724954	1.0150726	0.87379998	-0.194625	0.994937	1
LINC01332	1.0157309	1.01724954	1.0150726	0.87379998	-0.194625	0.994937	1
GUCA2A	1.0157309	1.01724954	1.0150726	0.87379998	-0.194625	0.994937	1
DLK2	1.0413915	1.04295268	1.0407148	0.947899061	-0.0771947	0.995023	1
MIR9-3HG	1.0290419	1.02717397	1.0298515	1.098532765	0.1355779	0.995037	1
CLEC4C	1.0178105	1.01932422	1.0171544	0.887715432	-0.1718308	0.99506	1

RP4-633O19__A.1	1.0178389	1.0193769	1.0171723	0.886225717	-0.1742539	0.99506	1
CTD-2013N17.7	1.0178337	1.01933584	1.0171826	0.88864004	-0.1703289	0.99506	1
KY	1.017814	1.01928849	1.0171748	0.890418399	-0.1674447	0.99506	1
RP11-403P17.4	1.0178324	1.01932422	1.0171858	0.88933979	-0.1691934	0.99506	1
CTD-3116E22.8	1.0178265	1.01934446	1.0171686	0.887518087	-0.1721516	0.99506	1
ASB8	1.2887587	1.29032076	1.2880816	0.992287357	-0.0111701	0.995079	1
RP11-791G15.2	1.0461075	1.04417178	1.0469465	1.062816809	0.0878929	0.995086	1
CLCN6	1.1487673	1.14684146	1.149602	1.018799607	0.0268703	0.995088	1
RP11-572M11.4	1.026577	1.02456635	1.0274485	1.117321266	0.1600441	0.995106	1
PRR36	1.1712052	1.16941321	1.171982	1.015162807	0.0217111	0.995113	1
IKZF3	1.0215816	1.01968348	1.0224043	1.138228275	0.1867899	0.995116	1
VAV2	1.1829361	1.18451315	1.1822526	0.987748455	-0.0177844	0.995208	1
RP11-620J15.2	1.028552	1.02666986	1.0293679	1.10116422	0.1390296	0.995226	1
AC068282.3	1.0285016	1.02657828	1.0293353	1.103732664	0.1423908	0.995226	1
OLAH	1.0203771	1.01849852	1.0211914	1.145573541	0.1960701	0.99527	1
U91328.19	1.0459209	1.04401919	1.0467452	1.061927164	0.0866848	0.995326	1
RP11-386I14.4	1.2032502	1.20116203	1.2041554	1.014880139	0.0213094	0.995332	1
CPM	1.0386686	1.03675748	1.0394969	1.074527715	0.1037027	0.995356	1
ARHGAP24	1.1425711	1.14414498	1.1418888	0.984348125	-0.0227595	0.995359	1
RP11-390F4.3	1.0513696	1.05283144	1.0507359	0.96033569	-0.0583893	0.995487	1
CCDC88C	1.1504643	1.1486665	1.1512435	1.017334227	0.0247937	0.99549	1
CEPT1	1.240263	1.23846337	1.241043	1.010817771	0.0155229	0.995557	1
ZNF250	1.1557419	1.15719158	1.1551135	0.986780049	-0.0191995	0.995562	1
MGMT	1.7901443	1.7923805	1.789175	0.99595454	-0.0058482	0.995596	1
MRPL30	1.3738476	1.37546304	1.3731474	0.993832498	-0.0089254	0.995611	1
EPM2A	1.0569237	1.05507311	1.0577259	1.048168254	0.0678703	0.99565	1
AP5Z1	1.0793252	1.08085547	1.0786619	0.972870476	-0.0396804	0.995663	1
RP11-111A21.1	1.0153755	1.01685285	1.0147351	0.874336889	-0.1937388	0.995694	1
RP11-415I12.3	1.0153751	1.01684363	1.0147385	0.875018245	-0.192615	0.995694	1
RP11-196G11.2	1.015381	1.01684335	1.0147471	0.875544873	-0.191747	0.995694	1
AGTR1	1.0153766	1.01685607	1.0147353	0.874182774	-0.1939931	0.995694	1
UBD	1.0153785	1.01686356	1.0147347	0.873761477	-0.1946886	0.995694	1
WASIR1	1.0153895	1.0168628	1.0147509	0.874761597	-0.1930382	0.995694	1
TCEAL6	1.015392	1.01685654	1.0147572	0.8754591	-0.1918883	0.995694	1
CTD-2574D22.7	1.0153816	1.01684684	1.0147465	0.875327484	-0.1921052	0.995694	1
LINC00891	1.0153907	1.01686526	1.0147515	0.874666508	-0.193195	0.995694	1
RP11-578F21.6	1.0153824	1.01686722	1.0147388	0.873813457	-0.1946028	0.995694	1
CD300A	1.0795329	1.07750783	1.0804107	1.037452529	0.0530453	0.995745	1
TDRKH	1.0533982	1.05497733	1.0527137	0.958826829	-0.0606578	0.9958	1
SH2D2A	1.0951424	1.09315562	1.0960036	1.030572331	0.0434458	0.995859	1
CTD-2320G14.2	1.026229	1.02766849	1.0256051	0.925423861	-0.1118138	0.995866	1
CFAP58	1.02617	1.02766258	1.0255231	0.922657076	-0.1161336	0.995866	1
INSM2	1.0248618	1.02629825	1.0242392	0.921704734	-0.1176234	0.995898	1
PABPC1L2A	1.0248601	1.02633658	1.0242201	0.919636293	-0.1208647	0.995898	1
AC010642.1	1.0248101	1.02626312	1.0241802	0.920692025	-0.1192094	0.995898	1
CRHBP	1.024826	1.02627668	1.0241972	0.920863014	-0.1189415	0.995898	1
AL356585.1	1.024499	1.02599815	1.0238493	0.917344119	-0.1244651	0.995933	1
RP11-70P17.1	1.0680437	1.06950065	1.0674122	0.969951314	-0.0440158	0.995973	1
SUSD5	1.0223011	1.02374383	1.0216758	0.912902815	-0.1314668	0.995978	1
ITPK1-AS1	1.0223368	1.02380044	1.0217024	0.911846739	-0.1331367	0.995978	1
PCDHAC2	1.0223212	1.02378284	1.0216876	0.911902239	-0.1330489	0.995978	1

RP11-177H2.2	1.0223213	1.02379419	1.0216829	0.911269883	-0.1340497	0.995978	1
LINC01085	1.0223234	1.02374517	1.0217072	0.914171474	-0.1294633	0.995978	1
TMEM220	1.0661504	1.06423278	1.0669816	1.042795136	0.0604558	0.995995	1
PRR26	1.020449	1.01859924	1.0212508	1.142560861	0.192271	0.99602	1
ALB	1.0204292	1.0185837	1.0212292	1.142355041	0.1920111	0.99602	1
TAF5	1.0931241	1.09466563	1.0924559	0.976657437	-0.0340755	0.99605	1
AHR	1.1918578	1.19006734	1.1926338	1.013503119	0.0193505	0.996095	1
AGPAT3	1.2586804	1.25698895	1.2594135	1.00943451	0.0135473	0.996112	1
FAM214B	1.3900871	1.3916455	1.3894116	0.994296145	-0.0082525	0.996113	1
CSF2RA	1.0264495	1.02786651	1.0258353	0.927107838	-0.1091909	0.996134	1
ESPL1	1.0700994	1.07153617	1.0694767	0.971210161	-0.0421446	0.996136	1
LL0XNC01-7P3.1	1.0227848	1.02424935	1.0221499	0.913423185	-0.1306447	0.99616	1
SPATA9	1.0198213	1.01802027	1.020602	1.143270289	0.1931665	0.996208	1
KCNE1	1.0198608	1.01801276	1.0206618	1.147065037	0.1979472	0.996208	1
TPBGL	1.0198708	1.01801944	1.0206732	1.147273624	0.1982095	0.996208	1
ZNF182	1.0863823	1.08782299	1.0857579	0.97648564	-0.0343293	0.996215	1
C3orf52	1.0371623	1.03537808	1.0379357	1.072294351	0.100701	0.996245	1
FLRT1	1.0462503	1.04438881	1.0470572	1.060113406	0.0842186	0.996292	1
HIST1H2BC	1.0403185	1.04181886	1.0396681	0.948569813	-0.0761741	0.996296	1
RP11-568A7.3	1.016302	1.01773736	1.0156798	0.884000998	-0.1778801	0.996359	1
LINC00443	1.0162721	1.01770814	1.0156496	0.883753688	-0.1782838	0.996359	1
C10orf53	1.0162758	1.01770451	1.0156566	0.884326363	-0.1773492	0.996359	1
SERPINB9P1	1.0162843	1.01772769	1.0156587	0.88328808	-0.1790441	0.996359	1
BX470102.3	1.0162775	1.01770632	1.0156582	0.884326637	-0.1773487	0.996359	1
RP11-290F24.6	1.0300475	1.02823647	1.0308325	1.091939436	0.1268928	0.996363	1
AFF3	1.0830699	1.08116548	1.0838954	1.033634079	0.0477255	0.996368	1
CFAP45	1.0275442	1.02573066	1.0283302	1.101030229	0.1388541	0.996445	1
ZNF600	1.0766503	1.07483106	1.0774389	1.034849332	0.0494207	0.99653	1
FUK	1.0560249	1.05741293	1.0554233	0.965344915	-0.0508836	0.996578	1
MRPL1	1.6269371	1.62872052	1.6261641	0.995933958	-0.005878	0.996579	1
PPM1M	1.0959035	1.09402767	1.0967165	1.028596592	0.0406773	0.99665	1
HHIP-AS1	1.0506689	1.05218387	1.0500123	0.958385804	-0.0613216	0.996736	1
ZFP90	1.6203636	1.6185145	1.6211651	1.004285443	0.0061694	0.99675	1
FAM120B	1.2108033	1.20891096	1.2116235	1.012984211	0.0186117	0.996766	1
CTD-2532N20.1	1.0195662	1.01775578	1.0203509	1.146157194	0.1968049	0.996791	1
GPR171	1.0195768	1.01775478	1.0203666	1.14710461	0.197997	0.996791	1
XKRX	1.0153834	1.01679675	1.0147708	0.879382653	-0.185437	0.99684	1
SPEM1	1.0153847	1.01679791	1.0147722	0.879406295	-0.1853982	0.99684	1
SAMD3	1.0153764	1.01678461	1.014766	0.879734405	-0.1848601	0.99684	1
RP11-567C2.1	1.0153853	1.0168044	1.0147702	0.878946619	-0.1861525	0.99684	1
ATP4B	1.0153768	1.01678797	1.0147651	0.879504533	-0.1852371	0.99684	1
ZNF30-AS1	1.0153762	1.01679133	1.0147628	0.879191089	-0.1857513	0.99684	1
RP4-809F18.1	1.0153759	1.01678797	1.0147638	0.87942791	-0.1853628	0.99684	1
LILRA2	1.0153812	1.01679539	1.0147682	0.879301721	-0.1855698	0.99684	1
HIST1H2AK	1.0153783	1.016786	1.0147682	0.879791012	-0.1847672	0.99684	1
FLT3LG	1.0490008	1.04707889	1.0498339	1.05851975	0.0820482	0.996901	1
RNF125	1.0309733	1.03238051	1.0303633	0.937702878	-0.0927972	0.996928	1
RP4-565E6.1	1.0347773	1.03617407	1.0341719	0.944651342	-0.0821461	0.996931	1
DNAH10	1.0287645	1.02695047	1.0295507	1.096483232	0.1328838	0.996935	1
LINC00592	1.0221477	1.02035824	1.0229234	1.12599968	0.1712064	0.996943	1
RP11-714M23.2	1.0175923	1.01899597	1.0169839	0.894077927	-0.1615275	0.996944	1

RP11-607P23.1	1.0175923	1.01899597	1.0169839	0.894077927	-0.1615275	0.996944	1
KCNE1B	1.0175923	1.01899597	1.0169839	0.894077927	-0.1615275	0.996944	1
RP11-1293J14.1	1.0175923	1.01899597	1.0169839	0.894077927	-0.1615275	0.996944	1
CYP2C18	1.0175923	1.01899597	1.0169839	0.894077927	-0.1615275	0.996944	1
NOX1	1.0175923	1.01899597	1.0169839	0.894077927	-0.1615275	0.996944	1
AP000302.58	1.0175923	1.01899597	1.0169839	0.894077927	-0.1615275	0.996944	1
CLPSL1	1.0175923	1.01899597	1.0169839	0.894077927	-0.1615275	0.996944	1
LINC01347	1.0175923	1.01899597	1.0169839	0.894077927	-0.1615275	0.996944	1
IL2RB	1.0175923	1.01899597	1.0169839	0.894077927	-0.1615275	0.996944	1
RP11-554A11.7	1.0175923	1.01899597	1.0169839	0.894077927	-0.1615275	0.996944	1
RP1-118J21.25	1.0175923	1.01899597	1.0169839	0.894077927	-0.1615275	0.996944	1
KLHDC7B	1.0175923	1.01899597	1.0169839	0.894077927	-0.1615275	0.996944	1
IL16	1.0175923	1.01899597	1.0169839	0.894077927	-0.1615275	0.996944	1
RP11-178L8.3	1.0175923	1.01899597	1.0169839	0.894077927	-0.1615275	0.996944	1
AC142293.3	1.0175923	1.01899597	1.0169839	0.894077927	-0.1615275	0.996944	1
RP11-829H16.3	1.0175923	1.01899597	1.0169839	0.894077927	-0.1615275	0.996944	1
MOB1B	1.2608905	1.26213971	1.2603491	0.993169163	-0.0098886	0.996997	1
NOL11	1.2519632	1.25032433	1.2526735	1.009384663	0.0134761	0.997044	1
NOP14-AS1	1.1174785	1.118851	1.1168836	0.983446876	-0.024081	0.997065	1
AC104653.1	1.0773962	1.07869869	1.0768316	0.976276019	-0.034639	0.997088	1
C2orf50	1.0249503	1.02310285	1.025751	1.114625893	0.1565596	0.997095	1
ALDH3B1	1.0314962	1.03300602	1.0308418	0.934428227	-0.0978442	0.99711	1
STUM	1.0224893	1.02385762	1.0218962	0.917788092	-0.123767	0.997116	1
AKNAD1	1.0224924	1.02387941	1.0218913	0.916741715	-0.1254128	0.997116	1
SLITRK3	1.0224696	1.02384772	1.0218723	0.917164244	-0.124748	0.997116	1
C12orf54	1.0188629	1.02025907	1.0182577	0.901211884	-0.1500618	0.997122	1
RP11-426C22.5	1.0188675	1.02025372	1.0182667	0.901892216	-0.1489731	0.997122	1
RP11-687F6.5	1.0188633	1.02025283	1.018261	0.901653181	-0.1493555	0.997122	1
RP11-635L1.3	1.0188578	1.02024263	1.0182576	0.901936002	-0.148903	0.997122	1
CDC20B	1.0188564	1.02025028	1.0182522	0.901330112	-0.1498725	0.997122	1
CTD-2349P21.6	1.0188629	1.0202515	1.018261	0.901710373	-0.149264	0.997122	1
EGOT	1.0188574	1.02025907	1.0182498	0.900821115	-0.1506875	0.997122	1
LA16c-390E6.5	1.0188742	1.02027757	1.0182659	0.900792506	-0.1507333	0.997122	1
CTB-152G17.6	1.0685234	1.06985437	1.0679465	0.972687848	-0.0399512	0.997136	1
FEZF2	1.1344578	1.13570619	1.1339167	0.986813766	-0.0191503	0.99716	1
PECAM1	1.028571	1.02677709	1.0293486	1.096032338	0.1322904	0.997173	1
LRRC4	1.0285964	1.0268192	1.0293668	1.094992088	0.1309204	0.997173	1
WNT10B	1.0375538	1.03582093	1.0383049	1.069343326	0.0967251	0.997208	1
FAM131B	1.1077846	1.10915508	1.1071906	0.98200291	-0.0262008	0.99727	1
RP4-650F12.2	1.0161365	1.01751032	1.0155411	0.887536996	-0.1721208	0.997275	1
AC098820.4	1.0161221	1.01751053	1.0155202	0.886338077	-0.174071	0.997275	1
AFAP1-AS1	1.0161304	1.01749977	1.0155368	0.88782834	-0.1716473	0.997275	1
RP11-360L9.7	1.01612	1.01751305	1.0155161	0.885974802	-0.1746624	0.997275	1
RP11-160H22.3	1.0161348	1.01751059	1.0155384	0.887373275	-0.172387	0.997275	1
SMC1B	1.0161246	1.0175167	1.0155212	0.88607923	-0.1744924	0.997275	1
SLC39A2	1.0236381	1.02184523	1.0244152	1.117643068	0.1604595	0.997295	1
KB-1980E6.3	1.0236549	1.02183518	1.0244437	1.119465487	0.1628101	0.997295	1
C8orf44-SGK3	1.0236723	1.02186553	1.0244555	1.118449031	0.1614995	0.997295	1
PTGER4P2-CDK2AP2P2	1.0236351	1.0218327	1.0244164	1.11834216	0.1613617	0.997295	1
HLA-G	1.0237298	1.02184923	1.0245449	1.123375483	0.1678402	0.997295	1
RP11-326I11.3	1.051605	1.05293816	1.0510271	0.963900867	-0.0530433	0.997316	1

RP11-380N8.7	1.0610615	1.06243857	1.0604646	0.96838495	-0.0463474	0.997316	1
RP11-2C24.9	1.0227096	1.02412274	1.0220971	0.916028972	-0.1265349	0.997353	1
RP11-362K2.2	1.0213927	1.01964474	1.0221504	1.127545937	0.1731862	0.997379	1
RP11-454P21.1	1.0189247	1.0171592	1.01969	1.147486564	0.1984773	0.997383	1
RP11-307N16.6	1.0189259	1.0171592	1.0196917	1.147590691	0.1986082	0.997383	1
STMND1	1.0189433	1.0171592	1.0197167	1.14904488	0.2004351	0.997383	1
RP11-569A11.1	1.0189593	1.0171592	1.0197396	1.150381057	0.2021118	0.997383	1
BDKRB2	1.0189239	1.0171592	1.0196888	1.147417882	0.1983909	0.997383	1
RP11-879F14.2	1.0189096	1.0171592	1.0196684	1.146227866	0.1968939	0.997383	1
C10orf142	1.0189282	1.0171592	1.0196949	1.14777683	0.1988422	0.997383	1
CRYGN	1.0248971	1.02628963	1.0242934	0.924069272	-0.1139271	0.9974	1
FGF23	1.0248931	1.02630004	1.0242833	0.923317671	-0.115101	0.9974	1
RP4-613B23.5	1.0135231	1.01490147	1.0129257	0.867408948	-0.2052158	0.997446	1
MYL2	1.0135231	1.01490147	1.0129257	0.867408948	-0.2052158	0.997446	1
IFNL3	1.0135231	1.01490147	1.0129257	0.867408948	-0.2052158	0.997446	1
LRMP	1.0135231	1.01490147	1.0129257	0.867408948	-0.2052158	0.997446	1
LGALS12	1.0135231	1.01490147	1.0129257	0.867408948	-0.2052158	0.997446	1
NPIPB3	1.0135231	1.01490147	1.0129257	0.867408948	-0.2052158	0.997446	1
RP11-545G3.1	1.0135231	1.01490147	1.0129257	0.867408948	-0.2052158	0.997446	1
LINC00704	1.0135231	1.01490147	1.0129257	0.867408948	-0.2052158	0.997446	1
CGB2	1.0135231	1.01490147	1.0129257	0.867408948	-0.2052158	0.997446	1
ABI3BP	1.0135231	1.01490147	1.0129257	0.867408948	-0.2052158	0.997446	1
LRIT1	1.0135231	1.01490147	1.0129257	0.867408948	-0.2052158	0.997446	1
DENND2D	1.0135231	1.01490147	1.0129257	0.867408948	-0.2052158	0.997446	1
RP11-43N5.1	1.0135231	1.01490147	1.0129257	0.867408948	-0.2052158	0.997446	1
RP11-3D4.2	1.0135231	1.01490147	1.0129257	0.867408948	-0.2052158	0.997446	1
RP1-140K8.5	1.0135231	1.01490147	1.0129257	0.867408948	-0.2052158	0.997446	1
RP3-393E18.2	1.0135231	1.01490147	1.0129257	0.867408948	-0.2052158	0.997446	1
AC004160.4	1.0135231	1.01490147	1.0129257	0.867408948	-0.2052158	0.997446	1
RP11-445P19.3	1.0135231	1.01490147	1.0129257	0.867408948	-0.2052158	0.997446	1
RP11-553L6.2	1.0135231	1.01490147	1.0129257	0.867408948	-0.2052158	0.997446	1
LRRIQ4	1.0135231	1.01490147	1.0129257	0.867408948	-0.2052158	0.997446	1
RAB44	1.0135231	1.01490147	1.0129257	0.867408948	-0.2052158	0.997446	1
RP11-354P11.4	1.0135231	1.01490147	1.0129257	0.867408948	-0.2052158	0.997446	1
C16orf89	1.0135231	1.01490147	1.0129257	0.867408948	-0.2052158	0.997446	1
RP4-816N1.7	1.0135231	1.01490147	1.0129257	0.867408948	-0.2052158	0.997446	1
GNAT3	1.0135231	1.01490147	1.0129257	0.867408948	-0.2052158	0.997446	1
GLRA2	1.0648572	1.06299248	1.0656654	1.042432508	0.059954	0.997451	1
INO80B	1.109373	1.11068531	1.1088041	0.983004046	-0.0247307	0.997471	1
CCDC141	1.021522	1.02287624	1.0209349	0.915138779	-0.1279376	0.997842	1
PITRM1-AS1	1.0215359	1.02288596	1.0209507	0.915438548	-0.1274651	0.997842	1
RP11-462P6.1	1.0215478	1.02290513	1.0209595	0.915054979	-0.1280697	0.997842	1
RP11-21L1.1	1.0145145	1.01587604	1.0139243	0.877063141	-0.1892474	0.997848	1
CNGB3	1.0145119	1.01586347	1.013926	0.877868764	-0.1879228	0.997848	1
CTC-251D13.1	1.0145059	1.01584852	1.0139239	0.878559857	-0.1867875	0.997848	1
LA16c-380H5.6	1.0145046	1.01586094	1.0139167	0.87742163	-0.1886578	0.997848	1
CTB-119C2.1	1.0145249	1.01588638	1.0139348	0.877153503	-0.1890988	0.997848	1
RP11-13P5.1	1.014513	1.01585983	1.0139292	0.878270135	-0.1872633	0.997848	1
CD79B	1.0145054	1.01585983	1.0139183	0.87758209	-0.188394	0.997848	1
CPZ	1.0145151	1.0158779	1.0139244	0.876968832	-0.1894025	0.997848	1
RP11-381P6.1	1.0145103	1.01586417	1.0139234	0.87766285	-0.1882613	0.997848	1

AFP	1.0222857	1.02055388	1.0230364	1.120782027	0.1645057	0.997861	1
CBR3	1.0222813	1.02055612	1.0230291	1.120304243	0.1638906	0.997861	1
GPR160	1.0223674	1.02055471	1.0231531	1.126411976	0.1717346	0.997861	1
RP11-406H21.2	1.0397666	1.04104085	1.0392143	0.955493225	-0.0656825	0.997869	1
CLC	1.0201617	1.01839445	1.0209277	1.137720436	0.1861461	0.997871	1
NTSR1	1.0201449	1.01839445	1.0209037	1.136411063	0.1844848	0.997871	1
SERPINB5	1.0201439	1.01839445	1.0209022	1.136330637	0.1843827	0.997871	1
CASQ1	1.0201493	1.01839445	1.0209099	1.136751867	0.1849174	0.997871	1
CAMP	1.0201937	1.01839445	1.0209735	1.140210282	0.1892999	0.997871	1
RP11-342D14.1	1.0201258	1.01839445	1.0208763	1.134921823	0.1825929	0.997871	1
CHIT1	1.0201646	1.01839445	1.0209319	1.137948948	0.1864358	0.997871	1
AZGP1	1.0201483	1.01839445	1.0209085	1.136671774	0.1848157	0.997871	1
AC001226.7	1.0201441	1.01839445	1.0209025	1.136349401	0.1844065	0.997871	1
RP11-235E17.2	1.0201444	1.01839445	1.020903	1.136373823	0.1844375	0.997871	1
RP11-627D16.1	1.0201686	1.01839445	1.0209376	1.138256983	0.1868263	0.997871	1
KCNV2	1.0201479	1.01839445	1.020908	1.136644522	0.1847811	0.997871	1
HOXD-AS2	1.0201821	1.01839445	1.0209569	1.13930626	0.1881556	0.997871	1
RXRG	1.0401639	1.03836181	1.040945	1.067336847	0.0940156	0.997927	1
CARD10	1.040181	1.03842659	1.0409415	1.065446998	0.0914588	0.997927	1
WEE2-AS1	1.026236	1.02759684	1.0256462	0.929314863	-0.1057606	0.997949	1
LINC01209	1.0262764	1.02766214	1.0256757	0.92819031	-0.1075075	0.997949	1
RP1-34B20.21	1.0262863	1.02768089	1.0256818	0.927780949	-0.1081439	0.997949	1
ZNF148	1.8013423	1.79978123	1.802019	1.00279795	0.004031	0.997983	1
AC069155.1	1.0138198	1.01516282	1.0132377	0.873035378	-0.195888	0.998028	1
SLC9A4	1.0138198	1.01516282	1.0132377	0.873035378	-0.195888	0.998028	1
RP5-1071N3.1	1.0138198	1.01516282	1.0132377	0.873035378	-0.195888	0.998028	1
CYP2C9	1.0138198	1.01516282	1.0132377	0.873035378	-0.195888	0.998028	1
CTD-2207P18.2	1.0138198	1.01516282	1.0132377	0.873035378	-0.195888	0.998028	1
KRT6A	1.0138198	1.01516282	1.0132377	0.873035378	-0.195888	0.998028	1
BOLL	1.0138198	1.01516282	1.0132377	0.873035378	-0.195888	0.998028	1
TRPM6	1.0138198	1.01516282	1.0132377	0.873035378	-0.195888	0.998028	1
LINC00964	1.0138198	1.01516282	1.0132377	0.873035378	-0.195888	0.998028	1
RP11-159D12.11	1.0138198	1.01516282	1.0132377	0.873035378	-0.195888	0.998028	1
TDRG1	1.0138198	1.01516282	1.0132377	0.873035378	-0.195888	0.998028	1
RP11-642D21.2	1.0138198	1.01516282	1.0132377	0.873035378	-0.195888	0.998028	1
RP11-158M2.2	1.0138198	1.01516282	1.0132377	0.873035378	-0.195888	0.998028	1
BTNL9	1.0138198	1.01516282	1.0132377	0.873035378	-0.195888	0.998028	1
GRIFIN	1.0138198	1.01516282	1.0132377	0.873035378	-0.195888	0.998028	1
TMEM71	1.0138198	1.01516282	1.0132377	0.873035378	-0.195888	0.998028	1
ARL14	1.0138198	1.01516282	1.0132377	0.873035378	-0.195888	0.998028	1
SHBG	1.0464551	1.04463319	1.0472448	1.05851349	0.0820397	0.998054	1
THSD7B	1.0311615	1.03248683	1.030587	0.941521378	-0.0869342	0.99807	1
CTC-505O3.3	1.0312011	1.03248691	1.0306437	0.943263563	-0.0842672	0.99807	1
TMEM204	1.0198978	1.01815945	1.0206512	1.137217018	0.1855076	0.998108	1
RP11-356N1.2	1.0198739	1.01815945	1.0206171	1.135336261	0.1831197	0.998108	1
CHRM3-AS2	1.0198809	1.01815945	1.0206271	1.135885689	0.1838177	0.998108	1
RP11-524D16__A.3	1.0198959	1.01815945	1.0206485	1.137067986	0.1853185	0.998108	1
RP11-798K3.2	1.0198708	1.01815945	1.0206126	1.135086861	0.1828027	0.998108	1
RP11-296I10.3	1.0199157	1.01815945	1.020677	1.138633667	0.1873037	0.998108	1
GRHL3	1.0198748	1.01815945	1.0206183	1.135403557	0.1832052	0.998108	1
RP11-435J9.2	1.0198978	1.01815945	1.0206513	1.137222051	0.185514	0.998108	1

RP11-93G5.1	1.0198868	1.01815945	1.0206355	1.136350362	0.1844077	0.998108	1
GSDMB	1.0610106	1.05915049	1.0618168	1.045077237	0.0636096	0.998115	1
KRBA1	1.1152485	1.1135176	1.1159988	1.021857678	0.0311943	0.99813	1
SEPSECS-AS1	1.0576464	1.05892575	1.0570919	0.968878145	-0.0456129	0.998142	1
RP1-199J3.7	1.0275362	1.02884942	1.0269669	0.934746836	-0.0973524	0.998167	1
LINC00934	1.0144129	1.01574718	1.0138345	0.878537899	-0.1868236	0.998215	1
LINC00911	1.0144129	1.01574718	1.0138345	0.878537899	-0.1868236	0.998215	1
LINC01163	1.0144129	1.01574718	1.0138345	0.878537899	-0.1868236	0.998215	1
MYOC	1.0144129	1.01574718	1.0138345	0.878537899	-0.1868236	0.998215	1
ANKRD7	1.0144129	1.01574718	1.0138345	0.878537899	-0.1868236	0.998215	1
LINC01033	1.0144129	1.01574718	1.0138345	0.878537899	-0.1868236	0.998215	1
ARSF	1.0144129	1.01574718	1.0138345	0.878537899	-0.1868236	0.998215	1
CTC-441N14.2	1.0144129	1.01574718	1.0138345	0.878537899	-0.1868236	0.998215	1
MNX1-AS2	1.0144129	1.01574718	1.0138345	0.878537899	-0.1868236	0.998215	1
ANKRD30A	1.0144129	1.01574718	1.0138345	0.878537899	-0.1868236	0.998215	1
RP11-157I4.4	1.0144129	1.01574718	1.0138345	0.878537899	-0.1868236	0.998215	1
GPR17	1.0144129	1.01574718	1.0138345	0.878537899	-0.1868236	0.998215	1
RP1-20B21.4	1.0144129	1.01574718	1.0138345	0.878537899	-0.1868236	0.998215	1
RP11-456O19.3	1.0144129	1.01574718	1.0138345	0.878537899	-0.1868236	0.998215	1
DLGAP1-AS5	1.0144129	1.01574718	1.0138345	0.878537899	-0.1868236	0.998215	1
ATXN2-AS	1.0144129	1.01574718	1.0138345	0.878537899	-0.1868236	0.998215	1
IGBP1-AS2	1.0144129	1.01574718	1.0138345	0.878537899	-0.1868236	0.998215	1
RP11-24D15.1	1.0144129	1.01574718	1.0138345	0.878537899	-0.1868236	0.998215	1
AC005253.4	1.0144129	1.01574718	1.0138345	0.878537899	-0.1868236	0.998215	1
RP11-314P15.2	1.0144129	1.01574718	1.0138345	0.878537899	-0.1868236	0.998215	1
KLF1	1.0144129	1.01574718	1.0138345	0.878537899	-0.1868236	0.998215	1
AC007192.6	1.0144129	1.01574718	1.0138345	0.878537899	-0.1868236	0.998215	1
AC114730.5	1.0144129	1.01574718	1.0138345	0.878537899	-0.1868236	0.998215	1
LRRC3	1.1116096	1.10976847	1.1124077	1.024043293	0.0342767	0.998272	1
RP11-996F15.6	1.0314251	1.03275006	1.0308508	0.942007081	-0.0861902	0.998308	1
LINC01152	1.0595769	1.0608684	1.059017	0.96958414	-0.044562	0.998322	1
RP11-191L17.1	1.0561666	1.05439646	1.0569339	1.046647005	0.065775	0.998377	1
HGFAC	1.0308848	1.02914721	1.031638	1.085456277	0.1183016	0.998394	1
CA3-AS1	1.1021104	1.10025995	1.1029126	1.026457287	0.0376736	0.998422	1
CBWD1	1.537215	1.5382406	1.5367705	0.997268727	-0.0039458	0.99845	1
LINC01315	1.2450525	1.24609618	1.2446001	0.993920807	-0.0087972	0.998497	1
ASH2L	1.2531435	1.25132478	1.2539318	1.01037323	0.0148883	0.998506	1
LL0XNC01-116E7.2	1.0594507	1.05772428	1.060199	1.04287223	0.0605624	0.998521	1
SYNJ1	1.076952	1.07503673	1.0777822	1.036588923	0.0518439	0.998525	1
ATG4B	1.5707154	1.56889801	1.5715031	1.004579265	0.0065914	0.998604	1
MTRNR2L12	1.3886212	1.38681794	1.3894029	1.006682541	0.0096088	0.998605	1
KCNE4	1.0508625	1.05215484	1.0503023	0.964479069	-0.0521782	0.998722	1
LINC00853	1.0508064	1.05212527	1.0502348	0.963731772	-0.0532964	0.998722	1
MMP25-AS1	1.1442102	1.14264752	1.1448876	1.015703428	0.0224792	0.998733	1
SGK223	1.0853838	1.08369257	1.0861169	1.02896717	0.041197	0.998743	1
MIS18A-AS1	1.0403754	1.03867245	1.0411136	1.063122742	0.0883082	0.99878	1
STK38	1.2654075	1.26379158	1.2661079	1.008781017	0.012613	0.998793	1
AKAP7	1.1423056	1.14064533	1.1430252	1.0169213	0.024208	0.998801	1
RP1-242N11.1	1.0231147	1.0244201	1.0225488	0.923372488	-0.1150153	0.998895	1
RLN2	1.1033622	1.10153235	1.1041554	1.025834592	0.0367981	0.998929	1
RP11-221N13.3	1.0143597	1.01564645	1.013802	0.882118112	-0.1809563	0.998943	1

AC018755.17	1.0143782	1.01565258	1.0138258	0.883289775	-0.1790413	0.998943	1
RP11-12K11.2	1.0143675	1.01565968	1.0138074	0.881718593	-0.1816098	0.998943	1
KRTAP5-AS1	1.0143609	1.01565391	1.0138004	0.881597231	-0.1818084	0.998943	1
LINC01361	1.0143661	1.01565293	1.0138082	0.882151378	-0.1809019	0.998943	1
AC015922.6	1.0143639	1.01565391	1.0138047	0.881871996	-0.1813588	0.998943	1
RSPH10B	1.0143638	1.01565063	1.0138061	0.882142199	-0.1809169	0.998943	1
RP11-369E15.3	1.0143669	1.01566168	1.0138057	0.881495641	-0.1819747	0.998943	1
PGM5P3-AS1	1.0143652	1.01565968	1.0138041	0.88150313	-0.1819624	0.998943	1
ANKRD62	1.0143713	1.0156558	1.0138145	0.882390893	-0.1805102	0.998943	1
LINC01563	1.0143716	1.01565413	1.0138157	0.882562688	-0.1802293	0.998943	1
SOX21-AS1	1.0143677	1.0156555	1.0138095	0.882086081	-0.1810086	0.998943	1
RP11-509J21.2	1.0349174	1.03615857	1.0343794	0.95079634	-0.0727917	0.998945	1
WNT3	1.0349143	1.0362523	1.0343343	0.947092026	-0.0784235	0.998945	1
TTLL1	1.2818751	1.28304949	1.2813661	0.994052597	-0.0086059	0.998982	1
LINC00648	1.0403542	1.03866222	1.0410876	1.062733144	0.0877794	0.999036	1
UNC79	1.0403527	1.0386481	1.0410916	1.063223704	0.0884452	0.999036	1
GPR20	1.0224531	1.02374908	1.0218913	0.921774742	-0.1175139	0.999037	1
CPA2	1.0224288	1.02369935	1.021878	0.923148037	-0.1153661	0.999037	1
KRT15	1.0224306	1.023697	1.0218817	0.923394317	-0.1149812	0.999037	1
YPEL4	1.0499806	1.04816424	1.050768	1.054059512	0.0759563	0.999039	1
NFIA-AS2	1.0201712	1.01850742	1.0208924	1.128863898	0.1748716	0.999063	1
PPP1R27	1.0202084	1.01850742	1.0209458	1.131749578	0.1785548	0.999063	1
RP11-43F13.4	1.020191	1.01850742	1.0209208	1.130398705	0.1768317	0.999063	1
KLF2	1.0202078	1.01850742	1.0209448	1.131696522	0.1784871	0.999063	1
H2BFM	1.0201956	1.01850742	1.0209273	1.130752629	0.1772834	0.999063	1
RP11-90L20.2	1.020196	1.01850742	1.0209279	1.130785133	0.1773248	0.999063	1
RP1-102G20.5	1.020206	1.01850742	1.0209422	1.131558813	0.1783116	0.999063	1
BFSP1	1.0522255	1.05045213	1.0529942	1.050385819	0.0709193	0.999087	1
AC106801.1	1.0155548	1.01683833	1.0149985	0.890733008	-0.166935	0.999116	1
RP11-120J1.1	1.0155548	1.01683833	1.0149985	0.890733008	-0.166935	0.999116	1
RP5-842K16.2	1.0155548	1.01683833	1.0149985	0.890733008	-0.166935	0.999116	1
RNF39	1.0155548	1.01683833	1.0149985	0.890733008	-0.166935	0.999116	1
RP11-29H23.4	1.0155548	1.01683833	1.0149985	0.890733008	-0.166935	0.999116	1
AC241377.1	1.0155548	1.01683833	1.0149985	0.890733008	-0.166935	0.999116	1
TMPRSS3	1.0155548	1.01683833	1.0149985	0.890733008	-0.166935	0.999116	1
PADI1	1.0155548	1.01683833	1.0149985	0.890733008	-0.166935	0.999116	1
RP11-849H4.4	1.0155548	1.01683833	1.0149985	0.890733008	-0.166935	0.999116	1
CST5	1.0155548	1.01683833	1.0149985	0.890733008	-0.166935	0.999116	1
CRYAA	1.0155548	1.01683833	1.0149985	0.890733008	-0.166935	0.999116	1
CTD-3088G3.8	1.0155548	1.01683833	1.0149985	0.890733008	-0.166935	0.999116	1
KPNA7	1.0155548	1.01683833	1.0149985	0.890733008	-0.166935	0.999116	1
TNK2-AS1	1.0155548	1.01683833	1.0149985	0.890733008	-0.166935	0.999116	1
DYSF	1.0155548	1.01683833	1.0149985	0.890733008	-0.166935	0.999116	1
AP001065.15	1.0155548	1.01683833	1.0149985	0.890733008	-0.166935	0.999116	1
CLCA2	1.0155548	1.01683833	1.0149985	0.890733008	-0.166935	0.999116	1
RP11-561B11.6	1.0155548	1.01683833	1.0149985	0.890733008	-0.166935	0.999116	1
AC012368.1	1.0155548	1.01683833	1.0149985	0.890733008	-0.166935	0.999116	1
DNAJB5-AS1	1.0155548	1.01683833	1.0149985	0.890733008	-0.166935	0.999116	1
POP5	1.7739129	1.77572737	1.7731265	0.99664714	-0.0048453	0.999119	1
SPRN	1.0238925	1.02222303	1.0246161	1.10768528	0.147548	0.999144	1
RP11-362K14.6	1.0239026	1.02216704	1.0246549	1.112233335	0.1534595	0.999144	1

ACE	1.0238558	1.02220463	1.0245715	1.106595311	0.1461277	0.999144	1
GZF1	1.086934	1.0882262	1.0863739	0.979004767	-0.0306122	0.999162	1
AC012074.2	1.0204578	1.02171836	1.0199115	0.916802848	-0.1253166	0.999198	1
RP11-433J8.2	1.0204722	1.02171164	1.019935	0.918172416	-0.123163	0.999198	1
KRT222	1.0230604	1.0243288	1.0225106	0.925266059	-0.1120598	0.999225	1
LINC01220	1.023061	1.02431843	1.022516	0.925882621	-0.1110988	0.999225	1
ELF5	1.0197817	1.01804205	1.0205358	1.138219585	0.1867789	0.999244	1
RP11-1C8.6	1.0197195	1.01804205	1.0204466	1.133276309	0.1804997	0.999244	1
DLX6-AS1	1.0197222	1.01804205	1.0204505	1.133489341	0.1807708	0.999244	1
SLAMF9	1.0197072	1.01804205	1.0204289	1.132294699	0.1792495	0.999244	1
TP53TG5	1.0197067	1.01804205	1.0204282	1.132257118	0.1792016	0.999244	1
TRMT5	1.1878514	1.18893162	1.1873832	0.991804526	-0.0118723	0.999265	1
DLL1	1.1231826	1.121412	1.1239501	1.02090449	0.0298479	0.999279	1
HEATR4	1.021784	1.02007909	1.0225229	1.121711878	0.1657022	0.999308	1
MS4A6E	1.0217867	1.02009879	1.0225184	1.120384158	0.1639935	0.999308	1
PIH1D3	1.0217691	1.02009416	1.0224952	1.119487811	0.1628388	0.999308	1
MSR1	1.0217724	1.02009638	1.0224989	1.119547877	0.1629162	0.999308	1
FAM151A	1.0222741	1.02056357	1.0230156	1.119239309	0.1625185	0.999324	1
IL1A	1.0222304	1.02056357	1.0229529	1.116194487	0.1585884	0.999324	1
RP11-354E11.2	1.022237	1.02056357	1.0229624	1.116653449	0.1591815	0.999324	1
GPR158-AS1	1.0222456	1.02056357	1.0229747	1.117252185	0.1599549	0.999324	1
RP11-164N3.2	1.0222433	1.02056357	1.0229714	1.117091005	0.1597467	0.999324	1
AC092535.3	1.0222413	1.02056357	1.0229685	1.11695305	0.1595685	0.999324	1
MIR3179-3	1.0222293	1.02056357	1.0229513	1.116115848	0.1584868	0.999324	1
RP11-257P3.3	1.0222301	1.02056357	1.0229525	1.116172648	0.1585602	0.999324	1
GPR156	1.023618	1.02194494	1.0243431	1.109282486	0.1496268	0.999411	1
GAS7	1.2049744	1.20621955	1.2044347	0.99134492	-0.012541	0.999421	1
HHLA3	1.4726433	1.47374619	1.4721652	0.996662777	-0.0048226	0.99946	1
ZBTB18	1.1752111	1.17340263	1.175995	1.014950071	0.0214088	0.999486	1
ZNF205	1.2447089	1.24618728	1.244068	0.991391721	-0.0124729	0.99951	1
PRSS16	1.077514	1.07580234	1.0782559	1.032368484	0.045958	0.999521	1
RP11-384O8.1	1.0149612	1.0162132	1.0144186	0.88930939	-0.1692427	0.999534	1
CTB-35F21.2	1.0149628	1.01621919	1.0144182	0.888958557	-0.1698119	0.999534	1
TRPC3	1.014961	1.01622158	1.0144146	0.888609323	-0.1703788	0.999534	1
RAB37	1.0149712	1.01623933	1.0144215	0.888060913	-0.1712695	0.999534	1
LINC01176	1.0149658	1.01623082	1.0144175	0.888280171	-0.1709133	0.999534	1
SRGAP3-AS2	1.0149827	1.01622158	1.0144457	0.890521829	-0.1672771	0.999534	1
ITGA11	1.0149736	1.01623082	1.0144287	0.888970768	-0.1697921	0.999534	1
ANKDD1A	1.1141312	1.11243576	1.1148661	1.02161576	0.0308527	0.999541	1
OSBP2	1.0699923	1.06836679	1.070697	1.034083384	0.0483525	0.999575	1
RP11-359B12.2	1.1071543	1.1084044	1.1066124	0.983469336	-0.024048	0.999739	1
IGIP	1.0388342	1.04006794	1.0382994	0.955862277	-0.0651253	0.999748	1
C14orf79	1.1239616	1.12534599	1.1233615	0.984167633	-0.023024	0.999755	1
LRRC57	1.2775957	1.27872265	1.2771073	0.994204319	-0.0083857	0.999764	1
RP11-439L18.1	1.01524	1.01647066	1.0147066	0.892895698	-0.1634364	0.9998	1
EQTN	1.0152335	1.01647611	1.0146949	0.891888448	-0.1650648	0.9998	1
RP11-177J6.1	1.0152427	1.0164844	1.0147044	0.892020084	-0.1648519	0.9998	1
RP11-680F20.10	1.0152332	1.01647627	1.0146944	0.891850647	-0.165126	0.9998	1
AC007292.3	1.0152315	1.01646494	1.0146969	0.892618909	-0.1638837	0.9998	1
RP11-711C17.2	1.0152383	1.01647529	1.0147021	0.892373508	-0.1642804	0.9998	1
CDH22	1.0152416	1.01648609	1.0147021	0.891788893	-0.1652259	0.9998	1

LINC01505	1.0152326	1.01647596	1.0146937	0.891824554	-0.1651682	0.9998	1
ADRA2B	1.0152334	1.0164757	1.014695	0.891918239	-0.1650166	0.9998	1
AC000068.10	1.0152409	1.01648539	1.0147015	0.891791983	-0.1652209	0.9998	1
TP73	1.0152296	1.01647948	1.0146879	0.891283865	-0.1660431	0.9998	1
RP11-96C23.15	1.0152455	1.01645797	1.01472	0.894401022	-0.1610063	0.9998	1
RP11-900F13.3	1.0152475	1.01646731	1.0147188	0.893819499	-0.1619446	0.9998	1
RYBP	1.5518127	1.55065011	1.5523167	1.003026537	0.0043598	0.999832	1
KLHL36	1.3103625	1.30852838	1.3111575	1.008521455	0.0122418	0.999839	1
LARGE2	1.0242032	1.022599	1.0248986	1.101755374	0.1398039	0.99987	1
RP11-212I21.4	1.0242062	1.02256133	1.0249192	1.104507861	0.1434037	0.99987	1
ULBP2	1.0291383	1.02762833	1.0297928	1.078344159	0.1088177	0.999878	1
CCNT2-AS1	1.0435544	1.04469765	1.0430588	0.96333438	-0.0538914	0.999933	1
TSPAN12	1.2642496	1.26558822	1.2636694	0.992775193	-0.010461	0.999977	1
STAT4	1.0229925	1.02429187	1.0224293	0.923324101	-0.1150909	0.999995	1
OLIG2	1.0230087	1.02423794	1.0224759	0.927304393	-0.1088851	0.999995	1
RP11-848P1.4	1.0229802	1.02421472	1.0224451	0.926918795	-0.1094851	0.999995	1
KCNQ1-AS1	1.024173	1.02372903	1.0243654	1.026818186	0.0381808	1	1
RP11-62H20.1	1.0190149	1.01801971	1.0194462	1.07916431	0.1099145	1	1
RP11-305O4.3	1.0146763	1.01516282	1.0144655	0.954008475	-0.067926	1	1
PRKCE	1.0552395	1.05513717	1.0552839	1.002660552	0.0038333	1	1
RP1-90G24.6	1.016387	1.01516282	1.0169176	1.115729841	0.1579877	1	1
PXDNL	1.0172578	1.01801845	1.0169281	0.939489277	-0.0900514	1	1
RP11-963H4.5	1.0146873	1.01516282	1.0144812	0.955045186	-0.0663591	1	1
NOXRED1	1.0215984	1.02090147	1.0219005	1.047796771	0.0673589	1	1
NR5A2	1.0155416	1.01516282	1.0157057	1.035806893	0.0507551	1	1
CTD-2600O9.2	1.0164063	1.01516282	1.0169452	1.117552748	0.1603429	1	1
RP13-991F5.2	1.0146827	1.01516282	1.0144745	0.954606472	-0.067022	1	1
ST6GALNAC4	1.1993494	1.19801449	1.199928	1.009663673	0.0138748	1	1
RP11-574O16.1	1.0146767	1.01516282	1.0144661	0.954048236	-0.0678659	1	1
SNX8	1.157785	1.15809231	1.1576518	0.9972133	-0.004026	1	1
LINC01260	1.0155401	1.01516282	1.0157036	1.035666338	0.0505593	1	1
RP11-571L19.8	1.0155416	1.01516282	1.0157057	1.035806893	0.0507551	1	1
AP003025.2	1.0155507	1.01516282	1.0157188	1.036668092	0.0519541	1	1
LINC00305	1.0198956	1.02090532	1.019458	0.930766074	-0.1035095	1	1
AC133644.2	1.0259032	1.02669463	1.0255602	0.957502718	-0.0626515	1	1
RP11-486L19.2	1.014677	1.01516282	1.0144665	0.954074354	-0.0678264	1	1
LHFPL4	1.0198591	1.0209164	1.0194007	0.92753724	-0.1085229	1	1
PCK1	1.0155475	1.01516282	1.0157142	1.036365832	0.0515334	1	1
AC097721.2	1.0189979	1.01801971	1.0194219	1.077812043	0.1081056	1	1
RP11-1223D19.4	1.0155563	1.01516282	1.0157268	1.037194937	0.0526871	1	1
RP11-866E20.3	1.0146741	1.01516282	1.0144623	0.953799469	-0.0682421	1	1
ASCL4	1.0164245	1.01516282	1.0169714	1.119274716	0.1625642	1	1
ZNF185	1.0896469	1.08953254	1.0896965	1.001831163	0.0026394	1	1
TMEM8C	1.0146815	1.01516282	1.0144729	0.954500225	-0.0671826	1	1
CHRNA4	1.017263	1.01801957	1.0169351	0.939819038	-0.0895451	1	1
UTAT33	1.0146797	1.01516282	1.0144703	0.954327978	-0.0674429	1	1
NLRP2	1.0155549	1.01516282	1.0157248	1.037063222	0.0525038	1	1
HMGCS2	1.0189886	1.01800737	1.0194139	1.078109751	0.1085041	1	1
TECTB	1.0164258	1.01516282	1.0169732	1.119399359	0.1627248	1	1
USP50	1.0155401	1.01516282	1.0157037	1.035671148	0.050566	1	1
LINC01114	1.0155634	1.01516282	1.015737	1.037868272	0.0536233	1	1

LINC01197	1.0163977	1.01516282	1.0169329	1.1167391	0.1592922	1	1
RP11-72I8.1	1.0146836	1.01516282	1.0144759	0.954696941	-0.0668853	1	1
RP11-529H2.1	1.0146765	1.01516282	1.0144657	0.954022459	-0.0679049	1	1
BEND4	1.0509157	1.04938192	1.0515805	1.044521636	0.0628424	1	1
CTD-2026K11.6	1.0198628	1.02089263	1.0194165	0.929344762	-0.1057142	1	1
TEX15	1.0250253	1.02372869	1.0255873	1.078326911	0.1087946	1	1
RASSF3	1.1337972	1.13231653	1.134439	1.01604088	0.0229585	1	1
RP11-554D15.3	1.0146747	1.01516282	1.0144631	0.953852281	-0.0681622	1	1
GATA6-AS1	1.0146827	1.01516282	1.0144746	0.954614656	-0.0670096	1	1
RP11-62J1.4	1.016408	1.01516282	1.0169477	1.117715277	0.1605527	1	1
HHIP	1.0172576	1.01799924	1.0169362	0.940939535	-0.0878261	1	1
DLL3	1.5201911	1.51824595	1.5210342	1.005380153	0.0077411	1	1
LINC00488	1.0189899	1.01801276	1.0194134	1.077759423	0.1080352	1	1
RP11-50D16.4	1.0146809	1.01516282	1.014472	0.954443427	-0.0672684	1	1
RP11-6D1.3	1.0146755	1.01516282	1.0144642	0.953926883	-0.0680494	1	1
RP11-867G2.6	1.0146749	1.01516282	1.0144634	0.953875083	-0.0681277	1	1
LINGO1-AS1	1.0164122	1.01516282	1.0169537	1.118109609	0.1610616	1	1
LIN28A	1.0181317	1.01802317	1.0181788	1.008635462	0.0124049	1	1
AC004490.1	1.0146929	1.01516282	1.0144893	0.955579537	-0.0655521	1	1
LYZL4	1.0155434	1.01516282	1.0157083	1.0359757	0.0509902	1	1
RP11-121G22.3	1.0146816	1.01516282	1.014473	0.95450612	-0.0671736	1	1
ADRA2A	1.0146782	1.01516282	1.0144681	0.954181846	-0.0676639	1	1
STARD8	1.0172813	1.01801142	1.0169648	0.941891705	-0.0863669	1	1
CCR9	1.0146818	1.01516282	1.0144733	0.954524278	-0.0671462	1	1
HS3ST5	1.0172838	1.01801957	1.0169649	0.941472902	-0.0870085	1	1
RP11-809N8.2	1.0146766	1.01516282	1.0144658	0.954032382	-0.0678899	1	1
U47924.27	1.0155465	1.01516282	1.0157128	1.036269939	0.0513999	1	1
RP11-521D12.1	1.0146749	1.01516282	1.0144634	0.953875083	-0.0681277	1	1
CABP4	1.0155423	1.01516282	1.0157068	1.035875609	0.0508508	1	1
HYI-AS1	1.0155405	1.01516282	1.0157042	1.035701706	0.0506086	1	1
AC009299.3	1.0164133	1.01516282	1.0169554	1.118221119	0.1612055	1	1
RP11-774D14.1	1.0155343	1.01516282	1.0156953	1.03511607	0.0497926	1	1
CTD-2589H19.6	1.0163973	1.01516282	1.0169324	1.116706988	0.1592507	1	1
RP11-284N8.3	1.0190105	1.01802078	1.0194396	1.078730373	0.1093343	1	1
RP11-863P13.4	1.0146789	1.01516282	1.0144692	0.954253353	-0.0675557	1	1
RP11-582J16.5	1.0277156	1.02667136	1.0281683	1.056123803	0.078779	1	1
AC073254.1	1.0155573	1.01516282	1.0157283	1.037297272	0.0528294	1	1
CD200R1	1.0155529	1.01516282	1.015722	1.036881503	0.052251	1	1
RP11-27G24.1	1.0198961	1.02096302	1.0194337	0.927044374	-0.1092897	1	1
CTD-2313J17.6	1.01468	1.01516282	1.0144707	0.954351979	-0.0674066	1	1
TMPRSS7	1.0164137	1.01516282	1.0169559	1.118257752	0.1612528	1	1
COL21A1	1.0155457	1.01516282	1.0157116	1.036193933	0.051294	1	1
CTD-2655K5.1	1.0155428	1.01516282	1.0157074	1.03591829	0.0509102	1	1
LA16c-385E7.1	1.017255	1.0179925	1.0169353	0.941244366	-0.0873588	1	1
LINC00930	1.0155455	1.01516282	1.0157114	1.036177565	0.0512713	1	1
GJA3	1.0189761	1.01799973	1.0193992	1.077752407	0.1080258	1	1
AC067959.1	1.0155374	1.01516282	1.0156998	1.035411969	0.0502049	1	1
AC010907.2	1.0155538	1.01516282	1.0157232	1.036958183	0.0523577	1	1
RP11-76E17.4	1.017275	1.01801276	1.0169552	0.941286864	-0.0872936	1	1
TSTD3	1.150156	1.14946985	1.1504535	1.006580639	0.0094628	1	1
PRR18	1.0189795	1.01802196	1.0193946	1.076164684	0.1058989	1	1

P2RX3	1.0164068	1.01516282	1.016946	1.117599354	0.1604031	1	1
RP5-963E22.6	1.020715	1.02089263	1.020638	0.987813535	-0.0176894	1	1
RP11-240B13.2	1.0146779	1.01516282	1.0144677	0.954156094	-0.0677028	1	1
CTD-2568P8.1	1.0146778	1.01516282	1.0144676	0.954150225	-0.0677117	1	1
LINC01268	1.0164224	1.01516282	1.0169684	1.119077144	0.1623095	1	1
LINC00463	1.0146844	1.01516282	1.014477	0.954768862	-0.0667766	1	1
FAM53B	1.1295369	1.12955063	1.1295309	0.999847696	-0.0002197	1	1
RP11-299J3.8	1.0993269	1.09798284	1.0999094	1.019662591	0.0280918	1	1
RXFP2	1.0146786	1.01516282	1.0144688	0.954227968	-0.0675941	1	1
CXCL13	1.014675	1.01516282	1.0144636	0.953884988	-0.0681128	1	1
GLTSCR1L	1.0759329	1.07511305	1.0762883	1.015646228	0.022398	1	1
RP11-408N14.1	1.0163912	1.01516282	1.0169236	1.116128245	0.1585028	1	1
RP11-572B2.1	1.015553	1.01516282	1.0157221	1.036884444	0.0522551	1	1
CTA-268H5.14	1.0146844	1.01516282	1.014477	0.954770715	-0.0667738	1	1
C22orf23	1.0527411	1.0523649	1.0529042	1.010299194	0.0147826	1	1
KEL	1.0146819	1.01516282	1.0144734	0.954534262	-0.0671311	1	1
CTD-3075F15.1	1.0146838	1.01516282	1.0144761	0.954712857	-0.0668612	1	1
PABPC3	1.0406609	1.04096562	1.0405288	0.989336424	-0.0154669	1	1
STH	1.0155399	1.01516282	1.0157033	1.035646693	0.0505319	1	1
RP11-202D1.2	1.0146747	1.01516282	1.0144631	0.953855276	-0.0681577	1	1
RP11-572M11.1	1.0146831	1.01516282	1.0144751	0.954644647	-0.0669643	1	1
TVP23C-CDRT4	1.0371451	1.03805775	1.0367495	0.965625141	-0.0504649	1	1
RP11-158K1.3	1.0224971	1.02086398	1.0232049	1.112199872	0.1534161	1	1
RP11-247L20.4	1.0172702	1.01802317	1.0169438	0.94011287	-0.0890941	1	1
POP1	1.1561287	1.15519326	1.1565342	1.008640547	0.0124121	1	1
RP11-526A4.1	1.0155532	1.01516282	1.0157223	1.036901579	0.052279	1	1
SVOP	1.0250697	1.0237312	1.0256498	1.080849078	0.1121651	1	1
CBLN2	1.1381389	1.13816907	1.1381259	0.999687241	-0.0004513	1	1
GSTM5	1.0146783	1.01516282	1.0144683	0.954194059	-0.0676454	1	1
CTD-3193O13.13	1.0155505	1.01516282	1.0157185	1.036648045	0.0519262	1	1
PAK6	1.0379927	1.03808662	1.0379519	0.996464193	-0.0051101	1	1
RP11-234K24.3	1.0146821	1.01516282	1.0144737	0.954552426	-0.0671037	1	1
RP11-616M22.11	1.0146827	1.01516282	1.0144746	0.954614656	-0.0670096	1	1
TMPRSS11B	1.0146826	1.01516282	1.0144745	0.95460238	-0.0670282	1	1
C1QTNF1-AS1	1.0155497	1.01516282	1.0157173	1.03657151	0.0518196	1	1
TBX18	1.0146794	1.01516282	1.0144699	0.954299906	-0.0674854	1	1
RP11-674P19.2	1.0164168	1.01516282	1.0169604	1.118551281	0.1616314	1	1
SULT4A1	1.0466427	1.04660708	1.0466582	1.001096839	0.0015815	1	1
SLC5A4	1.0189905	1.01800414	1.0194181	1.078535752	0.109074	1	1
ITFG1-AS1	1.0172552	1.01803162	1.0169186	0.938275548	-0.0919164	1	1
AC007365.3	1.0164215	1.01516282	1.016967	1.118988988	0.1621958	1	1
RP11-333I13.1	1.0172612	1.01800618	1.0169383	0.940695917	-0.0881997	1	1
CLIC5	1.0163997	1.01516282	1.0169359	1.116933709	0.1595436	1	1
FGD5	1.0135434	1.01466374	1.0130577	0.890477516	-0.1673489	1	1
RP11-115N4.1	1.0135434	1.01466374	1.0130577	0.890477516	-0.1673489	1	1
CALHM3	1.0144029	1.01466374	1.0142899	0.974506018	-0.037257	1	1
LINC01276	1.0161286	1.01466374	1.0167636	1.14320141	0.1930796	1	1
IFITM10	1.0255976	1.02611282	1.0253743	0.971718404	-0.0413898	1	1
SPANXA2-OT1	1.0144067	1.01466374	1.0142952	0.974870463	-0.0367176	1	1
NFE2	1.0221453	1.02320649	1.0216853	0.934450125	-0.0978104	1	1
RP11-304C12.5	1.0152661	1.01466374	1.0155272	1.058885898	0.0825471	1	1

RNF112	1.0230291	1.02321749	1.0229475	0.988369679	-0.0168773	1	1
RP11-141J13.5	1.015266	1.01466374	1.015527	1.058873569	0.0825303	1	1
RP11-574F21.3	1.0152596	1.01466374	1.0155179	1.05824736	0.0816769	1	1
RP5-1028K7.2	1.0144071	1.01466374	1.0142958	0.974911874	-0.0366563	1	1
GBP2	1.1171262	1.1174601	1.1169814	0.99592491	-0.0058911	1	1
LINC00858	1.0204458	1.02036083	1.0204827	1.005982929	0.0086058	1	1
RP11-385F5.4	1.0230209	1.0232416	1.0229252	0.986386259	-0.0197754	1	1
AICDA	1.0144102	1.01466374	1.0143003	0.975211961	-0.0362123	1	1
CTA-221G9.12	1.0187097	1.01751459	1.0192277	1.097808867	0.1346269	1	1
RP11-180P8.3	1.0161214	1.01466374	1.0167533	1.142496595	0.1921899	1	1
SH3RF3-AS1	1.0135434	1.01466374	1.0130577	0.890477516	-0.1673489	1	1
CST2	1.0152674	1.01466374	1.0155291	1.059016083	0.0827245	1	1
RP11-498C9.16	1.0143995	1.01466374	1.014285	0.974171564	-0.0377522	1	1
RP11-525G13.2	1.023969	1.02321231	1.024297	1.046727326	0.0658857	1	1
MOGAT3	1.0144086	1.01466374	1.0142981	0.975063885	-0.0364313	1	1
RP11-798K3.3	1.0135434	1.01466374	1.0130577	0.890477516	-0.1673489	1	1
AC093159.1	1.0144	1.01466374	1.0142857	0.974216792	-0.0376852	1	1
C2-AS1	1.0161632	1.01466374	1.0168131	1.146576704	0.1973329	1	1
ART3	1.0144005	1.01466374	1.0142864	0.974266247	-0.037612	1	1
RP11-212D19.4	1.0144051	1.01466374	1.014293	0.974716889	-0.0369449	1	1
RP11-335O13.7	1.0144041	1.01466374	1.0142916	0.974621696	-0.0370858	1	1
RP11-145M9.5	1.0144098	1.01466374	1.0142998	0.975178376	-0.036262	1	1
KL	1.0187465	1.01751032	1.0192824	1.101200609	0.1390773	1	1
FAM186B	1.0161272	1.01466374	1.0167616	1.14306267	0.1929045	1	1
AC142119.1	1.0152666	1.01466374	1.015528	1.058936277	0.0826158	1	1
VIPR1-AS1	1.0144105	1.01466374	1.0143008	0.975247364	-0.0361599	1	1
GABRR2	1.0135434	1.01466374	1.0130577	0.890477516	-0.1673489	1	1
TMC2	1.0144078	1.01466374	1.0142968	0.974978653	-0.0365575	1	1
UNC13C	1.0152617	1.01466374	1.0155209	1.058457798	0.0819637	1	1
RP11-36B15.1	1.0135434	1.01466374	1.0130577	0.890477516	-0.1673489	1	1
NAV2-AS2	1.0161606	1.01466374	1.0168095	1.14633013	0.1970226	1	1
AP003774.5	1.0161316	1.01466374	1.0167678	1.143489825	0.1934435	1	1
AC005237.4	1.0144005	1.01466374	1.0142864	0.974270451	-0.0376058	1	1
CMKLR1	1.0152573	1.01466374	1.0155145	1.058021503	0.0813689	1	1
AMER3	1.0161396	1.01466374	1.0167794	1.144276737	0.194436	1	1
FOLR1	1.0342308	1.03461516	1.0340642	0.984084234	-0.0231463	1	1
RNF185-AS1	1.0144016	1.01466374	1.014288	0.974373663	-0.037453	1	1
HOXC9	1.0135434	1.01466374	1.0130577	0.890477516	-0.1673489	1	1
RNASEH2B-AS1	1.0351667	1.03463879	1.0353955	1.021844836	0.0311761	1	1
EREG	1.0161181	1.01466374	1.0167485	1.142173923	0.1917824	1	1
RP3-508I15.22	1.0195796	1.02037319	1.0192356	0.944164712	-0.0828895	1	1
LINC01111	1.0152725	1.01466374	1.0155364	1.059510532	0.0833979	1	1
RP11-41O4.2	1.0135434	1.01466374	1.0130577	0.890477516	-0.1673489	1	1
DSG1-AS1	1.0144041	1.01466374	1.0142916	0.974621696	-0.0370858	1	1
SPG20-AS1	1.0169951	1.01753604	1.0167606	0.955782295	-0.0652461	1	1
RP4-647C14.2	1.0144012	1.01466374	1.0142875	0.974340486	-0.0375021	1	1
RP11-384C4.7	1.0135434	1.01466374	1.0130577	0.890477516	-0.1673489	1	1
PIK3R5	1.0144022	1.01466374	1.0142888	0.974432934	-0.0373652	1	1
RP11-91A18.4	1.0144016	1.01466374	1.014288	0.974373663	-0.037453	1	1
ZMYM4-AS1	1.0144029	1.01466374	1.0142898	0.974501803	-0.0372632	1	1
RP11-423H2.3	1.02736	1.02604162	1.0279314	1.072569066	0.1010705	1	1

LIN37	1.0566446	1.05745072	1.0562952	0.979887392	-0.0293121	1	1
FAM109B	1.0144041	1.01466374	1.0142916	0.974621696	-0.0370858	1	1
GPSM1	1.2190904	1.21766618	1.2197077	1.009379214	0.0134683	1	1
GPR68	1.0273689	1.02616933	1.0278889	1.065707506	0.0918115	1	1
LY6D	1.0144191	1.01466374	1.0143131	0.976089644	-0.0349144	1	1
AC131056.3	1.0144016	1.01466374	1.014288	0.974373663	-0.037453	1	1
HLX	1.0152739	1.01466374	1.0155384	1.059650255	0.0835882	1	1
RP11-130L8.2	1.0161135	1.01466374	1.0167419	1.141723775	0.1912137	1	1
STATH	1.0135434	1.01466374	1.0130577	0.890477516	-0.1673489	1	1
RP4-678D15.1	1.0161255	1.01466374	1.0167591	1.142891064	0.1926879	1	1
FCGBP	1.0152613	1.01466374	1.0155203	1.058414631	0.0819049	1	1
RP11-6O2.2	1.0144065	1.01466374	1.0142949	0.974849786	-0.0367482	1	1
AC083843.1	1.0558421	1.05464918	1.0563592	1.031290784	0.0444512	1	1
CCR1	1.0195741	1.02040045	1.0192159	0.941932935	-0.0863038	1	1
KB-1410C5.5	1.0152741	1.01466374	1.0155387	1.059665598	0.0836091	1	1
RTP4	1.0152716	1.01466374	1.0155351	1.059426147	0.083283	1	1
RP11-462L8.1	1.0204333	1.02037123	1.0204602	1.004366525	0.0062859	1	1
RGS9BP	1.0152643	1.01466374	1.0155246	1.058705994	0.082302	1	1
CTD-2270L9.4	1.1420931	1.14310435	1.1416548	0.989870804	-0.0146879	1	1
HACE1	1.1493584	1.14941477	1.1493339	0.999459062	-0.0007806	1	1
RP11-474G23.2	1.0144025	1.01466374	1.0142892	0.974461046	-0.0373236	1	1
GPAT2	1.0152579	1.01466374	1.0155154	1.058079761	0.0814484	1	1
CBWD3	1.2229384	1.22328113	1.2227899	0.997799732	-0.0031778	1	1
AGER	1.0585041	1.05757419	1.0589071	1.023151844	0.0330203	1	1
AC118754.4	1.0135434	1.01466374	1.0130577	0.890477516	-0.1673489	1	1
MGC15885	1.0144019	1.01466374	1.0142883	0.974399733	-0.0374144	1	1
HAGLROS	1.0161313	1.01466374	1.0167674	1.143457858	0.1934032	1	1
RP11-706P11.2	1.0161248	1.01466374	1.0167581	1.142827531	0.1926077	1	1
SATL1	1.0144017	1.01466374	1.0142881	0.974380829	-0.0374423	1	1
BDKRB1	1.0144037	1.01466374	1.0142909	0.974575805	-0.0371537	1	1
CTD-2334D19.1	1.0152761	1.01466374	1.0155415	1.059858633	0.0838718	1	1
RP11-613D13.5	1.016984	1.01750815	1.0167568	0.957086236	-0.0632792	1	1
RP11-334E6.10	1.0152633	1.01466374	1.0155232	1.058613036	0.0821753	1	1
C17orf107	1.0144052	1.01466374	1.0142931	0.974725341	-0.0369323	1	1
CTD-2545M3.2	1.0144017	1.01466374	1.0142881	0.974381636	-0.0374412	1	1
AC006076.1	1.0152728	1.01466374	1.0155368	1.059538169	0.0834356	1	1
RP11-45M22.5	1.0187137	1.01751515	1.0192332	1.098090311	0.1349967	1	1
DNAJC14	1.1748529	1.17472545	1.1749081	1.001045259	0.0015072	1	1
RTEL1-TNFRSF6B	1.014402	1.01466374	1.0142885	0.974410016	-0.0373991	1	1
RP3-508I15.18	1.0135434	1.01466374	1.0130577	0.890477516	-0.1673489	1	1
SSX5	1.0152638	1.01466374	1.0155239	1.058656725	0.0822349	1	1
CTB-60E11.9	1.0144053	1.01466374	1.0142933	0.974737575	-0.0369142	1	1
CTD-2014B16.3	1.0152591	1.01466374	1.0155172	1.058200322	0.0816128	1	1
SLC4A11	1.0152596	1.01466374	1.0155179	1.058247553	0.0816772	1	1
RP11-787I22.3	1.0239167	1.02325162	1.024205	1.041003168	0.0579745	1	1
CTD-2297D10.2	1.0144115	1.01466374	1.0143022	0.975347073	-0.0360124	1	1
LCN9	1.0204439	1.02037076	1.0204756	1.005148232	0.0074083	1	1
RP11-264E20.1	1.0144061	1.01466374	1.0142944	0.974810318	-0.0368066	1	1
CTB-178M22.1	1.0135434	1.01466374	1.0130577	0.890477516	-0.1673489	1	1
RP11-90B9.3	1.0161653	1.01466374	1.0168161	1.14678199	0.1975912	1	1
RP1-228P16.8	1.0152672	1.01466374	1.0155287	1.058987861	0.0826861	1	1

RP11-484D2.2	1.0144049	1.01466374	1.0142928	0.974702344	-0.0369664		1	1
AMACR	1.0618168	1.0604224	1.0624212	1.033080074	0.0469521		1	1
AF064860.7	1.0144013	1.01466374	1.0142876	0.9743489	-0.0374896		1	1
RP4-669H2.1	1.0152726	1.01466374	1.0155365	1.059521208	0.0834125		1	1
RP11-5O24.1	1.0213184	1.02036385	1.0217321	1.067190415	0.0938176		1	1
LA16c-60D12.1	1.0144006	1.01466374	1.0142866	0.974278414	-0.037594		1	1
CD84	1.0135434	1.01466374	1.0130577	0.890477516	-0.1673489		1	1
RP11-452C8.1	1.0161304	1.01466374	1.0167661	1.143374734	0.1932983		1	1
LA16c-349E10.1	1.0248037	1.02319334	1.0255017	1.09952528	0.1368808		1	1
PLCE1	1.2214212	1.220188	1.2219557	1.008028031	0.0115358		1	1
RP11-521D12.5	1.0169824	1.01751032	1.0167535	0.956779271	-0.063742		1	1
FSTL4	1.0273599	1.02607371	1.0279174	1.070708872	0.0985663		1	1
CDHR5	1.0152644	1.01466374	1.0155248	1.058720285	0.0823215		1	1
AF127577.8	1.0144054	1.01466374	1.0142934	0.974747899	-0.036899		1	1
MICALCL	1.0152608	1.01466374	1.0155196	1.058362885	0.0818344		1	1
TNFSF14	1.0135434	1.01466374	1.0130577	0.890477516	-0.1673489		1	1
TCF4-AS2	1.0152709	1.01466374	1.0155341	1.05935192	0.0831819		1	1
CYP51A1-AS1	1.0169866	1.01750444	1.0167621	0.957591975	-0.062517		1	1
RP11-159H22.2	1.0135434	1.01466374	1.0130577	0.890477516	-0.1673489		1	1
RP11-342M21.2	1.0144033	1.01466374	1.0142904	0.974542992	-0.0372023		1	1
PRPF3	1.223426	1.22298813	1.2236158	1.002814836	0.0040552		1	1
HOXA1	1.0152637	1.01466374	1.0155238	1.058650198	0.082226		1	1
ILDR1	1.0135434	1.01466374	1.0130577	0.890477516	-0.1673489		1	1
WNT11	1.0152637	1.01466374	1.0155238	1.058650198	0.082226		1	1
RP11-1143G9.4	1.0299488	1.02889287	1.0304065	1.052389037	0.0736681		1	1
SGCZ	1.018797	1.0175212	1.01935	1.104377646	0.1432336		1	1
POTEI	1.0593202	1.06034191	1.0588773	0.975727768	-0.0354494		1	1
RP11-492E3.2	1.0144021	1.01466374	1.0142886	0.974419	-0.0373858		1	1
RP11-23P13.6	1.0144014	1.01466374	1.0142877	0.974353107	-0.0374834		1	1
RP1-142L7.9	1.0144006	1.01466374	1.0142866	0.974278414	-0.037594		1	1
RP5-1113E3.3	1.0169909	1.01749375	1.0167729	0.958795872	-0.0607044		1	1
FBXW12	1.0221514	1.02324566	1.0216771	0.932522046	-0.1007903		1	1
SPATA3-AS1	1.014399	1.01466374	1.0142842	0.974117968	-0.0378316		1	1
ZNF787	1.0833839	1.08307583	1.0835174	1.005315662	0.0076486		1	1
RP11-890B15.2	1.0204515	1.0204288	1.0204614	1.001594279	0.0022982		1	1
RP11-1079K10.4	1.0178445	1.01750627	1.0179911	1.027695136	0.0394124		1	1
PRB3	1.0143991	1.01466374	1.0142843	0.97412636	-0.0378192		1	1
RP11-122A21.2	1.0144086	1.01466374	1.0142981	0.975063885	-0.0364313		1	1
LINC01307	1.020445	1.0203881	1.0204697	1.004003061	0.0057637		1	1
RP11-535M15.1	1.0144018	1.01466374	1.0142883	0.974394224	-0.0374225		1	1
ELOVL2-AS1	1.0152738	1.01466374	1.0155382	1.059633721	0.0835657		1	1
AC128709.3	1.0144008	1.01466374	1.0142868	0.974295191	-0.0375692		1	1
AC096669.1	1.0135434	1.01466374	1.0130577	0.890477516	-0.1673489		1	1
RP1-144F13.3	1.0152624	1.01466374	1.0155219	1.058521596	0.0820507		1	1
ZNF449	1.089576	1.08924358	1.0897201	1.005339936	0.0076834		1	1
LTB	1.080093	1.08091408	1.0797371	0.985453654	-0.0211401		1	1
RP11-287D1.3	1.0152599	1.01466374	1.0155183	1.058280172	0.0817216		1	1
MIR133A1HG	1.0195742	1.02037052	1.0192291	0.943967171	-0.0831914		1	1
CTB-25B13.5	1.037746	1.03767883	1.0377752	1.002557006	0.0036843		1	1
PCSK1	1.0152585	1.01466374	1.0155163	1.058141981	0.0815332		1	1
RP11-148B18.1	1.0186922	1.01750709	1.0192059	1.097036588	0.1336116		1	1

RP11-493L12.3	1.0144025	1.01466374	1.0142892	0.974458822	-0.0373269	1	1
FCMR	1.0143987	1.01466374	1.0142838	0.974089086	-0.0378744	1	1
RP11-409I10.2	1.0144018	1.01466374	1.0142883	0.974394224	-0.0374225	1	1
SLC38A11	1.0135434	1.01466374	1.0130577	0.890477516	-0.1673489	1	1
RP11-360L9.8	1.0143991	1.01466374	1.0142843	0.97412636	-0.0378192	1	1
RP11-357N13.3	1.0152761	1.01466374	1.0155415	1.059858633	0.0838718	1	1
AC002429.5	1.0204473	1.02039008	1.0204721	1.004023248	0.0057927	1	1
C4orf32	1.0187152	1.01750627	1.0192392	1.098986638	0.1361738	1	1
AC005009.2	1.0126491	1.01281342	1.0125779	0.981621467	-0.0267613	1	1
RP11-996F15.4	1.0135056	1.01281342	1.0138056	1.077431086	0.1075956	1	1
RP11-294N21.3	1.0169561	1.01565812	1.0175188	1.11882896	0.1619895	1	1
IL12A	1.0126451	1.01281342	1.0125721	0.981165704	-0.0274313	1	1
CNTD2	1.0221136	1.02136502	1.0224381	1.050224735	0.0706981	1	1
NELFA	1.4074424	1.40656439	1.4078229	1.003095581	0.0044591	1	1
ZNF750	1.0160875	1.01566119	1.0162724	1.039023967	0.0552289	1	1
RP3-380B8.4	1.0160755	1.0156614	1.016255	1.037900321	0.0536679	1	1
MSC	1.0135031	1.01281342	1.0138021	1.077157568	0.1072293	1	1
RP11-11N7.4	1.0160784	1.01564421	1.0162665	1.039780131	0.0562785	1	1
RP11-416N4.4	1.0152365	1.0156537	1.0150557	0.961799724	-0.0561916	1	1
TNFSF15	1.0135027	1.01281342	1.0138015	1.077114991	0.1071723	1	1
RP11-496H1.2	1.0195366	1.01852075	1.0199769	1.078622734	0.1091903	1	1
AC073046.25	1.0126443	1.01281342	1.012571	0.981080896	-0.027556	1	1
AF131216.6	1.0126447	1.01281342	1.0125715	0.98112306	-0.027494	1	1
CCR4	1.01436	1.01281342	1.0150303	1.173012964	0.230219	1	1
RP11-259O2.1	1.0135153	1.01281342	1.0138195	1.078520812	0.109054	1	1
XXbac-BPG252P9.10	1.1556861	1.1554702	1.1557796	1.001990378	0.0028687	1	1
AC007364.1	1.0117887	1.01281342	1.0113445	0.885362368	-0.17566	1	1
TM4SF19	1.0135011	1.01281342	1.0137992	1.076933369	0.106929	1	1
CLMN	1.0669591	1.06716079	1.0668716	0.995694432	-0.006225	1	1
RP5-893G23.1	1.0117887	1.01281342	1.0113445	0.885362368	-0.17566	1	1
RP11-367G6.3	1.0117887	1.01281342	1.0113445	0.885362368	-0.17566	1	1
KCNG4	1.0126443	1.01281342	1.012571	0.981080896	-0.027556	1	1
AQP6	1.0178351	1.01855524	1.017523	0.944369218	-0.0825771	1	1
RP5-994D16.3	1.0212852	1.02140081	1.0212351	0.992254655	-0.0112177	1	1
CLEC1A	1.0126496	1.01281342	1.0125786	0.981671347	-0.026688	1	1
C19orf54	1.2022967	1.2011144	1.2028091	1.008426693	0.0121062	1	1
INAFM2	1.0583567	1.05844782	1.0583172	0.997765932	-0.0032267	1	1
HSPC324	1.0126443	1.01281342	1.012571	0.981080896	-0.027556	1	1
PPP2R2C	1.0135043	1.01281342	1.0138038	1.077295159	0.1074136	1	1
ADAM32	1.0177985	1.01850681	1.0174914	0.945135155	-0.0814074	1	1
E4F1	1.156597	1.15551131	1.1570675	1.010007237	0.0143656	1	1
SP9	1.0126456	1.01281342	1.0125728	0.981222284	-0.0273481	1	1
DEFB135	1.0126546	1.01281342	1.0125857	0.982229133	-0.0258685	1	1
SRRM5	1.0161299	1.01565315	1.0163366	1.043662809	0.0616557	1	1
AC098823.3	1.0117887	1.01281342	1.0113445	0.885362368	-0.17566	1	1
EYA1	1.0212602	1.0213358	1.0212275	0.994923032	-0.0073432	1	1
LBHD1	1.015235	1.01567558	1.0150441	0.959712716	-0.0593255	1	1
STX16-NPEPL1	1.0195429	1.01848755	1.0200003	1.081825911	0.1134684	1	1
EPHA1-AS1	1.0376211	1.03846339	1.037256	0.968610492	-0.0460115	1	1
KERA	1.0126531	1.01281342	1.0125836	0.982065867	-0.0261083	1	1
RP11-321L2.1	1.0117887	1.01281342	1.0113445	0.885362368	-0.17566	1	1

RP11-82L20.1	1.0126485	1.01281342	1.0125771	0.981555508	-0.0268582	1	1
RP11-879F14.1	1.0152448	1.01564547	1.0150711	0.963290279	-0.0539575	1	1
HIST1H2AD	1.0126468	1.01281342	1.0125746	0.981358604	-0.0271477	1	1
RP11-436D10.3	1.0126477	1.01281342	1.0125758	0.981458581	-0.0270007	1	1
RP11-757A13.1	1.0126573	1.01281342	1.0125896	0.982532944	-0.0254223	1	1
RP11-770J1.5	1.0160796	1.0156705	1.0162569	1.037421093	0.0530016	1	1
P4HA3	1.0135002	1.01281342	1.0137979	1.076832488	0.1067938	1	1
CTC-1337H24.4	1.0152333	1.01566218	1.0150475	0.960752061	-0.0577639	1	1
RP3-460G2.2	1.0126494	1.01281342	1.0125783	0.981652417	-0.0267158	1	1
RP11-864I4.4	1.0126466	1.01281342	1.0125743	0.98134036	-0.0271745	1	1
RP11-46C24.3	1.0126441	1.01281342	1.0125707	0.981057443	-0.0275905	1	1
NHLH2	1.0875724	1.08685246	1.0878845	1.01188271	0.0170421	1	1
RP11-459O1.2	1.012646	1.01281342	1.0125735	0.981273443	-0.0272729	1	1
CCIN	1.0117887	1.01281342	1.0113445	0.885362368	-0.17566	1	1
APBB1IP	1.0135045	1.01281342	1.0138041	1.077315075	0.1074402	1	1
LY6K	1.0126443	1.01281342	1.012571	0.981080896	-0.027556	1	1
ATP13A5	1.0135198	1.01281342	1.013826	1.079026	0.1097296	1	1
TH2LCRR	1.0126469	1.01281342	1.0125747	0.981373005	-0.0271265	1	1
CD34	1.01439	1.01281342	1.0150734	1.176377946	0.2343516	1	1
RP11-109D20.2	1.0126454	1.01281342	1.0125726	0.981207905	-0.0273692	1	1
HPCAL4	1.0195185	1.01848778	1.0199653	1.079920286	0.1109248	1	1
AC140912.1	1.015235	1.015653	1.0150538	0.961720313	-0.0563107	1	1
AC106786.1	1.0143601	1.01281342	1.0150305	1.173027845	0.2302373	1	1
HIST4H4	1.0273115	1.02706682	1.0274175	1.01295682	0.0185727	1	1
RP11-295P9.12	1.0126505	1.01281342	1.0125799	0.981778116	-0.0265311	1	1
RP11-438L19.1	1.0256016	1.02418072	1.0262175	1.0842327	0.1166744	1	1
RP11-400K9.4	1.0126454	1.01281342	1.0125726	0.981207905	-0.0273692	1	1
HOXA-AS2	1.016968	1.01564923	1.0175396	1.12079899	0.1645276	1	1
U91328.22	1.0135028	1.01281342	1.0138016	1.077119614	0.1071785	1	1
RP11-681H18.2	1.0126478	1.01281342	1.012576	0.981471382	-0.0269819	1	1
AC005740.6	1.015232	1.01565343	1.0150494	0.961410574	-0.0567754	1	1
RP11-701H16.4	1.012648	1.01281342	1.0125762	0.981489469	-0.0269553	1	1
PRODH	1.0143719	1.01281342	1.0150474	1.174348208	0.2318602	1	1
ZNF341-AS1	1.0126477	1.01281342	1.0125759	0.981463401	-0.0269936	1	1
RP5-1009E24.8	1.0143612	1.01281342	1.015032	1.173148156	0.2303852	1	1
CTD-2521M24.6	1.0135043	1.01281342	1.0138037	1.077285262	0.1074003	1	1
KLF8	1.0126514	1.01281342	1.0125812	0.981873224	-0.0263913	1	1
CASS4	1.0117887	1.01281342	1.0113445	0.885362368	-0.17566	1	1
TBXA2R	1.0135078	1.01281342	1.0138088	1.077680468	0.1079295	1	1
RP11-131L12.3	1.0255624	1.0242239	1.0261426	1.079207602	0.1099724	1	1
RP11-348H3.8	1.0117887	1.01281342	1.0113445	0.885362368	-0.17566	1	1
RGPD1	1.0135196	1.01281342	1.0138258	1.079006571	0.1097037	1	1
AC005609.18	1.0143802	1.01281342	1.0150593	1.175278206	0.2330023	1	1
RP11-375A5.1	1.0126445	1.01281342	1.0125713	0.981104354	-0.0275215	1	1
BCL2L10	1.0117887	1.01281342	1.0113445	0.885362368	-0.17566	1	1
FAM24B	1.0117887	1.01281342	1.0113445	0.885362368	-0.17566	1	1
TSPAN18	1.2451714	1.24377786	1.2457754	1.008194174	0.0117735	1	1
PKD1L3	1.0143624	1.01281342	1.0150338	1.17328613	0.2305549	1	1
ERVV-1	1.0126445	1.01281342	1.0125713	0.981104354	-0.0275215	1	1
EML5	1.0169959	1.01566077	1.0175747	1.122210536	0.1663434	1	1
RP11-434E6.4	1.0143607	1.01281342	1.0150314	1.173100985	0.2303272	1	1

ATG4D	1.3892635	1.38944781	1.3891837	0.999321743	-0.0009788	1	1
MYZAP	1.0126454	1.01281342	1.0125726	0.981203605	-0.0273756	1	1
GTSF1	1.0126494	1.01281342	1.0125783	0.981647704	-0.0267227	1	1
SIGLEC10	1.0152284	1.01566357	1.0150398	0.96017938	-0.0586241	1	1
RP11-164P12.4	1.0548387	1.05560391	1.054507	0.980273003	-0.0287445	1	1
CTD-2562J17.7	1.0126445	1.01281342	1.0125712	0.981097426	-0.0275317	1	1
RP11-497G19.2	1.0126482	1.01281342	1.0125766	0.981520158	-0.0269102	1	1
RP11-370F5.4	1.0117887	1.01281342	1.0113445	0.885362368	-0.17566	1	1
RP11-6N17.10	1.0135186	1.01281342	1.0138243	1.078892275	0.1095508	1	1
PIGO	1.1031454	1.10414099	1.1027138	0.986295561	-0.0199081	1	1
FRMPD4	1.0126475	1.01281342	1.0125755	0.981435036	-0.0270353	1	1
RP11-173P15.3	1.0126458	1.01281342	1.0125731	0.981247034	-0.0273117	1	1
CTD-2589M5.4	1.0126454	1.01281342	1.0125726	0.981207905	-0.0273692	1	1
RP11-144G7.2	1.0126454	1.01281342	1.0125726	0.981203605	-0.0273756	1	1
ADGRG7	1.0117887	1.01281342	1.0113445	0.885362368	-0.17566	1	1
CTD-2515C13.2	1.0126501	1.01281342	1.0125793	0.981730875	-0.0266005	1	1
PP12613	1.0385625	1.03845751	1.038608	1.003914221	0.005636	1	1
HIST1H3J	1.0126462	1.01281342	1.0125737	0.981292826	-0.0272444	1	1
LINC00881	1.0143726	1.01281342	1.0150484	1.174426991	0.231957	1	1
HLA-DQB2	1.0126483	1.01281342	1.0125768	0.98153194	-0.0268929	1	1
RP11-131L23.2	1.0126494	1.01281342	1.0125783	0.981652417	-0.0267158	1	1
LINC01343	1.0135153	1.01281342	1.0138195	1.078520812	0.109054	1	1
AC003986.7	1.0126531	1.01281342	1.0125836	0.982065867	-0.0261083	1	1
PLEK2	1.0135118	1.01281342	1.0138146	1.078133269	0.1085355	1	1
RP11-845M18.6	1.013508	1.01281342	1.0138091	1.077704082	0.1079611	1	1
RP11-494O16.4	1.0135207	1.01281342	1.0138272	1.079120936	0.1098566	1	1
RP11-815J21.2	1.0126454	1.01281342	1.0125725	0.981198797	-0.0273826	1	1
S100Z	1.0135043	1.01281342	1.0138038	1.077290072	0.1074068	1	1
HLA-DOB	1.0126462	1.01281342	1.0125737	0.981292826	-0.0272444	1	1
RP11-338N10.2	1.0143594	1.01281342	1.0150295	1.172947106	0.230138	1	1
MMP17	1.0212533	1.02137698	1.0211997	0.991708194	-0.0120124	1	1
FABP9	1.0126451	1.01281342	1.0125721	0.98117051	-0.0274242	1	1
STAMBPL1	1.0832863	1.08410615	1.082931	0.986027281	-0.0203005	1	1
NPHP3-ACAD11	1.0117887	1.01281342	1.0113445	0.885362368	-0.17566	1	1
LDLRAD1	1.0195606	1.01852539	1.0200093	1.080101342	0.1111667	1	1
RP11-815J21.4	1.0135117	1.01281342	1.0138144	1.078122998	0.1085218	1	1
KLRF2	1.0126531	1.01281342	1.0125836	0.982065219	-0.0261093	1	1
MRM3	1.1452155	1.14386425	1.1458012	1.01346401	0.0192949	1	1
XACT	1.0126504	1.01281342	1.0125798	0.981765657	-0.0265494	1	1
AC003991.3	1.019564	1.01851213	1.0200199	1.081448768	0.1129653	1	1
FOXO4	1.0445054	1.04423695	1.0446218	1.008699261	0.0124961	1	1
RP11-33A14.1	1.0126454	1.01281342	1.0125726	0.981203605	-0.0273756	1	1
RP11-15K19.2	1.0135122	1.01281342	1.013815	1.078168946	0.1085833	1	1
PLCXD3	1.0135161	1.01281342	1.0138207	1.078608108	0.1091708	1	1
ASCL5	1.0152205	1.01566042	1.0150298	0.959732877	-0.0592952	1	1
TEX38	1.0126451	1.01281342	1.0125721	0.98117051	-0.0274242	1	1
RP11-445K13.2	1.0126451	1.01281342	1.0125721	0.98117051	-0.0274242	1	1
DDX4	1.0135175	1.01281342	1.0138227	1.078768069	0.1093847	1	1
RP11-805J14.5	1.014373	1.01281342	1.015049	1.174469082	0.2320087	1	1
XXyac-YX65C7_A.3	1.0126531	1.01281342	1.0125836	0.982065867	-0.0261083	1	1
CLRN3	1.0135025	1.01281342	1.0138011	1.077084943	0.107132	1	1

PLA2G4D	1.0126465	1.01281342	1.0125742	0.981328601	-0.0271918	1	1
NETO1	1.0126478	1.01281342	1.012576	0.981471382	-0.0269819	1	1
RP11-6I2.3	1.0126474	1.01281342	1.0125754	0.981425398	-0.0270495	1	1
HOXA10	1.0126504	1.01281342	1.0125798	0.981765657	-0.0265494	1	1
RP11-292D4.3	1.0126456	1.01281342	1.0125728	0.981222284	-0.0273481	1	1
RP11-431J17.1	1.0126471	1.01281342	1.0125751	0.981397043	-0.0270912	1	1
MIR29A	1.0126447	1.01281342	1.0125715	0.98112306	-0.027494	1	1
CTD-2184D3.5	1.0117887	1.01281342	1.0113445	0.885362368	-0.17566	1	1
RP11-171I2.2	1.016955	1.01566042	1.0175162	1.118500934	0.1615665	1	1
TNFRSF17	1.0143807	1.01281342	1.01506	1.175330385	0.2330664	1	1
WFDC10B	1.0117887	1.01281342	1.0113445	0.885362368	-0.17566	1	1
PRSS50	1.0317191	1.03292371	1.031197	0.947552946	-0.0777215	1	1
RP5-942I16.1	1.0221579	1.02136454	1.0225018	1.053229323	0.0748196	1	1
RP11-307L14.1	1.0161057	1.01565916	1.0162993	1.040878835	0.0578021	1	1
RP4-585I14.3	1.0135244	1.01281342	1.0138326	1.079536819	0.1104124	1	1
HEPHL1	1.017115	1.01804205	1.0167132	0.926348505	-0.110373	1	1
RP11-684B21.1	1.0179856	1.01804205	1.0179611	0.995511949	-0.0064895	1	1
RP11-511B23.1	1.0188409	1.01804205	1.0191871	1.063465517	0.0887733	1	1
DEFB134	1.018842	1.01804205	1.0191887	1.063553575	0.0888927	1	1
SLC26A4-AS1	1.0205734	1.02088122	1.0204399	0.978866644	-0.0308158	1	1
LINC01351	1.0171254	1.01804205	1.0167281	0.927170447	-0.1090935	1	1
LINC01289	1.0188462	1.01804205	1.0191948	1.063893547	0.0893538	1	1
RP11-415J8.7	1.0171173	1.01804205	1.0167165	0.926528016	-0.1100935	1	1
PEX26	1.2921159	1.29322825	1.2916338	0.994562313	-0.0078663	1	1
RP11-930O11.3	1.0214662	1.02091354	1.0217057	1.037877164	0.0536357	1	1
RP11-203B9.4	1.0171156	1.01804205	1.016714	0.926393356	-0.1103032	1	1
RP11-426A6.5	1.0171137	1.01804205	1.0167113	0.926244147	-0.1105356	1	1
AF131215.6	1.0188768	1.01804205	1.0192386	1.066319934	0.0926404	1	1
RP11-1C8.5	1.0171188	1.01804205	1.0167186	0.926644273	-0.1099125	1	1
SLC5A11	1.0171188	1.01804205	1.0167186	0.926644273	-0.1099125	1	1
RP11-709D24.5	1.0188613	1.01804205	1.0192164	1.065087598	0.0909721	1	1
TCEAL3-AS1	1.0240632	1.02373719	1.0242045	1.019686436	0.0281256	1	1
ICAM2	1.0179717	1.01804205	1.0179412	0.994411064	-0.0080857	1	1
OAZ3	1.0430192	1.04380317	1.0426794	0.974345788	-0.0374942	1	1
GRM2	1.02495	1.02379598	1.0254502	1.06951654	0.0969588	1	1
AC098617.1	1.0171178	1.01804205	1.0167172	0.926566878	-0.110033	1	1
TMEM171	1.0171118	1.01804205	1.0167086	0.926093724	-0.1107699	1	1
RP11-863K10.7	1.0179966	1.01804205	1.0179769	0.996391465	-0.0052154	1	1
WNT7A	1.0275656	1.02667497	1.0279516	1.047859284	0.067445	1	1
CTD-3214H19.6	1.0179781	1.01804205	1.0179504	0.994920036	-0.0073475	1	1
RP11-7O11.3	1.0197295	1.02092125	1.0192129	0.918344059	-0.1228933	1	1
AC008440.5	1.0171142	1.01804205	1.016712	0.926277886	-0.110483	1	1
LUADT1	1.0206288	1.02090648	1.0205084	0.980959808	-0.0277341	1	1
TAS2R43	1.0171194	1.01804205	1.0167194	0.926691235	-0.1098394	1	1
FAM129C	1.0171243	1.01804205	1.0167264	0.927080554	-0.1092334	1	1
RP11-50B3.2	1.0171164	1.01804205	1.0167151	0.926454178	-0.1102085	1	1
USP26	1.01799	1.01804205	1.0179675	0.99586668	-0.0059755	1	1
RP11-806L2.2	1.0258027	1.0266478	1.0254364	0.954540533	-0.0671216	1	1
ZCCHC8	1.2197226	1.21889847	1.2200798	1.005396529	0.0077646	1	1
CEP295NL	1.0214683	1.02091735	1.0217071	1.037756016	0.0534673	1	1
RP11-326C3.12	1.0257426	1.02666297	1.0253437	0.95052042	-0.0732105	1	1

RP11-645C24.5	1.0171178	1.01804205	1.0167172	0.926566878	-0.110033	1	1
RAD21-AS1	1.0240505	1.02378897	1.0241639	1.015759278	0.0225585	1	1
RP11-30K9.6	1.0379271	1.03816494	1.037824	0.991067051	-0.0129454	1	1
CLYBL-AS1	1.0171185	1.01804205	1.0167182	0.926622175	-0.1099469	1	1
RP11-213H15.1	1.0171155	1.01804205	1.0167139	0.926384996	-0.1103162	1	1
RP11-252A24.3	1.0266494	1.02660302	1.0266695	1.002500837	0.0036034	1	1
NR0B1	1.0992324	1.09824571	1.0996601	1.014395981	0.0206209	1	1
RP11-173P15.9	1.0171175	1.01804205	1.0167167	0.926539824	-0.1100751	1	1
SLC26A7	1.0534952	1.05238572	1.0539762	1.030360064	0.0431486	1	1
RP11-1006G14.2	1.0179856	1.01804205	1.0179612	0.995516628	-0.0064827	1	1
RP11-497D6.3	1.0179816	1.01804205	1.0179554	0.995196107	-0.0069473	1	1
AC114730.7	1.0171194	1.01804205	1.0167194	0.926691235	-0.1098394	1	1
RP5-1021I20.5	1.0240729	1.02380599	1.0241887	1.016074463	0.0230061	1	1
RP11-154D17.1	1.0171126	1.01804205	1.0167098	0.92615733	-0.1106708	1	1
GPC5	1.0188292	1.01804205	1.0191703	1.062535956	0.0875117	1	1
RP11-94B19.3	1.0214524	1.02090809	1.0216883	1.037316226	0.0528558	1	1
KCNS2	1.0171175	1.01804205	1.0167167	0.926539824	-0.1100751	1	1
PLN	1.0171179	1.01804205	1.0167173	0.926573379	-0.1100229	1	1
RP11-399E6.1	1.0171191	1.01804205	1.016719	0.926667931	-0.1098756	1	1
PRR5-ARHGAP8	1.0171139	1.01804205	1.0167115	0.926254362	-0.1105197	1	1
GRP	1.0310054	1.02945356	1.0316781	1.075525871	0.1050422	1	1
RP11-712B9.2	1.026635	1.02663432	1.0266353	1.000038234	5.52E-05	1	1
LINC01527	1.0171152	1.01804205	1.0167135	0.926361796	-0.1103523	1	1
PPFIA2	1.0854686	1.08387155	1.0861608	1.027295016	0.0388506	1	1
TLL1	1.0438996	1.04387183	1.0439116	1.000906636	0.0013074	1	1
RP11-838N2.3	1.0171135	1.01804205	1.0167109	0.92622205	-0.11057	1	1
ZCWPW2	1.0396503	1.03812244	1.0403126	1.057450657	0.0805903	1	1
RP11-996F15.5	1.0291928	1.02947828	1.029069	0.98611729	-0.0201688	1	1
UBASH3A	1.0179947	1.01804205	1.0179742	0.996239975	-0.0054348	1	1
LINC01447	1.0171142	1.01804205	1.016712	0.926277886	-0.110483	1	1
LINC01625	1.0171169	1.01804205	1.0167159	0.926494515	-0.1101457	1	1
CYP4F8	1.017986	1.01804205	1.0179618	0.995550591	-0.0064335	1	1
RP11-1078H9.5	1.01712	1.01804205	1.0167204	0.92674498	-0.1097557	1	1
LINC01629	1.0179805	1.01804205	1.0179538	0.995107516	-0.0070757	1	1
NLRP8	1.0171261	1.01804205	1.016729	0.927224213	-0.1090099	1	1
GS1-166A23.1	1.0171183	1.01804205	1.0167179	0.926609238	-0.109967	1	1
FCAR	1.0179796	1.01804205	1.0179525	0.995038265	-0.0071761	1	1
RASAL1	1.0188397	1.01804205	1.0191854	1.063370675	0.0886446	1	1
RP11-356C4.5	1.0171166	1.01804205	1.0167155	0.926475402	-0.1101754	1	1
IL18	1.0205686	1.02088581	1.0204311	0.978227395	-0.0317582	1	1
RP11-168O16.1	1.0370011	1.0380528	1.0365453	0.960383246	-0.0583179	1	1
AC000403.4	1.0171161	1.01804205	1.0167147	0.926428717	-0.1102481	1	1
RP11-255P5.2	1.0171226	1.01804205	1.0167241	0.926949784	-0.1094369	1	1
RP11-419I17.2	1.0197006	1.02090619	1.019178	0.917335175	-0.1244791	1	1
LZTS1-AS1	1.0179895	1.01804205	1.0179667	0.995826033	-0.0060344	1	1
CCDC87	1.0179792	1.01804205	1.017952	0.995007926	-0.0072201	1	1
LINC01350	1.017113	1.01804205	1.0167103	0.926185742	-0.1106265	1	1
RP11-262A16.1	1.0188401	1.01804205	1.019186	1.063406444	0.0886931	1	1
RP5-901A4.1	1.0197056	1.02090619	1.0191852	0.917680376	-0.1239363	1	1
BEND5	1.508907	1.50826706	1.5091844	1.001804781	0.0026014	1	1
CTB-129P6.11	1.0171161	1.01804205	1.0167147	0.926432142	-0.1102428	1	1

SPIB	1.0179725	1.01804205	1.0179423	0.994471223	-0.0079985	1	1
USP30-AS1	1.0171169	1.01804205	1.0167159	0.92649946	-0.110138	1	1
RP11-802E16.3	1.0309347	1.02954657	1.0315364	1.067345954	0.0940279	1	1
KLB	1.0171175	1.01804205	1.0167167	0.926543254	-0.1100698	1	1
CTD-3224K15.2	1.0171165	1.01804205	1.0167154	0.926467031	-0.1101885	1	1
TCF24	1.0171142	1.01804205	1.016712	0.926277886	-0.110483	1	1
C22orf42	1.0171162	1.01804205	1.0167149	0.926442385	-0.1102268	1	1
CST11	1.0179715	1.01804205	1.0179409	0.994394142	-0.0081103	1	1
MTUS2	1.0231442	1.02374568	1.0228835	0.963689675	-0.0533594	1	1
AC016738.4	1.0188367	1.01804205	1.0191812	1.063139287	0.0883306	1	1
LINC01396	1.0179748	1.01804205	1.0179457	0.994659484	-0.0077254	1	1
RP11-835E18.5	1.0179966	1.01804205	1.0179769	0.996391465	-0.0052154	1	1
RP11-298E9.5	1.0179772	1.01804205	1.017949	0.994844547	-0.007457	1	1
THRIL	1.0188517	1.01804205	1.0192027	1.064327909	0.0899427	1	1
RP11-806K15.1	1.0171194	1.01804205	1.0167194	0.926691235	-0.1098394	1	1
PNMA2	1.1467354	1.14688434	1.1466708	0.998546361	-0.0020987	1	1
DPP6	1.0231695	1.02374163	1.0229216	0.965458549	-0.0507138	1	1
RP5-1057I20.5	1.0171246	1.01804205	1.0167269	0.927105893	-0.109194	1	1
HULC	1.0240369	1.02376146	1.0241563	1.016617868	0.0237775	1	1
CLCNKB	1.0283501	1.02945182	1.0278726	0.94638038	-0.0795079	1	1
CYP1A1	1.0179836	1.01804205	1.0179582	0.995352817	-0.0067201	1	1
BCL6B	1.0171298	1.01804205	1.0167343	0.927517675	-0.1085533	1	1
PCDHA1	1.0205895	1.02088765	1.0204602	0.979537477	-0.0298274	1	1
AC099342.1	1.0171137	1.01804205	1.0167113	0.926244147	-0.1105356	1	1
RP11-157E16.1	1.0188586	1.01804205	1.0192125	1.0648726	0.0906808	1	1
CTD-2350C19.2	1.0656966	1.06690426	1.0651731	0.974124509	-0.0378219	1	1
RP11-78O7.3	1.0197175	1.02091551	1.0191982	0.917894715	-0.1235994	1	1
NRP1	1.1287196	1.1297517	1.1282722	0.988597244	-0.0165452	1	1
SCARA5	1.017993	1.01804205	1.0179717	0.99610077	-0.0056364	1	1
RP11-142C4.6	1.0231769	1.02378897	1.0229115	0.963115889	-0.0542187	1	1
MS4A2	1.0214939	1.02091969	1.0217427	1.039343514	0.0556726	1	1
P4HA2-AS1	1.0171211	1.01804205	1.0167218	0.926826294	-0.1096291	1	1
RP11-433J8.1	1.0171154	1.01804205	1.0167137	0.926373576	-0.110334	1	1
RP13-516M14.10	1.0179823	1.01804205	1.0179564	0.995252959	-0.0068648	1	1
SLC5A2	1.0362137	1.03517284	1.0366649	1.042419482	0.059936	1	1
FAM185A	1.154764	1.15583004	1.1543019	0.990193574	-0.0142175	1	1
RP11-290H9.5	1.0188355	1.01804205	1.0191794	1.06303654	0.0881912	1	1
RP11-248M19.1	1.017979	1.01804205	1.0179517	0.994990883	-0.0072448	1	1
NFKBID	1.0802401	1.08103451	1.0798957	0.985946951	-0.0204181	1	1
PLLP	1.0412634	1.0409397	1.0414037	1.011332581	0.0162575	1	1
MYRFL	1.0179768	1.01804205	1.0179485	0.994815885	-0.0074986	1	1
TMEM184A	1.0240388	1.02380201	1.0241414	1.014259307	0.0204265	1	1
TEX40	1.0188311	1.01804205	1.0191731	1.062689353	0.0877199	1	1
PABPC5	1.2096699	1.21023235	1.2094261	0.996164742	-0.0055437	1	1
LINC00868	1.0171184	1.01804205	1.0167181	0.926619179	-0.1099516	1	1
LINC01054	1.017978	1.01804205	1.0179502	0.994909765	-0.0073624	1	1
ERVH48-1	1.0171249	1.01804205	1.0167274	0.927134702	-0.1091491	1	1
NR1H4	1.0240347	1.02378968	1.0241409	1.014763909	0.0211441	1	1
AC011523.2	1.0197374	1.02091389	1.0192274	0.9193604	-0.1212976	1	1
ZNF160	1.4330073	1.43115081	1.433812	1.006172257	0.0088773	1	1
N4BP3	1.1435811	1.1444388	1.1432093	0.991488022	-0.0123328	1	1

RP11-274B21.9	1.0171139	1.01804205	1.0167115	0.926254362	-0.1105197	1	1
RP11-518L10.5	1.0188437	1.01804205	1.0191911	1.063690098	0.0890779	1	1
LRCOL1	1.0223582	1.02090725	1.0229871	1.099479267	0.1368204	1	1
CTC-559E9.4	1.0171174	1.01804205	1.0167166	0.926536393	-0.1100805	1	1
CACNG3	1.0171212	1.01804205	1.0167221	0.926839899	-0.1096079	1	1
TCP11L1	1.0794393	1.07820377	1.0799748	1.0226467	0.0323078	1	1
RP11-554E23.2	1.0179831	1.01804205	1.0179575	0.995315772	-0.0067738	1	1
RP5-1125A11.6	1.0171146	1.01804205	1.0167126	0.926314699	-0.1104257	1	1
RP11-72M17.1	1.0266342	1.02663661	1.0266331	0.999868642	-0.0001895	1	1
CTD-2078B5.2	1.018888	1.01804205	1.0192547	1.067212494	0.0938475	1	1
AC073934.6	1.0171139	1.01804205	1.0167115	0.926254362	-0.1105197	1	1
SYN3	1.0231578	1.02376148	1.0228961	0.963581239	-0.0535218	1	1
LINC00885	1.0171202	1.01804205	1.0167206	0.926756826	-0.1097373	1	1
TBC1D30	1.0577562	1.05822372	1.0575535	0.988489586	-0.0167023	1	1
RP11-358N2.2	1.0223256	1.02087759	1.0229532	1.099418605	0.1367408	1	1
CYTH4	1.0171194	1.01804205	1.0167194	0.926691235	-0.1098394	1	1
BCL2L15	1.0179949	1.01804205	1.0179745	0.996253346	-0.0054154	1	1
RP11-2H8.2	1.0171212	1.01804205	1.0167221	0.926839899	-0.1096079	1	1
MTCL1	1.1303513	1.12975964	1.1306077	1.006535641	0.0093983	1	1
ANKRD20A4	1.0179756	1.01804205	1.0179468	0.994719731	-0.007638	1	1
XXbac-BPG27H4.8	1.0247884	1.02431341	1.0249943	1.028002953	0.0398444	1	1
CTD-2621I17.6	1.0152712	1.01574718	1.0150648	0.956669231	-0.0639079	1	1
RP11-379F4.6	1.0187281	1.01859173	1.0187872	1.010514554	0.0150901	1	1
MAP4K1	1.0662692	1.06729135	1.0658261	0.978225704	-0.0317607	1	1
CTD-2060L22.1	1.0152715	1.01574718	1.0150653	0.956700103	-0.0638613	1	1
CLNK	1.0152788	1.01574718	1.0150758	0.957362503	-0.0628628	1	1
AP000857.3	1.0152781	1.01574718	1.0150748	0.957301959	-0.062954	1	1
RP11-33B1.3	1.0161451	1.01574718	1.0163175	1.036218668	0.0513285	1	1
C1orf210	1.0152764	1.01574718	1.0150724	0.957147629	-0.0631866	1	1
SLC5A8	1.0152708	1.01574718	1.0150643	0.956634829	-0.0639598	1	1
HSPA4L	1.2604526	1.26191292	1.2598196	0.992007588	-0.0115769	1	1
INHBE	1.0195769	1.01858502	1.0200069	1.076506257	0.1063567	1	1
FAM156A	1.0152713	1.01574718	1.015065	0.956677033	-0.0638961	1	1
Z83851.4	1.0178619	1.0185911	1.0175458	0.94377602	-0.0834836	1	1
CABYR	1.0835122	1.08442503	1.0831166	0.984501944	-0.022534	1	1
RP5-1103G7.10	1.0169949	1.01574718	1.0175358	1.113583954	0.1552103	1	1
FAM20C	1.1481423	1.14720793	1.1485473	1.009098241	0.0130666	1	1
RP11-434H14.1	1.0152705	1.01574718	1.0150639	0.956610045	-0.0639972	1	1
RP11-385D13.1	1.0152732	1.01574718	1.0150677	0.956850455	-0.0636346	1	1
PRICKLE2-AS1	1.058568	1.05862056	1.0585452	0.99871516	-0.0018548	1	1
RP11-30L15.6	1.0178759	1.01859389	1.0175647	0.944649061	-0.0821496	1	1
CTD-2023N9.1	1.0152691	1.01574718	1.0150618	0.956477522	-0.064197	1	1
RP11-471M2.3	1.0196168	1.01862213	1.0200479	1.076562188	0.1064317	1	1
SEC14L6	1.0746827	1.07565642	1.0742606	0.981551063	-0.0268648	1	1
ATP10A	1.0152775	1.01574718	1.015074	0.957248405	-0.0630347	1	1
FAM124B	1.0178571	1.01859026	1.0175393	0.943465191	-0.0839588	1	1
RP11-571I18.5	1.0152732	1.01574718	1.0150678	0.956854383	-0.0636287	1	1
IFNL1	1.0161408	1.01574718	1.0163114	1.035830963	0.0507886	1	1
CCDC63	1.0152717	1.01574718	1.0150656	0.956715788	-0.0638377	1	1
RP11-794M8.1	1.0170428	1.01574718	1.0176044	1.117938031	0.1608402	1	1
RP11-362A1.1	1.0152767	1.01574718	1.0150728	0.957173038	-0.0631483	1	1

HSPB6	1.0161362	1.01574718	1.0163049	1.035415971	0.0502105	1	1
AC004449.6	1.0152764	1.01574718	1.0150723	0.957141525	-0.0631958	1	1
APOC3	1.01527	1.01574718	1.0150632	0.956565696	-0.064064	1	1
RP11-576N17.5	1.0161428	1.01574718	1.0163143	1.036012459	0.0510414	1	1
RP11-840I19.5	1.0161367	1.01574718	1.0163055	1.035454513	0.0502642	1	1
EDC4	1.0178584	1.01858858	1.0175418	0.943689285	-0.0836162	1	1
RP11-205K6.1	1.0152785	1.01574718	1.0150754	0.95733745	-0.0629005	1	1
TMEM262	1.018732	1.01860703	1.0187862	1.009629806	0.0138264	1	1
RP4-647C14.3	1.0213077	1.02147448	1.0212355	0.988870585	-0.0161464	1	1
BSND	1.0152782	1.01574718	1.0150749	0.95731024	-0.0629416	1	1
LINC00494	1.0161576	1.01574718	1.0163355	1.037360653	0.0529176	1	1
PI15	1.0152688	1.01574718	1.0150614	0.956450609	-0.0642376	1	1
LA16c-360H6.3	1.0282303	1.0271484	1.0286993	1.057125582	0.0801468	1	1
RP11-405M12.3	1.0152722	1.01574718	1.0150663	0.956761959	-0.0637681	1	1
MEOX2	1.0152766	1.01574718	1.0150726	0.957164771	-0.0631608	1	1
RP11-439E19.7	1.0170027	1.01574718	1.0175469	1.114286604	0.1561204	1	1
MYT1L	1.0239706	1.0243528	1.0238049	0.977500137	-0.0328312	1	1
CTD-2377D24.6	1.0170106	1.01574718	1.0175583	1.115011712	0.1570589	1	1
TGS1	1.3535026	1.3532824	1.3535981	1.000893669	0.0012887	1	1
RP11-463J10.4	1.0152777	1.01574718	1.0150742	0.957263745	-0.0630116	1	1
GLRA1	1.0152728	1.01574718	1.0150672	0.956819486	-0.0636813	1	1
AC008074.1	1.0170028	1.01574718	1.017547	1.114298227	0.1561354	1	1
SYCP3	1.0204798	1.0214821	1.0200453	0.933118085	-0.0998684	1	1
TRIM72	1.0161347	1.01574718	1.0163027	1.035274843	0.0500138	1	1
CTB-113P19.5	1.0152715	1.01574718	1.0150653	0.956700103	-0.0638613	1	1
ITIH1	1.0161507	1.01574718	1.0163256	1.036730473	0.0520409	1	1
AC007566.10	1.0169999	1.01574718	1.0175429	1.114032237	0.155791	1	1
PCDHA8	1.0161424	1.01574718	1.0163137	1.035978199	0.0509936	1	1
MYL3	1.022188	1.02143265	1.0225154	1.050517203	0.0710998	1	1
CERKL	1.0394591	1.03852526	1.0398639	1.034746229	0.049277	1	1
AF067845.2	1.0161424	1.01574718	1.0163137	1.035978199	0.0509936	1	1
RP11-154H23.4	1.0169921	1.01574718	1.0175317	1.113320802	0.1548694	1	1
UBL4B	1.0152788	1.01574718	1.0150758	0.957362503	-0.0628628	1	1
C2CD3	1.0791641	1.07862387	1.0793983	1.009850156	0.0141412	1	1
RP11-196G11.6	1.0282492	1.02717078	1.0287167	1.056896634	0.0798343	1	1
RP11-96B5.3	1.0299887	1.03013417	1.0299257	0.993080292	-0.0100177	1	1
SPTBN5	1.0152695	1.01574718	1.0150624	0.956515729	-0.0641394	1	1
CTA-363E6.7	1.0161436	1.01574718	1.0163154	1.036086154	0.051144	1	1
CTD-2020K17.1	1.0230703	1.02150444	1.0237491	1.104380835	0.1432378	1	1
AC024592.12	1.0152757	1.01574718	1.0150713	0.957079822	-0.0632888	1	1
LRIF1	1.3763482	1.37647698	1.3762924	0.999509661	-0.0007076	1	1
FAM83C-AS1	1.0152729	1.01574718	1.0150673	0.956823413	-0.0636754	1	1
RP11-359J14.2	1.0161425	1.01574718	1.0163139	1.035989592	0.0510095	1	1
LINC00654	1.0152703	1.01574718	1.0150636	0.956588733	-0.0640293	1	1
AXDND1	1.0222351	1.02147008	1.0225667	1.05107581	0.0718667	1	1
CTA-398F10.2	1.0169921	1.01574718	1.0175317	1.113321895	0.1548708	1	1
CKMT2	1.0161372	1.01574718	1.0163062	1.035502887	0.0503316	1	1
FSD2	1.0273759	1.02723081	1.0274387	1.007635767	0.0109742	1	1
TRIM74	1.0161335	1.01574718	1.016301	1.035170868	0.0498689	1	1
FZD4	1.0558535	1.05585854	1.0558514	0.999871311	-0.0001857	1	1
RP11-328J14.1	1.0152715	1.01574718	1.0150653	0.956700103	-0.0638613	1	1

CTAGE1	1.016137	1.01574718	1.016306	1.035487023	0.0503095	1	1
RP11-508N12.2	1.0152732	1.01574718	1.0150678	0.956856122	-0.0636261	1	1
HCG22	1.0222075	1.0214491	1.0225362	1.050682299	0.0713265	1	1
RAB40AL	1.0231231	1.0214821	1.0238345	1.10950331	0.149914	1	1
PRR29	1.0411541	1.04142736	1.0410357	0.990546268	-0.0137037	1	1
BIRC6	1.6718524	1.67126667	1.6721063	1.001250852	0.0018035	1	1
AC003958.2	1.0178757	1.01860364	1.0175602	0.943909949	-0.0832789	1	1
CPT1A	1.2097565	1.21028965	1.2095254	0.996365747	-0.0052527	1	1
SH2D6	1.0213157	1.02146938	1.0212491	0.989739353	-0.0148795	1	1
LINC00598	1.0282092	1.02714864	1.0286689	1.055997386	0.0786063	1	1
AC024560.2	1.0187419	1.01861196	1.0187982	1.010005908	0.0143637	1	1
VDR	1.0195785	1.01860668	1.0199997	1.07486795	0.1041594	1	1
RP11-582J16.4	1.0195857	1.01860828	1.0200094	1.075293645	0.1047307	1	1
C6orf58	1.0187233	1.01859264	1.0187799	1.010073599	0.0144604	1	1
PCDHGB7	1.0221607	1.02149281	1.0224501	1.044542243	0.0628708	1	1
MAPK7	1.250927	1.2503906	1.2511594	1.003070599	0.0044231	1	1
CTC-537E7.3	1.0161262	1.01574718	1.0162905	1.034501391	0.0489356	1	1
ARHGAP40	1.0152735	1.01574718	1.0150681	0.956879245	-0.0635912	1	1
RP11-107F6.3	1.0152688	1.01574718	1.0150614	0.956450609	-0.0642376	1	1
CAPN8	1.0187065	1.01858872	1.0187576	1.009082345	0.0130439	1	1
SLC46A3	1.0696303	1.07013062	1.0694134	0.989773018	-0.0148304	1	1
FEV	1.0170117	1.01574718	1.0175598	1.115108913	0.1571846	1	1
CCDC13-AS1	1.0161614	1.01574718	1.0163409	1.03770292	0.0533935	1	1
HDAC7	1.5422211	1.54209939	1.5422739	1.000321863	0.0004643	1	1
KDM4D	1.0722856	1.07300337	1.0719745	0.985906114	-0.0204778	1	1
STEAP1	1.1300053	1.13025756	1.129896	0.997224068	-0.0040104	1	1
BTBD6	1.0480716	1.04721121	1.0484445	1.026122956	0.0372036	1	1
CTC-260F20.3	1.0152768	1.01574718	1.0150729	0.957182673	-0.0631338	1	1
RP11-148K1.10	1.0152723	1.01574718	1.0150664	0.956771544	-0.0637536	1	1
TMEM156	1.0152739	1.01574718	1.0150687	0.956915494	-0.0635366	1	1
SYPL2	1.0152729	1.01574718	1.0150673	0.956823413	-0.0636754	1	1
AL133493.2	1.016126	1.01574718	1.0162902	1.0344836	0.0489108	1	1
OLIG1	1.0152709	1.01574718	1.0150644	0.956642665	-0.063948	1	1
MYO1H	1.0178669	1.01859544	1.0175511	0.943838112	-0.0833887	1	1
ZIM2-AS1	1.0161436	1.01574718	1.0163154	1.036086154	0.051144	1	1
PRR34-AS1	1.0152762	1.01574718	1.0150721	0.957129735	-0.0632136	1	1
RP11-13E5.2	1.0170026	1.01574718	1.0175467	1.114276135	0.1561068	1	1
MGAM2	1.0152732	1.01574718	1.0150678	0.956854383	-0.0636287	1	1
NAT16	1.0369069	1.03574604	1.0374101	1.046553345	0.0656459	1	1
AC074212.6	1.0161365	1.01574718	1.0163053	1.035443539	0.0502489	1	1
HOXA9	1.0152788	1.01574718	1.0150758	0.957362503	-0.0628628	1	1
SLC35G3	1.0152708	1.01574718	1.0150643	0.956634829	-0.0639598	1	1
RP11-244F12.3	1.0152693	1.01574718	1.0150621	0.956496623	-0.0641682	1	1
RP11-768G7.2	1.0152741	1.01574718	1.015069	0.956935118	-0.063507	1	1
FAM180B	1.0152703	1.01574718	1.0150636	0.956588733	-0.0640293	1	1
CRHR2	1.0152738	1.01574718	1.0150687	0.956911978	-0.0635419	1	1
RP6-99M1.3	1.017001	1.01574718	1.0175445	1.114137952	0.1559279	1	1
RP11-219B4.5	1.0152732	1.01574718	1.0150678	0.956854383	-0.0636287	1	1
BLID	1.0152713	1.01574718	1.0150651	0.956682725	-0.0638875	1	1
FREM3	1.0152691	1.01574718	1.0150619	0.956484517	-0.0641865	1	1
SMCO2	1.0152709	1.01574718	1.0150644	0.956642665	-0.063948	1	1

ZNF575	1.0369225	1.0357556	1.0374283	1.046780476	0.0659589	1	1
Z69666.2	1.0149672	1.01338003	1.0156551	1.170038528	0.226556	1	1
AQP2	1.0132473	1.01338003	1.0131898	0.985785033	-0.020655	1	1
IZUMO1	1.012388	1.01338003	1.0119579	0.893715177	-0.162113	1	1
RP11-307C18.1	1.0201354	1.01906634	1.0205987	1.080371831	0.1115279	1	1
TRIML2	1.0175462	1.01621459	1.0181234	1.117720499	0.1605595	1	1
AP001172.3	1.013248	1.01338003	1.0131908	0.985854831	-0.0205529	1	1
ALDH3B2	1.0132439	1.01338003	1.0131848	0.985411937	-0.0212011	1	1
SLC6A1	1.0141033	1.01338003	1.0144168	1.077483751	0.1076661	1	1
RP11-1277A3.3	1.0167015	1.01622941	1.0169062	1.041699938	0.0589398	1	1
CTD-2376I20.1	1.0132484	1.01338003	1.0131913	0.985893328	-0.0204965	1	1
AC004754.3	1.0261696	1.02476102	1.0267802	1.081546759	0.113096	1	1
RP11-323J4.1	1.0149667	1.01338003	1.0156544	1.169984028	0.2264888	1	1
NR2E3	1.017538	1.01622324	1.018108	1.11617417	0.1585622	1	1
RAB33B	1.0891304	1.08767993	1.0897591	1.023713493	0.033812	1	1
RP4-777L9.3	1.0132529	1.01338003	1.0131978	0.986382876	-0.0197803	1	1
AL354828.2	1.012388	1.01338003	1.0119579	0.893715177	-0.162113	1	1
C3AR1	1.0261365	1.02477269	1.0267277	1.078917923	0.1095851	1	1
RP6-201G10.2	1.0132496	1.01338003	1.013193	0.986022808	-0.0203071	1	1
BIRC6-AS2	1.0132497	1.01338003	1.0131933	0.986041287	-0.02028	1	1
FNDC9	1.0141138	1.01338003	1.0144319	1.078612414	0.1091765	1	1
RP1-261D10.2	1.0218538	1.02189232	1.0218371	0.997479564	-0.0036408	1	1
DUOX2	1.0158451	1.01622088	1.0156822	0.966793693	-0.04872	1	1
TTC16	1.012388	1.01338003	1.0119579	0.893715177	-0.162113	1	1
FBXO46	1.1484796	1.14747243	1.1489161	1.009789653	0.0140548	1	1
LHFPL1	1.0365891	1.0362192	1.0367494	1.014637801	0.0209648	1	1
TRAPPC3L	1.012388	1.01338003	1.0119579	0.893715177	-0.162113	1	1
RP1-30M3.6	1.0175994	1.01621933	1.0181976	1.121968338	0.166032	1	1
RP11-170N16.3	1.0132453	1.01338003	1.0131869	0.985568789	-0.0209715	1	1
CTD-2165H16.4	1.0529736	1.05348002	1.0527541	0.986425805	-0.0197176	1	1
USH1G	1.0158358	1.01623264	1.0156638	0.964959181	-0.0514602	1	1
HIST2H2BF	1.0184191	1.01906498	1.0181391	0.95143697	-0.07182	1	1
TFF3	1.0149737	1.01338003	1.0156644	1.170732308	0.2274112	1	1
CTC-529P8.1	1.017574	1.01622885	1.0181571	1.118815229	0.1619718	1	1
PROSER2-AS1	1.0132465	1.01338003	1.0131886	0.985696387	-0.0207848	1	1
MTRNR2L11	1.0166887	1.01623082	1.0168872	1.040438261	0.0571914	1	1
TMPPE	1.0149639	1.01338003	1.0156504	1.169684539	0.2261195	1	1
RP11-173P15.5	1.0132501	1.01338003	1.0131938	0.986084496	-0.0202168	1	1
XXbac-BPG308K3.5	1.0425724	1.04192326	1.0428537	1.022194827	0.0316702	1	1
TMEM63C	1.0227269	1.02190635	1.0230826	1.053694221	0.0754563	1	1
RABIF	1.3320869	1.33282894	1.3317653	0.996804218	-0.0046179	1	1
RP11-535A19.1	1.012388	1.01338003	1.0119579	0.893715177	-0.162113	1	1
CTD-2358C21.5	1.0141211	1.01338003	1.0144423	1.079389289	0.1102153	1	1
OVCH2	1.0132451	1.01338003	1.0131866	0.985542778	-0.0210096	1	1
AP000431.2	1.012388	1.01338003	1.0119579	0.893715177	-0.162113	1	1
RP5-1074L1.1	1.0166908	1.01621389	1.0168976	1.042165816	0.0595848	1	1
SPATA25	1.012388	1.01338003	1.0119579	0.893715177	-0.162113	1	1
LINC00222	1.015822	1.01622207	1.0156486	0.964649545	-0.0519232	1	1
RP11-706O15.7	1.0184523	1.01913089	1.0181581	0.949153281	-0.075287	1	1
AC019186.1	1.014967	1.01338003	1.0156549	1.170018295	0.2265311	1	1
RP5-1172N10.2	1.0166991	1.01621389	1.0169094	1.042897504	0.0605974	1	1

MYLK3	1.0184217	1.01909123	1.0181315	0.949728499	-0.0744129	1	1
GABRG1	1.0132442	1.01338003	1.0131853	0.985443127	-0.0211555	1	1
FGL1	1.0132472	1.01338003	1.0131896	0.985764518	-0.020685	1	1
RP11-187O7.3	1.0132455	1.01338003	1.0131872	0.985590363	-0.0209399	1	1
PLA2G4F	1.0132447	1.01338003	1.013186	0.985497786	-0.0210755	1	1
RP11-404O13.5	1.0132494	1.01338003	1.0131928	0.98600482	-0.0203334	1	1
ZNF630	1.042498	1.04186594	1.042772	1.021642075	0.0308898	1	1
IL18RAP	1.0132532	1.01338003	1.0131982	0.986407688	-0.0197441	1	1
RP11-408O19.5	1.0141164	1.01338003	1.0144356	1.07889062	0.1095486	1	1
FOXP3	1.013259	1.01338003	1.0132066	0.987035003	-0.0188268	1	1
SLC7A14	1.0141172	1.01338003	1.0144367	1.078973171	0.109659	1	1
CTC-526N19.1	1.0278647	1.02763037	1.0279663	1.012156605	0.0174325	1	1
SHANK2-AS3	1.0132497	1.01338003	1.0131933	0.986041287	-0.02028	1	1
CNGB1	1.014982	1.01338003	1.0156764	1.171628144	0.2285148	1	1
TAS2R13	1.0132451	1.01338003	1.0131866	0.985546283	-0.0210045	1	1
RP11-88E10.4	1.012388	1.01338003	1.0119579	0.893715177	-0.162113	1	1
KCTD14	1.0348111	1.03343085	1.0354094	1.059183907	0.0829531	1	1
LINC00501	1.0149604	1.01338003	1.0156454	1.169309971	0.2256574	1	1
RP11-374A4.1	1.0132507	1.01338003	1.0131946	0.986141144	-0.0201339	1	1
OSBP	1.2260916	1.22736144	1.2255412	0.99199424	-0.0115964	1	1
RP11-259O18.5	1.0141049	1.01338003	1.0144191	1.077659694	0.1079017	1	1
RP11-135A1.2	1.0166948	1.01622458	1.0168986	1.041545572	0.058726	1	1
LINC01600	1.0149813	1.01338003	1.0156754	1.17154868	0.2284169	1	1
FAM87A	1.0141117	1.01338003	1.0144289	1.078390022	0.1088791	1	1
RP11-538D16.2	1.0158617	1.01622458	1.0157044	0.967941341	-0.0470085	1	1
DLG1-AS1	1.0149623	1.01338003	1.0156481	1.169513652	0.2259087	1	1
RP11-504P24.9	1.0149741	1.01338003	1.0156651	1.170781922	0.2274724	1	1
ZNF534	1.0132484	1.01338003	1.0131914	0.985900486	-0.0204861	1	1
FADS3	1.1915443	1.19043154	1.1920267	1.00837635	0.0120342	1	1
CTA-254O6.1	1.0141247	1.01338003	1.0144474	1.079774786	0.1107304	1	1
NXPE2	1.0132449	1.01338003	1.0131863	0.985520279	-0.0210425	1	1
MAOA	1.0408167	1.04191173	1.040342	0.962547214	-0.0550708	1	1
NKX3-1	1.0175501	1.01622088	1.0181263	1.11746508	0.1602297	1	1
ACAN	1.0132545	1.01338003	1.0132	0.986547158	-0.0195401	1	1
RP3-339A18.6	1.0132457	1.01338003	1.0131875	0.985610353	-0.0209107	1	1
SMG6	1.4243441	1.42429921	1.4243635	1.000151553	0.0002186	1	1
RP1-253P7.4	1.0141114	1.01338003	1.0144284	1.078353059	0.1088296	1	1
RP11-159K7.2	1.0132449	1.01338003	1.0131863	0.985520279	-0.0210425	1	1
AC006378.2	1.0132449	1.01338003	1.0131863	0.985524885	-0.0210358	1	1
LMAN1L	1.012388	1.01338003	1.0119579	0.893715177	-0.162113	1	1
AC108004.3	1.0141115	1.01338003	1.0144286	1.078367397	0.1088488	1	1
ADAMTSL3	1.0149851	1.01338003	1.0156809	1.171960095	0.2289234	1	1
SOWAHB	1.0141105	1.01338003	1.0144271	1.078258408	0.108703	1	1
RP11-389G6.5	1.012388	1.01338003	1.0119579	0.893715177	-0.162113	1	1
GBP5	1.0132477	1.01338003	1.0131903	0.985823014	-0.0205994	1	1
RP11-109N23.4	1.0270165	1.02760536	1.0267612	0.969419822	-0.0448065	1	1
RP1L1	1.0132443	1.01338003	1.0131854	0.985453751	-0.0211399	1	1
ABL1	1.3691242	1.37002895	1.3687321	0.996495255	-0.0050652	1	1
LINC01322	1.0132466	1.01338003	1.0131887	0.985703043	-0.020775	1	1
CAMTA1	4.2939471	4.29427446	4.2938051	0.999857532	-0.0002056	1	1
CTB-51A17.1	1.0132456	1.01338003	1.0131874	0.98560162	-0.0209235	1	1

ALS2CR11	1.012388	1.01338003	1.0119579	0.893715177	-0.162113	1	1
RP11-524C21.2	1.0192907	1.01907516	1.0193842	1.016200529	0.0231851	1	1
LRIT3	1.0141064	1.01338003	1.0144213	1.077822505	0.1081196	1	1
RP11-184A2.3	1.014107	1.01338003	1.0144221	1.077885169	0.1082035	1	1
CA6	1.0132532	1.01338003	1.0131982	0.986407688	-0.0197441	1	1
C20orf141	1.012388	1.01338003	1.0119579	0.893715177	-0.162113	1	1
NRSN1	1.0219424	1.02193389	1.0219461	1.00055897	0.0008062	1	1
LINC00518	1.012388	1.01338003	1.0119579	0.893715177	-0.162113	1	1
CTC-338M12.7	1.0132484	1.01338003	1.0131913	0.985893328	-0.0204965	1	1
LINC01449	1.0149754	1.01338003	1.0156669	1.17091761	0.2276396	1	1
RP11-676J12.9	1.0141042	1.01338003	1.0144181	1.077580998	0.1077963	1	1
RP3-406A7.7	1.02621	1.02480476	1.0268191	1.081207438	0.1126433	1	1
RP11-53B2.3	1.0149847	1.01338003	1.0156803	1.171916543	0.2288698	1	1
RP11-463O9.9	1.012388	1.01338003	1.0119579	0.893715177	-0.162113	1	1
RP11-69I8.3	1.0141064	1.01338003	1.0144213	1.077823094	0.1081204	1	1
IGFL3	1.0132446	1.01338003	1.0131859	0.985493182	-0.0210822	1	1
SLC17A9	1.0683549	1.06757414	1.0686934	1.016563336	0.0237001	1	1
HLA-DRB5	1.0132431	1.01338003	1.0131837	0.985326169	-0.0213267	1	1
PRR15L	1.0176104	1.01623082	1.0182083	1.121837766	0.1658641	1	1
CAGE1	1.0132484	1.01338003	1.0131913	0.985893328	-0.0204965	1	1
CH25H	1.0132447	1.01338003	1.013186	0.985497786	-0.0210755	1	1
ZBP1	1.0132454	1.01338003	1.013187	0.985574498	-0.0209632	1	1
PGM5-AS1	1.0175974	1.01622352	1.0181929	1.121391019	0.1652894	1	1
FAM46C	1.0149693	1.01338003	1.0156581	1.170261449	0.2268309	1	1
PPM1J	1.0915871	1.09044314	1.092083	1.018131338	0.0259237	1	1
FOXD4	1.0149662	1.01338003	1.0156537	1.169927908	0.2264196	1	1
PCCA-AS1	1.0219479	1.0219927	1.0219285	0.997080731	-0.0042178	1	1
C17orf99	1.0132468	1.01338003	1.013189	0.985723545	-0.020745	1	1
AC019330.1	1.0132452	1.01338003	1.0131868	0.985558637	-0.0209864	1	1
SPRR2E	1.0132472	1.01338003	1.0131896	0.985764518	-0.020685	1	1
ST20-MTHFS	1.0132512	1.01338003	1.0131953	0.986193633	-0.0200572	1	1
CTD-3222D19.11	1.012388	1.01338003	1.0119579	0.893715177	-0.162113	1	1
RP11-131L23.1	1.0227314	1.02190998	1.0230874	1.053739271	0.0755179	1	1
RP4-656G21.1	1.012388	1.01338003	1.0119579	0.893715177	-0.162113	1	1
HIST1H2BK	1.016678	1.01621919	1.0168769	1.040549349	0.0573454	1	1
CTD-2196E14.5	1.0132485	1.01338003	1.0131915	0.985906713	-0.0204769	1	1
DNAJC3-AS1	1.2953043	1.29617128	1.2949285	0.995803737	-0.0060667	1	1
RP11-227D13.5	1.0132442	1.01338003	1.0131854	0.985451844	-0.0211427	1	1
RP11-170M17.1	1.012388	1.01338003	1.0119579	0.893715177	-0.162113	1	1
DSG1	1.0132515	1.01338003	1.0131957	0.986225577	-0.0200104	1	1
RP11-423H2.4	1.0158277	1.01621933	1.0156579	0.965384164	-0.0508249	1	1
RP11-217B7.2	1.012388	1.01338003	1.0119579	0.893715177	-0.162113	1	1
SYT2	1.012388	1.01338003	1.0119579	0.893715177	-0.162113	1	1
C20orf166-AS1	1.0210186	1.02196463	1.0206086	0.938262189	-0.091937	1	1
TMED7-TICAM2	1.0158226	1.0162278	1.015647	0.9642067	-0.0525856	1	1
XIRP1	1.0132618	1.01338003	1.0132106	0.98733533	-0.0183879	1	1
RP11-302F12.3	1.012388	1.01338003	1.0119579	0.893715177	-0.162113	1	1
CTA-29F11.1	1.3385408	1.33905902	1.3383161	0.997808985	-0.0031644	1	1
VSTM1	1.0132455	1.01338003	1.0131872	0.985590363	-0.0209399	1	1
DENND6A	1.2088904	1.20758181	1.2094576	1.009036306	0.0129781	1	1
HP09025	1.0132575	1.01338003	1.0132044	0.986871477	-0.0190659	1	1

SOHLH2	1.0235942	1.02481579	1.0230647	0.929438247	-0.1055691	1	1
AC015969.3	1.012388	1.01338003	1.0119579	0.893715177	-0.162113	1	1
RP11-435O5.5	1.0218613	1.02197365	1.0218126	0.992672619	-0.0106101	1	1
RP11-544A12.4	1.016279	1.01740877	1.0157893	0.90697433	-0.1408664	1	1
AC010975.1	1.0188733	1.01740877	1.0195081	1.120590902	0.1642597	1	1
RP11-672A2.5	1.016279	1.01740877	1.0157893	0.90697433	-0.1408664	1	1
TMEM78	1.018016	1.01740877	1.0182792	1.050000557	0.0703901	1	1
NTN3	1.0171375	1.01740877	1.01702	0.97766568	-0.0325869	1	1
S100P	1.0197256	1.02024298	1.0195013	0.963361611	-0.0538507	1	1
LBP	1.0171396	1.01740877	1.0170229	0.977833201	-0.0323397	1	1
CTD-2329K10.1	1.0171457	1.01740877	1.0170316	0.978335056	-0.0315995	1	1
ABCA9-AS1	1.016279	1.01740877	1.0157893	0.90697433	-0.1408664	1	1
OXCT2	1.0171392	1.01740877	1.0170223	0.977801627	-0.0323863	1	1
RP11-497G19.7	1.017996	1.01740877	1.0182506	1.048354092	0.0681261	1	1
GALNT4	1.0171416	1.01740877	1.0170258	0.978000744	-0.0320925	1	1
IMPG1	1.0171371	1.01740877	1.0170193	0.977629442	-0.0326404	1	1
RP1-310O13.7	1.0206283	1.02026363	1.0207863	1.025795116	0.0367426	1	1
RP11-944L7.4	1.0171414	1.01740877	1.0170255	0.977984917	-0.0321159	1	1
RNASE7	1.016279	1.01740877	1.0157893	0.90697433	-0.1408664	1	1
CTD-2619J13.14	1.0275234	1.02600857	1.02818	1.083489048	0.1156846	1	1
CTD-3131K8.3	1.0171433	1.01740877	1.0170283	0.978142518	-0.0318834	1	1
RP11-737O24.5	1.016279	1.01740877	1.0157893	0.90697433	-0.1408664	1	1
AP001626.1	1.0180053	1.01740877	1.0182638	1.049117035	0.0691756	1	1
SYCP2	1.0223654	1.02314977	1.0220254	0.951432046	-0.0718275	1	1
NFE4	1.0179963	1.01740877	1.0182509	1.048375853	0.068156	1	1
IL2RG	1.0179961	1.01740877	1.0182507	1.048362987	0.0681383	1	1
UCHL1-AS1	1.0171416	1.01740877	1.0170257	0.977997181	-0.0320978	1	1
NOX5	1.0171393	1.01740877	1.0170224	0.977808733	-0.0323758	1	1
CD163L1	1.0180072	1.01740877	1.0182665	1.049272844	0.0693899	1	1
MEI1	1.0214877	1.02026075	1.0220195	1.086806403	0.120095	1	1
SERPINE3	1.0180044	1.01740877	1.0182626	1.049048435	0.0690813	1	1
RP11-373D23.3	1.0205839	1.02027034	1.0207198	1.022175137	0.0316424	1	1
RP11-144G16.1	1.0171414	1.01740877	1.0170256	0.97798848	-0.0321106	1	1
RP11-59E19.4	1.0171358	1.01740877	1.0170175	0.977526795	-0.0327918	1	1
MUC6	1.0171452	1.01740877	1.0170309	0.978294897	-0.0316587	1	1
SCN1A	1.0179998	1.01740877	1.0182559	1.048663717	0.0685521	1	1
HP	1.0188824	1.01740877	1.0195211	1.121338894	0.1652224	1	1
CFAP57	1.0257927	1.02598709	1.0257084	0.989274975	-0.0155565	1	1
WDR35	1.1720835	1.1721458	1.1720565	0.999481216	-0.0007486	1	1
RP11-724N1.1	1.016279	1.01740877	1.0157893	0.90697433	-0.1408664	1	1
SERPINB2	1.0180209	1.01740877	1.0182862	1.050401891	0.0709414	1	1
AC092839.3	1.0171405	1.01740877	1.0170243	0.977912889	-0.0322221	1	1
TMOD4	1.0171455	1.01740877	1.0170313	0.978319533	-0.0316223	1	1
MEG9	1.0258282	1.02601616	1.0257467	0.989643826	-0.0150187	1	1
LINC00659	1.0206124	1.02027518	1.0207586	1.023842573	0.0339939	1	1
ABCC9	1.016279	1.01740877	1.0157893	0.90697433	-0.1408664	1	1
RP4-789D17.5	1.020607	1.02025372	1.0207602	1.025005717	0.035632	1	1
GAGE10	1.0171392	1.01740877	1.0170223	0.977801627	-0.0323863	1	1
LINC01254	1.0197372	1.02026764	1.0195073	0.962487603	-0.0551601	1	1
CATSPER4	1.018877	1.01740877	1.0195134	1.120896328	0.1646528	1	1
CELSR3-AS1	1.0188765	1.01740877	1.0195126	1.120850943	0.1645944	1	1

BHLHE40-AS1	1.0267175	1.02601924	1.0270201	1.038467551	0.0544561	1	1
LINC01123	1.0171462	1.01740877	1.0170323	0.978375994	-0.0315391	1	1
ARHGEF7	1.3105934	1.30975477	1.3109569	1.003881024	0.0055883	1	1
CTC-338M12.6	1.0180018	1.01740877	1.0182589	1.048834642	0.0687872	1	1
CTA-384D8.33	1.0171483	1.01740877	1.0170354	0.978553589	-0.0312772	1	1
MAGEE2	1.0171425	1.01740877	1.017027	0.978072385	-0.0319869	1	1
AC093620.5	1.0180128	1.01740877	1.0182747	1.049738677	0.0700302	1	1
CTB-33G10.6	1.016279	1.01740877	1.0157893	0.90697433	-0.1408664	1	1
KRTAP2-3	1.0179961	1.01740877	1.0182507	1.048364826	0.0681409	1	1
CTC-250I14.3	1.0188715	1.01740877	1.0195055	1.120439984	0.1640654	1	1
SAG	1.0171397	1.01740877	1.0170231	0.977847012	-0.0323193	1	1
RP11-70F11.11	1.0171421	1.01740877	1.0170264	0.97803865	-0.0320366	1	1
VNN1	1.0171392	1.01740877	1.0170223	0.977803233	-0.0323839	1	1
PALB2	1.1140334	1.11485772	1.1136761	0.989712533	-0.0149185	1	1
AC007193.9	1.0180154	1.01740877	1.0182784	1.049952731	0.0703244	1	1
RP11-220C2.1	1.0171463	1.01740877	1.0170325	0.978389053	-0.0315198	1	1
PXYLP1	1.2392771	1.23774339	1.2399418	1.009247174	0.0132795	1	1
HRASLS5	1.0171419	1.01740877	1.0170262	0.978026379	-0.0320547	1	1
SOHLH1	1.0188688	1.01740877	1.0195017	1.120220555	0.1637828	1	1
CLDN12	1.3052862	1.30396538	1.3058587	1.006228677	0.0089582	1	1
C7	1.0171391	1.01740877	1.0170222	0.977796502	-0.0323939	1	1
RP11-523H24.3	1.0171359	1.01740877	1.0170177	0.977533873	-0.0327814	1	1
RP11-658F2.8	1.1575276	1.15770313	1.1574516	0.998404872	-0.0023031	1	1
SPATA32	1.016279	1.01740877	1.0157893	0.90697433	-0.1408664	1	1
CTD-3028N15.3	1.0171397	1.01740877	1.0170231	0.977847012	-0.0323193	1	1
SETD4	1.3417657	1.34115134	1.342032	1.002581435	0.0037194	1	1
RP11-173D14.3	1.0171369	1.01740877	1.0170191	0.977617241	-0.0326584	1	1
RP11-90D11.1	1.0171416	1.01740877	1.0170257	0.977997181	-0.0320978	1	1
POU2AF1	1.0171392	1.01740877	1.0170223	0.977801627	-0.0323863	1	1
HAR1B	1.0180267	1.01740877	1.0182945	1.050881141	0.0715995	1	1
RGAG1	1.0180128	1.01740877	1.0182747	1.049738677	0.0700302	1	1
FBXW10	1.0171381	1.01740877	1.0170208	0.977716471	-0.0325119	1	1
RP11-127O4.3	1.016279	1.01740877	1.0157893	0.90697433	-0.1408664	1	1
RP11-93I21.3	1.0171469	1.01740877	1.0170334	0.978438845	-0.0314464	1	1
SPX	1.0223142	1.02313626	1.0219579	0.949068557	-0.0754158	1	1
AC093110.3	1.0171353	1.01740877	1.0170168	0.977483214	-0.0328562	1	1
AC112715.2	1.016279	1.01740877	1.0157893	0.90697433	-0.1408664	1	1
RP5-1142A6.9	1.016279	1.01740877	1.0157893	0.90697433	-0.1408664	1	1
PROK2	1.0171383	1.01740877	1.0170211	0.977730259	-0.0324916	1	1
RP11-758P17.3	1.0180082	1.01740877	1.018268	1.049356742	0.0695052	1	1
ALOX15	1.016279	1.01740877	1.0157893	0.90697433	-0.1408664	1	1
FAM189A1	1.0257868	1.02596859	1.025708	0.989963873	-0.0145522	1	1
CATSPERD	1.0179979	1.01740877	1.0182533	1.048511286	0.0683424	1	1
RP11-435D7.3	1.0171391	1.01740877	1.0170222	0.977796502	-0.0323939	1	1
HTR2A	1.0258193	1.02596835	1.0257547	0.991771099	-0.0119209	1	1
GLP2R	1.016279	1.01740877	1.0157893	0.90697433	-0.1408664	1	1
RP11-692C24.1	1.0171469	1.01740877	1.0170334	0.978438845	-0.0314464	1	1
LINC00243	1.0180049	1.01740877	1.0182633	1.049088542	0.0691364	1	1
RP11-321N4.5	1.0171463	1.01740877	1.0170325	0.978389053	-0.0315198	1	1
RP11-164H13.1	1.018021	1.01740877	1.0182863	1.05040962	0.070952	1	1
RP11-94C24.13	1.0188924	1.01740877	1.0195354	1.122161088	0.1662798	1	1

RP11-886P16.6	1.0171501	1.01740877	1.017038	0.978703249	-0.0310566	1	1
CGB3	1.016279	1.01740877	1.0157893	0.90697433	-0.1408664	1	1
GFI1	1.0171353	1.01740877	1.0170168	0.977483214	-0.0328562	1	1
AC067956.1	1.0188724	1.01740877	1.0195068	1.120513602	0.1641602	1	1
TTLL6	1.016279	1.01740877	1.0157893	0.90697433	-0.1408664	1	1
AC002550.5	1.0197425	1.02027874	1.01951	0.962093804	-0.0557505	1	1
NOD1	1.0456988	1.04609362	1.0455277	0.987722521	-0.0178223	1	1
LINC00379	1.019731	1.02025087	1.0195056	0.96319783	-0.054096	1	1
LINC01324	1.016279	1.01740877	1.0157893	0.90697433	-0.1408664	1	1
TEX11	1.0171421	1.01740877	1.0170264	0.97803865	-0.0320366	1	1
WBP4	1.899192	1.89998231	1.8988494	0.998741224	-0.0018172	1	1
PHF20	1.6112189	1.61074994	1.6114222	1.001100771	0.0015872	1	1
RP11-76I14.1	1.0387381	1.03743796	1.0393016	1.049780588	0.0700878	1	1
CABP2	1.0171366	1.01740877	1.0170186	0.977587738	-0.0327019	1	1
RP11-259N19.1	1.0448028	1.04603755	1.0442675	0.961552551	-0.0565624	1	1
VAV3-AS1	1.016279	1.01740877	1.0157893	0.90697433	-0.1408664	1	1
PHOSPHO1	1.0180034	1.01740877	1.0182611	1.048961121	0.0689612	1	1
MATN4	1.056063	1.0546282	1.0566849	1.037648904	0.0533184	1	1
RP11-521C20.5	1.0362416	1.03468819	1.0369149	1.064192746	0.0897595	1	1
LINC00972	1.0171425	1.01740877	1.017027	0.978072385	-0.0319869	1	1
UCP3	1.0249527	1.02600928	1.0244948	0.941771122	-0.0865516	1	1
OMG	1.0171389	1.01740877	1.0170219	0.977775598	-0.0324247	1	1
NCR2	1.016279	1.01740877	1.0157893	0.90697433	-0.1408664	1	1
LINC01451	1.0205927	1.02026139	1.0207362	1.023436707	0.0334219	1	1
RP1-45C12.1	1.0171412	1.01740877	1.0170253	0.977970604	-0.032137	1	1
RNASE6	1.016279	1.01740877	1.0157893	0.90697433	-0.1408664	1	1
RP11-554A11.8	1.016279	1.01740877	1.0157893	0.90697433	-0.1408664	1	1
TMEM132A	1.4635716	1.46473504	1.4630673	0.996411495	-0.0051864	1	1
RP11-950C14.7	1.0180077	1.01740877	1.0182673	1.049313866	0.0694463	1	1
CD274	1.0240776	1.02312765	1.0244893	1.05887565	0.0825332	1	1
RP11-309L24.10	1.0171478	1.01740877	1.0170347	0.978515069	-0.031334	1	1
CCDC190	1.0188593	1.01740877	1.019488	1.11943453	0.1627702	1	1
CTD-3037G24.3	1.016279	1.01740877	1.0157893	0.90697433	-0.1408664	1	1
AC023469.1	1.016279	1.01740877	1.0157893	0.90697433	-0.1408664	1	1
RP11-713P17.4	1.0171369	1.01740877	1.0170191	0.977617241	-0.0326584	1	1
ADRA1B	1.0171429	1.01740877	1.0170276	0.97810408	-0.0319401	1	1
PRMT5-AS1	1.0171464	1.01740877	1.0170327	0.978399389	-0.0315046	1	1
OLR1	1.0188616	1.01740877	1.0194914	1.119629623	0.1630216	1	1
AJ239322.1	1.0179945	1.01740877	1.0182484	1.048227536	0.0679519	1	1
RP11-118H15.1	1.0171393	1.01740877	1.0170224	0.977808733	-0.0323758	1	1
AC138517.6	1.0171382	1.01740877	1.017021	0.97772429	-0.0325004	1	1
PEX5L	1.0932404	1.09189826	1.0938222	1.020935468	0.0298917	1	1
PGBD4	1.0568958	1.0575152	1.0566272	0.984561431	-0.0224469	1	1
FOXA3	1.0456625	1.04610027	1.0454727	0.986387801	-0.0197731	1	1
CMB9-22P13.2	1.0171411	1.01740877	1.0170251	0.977958821	-0.0321544	1	1
RPGRIP1	1.0171366	1.01740877	1.0170186	0.977587738	-0.0327019	1	1
RP11-967K21.1	1.031884	1.03172107	1.0319546	1.007363359	0.0105842	1	1
TSKS	1.0171382	1.01740877	1.017021	0.97772429	-0.0325004	1	1
CMPK2	1.0474297	1.0459881	1.0480546	1.044936388	0.0634151	1	1
CASP10	1.018002	1.01740877	1.0182591	1.048845619	0.0688023	1	1
RP11-274H24.1	1.016279	1.01740877	1.0157893	0.90697433	-0.1408664	1	1

ERICH3	1.0197221	1.02025802	1.0194898	0.962077755	-0.0557746	1	1
NRXN1	1.0318272	1.03167478	1.0318933	1.006899631	0.0099199	1	1
RP4-583P15.15	1.0171388	1.01740877	1.0170217	0.977766924	-0.0324375	1	1
SLC22A16	1.0180015	1.01740877	1.0182584	1.048803545	0.0687445	1	1
GATA6	1.0171369	1.01740877	1.0170191	0.977617241	-0.0326584	1	1
CTA-204B4.2	1.0855411	1.08610498	1.0852967	0.990612844	-0.0136068	1	1
TLR7	1.017297	1.01815945	1.0169232	0.93192367	-0.1017163	1	1
RAD9B	1.0285832	1.02959475	1.0281447	0.951002018	-0.0724797	1	1
AF038458.5	1.0198886	1.02099848	1.0194075	0.92423369	-0.1136704	1	1
RP11-905K4.1	1.0173013	1.01815945	1.0169294	0.932263365	-0.1011905	1	1
KLHL30	1.0424103	1.04119734	1.0429361	1.042204405	0.0596383	1	1
RP5-1101C3.1	1.0172992	1.01815945	1.0169264	0.932097667	-0.101447	1	1
C8orf49	1.0190681	1.01815945	1.0194619	1.071722293	0.0999311	1	1
RP11-502M1.2	1.0173042	1.01815945	1.0169335	0.932490004	-0.1008398	1	1
C7orf77	1.0172948	1.01815945	1.01692	0.931747115	-0.1019896	1	1
C17orf98	1.0172964	1.01815945	1.0169223	0.931870432	-0.1017987	1	1
RP11-565A3.2	1.0190365	1.01815945	1.0194167	1.069231981	0.0965749	1	1
RP11-732A21.2	1.0233647	1.02388	1.0231413	0.969068095	-0.04533	1	1
GOLGA6L1	1.0173041	1.01815945	1.0169333	0.932480109	-0.1008551	1	1
RP11-993B23.3	1.0172945	1.01815945	1.0169196	0.931723757	-0.1020258	1	1
RP11-501C14.6	1.0172994	1.01815945	1.0169266	0.932110919	-0.1014265	1	1
RP5-862P8.2	1.0545653	1.05540391	1.0542018	0.978302468	-0.0316475	1	1
RP11-56G10.2	1.0173058	1.01815945	1.0169358	0.932614676	-0.100647	1	1
C21orf62	1.0544601	1.05530975	1.0540919	0.97798086	-0.0321219	1	1
LINC01402	1.0173031	1.01815945	1.0169319	0.932399055	-0.1009806	1	1
PABPN1L	1.0181726	1.01815945	1.0181783	1.001037285	0.0014957	1	1
GHRHR	1.0198819	1.02102403	1.0193869	0.922129346	-0.116959	1	1
CTD-2192J16.11	1.0173105	1.01815945	1.0169426	0.932988163	-0.1000693	1	1
FAM106A	1.0198795	1.02100688	1.0193909	0.923073503	-0.1154826	1	1
AC006116.21	1.0172964	1.01815945	1.0169223	0.931870432	-0.1017987	1	1
RP11-686D22.5	1.0181644	1.01815945	1.0181666	1.000392333	0.0005659	1	1
RP4-751H13.7	1.0173045	1.01815945	1.0169339	0.932514022	-0.1008027	1	1
FAM72A	1.0276236	1.02669695	1.0280253	1.049756165	0.0700543	1	1
RP1-309F20.4	1.0181693	1.01815945	1.0181735	1.000776172	0.0011193	1	1
GACAT2	1.0207481	1.02100071	1.0206386	0.982758184	-0.0250916	1	1
RP11-134G8.5	1.0250844	1.02388434	1.0256046	1.072025441	0.1003391	1	1
GAS8-AS1	1.0173012	1.01815945	1.0169292	0.932255015	-0.1012034	1	1
EBF2	1.0190522	1.01815945	1.0194392	1.070474814	0.0982509	1	1
MRAP2	1.021608	1.02102238	1.0218619	1.039935089	0.0564935	1	1
RP11-223P11.3	1.0173026	1.01815945	1.0169312	0.932364045	-0.1010347	1	1
FRMPD3	1.0181609	1.01815945	1.0181616	1.000115955	0.0001673	1	1
MTMR9	1.4171908	1.41677231	1.4173722	1.00143947	0.0020752	1	1
RP11-398G24.2	1.0181582	1.01815945	1.0181576	0.999898676	-0.0001462	1	1
ZP2	1.0172967	1.01815945	1.0169227	0.931895345	-0.1017601	1	1
PHACTR1	1.2873343	1.28759916	1.2872195	0.998679756	-0.001906	1	1
CTD-2035E11.4	1.0173006	1.01815945	1.0169283	0.932201517	-0.1012862	1	1
PSCA	1.0207528	1.02102444	1.020635	0.981477051	-0.0269736	1	1
RP11-335L23.4	1.0173033	1.01815945	1.0169322	0.932420768	-0.100947	1	1
RP11-446E24.4	1.0181587	1.01815945	1.0181584	0.999940487	-8.59E-05	1	1
LGALS9C	1.0181644	1.01815945	1.0181666	1.000394066	0.0005684	1	1
AC092171.2	1.022544	1.02102966	1.0232003	1.103220008	0.1417205	1	1

PRAMEF12	1.0173032	1.01815945	1.0169321	0.932413914	-0.1009576	1	1
SLC25A30-AS1	1.0172943	1.01815945	1.0169193	0.931706521	-0.1020525	1	1
CTD-2008L17.1	1.0546391	1.05536272	1.0543254	0.981262767	-0.0272886	1	1
C9orf66	1.0207834	1.02101394	1.0206835	0.984275204	-0.0228663	1	1
SLX1B	1.0250682	1.02384719	1.0255974	1.073391999	0.102177	1	1
CTD-2023N9.3	1.0181735	1.01815945	1.0181796	1.001108177	0.0015979	1	1
TF	1.0224842	1.02101951	1.0231191	1.099888475	0.1373572	1	1
RP11-169K16.4	1.0181613	1.01815945	1.018162	1.00014224	0.0002052	1	1
RP1-80N2.3	1.0328597	1.03244146	1.033041	1.018481365	0.0264196	1	1
AIRN	1.0173027	1.01815945	1.0169314	0.932372408	-0.1010218	1	1
RP4-764O22.1	1.0181634	1.01815945	1.0181651	1.00031192	0.0004499	1	1
RP11-20E24.1	1.0251309	1.02390708	1.0256614	1.073379099	0.1021597	1	1
RP11-457M11.7	1.0173058	1.01815945	1.0169358	0.932614676	-0.100647	1	1
TM4SF19-TCTEX1D2	1.019016	1.01815945	1.0193873	1.067616892	0.094394	1	1
RP1-111B22.3	1.0172967	1.01815945	1.0169227	0.931895345	-0.1017601	1	1
AC133785.1	1.019895	1.02102621	1.0194046	0.922876617	-0.1157903	1	1
RP11-548P2.2	1.0172945	1.01815945	1.0169196	0.931723757	-0.1020258	1	1
NOXO1	1.0207571	1.0210289	1.0206392	0.981470112	-0.0269838	1	1
SLCO4A1-AS1	1.0173016	1.01815945	1.0169297	0.932280069	-0.1011647	1	1
RP11-680A11.5	1.0458752	1.0469132	1.0454253	0.968283461	-0.0464986	1	1
RP11-162D9.3	1.0172963	1.01815945	1.0169222	0.931867033	-0.101804	1	1
SPP2	1.0190193	1.01815945	1.0193921	1.067876827	0.0947453	1	1
RP11-116O18.3	1.0173035	1.01815945	1.0169324	0.932431378	-0.1009305	1	1
POU5F1	1.0190186	1.01815945	1.019391	1.067817795	0.0946655	1	1
RP11-268G12.3	1.0173023	1.01815945	1.0169307	0.932337445	-0.1010759	1	1
CTC-444N24.6	1.0424136	1.04104552	1.0430066	1.047778372	0.0673336	1	1
RP11-374M1.3	1.0173018	1.01815945	1.0169301	0.932299741	-0.1011342	1	1
IL12A-AS1	1.0172963	1.01815945	1.0169221	0.931863634	-0.1018092	1	1
LEO1	1.2898017	1.29067162	1.2894247	0.995710123	-0.0062023	1	1
CCDC13	1.0518352	1.05249367	1.0515497	0.982018326	-0.0261781	1	1
AC068490.2	1.0190327	1.01815945	1.0194113	1.068934049	0.0961728	1	1
RP11-155O18.6	1.0173035	1.01815945	1.0169324	0.932431378	-0.1009305	1	1
AKR1B15	1.0181568	1.01815945	1.0181557	0.999791015	-0.0003015	1	1
VSIG2	1.0181611	1.01815945	1.0181618	1.000127688	0.0001842	1	1
RP11-7F18.2	1.0468114	1.046909	1.0467691	0.997017962	-0.0043086	1	1
ZNF786	1.1238423	1.12432104	1.1236347	0.99447962	-0.0079863	1	1
RP11-179A16.2	1.0173045	1.01815945	1.0169339	0.932509408	-0.1008098	1	1
PFKFB1	1.0190376	1.01815945	1.0194183	1.069321314	0.0966954	1	1
RP11-531A24.5	1.0216352	1.02102732	1.0218987	1.041442001	0.0585825	1	1
RP11-195B3.1	1.0172968	1.01815945	1.0169229	0.931907053	-0.101742	1	1
AC026150.8	1.0181689	1.01815945	1.018173	1.000746235	0.0010762	1	1
RP11-107I14.4	1.0172968	1.01815945	1.0169228	0.931901788	-0.1017502	1	1
NMUR1	1.0172962	1.01815945	1.016922	0.931858373	-0.1018174	1	1
RP11-96O20.5	1.0259506	1.02668975	1.0256302	0.96030079	-0.0584417	1	1
OXT	1.0181639	1.01815945	1.0181659	1.000353662	0.0005101	1	1
AC090587.5	1.0198867	1.02101951	1.0193957	0.922748528	-0.1159906	1	1
HCG15	1.0216588	1.0209943	1.0219469	1.045372988	0.0640178	1	1
SLC22A11	1.0190403	1.01815945	1.0194221	1.069531693	0.0969792	1	1
RP1-16A9.1	1.0172968	1.01815945	1.0169229	0.931907053	-0.101742	1	1
RP11-98D18.16	1.0181808	1.01815945	1.0181901	1.001685593	0.0024297	1	1
ROBO4	1.0199054	1.02101564	1.0194242	0.924271714	-0.1136111	1	1

RP4-564M11.2	1.0173026	1.01815945	1.0169312	0.932364045	-0.1010347	1	1
LINC00299	1.0173026	1.01815945	1.0169312	0.932364045	-0.1010347	1	1
RP11-89C3.3	1.0173035	1.01815945	1.0169324	0.932431378	-0.1009305	1	1
RP11-63A1.1	1.0233779	1.0238886	1.0231566	0.969356577	-0.0449006	1	1
CFP	1.0190264	1.01815945	1.0194021	1.068431696	0.0954947	1	1
RP11-158D2.2	1.018177	1.01815945	1.0181846	1.00138476	0.0019964	1	1
C5AR2	1.0198869	1.02102444	1.0193939	0.922443142	-0.1164681	1	1
RP11-544D21.2	1.017301	1.01815945	1.016929	0.932239372	-0.1012277	1	1
RP11-12D24.10	1.0172992	1.01815945	1.0169264	0.932097667	-0.101447	1	1
AVP	1.0172982	1.01815945	1.0169249	0.93201366	-0.101577	1	1
RP11-301L8.2	1.017304	1.01815945	1.0169332	0.93247137	-0.1008687	1	1
RP11-127L20.3	1.0172972	1.01815945	1.0169234	0.931933876	-0.1017005	1	1
ALDH3A1	1.0224693	1.02101318	1.0231004	1.099331172	0.1366261	1	1
WDFY4	1.0173035	1.01815945	1.0169324	0.932431378	-0.1009305	1	1
RP11-2B6.3	1.0207753	1.02102525	1.020667	0.982961284	-0.0247935	1	1
TMPRSS6	1.0172953	1.01815945	1.0169208	0.931790101	-0.1019231	1	1
RP11-688I9.4	1.0181606	1.01815945	1.0181612	1.000093832	0.0001354	1	1
RP11-687M24.8	1.0190617	1.01815945	1.0194527	1.071217954	0.099252	1	1
NINJ2	1.0190187	1.01815945	1.0193911	1.067826809	0.0946777	1	1
RP11-554E23.4	1.0181712	1.01815945	1.0181762	1.000923655	0.0013319	1	1
HOMER1	1.2518723	1.24997398	1.2526951	1.010885738	0.0156199	1	1
CTD-2026K11.2	1.0173011	1.01815945	1.0169291	0.932247721	-0.1012147	1	1
THADA	1.1549098	1.15582951	1.1545111	0.991539682	-0.0122576	1	1
NPY2R	1.0172972	1.01815945	1.0169235	0.931938785	-0.1016929	1	1
RP11-665G4.1	1.0224623	1.02100491	1.023094	1.099456906	0.1367911	1	1
MAML3	1.2072226	1.20770858	1.2070119	0.996645849	-0.0048472	1	1
GS1-204I12.4	1.0172967	1.01815945	1.0169227	0.931895345	-0.1017601	1	1
NPS	1.1432939	1.14414671	1.1429243	0.991519324	-0.0122872	1	1
RP11-474O21.5	1.019042	1.01815945	1.0194246	1.069669627	0.0971653	1	1
AC091814.3	1.0173007	1.01815945	1.0169284	0.932211406	-0.1012709	1	1
RP11-434H6.6	1.0172968	1.01815945	1.0169229	0.931907053	-0.101742	1	1
RP11-27M24.1	1.0198813	1.02101564	1.0193897	0.92262961	-0.1161765	1	1
AC020571.3	1.0172979	1.01815945	1.0169245	0.931993615	-0.101608	1	1
PCDHB8	1.0172955	1.01815945	1.016921	0.931803651	-0.1019021	1	1
ANKRD61	1.0173008	1.01815945	1.0169286	0.932221263	-0.1012557	1	1
RP11-332H18.7	1.0190541	1.01815945	1.0194418	1.070618794	0.0984449	1	1
GPR37L1	1.0190361	1.01815945	1.0194162	1.069203613	0.0965366	1	1
ENDOU	1.0173069	1.01815945	1.0169373	0.932700619	-0.100514	1	1
RP11-122K13.7	1.0172998	1.01815945	1.0169271	0.932137823	-0.1013848	1	1
XXyac-YX65C7_A.2	1.0181521	1.01815945	1.0181489	0.999418414	-0.0008393	1	1
RP11-233G1.4	1.0136628	1.01400962	1.0135124	0.964509548	-0.0521326	1	1
MATN1	1.017977	1.016846	1.0184672	1.096236008	0.1325584	1	1
AC015933.2	1.0153942	1.01400962	1.0159943	1.141667174	0.1911421	1	1
RP11-515O17.3	1.017127	1.01687033	1.0172383	1.021811933	0.0311297	1	1
RP11-316I3.1	1.013669	1.01400962	1.0135213	0.965143327	-0.0511849	1	1
HDAC11-AS1	1.0128011	1.01400962	1.0122773	0.876347828	-0.1904245	1	1
LINC00624	1.0145289	1.01400962	1.014754	1.053129698	0.0746831	1	1
RFPL1S	1.0369765	1.03682204	1.0370435	1.006014077	0.0086505	1	1
RP1-154K9.2	1.0145202	1.01400962	1.0147415	1.052244405	0.0734698	1	1
KCNH1	1.0153785	1.01400962	1.0159718	1.14006002	0.1891098	1	1
RP4-550H1.7	1.0257398	1.02544268	1.0258686	1.016739344	0.0239499	1	1

GATA4	1.0145164	1.01400962	1.014736	1.051848775	0.0729273	1	1
CTC-435M10.3	1.0136609	1.01400962	1.0135097	0.964316775	-0.0524209	1	1
AC007906.1	1.0162798	1.01686221	1.0160274	0.950492521	-0.0732528	1	1
RP3-416H24.1	1.0136599	1.01400962	1.0135083	0.96421296	-0.0525763	1	1
LINC01179	1.0136582	1.01400962	1.0135059	0.964042322	-0.0528316	1	1
RP11-426L16.10	1.013657	1.01400962	1.0135042	0.963923707	-0.0530091	1	1
AC022154.7	1.0179713	1.01685488	1.0184553	1.094950894	0.1308662	1	1
RP13-580F15.2	1.0162415	1.01685285	1.0159765	0.948001458	-0.0770388	1	1
RP11-455O6.9	1.0292093	1.02835788	1.0295783	1.043037905	0.0607916	1	1
LINC00992	1.0136594	1.01400962	1.0135075	0.964161099	-0.0526539	1	1
NME9	1.0637275	1.06263242	1.0642022	1.025063884	0.0357138	1	1
SLC15A3	1.0145426	1.01400962	1.0147737	1.054537899	0.0766109	1	1
CTD-2506P8.6	1.0326206	1.03114092	1.033262	1.068113425	0.0950649	1	1
CLSTN2-AS1	1.0128011	1.01400962	1.0122773	0.876347828	-0.1904245	1	1
ADAMTS16	1.0524445	1.05119247	1.0529872	1.035058065	0.0497117	1	1
CTD-2373H9.5	1.013662	1.01400962	1.0135113	0.964430914	-0.0522502	1	1
ABHD1	1.0515956	1.05111749	1.0518029	1.013407577	0.0192145	1	1
LPP-AS2	1.0300483	1.0311308	1.029579	0.950152533	-0.073769	1	1
NRG4	1.0378524	1.03688478	1.0382719	1.037606726	0.0532597	1	1
KANK3	1.0291898	1.02831633	1.0295684	1.044218672	0.0624239	1	1
ATP6V0A4	1.0128011	1.01400962	1.0122773	0.876347828	-0.1904245	1	1
AC093323.3	1.3326881	1.33379625	1.3322077	0.995241013	-0.0068822	1	1
KIAA0922	1.0455741	1.04554517	1.0455866	1.000909056	0.0013109	1	1
RP11-240G22.5	1.0179857	1.01685215	1.018477	1.096416498	0.1327959	1	1
TMEM212	1.0162319	1.01684363	1.0159668	0.947941839	-0.0771295	1	1
RP11-290L1.5	1.0136607	1.01400962	1.0135095	0.964301586	-0.0524437	1	1
POU2F3	1.0170954	1.01685439	1.0171999	1.02049807	0.0292735	1	1
RP5-908M14.5	1.0136607	1.01400962	1.0135094	0.964295222	-0.0524532	1	1
PCDH9-AS4	1.0128011	1.01400962	1.0122773	0.876347828	-0.1904245	1	1
RP11-266K4.13	1.0145208	1.01400962	1.0147423	1.052298821	0.0735444	1	1
MEGF11	1.0300982	1.0311532	1.0296409	0.95145612	-0.071791	1	1
LLNLR-246C6.1	1.0145288	1.01400962	1.0147539	1.053122708	0.0746735	1	1
RP1-118J21.5	1.0145203	1.01400962	1.0147417	1.052254963	0.0734843	1	1
RP11-130C19.3	1.0136621	1.01400962	1.0135115	0.96444625	-0.0522273	1	1
RP11-65D17.1	1.0136597	1.01400962	1.013508	0.964197788	-0.052599	1	1
RP11-442N1.1	1.0154097	1.01400962	1.0160165	1.143249842	0.1931407	1	1
RP11-347I19.8	1.0154046	1.01400962	1.0160093	1.142734326	0.19249	1	1
RP11-173A6.3	1.0188598	1.01971325	1.0184899	0.937944798	-0.0924251	1	1
GNA15	1.0136602	1.01400962	1.0135088	0.964249672	-0.0525213	1	1
MTRNR2L6	1.0128011	1.01400962	1.0122773	0.876347828	-0.1904245	1	1
AP000473.8	1.0215027	1.02263111	1.0210136	0.928526791	-0.1069846	1	1
KRT81	1.0170983	1.01686575	1.0171991	1.019766699	0.0282391	1	1
GJC2	1.0136591	1.01400962	1.0135072	0.964134715	-0.0526934	1	1
HRCT1	1.0128011	1.01400962	1.0122773	0.876347828	-0.1904245	1	1
CTD-2561B21.7	1.0145265	1.01400962	1.0147505	1.052882668	0.0743447	1	1
INPP5J	1.0464791	1.04547365	1.0469148	1.031692933	0.0450136	1	1
RP1-37E16.12	1.015383	1.01400962	1.0159783	1.140523109	0.1896957	1	1
MMD2	1.0128011	1.01400962	1.0122773	0.876347828	-0.1904245	1	1
RP11-412H8.2	1.0136581	1.01400962	1.0135057	0.964031579	-0.0528477	1	1
SLFN14	1.0128011	1.01400962	1.0122773	0.876347828	-0.1904245	1	1
RP11-552M14.1	1.0128011	1.01400962	1.0122773	0.876347828	-0.1904245	1	1

IL1B	1.0145174	1.01400962	1.0147375	1.051956793	0.0730755	1	1
RP11-84A19.2	1.014517	1.01400962	1.014737	1.051918127	0.0730224	1	1
LIG4	1.1127983	1.11404792	1.1122566	0.984293104	-0.0228401	1	1
DRICH1	1.0128011	1.01400962	1.0122773	0.876347828	-0.1904245	1	1
PWRN3	1.01366	1.01400962	1.0135084	0.964223726	-0.0525602	1	1
CACNA1B	1.0145181	1.01400962	1.0147385	1.052026231	0.0731707	1	1
DOK7	1.0162459	1.01687265	1.0159742	0.946751132	-0.0789429	1	1
AP001626.2	1.0170955	1.01685654	1.0171991	1.020320369	0.0290222	1	1
RP11-665C14.2	1.0231572	1.0226012	1.0233981	1.035260627	0.049994	1	1
GRIN3A	1.0136621	1.01400962	1.0135115	0.96444625	-0.0522273	1	1
AC022201.5	1.0136588	1.01400962	1.0135067	0.964104865	-0.052738	1	1
RP11-968A15.2	1.0145163	1.01400962	1.0147359	1.05184436	0.0729212	1	1
REG1A	1.0128011	1.01400962	1.0122773	0.876347828	-0.1904245	1	1
GRM4	1.0136599	1.01400962	1.0135083	0.96421296	-0.0525763	1	1
CTD-3126B10.4	1.0153966	1.01400962	1.0159978	1.141913309	0.1914531	1	1
RP11-445O16.3	1.0154034	1.01400962	1.0160075	1.142608306	0.1923309	1	1
RP11-304M2.5	1.0136581	1.01400962	1.0135058	0.964035977	-0.0528411	1	1
RP11-500G22.5	1.0188247	1.01971905	1.0184371	0.934988539	-0.0969794	1	1
RP11-10O22.1	1.0145326	1.01400962	1.0147593	1.053511025	0.0752054	1	1
MIR142	1.0128011	1.01400962	1.0122773	0.876347828	-0.1904245	1	1
RP5-1142A6.10	1.0136623	1.01400962	1.0135118	0.964466351	-0.0521972	1	1
HAPLN2	1.0205727	1.01972679	1.0209394	1.061471578	0.0860657	1	1
CTD-2044J15.1	1.0136594	1.01400962	1.0135075	0.964161099	-0.0526539	1	1
ARHGAP22	1.0162398	1.01684949	1.0159756	0.948133638	-0.0768377	1	1
RP4-583P15.10	1.0205842	1.01970273	1.0209662	1.064127853	0.0896715	1	1
CABLES1	1.0420955	1.04252541	1.0419091	0.985508054	-0.0210604	1	1
PF4V1	1.0171029	1.01686041	1.017208	1.020613635	0.0294368	1	1
RP11-666A8.8	1.0179934	1.01687265	1.0184792	1.095218142	0.1312183	1	1
RP11-90P5.2	1.0153844	1.01400962	1.0159804	1.140670789	0.1898825	1	1
RP11-449P15.2	1.028303	1.02830815	1.0283008	0.999739588	-0.0003757	1	1
RP11-109A6.3	1.0231511	1.02257377	1.0234013	1.036658359	0.0519405	1	1
RP11-661A12.12	1.0153908	1.01400962	1.0159895	1.141325398	0.1907102	1	1
CTD-2196P11.2	1.0136597	1.01400962	1.013508	0.964191432	-0.0526085	1	1
RP5-902P8.12	1.0136582	1.01400962	1.0135059	0.964042322	-0.0528316	1	1
RP13-895J2.2	1.0136609	1.01400962	1.0135097	0.964316775	-0.0524209	1	1
RP11-86H7.6	1.0145223	1.01400962	1.0147445	1.052452157	0.0737547	1	1
ZNF28	1.1007906	1.09968701	1.1012689	1.015868691	0.0227139	1	1
CTD-2215E18.1	1.0136626	1.01400962	1.0135122	0.964492369	-0.0521583	1	1
RP11-6E9.5	1.0128011	1.01400962	1.0122773	0.876347828	-0.1904245	1	1
AC012358.7	1.0128011	1.01400962	1.0122773	0.876347828	-0.1904245	1	1
RP11-81H14.2	1.0153862	1.01400962	1.0159829	1.14085109	0.1901105	1	1
KCCAT211	1.0145364	1.01400962	1.0147647	1.053899255	0.075737	1	1
RP11-368I23.3	1.0387183	1.03972686	1.0382812	0.963608916	-0.0534804	1	1
SLC36A2	1.0136644	1.01400962	1.0135147	0.964672233	-0.0518893	1	1
C1QL3	1.0145187	1.01400962	1.0147394	1.052088859	0.0732566	1	1
FOXN1	1.0136594	1.01400962	1.0135075	0.964161099	-0.0526539	1	1
GJA8	1.0153798	1.01400962	1.0159738	1.140198439	0.1892849	1	1
RP11-650L12.2	1.0335274	1.03406607	1.033294	0.977334692	-0.0330754	1	1
RP11-77H9.6	1.0136607	1.01400962	1.0135094	0.964295222	-0.0524532	1	1
APOA5	1.0145326	1.01400962	1.0147593	1.053511025	0.0752054	1	1
AP003774.6	1.018835	1.01969726	1.0184613	0.937252715	-0.09349	1	1

FREM1	1.0179656	1.01684782	1.0184501	1.095100564	0.1310634	1	1
RP11-20J15.3	1.0265972	1.02544651	1.027096	1.064822062	0.0906124	1	1
COLCA2	1.0464691	1.04546766	1.0469031	1.031571506	0.0448438	1	1
RP11-517I3.1	1.0136595	1.01400962	1.0135078	0.964178213	-0.0526283	1	1
RP11-16C18.3	1.0128011	1.01400962	1.0122773	0.876347828	-0.1904245	1	1
CTD-2026K11.4	1.0136593	1.01400962	1.0135074	0.96415428	-0.0526641	1	1
ZNF662	1.0136587	1.01400962	1.0135066	0.964094114	-0.0527541	1	1
RP11-249L12.1	1.0145382	1.01400962	1.0147673	1.054083314	0.0759889	1	1
RP11-16L21.7	1.0128011	1.01400962	1.0122773	0.876347828	-0.1904245	1	1
NDP-AS1	1.0153965	1.01400962	1.0159976	1.14190099	0.1914376	1	1
RP11-282I1.1	1.0128011	1.01400962	1.0122773	0.876347828	-0.1904245	1	1
RP11-1078H9.1	1.0136617	1.01400962	1.0135109	0.964401088	-0.0522948	1	1
CTB-11I22.1	1.0136617	1.01400962	1.0135109	0.964401088	-0.0522948	1	1
TTC39C-AS1	1.0248574	1.02539558	1.0246241	0.969619824	-0.0445089	1	1
LCMT1-AS2	1.015378	1.01400962	1.0159711	1.140012542	0.1890497	1	1
RP11-290F5.1	1.013664	1.01400962	1.0135142	0.964637347	-0.0519414	1	1
AC007557.1	1.0136621	1.01400962	1.0135114	0.964437288	-0.0522407	1	1
PTGES3L-AARSD1	1.0145141	1.01400962	1.0147327	1.051613844	0.072605	1	1
HRH4	1.014519	1.01400962	1.0147398	1.052119969	0.0732992	1	1
DHRS12	1.1205922	1.11969827	1.1209797	1.010705593	0.0153628	1	1
AP003774.4	1.0162569	1.01686294	1.0159942	0.948480772	-0.0763096	1	1
KCNQ1	1.0136597	1.01400962	1.013508	0.964197788	-0.052599	1	1
KCNK3	1.0188092	1.01968007	1.0184317	0.936566274	-0.094547	1	1
SLC24A3	1.0136609	1.01400962	1.0135097	0.964316775	-0.0524209	1	1
TNNI3K	1.0205676	1.01971354	1.0209378	1.062104084	0.0869252	1	1
RP5-1160K1.6	1.0145305	1.01400962	1.0147562	1.053293072	0.0749069	1	1
CTA-407F11.7	1.0153962	1.01400962	1.0159972	1.141869641	0.191398	1	1
UTF1	1.0145169	1.01400962	1.0147368	1.051904917	0.0730043	1	1
AC092718.1	1.0136605	1.01400962	1.0135091	0.964275625	-0.0524825	1	1
SLC35F2	1.1662227	1.16513034	1.1666962	1.009482329	0.0136157	1	1
RP11-4B16.4	1.0145291	1.01400962	1.0147542	1.053149228	0.0747099	1	1
RP11-3G20.2	1.0136633	1.01400962	1.0135132	0.964565449	-0.052049	1	1
PCDHGA2	1.0136579	1.01400962	1.0135055	0.964015974	-0.052871	1	1
RP1-59D14.1	1.0145244	1.01400962	1.0147475	1.052671268	0.074055	1	1
AP001092.4	1.0162368	1.01685654	1.0159682	0.947298846	-0.0781085	1	1
CTD-3193O13.14	1.0145313	1.01400962	1.0147575	1.053379812	0.0750257	1	1
CTD-2649C14.3	1.0145165	1.01400962	1.0147362	1.051860008	0.0729427	1	1
MNDA	1.0136609	1.01400962	1.0135097	0.964316775	-0.0524209	1	1
DAZL	1.0145253	1.01400962	1.0147488	1.052759529	0.0741759	1	1
IL33	1.0136686	1.01400962	1.0135208	0.965105773	-0.051241	1	1
RP11-431K24.1	1.0179702	1.01685853	1.0184521	1.094524319	0.130304	1	1
SPANXD	1.0145124	1.01400962	1.0147303	1.051439104	0.0723653	1	1
AC116609.2	1.0184518	1.01850742	1.0184277	0.995692276	-0.0062282	1	1
LMF1-AS1	1.0184736	1.01850742	1.018459	0.997383461	-0.0037798	1	1
TPSD1	1.0175927	1.01850742	1.0171962	0.929153503	-0.1060111	1	1
RP3-454G6.2	1.0193302	1.01850742	1.0196868	1.063723174	0.0891227	1	1
RP11-442N24__B.1	1.0175923	1.01850742	1.0171956	0.929120651	-0.1060621	1	1
TRMO	1.070324	1.07021435	1.0703715	1.002238793	0.0032263	1	1
PAUPAR	1.0462354	1.04714161	1.0458426	0.972444835	-0.0403117	1	1
RP11-482D24.3	1.0175947	1.01850742	1.0171991	0.929306457	-0.1057737	1	1
PDC	1.0271316	1.02708555	1.0271516	1.00243857	0.0035138	1	1

BACH1-AS1	1.021935	1.02135955	1.0221844	1.038615825	0.0546621	1	1
CTC-273B12.5	1.0184715	1.01850742	1.0184559	0.997217352	-0.0040201	1	1
RP11-467C18.1	1.017594	1.01850742	1.017198	0.929251	-0.1058598	1	1
LINC00326	1.0175894	1.01850742	1.0171915	0.928896512	-0.1064102	1	1
PLA1A	1.0193209	1.01850742	1.0196736	1.063009273	0.0881542	1	1
GGT5	1.0227634	1.02136509	1.0233695	1.093817222	0.1293717	1	1
EXOC3L4	1.0175927	1.01850742	1.0171962	0.929153503	-0.1060111	1	1
EIF4E1B	1.0175992	1.01850742	1.0172055	0.929653842	-0.1052345	1	1
RP11-379P15.1	1.0184722	1.01850742	1.018457	0.997273762	-0.0039385	1	1
RP11-452I5.2	1.0175948	1.01850742	1.0171992	0.929316146	-0.1057586	1	1
NEUROG2	1.0193443	1.01850742	1.0197071	1.064820067	0.0906097	1	1
RP11-275N1.1	1.0175925	1.01850742	1.017196	0.929139327	-0.1060331	1	1
MAGEB10	1.0175907	1.01850742	1.0171933	0.928994439	-0.1062581	1	1
RP11-123K19.1	1.0184528	1.01850742	1.0184291	0.99576734	-0.0061194	1	1
AP000487.4	1.0175873	1.01850742	1.0171884	0.928732906	-0.1066643	1	1
RP11-525A16.4	1.0175907	1.01850742	1.0171933	0.928994439	-0.1062581	1	1
AC005757.6	1.0201904	1.02136649	1.0196806	0.921096179	-0.1185763	1	1
RP11-162D16.2	1.02453	1.02426622	1.0246444	1.015583613	0.022309	1	1
MAN2C1	1.2370591	1.236883	1.2371355	1.001065824	0.0015368	1	1
RP11-650P15.1	1.0175881	1.01850742	1.0171896	0.928795044	-0.1065678	1	1
RP11-325L12.7	1.0193293	1.01850742	1.0196856	1.063657998	0.0890343	1	1
ZFX-AS1	1.0175881	1.01850742	1.0171896	0.928795044	-0.1065678	1	1
RIIAD1	1.0391929	1.03853115	1.0394797	1.02461827	0.0350865	1	1
RP11-557H15.4	1.0193236	1.01850742	1.0196774	1.063217253	0.0884364	1	1
RP11-429E11.3	1.0193204	1.01850742	1.0196728	1.062970752	0.0881019	1	1
SDR9C7	1.0184496	1.01850742	1.0184246	0.995525061	-0.0064705	1	1
PPHLN1	2.0094421	2.00875067	2.0097418	1.000982515	0.0014168	1	1
RP11-710F7.3	1.026318	1.02712031	1.0259702	0.957593789	-0.0625143	1	1
TTLL11-IT1	1.0184564	1.01850742	1.0184343	0.996048866	-0.0057116	1	1
CELA1	1.0175916	1.01850742	1.0171946	0.929066828	-0.1061457	1	1
NADK2	1.1988103	1.1994505	1.1985328	0.995398905	-0.0066533	1	1
RDH12	1.0228546	1.02135835	1.0235032	1.100423022	0.1380582	1	1
RP11-573D15.8	1.0184692	1.01850742	1.0184526	0.997037098	-0.0042809	1	1
PPIAL4G	1.0202092	1.02137838	1.0197025	0.921606328	-0.1177775	1	1
CTD-2503I6.1	1.0175913	1.01850742	1.0171942	0.929044868	-0.1061798	1	1
RP11-793H13.10	1.0175888	1.01850742	1.0171907	0.928853553	-0.1064769	1	1
FAM210B	2.381963	2.3808305	2.3824539	1.001175698	0.0016952	1	1
CTB-191K22.6	1.0175893	1.01850742	1.0171913	0.9288865	-0.1064258	1	1
XXbac-B476C20.9	1.0270899	1.02709903	1.0270859	0.999516323	-0.000698	1	1
XXbac-BPG181M17.6	1.0201803	1.0213765	1.0196618	0.919785087	-0.1206313	1	1
TMPRSS13	1.0227825	1.02136577	1.0233966	1.095052021	0.1309994	1	1
IRAK3	1.0262146	1.02707385	1.0258422	0.954506402	-0.0671732	1	1
SNCAIP	1.0488033	1.04731066	1.0494503	1.045225116	0.0638137	1	1
AC114877.3	1.0175952	1.01850742	1.0171998	0.929344602	-0.1057144	1	1
AC093843.1	1.0175889	1.01850742	1.0171908	0.928857919	-0.1064702	1	1
RP1-45N11.1	1.021941	1.02136577	1.0221904	1.038595205	0.0546335	1	1
STK11IP	1.151879	1.1503387	1.1525467	1.014686837	0.0210345	1	1
TRAF5	1.1810629	1.18191924	1.1806917	0.993252023	-0.0097683	1	1
SIX2	1.0176006	1.01850742	1.0172075	0.929764879	-0.1050622	1	1
RASGRP2	1.0598726	1.0585047	1.0604655	1.033515652	0.0475602	1	1
LINC00959	1.0911275	1.0901075	1.0915696	1.016226405	0.0232219	1	1

RP11-99L13.2	1.0184583	1.01850742	1.018437	0.99619593	-0.0054986	1	1
CTC-453G23.4	1.0175912	1.01850742	1.0171941	0.929037489	-0.1061913	1	1
LRTM2	1.0175948	1.01850742	1.0171992	0.929316146	-0.1057586	1	1
RP11-313A24.1	1.0175891	1.01850742	1.0171911	0.928877269	-0.1064401	1	1
GLT6D1	1.0175935	1.01850742	1.0171974	0.929217424	-0.1059119	1	1
TBC1D10C	1.0227961	1.02138042	1.0234097	1.094911955	0.1308149	1	1
RP11-13A1.1	1.0175894	1.01850742	1.0171914	0.928893174	-0.1064154	1	1
RP11-231G3.1	1.0271183	1.02712981	1.0271132	0.999389124	-0.0008816	1	1
CTC-558O2.1	1.0193324	1.01850742	1.01969	1.063899284	0.0893616	1	1
HNRNPCL1	1.0184721	1.01850742	1.0184568	0.997266995	-0.0039483	1	1
PTF1A	1.0193344	1.01850742	1.0196928	1.064051366	0.0895678	1	1
PON1	1.0184613	1.01850742	1.0184414	0.996431062	-0.0051581	1	1
EIF2AK1	2.4138183	2.41273374	2.4142883	1.001100423	0.0015867	1	1
CTD-2033D15.3	1.0175945	1.01850742	1.0171988	0.929290504	-0.1057984	1	1
CTB-133P21.1	1.0175949	1.01850742	1.0171994	0.929323313	-0.1057475	1	1
RP11-109P14.10	1.0184733	1.01850742	1.0184585	0.99735715	-0.0038179	1	1
RP11-867G23.12	1.0175948	1.01850742	1.0171992	0.929316146	-0.1057586	1	1
RP11-297J22.1	1.0374773	1.03854833	1.037013	0.960172402	-0.0586346	1	1
ZNF526	1.1318295	1.13017375	1.1325472	1.018233207	0.026068	1	1
VMO1	1.0357985	1.03573823	1.0358247	1.002418582	0.0034851	1	1
HUNK	1.0982331	1.09897596	1.0979112	0.989241958	-0.0156047	1	1
RSPO4	1.0454195	1.0442661	1.0459194	1.037348647	0.0529009	1	1
CNBD1	1.0184543	1.01850742	1.0184312	0.995883583	-0.005951	1	1
CTD-2026J24.1	1.0193252	1.01850742	1.0196796	1.063337596	0.0885997	1	1
KCNH6	1.0201962	1.02135805	1.0196925	0.92201927	-0.1171312	1	1
ABCF3	1.139814	1.1391213	1.1401143	1.007137314	0.0102604	1	1
ELANE	1.0175912	1.01850742	1.0171941	0.929037489	-0.1061913	1	1
RP13-512J5.1	1.0175868	1.01850742	1.0171877	0.928693731	-0.1067252	1	1
XXbac-B444P24.8	1.0175889	1.01850742	1.0171908	0.928857919	-0.1064702	1	1
PCDHB6	1.0280156	1.0271189	1.0284042	1.047395199	0.0668059	1	1
MMP25	1.0175907	1.01850742	1.0171933	0.928994439	-0.1062581	1	1
ABCG4	1.0280138	1.02713035	1.0283967	1.04667731	0.0658167	1	1
CTD-2281M20.1	1.0175925	1.01850742	1.017196	0.929140361	-0.1060315	1	1
PLXNB3	1.0175909	1.01850742	1.0171936	0.92900929	-0.1062351	1	1
SSX1	1.0210396	1.02134394	1.0209076	0.979558716	-0.0297961	1	1
CTC-296K1.4	1.0175873	1.01850742	1.0171884	0.928732906	-0.1066643	1	1
CNPY1	1.0418668	1.04142148	1.0420599	1.015411921	0.0220651	1	1
HSD11B2	1.0271652	1.02706969	1.0272065	1.005055589	0.0072753	1	1
RP11-112J3.15	1.0184493	1.01850742	1.0184242	0.995502236	-0.0065035	1	1
AP006285.2	1.0262309	1.02707142	1.0258666	0.955493273	-0.0656824	1	1
RP11-9N12.2	1.0210621	1.02137902	1.0209247	0.978749032	-0.0309891	1	1
RP11-379L18.1	1.0175906	1.01850742	1.0171932	0.928991449	-0.1062628	1	1
MEDAG	1.0339697	1.03284171	1.0344586	1.049232562	0.0693345	1	1
RP11-63M22.2	1.018454	1.01850742	1.0184308	0.995860818	-0.005984	1	1
CD6	1.0175981	1.01850742	1.0172039	0.929569565	-0.1053653	1	1
RP11-293E1.2	1.017593	1.01850742	1.0171966	0.929174706	-0.1059782	1	1
LINC01132	1.0201829	1.02136306	1.0196713	0.920811535	-0.1190222	1	1
CTC-499J9.1	1.0184517	1.01850742	1.0184276	0.995688051	-0.0062343	1	1
PGAP3	1.0859069	1.0842778	1.0866131	1.027709345	0.0394323	1	1
M1AP	1.0193082	1.01850742	1.0196553	1.062023804	0.0868161	1	1
FAM228A	1.0322931	1.03284827	1.0320524	0.975771348	-0.035385	1	1

RP11-546B15.1	1.0175926	1.01850742	1.017196	0.929143485	-0.1060267	1	1
SLC25A31	1.0175881	1.01850742	1.0171896	0.928795044	-0.1065678	1	1
CTC-228N24.2	1.0175871	1.01850742	1.0171881	0.928716644	-0.1066896	1	1
DLGAP1-AS4	1.019331	1.01850742	1.019688	1.063788053	0.0892107	1	1
CRNKL1	1.2906345	1.29123828	1.2903728	0.997028248	-0.0042937	1	1
RP1-202O8.3	1.0557625	1.05588588	1.0557091	0.996835889	-0.0045721	1	1
C9orf147	1.02974	1.03004371	1.0296084	0.985509416	-0.0210584	1	1
CNTF	1.0175942	1.01850742	1.0171983	0.929267393	-0.1058343	1	1
PWRN4	1.0175939	1.01850742	1.0171979	0.929242805	-0.1058725	1	1
CTD-3099C6.7	1.0175893	1.01850742	1.0171914	0.928889837	-0.1064206	1	1
KCNS1	1.0193467	1.01850742	1.0197106	1.065008252	0.0908646	1	1
CTA-992D9.7	1.0175904	1.01850742	1.0171929	0.928971783	-0.1062933	1	1
AC005954.4	1.0237068	1.02420529	1.0234908	0.970481939	-0.0432267	1	1
AC004012.1	1.0184605	1.01850742	1.0184402	0.996365894	-0.0052525	1	1
RP11-10J21.6	1.0193124	1.01850742	1.0196613	1.062345632	0.0872532	1	1
MIATNB	1.0296993	1.02995214	1.0295898	0.987901263	-0.0175612	1	1
CTC-510F12.2	1.0184567	1.01850742	1.0184347	0.99607226	-0.0056777	1	1
ABCB5	1.0245543	1.02424275	1.0246894	1.018422641	0.0263364	1	1
RP11-108M12.3	1.0400769	1.03854475	1.040741	1.056978127	0.0799455	1	1
ZNF296	1.0384799	1.03854815	1.0384503	0.997462278	-0.0036658	1	1
FAM110A	1.1198333	1.1187612	1.120298	1.012940012	0.0185487	1	1
RP11-94I2.4	1.0262625	1.02710062	1.0258992	0.955668606	-0.0654177	1	1
AC079776.1	1.0193251	1.01899597	1.0194678	1.024835699	0.0353926	1	1
LINC00470	1.0193215	1.01899597	1.0194626	1.02456313	0.0350089	1	1
KB-1043D8.8	1.0184613	1.01899597	1.0182296	0.959655193	-0.059412	1	1
RP11-66B24.1	1.0184522	1.01899597	1.0182166	0.95896957	-0.0604431	1	1
STX11	1.0201663	1.01899597	1.0206735	1.088311219	0.1220912	1	1
XXbac-BCX196D17.5	1.0210888	1.02183836	1.0207639	0.950798926	-0.0727878	1	1
RP11-368P15.3	1.0202243	1.01899597	1.0207568	1.092692504	0.1278875	1	1
LINC01040	1.0193446	1.01899597	1.0194957	1.026304849	0.0374593	1	1
DCDC2C	1.0184557	1.01899597	1.0182215	0.959231173	-0.0600496	1	1
POU4F1	1.019352	1.01899597	1.0195063	1.026866549	0.0382487	1	1
LINC01583	1.0193084	1.01899597	1.0194438	1.023574605	0.0336163	1	1
LINC01470	1.020194	1.01899597	1.0207133	1.090404411	0.1248633	1	1
ARHGEF18	1.0236497	1.02470775	1.0231911	0.938616198	-0.0913927	1	1
RP11-461A8.5	1.0184495	1.01899597	1.0182127	0.958764213	-0.060752	1	1
RP11-192H23.5	1.0184522	1.01899597	1.0182166	0.95896957	-0.0604431	1	1
AC092614.2	1.0193227	1.01899597	1.0194643	1.024652977	0.0351354	1	1
C7orf25	1.0332124	1.03329906	1.0331749	0.996270791	-0.0053902	1	1
XXbac-BPGBPG55C20.1	1.025386	1.02472285	1.0256734	1.03845006	0.0544318	1	1
SAP30L-AS1	1.0202181	1.01899597	1.0207479	1.092223997	0.1272688	1	1
AC073343.13	1.0201868	1.01899597	1.020703	1.089863311	0.1241472	1	1
RP11-679B19.1	1.0686694	1.06786144	1.0690196	1.017066478	0.024414	1	1
RP11-498P14.3	1.0184553	1.01899597	1.0182209	0.959199855	-0.0600967	1	1
DGKK	1.0193343	1.01899597	1.0194809	1.025530138	0.0363699	1	1
CTC-366B18.2	1.025477	1.02470854	1.02581	1.044579193	0.0629219	1	1
CTD-3214H19.4	1.030577	1.03040326	1.0306523	1.008192287	0.0117708	1	1
RP11-1100L3.8	1.0184533	1.01899597	1.018218	0.959045897	-0.0603282	1	1
RP4-660H19.1	1.0271801	1.02762634	1.0269867	0.976845267	-0.033798	1	1
CD19	1.021058	1.02186987	1.0207061	0.94678669	-0.0788887	1	1
HNRNPD	3.2158223	3.2166602	3.2154591	0.999458145	-0.0007819	1	1

RP11-776H12.1	1.0262384	1.02473116	1.0268918	1.087363268	0.120834	1	1
CEACAM19	1.0470989	1.04769327	1.0468413	0.982136701	-0.0260043	1	1
CTD-2521M24.8	1.0193126	1.01899597	1.0194498	1.023891636	0.034063	1	1
VAMP1	1.1371972	1.13675462	1.137389	1.004639133	0.0066774	1	1
RP11-335O4.3	1.0193285	1.01899597	1.0194726	1.025090823	0.0357517	1	1
GTF2A1L	1.0202293	1.01899597	1.0207639	1.093067937	0.1283831	1	1
GLS2	1.0288719	1.02763753	1.029407	1.064024619	0.0895315	1	1
CTC-453G23.8	1.0184631	1.01899597	1.0182321	0.959789039	-0.0592108	1	1
LINC01068	1.020195	1.01899597	1.0207147	1.090478759	0.1249617	1	1
RP11-483L5.1	1.0201673	1.01899597	1.020675	1.08838857	0.1221937	1	1
CDKL1	1.0288016	1.02754303	1.0293472	1.065503687	0.0915356	1	1
DCDC2B	1.0184613	1.01899597	1.0182296	0.959655193	-0.059412	1	1
AC015849.16	1.0184498	1.01899597	1.0182131	0.958786557	-0.0607184	1	1
NAV2-AS4	1.0193144	1.01899597	1.0194525	1.024030897	0.0342592	1	1
TMEM244	1.0184513	1.01899597	1.0182153	0.958901274	-0.0605458	1	1
AC010884.1	1.0271375	1.02754535	1.0269607	0.97877667	-0.0309484	1	1
RP1-276E15.1	1.0219376	1.02184991	1.0219756	1.005750222	0.0082721	1	1
AC005387.3	1.0184507	1.01899597	1.0182144	0.958856513	-0.0606132	1	1
RP5-1182A14.5	1.0184543	1.01899597	1.0182195	0.959124791	-0.0602096	1	1
PPIF	1.4634426	1.46377601	1.4632981	0.998969499	-0.0014875	1	1
RP11-284F21.9	1.0270881	1.0275982	1.026867	0.973506751	-0.0387371	1	1
ADRB2	1.0322597	1.03337999	1.0317741	0.951889514	-0.071134	1	1
UCN2	1.0193256	1.01899597	1.0194685	1.024872521	0.0354445	1	1
RP11-182J1.3	1.0227624	1.02184804	1.0231588	1.059994005	0.0840561	1	1
KRT27	1.0184508	1.01899597	1.0182145	0.95885942	-0.0606088	1	1
TMEM221	1.0513892	1.05066006	1.0517053	1.020631996	0.0294628	1	1
RP11-626P14.2	1.0210435	1.02187451	1.0206833	0.945545014	-0.080782	1	1
PGA5	1.0184555	1.01899597	1.0182212	0.95921616	-0.0600721	1	1
SUSD3	1.0279291	1.02753558	1.0280997	1.020488516	0.0292599	1	1
RP11-17A1.3	1.0184507	1.01899597	1.0182144	0.958856513	-0.0606132	1	1
EFCAB9	1.0193217	1.01899597	1.0194629	1.02458185	0.0350352	1	1
RP11-19J5.2	1.0202015	1.01899597	1.0207241	1.090973798	0.1256165	1	1
AC034110.1	1.0184542	1.01899597	1.0182194	0.959120084	-0.0602166	1	1
CTC-232P5.1	1.0193332	1.01899597	1.0194793	1.02544457	0.0362495	1	1
RP11-428J1.4	1.0236452	1.02471132	1.0231831	0.938155201	-0.0921015	1	1
IRX4	1.0184564	1.01899597	1.0182226	0.959285133	-0.0599684	1	1
GPR88	1.0193366	1.01899597	1.0194842	1.025700882	0.0366101	1	1
RP11-293M10.6	1.0201779	1.01899597	1.0206902	1.089191024	0.123257	1	1
KREMEN2	1.0928577	1.09350946	1.0925752	0.990009358	-0.0144859	1	1
MIR3681HG	1.0279581	1.02756548	1.0281283	1.020415832	0.0291572	1	1
OSGEPL1-AS1	1.043484	1.04185756	1.044189	1.055698545	0.0781979	1	1
AC145124.2	1.2636623	1.26309262	1.2639092	1.003103778	0.0044709	1	1
C8G	1.0184487	1.01899597	1.0182115	0.958703709	-0.0608431	1	1
SPDEF	1.022792	1.0218327	1.0232078	1.06298355	0.0881193	1	1
YEATS2-AS1	1.0184543	1.01899597	1.0182196	0.959128054	-0.0602047	1	1
RP4-545C24.1	1.0288056	1.02756289	1.0293443	1.064631216	0.0903538	1	1
ADGRF1	1.0184516	1.01899597	1.0182157	0.958923666	-0.0605121	1	1
AP000442.4	1.0184492	1.01899597	1.0182122	0.958741876	-0.0607856	1	1
RNF32	1.0938816	1.09335911	1.0941081	1.008022576	0.011528	1	1
RP11-678G14.2	1.0262311	1.02472723	1.0268829	1.087179152	0.1205897	1	1
AC004895.4	1.0201904	1.01899597	1.0207081	1.090130692	0.1245011	1	1

RP11-146F11.5	1.0184597	1.01899597	1.0182272	0.959530684	-0.0595992	1	1
CTD-2616J11.11	1.0193434	1.01899597	1.019494	1.026217305	0.0373363	1	1
RP11-638L3.1	1.0184518	1.01899597	1.0182159	0.958933051	-0.060498	1	1
RP11-513N24.1	1.0193153	1.01899597	1.0194537	1.024096025	0.034351	1	1
RASSF10	1.0201809	1.01899597	1.0206945	1.089415909	0.1235548	1	1
ITGB2-AS1	1.0184564	1.01899597	1.0182226	0.959285133	-0.0599684	1	1
SP140	1.0193443	1.01899597	1.0194952	1.026281382	0.0374263	1	1
RP11-561B11.2	1.0193094	1.01899597	1.0194452	1.023651038	0.033724	1	1
AC002550.6	1.0227693	1.02185717	1.0231647	1.059820889	0.0838205	1	1
CCDC178	1.0201938	1.01899597	1.020713	1.090389874	0.1248441	1	1
RASEF	1.0470272	1.04766261	1.0467518	0.980890208	-0.0278364	1	1
SLC22A3	1.0193106	1.01899597	1.019447	1.023743447	0.0338542	1	1
RP5-955M13.4	1.0184498	1.01899597	1.018213	0.958783311	-0.0607233	1	1
ITPKB-AS1	1.0193223	1.01899597	1.0194637	1.024622141	0.035092	1	1
RP11-717F1.2	1.0288609	1.0275458	1.029431	1.068437516	0.0955025	1	1
OR7D2	1.0193316	1.01899597	1.0194771	1.025329729	0.0360879	1	1
AC006277.2	1.0184534	1.01899597	1.0182182	0.959054202	-0.0603157	1	1
RP11-552F3.10	1.0184495	1.01899597	1.0182127	0.958765702	-0.0607498	1	1
SLC4A10	1.0193446	1.01899597	1.0194957	1.026304849	0.0374593	1	1
RP1-151F17.1	1.0184506	1.01899597	1.0182142	0.958843547	-0.0606327	1	1
RP11-325L12.6	1.0193443	1.01899597	1.0194952	1.026281382	0.0374263	1	1
IGF2BP2-AS1	1.0184513	1.01899597	1.0182151	0.958894771	-0.0605556	1	1
MPZL1	1.9932525	1.99217873	1.993718	1.001551356	0.0022364	1	1
RP11-70C1.1	1.023661	1.02468831	1.0232156	0.940349496	-0.088731	1	1
SEC61A2	1.3076146	1.3088598	1.3070749	0.994220925	-0.0083616	1	1
FGF4	1.0184551	1.01899597	1.0182206	0.959184244	-0.0601201	1	1
RP1-273N12.4	1.0184487	1.01899597	1.0182115	0.958703709	-0.0608431	1	1
RNF138	1.3863822	1.38598912	1.3865526	1.001459721	0.0021044	1	1
AGRP	1.0219177	1.02183982	1.0219514	1.005111207	0.0073551	1	1
RP11-308D13.3	1.0184552	1.01899597	1.0182208	0.959195143	-0.0601037	1	1
C6orf3	1.0712105	1.07065292	1.0714522	1.011313121	0.0162298	1	1
PDZK1	1.0228184	1.02185013	1.0232381	1.063521141	0.0888487	1	1
GTF2E1	1.1509572	1.15093545	1.1509666	1.000206243	0.0002975	1	1
RP11-723O4.2	1.0184498	1.01899597	1.018213	0.958783311	-0.0607233	1	1
GUCY2F	1.0218911	1.02185013	1.0219088	1.002684876	0.0038683	1	1
AKR1D1	1.0193227	1.01899597	1.0194644	1.024657396	0.0351416	1	1
NFKB1	1.1424683	1.14242715	1.1424861	1.000413623	0.0005966	1	1
RP11-461L13.5	1.0184525	1.01899597	1.0182169	0.958985816	-0.0604186	1	1
PLEKHD1	1.0184591	1.01899597	1.0182265	0.959490345	-0.0596598	1	1
RP13-941N14.1	1.0201708	1.01899597	1.02068	1.088650223	0.1225405	1	1
RP11-222G7.2	1.0193099	1.01899597	1.0194459	1.023685971	0.0337732	1	1
F8A3	1.0184586	1.01899597	1.0182256	0.959445524	-0.0597272	1	1
NSUN3	1.1439854	1.14527266	1.1434275	0.987298502	-0.0184418	1	1
PDF	1.0184651	1.01899597	1.0182351	0.959943297	-0.0589789	1	1
AOX1	1.0184554	1.01899597	1.018221	0.959204912	-0.060089	1	1
CTD-3154N5.2	1.021072	1.02185779	1.0207314	0.948467422	-0.0763299	1	1
PPP1R42	1.0504364	1.05058998	1.0503699	0.995649245	-0.0062905	1	1
RP11-403A21.1	1.0210355	1.02185196	1.0206816	0.946440842	-0.0794158	1	1
SLC26A8	1.0193243	1.01899597	1.0194666	1.024776057	0.0353087	1	1
ANKS4B	1.0184579	1.01899597	1.0182246	0.95939273	-0.0598066	1	1
MAP1LC3C	1.0193105	1.01899597	1.0194468	1.023734017	0.0338409	1	1

AC007126.1	1.0184713	1.01899597	1.0182439	0.96040793	-0.0582808	1	1
ADRA1A	1.0184495	1.01899597	1.0182127	0.958765702	-0.0607498	1	1
PPP1R17	1.021989	1.02188106	1.0220357	1.00706793	0.010161	1	1
RP11-110I1.6	1.0184598	1.01899597	1.0182274	0.959539717	-0.0595856	1	1
STARD6	1.0270856	1.02761189	1.0268574	0.972675694	-0.0399692	1	1
BEST2	1.0184601	1.01899597	1.0182278	0.959560969	-0.0595536	1	1
SDS	1.0184539	1.01899597	1.0182189	0.959092576	-0.060258	1	1
FAM182B	1.0193166	1.01899597	1.0194556	1.024193693	0.0344886	1	1
OR13C3	1.0184577	1.01899597	1.0182244	0.959379657	-0.0598262	1	1
RP3-340B19.3	1.0184564	1.01899597	1.0182226	0.959285133	-0.0599684	1	1
AL022344.7	1.0184551	1.01899597	1.0182206	0.959184244	-0.0601201	1	1
RGPD8	1.0184627	1.01899597	1.0182316	0.959761248	-0.0592525	1	1
PAEP	1.0193292	1.01899597	1.0194737	1.025147206	0.0358311	1	1
RP11-762I7.5	1.0184507	1.01899597	1.0182144	0.958856513	-0.0606132	1	1
RP11-689C9.1	1.0210439	1.02184923	1.0206948	0.947164839	-0.0783126	1	1
PHYHIP	1.0384229	1.03920084	1.0380857	0.971553624	-0.0416345	1	1
MBOAT1	1.0227732	1.0218455	1.0231753	1.060872818	0.0852517	1	1
RP4-680D5.9	1.0184533	1.01899597	1.018218	0.959045897	-0.0603282	1	1
RP11-644N4.1	1.018452	1.01899597	1.0182162	0.958952199	-0.0604692	1	1
LYPD5	1.0172847	1.01683833	1.0174782	1.037999653	0.053806	1	1
LINC00330	1.0172887	1.01683833	1.0174839	1.038340453	0.0542796	1	1
GIMAP2	1.0181668	1.01683833	1.0187426	1.113089558	0.1545697	1	1
RP11-665C16.6	1.0164184	1.01683833	1.0162364	0.964253508	-0.0525156	1	1
SAA2	1.0164143	1.01683833	1.0162305	0.963900594	-0.0530437	1	1
RP11-93B14.4	1.01642	1.01683833	1.0162387	0.964390095	-0.0523113	1	1
LINC01333	1.0172898	1.01683833	1.0174855	1.038431688	0.0544063	1	1
ITGB2	1.0164182	1.01683833	1.0162361	0.964235495	-0.0525426	1	1
CD38	1.0172701	1.01683833	1.0174573	1.036757955	0.0520791	1	1
RP11-266N13.2	1.0233471	1.022599	1.0236713	1.047449604	0.0668808	1	1
RP4-736L20.3	1.0172907	1.01683833	1.0174868	1.038511504	0.0545172	1	1
MRPL38	1.0864555	1.08559114	1.0868301	1.014475648	0.0207342	1	1
RP4-724E16.2	1.0164104	1.01683833	1.0162249	0.963571669	-0.0535361	1	1
RP11-106M3.3	1.017283	1.01683833	1.0174758	1.03785694	0.0536076	1	1
RP11-214O1.2	1.0233268	1.02256394	1.0236575	1.04846395	0.0682773	1	1
RP11-77I22.2	1.0164166	1.01683833	1.0162338	0.964098586	-0.0527474	1	1
RP11-329B9.4	1.0363849	1.0368952	1.0361637	0.980172404	-0.0288926	1	1
KCNE3	1.0259715	1.02546015	1.0261931	1.02878803	0.0409458	1	1
LGALS4	1.021614	1.02256408	1.0212021	0.939640949	-0.0898185	1	1
GGT1	1.0233474	1.02257594	1.0236818	1.048982043	0.06899	1	1
RP11-44N11.3	1.017273	1.01683833	1.0174614	1.037000237	0.0524162	1	1
RP11-98D18.15	1.0164158	1.01683833	1.0162327	0.96403364	-0.0528446	1	1
RP11-463D19.2	1.0172841	1.01683833	1.0174773	1.037945856	0.0537312	1	1
LINC00239	1.0164168	1.01683833	1.0162341	0.964114902	-0.052723	1	1
RP11-29P20.1	1.0224732	1.02251996	1.022453	0.997025766	-0.0042973	1	1
CTC-241F20.4	1.0363166	1.03683274	1.0360929	0.979913959	-0.029273	1	1
AC005329.7	1.0164178	1.01683833	1.0162355	0.964196863	-0.0526004	1	1
USP29	1.0164146	1.01683833	1.016231	0.963929197	-0.0530009	1	1
RP11-21K12.3	1.0172737	1.01683833	1.0174625	1.037066964	0.0525091	1	1
RP11-834C11.6	1.0164151	1.01683833	1.0162316	0.963968051	-0.0529428	1	1
TMEM132C	1.0364185	1.03709194	1.0361265	0.973972621	-0.0380469	1	1
GMNC	1.0164151	1.01683833	1.0162317	0.963972391	-0.0529363	1	1

RP1-142L7.8	1.0242084	1.02550874	1.0236448	0.926928008	-0.1094708	1	1
LINC01048	1.0164242	1.01683833	1.0162447	0.964746249	-0.0517786	1	1
PCDHGB2	1.0164198	1.01683833	1.0162384	0.964371659	-0.0523388	1	1
AC131097.4	1.016411	1.01683833	1.0162257	0.963618343	-0.0534662	1	1
GLRA3	1.0164198	1.01683833	1.0162384	0.964371659	-0.0523388	1	1
RP4-633I8.4	1.0164125	1.01683833	1.0162279	0.963747555	-0.0532728	1	1
ZNF350-AS1	1.016426	1.01683833	1.0162473	0.964897278	-0.0515527	1	1
RP11-982M15.8	1.0181576	1.01683833	1.0187294	1.112307937	0.1535562	1	1
VWA7	1.0172777	1.01683833	1.0174682	1.037405783	0.0529803	1	1
DLGAP1-AS1	1.3537595	1.35465489	1.3533714	0.996381001	-0.0052306	1	1
RP11-51J9.4	1.0164151	1.01683833	1.0162317	0.963973351	-0.0529348	1	1
AC006372.4	1.0172801	1.01683833	1.0174715	1.037605631	0.0532582	1	1
ZSCAN25	1.0768409	1.07691015	1.076811	0.998710258	-0.0018619	1	1
MCTP2	1.0198563	1.01969179	1.0199276	1.011973929	0.0171721	1	1
FAM153B	1.0164178	1.01683833	1.0162355	0.964196863	-0.0526004	1	1
RP11-296A18.6	1.0164202	1.01683833	1.0162389	0.964402816	-0.0522922	1	1
C9orf92	1.0164169	1.01683833	1.0162343	0.964126624	-0.0527055	1	1
MOV10L1	1.0294974	1.02831444	1.0300101	1.059886848	0.0839103	1	1
RP11-132A1.4	1.0483816	1.04832877	1.0484046	1.001568356	0.0022609	1	1
DCAF17	1.1445559	1.14309717	1.1451882	1.01461271	0.0209291	1	1
EGFLAM	1.0181479	1.01683833	1.0187155	1.111483729	0.1524868	1	1
RP11-184E9.1	1.0164151	1.01683833	1.0162316	0.963967092	-0.0529442	1	1
SLC22A2	1.0164124	1.01683833	1.0162278	0.963739741	-0.0532845	1	1
HTR1D	1.0190213	1.01969967	1.0187273	0.950640344	-0.0730285	1	1
RP3-413H6.2	1.0164112	1.01683833	1.0162261	0.963639862	-0.053434	1	1
CTD-2192J16.26	1.0172791	1.01683833	1.0174702	1.037522714	0.0531429	1	1
AC010894.3	1.1226965	1.12259046	1.1227425	1.001240049	0.0017879	1	1
TKTL1	1.0379799	1.03686887	1.0384614	1.043195617	0.0610097	1	1
RP11-374M1.4	1.0172896	1.01683833	1.0174853	1.03841979	0.0543898	1	1
CTD-3193O13.1	1.0164129	1.01683833	1.0162285	0.963785391	-0.0532162	1	1
KCNK13	1.0164122	1.01683833	1.0162275	0.963726004	-0.0533051	1	1
AL354828.1	1.0172777	1.01683833	1.0174681	1.037399724	0.0529719	1	1
RP11-231E6.1	1.0164188	1.01683833	1.016237	0.964287916	-0.0524641	1	1
RP11-10K17.6	1.019013	1.01969984	1.0187153	0.95002439	-0.0739635	1	1
C1QTNF7	1.0172708	1.01683833	1.0174583	1.036817342	0.0521618	1	1
MYH6	1.0164157	1.01683833	1.0162326	0.96402466	-0.052858	1	1
CTB-161M19.4	1.0259321	1.02545389	1.0261394	1.026930092	0.038338	1	1
SERPINC1	1.0172822	1.01683833	1.0174745	1.037782803	0.0535045	1	1
TEX14	1.0198572	1.01967387	1.0199366	1.013354874	0.0191395	1	1
NFATC2	1.0431757	1.04252916	1.043456	1.021792071	0.0311016	1	1
RP11-849I19.1	1.0164167	1.01683833	1.016234	0.964109592	-0.0527309	1	1
TCF15	1.0198903	1.01969235	1.0199761	1.014411675	0.0206433	1	1
AGXT	1.0164164	1.01683833	1.0162336	0.964083762	-0.0527696	1	1
CTD-2619J13.23	1.0172838	1.01683833	1.0174768	1.037920042	0.0536953	1	1
SLC1A2	1.0190245	1.0196957	1.0187335	0.95114733	-0.0722593	1	1
AP003068.9	1.0216046	1.02257693	1.0211831	0.93826245	-0.0919366	1	1
LILRB3	1.0302907	1.03124798	1.0298757	0.956085368	-0.0647887	1	1
HACD4	1.0379887	1.03685512	1.0384801	1.044090734	0.0622471	1	1
RP13-57D9.3	1.0164117	1.01683833	1.0162268	0.963682921	-0.0533696	1	1
SHANK2-AS1	1.0172901	1.01683833	1.0174859	1.038457195	0.0544418	1	1
RP4-677H15.4	1.0164112	1.01683833	1.0162261	0.963639862	-0.053434	1	1

RP11-337N6.1	1.0172936	1.01683833	1.0174909	1.038754358	0.0548545	1	1
RP11-101E13.5	1.0310705	1.03108949	1.0310623	0.999124013	-0.0012643	1	1
MIR1539	1.0181636	1.01683833	1.0187381	1.112823364	0.1542246	1	1
CTD-2540F13.2	1.0475058	1.04828185	1.0471695	0.976960522	-0.0336278	1	1
FRMPD1	1.0164125	1.01683833	1.0162279	0.963747555	-0.0532728	1	1
C2orf71	1.0164188	1.01683833	1.0162369	0.964281455	-0.0524738	1	1
RP11-949J7.8	1.0172934	1.01683833	1.0174906	1.038739508	0.0548339	1	1
LBX1	1.0181611	1.01683833	1.0187344	1.112604141	0.1539404	1	1
TPH1	1.0251344	1.02544117	1.0250015	0.982717245	-0.0251517	1	1
HOXB1	1.0189985	1.01967387	1.0187058	0.950792142	-0.0727981	1	1
AMBN	1.0164241	1.01683833	1.0162445	0.964736258	-0.0517935	1	1
RP11-285F16.1	1.0216288	1.02254896	1.02123	0.941506849	-0.0869565	1	1
CTD-2325A15.5	1.0164135	1.01683833	1.0162294	0.963837491	-0.0531382	1	1
CTD-2026K11.3	1.0181645	1.01683833	1.0187393	1.112897652	0.1543209	1	1
RP11-809C18.3	1.01642	1.01683833	1.0162387	0.964386401	-0.0523168	1	1
SPINK8	1.0164188	1.01683833	1.0162369	0.964281455	-0.0524738	1	1
MUC22	1.0164179	1.01683833	1.0162357	0.964208976	-0.0525822	1	1
ARHGEF38	1.0172948	1.01683833	1.0174926	1.038856326	0.0549961	1	1
RP11-557L19.1	1.0181423	1.01683833	1.0187075	1.111007772	0.1518689	1	1
RP5-971N18.3	1.0164194	1.01683833	1.0162378	0.964335543	-0.0523929	1	1
TMOD1	1.0405311	1.03968179	1.0408992	1.030679579	0.0435959	1	1
HCFC1-AS1	1.019887	1.01970037	1.0199679	1.013579306	0.019459	1	1
RP11-855A2.3	1.0164113	1.01683833	1.0162262	0.963646792	-0.0534236	1	1
LINC01108	1.0164189	1.01683833	1.0162371	0.964292099	-0.0524579	1	1
RBM44	1.0216039	1.02257693	1.0211821	0.938218159	-0.0920047	1	1
MAN2A2	1.2766159	1.27738801	1.2762812	0.996010036	-0.0057678	1	1
RP5-832C2.5	1.0779012	1.07700474	1.0782898	1.016688019	0.023877	1	1
AP000462.3	1.0164202	1.01683833	1.016239	0.964408147	-0.0522843	1	1
MUC4	1.0242124	1.02542063	1.0236887	0.931869505	-0.1018002	1	1
RP11-713M15.2	1.0164189	1.01683833	1.0162371	0.964292099	-0.0524579	1	1
AF186192.1	1.0250998	1.0254491	1.0249484	0.980325079	-0.0286679	1	1
GMFG	1.0371668	1.03683086	1.0373124	1.013074542	0.0187403	1	1
RP3-522J7.7	1.0172868	1.01683833	1.0174812	1.038176875	0.0540523	1	1
LINC00487	1.0198641	1.01969699	1.0199366	1.012164414	0.0174437	1	1
CTD-2246P4.1	1.0172797	1.01683833	1.017471	1.037573232	0.0532132	1	1
TPCN2	1.1090117	1.10852663	1.1092219	1.006406863	0.0092137	1	1
XPNPEP2	1.01642	1.01683833	1.0162387	0.964391236	-0.0523096	1	1
KANTR	1.0198621	1.01968877	1.0199372	1.012617258	0.018089	1	1
CTD-2256P15.1	1.0190193	1.01968834	1.0187293	0.95128909	-0.0720443	1	1
CTD-2194D22.3	1.0172757	1.01683833	1.0174653	1.037232152	0.0527388	1	1
PDZRN3-AS1	1.0190079	1.0196965	1.0187095	0.94988808	-0.0741706	1	1
SRGAP3-AS4	1.0233113	1.02252572	1.0236519	1.04999357	0.0703805	1	1
TMPRSS15	1.0164143	1.01683833	1.0162304	0.963898592	-0.0530467	1	1
TCP11X2	1.018176	1.01683833	1.0187558	1.113875973	0.1555886	1	1
CEBPE	1.0164151	1.01683833	1.0162317	0.963973351	-0.0529348	1	1
RP11-245P10.8	1.0181687	1.01683833	1.0187454	1.113258126	0.1547881	1	1
PCDHA11	1.0302483	1.03111583	1.0298723	0.960035711	-0.05884	1	1
UBXN2B	1.3406372	1.3402384	1.34081	1.001680031	0.0024217	1	1
C6orf222	1.0164211	1.01683833	1.0162402	0.964480822	-0.0521755	1	1
BMP8A	1.0145639	1.01486911	1.0144316	0.97057555	-0.0430876	1	1
GJC3	1.0154406	1.01486911	1.0156883	1.055091196	0.0773677	1	1

TMPRSS11E	1.0154179	1.01486911	1.0156558	1.052907697	0.074379	1	1
CTC-254B4.1	1.0284376	1.02912323	1.0281404	0.966251516	-0.0495293	1	1
RP11-473E2.2	1.0145542	1.01486911	1.0144177	0.969638171	-0.0444816	1	1
RP3-337H4.10	1.0154175	1.01486911	1.0156551	1.05286341	0.0743183	1	1
CTD-2349P21.12	1.0309794	1.03196944	1.0305502	0.955606665	-0.0655112	1	1
RP11-47J17.2	1.0197245	1.02056585	1.0193598	0.941356753	-0.0871865	1	1
AC100830.5	1.0162853	1.01486911	1.0168992	1.136532139	0.1846385	1	1
ALOXE3	1.0154196	1.01486911	1.0156582	1.053067895	0.0745985	1	1
RP13-820C6.4	1.0145586	1.01486911	1.0144239	0.970060983	-0.0438526	1	1
URB1-AS1	1.0283867	1.02919329	1.028037	0.960392424	-0.0583041	1	1
FAM138C	1.0145602	1.01486911	1.0144263	0.970221123	-0.0436145	1	1
GALT	1.5449041	1.54377781	1.5453922	1.0029689	0.0042769	1	1
RP11-88E10.5	1.0197283	1.02058567	1.0193567	0.940301271	-0.088805	1	1
AC011247.3	1.0163198	1.01486911	1.0169486	1.139855848	0.1888514	1	1
FBXO40	1.0136996	1.01486911	1.0131926	0.887250313	-0.1725869	1	1
KB-1440D3.14	1.0145555	1.01486911	1.0144195	0.969764007	-0.0442944	1	1
FGFBP1	1.0162711	1.01486911	1.0168788	1.13515739	0.1828923	1	1
FUT6	1.017168	1.01770884	1.0169335	0.956217676	-0.064589	1	1
PYCARD-AS1	1.0240217	1.02346366	1.0242636	1.034094342	0.0483678	1	1
RP11-500G22.4	1.0215035	1.02058583	1.0219012	1.063898677	0.0893608	1	1
GJA4	1.0188624	1.01770982	1.019362	1.093292793	0.1286798	1	1
ACP7	1.0145552	1.01486911	1.0144191	0.969735514	-0.0443368	1	1
RP11-108H9.1	1.018013	1.01772173	1.0181392	1.023557839	0.0335926	1	1
IFNL2	1.0154421	1.01486911	1.0156905	1.055240898	0.0775724	1	1
LINC00843	1.0171467	1.0177222	1.0168972	0.953447112	-0.0687752	1	1
AC084082.3	1.0145554	1.01486911	1.0144195	0.969759868	-0.0443005	1	1
RP11-317N8.5	1.0145556	1.01486911	1.0144197	0.969774114	-0.0442793	1	1
RP11-273B20.1	1.0231835	1.02343074	1.0230763	0.984874061	-0.0219888	1	1
AE000662.93	1.0162975	1.01486911	1.0169166	1.137703054	0.1861241	1	1
RP11-108O10.2	1.014558	1.01486911	1.0144232	0.970007954	-0.0439315	1	1
GBP4	1.0525505	1.05202721	1.0527774	1.014418466	0.0206529	1	1
CH17-408M7.1	1.015431	1.01486911	1.0156746	1.054171989	0.0761103	1	1
RP11-622C24.2	1.0154294	1.01486911	1.0156723	1.054016741	0.0758978	1	1
CTD-2555C10.3	1.0188704	1.0177272	1.019366	1.092443654	0.1275589	1	1
RP5-966M1.6	1.0162931	1.01486911	1.0169103	1.137277526	0.1855844	1	1
CTC-218B8.3	1.014558	1.01486911	1.0144232	0.970007954	-0.0439315	1	1
SLC1A1	1.0404317	1.04065684	1.0403341	0.99206199	-0.0114978	1	1
RP11-91P24.7	1.0136996	1.01486911	1.0131926	0.887250313	-0.1725869	1	1
CTC-281F24.5	1.0145639	1.01486911	1.0144316	0.97057555	-0.0430876	1	1
RP11-309L24.4	1.0136996	1.01486911	1.0131926	0.887250313	-0.1725869	1	1
GPR62	1.0188581	1.01771261	1.0193547	1.092706923	0.1279065	1	1
LINC01136	1.0214872	1.02054627	1.0218951	1.065646716	0.0917292	1	1
DNAH8	1.0145655	1.01486911	1.0144339	0.9707339	-0.0428522	1	1
COL20A1	1.0145556	1.01486911	1.0144198	0.969780631	-0.0442697	1	1
SOX13	1.0550728	1.05481883	1.0551829	1.006641984	0.0095507	1	1
LINC01611	1.0162722	1.01486911	1.0168804	1.135264177	0.1830281	1	1
CADM3-AS1	1.0154236	1.01486911	1.0156639	1.053454837	0.0751285	1	1
LLNLR-304G9.1	1.0283751	1.02911383	1.0280549	0.963626597	-0.0534539	1	1
LDLRAD2	1.0136996	1.01486911	1.0131926	0.887250313	-0.1725869	1	1
GABRA6	1.0145629	1.01486911	1.0144301	0.970477057	-0.043234	1	1
CRYBA4	1.0440544	1.04349144	1.0442984	1.018554038	0.0265225	1	1

CTD-2540B15.9	1.0188754	1.01772039	1.0193761	1.093435001	0.1288675	1	1
TTLL9	1.040591	1.04057667	1.0405972	1.000505729	0.0007294	1	1
RP3-325F22.5	1.0145622	1.01486911	1.0144292	0.970412283	-0.0433303	1	1
RP11-214D15.2	1.0145575	1.01486911	1.0144225	0.969963256	-0.043998	1	1
RP11-624J6.2	1.0171533	1.01773672	1.0169005	0.952852731	-0.0696748	1	1
VSTM5	1.0180054	1.01770884	1.0181339	1.02400252	0.0342193	1	1
RP4-777O23.1	1.0145639	1.01486911	1.0144316	0.97057784	-0.0430842	1	1
RP11-368I7.4	1.0309831	1.0319911	1.0305461	0.95483137	-0.0666821	1	1
RP11-138B4.1	1.0162848	1.01486911	1.0168984	1.136475734	0.1845669	1	1
ACRC	1.0396604	1.0405979	1.0392541	0.966899344	-0.0485624	1	1
AF165138.7	1.0145547	1.01486911	1.0144184	0.969686828	-0.0444092	1	1
LINC01234	1.0171622	1.01772739	1.0169172	0.954296445	-0.0674906	1	1
RP11-281P23.1	1.0180126	1.0177334	1.0181336	1.022565925	0.0321939	1	1
RP11-805I24.1	1.0162871	1.01486911	1.0169017	1.136697548	0.1848484	1	1
TUBA4B	1.0180109	1.01771822	1.0181378	1.023682382	0.0337682	1	1
RPEL1	1.0145593	1.01486911	1.014425	0.970134355	-0.0437435	1	1
RP4-564F22.7	1.0136996	1.01486911	1.0131926	0.887250313	-0.1725869	1	1
CTC-459F4.1	1.0542657	1.0548242	1.0540237	0.985397948	-0.0212216	1	1
AC007204.2	1.0136996	1.01486911	1.0131926	0.887250313	-0.1725869	1	1
CIB3	1.0145553	1.01486911	1.0144192	0.96974379	-0.0443245	1	1
RP1-149A16.3	1.014557	1.01486911	1.0144217	0.969910289	-0.0440768	1	1
SMPX	1.0171407	1.01770451	1.0168964	0.954353726	-0.067404	1	1
RP11-544L8__B.4	1.0145654	1.01486911	1.0144337	0.970719525	-0.0428736	1	1
LINC00519	1.0136996	1.01486911	1.0131926	0.887250313	-0.1725869	1	1
LSM11	1.0826585	1.08337739	1.0823469	0.987640759	-0.0179417	1	1
SYNPO2L	1.0283843	1.02917254	1.0280427	0.961268661	-0.0569884	1	1
LINC01204	1.0205892	1.02057395	1.0205958	1.001060743	0.0015295	1	1
RP11-390K5.3	1.0145722	1.01486911	1.0144435	0.9713734	-0.0419021	1	1
SLC25A44	1.0991636	1.09789246	1.0997147	1.018614308	0.0266079	1	1
ALPK2	1.1068232	1.10632169	1.1070405	1.006760997	0.0097212	1	1
CTD-2292M16.8	1.0369687	1.03773995	1.0366344	0.970706469	-0.042893	1	1
ACTBL2	1.0154107	1.01486911	1.0156455	1.052216763	0.0734319	1	1
RP11-87C12.2	1.0180105	1.01772946	1.0181323	1.022720499	0.0324119	1	1
PTPRC	1.0154187	1.01486911	1.0156569	1.052979862	0.0744778	1	1
RP11-271M24.2	1.0145598	1.01486911	1.0144257	0.970181026	-0.0436741	1	1
RP1-101D8.1	1.0240532	1.02347177	1.0243052	1.035506022	0.0503359	1	1
RP11-357H14.17	1.016308	1.01486911	1.0169318	1.138720228	0.1874133	1	1
RP1-69D17.3	1.0145566	1.01486911	1.0144212	0.969875766	-0.0441281	1	1
RP11-92F20.1	1.0145549	1.01486911	1.0144187	0.969710218	-0.0443744	1	1
ELL3	1.0162807	1.01486911	1.0168925	1.13608029	0.1840648	1	1
RP11-382A20.4	1.0145606	1.01486911	1.0144269	0.970256785	-0.0435615	1	1
HAND2-AS1	1.0180106	1.01772187	1.0181358	1.023354405	0.0333059	1	1
ZC3H10	1.1768314	1.17789384	1.1763709	0.99143903	-0.012404	1	1
ABHD10	1.201813	1.200468	1.202396	1.009617574	0.0138089	1	1
MAB21L3	1.0145566	1.01486911	1.0144212	0.969875766	-0.0441281	1	1
KYNU	1.0214635	1.02055695	1.0218565	1.063215914	0.0884346	1	1
C9orf170	1.0136996	1.01486911	1.0131926	0.887250313	-0.1725869	1	1
UCP1	1.0145564	1.01486911	1.0144209	0.969853212	-0.0441617	1	1
RP11-575F12.3	1.0136996	1.01486911	1.0131926	0.887250313	-0.1725869	1	1
RP11-375I20.6	1.0162922	1.01486911	1.016909	1.137188406	0.1854713	1	1
AC012442.6	1.0145556	1.01486911	1.0144198	0.969780631	-0.0442697	1	1

FOCAD	1.234539	1.23490932	1.2343784	0.997740108	-0.003264	1	1
RP5-965G21.3	1.0205783	1.02055138	1.0205899	1.001875203	0.0027028	1	1
RP11-462B18.2	1.0162812	1.01486911	1.0168932	1.136127719	0.184125	1	1
RP11-165F24.3	1.0197929	1.02061648	1.0194359	0.942737955	-0.0850713	1	1
RAX2	1.0145714	1.01486911	1.0144423	0.971296305	-0.0420166	1	1
ZNF780A	1.1199108	1.12068166	1.1195767	0.990843932	-0.0132703	1	1
GALK2	1.335726	1.33503677	1.3360247	1.002948799	0.004248	1	1
RP11-161I2.1	1.0145602	1.01486911	1.0144263	0.970221123	-0.0436145	1	1
RP11-432I5.2	1.020615	1.02058583	1.0206276	1.002028707	0.0029238	1	1
AC011718.2	1.0154262	1.01486911	1.0156677	1.053706858	0.0754736	1	1
MEMO1	1.2271483	1.22644043	1.2274551	1.004481139	0.0064505	1	1
HTR5A-AS1	1.0136996	1.01486911	1.0131926	0.887250313	-0.1725869	1	1
RP11-162A12.4	1.0171353	1.01771135	1.0168856	0.953375932	-0.0688829	1	1
SERPINA3	1.015425	1.01486911	1.015666	1.053593362	0.0753182	1	1
RP11-90C1.1	1.0154298	1.01486911	1.0156728	1.054053026	0.0759474	1	1
LINC01182	1.0136996	1.01486911	1.0131926	0.887250313	-0.1725869	1	1
RP11-123I22.1	1.0136996	1.01486911	1.0131926	0.887250313	-0.1725869	1	1
ZRSR1	1.0154198	1.01486911	1.0156586	1.053093269	0.0746332	1	1
RNASEK-C17orf49	1.0249219	1.02342645	1.0255701	1.091506121	0.1263202	1	1
SOX30	1.0136996	1.01486911	1.0131926	0.887250313	-0.1725869	1	1
LINC01489	1.0145575	1.01486911	1.0144224	0.969959107	-0.0440042	1	1
LRRC36	1.019714	1.02057338	1.0193414	0.940119739	-0.0890836	1	1
RP11-449J1.1	1.0145552	1.01486911	1.0144191	0.969735514	-0.0443368	1	1
SH3RF3	1.0429728	1.04352804	1.0427322	0.981716263	-0.026622	1	1
ITGB3	1.0154113	1.01486911	1.0156463	1.05226555	0.0734988	1	1
RBP3	1.0154129	1.01486911	1.0156485	1.052420041	0.0737106	1	1
NCBP2-AS1	1.01456	1.01486911	1.014426	0.97020135	-0.0436439	1	1
COL5A1-AS1	1.0145576	1.01486911	1.0144226	0.96997339	-0.0439829	1	1
PDP1	1.1137804	1.11509767	1.1132094	0.98359435	-0.0238646	1	1
TRPC5OS	1.0145569	1.01486911	1.0144216	0.969906142	-0.044083	1	1
RP11-359D14.2	1.0154247	1.01486911	1.0156656	1.05356591	0.0752806	1	1
AC009133.17	1.014558	1.01486911	1.0144232	0.970007954	-0.0439315	1	1
RP11-715J22.4	1.0145639	1.01486911	1.0144316	0.97057326	-0.043091	1	1
RP11-983P16.4	1.1640869	1.16365694	1.1642732	1.003765567	0.0054224	1	1
NPIPB6	1.0387372	1.03773267	1.0391726	1.038160422	0.0540294	1	1
WT1	1.0145552	1.01486911	1.0144191	0.969735514	-0.0443368	1	1
PARP9	1.1716902	1.17200954	1.1715517	0.997338439	-0.0038449	1	1
AC006159.4	1.014557	1.01486911	1.0144217	0.969910289	-0.0440768	1	1
PLSCR2	1.0145544	1.01486911	1.014418	0.969658362	-0.0444516	1	1
RP11-474I16.8	1.0171519	1.01772004	1.0169056	0.95403666	-0.0678834	1	1
LINC00437	1.0145578	1.01486911	1.0144228	0.969983526	-0.0439678	1	1
RP11-336K24.12	1.0240477	1.02340319	1.024327	1.039475135	0.0558552	1	1
LINC00426	1.0136996	1.01486911	1.0131926	0.887250313	-0.1725869	1	1
C4orf45	1.0162892	1.01486911	1.0169047	1.136901678	0.1851075	1	1
CTD-3035K23.6	1.0154181	1.01486911	1.015656	1.052921838	0.0743983	1	1
SLCO2B1	1.0145559	1.01486911	1.0144201	0.969805435	-0.0442328	1	1
RP11-701H24.4	1.0154198	1.01486911	1.0156584	1.053084856	0.0746217	1	1
DEFB1	1.0145559	1.01486911	1.0144201	0.969805435	-0.0442328	1	1
CPA1	1.0317868	1.0320193	1.031686	0.989589634	-0.0150977	1	1
RP5-1051H14.2	1.0136996	1.01486911	1.0131926	0.887250313	-0.1725869	1	1
RP11-182J1.13	1.0136996	1.01486911	1.0131926	0.887250313	-0.1725869	1	1

AP003419.16	1.0188648	1.01772004	1.019361	1.092604414	0.1277712	1	1
ZNF701	1.128336	1.12905838	1.1280229	0.991976686	-0.0116219	1	1
CTC-529L17.2	1.0154332	1.01486911	1.0156777	1.054381304	0.0763967	1	1
RP11-408H20.3	1.0145586	1.01486911	1.0144239	0.970060983	-0.0438526	1	1
AC073316.1	1.0145595	1.01486911	1.0144253	0.970154667	-0.0437133	1	1
ELAC1	1.2121919	1.21206854	1.2122454	1.000833979	0.0012027	1	1
FAM90A26	1.0136996	1.01486911	1.0131926	0.887250313	-0.1725869	1	1
KLRG2	1.0136996	1.01486911	1.0131926	0.887250313	-0.1725869	1	1
AC107218.3	1.0154318	1.01486911	1.0156757	1.054246321	0.076212	1	1
AC013463.2	1.0154233	1.01486911	1.0156635	1.053426576	0.0750898	1	1
HUS1B	1.0163008	1.01486911	1.0169213	1.138020351	0.1865264	1	1
LINC01102	1.015425	1.01486911	1.015666	1.053591549	0.0753157	1	1
IL15RA	1.0145577	1.01486911	1.0144227	0.969979376	-0.043974	1	1
KRBOX1	1.0188734	1.01773105	1.0193686	1.092354023	0.1274405	1	1
RP1-69M21.2	1.0154294	1.01486911	1.0156723	1.054016741	0.0758978	1	1
MACC1-AS1	1.0154332	1.01486911	1.0156777	1.054381304	0.0763967	1	1
RP11-863P13.1	1.0145629	1.01486911	1.0144301	0.970477057	-0.043234	1	1
RP11-260E18.1	1.0145564	1.01486911	1.0144209	0.969857355	-0.0441555	1	1
RP11-84D1.2	1.0154172	1.01486911	1.0156547	1.052836453	0.0742813	1	1
SCRIB	1.2474707	1.246145	1.2480453	1.007720401	0.0110954	1	1
SIAH3	1.0145569	1.01486911	1.0144216	0.969906142	-0.044083	1	1
CTB-60B18.12	1.0136996	1.01486911	1.0131926	0.887250313	-0.1725869	1	1
HDDC3	1.5733011	1.57275122	1.5735394	1.001376171	0.001984	1	1
NUTM2D	1.0163369	1.0171592	1.0159805	0.93130668	-0.1026718	1	1
CXorf40A	1.1709873	1.17174482	1.1706589	0.993677122	-0.0091509	1	1
TNFRSF11A	1.0163334	1.0171592	1.0159755	0.931015856	-0.1031224	1	1
KDM5A	1.8392463	1.83948504	1.8391428	0.999592298	-0.0005883	1	1
RP11-392P7.6	1.0171961	1.0171592	1.017212	1.003078442	0.0044344	1	1
SOWAHA	1.0172042	1.0171592	1.0172237	1.003760906	0.0054157	1	1
PCDHB1	1.0180874	1.0171592	1.0184898	1.077541731	0.1077437	1	1
CCDC125	1.0163365	1.0171592	1.01598	0.931276707	-0.1027182	1	1
PTPN21	1.0915582	1.09158202	1.0915478	0.999626662	-0.0005387	1	1
RP11-650L12.1	1.0197988	1.02000583	1.019709	0.985164024	-0.0215642	1	1
FAM41C	1.0354144	1.03441874	1.035846	1.041466237	0.0586161	1	1
POU6F1	1.0327432	1.03143196	1.0333116	1.05979895	0.0837906	1	1
CTD-2089N3.1	1.0163367	1.0171592	1.0159802	0.931288716	-0.1026996	1	1
AC073641.2	1.0180838	1.0171592	1.0184845	1.077236661	0.1073352	1	1
RP11-114H24.6	1.016341	1.0171592	1.0159864	0.93165008	-0.1021399	1	1
RIN2	1.5946422	1.59313266	1.5952965	1.003648098	0.0052535	1	1
RP11-398A8.5	1.0163375	1.0171592	1.0159813	0.931352186	-0.1026013	1	1
RP11-621L6.3	1.0379553	1.03714222	1.0383078	1.031381254	0.0445777	1	1
LILRB1	1.0181049	1.0171592	1.0185148	1.079003008	0.1096989	1	1
RP11-357N13.2	1.016335	1.0171592	1.0159778	0.931151344	-0.1029124	1	1
CORT	1.0198249	1.02002731	1.0197372	0.985515668	-0.0210493	1	1
EEF1E1-BLOC1S5	1.0163451	1.0171592	1.0159922	0.93198988	-0.1016138	1	1
RP11-353N14.4	1.0163396	1.0171592	1.0159843	0.931527675	-0.1023295	1	1
AC007563.5	1.0232423	1.02288341	1.0233979	1.022483454	0.0320775	1	1
RP11-469L4.1	1.0163365	1.0171592	1.0159799	0.931273108	-0.1027238	1	1
RP11-114F3.4	1.0189584	1.02002935	1.0184942	0.923354552	-0.1150434	1	1
AC136352.2	1.0163361	1.0171592	1.0159794	0.931243149	-0.1027702	1	1
IFNE	1.0180518	1.0171592	1.0184386	1.074562472	0.1037494	1	1

RP11-297K8.2	1.0215246	1.02003202	1.0221715	1.106803746	0.1463994	1	1
CCDC84	1.2161715	1.21493245	1.2167085	1.008263364	0.0118725	1	1
RP11-211G3.2	1.0180985	1.0171592	1.0185056	1.078464951	0.1089793	1	1
ASB12	1.0180551	1.0171592	1.0184434	1.074842297	0.104125	1	1
LA16c-352F7.1	1.0163363	1.0171592	1.0159796	0.931255533	-0.102751	1	1
CTD-2659N19.10	1.0302095	1.02866164	1.0308805	1.077414068	0.1075728	1	1
RP11-802O23.3	1.0171962	1.0171592	1.0172123	1.003094836	0.004458	1	1
CTD-2515A14.1	1.0301868	1.02856893	1.0308881	1.081177918	0.112604	1	1
RP11-556N4.1	1.0171959	1.0171592	1.0172118	1.003062962	0.0044122	1	1
TSHR	1.017204	1.0171592	1.0172234	1.003743237	0.0053903	1	1
KIAA1671	1.1805994	1.18054111	1.1806247	1.000463071	0.0006679	1	1
RP1-111C20.4	1.0301633	1.02863616	1.0308252	1.076442646	0.1062715	1	1
PLA2G7	1.0180638	1.0171592	1.0184558	1.075564853	0.1050945	1	1
CFAP161	1.021519	1.02002223	1.0221678	1.107160795	0.1468648	1	1
RP11-247A12.8	1.1028045	1.1032512	1.1026108	0.993797772	-0.0089758	1	1
ASPDH	1.0172027	1.0171592	1.0172215	1.003633481	0.0052325	1	1
RP1-30M3.5	1.0223959	1.02286002	1.0221948	0.970899301	-0.0426064	1	1
MEIS1-AS3	1.0172145	1.0171592	1.0172385	1.004621511	0.0066521	1	1
LINC00309	1.0172017	1.0171592	1.0172202	1.003552214	0.0051157	1	1
RP11-602N24.3	1.028446	1.0285955	1.0283812	0.992504333	-0.0108547	1	1
CTA-126B4.7	1.0163374	1.0171592	1.0159811	0.931343869	-0.1026142	1	1
RP11-54G14.1	1.0163413	1.0171592	1.0159867	0.93167137	-0.1021069	1	1
C10orf55	1.0171996	1.0171592	1.0172171	1.00337525	0.0048613	1	1
E2F3	1.3649201	1.3636774	1.3654587	1.004898061	0.0070492	1	1
SHE	1.0215412	1.02000979	1.0222051	1.109710114	0.1501829	1	1
RP4-530I15.9	1.0163379	1.0171592	1.0159819	0.9313895	-0.1025435	1	1
MT1A	1.018978	1.02002609	1.0185237	0.924980282	-0.1125055	1	1
RP11-567N4.3	1.0163386	1.0171592	1.0159829	0.93144744	-0.1024537	1	1
RP11-61L19.2	1.0163375	1.0171592	1.0159813	0.931357382	-0.1025932	1	1
RP11-168P13.1	1.0163412	1.0171592	1.0159866	0.931662533	-0.1021206	1	1
RP11-106M3.5	1.0163426	1.0171592	1.0159886	0.931781542	-0.1019363	1	1
HOXC8	1.0163462	1.0171592	1.0159938	0.932080382	-0.1014737	1	1
IL3RA	1.0180758	1.0171592	1.018473	1.076567783	0.1064392	1	1
CTD-3065B20.3	1.0215342	1.02000374	1.0221976	1.109672722	0.1501342	1	1
INHBA-AS1	1.0172002	1.0171592	1.0172179	1.0034227	0.0049295	1	1
ZNF540	1.027623	1.0286694	1.0271695	0.94768219	-0.0775248	1	1
CALCA	1.0171928	1.0171592	1.0172074	1.002809177	0.0040471	1	1
RP11-348B17.1	1.0172168	1.0171592	1.0172418	1.00481472	0.0069295	1	1
HCN4	1.0180763	1.0171592	1.0184738	1.076610627	0.1064966	1	1
RP11-753C18.12	1.016338	1.0171592	1.0159821	0.931400279	-0.1025268	1	1
MS4A8	1.0163418	1.0171592	1.0159874	0.931711987	-0.102044	1	1
MATR3.1	1.1106064	1.1117779	1.1100986	0.984976039	-0.0218395	1	1
CD14	1.0284594	1.0286309	1.028385	0.991413015	-0.0124419	1	1
ZNF177	1.0171982	1.0171592	1.0172151	1.003259873	0.0046954	1	1
TMC5	1.0206522	1.02001737	1.0209273	1.045457853	0.0641349	1	1
RP11-452K12.7	1.0172008	1.0171592	1.0172188	1.003471361	0.0049994	1	1
RP11-1017G21.4	1.0163357	1.0171592	1.0159788	0.931207637	-0.1028252	1	1
RP11-304F15.3	1.0180767	1.0171592	1.0184744	1.076648864	0.1065478	1	1
AL022476.2	1.0171954	1.0171592	1.0172111	1.00302307	0.0043548	1	1
CYP2E1	1.0266905	1.02575772	1.0270948	1.051910484	0.0730119	1	1
AC083843.2	1.016337	1.0171592	1.0159806	0.931311873	-0.1026637	1	1

RP11-96K19.2	1.0172027	1.0171592	1.0172215	1.003633258	0.0052322	1	1
CTC-338M12.2	1.0163423	1.0171592	1.0159882	0.931754993	-0.1019774	1	1
CTD-2265O21.3	1.018948	1.0200266	1.0184805	0.922796227	-0.115916	1	1
GJB5	1.0171939	1.0171592	1.0172089	1.002897314	0.0041739	1	1
AL365181.1	1.0587987	1.05729448	1.0594508	1.037634957	0.053299	1	1
RBMXL3	1.0163364	1.0171592	1.0159798	0.93126432	-0.1027374	1	1
OAS2	1.017218	1.0171592	1.0172435	1.004910831	0.0070675	1	1
RP11-31K23.2	1.0163407	1.0171592	1.0159859	0.931625179	-0.1021785	1	1
BRSK1	1.1375004	1.13753163	1.1374868	0.999674219	-0.0004701	1	1
AC005487.2	1.0163365	1.0171592	1.01598	0.931276707	-0.1027182	1	1
RP11-863H1.1	1.016336	1.0171592	1.0159791	0.931227172	-0.1027949	1	1
TMEM249	1.0171948	1.0171592	1.0172103	1.002977002	0.0042885	1	1
ATL3	1.2429286	1.24361503	1.2426311	0.995961096	-0.0058387	1	1
RP11-59D5__B.2	1.0163443	1.0171592	1.0159911	0.931925744	-0.1017131	1	1
RP11-6O2.3	1.0172052	1.0171592	1.0172251	1.003840127	0.0055295	1	1
SNPH	1.0301308	1.02860671	1.0307915	1.07637336	0.1061786	1	1
FAF1	1.2020806	1.20331822	1.2015441	0.991274105	-0.0126441	1	1
KIAA1462	1.0267289	1.02584349	1.0271126	1.049108717	0.0691642	1	1
AFF4	2.1806162	2.18054406	2.1806475	1.00008762	0.0001264	1	1
AC079630.4	1.0171962	1.0171592	1.0172122	1.003087757	0.0044478	1	1
AC093642.3	1.023258	1.02285671	1.0234319	1.025166943	0.0358589	1	1
RP11-166N17.1	1.0163423	1.0171592	1.0159882	0.931754993	-0.1019774	1	1
HOXA7	1.0163443	1.0171592	1.0159911	0.931925744	-0.1017131	1	1
RP11-613M10.8	1.0172215	1.0171592	1.0172485	1.005201982	0.0074854	1	1
FOXD3-AS1	1.017231	1.0171592	1.0172621	1.005999319	0.0086293	1	1
LGALS9	1.0223927	1.02290453	1.0221709	0.967968933	-0.0469673	1	1
RP11-385E5.5	1.0172168	1.0171592	1.0172418	1.00481472	0.0069295	1	1
FAM72C	1.0422296	1.04289479	1.0419412	0.977770185	-0.0324327	1	1
CTC-513N18.7	1.0189587	1.02001801	1.0184995	0.924145211	-0.1138085	1	1
ZP4	1.017208	1.0171592	1.0172292	1.004079639	0.0058737	1	1
AF131216.5	1.0180686	1.0171592	1.0184629	1.075973709	0.1056428	1	1
LRRC2	1.0180626	1.0171592	1.0184543	1.075472754	0.104971	1	1
RP11-73M7.6	1.0163424	1.0171592	1.0159884	0.931767465	-0.1019581	1	1
RP11-589C21.5	1.0172239	1.0171592	1.0172519	1.00540251	0.0077732	1	1
CTD-2026D20.2	1.0163414	1.0171592	1.0159869	0.931683446	-0.1020882	1	1
GRIK4	1.0336198	1.03433328	1.0333105	0.97020958	-0.0436317	1	1
KCNAB1-AS1	1.0163334	1.0171592	1.0159755	0.931015856	-0.1031224	1	1
SORCS3	1.0301855	1.02858726	1.0308783	1.080141478	0.1112203	1	1
ZNF860	1.0172083	1.0171592	1.0172296	1.00410447	0.0059094	1	1
RP11-427M20.1	1.016338	1.0171592	1.015982	0.931394699	-0.1025354	1	1
ARL5C	1.0163374	1.0171592	1.0159811	0.931343869	-0.1026142	1	1
HSPB7	1.016336	1.0171592	1.0159791	0.931227172	-0.1027949	1	1
CLEC2B	1.0215127	1.01999955	1.0221686	1.108454424	0.1485495	1	1
UBE2F-SCLY	1.0163438	1.0171592	1.0159903	0.93187939	-0.1017849	1	1
AC002306.1	1.0163426	1.0171592	1.0159886	0.931781542	-0.1019363	1	1
RP11-360O19.4	1.0163368	1.0171592	1.0159803	0.931295535	-0.102689	1	1
CTB-102L5.4	1.016344	1.0171592	1.0159906	0.931897118	-0.1017574	1	1
LINC01592	1.0163395	1.0171592	1.0159842	0.931522467	-0.1023375	1	1
RP11-185E8.1	1.0206586	1.0200147	1.0209377	1.046114321	0.0650405	1	1
KB-173C10.2	1.016336	1.0171592	1.0159792	0.931230768	-0.1027894	1	1
HRAT92	1.0189252	1.02000979	1.0184551	0.922305241	-0.1166838	1	1

ASPN	1.0163355	1.0171592	1.0159785	0.931188453	-0.1028549	1	1
RP11-379B18.6	1.0171996	1.0171592	1.0172172	1.003377781	0.0048649	1	1
SERPINB11	1.0143808	1.01490147	1.0141551	0.949909603	-0.0741379	1	1
ACBD7	1.0264869	1.02630519	1.0265657	1.009901912	0.0142152	1	1
ZNF208	1.0143808	1.01490147	1.0141552	0.94991788	-0.0741253	1	1
RP11-955H22.1	1.0143824	1.01490147	1.0141574	0.950064153	-0.0739032	1	1
CSF3R	1.0161173	1.01490147	1.0166443	1.116958993	0.1595762	1	1
RP5-921G16.1	1.0160965	1.01490147	1.0166144	1.11495141	0.1569808	1	1
CHKB-AS1	1.0566467	1.05772376	1.0561798	0.973252311	-0.0391142	1	1
TEK	1.017815	1.01775323	1.0178418	1.004988894	0.0071796	1	1
SPDYE5	1.0152404	1.01490147	1.0153873	1.032605212	0.0462888	1	1
TDRD15	1.0143862	1.01490147	1.0141629	0.950435136	-0.0733399	1	1
RP11-227G15.12	1.0161087	1.01490147	1.0166319	1.116126658	0.1585008	1	1
C16orf46	1.1740607	1.17490972	1.1736927	0.993042259	-0.010073	1	1
RP11-319E12.1	1.0152422	1.01490147	1.0153899	1.032776462	0.046528	1	1
LINC00929	1.016997	1.01774806	1.0166715	0.939343104	-0.0902759	1	1
RP5-956O18.3	1.016973	1.01775578	1.0166337	0.936803491	-0.0941816	1	1
SAMSN1	1.0161283	1.01490147	1.0166601	1.118018301	0.1609438	1	1
STEAP4	1.0187071	1.01774939	1.0191222	1.077343909	0.1074789	1	1
AC243756.1	1.0152551	1.01490147	1.0154084	1.034018134	0.0482615	1	1
CHRM1	1.0152437	1.01490147	1.0153921	1.03292298	0.0467327	1	1
SPATA13.1	1.0152424	1.01490147	1.0153902	1.032797281	0.0465571	1	1
RP5-974N19.1	1.0161404	1.01490147	1.0166774	1.119177981	0.1624395	1	1
RP11-347I19.7	1.0290748	1.02925151	1.0289982	0.991339028	-0.0125496	1	1
CTD-2384B11.2	1.0161161	1.01490147	1.0166425	1.116837384	0.1594191	1	1
AP000318.2	1.0161152	1.01490147	1.0166414	1.116759443	0.1593185	1	1
AC012360.6	1.0143821	1.01490147	1.0141569	0.950035612	-0.0739465	1	1
NEB	1.0169663	1.01776095	1.0166218	0.935864266	-0.0956288	1	1
RP11-307P5.1	1.0152458	1.01490147	1.015395	1.033121013	0.0470093	1	1
GSTA1	1.0169707	1.01775345	1.0166314	0.936799634	-0.0941876	1	1
EBF3	1.0143851	1.01490147	1.0141612	0.950322835	-0.0735104	1	1
SEPT12	1.0161038	1.01490147	1.016625	1.115660877	0.1578986	1	1
ABRA	1.0143915	1.01490147	1.0141705	0.950945399	-0.0725656	1	1
REN	1.0161283	1.01490147	1.0166601	1.118018301	0.1609438	1	1
MLLT3	1.3085815	1.30923171	1.3082997	0.996985952	-0.0043549	1	1
AC002454.1	1.0229748	1.02345416	1.022767	0.970702496	-0.0428989	1	1
CH17-437K3.1	1.0178241	1.01775345	1.0178547	1.005704338	0.0082062	1	1
PCDHGB5	1.0143852	1.01490147	1.0141615	0.950339035	-0.0734858	1	1
RP11-379K17.12	1.0204214	1.02063232	1.02033	0.985346236	-0.0212973	1	1
RP4-537K23.4	1.0195487	1.02058317	1.0191003	0.927959676	-0.107866	1	1
CARD16	1.0152457	1.01490147	1.0153949	1.033114812	0.0470006	1	1
OLIG3	1.0161358	1.01490147	1.0166709	1.118740552	0.1618755	1	1
DPPA3	1.0143818	1.01490147	1.0141565	0.95000708	-0.0739898	1	1
AC004158.3	1.0152386	1.01490147	1.0153848	1.032431574	0.0460462	1	1
RP11-192P3.4	1.0143829	1.01490147	1.0141581	0.95011668	-0.0738234	1	1
NTN4	1.0247415	1.02346837	1.0252934	1.077763786	0.108041	1	1
H2BFS	1.0143844	1.01490147	1.0141603	0.950263744	-0.0736001	1	1
RP11-449L23.2	1.0143798	1.01490147	1.0141537	0.949816375	-0.0742795	1	1
TRAIP	1.0807087	1.0806408	1.0807381	1.001206478	0.0017395	1	1
RP4-601P9.2	1.0143874	1.01490147	1.0141645	0.950545744	-0.073172	1	1
ENAM	1.0143797	1.01490147	1.0141536	0.949812241	-0.0742857	1	1

RP4-794I6.4	1.0273489	1.02630012	1.0278035	1.057163779	0.0801989	1	1
ADAMTS15	1.0186957	1.01774722	1.0191068	1.076607282	0.1064921	1	1
RP3-402G11.25	1.0152572	1.01490147	1.0154115	1.034223305	0.0485477	1	1
GNA14	1.0152428	1.01490147	1.0153908	1.032837569	0.0466134	1	1
AC008269.2	1.0178346	1.01776147	1.0178663	1.00590253	0.0084905	1	1
MIR670HG	1.016973	1.01774131	1.01664	0.937923393	-0.092458	1	1
CTD-2616J11.9	1.0161146	1.01490147	1.0166405	1.116701301	0.1592433	1	1
SLA2	1.0143839	1.01490147	1.0141595	0.950210686	-0.0736807	1	1
KCNH7	1.0143817	1.01490147	1.0141564	0.950000666	-0.0739996	1	1
RP11-431J24.2	1.0143834	1.01490147	1.0141588	0.950159538	-0.0737583	1	1
FAM83F	1.0169659	1.01775073	1.0166257	0.936619338	-0.0944653	1	1
RP11-946L16.1	1.0152418	1.01490147	1.0153893	1.032733643	0.0464682	1	1
SLC22A14	1.0143808	1.01490147	1.0141551	0.949909603	-0.0741379	1	1
LINC00857	1.0161042	1.01490147	1.0166255	1.115695822	0.1579438	1	1
SMARCD3	1.6401054	1.64077353	1.6398157	0.998505247	-0.0021581	1	1
RP11-571I18.4	1.0169614	1.01774722	1.0166208	0.936531816	-0.0946001	1	1
RBMX	7.8589351	7.85697229	7.859786	1.000410338	0.0005919	1	1
RP11-11C20.3	1.0143814	1.01490147	1.014156	0.949970751	-0.074045	1	1
RP11-268P4.4	1.0152633	1.01490147	1.0154201	1.034802343	0.0493552	1	1
LYL1	1.0143804	1.01490147	1.0141546	0.949878848	-0.0741846	1	1
WWTR1-AS1	1.0213084	1.02064516	1.021596	1.046053943	0.0649573	1	1
RP5-1024G6.7	1.0221426	1.0206181	1.0228034	1.105991253	0.14534	1	1
CSAG1	1.0143829	1.01490147	1.0141581	0.95011668	-0.0738234	1	1
HLA-DRA	1.0152487	1.01490147	1.0153993	1.033405859	0.047407	1	1
RFPL1	1.0152502	1.01490147	1.0154014	1.033549204	0.0476071	1	1
RP5-994D16.12	1.0143797	1.01490147	1.0141536	0.949812241	-0.0742857	1	1
SHISA8	1.0143849	1.01490147	1.0141611	0.950312121	-0.0735267	1	1
RP11-756H20.1	1.014382	1.01490147	1.0141568	0.950025091	-0.0739625	1	1
RP11-370I10.10	1.0160998	1.01490147	1.0166192	1.11527369	0.1573978	1	1
HSPB3	1.0160941	1.01490147	1.0166111	1.114727105	0.1566906	1	1
RP11-418H16.1	1.0463662	1.04629846	1.0463955	1.002096377	0.0030213	1	1
RP11-406D1.2	1.0143827	1.01490147	1.0141578	0.950092705	-0.0738598	1	1
RP11-747D18.1	1.0143825	1.01490147	1.0141575	0.950074281	-0.0738878	1	1
JHDM1D-AS1	1.096365	1.09489167	1.0970036	1.022255727	0.0317561	1	1
PDZK1IP1	1.0143781	1.01490147	1.0141513	0.949656336	-0.0745226	1	1
LY75	1.015241	1.01490147	1.0153882	1.032665438	0.0463729	1	1
OPN4	1.0143829	1.01490147	1.0141581	0.95011668	-0.0738234	1	1
PDGFRL	1.0160991	1.01490147	1.0166183	1.115210454	0.157316	1	1
LRRC26	1.0238589	1.02346531	1.0240296	1.024045681	0.0342801	1	1
AC005281.1	1.0143866	1.01490147	1.0141635	0.950473065	-0.0732824	1	1
RP1-12G14.7	1.0161106	1.01490147	1.0166348	1.116317018	0.1587468	1	1
LINC01320	1.0264784	1.02636442	1.0265278	1.006196672	0.0089123	1	1
RP11-350N15.6	1.0247101	1.02345822	1.0252527	1.076496601	0.1063438	1	1
RP11-354K1.1	1.0143789	1.01490147	1.0141524	0.949732903	-0.0744063	1	1
APOBEC3C	1.2182285	1.21816388	1.2182566	1.000424759	0.0006127	1	1
VGLL2	1.0143849	1.01490147	1.0141611	0.950312121	-0.0735267	1	1
IDI2	1.0143835	1.01490147	1.014159	0.950176104	-0.0737332	1	1
KCNH5	1.016108	1.01490147	1.016631	1.116066336	0.1584228	1	1
RP11-256I23.3	1.0161115	1.01490147	1.016636	1.116398207	0.1588517	1	1
RP11-479J7.2	1.0152494	1.01490147	1.0154002	1.033470026	0.0474965	1	1
INS	1.0152502	1.01490147	1.0154014	1.033549204	0.0476071	1	1

PCDHB7	1.0186982	1.01774131	1.019113	1.077313796	0.1074385	1	1
PARVG	1.0143893	1.01490147	1.0141673	0.950733398	-0.0728873	1	1
SLC22A4	1.0221308	1.02061575	1.0227875	1.105344614	0.1444962	1	1
PRND	1.015255	1.01490147	1.0154082	1.034005198	0.0482434	1	1
WTH3DI	1.0143793	1.01490147	1.014153	0.949771866	-0.0743471	1	1
RP11-15E18.1	1.0273983	1.02636227	1.0278474	1.056335723	0.0790684	1	1
RP11-118E18.2	1.0143818	1.01490147	1.0141565	0.95000708	-0.0739898	1	1
CTD-2302E22.6	1.0204116	1.02058661	1.0203358	0.987815902	-0.0176859	1	1
TOR2A	1.3343885	1.33492629	1.3341554	0.997698318	-0.0033245	1	1
MC5R	1.0143847	1.01490147	1.0141607	0.950288207	-0.073563	1	1
PTN	1.5552781	1.55428015	1.5557106	1.002580734	0.0037184	1	1
ZNF410	1.0290832	1.02914881	1.0290547	0.996772288	-0.0046641	1	1
KLHL4	1.0255884	1.02635606	1.0252556	0.958246617	-0.0615311	1	1
RP11-596C23.6	1.0152516	1.01490147	1.0154034	1.03368254	0.0477932	1	1
LINC00266-1	1.0143793	1.01490147	1.014153	0.94977251	-0.0743461	1	1
MTMR12	1.1215609	1.12092068	1.1218385	1.007589842	0.0109085	1	1
ATP13A5-AS1	1.0161027	1.01490147	1.0166234	1.115555764	0.1577626	1	1
LINC01416	1.0143818	1.01490147	1.0141565	0.95000708	-0.0739898	1	1
CTD-2260A17.2	1.036841	1.03780845	1.0364216	0.963318848	-0.0539147	1	1
NDOR1	1.0186854	1.01775073	1.0190906	1.075481427	0.1049826	1	1
RPH3A	1.0152613	1.01490147	1.0154173	1.034616895	0.0490967	1	1
RP11-344B5.4	1.0143808	1.01490147	1.0141551	0.949909603	-0.0741379	1	1
RP11-159F24.6	1.0143881	1.01490147	1.0141656	0.950615231	-0.0730666	1	1
RP11-269F20.1	1.0143796	1.01490147	1.0141534	0.949796184	-0.0743101	1	1
APOB	1.0143816	1.01490147	1.0141563	0.949990548	-0.0740149	1	1
LHFPL3	1.0143847	1.01490147	1.0141606	0.950284049	-0.0735693	1	1
RP11-1057B8.2	1.0143797	1.01490147	1.0141536	0.949812241	-0.0742857	1	1
LA16c-321D4.2	1.0152517	1.01490147	1.0154036	1.033694572	0.04781	1	1
RP13-895J2.3	1.0152463	1.01490147	1.0153957	1.033167465	0.0470741	1	1
CTD-2193P3.2	1.0152386	1.01490147	1.0153848	1.032434098	0.0460497	1	1
P2RX7	1.0152594	1.01490147	1.0154145	1.034427062	0.0488319	1	1
BHMG1	1.0143873	1.01490147	1.0141644	0.950537404	-0.0731847	1	1
RP11-326C3.15	1.0195584	1.0206312	1.0190935	0.925465065	-0.1117496	1	1
IL4I1	1.017822	1.01775578	1.0178508	1.005349561	0.0076972	1	1
RP11-587P21.2	1.0256383	1.0263536	1.0253282	0.961092237	-0.0572532	1	1
CTB-22K21.2	1.0161182	1.01490147	1.0166456	1.117042689	0.1596843	1	1
AC004947.2	1.0143835	1.01490147	1.014159	0.950176104	-0.0737332	1	1
AC003088.1	1.0143829	1.01490147	1.0141581	0.95011668	-0.0738234	1	1
LINC01284	1.0143834	1.01490147	1.0141589	0.950165964	-0.0737486	1	1
RP11-444E17.6	1.0152509	1.01490147	1.0154023	1.03361235	0.0476952	1	1
GRIK3	1.0161083	1.01490147	1.0166314	1.116089847	0.1584532	1	1
RP11-17A4.2	1.0196317	1.02056357	1.0192277	0.935038729	-0.096902	1	1
XAGE3	1.0196418	1.02056357	1.0192422	0.935744085	-0.0958141	1	1
RP11-123K3.9	1.0196297	1.02056357	1.019225	0.934903577	-0.0971105	1	1
RP11-752G15.8	1.020499	1.02056357	1.020471	0.995496435	-0.0065119	1	1
CTD-2639E6.9	1.0205306	1.02056357	1.0205163	0.997700083	-0.0033219	1	1
RP11-524G24.2	1.0205142	1.02056357	1.0204928	0.996557833	-0.0049746	1	1
TEX35	1.0196323	1.02056357	1.0192286	0.935080074	-0.0968382	1	1
BAALC-AS1	1.0334995	1.03199903	1.0341499	1.067217731	0.0938545	1	1
RP11-92C4.6	1.0196323	1.02056357	1.0192286	0.935080074	-0.0968382	1	1
RP11-397A16.1	1.0439242	1.04346671	1.0441224	1.015085713	0.0216016	1	1

CTD-2525I3.3	1.019631	1.02056357	1.0192268	0.934994759	-0.0969698	1	1
AC016722.4	1.0205099	1.02056357	1.0204867	0.996259538	-0.0054065	1	1
SUV39H1	1.0750538	1.07499244	1.0750803	1.001172134	0.00169	1	1
AC004257.1	1.0205005	1.02056357	1.0204731	0.995600504	-0.0063611	1	1
RP11-70F11.8	1.0205064	1.02056357	1.0204816	0.996015702	-0.0057596	1	1
SRGN	1.0196388	1.02056357	1.019238	0.935538095	-0.0961317	1	1
BATF3	1.0352443	1.03491753	1.035386	1.013416312	0.019227	1	1
RP11-15I11.3	1.0204962	1.02056357	1.020467	0.995301654	-0.0067943	1	1
TSPAN8	1.0196365	1.02056357	1.0192347	0.935375627	-0.0963823	1	1
RP1-50J22.4	1.0213552	1.02056357	1.0216983	1.055183234	0.0774935	1	1
RP1-122P22.4	1.0257062	1.02630925	1.0254448	0.967143158	-0.0481986	1	1
ADGRD2	1.0248614	1.02343209	1.0254809	1.08743781	0.1209329	1	1
RP11-34F13.2	1.0196353	1.02056357	1.019233	0.935292559	-0.0965104	1	1
MCCC1-AS1	1.0196331	1.02056357	1.0192297	0.935136131	-0.0967517	1	1
CUL5	1.8578735	1.85737007	1.8580917	1.000841718	0.0012138	1	1
ZNF257	1.0213915	1.02056357	1.0217503	1.057710953	0.0809454	1	1
RP11-227H15.4	1.0222496	1.02344029	1.0217335	0.927184701	-0.1090713	1	1
LURAP1L-AS1	1.0196312	1.02056357	1.019227	0.935003413	-0.0969565	1	1
RP11-119H12.6	1.0204995	1.02056357	1.0204718	0.995534843	-0.0064563	1	1
RP11-350J20.12	1.0196316	1.02056357	1.0192276	0.935030071	-0.0969153	1	1
EN2	1.0196388	1.02056357	1.019238	0.935538733	-0.0961307	1	1
CTD-2047H16.5	1.0213918	1.02056357	1.0217508	1.057736275	0.08098	1	1
SFTPA2	1.0196406	1.02056357	1.0192405	0.935660508	-0.0959429	1	1
NCRNA00250	1.0274846	1.02635959	1.0279723	1.061181157	0.085671	1	1
AC009014.3	1.019632	1.02056357	1.0192282	0.935059398	-0.0968701	1	1
RP5-965G21.6	1.0205149	1.02056357	1.0204938	0.99660525	-0.0049059	1	1
SLC2A14	1.0205005	1.02056357	1.0204732	0.99560612	-0.006353	1	1
BOK	1.2673934	1.26764341	1.2672851	0.99866119	-0.0019328	1	1
AC006126.3	1.0196341	1.02056357	1.0192312	0.935206942	-0.0966425	1	1
BPI	1.0213559	1.02056357	1.0216994	1.055233909	0.0775628	1	1
EMCN	1.0248492	1.02342238	1.0254676	1.087320107	0.1207767	1	1
RP11-572C15.5	1.01963	1.02056357	1.0192254	0.934924199	-0.0970787	1	1
NEUROD2	1.0257432	1.02630641	1.0254991	0.969312062	-0.0449669	1	1
RP11-179A10.1	1.0205074	1.02056357	1.020483	0.996080997	-0.005665	1	1
CTD-2589M5.5	1.0196406	1.02056357	1.0192405	0.935660508	-0.0959429	1	1
CSMD1	1.0196368	1.02056357	1.0192351	0.935399084	-0.0963461	1	1
RP11-15J22.8	1.0213788	1.02056357	1.0217322	1.056830101	0.0797435	1	1
RP11-378A13.2	1.0239358	1.02343507	1.0241528	1.03062598	0.0435209	1	1
RP11-498C9.13	1.0196289	1.02056357	1.0192238	0.934847737	-0.0971967	1	1
RP11-517C16.4	1.0205235	1.02056357	1.0205062	0.997209914	-0.0040309	1	1
RP11-437J19.1	1.0214142	1.02056357	1.0217829	1.059294636	0.0831039	1	1
RP11-143A12.3	1.0196328	1.02056357	1.0192293	0.935115436	-0.0967836	1	1
CTD-2227E11.1	1.0283172	1.02919946	1.0279348	0.956688079	-0.0638795	1	1
SLC12A3	1.021363	1.02056357	1.0217095	1.055727878	0.078238	1	1
TTC29	1.0534338	1.05211949	1.0540035	1.036148417	0.0512307	1	1
CCNI2	1.0205097	1.02056357	1.0204863	0.996242028	-0.0054318	1	1
RP3-331H24.5	1.0196331	1.02056357	1.0192297	0.935136131	-0.0967517	1	1
RP11-596D21.1	1.0248531	1.02343139	1.0254694	1.086978105	0.1203229	1	1
KBTBD12	1.0196341	1.02056357	1.0192312	0.935206942	-0.0966425	1	1
CTC-297N7.9	1.0196297	1.02056357	1.019225	0.934903577	-0.0971105	1	1
C8B	1.0196302	1.02056357	1.0192256	0.934932842	-0.0970654	1	1

GRM5	1.0265678	1.02632405	1.0266734	1.013271806	0.0190212	1	1
CTD-2213F21.2	1.0204909	1.02056357	1.0204594	0.994935205	-0.0073255	1	1
BFSP2-AS1	1.0196364	1.02056357	1.0192345	0.935369591	-0.0963916	1	1
RP11-126K1.9	1.0204908	1.02056357	1.0204592	0.994926551	-0.0073381	1	1
RP11-569G13.2	1.0196343	1.02056357	1.0192316	0.935224369	-0.0966156	1	1
AGAP9	1.1143935	1.11532953	1.1139877	0.988365473	-0.0168835	1	1
MYOM3	1.0222344	1.02341788	1.0217214	0.927556184	-0.1084934	1	1
CYP2J2	1.0196364	1.02056357	1.0192345	0.935369591	-0.0963916	1	1
RP3-337H4.9	1.0230872	1.02343507	1.0229364	0.978720684	-0.0310309	1	1
RP11-309M7.1	1.0196331	1.02056357	1.0192297	0.935136131	-0.0967517	1	1
RP11-327J17.2	1.019631	1.02056357	1.0192267	0.934988761	-0.0969791	1	1
NXNL1	1.0196426	1.02056357	1.0192434	0.935800122	-0.0957277	1	1
CTC-510F12.7	1.0222275	1.02343197	1.0217054	0.926317774	-0.1104209	1	1
RP11-381N20.1	1.0213997	1.02056357	1.0217621	1.058284222	0.0817271	1	1
DCLK3	1.0213693	1.02056357	1.0217185	1.056165573	0.078836	1	1
CTD-2118P12.1	1.019632	1.02056357	1.0192282	0.935059398	-0.0968701	1	1
RP11-90C4.1	1.0196323	1.02056357	1.0192286	0.935080074	-0.0968382	1	1
TMEM72	1.0222268	1.02341788	1.0217105	0.927090036	-0.1092186	1	1
DHRS7C	1.0213654	1.02056357	1.0217129	1.055893465	0.0784643	1	1
RP11-131K5.2	1.0196313	1.02056357	1.0192271	0.935009412	-0.0969472	1	1
RP11-77K12.10	1.0213562	1.02056357	1.0216997	1.055250586	0.0775856	1	1
AC116366.6	1.0222723	1.02343647	1.0217677	0.928795241	-0.1065675	1	1
RP11-220I1.5	1.0231003	1.0234425	1.0229519	0.979072017	-0.0305131	1	1
ECD	1.1878053	1.18716137	1.1880844	1.004931517	0.0070972	1	1
ARAP1-AS1	1.0205186	1.02056357	1.0204992	0.996867999	-0.0045256	1	1
CTD-2544N14.3	1.0196324	1.02056357	1.0192287	0.935086081	-0.0968289	1	1
MB	1.0291465	1.02915695	1.029142	0.99948844	-0.0007382	1	1
PPP3R1	1.1609451	1.16128194	1.160799	0.99700583	-0.0043262	1	1
MRO	1.0239752	1.02340079	1.0242242	1.035187907	0.0498927	1	1
AC010226.4	1.0605092	1.06082414	1.0603726	0.992576673	-0.0107495	1	1
PRSS48	1.0196307	1.02056357	1.0192264	0.934974113	-0.0970017	1	1
TTC39C	1.358477	1.35686997	1.3591736	1.006455208	0.009283	1	1
FAM71F1	1.0196362	1.02056357	1.0192342	0.935354849	-0.0964143	1	1
LINC00348	1.0196313	1.02056357	1.0192271	0.935009412	-0.0969472	1	1
RP11-661A12.5	1.0343734	1.03490955	1.0341411	0.977986428	-0.0321137	1	1
RP11-432J24.2	1.0196388	1.02056357	1.019238	0.935538095	-0.0961317	1	1
RP11-671P2.1	1.019631	1.02056357	1.0192267	0.934988761	-0.0969791	1	1
RP11-1399P15.1	1.0196336	1.02056357	1.0192305	0.935171526	-0.0966971	1	1
RP11-43F13.3	1.0196394	1.02056357	1.0192388	0.935577046	-0.0960716	1	1
SPRR2A	1.0196353	1.02056357	1.019233	0.935292559	-0.0965104	1	1
RP4-778K6.3	1.0196351	1.02056357	1.0192327	0.935277837	-0.0965331	1	1
GAST	1.0196294	1.02056357	1.0192245	0.934882962	-0.0971423	1	1
ROPN1B	1.0204971	1.02056357	1.0204683	0.995364613	-0.006703	1	1
CXorf65	1.020519	1.02056357	1.0204997	0.996892296	-0.0044904	1	1
RP11-456O19.5	1.0196365	1.02056357	1.0192347	0.935375627	-0.0963823	1	1
TRAF3IP3	1.0196367	1.02056357	1.019235	0.935390374	-0.0963595	1	1
KIF4B	1.0204981	1.02056357	1.0204697	0.995433881	-0.0066026	1	1
SIGLEC14	1.0196372	1.02056357	1.0192357	0.935425919	-0.0963047	1	1
AOAH	1.0222281	1.02343938	1.021703	0.925922304	-0.111037	1	1
LRRC55	1.0231333	1.02343168	1.023004	0.981748522	-0.0265746	1	1
CTC-436K13.1	1.0196328	1.02056357	1.0192293	0.935115436	-0.0967836	1	1

CTD-3193O13.11	1.0230938	1.02343705	1.022945	0.979005477	-0.0306112	1	1
IGSF22	1.0196294	1.02056357	1.0192245	0.934882962	-0.0971423	1	1
RP1-20C7.6	1.0274173	1.026287	1.0279072	1.061634956	0.0862878	1	1
SYP-AS1	1.0205183	1.02056357	1.0204987	0.996844876	-0.0045591	1	1
RP11-197K3.1	1.0196388	1.02056357	1.019238	0.935538733	-0.0961307	1	1
SALL3	1.0205046	1.02056357	1.020479	0.995888317	-0.0059441	1	1
RP11-182N22.8	1.0196377	1.02056357	1.0192363	0.935457364	-0.0962562	1	1
RP4-760C5.3	1.0196323	1.02056357	1.0192286	0.935080074	-0.0968382	1	1
ZNF335	1.0967385	1.0952988	1.0973625	1.021655327	0.0309086	1	1
AC145343.2	1.0291429	1.02917199	1.0291302	0.998568568	-0.0020666	1	1
FLI1	1.0205026	1.02056357	1.0204762	0.995752616	-0.0061407	1	1
AC010987.6	1.0205269	1.02056357	1.020511	0.997444014	-0.0036922	1	1
GRIK2	1.0343542	1.0348671	1.0341319	0.978913854	-0.0307462	1	1
HCLS1	1.0204943	1.02056357	1.0204642	0.995168294	-0.0069876	1	1
ZNF559-ZNF177	1.0222196	1.02340107	1.0217075	0.927629102	-0.10838	1	1
RRS1-AS1	1.026611	1.02630487	1.0267437	1.016682152	0.0238687	1	1
INSL4	1.0196406	1.02056357	1.0192405	0.935660508	-0.0959429	1	1
PHOX2A	1.0204867	1.02056357	1.0204533	0.994638158	-0.0077563	1	1
AJ239322.3	1.0196338	1.02056357	1.0192308	0.935186222	-0.0966744	1	1
SP6	1.021764	1.02296159	1.0212449	0.925237673	-0.1121041	1	1
RP11-428G5.5	1.0174565	1.01724954	1.0175462	1.017199935	0.0246033	1	1
CAPRIN2	1.4288505	1.42943171	1.4285986	0.998059914	-0.0028017	1	1
ZNF710	1.0806085	1.08022015	1.0807768	1.006939189	0.0099766	1	1
RP11-120C12.3	1.0200894	1.02010187	1.020084	0.99910946	-0.0012853	1	1
RP11-543D5.1	1.0183477	1.01724954	1.0188237	1.091257183	0.1259912	1	1
RP11-141M1.3	1.0365302	1.03734916	1.0361752	0.968567122	-0.0460761	1	1
RP5-894D12.5	1.0165893	1.01724954	1.0163031	0.945129931	-0.0814154	1	1
LGSN	1.0183211	1.01724954	1.0187856	1.089051554	0.1230723	1	1
CITF22-1A6.3	1.0226765	1.02302653	1.0225247	0.978207414	-0.0317877	1	1
RP11-298I3.1	1.0218295	1.02297517	1.0213329	0.928520661	-0.1069941	1	1
RP5-1050D4.5	1.0235308	1.02301631	1.0237538	1.03204241	0.0455023	1	1
ZNF284	1.0866609	1.08608695	1.0869097	1.00955739	0.0137229	1	1
RP11-164P12.3	1.0165917	1.01724954	1.0163065	0.945330461	-0.0811094	1	1
KCNJ5	1.0312835	1.03154241	1.0311713	0.988233845	-0.0170756	1	1
NLRP7	1.0165993	1.01724954	1.0163175	0.945966764	-0.0801386	1	1
RP11-122M14.1	1.0296503	1.02868837	1.0300672	1.048062829	0.0677252	1	1
FAM78B	1.0330462	1.03155119	1.0336942	1.067921592	0.0948057	1	1
SCGB1A1	1.0165943	1.01724954	1.0163103	0.945549134	-0.0807757	1	1
GBX2	1.0165974	1.01724954	1.0163147	0.945803755	-0.0803872	1	1
RP11-138E2.1	1.0200256	1.02008312	1.0200006	0.995893347	-0.0059368	1	1
RP11-728G15.1	1.0183323	1.01724954	1.0188016	1.089976269	0.1242967	1	1
VIPR1	1.0165932	1.01724954	1.0163087	0.945457271	-0.0809158	1	1
NRIP3	1.0978943	1.09745637	1.0980841	1.006440959	0.0092625	1	1
RP11-370A5.1	1.0183281	1.01724954	1.0187956	1.089630913	0.1238395	1	1
USP30	1.1525423	1.15184578	1.1528442	1.006574929	0.0094546	1	1
RP11-557H15.3	1.0226331	1.02296773	1.0224881	0.979117931	-0.0304455	1	1
TSPEAR	1.0165882	1.01724954	1.0163016	0.945042589	-0.0815487	1	1
IL6R	1.0200289	1.02009638	1.0199997	0.995189356	-0.006957	1	1
RP4-693M11.3	1.0165896	1.01724954	1.0163036	0.945161729	-0.0813669	1	1
LY6G6E	1.0174614	1.01724954	1.0175532	1.01760234	0.0251739	1	1
CTD-3194G12.2	1.0174925	1.01724954	1.0175978	1.020190204	0.0288382	1	1

RP11-48G14.1	1.0165926	1.01724954	1.0163078	0.945403855	-0.0809973	1	1
CXCR6	1.0165972	1.01724954	1.0163144	0.945786113	-0.0804141	1	1
RANBP10	1.1005327	1.10033204	1.1006197	1.002866835	0.0041301	1	1
RP11-122K13.12	1.0165919	1.01724954	1.0163068	0.945347991	-0.0810826	1	1
CTD-2126E3.3	1.0174678	1.01724954	1.0175623	1.018133548	0.0259268	1	1
PCDH20	1.0165885	1.01724954	1.016302	0.945069406	-0.0815078	1	1
CXorf58	1.0260824	1.02577532	1.0262155	1.017075997	0.0244275	1	1
LINC00602	1.0165986	1.01724954	1.0163165	0.945906497	-0.0802305	1	1
TULP2	1.0183656	1.01724954	1.0188494	1.092745209	0.1279571	1	1
RP11-310E22.5	1.0165911	1.01724954	1.0163057	0.945281069	-0.0811847	1	1
AC012314.8	1.0174658	1.01724954	1.0175595	1.017969754	0.0256947	1	1
RP11-442N1.2	1.0165934	1.01724954	1.016309	0.945474832	-0.080889	1	1
CTD-2033D15.1	1.0165967	1.01724954	1.0163137	0.945748136	-0.0804721	1	1
RP11-401P9.5	1.0165946	1.01724954	1.0163107	0.945573159	-0.080739	1	1
RP11-789C17.3	1.0165885	1.01724954	1.016302	0.945069406	-0.0815078	1	1
CLEC4G	1.0174432	1.01724954	1.0175272	1.016095661	0.0230362	1	1
SUN3	1.0165899	1.01724954	1.016304	0.94518638	-0.0813293	1	1
RP1-178F10.1	1.0183065	1.01724954	1.0187646	1.087832408	0.1214563	1	1
RP11-455J20.3	1.0243844	1.02301874	1.0249763	1.085042456	0.1177515	1	1
DEFB136	1.0165925	1.01724954	1.0163077	0.945397429	-0.0810072	1	1
AC007292.4	1.0165909	1.01724954	1.0163054	0.945263555	-0.0812115	1	1
AMZ1	1.01659	1.01724954	1.0163041	0.945192787	-0.0813195	1	1
PIK3IP1-AS1	1.0191678	1.02009109	1.0187676	0.934125174	-0.0983122	1	1
TSSK4	1.0183392	1.01724954	1.0188116	1.090554438	0.1250618	1	1
RP11-45A17.2	1.0322005	1.03159472	1.0324631	1.027486057	0.0391188	1	1
PHEX-AS1	1.0183099	1.01724954	1.0187696	1.088119346	0.1218368	1	1
RP11-379K17.4	1.0191944	1.0201277	1.0187899	0.933533226	-0.0992267	1	1
SAP30	1.2085236	1.20944673	1.2081234	0.993682013	-0.0091438	1	1
CLDN14	1.0165993	1.01724954	1.0163175	0.945966764	-0.0801386	1	1
RP11-62I21.1	1.0165876	1.01724954	1.0163007	0.944993396	-0.0816238	1	1
CTC-297N7.1	1.0183207	1.01724954	1.0187851	1.089018394	0.1230283	1	1
IL25	1.0165994	1.01724954	1.0163176	0.945974005	-0.0801276	1	1
QRFP	1.0174715	1.01724954	1.0175678	1.018447461	0.0263716	1	1
AP002954.3	1.0165888	1.01724954	1.0163025	0.945094981	-0.0814688	1	1
RP1-122K4.2	1.0174829	1.01724954	1.017584	1.019389941	0.027706	1	1
LINC00269	1.0174732	1.01724954	1.0175702	1.018589311	0.0265725	1	1
RP11-120K24.3	1.018355	1.01724954	1.0188342	1.091864183	0.1267934	1	1
RP11-659P15.1	1.0165866	1.01724954	1.0162993	0.944909378	-0.0817521	1	1
XXbac-BPG249D20.9	1.018315	1.01724954	1.0187768	1.088537601	0.1223912	1	1
LINC01214	1.0165865	1.01724954	1.0162991	0.944902245	-0.081763	1	1
RP11-818O24.3	1.0174662	1.01724954	1.0175601	1.018005086	0.0257448	1	1
SLC17A6	1.018367	1.01724954	1.0188513	1.092858447	0.1281065	1	1
C4orf51	1.0174663	1.01724954	1.0175603	1.018013014	0.025756	1	1
RP11-149B9.2	1.0165885	1.01724954	1.016302	0.945069406	-0.0815078	1	1
C11orf72	1.0226654	1.02293839	1.0225471	0.982940043	-0.0248247	1	1
AP001046.6	1.0165881	1.01724954	1.0163014	0.945035442	-0.0815597	1	1
HOXC4	1.0165966	1.01724954	1.0163136	0.945743624	-0.0804789	1	1
AC009312.1	1.0183306	1.01724954	1.0187992	1.089836605	0.1241119	1	1
CFAP99	1.01659	1.01724954	1.0163041	0.945192787	-0.0813195	1	1
AP004372.1	1.0174638	1.01724954	1.0175567	1.017807531	0.0254648	1	1
RP11-90K6.1	1.0200697	1.02011228	1.0200512	0.996964117	-0.0043865	1	1

VWA5B1	1.0165865	1.01724954	1.0162991	0.944902245	-0.081763	1	1
RP11-307O13.1	1.0165893	1.01724954	1.0163031	0.945129931	-0.0814154	1	1
HECTD2	1.2512247	1.25211483	1.2508388	0.994938796	-0.0073203	1	1
IFI30	1.028675	1.02874285	1.0286455	0.99661404	-0.0048932	1	1
ALDH1L1-AS2	1.0183278	1.01724954	1.0187952	1.089606528	0.1238073	1	1
LDB2	1.0659289	1.06600318	1.0658967	0.998386616	-0.0023295	1	1
PTPN14	1.1409933	1.14047729	1.141217	1.00526573	0.0075769	1	1
RP11-91I20.3	1.0174474	1.01724954	1.0175331	1.016439382	0.0235242	1	1
CTD-2017F17.1	1.0165909	1.01724954	1.0163054	0.945263555	-0.0812115	1	1
AC104654.2	1.0226491	1.02293673	1.0225244	0.982023769	-0.0261702	1	1
RP11-556I14.1	1.0166021	1.01724954	1.0163214	0.946194645	-0.0797911	1	1
RP11-3N13.2	1.0226898	1.02301259	1.0225499	0.979894658	-0.0293014	1	1
NKPD1	1.0174507	1.01724954	1.0175379	1.016716329	0.0239172	1	1
ADGRG2	1.0174514	1.01724954	1.017539	1.016778596	0.0240056	1	1
ANKRD34C	1.018313	1.01724954	1.0187739	1.088373776	0.1221741	1	1
SLC22A9	1.0174571	1.01724954	1.0175471	1.017248736	0.0246725	1	1
RP11-707G14.1	1.0165878	1.01724954	1.016301	0.945010844	-0.0815972	1	1
RP13-143G15.4	1.0485526	1.04872451	1.0484782	0.994943956	-0.0073128	1	1
RP3-468B3.2	1.017446	1.01724954	1.0175311	1.016323285	0.0233594	1	1
POMZP3	1.1435966	1.14309329	1.1438148	1.00504241	0.0072564	1	1
POLR3GL	2.3917253	2.39312865	2.391117	0.998556012	-0.0020847	1	1
TSNAX-DISC1	1.0191985	1.02011088	1.0188031	0.934971244	-0.0970061	1	1
RP11-1C8.4	1.0183501	1.01724954	1.0188272	1.091461597	0.1262614	1	1
AC015691.13	1.0391193	1.04029727	1.0386087	0.958097713	-0.0617553	1	1
LSAMP-AS1	1.0226325	1.02299393	1.0224759	0.977470098	-0.0328755	1	1
RP11-865I6.2	1.0183107	1.01724954	1.0187707	1.088183156	0.1219214	1	1
KRT79	1.0165919	1.01724954	1.0163068	0.945347991	-0.0810826	1	1
TBC1D26.1	1.0165901	1.01724954	1.0163042	0.945196712	-0.0813135	1	1
RP11-332K15.1	1.018321	1.01724954	1.0187854	1.089038347	0.1230548	1	1
RP11-428K3.1	1.0183181	1.01724954	1.0187812	1.088794621	0.1227318	1	1
USP38	1.153875	1.15448508	1.1536105	0.994338961	-0.0081904	1	1
RFPL2	1.0183198	1.01724954	1.0187837	1.088937953	0.1229218	1	1
RP11-88G17.6	1.0174513	1.01724954	1.0175388	1.016769834	0.0239931	1	1
RP11-123B3.2	1.0165893	1.01724954	1.0163031	0.945129931	-0.0814154	1	1
ALS2CR12	1.0165986	1.01724954	1.0163164	0.945902533	-0.0802366	1	1
RP11-104H15.8	1.0165917	1.01724954	1.0163065	0.945330461	-0.0811094	1	1
RHCG	1.0226472	1.02301631	1.0224872	0.977013092	-0.0335502	1	1
LINC00434	1.0174614	1.01724954	1.0175532	1.01760234	0.0251739	1	1
RP11-365H22.2	1.0183118	1.01724954	1.0187723	1.088277105	0.122046	1	1
RP11-422P24.10	1.0252736	1.02577084	1.0250581	0.972342607	-0.0404634	1	1
RP11-160E2.19	1.0174484	1.01724954	1.0175346	1.016522911	0.0236427	1	1
RP11-1L9.1	1.0183135	1.01724954	1.0187747	1.088414706	0.1222284	1	1
RP11-116D17.5	1.0165965	1.01724954	1.0163134	0.94572919	-0.080501	1	1
RP4-655J12.4	1.0165966	1.01724954	1.0163135	0.945736407	-0.08049	1	1
LHFPL5	1.0166078	1.01724954	1.0163297	0.946673658	-0.0790609	1	1
RP5-1061H20.4	1.0183075	1.01724954	1.018766	1.08791397	0.1215645	1	1
RP11-613D13.10	1.0174426	1.01724954	1.0175263	1.016046706	0.0229667	1	1
AL121578.2	1.0174431	1.01724954	1.0175271	1.016088593	0.0230262	1	1
SYNGR4	1.0217654	1.02297362	1.0212417	0.924612592	-0.1130791	1	1
RP11-549B18.1	1.0191738	1.02008243	1.01878	0.935146542	-0.0967356	1	1
RP11-923I11.3	1.0200396	1.02011235	1.0200081	0.994817326	-0.0074965	1	1

SYBU	1.0545724	1.05440632	1.0546444	1.004375468	0.0062987	1	1
RP11-1085N6.5	1.0191736	1.02008439	1.0187788	0.934993795	-0.0969713	1	1
GJB6	1.0165993	1.01724954	1.0163174	0.945962797	-0.0801446	1	1
CTD-2017C7.1	1.0174591	1.01724954	1.01755	1.017418225	0.0249128	1	1
RP11-2E11.5	1.0191916	1.02012004	1.0187892	0.933852929	-0.0987327	1	1
LINC01228	1.0174632	1.01724954	1.0175559	1.017757803	0.0253943	1	1
MPHOSPH10	1.4219843	1.42105091	1.4223888	1.003177591	0.004577	1	1
OR4D10	1.0165946	1.01724954	1.0163108	0.945576081	-0.0807346	1	1
RP11-874J12.4	1.0192804	1.01839445	1.0196644	1.069037677	0.0963127	1	1
TMEM256-PLSCR3.1	1.0175554	1.01839445	1.0171916	0.934610221	-0.0975633	1	1
RP11-864J10.4	1.0175465	1.01839445	1.017179	0.933920614	-0.0986282	1	1
RP11-16E23.5	1.0175418	1.01839445	1.0171722	0.9335517	-0.0991982	1	1
PXT1	1.018412	1.01839445	1.0184196	1.00136855	0.0019731	1	1
HEXA-AS1	1.0244881	1.02410643	1.0246535	1.022694078	0.0323747	1	1
LINC01539	1.0175465	1.01839445	1.017179	0.933920614	-0.0986282	1	1
OSTN-AS1	1.0175418	1.01839445	1.0171722	0.9335517	-0.0991982	1	1
RP11-353N4.6	1.0175414	1.01839445	1.0171716	0.933519011	-0.0992487	1	1
CTA-989H11.1	1.0175505	1.01839445	1.0171847	0.934230562	-0.0981495	1	1
LINC01603	1.0175541	1.01839445	1.0171898	0.934509932	-0.0977181	1	1
CTD-2263F21.1	1.0175421	1.01839445	1.0171726	0.933574745	-0.0991626	1	1
AGTR2	1.0192954	1.01839445	1.0196859	1.070206538	0.0978892	1	1
RP11-445O3.2	1.0175439	1.01839445	1.0171752	0.933716156	-0.098944	1	1
RP3-355L5.5	1.0184305	1.01839445	1.0184461	1.002806686	0.0040435	1	1
RAMP3	1.0184118	1.01839445	1.0184193	1.001348183	0.0019437	1	1
HCN2	1.0175499	1.01839445	1.0171838	0.934181939	-0.0982245	1	1
RP11-568N6.1	1.0227898	1.02125283	1.023456	1.103663621	0.1423005	1	1
CST1	1.018417	1.01839445	1.0184267	1.00175356	0.0025276	1	1
RAB39B	1.2011988	1.20212243	1.2007985	0.993449912	-0.0094809	1	1
UPP2	1.0201333	1.02123502	1.0196557	0.925626033	-0.1114987	1	1
GDNF	1.0175468	1.01839445	1.0171794	0.933943077	-0.0985935	1	1
GJB3	1.0192753	1.01839445	1.019657	1.068639951	0.0957759	1	1
RP11-83A16.1	1.0184234	1.01839445	1.018436	1.002259476	0.0032561	1	1
RP1-41C23.4	1.0175416	1.01839445	1.0171719	0.933535353	-0.0992234	1	1
RP11-733O18.1	1.0175515	1.01839445	1.0171862	0.934313442	-0.0980215	1	1
TNFSF18	1.0175463	1.01839445	1.0171787	0.933907155	-0.098649	1	1
AC026471.6	1.0288213	1.02983557	1.0283817	0.951269116	-0.0720746	1	1
RP11-295H24.4	1.0496231	1.05008113	1.0494246	0.986891327	-0.0190369	1	1
SLC39A12	1.0201387	1.02125031	1.0196568	0.925013527	-0.1124536	1	1
L3MBTL3	1.2080649	1.20795541	1.2081124	1.000754839	0.0010886	1	1
ZNRF2	1.0330924	1.03268103	1.0332707	1.018044581	0.0258007	1	1
CTB-191K22.5	1.0184239	1.01839445	1.0184366	1.002291232	0.0033018	1	1
VSIG10L	1.2184165	1.21892103	1.2181978	0.996696471	-0.0047739	1	1
RP11-25I15.2	1.0175425	1.01839445	1.0171732	0.933606566	-0.0991134	1	1
GIPC2	1.0184118	1.01839445	1.0184193	1.001350999	0.0019478	1	1
RP11-799M12.2	1.0192861	1.01839445	1.0196726	1.069486866	0.0969188	1	1
ZFPM2	1.0192741	1.01839445	1.0196555	1.068553575	0.0956592	1	1
CD3D	1.0175421	1.01839445	1.0171726	0.933574745	-0.0991626	1	1
CCDC183-AS1	1.0227779	1.02125958	1.023436	1.102372553	0.1406119	1	1
NR4A3	1.0313638	1.02985685	1.0320171	1.072352035	0.1007786	1	1
RP11-495O11.1	1.0175475	1.01839445	1.0171804	0.933999877	-0.0985057	1	1
PDE11A	1.0886567	1.08739817	1.0892022	1.020641361	0.029476	1	1

TAP2	1.0581683	1.05854148	1.0580065	0.99086203	-0.0132439	1	1
CTD-2547L24.4	1.01755	1.01839445	1.0171839	0.934191686	-0.0982095	1	1
RP11-218I7.2	1.0175499	1.01839445	1.0171838	0.934181939	-0.0982245	1	1
RP4-800J21.3	1.0201531	1.02126454	1.0196713	0.925077408	-0.112354	1	1
RP4-568C11.4	1.0193336	1.01839445	1.0197406	1.073185014	0.1018988	1	1
AP000692.10	1.0305664	1.02990793	1.0308518	1.031557523	0.0448243	1	1
RP11-242J7.1	1.0192726	1.01839445	1.0196532	1.068431506	0.0954944	1	1
RP11-367G18.1	1.020983	1.02123502	1.0208737	0.982987081	-0.0247556	1	1
RNF222	1.0175503	1.01839445	1.0171844	0.934214953	-0.0981736	1	1
CD5	1.0184005	1.01839445	1.0184031	1.000468001	0.000675	1	1
PLA2G2C	1.0175457	1.01839445	1.0171778	0.933857866	-0.0987251	1	1
ZSCAN5B	1.0175436	1.01839445	1.0171748	0.933693062	-0.0989797	1	1
SEMA3F	1.1352645	1.13613502	1.1348872	0.990833962	-0.0132848	1	1
RTEL1	1.0175531	1.01839445	1.0171885	0.934437499	-0.0978299	1	1
RP11-57H12.3	1.0322742	1.03281232	1.0320409	0.97649004	-0.0343228	1	1
INE2	1.0193127	1.01839445	1.0197107	1.071557092	0.0997087	1	1
RP11-707P17.2	1.0210092	1.02126975	1.0208963	0.982441759	-0.0255562	1	1
AC012456.4	1.0184132	1.01839445	1.0184214	1.001464488	0.0021113	1	1
PKN2-AS1	1.0201334	1.02125467	1.0196474	0.924382422	-0.1134383	1	1
HMSD	1.0348309	1.0355463	1.0345209	0.971152494	-0.0422302	1	1
RHOH	1.0210034	1.02127404	1.0208861	0.981765042	-0.0265503	1	1
TRIM60	1.0192874	1.01839445	1.0196745	1.069589559	0.0970573	1	1
NPC1L1	1.0175495	1.01839445	1.0171832	0.93415193	-0.0982709	1	1
SESTD1	1.554101	1.55420139	1.5540576	0.999740459	-0.0003745	1	1
AC079586.1	1.0236113	1.02411587	1.0233926	0.97000667	-0.0439334	1	1
PHF24	1.0192666	1.01839445	1.0196447	1.067968914	0.0948697	1	1
RP11-166N6.2	1.017543	1.01839445	1.0171739	0.93364393	-0.0990556	1	1
CYP4F26P	1.0175454	1.01839445	1.0171774	0.933834723	-0.0987609	1	1
AMBP	1.0175465	1.01839445	1.017179	0.933920614	-0.0986282	1	1
EGR3	1.0218639	1.02128211	1.0221161	1.03918943	0.0554587	1	1
IGDCC4	1.2870646	1.28810087	1.2866154	0.994843882	-0.0074579	1	1
MCMDC2	1.0253179	1.02413206	1.025832	1.070441477	0.0982059	1	1
RP11-86A5.1	1.0175434	1.01839445	1.0171745	0.933676681	-0.099005	1	1
SYN2	1.0235909	1.02411515	1.0233637	0.968837678	-0.0456731	1	1
AC073133.1	1.0184215	1.01839445	1.0184332	1.002105663	0.0030346	1	1
LINC00458	1.0210058	1.02124228	1.0209032	0.984039279	-0.0232122	1	1
ACOT6	1.0175431	1.01839445	1.017174	0.9336536	-0.0990407	1	1
SYN1	1.0771648	1.07556182	1.0778596	1.030409543	0.0432179	1	1
TBL1XR1-AS1	1.0175421	1.01839445	1.0171726	0.933574745	-0.0991626	1	1
PLPP5	1.414778	1.41467645	1.4148221	1.000351149	0.0005065	1	1
NPY1R	1.0652465	1.06422923	1.0656875	1.022703575	0.032388	1	1
ITGAM	1.0175492	1.01839445	1.0171828	0.934128685	-0.0983068	1	1
RNF141	1.4693942	1.46887911	1.4696175	1.001574861	0.0022703	1	1
PIK3C2B	1.0659583	1.06712241	1.0654537	0.975139755	-0.0363191	1	1
HAND2	1.0184462	1.01839445	1.0184687	1.00403621	0.0058113	1	1
RP13-216E22.4	1.0244944	1.02411125	1.0246604	1.022777063	0.0324917	1	1
CTD-2369P2.8	1.0192918	1.01839445	1.0196807	1.069928179	0.097514	1	1
RP1-56K13.2	1.0184152	1.01839445	1.0184242	1.001617583	0.0023318	1	1
GRIN1	1.0201435	1.02127485	1.0196531	0.923769645	-0.114395	1	1
WWC2-AS1	1.0175448	1.01839445	1.0171765	0.93378846	-0.0988323	1	1
RP11-103H7.1	1.0175411	1.01839445	1.0171711	0.933495984	-0.0992843	1	1

CTB-107G13.1	1.0184161	1.01839445	1.0184255	1.001687407	0.0024324	1	1
PTAFR	1.0175495	1.01839445	1.0171832	0.93415193	-0.0982709	1	1
AC005013.5	1.0175527	1.01839445	1.0171878	0.934403661	-0.0978822	1	1
AL121656.5	1.0175493	1.01839445	1.0171829	0.934135436	-0.0982964	1	1
EXOSC6	1.2738673	1.27351793	1.2740187	1.001830975	0.0026391	1	1
RP11-342C20.2	1.0175492	1.01839445	1.0171828	0.934128685	-0.0983068	1	1
PNLIPRP3	1.0175445	1.01839445	1.0171761	0.93376534	-0.0988681	1	1
CTRL	1.0192662	1.01839445	1.0196441	1.067937978	0.0948279	1	1
RP11-235E17.6	1.0175418	1.01839445	1.0171722	0.9335517	-0.0991982	1	1
RP11-407N8.6	1.0175446	1.01839445	1.0171762	0.933772055	-0.0988577	1	1
RUBCNL	1.0192698	1.01839445	1.0196493	1.068216196	0.0952037	1	1
RP11-434D9.2	1.0227714	1.02126326	1.0234252	1.10167347	0.1396967	1	1
TDRD6	1.0296317	1.02986224	1.0295317	0.988931985	-0.0160568	1	1
RP5-1056L3.1	1.0175461	1.01839445	1.0171784	0.933890721	-0.0986744	1	1
CFC1B	1.0175463	1.01839445	1.0171787	0.933907155	-0.098649	1	1
RP11-281A20.2	1.0175476	1.01839445	1.0171805	0.934005905	-0.0984964	1	1
UGT2A3	1.0175505	1.01839445	1.0171847	0.934230562	-0.0981495	1	1
C5orf51	1.2226562	1.22228477	1.2228172	1.002395078	0.0034512	1	1
RP11-396C23.2	1.0558435	1.05588312	1.0558264	0.998984186	-0.0014663	1	1
C1orf61	1.0460708	1.04709069	1.0456287	0.968953227	-0.0455011	1	1
HPS6	1.143902	1.14485612	1.1434884	0.990558103	-0.0136865	1	1
RP11-77H9.8	1.0201366	1.02124005	1.0196583	0.92552897	-0.1116499	1	1
HEATR5A	1.1378738	1.13888567	1.1374352	0.98955661	-0.0151459	1	1
RP11-258F1.1	1.0279388	1.02700257	1.0283447	1.04970277	0.0699809	1	1
SOX3	1.083373	1.08432704	1.0829594	0.983782197	-0.0235891	1	1
KCNMA1-AS3	1.0192724	1.01839445	1.0196529	1.068414991	0.0954721	1	1
DUOXA2	1.0193171	1.01839445	1.019717	1.071899451	0.1001696	1	1
SCGB1D2	1.0175544	1.01839445	1.0171903	0.93453703	-0.0976763	1	1
RP1-166H4.2	1.0184152	1.01839445	1.0184242	1.001619908	0.0023351	1	1
RP11-2E11.9	1.1464489	1.14710055	1.1461664	0.993649376	-0.0091912	1	1
RP11-881M11.2	1.0175475	1.01839445	1.0171804	0.933999877	-0.0985057	1	1
ZNF668	1.1343552	1.13295339	1.1349629	1.015114132	0.0216419	1	1
CMB9-55A18.1	1.0175507	1.01839445	1.0171849	0.93424499	-0.0981272	1	1
PTPRT	1.0244924	1.02414774	1.0246418	1.020460249	0.02922	1	1
FOXA1	1.0192659	1.01839445	1.0196437	1.067913661	0.094795	1	1
EPHA1	1.0192813	1.01839445	1.0196657	1.069109668	0.0964099	1	1
PARP12	1.0236098	1.02415981	1.0233714	0.967366203	-0.047866	1	1
HM13-AS1	1.0262288	1.02697097	1.0259071	0.960556659	-0.0580574	1	1
RP11-111K18.2	1.021012	1.02125308	1.0209075	0.983738185	-0.0236537	1	1
TP53INP2	1.1056589	1.10422435	1.1062807	1.019729938	0.0281871	1	1
RP11-461L13.3	1.0192708	1.01839445	1.0196507	1.068293586	0.0953082	1	1
RP11-218C14.5	1.0175491	1.01839445	1.0171827	0.934122795	-0.0983159	1	1
USP27X	1.1647843	1.16479424	1.16478	0.999913757	-0.0001244	1	1
RP11-17E3.1	1.0184093	1.01839445	1.0184157	1.001154509	0.0016646	1	1
RP11-60H5.1	1.0192832	1.01839445	1.0196684	1.069259295	0.0966117	1	1
PPP1R2P3	1.023631	1.02408163	1.0234357	0.973177638	-0.0392249	1	1
CTC-487M23.7	1.0175504	1.01839445	1.0171846	0.934227761	-0.0981538	1	1
RP11-818F20.5	1.0184082	1.01839445	1.0184141	1.001069827	0.0015426	1	1
MEPCE	1.1872102	1.18780521	1.1869524	0.995458828	-0.0065664	1	1
EPGN	1.017854	1.01886772	1.0174147	0.922987327	-0.1156173	1	1
SHOX	1.0187131	1.01886772	1.0186461	0.988251575	-0.0170497	1	1

MAGEA10	1.0178586	1.01886772	1.0174212	0.923332904	-0.1150772	1	1
TELO2	1.1734973	1.17370053	1.1734092	0.998323061	-0.0024213	1	1
RP5-1029F21.3	1.0187584	1.01886772	1.018711	0.991692056	-0.0120359	1	1
PCDHA2	1.0204499	1.01886772	1.0211356	1.120201098	0.1637577	1	1
NHS-AS1	1.017854	1.01886772	1.0174146	0.922985306	-0.1156204	1	1
ELFN2	1.0204991	1.01886772	1.0212062	1.123939862	0.1685648	1	1
ITGB1BP2	1.0316868	1.0303129	1.0322824	1.064971466	0.0908148	1	1
LINC01411	1.0204309	1.01886772	1.0211085	1.118763099	0.1619046	1	1
OSR2	1.0195818	1.01886772	1.0198913	1.054248187	0.0762145	1	1
PLAC1	1.0178609	1.01886772	1.0174245	0.923510696	-0.1147994	1	1
AC007950.2	1.0204699	1.01886772	1.0211644	1.121727769	0.1657226	1	1
RP11-57H14.2	1.0300204	1.0303093	1.0298952	0.986336744	-0.0198478	1	1
RP11-673F18.1	1.0178526	1.01886772	1.0174126	0.922876383	-0.1157907	1	1
RP11-89G4.1	1.0178609	1.01886772	1.0174245	0.923510696	-0.1147994	1	1
HPN	1.0264906	1.02745892	1.0260709	0.949449877	-0.0748363	1	1
RP11-114G22.1	1.0196043	1.01886772	1.0199235	1.055959779	0.0785549	1	1
TDRD10	1.0187248	1.01886772	1.0186629	0.989145522	-0.0157453	1	1
RP11-12L8.1	1.0178522	1.01886772	1.017412	0.92284736	-0.115836	1	1
RP4-657D16.3	1.0187137	1.01886772	1.0186469	0.988296427	-0.0169843	1	1
LDHC	1.017852	1.01886772	1.0174117	0.922831409	-0.115861	1	1
RP11-463C8.7	1.0178545	1.01886772	1.0174154	0.923023836	-0.1155602	1	1
RP11-65L3.2	1.023044	1.02170759	1.0236233	1.08824897	0.1220087	1	1
LLNLR-307A6.1	1.017857	1.01886772	1.0174189	0.923213233	-0.1152642	1	1
RP11-89K10.1	1.023038	1.02171963	1.0236095	1.087009575	0.1203646	1	1
ZNF474	1.0239394	1.02455587	1.0236722	0.964015097	-0.0528724	1	1
RP11-25G10.2	1.0178556	1.01886772	1.0174169	0.923107524	-0.1154294	1	1
RP11-15B17.1	1.0204367	1.01886772	1.0211168	1.119205256	0.1624746	1	1
RP1-155D22.2	1.0178529	1.01886772	1.017413	0.922901775	-0.115751	1	1
KCNU1	1.0178552	1.01886772	1.0174164	0.923078398	-0.1154749	1	1
CTD-2553L13.10	1.0776966	1.07623724	1.0783292	1.027440596	0.039055	1	1
RP3-329E20.2	1.0178583	1.01886772	1.0174207	0.923307345	-0.1151171	1	1
CTC-436P18.1	1.0178528	1.01886772	1.0174129	0.922892346	-0.1157657	1	1
USP2-AS1	1.0238995	1.02459658	1.0235974	0.959376939	-0.0598303	1	1
PCDHGA9	1.0214044	1.021723	1.0212663	0.978975069	-0.030656	1	1
RP11-700J17.1	1.0239072	1.02457303	1.0236186	0.961157645	-0.057155	1	1
FAM131C	1.139022	1.13915933	1.1389625	0.998585566	-0.002042	1	1
RP11-499A10.3	1.0178539	1.01886772	1.0174145	0.92297876	-0.1156306	1	1
EPHB3	1.1494164	1.14799038	1.1500345	1.013812512	0.0197909	1	1
RP11-231C14.7	1.0204336	1.01886772	1.0211123	1.118966562	0.1621669	1	1
RP3-402G11.28	1.0187209	1.01886772	1.0186572	0.988845075	-0.0161836	1	1
WARS2-IT1	1.017857	1.01886772	1.0174189	0.923213233	-0.1152642	1	1
PDGFRA	1.8072606	1.8086528	1.8066572	0.997532139	-0.0035648	1	1
VRTN	1.0178546	1.01886772	1.0174155	0.923030386	-0.11555	1	1
RP3-395M20.2	1.0204525	1.01886772	1.0211395	1.12040326	0.1640181	1	1
DNAH12	1.0178573	1.01886772	1.0174193	0.923232878	-0.1152335	1	1
LINC00642	1.01786	1.01886772	1.0174232	0.923439673	-0.1149104	1	1
AC068492.1	1.0377681	1.03901441	1.0372279	0.954208522	-0.0676235	1	1
GPX2	1.0178545	1.01886772	1.0174154	0.923023836	-0.1155602	1	1
ERVK-28	1.0178515	1.01886772	1.017411	0.922792992	-0.115921	1	1
ANKRD13A	1.2006289	1.19960208	1.201074	1.007374475	0.0106001	1	1
RP11-89M20.2	1.0178594	1.01886772	1.0174223	0.923390701	-0.1149869	1	1

GOLGA8K	1.0178563	1.01886772	1.017418	0.923162155	-0.115344	1	1
C9orf64	1.017854	1.01886772	1.0174146	0.922985306	-0.1156204	1	1
RPA1	1.5724396	1.57219458	1.5725458	1.000613858	0.0008853	1	1
PLPPR4	1.0187388	1.01886772	1.0186829	0.990206375	-0.0141989	1	1
ANGPT4	1.0300016	1.03035131	1.0298499	0.98348131	-0.0240305	1	1
CECR3	1.0187303	1.01886772	1.0186707	0.989556254	-0.0151464	1	1
RP11-1228E12.2	1.0247697	1.02462287	1.0248334	1.008548246	0.0122801	1	1
CTD-3064H18.2	1.0213033	1.02172272	1.0211216	0.972326136	-0.0404878	1	1
CNTNAP4	1.0204499	1.01886772	1.0211357	1.120207126	0.1637655	1	1
RP11-506M12.1	1.0178571	1.01886772	1.017419	0.923216833	-0.1152586	1	1
RP11-863P13.3	1.0178522	1.01886772	1.017412	0.92284736	-0.115836	1	1
RP1-293L6.1	1.0178536	1.01886772	1.017414	0.922952649	-0.1156715	1	1
ZNF763	1.0395504	1.03898903	1.0397937	1.020638821	0.0294724	1	1
RP11-179K3.2	1.0213287	1.02171824	1.0211599	0.974290415	-0.0375762	1	1
CTD-2233C11.2	1.0178579	1.01886772	1.0174201	0.92327813	-0.1151628	1	1
ZNF585B	1.0377122	1.0389286	1.0371849	0.955207722	-0.0661136	1	1
AL023806.1	1.0221508	1.02172033	1.0223373	1.028407259	0.0404117	1	1
PLG	1.0178556	1.01886772	1.0174169	0.923107524	-0.1154294	1	1
FBXL14	1.0230223	1.02170465	1.0235935	1.087023441	0.1203831	1	1
TMED6	1.0178639	1.01886772	1.0174288	0.923736832	-0.1144462	1	1
HBA2	1.0187229	1.01886772	1.0186602	0.988999464	-0.0159584	1	1
ARMS2	1.0178532	1.01886772	1.0174135	0.922924282	-0.1157158	1	1
RP11-636O21.1	1.0187359	1.01886772	1.0186787	0.989984346	-0.0145224	1	1
RP11-794G24.1	1.0275249	1.02748757	1.0275411	1.001947818	0.0028074	1	1
RP11-424D14.1	1.0178537	1.01886772	1.0174142	0.922962777	-0.1156556	1	1
CBFB	1.4694052	1.46848035	1.4698061	1.002829887	0.0040769	1	1
LAG3	1.0379153	1.03905107	1.037423	0.958309456	-0.0614365	1	1
RP11-568J23.4	1.0178531	1.01886772	1.0174133	0.922917743	-0.115726	1	1
RP11-295K2.3	1.018719	1.01886772	1.0186546	0.988702272	-0.0163919	1	1
TPO	1.0238871	1.02457714	1.023588	0.959753744	-0.0592638	1	1
RP1-206D15.6	1.0187298	1.01886772	1.0186701	0.989523958	-0.0151935	1	1
AC016999.2	1.0178546	1.01886772	1.0174155	0.923032409	-0.1155468	1	1
ERICH6	1.0187099	1.01886772	1.0186415	0.98801182	-0.0173998	1	1
RP3-431P23.5	1.0187147	1.01886772	1.0186484	0.98837397	-0.0168711	1	1
TACR2	1.0187185	1.01886772	1.0186539	0.988667244	-0.0164431	1	1
MIR222HG	1.1433363	1.14220209	1.1438279	1.011432809	0.0164005	1	1
IL23A	1.0213197	1.02172346	1.0211447	0.973359534	-0.0389553	1	1
AC016751.3	1.0178536	1.01886772	1.017414	0.922952649	-0.1156715	1	1
RP11-81H14.1	1.0187186	1.01886772	1.0186539	0.988668614	-0.0164411	1	1
CTC-490E21.14	1.0187228	1.01886772	1.01866	0.988992226	-0.0159689	1	1
INF2	1.2435805	1.24242154	1.2440828	1.006852765	0.0098527	1	1
RP11-575I8.1	1.0178528	1.01886772	1.0174129	0.922892346	-0.1157657	1	1
RP11-659E9.2	1.0204385	1.01886772	1.0211193	1.119336459	0.1626438	1	1
SLC13A4	1.0411839	1.0418892	1.0408781	0.975863229	-0.0352491	1	1
LINC00626	1.0195797	1.01886772	1.0198883	1.054090027	0.0759981	1	1
AC007040.6	1.0178574	1.01886772	1.0174195	0.92324602	-0.115213	1	1
SLC39A5	1.0195923	1.01886772	1.0199064	1.055050024	0.0773114	1	1
TUBB1	1.0187437	1.01886772	1.01869	0.990579289	-0.0136556	1	1
RP5-1050D4.4	1.01786	1.01886772	1.0174232	0.923439673	-0.1149104	1	1
ALS2CL	1.0273312	1.02741589	1.0272945	0.995572151	-0.0064022	1	1
SCNN1A	1.0282225	1.02746161	1.0285523	1.039717479	0.0561916	1	1

RNU11	1.0187136	1.01886772	1.0186467	0.988287898	-0.0169967	1	1
RP11-85L21.4	1.0178568	1.01886772	1.0174187	0.92319922	-0.1152861	1	1
RP11-220I1.2	1.0178567	1.01886772	1.0174185	0.923190626	-0.1152995	1	1
FAM151B	1.0377386	1.03889389	1.0372378	0.957419429	-0.062777	1	1
C1QTNF1	1.0195789	1.01886772	1.0198872	1.05403418	0.0759217	1	1
PCP2	1.02744	1.02746213	1.0274304	0.998844397	-0.0016681	1	1
RP11-243A14.1	1.0195708	1.01886772	1.0198755	1.053413809	0.0750723	1	1
AC007009.1	1.0222126	1.02171652	1.0224277	1.032746987	0.0464869	1	1
CTF1	1.0351691	1.03615961	1.0347397	0.960732279	-0.0577936	1	1
RP11-13J8.1	1.018746	1.01886772	1.0186933	0.990755485	-0.013399	1	1
MPL	1.018727	1.01886772	1.018666	0.989309788	-0.0155057	1	1
UBE4A	1.3486372	1.3490705	1.3484495	0.998220851	-0.0025691	1	1
CYS1	1.0377132	1.03891149	1.0371938	0.955855978	-0.0651348	1	1
MROH7	1.0256471	1.0245932	1.026104	1.061431083	0.0860107	1	1
CORO1A	1.0766605	1.07611628	1.0768963	1.010248303	0.0147099	1	1
ADGRF5	1.0178522	1.01886772	1.017412	0.922843782	-0.1158416	1	1
CEMIP	1.0196176	1.01886772	1.0199427	1.056974781	0.079941	1	1
CTD-2587H19.3	1.0248375	1.02463082	1.0249271	1.0120289	0.0172505	1	1
LINC00520	1.0195837	1.01886772	1.019894	1.054394938	0.0764153	1	1
RP11-96A15.1	1.0187361	1.01886772	1.0186791	0.99000311	-0.014495	1	1
CTD-3064M3.4	1.0187348	1.01886772	1.0186772	0.989900298	-0.0146449	1	1
RP11-260M19.1	1.0178545	1.01886772	1.0174154	0.923023836	-0.1155602	1	1
ENPP6	1.0187222	1.01886772	1.0186591	0.988941052	-0.0160436	1	1
SLC15A1	1.0178545	1.01886772	1.0174154	0.923023836	-0.1155602	1	1
CTC-241N9.1	1.2128149	1.21099852	1.2136023	1.012340161	0.0176941	1	1
C9orf84	1.0178563	1.01886772	1.017418	0.923162155	-0.115344	1	1
RP3-461P17.9	1.0178581	1.01886772	1.0174205	0.923294191	-0.1151377	1	1
RP11-164O23.8	1.017855	1.01886772	1.0174161	0.92306239	-0.1154999	1	1
DACT3-AS1	1.0187584	1.01886772	1.018711	0.991692056	-0.0120359	1	1
CACNA1I	1.0178534	1.01886772	1.0174138	0.922940256	-0.1156908	1	1
RNF219-AS1	1.0187144	1.01886772	1.0186479	0.988349541	-0.0169067	1	1
PLA2G4B	1.0178661	1.01886772	1.017432	0.92390678	-0.1141808	1	1
ELDR	1.0204353	1.01886772	1.0211148	1.119096096	0.1623339	1	1
MZB1	1.0178573	1.01886772	1.0174193	0.923232	-0.1152349	1	1
TACR1	1.0178587	1.01886772	1.0174213	0.923339485	-0.1150669	1	1
ARX	1.0404217	1.03924482	1.0409318	1.042986394	0.0607203	1	1
MSH2	1.3823898	1.38345806	1.3819268	0.996006666	-0.0057727	1	1
SLC25A41	1.0187169	1.01886772	1.0186515	0.98854207	-0.0166257	1	1
RP1-244F24.1	1.023924	1.02462478	1.0236203	0.959208765	-0.0600833	1	1
RP11-320N7.2	1.0178536	1.01886772	1.017414	0.922952649	-0.1156715	1	1
RP11-407G23.1	1.0178545	1.01886772	1.0174154	0.923023836	-0.1155602	1	1
RP4-756G23.5	1.0230621	1.02173342	1.023638	1.087634816	0.1211942	1	1
RP11-62C3.6	1.0196032	1.01886772	1.019922	1.055879249	0.0784449	1	1
FPR1	1.0204494	1.01886772	1.021135	1.120168538	0.1637158	1	1
CYP51A1	1.0567301	1.05623002	1.0569469	1.012749299	0.0182771	1	1
LINC01415	1.0204497	1.01886772	1.0211354	1.120187686	0.1637405	1	1
RP11-543C4.1	1.0187115	1.01886772	1.0186438	0.988133812	-0.0172217	1	1
MASP1	1.025652	1.02458351	1.0261151	1.062300891	0.0871925	1	1
KRT6B	1.0187186	1.01886772	1.0186539	0.988668614	-0.0164411	1	1
PACS1	1.3487006	1.34873934	1.3486838	0.999840742	-0.0002298	1	1
FHOD3	1.0689305	1.06751697	1.0695432	1.030010226	0.0426587	1	1

FLT4	1.0300399	1.03101909	1.0296154	0.954748514	-0.0668073	1	1
U52111.14	1.0128003	1.01394208	1.0123053	0.882604659	-0.1801607	1	1
AC068831.16	1.0145248	1.01394208	1.0147775	1.059917601	0.0839521	1	1
FO538757.2	1.0136575	1.01394208	1.0135341	0.970739231	-0.0428443	1	1
RP11-322E11.2	1.0171115	1.01678432	1.0172533	1.027939194	0.0397549	1	1
TARDBP	1.9666963	1.96755651	1.9663235	0.998725645	-0.0018397	1	1
RP11-1263C18.1	1.0145299	1.01394208	1.0147847	1.060433921	0.0846547	1	1
THEGL	1.01366	1.01394208	1.0135378	0.970999795	-0.0424571	1	1
AF001548.5	1.018813	1.01963386	1.0184571	0.940067423	-0.0891639	1	1
PRRT4	1.0171056	1.01680201	1.0172371	1.025896546	0.0368853	1	1
MC2R	1.0136566	1.01394208	1.0135328	0.970647961	-0.0429799	1	1
CH17-125A10.2	1.0136593	1.01394208	1.0135367	0.970925976	-0.0425668	1	1
MB21D2	1.0817606	1.08250439	1.0814383	0.987077888	-0.0187642	1	1
TMPRSS4	1.0145253	1.01394208	1.0147781	1.059961056	0.0840113	1	1
POMT1	1.2196825	1.21954306	1.2197429	1.000910233	0.0013126	1	1
FAM221B	1.016239	1.01678027	1.0160044	0.953760682	-0.0683008	1	1
RP11-823E8.3	1.0136585	1.01394208	1.0135356	0.970843364	-0.0426895	1	1
LINC00997	1.0593406	1.05956963	1.0592413	0.994488368	-0.0079736	1	1
KLRB1	1.0136615	1.01394208	1.0135399	0.971156503	-0.0422243	1	1
LINC01433	1.0128003	1.01394208	1.0123053	0.882604659	-0.1801607	1	1
MYOZ2	1.0128003	1.01394208	1.0123053	0.882604659	-0.1801607	1	1
RP11-138H10.2	1.0153908	1.01394208	1.0160188	1.148953581	0.2003205	1	1
TRAF6	1.1128533	1.11429295	1.1122293	0.981944424	-0.0262867	1	1
AK5	1.0206092	1.01963776	1.0210302	1.070908953	0.0988358	1	1
HHIPL2	1.0128003	1.01394208	1.0123053	0.882604659	-0.1801607	1	1
RP4-539M6.14	1.0136602	1.01394208	1.0135381	0.971021459	-0.0424249	1	1
C10orf90	1.015421	1.01394208	1.016062	1.152051317	0.204205	1	1
AC025442.3	1.0136566	1.01394208	1.0135328	0.970647961	-0.0429799	1	1
CTB-111F10.1	1.0136598	1.01394208	1.0135375	0.970978137	-0.0424893	1	1
BTN3A2	1.2398142	1.23935643	1.2400126	1.002741269	0.0039494	1	1
ARSD-AS1	1.0145232	1.01394208	1.0147751	1.059751752	0.0837264	1	1
CTXN3	1.0188305	1.01963755	1.0184807	0.941089033	-0.0875969	1	1
EFCAB6-AS1	1.0136579	1.01394208	1.0135347	0.970782435	-0.0427801	1	1
ARL14EPL	1.0136569	1.01394208	1.0135333	0.970678374	-0.0429347	1	1
RP11-10A14.3	1.01366	1.01394208	1.0135378	0.970999795	-0.0424571	1	1
RP1-232L24.3	1.0136593	1.01394208	1.0135367	0.970925976	-0.0425668	1	1
RP11-849F2.4	1.0136603	1.01394208	1.0135382	0.97103033	-0.0424117	1	1
AC025165.8	1.0145196	1.01394208	1.0147699	1.059373446	0.0832113	1	1
PRKG1-AS1	1.0136602	1.01394208	1.0135381	0.971021459	-0.0424249	1	1
NAV2-AS1	1.0136615	1.01394208	1.0135398	0.97114762	-0.0422375	1	1
RP11-440G5.2	1.0145177	1.01394208	1.0147672	1.059178738	0.0829461	1	1
LINC00462	1.014543	1.01394208	1.0148035	1.061786105	0.0864932	1	1
ZNF625-ZNF20	1.0153858	1.01394208	1.0160116	1.14843912	0.1996744	1	1
HR	1.0145143	1.01394208	1.0147624	1.0588347	0.0824774	1	1
RP11-108K3.1	1.0145262	1.01394208	1.0147794	1.060056045	0.0841405	1	1
GPR158	1.0369314	1.03682587	1.0369771	1.004106396	0.0059121	1	1
TMEM266	1.0335568	1.03391396	1.033402	0.984903464	-0.0219458	1	1
APOD	1.0145241	1.01394208	1.0147764	1.059844413	0.0838525	1	1
ATP2B2	1.013662	1.01394208	1.0135407	0.971208801	-0.0421466	1	1
RP11-566J3.2	1.0153865	1.01394208	1.0160126	1.148505494	0.1997578	1	1
LINC00265	1.018831	1.01967292	1.0184661	0.938657107	-0.0913299	1	1

RP4-536B24.4	1.0145243	1.01394208	1.0147766	1.059856373	0.0838688	1	1
RP11-776A13.3	1.0145384	1.01394208	1.0147969	1.061309816	0.0858459	1	1
RP4-564F22.5	1.0162309	1.01679104	1.0159881	0.952181687	-0.0706912	1	1
RP11-367H1.1	1.0153862	1.01394208	1.0160121	1.148476146	0.1997209	1	1
AP2A1	1.7823599	1.7821121	1.7824673	1.000454115	0.000655	1	1
NEURL1B	1.054942	1.05380758	1.0554337	1.030221592	0.0429547	1	1
XXbac-B33L19.4	1.0136608	1.01394208	1.0135389	0.971082553	-0.0423341	1	1
RP11-133F8.2	1.0145135	1.01394208	1.0147612	1.058752365	0.0823652	1	1
GTF3C2-AS1	1.0136551	1.01394208	1.0135307	0.970492153	-0.0432115	1	1
DUSP18	1.2707631	1.27110146	1.2706164	0.998210835	-0.0025835	1	1
RP11-407N8.5	1.0136574	1.01394208	1.013534	0.970730389	-0.0428574	1	1
CACNG2	1.0136618	1.01394208	1.0135402	0.971178205	-0.042192	1	1
RP5-857K21.4	1.0136602	1.01394208	1.0135381	0.971021459	-0.0424249	1	1
RP11-1M18.1	1.0128003	1.01394208	1.0123053	0.882604659	-0.1801607	1	1
RP4-784A16.2	1.0128003	1.01394208	1.0123053	0.882604659	-0.1801607	1	1
RP11-38L15.8	1.0145323	1.01394208	1.0147882	1.060687664	0.0849999	1	1
RP11-329N22.1	1.0136585	1.01394208	1.0135356	0.970843364	-0.0426895	1	1
LRRK2	1.0223189	1.02260882	1.0221932	0.98161586	-0.0267695	1	1
RP11-388P9.2	1.0136621	1.01394208	1.0135408	0.971217691	-0.0421334	1	1
RP11-367J11.2	1.0145199	1.01394208	1.0147703	1.059403808	0.0832526	1	1
CTB-167B5.1	1.0145167	1.01394208	1.0147658	1.059082717	0.0828153	1	1
DPEP1	1.0136579	1.01394208	1.0135347	0.970782435	-0.0427801	1	1
NAALADL2	1.0869092	1.08815054	1.0863712	0.979814596	-0.0294193	1	1
RP11-894P9.2	1.0136597	1.01394208	1.0135373	0.970969271	-0.0425025	1	1
MC1R	1.0679189	1.0682088	1.0677932	0.993907473	-0.0088165	1	1
RP11-797D24.3	1.0128003	1.01394208	1.0123053	0.882604659	-0.1801607	1	1
LINC00539	1.0136616	1.01394208	1.0135401	0.971164451	-0.0422125	1	1
RP11-313P22.1	1.0136658	1.01394208	1.013546	0.971593606	-0.0415751	1	1
STPG3	1.0136597	1.01394208	1.0135373	0.970969271	-0.0425025	1	1
SLC6A7	1.0136569	1.01394208	1.0135333	0.970678374	-0.0429347	1	1
RP11-123O22.1	1.0136562	1.01394208	1.0135322	0.970604823	-0.0430441	1	1
ACVRL1	1.0153749	1.01394208	1.0159959	1.147312183	0.198258	1	1
TCN1	1.0128003	1.01394208	1.0123053	0.882604659	-0.1801607	1	1
LINC00407	1.0136554	1.01394208	1.0135311	0.970522512	-0.0431664	1	1
RP13-379L11.2	1.0136589	1.01394208	1.0135362	0.97088662	-0.0426253	1	1
SATB2-AS1	1.0136554	1.01394208	1.0135311	0.970522512	-0.0431664	1	1
DNAH17	1.0136587	1.01394208	1.0135358	0.970860138	-0.0426646	1	1
TUBB8	1.0153793	1.01394208	1.0160023	1.147767743	0.1988307	1	1
GNAT2	1.0145381	1.01394208	1.0147965	1.061283784	0.0858105	1	1
XX-C00717C00720L.1	1.0136598	1.01394208	1.0135375	0.970978137	-0.0424893	1	1
AC007292.7	1.0128003	1.01394208	1.0123053	0.882604659	-0.1801607	1	1
RP11-317N12.1	1.0153958	1.01394208	1.0160259	1.149459766	0.200956	1	1
USH1C	1.0145283	1.01394208	1.0147824	1.060271577	0.0844338	1	1
RP11-466I1.1	1.01366	1.01394208	1.0135378	0.970999795	-0.0424571	1	1
PTPRS	1.0542359	1.05398761	1.0543435	1.00659239	0.0094796	1	1
GPRIN2	1.0222661	1.02252032	1.0221559	0.983816383	-0.023539	1	1
RP11-745C15.2	1.0136623	1.01394208	1.013541	0.971230516	-0.0421143	1	1
AP000235.3	1.0136562	1.01394208	1.0135322	0.970604823	-0.0430441	1	1
RP11-365N19.2	1.015373	1.01394208	1.0159932	1.147116384	0.1980118	1	1
CDH26	1.014512	1.01394208	1.014759	1.058596227	0.0821524	1	1
CITF22-49E9.3	1.0136549	1.01394208	1.0135304	0.970470619	-0.0432436	1	1

ADARB2-AS1	1.0188256	1.01965208	1.0184674	0.939714869	-0.089705	1	1
IDO1	1.0128003	1.01394208	1.0123053	0.882604659	-0.1801607	1	1
RIPPLY3	1.0205492	1.01967151	1.0209297	1.06395899	0.0894425	1	1
MRAP	1.0136602	1.01394208	1.0135381	0.971021459	-0.0424249	1	1
RP11-105N14.2	1.0136627	1.01394208	1.0135416	0.971277983	-0.0420438	1	1
PSORS1C2	1.0145385	1.01394208	1.014797	1.06131964	0.0858592	1	1
RP1-102E24.8	1.0136616	1.01394208	1.0135401	0.971164451	-0.0422125	1	1
RP5-887A10.1	1.01366	1.01394208	1.0135378	0.970999795	-0.0424571	1	1
LINC00939	1.0265477	1.02533965	1.0270714	1.068341225	0.0953725	1	1
LRRC38	1.0162445	1.01679203	1.0160072	0.953260852	-0.069057	1	1
AC034228.2	1.0136618	1.01394208	1.0135402	0.971178205	-0.042192	1	1
LRRN4CL	1.0188269	1.01968762	1.0184538	0.937329363	-0.093372	1	1
RP4-635E18.9	1.0136615	1.01394208	1.0135399	0.971156503	-0.0422243	1	1
TBX5	1.0473464	1.04820172	1.0469756	0.974563446	-0.037172	1	1
RP11-686O6.2	1.0516446	1.05114326	1.0518619	1.014050624	0.0201297	1	1
UTS2	1.0136602	1.01394208	1.0135381	0.971021459	-0.0424249	1	1
RP11-22N19.2	1.0577015	1.05681349	1.0580863	1.02240417	0.0319656	1	1
FAM196B	1.0128003	1.01394208	1.0123053	0.882604659	-0.1801607	1	1
RP11-1100L3.7	1.013659	1.01394208	1.0135363	0.970895477	-0.0426121	1	1
RP11-214K3.25	1.0153787	1.01394208	1.0160014	1.147704036	0.1987507	1	1
STX19	1.0145208	1.01394208	1.0147716	1.05949962	0.0833831	1	1
RP11-17E13.2	1.0145141	1.01394208	1.0147621	1.058812978	0.0824478	1	1
CTD-2201E9.1	1.0136597	1.01394208	1.0135373	0.970969271	-0.0425025	1	1
RP11-103H7.2	1.0128003	1.01394208	1.0123053	0.882604659	-0.1801607	1	1
WFDC3	1.0300687	1.03109546	1.0296237	0.952670175	-0.0699513	1	1
RP11-505P4.6	1.0136572	1.01394208	1.0135337	0.970708797	-0.0428895	1	1
BACH1-IT2	1.0136562	1.01394208	1.0135322	0.970604823	-0.0430441	1	1
C14orf105	1.0162381	1.01678432	1.0160013	0.953348151	-0.0689249	1	1
ADCY10	1.0136578	1.01394208	1.0135345	0.970769675	-0.0427991	1	1
PSAPL1	1.0136567	1.01394208	1.013533	0.970656795	-0.0429668	1	1
CTC-523E23.4	1.017975	1.01678895	1.0184891	1.101263458	0.1391597	1	1
RP11-555G19.1	1.0128003	1.01394208	1.0123053	0.882604659	-0.1801607	1	1
AP001891.1	1.0128003	1.01394208	1.0123053	0.882604659	-0.1801607	1	1
FYB	1.0128003	1.01394208	1.0123053	0.882604659	-0.1801607	1	1
NR1I3	1.0145323	1.01394208	1.0147882	1.060687664	0.0849999	1	1
RP1-278O22.1	1.0145287	1.01394208	1.0147829	1.060311129	0.0844877	1	1
RP11-10N23.2	1.01885	1.01967348	1.018493	0.939998268	-0.08927	1	1
RP11-10L7.1	1.0128003	1.01394208	1.0123053	0.882604659	-0.1801607	1	1
DVL2	1.5943504	1.59380446	1.5945871	1.001317988	0.0019002	1	1
APOC2	1.0136587	1.01394208	1.0135359	0.970864989	-0.0426574	1	1
ZNF720	1.552901	1.55369243	1.5525579	0.997951071	-0.002959	1	1
KLF16	1.1153732	1.11384596	1.1160352	1.019229768	0.0274793	1	1
RP11-92A5.2	1.0153802	1.01394208	1.0160036	1.147860061	0.1989468	1	1
RP11-447L10.1	1.0145376	1.01394208	1.0147958	1.061232075	0.0857402	1	1
PELI3	1.0817236	1.08244802	1.0814096	0.987404839	-0.0182864	1	1
AC113608.1	1.0128003	1.01394208	1.0123053	0.882604659	-0.1801607	1	1
CH507-42P11.7	1.0136549	1.01394208	1.0135304	0.970470619	-0.0432436	1	1
BAIAP2L2	1.0266127	1.02531913	1.0271734	1.073234416	0.1019652	1	1
DHH	1.0153977	1.01394208	1.0160286	1.149656527	0.2012029	1	1
NOCT	1.0309569	1.03112164	1.0308855	0.992412182	-0.0109886	1	1
AC068535.2	1.0154094	1.01394208	1.0160454	1.150862363	0.2027153	1	1

IKZF1	1.0153935	1.01394208	1.0160226	1.1492255	0.2006619	1	1
ZBTB45	1.1655743	1.16552952	1.1655937	1.000387591	0.0005591	1	1
RP11-486O13.2	1.0136611	1.01394208	1.0135393	0.971113116	-0.0422887	1	1
PTGES3L	1.1033835	1.10239707	1.1038111	1.013809134	0.0197861	1	1
EIF2B5-AS1	1.0153718	1.01394208	1.0159916	1.147000153	0.1978656	1	1
LINC00538	1.0145481	1.01394208	1.0148108	1.062307866	0.0872019	1	1
RP11-31I22.3	1.0145253	1.01394208	1.0147781	1.059961056	0.0840113	1	1
CTA-243E7.4	1.0179915	1.01680144	1.0185074	1.101535127	0.1395155	1	1
RP11-699A5.2	1.01538	1.01394208	1.0160033	1.147842643	0.1989249	1	1
RP11-1277A3.1	1.0206076	1.01965208	1.0210217	1.069695135	0.0971997	1	1
CTD-2631K10.1	1.0136575	1.01394208	1.0135341	0.970739231	-0.0428443	1	1
MMP10	1.0145323	1.01394208	1.0147882	1.060687664	0.0849999	1	1
AWAT2	1.0128003	1.01394208	1.0123053	0.882604659	-0.1801607	1	1
GGT2	1.0128003	1.01394208	1.0123053	0.882604659	-0.1801607	1	1
RP11-271F18.1	1.0136613	1.01394208	1.0135396	0.971134807	-0.0422565	1	1
PKHD1	1.0128003	1.01394208	1.0123053	0.882604659	-0.1801607	1	1
DSG3	1.0128003	1.01394208	1.0123053	0.882604659	-0.1801607	1	1
CTD-2349P21.5	1.0153779	1.01394208	1.0160002	1.147620838	0.1986461	1	1
FHDC1	1.0257321	1.02539686	1.0258774	1.018922083	0.0270437	1	1
RP11-229E13.4	1.0145253	1.01394208	1.0147781	1.059961056	0.0840113	1	1
C9orf41-AS1	1.0154147	1.01394208	1.016053	1.151403794	0.2033939	1	1
ACSM2B	1.0136587	1.01394208	1.0135358	0.970860138	-0.0426646	1	1
CSN1S1	1.0136575	1.01394208	1.0135341	0.970739231	-0.0428443	1	1
RP11-637O19.3	1.0127909	1.01301898	1.012692	0.974884669	-0.0366965	1	1
CTD-2105E13.6	1.017973	1.0187273	1.017646	0.942262043	-0.0857998	1	1
PLA2G4E-AS1	1.0127939	1.01301898	1.0126964	0.975220093	-0.0362002	1	1
RP11-251G23.5	1.0171048	1.0158758	1.0176375	1.11096724	0.1518163	1	1
NPR1	1.0119324	1.01301898	1.0114615	0.880366329	-0.1838241	1	1
LRP1-AS	1.0136592	1.01301898	1.0139368	1.070494895	0.0982779	1	1
LINC00678	1.0127927	1.01301898	1.0126946	0.97508494	-0.0364002	1	1
CTD-2293H3.2	1.0127896	1.01301898	1.0126901	0.974740525	-0.0369099	1	1
RP11-1060J15.9	1.0127881	1.01301898	1.0126881	0.974582965	-0.0371431	1	1
ATP2C2	1.0162289	1.01586557	1.0163863	1.032823661	0.046594	1	1
RP11-191L9.4	1.0136607	1.01301898	1.0139388	1.070653806	0.0984921	1	1
CTD-2201E18.5	1.0136661	1.01301898	1.0139466	1.071254677	0.0993015	1	1
ZAP70	1.0136557	1.01301898	1.0139316	1.070102465	0.0977489	1	1
LINC01039	1.0127912	1.01301898	1.0126924	0.974917319	-0.0366482	1	1
RP11-1149O23.4	1.0119324	1.01301898	1.0114615	0.880366329	-0.1838241	1	1
HIST1H2BF	1.017096	1.01585117	1.0176356	1.112571954	0.1538986	1	1
RP11-345P4.10	1.0153689	1.01586893	1.0151522	0.954832389	-0.0666806	1	1
RP11-527F13.1	1.0127931	1.01301898	1.0126952	0.975131372	-0.0363315	1	1
RP11-656E20.5	1.0127898	1.01301898	1.0126904	0.974763654	-0.0368756	1	1
ARHGAP30	1.0136539	1.01301898	1.0139291	1.069908019	0.0974868	1	1
CCL26	1.0145231	1.01301898	1.015175	1.165606552	0.2210809	1	1
TDRD12	1.0153755	1.01587427	1.0151593	0.954958072	-0.0664907	1	1
RP11-472G21.2	1.0127953	1.01301898	1.0126984	0.975374501	-0.0359718	1	1
RP11-131N11.4	1.0145183	1.01301898	1.0151682	1.16508513	0.2204354	1	1
RESP18	1.0127948	1.01301898	1.0126977	0.975318423	-0.0360548	1	1
TUBA3D	1.0136721	1.01301898	1.0139552	1.071912878	0.1001877	1	1
CCL25	1.0170975	1.01584852	1.0176388	1.11296425	0.1544073	1	1
RP11-803P9.1	1.0119324	1.01301898	1.0114615	0.880366329	-0.1838241	1	1

RP11-65N13.8	1.0127912	1.01301898	1.0126924	0.974917319	-0.0366482	1	1
AC016831.7	1.0283235	1.02726945	1.0287803	1.055405505	0.0777974	1	1
SHC4	1.0421053	1.04151043	1.0423631	1.020542099	0.0293357	1	1
TPTE2	1.0136614	1.01301898	1.0139399	1.070736621	0.0986037	1	1
WISP1	1.0145222	1.01301898	1.0151737	1.165508141	0.2209591	1	1
KRT72	1.013648	1.01301898	1.0139207	1.069258866	0.0966112	1	1
RP11-313P13.5	1.0274284	1.02727705	1.027494	1.007953743	0.0114294	1	1
KLHL1	1.0127893	1.01301898	1.0126897	0.97470705	-0.0369594	1	1
BARX1	1.0197009	1.01872253	1.020125	1.074907987	0.1042132	1	1
GRM5-AS1	1.0136553	1.01301898	1.0139311	1.070063429	0.0976963	1	1
RP11-116D17.1	1.0153715	1.01585424	1.0151622	0.956351311	-0.0643874	1	1
LINC01117	1.0136668	1.01301898	1.0139476	1.071329093	0.0994017	1	1
GABRG3	1.0506913	1.05002853	1.0509786	1.018990727	0.0271409	1	1
EBI3	1.0213587	1.02152994	1.0212845	0.988597933	-0.0165442	1	1
RP11-469N6.1	1.0136572	1.01301898	1.0139338	1.070270518	0.0979755	1	1
HIST3H2BB	1.0344433	1.03298551	1.0350752	1.063351777	0.0886189	1	1
RP11-98D18.17	1.0145225	1.01301898	1.0151743	1.165548789	0.2210094	1	1
CHRD	1.0291308	1.03009223	1.028714	0.954201131	-0.0676347	1	1
PZP	1.0127891	1.01301898	1.0126895	0.974694285	-0.0369783	1	1
GRM1	1.0205454	1.02156645	1.0201029	0.932136206	-0.1013873	1	1
CTD-2008P7.1	1.0119324	1.01301898	1.0114615	0.880366329	-0.1838241	1	1
VWC2	1.043923	1.04435753	1.0437346	0.985956278	-0.0204044	1	1
RP11-225H22.7	1.0145312	1.01301898	1.0151866	1.16649811	0.222184	1	1
C3orf70	1.0309231	1.03009258	1.0312832	1.039563736	0.0559782	1	1
AC074183.4	1.0145296	1.01301898	1.0151844	1.166328413	0.2219741	1	1
TBX1	1.0127928	1.01301898	1.0126947	0.975094443	-0.0363861	1	1
LINC00845	1.0145065	1.01301898	1.0151513	1.163788155	0.2188285	1	1
ATP2B3	1.0136511	1.01301898	1.0139251	1.069603281	0.0970758	1	1
LRG1	1.0119324	1.01301898	1.0114615	0.880366329	-0.1838241	1	1
ZPLD1	1.0136539	1.01301898	1.0139291	1.069906066	0.0974841	1	1
F10	1.1773955	1.17872438	1.1768194	0.989341427	-0.0154596	1	1
RP4-545L17.11	1.0188154	1.01873433	1.0188505	1.006201136	0.0089187	1	1
IL36RN	1.0127896	1.01301898	1.0126901	0.974740525	-0.0369099	1	1
RP11-18C24.8	1.0145158	1.01301898	1.0151645	1.164802456	0.2200853	1	1
RP11-5C23.1	1.0136481	1.01301898	1.0139208	1.069269097	0.096625	1	1
RP5-894D12.4	1.0119324	1.01301898	1.0114615	0.880366329	-0.1838241	1	1
RP11-182J1.18	1.0162375	1.01585521	1.0164032	1.034564145	0.0490231	1	1
LINC00880	1.0153754	1.01586725	1.0151622	0.955567383	-0.0655705	1	1
RORC	1.0136511	1.01301898	1.0139251	1.069603187	0.0970757	1	1
RP11-297N6.4	1.041303	1.04155671	1.0411931	0.991249649	-0.0126796	1	1
RP11-407N17.4	1.0153712	1.01587427	1.0151531	0.954568182	-0.0670798	1	1
C2CD4B	1.0127931	1.01301898	1.0126952	0.975131372	-0.0363315	1	1
RP11-70L8.4	1.0136535	1.01301898	1.0139285	1.069861544	0.0974241	1	1
OR51E2	1.0136625	1.01301898	1.0139415	1.070859523	0.0987692	1	1
RP11-571M6.18	1.0171168	1.01586779	1.0176582	1.112830684	0.1542341	1	1
TAS2R30	1.0291851	1.03019359	1.028748	0.952122959	-0.0707802	1	1
RP11-353N14.2	1.0145126	1.01301898	1.0151599	1.164449658	0.2196483	1	1
CD3E	1.0136653	1.01301898	1.0139455	1.071167058	0.0991835	1	1
SLC35D2	1.2349307	1.23542669	1.2347156	0.99697973	-0.0043639	1	1
RP11-326A19.3	1.0136586	1.01301898	1.0139359	1.070429241	0.0981894	1	1
SLC25A19	1.1203939	1.12127951	1.1200101	0.989532914	-0.0151804	1	1

SNX25	1.1541286	1.15281542	1.1546978	1.012318137	0.0176628	1	1
AC009495.2	1.0127943	1.01301898	1.0126969	0.975262379	-0.0361377	1	1
RP11-379B8.1	1.0136473	1.01301898	1.0139196	1.069180141	0.0965049	1	1
AMY1C	1.0119324	1.01301898	1.0114615	0.880366329	-0.1838241	1	1
FAM138E	1.0145074	1.01301898	1.0151526	1.163884755	0.2189482	1	1
AC104134.2	1.0145108	1.01301898	1.0151574	1.164253187	0.2194048	1	1
CILP	1.0136529	1.01301898	1.0139276	1.069794077	0.0973331	1	1
AC007182.6	1.0119324	1.01301898	1.0114615	0.880366329	-0.1838241	1	1
RP11-508N22.12	1.0136625	1.01301898	1.0139415	1.070859523	0.0987692	1	1
RP1-10C16.1	1.0127881	1.01301898	1.012688	0.974573513	-0.0371571	1	1
F13A1	1.0145008	1.01301898	1.0151431	1.163155223	0.2180436	1	1
USP35	1.0343482	1.03301609	1.0349256	1.057837211	0.0811176	1	1
RHBG	1.0119324	1.01301898	1.0114615	0.880366329	-0.1838241	1	1
RP11-239H6.2	1.0127896	1.01301898	1.0126901	0.974740525	-0.0369099	1	1
RP11-157F20.3	1.0145061	1.01301898	1.0151507	1.163740839	0.2187698	1	1
RP11-6F2.5	1.0136519	1.01301898	1.0139262	1.069682362	0.0971825	1	1
STAU2-AS1	1.0136614	1.01301898	1.0139399	1.070736621	0.0986037	1	1
NCF1	1.0231263	1.02156379	1.0238036	1.103869671	0.1425698	1	1
MFRP	1.0119324	1.01301898	1.0114615	0.880366329	-0.1838241	1	1
SERTM1	1.0153706	1.01586389	1.0151567	0.955422591	-0.0657891	1	1
RP11-380O24.1	1.0136566	1.01301898	1.013933	1.070204942	0.0978871	1	1
LINC01597	1.0127934	1.01301898	1.0126956	0.975164107	-0.0362831	1	1
RP13-514E23.2	1.0153841	1.01587497	1.0151713	0.955674856	-0.0654082	1	1
RP11-196H14.2	1.0127878	1.01301898	1.0126875	0.974540983	-0.0372052	1	1
AC144449.1	1.0196657	1.01871413	1.0200781	1.072885965	0.1014967	1	1
RP11-1246C19.1	1.0464103	1.04719859	1.0460686	0.976057864	-0.0349614	1	1
[truncated: 19,036 more chars]
