# Supplementary material for: Single cell RNA sequencing of stem cell-derived retinal ganglion cells
Source: Sci Data. 2018 Feb 13;5:180013. doi: 10.1038/sdata.2018.13 (PMC5810423; doi:10.1038/sdata.2018.13)
Supplement: Supplementary Tables [file sdata201813-s2.zip › Supplementary tables/table s3.pdf]

id	baseMean	baseMeanA	baseMeanB	foldChange	log2FoldChange	pval	padj
IGFBP7	5.601269	16.8799	1.935077	0.05888	-4.086	6.60E-242	6.63E-239
ELN	2.712724	7.000209	1.31905	0.05317	-4.2332	1.43E-189	1.44E-186
CTGF	2.798627	7.441898	1.289301	0.04491	-4.4768	3.61E-173	3.63E-170
FN1	3.034981	8.118976	1.382396	0.05372	-4.2185	7.84E-163	3.94E-160
DCT	8.904439	30.88327	1.760078	0.02543	-5.297	5.90E-154	5.93E-151
SERPINF1	4.116021	9.834874	2.257071	0.14229	-2.8131	1.46E-130	1.47E-127
KRT19	3.617095	9.11733	1.829208	0.10215	-3.2912	5.64E-116	5.67E-113
PMEL	5.187152	12.97177	2.656711	0.13838	-2.8532	1.54E-109	1.54E-106
MGP	1.90409	4.61518	1.022833	0.00632	-7.3068	9.46E-100	9.51E-97
TMEM98	5.535272	11.6191	3.557686	0.24086	-2.0538	2.63E-90	2.65E-87
FABP7	4.374693	1.333218	5.363344	13.0946	3.7109	2.28E-83	1.15E-80
GJA1	3.472938	7.131228	2.283787	0.20938	-2.2558	1.10E-82	1.10E-79
SIX6	4.677512	1.685322	5.650142	6.78534	2.7624	8.60E-82	2.88E-79
PTGDS	3.572607	9.243818	1.729144	0.08845	-3.499	1.18E-81	5.95E-79
ATP6V1C2	1.804722	3.796046	1.157429	0.0563	-4.1506	7.68E-81	3.86E-78
TPM1	8.67694	20.66472	4.780234	0.19223	-2.3791	4.88E-74	2.45E-71
TYRP1	1.703217	3.642974	1.072686	0.0275	-5.1843	2.89E-73	9.69E-71
TOP2A	3.1728	1.338187	3.769153	8.18823	3.0336	7.62E-72	7.66E-69
ITM2C	4.098314	7.186694	3.094417	0.33854	-1.5626	1.11E-69	1.11E-66
KRT7	1.970263	4.24166	1.23193	0.07155	-3.805	6.48E-69	6.52E-66
ATP1B1	3.003385	5.802132	2.093634	0.22774	-2.1345	2.69E-67	2.71E-64
SPARC	8.037737	13.82906	6.155231	0.40184	-1.3153	1.55E-66	1.56E-63
IGFBP5	13.50654	32.3356	7.386027	0.20379	-2.2948	1.78E-65	5.95E-63
LEFTY2	3.851251	1.205887	4.711144	18.0251	4.1719	1.63E-63	5.47E-61
FTH1	111.8545	174.0249	91.6456	0.52389	-0.9327	5.46E-63	5.48E-60
TRPM1	1.715868	3.446392	1.15335	0.06268	-3.9958	2.14E-62	2.15E-59
SPATS2L	1.72846	3.054082	1.297559	0.14486	-2.7872	5.59E-62	1.40E-59
PTH2	7.974922	1.786291	9.986577	11.4291	3.5146	5.51E-61	2.77E-58
S100A9	1.70409	3.736274	1.043516	0.0159	-5.9745	1.47E-60	7.39E-58
SOX2	3.662195	1.394593	4.399293	8.61468	3.1068	3.50E-60	1.76E-57
HMGB2	6.181981	2.396504	7.412475	4.5918	2.1991	4.12E-60	4.15E-57
KRT17	1.754374	3.699113	1.122225	0.04528	-4.4649	1.22E-58	3.06E-56
APOE	6.574608	12.27327	4.722223	0.33018	-1.5987	1.71E-57	5.75E-55
COL1A1	4.025737	8.618977	2.532675	0.20117	-2.3135	2.31E-57	1.16E-54
KRT18	5.236058	11.7401	3.121878	0.19757	-2.3396	6.74E-56	1.70E-53
SLC7A8	1.810348	3.271125	1.335513	0.14773	-2.759	7.50E-56	2.51E-53
KRT8	5.610781	10.94802	3.875878	0.28909	-1.7904	9.93E-55	4.99E-52
WLS	1.531049	2.773214	1.127275	0.07178	-3.8003	1.05E-52	3.50E-50
ITGB1	4.581598	7.66012	3.580905	0.38752	-1.3677	3.09E-52	7.76E-50
COL3A1	1.602309	3.357448	1.031789	0.01348	-6.2125	5.33E-52	2.68E-49
MAB21L1	5.345335	2.240752	6.354499	4.31553	2.1095	1.39E-51	1.40E-48
MIAT	7.384079	2.483441	8.977063	5.3774	2.4269	1.01E-50	5.06E-48

TNFRSF12A	2.108731	3.874593	1.534726	0.18602	-2.4265	4.45E-50	4.48E-47
IGFBP3	1.745576	3.454548	1.190063	0.07743	-3.6909	6.40E-50	2.15E-47
CRABP1	26.44976	7.478802	32.61639	4.87998	2.2869	5.00E-49	2.52E-46
MFGE8	3.020437	4.920375	2.402849	0.35784	-1.4826	3.43E-47	1.15E-44
DAPL1	13.743	4.727899	16.67342	4.20436	2.0719	4.63E-47	4.65E-44
PLIN2	3.505191	6.710192	2.463385	0.25628	-1.9642	2.89E-46	5.80E-44
TMSB15A	4.694445	2.168042	5.515668	3.86601	1.9508	4.50E-46	4.52E-43
RAB31	3.140863	1.538254	3.661801	4.94525	2.306	2.24E-45	5.64E-43
HMX1	2.62705	1.33299	3.047693	6.14941	2.6204	3.29E-45	8.28E-43
TGM2	1.70045	3.161064	1.225668	0.10442	-3.2595	4.84E-45	2.43E-42
CENPF	3.628055	1.497881	4.320482	6.66922	2.7375	1.42E-44	2.86E-42
CYP1B1	11.93356	3.409382	14.70439	5.68793	2.5079	2.61E-44	8.76E-42
TMEFF2	1.832765	3.231948	1.377951	0.16934	-2.562	3.22E-44	5.39E-42
TIMP3	1.752726	3.101072	1.314437	0.14966	-2.7403	1.00E-43	5.03E-41
SEZ6L2	1.575655	2.543465	1.261062	0.16914	-2.5637	1.44E-43	7.25E-41
SLC2A1	11.06684	18.21728	8.742543	0.4497	-1.153	2.02E-42	6.79E-40
UBE2C	2.623051	1.300992	3.052795	6.8201	2.7698	6.87E-42	1.73E-39
CTSH	1.900304	3.309982	1.442079	0.19138	-2.3855	7.34E-42	2.46E-39
TBX2-AS1	2.506593	1.408193	2.863635	4.56557	2.1908	2.59E-41	8.67E-39
GPNMB	1.51841	2.740269	1.121236	0.06967	-3.8434	1.11E-40	5.59E-38
ITGAV	2.218818	3.453934	1.817336	0.33307	-1.5861	3.97E-40	1.99E-37
B2M	10.84644	16.61653	8.970829	0.51041	-0.9703	6.30E-40	1.27E-37
FZD5	2.066686	1.113875	2.376403	12.0869	3.5954	9.23E-40	3.10E-37
SFRP2	35.90345	11.37562	43.87638	4.13242	2.047	3.29E-39	1.65E-36
TIMP2	1.947842	3.001501	1.605344	0.30244	-1.7253	3.33E-39	6.70E-37
FGF19	2.213944	1.222821	2.536115	6.89394	2.7853	1.06E-38	5.35E-36
CD63	12.23492	18.25131	10.27926	0.53789	-0.8946	1.24E-38	3.12E-36
HES6	2.491911	1.364698	2.858319	5.09551	2.3492	5.34E-38	2.69E-35
MSI2	6.483071	3.478536	7.459714	2.60626	1.382	1.23E-37	3.09E-35
MYL9	7.480745	12.58609	5.82122	0.41612	-1.2649	2.13E-37	5.35E-35
CSTB	6.645434	10.17814	5.497104	0.48998	-1.0292	1.35E-36	2.72E-34
LGALS1	4.354816	10.4115	2.38605	0.14727	-2.7634	3.47E-36	1.16E-33
BMP4	1.591418	2.684707	1.236037	0.14011	-2.8354	6.91E-36	1.39E-33
IL32	1.69624	3.057808	1.253653	0.12326	-3.0202	1.17E-35	3.93E-33
COL4A2	2.292283	3.537882	1.887393	0.34966	-1.516	3.86E-35	9.71E-33
ANXA1	4.420578	7.575018	3.395207	0.36429	-1.4568	8.64E-35	1.45E-32
MKI67	2.28797	1.25825	2.622686	6.2834	2.6515	9.45E-35	1.58E-32
S100A10	6.710315	12.6312	4.785693	0.32548	-1.6194	1.30E-34	6.53E-32
VSX2	1.910883	1.088732	2.178128	13.2773	3.7309	6.59E-34	1.66E-31
FSTL1	2.520187	3.925155	2.063494	0.36357	-1.4597	7.44E-34	1.87E-31
APOC1	1.9502	3.276743	1.518999	0.22796	-2.1332	8.12E-34	2.72E-31
GAP43	2.823529	1.596719	3.222312	3.72422	1.8969	1.34E-33	4.50E-31
CKS1B	4.373549	2.485748	4.987191	2.68363	1.4242	1.61E-33	3.04E-31
ACTN4	2.452079	3.746275	2.031392	0.37556	-1.4129	2.00E-33	3.04E-31
LY6E	2.664418	4.413481	2.095874	0.32104	-1.6392	2.12E-33	3.04E-31

C9orf135	1.998444	1.148582	2.274696	8.57906	3.1008	2.24E-33	5.62E-31
LY6H	1.953057	3.223003	1.540253	0.24303	-2.0408	4.11E-33	8.25E-31
IER3	1.639523	2.695081	1.296407	0.17486	-2.5157	5.91E-33	1.19E-30
NRP2	1.513812	2.452169	1.208794	0.14378	-2.7981	7.98E-33	1.34E-30
S100A11	10.67896	18.57099	8.113603	0.40485	-1.3045	1.46E-32	4.89E-30
FIGN	2.916925	1.643077	3.330998	3.62476	1.8579	2.12E-32	3.55E-30
CRB1	1.966446	1.131111	2.237977	9.44224	3.2391	2.52E-32	5.07E-30
SMC4	2.497638	1.504934	2.820323	3.60507	1.85	3.81E-32	1.28E-29
DSP	1.697247	2.720364	1.364676	0.21198	-2.238	4.03E-32	5.79E-30
BIRC5	2.363441	1.263285	2.721054	6.53686	2.7086	5.13E-32	6.45E-30
TRPM3	4.545056	7.71727	3.513907	0.37425	-1.4179	6.45E-32	9.26E-30
ACTN1	2.000631	3.213141	1.606496	0.27404	-1.8675	1.26E-31	3.16E-29
S100A4	10.18826	22.79763	6.089504	0.23349	-2.0986	1.42E-31	2.85E-29
COL4A1	1.791397	2.828865	1.454162	0.24833	-2.0097	2.35E-31	5.91E-29
PLPP3	2.242824	1.361473	2.529312	4.23077	2.0809	3.02E-31	7.60E-29
LHX2	2.483289	1.460078	2.81589	3.94692	1.9807	3.92E-31	1.31E-28
ATP6V1B1	2.607975	1.298642	3.033582	6.80944	2.7675	4.16E-31	8.35E-29
TFPI2	1.483107	2.692125	1.090108	0.05325	-4.231	5.30E-31	5.93E-29
KIAA0101	3.400487	1.861243	3.900829	3.36819	1.752	5.62E-31	8.06E-29
STMN1	13.95965	8.109421	15.8613	2.09037	1.0638	1.48E-30	2.98E-28
C12orf75	2.137209	3.453552	1.709323	0.2891	-1.7904	4.36E-30	8.76E-28
PCCA	2.032427	1.228026	2.293903	5.67437	2.5045	5.83E-30	1.47E-27
DHRS3	1.526305	2.478868	1.216668	0.14651	-2.7709	8.05E-30	1.62E-27
CD164	2.957817	4.189362	2.557496	0.48834	-1.034	1.16E-29	1.94E-27
ZNF503	1.57127	2.396408	1.303053	0.21702	-2.2041	1.35E-29	2.26E-27
PTTG1	4.621397	2.481	5.317147	2.91502	1.5435	1.87E-29	2.68E-27
HSPD1	10.56728	7.383091	11.60232	1.661	0.7321	3.54E-29	7.11E-27
ALCAM	1.589105	2.380842	1.331746	0.24025	-2.0574	5.66E-29	1.14E-26
SLC6A15	1.565421	2.443622	1.279957	0.19393	-2.3664	1.14E-28	1.43E-26
STC2	2.366687	4.124612	1.795261	0.25452	-1.9742	2.22E-28	3.72E-26
GAD2	2.039781	1.222728	2.30537	5.86081	2.5511	2.25E-28	3.78E-26
IMPDH2	6.191982	3.946375	6.921931	2.0099	1.0071	2.74E-28	4.60E-26
DIO3	3.154498	1.512224	3.688329	5.24834	2.3919	3.14E-28	5.27E-26
WNT2B	1.56493	2.666067	1.206999	0.12424	-3.0088	7.88E-28	1.59E-25
COL8A1	1.363926	2.192259	1.094671	0.0794	-3.6546	8.61E-28	1.08E-25
PSIP1	4.425719	2.715518	4.981631	2.32095	1.2147	1.28E-27	2.15E-25
SNCA	3.027411	4.499152	2.549012	0.44268	-1.1757	1.80E-27	4.53E-25
MIS18BP1	2.12854	1.327963	2.388773	4.23455	2.0822	2.74E-27	4.59E-25
COL9A3	1.559625	2.342142	1.305262	0.22744	-2.1364	4.05E-27	5.82E-25
HOMER2	3.286635	2.095016	3.673979	2.44195	1.288	4.54E-27	5.70E-25
ARPC2	6.248985	9.024767	5.3467	0.54166	-0.8845	5.03E-27	1.01E-24
PTMS	12.3391	8.872071	13.46608	1.58358	0.6632	7.92E-27	1.14E-24
OTX2	1.611874	2.535705	1.311577	0.20289	-2.3012	1.17E-26	1.69E-24
TPPP3	2.657507	1.62775	2.992236	3.17361	1.6661	1.48E-26	1.86E-24
CKB	9.249314	3.292716	11.18554	4.44257	2.1514	1.94E-26	4.89E-24

TSPAN10	1.543126	2.38645	1.268997	0.19402	-2.3657	4.27E-26	7.15E-24
TNRC6B	4.259657	2.692198	4.769169	2.22738	1.1553	4.36E-26	6.27E-24
CRIM1	1.649116	2.472364	1.381514	0.25912	-1.9483	4.49E-26	1.13E-23
RBMS1	2.236527	3.176765	1.930896	0.42765	-1.2255	5.57E-26	7.00E-24
CXCL14	2.466849	4.498118	1.806572	0.23057	-2.1167	6.05E-26	8.70E-24
DNAJC1	4.423465	2.627387	5.007292	2.46241	1.3001	2.22E-25	3.19E-23
CALU	3.409631	4.734131	2.979094	0.53	-0.9159	2.43E-25	3.06E-23
CENPV	6.188917	3.302697	7.127102	2.66084	1.4119	3.17E-25	3.99E-23
MYH9	2.450935	3.635588	2.065855	0.40441	-1.3061	4.36E-25	7.31E-23
ASAH1	2.851179	4.081727	2.451182	0.4709	-1.0865	6.55E-25	6.59E-23
CDON	2.362134	1.495964	2.643688	3.31413	1.7286	7.13E-25	1.43E-22
TMEM97	2.40484	1.573169	2.67518	2.92267	1.5473	7.71E-25	1.29E-22
COL5A1	1.496884	2.303461	1.234701	0.18006	-2.4735	8.32E-25	9.30E-23
RP11-247C2.2	1.689044	1.024498	1.905059	36.9449	5.2073	1.46E-24	1.34E-22
TYMS	3.177232	2.0618	3.53981	2.39199	1.2582	1.70E-24	1.89E-22
FAM84B	1.477474	2.231668	1.232318	0.18862	-2.4064	3.09E-24	4.43E-22
PRRT2	2.448345	1.551081	2.740007	3.15744	1.6588	3.68E-24	4.62E-22
CHPF	2.736312	3.871223	2.367402	0.47624	-1.0702	9.27E-24	1.33E-21
PLS3	1.888191	2.662822	1.636392	0.38272	-1.3856	1.28E-23	1.44E-21
HSD17B2	2.57913	4.173199	2.060967	0.33435	-1.5806	1.75E-23	2.94E-21
CP	1.939714	1.216456	2.174814	5.4275	2.4403	1.79E-23	2.25E-21
MYL12B	4.956481	6.962754	4.304329	0.55416	-0.8516	2.96E-23	4.26E-21
SELENOP	2.456326	1.503759	2.765965	3.50558	1.8097	5.62E-23	1.60E-20
CRYAB	4.116092	10.36041	2.086336	0.11606	-3.1071	6.18E-23	6.22E-21
ASPM	1.959857	1.204743	2.205312	5.88696	2.5575	6.36E-23	1.60E-20
HLA-C	2.560704	3.786549	2.162235	0.41709	-1.2616	1.10E-22	1.58E-20
FAM132A	1.674954	2.427478	1.430342	0.30147	-1.7299	1.31E-22	1.65E-20
CHD7	2.837945	1.87898	3.149663	2.44563	1.2902	1.42E-22	1.59E-20
NUSAP1	1.915444	1.206135	2.146009	5.55951	2.475	1.65E-22	2.37E-20
CLRN1	1.781711	1.140155	1.990253	7.06544	2.8208	1.80E-22	2.27E-20
YBX1	37.12853	30.20202	39.38004	1.31429	0.3943	2.01E-22	1.84E-20
GSTP1	29.32064	38.78048	26.24565	0.66822	-0.5816	2.33E-22	2.34E-20
COL2A1	2.715604	1.579927	3.084763	3.59487	1.8459	2.47E-22	3.55E-20
TGFBI	1.43323	2.28329	1.156913	0.12227	-3.0318	2.71E-22	3.03E-20
RBPMS	1.606969	2.26424	1.393318	0.31111	-1.6845	3.64E-22	7.31E-20
PTMA	80.67723	62.57413	86.56176	1.38957	0.4746	4.97E-22	8.33E-20
LHFP	1.408692	2.022907	1.209037	0.20436	-2.2908	5.77E-22	5.80E-20
EEF1B2	19.90031	15.70908	21.2627	1.37756	0.4621	5.93E-22	6.63E-20
SFRP1	4.773819	7.175086	3.993272	0.48473	-1.0447	6.01E-22	6.71E-20
CAPN2	2.811475	3.93736	2.445499	0.49211	-1.023	6.16E-22	7.75E-20
WDR54	4.240111	2.960515	4.656052	1.86484	0.8991	6.52E-22	5.97E-20
DEK	4.995175	3.389966	5.516959	1.88997	0.9184	7.15E-22	7.99E-20
ARHGDIB	1.90801	2.908136	1.582912	0.30549	-1.7108	7.89E-22	7.21E-20
RACK1	58.99207	47.05664	62.87175	1.34338	0.4259	9.17E-22	1.15E-19
C4orf48	7.36429	9.552261	6.653076	0.661	-0.5973	9.49E-22	1.49E-19

TAGLN	12.32001	22.65235	8.961417	0.36769	-1.4434	1.03E-21	1.29E-19
SCG5	2.16196	3.076604	1.864649	0.41638	-1.264	1.03E-21	1.15E-19
BEX1	4.801705	3.030707	5.377379	2.15559	1.1081	1.04E-21	1.49E-19
OPTN	1.857825	2.569813	1.626389	0.39902	-1.3255	1.58E-21	2.26E-19
BHLHE40	1.836021	2.802344	1.521912	0.28957	-1.788	1.64E-21	1.38E-19
NUDT4	5.136292	3.147223	5.782851	2.22746	1.1554	1.69E-21	1.70E-19
NACA	32.7749	26.12259	34.93727	1.35087	0.4339	1.82E-21	1.52E-19
ERICH5	2.611489	3.636679	2.278245	0.48479	-1.0446	1.98E-21	2.22E-19
HNRNPA1	49.03652	31.90218	54.60615	1.7347	0.7947	2.06E-21	2.58E-19
HSP90AB1	24.82443	18.22995	26.96801	1.50714	0.5918	2.53E-21	2.55E-19
WDR1	2.473345	3.532737	2.128983	0.44576	-1.1657	2.60E-21	3.73E-19
MYL12A	4.028619	5.877178	3.427733	0.49777	-1.0064	2.73E-21	2.68E-19
BMPR1B	1.729946	1.102539	1.933889	9.10763	3.1871	2.94E-21	2.68E-19
EEF1A1	200.3231	161.5363	212.931	1.32014	0.4007	3.55E-21	3.57E-19
ARPC1B	2.273823	3.159633	1.985884	0.45651	-1.1313	4.61E-21	4.63E-19
MARCH3	1.838413	1.20469	2.044408	5.10239	2.3512	4.65E-21	4.67E-19
SHISA5	2.485579	3.417177	2.182758	0.48931	-1.0312	6.43E-21	4.98E-19
AHNAK	1.386863	1.980915	1.193762	0.19753	-2.3398	6.69E-21	6.11E-19
HMGN2	12.48244	8.023282	13.93191	1.84129	0.8807	9.28E-21	1.04E-18
STXBP6	1.438478	2.04703	1.240665	0.22985	-2.1212	1.45E-20	1.33E-18
RP11-71N10.1	2.11902	1.380189	2.359182	3.57502	1.838	1.80E-20	2.27E-18
NPM1	26.23858	19.69562	28.36541	1.46373	0.5497	1.84E-20	2.05E-18
CPAMD8	1.994116	1.339288	2.206973	3.55737	1.8308	2.32E-20	1.94E-18
CENPH	2.020205	1.314963	2.249448	3.96697	1.988	2.48E-20	2.77E-18
RBMX	7.858935	5.380864	8.664448	1.74953	0.807	3.54E-20	3.56E-18
PLK2	2.339952	3.611722	1.926555	0.35477	-1.4951	4.15E-20	4.64E-18
PRSS33	1.298261	1.974244	1.078528	0.0806	-3.633	4.92E-20	4.95E-18
MYRF	1.311811	1.884017	1.125812	0.14232	-2.8128	5.36E-20	4.14E-18
CALB1	3.610691	1.53793	4.284456	6.10573	2.6102	5.79E-20	6.47E-18
PERP	2.626423	3.94416	2.198084	0.40694	-1.2971	7.25E-20	5.21E-18
MAD2L1	2.000503	1.332765	2.217555	3.6589	1.8714	8.32E-20	7.61E-18
H2AFZ	18.06459	12.42933	19.89636	1.65332	0.7254	8.59E-20	6.16E-18
PCSK1N	3.091968	4.285386	2.70404	0.51867	-0.9471	8.76E-20	1.47E-17
DSG2	1.610702	2.286906	1.390897	0.30375	-1.719	1.55E-19	1.30E-17
FLNA	3.192801	4.385912	2.804972	0.53308	-0.9076	1.57E-19	1.58E-17
ASCL1	1.62593	1.043177	1.815357	18.8839	4.2391	2.41E-19	2.20E-17
RLBP1	1.292469	1.942081	1.081309	0.08631	-3.5344	2.49E-19	2.09E-17
PSAP	4.183466	5.658181	3.7041	0.58051	-0.7846	2.83E-19	2.59E-17
SAMD11	2.192177	1.456848	2.4312	3.13277	1.6474	2.93E-19	2.46E-17
TMEM176A	2.429905	3.611962	2.04567	0.40034	-1.3207	4.28E-19	6.15E-17
CKS2	4.991044	2.52576	5.7924	3.14099	1.6512	5.13E-19	5.16E-17
TPM4	6.243764	8.70621	5.44333	0.57659	-0.7944	5.17E-19	5.20E-17
SPAG4	2.490754	1.671837	2.756948	2.61514	1.3869	5.38E-19	4.92E-17
SRGAP3	2.355519	1.662346	2.58084	2.38673	1.255	5.53E-19	4.28E-17
TPX2	1.790015	1.166417	1.992719	5.96526	2.5766	5.62E-19	3.76E-17

COLGALT2	1.812824	2.523313	1.581875	0.38198	-1.3884	7.18E-19	6.02E-17
CRTAC1	1.901695	1.288612	2.100981	3.81475	1.9316	7.69E-19	6.45E-17
CLU	7.490653	10.67584	6.455287	0.56381	-0.8267	1.00E-18	9.16E-17
GNG11	1.741724	2.549576	1.479126	0.3092	-1.6934	1.12E-18	9.40E-17
FDCSP	1.366775	2.340502	1.050258	0.03749	-4.7373	1.25E-18	1.14E-16
GABPB1-AS1	3.679346	2.42448	4.087249	2.16728	1.1159	1.52E-18	1.70E-16
SLF1	1.86136	1.266106	2.054851	3.96403	1.987	1.54E-18	1.19E-16
HIST1H4C	12.79828	5.072605	15.30955	3.51361	1.813	1.69E-18	2.13E-16
DCBLD2	8.724311	12.04874	7.643685	0.60131	-0.7338	2.06E-18	1.73E-16
P3H2	1.819361	2.447682	1.615121	0.4249	-1.2348	2.68E-18	2.25E-16
IGFBP6	1.704212	2.480635	1.45183	0.30516	-1.7124	2.77E-18	2.15E-16
METRN	2.899901	2.037323	3.180288	2.10184	1.0717	2.81E-18	2.18E-16
CCNB1IP1	3.69386	2.396854	4.11546	2.23034	1.1573	4.11E-18	4.58E-16
CLTB	2.494747	3.463442	2.179866	0.47895	-1.0621	7.54E-18	7.58E-16
FRZB	1.431648	2.122774	1.206993	0.18436	-2.4394	9.57E-18	7.41E-16
SERPINB9	1.405556	2.06034	1.192714	0.18175	-2.46	9.84E-18	7.07E-16
TTYH1	2.973845	1.875301	3.330933	2.66301	1.4131	1.24E-17	8.35E-16
PRPH	1.295159	1.906836	1.09633	0.10623	-3.2348	1.26E-17	9.76E-16
CCNB1	1.798501	1.181481	1.999068	5.50509	2.4608	1.27E-17	1.07E-15
CD59	2.669167	3.548982	2.383177	0.54264	-0.8819	1.28E-17	1.17E-15
APLP1	2.28167	3.130596	2.005721	0.47204	-1.083	1.43E-17	1.10E-15
SLC3A2	2.399024	3.454927	2.055796	0.43007	-1.2173	1.51E-17	1.26E-15
LAYN	1.417084	2.001734	1.22704	0.22665	-2.1415	1.55E-17	1.20E-15
PLP2	2.58237	3.577579	2.258871	0.48839	-1.0339	1.78E-17	1.78E-15
ASS1	2.00792	1.393288	2.207711	3.0708	1.6186	1.82E-17	1.66E-15
GPM6B	3.070292	2.047117	3.402882	2.29476	1.1983	1.97E-17	1.53E-15
SHISA2	1.980777	1.331161	2.19194	3.59928	1.8477	2.30E-17	2.10E-15
HLA-B	2.69565	3.914664	2.299402	0.44582	-1.1655	2.58E-17	1.62E-15
ITIH5	1.20885	1.709164	1.046219	0.06517	-3.9396	2.70E-17	1.94E-15
TMEM176B	2.331433	3.315329	2.011612	0.43692	-1.1946	3.03E-17	2.54E-15
MIR4435-2HG	2.189419	3.334718	1.817133	0.34999	-1.5146	3.33E-17	3.04E-15
S100A16	4.312459	6.438512	3.621372	0.482	-1.0529	4.04E-17	4.07E-15
H3F3A	35.95869	27.66063	38.65602	1.41242	0.4982	4.19E-17	3.01E-15
RPA3	2.413192	1.738752	2.632423	2.2097	1.1439	4.36E-17	8.77E-15
MUM1	3.256348	2.217845	3.59392	2.12993	1.0908	4.50E-17	3.23E-15
COL18A1	2.731936	3.652825	2.432595	0.54003	-0.8889	4.57E-17	3.28E-15
CTSB	4.335317	6.270285	3.706343	0.51351	-0.9615	5.05E-17	4.23E-15
ANXA3	1.326048	1.909698	1.136329	0.14986	-2.7383	6.06E-17	1.02E-14
NCCRP1	1.307597	1.999907	1.082557	0.08256	-3.5983	8.77E-17	6.78E-15
AKAP12	5.401181	8.404422	4.424958	0.46256	-1.1123	9.06E-17	8.29E-15
SRSF5	6.560237	4.478573	7.236895	1.79295	0.8423	9.19E-17	6.60E-15
ZIC2	1.681418	1.146606	1.855262	5.83376	2.5444	9.92E-17	7.13E-15
PARP1	3.614042	2.476703	3.983742	2.02054	1.0147	1.03E-16	6.91E-15
CCDC80	1.795553	2.530107	1.556782	0.36388	-1.4584	1.28E-16	1.07E-14
GIPC1	1.938052	2.551562	1.738627	0.47605	-1.0708	1.38E-16	8.18E-15

TFDP2	3.034577	2.151248	3.321709	2.01669	1.012	1.68E-16	1.06E-14
HMGB3	4.578773	3.244094	5.012619	1.78808	0.8384	1.69E-16	1.06E-14
LAPTM4B	3.245219	4.377661	2.877112	0.55574	-0.8475	1.83E-16	1.22E-14
SRP9	10.65359	7.569019	11.65625	1.6222	0.698	2.11E-16	1.42E-14
SQSTM1	2.339225	3.089547	2.095328	0.52419	-0.9318	2.30E-16	1.44E-14
FLRT3	2.065463	1.431616	2.271499	2.9459	1.5587	2.50E-16	1.80E-14
LAMP5	1.632262	1.111279	1.801611	7.20359	2.8487	3.08E-16	2.21E-14
TLE4	3.372192	4.57516	2.981159	0.55415	-0.8517	3.11E-16	2.41E-14
MEIS1	2.08378	1.487335	2.277659	2.62173	1.3905	3.42E-16	4.30E-14
ANP32E	3.172959	2.280016	3.463216	1.92436	0.9444	3.46E-16	4.97E-14
COL9A1	1.794176	1.252084	1.970386	3.84945	1.9447	4.15E-16	2.78E-14
CASP7	1.881232	1.318246	2.064234	3.34406	1.7416	4.38E-16	3.38E-14
GTSE1	1.672459	1.130942	1.848483	6.47985	2.696	4.50E-16	2.66E-14
ANXA2	12.46781	18.26512	10.58335	0.55507	-0.8493	4.71E-16	3.38E-14
PAIP2	5.806315	3.997209	6.394377	1.7998	0.8478	4.78E-16	6.01E-14
SEMA5A	1.847723	1.294361	2.027597	3.49095	1.8036	7.80E-16	8.71E-14
LSM4	7.620229	5.33949	8.361598	1.69642	0.7625	8.01E-16	4.74E-14
SSBP2	2.549475	1.840775	2.779842	2.11691	1.082	9.06E-16	6.07E-14
C5orf46	1.180656	1.652142	1.027397	0.04201	-4.5731	1.22E-15	1.23E-13
SRSF10	3.212603	2.332717	3.498616	1.87483	0.9068	1.25E-15	7.83E-14
SSNA1	3.413045	4.501658	3.059184	0.58806	-0.766	1.30E-15	1.64E-13
PDLIM1	1.964572	2.802862	1.69208	0.38388	-1.3813	1.36E-15	1.14E-13
LINC01508	1.615445	1.109967	1.779753	7.09077	2.8259	1.39E-15	9.33E-14
FLNB	1.483463	2.001978	1.314917	0.31429	-1.6698	1.47E-15	1.65E-13
EVA1A	1.386924	1.888869	1.223763	0.25174	-1.99	1.51E-15	9.47E-14
PDLIM3	1.581767	2.175134	1.38889	0.33093	-1.5954	1.63E-15	1.49E-13
GPM6A	2.178381	1.571716	2.375582	2.40606	1.2667	1.73E-15	9.66E-14
PDGFC	1.308451	1.795417	1.15016	0.18878	-2.4052	1.94E-15	1.22E-13
CCNA2	1.632289	1.102827	1.804394	7.82282	2.9677	2.00E-15	1.55E-13
HELLS	2.056469	1.473979	2.245812	2.62841	1.3942	2.08E-15	1.39E-13
HSPB1	8.912559	12.45139	7.762239	0.59052	-0.7599	2.30E-15	1.92E-13
HES1	5.633512	3.082397	6.462768	2.62331	1.3914	2.98E-15	2.00E-13
DSC2	1.699924	2.247761	1.521846	0.41823	-1.2576	3.05E-15	2.19E-13
TRH	3.442689	1.865901	3.955234	3.4129	1.771	3.20E-15	1.89E-13
PTRF	1.418434	1.952453	1.244847	0.25707	-1.9598	3.34E-15	1.87E-13
HACD1	1.605025	2.135875	1.432469	0.38074	-1.3931	4.30E-15	4.81E-13
CDKN1A	2.289847	3.41579	1.923852	0.38242	-1.3868	5.36E-15	3.37E-13
CDK1	1.737449	1.201005	1.911824	4.53632	2.1815	5.45E-15	3.66E-13
CDKN1C	1.775385	2.608475	1.504584	0.3137	-1.6725	5.74E-15	3.61E-13
ATP6V0E1	4.060226	5.383445	3.630105	0.60001	-0.7369	5.93E-15	3.73E-13
SDC2	2.356022	3.144448	2.09974	0.51283	-0.9634	6.16E-15	4.77E-13
ARL4A	3.512835	5.047251	3.014063	0.49764	-1.0068	6.40E-15	4.02E-13
GINS2	1.900334	1.352862	2.078293	3.05585	1.6116	7.54E-15	4.46E-13
CLNS1A	3.646119	2.732115	3.943223	1.69921	0.7649	8.38E-15	5.27E-13
CIRBP	11.70404	9.547603	12.40501	1.33429	0.4161	8.96E-15	1.13E-12

ZCCHC11	2.918271	2.14323	3.170203	1.89831	0.9247	1.12E-14	6.61E-13
NASP	3.171457	2.216065	3.482013	2.04102	1.0293	1.12E-14	5.95E-13
PARD6B	1.536136	2.05502	1.367469	0.34831	-1.5216	1.19E-14	7.02E-13
MAP1B	7.777296	5.154866	8.629734	1.83634	0.8768	1.25E-14	7.38E-13
APLP2	7.279165	10.57291	6.208511	0.54409	-0.8781	1.61E-14	1.01E-12
SDCBP	4.436821	6.120821	3.889426	0.56425	-0.8256	1.64E-14	9.15E-13
CPLX3	1.231325	1.794326	1.048318	0.06083	-4.0391	1.72E-14	1.33E-12
IVNS1ABP	2.407102	3.451593	2.067584	0.43547	-1.1994	1.72E-14	1.02E-12
DMKN	1.522175	2.027298	1.357982	0.34847	-1.5209	2.03E-14	1.36E-12
CD74	1.702132	2.264494	1.519332	0.4107	-1.2838	2.10E-14	1.24E-12
APP	3.519465	4.740417	3.122587	0.56747	-0.8174	2.11E-14	1.51E-12
RAN	10.07196	7.288353	10.97679	1.58655	0.6659	2.27E-14	1.63E-12
CAST	2.759774	3.71137	2.450452	0.53495	-0.9025	2.34E-14	1.38E-12
RARRES2	3.494702	2.453676	3.833095	1.94892	0.9627	2.40E-14	1.27E-12
TUBA1B	21.56051	12.07281	24.64455	2.13537	1.0945	2.49E-14	1.39E-12
FABP3	1.365883	1.840918	1.21147	0.25148	-1.9915	2.72E-14	1.52E-12
AHNAK2	1.780864	2.368618	1.58981	0.43095	-1.2144	3.08E-14	1.72E-12
EML1	1.817557	1.307415	1.983382	3.19887	1.6776	4.18E-14	2.47E-12
PCP4	1.377327	2.029327	1.16539	0.16068	-2.6378	4.41E-14	2.33E-12
FAM64A	1.598556	1.114876	1.75578	6.57908	2.7179	4.43E-14	2.26E-12
TPRN	1.249305	1.70686	1.100574	0.14228	-2.8132	4.49E-14	2.26E-12
TUBB	23.84702	17.9723	25.75663	1.45865	0.5446	5.27E-14	4.42E-12
CENPA	1.530336	1.044721	1.688188	15.3885	3.9438	6.08E-14	4.37E-12
PHYHIPL	1.80772	1.297621	1.973531	3.27104	1.7098	6.34E-14	4.90E-12
LRRC17	3.125108	4.666895	2.62394	0.44286	-1.1751	6.42E-14	3.59E-12
LUC7L3	4.427223	3.240813	4.812873	1.70156	0.7669	7.22E-14	3.46E-12
AURKB	1.595783	1.114467	1.752238	6.57165	2.7163	7.36E-14	3.89E-12
DEF8	1.921951	2.472607	1.742956	0.50452	-0.987	7.52E-14	4.02E-12
VCL	1.79507	2.356137	1.612691	0.45179	-1.1463	7.60E-14	4.02E-12
CBX1	4.55697	3.313998	4.961006	1.71176	0.7755	9.22E-14	9.28E-12
LINC01474	1.408805	2.139271	1.171363	0.15041	-2.733	9.43E-14	4.75E-12
LSM5	4.939297	3.690189	5.345327	1.61525	0.6918	9.49E-14	5.97E-12
SPSB1	1.262486	1.71036	1.116901	0.16457	-2.6033	9.57E-14	5.07E-12
ERO1A	2.200959	2.998624	1.941673	0.47116	-1.0857	1.03E-13	5.18E-12
PRCD	1.395009	1.941211	1.217463	0.23105	-2.1138	1.06E-13	5.35E-12
SCARB2	3.43872	4.379487	3.132917	0.63114	-0.664	1.14E-13	8.08E-12
SNRPF	6.082596	4.552661	6.579911	1.57063	0.6513	1.14E-13	6.74E-12
LPP	2.213564	2.878605	1.997389	0.53092	-0.9134	1.21E-13	8.08E-12
ITM2B	6.542578	8.488699	5.909979	0.65565	-0.609	1.40E-13	6.40E-12
SNRPD1	4.79455	3.53605	5.203634	1.65755	0.7291	1.70E-13	7.44E-12
SRSF7	3.970273	2.920481	4.311515	1.72432	0.786	1.71E-13	1.44E-11
CEBPB	1.466854	1.969193	1.303565	0.31321	-1.6748	1.71E-13	1.44E-11
ZNF436-AS1	2.03872	1.457841	2.227538	2.68115	1.4229	1.90E-13	1.36E-11
EIF4G2	7.387281	9.053095	6.845797	0.72591	-0.4621	1.96E-13	2.00E-11
CAPG	1.470533	1.969247	1.308423	0.31821	-1.652	1.99E-13	2.00E-11

ABHD2	1.369738	1.828449	1.220631	0.26632	-1.9088	2.04E-13	8.56E-12
PPIC	1.858911	2.39009	1.686248	0.49367	-1.0184	2.36E-13	1.32E-11
C8orf4	1.916871	2.679958	1.668824	0.39812	-1.3287	2.40E-13	1.61E-11
CDT1	1.613219	1.150109	1.763756	5.08801	2.3471	2.61E-13	1.31E-11
RHOD	1.268562	1.707649	1.125834	0.17782	-2.4915	2.65E-13	1.33E-11
ARSI	1.398745	1.858972	1.249145	0.29005	-1.7856	2.70E-13	1.81E-11
VEGFA	1.981229	2.690251	1.750756	0.44417	-1.1708	2.72E-13	1.30E-11
PTP4A3	1.728824	2.362971	1.52269	0.38349	-1.3827	2.80E-13	1.87E-11
EFHD2	2.130193	1.586077	2.307061	2.23019	1.1572	2.90E-13	1.82E-11
CYR61	1.979583	3.098036	1.616023	0.29362	-1.768	2.98E-13	1.58E-11
ANKRD1	1.315537	2.076824	1.068075	0.06322	-3.9835	3.03E-13	2.03E-11
MZT1	2.227133	1.655108	2.413074	2.15701	1.109	3.27E-13	1.73E-11
LSM7	5.654382	4.130336	6.149783	1.64512	0.7182	3.83E-13	1.83E-11
CTSD	2.243967	2.873411	2.039362	0.5548	-0.85	3.91E-13	1.87E-11
RHOC	4.903801	6.350552	4.433525	0.64171	-0.64	4.65E-13	3.60E-11
SMAD2	2.815741	1.995281	3.082437	2.09231	1.0651	5.12E-13	3.21E-11
RP11-490M8.1	1.664878	2.179691	1.497535	0.42175	-1.2455	5.19E-13	2.61E-11
FGFBP3	1.702344	1.23172	1.855324	3.6912	1.8841	5.61E-13	2.69E-11
IMMP1L	2.864314	2.113129	3.108492	1.8942	0.9216	6.04E-13	3.80E-11
RSPH9	1.798749	1.3167	1.955442	3.01687	1.5931	6.37E-13	5.83E-11
RP4-614C10.2	2.474832	3.153364	2.254271	0.58247	-0.7797	6.77E-13	4.87E-11
TMSB4X	411.5709	508.5088	380.0606	0.7469	-0.421	6.97E-13	4.38E-11
CDKN3	1.713916	1.218725	1.874881	3.99992	2	7.21E-13	3.45E-11
DNPH1	3.312885	2.49391	3.579098	1.72641	0.7878	7.34E-13	7.32E-11
RP11-96L14.7	2.42836	3.206064	2.175562	0.53288	-0.9081	7.75E-13	3.90E-11
PRNP	1.655218	2.145002	1.49601	0.4332	-1.2069	7.88E-13	6.61E-11
C19orf33	1.27723	1.850493	1.090888	0.10686	-3.2261	8.01E-13	7.32E-11
CENPK	1.734396	1.263876	1.887342	3.36273	1.7496	8.63E-13	5.10E-11
HLA-A	3.550085	4.731268	3.166134	0.58054	-0.7845	1.02E-12	7.88E-11
CYTOR	2.62822	3.796059	2.248606	0.44656	-1.1631	1.10E-12	6.48E-11
S1PR3	1.65414	1.194621	1.80351	4.12858	2.0456	1.23E-12	7.70E-11
LRP2	1.796866	1.314338	1.953715	3.03404	1.6012	1.40E-12	8.26E-11
THUMPD3-AS1	2.484411	1.843167	2.692852	2.00773	1.0056	1.56E-12	7.13E-11
DLK1	5.839202	2.557959	6.905791	3.79072	1.9225	1.66E-12	9.25E-11
RAX	1.574917	1.1333	1.718467	5.38984	2.4302	1.81E-12	1.01E-10
SLC12A8	1.201614	1.613749	1.067647	0.11022	-3.1816	1.82E-12	1.02E-10
SAT1	6.43535	8.913554	5.629793	0.58505	-0.7734	1.93E-12	1.29E-10
FCGRT	1.343656	1.77819	1.202409	0.2601	-1.9429	2.09E-12	1.17E-10
DARS	3.214924	2.355966	3.494134	1.83938	0.8792	2.10E-12	1.00E-10
ILF3	3.57655	2.698024	3.86212	1.68556	0.7532	2.27E-12	1.90E-10
ZYX	1.605668	2.096837	1.446011	0.40663	-1.2982	2.27E-12	1.20E-10
FZD3	2.396044	1.811066	2.586195	1.95569	0.9677	2.41E-12	1.35E-10
TMEM258	9.349573	7.638364	9.905813	1.34157	0.4239	2.46E-12	1.77E-10
NPM3	2.99813	2.172959	3.266357	1.93217	0.9502	2.76E-12	1.63E-10
SYT7	1.642677	1.195217	1.788127	4.03718	2.0133	3.11E-12	1.74E-10

EIF2S3	4.912311	3.687034	5.310595	1.60422	0.6819	3.19E-12	1.28E-10
TMED3	2.806828	3.534664	2.570241	0.61951	-0.6908	3.33E-12	1.76E-10
CCDC34	2.883315	2.171042	3.114844	1.80595	0.8528	3.52E-12	2.21E-10
DNAJC3	1.876171	2.531096	1.663283	0.43321	-1.2069	3.56E-12	1.79E-10
TTR	4.275116	12.42337	1.626472	0.05484	-4.1886	3.72E-12	1.70E-10
CLDN6	1.667152	2.348717	1.445604	0.33039	-1.5978	3.83E-12	1.93E-10
ATP5E	24.80436	28.05026	23.74926	0.841	-0.2498	4.39E-12	1.92E-10
ABAT	1.806737	1.340487	1.958294	2.81448	1.4929	4.46E-12	1.73E-10
FHL2	1.439373	1.996646	1.258228	0.2591	-1.9484	5.16E-12	1.92E-10
CHRNA3	1.417036	1.922321	1.25279	0.27408	-1.8673	5.33E-12	2.82E-10
BAZ2B	2.809489	2.079794	3.046681	1.89544	0.9225	5.64E-12	2.36E-10
CKAP2	1.872616	1.392304	2.028745	2.62232	1.3908	5.87E-12	3.28E-10
IFITM2	5.607067	4.140867	6.083665	1.61855	0.6947	6.35E-12	3.76E-10
C19orf48	2.467931	1.890503	2.655628	1.85921	0.8947	7.25E-12	3.84E-10
BIRC7	2.130752	2.859011	1.894027	0.48092	-1.0561	7.90E-12	2.84E-10
TNC	1.530344	2.149166	1.329192	0.28646	-1.8036	8.60E-12	4.12E-10
ADAM28	1.172712	1.597291	1.0347	0.0581	-4.1054	8.75E-12	5.87E-10
ARGLU1	4.920725	3.687796	5.321496	1.60782	0.6851	8.89E-12	4.47E-10
PNRC2	2.734181	2.075329	2.948344	1.81186	0.8575	9.03E-12	4.12E-10
FBXO2	1.402781	1.833019	1.26293	0.31563	-1.6637	9.31E-12	4.25E-10
H2AFX	2.087542	1.562268	2.258285	2.23787	1.1621	9.90E-12	4.33E-10
ACTC1	1.836642	3.1934	1.39562	0.18037	-2.471	1.02E-11	4.11E-10
UBE2T	1.831501	1.361499	1.984278	2.72277	1.4451	1.04E-11	4.98E-10
ASPH	2.734532	3.504529	2.48424	0.59262	-0.7548	1.04E-11	4.84E-10
DIO3OS	1.81082	1.351698	1.960061	2.72979	1.4488	1.06E-11	4.84E-10
HTRA1	1.869609	2.501354	1.664256	0.44244	-1.1765	1.06E-11	6.30E-10
MSI1	2.250879	1.682153	2.435747	2.10473	1.0736	1.30E-11	7.24E-10
LRP10	1.709925	2.178639	1.557567	0.47306	-1.0799	1.45E-11	6.32E-10
RRAS	1.529827	1.97189	1.386132	0.3973	-1.3317	1.63E-11	7.46E-10
CXADR	2.401675	3.007765	2.204662	0.6	-0.737	1.68E-11	1.30E-09
CCNB2	1.59947	1.148894	1.745932	5.00983	2.3248	1.72E-11	1.33E-09
SELENOM	2.665874	3.373854	2.43574	0.60481	-0.7254	1.73E-11	7.58E-10
ARL6IP6	2.017496	1.536246	2.17393	2.18916	1.1304	1.88E-11	8.61E-10
PDLIM2	2.186307	2.723591	2.01166	0.58695	-0.7687	2.23E-11	1.60E-09
MITF	1.470666	1.924787	1.323051	0.34933	-1.5174	2.28E-11	9.97E-10
DLGAP5	1.491421	1.075424	1.626644	8.30829	3.0546	2.35E-11	1.13E-09
IFI16	1.997328	1.485166	2.163809	2.39878	1.2623	2.42E-11	1.10E-09
MEST	9.043304	6.39715	9.903454	1.64966	0.7222	2.42E-11	1.28E-09
KREMEN1	1.384885	1.815608	1.244876	0.30024	-1.7358	2.43E-11	1.22E-09
IFITM3	9.241149	6.761939	10.04703	1.57014	0.6509	2.47E-11	1.77E-09
DNER	1.498543	1.090955	1.631032	6.93788	2.7945	2.71E-11	1.19E-09
SCARA3	1.303271	1.707608	1.171838	0.24284	-2.0419	2.92E-11	1.33E-09
HMGB1	19.63298	15.46192	20.98882	1.38217	0.4669	3.07E-11	1.29E-09
SNRPD2	10.59507	8.311359	11.33741	1.41388	0.4997	3.12E-11	1.21E-09
NUDCD2	2.951927	2.273688	3.172394	1.70559	0.7703	3.18E-11	1.39E-09

EZR	1.647201	2.143987	1.485717	0.42458	-1.2359	3.20E-11	1.40E-09
SH3BP5	1.418527	1.839953	1.28154	0.33519	-1.577	3.29E-11	1.38E-09
SNRPE	8.603666	6.505942	9.285545	1.50484	0.5896	3.34E-11	1.34E-09
DTYMK	2.411335	1.875144	2.585627	1.81185	0.8575	3.67E-11	2.46E-09
PGK1	11.07602	8.304759	11.97683	1.5027	0.5876	3.69E-11	1.55E-09
TTYH3	1.938503	2.425957	1.780053	0.54704	-0.8703	3.82E-11	2.13E-09
COL8A2	1.154548	1.535666	1.030663	0.05724	-4.1268	3.87E-11	1.56E-09
NR2F2	1.849467	2.372359	1.679498	0.49513	-1.0141	4.34E-11	1.82E-09
LMAN1	4.046216	3.084532	4.358818	1.61131	0.6882	4.61E-11	1.60E-09
SERPINE1	1.309646	1.956435	1.099403	0.10393	-3.2663	4.66E-11	1.80E-09
MGST1	1.691378	2.226525	1.517425	0.42186	-1.2452	5.04E-11	2.03E-09
CALD1	8.316254	10.73359	7.530482	0.67092	-0.5758	5.08E-11	1.97E-09
ELP4	1.594043	2.084504	1.434615	0.40075	-1.3192	5.32E-11	1.78E-09
CCNG1	6.456437	4.899171	6.962637	1.52921	0.6128	5.57E-11	2.95E-09
MYH14	1.328479	1.730457	1.197814	0.27081	-1.8847	5.84E-11	3.09E-09
SRSF3	5.468587	3.999116	5.946247	1.64924	0.7218	6.12E-11	2.56E-09
PKM	19.56027	15.46636	20.89103	1.37498	0.4594	6.48E-11	2.41E-09
HNRNPR	4.013233	3.065014	4.321457	1.60844	0.6857	7.40E-11	4.96E-09
ARPC5	3.360381	4.186907	3.091714	0.65635	-0.6075	7.80E-11	3.14E-09
RP11-1260E13.1	1.565271	1.144082	1.702181	4.87347	2.2849	7.87E-11	3.44E-09
TINAGL1	1.233479	1.840827	1.036057	0.04288	-4.5434	8.02E-11	4.48E-09
HSBP1L1	1.417435	1.829522	1.283484	0.34174	-1.549	8.16E-11	4.32E-09
COL6A1	2.572856	3.212865	2.364817	0.61676	-0.6972	8.39E-11	3.02E-09
ABR	1.638717	2.081726	1.494715	0.45734	-1.1287	8.53E-11	4.29E-09
MAP2	2.217133	1.681607	2.391209	2.04107	1.0293	9.04E-11	4.54E-09
APEX1	4.752964	3.605511	5.12595	1.58355	0.6632	9.57E-11	6.02E-09
FAM198B	1.550095	1.977456	1.411179	0.42066	-1.2493	1.07E-10	4.07E-09
SNHG15	2.033201	1.562191	2.186306	2.11015	1.0773	1.09E-10	4.07E-09
POLR2J3	4.079815	2.856484	4.477466	1.87315	0.9055	1.10E-10	5.01E-09
IFITM1	1.723443	1.286133	1.865593	3.02514	1.597	1.11E-10	4.65E-09
TPD52L1	4.060137	5.254059	3.672045	0.62812	-0.6709	1.14E-10	5.48E-09
UPP1	1.57286	2.001667	1.433474	0.43275	-1.2084	1.19E-10	4.11E-09
DDB2	2.073833	1.594719	2.229572	2.06748	1.0479	1.19E-10	5.99E-09
C20orf27	2.587569	1.984323	2.783657	1.81206	0.8576	1.19E-10	4.61E-09
XKR4	1.58935	1.178091	1.723033	4.05991	2.0214	1.23E-10	3.99E-09
TGIF1	1.993898	2.483966	1.834599	0.56241	-0.8303	1.31E-10	5.26E-09
DUSP6	1.261276	1.693983	1.120622	0.17381	-2.5244	1.34E-10	6.41E-09
RORB	1.518059	1.112331	1.649944	5.786	2.5326	1.34E-10	4.51E-09
LRPAP1	2.009812	2.499056	1.85078	0.56754	-0.8172	1.42E-10	5.47E-09
SNHG7	5.014655	3.995991	5.345779	1.45053	0.5366	1.47E-10	7.02E-09
BGN	1.284162	1.792405	1.118955	0.15012	-2.7358	1.57E-10	5.64E-09
RANBP1	7.121175	5.242362	7.731895	1.58683	0.6661	1.65E-10	7.55E-09
MARCKSL1	16.77148	13.26745	17.91049	1.37848	0.4631	1.66E-10	5.39E-09
DPYSL3	1.330957	1.722409	1.203713	0.28199	-1.8263	1.67E-10	7.10E-09
GNB3	1.334591	1.787846	1.187258	0.23768	-2.0729	1.69E-10	7.10E-09

PABPC1	13.70969	10.67738	14.69536	1.41519	0.501	1.76E-10	5.54E-09
GCHFR	4.840785	3.620988	5.237287	1.61668	0.693	1.98E-10	8.30E-09
TMEM107	2.083184	1.614183	2.235636	2.01184	1.0085	2.42E-10	9.73E-09
RAD51AP1	1.513723	1.124231	1.64033	5.15437	2.3658	2.53E-10	1.27E-08
FNDC3B	1.388282	1.783393	1.259849	0.3317	-1.5921	2.60E-10	1.37E-08
H2AFV	3.718963	2.885907	3.989753	1.58531	0.6648	2.64E-10	8.31E-09
ANXA11	1.674584	2.108982	1.53338	0.48096	-1.056	2.77E-10	1.16E-08
LYPD6B	1.368872	1.776112	1.236496	0.30472	-1.7145	2.91E-10	1.08E-08
PEBP1	10.10392	8.027253	10.77896	1.39158	0.4767	2.97E-10	1.03E-08
RAB32	1.673637	2.105046	1.533405	0.4827	-1.0508	3.09E-10	1.15E-08
DNAJC19	2.817115	2.177952	3.024879	1.71898	0.7816	3.45E-10	1.22E-08
RASL11B	1.262534	1.638044	1.140472	0.22016	-2.1834	3.50E-10	1.22E-08
SLC16A3	3.179108	4.028023	2.903162	0.62852	-0.67	3.51E-10	1.26E-08
CPE	3.766224	2.828526	4.071028	1.67951	0.748	3.53E-10	1.22E-08
SRSF2	4.557276	3.499877	4.90099	1.56047	0.642	3.82E-10	2.26E-08
RAB38	1.445337	1.891647	1.30026	0.33675	-1.5703	4.19E-10	1.40E-08
CENPU	1.606087	1.208942	1.735182	3.5186	1.815	4.50E-10	1.97E-08
HMGN5	1.876735	1.439398	2.018894	2.31884	1.2134	4.68E-10	1.88E-08
ATP1A1	2.135365	2.679668	1.958436	0.57061	-0.8094	5.00E-10	2.51E-08
CBX5	4.708633	3.610225	5.065677	1.5576	0.6393	5.01E-10	3.15E-08
NR2F1	5.499608	3.796132	6.053335	1.80726	0.8538	5.39E-10	2.08E-08
JAG1	1.284434	1.658589	1.162812	0.24721	-2.0162	5.40E-10	2.17E-08
COL6A2	1.917944	2.437688	1.748998	0.52097	-0.9407	5.73E-10	2.13E-08
HNRNPDL	11.76466	8.196054	12.92466	1.65711	0.7287	5.73E-10	2.40E-08
CGNL1	1.219431	1.584364	1.100807	0.17251	-2.5353	5.84E-10	1.78E-08
CLTC	2.020728	2.49309	1.867184	0.5808	-0.7839	5.84E-10	1.90E-08
CST3	7.067367	9.508926	6.273722	0.61979	-0.6902	5.88E-10	2.36E-08
H1FX	3.019838	2.238718	3.273746	1.83556	0.8762	5.92E-10	2.83E-08
FBN1	1.518355	1.922085	1.38712	0.41983	-1.2521	6.31E-10	2.19E-08
SRSF1	3.031828	2.395285	3.23874	1.6045	0.6821	6.36E-10	3.05E-08
PRC1	1.526897	1.141238	1.652258	4.61813	2.2073	6.37E-10	2.46E-08
TMSB10	80.68803	98.53042	74.88824	0.75759	-0.4005	6.38E-10	3.21E-08
MALAT1	648.8266	539.6306	684.3214	1.26863	0.3433	6.42E-10	1.96E-08
CDH6	4.011964	2.986767	4.345211	1.68375	0.7517	6.58E-10	2.21E-08
CDC20	1.498246	1.096888	1.628711	6.48907	2.698	7.32E-10	2.30E-08
PAICS	2.847208	2.176332	3.065281	1.7557	0.812	7.34E-10	2.73E-08
PDXK	1.722873	2.151848	1.583432	0.50652	-0.9813	7.38E-10	3.37E-08
LMNA	2.086757	2.756955	1.868904	0.49455	-1.0158	8.36E-10	4.01E-08
MT1G	1.396873	2.166969	1.146548	0.12558	-2.9933	8.44E-10	4.04E-08
NONO	5.267737	4.03613	5.668079	1.53751	0.6206	8.58E-10	3.08E-08
ACP1	4.489601	3.404041	4.842469	1.59834	0.6766	8.86E-10	4.05E-08
LINC01551	1.444852	1.075394	1.564947	7.49324	2.9056	9.42E-10	3.79E-08
MRPL41	3.351681	4.098676	3.108866	0.68057	-0.5552	1.03E-09	4.49E-08
PHGDH	2.241102	1.762255	2.396755	1.8324	0.8737	1.06E-09	4.84E-08
HSPA5	4.136696	5.519184	3.68731	0.59464	-0.7499	1.11E-09	5.09E-08

LBH	1.424617	1.811122	1.298981	0.3686	-1.4399	1.12E-09	5.74E-08
SVIL	1.512033	1.908939	1.383016	0.42139	-1.2468	1.13E-09	4.96E-08
PBK	1.513301	1.130739	1.637655	4.87731	2.2861	1.14E-09	5.74E-08
MCM7	1.903737	1.480551	2.041296	2.16688	1.1156	1.17E-09	4.21E-08
GOLGA4	3.174037	3.849996	2.954312	0.68572	-0.5443	1.19E-09	5.43E-08
GCSH	4.4906	3.475284	4.820634	1.54351	0.6262	1.19E-09	7.03E-08
CAV2	1.253839	1.615286	1.136348	0.2216	-2.174	1.23E-09	6.85E-08
CAP1	2.487019	3.105005	2.286138	0.61099	-0.7108	1.26E-09	4.54E-08
CCDC151	2.092635	1.6337	2.241815	1.95962	0.9706	1.29E-09	4.47E-08
DHFR	1.954104	1.520451	2.095066	2.10407	1.0732	1.34E-09	7.09E-08
AQP1	1.179215	1.551533	1.058191	0.10551	-3.2446	1.39E-09	4.24E-08
MAGED1	3.030954	3.788955	2.78456	0.63987	-0.6442	1.42E-09	4.75E-08
SGO2	1.474727	1.104594	1.595041	5.68905	2.5082	1.45E-09	5.40E-08
NPC2	7.861868	10.04083	7.153582	0.68064	-0.555	1.47E-09	6.71E-08
FSTL3	1.356467	1.730105	1.235014	0.32189	-1.6354	1.47E-09	5.68E-08
NRCAM	1.424514	1.061915	1.54238	8.76	3.1309	1.53E-09	6.39E-08
INHBA	1.187713	1.607678	1.051201	0.08426	-3.5691	1.57E-09	5.85E-08
RSL1D1	3.38083	2.660136	3.615096	1.57523	0.6556	1.58E-09	4.68E-08
SPINT2	4.84487	6.099353	4.437092	0.67403	-0.5691	1.62E-09	7.76E-08
DUSP4	1.394821	1.90001	1.230606	0.25623	-1.9645	1.63E-09	5.28E-08
ITGB3BP	2.141788	1.696695	2.286469	1.84653	0.8848	1.68E-09	7.35E-08
CCT3	4.098611	3.199407	4.390903	1.54174	0.6246	1.85E-09	7.17E-08
KIF20B	1.557483	1.181032	1.679851	3.75543	1.909	1.94E-09	7.24E-08
LMNB1	1.599804	1.219373	1.723465	3.29787	1.7215	1.98E-09	8.28E-08
FBLIM1	1.411537	1.788647	1.288955	0.36639	-1.4485	2.11E-09	7.08E-08
SLC7A6	1.337062	1.704249	1.217705	0.30913	-1.6937	2.30E-09	1.16E-07
TMPO	2.046743	1.608775	2.189107	1.95328	0.9659	2.43E-09	6.97E-08
LYPD1	1.669277	2.21063	1.493307	0.40748	-1.2952	2.48E-09	8.89E-08
GDE1	1.898463	2.333822	1.756946	0.5675	-0.8173	2.53E-09	8.77E-08
SLC16A1-AS1	1.62593	1.243532	1.750231	3.08063	1.6232	2.66E-09	1.27E-07
TGFB1I1	1.558326	1.961755	1.427189	0.44418	-1.1708	2.69E-09	1.18E-07
C21orf58	1.525732	1.157062	1.64557	4.11028	2.0392	2.79E-09	1.17E-07
ATP6V0B	3.064419	3.770183	2.835006	0.66241	-0.5942	2.83E-09	8.38E-08
UBC	6.367864	8.49205	5.677383	0.62431	-0.6797	2.91E-09	9.14E-08
SERPINH1	2.733437	3.414489	2.512057	0.62624	-0.6752	3.15E-09	1.09E-07
HSPG2	1.262659	1.615512	1.147962	0.24039	-2.0566	3.30E-09	1.51E-07
SERPING1	1.700552	1.311784	1.826923	2.65223	1.4072	3.41E-09	1.22E-07
FUS	5.546448	4.24159	5.9706	1.53338	0.6167	3.48E-09	1.59E-07
RP11-620J15.3	2.353174	1.87605	2.508266	1.72167	0.7838	3.60E-09	1.07E-07
DNAJB11	1.806662	2.231039	1.668715	0.54321	-0.8804	3.65E-09	1.59E-07
MAD2L2	2.777674	2.162732	2.977566	1.70079	0.7662	3.66E-09	1.19E-07
CTSC	1.597388	1.991301	1.469344	0.47346	-1.0787	4.28E-09	2.69E-07
STOML2	4.211603	3.342477	4.494118	1.49163	0.5769	4.32E-09	1.40E-07
ARID5B	1.370293	1.734946	1.251761	0.34256	-1.5456	4.32E-09	1.67E-07
GRN	1.971334	2.407994	1.829395	0.58906	-0.7635	4.34E-09	1.56E-07

BNIP3	13.52632	10.74149	14.43155	1.3788	0.4634	4.59E-09	1.32E-07
SPTAN1	1.667519	2.067889	1.537377	0.50321	-0.9908	4.69E-09	1.96E-07
FAM60A	3.028619	2.404938	3.23135	1.58822	0.6674	4.75E-09	2.65E-07
HNRNPA2B1	10.68478	8.100272	11.52489	1.48232	0.5679	4.81E-09	1.51E-07
PRMT1	3.437837	2.746045	3.662709	1.52499	0.6088	4.87E-09	1.94E-07
ELK3	1.464241	1.839559	1.342241	0.40764	-1.2946	5.02E-09	1.74E-07
CXXC5	3.280851	4.017995	3.041237	0.67636	-0.5641	5.19E-09	1.94E-07
BRI3	2.675325	3.232228	2.4943	0.66942	-0.579	5.22E-09	1.94E-07
SHMT2	2.109456	1.663763	2.254332	1.88973	0.9182	5.26E-09	1.60E-07
CRABP2	6.627125	8.84439	5.906389	0.62546	-0.677	5.27E-09	1.66E-07
NOB1	1.936739	1.526197	2.070189	2.03382	1.0242	5.35E-09	2.15E-07
SNRPA	2.142953	1.710117	2.283649	1.80766	0.8541	5.62E-09	2.17E-07
SOD3	1.307292	1.661731	1.192079	0.29027	-1.7845	5.63E-09	1.89E-07
TAGLN2	4.416222	5.790974	3.96935	0.61978	-0.6902	5.65E-09	2.47E-07
S100A2	2.328821	3.755404	1.865101	0.31397	-1.6713	5.77E-09	2.00E-07
SMC2	1.792271	1.398105	1.920398	2.31195	1.2091	5.89E-09	2.47E-07
GPC1	1.838186	2.254543	1.702846	0.56024	-0.8359	6.04E-09	3.20E-07
EIF3E	10.31344	8.049668	11.0493	1.4255	0.5115	6.33E-09	2.77E-07
FAM13C	1.621797	1.249799	1.742718	2.97327	1.572	6.42E-09	3.80E-07
GPX1	8.146334	9.613014	7.66958	0.77436	-0.3689	6.86E-09	2.76E-07
SPTBN1	2.001108	2.437874	1.859135	0.5975	-0.743	7.16E-09	2.88E-07
ETFB	3.865822	3.074125	4.123168	1.50578	0.5905	7.18E-09	2.49E-07
RAMP2	2.159287	1.706121	2.306591	1.85038	0.8878	7.56E-09	2.01E-07
EFEMP2	1.998129	2.430211	1.857678	0.59969	-0.7377	7.56E-09	2.01E-07
NUF2	1.43409	1.086859	1.546959	6.29713	2.6547	7.59E-09	2.01E-07
HNRNPH1	3.942196	2.886891	4.28523	1.74108	0.8	7.80E-09	3.02E-07
TMEM30A	1.652148	2.044292	1.524679	0.50243	-0.993	8.25E-09	3.61E-07
TECR	3.616705	2.872245	3.858696	1.52688	0.6106	8.36E-09	2.71E-07
ZFAND5	2.655581	3.256126	2.46037	0.64729	-0.6275	8.37E-09	2.55E-07
CD46	1.93128	2.355401	1.793416	0.58537	-0.7726	8.40E-09	3.13E-07
CENPM	1.503167	1.148206	1.61855	4.17359	2.0613	8.65E-09	3.63E-07
LGR4	2.462395	1.984252	2.617818	1.6437	0.7169	8.66E-09	3.63E-07
TSPO	2.158839	2.704778	1.981378	0.57566	-0.7967	9.17E-09	3.69E-07
CD81	1.850362	2.263222	1.716159	0.56693	-0.8188	9.34E-09	2.85E-07
STK17A	1.340846	1.763682	1.2034	0.26634	-1.9087	9.36E-09	3.13E-07
IFRD1	2.518001	1.93533	2.707402	1.82545	0.8683	9.54E-09	4.00E-07
ADARB1	1.614881	1.246798	1.734529	2.97623	1.5735	9.90E-09	2.93E-07
RNF19A	1.458893	1.8253	1.33979	0.41172	-1.2803	1.00E-08	5.59E-07
HMGN1	15.27596	11.13914	16.62066	1.54063	0.6235	1.03E-08	2.97E-07
DACH1	2.496889	1.900557	2.690731	1.87743	0.9088	1.05E-08	4.05E-07
SLC2A3	2.440414	3.225046	2.185364	0.53274	-0.9085	1.05E-08	3.03E-07
ZNF883	1.641065	1.271837	1.761085	2.79979	1.4853	1.10E-08	3.69E-07
RP11-390P2.4	1.597536	1.233171	1.715976	3.07061	1.6185	1.11E-08	3.10E-07
S100A6	1.35904	2.043608	1.136516	0.13081	-2.9344	1.13E-08	4.19E-07
GNG12	1.450088	1.814332	1.331688	0.40731	-1.2958	1.14E-08	4.23E-07

SMARCE1	3.346911	2.671121	3.566581	1.53584	0.619	1.15E-08	3.12E-07
ATOX1	5.268991	6.50168	4.868298	0.70311	-0.5082	1.18E-08	3.48E-07
ITGA1	1.203775	1.557383	1.088832	0.15937	-2.6495	1.19E-08	5.97E-07
NUDT1	1.787169	1.40303	1.912036	2.26295	1.1782	1.20E-08	4.03E-07
SPP1	2.06648	2.914173	1.790932	0.4132	-1.2751	1.28E-08	4.16E-07
NIPSNAP1	2.931533	2.334161	3.125713	1.5933	0.672	1.30E-08	4.37E-07
GRB10	1.652408	1.283871	1.772204	2.72026	1.4437	1.32E-08	4.75E-07
NMB	1.771203	2.229328	1.622287	0.5062	-0.9822	1.35E-08	4.84E-07
CD151	4.344201	5.392741	4.003367	0.68371	-0.5485	1.38E-08	4.66E-07
FBLN1	2.61102	2.075301	2.785159	1.66015	0.7313	1.38E-08	5.57E-07
SLC25A4	1.983608	2.42204	1.841093	0.59147	-0.7576	1.39E-08	4.81E-07
TMED9	4.119555	5.111207	3.797212	0.68039	-0.5556	1.40E-08	4.66E-07
ODF2L	2.305652	1.843313	2.455939	1.72645	0.7878	1.40E-08	4.66E-07
PROX1	1.304989	1.648578	1.193304	0.29804	-1.7464	1.43E-08	4.66E-07
POLE3	1.86588	1.470594	1.99437	2.11301	1.0793	1.44E-08	4.45E-07
POLD2	3.651505	2.676393	3.968472	1.77075	0.8244	1.46E-08	4.45E-07
ZFAS1	16.45838	13.84544	17.30773	1.26953	0.3443	1.48E-08	5.74E-07
TTC3	9.171796	7.445144	9.733056	1.35498	0.4383	1.54E-08	4.31E-07
EIF3L	7.626006	5.851646	8.202773	1.4846	0.5701	1.66E-08	5.23E-07
FAM161A	1.639811	1.275308	1.758295	2.75435	1.4617	1.75E-08	4.62E-07
KIF5C	2.259284	2.86027	2.063929	0.57192	-0.8061	1.79E-08	7.18E-07
PABPN1	4.173098	3.316115	4.451665	1.49028	0.5756	1.80E-08	4.65E-07
HMGA1	15.20249	11.77596	16.31631	1.42134	0.5073	1.84E-08	5.77E-07
GYG1	2.917474	2.248297	3.134994	1.71033	0.7743	1.88E-08	6.09E-07
MMP14	1.383782	1.733882	1.269979	0.36788	-1.4427	1.92E-08	5.37E-07
DCLK1	1.522913	1.163029	1.639895	3.92503	1.9727	1.95E-08	7.26E-07
CTSZ	1.29275	1.630593	1.182932	0.2901	-1.7854	2.01E-08	5.05E-07
CDKN2B	1.137471	1.475298	1.027658	0.05819	-4.1031	2.02E-08	7.24E-07
LGI1	1.153379	1.474589	1.048968	0.10318	-3.2768	2.02E-08	5.97E-07
UBE2L6	2.180564	1.734647	2.325513	1.80429	0.8514	2.02E-08	5.50E-07
VAMP8	1.333742	1.814737	1.177391	0.21773	-2.1994	2.04E-08	5.26E-07
SPINT1	1.256622	1.593001	1.147281	0.24836	-2.0095	2.06E-08	6.29E-07
KDELR2	3.270999	3.975464	3.042008	0.68628	-0.5431	2.18E-08	6.63E-07
NUDT14	1.589107	1.962544	1.467719	0.48592	-1.0412	2.23E-08	5.47E-07
EBPL	3.364111	2.606339	3.61043	1.62508	0.7005	2.34E-08	9.05E-07
BZW2	2.220689	1.795014	2.359058	1.70948	0.7736	2.39E-08	8.58E-07
PRDX2	14.45734	11.08701	15.55288	1.44273	0.5288	2.39E-08	7.07E-07
NEIL2	1.988513	1.589613	2.118179	1.89646	0.9233	2.40E-08	7.10E-07
HIST1H1D	1.601776	1.234294	1.721229	3.07831	1.6221	2.46E-08	7.72E-07
MFAP2	2.336246	2.839433	2.172682	0.63752	-0.6495	2.50E-08	7.40E-07
NQO1	1.991329	2.506099	1.823999	0.54711	-0.8701	2.52E-08	7.25E-07
RNASEH2A	1.822968	1.443846	1.946205	2.13183	1.0921	2.55E-08	1.02E-06
SULT1A1	1.522188	1.176694	1.634493	3.59092	1.8444	2.70E-08	1.00E-06
SET	7.917051	6.595609	8.346594	1.31292	0.3928	2.75E-08	9.56E-07
ZFP36L2	5.408747	3.834995	5.920305	1.73556	0.7954	2.84E-08	8.59E-07

LSM2	4.531945	3.610303	4.83153	1.46785	0.5537	2.85E-08	9.25E-07
PDLIM4	2.499644	3.099366	2.3047	0.62147	-0.6862	2.90E-08	8.59E-07
VAX2	1.372983	1.046348	1.479158	10.3382	3.3699	2.99E-08	1.58E-06
NGFR	1.429787	1.096484	1.538129	5.57736	2.4796	3.13E-08	8.98E-07
RFK	1.594762	1.963876	1.47478	0.49257	-1.0216	3.24E-08	1.02E-06
BMF	1.171072	1.48862	1.067851	0.13886	-2.8483	3.30E-08	1.66E-06
DPM3	2.701043	2.195526	2.865365	1.56029	0.6418	3.42E-08	1.32E-06
SLC35G1	1.497411	1.855735	1.380935	0.44516	-1.1676	3.54E-08	9.88E-07
CMBL	1.842866	1.464461	1.965868	2.07955	1.0563	3.54E-08	1.19E-06
PTAR1	1.895666	1.510537	2.020855	1.99957	0.9997	3.58E-08	1.29E-06
SMC3	2.586593	2.077503	2.752075	1.62605	0.7014	3.60E-08	1.39E-06
SOX9	1.791315	2.254478	1.64076	0.51078	-0.9692	3.60E-08	1.72E-06
VAT1	2.368092	2.819716	2.221289	0.67114	-0.5753	3.79E-08	1.82E-06
ILF3-AS1	1.861335	1.482121	1.984601	2.04223	1.0301	3.97E-08	1.29E-06
CECR5	2.247021	1.82867	2.383009	1.66895	0.7389	4.03E-08	1.84E-06
TGFB2	2.001451	2.506396	1.837315	0.55584	-0.8473	4.07E-08	1.17E-06
PHF14	3.525403	2.843659	3.747008	1.48998	0.5753	4.08E-08	1.08E-06
TKT	13.53211	10.63559	14.47365	1.39832	0.4837	4.11E-08	1.88E-06
RP3-525N10.2	1.530928	1.187146	1.642676	3.43409	1.7799	4.12E-08	1.04E-06
SLITRK5	1.582998	1.234054	1.696424	2.97548	1.5731	4.12E-08	1.12E-06
BRCA2	1.393582	1.068242	1.499336	7.31709	2.8713	4.31E-08	1.49E-06
RBP1	23.77806	19.94773	25.02314	1.26786	0.3424	4.34E-08	1.45E-06
ST13	8.886361	7.060467	9.479879	1.39921	0.4846	4.46E-08	1.66E-06
ERH	7.973533	6.462519	8.464698	1.36653	0.4505	4.48E-08	1.55E-06
NAP1L1	18.43816	13.08024	20.17979	1.5877	0.6669	4.49E-08	1.29E-06
PFN1	21.55027	26.46591	19.95241	0.74423	-0.4262	4.54E-08	1.54E-06
DAB2	1.244688	1.568531	1.139421	0.24523	-2.0278	4.59E-08	1.54E-06
COL5A2	1.406024	1.813322	1.27363	0.33643	-1.5716	4.68E-08	1.43E-06
CCL2	2.946343	5.36287	2.160835	0.26607	-1.9101	5.02E-08	1.80E-06
TPBG	1.368099	1.7062	1.258197	0.36561	-1.4516	5.18E-08	1.49E-06
ADAM9	1.317255	1.649507	1.209254	0.32217	-1.6341	5.20E-08	1.28E-06
LINC00908	1.493196	1.156968	1.602489	3.83828	1.9405	5.36E-08	1.74E-06
NHP2	5.0487	3.945034	5.407453	1.49657	0.5817	5.39E-08	1.87E-06
TAX1BP1	2.624756	3.126601	2.461628	0.68731	-0.541	5.42E-08	1.70E-06
CNBP	5.553707	4.203177	5.992706	1.55867	0.6403	5.60E-08	1.82E-06
HADH	1.913585	1.533665	2.037081	1.94332	0.9585	6.21E-08	1.57E-06
EIF4B	4.695964	3.888041	4.958584	1.37068	0.4549	6.23E-08	1.90E-06
C12orf57	4.292719	3.488246	4.554217	1.4284	0.5144	6.23E-08	1.57E-06
LSAMP	2.539899	2.015338	2.710411	1.68457	0.7524	6.27E-08	1.97E-06
HNRNPA0	6.200884	4.978238	6.598313	1.40723	0.4929	6.38E-08	2.14E-06
PBX3	1.314232	1.643184	1.207304	0.32231	-1.6335	6.48E-08	2.04E-06
GPX3	1.696475	1.329702	1.815696	2.47404	1.3069	6.50E-08	2.84E-06
INAFM1	1.950767	1.560227	2.077715	1.92371	0.9439	6.53E-08	2.16E-06
FAM3C	2.676482	3.230001	2.496557	0.6711	-0.5754	6.56E-08	1.55E-06
ODC1	4.136707	3.351517	4.391938	1.44245	0.5285	6.64E-08	1.55E-06

DNMT1	2.019501	1.630189	2.14605	1.81858	0.8628	6.67E-08	2.16E-06
PITPNA	1.402919	1.741921	1.292724	0.39455	-1.3417	6.77E-08	2.96E-06
TMEM59	3.278787	4.106326	3.009789	0.647	-0.6282	6.86E-08	1.92E-06
NET1	1.399184	1.737765	1.289126	0.39189	-1.3515	6.90E-08	2.10E-06
CTD-2034I4.2	1.958958	1.57208	2.084715	1.89609	0.923	7.03E-08	1.68E-06
REEP2	1.97145	1.585552	2.096889	1.87326	0.9055	7.09E-08	2.10E-06
GPR143	1.33411	1.709113	1.212213	0.29926	-1.7405	7.27E-08	2.03E-06
ATP6V1D	2.180772	2.626762	2.0358	0.63672	-0.6513	7.51E-08	2.10E-06
TMEM14A	2.267827	2.805252	2.093134	0.60553	-0.7237	7.67E-08	2.08E-06
TCEAL9	8.609863	10.35885	8.041343	0.75237	-0.4105	8.01E-08	1.87E-06
CDCA7L	2.29119	1.784513	2.455889	1.85579	0.892	8.01E-08	2.69E-06
HPRT1	2.322619	1.903215	2.458949	1.61528	0.6918	8.04E-08	2.38E-06
VIM	95.26241	114.8267	88.90292	0.77225	-0.3729	8.04E-08	2.19E-06
PRKDC	3.214442	2.61322	3.409873	1.49383	0.579	8.18E-08	2.42E-06
SDF4	2.237728	2.670335	2.097107	0.65682	-0.6064	8.32E-08	2.61E-06
CNN3	5.206491	6.47002	4.795772	0.69392	-0.5272	8.55E-08	2.61E-06
TPI1	28.62179	23.00754	30.44674	1.33803	0.4201	8.56E-08	2.46E-06
NCL	5.564756	4.462354	5.923099	1.42189	0.5078	8.58E-08	2.61E-06
ATF6B	2.231259	1.811197	2.367803	1.68615	0.7537	8.84E-08	2.40E-06
NBEAL1	3.976397	3.205837	4.226873	1.46288	0.5488	9.05E-08	2.39E-06
GPI	4.085113	3.226034	4.364362	1.51137	0.5959	9.30E-08	3.02E-06
RP13-942N8.1	1.565176	1.226792	1.67517	2.97705	1.5739	9.54E-08	3.10E-06
NECTIN2	2.280433	2.720724	2.137313	0.66095	-0.5974	9.58E-08	4.01E-06
PCBP2	7.146865	5.793936	7.586643	1.37395	0.4583	1.00E-07	3.14E-06
UNC5D	1.164157	1.469114	1.065029	0.13862	-2.8508	1.01E-07	2.60E-06
FILIP1L	1.289575	1.676626	1.163762	0.24203	-2.0468	1.02E-07	2.70E-06
DECR1	2.149056	1.73154	2.284773	1.75626	0.8125	1.03E-07	2.89E-06
SSRP1	2.706623	2.166035	2.882344	1.61431	0.6909	1.05E-07	3.01E-06
FBL	3.658373	3.022978	3.864913	1.41619	0.502	1.05E-07	3.03E-06
CBR4	2.033467	1.646389	2.159289	1.79349	0.8428	1.07E-07	2.68E-06
COX17	3.202064	3.861128	2.987831	0.69477	-0.5254	1.07E-07	2.45E-06
LINC00685	2.237471	1.788209	2.383506	1.75525	0.8117	1.08E-07	2.93E-06
HNRNPA1L2	1.770485	1.413887	1.886399	2.14165	1.0987	1.21E-07	2.98E-06
TRAPPC2L	2.266853	2.691016	2.128976	0.66763	-0.5829	1.24E-07	3.46E-06
FOSB	1.699968	1.346883	1.81474	2.34875	1.2319	1.24E-07	2.77E-06
OSBPL1A	2.404319	2.966787	2.221485	0.62106	-0.6872	1.27E-07	4.11E-06
CD24	9.089113	6.814971	9.828337	1.51821	0.6024	1.28E-07	3.67E-06
MRPL11	2.810237	2.205675	3.006754	1.66442	0.735	1.33E-07	4.04E-06
AK4	2.22479	1.804946	2.361263	1.69112	0.758	1.34E-07	3.21E-06
FAM177A1	2.04532	2.490243	1.900695	0.60439	-0.7264	1.36E-07	3.59E-06
WBP2	1.689551	2.052499	1.571572	0.54306	-0.8808	1.39E-07	4.35E-06
FAM216A	1.811081	1.452467	1.927651	2.05021	1.0358	1.39E-07	3.89E-06
NDUFS2	2.422075	1.972483	2.568218	1.61259	0.6894	1.40E-07	4.14E-06
NFIB	1.209011	1.516892	1.108933	0.21075	-2.2464	1.42E-07	4.21E-06
PFN2	7.260481	5.490986	7.835667	1.52209	0.606	1.44E-07	3.71E-06

IGFBP2	8.085889	6.422474	8.626593	1.40648	0.4921	1.53E-07	4.54E-06
PRPSAP1	2.398805	1.973736	2.536976	1.57843	0.6585	1.55E-07	5.76E-06
SSFA2	1.564243	1.912668	1.450985	0.49414	-1.017	1.56E-07	4.48E-06
SPON2	1.145874	1.467601	1.041294	0.08831	-3.5013	1.59E-07	4.00E-06
GMPR	1.141214	1.438417	1.044606	0.10174	-3.297	1.61E-07	4.39E-06
RNF11	2.02144	2.443698	1.884183	0.61244	-0.7074	1.64E-07	6.11E-06
OARD1	2.255681	1.771254	2.413147	1.83227	0.8736	1.64E-07	5.15E-06
FOS	3.926178	2.706499	4.322642	1.94705	0.9613	1.66E-07	4.63E-06
MARCKS	6.850932	5.348858	7.339191	1.45767	0.5437	1.67E-07	4.66E-06
EXOSC8	1.980166	1.607945	2.101159	1.81128	0.857	1.70E-07	6.85E-06
CAV1	1.677154	2.169717	1.517043	0.44202	-1.1778	1.72E-07	4.68E-06
LEPROT	2.537786	3.032331	2.377031	0.67756	-0.5616	1.82E-07	5.22E-06
RP11-798M19.6	2.326875	1.883906	2.470864	1.66405	0.7347	1.86E-07	4.25E-06
CYSTM1	3.090095	3.789193	2.862848	0.66788	-0.5823	1.86E-07	5.35E-06
SLC4A2	1.320632	1.6386	1.217275	0.34024	-1.5554	1.92E-07	5.36E-06
DUT	5.15201	4.188129	5.465325	1.40061	0.4861	1.97E-07	4.41E-06
LSM6	2.566905	2.061667	2.731135	1.63058	0.7054	2.02E-07	4.95E-06
DHRS4-AS1	1.772153	1.422592	1.88578	2.09606	1.0677	2.07E-07	5.78E-06
ALDH1A1	4.683427	5.956222	4.269697	0.65972	-0.6001	2.10E-07	6.39E-06
DHCR24	1.317781	1.633431	1.215176	0.3397	-1.5577	2.11E-07	7.56E-06
KLF6	1.75416	2.18177	1.615163	0.52054	-0.9419	2.11E-07	4.62E-06
BACE2	1.228245	1.533581	1.128994	0.24175	-2.0484	2.13E-07	4.65E-06
PAPPA	1.179973	1.564646	1.054932	0.09729	-3.3616	2.14E-07	5.67E-06
PLAU	1.190857	1.548484	1.074607	0.13602	-2.8781	2.16E-07	6.39E-06
SEMA3E	1.640602	1.304764	1.749768	2.46016	1.2988	2.30E-07	9.65E-06
FBN2	1.880539	2.283229	1.749642	0.58418	-0.7755	2.31E-07	8.02E-06
SGO1	1.428688	1.118297	1.529582	4.47671	2.1624	2.67E-07	7.25E-06
HMGN3	9.309426	7.386342	9.934537	1.39901	0.4844	2.70E-07	1.07E-05
GMNN	1.781924	1.435107	1.894659	2.05618	1.04	2.77E-07	1.07E-05
PRDX1	10.03516	8.165649	10.64286	1.34571	0.4284	2.84E-07	7.51E-06
BRCA1	1.432125	1.122559	1.532752	4.34688	2.12	2.97E-07	7.67E-06
SRP14	20.42887	23.81088	19.32953	0.80354	-0.3156	2.98E-07	7.67E-06
PLEKHB1	1.716399	2.087058	1.595914	0.54819	-0.8673	3.00E-07	8.16E-06
CTHRC1	1.27353	1.597341	1.168273	0.2817	-1.8277	3.07E-07	7.92E-06
PLEKHA1	2.914503	2.327195	3.105412	1.58636	0.6657	3.08E-07	8.31E-06
HERPUD1	2.095535	2.772633	1.875441	0.49386	-1.0178	3.11E-07	8.24E-06
RRBP1	1.812724	2.185312	1.691611	0.58348	-0.7772	3.14E-07	8.31E-06
NOP56	2.206117	1.746449	2.355536	1.81598	0.8607	3.26E-07	9.36E-06
FUNDC2	2.852902	2.324619	3.024624	1.52846	0.6121	3.42E-07	7.32E-06
DTNA	2.044505	1.664368	2.168072	1.75817	0.8141	3.74E-07	1.45E-05
NAE1	2.51687	2.057908	2.666058	1.57486	0.6552	3.78E-07	1.15E-05
SPOCD1	1.201377	1.496398	1.105478	0.21249	-2.2345	3.84E-07	1.29E-05
KCNQ1OT1	7.25591	8.953158	6.704208	0.71723	-0.4795	3.89E-07	1.09E-05
ZNF292	3.230459	2.682896	3.408448	1.43113	0.5172	3.95E-07	1.47E-05
NUCKS1	7.492754	5.912911	8.006292	1.4261	0.5121	4.04E-07	1.10E-05

CDKN2A	1.190412	1.555498	1.071738	0.12914	-2.953	4.05E-07	1.10E-05
SKA2	2.406622	1.889533	2.574705	1.77026	0.824	4.14E-07	1.04E-05
TUBB6	1.997393	2.463202	1.845979	0.57817	-0.7904	4.17E-07	1.02E-05
SPHK1	2.985112	2.368946	3.185401	1.59641	0.6748	4.24E-07	1.18E-05
ZNF302	1.862415	1.511923	1.976345	1.90721	0.9315	4.32E-07	1.11E-05
KTN1	4.483953	5.371145	4.195565	0.73106	-0.4519	4.33E-07	1.14E-05
CDCA7	1.427445	1.121565	1.526874	4.33409	2.1157	4.34E-07	1.06E-05
TAF7	4.011038	3.293576	4.244254	1.4145	0.5003	4.42E-07	1.11E-05
LOXL2	1.435262	1.757821	1.330412	0.436	-1.1976	4.59E-07	1.07E-05
RAB27A	1.268131	1.569932	1.170029	0.29833	-1.745	4.65E-07	1.73E-05
ZBTB16	1.380866	1.08207	1.477992	5.82419	2.5421	4.67E-07	1.27E-05
TMA7	16.02746	18.76932	15.13619	0.79554	-0.33	4.97E-07	1.31E-05
TMEM91	1.490655	1.815911	1.384928	0.47178	-1.0838	5.01E-07	1.14E-05
PHLDA2	1.541803	1.974671	1.401097	0.41152	-1.281	5.04E-07	1.81E-05
GRIA4	1.903722	1.550511	2.018535	1.85016	0.8877	5.08E-07	1.31E-05
FAM162A	7.947525	6.218122	8.509679	1.43915	0.5252	5.11E-07	1.51E-05
RAI14	1.940249	2.308444	1.820565	0.62713	-0.6732	5.15E-07	1.26E-05
KHDRBS1	4.422453	3.367666	4.765318	1.59031	0.6693	5.19E-07	1.24E-05
ANGPTL4	1.614638	2.183325	1.429782	0.3632	-1.4612	5.59E-07	1.20E-05
UCP2	2.210117	1.796644	2.344519	1.68773	0.7551	5.80E-07	1.36E-05
TMEM161B-AS1	1.832016	1.488431	1.943701	1.93211	0.9502	5.85E-07	1.68E-05
HNRNPD	3.215822	2.641373	3.402551	1.46374	0.5497	5.90E-07	1.24E-05
MAGI3	1.538604	1.225592	1.640351	2.83853	1.5051	5.98E-07	2.15E-05
CXCL12	1.156083	1.440772	1.063542	0.14416	-2.7942	6.20E-07	1.27E-05
LRRC4C	1.356223	1.062462	1.451712	7.2318	2.8544	6.25E-07	1.43E-05
LINC00632	2.298795	1.855366	2.442935	1.68692	0.7544	6.45E-07	1.71E-05
HAUS1	2.17678	1.798993	2.299582	1.62652	0.7018	6.45E-07	1.62E-05
ENO1	32.92379	22.38827	36.34843	1.6527	0.7248	6.50E-07	1.82E-05
OLA1	4.090658	3.36603	4.326204	1.40582	0.4914	6.60E-07	1.58E-05
LDHB	14.75122	12.3751	15.52359	1.27679	0.3525	6.66E-07	1.72E-05
GREM1	1.179771	1.502599	1.074834	0.14889	-2.7476	6.79E-07	1.80E-05
PHLDA3	2.064009	2.472451	1.931242	0.63244	-0.661	6.95E-07	1.73E-05
ZNF516	1.83359	1.488058	1.945907	1.9381	0.9546	6.98E-07	1.80E-05
AKAP6	1.677143	1.345709	1.784878	2.27034	1.1829	7.02E-07	1.91E-05
PDPN	1.834958	2.211092	1.712693	0.58847	-0.765	7.03E-07	1.73E-05
PCNA	1.887108	1.538822	2.00032	1.8565	0.8926	7.16E-07	1.85E-05
KCTD12	1.216835	1.605841	1.090387	0.14919	-2.7448	7.27E-07	1.83E-05
RAB2A	3.287106	3.885646	3.092546	0.72516	-0.4636	7.40E-07	1.81E-05
FGF9	1.369727	1.077053	1.464862	6.03302	2.5929	7.59E-07	1.82E-05
CITED2	2.004406	2.420144	1.869268	0.6121	-0.7082	7.60E-07	1.91E-05
SMAD3	1.393503	1.702158	1.293173	0.41753	-1.26	7.80E-07	1.74E-05
ICE2	1.717746	1.388885	1.824644	2.12053	1.0844	7.86E-07	2.14E-05
IP6K2	2.720942	2.202164	2.889574	1.57181	0.6524	7.88E-07	1.93E-05
DICER1-AS1	1.502227	1.196128	1.601727	3.06803	1.6173	8.39E-07	2.01E-05
SPNS2	1.119562	1.397387	1.029253	0.07361	-3.7639	8.39E-07	2.22E-05

PFKP	2.011903	2.406144	1.883752	0.62849	-0.67	8.44E-07	2.74E-05
FAM114A1	1.53205	1.853541	1.427548	0.50091	-0.9974	8.46E-07	3.04E-05
TRAPPC3	2.401201	2.813159	2.267291	0.69894	-0.5168	8.53E-07	2.26E-05
DCXR	2.676463	2.214752	2.826546	1.50364	0.5885	8.75E-07	2.09E-05
FANCL	1.652272	1.332043	1.756364	2.27791	1.1877	8.81E-07	2.22E-05
PSMA4	4.37448	3.587123	4.630415	1.40326	0.4888	9.06E-07	2.12E-05
SMS	5.821849	7.086437	5.410787	0.72469	-0.4646	9.09E-07	2.41E-05
PPA1	5.038831	3.772955	5.450313	1.6049	0.6825	9.09E-07	2.13E-05
LAMP1	1.919795	2.27866	1.803144	0.62811	-0.6709	9.30E-07	2.28E-05
ATAD5	1.491777	1.189076	1.590172	3.12134	1.6422	9.52E-07	2.17E-05
FGFR2	1.141749	1.419846	1.051351	0.12231	-3.0314	9.53E-07	2.34E-05
TNNI3	1.522591	1.217413	1.621791	2.85995	1.516	1.04E-06	2.33E-05
ZNF644	2.086683	1.728598	2.203081	1.65123	0.7235	1.11E-06	2.58E-05
RGS16	1.425251	1.110459	1.527576	4.77621	2.2559	1.13E-06	2.58E-05
MSMO1	1.868707	2.356256	1.710226	0.52367	-0.9333	1.17E-06	2.79E-05
PALLD	3.327494	2.701212	3.531071	1.4878	0.5732	1.19E-06	2.85E-05
LAMP2	1.60707	1.932067	1.501428	0.53797	-0.8944	1.20E-06	3.17E-05
SRSF9	7.143102	5.712231	7.608216	1.40235	0.4879	1.23E-06	3.10E-05
OLFM2	1.530547	1.847126	1.427641	0.50481	-0.9862	1.24E-06	3.20E-05
STMN3	1.846139	1.509692	1.955504	1.87467	0.9066	1.28E-06	4.45E-05
TAX1BP3	2.918448	3.48998	2.732668	0.69586	-0.5231	1.29E-06	3.09E-05
CA9	1.49093	1.190853	1.588472	3.08338	1.6245	1.40E-06	3.51E-05
BANF1	6.082936	4.919046	6.461266	1.39352	0.4787	1.46E-06	3.78E-05
WDR18	1.6707	1.355978	1.773002	2.17149	1.1187	1.57E-06	3.51E-05
CYBA	3.073842	3.679477	2.876976	0.7005	-0.5135	1.60E-06	3.74E-05
GABPB1	2.331288	1.953781	2.453998	1.52446	0.6083	1.64E-06	4.22E-05
CDC25B	1.586639	1.280852	1.686038	2.4427	1.2885	1.68E-06	5.63E-05
RFC4	1.540517	1.240062	1.638182	2.6584	1.4106	1.70E-06	4.27E-05
ORC6	1.635253	1.325036	1.736091	2.26465	1.1793	1.73E-06	3.79E-05
CAPNS1	2.265168	2.701605	2.123302	0.66014	-0.5992	1.74E-06	5.65E-05
CSTF3	2.010688	1.647087	2.128879	1.74456	0.8029	1.79E-06	4.40E-05
PSRC1	1.418449	1.131669	1.511669	3.88603	1.9583	1.85E-06	4.03E-05
CTTN	2.49379	2.958527	2.342724	0.68558	-0.5446	1.85E-06	3.87E-05
CMSS1	2.313352	1.916339	2.442404	1.57409	0.6545	1.85E-06	3.96E-05
ATP5G2	16.90747	12.88906	18.21367	1.44786	0.5339	1.86E-06	3.74E-05
CDK4	4.316041	3.600455	4.548647	1.36463	0.4485	1.86E-06	4.36E-05
LRRC39	1.13442	1.404508	1.046626	0.11527	-3.117	1.92E-06	4.94E-05
FOSL2	1.268633	1.552332	1.176415	0.3194	-1.6466	1.93E-06	4.61E-05
SDHAF3	2.103362	1.752559	2.217393	1.61767	0.6939	1.93E-06	4.41E-05
CCT8	4.288367	3.624609	4.504126	1.3351	0.417	1.98E-06	6.85E-05
SESN3	3.381602	4.153163	3.130801	0.67577	-0.5654	1.99E-06	4.08E-05
ARL6IP1	4.009789	3.219855	4.266562	1.47152	0.5573	2.00E-06	4.57E-05
CSN3	1.203992	1.680823	1.048995	0.07196	-3.7966	2.00E-06	4.94E-05
ADGRG6	1.121508	1.38773	1.034971	0.09019	-3.4708	2.01E-06	4.94E-05
C9orf116	1.631867	1.32455	1.731763	2.2547	1.1729	2.10E-06	5.42E-05

TROAP	1.400623	1.117067	1.492795	4.2095	2.0736	2.12E-06	4.96E-05
HNRNPH3	3.815229	3.015128	4.075307	1.52611	0.6099	2.16E-06	5.18E-05
FXYD5	1.642258	1.962078	1.538298	0.55952	-0.8378	2.19E-06	4.89E-05
CENPE	1.405603	1.122114	1.497752	4.07612	2.0272	2.20E-06	4.42E-05
QTRT1	2.006263	1.661631	2.118288	1.6902	0.7572	2.20E-06	5.02E-05
AMOTL2	1.291303	1.575337	1.198976	0.34584	-1.5318	2.30E-06	4.53E-05
HNRNPA3	5.870529	4.85597	6.200318	1.34864	0.4315	2.47E-06	4.87E-05
NDP	1.304676	1.032428	1.393172	12.1244	3.5998	2.56E-06	5.84E-05
TES	1.755974	2.083817	1.649407	0.59918	-0.7389	2.70E-06	6.78E-05
FMC1	2.329922	1.918898	2.463528	1.5927	0.6715	2.71E-06	6.06E-05
LTA4H	2.829402	2.323734	2.993773	1.50617	0.5909	2.75E-06	6.14E-05
MGAT4B	1.596792	1.909226	1.495233	0.54468	-0.8765	2.75E-06	6.43E-05
CTSV	1.218072	1.51031	1.123078	0.24118	-2.0518	2.79E-06	5.39E-05
HN1	6.096708	4.990677	6.456231	1.36724	0.4513	2.87E-06	6.29E-05
OST4	14.1061	15.64058	13.60731	0.86112	-0.2157	2.95E-06	9.88E-05
PTP4A1	2.059905	2.419236	1.943103	0.66451	-0.5896	2.95E-06	9.90E-05
PCDH19	1.230481	1.504522	1.141402	0.28027	-1.8351	2.95E-06	9.90E-05
RCN1	5.081459	4.220211	5.361414	1.35439	0.4376	2.96E-06	5.71E-05
ZNF518A	2.340951	1.942148	2.470585	1.56088	0.6424	3.01E-06	5.71E-05
ITGA3	1.357955	1.645013	1.264645	0.41029	-1.2853	3.04E-06	7.06E-05
SLC7A2	1.15485	1.421322	1.068231	0.16195	-2.6264	3.06E-06	7.33E-05
PTPRA	2.802174	2.355595	2.947337	1.43652	0.5226	3.09E-06	7.06E-05
SYTL2	1.405208	1.125475	1.496137	3.95408	1.9833	3.16E-06	7.08E-05
ADAMTS18	1.519184	1.841791	1.414318	0.49219	-1.0227	3.17E-06	7.08E-05
TBX2	1.452547	1.167621	1.545164	3.25236	1.7015	3.21E-06	7.17E-05
CERK	1.798666	1.481408	1.901792	1.87324	0.9055	3.40E-06	7.59E-05
EIF5	5.116108	4.220122	5.407354	1.36869	0.4528	3.43E-06	7.18E-05
AURKA	1.377597	1.10186	1.467227	4.58693	2.1975	3.43E-06	6.52E-05
PTGES	1.547894	1.935427	1.421924	0.45105	-1.1486	3.48E-06	8.75E-05
CENPN	1.48177	1.195248	1.574906	2.9445	1.558	3.48E-06	7.77E-05
IDS	2.35157	2.770905	2.215263	0.68624	-0.5432	3.53E-06	7.71E-05
CADM1	2.153275	2.514629	2.035815	0.68387	-0.5482	3.54E-06	8.68E-05
FBXO5	1.468133	1.183276	1.560728	3.05947	1.6133	3.65E-06	7.98E-05
SLC9A3R1	1.319057	1.600387	1.227608	0.3791	-1.3993	3.68E-06	6.85E-05
MIF	15.15873	13.26549	15.77414	1.20453	0.2685	3.81E-06	9.35E-05
GADD45B	1.513121	1.959093	1.368154	0.38386	-1.3814	3.81E-06	8.16E-05
ALDOA	31.22104	25.97938	32.92488	1.27805	0.3539	3.93E-06	8.60E-05
RAC3	2.302427	1.942177	2.419529	1.50665	0.5913	3.94E-06	9.43E-05
C1orf122	3.119689	3.711939	2.927175	0.71063	-0.4928	3.95E-06	8.65E-05
NMRK2	1.16116	1.424952	1.075413	0.17746	-2.4944	3.98E-06	7.28E-05
CCDC171	1.409019	1.130617	1.499515	3.82426	1.9352	3.98E-06	7.42E-05
TMEM151A	1.123343	1.383506	1.038775	0.10111	-3.3061	4.00E-06	8.53E-05
ATP6V1F	5.967991	7.077127	5.60746	0.75816	-0.3994	4.01E-06	8.40E-05
DCTN2	3.032264	2.568889	3.182887	1.39136	0.4765	4.06E-06	0.000131
CERCAM	1.419063	1.709314	1.324715	0.45779	-1.1273	4.06E-06	8.69E-05

HNRNPK	7.831877	6.604678	8.230786	1.29013	0.3675	4.07E-06	8.53E-05
NRN1	1.380185	1.106441	1.469168	4.40778	2.1401	4.16E-06	0.000131
CCDC167	3.07782	2.539522	3.252797	1.46331	0.5492	4.32E-06	0.000106
HINT2	2.142643	1.799531	2.254173	1.56864	0.6495	4.39E-06	9.39E-05
ELOVL1	1.400531	1.687496	1.307251	0.44691	-1.1619	4.43E-06	7.97E-05
NDRG1	2.029376	2.556495	1.858032	0.55126	-0.8592	4.46E-06	9.55E-05
TMA16	1.993913	1.65989	2.102489	1.67072	0.7405	4.57E-06	9.99E-05
MET	1.148509	1.408669	1.063943	0.15647	-2.6761	4.58E-06	0.000107
TCEAL5	2.050056	1.712178	2.159885	1.62865	0.7037	4.70E-06	9.85E-05
PSMA3-AS1	1.970971	1.64389	2.07729	1.6731	0.7425	4.77E-06	0.000109
HLTF	2.04772	1.709792	2.157566	1.63085	0.7056	4.80E-06	0.000103
LBR	1.709338	1.406952	1.807631	1.98458	0.9888	4.92E-06	0.000101
ARHGAP29	1.591349	2.044287	1.444118	0.42528	-1.2335	4.99E-06	0.000104
CCDC51	1.351897	1.633234	1.260446	0.4113	-1.2818	5.00E-06	0.000109
CDCA3	1.337006	1.070804	1.423537	5.98178	2.5806	5.28E-06	0.000161
CCDC88A	1.879062	1.560821	1.982509	1.75191	0.8089	5.37E-06	9.83E-05
SEMA3C	1.166463	1.426657	1.081885	0.19192	-2.3814	5.54E-06	9.96E-05
HSPE1	10.25988	8.549109	10.81598	1.30028	0.3788	5.56E-06	0.000175
RIDA	1.778698	1.470701	1.878814	1.86703	0.9007	5.62E-06	0.00012
RIC3	1.892804	1.573929	1.996456	1.7362	0.7959	5.64E-06	0.000132
FAM133B	2.358233	1.995519	2.476135	1.48278	0.5683	5.72E-06	0.00018
TMEM167A	2.288279	2.651373	2.170253	0.70865	-0.4968	5.87E-06	0.000174
CSRP2	3.335001	2.79506	3.510513	1.39857	0.484	5.89E-06	0.000104
TMEM147	4.66388	5.531989	4.381695	0.74618	-0.4224	5.89E-06	0.000134
RP11-486G15.2	1.572879	1.284309	1.666681	2.34492	1.2295	5.94E-06	0.000125
USP1	1.964621	1.622256	2.075909	1.72904	0.79	5.96E-06	0.000182
ZEB2	1.719841	1.417516	1.818114	1.95948	0.9705	6.00E-06	0.000121
FTL	103.3532	119.8908	97.97752	0.81569	-0.2939	6.01E-06	0.000147
NME1	5.443532	4.482431	5.755944	1.3657	0.4496	6.02E-06	0.000141
ABCD4	2.741299	2.187269	2.92139	1.61833	0.6945	6.06E-06	0.000132
DCAF7	2.533055	2.112041	2.669908	1.50166	0.5866	6.25E-06	0.000108
TGIF2	1.658207	1.363607	1.753969	2.07358	1.0521	6.47E-06	0.000197
MRPL23	2.470661	2.074483	2.599441	1.48857	0.5739	6.50E-06	0.000156
WSB1	9.281884	7.352802	9.908945	1.40236	0.4879	6.50E-06	0.000133
STT3B	1.922857	2.259264	1.813505	0.64602	-0.6304	6.52E-06	0.000146
SERBP1	8.038561	6.648423	8.490434	1.32611	0.4072	6.84E-06	0.000202
ENC1	1.3998	1.681111	1.308358	0.45273	-1.1433	6.85E-06	0.000222
LIMA1	1.646303	1.962769	1.543434	0.56445	-0.8251	6.88E-06	0.000138
FAM111A	1.427088	1.154862	1.515577	3.32926	1.7352	7.06E-06	0.000203
PHLDA1	2.041155	2.50439	1.890577	0.59199	-0.7564	7.12E-06	0.000163
KIAA1143	1.949038	1.630127	2.052702	1.67062	0.7404	7.34E-06	0.000151
COTL1	5.287091	6.562909	4.872378	0.69611	-0.5226	7.36E-06	0.000157
GRID2	1.114237	1.36562	1.032524	0.08896	-3.4908	7.55E-06	0.000237
RPA2	1.733482	1.433977	1.830837	1.91447	0.9369	7.70E-06	0.000152
MIR210HG	1.972132	1.652508	2.076028	1.64906	0.7216	7.73E-06	0.000162

BCL11A	1.554065	1.270832	1.646131	2.38573	1.2544	7.74E-06	0.000216
EZH2	1.71871	1.420782	1.815553	1.93819	0.9547	7.81E-06	0.000138
POLR2L	11.97873	13.802	11.38606	0.81128	-0.3017	7.81E-06	0.00016
IDH2	3.368513	2.828073	3.544187	1.39173	0.4769	7.93E-06	0.000242
MTFP1	1.733935	1.434848	1.831155	1.91137	0.9346	7.94E-06	0.00019
XRCC2	1.371502	1.106487	1.457647	4.29769	2.1036	8.05E-06	0.000188
YEATS4	2.073815	1.740967	2.182009	1.59522	0.6738	8.19E-06	0.00014
ACTG2	1.153105	1.532196	1.029879	0.05614	-4.1548	8.21E-06	0.000172
CDK5RAP3	2.171695	1.79139	2.295316	1.63676	0.7108	8.23E-06	0.00018
RABAC1	4.03873	4.711065	3.820184	0.75994	-0.396	8.38E-06	0.000179
RNF175	1.57705	1.292841	1.669434	2.286	1.1928	8.38E-06	0.000248
PTOV1	3.069067	2.590141	3.224744	1.39909	0.4845	8.40E-06	0.000169
VASP	1.700822	2.010447	1.600177	0.59397	-0.7515	8.45E-06	0.000147
ZCCHC7	1.803833	1.499199	1.902856	1.80861	0.8549	8.57E-06	0.000179
JUP	1.503083	1.790956	1.409508	0.51774	-0.9497	8.60E-06	0.000173
HES4	2.782923	3.304432	2.613403	0.70013	-0.5143	8.60E-06	0.000192
MND1	1.36346	1.100353	1.448985	4.47405	2.1616	8.68E-06	0.000257
FOXN4	1.268748	1.015231	1.351156	23.056	4.5271	8.81E-06	0.000185
CDC42EP1	1.298493	1.565741	1.211622	0.37406	-1.4187	8.83E-06	0.000185
ATF7IP	1.990302	1.67076	2.094171	1.63124	0.706	8.87E-06	0.000241
TTC19	2.364301	1.918177	2.509316	1.64382	0.7171	8.98E-06	0.000187
BTF3L4	3.362971	2.827952	3.536883	1.38783	0.4728	9.01E-06	0.000178
AGPAT2	2.024084	2.391957	1.904504	0.64981	-0.6219	9.10E-06	0.000187
NDUFA4L2	3.245663	2.209707	3.582407	2.13474	1.0941	9.26E-06	0.000222
TOX	1.699274	1.404988	1.794934	1.96286	0.973	9.26E-06	0.000203
PLEKHA5	1.471609	1.755317	1.379388	0.50229	-0.9934	9.38E-06	0.000248
VRK1	1.428999	1.160284	1.516347	3.22145	1.6877	9.39E-06	0.000189
LPAR6	1.431797	1.162092	1.519466	3.20475	1.6802	9.43E-06	0.000158
REEP5	2.236981	2.615944	2.113797	0.68925	-0.5369	9.50E-06	0.000195
PFKM	1.827623	1.523022	1.926635	1.7717	0.8251	9.59E-06	0.000224
GATA3	1.142384	1.392765	1.060996	0.1553	-2.6869	9.70E-06	0.000199
S100A3	1.23831	1.524277	1.145355	0.27725	-1.8508	9.71E-06	0.000199
CHST11	1.194294	1.449854	1.111223	0.24724	-2.016	9.92E-06	0.000204
DTX4	1.404358	1.138695	1.490713	3.53806	1.823	1.06E-05	0.000236
CKAP4	2.407288	2.810955	2.276073	0.70464	-0.505	1.07E-05	0.000177
ELP6	1.809469	1.507497	1.907627	1.78844	0.8387	1.07E-05	0.000246
BCAS4	1.452277	1.731768	1.361427	0.49391	-1.0177	1.08E-05	0.000231
H2AFY2	2.821903	2.295045	2.993161	1.53907	0.6221	1.09E-05	0.000254
CADPS	1.191232	1.445846	1.108468	0.24329	-2.0393	1.09E-05	0.000238
PAPPA2	1.316795	1.632079	1.214309	0.33905	-1.5604	1.11E-05	0.000318
GOLM1	2.61914	2.196714	2.756452	1.46773	0.5536	1.12E-05	0.000181
PLK1	1.346401	1.086113	1.43101	5.00519	2.3234	1.17E-05	0.00025
ZNF106	1.945472	2.267346	1.840845	0.66347	-0.5919	1.19E-05	0.000234
SELENOT	2.366551	2.758025	2.2393	0.70494	-0.5044	1.21E-05	0.000239
ILF2	3.395307	2.870957	3.56575	1.37136	0.4556	1.21E-05	0.000339

COA1	3.678926	3.14989	3.850892	1.32606	0.4072	1.25E-05	0.000321
DBF4	1.597323	1.317607	1.688247	2.16698	1.1157	1.25E-05	0.000263
ORAI3	1.788506	2.09643	1.688414	0.62787	-0.6715	1.25E-05	0.000286
TUBA1A	15.99203	12.12185	17.25005	1.46109	0.547	1.26E-05	0.000201
PDE4D	1.269405	1.530185	1.184637	0.34825	-1.5218	1.27E-05	0.00026
MGST3	4.852608	4.05954	5.1104	1.34347	0.426	1.27E-05	0.000246
ZBTB41	1.524848	1.250961	1.613877	2.44611	1.2905	1.28E-05	0.000349
EMC7	1.607474	1.899289	1.512618	0.57003	-0.8109	1.29E-05	0.000325
PIF1	1.285722	1.035234	1.367145	10.4201	3.3813	1.31E-05	0.000205
GSTA4	2.12305	1.795123	2.229645	1.54648	0.629	1.35E-05	0.00027
ID3	10.87907	8.117248	11.77682	1.51418	0.5985	1.37E-05	0.000386
ESPN	1.502205	1.231355	1.590246	2.55125	1.3512	1.38E-05	0.000386
ERV3-1	1.758609	1.465253	1.853966	1.83549	0.8762	1.38E-05	0.000367
CNTN5	1.365571	1.107306	1.449522	4.18917	2.0667	1.39E-05	0.000292
ERRFI1	1.296532	1.558521	1.21137	0.37845	-1.4018	1.40E-05	0.000281
CD44	1.218522	1.471272	1.136364	0.28935	-1.7891	1.41E-05	0.000273
CHMP4B	2.423896	2.803127	2.300624	0.72132	-0.4713	1.43E-05	0.00039
CDV3	1.784415	2.088416	1.685598	0.6299	-0.6668	1.47E-05	0.00028
IPO5	2.448538	2.058625	2.575282	1.48805	0.5734	1.47E-05	0.000228
S100A14	1.141332	1.501968	1.024105	0.04802	-4.3802	1.50E-05	0.00028
ING4	1.94427	1.635834	2.044529	1.64277	0.7161	1.50E-05	0.000303
DNAJC15	1.697619	1.994314	1.601177	0.60461	-0.7259	1.51E-05	0.00028
CD99	3.925405	4.795498	3.642575	0.69624	-0.5223	1.51E-05	0.000298
NCAM1	1.668907	1.384308	1.761417	1.98127	0.9864	1.57E-05	0.000316
TMEM256	6.005283	4.970197	6.341744	1.34546	0.4281	1.57E-05	0.000452
RBBP4	3.518093	2.975766	3.694379	1.36371	0.4475	1.57E-05	0.000311
CDCA8	1.333311	1.080921	1.415352	5.13278	2.3597	1.58E-05	0.00024
EIF3H	6.172016	5.124474	6.512526	1.33654	0.4185	1.61E-05	0.000317
SLC35E2B	2.158298	1.83254	2.264188	1.51847	0.6026	1.62E-05	0.000363
DZIP3	1.731331	1.441779	1.825452	1.86847	0.9019	1.63E-05	0.00032
PTRHD1	2.40146	2.021759	2.524884	1.49241	0.5776	1.63E-05	0.000297
A2M	1.39853	1.140052	1.48255	3.44551	1.7847	1.63E-05	0.000327
EPCAM	1.229632	1.603127	1.108225	0.17944	-2.4784	1.68E-05	0.000469
BAALC	1.71005	2.021192	1.608911	0.59627	-0.746	1.68E-05	0.000325
PARVB	1.316863	1.577244	1.232224	0.4023	-1.3137	1.69E-05	0.00037
ANP32B	4.413054	3.64101	4.664011	1.38735	0.4723	1.69E-05	0.00037
TPGS1	1.720526	1.43371	1.813757	1.87627	0.9079	1.72E-05	0.000444
TUBB2A	2.186002	2.640887	2.038139	0.63267	-0.6605	1.72E-05	0.000373
PPP1CC	4.194172	3.497504	4.420628	1.36962	0.4538	1.74E-05	0.000337
KC6	2.1829	1.846244	2.292332	1.52714	0.6108	1.74E-05	0.000373
LINC00973	1.261206	1.526524	1.174963	0.3323	-1.5894	1.79E-05	0.000321
ARPC5L	1.942629	2.256802	1.840505	0.66877	-0.5804	1.80E-05	0.000338
SMARCC1	2.851934	2.446094	2.983855	1.37187	0.4561	1.81E-05	0.000444
SLC38A2	2.018529	2.337762	1.91476	0.6838	-0.5484	1.82E-05	0.000338
RRM2	1.340267	1.088829	1.421998	4.75065	2.2481	1.82E-05	0.000375

MIR124-2HG	1.296216	1.048215	1.37683	7.81556	2.9663	1.83E-05	0.00036
EFHD1	1.239836	1.492993	1.157545	0.31957	-1.6458	1.84E-05	0.000359
GAPDH	128.8507	114.1718	133.6222	1.17187	0.2288	1.85E-05	0.000351
ZBTB38	1.925492	1.621827	2.0242	1.64708	0.7199	1.85E-05	0.000359
ALKBH2	1.635165	1.355985	1.725914	2.03917	1.028	1.87E-05	0.000281
SNHG18	2.444095	2.05676	2.570001	1.48567	0.5711	1.93E-05	0.000373
PNN	3.555664	2.942486	3.754981	1.41828	0.5041	1.95E-05	0.000356
HNRNPC	5.743711	4.980417	5.991825	1.2541	0.3266	1.95E-05	0.000393
ATP2B4	1.939372	1.635651	2.038099	1.63313	0.7076	1.97E-05	0.000403
GLIPR1	1.149466	1.391623	1.070752	0.18066	-2.4686	2.00E-05	0.000371
ECE1	1.435885	1.705218	1.348337	0.49394	-1.0176	2.00E-05	0.00038
NINJ1	2.228183	2.563804	2.119087	0.71562	-0.4827	2.00E-05	0.000388
DOC2B	1.458173	1.195753	1.543474	2.77633	1.4732	2.04E-05	0.000387
FLNC	1.358905	1.621439	1.273567	0.44022	-1.1837	2.07E-05	0.000434
ASF1B	1.347979	1.097366	1.429442	4.41057	2.141	2.08E-05	0.000368
NAP1L4	2.356524	2.00056	2.472233	1.47141	0.5572	2.09E-05	0.000375
BRD8	1.531041	1.263105	1.618136	2.34939	1.2323	2.13E-05	0.000397
DSTN	8.219998	9.616084	7.766191	0.7853	-0.3487	2.14E-05	0.000457
PAFAH1B3	4.605192	3.617704	4.926181	1.49986	0.5848	2.15E-05	0.000415
RDX	3.722855	4.472867	3.479059	0.71384	-0.4863	2.17E-05	0.000412
TIMELESS	1.443889	1.185095	1.528011	2.85265	1.5123	2.25E-05	0.000427
ZIC3	1.281353	1.037674	1.360562	9.57055	3.2586	2.27E-05	0.000387
DNAJB9	1.710575	2.001301	1.616072	0.61527	-0.7007	2.42E-05	0.000569
CTD-3184A7.4	1.452349	1.193897	1.536361	2.76622	1.4679	2.43E-05	0.000569
ATP6AP1	1.607516	1.890239	1.515615	0.57919	-0.7879	2.46E-05	0.000458
UBE2H	2.002422	2.316576	1.900304	0.68382	-0.5483	2.47E-05	0.000497
TNIK	1.337227	1.594379	1.253638	0.42673	-1.2286	2.47E-05	0.000487
HOMER3	1.835148	2.135178	1.737622	0.64978	-0.622	2.49E-05	0.000626
DCLRE1C	1.624279	1.351066	1.713088	2.03121	1.0223	2.51E-05	0.000422
FOXP2	1.673179	1.395785	1.763348	1.92869	0.9476	2.53E-05	0.000531
STMN4	1.318666	1.066384	1.400672	6.03572	2.5935	2.55E-05	0.000484
UBE2E3	4.896173	4.119129	5.148756	1.3301	0.4115	2.56E-05	0.000476
GAS2L3	1.3564	1.107419	1.437333	4.07129	2.0255	2.57E-05	0.000507
SCHIP1.1	2.296332	1.890386	2.428288	1.60412	0.6818	2.61E-05	0.000485
SLC37A4	1.777814	1.492363	1.870602	1.76821	0.8223	2.62E-05	0.000712
MMP2	1.157143	1.420803	1.071438	0.16977	-2.5584	2.64E-05	0.0005
DAAM2	1.121149	1.357397	1.044355	0.12411	-3.0104	2.68E-05	0.00055
HNRNPU	5.166722	4.329748	5.438786	1.33307	0.4148	2.77E-05	0.000535
PHIP	2.708184	2.292045	2.843453	1.42677	0.5128	2.83E-05	0.000419
LINC00665	1.943243	1.643092	2.040809	1.61845	0.6946	2.84E-05	0.000492
LINC01003	1.598319	1.32881	1.685924	2.08608	1.0608	2.99E-05	0.000547
DBP	1.632479	1.913916	1.540996	0.59195	-0.7564	3.01E-05	0.000497
ACTB	60.32267	70.39514	57.04856	0.80767	-0.3082	3.07E-05	0.000498
PABPC4	2.771694	2.324944	2.916913	1.44679	0.5329	3.08E-05	0.000595
ACTR3	2.458414	2.841616	2.333852	0.72428	-0.4654	3.10E-05	0.000761

EGR1	2.181158	1.77591	2.312887	1.69206	0.7588	3.13E-05	0.000642
FBXO32	1.397346	1.658596	1.312425	0.47438	-1.0759	3.16E-05	0.0006
NHLRC3	2.04134	1.738725	2.139706	1.5428	0.6256	3.17E-05	0.000725
TMEM59L	1.312785	1.56377	1.231201	0.4101	-1.286	3.21E-05	0.000587
TET1	2.306926	1.956446	2.420852	1.48555	0.571	3.22E-05	0.000514
KIFC1	1.30547	1.064663	1.383746	5.93458	2.5691	3.24E-05	0.000603
SURF4	1.97826	2.290608	1.876729	0.67931	-0.5578	3.29E-05	0.000561
C1orf43	3.700012	3.1387	3.882471	1.34777	0.4306	3.31E-05	0.000875
ARL4C	1.954951	2.321206	1.835897	0.63268	-0.6605	3.31E-05	0.000604
UBE2S	2.17356	1.793289	2.297169	1.63518	0.7094	3.32E-05	0.000667
KPNA2	2.415188	1.953729	2.565188	1.64112	0.7147	3.33E-05	0.000761
KNOP1	2.208131	1.878453	2.315296	1.49729	0.5824	3.34E-05	0.000593
BTG2	1.933661	1.638341	2.029657	1.61302	0.6898	3.36E-05	0.000593
PLEKHA2	1.180155	1.418682	1.10262	0.2451	-2.0285	3.37E-05	0.000893
CLDN4	1.316083	1.687558	1.195332	0.2841	-1.8156	3.44E-05	0.000541
NCKAP1	1.951859	2.254029	1.853637	0.68072	-0.5549	3.51E-05	0.000641
SOCS2	1.496306	1.763063	1.409595	0.53678	-0.8976	3.53E-05	0.000634
C19orf81	2.057795	1.731564	2.163838	1.59089	0.6698	3.57E-05	0.000921
BTF3	17.67848	15.29607	18.45289	1.22082	0.2878	3.62E-05	0.000608
HTN1	1.455179	2.241992	1.199421	0.16057	-2.6388	3.65E-05	0.000874
NDUFS4	3.373559	2.912252	3.523509	1.31965	0.4002	3.83E-05	0.00074
MINCR	1.842759	1.55761	1.935449	1.6776	0.7464	3.89E-05	0.000798
BCHE	1.654297	1.384477	1.742004	1.9299	0.9485	3.96E-05	0.000577
CDK6	1.533815	1.801902	1.446672	0.55702	-0.8442	3.97E-05	0.000754
CLSTN2	1.448675	1.708872	1.364096	0.51363	-0.9612	4.00E-05	0.000659
LAMC1	1.604719	1.879117	1.515525	0.58641	-0.77	4.02E-05	0.000734
GALNT10	1.20471	1.443216	1.127182	0.28695	-1.8011	4.04E-05	0.000753
RBBP7	3.752143	3.132178	3.953667	1.38528	0.4702	4.04E-05	0.000783
CTNNBIP1	2.259132	1.889784	2.379191	1.55003	0.6323	4.05E-05	0.000783
CEP41	1.517303	1.260372	1.600821	2.30755	1.2064	4.05E-05	0.000905
HSPB11	2.439027	2.077685	2.556484	1.44428	0.5304	4.06E-05	0.000716
EEF2	28.75527	23.85347	30.34864	1.28421	0.3609	4.08E-05	0.00082
DCTPP1	2.298929	1.978288	2.403155	1.4343	0.5203	4.08E-05	0.000732
CRLF1	1.180521	1.415927	1.104001	0.25005	-1.9997	4.14E-05	0.000734
MFAP3L	1.114649	1.344639	1.03989	0.11574	-3.111	4.14E-05	0.00073
TULP4	1.724762	2.008296	1.632597	0.62739	-0.6726	4.14E-05	0.000834
CACNA2D1	1.411157	1.162783	1.491893	3.02177	1.5954	4.17E-05	0.000734
UGCG	1.490686	1.75368	1.405197	0.53762	-0.8953	4.25E-05	0.000837
PHF6	3.116827	2.692724	3.254685	1.33199	0.4136	4.30E-05	0.00094
ACBD3	1.327787	1.577444	1.246635	0.42711	-1.2273	4.30E-05	0.001082
NUDT15	1.62283	1.357385	1.709115	1.98417	0.9885	4.33E-05	0.00067
RP11-352M15.2	1.796813	1.516748	1.88785	1.71815	0.7809	4.41E-05	0.000822
XAGE2	1.153118	1.540602	1.027164	0.05025	-4.3148	4.53E-05	0.000785
LINC01021	3.038041	2.403067	3.244443	1.59967	0.6778	4.62E-05	0.000664
ZFP36L1	9.161817	7.798005	9.605133	1.26583	0.3401	4.68E-05	0.000868

C16orf45	2.811332	2.37237	2.95402	1.42383	0.5098	4.69E-05	0.000908
SLC25A3	11.52922	9.996386	12.02748	1.22577	0.2937	4.71E-05	0.000862
TRIOBP	2.080723	2.387444	1.981021	0.70707	-0.5001	4.75E-05	0.000868
PHACTR2	1.48517	1.746116	1.400348	0.53658	-0.8981	4.77E-05	0.001065
LSM3	3.510974	3.007003	3.674793	1.33273	0.4144	4.78E-05	0.000849
CASZ1	1.273671	1.040221	1.349556	8.69085	3.1195	4.82E-05	0.000849
RRM1	1.61696	1.353963	1.702449	1.98453	0.9888	4.85E-05	0.001227
TPM2	7.765399	9.203788	7.297842	0.76767	-0.3814	4.88E-05	0.001227
PEA15	1.93191	2.229334	1.83523	0.67942	-0.5576	4.88E-05	0.000832
AASDHPPT	2.257907	1.875945	2.382066	1.5778	0.6579	4.89E-05	0.000848
PTGR1	2.070553	1.76863	2.168696	1.52049	0.6045	4.91E-05	0.000969
HMGXB4	1.997136	1.689327	2.097192	1.59169	0.6706	4.98E-05	0.000944
INSIG1	1.588453	1.963054	1.466686	0.48459	-1.0452	5.02E-05	0.000808
COPS9	4.789348	5.501026	4.558012	0.79049	-0.3392	5.02E-05	0.000902
RAB18	1.846507	2.1365	1.752243	0.66189	-0.5953	5.05E-05	0.000875
GNL3	2.022093	1.723585	2.119125	1.54664	0.6291	5.06E-05	0.000808
SLC24A5	1.12541	1.354356	1.050989	0.14389	-2.7969	5.07E-05	0.000718
PARK7	7.271528	6.083423	7.657729	1.30969	0.3892	5.12E-05	0.001257
SLC7A5	1.197416	1.432475	1.121009	0.27981	-1.8375	5.18E-05	0.000983
AP1S1	2.965445	2.552317	3.099735	1.35265	0.4358	5.19E-05	0.000915
MT1E	1.309428	1.726893	1.173728	0.239	-2.0649	5.23E-05	0.001282
SORCS1	1.449922	1.201893	1.530546	2.62786	1.3939	5.23E-05	0.001013
CEP85L	2.230358	1.861633	2.350214	1.56704	0.648	5.31E-05	0.000988
PSAT1	2.218917	2.6402	2.081977	0.65966	-0.6002	5.35E-05	0.00128
TRIM59	1.38304	1.141987	1.461396	3.24957	1.7002	5.45E-05	0.000978
JAK1	1.834135	2.121321	1.740783	0.66063	-0.5981	5.46E-05	0.000763
KRT10	4.290364	4.997386	4.060542	0.76564	-0.3853	5.52E-05	0.000925
ARL6IP5	3.495057	4.07555	3.306365	0.7499	-0.4152	5.56E-05	0.001055
DBI	4.75042	4.1374	4.949686	1.2589	0.3322	5.58E-05	0.000985
TRIM36	1.245222	1.482963	1.167942	0.34773	-1.524	5.58E-05	0.000769
TXN2	2.975295	2.577761	3.104516	1.33386	0.4156	5.68E-05	0.001216
EIF4A2	11.48243	9.737251	12.04972	1.26467	0.3388	5.81E-05	0.001003
AFF4	2.180616	2.509534	2.073699	0.71128	-0.4915	5.82E-05	0.001105
N4BP2	2.054718	1.759118	2.150805	1.51598	0.6002	5.89E-05	0.001003
REEP3	1.735074	2.014163	1.644354	0.63536	-0.6544	5.98E-05	0.000986
URI1	1.715237	1.446985	1.802434	1.79521	0.8442	5.99E-05	0.001038
SUMO2	16.926	13.25656	18.11878	1.3967	0.482	6.00E-05	0.001023
DDA1	1.643556	1.916384	1.554872	0.6055	-0.7238	6.01E-05	0.001041
LOX	1.414737	1.666053	1.333045	0.50003	-0.9999	6.05E-05	0.001128
HDAC2	4.12933	3.536252	4.322114	1.30985	0.3894	6.14E-05	0.001046
LSM10	2.299874	2.70475	2.168267	0.6853	-0.5452	6.20E-05	0.001038
RP11-792A8.4	1.706763	1.439511	1.793634	1.80572	0.8526	6.25E-05	0.001497
UBE2E2	1.721205	1.997522	1.631386	0.63295	-0.6598	6.27E-05	0.000956
MSANTD4	1.908852	1.626073	2.000772	1.59849	0.6767	6.29E-05	0.001151
RASGEF1B	1.376128	1.136826	1.453915	3.31746	1.7301	6.36E-05	0.001185

GLRX5	2.48861	2.149948	2.598694	1.39023	0.4753	6.38E-05	0.001493
NDUFAF3	3.476137	3.994111	3.307766	0.77077	-0.3756	6.41E-05	0.000872
TM9SF3	2.137063	2.443469	2.037464	0.71873	-0.4765	6.48E-05	0.001357
ELF3	1.138103	1.393431	1.055107	0.14007	-2.8358	6.57E-05	0.001202
CSRP1	1.361628	1.607016	1.281863	0.46434	-1.1067	6.59E-05	0.001163
PLXNB2	1.680471	1.952693	1.591984	0.62138	-0.6865	6.59E-05	0.001542
WDR34	1.838453	1.561749	1.928398	1.65269	0.7248	6.62E-05	0.001109
CALM1	4.239642	4.9503	4.008638	0.76162	-0.3929	6.62E-05	0.001211
TMEM179B	1.98086	2.27567	1.88503	0.69378	-0.5275	6.69E-05	0.001121
ANKRD45	4.163238	3.485766	4.383454	1.36113	0.4448	6.69E-05	0.001085
TTF2	1.507091	1.256921	1.588411	2.29024	1.1955	6.81E-05	0.000913
MYCN	1.330042	1.096601	1.405923	4.20205	2.0711	6.85E-05	0.001496
LINC00467	1.876758	1.594191	1.968608	1.63013	0.705	6.89E-05	0.001135
BCLAF1	2.924645	2.524225	3.054804	1.3481	0.4309	6.90E-05	0.001137
MXD3	1.344383	1.10985	1.42062	3.82905	1.937	6.93E-05	0.001292
DEPDC1	1.291801	1.0624	1.366369	5.87132	2.5537	6.97E-05	0.001096
PLOD3	1.320722	1.561809	1.242355	0.43138	-1.213	6.99E-05	0.001237
RBM23	2.157095	1.85808	2.254291	1.46174	0.5477	7.01E-05	0.001237
MGARP	4.148376	3.368621	4.40184	1.43621	0.5223	7.14E-05	0.001465
EGLN3	1.581748	1.975299	1.453821	0.46532	-1.1037	7.24E-05	0.001174
MIS18A	1.514514	1.265883	1.595334	2.23908	1.1629	7.25E-05	0.001302
GABRP	1.135325	1.496014	1.018081	0.03645	-4.7778	7.27E-05	0.001261
BMPR2	1.558763	1.819487	1.474014	0.57843	-0.7898	7.34E-05	0.001251
PIH1D1	2.80989	2.358475	2.956625	1.44031	0.5264	7.35E-05	0.001138
CCDC66	2.391932	2.055755	2.501208	1.42193	0.5078	7.42E-05	0.001263
HIF1A	2.519207	2.921578	2.388414	0.72254	-0.4689	7.47E-05	0.001697
XRCC6	3.086588	2.63798	3.232411	1.36291	0.4467	7.48E-05	0.001122
UNG	1.472023	1.226855	1.551716	2.43202	1.2822	7.50E-05	0.001715
C15orf40	1.852106	1.577319	1.941427	1.63069	0.7055	7.59E-05	0.001697
NOG	1.102033	1.322673	1.030313	0.09394	-3.4121	7.65E-05	0.0014
FKBP3	3.038267	2.487859	3.21718	1.49018	0.5755	7.82E-05	0.001248
GLIPR2	1.432192	1.681474	1.351161	0.5153	-0.9565	7.83E-05	0.00175
FLJ37453	1.539097	1.289407	1.62026	2.14321	1.0998	7.84E-05	0.001714
BCO2	1.416944	1.177235	1.494863	2.79212	1.4814	7.87E-05	0.001317
NEK2	1.26442	1.036794	1.338411	9.1975	3.2012	7.87E-05	0.00144
EID1	6.408729	5.494821	6.7058	1.26942	0.3442	7.88E-05	0.001165
PPP1R14C	1.234199	1.46591	1.15888	0.34101	-1.5521	7.90E-05	0.001301
PXN-AS1	1.390822	1.153601	1.467932	3.0464	1.6071	7.90E-05	0.001395
ARHGAP11B	1.288454	1.060609	1.362516	5.9812	2.5804	7.97E-05	0.001457
ACTA2	2.435814	3.11589	2.214751	0.57411	-0.8006	8.03E-05	0.001443
TSHZ2	1.266568	1.501129	1.190322	0.37979	-1.3967	8.04E-05	0.00137
TEKT2	1.302268	1.072932	1.376815	5.16669	2.3692	8.04E-05	0.001225
TLN1	1.946783	2.235873	1.852813	0.69005	-0.5352	8.05E-05	0.001285
KLHDC8B	2.550171	2.166943	2.674741	1.43515	0.5212	8.17E-05	0.001227
PNISR	7.209827	6.121797	7.563498	1.28148	0.3578	8.19E-05	0.001079

CDCA5	1.315786	1.086069	1.390456	4.53656	2.1816	8.22E-05	0.001797
FRMD4A	1.834032	1.561128	1.922742	1.64444	0.7176	8.26E-05	0.001079
TNNT1	1.509181	1.763632	1.42647	0.55848	-0.8404	8.33E-05	0.001782
PRELP	1.268638	1.042588	1.342117	8.03319	3.006	8.35E-05	0.001311
SMC1A	1.818753	1.548337	1.906654	1.65346	0.7255	8.39E-05	0.001506
HSPH1	1.925474	2.230551	1.826307	0.67149	-0.5746	8.40E-05	0.001408
HSD17B14	1.467334	1.718425	1.385715	0.53689	-0.8973	8.41E-05	0.001385
ELOVL5	1.65133	1.917634	1.564766	0.61546	-0.7003	8.48E-05	0.001375
ENO2	3.114946	3.63785	2.944973	0.73733	-0.4396	8.48E-05	0.001985
NR2F1-AS1	1.783175	1.514365	1.870553	1.69248	0.7591	8.58E-05	0.001415
DCX	1.277608	1.03651	1.355979	9.75026	3.2854	8.69E-05	0.001859
PTGES3	7.260695	5.700752	7.767765	1.43972	0.5258	8.72E-05	0.001369
SOX6	1.164454	1.388932	1.091486	0.23522	-2.0879	8.80E-05	0.001428
FAM215B	1.551547	1.302445	1.632519	2.09136	1.0644	8.88E-05	0.001786
HP1BP3	3.070319	2.611466	3.219472	1.3773	0.4618	8.93E-05	0.001605
ZWINT	1.424659	1.186301	1.502139	2.6953	1.4304	8.96E-05	0.001156
SUCO	2.024127	2.338312	1.921999	0.68893	-0.5376	8.98E-05	0.001881
TOP2B	2.364573	2.030652	2.473116	1.42931	0.5153	9.08E-05	0.00163
GDF15	1.178194	1.494876	1.075254	0.15207	-2.7172	9.16E-05	0.001417
LASP1	1.798952	2.074196	1.709482	0.66048	-0.5984	9.27E-05	0.001481
LCA5	1.661844	1.404669	1.745441	1.8421	0.8814	9.30E-05	0.00164
MYL6	25.95646	29.00372	24.96592	0.85581	-0.2246	9.38E-05	0.001388
HK2	1.772459	2.181264	1.639575	0.54143	-0.8851	9.39E-05	0.001658
TMED7	1.750538	2.02251	1.662132	0.64756	-0.6269	9.56E-05	0.001658
CREB3L4	1.405488	1.169886	1.482072	2.83762	1.5047	9.89E-05	0.001603
KNSTRN	1.386502	1.153539	1.462229	3.0105	1.59	9.90E-05	0.001717
EMP3	3.725889	4.334438	3.528076	0.75817	-0.3994	0.000101	0.00231
CD53	1.101758	1.318003	1.031466	0.09895	-3.3372	0.000102	0.00176
OSTM1	1.240107	1.46854	1.165853	0.35398	-1.4983	0.000102	0.001705
C8orf33	2.673311	2.318223	2.788735	1.35693	0.4403	0.000102	0.001652
LSM8	2.737985	2.343705	2.866149	1.38881	0.4738	0.000102	0.001766
ANK3	1.458626	1.706728	1.377979	0.53483	-0.9028	0.000103	0.001822
MIR325HG	1.309855	1.083215	1.383526	4.60885	2.2044	0.000104	0.001766
CHCHD10	2.500482	2.869535	2.380519	0.73843	-0.4375	0.000104	0.001657
PXDN	2.198098	2.525458	2.091687	0.71565	-0.4827	0.000104	0.001608
PCNP	3.817413	3.213063	4.013861	1.36185	0.4456	0.000105	0.001645
BCAP31	2.693011	3.092943	2.56301	0.7468	-0.4212	0.000106	0.001346
CNN1	1.319785	1.577738	1.235936	0.40838	-1.292	0.000106	0.001691
SLC45A2	1.097295	1.312332	1.027396	0.08771	-3.5111	0.000107	0.001557
DNAJC12	1.448655	1.211587	1.525715	2.48463	1.313	0.000108	0.002092
EHMT2	2.015306	1.734268	2.10666	1.50716	0.5918	0.000108	0.002092
CTDSP2	1.969345	1.665442	2.068131	1.60515	0.6827	0.000109	0.002274
JUNB	2.408357	2.992761	2.218393	0.61141	-0.7098	0.000109	0.001925
GGH	2.171969	1.875517	2.268332	1.44867	0.5347	0.00011	0.001733
RSRC2	3.363428	2.910599	3.510622	1.31405	0.394	0.00011	0.001733

CRYZL1	1.997618	1.703661	2.093171	1.55355	0.6356	0.000111	0.001866
PAXIP1-AS1	1.765406	1.503694	1.850477	1.68848	0.7557	0.000111	0.001866
SFN	1.106073	1.373516	1.019138	0.05124	-4.2866	0.000112	0.002495
PCMTD1	2.190113	1.889365	2.287873	1.44808	0.5341	0.000112	0.001633
BAMBI	1.465206	1.710495	1.385473	0.54254	-0.8822	0.000117	0.001836
MDK	26.78933	23.43888	27.87841	1.19785	0.2604	0.00012	0.002087
PTGFRN	1.379577	1.61881	1.301813	0.48773	-1.0358	0.000121	0.002093
MRPL9	2.089683	1.803514	2.182705	1.47191	0.5577	0.000122	0.002075
NIN	1.489293	1.250142	1.567031	2.26684	1.1807	0.000122	0.002011
ANKRD37	1.595173	1.998297	1.464134	0.46493	-1.1049	0.000122	0.00251
SNRPA1	1.937954	1.666301	2.026256	1.54023	0.6231	0.000125	0.001926
TMEM123	3.370602	2.761129	3.568715	1.45856	0.5445	0.000126	0.001924
JPX	2.349684	2.021815	2.45626	1.42517	0.5111	0.000127	0.001827
ZIC1	1.400318	1.169678	1.475289	2.80113	1.486	0.000128	0.002073
PNRC1	4.313799	3.771903	4.489945	1.25904	0.3323	0.000128	0.002118
PLCD3	1.305012	1.535803	1.229992	0.42925	-1.2201	0.00013	0.002018
LIMS1	1.905728	2.202064	1.809402	0.67334	-0.5706	0.000131	0.002192
RNPS1	4.042944	3.47655	4.227054	1.30304	0.3819	0.000132	0.002251
IER5L	1.362155	1.597878	1.285531	0.47757	-1.0662	0.000132	0.001964
TK1	1.366357	1.138766	1.440336	3.17322	1.6659	0.000133	0.001964
LAMB1	1.201358	1.422059	1.129618	0.30711	-1.7032	0.000134	0.002132
CADM2	1.606168	1.358223	1.686764	1.91714	0.939	0.000136	0.002097
GLTSCR2	5.338254	4.521923	5.603608	1.30713	0.3864	0.000136	0.002077
MCRIP1	4.132633	3.519038	4.332086	1.32276	0.4036	0.000137	0.002085
PCDH11X	1.281966	1.061579	1.353604	5.74225	2.5216	0.00014	0.002305
NCBP2	3.096237	2.609481	3.25446	1.40074	0.4862	0.000141	0.002401
IFT81	1.923186	1.65315	2.010963	1.54783	0.6302	0.000143	0.001794
ADAMTS6	1.62607	1.377812	1.706768	1.87069	0.9036	0.000144	0.002412
DESI2	1.790622	2.05815	1.70366	0.66499	-0.5886	0.000145	0.002274
OXTR	1.124816	1.347242	1.052515	0.15123	-2.7251	0.000146	0.002453
ADSS	1.856929	2.128642	1.768607	0.681	-0.5543	0.00015	0.002361
MRPL45	1.855225	1.591392	1.940986	1.59114	0.6701	0.000151	0.002444
EPB41L4A-AS1	2.955241	2.506366	3.101151	1.39485	0.4801	0.000153	0.002516
FERMT2	1.423317	1.661492	1.345896	0.5229	-0.9354	0.000153	0.002444
LTBP1	1.299329	1.52744	1.22518	0.42693	-1.2279	0.000156	0.003337
KLF5	1.149641	1.363483	1.08013	0.22045	-2.1815	0.000156	0.002412
GPBP1	3.976388	3.423489	4.156111	1.3023	0.3811	0.000158	0.003192
ABCC5	1.272199	1.497535	1.198952	0.39988	-1.3224	0.000158	0.002606
NME7	1.687369	1.437037	1.768742	1.75898	0.8147	0.000159	0.003192
CRACR2B	2.529183	2.17522	2.644241	1.39909	0.4845	0.00016	0.002772
GAL	2.571026	1.868036	2.799537	2.07311	1.0518	0.000161	0.002485
CNOT10	1.456637	1.224529	1.532085	2.36978	1.2448	0.000161	0.002278
HIF3A	1.619862	1.872629	1.537699	0.61618	-0.6986	0.000161	0.003376
SLC5A3	1.956265	2.259094	1.857828	0.68131	-0.5536	0.000162	0.003253
CXCL16	1.105925	1.315848	1.037689	0.11933	-3.067	0.000164	0.002615

KCNG1	1.11787	1.357558	1.039958	0.11175	-3.1616	0.000164	0.002615
POGZ	2.259089	1.960323	2.356205	1.41224	0.498	0.000164	0.00275
THOC1	1.505612	1.269249	1.582443	2.16322	1.1132	0.000164	0.002501
LINC01089	1.638573	1.391606	1.718851	1.83565	0.8763	0.000164	0.002669
RBP7	1.141021	1.353641	1.071907	0.20333	-2.2981	0.000165	0.002807
MARCH6	2.621815	2.238663	2.746362	1.40988	0.4956	0.000166	0.002326
ADSL	2.133707	1.853652	2.22474	1.43471	0.5208	0.000168	0.003184
OIP5	1.289661	1.072249	1.360332	4.98739	2.3183	0.00017	0.00248
CCT4	4.55623	3.919143	4.763319	1.28919	0.3665	0.00017	0.002808
SLC35D3	1.095811	1.30437	1.028017	0.09205	-3.4415	0.00017	0.002904
SEMA6A	1.592892	1.350387	1.671719	1.91708	0.9389	0.000171	0.002456
ATP5O	7.041199	6.015133	7.374729	1.2711	0.3461	0.000172	0.002573
PXMP2	1.964738	1.695787	2.052162	1.51219	0.5966	0.000174	0.00282
PAWR	2.001097	2.279649	1.910552	0.71156	-0.4909	0.000174	0.002579
NUP37	1.495002	1.260811	1.571127	2.18981	1.1308	0.000175	0.002702
GAMT	2.636264	2.300614	2.745369	1.34196	0.4243	0.000175	0.002702
VPS28	3.247567	2.817507	3.387361	1.31354	0.3935	0.000175	0.002637
SMARCA5	2.69942	2.340565	2.816068	1.3547	0.438	0.000176	0.002637
STOM	1.206846	1.424372	1.136138	0.3208	-1.6403	0.000176	0.002427
INPP1	1.28691	1.511712	1.213837	0.41789	-1.2588	0.000179	0.002225
YTHDC1	2.091608	1.809108	2.183437	1.46264	0.5486	0.000181	0.003712
SRRM1	3.783274	3.269274	3.950353	1.30013	0.3787	0.000184	0.002931
FAM175A	1.820194	1.562068	1.9041	1.60852	0.6857	0.000184	0.00308
TNFRSF19	1.141329	1.35223	1.072775	0.20661	-2.275	0.000186	0.002286
CCDC6	1.618711	1.868836	1.537407	0.61854	-0.6931	0.000188	0.002857
CLDN7	1.112284	1.3931	1.021004	0.05343	-4.2262	0.000188	0.003776
TCN2	1.562697	1.325335	1.639853	1.96675	0.9758	0.000191	0.002869
PTK7	1.569789	1.816017	1.489751	0.60017	-0.7366	0.000192	0.003022
DYNLRB1	4.094591	4.716621	3.892397	0.77823	-0.3617	0.000192	0.002885
GPRC5A	1.120085	1.329217	1.052105	0.15827	-2.6595	0.000193	0.003187
FURIN	1.260142	1.481075	1.188326	0.39147	-1.353	0.000193	0.002946
AKR1A1	3.350424	2.909781	3.493658	1.30573	0.3849	0.000194	0.00383
RSU1	1.540676	1.783358	1.46179	0.5895	-0.7624	0.000197	0.002953
PIM3	1.412216	1.659782	1.331743	0.50281	-0.9919	0.000198	0.003903
ECSIT	1.673961	1.427727	1.754001	1.76281	0.8179	0.000199	0.002699
AAK1	1.743187	2.001739	1.659143	0.658	-0.6038	0.000199	0.003146
FGFR3	1.142739	1.353545	1.074215	0.20992	-2.2521	0.0002	0.002956
BCL2L1	1.438272	1.673425	1.361834	0.5373	-0.8962	0.0002	0.003146
UBAC2	2.077328	1.800393	2.167347	1.45847	0.5445	0.000201	0.002933
GALE	1.61626	1.86531	1.535304	0.61863	-0.6929	0.000202	0.003075
P4HA1	3.234907	3.702421	3.082939	0.77077	-0.3756	0.000203	0.002999
TRA2B	2.749407	2.38065	2.869274	1.35391	0.4371	0.000207	0.003469
PCM1	3.163194	2.738893	3.301116	1.32332	0.4042	0.000208	0.002519
RP11-12G12.7	1.479297	1.248509	1.554315	2.23056	1.1574	0.000209	0.003384
UBTD1	1.383735	1.613914	1.308914	0.50319	-0.9908	0.000209	0.003384

EIF3J-AS1	2.015174	1.7212	2.110732	1.54012	0.623	0.00021	0.003009
TUBB2B	10.95661	9.464872	11.44151	1.23351	0.3028	0.00021	0.003099
FRMD4B	1.85642	1.597806	1.940483	1.57322	0.6537	0.000211	0.003099
AKR1B1	2.707497	2.338179	2.827546	1.3657	0.4496	0.000212	0.003001
CYB5R3	2.746757	3.200075	2.599403	0.72698	-0.46	0.000213	0.004111
ZMAT3	2.277763	2.608467	2.170266	0.72757	-0.4588	0.000213	0.003099
WASF3	1.718269	1.468893	1.79933	1.70472	0.7695	0.000215	0.002579
SERINC2	2.223447	2.531704	2.123246	0.73333	-0.4475	0.000218	0.003041
CNTRL	1.355006	1.135087	1.426492	3.15718	1.6586	0.000221	0.003173
CDC42EP3	1.80594	2.080752	1.716611	0.66307	-0.5928	0.000221	0.002967
SPC24	1.251418	1.040621	1.319939	7.87628	2.9775	0.000223	0.003566
AGRN	1.482189	1.718292	1.405442	0.56445	-0.8251	0.000227	0.003301
AKR7A2	2.591061	2.198608	2.71863	1.43385	0.5199	0.000228	0.003373
RNF24	1.594313	1.356638	1.671571	1.88306	0.9131	0.000229	0.004337
ERI3	2.349434	2.05495	2.445159	1.36988	0.4541	0.000229	0.004435
ASPHD1	1.134824	1.342153	1.06743	0.19708	-2.3432	0.000231	0.003466
MAL2	1.113643	1.319282	1.046798	0.14657	-2.7703	0.000232	0.003201
ZNHIT3	2.545765	2.188718	2.661825	1.398	0.4834	0.000233	0.003085
MYO10	1.504001	1.74172	1.426729	0.57532	-0.7976	0.000233	0.003609
SRI	4.203528	3.55089	4.415672	1.33901	0.4212	0.000234	0.004353
MTERF2	1.527362	1.294931	1.602916	2.04426	1.0316	0.000236	0.003713
LOXL1	2.654407	3.040524	2.528897	0.74927	-0.4164	0.000237	0.003223
CNTFR	1.631633	1.392135	1.709483	1.80928	0.8554	0.000237	0.003409
MCM4	1.369111	1.149611	1.440461	2.94405	1.5578	0.000239	0.004719
ID2	4.157657	3.26335	4.448357	1.52356	0.6074	0.000242	0.004405
NT5C	2.395609	2.094786	2.493394	1.3641	0.4479	0.000242	0.003477
PPP1R3E	1.513162	1.2829	1.588011	2.07851	1.0555	0.000245	0.004405
EGFL6	1.12902	1.391708	1.043632	0.11139	-3.1663	0.000246	0.003484
UBL7-AS1	1.391089	1.169722	1.463045	2.72826	1.448	0.000247	0.003643
ARHGAP11A	1.308727	1.094777	1.378273	3.99117	1.9968	0.000247	0.002918
RAB11A	3.832299	3.321424	3.998362	1.2916	0.3692	0.000248	0.003785
EMC6	2.846796	3.234268	2.720846	0.77021	-0.3767	0.000248	0.003516
IDI1	1.991882	2.38241	1.864938	0.62567	-0.6765	0.000249	0.005443
CCDC14	2.457452	2.077446	2.580976	1.46734	0.5532	0.000251	0.00443
RDH11	1.616997	1.861937	1.537377	0.62345	-0.6816	0.000252	0.003522
ATAD2	1.408375	1.187378	1.480211	2.5628	1.3577	0.000253	0.003744
ZNF423	1.322515	1.107814	1.392305	3.63872	1.8634	0.000256	0.004016
CHEK1	1.406281	1.184497	1.478373	2.59285	1.3745	0.000256	0.004876
ELFN1	1.376197	1.157177	1.44739	2.84641	1.5091	0.000257	0.003872
AGO3	1.715911	1.470624	1.795644	1.69061	0.7575	0.000257	0.004876
PTTG1IP	3.445178	3.914944	3.292478	0.78646	-0.3466	0.000257	0.003872
GRINA	2.510134	2.864777	2.394855	0.748	-0.4189	0.000258	0.003872
RP11-111M22.3	1.435027	1.211718	1.507616	2.39761	1.2616	0.000259	0.003778
LRRFIP1	1.673087	1.920963	1.592514	0.64336	-0.6363	0.000261	0.004957
S100A13	6.826461	7.875993	6.485305	0.79775	-0.326	0.000262	0.005603

SUCLG2	1.618258	1.380881	1.695419	1.82581	0.8685	0.000263	0.0045
AHI1	2.126855	1.840924	2.219798	1.45055	0.5366	0.000264	0.003839
KLHDC3	2.538234	2.202826	2.64726	1.36949	0.4536	0.000264	0.004348
KLHL5	1.367069	1.591581	1.29409	0.49713	-1.0083	0.000264	0.0045
UPF3B	1.736867	1.49091	1.816817	1.66388	0.7346	0.000266	0.003706
FKBP7	1.746773	1.501216	1.826593	1.64917	0.7217	0.000269	0.00392
LGMN	1.577834	1.819476	1.499286	0.60927	-0.7148	0.000269	0.003984
AGGF1	1.972935	1.71042	2.058268	1.48964	0.575	0.00027	0.003826
F2R	1.643306	1.887911	1.563795	0.63497	-0.6552	0.000271	0.004076
ARL4D	1.433638	1.662426	1.359269	0.54235	-0.8827	0.000271	0.003642
SOX11	3.338599	2.891029	3.484085	1.31362	0.3935	0.000276	0.005139
LRCH2	1.301223	1.519116	1.230396	0.44382	-1.1719	0.000281	0.003677
ROBO1	1.300003	1.518781	1.228888	0.4412	-1.1805	0.000285	0.00404
LRRIQ1	1.353879	1.137648	1.424167	3.08154	1.6237	0.000285	0.00404
SSB	3.704228	3.218192	3.862217	1.29034	0.3677	0.000285	0.004221
ANOS1	1.332669	1.553691	1.260824	0.47106	-1.086	0.000288	0.004824
PPIA	17.7728	15.78192	18.41996	1.17846	0.2369	0.000288	0.004199
EPB41L3	1.625299	1.868215	1.546337	0.62926	-0.6683	0.000289	0.00539
SLC25A11	2.199684	2.483951	2.107281	0.74617	-0.4224	0.000292	0.004074
HDHD2	1.632562	1.395862	1.709503	1.7923	0.8418	0.000299	0.004856
FOXM1	1.24903	1.043423	1.315864	7.2741	2.8628	0.0003	0.004376
RHOF	1.34164	1.561922	1.270036	0.48056	-1.0572	0.000304	0.004189
GINM1	1.961393	2.229844	1.874131	0.71077	-0.4926	0.000305	0.003939
CDH20	1.353693	1.139696	1.423254	3.02982	1.5992	0.000315	0.004167
CENPX	3.038699	2.620748	3.174557	1.3417	0.4241	0.000316	0.004884
TMEM106C	3.178577	2.680244	3.340564	1.39299	0.4782	0.000316	0.004027
TRAPPC6A	1.803347	1.556077	1.883724	1.58921	0.6683	0.000319	0.006678
GNG3	1.456114	1.233681	1.528418	2.26128	1.1771	0.000319	0.00437
RP11-660L16.2	1.428203	1.208186	1.49972	2.40035	1.2632	0.00032	0.004606
PITPNA-AS1	1.646171	1.40983	1.722995	1.76413	0.819	0.000322	0.00437
TAGLN3	1.408161	1.17321	1.484533	2.79738	1.4841	0.000325	0.004947
POLR3GL	2.391725	2.080029	2.493044	1.38241	0.4672	0.000328	0.004391
GNB1	2.449082	2.75733	2.348885	0.76758	-0.3816	0.000328	0.004455
NUP107	1.514136	1.288434	1.587502	2.03687	1.0264	0.000329	0.006016
PRKAA2	1.745284	1.501346	1.824577	1.64473	0.7178	0.000331	0.004753
PDE4DIP	1.345921	1.565483	1.274551	0.48552	-1.0424	0.000332	0.006184
MAPK10	1.443274	1.22283	1.514931	2.31087	1.2084	0.000333	0.005486
MAT2A	2.218007	1.887285	2.32551	1.49389	0.5791	0.000335	0.004739
TRIB1	1.368576	1.588572	1.297064	0.50472	-0.9864	0.000338	0.004469
ATP6V1E1	1.673345	1.916135	1.594424	0.64884	-0.6241	0.000342	0.006151
BUB3	2.192442	1.915377	2.282504	1.40107	0.4865	0.000344	0.004798
BCL2L13	1.608633	1.848265	1.530739	0.62568	-0.6765	0.000344	0.005498
PA2G4	3.574406	3.115308	3.723639	1.28759	0.3647	0.000344	0.006299
RND3	2.046009	2.372021	1.940036	0.68515	-0.5455	0.000344	0.00495
PTBP3	1.382258	1.604614	1.30998	0.51269	-0.9638	0.000348	0.004861

RP11-89K21.1	2.231777	1.932502	2.329058	1.42526	0.5112	0.000348	0.004861
NKTR	3.430805	2.989906	3.574122	1.29359	0.3714	0.00035	0.004096
CCDC107	2.119633	2.452448	2.01145	0.69638	-0.5221	0.000353	0.005468
CDIPT	2.010671	2.295211	1.91818	0.7089	-0.4963	0.000355	0.007298
IQGAP1	1.623973	1.863078	1.54625	0.63291	-0.6599	0.000362	0.004985
FDPS	2.769104	2.404354	2.887668	1.34415	0.4267	0.000366	0.004776
CHD9	3.062611	2.676051	3.188265	1.30561	0.3847	0.000368	0.005359
EFEMP1	1.198578	1.403931	1.131827	0.32636	-1.6155	0.000369	0.006509
PRR13	2.954721	3.367435	2.820566	0.769	-0.3789	0.000371	0.005574
BIN1	2.384843	2.083247	2.482878	1.36892	0.453	0.000372	0.005122
GPRC5C	1.247889	1.457126	1.179875	0.39349	-1.3456	0.000373	0.005356
PLXND1	1.272373	1.483728	1.20367	0.42104	-1.248	0.000377	0.00581
FOXP1	2.810923	2.440529	2.931322	1.3407	0.423	0.000378	0.004875
SCAF11	3.00349	2.588811	3.138283	1.34584	0.4285	0.000378	0.005932
ISG15	1.474337	1.762246	1.38075	0.49951	-1.0014	0.000379	0.005359
RHBDD2	2.267251	2.561995	2.171443	0.74997	-0.4151	0.000379	0.005932
C4orf3	10.7017	9.248247	11.17416	1.23349	0.3027	0.000379	0.004948
TMEM80	1.464863	1.244663	1.53644	2.19257	1.1326	0.000379	0.00581
CHRAC1	1.664962	1.430401	1.741207	1.72213	0.7842	0.00038	0.005168
HLA-E	1.777905	2.026924	1.69696	0.67869	-0.5592	0.000381	0.00581
VDAC3	2.738103	2.368945	2.8581	1.35732	0.4408	0.000383	0.005932
MRPL17	2.127575	2.407805	2.036484	0.73624	-0.4417	0.000388	0.004485
OTUB1	2.242688	1.960386	2.334453	1.3895	0.4746	0.000391	0.007144
CCNI	12.57524	11.10306	13.05378	1.19308	0.2547	0.000394	0.005505
BHLHE41	1.218756	1.426123	1.151351	0.35518	-1.4934	0.000401	0.005678
GSTM3	2.183721	1.907265	2.273585	1.40376	0.4893	0.000401	0.007201
GTF2H2	1.844063	1.597719	1.924139	1.54611	0.6286	0.000404	0.005803
CA3	2.078619	1.69818	2.202284	1.72203	0.7841	0.000405	0.007022
DLC1	1.103554	1.299206	1.039956	0.13354	-2.9047	0.000409	0.005798
PPIL1	1.766956	1.527059	1.844936	1.60311	0.6809	0.000414	0.005866
FAM92A	2.486443	2.161943	2.591923	1.37005	0.4542	0.000417	0.004766
EMP2	1.432008	1.654638	1.359641	0.54937	-0.8641	0.000417	0.005826
RAB25	1.092404	1.33299	1.014199	0.04264	-4.5516	0.000421	0.007504
STAG2	2.174861	1.902268	2.263469	1.40033	0.4858	0.000424	0.006458
MCM3	1.582215	1.356117	1.655709	1.84127	0.8807	0.000427	0.005805
CENPW	1.818257	1.57174	1.898389	1.57133	0.652	0.000428	0.00538
SERINC1	1.835676	2.086394	1.754178	0.6942	-0.5266	0.000429	0.005904
CXXC4	1.598108	1.370304	1.672157	1.81515	0.8601	0.000432	0.007504
SNF8	3.095103	3.487659	2.9675	0.7909	-0.3384	0.000433	0.007504
UBXN11	1.43678	1.221089	1.506892	2.29271	1.1971	0.000433	0.006048
DPYSL2	3.183122	2.793195	3.30987	1.28813	0.3653	0.000434	0.005819
ZDHHC3	1.556712	1.78817	1.481474	0.61088	-0.711	0.000436	0.006088
C17orf58	1.517514	1.296166	1.589464	1.99032	0.993	0.000438	0.00603
MT2A	4.513595	6.502522	3.867081	0.52105	-0.9405	0.000443	0.006103
LINC00116	1.882282	2.134545	1.800282	0.70538	-0.5035	0.000451	0.007693

MMS22L	1.429893	1.214747	1.499827	2.32751	1.2188	0.000452	0.005107
CEP57	2.221574	1.898053	2.326737	1.47735	0.563	0.000452	0.006137
B4GALT7	1.725302	1.967227	1.646663	0.66857	-0.5808	0.000462	0.006449
NDC80	1.230363	1.032413	1.294707	9.09217	3.1846	0.000464	0.006159
CDH2	3.315289	3.748543	3.174457	0.79113	-0.338	0.000464	0.005979
ACTR3B	1.684223	1.451461	1.759884	1.68317	0.7512	0.000465	0.006159
TSC22D3	2.000968	2.27696	1.911255	0.71361	-0.4868	0.00047	0.007047
KPNB1	4.024924	3.476221	4.203283	1.29362	0.3714	0.000475	0.006544
CAP2	1.366255	1.581323	1.296345	0.50978	-0.9721	0.000476	0.005324
CREB3	1.744151	1.98682	1.66527	0.67416	-0.5688	0.000476	0.007047
STAT3	1.830293	1.588947	1.908744	1.543	0.6257	0.000479	0.008175
GNS	1.463602	1.686125	1.39127	0.57026	-0.8103	0.000481	0.00967
TNFAIP1	1.511258	1.736829	1.437935	0.59435	-0.7506	0.000481	0.005975
PSMD2	2.260883	2.587888	2.154588	0.72712	-0.4597	0.000482	0.006525
DICER1	2.053794	2.317134	1.968194	0.73508	-0.444	0.000483	0.006389
MICA	1.296146	1.506025	1.227924	0.45042	-1.1507	0.000485	0.006496
KLHL23	1.839893	1.597852	1.91857	1.53645	0.6196	0.000487	0.006525
EPPK1	1.128416	1.348149	1.056991	0.1637	-2.6109	0.000494	0.007315
EVL	1.863999	2.11454	1.78256	0.70214	-0.5102	0.000498	0.006506
RP11-146F11.1	1.335008	1.128991	1.401976	3.11631	1.6398	0.000498	0.007261
ANAPC16	5.984398	5.144673	6.257356	1.26846	0.3431	0.000506	0.007374
SFXN1	2.349156	2.057426	2.443984	1.36557	0.4495	0.000508	0.006557
MTDH	3.537494	3.958815	3.40054	0.81132	-0.3017	0.000509	0.008531
TAPBP	1.736323	1.976826	1.658145	0.67376	-0.5697	0.000511	0.006265
KNL1	1.240928	1.043508	1.305101	7.01256	2.8099	0.000515	0.006549
NFE2L1	1.708205	1.946258	1.630824	0.66665	-0.585	0.000529	0.007184
ABI2	2.954016	2.553009	3.084366	1.34215	0.4245	0.000539	0.010633
LINC01224	1.260081	1.061569	1.324609	5.27226	2.3984	0.00054	0.009529
HPCAL1	1.377391	1.590904	1.307987	0.52121	-0.9401	0.00054	0.007147
THY1	1.147803	1.3447	1.0838	0.24311	-2.0403	0.000544	0.008978
SHPRH	1.833256	1.591351	1.911888	1.54204	0.6248	0.000549	0.007256
VAV3	1.128276	1.321896	1.065339	0.20298	-2.3006	0.000551	0.007188
HEXB	1.629344	1.861292	1.553948	0.64316	-0.6368	0.000553	0.007223
RPH3AL	1.445368	1.233549	1.514221	2.20176	1.1387	0.000555	0.00746
KIF1C	1.394101	1.608468	1.32442	0.53317	-0.9073	0.000556	0.00746
UXT	3.787604	3.311077	3.942502	1.27322	0.3485	0.000557	0.007677
TEX2	1.164665	1.361235	1.100769	0.27896	-1.8419	0.000558	0.008026
MANF	3.32957	3.745011	3.194528	0.79946	-0.3229	0.000571	0.007563
CALM3	4.924643	4.26721	5.138345	1.26663	0.341	0.000573	0.009934
GNAS	6.258693	7.117131	5.979652	0.81405	-0.2968	0.000574	0.007695
ACYP1	1.95057	1.693098	2.034263	1.49223	0.5775	0.00058	0.011217
ALDH7A1	1.949781	1.702956	2.030013	1.46526	0.5512	0.000589	0.007791
LECT1	1.111331	1.303256	1.048944	0.1614	-2.6313	0.000593	0.011248
SIVA1	3.636409	3.058212	3.824355	1.37224	0.4565	0.000593	0.007187
UNC5B	1.266966	1.471722	1.200408	0.42484	-1.235	0.000602	0.007853

RNF165	1.433053	1.223354	1.501217	2.24404	1.1661	0.000602	0.008067
BORCS7	1.998337	1.749974	2.079069	1.43881	0.5249	0.000603	0.010108
SLC25A5	8.349455	7.142972	8.74163	1.26024	0.3337	0.000605	0.009228
PLEC	1.423432	1.638595	1.353492	0.55355	-0.8532	0.000606	0.007712
KIF23	1.248643	1.052976	1.312246	5.89407	2.5593	0.000606	0.007912
CSAD	1.578612	1.358187	1.650263	1.81543	0.8603	0.000606	0.008517
LGALS3	2.89546	3.411413	2.727747	0.71649	-0.481	0.00061	0.008517
FRMD6	1.170673	1.366594	1.106987	0.29184	-1.7768	0.000612	0.00677
TGFB1	1.58781	1.814518	1.514117	0.63119	-0.6638	0.000614	0.007906
U2SURP	2.390681	2.105055	2.483526	1.34249	0.4249	0.000619	0.010547
DIXDC1	1.785084	1.550322	1.861395	1.56526	0.6464	0.00062	0.007791
CKAP2L	1.245297	1.050559	1.308597	6.10367	2.6097	0.000621	0.010241
SUMO1	6.215932	5.362884	6.49322	1.25908	0.3324	0.000626	0.008832
PEX7	1.249818	1.452086	1.18407	0.40716	-1.2963	0.000629	0.006862
MORF4L2	10.05405	8.768348	10.47197	1.2193	0.2861	0.00063	0.008832
ELAVL2	1.29545	1.09571	1.360377	3.76529	1.9128	0.000632	0.008832
LCNL1	1.086281	1.275321	1.024832	0.09019	-3.4708	0.000633	0.011787
NCAPG	1.255219	1.059689	1.318777	5.34061	2.417	0.000637	0.006862
RPP25L	1.70906	1.480222	1.783445	1.63142	0.7061	0.000639	0.007937
ZDHHC12	2.174661	2.439613	2.088536	0.75613	-0.4033	0.00064	0.008236
ENY2	4.655667	4.09689	4.837302	1.23908	0.3093	0.000641	0.008827
GPC4	1.462432	1.250899	1.531192	2.11715	1.0821	0.000641	0.006862
TP53	1.535733	1.31823	1.606433	1.90564	0.9303	0.000647	0.010847
ANAPC15	2.014982	1.766499	2.095754	1.42956	0.5156	0.000647	0.008236
POLR2I	4.879417	4.176248	5.107986	1.29335	0.3711	0.000651	0.01056
PRAF2	2.199368	2.465129	2.11298	0.75965	-0.3966	0.000652	0.010436
CPEB4	1.462155	1.679574	1.391481	0.57607	-0.7957	0.000656	0.008341
ALOX5AP	1.093316	1.280387	1.032507	0.11594	-3.1086	0.000663	0.010436
KMT2A	1.951717	1.700051	2.033523	1.47635	0.562	0.000664	0.008551
NTM	1.14852	1.340456	1.08613	0.25298	-1.9829	0.000664	0.010436
RAB5C	2.953223	3.300455	2.840353	0.8	-0.3219	0.000664	0.008678
ZNF605	1.459641	1.248908	1.528142	2.12184	1.0853	0.000666	0.009069
TMTC4	1.667043	1.441975	1.740202	1.67476	0.744	0.000667	0.009069
CYCS	3.618518	4.133625	3.451079	0.78219	-0.3544	0.00067	0.009237
MSH6	1.618007	1.396147	1.690124	1.74209	0.8008	0.000672	0.010091
SYNCRIP	2.226257	1.965381	2.311056	1.35807	0.4416	0.000676	0.010467
TSPAN4	2.35945	2.657938	2.262425	0.76144	-0.3932	0.000683	0.009161
ACP5	1.198833	1.39559	1.134876	0.34095	-1.5524	0.000684	0.008806
FAM103A1	2.699871	2.363735	2.809134	1.3266	0.4077	0.000684	0.009295
CLMP	1.115384	1.305036	1.053736	0.17616	-2.505	0.000686	0.00933
EHD2	1.153127	1.344931	1.090779	0.26318	-1.9259	0.00069	0.011192
C1orf21	2.047337	2.31902	1.959025	0.72707	-0.4598	0.000692	0.008806
RHNO1	1.86661	1.629456	1.943698	1.49923	0.5842	0.000693	0.009427
NEDD4L	1.323412	1.529412	1.256451	0.48441	-1.0457	0.000695	0.01109
NEAT1	53.20754	60.91546	50.70203	0.82954	-0.2696	0.000698	0.008763

RP11-128P17.4	1.225998	1.03438	1.288285	8.38527	3.0679	0.000706	0.008455
PIGX	2.150071	1.894786	2.233053	1.37804	0.4626	0.000713	0.009082
C5orf24	2.668095	2.986481	2.564601	0.78762	-0.3444	0.000717	0.011454
HYI	1.596367	1.821215	1.523278	0.6372	-0.6502	0.00072	0.009654
SMAD9	1.785555	1.549141	1.862402	1.57046	0.6512	0.000721	0.009528
TMEM67	1.514245	1.301104	1.583527	1.93796	0.9545	0.000728	0.009638
RP11-1338A24.1	1.332583	1.13321	1.39739	2.98317	1.5768	0.000729	0.008631
CCDC12	1.968184	1.725463	2.047082	1.44333	0.5294	0.000732	0.011498
SNRPB	5.176596	4.465732	5.407667	1.27179	0.3469	0.000736	0.009738
CFAP97	2.132738	1.879258	2.215133	1.382	0.4668	0.000737	0.009266
LAMB2	1.340444	1.546406	1.273495	0.50053	-0.9985	0.000737	0.011572
ZNF638	2.641208	2.323741	2.744403	1.31778	0.3981	0.000738	0.009636
CBX3	5.439129	4.727061	5.670592	1.25316	0.3256	0.000741	0.009548
CCND1	3.656554	4.29316	3.449622	0.74385	-0.4269	0.000743	0.008649
UXS1	1.410307	1.621245	1.341741	0.55009	-0.8623	0.000748	0.008649
TCF12	1.962612	1.720194	2.041412	1.44602	0.5321	0.000748	0.011231
EGFL7	1.792163	2.029583	1.714988	0.69444	-0.5261	0.000758	0.011558
SLC35B1	1.817233	2.055172	1.73989	0.7012	-0.5121	0.000767	0.009877
MXRA7	1.681578	1.910403	1.607197	0.66695	-0.5843	0.000767	0.009745
SNRNP70	3.510892	3.027971	3.667868	1.31554	0.3957	0.000776	0.009745
ZNF22	2.327875	1.999185	2.434718	1.43589	0.5219	0.000776	0.011999
UTRN	2.077576	1.823216	2.160257	1.40942	0.4951	0.000779	0.012846
COL27A1	1.310101	1.512866	1.244191	0.47613	-1.0706	0.000785	0.01053
SIGIRR	2.0612	1.813101	2.141846	1.40431	0.4899	0.000789	0.009796
MTF2	2.623765	2.312886	2.724818	1.31376	0.3937	0.000801	0.010738
LINC01481	1.611941	1.393685	1.682886	1.7346	0.7946	0.000804	0.011938
CLDND2	1.348304	1.149158	1.413037	2.76912	1.4694	0.000805	0.012254
MSX2	1.099635	1.284954	1.039396	0.13825	-2.8546	0.000807	0.011938
GPSM2	1.265566	1.072939	1.32818	4.49937	2.1697	0.000809	0.009245
CPXM1	1.719852	1.495815	1.792677	1.59873	0.6769	0.000817	0.012088
FAM107A	1.116615	1.303675	1.05581	0.18378	-2.4439	0.000818	0.010289
ID4	1.487775	1.278342	1.555852	1.99701	0.9978	0.000819	0.010159
MYDGF	3.016572	3.397177	2.892854	0.78962	-0.3408	0.000828	0.008765
NLRP1	2.829113	2.475089	2.944191	1.31802	0.3984	0.000833	0.010889
VPS37A	1.518866	1.735223	1.448537	0.61007	-0.713	0.000836	0.010252
RRAGD	1.325887	1.529018	1.259858	0.49121	-1.0256	0.000842	0.013038
WSB2	1.51871	1.734921	1.448429	0.61017	-0.7127	0.000846	0.015481
GUCA1A	1.620696	1.843562	1.548252	0.64993	-0.6217	0.00085	0.010549
ODF2	1.471853	1.264662	1.539201	2.03732	1.0267	0.00086	0.011097
CCNDBP1	1.850342	1.609492	1.928632	1.52362	0.6075	0.000862	0.01285
OPN3	1.688645	1.915682	1.614846	0.67146	-0.5746	0.000864	0.012781
HIST2H2AC	1.324959	1.128544	1.388805	3.02468	1.5968	0.000865	0.010868
AP3M2	1.399811	1.198267	1.465325	2.34696	1.2308	0.000869	0.01285
DEAF1	2.099681	1.846494	2.181981	1.39633	0.4816	0.000872	0.009817
WDR73	1.692266	1.469959	1.764528	1.6268	0.702	0.000872	0.011395

TMED2	4.23287	4.933836	4.005016	0.76389	-0.3886	0.000878	0.009817
P4HA2	1.530362	1.747174	1.459885	0.6155	-0.7002	0.000881	0.011221
ISYNA1	3.933747	3.462092	4.087062	1.25384	0.3263	0.000882	0.011215
NREP	4.645134	5.267657	4.442779	0.80671	-0.3099	0.000883	0.014332
MAP4	1.951687	2.194296	1.872825	0.73083	-0.4524	0.000886	0.012914
TTC14	2.424213	2.147461	2.514173	1.31959	0.4001	0.000887	0.013514
SYNGR2	1.483846	1.696333	1.414776	0.59566	-0.7474	0.000894	0.013028
MS4A6A	1.111797	1.359009	1.031439	0.08757	-3.5134	0.000898	0.009414
BLVRB	2.145095	2.43362	2.051308	0.73332	-0.4475	0.000905	0.010961
TCEAL1	2.156233	1.905489	2.237739	1.36693	0.4509	0.000908	0.011266
SDK2	1.973635	2.237565	1.887843	0.71741	-0.4791	0.000909	0.011147
SLC16A1	2.289068	2.011612	2.379257	1.36343	0.4472	0.000914	0.009477
GLUL	2.92248	2.519673	3.053414	1.35122	0.4343	0.000921	0.010103
QARS	3.112939	2.704678	3.245646	1.31734	0.3976	0.000922	0.016564
TOMM7	10.73549	12.26345	10.23881	0.82025	-0.2859	0.000924	0.010103
ST8SIA2	1.1938	1.385347	1.131536	0.34134	-1.5507	0.000928	0.01181
SPC25	1.250142	1.061196	1.31156	5.09117	2.348	0.000929	0.013342
ECHDC2	1.301982	1.501823	1.237022	0.47232	-1.0822	0.000933	0.009574
SPATC1L	2.0267	1.78258	2.106053	1.41334	0.4991	0.00095	0.012249
RBM4	1.880556	1.647683	1.956252	1.47642	0.5621	0.00096	0.011766
DDX11	1.315528	1.12158	1.378573	3.11378	1.6387	0.000978	0.012939
ATF4	2.757621	2.447789	2.858334	1.28357	0.3602	0.000978	0.011993
C21orf59	1.965982	1.704078	2.051116	1.4929	0.5781	0.000988	0.013982
DUSP23	2.079318	1.831841	2.159762	1.39421	0.4794	0.000989	0.012591
SLC20A2	1.235551	1.428192	1.172933	0.40387	-1.308	0.000989	0.013091
SRP19	1.663225	1.886348	1.590697	0.66644	-0.5855	0.000998	0.012941
TMBIM1	1.290717	1.488108	1.226554	0.46415	-1.1073	0.001001	0.012269
PTK2B	1.114553	1.298259	1.054839	0.18386	-2.4433	0.001003	0.012609
ATP6V1G1	7.566079	8.455982	7.276811	0.84185	-0.2484	0.001003	0.012941
MRPL33	3.998752	4.554038	3.818253	0.79297	-0.3347	0.001006	0.012644
SLC52A2	1.444572	1.652455	1.376999	0.57782	-0.7913	0.001016	0.010326
DPYSL4	1.621618	1.406831	1.691436	1.69957	0.7652	0.00102	0.01103
DHRSX	1.560865	1.77719	1.490548	0.63118	-0.6639	0.001021	0.012828
ANKRD26	1.829816	1.601979	1.903876	1.50151	0.5864	0.001035	0.012385
SPATA33	1.738095	1.51672	1.810055	1.56769	0.6486	0.001037	0.015115
RSBN1L	1.855365	1.626828	1.929653	1.48311	0.5686	0.001054	0.012459
DHX32	1.557843	1.348338	1.625944	1.79695	0.8455	0.001065	0.012909
RP11-1094M14.11	1.579256	1.368026	1.647917	1.76052	0.816	0.001066	0.015544
PYCR2	1.660481	1.444842	1.730576	1.64233	0.7157	0.001069	0.013276
ITGB6	1.172043	1.402796	1.097035	0.2409	-2.0535	0.001074	0.01334
PRIM1	1.313209	1.121259	1.375603	3.09752	1.6311	0.001083	0.015558
CETN2	2.724434	2.424862	2.821812	1.27859	0.3546	0.001086	0.015609
POMP	6.202022	7.041432	5.929166	0.81589	-0.2935	0.001089	0.01336
OAZ2	4.997891	4.334768	5.213443	1.26349	0.3374	0.001109	0.013447
RP11-553A10.1	1.766477	1.993765	1.692595	0.69694	-0.5209	0.001109	0.013447

MRPL42	2.951632	2.573792	3.074452	1.31812	0.3985	0.001111	0.019604
MAP1LC3B	2.892078	3.236094	2.780254	0.79614	-0.3289	0.001111	0.014513
DNAJC7	2.270637	1.960657	2.371398	1.42756	0.5136	0.001114	0.015556
LAMC2	1.096001	1.275621	1.037615	0.13647	-2.8733	0.001117	0.014221
PHF3	2.48899	2.204011	2.581625	1.31363	0.3936	0.001124	0.017861
NETO2	1.581304	1.371461	1.649515	1.74854	0.8062	0.001132	0.014059
MATN2	1.129246	1.3119	1.069873	0.22402	-2.1583	0.001135	0.014272
MXRA5	1.189494	1.395166	1.12264	0.31035	-1.688	0.001135	0.017043
PIFO	1.256958	1.45008	1.194183	0.43144	-1.2128	0.001136	0.017861
PMVK	2.127276	1.884482	2.206198	1.36373	0.4476	0.00114	0.013798
SIX3-AS1	1.826167	1.597801	1.900398	1.50618	0.5909	0.001147	0.014789
SNRPG	8.486478	7.670379	8.751756	1.16212	0.2168	0.001151	0.013789
SYNGR1	1.727531	1.951694	1.654666	0.68789	-0.5397	0.001154	0.016347
ICMT	1.451316	1.657327	1.384351	0.58472	-0.7742	0.001171	0.01401
FOXG1	1.257822	1.071682	1.318328	4.44085	2.1508	0.001182	0.017482
NAV2	1.622479	1.410283	1.691454	1.68531	0.753	0.001184	0.014523
PMPCB	2.17282	1.925697	2.253149	1.35374	0.4369	0.001186	0.017048
PKN2	2.149509	1.897355	2.231473	1.37234	0.4566	0.001198	0.016977
SBF2	3.127027	2.64488	3.283753	1.3884	0.4734	0.001207	0.012142
DNAJB1	1.757721	2.027856	1.669912	0.65176	-0.6176	0.001207	0.014976
RFC5	1.44406	1.245884	1.508478	2.06796	1.0482	0.001219	0.014777
SRSF6	2.017126	1.781058	2.093862	1.40049	0.4859	0.001226	0.012214
RASSF4	1.835025	1.60752	1.908977	1.49621	0.5813	0.00123	0.014888
FGFR1	1.839264	2.069675	1.764367	0.71458	-0.4848	0.001236	0.014629
ADRM1	1.924504	2.1596	1.848085	0.73136	-0.4513	0.001253	0.015351
TMEM170A	1.776091	1.55523	1.847883	1.52709	0.6108	0.001259	0.015631
SMARCB1	3.34389	2.914013	3.483625	1.2976	0.3758	0.001264	0.021926
NRL	1.134409	1.315751	1.075463	0.23899	-2.065	0.001275	0.015197
BCAR1	1.627779	1.8433	1.557723	0.66136	-0.5965	0.001278	0.014937
MCFD2	2.117469	2.370388	2.035256	0.75545	-0.4046	0.001279	0.015197
MYOF	1.257342	1.44698	1.195699	0.43783	-1.1916	0.001283	0.017666
CLEC19A	1.212286	1.030621	1.271337	8.86106	3.1475	0.001285	0.015197
LARS	2.433082	2.1404	2.52822	1.34007	0.4223	0.001287	0.015788
LAMA5	1.405283	1.605353	1.340249	0.56207	-0.8312	0.001295	0.016269
SULF1	1.261262	1.451785	1.199331	0.44121	-1.1805	0.001296	0.015705
DYNLT3	1.785976	2.011772	1.712579	0.70429	-0.5058	0.001299	0.015197
SDC4	1.43369	1.636365	1.36781	0.57799	-0.7909	0.0013	0.020128
TPM3	3.166427	3.563913	3.037222	0.79458	-0.3317	0.001306	0.015282
PCBP4	2.076168	2.346555	1.988277	0.73393	-0.4463	0.001321	0.015908
SNRPD3	2.602533	2.324555	2.692892	1.27808	0.354	0.001322	0.018466
C19orf24	2.077621	2.320239	1.998756	0.7565	-0.4026	0.001326	0.015676
GS1-600G8.5	1.131404	1.423518	1.03645	0.08607	-3.5384	0.001328	0.015908
RBFA	1.534058	1.330843	1.600114	1.81389	0.8591	0.001351	0.018612
RNF5	3.036697	2.652917	3.161447	1.30766	0.387	0.00136	0.022959
ATP2A1-AS1	1.419315	1.223761	1.482881	2.15802	1.1097	0.001361	0.020737

MYO1C	1.205699	1.390865	1.14551	0.37228	-1.4255	0.001364	0.01989
CEBPD	1.431394	1.633732	1.365623	0.57694	-0.7935	0.001369	0.022959
C1QTNF4	1.392767	1.199466	1.455601	2.2841	1.1916	0.001383	0.020761
NT5C3A	1.730945	1.51509	1.80111	1.55528	0.6372	0.001384	0.016764
SLC39A13	1.437118	1.638701	1.371592	0.58179	-0.7814	0.001394	0.016116
SCD	2.315088	2.66944	2.199903	0.71875	-0.4764	0.001395	0.019486
PIDD1	1.403832	1.208922	1.467189	2.23619	1.161	0.001399	0.019829
RMI2	1.416763	1.222241	1.479993	2.15979	1.1109	0.001411	0.016296
TRIM8	1.289689	1.480242	1.227748	0.47424	-1.0763	0.001413	0.016923
NUBP2	2.193876	1.947444	2.27398	1.34465	0.4272	0.001415	0.01688
POLR2G	3.063017	2.676474	3.188665	1.30552	0.3846	0.001415	0.016943
OSTC	5.981285	5.212525	6.231175	1.24181	0.3124	0.001426	0.01688
DKC1	1.675483	1.463888	1.744263	1.6044	0.682	0.001429	0.02357
C14orf39	1.277479	1.092952	1.337461	3.63049	1.8602	0.001434	0.019243
RBM24	1.145885	1.326121	1.087298	0.26769	-1.9014	0.001434	0.016581
TSPAN9	1.191763	1.375761	1.131953	0.35116	-1.5098	0.001435	0.016581
MAP3K13	1.88487	1.64808	1.96184	1.48414	0.5696	0.001451	0.019243
DCN	1.081021	1.283869	1.015084	0.05314	-4.2341	0.001455	0.019243
ENSA	3.263176	2.855189	3.395795	1.2914	0.3689	0.00146	0.018339
PKDCC	1.343497	1.153673	1.4052	2.63677	1.3988	0.001468	0.016672
UQCR11	10.13926	11.36843	9.739714	0.84292	-0.2465	0.001476	0.016672
DDX3X	2.121037	2.364766	2.041811	0.76336	-0.3896	0.00148	0.018358
MFF	2.417341	2.685311	2.330235	0.78931	-0.3413	0.001485	0.017168
TBC1D14	1.40556	1.604291	1.340962	0.56423	-0.8256	0.00149	0.018489
ZNF536	1.210012	1.394097	1.150175	0.38106	-1.3919	0.001504	0.020236
KIF14	1.218938	1.03996	1.277115	6.93477	2.7938	0.001505	0.017202
AHCY	3.577483	3.092753	3.735047	1.30691	0.3862	0.001505	0.019648
RRM2B	1.410918	1.609144	1.346484	0.5688	-0.814	0.001506	0.017619
TMEM92	1.118373	1.29488	1.060998	0.20686	-2.2733	0.001509	0.020236
CCDC74A	1.745855	1.530524	1.815849	1.53782	0.6209	0.001509	0.019148
RAB29	1.243499	1.430483	1.182719	0.42445	-1.2363	0.001512	0.014915
TRPT1	2.330107	2.06049	2.417747	1.33688	0.4189	0.001518	0.016247
PRR4	1.869992	1.646925	1.942501	1.45689	0.5429	0.001523	0.019148
STRIP2	1.137112	1.316338	1.078854	0.24927	-2.0042	0.001523	0.017947
TCIRG1	1.153488	1.333677	1.094917	0.28446	-1.8137	0.00153	0.017947
FAM213A	3.874119	4.495923	3.671998	0.76432	-0.3878	0.001536	0.017947
SDF2L1	2.257742	2.542398	2.165213	0.75546	-0.4046	0.001537	0.017774
PBX1	3.410634	3.031278	3.533946	1.24746	0.319	0.001543	0.017232
PPP1R10	1.59923	1.393355	1.666151	1.69351	0.76	0.001555	0.025234
UBA2	2.401038	2.120125	2.492351	1.33231	0.4139	0.001555	0.01647
KHDC1	1.397091	1.204785	1.459601	2.24432	1.1663	0.001558	0.017808
RALYL	1.344665	1.156469	1.405839	2.59374	1.375	0.001562	0.021831
HIST3H2A	1.630468	1.423158	1.697856	1.64916	0.7217	0.001567	0.020196
OXR1	1.819342	1.600474	1.890487	1.48297	0.5685	0.001583	0.0181
HSPA8	11.21542	9.67221	11.71705	1.23579	0.3054	0.001592	0.015546

SCG3	1.215523	1.034711	1.274297	7.90239	2.9823	0.001595	0.019808
LINC01503	1.216556	1.40026	1.156841	0.39185	-1.3516	0.0016	0.016763
HIST1H1C	1.396742	1.206222	1.458672	2.22416	1.1533	0.001608	0.019034
UBE2I	4.800841	4.110215	5.025333	1.29423	0.3721	0.001612	0.019081
SMIM20	2.690181	2.326166	2.808506	1.36371	0.4475	0.001616	0.020555
EID2B	1.392496	1.201811	1.454479	2.252	1.1712	0.00163	0.01998
AC004556.1	1.648532	1.440868	1.716035	1.62415	0.6997	0.001631	0.019303
ETFRF1	1.880984	1.654256	1.954684	1.45919	0.5452	0.001638	0.026153
SPAG16	2.352773	2.082241	2.440712	1.33123	0.4128	0.001647	0.017078
LINC00969	2.253513	2.007887	2.333355	1.32292	0.4037	0.001651	0.018231
SYMPK	2.051924	1.82017	2.127257	1.37442	0.4588	0.001663	0.020134
PSMC3IP	1.34209	1.155726	1.402669	2.58575	1.3706	0.001673	0.019567
LIG1	1.327437	1.141794	1.387781	2.73482	1.4514	0.001677	0.023109
C3orf14	1.612387	1.407748	1.678907	1.66502	0.7355	0.001677	0.022204
PDLIM5	2.234533	2.492418	2.150706	0.77103	-0.3751	0.001678	0.023124
FANCD2	1.245947	1.066419	1.304304	4.58157	2.1958	0.00169	0.020674
THOC7	2.492589	2.204996	2.586073	1.31625	0.3964	0.001694	0.019592
TMEM47	2.111795	2.383269	2.023551	0.73995	-0.4345	0.001694	0.019153
RAB3IP	1.603132	1.812403	1.535108	0.65867	-0.6024	0.001698	0.02062
OC90	1.078749	1.251948	1.02245	0.0891	-3.4884	0.001703	0.02062
TRAM2	1.187737	1.368016	1.129136	0.3509	-1.5109	0.001705	0.01994
RAD21	2.633109	2.344271	2.726998	1.28471	0.3614	0.001706	0.020674
ITGA2	1.253615	1.437637	1.193797	0.44283	-1.1752	0.001709	0.017539
SYNC	1.613546	1.406373	1.680889	1.67553	0.7446	0.001711	0.0215
MSX1	1.13613	1.327919	1.073788	0.22502	-2.1519	0.001713	0.019564
SIPA1L2	1.174776	1.354104	1.116484	0.32895	-1.604	0.001721	0.019458
BBS4	1.441769	1.248451	1.504609	2.03102	1.0222	0.001724	0.023441
NUCB1	2.209447	2.454979	2.129635	0.77639	-0.3651	0.001727	0.02256
NIFK-AS1	1.569815	1.368288	1.635323	1.72507	0.7867	0.001742	0.017704
PRSS8	1.13133	1.307487	1.07407	0.24089	-2.0536	0.001744	0.019715
PCDH7	3.280485	2.848287	3.420973	1.30985	0.3894	0.001744	0.019695
HRASLS	1.355961	1.168551	1.416879	2.47331	1.3064	0.001747	0.027464
SUPT16H	2.196914	1.944713	2.278894	1.35374	0.4369	0.001756	0.016984
RAP2B	1.469675	1.668311	1.405108	0.60617	-0.7222	0.001757	0.021804
STOX2	1.419135	1.22742	1.481453	2.11702	1.082	0.001763	0.019262
SNAI2	1.118487	1.293803	1.061499	0.20932	-2.2562	0.001765	0.021116
UFC1	3.452923	3.060979	3.580327	1.25199	0.3242	0.001795	0.02148
RBMS3	1.154766	1.331375	1.097357	0.2938	-1.7671	0.001796	0.022013
RTN3	3.222727	2.855209	3.342191	1.26249	0.3363	0.001797	0.024107
PLAC9	1.083871	1.256117	1.027882	0.10886	-3.1994	0.001803	0.026676
XRN2	2.993068	2.629287	3.111318	1.29585	0.3739	0.001812	0.024634
GMCL1	1.637231	1.431868	1.703986	1.6301	0.705	0.001819	0.019656
PGAM1	9.942629	11.42595	9.460466	0.81148	-0.3014	0.001829	0.021144
QSOX1	1.382964	1.576372	1.320096	0.55536	-0.8485	0.001834	0.026734
SPOCK1	2.502646	2.817033	2.400452	0.77074	-0.3757	0.001837	0.021724

ZNF146	2.167596	1.90446	2.25313	1.3855	0.4704	0.001839	0.028455
PMAIP1	1.580414	1.792031	1.511626	0.64597	-0.6305	0.00184	0.02176
FXYD6	3.080511	2.634898	3.22536	1.36116	0.4448	0.001857	0.02145
YWHAQ	7.949211	6.906673	8.288094	1.23387	0.3032	0.001864	0.025004
XRCC1	1.591891	1.390813	1.657253	1.68176	0.75	0.00189	0.021102
TLE6	1.231209	1.054456	1.288664	5.30091	2.4062	0.001895	0.022812
PJA1	1.692466	1.485457	1.759755	1.56503	0.6462	0.001905	0.022818
TMEM2	1.697263	1.490321	1.764531	1.55924	0.6408	0.001907	0.022812
NHSL1	1.537612	1.339373	1.60205	1.77401	0.827	0.001908	0.022297
ZNF207	2.855362	2.52251	2.963558	1.28968	0.367	0.001932	0.021596
GGCT	2.553646	2.226652	2.659938	1.35323	0.4364	0.001936	0.021377
EHMT1	1.75543	1.544417	1.824021	1.51358	0.598	0.001937	0.024981
HOXB5	1.232585	1.036866	1.296205	8.03469	3.0062	0.00194	0.025676
MZT2A	6.128639	5.247838	6.414949	1.27475	0.3502	0.001944	0.022226
CLPTM1	1.46254	1.659708	1.398449	0.60398	-0.7274	0.001951	0.02582
CLN8	1.415659	1.609261	1.352727	0.57894	-0.7885	0.001953	0.022322
HNRNPM	3.418147	3.004121	3.552728	1.27374	0.3491	0.001956	0.02286
BNIP2	1.473673	1.672072	1.409182	0.60884	-0.7159	0.001959	0.022378
PSMB8	1.354791	1.545481	1.292805	0.53678	-0.8976	0.001974	0.019859
PGD	2.793185	2.421461	2.914016	1.34651	0.4292	0.001981	0.023419
PTPA	1.883371	2.105302	1.811231	0.73395	-0.4463	0.001982	0.022403
CEP70	1.445403	1.254785	1.507365	1.99134	0.9937	0.002008	0.02623
SLFN5	1.240548	1.42175	1.181647	0.4307	-1.2153	0.002009	0.030621
PTPRN2	1.376708	1.568152	1.314477	0.55351	-0.8533	0.002011	0.022235
PRKAG2	1.224443	1.404308	1.165976	0.41052	-1.2845	0.002034	0.025902
ITPKB	1.151304	1.326162	1.094465	0.28963	-1.7877	0.002045	0.028972
HTATIP2	1.088846	1.258883	1.033574	0.12969	-2.9469	0.002045	0.028972
PGM5	1.166953	1.342264	1.109967	0.32129	-1.638	0.002073	0.024223
ACTR2	2.456409	2.742108	2.363541	0.7827	-0.3535	0.002076	0.023465
SCN3A	1.210311	1.036363	1.266854	7.33863	2.8755	0.002081	0.026846
ENDOV	1.72041	1.512612	1.787956	1.53714	0.6202	0.002082	0.027177
SELENOS	2.160915	2.397328	2.084067	0.77581	-0.3662	0.002092	0.023388
ITGA6	1.336159	1.523259	1.275341	0.5262	-0.9263	0.002101	0.024578
NAA38	3.946237	3.494665	4.093023	1.23986	0.3102	0.002107	0.027177
USP28	1.490672	1.297519	1.553458	1.86024	0.8955	0.002122	0.029656
PANK3	1.499235	1.696888	1.434986	0.62418	-0.68	0.002127	0.030571
CERS2	1.593498	1.797521	1.527179	0.66102	-0.5972	0.002144	0.02054
AFAP1	1.363384	1.552219	1.302002	0.54689	-0.8707	0.002154	0.024909
H2AFY	5.108722	4.462794	5.318685	1.24717	0.3187	0.00216	0.027506
EIF2AK4	2.385717	2.129115	2.469127	1.30113	0.3798	0.002174	0.02986
APOLD1	1.232312	1.05818	1.288915	4.96592	2.3121	0.002196	0.02986
MAP1LC3A	1.71012	1.920606	1.6417	0.69704	-0.5207	0.002205	0.024651
SEPT8	1.358808	1.547076	1.29761	0.544	-0.8783	0.00221	0.024171
MRPL13	2.099753	1.840058	2.184169	1.40963	0.4953	0.002213	0.024986
MAMDC2	1.151839	1.324479	1.095721	0.295	-1.7612	0.002242	0.028199

THAP9-AS1	1.814664	1.602893	1.883501	1.46544	0.5513	0.002256	0.024399
DKK2	1.096059	1.341041	1.016426	0.04816	-4.3759	0.002261	0.030325
TRIO	1.4732	1.668053	1.409862	0.61352	-0.7048	0.002266	0.026814
MTHFD2	1.71892	1.513949	1.785547	1.52845	0.6121	0.002277	0.025451
RP11-25K19.1	1.498667	1.306512	1.561128	1.83069	0.8724	0.0023	0.024619
RAB30-AS1	1.654873	1.453891	1.720203	1.58673	0.6661	0.002309	0.029038
ACSL4	1.575838	1.378988	1.639825	1.68824	0.7555	0.00232	0.02714
BMP1	1.218633	1.395429	1.161164	0.40757	-1.2949	0.002331	0.029685
STARD3NL	2.048857	2.276	1.975023	0.76412	-0.3881	0.002334	0.027919
ZIC5	1.237804	1.063965	1.294311	4.60111	2.202	0.002349	0.025963
FEZ2	1.952915	2.175784	1.88047	0.74884	-0.4173	0.002369	0.026479
FAT1	1.676674	1.883092	1.609576	0.69028	-0.5348	0.00237	0.026463
WHSC1	2.07966	1.854942	2.152706	1.34829	0.4311	0.002376	0.026262
MMAB	1.85867	1.645725	1.92789	1.43697	0.523	0.002399	0.026524
TMEM45A	2.160178	1.890479	2.247845	1.40132	0.4868	0.002419	0.026711
BMP7	1.353556	1.539488	1.293117	0.54332	-0.8801	0.002423	0.028645
UBE2F	1.507084	1.702627	1.443521	0.63123	-0.6638	0.002428	0.030018
LUM	1.117572	1.347819	1.042728	0.12285	-3.0251	0.002432	0.032194
PGLS	4.013166	3.495934	4.181296	1.27459	0.35	0.002452	0.0281
TXNIP	4.071495	3.55273	4.240123	1.26928	0.344	0.002459	0.030207
TRMT11	1.479458	1.289975	1.54105	1.86585	0.8998	0.002462	0.030207
PARM1	1.172039	1.345294	1.115722	0.33514	-1.5772	0.002465	0.030018
CDCP1	1.078924	1.244642	1.025057	0.10242	-3.2874	0.002468	0.027281
SYPL1	1.873806	2.094965	1.801917	0.73237	-0.4494	0.002469	0.026971
TPRKB	2.058478	1.80562	2.140671	1.41589	0.5017	0.002471	0.0281
CUBN	1.140021	1.310297	1.084671	0.27287	-1.8737	0.002476	0.027077
UQCRQ	11.58827	13.20137	11.06392	0.82482	-0.2779	0.002488	0.0281
ARMCX1	1.697469	1.494398	1.763478	1.54426	0.6269	0.00249	0.023634
DDIT3	1.549479	1.807434	1.465629	0.57668	-0.7942	0.002493	0.029133
POLR2H	2.545305	2.289401	2.628488	1.26298	0.3368	0.002494	0.030018
FRG1	1.891468	1.678247	1.960777	1.41656	0.5024	0.002507	0.030018
CTNNA1	2.357969	2.623607	2.271622	0.78321	-0.3525	0.00251	0.027443
RP11-499F3.2	1.601439	1.403851	1.665666	1.6483	0.721	0.002523	0.027268
PRKAR1A	2.756056	3.071938	2.653376	0.79799	-0.3256	0.002548	0.023958
NAPEPLD	1.373201	1.190899	1.432459	2.26538	1.1798	0.002566	0.029639
ARRB2	1.51434	1.323987	1.576215	1.77851	0.8307	0.002597	0.028367
CYP27A1	1.263806	1.442568	1.205698	0.46478	-1.1054	0.002597	0.033758
THYN1	2.373745	2.113873	2.458218	1.30914	0.3886	0.002606	0.028194
CRX	1.140872	1.311767	1.085321	0.27367	-1.8695	0.002612	0.024333
G2E3	1.504959	1.315214	1.566637	1.79763	0.8461	0.002617	0.033758
ZC3H8	1.67446	1.474301	1.739523	1.55919	0.6408	0.00266	0.033878
TRA2A	2.101117	1.845277	2.184279	1.40106	0.4865	0.002661	0.02845
FAM171B	1.527189	1.335864	1.589381	1.75482	0.8113	0.002663	0.030516
TMEM50A	2.699364	2.987157	2.605816	0.8081	-0.3074	0.002669	0.028541
CEP290	2.161605	1.924569	2.238655	1.33971	0.4219	0.002672	0.030516

MTHFD2L	1.71406	1.511213	1.779997	1.52578	0.6095	0.002674	0.028926
SLC35F3	1.267389	1.094011	1.323747	3.44373	1.784	0.002686	0.028745
FUZ	1.4893	1.301304	1.55041	1.82676	0.8693	0.002735	0.031272
ACTL6A	1.610028	1.41543	1.673284	1.62069	0.6966	0.002755	0.030125
C2orf40	1.25554	1.432408	1.198048	0.45801	-1.1265	0.002757	0.031876
CEP57L1	1.353315	1.174325	1.411496	2.36052	1.2391	0.002773	0.029337
USMG5	9.879455	10.96884	9.525344	0.8552	-0.2257	0.002778	0.031724
PLEKHA3	1.735853	1.943183	1.668459	0.70873	-0.4967	0.002796	0.0316
SNHG19	1.566096	1.373974	1.628546	1.68072	0.7491	0.002802	0.0397
SSC4D	1.322196	1.502325	1.263644	0.52485	-0.93	0.002809	0.033242
HAUS6	1.473083	1.287155	1.53352	1.85795	0.8937	0.002815	0.035397
YWHAZ	5.641624	6.277754	5.434846	0.84029	-0.251	0.002845	0.031107
MYEF2	2.686777	2.409702	2.776842	1.26044	0.3339	0.002847	0.039777
ARL16	2.530303	2.263811	2.616928	1.27941	0.3555	0.002847	0.035441
SCNM1	1.829966	1.621343	1.897781	1.4449	0.531	0.002854	0.035441
AMOTL1	1.369438	1.553082	1.309743	0.56003	-0.8364	0.002875	0.032104
FAM110B	1.423231	1.24067	1.482573	2.00512	1.0037	0.002877	0.030787
REPIN1	2.131953	1.899884	2.207388	1.34171	0.4241	0.002877	0.026552
TSKU	1.299405	1.478553	1.241172	0.50396	-0.9886	0.002882	0.043266
NDFIP2	1.369393	1.552798	1.309777	0.56038	-0.8355	0.002885	0.031136
TMEM63A	1.361175	1.182247	1.419337	2.30093	1.2022	0.002905	0.033215
NORAD	2.990777	3.298361	2.890795	0.82267	-0.2816	0.002909	0.031136
HJURP	1.200526	1.033518	1.254813	7.60223	2.9264	0.002925	0.030622
RSBN1	1.60612	1.412366	1.669101	1.62259	0.6983	0.002964	0.032673
KIF20A	1.20382	1.036698	1.258144	7.03421	2.8144	0.002971	0.036903
KIF22	1.496782	1.310016	1.557492	1.79827	0.8466	0.002973	0.027193
SOX4	10.1746	11.30876	9.805936	0.85422	-0.2273	0.002976	0.032158
LRRFIP2	1.424004	1.610332	1.363437	0.59547	-0.7479	0.002979	0.031541
USP46	1.562878	1.757512	1.499611	0.65954	-0.6005	0.002987	0.031628
TSPAN2	1.223994	1.396954	1.167772	0.42265	-1.2425	0.002989	0.044217
TRMT1	1.521566	1.333209	1.582793	1.74903	0.8066	0.002995	0.029836
FANCB	1.274878	1.102761	1.330826	3.21938	1.6868	0.002996	0.031723
BOD1	2.944657	2.621306	3.049764	1.26427	0.3383	0.002999	0.03216
FEN1	1.342694	1.165916	1.400157	2.41181	1.2701	0.003004	0.033552
UBA52	27.79234	25.18406	28.64018	1.14291	0.1927	0.003005	0.033552
IGF2	5.961897	7.618668	5.423352	0.66831	-0.5814	0.003012	0.041514
PTN	1.555278	1.358226	1.619331	1.72888	0.7898	0.003025	0.03216
CRTAP	2.77695	3.102144	2.671243	0.79502	-0.3309	0.003031	0.036734
C6orf1	1.636608	1.835579	1.571932	0.68447	-0.5469	0.003037	0.03216
STK32C	1.458375	1.274862	1.518027	1.88468	0.9143	0.003041	0.035575
HDGF	2.49239	2.226156	2.578931	1.28771	0.3648	0.003042	0.031873
YPEL1	1.549367	1.360078	1.610896	1.69656	0.7626	0.003082	0.027932
EIF3M	3.276719	2.951542	3.382419	1.22079	0.2878	0.003096	0.032781
TPP1	1.400515	1.584986	1.340551	0.58215	-0.7805	0.0031	0.032673
C4orf47	1.350288	1.173503	1.407753	2.35011	1.2327	0.003104	0.03428

ENOSF1	1.507931	1.321291	1.568599	1.76973	0.8235	0.003112	0.032673
ACAA2	2.722035	2.407486	2.824282	1.29613	0.3742	0.003121	0.045502
ARL2	3.915234	3.490118	4.053421	1.22622	0.2942	0.003123	0.035297
RAMP1	1.482631	1.670962	1.421413	0.62807	-0.671	0.003131	0.032673
ARID3A	2.100075	1.841509	2.184123	1.40714	0.4928	0.003132	0.032673
DPCD	2.773387	2.421559	2.887751	1.32794	0.4092	0.003134	0.032673
NME4	6.159684	5.406626	6.404471	1.22644	0.2945	0.003146	0.036341
SNRPC	3.255684	2.843398	3.3897	1.29636	0.3745	0.003153	0.032673
RP11-242D8.1	1.479154	1.294513	1.539173	1.83073	0.8724	0.003157	0.035291
BNIP3L	4.633546	4.111869	4.80312	1.22213	0.2894	0.003172	0.042657
LINC01473	1.368686	1.1912	1.426379	2.23001	1.1571	0.00318	0.042657
SNRNP40	1.62501	1.43274	1.687508	1.58873	0.6679	0.003216	0.037189
COQ8B	1.452093	1.26933	1.511501	1.89917	0.9254	0.003228	0.03383
ADM	2.346304	2.946245	2.151289	0.59154	-0.7574	0.003233	0.034238
FABP4	1.097863	1.260318	1.045057	0.17308	-2.5305	0.003236	0.034238
DLG1	1.695167	1.896467	1.629734	0.70246	-0.5095	0.003238	0.038779
BNC2	1.138417	1.304176	1.084536	0.27792	-1.8473	0.003242	0.036606
THAP2	1.57551	1.385266	1.63735	1.65431	0.7262	0.003268	0.034156
ZNF215	1.239828	1.071989	1.294385	4.0893	2.0319	0.003277	0.043373
KMO	1.11973	1.283741	1.066417	0.23408	-2.095	0.00328	0.03402
COIL	1.515858	1.32982	1.57633	1.74741	0.8052	0.003287	0.036739
TRAP1	1.888376	1.681625	1.955582	1.40192	0.4874	0.003293	0.034156
FAM134B	1.22125	1.392271	1.165659	0.42231	-1.2436	0.003315	0.040663
ANKS1B	1.193838	1.02924	1.247341	8.4589	3.0805	0.00333	0.034856
LSM1	2.142594	2.381918	2.0648	0.77052	-0.3761	0.00334	0.03649
CADM4	1.489888	1.678002	1.42874	0.63236	-0.6612	0.003343	0.034633
CTA-392E5.1	1.075303	1.23522	1.02332	0.09914	-3.3343	0.003361	0.038225
RGS3	1.496822	1.685173	1.435598	0.63575	-0.6535	0.003382	0.038225
LIAS	1.337305	1.162654	1.394076	2.42278	1.2767	0.003386	0.036586
FGF14-AS2	1.42051	1.241165	1.478807	1.98539	0.9894	0.00339	0.03786
NOVA1	2.730548	2.419806	2.831556	1.29	0.3674	0.003396	0.034864
PDAP1	3.943361	3.522333	4.080219	1.22118	0.2883	0.003398	0.038812
RAD9A	1.507721	1.322637	1.567883	1.76013	0.8157	0.003419	0.035066
SIRT2	1.434275	1.619663	1.374014	0.60358	-0.7284	0.003425	0.036152
SMIM15	1.974882	1.763047	2.04374	1.36786	0.4519	0.003431	0.041583
CSNK1D	1.967912	2.191499	1.895234	0.75135	-0.4124	0.003447	0.03571
GINS4	1.266171	1.097297	1.321065	3.29986	1.7224	0.00345	0.035054
DEPDC1B	1.257376	1.089542	1.311932	3.48365	1.8006	0.003452	0.038976
CCM2	1.456993	1.642784	1.3966	0.617	-0.6967	0.003453	0.036152
TMEM54	1.103139	1.265231	1.05045	0.19021	-2.3943	0.003467	0.035557
FAM96B	3.767885	4.142153	3.646227	0.84217	-0.2478	0.003518	0.039322
PCYOX1	1.793162	1.998303	1.72648	0.72772	-0.4586	0.003526	0.036155
DAD1	4.197203	4.695534	4.035217	0.82132	-0.284	0.003529	0.037733
BEX2	5.119862	4.592559	5.291265	1.19449	0.2564	0.003532	0.039051
HNRNPUL1	2.665095	2.382217	2.757047	1.27118	0.3462	0.00354	0.039094

FUBP1	2.115472	1.897438	2.186346	1.32192	0.4026	0.003544	0.046308
OFD1	1.763799	1.564202	1.828679	1.46876	0.5546	0.003554	0.036861
RFC1	2.677913	2.388435	2.77201	1.27626	0.3519	0.003565	0.036599
FAM228B	1.492235	1.308865	1.551841	1.78667	0.8373	0.003569	0.039458
ACADM	1.724441	1.528524	1.788126	1.49118	0.5765	0.003594	0.037665
ACVR2B	1.592288	1.403123	1.653778	1.62178	0.6976	0.003597	0.037989
AGTPBP1	1.524649	1.339646	1.584786	1.72175	0.7839	0.003599	0.0426
RP11-332H14.2	1.373562	1.197859	1.430675	2.17667	1.1221	0.003629	0.037989
PPT1	1.970468	1.759511	2.039041	1.36804	0.4521	0.003647	0.035966
SMKR1	1.222351	1.057398	1.27597	4.80804	2.2654	0.003658	0.037138
PIGS	1.448637	1.63322	1.388637	0.61375	-0.7043	0.003659	0.04001
RAB1A	3.053213	3.433319	2.929657	0.79301	-0.3346	0.003661	0.036835
GAR1	1.624377	1.433416	1.68645	1.58381	0.6634	0.003669	0.038451
SNHG8	3.195954	2.821321	3.317731	1.27255	0.3477	0.003693	0.053075
G3BP2	2.04008	2.257908	1.969274	0.77054	-0.376	0.003697	0.038301
RFXAP	1.303201	1.132956	1.358541	2.69669	1.4312	0.003721	0.036347
CRY1	1.185862	1.352283	1.131766	0.37403	-1.4188	0.003744	0.037631
CCDC115	1.852412	1.64991	1.918236	1.41287	0.4986	0.003753	0.041494
ETNK2	1.147127	1.310465	1.094033	0.30288	-1.7232	0.003757	0.037981
SLC1A3	1.283891	1.11517	1.338735	2.94118	1.5564	0.00377	0.04078
CEP135	1.368482	1.194618	1.424997	2.18375	1.1268	0.003779	0.037981
ZNF519	1.312251	1.141635	1.367711	2.59619	1.3764	0.0038	0.039003
LPGAT1	1.658628	1.855083	1.594769	0.69557	-0.5237	0.003804	0.045553
RNASEH2C	2.907709	2.625857	2.999327	1.22971	0.2983	0.003834	0.041927
XXbac-BPGBPG55C20.2	1.342912	1.169962	1.39913	2.34834	1.2316	0.003838	0.044894
KIF18A	1.217595	1.054369	1.270653	4.97804	2.3156	0.003863	0.041799
AGO1	1.711457	1.516046	1.774977	1.50176	0.5867	0.003867	0.041303
ARL2BP	2.47997	2.234096	2.559893	1.264	0.338	0.003867	0.049876
SSBP1	3.366208	3.013845	3.480746	1.23185	0.3008	0.003868	0.041799
CEP83	1.388406	1.21338	1.4453	2.08689	1.0614	0.003886	0.039847
CD47	2.483392	2.212193	2.571546	1.29645	0.3746	0.003901	0.039637
H1F0	4.040642	3.612806	4.179712	1.21697	0.2833	0.003924	0.041303
CEP126	1.411189	1.234409	1.468653	1.9993	0.9995	0.003941	0.041303
ST3GAL1	1.112333	1.272702	1.060204	0.22077	-2.1794	0.00397	0.046982
KIF5A	1.245444	1.079294	1.299453	3.77651	1.9171	0.003974	0.040344
GOLGA2	1.458978	1.642326	1.399379	0.62177	-0.6855	0.003978	0.046
DDX46	2.797283	2.528062	2.884796	1.23345	0.3027	0.003982	0.043078
ZNF714	1.674157	1.482909	1.736324	1.52477	0.6086	0.003989	0.044544
CACYBP	2.777308	2.415019	2.895072	1.33926	0.4214	0.004038	0.040807
PRRX1	1.075718	1.242513	1.0215	0.08865	-3.4957	0.00406	0.040807
GREB1	1.238905	1.073992	1.292512	3.95328	1.9831	0.004069	0.036391
OBSL1	3.361607	3.02381	3.47141	1.22117	0.2883	0.004075	0.043608
C1orf174	1.624591	1.436337	1.685784	1.57169	0.6523	0.004078	0.040125
NARF	2.841846	2.545507	2.938173	1.25407	0.3266	0.004081	0.043631
RP11-85O21.2	1.34441	1.173331	1.40002	2.30783	1.2065	0.004088	0.036391

AKIRIN1	2.364523	2.649496	2.27189	0.77108	-0.3751	0.004091	0.040125
CCT7	3.248322	2.882088	3.367369	1.25784	0.331	0.004108	0.042564
HRAS	1.454488	1.636197	1.395422	0.62154	-0.6861	0.004112	0.040125
LMNB2	1.423826	1.247731	1.481067	1.94189	0.9575	0.004122	0.047125
LINC01355	1.285071	1.117531	1.339531	2.88887	1.5305	0.004151	0.043918
SNHG21	1.344406	1.173096	1.400092	2.3114	1.2088	0.004153	0.04243
THOC3	2.075248	1.859955	2.145231	1.33173	0.4133	0.004158	0.041417
IRS2	1.348233	1.523198	1.29136	0.55688	-0.8446	0.004168	0.043223
PTS	1.762373	1.962359	1.697366	0.72464	-0.4647	0.004169	0.042366
FAM129A	1.112357	1.272105	1.06043	0.22208	-2.1708	0.004172	0.04243
AC090498.1	4.232224	3.721728	4.398163	1.24853	0.3202	0.004176	0.04243
ARCN1	1.513908	1.699052	1.453726	0.64906	-0.6236	0.004183	0.041624
SLC27A5	1.664589	1.474259	1.726458	1.53177	0.6152	0.004213	0.043244
SNAPC3	1.977819	1.770359	2.045255	1.35684	0.4403	0.00422	0.048937
ACTR1B	2.216865	1.987864	2.291303	1.30717	0.3864	0.004232	0.048937
PEMT	2.160253	2.380261	2.088738	0.78879	-0.3423	0.004246	0.044958
MAD1L1	1.416752	1.241538	1.473706	1.96121	0.9717	0.00426	0.041971
XPC	1.648952	1.459809	1.710434	1.54506	0.6277	0.004274	0.052436
GABARAPL1	1.819864	2.022066	1.754137	0.73786	-0.4386	0.004275	0.042588
AASDH	1.459993	1.281886	1.517888	1.83723	0.8775	0.004276	0.042588
CTD-2547G23.4	1.281564	1.11505	1.33569	2.91776	1.5449	0.0043	0.041592
BRD4	1.774195	1.974157	1.709195	0.72801	-0.458	0.004307	0.044857
THAP7	1.741818	1.547256	1.805062	1.47109	0.5569	0.004329	0.044857
IQCB1	1.453891	1.276483	1.511559	1.85024	0.8877	0.004334	0.042287
RPN1	1.487906	1.671065	1.428369	0.63834	-0.6476	0.004335	0.049559
ELL2	1.329828	1.159978	1.385039	2.40683	1.2671	0.004342	0.052631
STON2	1.505273	1.324777	1.563945	1.73641	0.7961	0.004369	0.044807
MAB21L2	1.572687	1.760399	1.51167	0.6729	-0.5715	0.004385	0.055839
METTL26	3.398405	2.957438	3.541744	1.29851	0.3769	0.004421	0.042153
DNAJC4	2.313195	2.083117	2.387983	1.28147	0.3578	0.004426	0.048393
BEND5	1.508907	1.329388	1.567261	1.72217	0.7842	0.004436	0.05014
RASSF6	1.121238	1.279387	1.069831	0.24994	-2.0003	0.004442	0.042153
NFKBIA	1.631679	1.896675	1.545541	0.6084	-0.7169	0.004454	0.045258
GOLIM4	2.252491	2.024126	2.326723	1.29547	0.3735	0.004492	0.056488
SH3GLB1	2.015241	2.241001	1.941856	0.75895	-0.3979	0.004492	0.06365
OCIAD2	5.094956	5.673804	4.906798	0.83589	-0.2586	0.004502	0.050892
SCRN2	1.766404	1.571938	1.829617	1.45054	0.5366	0.004528	0.045282
C12orf29	1.415316	1.241217	1.471907	1.95636	0.9682	0.004532	0.054273
SDHD	1.669688	1.481695	1.730796	1.51714	0.6013	0.004537	0.047541
APRT	3.455898	3.051854	3.587235	1.26093	0.3345	0.004542	0.043889
PBXIP1	1.366423	1.541107	1.309641	0.57224	-0.8053	0.004544	0.045715
TMED10	3.087645	3.422656	2.978748	0.81677	-0.292	0.004546	0.045282
RAB40B	1.587015	1.402497	1.646994	1.60745	0.6848	0.004597	0.051384
PDDC1	1.583416	1.399881	1.643076	1.60817	0.6854	0.004598	0.045349
EPM2AIP1	2.085621	1.871613	2.155186	1.32534	0.4064	0.004601	0.045378

EIF4G1	1.892588	2.097335	1.826034	0.75276	-0.4097	0.004605	0.050859
MZT2B	9.53733	8.509791	9.871338	1.1813	0.2404	0.004609	0.046371
MGME1	1.542301	1.361448	1.601088	1.663	0.7338	0.004632	0.046554
BTBD3	1.618937	1.433655	1.679164	1.56614	0.6472	0.004636	0.046131
B4GAT1	1.636351	1.827452	1.574233	0.69398	-0.527	0.004638	0.048097
PIEZO2	1.287025	1.122165	1.340614	2.78814	1.4793	0.004642	0.043648
PRDX6	10.11144	9.039067	10.46003	1.17676	0.2348	0.004652	0.064992
LHFPL2	1.164099	1.325193	1.111734	0.34359	-1.5412	0.004668	0.051604
CACNA2D3	1.262923	1.0996	1.316012	3.17279	1.6658	0.004679	0.044783
NEUROD1	1.195761	1.023603	1.251722	10.6647	3.4148	0.004683	0.045735
TMEM144	1.330263	1.502268	1.274352	0.54623	-0.8724	0.004685	0.055443
HIPK3	1.265673	1.433417	1.211147	0.48717	-1.0375	0.004689	0.048131
MYBL2	1.310589	1.144119	1.364701	2.53055	1.3395	0.004702	0.041492
NEU1	1.592108	1.779829	1.531089	0.68103	-0.5542	0.004712	0.048327
MCM3AP-AS1	1.43649	1.261971	1.493218	1.88272	0.9128	0.004746	0.048177
FHL3	1.364092	1.538094	1.307531	0.57152	-0.8071	0.004758	0.051015
ELF2	1.762422	1.5695	1.825132	1.44887	0.5349	0.004767	0.051015
LIMD2	2.747531	2.413245	2.856193	1.31343	0.3933	0.004802	0.047315
CEP55	1.202051	1.043512	1.253586	5.82796	2.543	0.004845	0.049183
TMCO1	2.771731	3.053952	2.679993	0.81793	-0.2899	0.004846	0.051313
PTPMT1	2.104574	2.328172	2.031893	0.77693	-0.3641	0.004871	0.048514
CENPC	1.434551	1.261426	1.490826	1.8775	0.9088	0.00489	0.06073
PRPF40A	2.69363	2.400919	2.788778	1.27686	0.3526	0.004906	0.047915
CTDSPL2	1.497581	1.320844	1.555031	1.72991	0.7907	0.004921	0.050002
MRPL50	1.821295	1.625378	1.884979	1.41511	0.5009	0.004942	0.051788
KLHDC9	1.476958	1.301119	1.534116	1.77377	0.8268	0.004946	0.047843
MLST8	2.193617	1.977724	2.263795	1.29259	0.3703	0.004956	0.049315
PIGF	1.936637	1.735452	2.002033	1.36247	0.4462	0.004977	0.060797
RHOB	1.527702	1.709291	1.468675	0.66077	-0.5978	0.004989	0.058362
PUS7L	1.447061	1.273184	1.503581	1.84337	0.8823	0.005014	0.048039
VRK3	1.519898	1.341579	1.577862	1.69174	0.7585	0.005016	0.060797
SH3KBP1	1.435134	1.611633	1.377761	0.61763	-0.6952	0.00506	0.052481
TM2D1	2.025183	2.238425	1.955868	0.77184	-0.3736	0.005068	0.047205
C11orf54	1.426405	1.254373	1.482325	1.89613	0.9231	0.005075	0.049521
TMEM9B	1.540425	1.723729	1.48084	0.66439	-0.5899	0.005106	0.055835
DGCR2	1.447253	1.625246	1.389395	0.62279	-0.6832	0.005114	0.04474
PROM1	1.276552	1.113986	1.329396	2.8898	1.531	0.005126	0.049539
CEP162	1.377641	1.208307	1.432684	2.07715	1.0546	0.00515	0.051757
ARL14EP	1.696679	1.509011	1.757681	1.48853	0.5739	0.005165	0.047669
IQCK	1.478394	1.303216	1.535336	1.76553	0.8201	0.005165	0.056423
DAPK3	1.455564	1.633022	1.39788	0.62854	-0.6699	0.005169	0.071229
HS3ST3B1	1.085802	1.23922	1.035933	0.15021	-2.7349	0.005184	0.053232
ARHGDIA	2.213847	2.43156	2.143078	0.79848	-0.3247	0.005186	0.053232
PARPBP	1.291086	1.127887	1.344135	2.69093	1.4281	0.005204	0.052296
ECT2	1.346719	1.179949	1.400929	2.22801	1.1558	0.005209	0.051836

RNF207	1.27625	1.441965	1.222384	0.50317	-0.9909	0.005213	0.047675
HPF1	2.075651	1.869307	2.142725	1.31452	0.3945	0.005234	0.053728
SMIM7	2.280762	2.042799	2.358113	1.30237	0.3811	0.005235	0.051632
KIF2C	1.219488	1.06147	1.270853	4.40623	2.1395	0.005283	0.050567
CASP6	1.620911	1.438409	1.680234	1.55159	0.6338	0.005295	0.050255
PRSS35	1.30829	1.144397	1.361564	2.50397	1.3242	0.005311	0.072201
SMARCA4	2.668131	2.39585	2.756638	1.25847	0.3317	0.005325	0.059523
RP11-14N7.2	1.533377	1.713465	1.474838	0.66554	-0.5874	0.005337	0.053689
MCC	1.162854	1.321191	1.111386	0.34679	-1.5279	0.005357	0.064154
JUN	2.967549	2.511554	3.115773	1.39973	0.4852	0.005375	0.052502
FAM172A	1.754902	1.564254	1.816873	1.4477	0.5338	0.005376	0.046621
TRMT10C	1.550876	1.37201	1.609018	1.6371	0.7111	0.005397	0.054842
TNFAIP2	1.100238	1.25416	1.050205	0.19753	-2.3398	0.005416	0.053415
TSPAN14	1.692075	1.504923	1.75291	1.49114	0.5764	0.005435	0.062844
C11orf57	1.584033	1.403757	1.642633	1.59163	0.6705	0.005478	0.052988
DDX24	2.691705	2.956072	2.605771	0.82092	-0.2847	0.005486	0.064931
AP000345.1	1.182961	1.027797	1.233398	8.3964	3.0698	0.005496	0.055285
NABP2	1.792907	1.601119	1.855249	1.42276	0.5087	0.005499	0.054183
NR2E1	1.166957	1.013073	1.216978	16.5981	4.0529	0.005523	0.051929
IGF1R	2.030561	2.237509	1.963291	0.77841	-0.3614	0.005532	0.055105
MAP2K1	1.790223	1.985122	1.72687	0.73785	-0.4386	0.00556	0.060082
IRF3	1.6051	1.790298	1.5449	0.68949	-0.5364	0.00557	0.047893
MRPS34	3.025631	2.693709	3.133524	1.25968	0.3331	0.005584	0.054543
PDCL3	1.485198	1.311301	1.541725	1.7402	0.7992	0.005594	0.052109
GEM	1.34384	1.575024	1.268693	0.46727	-1.0977	0.005597	0.053069
TNFRSF1B	1.170162	1.016045	1.220259	13.7276	3.779	0.005606	0.054703
TPD52	1.816911	2.012621	1.753294	0.74391	-0.4268	0.005613	0.053214
C1orf54	1.843329	1.649696	1.90627	1.39491	0.4802	0.005654	0.048199
NUTF2	2.976199	2.640122	3.085443	1.27152	0.3466	0.005685	0.05499
MYO3A	1.248111	1.089365	1.299712	3.35381	1.7458	0.005717	0.056884
UBAC1	1.743133	1.554918	1.804314	1.44943	0.5355	0.005719	0.065381
PQBP1	3.037094	2.737675	3.134422	1.22832	0.2967	0.005749	0.057265
DHPS	1.970703	1.770904	2.035649	1.34342	0.4259	0.005751	0.055579
MBP	1.197231	1.356357	1.145506	0.40831	-1.2922	0.005775	0.061742
MRPL10	2.105085	1.890622	2.174797	1.31908	0.3995	0.005776	0.05534
MAF1	3.025231	2.636994	3.15143	1.31426	0.3942	0.005815	0.056195
THRA	1.724947	1.538008	1.785712	1.46041	0.5464	0.00582	0.065788
LINC00662	1.589895	1.410762	1.648123	1.57785	0.658	0.005843	0.052956
DNMT3A	1.814825	1.621933	1.877526	1.41097	0.4967	0.005876	0.057954
RIN2	1.594642	1.412719	1.653777	1.58407	0.6636	0.005879	0.058553
PPHLN1	2.009442	1.806231	2.075497	1.33398	0.4157	0.005907	0.056058
NPRL2	1.491579	1.317689	1.548103	1.72528	0.7868	0.005912	0.058248
HACD3	3.177081	2.861248	3.279745	1.22485	0.2926	0.00594	0.058526
FAR2	1.300924	1.139352	1.353443	2.53633	1.3427	0.005943	0.056882
GPD1	1.082979	1.234197	1.033824	0.14443	-2.7916	0.005977	0.06607

ZNF512	1.509021	1.334937	1.565609	1.6887	0.7559	0.005985	0.070005
B4GALT1	1.382764	1.555015	1.326772	0.58876	-0.7642	0.006001	0.05861
HSP90B1	7.401954	8.278859	7.116911	0.84037	-0.2509	0.006076	0.067916
KHSRP	1.808844	1.618629	1.870675	1.40743	0.4931	0.006105	0.081888
XBP1	1.792654	1.985195	1.730067	0.74104	-0.4324	0.006106	0.070607
INTS10	1.68534	1.501221	1.745189	1.48675	0.5722	0.006121	0.051743
NECAB2	1.231946	1.075259	1.282878	3.75871	1.9102	0.006127	0.066208
ECM1	1.248348	1.090921	1.29952	3.29428	1.72	0.006127	0.059052
UBXN4	3.521015	3.878917	3.404676	0.83527	-0.2597	0.006145	0.057778
ATXN7L3B	2.383606	2.142136	2.462097	1.28014	0.3563	0.00616	0.056616
GPR180	1.279679	1.442949	1.226607	0.51159	-0.9669	0.006169	0.059052
FAM118A	1.771979	1.963758	1.709641	0.73633	-0.4416	0.006186	0.066208
CDCA2	1.196604	1.042915	1.246562	5.74542	2.5224	0.006188	0.059853
CACNA1D	1.255248	1.097409	1.306555	3.14709	1.654	0.006191	0.056616
ARL3	3.411533	3.043083	3.5313	1.23896	0.3091	0.006208	0.060569
ERLEC1	2.097456	2.304348	2.030205	0.78982	-0.3404	0.006222	0.059052
NKAIN4	3.848864	4.260801	3.714961	0.83261	-0.2643	0.006244	0.061582
CPQ	1.165985	1.3214	1.115466	0.35926	-1.4769	0.006254	0.061023
RP11-83A24.2	1.634124	1.453985	1.692679	1.52578	0.6095	0.006262	0.058334
CASP8AP2	1.481971	1.310353	1.537756	1.73273	0.793	0.006314	0.052931
ITPR2	1.159092	1.314421	1.108602	0.3454	-1.5337	0.006323	0.060424
ARF6	1.977407	2.199357	1.905261	0.75479	-0.4059	0.006324	0.059398
SEPT1	1.528877	1.354485	1.585565	1.65187	0.7241	0.006336	0.060601
CFI	1.32359	1.489427	1.269683	0.55102	-0.8598	0.00634	0.08392
CELF5	1.317501	1.156062	1.369977	2.3707	1.2453	0.006354	0.06722
CCDC77	1.267964	1.10998	1.319318	2.90342	1.5378	0.006367	0.060424
MOB3B	1.495278	1.323702	1.55105	1.70234	0.7675	0.00638	0.064827
TIMM17A	1.92655	2.132096	1.859736	0.75942	-0.397	0.006392	0.060601
SMPD1	1.264984	1.42632	1.212541	0.49855	-1.0042	0.006415	0.06327
ANAPC11	6.974481	6.24483	7.211659	1.18434	0.2441	0.006426	0.053425
RNASEH2B	1.734642	1.549157	1.794936	1.44756	0.5336	0.00644	0.060549
CHD1	1.893629	1.700172	1.956514	1.36611	0.4501	0.006444	0.061095
SLC20A1	1.425015	1.596069	1.369412	0.61975	-0.6902	0.00645	0.062333
CREB3L2	1.372329	1.54105	1.317485	0.58679	-0.7691	0.006516	0.062965
PDLIM7	4.614632	5.161215	4.436962	0.82595	-0.2759	0.006526	0.060723
TMEM88	1.162134	1.316606	1.111922	0.35351	-1.5002	0.006589	0.08608
SYNPR	1.962733	1.766743	2.026441	1.3387	0.4208	0.006593	0.061983
LPIN2	1.595777	1.775997	1.537196	0.69227	-0.5306	0.006636	0.070276
EPHX2	1.608661	1.430899	1.666443	1.54663	0.6291	0.006637	0.064822
TMEM134	2.775203	3.071903	2.678758	0.81025	-0.3036	0.006691	0.06192
TMEM18	1.749793	1.565314	1.809759	1.43241	0.5184	0.006704	0.063664
B4GALT5	1.305829	1.468937	1.25281	0.53911	-0.8913	0.006706	0.064869
UBE2A	1.776398	1.966924	1.714466	0.73891	-0.4365	0.006727	0.07049
SPOP	1.691324	1.509877	1.750305	1.47154	0.5573	0.006741	0.062215
SLC29A4	1.340373	1.505783	1.286606	0.56666	-0.8195	0.006743	0.064361

RAC1	7.150249	7.914138	6.901941	0.8536	-0.2284	0.006747	0.062849
ARID1A	2.179683	1.972665	2.246975	1.28202	0.3584	0.006752	0.06192
APOO	1.921124	1.727623	1.984022	1.35238	0.4355	0.006755	0.063451
TMEM158	1.079149	1.227838	1.030816	0.13526	-2.8862	0.006761	0.063664
DPYSL5	1.4201	1.254105	1.474058	1.8656	0.8996	0.006765	0.066076
VBP1	2.232483	2.02336	2.30046	1.27077	0.3457	0.006766	0.077342
RSL24D1	3.73136	3.317045	3.866036	1.23694	0.3068	0.006771	0.06192
POLR2D	1.559862	1.385564	1.616519	1.599	0.6772	0.006771	0.063664
CREB3L1	1.15511	1.308382	1.105288	0.34142	-1.5504	0.006784	0.060939
DPY19L1	1.398836	1.233708	1.452512	1.93623	0.9532	0.006788	0.064361
LEMD1	1.411914	1.246318	1.465743	1.89082	0.919	0.006793	0.087607
RBM39	4.928397	4.380409	5.106524	1.2148	0.2807	0.00688	0.071354
HEBP2	2.53869	2.797917	2.454427	0.80895	-0.3059	0.006887	0.056788
MPPED2	1.339352	1.17804	1.391787	2.20055	1.1379	0.006899	0.07627
STX3	1.449218	1.620139	1.39366	0.63479	-0.6556	0.006958	0.07284
NDUFB2	6.897221	7.802575	6.60293	0.82365	-0.2799	0.006998	0.064956
ADAM19	1.09995	1.248842	1.051552	0.20717	-2.2711	0.007002	0.064368
STON1	1.344756	1.183601	1.397141	2.16307	1.1131	0.00701	0.065838
KIF11	1.230051	1.076703	1.279898	3.64913	1.8676	0.007038	0.064956
HEG1	1.139806	1.291325	1.090554	0.31084	-1.6858	0.007038	0.064368
GRSF1	2.097486	2.304349	2.030244	0.78985	-0.3403	0.007064	0.067678
GLMN	1.372903	1.209764	1.425933	2.03054	1.0219	0.007067	0.066373
MED4	2.246003	2.031286	2.315798	1.27588	0.3515	0.007077	0.073326
PAX6	10.13785	9.150765	10.45871	1.16047	0.2147	0.007088	0.064241
CD9	2.465179	2.043404	2.602279	1.53563	0.6188	0.007125	0.058175
PRKCDBP	2.422531	2.173504	2.503479	1.28119	0.3575	0.007153	0.080848
HDAC7	1.542221	1.71797	1.485093	0.67565	-0.5657	0.007154	0.091102
RAB11FIP1	1.404545	1.572065	1.350092	0.61198	-0.7084	0.007155	0.064268
RIT1	1.485306	1.657263	1.42941	0.65333	-0.6141	0.007159	0.064878
SFI1	1.367023	1.204423	1.419878	2.05396	1.0384	0.007171	0.058175
ADIPOR1	1.98974	2.188167	1.92524	0.77871	-0.3608	0.007208	0.073992
HMMR	1.231286	1.078143	1.281066	3.59682	1.8467	0.007225	0.065483
THOC6	1.437713	1.271831	1.491634	1.8086	0.8549	0.007253	0.066872
RP11-115C21.2	1.479322	1.310423	1.534224	1.72095	0.7832	0.007254	0.068841
SPCS2	2.999517	2.700728	3.09664	1.23279	0.3019	0.007277	0.064783
TRAK1	1.298892	1.459953	1.246538	0.53601	-0.8997	0.007282	0.066598
WIPI2	2.163653	1.959206	2.23011	1.28242	0.3589	0.007291	0.070525
ILDR2	1.323825	1.165112	1.375415	2.27371	1.185	0.007347	0.065991
PXDC1	1.126528	1.276632	1.077736	0.28101	-1.8313	0.007347	0.068369
SFRP5	1.068719	1.21531	1.021069	0.09785	-3.3532	0.007354	0.059186
AC009005.2	1.341613	1.181893	1.39353	2.16353	1.1134	0.007413	0.065997
LMAN2	2.602785	2.900188	2.506113	0.79261	-0.3353	0.007426	0.069106
MAPRE2	1.420271	1.255425	1.473855	1.85516	0.8915	0.007441	0.068606
SPATA6	1.433586	1.267894	1.487445	1.81954	0.8636	0.00745	0.059486
MLANA	1.079617	1.227002	1.031709	0.13968	-2.8398	0.007512	0.0719

RP11-182L21.6	1.459684	1.293336	1.513757	1.75143	0.8085	0.007531	0.068803
FAM53C	1.650742	1.472906	1.708548	1.49829	0.5833	0.007551	0.059811
RP11-736K20.5	1.104299	1.252501	1.056125	0.22228	-2.1696	0.007557	0.066689
CCT5	2.537791	2.285439	2.619819	1.26013	0.3336	0.007599	0.068253
NEDD1	1.374306	1.212611	1.426866	2.00773	1.0056	0.007601	0.083119
SNHG10	1.315862	1.15809	1.367146	2.32238	1.2156	0.007603	0.095613
ATM	1.882393	1.693643	1.943747	1.36057	0.4442	0.007635	0.069198
GDI2	4.413321	3.814752	4.60789	1.28178	0.3581	0.007689	0.070252
EEF1G	1.553489	1.381061	1.609538	1.59958	0.6777	0.007724	0.073233
VDAC1	5.745015	5.034451	5.975988	1.23337	0.3026	0.007734	0.070734
F3	1.103478	1.25075	1.055606	0.22176	-2.1729	0.007755	0.078804
NUMA1	1.647428	1.827107	1.589022	0.71215	-0.4898	0.007787	0.084238
DDT	3.805983	3.432592	3.927356	1.20339	0.2671	0.007816	0.073412
ZNF704	1.245659	1.092892	1.295317	3.17912	1.6686	0.007835	0.080349
ZNF90	1.590043	1.417767	1.646043	1.54642	0.6289	0.007844	0.069221
JAM2	1.447205	1.282467	1.500753	1.77278	0.826	0.00785	0.071078
CA2	1.826424	2.06105	1.750158	0.707	-0.5002	0.007874	0.061769
SMIM3	1.365779	1.529474	1.312569	0.59034	-0.7604	0.007926	0.061769
PRKD3	1.534235	1.364956	1.58926	1.61461	0.6912	0.007948	0.069524
MPST	2.865235	2.550636	2.967497	1.26883	0.3435	0.007984	0.069846
RPP25	1.202943	1.356407	1.153058	0.42945	-1.2194	0.007991	0.074969
WDR60	2.059219	1.858425	2.124488	1.30994	0.3895	0.008022	0.061769
NDRG4	1.465667	1.299724	1.519608	1.73362	0.7938	0.008043	0.061769
SHISA4	1.48899	1.658522	1.433882	0.65887	-0.6019	0.008048	0.074969
RHOQ	1.403831	1.568931	1.350165	0.61548	-0.7002	0.00805	0.086149
DDX21	1.912514	2.118925	1.845419	0.75556	-0.4044	0.008067	0.072144
LYPLA1	3.061983	2.723626	3.171968	1.26012	0.3336	0.008074	0.073171
GSX2	1.0708	1.215951	1.023617	0.10936	-3.1928	0.00808	0.075888
FAM57B	1.207496	1.057491	1.256255	4.4573	2.1562	0.008083	0.077445
DCAF16	1.411717	1.249484	1.464452	1.86165	0.8966	0.008103	0.100641
ASGR1	1.398409	1.236728	1.450964	1.90499	0.9298	0.008112	0.072144
KANK4	1.071393	1.215928	1.024412	0.11305	-3.1449	0.008123	0.08246
ORMDL2	1.471988	1.640974	1.417059	0.65066	-0.62	0.008146	0.075185
SEC31A	1.884494	2.077989	1.821597	0.76216	-0.3918	0.008147	0.073355
ILK	2.230947	2.445369	2.161248	0.80343	-0.3158	0.008148	0.073183
KIF15	1.16161	1.015211	1.209198	13.7528	3.7817	0.008172	0.075343
RP11-61K9.3	1.422196	1.25897	1.475253	1.83517	0.8759	0.008175	0.073355
HSPA9	2.100807	1.902557	2.16525	1.29105	0.3685	0.008192	0.072933
MAP3K2	1.604876	1.780823	1.547683	0.70142	-0.5117	0.008195	0.086781
RP11-119B16.2	1.278827	1.123939	1.329174	2.65595	1.4092	0.008197	0.076279
PYCR1	2.10286	1.899827	2.168857	1.29898	0.3774	0.008223	0.062668
TMEM218	1.520221	1.352045	1.574888	1.633	0.7075	0.008245	0.078253
COL13A1	1.240395	1.089358	1.289491	3.23968	1.6959	0.008263	0.075493
THRAP3	2.389938	2.160206	2.464614	1.26237	0.3361	0.008338	0.074227
YIF1A	2.398477	2.679605	2.307094	0.77821	-0.3618	0.008347	0.074237

NELFCD	2.218319	2.015211	2.28434	1.2651	0.3392	0.008355	0.084053
CLINT1	1.544775	1.716932	1.488814	0.68181	-0.5525	0.008386	0.087875
WNK2	1.326867	1.487118	1.274777	0.56409	-0.826	0.00839	0.074036
RUVBL2	1.992277	1.791118	2.057665	1.33693	0.4189	0.008413	0.076176
AGPAT1	1.739056	1.92116	1.679862	0.73805	-0.4382	0.008448	0.092377
MYO5A	1.240478	1.394891	1.190285	0.48187	-1.0533	0.008462	0.094586
FGF13	1.444929	1.28213	1.497847	1.7646	0.8193	0.008475	0.079684
HIST1H1E	1.264533	1.112237	1.314038	2.79799	1.4844	0.008501	0.104082
RAB7A	2.892361	3.178138	2.799467	0.82615	-0.2755	0.008623	0.093275
VPS41	1.382802	1.545136	1.330035	0.60542	-0.724	0.008632	0.076846
CAPN1	1.592463	1.766277	1.535964	0.69944	-0.5157	0.008638	0.07751
VMA21	1.923299	1.721521	1.988888	1.37056	0.4548	0.008669	0.076421
ANXA5	4.049167	4.512412	3.898586	0.82524	-0.2771	0.00868	0.104082
TMEM259	1.639855	1.816353	1.582483	0.71352	-0.487	0.008691	0.104082
NIPBL	2.196962	1.985475	2.265707	1.28436	0.3611	0.008704	0.065836
PLPP4	1.177703	1.327442	1.129029	0.39405	-1.3435	0.008713	0.076219
PIK3CD-AS2	1.434523	1.272584	1.487162	1.7872	0.8377	0.008784	0.080989
DDAH1	1.514263	1.683478	1.459259	0.67194	-0.5736	0.008817	0.076462
SERGEF	1.699429	1.522921	1.756804	1.44726	0.5333	0.008819	0.076483
LAMA1	1.139987	1.287676	1.09198	0.31974	-1.645	0.008828	0.082151
VGF	1.489832	1.325801	1.543151	1.66712	0.7374	0.008831	0.094513
PPP1R15A	1.663097	1.847218	1.603247	0.71203	-0.49	0.00884	0.088046
PTPRF	2.036441	2.252934	1.966069	0.77105	-0.3751	0.008855	0.078061
SHC1	2.205522	2.409363	2.139263	0.80835	-0.3069	0.008879	0.098159
PCNX1	1.395126	1.557646	1.342298	0.61383	-0.7041	0.008929	0.089823
CSRNP1	1.129455	1.276306	1.081721	0.29576	-1.7575	0.008931	0.089752
SEC62	3.506095	3.847237	3.395205	0.84124	-0.2494	0.009066	0.08
ZNF397	1.929593	1.72592	1.995798	1.37177	0.456	0.009102	0.079539
CCDC160	1.395175	1.236858	1.446637	1.88567	0.9151	0.009127	0.085018
GDF7	1.065997	1.207563	1.01998	0.09626	-3.377	0.009186	0.0906
TAF1D	2.654429	2.398917	2.737485	1.24202	0.3127	0.009222	0.084344
HDAC3	2.135106	1.938494	2.199015	1.27759	0.3534	0.009247	0.080894
OSER1-AS1	1.354717	1.198073	1.405635	2.04791	1.0342	0.00925	0.084514
PVRL3-AS1	1.52062	1.688988	1.465891	0.6762	-0.5645	0.009264	0.096405
EIF1AX	3.489951	3.155658	3.598615	1.20549	0.2696	0.009272	0.096405
C2orf68	1.542399	1.375241	1.596735	1.59027	0.6693	0.009296	0.096405
ESRRG	1.301894	1.148941	1.351613	2.36075	1.2392	0.009301	0.100804
NFIC	1.421165	1.583762	1.368312	0.63093	-0.6645	0.009319	0.100804
ZNF326	2.245973	2.451408	2.179196	0.81245	-0.2997	0.009322	0.091044
PRKRA	2.262881	2.058569	2.329294	1.25575	0.3285	0.009361	0.081179
ZNF721	1.636746	1.465267	1.692486	1.48836	0.5737	0.009448	0.093275
MACF1	2.193697	2.394669	2.12837	0.80906	-0.3057	0.009467	0.081399
ATP13A2	1.415569	1.577246	1.363014	0.62887	-0.6692	0.009484	0.093275
NFIA	1.198743	1.348511	1.15006	0.43058	-1.2157	0.009484	0.091736
HBEGF	1.082526	1.224703	1.03631	0.16159	-2.6296	0.00949	0.112321

PTPRD	1.728221	1.55175	1.785584	1.42381	0.5098	0.00951	0.084581
CDKN2AIPNL	1.885897	1.699489	1.946491	1.35312	0.4363	0.009521	0.08258
COMMD4	1.768794	1.590304	1.826814	1.40066	0.4861	0.009532	0.08258
SGCD	1.095056	1.238941	1.048285	0.20208	-2.307	0.009536	0.087924
DHX40	1.775769	1.597062	1.833859	1.3966	0.4819	0.009542	0.097949
UBL3	1.695982	1.872494	1.638605	0.73193	-0.4502	0.00956	0.093275
HINT1	22.90186	20.9872	23.52423	1.12693	0.1724	0.009573	0.089079
ZNF677	1.764765	1.586603	1.822678	1.40244	0.4879	0.009579	0.081598
CORO1B	1.768076	1.948575	1.709404	0.74786	-0.4192	0.009633	0.081598
FNIP2	1.387682	1.548441	1.335426	0.6116	-0.7093	0.009652	0.081598
MLH3	1.530954	1.365072	1.584875	1.60208	0.6799	0.009668	0.086569
GALNT2	1.610424	1.78245	1.554505	0.70868	-0.4968	0.00971	0.083598
SDHC	3.152502	2.831794	3.256751	1.23199	0.301	0.009718	0.087988
METTL23	1.794282	1.614649	1.852673	1.38725	0.4722	0.009721	0.0835
MGST2	1.367135	1.52599	1.315499	0.59982	-0.7374	0.009723	0.083598
COL4A5	1.652577	1.826125	1.596164	0.72164	-0.4707	0.009724	0.086569
WEE1	1.660476	1.489541	1.71604	1.46268	0.5486	0.009748	0.085936
MLLT1	1.388331	1.548709	1.336199	0.61271	-0.7067	0.009749	0.093407
PLEKHA4	1.777642	1.599611	1.835513	1.39342	0.4786	0.009758	0.087943
XRCC5	3.720235	3.373915	3.832808	1.19331	0.255	0.00976	0.073272
RIMKLB	1.455845	1.29477	1.508204	1.72407	0.7858	0.009766	0.083973
GTF2H5	3.480143	3.082082	3.609536	1.25333	0.3258	0.009784	0.08634
RBX1	6.51164	5.836236	6.731185	1.18505	0.2449	0.009791	0.087943
GPATCH8	1.779242	1.601182	1.837122	1.39246	0.4776	0.00986	0.102255
LYRM1	1.785478	1.606612	1.843619	1.39071	0.4758	0.009877	0.073601
TMX1	1.676461	1.50441	1.732388	1.45197	0.538	0.009883	0.097494
PRR11	1.210571	1.064386	1.258089	4.00845	2.003	0.009885	0.097494
LINC00094	1.782597	1.604495	1.84049	1.3904	0.4755	0.009945	0.084448
WNT6	1.070198	1.233978	1.016961	0.07249	-3.7861	0.009971	0.09176
VAMP2	3.744818	3.387524	3.860959	1.1983	0.261	0.010043	0.09176
PSMA3	2.717471	2.47048	2.797756	1.22256	0.2899	0.010068	0.084448
NUDT3	2.027856	1.836782	2.089965	1.30257	0.3814	0.010105	0.084448
SUB1	6.606048	5.951463	6.818825	1.17517	0.2329	0.010163	0.084448
ENDOD1	1.175705	1.322661	1.127936	0.3965	-1.3346	0.010239	0.089572
PGF	1.206376	1.354755	1.158144	0.44578	-1.1656	0.010241	0.084448
TACC1	1.278472	1.431539	1.228716	0.53	-0.9159	0.010291	0.104572
LTBP3	1.570242	1.738321	1.515606	0.69835	-0.518	0.010331	0.095146
RNASEH1-AS1	1.457776	1.297529	1.509865	1.71367	0.7771	0.010335	0.106094
ASRGL1	1.489405	1.328411	1.541737	1.64957	0.7221	0.010362	0.089776
LINC01315	1.245053	1.097628	1.292974	3.00092	1.5854	0.010374	0.086967
TMEM39B	1.338222	1.184879	1.388067	2.09903	1.0697	0.010385	0.09488
RGS12	1.529569	1.365566	1.582879	1.59446	0.6731	0.010388	0.121512
GPR162	1.589931	1.423329	1.644086	1.52148	0.6055	0.010404	0.095146
COL1A2	4.209665	4.891579	3.988005	0.76781	-0.3812	0.01042	0.085227
LAMTOR1	3.825054	4.254804	3.685361	0.82505	-0.2775	0.010468	0.089916

ARF4	3.075775	3.470295	2.947534	0.78838	-0.343	0.010507	0.089486
CANX	4.92489	5.399601	4.770582	0.85703	-0.2226	0.010535	0.077768
SKP2	1.390043	1.234275	1.440676	1.88102	0.9115	0.010567	0.095676
RBM41	1.758279	1.581929	1.815603	1.40155	0.487	0.010591	0.077768
SEC11C	2.374484	2.637668	2.288935	0.78705	-0.3455	0.010617	0.090421
RFLNB	1.258294	1.409846	1.209031	0.51002	-0.9714	0.010687	0.094554
NRBP1	1.767239	1.945628	1.709253	0.75003	-0.415	0.010689	0.090273
MRE11	1.370503	1.216354	1.42061	1.94408	0.9591	0.010696	0.095978
COL25A1	1.201169	1.057023	1.248025	4.34957	2.1209	0.010715	0.094554
UBXN2A	1.995095	1.807906	2.055942	1.30701	0.3863	0.010753	0.093252
CDCA4	1.648919	1.481043	1.703488	1.46242	0.5484	0.010756	0.097382
PIN4	2.831021	2.551201	2.921978	1.23903	0.3092	0.010762	0.091749
ZKSCAN1	2.953865	2.674608	3.044639	1.22097	0.288	0.010777	0.0951
TUBB4B	3.14963	2.800672	3.263061	1.25679	0.3297	0.010784	0.123526
PFDN2	2.688126	2.96365	2.598565	0.81408	-0.2968	0.010805	0.123526
FYN	1.601546	1.43465	1.655796	1.50879	0.5934	0.010852	0.108394
VCAN	2.005364	2.275412	1.917584	0.71944	-0.4751	0.010864	0.097487
NLGN2	1.448841	1.610726	1.396219	0.64877	-0.6242	0.010879	0.091966
VPS25	2.330685	2.114642	2.400912	1.25683	0.3298	0.010882	0.108394
FAM227A	1.527752	1.364614	1.580781	1.59286	0.6716	0.010886	0.09117
INPP5F	1.673742	1.503265	1.729157	1.44885	0.5349	0.010887	0.116514
LLNLR-245B6.1	2.569512	2.323332	2.649535	1.2465	0.3179	0.010893	0.106394
C15orf61	1.505463	1.670035	1.451968	0.67454	-0.568	0.010918	0.095505
RP11-841O20.2	1.463522	1.625519	1.410864	0.65684	-0.6064	0.010967	0.091183
SOD1	9.447725	8.330516	9.810881	1.20195	0.2654	0.010968	0.094306
SCLT1	1.277794	1.129862	1.32588	2.50943	1.3274	0.010991	0.089171
ERGIC1	1.987574	1.802213	2.047826	1.30617	0.3853	0.010997	0.105288
UBE2V2	2.841083	2.583684	2.924753	1.21536	0.2814	0.011	0.105288
CWC27	1.708386	1.536574	1.764235	1.42429	0.5102	0.011006	0.093832
FOPNL	1.508199	1.34655	1.560745	1.61808	0.6943	0.011061	0.10909
ATG101	1.408317	1.566622	1.356859	0.6298	-0.667	0.011155	0.11813
HNRNPF	2.835193	2.594116	2.913557	1.20039	0.2635	0.011171	0.106019
PPP2CB	1.553668	1.71919	1.499864	0.69504	-0.5248	0.011213	0.092457
SMIM19	2.113632	1.923328	2.175492	1.2731	0.3484	0.01122	0.094064
TWF1	1.809952	1.988097	1.752044	0.7611	-0.3938	0.011268	0.113842
FKBP9	1.34563	1.499965	1.295462	0.59096	-0.7589	0.011302	0.118436
PCSK7	1.511373	1.675005	1.458183	0.67878	-0.559	0.011316	0.113842
CETN3	1.82213	1.646	1.879382	1.36127	0.445	0.011339	0.095858
FAM229B	2.764787	2.488968	2.854444	1.24546	0.3167	0.011356	0.128357
FSCN1	3.477899	3.159433	3.581419	1.19542	0.2575	0.011356	0.09934
C6orf106	1.280917	1.431612	1.231933	0.53736	-0.896	0.011366	0.101992
RFX2	1.2858	1.137879	1.333882	2.42156	1.2759	0.01138	0.099449
ARHGEF26	1.255739	1.109554	1.303258	2.76811	1.4689	0.011381	0.106998
TMCC1-AS1	1.256772	1.110483	1.304324	2.75449	1.4618	0.011392	0.096212
RAF1	1.681833	1.512518	1.73687	1.43775	0.5238	0.011413	0.093349

MRPS21	5.17252	4.480695	5.397402	1.26337	0.3373	0.011445	0.095057
SCAMP3	1.363099	1.518449	1.312602	0.60296	-0.7299	0.011465	0.082743
ZDHHC2	1.226021	1.373328	1.178138	0.47716	-1.0675	0.011488	0.082743
BUB1	1.180228	1.038742	1.226219	5.83909	2.5457	0.011515	0.082743
KCTD20	1.616807	1.450592	1.670837	1.48879	0.5741	0.011548	0.119763
RFC2	1.434748	1.279113	1.485339	1.73886	0.7981	0.011612	0.09654
VDAC2	4.978289	5.482644	4.814345	0.85091	-0.2329	0.011685	0.101236
PTBP2	1.392001	1.238094	1.442029	1.85653	0.8926	0.011713	0.113637
TERF1	1.97049	1.788292	2.029715	1.30626	0.3854	0.011716	0.111079
CEP63	1.505878	1.345992	1.557849	1.61232	0.6891	0.011747	0.104477
OCEL1	1.551357	1.388848	1.604182	1.55377	0.6358	0.011748	0.113637
SLC4A8	1.302036	1.153713	1.35025	2.2786	1.1881	0.011788	0.104837
TMEM8A	1.256844	1.40549	1.208526	0.51426	-0.9594	0.011822	0.121356
PPIH	1.517595	1.35744	1.569655	1.59371	0.6724	0.011861	0.102867
ATP6V0D1	1.615618	1.784257	1.560801	0.71507	-0.4838	0.012001	0.114978
PPID	1.703664	1.534293	1.758719	1.42004	0.5059	0.012012	0.101974
SPDL1	1.466373	1.309868	1.517245	1.66924	0.7392	0.012027	0.101974
GNAQ	1.559844	1.724029	1.506474	0.69952	-0.5156	0.012074	0.101974
CENPJ	1.238041	1.093947	1.284879	3.03232	1.6004	0.012085	0.098043
RAB9A	1.794067	1.971566	1.73637	0.75792	-0.3999	0.012099	0.099668
LITAF	2.456475	2.691136	2.380197	0.81614	-0.2931	0.012104	0.109696
ADGRA2	1.38047	1.228961	1.429719	1.87682	0.9083	0.012245	0.113791
TPR	3.09982	2.813485	3.192895	1.20922	0.2741	0.012258	0.105398
HMG20B	2.613126	2.370918	2.691857	1.2341	0.3035	0.012269	0.087537
FAM174A	1.407619	1.56389	1.356822	0.63279	-0.6602	0.012285	0.10289
PIGM	1.301022	1.153422	1.349	2.27477	1.1857	0.012312	0.104561
ZBTB4	1.243443	1.390152	1.195754	0.50174	-0.995	0.012329	0.113791
GLO1	3.617357	3.210689	3.749547	1.24375	0.3147	0.012369	0.104561
MYO1E	1.221296	1.366471	1.174106	0.47509	-1.0737	0.012402	0.116488
FBXO17	1.756545	1.58552	1.812138	1.38704	0.472	0.012417	0.107686
MELK	1.202837	1.061463	1.248791	4.04783	2.0172	0.012434	0.123843
TCERG1	1.884495	1.707796	1.941932	1.3308	0.4123	0.012462	0.118274
DRAM2	1.857087	1.681273	1.914236	1.34195	0.4243	0.012479	0.104614
PCAT6	1.778562	1.601287	1.836186	1.39066	0.4758	0.012486	0.110072
AC114803.3	1.336615	1.18749	1.38509	2.05392	1.0384	0.01252	0.102877
RTKN2	1.281673	1.13597	1.329035	2.41992	1.275	0.012526	0.100811
EIF4A1	4.007888	3.590573	4.143539	1.21345	0.2791	0.012578	0.102877
EP400	1.475528	1.319017	1.526402	1.65008	0.7225	0.012596	0.104727
NFE2L3	1.161886	1.303297	1.115919	0.3822	-1.3876	0.01268	0.12885
HAT1	1.599952	1.438001	1.652595	1.48994	0.5753	0.012735	0.103243
ZMYM2	2.486903	2.268074	2.558035	1.22866	0.2971	0.012755	0.125799
ADGRV1	1.357463	1.207725	1.406137	1.95517	0.9673	0.0128	0.117061
ADAMTS7	1.256842	1.404062	1.208987	0.51722	-0.9512	0.012846	0.112263
PLEKHG3	1.136856	1.276488	1.091468	0.33082	-1.5959	0.012847	0.105934
PLXNB1	1.280488	1.428803	1.232278	0.54169	-0.8845	0.012849	0.103411

CYB5B	2.00338	1.821313	2.062562	1.29374	0.3715	0.012886	0.103243
WBSCR22	1.886594	2.06597	1.828287	0.77703	-0.364	0.012905	0.115919
CD70	1.112963	1.251403	1.067962	0.27033	-1.8872	0.012913	0.120161
OTX2-AS1	1.072683	1.208883	1.02841	0.13601	-2.8782	0.012931	0.103243
PHYKPL	1.868643	1.689594	1.926844	1.34404	0.4266	0.012942	0.144301
PRPF4B	2.167043	1.969967	2.231104	1.26922	0.3439	0.012963	0.108671
NSD1	2.319947	2.110651	2.38798	1.2497	0.3216	0.012984	0.125595
UHRF1	1.2799	1.135198	1.326937	2.41821	1.2739	0.01301	0.108056
CAPRIN1	2.433974	2.196404	2.511198	1.26312	0.337	0.013053	0.144301
C20orf96	1.727151	1.557759	1.782212	1.40242	0.4879	0.013072	0.092609
PALMD	1.375702	1.225422	1.424552	1.88336	0.9133	0.013079	0.118535
MRPS9	1.585691	1.424276	1.638161	1.50412	0.5889	0.013092	0.123085
DYNC1H1	2.406818	2.624805	2.335959	0.82223	-0.2824	0.013099	0.112632
CTC-338M12.5	1.411797	1.259786	1.461209	1.77534	0.8281	0.013115	0.107159
MEF2A	1.480965	1.639629	1.42939	0.67131	-0.5749	0.013119	0.128129
CLVS1	1.233218	1.091111	1.279411	3.06672	1.6167	0.013175	0.105192
RBM12B	1.552165	1.392617	1.604026	1.53846	0.6215	0.013205	0.117438
PRDX3	2.918615	2.666239	3.000652	1.2007	0.2639	0.013208	0.132873
VEPH1	1.112604	1.25026	1.067858	0.27115	-1.8828	0.013216	0.11068
ABCA1	1.086286	1.22247	1.042018	0.18887	-2.4045	0.013237	0.11285
CLPTM1L	1.282094	1.429518	1.234172	0.5452	-0.8751	0.013238	0.117856
PAG1	1.190248	1.3326	1.143975	0.43288	-1.208	0.013276	0.123664
ANTXR1	1.489409	1.647457	1.438035	0.67655	-0.5637	0.013401	0.109515
MRPL2	1.632534	1.468845	1.685743	1.46262	0.5486	0.013414	0.118255
STAP2	1.333852	1.484046	1.28503	0.58885	-0.764	0.01343	0.123954
BST2	1.678164	1.882885	1.611618	0.69275	-0.5296	0.013444	0.111663
TDG	1.835822	2.016253	1.777172	0.76474	-0.387	0.013493	0.109515
ALDH6A1	1.333187	1.186209	1.380963	2.04589	1.0327	0.013506	0.106981
COMTD1	1.159916	1.299928	1.114404	0.38144	-1.3905	0.013512	0.109515
RCC1	1.472233	1.317855	1.522414	1.64356	0.7168	0.013517	0.119281
KIF4A	1.198113	1.058828	1.243388	4.13728	2.0487	0.013534	0.10806
CNTLN	1.473955	1.318696	1.524422	1.64553	0.7185	0.01357	0.11859
UBE2Z	1.518173	1.677927	1.466244	0.68775	-0.54	0.013594	0.119844
CYB5R2	1.142591	1.281176	1.097543	0.34691	-1.5274	0.013668	0.122772
NSRP1	1.88498	1.709677	1.941963	1.32731	0.4085	0.013751	0.132609
FBXO22	1.73329	1.56481	1.788056	1.39526	0.4805	0.013805	0.111277
HEY1	1.416921	1.265225	1.466231	1.75787	0.8138	0.013806	0.119613
ABT1	1.812782	1.639816	1.869005	1.35821	0.4417	0.01384	0.111277
FIRRE	1.313235	1.167264	1.360683	2.15636	1.1086	0.013841	0.132609
STUB1	2.979148	3.292423	2.877316	0.81892	-0.2882	0.01387	0.113803
EPHB2	1.574778	1.414889	1.62675	1.51065	0.5952	0.013928	0.113803
FAM127B	2.858672	2.623124	2.935238	1.19229	0.2537	0.014024	0.124847
SOCS1	1.081506	1.2169	1.037495	0.17287	-2.5323	0.01406	0.12077
HCFC1R1	3.632341	4.081726	3.486266	0.80678	-0.3098	0.01409	0.112381
PTGES2	1.791465	1.963579	1.735519	0.76332	-0.3896	0.014109	0.133906

MPHOSPH9	1.500054	1.344901	1.550488	1.59607	0.6745	0.014121	0.154408
ZMYM4	1.691276	1.526452	1.744853	1.41486	0.5007	0.01416	0.117727
LRRC59	1.737246	1.907146	1.682019	0.75183	-0.4115	0.014205	0.123065
SECISBP2	2.079499	1.896744	2.138904	1.27004	0.3449	0.014284	0.112264
UBXN6	1.502505	1.660141	1.451265	0.68359	-0.5488	0.014353	0.130661
UBE2J1	1.822084	1.994238	1.766124	0.77056	-0.376	0.014417	0.130661
PGAP1	1.587921	1.428516	1.639736	1.49291	0.5781	0.014515	0.135911
TSC22D1	11.26575	9.686771	11.77901	1.24085	0.3113	0.014573	0.13789
MRPL1	1.626937	1.465777	1.679323	1.45847	0.5445	0.01459	0.13789
EMC8	1.640456	1.804967	1.586981	0.7292	-0.4556	0.014591	0.135911
SMOX	1.272399	1.417313	1.225294	0.53987	-0.8893	0.014618	0.119558
IARS	1.616011	1.45518	1.66829	1.46819	0.554	0.014666	0.13789
SRM	1.918276	2.095018	1.860826	0.78613	-0.3472	0.014674	0.121001
ACP6	1.598698	1.43931	1.650508	1.48075	0.5663	0.014716	0.12986
DNAJB14	1.692298	1.859129	1.638068	0.74269	-0.4292	0.014719	0.116165
IMP3	2.034081	1.840592	2.096976	1.305	0.3841	0.014778	0.159859
PIEZO1	1.159299	1.29758	1.11435	0.38427	-1.3798	0.014795	0.116165
SLITRK4	1.189574	1.329358	1.144136	0.43763	-1.1922	0.014875	0.120679
ZCCHC9	1.3474	1.200952	1.395004	1.96566	0.975	0.014901	0.125969
SF3A2	1.742932	1.577111	1.796834	1.38073	0.4654	0.014988	0.128741
KDM1A	1.85205	1.681007	1.907649	1.3328	0.4145	0.015023	0.139936
ADAMTS9	1.188753	1.327947	1.143508	0.43759	-1.1923	0.015025	0.138534
MRPS18B	1.89584	1.72375	1.951779	1.31507	0.3951	0.015068	0.126324
MED30	1.538654	1.383302	1.589152	1.53705	0.6202	0.015099	0.117752
FUNDC1	1.601905	1.443126	1.653517	1.47479	0.5605	0.015134	0.122658
TM7SF2	2.714571	2.47729	2.791701	1.21283	0.2784	0.015151	0.139834
ITGA5	1.149786	1.287209	1.105116	0.36599	-1.4501	0.015159	0.118102
EVA1B	1.654726	1.818498	1.601491	0.73487	-0.4444	0.015161	0.136183
DHCR7	1.367965	1.517258	1.319436	0.61756	-0.6954	0.015277	0.133133
CEBPZOS	1.952837	1.771523	2.011774	1.3114	0.3911	0.015292	0.122947
DVL3	1.475574	1.630083	1.42535	0.67507	-0.5669	0.015298	0.122994
BAZ1A	1.919573	1.745292	1.976224	1.30985	0.3894	0.015334	0.130727
NENF	3.549336	3.843141	3.453833	0.86307	-0.2124	0.015351	0.133133
ECHS1	2.937223	2.672034	3.023424	1.21016	0.2752	0.015376	0.12576
JAZF1	1.143342	1.280045	1.098906	0.35318	-1.5015	0.015399	0.14083
MAPKAPK3	1.880673	1.707004	1.937125	1.32549	0.4065	0.015426	0.119254
COL11A1	2.225535	2.020662	2.292131	1.26597	0.3402	0.015465	0.135152
GEMIN8	1.475376	1.323428	1.524767	1.62251	0.6982	0.01548	0.135413
BDP1	2.030047	1.837453	2.092651	1.30473	0.3838	0.015551	0.166427
ELP5	2.039475	1.858361	2.098347	1.27959	0.3557	0.015711	0.135959
RACGAP1	1.235181	1.096865	1.280141	2.89208	1.5321	0.015733	0.133006
AMMECR1	1.309402	1.166606	1.355819	2.13568	1.0947	0.015751	0.125631
COQ4	2.020051	1.830508	2.081664	1.30241	0.3812	0.015799	0.167305
LLNLF-187D8.1	1.19326	1.056708	1.237647	4.19072	2.0672	0.015828	0.135959
FLRT2	1.443092	1.292797	1.491946	1.68016	0.7486	0.015854	0.124687

ORMDL1	2.726039	2.473544	2.808114	1.22705	0.2952	0.015865	0.124687
TMEM184B	1.44687	1.599708	1.397188	0.6623	-0.5944	0.015881	0.131044
UQCRB	10.5922	11.33767	10.34988	0.90445	-0.1449	0.015885	0.136585
VTI1B	2.78403	3.040389	2.700699	0.83352	-0.2627	0.015892	0.131044
FUCA2	1.502959	1.658751	1.452317	0.68663	-0.5424	0.015895	0.12578
TAF10	1.893776	2.067709	1.837238	0.78414	-0.3508	0.015911	0.13799
ALDH1A3	1.373358	1.53213	1.321748	0.60464	-0.7258	0.015954	0.158911
RARB	1.170345	1.307865	1.125644	0.40811	-1.293	0.016023	0.14788
NDUFA7	2.281615	2.078204	2.347735	1.24998	0.3219	0.016026	0.128649
ZC3H6	1.828893	1.660526	1.883622	1.33775	0.4198	0.016058	0.138069
HPS1	1.843779	2.015278	1.788033	0.77617	-0.3655	0.01607	0.128649
YBX3	3.10026	2.846352	3.182794	1.18222	0.2415	0.01611	0.137344
NDUFC1	6.110148	5.570139	6.285682	1.15657	0.2099	0.016113	0.128649
ZNF608	1.805346	1.637064	1.860047	1.35002	0.433	0.016131	0.123749
ERCC6L2	1.353124	1.207941	1.400317	1.92514	0.945	0.016161	0.113691
FAM199X	1.541515	1.698721	1.490414	0.70187	-0.5107	0.016291	0.127043
OAZ1	12.8179	13.54171	12.58262	0.92353	-0.1148	0.016352	0.139289
ATP1A2	1.157076	1.023768	1.200409	8.43201	3.0759	0.016472	0.130479
FBXW5	1.79738	1.965653	1.742681	0.7691	-0.3788	0.016476	0.139289
COX7A2	10.67762	12.00994	10.24455	0.83965	-0.2521	0.016502	0.150766
SEMA3B	1.281112	1.424252	1.234583	0.55293	-0.8548	0.016517	0.140672
ADD2	1.235774	1.097851	1.280606	2.86769	1.5199	0.016551	0.128076
ST14	1.069751	1.200724	1.027177	0.13539	-2.8848	0.016592	0.173872
OBSCN	1.19039	1.328267	1.145572	0.44346	-1.1731	0.016726	0.133412
DNAJC8	2.831061	2.589185	2.909684	1.20167	0.265	0.016752	0.151678
TCTEX1D2	2.285204	2.080299	2.351809	1.25133	0.3235	0.016759	0.140494
ABHD17A	1.540073	1.695912	1.489417	0.70327	-0.5078	0.016888	0.150347
C20orf24	2.266311	2.466626	2.201198	0.81902	-0.288	0.016905	0.142773
FAM192A	2.012342	1.836363	2.069545	1.27881	0.3548	0.017008	0.130614
MAML2	1.56094	1.406796	1.611045	1.50209	0.587	0.017087	0.135354
RPIA	1.590346	1.434309	1.641067	1.47606	0.5618	0.0171	0.145639
BCAM	1.550222	1.705643	1.499701	0.70815	-0.4979	0.017117	0.151048
USP16	1.855341	1.687494	1.909901	1.3235	0.4044	0.017206	0.133148
FAHD2A	1.755629	1.5924	1.808687	1.3651	0.449	0.017207	0.140367
CHCHD2	14.29653	12.8272	14.77415	1.16462	0.2199	0.017224	0.15752
RASSF8	1.342807	1.487481	1.29578	0.60675	-0.7208	0.017324	0.144031
LETMD1	1.754588	1.591296	1.807667	1.36593	0.4499	0.01736	0.180048
ARHGEF3	1.217843	1.35611	1.172898	0.48552	-1.0424	0.017402	0.171633
FAM219B	2.010876	1.835367	2.067926	1.27839	0.3543	0.017406	0.140367
RPRM	1.547256	1.703571	1.496445	0.70561	-0.5031	0.017418	0.132747
CCNF	1.224531	1.088951	1.268602	3.01965	1.5944	0.017495	0.140367
IFT57	2.52244	2.259665	2.607857	1.27642	0.3521	0.017496	0.137066
MYLK-AS1	1.308079	1.167503	1.353775	2.11205	1.0786	0.0175	0.157252
DERL3	1.172563	1.039915	1.215681	5.40355	2.4339	0.017502	0.147959
TJP1	1.982127	2.158427	1.924819	0.79834	-0.3249	0.017507	0.157252

ZNF770	1.797306	1.63226	1.850956	1.3459	0.4286	0.017553	0.137956
SNX5	1.877722	1.709692	1.932342	1.31373	0.3937	0.017564	0.14659
GOLT1B	1.335198	1.47976	1.288207	0.60073	-0.7352	0.017594	0.137066
PAIP2B	1.268589	1.130694	1.313412	2.39806	1.2619	0.017632	0.14659
MAN1A1	1.946786	1.775957	2.002316	1.29172	0.3693	0.017689	0.140367
STXBP2	1.378249	1.524652	1.330659	0.63025	-0.666	0.017728	0.173147
EPAS1	1.108335	1.239747	1.065618	0.2737	-1.8693	0.017762	0.140561
SAMD4A	1.125525	1.258056	1.082445	0.31949	-1.6462	0.017804	0.137641
CTNNAL1	2.053686	1.87784	2.110846	1.26543	0.3396	0.01781	0.140367
GGA2	1.779818	1.946695	1.725574	0.76643	-0.3838	0.017831	0.124569
BACE1	1.395859	1.543393	1.347902	0.64024	-0.6433	0.01786	0.140367
RSRC1	1.722271	1.561349	1.77458	1.37985	0.4645	0.017902	0.149301
NPTN	1.164032	1.298714	1.120253	0.40257	-1.3127	0.017928	0.135492
HM13	2.050126	2.227172	1.992576	0.80883	-0.3061	0.017961	0.161166
NCAPD2	1.336154	1.194815	1.382097	1.96133	0.9718	0.017976	0.149301
H3F3B	18.10379	19.53761	17.63772	0.89751	-0.156	0.018048	0.135492
CDK5	1.487184	1.639451	1.437688	0.68447	-0.5469	0.018106	0.125621
NDUFAF2	2.077652	1.89918	2.135665	1.263	0.3369	0.01811	0.145752
PPA2	1.919286	2.097581	1.86133	0.78475	-0.3497	0.018118	0.185988
INF2	1.24358	1.382194	1.198523	0.51943	-0.945	0.018179	0.154828
SPNS1	1.579565	1.735582	1.528851	0.71895	-0.476	0.018198	0.139162
SLC25A22	1.401319	1.257519	1.448062	1.73992	0.799	0.018212	0.150171
HHLA3	1.472643	1.324438	1.520818	1.60529	0.6828	0.018217	0.176215
ERP44	1.658998	1.819347	1.606876	0.74068	-0.4331	0.018264	0.150406
RABGGTB	2.089247	2.272889	2.029554	0.80883	-0.3061	0.018271	0.142484
PFKFB4	1.609697	1.455087	1.659955	1.45017	0.5362	0.018278	0.139162
ESD	4.272627	3.854809	4.408441	1.19393	0.2557	0.018305	0.140573
DPH5	1.745728	1.584508	1.798133	1.36548	0.4494	0.018329	0.151583
HSD17B12	2.233341	2.419538	2.172816	0.8262	-0.2754	0.018352	0.154994
XRRA1	1.760141	1.598047	1.812831	1.35914	0.4427	0.018379	0.151583
MT1H	1.140489	1.358006	1.069783	0.19492	-2.359	0.018383	0.151583
SAT2	5.522136	5.016294	5.686563	1.16689	0.2227	0.018408	0.150406
RP5-940J5.9	1.2588	1.122102	1.303235	2.48346	1.3123	0.01845	0.142776
MAPKAPK5-AS1	1.734799	1.574149	1.787019	1.37076	0.455	0.018503	0.139819
GRAMD1B	1.120546	1.252242	1.077737	0.30818	-1.6981	0.018508	0.140913
LHX1	1.080824	1.20997	1.038844	0.185	-2.4344	0.018528	0.145616
EXT1	1.223706	1.361032	1.179067	0.49599	-1.0116	0.018559	0.165223
RSPO3	1.078999	1.207761	1.037143	0.17878	-2.4838	0.018563	0.151825
MRPL20	3.333842	3.625688	3.238976	0.85272	-0.2299	0.018644	0.151108
MPLKIP	2.581039	2.353316	2.655062	1.22297	0.2904	0.018655	0.153828
RHOG	1.451995	1.601073	1.403536	0.67136	-0.5748	0.018707	0.141359
AJUBA	1.304383	1.445503	1.258511	0.58027	-0.7852	0.018729	0.165272
PICALM	1.446555	1.59462	1.398426	0.67005	-0.5777	0.018799	0.180111
RER1	1.901183	2.07093	1.846005	0.78997	-0.3401	0.018903	0.153356
COLEC12	1.383374	1.528635	1.336156	0.63589	-0.6531	0.018946	0.154957

PDRG1	1.59712	1.444062	1.646872	1.45672	0.5427	0.019029	0.142716
C9orf142	2.535859	2.326563	2.603892	1.20906	0.2739	0.019086	0.142227
NGDN	1.496753	1.349091	1.544751	1.56048	0.642	0.019092	0.194003
EMILIN2	1.0879	1.216808	1.045997	0.21216	-2.2368	0.01916	0.159933
ADI1	2.023107	1.830727	2.085641	1.30686	0.3861	0.019186	0.142831
POSTN	1.059572	1.199139	1.014204	0.07133	-3.8094	0.019207	0.182288
UBA1	2.590451	2.379871	2.658901	1.20221	0.2657	0.019256	0.159933
ARL8A	1.31468	1.455475	1.268914	0.5904	-0.7602	0.019379	0.169525
HMGA2	3.626969	4.056693	3.487285	0.81372	-0.2974	0.019438	0.14818
COPG2	1.336071	1.196438	1.381459	1.94188	0.9575	0.019443	0.14818
DDX18	2.221384	2.00307	2.292349	1.28839	0.3656	0.019644	0.146468
PIK3R1	1.943234	1.767081	2.000494	1.30429	0.3833	0.019645	0.197633
GCA	1.607895	1.764408	1.557019	0.72869	-0.4566	0.019675	0.146468
TOMM20	4.54362	4.104383	4.686397	1.18748	0.2479	0.019693	0.150084
SPECC1	1.396985	1.541482	1.350016	0.6464	-0.6295	0.01972	0.15415
NELFE	2.352485	2.127782	2.425526	1.26401	0.338	0.019786	0.15415
GPR37	1.496782	1.348945	1.544838	1.56139	0.6428	0.019803	0.175566
MCM5	1.303398	1.165864	1.348104	2.09873	1.0695	0.019826	0.13661
SLC35B2	1.644015	1.800847	1.593035	0.74051	-0.4334	0.019873	0.171323
ERI1	1.252474	1.117711	1.296279	2.51701	1.3317	0.019908	0.147115
NFE2L2	1.982687	2.154592	1.926808	0.80271	-0.317	0.019925	0.171323
CRIPT	1.770446	1.609533	1.822752	1.34981	0.4328	0.020023	0.175566
AP4M1	1.515793	1.36706	1.56414	1.53692	0.62	0.020132	0.182456
FLOT1	2.564978	2.781298	2.494662	0.83909	-0.2531	0.020134	0.175566
MCM6	1.284693	1.148713	1.328894	2.2116	1.1451	0.020168	0.163234
ISOC2	2.250362	2.460164	2.182165	0.80961	-0.3047	0.02023	0.2015
ZC3H7A	1.561194	1.41099	1.610018	1.48426	0.5697	0.020268	0.149924
YAP1	1.837876	2.002788	1.784271	0.78209	-0.3546	0.020296	0.163234
ZNF891	1.299503	1.162447	1.344055	2.11795	1.0827	0.02036	0.139332
DIS3L	1.277938	1.142245	1.322045	2.26401	1.1789	0.020361	0.175566
NSMCE1	1.416238	1.273171	1.462743	1.69397	0.7604	0.020427	0.165942
LIX1	1.303354	1.166641	1.347793	2.08708	1.0615	0.020439	0.175566
ATP5I	11.20039	11.94425	10.95859	0.90994	-0.1362	0.020445	0.163234
LEPROTL1	1.723697	1.883786	1.67166	0.75998	-0.396	0.020447	0.165942
CHAF1A	1.271445	1.13645	1.315326	2.31092	1.2085	0.020449	0.150156
MLXIP	1.243026	1.378901	1.19886	0.52483	-0.9301	0.020474	0.165942
HSD17B10	2.380272	2.140529	2.458202	1.27853	0.3545	0.020532	0.150615
KCNIP4	1.113914	1.242971	1.071963	0.29618	-1.7555	0.020545	0.202634
MMADHC	2.284844	2.479325	2.221627	0.8258	-0.2761	0.020603	0.155836
CYB5R1	1.403303	1.547648	1.356383	0.65075	-0.6198	0.02068	0.18575
METTL21A	1.768596	1.609581	1.820284	1.34565	0.4283	0.020689	0.176203
POC1A	1.196239	1.065622	1.238697	3.63745	1.8629	0.020779	0.162041
MUC12	1.19642	1.065154	1.239089	3.66958	1.8756	0.020781	0.16708
XRCC4	1.281919	1.14702	1.325769	2.21582	1.1478	0.020818	0.156261
HOOK2	1.933845	1.767244	1.988	1.28773	0.3648	0.020886	0.196364

MALL	1.071812	1.198732	1.030556	0.15375	-2.7013	0.020901	0.176514
VTCN1	1.07534	1.217321	1.029188	0.13431	-2.8964	0.020948	0.186497
SMIM10	1.259473	1.125496	1.303023	2.41461	1.2718	0.020969	0.156261
DSN1	1.235834	1.103447	1.278868	2.69576	1.4307	0.021037	0.167492
KRCC1	1.938755	2.106399	1.884261	0.79922	-0.3233	0.021059	0.15929
FXN	1.396311	1.255134	1.442202	1.73321	0.7934	0.021076	0.17968
PLEKHO1	1.899467	1.734598	1.953059	1.29739	0.3756	0.021101	0.16715
CYTH2	1.913474	1.7472	1.967522	1.29486	0.3728	0.021127	0.167492
GNPAT	1.844119	1.681243	1.897062	1.3168	0.397	0.021166	0.167492
A2M-AS1	1.243301	1.110336	1.286522	2.59681	1.3767	0.021167	0.197165
FAM136A	2.562321	2.330315	2.637736	1.23109	0.2999	0.02119	0.206962
F2RL1	1.078991	1.204915	1.038059	0.18573	-2.4287	0.021216	0.179176
CALCOCO1	1.645614	1.491587	1.695681	1.41517	0.501	0.021372	0.170466
CCT2	3.407703	3.144457	3.493272	1.16266	0.2174	0.02144	0.168502
PARVA	1.613591	1.766791	1.563792	0.73526	-0.4437	0.021455	0.165863
UPF2	1.55598	1.407697	1.604181	1.48193	0.5675	0.021513	0.156825
TRIM37	1.465327	1.611966	1.41766	0.68249	-0.5511	0.021552	0.170552
BCAS1	1.059553	1.184753	1.018856	0.10206	-3.2925	0.021616	0.190749
SEC24D	1.293904	1.431203	1.249274	0.57809	-0.7906	0.021661	0.16762
ZNF428	5.220665	4.745228	5.375209	1.16821	0.2243	0.021716	0.183578
TMEM42	1.755285	1.597602	1.80654	1.34963	0.4326	0.021802	0.158777
TSPAN5	1.433767	1.578024	1.386875	0.66931	-0.5793	0.021858	0.169835
CSNK1E	2.263948	2.461857	2.199616	0.82061	-0.2852	0.02186	0.178787
BBIP1	1.953931	1.788452	2.007722	1.2781	0.354	0.021897	0.202091
VCP	2.449604	2.675368	2.376218	0.82144	-0.2838	0.021907	0.211904
REXO2	2.043604	2.216533	1.987393	0.81164	-0.3011	0.021933	0.183686
ISCA2	2.19592	2.018267	2.253667	1.23118	0.3	0.021947	0.169835
PJA2	2.289802	2.481307	2.227552	0.82869	-0.2711	0.021994	0.192396
AC069277.2	1.307637	1.172408	1.351594	2.03931	1.0281	0.022066	0.159699
BRIP1	1.165402	1.037728	1.206904	5.4841	2.4553	0.022095	0.163441
RNF212	1.184337	1.316182	1.141481	0.44747	-1.1602	0.022158	0.193831
STAT6	1.276545	1.413229	1.232114	0.56171	-0.8321	0.022178	0.150747
EXT2	1.285143	1.421569	1.240796	0.57119	-0.808	0.022224	0.212927
COX14	3.037723	3.297092	2.953413	0.85039	-0.2338	0.022314	0.187066
CEP19	1.351028	1.21357	1.395709	1.85283	0.8897	0.022333	0.161472
CEP164	1.466938	1.324183	1.513341	1.58349	0.6631	0.022578	0.173214
OPTC	1.171182	1.043914	1.212551	4.84017	2.2751	0.022592	0.166945
SEC13	1.751748	1.91079	1.700051	0.76862	-0.3797	0.02262	0.180602
HN1L	1.949643	1.776317	2.005984	1.29584	0.3739	0.02267	0.215155
BAG1	2.541222	2.333253	2.608824	1.20669	0.2711	0.022717	0.190254
MMP23B	1.302031	1.438437	1.257691	0.58775	-0.7667	0.022767	0.17324
HCG18	1.584321	1.435217	1.632788	1.45396	0.54	0.022784	0.167301
SAMD5	1.069469	1.193671	1.029096	0.15024	-2.7347	0.022801	0.153947
NHLRC2	1.408457	1.267971	1.454123	1.69467	0.761	0.022841	0.171474
AZI2	1.919234	1.754943	1.972638	1.28836	0.3655	0.022849	0.179398

FAM89A	1.305015	1.441857	1.260533	0.58963	-0.7621	0.022879	0.17324
CD276	1.893405	2.058568	1.839718	0.79326	-0.3341	0.02288	0.173988
ABHD8	1.505739	1.361097	1.552755	1.53077	0.6143	0.022903	0.17324
SREK1IP1	2.657062	2.383445	2.746002	1.26207	0.3358	0.022963	0.164843
CREB1	1.842164	1.682489	1.894067	1.31001	0.3896	0.023025	0.173988
SRPRA	1.30699	1.443395	1.262651	0.59236	-0.7554	0.023079	0.179984
FARP1	2.040248	2.211778	1.984491	0.81243	-0.2997	0.023092	0.191799
IGFBP4	1.719117	1.887236	1.664469	0.74892	-0.4171	0.023124	0.21148
SGF29	1.227585	1.097373	1.269912	2.77194	1.4709	0.023126	0.173617
RANGRF	2.032287	1.864084	2.086963	1.25794	0.3311	0.023184	0.186549
PPFIBP1	1.645959	1.798107	1.596502	0.7474	-0.4201	0.023268	0.21876
FAM221A	1.356428	1.219247	1.401019	1.82907	0.8711	0.023285	0.191811
PMEPA1	1.48655	1.631459	1.439447	0.69592	-0.523	0.023307	0.166127
WDR27	1.348958	1.212235	1.393401	1.85361	0.8903	0.023342	0.179253
ADPRHL2	1.644728	1.492826	1.694104	1.40842	0.4941	0.023346	0.156575
DPH3	1.665602	1.819039	1.615726	0.75177	-0.4116	0.023369	0.18843
CCDC25	2.425123	2.218276	2.49236	1.22498	0.2928	0.023413	0.18843
SNW1	1.881871	1.721563	1.93398	1.29439	0.3723	0.023431	0.212357
ATRX	3.587767	3.314634	3.676551	1.15636	0.2096	0.023518	0.186549
MLLT11	5.634186	5.209047	5.772381	1.13384	0.1812	0.02355	0.186549
ZNF43	1.43885	1.298433	1.484493	1.62345	0.6991	0.023586	0.185185
PAAF1	1.52929	1.384114	1.57648	1.50081	0.5857	0.023738	0.15815
C16orf59	1.284911	1.152269	1.328027	2.15426	1.1072	0.023791	0.174528
EIF3F	8.056569	7.203057	8.334008	1.18232	0.2416	0.023825	0.17368
UBE2N	2.73943	2.519426	2.810944	1.19186	0.2532	0.023825	0.170422
SLC38A1	2.25785	2.436456	2.199794	0.83525	-0.2597	0.023879	0.186031
ING5	1.435428	1.295487	1.480916	1.62754	0.7027	0.023886	0.170422
SF3B5	4.405333	4.051444	4.520367	1.15367	0.2062	0.023913	0.190927
RABL2A	1.407714	1.269055	1.452786	1.68288	0.7509	0.024151	0.20945
FAM69C	1.147557	1.022518	1.188202	8.35792	3.0631	0.024162	0.21703
C19orf60	3.100717	2.82445	3.19052	1.20065	0.2638	0.02428	0.198387
PXN	1.474739	1.622913	1.426574	0.68481	-0.5462	0.024292	0.190917
PLTP	1.870476	2.038908	1.815726	0.78518	-0.3489	0.024293	0.18103
CACUL1	1.942528	1.779652	1.995472	1.27682	0.3526	0.024317	0.192618
GLB1	1.43262	1.574731	1.386426	0.67236	-0.5727	0.024365	0.185689
LSR	1.515063	1.661056	1.467607	0.70736	-0.4995	0.024369	0.172642
APOA1	1.176362	1.050253	1.217354	4.32522	2.1128	0.024392	0.18294
POLDIP3	1.558067	1.412478	1.605392	1.4677	0.5536	0.024402	0.202675
ASPSCR1	1.777211	1.622037	1.827651	1.33055	0.412	0.024478	0.18935
DNAJC24	1.438663	1.299138	1.484017	1.61804	0.6942	0.024507	0.228279
OGG1	1.437162	1.297724	1.482488	1.62059	0.6965	0.02454	0.19287
GGPS1	2.024562	1.857487	2.078871	1.25818	0.3313	0.024637	0.18935
ZFP36	1.173451	1.302051	1.131649	0.43585	-1.1981	0.024648	0.178385
RNF26	1.779654	1.625136	1.829881	1.32752	0.4087	0.024681	0.18935
LDHA	26.03806	23.8097	26.76241	1.12945	0.1756	0.024757	0.187262

SKIL	2.498936	2.293134	2.565833	1.21088	0.2761	0.024804	0.174496
SASS6	1.236853	1.107959	1.278751	2.58202	1.3685	0.024875	0.193987
IK	2.14346	1.973062	2.198849	1.23204	0.301	0.024915	0.185665
DPP7	2.143296	2.319408	2.08605	0.82313	-0.2808	0.024957	0.205589
TFDP1	1.67288	1.522683	1.721702	1.38077	0.4655	0.024962	0.179372
DCP2	1.665268	1.515588	1.713922	1.38467	0.4695	0.02505	0.215389
FILIP1	1.125026	1.250747	1.084159	0.33563	-1.575	0.02509	0.166058
MID1IP1	1.821555	1.662158	1.873368	1.31897	0.3994	0.025165	0.183265
EMD	1.603549	1.752025	1.555286	0.73839	-0.4375	0.025232	0.187841
IFT52	1.838822	1.682171	1.889742	1.30428	0.3833	0.025556	0.212473
PAFAH1B1	2.331037	2.512348	2.272101	0.84114	-0.2496	0.025574	0.22768
SLC30A7	1.372511	1.510049	1.327803	0.64269	-0.6378	0.025627	0.198114
DDX39A	1.501011	1.359799	1.546912	1.52005	0.6041	0.025631	0.189597
WHRN	1.250759	1.382261	1.208013	0.54416	-0.8779	0.025649	0.18973
ZNF738	1.464802	1.325097	1.510214	1.56942	0.6502	0.025671	0.168791
TSGA10	1.311637	1.179168	1.354697	1.97968	0.9853	0.025672	0.236932
PPP1R14A	1.6061	1.786924	1.547321	0.69552	-0.5238	0.025692	0.202509
ADGRB3	1.195202	1.068795	1.236291	3.4347	1.7802	0.025767	0.202509
CRYBB2	1.097762	1.314782	1.027218	0.08647	-3.5317	0.025781	0.182263
PCDH18	1.119357	1.244335	1.078732	0.32223	-1.6338	0.025792	0.20904
COL6A3	1.066936	1.21019	1.02037	0.09691	-3.3672	0.025838	0.196722
NPLOC4	1.231174	1.361055	1.188955	0.52334	-0.9342	0.025871	0.184586
HSD17B8	1.285974	1.155069	1.328525	2.11857	1.0831	0.025886	0.201873
GALNT1	1.853921	2.013126	1.80217	0.79178	-0.3368	0.025909	0.180149
HIBADH	1.813839	1.657849	1.864544	1.3142	0.3942	0.025934	0.182263
ANP32A	2.69245	2.479901	2.76154	1.19031	0.2513	0.025966	0.180149
YWHAE	10.96609	10.1382	11.2352	1.12005	0.1636	0.025994	0.192085
ADAM17	1.716916	1.564789	1.766366	1.35691	0.4403	0.026046	0.170147
GABARAP	1.844919	1.683272	1.897463	1.31348	0.3934	0.026047	0.188327
RP5-1172A22.1	1.087223	1.209742	1.047398	0.22598	-2.1457	0.026129	0.226599
CUEDC2	2.933891	2.67797	3.01708	1.2021	0.2656	0.026143	0.22288
NSL1	1.6543	1.505876	1.702547	1.38877	0.4738	0.026148	0.200598
PIP5K1C	1.258183	1.389694	1.215435	0.55283	-0.8551	0.026175	0.182677
ZNF75A	1.494116	1.353119	1.539948	1.52908	0.6127	0.026229	0.195488
NEMP1	1.208115	1.081747	1.249191	3.04831	1.608	0.026234	0.195488
TFAM	1.643137	1.496307	1.690865	1.39201	0.4772	0.026258	0.192623
ARHGEF1	1.267075	1.399001	1.224192	0.56188	-0.8317	0.026311	0.188875
TMEM165	2.226089	2.407065	2.167262	0.82957	-0.2696	0.026324	0.217066
RCN2	6.027166	5.374147	6.239435	1.19782	0.2604	0.026332	0.186547
FAM129B	1.189828	1.317201	1.148425	0.46792	-1.0957	0.026477	0.19585
DPF2	1.933929	1.773759	1.985994	1.27429	0.3497	0.026506	0.18264
JAGN1	1.925633	1.765875	1.977563	1.2764	0.3521	0.026584	0.172542
RTCA	1.583741	1.730344	1.536087	0.73402	-0.4461	0.026663	0.225399
NYNRIN	1.298334	1.431598	1.255015	0.59086	-0.7591	0.026691	0.213952
CNOT9	1.494558	1.353499	1.540411	1.52874	0.6123	0.026727	0.201959

TRIP10	1.390686	1.527675	1.346157	0.656	-0.6082	0.026757	0.205907
EXD3	1.38905	1.253847	1.432998	1.70574	0.7704	0.026813	0.205907
CAB39	1.350728	1.485697	1.306856	0.63178	-0.6625	0.026814	0.236619
MRPS14	2.012846	1.849074	2.066081	1.25558	0.3284	0.026824	0.213952
ELAVL1	2.466204	2.231926	2.542357	1.25199	0.3242	0.026892	0.191679
CH17-189H20.1	1.514459	1.657297	1.468029	0.71205	-0.4899	0.027066	0.211071
ANXA7	1.56762	1.712903	1.520395	0.72997	-0.4541	0.027209	0.191411
HDHD3	1.430556	1.569405	1.385422	0.67689	-0.563	0.027215	0.188628
RASA4	1.428843	1.291437	1.473508	1.62474	0.7002	0.027227	0.186327
GPN1	1.41466	1.27863	1.458878	1.64691	0.7198	0.027339	0.20075
RCC1L	1.52122	1.38019	1.567062	1.49152	0.5768	0.027693	0.210564
PIM1	1.220171	1.348608	1.178422	0.51181	-0.9663	0.027694	0.214309
IKBIP	1.988191	1.824909	2.041267	1.26228	0.336	0.027705	0.193548
MGLL	1.165474	1.291281	1.12458	0.42769	-1.2253	0.027728	0.190864
NAT14	2.295856	2.121014	2.35269	1.20667	0.271	0.027838	0.210564
CAAP1	1.507701	1.367415	1.553302	1.50593	0.5907	0.027895	0.228152
ANKRD36C	1.606609	1.462389	1.653488	1.41329	0.4991	0.027921	0.197098
LRRC69	1.26297	1.135086	1.304539	2.2544	1.1727	0.027939	0.228284
RORA	1.36097	1.496123	1.317038	0.63903	-0.646	0.027954	0.216323
PTBP1	2.502358	2.266393	2.57906	1.2469	0.3183	0.028045	0.197098
CNOT3	1.378509	1.244707	1.422002	1.72452	0.7862	0.028051	0.217074
RFC3	1.332479	1.20127	1.375129	1.86381	0.8983	0.028102	0.245832
RASAL2	1.611287	1.467149	1.65814	1.40884	0.4945	0.028236	0.195902
TMEM128	1.601979	1.457962	1.648792	1.41669	0.5025	0.028349	0.229761
LRRCC1	1.612603	1.469178	1.659224	1.40506	0.4906	0.02841	0.198275
UNC5B-AS1	1.104634	1.226055	1.065166	0.28827	-1.7945	0.028444	0.208865
C14orf159	1.438699	1.302468	1.482982	1.5968	0.6752	0.028464	0.260314
PLXNC1	1.226288	1.100775	1.267086	2.65032	1.4062	0.028611	0.195604
C12orf65	1.504372	1.365514	1.549509	1.50339	0.5882	0.028617	0.219763
ARPP21	1.155012	1.032778	1.194745	5.94138	2.5708	0.028628	0.24
PPP2R1A	3.611977	3.271548	3.722636	1.19858	0.2613	0.028676	0.198755
NSFL1C	1.728473	1.879431	1.679403	0.77255	-0.3723	0.028682	0.246619
MRPS7	2.354628	2.166803	2.415682	1.2133	0.2789	0.028712	0.220492
DOT1L	1.335082	1.204303	1.377592	1.8482	0.8861	0.028895	0.220211
SPHK2	1.311775	1.181696	1.354059	1.94863	0.9625	0.028923	0.220212
SLC35E3	1.658126	1.512081	1.705599	1.37791	0.4625	0.029228	0.242287
EEF1E1	1.938249	1.777418	1.990528	1.27413	0.3495	0.029283	0.213256
WBP1L	1.303327	1.434381	1.260727	0.60023	-0.7364	0.029383	0.242287
COMMD8	1.556816	1.415876	1.602629	1.44906	0.5351	0.029383	0.20226
SEMA4C	1.230209	1.358033	1.188659	0.52693	-0.9243	0.029394	0.238471
SNRNP25	2.412038	2.207998	2.478362	1.22381	0.2914	0.029454	0.23308
RBFOX1	1.10254	1.223344	1.063272	0.28329	-1.8196	0.029491	0.237111
PSMG2	2.506254	2.279012	2.58012	1.23542	0.305	0.029521	0.213446
GPR137	1.547272	1.689825	1.500935	0.72618	-0.4616	0.029549	0.267064
LIPG	1.060086	1.17829	1.021664	0.12151	-3.0409	0.029549	0.225201

SMIM1	1.862597	2.030241	1.808103	0.78438	-0.3504	0.02957	0.215561
CHURC1	2.92271	2.690475	2.9982	1.18203	0.2413	0.029696	0.222723
C9orf16	6.606089	7.196084	6.414307	0.87383	-0.1946	0.029733	0.267064
HS3ST1	1.0904	1.210002	1.051523	0.24535	-2.0271	0.02981	0.225478
BBC3	1.535958	1.3961	1.58142	1.46786	0.5537	0.029849	0.205673
RBMXL1	1.485183	1.347933	1.529797	1.5227	0.6066	0.02992	0.226088
RSAD1	1.469171	1.332517	1.513591	1.54455	0.6272	0.029977	0.223159
SFR1	1.283246	1.156388	1.324482	2.07485	1.053	0.030003	0.204429
FAM160A2	1.306966	1.177732	1.348974	1.96349	0.9734	0.030045	0.204018
FAM183A	1.220353	1.096273	1.260686	2.70778	1.4371	0.030105	0.204429
TDRD3	1.412804	1.279292	1.456203	1.63343	0.7079	0.03014	0.219716
BOC	1.466213	1.330616	1.510289	1.54345	0.6262	0.030235	0.205516
SORBS3	1.767393	1.918752	1.718193	0.7817	-0.3553	0.030281	0.235936
ZSCAN16-AS1	2.126527	1.954168	2.182553	1.23936	0.3096	0.030284	0.235936
GTF2IRD1	1.270767	1.399747	1.228841	0.57246	-0.8047	0.030308	0.247887
IGSF3	1.202858	1.327773	1.162254	0.49502	-1.0144	0.030341	0.227786
RNF130	2.114635	1.935554	2.172846	1.25364	0.3261	0.030383	0.270492
KMT2E	3.528086	3.171721	3.643924	1.21743	0.2838	0.030424	0.206929
DKK1	1.306359	1.167484	1.351501	2.09872	1.0695	0.030535	0.206929
TNFRSF9	1.15653	1.27867	1.116828	0.41923	-1.2542	0.030578	0.206245
FNDC3A	2.373699	2.197769	2.430886	1.19463	0.2566	0.030613	0.230845
ITGAE	2.390417	2.19734	2.453178	1.21367	0.2794	0.030648	0.206929
CHD1L	1.359029	1.228215	1.40155	1.75953	0.8152	0.030711	0.271012
COL23A1	1.227528	1.103465	1.267855	2.58885	1.3723	0.030715	0.249191
TRAF4	1.821516	1.973388	1.772149	0.79326	-0.3341	0.030738	0.236053
RNF180	1.436594	1.302441	1.480202	1.58775	0.667	0.030749	0.230845
NDUFA5	3.236678	2.944664	3.331599	1.19897	0.2618	0.0308	0.221097
TRIM2	1.566386	1.426244	1.61194	1.43566	0.5217	0.030903	0.246492
ATXN2	1.833872	1.987456	1.783949	0.79391	-0.333	0.030941	0.223936
ZNF511	1.890687	1.734186	1.941559	1.28245	0.3589	0.031019	0.20922
RAD50	2.241761	2.037868	2.308037	1.26031	0.3338	0.031151	0.207945
KDM4B	1.552857	1.413573	1.598132	1.44626	0.5323	0.031164	0.232886
MAP3K7	1.677961	1.532658	1.725193	1.36146	0.4452	0.031207	0.200178
UBL7	1.692218	1.546479	1.739591	1.35338	0.4366	0.031241	0.200178
ITPA	1.767466	1.618961	1.815738	1.31791	0.3983	0.031241	0.232802
FANCA	1.219491	1.096266	1.259546	2.69614	1.4309	0.031243	0.207945
ZC3H13	2.098684	1.93392	2.152242	1.23377	0.3031	0.031252	0.232886
LUC7L	1.696545	1.550565	1.743997	1.35133	0.4344	0.031375	0.23185
CLEC2D	1.309104	1.181653	1.350533	1.92969	0.9484	0.031398	0.250682
RCOR2	1.309121	1.181638	1.35056	1.92999	0.9486	0.031398	0.250682
GADD45G	1.235648	1.111242	1.276087	2.48185	1.3114	0.031458	0.235934
MRPL36	2.0753	2.24275	2.02087	0.82146	-0.2837	0.031623	0.241002
HEMK1	1.407836	1.275595	1.450822	1.63581	0.71	0.031651	0.209274
FAM98B	1.345938	1.21643	1.388035	1.79289	0.8423	0.031704	0.27029
STK25	2.3452	2.146419	2.409814	1.22975	0.2984	0.031729	0.277562

CEP44	1.275241	1.149368	1.316156	2.11662	1.0818	0.031923	0.252868
PHLDB2	1.107345	1.226063	1.068755	0.30414	-1.7172	0.031962	0.209378
CCDC30	1.368108	1.237758	1.410479	1.72646	0.7878	0.031972	0.213967
EBAG9	1.552421	1.413442	1.597597	1.44542	0.5315	0.031984	0.247262
RAB3B	1.27602	1.402956	1.234758	0.58259	-0.7794	0.032035	0.236961
TNFAIP3	1.084144	1.201836	1.045888	0.22735	-2.137	0.032084	0.209378
LMBRD1	1.406858	1.274804	1.449782	1.63674	0.7108	0.032103	0.228819
ESCO2	1.156254	1.036854	1.195066	5.29301	2.4041	0.032136	0.271389
MZF1-AS1	1.212708	1.090012	1.252591	2.80621	1.4886	0.032148	0.213967
N4BP2L2	4.479138	4.128466	4.593126	1.14853	0.1998	0.03216	0.204764
BOLA1	1.500229	1.364239	1.544434	1.49472	0.5799	0.032272	0.259724
ADGRL2	1.50708	1.370626	1.551436	1.48785	0.5732	0.032279	0.231947
DNALI1	1.567479	1.428495	1.612656	1.42979	0.5158	0.03237	0.243869
CCDC181	1.393339	1.262343	1.43592	1.66164	0.7326	0.032372	0.271389
C9orf3	1.287282	1.414592	1.245899	0.59311	-0.7536	0.032497	0.281096
ZNF791	1.573923	1.434506	1.619242	1.42516	0.5111	0.032531	0.259729
PCF11	1.670241	1.52667	1.71691	1.36121	0.4449	0.032561	0.218373
LGALS3BP	2.054216	2.215935	2.001648	0.82377	-0.2797	0.032615	0.234418
CTNND2	1.318214	1.190976	1.359573	1.88281	0.9129	0.032646	0.234418
KIF21A	1.820629	1.971819	1.771484	0.79386	-0.3331	0.032671	0.243869
RP11-245D16.4	1.172479	1.0525	1.211479	4.02818	2.0101	0.032692	0.281096
CA8	1.245176	1.121892	1.28525	2.34019	1.2266	0.032726	0.243869
ZEB1-AS1	1.309091	1.182452	1.350256	1.91971	0.9409	0.032816	0.221566
RP11-398K22.12	1.316253	1.188429	1.357803	1.89887	0.9251	0.032864	0.244658
SFXN3	1.411469	1.544523	1.368218	0.67622	-0.5644	0.032943	0.217813
PPP2R3C	1.898413	1.746078	1.947931	1.27055	0.3455	0.033005	0.244139
AASS	1.252203	1.128303	1.292478	2.27959	1.1888	0.0331	0.242817
ATP5G1	4.238653	3.874458	4.357037	1.16789	0.2239	0.033104	0.233631
KIAA1586	1.344958	1.216709	1.386647	1.78417	0.8353	0.033182	0.243654
ARHGAP23	1.13111	1.250314	1.092362	0.36898	-1.4384	0.033188	0.234418
DHRS2	1.067897	1.183682	1.03026	0.16474	-2.6018	0.03323	0.263226
XPO1	2.16938	1.98419	2.229577	1.24933	0.3212	0.033243	0.233631
MCM2	1.289099	1.163722	1.329853	2.01472	1.0106	0.033312	0.218815
MDM4	2.007553	1.845119	2.060353	1.25468	0.3273	0.033313	0.25557
NTRK2	1.580653	1.734659	1.530592	0.72223	-0.4695	0.033338	0.244803
MPP1	1.280324	1.155674	1.320843	2.061	1.0433	0.033347	0.245548
PRPF38A	1.826707	1.678772	1.874794	1.28879	0.366	0.033384	0.222098
TAF1B	1.311216	1.184751	1.352325	1.90702	0.9313	0.033416	0.27782
SLC8A1	1.235646	1.360237	1.195147	0.54172	-0.8844	0.033422	0.289851
TNKS1BP1	1.21823	1.341673	1.178104	0.52127	-0.9399	0.033439	0.245548
MEIS2	3.223919	3.525005	3.126048	0.842	-0.2481	0.033445	0.24381
NDUFB3	3.37979	3.091645	3.473453	1.18254	0.2419	0.033505	0.234418
ETF1	1.497737	1.635204	1.453052	0.71324	-0.4875	0.033517	0.234418
MTMR4	1.704773	1.56049	1.751673	1.3411	0.4234	0.033555	0.234418
EXOC5	1.68429	1.829892	1.636961	0.76752	-0.3817	0.033558	0.222098

ME3	1.35891	1.230365	1.400695	1.73939	0.7986	0.033563	0.263784
ZNF830	1.342925	1.215302	1.38441	1.78544	0.8363	0.033594	0.23446
BCCIP	1.586314	1.447379	1.631476	1.4115	0.4972	0.033596	0.265858
GABPA	1.36245	1.233431	1.404388	1.73237	0.7927	0.033604	0.244547
CNTNAP3B	1.291633	1.166082	1.332443	2.00168	1.0012	0.033652	0.240099
ABHD12	1.579269	1.719578	1.533661	0.74163	-0.4312	0.03373	0.244117
MPHOSPH8	2.163984	1.99562	2.218711	1.22407	0.2917	0.033823	0.244547
MARCH1	1.108386	1.225764	1.070231	0.31108	-1.6846	0.033864	0.291172
PCGF2	2.013538	2.170788	1.962422	0.82203	-0.2827	0.033916	0.245623
CCND2	2.607634	2.798895	2.545463	0.85912	-0.2191	0.033938	0.245623
SARAF	4.830788	5.240975	4.697454	0.87184	-0.1979	0.033985	0.280239
MALSU1	1.688664	1.832296	1.641976	0.77133	-0.3746	0.034185	0.223024
EBLN3P	1.995669	1.840521	2.0461	1.24459	0.3157	0.03422	0.229505
AIMP1	2.37925	2.209085	2.434563	1.18649	0.2467	0.034258	0.254104
CISD2	2.510603	2.723763	2.441314	0.83614	-0.2582	0.034292	0.292355
SUGP2	2.005418	1.850221	2.055865	1.24187	0.3125	0.034352	0.254104
NUDT11	1.606554	1.467519	1.651748	1.39406	0.4793	0.034363	0.252332
DENND3	1.180166	1.301404	1.140756	0.467	-1.0985	0.034397	0.223024
TMEM231	1.331822	1.204986	1.373051	1.81989	0.8639	0.034401	0.261913
CBFB	1.469405	1.336338	1.512659	1.52424	0.6081	0.034467	0.254703
MDFI	1.948991	2.103965	1.898616	0.81399	-0.2969	0.034475	0.239186
SCYL2	1.415825	1.548084	1.372833	0.68025	-0.5559	0.034477	0.229691
COA4	2.584357	2.383255	2.649726	1.19264	0.2542	0.034556	0.267726
RGS20	1.425311	1.294508	1.46783	1.58852	0.6677	0.034597	0.267726
GBP1	1.057523	1.172142	1.020265	0.11772	-3.0865	0.034602	0.292515
CKAP5	1.503572	1.369518	1.547147	1.4807	0.5663	0.034658	0.240216
ARHGAP21	1.712097	1.568773	1.758685	1.3339	0.4156	0.034711	0.228233
CCDC148	1.26885	1.14526	1.309023	2.12738	1.0891	0.03474	0.262509
AKAP8L	1.596607	1.458033	1.641652	1.40088	0.4863	0.034744	0.225273
RPA1	1.57244	1.435929	1.616813	1.41494	0.5007	0.034815	0.255391
FZD6	1.222833	1.346084	1.18277	0.52811	-0.9211	0.034904	0.240504
ERCC1	2.639344	2.42893	2.707741	1.19512	0.2572	0.034994	0.255104
CTA-29F11.1	1.338541	1.212121	1.379634	1.78971	0.8397	0.035101	0.252228
HIGD1A	2.672477	2.87716	2.605944	0.85552	-0.2251	0.035168	0.287634
DLEU1	1.339637	1.213339	1.38069	1.78444	0.8355	0.035227	0.256798
NRM	1.371276	1.243985	1.412653	1.69131	0.7581	0.035471	0.277579
MAN1B1-AS1	1.196804	1.077273	1.235658	3.04968	1.6087	0.035565	0.225021
NAT9	1.640558	1.500117	1.68621	1.3721	0.4564	0.03563	0.277579
RP11-178L8.7	1.199033	1.079505	1.237887	2.99209	1.5812	0.035689	0.259909
RP11-54H7.4	1.062924	1.177152	1.025793	0.1456	-2.7799	0.035709	0.280648
PUF60	2.170643	2.002854	2.225185	1.2217	0.2889	0.035814	0.225182
SH3BGRL3	14.17534	15.27028	13.81942	0.89833	-0.1547	0.035853	0.300105
DDAH2	5.399123	4.961853	5.541261	1.14625	0.1969	0.036008	0.238318
COPS7A	1.684699	1.543732	1.730522	1.34353	0.426	0.036019	0.260686
FGFRL1	1.140723	1.259051	1.10226	0.39475	-1.341	0.036052	0.292485

PCAT7	1.474754	1.342359	1.51779	1.51242	0.5969	0.036058	0.278758
POLD3	1.336262	1.210758	1.377058	1.78905	0.8392	0.036096	0.300105
MBD4	1.699671	1.55767	1.745829	1.3374	0.4194	0.036118	0.259279
TULP1	1.110053	1.227395	1.071911	0.31624	-1.6609	0.036152	0.247406
NPS	1.143294	1.019177	1.183639	9.57605	3.2594	0.036155	0.277648
EDNRA	1.080839	1.196315	1.043303	0.22058	-2.1806	0.036182	0.233097
NUP160	1.431722	1.302039	1.473876	1.56892	0.6498	0.036219	0.236597
ANO6	1.4202	1.551438	1.377541	0.68465	-0.5466	0.036386	0.292837
HDAC5	1.470301	1.338816	1.513041	1.51422	0.5986	0.036407	0.257925
SNHG25	1.720024	1.577695	1.766288	1.32646	0.4076	0.036541	0.235408
HERC2	1.862218	1.713163	1.910669	1.27694	0.3527	0.036626	0.274698
TTC32	1.256965	1.135328	1.296504	2.191	1.1316	0.036699	0.279692
EIF5B	3.247933	3.50131	3.165572	0.86577	-0.2079	0.036717	0.265734
BPGM	1.519297	1.385405	1.56282	1.46033	0.5463	0.03688	0.23608
SELENOW	6.770529	7.363746	6.5777	0.87648	-0.1902	0.036886	0.294506
PIGP	2.041254	1.887026	2.091387	1.23039	0.2991	0.03699	0.315353
SELENOH	7.73354	7.092582	7.941887	1.1394	0.1883	0.036994	0.303059
PEX13	1.508649	1.374825	1.552149	1.47309	0.5588	0.037003	0.265893
LRR1	1.265398	1.144059	1.304841	2.11609	1.0814	0.037054	0.303059
GPX7	2.466856	2.277219	2.528499	1.19674	0.2591	0.037094	0.280579
EXOSC5	1.358721	1.232993	1.39959	1.71503	0.7782	0.037145	0.263653
SEC23IP	1.30472	1.43081	1.263734	0.61218	-0.708	0.037215	0.263653
CPSF4	1.415184	1.286745	1.456935	1.59353	0.6722	0.037337	0.264337
BDH2	2.117218	1.95767	2.16908	1.22075	0.2878	0.037349	0.264337
PPIL3	1.707065	1.565773	1.752993	1.33091	0.4124	0.037456	0.278836
GTF2E2	1.728868	1.872412	1.682208	0.78198	-0.3548	0.037633	0.239243
MRPL16	1.559179	1.424166	1.603066	1.42177	0.5077	0.03774	0.269269
KIAA1524	1.212845	1.094667	1.251259	2.65412	1.4082	0.037778	0.248398
ISCU	2.774598	3.014622	2.696577	0.84213	-0.2479	0.03778	0.245207
C1orf52	2.003736	1.850824	2.053441	1.23814	0.3082	0.037791	0.294714
TIMM17B	4.386251	4.044026	4.497494	1.14897	0.2003	0.037846	0.288389
KLHL28	1.390122	1.26269	1.431545	1.64279	0.7161	0.03785	0.239243
GORASP2	1.629995	1.768904	1.584842	0.76062	-0.3948	0.037853	0.277957
MPHOSPH10	1.421984	1.293775	1.46366	1.57828	0.6584	0.038013	0.267001
GABRB3	1.1145	1.229909	1.076986	0.33485	-1.5784	0.038038	0.288389
SDR39U1	1.97492	1.823487	2.024145	1.24367	0.3146	0.03806	0.275184
EPRS	1.83521	1.68969	1.882513	1.27958	0.3557	0.038105	0.30099
SELENOK	4.211137	4.545657	4.1024	0.87499	-0.1927	0.038117	0.273895
C8orf76	1.448544	1.319105	1.49062	1.53749	0.6206	0.038159	0.239687
ZMAT1	1.399453	1.272214	1.440813	1.61936	0.6954	0.038165	0.288389
ZNF581	2.250775	2.081719	2.305728	1.20709	0.2715	0.038326	0.275399
CPSF6	1.787063	1.643201	1.833826	1.29637	0.3745	0.038332	0.267001
TCTN1	1.758851	1.616676	1.805066	1.30549	0.3846	0.038384	0.270031
MAP6	1.485007	1.353649	1.527706	1.49217	0.5774	0.038392	0.283708
QDPR	1.617022	1.755425	1.572034	0.75723	-0.4012	0.038411	0.246845

BROX	1.742946	1.60071	1.789181	1.31375	0.3937	0.038421	0.288442
MAP9	1.875749	2.025083	1.827208	0.80697	-0.3094	0.038464	0.272496
JKAMP	1.620135	1.758493	1.575161	0.75829	-0.3992	0.038523	0.246845
SNHG9	1.764889	1.622191	1.811274	1.3039	0.3828	0.03853	0.30099
SMIM8	1.742348	1.600218	1.788548	1.31377	0.3937	0.038553	0.240894
EXOSC1	1.479237	1.611574	1.43622	0.71327	-0.4875	0.038596	0.30099
PTPRO	1.148223	1.033048	1.185661	5.61791	2.49	0.038676	0.267001
THRB	1.240217	1.120935	1.27899	2.30695	1.206	0.038744	0.270673
DDX19B	1.403774	1.276756	1.445062	1.60813	0.6854	0.038856	0.27723
KXD1	2.575232	2.379492	2.638859	1.18802	0.2486	0.038858	0.267001
HDDC3	1.573301	1.438347	1.617169	1.40794	0.4936	0.03889	0.247614
WFS1	1.158347	1.275794	1.12017	0.43573	-1.1985	0.03894	0.285654
ZNF660	1.29611	1.17371	1.335897	1.93366	0.9513	0.039054	0.267001
MGAT2	1.429529	1.55966	1.38723	0.6919	-0.5314	0.039062	0.268885
PPFIA3	1.446602	1.317932	1.488427	1.53626	0.6194	0.039189	0.243362
ALAD	1.259203	1.381402	1.219481	0.57546	-0.7972	0.039201	0.250938
DTL	1.20984	1.092057	1.248126	2.69535	1.4305	0.039258	0.26685
NTHL1	1.508467	1.377111	1.551165	1.46155	0.5475	0.039268	0.294513
APBA2	1.442633	1.314225	1.484373	1.54148	0.6243	0.039274	0.332011
MALT1	1.42892	1.55848	1.386805	0.6926	-0.5299	0.03939	0.249224
NT5C3B	2.75382	2.492467	2.838774	1.23204	0.301	0.039414	0.246035
VAT1L	1.183203	1.301249	1.144832	0.48077	-1.0566	0.039627	0.304542
PPP2R5E	1.885063	1.73835	1.932753	1.26329	0.3372	0.039657	0.304542
DDX6	2.021331	1.868313	2.071071	1.23351	0.3028	0.039668	0.305704
DBF4B	1.15847	1.043736	1.195765	4.47603	2.1622	0.039685	0.295436
ZBTB10	1.524058	1.392286	1.566891	1.44509	0.5312	0.039731	0.269794
ST6GAL2	1.118468	1.233251	1.081156	0.34794	-1.5231	0.039788	0.279907
SIMC1	1.415418	1.288512	1.45667	1.58285	0.6625	0.03987	0.321444
HSP90AA1	18.59744	17.16983	19.06149	1.11699	0.1596	0.039902	0.289383
PLEKHF2	1.157687	1.274233	1.119803	0.43687	-1.1947	0.039941	0.321444
MAPK1	1.608381	1.745625	1.563769	0.7561	-0.4033	0.039949	0.260964
ATP5F1	3.564048	3.298496	3.650368	1.15309	0.2055	0.039954	0.246586
ISLR2	1.13359	1.020072	1.17049	8.49381	3.0864	0.040002	0.283395
MSL3	1.922164	1.773614	1.970451	1.25444	0.327	0.040024	0.289383
MRPS12	2.087561	2.24408	2.036684	0.83329	-0.2631	0.040025	0.268969
AC074289.1	1.15418	1.03977	1.19137	4.81186	2.2666	0.040051	0.273815
PPWD1	1.335484	1.212159	1.375572	1.77024	0.8239	0.040086	0.296222
STARD4-AS1	1.43246	1.304735	1.473978	1.55538	0.6373	0.040105	0.305704
CHID1	2.70798	2.925345	2.637324	0.85041	-0.2338	0.040112	0.305704
ATAT1	1.61164	1.476241	1.655653	1.37673	0.4612	0.040116	0.280254
ETNK1	1.743902	1.885874	1.697753	0.78764	-0.3444	0.040138	0.292603
LARP7	1.99187	1.839732	2.041324	1.24007	0.3104	0.040142	0.252395
SC5D	1.369144	1.495843	1.32796	0.66142	-0.5964	0.040168	0.249188
PLPP1	1.249514	1.369835	1.210402	0.56891	-0.8137	0.040297	0.268969
CHST15	1.320061	1.19748	1.359907	1.8225	0.8659	0.040344	0.247474

RNF213	1.482688	1.613378	1.440206	0.71767	-0.4786	0.040398	0.268969
CLCN3	2.361575	2.537614	2.304353	0.8483	-0.2374	0.040408	0.296421
CFDP1	4.10431	3.742509	4.221916	1.17481	0.2324	0.040461	0.257362
PCGF6	1.344302	1.221137	1.384337	1.73801	0.7974	0.040486	0.262769
CDC5L	1.738091	1.598144	1.783581	1.31002	0.3896	0.04049	0.279175
STARD7	2.264515	2.066688	2.32882	1.24574	0.317	0.0405	0.249709
KIF3C	1.175568	1.292351	1.137607	0.47069	-1.0872	0.040504	0.275046
TSPAN13	1.595944	1.731604	1.551846	0.7543	-0.4068	0.040517	0.279175
SLC25A38	1.427084	1.299607	1.468521	1.56378	0.645	0.040639	0.268969
TMEM132A	1.463572	1.59372	1.421266	0.70954	-0.4951	0.040663	0.309901
ALDOC	1.564234	1.431956	1.607232	1.40577	0.4914	0.04077	0.254747
LARP1	1.986958	2.145907	1.935291	0.8162	-0.293	0.040916	0.275978
NOSIP	2.322149	2.158696	2.375281	1.18692	0.2472	0.040961	0.292968
CAMK4	1.548561	1.415742	1.591735	1.42332	0.5093	0.041028	0.28465
HAGHL	1.379302	1.505707	1.338213	0.66879	-0.5804	0.041103	0.292968
ZMAT2	2.067089	1.915084	2.116499	1.22011	0.287	0.041162	0.345077
RAB8A	1.49704	1.367516	1.539143	1.46699	0.5529	0.041285	0.277579
UNC13D	1.090204	1.203364	1.053421	0.26269	-1.9286	0.041325	0.307945
SMARCA2	1.244772	1.364855	1.205738	0.56389	-0.8265	0.041338	0.290815
USP47	1.915922	1.76867	1.963787	1.25384	0.3263	0.04143	0.277579
NMT1	1.953724	2.102621	1.905324	0.82107	-0.2844	0.041601	0.286647
ZNF385A	1.5481	1.416924	1.59074	1.4169	0.5027	0.041612	0.300857
IREB2	1.880132	1.734694	1.927407	1.2623	0.3361	0.041657	0.268632
VWA9	1.36103	1.237858	1.401068	1.68616	0.7537	0.041657	0.291023
POP5	1.773913	1.632443	1.819899	1.2964	0.3745	0.041681	0.253902
KMT5C	1.557438	1.424714	1.60058	1.41408	0.4999	0.041685	0.253902
B3GALNT1	1.458245	1.330615	1.499732	1.51152	0.596	0.041712	0.301885
COX6A1	7.857874	8.440917	7.668352	0.89617	-0.1582	0.041806	0.278309
LINC00998	4.188672	3.842536	4.301186	1.16135	0.2158	0.041816	0.278309
PIR	1.627874	1.493487	1.671558	1.36084	0.4445	0.041826	0.34522
MIEN1	2.812419	3.014002	2.746894	0.86737	-0.2053	0.041866	0.34522
TRIM16L	1.268287	1.149068	1.30704	2.05973	1.0425	0.041912	0.317022
SEC61A1	1.837443	1.981779	1.790525	0.8052	-0.3126	0.041936	0.300857
SHFM1	6.321734	5.787538	6.495378	1.14785	0.1989	0.04208	0.311271
MPG	2.686551	2.89489	2.618829	0.85431	-0.2272	0.042088	0.296086
RP11-445F12.1	1.094859	1.206618	1.058531	0.28328	-1.8197	0.042098	0.269746
JPH4	1.464318	1.336243	1.50595	1.50471	0.5895	0.042188	0.280785
NDUFB7	5.111195	5.501366	4.984368	0.88515	-0.176	0.042279	0.257771
SYT1	4.092151	4.405814	3.990193	0.87797	-0.1878	0.04229	0.345881
STK33	1.422044	1.296283	1.462923	1.56244	0.6438	0.042296	0.267344
WIPI1	1.279211	1.399878	1.239988	0.60015	-0.7366	0.042382	0.299954
NALT1	1.168089	1.054233	1.205098	3.78179	1.9191	0.042452	0.300857
LYPLAL1	1.719498	1.580995	1.76452	1.31588	0.396	0.042462	0.300857
LZTS2	1.570468	1.704335	1.526954	0.74816	-0.4186	0.042509	0.300857
ZNF730	1.192755	1.078174	1.230001	2.94216	1.5569	0.042662	0.306557

TTF1	1.526278	1.395951	1.568642	1.43614	0.5222	0.042704	0.281054
RRAS2	1.500299	1.630386	1.458013	0.72656	-0.4608	0.042766	0.29267
IQSEC2	1.312582	1.191582	1.351913	1.83688	0.8773	0.042769	0.314059
FNDC4	1.628952	1.494741	1.672579	1.35946	0.443	0.042787	0.281054
KRIT1	1.674573	1.538525	1.718796	1.33475	0.4166	0.042801	0.291319
MTA3	1.608921	1.47579	1.652196	1.37077	0.455	0.042821	0.347407
ORC4	1.557316	1.426175	1.599944	1.40774	0.4934	0.042858	0.291319
SEC63	2.271953	2.437699	2.218076	0.84724	-0.2392	0.042915	0.282175
IDH1	1.762025	1.903339	1.716089	0.79271	-0.3351	0.043059	0.32048
GALT	1.544904	1.413918	1.587482	1.41932	0.5052	0.043061	0.267406
HIST1H1A	1.231618	1.115618	1.269325	2.32944	1.22	0.043083	0.273591
FANCI	1.305016	1.184973	1.344037	1.85993	0.8952	0.043085	0.307404
PIGT	1.684738	1.823065	1.639773	0.77731	-0.3634	0.043137	0.326284
TYR	1.053734	1.163332	1.018108	0.11087	-3.1731	0.043146	0.281569
RRAD	1.070031	1.180169	1.03423	0.18999	-2.396	0.043164	0.29143
KCNMA1	1.300568	1.422896	1.260805	0.61671	-0.6973	0.043242	0.273591
CPNE1	2.19291	2.007277	2.253252	1.2442	0.3152	0.043261	0.261914
TPRA1	1.427404	1.554481	1.386096	0.69632	-0.5222	0.043279	0.32048
TOR2A	1.334389	1.213261	1.373762	1.7526	0.8095	0.043283	0.300292
ZC3H14	1.624461	1.490652	1.667956	1.36137	0.4451	0.043325	0.32048
SACS	1.583667	1.71661	1.540453	0.75418	-0.407	0.043409	0.287016
PRPS2	1.531589	1.401727	1.573801	1.42834	0.5143	0.043521	0.263746
KIFC3	1.260523	1.380233	1.221611	0.58283	-0.7789	0.04361	0.348187
ITGA7	1.224466	1.342005	1.18626	0.54461	-0.8767	0.04361	0.284884
YWHAB	4.559303	4.945106	4.433896	0.87042	-0.2002	0.043637	0.294208
RBM4B	1.492483	1.364149	1.534198	1.46697	0.5528	0.043663	0.283106
RP11-390E23.6	1.401735	1.278033	1.441945	1.58954	0.6686	0.043682	0.274652
GON7	1.402533	1.278788	1.442757	1.58815	0.6673	0.043741	0.294208
MED28	2.888064	2.670575	2.958761	1.17251	0.2296	0.043847	0.302124
ZDHHC7	1.352773	1.476093	1.312687	0.65678	-0.6065	0.043868	0.294208
GPALPP1	1.411134	1.286371	1.451689	1.57729	0.6574	0.043878	0.314978
AC004540.4	1.32768	1.206644	1.367024	1.77611	0.8287	0.044025	0.271711
NDUFB5	3.290784	3.027301	3.376431	1.17221	0.2292	0.044141	0.286487
SPCS3	1.862526	2.006496	1.815728	0.81046	-0.3032	0.044214	0.308884
MOSPD2	1.643148	1.509193	1.686691	1.34859	0.4314	0.044223	0.322381
CTTNBP2NL	1.479987	1.608761	1.438128	0.71971	-0.4745	0.04425	0.348251
GSE1	1.515581	1.386821	1.557435	1.44107	0.5271	0.04431	0.348251
DCTN3	2.63756	2.455147	2.696855	1.16611	0.2217	0.04432	0.297242
PLRG1	1.484476	1.356789	1.525982	1.47421	0.5599	0.04435	0.285714
UACA	1.760351	1.622019	1.805317	1.29468	0.3726	0.044578	0.278546
WDR61	1.888623	1.745777	1.935056	1.2538	0.3263	0.044715	0.359863
ARHGEF9	1.306149	1.427329	1.266758	0.62425	-0.6798	0.044717	0.269372
MBOAT7	1.708837	1.846602	1.664056	0.78438	-0.3504	0.044744	0.323831
FAM83D	1.148176	1.036889	1.184351	4.99747	2.3212	0.044871	0.336866
MRPL52	4.004252	4.275022	3.916237	0.89045	-0.1674	0.044909	0.319441

HSPA4	1.899223	1.749637	1.947847	1.26441	0.3385	0.044916	0.299241
CACNA1A	1.429566	1.305197	1.469993	1.53997	0.6229	0.044919	0.270319
EXOSC2	1.252407	1.136077	1.290221	2.13278	1.0927	0.044923	0.318258
NNT-AS1	1.788893	1.650022	1.834034	1.28308	0.3596	0.044924	0.307439
SKIDA1	1.266163	1.148182	1.304513	2.05499	1.0391	0.045025	0.269611
WDR90	1.198816	1.085045	1.235798	2.77263	1.4713	0.04509	0.319441
FAM213B	1.419212	1.544948	1.37834	0.69427	-0.5264	0.045217	0.270494
PDK1	2.225525	2.03278	2.288178	1.24729	0.3188	0.045428	0.35427
CSRNP2	1.173153	1.287365	1.136028	0.47336	-1.079	0.045542	0.30956
HOOK1	1.289814	1.410086	1.250719	0.61138	-0.7099	0.045557	0.303509
PACS1	1.348701	1.470944	1.308965	0.65605	-0.6081	0.045662	0.320913
RNF170	1.598622	1.46733	1.641299	1.37226	0.4566	0.045678	0.32134
NOLC1	1.419287	1.295948	1.459379	1.55223	0.6343	0.045774	0.300671
LRRC4B	1.296532	1.416338	1.257588	0.6187	-0.6927	0.045895	0.30375
JARID2	1.436501	1.562435	1.395566	0.70331	-0.5078	0.045957	0.273292
MTHFD1	1.347568	1.227873	1.386476	1.69601	0.7621	0.0464	0.326425
ATE1	1.335606	1.456712	1.29624	0.64864	-0.6245	0.046429	0.288316
LAMA4	1.121769	1.233056	1.085594	0.36727	-1.4451	0.046503	0.305764
PREX2	1.140686	1.029988	1.176669	5.89122	2.5586	0.046512	0.322697
UPF3A	2.630473	2.424063	2.697568	1.19206	0.2535	0.046759	0.286828
HERC4	1.613221	1.481628	1.655996	1.36204	0.4458	0.046775	0.36197
TMEM246	1.615446	1.484441	1.65803	1.35833	0.4418	0.0469	0.334289
AUTS2	1.310003	1.429608	1.271124	0.6311	-0.6641	0.047156	0.307739
SREK1	2.151076	1.99629	2.20139	1.20586	0.2701	0.047298	0.352273
TMCO3	1.894456	2.037056	1.848104	0.8178	-0.2902	0.047373	0.292377
GGT7	1.258764	1.142761	1.296471	2.0767	1.0543	0.047376	0.330646
CHMP2B	1.982218	2.128523	1.934661	0.82822	-0.2719	0.047433	0.303633
LINC01164	1.125679	1.23608	1.089793	0.38035	-1.3946	0.047562	0.308387
TSPAN6	2.183546	2.026251	2.234675	1.20309	0.2667	0.047598	0.342029
TIMM13	5.544234	5.102022	5.687978	1.14285	0.1926	0.047623	0.352273
NDUFB11	6.599954	6.070372	6.772098	1.1384	0.187	0.047637	0.365822
ERI2	1.207155	1.09416	1.243885	2.59012	1.373	0.047755	0.291163
PHF5A	1.862835	1.722908	1.90832	1.25648	0.3294	0.047756	0.311968
FBXW11	1.668267	1.802003	1.624795	0.77904	-0.3602	0.047893	0.382387
CCHCR1	1.258309	1.142593	1.295923	2.0753	1.0533	0.047904	0.283196
FBXW7	1.460721	1.336345	1.501151	1.48999	0.5753	0.047936	0.32365
SHTN1	1.351167	1.471924	1.311914	0.66094	-0.5974	0.048192	0.318957
EIF2A	1.798766	1.661345	1.843435	1.27533	0.3509	0.04825	0.341488
TAP1	1.159932	1.271616	1.123629	0.45516	-1.1356	0.048337	0.303619
RP11-149I23.3	1.093346	1.202149	1.057979	0.28681	-1.8018	0.048356	0.345005
NDC1	1.286039	1.170022	1.323751	1.90417	0.9292	0.048361	0.383084
NXT2	1.352999	1.233783	1.39175	1.6757	0.7448	0.048603	0.354441
IARS2	1.908145	1.766011	1.954347	1.24587	0.3171	0.048677	0.32006
GAS1	2.112591	2.305612	2.049849	0.80411	-0.3145	0.048781	0.314574
FZD7	1.413526	1.537288	1.373296	0.69478	-0.5254	0.048809	0.354441

PHB2	3.698093	3.384613	3.799991	1.17419	0.2317	0.048899	0.30524
TMEM214	1.225044	1.339909	1.187706	0.55222	-0.8567	0.048922	0.345891
ANKS3	1.400146	1.278843	1.439576	1.57643	0.6567	0.048941	0.341498
TOM1L1	1.539794	1.411851	1.581382	1.41163	0.4974	0.048961	0.291447
ANXA4	1.410218	1.533528	1.370135	0.69375	-0.5275	0.048967	0.296752
GNPTAB	1.184041	1.297212	1.147254	0.49545	-1.0132	0.048973	0.354441
NR1H2	1.603189	1.733464	1.560842	0.76465	-0.3871	0.048979	0.326048
RAD23A	3.449462	3.723279	3.360456	0.86677	-0.2063	0.04906	0.344791
CCDC138	1.229878	1.116688	1.26667	2.28532	1.1924	0.049129	0.343221
PSMC4	1.671338	1.805948	1.627583	0.77869	-0.3609	0.049167	0.345891
INTS8	1.406724	1.285364	1.446173	1.56352	0.6448	0.049198	0.316949
FANCG	1.2863	1.17071	1.323874	1.89722	0.9239	0.049211	0.313018
PTX3	1.11303	1.2224	1.077479	0.34838	-1.5213	0.049258	0.315631
PLEKHG1	1.374239	1.254245	1.413244	1.62538	0.7008	0.049261	0.341498
FNBP1L	1.946357	1.803497	1.992795	1.23559	0.3052	0.049269	0.326048
PGAM2	1.319814	1.195901	1.360092	1.83814	0.8782	0.049271	0.326048
HSCB	1.727252	1.593248	1.77081	1.29931	0.3777	0.049332	0.344636
AP001347.6	1.248846	1.135191	1.28579	2.11398	1.08	0.049392	0.341498
LMCD1	1.197111	1.310052	1.160399	0.51733	-0.9508	0.049485	0.388918
LINC00545	1.069526	1.176369	1.034796	0.19729	-2.3416	0.049584	0.317403
EPHB4	1.244003	1.359482	1.206466	0.57434	-0.8	0.04961	0.322146
C10orf10	1.056675	1.162934	1.022135	0.13585	-2.8799	0.049611	0.341498
PTPRM	1.180373	1.292735	1.143848	0.49139	-1.025	0.049635	0.322146
TRAM1	2.204994	2.360779	2.154355	0.8483	-0.2373	0.049693	0.357082
TGFBR3L	1.207046	1.320477	1.170175	0.531	-0.9132	0.049734	0.294305
AP1S3	1.1248	1.234134	1.08926	0.38124	-1.3912	0.049756	0.326048
KANK1	1.202169	1.315982	1.165173	0.52273	-0.9359	0.049838	0.345425
C8orf46	1.158866	1.041239	1.197102	4.77947	2.2569	0.049912	0.326048
MCM10	1.164339	1.054287	1.200112	3.68622	1.8821	0.049921	0.348756
MPRIP	1.352818	1.47306	1.313733	0.6632	-0.5925	0.049934	0.317937
PPP1R9A	1.530613	1.403942	1.571788	1.41552	0.5013	0.049987	0.305629
AFMID	1.39983	1.279036	1.439095	1.57361	0.6541	0.050056	0.390356
EPC1	2.02098	1.876091	2.068077	1.21914	0.2859	0.05008	0.322952
GPR85	1.302447	1.186201	1.340233	1.82723	0.8697	0.050128	0.305629
TTC9	1.080597	1.188099	1.045653	0.24271	-2.0427	0.050173	0.315485
MRPL57	4.883751	4.487882	5.012431	1.15039	0.2021	0.050182	0.380457
WNK3	1.40756	1.286824	1.446806	1.55777	0.6395	0.050226	0.315485
KCMF1	1.426532	1.54918	1.386665	0.70408	-0.5062	0.050236	0.34345
EAPP	1.738913	1.604648	1.782557	1.29424	0.3721	0.050299	0.380457
ANKRD52	1.166773	1.277712	1.130712	0.47067	-1.0872	0.050623	0.351219
PLK3	1.186293	1.29833	1.149875	0.50238	-0.9931	0.050732	0.30745
SAE1	1.778266	1.643654	1.822022	1.27712	0.3529	0.050792	0.321967
CDC34	1.692286	1.824378	1.649348	0.78768	-0.3443	0.050886	0.299367
HAUS5	1.267973	1.153317	1.305243	1.99093	0.9934	0.050935	0.299354
RB1CC1	2.176645	2.005343	2.232327	1.22578	0.2937	0.050938	0.321967

GADD45A	2.182699	1.933568	2.26368	1.3536	0.4368	0.050989	0.355858
GPC3	1.359367	1.504358	1.312237	0.61908	-0.6918	0.051035	0.3513
WDR25	1.667119	1.523279	1.713875	1.36423	0.4481	0.051378	0.353701
RNASET2	1.782125	1.919191	1.73757	0.80241	-0.3176	0.051508	0.357003
KLC1	1.719498	1.853446	1.675958	0.79203	-0.3364	0.051581	0.357865
FAM111B	1.155819	1.047284	1.191099	4.04155	2.0149	0.051582	0.348906
PLEKHJ1	2.754111	2.572856	2.813028	1.1527	0.205	0.051684	0.353701
VEZT	1.934621	1.793362	1.980538	1.23593	0.3056	0.051722	0.32604
RP11-527L4.2	1.130261	1.022739	1.165211	7.26563	2.8611	0.051728	0.348906
TSPAN31	1.737335	1.603724	1.780766	1.29325	0.371	0.051826	0.33637
AC007040.11	1.136936	1.029316	1.171919	5.8644	2.552	0.051868	0.325799
TRMT112	5.94395	6.423069	5.78821	0.88293	-0.1796	0.051882	0.32604
PDE4C	1.219641	1.1077	1.256029	2.37725	1.2493	0.051893	0.301813
PSMA2	3.907508	3.530268	4.030132	1.19755	0.2601	0.051954	0.301813
SMYD4	1.33771	1.220514	1.375806	1.70423	0.7691	0.051999	0.35438
EXTL2	1.76967	1.635531	1.813273	1.27968	0.3558	0.052066	0.313277
AKT2	1.553839	1.427314	1.594966	1.39234	0.4775	0.052116	0.361493
HADHB	2.02638	2.181494	1.97596	0.82604	-0.2757	0.052147	0.387211
PNO1	1.197398	1.309288	1.161028	0.52064	-0.9416	0.052166	0.387211
ZFHX4	1.984313	2.127766	1.937683	0.83145	-0.2663	0.052179	0.32604
ETHE1	2.071022	2.221503	2.022107	0.83676	-0.2571	0.052187	0.35438
USP49	1.184007	1.074427	1.219627	2.95089	1.5612	0.05222	0.354955
PKIB	2.635598	2.854218	2.564534	0.84377	-0.2451	0.052317	0.313277
AKAP13	1.789509	1.925606	1.74527	0.80517	-0.3126	0.052396	0.387211
CAT	1.486779	1.363701	1.526787	1.44841	0.5345	0.052399	0.373854
AHSA2	1.696581	1.565572	1.739166	1.30694	0.3862	0.052433	0.338124
TGOLN2	1.447974	1.570703	1.40808	0.71505	-0.4839	0.052456	0.32542
EHD1	1.317476	1.434406	1.279468	0.64333	-0.6364	0.052553	0.328051
ZFP14	1.528815	1.40384	1.56944	1.41006	0.4958	0.05258	0.361493
SF1	2.473863	2.308064	2.527757	1.16795	0.224	0.052653	0.355145
THUMPD2	1.3684	1.250456	1.406738	1.62399	0.6995	0.052692	0.308186
DERA	1.624071	1.49567	1.665808	1.34325	0.4257	0.052731	0.387211
SALL1	1.19752	1.087414	1.23331	2.66902	1.4163	0.052769	0.316335
CDC42BPB	1.460079	1.582673	1.420229	0.72121	-0.4715	0.052792	0.314254
SORT1	1.257956	1.372754	1.220641	0.59192	-0.7565	0.052827	0.316335
GALNT6	1.050855	1.155601	1.016806	0.10801	-3.2108	0.052871	0.328321
RCAN3	1.174504	1.284905	1.138618	0.48654	-1.0394	0.052937	0.357415
TANC2	1.301075	1.416994	1.263395	0.63165	-0.6628	0.052975	0.361493
VASN	1.085075	1.191637	1.050436	0.26318	-1.9259	0.053061	0.326147
GTF3C5	1.485998	1.36294	1.525999	1.44927	0.5353	0.053174	0.345114
LDLRAP1	1.236752	1.350022	1.199933	0.5712	-0.8079	0.053182	0.361493
MRPS27	1.601488	1.474477	1.642774	1.3547	0.438	0.05326	0.391094
KIF1A	2.135806	2.29152	2.08519	0.84024	-0.2511	0.053325	0.310087
CENPS	1.360727	1.243783	1.39874	1.63563	0.7098	0.053373	0.326147
TOMM34	1.613425	1.486238	1.654768	1.3466	0.4293	0.053546	0.326147

NCSTN	1.38327	1.502598	1.344481	0.6854	-0.545	0.053675	0.317656
DNASE2	1.541694	1.667806	1.500701	0.74977	-0.4155	0.053679	0.317656
LRP8	1.149055	1.258395	1.113514	0.4393	-1.1867	0.053685	0.369541
NEMF	1.887203	1.746084	1.933075	1.25063	0.3227	0.053718	0.391594
PPCS	1.95703	1.81194	2.004192	1.23678	0.3066	0.053743	0.334247
COMT	1.306437	1.422689	1.268648	0.63557	-0.6539	0.053879	0.334247
HADHA	2.647541	2.452409	2.710971	1.17802	0.2364	0.053914	0.347674
NSMCE4A	1.528483	1.404782	1.568693	1.40494	0.4905	0.054093	0.346607
PDCD4	1.720311	1.588587	1.763129	1.29655	0.3747	0.054177	0.334368
CHD4	3.568759	3.291205	3.658979	1.16052	0.2148	0.054179	0.383834
RTN1	1.202784	1.08632	1.240642	2.78779	1.4791	0.05427	0.36361
VEGFB	1.534749	1.660085	1.494007	0.7484	-0.4181	0.05433	0.373536
PIBF1	1.383807	1.266132	1.422058	1.5859	0.6653	0.054344	0.347183
KLF3	1.874644	1.738515	1.918894	1.24424	0.3153	0.054528	0.347183
LTB	1.080093	1.185224	1.04592	0.24791	-2.0121	0.054596	0.373536
RP1-39G22.7	1.460593	1.3393	1.50002	1.47368	0.5594	0.0546	0.373286
EFNB1	2.033216	1.886207	2.081003	1.21981	0.2867	0.054689	0.398672
PRDX5	8.046229	8.767226	7.811864	0.877	-0.1893	0.054755	0.324019
PPP1R12A	1.992801	2.135473	1.946424	0.83351	-0.2627	0.054827	0.351309
C12orf76	2.512187	2.336438	2.569315	1.17425	0.2317	0.054865	0.33655
AGA	1.341578	1.458543	1.303557	0.662	-0.5951	0.054868	0.367615
HSD17B11	1.424588	1.305771	1.463211	1.51489	0.5992	0.054868	0.3216
COL9A2	2.435903	2.617207	2.376969	0.85145	-0.232	0.054954	0.373536
ABCC4	1.199535	1.089841	1.235192	2.61788	1.3884	0.054956	0.317419
SDHAF4	1.31106	1.196417	1.348325	1.77339	0.8265	0.054985	0.3216
CRYL1	1.735392	1.603917	1.778129	1.28847	0.3657	0.055072	0.341648
FXYD3	1.055587	1.1598	1.021712	0.13587	-2.8797	0.055241	0.351727
SLC22A17	1.52976	1.653816	1.489435	0.74858	-0.4178	0.055249	0.340648
CNOT7	3.081388	2.850622	3.156401	1.16523	0.2206	0.055286	0.337076
DNA2	1.162178	1.05457	1.197157	3.61292	1.8532	0.055397	0.379606
RHBDF2	1.113433	1.219805	1.078855	0.35875	-1.4789	0.055469	0.379606
ALG8	1.5891	1.463303	1.629991	1.35978	0.4434	0.055501	0.369393
GTF3A	3.188403	3.43761	3.107396	0.86453	-0.21	0.055519	0.401812
AAAS	1.435555	1.316697	1.47419	1.4973	0.5824	0.055591	0.372827
COQ7	1.390752	1.273586	1.428838	1.56747	0.6484	0.055776	0.320313
TRIM9	1.22436	1.114066	1.260212	2.28124	1.1898	0.055875	0.344503
ITSN2	1.310764	1.425743	1.273389	0.64215	-0.639	0.055949	0.342861
USP5	1.385341	1.268488	1.423325	1.5767	0.6569	0.05609	0.373317
UNC119B	1.362638	1.246627	1.400348	1.62329	0.6989	0.056299	0.342909
CDS1	1.193652	1.30354	1.157932	0.5203	-0.9426	0.056374	0.356431
MARCH2	1.227142	1.338543	1.19093	0.56397	-0.8263	0.056441	0.32632
TRMU	1.522161	1.399084	1.562168	1.40865	0.4943	0.0566	0.381855
HNRNPL	1.625406	1.498842	1.666547	1.33619	0.4181	0.056602	0.384743
GDPD1	1.187862	1.079346	1.223136	2.8122	1.4917	0.05668	0.374759
SBDS	2.135753	2.287203	2.086523	0.8441	-0.2445	0.056696	0.347436

HAS1	1.102774	1.208191	1.068507	0.32906	-1.6036	0.056702	0.356431
KIAA1217	1.47652	1.35556	1.515839	1.45078	0.5368	0.056762	0.359137
NOL12	1.305431	1.192253	1.342221	1.78005	0.8319	0.05681	0.411158
CEP152	1.230166	1.120084	1.265949	2.2147	1.1471	0.056814	0.381855
C14orf1	2.438245	2.237931	2.503359	1.21441	0.2803	0.056881	0.344371
NCBP2-AS2	2.318709	2.159319	2.370519	1.18218	0.2414	0.056928	0.356431
PPAT	1.34806	1.233389	1.385334	1.65103	0.7234	0.056951	0.375764
C10orf11	1.076072	1.180994	1.041966	0.23187	-2.1086	0.056993	0.381855
TPP2	1.383456	1.266607	1.421438	1.58075	0.6606	0.057034	0.331654
CNPY3	1.962697	2.102766	1.917167	0.8317	-0.2659	0.057043	0.356431
NCLN	1.205136	1.315168	1.16937	0.53739	-0.8959	0.05719	0.386128
MAPKAPK2	1.278136	1.391171	1.241394	0.6171	-0.6964	0.057206	0.375764
C8orf82	1.508187	1.386829	1.547635	1.4157	0.5015	0.057208	0.381133
KIF21B	1.066878	1.171066	1.033011	0.19297	-2.3735	0.057266	0.3488
MRPL39	1.406142	1.289305	1.44412	1.53513	0.6184	0.057285	0.360176
ZNF467	1.25621	1.368496	1.219711	0.59624	-0.746	0.057397	0.363152
MPI	1.504214	1.383069	1.543593	1.41905	0.5049	0.057434	0.332059
TRIP13	1.193481	1.085665	1.228527	2.66768	1.4156	0.05757	0.34599
LYRM2	2.154733	2.007604	2.202558	1.19348	0.2552	0.057721	0.349803
CYC1	3.157269	2.935935	3.229215	1.15149	0.2035	0.057738	0.389829
VPS39	1.177427	1.285917	1.142161	0.49721	-1.0081	0.057814	0.446788
TMPO-AS1	1.16786	1.061226	1.202522	3.30776	1.7259	0.057837	0.34599
FSIP2	1.366897	1.250997	1.404571	1.61185	0.6887	0.057948	0.349114
QKI	2.253922	2.096964	2.304942	1.18959	0.2505	0.058012	0.349114
DTD1	2.319231	2.139172	2.377761	1.20944	0.2743	0.058034	0.35135
SUZ12	1.717074	1.58773	1.759119	1.29161	0.3692	0.058189	0.346033
MRNIP	1.660444	1.533365	1.701752	1.31571	0.3958	0.05822	0.409578
ESF1	2.523692	2.344852	2.581825	1.17621	0.2341	0.05825	0.361728
PAK3	1.347723	1.232832	1.385069	1.65385	0.7258	0.058261	0.446788
LIMK1	1.365101	1.48128	1.327336	0.68014	-0.5561	0.058313	0.335215
PACSIN3	1.485613	1.606552	1.446301	0.7358	-0.4426	0.058358	0.388412
GM2A	1.20627	1.316372	1.170481	0.53886	-0.892	0.058437	0.343788
DCAF11	1.559602	1.436984	1.59946	1.37181	0.4561	0.058624	0.446788
RGS2	1.199387	1.309011	1.163753	0.52993	-0.9161	0.058655	0.334367
MIF4GD	1.696637	1.568516	1.738284	1.29862	0.377	0.058757	0.410486
LDLRAD3	1.333065	1.219503	1.36998	1.68554	0.7532	0.058792	0.381273
CBY1	1.855567	1.722261	1.898898	1.24456	0.3156	0.058803	0.381273
PDHA1	1.777955	1.646458	1.820699	1.26953	0.3443	0.058889	0.334367
RYK	1.517866	1.639896	1.478199	0.74731	-0.4202	0.059128	0.391337
CCPG1	1.486515	1.606713	1.447443	0.73749	-0.4393	0.059302	0.345553
STK16	1.766585	1.636012	1.809028	1.27203	0.3471	0.05933	0.339125
TIAM1	1.354609	1.240063	1.391843	1.63225	0.7069	0.059345	0.357493
FAM46A	1.331418	1.445405	1.294366	0.6609	-0.5975	0.059424	0.345553
RTF1	3.618234	3.309327	3.718646	1.17725	0.2354	0.05943	0.401254
SST	1.072211	1.226046	1.022206	0.09824	-3.3476	0.059485	0.449096

ZBED6	1.495924	1.375623	1.535029	1.42438	0.5103	0.059649	0.336784
SETD3	1.353157	1.468515	1.315659	0.67374	-0.5697	0.05982	0.449096
BLOC1S2	2.058912	2.203656	2.011861	0.84066	-0.2504	0.059923	0.401885
TRMT13	1.394934	1.278708	1.432715	1.55257	0.6347	0.059956	0.344663
HIRIP3	1.265905	1.15604	1.301618	1.93296	0.9508	0.060035	0.357466
GTF2A2	3.238744	3.484043	3.159008	0.86915	-0.2023	0.060169	0.360297
SZRD1	1.803633	1.672388	1.846296	1.25864	0.3319	0.060186	0.387737
SAPCD2	1.155692	1.050894	1.189758	3.72849	1.8986	0.060249	0.357466
OTX1	1.102394	1.206271	1.068628	0.33271	-1.5877	0.060312	0.404491
RFXANK	1.872064	1.737916	1.915669	1.24089	0.3114	0.060465	0.434483
DSC3	1.101043	1.204701	1.067348	0.32901	-1.6038	0.060515	0.377924
VHL	1.299524	1.18794	1.335795	1.78672	0.8373	0.06066	0.357466
MRPL34	2.93755	3.13298	2.874024	0.87859	-0.1867	0.060794	0.453031
EDN2	1.098786	1.202391	1.065109	0.3217	-1.6362	0.060809	0.405124
TMEM51	1.152209	1.258584	1.117632	0.45491	-1.1364	0.060823	0.357466
MYC	1.074602	1.177529	1.041144	0.23176	-2.1093	0.060858	0.377924
PDS5B	1.568715	1.446534	1.60843	1.36256	0.4463	0.060916	0.400533
POLE4	2.696899	2.92974	2.621212	0.84012	-0.2513	0.061062	0.341257
RIF1	1.877193	1.741931	1.921161	1.24157	0.3122	0.061121	0.341257
ETFA	2.126653	1.984034	2.173012	1.19204	0.2534	0.061129	0.346641
MCM8	1.203202	1.096285	1.237957	2.47138	1.3053	0.061176	0.36027
SGCB	1.52746	1.648865	1.487996	0.75208	-0.411	0.061182	0.407607
SSR4	7.108806	7.628538	6.939864	0.8961	-0.1583	0.061201	0.376346
CHMP4A	2.03318	1.892233	2.078996	1.20932	0.2742	0.061221	0.347269
VGLL3	1.070457	1.173046	1.03711	0.21445	-2.2213	0.06123	0.347269
PAK2	2.678371	2.459756	2.749433	1.19844	0.2612	0.0613	0.36027
NNT	1.466702	1.348566	1.505103	1.44909	0.5351	0.061314	0.378415
EIF3D	3.444233	3.210264	3.520286	1.14026	0.1894	0.061334	0.346641
RICTOR	1.647016	1.773049	1.606049	0.78397	-0.3511	0.061353	0.376346
SF3B6	5.203602	4.842016	5.321138	1.12471	0.1695	0.061469	0.347269
PRPF19	1.464175	1.346313	1.502486	1.45096	0.537	0.061497	0.412438
SARS	2.142023	1.996727	2.189252	1.19316	0.2548	0.061677	0.408202
AKAP17A	1.51327	1.633944	1.474044	0.74777	-0.4193	0.06176	0.456842
SCN5A	1.178908	1.286531	1.143924	0.5023	-0.9934	0.06179	0.347269
PSMD10	1.806938	1.676672	1.849282	1.25509	0.3278	0.061941	0.367082
PRR14L	1.555023	1.433906	1.594393	1.36987	0.454	0.062032	0.367082
ARF1	4.174752	4.457323	4.082901	0.8917	-0.1654	0.062102	0.406509
SUV39H2	1.324873	1.213141	1.361192	1.69462	0.761	0.062172	0.406443
SMC6	1.437235	1.555565	1.398771	0.71777	-0.4784	0.062281	0.406443
ZNF330	1.715813	1.843462	1.67432	0.79947	-0.3229	0.062322	0.413258
IVD	1.444268	1.327793	1.482129	1.47083	0.5566	0.062336	0.380635
MAFF	1.147688	1.252791	1.113524	0.44908	-1.155	0.062402	0.350704
TEAD2	2.128317	1.983103	2.175519	1.19572	0.2579	0.062428	0.360935
BCL2L12	1.597378	1.475747	1.636915	1.33877	0.4209	0.06243	0.380635
TRIM38	1.156684	1.262366	1.122331	0.46626	-1.1008	0.062441	0.413258

FDFT1	2.322532	2.496242	2.266067	0.84616	-0.241	0.062499	0.406509
TMEM68	1.542059	1.421222	1.581338	1.38012	0.4648	0.06259	0.446567
COL12A1	1.092768	1.195392	1.05941	0.30406	-1.7176	0.062613	0.406509
PSTK	1.310091	1.199014	1.346197	1.73956	0.7987	0.062633	0.406509
ASNSD1	1.54587	1.425447	1.585014	1.37506	0.4595	0.062852	0.378239
CLVS2	1.125765	1.022959	1.159182	6.93327	2.7935	0.06297	0.349637
AAMDC	2.154514	2.008781	2.201885	1.19142	0.2527	0.063064	0.382181
ANKRA2	1.696755	1.570588	1.737767	1.29299	0.3707	0.063093	0.368654
CNTN1	1.069198	1.171045	1.036091	0.211	-2.2447	0.063124	0.41505
AC062017.1	1.224235	1.11704	1.259079	2.2136	1.1464	0.063138	0.384952
ZRANB3	1.171056	1.066436	1.205063	3.08661	1.626	0.063486	0.354818
DET1	1.253441	1.145434	1.288549	1.98406	0.9885	0.063538	0.464121
TMEM191B	1.143216	1.247748	1.109237	0.44092	-1.1814	0.063593	0.407079
FAU	31.1044	28.94644	31.80586	1.10232	0.1405	0.06366	0.418576
ARL1	1.957908	2.094423	1.913533	0.83472	-0.2606	0.063917	0.464121
GCK	2.117925	1.76661	2.232123	1.60723	0.6846	0.064076	0.458882
NEO1	1.408022	1.523917	1.370349	0.70688	-0.5005	0.064093	0.353922
C1RL	1.330536	1.219236	1.366715	1.6727	0.7422	0.064128	0.464121
TPT1	53.40383	55.51941	52.71614	0.94858	-0.0762	0.064444	0.376148
DIAPH3	1.240418	1.133259	1.27525	2.06552	1.0465	0.064515	0.458882
IGLON5	1.197213	1.303925	1.162525	0.53475	-0.9031	0.064616	0.380136
PLA2G16	2.068409	2.211757	2.021812	0.84325	-0.246	0.06475	0.376148
RP11-66N11.8	1.185494	1.080763	1.219537	2.71828	1.4427	0.064773	0.458882
PLEKHM2	1.216939	1.324283	1.182046	0.56138	-0.8329	0.064944	0.38561
SLC48A1	1.331375	1.443216	1.29502	0.66564	-0.5872	0.065012	0.38561
SYT5	1.242926	1.135927	1.277706	2.04305	1.0307	0.065109	0.409373
FRAS1	1.235586	1.343192	1.200608	0.58454	-0.7746	0.065111	0.357579
MED10	2.29157	2.14411	2.339503	1.17078	0.2275	0.065161	0.422495
SRA1	1.547841	1.668313	1.508681	0.76114	-0.3938	0.065227	0.38561
FAN1	1.252869	1.145054	1.287915	1.98487	0.989	0.06536	0.396099
CDK10	1.423765	1.309213	1.461	1.49088	0.5762	0.065519	0.460926
MED31	1.674625	1.550102	1.715102	1.29994	0.3784	0.065595	0.455095
TMEM251	1.498179	1.380896	1.536303	1.40801	0.4937	0.065631	0.381265
FAM104B	1.558401	1.438579	1.59735	1.36201	0.4457	0.065817	0.359491
SRD5A1	1.452113	1.336432	1.489716	1.45562	0.5416	0.065819	0.42445
TCAF1	2.518499	2.351267	2.572859	1.16399	0.2191	0.066001	0.419818
C11orf49	1.641959	1.519176	1.68187	1.31337	0.3933	0.066018	0.431258
LZTFL1	1.335861	1.225307	1.371798	1.65019	0.7226	0.066167	0.432237
CRK	1.64728	1.77092	1.60709	0.78749	-0.3447	0.066318	0.383044
RALGPS2	1.537005	1.418043	1.575674	1.37707	0.4616	0.066373	0.463692
MAGOHB	1.543476	1.424692	1.582087	1.37061	0.4548	0.066509	0.369659
TBCB	3.690375	3.440364	3.771643	1.13575	0.1836	0.066532	0.478082
PSMD11	1.914639	2.048216	1.871219	0.83114	-0.2668	0.066754	0.402125
NAA15	1.601368	1.480777	1.640567	1.33236	0.414	0.066852	0.417723
KIAA1191	1.982166	2.116781	1.938409	0.84028	-0.2511	0.066953	0.434544

TMEM141	2.558497	2.396481	2.611162	1.15373	0.2063	0.067169	0.479231
PLAT	1.402914	1.517448	1.365684	0.70671	-0.5008	0.067292	0.466865
ATOH8	1.156664	1.054425	1.189898	3.48916	1.8029	0.067378	0.386943
SKA3	1.137541	1.036215	1.170478	4.70734	2.2349	0.06743	0.396301
NDUFA4	17.73083	18.87213	17.35984	0.91538	-0.1276	0.067446	0.388761
MTIF2	1.496594	1.38006	1.534475	1.40629	0.4919	0.067452	0.403911
NFKBIE	1.175635	1.28024	1.141632	0.5054	-0.9845	0.067476	0.419018
DCAKD	1.591045	1.471062	1.630046	1.3375	0.4195	0.067523	0.387169
SKI	1.316435	1.427427	1.280357	0.65592	-0.6084	0.067573	0.427113
EIF4EBP1	3.233456	2.978033	3.316483	1.1711	0.2279	0.06762	0.437671
NDUFS5	10.82147	9.697964	11.18668	1.17116	0.2279	0.067655	0.367533
COPE	4.114494	4.401154	4.021313	0.88832	-0.1708	0.067695	0.388761
ZFYVE21	1.639445	1.761371	1.599812	0.78781	-0.3441	0.067735	0.387169
TIMM9	2.153952	2.012081	2.200068	1.18574	0.2458	0.067869	0.437671
NOVA2	1.30288	1.193946	1.33829	1.74424	0.8026	0.068	0.380045
NELL2	2.041175	2.19698	1.99053	0.82752	-0.2731	0.068061	0.468965
KRAS	1.803112	1.932024	1.761208	0.81673	-0.2921	0.068087	0.437993
APPL1	2.221215	2.078424	2.26763	1.17545	0.2332	0.068203	0.408831
ZFHX3	1.61713	1.738749	1.577597	0.78186	-0.355	0.068274	0.483687
C10orf35	1.497278	1.380623	1.535198	1.40611	0.4917	0.068274	0.408831
MSH3	1.369054	1.258011	1.405149	1.57028	0.651	0.068301	0.427307
CNRIP1	1.219645	1.114812	1.253721	2.20989	1.144	0.068423	0.437993
MGMT	1.790144	1.66354	1.831298	1.25282	0.3252	0.068451	0.438609
B9D1	1.66435	1.542123	1.70408	1.29875	0.3771	0.068454	0.427307
PCOLCE	1.692683	1.569455	1.73274	1.28674	0.3637	0.068587	0.442298
SLC35A2	1.345961	1.456897	1.3099	0.67827	-0.5601	0.068602	0.472694
TBRG1	1.521305	1.639658	1.482834	0.75483	-0.4058	0.068713	0.390536
ATN1	1.545639	1.6647	1.506937	0.76266	-0.3909	0.068772	0.449249
EYA3	1.376439	1.264974	1.412671	1.55741	0.6391	0.068883	0.402885
DRAP1	4.077901	4.35113	3.989087	0.89196	-0.1649	0.068929	0.383109
TNFAIP8	1.194991	1.300004	1.160857	0.53618	-0.8992	0.069031	0.410921
LRRC1	1.217382	1.323041	1.183037	0.56661	-0.8196	0.069094	0.380866
TOLLIP	1.498911	1.615805	1.460915	0.74848	-0.418	0.069109	0.484058
C14orf119	2.033178	2.169244	1.988949	0.8458	-0.2416	0.069274	0.412364
AMZ2	2.327402	2.178944	2.375659	1.16686	0.2226	0.069283	0.380866
SHC2	1.216343	1.321527	1.182152	0.56652	-0.8198	0.069362	0.396072
REEP4	1.305796	1.198036	1.340824	1.72102	0.7833	0.069457	0.484058
SERTAD4-AS1	1.086638	1.186606	1.054142	0.29014	-1.7852	0.069477	0.384034
PMP22	1.15899	1.261699	1.125604	0.47996	-1.059	0.069491	0.451019
ZHX1	1.66039	1.538786	1.699919	1.29907	0.3775	0.0695	0.40609
TOB2	1.641716	1.519667	1.681389	1.3112	0.3909	0.069526	0.442238
FAM174B	1.105346	1.205973	1.072637	0.35265	-1.5037	0.0696	0.493085
POLQ	1.142006	1.041108	1.174804	4.2523	2.0882	0.06967	0.443598
ZDHHC21	1.507949	1.391505	1.5458	1.39411	0.4793	0.06972	0.412577
POLH	1.148167	1.047088	1.181023	3.84438	1.9428	0.06977	0.484058

PSME2	2.43125	2.272826	2.482746	1.16493	0.2202	0.070058	0.43462
ABCE1	1.753743	1.628691	1.794392	1.26357	0.3375	0.070166	0.449601
DEGS1	1.617483	1.738366	1.578188	0.78306	-0.3528	0.070185	0.40772
ATAD1	1.65051	1.773164	1.610641	0.78979	-0.3405	0.070191	0.394475
MDC1	1.211433	1.107323	1.245275	2.28539	1.1924	0.070255	0.379514
KIAA1522	1.220951	1.325672	1.186911	0.57392	-0.8011	0.070281	0.394475
RP11-244M2.1	1.159174	1.05783	1.192116	3.3221	1.7321	0.070296	0.401409
CENPQ	1.253974	1.148254	1.288339	1.94489	0.9597	0.070326	0.413728
GNG4	1.388646	1.278167	1.424558	1.52627	0.61	0.070425	0.384946
TRIM13	1.581649	1.463022	1.62021	1.33948	0.4217	0.070582	0.394475
RAB6B	1.329146	1.438992	1.29344	0.66844	-0.5811	0.070616	0.379514
AC002310.12	1.23393	1.129031	1.268028	2.07724	1.0547	0.070628	0.4359
SPICE1	1.321046	1.21255	1.356313	1.67637	0.7453	0.070644	0.401114
FAM210B	2.381963	2.218436	2.435119	1.17784	0.2361	0.070707	0.453065
TUFM	3.831956	3.551611	3.923084	1.14558	0.1961	0.070921	0.485347
HMGXB3	1.32043	1.429535	1.284965	0.66343	-0.592	0.070983	0.409131
NDN	3.09269	3.305978	3.023359	0.87744	-0.1886	0.071105	0.436169
PITRM1	1.248314	1.354809	1.213696	0.60228	-0.7315	0.071132	0.449607
THNSL2	1.301823	1.194658	1.336658	1.72949	0.7903	0.071133	0.490137
TMEM216	1.378403	1.26818	1.414231	1.5446	0.6272	0.071242	0.409131
PAIP1	2.757728	2.937136	2.699411	0.87728	-0.1889	0.07125	0.384946
WNK1	2.09105	2.227965	2.046544	0.85226	-0.2306	0.071353	0.43994
UBE2Q2	1.57107	1.689394	1.532608	0.77257	-0.3723	0.071369	0.422336
C18orf32	1.536555	1.654643	1.498169	0.76098	-0.3941	0.071429	0.502498
MAFG-AS1	1.40194	1.290737	1.438087	1.50682	0.5915	0.071435	0.451974
POLR2J2	1.189982	1.087401	1.223327	2.55518	1.3534	0.07153	0.384946
ATG4B	1.570715	1.452786	1.609049	1.34511	0.4277	0.071678	0.38317
LIN7B	1.221816	1.326565	1.187767	0.57498	-0.7984	0.07172	0.384946
TMEM35A	1.265954	1.160135	1.300351	1.87561	0.9074	0.071905	0.463694
ISPD	1.080233	1.179109	1.048092	0.26851	-1.897	0.071938	0.384946
MAGOH	1.961101	1.8309	2.003424	1.20764	0.2722	0.072008	0.452299
FHAD1	1.170839	1.06968	1.203721	2.92364	1.5478	0.072269	0.401674
KIZ	1.318018	1.210262	1.353045	1.67907	0.7477	0.072328	0.454764
EIF5A	7.393059	6.648218	7.635175	1.17474	0.2323	0.072673	0.385379
UQCC1	1.411825	1.300382	1.448051	1.4916	0.5769	0.072704	0.494189
GRHPR	2.467483	2.282367	2.527656	1.19128	0.2525	0.072761	0.415481
OXCT1	1.302597	1.409909	1.267714	0.65311	-0.6146	0.072793	0.50854
LRRC75A	1.350308	1.240913	1.385868	1.60169	0.6796	0.07281	0.411089
ADAM10	1.745102	1.869164	1.704775	0.81086	-0.3025	0.072858	0.385379
LNP1	1.188561	1.086827	1.22163	2.55254	1.3519	0.072887	0.45543
RP11-136K7.2	1.118179	1.019163	1.150365	7.84677	2.9721	0.073057	0.414816
HIBCH	2.038005	1.904999	2.081239	1.19474	0.2567	0.073154	0.448291
FEZF2	1.134458	1.035086	1.166759	4.75284	2.2488	0.073163	0.497961
ATP5A1	4.644255	4.333872	4.745147	1.12336	0.1678	0.073252	0.446615
MOXD1	1.40424	1.515854	1.367959	0.7133	-0.4874	0.073259	0.497961

ACSL3	1.471093	1.585378	1.433943	0.7413	-0.4319	0.073274	0.426093
SPIDR	1.377969	1.268339	1.413605	1.54135	0.6242	0.073353	0.429028
LATS2	1.179544	1.281968	1.146251	0.51868	-0.9471	0.073434	0.403687
NUDT21	1.742034	1.619338	1.781917	1.26251	0.3363	0.073486	0.502904
CALR	6.811507	7.319621	6.646341	0.89346	-0.1625	0.073669	0.413616
FAM96A	2.486249	2.318944	2.540633	1.16808	0.2241	0.073686	0.497506
C8orf44	1.267902	1.162797	1.302067	1.85548	0.8918	0.073899	0.434753
RAB36	1.196694	1.094986	1.229755	2.41882	1.2743	0.074037	0.499872
POU2F2	1.224119	1.120957	1.257652	2.13012	1.0909	0.074074	0.418229
ZNF688	1.481219	1.367868	1.518064	1.40829	0.4939	0.074119	0.46027
PDCD7	1.580413	1.463361	1.618461	1.33473	0.4165	0.074149	0.514444
CTSL	1.731061	1.855399	1.690644	0.80739	-0.3087	0.074411	0.391532
ATP5B	5.376131	5.000695	5.498168	1.12435	0.1691	0.074702	0.396355
FBXO3	1.378289	1.268757	1.413894	1.54003	0.623	0.074707	0.492478
KIAA1958	1.387135	1.277717	1.422702	1.52206	0.606	0.074858	0.396355
SLC6A8	1.279353	1.385649	1.244801	0.63478	-0.6557	0.074871	0.479747
ARL8B	1.350279	1.459375	1.314817	0.68532	-0.5452	0.074925	0.418329
ATP5SL	1.747931	1.624664	1.788	1.26148	0.3351	0.074974	0.492478
GFY	1.124071	1.025713	1.156043	6.06856	2.6014	0.075177	0.42685
ADAM12	1.182939	1.284936	1.149784	0.52568	-0.9278	0.075191	0.504284
SLITRK2	1.157185	1.057439	1.189607	3.30103	1.7229	0.075237	0.504591
ZNF620	1.245568	1.142061	1.279214	1.96545	0.9749	0.075276	0.42264
RBM28	1.396721	1.287387	1.432261	1.50411	0.5889	0.075355	0.463384
ZNF24	1.773816	1.650039	1.81405	1.25231	0.3246	0.075691	0.396196
PHF1	1.380599	1.490733	1.344799	0.70262	-0.5092	0.075718	0.41398
ELMOD2	1.483494	1.370812	1.520122	1.40266	0.4882	0.075745	0.437927
TADA3	2.679914	2.499576	2.738534	1.15935	0.2133	0.07587	0.483074
PSMC3	2.446369	2.281798	2.499863	1.17012	0.2267	0.075876	0.418713
MFSD12	1.270767	1.376063	1.23654	0.62899	-0.6689	0.07588	0.505532
MUTYH	1.304597	1.199132	1.338879	1.70178	0.767	0.075895	0.483229
RP11-672L10.6	1.220987	1.118518	1.254295	2.14562	1.1014	0.075976	0.463384
CEP95	1.385497	1.276841	1.420816	1.52006	0.6041	0.076002	0.463384
DEXI	1.56549	1.682162	1.527565	0.77337	-0.3708	0.076009	0.40034
MPDZ	1.70347	1.582711	1.742723	1.2746	0.35	0.076168	0.418713
MEX3A	2.155563	2.019498	2.199792	1.17685	0.2349	0.076259	0.446026
RBPMS2	1.231764	1.335957	1.197896	0.58905	-0.7635	0.076271	0.43845
CTC-260E6.6	1.135018	1.036465	1.167053	4.5812	2.1957	0.076282	0.423552
SNX3	5.522124	5.904568	5.397808	0.89668	-0.1573	0.076321	0.430912
TSR3	1.520723	1.635559	1.483395	0.76058	-0.3948	0.076395	0.483357
EMB	1.080364	1.177328	1.048845	0.27545	-1.8601	0.076406	0.483426
ATP11A	1.155231	1.256215	1.122405	0.47774	-1.0657	0.076546	0.398594
ADD1	1.76652	1.890038	1.726369	0.81611	-0.2932	0.076914	0.509047
INPP5K	1.15888	1.25991	1.12604	0.48494	-1.0441	0.077028	0.484312
LUC7L2	1.859459	1.733695	1.900339	1.22713	0.2953	0.077057	0.4213
RP11-32K4.1	1.130766	1.032515	1.162703	5.00395	2.3231	0.077122	0.440825

TRIM16	1.546805	1.431435	1.584306	1.35433	0.4376	0.077148	0.485065
RP11-386G11.10	1.245679	1.143016	1.279051	1.95118	0.9644	0.077154	0.528009
PROSER3	1.298965	1.193576	1.333223	1.7214	0.7836	0.077154	0.418652
IRF2BPL	1.906435	2.035706	1.864414	0.83461	-0.2608	0.077154	0.528009
RASL10B	1.133198	1.23238	1.100959	0.43445	-1.2027	0.077159	0.491129
LINC01184	1.600999	1.483994	1.639032	1.32033	0.4009	0.077289	0.50393
PIGL	1.258167	1.155082	1.291676	1.88079	0.9113	0.077624	0.491129
HCRT	1.128373	1.030687	1.160126	5.21803	2.3835	0.07764	0.46825
GOLGA6L10	1.274057	1.170312	1.30778	1.80715	0.8537	0.077645	0.433518
CDK19	1.608779	1.492645	1.646529	1.31236	0.3922	0.077698	0.407104
GPR137B	1.356815	1.464842	1.3217	0.69206	-0.531	0.077707	0.436286
RAI1	1.182553	1.283886	1.149614	0.52702	-0.9241	0.077731	0.46825
CACNB4	1.178512	1.0786	1.210989	2.68434	1.4246	0.077828	0.526922
RNF44	1.505318	1.391799	1.542219	1.38392	0.4688	0.077856	0.429918
LLGL2	1.096338	1.193941	1.064611	0.33315	-1.5858	0.077944	0.418652
TMEM43	1.435988	1.547106	1.399868	0.73088	-0.4523	0.077978	0.470843
EPB41	1.482622	1.370551	1.519052	1.40076	0.4862	0.077996	0.443224
LMO1	1.313064	1.438666	1.272236	0.6206	-0.6883	0.078029	0.50393
CCT6A	3.547614	3.336429	3.616261	1.11977	0.1632	0.078036	0.453784
ABI1	1.367226	1.475462	1.332043	0.69836	-0.518	0.078043	0.526922
TAB2	1.425905	1.53611	1.390083	0.72762	-0.4587	0.078082	0.418652
ANGPTL2	1.101536	1.199453	1.069708	0.34949	-1.5167	0.078115	0.485558
C6orf62	2.214116	2.0677	2.261709	1.18171	0.2409	0.078162	0.470843
NOL4	1.115476	1.017948	1.147178	8.20042	3.0357	0.078222	0.50393
OXA1L	2.356655	2.502466	2.309258	0.87141	-0.1986	0.078273	0.488599
GCFC2	1.361245	1.253976	1.396113	1.55965	0.6412	0.078545	0.443224
EPB41L4A	1.117849	1.215704	1.086041	0.39888	-1.326	0.078561	0.436207
RP11-348J24.2	1.18533	1.085563	1.21776	2.54503	1.3477	0.078609	0.438556
MRRF	1.467546	1.356316	1.503703	1.41364	0.4994	0.078618	0.474289
PTPN18	1.399535	1.291077	1.43479	1.49373	0.5789	0.078685	0.485558
TBCD	1.371949	1.264888	1.406751	1.53556	0.6188	0.078736	0.45468
CLDN1	2.127397	1.987404	2.172903	1.18787	0.2484	0.07875	0.474289
TFAP2A	1.162325	1.262476	1.12977	0.49441	-1.0162	0.078775	0.535459
NCOR2	1.486837	1.599515	1.45021	0.75096	-0.4132	0.07879	0.418652
MAPK13	1.114449	1.212281	1.082648	0.38934	-1.3609	0.078812	0.474289
VMP1	2.474316	2.701754	2.400386	0.82291	-0.2812	0.078864	0.443224
FUT8	1.240657	1.343753	1.207145	0.6026	-0.7307	0.07892	0.429154
KIAA0586	1.29264	1.188428	1.326515	1.73284	0.7931	0.078968	0.493189
TIPIN	1.280338	1.17622	1.314182	1.78289	0.8342	0.079034	0.418652
MELTF-AS1	1.229283	1.127741	1.26229	2.05329	1.0379	0.079046	0.530135
PPIF	1.463443	1.352606	1.499471	1.41651	0.5023	0.07907	0.418652
TIAL1	2.178306	2.016945	2.230758	1.21025	0.2753	0.079097	0.436496
CYP24A1	1.046582	1.14131	1.01579	0.11174	-3.1618	0.079115	0.458653
FAM89B	1.586449	1.702452	1.548741	0.78118	-0.3563	0.079202	0.410297
EIF2AK1	2.413818	2.252621	2.466216	1.17052	0.2271	0.079292	0.485558

CLSTN3	1.232712	1.335395	1.199334	0.59433	-0.7507	0.07933	0.458653
CNOT1	1.532379	1.419267	1.569147	1.35748	0.4409	0.079346	0.45468
ZNF606	1.29675	1.192021	1.330793	1.72269	0.7847	0.079454	0.485558
IKZF4	1.331314	1.225803	1.365611	1.61916	0.6952	0.079627	0.45468
SPRR2F	1.051359	1.146492	1.020436	0.1395	-2.8417	0.079639	0.485558
RP11-54O7.1	1.181898	1.08242	1.214235	2.5993	1.3781	0.079662	0.437486
SDC1	1.262547	1.36637	1.228798	0.6245	-0.6792	0.079667	0.438556
ZFP62	1.262022	1.159194	1.295448	1.8559	0.8921	0.079705	0.445463
FRRS1L	1.241548	1.139634	1.274675	1.9671	0.9761	0.079746	0.431317
SCMH1	1.358485	1.25189	1.393134	1.56073	0.6422	0.079751	0.493189
AAMP	1.786359	1.664457	1.825984	1.2431	0.3139	0.079752	0.438556
FNBP4	1.897897	1.764591	1.941228	1.23102	0.2999	0.079778	0.436496
URM1	1.986601	1.857837	2.028457	1.1989	0.2617	0.079799	0.47617
KAT2A	1.411164	1.302723	1.446413	1.47466	0.5604	0.079804	0.524727
ZNF253	1.250692	1.148693	1.283847	1.90894	0.9328	0.079857	0.438556
FAM98A	1.293498	1.398519	1.25936	0.65081	-0.6197	0.079916	0.436496
AGPAT5	1.31823	1.213146	1.352388	1.65327	0.7253	0.079993	0.47617
ZNF300	1.257966	1.155647	1.291225	1.87106	0.9039	0.079998	0.45468
MSRA	1.371549	1.479439	1.336478	0.70182	-0.5108	0.080009	0.478238
SP100	1.085292	1.181905	1.053887	0.29624	-1.7552	0.080101	0.496269
MGEA5	2.299374	2.159771	2.344753	1.1595	0.2135	0.08011	0.50369
SAAL1	1.328959	1.224147	1.363029	1.6196	0.6956	0.080116	0.412906
ENKUR	1.141035	1.043575	1.172715	3.96364	1.9868	0.080214	0.438127
SLC9A7	1.220602	1.321928	1.187666	0.58294	-0.7786	0.080223	0.431573
GARS	1.57955	1.694679	1.542127	0.7804	-0.3577	0.080236	0.480463
SLC40A1	1.095219	1.192199	1.063696	0.3314	-1.5933	0.080278	0.493189
ATP6V1G2	1.265539	1.162882	1.298908	1.83512	0.8759	0.08032	0.44642
PLPPR3	1.397915	1.289865	1.433037	1.49392	0.5791	0.080388	0.514586
PLEKHM3	1.174017	1.075325	1.206097	2.73611	1.4521	0.08042	0.478238
GLI3	1.37036	1.478331	1.335263	0.7009	-0.5127	0.080451	0.419348
LIX1L	1.640357	1.523222	1.678432	1.29664	0.3748	0.080489	0.496269
SRGAP2B	1.312927	1.20864	1.346826	1.66232	0.7332	0.080516	0.536418
CDC42EP4	1.318238	1.213053	1.352428	1.65418	0.7261	0.080554	0.488176
NUP62	1.493136	1.382261	1.529177	1.38433	0.4692	0.0808	0.432366
ZADH2	1.431476	1.322198	1.466997	1.44941	0.5355	0.080865	0.47853
TRIP6	1.639038	1.755622	1.601142	0.79556	-0.33	0.080919	0.455642
AK1	2.270426	2.121172	2.318941	1.1764	0.2344	0.080986	0.425719
RAD23B	2.381616	2.529552	2.333529	0.87184	-0.1979	0.081006	0.420063
TAF11	2.316341	2.168407	2.364427	1.16777	0.2238	0.081028	0.493189
TM7SF3	1.672726	1.790881	1.634319	0.80204	-0.3183	0.081068	0.442789
MRPL40	1.935118	2.062029	1.893865	0.84166	-0.2487	0.081074	0.455642
EEF1AKMT1	1.338932	1.233765	1.373118	1.59613	0.6746	0.081114	0.440647
IDH3B	1.765966	1.645694	1.805061	1.24681	0.3182	0.08125	0.449107
TLE3	1.415772	1.307147	1.451081	1.46861	0.5545	0.081289	0.425719
TTK	1.141098	1.04403	1.172651	3.92125	1.9713	0.081316	0.415648

SIX3	3.297278	3.075917	3.369232	1.14129	0.1907	0.081375	0.508468
CHCHD1	2.483409	2.320666	2.53631	1.16328	0.2182	0.081412	0.484616
NR1D1	1.163376	1.262616	1.131118	0.49928	-1.0021	0.081523	0.550419
CCDC18	1.206717	1.106777	1.239203	2.24022	1.1636	0.081623	0.441029
LIF	1.079006	1.17478	1.047874	0.27391	-1.8682	0.081649	0.415648
CAMK1	1.355007	1.461285	1.32046	0.69471	-0.5255	0.081674	0.425719
FAM127A	2.507674	2.668895	2.455269	0.872	-0.1976	0.081746	0.493189
TRDMT1	1.218193	1.117968	1.250772	2.12575	1.088	0.081797	0.493189
BUB1B	1.158921	1.061371	1.19063	3.10617	1.6351	0.081871	0.493189
DENND4A	1.339948	1.234244	1.374307	1.59793	0.6762	0.081889	0.415648
PTH1R	1.081312	1.177137	1.050163	0.28319	-1.8202	0.081916	0.470467
RP11-108M9.4	2.078163	1.93649	2.124214	1.20046	0.2636	0.081931	0.53521
NGLY1	1.547456	1.66115	1.5105	0.77214	-0.3731	0.082084	0.42298
COL26A1	1.105025	1.201449	1.073682	0.36576	-1.451	0.082266	0.446905
LRRC49	1.332054	1.227644	1.365993	1.60775	0.685	0.082351	0.487327
RP11-110G21.1	1.181768	1.08321	1.213805	2.56947	1.3615	0.082358	0.484513
CAMK1D	1.33206	1.227863	1.36593	1.60592	0.6834	0.082442	0.438818
PTPRU	1.408345	1.516564	1.373167	0.7224	-0.4691	0.08251	0.42298
SETD9	1.379071	1.272593	1.413682	1.51758	0.6018	0.082655	0.44306
MARC1	1.193251	1.094611	1.225314	2.38149	1.2519	0.08283	0.42298
RTCB	1.685696	1.568284	1.723861	1.27377	0.3491	0.082842	0.484529
PAM	1.965755	2.093679	1.924172	0.84501	-0.243	0.082881	0.44306
NPAS2	1.081868	1.177114	1.050908	0.28743	-1.7987	0.082888	0.552222
RASGRP1	1.132589	1.229875	1.100966	0.43922	-1.187	0.082916	0.490178
CRBN	1.62099	1.50549	1.658534	1.30276	0.3816	0.082949	0.439192
SPR	1.088675	1.184105	1.057654	0.31316	-1.675	0.083028	0.549515
NBN	1.303728	1.200819	1.337179	1.67902	0.7476	0.083033	0.515622
OGT	1.45675	1.347413	1.492291	1.41702	0.5029	0.083082	0.419584
EXOC3-AS1	1.330746	1.226134	1.36475	1.61298	0.6897	0.083217	0.470467
MIR1302-9	1.150641	1.053481	1.182223	3.40722	1.7686	0.083363	0.470467
RFTN1	1.064824	1.159122	1.034172	0.21475	-2.2192	0.083455	0.470467
DCTD	2.085732	1.955686	2.128004	1.18031	0.2392	0.083711	0.470467
MAP2K7	1.515076	1.403724	1.551271	1.36547	0.4494	0.083727	0.513084
EPB41L5	1.217526	1.318485	1.184708	0.57996	-0.786	0.083744	0.532678
ADAL	1.513106	1.402076	1.549197	1.36591	0.4499	0.083874	0.455642
POLR2E	3.362812	3.143585	3.434073	1.13552	0.1833	0.083954	0.493364
KRT4	1.045168	1.138481	1.014836	0.10714	-3.2225	0.084287	0.471073
CLIC4	1.730803	1.850163	1.692004	0.81397	-0.297	0.084346	0.558234
SDK1	1.148244	1.051656	1.17964	3.47762	1.7981	0.084353	0.493364
IGSF10	1.324074	1.220255	1.357821	1.62458	0.7001	0.0844	0.505397
MFHAS1	1.183491	1.282302	1.151372	0.53621	-0.8991	0.084541	0.555869
CIT	1.132674	1.037061	1.163754	4.41845	2.1435	0.08461	0.51535
BPTF	2.649745	2.481085	2.704569	1.15089	0.2028	0.084685	0.450308
VPS36	1.547484	1.435775	1.583795	1.33967	0.4219	0.084724	0.425737
ONECUT2	1.128344	1.032405	1.159529	4.92294	2.2995	0.084726	0.473523

C6orf118	1.135305	1.038939	1.16663	4.27929	2.0974	0.085054	0.537606
SMYD3	1.524111	1.639704	1.486537	0.76057	-0.3949	0.085149	0.571067
SLC25A21-AS1	1.266077	1.165266	1.298846	1.80827	0.8546	0.08529	0.433344
PEX2	2.180032	2.039939	2.22557	1.1785	0.237	0.085303	0.51386
TACSTD2	1.045543	1.138455	1.015341	0.1108	-3.174	0.085312	0.557295
TCF19	1.168483	1.071419	1.200035	2.80085	1.4859	0.085766	0.563926
ZNF451	1.609959	1.495763	1.647079	1.30522	0.3843	0.085883	0.55741
TMEM248	1.714329	1.832166	1.676025	0.81237	-0.2998	0.085889	0.452379
ZNF514	1.356664	1.252046	1.390671	1.55	0.6323	0.085956	0.572657
ANKRD36	1.385966	1.280136	1.420366	1.50058	0.5855	0.086191	0.519748
PLPP2	1.066292	1.159768	1.035908	0.22475	-2.1536	0.086359	0.50755
DNAAF2	1.631016	1.515989	1.668407	1.29539	0.3734	0.086366	0.519748
LGR5	1.14149	1.045816	1.172589	3.76696	1.9134	0.086377	0.447912
P4HB	4.82351	5.150392	4.717256	0.89564	-0.159	0.086443	0.559002
RNF20	1.370059	1.265368	1.404089	1.52275	0.6067	0.086514	0.457614
RBM7	1.829006	1.707105	1.868631	1.22843	0.2968	0.086586	0.432932
HTRA2	1.498396	1.388897	1.533989	1.37309	0.4574	0.0866	0.503584
COL16A1	1.093257	1.187856	1.062507	0.33274	-1.5875	0.086705	0.566397
RCCD1	1.440282	1.33311	1.475119	1.42631	0.5123	0.086866	0.574918
TMEM168	1.219711	1.12097	1.251807	2.08157	1.0577	0.086884	0.469454
RBM47	1.144824	1.24149	1.113402	0.46959	-1.0905	0.087006	0.516336
DHRS7B	1.358826	1.254159	1.392848	1.54568	0.6282	0.087048	0.546767
AKAP9	3.016513	3.23882	2.94425	0.86843	-0.2035	0.087052	0.456119
ATP6V1C1	1.44166	1.550073	1.406419	0.73885	-0.4367	0.08716	0.559002
ZNF793	1.438814	1.331489	1.473701	1.42901	0.515	0.087187	0.482931
HIST1H1B	1.138525	1.043499	1.169414	3.89466	1.9615	0.08724	0.559002
ALDH18A1	1.358874	1.254874	1.39268	1.54069	0.6236	0.087276	0.459151
LPCAT2	1.111026	1.206235	1.080078	0.38828	-1.3648	0.087326	0.516336
EXOG	1.314357	1.211843	1.34768	1.64121	0.7148	0.087369	0.482931
ITPR3	1.059167	1.151673	1.029098	0.19184	-2.382	0.087394	0.472211
MIR762HG	1.281038	1.179681	1.313985	1.74746	0.8053	0.087407	0.450928
TYW3	1.326527	1.22344	1.360036	1.61133	0.6883	0.087502	0.516336
CHCHD5	2.95535	2.772158	3.014897	1.13697	0.1852	0.087521	0.575463
GPAA1	2.248554	2.384154	2.204477	0.87019	-0.2006	0.087557	0.540385
NRBP2	1.209523	1.308476	1.177358	0.57495	-0.7985	0.087643	0.456834
LAMTOR4	4.754227	5.095659	4.643243	0.88954	-0.1689	0.087687	0.56547
UNC5C	1.19374	1.096331	1.225403	2.33988	1.2264	0.087718	0.459151
TUSC3	3.633895	3.906973	3.545129	0.87553	-0.1918	0.08773	0.471492
CTA-445C9.14	1.178671	1.081465	1.210269	2.5811	1.368	0.087788	0.547996
PPIG	3.091858	2.895557	3.155667	1.13722	0.1855	0.087996	0.516336
CDH1	1.043002	1.135111	1.013062	0.09667	-3.3707	0.088105	0.560974
ANLN	1.211131	1.113282	1.242938	2.14454	1.1007	0.08811	0.484365
WDHD1	1.238079	1.139239	1.270208	1.94061	0.9565	0.08828	0.516336
RRNAD1	1.319185	1.216635	1.35252	1.62725	0.7024	0.088289	0.528158
STX4	1.452994	1.560832	1.41794	0.74521	-0.4243	0.088369	0.572997

COX11	1.887813	1.76585	1.927457	1.21102	0.2762	0.088489	0.458864
CHTF18	1.181405	1.084728	1.21283	2.51191	1.3288	0.088495	0.454214
FIBCD1	3.383011	3.654414	3.294789	0.86452	-0.21	0.088621	0.549778
CCNJ	1.26044	1.160519	1.29292	1.82483	0.8678	0.088768	0.513224
MYO6	1.811819	1.931699	1.772852	0.82951	-0.2697	0.088786	0.46086
TIAM2	1.172613	1.270076	1.140932	0.52182	-0.9384	0.088802	0.441811
IQSEC1	1.368571	1.472845	1.334675	0.70779	-0.4986	0.088854	0.572997
PTPRD-AS1	1.200561	1.103168	1.232219	2.25089	1.1705	0.088859	0.485829
DONSON	1.32152	1.219222	1.354772	1.61833	0.6945	0.088962	0.46086
PINLYP	1.313517	1.211886	1.346553	1.63557	0.7098	0.089084	0.487164
CLDN3	1.841559	1.963179	1.802026	0.83269	-0.2642	0.089104	0.487164
ZNF681	1.190282	1.093294	1.221809	2.37751	1.2495	0.089161	0.582442
SRSF8	2.066591	1.939576	2.107877	1.17912	0.2377	0.089167	0.44869
PDE7A	1.458412	1.351136	1.493283	1.40482	0.4904	0.089203	0.44869
CAHM	1.225939	1.127574	1.257913	2.02167	1.0155	0.089212	0.479457
NUDT12	1.249618	1.150592	1.281807	1.87133	0.9041	0.08963	0.4624
EFR3B	1.420211	1.526481	1.385668	0.73254	-0.449	0.089635	0.520791
WDYHV1	1.515612	1.406897	1.550951	1.35403	0.4373	0.090162	0.446367
PSMA1	4.470262	4.18182	4.564022	1.12012	0.1637	0.090182	0.463036
TMEM245	1.750497	1.867961	1.712315	0.82068	-0.2851	0.090203	0.460632
FABP5	11.8644	13.16319	11.44222	0.85851	-0.2201	0.090518	0.58001
EIF2B1	1.438912	1.332989	1.473343	1.42149	0.5074	0.09056	0.526611
SH3PXD2A	1.267248	1.367514	1.234655	0.63849	-0.6473	0.090729	0.463036
PLCB4	1.254333	1.353919	1.221962	0.62715	-0.6731	0.090729	0.520791
TFE3	1.457667	1.564761	1.422855	0.74873	-0.4175	0.090764	0.463036
SLC2A6	1.127911	1.222783	1.097072	0.43572	-1.1985	0.090974	0.494701
C3orf33	1.18378	1.087826	1.214971	2.4477	1.2914	0.091276	0.584861
SEPT6	1.727829	1.611815	1.76554	1.25126	0.3234	0.091426	0.528592
IPO9	1.919097	1.796951	1.958802	1.20309	0.2667	0.091549	0.520791
NEK4	1.259465	1.160753	1.291551	1.81366	0.8589	0.091571	0.520791
PTHLH	1.065305	1.157118	1.035461	0.22569	-2.1476	0.09167	0.520791
CRB2	2.786899	2.586236	2.852125	1.16762	0.2236	0.091671	0.593288
TMEM164	1.307238	1.40895	1.274176	0.67044	-0.5768	0.091679	0.498533
ARHGAP10	1.165195	1.260953	1.134069	0.51377	-0.9608	0.091871	0.581275
NIPA2	1.543267	1.653834	1.507326	0.77592	-0.366	0.091985	0.547557
PRKCB	1.071672	1.164124	1.04162	0.25359	-1.9794	0.092001	0.593288
RHBDF1	1.138765	1.233527	1.107961	0.46231	-1.1131	0.092112	0.53821
IFT43	1.683719	1.569661	1.720795	1.26531	0.3395	0.092123	0.586558
PILRB	1.27404	1.17426	1.306475	1.75872	0.8145	0.092137	0.529047
QSER1	1.631852	1.519784	1.668281	1.28569	0.3625	0.092148	0.520791
TTLL4	1.216461	1.314749	1.184512	0.58622	-0.7705	0.092192	0.56842
AC116614.1	1.133144	1.039494	1.163585	4.14206	2.0503	0.092408	0.499797
KATNAL2	1.229567	1.132456	1.261134	1.97147	0.9793	0.092557	0.529047
ZNF385C	1.203204	1.106764	1.234553	2.19694	1.1355	0.092665	0.590009
CWC25	1.399019	1.29493	1.432854	1.46765	0.5535	0.092667	0.463795

SYTL1	1.241535	1.340344	1.209417	0.61531	-0.7006	0.092692	0.470903
RP11-488L18.10	1.174006	1.078725	1.204977	2.6037	1.3806	0.092756	0.49585
CXorf57	1.133822	1.040476	1.164165	4.05591	2.02	0.092963	0.593367
ETV1	1.317871	1.419354	1.284883	0.67934	-0.5578	0.092992	0.51685
IAH1	2.059224	1.921038	2.104142	1.1988	0.2616	0.093013	0.497221
PPM1B	1.525675	1.635092	1.490108	0.77171	-0.3739	0.093034	0.500493
PRRC2A	1.999116	1.873816	2.039846	1.19001	0.251	0.093048	0.522121
BSDC1	1.777991	1.660281	1.816253	1.23622	0.3059	0.093075	0.57037
PAX2	1.12245	1.029607	1.152629	5.15518	2.366	0.093118	0.58548
CRB3	1.048279	1.13923	1.018714	0.13441	-2.8953	0.093193	0.593367
SIPA1	1.291001	1.39113	1.258453	0.66079	-0.5977	0.093295	0.496094
CHPF2	1.135759	1.230026	1.105116	0.45697	-1.1298	0.093364	0.472083
MKKS	2.374783	2.225993	2.423148	1.16081	0.2151	0.093408	0.470903
HPS4	1.827723	1.709338	1.866205	1.22115	0.2882	0.093421	0.522121
KLHL8	1.297636	1.197695	1.330122	1.66986	0.7397	0.093455	0.473971
C11orf71	1.367642	1.264919	1.401033	1.5138	0.5982	0.093708	0.544371
UQCC3	2.385663	2.234826	2.434694	1.16186	0.2164	0.093758	0.473971
ACAT1	1.525368	1.417519	1.560425	1.34227	0.4247	0.0938	0.470903
DPM2	2.208408	2.075136	2.251729	1.16425	0.2194	0.093802	0.470903
DUSP26	1.255514	1.157003	1.287536	1.8314	0.8729	0.09383	0.557985
ARMC6	1.301447	1.201711	1.333867	1.65517	0.727	0.093831	0.496315
EDN1	1.080041	1.181606	1.047026	0.25895	-1.9493	0.093937	0.470903
CREBZF	1.52719	1.419507	1.562193	1.34013	0.4224	0.093967	0.508233
EXOC4	1.511086	1.403877	1.545935	1.35174	0.4348	0.094087	0.470903
TMEM237	1.543129	1.434691	1.578377	1.33055	0.412	0.094189	0.472083
ZNF273	1.257915	1.15996	1.289756	1.81142	0.8571	0.0943	0.596641
TANC1	1.326024	1.427895	1.29291	0.68454	-0.5468	0.094413	0.472083
NAA40	1.25784	1.159774	1.289717	1.8133	0.8586	0.094417	0.472083
H1FX-AS1	1.162626	1.06855	1.193206	2.81848	1.4949	0.094624	0.559954
NDE1	1.215256	1.119553	1.246365	2.06072	1.0431	0.094715	0.599263
LRRC32	1.052159	1.142759	1.022709	0.15907	-2.6522	0.094806	0.53634
LTBP2	1.130061	1.223922	1.099551	0.44458	-1.1695	0.094899	0.53634
PMS1	1.417895	1.314406	1.451534	1.43615	0.5222	0.094903	0.499359
POLR2M	1.466246	1.360767	1.500532	1.38741	0.4724	0.094987	0.472583
TSC22D2	1.371059	1.474002	1.337596	0.71223	-0.4896	0.095039	0.471604
SS18	1.56948	1.459584	1.605202	1.31685	0.3971	0.095082	0.562102
SLC12A2	1.432109	1.53874	1.397448	0.73774	-0.4388	0.095159	0.594595
ENO3	1.870531	1.751313	1.909284	1.21026	0.2753	0.095165	0.471604
CHRDL1	1.176379	1.081768	1.207133	2.53319	1.341	0.095209	0.509472
SNRNP200	1.875864	1.756168	1.914772	1.20975	0.2747	0.095345	0.560918
ZBTB8OS	1.660386	1.547557	1.697062	1.27304	0.3483	0.09549	0.475559
MME	1.06532	1.156227	1.03577	0.22896	-2.1268	0.095592	0.551527
TMED4	2.915398	2.75008	2.969136	1.12517	0.1701	0.095617	0.586526
QPRT	4.249864	4.544213	4.154183	0.88995	-0.1682	0.095732	0.601917
ZNF124	1.327574	1.226779	1.360337	1.58894	0.6681	0.095785	0.473502

MSANTD3	1.373444	1.475504	1.340269	0.7156	-0.4828	0.096088	0.551527
C10orf54	1.051895	1.141674	1.022712	0.16031	-2.6411	0.096114	0.473502
USP11	2.577551	2.419649	2.628878	1.14738	0.1983	0.096147	0.562349
SND1	2.281678	2.137567	2.328521	1.16786	0.2239	0.09616	0.540429
DPY19L3	1.284	1.185024	1.316172	1.70882	0.773	0.096361	0.566334
GNPNAT1	1.378628	1.276782	1.411734	1.48757	0.573	0.096376	0.535254
PRPSAP2	1.79386	1.677925	1.831546	1.2266	0.2947	0.096419	0.587863
EIF4G3	1.78038	1.663799	1.818275	1.23271	0.3018	0.096703	0.54046
ZNF771	1.351695	1.250612	1.384553	1.53445	0.6177	0.096726	0.551527
NT5C2	1.349669	1.248789	1.38246	1.53729	0.6204	0.096835	0.535254
METTL2B	1.349651	1.248914	1.382396	1.53626	0.6194	0.096907	0.477886
CDH23	1.216586	1.121115	1.24762	2.0445	1.0317	0.096983	0.598648
KIF3A	1.557761	1.449192	1.593053	1.32027	0.4008	0.097043	0.478081
MXD4	1.58821	1.478618	1.623834	1.30341	0.3823	0.097121	0.598648
MRPS17	1.369691	1.268416	1.402611	1.49995	0.5849	0.097135	0.551527
EIF2D	1.597073	1.487301	1.632755	1.29849	0.3768	0.097371	0.535273
OXLD1	1.994849	2.117645	1.954933	0.85441	-0.227	0.097422	0.538499
EFCAB2	1.2126	1.117122	1.243635	2.08018	1.0567	0.097479	0.524256
EML2	1.386826	1.285042	1.419912	1.47316	0.5589	0.097557	0.613392
GULP1	2.864364	3.052312	2.803271	0.87865	-0.1866	0.097593	0.598648
PLCH1	1.274898	1.177205	1.306653	1.73049	0.7912	0.097743	0.511621
WAC-AS1	1.681755	1.568901	1.718438	1.26285	0.3367	0.097864	0.479772
ZC3H4	1.320184	1.220304	1.352651	1.60075	0.6787	0.097949	0.544402
C2orf69	1.387174	1.285964	1.420073	1.46897	0.5548	0.097972	0.524256
CEP170	1.840931	1.957796	1.802943	0.83832	-0.2544	0.09807	0.481261
CHAMP1	1.362133	1.261176	1.39495	1.5122	0.5966	0.098114	0.522239
PTPRE	1.188377	1.283132	1.157576	0.55655	-0.8454	0.098155	0.53665
TSC22D4	1.680352	1.792757	1.643813	0.81212	-0.3002	0.098215	0.539915
SPPL2B	1.487286	1.38194	1.52153	1.36548	0.4494	0.098221	0.598256
TRIB3	1.151367	1.245306	1.120832	0.49258	-1.0216	0.098335	0.619287
PTGR2	1.282952	1.185103	1.314758	1.70045	0.7659	0.098495	0.619287
ATF2	1.739436	1.62552	1.776466	1.24131	0.3119	0.098627	0.573516
SH3YL1	1.625887	1.516091	1.661576	1.2819	0.3583	0.098628	0.616274
RBM33	1.4401	1.336622	1.473736	1.40733	0.493	0.098727	0.567538
ARSB	1.18799	1.283209	1.157039	0.5545	-0.8507	0.098893	0.558354
ZNF214	1.142994	1.05074	1.172982	3.40915	1.7694	0.098982	0.51208
ARPC3	5.361442	5.678771	5.258293	0.91013	-0.1359	0.099242	0.548401
TP53INP1	1.311711	1.410727	1.279525	0.68056	-0.5552	0.099291	0.58016
TUBA1C	2.881456	3.099167	2.810687	0.86257	-0.2133	0.099398	0.51208
EIF2S2	3.388769	3.600944	3.3198	0.89191	-0.165	0.099652	0.569602
TSPAN19	1.146465	1.054344	1.176409	3.24613	1.6987	0.099745	0.51208
PLEKHG4B	1.297341	1.199188	1.329247	1.65294	0.725	0.099759	0.548401
MED11	1.511058	1.405337	1.545424	1.34561	0.4283	0.099868	0.51208
YARS2	1.298947	1.20079	1.330854	1.64776	0.7205	0.09993	0.502648
CEP68	1.342909	1.243409	1.375252	1.54165	0.6245	0.10007	0.527315

MOK	1.266245	1.169755	1.29761	1.75317	0.81	0.100428	0.527315
CASK	1.443943	1.548038	1.410107	0.74832	-0.4183	0.100538	0.578498
SPTY2D1	1.270663	1.368404	1.238892	0.64845	-0.6249	0.100559	0.62834
DNAH14	1.641455	1.531812	1.677095	1.27318	0.3484	0.100622	0.527315
PGM1	2.152552	2.023026	2.194656	1.16777	0.2238	0.100633	0.578498
MGA	1.426535	1.3242	1.4598	1.41826	0.5041	0.100741	0.527315
CAND1	2.337394	2.198863	2.382424	1.15311	0.2055	0.100843	0.551348
SYNE4	1.099101	1.189784	1.069624	0.36686	-1.4467	0.100928	0.630644
BCAS2	1.694695	1.582937	1.731023	1.25403	0.3266	0.101032	0.500679
GSTK1	2.068151	2.206577	2.023155	0.84798	-0.2379	0.101042	0.492947
ANAPC5	2.213389	2.082062	2.256077	1.16082	0.2151	0.101154	0.550061
FMR1	1.443722	1.547551	1.409972	0.74874	-0.4175	0.101172	0.494745
ING2	1.799865	1.684751	1.837283	1.22276	0.2901	0.101294	0.629023
JAKMIP2	1.453058	1.349633	1.486677	1.39196	0.4771	0.101426	0.494745
GFOD2	1.412576	1.310374	1.445797	1.43632	0.5224	0.101472	0.537268
C1orf109	1.317083	1.218953	1.348981	1.59386	0.6725	0.101525	0.618996
SKA1	1.124752	1.034096	1.15422	4.52308	2.1773	0.101552	0.574191
ECHDC1	1.991229	1.871248	2.03023	1.18248	0.2418	0.101596	0.574191
TCEB3-AS1	1.241695	1.14615	1.272753	1.86625	0.9001	0.101615	0.493346
TMEM70	1.419478	1.522819	1.385886	0.73809	-0.4381	0.10171	0.509056
PRPF4	1.358739	1.259075	1.391136	1.50974	0.5943	0.101717	0.550147
DCAF13	1.777459	1.664266	1.814253	1.22579	0.2937	0.101903	0.494745
CDKL3	1.204195	1.110744	1.234573	2.11816	1.0828	0.102371	0.522248
AARSD1	1.220171	1.125809	1.250844	1.99385	0.9956	0.102385	0.499995
GOLGA6L9	1.229313	1.134613	1.260096	1.93217	0.9502	0.102404	0.533245
ECI2	2.177228	2.051507	2.218094	1.15843	0.2122	0.102448	0.505209
TATDN1	1.918426	1.800463	1.95677	1.19527	0.2573	0.102718	0.541016
KANK2	1.198866	1.292785	1.168337	0.57495	-0.7985	0.102905	0.577765
DGCR6L	2.953814	2.74685	3.021089	1.15699	0.2104	0.102984	0.500492
HOTAIRM1	1.06045	1.149671	1.031448	0.21011	-2.2508	0.103072	0.636138
EPB41L2	1.515612	1.411321	1.549513	1.33597	0.4179	0.103123	0.599066
TP53TG1	2.615933	2.765664	2.567262	0.88763	-0.172	0.103144	0.625078
USP18	1.137703	1.229496	1.107865	0.47001	-1.0892	0.103163	0.533508
FAM32A	3.164951	2.97283	3.227401	1.12904	0.1751	0.103212	0.564301
ROGDI	1.641721	1.75125	1.606117	0.80681	-0.3097	0.103347	0.555973
ZWILCH	1.315992	1.218741	1.347603	1.58911	0.6682	0.103424	0.515072
GKAP1	1.53174	1.426903	1.565818	1.3254	0.4064	0.103523	0.522919
ST3GAL3	1.28778	1.191299	1.319142	1.66829	0.7384	0.103543	0.522919
SIX5	1.320616	1.222574	1.352485	1.58367	0.6633	0.103754	0.628152
PDCD11	1.45146	1.348688	1.484867	1.39055	0.4757	0.103796	0.55248
IFT74	1.369387	1.270153	1.401644	1.48672	0.5721	0.10381	0.502081
SS18L2	1.886207	1.769905	1.924011	1.20016	0.2632	0.103826	0.533508
NDRG3	1.428262	1.326496	1.461342	1.41301	0.4988	0.104038	0.510549
SNAP47	1.559799	1.453777	1.594262	1.30959	0.3891	0.104047	0.533508
SV2A	1.342465	1.441301	1.310337	0.70323	-0.5079	0.104375	0.504312

HYAL2	2.222267	2.349346	2.18096	0.87521	-0.1923	0.104477	0.629362
PPP2R5A	1.189892	1.283211	1.159559	0.56339	-0.8278	0.104497	0.648911
PARP2	1.341595	1.244029	1.37331	1.52978	0.6133	0.104637	0.554028
COQ5	1.589539	1.48376	1.623923	1.28974	0.3671	0.104789	0.526566
TRNAU1AP	1.606531	1.499291	1.64139	1.2846	0.3613	0.104892	0.635668
RP11-421L21.3	1.310548	1.213583	1.342067	1.60157	0.6795	0.1049	0.504925
CLSPN	1.246547	1.151869	1.277323	1.82606	0.8687	0.105018	0.631992
SNX10	1.405323	1.304831	1.437989	1.43683	0.5229	0.105094	0.565886
CMIP	1.385541	1.485573	1.353025	0.72703	-0.4599	0.105279	0.554504
ZNF793-AS1	1.229707	1.135717	1.260259	1.91767	0.9394	0.105386	0.565886
ZNF785	1.391316	1.291287	1.423831	1.45503	0.541	0.105424	0.565886
ZNF217	1.221798	1.316033	1.191166	0.60489	-0.7253	0.10551	0.515257
ING3	1.331407	1.233795	1.363136	1.55322	0.6353	0.105721	0.64851
RDM1	1.122967	1.033533	1.152038	4.53404	2.1808	0.105868	0.565886
DESI1	1.384388	1.484104	1.351975	0.72706	-0.4598	0.105888	0.511622
PSME4	1.351301	1.450181	1.319159	0.70896	-0.4962	0.105903	0.653762
CTNNA2	1.200171	1.107505	1.230293	2.14216	1.0991	0.105945	0.604518
CBWD5	1.464701	1.362288	1.497991	1.37457	0.459	0.105972	0.653762
CPXM2	1.211151	1.304604	1.180774	0.59347	-0.7527	0.106134	0.508433
TROVE2	2.494097	2.353766	2.539712	1.13735	0.1857	0.106215	0.636026
EFR3A	1.309457	1.406678	1.277854	0.68323	-0.5496	0.106239	0.655683
ANKZF1	1.590114	1.483929	1.62463	1.29075	0.3682	0.106341	0.557182
WFDC1	1.213511	1.322042	1.178232	0.55344	-0.8535	0.106361	0.604518
USP32	1.257674	1.352886	1.226725	0.64249	-0.6383	0.106377	0.531885
TMEM37	1.107631	1.197654	1.078368	0.39649	-1.3346	0.106506	0.52781
SFPQ	2.932511	2.744242	2.993709	1.14302	0.1929	0.106578	0.653762
ANKRD10	2.027526	1.908743	2.066137	1.1732	0.2304	0.106623	0.558657
BRE	1.46322	1.360741	1.496531	1.37642	0.4609	0.106647	0.565886
NDUFS3	2.401126	2.266952	2.444741	1.14033	0.1894	0.10667	0.616111
CAMSAP2	1.504429	1.609202	1.470371	0.77211	-0.3731	0.106877	0.565886
NECAP2	1.532212	1.637116	1.498112	0.78182	-0.3551	0.106981	0.532258
ARHGEF39	1.152013	1.061603	1.181401	2.94466	1.5581	0.107068	0.656774
COPZ1	2.563556	2.402496	2.615909	1.15217	0.2043	0.107157	0.510902
FXR1	2.997144	2.791867	3.063871	1.1518	0.2039	0.107356	0.54768
CMTR2	1.506272	1.402713	1.539934	1.34074	0.423	0.107388	0.654743
AIG1	1.791842	1.679357	1.828406	1.2194	0.2862	0.107598	0.640494
KCNJ3	1.084773	1.173922	1.055795	0.3208	-1.6402	0.107723	0.642821
CAPS2	1.162985	1.072323	1.192455	2.66104	1.412	0.107937	0.568506
GOLPH3	1.495399	1.598804	1.461787	0.77118	-0.3749	0.10807	0.535024
HSD11B1L	1.915268	1.798076	1.953362	1.19458	0.2565	0.108096	0.642821
LINC01560	1.279183	1.184187	1.310062	1.68341	0.7514	0.108136	0.562251
MCMBP	1.482349	1.380305	1.515519	1.35554	0.4389	0.108155	0.548787
WDR6	1.881497	1.765938	1.919061	1.19992	0.2629	0.108181	0.655601
PTCD3	1.519124	1.415939	1.552665	1.32872	0.41	0.108595	0.642626
C6orf141	1.078335	1.166507	1.049674	0.29833	-1.745	0.108665	0.548787

FST	1.197214	1.289854	1.1671	0.5765	-0.7946	0.108726	0.535633
KMT2E-AS1	1.161712	1.071053	1.191181	2.69068	1.428	0.10882	0.567219
PAXBP1	1.562922	1.458299	1.596931	1.30249	0.3813	0.108822	0.562251
LAS1L	1.250787	1.157157	1.281223	1.78944	0.8395	0.108963	0.612385
LMTK3	1.109149	1.198176	1.08021	0.40474	-1.3049	0.108985	0.562251
SERF2	25.02224	27.00056	24.37918	0.89918	-0.1533	0.109091	0.524577
AGFG1	1.269597	1.364044	1.238897	0.65623	-0.6077	0.10912	0.626661
METTL10	1.330433	1.233597	1.36191	1.54929	0.6316	0.109146	0.52484
PROSC	1.586723	1.48119	1.621027	1.29061	0.3681	0.109338	0.666629
ELOF1	2.284299	2.418944	2.240531	0.87426	-0.1939	0.109419	0.659136
OSGEPL1	1.224039	1.131633	1.254076	1.93019	0.9487	0.10944	0.611038
MAPKAP1	1.685356	1.79356	1.650183	0.81932	-0.2875	0.10962	0.61954
GPX4	13.90558	14.43911	13.73216	0.9474	-0.078	0.109654	0.653955
GINS1	1.190073	1.099434	1.219536	2.20787	1.1427	0.109721	0.653955
CCDC15	1.130916	1.04205	1.159803	3.80027	1.9261	0.109741	0.668429
PRIM2	1.198225	1.106653	1.227991	2.13769	1.0961	0.109814	0.646039
LIMS2	1.078011	1.165471	1.049581	0.29964	-1.7387	0.109859	0.653955
SLC16A10	1.082545	1.170493	1.053957	0.31648	-1.6598	0.110079	0.539654
CDK8	1.6087	1.502313	1.643282	1.28064	0.3569	0.110126	0.667393
NTAN1	1.675208	1.78347	1.640017	0.8169	-0.2918	0.110179	0.629145
SYP	1.228227	1.135986	1.25821	1.89879	0.9251	0.110201	0.611892
SCCPDH	2.216344	2.075926	2.261988	1.17293	0.2301	0.110297	0.668429
VRK2	1.272614	1.178529	1.303197	1.6983	0.7641	0.110304	0.523422
RP3-510D11.2	1.222833	1.130153	1.252959	1.94355	0.9587	0.110371	0.544282
AMD1	2.205942	2.075639	2.248298	1.16052	0.2148	0.110393	0.526143
COX5A	6.378946	5.979334	6.508843	1.10634	0.1458	0.110464	0.526143
TMEM160	3.468377	3.266148	3.534113	1.11825	0.1612	0.110572	0.646719
BLMH	1.470896	1.370093	1.503662	1.36091	0.4446	0.110655	0.567954
QTRT2	1.184009	1.093187	1.213531	2.29142	1.1962	0.110861	0.667824
CDC25C	1.124145	1.036019	1.15279	4.24197	2.0847	0.110958	0.57365
NSMCE2	1.536943	1.640724	1.503208	0.78537	-0.3485	0.110969	0.61277
PRR7	2.13292	2.007984	2.173532	1.16424	0.2194	0.111028	0.524385
SSH1	1.184314	1.275745	1.154593	0.56064	-0.8349	0.111185	0.54243
YTHDF3-AS1	1.150797	1.061204	1.17992	2.93967	1.5557	0.111239	0.526897
RP11-345P4.9	1.416169	1.317367	1.448286	1.41252	0.4983	0.111313	0.558357
NEXN	1.174921	1.265665	1.145424	0.54739	-0.8693	0.111338	0.531239
LRP1	1.569099	1.673578	1.535137	0.79447	-0.3319	0.111346	0.66675
SAP30L	1.217607	1.309996	1.187575	0.60509	-0.7248	0.111386	0.546607
METAP2	3.505581	3.29644	3.573564	1.12068	0.1644	0.111479	0.632972
NBL1	1.887359	1.767651	1.92627	1.20663	0.271	0.111534	0.531239
UBE2G1	1.602254	1.708281	1.567788	0.80164	-0.319	0.111547	0.606573
TCTN2	1.183907	1.092787	1.213526	2.30125	1.2024	0.111671	0.526897
DUSP14	1.401233	1.302817	1.433224	1.43065	0.5167	0.111671	0.558357
OLFML2A	1.05824	1.144314	1.030261	0.20969	-2.2537	0.111759	0.661351
ZNF480	1.344216	1.247349	1.375703	1.51892	0.603	0.1118	0.654475

TMEM190	1.133108	1.2222	1.104148	0.46871	-1.0932	0.111811	0.654475
FOXK1	1.44262	1.543461	1.409841	0.75413	-0.4071	0.111822	0.57365
LINC01122	1.132655	1.043832	1.161528	3.68512	1.8817	0.111857	0.621548
RP11-499E18.1	1.216341	1.124697	1.246131	1.97382	0.981	0.11191	0.543331
RP11-51J9.5	1.275436	1.181256	1.30605	1.68849	0.7557	0.111959	0.621548
TXN	10.91859	10.2813	11.12574	1.09098	0.1256	0.11201	0.654475
P3H1	1.500885	1.603064	1.46767	0.77549	-0.3668	0.112094	0.547022
TEX9	1.425677	1.326281	1.457987	1.40366	0.4892	0.112167	0.586939
APTR	1.402459	1.303836	1.434518	1.43011	0.5161	0.112187	0.57365
METTL6	1.208295	1.116596	1.238103	2.04212	1.0301	0.112335	0.57365
C7orf26	1.223307	1.315902	1.193209	0.61161	-0.7093	0.112337	0.545947
SPEG	1.232838	1.3256	1.202685	0.62249	-0.6839	0.112388	0.621548
RNF8	1.595809	1.49075	1.62996	1.28367	0.3603	0.112558	0.547022
ANXA6	1.75408	1.864852	1.718072	0.83028	-0.2683	0.112565	0.621548
AGPAT4	1.367656	1.270844	1.399126	1.47364	0.5594	0.112882	0.677918
SLC36A4	2.010273	1.892105	2.048684	1.17552	0.2333	0.112885	0.599934
BCKDK	2.671158	2.510662	2.723329	1.14078	0.19	0.112903	0.586939
WWTR1	1.546949	1.650267	1.513365	0.78947	-0.341	0.112934	0.634139
CACHD1	1.138162	1.226962	1.109297	0.48157	-1.0542	0.112946	0.634139
ADK	1.399082	1.301107	1.430929	1.43115	0.5172	0.112967	0.599934
EHD3	1.107644	1.196055	1.078905	0.40246	-1.3131	0.113003	0.5357
LRTOMT	1.204726	1.113669	1.234325	2.06147	1.0437	0.113056	0.672984
LPCAT4	1.530877	1.42782	1.564377	1.31919	0.3997	0.113065	0.621548
CSPG5	1.147024	1.05818	1.175904	3.02346	1.5962	0.113079	0.677126
DPP8	1.291097	1.386002	1.260247	0.67421	-0.5687	0.113187	0.586939
STEAP1	1.130005	1.041991	1.158615	3.77733	1.9174	0.113211	0.677918
WDR76	1.191281	1.100984	1.220632	2.18483	1.1275	0.113266	0.630691
VSIG10	1.319444	1.224414	1.350334	1.5611	0.6426	0.113308	0.599934
CATSPER2	1.271925	1.178289	1.302362	1.69591	0.7621	0.113474	0.630691
RP11-655M14.13	1.182489	1.092195	1.211839	2.29772	1.2002	0.113614	0.577253
METTL16	1.378994	1.28202	1.410516	1.45563	0.5416	0.113787	0.566119
UMPS	1.20944	1.118617	1.238962	2.01457	1.0105	0.113925	0.535556
TRADD	1.209696	1.300864	1.180062	0.59848	-0.7406	0.113959	0.53698
FBXO18	1.306849	1.402186	1.27586	0.6859	-0.5439	0.113989	0.61652
ZNF3	1.541322	1.438091	1.574877	1.31223	0.392	0.11402	0.659508
CRISPLD1	1.397036	1.494883	1.36523	0.73801	-0.4383	0.114087	0.636983
DDX55	1.225442	1.13412	1.255127	1.90223	0.9277	0.114165	0.671027
EHD4	1.208269	1.299706	1.178547	0.59574	-0.7472	0.114183	0.659508
IGF2BP2	3.471828	3.203449	3.559067	1.16139	0.2159	0.114342	0.53698
ARHGAP33	1.164292	1.075425	1.193179	2.5612	1.3568	0.114446	0.552972
CCNL2	1.71891	1.827731	1.683536	0.82579	-0.2761	0.114449	0.663093
TMEM208	2.140859	2.26221	2.101413	0.87261	-0.1966	0.114468	0.584542
WBP11	2.308046	2.171871	2.35231	1.15398	0.2066	0.114517	0.55294
DNAJB6	3.146316	3.329534	3.086759	0.89578	-0.1588	0.114546	0.536524
UQCC2	4.797853	4.499165	4.894944	1.11311	0.1546	0.114604	0.682201

NFIX	1.0542	1.139763	1.026387	0.1888	-2.4051	0.114671	0.536553
SYNGAP1	1.460843	1.360712	1.493392	1.36783	0.4519	0.11474	0.678991
TMEM14C	5.677445	6.029338	5.56306	0.90729	-0.1404	0.114779	0.536524
NISCH	1.378734	1.475986	1.347122	0.72927	-0.4555	0.114855	0.629487
ATP5J2	7.38927	6.929638	7.538677	1.10271	0.1411	0.114875	0.55294
UBE2M	1.313663	1.408433	1.282857	0.69254	-0.53	0.115081	0.663093
HDLBP	2.764397	2.939012	2.707638	0.88067	-0.1833	0.115105	0.679105
DLL3	1.520191	1.418056	1.553391	1.32372	0.4046	0.115149	0.556925
C15orf57	1.393683	1.296514	1.425268	1.43423	0.5203	0.115322	0.55318
PHTF1	1.832983	1.721785	1.869129	1.20414	0.268	0.115328	0.629487
SAFB	1.607888	1.50354	1.641807	1.27459	0.35	0.115349	0.663093
SETD5	2.537238	2.382756	2.587454	1.14804	0.1992	0.115575	0.621758
PPIE	1.633291	1.528434	1.667376	1.26293	0.3368	0.11559	0.55318
PMF1	1.943217	1.82388	1.982008	1.19193	0.2533	0.115638	0.679105
GMPS	1.885255	1.772664	1.921853	1.19308	0.2547	0.115764	0.609732
FKBP5	1.23691	1.145622	1.266583	1.83066	0.8724	0.115876	0.629487
COPS6	3.43167	3.21199	3.503079	1.1316	0.1784	0.115878	0.679105
TSN	1.736073	1.627703	1.771299	1.22876	0.2972	0.115936	0.555388
SGSM3	1.562652	1.459322	1.596241	1.29809	0.3764	0.116013	0.540291
SLIT3	1.112805	1.200295	1.084365	0.42121	-1.2474	0.116109	0.679105
APH1B	1.145585	1.234596	1.116651	0.49724	-1.008	0.116146	0.540291
ETS1	1.057328	1.14258	1.029617	0.20772	-2.2673	0.116349	0.600239
ZMPSTE24	1.242674	1.335198	1.212599	0.63425	-0.6569	0.116468	0.623227
PSMC6	2.149293	2.029756	2.188149	1.15382	0.2064	0.11647	0.671027
TNFRSF21	1.255696	1.348298	1.225595	0.64771	-0.6266	0.116495	0.671027
CTSS	1.064139	1.149387	1.036428	0.24385	-2.0359	0.11666	0.540291
FAM184B	1.244205	1.152473	1.274023	1.79719	0.8457	0.116749	0.588436
IMPAD1	1.419187	1.517602	1.387196	0.74806	-0.4188	0.116769	0.59081
RP11-567M16.6	1.126107	1.03868	1.154526	3.99499	1.9982	0.116845	0.557093
SLTM	2.347494	2.220237	2.38886	1.13819	0.1867	0.11687	0.59081
KIRREL	1.199768	1.290555	1.170257	0.58597	-0.7711	0.116972	0.63731
AC093673.5	1.344967	1.440403	1.313945	0.71286	-0.4883	0.117091	0.66928
SLC35F5	1.307803	1.402307	1.277083	0.68874	-0.538	0.117127	0.632866
TUBB4A	1.271635	1.364807	1.241349	0.66158	-0.596	0.117145	0.543419
FAM208B	1.30207	1.396837	1.271265	0.68357	-0.5488	0.117199	0.63731
SPG21	3.098783	3.307216	3.031031	0.88029	-0.1839	0.117201	0.612219
CYB5D2	1.51637	1.406872	1.551963	1.3566	0.44	0.117391	0.543419
SGSM2	1.18857	1.278614	1.159301	0.57176	-0.8065	0.117424	0.581334
REV3L	1.878232	1.766133	1.91467	1.19388	0.2557	0.117453	0.612219
XPO6	1.253351	1.345665	1.223343	0.64613	-0.6301	0.117475	0.674192
SCGB3A1	1.112961	1.026622	1.141026	5.29725	2.4052	0.117504	0.652204
TMEM11	1.686199	1.793314	1.65138	0.82109	-0.2844	0.117673	0.588436
CNTN2	1.10831	1.021859	1.136411	6.24046	2.6417	0.117693	0.626452
CUTC	1.372693	1.276708	1.403894	1.45964	0.5456	0.117759	0.543419
DUSP12	1.609815	1.506285	1.643468	1.27096	0.3459	0.117759	0.671027

DDIT4	12.81026	14.46255	12.27317	0.83737	-0.2561	0.117848	0.637395
HSPA4L	1.260453	1.168723	1.29027	1.72039	0.7827	0.117856	0.685338
SLX4IP	1.272526	1.179804	1.302665	1.6833	0.7513	0.117886	0.559404
NCS1	1.175276	1.264768	1.146186	0.55213	-0.8569	0.117984	0.588436
FAM104A	1.540571	1.642721	1.507366	0.7894	-0.3412	0.118088	0.674192
RP11-390F4.6	1.217903	1.127641	1.247243	1.93702	0.9538	0.118155	0.588436
CTB-12O2.1	1.046293	1.130901	1.018791	0.14355	-2.8004	0.118205	0.671027
CNTROB	1.21215	1.121796	1.241521	1.983	0.9877	0.118345	0.652204
SORD	1.468961	1.370294	1.501033	1.35307	0.4362	0.118483	0.673411
RNFT1	1.27378	1.181458	1.30379	1.67416	0.7434	0.118535	0.6061
CDKL2	1.22931	1.138303	1.258892	1.87192	0.9045	0.118564	0.697519
RDH10	2.109125	2.277143	2.05451	0.82568	-0.2763	0.118643	0.596774
RP11-430H10.1	1.181108	1.09194	1.210092	2.2851	1.1923	0.118738	0.674192
IL13RA1	1.735729	1.844165	1.700481	0.82979	-0.2692	0.118759	0.652204
TRIM24	1.409868	1.312892	1.441391	1.41068	0.4964	0.118771	0.671027
THBS1	1.102326	1.188624	1.074274	0.39377	-1.3446	0.11898	0.657658
SCAI	1.409605	1.312363	1.441214	1.41251	0.4983	0.118991	0.6061
LYAR	1.311531	1.218677	1.341713	1.56264	0.644	0.119001	0.671027
ZNF507	1.213345	1.123052	1.242695	1.9723	0.9799	0.119058	0.56231
ATIC	1.670396	1.565177	1.704598	1.24668	0.3181	0.119302	0.671027
NKAP	1.834614	1.724402	1.870439	1.2016	0.265	0.119393	0.6061
GADD45GIP1	4.811562	5.102826	4.716884	0.90593	-0.1425	0.119441	0.574915
RBBP6	1.997369	1.882629	2.034666	1.17225	0.2293	0.119462	0.555831
SNRPB2	3.337788	3.131236	3.404929	1.12842	0.1743	0.11952	0.699054
RP11-758H9.2	1.220226	1.130028	1.249545	1.91916	0.9405	0.119604	0.690099
ZNF692	1.389501	1.293046	1.420855	1.43614	0.5222	0.11976	0.59349
SYT6	1.118623	1.032783	1.146526	4.46954	2.1601	0.119877	0.590572
FUT11	1.571037	1.687484	1.533185	0.77556	-0.3667	0.119878	0.644903
MLLT10	1.55087	1.449396	1.583856	1.2992	0.3776	0.119894	0.6061
APBB1	1.505044	1.40539	1.537437	1.32573	0.4068	0.119902	0.642197
PPARA	1.146644	1.23463	1.118044	0.5031	-0.9911	0.11991	0.550874
CA14	1.361782	1.457304	1.330732	0.72322	-0.4675	0.119949	0.671027
BTN3A2	1.239814	1.149468	1.269182	1.80093	0.8487	0.120132	0.642197
TMEM130	1.067571	1.152474	1.039973	0.26216	-1.9315	0.12021	0.601648
SLC39A3	1.592983	1.696098	1.559465	0.80372	-0.3152	0.12029	0.550874
LRRC41	1.767858	1.659972	1.802927	1.21661	0.2829	0.120335	0.661513
SVIP	2.40086	2.56527	2.347418	0.86082	-0.2162	0.120589	0.550874
STEAP2	1.143752	1.056957	1.171965	3.0192	1.5942	0.120934	0.671027
LIG3	1.38918	1.293839	1.420171	1.42994	0.516	0.12096	0.703384
THOC2	1.91672	1.804935	1.953057	1.18402	0.2437	0.121051	0.671027
DDX56	1.406324	1.310218	1.437564	1.4105	0.4962	0.121191	0.603555
CTDSP1	1.597009	1.494962	1.63018	1.27319	0.3484	0.121574	0.664695
CCDC8	1.623613	1.520058	1.657274	1.26385	0.3378	0.121716	0.583075
PLA2R1	1.115426	1.201614	1.08741	0.43355	-1.2057	0.12186	0.688716
HMBOX1	1.410889	1.314745	1.442141	1.40476	0.4903	0.121924	0.704914

FKBP1A	6.172622	6.566625	6.044549	0.90621	-0.1421	0.121952	0.721556
TOP1MT	1.292543	1.200557	1.322444	1.60774	0.685	0.122058	0.671027
ACVR1B	1.179895	1.091897	1.208499	2.26882	1.1819	0.122066	0.671027
VAV2	1.182936	1.271522	1.154141	0.56769	-0.8168	0.122134	0.605257
TSPAN15	1.188921	1.277763	1.160042	0.57618	-0.7954	0.122142	0.690099
CYB5A	1.846807	1.957807	1.810726	0.84644	-0.2405	0.122247	0.614902
NBEA	2.120436	1.988892	2.163195	1.17626	0.2342	0.122545	0.690099
CCDC90B	2.523804	2.378502	2.571036	1.13967	0.1886	0.122552	0.567581
LINC00649	1.241106	1.150865	1.270439	1.79259	0.842	0.122592	0.584489
SCAMP2	1.406889	1.502974	1.375656	0.74687	-0.4211	0.122798	0.605563
AK6	2.003557	1.888738	2.04088	1.17119	0.228	0.122927	0.637444
PPP3CC	1.248166	1.338511	1.218799	0.64636	-0.6296	0.122944	0.585585
PLD1	1.106744	1.192274	1.078943	0.41057	-1.2843	0.123061	0.565295
BPHL	1.662647	1.558463	1.696513	1.2472	0.3187	0.123107	0.721556
APCDD1L	1.202432	1.291209	1.173575	0.59605	-0.7465	0.123181	0.690099
PNKP	1.732191	1.626273	1.76662	1.2241	0.2917	0.123208	0.690099
OSGIN1	1.05788	1.141984	1.030541	0.2151	-2.2169	0.123355	0.608308
TTC5	1.320054	1.227272	1.350214	1.54094	0.6238	0.123367	0.721556
C2CD5	1.204644	1.116048	1.233442	2.01161	1.0083	0.123516	0.690099
ZFP1	1.234159	1.144596	1.263273	1.82075	0.8645	0.123549	0.67549
NUP88	1.203356	1.114948	1.232093	2.01912	1.0137	0.123595	0.67209
CLIP1	1.472425	1.570791	1.440451	0.77165	-0.374	0.123598	0.657228
OCRL	1.161384	1.24912	1.132864	0.53333	-0.9069	0.123642	0.565382
SMAD4	1.447372	1.350632	1.478818	1.36559	0.4495	0.12365	0.661658
CCSAP	1.194745	1.283976	1.16574	0.58364	-0.7768	0.123728	0.562655
CDR1-AS	1.26851	1.177633	1.29805	1.6779	0.7467	0.12389	0.571143
KLF10	1.45028	1.547359	1.418724	0.76499	-0.3865	0.123933	0.674079
GLI4	1.376757	1.282202	1.407493	1.44398	0.53	0.123933	0.696519
FANCM	1.155761	1.068922	1.183989	2.66953	1.4166	0.124084	0.674079
NCAPH2	1.255055	1.164839	1.284381	1.7252	0.7868	0.124092	0.690099
PAPSS1	1.567383	1.669249	1.534271	0.79831	-0.325	0.124163	0.690099
CELSR1	1.171038	1.258929	1.142469	0.55022	-0.8619	0.124322	0.722935
TXNDC15	1.508665	1.608507	1.47621	0.78259	-0.3537	0.124352	0.566054
TBC1D5	1.596948	1.494452	1.630265	1.27467	0.3501	0.124427	0.702525
TNFRSF14	1.075743	1.160133	1.048311	0.30169	-1.7288	0.124588	0.590617
CBLN2	1.138139	1.052256	1.166056	3.17773	1.668	0.12467	0.591592
HOOK3	1.828614	1.719675	1.864025	1.20058	0.2637	0.124826	0.678786
CDC6	1.177312	1.090204	1.205627	2.27958	1.1888	0.125085	0.676533
BLOC1S4	1.83293	1.724436	1.868197	1.19844	0.2612	0.125222	0.611545
PLEKHG5	1.126229	1.212615	1.098149	0.46163	-1.1152	0.125234	0.611545
SCAPER	1.455362	1.358086	1.486982	1.35996	0.4436	0.125283	0.567158
RPP30	1.430868	1.335127	1.461989	1.37855	0.4631	0.125694	0.664857
FGFR1OP	1.277792	1.187324	1.307199	1.63994	0.7136	0.125835	0.611545
WDR12	1.355768	1.262601	1.386052	1.47011	0.5559	0.125846	0.669847
PNMA2	1.146735	1.061013	1.1746	2.86168	1.5169	0.125882	0.723639

LRRC27	1.374289	1.280182	1.404879	1.44505	0.5311	0.125953	0.625861
LOXL1-AS1	1.314433	1.406807	1.284406	0.69912	-0.5164	0.126073	0.676768
POU2F1	1.566609	1.4664	1.599183	1.2847	0.3614	0.126191	0.579094
RNF217	1.386052	1.480696	1.355288	0.73911	-0.4361	0.126235	0.625861
AK2	2.125529	2.009447	2.163262	1.15238	0.2046	0.126245	0.596255
BOP1	1.307993	1.216458	1.337747	1.56033	0.6419	0.126292	0.625861
MAPRE1	2.123327	2.243537	2.084252	0.87191	-0.1977	0.126348	0.67971
SNX9	1.450609	1.546963	1.419289	0.76658	-0.3835	0.126536	0.710439
TMEM129	1.796879	1.689776	1.831693	1.20574	0.2699	0.126537	0.706146
ILVBL	1.519669	1.421459	1.551593	1.30877	0.3882	0.126581	0.676768
TMEM243	1.38114	1.286715	1.411834	1.43639	0.5224	0.126599	0.676768
TSEN15	1.707705	1.603215	1.74167	1.22953	0.2981	0.126722	0.595711
CMC1	1.874302	1.764455	1.910008	1.1904	0.2514	0.126762	0.671171
ANKRD50	1.236557	1.326204	1.207417	0.63585	-0.6532	0.12683	0.569595
INA	1.140224	1.05449	1.168092	3.08481	1.6252	0.126857	0.729379
DENND2A	1.154778	1.241712	1.12652	0.52343	-0.9339	0.12688	0.729379
PCID2	1.594112	1.493851	1.626703	1.26901	0.3437	0.126989	0.666624
TNS3	1.336951	1.429774	1.306779	0.71382	-0.4864	0.127104	0.706146
PIPOX	1.131975	1.046883	1.159635	3.40494	1.7676	0.127167	0.620298
GLIS2	1.258241	1.348224	1.228992	0.6576	-0.6047	0.127169	0.702924
KLHL14	1.209221	1.297547	1.18051	0.60666	-0.721	0.127411	0.657111
CTD-2008L17.2	1.279297	1.18897	1.308658	1.63337	0.7079	0.127539	0.569595
NECAB3	1.361051	1.267832	1.391353	1.46119	0.5471	0.127622	0.620298
GLRX3	2.482861	2.620206	2.438217	0.88768	-0.1719	0.127674	0.666624
NSUN5	1.624554	1.521703	1.657986	1.26123	0.3348	0.12768	0.597426
PCNX4	1.799846	1.693255	1.834494	1.20373	0.2675	0.127742	0.597508
PHYHD1	1.238708	1.328351	1.209569	0.63825	-0.6478	0.127752	0.706146
EIF3I	3.62181	3.392878	3.696226	1.12677	0.1722	0.127763	0.620298
CYHR1	2.425136	2.285889	2.470399	1.14349	0.1934	0.12782	0.683973
ABHD17B	1.31825	1.226769	1.347987	1.53455	0.6178	0.127846	0.584024
PRKAG1	1.765316	1.659143	1.799828	1.21344	0.2791	0.127932	0.627802
MRPL47	2.099378	1.984177	2.136825	1.1551	0.208	0.127993	0.597508
TRIM45	1.17332	1.086881	1.201417	2.3183	1.2131	0.128026	0.657111
TARDBP	1.966696	1.855364	2.002886	1.17247	0.2295	0.128057	0.666624
TCTE3	1.151025	1.065359	1.178871	2.73673	1.4525	0.128128	0.569595
SNU13	4.712378	4.984374	4.623964	0.90954	-0.1368	0.128256	0.680987
PLP1	1.265424	1.175463	1.294666	1.67936	0.7479	0.128292	0.597508
SLC39A10	2.713068	2.556128	2.764082	1.13364	0.181	0.128324	0.597655
APTX	1.559469	1.459763	1.591879	1.28736	0.3644	0.128373	0.633055
TMTC2	1.249686	1.160539	1.278664	1.7358	0.7956	0.128394	0.569595
DNAJA3	1.421732	1.327068	1.452503	1.38351	0.4683	0.128397	0.690212
MEGF8	1.271888	1.362454	1.242448	0.66891	-0.5801	0.128469	0.690212
CENPL	1.168723	1.082711	1.196681	2.37793	1.2497	0.128655	0.569595
ZGRF1	1.15818	1.072443	1.18605	2.56823	1.3608	0.128744	0.680987
KNTC1	1.17559	1.08924	1.203659	2.28214	1.1904	0.128979	0.733466

SAMD8	1.246471	1.335787	1.217438	0.64755	-0.6269	0.129049	0.733466
CTXN1	1.977896	1.860852	2.015942	1.18016	0.239	0.129076	0.62365
GALNT18	1.134653	1.220083	1.106884	0.48565	-1.042	0.129214	0.738577
EGFR	1.617815	1.51501	1.651232	1.2645	0.3386	0.129237	0.634209
COL4A3BP	1.514523	1.61254	1.482661	0.78797	-0.3438	0.12927	0.666624
APBB3	1.19763	1.110411	1.225981	2.04674	1.0333	0.129345	0.666624
WWC1	1.148108	1.233791	1.120256	0.51437	-0.9591	0.129423	0.588551
TRAPPC2	1.284868	1.194495	1.314245	1.61569	0.6922	0.129521	0.71201
CYP46A1	1.147511	1.062305	1.175208	2.81208	1.4916	0.129534	0.689476
PRRT1	1.243991	1.15502	1.272911	1.76049	0.816	0.129672	0.690212
TK2	1.243619	1.155233	1.27235	1.75445	0.811	0.129672	0.690212
AVL9	1.404061	1.310121	1.434597	1.40138	0.4868	0.129694	0.62365
TMEM147-AS1	1.239374	1.150913	1.268129	1.77671	0.8292	0.129906	0.602235
CLUAP1	1.542699	1.44436	1.574665	1.29324	0.371	0.130139	0.635252
STAT1	1.372459	1.466087	1.342024	0.73382	-0.4465	0.130182	0.735746
ARL13B	1.331574	1.240105	1.361307	1.50478	0.5896	0.130472	0.62817
RBM22	1.520121	1.422889	1.551727	1.30466	0.3837	0.130505	0.62817
CHMP1B	1.966179	2.077724	1.929921	0.86286	-0.2128	0.13051	0.684771
A1BG	2.768744	2.935727	2.714465	0.8857	-0.1751	0.130538	0.717604
SEC11A	5.865233	5.550387	5.967576	1.09168	0.1266	0.130633	0.637944
UBL5	14.08244	14.84491	13.8346	0.92703	-0.1093	0.130713	0.635252
RP5-1092A3.4	1.16271	1.077058	1.190551	2.47284	1.3062	0.130855	0.603855
PDPK1	1.276282	1.366224	1.247046	0.67458	-0.5679	0.131047	0.627154
PLK4	1.124219	1.040384	1.15147	3.75076	1.9072	0.131058	0.744885
BUD31	2.926935	2.771742	2.977382	1.11607	0.1584	0.131088	0.684771
CHRNA5	1.179371	1.093356	1.207331	2.22087	1.1511	0.131263	0.578593
FAM19A5	1.230943	1.143137	1.259485	1.81285	0.8583	0.131374	0.638465
ZFAND1	1.562526	1.463019	1.594871	1.28476	0.3615	0.131534	0.733983
UBE2D1	1.791574	1.896645	1.75742	0.84473	-0.2434	0.1317	0.675298
FNBP1	1.584894	1.684362	1.552561	0.80741	-0.3086	0.131701	0.697324
ADH5	2.20749	2.090533	2.245508	1.14211	0.1917	0.131754	0.605226
DERL2	1.542225	1.640616	1.510242	0.79649	-0.3283	0.131835	0.720793
FRYL	1.565228	1.465968	1.597494	1.28226	0.3587	0.131865	0.737456
MDH1	2.579366	2.434797	2.626359	1.13351	0.1808	0.131901	0.684771
RGS17	1.236874	1.149343	1.265327	1.77663	0.8291	0.13195	0.737456
VAMP7	1.372981	1.280798	1.402946	1.435	0.5211	0.132185	0.684771
CYTL1	1.056166	1.138451	1.029418	0.21248	-2.2346	0.13219	0.733983
TMEM145	1.111381	1.028026	1.138476	4.941	2.3048	0.132232	0.722962
ZNF138	1.268897	1.180077	1.297769	1.65357	0.7256	0.132251	0.613109
SMARCD1	1.813536	1.707675	1.847947	1.19821	0.2609	0.132668	0.721428
KIF27	1.219358	1.13235	1.247641	1.87111	0.9039	0.133024	0.583793
RYBP	1.551813	1.650444	1.519752	0.79907	-0.3236	0.133389	0.753872
CDC7	1.191606	1.105664	1.219541	2.07773	1.055	0.133396	0.613651
AURKAIP1	3.875694	4.155467	3.784753	0.88252	-0.1803	0.133487	0.604903
ZNF385D	1.48064	1.576008	1.44964	0.78062	-0.3573	0.133489	0.72589

DOLK	1.168215	1.254084	1.140303	0.55219	-0.8568	0.133821	0.613651
PRDM16	1.076849	1.159204	1.050079	0.31456	-1.6686	0.133888	0.705188
CARD8	1.373043	1.281077	1.402938	1.43355	0.5196	0.134012	0.612799
NUDT2	1.740484	1.63669	1.774223	1.21601	0.2822	0.13403	0.690768
FOXN3	1.503288	1.599993	1.471853	0.78643	-0.3466	0.134221	0.746003
SALL2	1.329999	1.239585	1.359389	1.50005	0.585	0.13431	0.711138
MIA3	1.503578	1.406812	1.535033	1.31518	0.3953	0.134364	0.606146
TDP1	1.257552	1.169745	1.286094	1.68544	0.7531	0.134477	0.640229
MARVELD1	1.46669	1.561493	1.435874	0.77628	-0.3654	0.134525	0.742844
CLTA	5.831089	6.195293	5.712702	0.90711	-0.1407	0.134535	0.686332
TOP3A	1.180474	1.094992	1.208261	2.19239	1.1325	0.134658	0.613651
DLEU2	1.132717	1.049325	1.159824	3.24021	1.6961	0.134808	0.613651
ALMS1	1.298551	1.209135	1.327616	1.56653	0.6476	0.134856	0.610497
PHPT1	6.418453	6.056964	6.535957	1.09472	0.1306	0.135053	0.640229
TMEM219	3.378408	3.571051	3.315788	0.90072	-0.1509	0.135055	0.711334
RBM15B	1.612115	1.512371	1.644537	1.25795	0.3311	0.135239	0.654089
PLCB1	1.301845	1.21217	1.330995	1.56004	0.6416	0.135394	0.748386
ITFG1	1.771691	1.875482	1.737954	0.84291	-0.2465	0.135441	0.732545
COA3	2.944399	2.769328	3.001307	1.13111	0.1777	0.135572	0.710339
RC3H1	1.422706	1.329589	1.452975	1.37437	0.4588	0.135725	0.693095
PSMD1	1.67564	1.777405	1.642561	0.82655	-0.2748	0.135861	0.734818
DDRGK1	2.046384	1.934142	2.08287	1.15921	0.2131	0.135957	0.713909
RABGEF1	1.114246	1.197017	1.087341	0.44332	-1.1736	0.136253	0.713909
ARMCX5	1.251281	1.163861	1.279697	1.70692	0.7714	0.13643	0.656689
TONSL	1.144779	1.061283	1.17192	2.80537	1.4882	0.136591	0.693301
GPX8	1.913834	2.022009	1.878671	0.85975	-0.218	0.1367	0.71254
MAZ	2.133137	2.019469	2.170085	1.14774	0.1988	0.136721	0.622358
EMILIN1	1.078562	1.160401	1.05196	0.32394	-1.6262	0.1368	0.764456
EPDR1	1.177399	1.26229	1.149805	0.57114	-0.8081	0.136841	0.69526
BDNF	1.118156	1.201083	1.091201	0.45355	-1.1407	0.136848	0.661869
SYNE1	1.350958	1.44207	1.321342	0.7269	-0.4602	0.136881	0.764456
TIGAR	1.214439	1.300625	1.186424	0.62012	-0.6894	0.137133	0.726081
LL22NC03-2H8.5	1.395511	1.303709	1.425352	1.40052	0.486	0.137213	0.657316
ARL5A	1.735276	1.838541	1.701709	0.83682	-0.257	0.13728	0.738522
NCAPG2	1.171531	1.087297	1.198912	2.27857	1.1881	0.137497	0.623071
TTC26	1.214532	1.128804	1.242398	1.88191	0.9122	0.137541	0.764456
PIGBOS1	1.640759	1.541454	1.673038	1.24302	0.3139	0.137713	0.59778
KHDRBS2	1.1562	1.072306	1.18347	2.5374	1.3433	0.137821	0.59778
ASXL2	1.239888	1.327304	1.211473	0.64611	-0.6302	0.13785	0.715605
TP53I13	1.846544	1.953426	1.811801	0.85146	-0.232	0.137995	0.59778
PDGFB	1.07415	1.155282	1.047777	0.30768	-1.7005	0.138134	0.758604
ADSSL1	1.220608	1.306957	1.19254	0.62726	-0.6729	0.13814	0.739147
KMT5A	1.286114	1.197965	1.314768	1.59002	0.669	0.138201	0.665218
PODXL2	1.487394	1.392878	1.518117	1.31877	0.3992	0.13838	0.725054
ACVR1	1.115594	1.198066	1.088786	0.44826	-1.1576	0.138713	0.762542

DKFZP434L187	1.191151	1.106683	1.218608	2.04913	1.035	0.138743	0.716363
METTL15	1.326336	1.236954	1.35539	1.49983	0.5848	0.138858	0.716363
CARNS1	1.047156	1.127347	1.021089	0.16561	-2.5942	0.138858	0.715605
RP11-539L10.3	1.296934	1.207783	1.325913	1.56852	0.6494	0.138946	0.629638
PARP11	1.21673	1.131148	1.244549	1.86467	0.8989	0.138948	0.661307
KCNK1	1.248606	1.162139	1.276712	1.70664	0.7712	0.13901	0.715605
MED15	1.416097	1.509021	1.385892	0.75811	-0.3995	0.139039	0.759425
CTBP2	1.69733	1.798054	1.664589	0.83276	-0.264	0.139133	0.739147
KCTD6	1.582623	1.485819	1.61409	1.26403	0.338	0.139357	0.749697
ZNF414	1.387184	1.296061	1.416805	1.40783	0.4935	0.139361	0.661307
MYL6B	6.577476	6.279057	6.674479	1.0749	0.1042	0.139422	0.715605
VPS51	1.654013	1.5542	1.686457	1.23865	0.3088	0.139526	0.705341
TYRO3	1.194448	1.109786	1.221969	2.02184	1.0157	0.139633	0.716687
MRPS2	1.802732	1.699389	1.836324	1.19579	0.258	0.139773	0.625516
IMPA2	1.685928	1.585965	1.718422	1.22605	0.294	0.139802	0.62965
S100B	1.234131	1.320308	1.206118	0.6435	-0.636	0.139912	0.660147
SLC26A11	1.293213	1.381859	1.264398	0.6924	-0.5303	0.139983	0.630867
KIF2A	1.875101	1.76891	1.909619	1.183	0.2424	0.140044	0.625516
GLYATL2	1.120783	1.03865	1.147481	3.81581	1.932	0.1402	0.62965
MPHOSPH6	1.71717	1.615659	1.750166	1.21848	0.2851	0.140257	0.708335
PDE3B	1.172647	1.089036	1.199826	2.24434	1.1663	0.14034	0.662828
SRP72	2.208639	2.083792	2.249221	1.15264	0.2049	0.14044	0.762931
PDCL	1.398433	1.307281	1.428063	1.39307	0.4783	0.14045	0.672824
ZNF487	1.250214	1.163485	1.278406	1.70295	0.768	0.140523	0.740138
MPZL2	1.055729	1.136275	1.029548	0.21682	-2.2054	0.140811	0.674554
CCDC85B	3.097855	3.300676	3.031926	0.88319	-0.1792	0.1411	0.625516
IGSF9	1.308819	1.220712	1.337458	1.52895	0.6125	0.141145	0.625516
DTD2	1.19217	1.107745	1.219613	2.03827	1.0273	0.14115	0.735737
STARD3	1.630645	1.532255	1.662627	1.24494	0.3161	0.141194	0.708898
MRC2	1.327128	1.416049	1.298224	0.7168	-0.4804	0.141252	0.763216
TMEM132D	1.075466	1.155868	1.049331	0.31649	-1.6598	0.141278	0.666594
DDR2	1.092152	1.17352	1.065702	0.37864	-1.4011	0.141419	0.66414
SERTAD2	1.372038	1.462636	1.342588	0.74051	-0.4334	0.141439	0.771019
ELMOD3	1.320284	1.231871	1.349023	1.50525	0.59	0.141504	0.636308
PPP1R3B	1.211176	1.296378	1.183481	0.61908	-0.6918	0.141514	0.739147
MAT2B	1.389139	1.298576	1.418578	1.40191	0.4874	0.141683	0.636308
VPS72	1.807277	1.703961	1.84086	1.19447	0.2564	0.141766	0.739147
IQGAP3	1.110921	1.029358	1.137434	4.68132	2.2269	0.141788	0.771019
LEO1	1.289802	1.202681	1.318121	1.56956	0.6504	0.141978	0.743903
SNRNP48	1.349578	1.260132	1.378653	1.45562	0.5416	0.142239	0.758168
SAMD13	1.186986	1.103307	1.214186	2.0733	1.0519	0.142356	0.739147
SPARCL1	1.079245	1.159468	1.053168	0.33341	-1.5846	0.142383	0.708898
DNAAF3	1.121428	1.040092	1.147866	3.68819	1.8829	0.142402	0.739147
FOXRED2	1.182083	1.098618	1.209214	2.12145	1.0851	0.142439	0.758168
ADAR	1.937708	1.830438	1.972577	1.17116	0.2279	0.142485	0.708898

HAUS4	1.212879	1.127942	1.240489	1.87967	0.9105	0.142535	0.639499
YPEL2	1.170619	1.25418	1.143456	0.56439	-0.8252	0.142539	0.739147
PSMA6	1.450735	1.358176	1.480822	1.34242	0.4248	0.142617	0.679966
BCKDHB	1.275324	1.188299	1.303613	1.6124	0.6892	0.142764	0.615785
FBRS	1.25577	1.342391	1.227613	0.66478	-0.5891	0.142837	0.670799
PDXDC1	1.592008	1.68916	1.560428	0.8132	-0.2983	0.142976	0.730122
PDE9A	1.19762	1.282223	1.170119	0.60278	-0.7303	0.143046	0.672451
BTN2A1	1.359303	1.44934	1.330036	0.73449	-0.4452	0.143296	0.679982
GBF1	1.233658	1.319123	1.205877	0.64513	-0.6323	0.143333	0.638626
RCBTB2	1.216252	1.131739	1.243724	1.85006	0.8876	0.143412	0.775661
LRRN1	1.414563	1.323465	1.444175	1.37318	0.4575	0.143469	0.638626
DDX17	5.476655	5.074283	5.607448	1.13086	0.1774	0.143646	0.631125
ZNF561	1.493787	1.399812	1.524335	1.31145	0.3912	0.143666	0.631125
PP7080	1.341309	1.2524	1.370209	1.46675	0.5526	0.143724	0.784396
R3HDM1	1.547746	1.452325	1.578763	1.27953	0.3556	0.143728	0.721176
RFX5	1.387941	1.297215	1.417433	1.40448	0.49	0.143786	0.641802
PEX11A	1.255881	1.170175	1.283741	1.66735	0.7376	0.143965	0.711347
TEX264	1.742523	1.843507	1.709697	0.84137	-0.2492	0.14397	0.672976
CHMP1A	1.609852	1.707251	1.578192	0.81752	-0.2907	0.14398	0.673691
RMI1	1.256763	1.17088	1.28468	1.66596	0.7364	0.144034	0.784396
PSMD4	2.91748	2.741914	2.974549	1.13355	0.1809	0.144092	0.721176
SLC44A1	1.438507	1.531275	1.408352	0.76863	-0.3796	0.144134	0.680747
FAM65A	1.185877	1.2698	1.158597	0.58783	-0.7665	0.144186	0.73932
C22orf39	2.069432	1.957922	2.105679	1.15425	0.207	0.144291	0.784396
RP11-434H6.7	1.121377	1.040112	1.147793	3.68455	1.8815	0.144326	0.641802
RP11-440L14.1	1.181359	1.097962	1.208468	2.12806	1.0895	0.144329	0.639623
KCTD3	1.430266	1.338644	1.460048	1.3585	0.442	0.144393	0.711347
FKBP2	4.407445	4.657114	4.326289	0.90954	-0.1368	0.144488	0.748835
SEMA3A	1.674146	1.77319	1.641952	0.83026	-0.2684	0.144628	0.748835
APCDD1	1.17932	1.096271	1.206316	2.14308	1.0997	0.144678	0.745209
TFEC	1.079107	1.159161	1.053085	0.33353	-1.5841	0.144698	0.673245
ST20	1.719843	1.820503	1.687123	0.83744	-0.2559	0.144776	0.676746
RP11-128M1.1	1.14029	1.058788	1.166782	2.83699	1.5044	0.144849	0.751125
TMEM5	1.605713	1.702202	1.574349	0.81793	-0.29	0.145152	0.748835
ZNF682	1.164557	1.08224	1.191314	2.32628	1.218	0.14519	0.745209
DHTKD1	1.278429	1.191694	1.306623	1.59954	0.6777	0.145213	0.784396
CECR5-AS1	1.128936	1.047502	1.155406	3.2716	1.71	0.145278	0.784396
THSD4	1.058659	1.13782	1.032927	0.23891	-2.0654	0.145299	0.741982
GSTM4	1.448838	1.356868	1.478733	1.34148	0.4238	0.145412	0.78149
NAA50	1.815313	1.711324	1.849116	1.19371	0.2555	0.145607	0.677478
NDUFA2	4.650002	4.367851	4.741717	1.11101	0.1519	0.145778	0.771859
RNF182	1.260401	1.174751	1.288242	1.64944	0.722	0.145804	0.75183
GMPR2	1.900644	1.795716	1.934751	1.17473	0.2323	0.14584	0.744004
TCTN3	1.61202	1.51552	1.643388	1.24804	0.3197	0.145904	0.726632
ZNF213-AS1	1.282222	1.195429	1.310435	1.58848	0.6676	0.146077	0.784396

RP11-1042B17.3	1.160712	1.078685	1.187375	2.38134	1.2518	0.146464	0.787931
UFL1	1.488715	1.39635	1.518739	1.30879	0.3882	0.14648	0.75183
PHKB	1.584076	1.488002	1.615306	1.26087	0.3344	0.146587	0.784396
ZNF431	1.428754	1.337909	1.458284	1.35623	0.4396	0.146646	0.647044
PARL	1.760272	1.659447	1.793046	1.20259	0.2661	0.146815	0.74594
THAP3	1.39333	1.303301	1.422595	1.39332	0.4785	0.146816	0.74973
ADGRG1	1.216189	1.300411	1.188812	0.62851	-0.67	0.146979	0.650721
NRDC	2.157582	2.045434	2.194036	1.14214	0.1917	0.147025	0.718544
CRNDE	1.602564	1.506915	1.633656	1.25003	0.322	0.147215	0.775384
RBM42	1.465527	1.557985	1.435472	0.78044	-0.3576	0.147539	0.731153
RBM17	2.559346	2.419748	2.604723	1.13029	0.1767	0.147695	0.754219
CNN2	3.423677	3.60382	3.36512	0.90833	-0.1387	0.147724	0.750558
ZNF720	1.552901	1.457971	1.583759	1.27466	0.3501	0.147737	0.718544
PIK3R3	1.214398	1.130199	1.241768	1.85692	0.8929	0.147779	0.660738
FBXO10	1.094109	1.174232	1.068064	0.39065	-1.356	0.148135	0.718544
ANKH	1.199833	1.116516	1.226916	1.94752	0.9616	0.148212	0.788122
GOSR2	1.483534	1.576273	1.453389	0.78676	-0.346	0.148349	0.789627
SMYD2	1.520665	1.614106	1.490291	0.79838	-0.3248	0.148472	0.762056
SLBP	1.950062	2.056778	1.915373	0.86619	-0.2072	0.148485	0.788122
PSMD12	1.689316	1.788663	1.657023	0.83308	-0.2635	0.148624	0.63832
MED23	1.237065	1.152836	1.264445	1.73025	0.791	0.148737	0.795902
MFAP1	1.668413	1.571361	1.69996	1.22508	0.2929	0.14885	0.718544
RTN4	7.320447	6.915397	7.452111	1.09073	0.1253	0.148998	0.788122
AC069513.4	1.172703	1.090443	1.199443	2.20517	1.1409	0.149073	0.754659
LMO4	2.381497	2.518692	2.336902	0.8803	-0.1839	0.149096	0.694401
FGD4	1.525479	1.618902	1.495112	0.79998	-0.322	0.149229	0.691128
EFCAB11	1.223014	1.139456	1.250175	1.79394	0.8431	0.149281	0.754659
PFKL	2.260226	2.141296	2.298885	1.13808	0.1866	0.149476	0.737122
RAB39B	1.201199	1.118217	1.228173	1.93012	0.9487	0.149713	0.64026
ABCB7	1.288524	1.202358	1.316533	1.56423	0.6454	0.149842	0.704397
ZNRD1	1.563035	1.469306	1.593503	1.26464	0.3387	0.149955	0.718544
SMG1	1.709964	1.809411	1.677638	0.8372	-0.2564	0.150017	0.660977
HARS	1.491992	1.584715	1.461852	0.78987	-0.3403	0.150144	0.718544
MED19	1.514521	1.421708	1.54469	1.29163	0.3692	0.150154	0.659627
CALCRL	1.109001	1.029321	1.134902	4.60091	2.2019	0.150457	0.800848
PDCD6IP	1.785561	1.68457	1.818389	1.19548	0.2576	0.150598	0.757506
FAM76B	1.295197	1.209088	1.323188	1.5457	0.6283	0.150611	0.660977
ZFX	1.508528	1.415591	1.538739	1.29632	0.3744	0.150636	0.698338
CCNO	1.076831	1.155629	1.051217	0.3291	-1.6034	0.150819	0.716603
CPSF3	1.468996	1.377939	1.498595	1.31925	0.3997	0.151029	0.670769
NBPF1	1.287118	1.201712	1.31488	1.56104	0.6425	0.151043	0.716603
GCDH	1.228084	1.144501	1.255254	1.76645	0.8209	0.151454	0.670769
SMUG1	1.55067	1.456852	1.581166	1.27211	0.3472	0.151521	0.769082
MAN2A1	1.330089	1.417397	1.30171	0.72284	-0.4683	0.151555	0.802442
ZRANB2	2.648332	2.487295	2.700678	1.14347	0.1934	0.15167	0.69921

MECOM	1.221143	1.138152	1.248119	1.79598	0.8448	0.151726	0.716603
ICAM1	1.127588	1.207692	1.101549	0.48894	-1.0323	0.152023	0.670769
YTHDF1	1.245071	1.329428	1.21765	0.66069	-0.598	0.152091	0.708287
GNAI3	2.172562	2.062449	2.208355	1.13733	0.1857	0.152143	0.769125
FHL1	2.296566	2.153804	2.342972	1.16395	0.219	0.152326	0.648675
KANSL3	1.32082	1.234713	1.348809	1.48611	0.5715	0.152527	0.667141
TNIP2	1.378489	1.46695	1.349734	0.74898	-0.417	0.152552	0.708287
DNAJC2	1.564013	1.658323	1.533356	0.81017	-0.3037	0.152594	0.795591
GLCCI1	1.358333	1.270968	1.386732	1.42722	0.5132	0.152633	0.795591
TOMM22	2.888459	2.73563	2.938137	1.11668	0.1592	0.152758	0.665181
SFT2D3	1.184917	1.103189	1.211483	2.04946	1.0352	0.152996	0.808429
SLC25A24	1.528421	1.621566	1.498143	0.80143	-0.3193	0.152998	0.805039
POLR3D	1.465942	1.375401	1.495373	1.31958	0.4001	0.153365	0.749941
DYRK4	1.87647	1.77438	1.909655	1.17469	0.2323	0.153375	0.708287
PAK1IP1	1.32697	1.414335	1.298571	0.7206	-0.4727	0.153486	0.708287
MAGI1	1.541889	1.448508	1.572244	1.27588	0.3515	0.153489	0.808429
SYCE1L	1.949035	1.844491	1.983018	1.16404	0.2191	0.153501	0.772112
CBWD3	1.222938	1.139878	1.249938	1.78682	0.8374	0.153567	0.749941
RPAP2	1.465609	1.374755	1.495142	1.32124	0.4019	0.153654	0.665181
TOR1A	1.335724	1.422447	1.307534	0.72798	-0.458	0.153705	0.777023
NMT2	1.512189	1.604469	1.482192	0.79771	-0.3261	0.153749	0.809799
LYPD2	1.05121	1.128516	1.026082	0.20295	-2.3008	0.153753	0.651992
PANX1	1.146826	1.228178	1.120382	0.52758	-0.9225	0.154203	0.777214
GPR108	1.573839	1.47962	1.604465	1.2603	0.3338	0.154308	0.67759
ZNF678	1.191636	1.109994	1.218174	1.9835	0.988	0.154324	0.707466
LARP6	1.548927	1.641747	1.518756	0.80835	-0.3069	0.154416	0.665181
NDUFAF8	3.685231	3.490048	3.748677	1.10386	0.1426	0.154541	0.675527
MRPS28	1.658549	1.562136	1.689889	1.22726	0.2954	0.15467	0.777214
LAPTM4A	4.575201	4.849776	4.485949	0.90549	-0.1432	0.154746	0.810806
RALB	1.402481	1.491711	1.373476	0.75954	-0.3968	0.1548	0.665181
NHLH1	1.117531	1.036595	1.143839	3.93055	1.9747	0.154878	0.665181
SMARCC2	1.763241	1.663156	1.795774	1.19998	0.263	0.154916	0.67759
ARHGAP1	1.22855	1.311504	1.201585	0.64714	-0.6279	0.155065	0.781262
LIN52	1.204387	1.122583	1.230978	1.88426	0.914	0.155116	0.675527
CXCL10	1.041651	1.120583	1.015993	0.13263	-2.9145	0.155128	0.707466
RP1-102K2.8	1.064888	1.142457	1.039673	0.27849	-1.8443	0.155261	0.781262
NCAPH	1.104329	1.026205	1.129724	4.95028	2.3075	0.155301	0.781262
MPPE1	1.231899	1.315125	1.204846	0.65005	-0.6214	0.15532	0.781262
ARRDC1-AS1	1.419443	1.330822	1.44825	1.35496	0.4382	0.155329	0.789195
DPP10	1.137808	1.21777	1.111816	0.51346	-0.9617	0.155401	0.805841
MLEC	3.196878	3.041952	3.247237	1.10053	0.1382	0.155404	0.707466
SNTA1	1.13216	1.212058	1.106188	0.50075	-0.9978	0.155417	0.707466
RAPH1	1.210718	1.12832	1.237502	1.85085	0.8882	0.155464	0.777322
RNF145	2.237821	2.107433	2.280204	1.15601	0.2092	0.155519	0.713683
ZNF91	1.511264	1.4197	1.541028	1.28908	0.3663	0.155584	0.72056

PELO	1.424919	1.514026	1.395954	0.7703	-0.3765	0.155702	0.779283
GLYR1	1.377456	1.465209	1.348931	0.75005	-0.4149	0.1558	0.742083
CASP3	1.549126	1.456289	1.579303	1.2696	0.3444	0.156177	0.78952
IFT20	1.888261	1.989997	1.855191	0.86383	-0.2112	0.156219	0.785783
SAYSD1	1.283644	1.199652	1.310946	1.55744	0.6392	0.156283	0.68061
DIP2A	1.44449	1.354346	1.473792	1.33709	0.4191	0.15633	0.77778
TP53BP2	1.354754	1.441827	1.326451	0.73887	-0.4366	0.156592	0.814539
ZNHIT1	4.149742	4.425305	4.060168	0.8934	-0.1626	0.156679	0.761445
EIF4EBP2	2.038692	1.933082	2.073022	1.14998	0.2016	0.156833	0.743478
SIAH2	1.314381	1.399739	1.286635	0.71706	-0.4798	0.156893	0.717633
TRAF3IP2-AS1	1.334996	1.249287	1.362857	1.45558	0.5416	0.156938	0.717633
PMS2	1.373577	1.28593	1.402067	1.40617	0.4918	0.156949	0.818002
RAB11FIP5	1.069911	1.147259	1.044769	0.30402	-1.7178	0.157048	0.677562
NUP35	1.165976	1.085507	1.192133	2.247	1.168	0.157078	0.814539
RBM6	2.438495	2.301864	2.482908	1.13907	0.1879	0.157089	0.818002
RBM15	1.178691	1.09815	1.204871	2.08733	1.0617	0.157231	0.677562
ALPK2	1.106823	1.185448	1.081266	0.43821	-1.1903	0.157283	0.682012
TSPAN1	1.052209	1.128764	1.027325	0.21221	-2.2365	0.15735	0.785921
PDZRN4	1.067928	1.145188	1.042814	0.29489	-1.7618	0.157548	0.71971
RP11-521B24.3	1.196854	1.115526	1.22329	1.93282	0.9507	0.157604	0.677562
ZNF33B	1.388748	1.301255	1.417188	1.38483	0.4697	0.157679	0.663596
PCOLCE2	1.150858	1.23106	1.124788	0.54007	-0.8888	0.15781	0.663596
NDUFV1	2.272662	2.136544	2.316908	1.1587	0.2125	0.157836	0.725107
LAMTOR5	6.418801	6.767415	6.305482	0.91991	-0.1204	0.157974	0.782088
HMCES	1.307519	1.222756	1.335072	1.50421	0.589	0.157979	0.815009
NFATC3	1.432698	1.522109	1.403634	0.77308	-0.3713	0.158008	0.725107
GPATCH11	1.1898	1.10888	1.216104	1.98478	0.989	0.158081	0.80657
ACAD10	1.213075	1.13146	1.239604	1.82265	0.866	0.158204	0.787889
FOXJ3	1.334369	1.420642	1.306326	0.72823	-0.4575	0.158232	0.819666
UROS	2.596984	2.449552	2.644908	1.13477	0.1824	0.158379	0.80657
YME1L1	1.844616	1.744033	1.877311	1.17913	0.2377	0.158426	0.785921
CEP128	1.123028	1.044702	1.148488	3.32176	1.7319	0.15859	0.785921
APIP	1.52708	1.436228	1.556611	1.27596	0.3516	0.158718	0.80657
NAIP	1.253936	1.171031	1.280885	1.64231	0.7157	0.158748	0.722223
BOD1L1	1.448929	1.538324	1.419871	0.77996	-0.3585	0.158748	0.80657
AGL	1.294188	1.209799	1.32162	1.53299	0.6163	0.15904	0.783505
NRAV	1.168817	1.08907	1.194739	2.18636	1.1285	0.159243	0.724156
PNPLA6	1.218055	1.299797	1.191484	0.63871	-0.6468	0.159334	0.789605
RAB6A	2.18036	2.289934	2.144742	0.88744	-0.1723	0.159589	0.802734
GID8	1.86349	1.965184	1.830434	0.86039	-0.2169	0.159609	0.819666
DYNC2H1	1.529606	1.438415	1.559249	1.27562	0.3512	0.159658	0.772194
DMTF1	1.515219	1.424533	1.544697	1.28305	0.3596	0.159842	0.820414
RUVBL1	1.681178	1.586048	1.7121	1.21509	0.2811	0.159855	0.819666
LYPD6	1.137799	1.216788	1.112122	0.5172	-0.9512	0.159871	0.690259
CH507-254M2.3	1.155382	1.075933	1.181208	2.38641	1.2548	0.159907	0.722223

C7orf50	3.52566	3.714701	3.46421	0.90773	-0.1397	0.159916	0.788604
PITX1	1.041351	1.117623	1.016558	0.14077	-2.8286	0.159963	0.724156
TMEM63B	1.291777	1.375738	1.264485	0.70391	-0.5065	0.160068	0.685226
KMT2D	1.153766	1.233578	1.127823	0.54724	-0.8698	0.160075	0.682118
UGDH	1.643782	1.549248	1.674511	1.22806	0.2964	0.160075	0.725387
UBR7	1.173305	1.093165	1.199354	2.13979	1.0975	0.160243	0.722223
MED6	1.6364	1.542616	1.666886	1.22902	0.2975	0.160357	0.790778
RALY	3.228408	3.059473	3.283321	1.10869	0.1489	0.160441	0.803004
TBC1D10A	1.118814	1.197655	1.093186	0.47146	-1.0848	0.160603	0.699423
NUDT22	1.896505	1.79437	1.929705	1.17037	0.227	0.160813	0.722223
WFIKKN1	1.111525	1.033592	1.136858	4.07416	2.0265	0.160847	0.788542
IMPACT	1.496516	1.40615	1.52589	1.29482	0.3727	0.160858	0.682118
WNT5A	1.497107	1.407069	1.526374	1.29308	0.3708	0.160858	0.682118
RP4-639F20.1	1.271957	1.355318	1.24486	0.68913	-0.5372	0.160871	0.809192
DAG1	1.411449	1.499441	1.382847	0.76655	-0.3835	0.160873	0.809192
CCDC117	1.364808	1.278593	1.392833	1.41006	0.4958	0.160941	0.805508
RSF1	2.574629	2.444926	2.61679	1.11894	0.1621	0.161179	0.687061
ZNF277	1.545615	1.454596	1.575201	1.2653	0.3395	0.161198	0.725968
PDGFA	1.07219	1.148684	1.047326	0.3183	-1.6516	0.16133	0.791699
C14orf37	1.248202	1.16566	1.275033	1.66022	0.7314	0.161358	0.699683
FXR2	1.201427	1.282443	1.175092	0.61992	-0.6898	0.161471	0.758966
ANKRD18B	1.136174	1.057634	1.161703	2.80568	1.4884	0.161611	0.758966
NPHP1	1.264255	1.181181	1.291258	1.60755	0.6849	0.161686	0.805227
POLL	1.367938	1.282176	1.395815	1.40272	0.4882	0.161808	0.725968
ASB8	1.288759	1.205416	1.31585	1.53761	0.6207	0.162152	0.821852
SCAMP1-AS1	1.305811	1.221808	1.333117	1.50183	0.5867	0.162165	0.811631
PRDM2	1.525168	1.434166	1.554749	1.27773	0.3536	0.162188	0.821852
NKD2	1.089113	1.166763	1.063873	0.38301	-1.3845	0.162399	0.82848
ZNF593	1.885744	1.986486	1.852998	0.86468	-0.2098	0.162462	0.763725
AC005076.5	1.117456	1.03982	1.142693	3.58345	1.8414	0.162573	0.821852
MCAM	1.106192	1.183499	1.081063	0.44176	-1.1787	0.162646	0.733732
RP11-410L14.2	1.218788	1.137389	1.245247	1.78505	0.836	0.162677	0.702374
AFDN-AS1	1.26678	1.184331	1.293581	1.59269	0.6715	0.162861	0.811079
ACTR1A	1.743816	1.840356	1.712435	0.84778	-0.2382	0.162956	0.795796
NPPB	1.045615	1.133204	1.017143	0.1287	-2.9579	0.163217	0.782677
TMEM116	1.224171	1.143199	1.250492	1.74926	0.8067	0.163236	0.798349
SLC22A18	1.243761	1.325578	1.217166	0.66702	-0.5842	0.163382	0.782677
UQCR10	8.603195	8.077131	8.774196	1.0985	0.1355	0.163475	0.741807
EFNA4	1.73466	1.638684	1.765858	1.19912	0.262	0.163479	0.798349
CTBS	1.457595	1.546446	1.428713	0.78455	-0.3501	0.163547	0.741807
CERS4	1.582531	1.675467	1.552321	0.81769	-0.2904	0.163647	0.830632
PYCARD	1.062025	1.137907	1.037358	0.2709	-1.8842	0.163804	0.766139
PIK3C2A	1.546759	1.637711	1.517195	0.81102	-0.3022	0.163862	0.741807
DYRK2	1.353517	1.43895	1.325746	0.7421	-0.4303	0.163909	0.691201
NOTCH2	1.432318	1.520327	1.40371	0.77588	-0.3661	0.163998	0.691201

NRIP1	2.320308	2.200453	2.359267	1.13229	0.1792	0.164087	0.692888
LINC00526	1.25879	1.176719	1.285468	1.61538	0.6919	0.164175	0.705813
CHTOP	2.154867	2.048493	2.189444	1.13443	0.182	0.164212	0.691201
FBXW2	1.395138	1.481402	1.367097	0.76256	-0.3911	0.164215	0.821891
TMSB15B	1.493351	1.404247	1.522315	1.29207	0.3697	0.164279	0.818065
PPP6R1	1.320833	1.404688	1.293575	0.72543	-0.4631	0.164608	0.739266
XPNPEP1	1.799716	1.70247	1.831327	1.18343	0.243	0.164944	0.786416
RABGAP1L	1.179191	1.258965	1.15326	0.59182	-0.7568	0.165004	0.817705
C2orf81	1.178147	1.099061	1.203854	2.05787	1.0411	0.165077	0.818065
IL17RC	1.236885	1.155505	1.263338	1.69343	0.7599	0.165144	0.766139
SF3A3	1.603481	1.512383	1.633093	1.23558	0.3052	0.165172	0.818538
PRELID2	1.451714	1.36394	1.480246	1.31957	0.4001	0.165261	0.766139
CNKSR3	1.161011	1.240036	1.135324	0.56376	-0.8268	0.165268	0.738197
UBN1	1.588766	1.680959	1.558799	0.8206	-0.2852	0.165406	0.831994
ENTPD1-AS1	1.166642	1.087811	1.192267	2.18957	1.1306	0.165877	0.826098
SORL1	1.110137	1.187144	1.085105	0.45476	-1.1368	0.166007	0.738218
ZNF853	1.080704	1.157003	1.055902	0.35606	-1.4898	0.166155	0.695773
TM9SF2	1.737729	1.833707	1.706531	0.84746	-0.2388	0.166257	0.743355
MEA1	2.744499	2.886917	2.698204	0.89999	-0.152	0.166329	0.71203
COX7B	7.710076	8.134297	7.57218	0.92121	-0.1184	0.166689	0.700931
FAM208A	1.864148	1.764823	1.896434	1.17208	0.2291	0.166984	0.823461
DYRK1B	1.361531	1.276558	1.389152	1.40713	0.4928	0.167045	0.80994
ANKRD36B	1.339943	1.255648	1.367343	1.43691	0.523	0.167256	0.744128
SLC25A36	2.854523	2.703223	2.903704	1.11771	0.1605	0.167518	0.773043
SNTB1	1.0604	1.135716	1.035918	0.26465	-1.9178	0.167696	0.878657
TSR2	1.569697	1.479225	1.599106	1.25015	0.3221	0.16781	0.784415
ATP6V0E2	2.163373	2.05556	2.198418	1.13534	0.1831	0.167875	0.819
POLA1	1.288346	1.206549	1.314935	1.52474	0.6086	0.16791	0.744128
SPG7	1.737969	1.642614	1.768965	1.19662	0.259	0.168024	0.80994
SLC5A6	1.351091	1.435522	1.323647	0.74312	-0.4283	0.168185	0.704976
DPY19L2	1.306632	1.223648	1.333607	1.49166	0.5769	0.168213	0.828505
MAPK14	1.316397	1.400207	1.289153	0.72251	-0.4689	0.168268	0.80994
JUND	2.084279	2.188091	2.050535	0.88422	-0.1775	0.168747	0.84129
IQGAP2	1.108249	1.185057	1.083283	0.45004	-1.1519	0.16883	0.828505
SEC22B	1.649731	1.74278	1.619485	0.83401	-0.2619	0.168831	0.753384
MRPL54	2.102645	1.998246	2.136581	1.13858	0.1872	0.168927	0.84129
CHN2	1.060272	1.135223	1.035908	0.26555	-1.9129	0.168997	0.786307
LRP4-AS1	1.108861	1.032905	1.133551	4.05872	2.021	0.169249	0.753384
MAX	1.56218	1.65336	1.532541	0.81508	-0.295	0.16948	0.778524
PM20D2	1.460928	1.373614	1.48931	1.30967	0.3892	0.169492	0.761694
ZNF93	1.212013	1.132553	1.237842	1.79432	0.8434	0.16953	0.804469
PPP2CA	2.027318	2.129858	1.993987	0.87974	-0.1848	0.16953	0.819319
RNASEH1	1.433049	1.519615	1.40491	0.77925	-0.3598	0.16957	0.819319
MRPL37	2.077562	2.191651	2.040477	0.87314	-0.1957	0.169606	0.748517
METTL22	1.316203	1.233117	1.343211	1.47227	0.558	0.169614	0.855824

LIM2	1.150504	1.259526	1.115065	0.44337	-1.1734	0.169683	0.723311
RGS5	1.247183	1.16695	1.273263	1.6368	0.7109	0.169771	0.761694
GDI1	1.82797	1.92511	1.796395	0.86086	-0.2161	0.169902	0.822915
RP11-141B14.1	1.13889	1.06162	1.164008	2.66159	1.4123	0.169938	0.749814
C17orf75	1.447391	1.360811	1.475534	1.31796	0.3983	0.170056	0.842743
WDR13	1.897773	1.798321	1.9301	1.16507	0.2204	0.170113	0.748517
MRPL51	4.954141	4.68768	5.040756	1.09574	0.1319	0.170146	0.822915
ZNF680	1.206842	1.127338	1.232685	1.8273	0.8697	0.170425	0.836328
LRIF1	1.376348	1.29149	1.403932	1.38575	0.4707	0.170459	0.844737
ITGB8	1.429721	1.343101	1.457878	1.33453	0.4163	0.170554	0.817034
IFI27L1	1.947712	1.847795	1.98019	1.15616	0.2093	0.170631	0.756188
ELOVL2	1.167765	1.089735	1.193129	2.15222	1.1058	0.170658	0.885287
SPAG5	1.15766	1.080527	1.182733	2.26922	1.1822	0.170691	0.712509
PPP4R3B	1.69052	1.596614	1.721045	1.20856	0.2733	0.170771	0.715105
PUDP	1.532211	1.443412	1.561076	1.26536	0.3395	0.170993	0.748517
MACROD1	1.604064	1.513073	1.633641	1.23499	0.3045	0.171189	0.885287
GLT8D1	1.87521	1.776768	1.907208	1.16793	0.2239	0.171278	0.855824
LDOC1	1.741678	1.647094	1.772423	1.19368	0.2554	0.171302	0.748517
EMC10	3.193737	3.382931	3.132238	0.8948	-0.1604	0.171307	0.809086
PDHB	2.087648	1.981396	2.122185	1.14346	0.1934	0.171342	0.727299
MRPS15	2.488138	2.604971	2.45016	0.90354	-0.1463	0.171464	0.845554
SEC24A	1.161151	1.239166	1.135791	0.56777	-0.8166	0.171491	0.845685
WNT10A	1.04738	1.121415	1.023314	0.19202	-2.3807	0.171601	0.885287
PACRGL	1.217173	1.137918	1.242935	1.76145	0.8168	0.171709	0.855824
EEF1D	11.43391	10.82602	11.6315	1.08197	0.1137	0.171779	0.71634
DSEL	1.655219	1.562956	1.68521	1.21716	0.2835	0.171801	0.71528
SMURF2	1.360379	1.444076	1.333173	0.75026	-0.4145	0.171843	0.785793
PQLC3	1.25427	1.173201	1.280622	1.62021	0.6962	0.172016	0.855824
TMEM189	1.430211	1.516606	1.402128	0.7784	-0.3614	0.172237	0.71528
MEAF6	2.99377	2.842377	3.042982	1.10888	0.1491	0.172324	0.841545
SS18L1	1.238943	1.319365	1.212802	0.66633	-0.5857	0.172332	0.810122
MED7	1.282805	1.201618	1.309196	1.53357	0.6169	0.172369	0.829682
TP53I3	1.441926	1.528096	1.413916	0.78379	-0.3515	0.172389	0.798393
FDX2	1.488537	1.575755	1.460186	0.79927	-0.3232	0.172607	0.830001
NHSL2	1.237384	1.317613	1.211305	0.66529	-0.5879	0.172826	0.762556
SMAGP	1.152052	1.229227	1.126966	0.55389	-0.8523	0.17293	0.730957
ANGPT2	1.109631	1.190631	1.083301	0.43698	-1.1944	0.173155	0.788207
UBE2G2	1.534196	1.444847	1.56324	1.26614	0.3404	0.173258	0.857756
SMDT1	4.211835	3.997446	4.281523	1.09477	0.1306	0.173374	0.799268
RP3-512B11.3	1.155762	1.233044	1.13064	0.56058	-0.835	0.173417	0.830748
PSMB2	3.324043	3.153243	3.379562	1.10511	0.1442	0.173606	0.822637
EIF4E2	2.170065	2.294668	2.129562	0.87247	-0.1968	0.173619	0.775499
NCOA3	1.234369	1.314357	1.208368	0.66284	-0.5933	0.173728	0.843811
INTS3	1.377321	1.293455	1.404582	1.37869	0.4633	0.173963	0.788284
ABHD14B	1.429371	1.343734	1.457208	1.33012	0.4116	0.174003	0.892786

ARSJ	1.051275	1.12509	1.027282	0.21809	-2.197	0.174028	0.763785
FYTTD1	1.986839	1.885789	2.019687	1.15116	0.2031	0.174219	0.765348
NID2	1.053468	1.127497	1.029405	0.23063	-2.1164	0.174225	0.858316
PSPH	1.498346	1.41123	1.526664	1.2807	0.3569	0.174272	0.855209
FLOT2	1.465047	1.552173	1.436726	0.79092	-0.3384	0.174623	0.763785
SELENBP1	1.264421	1.184203	1.290496	1.57705	0.6572	0.174657	0.861298
RRP36	1.193306	1.271342	1.16794	0.61892	-0.6922	0.174739	0.788284
GAS7	1.204974	1.283544	1.179435	0.63283	-0.6601	0.174786	0.822637
APAF1	1.312521	1.230984	1.339025	1.46774	0.5536	0.174821	0.722598
EIF5AL1	1.305043	1.223332	1.331604	1.4848	0.5703	0.17483	0.892786
PPFIA4	1.384038	1.299839	1.411407	1.3721	0.4564	0.174844	0.843811
RFFL	1.223995	1.14529	1.249579	1.7178	0.7806	0.174943	0.761117
ZNF331	1.214601	1.136201	1.240086	1.76273	0.8178	0.175164	0.822637
ESCO1	1.636807	1.54538	1.666526	1.22213	0.2894	0.175177	0.766207
TFPI	1.498472	1.585824	1.470077	0.80242	-0.3176	0.175388	0.738244
KIAA1324L	1.243262	1.163829	1.269082	1.64246	0.7159	0.175437	0.722598
TMUB1	2.05212	2.155772	2.018428	0.88117	-0.1825	0.175488	0.818601
RPF1	1.411446	1.327085	1.438868	1.34176	0.4241	0.175568	0.857386
CENPO	1.112719	1.03766	1.137117	3.64094	1.8643	0.175628	0.822637
TMUB2	1.536297	1.448086	1.564971	1.26085	0.3344	0.175644	0.843811
GATM	1.268649	1.349006	1.242529	0.69491	-0.5251	0.175646	0.806044
BAG3	1.287141	1.206407	1.313384	1.51828	0.6024	0.175763	0.818601
APBB2	1.222241	1.300952	1.196656	0.65345	-0.6139	0.176032	0.822637
LOXL4	1.077657	1.151653	1.053604	0.35346	-1.5004	0.176121	0.843811
GNAI2	2.503413	2.627777	2.462988	0.89876	-0.154	0.176125	0.855952
ATG16L2	1.209376	1.130995	1.234854	1.79285	0.8423	0.176144	0.843811
TOP3B	1.341091	1.258325	1.367994	1.42454	0.5105	0.176165	0.725096
CCDC137	1.470471	1.384346	1.498466	1.29692	0.3751	0.176308	0.784023
HMGCS1	1.757526	1.880344	1.717604	0.81514	-0.2949	0.176331	0.86369
LNPK	1.943518	1.844215	1.975797	1.15586	0.209	0.176627	0.897406
FBXO21	1.739363	1.645576	1.76985	1.1925	0.254	0.17663	0.822637
CCDC150	1.204523	1.126892	1.229757	1.81065	0.8565	0.176859	0.86369
PAPLN	1.099706	1.025307	1.12389	4.89547	2.2914	0.177194	0.859597
DNAJC9	1.706807	1.615356	1.736534	1.19692	0.2593	0.177322	0.848612
CDC42	5.387422	5.681169	5.291937	0.91685	-0.1252	0.17737	0.725096
LCORL	1.54235	1.45491	1.570773	1.2547	0.3273	0.177475	0.846161
AFG3L2	1.530692	1.618876	1.502027	0.81119	-0.3019	0.177503	0.737885
PIGU	1.321507	1.240014	1.347997	1.4499	0.536	0.177612	0.845036
TMEM126B	1.994157	1.883675	2.03007	1.16566	0.2212	0.177631	0.785668
BTN2A2	1.240872	1.319962	1.215163	0.67246	-0.5725	0.17773	0.859597
RRP8	1.275346	1.195418	1.301327	1.54196	0.6248	0.177744	0.725096
MFSD4B	1.275342	1.195043	1.301444	1.54553	0.6281	0.177744	0.725096
KALRN	1.153382	1.23001	1.128473	0.55856	-0.8402	0.177789	0.871365
IDE	1.269735	1.349748	1.243727	0.69686	-0.5211	0.17793	0.725096
NDUFB6	2.392937	2.270424	2.432761	1.12778	0.1735	0.178206	0.773809

CDR2	1.136533	1.212357	1.111885	0.52687	-0.9245	0.178208	0.725096
PI4K2A	1.123529	1.199198	1.098933	0.49666	-1.0097	0.178243	0.897616
CSPP1	1.260184	1.180955	1.285938	1.58016	0.6601	0.178263	0.785668
WRNIP1	1.256895	1.336295	1.231085	0.68715	-0.5413	0.17838	0.826962
SLC18B1	1.279316	1.199441	1.30528	1.53068	0.6142	0.17848	0.827424
MED25	1.387084	1.304552	1.413911	1.35908	0.4426	0.178608	0.871365
TRMT10A	1.222886	1.144416	1.248394	1.71999	0.7824	0.17861	0.741436
SBNO1	1.843517	1.747083	1.874863	1.17104	0.2278	0.178823	0.845036
ZBED5	1.560829	1.649469	1.532016	0.81916	-0.2878	0.178919	0.845036
C19orf68	1.171648	1.094806	1.196626	2.07399	1.0524	0.178929	0.741436
SLC15A4	1.262616	1.342139	1.236766	0.69202	-0.5311	0.179023	0.785668
RRAGC	1.392769	1.475726	1.365803	0.76894	-0.3791	0.179024	0.741436
PWAR6	1.795166	1.700707	1.82587	1.17862	0.2371	0.179028	0.814174
FAM189B	1.66696	1.575402	1.696722	1.21084	0.276	0.179094	0.741436
PABPC5	1.20967	1.131535	1.235068	1.78712	0.8376	0.179125	0.879025
RNPEPL1	1.158059	1.234587	1.133183	0.56773	-0.8167	0.179164	0.8498
MRM2	1.436708	1.352516	1.464075	1.31646	0.3967	0.179214	0.773714
GUK1	7.313712	7.658225	7.201726	0.93144	-0.1025	0.179255	0.773809
ARMCX3	2.383265	2.492825	2.347652	0.90275	-0.1476	0.179259	0.862845
TMEM87A	1.522657	1.435489	1.550991	1.26522	0.3394	0.179261	0.8498
HIVEP3	1.302946	1.222423	1.32912	1.4797	0.5653	0.179279	0.814174
VWA5A	1.268025	1.188348	1.293925	1.56054	0.642	0.179328	0.897616
SLC37A1	1.162422	1.086048	1.187248	2.1761	1.1217	0.179345	0.897616
DNAJA1	3.494536	3.311019	3.55419	1.10522	0.1443	0.17935	0.773809
CD68	1.117154	1.19231	1.092724	0.48216	-1.0524	0.179448	0.733895
ISCA1	2.227744	2.122479	2.261961	1.12426	0.169	0.179461	0.742952
CRYM	1.100415	1.026582	1.124414	4.68045	2.2266	0.17964	0.733895
DSCC1	1.132392	1.057574	1.156712	2.72193	1.4446	0.179694	0.852699
PLCXD1	1.494611	1.408468	1.522613	1.27945	0.3555	0.179763	0.773714
SART3	1.216554	1.138623	1.241886	1.74492	0.8032	0.179847	0.814174
MRPS31	1.558985	1.470968	1.587595	1.24763	0.3192	0.179991	0.773809
ITGB5	1.119135	1.194203	1.094734	0.48781	-1.0356	0.180106	0.874426
TMEM40	1.037805	1.110171	1.014283	0.12964	-2.9474	0.180148	0.773714
MTERF3	1.248895	1.170251	1.274459	1.61209	0.6889	0.180428	0.729007
PENK	1.041757	1.124073	1.014999	0.12089	-3.0483	0.180563	0.744451
PRKG1	1.103011	1.177711	1.078729	0.44301	-1.1746	0.18062	0.729007
C14orf2	11.77064	11.05504	12.00325	1.0943	0.13	0.180799	0.834331
POLR2C	1.968965	1.870876	2.00085	1.14925	0.2007	0.180846	0.809426
UCK1	1.554655	1.643294	1.525842	0.81742	-0.2908	0.181069	0.835575
SCAMP1	1.561906	1.650386	1.533145	0.81974	-0.2868	0.181195	0.777276
ZCCHC17	2.47493	2.362578	2.51145	1.10926	0.1496	0.181249	0.868267
GFPT1	1.409167	1.492668	1.382024	0.77542	-0.367	0.181298	0.775339
DDHD2	1.484357	1.398876	1.512143	1.28397	0.3606	0.181347	0.852501
WSCD2	1.09747	1.023878	1.121391	5.0837	2.3459	0.18157	0.777276
GBE1	1.801971	1.898112	1.77072	0.85816	-0.2207	0.182095	0.903545

AUP1	1.950675	2.048598	1.918845	0.87626	-0.1906	0.182326	0.903545
TUBGCP4	1.545939	1.459259	1.574115	1.25009	0.322	0.182408	0.8535
DRG2	1.374986	1.292829	1.401692	1.37176	0.456	0.182438	0.858324
ZNF493	1.439376	1.355334	1.466694	1.3134	0.3933	0.182569	0.870445
FAM122B	1.338755	1.25787	1.365047	1.41562	0.5014	0.182586	0.858324
ANAPC13	3.268037	3.091296	3.325489	1.11198	0.1531	0.182653	0.891987
ZNF563	1.119794	1.045519	1.143938	3.16219	1.6609	0.182712	0.887963
PDIA6	5.418215	5.156811	5.503186	1.08333	0.1155	0.182818	0.782616
MIOS	1.205927	1.129053	1.230915	1.7893	0.8394	0.182843	0.837855
MIR217HG	1.098773	1.025092	1.122724	4.89095	2.2901	0.183042	0.884408
TBCC	1.299119	1.219292	1.325068	1.48235	0.5679	0.183046	0.824937
GATC	1.349928	1.268571	1.376373	1.40139	0.4869	0.183113	0.800125
FAM227B	1.278641	1.199259	1.304445	1.52788	0.6115	0.183229	0.837855
RP11-1275H24.1	1.204278	1.127272	1.229309	1.80172	0.8494	0.183298	0.755727
RP3-402G11.26	1.176449	1.100516	1.201131	2.00099	1.0007	0.183503	0.904922
PCED1A	1.433239	1.349597	1.460428	1.31702	0.3973	0.183628	0.809426
PTPN14	1.140993	1.216028	1.116603	0.53976	-0.8896	0.183697	0.737316
MYBL1	1.114124	1.040287	1.138126	3.42854	1.7776	0.183723	0.885855
ZNF446	1.197053	1.12038	1.221976	1.84397	0.8828	0.183734	0.809426
GNA13	1.258987	1.3375	1.233466	0.69175	-0.5317	0.183867	0.872502
FGF11	1.638837	1.548879	1.668079	1.21717	0.2835	0.183937	0.889618
TSEN2	1.287896	1.208328	1.313761	1.50609	0.5908	0.184125	0.809426
HAUS2	1.207855	1.13087	1.232879	1.77947	0.8314	0.184204	0.885152
LDLR	1.167275	1.243121	1.14262	0.58662	-0.7695	0.184225	0.756449
APMAP	1.905661	2.002071	1.874323	0.87252	-0.1967	0.184315	0.859641
MFSD8	1.179295	1.10337	1.203974	1.97324	0.9806	0.184484	0.826885
GNAL	1.273468	1.194773	1.299048	1.53537	0.6186	0.184575	0.859641
PTPRZ1	1.194061	1.117563	1.218927	1.8622	0.897	0.184611	0.803178
RNPC3	1.404288	1.32158	1.431173	1.34079	0.4231	0.184726	0.860345
MNAT1	1.774616	1.681094	1.805016	1.18194	0.2412	0.184813	0.885855
WWC3	1.206697	1.283405	1.181763	0.64136	-0.6408	0.184861	0.841493
THEM4	1.466695	1.381489	1.494392	1.29596	0.374	0.184867	0.737316
RP11-192H23.6	1.194118	1.117478	1.21903	1.86444	0.8987	0.184879	0.737316
TCEB2	13.62546	14.5855	13.31339	0.90636	-0.1418	0.18492	0.885855
FAM91A1	1.322776	1.403421	1.296561	0.73512	-0.444	0.184957	0.885152
C19orf53	5.835395	6.151981	5.732487	0.91858	-0.1225	0.185018	0.809426
BRAT1	1.273203	1.194464	1.298797	1.53652	0.6197	0.185071	0.809426
MANBAL	2.028021	1.928737	2.060294	1.14165	0.1911	0.185123	0.826885
MIR1302-10	1.106351	1.032801	1.130259	3.97119	1.9896	0.185181	0.899963
CLIP4	1.169385	1.244916	1.144833	0.59136	-0.7579	0.185184	0.873758
IBTK	1.894951	1.99072	1.86382	0.87191	-0.1977	0.185301	0.873712
EI24	3.63997	3.80205	3.587285	0.92335	-0.115	0.185373	0.807294
LINC00954	1.268826	1.190316	1.294346	1.54661	0.6291	0.185859	0.873712
ACADS	1.251479	1.173812	1.276725	1.59209	0.6709	0.185933	0.805441
FAM76A	1.251242	1.173111	1.276639	1.59804	0.6763	0.186344	0.887561

PPP1R16A	1.525437	1.611118	1.497585	0.81422	-0.2965	0.186445	0.886458
CCDC191	1.171205	1.096031	1.19564	2.03727	1.0266	0.186536	0.809426
ZCCHC12	1.170901	1.095768	1.195323	2.03954	1.0282	0.186536	0.809426
TPGS2	3.03853	2.881111	3.0897	1.11089	0.1517	0.186667	0.809426
RALBP1	2.130704	2.013347	2.168852	1.15346	0.206	0.187284	0.762027
KCNAB3	1.131472	1.057801	1.155419	2.68884	1.427	0.187549	0.905001
CCDC28A	1.270055	1.191387	1.295627	1.54465	0.6273	0.187785	0.886458
LINC01420	5.15917	4.903857	5.242162	1.08666	0.1199	0.187883	0.768335
EIF1	20.146	21.29078	19.77389	0.92524	-0.1121	0.188017	0.905001
CCNH	1.437118	1.354195	1.464072	1.31021	0.3898	0.188127	0.762368
NKAIN1	1.094615	1.022221	1.118147	5.317	2.4106	0.188161	0.797943
ANKRD39	1.474015	1.558303	1.446617	0.79995	-0.322	0.188539	0.886458
CENPB	1.175271	1.250731	1.150742	0.60121	-0.7341	0.188549	0.81274
UFD1L	1.882677	1.976974	1.852026	0.87211	-0.1974	0.188562	0.921873
PECR	1.208515	1.132568	1.233201	1.75911	0.8148	0.18885	0.855781
DLGAP1	1.166168	1.241013	1.14184	0.58852	-0.7648	0.188916	0.886458
RP1-315G1.3	1.1204	1.047313	1.144158	3.04686	1.6073	0.188954	0.815015
CMTM4	1.240273	1.317202	1.215267	0.67864	-0.5593	0.188997	0.876181
ACSS3	1.334706	1.253961	1.360952	1.42129	0.5072	0.18912	0.896538
LMF2	1.383216	1.464673	1.356739	0.76772	-0.3813	0.189369	0.797943
PIM2	1.310592	1.231518	1.336295	1.45257	0.5386	0.189431	0.773169
STK35	1.172983	1.248478	1.148443	0.59741	-0.7432	0.189452	0.886458
SLC9A3R2	1.165332	1.240012	1.141056	0.5877	-0.7668	0.189469	0.878367
GPR153	1.16057	1.235438	1.136234	0.57864	-0.7893	0.189564	0.773169
MIR22HG	1.059542	1.130812	1.036376	0.27808	-1.8464	0.189721	0.81274
AGTRAP	2.013761	1.910455	2.047342	1.15035	0.2021	0.189724	0.797943
DDX5	6.118116	6.458937	6.00733	0.91727	-0.1246	0.189739	0.843752
DST	2.562507	2.702999	2.516839	0.89069	-0.167	0.18976	0.797943
DKK3	5.900725	5.582125	6.004287	1.09213	0.1271	0.189834	0.773169
MIGA1	1.326892	1.247069	1.352838	1.4281	0.5141	0.189855	0.81274
HNRNPAB	2.612607	2.482206	2.654995	1.11658	0.1591	0.189978	0.809823
FBXO25	1.207938	1.283806	1.183276	0.64578	-0.6309	0.19	0.872788
TAOK3	1.531639	1.446138	1.559432	1.25394	0.3265	0.190027	0.921873
ELAC1	1.212192	1.136327	1.236852	1.73739	0.7969	0.190045	0.810106
DNAJC17	1.23019	1.153761	1.255034	1.65863	0.73	0.190437	0.894343
TYSND1	1.257829	1.180478	1.282973	1.56791	0.6488	0.190462	0.878922
NLN	1.396502	1.315233	1.422919	1.34161	0.424	0.190537	0.858994
CRELD2	1.858923	1.953514	1.828176	0.86855	-0.2033	0.190782	0.770025
SUPT3H	1.305879	1.226556	1.331664	1.46394	0.5499	0.190897	0.914487
GLRX	1.513593	1.428783	1.541162	1.26209	0.3358	0.19104	0.919549
SLC38A10	1.303185	1.382106	1.277531	0.72632	-0.4613	0.191128	0.921873
PLPPR1	1.128181	1.05519	1.151908	2.75246	1.4607	0.191267	0.858994
LRRC42	1.536718	1.62229	1.508902	0.81779	-0.2902	0.191275	0.775899
TRIM22	1.156373	1.230451	1.132294	0.57406	-0.8007	0.19131	0.921873
RP11-112J3.16	1.170561	1.0959	1.19483	2.03159	1.0226	0.191668	0.814581

TNNI1	1.38748	1.46804	1.361293	0.77193	-0.3735	0.19186	0.831943
COX15	1.297381	1.21956	1.322677	1.46965	0.5555	0.191904	0.814581
IFT172	1.266615	1.188693	1.291945	1.5472	0.6297	0.192022	0.77193
SCPEP1	1.482466	1.399282	1.509506	1.27605	0.3517	0.192083	0.850413
FARSB	1.303049	1.22507	1.328397	1.45909	0.5451	0.192369	0.7772
ZNF711	1.385158	1.304146	1.411492	1.35294	0.4361	0.192604	0.901638
PPCDC	1.171124	1.096741	1.195303	2.01882	1.0135	0.192662	0.921873
PRKX	1.577638	1.490868	1.605843	1.23423	0.3036	0.192664	0.901638
GRIK1	1.044018	1.11481	1.021007	0.18297	-2.4503	0.192861	0.921873
NME6	1.416639	1.334974	1.443185	1.32304	0.4039	0.19302	0.890727
CDKN2C	1.101547	1.029813	1.124865	4.18832	2.0664	0.193026	0.850839
MFNG	1.098295	1.026004	1.121794	4.68356	2.2276	0.193065	0.763935
NME2	1.203398	1.128198	1.227841	1.77726	0.8297	0.193074	0.763935
DHRS7	1.697439	1.607812	1.726573	1.19539	0.2575	0.19311	0.92509
EHBP1	1.487474	1.40339	1.514806	1.2762	0.3519	0.193355	0.921873
EBNA1BP2	1.942022	2.036786	1.911218	0.87889	-0.1863	0.193534	0.88902
ERAL1	1.463983	1.38076	1.491035	1.28962	0.3669	0.19357	0.901638
SMG7	1.352103	1.271811	1.378203	1.39142	0.4766	0.193854	0.901638
C15orf65	1.170423	1.096309	1.194514	2.01968	1.0141	0.193892	0.764161
IRGQ	1.222611	1.298331	1.197998	0.66369	-0.5914	0.193964	0.78051
SH3BGR	1.261898	1.185259	1.28681	1.54816	0.6306	0.194027	0.867518
VSIG10L	1.218417	1.142524	1.243086	1.70557	0.7703	0.194047	0.776963
MRPL27	2.11496	2.012813	2.148163	1.13364	0.181	0.19406	0.790383
SLC25A29	1.362193	1.442021	1.336245	0.7607	-0.3946	0.194598	0.820099
STIL	1.101064	1.029307	1.124389	4.24436	2.0855	0.194687	0.928223
APH1A	2.676854	2.537699	2.722087	1.11991	0.1634	0.194765	0.928595
HBD	1.046657	1.11703	1.023782	0.20321	-2.299	0.194988	0.8957
GALNT16	1.136646	1.063849	1.16031	2.51075	1.3281	0.195079	0.901638
ACAP1	1.367876	1.447318	1.342052	0.76467	-0.3871	0.195308	0.838821
C8orf59	4.005912	3.816385	4.067519	1.08917	0.1232	0.195317	0.902096
RBMS2	1.180879	1.255255	1.156703	0.61391	-0.7039	0.195348	0.909814
ARMCX4	1.209538	1.134095	1.234061	1.74549	0.8036	0.195515	0.793096
TCEA2	2.48433	2.358049	2.525379	1.12321	0.1676	0.195579	0.901638
FOXN2	1.142244	1.069405	1.165921	2.39062	1.2574	0.195812	0.820099
C1orf27	1.379827	1.299956	1.405789	1.35283	0.436	0.1959	0.845818
UQCRHL	1.268088	1.19131	1.293045	1.53178	0.6152	0.196061	0.930367
CD83	1.106122	1.178121	1.082719	0.4644	-1.1066	0.196063	0.902096
SCUBE1	1.050737	1.120987	1.027902	0.23062	-2.1164	0.196173	0.897045
RP11-195F19.5	1.493407	1.410097	1.520488	1.26918	0.3439	0.19622	0.820099
CCNA1	1.125386	1.053466	1.148765	2.78242	1.4763	0.196341	0.914467
KCNMB4	1.358556	1.438194	1.332669	0.75918	-0.3975	0.196422	0.793575
MYH10	2.170963	2.27555	2.136966	0.89135	-0.1659	0.196429	0.87437
FAXDC2	1.26931	1.192074	1.294415	1.53282	0.6162	0.196432	0.902096
MICAL2	1.048032	1.11795	1.025304	0.21453	-2.2207	0.196514	0.902096
MEIS1-AS2	1.109759	1.037839	1.133138	3.51856	1.815	0.196563	0.820099

C14orf28	1.196135	1.121559	1.220376	1.81291	0.8583	0.196576	0.902096
ALDH5A1	1.174733	1.100715	1.198793	1.97382	0.981	0.196578	0.834421
ACOT11	1.229696	1.305315	1.205115	0.67182	-0.5739	0.196606	0.820099
PINK1	1.309002	1.386695	1.283747	0.73377	-0.4466	0.196757	0.933669
DHRS4	1.263684	1.187325	1.288506	1.54014	0.6231	0.19676	0.823934
KMT2C	1.952951	1.857606	1.983944	1.14732	0.1983	0.196791	0.772558
SPACA9	1.25215	1.176226	1.27683	1.57088	0.6516	0.196817	0.914467
GNGT1	1.064874	1.135519	1.04191	0.30925	-1.6931	0.196834	0.911196
FKBP4	2.173581	2.072115	2.206563	1.1254	0.1704	0.197434	0.820099
ZFAND6	2.687791	2.822174	2.644109	0.90228	-0.1484	0.197535	0.93296
CTD-2517O10.6	1.147118	1.074473	1.170732	2.29254	1.1969	0.197589	0.914467
UBE3C	1.24626	1.322298	1.221543	0.68739	-0.5408	0.197739	0.938327
COQ8A	1.177238	1.103883	1.201082	1.93566	0.9528	0.197865	0.911196
GAPVD1	1.335852	1.414236	1.310373	0.74927	-0.4165	0.198095	0.820099
HOXB8	1.083488	1.011304	1.106951	9.46111	3.242	0.198165	0.914467
DCLRE1A	1.125705	1.053929	1.149036	2.76356	1.4665	0.198227	0.824447
COMMD5	1.392241	1.47208	1.366289	0.7759	-0.366	0.198263	0.797811
AQP3	1.082288	1.153328	1.059195	0.38607	-1.3731	0.198362	0.911196
ANGPTL7	1.03757	1.106726	1.01509	0.14139	-2.8222	0.198421	0.848566
NAA20	1.853363	1.945718	1.823342	0.8706	-0.1999	0.198524	0.824447
RANBP9	1.277786	1.201136	1.302702	1.50496	0.5897	0.198562	0.853646
MCL1	1.946354	2.045047	1.914273	0.87486	-0.1929	0.198584	0.937915
PWWP2B	1.101963	1.17341	1.078738	0.45406	-1.1391	0.198961	0.840984
SRSF11	3.680924	3.463239	3.751684	1.1171	0.1598	0.199156	0.910686
RNF138	1.386382	1.307347	1.412073	1.34074	0.423	0.199247	0.798578
ZNF7	1.329808	1.251445	1.35528	1.41295	0.4987	0.199425	0.911918
KLHL21	1.112469	1.184339	1.089107	0.48339	-1.0488	0.199527	0.854145
HILPDA	6.656417	5.807146	6.932477	1.2341	0.3035	0.199542	0.938032
NOC2L	1.645109	1.558672	1.673206	1.20501	0.269	0.199856	0.823997
ZMAT5	1.656972	1.744184	1.628623	0.84471	-0.2435	0.199907	0.84145
RGCC	1.040617	1.109957	1.018078	0.16441	-2.6046	0.200166	0.915306
FBXO34	1.307782	1.38528	1.282591	0.73347	-0.4472	0.200316	0.783338
EXOC7	1.883234	1.789766	1.913616	1.15682	0.2102	0.200606	0.800834
UBQLN1	1.724872	1.812723	1.696315	0.85677	-0.223	0.200796	0.917272
CCDC33	1.416781	1.336724	1.442805	1.31504	0.3951	0.201077	0.825645
FLJ16779	1.235839	1.310753	1.211487	0.68056	-0.5552	0.201101	0.943253
GNPDA1	2.144234	2.033854	2.180114	1.14147	0.1909	0.201193	0.830581
DTNB	1.235558	1.160512	1.259951	1.61951	0.6956	0.201213	0.946366
MCAT	1.242754	1.167663	1.267163	1.59346	0.6722	0.201272	0.801661
MBD5	1.418923	1.338398	1.445099	1.31531	0.3954	0.201293	0.858054
CCDC50	1.867141	1.957866	1.837651	0.8745	-0.1935	0.201373	0.924651
CCK	1.395146	1.315596	1.421004	1.334	0.4158	0.201431	0.916381
AL132709.1	1.121106	1.049623	1.144342	2.90875	1.5404	0.201486	0.924629
ALG3	1.636624	1.550365	1.664663	1.20768	0.2722	0.20159	0.943253
DTWD1	2.049724	1.945114	2.083728	1.14666	0.1974	0.201653	0.830581

KDM3B	1.467866	1.386601	1.494282	1.27854	0.3545	0.201819	0.806228
UNC45A	1.469373	1.3878	1.495889	1.27873	0.3547	0.201822	0.918701
CHCHD3	2.273345	2.168919	2.30729	1.11837	0.1614	0.201829	0.946366
LPAR4	1.288835	1.212793	1.313553	1.47351	0.5593	0.201938	0.856601
TDRP	1.265266	1.341285	1.240556	0.70485	-0.5046	0.201958	0.806228
SLCO5A1	1.143674	1.071873	1.167014	2.32372	1.2164	0.202004	0.856601
ZNF274	1.271474	1.195632	1.296127	1.51369	0.5981	0.202223	0.916381
KCTD18	1.238827	1.163899	1.263183	1.60577	0.6833	0.202255	0.946366
BET1	1.855613	1.763453	1.88557	1.15995	0.2141	0.202355	0.801661
PAPOLA	4.03504	3.845329	4.096707	1.08835	0.1221	0.202486	0.920808
RP11-793J2.1	1.142405	1.070431	1.165801	2.3541	1.2352	0.202522	0.805286
CTDNEP1	1.971063	2.069654	1.939015	0.87787	-0.1879	0.202575	0.947861
GBAS	2.300298	2.194624	2.334648	1.11721	0.1599	0.20258	0.849146
TRAF7	1.843637	1.751817	1.873484	1.16183	0.2164	0.20272	0.925647
ABLIM1	1.593848	1.677495	1.566658	0.8364	-0.2577	0.202949	0.806981
ATP2A2	1.735399	1.823402	1.706793	0.85838	-0.2203	0.202998	0.890888
EMX1	1.086089	1.015961	1.108885	6.82191	2.7702	0.20305	0.801661
ATL1	1.200434	1.126824	1.224362	1.76908	0.823	0.203113	0.924651
GLTP	1.671314	1.584389	1.699569	1.19709	0.2595	0.203151	0.924751
RGL2	1.464126	1.545365	1.437719	0.80262	-0.3172	0.203207	0.833565
MVP	1.143445	1.215376	1.120063	0.55746	-0.8431	0.203215	0.920876
E2F4	1.431979	1.512412	1.405833	0.79201	-0.3364	0.203286	0.831325
CCKBR	1.223881	1.150109	1.247861	1.65121	0.7235	0.203334	0.947008
ZNF107	1.140461	1.068781	1.163761	2.38091	1.2515	0.203407	0.801661
GNA11	1.215832	1.289952	1.191739	0.66128	-0.5967	0.20355	0.925647
PIK3C3	1.464292	1.383209	1.490648	1.28037	0.3566	0.203755	0.924651
METAP1D	1.23496	1.160632	1.259121	1.61314	0.6899	0.203946	0.862056
XPA	1.445962	1.366044	1.47194	1.2893	0.3666	0.204048	0.924651
SMIM14	1.817733	1.726546	1.847373	1.1663	0.2219	0.204106	0.805931
NUP155	1.204887	1.1313	1.228807	1.74262	0.8013	0.204308	0.860877
ST3GAL4	1.242945	1.317719	1.218639	0.68815	-0.5392	0.204336	0.891754
CHKA	1.171048	1.244057	1.147316	0.60361	-0.7283	0.204394	0.852313
ADAMTS9-AS1	1.259874	1.185233	1.284137	1.53394	0.6172	0.204565	0.796853
TERF2IP	2.487376	2.34825	2.532599	1.13673	0.1849	0.204726	0.860877
SCN9A	1.153621	1.225631	1.130214	0.57711	-0.7931	0.204734	0.805931
FALEC	1.168799	1.096339	1.192352	1.99661	0.9976	0.20476	0.950248
PIGC	1.63061	1.544343	1.658651	1.20999	0.275	0.204967	0.927893
ZCCHC18	1.284905	1.20938	1.309455	1.47796	0.5636	0.20497	0.891754
SRPK1	2.01204	1.910899	2.044916	1.14713	0.198	0.204981	0.954679
RNF13	1.837324	1.927837	1.807902	0.87074	-0.1997	0.205033	0.950248
MAPK1IP1L	2.292298	2.183367	2.327706	1.12197	0.166	0.205088	0.805931
VEZF1	1.88979	1.798049	1.919611	1.15232	0.2045	0.205136	0.930584
RAD54L	1.100541	1.030615	1.123271	4.0265	2.0095	0.205203	0.925714
RP11-197K6.1	1.147438	1.075577	1.170797	2.25989	1.1763	0.205358	0.930584
TMEM261	2.967233	2.789911	3.024873	1.13127	0.1779	0.205412	0.806404

RARA-AS1	1.230835	1.156582	1.254971	1.62836	0.7034	0.205445	0.947798
PSMB3	4.077223	3.883271	4.140269	1.08913	0.1232	0.205469	0.797284
MMRN1	1.237457	1.162877	1.2617	1.60674	0.6841	0.205522	0.925698
SNX24	1.422049	1.342534	1.447896	1.3076	0.3869	0.205571	0.852313
KYAT3	1.296512	1.220606	1.321186	1.45593	0.5419	0.205615	0.865474
SLC12A4	1.128157	1.199082	1.105103	0.52794	-0.9216	0.205853	0.910126
CNPY2	5.614358	5.904114	5.520171	0.92171	-0.1176	0.205877	0.852313
PHF10	1.53477	1.452203	1.561608	1.24194	0.3126	0.205919	0.950248
NAALAD2	1.248354	1.174017	1.272517	1.56604	0.6471	0.205954	0.87422
CFAP74	1.089093	1.019332	1.111769	5.78147	2.5314	0.206122	0.947798
CBX2	1.22209	1.148686	1.245951	1.65416	0.7261	0.206177	0.84231
MANEA	1.270586	1.195334	1.295047	1.51047	0.595	0.20633	0.947798
PDIA5	1.192699	1.120036	1.216319	1.80213	0.8497	0.206341	0.930848
FBXO4	1.122308	1.051804	1.145225	2.80335	1.4872	0.206425	0.910126
RP5-1159O4.1	1.122552	1.051702	1.145582	2.81577	1.4935	0.206477	0.927305
FAM92B	1.095059	1.025357	1.117716	4.64243	2.2149	0.206539	0.925698
RTKN	1.350321	1.427659	1.325182	0.76038	-0.3952	0.206545	0.93177
MBTD1	1.299838	1.223648	1.324604	1.45141	0.5375	0.20662	0.866082
ACOT9	1.260481	1.334908	1.236288	0.70553	-0.5032	0.206637	0.935451
SYNM	1.157392	1.085806	1.180662	2.10548	1.0742	0.206715	0.799034
SOBP	1.142237	1.213659	1.119022	0.55706	-0.8441	0.206741	0.84203
SLC38A7	1.145548	1.217298	1.122225	0.56248	-0.8301	0.207176	0.910126
PTDSS1	1.393452	1.471711	1.368013	0.78017	-0.3581	0.207201	0.874433
NOP58	1.539859	1.457995	1.566469	1.23685	0.3067	0.207377	0.96139
LARP1B	1.37332	1.451345	1.347958	0.77093	-0.3753	0.207584	0.925698
RP5-908M14.9	1.125781	1.055269	1.148701	2.6905	1.4279	0.207616	0.928273
RP11-532F12.5	1.097135	1.167712	1.074193	0.44238	-1.1766	0.207683	0.7997
JMJD1C	2.431834	2.31472	2.469903	1.11804	0.161	0.207684	0.842092
MTFR1	1.882463	1.791275	1.912104	1.1527	0.205	0.20769	0.866955
CAB39L	1.108877	1.179029	1.086074	0.48078	-1.0565	0.207695	0.822605
COG4	1.290917	1.215886	1.315307	1.46052	0.5465	0.207765	0.874433
IMMT	1.75384	1.666535	1.78222	1.17356	0.2309	0.20796	0.925698
TMX4	1.809886	1.721231	1.838704	1.16288	0.2177	0.208088	0.845301
LINC01170	1.109398	1.039483	1.132124	3.34633	1.7426	0.208355	0.931653
CPS1	1.235902	1.162198	1.259861	1.60212	0.68	0.208372	0.931653
GTPBP6	1.386313	1.308275	1.41168	1.33543	0.4173	0.20843	0.842092
SMURF1	1.110135	1.180283	1.087332	0.48442	-1.0457	0.208592	0.845301
BAIAP2	1.4212	1.499798	1.395651	0.79162	-0.3371	0.208612	0.874433
ATF3	1.133628	1.204661	1.110539	0.54011	-0.8887	0.208942	0.817882
RAD51	1.13803	1.067594	1.160925	2.38078	1.2514	0.208972	0.925809
MED16	1.301577	1.226102	1.32611	1.44231	0.5284	0.209167	0.862384
IL4R	1.100895	1.170734	1.078193	0.45798	-1.1266	0.209239	0.954735
HCG11	1.234086	1.160115	1.258131	1.61216	0.689	0.209289	0.870018
TOPBP1	1.449187	1.366314	1.476125	1.29977	0.3783	0.209371	0.943578
AKAP11	1.381636	1.459924	1.356188	0.77445	-0.3688	0.209587	0.800554

PCNX2	1.183205	1.255475	1.159713	0.62516	-0.6777	0.209738	0.954735
PRPF38B	2.100613	1.99612	2.134579	1.139	0.1878	0.209888	0.968566
RP5-1120P11.1	1.131687	1.061286	1.154572	2.52212	1.3346	0.209953	0.800554
MAN1C1	1.177952	1.249877	1.154573	0.61859	-0.6929	0.209975	0.934669
MFAP4	1.329297	1.408514	1.303547	0.74305	-0.4285	0.210176	0.821896
DERL1	1.480496	1.56073	1.454416	0.8104	-0.3033	0.210247	0.961401
CDS2	1.480886	1.561081	1.454819	0.81061	-0.3029	0.210247	0.961401
PYURF	3.721541	3.54646	3.778452	1.0911	0.1258	0.210295	0.800554
CCDC157	1.217718	1.14547	1.241203	1.6581	0.7295	0.210499	0.849602
FJX1	1.348564	1.272027	1.373443	1.37282	0.4571	0.210715	0.925809
PML	1.254224	1.180302	1.278253	1.54327	0.626	0.210746	0.925809
PTK2	1.954342	1.862324	1.984253	1.1414	0.1908	0.210862	0.950756
CEP250	1.434718	1.355687	1.460407	1.29442	0.3723	0.210917	0.880426
PLAGL1	1.426972	1.505712	1.401378	0.79369	-0.3334	0.211008	0.944901
PRKACB	1.42661	1.505531	1.400957	0.79314	-0.3344	0.211008	0.944901
EIF4H	3.776547	3.59946	3.83411	1.09027	0.1247	0.211247	0.801144
POLR2J	3.743153	3.509926	3.818965	1.12313	0.1675	0.211375	0.823381
HIST1H2BD	1.151978	1.081152	1.175001	2.15645	1.1087	0.211461	0.824536
PRUNE2	1.093917	1.163279	1.07137	0.43711	-1.1939	0.211516	0.950756
MRPS22	1.36126	1.284592	1.386181	1.35696	0.4404	0.211543	0.971745
PLA2G15	1.172079	1.243592	1.148833	0.61099	-0.7108	0.211545	0.944901
DACT2	1.083377	1.014808	1.105666	7.13567	2.835	0.211625	0.937173
TMC6	1.117377	1.187329	1.094639	0.5052	-0.9851	0.211672	0.869151
COMMD1	1.668018	1.753367	1.640275	0.84988	-0.2347	0.211699	0.950756
RAB4B	1.206603	1.279371	1.182949	0.65486	-0.6107	0.211767	0.886776
STIM2	1.360685	1.283764	1.385688	1.35919	0.4427	0.21186	0.852524
ICA1	1.230214	1.15726	1.253928	1.6147	0.6913	0.211922	0.926927
L1CAM	1.199723	1.271369	1.176433	0.65016	-0.6211	0.212428	0.920216
MAGED2	3.136155	2.970258	3.190081	1.11157	0.1526	0.212454	0.945703
TBC1D2B	1.165855	1.237169	1.142674	0.60157	-0.7332	0.212595	0.967741
CDC26	2.348342	2.22656	2.387929	1.13156	0.1783	0.212721	0.852379
DYNLL1	8.916629	9.364116	8.77117	0.92911	-0.1061	0.212875	0.804285
ARSA	1.121063	1.190724	1.098419	0.51603	-0.9545	0.212887	0.931149
UGGT2	1.394714	1.317325	1.41987	1.32316	0.404	0.213052	0.852379
ZNF131	1.603805	1.520451	1.6309	1.21222	0.2776	0.21321	0.949878
NAT10	1.261352	1.187943	1.285214	1.51755	0.6017	0.213245	0.953441
AP2M1	5.998841	6.322095	5.893765	0.91952	-0.121	0.213246	0.889261
TMEM159	1.462595	1.383161	1.488416	1.2747	0.3502	0.213629	0.937173
ADAT2	1.141786	1.071646	1.164585	2.29721	1.1999	0.213716	0.968461
AGK	1.213421	1.140971	1.236971	1.68099	0.7493	0.213724	0.826737
PCNT	1.228038	1.155617	1.251578	1.61665	0.693	0.213731	0.852379
ECI1	1.443772	1.365295	1.469281	1.28466	0.3614	0.213994	0.937173
PSMB10	1.243421	1.316925	1.219528	0.69268	-0.5297	0.214024	0.978674
VPS13B	1.206587	1.278961	1.183062	0.65623	-0.6077	0.21403	0.826737
TNS1	1.087955	1.156618	1.065636	0.41909	-1.2547	0.214079	0.970104

FOXC2	1.047201	1.114464	1.025336	0.22135	-2.1756	0.214105	0.949878
TBC1D1	1.447408	1.368573	1.473033	1.28342	0.36	0.214237	0.949878
HAPLN3	1.076823	1.145299	1.054565	0.37553	-1.413	0.214267	0.876228
TPCN1	1.13104	1.200972	1.108309	0.53892	-0.8918	0.214553	0.826737
RPGRIP1L	1.322407	1.247354	1.346803	1.40205	0.4875	0.214581	0.955172
USP9X	1.761398	1.84789	1.733283	0.86483	-0.2095	0.214602	0.831984
RAP1A	1.786907	1.87407	1.758575	0.86786	-0.2045	0.214669	0.934878
ZBTB17	1.224843	1.152425	1.248383	1.62955	0.7045	0.21505	0.927122
KPNA6	1.571913	1.65348	1.545399	0.83461	-0.2608	0.215051	0.937173
GALNT11	1.716136	1.801625	1.688347	0.85869	-0.2198	0.215196	0.937173
C19orf70	3.028902	3.188214	2.977116	0.90353	-0.1464	0.215221	0.85493
CHSY3	1.109613	1.040794	1.131983	3.23534	1.6939	0.215279	0.949878
TNFRSF18	1.14127	1.211305	1.118505	0.56082	-0.8344	0.215313	0.826737
ZNF76	1.278908	1.204906	1.302963	1.47855	0.5642	0.215316	0.895074
KIAA0100	1.374048	1.297555	1.398912	1.34063	0.4229	0.215606	0.93896
KCNQ2	1.105891	1.037045	1.128269	3.46253	1.7918	0.215754	0.831984
KATNB1	1.398168	1.321067	1.423231	1.3182	0.3986	0.215867	0.927122
APOL2	1.279329	1.204839	1.303542	1.48186	0.5674	0.216197	0.960913
TXNDC16	1.279981	1.206019	1.304023	1.4757	0.5614	0.216261	0.949878
CDC40	1.481432	1.402177	1.507194	1.26112	0.3347	0.216397	0.881358
AMH	1.113259	1.044272	1.135684	3.06481	1.6158	0.21644	0.949878
MBLAC2	1.146969	1.077252	1.169631	2.19581	1.1348	0.216795	0.855279
RELL2	1.174184	1.245453	1.151018	0.61526	-0.7007	0.216848	0.831984
PRMT2	3.731283	3.958801	3.657327	0.89811	-0.155	0.216895	0.831984
TIMM10	2.226472	2.124431	2.259641	1.12025	0.1638	0.217464	0.818544
ATP13A3	1.302037	1.376557	1.277814	0.73777	-0.4388	0.217596	0.988798
FAHD2B	1.372645	1.296304	1.39746	1.3414	0.4237	0.217609	0.936867
ACBD6	2.045305	1.952561	2.075452	1.12901	0.1751	0.217626	0.981756
UBE3D	1.266892	1.193734	1.290673	1.50037	0.5853	0.217856	0.943782
TCEAL7	4.081497	3.89233	4.142988	1.08666	0.1199	0.21787	0.833373
MAP2K2	1.461827	1.540052	1.4364	0.80807	-0.3074	0.217962	0.960913
RAB9B	1.252074	1.179259	1.275743	1.53824	0.6213	0.218204	0.988798
GOLGA3	1.207349	1.278838	1.184112	0.66028	-0.5988	0.218268	0.884539
HERPUD2	1.402899	1.325817	1.427956	1.31349	0.3934	0.218416	0.875406
DXO	1.242401	1.16986	1.265981	1.56589	0.647	0.218495	0.936867
ZNF181	1.17654	1.106195	1.199406	1.87774	0.909	0.21859	0.943782
RAP1GAP	1.060316	1.1273	1.038543	0.30277	-1.7237	0.218937	0.884539
IER3IP1	2.674696	2.799756	2.634045	0.90793	-0.1394	0.219106	0.907082
AMIGO2	1.141275	1.071666	1.163902	2.28703	1.1935	0.219122	0.818696
MTR	1.481836	1.40303	1.507453	1.2591	0.3324	0.219133	0.818696
RAPGEF1	1.154203	1.22401	1.131512	0.58708	-0.7684	0.219312	0.969848
DNM1	1.741014	1.655397	1.768844	1.1731	0.2303	0.21943	0.966994
SLC4A3	1.132368	1.201601	1.109863	0.54495	-0.8758	0.219667	0.936867
TENM2	1.082511	1.149965	1.060584	0.40399	-1.3076	0.219807	0.969848
KIAA1328	1.210748	1.13937	1.23395	1.67862	0.7473	0.219923	0.840389

PARD6A	1.31096	1.236286	1.335233	1.41876	0.5046	0.220064	0.960913
RP11-983P16.4	1.164087	1.094309	1.186769	1.98039	0.9858	0.220249	0.879249
SNUPN	1.293119	1.219601	1.317016	1.4436	0.5297	0.220355	0.819858
CPNE5	1.092445	1.160383	1.070361	0.43871	-1.1887	0.220449	0.991016
ZCCHC10	1.614438	1.532494	1.641075	1.20391	0.2677	0.220507	0.943021
XXYLT1	1.787761	1.701105	1.81593	1.16378	0.2188	0.220531	0.936867
RP11-395G23.3	1.908233	1.803567	1.942255	1.17259	0.2297	0.22055	0.966994
CARNMT1	1.314441	1.240458	1.33849	1.40769	0.4933	0.220635	0.908903
SSBP4	3.078613	2.942025	3.123012	1.0932	0.1286	0.220706	0.863189
CFAP36	2.364214	2.261452	2.397617	1.10794	0.1479	0.220798	0.991016
RP11-179B2.2	1.137112	1.06816	1.159525	2.34044	1.2268	0.22094	0.966994
MRPS5	2.060641	1.960427	2.093216	1.13826	0.1868	0.22096	0.996797
HSPBP1	1.539937	1.459467	1.566094	1.23207	0.3011	0.221012	0.863189
NDUFS7	2.961477	2.801169	3.013585	1.11793	0.1608	0.221037	0.908903
XKR8	1.294072	1.367246	1.270287	0.73598	-0.4423	0.221076	0.819858
CUL4A	1.412705	1.48965	1.387694	0.79178	-0.3368	0.221082	0.966994
NDUFB10	4.365887	4.58987	4.293079	0.91733	-0.1245	0.221191	0.889711
POLE2	1.111661	1.04371	1.13375	3.05996	1.6135	0.221198	0.908903
NPB	1.41236	1.335804	1.437245	1.30209	0.3808	0.221258	0.960913
ZFC3H1	1.412527	1.33547	1.437575	1.30436	0.3833	0.221258	0.960913
PPP1R2	1.857018	1.944251	1.828663	0.87759	-0.1884	0.221269	0.936867
STAM-AS1	1.106307	1.038314	1.128408	3.3515	1.7448	0.221374	0.863189
METTL14	1.296476	1.222461	1.320535	1.44086	0.5269	0.221395	0.960913
BCL7B	1.532164	1.452195	1.558159	1.23433	0.3037	0.22144	0.970669
MRPS18A	1.618928	1.70151	1.592085	0.84401	-0.2447	0.221573	0.908903
ST20-AS1	1.167149	1.236814	1.144504	0.6102	-0.7126	0.22165	0.943455
RP11-161M6.2	1.253971	1.181726	1.277455	1.52678	0.6105	0.221682	0.843902
ZNF100	1.198198	1.127509	1.221176	1.73459	0.7946	0.221707	0.877228
DNAL4	1.466172	1.38748	1.491751	1.2691	0.3438	0.221784	0.936867
PCSK5	1.077647	1.144833	1.055807	0.38532	-1.3759	0.221823	0.960913
RP11-834C11.4	1.08498	1.153116	1.062831	0.41035	-1.2851	0.221917	0.936867
SNX18	1.215183	1.286088	1.192134	0.67159	-0.5743	0.221929	0.993977
RAE1	1.391508	1.315571	1.416192	1.31885	0.3993	0.221932	0.912796
E2F3	1.36492	1.289184	1.389538	1.34702	0.4298	0.221986	0.889711
PSMD13	1.838307	1.751346	1.866574	1.15336	0.2058	0.22207	0.820516
OPRK1	1.10865	1.04073	1.130728	3.2096	1.6824	0.222301	0.912796
TAZ	1.281745	1.208697	1.30549	1.46379	0.5497	0.222377	0.993977
DLGAP4	1.759591	1.844594	1.731961	0.86664	-0.2065	0.222486	0.943455
SSH2	1.340466	1.266029	1.364663	1.37077	0.455	0.222535	0.964235
ARFGEF1	1.549263	1.468768	1.575428	1.22753	0.2958	0.222576	0.936867
PAN3	1.373269	1.297862	1.397781	1.33545	0.4173	0.22258	0.997973
SPAG17	1.108518	1.040701	1.130562	3.20787	1.6816	0.222817	0.845024
MLF2	5.210762	4.943089	5.297771	1.08995	0.1243	0.222826	0.997219
RUFY3	2.127355	2.023542	2.1611	1.13439	0.1819	0.223036	0.997219
USP42	1.266886	1.194411	1.290444	1.49397	0.5791	0.223126	0.923724

SUGT1	2.108625	2.013647	2.139499	1.12416	0.1688	0.223129	0.991016
NBAS	1.412082	1.488712	1.387173	0.79223	-0.336	0.223145	0.991016
IRS1	1.240677	1.312538	1.217318	0.69533	-0.5242	0.223156	0.975093
HGS	1.470107	1.548059	1.444768	0.81153	-0.3013	0.223205	0.997973
ACYP2	1.675689	1.758714	1.648701	0.855	-0.226	0.223299	0.993977
ZNF780B	1.136001	1.067422	1.158293	2.34781	1.2313	0.223378	0.958837
RP11-326C3.11	1.140994	1.072364	1.163303	2.25667	1.1742	0.223619	0.991016
MTERF4	1.401313	1.325168	1.426065	1.31029	0.3899	0.223625	0.964491
NUP85	1.277375	1.204741	1.300985	1.47007	0.5559	0.223959	0.972084
GLG1	1.976288	2.065952	1.947142	0.88854	-0.1705	0.224008	0.824645
TACC3	1.612477	1.531281	1.63887	1.20251	0.266	0.224159	0.883449
DCDC1	1.604928	1.523324	1.631453	1.20662	0.271	0.224177	0.997884
BORCS8	1.269651	1.342019	1.246127	0.71963	-0.4747	0.224178	0.972084
SPAG9	2.1532	2.057418	2.184334	1.12002	0.1635	0.224358	0.970669
VLDLR	1.280494	1.353521	1.256756	0.72628	-0.4614	0.224568	0.964491
VPS29	2.313565	2.417372	2.279822	0.90295	-0.1473	0.224626	0.999885
RARG	1.097463	1.164801	1.075575	0.45858	-1.1247	0.224697	0.87276
MOV10	1.309127	1.235988	1.332901	1.41067	0.4964	0.224925	0.958837
RAB5B	1.717494	1.800868	1.690392	0.86205	-0.2141	0.224936	0.958837
ANKRD46	1.387587	1.311847	1.412206	1.32182	0.4025	0.225051	0.894868
SNN	1.466789	1.389325	1.491969	1.26365	0.3376	0.225176	0.970669
GIGYF1	1.369586	1.294288	1.394062	1.33904	0.4212	0.225227	0.964235
ZCCHC14	1.220413	1.149592	1.243434	1.62732	0.7025	0.225373	0.994409
VAMP5	1.997756	2.087776	1.968494	0.89034	-0.1676	0.225389	0.970669
RHOJ	1.110533	1.178043	1.088588	0.49757	-1.007	0.225608	0.978051
CTD-2017D11.1	1.226791	1.155971	1.249811	1.60165	0.6796	0.225861	0.975178
NIF3L1	1.22985	1.159037	1.252868	1.58999	0.669	0.225994	0.887203
PKP3	1.037858	1.103314	1.016581	0.16049	-2.6394	0.226115	1
RNF208	1.214763	1.285215	1.191862	0.67269	-0.572	0.226131	0.85911
TRIM21	1.086755	1.153532	1.065048	0.42368	-1.239	0.226296	0.970669
VPS8	1.368139	1.292876	1.392604	1.34051	0.4228	0.226306	0.85911
TRIM56	1.839489	1.752292	1.867832	1.15358	0.2061	0.226336	0.925587
FAM222B	1.367544	1.441806	1.343405	0.77728	-0.3635	0.226448	0.994787
ABCF2	1.264511	1.336351	1.24116	0.71699	-0.48	0.226558	0.970669
BAG5	1.374281	1.44868	1.350097	0.78028	-0.3579	0.22673	0.897992
UHMK1	1.398872	1.474068	1.374429	0.78982	-0.3404	0.226745	0.978051
RND2	1.514034	1.435382	1.5396	1.23937	0.3096	0.226752	0.978051
RWDD1	3.553946	3.709952	3.503235	0.92372	-0.1145	0.22683	0.957832
ISG20	1.07294	1.139235	1.05139	0.36909	-1.438	0.227056	0.857862
SPRY2	1.173619	1.242645	1.151182	0.62306	-0.6826	0.227101	0.963984
PSMB7	3.722549	3.890611	3.667919	0.92296	-0.1157	0.227151	0.830741
GTF3C2	1.312842	1.239418	1.336709	1.40637	0.492	0.227215	0.859316
FBLN5	1.041328	1.106934	1.020002	0.18705	-2.4185	0.227255	0.888681
ANKRD54	1.393836	1.318737	1.418247	1.3122	0.392	0.227317	0.830741
HBP1	1.57847	1.498447	1.604482	1.21273	0.2783	0.227952	0.9721

ZNF512B	1.165643	1.234575	1.143236	0.61062	-0.7116	0.228053	0.858402
ZNF827	1.528764	1.607198	1.503268	0.82884	-0.2708	0.228262	0.970669
TMEM260	1.338714	1.265017	1.36267	1.36847	0.4526	0.228274	0.9721
GNG8	1.100202	1.033284	1.121953	3.66399	1.8734	0.228475	0.960742
DRAIC	1.145168	1.077107	1.167292	2.16962	1.1174	0.228477	0.884029
ICK	1.24581	1.174675	1.268932	1.53961	0.6226	0.228537	0.970669
EDRF1	1.244788	1.173879	1.267837	1.54037	0.6233	0.228633	1
RNF10	1.9754	1.885172	2.004729	1.13507	0.1828	0.228717	0.832828
SPTY2D1-AS1	1.097421	1.030798	1.119077	3.8664	1.951	0.228718	0.902315
ATXN1	1.256698	1.327736	1.233607	0.71279	-0.4884	0.229141	0.985112
SMAD5	1.997955	1.90809	2.027166	1.13113	0.1778	0.229365	1
CCDC102B	1.173129	1.104463	1.195449	1.87098	0.9038	0.229375	0.970669
TATDN3	1.225519	1.154842	1.248492	1.60481	0.6824	0.229467	0.863294
ATP1B2	1.433112	1.509021	1.408437	0.8024	-0.3176	0.229641	0.970669
MIR503HG	1.103799	1.03701	1.12551	3.39125	1.7618	0.229716	1
FLJ20021	1.231139	1.160559	1.254081	1.58248	0.6622	0.229827	0.964235
NCEH1	1.100679	1.167801	1.078861	0.46997	-1.0894	0.229837	0.931598
ESYT2	1.281058	1.352685	1.257775	0.73089	-0.4523	0.229983	0.863294
C18orf21	1.458603	1.382305	1.483404	1.26444	0.3385	0.230013	1
UBR5-AS1	1.109999	1.043037	1.131765	3.06168	1.6143	0.230045	0.964272
FDX1	2.368515	2.26188	2.403178	1.11197	0.1531	0.230167	0.964235
IWS1	1.516153	1.594168	1.490793	0.82602	-0.2758	0.230201	0.885215
POLR3E	1.24058	1.169966	1.263534	1.55051	0.6327	0.230223	0.985549
B3GNT7	1.060157	1.125387	1.038953	0.31066	-1.6866	0.230472	0.964235
TIA1	2.20196	2.107459	2.232678	1.11307	0.1545	0.230543	0.885215
SLC30A1	1.109643	1.176626	1.087869	0.49749	-1.0073	0.230632	0.977997
RASSF9	1.085215	1.019192	1.106677	5.55826	2.4746	0.230866	0.943171
ZNF169	1.122306	1.055178	1.144126	2.61203	1.3852	0.230876	0.964235
SPA17	1.272505	1.201332	1.29564	1.46842	0.5543	0.230895	0.949389
RGS4	1.066972	1.132545	1.045657	0.34447	-1.5376	0.231035	0.964235
DCTN1	1.463936	1.54008	1.439184	0.81318	-0.2983	0.231059	0.964235
HDAC11	1.183215	1.252209	1.160788	0.63752	-0.6495	0.231071	0.992419
PPL	1.152575	1.220692	1.130433	0.59102	-0.7587	0.23125	0.964235
DUSP3	1.393753	1.468002	1.369617	0.78978	-0.3405	0.231465	0.901638
HDAC1	1.443572	1.368078	1.468112	1.27177	0.3468	0.231497	1
ROCK1	2.008063	2.096844	1.979205	0.89275	-0.1637	0.231783	0.949389
LDAH	1.370852	1.296863	1.394903	1.33025	0.4117	0.231922	0.979333
MEGF10	1.081618	1.015934	1.102969	6.46215	2.692	0.232094	0.931598
DGUOK	3.921523	3.75173	3.976715	1.08176	0.1134	0.23212	0.931598
LINC00327	1.041049	1.106029	1.019926	0.18793	-2.4117	0.232162	0.868234
SEMA6C	1.194308	1.124974	1.216846	1.73513	0.795	0.232259	0.931598
FGF8	1.081342	1.015676	1.102687	6.55081	2.7117	0.232329	1
TSR1	1.29766	1.225797	1.32102	1.42172	0.5076	0.232691	0.949389
RP11-382A20.3	2.316405	2.415602	2.28416	0.90715	-0.1406	0.232789	0.928848
INTU	1.517248	1.440099	1.542326	1.23228	0.3013	0.232899	0.994065

VPS13D	1.180313	1.249168	1.157932	0.63384	-0.6578	0.232947	0.915408
COPS5	1.848587	1.762325	1.876628	1.14994	0.2016	0.233101	0.949389
CTD-2325M2.1	1.147258	1.079772	1.169195	2.12097	1.0847	0.23312	0.964235
SMNDC1	1.520129	1.597992	1.494819	0.82747	-0.2732	0.23319	1
FEM1B	1.438587	1.513761	1.414152	0.80612	-0.3109	0.233191	0.994027
ORAI1	1.178008	1.246266	1.15582	0.63273	-0.6603	0.233428	0.986675
RNF128	1.053249	1.118093	1.032171	0.27242	-1.8761	0.233432	0.994065
HSPA1B	1.101589	1.168165	1.079949	0.47542	-1.0727	0.233745	0.964235
RLN2	1.103362	1.037248	1.124853	3.3519	1.745	0.233746	0.876547
ANKRD49	1.472498	1.39619	1.497302	1.25521	0.3279	0.233852	1
ATP6V1B2	1.546339	1.624912	1.520798	0.8334	-0.2629	0.233947	1
TTC31	1.168928	1.101004	1.191008	1.8911	0.9192	0.234171	0.977649
BIVM	1.384744	1.311191	1.408653	1.31319	0.3931	0.234295	0.981111
PPP2R2B	1.182185	1.250609	1.159943	0.63822	-0.6479	0.234338	0.928848
AXL	1.960878	2.07985	1.922206	0.85401	-0.2277	0.234357	0.931598
RABL2B	1.685261	1.603545	1.711824	1.1794	0.2381	0.234484	0.964235
RP11-214N9.1	1.124788	1.057943	1.146516	2.52862	1.3383	0.23451	1
BACH1	1.391482	1.465552	1.367405	0.78918	-0.3416	0.23452	0.928848
ATXN3	1.564043	1.486098	1.58938	1.21247	0.278	0.234609	1
PLPP5	1.414778	1.339895	1.439119	1.29193	0.3695	0.23483	0.877338
MTPAP	1.267375	1.196791	1.290319	1.47527	0.561	0.234889	1
MSH2	1.38239	1.308827	1.406302	1.31563	0.3958	0.234895	0.996073
TRNP1	1.075545	1.140839	1.054321	0.38569	-1.3745	0.234915	0.875276
ACAT2	1.558731	1.480676	1.584104	1.21517	0.2812	0.234926	0.931598
PRPF39	1.26668	1.195829	1.28971	1.47941	0.565	0.235021	0.964235
RFX7	1.266593	1.19573	1.289628	1.47973	0.5653	0.235082	0.977649
SLFN12	1.171402	1.103296	1.193541	1.87366	0.9059	0.235125	0.956682
FBXO7	2.062864	1.970725	2.092815	1.12577	0.1709	0.235162	0.931598
GNA12	1.214619	1.284075	1.192042	0.67602	-0.5649	0.23517	0.920549
PATZ1	1.418869	1.344234	1.44313	1.28729	0.3643	0.235215	0.931598
ZFAND2B	1.482929	1.406325	1.50783	1.24981	0.3217	0.235244	0.977649
TTPAL	1.183856	1.11555	1.206059	1.78329	0.8345	0.235353	0.896532
TNFRSF1A	2.152879	2.059529	2.183223	1.11674	0.1593	0.235488	1
DHX33	1.259784	1.1894	1.282663	1.49241	0.5776	0.23552	0.896532
OSGEP	1.566849	1.48856	1.592297	1.21233	0.2778	0.235529	1
KBTBD3	1.346077	1.273001	1.36983	1.35469	0.438	0.235619	0.964235
ARL17B	1.117945	1.051713	1.139474	2.69706	1.4314	0.235787	0.964235
NUAK2	1.175741	1.243923	1.153577	0.62961	-0.6675	0.235807	0.999063
AGAP1	1.389465	1.463086	1.365534	0.78934	-0.3413	0.235839	1
SFXN5	1.327804	1.255725	1.351233	1.37348	0.4578	0.236101	0.977649
MRPL22	2.145293	2.053058	2.175274	1.11606	0.1584	0.236107	1
AACS	1.319371	1.247234	1.34282	1.38662	0.4716	0.236212	0.979303
RANBP17	1.279413	1.20884	1.302353	1.44777	0.5338	0.236359	0.999063
NQO2	1.962175	1.874501	1.990674	1.13284	0.1799	0.236582	0.977649
NSMCE3	1.417235	1.491184	1.393197	0.80051	-0.321	0.236818	0.88149

IFI6	1.830305	1.738219	1.860237	1.16529	0.2207	0.236868	1
PLXNA1	1.292892	1.364117	1.269739	0.74081	-0.4328	0.236883	0.996098
PIP4K2A	1.172258	1.239854	1.150285	0.62657	-0.6745	0.236943	0.919414
ATP5S	1.494045	1.41794	1.518783	1.24129	0.3118	0.237062	1
TUBGCP3	1.215447	1.146538	1.237846	1.6231	0.6988	0.237124	0.977649
PRRC2C	3.674404	3.515078	3.726194	1.08394	0.1163	0.237143	0.880317
CAPZB	4.372254	4.590207	4.301407	0.91956	-0.121	0.237324	0.979303
ATP6V1H	1.703387	1.785164	1.676804	0.86199	-0.2143	0.237352	0.936379
RPRD1A	1.717391	1.635502	1.74401	1.17074	0.2274	0.237474	0.925966
S100A1	1.068776	1.133296	1.047804	0.35863	-1.4794	0.237642	1
KDSR	1.598315	1.519308	1.623997	1.20159	0.2649	0.237757	1
PIGQ	1.392314	1.319109	1.416111	1.30398	0.3829	0.23778	0.968448
DAZAP2	2.318647	2.41865	2.28614	0.90659	-0.1415	0.237816	0.938208
MICU2	1.584277	1.505996	1.609722	1.20499	0.269	0.238089	0.896532
DFFA	1.906755	1.820969	1.93464	1.13846	0.1871	0.238145	0.979303
DYX1C1	1.219024	1.149943	1.241479	1.61047	0.6875	0.238254	0.978299
RAB11B-AS1	1.445204	1.370502	1.469486	1.26716	0.3416	0.238259	0.979303
TINF2	1.544509	1.467138	1.569659	1.21947	0.2862	0.238346	0.896532
SNAPC1	1.752365	1.668695	1.779562	1.1658	0.2213	0.23842	0.926065
BCL7A	1.703344	1.622243	1.729707	1.1727	0.2298	0.238452	0.998516
NBPF9	1.210282	1.141489	1.232644	1.64425	0.7174	0.238498	0.979303
INTS4	1.155804	1.088871	1.177561	1.99796	0.9985	0.238545	0.922067
MCU	1.132452	1.199081	1.110794	0.55653	-0.8455	0.238569	0.937501
C14orf80	1.145116	1.078637	1.166725	2.12019	1.0842	0.238677	0.960177
RFWD3	1.242977	1.173521	1.265554	1.53039	0.6139	0.238717	0.995479
FBXW9	1.270379	1.20007	1.293234	1.46566	0.5515	0.238827	0.896532
RP5-1074L1.4	1.165429	1.098398	1.187218	1.90266	0.928	0.238866	1
KITLG	1.337427	1.265412	1.360836	1.35953	0.4431	0.239055	1
UHRF1BP1L	1.198608	1.130366	1.220791	1.69363	0.7601	0.239205	0.884708
ZMYM5	1.609945	1.531441	1.635463	1.19574	0.2579	0.239306	0.896532
ZNF420	1.222497	1.153746	1.244845	1.59253	0.6713	0.239323	0.970803
WHAMM	1.358476	1.286236	1.381958	1.33441	0.4162	0.239611	0.971004
RGL3	1.21632	1.147094	1.238823	1.62361	0.6992	0.239729	0.896532
DOK4	1.282544	1.352382	1.259843	0.73739	-0.4395	0.239984	0.960177
ARMT1	1.364883	1.437802	1.341181	0.7793	-0.3597	0.240052	0.981677
RP11-448A19.1	1.24778	1.178254	1.27038	1.51682	0.6011	0.240079	1
BBX	3.214287	3.083864	3.256681	1.08293	0.1149	0.240183	0.960177
KCNC3	1.229084	1.297935	1.206704	0.69379	-0.5274	0.240214	1
STRA6	1.9778	1.890373	2.006219	1.13011	0.1765	0.240237	0.885269
NCALD	1.405696	1.479059	1.381849	0.79708	-0.3272	0.240305	0.960177
ZNF85	1.155666	1.08873	1.177424	1.9996	0.9997	0.240392	0.94223
ZNF268	1.334571	1.262888	1.357872	1.36131	0.445	0.240395	0.891502
ASL	1.195539	1.127739	1.217577	1.7033	0.7683	0.240428	1
PEX19	1.533368	1.457186	1.558131	1.2208	0.2878	0.240829	0.89731
OLMALINC	1.119401	1.053534	1.140811	2.63031	1.3952	0.24083	0.94223

HEBP1	2.337568	2.22805	2.373167	1.11817	0.1611	0.241006	1
CCDC136	1.280215	1.20975	1.30312	1.44515	0.5312	0.241188	0.939315
RNF216	1.439134	1.513716	1.414891	0.80763	-0.3082	0.241192	1
PEAK1	1.30664	1.377522	1.2836	0.75121	-0.4127	0.241287	0.960177
KCTD11	1.238535	1.307438	1.216137	0.70303	-0.5083	0.241298	1
PDE4B	1.115795	1.050523	1.137011	2.71184	1.4393	0.241476	0.960177
FIGNL1	1.207832	1.139921	1.229907	1.64312	0.7164	0.241507	1
SPEF2	1.171764	1.104795	1.193533	1.84678	0.885	0.241534	0.926805
SLC44A2	1.387209	1.460137	1.363503	0.78999	-0.3401	0.241705	0.939315
KSR2	1.105164	1.040168	1.126291	3.14407	1.6526	0.2418	1
PTPN4	1.446275	1.371526	1.470572	1.26659	0.341	0.241949	0.926805
SESN1	1.327023	1.256006	1.350107	1.36758	0.4516	0.241962	0.926805
TCEAL2	1.529217	1.452517	1.554149	1.22459	0.2923	0.241972	1
SMAD6	1.083294	1.147846	1.062312	0.42146	-1.2465	0.241976	0.888422
CDC45	1.108926	1.043834	1.130085	2.96768	1.5693	0.242126	1
RP11-538P18.2	1.199343	1.131206	1.221492	1.68812	0.7554	0.242155	0.94223
NDFIP1	3.024407	2.868767	3.074998	1.11036	0.151	0.242296	0.926805
ARRDC4	1.230184	1.298462	1.207989	0.69687	-0.521	0.242478	1
CCDC58	1.336536	1.264911	1.359818	1.35826	0.4418	0.242582	0.94223
ASNS	1.369368	1.297003	1.39289	1.32285	0.4036	0.242648	1
EIF2B4	1.477456	1.552613	1.453026	0.81979	-0.2867	0.242734	1
LRRC73	1.207307	1.139178	1.229452	1.64863	0.7213	0.242796	0.939315
ZNF185	1.089647	1.153913	1.068757	0.44672	-1.1625	0.243107	0.902276
POU6F2	1.177141	1.110381	1.198842	1.80142	0.8491	0.243133	0.960956
RGMA	1.129204	1.194877	1.107856	0.55346	-0.8535	0.243535	1
POLR2K	2.887234	3.006107	2.848593	0.92148	-0.118	0.243582	0.960956
WDR11	1.663741	1.5837	1.689759	1.1817	0.2409	0.243912	1
GNG5	10.18754	10.83629	9.976661	0.91261	-0.1319	0.243913	0.901224
IL6ST	1.780008	1.697984	1.806671	1.15572	0.2088	0.243932	0.989671
PCDH9	4.199958	4.429692	4.125282	0.91124	-0.1341	0.24394	1
OSBPL9	1.831047	1.914331	1.803976	0.87931	-0.1856	0.243955	0.902276
OTULIN	1.28517	1.21527	1.307892	1.43026	0.5163	0.243975	0.989671
ESRP1	1.037515	1.10066	1.016989	0.16878	-2.5668	0.244046	0.939315
SPRR3	1.037749	1.101616	1.016989	0.16719	-2.5804	0.244046	0.939315
BRD7	1.741518	1.660519	1.767847	1.16249	0.2172	0.244128	0.885736
CEP104	1.373698	1.301234	1.397252	1.31875	0.3992	0.244132	0.930291
PITPNB	1.497259	1.421811	1.521784	1.23701	0.3069	0.244306	1
TIMM44	1.666308	1.587023	1.692081	1.17897	0.2375	0.244486	1
NGF	1.044336	1.107299	1.023869	0.22246	-2.1684	0.244633	0.939315
ZC3H12B	1.111584	1.046544	1.132726	2.8516	1.5118	0.244694	0.946779
AC007246.3	1.479032	1.404066	1.503399	1.24583	0.3171	0.244933	1
NUP54	1.376728	1.304645	1.400159	1.31353	0.3934	0.244957	0.989666
G0S2	1.079966	1.143949	1.059168	0.41104	-1.2827	0.245051	1
PCCB	1.222251	1.154234	1.24436	1.58435	0.6639	0.245204	1
SLC43A3	1.137597	1.071855	1.158967	2.21234	1.1456	0.245518	1

WDR19	1.278883	1.209458	1.301451	1.43919	0.5253	0.245646	0.888036
AMPD2	1.322505	1.393165	1.299536	0.76186	-0.3924	0.245673	1
TMEM206	1.248769	1.317564	1.226408	0.71295	-0.4881	0.245722	0.991711
ZNF334	1.278537	1.209117	1.301103	1.43988	0.5259	0.245737	1
RAD51C	1.579398	1.502382	1.604433	1.20313	0.2668	0.245752	0.940023
EVI5	1.587722	1.664696	1.562702	0.84656	-0.2403	0.245756	1
MTHFD1L	1.28667	1.355844	1.264184	0.74241	-0.4297	0.2458	0.992085
MMD	1.800204	1.718346	1.826812	1.15099	0.2029	0.245889	1
DNMT3B	1.147847	1.082209	1.169183	2.05798	1.0412	0.246073	0.966991
YIF1B	1.716152	1.796477	1.690042	0.86637	-0.2069	0.246201	0.948877
CHML	1.622549	1.5438	1.648147	1.19188	0.2532	0.246386	1
PEX1	1.313611	1.243253	1.336482	1.38326	0.4681	0.246425	0.948877
MBIP	1.441028	1.367291	1.464996	1.26602	0.3403	0.246445	1
AHDC1	1.367923	1.439333	1.344711	0.78462	-0.3499	0.246597	0.908704
LAGE3	3.521272	3.372952	3.569484	1.08282	0.1148	0.246989	1
DRC3	1.115502	1.050988	1.136473	2.67657	1.4204	0.246995	1
YAF2	1.915065	1.830355	1.9426	1.13518	0.1829	0.247023	1
DOK5	1.194967	1.128179	1.216676	1.69042	0.7574	0.247123	0.948877
MAN2A2	1.276616	1.346065	1.254041	0.73409	-0.446	0.247303	0.991711
MASTL	1.153226	1.087583	1.174564	1.99314	0.995	0.247691	1
COX6C	11.07139	10.33685	11.31016	1.10424	0.1431	0.24781	0.912269
ACAP2	1.741715	1.660775	1.768025	1.16231	0.217	0.247931	1
CTD-2095E4.5	1.169844	1.103255	1.191489	1.85454	0.8911	0.247993	1
CD200	1.488615	1.413617	1.512994	1.24026	0.3106	0.248069	1
PHF7	1.108418	1.044069	1.129334	2.93478	1.5533	0.248252	0.943952
MUC16	1.034248	1.096713	1.013943	0.14417	-2.7942	0.248276	0.993907
TOPORS	1.440213	1.366815	1.464072	1.26514	0.3393	0.248396	0.991711
LINC01139	1.230367	1.298169	1.208327	0.69869	-0.5173	0.248411	1
RHOU	1.448438	1.522587	1.424336	0.81199	-0.3005	0.24844	0.950114
RARS2	1.712163	1.632079	1.738195	1.16788	0.2239	0.248487	0.907996
WTAP	2.021016	1.932915	2.049654	1.12513	0.1701	0.248655	0.943952
ERGIC3	4.045893	4.20217	3.995094	0.93533	-0.0964	0.248677	1
WDPCP	1.136687	1.071633	1.157834	2.20336	1.1397	0.248765	0.973764
VAPB	1.470322	1.396375	1.494359	1.2472	0.3187	0.248889	0.94484
CARS2	1.38944	1.317585	1.412798	1.2998	0.3783	0.248931	0.993907
ZNRF3	1.161055	1.226847	1.139669	0.6157	-0.6997	0.24907	0.897189
TMEM155	1.19036	1.123407	1.212124	1.7189	0.7815	0.249112	0.907996
EDA2R	1.21381	1.14639	1.235725	1.61026	0.6873	0.249217	1
PPP6R2	1.41334	1.485638	1.389839	0.80274	-0.317	0.249424	0.95676
LINC00869	1.412342	1.339769	1.435932	1.28302	0.3595	0.249535	1
XPOT	1.674442	1.595175	1.700208	1.17647	0.2345	0.249705	0.991711
BLM	1.129883	1.065264	1.150888	2.31195	1.2091	0.249755	0.912732
EVA1C	1.043931	1.106149	1.023706	0.22333	-2.1627	0.24987	0.950114
LRRC28	1.43787	1.364928	1.46158	1.26485	0.339	0.2499	1
GPBP1L1	1.725777	1.645952	1.751725	1.16375	0.2188	0.250115	1

NMRAL1	1.735198	1.655472	1.761113	1.16117	0.2156	0.250279	0.950114
NECTIN4	1.037188	1.099493	1.016936	0.17022	-2.5545	0.25032	0.991711
ANKRD13B	1.140287	1.0751	1.161476	2.15015	1.1044	0.250376	0.993907
PDIK1L	1.151772	1.086454	1.173004	2.00112	1.0008	0.250642	0.912732
PCSK4	1.20984	1.142866	1.231611	1.62117	0.697	0.250661	0.912732
ARHGEF5	1.072427	1.135366	1.051968	0.38391	-1.3812	0.250889	1
KIAA1109	1.619346	1.696552	1.594249	0.85313	-0.2292	0.250963	1
SLFN13	1.132123	1.067503	1.153128	2.26847	1.1817	0.250995	0.991711
SEPHS1	1.498563	1.424514	1.522633	1.23113	0.3	0.251024	1
MRPL15	1.707807	1.628835	1.733478	1.16641	0.2221	0.251094	0.949626
SCN1B	1.07235	1.135269	1.051898	0.38367	-1.3821	0.251268	0.993907
CXorf23	1.163111	1.097344	1.184489	1.89523	0.9224	0.25148	1
HDAC9	1.293017	1.223387	1.315651	1.41303	0.4988	0.251493	0.951137
BNIP1	1.198643	1.132126	1.220265	1.66709	0.7373	0.251505	1
ACER3	1.242893	1.310335	1.220971	0.71204	-0.49	0.251573	0.991711
RHPN1	1.345933	1.275296	1.368894	1.33999	0.4222	0.251593	0.951512
HAUS8	1.156072	1.091271	1.177136	1.94077	0.9566	0.251711	0.924165
PHF19	1.371638	1.301176	1.394542	1.31001	0.3896	0.251929	0.913773
ST6GAL1	1.157704	1.223073	1.136455	0.61171	-0.7091	0.251938	1
NUP50	1.440629	1.368171	1.464182	1.26078	0.3343	0.252028	1
REEP6	1.626861	1.549681	1.651949	1.18605	0.2462	0.252053	0.993907
AP1M2	1.040153	1.102413	1.019914	0.19445	-2.3625	0.252076	0.915483
ASUN	1.360334	1.289532	1.383348	1.32403	0.4049	0.252185	0.993907
TSHZ1	1.26268	1.19436	1.284887	1.46577	0.5517	0.252253	0.982936
MLYCD	1.256636	1.325049	1.234398	0.72112	-0.4717	0.252364	0.991711
TAF9	2.010254	2.1016	1.980562	0.89013	-0.1679	0.252505	1
NFU1	1.840026	1.92144	1.813562	0.88293	-0.1796	0.252598	1
EMP1	1.299364	1.369157	1.276677	0.74948	-0.416	0.252637	0.951882
VEGFC	1.054942	1.117127	1.034729	0.29651	-1.7539	0.252672	1
C14orf132	1.581993	1.50599	1.606698	1.19903	0.2619	0.252765	0.913773
AGAP3	1.25365	1.321472	1.231604	0.72045	-0.473	0.252882	1
ERBB4	1.151434	1.086426	1.172565	1.99669	0.9976	0.252922	1
DKKL1	1.148336	1.083299	1.169476	2.03456	1.0247	0.253017	1
C11orf68	1.188139	1.254393	1.166603	0.6549	-0.6106	0.253062	0.982936
PXMP4	1.141554	1.076799	1.162602	2.11725	1.0822	0.253213	0.926298
ARFGEF3	1.256761	1.32441	1.234771	0.72369	-0.4666	0.253281	1
ZNF521	1.355972	1.285218	1.378971	1.32871	0.41	0.253442	0.966257
COMMD6	12.53081	13.04872	12.36246	0.94304	-0.0846	0.253597	1
SEC22A	1.186692	1.120875	1.208086	1.72149	0.7837	0.253859	1
NKAIN3	1.197426	1.130991	1.219021	1.67202	0.7416	0.253906	1
ZC3H12C	1.161992	1.096527	1.183271	1.89866	0.925	0.253922	1
NVL	1.212268	1.145829	1.233865	1.6037	0.6814	0.253925	1
GCH1	1.058539	1.120751	1.038317	0.31732	-1.656	0.254265	1
ZNF587	1.292855	1.361404	1.270573	0.74867	-0.4176	0.254273	0.915928
GALNT3	1.060532	1.123175	1.040169	0.32612	-1.6165	0.254285	0.926852

MKLN1	1.662816	1.585923	1.687811	1.17389	0.2313	0.254309	1
ZNF566	1.281589	1.212967	1.303895	1.42696	0.5129	0.254516	1
PDGFD	1.106199	1.169961	1.085472	0.50289	-0.9917	0.254625	0.959374
YAE1D1	1.288916	1.220581	1.311129	1.4105	0.4962	0.254631	1
RP4-816N1.6	1.118029	1.054257	1.138758	2.55741	1.3547	0.254668	1
LIPE	1.191515	1.125626	1.212932	1.69496	0.7613	0.254818	0.954701
IPO13	1.119344	1.183148	1.098604	0.53838	-0.8933	0.254899	1
NTS	1.15631	1.088388	1.178389	2.01824	1.0131	0.254957	1
SERPINA1	1.038711	1.100191	1.018726	0.1869	-2.4196	0.255082	1
TLE2	1.132273	1.068045	1.15315	2.25071	1.1704	0.255142	1
RP11-77P6.2	1.09741	1.034332	1.117914	3.43454	1.7801	0.255283	0.954701
WARS	1.277026	1.34479	1.254999	0.73958	-0.4352	0.255374	0.966257
PGGHG	1.28516	1.217404	1.307184	1.41297	0.4987	0.255624	1
PSMG1	1.526378	1.600765	1.502199	0.83593	-0.2585	0.255635	1
TTN-AS1	1.304158	1.235578	1.32645	1.38574	0.4707	0.255828	0.929108
CH507-154B10.1	1.10683	1.043581	1.127389	2.92303	1.5475	0.255904	1
TMEM64	1.251654	1.31903	1.229753	0.72016	-0.4736	0.255908	1
UBR4	1.366531	1.436417	1.343814	0.78781	-0.3441	0.256182	0.98791
SORBS2	1.611354	1.534716	1.636265	1.18991	0.2509	0.256299	0.966257
FASTK	1.723895	1.801505	1.698668	0.8717	-0.1981	0.256307	0.98791
TRIM44	2.182334	2.092509	2.211532	1.10894	0.1492	0.256309	0.962116
CSTF1	1.298179	1.230148	1.320293	1.39168	0.4768	0.256389	1
STK38	1.265408	1.332805	1.2435	0.73166	-0.4508	0.256647	1
PPP1R3C	1.185109	1.250485	1.163858	0.65416	-0.6123	0.256741	0.966257
DDI2	1.226744	1.293415	1.205072	0.69891	-0.5168	0.25695	1
TSPYL2	1.230639	1.163814	1.25236	1.54053	0.6234	0.256977	0.968235
RP11-314A20.2	1.095554	1.032775	1.11596	3.53804	1.823	0.257036	0.966257
COMMD7	2.041307	2.13274	2.011586	0.89304	-0.1632	0.257058	1
RERG	1.122205	1.058834	1.142805	2.42724	1.2793	0.257296	1
ARSD	1.27588	1.343374	1.25394	0.73954	-0.4353	0.257329	1
PKIG	2.285768	2.188217	2.317478	1.10879	0.149	0.25733	0.923389
KCTD16	1.31752	1.248244	1.340038	1.36978	0.4539	0.257384	1
USP12	1.203795	1.269564	1.182417	0.67671	-0.5634	0.257486	1
SHARPIN	1.977146	2.069201	1.947223	0.88592	-0.1748	0.257517	1
STOX1	1.107096	1.043965	1.127617	2.90269	1.5374	0.257577	1
CD40	1.135007	1.198657	1.114317	0.57545	-0.7972	0.257658	0.924807
C11orf80	1.250243	1.183505	1.271937	1.48191	0.5675	0.257669	0.966257
SUFU	1.131733	1.06799	1.152453	2.24227	1.165	0.258006	1
ZNF324	1.213476	1.147374	1.234964	1.59434	0.673	0.25817	0.934241
CHIC2	1.331246	1.39991	1.308927	0.77249	-0.3724	0.258269	1
DGCR6	1.438684	1.510555	1.415322	0.81347	-0.2978	0.258539	1
NDEL1	1.17909	1.24408	1.157965	0.64718	-0.6278	0.258546	1
KDM4A	1.268743	1.201357	1.290647	1.44344	0.5295	0.2588	0.967853
SUPT20H	1.920514	1.837485	1.947503	1.13137	0.1781	0.259114	1
TRIQK	1.539243	1.465165	1.563323	1.21102	0.2762	0.259138	1

FAM204A	2.440923	2.330981	2.476661	1.10945	0.1498	0.259242	1
MDGA1	1.089679	1.027343	1.109942	4.0208	2.0075	0.259371	0.966397
KIAA1841	1.355164	1.285787	1.377716	1.32167	0.4024	0.259384	0.969075
TRAM2-AS1	1.179802	1.114756	1.200946	1.75107	0.8082	0.259636	0.974605
KDM5C	1.555516	1.481086	1.57971	1.205	0.269	0.259892	1
GTF2H3	1.284012	1.216013	1.306115	1.41711	0.503	0.259999	0.923389
TRIM71	1.099005	1.036524	1.119314	3.26673	1.7078	0.260012	1
WDR35	1.172083	1.107253	1.193157	1.80095	0.8488	0.260186	1
DCK	1.504123	1.430864	1.527936	1.2253	0.2931	0.260186	1
ITPRIPL2	1.252399	1.319129	1.230708	0.72293	-0.4681	0.260363	1
POLD1	1.225474	1.159762	1.246833	1.545	0.6276	0.260376	0.923389
SNX16	1.172933	1.238032	1.151773	0.63761	-0.6492	0.260494	1
INO80C	1.554301	1.628358	1.530228	0.84383	-0.245	0.260502	0.923389
CCP110	1.504735	1.432256	1.528295	1.22218	0.2895	0.260558	0.931888
ALKBH4	1.413019	1.342343	1.435993	1.27355	0.3489	0.260619	1
GDPD5	1.052636	1.113976	1.032698	0.28688	-1.8015	0.260678	1
HNRNPUL2	1.408434	1.479357	1.385381	0.80395	-0.3148	0.260705	1
ZNF362	1.224774	1.290479	1.203416	0.70028	-0.514	0.260832	1
MYBPHL	1.042436	1.103244	1.02267	0.21958	-2.1872	0.260938	0.923389
RP11-867G23.8	1.135774	1.072198	1.15644	2.16683	1.1156	0.260988	1
ZMAT4	1.206237	1.140746	1.227525	1.61656	0.6929	0.261369	0.945816
ACD	1.472645	1.400857	1.49598	1.2373	0.3072	0.261702	1
PMM1	1.519525	1.446583	1.543235	1.21643	0.2826	0.262335	1
ELF1	1.396854	1.466412	1.374243	0.80239	-0.3176	0.26259	0.971421
EWSR1	2.07036	1.983944	2.098451	1.11638	0.1588	0.262651	0.971421
TIMM22	1.466426	1.538416	1.443024	0.82283	-0.2813	0.262665	0.982307
PFDN5	14.17556	13.59424	14.36452	1.06116	0.0856	0.262833	1
TRMT10B	1.289698	1.221815	1.311764	1.40552	0.4911	0.262903	0.94796
TMCO6	1.175034	1.11074	1.195933	1.76931	0.8232	0.262926	0.927159
TIMM29	1.258244	1.324767	1.23662	0.72858	-0.4568	0.262997	1
ZNF789	1.145176	1.081646	1.165827	2.03105	1.0222	0.26347	0.974498
ANKRD65	1.139908	1.076464	1.160531	2.09944	1.07	0.263705	0.971748
TUBGCP5	1.235969	1.17	1.257412	1.51419	0.5985	0.263765	1
CDC123	1.756579	1.679625	1.781593	1.15004	0.2017	0.263971	0.983168
AFF1	1.2518	1.317959	1.230294	0.72429	-0.4654	0.263986	1
RSRP1	2.596852	2.487201	2.632494	1.0977	0.1345	0.264037	0.974498
CLIC3	1.049161	1.1098	1.02945	0.26821	-1.8986	0.264123	0.974498
GSK3A	1.371749	1.440271	1.349476	0.79377	-0.3332	0.264233	0.947985
RCAN1	1.106106	1.168447	1.085841	0.5096	-0.9726	0.264321	0.95025
CUTA	7.002492	7.30896	6.902873	0.93563	-0.096	0.264483	0.95025
MYCBP	1.272323	1.206014	1.293877	1.42649	0.5125	0.264653	1
MLH1	1.347597	1.2789	1.369928	1.32638	0.4075	0.264668	0.974498
AC009403.2	1.294937	1.227658	1.316807	1.39159	0.4767	0.264849	0.983168
MAMDC4	1.123019	1.060431	1.143363	2.37233	1.2463	0.26485	1
EPHA2	1.268717	1.334415	1.247362	0.73969	-0.435	0.264886	1

PHLDB1	1.241214	1.306522	1.219985	0.71768	-0.4786	0.265156	1
CTDSPL	1.184323	1.248298	1.163527	0.65859	-0.6025	0.265174	1
PNPT1	1.357917	1.28877	1.380393	1.31729	0.3976	0.265581	0.947985
RNF34	1.377365	1.308233	1.399837	1.29719	0.3754	0.265591	1
RAD54L2	1.254772	1.188833	1.276205	1.46269	0.5486	0.26562	0.946625
SLC2A13	1.194982	1.259587	1.173981	0.67022	-0.5773	0.265626	0.975255
WDR48	1.405569	1.335512	1.428342	1.27668	0.3524	0.265722	1
MOCS2	1.910553	1.828721	1.937153	1.13084	0.1774	0.265737	0.947985
PLCG2	1.196092	1.261075	1.174969	0.67019	-0.5774	0.265979	1
CHMP5	2.636721	2.73995	2.603166	0.92139	-0.1181	0.266036	1
CLIC1	6.982772	7.303133	6.878637	0.93265	-0.1006	0.266067	0.974498
DHX57	1.213803	1.14909	1.234839	1.57515	0.6555	0.26611	1
NDUFA1	5.384534	5.667053	5.292699	0.91979	-0.1206	0.266333	1
NECTIN3	1.196174	1.260458	1.175278	0.67296	-0.5714	0.266354	1
AC084219.4	1.134103	1.071116	1.154577	2.17359	1.1201	0.266389	0.974498
KCNB2	1.146062	1.083021	1.166554	2.00617	1.0044	0.267043	1
TMED5	1.520554	1.592652	1.497118	0.8388	-0.2536	0.267096	1
LONRF2	1.142084	1.07918	1.162531	2.05269	1.0375	0.267421	1
RERE	2.079123	2.163726	2.051622	0.90367	-0.1461	0.267718	1
DNAJC30	1.34502	1.2769	1.367162	1.32598	0.4071	0.267854	0.990665
TRMT61B	1.291681	1.225177	1.313299	1.39135	0.4765	0.267891	1
IFI44L	1.076862	1.138009	1.056986	0.41292	-1.2761	0.267896	1
DDX39B	1.434439	1.363912	1.457364	1.2568	0.3298	0.268004	1
LUZP1	1.565677	1.638154	1.542118	0.84951	-0.2353	0.26818	1
RNF214	1.355597	1.286954	1.37791	1.31697	0.3972	0.268189	1
EPSTI1	1.06034	1.120999	1.040623	0.33573	-1.5746	0.268324	1
DAPK1	1.303352	1.370191	1.281626	0.76076	-0.3945	0.268372	1
FAM169A	1.377096	1.308199	1.399492	1.29622	0.3743	0.268553	1
RP11-452L6.1	1.104902	1.042936	1.125045	2.91239	1.5422	0.2687	1
THNSL1	1.169864	1.106266	1.190536	1.79301	0.8424	0.268811	1
TBL1X	1.572493	1.498956	1.596397	1.19529	0.2574	0.269003	0.961602
CRADD	1.241581	1.306866	1.22036	0.7181	-0.4777	0.269076	1
B3GNTL1	1.134079	1.071683	1.154361	2.15337	1.1066	0.269157	0.945813
PER3	1.091238	1.152632	1.071281	0.46701	-1.0985	0.269218	1
FLYWCH1	1.235145	1.300114	1.214027	0.71315	-0.4877	0.269274	0.979625
USP33	1.512231	1.584005	1.488901	0.83715	-0.2564	0.269555	0.961602
MTMR11	1.319357	1.251718	1.341344	1.35605	0.4394	0.269578	1
TOLLIP-AS1	1.130055	1.06781	1.150288	2.2163	1.1482	0.269708	1
ZNF84	1.531257	1.458667	1.554853	1.20971	0.2747	0.269738	0.979625
TIMM50	1.541344	1.613839	1.51778	0.84351	-0.2455	0.269748	0.958892
ABCB4	1.136716	1.074385	1.156977	2.11034	1.0775	0.269854	1
GLE1	1.166368	1.103331	1.186859	1.80835	0.8547	0.270219	1
MFSD1	1.187815	1.251601	1.16708	0.66407	-0.5906	0.270437	0.947001
RHOBTB1	1.542553	1.614844	1.519055	0.84421	-0.2443	0.270913	1
TRPC4	1.05748	1.117505	1.037969	0.32312	-1.6298	0.270964	1

COPB1	1.68381	1.7591	1.659336	0.86858	-0.2033	0.271026	0.990044
ANO5	1.133362	1.195591	1.113135	0.57842	-0.7898	0.271453	1
OCLN	1.214456	1.14967	1.235515	1.57356	0.654	0.27152	0.996755
SEPT9	2.15259	2.251957	2.12029	0.89483	-0.1603	0.271614	1
RP11-128I7.1	1.118629	1.057139	1.138617	2.42594	1.2785	0.27162	1
AKAP7	1.142306	1.079908	1.162588	2.03469	1.0248	0.271622	0.990044
ENOX2	1.26024	1.194774	1.281521	1.44537	0.5314	0.271792	1
RSPRY1	1.545554	1.618081	1.521979	0.84452	-0.2438	0.271945	0.948974
ARL5B	1.488246	1.41747	1.511252	1.22464	0.2924	0.271946	1
FGF2	1.137017	1.199417	1.116734	0.58537	-0.7726	0.27195	0.996755
ISOC1	1.406296	1.337507	1.428656	1.27006	0.3449	0.271953	1
C15orf52	1.057638	1.117439	1.038199	0.32527	-1.6203	0.27223	1
DUSP22	1.355718	1.288048	1.377714	1.31129	0.391	0.272393	0.968296
ITFG2	1.26264	1.197223	1.283904	1.43951	0.5256	0.272429	1
WDR59	1.380539	1.31187	1.40286	1.29176	0.3693	0.272473	0.996755
MCHR1	1.146422	1.08435	1.166599	1.97509	0.9819	0.272524	1
RFTN2	1.162765	1.10007	1.183144	1.83017	0.872	0.272726	1
LINC00672	1.273976	1.208561	1.295239	1.4156	0.5014	0.272888	1
FBXL2	1.304445	1.237619	1.326168	1.37265	0.457	0.273047	1
ZNF765	1.201853	1.137876	1.222649	1.61485	0.6914	0.273051	1
DTNBP1	1.391027	1.459365	1.368813	0.80288	-0.3167	0.273176	0.949972
ARPIN	1.223838	1.159294	1.244819	1.5369	0.62	0.273189	1
FAM212A	1.058016	1.118142	1.038472	0.32564	-1.6186	0.27325	1
NDUFA8	2.277427	2.183005	2.308119	1.10576	0.145	0.273357	1
TMEM199	1.277107	1.343608	1.255491	0.74355	-0.4275	0.273386	1
STK24	1.459686	1.529309	1.437054	0.82571	-0.2763	0.273436	0.965998
MYO9B	1.430956	1.361377	1.453573	1.25512	0.3278	0.273489	1
CCDC186	1.55633	1.627925	1.533058	0.84892	-0.2363	0.273781	1
POP4	1.432515	1.362783	1.455182	1.2547	0.3273	0.273802	1
AMHR2	1.038892	1.098323	1.019573	0.19907	-2.3286	0.27382	0.96994
SAR1B	1.364069	1.431307	1.342213	0.79343	-0.3338	0.273846	1
DOCK10	1.057496	1.117087	1.038125	0.32561	-1.6188	0.273859	1
YBEY	1.703332	1.627667	1.727927	1.15973	0.2138	0.274073	1
STC1	1.557092	1.628528	1.533871	0.8494	-0.2355	0.274103	1
ATP5J	7.419622	7.806977	7.29371	0.9246	-0.1131	0.27418	1
C10orf88	1.164947	1.101846	1.185458	1.82097	0.8647	0.274183	0.965998
PDP1	1.11378	1.174748	1.093963	0.5377	-0.8951	0.274365	0.996431
PIAS3	1.29372	1.359567	1.272316	0.75734	-0.401	0.274367	1
RP11-15A1.3	1.140077	1.078269	1.160167	2.04637	1.0331	0.274515	1
C17orf62	1.75463	1.677941	1.779558	1.14989	0.2015	0.274519	1
LEFTY1	1.088593	1.028059	1.108269	3.85866	1.9481	0.274628	0.965998
C11orf70	1.190967	1.127833	1.211489	1.65442	0.7263	0.274657	1
CDH11	1.587815	1.505994	1.614412	1.21427	0.2801	0.274699	1
KIF18B	1.082032	1.021819	1.101605	4.65661	2.2193	0.274754	1
RHOA	5.999425	5.741367	6.083308	1.07212	0.1005	0.274777	0.952245

PGP	1.950885	2.037788	1.922637	0.88904	-0.1697	0.274865	1
NOMO1	1.123885	1.185281	1.103927	0.56092	-0.8341	0.275166	1
ZNF195	1.438624	1.369498	1.461094	1.24789	0.3195	0.275352	1
SSX2IP	1.633625	1.707588	1.609582	0.86149	-0.2151	0.27538	1
STXBP5	1.163457	1.225685	1.143229	0.63464	-0.656	0.275567	1
CHDH	1.137375	1.075699	1.157423	2.0796	1.0563	0.275648	1
MYO1D	1.092486	1.152867	1.072859	0.47662	-1.0691	0.275737	1
TMTC1	1.19032	1.127086	1.210874	1.6593	0.7306	0.276093	1
C2orf74	1.411914	1.34314	1.434269	1.26557	0.3398	0.276192	1
OPA1	1.439799	1.50929	1.41721	0.8192	-0.2877	0.276467	1
PTCH1	1.13262	1.070801	1.152714	2.15694	1.109	0.276673	1
PROCA1	1.213907	1.150355	1.234565	1.56007	0.6416	0.276702	1
GPR87	1.037753	1.096908	1.018524	0.19115	-2.3872	0.276733	1
POP7	2.091942	2.007481	2.119397	1.11108	0.152	0.276804	1
IRAK1BP1	1.20839	1.14512	1.228956	1.5777	0.6578	0.276813	1
WDR86	1.324186	1.390587	1.302602	0.77474	-0.3682	0.276891	1
FAM185A	1.154764	1.092548	1.174988	1.89078	0.919	0.277026	1
ENPP2	1.087264	1.147484	1.06769	0.45896	-1.1235	0.277104	0.978129
HKDC1	1.048169	1.107141	1.028999	0.27067	-1.8854	0.277115	1
KLHDC2	2.299317	2.211141	2.32798	1.09647	0.1329	0.277371	1
PLD5	1.049871	1.108678	1.030756	0.283	-1.8211	0.277465	1
MYOM2	1.119822	1.058737	1.139678	2.37802	1.2498	0.277468	1
ZNF670	1.130141	1.068902	1.150047	2.17769	1.1228	0.277562	0.98569
PRR3	1.444234	1.375327	1.466633	1.24327	0.3141	0.277601	1
CPB1	1.041034	1.102488	1.021058	0.20547	-2.283	0.277618	0.973113
ZFYVE16	1.555158	1.626571	1.531945	0.84898	-0.2362	0.277623	1
FASTKD1	1.159734	1.097641	1.179918	1.84265	0.8818	0.277861	1
MTUS1	1.085267	1.145379	1.065727	0.45211	-1.1453	0.277864	1
DOCK5	1.050552	1.109724	1.031318	0.28543	-1.8088	0.277885	1
GLS	1.376613	1.443846	1.354759	0.79928	-0.3232	0.277995	1
LDLRAD4	1.212948	1.14958	1.233546	1.56135	0.6428	0.278123	1
GS1-124K5.4	1.352752	1.28587	1.374493	1.31001	0.3896	0.278349	1
MOSPD1	1.260166	1.325104	1.239057	0.73533	-0.4435	0.278381	1
DCUN1D5	1.936795	1.857382	1.962609	1.12273	0.167	0.278399	1
TUFT1	1.129863	1.191334	1.109882	0.57429	-0.8001	0.278604	1
MRPS25	1.79127	1.867908	1.766359	0.883	-0.1795	0.278786	1
INIP	1.427851	1.359159	1.450179	1.25342	0.3259	0.278851	1
TWSG1	1.712208	1.637073	1.736631	1.15627	0.2095	0.279037	1
TRAPPC11	1.222082	1.158653	1.2427	1.52976	0.6133	0.279113	1
RP11-544A12.8	1.182495	1.120278	1.202719	1.68542	0.7531	0.279199	1
DBX2	1.115213	1.054733	1.134872	2.46421	1.3011	0.279394	0.986418
FAM179B	1.465076	1.395634	1.487649	1.23258	0.3017	0.279411	0.975997
TM2D2	1.530796	1.459356	1.554017	1.20607	0.2703	0.279471	1
DYNC1I1	1.052124	1.111139	1.032941	0.2964	-1.7544	0.279731	0.986418
SPRYD4	1.251345	1.187489	1.272101	1.45129	0.5373	0.279738	1

MYADM	1.556908	1.628508	1.533633	0.84905	-0.2361	0.279796	1
MAPK8IP1	1.197399	1.134411	1.217874	1.62095	0.6968	0.279844	1
ESRRA	1.206764	1.269489	1.186375	0.69159	-0.532	0.27997	1
TEAD3	1.184761	1.247103	1.164497	0.6657	-0.5871	0.280036	1
MICAL1	1.126891	1.187779	1.107098	0.57034	-0.8101	0.280166	1
NANOS1	1.059099	1.11828	1.039862	0.33702	-1.5691	0.280835	1
INTS6	1.53745	1.465393	1.560872	1.20516	0.2692	0.2809	0.970118
CORO1C	1.583596	1.655653	1.560174	0.85438	-0.2271	0.280977	1
ZNF439	1.36286	1.296437	1.384452	1.29691	0.3751	0.281122	1
RP11-563K23.1	1.090462	1.030826	1.109847	3.56347	1.8333	0.281274	1
DSE	1.23785	1.301321	1.217218	0.72089	-0.4722	0.281463	1
HK1	1.723582	1.798222	1.699319	0.8761	-0.1908	0.281488	1
RPUSD4	1.265323	1.200937	1.286252	1.42459	0.5105	0.28154	0.980032
C5orf38	1.072736	1.132253	1.053389	0.40369	-1.3087	0.281553	1
TRAM1L1	1.147863	1.086514	1.167804	1.93961	0.9558	0.281851	1
LYRM7	1.406302	1.338933	1.428201	1.26338	0.3373	0.28198	1
RWDD4	1.668373	1.741097	1.644733	0.86997	-0.201	0.282074	1
AC013461.1	1.475001	1.544157	1.452521	0.8316	-0.266	0.282155	0.971114
STAM	1.249282	1.312947	1.228588	0.73044	-0.4532	0.282179	1
SLC22A23	1.13151	1.070948	1.151196	2.13108	1.0916	0.282254	1
SLITRK1	1.074537	1.015372	1.093769	6.10012	2.6088	0.282298	1
EPHA6	1.086184	1.026479	1.105591	3.98771	1.9956	0.282488	1
ARHGEF10	1.388217	1.455384	1.366384	0.80456	-0.3137	0.282612	1
ACTA1	1.05899	1.135334	1.034174	0.25251	-1.9856	0.282671	1
BORA	1.110369	1.050365	1.129873	2.57862	1.3666	0.28268	1
AP2A2	1.657059	1.583939	1.680828	1.16592	0.2215	0.282745	1
AMOT	1.233429	1.296561	1.212908	0.71792	-0.4781	0.28294	1
SYT11	1.678823	1.604932	1.702842	1.16185	0.2164	0.283024	1
LRRC20	1.26685	1.202686	1.287708	1.41947	0.5054	0.283182	0.995099
ASF1A	1.618071	1.545398	1.641693	1.17656	0.2346	0.283191	1
ARFGAP2	1.545014	1.474591	1.567905	1.19662	0.259	0.28345	1
BBS9	1.255406	1.191399	1.276212	1.44312	0.5292	0.283707	1
KAT14	1.1796	1.1177	1.199721	1.69686	0.7629	0.283818	1
MSRB3	1.299982	1.364436	1.279031	0.76565	-0.3852	0.284234	1
BAG4	1.313408	1.248478	1.334514	1.34625	0.4289	0.284321	1
ZNF500	1.190799	1.128434	1.211071	1.64342	0.7167	0.284359	1
ADAMTS3	1.106787	1.047104	1.126187	2.67889	1.4216	0.28442	1
SNX19	1.166551	1.227743	1.14666	0.64397	-0.6349	0.284518	1
STIM1	1.166277	1.227452	1.146392	0.64362	-0.6357	0.284737	1
IFT88	1.387902	1.32094	1.409669	1.27646	0.3522	0.284788	1
USP37	1.258706	1.195013	1.27941	1.43278	0.5188	0.284871	1
H2AFJ	2.437831	2.338735	2.470042	1.09808	0.135	0.284887	1
ATXN7	1.284674	1.220218	1.305626	1.38783	0.4728	0.285008	1
NBPF15	1.180101	1.118603	1.200091	1.68707	0.7545	0.285094	1
CXCR4	2.007123	2.226355	1.935861	0.76312	-0.39	0.285158	0.996032

RAB21	1.46035	1.528203	1.438295	0.82979	-0.2692	0.28536	1
KCTD5	1.247511	1.310938	1.226894	0.72971	-0.4546	0.285434	1
VIPR2	1.082595	1.023604	1.101771	4.3115	2.1082	0.285536	1
BOLA3-AS1	1.172502	1.111377	1.192371	1.72721	0.7884	0.2857	0.996032
SAMD4B	1.555523	1.485035	1.578436	1.19257	0.2541	0.285713	1
HMG20A	1.282169	1.21812	1.302988	1.38909	0.4741	0.285863	1
AKIRIN2	1.550837	1.621315	1.527928	0.8497	-0.235	0.285967	1
SFT2D1	1.957192	2.03607	1.931552	0.89912	-0.1534	0.285982	1
HTT	1.301728	1.23668	1.322872	1.36417	0.448	0.286003	1
RP11-545E17.3	1.147676	1.208239	1.127989	0.61463	-0.7022	0.286094	1
BACE1-AS	1.155603	1.094479	1.175471	1.85724	0.8932	0.28618	1
SIRT5	1.284409	1.220294	1.30525	1.38565	0.4706	0.286277	1
IGF2BP1	2.02846	1.948188	2.054552	1.11218	0.1534	0.286348	1
PATJ	1.193113	1.254858	1.173042	0.67897	-0.5586	0.286382	1
MORC2-AS1	1.16442	1.103191	1.184323	1.78623	0.8369	0.286392	1
IGFBPL1	1.081131	1.022261	1.100267	4.50414	2.1713	0.286513	1
SLC25A1	1.940505	2.018125	1.915274	0.89898	-0.1536	0.286658	1
TRIAP1	2.086975	2.003783	2.114017	1.10982	0.1503	0.286727	1
FAM222A	1.091657	1.032526	1.110877	3.40891	1.7693	0.286872	1
ELOVL7	1.048379	1.106195	1.029585	0.27859	-1.8438	0.286915	1
MPC2	2.746949	2.647303	2.779339	1.08015	0.1112	0.287023	1
DNAJC9-AS1.1	1.141486	1.081013	1.161143	1.98911	0.9921	0.287184	0.996232
HMGCL	1.51056	1.441046	1.533156	1.20885	0.2736	0.287247	1
RHEB	5.018643	5.241514	4.946198	0.93037	-0.1041	0.28733	1
JADE2	1.085782	1.144536	1.066683	0.46136	-1.116	0.287588	1
PDCD6	2.698109	2.58549	2.734717	1.09412	0.1298	0.287665	1
PKN3	1.117856	1.058312	1.137211	2.35305	1.2345	0.28771	0.996032
CTD-2366F13.1	1.116711	1.056996	1.136122	2.38825	1.256	0.287733	1
NCAPD3	1.204649	1.142482	1.224857	1.57814	0.6582	0.287781	1
E2F5	1.28657	1.222881	1.307273	1.37864	0.4632	0.28801	1
TGS1	1.353503	1.287954	1.37481	1.30163	0.3803	0.288075	1
TNFRSF10D	1.183607	1.244535	1.163802	0.66985	-0.5781	0.288082	0.996032
CEP78	1.767301	1.69269	1.791554	1.14273	0.1925	0.288335	1
CLIP3	1.531901	1.601059	1.509421	0.84754	-0.2387	0.288403	0.996032
CHST9	1.102424	1.043405	1.121609	2.80172	1.4863	0.28842	1
ZC3HC1	1.207393	1.145408	1.227541	1.56484	0.646	0.288515	1
SEPT11	2.359057	2.268072	2.388633	1.09507	0.131	0.28857	1
CCSER2	1.58972	1.659904	1.566907	0.85907	-0.2191	0.288867	0.998628
ZNF114	1.08042	1.021823	1.099468	4.55799	2.1884	0.28897	1
CCDC144NL-AS1	2.833727	2.699299	2.877423	1.10482	0.1438	0.289039	1
SYT14	1.066288	1.124248	1.047448	0.38188	-1.3888	0.289094	1
ID1	5.38396	5.022289	5.501523	1.11914	0.1624	0.28925	1
GPR161	1.304335	1.368287	1.283547	0.76991	-0.3772	0.289265	1
NME3	2.371339	2.47167	2.338726	0.90966	-0.1366	0.289341	1
ENOPH1	1.928156	1.844052	1.955495	1.13203	0.1789	0.289343	1

ETV2	1.133601	1.073615	1.1531	2.07973	1.0564	0.289439	1
SPEN	1.495401	1.426618	1.517759	1.21364	0.2793	0.289466	1
RASSF8-AS1	1.513631	1.444269	1.536178	1.20687	0.2713	0.289604	1
ABCB1	1.085109	1.026574	1.104136	3.91868	1.9704	0.28966	1
SNTB2	1.200834	1.262252	1.180869	0.68968	-0.536	0.289661	1
FBLN7	1.131477	1.071823	1.150868	2.10056	1.0708	0.289869	0.994259
FAM160B1	1.208079	1.269603	1.188081	0.69762	-0.5195	0.290023	1
BSN	1.080368	1.021914	1.099368	4.53455	2.181	0.290103	1
NOTCH3	1.473168	1.541154	1.451069	0.83353	-0.2627	0.29022	1
SHISA9	1.15416	1.093747	1.173797	1.85389	0.8906	0.290264	1
HTATSF1	1.852217	1.776476	1.876838	1.12925	0.1754	0.2903	1
MLX	1.387191	1.452931	1.365821	0.80768	-0.3082	0.290325	0.999234
SLC23A2	1.100292	1.159287	1.081115	0.50924	-0.9736	0.290415	1
DANT2	1.267965	1.204937	1.288452	1.40751	0.4931	0.290451	1
CAMK2A	1.066481	1.124542	1.047608	0.38227	-1.3874	0.290607	1
HUNK	1.098233	1.157259	1.079046	0.50265	-0.9924	0.290844	1
MAP3K12	1.194066	1.132245	1.214162	1.61943	0.6955	0.291013	1
WRN	1.278768	1.21581	1.299232	1.38655	0.4715	0.291033	1
TNFRSF4	1.073395	1.131207	1.054603	0.41616	-1.2648	0.291089	1
TCEANC2	1.198369	1.137162	1.218264	1.59128	0.6702	0.291119	1
ALDH2	1.553295	1.483641	1.575936	1.19083	0.252	0.291122	1
RP11-93H24.3	1.155629	1.095469	1.175185	1.835	0.8758	0.291133	1
CREBL2	1.305506	1.369741	1.284625	0.7698	-0.3775	0.291385	1
MYCBP2	1.645399	1.717478	1.621969	0.86688	-0.2061	0.291445	1
TMEM161B	1.228277	1.166231	1.248446	1.49458	0.5797	0.291528	1
AES	2.902092	2.789556	2.938673	1.08333	0.1155	0.291552	1
B3GNT2	1.10409	1.162985	1.084945	0.52119	-0.9401	0.291597	1
AP5M1	1.425038	1.491132	1.403554	0.82168	-0.2834	0.291621	1
C1GALT1	1.563882	1.633821	1.541148	0.85379	-0.2281	0.291624	1
TCF25	3.340792	3.209259	3.383548	1.07889	0.1095	0.291666	1
ZNF429	1.17049	1.109945	1.190171	1.72969	0.7905	0.291705	1
APC	1.42251	1.488865	1.400941	0.82015	-0.286	0.291903	1
PLXDC1	1.076436	1.134306	1.057625	0.42906	-1.2208	0.291926	1
ZNF488	1.134075	1.074285	1.15351	2.06651	1.0472	0.29194	1
TWF2	1.541874	1.611443	1.51926	0.84924	-0.2358	0.291971	1
EPS15	1.406039	1.472104	1.384564	0.81458	-0.2959	0.291979	1
KLF7	1.292813	1.229043	1.313542	1.36892	0.453	0.29199	1
SUSD1	1.049918	1.107335	1.031254	0.29118	-1.78	0.292079	1
ZSCAN1	1.243956	1.181665	1.264204	1.45435	0.5404	0.292107	1
ZNF592	1.118825	1.178028	1.099581	0.55936	-0.8382	0.29215	1
PAN2	1.120317	1.061007	1.139597	2.2882	1.1942	0.292211	1
GRIA1	1.098373	1.156904	1.079347	0.5057	-0.9836	0.292258	1
MANEA-AS1	1.210407	1.148976	1.230375	1.5464	0.6289	0.292343	1
MAP7D1	1.57267	1.642176	1.550076	0.85658	-0.2233	0.292605	1
NUP62CL	1.090759	1.03251	1.109694	3.3742	1.7545	0.292946	1

TAMM41	1.173936	1.113311	1.193642	1.70895	0.7731	0.292965	1
STAG1	1.513829	1.44494	1.536222	1.20516	0.2692	0.292994	1
ZBTB25	1.317117	1.252814	1.338019	1.33703	0.419	0.293165	1
NF2	1.337159	1.401498	1.316245	0.78766	-0.3444	0.293216	1
TMEM181	1.320977	1.384965	1.300177	0.77975	-0.3589	0.293246	1
EARS2	1.148564	1.088645	1.168041	1.89567	0.9227	0.293345	1
RFX3	1.552036	1.482623	1.574599	1.19057	0.2517	0.293373	1
TMEM191C	1.146248	1.205697	1.126923	0.61704	-0.6966	0.293508	1
MED9	1.215686	1.15428	1.235647	1.5274	0.6111	0.293581	1
ZNF664	1.487563	1.554868	1.465685	0.83927	-0.2528	0.293588	1
ARID4A	1.551651	1.482276	1.574202	1.19061	0.2517	0.293664	1
SFT2D2	1.373725	1.308371	1.394969	1.28082	0.3571	0.293757	1
C2orf76	1.296313	1.232954	1.316908	1.36039	0.444	0.293983	1
NLE1	1.16456	1.104435	1.184105	1.76286	0.8179	0.294213	1
SH3PXD2B	1.219061	1.280114	1.199215	0.71119	-0.4917	0.294221	1
PGAP2	1.369044	1.304016	1.390182	1.28343	0.36	0.294263	1
RP11-796E2.4	1.196947	1.135834	1.216812	1.59616	0.6746	0.294352	1
ANAPC4	1.204169	1.143035	1.224041	1.56634	0.6474	0.294679	1
MRTO4	1.487059	1.419776	1.50893	1.21239	0.2778	0.294782	1
CCNC	1.932401	1.855434	1.957419	1.11922	0.1625	0.294841	1
SSC5D	1.139751	1.199042	1.120478	0.60529	-0.7243	0.294844	1
JADE1	1.373117	1.3081	1.394251	1.27962	0.3557	0.294983	1
BLOC1S5	1.321657	1.257675	1.342454	1.32902	0.4104	0.294997	1
DNAJA2	1.610114	1.680691	1.587173	0.86261	-0.2132	0.295008	1
MIR222HG	1.143336	1.202859	1.123988	0.6112	-0.7103	0.295049	1
CIAO1	1.482398	1.414865	1.50435	1.2157	0.2818	0.295132	1
RECQL4	1.130658	1.071696	1.149823	2.08971	1.0633	0.295172	1
C5orf42	1.529061	1.460469	1.551358	1.19738	0.2599	0.295221	1
ZNF609	1.41604	1.481774	1.394673	0.81921	-0.2877	0.295538	1
CCDC159	1.209918	1.14898	1.229726	1.542	0.6248	0.295548	1
TMEM200A	1.05729	1.114285	1.038764	0.33918	-1.5599	0.295622	1
ZBTB44	1.731764	1.803892	1.708318	0.88111	-0.1826	0.295678	1
TRIM5	1.411737	1.345456	1.433282	1.25423	0.3268	0.295683	1
ACLY	1.540097	1.608144	1.517978	0.85174	-0.2315	0.295719	1
SFSWAP	1.572378	1.503164	1.594876	1.18227	0.2416	0.29578	1
COPS8	2.35404	2.262447	2.383813	1.09614	0.1324	0.295793	1
ERBIN	1.769476	1.695719	1.793452	1.14048	0.1896	0.29589	1
SPATA13	1.160446	1.100716	1.179862	1.78582	0.8366	0.295943	1
MAGEF1	2.421392	2.328372	2.451628	1.09279	0.128	0.295998	1
BCAS3	1.210359	1.149446	1.230159	1.54009	0.623	0.29601	1
PDIA4	2.780734	2.88199	2.74782	0.92871	-0.1067	0.296032	1
SPIN1	1.998368	2.076116	1.973095	0.90427	-0.1452	0.296065	1
GOSR1	1.421201	1.355135	1.442676	1.2465	0.3179	0.296073	1
PRPF31	1.978098	1.900336	2.003374	1.11444	0.1563	0.296153	1
ETS2	1.10746	1.166138	1.088386	0.53201	-0.9105	0.29628	1

TMEM117	1.180915	1.120292	1.200621	1.66779	0.7379	0.296427	1
BTG1	10.59957	11.01627	10.46412	0.94488	-0.0818	0.296454	1
DDX50	1.755162	1.682407	1.778812	1.14127	0.1906	0.296584	1
TMEM131	1.252086	1.190188	1.272206	1.43125	0.5173	0.296586	1
HELB	1.210215	1.14931	1.230012	1.5405	0.6234	0.296632	1
TBC1D31	1.142096	1.082779	1.161377	1.94949	0.9631	0.296714	1
NFYB	1.596534	1.526537	1.619287	1.17615	0.2341	0.296957	1
PIP4K2C	1.101523	1.159658	1.082625	0.51751	-0.9503	0.297084	1
ZNF786	1.123842	1.064979	1.142976	2.20033	1.1377	0.297116	1
AC006262.5	1.036632	1.09359	1.018118	0.19359	-2.3689	0.297249	1
CNTNAP1	1.10507	1.163127	1.086198	0.52841	-0.9203	0.297291	1
GNB4	1.500769	1.567903	1.478947	0.84336	-0.2458	0.297377	1
ZNF813	1.122787	1.064028	1.141888	2.21602	1.148	0.297491	1
RP11-37B2.1	1.174975	1.114982	1.194476	1.69136	0.7582	0.297775	1
LIMD1	1.132161	1.19095	1.113051	0.59204	-0.7562	0.297944	1
PTPN9	1.308673	1.371608	1.288215	0.77559	-0.3666	0.298116	1
METTL8	1.31187	1.248113	1.332594	1.3405	0.4228	0.298219	1
PEF1	1.891209	1.815622	1.915779	1.1228	0.1671	0.298294	1
RAPGEF6	1.13415	1.075162	1.153324	2.03991	1.0285	0.298317	1
PYCRL	1.173663	1.113646	1.193171	1.69976	0.7653	0.298382	1
MMP16	1.274485	1.212183	1.294737	1.38907	0.4741	0.298503	1
UFM1	2.217851	2.131638	2.245875	1.10095	0.1387	0.298714	1
TBC1D9B	1.527196	1.594653	1.505269	0.84969	-0.235	0.298736	1
PCYT1B	1.156848	1.097682	1.17608	1.80258	0.8501	0.29877	1
BOLA3	2.284391	2.370213	2.256495	0.91701	-0.125	0.298831	1
MIR4500HG	1.090434	1.032795	1.109169	3.3288	1.735	0.298921	1
OTOS	1.205678	1.30935	1.171979	0.55594	-0.847	0.298948	1
SLC35F2	1.166223	1.106533	1.185625	1.74243	0.8011	0.298982	1
SGCG	1.041288	1.097412	1.023044	0.23656	-2.0797	0.299049	1
NUTM2B-AS1	2.414122	2.327402	2.44231	1.08657	0.1198	0.299054	1
LINC01572	1.09334	1.035523	1.112134	3.15665	1.6584	0.299331	1
GTF3C1	1.248958	1.310223	1.229043	0.73832	-0.4377	0.299364	1
C6orf52	1.151497	1.092425	1.170699	1.84688	0.8851	0.299489	1
SYNJ2BP	1.719343	1.64661	1.742986	1.14905	0.2004	0.299567	1
RP11-16E12.1	1.132129	1.073471	1.151196	2.05791	1.0412	0.299714	1
KAT6A	1.49807	1.430925	1.519896	1.20646	0.2708	0.299867	1
MVD	1.283398	1.345732	1.263136	0.7611	-0.3938	0.29989	1
PGRMC1	4.523668	4.2793	4.603102	1.09874	0.1359	0.30002	1
JRK	1.221689	1.160823	1.241474	1.50149	0.5864	0.300092	1
RP11-732A21.3	1.10868	1.05063	1.12755	2.51924	1.333	0.300102	1
KDM4A-AS1	1.101213	1.043416	1.120001	2.76398	1.4667	0.300143	1
MDH2	3.584333	3.442786	3.630343	1.07678	0.1067	0.300249	1
PITX3	1.08471	1.141824	1.066144	0.46638	-1.1004	0.300272	1
STXBP1	1.404826	1.470863	1.38336	0.81416	-0.2966	0.300356	1
MORC2	1.181856	1.122029	1.201303	1.64964	0.7221	0.300361	1

BMP2	1.062009	1.11891	1.043512	0.36593	-1.4504	0.300383	1
PTPRB	1.05926	1.115749	1.040898	0.35334	-1.5009	0.300527	1
TRIM69	1.338988	1.275401	1.359657	1.30594	0.3851	0.300679	1
C16orf72	1.533459	1.601407	1.511372	0.85029	-0.234	0.300922	1
TSPAN7	1.102681	1.044907	1.121461	2.70474	1.4355	0.300976	1
ADAMTS8	1.064406	1.121279	1.045919	0.37863	-1.4012	0.300986	1
AC073283.4	1.21792	1.156902	1.237754	1.5153	0.5996	0.301053	1
CLMN	1.066959	1.12363	1.048538	0.39261	-1.3488	0.301359	1
NAA16	1.324352	1.260837	1.344998	1.32266	0.4034	0.301463	1
ZMIZ2	1.207322	1.267694	1.187698	0.70117	-0.5122	0.301473	1
MMAA	1.084499	1.027123	1.103149	3.80302	1.9271	0.301523	1
SF3A1	1.408478	1.343479	1.429607	1.25075	0.3228	0.30156	1
ADD3	1.68905	1.760283	1.665895	0.87585	-0.1912	0.301563	1
RP11-1055B8.4	1.140443	1.081445	1.159621	1.95985	0.9707	0.301706	1
USP10	1.701712	1.63095	1.724714	1.14861	0.1999	0.301824	1
PAPSS2	1.122262	1.179877	1.103534	0.57558	-0.7969	0.301985	1
SATB1	1.240103	1.178852	1.260013	1.45378	0.5398	0.30216	1
C19orf66	1.506723	1.438629	1.528858	1.20571	0.2699	0.302476	1
RP11-140K17.3	1.354557	1.290849	1.375265	1.29024	0.3676	0.302479	1
ZC3H15	2.865083	2.745493	2.903956	1.09078	0.1254	0.302779	1
CXXC1	1.33287	1.269554	1.353452	1.31124	0.3909	0.302815	1
ZNF346	1.25038	1.189141	1.270286	1.42902	0.515	0.302856	1
SYNE2	2.108083	2.196677	2.079285	0.9019	-0.149	0.303137	1
HMGCR	1.380333	1.444416	1.359502	0.80893	-0.3059	0.303379	1
MIR4453	1.118543	1.060518	1.137404	2.27048	1.183	0.303482	1
GDF10	1.064802	1.121122	1.046495	0.38387	-1.3813	0.303501	1
NDUFA9	1.598268	1.667184	1.575867	0.86313	-0.2123	0.303652	1
AR	1.194788	1.134841	1.214274	1.58909	0.6682	0.303692	1
COMMD9	1.469553	1.402898	1.49122	1.21922	0.286	0.303734	1
UBL4A	1.518714	1.585928	1.496866	0.848	-0.2379	0.303738	1
ALX3	1.037044	1.092913	1.018884	0.20324	-2.2987	0.303803	1
RNF157	1.195312	1.135698	1.214689	1.58211	0.6618	0.304016	1
GOLPH3L	1.266051	1.204677	1.286001	1.39733	0.4827	0.304055	1
NUMB	1.613394	1.543763	1.636028	1.16968	0.2261	0.30411	1
ARHGAP5	2.324972	2.231228	2.355444	1.10089	0.1387	0.304118	1
POLI	1.311103	1.24894	1.331309	1.33088	0.4124	0.304292	1
BTBD10	1.383494	1.447597	1.362656	0.81023	-0.3036	0.30452	1
COX7A2L	6.743587	7.000251	6.660157	0.94332	-0.0842	0.304736	1
HAP1	1.144655	1.086203	1.163655	1.89848	0.9248	0.304874	1
LNX1	1.137695	1.195513	1.1189	0.60815	-0.7175	0.304885	1
C16orf46	1.174061	1.115136	1.193215	1.67815	0.7469	0.304909	1
YIPF6	1.564262	1.632289	1.542149	0.85744	-0.2219	0.304994	1
TSEN34	2.800301	2.700218	2.832834	1.078	0.1084	0.305155	1
STS	1.084727	1.141854	1.066158	0.46638	-1.1004	0.305179	1
KDELR1	6.043914	5.782149	6.129002	1.07253	0.101	0.305189	1

UTP23	1.348622	1.418172	1.326014	0.77962	-0.3592	0.305551	1
FOSL1	1.04769	1.103639	1.029503	0.28467	-1.8126	0.305712	1
CYB561A3	1.237744	1.177014	1.257484	1.4546	0.5406	0.305785	1
AC012123.1	1.117564	1.059803	1.13634	2.27983	1.1889	0.305911	1
THTPA	1.455142	1.38978	1.476388	1.2222	0.2895	0.305912	1
MIPOL1	1.238997	1.178315	1.258722	1.45093	0.537	0.305967	1
DIRAS2	1.156284	1.097762	1.175307	1.7932	0.8425	0.305975	1
KCNAB2	1.095514	1.152434	1.077012	0.50522	-0.985	0.306029	1
SLMAP	1.498438	1.564931	1.476824	0.84404	-0.2446	0.306113	1
TMTC3	1.318387	1.380803	1.298099	0.78282	-0.3533	0.306194	1
GLYATL1	1.076542	1.020112	1.094885	4.71775	2.2381	0.30629	1
UBE2Q1	1.246289	1.306832	1.226609	0.73855	-0.4372	0.306343	1
ATP6V1A	1.44915	1.514278	1.427979	0.83219	-0.265	0.30646	1
LINC01011	1.133983	1.075818	1.15289	2.01653	1.0119	0.306562	1
TM2D3	1.700293	1.630073	1.723118	1.14767	0.1987	0.306885	1
GTF2H2C	1.303725	1.241817	1.323849	1.33923	0.4214	0.306887	1
FAM217B	1.541403	1.473975	1.56332	1.1885	0.2491	0.306908	1
ZNF781	1.198131	1.138806	1.217415	1.56632	0.6474	0.307085	1
PTCHD4	1.147333	1.205354	1.128473	0.62562	-0.6766	0.307093	1
SCYL1	1.36206	1.425268	1.341514	0.80306	-0.3164	0.307326	1
SRRM2	3.834879	3.668561	3.888942	1.08258	0.1145	0.307585	1
SPINK1	1.033536	1.088493	1.015672	0.1771	-2.4974	0.307769	1
TRIM73	1.235923	1.175301	1.255629	1.45822	0.5442	0.307941	1
FBXO9	1.625249	1.693705	1.602997	0.86924	-0.2022	0.307995	1
CNIH2	1.157361	1.098942	1.176351	1.78237	0.8338	0.308131	1
ZNF766	1.364581	1.300822	1.385307	1.28085	0.3571	0.30817	1
MTOR	1.207796	1.266999	1.188552	0.70619	-0.5019	0.308405	1
POLR1E	1.216316	1.156786	1.235667	1.50312	0.588	0.308429	1
PDE7B	1.122181	1.064842	1.14082	2.17173	1.1188	0.308526	1
B4GALT4	1.423062	1.487244	1.4022	0.82546	-0.2767	0.308586	1
PPP1R1C	1.113045	1.169776	1.094605	0.55723	-0.8437	0.308701	1
ZNF444	1.579282	1.511098	1.601446	1.17677	0.2348	0.308892	1
CLK4	1.330962	1.268633	1.351223	1.30744	0.3867	0.308917	1
POLR3F	1.209226	1.149477	1.228648	1.52965	0.6132	0.309016	1
SLC39A14	1.230773	1.290275	1.211431	0.72838	-0.4572	0.309031	1
AC004540.5	1.275517	1.336144	1.25581	0.76101	-0.394	0.309106	1
SKAP2	1.076523	1.13289	1.058201	0.43796	-1.1911	0.309114	1
TSC2	1.432678	1.368147	1.453654	1.23226	0.3013	0.309173	1
PLEKHA8	1.474683	1.408966	1.496045	1.21292	0.2785	0.309399	1
SRSF12	1.260803	1.200124	1.280527	1.40177	0.4872	0.309468	1
MTCL1	1.130351	1.187851	1.11166	0.59441	-0.7505	0.309695	1
PTPRG	1.593322	1.661703	1.571094	0.86307	-0.2125	0.309729	1
SIK3	1.193601	1.134327	1.212868	1.58471	0.6642	0.30979	1
CHCHD7	1.889351	1.815653	1.913307	1.11973	0.1631	0.309922	1
WDR83	1.396509	1.332248	1.417397	1.25628	0.3292	0.30999	1

KANSL1	1.486741	1.42097	1.50812	1.20702	0.2715	0.310238	1
PWP1	1.687775	1.617512	1.710615	1.15077	0.2026	0.310522	1
LMO3	1.059025	1.114609	1.040957	0.35736	-1.4845	0.310529	1
ANKRD11	2.258906	2.176994	2.285532	1.09222	0.1273	0.310698	1
AHSA1	1.992242	1.916288	2.016932	1.10984	0.1503	0.310759	1
MAP7	1.312908	1.250865	1.333075	1.32771	0.4089	0.310831	1
ATG4C	1.274129	1.213461	1.293849	1.37659	0.4611	0.31103	1
DOCK4	1.129125	1.071803	1.147757	2.05781	1.0411	0.31116	1
ANO4	1.038633	1.093627	1.020757	0.2217	-2.1733	0.311262	1
MT1X	2.324063	2.599684	2.234471	0.7717	-0.3739	0.311336	1
STARD9	1.211955	1.152552	1.231265	1.51598	0.6002	0.311366	1
PROCR	1.061842	1.11734	1.043802	0.37329	-1.4216	0.311701	1
WDFY2	1.307083	1.36818	1.287223	0.78012	-0.3582	0.311831	1
APPL2	1.229876	1.289295	1.210561	0.72784	-0.4583	0.311893	1
LINC00941	1.127566	1.070436	1.146136	2.07473	1.0529	0.312209	1
ICA1L	1.248494	1.188378	1.268036	1.42286	0.5088	0.312307	1
POLG2	1.195659	1.136945	1.214744	1.5681	0.649	0.312343	1
BICDL1	1.060978	1.116515	1.042925	0.36841	-1.4406	0.312388	1
MKS1	1.171991	1.113989	1.190845	1.67424	0.7435	0.312563	1
DDX42	1.540613	1.474336	1.562157	1.18515	0.2451	0.312758	1
JMJD8	1.38604	1.448918	1.3656	0.8144	-0.2962	0.312809	1
DYNC2LI1	1.601717	1.53411	1.623693	1.16772	0.2237	0.313012	1
SCOC	3.854059	3.689398	3.907583	1.08113	0.1125	0.313025	1
SPOCK2	1.238195	1.178818	1.257496	1.43999	0.5261	0.313116	1
TOM1L2	1.252266	1.192223	1.271783	1.41389	0.4997	0.313207	1
P3H3	1.348297	1.410309	1.328139	0.79974	-0.3224	0.313319	1
WWC2	1.302613	1.363634	1.282778	0.77764	-0.3628	0.31338	1
ACACA	1.273478	1.213146	1.293089	1.37506	0.4595	0.31365	1
ZRANB1	1.354182	1.416211	1.334019	0.80252	-0.3174	0.313776	1
KHDRBS3	1.147308	1.089789	1.166005	1.84884	0.8866	0.313894	1
ZSCAN26	1.240514	1.180747	1.259941	1.43815	0.5242	0.314083	1
ZNF219	1.543496	1.476855	1.565158	1.18518	0.2451	0.31412	1
NOL4L	1.493286	1.558134	1.472207	0.84605	-0.2412	0.314233	1
CEP295	1.330093	1.268979	1.349958	1.30106	0.3797	0.314235	1
RGS9	1.279295	1.340002	1.259562	0.76341	-0.3895	0.314249	1
COL14A1	1.036109	1.090611	1.018392	0.20298	-2.3006	0.31439	1
SETD6	1.199641	1.141011	1.218699	1.55094	0.6331	0.314663	1
BCL9L	1.113681	1.169912	1.095403	0.56149	-0.8327	0.314723	1
PDE12	1.186962	1.245031	1.168086	0.68598	-0.5438	0.314765	1
CRYGD	1.129204	1.072481	1.147643	2.03697	1.0264	0.314826	1
C1orf123	1.749781	1.679506	1.772624	1.13704	0.1853	0.315031	1
LYNX1	1.076209	1.131448	1.058254	0.44317	-1.1741	0.315199	1
SEC22C	1.793462	1.7215	1.816854	1.13216	0.1791	0.315301	1
ZMYND8	1.975529	1.899623	2.000203	1.1118	0.1529	0.315417	1
NEK9	1.285723	1.225445	1.305316	1.35428	0.4375	0.315442	1

AP2A1	1.78236	1.711225	1.805483	1.13253	0.1795	0.315617	1
GABRA5	1.041641	1.096031	1.023961	0.24952	-2.0028	0.315658	1
AADAT	1.266291	1.206351	1.285775	1.38489	0.4698	0.315671	1
WNT7B	1.086593	1.141903	1.068615	0.48353	-1.0483	0.31576	1
SOAT1	1.175782	1.233478	1.157028	0.67256	-0.5723	0.315965	1
NFYC	1.500808	1.435747	1.521956	1.19784	0.2604	0.316012	1
ROCK2	1.417362	1.353883	1.437996	1.23769	0.3076	0.316191	1
RP11-617F23.1	1.142035	1.085043	1.16056	1.888	0.9169	0.31624	1
MAP2K6	1.214638	1.156	1.233698	1.49806	0.5831	0.316449	1
SF3B1	2.384608	2.30182	2.411518	1.08427	0.1167	0.316477	1
CELF4	1.345769	1.40682	1.325925	0.80115	-0.3199	0.31652	1
LEMD2	1.35918	1.420782	1.339156	0.80601	-0.3111	0.316541	1
U2AF2	1.599892	1.532547	1.621783	1.16757	0.2235	0.316614	1
MRPL30	1.373848	1.311283	1.394185	1.26632	0.3406	0.31672	1
PURA	1.344118	1.282283	1.364218	1.29026	0.3677	0.316839	1
YY1	2.699127	2.804131	2.664994	0.92288	-0.1158	0.3171	1
EDNRB	1.065605	1.120564	1.04774	0.39597	-1.3365	0.317658	1
VWA1	1.402805	1.46593	1.382285	0.82048	-0.2855	0.317787	1
GLIS3	1.257179	1.31617	1.238003	0.75277	-0.4097	0.317995	1
DENR	2.627243	2.722863	2.596161	0.92646	-0.1102	0.318038	1
C1S	1.104564	1.160428	1.086406	0.5386	-0.8927	0.318371	1
OGDH	1.200678	1.259005	1.181719	0.7016	-0.5113	0.318387	1
U2AF1L4	1.190677	1.132701	1.209522	1.5789	0.6589	0.318771	1
C3orf67	1.232472	1.173584	1.251615	1.44953	0.5356	0.318806	1
SH3D19	1.315375	1.254914	1.335028	1.31428	0.3943	0.318968	1
HAS2	1.315563	1.255098	1.335218	1.31408	0.394	0.318968	1
TTLL12	1.307841	1.368563	1.288102	0.78169	-0.3553	0.318983	1
DAB1	1.208089	1.266837	1.188993	0.70827	-0.4976	0.318999	1
TMEM14B	4.112497	4.268446	4.061804	0.93678	-0.0942	0.319007	1
UHRF1BP1	1.211201	1.152669	1.230228	1.50802	0.5927	0.319265	1
IPP	1.163511	1.106522	1.182036	1.70889	0.7731	0.319276	1
SLC35F6	1.309071	1.369736	1.289352	0.78259	-0.3537	0.3194	1
UCHL3	1.498764	1.433364	1.520022	1.19997	0.263	0.319508	1
VTN	1.062806	1.117438	1.045047	0.38358	-1.3824	0.319649	1
USP34	2.286849	2.374969	2.258204	0.91508	-0.128	0.319678	1
SVBP	2.657038	2.543653	2.693895	1.09733	0.134	0.319819	1
WASH1	1.565312	1.498808	1.586929	1.17666	0.2347	0.319911	1
ZNF280C	1.111148	1.055332	1.129291	2.33664	1.2244	0.319976	1
COASY	1.667836	1.599266	1.690125	1.15162	0.2037	0.320071	1
AP1G1	1.345943	1.40689	1.326132	0.80152	-0.3192	0.320345	1
EPB41L4B	1.061772	1.116549	1.043966	0.37724	-1.4065	0.320367	1
THOP1	1.343501	1.282763	1.363244	1.28463	0.3613	0.320424	1
CXCL1	1.049163	1.103501	1.031501	0.30435	-1.7162	0.320452	1
KAT8	1.39961	1.337747	1.419719	1.2427	0.3135	0.320601	1
TNNC1	1.140854	1.197028	1.122594	0.62222	-0.6845	0.320719	1

VAC14	1.244482	1.303037	1.225448	0.74396	-0.4267	0.320802	1
UQCRH	12.47436	11.94136	12.64762	1.06455	0.0902	0.320823	1
DEC1	1.042005	1.095691	1.024554	0.2566	-1.9624	0.320867	1
RNF139-AS1	1.117756	1.061853	1.135928	2.19761	1.1359	0.32105	1
PDIA3	3.911934	4.077913	3.857982	0.92855	-0.107	0.321146	1
MRPL32	1.929149	2.002054	1.905451	0.9036	-0.1463	0.321238	1
DIP2C	1.277082	1.336758	1.257683	0.76519	-0.3861	0.321404	1
FAM212B	1.090259	1.035084	1.108194	3.08388	1.6247	0.321438	1
DCP1B	1.293135	1.233053	1.312665	1.3416	0.424	0.321552	1
HMOX2	1.546477	1.611772	1.525252	0.85857	-0.22	0.321647	1
LST1	1.095487	1.040338	1.113414	2.81161	1.4914	0.321834	1
SLC7A11	1.157966	1.100858	1.176529	1.75028	0.8076	0.322047	1
TRAF3IP1	1.254557	1.195327	1.27381	1.4018	0.4873	0.322294	1
NRXN2	1.242022	1.183387	1.261082	1.42367	0.5096	0.322331	1
CHAF1B	1.133485	1.077664	1.151631	1.95239	0.9652	0.322422	1
TLK2	1.347801	1.286604	1.367694	1.28294	0.3594	0.322461	1
MINA	1.199763	1.142343	1.218428	1.53452	0.6178	0.322813	1
YIPF5	1.623413	1.690054	1.601751	0.87203	-0.1975	0.322983	1
ZNF197	1.258063	1.19882	1.277321	1.39484	0.4801	0.323119	1
C9orf47	1.108994	1.05368	1.126974	2.36538	1.2421	0.323408	1
GPSM1	1.21909	1.277174	1.20021	0.72233	-0.4693	0.323465	1
KLHL20	1.950533	2.021817	1.927362	0.90756	-0.1399	0.323745	1
CTD-2186M15.3	1.141031	1.084947	1.159261	1.87483	0.9068	0.323766	1
CDC23	1.278349	1.219246	1.297561	1.3572	0.4406	0.323931	1
CABIN1	1.565039	1.499671	1.586287	1.17335	0.2306	0.323989	1
LINC01515	1.167302	1.110619	1.185727	1.67898	0.7476	0.324048	1
GPRIN3	1.063923	1.118205	1.046279	0.39151	-1.3529	0.324124	1
GLIS1	1.122891	1.178676	1.104758	0.5863	-0.7703	0.324138	1
ADAM22	1.131083	1.075291	1.149219	1.98191	0.9869	0.324259	1
TNFSF12	1.064402	1.118504	1.046816	0.39506	-1.3399	0.324274	1
DDX19A	1.326134	1.265552	1.345827	1.30229	0.3811	0.324306	1
LIMK2	1.438327	1.50084	1.418007	0.83461	-0.2608	0.324434	1
CCDC189	1.191971	1.134412	1.210681	1.56742	0.6484	0.324456	1
MAN1A2	1.974659	1.900998	1.998603	1.10833	0.1484	0.324494	1
CABP7	1.069968	1.015529	1.087664	5.64525	2.497	0.324567	1
SCFD1	1.78629	1.716465	1.808986	1.12914	0.1752	0.32463	1
BEX3	23.39936	24.01228	23.20012	0.96471	-0.0518	0.32471	1
ITPK1	1.155358	1.211815	1.137006	0.64682	-0.6286	0.324745	1
ZFP82	1.290623	1.231178	1.309946	1.34073	0.423	0.324797	1
DPY19L4	1.341168	1.40198	1.321401	0.79954	-0.3228	0.32486	1
DHX30	2.02097	1.946036	2.045327	1.10496	0.144	0.324932	1
C18orf54	1.158063	1.101942	1.176305	1.72947	0.7903	0.325016	1
TMEM50B	2.060562	1.985281	2.085033	1.10124	0.1391	0.325346	1
SH3BGRL	3.228754	3.113719	3.266147	1.07211	0.1005	0.325609	1
ITPR1	1.066552	1.120636	1.048971	0.40594	-1.3007	0.325699	1

HOXB6	1.105393	1.048328	1.123942	2.56459	1.3587	0.325893	1
TOPORS-AS1	1.669172	1.601789	1.691076	1.14837	0.1996	0.325957	1
GDF11	1.549534	1.614039	1.528567	0.8608	-0.2162	0.326086	1
ATPIF1	5.95366	6.176601	5.881191	0.94293	-0.0848	0.326149	1
CALB2	1.039592	1.093087	1.022203	0.23852	-2.0678	0.326312	1
SP4	1.152611	1.096414	1.170878	1.77234	0.8257	0.326403	1
CRNKL1	1.290635	1.231095	1.309988	1.34139	0.4237	0.326429	1
CLEC16A	1.112289	1.167481	1.094349	0.56334	-0.8279	0.326616	1
POLR1C	1.296825	1.237893	1.315981	1.32824	0.4095	0.326637	1
WWOX	1.376617	1.3157	1.396418	1.25568	0.3285	0.326816	1
RNF103	1.123236	1.178214	1.105364	0.59123	-0.7582	0.326991	1
PPM1G	2.871909	2.766337	2.906226	1.0792	0.11	0.327062	1
ALG14	1.211232	1.153691	1.229936	1.4961	0.5812	0.327178	1
KLHDC8A	1.209563	1.151575	1.228412	1.50693	0.5916	0.327182	1
NOC3L	1.380084	1.318541	1.400089	1.25601	0.3288	0.327202	1
LIN7A	1.512868	1.449445	1.533484	1.18698	0.2473	0.327266	1
PPP2R2A	2.024789	1.950033	2.049089	1.10427	0.1431	0.327402	1
PCBD2	1.193127	1.136358	1.211581	1.55165	0.6338	0.327467	1
CTC-378H22.2	1.068826	1.014816	1.086383	5.83044	2.5436	0.327647	1
POM121	1.36574	1.304721	1.385575	1.26534	0.3395	0.327805	1
CCNT2	1.476674	1.413897	1.49708	1.20097	0.2642	0.328142	1
PKMYT1	1.094568	1.040215	1.112236	2.79086	1.4807	0.328162	1
MFSD7	1.044923	1.098034	1.027659	0.28213	-1.8256	0.328204	1
SPATA18	1.152366	1.096387	1.170562	1.76955	0.8234	0.328475	1
IRF6	1.032597	1.085449	1.015417	0.18042	-2.4706	0.328476	1
WASF2	2.799958	2.926425	2.758849	0.91301	-0.1313	0.328551	1
HRC	1.088194	1.033876	1.105851	3.12465	1.6437	0.328653	1
P2RX6	1.087999	1.033645	1.105667	3.14067	1.6511	0.328653	1
SORCS2	1.136237	1.080859	1.154238	1.90748	0.9317	0.328952	1
USP6	1.073055	1.127382	1.055396	0.43488	-1.2013	0.328988	1
SAMD15	1.129808	1.074449	1.147802	1.98528	0.9893	0.329079	1
GXYLT1	1.436418	1.498741	1.416159	0.83442	-0.2612	0.3292	1
CHD6	1.731722	1.663953	1.75375	1.13525	0.183	0.329204	1
ARID3B	1.203169	1.146223	1.22168	1.51604	0.6003	0.329739	1
RAB34	3.702993	3.566221	3.747452	1.07062	0.0984	0.329773	1
LAMTOR2	3.112562	3.242837	3.070216	0.92303	-0.1155	0.329884	1
PPP2R3A	1.33316	1.272967	1.352727	1.2922	0.3698	0.329987	1
LRIG3	1.074073	1.127648	1.056657	0.44386	-1.1718	0.330021	1
CLCN4	1.249794	1.307366	1.23108	0.75181	-0.4116	0.330225	1
SPATA7	1.485737	1.422537	1.506281	1.19819	0.2609	0.330318	1
LLGL1	1.300128	1.240958	1.319362	1.32538	0.4064	0.330336	1
DAXX	1.393484	1.332483	1.413312	1.24311	0.314	0.33048	1
NFKBIL1	1.710874	1.643372	1.732815	1.13902	0.1878	0.33052	1
MIER1	1.790873	1.721347	1.813473	1.12771	0.1734	0.330655	1
SLU7	1.414553	1.476211	1.39451	0.82843	-0.2715	0.330658	1

PITX2	1.031739	1.084575	1.014564	0.17221	-2.5378	0.330701	1
DUSP18	1.270763	1.21211	1.289829	1.36641	0.4504	0.330753	1
HECA	1.129896	1.184918	1.11201	0.60573	-0.7233	0.330854	1
AP006621.5	1.310417	1.251366	1.329612	1.31128	0.391	0.331072	1
SIL1	1.374994	1.435991	1.355166	0.81462	-0.2958	0.331168	1
GRIN2B	1.094976	1.040712	1.112615	2.76613	1.4679	0.331377	1
MRPS16	2.670311	2.565179	2.704484	1.089	0.123	0.33138	1
METRNL	1.131856	1.18679	1.113999	0.61031	-0.7124	0.331622	1
NOL10	1.245323	1.187374	1.26416	1.4098	0.4955	0.33163	1
ADGRB1	1.046252	1.099094	1.029076	0.29342	-1.769	0.331852	1
STN1	1.482435	1.419883	1.502768	1.1974	0.2599	0.332022	1
SPTBN2	1.17374	1.229499	1.155615	0.67806	-0.5605	0.332131	1
FAHD1	1.379182	1.318805	1.398808	1.25095	0.323	0.332143	1
C16orf87	1.909563	1.980688	1.886444	0.9039	-0.1458	0.332176	1
TMEM263	1.861731	1.931545	1.839037	0.90069	-0.1509	0.332329	1
YPEL5	2.362747	2.45261	2.333536	0.91803	-0.1234	0.33247	1
TAF8	1.249923	1.192338	1.268641	1.39672	0.482	0.332567	1
CTB-31O20.2	1.227968	1.17062	1.246609	1.44537	0.5314	0.332968	1
PPP1CA	2.813943	2.92311	2.778457	0.92478	-0.1128	0.333012	1
NAP1L3	1.440002	1.502038	1.419838	0.83627	-0.258	0.333048	1
ZDHHC6	1.292507	1.233562	1.311667	1.33441	0.4162	0.33314	1
ETV3L	1.032832	1.085263	1.01579	0.18519	-2.4329	0.333197	1
VPS54	1.185383	1.241301	1.167207	0.69294	-0.5292	0.333294	1
ZFHX4-AS1	1.054171	1.107111	1.036963	0.34509	-1.535	0.333502	1
SLC35C1	1.184219	1.23979	1.166155	0.69292	-0.5292	0.33354	1
KIAA0907	1.588771	1.524244	1.609746	1.1631	0.218	0.33357	1
MIR7-3HG	1.084546	1.030825	1.102008	3.30928	1.7265	0.333778	1
TMCC3	1.097414	1.151063	1.079975	0.52942	-0.9175	0.334106	1
ABHD6	1.084713	1.138121	1.067353	0.48763	-1.0361	0.334114	1
PLEKHH1	1.20951	1.152527	1.228032	1.49503	0.5802	0.334149	1
MBOAT2	1.699802	1.767044	1.677945	0.88384	-0.1781	0.334156	1
ARIH1	1.56927	1.632633	1.548673	0.86728	-0.2054	0.334589	1
TNKS2	1.493647	1.556126	1.473338	0.85113	-0.2325	0.334605	1
GGA1	1.800567	1.731922	1.822881	1.12427	0.169	0.334613	1
RNF185	1.231738	1.174535	1.250332	1.43428	0.5203	0.334662	1
RGL1	1.11124	1.165452	1.093618	0.56583	-0.8215	0.334744	1
PPP1R32	1.111954	1.057899	1.129525	2.2371	1.1616	0.334952	1
CIB1	2.495588	2.588297	2.465452	0.92266	-0.1161	0.335186	1
HYOU1	1.371945	1.431858	1.352469	0.81617	-0.2931	0.335286	1
USO1	1.936447	1.862926	1.960345	1.11289	0.1543	0.335712	1
AHR	1.191858	1.247498	1.173771	0.70211	-0.5102	0.335713	1
KIAA2022	1.152487	1.097365	1.170405	1.75016	0.8075	0.335744	1
PTPN11	1.676292	1.741529	1.655087	0.88343	-0.1788	0.335775	1
FADS1	3.089223	2.982923	3.123776	1.07103	0.099	0.335786	1
NRAP	1.070628	1.017436	1.087918	5.04245	2.3341	0.335802	1

ZNF629	1.312949	1.254052	1.332094	1.30719	0.3865	0.335836	1
ASCC2	1.246306	1.30362	1.227675	0.74987	-0.4153	0.335841	1
PPY	1.079915	1.026539	1.097265	3.665	1.8738	0.336026	1
NPHP4	1.093833	1.040266	1.111245	2.7628	1.4661	0.336041	1
ZNF718	1.132899	1.078503	1.150581	1.91816	0.9397	0.336191	1
JTB	4.201916	4.35288	4.152843	0.94034	-0.0887	0.33628	1
SHD	1.091304	1.037704	1.108727	2.88366	1.5279	0.336312	1
C12orf45	1.612979	1.547873	1.634142	1.15746	0.211	0.336332	1
PLAUR	1.298083	1.35633	1.279149	0.7834	-0.3522	0.336468	1
RAB8B	1.353607	1.412875	1.334341	0.80979	-0.3044	0.3365	1
USF2	1.707807	1.774276	1.686201	0.88625	-0.1742	0.336552	1
PCIF1	1.324056	1.265424	1.343114	1.2927	0.3704	0.336717	1
RP11-451G4.2	1.03357	1.085879	1.016567	0.19291	-2.374	0.336807	1
MRPL46	1.294622	1.236194	1.313614	1.32778	0.409	0.33685	1
PRKCH	1.050859	1.103399	1.03378	0.3267	-1.614	0.336893	1
CYB5R4	1.349694	1.289671	1.369205	1.27457	0.35	0.337013	1
DHRS4L2	1.74186	1.674453	1.763771	1.13243	0.1794	0.337076	1
ARHGAP19	1.106055	1.052218	1.123556	2.36615	1.2425	0.337095	1
AC000068.5	1.075925	1.022666	1.093237	4.11358	2.0404	0.337116	1
LINC00982	1.077357	1.130412	1.06011	0.46093	-1.1174	0.337345	1
SF3B2	3.005421	2.889189	3.043203	1.08152	0.1131	0.337413	1
PSMG4	1.522721	1.459756	1.543189	1.18147	0.2406	0.337516	1
CTC-459F4.3	1.150767	1.096031	1.16856	1.75526	0.8117	0.338147	1
FKBP11	1.267895	1.324973	1.249341	0.76727	-0.3822	0.338299	1
EXOC3	1.157813	1.213117	1.139836	0.65615	-0.6079	0.338337	1
ZNF71	1.331977	1.273087	1.351119	1.28574	0.3626	0.338397	1
TKFC	1.240773	1.183646	1.259343	1.41219	0.4979	0.338446	1
C4orf46	1.346038	1.287609	1.36503	1.26919	0.3439	0.338455	1
UTP11	1.640254	1.574777	1.661538	1.15095	0.2028	0.338617	1
ZNF776	1.218886	1.162836	1.237106	1.4561	0.5421	0.338717	1
RAMP2-AS1	1.131409	1.077022	1.149087	1.93564	0.9528	0.338775	1
MEGF6	1.123218	1.177209	1.105668	0.59629	-0.7459	0.338816	1
AC009506.1	1.158653	1.103445	1.176598	1.70717	0.7716	0.338828	1
ITGA8	1.034552	1.086819	1.017563	0.20229	-2.3055	0.338969	1
TMEM232	1.122531	1.068412	1.140123	2.04821	1.0344	0.339003	1
PDE8A	1.140951	1.195321	1.123277	0.63115	-0.6639	0.339009	1
RAD51B	1.16149	1.106651	1.179316	1.68134	0.7496	0.339019	1
PWWP2A	1.360291	1.419719	1.340974	0.81239	-0.2998	0.339074	1
RIPK4	1.035142	1.087169	1.018231	0.20915	-2.2574	0.339113	1
AXIN1	1.141335	1.1959	1.123598	0.63092	-0.6645	0.339325	1
ZNF322	1.849846	1.780641	1.872342	1.11747	0.1602	0.339382	1
DIRAS3	1.041088	1.092772	1.024287	0.26179	-1.9335	0.339656	1
USP39	1.426335	1.365146	1.446225	1.22205	0.2893	0.339704	1
FAH	1.44648	1.507352	1.426693	0.84102	-0.2498	0.339808	1
SOS1	1.370381	1.429437	1.351184	0.81778	-0.2902	0.339952	1

FOXO1	1.120916	1.174203	1.103595	0.59468	-0.7498	0.339975	1
MMP7	1.050231	1.120013	1.027547	0.22954	-2.1232	0.340039	1
OSGIN2	1.131993	1.185892	1.114473	0.6158	-0.6995	0.340243	1
OGFRL1	1.45535	1.515075	1.435936	0.84635	-0.2407	0.340395	1
NIPAL4	1.030853	1.082241	1.014149	0.17204	-2.5392	0.340558	1
MESDC1	1.479183	1.540878	1.459129	0.84886	-0.2364	0.340777	1
C16orf74	1.06714	1.119769	1.050033	0.41774	-1.2593	0.340844	1
ZNF320	1.171428	1.226372	1.153568	0.67839	-0.5598	0.340858	1
EDEM1	1.14149	1.195943	1.12379	0.63176	-0.6625	0.34092	1
GATB	1.183857	1.128393	1.201887	1.57242	0.653	0.341035	1
PSMA5	2.237015	2.313076	2.212291	0.92325	-0.1152	0.341161	1
SBSPON	1.061523	1.113853	1.044514	0.39098	-1.3549	0.341182	1
ARHGEF35	1.079368	1.131979	1.062267	0.4718	-1.0838	0.341221	1
RANGAP1	1.329905	1.271556	1.348872	1.28471	0.3614	0.341738	1
BACH2	1.09025	1.142946	1.073121	0.51152	-0.9671	0.34174	1
CWF19L1	1.125594	1.071993	1.143018	1.98654	0.9903	0.341851	1
SCLY	1.132177	1.078124	1.149748	1.9168	0.9387	0.341886	1
PTPRK	1.3557	1.414169	1.336694	0.81294	-0.2988	0.342024	1
CNFN	1.201834	1.146248	1.219902	1.50363	0.5884	0.342085	1
ELOVL6	1.43242	1.371513	1.452218	1.21723	0.2836	0.342216	1
DNM1L	1.683989	1.618584	1.705249	1.1401	0.1892	0.342222	1
ZDHHC17	1.344409	1.285886	1.363432	1.27125	0.3462	0.342382	1
GPR155	1.278092	1.220529	1.296803	1.34587	0.4285	0.342428	1
CAMK2N1	1.295566	1.353293	1.276802	0.78349	-0.352	0.342517	1
ZNF282	1.104963	1.157907	1.087753	0.55572	-0.8476	0.342582	1
HRH1	1.044917	1.096563	1.02813	0.29131	-1.7794	0.342585	1
SOCS3	1.8946	1.975828	1.868197	0.8897	-0.1686	0.342682	1
SF3B4	1.434832	1.37453	1.454433	1.21334	0.279	0.342843	1
PCDHB2	1.32235	1.264172	1.341262	1.29182	0.3694	0.34291	1
WRAP53	1.190205	1.135194	1.208087	1.53917	0.6222	0.34292	1
SSR1	2.432476	2.517014	2.404996	0.92616	-0.1107	0.343104	1
AEBP1	2.07693	2.150471	2.053025	0.9153	-0.1277	0.343171	1
PIH1D2	1.108485	1.055136	1.125827	2.28211	1.1904	0.343264	1
CFD	1.125046	1.178589	1.107641	0.60273	-0.7304	0.343285	1
MEGF9	1.3298	1.387611	1.311008	0.80237	-0.3177	0.343356	1
DNASE1	1.384261	1.324506	1.403685	1.244	0.315	0.34336	1
C16orf86	1.182589	1.127498	1.200497	1.57255	0.6531	0.343392	1
STK4	1.637086	1.572407	1.658111	1.14972	0.2013	0.343416	1
SFXN4	1.34817	1.289556	1.367223	1.26823	0.3428	0.343557	1
DPF3	1.107335	1.053984	1.124678	2.30953	1.2076	0.343558	1
CD82	1.13157	1.18502	1.114196	0.61721	-0.6962	0.34356	1
SLF2	1.630159	1.566089	1.650986	1.14997	0.2016	0.343611	1
COQ10B	1.27255	1.328978	1.254208	0.77272	-0.372	0.343698	1
PPP4R4	1.069206	1.121371	1.05225	0.43049	-1.2159	0.343839	1
SULT1C2	1.13324	1.187002	1.115764	0.61905	-0.6919	0.344064	1

CSF1R	1.032771	1.083913	1.016146	0.19242	-2.3777	0.344245	1
PNPLA3	1.086946	1.139549	1.069847	0.50051	-0.9985	0.344298	1
BTG3	2.607483	2.705863	2.575504	0.92358	-0.1147	0.344345	1
RECQL5	1.111023	1.057786	1.128328	2.22076	1.1511	0.344374	1
SQLE	2.145314	2.226932	2.118784	0.91185	-0.1331	0.344482	1
UBALD2	1.229046	1.17295	1.24728	1.42978	0.5158	0.344613	1
LRRC6	1.089295	1.036584	1.106429	2.90916	1.5406	0.34467	1
SLC10A3	1.169723	1.223853	1.152127	0.67959	-0.5573	0.344972	1
AP3M1	1.423065	1.363289	1.442495	1.21802	0.2845	0.345037	1
RIC8A	1.686083	1.750301	1.665208	0.88659	-0.1737	0.345048	1
TMEM126A	1.718577	1.652877	1.739933	1.13334	0.1806	0.34506	1
CPNE3	2.505986	2.415351	2.535448	1.08485	0.1175	0.345251	1
FLJ46066	1.071346	1.019192	1.0883	4.60087	2.2019	0.345278	1
DRG1	1.612341	1.676118	1.591609	0.87501	-0.1926	0.345418	1
DBT	1.297677	1.2401	1.316392	1.31775	0.3981	0.345441	1
LRRC37B	1.315427	1.257804	1.334158	1.29617	0.3743	0.345444	1
CNP	1.702266	1.767424	1.681087	0.8875	-0.1722	0.345583	1
TPMT	1.330983	1.273126	1.34979	1.28069	0.3569	0.345689	1
SLC39A8	1.272343	1.215675	1.290763	1.34816	0.431	0.345773	1
RP11-343J3.2	1.068025	1.016045	1.084922	5.29272	2.404	0.345844	1
FKBP14	1.330978	1.388734	1.312204	0.80313	-0.3163	0.345853	1
GRWD1	1.212928	1.157562	1.230925	1.46562	0.5515	0.345869	1
ERVK3-1	1.276015	1.219428	1.294409	1.34172	0.4241	0.345951	1
FAM122C	1.111468	1.058322	1.128744	2.20748	1.1424	0.346139	1
GEMIN7	1.725942	1.791432	1.704654	0.89035	-0.1675	0.346186	1
MCPH1	1.196483	1.141461	1.214368	1.51538	0.5997	0.346187	1
WDR89	1.19267	1.137976	1.210449	1.52526	0.6091	0.346193	1
ZNF136	1.196039	1.14114	1.213884	1.5154	0.5997	0.346281	1
CDKN1B	1.412497	1.353232	1.431762	1.22232	0.2896	0.346398	1
ARHGAP42	1.113685	1.166748	1.096437	0.57834	-0.79	0.346517	1
SERPINB6	2.785165	2.671949	2.821967	1.08973	0.124	0.346575	1
PPARD	1.145794	1.199157	1.128448	0.64496	-0.6327	0.346848	1
DYRK1A	1.343642	1.40178	1.324744	0.80826	-0.3071	0.346878	1
IPO7	2.439307	2.353988	2.467041	1.0835	0.1157	0.346889	1
MAP4K4	1.811299	1.877473	1.789788	0.90007	-0.1519	0.347055	1
LTN1	1.445954	1.385716	1.465535	1.20694	0.2713	0.347071	1
GRPEL1	1.557878	1.495364	1.578199	1.16722	0.2231	0.347263	1
ARSG	1.201207	1.146037	1.219141	1.50059	0.5855	0.347451	1
NME8	1.140541	1.086944	1.157963	1.81684	0.8614	0.347507	1
TMED1	1.932068	1.860813	1.95523	1.10968	0.1501	0.347589	1
DENND1B	1.326298	1.268369	1.345128	1.28602	0.3629	0.347616	1
LRRC14	1.268971	1.212356	1.287374	1.35327	0.4364	0.347686	1
IFRD2	1.508258	1.568892	1.488548	0.85877	-0.2197	0.347691	1
PODXL	1.10012	1.152714	1.083024	0.54366	-0.8792	0.347743	1
POPDC3	1.030793	1.081775	1.014221	0.17391	-2.5236	0.347792	1

PPM1E	1.139345	1.085926	1.15671	1.82378	0.8669	0.347864	1
PDE10A	1.089216	1.036872	1.106231	2.88109	1.5266	0.347889	1
C19orf25	1.974334	1.904053	1.99718	1.10301	0.1414	0.347904	1
TSTD1	2.637827	2.74168	2.604068	0.92099	-0.1187	0.348003	1
ZNF674-AS1	1.132423	1.079236	1.149712	1.88945	0.918	0.348123	1
ZNF790	1.270007	1.213664	1.288322	1.34942	0.4323	0.348237	1
MYO5B	1.254338	1.310069	1.236222	0.76184	-0.3924	0.348318	1
PRR14	1.495672	1.4342	1.515654	1.1876	0.248	0.348382	1
C17orf82	1.073472	1.021443	1.090385	4.21514	2.0756	0.348477	1
PPME1	1.519629	1.458343	1.53955	1.17718	0.2353	0.348493	1
SERPINE2	1.609614	1.694943	1.581877	0.8373	-0.2562	0.348528	1
AC093375.1	1.131661	1.078288	1.14901	1.90336	0.9285	0.348541	1
LL21NC02-21A1.1	1.063739	1.115372	1.046955	0.40699	-1.2969	0.348686	1
ACTR6	1.294832	1.238375	1.313184	1.31383	0.3938	0.348724	1
PHACTR1	1.287334	1.230919	1.305672	1.32372	0.4046	0.348836	1
RP11-60L3.6	1.09286	1.040695	1.109817	2.69853	1.4322	0.348963	1
CFAP65	1.062687	1.114017	1.046002	0.40347	-1.3095	0.349111	1
GBA2	1.211272	1.26626	1.193397	0.72635	-0.4613	0.349198	1
HECTD2	1.251225	1.195119	1.269462	1.38101	0.4657	0.34935	1
MRPL21	2.135784	2.062724	2.159533	1.09109	0.1258	0.349363	1
PFKFB2	1.076775	1.128491	1.059965	0.46669	-1.0995	0.349431	1
TBL1XR1	2.202691	2.27664	2.178653	0.92325	-0.1152	0.349458	1
M6PR	1.782459	1.716661	1.803847	1.12166	0.1656	0.349622	1
SNAPIN	2.18217	2.105184	2.207195	1.0923	0.1274	0.34974	1
PMPCA	1.452078	1.392113	1.47157	1.20264	0.2662	0.349749	1
ZFP64	1.230365	1.174316	1.248584	1.42605	0.512	0.349858	1
ARIH2OS	1.104167	1.051717	1.121216	2.34382	1.2289	0.349873	1
SLC6A9	1.104089	1.051718	1.121113	2.34181	1.2276	0.349873	1
MAPK6	2.036109	2.110832	2.011819	0.91087	-0.1347	0.3501	1
C17orf49	1.185384	1.130839	1.203114	1.55239	0.6345	0.350121	1
HS2ST1	1.571149	1.50877	1.591426	1.16246	0.2172	0.350183	1
NOP14	1.228003	1.173088	1.245853	1.4204	0.5063	0.350225	1
CEP350	1.736692	1.671215	1.757976	1.12926	0.1754	0.350253	1
DENND6B	1.204445	1.149785	1.222213	1.48355	0.569	0.35038	1
B3GALNT2	1.366994	1.30872	1.385936	1.25012	0.3221	0.350442	1
LDOC1L	1.289799	1.232885	1.3083	1.32383	0.4047	0.350502	1
SSTR2	1.074486	1.022666	1.091331	4.0294	2.0106	0.350509	1
CYBRD1	1.227452	1.282332	1.209613	0.74244	-0.4297	0.350702	1
FAAP24	1.182336	1.128198	1.199934	1.55957	0.6412	0.350782	1
BRD2	2.356839	2.275356	2.383326	1.08466	0.1172	0.35117	1
MPZL1	1.993253	2.063274	1.970492	0.91274	-0.1317	0.351182	1
CBLL1	1.218803	1.163892	1.236652	1.44395	0.53	0.351393	1
MED24	1.393319	1.334995	1.412277	1.2307	0.2995	0.351421	1
PTEN	1.508232	1.56815	1.488755	0.86026	-0.2172	0.351485	1
PDGFRA	1.807261	1.879435	1.7838	0.89125	-0.1661	0.3516	1

PNPLA2	1.298003	1.354529	1.279628	0.78873	-0.3424	0.351602	1
SRPX	1.37901	1.437524	1.35999	0.82279	-0.2814	0.351785	1
ACOT13	1.861268	1.793762	1.883212	1.11269	0.1541	0.3518	1
KCNMB2	1.088111	1.036489	1.104892	2.87461	1.5234	0.3518	1
NACC2	1.133381	1.185883	1.116315	0.62574	-0.6764	0.351911	1
NHLH2	1.087572	1.035836	1.10439	2.91302	1.5425	0.352115	1
MON2	1.238467	1.18322	1.256426	1.39955	0.485	0.35223	1
MRPS26	3.166903	3.036621	3.209252	1.08476	0.1174	0.352266	1
BIRC6	1.671852	1.735171	1.65127	0.88588	-0.1748	0.35227	1
CLUL1	1.137542	1.190044	1.120476	0.63394	-0.6576	0.352369	1
SCML1	1.265456	1.209752	1.283563	1.3519	0.435	0.352437	1
EAF1	1.214044	1.15893	1.231959	1.45951	0.5455	0.352601	1
FAM127C	1.32776	1.270886	1.346247	1.2782	0.3541	0.352682	1
CBX8	1.135662	1.082694	1.152879	1.84872	0.8865	0.352692	1
MTSS1L	1.111011	1.163166	1.094057	0.57645	-0.7947	0.352694	1
METTL13	1.193372	1.139108	1.211011	1.51689	0.6011	0.352698	1
GTPBP10	1.17033	1.116547	1.187813	1.61148	0.6884	0.352718	1
SPIN3	1.219018	1.16418	1.236843	1.44258	0.5287	0.352719	1
CES2	1.66174	1.597561	1.682602	1.14231	0.192	0.352825	1
NOV	1.047909	1.098433	1.031486	0.31987	-1.6444	0.352825	1
FCHO2	1.197436	1.143083	1.215103	1.50335	0.5882	0.352861	1
MICALL1	1.080611	1.131916	1.063934	0.48466	-1.045	0.353076	1
CLYBL	1.103408	1.051195	1.12038	2.35139	1.2335	0.353103	1
C1R	1.098031	1.150049	1.081122	0.54063	-0.8873	0.35313	1
BAHD1	1.104974	1.157153	1.088013	0.56005	-0.8364	0.353422	1
RP11-505K9.1	1.127404	1.074942	1.144458	1.92759	0.9468	0.353826	1
TCF7L2	1.599398	1.536636	1.619799	1.15497	0.2079	0.353919	1
ZNF529	1.222612	1.167806	1.240427	1.43277	0.5188	0.353999	1
KIAA1211	1.114628	1.062199	1.13167	2.11691	1.082	0.354006	1
MARK1	1.125781	1.178018	1.108801	0.61118	-0.7103	0.354261	1
MAPK9	1.196161	1.24955	1.178807	0.71652	-0.4809	0.354268	1
POLG	1.286784	1.342483	1.268678	0.7845	-0.3502	0.354419	1
HES7	1.10668	1.054511	1.123638	2.26811	1.1815	0.354479	1
HAX1	2.195682	2.122457	2.219484	1.08644	0.1196	0.354538	1
SUN2	1.228395	1.173671	1.246183	1.41753	0.5034	0.354742	1
SLC37A3	1.339716	1.282378	1.358354	1.26906	0.3438	0.354777	1
CCDC59	1.87531	1.80869	1.896966	1.10916	0.1495	0.35512	1
RP11-1000B6.3	1.205467	1.151177	1.223114	1.47585	0.5615	0.355207	1
RAB5A	1.815315	1.880779	1.794036	0.90152	-0.1496	0.355221	1
GLT1D1	1.056306	1.1068	1.039893	0.37353	-1.4207	0.355253	1
KAT5	1.296325	1.240334	1.314525	1.3087	0.3881	0.355359	1
KLF9	1.102735	1.154195	1.086008	0.55778	-0.8422	0.355591	1
SGCE	1.274832	1.219279	1.29289	1.3357	0.4176	0.355607	1
JMY	1.204164	1.258158	1.186613	0.72286	-0.4682	0.355889	1
WDR26	1.527556	1.587561	1.508051	0.86468	-0.2098	0.355936	1

FASN	1.579022	1.517895	1.598892	1.1564	0.2096	0.355958	1
HSPA6	1.033409	1.083632	1.017084	0.20428	-2.2914	0.356076	1
TMEM44	1.130655	1.078115	1.147734	1.89124	0.9193	0.35661	1
NSUN6	1.340837	1.28393	1.359335	1.26557	0.3398	0.35667	1
REST	2.057922	2.136404	2.032411	0.90849	-0.1385	0.357003	1
EGLN1	1.995315	2.064342	1.972878	0.91406	-0.1296	0.357016	1
ACOX2	1.113572	1.061758	1.130415	2.11172	1.0784	0.357223	1
COL22A1	1.10824	1.159554	1.091561	0.57385	-0.8012	0.357278	1
ZNF436	1.217281	1.163035	1.234914	1.44088	0.527	0.357651	1
RP11-801F7.1	1.084703	1.033416	1.101375	3.03369	1.6011	0.357892	1
KIF26A	1.189659	1.136129	1.207059	1.52105	0.6051	0.358063	1
GMEB1	1.2567	1.201474	1.274652	1.36322	0.447	0.358178	1
RBM25	2.80126	2.899934	2.769186	0.93118	-0.1029	0.35854	1
PARP16	1.210111	1.155957	1.227714	1.46011	0.5461	0.358575	1
SYNJ2	1.083279	1.134253	1.06671	0.4969	-1.009	0.358669	1
TXNRD2	1.09836	1.14934	1.081789	0.54767	-0.8686	0.358742	1
C5	1.07912	1.028164	1.095684	3.39732	1.7644	0.358866	1
SMIM13	1.097878	1.14893	1.081283	0.54578	-0.8736	0.359022	1
STK19	1.391596	1.33425	1.410237	1.22734	0.2955	0.359083	1
ZNF573	1.153052	1.100387	1.170172	1.69516	0.7614	0.359447	1
VPS13A	1.493554	1.552752	1.474312	0.85809	-0.2208	0.359486	1
S100PBP	1.433931	1.374696	1.453185	1.20947	0.2744	0.35956	1
MIIP	1.425486	1.36663	1.444617	1.21271	0.2782	0.35964	1
PARP9	1.17169	1.118594	1.18895	1.59325	0.672	0.359705	1
HIF1AN	1.349859	1.293306	1.368242	1.25549	0.3282	0.360033	1
RALGPS1	1.11826	1.169712	1.101535	0.59828	-0.7411	0.360283	1
RP11-344P13.6	1.086811	1.035787	1.103396	2.88917	1.5307	0.360382	1
ABCD1	1.151294	1.098869	1.168334	1.7026	0.7677	0.360457	1
ZDHHC1	1.227566	1.173325	1.245197	1.41467	0.5005	0.360477	1
VEGFD	1.127119	1.075561	1.143878	1.90412	0.9291	0.360522	1
FNDC5	1.087872	1.036794	1.104475	2.83947	1.5056	0.36056	1
STAMBP	1.582643	1.521151	1.602631	1.15635	0.2096	0.360752	1
SPATS2	2.390794	2.307084	2.418004	1.08486	0.1175	0.360802	1
ZNF287	1.134878	1.082678	1.151846	1.83659	0.877	0.360809	1
CTB-50L17.10	1.609949	1.548675	1.629867	1.14798	0.1991	0.360958	1
DYM	1.640432	1.578136	1.660681	1.14278	0.1925	0.360971	1
CFAP20	1.737079	1.673554	1.757729	1.12497	0.1699	0.361157	1
C1orf131	1.424424	1.366296	1.443319	1.21028	0.2753	0.361736	1
LIN54	1.154743	1.102487	1.17173	1.67562	0.7447	0.361798	1
GDAP1	1.22205	1.168002	1.239618	1.42628	0.5123	0.362091	1
ZNF92	1.21412	1.160549	1.231533	1.44213	0.5282	0.362095	1
AP001258.4	1.094975	1.043878	1.111584	2.54303	1.3466	0.362112	1
ICOSLG	1.046505	1.096284	1.030324	0.31495	-1.6668	0.36218	1
ATOH7	1.088439	1.037257	1.105076	2.82031	1.4959	0.362525	1
TMEM55B	1.421007	1.478678	1.40226	0.84036	-0.2509	0.362746	1

ULK1	1.201322	1.254242	1.18412	0.72419	-0.4656	0.362802	1
RABGAP1	1.489429	1.548493	1.470229	0.85731	-0.2221	0.362829	1
XRN1	1.428083	1.369767	1.447039	1.20898	0.2738	0.362876	1
CYP2U1	1.177081	1.229601	1.160009	0.6969	-0.521	0.362927	1
EBF4	1.107995	1.159075	1.091391	0.57452	-0.7996	0.36293	1
CDC37	2.963311	2.853693	2.998943	1.07836	0.1088	0.362977	1
RB1	1.504336	1.563183	1.485207	0.86154	-0.215	0.363102	1
COBLL1	1.049808	1.099465	1.033667	0.33848	-1.5629	0.363127	1
OCIAD1	2.942106	3.040156	2.910234	0.93632	-0.0949	0.363181	1
R3HDM2	1.716925	1.653446	1.737559	1.12872	0.1747	0.363315	1
SCP2	3.277163	3.149919	3.318525	1.07842	0.1089	0.363363	1
INPPL1	1.385855	1.442902	1.367311	0.82933	-0.27	0.363513	1
RP11-629G13.1	1.095511	1.044803	1.111994	2.49968	1.3217	0.363633	1
C16orf62	1.289012	1.34425	1.271056	0.78738	-0.3449	0.363647	1
LIPE-AS1	1.202744	1.149526	1.220043	1.47161	0.5574	0.363865	1
TESMIN	1.152676	1.100783	1.169544	1.68226	0.7504	0.363937	1
KRBA2	1.13778	1.085671	1.154718	1.80596	0.8528	0.364044	1
ZNF544	1.477965	1.419092	1.497102	1.18614	0.2463	0.364105	1
VCPIP1	1.20475	1.151522	1.222052	1.46548	0.5514	0.364199	1
RP11-262H14.4	1.172925	1.120674	1.18991	1.57375	0.6542	0.364558	1
DHFR2	1.133406	1.081919	1.150142	1.83282	0.8741	0.364718	1
CLEC18B	1.078292	1.027961	1.094653	3.38516	1.7592	0.364735	1
RP11-469A15.2	1.13618	1.084488	1.152983	1.81072	0.8566	0.364748	1
SMIM5	1.034267	1.083513	1.01826	0.21865	-2.1933	0.364767	1
UPK1B	1.03433	1.083753	1.018265	0.21808	-2.197	0.364767	1
AC009501.4	1.127923	1.179011	1.111317	0.62185	-0.6854	0.3648	1
CADPS2	1.284357	1.22988	1.302065	1.31401	0.394	0.36486	1
C5orf15	1.804649	1.869243	1.783653	0.90154	-0.1495	0.364906	1
HEATR6	1.151627	1.099725	1.168498	1.68962	0.7567	0.365166	1
SMPDL3A	1.254443	1.200378	1.272017	1.35752	0.441	0.365266	1
RAP2C	1.723546	1.660153	1.744152	1.12724	0.1728	0.365276	1
GFM1	1.273971	1.328395	1.25628	0.7804	-0.3577	0.365276	1
CORO2A	1.044572	1.093799	1.02857	0.30459	-1.715	0.36528	1
GATA3-AS1	1.042113	1.091342	1.02611	0.28585	-1.8067	0.365298	1
LHX5-AS1	1.073838	1.023065	1.090342	3.91684	1.9697	0.365342	1
EEPD1	1.122589	1.173887	1.105914	0.60909	-0.7153	0.365439	1
ZNF654	1.274158	1.219235	1.292011	1.33196	0.4135	0.365492	1
PREX1	1.113829	1.0628	1.130417	2.0767	1.0543	0.365597	1
PRRC2B	2.464845	2.381843	2.491826	1.07959	0.1105	0.365618	1
ZNF254	1.424605	1.366904	1.443361	1.20838	0.2731	0.365624	1
CTCF	1.624091	1.563254	1.643866	1.14312	0.193	0.365766	1
CGGBP1	2.002511	1.934208	2.024713	1.09688	0.1334	0.365868	1
DDX59	1.191923	1.139122	1.209086	1.50289	0.5877	0.366016	1
AC159540.1	1.198329	1.14553	1.215492	1.48074	0.5663	0.366088	1
N4BP3	1.143581	1.19481	1.126929	0.65155	-0.618	0.366196	1

CGA	1.03285	1.082192	1.016811	0.20454	-2.2896	0.366203	1
PRMT5	1.341991	1.286613	1.359992	1.25602	0.3289	0.366213	1
THAP8	1.416016	1.358401	1.434744	1.21301	0.2786	0.366251	1
RMDN1	1.446156	1.387727	1.465149	1.19968	0.2627	0.366448	1
GJB2	1.033463	1.082258	1.017602	0.21399	-2.2244	0.366551	1
ZDHHC14	1.079038	1.128896	1.062831	0.48746	-1.0367	0.366632	1
ZNF558	1.117395	1.066334	1.133993	2.01998	1.0143	0.366991	1
NTPCR	2.32518	2.236698	2.353942	1.0948	0.1307	0.367021	1
CLDN11	1.166051	1.114081	1.182944	1.60363	0.6813	0.36705	1
DUSP11	1.324191	1.268793	1.342199	1.27309	0.3483	0.36706	1
SCD5	1.439762	1.382483	1.458381	1.19843	0.2611	0.367372	1
GREB1L	1.121724	1.070444	1.138392	1.96456	0.9742	0.367477	1
ORC3	1.349236	1.293841	1.367242	1.2498	0.3217	0.367566	1
DSCR3	1.479691	1.420922	1.498794	1.185	0.2449	0.367666	1
TTLL1	1.281875	1.227709	1.299482	1.3152	0.3953	0.367745	1
MFSD6	1.043152	1.092149	1.027225	0.29544	-1.7591	0.36797	1
GALK1	1.62242	1.682951	1.602744	0.88256	-0.1802	0.368119	1
LRP6	1.447609	1.38931	1.466559	1.19842	0.2611	0.368176	1
AKTIP	1.330471	1.385357	1.31263	0.81127	-0.3017	0.368192	1
FAM110A	1.119833	1.069188	1.136296	1.96994	0.9781	0.36824	1
POPDC2	1.050078	1.099146	1.034128	0.34422	-1.5386	0.368267	1
ZNF689	1.220543	1.167248	1.237866	1.42223	0.5082	0.368437	1
KDELR3	1.470877	1.528321	1.452204	0.85593	-0.2244	0.368765	1
OS9	1.634806	1.695844	1.614965	0.88377	-0.1783	0.36881	1
KRI1	1.146653	1.094885	1.163481	1.72293	0.7849	0.368875	1
IER2	2.437344	2.346845	2.466762	1.08904	0.1231	0.368896	1
EPHX1	1.25831	1.312542	1.240682	0.77008	-0.3769	0.368927	1
ZNF568	1.108186	1.057673	1.124606	2.16057	1.1114	0.370313	1
ATG12	2.206349	2.282445	2.181613	0.92138	-0.1181	0.370381	1
DZIP1L	1.134354	1.08339	1.15092	1.8098	0.8558	0.370461	1
TARBP1	1.303576	1.24913	1.321274	1.28958	0.3669	0.370666	1
DLG4	1.309396	1.25456	1.327221	1.28544	0.3623	0.370731	1
DHRS1	1.527636	1.586555	1.508484	0.8669	-0.2061	0.370754	1
DCP1A	1.536944	1.478057	1.556085	1.16322	0.2181	0.370756	1
FBF1	1.158125	1.106448	1.174923	1.64326	0.7166	0.370971	1
TMEM44-AS1	1.119026	1.068313	1.13551	1.98367	0.9882	0.371526	1
ASIC4	1.092962	1.042805	1.109266	2.55265	1.352	0.371593	1
FAM27C	1.111433	1.061102	1.127793	2.09145	1.0645	0.37169	1
INTS7	1.121733	1.071105	1.13819	1.94347	0.9586	0.371729	1
FAM102A	1.123974	1.174548	1.107535	0.61608	-0.6988	0.371768	1
TMEM254	1.407874	1.351173	1.426306	1.21395	0.2797	0.371897	1
UBXN8	1.201951	1.149179	1.219104	1.46873	0.5546	0.371908	1
LRFN4	1.12297	1.173114	1.106671	0.61619	-0.6986	0.371992	1
DNM2	1.303398	1.357741	1.285733	0.79871	-0.3242	0.37205	1
ARHGAP4	1.180148	1.128392	1.196972	1.53415	0.6174	0.372082	1

CCDC84	1.216171	1.268405	1.199193	0.74213	-0.4302	0.37215	1
EIF3A	3.297822	3.188537	3.333345	1.06617	0.0924	0.372162	1
NT5DC1	1.294491	1.348784	1.276843	0.79374	-0.3333	0.372368	1
ZC3H7B	1.314917	1.368887	1.297374	0.80614	-0.3109	0.37237	1
RUNDC3B	1.068951	1.019532	1.085015	4.35261	2.1219	0.372464	1
POLDIP2	1.888979	1.953142	1.868123	0.9108	-0.1348	0.372747	1
RP11-456O19.2	1.18043	1.128669	1.197255	1.53304	0.6164	0.372877	1
MTFR2	1.08628	1.036765	1.102375	2.78459	1.4775	0.372921	1
SLC25A28	1.088579	1.137884	1.072552	0.52618	-0.9264	0.373175	1
NCOR1	2.655038	2.562905	2.684986	1.07811	0.1085	0.373288	1
FBXL15	1.755458	1.691895	1.77612	1.12173	0.1657	0.373303	1
PRRG3	1.119692	1.069416	1.136035	1.95969	0.9706	0.373307	1
ARRDC3	2.025553	2.136421	1.989514	0.87073	-0.1997	0.373323	1
DPYD	1.098183	1.048283	1.114404	2.36945	1.2446	0.373376	1
RASSF2	1.132139	1.182871	1.115648	0.6324	-0.6611	0.373397	1
PHACTR4	1.683942	1.622594	1.703883	1.13057	0.177	0.373403	1
XPO5	1.29825	1.243894	1.315919	1.29531	0.3733	0.373648	1
POP1	1.156129	1.104994	1.17275	1.64533	0.7184	0.373848	1
HIVEP1	1.210256	1.262129	1.193394	0.73778	-0.4387	0.373877	1
POLR1D	3.731719	3.607063	3.77224	1.06336	0.0886	0.374192	1
YWHAG	2.23656	2.3179	2.21012	0.91822	-0.1231	0.374231	1
NR2C1	1.362775	1.307369	1.380785	1.23885	0.309	0.374505	1
PATL1	1.244792	1.297474	1.227667	0.76533	-0.3858	0.374637	1
DPP10-AS1	1.060129	1.108808	1.044306	0.40719	-1.2962	0.374812	1
TOE1	1.144735	1.094401	1.161096	1.70651	0.7711	0.374814	1
ZNF48	1.283469	1.22974	1.300933	1.30989	0.3894	0.374828	1
SYT13	1.073351	1.122648	1.057327	0.46741	-1.0972	0.374878	1
RP11-1275H24.3	1.080269	1.030643	1.0964	3.14592	1.6535	0.374903	1
CARMIL1	1.1547	1.205528	1.138178	0.67231	-0.5728	0.375005	1
BCS1L	1.24819	1.195253	1.265398	1.35925	0.4428	0.375197	1
LONP2	1.61345	1.672923	1.594118	0.88289	-0.1797	0.375274	1
ZBTB7B	1.087111	1.13623	1.071145	0.52224	-0.9372	0.375334	1
STAU2	1.609141	1.549133	1.628647	1.1448	0.1951	0.375536	1
SHB	1.185064	1.236251	1.168425	0.71291	-0.4882	0.375586	1
PHLDB3	1.164017	1.113017	1.180594	1.59794	0.6762	0.375711	1
ACO2	1.587034	1.527508	1.606383	1.14952	0.201	0.37587	1
GSTO2	1.40871	1.352994	1.426821	1.20915	0.274	0.37594	1
PDE1C	1.08498	1.133981	1.069051	0.51538	-0.9563	0.375968	1
SNCG	1.731032	1.822152	1.701413	0.85314	-0.2291	0.376067	1
SUGCT	1.104159	1.153903	1.087989	0.57172	-0.8066	0.376126	1
ABHD4	1.199394	1.251258	1.182535	0.72648	-0.461	0.376277	1
TMX2	1.473168	1.415987	1.491754	1.18214	0.2414	0.376341	1
RPAP1	1.205274	1.153349	1.222153	1.44868	0.5347	0.37635	1
MRPS35	1.707509	1.645681	1.727606	1.12688	0.1723	0.376458	1
GTF2I	4.463764	4.313346	4.512658	1.06015	0.0843	0.376584	1

SEMA4D	1.168638	1.11739	1.185296	1.57847	0.6585	0.376596	1
ZBTB24	1.183799	1.132232	1.200561	1.51673	0.601	0.376662	1
ITPKC	1.075143	1.124165	1.059208	0.47685	-1.0684	0.376697	1
CMAS	1.931342	1.995497	1.910488	0.91461	-0.1288	0.377021	1
STX2	1.291903	1.345105	1.27461	0.79573	-0.3297	0.37717	1
COA5	1.829614	1.765959	1.850306	1.11012	0.1507	0.377456	1
CCND3	1.351945	1.297091	1.369776	1.24466	0.3157	0.377456	1
C15orf48	1.037754	1.085483	1.022239	0.26016	-1.9425	0.377597	1
SNRNP35	1.343949	1.289506	1.361647	1.24919	0.321	0.377718	1
ZEB1	1.414311	1.358394	1.432487	1.20673	0.2711	0.377781	1
ZNF426	1.356238	1.30098	1.3742	1.24327	0.3141	0.377816	1
RP11-158I9.8	1.108718	1.059047	1.124863	2.11466	1.0804	0.377837	1
PCDHGB6	1.183734	1.234821	1.167128	0.71172	-0.4906	0.377891	1
TMEM143	1.132246	1.082108	1.148544	1.80913	0.8553	0.378103	1
RRP15	1.533128	1.47477	1.552097	1.16287	0.2177	0.37829	1
PROSER2	1.057718	1.106003	1.042022	0.39643	-1.3349	0.378377	1
AC004381.6	1.089161	1.039954	1.105156	2.6319	1.3961	0.37838	1
ZBTB20	2.269874	2.181649	2.298552	1.09893	0.1361	0.378452	1
NAA35	1.308347	1.254284	1.325921	1.28172	0.3581	0.378545	1
TNFRSF10B	1.792997	1.854331	1.77306	0.90487	-0.1442	0.378578	1
ZNF691	1.176951	1.12577	1.193588	1.53922	0.6222	0.378664	1
TXLNG	1.417736	1.361634	1.435972	1.20556	0.2697	0.37895	1
ZNF141	1.386051	1.330914	1.403973	1.22078	0.2878	0.37947	1
SETD4	1.341766	1.287576	1.35938	1.24969	0.3216	0.379478	1
CDKAL1	1.337921	1.283606	1.355577	1.25377	0.3263	0.379526	1
LIN9	1.12672	1.076851	1.142931	1.85985	0.8952	0.379561	1
RANBP2	1.491355	1.548672	1.472723	0.86158	-0.2149	0.379617	1
WDR55	1.276982	1.330425	1.25961	0.78569	-0.348	0.379643	1
ABHD10	1.201813	1.150446	1.21851	1.45241	0.5385	0.379716	1
MPV17L2	1.182118	1.232971	1.165588	0.71077	-0.4926	0.379734	1
ARHGEF12	1.729467	1.789879	1.70983	0.89866	-0.1542	0.379774	1
SKP1	9.167784	8.782909	9.29289	1.06553	0.0916	0.379836	1
RP13-1032I1.7	1.146251	1.095755	1.162665	1.69876	0.7645	0.379928	1
MTRF1L	1.567723	1.508821	1.586869	1.15339	0.2059	0.379943	1
CRYGC	1.05155	1.099443	1.035982	0.36184	-1.4666	0.380079	1
RCOR3	1.338797	1.392618	1.321303	0.81836	-0.2892	0.380282	1
SYT4	1.089048	1.039887	1.105028	2.63314	1.3968	0.380405	1
NUP210	1.210982	1.1593	1.227782	1.42989	0.5159	0.380414	1
NEIL3	1.073052	1.024479	1.088841	3.62932	1.8597	0.380426	1
CBLN1	1.07768	1.028889	1.09354	3.23798	1.6951	0.380461	1
ZNF585A	1.157629	1.107007	1.174084	1.62685	0.7021	0.38053	1
HINT3	1.496582	1.439794	1.515041	1.1711	0.2279	0.38053	1
SLK	1.449514	1.505578	1.43129	0.85306	-0.2293	0.380552	1
PPIL6	1.19429	1.142642	1.211079	1.47978	0.5654	0.380621	1
SKIV2L2	1.529568	1.471484	1.548449	1.16324	0.2181	0.380631	1

PCGF3	1.435658	1.491587	1.417479	0.84925	-0.2357	0.380828	1
THAP11	1.437662	1.493639	1.419466	0.84974	-0.2349	0.381062	1
PANK4	1.13188	1.081861	1.148139	1.80964	0.8557	0.381398	1
PTDSS2	1.366542	1.311392	1.384469	1.23468	0.3041	0.381404	1
RGS10	1.197284	1.146215	1.213884	1.4628	0.5487	0.38151	1
SDCCAG3	1.165553	1.215605	1.149283	0.69239	-0.5303	0.381747	1
KDM2A	1.303394	1.356536	1.28612	0.8025	-0.3174	0.381753	1
COQ10A	1.274633	1.221956	1.291756	1.31448	0.3945	0.381768	1
NPIPA1	1.222638	1.170643	1.239539	1.40374	0.4893	0.382065	1
NT5M	1.139294	1.089215	1.155572	1.7438	0.8022	0.382087	1
MIR24-2	1.106996	1.156112	1.091031	0.58311	-0.7782	0.382109	1
RP11-386I14.4	1.20325	1.151876	1.21995	1.44822	0.5343	0.382118	1
TIMP1	6.665133	6.885495	6.593503	0.95039	-0.0734	0.38233	1
TFR2	1.093385	1.044294	1.109342	2.46852	1.3036	0.382349	1
C1D	1.8341	1.896577	1.813791	0.90766	-0.1398	0.382364	1
CXorf40B	1.207024	1.258567	1.19027	0.73587	-0.4425	0.382791	1
GTF3C6	3.072538	2.963003	3.108144	1.07394	0.1029	0.382887	1
LMBR1	1.276024	1.223206	1.293193	1.31355	0.3935	0.382922	1
AKR1C2	1.117649	1.068325	1.133682	1.95656	0.9683	0.382932	1
BIK	1.046396	1.094187	1.030861	0.32766	-1.6097	0.38304	1
LRPPRC	1.623908	1.564872	1.643097	1.13848	0.1871	0.383045	1
IP6K3	1.049758	1.097388	1.034276	0.35195	-1.5066	0.383156	1
CCDC112	1.441608	1.385567	1.459824	1.19259	0.2541	0.383194	1
CTD-2147F2.1	1.148524	1.198523	1.132272	0.66628	-0.5858	0.383378	1
CTD-2256P15.2	1.136152	1.086226	1.152381	1.76722	0.8215	0.38351	1
MKRN1	2.394714	2.31448	2.420795	1.08088	0.1122	0.383594	1
TMEM121	1.297618	1.350404	1.28046	0.80039	-0.3212	0.383652	1
SLC2A11	1.420318	1.364308	1.438524	1.20372	0.2675	0.383746	1
ZNF616	1.149996	1.099839	1.1663	1.66568	0.7361	0.383833	1
GTPBP4	1.473466	1.530126	1.455048	0.85838	-0.2203	0.383843	1
KCNK6	1.066134	1.113966	1.050586	0.44387	-1.1718	0.38399	1
ACOX1	1.264052	1.315988	1.247169	0.78221	-0.3544	0.383993	1
FXYD1	1.38006	1.433916	1.362553	0.83554	-0.2592	0.383998	1
LAD1	1.031044	1.078333	1.015672	0.20006	-2.3215	0.384063	1
LAMTOR3	1.761727	1.6996	1.781922	1.11767	0.1605	0.384263	1
DIRC3	1.135007	1.085101	1.151229	1.77705	0.8295	0.384292	1
REXO1	1.108516	1.157164	1.092703	0.58985	-0.7616	0.38472	1
ABCC1	1.190601	1.240895	1.174252	0.72335	-0.4672	0.384742	1
CCNK	1.443531	1.498898	1.425534	0.85295	-0.2295	0.384758	1
SMARCAL1	1.135158	1.085604	1.151266	1.76704	0.8213	0.384765	1
POLR2B	1.84671	1.910694	1.825912	0.9069	-0.141	0.384843	1
TRPV1	1.085722	1.03695	1.101576	2.74899	1.4589	0.384849	1
CNOT6	1.423474	1.368105	1.441472	1.19931	0.2622	0.384906	1
KB-1125A3.11	1.100121	1.051058	1.11607	2.27328	1.1848	0.38515	1
NUBP1	1.309678	1.256404	1.326996	1.27532	0.3509	0.385254	1

MESDC2	2.286687	2.363263	2.261795	0.92557	-0.1116	0.385575	1
ZNF503-AS2	1.139175	1.188624	1.123101	0.65263	-0.6157	0.385702	1
XRCC3	1.110192	1.061448	1.126036	2.0511	1.0364	0.385735	1
RP11-392O17.2	1.123386	1.073924	1.139464	1.88658	0.9158	0.385778	1
NID1	1.519611	1.461826	1.538394	1.16579	0.2213	0.385877	1
GPSM3	1.160282	1.209855	1.144168	0.68699	-0.5416	0.385914	1
LSM14A	3.106437	2.995361	3.142544	1.07376	0.1027	0.385956	1
ZNF784	1.108531	1.059653	1.12442	2.08574	1.0606	0.385975	1
TFG	2.269544	2.353674	2.242197	0.91765	-0.124	0.386062	1
CENPP	1.085053	1.03681	1.100735	2.7366	1.4524	0.386123	1
IGF2R	1.279995	1.331944	1.263109	0.79263	-0.3353	0.386199	1
SLC9A6	1.11745	1.166409	1.101536	0.61016	-0.7127	0.386782	1
SHCBP1	1.081847	1.033545	1.097548	2.90794	1.54	0.386818	1
NOTCH4	1.100917	1.052251	1.116736	2.23414	1.1597	0.386821	1
ZNF506	1.260266	1.208102	1.277223	1.33215	0.4138	0.386868	1
HDAC6	1.380046	1.326067	1.397592	1.21936	0.2861	0.38707	1
RAB15	1.084129	1.132206	1.068502	0.51814	-0.9486	0.387219	1
AUH	1.266749	1.214854	1.283617	1.32005	0.4006	0.387278	1
C16orf91	1.406297	1.351817	1.424006	1.20519	0.2693	0.38745	1
THBS2	1.080556	1.128796	1.064875	0.50371	-0.9893	0.387524	1
POLE	1.146542	1.096798	1.162711	1.68094	0.7493	0.387602	1
RP3-473L9.4	1.068987	1.020965	1.084597	4.03526	2.0127	0.38773	1
TEAD4	1.103719	1.151962	1.088038	0.57934	-0.7875	0.387806	1
CTD-2287O16.5	1.107191	1.058276	1.123091	2.11223	1.0788	0.387941	1
ZBTB6	1.091052	1.139254	1.075383	0.54134	-0.8854	0.388181	1
CCDC130	1.43521	1.379984	1.453162	1.19258	0.2541	0.388182	1
MCOLN3	1.042936	1.090076	1.027613	0.30655	-1.7058	0.388206	1
RP11-212P7.2	1.148921	1.099544	1.164972	1.65728	0.7288	0.388295	1
ALKBH5	2.76873	2.685943	2.795641	1.06507	0.0909	0.388312	1
ZNF626	1.339891	1.39344	1.322485	0.81965	-0.2869	0.388367	1
CEP89	1.44693	1.391824	1.464843	1.18636	0.2465	0.388401	1
NPW	3.930624	4.085765	3.880194	0.93338	-0.0995	0.38882	1
CBFA2T2	1.4248	1.478781	1.407253	0.85061	-0.2334	0.389394	1
MTFR1L	1.807468	1.868533	1.787618	0.90684	-0.1411	0.389698	1
EPHB6	1.160956	1.111348	1.177082	1.59034	0.6693	0.389837	1
UBA6-AS1	1.188803	1.138873	1.205033	1.47641	0.5621	0.390185	1
TOX4	1.716146	1.775724	1.696779	0.89823	-0.1548	0.390245	1
FOXRED1	1.196605	1.146462	1.212905	1.45365	0.5397	0.39025	1
CHST10	1.153798	1.104036	1.169974	1.6338	0.7082	0.390318	1
NDUFA3	4.79466	4.964702	4.739387	0.94317	-0.0844	0.39058	1
UTP18	1.432301	1.377468	1.450125	1.19249	0.254	0.39063	1
NUS1	1.428479	1.482982	1.410762	0.85047	-0.2337	0.390645	1
POM121C	1.454386	1.398969	1.4724	1.18405	0.2437	0.390679	1
KBTBD7	1.242329	1.191025	1.259005	1.35587	0.4392	0.390937	1
DEPDC4	1.090792	1.042638	1.106445	2.49647	1.3199	0.390982	1

ZC4H2	1.288929	1.236844	1.305859	1.2914	0.3689	0.391031	1
THG1L	1.300318	1.248069	1.317301	1.27908	0.3551	0.391186	1
KRT23	1.030049	1.076396	1.014984	0.19613	-2.3501	0.391431	1
MAEA	1.490382	1.434402	1.508578	1.17075	0.2274	0.391568	1
COG8	1.200203	1.149749	1.216604	1.44644	0.5325	0.391569	1
TBC1D13	1.188968	1.238936	1.172726	0.7229	-0.4681	0.391578	1
SLC6A17	1.029891	1.076245	1.014824	0.19442	-2.3627	0.391606	1
CYB561D2.1	1.139343	1.090141	1.155337	1.72327	0.7852	0.391713	1
CDH24	1.137478	1.088424	1.153424	1.73509	0.795	0.391713	1
MOB1B	1.260891	1.31255	1.244098	0.78099	-0.3566	0.391766	1
TXLNA	1.428997	1.483939	1.411138	0.84957	-0.2352	0.391777	1
ENG	1.033309	1.079693	1.018231	0.22877	-2.1281	0.391795	1
GALNT14	1.033398	1.079891	1.018285	0.22888	-2.1273	0.391795	1
GEMIN5	1.105316	1.056992	1.121025	2.12355	1.0865	0.391803	1
EML6	1.108911	1.060467	1.124658	2.06159	1.0438	0.391812	1
SORBS1	1.100679	1.052514	1.116336	2.21531	1.1475	0.391833	1
CST6	1.030777	1.077155	1.015702	0.20352	-2.2968	0.391852	1
FIBIN	1.043964	1.09075	1.028757	0.31688	-1.658	0.391864	1
DIS3L2	1.281298	1.229249	1.298216	1.30084	0.3794	0.391958	1
SWI5	1.859563	1.920896	1.839626	0.91175	-0.1333	0.392004	1
IRX2	1.050048	1.097225	1.034712	0.35703	-1.4859	0.392267	1
BIRC3	1.046287	1.093014	1.031098	0.33433	-1.5806	0.392421	1
C5orf34	1.113579	1.065228	1.129296	1.98223	0.9871	0.392425	1
RASSF1	1.252138	1.200899	1.268794	1.33795	0.42	0.392445	1
ABLIM2	1.114846	1.066282	1.130632	1.97085	0.9788	0.392494	1
ADAM15	1.333413	1.38571	1.316414	0.82034	-0.2857	0.392501	1
SRSF4	2.408602	2.321009	2.437074	1.08786	0.1215	0.392504	1
ERVMER34-1	1.061804	1.108738	1.046548	0.42808	-1.2241	0.392798	1
CTD-3193K9.4	1.146543	1.097528	1.162476	1.66595	0.7363	0.392902	1
WDFY1	1.394041	1.447816	1.376562	0.84089	-0.25	0.392964	1
ADCY6	1.191097	1.240391	1.175073	0.72828	-0.4574	0.393013	1
NAV3	1.052893	1.09966	1.037691	0.3782	-1.4028	0.393149	1
SNX32	1.113545	1.065184	1.129265	1.98308	0.9877	0.393182	1
ASCC1	1.394039	1.340158	1.411554	1.20989	0.2749	0.39324	1
RSPH3	1.303344	1.250657	1.32047	1.27852	0.3545	0.393252	1
AC009133.12	1.125135	1.076616	1.140906	1.83913	0.879	0.393402	1
PEX6	1.207234	1.156944	1.223581	1.42459	0.5105	0.39341	1
AP000344.3	1.090796	1.042935	1.106353	2.47708	1.3086	0.393522	1
HAGH	1.994899	2.058094	1.974357	0.92086	-0.1189	0.393565	1
LINC00159	1.032621	1.078885	1.017583	0.2229	-2.1656	0.393578	1
AC137932.4	1.084751	1.036943	1.100291	2.71476	1.4408	0.393617	1
RAB30	1.220776	1.170233	1.237206	1.39341	0.4786	0.393664	1
DIDO1	1.473021	1.417493	1.49107	1.17624	0.2342	0.393674	1
HDDC2	4.392007	4.239273	4.441655	1.06248	0.0874	0.393757	1
SLC25A25	1.090206	1.137655	1.074782	0.54326	-0.8803	0.393837	1

GHITM	3.204231	3.10942	3.23505	1.05956	0.0835	0.393874	1
C2orf82	1.155588	1.106179	1.171649	1.6166	0.693	0.3939	1
NEK7	1.288566	1.340238	1.271769	0.79876	-0.3242	0.393948	1
DRAM1	1.08394	1.131109	1.068608	0.52329	-0.9343	0.394005	1
RIOK2	1.239963	1.189401	1.256398	1.35373	0.4369	0.394072	1
HID1	1.126499	1.174955	1.110748	0.63301	-0.6597	0.394136	1
UBE2L3	3.207497	3.331269	3.167265	0.92965	-0.1052	0.39422	1
KCTD2	1.140642	1.189105	1.124888	0.66042	-0.5985	0.394222	1
C14orf166	4.883802	4.748087	4.927918	1.04798	0.0676	0.394635	1
DNAJC21	1.523335	1.467187	1.541586	1.15925	0.2132	0.394672	1
MAATS1	1.170147	1.120949	1.18614	1.53899	0.622	0.394734	1
RP11-696N14.1	1.076715	1.029203	1.092159	3.1558	1.658	0.394787	1
AIFM1	1.31546	1.367224	1.298634	0.81322	-0.2983	0.394849	1
PBDC1	1.539148	1.482698	1.557497	1.15496	0.2078	0.394864	1
KLC3	1.040061	1.086652	1.024917	0.28755	-1.7981	0.394884	1
CCDC82	1.511056	1.455166	1.529224	1.16271	0.2175	0.395173	1
KSR1	1.091331	1.138828	1.075891	0.54666	-0.8713	0.395282	1
SSH3	1.195707	1.24517	1.179628	0.73267	-0.4488	0.395298	1
KIF1B	1.956465	1.887027	1.979036	1.10373	0.1424	0.395452	1
SEC24B	1.212973	1.262957	1.196726	0.74813	-0.4186	0.395517	1
SEPT2	4.423072	4.280169	4.469524	1.05773	0.081	0.395523	1
UBR5	1.846043	1.784883	1.865924	1.10325	0.1418	0.395589	1
C19orf73	1.140994	1.092226	1.156847	1.70068	0.7661	0.395606	1
RASSF7	1.513427	1.568161	1.495635	0.87235	-0.197	0.395627	1
CDK12	1.690961	1.631864	1.710171	1.12393	0.1686	0.396143	1
EDEM3	1.227991	1.277973	1.211745	0.76175	-0.3926	0.396212	1
OTUD4	1.248712	1.298944	1.232384	0.77735	-0.3634	0.396413	1
SNX14	1.459248	1.513575	1.441589	0.85983	-0.2179	0.396482	1
TWISTNB	1.339759	1.287301	1.35681	1.24194	0.3126	0.396494	1
NANS	1.465432	1.520111	1.447658	0.8607	-0.2164	0.396684	1
MGAT1	1.616774	1.559494	1.635393	1.13566	0.1835	0.396999	1
BARX2	1.035114	1.081918	1.019901	0.24293	-2.0414	0.397004	1
TMEM55A	1.669065	1.609625	1.688386	1.1292	0.1753	0.397028	1
RP11-77P16.4	1.084597	1.037128	1.100026	2.69408	1.4298	0.397055	1
ZER1	1.119628	1.167396	1.104101	0.62188	-0.6853	0.397101	1
LRBA	1.621549	1.564235	1.64018	1.1346	0.1822	0.397392	1
FOXO3	1.808569	1.746916	1.828609	1.10937	0.1497	0.397478	1
SLC25A32	1.112748	1.160354	1.097273	0.60662	-0.7211	0.397708	1
NFXL1	1.218395	1.168576	1.234589	1.39159	0.4767	0.398018	1
CHL1	1.112798	1.064696	1.128433	1.98517	0.9893	0.398154	1
NOL11	1.251963	1.201537	1.268355	1.33154	0.4131	0.398166	1
KIN	1.321434	1.269386	1.338353	1.25601	0.3289	0.398376	1
ETFDH	1.228485	1.178585	1.244705	1.37024	0.4544	0.398481	1
AP000769.1	1.156333	1.10753	1.172197	1.60138	0.6793	0.398505	1
ZNF318	1.207949	1.158433	1.224044	1.41413	0.4999	0.398533	1

ALG13	1.401848	1.454949	1.384587	0.84534	-0.2424	0.398558	1
RP11-544I20.2	1.086957	1.039796	1.102287	2.57027	1.3619	0.399118	1
GSTO1	2.782516	2.69327	2.811525	1.06984	0.0974	0.399528	1
FERMT1	1.050267	1.096433	1.03526	0.36564	-1.4515	0.399569	1
CCDC71L	1.138178	1.186382	1.122508	0.6573	-0.6054	0.399631	1
TSHZ3	1.105087	1.0576	1.120523	2.0924	1.0652	0.399676	1
OSBPL6	1.16376	1.114899	1.179643	1.56348	0.6448	0.399733	1
KAT2B	1.087799	1.134653	1.072568	0.53893	-0.8918	0.399794	1
SH2B3	1.060549	1.106655	1.045562	0.42719	-1.2271	0.399822	1
B4GALT2	1.301251	1.249641	1.318027	1.27394	0.3493	0.399837	1
GAS2L1	1.130245	1.177892	1.114757	0.64509	-0.6324	0.399862	1
COA6	1.638605	1.580998	1.65733	1.13138	0.1781	0.399923	1
AVPI1	1.24443	1.29438	1.228193	0.77517	-0.3674	0.399992	1
RMND5B	1.421182	1.367386	1.438669	1.19403	0.2558	0.399993	1
DHX9	2.15087	2.080953	2.173596	1.08571	0.1186	0.400105	1
ACO1	1.317277	1.265714	1.334038	1.25713	0.3301	0.400172	1
MXD1	1.108386	1.155574	1.093047	0.59809	-0.7416	0.400176	1
EPHA3	1.088126	1.040652	1.103558	2.5474	1.349	0.400177	1
CWC15	2.776727	2.686319	2.806115	1.07104	0.099	0.400669	1
TIGD4	1.120615	1.07296	1.136105	1.86547	0.8995	0.40072	1
ZBTB18	1.175211	1.22383	1.159407	0.71218	-0.4897	0.400792	1
FOXK2	1.196057	1.244734	1.180234	0.73645	-0.4413	0.401151	1
DGKB	1.041174	1.087061	1.026258	0.3016	-1.7293	0.40127	1
TARBP2	1.412911	1.359672	1.430216	1.19613	0.2584	0.401295	1
EPC2	1.237396	1.187259	1.253694	1.35478	0.4381	0.401323	1
CCDC97	1.261358	1.210905	1.277759	1.31699	0.3972	0.40133	1
C6orf203	1.40187	1.348652	1.419169	1.20225	0.2657	0.401422	1
RRP9	1.216764	1.266459	1.20061	0.75287	-0.4095	0.40147	1
TOB1	1.377242	1.324434	1.394407	1.21568	0.2818	0.401605	1
NOP10	5.442678	5.613202	5.387247	0.95102	-0.0725	0.401791	1
FSD1	1.489731	1.434383	1.507722	1.16883	0.2251	0.401951	1
MCRS1	1.652815	1.595804	1.671347	1.12679	0.1722	0.402091	1
CCND2-AS1	1.078608	1.125	1.063527	0.50822	-0.9765	0.402169	1
PRELID3A	1.120156	1.072312	1.135707	1.87669	0.9082	0.402244	1
NLGN4X	1.236853	1.28675	1.220633	0.76943	-0.3781	0.402351	1
SCN3B	1.078199	1.124585	1.063121	0.50665	-0.9809	0.402405	1
CMTM6	2.071314	2.137212	2.049893	0.92322	-0.1153	0.402515	1
GPRC5B	1.357104	1.30479	1.374109	1.22743	0.2956	0.402664	1
NTRK1	1.06031	1.013947	1.075381	5.40496	2.4343	0.402673	1
RP11-148L24.1	1.075254	1.028528	1.090443	3.17027	1.6646	0.403064	1
DGKZ	1.312944	1.363795	1.296415	0.81479	-0.2955	0.40309	1
PRKCA	1.063859	1.10988	1.048899	0.44502	-1.168	0.403427	1
FBXO16	1.173018	1.12446	1.188803	1.51697	0.6012	0.403509	1
NDRG2	1.161976	1.210388	1.14624	0.6951	-0.5247	0.403568	1
GALK2	1.335726	1.28374	1.352624	1.24277	0.3136	0.403588	1

MTMR3	1.158183	1.206039	1.142627	0.69223	-0.5307	0.403606	1
MRPS30	1.572814	1.516779	1.591029	1.14368	0.1937	0.40369	1
ATP13A1	1.213833	1.26313	1.197809	0.75175	-0.4117	0.403931	1
RAB23	1.196287	1.245055	1.180434	0.7363	-0.4416	0.404051	1
ARMC1	1.755428	1.696153	1.774695	1.11282	0.1542	0.404121	1
MSRB1	1.483288	1.429029	1.500925	1.16758	0.2235	0.404204	1
FRS2	1.24343	1.193887	1.259535	1.33859	0.4207	0.404277	1
RANBP6	1.177154	1.225484	1.161444	0.71599	-0.482	0.40428	1
RPP21	1.337774	1.286247	1.354523	1.23852	0.3086	0.404297	1
ZNF607	1.093772	1.047176	1.108918	2.30876	1.2071	0.404323	1
PYGM	1.092267	1.13902	1.07707	0.55438	-0.8511	0.40436	1
RP11-299J3.8	1.099327	1.052294	1.114615	2.19176	1.1321	0.404386	1
TANK	1.413845	1.466438	1.396749	0.85059	-0.2335	0.40442	1
ITGB4	1.058324	1.104076	1.043452	0.41751	-1.2601	0.40468	1
CAPN10-AS1	1.19415	1.145405	1.209995	1.44421	0.5303	0.404718	1
GPATCH4	1.282074	1.231587	1.298485	1.28887	0.3661	0.404776	1
C3orf62	1.15151	1.103393	1.167151	1.61666	0.693	0.40479	1
PHC1	1.292429	1.241752	1.308902	1.27776	0.3536	0.404973	1
TMEM106B	2.144306	2.208366	2.123483	0.92975	-0.1051	0.405104	1
CDPF1	1.294539	1.243747	1.311049	1.27611	0.3518	0.405137	1
RNF115	1.731394	1.672444	1.750557	1.11616	0.1585	0.405141	1
AKIP1	1.582507	1.525989	1.600878	1.14238	0.192	0.405271	1
COPS2	2.09132	2.019109	2.114793	1.09389	0.1295	0.405284	1
SUDS3	1.869389	1.807881	1.889383	1.10088	0.1387	0.405621	1
GUCD1	1.342213	1.2908	1.358925	1.23427	0.3037	0.405748	1
GALNT12	1.078903	1.125195	1.063855	0.51005	-0.9713	0.405973	1
RBM10	1.390169	1.337952	1.407142	1.20473	0.2687	0.40616	1
CAPN3	1.078392	1.124413	1.063432	0.50985	-0.9718	0.40617	1
ZNF383	1.113854	1.066868	1.129127	1.93107	0.9494	0.406291	1
BTBD2	1.352108	1.300005	1.369044	1.23013	0.2988	0.406361	1
DDB1	1.815857	1.875191	1.79657	0.91017	-0.1358	0.406416	1
ZNF140	1.181047	1.132733	1.196751	1.48231	0.5678	0.406422	1
ANTXR2	1.243668	1.193884	1.25985	1.34024	0.4225	0.406466	1
FNTA	1.847646	1.787616	1.867159	1.10099	0.1388	0.406686	1
SESTD1	1.554101	1.498721	1.572103	1.14714	0.198	0.406687	1
CDR2L	1.130676	1.177594	1.115425	0.64994	-0.6216	0.406732	1
MRI1	1.297218	1.246488	1.313708	1.27271	0.3479	0.406767	1
POLK	1.518531	1.463707	1.536351	1.15666	0.21	0.406838	1
PIAS4	1.206998	1.158118	1.222887	1.40963	0.4953	0.407018	1
NBR1	1.518813	1.572796	1.501266	0.87512	-0.1924	0.407081	1
ZNF672	1.466096	1.412127	1.48364	1.17352	0.2308	0.407329	1
CLASRP	1.292154	1.241538	1.308608	1.27768	0.3535	0.407491	1
WDR33	1.836359	1.896296	1.816877	0.91139	-0.1339	0.407736	1
PES1	1.399444	1.347101	1.416458	1.19982	0.2628	0.407746	1
STEAP3	1.092682	1.04631	1.107755	2.32683	1.2184	0.407898	1

SDHB	1.84073	1.900723	1.821229	0.91174	-0.1333	0.407979	1
LPCAT1	1.401635	1.349235	1.418667	1.19881	0.2616	0.408061	1
MSRB2	2.265483	2.18944	2.290201	1.08471	0.1173	0.408306	1
FOXP4-AS1	1.094814	1.048292	1.109937	2.27649	1.1868	0.408377	1
TRIT1	1.450143	1.397198	1.467353	1.17663	0.2347	0.408395	1
SPG20	1.706699	1.765097	1.687716	0.89886	-0.1538	0.408483	1
RP11-128P17.2	1.073116	1.027277	1.088016	3.22669	1.6901	0.408757	1
PHTF2	1.301435	1.351635	1.285118	0.81083	-0.3025	0.40908	1
ECH1	2.638728	2.722429	2.61152	0.93561	-0.096	0.409147	1
HYDIN	1.08683	1.040509	1.101886	2.51513	1.3306	0.409151	1
LANCL1	1.448763	1.395238	1.466161	1.17944	0.2381	0.409176	1
SPSB2	1.155665	1.203254	1.140196	0.68976	-0.5358	0.409297	1
ALDH3A2	1.401909	1.453609	1.385103	0.84898	-0.2362	0.409541	1
SPRED3	1.092599	1.138376	1.077719	0.56165	-0.8323	0.409657	1
CNOT2	1.876313	1.816869	1.895636	1.09643	0.1328	0.409679	1
RP11-710C12.1	1.085562	1.039588	1.100506	2.53878	1.3441	0.409947	1
GUF1	1.383263	1.331569	1.400066	1.20658	0.2709	0.410109	1
ALDH9A1	1.535854	1.590372	1.518133	0.87764	-0.1883	0.410121	1
SLC12A7	1.141097	1.187583	1.125987	0.67163	-0.5743	0.410304	1
PAX8	1.065152	1.019448	1.080008	4.11394	2.0405	0.410355	1
YIPF2	1.580547	1.635112	1.56281	0.88616	-0.1744	0.410479	1
PGM5P4-AS1	1.029121	1.073904	1.014564	0.19707	-2.3432	0.4105	1
KTN1-AS1	1.090313	1.04398	1.105374	2.39593	1.2606	0.410579	1
FUT9	1.075489	1.029494	1.09044	3.06642	1.6166	0.41059	1
ZNF135	1.168753	1.121028	1.184266	1.5225	0.6064	0.410818	1
FMN1	1.051695	1.096571	1.037108	0.38426	-1.3799	0.410852	1
PYY	1.137133	1.090232	1.152378	1.68875	0.756	0.41092	1
VSTM2B	1.06457	1.018986	1.079387	4.1813	2.064	0.410933	1
HMBS	1.470112	1.416965	1.487388	1.16889	0.2251	0.411192	1
SELENON	1.515102	1.568813	1.497643	0.87488	-0.1928	0.411318	1
CPEB2	1.079951	1.125399	1.065179	0.51977	-0.9441	0.411344	1
DAZAP1	1.683518	1.626781	1.70196	1.11994	0.1634	0.411408	1
STARD4	1.575568	1.629943	1.557893	0.88562	-0.1752	0.411437	1
NEBL-AS1	1.082931	1.03689	1.097897	2.65373	1.408	0.411528	1
SLC12A6	1.151433	1.103892	1.166887	1.60635	0.6838	0.41153	1
RPE	1.365479	1.417397	1.348603	0.83518	-0.2598	0.411562	1
HLCS	1.17179	1.123952	1.18734	1.51139	0.5959	0.411614	1
ALPK3	1.104254	1.149975	1.089391	0.59604	-0.7465	0.411747	1
HEXDC	1.241487	1.192465	1.257421	1.3375	0.4195	0.411917	1
TSPAN11	1.085101	1.130985	1.070187	0.53584	-0.9001	0.411951	1
PSMA7	8.077288	8.35947	7.985563	0.94919	-0.0752	0.412051	1
GTF2IRD2	1.287097	1.237263	1.303295	1.27831	0.3542	0.412051	1
RP11-126K1.6	1.182865	1.134858	1.19847	1.4717	0.5575	0.412272	1
DDHD1	1.608179	1.552227	1.626367	1.13426	0.1817	0.41229	1
SMCHD1	1.930809	1.869059	1.950882	1.09415	0.1298	0.41248	1

HIPK1	1.357178	1.407681	1.340762	0.83586	-0.2587	0.413312	1
ABLIM3	1.033628	1.077991	1.019208	0.24628	-2.0216	0.413438	1
NPC1	1.113708	1.15977	1.098736	0.61799	-0.6943	0.413892	1
RCC2	1.640184	1.696233	1.621964	0.89333	-0.1627	0.413938	1
CDK2	1.357894	1.307158	1.374386	1.21887	0.2855	0.414014	1
ZC3H10	1.176831	1.12934	1.192269	1.48654	0.572	0.414023	1
TMEM200B	1.041467	1.086037	1.026979	0.31358	-1.6731	0.414061	1
LY96	1.034876	1.079292	1.020438	0.25776	-1.9559	0.414226	1
ITPRIPL1	1.086802	1.041163	1.101637	2.4691	1.304	0.41446	1
SAC3D1	1.686511	1.630249	1.7048	1.11829	0.1613	0.414512	1
MECR	1.379309	1.327813	1.396048	1.20815	0.2728	0.414564	1
RELA	1.389493	1.440437	1.372934	0.84673	-0.24	0.414583	1
SIPA1L3	1.095856	1.141455	1.081033	0.57286	-0.8038	0.414787	1
LINC01023	1.143054	1.096408	1.158216	1.64112	0.7147	0.414934	1
SGTA	1.541196	1.48695	1.558829	1.14761	0.1986	0.414957	1
BHLHE22	1.060537	1.01526	1.075254	4.93156	2.302	0.415047	1
DPH7	1.331062	1.381168	1.314775	0.82582	-0.2761	0.415071	1
SLC1A5	1.370994	1.320345	1.387458	1.2095	0.2744	0.415277	1
DCUN1D3	1.072289	1.117297	1.057659	0.49156	-1.0246	0.415309	1
RP11-160E2.6	1.114901	1.068527	1.129975	1.8967	0.9235	0.415363	1
CEP97	1.265931	1.217007	1.281834	1.29873	0.3771	0.415382	1
BIRC2	1.589133	1.534288	1.606961	1.13602	0.184	0.415426	1
NNMT	1.159685	1.112634	1.17498	1.55352	0.6355	0.415521	1
SETD2	1.59065	1.536205	1.608347	1.13454	0.1821	0.415661	1
MMACHC	1.096111	1.050011	1.111097	2.22146	1.1515	0.415666	1
EIF3B	1.516455	1.462544	1.533979	1.15444	0.2072	0.415784	1
PPFIA2	1.085469	1.03973	1.100336	2.52546	1.3365	0.415932	1
HLA-DRB1	1.104233	1.058227	1.119187	2.04694	1.0335	0.41626	1
METTL18	1.160688	1.113912	1.175893	1.54412	0.6268	0.416299	1
RP11-315A16.1	1.071799	1.02642	1.086549	3.27589	1.7119	0.416306	1
TMEM101	1.671473	1.728095	1.653067	0.89695	-0.1569	0.416409	1
MRAS	1.31049	1.359861	1.294442	0.81821	-0.2895	0.41647	1
RGN	1.102712	1.148446	1.087846	0.59177	-0.7569	0.416766	1
C12orf4	1.150589	1.10372	1.165824	1.59877	0.677	0.41678	1
AC142528.1	1.082598	1.03709	1.097391	2.62584	1.3928	0.416815	1
NCKAP5L	1.256259	1.304786	1.240485	0.78903	-0.3419	0.416897	1
LINC00342	1.124887	1.078532	1.139955	1.78215	0.8336	0.416947	1
CPSF3L	1.504631	1.451255	1.521982	1.15673	0.2101	0.416983	1
GINS3	1.094552	1.049093	1.109329	2.227	1.1551	0.417156	1
RP11-499P20.2	1.082253	1.036787	1.097032	2.63767	1.3993	0.417161	1
TMEM25	1.403517	1.454472	1.386954	0.85144	-0.232	0.417165	1
SEPT10	1.657239	1.601757	1.675274	1.12217	0.1663	0.417213	1
RAD1	1.38261	1.331445	1.399241	1.20455	0.2685	0.417216	1
POLA2	1.150536	1.103777	1.165735	1.59703	0.6754	0.417221	1
RP11-966I7.2	1.056823	1.011999	1.071393	5.9498	2.5728	0.417221	1

DLG5	1.267071	1.315996	1.251167	0.79484	-0.3313	0.417307	1
TST	1.222423	1.174487	1.238005	1.36403	0.4479	0.417329	1
SNRNP27	2.185749	2.261338	2.161179	0.92059	-0.1194	0.417376	1
AGPAT3	1.25868	1.307036	1.242962	0.79132	-0.3377	0.417421	1
ZIC4	1.135695	1.089005	1.150872	1.69509	0.7614	0.417464	1
TULP3	1.296223	1.24692	1.312249	1.26458	0.3387	0.417486	1
MAP3K4	1.317799	1.267653	1.334099	1.24825	0.3199	0.417626	1
FBXW4	1.270361	1.220949	1.286423	1.29633	0.3744	0.417633	1
SEC16A	1.198312	1.245257	1.183052	0.74637	-0.422	0.417842	1
MIR34A	1.128029	1.081732	1.143078	1.75058	0.8078	0.417884	1
XPNPEP3	1.358021	1.307607	1.374409	1.21717	0.2835	0.41789	1
SMOC1	1.088244	1.042641	1.103068	2.41711	1.2733	0.417891	1
MAP3K9	1.128722	1.175095	1.113649	0.64907	-0.6236	0.417916	1
ZNF703	1.30096	1.251373	1.317079	1.26139	0.335	0.417933	1
C2orf88	1.034746	1.07893	1.020383	0.25825	-1.9532	0.41804	1
RSPH1	1.118874	1.072781	1.133856	1.83916	0.879	0.418236	1
GPR89A	1.201948	1.154442	1.21739	1.40758	0.4932	0.418292	1
ZBTB21	1.113656	1.159373	1.098796	0.6199	-0.6899	0.418453	1
HEXA	1.689073	1.744619	1.671018	0.90116	-0.1502	0.418459	1
CRYBB1	1.035867	1.084812	1.019957	0.23531	-2.0874	0.418762	1
ABCC10	1.161956	1.208517	1.146821	0.70412	-0.5061	0.418775	1
ZNF501	1.092405	1.046961	1.107176	2.28222	1.1904	0.418777	1
COG3	1.168808	1.21541	1.15366	0.71334	-0.4873	0.418804	1
ASB16-AS1	1.257848	1.208719	1.273818	1.3119	0.3917	0.418824	1
TASP1	1.1428	1.096367	1.157893	1.63846	0.7123	0.418846	1
IKBKE	1.093369	1.138306	1.078762	0.56947	-0.8123	0.418931	1
FEZF1-AS1	1.167592	1.120484	1.182905	1.51808	0.6023	0.419028	1
SDF2	1.762613	1.819368	1.744165	0.90822	-0.1389	0.419061	1
RP13-766D20.4	1.111054	1.065261	1.12594	1.92977	0.9484	0.419083	1
BAG2	1.386204	1.33493	1.402871	1.20285	0.2665	0.419367	1
USP30	1.152542	1.198871	1.137483	0.69132	-0.5326	0.419451	1
DLL1	1.123183	1.077479	1.138039	1.78163	0.8332	0.419463	1
TNIP1	2.050092	1.987798	2.070341	1.08356	0.1158	0.419497	1
UBE2D4	1.405593	1.3538	1.422428	1.19397	0.2558	0.419676	1
TRAPPC1	4.634038	4.794805	4.58178	0.94386	-0.0833	0.419682	1
ANKIB1	1.478539	1.530147	1.461764	0.87101	-0.1992	0.419954	1
SHF	1.156466	1.109715	1.171663	1.56462	0.6458	0.419992	1
PNKD	2.853542	2.93288	2.827753	0.94561	-0.0807	0.420122	1
NR4A1	1.056056	1.100393	1.041643	0.41481	-1.2695	0.420387	1
MADCAM1	1.11469	1.068638	1.129659	1.88902	0.9176	0.42049	1
MAST4	1.433875	1.382045	1.450722	1.17976	0.2385	0.420871	1
SUCLG1	1.950793	2.012612	1.930699	0.91911	-0.1217	0.420903	1
NIFK	1.723698	1.667358	1.742011	1.11186	0.153	0.420953	1
ALG6	1.171213	1.124445	1.186416	1.49798	0.583	0.420957	1
ZNF665	1.119934	1.073969	1.134875	1.82339	0.8666	0.421047	1

TMX3	1.410527	1.461553	1.39394	0.85351	-0.2285	0.421552	1
ANKRD6	1.138566	1.184414	1.123663	0.67057	-0.5765	0.421621	1
ACOT8	1.311199	1.261197	1.327452	1.25366	0.3261	0.421675	1
KCNQ4	1.037992	1.082007	1.023685	0.28881	-1.7918	0.421677	1
KRTCAP3	1.041454	1.085541	1.027124	0.31709	-1.657	0.421745	1
AREL1	1.15923	1.205674	1.144133	0.70078	-0.513	0.421766	1
MCCC1	1.239097	1.19048	1.2549	1.3382	0.4203	0.421793	1
NECTIN1	1.137558	1.183381	1.122663	0.66889	-0.5801	0.421823	1
TMEM120B	1.182183	1.228697	1.167063	0.7305	-0.453	0.421841	1
SGK1	1.17993	1.22654	1.164779	0.72737	-0.4592	0.421872	1
CLDN10	1.081593	1.036762	1.096166	2.61594	1.3873	0.421957	1
RP11-644F5.11	1.171094	1.124305	1.186303	1.49876	0.5838	0.422171	1
STRADB	2.057487	2.122244	2.036437	0.92354	-0.1148	0.422258	1
TCF3	1.710218	1.65379	1.72856	1.11437	0.1562	0.422349	1
CELF3	1.066678	1.021902	1.081233	3.70899	1.891	0.422478	1
MAF	1.804616	1.861217	1.786218	0.91291	-0.1314	0.422703	1
WDR37	1.153235	1.106968	1.168275	1.57314	0.6536	0.422993	1
TXLNB	1.074672	1.029989	1.089196	2.9743	1.5726	0.423145	1
RP11-658F2.8	1.157528	1.111004	1.17265	1.55535	0.6372	0.423161	1
PTPN12	1.599935	1.653473	1.582532	0.89144	-0.1658	0.42321	1
DNTTIP1	1.461689	1.409918	1.478517	1.16735	0.2232	0.423228	1
BBOF1	1.148305	1.102087	1.163328	1.59989	0.678	0.42335	1
VOPP1	1.69631	1.751313	1.678431	0.90299	-0.1472	0.423372	1
OSER1	1.391946	1.34137	1.408387	1.19632	0.2586	0.423438	1
PISD	1.293874	1.34233	1.278123	0.81244	-0.2997	0.423473	1
LRRC8A	1.231415	1.278633	1.216066	0.77545	-0.3669	0.423556	1
SERINC3	1.577977	1.523873	1.595564	1.13685	0.185	0.423559	1
TOM1	1.242134	1.29027	1.226487	0.78026	-0.358	0.423561	1
PGBD2	1.102153	1.05667	1.116937	2.06349	1.0451	0.423596	1
ASIP	1.101919	1.056924	1.116545	2.04738	1.0338	0.423596	1
WRB	2.161929	2.094982	2.183691	1.08101	0.1124	0.423926	1
RSPO1	1.042706	1.086287	1.028539	0.33075	-1.5962	0.423976	1
ERCC5	1.16042	1.114042	1.175495	1.53887	0.6219	0.423998	1
FLRT1	1.04625	1.090235	1.031953	0.35411	-1.4977	0.424047	1
C19orf54	1.202297	1.155302	1.217573	1.40097	0.4864	0.424084	1
BECN1	1.421709	1.471606	1.40549	0.85981	-0.2179	0.424205	1
TBC1D22A	1.250941	1.202671	1.266631	1.31558	0.3957	0.424358	1
RBM3	3.838693	3.700888	3.883488	1.06761	0.0944	0.424651	1
SMCR5	1.38036	1.43063	1.364019	0.84532	-0.2424	0.424664	1
CNTN3	1.155698	1.20174	1.140732	0.69759	-0.5195	0.424756	1
MYLK	1.118403	1.163165	1.103852	0.63649	-0.6518	0.424855	1
NOTCH2NL	1.105439	1.060334	1.120101	1.99059	0.9932	0.425014	1
TMEM183A	2.50008	2.580034	2.47409	0.93295	-0.1001	0.425117	1
CNTNAP3	1.099211	1.054109	1.113872	2.10449	1.0735	0.425263	1
RBBP5	1.251444	1.20271	1.267285	1.31855	0.399	0.42546	1

FAM3A	1.586388	1.640228	1.568886	0.88857	-0.1704	0.425538	1
TRAF5	1.181063	1.227313	1.166029	0.7304	-0.4532	0.425778	1
DNAJC10	2.016642	2.076701	1.99712	0.92609	-0.1108	0.425818	1
MAFG	1.187966	1.234446	1.172857	0.7373	-0.4397	0.425819	1
ST3GAL5	1.169781	1.215766	1.154833	0.7176	-0.4788	0.425855	1
PHLPP1	1.114465	1.069308	1.129144	1.86334	0.8979	0.42589	1
TRIM47	1.075439	1.119324	1.061174	0.51267	-0.9639	0.425897	1
CRTC2	1.152304	1.198089	1.137421	0.69374	-0.5275	0.425919	1
MARK2	1.233036	1.279954	1.217785	0.77793	-0.3623	0.425987	1
NPDC1	2.625747	2.701284	2.601193	0.94117	-0.0875	0.426282	1
NSUN7	1.209863	1.163057	1.225078	1.38036	0.465	0.426375	1
PODNL1	1.10902	1.063891	1.123689	1.93594	0.953	0.42643	1
KDM2B	1.292292	1.24393	1.308013	1.26271	0.3365	0.426638	1
SIN3A	1.401296	1.350675	1.417751	1.19128	0.2525	0.426757	1
SLC30A4	1.112246	1.157084	1.097671	0.62178	-0.6855	0.426934	1
ADNP	1.96655	1.904152	1.986833	1.09145	0.1262	0.427166	1
GPT2	1.347432	1.297656	1.363612	1.22158	0.2888	0.427264	1
AGBL5	1.347183	1.29755	1.363317	1.22103	0.2881	0.427264	1
PPP1R35	1.403578	1.353298	1.419922	1.18858	0.2492	0.427278	1
AHCTF1	1.321831	1.272782	1.337774	1.23826	0.3083	0.427426	1
SGK494	1.113873	1.068972	1.128469	1.86263	0.8973	0.427825	1
TMCC2	1.063234	1.107016	1.049003	0.45791	-1.1269	0.428033	1
FAM173B	1.228783	1.181364	1.244197	1.34644	0.4292	0.428259	1
ATP6V1E2	1.139057	1.093732	1.15379	1.64074	0.7143	0.428444	1
FAM13A	1.697201	1.630322	1.71894	1.14059	0.1898	0.428543	1
STRN4	1.284053	1.331598	1.268598	0.81001	-0.304	0.428566	1
KIAA0141	1.280963	1.32857	1.265488	0.80801	-0.3076	0.428568	1
TRMT1L	1.342072	1.292632	1.358144	1.22387	0.2915	0.428569	1
EME1	1.074682	1.030618	1.089005	2.9069	1.5395	0.42861	1
KLHL35	1.08984	1.045194	1.104353	2.30901	1.2073	0.428749	1
NOL7	2.167683	2.229663	2.147537	0.93321	-0.0997	0.428822	1
FKBP15	1.191956	1.145583	1.20703	1.42208	0.508	0.429081	1
PSMD7	2.415383	2.486827	2.392159	0.93633	-0.0949	0.429082	1
HSF2	1.384244	1.334505	1.400412	1.19703	0.2595	0.429224	1
RBMX2	1.397847	1.347544	1.414198	1.19179	0.2531	0.429281	1
ATP5C1	3.850749	3.696157	3.901	1.07598	0.1056	0.42932	1
SURF1	1.988021	2.046852	1.968898	0.92553	-0.1116	0.429381	1
SLC25A40	1.192988	1.147011	1.207933	1.41441	0.5002	0.42939	1
RBM26-AS1	1.09996	1.055412	1.114441	2.06526	1.0463	0.429451	1
GEMIN6	1.387409	1.337568	1.40361	1.19564	0.2578	0.429482	1
STRBP	1.590214	1.643171	1.573	0.8909	-0.1667	0.429736	1
B3GALT5-AS1	1.035922	1.078843	1.02197	0.27866	-1.8434	0.429894	1
SENP2	1.392383	1.342293	1.408665	1.1939	0.2557	0.429921	1
CTD-3131K8.2	1.162748	1.116964	1.17763	1.51868	0.6028	0.430138	1
FRA10AC1	2.113145	2.050269	2.133583	1.07933	0.1101	0.430146	1

KIF13B	1.085154	1.129446	1.070757	0.54661	-0.8714	0.430179	1
MTPN	2.504316	2.574937	2.481361	0.94058	-0.0884	0.430329	1
SLC35E4	1.112775	1.157269	1.098312	0.62512	-0.6778	0.430395	1
SLC4A5	1.077384	1.120973	1.063215	0.52255	-0.9364	0.430409	1
ST6GALNAC3	1.28173	1.328745	1.266447	0.8105	-0.3031	0.430432	1
RP11-259K5.2	1.035626	1.078629	1.021648	0.27532	-1.8608	0.43044	1
AC018816.3	1.094466	1.138289	1.080221	0.5801	-0.7856	0.430568	1
HINFP	1.265254	1.217626	1.280736	1.28999	0.3674	0.430581	1
COX5B	9.033483	8.783251	9.114823	1.0426	0.0602	0.430653	1
MCRIP2	1.852645	1.90925	1.834246	0.91751	-0.1242	0.430789	1
AC012354.6	1.132487	1.177272	1.117929	0.66525	-0.588	0.430812	1
LINC00909	1.289687	1.241255	1.30543	1.266	0.3403	0.430841	1
CHMP6	1.377578	1.327765	1.39377	1.20138	0.2647	0.430917	1
SMARCD3	1.640105	1.586131	1.65765	1.12202	0.1661	0.430928	1
DOHH	1.193383	1.239106	1.178521	0.74662	-0.4216	0.431138	1
CRCP	1.270082	1.222218	1.285641	1.28541	0.3622	0.431188	1
TSG101	1.875276	1.931802	1.856902	0.91962	-0.1209	0.431234	1
PDE5A	1.10808	1.152534	1.09363	0.61383	-0.7041	0.431239	1
TIGD1	1.23566	1.188742	1.250911	1.32938	0.4108	0.431248	1
MOB4	1.777773	1.721525	1.796056	1.1033	0.1418	0.431489	1
NOC4L	1.201057	1.247184	1.186063	0.75273	-0.4098	0.43151	1
PAQR4	1.318206	1.270077	1.33385	1.23613	0.3058	0.431716	1
MTA1	1.645122	1.590997	1.662716	1.12135	0.1652	0.431767	1
ZNF701	1.128336	1.083442	1.142929	1.71293	0.7765	0.431814	1
MON1B	1.271591	1.223595	1.287192	1.28443	0.3611	0.432018	1
PLOD1	2.151129	2.219041	2.129054	0.92618	-0.1106	0.432125	1
NHS	1.127245	1.08219	1.14189	1.72637	0.7877	0.432149	1
BTBD7	1.542024	1.489675	1.559041	1.14166	0.1911	0.432149	1
GALM	1.284426	1.331983	1.268967	0.81018	-0.3037	0.432434	1
B3GNT9	1.118695	1.162912	1.104323	0.64036	-0.643	0.432486	1
TSSC1	1.46843	1.417083	1.48512	1.16313	0.218	0.4325	1
UBXN7	1.739311	1.68409	1.75726	1.10696	0.1466	0.432528	1
SETX	1.435017	1.485197	1.418706	0.86296	-0.2126	0.4326	1
BSG	10.01403	10.302	9.920421	0.95898	-0.0604	0.432707	1
TJP2	1.198146	1.244097	1.18321	0.75056	-0.414	0.432732	1
TBCK	1.302531	1.254627	1.318102	1.24928	0.3211	0.432788	1
PPP2R5D	1.208942	1.162389	1.224074	1.37986	0.4645	0.432876	1
ATRAID	3.749209	3.605484	3.795927	1.07309	0.1018	0.433171	1
DUS4L	1.371485	1.322118	1.387532	1.20307	0.2667	0.433219	1
CPA4	1.083771	1.127756	1.069473	0.5438	-0.8789	0.433416	1
ZMIZ1	1.389501	1.438096	1.373705	0.85302	-0.2293	0.433449	1
SMU1	1.446897	1.396465	1.46329	1.16855	0.2247	0.433504	1
BTAF1	1.299537	1.251558	1.315133	1.25273	0.3251	0.433964	1
GTF3C4	1.292654	1.244982	1.308151	1.25785	0.331	0.434047	1
MTX3	1.291354	1.243794	1.306814	1.2585	0.3317	0.434069	1

YTHDF3	1.313051	1.360575	1.297603	0.82536	-0.2769	0.434163	1
ISG20L2	1.539043	1.487573	1.555773	1.13988	0.1889	0.43426	1
ETV5	1.378214	1.427444	1.362211	0.84739	-0.2389	0.434421	1
RELB	1.102282	1.146099	1.088038	0.60259	-0.7307	0.43443	1
NDUFAF7	1.262746	1.215961	1.277953	1.28705	0.3641	0.434976	1
PIGA	1.147996	1.102648	1.162737	1.58539	0.6648	0.435259	1
GSN	2.171598	2.240135	2.149319	0.92677	-0.1097	0.435406	1
RP1-193H18.2	1.127355	1.082635	1.141892	1.7171	0.78	0.435602	1
PPP1R1A	1.24149	1.194295	1.256831	1.32186	0.4026	0.435926	1
P2RY6	1.046411	1.088972	1.032576	0.36614	-1.4495	0.436079	1
PDK2	1.487954	1.437139	1.504471	1.15403	0.2067	0.436153	1
DBR1	1.152095	1.107041	1.16674	1.55772	0.6394	0.436154	1
C9orf69	1.269784	1.222536	1.285142	1.28133	0.3576	0.436213	1
PAK5	1.064441	1.021032	1.078551	3.73489	1.9011	0.436246	1
SCARB1	1.293221	1.341037	1.277677	0.81421	-0.2965	0.436662	1
PFKFB3	1.567637	1.622944	1.549659	0.88236	-0.1806	0.43667	1
GTPBP8	1.283123	1.235902	1.298472	1.26524	0.3394	0.436691	1
CASC9	1.307348	1.259309	1.322963	1.24547	0.3167	0.4371	1
PRELID1	5.212362	5.341798	5.170288	0.9605	-0.0581	0.437178	1
PRSS22	1.029642	1.072161	1.015821	0.21925	-2.1894	0.437387	1
TET2	1.195521	1.240833	1.180792	0.75069	-0.4137	0.437442	1
ZNF574	1.131002	1.086526	1.145459	1.68111	0.7494	0.43745	1
XKR6	1.202635	1.156999	1.21747	1.38517	0.4701	0.437529	1
KDM6B	1.560011	1.507399	1.577113	1.1374	0.1857	0.437692	1
WDR66	1.115903	1.071381	1.130375	1.82645	0.869	0.437866	1
ZNF180	1.120525	1.076174	1.134941	1.77148	0.825	0.437911	1
RP11-258C19.7	1.18211	1.136848	1.196822	1.43825	0.5243	0.438055	1
B3GAT2	1.410895	1.3617	1.426887	1.18022	0.2391	0.438166	1
B9D2	1.218142	1.172193	1.233078	1.35359	0.4368	0.438478	1
EIF2B3	1.245404	1.198912	1.260516	1.30971	0.3892	0.439013	1
FBXO43	1.082821	1.039514	1.096899	2.45227	1.2941	0.439118	1
PPP1R13L	1.299141	1.345612	1.284035	0.82183	-0.2831	0.439189	1
SMAP1	2.432287	2.36065	2.455573	1.06976	0.0973	0.439272	1
ZNF747	1.15167	1.107123	1.16615	1.55101	0.6332	0.439312	1
MRPS6	5.203506	5.411755	5.135813	0.93745	-0.0932	0.439331	1
SGMS2	1.058464	1.100834	1.044691	0.44321	-1.1739	0.439376	1
MRPS33	2.564065	2.489482	2.588309	1.06635	0.0927	0.439432	1
DCAF15	1.19875	1.153092	1.213592	1.39518	0.4805	0.439522	1
YPEL3	1.581124	1.528747	1.598149	1.13126	0.1779	0.439591	1
CTTNBP2	1.061841	1.104371	1.048016	0.46005	-1.1201	0.439592	1
DNAJC3-AS1	1.295304	1.342311	1.280024	0.81804	-0.2898	0.439846	1
SOS2	1.199546	1.244714	1.184863	0.75543	-0.4046	0.439863	1
FAM220A	1.189335	1.234761	1.174569	0.7436	-0.4274	0.439907	1
AMT	1.105547	1.061734	1.119789	1.94042	0.9564	0.440044	1
TAF13	1.118101	1.161682	1.103934	0.64283	-0.6375	0.44012	1

AP1AR	1.143297	1.098681	1.1578	1.59909	0.6772	0.440121	1
ARPC1A	3.095397	3.178042	3.068533	0.94972	-0.0744	0.440131	1
PNMAL1	1.35861	1.310631	1.374206	1.20466	0.2686	0.440151	1
SIRT3	1.342351	1.294295	1.357971	1.21637	0.2826	0.440215	1
TTC21A	1.136738	1.092351	1.151167	1.63688	0.7109	0.440271	1
RNFT2	1.266339	1.219923	1.281426	1.27966	0.3558	0.440276	1
MAD2L1BP	1.273882	1.226783	1.289191	1.27519	0.3507	0.44064	1
HIST1H2BH	1.074725	1.031714	1.088706	2.79708	1.4839	0.4407	1
RP11-795F19.5	1.069224	1.026199	1.08321	3.17607	1.6672	0.440837	1
SFMBT1	1.075828	1.032809	1.089811	2.73741	1.4528	0.441003	1
ATMIN	1.27804	1.324707	1.26287	0.80956	-0.3048	0.441093	1
DCTN6	1.744347	1.798701	1.726679	0.90983	-0.1363	0.441174	1
SDCCAG8	1.782922	1.727295	1.801004	1.10135	0.1393	0.441213	1
ACAP3	1.362939	1.314769	1.378597	1.20278	0.2664	0.441392	1
PYGL	1.269814	1.223025	1.285023	1.27798	0.3539	0.441574	1
EPS8	1.202138	1.24729	1.187462	0.75806	-0.3996	0.441617	1
ZNF655	1.430312	1.479242	1.414407	0.86471	-0.2097	0.441778	1
SETD7	1.164385	1.208545	1.150031	0.71942	-0.4751	0.441948	1
AKT3	1.565227	1.513307	1.582104	1.13403	0.1815	0.44195	1
NOL9	1.177769	1.132845	1.192372	1.44809	0.5342	0.442105	1
AARS2	1.102009	1.058274	1.116225	1.99444	0.996	0.44216	1
ZNF543	1.10797	1.064428	1.122123	1.89552	0.9226	0.442167	1
DAAM1	2.512813	2.587101	2.488665	0.93798	-0.0924	0.442459	1
ERG	1.029421	1.071355	1.01579	0.22128	-2.176	0.442496	1
HOXB2	1.153649	1.106388	1.169012	1.58864	0.6678	0.442496	1
LINGO2	1.039691	1.08164	1.026055	0.31914	-1.6477	0.442623	1
HABP4	1.400101	1.448323	1.384426	0.85748	-0.2218	0.442664	1
DCHS1	1.259091	1.305256	1.244085	0.79961	-0.3226	0.442917	1
ZNF438	1.109272	1.065751	1.123418	1.87704	0.9085	0.442953	1
SLC16A12	1.033642	1.075204	1.020132	0.2677	-1.9013	0.443006	1
GDPD2	1.089433	1.132357	1.075481	0.57028	-0.8102	0.443089	1
SOX7	1.044156	1.086246	1.030475	0.35335	-1.5008	0.443131	1
ATP11B	1.201478	1.155804	1.216325	1.38845	0.4735	0.443149	1
RCAN2	1.167523	1.211635	1.153184	0.72381	-0.4663	0.44327	1
RP5-882C2.2	1.135548	1.091404	1.149897	1.63995	0.7136	0.44336	1
PCMTD2	1.546553	1.495207	1.563244	1.13739	0.1857	0.443405	1
IL11	1.071716	1.114232	1.057896	0.50683	-0.9804	0.443407	1
ADORA2B	1.105306	1.06186	1.119429	1.93062	0.9491	0.443578	1
BLACAT1	1.103741	1.060164	1.117906	1.95975	0.9707	0.443609	1
CCDC40	1.128628	1.084675	1.142915	1.6878	0.7551	0.443696	1
ZCCHC2	1.151646	1.195745	1.137311	0.70148	-0.5115	0.443764	1
TMEM177	1.244796	1.198671	1.259789	1.30764	0.387	0.443965	1
CHSY1	1.20348	1.248079	1.188983	0.76179	-0.3925	0.444078	1
SLC25A17	1.226569	1.272034	1.21179	0.77854	-0.3612	0.444088	1
MED1	1.269759	1.223103	1.284925	1.2771	0.3529	0.444178	1

ZBTB7A	1.157528	1.201178	1.143339	0.7125	-0.489	0.444267	1
GNB5	1.397867	1.446093	1.382191	0.85675	-0.223	0.44431	1
CTPS1	1.277145	1.230503	1.292307	1.26813	0.3427	0.444376	1
EXO1	1.081862	1.039006	1.095793	2.45583	1.2962	0.444468	1
PCSK6	1.06627	1.023659	1.080121	3.38644	1.7598	0.444488	1
TM9SF4	1.362429	1.409787	1.347034	0.84687	-0.2398	0.444514	1
DYNC1LI1	1.480642	1.430525	1.496933	1.15425	0.207	0.444543	1
PPP6R3	1.532464	1.481124	1.549152	1.14139	0.1908	0.444799	1
AP001372.2	1.212713	1.167247	1.227492	1.36022	0.4438	0.444894	1
TMEM139	1.136192	1.179414	1.122142	0.68078	-0.5547	0.444975	1
MBD3	1.385027	1.33692	1.400665	1.1892	0.25	0.44508	1
MAP3K11	1.427423	1.377733	1.443576	1.17431	0.2318	0.445134	1
ZNF567	1.172282	1.127954	1.186692	1.45905	0.545	0.445225	1
NAXE	2.729985	2.827286	2.698357	0.92944	-0.1056	0.445464	1
MARVELD3	1.038906	1.080697	1.025321	0.31378	-1.6722	0.445626	1
LENG8	2.001466	1.942863	2.020515	1.08236	0.1142	0.445653	1
DRD2	1.058333	1.015934	1.072115	4.52582	2.1782	0.445666	1
AMBRA1	1.090689	1.13361	1.076738	0.57434	-0.8	0.445674	1
CTD-2089N3.2	1.063494	1.021029	1.077297	3.67567	1.878	0.445794	1
EXOSC6	1.273867	1.227536	1.288928	1.26981	0.3446	0.445941	1
GYPC	2.353188	2.419543	2.33162	0.93806	-0.0922	0.446009	1
DIAPH2	1.578502	1.527303	1.595144	1.12866	0.1746	0.446332	1
ZKSCAN2	1.118453	1.075154	1.132527	1.76342	0.8184	0.446427	1
SGTB	1.237791	1.282989	1.2231	0.78837	-0.3431	0.446514	1
FAM234B	1.194079	1.149327	1.208626	1.39711	0.4824	0.446553	1
DENND5A	1.369801	1.417596	1.354265	0.84834	-0.2373	0.446606	1
EAF2	1.094684	1.051703	1.108656	2.10155	1.0715	0.446626	1
RIC8B	1.180345	1.13546	1.194935	1.43906	0.5251	0.446704	1
CTD-3001H11.2	1.108593	1.065413	1.122629	1.8747	0.9067	0.446829	1
RP11-73K9.2	1.09507	1.051909	1.1091	2.10176	1.0716	0.446905	1
RP11-495P10.6	1.071251	1.028851	1.085034	2.94732	1.5594	0.447007	1
SGSM1	1.078646	1.035971	1.092518	2.57202	1.3629	0.447159	1
IPO4	1.214162	1.169102	1.228809	1.35308	0.4362	0.447172	1
WASF1	1.450766	1.401321	1.466839	1.16326	0.2182	0.447194	1
LINC01116	1.142859	1.09907	1.157093	1.58568	0.6651	0.447317	1
GEMIN2	1.268871	1.22304	1.283768	1.27228	0.3474	0.447349	1
BCL10	1.288486	1.242181	1.303537	1.25335	0.3258	0.447408	1
MEPCE	1.18721	1.142574	1.20172	1.41485	0.5006	0.4475	1
CD99L2	1.671828	1.724189	1.654807	0.90419	-0.1453	0.447539	1
NDUFAB1	3.558776	3.682787	3.518465	0.93875	-0.0912	0.447619	1
TARSL2	1.539243	1.488672	1.555682	1.13713	0.1854	0.447746	1
RP3-395M20.12	1.097128	1.054332	1.111039	2.04369	1.0312	0.44785	1
THAP7-AS1	1.139037	1.095107	1.153317	1.61204	0.6889	0.447965	1
ADHFE1	1.098254	1.055239	1.112236	2.03181	1.0228	0.448068	1
TARS	1.70737	1.759779	1.690334	0.9086	-0.1383	0.448397	1

MEN1	1.342547	1.295398	1.357874	1.2115	0.2768	0.448448	1
PPP1R15B	1.26387	1.309169	1.249146	0.80586	-0.3114	0.448513	1
CLCN7	1.244446	1.289422	1.229827	0.79409	-0.3326	0.448759	1
HSPA13	1.498507	1.54846	1.482269	0.87932	-0.1855	0.448771	1
DMC1	1.070372	1.028034	1.084135	3.00121	1.5855	0.448773	1
SEC14L1	1.915868	1.970791	1.898015	0.92503	-0.1124	0.448837	1
ARHGEF19	1.110576	1.067469	1.124588	1.84659	0.8849	0.449095	1
RP11-521O16.2	1.071776	1.029294	1.085586	2.92161	1.5468	0.449112	1
PGM5-AS1.1	1.114657	1.157341	1.100782	0.64053	-0.6427	0.449195	1
RBM43	1.208964	1.164241	1.223501	1.36081	0.4445	0.449198	1
CCDC22	1.409443	1.361417	1.425054	1.17608	0.234	0.449331	1
TMEM127	1.184682	1.228668	1.170384	0.74512	-0.4245	0.449333	1
GTPBP2	1.141659	1.184763	1.127647	0.69087	-0.5335	0.449454	1
RP11-271F18.4	1.054036	1.012187	1.067639	5.55023	2.4725	0.449463	1
EIF3J	2.263971	2.336948	2.240249	0.92767	-0.1083	0.449647	1
MYL7	1.067003	1.108779	1.053423	0.49111	-1.0259	0.449659	1
NDUFV3	2.541688	2.468901	2.565348	1.06566	0.0917	0.449755	1
POMGNT2	1.289261	1.334898	1.274427	0.81943	-0.2873	0.449886	1
VAPA	3.800333	3.699155	3.833222	1.04967	0.0699	0.449927	1
ZCWPW1	1.157443	1.113462	1.17174	1.51364	0.598	0.450123	1
ZNF234	1.15728	1.113553	1.171493	1.51025	0.5948	0.450123	1
RP11-651L5.3	1.115315	1.072321	1.12929	1.78773	0.8381	0.450482	1
CTA-243E7.1	1.105948	1.062966	1.11992	1.90453	0.9294	0.450752	1
CARF	1.302114	1.255929	1.317126	1.23912	0.3093	0.450874	1
MN1	1.189203	1.233271	1.174879	0.74968	-0.4157	0.450944	1
KIAA1257	1.105031	1.06221	1.11895	1.91206	0.9351	0.451027	1
WHSC1L1	2.91362	2.831967	2.940162	1.05906	0.0828	0.451037	1
KIF13A	1.193082	1.236967	1.178818	0.75461	-0.4062	0.45113	1
HSDL1	1.501658	1.550894	1.485653	0.88157	-0.1818	0.451566	1
KLHL36	1.310362	1.356112	1.295491	0.82977	-0.2692	0.451803	1
MAP4K5	1.594148	1.644033	1.577932	0.89736	-0.1562	0.451827	1
TNPO1	2.03897	1.981331	2.057706	1.07783	0.1081	0.451917	1
MORN4	1.3292	1.375294	1.314216	0.83725	-0.2563	0.451937	1
LGALS8	1.365112	1.41173	1.349959	0.84997	-0.2345	0.452193	1
MYO19	1.242673	1.19749	1.25736	1.30316	0.382	0.452217	1
TCOF1	1.397395	1.444462	1.382096	0.85968	-0.2181	0.4524	1
SAP30BP	1.520784	1.56985	1.504835	0.88591	-0.1748	0.452433	1
CHD8	1.402181	1.354451	1.417695	1.17843	0.2369	0.45269	1
EFTUD2	1.29269	1.24675	1.307622	1.2467	0.3181	0.4527	1
MFSD14C	1.253309	1.207721	1.268127	1.29081	0.3683	0.452701	1
TNFAIP8L1	1.174219	1.130629	1.188388	1.44216	0.5282	0.452719	1
ZNF281	1.443097	1.491262	1.427441	0.87009	-0.2008	0.452756	1
VPS9D1	1.102827	1.144868	1.089161	0.61547	-0.7002	0.452795	1
LYST	1.116534	1.158908	1.10276	0.64666	-0.6289	0.452795	1
SEMA3F	1.135265	1.178045	1.121358	0.68161	-0.553	0.452803	1

EXPH5	1.093769	1.135959	1.080054	0.58881	-0.7641	0.452814	1
POGLUT1	1.23093	1.274918	1.216631	0.78798	-0.3438	0.453278	1
G3BP1	2.038463	1.982118	2.056778	1.07602	0.1057	0.453529	1
WDR24	1.152615	1.109414	1.166658	1.52318	0.6071	0.453616	1
AP1S2	5.74977	5.91567	5.695843	0.95528	-0.066	0.453669	1
ARHGEF25	1.183567	1.13967	1.197836	1.41646	0.5023	0.453767	1
AEBP2	1.432463	1.383916	1.448243	1.16755	0.2235	0.45378	1
CTSO	1.175729	1.21925	1.161582	0.73698	-0.4403	0.45391	1
NUCB2	2.379147	2.445341	2.357631	0.93932	-0.0903	0.454025	1
CTC-471J1.11	1.080353	1.038376	1.093998	2.44938	1.2924	0.454149	1
METTL5	2.190163	2.249927	2.170736	0.93664	-0.0944	0.454214	1
RP11-379H18.1	1.125625	1.082426	1.139667	1.69444	0.7608	0.454258	1
ZNF700	1.100601	1.058156	1.114398	1.96708	0.9761	0.454285	1
LIMCH1	1.498291	1.547394	1.482329	0.88114	-0.1826	0.454306	1
TCEA1	3.786594	3.677951	3.821908	1.05376	0.0755	0.454487	1
RNH1	2.452902	2.517062	2.432047	0.94396	-0.0832	0.454625	1
POLB	1.283912	1.237897	1.29887	1.2563	0.3292	0.454921	1
KCNJ8	1.053344	1.011999	1.066783	5.56565	2.4766	0.455241	1
KIAA0753	1.181918	1.137983	1.1962	1.42192	0.5078	0.455504	1
F11R	1.152802	1.195665	1.138869	0.70973	-0.4947	0.455506	1
NARFL	1.261026	1.215816	1.275722	1.27758	0.3534	0.455521	1
MAPKAPK5	1.720269	1.667383	1.73746	1.105	0.144	0.455561	1
USF1	1.311061	1.26502	1.326027	1.23019	0.2989	0.455751	1
BMP2K	1.228529	1.272454	1.214251	0.78638	-0.3467	0.455765	1
SNRPN	5.432729	5.596476	5.379502	0.9528	-0.0698	0.45577	1
R3HDM4	1.346607	1.392792	1.331594	0.8442	-0.2443	0.4558	1
MED12	1.186364	1.142264	1.200699	1.41075	0.4965	0.455814	1
GEMIN4	1.114198	1.071629	1.128036	1.78747	0.8379	0.455873	1
PRIMPOL	1.228223	1.183857	1.242644	1.31975	0.4003	0.455907	1
ATP5G3	9.708028	10.01404	9.608555	0.95502	-0.0664	0.456098	1
ZCCHC4	1.09373	1.05172	1.107385	2.0763	1.054	0.456154	1
TMEM60	1.777815	1.725042	1.794969	1.09644	0.1328	0.456232	1
L3MBTL1	1.229377	1.184426	1.243989	1.32296	0.4038	0.456667	1
PHOSPHO2	1.149714	1.106546	1.163747	1.53687	0.62	0.456669	1
BCDIN3D	1.133776	1.09098	1.147687	1.62329	0.6989	0.45678	1
NT5DC2	1.920665	1.865707	1.93853	1.08412	0.1165	0.456859	1
AC009948.5	1.144807	1.101763	1.158799	1.56048	0.642	0.456885	1
SPIN4	1.144963	1.101925	1.158952	1.55951	0.6411	0.456885	1
CLEC11A	1.9941	1.932953	2.013977	1.08685	0.1201	0.457158	1
CELF2	2.065153	2.121243	2.046921	0.93372	-0.0989	0.457172	1
FDXR	1.231565	1.186927	1.246075	1.31642	0.3966	0.457238	1
LINC01006	1.267543	1.222243	1.282268	1.27009	0.3449	0.457491	1
CDK5RAP2	1.371463	1.325161	1.386514	1.18868	0.2494	0.457495	1
RAB10	2.249308	2.311459	2.229105	0.9372	-0.0936	0.457533	1
CSNK1G1	1.250969	1.295094	1.236626	0.80187	-0.3186	0.457799	1

GRK6	1.28478	1.23927	1.299573	1.25203	0.3243	0.458084	1
ATP5L2	1.13618	1.093224	1.150143	1.61057	0.6876	0.458087	1
C9orf40	1.243716	1.199192	1.258189	1.29618	0.3743	0.4582	1
AMN1	1.317836	1.272157	1.332684	1.22239	0.2897	0.458268	1
PKN1	1.409457	1.455964	1.39434	0.86485	-0.2095	0.458274	1
ROR1	1.177563	1.220599	1.163574	0.7415	-0.4315	0.458293	1
STK39	1.214335	1.257827	1.200198	0.77648	-0.365	0.458344	1
TENM3	1.697623	1.749724	1.680687	0.90792	-0.1394	0.458413	1
UBR3	1.223666	1.267488	1.209422	0.78292	-0.3531	0.458418	1
IGDCC3	1.373072	1.326457	1.388225	1.18921	0.25	0.45845	1
LRSAM1	1.138509	1.095913	1.152356	1.58849	0.6677	0.458557	1
BHMT	1.061761	1.020071	1.075312	3.7522	1.9077	0.458563	1
C19orf43	4.287968	4.449293	4.235529	0.93803	-0.0923	0.458671	1
ZNF226	1.607304	1.55653	1.623808	1.12089	0.1646	0.458794	1
CDIP1	1.651957	1.600693	1.668621	1.11308	0.1546	0.458873	1
PALM	1.559395	1.608232	1.54352	0.89361	-0.1623	0.458923	1
ARMC10	2.272875	2.207338	2.294178	1.07193	0.1002	0.459044	1
ZNF595	1.161304	1.118357	1.175264	1.48081	0.5664	0.459222	1
SSU72	3.7697	3.87621	3.735078	0.95093	-0.0726	0.459222	1
MRPL4	2.088908	2.023177	2.110275	1.08512	0.1179	0.459256	1
HMCN1	1.042942	1.083763	1.029673	0.35425	-1.4972	0.459297	1
CYP11A1	1.100743	1.058804	1.114376	1.94504	0.9598	0.459322	1
CHN1	1.60868	1.657957	1.592663	0.90076	-0.1508	0.45947	1
ERMP1	1.088623	1.130034	1.075162	0.57802	-0.7908	0.459484	1
CES4A	1.178686	1.135129	1.192844	1.42711	0.5131	0.459484	1
RILP	1.169265	1.212318	1.15527	0.73131	-0.4514	0.459497	1
SIGMAR1	2.215453	2.151616	2.236204	1.07345	0.1023	0.459535	1
POMC	1.078087	1.036712	1.091536	2.49334	1.3181	0.459765	1
ACP2	1.329757	1.375016	1.315045	0.84008	-0.2514	0.459904	1
TUBGCP2	1.6954	1.643726	1.712197	1.10637	0.1458	0.460023	1
RP11-111F5.4	1.102283	1.060445	1.115883	1.91717	0.939	0.460046	1
FAM219A	1.303529	1.257863	1.318373	1.23466	0.3041	0.460084	1
C8orf58	1.217988	1.261045	1.203992	0.78144	-0.3558	0.460183	1
CTD-2561B21.11	1.074304	1.032782	1.087801	2.67832	1.4213	0.460236	1
MIF-AS1	1.170425	1.127014	1.184536	1.45288	0.5389	0.460614	1
DNAJC27	1.143175	1.100542	1.157034	1.56187	0.6433	0.460871	1
FANCF	1.376139	1.329632	1.391257	1.18695	0.2473	0.460947	1
SLC35A5	1.35494	1.30858	1.370009	1.19907	0.2619	0.460948	1
RP11-386G11.5	1.123149	1.081018	1.136844	1.68905	0.7562	0.461108	1
KCNS3	1.048293	1.088683	1.035164	0.39651	-1.3346	0.461127	1
POU4F2	1.07147	1.030014	1.084945	2.83016	1.5009	0.461223	1
TBX6	1.083599	1.042009	1.097118	2.31181	1.209	0.461254	1
UBAP1L	1.083527	1.124734	1.070132	0.56225	-0.8307	0.461311	1
PID1	1.035213	1.075535	1.022106	0.29266	-1.7727	0.461338	1
AZIN1	1.991701	2.047426	1.973588	0.9295	-0.1055	0.461405	1

ENTPD1	1.14859	1.105701	1.162532	1.53765	0.6207	0.461589	1
ZNF837	1.11051	1.068573	1.124142	1.81036	0.8563	0.46186	1
RIN1	1.121355	1.079101	1.13509	1.70783	0.7722	0.461978	1
PAQR7	1.181611	1.138053	1.19577	1.41807	0.5039	0.46209	1
WAPL	1.737249	1.685006	1.754231	1.10106	0.1389	0.4621	1
BCR	1.170812	1.213251	1.157017	0.7363	-0.4416	0.462165	1
PAQR5	1.163439	1.120445	1.177415	1.473	0.5588	0.462202	1
ARHGEF17	1.212106	1.255193	1.198101	0.77628	-0.3654	0.46221	1
LSM12	1.701626	1.752366	1.685133	0.91064	-0.1351	0.462287	1
PPIP5K2	1.621926	1.571646	1.63827	1.11655	0.159	0.462311	1
FAM71E1	1.264984	1.309382	1.250552	0.80985	-0.3043	0.462315	1
PRR5L	1.131332	1.173184	1.117728	0.67979	-0.5568	0.462425	1
HPS5	1.152961	1.110259	1.166841	1.51318	0.5976	0.462486	1
YTHDC2	1.328404	1.28237	1.343368	1.21602	0.2822	0.462501	1
ANKRD33B	1.052959	1.093472	1.03979	0.42569	-1.2321	0.462669	1
ASH1L	2.148736	2.207652	2.129586	0.93536	-0.0964	0.462791	1
CFAP46	1.134721	1.092715	1.148375	1.60034	0.6784	0.4629	1
APC2	1.095595	1.054191	1.109054	2.01242	1.0089	0.462944	1
MIR181A2HG	1.182926	1.140112	1.196843	1.4049	0.4905	0.463013	1
RIBC2	1.063259	1.022346	1.076558	3.42604	1.7765	0.463072	1
RP4-621F18.2	1.090814	1.131609	1.077553	0.58927	-0.763	0.463099	1
C10orf107	1.04582	1.086232	1.032684	0.37902	-1.3996	0.46311	1
ZNF649	1.170415	1.127543	1.184351	1.44541	0.5315	0.463367	1
UBR2	1.305599	1.350514	1.290999	0.83021	-0.2685	0.463419	1
PDE6H	1.039302	1.079494	1.026237	0.33005	-1.5993	0.463457	1
RP5-1136G13.2	1.149991	1.107208	1.163898	1.52879	0.6124	0.463643	1
DCAF6	1.281762	1.325996	1.267383	0.8202	-0.2859	0.463679	1
SERTAD3	1.31887	1.363844	1.304251	0.83621	-0.2581	0.463739	1
RP11-159H10.3	1.080425	1.039051	1.093874	2.40392	1.2654	0.463754	1
PDZRN3	1.274936	1.318977	1.26062	0.81705	-0.2915	0.463782	1
RP11-412D9.4	1.156496	1.113877	1.170349	1.4959	0.581	0.463857	1
STX8	1.804023	1.751608	1.821061	1.09241	0.1275	0.464288	1
TIMM8A	1.283912	1.239317	1.298408	1.24692	0.3184	0.464323	1
RP5-1042K10.10	1.112629	1.070969	1.126171	1.77783	0.8301	0.464335	1
SNX8	1.157785	1.19996	1.144076	0.72052	-0.4729	0.464362	1
ORC2	1.188124	1.144943	1.20216	1.39476	0.48	0.464808	1
TLCD2	1.123469	1.16483	1.110024	0.6675	-0.5832	0.464865	1
RP5-1198O20.4	1.103106	1.061611	1.116594	1.89244	0.9202	0.464867	1
SH3GL1	1.476322	1.428572	1.491844	1.14764	0.1987	0.46489	1
SEMA5B	1.078163	1.037035	1.091531	2.47147	1.3054	0.464956	1
ATXN7L3	1.232806	1.275968	1.218776	0.79276	-0.335	0.464978	1
RP11-666O2.2	1.077212	1.035943	1.090627	2.52139	1.3342	0.464986	1
SEPT7	4.973153	4.793856	5.031435	1.06262	0.0876	0.464988	1
SCRN3	1.338376	1.292391	1.353324	1.20839	0.2731	0.465125	1
LINC01311	1.070835	1.029944	1.084128	2.8095	1.4903	0.465289	1

AGT	1.039539	1.079753	1.026467	0.33187	-1.5913	0.46529	1
SGK223	1.085384	1.126089	1.072152	0.57224	-0.8053	0.465312	1
SLC47A1	1.053396	1.09389	1.040234	0.42852	-1.2226	0.465333	1
PAK4	1.489922	1.537697	1.474393	0.88227	-0.1807	0.465358	1
ALDH16A1	1.153154	1.110811	1.166918	1.50633	0.591	0.465406	1
PCGF5	1.317512	1.362178	1.302994	0.83659	-0.2574	0.465601	1
COL17A1	1.026746	1.066383	1.013862	0.20881	-2.2597	0.465759	1
SLC43A2	1.102408	1.061119	1.115829	1.89513	0.9223	0.465798	1
SMARCA1	2.071156	2.009724	2.091125	1.08062	0.1119	0.465813	1
SEC14L2	1.292065	1.33619	1.277721	0.82608	-0.2756	0.46584	1
ACSM3	1.049084	1.089339	1.035999	0.40294	-1.3114	0.465926	1
FUT4	1.055959	1.096058	1.042925	0.44686	-1.1621	0.465944	1
TTC38	1.170413	1.127876	1.18424	1.44077	0.5268	0.466084	1
UBE2D2	4.878666	5.051244	4.822569	0.94355	-0.0838	0.466159	1
SOCS7	1.202309	1.159341	1.216276	1.35732	0.4408	0.466226	1
MYSM1	1.265086	1.220668	1.279525	1.26672	0.3411	0.466341	1
RLF	1.559538	1.510496	1.57548	1.1273	0.1729	0.466394	1
CACFD1	1.288795	1.24381	1.303417	1.24448	0.3155	0.466521	1
MTRNR2L8	1.052474	1.09285	1.039349	0.42379	-1.2386	0.466621	1
KIAA0232	1.588062	1.538654	1.604123	1.12154	0.1655	0.466629	1
NACA2	1.162317	1.119973	1.176081	1.46767	0.5535	0.467214	1
CHEK2	1.148884	1.107305	1.1624	1.51344	0.5978	0.46725	1
PPP5C	1.338595	1.292928	1.353439	1.20657	0.2709	0.467283	1
METTL3	1.402645	1.356297	1.417711	1.17237	0.2294	0.467411	1
METTL2A	1.306163	1.261552	1.320664	1.226	0.294	0.467713	1
SYF2	2.583093	2.509438	2.607035	1.06466	0.0904	0.467762	1
ATG3	1.575514	1.526213	1.59154	1.12415	0.1688	0.467771	1
RBSN	1.281148	1.236833	1.295553	1.24793	0.3195	0.467815	1
CHKB	1.242115	1.198596	1.256261	1.29036	0.3678	0.467987	1
GANAB	1.787354	1.734244	1.804618	1.09585	0.132	0.46812	1
GAA	1.277316	1.321307	1.263017	0.81858	-0.2888	0.468249	1
CAMLG	4.047523	3.925885	4.087062	1.05509	0.0774	0.468357	1
FAM171A1	1.41003	1.455909	1.395116	0.86666	-0.2065	0.468438	1
PEX16	1.341901	1.296431	1.356682	1.20325	0.2669	0.468518	1
TJAP1	1.191032	1.233422	1.177252	0.75936	-0.3971	0.468721	1
LPIN1	1.310025	1.265299	1.324564	1.22339	0.2909	0.468792	1
SLC25A44	1.099164	1.139623	1.086012	0.61603	-0.6989	0.468811	1
NUP153	1.266779	1.222701	1.281107	1.26226	0.336	0.468884	1
PIK3CB	1.127512	1.168546	1.114173	0.6774	-0.5619	0.469	1
CEP131	1.22741	1.184371	1.2414	1.30932	0.3888	0.469014	1
SLC30A5	1.390849	1.436555	1.375992	0.86127	-0.2155	0.46903	1
RP11-496I9.1	1.180867	1.138067	1.19478	1.41076	0.4965	0.469053	1
LRRN3	1.091892	1.132371	1.078734	0.5948	-0.7495	0.469077	1
SH2D4A	1.061062	1.10098	1.048086	0.47619	-1.0704	0.469121	1
ATCAY	1.131638	1.172949	1.11821	0.6835	-0.549	0.469163	1

ZBTB33	1.188921	1.146122	1.202833	1.38811	0.4731	0.469264	1
ZMYND10	1.088349	1.047595	1.101597	2.13462	1.094	0.469305	1
DNAH9	1.076318	1.035939	1.089444	2.48879	1.3154	0.469312	1
GAL3ST3	1.213919	1.170852	1.227919	1.33401	0.4158	0.469323	1
FKRP	1.142682	1.18416	1.129199	0.70156	-0.5114	0.46942	1
FREM2	1.040298	1.079967	1.027404	0.34268	-1.545	0.469517	1
TWIST1	1.029967	1.069554	1.017099	0.24583	-2.0243	0.469557	1
RP11-763B22.4	1.072427	1.031562	1.08571	2.7156	1.4413	0.469739	1
TCFL5	1.101241	1.142043	1.087978	0.61937	-0.6911	0.469884	1
KPNA4	1.925111	1.868971	1.94336	1.08561	0.1185	0.469898	1
NSDHL	1.180405	1.222572	1.166699	0.74897	-0.417	0.469963	1
ANGEL2	1.250084	1.206375	1.264292	1.28064	0.3569	0.469989	1
CEP192	1.195911	1.153222	1.209788	1.36917	0.4533	0.469991	1
GDF5	1.176741	1.218357	1.163213	0.74746	-0.4199	0.470013	1
TMEM150A	1.292729	1.336614	1.278464	0.82725	-0.2736	0.470187	1
ST6GALNAC2	1.039137	1.078847	1.026229	0.33266	-1.5879	0.470311	1
AC007163.3	1.05496	1.014806	1.068012	4.59359	2.1996	0.470346	1
USP25	1.149462	1.191107	1.135925	0.71125	-0.4916	0.470388	1
SLC35A4	1.428624	1.382057	1.44376	1.1615	0.216	0.470747	1
GHRL	1.079891	1.039297	1.093087	2.36884	1.2442	0.470772	1
MINOS1	4.515827	4.379093	4.560274	1.05362	0.0754	0.470782	1
HLA-DQB1	1.073521	1.113622	1.060486	0.53234	-0.9096	0.47112	1
COPG1	1.284831	1.328352	1.270683	0.82437	-0.2786	0.471223	1
NOTCH1	1.281563	1.325417	1.267307	0.82143	-0.2838	0.47129	1
CPED1	1.028503	1.067978	1.015672	0.23054	-2.1169	0.471316	1
TRIM26	1.132782	1.091144	1.146316	1.60533	0.6829	0.471666	1
NXPH4	1.12314	1.081991	1.136516	1.665	0.7355	0.471938	1
PFDN1	2.463318	2.523244	2.443839	0.94787	-0.0772	0.472205	1
DOCK7	1.574386	1.62296	1.558596	0.89668	-0.1573	0.472226	1
ZNF768	1.25783	1.214352	1.271963	1.26877	0.3434	0.472304	1
UBR1	1.218935	1.261441	1.205118	0.78457	-0.35	0.472384	1
FGD1	1.145575	1.103921	1.159115	1.53112	0.6146	0.472624	1
PVALB	1.126427	1.167553	1.113058	0.67476	-0.5675	0.472755	1
MIR193BHG	1.04924	1.088702	1.036413	0.41051	-1.2845	0.472776	1
AMN	1.043577	1.083213	1.030693	0.36884	-1.4389	0.472829	1
XAB2	1.252046	1.208473	1.26621	1.27695	0.3527	0.472835	1
VGLL1	1.029696	1.068998	1.016921	0.24524	-2.0277	0.472858	1
GABRA2	1.10545	1.064498	1.118762	1.84134	0.8808	0.473125	1
MAL	1.411857	1.478645	1.390147	0.81511	-0.2949	0.473149	1
LTBR	1.067497	1.107319	1.054552	0.50832	-0.9762	0.473193	1
LRRC40	1.205759	1.163321	1.219553	1.3443	0.4269	0.473259	1
PRPF8	1.677968	1.627951	1.694226	1.10554	0.1448	0.473264	1
PIAS2	2.172705	2.114954	2.191478	1.06863	0.0958	0.473276	1
GRB2	1.909696	1.856478	1.926994	1.08233	0.1141	0.473351	1
RP11-247A12.2	1.085013	1.125029	1.072005	0.57591	-0.7961	0.47347	1

UIMC1	1.327103	1.282257	1.34168	1.21053	0.2756	0.473499	1
SLC50A1	1.637123	1.587337	1.653307	1.11232	0.1536	0.473641	1
BRCC3	1.248849	1.205824	1.262834	1.27698	0.3527	0.473648	1
CYP2S1	1.032575	1.071603	1.019889	0.27777	-1.848	0.473788	1
NPTXR	1.080348	1.120512	1.067293	0.55839	-0.8406	0.473899	1
CTBP1-AS2	1.210436	1.167822	1.224288	1.33646	0.4184	0.474051	1
P2RY1	1.114681	1.073794	1.127972	1.73418	0.7943	0.474232	1
HS6ST1	1.212349	1.169759	1.226194	1.33244	0.4141	0.474233	1
OMA1	1.144406	1.102967	1.157875	1.53326	0.6166	0.47424	1
SRGAP1	1.613042	1.563766	1.629059	1.11582	0.1581	0.474329	1
CSRNP3	1.414573	1.368594	1.429519	1.16529	0.2207	0.474337	1
EIF4E	2.214102	2.157939	2.232358	1.06427	0.0899	0.474379	1
MARK3	1.691109	1.740811	1.674953	0.9111	-0.1343	0.474525	1
CTNNB1	3.354718	3.223351	3.397419	1.07829	0.1087	0.474564	1
MTRR	1.132947	1.091504	1.146418	1.60012	0.6782	0.475001	1
SLC1A4	1.082897	1.042579	1.096003	2.25469	1.1729	0.475029	1
NMD3	1.477504	1.430694	1.492721	1.14402	0.1941	0.47508	1
TMEM254-AS1	1.080747	1.040602	1.093796	2.31015	1.208	0.475414	1
RASGEF1A	1.071614	1.111402	1.05868	0.52674	-0.9248	0.475432	1
FAM83G	1.065614	1.10547	1.052658	0.49927	-1.0021	0.475589	1
OTUD5	1.55778	1.604888	1.542467	0.89681	-0.1571	0.475628	1
C1orf198	1.409899	1.45478	1.39531	0.86923	-0.2022	0.475698	1
CDH3	1.060437	1.100198	1.047513	0.47419	-1.0765	0.476083	1
RD3	1.032736	1.071915	1.020001	0.27813	-1.8462	0.476126	1
EFCAB7	1.157169	1.115849	1.1706	1.47261	0.5584	0.476139	1
RP11-1008C21.2	1.068611	1.028803	1.081551	2.8313	1.5015	0.476252	1
NSUN2	1.140342	1.180929	1.127149	0.70276	-0.5089	0.476296	1
REXO4	1.711144	1.660352	1.727655	1.10192	0.14	0.476297	1
RFWD2	1.382175	1.336676	1.396964	1.17907	0.2376	0.47646	1
ERF	1.220768	1.262821	1.207099	0.78798	-0.3438	0.47652	1
RP11-274B21.10	1.062048	1.022211	1.074998	3.37657	1.7556	0.476574	1
CISH	1.113133	1.072371	1.126384	1.74633	0.8043	0.476576	1
CASP9	1.144473	1.103475	1.157799	1.525	0.6088	0.476646	1
B3GNT5	1.188375	1.146353	1.202035	1.38046	0.4651	0.476707	1
IRX3	1.053335	1.092682	1.040545	0.43747	-1.1928	0.476768	1
TMEM209	1.179761	1.138002	1.193335	1.40096	0.4864	0.476879	1
PACSIN2	1.334586	1.290187	1.349018	1.20274	0.2663	0.476992	1
BUD13	1.166089	1.124377	1.179648	1.44437	0.5304	0.477042	1
CDADC1	1.27673	1.233166	1.29089	1.24757	0.3191	0.477055	1
ZNF367	1.069307	1.029508	1.082244	2.78718	1.4788	0.477292	1
NPEPPS	2.554724	2.47578	2.580385	1.07088	0.0988	0.477316	1
ADCK5	1.140963	1.099582	1.154414	1.55061	0.6328	0.47743	1
MID1	2.321982	2.256972	2.343114	1.06853	0.0956	0.477532	1
C11orf74	1.897789	1.844405	1.915142	1.08377	0.1161	0.477533	1
NUDT6	1.221564	1.179396	1.23527	1.31146	0.3912	0.477559	1

HSBP1	11.05428	10.70495	11.16783	1.0477	0.0672	0.477619	1
RRAGB	1.310202	1.265912	1.324599	1.2207	0.2877	0.477723	1
GPC2	1.477647	1.431144	1.492764	1.14292	0.1927	0.477818	1
ZNF529-AS1	1.17875	1.219966	1.165352	0.75171	-0.4117	0.47782	1
PDZD11	1.892323	1.839465	1.909505	1.08343	0.1156	0.477873	1
HBQ1	1.109145	1.068616	1.122319	1.78266	0.834	0.477913	1
DGCR14	1.164922	1.123594	1.178356	1.44308	0.5292	0.478118	1
NES	1.921732	1.866558	1.939667	1.08437	0.1169	0.478425	1
AK9	1.194871	1.152815	1.208541	1.36466	0.4485	0.478568	1
MTIF3	2.386354	2.450311	2.365564	0.94157	-0.0869	0.478631	1
ABCD3	1.298042	1.254461	1.312208	1.22694	0.2951	0.478651	1
CUL5	1.857874	1.806107	1.874701	1.08509	0.1178	0.478782	1
RP11-708J19.1	1.124162	1.083377	1.13742	1.64817	0.7209	0.478786	1
GLRB	1.146942	1.187714	1.133688	0.71219	-0.4897	0.479128	1
DARS-AS1	1.087892	1.047636	1.100977	2.11976	1.0839	0.479147	1
BBS2	1.269564	1.226091	1.283696	1.25479	0.3274	0.479303	1
TICAM1	1.091951	1.131679	1.079037	0.60023	-0.7364	0.47939	1
CTB-113I20.2	1.186801	1.145031	1.200379	1.38163	0.4664	0.479593	1
ARF5	2.498244	2.431161	2.52005	1.06211	0.0869	0.479697	1
KEAP1	1.593789	1.54565	1.609437	1.1169	0.1595	0.479725	1
ACTR10	1.942071	1.994888	1.924903	0.92965	-0.1052	0.479736	1
MYO3B	1.034269	1.072848	1.021728	0.29827	-1.7453	0.479743	1
SLC25A21	1.091309	1.051257	1.104328	2.03539	1.0253	0.479749	1
HES2	1.041095	1.079875	1.028489	0.35666	-1.4874	0.479767	1
LSP1	1.034178	1.072646	1.021674	0.29835	-1.7449	0.47983	1
NEIL1	1.352247	1.307615	1.366755	1.19225	0.2537	0.479938	1
THEM6	1.228644	1.186353	1.242391	1.30071	0.3793	0.48022	1
ANO1	1.058525	1.019218	1.071303	3.71024	1.8915	0.480231	1
TSFM	1.315145	1.271585	1.329305	1.21253	0.278	0.480271	1
MED22	1.255952	1.213203	1.269848	1.26569	0.3399	0.480329	1
FZD8	1.101408	1.061094	1.114512	1.87436	0.9064	0.480577	1
ZFP30	1.22784	1.185471	1.241613	1.3027	0.3815	0.480734	1
ZZZ3	1.458368	1.41244	1.473298	1.14755	0.1986	0.480769	1
ASNA1	1.817613	1.868547	1.801056	0.9223	-0.1167	0.480945	1
TRAIP	1.080709	1.041174	1.09356	2.27231	1.1842	0.48108	1
AC142472.6	1.104977	1.064878	1.118011	1.81896	0.8631	0.481107	1
RBKS	1.094662	1.054653	1.107667	1.97	0.9782	0.481187	1
C20orf194	1.229891	1.187628	1.243629	1.29847	0.3768	0.481514	1
MSL2	1.181989	1.140414	1.195503	1.39233	0.4775	0.48154	1
KCNH2	1.172179	1.213169	1.158854	0.74521	-0.4243	0.481933	1
C8orf37	1.108203	1.068206	1.121204	1.77702	0.8295	0.481943	1
SLC16A2	1.514065	1.467699	1.529137	1.13136	0.1781	0.482009	1
RILPL1	1.189836	1.231062	1.176435	0.76358	-0.3891	0.482018	1
SLC2A4RG	1.363214	1.40671	1.349075	0.85829	-0.2205	0.482174	1
RND1	1.062155	1.022851	1.074932	3.2792	1.7133	0.482195	1

GSAP	1.059341	1.020065	1.072107	3.59361	1.8454	0.482244	1
ZFAND3	1.866148	1.814064	1.883078	1.08478	0.1174	0.482251	1
KIAA1024L	1.059304	1.020132	1.072037	3.57829	1.8393	0.482332	1
PRDM4	1.305538	1.262046	1.319675	1.21992	0.2868	0.482485	1
SNX13	1.361783	1.317323	1.376235	1.18565	0.2457	0.482592	1
KRR1	1.5416	1.494778	1.556819	1.12539	0.1704	0.482668	1
ERMARD	1.135336	1.094692	1.148548	1.56875	0.6496	0.482739	1
TRO	1.235704	1.193707	1.249355	1.28728	0.3643	0.482872	1
PET100	3.431595	3.317277	3.468754	1.06537	0.0914	0.482927	1
PTGER4	1.050062	1.088706	1.0375	0.42275	-1.2421	0.483032	1
NFATC2	1.043176	1.081711	1.030649	0.37509	-1.4147	0.483089	1
LRIG1	1.120528	1.160081	1.107671	0.67261	-0.5722	0.483137	1
ST6GALNAC4	1.199349	1.157767	1.212866	1.34924	0.4321	0.483222	1
CSTF2T	1.175643	1.134646	1.188969	1.40345	0.489	0.483385	1
NOA1	1.463229	1.417263	1.47817	1.14597	0.1966	0.483484	1
KCNAB1	1.040738	1.079431	1.028161	0.35454	-1.496	0.483606	1
CEP85	1.136618	1.096388	1.149695	1.55304	0.6351	0.484113	1
STK11IP	1.151879	1.111312	1.165066	1.48291	0.5684	0.484164	1
SLC30A9	1.727421	1.776422	1.711493	0.91637	-0.126	0.484383	1
NEPRO	1.388094	1.344069	1.402404	1.16955	0.2259	0.484401	1
DTX2	1.108467	1.147877	1.095657	0.64687	-0.6285	0.48446	1
ANKMY2	1.227834	1.185895	1.241467	1.29894	0.3773	0.48463	1
PNMAL2	1.086669	1.047113	1.099526	2.11249	1.0789	0.484655	1
INSM1	1.056297	1.017199	1.069006	4.01224	2.0044	0.484659	1
NANP	1.10163	1.061764	1.114588	1.85525	0.8916	0.484904	1
EPG5	1.189675	1.230519	1.176399	0.76523	-0.386	0.485001	1
KIAA0895L	1.341633	1.297961	1.355829	1.19421	0.2561	0.485104	1
ARPP19	3.173662	3.092782	3.199953	1.05121	0.0721	0.485132	1
LHCGR	1.054844	1.015934	1.067492	4.23563	2.0826	0.485182	1
CTB-193M12.5	1.06003	1.021087	1.072688	3.44712	1.7854	0.485216	1
RALGAPA1	1.541789	1.587886	1.526805	0.8961	-0.1583	0.485349	1
GK5	1.295219	1.252453	1.30912	1.22447	0.2922	0.485383	1
CTSA	1.427059	1.471584	1.412586	0.87489	-0.1928	0.485455	1
CAMK2G	1.245651	1.286952	1.232226	0.80928	-0.3053	0.485705	1
RP11-97C16.1	1.086344	1.046961	1.099146	2.11122	1.0781	0.485726	1
ETAA1	1.395046	1.350577	1.409501	1.16808	0.2241	0.485751	1
MYLIP	1.113179	1.152742	1.100319	0.65679	-0.6065	0.485806	1
AGBL3	1.104748	1.064791	1.117736	1.81715	0.8617	0.485863	1
ZNF382	1.166246	1.12545	1.179507	1.43091	0.5169	0.485908	1
ORAI2	1.862883	1.809035	1.880387	1.08819	0.1219	0.486216	1
MAN1B1	1.387624	1.431482	1.373368	0.86532	-0.2087	0.486332	1
STMN2	2.256205	2.407273	2.207099	0.85776	-0.2214	0.486344	1
KMT5B	1.667225	1.618486	1.683068	1.10442	0.1433	0.486411	1
DCUN1D1	1.488744	1.442931	1.503636	1.13705	0.1853	0.486444	1
CACNA2D2	1.084618	1.045365	1.097377	2.14654	1.102	0.486498	1

AC093323.3	1.332688	1.289077	1.346864	1.1999	0.2629	0.486644	1
CTSK	1.071383	1.110155	1.058779	0.5336	-0.9062	0.486722	1
WDR78	1.158982	1.118025	1.172296	1.45983	0.5458	0.486924	1
MBNL1	1.486648	1.440362	1.501694	1.13928	0.1881	0.48705	1
RAB11FIP2	1.424694	1.379808	1.439284	1.15659	0.2099	0.487186	1
G6PC3	2.340231	2.280761	2.359562	1.06153	0.0861	0.487255	1
UBB	8.035811	8.255405	7.96443	0.9599	-0.0591	0.487279	1
SYDE1	1.173095	1.213277	1.160034	0.75035	-0.4144	0.487573	1
WDR46	1.327601	1.370788	1.313563	0.84567	-0.2418	0.487639	1
ZSWIM8	1.156046	1.19588	1.143098	0.73054	-0.453	0.487673	1
RAPGEF2	1.202972	1.24354	1.189785	0.77927	-0.3598	0.487677	1
CNOT11	1.303183	1.345285	1.289498	0.83843	-0.2542	0.487841	1
GOLGA8B	1.659054	1.706745	1.643551	0.91058	-0.1351	0.487869	1
VARS2	1.139161	1.099101	1.152183	1.53563	0.6188	0.487939	1
TICRR	1.057531	1.018989	1.070059	3.68937	1.8834	0.488004	1
PRR5	1.241357	1.282533	1.227972	0.80689	-0.3096	0.48801	1
TBC1D8B	1.151375	1.191218	1.138424	0.72391	-0.4661	0.48829	1
SLC35G2	1.197134	1.156023	1.210498	1.34915	0.4321	0.488305	1
ZNF814	1.348963	1.305432	1.363113	1.18885	0.2496	0.488354	1
IPMK	1.13804	1.097871	1.151098	1.54385	0.6265	0.488409	1
FAM72D	1.061308	1.022685	1.073862	3.25594	1.7031	0.488503	1
CALM2	13.08121	13.40569	12.97573	0.96534	-0.0509	0.488506	1
ZNF582	1.118332	1.078568	1.131257	1.67063	0.7404	0.488582	1
VPS37C	1.158034	1.197974	1.145051	0.73267	-0.4488	0.488753	1
RNF219	1.323505	1.280176	1.337589	1.20492	0.2689	0.488755	1
CH17-264L24.1	1.117415	1.077517	1.130384	1.682	0.7502	0.488792	1
THAP4	1.321328	1.363866	1.307501	0.8451	-0.2428	0.488853	1
KLHL2	1.097226	1.136085	1.084595	0.62164	-0.6859	0.489113	1
S1PR1	1.097244	1.135931	1.084668	0.62287	-0.683	0.489204	1
CPT1C	1.316453	1.273541	1.330401	1.20787	0.2725	0.489302	1
VPS37D	1.253362	1.294722	1.239918	0.81405	-0.2968	0.489304	1
RBAK-RBAKDN	1.24729	1.205342	1.260925	1.27069	0.3456	0.489317	1
ASTN1	1.182153	1.141157	1.195479	1.38483	0.4697	0.489411	1
RP11-480A16.1	1.100672	1.061394	1.11344	1.84774	0.8858	0.489552	1
RP11-572O17.1	1.079522	1.040556	1.092188	2.27311	1.1847	0.489563	1
SYNGR3	1.176488	1.135906	1.18968	1.39567	0.481	0.490037	1
RP11-53O19.1	1.165353	1.12494	1.178489	1.4286	0.5146	0.490065	1
PSORS1C1	1.045372	1.083066	1.03312	0.39872	-1.3266	0.490081	1
RNF2	1.443635	1.398734	1.458231	1.14921	0.2006	0.490332	1
ATPAF1	1.541164	1.49496	1.556183	1.12369	0.1683	0.49038	1
ALKBH7	3.487665	3.371421	3.525451	1.06495	0.0908	0.490413	1
RWDD2A	1.172596	1.131948	1.185809	1.4082	0.4939	0.490414	1
CCDC71	1.262356	1.303876	1.24886	0.81895	-0.2881	0.490468	1
PSENEN	2.56593	2.631102	2.544745	0.94706	-0.0785	0.490598	1
CD58	1.200654	1.240761	1.187617	0.77927	-0.3598	0.490605	1

MSLN	1.029877	1.067484	1.017652	0.26158	-1.9347	0.490712	1
ELP3	1.1756	1.135197	1.188734	1.396	0.4813	0.490788	1
NXT1	1.738749	1.689877	1.754635	1.09387	0.1294	0.490911	1
RP11-247A12.8	1.102804	1.141452	1.090242	0.63797	-0.6484	0.490988	1
ACBD5	1.389856	1.432684	1.375935	0.86884	-0.2028	0.491174	1
CSNK2B	3.77687	3.871764	3.746024	0.95621	-0.0646	0.491315	1
OR2A1-AS1	1.068165	1.106452	1.05572	0.52343	-0.9339	0.491578	1
ARMCX6	1.756151	1.707143	1.772081	1.09183	0.1267	0.49164	1
RGP1	1.531746	1.485897	1.54665	1.12503	0.17	0.491668	1
TBCA	9.280246	8.999266	9.37158	1.04654	0.0656	0.491874	1
DDX1	1.951041	1.896705	1.968703	1.08029	0.1114	0.491898	1
ANKRD35	1.169187	1.128573	1.182389	1.41856	0.5044	0.491924	1
CUL1	1.679803	1.727951	1.664152	0.91236	-0.1323	0.491969	1
GS1-124K5.3	1.05753	1.019215	1.069985	3.64224	1.8648	0.49213	1
NBPF20	1.204149	1.163398	1.217396	1.33046	0.4119	0.492142	1
GCC2	2.051218	2.10712	2.033046	0.93309	-0.0999	0.492152	1
TEX30	1.376487	1.332902	1.390655	1.17348	0.2308	0.492247	1
C4orf36	1.084272	1.04558	1.096849	2.12483	1.0873	0.49234	1
FAM118B	1.179046	1.219291	1.165964	0.75682	-0.402	0.492468	1
ZNF394	1.179226	1.218967	1.166308	0.75951	-0.3969	0.492468	1
ZNF658	1.078731	1.039813	1.091382	2.29525	1.1987	0.492887	1
RNF168	1.421375	1.377234	1.435724	1.15505	0.208	0.492898	1
PRLR	1.057398	1.018986	1.069885	3.68081	1.88	0.492977	1
COX4I2	1.08235	1.043677	1.094921	2.17326	1.1199	0.493209	1
PLEKHO2	1.090039	1.128632	1.077494	0.60245	-0.7311	0.493333	1
ZNF10	1.089589	1.050794	1.1022	2.01204	1.0087	0.493371	1
FAM133A	1.146075	1.106268	1.159014	1.49635	0.5814	0.493423	1
SMC5	1.794224	1.744957	1.810238	1.08763	0.1212	0.4935	1
RP11-107N15.1	1.079631	1.040789	1.092257	2.26181	1.1775	0.493512	1
WDR47	1.120181	1.15914	1.107518	0.67562	-0.5657	0.493644	1
CCDC92	1.192662	1.232448	1.17973	0.7732	-0.3711	0.493713	1
KCTD9	1.187357	1.227655	1.174258	0.76545	-0.3856	0.493847	1
G6PD	1.822913	1.872055	1.80694	0.92533	-0.112	0.493891	1
HOXB4	1.123704	1.082944	1.136954	1.65117	0.7235	0.494031	1
AMFR	1.343004	1.38432	1.329574	0.85755	-0.2217	0.494292	1
CSE1L	1.460429	1.41639	1.474744	1.14014	0.1892	0.49436	1
NFRKB	1.19603	1.155439	1.209225	1.34602	0.4287	0.494838	1
KIFC2	1.141809	1.180765	1.129147	0.71444	-0.4851	0.495416	1
CYP20A1	1.229504	1.188801	1.242735	1.28566	0.3625	0.495603	1
FN3KRP	1.369993	1.326786	1.384038	1.1752	0.2329	0.495624	1
SLC35B3	1.255205	1.296324	1.241839	0.81613	-0.2931	0.495796	1
RP11-793H13.11	1.067454	1.029433	1.079813	2.71165	1.4392	0.495965	1
CAMKMT	1.224698	1.183764	1.238004	1.29516	0.3731	0.495995	1
TMEM132B	1.092447	1.053922	1.10497	1.94669	0.961	0.49606	1
C1orf50	1.184936	1.144526	1.198071	1.37049	0.4547	0.496081	1

RP5-832C2.5	1.077901	1.039425	1.090408	2.29317	1.1973	0.496314	1
EPHA7	1.086316	1.124575	1.07388	0.59306	-0.7537	0.496348	1
SCEL	1.029836	1.067421	1.017618	0.26132	-1.9361	0.496438	1
PSTPIP2	1.149582	1.109933	1.16247	1.4779	0.5635	0.496439	1
ZNF483	1.099381	1.060642	1.111974	1.84647	0.8848	0.49649	1
WDSUB1	1.189322	1.149084	1.202401	1.35764	0.4411	0.496511	1
THAP1	1.281864	1.240173	1.295416	1.23001	0.2987	0.496514	1
IQCH	1.06966	1.031499	1.082064	2.60528	1.3814	0.496593	1
TBP	1.246014	1.204768	1.259421	1.2669	0.3413	0.496841	1
ARHGAP8	1.04829	1.085748	1.036114	0.42116	-1.2476	0.496854	1
TTC23	1.220951	1.180413	1.234129	1.29773	0.376	0.497064	1
SNX33	1.096943	1.135148	1.084524	0.62542	-0.6771	0.497303	1
GRK4	1.188382	1.148036	1.201496	1.36113	0.4448	0.497462	1
ANO10	1.305576	1.346864	1.292155	0.84228	-0.2476	0.497741	1
E2F8	1.062051	1.02419	1.074359	3.07393	1.6201	0.497804	1
BAG6	2.028187	1.976193	2.045088	1.07058	0.0984	0.497834	1
RP11-486O12.2	1.077836	1.039599	1.090266	2.27951	1.1887	0.497892	1
STK38L	1.430894	1.473662	1.416992	0.88036	-0.1838	0.497932	1
TMEM119	1.02405	1.061114	1.012002	0.19639	-2.3482	0.498053	1
PTPN23	1.146396	1.185456	1.133699	0.72092	-0.4721	0.498174	1
PANK1	1.176065	1.215484	1.163252	0.75761	-0.4005	0.498186	1
RAD54B	1.150473	1.11089	1.163339	1.47298	0.5587	0.498277	1
CYB5D1	1.113667	1.074919	1.126262	1.68532	0.753	0.498495	1
DLGAP1-AS2	1.129169	1.167594	1.116679	0.6962	-0.5224	0.498535	1
ANKLE2	1.663165	1.70903	1.648257	0.91429	-0.1293	0.498898	1
TMEM223	1.610272	1.563825	1.62537	1.10916	0.1495	0.498964	1
DIRC2	1.111898	1.149938	1.099534	0.66383	-0.5911	0.498972	1
C21orf62-AS1	1.08599	1.047623	1.098462	2.06752	1.0479	0.499183	1
PDSS1	1.100581	1.138724	1.088182	0.63567	-0.6537	0.49955	1
NSA2	3.405516	3.309866	3.436608	1.05487	0.0771	0.499718	1
TMEM192	1.383054	1.339864	1.397094	1.16839	0.2245	0.499788	1
GPER1	1.092069	1.053797	1.10451	1.94266	0.958	0.499849	1
SEC14L6	1.074683	1.112205	1.062486	0.55689	-0.8445	0.500154	1
PRELID3B	1.27929	1.237897	1.292745	1.23056	0.2993	0.500161	1
TINCR	1.056803	1.094272	1.044624	0.47335	-1.079	0.500238	1
KIF9	1.429618	1.38581	1.443858	1.15046	0.2022	0.50034	1
UROD	1.732317	1.779578	1.716955	0.91967	-0.1208	0.500396	1
SLC25A53	1.188792	1.148937	1.201748	1.35459	0.4379	0.500408	1
AC010729.2	1.06322	1.025373	1.075522	2.97651	1.5736	0.500662	1
RP13-436F16.1	1.039447	1.076478	1.02741	0.35841	-1.4803	0.500678	1
ZNF469	1.047167	1.084204	1.035128	0.41718	-1.2613	0.500736	1
DRAXIN	1.070019	1.032029	1.082368	2.57163	1.3627	0.50085	1
C16orf52	1.22479	1.264655	1.211831	0.8004	-0.3212	0.500894	1
CIB2	1.457993	1.414434	1.472152	1.13927	0.1881	0.500901	1
SENP5	1.563621	1.608554	1.549015	0.90216	-0.1485	0.500924	1

PLEKHG6	1.033418	1.070268	1.021439	0.3051	-1.7126	0.501005	1
SIPA1L1	1.210957	1.250757	1.19802	0.78969	-0.3406	0.501033	1
GTDC1	1.220392	1.180163	1.233469	1.29588	0.3739	0.501075	1
E2F1	1.117971	1.079052	1.130622	1.65235	0.7245	0.501115	1
LAP3	1.439616	1.39607	1.453771	1.14568	0.1962	0.501209	1
TET3	1.104095	1.142154	1.091724	0.64524	-0.6321	0.501349	1
CLPX	1.441762	1.398558	1.455805	1.14363	0.1936	0.50143	1
LRP1B	1.074375	1.036305	1.08675	2.38945	1.2567	0.501599	1
PITPNC1	1.20414	1.243875	1.191224	0.78411	-0.3509	0.501708	1
WIF1	1.024535	1.061387	1.012556	0.20454	-2.2896	0.501757	1
PRDX4	5.950437	6.118651	5.895758	0.95645	-0.0642	0.501907	1
ADAMTS9-AS2	1.125351	1.086875	1.137857	1.58684	0.6662	0.501958	1
TBC1D32	1.203054	1.24264	1.190187	0.78382	-0.3514	0.502132	1
TMEM8B	1.302519	1.261068	1.315993	1.21039	0.2755	0.502222	1
TOR4A	1.062873	1.100011	1.050801	0.50795	-0.9772	0.50226	1
INCENP	1.072348	1.034751	1.084569	2.43356	1.2831	0.502264	1
CARMN	1.051784	1.08911	1.039651	0.44496	-1.1682	0.5023	1
FAM200A	1.238767	1.198322	1.251914	1.27023	0.3451	0.502346	1
TSC1	1.252674	1.211861	1.265941	1.25526	0.328	0.502669	1
SLC16A6	1.057645	1.020122	1.069842	3.47091	1.7953	0.502674	1
CASP4	1.031091	1.067962	1.019106	0.28113	-1.8307	0.502688	1
DOCK9	1.142986	1.181343	1.130517	0.71972	-0.4745	0.502826	1
EMG1.1	1.076646	1.03863	1.089004	2.30398	1.2041	0.502836	1
TTL	1.292254	1.333025	1.279001	0.83778	-0.2554	0.50287	1
FAAP100	1.231841	1.191216	1.245047	1.28152	0.3579	0.502907	1
SOX10	1.028342	1.06498	1.016433	0.25289	-1.9834	0.502922	1
L2HGDH	1.151866	1.11262	1.164623	1.46176	0.5477	0.503066	1
RP11-2E11.9	1.146449	1.107579	1.159084	1.47876	0.5644	0.503245	1
KPNA1	1.400552	1.442178	1.387021	0.87526	-0.1922	0.503474	1
RP11-727F15.11	1.10373	1.065394	1.116191	1.77678	0.8293	0.503542	1
TRIL	1.070343	1.107732	1.05819	0.54013	-0.8886	0.503577	1
ZNF35	1.108503	1.146339	1.096204	0.6574	-0.6051	0.503638	1
AC245100.1	1.127933	1.16586	1.115604	0.697	-0.5208	0.50365	1
RP11-495P10.1	1.078693	1.040747	1.091027	2.23398	1.1596	0.503675	1
UBE2O	1.249279	1.208622	1.262495	1.25823	0.3314	0.503713	1
IFNAR2	1.386349	1.343245	1.400361	1.1664	0.2221	0.503897	1
FKBPL	1.139112	1.100558	1.151645	1.50803	0.5927	0.503961	1
PALB2	1.114033	1.075652	1.12651	1.67227	0.7418	0.504034	1
STAC	1.088935	1.050966	1.101277	1.98717	0.9907	0.504053	1
C9orf172	1.131643	1.092802	1.144269	1.55459	0.6365	0.504104	1
MAP4K3	1.254452	1.294325	1.241491	0.82049	-0.2854	0.504277	1
ODAM	1.028809	1.065381	1.016921	0.25881	-1.95	0.504345	1
EXOC2	1.073718	1.110979	1.061606	0.55511	-0.8492	0.504371	1
DMAP1	1.476133	1.4324	1.490349	1.13402	0.1814	0.504422	1
FAM86B1	1.173796	1.134419	1.186596	1.38817	0.4732	0.504439	1

KLHL30	1.04241	1.07904	1.030504	0.38592	-1.3736	0.504575	1
MTBP	1.088285	1.050604	1.100534	1.98667	0.9904	0.504718	1
MTMR14	1.235771	1.195329	1.248917	1.27435	0.3498	0.50478	1
RP11-91G21.1	1.125006	1.086498	1.137524	1.58992	0.669	0.504813	1
CC2D2A	1.472983	1.516103	1.458967	0.88929	-0.1693	0.504851	1
AP001462.6	1.099944	1.061792	1.112346	1.81813	0.8625	0.504943	1
MORC4	1.366092	1.407931	1.352492	0.8641	-0.2107	0.50504	1
FAM207A	1.516632	1.560396	1.502407	0.89652	-0.1576	0.505151	1
EIF4A3	1.606566	1.561736	1.621138	1.10575	0.145	0.505624	1
GCAT	1.346631	1.305175	1.360106	1.18	0.2388	0.505721	1
ONECUT1	1.052822	1.015676	1.064897	4.14001	2.0496	0.506049	1
RGS8	1.052874	1.015676	1.064966	4.14441	2.0512	0.506049	1
WDR5	1.255891	1.215534	1.26901	1.24811	0.3197	0.506112	1
UTP14A	1.120584	1.082348	1.133013	1.61525	0.6918	0.506229	1
DDR1	2.287704	2.228708	2.306882	1.06362	0.089	0.506468	1
RP11-44N21.1	1.067095	1.02969	1.079253	2.66935	1.4165	0.506788	1
WDR3	1.184235	1.145164	1.196935	1.35663	0.44	0.506824	1
SPATA2	1.08028	1.117474	1.06819	0.58047	-0.7847	0.506827	1
GAD1	1.118795	1.156569	1.106516	0.68031	-0.5557	0.506973	1
ATRNL1	1.115135	1.152575	1.102965	0.67485	-0.5674	0.507079	1
FAF1	1.202081	1.162611	1.21491	1.32162	0.4023	0.507195	1
RP5-1068E13.7	1.11824	1.080163	1.130617	1.6294	0.7043	0.507241	1
TBC1D20	1.361349	1.402171	1.34808	0.8655	-0.2084	0.507475	1
COX18	1.270725	1.230429	1.283823	1.23172	0.3007	0.507504	1
PPP1R14B	2.11212	2.16336	2.095464	0.94164	-0.0868	0.50758	1
COPB2	1.68907	1.734772	1.674215	0.91758	-0.1241	0.507604	1
TMEM38B	1.518096	1.562464	1.503674	0.89548	-0.1593	0.507663	1
GTF3C3	1.303715	1.263055	1.316932	1.20481	0.2688	0.507699	1
AC007773.2	1.116977	1.078956	1.129335	1.63808	0.712	0.507715	1
UTP6	1.265886	1.225707	1.278946	1.23588	0.3055	0.507957	1
FAM13B	1.216611	1.176536	1.229638	1.3008	0.3794	0.508145	1
ZNF740	1.149438	1.110751	1.162013	1.46286	0.5488	0.508166	1
STAU1	2.703842	2.773316	2.681259	0.94809	-0.0769	0.508357	1
TOX2	1.12139	1.083259	1.133785	1.60686	0.6842	0.508361	1
AP5S1	1.167397	1.128509	1.180038	1.40098	0.4864	0.508596	1
SEL1L3	1.346265	1.304062	1.359983	1.18391	0.2436	0.508708	1
PRMT9	1.334878	1.375522	1.321666	0.85658	-0.2233	0.508769	1
ANKS1A	1.194824	1.233415	1.18228	0.78093	-0.3567	0.508876	1
AKAP8	1.201013	1.16152	1.213851	1.32399	0.4049	0.508941	1
MAN2B2	1.119173	1.156842	1.106928	0.68175	-0.5527	0.509247	1
ZNF576	1.309791	1.268375	1.323254	1.20449	0.2684	0.509255	1
FKBP1B	1.728003	1.77305	1.71336	0.92279	-0.1159	0.509288	1
PPTC7	1.112611	1.150071	1.100435	0.66925	-0.5794	0.509297	1
B3GALT6	1.109214	1.146503	1.097093	0.66273	-0.5935	0.509306	1
SRRM4	1.085271	1.047615	1.097512	2.0479	1.0341	0.509375	1

MIR181A1HG	1.16503	1.126237	1.17764	1.4072	0.4928	0.509482	1
SP8	1.072923	1.035812	1.084986	2.37311	1.2468	0.509506	1
SEMA6D	1.125432	1.163125	1.11318	0.69383	-0.5274	0.509745	1
PDP2	1.095427	1.132523	1.083369	0.62909	-0.6687	0.509801	1
MFSD13A	1.074719	1.037582	1.086791	2.30934	1.2075	0.509954	1
IFT140	1.170979	1.132388	1.183523	1.38625	0.4712	0.509956	1
C12orf73	1.83942	1.886217	1.824208	0.93003	-0.1047	0.509975	1
PLPPR5	1.081866	1.119021	1.069789	0.58636	-0.7701	0.51019	1
ZNF579	1.235701	1.274387	1.223126	0.81318	-0.2984	0.510246	1
TFB1M	1.27113	1.230461	1.284349	1.23382	0.3031	0.510309	1
DUSP19	1.088296	1.050935	1.10044	1.97195	0.9796	0.510339	1
A1BG-AS1	1.082547	1.119387	1.070572	0.59111	-0.7585	0.510391	1
TH	1.241324	1.201535	1.254258	1.26161	0.3353	0.51044	1
EFCAB1	1.070668	1.107473	1.058704	0.54622	-0.8724	0.510619	1
BET1L	1.218905	1.257703	1.206294	0.80051	-0.321	0.510623	1
FNIP1	1.801106	1.754359	1.816302	1.08211	0.1139	0.510634	1
ZNF510	1.098766	1.061214	1.110972	1.81284	0.8583	0.51064	1
UFSP2	1.685678	1.640103	1.700493	1.09434	0.1301	0.510715	1
ADGRL1	1.460208	1.502273	1.446535	0.88903	-0.1697	0.510723	1
MROH1	1.12868	1.166138	1.116504	0.70125	-0.512	0.510794	1
UBAC2-AS1	1.154535	1.116165	1.167007	1.43768	0.5237	0.510801	1
CNIH4	2.481771	2.555169	2.457912	0.93746	-0.0932	0.510814	1
MRPL18	2.311714	2.245373	2.333279	1.07059	0.0984	0.51102	1
NDUFC2	4.761906	4.88274	4.722629	0.95876	-0.0608	0.511159	1
TMEM56	1.272859	1.2327	1.285913	1.22868	0.2971	0.511387	1
RP11-872J21.3	1.094731	1.057489	1.106837	1.85839	0.8941	0.511447	1
LAMA3	1.026436	1.062215	1.014806	0.23798	-2.0711	0.511622	1
RDH14	1.519415	1.562385	1.505447	0.89876	-0.154	0.511803	1
RP11-47I22.2	1.035007	1.070884	1.023345	0.32934	-1.6023	0.511814	1
CHMP7	1.303392	1.343514	1.290351	0.84524	-0.2426	0.512184	1
RNF150	1.259377	1.219444	1.272358	1.24112	0.3116	0.512195	1
FAM57A	1.311362	1.351472	1.298324	0.84878	-0.2365	0.512216	1
GOLGA5	1.254071	1.293271	1.241328	0.82288	-0.2812	0.512247	1
ABHD14A	1.865285	1.91298	1.849782	0.93078	-0.1035	0.512452	1
COA7	1.181979	1.143367	1.19453	1.35687	0.4403	0.512455	1
ATP11C	1.491576	1.533559	1.477929	0.89574	-0.1588	0.512576	1
IFNGR2	2.331703	2.384353	2.314588	0.9496	-0.0746	0.5129	1
CREM	1.990713	2.039738	1.974777	0.93752	-0.0931	0.513213	1
RBL1	1.108698	1.071356	1.120836	1.69341	0.7599	0.513343	1
SIGLEC15	1.031845	1.067542	1.020242	0.29969	-1.7385	0.513372	1
SMIM22	1.025823	1.061765	1.01414	0.22893	-2.127	0.513688	1
NLRX1	1.102655	1.065273	1.114806	1.75886	0.8146	0.513702	1
ADORA1	1.062807	1.026165	1.074718	2.85562	1.5138	0.513797	1
BCL3	1.219705	1.180251	1.23253	1.29003	0.3674	0.513886	1
ATP6V0A1	1.506752	1.462908	1.521003	1.1255	0.1706	0.513922	1

WBP4	1.899192	1.850519	1.915013	1.07583	0.1054	0.513951	1
ZNF117	1.280099	1.24004	1.29312	1.22113	0.2882	0.513985	1
MYL4	1.09161	1.127952	1.079796	0.62365	-0.6812	0.514024	1
EMSY	1.259203	1.219355	1.272155	1.24071	0.3112	0.514248	1
AZIN1-AS1	1.202927	1.1639	1.215614	1.31552	0.3956	0.514307	1
ABHD11	1.58096	1.624827	1.566701	0.90697	-0.1409	0.514633	1
S100A5	1.045702	1.081453	1.034081	0.41842	-1.257	0.514636	1
SLCO3A1	1.084287	1.120731	1.07244	0.60001	-0.7369	0.514637	1
FOCAD	1.234539	1.273076	1.222012	0.813	-0.2987	0.51479	1
BMP6	1.031955	1.067571	1.020378	0.30157	-1.7294	0.514956	1
CXCL8	1.028537	1.064253	1.016927	0.26344	-1.9244	0.515034	1
MIS12	1.341448	1.300241	1.354843	1.18186	0.2411	0.515039	1
PEX5	1.233109	1.19352	1.245978	1.27107	0.346	0.51515	1
TTC30A	1.139698	1.10178	1.152023	1.49364	0.5788	0.515212	1
SRCIN1	1.060251	1.096475	1.048476	0.50247	-0.9929	0.515247	1
RP11-268J15.5	1.163748	1.12553	1.176171	1.40342	0.4889	0.51526	1
BAZ1B	2.045091	1.995408	2.061241	1.06614	0.0924	0.515337	1
FAM49A	1.100727	1.137151	1.088887	0.6481	-0.6257	0.515477	1
XK	1.047051	1.083119	1.035326	0.42501	-1.2344	0.515531	1
ARL9	1.08957	1.052949	1.101474	1.91644	0.9384	0.515583	1
TMEM238	1.056106	1.019546	1.067991	3.47857	1.7985	0.515622	1
RNF114	1.761544	1.807648	1.746558	0.92436	-0.1135	0.515653	1
RP11-426L16.3	1.055075	1.018713	1.066895	3.57489	1.8379	0.515885	1
JOSD1	1.314243	1.353882	1.301358	0.85158	-0.2318	0.515886	1
USP38	1.153875	1.191261	1.141722	0.74099	-0.4325	0.516003	1
RBM48	1.151371	1.113386	1.163718	1.4439	0.53	0.516013	1
TMEM167B	1.425484	1.466654	1.412102	0.8831	-0.1794	0.516028	1
EIF6	2.36048	2.418137	2.341738	0.94613	-0.0799	0.516269	1
COQ2	1.222854	1.183955	1.235498	1.2802	0.3564	0.516286	1
PTPN2	1.596713	1.552632	1.611042	1.10569	0.145	0.516443	1
ARFIP2	1.596586	1.552013	1.611075	1.10699	0.1466	0.516497	1
SGMS1	1.203251	1.241751	1.190736	0.78898	-0.3419	0.51652	1
INPP5D	1.244959	1.205082	1.257921	1.25765	0.3307	0.51654	1
ARID4B	2.657436	2.726495	2.634987	0.947	-0.0786	0.51679	1
TUBG2	1.260846	1.221253	1.273717	1.23712	0.307	0.516856	1
NEK6	1.584576	1.62821	1.570393	0.90797	-0.1393	0.516926	1
RP11-701H24.9	1.258681	1.219293	1.271484	1.238	0.308	0.516953	1
RNF220	1.311546	1.351017	1.298716	0.851	-0.2328	0.516986	1
EPHA4	1.22156	1.182734	1.234181	1.28154	0.3579	0.516991	1
SH3BP5L	1.126274	1.163236	1.114259	0.69996	-0.5147	0.517019	1
CCDC169	1.068008	1.031519	1.079869	2.534	1.3414	0.517163	1
PPM1D	1.163196	1.125023	1.175604	1.40457	0.4901	0.517164	1
DLG2	1.163416	1.125148	1.175855	1.40518	0.4908	0.517164	1
CGN	1.236752	1.197726	1.249438	1.26153	0.3352	0.517361	1
CTB-58E17.3	1.0584	1.022259	1.070147	3.15135	1.656	0.517376	1

ROM1	1.136351	1.098608	1.14862	1.50717	0.5918	0.51738	1
SOCS5	1.179007	1.216712	1.166751	0.76946	-0.3781	0.51744	1
CDH13	1.038293	1.073796	1.026752	0.36252	-1.4639	0.517588	1
CEBPG	1.382143	1.340911	1.395545	1.16026	0.2144	0.517701	1
FER	1.37063	1.329352	1.384048	1.16607	0.2217	0.517718	1
SOX12	1.522651	1.479491	1.53668	1.11927	0.1626	0.517749	1
ARFGAP1	1.265869	1.304881	1.253187	0.83045	-0.268	0.517826	1
RP11-399K21.13	1.053497	1.089292	1.041861	0.46881	-1.0929	0.517961	1
RTTN	1.105788	1.068755	1.117825	1.71368	0.7771	0.51799	1
THAP12	1.449745	1.4077	1.463412	1.13665	0.1848	0.518348	1
GSTM2	1.076122	1.039557	1.088007	2.22483	1.1537	0.518427	1
MEG3	1.05451	1.018361	1.066261	3.60876	1.8515	0.518459	1
FAAH	1.15815	1.120322	1.170447	1.41659	0.5024	0.518772	1
RIPK2	1.273473	1.312081	1.260923	0.83607	-0.2583	0.518783	1
SNX29	1.157239	1.194575	1.145103	0.74574	-0.4233	0.51891	1
KLHL24	1.75067	1.795913	1.735964	0.92468	-0.113	0.518948	1
PGS1	1.236855	1.198036	1.249474	1.25974	0.3331	0.518973	1
SPSB3	1.760744	1.714623	1.775735	1.08552	0.1184	0.519125	1
CLCF1	1.035987	1.071244	1.024526	0.34426	-1.5384	0.519137	1
DDOST	2.549522	2.491884	2.568257	1.05119	0.072	0.519293	1
CCZ1B	1.360644	1.401005	1.347525	0.86664	-0.2065	0.519501	1
ZNF695	1.069225	1.032996	1.081001	2.45486	1.2956	0.519542	1
HACE1	1.149358	1.111483	1.16167	1.45018	0.5362	0.519708	1
PRTFDC1	1.252607	1.213452	1.265335	1.24307	0.3139	0.519738	1
PRICKLE2	1.170649	1.132215	1.183142	1.38518	0.4701	0.519887	1
BEND6	1.054268	1.018393	1.065929	3.58449	1.8418	0.520056	1
SCAMP5	1.161159	1.198627	1.14898	0.75005	-0.4149	0.520111	1
TTC30B	1.099047	1.062429	1.110949	1.7772	0.8296	0.520118	1
PPP6C	1.443207	1.484659	1.429733	0.88667	-0.1735	0.520119	1
STARD10	1.765832	1.811643	1.750941	0.92521	-0.1121	0.520139	1
RP11-127B20.3	1.175747	1.137675	1.188123	1.36643	0.4504	0.52019	1
RP11-966I7.4	1.049892	1.013947	1.061576	4.41511	2.1424	0.520225	1
FLYWCH2	1.651246	1.606434	1.665812	1.09791	0.1348	0.520238	1
BCOR	1.240436	1.201674	1.253035	1.25467	0.3273	0.520278	1
ETNPPL	1.048965	1.013102	1.060622	4.62714	2.2101	0.520515	1
NEK5	1.136718	1.099336	1.148869	1.49864	0.5837	0.520528	1
CYYR1	1.397484	1.356663	1.410753	1.15166	0.2037	0.520649	1
JADE3	1.088451	1.124554	1.076716	0.61592	-0.6992	0.520692	1
SIN3B	1.210558	1.248301	1.198289	0.79858	-0.3245	0.520881	1
CBX4	1.080036	1.115817	1.068405	0.59063	-0.7597	0.521001	1
LTV1	1.197253	1.158915	1.209715	1.31967	0.4002	0.521041	1
SAP130	1.193076	1.230999	1.180748	0.78246	-0.3539	0.521101	1
TTC33	1.201486	1.163481	1.21384	1.30804	0.3874	0.521248	1
C6orf48	5.368718	5.502208	5.325326	0.96071	-0.0578	0.521419	1
MLF1	1.503647	1.545479	1.490049	0.89838	-0.1546	0.521424	1

GUSBP1	1.067309	1.031238	1.079034	2.53009	1.3392	0.521438	1
ZNF580	3.381559	3.300791	3.407812	1.04651	0.0656	0.521478	1
TCEAL8	5.294607	5.429823	5.250654	0.95955	-0.0596	0.521646	1
ZNF577	1.091162	1.054621	1.10304	1.88646	0.9157	0.521685	1
EPB42	1.068968	1.03275	1.08074	2.46533	1.3018	0.521823	1
ARF4-AS1	1.068789	1.032771	1.080497	2.45633	1.2965	0.521823	1
SLC16A8	1.050903	1.086402	1.039364	0.45559	-1.1342	0.52184	1
PLXNA3	1.204838	1.242172	1.192702	0.79572	-0.3297	0.521954	1
GUCY1A2	1.048916	1.084281	1.037421	0.444	-1.1714	0.521988	1
OSMR	1.037656	1.072756	1.026246	0.36074	-1.471	0.522105	1
POU5F1B	1.042047	1.077252	1.030603	0.39614	-1.3359	0.522199	1
NCF2	1.02729	1.06211	1.015972	0.25715	-1.9593	0.522323	1
DDIAS	1.065663	1.029696	1.077355	2.60484	1.3812	0.522351	1
RNPEP	1.696403	1.651485	1.711004	1.09136	0.1261	0.522416	1
SLCO4A1	1.050294	1.085727	1.038776	0.45233	-1.1446	0.522525	1
RP11-1398P2.1	1.029859	1.064751	1.018518	0.28598	-1.806	0.522543	1
R3HCC1L	1.190908	1.228557	1.17867	0.78173	-0.3553	0.52257	1
TMEM187	1.321969	1.282026	1.334952	1.18766	0.2481	0.522659	1
SLC25A51	1.174789	1.212206	1.162626	0.76636	-0.3839	0.522684	1
WDR74	1.3543	1.313726	1.367488	1.17137	0.2282	0.522722	1
ORC1	1.058463	1.022671	1.070097	3.09195	1.6285	0.522768	1
GLRX2	1.214154	1.251942	1.201871	0.80126	-0.3197	0.522861	1
LINC01504	1.036578	1.071786	1.025134	0.35012	-1.5141	0.522944	1
SLIRP	6.29966	6.131516	6.354317	1.04342	0.0613	0.523013	1
CEP83-AS1	1.071779	1.035581	1.083546	2.34803	1.2315	0.523017	1
TCF4	2.262596	2.203003	2.281967	1.06564	0.0917	0.523073	1
NAGA	1.276532	1.315273	1.263939	0.83717	-0.2564	0.523117	1
PIAS1	1.881054	1.833938	1.89637	1.07486	0.1042	0.523361	1
TNRC18	1.197197	1.159054	1.209596	1.31776	0.3981	0.523767	1
LINC00893	1.110426	1.073752	1.122347	1.6589	0.7302	0.524215	1
RP11-469H8.6	1.089954	1.053672	1.101747	1.89572	0.9227	0.524229	1
SUV39H1	1.075054	1.039063	1.086753	2.22081	1.1511	0.52447	1
CLASP1	1.350496	1.390208	1.337588	0.86515	-0.209	0.524583	1
RCE1	1.212893	1.174914	1.225239	1.28771	0.3648	0.524633	1
SOX5	1.219309	1.181106	1.231727	1.27951	0.3556	0.524719	1
HELQ	1.187817	1.150043	1.200096	1.33359	0.4153	0.524852	1
ACSF2	1.135805	1.098994	1.14777	1.49272	0.5779	0.525057	1
JAG2	1.057026	1.092337	1.045547	0.49327	-1.0195	0.525066	1
TBXAS1	1.079468	1.043571	1.091136	2.09165	1.0646	0.525143	1
NPAS3	1.275779	1.23688	1.288424	1.21759	0.284	0.525163	1
NRSN2	1.48767	1.445381	1.501417	1.12581	0.171	0.52523	1
HS6ST2	1.207466	1.169183	1.21991	1.29984	0.3783	0.525244	1
TRIM65	1.175544	1.138094	1.187717	1.35935	0.4429	0.525614	1
TMEM132C	1.036418	1.071418	1.025042	0.35063	-1.512	0.525669	1
BTBD8	1.129154	1.092406	1.141099	1.52694	0.6106	0.5258	1

C22orf31	1.0305	1.065267	1.019199	0.29417	-1.7653	0.525896	1
ZMYM3	1.464697	1.423259	1.478166	1.12973	0.176	0.525934	1
CMTR1	1.216708	1.178303	1.229192	1.28541	0.3622	0.525971	1
AUNIP	1.054089	1.018714	1.065588	3.50483	1.8093	0.526379	1
LINC00240	1.08303	1.046962	1.094754	2.01768	1.0127	0.526578	1
SLC39A6	2.271008	2.323162	2.254055	0.94777	-0.0774	0.526799	1
SLC16A5	1.096603	1.132557	1.084917	0.6406	-0.6425	0.526826	1
GOLGA6L4	1.10159	1.065349	1.113371	1.73484	0.7948	0.526864	1
PART1	1.026597	1.061202	1.015348	0.25079	-1.9955	0.526889	1
DPH6	1.323828	1.284022	1.336767	1.1857	0.2457	0.527093	1
EXOSC9	1.315598	1.275963	1.328481	1.19031	0.2513	0.52723	1
MDH1B	1.075216	1.039513	1.086821	2.1973	1.1357	0.527238	1
UAP1L1	1.075724	1.111149	1.064208	0.57767	-0.7917	0.527452	1
AIF1	1.161168	1.124042	1.173237	1.3966	0.4819	0.52747	1
PRKCSH	2.479028	2.406859	2.502487	1.06797	0.0949	0.527523	1
WDR17	1.082653	1.046898	1.094275	2.01019	1.0073	0.527701	1
CA12	1.065337	1.100404	1.053938	0.53722	-0.8964	0.527755	1
DYNLL2	1.324898	1.363909	1.312217	0.85795	-0.221	0.528168	1
TSPAN17	1.454947	1.495498	1.441766	0.89156	-0.1656	0.528185	1
INPP5E	1.161899	1.198213	1.150095	0.75724	-0.4012	0.528344	1
PLAC8	1.041306	1.076352	1.029914	0.39179	-1.3519	0.528436	1
LINC01273	1.100313	1.0641	1.112084	1.74857	0.8062	0.528543	1
PLEKHA7	1.064984	1.029504	1.076518	2.59349	1.3749	0.528545	1
KAT6B	1.478845	1.520022	1.465461	0.89508	-0.1599	0.528649	1
VSX1	1.050277	1.014881	1.061783	4.15173	2.0537	0.529138	1
HIST2H2BE	1.132464	1.095924	1.144341	1.50475	0.5895	0.529312	1
TNNT2	1.0301	1.06471	1.018849	0.29129	-1.7795	0.529354	1
ADAMTS1	1.121585	1.157103	1.110039	0.70043	-0.5137	0.529482	1
TIPARP	1.231973	1.269808	1.219675	0.81419	-0.2966	0.529622	1
DHX15	1.761018	1.716151	1.775602	1.08301	0.1151	0.529691	1
MMP15	1.12734	1.16315	1.115699	0.70916	-0.4958	0.529759	1
GLB1L2	1.114167	1.078343	1.125811	1.60591	0.6834	0.529819	1
MDM1	1.226766	1.18881	1.239103	1.26637	0.3407	0.529968	1
SYT10	1.03348	1.067983	1.022265	0.32751	-1.6104	0.530075	1
SOX3	1.083373	1.047721	1.094962	1.98995	0.9927	0.530101	1
RP11-420A23.1	1.058282	1.022991	1.069753	3.03396	1.6012	0.530443	1
LPAR2	1.225454	1.187802	1.237694	1.26566	0.3399	0.530614	1
MYCNOS	1.087132	1.051452	1.09873	1.91888	0.9403	0.530656	1
RP11-351D16.3	1.098504	1.062498	1.110208	1.76337	0.8183	0.530761	1
C9orf163	1.05091	1.015826	1.062315	3.93745	1.9773	0.530954	1
MINK1	1.18744	1.224433	1.175415	0.78159	-0.3555	0.530978	1
DCTN4	1.44538	1.404388	1.458704	1.13432	0.1818	0.531053	1
GPLD1	1.073319	1.037936	1.08482	2.23587	1.1608	0.531058	1
MX2	1.137389	1.173484	1.125656	0.72431	-0.4653	0.531194	1
RNF126	1.71749	1.760494	1.703511	0.92507	-0.1124	0.531247	1

COX8A	8.610922	8.397051	8.680442	1.03831	0.0542	0.53143	1
DES	1.142845	1.106625	1.154619	1.45013	0.5362	0.531445	1
RABEPK	1.356812	1.317022	1.369746	1.16631	0.222	0.531889	1
CD55	1.071143	1.105909	1.059843	0.56504	-0.8236	0.532137	1
ASMTL	1.238422	1.275607	1.226335	0.82122	-0.2842	0.532603	1
SEC24B-AS1	1.121184	1.085034	1.132934	1.5633	0.6446	0.532637	1
UBN2	1.372972	1.333261	1.385881	1.1579	0.2115	0.532693	1
UGDH-AS1	1.097613	1.061963	1.109202	1.76237	0.8175	0.532939	1
C9orf85	1.265573	1.227529	1.27794	1.22156	0.2887	0.532944	1
SPATA20	1.454887	1.49485	1.441897	0.89299	-0.1633	0.533028	1
C5orf51	1.222656	1.185065	1.234875	1.26915	0.3439	0.533057	1
NKIRAS2	1.76248	1.717326	1.777158	1.08341	0.1156	0.533066	1
RAB28	1.497	1.455705	1.510423	1.12007	0.1636	0.53317	1
RP11-1191J2.5	1.078176	1.042874	1.089651	2.09102	1.0642	0.533212	1
TRPV2	1.034394	1.068475	1.023315	0.34049	-1.5543	0.533245	1
SLC6A16	1.105276	1.069376	1.116945	1.68566	0.7533	0.533417	1
TM4SF18	1.024689	1.05859	1.01367	0.23331	-2.0997	0.533436	1
FAM184A	1.132415	1.09623	1.144177	1.49825	0.5833	0.533454	1
TNKS	1.497781	1.455692	1.511462	1.12239	0.1666	0.533483	1
ANKHD1	1.142705	1.106326	1.15453	1.45336	0.5394	0.533489	1
ACSL1	1.075066	1.109731	1.063798	0.58141	-0.7824	0.533584	1
TBC1D2	1.040813	1.074993	1.029702	0.39607	-1.3362	0.533672	1
ZNF624	1.100426	1.064804	1.112005	1.72836	0.7894	0.5337	1
GK	1.099035	1.063525	1.110578	1.7407	0.7997	0.533787	1
PGM2L1	1.445397	1.484775	1.432597	0.89237	-0.1643	0.53395	1
ENPP4	1.135226	1.170961	1.12361	0.72303	-0.4679	0.53397	1
PABPC1L	1.19274	1.15587	1.204724	1.31343	0.3933	0.534099	1
PPM1A	1.799637	1.843806	1.785279	0.93064	-0.1037	0.534136	1
ABCF1	1.87596	1.92138	1.861196	0.93468	-0.0975	0.53434	1
MAPK7	1.250927	1.288207	1.238809	0.8286	-0.2712	0.534473	1
CCDC127	1.320868	1.28227	1.333414	1.18119	0.2402	0.534525	1
ATG4A	1.173425	1.20965	1.16165	0.77105	-0.3751	0.534566	1
RAP2A	1.325077	1.363303	1.312651	0.86058	-0.2166	0.534581	1
UBASH3B	1.047433	1.081718	1.036288	0.44406	-1.1712	0.534583	1
ORC5	1.133561	1.097407	1.145313	1.49182	0.5771	0.534648	1
DLX1	1.050224	1.01526	1.061589	4.03606	2.0129	0.534664	1
VPS16	1.169963	1.205966	1.158259	0.76837	-0.3801	0.534702	1
MRFAP1L1	1.530141	1.488083	1.543813	1.11418	0.156	0.534778	1
ENTPD4	1.538538	1.578904	1.525417	0.90761	-0.1399	0.534835	1
CEND1	1.108624	1.072774	1.120277	1.65273	0.7249	0.534847	1
PXYLP1	1.239277	1.276633	1.227134	0.82107	-0.2844	0.534984	1
RP11-18H7.1	1.056586	1.021844	1.067879	3.10744	1.6357	0.534996	1
IRF9	1.22966	1.192055	1.241884	1.25945	0.3328	0.535171	1
SLC39A9	1.237059	1.198899	1.249464	1.25422	0.3268	0.535224	1
FAM126B	1.268052	1.229821	1.280479	1.22043	0.2874	0.535337	1

SYCE2	1.061181	1.026323	1.072512	2.75464	1.4619	0.535399	1
RP11-839D17.3	1.072011	1.037081	1.083365	2.24821	1.1688	0.535404	1
TGDS	1.233958	1.271127	1.221876	0.81835	-0.2892	0.535423	1
RP11-501C14.5	1.077278	1.042105	1.088712	2.10694	1.0752	0.535488	1
CSK	1.215383	1.252306	1.203381	0.80609	-0.311	0.535605	1
SCAF1	1.208715	1.245321	1.196816	0.80228	-0.3178	0.535938	1
CXorf40A	1.170987	1.134483	1.182853	1.35967	0.4433	0.536024	1
MROH8	1.150823	1.114781	1.162539	1.41608	0.5019	0.536222	1
NFYC-AS1	1.070907	1.03603	1.082245	2.28268	1.1907	0.536321	1
SQRDL	1.045164	1.079149	1.034117	0.43105	-1.2141	0.536364	1
INTS9	1.076775	1.041741	1.088163	2.11211	1.0787	0.53637	1
MYOCD	1.13111	1.0953	1.142751	1.49791	0.5829	0.536373	1
BLOC1S1	3.605073	3.520055	3.632709	1.0447	0.0631	0.536388	1
KIF16B	1.186877	1.149875	1.198904	1.32713	0.4083	0.536667	1
PLAG1	1.581715	1.539419	1.595463	1.1039	0.1426	0.536708	1
TMEM17	1.117987	1.082307	1.129584	1.5744	0.6548	0.536823	1
ZC3H12A	1.044909	1.078998	1.033828	0.42821	-1.2236	0.536851	1
ZSCAN5A	1.139448	1.103443	1.151152	1.46122	0.5472	0.536892	1
IFIT2	1.041439	1.075404	1.030398	0.40314	-1.3106	0.536969	1
GPR82	1.143629	1.107475	1.155381	1.44574	0.5318	0.537082	1
ZBED8	1.130267	1.094344	1.141944	1.50454	0.5893	0.537189	1
CTA-392C11.1	1.03451	1.068276	1.023534	0.34469	-1.5366	0.537203	1
SMPDL3B	1.044684	1.078748	1.033611	0.42682	-1.2283	0.537257	1
ANK2	1.267929	1.229654	1.280371	1.22084	0.2879	0.537468	1
RP11-108L7.15	1.064011	1.029425	1.075254	2.55745	1.3547	0.537539	1
CLRN1-AS1	1.050199	1.015676	1.061422	3.91832	1.9702	0.537674	1
FAP	1.031402	1.065173	1.020424	0.31338	-1.674	0.53771	1
USB1	1.572021	1.613361	1.558583	0.91069	-0.135	0.537758	1
COL4A4	1.027963	1.061722	1.016989	0.27525	-1.8612	0.537824	1
KPNA5	1.458569	1.417916	1.471783	1.1289	0.1749	0.53783	1
FLCN	1.340258	1.378265	1.327903	0.86686	-0.2061	0.537909	1
RP11-579D7.4	1.090376	1.055228	1.101801	1.84331	0.8823	0.538019	1
UBE2E1	2.230196	2.279626	2.214129	0.94882	-0.0758	0.53804	1
CHRNA7	1.084396	1.049176	1.095844	1.94901	0.9627	0.538434	1
GRAMD1A	2.05462	2.101234	2.039468	0.94391	-0.0833	0.538475	1
C5orf49	1.132515	1.096692	1.144159	1.4909	0.5762	0.538654	1
RAB22A	1.390233	1.350711	1.40308	1.14932	0.2008	0.538792	1
DUSP9	1.119681	1.084414	1.131145	1.5536	0.6356	0.538809	1
TM9SF1	1.135132	1.099567	1.146693	1.47331	0.5591	0.538872	1
RASEF	1.047027	1.080936	1.036005	0.44486	-1.1686	0.539016	1
DOPEY1	1.146089	1.110126	1.15778	1.43272	0.5188	0.539034	1
SPIRE2	1.066969	1.032307	1.078235	2.42162	1.276	0.539402	1
CREBBP	1.448365	1.488025	1.435474	0.89232	-0.1644	0.539484	1
SNX17	2.135677	2.08648	2.151669	1.06	0.0841	0.539581	1
RASSF5	1.078344	1.043634	1.089627	2.05407	1.0385	0.539607	1

MBD1	1.330039	1.367762	1.317778	0.86409	-0.2108	0.53982	1
GPR146	1.135006	1.170289	1.123538	0.72546	-0.463	0.539912	1
HACL1	1.142556	1.106942	1.154133	1.44128	0.5274	0.540071	1
GSPT2	1.256339	1.219272	1.268388	1.22399	0.2916	0.540536	1
SCO1	1.32086	1.358312	1.308686	0.8615	-0.2151	0.54072	1
SLC2A4	1.117717	1.082414	1.129192	1.56761	0.6486	0.54073	1
RNASEK	1.164925	1.128819	1.176662	1.3714	0.4556	0.540789	1
VPS4A	1.728275	1.684345	1.742555	1.08506	0.1178	0.540854	1
PCDHA9	1.073287	1.03851	1.084591	2.19662	1.1353	0.541119	1
CLHC1	1.235474	1.198326	1.247549	1.24819	0.3198	0.541168	1
IL15	1.027397	1.060745	1.016557	0.27256	-1.8753	0.541304	1
TMEM87B	1.184422	1.219959	1.17287	0.78592	-0.3475	0.541366	1
ELAVL3	1.151296	1.115436	1.162953	1.41163	0.4974	0.541411	1
LRRC45	1.171946	1.135699	1.183728	1.35393	0.4372	0.541413	1
NEK10	1.06367	1.029222	1.074867	2.56204	1.3573	0.541437	1
PPFIBP2	1.045057	1.078855	1.034071	0.43207	-1.2106	0.54153	1
CARM1	1.396547	1.436053	1.383706	0.87995	-0.1845	0.541624	1
NAGLU	1.138092	1.173188	1.126684	0.73148	-0.4511	0.541643	1
TMEM39A	1.187638	1.22355	1.175965	0.78714	-0.3453	0.541729	1
NEK3	1.210007	1.173688	1.221812	1.27707	0.3528	0.541879	1
SEMA4F	1.204386	1.240297	1.192713	0.80198	-0.3184	0.541957	1
CUX2	1.047344	1.013274	1.058419	4.40094	2.1378	0.542006	1
HS3ST3A1	1.088493	1.122797	1.077342	0.62984	-0.6669	0.542038	1
RP11-129M6.1	1.071148	1.036488	1.082414	2.25866	1.1755	0.542091	1
NPRL3	1.647295	1.604973	1.661052	1.0927	0.1279	0.542109	1
GNPTG	1.197389	1.233108	1.185778	0.79696	-0.3274	0.542272	1
TMEM185A	1.314703	1.276656	1.327071	1.18223	0.2415	0.542528	1
PDS5A	1.6614	1.618531	1.675335	1.09184	0.1268	0.542532	1
GTF2F2	1.728268	1.68485	1.742381	1.08401	0.1164	0.542638	1
FAM200B	2.700789	2.759298	2.68177	0.95593	-0.065	0.542776	1
POT1-AS1	1.144369	1.108749	1.155947	1.43401	0.5201	0.542841	1
PPARGC1A	1.083104	1.117329	1.071979	0.61348	-0.7049	0.542855	1
B3GALT4	1.06649	1.100401	1.055467	0.55245	-0.8561	0.54293	1
STYX	1.232402	1.268886	1.220543	0.82021	-0.2859	0.542947	1
DBN1	2.101183	2.148496	2.085803	0.94541	-0.081	0.543022	1
DENND1A	1.263265	1.226078	1.275353	1.21795	0.2845	0.543128	1
GPKOW	1.203833	1.167333	1.215698	1.28903	0.3663	0.543333	1
WDFY3	1.261585	1.298072	1.249725	0.8378	-0.2553	0.54334	1
PRSS23	3.012743	3.122162	2.977176	0.93168	-0.1021	0.543434	1
LPAR1	1.171784	1.136099	1.183384	1.34743	0.4302	0.543512	1
ZNF266	1.155626	1.119929	1.16723	1.39441	0.4797	0.543601	1
MREG	1.10434	1.069622	1.115625	1.66076	0.7318	0.54386	1
TNIP3	1.049479	1.015453	1.060539	3.91752	1.9699	0.543913	1
CEBPZ	1.828979	1.784947	1.843292	1.07433	0.1034	0.54396	1
BORCS5	1.157568	1.122174	1.169073	1.38387	0.4687	0.544082	1

ARHGEF40	1.465006	1.504527	1.452159	0.8962	-0.1581	0.544095	1
INTS6-AS1	1.130588	1.095445	1.142011	1.48789	0.5733	0.544179	1
ABCF3	1.139814	1.174785	1.128447	0.73489	-0.4444	0.544312	1
DMBX1	1.031876	1.065301	1.021012	0.32177	-1.6359	0.544375	1
PPP3CB	1.425264	1.463667	1.41278	0.89025	-0.1677	0.544445	1
RP11-513M16.7	1.063657	1.02951	1.074757	2.5333	1.341	0.544462	1
RNF139	1.341573	1.378904	1.329438	0.86945	-0.2018	0.544586	1
STX7	1.704237	1.746194	1.690599	0.9255	-0.1117	0.544715	1
GMDS-AS1	1.132659	1.097386	1.144125	1.47993	0.5655	0.544752	1
UMAD1	1.266147	1.229528	1.27805	1.2114	0.2767	0.544857	1
SCRG1	1.153687	1.118312	1.165186	1.39619	0.4815	0.544861	1
CROT	1.332842	1.369987	1.320768	0.86697	-0.2059	0.544918	1
C2orf42	1.092766	1.058134	1.104024	1.78938	0.8395	0.544941	1
ADAMTSL5	1.031279	1.064339	1.020533	0.31914	-1.6478	0.545035	1
LRCH1	1.122238	1.156974	1.110946	0.70678	-0.5007	0.545064	1
ARG2	1.327417	1.289777	1.339652	1.17211	0.2291	0.545138	1
ATG5	1.472484	1.512539	1.459464	0.89645	-0.1577	0.545264	1
CCDC85C	1.48058	1.519737	1.467851	0.90017	-0.1517	0.545269	1
HNRNPLL	1.637446	1.595575	1.651056	1.09315	0.1285	0.545403	1
RP11-368I23.2	1.053008	1.018986	1.064067	3.3744	1.7546	0.545403	1
SGSH	1.108548	1.143226	1.097276	0.67918	-0.5581	0.545656	1
PPIB	5.771615	5.917732	5.724118	0.96063	-0.0579	0.545668	1
PFDN6	1.921706	1.873338	1.937428	1.07338	0.1022	0.545798	1
CTNND1	1.634606	1.675172	1.621421	0.92039	-0.1197	0.545948	1
STK40	1.232144	1.268376	1.220367	0.82111	-0.2843	0.546088	1
FO538757.1	1.425336	1.385618	1.438247	1.13648	0.1846	0.546144	1
GTF2F1	1.834142	1.789744	1.848574	1.07449	0.1037	0.546162	1
SDPR	1.035145	1.068091	1.024436	0.35887	-1.4785	0.546178	1
PRTG	1.518723	1.478101	1.531928	1.11258	0.1539	0.546297	1
BRD9	1.465379	1.425498	1.478342	1.12419	0.1689	0.546316	1
GRHL1	1.031808	1.06515	1.020969	0.32187	-1.6355	0.546317	1
MID2	1.031742	1.064778	1.021004	0.32425	-1.6248	0.546317	1
SULT1A3	1.03178	1.064999	1.020982	0.32281	-1.6313	0.546317	1
UCK2	1.215236	1.25084	1.203663	0.81192	-0.3006	0.546325	1
TMOD2	1.113842	1.148324	1.102633	0.69195	-0.5313	0.546666	1
LL0XNC01-237H1.2	1.078585	1.044258	1.089744	2.02774	1.0199	0.54691	1
ZC3H18	1.371158	1.333209	1.383493	1.15091	0.2028	0.546949	1
RP11-256I23.2	1.077415	1.111442	1.066354	0.59541	-0.748	0.54696	1
RP11-479G22.8	1.066474	1.099869	1.055619	0.55692	-0.8445	0.546984	1
KLRG1	1.106341	1.07175	1.117585	1.63882	0.7127	0.547004	1
ST8SIA4	1.089033	1.054628	1.100217	1.83454	0.8754	0.547081	1
GFER	1.585339	1.626081	1.572095	0.91377	-0.1301	0.547256	1
PLGRKT	1.656623	1.614277	1.670389	1.09135	0.1261	0.547269	1
SLC4A4	1.208436	1.172263	1.220195	1.27825	0.3542	0.547281	1
TRIM52	1.15338	1.117953	1.164896	1.39798	0.4833	0.547364	1

SLIT2	1.457775	1.496005	1.445348	0.89787	-0.1554	0.547485	1
TIPARP-AS1	1.060976	1.027134	1.071977	2.65267	1.4074	0.547707	1
KREMEN2	1.092858	1.12688	1.081799	0.64469	-0.6333	0.547735	1
GSTT2B	1.099204	1.133117	1.08818	0.66243	-0.5942	0.547811	1
PVR	1.105293	1.139103	1.094303	0.67793	-0.5608	0.547863	1
GMDS	1.254417	1.290575	1.242664	0.83512	-0.26	0.548152	1
COQ9	1.699853	1.65664	1.713899	1.0872	0.1206	0.548251	1
MST1	1.275519	1.238238	1.287637	1.20735	0.2718	0.548277	1
RP11-333E1.1	1.059912	1.026029	1.070926	2.72487	1.4462	0.548372	1
PPDPF	11.75344	11.52153	11.82882	1.02921	0.0415	0.548396	1
TFAP2B	1.02887	1.061913	1.018129	0.29281	-1.7719	0.548898	1
SLC25A26	1.728798	1.685957	1.742723	1.08275	0.1147	0.549013	1
ZNF526	1.13183	1.096895	1.143185	1.47774	0.5634	0.549038	1
SNAP29	1.575444	1.615135	1.562542	0.9145	-0.1289	0.549099	1
CC2D1A	1.309679	1.345717	1.297965	0.86188	-0.2144	0.54921	1
BYSL	1.196621	1.160754	1.20828	1.29564	0.3737	0.549213	1
GPS1	1.761254	1.803868	1.747402	0.92976	-0.1051	0.549238	1
FAM117B	1.186471	1.150786	1.19807	1.31359	0.3935	0.549246	1
PREPL	1.735759	1.777961	1.722041	0.92812	-0.1076	0.549327	1
FAM171A2	1.147519	1.182078	1.136285	0.7485	-0.4179	0.549341	1
CD320	2.428423	2.364184	2.449304	1.0624	0.0873	0.549377	1
SULT1A2	1.064557	1.030791	1.075533	2.45309	1.2946	0.549388	1
VAMP1	1.137197	1.102166	1.148584	1.45434	0.5404	0.549582	1
FAM131C	1.139022	1.103935	1.150427	1.44731	0.5334	0.549728	1
STXBP4	1.197849	1.162191	1.20944	1.29131	0.3688	0.549741	1
ARL10	1.316266	1.279077	1.328355	1.17657	0.2346	0.550053	1
IBA57	1.112182	1.077321	1.123514	1.59741	0.6757	0.550066	1
NTF4	1.066474	1.099832	1.055631	0.55725	-0.8436	0.550074	1
ERLIN1	1.296025	1.258791	1.308129	1.19064	0.2517	0.550359	1
ZNF473	1.095247	1.061056	1.106361	1.74202	0.8008	0.550573	1
SLC35E1	1.310398	1.346838	1.298553	0.86079	-0.2163	0.550759	1
GCLM	1.283973	1.247155	1.295941	1.19739	0.2599	0.550836	1
TMEM222	1.401727	1.363179	1.414258	1.14064	0.1898	0.550856	1
SRD5A3	1.201238	1.165404	1.212887	1.28707	0.3641	0.551082	1
EXOSC4	1.60543	1.645739	1.592328	0.91729	-0.1246	0.551212	1
H6PD	1.257219	1.22055	1.269138	1.2203	0.2872	0.551437	1
ZNF354B	1.12875	1.162734	1.117704	0.72329	-0.4674	0.55144	1
LONP1	1.371168	1.332634	1.383694	1.1535	0.206	0.551535	1
HEXIM1	1.397368	1.434749	1.385218	0.88607	-0.1745	0.551814	1
RP4-798A10.7	1.071472	1.037647	1.082466	2.19052	1.1313	0.552099	1
ALKBH8	1.116063	1.081413	1.127327	1.56397	0.6452	0.552118	1
HPCA	1.116031	1.081677	1.127198	1.55733	0.6391	0.552118	1
CDKN2AIP	1.246078	1.209556	1.25795	1.23094	0.2998	0.552167	1
CLEC4F	1.045534	1.012187	1.056373	4.62578	2.2097	0.552276	1
ZNF148	1.801342	1.843984	1.787481	0.93305	-0.1	0.552353	1

CNBD2	1.264127	1.227431	1.276055	1.21379	0.2795	0.552574	1
TFRC	1.357832	1.320017	1.370124	1.15658	0.2099	0.552649	1
PRKCZ	1.377957	1.415123	1.365875	0.88137	-0.1822	0.552855	1
SEC24C	1.256967	1.220642	1.268775	1.21815	0.2847	0.55288	1
RP4-605O3.4	1.195962	1.160475	1.207497	1.29302	0.3707	0.552937	1
KIAA0895	1.174425	1.139249	1.18586	1.33473	0.4165	0.553	1
IGBP1	2.13432	2.087794	2.149443	1.05667	0.0795	0.553081	1
ATG9A	1.272447	1.308197	1.260827	0.8463	-0.2408	0.55309	1
NUDT10	1.153264	1.118751	1.164483	1.38511	0.47	0.553106	1
NT5DC3	1.080742	1.114049	1.069916	0.61303	-0.706	0.553193	1
SLITRK6	1.153478	1.118675	1.164791	1.38859	0.4736	0.553328	1
RP11-545I5.3	1.153788	1.118874	1.165137	1.38917	0.4742	0.553328	1
RP11-66N24.3	1.093965	1.059852	1.105054	1.75522	0.8117	0.553427	1
ARID5A	1.2266	1.261671	1.2152	0.82241	-0.2821	0.553465	1
FLJ31104	1.047998	1.014797	1.058791	3.97313	1.9903	0.553532	1
KLF4	1.076339	1.109549	1.065544	0.59831	-0.741	0.55359	1
UVRAG	1.373134	1.334928	1.385553	1.15115	0.2031	0.553765	1
ZNF839	1.149639	1.114692	1.160999	1.40374	0.4893	0.553846	1
TCAIM	1.429923	1.391011	1.442572	1.13187	0.1787	0.554162	1
SDAD1	1.57121	1.530757	1.584359	1.10099	0.1388	0.554175	1
RP11-354P11.3	1.093313	1.059385	1.104341	1.75701	0.8131	0.554273	1
RP1-40E16.12	1.076416	1.04297	1.087288	2.03136	1.0224	0.554443	1
TRIM27	1.638775	1.679519	1.62553	0.92055	-0.1194	0.554529	1
EFL1	1.129908	1.095407	1.141123	1.47918	0.5648	0.554599	1
TESC	1.048716	1.015453	1.059529	3.85214	1.9457	0.554744	1
IRF2BP2	1.684419	1.725915	1.670931	0.92426	-0.1136	0.554748	1
APOOL	1.326783	1.289476	1.33891	1.17077	0.2275	0.55477	1
RP11-406A9.2	1.063457	1.030125	1.074292	2.46609	1.3022	0.554792	1
SERTAD4	1.037033	1.069695	1.026416	0.37903	-1.3996	0.554802	1
ERP29	4.199982	4.093882	4.23447	1.04544	0.0641	0.554949	1
MZF1	1.291483	1.2543	1.30357	1.19375	0.2555	0.554958	1
PLEKHG4	1.078268	1.044841	1.089134	1.98779	0.9912	0.555007	1
LINC00471	1.051414	1.018306	1.062176	3.39647	1.764	0.555008	1
FTSJ3	1.17877	1.213274	1.167555	0.78563	-0.3481	0.555141	1
PFAS	1.121557	1.087518	1.132621	1.51536	0.5997	0.555177	1
GTF2E1	1.150957	1.11599	1.162323	1.39945	0.4849	0.555219	1
PROSER1	1.158197	1.123385	1.169513	1.37386	0.4582	0.555278	1
VPS53	1.402362	1.440116	1.39009	0.88634	-0.1741	0.55531	1
DDX60	1.066967	1.033567	1.077824	2.31849	1.2132	0.555347	1
TGM1	1.03847	1.070948	1.027912	0.39342	-1.3459	0.555409	1
PSMB5	4.335779	4.236538	4.368038	1.04063	0.0575	0.555648	1
WNT9A	1.031561	1.063842	1.021067	0.32999	-1.5995	0.555734	1
TTC39B	1.106825	1.072816	1.11788	1.61889	0.695	0.556053	1
SPOPL	1.171038	1.205603	1.159802	0.77723	-0.3636	0.556178	1
ZNF121	1.242315	1.206369	1.253999	1.2308	0.2996	0.55618	1

RNF135	1.229608	1.264792	1.218172	0.82394	-0.2794	0.55644	1
EEF2K	1.24433	1.208565	1.255955	1.22722	0.2954	0.556443	1
ZBTB14	1.169458	1.134615	1.180784	1.34297	0.4254	0.556486	1
BLOC1S3	1.095195	1.061421	1.106174	1.72863	0.7896	0.556495	1
ACTG1	66.84634	67.84467	66.52182	0.98021	-0.0288	0.556668	1
RP11-783K16.5	1.102331	1.135531	1.091539	0.67541	-0.5662	0.55672	1
SUMO3	3.168321	3.082006	3.196379	1.05493	0.0772	0.556727	1
DCC	1.103102	1.069084	1.11416	1.65248	0.7246	0.55686	1
PRKAB2	1.315346	1.27898	1.327167	1.17272	0.2299	0.556874	1
RUNDC3A	1.059632	1.026319	1.070461	2.67723	1.4207	0.55689	1
RPUSD3	1.829298	1.871652	1.81553	0.93561	-0.096	0.557146	1
TIMM21	1.299371	1.26315	1.311145	1.18239	0.2417	0.557225	1
SLC39A7	1.549858	1.589053	1.537118	0.91183	-0.1332	0.557272	1
ZMYM1	1.234816	1.199251	1.246376	1.23651	0.3063	0.557318	1
NUP43	1.153585	1.119019	1.164821	1.38483	0.4697	0.557391	1
CHP1	1.739246	1.696189	1.753242	1.08195	0.1136	0.558005	1
POMZP3	1.143597	1.177676	1.132519	0.74585	-0.423	0.558259	1
PUS10	1.129875	1.095938	1.140907	1.46874	0.5546	0.558262	1
MRPL19	1.841536	1.799066	1.855341	1.07043	0.0982	0.558306	1
NDUFA12	3.325772	3.403443	3.300525	0.95718	-0.0631	0.558335	1
ZNF45	1.091163	1.057769	1.102019	1.76599	0.8205	0.558469	1
ERBB3	1.042333	1.074699	1.031812	0.42587	-1.2315	0.558523	1
RP11-355B11.2	1.073587	1.040408	1.084373	2.08803	1.0621	0.558642	1
FBXL7	1.180446	1.21475	1.169295	0.78834	-0.3431	0.558768	1
RIMKLA	1.255242	1.290519	1.243775	0.8391	-0.2531	0.558798	1
SMARCAD1	1.393977	1.356502	1.406159	1.13929	0.1881	0.558808	1
TIMM8B	4.480095	4.363259	4.518073	1.04603	0.0649	0.55887	1
PAPD4	1.388205	1.350463	1.400473	1.1427	0.1924	0.558895	1
IFFO2	1.0539	1.086437	1.043324	0.50122	-0.9965	0.558981	1
TBC1D17	1.268414	1.304046	1.256832	0.84471	-0.2435	0.55911	1
TCHP	1.205496	1.240042	1.194266	0.8093	-0.3052	0.559155	1
GTF2H1	1.319122	1.28237	1.331068	1.17246	0.2295	0.559175	1
SETDB2	1.233478	1.268419	1.22212	0.82751	-0.2731	0.559389	1
XXbac-BPG299F13.17	1.073209	1.040138	1.083959	2.09175	1.0647	0.559544	1
SOX15	1.069572	1.102268	1.058944	0.57637	-0.7949	0.559928	1
ABCB8	1.151209	1.116845	1.162379	1.3897	0.4748	0.560023	1
MANSC1	1.220285	1.185368	1.231635	1.24959	0.3215	0.560029	1
CCL28	1.029215	1.061282	1.018791	0.30663	-1.7054	0.560053	1
REEP1	1.077115	1.043855	1.087926	2.0049	1.0035	0.560083	1
RP13-582O9.7	1.087499	1.054211	1.098319	1.81362	0.8589	0.560087	1
FAM188A	1.189902	1.154868	1.201291	1.29975	0.3782	0.560109	1
AC074117.10	1.183178	1.148464	1.194461	1.30982	0.3894	0.56015	1
CRTC1	1.188782	1.223063	1.177639	0.79636	-0.3285	0.5602	1
THBS4	1.08111	1.113852	1.070467	0.61894	-0.6921	0.560432	1
ZNF610	1.093	1.059723	1.103817	1.73831	0.7977	0.56047	1

KIAA1549	1.313151	1.276684	1.325004	1.17464	0.2322	0.560615	1
C1QBP	3.517283	3.431181	3.545271	1.04693	0.0662	0.560758	1
HIKESHI	2.511503	2.443816	2.533506	1.06212	0.0869	0.560965	1
TTI1	1.172882	1.138396	1.184091	1.33018	0.4116	0.560984	1
CMTM7	1.49397	1.532249	1.481527	0.9047	-0.1445	0.561001	1
NUDT7	1.147578	1.113504	1.158654	1.39779	0.4831	0.561126	1
RNF187	1.373887	1.409936	1.362169	0.88348	-0.1787	0.561191	1
RP11-1348G14.4	1.045259	1.077353	1.034826	0.45023	-1.1513	0.561274	1
IGSF1	1.174246	1.139704	1.185474	1.32762	0.4088	0.561338	1
USF3	1.262021	1.226167	1.273675	1.21006	0.2751	0.561496	1
TRIM41	1.174234	1.139747	1.185445	1.32701	0.4082	0.561564	1
GBGT1	1.128463	1.161746	1.117644	0.72733	-0.4593	0.561604	1
STAM2	1.127107	1.160599	1.11622	0.72367	-0.4666	0.561637	1
RP11-798L4.1	1.051644	1.019034	1.062245	3.27011	1.7093	0.561722	1
POFUT2	1.222229	1.257178	1.210869	0.81993	-0.2864	0.561816	1
ZFAND2A	1.237	1.271684	1.225726	0.83084	-0.2674	0.561941	1
DOCK11	1.077973	1.11049	1.067403	0.61004	-0.713	0.561946	1
VILL	1.073324	1.040252	1.084074	2.0887	1.0626	0.562046	1
PAK6	1.037993	1.069866	1.027632	0.3955	-1.3382	0.562064	1
GPAT4	1.36685	1.329224	1.379081	1.15144	0.2034	0.562079	1
CYLD	1.18508	1.219022	1.174047	0.79466	-0.3316	0.562097	1
CCDC180	1.095579	1.062223	1.106422	1.71032	0.7743	0.562113	1
EMILIN3	1.113164	1.146203	1.102425	0.70057	-0.5134	0.562403	1
TCP1	3.039474	2.972513	3.06124	1.04498	0.0635	0.562442	1
PRCP	1.591053	1.5517	1.603845	1.09452	0.1303	0.56251	1
LRRC75B	1.101651	1.06831	1.112488	1.64673	0.7196	0.562737	1
NACC1	1.282333	1.317252	1.270982	0.85415	-0.2274	0.562762	1
F12	1.351848	1.388329	1.339989	0.87552	-0.1918	0.562898	1
FAM50B	1.112743	1.079416	1.123576	1.55607	0.6379	0.563149	1
RP5-1050D4.3	1.064394	1.031732	1.075011	2.36388	1.2412	0.563237	1
SPCS1	4.673907	4.787295	4.63705	0.96033	-0.0584	0.56324	1
MRFAP1	4.997397	4.871423	5.038345	1.04312	0.0609	0.563269	1
ATP8A1	1.126155	1.092478	1.137102	1.48254	0.5681	0.563305	1
TELO2	1.173497	1.139094	1.18468	1.32774	0.409	0.563335	1
MCF2L	1.191651	1.22574	1.180571	0.79991	-0.3221	0.563379	1
OGFOD2	1.225492	1.190325	1.236923	1.24484	0.316	0.563418	1
ZMYND11	1.623547	1.662773	1.610796	0.92158	-0.1178	0.563465	1
GPN3	1.485495	1.447706	1.497779	1.11184	0.153	0.563566	1
WDR62	1.069172	1.036549	1.079777	2.18276	1.1262	0.563608	1
ARFGEF2	1.157346	1.190462	1.146582	0.76961	-0.3778	0.563623	1
ZNF599	1.240572	1.20514	1.25209	1.22886	0.2973	0.563767	1
MTO1	1.122576	1.155864	1.111756	0.71701	-0.4799	0.563776	1
C14orf93	1.128361	1.094873	1.139247	1.46772	0.5536	0.563993	1
PROS1	1.631948	1.67106	1.619235	0.92277	-0.116	0.564161	1
CLGN	1.318493	1.282522	1.330186	1.16871	0.2249	0.564172	1

SYT9	1.073992	1.041186	1.084655	2.05542	1.0394	0.564246	1
PTPN1	1.292854	1.327798	1.281495	0.85875	-0.2197	0.564271	1
DDIT4L	1.074827	1.107022	1.064362	0.60139	-0.7336	0.564487	1
NADK2	1.19881	1.164177	1.210068	1.27952	0.3556	0.564508	1
XYLT2	1.173615	1.207046	1.162748	0.78605	-0.3473	0.564899	1
SLAIN2	1.465191	1.501776	1.453299	0.90339	-0.1466	0.565014	1
FUCA1	1.279143	1.31464	1.267605	0.85051	-0.2336	0.56513	1
ZNF19	1.147272	1.113523	1.158242	1.39392	0.4792	0.565262	1
ZNF212	1.147202	1.113701	1.158091	1.39041	0.4755	0.565262	1
TGFBR2	1.063582	1.095657	1.053156	0.55569	-0.8476	0.565276	1
ASB13	1.196229	1.230142	1.185205	0.80474	-0.3134	0.565404	1
RBM26	1.820245	1.777226	1.834228	1.07334	0.1021	0.565714	1
RBM14	1.493013	1.454959	1.505383	1.11083	0.1516	0.565863	1
TCP11L2	1.081721	1.114125	1.071188	0.62377	-0.6809	0.566092	1
C2orf49	1.511634	1.473039	1.52418	1.10811	0.1481	0.566173	1
CIZ1	1.593585	1.632803	1.580837	0.91788	-0.1236	0.566255	1
NGEF	1.095853	1.062834	1.106586	1.69631	0.7624	0.56642	1
SGCA	1.036821	1.068344	1.026574	0.38882	-1.3628	0.566523	1
SH3TC1	1.036821	1.068441	1.026543	0.38783	-1.3665	0.566523	1
TMCC1	1.302146	1.337192	1.290754	0.86228	-0.2138	0.566679	1
PITHD1	1.583436	1.543739	1.59634	1.09674	0.1332	0.566791	1
COX6B1	12.2573	12.4911	12.1813	0.97304	-0.0394	0.566977	1
SLC35F1	1.236537	1.202163	1.247711	1.2253	0.2931	0.566984	1
TRAF3	1.175723	1.209298	1.164809	0.78744	-0.3448	0.566987	1
ORAOV1	1.188041	1.153836	1.19916	1.29463	0.3725	0.566988	1
CDK14	1.177797	1.143954	1.188798	1.31151	0.3912	0.567065	1
ZDHHC15	1.139285	1.172869	1.128369	0.74258	-0.4294	0.567084	1
COMMD10	1.439832	1.402406	1.451998	1.12324	0.1677	0.567101	1
ST18	1.04756	1.01526	1.058059	3.80471	1.9278	0.56723	1
AF003626.1	1.025601	1.056941	1.015414	0.2707	-1.8852	0.567295	1
HIVEP2	1.088369	1.120565	1.077903	0.64615	-0.6301	0.567359	1
EGLN2	1.857449	1.899225	1.843869	0.93844	-0.0917	0.567407	1
EIF4ENIF1	1.182124	1.147816	1.193276	1.30754	0.3869	0.567702	1
PRICKLE4	1.112413	1.0793	1.123177	1.55331	0.6353	0.567751	1
ST6GALNAC1	1.022774	1.054209	1.012556	0.23162	-2.1101	0.56788	1
RP11-533E19.7	1.079743	1.04713	1.090344	1.91691	0.9388	0.567985	1
BFAR	1.937145	1.890785	1.952215	1.06896	0.0962	0.568164	1
OSCP1	1.17194	1.13795	1.182988	1.32648	0.4076	0.568224	1
KIAA0556	1.121992	1.088725	1.132806	1.49681	0.5819	0.568307	1
RP11-456H18.2	1.209171	1.174687	1.220381	1.26157	0.3352	0.568482	1
OAT	1.563474	1.602072	1.550928	0.91505	-0.1281	0.568575	1
ALKBH3	1.19935	1.232816	1.188472	0.80953	-0.3048	0.568622	1
INPP5A	1.081714	1.113662	1.07133	0.62756	-0.6722	0.568635	1
BBS12	1.169417	1.135161	1.180552	1.33583	0.4177	0.568849	1
IPO8	1.256181	1.290438	1.245046	0.84371	-0.2452	0.568989	1

SLC19A1	1.173387	1.206807	1.162524	0.78587	-0.3476	0.569064	1
LINC00524	1.060233	1.028058	1.070692	2.51947	1.3331	0.569111	1
HUWE1	1.764312	1.804328	1.751305	0.93408	-0.0984	0.56915	1
ZNF530	1.097893	1.065127	1.108544	1.66665	0.737	0.569154	1
UBQLN2	1.455043	1.491545	1.443178	0.9016	-0.1494	0.569168	1
ROPN1L	1.079468	1.04693	1.090044	1.91871	0.9401	0.569223	1
ZDBF2	1.210001	1.243634	1.199068	0.81708	-0.2915	0.569341	1
DNAJC27-AS1	1.168387	1.134746	1.179322	1.33081	0.4123	0.569345	1
PCDHA12	1.116529	1.148961	1.105987	0.71151	-0.491	0.569646	1
BRAF	1.230848	1.264791	1.219814	0.83014	-0.2686	0.569883	1
CUL2	1.297284	1.332176	1.285942	0.86081	-0.2162	0.569913	1
POC1B	1.242675	1.208475	1.253792	1.21737	0.2838	0.570072	1
ZNF66	1.054306	1.022269	1.06472	2.90628	1.5392	0.57013	1
DNAH7	1.072364	1.040097	1.082852	2.06628	1.047	0.570395	1
DGKI	1.077281	1.109123	1.066931	0.61335	-0.7052	0.570563	1
CYP2R1	1.28214	1.246953	1.293578	1.1888	0.2495	0.570611	1
JPH3	1.040321	1.071626	1.030145	0.42087	-1.2486	0.570785	1
RP11-636O21.2	1.079269	1.111182	1.068896	0.61967	-0.6904	0.570937	1
HSF2BP	1.076228	1.04404	1.086691	1.96846	0.9771	0.570938	1
MLLT3	1.308582	1.343434	1.297253	0.86553	-0.2083	0.570955	1
FAM196A	1.053475	1.021453	1.063884	2.97789	1.5743	0.570964	1
SLC27A1	1.226615	1.192299	1.237769	1.23646	0.3062	0.571044	1
PYROXD1	1.130007	1.096971	1.140745	1.45141	0.5375	0.571054	1
MAFB	1.43131	1.384412	1.446554	1.16165	0.2162	0.571124	1
RGR	1.026773	1.057807	1.016686	0.28865	-1.7926	0.571127	1
SERTAD1	1.276632	1.310545	1.265608	0.8553	-0.2255	0.571161	1
CELSR2	1.307646	1.342019	1.296473	0.86683	-0.2062	0.571227	1
CASP2	1.28486	1.249646	1.296306	1.1869	0.2472	0.57135	1
RTN4RL1	1.030611	1.061905	1.020439	0.33016	-1.5988	0.571355	1
B3GALT5	1.039489	1.070724	1.029336	0.4148	-1.2695	0.571422	1
C6orf136	1.255179	1.220615	1.266414	1.2076	0.2721	0.571444	1
LYPD3	1.135282	1.16795	1.124663	0.74226	-0.43	0.571632	1
CFAP69	1.071652	1.039458	1.082117	2.08111	1.0574	0.571729	1
SLC1A6	1.221766	1.187626	1.232864	1.24111	0.3116	0.571977	1
ZNF69	1.085093	1.052644	1.095641	1.81675	0.8614	0.572048	1
ABCC6	1.097519	1.064747	1.108171	1.67067	0.7404	0.572061	1
CTD-3222D19.12	1.058623	1.026594	1.069035	2.59585	1.3762	0.572242	1
MRPL24	1.593576	1.555023	1.606107	1.09204	0.127	0.57255	1
PCK2	1.167393	1.133632	1.178368	1.33477	0.4166	0.572633	1
SLC13A4	1.041184	1.072202	1.031101	0.43075	-1.2151	0.57287	1
NEBL	1.373976	1.338008	1.385667	1.141	0.1903	0.572976	1
NT5E	1.042187	1.073244	1.032092	0.43816	-1.1905	0.573085	1
PRDM5	1.094789	1.062415	1.105312	1.68729	0.7547	0.573197	1
SMOC2	1.032432	1.063897	1.022205	0.34751	-1.5249	0.573322	1
ZSCAN30	1.218119	1.183844	1.229261	1.24704	0.3185	0.57344	1

SLC39A4	1.110114	1.142067	1.099728	0.70198	-0.5105	0.573457	1
CTR9	1.339518	1.30382	1.351121	1.15569	0.2088	0.573621	1
RP3-414A15.10	1.072598	1.040569	1.083009	2.0461	1.0329	0.573635	1
ZNF724	1.10376	1.071278	1.114318	1.60382	0.6815	0.573732	1
TATDN2	1.100646	1.132463	1.090304	0.68173	-0.5527	0.573736	1
PFDN4	2.811729	2.752606	2.830947	1.0447	0.0631	0.573765	1
USP4	1.178022	1.144611	1.188882	1.30614	0.3853	0.573842	1
LRCH4	1.260298	1.294481	1.249187	0.84619	-0.2409	0.573871	1
RAD18	1.151599	1.118547	1.162343	1.36944	0.4536	0.574025	1
CD7	1.072055	1.039954	1.08249	2.06463	1.0459	0.57425	1
AP1G2	1.3191	1.283758	1.330589	1.16504	0.2204	0.574272	1
HGH1	1.153761	1.120363	1.164617	1.36767	0.4517	0.574377	1
UNC119	2.460812	2.519699	2.441671	0.94866	-0.076	0.574414	1
CUL3	1.433247	1.396376	1.445232	1.12326	0.1677	0.574529	1
CEP170B	1.102895	1.134593	1.092592	0.68794	-0.5397	0.574532	1
C17orf96	1.07863	1.046466	1.089084	1.91718	0.939	0.574611	1
SLC9A5	1.058067	1.02639	1.068363	2.59049	1.3732	0.574697	1
FAM167B	1.058135	1.026228	1.068507	2.61202	1.3852	0.574697	1
GLA	1.253184	1.218721	1.264386	1.20879	0.2736	0.57474	1
DDX28	1.086639	1.054593	1.097056	1.77781	0.8301	0.574813	1
PHF12	1.251187	1.284891	1.240232	0.84324	-0.246	0.575197	1
RP11-481J2.3	1.050715	1.01918	1.060965	3.17859	1.6684	0.575209	1
RFX3-AS1	1.057148	1.025384	1.067474	2.65815	1.4104	0.575234	1
DLGAP4-AS1	1.063863	1.03199	1.074223	2.3202	1.2143	0.575316	1
PHLPP2	1.081531	1.112979	1.071309	0.63117	-0.6639	0.575358	1
CLDN19	1.068671	1.100085	1.05846	0.58411	-0.7757	0.575953	1
LACTB	1.307728	1.342449	1.296441	0.86565	-0.2081	0.576051	1
BRINP2	1.07475	1.04284	1.085122	1.98695	0.9906	0.576107	1
RPF2	1.553697	1.515508	1.566111	1.09816	0.1351	0.576197	1
CDC73	1.455587	1.492204	1.443684	0.90142	-0.1497	0.576286	1
ZNF286A	1.172357	1.139132	1.183157	1.31643	0.3966	0.576357	1
NCBP1	1.214103	1.180054	1.22517	1.25057	0.3226	0.576395	1
ZNF32	2.206091	2.161178	2.22069	1.05125	0.0721	0.57646	1
SLC29A3	1.089879	1.057895	1.100276	1.73202	0.7925	0.576646	1
FAM26F	1.136207	1.103254	1.146918	1.42288	0.5088	0.576723	1
FBXL3	1.226988	1.259865	1.216301	0.83236	-0.2647	0.576767	1
MARVELD2	1.086097	1.117383	1.075928	0.64684	-0.6285	0.57692	1
MPP6	1.364792	1.399584	1.353482	0.88462	-0.1769	0.576942	1
RNF146	1.636445	1.59805	1.648926	1.08507	0.1178	0.577009	1
RSPH14	1.078703	1.046907	1.089038	1.8982	0.9246	0.577131	1
SBF1	1.219871	1.253138	1.209058	0.82586	-0.276	0.577231	1
FAM21A	1.163297	1.195202	1.152926	0.78342	-0.3521	0.577233	1
RP11-42I10.1	1.042047	1.072943	1.032004	0.43875	-1.1885	0.577254	1
ARHGAP12	1.296509	1.261266	1.307965	1.17874	0.2372	0.577285	1
TRAFD1	1.199874	1.166616	1.210685	1.26449	0.3386	0.577344	1

CH507-154B10.2	1.054358	1.022868	1.064595	2.82469	1.4981	0.577355	1
HOMEZ	1.228086	1.193972	1.239176	1.23304	0.3022	0.577365	1
ST7L	1.239779	1.206059	1.25074	1.21683	0.2831	0.577454	1
TIMM23	1.280852	1.314874	1.269793	0.85683	-0.2229	0.577671	1
LRRN2	1.154941	1.187119	1.144481	0.77214	-0.3731	0.577869	1
ABHD17C	1.068772	1.099968	1.058631	0.5865	-0.7698	0.577941	1
CLP1	1.107502	1.075334	1.117958	1.5658	0.6469	0.578307	1
LZTS1	1.060395	1.028945	1.070618	2.43972	1.2867	0.578374	1
LRRC58	1.60694	1.569787	1.619017	1.0864	0.1196	0.578471	1
LMX1A	1.025614	1.056097	1.015705	0.27996	-1.8367	0.578576	1
BRMS1L	1.218859	1.185191	1.229803	1.2409	0.3114	0.578664	1
RIOK3	1.597984	1.635386	1.585826	0.922	-0.1172	0.578788	1
KLC2	1.191259	1.223899	1.180649	0.80683	-0.3097	0.578859	1
ZNF788	1.09298	1.061307	1.103275	1.68457	0.7524	0.578924	1
HOXA2	1.027179	1.057952	1.017176	0.29638	-1.7545	0.579043	1
LDB1	1.190243	1.222594	1.179727	0.80742	-0.3086	0.57912	1
DHRS13	1.329338	1.294791	1.340568	1.15529	0.2082	0.579194	1
GNL2	1.448493	1.413026	1.460021	1.11378	0.1555	0.579246	1
SEPSECS	1.121474	1.089237	1.131952	1.47868	0.5643	0.579287	1
CYP26A1	1.107385	1.074679	1.118017	1.58032	0.6602	0.57941	1
CPSF7	1.442982	1.406628	1.454799	1.11847	0.1615	0.579445	1
PLEKHN1	1.040541	1.071285	1.030548	0.42853	-1.2225	0.579509	1
TRMT12	1.095759	1.127058	1.085585	0.67359	-0.5701	0.579584	1
N4BP2L1	1.197131	1.163879	1.20794	1.26886	0.3435	0.579664	1
NANOS3	1.131368	1.163141	1.121039	0.74193	-0.4306	0.579756	1
PIK3C2B	1.065958	1.097074	1.055844	0.57528	-0.7977	0.579833	1
MTCH1	4.485632	4.578185	4.455547	0.96573	-0.0503	0.579872	1
CBX6	1.970264	2.011154	1.956972	0.94642	-0.0795	0.579888	1
YIPF3	2.484343	2.54226	2.465516	0.95024	-0.0736	0.579898	1
KATNAL1	1.258032	1.291111	1.24728	0.84944	-0.2354	0.580034	1
NPHP3	1.319819	1.353869	1.30875	0.8725	-0.1968	0.580164	1
SURF6	1.444889	1.481317	1.433048	0.89971	-0.1525	0.580214	1
RP1-223E5.4	1.058464	1.027071	1.068668	2.53656	1.3429	0.580335	1
USP8	1.784134	1.823102	1.771467	0.93727	-0.0935	0.580343	1
EZH1	1.304032	1.269376	1.315297	1.17047	0.2271	0.580462	1
PRRG1	1.378086	1.412809	1.366799	0.88854	-0.1705	0.580495	1
TMEM120A	1.252949	1.218878	1.264024	1.20626	0.2705	0.580513	1
INSR	1.543246	1.579863	1.531343	0.91633	-0.1261	0.580524	1
NRK	1.062256	1.093167	1.052208	0.56037	-0.8355	0.580544	1
CYP2W1	1.024009	1.054394	1.014133	0.25983	-1.9444	0.58056	1
CECR2	1.270887	1.236728	1.281991	1.1912	0.2524	0.580562	1
RRP1B	1.584493	1.54674	1.596765	1.0915	0.1263	0.580565	1
CLDN15	1.098585	1.129816	1.088433	0.68122	-0.5538	0.580666	1
CSDE1	5.057829	4.949317	5.093102	1.03641	0.0516	0.580681	1
CYGB	1.114057	1.145699	1.103772	0.71224	-0.4896	0.580756	1

AC091729.9	1.146614	1.114157	1.157164	1.37673	0.4612	0.580848	1
FAM214B	1.390087	1.424689	1.37884	0.89204	-0.1648	0.58086	1
LENG1	1.252295	1.218265	1.263356	1.20659	0.2709	0.580962	1
MAN2C1	1.237059	1.270329	1.226245	0.83692	-0.2568	0.581069	1
POLR3H	1.528038	1.489919	1.540429	1.1031	0.1416	0.581128	1
ZBED3-AS1	1.141887	1.109621	1.152375	1.39002	0.4751	0.581287	1
ZNF491	1.06276	1.031512	1.072917	2.31396	1.2104	0.581581	1
IGF2-AS	1.04827	1.079096	1.03825	0.48359	-1.0481	0.581687	1
CITED1	1.057748	1.088557	1.047733	0.53901	-0.8916	0.581778	1
PRKD2	1.142963	1.174608	1.132677	0.75985	-0.3962	0.58199	1
BLNK	1.041908	1.072613	1.031928	0.4397	-1.1854	0.582144	1
FAM83B	1.031115	1.061409	1.021268	0.34634	-1.5298	0.582327	1
EGF	1.075509	1.044056	1.085733	1.94601	0.9605	0.58241	1
CH507-9B2.5	1.07501	1.043651	1.085203	1.95191	0.9649	0.582779	1
PCBP1	4.171824	4.271701	4.139358	0.95955	-0.0596	0.582877	1
KLHL3	1.201759	1.234571	1.191094	0.81465	-0.2957	0.582889	1
HSF1	1.882606	1.840909	1.89616	1.0657	0.0918	0.58291	1
ALX1	1.028008	1.058401	1.018129	0.31043	-1.6877	0.582929	1
SPIRE1	1.214046	1.24718	1.203276	0.82238	-0.2821	0.582939	1
HERC1	1.320817	1.286464	1.331983	1.1589	0.2128	0.582949	1
FBXL8	1.108639	1.076816	1.118983	1.54893	0.6313	0.582949	1
AC108142.1	1.17367	1.140898	1.184323	1.3082	0.3876	0.582971	1
TOR1AIP2	1.869098	1.826908	1.882813	1.06761	0.0944	0.583146	1
RAD52	1.099442	1.067544	1.109811	1.62578	0.7011	0.583164	1
TUB	1.131676	1.099232	1.142222	1.43323	0.5193	0.58317	1
LYSMD1	1.114334	1.082097	1.124813	1.52031	0.6044	0.58325	1
MAGT1	1.490842	1.527074	1.479064	0.90891	-0.1378	0.583352	1
GMFB	1.504353	1.540549	1.492588	0.91127	-0.134	0.583508	1
SOGA1	1.268358	1.301538	1.257572	0.85419	-0.2274	0.583652	1
AP4E1	1.096787	1.065163	1.107066	1.64306	0.7164	0.583692	1
IFT80	1.275229	1.241569	1.286171	1.18463	0.2444	0.583839	1
SLC16A9	1.178152	1.145503	1.188764	1.29732	0.3755	0.583883	1
TADA2A	1.12325	1.091225	1.133659	1.46516	0.5511	0.583958	1
CDK7	1.293201	1.258995	1.30432	1.175	0.2327	0.584085	1
ZP1	1.064085	1.032924	1.074214	2.25411	1.1726	0.584096	1
ASAH2	1.07434	1.043052	1.084511	1.96301	0.9731	0.584153	1
TMEM19	1.2669	1.300447	1.255996	0.85205	-0.231	0.58427	1
RP11-79P5.9	1.071681	1.040477	1.081824	2.0215	1.0154	0.584638	1
RP11-319G6.1	1.095493	1.063959	1.105743	1.65328	0.7253	0.584684	1
TMEM173	1.041278	1.071446	1.031472	0.4405	-1.1828	0.584879	1
SLC41A3	1.566775	1.603874	1.554716	0.9186	-0.1225	0.584975	1
THUMPD3	1.585451	1.547786	1.597695	1.09111	0.1258	0.584979	1
GCNT1	1.028256	1.058431	1.018447	0.3157	-1.6634	0.584979	1
RP4-591C20.9	1.089605	1.058229	1.099804	1.714	0.7774	0.585073	1
PLA2G6	1.110902	1.079178	1.121214	1.53091	0.6144	0.585092	1

WDR44	1.166965	1.198477	1.156722	0.78962	-0.3408	0.585145	1
ZNF81	1.044662	1.075018	1.034794	0.46381	-1.1084	0.585316	1
C9orf24	1.097529	1.12858	1.087436	0.68001	-0.5564	0.585355	1
SLC30A2	1.031104	1.061697	1.02116	0.34297	-1.5438	0.585457	1
KIAA1211L	1.030958	1.061066	1.021171	0.34669	-1.5283	0.585457	1
AC002117.1	1.125726	1.093904	1.13607	1.44903	0.5351	0.58557	1
SURF2	1.441402	1.47603	1.430146	0.90361	-0.1462	0.585646	1
ZNF227	1.180571	1.147972	1.191168	1.29192	0.3695	0.585666	1
MARCH5	1.88726	1.929115	1.873655	0.94031	-0.0888	0.585786	1
CDH8	1.173157	1.204924	1.162831	0.79459	-0.3317	0.585803	1
RP1-198K11.5	1.091261	1.059972	1.101432	1.69133	0.7582	0.585873	1
PDZD4	1.073103	1.042085	1.083186	1.97663	0.983	0.585924	1
BRSK2	1.073088	1.042009	1.083191	1.98028	0.9857	0.585924	1
ASTN2	1.098749	1.067429	1.10893	1.61548	0.692	0.5861	1
ENKD1	1.185662	1.152983	1.196284	1.28304	0.3596	0.586111	1
UPK2	1.045164	1.075426	1.035327	0.46836	-1.0943	0.586393	1
ARHGAP44	1.145474	1.113457	1.155882	1.37393	0.4583	0.586439	1
LRRC10B	1.085942	1.054734	1.096086	1.75552	0.8119	0.586688	1
SLC25A20	1.076765	1.107454	1.066789	0.62156	-0.686	0.58672	1
CDK5R1	1.084732	1.053512	1.09488	1.77307	0.8262	0.586723	1
ZNF709	1.046299	1.015703	1.056245	3.58171	1.8406	0.586832	1
SELENOF	2.979602	2.901415	3.005017	1.05449	0.0765	0.587113	1
C1orf53	1.354897	1.32054	1.366064	1.14202	0.1916	0.587317	1
ZNF713	1.078683	1.047598	1.088788	1.86537	0.8995	0.587522	1
FUT10	1.175097	1.142806	1.185594	1.29962	0.3781	0.587817	1
LIN28B	1.303737	1.269823	1.314761	1.16654	0.2222	0.587871	1
EFNA3	1.567611	1.604698	1.555555	0.91873	-0.1223	0.587891	1
ABHD14A-ACY1	1.104242	1.072712	1.114491	1.57458	0.655	0.588	1
SENP1	1.121899	1.152742	1.111873	0.73243	-0.4492	0.588059	1
DDX41	1.620306	1.582224	1.632684	1.08667	0.1199	0.588085	1
FAM149B1	1.131719	1.099921	1.142056	1.42168	0.5076	0.588095	1
AKAP1	1.146124	1.114192	1.156503	1.37053	0.4547	0.588095	1
CTBP1	1.60728	1.644225	1.595271	0.92401	-0.114	0.588219	1
SPOUT1	1.239353	1.205882	1.250232	1.21542	0.2815	0.588282	1
ZNF550	1.152035	1.119849	1.162497	1.35584	0.4392	0.588283	1
TGFBR1	1.406684	1.440489	1.395695	0.89831	-0.1547	0.588444	1
PHKG1	1.216753	1.183698	1.227497	1.23843	0.3085	0.588503	1
NR0B1	1.099232	1.067991	1.109388	1.60886	0.686	0.588589	1
UGT3A2	1.064244	1.033212	1.074332	2.2381	1.1623	0.588671	1
THAP10	1.127898	1.096232	1.138192	1.43603	0.5221	0.588755	1
CDNF	1.040445	1.070528	1.030667	0.43481	-1.2015	0.589136	1
AP2B1	2.903953	2.963369	2.88464	0.9599	-0.059	0.589188	1
DUS2	1.121025	1.089455	1.131287	1.46762	0.5535	0.589263	1
LIFR	1.143252	1.174356	1.133141	0.76362	-0.3891	0.589315	1
MT1F	1.210202	1.243651	1.19933	0.81809	-0.2897	0.589381	1

CMTM5	1.059562	1.028938	1.069517	2.40232	1.2644	0.589478	1
EEA1	1.640056	1.676822	1.628105	0.92802	-0.1078	0.589512	1
ANKDD1A	1.114131	1.144883	1.104135	0.71876	-0.4764	0.589592	1
MRGBP	1.437825	1.402151	1.449421	1.11754	0.1603	0.589665	1
BICD2	1.263705	1.295993	1.25321	0.85546	-0.2252	0.589696	1
GTPBP3	1.361621	1.326995	1.372876	1.14031	0.1894	0.58975	1
MARCH10	1.053114	1.022669	1.06301	2.77956	1.4749	0.589755	1
PPAN	1.361548	1.395981	1.350355	0.88478	-0.1766	0.589757	1
RET	1.030114	1.06033	1.020292	0.33636	-1.5719	0.589808	1
RP4-555D20.2	1.025559	1.055137	1.015944	0.28918	-1.79	0.589822	1
LINC01456	1.088139	1.05718	1.098202	1.71742	0.7802	0.589869	1
TALDO1	2.952659	2.890713	2.972796	1.04341	0.0613	0.58988	1
IQCE	1.247979	1.214971	1.258708	1.20346	0.2672	0.589896	1
TRIM55	1.024709	1.05436	1.015071	0.27725	-1.8507	0.590066	1
GJC1	1.258227	1.290899	1.247607	0.85118	-0.2325	0.590068	1
YARS	1.395712	1.360932	1.407017	1.12768	0.1734	0.590097	1
ZNF280D	1.131347	1.099588	1.14167	1.42256	0.5085	0.590123	1
DCUN1D4	1.320739	1.354462	1.309778	0.87394	-0.1944	0.590256	1
RAB35	1.185041	1.216837	1.174706	0.8057	-0.3117	0.590282	1
SAXO2	1.109136	1.077926	1.119281	1.5307	0.6142	0.590325	1
SAMHD1	1.075015	1.044064	1.085075	1.93072	0.9491	0.590455	1
GTF2B	1.267769	1.234507	1.278581	1.18795	0.2485	0.590474	1
PIGH	1.335262	1.301129	1.346357	1.1502	0.2019	0.590497	1
KLF12	1.1748	1.206161	1.164606	0.79843	-0.3248	0.590619	1
ZNF335	1.096738	1.127324	1.086797	0.6817	-0.5528	0.590697	1
C2orf48	1.05284	1.022439	1.062722	2.79528	1.483	0.590845	1
AIF1L	1.46224	1.426911	1.473723	1.10965	0.1501	0.590945	1
GRB14	1.06717	1.036669	1.077084	2.10214	1.0719	0.590987	1
HECW2	1.08083	1.111099	1.070991	0.63899	-0.6461	0.591145	1
PRR36	1.171205	1.139208	1.181606	1.30457	0.3836	0.591291	1
GYG2	1.245054	1.277325	1.234564	0.84581	-0.2416	0.591456	1
TMF1	1.839268	1.87869	1.826454	0.94055	-0.0884	0.591535	1
CALHM2	1.145622	1.11387	1.155943	1.36948	0.4536	0.591536	1
FMO1	1.031817	1.061541	1.022155	0.36	-1.4739	0.591593	1
ARNTL2	1.066467	1.096418	1.056731	0.58839	-0.7651	0.591609	1
ALG12	1.338641	1.304635	1.349695	1.14792	0.199	0.59162	1
CLEC18C	1.048991	1.018715	1.058833	3.14355	1.6524	0.591694	1
MYO1B	1.51556	1.550177	1.504308	0.91663	-0.1256	0.591806	1
LINC00461	1.048434	1.017948	1.058344	3.25081	1.7008	0.591807	1
ZNF143	1.213998	1.181561	1.224542	1.23673	0.3065	0.591843	1
NUBPL	1.328228	1.29489	1.339065	1.1498	0.2014	0.59192	1
SLC24A4	1.024897	1.054499	1.015275	0.28027	-1.8351	0.592035	1
TMEM109	1.432159	1.466671	1.42094	0.90201	-0.1488	0.592036	1
INPP4B	1.059313	1.089259	1.049579	0.55545	-0.8483	0.592382	1
PRKAR1B	1.155233	1.186478	1.145077	0.77798	-0.3622	0.592438	1

MED14	1.208291	1.240253	1.197901	0.82372	-0.2798	0.592439	1
NOP16	1.297432	1.330296	1.286749	0.86816	-0.204	0.592482	1
PDK4	1.127003	1.095961	1.137093	1.42864	0.5146	0.592588	1
EMC2	1.495221	1.53058	1.483728	0.9117	-0.1334	0.592617	1
GNAZ	1.143851	1.174831	1.133781	0.7652	-0.3861	0.592629	1
RNF152	1.042634	1.072282	1.032997	0.4565	-1.1313	0.592635	1
KIF1BP	1.204653	1.23608	1.194437	0.82361	-0.28	0.592683	1
EOGT	1.049351	1.079364	1.039595	0.49891	-1.0032	0.592707	1
MNS1	1.109724	1.078716	1.119803	1.52197	0.6059	0.592754	1
GART	1.338842	1.304249	1.350086	1.15066	0.2025	0.592782	1
PEG10	2.531679	2.588598	2.513177	0.95252	-0.0702	0.592816	1
ZFHX2	1.184163	1.151842	1.19467	1.28206	0.3585	0.592832	1
LINC00339	1.200138	1.168053	1.210567	1.25298	0.3254	0.592957	1
GPR19	1.046098	1.015961	1.055894	3.50191	1.8081	0.593149	1
ZBED9	1.089525	1.05871	1.099542	1.69547	0.7617	0.593298	1
LAMB3	1.042036	1.071642	1.032413	0.45243	-1.1442	0.593475	1
RP3-467N11.1	1.172891	1.140987	1.183262	1.29985	0.3783	0.593547	1
RGS22	1.083522	1.052669	1.09355	1.7762	0.8288	0.594007	1
ZNF112	1.083465	1.052715	1.09346	1.77292	0.8261	0.594007	1
DGKD	1.225573	1.257302	1.215259	0.8366	-0.2574	0.594071	1
ZNF365	1.032329	1.061881	1.022723	0.36721	-1.4453	0.59421	1
LINC00882	1.090967	1.060409	1.100901	1.67029	0.7401	0.594231	1
KCTD21-AS1	1.067259	1.03697	1.077104	2.08559	1.0605	0.5943	1
PPFIA1	1.524214	1.559185	1.512847	0.91713	-0.1248	0.594334	1
FKBP8	4.240001	4.331722	4.210187	0.96352	-0.0536	0.59446	1
CPLX2	1.045057	1.014881	1.054865	3.6869	1.8824	0.594882	1
VPS33A	1.184177	1.215612	1.173959	0.80681	-0.3097	0.595071	1
MX1	1.066229	1.096101	1.056519	0.58812	-0.7658	0.595201	1
RP11-206L10.9	1.169774	1.137982	1.180108	1.30529	0.3844	0.59533	1
MTHFS	1.41747	1.45126	1.406486	0.90078	-0.1508	0.595374	1
NEURL1	1.052584	1.082169	1.042967	0.52292	-0.9354	0.595675	1
MICALL2	1.135655	1.166224	1.125719	0.75632	-0.4029	0.595774	1
CHD2	1.738673	1.700499	1.751082	1.07221	0.1006	0.595774	1
RP11-181C3.1	1.060402	1.03007	1.070262	2.33657	1.2244	0.595917	1
TBC1D9	1.443328	1.477027	1.432373	0.90639	-0.1418	0.596125	1
FAM102B	1.11769	1.086728	1.127755	1.47305	0.5588	0.596212	1
MMP25-AS1	1.14421	1.112591	1.154488	1.37212	0.4564	0.596255	1
JMJD6	1.167068	1.198126	1.156973	0.79229	-0.3359	0.596283	1
PIP5K1B	1.308765	1.275786	1.319485	1.15845	0.2122	0.596427	1
USP53	1.366758	1.40024	1.355875	0.88915	-0.1695	0.596559	1
GSTCD	1.234385	1.201991	1.244915	1.21251	0.278	0.596631	1
RP11-1017G21.5	1.115045	1.084123	1.125097	1.48707	0.5725	0.596669	1
NCOA2	1.270669	1.237822	1.281347	1.18302	0.2425	0.596778	1
TENM4	1.242297	1.209548	1.252942	1.20708	0.2715	0.596796	1
LMO7	1.059819	1.089468	1.050181	0.56088	-0.8342	0.596816	1

SH3RF2	1.042095	1.071385	1.032574	0.45632	-1.1319	0.59696	1
ATP6AP2	2.390915	2.438508	2.375444	0.95616	-0.0647	0.597131	1
PTP4A2	2.751136	2.806504	2.733138	0.95939	-0.0598	0.597237	1
ACAD11	1.063751	1.033598	1.073553	2.18919	1.1304	0.597345	1
EYA2	1.111622	1.141993	1.10175	0.71659	-0.4808	0.597493	1
TUBB3	1.141972	1.110977	1.152047	1.37008	0.4543	0.597506	1
DUS3L	1.127179	1.096397	1.137184	1.42312	0.5091	0.597531	1
NFKBIZ	1.079186	1.109092	1.069465	0.63675	-0.6512	0.597891	1
VWA8	1.11249	1.08217	1.122346	1.48894	0.5743	0.597959	1
CRIP2	1.697423	1.733426	1.68572	0.93495	-0.097	0.598013	1
CBR1	1.79738	1.835035	1.78514	0.94025	-0.0889	0.598056	1
AC002451.3	1.045486	1.015688	1.055172	3.51675	1.8142	0.598071	1
RP11-473I1.5	1.049246	1.019349	1.058964	3.04741	1.6076	0.598263	1
PNPLA8	1.522758	1.486421	1.534569	1.09899	0.1362	0.598625	1
LSM14B	1.212643	1.244037	1.202438	0.82954	-0.2696	0.598846	1
RALA	1.748065	1.710043	1.760424	1.07096	0.0989	0.598848	1
KLC4	1.272231	1.239388	1.282906	1.18179	0.241	0.598858	1
SGPP1	1.090746	1.1205	1.081075	0.67282	-0.5717	0.598963	1
TAF5L	1.066297	1.036188	1.076084	2.10246	1.0721	0.598996	1
RP11-373I8.1	1.065549	1.035574	1.075292	2.11649	1.0817	0.59901	1
TMBIM6	4.88415	4.786211	4.915986	1.03428	0.0486	0.599079	1
COQ3	1.116856	1.086209	1.126818	1.47105	0.5569	0.59931	1
PAPD7	1.184262	1.152721	1.194515	1.27366	0.349	0.599505	1
PRKACA	1.304591	1.271777	1.315258	1.15999	0.2141	0.599533	1
PTRH2	1.190784	1.159583	1.200926	1.25907	0.3324	0.599794	1
CHORDC1	1.565842	1.529778	1.577564	1.0902	0.1246	0.599861	1
BRMS1	1.454501	1.488395	1.443484	0.90804	-0.1392	0.600032	1
TSPYL4	1.320377	1.287214	1.331156	1.153	0.2054	0.600079	1
UNKL	1.208424	1.176579	1.218775	1.23896	0.3091	0.600232	1
SPPL2A	1.303438	1.335769	1.292929	0.87241	-0.1969	0.600236	1
CINP	1.386816	1.352655	1.39792	1.12836	0.1742	0.600352	1
NAB1	1.321533	1.354042	1.310966	0.87833	-0.1872	0.600424	1
PLBD1	1.189444	1.220602	1.179316	0.81285	-0.2989	0.600445	1
TRMT5	1.187851	1.156408	1.198072	1.26638	0.3407	0.600547	1
ZNF485	1.058701	1.028974	1.068364	2.35951	1.2385	0.600687	1
ALAS1	1.296525	1.328599	1.286099	0.87066	-0.1998	0.600745	1
B3GNT8	1.09177	1.121349	1.082156	0.67702	-0.5627	0.600753	1
SMIM12	1.58842	1.623225	1.577106	0.926	-0.1109	0.600767	1
TTPA	1.0448	1.01526	1.054403	3.56512	1.834	0.600782	1
EFHB	1.072161	1.042067	1.081944	1.94791	0.9619	0.600793	1
KCNJ2	1.030078	1.059051	1.020661	0.34988	-1.5151	0.600809	1
FNTB	1.187318	1.155985	1.197503	1.26616	0.3405	0.600821	1
AF127936.9	1.087187	1.057054	1.096982	1.69983	0.7654	0.600933	1
DUSP1	1.763289	1.809161	1.748377	0.92488	-0.1127	0.601107	1
CHST3	1.098893	1.128339	1.089321	0.69598	-0.5229	0.60122	1

FLII	1.37281	1.405159	1.362295	0.8942	-0.1613	0.60126	1
PRAP1	1.02649	1.055242	1.017144	0.31034	-1.6881	0.601274	1
SNX27	1.516692	1.551243	1.505461	0.91695	-0.1251	0.601329	1
KIAA2013	1.313606	1.345656	1.303187	0.87714	-0.1891	0.601371	1
ATP2B1	1.984689	2.027895	1.970644	0.9443	-0.0827	0.601536	1
WDR75	1.268719	1.236566	1.27917	1.1801	0.2389	0.601609	1
CTC-367J11.1	1.071776	1.041781	1.081526	1.95128	0.9644	0.60162	1
TRMT44	1.081815	1.051687	1.091609	1.77238	0.8257	0.601631	1
TBCE	1.159731	1.128709	1.169815	1.31937	0.3999	0.601644	1
PRPS1	1.759575	1.796454	1.747588	0.93864	-0.0913	0.601735	1
ZNF559	1.162862	1.131748	1.172976	1.31293	0.3928	0.601766	1
ADNP2	1.225146	1.193444	1.235451	1.21715	0.2835	0.601844	1
KRT5	1.022131	1.05082	1.012806	0.25198	-1.9886	0.601863	1
METTL7A	1.049958	1.07904	1.040505	0.51245	-0.9645	0.601906	1
ZCCHC8	1.219723	1.187853	1.230082	1.2248	0.2925	0.601953	1
ZHX2	1.186927	1.155337	1.197196	1.26947	0.3442	0.601989	1
AGR2	1.021327	1.050186	1.011946	0.23804	-2.0707	0.602094	1
AC010894.3	1.122697	1.092176	1.132617	1.43875	0.5248	0.602109	1
CSNK1A1	3.795342	3.86612	3.772336	0.96728	-0.048	0.602169	1
DOLPP1	1.101537	1.071314	1.111361	1.56156	0.643	0.602214	1
CBFA2T3	1.067759	1.097047	1.058239	0.60011	-0.7367	0.602446	1
PEX11B	1.302156	1.334215	1.291735	0.87289	-0.1961	0.602469	1
TBL2	1.365762	1.332433	1.376595	1.13284	0.18	0.602821	1
RP11-297D21.4	1.137738	1.107106	1.147695	1.37896	0.4636	0.602969	1
TTC34	1.077538	1.047731	1.087227	1.82746	0.8698	0.603014	1
CAMK1G	1.076662	1.046854	1.086352	1.84299	0.882	0.603328	1
CCDC149	1.17056	1.139417	1.180683	1.29599	0.3741	0.603379	1
WDR92	1.194443	1.163263	1.204578	1.25305	0.3254	0.603442	1
TM4SF1	1.025706	1.054284	1.016417	0.30242	-1.7254	0.603605	1
TRRAP	1.203833	1.234809	1.193764	0.8252	-0.2772	0.603663	1
KIAA0319L	1.473429	1.506881	1.462555	0.91255	-0.132	0.603756	1
KAZN	1.398039	1.364448	1.408958	1.12213	0.1662	0.603813	1
CTD-2132N18.2	1.086832	1.057013	1.096525	1.69303	0.7596	0.603884	1
CHMP3	1.75888	1.721685	1.77097	1.06829	0.0953	0.603895	1
NUPL2	1.304073	1.271749	1.31458	1.15761	0.2111	0.604084	1
RP11-418J17.1	1.073595	1.043859	1.083261	1.8984	0.9248	0.604135	1
ZNF316	1.085244	1.114384	1.075771	0.66243	-0.5942	0.60432	1
PKD2	1.216346	1.247476	1.206227	0.83332	-0.2631	0.604345	1
PAXBP1-AS1	1.051638	1.022253	1.061189	2.74975	1.4593	0.60442	1
L3MBTL2	1.167916	1.198274	1.158048	0.79712	-0.3271	0.60443	1
ZCCHC3	1.395146	1.427849	1.384516	0.89872	-0.1541	0.604528	1
MAP2K4	1.506781	1.540283	1.495891	0.91784	-0.1237	0.604656	1
ZNF497	1.072661	1.043001	1.082301	1.91393	0.9365	0.604688	1
RP11-120I21.2	1.241503	1.273181	1.231205	0.84634	-0.2407	0.604725	1
TBCCD1	1.136758	1.106177	1.146698	1.38164	0.4664	0.604778	1

ABHD13	1.266266	1.297929	1.255974	0.85918	-0.219	0.604908	1
STYXL1	1.330818	1.363055	1.320339	0.88234	-0.1806	0.60514	1
FBLN2	1.084209	1.113292	1.074756	0.65985	-0.5998	0.6052	1
TC2N	1.02618	1.054663	1.016921	0.30956	-1.6917	0.605334	1
E2F2	1.064479	1.034992	1.074064	2.11664	1.0818	0.605355	1
LRRTM1	1.036122	1.064699	1.026833	0.41474	-1.2697	0.60544	1
DNAJB4	1.395384	1.361377	1.406439	1.1247	0.1695	0.605466	1
SP3	1.555537	1.520352	1.566974	1.0896	0.1238	0.605488	1
RP11-260M2.1	1.093336	1.063415	1.103061	1.62518	0.7006	0.605571	1
NDUFAF6	1.297055	1.264489	1.307641	1.16315	0.218	0.605707	1
VIM-AS1	1.226082	1.194499	1.236349	1.21517	0.2812	0.605772	1
SMTNL2	1.148154	1.117849	1.158005	1.34075	0.423	0.605773	1
PGM2	1.229706	1.198462	1.239863	1.20861	0.2733	0.605806	1
DLG3	1.185193	1.215759	1.175258	0.81229	-0.2999	0.605851	1
SIRT1	1.168236	1.137542	1.178213	1.2957	0.3737	0.605912	1
LRRC24	1.120614	1.090436	1.130424	1.44218	0.5282	0.605942	1
PRKD1	1.082873	1.052976	1.092591	1.74777	0.8055	0.606109	1
STX18-AS1	1.058761	1.087764	1.049333	0.56211	-0.8311	0.606198	1
ADCK2	1.22343	1.191697	1.233745	1.21935	0.2861	0.606236	1
SETMAR	1.190426	1.159475	1.200487	1.25717	0.3302	0.606256	1
EFCAB14	1.547516	1.581687	1.536408	0.92216	-0.1169	0.60633	1
CACNG7	1.149233	1.118854	1.159107	1.33868	0.4208	0.606367	1
NEURL2	1.105079	1.075427	1.114718	1.52091	0.6049	0.606455	1
ENTPD6	1.282556	1.250274	1.29305	1.17092	0.2276	0.606714	1
RHBDD3	1.430377	1.396201	1.441487	1.1143	0.1561	0.606782	1
RP11-513G11.4	1.080522	1.051088	1.09009	1.76342	0.8184	0.606967	1
RUNX1	1.133342	1.162915	1.123729	0.75947	-0.3969	0.607072	1
SWT1	1.099825	1.128995	1.090343	0.70036	-0.5138	0.607294	1
KLHL9	1.510541	1.476279	1.521677	1.09532	0.1313	0.607693	1
MCUB	1.697157	1.659745	1.709319	1.07514	0.1045	0.607878	1
LDB2	1.065929	1.036589	1.075466	2.06256	1.0444	0.60792	1
MSH5	1.062086	1.032805	1.071604	2.18272	1.1261	0.607923	1
POLM	1.257679	1.226171	1.267921	1.18459	0.2444	0.608082	1
LINC01410	1.069623	1.040421	1.079115	1.95729	0.9689	0.60819	1
RRP12	1.155098	1.185266	1.145291	0.78423	-0.3507	0.608311	1
LINC00960	1.25568	1.224082	1.265951	1.18685	0.2471	0.608313	1
DNM3OS	1.028063	1.056702	1.018754	0.33076	-1.5962	0.608318	1
RP11-234B24.2	1.02798	1.056301	1.018774	0.33346	-1.5844	0.608318	1
UBIAD1	1.139963	1.16947	1.130371	0.76929	-0.3784	0.608343	1
PGGT1B	1.271848	1.240089	1.282172	1.17528	0.233	0.608683	1
ZNF337	1.141283	1.111064	1.151106	1.36052	0.4442	0.608915	1
LYSMD2	1.112795	1.082825	1.122537	1.47946	0.5651	0.608937	1
LRFN1	1.081598	1.110449	1.07222	0.65388	-0.6129	0.608988	1
SYS1	1.476365	1.442058	1.487517	1.10283	0.1412	0.609014	1
UQCRFS1	3.270928	3.201664	3.293443	1.04169	0.0589	0.609023	1

RFESD	1.127676	1.097563	1.137465	1.40898	0.4947	0.609042	1
FBXW8	1.122807	1.092839	1.132548	1.42772	0.5137	0.609223	1
POC5	1.164931	1.134661	1.17477	1.29786	0.3761	0.609351	1
ZNF224	1.389671	1.356619	1.400415	1.12281	0.1671	0.609502	1
RP11-35G9.3	1.076842	1.047516	1.086374	1.8178	0.8622	0.609815	1
EHBP1L1	1.077827	1.106375	1.068547	0.64439	-0.634	0.609922	1
SLC17A7	1.047645	1.018869	1.056999	3.02075	1.5949	0.610213	1
EBP	1.660983	1.6249	1.672712	1.07651	0.1064	0.610257	1
SBK1	1.412291	1.378843	1.423164	1.11699	0.1596	0.610268	1
HOGA1	1.051572	1.022692	1.060959	2.68635	1.4256	0.610302	1
PPP1R21	1.218242	1.187247	1.228317	1.21933	0.2861	0.610318	1
CHCHD4	1.241201	1.209795	1.251409	1.19836	0.2611	0.610374	1
COG7	1.188052	1.157227	1.198072	1.25979	0.3332	0.610413	1
RHOV	1.033016	1.061315	1.023817	0.38844	-1.3642	0.610413	1
METTL25	1.08007	1.05085	1.089569	1.76142	0.8167	0.610425	1
AC100830.3	1.058432	1.086967	1.049157	0.56523	-0.8231	0.610508	1
POLR2F	3.69601	3.766167	3.673205	0.96639	-0.0493	0.610543	1
SOX13	1.055073	1.083607	1.045798	0.54777	-0.8683	0.610552	1
COPZ2	1.197414	1.227539	1.187622	0.82457	-0.2783	0.610675	1
LINC00115	1.178131	1.147478	1.188095	1.27541	0.351	0.610692	1
SMN1	1.116804	1.145857	1.10736	0.73607	-0.4421	0.61075	1
PTOV1-AS1	1.069179	1.039977	1.078672	1.96795	0.9767	0.610751	1
AC006942.4	1.168897	1.198662	1.159221	0.80147	-0.3193	0.610757	1
GRASP	1.054906	1.083341	1.045663	0.5479	-0.868	0.610776	1
FAM114A2	1.207518	1.176378	1.21764	1.23394	0.3033	0.610797	1
TRIM11	1.256136	1.286853	1.246151	0.85811	-0.2208	0.610836	1
MAPK4	1.054943	1.083322	1.045718	0.54869	-0.8659	0.610875	1
IKZF5	1.120723	1.150426	1.111067	0.73835	-0.4376	0.610921	1
ZNF280B	1.241155	1.271681	1.231233	0.85112	-0.2326	0.611341	1
PEX3	1.204247	1.172902	1.214436	1.24022	0.3106	0.611367	1
ERAP1	1.105899	1.076494	1.115457	1.50936	0.5939	0.611512	1
PYGO1	1.265969	1.234259	1.276276	1.17936	0.238	0.611547	1
ZNF443	1.062494	1.033581	1.071892	2.14086	1.0982	0.611662	1
BISPR	1.068318	1.039157	1.077796	1.98677	0.9904	0.611712	1
GATSL3	1.259032	1.289575	1.249104	0.86024	-0.2172	0.611769	1
NUDT19	1.183915	1.2138	1.174201	0.81478	-0.2955	0.611814	1
CILP2	1.041146	1.069462	1.031942	0.45984	-1.1208	0.611946	1
MC1R	1.067919	1.096348	1.058678	0.60902	-0.7154	0.612097	1
DAB2IP	1.246112	1.276643	1.236187	0.85376	-0.2281	0.612123	1
SH2D3A	1.042062	1.070342	1.03287	0.46728	-1.0976	0.61216	1
ALKBH6	1.264683	1.23355	1.274803	1.17663	0.2347	0.612196	1
GATA2	1.037781	1.066049	1.028592	0.4329	-1.2079	0.612213	1
SAMM50	1.825674	1.788983	1.837601	1.06162	0.0863	0.612343	1
TPPP	1.036867	1.065006	1.027721	0.42643	-1.2296	0.612463	1
SLC45A1	1.03425	1.062441	1.025086	0.40175	-1.3156	0.61248	1

EPS8L1	1.084743	1.113182	1.075499	0.66706	-0.5841	0.612541	1
TMEM257	1.052382	1.023625	1.06173	2.61293	1.3857	0.612565	1
COPS4	1.627661	1.592133	1.63921	1.0795	0.1104	0.612795	1
AARD	1.033621	1.06177	1.02447	0.39615	-1.3359	0.612803	1
MTRNR2L12	1.388621	1.420639	1.378214	0.89914	-0.1534	0.61284	1
CTD-2006H14.2	1.070893	1.041812	1.080346	1.92161	0.9423	0.612859	1
RBFOX3	1.043955	1.015231	1.053292	3.4988	1.8069	0.61286	1
CD109	1.056918	1.08521	1.047722	0.56005	-0.8364	0.612895	1
PORCN	1.150563	1.120494	1.160338	1.33067	0.4122	0.613013	1
PCP4L1	1.05468	1.025977	1.064011	2.46413	1.3011	0.613101	1
VSTM4	1.028963	1.056768	1.019925	0.35099	-1.5105	0.61323	1
GABBR2	1.028988	1.056836	1.019935	0.35075	-1.5115	0.61323	1
ZNF213	1.126795	1.097046	1.136466	1.40619	0.4918	0.613473	1
RAP1B	1.712332	1.676018	1.724136	1.07118	0.0992	0.61356	1
P2RY2	1.022181	1.050172	1.013082	0.26074	-1.9393	0.613763	1
KBTBD6	1.199127	1.168662	1.20903	1.23934	0.3096	0.613816	1
TMEM170B	1.162493	1.192118	1.152863	0.79568	-0.3297	0.61387	1
RIN3	1.065061	1.093327	1.055873	0.59868	-0.7401	0.61415	1
TOP1	2.249522	2.291651	2.235828	0.95678	-0.0637	0.614244	1
NBPF19	1.235723	1.204791	1.245778	1.20014	0.2632	0.614309	1
FAM218A	1.047242	1.018736	1.056509	3.01608	1.5927	0.614322	1
ZNF384	1.153885	1.124117	1.163561	1.3178	0.3981	0.614359	1
FZR1	1.237715	1.268223	1.227798	0.84929	-0.2357	0.614393	1
TRAPPC9	1.109255	1.138134	1.099867	0.72297	-0.468	0.614499	1
PPP4R2	2.338871	2.388179	2.322843	0.95293	-0.0696	0.614563	1
COG6	1.245537	1.21418	1.255729	1.19399	0.2558	0.614623	1
RP11-89F3.2	1.049673	1.021025	1.058986	2.80557	1.4883	0.614656	1
ZNF594	1.150402	1.120474	1.16013	1.32917	0.4105	0.614823	1
NCBP3	1.554818	1.520413	1.566001	1.0876	0.1211	0.61484	1
GLRA2	1.064857	1.035943	1.074256	2.06594	1.0468	0.614864	1
FBXO33	1.150052	1.179067	1.14062	0.78529	-0.3487	0.614896	1
NAA25	1.337246	1.368811	1.326985	0.88659	-0.1737	0.61522	1
YJEFN3	1.1298	1.09991	1.139516	1.39641	0.4817	0.615271	1
BCL9	1.317822	1.286154	1.328116	1.14664	0.1974	0.615385	1
L1TD1	1.046757	1.074914	1.037605	0.50197	-0.9943	0.615426	1
RP11-115D19.3	1.047879	1.075915	1.038765	0.51064	-0.9696	0.615461	1
ZBTB46	1.057631	1.08577	1.048484	0.56528	-0.823	0.615498	1
PARD3-AS1	1.051639	1.023052	1.060931	2.64317	1.4023	0.615721	1
CNTN6	1.054827	1.08276	1.045747	0.55276	-0.8553	0.61588	1
LRRC37A2	1.100273	1.071138	1.109744	1.54269	0.6254	0.615932	1
TGFB3	1.047631	1.075726	1.038499	0.5084	-0.976	0.615981	1
MTA2	1.134949	1.105076	1.144659	1.37671	0.4612	0.61602	1
CAMTA2	1.134073	1.163028	1.124661	0.76466	-0.3871	0.616077	1
SLIT1	1.053311	1.024514	1.062671	2.55658	1.3542	0.616122	1
MAPK8IP2	1.058148	1.029488	1.067464	2.28784	1.194	0.616365	1

CORO2B	1.099496	1.12806	1.090212	0.70445	-0.5054	0.616556	1
GNB2	3.899769	3.972925	3.875989	0.96739	-0.0478	0.616834	1
BVES	1.046511	1.074503	1.037412	0.50216	-0.9938	0.617096	1
TGFBRAP1	1.122308	1.150918	1.113009	0.74881	-0.4173	0.617187	1
PCDH9-AS1	1.063139	1.091322	1.053978	0.59107	-0.7586	0.617404	1
FLJ37035	1.093317	1.064444	1.102702	1.59365	0.6723	0.617436	1
ST6GALNAC5	1.139384	1.109654	1.149048	1.35926	0.4428	0.617552	1
ELAVL4	1.043296	1.014881	1.052532	3.53012	1.8197	0.617656	1
FAM72B	1.043067	1.014881	1.052229	3.50975	1.8114	0.617656	1
NCK1	1.500105	1.466763	1.510942	1.09465	0.1305	0.617743	1
LRRIQ3	1.061593	1.033014	1.070883	2.14703	1.1023	0.617811	1
MFSD11	1.265634	1.234486	1.275759	1.17601	0.2339	0.617876	1
RPRD1B	1.286934	1.317373	1.27704	0.87291	-0.1961	0.61819	1
PLBD2	1.116569	1.145121	1.107288	0.7393	-0.4358	0.618193	1
FHIT	1.254767	1.223931	1.264791	1.18247	0.2418	0.618202	1
TAS2R4	1.171478	1.142202	1.180994	1.27279	0.348	0.618225	1
MAP3K7CL	1.029193	1.056907	1.020185	0.35471	-1.4953	0.618395	1
GPC6	2.698449	2.647797	2.714913	1.04073	0.0576	0.618418	1
MISP3	1.164553	1.134821	1.174218	1.29221	0.3698	0.618537	1
DDX27	1.354651	1.385887	1.344498	0.89274	-0.1637	0.618679	1
IGFL2	1.044666	1.072282	1.035689	0.49374	-1.0182	0.61878	1
MIR99AHG	1.58565	1.550786	1.596983	1.08387	0.1162	0.618816	1
AP3B2	1.043814	1.015684	1.052958	3.37662	1.7556	0.618843	1
IQCH-AS1	1.11831	1.08934	1.127727	1.42968	0.5157	0.618927	1
TUG1	2.02309	2.061429	2.010627	0.95214	-0.0708	0.618986	1
LHPP	1.246478	1.215576	1.256523	1.18994	0.2509	0.619149	1
RFNG	1.298196	1.32876	1.288261	0.87681	-0.1897	0.619176	1
CA5B	1.182631	1.15291	1.192292	1.25755	0.3306	0.619222	1
PARD6G	1.174817	1.144949	1.184526	1.27304	0.3483	0.619551	1
DCLK2	1.155194	1.125667	1.164791	1.31133	0.391	0.619633	1
MRPL55	2.635882	2.588127	2.651405	1.03984	0.0564	0.61965	1
CAMK2D	1.574679	1.540032	1.585942	1.08501	0.1177	0.619751	1
ZNF486	1.098174	1.069306	1.107558	1.55192	0.6341	0.619765	1
LINC01152	1.059577	1.031246	1.068786	2.20142	1.1384	0.619866	1
BRK1	8.34729	8.162107	8.407485	1.03426	0.0486	0.61992	1
ULK3	1.303013	1.271956	1.313109	1.15132	0.2033	0.619949	1
LMBR1L	1.293679	1.324091	1.283794	0.87566	-0.1916	0.619973	1
MAP1S	1.192546	1.162692	1.202251	1.24315	0.314	0.619991	1
UBE3A	2.296708	2.252921	2.310941	1.04631	0.0653	0.620454	1
RHOBTB2	1.154442	1.183407	1.145026	0.79074	-0.3387	0.620465	1
B3GLCT	1.126961	1.155366	1.117728	0.75774	-0.4002	0.620579	1
ERCC6L	1.042768	1.014798	1.05186	3.50453	1.8092	0.620625	1
C4orf33	1.182843	1.153049	1.192528	1.25795	0.3311	0.620657	1
SBNO2	1.085625	1.113621	1.076525	0.67351	-0.5702	0.620771	1
CRTC3	1.26946	1.299395	1.25973	0.86752	-0.205	0.620836	1

RP11-115C10.1	1.10245	1.073641	1.111814	1.51838	0.6025	0.620941	1
CXCL2	1.044631	1.016586	1.053747	3.24051	1.6962	0.621111	1
PPP1R18	1.499393	1.53168	1.488898	0.91954	-0.121	0.621117	1
BDH1	1.073073	1.044644	1.082314	1.8438	0.8827	0.621168	1
APOL4	1.030042	1.057511	1.021113	0.36711	-1.4457	0.62124	1
MORN1	1.101576	1.072757	1.110944	1.52486	0.6087	0.621264	1
MORN3	1.073117	1.044667	1.082365	1.84397	0.8828	0.621268	1
CCDC113	1.158666	1.129316	1.168206	1.30074	0.3793	0.621322	1
USP24	1.271889	1.240713	1.282023	1.17162	0.2285	0.621381	1
RNASE1	1.071228	1.099225	1.062127	0.62612	-0.6755	0.621488	1
MKRN3	1.073483	1.04475	1.082823	1.85081	0.8882	0.621495	1
TMEM184C	1.276259	1.244994	1.286422	1.1691	0.2254	0.621671	1
SDCBP2-AS1	1.139148	1.109931	1.148645	1.35217	0.4353	0.621732	1
SSR2	4.821033	4.730877	4.850339	1.03202	0.0455	0.621774	1
MOCS3	1.089482	1.117348	1.080423	0.68534	-0.5451	0.621883	1
SRF	1.120466	1.148736	1.111277	0.74815	-0.4186	0.622019	1
JRKL	1.194546	1.223767	1.185048	0.82697	-0.2741	0.622183	1
CNTN4	1.208287	1.17871	1.217901	1.2193	0.2861	0.622315	1
CNST	1.145901	1.174384	1.136643	0.78357	-0.3519	0.622417	1
PRRG4	1.026992	1.05437	1.018092	0.33276	-1.5875	0.622691	1
WIPF3	1.055339	1.082998	1.046348	0.55843	-0.8406	0.622781	1
MYOZ1	1.100732	1.129074	1.091519	0.70904	-0.4961	0.622872	1
DOC2A	1.090292	1.061647	1.099604	1.61572	0.6922	0.622952	1
ETV6	1.163334	1.134066	1.172848	1.28927	0.3666	0.623155	1
SMIM10L2B	1.123365	1.151637	1.114176	0.75296	-0.4094	0.623283	1
PSMD8	3.606953	3.680131	3.583165	0.96382	-0.0532	0.623308	1
SPRYD7	1.255604	1.285107	1.246013	0.86288	-0.2128	0.623472	1
PPP2R3B	1.179119	1.149488	1.188751	1.26265	0.3365	0.623648	1
CCDC170	1.050585	1.022722	1.059642	2.62482	1.3922	0.623747	1
CD52	1.061923	1.089389	1.052995	0.59285	-0.7543	0.624164	1
FMNL3	1.099266	1.12711	1.090215	0.70973	-0.4946	0.624569	1
DUOX1	1.200708	1.170693	1.210465	1.233	0.3022	0.624714	1
FAM47E	1.154105	1.124858	1.163613	1.31039	0.39	0.624724	1
ARC	1.028319	1.055576	1.019459	0.35013	-1.514	0.624749	1
COQ6	1.172129	1.142639	1.181714	1.27394	0.3493	0.624948	1
ZNF44	1.161646	1.19017	1.152374	0.80125	-0.3197	0.625069	1
ATG7	1.197705	1.168092	1.207331	1.23344	0.3027	0.625121	1
LOH12CR2	1.096975	1.068675	1.106173	1.54602	0.6286	0.625202	1
AREG	1.023842	1.050757	1.015093	0.29735	-1.7498	0.625289	1
C1orf159	1.152923	1.123693	1.162424	1.31312	0.393	0.625307	1
KLHL12	1.275119	1.244456	1.285086	1.16621	0.2218	0.625741	1
SPACA3	1.055174	1.02733	1.064224	2.34995	1.2326	0.625762	1
TSNAX	1.49825	1.465824	1.50879	1.09224	0.1273	0.625859	1
CTB-129O4.1	1.074928	1.04701	1.084003	1.78692	0.8375	0.625861	1
GRTP1	1.110566	1.138622	1.101446	0.73182	-0.4504	0.625908	1

SLC41A2	1.072295	1.099598	1.063419	0.63675	-0.6512	0.625952	1
COLCA1	1.057233	1.02949	1.066251	2.24659	1.1677	0.625953	1
SNX6	2.360056	2.31551	2.374536	1.04487	0.0633	0.62599	1
THBS3	1.288564	1.257778	1.298571	1.15825	0.2119	0.62605	1
IL17RA	1.072983	1.100528	1.064029	0.63693	-0.6508	0.626092	1
CARHSP1	3.224647	3.168614	3.24286	1.03424	0.0486	0.626096	1
STAT2	1.238562	1.208642	1.248287	1.19002	0.251	0.626166	1
MXRA8	2.177172	2.216998	2.164226	0.95664	-0.064	0.626208	1
PASK	1.110695	1.082236	1.119946	1.45856	0.5445	0.626376	1
SLC41A1	1.2569	1.22648	1.266788	1.17797	0.2363	0.626768	1
RP11-15A1.7	1.05846	1.030616	1.067511	2.2051	1.1408	0.62697	1
GDAP1L1	1.076872	1.04889	1.085967	1.7584	0.8143	0.627062	1
GPR157	1.058514	1.085938	1.049599	0.57715	-0.793	0.627079	1
NDUFAF4	1.431745	1.399503	1.442226	1.10694	0.1466	0.627086	1
CYP7B1	1.050737	1.023119	1.059714	2.58295	1.369	0.627208	1
SEMA7A	1.044067	1.071252	1.03523	0.49444	-1.0161	0.627366	1
ZNF708	1.204476	1.174795	1.214124	1.22501	0.2928	0.627397	1
TTC9C	1.582739	1.549579	1.593517	1.07995	0.111	0.627549	1
EXOSC10	1.278995	1.248773	1.288819	1.16097	0.2153	0.627562	1
KANSL1-AS1	1.116915	1.088438	1.126172	1.42668	0.5127	0.627586	1
C1QTNF3	1.103944	1.131373	1.095027	0.72334	-0.4673	0.627621	1
ZNF652	1.442599	1.410241	1.453117	1.10452	0.1434	0.627773	1
TAOK2	1.207442	1.236377	1.198036	0.8378	-0.2553	0.627976	1
AGO2	1.56764	1.5344	1.578445	1.08242	0.1143	0.6281	1
CITF22-92A6.1	1.090408	1.062446	1.099497	1.59334	0.672	0.628325	1
SMPD4	1.252674	1.28213	1.2431	0.86166	-0.2148	0.628353	1
PRSS36	1.061907	1.034185	1.070918	2.07455	1.0528	0.6284	1
MIB1	1.879628	1.843877	1.891249	1.05614	0.0788	0.62851	1
TIPRL	1.756147	1.720827	1.767628	1.06493	0.0908	0.62854	1
UCN	1.062113	1.034316	1.071148	2.07333	1.052	0.628626	1
FAM66C	1.081278	1.053129	1.090428	1.70206	0.7673	0.628707	1
RP5-894A10.2	1.068028	1.040165	1.077085	1.91919	0.9405	0.628835	1
APEH	1.487447	1.454818	1.498054	1.09506	0.131	0.628916	1
RNF4	1.481827	1.44928	1.492407	1.09599	0.1322	0.628996	1
THAP9	1.092124	1.064366	1.101146	1.57142	0.6521	0.629122	1
ZFAND4	1.113035	1.08456	1.122291	1.44621	0.5323	0.629139	1
ZNF350	1.117744	1.089409	1.126955	1.41994	0.5058	0.629156	1
AP3B1	1.439503	1.470386	1.429464	0.913	-0.1313	0.629285	1
XXbac-BPG252P9.9	1.107374	1.079265	1.116511	1.46989	0.5557	0.629341	1
EMX2	1.112686	1.083059	1.122317	1.47265	0.5584	0.629383	1
MFN1	1.230106	1.259153	1.220664	0.85148	-0.232	0.629457	1
CFL1	24.01326	24.35253	23.90298	0.98075	-0.028	0.629548	1
SUMF1	1.204943	1.234195	1.195434	0.83449	-0.261	0.629562	1
TMEM133	1.056925	1.083971	1.048134	0.57323	-0.8028	0.629604	1
NKD1	1.079715	1.107073	1.070822	0.66144	-0.5963	0.629805	1

ROBO3	1.14088	1.112145	1.150221	1.33953	0.4217	0.629809	1
WDR7	1.113327	1.084923	1.12256	1.44319	0.5293	0.629829	1
RP11-422P24.11	1.079036	1.051164	1.088096	1.72185	0.784	0.630107	1
FNDC10	1.194506	1.223253	1.185162	0.82938	-0.2699	0.630231	1
NCK2	1.232258	1.261241	1.222837	0.85299	-0.2294	0.630246	1
HECTD4	1.272038	1.301303	1.262525	0.8713	-0.1988	0.630296	1
FPGS	1.320184	1.289165	1.330267	1.14214	0.1917	0.630335	1
TRAPPC13	1.146488	1.174751	1.137301	0.7857	-0.348	0.630379	1
RP11-95D17.1	1.078336	1.050538	1.087372	1.72885	0.7898	0.630404	1
NFX1	1.228583	1.198914	1.238227	1.19764	0.2602	0.630489	1
RP11-154H23.3	1.059177	1.031579	1.068148	2.15802	1.1097	0.630508	1
SLC9A9	1.053969	1.026525	1.06289	2.37097	1.2455	0.630759	1
AP001469.9	1.071913	1.044175	1.08093	1.83204	0.8734	0.631088	1
FAM81B	1.036591	1.063139	1.027962	0.44286	-1.1751	0.631195	1
DCPS	1.244971	1.215837	1.254442	1.17886	0.2374	0.631202	1
VKORC1L1	1.306758	1.276059	1.316737	1.14735	0.1983	0.631236	1
RP11-637A17.2	1.087166	1.059302	1.096224	1.62261	0.6983	0.63133	1
FAM135A	1.321278	1.290759	1.331198	1.13908	0.1879	0.631348	1
XXbac-BPG252P9.10	1.155686	1.127167	1.164957	1.29717	0.3754	0.631432	1
NIPAL2	1.088222	1.060306	1.097296	1.61336	0.6901	0.631492	1
GOT1	1.275752	1.245947	1.285441	1.16058	0.2148	0.631526	1
ACAD8	1.2126	1.241572	1.203182	0.84108	-0.2497	0.631538	1
MATR3.1	1.110606	1.138201	1.101636	0.73542	-0.4434	0.631545	1
CEP112	1.262756	1.2332	1.272363	1.16794	0.224	0.631551	1
ARID1B	2.460461	2.417421	2.474452	1.04024	0.0569	0.631555	1
RALGAPA2	1.261845	1.231914	1.271574	1.17101	0.2278	0.631581	1
AC023590.1	1.071042	1.043428	1.080018	1.84254	0.8817	0.631634	1
NPHS1	1.029763	1.056574	1.021047	0.37203	-1.4265	0.631833	1
CALY	1.254832	1.283896	1.245385	0.86435	-0.2103	0.631849	1
WBSCR17	1.181291	1.152359	1.190695	1.25161	0.3238	0.631856	1
MAPK15	1.101739	1.073848	1.110805	1.50045	0.5854	0.632185	1
CHFR	1.201454	1.229839	1.192228	0.83636	-0.2578	0.63239	1
MAK16	1.141959	1.170136	1.1328	0.78055	-0.3574	0.63243	1
GPCPD1	1.199301	1.22778	1.190044	0.83433	-0.2613	0.632438	1
IFI44	1.027552	1.054295	1.018859	0.34734	-1.5256	0.632573	1
C12orf43	1.308822	1.278954	1.318531	1.14188	0.1914	0.632739	1
SLC38A6	1.270566	1.29978	1.261069	0.87087	-0.1995	0.632795	1
ARMC9	1.266016	1.23637	1.275653	1.1662	0.2218	0.632821	1
NBPF10	1.092109	1.06437	1.101126	1.57101	0.6517	0.632939	1
RABL3	1.117914	1.089809	1.127049	1.41466	0.5005	0.633008	1
TBRG4	1.427757	1.396302	1.437982	1.10517	0.1443	0.63302	1
SH3BP1	1.180657	1.151499	1.190135	1.25502	0.3277	0.633037	1
MUC20	1.063012	1.035583	1.071928	2.02141	1.0154	0.63315	1
PDCD2	2.182037	2.221714	2.16914	0.95697	-0.0635	0.633207	1
GGACT	1.140738	1.168531	1.131703	0.78148	-0.3557	0.633262	1

LZTS3	1.073782	1.046473	1.082659	1.77866	0.8308	0.633477	1
MAML3	1.207223	1.177786	1.216791	1.21939	0.2862	0.633537	1
BRWD1-AS2	1.067333	1.039884	1.076256	1.91197	0.9351	0.633546	1
ADA	1.61734	1.649583	1.606859	0.93423	-0.0982	0.633557	1
SSR3	3.230778	3.287067	3.212481	0.96739	-0.0478	0.633571	1
LRRTM2	1.045412	1.018301	1.054224	2.96286	1.567	0.633607	1
RP11-464F9.20	1.074719	1.047246	1.083649	1.77051	0.8242	0.633717	1
SNIP1	1.083482	1.110758	1.074615	0.67368	-0.5699	0.63395	1
POLRMT	1.205663	1.176625	1.215102	1.21785	0.2843	0.634136	1
CCDC3	1.182943	1.211026	1.173814	0.82366	-0.2799	0.634276	1
RP11-164P12.4	1.054839	1.08137	1.046215	0.56796	-0.8161	0.634579	1
DNAJC14	1.174853	1.203141	1.165658	0.81548	-0.2943	0.634581	1
TMEM108	1.249915	1.278665	1.24057	0.8633	-0.2121	0.634813	1
C1QL2	1.02446	1.051036	1.015821	0.31	-1.6897	0.634831	1
ANG	1.237845	1.208412	1.247413	1.18713	0.2475	0.634915	1
MARCH7	1.843592	1.878636	1.832201	0.94715	-0.0783	0.635166	1
C11orf58	5.863684	5.727138	5.908069	1.03828	0.0542	0.635294	1
CERS5	1.390657	1.420586	1.380929	0.90571	-0.1429	0.635379	1
KDM5B	2.204978	2.249114	2.190631	0.95318	-0.0692	0.635451	1
TTC7B	1.098011	1.125087	1.08921	0.71319	-0.4876	0.635578	1
U2AF1L5	1.069042	1.041859	1.077878	1.86047	0.8957	0.63563	1
CREB5	1.305741	1.335051	1.296214	0.88409	-0.1777	0.63568	1
NFYA	1.169017	1.140445	1.178304	1.26957	0.3443	0.635725	1
FAM46B	1.035919	1.062195	1.027378	0.4402	-1.1838	0.635848	1
CRLS1	1.748184	1.713492	1.75946	1.06443	0.0901	0.635918	1
SH3TC2	1.036003	1.062401	1.027423	0.43946	-1.1862	0.635948	1
CTD-2165H16.4	1.052974	1.025937	1.061762	2.38124	1.2517	0.63604	1
PEBP4	1.032541	1.058945	1.023959	0.40645	-1.2988	0.63616	1
SLC2A1-AS1	1.460404	1.429068	1.47059	1.09677	0.1333	0.636209	1
INVS	1.101738	1.073966	1.110766	1.49752	0.5826	0.63621	1
NIT1	1.616482	1.583521	1.627196	1.07485	0.1041	0.636227	1
PRR15	1.032494	1.058734	1.023964	0.40802	-1.2933	0.63626	1
VPS9D1-AS1	1.063687	1.036591	1.072495	1.98122	0.9864	0.636264	1
RORA-AS1	1.044509	1.017512	1.053285	3.04282	1.6054	0.636271	1
MEIG1	1.059548	1.032404	1.068372	2.10999	1.0772	0.636342	1
SRPX2	1.029075	1.055411	1.020515	0.37024	-1.4335	0.636471	1
GOLGA8A	1.795103	1.760227	1.806439	1.06079	0.0851	0.636682	1
IDH1-AS1	1.067542	1.04029	1.076401	1.8963	0.9232	0.636833	1
FBN3	1.067532	1.040391	1.076355	1.89039	0.9187	0.636833	1
ARHGAP9	1.02839	1.054681	1.019843	0.36289	-1.4624	0.636939	1
PDSS2	1.17051	1.142308	1.179677	1.26259	0.3364	0.637015	1
GMPPA	1.268723	1.2974	1.259402	0.87223	-0.1972	0.637124	1
SNHG23	1.085099	1.057528	1.094061	1.63504	0.7093	0.63725	1
GAPLINC	1.024922	1.051058	1.016426	0.32172	-1.6361	0.637251	1
RHPN2	1.122219	1.149366	1.113395	0.75917	-0.3975	0.637405	1

AMDHD2	1.230718	1.201935	1.240074	1.18887	0.2496	0.637479	1
APOL1	1.027886	1.053973	1.019406	0.35956	-1.4757	0.637488	1
FAM20C	1.148142	1.175791	1.139155	0.79159	-0.3372	0.637506	1
LANCL2	1.14628	1.118188	1.155412	1.31496	0.395	0.637571	1
FGFR1OP2	1.678804	1.644963	1.689804	1.06953	0.097	0.637666	1
BAX	3.713376	3.643465	3.736101	1.03504	0.0497	0.637866	1
PIP5K1A	1.414218	1.444802	1.404276	0.90889	-0.1378	0.637917	1
HNRNPH2	1.733244	1.768077	1.721921	0.93991	-0.0894	0.637988	1
AATK	1.09501	1.121647	1.086351	0.70985	-0.4944	0.638077	1
PRICKLE2-AS1	1.058568	1.031409	1.067396	2.1458	1.1015	0.638169	1
SCAND1	3.521036	3.587091	3.499564	0.96617	-0.0497	0.638185	1
MEF2D	1.100996	1.127593	1.092351	0.72379	-0.4664	0.638199	1
FBXO42	1.288876	1.259202	1.298522	1.1517	0.2038	0.638343	1
PIN1	3.274081	3.196584	3.299271	1.04675	0.0659	0.638598	1
PRMT7	1.306383	1.335269	1.296993	0.88584	-0.1749	0.638612	1
RNF38	1.406426	1.375188	1.41658	1.11032	0.151	0.638952	1
GRK3	1.175162	1.146759	1.184395	1.25645	0.3293	0.638956	1
TANGO2	1.090629	1.117597	1.081863	0.69613	-0.5226	0.639092	1
TTC37	2.092218	2.055184	2.104257	1.04651	0.0656	0.639135	1
LTC4S	1.084823	1.057634	1.09366	1.62507	0.7005	0.63921	1
VASH2	1.137713	1.109644	1.146837	1.33922	0.4214	0.639309	1
MCUR1	1.892435	1.926631	1.881319	0.9511	-0.0723	0.63934	1
MRPL53	1.226625	1.197191	1.236193	1.19779	0.2604	0.639385	1
PPP4R3A	1.628334	1.660522	1.617871	0.93543	-0.0963	0.639461	1
ALOX5	1.042573	1.068659	1.034094	0.49657	-1.0099	0.639473	1
COX16	1.386732	1.356357	1.396606	1.11294	0.1544	0.639506	1
SMCO4	1.466908	1.435424	1.477142	1.09581	0.132	0.639591	1
ZKSCAN5	1.137591	1.109847	1.146609	1.33467	0.4165	0.639679	1
SLC25A43	1.135284	1.107306	1.144378	1.34548	0.4281	0.639766	1
HMOX1	1.039208	1.06543	1.030684	0.46896	-1.0925	0.639778	1
BEST1	1.282089	1.311133	1.272648	0.87631	-0.1905	0.639953	1
PPM1J	1.091587	1.064427	1.100416	1.55859	0.6402	0.640033	1
CA13	1.080947	1.053807	1.089769	1.66833	0.7384	0.640102	1
TADA1	1.134945	1.107202	1.143962	1.3429	0.4254	0.640209	1
NCKAP5	1.076802	1.10318	1.068227	0.66125	-0.5967	0.640285	1
ARHGAP17	1.235546	1.263833	1.226351	0.85794	-0.2211	0.640302	1
RP13-131K19.7	1.044994	1.018345	1.053656	2.92491	1.5484	0.640505	1
NAT6	1.283568	1.254033	1.293168	1.15406	0.2067	0.64058	1
VPS26B	1.483731	1.451669	1.494153	1.09406	0.1297	0.640681	1
NR1D2	1.492911	1.523607	1.482933	0.92232	-0.1167	0.64069	1
CRISPLD2	1.028766	1.054675	1.020345	0.3721	-1.4262	0.640696	1
TUBG1	1.882306	1.91678	1.871099	0.95017	-0.0737	0.640803	1
FBXO15	1.077894	1.050958	1.08665	1.70042	0.7659	0.641027	1
COL7A1	1.11141	1.083851	1.120368	1.4355	0.5215	0.641147	1
SH2D2A	1.095142	1.068013	1.103961	1.52854	0.6122	0.641419	1

SOWAHC	1.125652	1.152586	1.116897	0.76611	-0.3844	0.641631	1
ITSN1	1.72357	1.756043	1.713015	0.94309	-0.0845	0.641739	1
PTPN6	1.06272	1.08887	1.054219	0.6101	-0.7129	0.641835	1
C1orf112	1.124832	1.097449	1.133733	1.37233	0.4566	0.642035	1
TRUB2	1.394913	1.3649	1.404669	1.10899	0.1492	0.642154	1
TNK2	1.147113	1.174197	1.138309	0.79398	-0.3328	0.642183	1
BRAP	1.184165	1.156283	1.193229	1.2364	0.3061	0.642293	1
RP11-315D16.4	1.037688	1.011304	1.046264	4.09261	2.033	0.642376	1
SCAP	1.33451	1.304754	1.344182	1.12938	0.1755	0.642424	1
MFSD5	1.136898	1.163692	1.128188	0.7831	-0.3527	0.64259	1
ARAF	1.270857	1.241969	1.280247	1.15819	0.2119	0.642755	1
SGPL1	1.290718	1.319004	1.281524	0.88251	-0.1803	0.642793	1
ISY1	1.350895	1.321009	1.360609	1.12336	0.1678	0.642804	1
MAPK11	1.08718	1.060093	1.095985	1.59727	0.6756	0.642921	1
RP5-991G20.1	1.080423	1.053498	1.089176	1.6669	0.7372	0.642987	1
SIKE1	1.710432	1.743833	1.699575	0.9405	-0.0885	0.64303	1
RAB3A	1.423593	1.393658	1.433324	1.10076	0.1385	0.643055	1
SLC29A2	1.203168	1.230631	1.19424	0.84221	-0.2477	0.643093	1
DNAJB12	1.273135	1.244299	1.282509	1.1564	0.2096	0.643214	1
UPF1	1.165056	1.19187	1.15634	0.81482	-0.2954	0.643315	1
AC008079.10	1.058894	1.032049	1.067621	2.10991	1.0772	0.643321	1
CDC25A	1.095224	1.068262	1.103989	1.52339	0.6073	0.643416	1
PLXNA4	1.098799	1.071777	1.107582	1.49884	0.5838	0.643485	1
TRNT1	1.340135	1.310269	1.349843	1.12755	0.1732	0.643681	1
ANKRD12	2.609038	2.5611	2.624621	1.04069	0.0575	0.643815	1
MDP1	1.24803	1.219174	1.25741	1.17445	0.232	0.643844	1
ZNF333	1.13747	1.109922	1.146425	1.33208	0.4137	0.643972	1
HOXA3	1.027113	1.052817	1.018757	0.35514	-1.4935	0.644065	1
FAM21C	1.255326	1.226357	1.264742	1.16958	0.226	0.644117	1
SLC22A5	1.142186	1.169037	1.133458	0.78952	-0.341	0.644225	1
PCDHB16	1.194511	1.222219	1.185504	0.83478	-0.2605	0.644301	1
ANAPC7	1.266114	1.23745	1.275432	1.15996	0.2141	0.644349	1
ASXL1	1.30433	1.332941	1.29503	0.88613	-0.1744	0.644387	1
RLN1	1.042983	1.016599	1.051559	3.10615	1.6351	0.644404	1
DFNB59	1.062235	1.035562	1.070905	1.99385	0.9956	0.644726	1
ARL6IP4	5.987798	6.097049	5.952285	0.9716	-0.0416	0.644873	1
ZNF783	1.188144	1.15978	1.197365	1.23523	0.3048	0.64511	1
MYCBPAP	1.051879	1.025421	1.060479	2.37912	1.2504	0.645111	1
SPSB4	1.103264	1.076426	1.111988	1.46531	0.5512	0.645126	1
GSPT1	1.947526	1.909913	1.959752	1.05477	0.0769	0.64521	1
MYNN	1.192121	1.164235	1.201186	1.22498	0.2928	0.645292	1
MTERF1	1.14085	1.113605	1.149707	1.31779	0.3981	0.645315	1
P2RX4	1.183642	1.210645	1.174864	0.83014	-0.2686	0.645364	1
SLC27A4	1.065279	1.091263	1.056833	0.62274	-0.6833	0.645373	1
FTSJ1	1.233437	1.261257	1.224394	0.8589	-0.2194	0.64545	1

NAGK	1.64327	1.674762	1.633033	0.93816	-0.0921	0.645491	1
KLF11	1.14076	1.167474	1.132076	0.78863	-0.3426	0.645521	1
MYOM1	1.024853	1.050327	1.016572	0.32929	-1.6026	0.645549	1
STX18	1.201969	1.229519	1.193014	0.84095	-0.2499	0.645633	1
RP11-196G18.23	1.088438	1.06175	1.097113	1.57268	0.6532	0.645721	1
VLDLR-AS1	1.112509	1.085314	1.121349	1.42239	0.5083	0.645764	1
ALG10B	1.163664	1.136024	1.172649	1.26926	0.344	0.645769	1
MCCC2	1.344966	1.315571	1.354521	1.12343	0.1679	0.64593	1
ATF6	1.460943	1.49066	1.451284	0.91975	-0.1207	0.646319	1
ZNF816	1.076378	1.049804	1.085016	1.70702	0.7715	0.646354	1
LRRC8C	1.053195	1.078928	1.04483	0.56798	-0.8161	0.646404	1
PYROXD2	1.155037	1.127547	1.163972	1.28558	0.3624	0.646441	1
RP11-196G18.24	1.050651	1.076379	1.042288	0.55366	-0.8529	0.646479	1
ZSWIM7	2.055167	2.018327	2.067142	1.04794	0.0676	0.646743	1
TLE1	1.539619	1.569992	1.529746	0.92939	-0.1056	0.646782	1
HUS1	1.162745	1.135021	1.171756	1.27207	0.3472	0.646854	1
RP11-21L23.3	1.07831	1.051693	1.086962	1.68228	0.7504	0.646856	1
ZNF354C	1.05071	1.02444	1.059249	2.42424	1.2775	0.646942	1
DMRTA1	1.166914	1.193743	1.158193	0.81651	-0.2925	0.64702	1
ATG10	1.580737	1.548856	1.5911	1.07697	0.107	0.647065	1
RP11-563N4.1	1.068235	1.041699	1.076861	1.84325	0.8823	0.647159	1
CDC42SE1	1.52098	1.550296	1.511451	0.92941	-0.1056	0.647183	1
ROBO2	1.10833	1.081422	1.117077	1.43791	0.524	0.647364	1
FAM43A	1.16315	1.190069	1.154399	0.81233	-0.2999	0.647411	1
EIF1AD	1.215673	1.243144	1.206743	0.85029	-0.234	0.647885	1
TRABD	1.654125	1.622194	1.664505	1.068	0.0949	0.648259	1
HAUS7	1.12736	1.100445	1.13611	1.35507	0.4384	0.648328	1
MAP10	1.081704	1.055238	1.090307	1.63488	0.7092	0.648356	1
SAMD12	1.126505	1.152984	1.117898	0.77066	-0.3758	0.64847	1
KRT80	1.029889	1.055111	1.02169	0.39357	-1.3453	0.648479	1
TMEM161A	1.291127	1.262312	1.300493	1.14556	0.1961	0.648509	1
CTD-2270L9.4	1.142093	1.115125	1.150859	1.3104	0.39	0.648902	1
SNX30	1.155045	1.127836	1.163889	1.28203	0.3584	0.648922	1
OTUD7B	1.323692	1.294553	1.333163	1.13108	0.1777	0.649161	1
CSMD2	1.02583	1.051227	1.017575	0.34308	-1.5434	0.649204	1
ZSWIM5	1.080777	1.054047	1.089466	1.65534	0.7271	0.649291	1
SHC3	1.0807	1.054181	1.08932	1.64855	0.7212	0.649291	1
OTUD6B-AS1	2.507309	2.462484	2.52188	1.04061	0.0574	0.649396	1
KIAA1456	1.17517	1.147602	1.184132	1.24749	0.319	0.649397	1
NEFM	1.203244	1.172404	1.213268	1.23702	0.3069	0.649504	1
FARSA	1.501197	1.470189	1.511276	1.08739	0.1209	0.649615	1
KRT13	1.025322	1.050526	1.017129	0.33901	-1.5606	0.649736	1
TXNL4B	1.165148	1.137722	1.174063	1.26387	0.3378	0.649919	1
LMOD1	1.134231	1.160757	1.125608	0.78135	-0.356	0.649932	1
TSTA3	1.760091	1.792203	1.749653	0.94629	-0.0796	0.649992	1

SPOCK3	1.091275	1.06499	1.099819	1.5359	0.6191	0.65005	1
NFAT5	1.572669	1.602892	1.562845	0.93357	-0.0992	0.650176	1
TBKBP1	1.133983	1.160472	1.125373	0.78127	-0.3561	0.650337	1
NUDT13	1.062758	1.036757	1.07121	1.93734	0.9541	0.650488	1
PIGG	1.14778	1.120488	1.156652	1.30014	0.3787	0.650503	1
FRAT2	1.050406	1.075913	1.042115	0.55479	-0.85	0.650689	1
TYK2	1.290146	1.261418	1.299484	1.14561	0.1961	0.650786	1
MURC	1.162891	1.135662	1.171742	1.26596	0.3402	0.650836	1
SYVN1	1.128355	1.15443	1.11988	0.77627	-0.3654	0.650853	1
COPS3	1.658379	1.689883	1.648138	0.93949	-0.09	0.650898	1
ZBTB43	1.287334	1.315398	1.278212	0.8821	-0.181	0.650933	1
CYFIP1	1.390629	1.36124	1.400182	1.1078	0.1477	0.651122	1
GRIN2D	1.053918	1.079307	1.045665	0.5758	-0.7964	0.651326	1
AC010883.5	1.060144	1.034155	1.068592	2.00826	1.0059	0.651416	1
CRYBB3	1.106644	1.079728	1.115393	1.44734	0.5334	0.651418	1
SERPINA5	1.049825	1.075071	1.041618	0.55439	-0.851	0.651469	1
MUM1L1	1.149177	1.12187	1.158054	1.29691	0.3751	0.651581	1
FDXACB1	1.052399	1.02657	1.060795	2.28807	1.1941	0.651878	1
LINC00475	1.123715	1.096819	1.132458	1.36809	0.4522	0.651908	1
ABCA7	1.069033	1.094677	1.060697	0.6411	-0.6414	0.651997	1
SLC17A5	1.199842	1.22644	1.191196	0.84436	-0.2441	0.652268	1
USP14	1.895316	1.929595	1.884173	0.95114	-0.0723	0.652378	1
ACAD9	1.236808	1.208736	1.245933	1.1782	0.2366	0.652396	1
MNT	1.258534	1.230175	1.267753	1.16326	0.2182	0.652542	1
AC005754.8	1.064671	1.038615	1.07314	1.89408	0.9215	0.652604	1
RETSAT	1.109737	1.135631	1.10132	0.74702	-0.4208	0.652768	1
KIAA1429	1.273765	1.30132	1.264808	0.87883	-0.1864	0.652776	1
PRNCR1	1.070504	1.095932	1.062238	0.64877	-0.6242	0.652863	1
DGAT1	1.21939	1.24627	1.210653	0.85537	-0.2254	0.652903	1
SSBP3	1.774351	1.74102	1.785185	1.0596	0.0835	0.653048	1
LMCD1-AS1	1.083144	1.056804	1.091706	1.61442	0.691	0.653066	1
ZNF404	1.100044	1.125647	1.091721	0.72999	-0.454	0.653067	1
TIMM10B	1.403578	1.432189	1.394278	0.91228	-0.1324	0.653116	1
C3orf58	1.318029	1.346072	1.308914	0.89263	-0.1639	0.653443	1
MEIS3	1.739591	1.70689	1.75022	1.0613	0.0858	0.653643	1
CATIP	1.0374	1.011871	1.045699	3.84967	1.9447	0.653691	1
NLRC5	1.025794	1.050844	1.017651	0.34717	-1.5263	0.653753	1
STAR	1.048125	1.022541	1.056441	2.5039	1.3242	0.653901	1
UTP15	1.097203	1.123012	1.088813	0.72199	-0.47	0.653983	1
SLC26A7	1.053495	1.078925	1.045229	0.57307	-0.8032	0.654214	1
TMEM241	1.144823	1.170827	1.13637	0.79829	-0.325	0.654303	1
ZNF449	1.089576	1.115081	1.081286	0.70634	-0.5016	0.654353	1
KIAA0408	1.039434	1.013947	1.047719	3.42158	1.7747	0.654397	1
NBPF14	1.276958	1.249301	1.285948	1.147	0.1979	0.654515	1
NSMAF	1.326268	1.297451	1.335635	1.12837	0.1742	0.654547	1

PRDM10	1.090499	1.116476	1.082055	0.70447	-0.5054	0.654683	1
DUBR	1.140217	1.113401	1.148933	1.31334	0.3932	0.654707	1
CCDC155	1.038674	1.013102	1.046986	3.58633	1.8425	0.654718	1
TTC12	1.112469	1.086091	1.121043	1.40599	0.4916	0.654741	1
PSMD9	1.40712	1.43556	1.397876	0.91348	-0.1306	0.654764	1
ARX	1.040422	1.065165	1.032379	0.49687	-1.0091	0.654796	1
PLCG1	1.248439	1.220609	1.257485	1.16715	0.223	0.654851	1
PDCD10	1.721087	1.688589	1.731651	1.06254	0.0875	0.655035	1
CNOT8	1.446824	1.475973	1.437349	0.91885	-0.1221	0.655109	1
AC144652.1	1.060947	1.035226	1.069307	1.96752	0.9764	0.655161	1
PTCD1	1.118466	1.091943	1.127088	1.38224	0.467	0.65519	1
HSD17B4	1.466861	1.436884	1.476606	1.09092	0.1255	0.655206	1
WNT3	1.034914	1.059819	1.026819	0.44834	-1.1573	0.655331	1
TEX10	1.172266	1.14545	1.180982	1.24429	0.3153	0.655457	1
MXI1	1.591367	1.560415	1.601428	1.07318	0.1019	0.655459	1
RBMS3-AS3	1.034509	1.05942	1.026412	0.4445	-1.1698	0.655608	1
PLOD2	2.226018	2.18498	2.239357	1.04589	0.0647	0.655687	1
SNX25	1.154129	1.180196	1.145655	0.80831	-0.307	0.655692	1
ZNF276	1.178095	1.150975	1.18691	1.23802	0.308	0.655715	1
SUPT6H	1.315802	1.286903	1.325195	1.13347	0.1807	0.655801	1
AKR1C1	1.08995	1.063813	1.098446	1.54274	0.6255	0.655812	1
YDJC	1.720251	1.687575	1.730873	1.06297	0.0881	0.655956	1
PPP1R3G	1.134042	1.107474	1.142677	1.32755	0.4088	0.655966	1
TAF1A	1.115843	1.089719	1.124335	1.38582	0.4707	0.656091	1
CHST14	1.228562	1.255824	1.2197	0.85879	-0.2196	0.656216	1
LINC01186	1.029208	1.053904	1.02118	0.39291	-1.3477	0.656471	1
UEVLD	1.132708	1.158199	1.124422	0.78649	-0.3465	0.656714	1
RGS5.1	1.05087	1.025442	1.059136	2.32436	1.2168	0.65682	1
INMT	1.043758	1.018295	1.052035	2.84428	1.5081	0.656969	1
PLPP6	1.13336	1.106876	1.141968	1.32835	0.4096	0.657102	1
RPP40	1.135377	1.10879	1.14402	1.32383	0.4047	0.65714	1
TBC1D10B	1.218237	1.244993	1.209539	0.85529	-0.2255	0.657265	1
FAM161B	1.086579	1.060521	1.09505	1.57053	0.6513	0.65728	1
LCOR	1.660808	1.629181	1.671089	1.06661	0.093	0.657312	1
FOXO6	1.039881	1.064761	1.031793	0.49094	-1.0264	0.65734	1
PAFAH1B2	2.094348	2.058382	2.106039	1.04503	0.0635	0.657383	1
PCDHB9	1.104988	1.078779	1.113507	1.44083	0.5269	0.657651	1
CISD3	1.424375	1.452322	1.415291	0.91813	-0.1232	0.657658	1
DGCR8	1.189996	1.163147	1.198723	1.21806	0.2846	0.657707	1
ATP10D	1.193045	1.219314	1.184506	0.84129	-0.2493	0.65771	1
FAM19A2	1.082983	1.057313	1.091327	1.59347	0.6722	0.657713	1
ANKLE1	1.057884	1.032478	1.066143	2.03658	1.0261	0.657844	1
CACNB1	1.075426	1.049851	1.083739	1.6798	0.7483	0.657916	1
FGD6	1.118544	1.092313	1.127071	1.37652	0.461	0.658023	1
TRAPPC8	1.169383	1.195783	1.160801	0.82133	-0.284	0.658151	1

FZD4	1.055854	1.080801	1.047744	0.59088	-0.7591	0.658203	1
CASC15	1.961007	1.925542	1.972535	1.05077	0.0715	0.658561	1
SH3BGRL2	1.315049	1.286742	1.32425	1.13081	0.1774	0.658567	1
CAMTA1	4.293947	4.229411	4.314925	1.02648	0.0377	0.658712	1
KIAA0430	1.347705	1.318626	1.357157	1.12093	0.1647	0.658738	1
DCAF1	1.130417	1.104206	1.138938	1.3333	0.415	0.65875	1
TFEB	1.089349	1.114629	1.081132	0.70777	-0.4986	0.658775	1
FER1L5	1.049008	1.023642	1.057254	2.4217	1.276	0.658885	1
PSMC1	2.44782	2.489627	2.43423	0.96281	-0.0547	0.658915	1
ZNF496	1.18977	1.162534	1.198623	1.22204	0.2893	0.659014	1
NAV1	1.766514	1.797528	1.756433	0.94847	-0.0763	0.659026	1
ERBB2	1.366027	1.393812	1.356996	0.90651	-0.1416	0.659138	1
SYDE2	1.147016	1.120258	1.155714	1.29484	0.3728	0.659222	1
PURG	1.172981	1.146192	1.181689	1.24281	0.3136	0.659362	1
DYRK3	1.10947	1.083448	1.117928	1.4132	0.499	0.659385	1
MSTO1	1.156939	1.183012	1.148464	0.81123	-0.3018	0.65941	1
PAX5	1.042711	1.017512	1.050902	2.9067	1.5394	0.659746	1
KLHL31	1.039435	1.064039	1.031437	0.4909	-1.0265	0.6599	1
ECEL1	1.153884	1.127551	1.162444	1.27356	0.3489	0.659997	1
OSBPL3	1.231428	1.203951	1.240359	1.17851	0.237	0.660075	1
HES5	1.043964	1.018783	1.05215	2.77639	1.4732	0.660151	1
C1orf74	1.073175	1.047653	1.081471	1.70969	0.7737	0.660192	1
ENTPD3-AS1	1.158669	1.132193	1.167275	1.26538	0.3396	0.66026	1
LPIN3	1.158738	1.132154	1.167379	1.26655	0.3409	0.66026	1
INO80	1.21883	1.191295	1.22778	1.19073	0.2518	0.660418	1
C17orf97	1.103304	1.077448	1.111709	1.44237	0.5284	0.660454	1
RP1-267D11.6	1.172363	1.145918	1.180959	1.24015	0.3105	0.660462	1
RADIL	1.042287	1.017212	1.050437	2.9303	1.551	0.660544	1
RP11-422P24.12	1.071771	1.046127	1.080107	1.73667	0.7963	0.660559	1
RP11-419I17.1	1.071554	1.04608	1.079835	1.73253	0.7929	0.660559	1
MMS19	1.302144	1.329298	1.293318	0.89074	-0.1669	0.660734	1
VWC2	1.043923	1.018772	1.052098	2.77528	1.4726	0.660768	1
IMP4	1.777993	1.809576	1.767726	0.94831	-0.0766	0.660822	1
RP11-441O15.3	1.065497	1.040098	1.073754	1.83936	0.8792	0.660865	1
CCDC142	1.130196	1.104138	1.138666	1.33156	0.4131	0.661003	1
SETBP1	1.121735	1.09591	1.13013	1.3568	0.4402	0.66121	1
ARID2	1.565874	1.535992	1.575588	1.07387	0.1028	0.661384	1
TOR3A	1.290193	1.317134	1.281435	0.88743	-0.1723	0.661605	1
PLEKHM1	1.093202	1.118359	1.085024	0.71836	-0.4772	0.661645	1
UBP1	1.262073	1.234552	1.271018	1.15547	0.2085	0.66168	1
CCDC74B	1.222306	1.195115	1.231144	1.18466	0.2445	0.661682	1
ZNF260	1.353947	1.325558	1.363175	1.11555	0.1577	0.661872	1
CHRNB4	1.069429	1.044185	1.077635	1.75702	0.8131	0.661982	1
AQR	1.349341	1.32085	1.358602	1.11766	0.1605	0.662126	1
METTL12	1.517805	1.487973	1.527503	1.08101	0.1124	0.662155	1

AATF	1.335838	1.363158	1.326957	0.90032	-0.1515	0.66219	1
DRD4	1.061084	1.085977	1.052993	0.61636	-0.6982	0.662323	1
SLC12A1	1.032852	1.057199	1.024938	0.43599	-1.1976	0.662531	1
RP3-403A15.5	1.040991	1.015961	1.049127	3.0779	1.6219	0.662581	1
CLSTN1	1.76353	1.794849	1.75335	0.94779	-0.0774	0.662783	1
ZFP28	1.099709	1.124805	1.091551	0.73355	-0.447	0.662918	1
IQCD	1.100646	1.075207	1.108916	1.44822	0.5343	0.66292	1
ASIC1	1.059332	1.034118	1.067527	1.97923	0.9849	0.663114	1
BMP5	1.026633	1.050883	1.01875	0.3685	-1.4403	0.66315	1
AAGAB	1.425153	1.45338	1.415978	0.9175	-0.1242	0.66321	1
RP11-398C13.6	1.059247	1.034163	1.0674	1.97292	0.9803	0.663217	1
CDH18	1.047535	1.07188	1.039622	0.55123	-0.8593	0.663482	1
TTLL5	1.224911	1.19806	1.233639	1.17963	0.2383	0.663578	1
RUSC1	1.469684	1.498133	1.460437	0.92432	-0.1135	0.663744	1
GSTZ1	1.304305	1.276617	1.313305	1.13263	0.1797	0.663983	1
RP11-497E19.1	1.053134	1.028079	1.061279	2.18234	1.1259	0.664053	1
JAM3	1.571883	1.541799	1.581663	1.07358	0.1024	0.664061	1
GPR158	1.036931	1.012186	1.044975	3.69059	1.8839	0.66413	1
ABCA2	1.170906	1.196806	1.162487	0.82562	-0.2764	0.664218	1
CBARP	1.063577	1.088196	1.055574	0.63012	-0.6663	0.66428	1
PCMT1	2.364811	2.409233	2.350371	0.95823	-0.0616	0.664401	1
C3orf52	1.037162	1.061358	1.029298	0.47749	-1.0665	0.664531	1
ATP1B3	3.142438	3.193081	3.125976	0.9694	-0.0448	0.664565	1
SMAD7	1.151602	1.176873	1.143388	0.81068	-0.3028	0.664808	1
MTCH2	3.060723	3.002205	3.079744	1.03873	0.0548	0.66481	1
RP11-465N4.4	1.033733	1.057983	1.025851	0.44583	-1.1654	0.664881	1
SUPV3L1	1.174324	1.14783	1.182936	1.23747	0.3074	0.664884	1
PPP1R9B	1.205463	1.231243	1.197083	0.85228	-0.2306	0.664901	1
SMC5-AS1	1.075843	1.050624	1.08404	1.66009	0.7313	0.664993	1
CTD-2020K17.4	1.064628	1.089213	1.056637	0.63485	-0.6555	0.665101	1
ADCY2	1.03025	1.054366	1.022411	0.41222	-1.2785	0.665232	1
NIT2	1.648417	1.67867	1.638584	0.94093	-0.0878	0.665338	1
ADCY7	1.12007	1.144969	1.111977	0.77242	-0.3726	0.665346	1
OTUD7A	1.053573	1.028543	1.061709	2.162	1.1124	0.665378	1
BMPR1B-AS1	1.036644	1.011871	1.044696	3.76521	1.9127	0.665527	1
AL356053.1	1.047345	1.022505	1.05542	2.46252	1.3001	0.665683	1
ATF5	1.199737	1.173114	1.20839	1.20377	0.2676	0.665684	1
TIFA	1.249471	1.222744	1.258158	1.15899	0.2129	0.665884	1
AC114271.2	1.066107	1.041114	1.074231	1.80548	0.8524	0.665912	1
POT1	1.182533	1.156132	1.191114	1.22406	0.2917	0.665921	1
TOMM40	1.976046	1.94239	1.986986	1.04732	0.0667	0.665957	1
AC046143.3	1.054381	1.078686	1.04648	0.5907	-0.7595	0.66613	1
SCO2	1.300196	1.32645	1.291662	0.89344	-0.1626	0.666276	1
ERAP2	1.044624	1.068783	1.036771	0.5346	-0.9035	0.666449	1
RP4-548D19.3	1.045729	1.069958	1.037853	0.54108	-0.8861	0.666466	1

LRRC47	1.473515	1.502274	1.464167	0.92413	-0.1138	0.666488	1
TSEN54	1.268618	1.241695	1.27737	1.1476	0.1986	0.666525	1
HIP1R	1.195853	1.221346	1.187567	0.84739	-0.2389	0.666625	1
COLGALT1	1.292212	1.318852	1.283553	0.88929	-0.1693	0.666671	1
UVSSA	1.149357	1.123088	1.157897	1.2828	0.3593	0.666791	1
DNAL1	1.270519	1.243304	1.279365	1.14821	0.1994	0.666798	1
PEX10	1.7138	1.683212	1.723743	1.05932	0.0831	0.666866	1
GAS2	1.039246	1.063397	1.031395	0.49522	-1.0139	0.666975	1
PURB	1.365072	1.392187	1.356258	0.90839	-0.1386	0.667002	1
GSKIP	1.374851	1.346624	1.384026	1.1079	0.1478	0.667065	1
LRP12	1.242047	1.2679	1.233643	0.87213	-0.1974	0.667258	1
TXNDC17	4.634894	4.718669	4.607663	0.97015	-0.0437	0.667308	1
OXNAD1	1.130078	1.104393	1.138427	1.32602	0.4071	0.667385	1
UBFD1	1.382771	1.410047	1.373905	0.91186	-0.1331	0.667487	1
DZANK1	1.122474	1.097238	1.130676	1.34388	0.4264	0.667511	1
RP11-337C18.8	1.069833	1.044867	1.077948	1.73733	0.7969	0.667641	1
UBAP1	1.478541	1.506597	1.469421	0.92662	-0.11	0.667705	1
CCDC24	1.139776	1.164993	1.131579	0.79749	-0.3265	0.667717	1
DDX54	1.591168	1.620557	1.581615	0.93725	-0.0935	0.667762	1
CGRRF1	1.299077	1.271669	1.307987	1.13368	0.181	0.667803	1
RP11-589C21.6	1.043956	1.019335	1.05196	2.6874	1.4262	0.66782	1
NUPR1	1.030928	1.05495	1.023119	0.42072	-1.2491	0.667834	1
PKP4	1.618919	1.588654	1.628757	1.06813	0.0951	0.667941	1
IGSF8	1.303875	1.276625	1.312733	1.13053	0.177	0.667962	1
TRIP12	1.734874	1.703021	1.745228	1.06004	0.0841	0.667992	1
PARD3	1.388389	1.359743	1.397701	1.10551	0.1447	0.668064	1
C2orf15	1.181798	1.15579	1.190252	1.2212	0.2883	0.668178	1
TWIST2	1.027458	1.051706	1.019576	0.3786	-1.4012	0.668181	1
EAF1-AS1	1.047417	1.022718	1.055446	2.44064	1.2873	0.668354	1
ZNF562	1.068586	1.043709	1.076672	1.75416	0.8108	0.668362	1
SLC25A45	1.064697	1.039829	1.072781	1.82732	0.8697	0.668452	1
TMEM125	1.036961	1.0609	1.02918	0.47915	-1.0615	0.668452	1
IRF2	1.242051	1.267831	1.233671	0.87246	-0.1968	0.668509	1
ITIH3	1.030089	1.053939	1.022337	0.41411	-1.2719	0.668543	1
RAB14	1.875465	1.907314	1.865113	0.95349	-0.0687	0.668548	1
HCFC1	1.569956	1.540162	1.579641	1.07309	0.1018	0.668707	1
FEM1A	1.133343	1.10785	1.14163	1.31322	0.3931	0.668722	1
AC002467.7	1.092412	1.067139	1.100627	1.49879	0.5838	0.668773	1
ATP6V0D2	1.026592	1.050388	1.018856	0.37422	-1.4181	0.668889	1
CHPT1	1.47193	1.500497	1.462644	0.92437	-0.1135	0.668938	1
MIPEP	1.164465	1.138616	1.172867	1.24709	0.3186	0.669035	1
OSBPL7	1.063026	1.03831	1.071061	1.8549	0.8913	0.669084	1
ABCB9	1.107905	1.082571	1.11614	1.40655	0.4922	0.669357	1
TOR1B	1.081942	1.106361	1.074004	0.69578	-0.5233	0.669389	1
PGRMC2	1.815407	1.783515	1.825774	1.05394	0.0758	0.66939	1

ZNF441	1.124798	1.099545	1.133007	1.33614	0.4181	0.669489	1
DYNC1LI2	1.997345	2.029039	1.987043	0.95919	-0.0601	0.669532	1
CEP76	1.134945	1.109542	1.143202	1.30728	0.3866	0.669532	1
RAB12	1.061005	1.084837	1.053258	0.62777	-0.6717	0.669649	1
LINS1	1.156798	1.13111	1.165148	1.25961	0.333	0.669759	1
RP11-442H21.2	1.068116	1.043625	1.076077	1.74388	0.8023	0.66982	1
SLC9B2	1.246906	1.220529	1.255479	1.15848	0.2122	0.669965	1
LINC00958	1.090302	1.065488	1.098367	1.50207	0.5869	0.670218	1
MAU2	1.262487	1.235642	1.271214	1.15096	0.2028	0.6704	1
C1orf216	1.159146	1.184053	1.15105	0.82069	-0.2851	0.670452	1
LRCH3	1.385558	1.357387	1.394715	1.10445	0.1433	0.670455	1
ALPL	1.090146	1.114614	1.082192	0.71712	-0.4797	0.670522	1
RP11-406H21.2	1.039767	1.01526	1.047733	3.12802	1.6452	0.670634	1
VSTM2L	1.120976	1.095733	1.129182	1.3494	0.4323	0.670808	1
NPR2	1.06814	1.092211	1.060315	0.6541	-0.6124	0.670873	1
CCDC120	1.086743	1.111045	1.078843	0.71	-0.4941	0.670889	1
HARBI1	1.061728	1.037044	1.069751	1.8829	0.913	0.670966	1
DCLRE1B	1.125711	1.100327	1.133962	1.33525	0.4171	0.671097	1
PUS1	1.139405	1.114103	1.14763	1.29383	0.3716	0.671115	1
CLK1	1.877211	1.842773	1.888405	1.05415	0.0761	0.671149	1
AHCYL1	2.055888	2.022267	2.066817	1.04358	0.0615	0.671397	1
CPNE2	1.291434	1.317559	1.282942	0.89099	-0.1665	0.671479	1
RP11-359I18.5	1.046875	1.022457	1.054812	2.44076	1.2873	0.67172	1
SERP1	3.59723	3.539132	3.616116	1.03032	0.0431	0.671733	1
CTA-293F17.1	1.140617	1.11527	1.148857	1.29137	0.3689	0.671733	1
MAEL	1.064984	1.040275	1.073016	1.81291	0.8583	0.671808	1
ERCC8	1.172335	1.146901	1.180603	1.22941	0.298	0.672029	1
NFKB2	1.142898	1.167685	1.134841	0.80413	-0.3145	0.672094	1
NFASC	1.051623	1.02717	1.059572	2.19258	1.1326	0.672105	1
FAM45A	1.781359	1.811405	1.771592	0.95093	-0.0726	0.672175	1
PPM1L	1.221886	1.195756	1.23038	1.17687	0.235	0.672197	1
ZNF565	1.065191	1.040515	1.073212	1.80705	0.8536	0.672348	1
ALG2	1.196947	1.222057	1.188785	0.85017	-0.2342	0.672443	1
SCOC-AS1	1.112195	1.087226	1.120311	1.37931	0.4639	0.672469	1
TEX261	1.280986	1.253897	1.289791	1.14137	0.1908	0.672519	1
KCND2	1.03739	1.061057	1.029697	0.48638	-1.0398	0.672721	1
FHOD1	1.159084	1.184054	1.150967	0.82023	-0.2859	0.672758	1
SMAP2	1.195059	1.168948	1.203546	1.20478	0.2688	0.672777	1
THUMPD1	1.60164	1.572086	1.611246	1.06845	0.0955	0.672822	1
ZNF778	1.083614	1.058749	1.091696	1.5608	0.6423	0.672937	1
PRKAR2A	1.803384	1.834283	1.79334	0.95092	-0.0726	0.67304	1
ZKSCAN8	1.171427	1.14617	1.179637	1.22896	0.2974	0.673188	1
UTP14C	1.211726	1.237027	1.203502	0.85856	-0.22	0.673274	1
DENND5B	1.327835	1.354189	1.319269	0.90141	-0.1497	0.673282	1
DUSP15	1.220432	1.194303	1.228925	1.17818	0.2366	0.673283	1

RP11-267M23.4	1.0362	1.011999	1.044066	3.67245	1.8767	0.67338	1
CNKSR1	1.044738	1.068473	1.037023	0.54069	-0.8871	0.673383	1
CTD-2369P2.10	1.118377	1.093256	1.126543	1.35694	0.4404	0.673414	1
MFAP5	1.026433	1.049958	1.018786	0.37603	-1.4111	0.67342	1
UBAP2L	1.474139	1.502503	1.464919	0.92521	-0.1122	0.673495	1
MSANTD2	1.177964	1.152327	1.186297	1.22301	0.2904	0.67352	1
AC053503.4	1.040157	1.015934	1.048031	3.01431	1.5918	0.673522	1
AC098973.2	1.252441	1.225949	1.261052	1.15536	0.2083	0.673537	1
CCDC7	1.061115	1.03677	1.069028	1.87732	0.9087	0.673925	1
DDX10	1.328017	1.301085	1.336771	1.11852	0.1616	0.673974	1
UBTF	1.517611	1.546516	1.508216	0.92992	-0.1048	0.673986	1
HCST	1.062302	1.086313	1.054497	0.63139	-0.6634	0.674013	1
BLOC1S6	1.878689	1.909883	1.868549	0.95457	-0.0671	0.674201	1
CLIP2	1.195386	1.220481	1.187229	0.84918	-0.2359	0.674335	1
MTF1	1.174868	1.149084	1.183249	1.22916	0.2977	0.674499	1
AP4S1	1.089467	1.113414	1.081683	0.72022	-0.4735	0.674501	1
GRIK4	1.03362	1.057119	1.025981	0.45486	-1.1365	0.674617	1
MOB3A	1.230986	1.204994	1.239435	1.16801	0.2241	0.674623	1
PDGFRB	1.569547	1.539999	1.579152	1.07251	0.101	0.674678	1
MYH7B	1.076925	1.052362	1.08491	1.62159	0.6974	0.674691	1
ZNF549	1.095996	1.071131	1.104078	1.46319	0.5491	0.674804	1
CTD-2540F13.2	1.047506	1.071184	1.039809	0.55924	-0.8385	0.674919	1
DNAI1	1.043717	1.019441	1.051608	2.65462	1.4085	0.674919	1
PTRH1	1.395823	1.368239	1.404789	1.09926	0.1365	0.674935	1
TMEM186	1.262779	1.236613	1.271284	1.14653	0.1973	0.6751	1
PAFAH2	1.119184	1.093915	1.127398	1.35653	0.4399	0.675164	1
RUSC1-AS1	1.058594	1.0343	1.066491	1.9385	0.9549	0.675238	1
ZNF445	1.3989	1.371159	1.407917	1.09904	0.1362	0.675247	1
SSPO	1.086092	1.061485	1.094091	1.5303	0.6138	0.675312	1
FAM50A	2.285254	2.319109	2.27425	0.96599	-0.0499	0.675315	1
SLC11A1	1.049863	1.025753	1.0577	2.24051	1.1638	0.675446	1
MTM1	1.109816	1.08493	1.117905	1.38826	0.4733	0.675506	1
RRS1	1.166076	1.191161	1.157922	0.82612	-0.2756	0.675821	1
DYDC2	1.043474	1.019454	1.051282	2.63609	1.3984	0.675888	1
CPLX1	1.042418	1.018355	1.05024	2.73716	1.4527	0.675924	1
TMEM102	1.082777	1.05823	1.090756	1.55859	0.6402	0.675979	1
APITD1-CORT	1.042975	1.018997	1.05077	2.67254	1.4182	0.676167	1
LYG2	1.043082	1.019017	1.050905	2.67687	1.4205	0.676167	1
RP13-401N8.1	1.043019	1.019029	1.050817	2.67057	1.4171	0.676167	1
LIFR-AS1	1.061855	1.037679	1.069714	1.85018	0.8877	0.676294	1
MFSD2B	1.092837	1.068363	1.100793	1.47438	0.5601	0.67667	1
HIST1H2AH	1.051767	1.027773	1.059566	2.14477	1.1008	0.67667	1
UCHL5	1.380079	1.353029	1.388872	1.10153	0.1395	0.676941	1
U73166.2	1.093889	1.069426	1.101841	1.4669	0.5528	0.677041	1
SPATA17	1.09371	1.06926	1.101657	1.46776	0.5536	0.677041	1

EFCAB3	1.039525	1.015699	1.04727	3.01095	1.5902	0.677452	1
TTC17	1.854402	1.822543	1.864758	1.05132	0.0722	0.677471	1
APPBP2	1.592178	1.620441	1.582991	0.93964	-0.0898	0.677509	1
NCOA4	1.591372	1.562455	1.600772	1.06813	0.0951	0.677572	1
A4GALT	1.027686	1.050957	1.020122	0.39488	-1.3405	0.677732	1
C5orf56	1.077794	1.053541	1.085678	1.60024	0.6783	0.677872	1
URGCP	1.17522	1.149694	1.183518	1.22595	0.2939	0.677994	1
FAM149A	1.078364	1.054295	1.086188	1.5874	0.6667	0.678062	1
AFTPH	1.306254	1.279288	1.315019	1.12794	0.1737	0.678136	1
AC016723.4	1.027964	1.051175	1.020419	0.39899	-1.3256	0.678165	1
TIMP4	1.036773	1.013073	1.044477	3.4023	1.7665	0.678393	1
LINC00511	1.110082	1.085587	1.118044	1.37923	0.4639	0.678488	1
SULT4A1	1.046643	1.070022	1.039043	0.55758	-0.8427	0.678488	1
OSTN	1.024504	1.047624	1.016989	0.35673	-1.4871	0.678539	1
FAM126A	1.381577	1.408653	1.372776	0.91221	-0.1326	0.678592	1
HSD17B7	1.313905	1.287186	1.32259	1.12328	0.1677	0.678609	1
RP5-827C21.4	1.097436	1.072912	1.105408	1.44568	0.5318	0.678639	1
TNPO3	1.473335	1.445379	1.482423	1.08317	0.1153	0.678673	1
BAIAP2-AS1	1.252287	1.226119	1.260794	1.15335	0.2058	0.678739	1
GALNT8	1.043114	1.0192	1.050888	2.65042	1.4062	0.67885	1
C1orf145	1.042009	1.065312	1.034434	0.52721	-0.9235	0.678871	1
LZIC	1.678011	1.648216	1.687696	1.06091	0.0853	0.678893	1
MBTPS1	1.611361	1.639765	1.602129	0.94117	-0.0875	0.679075	1
TMEM200C	1.080756	1.10434	1.07309	0.7005	-0.5135	0.679105	1
MACROD2	1.092483	1.068014	1.100437	1.47672	0.5624	0.679284	1
ZNF12	1.380003	1.352769	1.388855	1.10229	0.1405	0.67935	1
KHNYN	1.294714	1.268347	1.303285	1.1302	0.1766	0.679364	1
ATXN1L	1.187284	1.2117	1.179348	0.84718	-0.2393	0.67943	1
CACNG8	1.15181	1.12693	1.159898	1.25974	0.3331	0.679489	1
PLS1	1.074065	1.097516	1.066442	0.68135	-0.5535	0.679554	1
RP11-504A18.1	1.041179	1.017447	1.048894	2.80244	1.4867	0.679578	1
CTNS	1.114066	1.13813	1.106244	0.76915	-0.3787	0.679604	1
PLEKHH3	1.17035	1.145204	1.178524	1.22947	0.298	0.67968	1
UG0898H09	1.179319	1.203532	1.171449	0.84237	-0.2475	0.679851	1
RP3-522D1.1	1.070341	1.046315	1.078151	1.68736	0.7548	0.679934	1
UPK3A	1.031683	1.0547	1.024202	0.44244	-1.1764	0.679979	1
IRF2BP1	1.162538	1.137408	1.170707	1.24234	0.3131	0.680056	1
SNTG2	1.04047	1.016767	1.048175	2.87318	1.5226	0.680136	1
RP11-285F7.2	1.062627	1.038682	1.070411	1.82028	0.8642	0.680144	1
TFAP4	1.109708	1.085408	1.117606	1.37699	0.4615	0.680198	1
RP11-37C7.3	1.074516	1.050505	1.082321	1.62998	0.7049	0.680862	1
CTD-2017F17.2	1.06217	1.038276	1.069937	1.82718	0.8696	0.680893	1
LIPT2	1.107002	1.082925	1.114829	1.38474	0.4696	0.680943	1
ACSF3	1.196435	1.171202	1.204637	1.1953	0.2574	0.681064	1
SH3GL3	1.065264	1.088705	1.057645	0.64984	-0.6218	0.681201	1

JPH2	1.030879	1.053717	1.023455	0.43665	-1.1955	0.681344	1
EDF1	7.086414	7.208425	7.046754	0.97396	-0.0381	0.681393	1
AC003075.4	1.041669	1.064767	1.034161	0.52745	-0.9229	0.68143	1
ITGB1BP1	3.641994	3.693393	3.625286	0.97471	-0.037	0.681536	1
DARS2	1.070982	1.047044	1.078763	1.67426	0.7435	0.681537	1
ABTB1	1.104112	1.127893	1.096382	0.75361	-0.4081	0.681566	1
PTPDC1	1.182144	1.156964	1.190329	1.21257	0.2781	0.681589	1
FAM110D	1.049857	1.026274	1.057522	2.18932	1.1305	0.681633	1
GOLGB1	2.353398	2.390408	2.341367	0.96473	-0.0518	0.681642	1
RP11-118F19.1	1.046056	1.022365	1.053756	2.40358	1.2652	0.68166	1
PLCXD2	1.056629	1.079936	1.049053	0.61366	-0.7045	0.681882	1
CH507-9B2.3	1.052733	1.028962	1.06046	2.08758	1.0618	0.681903	1
PRKRIP1	1.419905	1.392454	1.428829	1.09268	0.1279	0.681977	1
EIF5A2	1.157627	1.181532	1.149856	0.82551	-0.2766	0.682014	1
NEK1	1.473533	1.445674	1.482589	1.08283	0.1148	0.682219	1
CYP1B1-AS1	1.0929	1.068775	1.100742	1.4648	0.5507	0.68226	1
CAMK2B	1.09296	1.068926	1.100772	1.46204	0.548	0.68226	1
C19orf57	1.06564	1.041812	1.073386	1.75515	0.8116	0.682292	1
TUBA4A	1.049895	1.072874	1.042426	0.58218	-0.7805	0.682342	1
PIKFYVE	1.269873	1.244136	1.278239	1.13969	0.1886	0.682581	1
IKBKAP	1.191164	1.216065	1.183069	0.84729	-0.2391	0.68259	1
RPN2	2.816896	2.772997	2.831166	1.03281	0.0466	0.682662	1
PPP1R12B	1.18422	1.159102	1.192384	1.20919	0.274	0.682905	1
KLK8	1.050751	1.074039	1.043181	0.58322	-0.7779	0.682906	1
GTF2IRD2B	1.184671	1.159869	1.192733	1.20557	0.2697	0.682946	1
USP13	1.194705	1.219098	1.186776	0.85248	-0.2303	0.68305	1
MVK	1.191915	1.216149	1.184038	0.85144	-0.232	0.683207	1
FGF7	1.171795	1.146731	1.179942	1.22634	0.2944	0.683295	1
SLC16A14	1.112308	1.13614	1.104561	0.76804	-0.3807	0.683309	1
RP11-54O7.3	1.112137	1.08796	1.119995	1.3642	0.4481	0.683462	1
GLB1L	1.106541	1.082129	1.114476	1.39386	0.4791	0.683517	1
PSMD14	2.124635	2.156201	2.114374	0.96382	-0.0532	0.683536	1
CDH12	1.039585	1.062457	1.032151	0.51476	-0.958	0.683546	1
POLD4	1.208292	1.232774	1.200334	0.86064	-0.2165	0.683661	1
ARHGEF28	1.124166	1.099824	1.132078	1.32311	0.4039	0.683775	1
CABLES2	1.077123	1.100336	1.069577	0.69344	-0.5281	0.683899	1
TNRC6A	1.788808	1.817897	1.779352	0.95287	-0.0696	0.68419	1
DUSP28	1.184315	1.208495	1.176455	0.84633	-0.2407	0.684219	1
MED8	1.42769	1.454018	1.419132	0.92316	-0.1153	0.68425	1
RP11-159D12.5	1.081644	1.057854	1.089377	1.54487	0.6275	0.684291	1
PGPEP1	1.292618	1.318043	1.284354	0.89407	-0.1615	0.684356	1
PON2	2.20185	2.164068	2.214132	1.04301	0.0607	0.684624	1
MTG2	1.210116	1.184776	1.218352	1.18171	0.2409	0.68471	1
FLJ22447	1.035249	1.057858	1.027899	0.4822	-1.0523	0.684745	1
OXSM	1.165761	1.140899	1.173843	1.23381	0.3031	0.68479	1

SLC2A12	1.095627	1.07161	1.103434	1.4444	0.5305	0.684793	1
ZYG11B	1.436045	1.408962	1.444848	1.08775	0.1213	0.684867	1
LSM11	1.082659	1.058798	1.090415	1.53772	0.6208	0.684899	1
ACY1	1.082828	1.058877	1.090614	1.53905	0.622	0.684899	1
RP11-357K6.1	1.044536	1.021006	1.052185	2.48424	1.3128	0.685013	1
IL34	1.0258	1.048504	1.018419	0.37975	-1.3969	0.685159	1
WTIP	1.126735	1.150082	1.119145	0.79387	-0.333	0.685307	1
EEF2KMT	1.184077	1.208183	1.176241	0.84657	-0.2403	0.685329	1
RAB4A	2.963553	3.013392	2.947352	0.9672	-0.0481	0.685359	1
DRP2	1.045763	1.022291	1.053392	2.39523	1.2602	0.685361	1
LRRC8B	1.166249	1.141502	1.174293	1.23174	0.3007	0.68543	1
ZRSR2	1.350851	1.324485	1.359422	1.10767	0.1475	0.685519	1
C2orf54	1.022353	1.045062	1.014971	0.33224	-1.5897	0.685564	1
HIPK2	1.935927	1.904373	1.946184	1.04623	0.0652	0.685704	1
TCEAL3	2.761597	2.806035	2.747152	0.9674	-0.0478	0.68573	1
CABS1	1.024743	1.047416	1.017373	0.3664	-1.4485	0.685753	1
TCP10L	1.06002	1.082951	1.052565	0.63369	-0.6581	0.685858	1
CLDN9	1.022143	1.04471	1.014807	0.33119	-1.5943	0.685898	1
PHF21B	1.09849	1.074534	1.106277	1.42589	0.5119	0.685979	1
KBTBD11-OT1	1.041453	1.064481	1.033968	0.52678	-0.9247	0.685981	1
AF003625.3	1.027885	1.050492	1.020536	0.40672	-1.2979	0.686033	1
HPGD	1.023011	1.045494	1.015702	0.34515	-1.5347	0.686121	1
CCAR1	1.873878	1.84301	1.883912	1.04852	0.0684	0.686121	1
PEX11G	1.099092	1.075123	1.106883	1.42279	0.5087	0.686127	1
CTD-3222D19.8	1.071052	1.047504	1.078706	1.65682	0.7284	0.686376	1
RP11-467P9.1	1.038033	1.014833	1.045575	3.07261	1.6195	0.686524	1
NRIP3	1.097894	1.121034	1.090373	0.74667	-0.4214	0.686829	1
SYNJ1	1.076952	1.053371	1.084617	1.58545	0.6649	0.686833	1
FAM35A	1.199557	1.174795	1.207606	1.18772	0.2482	0.686892	1
RNF113A	1.322517	1.296625	1.330934	1.11566	0.1579	0.686904	1
MORN5	1.070282	1.046442	1.078031	1.6802	0.7486	0.686959	1
RP4-773N10.4	1.048351	1.071084	1.040962	0.57625	-0.7952	0.687003	1
FAM201A	1.079877	1.056121	1.087599	1.56089	0.6424	0.687125	1
RP11-290L1.2	1.047516	1.024198	1.055095	2.27682	1.187	0.687188	1
ATRN	1.213914	1.189023	1.222005	1.17449	0.232	0.687302	1
IPPK	1.072127	1.095076	1.064667	0.68017	-0.556	0.687357	1
GCC1	1.072097	1.095082	1.064625	0.67968	-0.5571	0.687357	1
MLIP	1.028271	1.050743	1.020966	0.41317	-1.2752	0.687448	1
RPAIN	3.321529	3.269309	3.338504	1.03049	0.0433	0.687674	1
CDK5RAP1	1.167212	1.142587	1.175217	1.22884	0.2973	0.687781	1
LINC00638	1.048346	1.025019	1.055929	2.23543	1.1606	0.68783	1
TTC1	1.65465	1.682207	1.645692	0.94647	-0.0794	0.68796	1
LINC01621	1.035233	1.012186	1.042724	3.5059	1.8098	0.688057	1
ZZEF1	1.14388	1.167349	1.136251	0.81417	-0.2966	0.688167	1
CLCN5	1.462286	1.488748	1.453684	0.92826	-0.1074	0.688367	1

ERICH2	1.046063	1.068659	1.038718	0.56393	-0.8264	0.688416	1
APEX2	1.156228	1.179888	1.148537	0.82572	-0.2763	0.688478	1
LACE1	1.067115	1.043767	1.074704	1.70687	0.7714	0.688516	1
ALYREF	1.095728	1.072167	1.103387	1.43262	0.5187	0.688573	1
SCX	1.105408	1.128738	1.097825	0.75987	-0.3962	0.688587	1
TRIM25	1.217367	1.192444	1.225469	1.17161	0.2285	0.688673	1
LINC00844	1.046018	1.02267	1.053607	2.3647	1.2417	0.68872	1
ZNF285	1.052417	1.029216	1.059959	2.05224	1.0372	0.688762	1
FEZF1	1.110203	1.086279	1.11798	1.36743	0.4515	0.688932	1
TMEM99	1.462368	1.435917	1.470967	1.0804	0.1116	0.689048	1
NRG1	1.051784	1.028462	1.059365	2.08575	1.0606	0.689207	1
RP11-465L10.10	1.051754	1.028598	1.059281	2.07295	1.0517	0.689207	1
MRPS24	1.059837	1.03666	1.067371	1.83773	0.8779	0.689247	1
FOXI3	1.05479	1.031543	1.062347	1.97655	0.983	0.68925	1
ERN2	1.054893	1.031579	1.062471	1.97822	0.9842	0.68925	1
GABBR1	1.159799	1.13546	1.167711	1.23808	0.3081	0.689293	1
CD4	1.065424	1.04205	1.073021	1.73652	0.7962	0.689312	1
ZNF391	1.084407	1.060846	1.092065	1.5131	0.5975	0.689341	1
CCDC68	1.035222	1.05763	1.027938	0.48479	-1.0446	0.68935	1
RBP4	1.027653	1.050012	1.020385	0.40761	-1.2947	0.689382	1
NEMP2	1.054787	1.077684	1.047344	0.60945	-0.7144	0.689498	1
RP5-1085F17.3	1.15189	1.175594	1.144185	0.82112	-0.2843	0.689533	1
RP11-115D19.1	3.244376	3.176801	3.266342	1.04113	0.0582	0.689562	1
ARRDC2	1.115916	1.092074	1.123666	1.34312	0.4256	0.689788	1
AP000251.3	1.086887	1.063693	1.094427	1.48254	0.5681	0.689888	1
GLT8D2	1.093027	1.116114	1.085522	0.73654	-0.4412	0.689998	1
SRR	1.211915	1.186952	1.220029	1.17693	0.235	0.690166	1
NAMPT	1.792819	1.763008	1.80251	1.05177	0.0728	0.690298	1
SCAF4	1.400908	1.374525	1.409485	1.09335	0.1287	0.690329	1
DRC7	1.025127	1.047594	1.017824	0.37451	-1.4169	0.690355	1
SGIP1	1.333156	1.306819	1.341718	1.11374	0.1554	0.690435	1
ZNF470	1.153004	1.128719	1.160898	1.24999	0.3219	0.690713	1
BEX5	1.404619	1.430043	1.396355	0.92166	-0.1177	0.690938	1
SARS2	1.192168	1.167806	1.200086	1.19237	0.2538	0.691002	1
CDO1	1.562363	1.534525	1.571412	1.06901	0.0963	0.691122	1
MKRN2	1.476426	1.44893	1.485363	1.08116	0.1126	0.691288	1
GPRASP1	1.099967	1.076401	1.107628	1.40873	0.4944	0.691329	1
TNFSF10	1.045581	1.067876	1.038334	0.56476	-0.8243	0.691356	1
EML4	1.732937	1.70395	1.742359	1.05456	0.0766	0.691421	1
TXNDC9	1.402751	1.42825	1.394463	0.9211	-0.1186	0.691522	1
CTD-2371O3.3	1.150333	1.126534	1.158069	1.24922	0.321	0.691559	1
ZDHHC11B	1.037531	1.059962	1.030239	0.50431	-0.9876	0.691657	1
ZNF222	1.069523	1.046416	1.077034	1.65966	0.7309	0.691767	1
NCDN	1.094673	1.117364	1.087298	0.74382	-0.427	0.691792	1
PCDH1	1.045556	1.067886	1.038297	0.56414	-0.8259	0.691824	1

CUEDC1	1.267721	1.292261	1.259744	0.88874	-0.1702	0.691934	1
AZIN2	1.156371	1.132372	1.164172	1.24023	0.3106	0.691994	1
GNRH1	1.052488	1.029572	1.059937	2.02685	1.0192	0.692257	1
RP5-1112D6.7	1.084111	1.060625	1.091746	1.51333	0.5977	0.692375	1
CLPP	2.286668	2.319444	2.276014	0.96708	-0.0483	0.69238	1
ZBTB34	1.076913	1.099522	1.069564	0.69898	-0.5167	0.692398	1
ZDHHC5	1.174388	1.197877	1.166753	0.84271	-0.2469	0.692445	1
RBM45	1.126871	1.103099	1.134598	1.30553	0.3846	0.692462	1
CTD-3064M3.7	1.03061	1.052924	1.023357	0.44134	-1.18	0.692525	1
GALNT7	1.719983	1.748099	1.710843	0.9502	-0.0737	0.692567	1
RP11-1275H24.2	1.063903	1.040681	1.071451	1.75637	0.8126	0.692897	1
ZNF782	1.068876	1.09158	1.061496	0.67149	-0.5746	0.692912	1
RP11-57A19.2	1.041971	1.019197	1.049374	2.57191	1.3628	0.692946	1
RP11-172H24.4	1.066965	1.089465	1.059651	0.66675	-0.5848	0.69295	1
CTB-47B11.3	1.025703	1.048045	1.018441	0.38382	-1.3815	0.692987	1
SLC35A3	1.252831	1.277108	1.244939	0.88391	-0.178	0.6931	1
DDX23	1.347831	1.373098	1.339618	0.91026	-0.1356	0.693178	1
ESPL1	1.070099	1.047009	1.077605	1.65086	0.7232	0.693399	1
UBE4B	1.35896	1.333283	1.367307	1.10209	0.1402	0.693622	1
TMEM154	1.028284	1.050296	1.021129	0.42009	-1.2512	0.693973	1
CSPG4	1.028284	1.050262	1.021139	0.42058	-1.2495	0.693973	1
CASC2	1.077076	1.053952	1.084593	1.56792	0.6489	0.694042	1
TLDC1	1.295088	1.269633	1.303362	1.12509	0.17	0.69406	1
PPP3CA	1.755301	1.78326	1.746213	0.9527	-0.0699	0.694233	1
TWNK	1.067284	1.044107	1.074818	1.69628	0.7624	0.694248	1
METTL9	4.169223	4.105092	4.19007	1.02737	0.039	0.69433	1
GALNT13	1.056955	1.079363	1.049672	0.62588	-0.676	0.694424	1
DRC1	1.035604	1.057709	1.028418	0.49244	-1.022	0.694446	1
PC	1.058699	1.035912	1.066106	1.84081	0.8803	0.694791	1
RP11-401F2.3	1.09088	1.067896	1.098351	1.44856	0.5346	0.695057	1
MPV17	2.164195	2.132311	2.174558	1.03731	0.0528	0.695148	1
DNAH11	1.113293	1.089968	1.120876	1.34354	0.426	0.695283	1
USP48	1.589255	1.562014	1.59811	1.06423	0.0898	0.695469	1
RP11-111M22.2	1.22581	1.201191	1.233812	1.16214	0.2168	0.695496	1
ACOX3	1.109663	1.13235	1.102288	0.77286	-0.3717	0.695498	1
INTS2	1.161927	1.138153	1.169655	1.22802	0.2963	0.695503	1
NDUFB4	4.999487	4.919603	5.025454	1.02701	0.0384	0.69555	1
DPF1	1.068152	1.045278	1.075587	1.66938	0.7393	0.695562	1
TMEM178B	1.063221	1.040288	1.070675	1.75422	0.8108	0.695569	1
ZBTB7C	1.040171	1.017512	1.047536	2.71453	1.4407	0.695647	1
STRADA	1.050761	1.027945	1.058178	2.08188	1.0579	0.695756	1
RP1-120G22.11	1.052621	1.029888	1.06001	2.00784	1.0056	0.695924	1
HECTD3	1.119361	1.141793	1.112069	0.79037	-0.3394	0.696085	1
CAPN6	1.043136	1.065175	1.035972	0.55192	-0.8575	0.696116	1
IFNLR1	1.041981	1.063824	1.034881	0.54653	-0.8716	0.696144	1

RP11-436D23.1	1.073411	1.050423	1.080884	1.60413	0.6818	0.696205	1
XPO7	1.303626	1.278358	1.311839	1.12028	0.1639	0.696217	1
CLIC6	1.233018	1.256296	1.225452	0.87965	-0.185	0.696259	1
SYNPO	1.039417	1.06159	1.032209	0.52296	-0.9352	0.696321	1
ADM5	1.039719	1.017203	1.047038	2.73424	1.4511	0.696403	1
SLC44A3	1.105318	1.127943	1.097964	0.76568	-0.3852	0.696645	1
C18orf25	1.158119	1.134326	1.165853	1.2347	0.3042	0.696673	1
C2CD2	1.19544	1.218754	1.187861	0.85878	-0.2196	0.696678	1
AJ011932.1	1.0527	1.075014	1.045446	0.60584	-0.723	0.696909	1
CAPS	1.093316	1.115574	1.08608	0.74481	-0.4251	0.69694	1
ZNF597	1.03911	1.061001	1.031994	0.52448	-0.931	0.697011	1
OSGEPL1-AS1	1.043484	1.021066	1.050771	2.41007	1.2691	0.697058	1
TGFB2-AS1	1.048599	1.025988	1.055949	2.15289	1.1063	0.697064	1
SOGA3	1.040536	1.017948	1.047878	2.66764	1.4156	0.697141	1
MRPS18C	2.229317	2.261475	2.218864	0.96622	-0.0496	0.697149	1
RP11-707G18.1	1.072563	1.049751	1.079978	1.60757	0.6849	0.697374	1
LIPA	1.396942	1.370853	1.405422	1.09321	0.1286	0.697527	1
ZNF583	1.201078	1.176917	1.208932	1.18096	0.24	0.697659	1
TDRKH	1.053398	1.030824	1.060736	1.97041	0.9785	0.697703	1
LSS	1.261933	1.285688	1.254211	0.88982	-0.1684	0.697826	1
ZFYVE9	1.269987	1.245622	1.277908	1.13145	0.1782	0.697946	1
USP21	1.130108	1.106979	1.137626	1.28647	0.3634	0.697958	1
UNK	1.364883	1.339126	1.373255	1.10064	0.1383	0.698043	1
CTD-2553L13.10	1.077697	1.054821	1.085132	1.55291	0.635	0.698275	1
SERPINF2	1.023921	1.045654	1.016856	0.36922	-1.4375	0.698414	1
SLC38A4	1.055766	1.033236	1.06309	1.89821	0.9246	0.698475	1
FBXO11	1.474926	1.500482	1.466619	0.93234	-0.1011	0.698662	1
AIM1	1.021241	1.042817	1.014227	0.33229	-1.5895	0.698696	1
ESRP2	1.025297	1.046978	1.018249	0.38845	-1.3642	0.69871	1
RASGRF2	1.113853	1.136253	1.106571	0.78216	-0.3545	0.698727	1
SCNN1D	1.056807	1.03413	1.064178	1.88039	0.911	0.69874	1
RP1-313I6.12	1.176392	1.153113	1.183959	1.20146	0.2648	0.698838	1
LARP4	1.513398	1.539134	1.505032	0.93675	-0.0943	0.69888	1
SEH1L	1.244763	1.220368	1.252693	1.14669	0.1975	0.698889	1
ATP9B	1.189835	1.213011	1.182301	0.85583	-0.2246	0.698966	1
ELOVL4	1.262316	1.237609	1.270347	1.13778	0.1862	0.699059	1
IQUB	1.05685	1.034296	1.064181	1.87138	0.9041	0.69908	1
KHK	1.214041	1.190113	1.22182	1.16678	0.2225	0.699173	1
TRIP11	1.81685	1.845072	1.807676	0.95575	-0.0653	0.699446	1
SLC35A1	1.242529	1.266728	1.234663	0.87979	-0.1848	0.699498	1
RAB3GAP2	1.505264	1.530329	1.497117	0.93737	-0.0933	0.699575	1
LXN	1.176797	1.153433	1.184391	1.20177	0.2652	0.699605	1
ZBTB5	1.176937	1.153524	1.184548	1.20208	0.2655	0.699605	1
PAN3-AS1	1.081157	1.058466	1.088533	1.51427	0.5986	0.699608	1
GLUD1	1.714353	1.742134	1.705322	0.9504	-0.0734	0.699622	1

SLC16A7	1.10933	1.132014	1.101957	0.77232	-0.3727	0.699782	1
PPP1R42	1.050436	1.028065	1.057708	2.05622	1.04	0.699867	1
RP1-40E16.9	1.152882	1.129646	1.160435	1.23749	0.3074	0.69987	1
SLC43A1	1.047406	1.024942	1.054708	2.19338	1.1332	0.699885	1
PHF11	1.271887	1.2471	1.279945	1.13292	0.18	0.699887	1
BASP1	3.530742	3.478142	3.547839	1.02812	0.04	0.700063	1
ZNF844	1.112501	1.135194	1.105125	0.77758	-0.3629	0.700066	1
PSD	1.140126	1.162581	1.132827	0.81699	-0.2916	0.700196	1
PHAX	1.528796	1.501891	1.537542	1.07103	0.099	0.700201	1
MFN2	1.235298	1.258949	1.22761	0.87898	-0.1861	0.700338	1
LINC01569	1.066259	1.04378	1.073566	1.68037	0.7488	0.700432	1
RP1-168L15.5	1.062389	1.03992	1.069693	1.74581	0.8039	0.700517	1
ELMO3	1.04713	1.069105	1.039987	0.57865	-0.7892	0.700603	1
WDCP	1.109491	1.08661	1.116929	1.35006	0.433	0.700642	1
ITPRIP	1.173506	1.149733	1.181233	1.21037	0.2755	0.700695	1
RP11-326I11.3	1.051605	1.029283	1.058861	2.01008	1.0073	0.700754	1
SLC26A6	1.180964	1.157009	1.188751	1.20217	0.2656	0.700861	1
TCF7L1	1.409185	1.433945	1.401137	0.9244	-0.1134	0.701031	1
UBE3B	1.326208	1.35045	1.318328	0.90834	-0.1387	0.701068	1
AC104655.3	1.141105	1.117815	1.148675	1.26194	0.3356	0.701157	1
LINC-PINT	1.067096	1.089023	1.059968	0.67363	-0.57	0.701311	1
ERCC3	1.18335	1.159618	1.191065	1.19701	0.2594	0.701343	1
TXNDC12	2.611989	2.652023	2.598976	0.96789	-0.0471	0.701343	1
ADAP2	1.036285	1.057794	1.029293	0.50685	-0.9804	0.701416	1
SPATA24	1.119115	1.096325	1.126523	1.31349	0.3934	0.70152	1
TTC28	1.639388	1.611785	1.64836	1.05978	0.0838	0.701698	1
RP11-350J20.5	1.048813	1.026533	1.056055	2.1127	1.0791	0.701707	1
IFI27L2	3.721267	3.775578	3.703613	0.97407	-0.0379	0.701709	1
ADRB1	1.140078	1.117017	1.147575	1.26114	0.3347	0.701764	1
RP5-1112D6.4	1.112953	1.09006	1.120395	1.33683	0.4188	0.701955	1
GRIA2	1.053683	1.031254	1.060974	1.95088	0.9641	0.701997	1
SLC25A37	2.11434	2.144399	2.104569	0.9652	-0.0511	0.702069	1
CTC-529I10.2	1.034286	1.012187	1.04147	3.40289	1.7668	0.702263	1
SLC25A46	1.398576	1.423268	1.39055	0.9227	-0.1161	0.70229	1
MOB2	1.360084	1.384286	1.352218	0.91655	-0.1257	0.702319	1
RP11-139K4.2	1.035124	1.013102	1.042283	3.2273	1.6903	0.702629	1
ELMSAN1	1.237788	1.261038	1.23023	0.88198	-0.1812	0.702668	1
MFSD2A	1.067606	1.089334	1.060543	0.67772	-0.5612	0.70268	1
RWDD3	1.090654	1.06807	1.097995	1.43961	0.5257	0.702845	1
KCNMB3	1.090683	1.067872	1.098098	1.44534	0.5314	0.702845	1
PSEN2	1.179765	1.202467	1.172386	0.85143	-0.232	0.702983	1
ACIN1	1.653709	1.626553	1.662537	1.05743	0.0806	0.703171	1
TFCP2	1.130934	1.108233	1.138313	1.27792	0.3538	0.703267	1
AC062029.1	1.076114	1.053688	1.083404	1.55348	0.6355	0.703411	1
SGMS1-AS1	1.076136	1.053698	1.08343	1.55368	0.6357	0.703411	1

ZNF337-AS1	1.101357	1.078602	1.108753	1.3836	0.4684	0.703439	1
CRLF3	1.123463	1.100343	1.130978	1.30531	0.3844	0.703566	1
TAF3	1.260311	1.283903	1.252642	0.88989	-0.1683	0.703616	1
SRFBP1	1.355452	1.330626	1.363521	1.09949	0.1368	0.703634	1
UNC50	1.651969	1.624617	1.66086	1.05802	0.0814	0.703782	1
NECAP1	1.280107	1.255685	1.288046	1.12657	0.1719	0.70398	1
DHX16	1.185821	1.162594	1.193371	1.18929	0.2501	0.703992	1
RP1-17K7.2	1.038633	1.01661	1.045791	2.75679	1.463	0.704017	1
ZNF619	1.061058	1.038859	1.068274	1.75694	0.8131	0.704046	1
SNAP23	1.513572	1.539357	1.50519	0.93665	-0.0944	0.704166	1
BAHCC1	1.136899	1.159206	1.129648	0.81434	-0.2963	0.70423	1
AP4B1	1.083032	1.060535	1.090344	1.49243	0.5777	0.70424	1
ARHGAP28	1.185807	1.20892	1.178294	0.85341	-0.2287	0.704307	1
KLHDC1	1.070488	1.048217	1.077727	1.61202	0.6889	0.704307	1
ZFP42	1.033477	1.054998	1.026481	0.48149	-1.0544	0.704493	1
CEACAM1	1.070615	1.048388	1.07784	1.60867	0.6859	0.704546	1
DIABLO	1.215342	1.191727	1.223018	1.1632	0.2181	0.704831	1
RUNX2	1.055491	1.03326	1.062717	1.88568	0.9151	0.704871	1
CCNL1	2.271522	2.238846	2.282144	1.03495	0.0496	0.705012	1
C16orf82	1.042938	1.020977	1.050077	2.38725	1.2553	0.705069	1
CAMSAP1	1.34689	1.321775	1.355054	1.10342	0.142	0.705104	1
VAMP4	1.158424	1.18101	1.151082	0.83466	-0.2607	0.705398	1
RP11-396C23.2	1.055844	1.033644	1.06306	1.87433	0.9064	0.705419	1
AP3D1	2.122172	2.151206	2.112734	0.96658	-0.049	0.705474	1
SPTBN4	1.065537	1.043385	1.072738	1.67655	0.7455	0.705495	1
CTD-2350C19.2	1.065697	1.043367	1.072955	1.68227	0.7504	0.705495	1
MANBA	1.312393	1.335662	1.30483	0.90815	-0.139	0.705806	1
ARMC8	1.418903	1.44402	1.410739	0.92505	-0.1124	0.705928	1
LINC01588	1.043648	1.021822	1.050743	2.3253	1.2174	0.706163	1
THADA	1.15491	1.131775	1.16243	1.23264	0.3017	0.706291	1
UTP4	1.215309	1.238078	1.207908	0.87328	-0.1955	0.706349	1
LL22NC03-32F9.1	1.044567	1.022717	1.051669	2.27444	1.1855	0.70641	1
APBA3	1.119588	1.141421	1.112491	0.79543	-0.3302	0.706496	1
PPIL2	1.254761	1.231003	1.262483	1.13628	0.1843	0.706608	1
RUSC2	1.096353	1.117972	1.089326	0.75718	-0.4013	0.70678	1
HSPA14	1.270001	1.246056	1.277784	1.12894	0.175	0.706813	1
SMN2	1.080322	1.058161	1.087525	1.50488	0.5896	0.706848	1
GAB1	1.153914	1.131006	1.161361	1.23171	0.3007	0.706903	1
FAS	1.091923	1.113583	1.084883	0.74732	-0.4202	0.706929	1
DISP1	1.112424	1.089636	1.119832	1.33687	0.4189	0.707013	1
CYB561D2	1.292565	1.268479	1.300394	1.11887	0.162	0.707037	1
NABP1	1.146542	1.168398	1.139438	0.82803	-0.2722	0.70713	1
ZNF557	1.146725	1.168933	1.139506	0.82581	-0.2761	0.70713	1
PHC3	1.402213	1.376971	1.410417	1.08872	0.1226	0.707438	1
FAM120A	1.492407	1.517364	1.484295	0.93608	-0.0953	0.707592	1

MORC3	1.254851	1.230983	1.262609	1.13692	0.1851	0.70769	1
TIGD2	1.113125	1.135024	1.106006	0.78509	-0.3491	0.707703	1
ASH2L	1.253143	1.229442	1.260848	1.13688	0.1851	0.70773	1
BIN3	1.299938	1.276203	1.307653	1.11387	0.1556	0.707784	1
CTC-444N24.8	1.077683	1.099357	1.070637	0.71095	-0.4922	0.707888	1
ZSCAN12	1.126695	1.104114	1.134035	1.28739	0.3644	0.708193	1
CTB-178M22.2	1.093429	1.071391	1.100593	1.40904	0.4947	0.708228	1
IZUMO4	1.061974	1.040155	1.069067	1.72	0.7824	0.708329	1
DCDC2	1.042862	1.063995	1.035992	0.56242	-0.8303	0.708371	1
NOP2	1.097508	1.119171	1.090466	0.75913	-0.3976	0.708424	1
FAM49B	1.970827	1.999564	1.961486	0.96191	-0.056	0.708637	1
AMMECR1L	1.131724	1.15361	1.124609	0.8112	-0.3019	0.708677	1
SOCS6	1.329222	1.352708	1.321588	0.91177	-0.1333	0.70878	1
ZNF684	1.073136	1.051137	1.080287	1.57005	0.6508	0.708895	1
RP11-712L6.5	1.072274	1.050403	1.079383	1.57498	0.6553	0.708995	1
C18orf8	1.074834	1.096188	1.067892	0.70583	-0.5026	0.709082	1
HEXIM2	1.193962	1.170808	1.201488	1.17962	0.2383	0.709285	1
MAFA-AS1	1.104	1.081799	1.111217	1.35964	0.4432	0.709344	1
ZNF77	1.085236	1.063139	1.092419	1.46374	0.5497	0.709365	1
LINC00271	1.066057	1.044043	1.073213	1.66232	0.7332	0.709474	1
MEX3B	1.285427	1.261313	1.293265	1.12227	0.1664	0.709524	1
ZSCAN32	1.205181	1.181358	1.212924	1.17405	0.2315	0.709555	1
TMEM179	1.033549	1.011999	1.040554	3.3797	1.7569	0.709612	1
CREG2	1.030839	1.051975	1.023969	0.46115	-1.1167	0.70965	1
AIDA	1.903486	1.87425	1.912989	1.04431	0.0626	0.709727	1
ZSCAN29	1.148236	1.125575	1.155602	1.23911	0.3093	0.709764	1
PDCD2L	1.172067	1.193976	1.164946	0.85034	-0.2339	0.709998	1
CDH7	1.377205	1.353162	1.38502	1.09021	0.1246	0.710019	1
PELI2	1.150405	1.127735	1.157774	1.23516	0.3047	0.710046	1
ZNF134	1.379981	1.403227	1.372425	0.92361	-0.1146	0.710116	1
KIAA0355	1.551187	1.576116	1.543084	0.94267	-0.0852	0.710123	1
GATA2-AS1	1.030115	1.051066	1.023304	0.45635	-1.1318	0.710159	1
CORO1A	1.07666	1.054818	1.083761	1.52798	0.6116	0.710223	1
HCN3	1.076723	1.054698	1.083882	1.53354	0.6169	0.710223	1
AC004076.5	1.101226	1.07886	1.108496	1.37581	0.4603	0.710267	1
FAM43B	1.053935	1.032323	1.060961	1.88601	0.9153	0.710268	1
TSPAN18	1.245171	1.221638	1.252821	1.14069	0.1899	0.710278	1
BTBD19	1.08379	1.104931	1.076918	0.73304	-0.448	0.710283	1
MED12L	1.213456	1.190134	1.221037	1.16253	0.2173	0.710403	1
SENP8	1.085892	1.064048	1.092993	1.45192	0.538	0.710412	1
COL15A1	1.026678	1.047698	1.019845	0.41606	-1.2651	0.710611	1
LINC00473	1.026649	1.047572	1.019848	0.41721	-1.2612	0.710611	1
EIF2B5	1.346307	1.369662	1.338715	0.91628	-0.1261	0.710666	1
GCGR	1.089014	1.110721	1.081957	0.74022	-0.434	0.710668	1
TMEM74B	1.065262	1.086577	1.058334	0.67378	-0.5696	0.71067	1

CSF1	1.088743	1.066607	1.095939	1.44038	0.5264	0.710703	1
RP11-96D1.11	1.024755	1.045585	1.017985	0.39453	-1.3418	0.710778	1
PTPN7	1.05582	1.034181	1.062854	1.83887	0.8788	0.710889	1
LRRTM4	1.117579	1.139422	1.110478	0.79241	-0.3357	0.710989	1
RP11-1069G10.2	1.020399	1.041101	1.01367	0.33259	-1.5882	0.711004	1
TLX2	1.026184	1.046925	1.019442	0.41431	-1.2712	0.711055	1
CLDN5	1.041035	1.019453	1.04805	2.46998	1.3045	0.711122	1
UBA3	1.460933	1.435989	1.469041	1.07581	0.1054	0.711179	1
SCRIB	1.247471	1.223712	1.255194	1.14073	0.19	0.71121	1
TRPM4	1.153808	1.131249	1.161141	1.22775	0.296	0.711328	1
DMWD	1.243193	1.26609	1.23575	0.88598	-0.1747	0.711333	1
NOX4	1.081915	1.103482	1.074904	0.72384	-0.4663	0.711522	1
LINC00599	1.04837	1.026575	1.055455	2.08672	1.0612	0.711603	1
NDUFS8	4.434524	4.488598	4.416947	0.97946	-0.0299	0.711751	1
CTIF	1.180127	1.202219	1.172945	0.85524	-0.2256	0.711817	1
RP11-299G20.2	1.046616	1.024956	1.053656	2.15001	1.1043	0.712024	1
RP11-111F5.3	1.046603	1.024968	1.053636	2.14821	1.1031	0.712024	1
EIF2S3L	1.083011	1.061269	1.090079	1.47022	0.556	0.712124	1
ESYT1	1.513907	1.488392	1.522202	1.06923	0.0966	0.712219	1
TMEM266	1.033557	1.012186	1.040503	3.32367	1.7328	0.712331	1
MAOB	1.10709	1.128585	1.100103	0.7785	-0.3612	0.71235	1
GAN	1.385833	1.409286	1.378209	0.92407	-0.1139	0.712392	1
ZNF74	1.242723	1.219499	1.250272	1.1402	0.1893	0.712411	1
STKLD1	1.065379	1.043764	1.072406	1.65448	0.7264	0.71243	1
RNF183	1.02659	1.047319	1.019852	0.41954	-1.2531	0.712488	1
HMGN3-AS1	1.050803	1.029212	1.057821	1.97932	0.985	0.712829	1
SV2B	1.023142	1.043839	1.016415	0.37444	-1.4172	0.712936	1
ZBTB8A	1.253245	1.230045	1.260787	1.13363	0.181	0.712982	1
STK3	1.419211	1.394825	1.427138	1.08184	0.1135	0.713051	1
CYB5RL	1.112965	1.090988	1.120108	1.32004	0.4006	0.713184	1
ZFAT	1.061818	1.040239	1.068832	1.71056	0.7745	0.713235	1
BTBD1	1.249826	1.226404	1.257439	1.13708	0.1853	0.713286	1
RP6-24A23.3	1.049994	1.028604	1.056947	1.99089	0.9934	0.713379	1
IFIT3	1.07652	1.054694	1.083615	1.52879	0.6124	0.713491	1
ZNF17	1.062723	1.04115	1.069735	1.69466	0.761	0.713502	1
NDUFB1	5.076983	5.15278	5.052345	0.97582	-0.0353	0.71362	1
RP11-87C12.5	1.036998	1.015698	1.043921	2.79788	1.4843	0.713702	1
PTPRR	1.090206	1.111145	1.0834	0.75037	-0.4143	0.714115	1
NDUFAF1	1.181195	1.158523	1.188564	1.18951	0.2504	0.714124	1
SNAI3-AS1	1.087055	1.065326	1.094118	1.44075	0.5268	0.71418	1
SLC35C2	1.635739	1.661225	1.627455	0.94893	-0.0756	0.714195	1
OGDHL	1.043001	1.063861	1.03622	0.56717	-0.8181	0.714199	1
B4GALT3	1.491586	1.516209	1.483582	0.93679	-0.0942	0.714261	1
EIF3C	1.079556	1.057846	1.086614	1.49733	0.5824	0.714265	1
CKLF	1.814897	1.787434	1.823825	1.04621	0.0652	0.714486	1

RP11-156E8.1	1.052588	1.073451	1.045806	0.62363	-0.6812	0.714518	1
GPD1L	1.057824	1.078861	1.050986	0.64653	-0.6292	0.714534	1
PSMB1	5.028892	5.091866	5.008422	0.97961	-0.0297	0.714575	1
MEDAG	1.03397	1.054483	1.027302	0.5011	-0.9968	0.714624	1
ALKBH1	1.129062	1.106942	1.136252	1.27408	0.3495	0.714662	1
PAXIP1	1.143057	1.121042	1.150213	1.241	0.3115	0.714719	1
PTGES3L	1.103384	1.125014	1.096352	0.77073	-0.3757	0.714766	1
TUSC1	1.225861	1.202613	1.233417	1.15203	0.2042	0.714779	1
SRGAP2	1.167938	1.14571	1.175163	1.20213	0.2656	0.714897	1
IST1	1.873725	1.900916	1.864887	0.96001	-0.0589	0.714908	1
LBX2	1.098064	1.076298	1.105139	1.37801	0.4626	0.714926	1
ZNF787	1.083384	1.104568	1.076498	0.73156	-0.451	0.714926	1
BMP3	1.022453	1.042952	1.01579	0.36761	-1.4438	0.714934	1
SMCO3	1.022435	1.042877	1.01579	0.36825	-1.4412	0.714934	1
ARMC7	1.130144	1.108059	1.137323	1.27081	0.3457	0.714937	1
PSMB4	1.965884	1.993656	1.956856	0.96296	-0.0544	0.715101	1
IRF7	1.20899	1.231078	1.20181	0.87334	-0.1954	0.715147	1
ROR2	1.084289	1.105384	1.077431	0.73475	-0.4447	0.715266	1
RP11-22A3.2	1.043177	1.021905	1.050092	2.28681	1.1933	0.715341	1
B4GALNT4	1.324495	1.347505	1.317015	0.91226	-0.1325	0.715408	1
LINC01099	1.056156	1.034794	1.0631	1.81356	0.8588	0.715455	1
AP001062.7	1.045723	1.066446	1.038986	0.58673	-0.7692	0.715469	1
RP13-463N16.6	1.023348	1.043842	1.016686	0.38059	-1.3937	0.715516	1
BNC1	1.023377	1.043963	1.016686	0.37954	-1.3977	0.715516	1
RNF41	1.426491	1.450148	1.418801	0.93036	-0.1041	0.715557	1
VWCE	1.083091	1.061318	1.090168	1.4705	0.5563	0.71559	1
BSPRY	1.023114	1.043536	1.016476	0.37846	-1.4018	0.715657	1
FZD10	1.023154	1.043675	1.016484	0.37742	-1.4058	0.715657	1
GOPC	1.582045	1.606563	1.574075	0.94644	-0.0794	0.715675	1
SACM1L	1.300555	1.323202	1.293193	0.90715	-0.1406	0.715736	1
PITPNM2	1.042675	1.063321	1.035964	0.56796	-0.8161	0.715761	1
INSL3	1.047446	1.068094	1.040735	0.59821	-0.7413	0.715837	1
ALDH1B1	1.170104	1.191882	1.163024	0.8496	-0.2351	0.715906	1
ZNF773	1.064816	1.043537	1.071733	1.64762	0.7204	0.716012	1
AMPD3	1.102499	1.080676	1.109593	1.35843	0.4419	0.716013	1
XXbac-BPG32J3.19	1.040549	1.019351	1.047439	2.45151	1.2937	0.716036	1
ANKRD22	1.019739	1.040283	1.013062	0.32425	-1.6248	0.716101	1
RP3-428L16.2	1.123774	1.144828	1.11693	0.80737	-0.3087	0.716135	1
FGF12	1.039284	1.060092	1.03252	0.54116	-0.8859	0.71624	1
SGPP2	1.038881	1.059267	1.032254	0.54421	-0.8778	0.716424	1
CTD-2035E11.5	1.038041	1.016781	1.044952	2.67867	1.4215	0.716435	1
NCKIPSD	1.19703	1.218683	1.189992	0.8688	-0.2029	0.716447	1
ZNF20	1.054957	1.033556	1.061914	1.84506	0.8837	0.716535	1
LENG8-AS1	1.071911	1.050413	1.078899	1.56507	0.6462	0.716572	1
ULK2	1.26584	1.242653	1.273378	1.12662	0.172	0.716649	1

MMP24	1.035764	1.056179	1.029128	0.51849	-0.9476	0.716721	1
PIK3R2	1.094075	1.072248	1.10117	1.40031	0.4857	0.716798	1
PRR16	1.034349	1.054944	1.027655	0.50332	-0.9904	0.716856	1
PRR7-AS1	1.086503	1.065172	1.093437	1.43369	0.5197	0.716911	1
ZNF669	1.156741	1.134563	1.16395	1.21839	0.285	0.716928	1
QRSL1	1.250207	1.227114	1.257713	1.13473	0.1824	0.717013	1
TSPOAP1	1.156778	1.178189	1.149819	0.84078	-0.2502	0.717116	1
RP11-141M1.3	1.03653	1.015453	1.043381	2.80722	1.4891	0.717196	1
STRN	1.154153	1.131861	1.161399	1.22401	0.2916	0.71741	1
CRYBG3	1.201176	1.178284	1.208618	1.17014	0.2267	0.717414	1
MTG1	1.196368	1.173725	1.203728	1.17271	0.2298	0.717466	1
RP11-55K13.1	1.041164	1.020062	1.048024	2.39377	1.2593	0.717466	1
WDR31	1.100558	1.078838	1.107619	1.36506	0.449	0.717479	1
RANBP10	1.100533	1.078846	1.107582	1.36445	0.4483	0.717479	1
SLC24A1	1.07599	1.054597	1.082944	1.51919	0.6033	0.717487	1
ZBTB11	1.155701	1.177353	1.148663	0.83823	-0.2546	0.717508	1
VPS26A	1.7124	1.739455	1.703606	0.95152	-0.0717	0.717559	1
RP11-586K2.1	1.053638	1.032503	1.060508	1.86161	0.8966	0.717569	1
RAB17	1.033586	1.054245	1.026871	0.49537	-1.0134	0.717585	1
GS1-124K5.11	1.086163	1.064682	1.093145	1.44005	0.5261	0.717625	1
RP11-382D12.2	1.05423	1.033085	1.061103	1.84687	0.8851	0.71773	1
PDZD2	1.027355	1.047727	1.020733	0.43442	-1.2029	0.717737	1
DCAF8	1.48524	1.460008	1.493441	1.07268	0.1012	0.717747	1
NFATC4	1.417286	1.393227	1.425107	1.08107	0.1125	0.717749	1
VARS	1.417426	1.393137	1.425322	1.08187	0.1135	0.717749	1
CSGALNACT2	1.169165	1.146777	1.176443	1.20211	0.2656	0.7178	1
SLC17A9	1.068355	1.089014	1.061639	0.69247	-0.5302	0.717858	1
ARHGAP26	1.075252	1.053876	1.0822	1.52573	0.6095	0.71791	1
LPCAT3	1.200331	1.222122	1.193248	0.87001	-0.2009	0.71798	1
AC240274.1	1.097475	1.075888	1.104492	1.37693	0.4615	0.718101	1
KIAA0930	1.190471	1.211861	1.183518	0.86622	-0.2072	0.718157	1
CCDC86	1.19938	1.221204	1.192286	0.86927	-0.2021	0.718215	1
WASIR2	1.064469	1.043226	1.071375	1.65121	0.7235	0.718296	1
SH2D3C	1.042811	1.021779	1.049647	2.27953	1.1887	0.718319	1
ANKRD16	1.127913	1.105885	1.135073	1.27566	0.3512	0.718428	1
ADGRB2	1.191958	1.169402	1.19929	1.17644	0.2344	0.718446	1
LINC01106	1.026593	1.046787	1.020029	0.42809	-1.224	0.718464	1
MTX2	1.890987	1.862802	1.900149	1.04329	0.0611	0.718821	1
RP11-70P17.1	1.068044	1.046858	1.07493	1.5991	0.6773	0.718875	1
RP11-446N19.1	1.068251	1.046857	1.075206	1.605	0.6826	0.718875	1
RBAK	1.257822	1.234703	1.265337	1.13052	0.177	0.718923	1
DENND4B	1.181447	1.158989	1.188747	1.18717	0.2475	0.718961	1
SF3B3	1.650229	1.624505	1.658591	1.05458	0.0767	0.719032	1
FPGT-TNNI3K	1.044764	1.023786	1.051583	2.16859	1.1168	0.719175	1
RP11-218F10.3	1.046907	1.026016	1.053697	2.06397	1.0454	0.719232	1

C9orf43	1.034563	1.013691	1.041347	3.02009	1.5946	0.719351	1
BCL2L11	1.208802	1.186518	1.216046	1.15831	0.212	0.719541	1
FRMD5	1.08613	1.106793	1.079413	0.74362	-0.4274	0.719843	1
TRAPPC10	1.10177	1.122703	1.094966	0.77394	-0.3697	0.719931	1
ZNF532	1.475093	1.498986	1.467326	0.93655	-0.0946	0.719932	1
ADAMTS20	1.048959	1.028004	1.055771	1.99154	0.9939	0.719967	1
TBPL1	1.670801	1.69539	1.662808	0.95315	-0.0692	0.720021	1
RP11-395B7.4	1.075296	1.054012	1.082215	1.52217	0.6061	0.720074	1
TMEM135	1.176024	1.15401	1.18318	1.18941	0.2502	0.720079	1
PNPO	1.067282	1.046035	1.074188	1.61156	0.6885	0.720086	1
ARHGEF7	1.310593	1.333226	1.303237	0.91	-0.1361	0.720093	1
TMEM106A	1.092566	1.071129	1.099535	1.39936	0.4848	0.720141	1
MFAP3	1.071643	1.050486	1.078521	1.5553	0.6372	0.720194	1
NRXN3	1.048269	1.068526	1.041685	0.60831	-0.7171	0.720207	1
PIGW	1.0799	1.058636	1.086812	1.48053	0.5661	0.720222	1
FCF1	1.222883	1.244845	1.215744	0.88114	-0.1825	0.720483	1
NELFA	1.407442	1.383917	1.415089	1.08119	0.1126	0.720698	1
SLC25A33	1.358475	1.335315	1.366003	1.09152	0.1263	0.720845	1
MRM3	1.145216	1.12362	1.152235	1.23148	0.3004	0.720845	1
C6orf120	1.422555	1.445478	1.415104	0.93182	-0.1019	0.720878	1
BOK	1.267393	1.28915	1.260321	0.9003	-0.1515	0.720913	1
C1RL-AS1	1.0717	1.092229	1.065027	0.70505	-0.5042	0.720915	1
CUL7	1.204174	1.181638	1.2115	1.16441	0.2196	0.720922	1
BBS7	1.388776	1.41168	1.381331	0.92628	-0.1105	0.720945	1
CARD10	1.040181	1.060381	1.033615	0.55672	-0.845	0.721134	1
TCEANC	1.050573	1.029702	1.057357	1.93111	0.9494	0.721288	1
FBXO31	1.097067	1.075847	1.103964	1.3707	0.4549	0.721413	1
MBD6	1.209596	1.231232	1.202563	0.87602	-0.191	0.721451	1
NUP133	1.253207	1.275357	1.246007	0.89341	-0.1626	0.721467	1
IDNK	1.147367	1.125513	1.15447	1.23071	0.2995	0.721496	1
RP11-977G19.11	1.07379	1.052766	1.080624	1.52796	0.6116	0.721589	1
KLK1	1.037913	1.058029	1.031374	0.54065	-0.8872	0.72162	1
DCBLD1	1.107597	1.128169	1.100911	0.78733	-0.345	0.721686	1
SHOC2	1.769659	1.795466	1.76127	0.95701	-0.0634	0.72179	1
PHC2	1.447197	1.422536	1.455213	1.07734	0.1075	0.721901	1
CTB-186G2.1	1.034433	1.054505	1.027908	0.51202	-0.9657	0.722093	1
TMCO4	1.028379	1.048507	1.021837	0.45017	-1.1514	0.722167	1
FLVCR1	1.328417	1.350669	1.321184	0.91592	-0.1267	0.72226	1
AP001596.6	1.032555	1.052615	1.026034	0.49481	-1.015	0.722263	1
RP11-467J12.4	1.032504	1.052487	1.026008	0.49551	-1.013	0.722263	1
NDNF	1.030761	1.050868	1.024224	0.47622	-1.0703	0.7223	1
RASAL2-AS1	1.053203	1.032461	1.059946	1.84671	0.885	0.72239	1
GHRH	1.036388	1.015676	1.043121	2.75083	1.4599	0.722447	1
CDK9	1.3521	1.374781	1.344728	0.91981	-0.1206	0.722534	1
TDP2	1.333264	1.309658	1.340937	1.10101	0.1388	0.722537	1

MATN3	1.030907	1.050872	1.024417	0.47998	-1.059	0.722567	1
RP11-502I4.3	1.050357	1.029495	1.057138	1.93723	0.954	0.722697	1
SCRN1	1.768608	1.794203	1.760289	0.9573	-0.063	0.722745	1
RP11-467L13.7	1.08986	1.068636	1.096759	1.40975	0.4954	0.722821	1
CTAGE5	1.150082	1.171047	1.143267	0.83759	-0.2557	0.722838	1
GIGYF2	1.490099	1.465152	1.498208	1.07106	0.099	0.722879	1
CCDC69	1.062267	1.082557	1.055672	0.67435	-0.5684	0.722887	1
RP11-481J2.4	1.068013	1.047035	1.074833	1.59101	0.6699	0.723056	1
UBE2K	2.031046	2.058419	2.022148	0.96573	-0.0503	0.723143	1
RAB33B	1.08913	1.067974	1.096007	1.41241	0.4982	0.723219	1
SENP3	1.089109	1.067935	1.095992	1.41301	0.4988	0.723219	1
NAA30	1.324496	1.346769	1.317256	0.91489	-0.1283	0.723335	1
PTGS2	1.021284	1.041148	1.014827	0.36035	-1.4725	0.723355	1
FABP6	1.040086	1.019458	1.046791	2.40479	1.2659	0.723357	1
LINC00575	1.040144	1.019448	1.046871	2.41011	1.2691	0.723357	1
SPG11	1.346317	1.322561	1.354039	1.09759	0.1343	0.723556	1
NCOA6	1.282668	1.304593	1.275541	0.90462	-0.1446	0.723816	1
NCOA5	1.135319	1.113711	1.142343	1.2518	0.324	0.723862	1
GPANK1	1.679323	1.653909	1.687584	1.0515	0.0724	0.72395	1
HHAT	1.145572	1.166486	1.138773	0.83354	-0.2627	0.724041	1
TRIB2	1.597746	1.62247	1.58971	0.94737	-0.078	0.724052	1
MCPH1-AS1	1.081919	1.061023	1.088711	1.45375	0.5398	0.724125	1
MAG	1.045633	1.024972	1.052349	2.0963	1.0678	0.724244	1
DAP3	1.758807	1.732476	1.767367	1.04763	0.0671	0.724284	1
HENMT1	1.107489	1.086317	1.114372	1.32502	0.406	0.724295	1
FMOD	1.020138	1.039967	1.013692	0.34258	-1.5455	0.724418	1
MRPL14	3.95516	3.893715	3.975133	1.02814	0.04	0.724434	1
RGAG4	1.139354	1.117857	1.146341	1.24169	0.3123	0.72448	1
ATF7	1.227612	1.205476	1.234808	1.14275	0.1925	0.724543	1
LINC00664	1.032682	1.012186	1.039345	3.22857	1.6909	0.724592	1
ATF7IP2	1.314357	1.336564	1.307139	0.91257	-0.132	0.724698	1
ZNF667-AS1	1.53214	1.50768	1.540091	1.06384	0.0893	0.724715	1
PNMT	1.048588	1.068992	1.041956	0.60813	-0.7176	0.724733	1
PIGO	1.103145	1.12374	1.096451	0.77946	-0.3594	0.724799	1
GFPT2	1.104494	1.125009	1.097826	0.78255	-0.3537	0.724804	1
RP11-977B10.2	1.035427	1.014881	1.042105	2.82942	1.5005	0.724866	1
KLK10	1.027444	1.047301	1.02099	0.44374	-1.1722	0.724911	1
CTD-3074O7.5	1.1247	1.103407	1.131622	1.27285	0.3481	0.725115	1
GRIPAP1	1.590286	1.565186	1.598445	1.05885	0.0825	0.725146	1
SNCAIP	1.048803	1.068914	1.042266	0.61332	-0.7053	0.725181	1
UTP20	1.098064	1.118708	1.091353	0.76956	-0.3779	0.72522	1
TAC1	1.024017	1.043846	1.017572	0.40076	-1.3192	0.725382	1
RP11-347C12.10	1.064697	1.043917	1.071452	1.62697	0.7022	0.725544	1
WDR83OS	4.396195	4.325805	4.419076	1.02804	0.0399	0.725544	1
TUSC2	2.09379	2.064755	2.103228	1.03613	0.0512	0.725688	1

NUB1	1.426064	1.449118	1.41857	0.93198	-0.1016	0.725713	1
C9orf135-AS1	1.042341	1.021883	1.048992	2.23877	1.1627	0.725737	1
DMPK	1.163713	1.184784	1.156863	0.8489	-0.2363	0.725822	1
C19orf44	1.079061	1.058085	1.08588	1.47852	0.5642	0.725843	1
CDH4	1.093987	1.073236	1.100733	1.37546	0.4599	0.725875	1
LOXL3	1.228161	1.206243	1.235285	1.14081	0.1901	0.725876	1
C2orf73	1.042976	1.022509	1.049628	2.20486	1.1407	0.725901	1
RP11-352G9.1	1.036137	1.015691	1.042784	2.72663	1.4471	0.725951	1
PHRF1	1.143973	1.164609	1.137265	0.83389	-0.2621	0.725975	1
HSF4	1.194327	1.172618	1.201384	1.16665	0.2224	0.725984	1
TACC2	1.22553	1.246655	1.218664	0.88652	-0.1738	0.726049	1
KATNBL1	2.218885	2.249727	2.20886	0.9673	-0.048	0.726088	1
CA11	1.823864	1.796882	1.832634	1.04486	0.0633	0.726247	1
ZSCAN20	1.053273	1.032804	1.059927	1.82682	0.8693	0.726346	1
BCAR3	1.152955	1.173901	1.146147	0.8404	-0.2508	0.726569	1
PRRT3-AS1	1.032519	1.012187	1.039128	3.21068	1.6829	0.726728	1
IQCC	1.097329	1.076447	1.104117	1.36194	0.4457	0.726839	1
LETM1	1.335996	1.358087	1.328815	0.91826	-0.123	0.726876	1
RP1-101K10.6	1.097366	1.076359	1.104194	1.36453	0.4484	0.726947	1
ATP6V0A2	1.137157	1.157605	1.13051	0.82809	-0.2721	0.726962	1
ZNF398	1.10327	1.082264	1.110098	1.33836	0.4205	0.726973	1
DFFB	1.058418	1.078452	1.051906	0.66163	-0.5959	0.727073	1
CS	1.687666	1.661468	1.696182	1.05248	0.0738	0.727132	1
COLCA2	1.046469	1.026037	1.053111	2.03982	1.0284	0.72724	1
P4HTM	1.779199	1.753368	1.787596	1.04543	0.0641	0.727252	1
VANGL1	1.121342	1.141774	1.114701	0.80904	-0.3057	0.72727	1
AC006994.2	1.033591	1.013274	1.040196	3.02812	1.5984	0.727335	1
NFKBIB	1.181976	1.160286	1.189027	1.17931	0.2379	0.727418	1
FAM72C	1.04223	1.021935	1.048827	2.22598	1.1544	0.727579	1
BRIX1	1.448511	1.425728	1.455916	1.07091	0.0988	0.727621	1
LCLAT1	1.131577	1.110538	1.138416	1.25221	0.3245	0.727691	1
STPG1	1.131623	1.110479	1.138496	1.25359	0.3261	0.727691	1
ATL2	1.220247	1.198743	1.227238	1.14338	0.1933	0.727706	1
FGF5	1.033576	1.053257	1.027178	0.51032	-0.9705	0.727966	1
CAPN5	1.077349	1.097443	1.070817	0.72676	-0.4605	0.727999	1
APLF	1.077269	1.097177	1.070798	0.72855	-0.4569	0.727999	1
GRPEL2	1.118903	1.139258	1.112287	0.80632	-0.3106	0.728048	1
KIAA1024	1.035962	1.015685	1.042553	2.71297	1.4399	0.728091	1
PIK3CA	1.253573	1.275206	1.246541	0.89584	-0.1587	0.728118	1
PAGR1	1.237703	1.215675	1.244863	1.13533	0.1831	0.72817	1
HVCN1	1.06874	1.048143	1.075436	1.56691	0.6479	0.728388	1
LCP1	1.024484	1.044042	1.018127	0.41158	-1.2808	0.728443	1
TMEM33	1.643176	1.667188	1.635371	0.95231	-0.0705	0.728523	1
SULF2	1.324966	1.346981	1.31781	0.91593	-0.1267	0.728566	1
DROSHA	1.241492	1.262733	1.234588	0.89287	-0.1635	0.728587	1

FAM65B	1.036991	1.016781	1.04356	2.59578	1.3762	0.728699	1
CNOT4	1.727337	1.701563	1.735716	1.04868	0.0686	0.728784	1
SWAP70	1.19825	1.218964	1.191517	0.87465	-0.1932	0.728788	1
ZNF440	1.089899	1.110045	1.08335	0.75742	-0.4008	0.728852	1
MAST4-AS1	1.045739	1.025401	1.052351	2.06098	1.0433	0.72893	1
ANXA8L1	1.045707	1.025389	1.052312	2.06046	1.043	0.72893	1
MPRIP-AS1	1.058877	1.038325	1.065558	1.71058	0.7745	0.728966	1
SPTSSA	2.353182	2.387386	2.342064	0.96733	-0.0479	0.729154	1
TNFRSF13C	1.053665	1.033277	1.060293	1.81182	0.8574	0.72917	1
TCEAL4	7.468307	7.363441	7.502394	1.02184	0.0312	0.729315	1
DNTTIP2	1.366559	1.343822	1.37395	1.08763	0.1212	0.729406	1
TBC1D12	1.188929	1.210008	1.182077	0.867	-0.2059	0.729426	1
ZDHHC16	1.498311	1.521461	1.490786	0.94117	-0.0875	0.729446	1
AC068499.10	1.039499	1.019183	1.046103	2.40328	1.265	0.72945	1
GEN1	1.138059	1.117302	1.144806	1.23448	0.3039	0.72952	1
PRDM1	1.063931	1.043553	1.070555	1.61998	0.696	0.729687	1
TMEM255B	1.067815	1.087634	1.061373	0.70034	-0.5139	0.729796	1
STIP1	1.765505	1.740769	1.773545	1.04425	0.0625	0.729856	1
NUTM2A-AS1	1.30352	1.325764	1.29629	0.90952	-0.1368	0.729911	1
REC8	1.97833	1.949385	1.987738	1.0404	0.0571	0.729912	1
ZFYVE19	1.272674	1.294148	1.265693	0.90326	-0.1468	0.730224	1
PLD3	5.784213	5.700766	5.811338	1.02352	0.0335	0.730249	1
THEMIS2	1.049249	1.028929	1.055854	1.93073	0.9491	0.730275	1
THSD7A	1.231015	1.252044	1.224179	0.88944	-0.169	0.730288	1
PAF1	1.403836	1.380959	1.411273	1.07957	0.1105	0.730424	1
SPRED2	1.150354	1.170737	1.143729	0.84181	-0.2484	0.730468	1
RP11-802E16.3	1.030935	1.0504	1.024607	0.48825	-1.0343	0.730481	1
WDR91	1.060135	1.079962	1.05369	0.67144	-0.5747	0.730539	1
RUNDC3A-AS1	1.026265	1.04581	1.019912	0.43467	-1.202	0.730658	1
RSG1	1.074726	1.054312	1.081362	1.49804	0.5831	0.730671	1
CDHR1	1.024815	1.044409	1.018446	0.41538	-1.2675	0.730679	1
SYCE3	1.04478	1.024504	1.05137	2.09638	1.0679	0.730842	1
KIAA1683	1.044711	1.024496	1.051282	2.09346	1.0659	0.730842	1
PBRM1	1.824947	1.799165	1.833327	1.04275	0.0604	0.731207	1
ARHGEF6	1.100131	1.120068	1.09365	0.77998	-0.3585	0.731338	1
DENND6A	1.20889	1.187196	1.215942	1.15356	0.2061	0.73136	1
CDK2AP1	1.202317	1.180601	1.209375	1.15933	0.2133	0.731396	1
ZNF165	1.045943	1.025913	1.052454	2.0242	1.0174	0.731453	1
PPM1F	1.187992	1.208331	1.18138	0.87064	-0.1999	0.731458	1
SLC25A30	1.05488	1.074471	1.048512	0.65142	-0.6183	0.731551	1
FAM110C	1.054209	1.073898	1.047809	0.64696	-0.6283	0.73175	1
UNC80	1.060613	1.04056	1.067131	1.65509	0.7269	0.731829	1
KCNK10	1.045339	1.025317	1.051848	2.04791	1.0341	0.731904	1
ATP8B3	1.078172	1.057947	1.084747	1.46247	0.5484	0.731995	1
OSBPL10	1.049753	1.069279	1.043406	0.62653	-0.6745	0.73201	1

CCDC43	1.454696	1.477546	1.447269	0.9366	-0.0945	0.732211	1
CBS	1.04252	1.02243	1.049051	2.18681	1.1288	0.732219	1
IFI27	1.107556	1.127666	1.10102	0.79128	-0.3377	0.732253	1
BMPR1A	1.379062	1.356126	1.386517	1.08534	0.1181	0.732421	1
RP11-806H10.4	1.179356	1.158268	1.186211	1.17655	0.2346	0.732516	1
RP11-273G15.2	1.020571	1.039931	1.014279	0.35758	-1.4837	0.732568	1
GRK2	1.134452	1.154433	1.127957	0.82856	-0.2713	0.732596	1
CIR1	1.926656	1.952177	1.918361	0.96449	-0.0522	0.732693	1
CAPN15	1.130027	1.150111	1.123498	0.82271	-0.2815	0.732846	1
RP11-615I2.6	1.056146	1.036007	1.062692	1.74111	0.8	0.732954	1
MPP2	1.180372	1.159363	1.187201	1.17468	0.2323	0.732975	1
ANK1	1.069076	1.048793	1.075669	1.55083	0.633	0.733021	1
TSPAN33	1.044537	1.063924	1.038236	0.59814	-0.7414	0.733024	1
HOMER1	1.251872	1.27313	1.244962	0.89687	-0.157	0.733035	1
FOXA3	1.045663	1.065035	1.039365	0.6053	-0.7243	0.733056	1
CLPB	1.070593	1.050218	1.077216	1.53762	0.6207	0.73309	1
AGAP6	1.070482	1.050212	1.07707	1.53488	0.6181	0.73309	1
TGFBR3	1.072673	1.092273	1.066302	0.71854	-0.4769	0.733136	1
DNAJC5	1.357405	1.378891	1.35042	0.92486	-0.1127	0.733232	1
TLCD1	1.129445	1.149461	1.122939	0.82255	-0.2818	0.733399	1
LINC01127	1.024354	1.043596	1.018099	0.41515	-1.2683	0.733485	1
SLC45A4	1.039418	1.058839	1.033105	0.56264	-0.8297	0.733491	1
SPEF1	1.074304	1.053946	1.080922	1.50005	0.585	0.733608	1
NEU3	1.073016	1.052605	1.07965	1.51411	0.5985	0.733697	1
OLFM3	1.180788	1.159705	1.187641	1.17492	0.2326	0.733721	1
YIPF4	1.927209	1.900419	1.935918	1.03943	0.0558	0.733732	1
NME9	1.063728	1.043674	1.070246	1.60843	0.6857	0.73387	1
RP3-467K16.2	1.03791	1.017948	1.044399	2.47378	1.3067	0.73387	1
VPS4B	1.434114	1.456382	1.426876	0.93535	-0.0964	0.733921	1
RP1-197B17.3	1.067707	1.047515	1.07427	1.56308	0.6444	0.734019	1
TXNL4A	2.497123	2.464822	2.507622	1.02922	0.0415	0.734027	1
NAXD	1.281158	1.259198	1.288296	1.11226	0.1535	0.734072	1
CEP120	1.150888	1.129776	1.157751	1.21557	0.2816	0.734105	1
RP11-285J16.1	1.032757	1.052222	1.02643	0.50612	-0.9825	0.73414	1
COL19A1	1.031782	1.011999	1.038213	3.18462	1.6711	0.734184	1
CTD-2623N2.11	1.035874	1.015934	1.042356	2.65816	1.4104	0.73433	1
CDAN1	1.084197	1.063875	1.090803	1.42158	0.5075	0.734374	1
ABL1	1.369124	1.3909	1.362046	0.92619	-0.1106	0.734502	1
AIFM3	1.042079	1.022202	1.048541	2.18636	1.1285	0.734504	1
NTNG2	1.08165	1.061363	1.088244	1.43806	0.5241	0.734819	1
ST3GAL4-AS1	1.140576	1.119445	1.147445	1.23442	0.3038	0.734839	1
ZNF264	1.201546	1.221919	1.194923	0.87835	-0.1871	0.734847	1
ZNF343	1.09076	1.110643	1.084297	0.76189	-0.3924	0.734875	1
AGAP5	1.055038	1.035037	1.06154	1.75643	0.8126	0.73488	1
GPATCH2	1.40221	1.379274	1.409665	1.08013	0.1112	0.735052	1

NEK8	1.110396	1.090002	1.117025	1.30025	0.3788	0.735187	1
SLC19A2	1.115764	1.135574	1.109325	0.80638	-0.3105	0.735372	1
CFAP54	1.056333	1.036554	1.062763	1.71701	0.7799	0.735472	1
NAA60	1.471222	1.493545	1.463966	0.94007	-0.0892	0.73549	1
SNAPC2	1.49939	1.475484	1.507161	1.06662	0.093	0.735603	1
ERO1B	1.153298	1.132512	1.160054	1.20785	0.2724	0.735611	1
KLF13	1.168424	1.18845	1.161914	0.85919	-0.219	0.735715	1
LIMS3	1.024722	1.043808	1.018518	0.42271	-1.2423	0.735747	1
TRIM33	1.660681	1.635996	1.668705	1.05143	0.0724	0.735827	1
CREG1	1.765893	1.740697	1.774084	1.04508	0.0636	0.736011	1
CRIPAK	1.1021	1.081704	1.10873	1.33077	0.4123	0.736015	1
RP11-279O9.4	1.075073	1.094605	1.068724	0.72643	-0.4611	0.736038	1
RASGRP2	1.059873	1.07924	1.053577	0.67614	-0.5646	0.736114	1
FXYD2	1.077017	1.05675	1.083605	1.47322	0.559	0.736172	1
ZNF358	1.431643	1.45413	1.424333	0.93439	-0.0979	0.736222	1
RP11-293A21.1	1.052975	1.033225	1.059396	1.78769	0.8381	0.736289	1
ZSCAN21	1.121596	1.101108	1.128255	1.26849	0.3431	0.736292	1
TRPC1	1.194655	1.173654	1.201482	1.16025	0.2144	0.736357	1
SPATA4	1.03452	1.053763	1.028265	0.52573	-0.9276	0.736628	1
TAF4B	1.063747	1.043762	1.070243	1.6051	0.6827	0.73666	1
RIC1	1.149798	1.170088	1.143202	0.84193	-0.2482	0.73667	1
TMPRSS5	1.038022	1.018364	1.044411	2.41833	1.274	0.736706	1
FADS3	1.191544	1.211684	1.184998	0.87393	-0.1944	0.736802	1
RTFDC1	2.184114	2.156516	2.193085	1.03162	0.0449	0.736853	1
CCDC121	1.082053	1.061839	1.088624	1.43316	0.5192	0.73695	1
ZNF347	1.125848	1.105362	1.132508	1.25765	0.3307	0.736979	1
LINC00863	1.079499	1.059395	1.086033	1.44849	0.5345	0.737038	1
HIF1A-AS2	1.074525	1.054377	1.081074	1.49096	0.5762	0.737096	1
ENAH	2.879299	2.9121	2.868637	0.97727	-0.0332	0.737328	1
ZNF646	1.088427	1.068371	1.094946	1.38869	0.4737	0.737467	1
RP11-378J18.8	1.084623	1.064665	1.09111	1.40897	0.4946	0.737491	1
GPATCH1	1.097979	1.11744	1.091653	0.78043	-0.3577	0.737577	1
DOCK8	1.027553	1.046462	1.021406	0.46073	-1.118	0.737618	1
SPRED1	1.498009	1.520591	1.490668	0.94252	-0.0854	0.737628	1
CDR1	1.074698	1.054694	1.081201	1.48465	0.5701	0.737658	1
CCDC173	1.060961	1.041214	1.067379	1.63485	0.7092	0.737782	1
RP11-209D14.2	1.051456	1.031594	1.057913	1.83305	0.8742	0.737793	1
TSNAXIP1	1.074055	1.054279	1.080483	1.48278	0.5683	0.737797	1
SRPK2	1.946902	1.919949	1.955663	1.03882	0.0549	0.737884	1
ZP3	1.125285	1.145433	1.118736	0.81643	-0.2926	0.737995	1
RALGAPB	1.297564	1.318745	1.290679	0.91195	-0.133	0.738123	1
FAM65C	1.061185	1.041585	1.067556	1.62454	0.7	0.738129	1
LPL	1.066271	1.085647	1.059973	0.70024	-0.5141	0.738187	1
PRKCD	1.091959	1.111471	1.085616	0.76806	-0.3807	0.738194	1
SREBF1	1.092051	1.111438	1.085749	0.76947	-0.3781	0.738194	1

TCAP	1.034611	1.053747	1.028391	0.52823	-0.9208	0.73848	1
KCNIP2	1.048306	1.067437	1.042087	0.6241	-0.6801	0.738519	1
TPCN2	1.109012	1.08879	1.115585	1.30178	0.3805	0.738585	1
C9orf78	1.812851	1.787673	1.821035	1.04236	0.0598	0.738625	1
ARPC4	2.694339	2.732877	2.681812	0.97053	-0.0432	0.738684	1
KCTD1	1.172264	1.192196	1.165785	0.86258	-0.2133	0.738734	1
KIF19	1.045992	1.026233	1.052415	1.99804	0.9986	0.738785	1
BTRC	1.201533	1.180533	1.208359	1.15413	0.2068	0.7388	1
ZNF225	1.063538	1.04384	1.069942	1.59538	0.6739	0.73884	1
AVEN	1.138044	1.158092	1.131528	0.83197	-0.2654	0.73884	1
BRF1	1.176349	1.155825	1.183021	1.17453	0.2321	0.738867	1
FBXO48	1.078085	1.058091	1.084585	1.45608	0.5421	0.738965	1
CTPS2	1.395926	1.417941	1.38877	0.9302	-0.1044	0.738984	1
CH17-353B19.1	1.03162	1.012187	1.037936	3.11293	1.6383	0.739079	1
CITED4	1.054414	1.07347	1.04822	0.65632	-0.6075	0.739142	1
SYTL5	1.035201	1.05422	1.029019	0.5352	-0.9018	0.739177	1
N6AMT1	1.150158	1.12973	1.156798	1.20865	0.2734	0.739277	1
B3GAT3	2.088856	2.061234	2.097835	1.03449	0.0489	0.739303	1
C1orf61	1.046071	1.026538	1.05242	1.9753	0.9821	0.73934	1
RP11-389G6.3	1.03256	1.013073	1.038895	2.97531	1.573	0.739346	1
CBX7	1.134649	1.154228	1.128285	0.83179	-0.2657	0.739356	1
RAD51D	1.21861	1.197562	1.225452	1.14117	0.1905	0.739414	1
PDCD4-AS1	1.119614	1.099577	1.126127	1.26663	0.341	0.7395	1
KIAA0922	1.045574	1.026066	1.051915	1.99165	0.994	0.739515	1
COG1	1.224135	1.202817	1.231065	1.13928	0.1881	0.739563	1
ZNF160	1.433007	1.410618	1.440285	1.07225	0.1006	0.739593	1
TRPM7	1.32706	1.304962	1.334243	1.09602	0.1323	0.739659	1
NPIPA5	1.054382	1.034737	1.060768	1.74939	0.8069	0.739832	1
ENTPD7	1.04986	1.069003	1.043637	0.63239	-0.6611	0.73984	1
MUC1	1.083295	1.102492	1.077055	0.75181	-0.4116	0.739841	1
HACD2	1.248949	1.269481	1.242275	0.89904	-0.1535	0.739907	1
FBXL17	1.226573	1.205572	1.2334	1.13537	0.1832	0.739953	1
MMP28	1.034244	1.014812	1.04056	2.73829	1.4533	0.740179	1
THRA1/BTR	1.034221	1.014803	1.040533	2.73822	1.4532	0.740179	1
RP11-770J1.3	1.068913	1.049059	1.075367	1.53624	0.6194	0.740203	1
ASXL3	1.094101	1.113472	1.087804	0.77379	-0.37	0.740512	1
ARF3	1.912306	1.937347	1.904166	0.9646	-0.052	0.740522	1
LINC00205	1.149162	1.128668	1.155824	1.21106	0.2763	0.740596	1
E2F6	1.457788	1.434603	1.465325	1.07069	0.0985	0.740647	1
TP53INP2	1.105659	1.124963	1.099384	0.79531	-0.3304	0.740974	1
PCYT1A	1.188582	1.20844	1.182127	0.87377	-0.1947	0.741035	1
POLR1A	1.137427	1.116984	1.144073	1.23156	0.3005	0.741299	1
FBXL5	1.536436	1.558616	1.529226	0.94739	-0.078	0.741499	1
ATPAF2	1.300519	1.321567	1.293678	0.91327	-0.1309	0.741506	1
EDC3	1.185003	1.205053	1.178485	0.87043	-0.2002	0.741525	1

ZBTB2	1.074201	1.054498	1.080606	1.47905	0.5647	0.741696	1
ST6GALNAC6	1.504974	1.5272	1.49775	0.94414	-0.0829	0.741741	1
RP11-324E6.10	1.038522	1.019213	1.044799	2.33174	1.2214	0.741776	1
DEDD	1.197548	1.217448	1.191079	0.87874	-0.1865	0.741824	1
ABRACL	3.048432	3.009712	3.061018	1.02553	0.0364	0.741895	1
FGF13-AS1	1.0519	1.032476	1.058214	1.79254	0.842	0.742031	1
RP11-350N15.5	1.039448	1.020093	1.045739	2.27636	1.1867	0.742044	1
KIT	1.039476	1.020111	1.045771	2.27591	1.1864	0.74215	1
HSD17B6	1.039413	1.020121	1.045684	2.27042	1.183	0.74215	1
RAB3IL1	1.061105	1.079946	1.05498	0.68772	-0.5401	0.742514	1
ELP2	1.614225	1.590367	1.62198	1.05355	0.0753	0.742537	1
TMEM41A	1.421479	1.443574	1.414297	0.934	-0.0985	0.742544	1
AC007228.11	1.072931	1.053344	1.079298	1.48655	0.572	0.742545	1
FAM229A	1.179525	1.199106	1.173161	0.86969	-0.2014	0.742547	1
RBBP9	1.205924	1.185394	1.212597	1.14673	0.1975	0.742629	1
GALNT5	1.030567	1.049161	1.024522	0.49882	-1.0034	0.742677	1
DISP3	1.03089	1.011717	1.037122	3.16813	1.6636	0.742772	1
RP11-38L15.2	1.087335	1.067622	1.093742	1.38628	0.4712	0.742901	1
EDA	1.046009	1.064821	1.039894	0.61546	-0.7003	0.742906	1
SLC25A14	1.260987	1.239946	1.267827	1.1162	0.1586	0.74298	1
COMMD2	1.6535	1.629438	1.661321	1.05065	0.0713	0.742992	1
AC016549.1	1.098891	1.117982	1.092685	0.78559	-0.3482	0.742996	1
CNKSR2	1.115793	1.095675	1.122332	1.27862	0.3546	0.743059	1
IGHMBP2	1.115898	1.095954	1.12238	1.27541	0.351	0.743059	1
SREBF2	1.512964	1.490058	1.520409	1.06193	0.0867	0.743149	1
NUDT8	1.410014	1.388236	1.417093	1.07433	0.1034	0.743243	1
HEY2	1.025684	1.044453	1.019583	0.44053	-1.1827	0.743243	1
NSMF	1.468542	1.445727	1.475958	1.06782	0.0947	0.743314	1
MATR3	1.045691	1.026327	1.051985	1.97459	0.9816	0.743327	1
RBM11	1.099325	1.079437	1.105789	1.33173	0.4133	0.74337	1
TYW1	1.142059	1.122261	1.148495	1.21458	0.2805	0.74358	1
ZNF525	1.109176	1.089327	1.115629	1.29444	0.3723	0.743725	1
CDA	1.023719	1.042488	1.017618	0.41467	-1.27	0.743742	1
AP000487.5	1.076451	1.056919	1.0828	1.45471	0.5407	0.743856	1
RP11-324I22.4	1.064702	1.045257	1.071022	1.56929	0.6501	0.743864	1
OSBPL11	1.310126	1.288586	1.317127	1.0989	0.1361	0.743874	1
RP11-296O14.3	1.066282	1.046769	1.072625	1.55286	0.6349	0.743922	1
MUS81	1.429867	1.407356	1.437185	1.07323	0.102	0.743981	1
MBNL3	1.032783	1.013691	1.038989	2.84784	1.5099	0.744028	1
RP11-20D14.6	1.034366	1.015203	1.040596	2.67023	1.417	0.744131	1
C2CD3	1.079164	1.059604	1.085522	1.43484	0.5209	0.744151	1
COPRS	2.143661	2.117326	2.152221	1.03123	0.0444	0.744342	1
CBWD2	1.413596	1.391439	1.420798	1.075	0.1043	0.744343	1
ENGASE	1.110323	1.09054	1.116753	1.28952	0.3668	0.744359	1
GSC	1.019915	1.038536	1.013862	0.3597	-1.4751	0.744408	1

PTPRN	1.095524	1.075736	1.101956	1.3462	0.4289	0.744412	1
TNNT3	1.035631	1.054346	1.029547	0.54368	-0.8792	0.744474	1
KRBOX4	1.211368	1.231425	1.204849	0.88516	-0.176	0.744481	1
RP11-165A20.3	1.041641	1.022489	1.047866	2.12842	1.0898	0.74456	1
GSS	1.397521	1.375319	1.404738	1.07838	0.1089	0.744577	1
MOGS	1.213571	1.23322	1.207184	0.88837	-0.1708	0.74464	1
PSMD5	1.240013	1.219161	1.246791	1.12607	0.1713	0.744672	1
FAM124A	1.049697	1.068262	1.043663	0.63964	-0.6447	0.744675	1
RP11-462G2.2	1.036652	1.017512	1.042873	2.44824	1.2917	0.744696	1
AC008088.4	1.036669	1.017512	1.042897	2.44958	1.2925	0.744696	1
TMEM69	1.464897	1.487109	1.457677	0.93958	-0.0899	0.744726	1
ZBBX	1.046345	1.027106	1.052598	1.94046	0.9564	0.744831	1
CHM	1.218795	1.19821	1.225487	1.13762	0.186	0.744849	1
PKIA	1.451913	1.429256	1.459278	1.06994	0.0975	0.744937	1
RP11-44N11.2	1.04853	1.029408	1.054746	1.86158	0.8965	0.745082	1
BTBD6	1.048072	1.066735	1.042005	0.62942	-0.6679	0.745153	1
IFIT5	1.227362	1.247536	1.220805	0.89201	-0.1649	0.745181	1
PRKCG	1.068316	1.04911	1.074559	1.51821	0.6024	0.7452	1
TAPT1	1.112554	1.131785	1.106302	0.80664	-0.31	0.745268	1
BEND7	1.12899	1.148095	1.12278	0.82906	-0.2705	0.745337	1
MYH15	1.037815	1.01874	1.044015	2.34875	1.2319	0.745486	1
ZKSCAN3	1.094775	1.075043	1.101189	1.34841	0.4313	0.745541	1
FBXO30	1.129667	1.148781	1.123454	0.82977	-0.2692	0.74581	1
LINC00449	1.045143	1.025964	1.051377	1.97877	0.9846	0.745908	1
STRAP	4.298981	4.245178	4.31647	1.02197	0.0314	0.745975	1
CARS	1.31168	1.290253	1.318644	1.09782	0.1346	0.746139	1
SLC35E2	1.130904	1.111075	1.13735	1.23655	0.3063	0.746163	1
PRMT3	1.11801	1.09841	1.124381	1.2639	0.3379	0.746327	1
SULT1C4	1.474168	1.451117	1.481661	1.06771	0.0945	0.746342	1
CDC16	1.578091	1.600301	1.570871	0.95098	-0.0725	0.746369	1
ZNF564	1.083342	1.063927	1.089653	1.40243	0.4879	0.746466	1
BTBD9	1.071729	1.052384	1.078018	1.48933	0.5747	0.746531	1
PSEN1	1.350019	1.328232	1.357101	1.08795	0.1216	0.746659	1
ARHGAP18	1.199558	1.219255	1.193156	0.88096	-0.1828	0.746669	1
VPS18	1.122759	1.141614	1.11663	0.82357	-0.28	0.746833	1
RP11-128A17.1	1.051436	1.032347	1.057641	1.78196	0.8335	0.746864	1
OGFOD1	1.240918	1.261573	1.234204	0.89537	-0.1594	0.746905	1
LURAP1	1.076979	1.095581	1.070933	0.74213	-0.4303	0.746951	1
POMT2	1.180159	1.15995	1.186728	1.16741	0.2233	0.747041	1
TNFRSF11B	1.036153	1.054355	1.030236	0.55626	-0.8462	0.747054	1
HLA-DPA1	1.07682	1.057359	1.083146	1.44958	0.5356	0.747068	1
COL24A1	1.042139	1.023107	1.048326	2.09139	1.0645	0.747117	1
MTSS1	1.15879	1.138699	1.165321	1.19195	0.2533	0.747278	1
MIEF1	1.349725	1.370346	1.343021	0.92622	-0.1106	0.747525	1
RP11-88I18.2	1.119561	1.099945	1.125937	1.26007	0.3335	0.747564	1

EFCAB5	1.044892	1.025781	1.051104	1.9822	0.9871	0.74769	1
TRIM23	1.152785	1.132673	1.159323	1.20086	0.2641	0.747715	1
MRPS23	1.737058	1.760833	1.72933	0.95859	-0.061	0.747752	1
COLEC11	1.033795	1.014833	1.039958	2.69398	1.4297	0.747772	1
HSD17B1	1.115858	1.096374	1.122192	1.26788	0.3424	0.747894	1
MDGA2	1.032198	1.050451	1.026265	0.5206	-0.9417	0.747968	1
ODF3B	1.14798	1.12778	1.154546	1.20948	0.2744	0.748192	1
GLTSCR1L	1.075933	1.09452	1.069891	0.73943	-0.4355	0.748305	1
BCDIN3D-AS1	1.055793	1.036901	1.061933	1.67835	0.747	0.748442	1
SLC4A7	1.882265	1.857457	1.890329	1.03834	0.0543	0.748471	1
CRMP1	1.299862	1.278171	1.306913	1.10333	0.1419	0.748497	1
ABCA4	1.023035	1.041253	1.017113	0.41483	-1.2694	0.748563	1
PIANP	1.290694	1.269838	1.297474	1.10242	0.1407	0.748588	1
IGIP	1.038834	1.057262	1.032844	0.57358	-0.8019	0.748634	1
SEMA6B	1.105733	1.124493	1.099635	0.80033	-0.3213	0.748678	1
CHKB-AS1	1.056647	1.037621	1.062831	1.67009	0.7399	0.748713	1
YOD1	1.165077	1.184331	1.158819	0.8616	-0.2149	0.748777	1
PRPF3	1.223426	1.202781	1.230137	1.1349	0.1826	0.748802	1
LINC01125	1.056609	1.037605	1.062786	1.66964	0.7395	0.74882	1
RP11-397A16.1	1.043924	1.024984	1.050081	2.00451	1.0033	0.748918	1
KDF1	1.019608	1.037823	1.013687	0.36187	-1.4665	0.748982	1
SYAP1	2.906878	2.941174	2.895729	0.97659	-0.0342	0.749052	1
ZFYVE1	1.147089	1.166143	1.140896	0.84804	-0.2378	0.749066	1
AMTN	1.019534	1.037575	1.01367	0.36379	-1.4588	0.749088	1
FAM63A	1.124009	1.104524	1.130343	1.24701	0.3185	0.749115	1
PIK3C2G	1.02236	1.040613	1.016426	0.40446	-1.3059	0.749147	1
FLJ35934	1.078686	1.059599	1.084891	1.42435	0.5103	0.749181	1
CACNA1C	1.06966	1.088208	1.063631	0.72137	-0.4712	0.749237	1
LINC00457	1.020456	1.038582	1.014564	0.37749	-1.4055	0.749318	1
C1orf116	1.020438	1.038508	1.014564	0.37822	-1.4027	0.749318	1
CCL20	1.018659	1.036665	1.012806	0.34927	-1.5176	0.749355	1
WDR45B	2.780264	2.74171	2.792796	1.02933	0.0417	0.74978	1
LRFN5	1.068158	1.086591	1.062167	0.71793	-0.4781	0.749785	1
RNF122	1.072734	1.053537	1.078975	1.47514	0.5609	0.74989	1
RNF144B	1.056183	1.074425	1.050254	0.67523	-0.5665	0.749975	1
KIAA1033	1.396215	1.374329	1.403329	1.07747	0.1076	0.75009	1
FCHO1	1.193636	1.173468	1.200191	1.15405	0.2067	0.750214	1
STXBP3	1.470645	1.492267	1.463617	0.9418	-0.0865	0.750399	1
CFAP126	1.038844	1.05698	1.032949	0.57826	-0.7902	0.750499	1
LCAT	1.042931	1.061058	1.037038	0.60661	-0.7212	0.750673	1
RP5-943J3.2	1.034405	1.015681	1.040491	2.58212	1.3686	0.750681	1
LRRC29	1.09188	1.110273	1.085901	0.77899	-0.3603	0.750762	1
KLHL29	1.09181	1.110304	1.085799	0.77784	-0.3625	0.750762	1
RAVER1	1.050089	1.031288	1.0562	1.79619	0.8449	0.750793	1
ARV1	1.248676	1.228817	1.255132	1.115	0.157	0.750856	1

PLA2G12A	2.154796	2.180072	2.14658	0.97162	-0.0415	0.750864	1
ANAPC10	1.419584	1.398311	1.426499	1.07077	0.0986	0.750884	1
SYN1	1.077165	1.058244	1.083315	1.43046	0.5165	0.751147	1
SLC6A20	1.02152	1.039513	1.015672	0.39662	-1.3342	0.751232	1
FAM120AOS	1.566478	1.588154	1.559432	0.95117	-0.0722	0.751252	1
CLEC7A	1.018833	1.036779	1.013	0.35346	-1.5004	0.751508	1
LINC01134	1.032644	1.013947	1.038721	2.77641	1.4732	0.751544	1
SRP54	1.67339	1.696172	1.665985	0.95664	-0.064	0.751802	1
BEST4	1.086914	1.067903	1.093094	1.37099	0.4552	0.75183	1
FOXD2-AS1	1.031664	1.013102	1.037698	2.87737	1.5248	0.75188	1
PRSS56	1.031639	1.013102	1.037665	2.87484	1.5235	0.75188	1
GLDC	1.097974	1.078863	1.104186	1.3211	0.4017	0.751897	1
SIX4	1.112331	1.130866	1.106306	0.81233	-0.2999	0.752048	1
PHF21A	1.506494	1.484193	1.513744	1.06103	0.0855	0.752077	1
CPA1	1.031787	1.013274	1.037804	2.84797	1.5099	0.752119	1
IL7R	1.053556	1.034708	1.059683	1.71954	0.782	0.752133	1
SLC35B4	1.363239	1.383424	1.356678	0.93024	-0.1043	0.752167	1
ZNF230	1.123431	1.104101	1.129715	1.24605	0.3174	0.752191	1
RAPGEF4	1.055039	1.036311	1.061127	1.68344	0.7514	0.752194	1
ZNF641	1.104962	1.085769	1.1112	1.29651	0.3746	0.752223	1
ZCRB1	3.249854	3.210288	3.262715	1.02372	0.0338	0.752326	1
LINC00894	1.056075	1.037233	1.062199	1.67051	0.7403	0.752343	1
CCDC94	1.147237	1.127799	1.153556	1.20155	0.2649	0.752427	1
PTGIR	1.026213	1.044192	1.020369	0.46091	-1.1174	0.752602	1
GTPBP1	1.233791	1.213649	1.240339	1.12492	0.1698	0.752659	1
ZNF589	1.126318	1.106945	1.132615	1.24003	0.3104	0.752666	1
PRKAB1	1.203376	1.222662	1.197107	0.88523	-0.1759	0.752842	1
TAL2	1.034295	1.015674	1.040348	2.57426	1.3642	0.752844	1
GPATCH3	1.125209	1.143771	1.119175	0.82892	-0.2707	0.752929	1
MAP3K1	1.140997	1.159671	1.134927	0.84503	-0.2429	0.753077	1
RAB3GAP1	1.386541	1.407148	1.379842	0.93293	-0.1002	0.753084	1
SPNS3	1.029691	1.047533	1.023892	0.50263	-0.9924	0.753088	1
E2F7	1.158836	1.177715	1.152699	0.85924	-0.2189	0.753161	1
RP11-15A1.2	1.035118	1.016591	1.04114	2.47976	1.3102	0.753219	1
PTCD2	1.119734	1.100476	1.125994	1.25397	0.3265	0.753295	1
ALG5	1.6797	1.655851	1.687452	1.04818	0.0679	0.753421	1
ACACB	1.126902	1.107683	1.13315	1.23649	0.3063	0.753436	1
CLCN2	1.127998	1.147056	1.121804	0.82828	-0.2718	0.753466	1
WSCD1	1.058362	1.039688	1.064433	1.6235	0.6991	0.753522	1
RP11-298I3.4	1.058446	1.039791	1.06451	1.62121	0.6971	0.753522	1
IFT22	2.460278	2.423602	2.4722	1.03414	0.0484	0.753529	1
FAM178B	1.046598	1.064615	1.040741	0.63052	-0.6654	0.753551	1
ARSE	1.026263	1.044244	1.020418	0.4615	-1.1156	0.753605	1
EIF2B2	1.384556	1.363158	1.391511	1.07807	0.1085	0.75365	1
RAC2	1.022468	1.040258	1.016686	0.41447	-1.2707	0.753675	1

RP11-436K8.1	1.022511	1.04043	1.016686	0.4127	-1.2768	0.753675	1
AC092652.1	1.022328	1.040213	1.016515	0.41069	-1.2839	0.753753	1
ICAM3	1.165152	1.145392	1.171575	1.18009	0.2389	0.753762	1
CCDC13	1.051835	1.033168	1.057903	1.74575	0.8039	0.753776	1
CPD	1.487532	1.464546	1.495003	1.06556	0.0916	0.753839	1
SLC12A9	1.151477	1.170175	1.145399	0.85441	-0.227	0.753907	1
CMTM3	1.823491	1.846033	1.816163	0.96469	-0.0519	0.754082	1
LINC00862	1.022756	1.040497	1.016989	0.41952	-1.2532	0.754123	1
CERKL	1.039459	1.020958	1.045473	2.16973	1.1175	0.754215	1
UPK3B	1.018845	1.036635	1.013062	0.35654	-1.4879	0.75427	1
RIMBP2	1.052192	1.033519	1.058262	1.73818	0.7976	0.754336	1
ZNF283	1.079454	1.060666	1.085561	1.41037	0.4961	0.754374	1
RP11-390P24.1	1.051557	1.033018	1.057583	1.74395	0.8024	0.754479	1
ABHD1	1.051596	1.033116	1.057603	1.73944	0.7986	0.754479	1
SMAD1	1.1826	1.201506	1.176455	0.87568	-0.1915	0.754524	1
STOML1	1.137784	1.118301	1.144117	1.21822	0.2848	0.754529	1
MEX3C	1.272959	1.292312	1.266668	0.91227	-0.1325	0.754637	1
ALDH1L2	1.04472	1.026233	1.05073	1.93378	0.9514	0.754653	1
ZNF808	1.112086	1.093156	1.118239	1.26926	0.344	0.75476	1
LINC00864	1.038892	1.020333	1.044925	2.20948	1.1437	0.754793	1
CDHR3	1.060532	1.041783	1.066627	1.59458	0.6732	0.7548	1
RHOT1	1.681032	1.65788	1.688558	1.04663	0.0658	0.75485	1
PSMD3	1.875417	1.899339	1.867641	0.96475	-0.0518	0.75486	1
RP11-380L11.4	1.062071	1.079956	1.056258	0.70361	-0.5072	0.754896	1
MBD2	1.146592	1.165016	1.140603	0.85206	-0.231	0.754919	1
MAP4K1	1.066269	1.084219	1.060435	0.71759	-0.4788	0.754949	1
ORMDL3	1.261642	1.241494	1.268191	1.11055	0.1513	0.754969	1
PLIN3	1.77127	1.747471	1.779006	1.04219	0.0596	0.754984	1
SLC5A2	1.036214	1.054364	1.030314	0.5576	-0.8427	0.755073	1
AMACR	1.061817	1.079824	1.055963	0.70108	-0.5123	0.75514	1
NBPF3	1.105398	1.123844	1.099402	0.80264	-0.3172	0.755151	1
APOPT1	2.030505	2.054412	2.022735	0.96996	-0.044	0.755157	1
MYH3	1.04033	1.021878	1.046328	2.11753	1.0824	0.755253	1
FBXO41	1.064717	1.082646	1.058889	0.71254	-0.489	0.755309	1
SLC25A35	1.128888	1.109855	1.135075	1.22957	0.2982	0.755313	1
PIGV	1.128987	1.109617	1.135284	1.23415	0.3035	0.755313	1
LINC01521	1.080084	1.061365	1.086168	1.40418	0.4897	0.755381	1
KBTBD4	1.194149	1.174439	1.200556	1.14972	0.2013	0.755467	1
TACO1	1.32961	1.308898	1.336343	1.08885	0.1228	0.755529	1
TMEM242	1.48596	1.507169	1.479066	0.94459	-0.0822	0.755539	1
SPTLC2	1.311234	1.290955	1.317826	1.09235	0.1274	0.755629	1
DNAJC25	1.265994	1.285636	1.25961	0.90888	-0.1378	0.755695	1
RP11-259N19.1	1.044803	1.026358	1.050798	1.92724	0.9465	0.755716	1
PHF2	1.141772	1.160574	1.135661	0.84485	-0.2432	0.755795	1
ACOXL	1.042026	1.023638	1.048004	2.03077	1.022	0.755882	1

SNAI1	1.027625	1.045397	1.021848	0.48126	-1.0551	0.755937	1
KIF12	1.048189	1.029755	1.05418	1.82086	0.8646	0.755968	1
ELK4	1.265357	1.245161	1.271921	1.10915	0.1495	0.756128	1
KIDINS220	1.853214	1.876524	1.845637	0.96476	-0.0518	0.756129	1
CHD3	1.481384	1.502033	1.474672	0.9455	-0.0809	0.75623	1
KDR	1.05492	1.072871	1.049085	0.67359	-0.5701	0.75632	1
HELZ	1.559268	1.581129	1.552162	0.95016	-0.0738	0.756425	1
CNNM3	1.077465	1.059013	1.083463	1.41432	0.5001	0.756575	1
KCNN4	1.025743	1.04335	1.020019	0.46181	-1.1146	0.756606	1
DNAJB2	2.186188	2.159983	2.194706	1.02993	0.0426	0.756846	1
SMIM4	1.771278	1.748215	1.778775	1.04084	0.0578	0.756932	1
RP11-395A13.2	1.233267	1.213473	1.239701	1.12287	0.1672	0.757005	1
CTD-3193O13.12	1.048315	1.029959	1.054282	1.81188	0.8575	0.75704	1
CDKN2B-AS1	1.02234	1.040058	1.016581	0.41392	-1.2726	0.757119	1
HIC2	1.249573	1.229757	1.256014	1.11428	0.1561	0.757136	1
RNASEL	1.064566	1.082566	1.058716	0.71114	-0.4918	0.757193	1
TBC1D8	1.062587	1.044112	1.068593	1.55498	0.6369	0.757226	1
HFM1	1.062604	1.044079	1.068625	1.55686	0.6386	0.757226	1
SERF1A	1.079015	1.096948	1.073185	0.7549	-0.4056	0.757269	1
ZW10	1.135311	1.116277	1.141499	1.21691	0.2832	0.757277	1
RP11-686O6.2	1.051645	1.033315	1.057603	1.72901	0.7899	0.757279	1
MUT	1.278426	1.258667	1.284848	1.10122	0.1391	0.757389	1
UNC13B	1.172242	1.191007	1.166142	0.86982	-0.2012	0.757416	1
CCNY	1.32108	1.340444	1.314786	0.92463	-0.113	0.757545	1
TMEM9	2.737185	2.76694	2.727513	0.97769	-0.0326	0.757574	1
SLC44A5	1.126387	1.144664	1.120446	0.83259	-0.2643	0.757874	1
OCA2	1.046263	1.06413	1.040455	0.63083	-0.6647	0.757923	1
CRYZ	1.162882	1.181403	1.156862	0.86472	-0.2097	0.75795	1
ZNF799	1.068274	1.049801	1.074279	1.49151	0.5768	0.758047	1
BBOX1-AS1	1.27553	1.294534	1.269353	0.91451	-0.1289	0.758097	1
KLK4	1.041321	1.059266	1.035488	0.59878	-0.7399	0.758129	1
TNFRSF10C	1.047935	1.029734	1.053851	1.81108	0.8568	0.75815	1
CCNJL	1.071899	1.089819	1.066074	0.73563	-0.4429	0.758319	1
CHRM3	1.16542	1.184154	1.159331	0.8652	-0.2089	0.758445	1
RP11-35O15.1	1.04045	1.022273	1.046359	2.08138	1.0575	0.758814	1
CAMSAP3	1.087691	1.105604	1.081869	0.77524	-0.3673	0.758821	1
RP11-554J4.1	1.085246	1.066652	1.091289	1.36964	0.4538	0.758925	1
RP11-680F20.4	1.03006	1.011999	1.03593	2.9944	1.5823	0.759062	1
CCL27	1.038834	1.020674	1.044737	2.16387	1.1136	0.759173	1
ASIC3	1.036818	1.054507	1.031068	0.56998	-0.811	0.759291	1
CCDC53	1.55346	1.531607	1.560564	1.05447	0.0765	0.759747	1
SEMA4A	1.036542	1.054066	1.030845	0.57051	-0.8097	0.760001	1
CPPED1	1.142009	1.160198	1.136097	0.84955	-0.2352	0.760006	1
RP11-686F15.2	1.034039	1.015961	1.039915	2.50079	1.3224	0.760217	1
RBM18	1.326858	1.346523	1.320466	0.92481	-0.1128	0.760414	1

CTD-2574D22.2	1.058309	1.076188	1.052497	0.68905	-0.5373	0.760434	1
AFF2	1.05835	1.076066	1.052591	0.69138	-0.5324	0.760434	1
RP11-46C24.7	1.087649	1.069211	1.093642	1.35298	0.4361	0.76045	1
GUSB	1.302781	1.322607	1.296336	0.91857	-0.1225	0.760539	1
SOST	1.027404	1.044756	1.021764	0.48627	-1.0402	0.76055	1
RP11-596C23.2	1.079422	1.061096	1.085379	1.39747	0.4828	0.76057	1
SNAP25	1.151044	1.132187	1.157174	1.18903	0.2498	0.760578	1
MTMR10	1.08787	1.069539	1.093828	1.34929	0.4322	0.760805	1
PEX14	1.21442	1.233209	1.208313	0.89324	-0.1629	0.760819	1
LRRC9	1.069589	1.051159	1.07558	1.47734	0.563	0.760831	1
SNX21	1.215809	1.234526	1.209726	0.89425	-0.1612	0.76086	1
SMYD5	1.118518	1.099802	1.124601	1.24849	0.3202	0.761179	1
ARAP3	1.119991	1.137746	1.11422	0.82921	-0.2702	0.761187	1
FIG4	1.254346	1.273259	1.248198	0.90829	-0.1388	0.761262	1
RP11-531A24.3	1.080381	1.062176	1.086299	1.38798	0.473	0.761327	1
LINC00311	1.047431	1.029296	1.053326	1.82029	0.8642	0.761405	1
RP11-219A15.1	1.020586	1.037847	1.014975	0.39568	-1.3376	0.761408	1
NFATC1	1.050024	1.067638	1.044299	0.65494	-0.6106	0.761461	1
RP5-862P8.2	1.054565	1.072138	1.048853	0.67722	-0.5623	0.76153	1
PPP2R5B	1.239732	1.258195	1.23373	0.90525	-0.1436	0.761535	1
CTC-241F20.4	1.036317	1.01834	1.04216	2.29887	1.2009	0.76157	1
RP11-329B9.4	1.036385	1.018361	1.042244	2.30079	1.2021	0.76157	1
IL13RA2	1.026195	1.043383	1.020608	0.47501	-1.074	0.761633	1
YIPF1	1.315946	1.296058	1.322411	1.08901	0.123	0.761696	1
SAP18	4.616109	4.567297	4.631976	1.01813	0.0259	0.761706	1
DDX31	1.091114	1.072737	1.097087	1.33477	0.4166	0.761733	1
TMEM229B	1.020474	1.037899	1.01481	0.39077	-1.3556	0.761754	1
ELSPBP1	1.023184	1.040477	1.017563	0.4339	-1.2046	0.761814	1
CORIN	1.023208	1.040532	1.017576	0.43365	-1.2054	0.761814	1
C19orf45	1.057506	1.03935	1.063408	1.6114	0.6883	0.761829	1
LINC00683	1.045751	1.027709	1.051615	1.86273	0.8974	0.761848	1
MED13L	1.434923	1.41391	1.441753	1.06727	0.0939	0.761928	1
ASB6	1.128384	1.146446	1.122512	0.83657	-0.2574	0.762112	1
C22orf29	1.23047	1.21106	1.23678	1.12186	0.1659	0.76228	1
WDR36	1.180203	1.161088	1.186416	1.15723	0.2107	0.762294	1
AVIL	1.06219	1.044013	1.068098	1.54725	0.6297	0.762297	1
BEGAIN	1.066418	1.084023	1.060696	0.72238	-0.4692	0.76231	1
RHOT2	1.612695	1.590375	1.61995	1.05009	0.0705	0.762414	1
EPHB3	1.149416	1.167737	1.143461	0.85527	-0.2255	0.762424	1
PIGN	1.160984	1.141891	1.16719	1.1783	0.2367	0.762577	1
RP11-54O7.2	1.046555	1.028591	1.052394	1.83253	0.8738	0.762669	1
ARNTL	1.126681	1.107972	1.132762	1.2296	0.2982	0.762697	1
LL0XNC01-116E7.2	1.059451	1.041323	1.065343	1.58127	0.6611	0.762708	1
RP11-680F8.1	1.093737	1.075167	1.099774	1.32736	0.4086	0.762735	1
EXOC6	1.245389	1.226102	1.251659	1.11303	0.1545	0.76283	1

RP13-516M14.1	1.041018	1.022965	1.046886	2.04159	1.0297	0.762843	1
PAQR3	1.250554	1.230515	1.257068	1.11519	0.1573	0.762844	1
LINC01356	1.03977	1.021856	1.045594	2.08613	1.0608	0.762881	1
IDH3G	1.877546	1.854519	1.885031	1.03571	0.0506	0.762955	1
SIK2	1.169581	1.187696	1.163692	0.87211	-0.1974	0.763127	1
RAI2	1.043884	1.061111	1.038284	0.62646	-0.6747	0.763201	1
RXRA	1.220779	1.20109	1.227179	1.12974	0.176	0.763292	1
LRRC3	1.11161	1.092954	1.117674	1.26594	0.3402	0.763297	1
ZNF70	1.061847	1.043757	1.067727	1.5478	0.6302	0.763387	1
ARRB1	1.061701	1.043769	1.06753	1.54287	0.6256	0.763387	1
ANKRD13C	1.245144	1.263689	1.239117	0.90681	-0.1411	0.763496	1
RTN4R	1.091183	1.10899	1.085394	0.78351	-0.352	0.763508	1
RP11-506H21.5	1.044144	1.026254	1.04996	1.90293	0.9282	0.76359	1
CRYBA1	1.044078	1.026236	1.049878	1.90116	0.9269	0.76359	1
DIEXF	1.125661	1.106919	1.131754	1.23228	0.3013	0.763715	1
AC021188.4	1.029024	1.011304	1.034784	3.07706	1.6216	0.763754	1
ADAMTS5	1.02238	1.039516	1.016809	0.42538	-1.2332	0.76391	1
HOXA4	1.022409	1.039628	1.016811	0.42422	-1.2371	0.76391	1
RAPGEF5	1.049927	1.032015	1.05575	1.74136	0.8002	0.763968	1
ACTR8	1.14867	1.166601	1.142842	0.85739	-0.222	0.76397	1
ZBTB48	1.084614	1.06654	1.090489	1.35992	0.4435	0.764001	1
PSPN	1.056058	1.07334	1.05044	0.68775	-0.54	0.764005	1
CSDC2	1.179514	1.197627	1.173626	0.87856	-0.1868	0.764078	1
RP11-473M20.16	1.043298	1.025328	1.049139	1.94007	0.9561	0.764187	1
NARS	2.354873	2.326793	2.364001	1.02804	0.0399	0.764261	1
USP3-AS1	1.043887	1.026005	1.0497	1.91114	0.9344	0.764287	1
FAM66A	1.033164	1.015382	1.038944	2.53183	1.3402	0.764623	1
WNT10B	1.037554	1.054951	1.031899	0.58049	-0.7847	0.764657	1
RP11-138A9.1	1.037472	1.05475	1.031856	0.58184	-0.7813	0.764657	1
HIP1	1.515452	1.494286	1.522333	1.05674	0.0796	0.764658	1
CROCC	1.193759	1.175152	1.199807	1.14076	0.19	0.764684	1
NDUFS6	4.745947	4.802923	4.727426	0.98015	-0.0289	0.764741	1
NUP50-AS1	1.081481	1.098997	1.075788	0.76556	-0.3854	0.764903	1
GMEB2	1.142722	1.124123	1.148768	1.19856	0.2613	0.76505	1
IFT27	2.046155	2.021813	2.054068	1.03157	0.0448	0.765208	1
AIFM2	1.120027	1.137621	1.114308	0.8306	-0.2678	0.765243	1
TTC29	1.053434	1.035557	1.059245	1.66621	0.7366	0.765263	1
C10orf25	1.066394	1.048461	1.072223	1.49032	0.5756	0.765313	1
ZNF737	1.066308	1.048374	1.072137	1.49125	0.5765	0.765313	1
KIF6	1.048123	1.065466	1.042485	0.64897	-0.6238	0.765346	1
YTHDF2	2.340425	2.369765	2.330887	0.97162	-0.0415	0.765451	1
GAREM2	1.078163	1.095737	1.072451	0.75677	-0.4021	0.765452	1
ABCB10	1.078064	1.095389	1.072432	0.75933	-0.3972	0.765452	1
AP3S2	1.205384	1.186207	1.211618	1.13646	0.1846	0.765476	1
TMEM31	1.046667	1.028899	1.052443	1.8147	0.8597	0.76549	1

PRSS16	1.077514	1.095016	1.071825	0.75592	-0.4037	0.765705	1
CAMKK1	1.051307	1.033493	1.057098	1.70477	0.7696	0.765733	1
MGAT4A	1.118429	1.100301	1.124321	1.23949	0.3097	0.765793	1
GABRG3	1.050691	1.068056	1.045047	0.66191	-0.5953	0.765856	1
LHFPL1	1.036589	1.018903	1.042338	2.23971	1.1633	0.76594	1
DCAF12	1.660872	1.639046	1.667967	1.04526	0.0639	0.765962	1
SLC9A1	1.106705	1.124245	1.101003	0.81294	-0.2988	0.765976	1
ALS2	1.230104	1.248813	1.224023	0.90037	-0.1514	0.765991	1
ATL3	1.242929	1.261664	1.236839	0.90512	-0.1438	0.766155	1
CYB561D1	1.069585	1.051632	1.075421	1.46073	0.5467	0.766255	1
MEX3D	1.192433	1.210407	1.186591	0.88681	-0.1733	0.766278	1
UBE2B	2.542562	2.575041	2.532005	0.97268	-0.04	0.766294	1
RP11-480C16.1	1.071901	1.054063	1.077699	1.4372	0.5233	0.766549	1
RP11-390F4.3	1.05137	1.033618	1.05714	1.69969	0.7653	0.76675	1
DGAT2	1.039254	1.056499	1.033649	0.59557	-0.7477	0.766754	1
F8	1.039149	1.056188	1.033611	0.59818	-0.7414	0.766754	1
HESX1	1.107972	1.089813	1.113875	1.26792	0.3425	0.766765	1
CTD-2325P2.4	1.068748	1.086125	1.063099	0.73265	-0.4488	0.76685	1
TIGD3	1.040496	1.022831	1.046238	2.02525	1.0181	0.76686	1
CTD-2308L22.1	1.040543	1.022828	1.046302	2.02828	1.0203	0.76686	1
TAS2R14	1.050747	1.033034	1.056504	1.71051	0.7744	0.766874	1
EP300	1.368893	1.38804	1.36267	0.93462	-0.0975	0.76689	1
RP1-317E23.3	1.037697	1.020097	1.043418	2.16041	1.1113	0.76692	1
RP11-479O16.1	1.037723	1.054728	1.032196	0.58828	-0.7654	0.766942	1
INTS6L	1.100301	1.117821	1.094606	0.80297	-0.3166	0.766961	1
CTB-25B13.5	1.037746	1.020085	1.043487	2.1651	1.1144	0.767027	1
FZD1	1.302393	1.282899	1.308729	1.0913	0.1261	0.767028	1
SPTLC1	1.128293	1.110058	1.13422	1.21954	0.2863	0.76721	1
KIAA1551	1.27733	1.257943	1.283632	1.09959	0.137	0.767226	1
GLMP	1.783377	1.80457	1.776488	0.9651	-0.0513	0.767243	1
RP11-563J2.2	1.040089	1.022456	1.045821	2.04051	1.0289	0.767254	1
ABHD5	1.14341	1.124902	1.149426	1.19634	0.2586	0.767261	1
TAF15	1.747451	1.725343	1.754637	1.04039	0.0571	0.767276	1
MLXIPL	1.046643	1.028998	1.052378	1.80625	0.853	0.767368	1
ARHGAP35	1.403256	1.422762	1.396915	0.93886	-0.091	0.767464	1
MAP2K5	1.280571	1.261125	1.286892	1.09867	0.1358	0.767587	1
SUOX	1.121393	1.103255	1.127289	1.23276	0.3019	0.767648	1
ENPEP	1.06269	1.079787	1.057133	0.71607	-0.4818	0.767687	1
AC013271.5	1.03702	1.054128	1.031459	0.58119	-0.7829	0.767688	1
RAB3D	1.248031	1.266232	1.242115	0.90941	-0.137	0.767721	1
RP11-26J3.1	1.029216	1.011717	1.034905	2.9789	1.5748	0.767735	1
C19orf12	1.572965	1.593373	1.566332	0.95443	-0.0673	0.767744	1
TAF1C	1.16364	1.145277	1.16961	1.16749	0.2234	0.767769	1
TPRG1L	1.108692	1.126161	1.103014	0.81653	-0.2924	0.767827	1
FAF2	1.411504	1.430594	1.405299	0.94126	-0.0873	0.767836	1

HEATR1	1.234917	1.21602	1.24106	1.11591	0.1582	0.767976	1
AP000254.8	1.031893	1.04879	1.026401	0.54111	-0.886	0.768008	1
DECR2	1.171272	1.152689	1.177312	1.16127	0.2157	0.768009	1
STPG2-AS1	1.030896	1.047791	1.025404	0.53157	-0.9117	0.768012	1
SNX4	1.502736	1.522964	1.496161	0.94875	-0.0759	0.768128	1
CTD-2270P14.5	1.036471	1.018953	1.042165	2.22471	1.1536	0.768135	1
GVQW2	1.043946	1.026382	1.049655	1.88212	0.9124	0.768175	1
TAF1	1.213387	1.194705	1.21946	1.12714	0.1727	0.768247	1
S1PR2	1.144023	1.125644	1.149997	1.19383	0.2556	0.768562	1
DIO2	1.033497	1.050233	1.028057	0.55855	-0.8402	0.768706	1
RP11-1E4.1	1.032795	1.01526	1.038495	2.52268	1.335	0.76874	1
CD22	1.031121	1.013691	1.036787	2.68702	1.426	0.769003	1
C8orf88	1.269447	1.250388	1.275643	1.10086	0.1386	0.76906	1
SCIN	1.025408	1.042358	1.019898	0.46976	-1.09	0.769071	1
DDX52	1.63568	1.656392	1.628948	0.95819	-0.0616	0.769124	1
TIGD5	1.093438	1.075563	1.099248	1.31344	0.3934	0.769174	1
PNOC	1.026626	1.043483	1.021147	0.48632	-1.04	0.769292	1
RP11-560J1.2	1.045477	1.02797	1.051168	1.8294	0.8714	0.769313	1
GOLGA8R	1.045604	1.028027	1.051317	1.83101	0.8726	0.769313	1
DIAPH2-AS1	1.068147	1.08512	1.06263	0.73579	-0.4426	0.769339	1
NDUFA13	5.649591	5.57685	5.673237	1.02106	0.0301	0.769396	1
SP2-AS1	1.033441	1.016045	1.039096	2.43662	1.2849	0.769482	1
IQCG	1.17177	1.189624	1.165966	0.87524	-0.1923	0.76965	1
ZDHHC4	1.803606	1.781016	1.810949	1.03833	0.0543	0.769668	1
PI4KA	1.324127	1.342553	1.318137	0.92872	-0.1067	0.769712	1
MCTP1	1.082619	1.099628	1.07709	0.77378	-0.37	0.76974	1
ZNF584	1.209989	1.191282	1.21607	1.12959	0.1758	0.769769	1
RASSF3	1.133797	1.115445	1.139763	1.21064	0.2758	0.769796	1
TRIM29	1.023167	1.039932	1.017717	0.44368	-1.1724	0.769829	1
ZNF696	1.078953	1.061261	1.084703	1.38266	0.4674	0.769839	1
EPN1	2.223359	2.197117	2.231889	1.02905	0.0413	0.76985	1
ZNF772	1.103591	1.120765	1.098008	0.81156	-0.3012	0.77002	1
RPRD2	1.322677	1.3034	1.328943	1.08419	0.1166	0.770026	1
GNG7	1.141763	1.159436	1.136018	0.85312	-0.2292	0.770028	1
AKR1C3	1.061474	1.043958	1.067168	1.52802	0.6117	0.770053	1
ERICH6-AS1	1.095028	1.112144	1.089464	0.79776	-0.326	0.770114	1
MAMDC2-AS1	1.075422	1.057581	1.081221	1.41056	0.4963	0.77013	1
DPPA4	1.045722	1.062597	1.040237	0.64279	-0.6376	0.77014	1
ZNF189	1.147486	1.129201	1.15343	1.18753	0.248	0.770163	1
CEP72	1.047999	1.065054	1.042455	0.65261	-0.6157	0.770201	1
PPRC1	1.079601	1.096595	1.074077	0.76688	-0.3829	0.770211	1
RNF121	1.215343	1.233413	1.209469	0.89742	-0.1562	0.770212	1
AP001505.10	1.080032	1.062491	1.085733	1.37193	0.4562	0.770225	1
AC010136.2	1.034577	1.017178	1.040233	2.3421	1.2278	0.770338	1
USP27X	1.164784	1.146283	1.170798	1.16758	0.2235	0.770371	1

RIPK1	1.103236	1.085351	1.10905	1.27767	0.3535	0.770413	1
KLHL32	1.04716	1.029715	1.05283	1.77792	0.8302	0.770582	1
POLR3K	1.51767	1.496989	1.524393	1.05514	0.0774	0.770635	1
METTL17	1.497959	1.4772	1.504706	1.05764	0.0809	0.770743	1
SNX11	1.180914	1.19861	1.175162	0.88194	-0.1812	0.77088	1
NPY	1.019676	1.036304	1.014271	0.39308	-1.3471	0.771011	1
RP11-318E3.9	1.038437	1.021006	1.044103	2.09958	1.0701	0.771013	1
IGSF21	1.041987	1.058662	1.036566	0.62334	-0.6819	0.771056	1
STX10	1.783587	1.804506	1.776787	0.96555	-0.0506	0.771078	1
ZFP2	1.071428	1.05392	1.077119	1.43026	0.5163	0.771081	1
UNC5CL	1.048247	1.030856	1.053901	1.74687	0.8048	0.771204	1
ZNF706	4.608057	4.556023	4.624971	1.01939	0.0277	0.77124	1
SLC38A8	1.035199	1.017948	1.040807	2.27365	1.185	0.771292	1
ATAD2B	1.186064	1.167643	1.192051	1.1456	0.1961	0.771383	1
MTHFR	1.18891	1.206603	1.183159	0.88653	-0.1738	0.771561	1
CTC-479C5.12	1.040238	1.056856	1.034836	0.6127	-0.7068	0.771584	1
BABAM1	2.291469	2.264894	2.300107	1.02784	0.0396	0.771604	1
RP11-159D12.2	1.166898	1.148424	1.172903	1.16493	0.2202	0.771619	1
RCN3	1.115254	1.13267	1.109593	0.82606	-0.2757	0.77163	1
CTD-2555O16.4	1.033241	1.015934	1.038867	2.4392	1.2864	0.771759	1
MKL2	1.264349	1.282744	1.25837	0.91379	-0.1301	0.771772	1
SLC31A2	1.06596	1.082874	1.060461	0.72956	-0.4549	0.771835	1
NPY1R	1.065247	1.047665	1.070961	1.48874	0.5741	0.7719	1
FKBP10	2.868798	2.898574	2.859119	0.97922	-0.0303	0.772146	1
XXbac-B135H6.18	1.100619	1.082755	1.106426	1.28603	0.3629	0.77226	1
BSCL2	1.057609	1.040217	1.063263	1.57304	0.6536	0.772269	1
CCNE1	1.081734	1.098849	1.07617	0.77057	-0.376	0.772311	1
MYF6	1.096954	1.114138	1.091369	0.80051	-0.321	0.772389	1
CPNE8	1.381211	1.361787	1.387525	1.07114	0.0991	0.772445	1
ARHGEF2	1.25864	1.276712	1.252765	0.91346	-0.1306	0.772446	1
RP11-420L9.5	1.065328	1.082175	1.059851	0.72834	-0.4573	0.772467	1
CSNK1G2	1.335456	1.354173	1.329372	0.92997	-0.1047	0.772521	1
RP11-127B20.2	1.086962	1.069431	1.09266	1.33455	0.4164	0.772669	1
F10	1.177395	1.15914	1.18333	1.152	0.2041	0.772674	1
TUBD1	1.189672	1.17147	1.195589	1.14066	0.1899	0.772701	1
LINC00890	1.033132	1.015961	1.038713	2.42548	1.2783	0.772769	1
AC019172.2	1.033184	1.015961	1.038783	2.42985	1.2809	0.772769	1
RP11-11N9.4	2.209455	2.184608	2.217531	1.02779	0.0395	0.772789	1
PNCK	1.123288	1.105475	1.129078	1.22378	0.2913	0.772865	1
ZBED5-AS1	1.217969	1.199491	1.223975	1.12273	0.167	0.772905	1
NHLRC4	1.041396	1.024181	1.046992	1.94337	0.9586	0.772994	1
CTD-2012K14.8	1.080185	1.097193	1.074657	0.76813	-0.3806	0.773011	1
NEUROD4	1.036651	1.019453	1.042241	2.17136	1.1186	0.773058	1
PLCD1	1.121461	1.138689	1.115861	0.8354	-0.2595	0.773074	1
EFHC2	1.068652	1.051255	1.074307	1.44974	0.5358	0.773177	1

RPPH1	1.067453	1.050094	1.073096	1.45917	0.5452	0.773215	1
TMEM41B	1.569567	1.589775	1.562999	0.9546	-0.067	0.773319	1
MYEOV	1.030907	1.047367	1.025556	0.53954	-0.8902	0.77339	1
TBC1D25	1.103953	1.086191	1.109727	1.27306	0.3483	0.77351	1
RP11-380N8.7	1.061061	1.043733	1.066694	1.52502	0.6088	0.773553	1
CDC42EP2	1.07404	1.090768	1.068602	0.75579	-0.4039	0.773585	1
COX4I1	13.08624	13.25014	13.03296	0.98227	-0.0258	0.773606	1
ANKRD18A	1.089456	1.071949	1.095146	1.32241	0.4032	0.773661	1
GABPB2	1.135904	1.153234	1.130271	0.85014	-0.2342	0.77372	1
LRAT	1.024795	1.041392	1.0194	0.4687	-1.0933	0.773795	1
C17orf53	1.063903	1.046715	1.06949	1.48753	0.5729	0.773836	1
IQSEC3	1.026417	1.042934	1.021048	0.49025	-1.0284	0.773855	1
RP11-286N22.8	1.042297	1.024951	1.047935	1.92118	0.942	0.773883	1
HNF1A	1.046581	1.02938	1.052172	1.7758	0.8285	0.773883	1
PCSK9	1.027529	1.044118	1.022137	0.50177	-0.9949	0.773962	1
SIAH1	1.465874	1.485381	1.459533	0.94675	-0.0789	0.773991	1
RASA2	1.237712	1.219311	1.243693	1.11117	0.1521	0.774006	1
NMNAT2	1.050947	1.067484	1.045572	0.67531	-0.5664	0.774014	1
RP3-466P17.1	1.0366	1.019486	1.042163	2.1637	1.1135	0.774071	1
FAM193A	1.205132	1.222854	1.199371	0.89463	-0.1606	0.774107	1
CD69	1.023857	1.040321	1.018506	0.45896	-1.1236	0.774173	1
ETV3	1.144234	1.161665	1.138567	0.85713	-0.2224	0.774219	1
PALD1	1.080884	1.063443	1.086553	1.36426	0.4481	0.774363	1
LINC01144	1.029221	1.012186	1.034759	2.85227	1.5121	0.774399	1
TBC1D22B	1.099801	1.116616	1.094336	0.80894	-0.3059	0.774423	1
STAT4	1.022992	1.039326	1.017683	0.44966	-1.1531	0.774426	1
SAMD14	1.352968	1.333211	1.359391	1.07857	0.1091	0.774467	1
FAM159A	1.07146	1.054022	1.077129	1.42772	0.5137	0.774533	1
RPP38	1.39074	1.370871	1.397198	1.07099	0.0989	0.774624	1
CSNK2A2	1.402467	1.382564	1.408937	1.06894	0.0962	0.774652	1
MYL3	1.022188	1.038633	1.016842	0.43596	-1.1977	0.774708	1
MPV17L	1.053911	1.03657	1.059547	1.62828	0.7034	0.774718	1
OVOS2	1.039883	1.022734	1.045458	1.99952	0.9997	0.774806	1
KLHL7-AS1	1.082903	1.065624	1.08852	1.3489	0.4318	0.774871	1
RP11-295P9.13	1.019546	1.035891	1.014234	0.39658	-1.3343	0.774999	1
ARHGAP39	1.093714	1.076238	1.099395	1.30375	0.3827	0.775206	1
CPNE4	1.030854	1.047088	1.025577	0.54317	-0.8805	0.775216	1
U2AF1	1.089414	1.072234	1.094999	1.31516	0.3952	0.775274	1
TP53BP1	1.634668	1.654725	1.628149	0.95941	-0.0598	0.775282	1
JMJD4	1.231996	1.213311	1.238069	1.11606	0.1584	0.775328	1
TXNL1	2.939144	2.902925	2.950917	1.02522	0.0359	0.775492	1
RP1-111C20.4	1.030163	1.046565	1.024832	0.53328	-0.907	0.775504	1
SASH1	1.296933	1.314615	1.291185	0.92553	-0.1116	0.77561	1
PBLD	1.049828	1.032748	1.05538	1.69109	0.758	0.775661	1
LEPR	1.134478	1.151621	1.128906	0.85019	-0.2341	0.775827	1

KIAA1671	1.180599	1.197463	1.175118	0.88684	-0.1733	0.775927	1
SPON1	1.079067	1.061763	1.084692	1.37125	0.4555	0.775948	1
SPANXB1	1.026787	1.043118	1.021479	0.49813	-1.0054	0.776073	1
MTX1	1.769659	1.74827	1.776612	1.03788	0.0536	0.776099	1
MCOLN1	1.19991	1.181464	1.205906	1.13469	0.1823	0.776175	1
BFSP1	1.052226	1.035253	1.057743	1.63795	0.7119	0.776314	1
PREB	1.59235	1.571422	1.599153	1.04853	0.0684	0.776319	1
MAML1	1.192413	1.209745	1.186779	0.89051	-0.1673	0.776328	1
TMEM203	1.700158	1.720757	1.693463	0.96213	-0.0557	0.776426	1
HLA-DPB1	1.574178	1.553248	1.580982	1.05013	0.0706	0.776439	1
CDC14A	1.097185	1.079752	1.102851	1.28964	0.367	0.776469	1
C21orf91	1.235429	1.217021	1.241413	1.1124	0.1537	0.776499	1
VPS52	1.195507	1.177175	1.201466	1.1371	0.1854	0.776517	1
KCTD10	1.273415	1.290739	1.267784	0.92105	-0.1187	0.776592	1
ASCC3	1.79196	1.81193	1.785469	0.96741	-0.0478	0.776629	1
RP11-493L12.4	1.04241	1.025383	1.047944	1.88885	0.9175	0.776698	1
CTC-273B12.10	1.042436	1.025444	1.047959	1.88489	0.9145	0.776698	1
LA16c-325D7.2	1.042371	1.025359	1.0479	1.88888	0.9175	0.776698	1
ZNF880	1.084708	1.067272	1.090375	1.34343	0.4259	0.776727	1
AK5	1.020609	1.036998	1.015282	0.41305	-1.2756	0.776922	1
SPRYD3	1.12765	1.144266	1.122248	0.84738	-0.2389	0.776995	1
ZNF211	1.083412	1.100088	1.077991	0.77922	-0.3599	0.777	1
CRIP1	1.053923	1.070371	1.048576	0.69029	-0.5347	0.777017	1
RNF141	1.469394	1.44954	1.475848	1.05852	0.0821	0.777089	1
RP11-384P7.7	1.038896	1.021895	1.044422	2.02891	1.0207	0.777284	1
NR4A3	1.031364	1.047596	1.026087	0.5481	-0.8675	0.777418	1
C21orf2	1.409571	1.428307	1.403481	0.94204	-0.0861	0.777617	1
BANP	1.240928	1.222738	1.246841	1.10822	0.1482	0.777637	1
SLC25A12	1.103677	1.086241	1.109345	1.2679	0.3424	0.777665	1
TSPOAP1-AS1	1.082174	1.064925	1.087781	1.35204	0.4351	0.77767	1
ZNF582-AS1	1.190043	1.171845	1.195959	1.14032	0.1894	0.777681	1
UBE2E1-AS1	1.032498	1.015679	1.037965	2.42144	1.2759	0.777886	1
HCG14	1.032489	1.015703	1.037945	2.41643	1.2729	0.777886	1
ARAP2	1.080357	1.096825	1.075004	0.77464	-0.3684	0.77793	1
RP11-727F15.9	1.088109	1.070745	1.093753	1.32523	0.4062	0.777981	1
KDM7A	1.139149	1.121413	1.144914	1.19356	0.2553	0.777999	1
RP11-713P17.3	1.046414	1.029611	1.051875	1.75193	0.8089	0.77806	1
LRRK1	1.036268	1.019359	1.041765	2.15733	1.1092	0.778097	1
MTURN	1.105014	1.121577	1.09963	0.81948	-0.2872	0.778112	1
COG5	1.209627	1.226706	1.204075	0.90017	-0.1517	0.778153	1
DGKG	1.050437	1.033496	1.055943	1.67014	0.74	0.77821	1
C22orf15	1.056737	1.03972	1.062269	1.5677	0.6487	0.778269	1
KCTD15	1.159183	1.141312	1.164991	1.16757	0.2235	0.778277	1
RP11-490B18.5	1.039495	1.022676	1.044962	1.98285	0.9876	0.7783	1
WDR93	1.039557	1.022805	1.045002	1.97337	0.9807	0.7783	1

IGSF11	1.033615	1.016797	1.039082	2.32666	1.2183	0.778506	1
PTPRT	1.024492	1.040693	1.019226	0.47248	-1.0817	0.778552	1
MYB	1.042266	1.025439	1.047736	1.87654	0.9081	0.77858	1
LINC00323	1.027482	1.043619	1.022236	0.50978	-0.972	0.778583	1
AC007325.4	1.314198	1.295791	1.320182	1.08246	0.1143	0.778639	1
FANCE	1.074846	1.091237	1.069519	0.76196	-0.3922	0.778682	1
ZNF461	1.068752	1.051675	1.074303	1.43791	0.524	0.778715	1
GJD2	1.033522	1.049651	1.028279	0.56955	-0.8121	0.778903	1
MCM9	1.107202	1.089778	1.112866	1.25716	0.3302	0.778919	1
GFM2	1.217206	1.23455	1.211568	0.90201	-0.1488	0.779104	1
RNMT	3.349726	3.310982	3.36232	1.02221	0.0317	0.779182	1
DDX60L	1.091576	1.074355	1.097173	1.30688	0.3861	0.77923	1
TRAPPC5	1.139552	1.121976	1.145265	1.19092	0.2521	0.779291	1
HEATR5B	1.10082	1.117389	1.095435	0.81298	-0.2987	0.779318	1
RP1-138B7.7	1.039714	1.022893	1.045182	1.97366	0.9809	0.779402	1
LINC01547	1.03971	1.022865	1.045185	1.97614	0.9827	0.779402	1
STT3A	1.287168	1.304672	1.281479	0.92388	-0.1142	0.779453	1
IL17RE	1.141733	1.124065	1.147476	1.1887	0.2494	0.779483	1
CLN3	1.061108	1.044217	1.066599	1.50618	0.5909	0.779714	1
RP11-22P6.2	1.075251	1.05808	1.080832	1.39175	0.4769	0.779822	1
LYSMD3	1.18775	1.20507	1.18212	0.88809	-0.1712	0.780009	1
KIF26B	1.07948	1.095901	1.074143	0.77312	-0.3712	0.780097	1
ATP5L	13.16998	13.04016	13.21218	1.01429	0.0205	0.780229	1
RP13-131K19.1	1.059608	1.042598	1.065138	1.52914	0.6127	0.780287	1
RP11-666A20.4	1.053932	1.036949	1.059453	1.60905	0.6862	0.780512	1
RP11-104N10.1	1.043201	1.0264	1.048663	1.84332	0.8823	0.780703	1
ARNT	1.281583	1.299203	1.275856	0.92197	-0.1172	0.780717	1
GNAS-AS1	1.057432	1.040696	1.062873	1.54493	0.6275	0.780761	1
C7orf49	1.588606	1.56822	1.595233	1.04754	0.067	0.780968	1
WFDC2	2.349603	2.380414	2.339587	0.97042	-0.0433	0.780989	1
SLC8B1	1.090335	1.106753	1.084999	0.79622	-0.3288	0.780997	1
XXbac-BPG308K3.5	1.042572	1.025872	1.048001	1.85532	0.8917	0.781073	1
MB21D2	1.081761	1.064831	1.087264	1.34602	0.4287	0.781174	1
LA16c-313D11.9	1.029344	1.045354	1.024139	0.53224	-0.9099	0.781287	1
OSBPL2	1.333256	1.351103	1.327454	0.93264	-0.1006	0.781396	1
MED18	1.0925	1.075371	1.098068	1.30114	0.3798	0.781424	1
OSBPL8	2.468509	2.442016	2.477121	1.02434	0.0347	0.781438	1
BEND3	1.066127	1.049186	1.071634	1.45637	0.5424	0.781546	1
CTA-246H3.12	1.030273	1.013691	1.035663	2.60487	1.3812	0.781595	1
RP11-31F15.2	1.042881	1.02622	1.048296	1.84197	0.8813	0.781788	1
FANK1	1.258455	1.275737	1.252838	0.91695	-0.1251	0.781804	1
REPS2	1.093326	1.109724	1.087995	0.80197	-0.3184	0.781888	1
ZNF614	1.148604	1.131008	1.154324	1.17798	0.2363	0.781919	1
TDRD7	1.030854	1.046746	1.025689	0.54953	-0.8637	0.781965	1
DNAAF1	1.04658	1.029907	1.052	1.73874	0.798	0.782	1

MORF4L1	8.235658	8.12494	8.271648	1.02059	0.0294	0.782058	1
PCDH15	1.043751	1.027174	1.049139	1.80829	0.8546	0.782061	1
CTNNBL1	1.483992	1.502703	1.47791	0.95068	-0.073	0.782118	1
MFSD9	1.127189	1.109738	1.132862	1.21072	0.2759	0.782192	1
TTC9B	1.034132	1.017512	1.039534	2.25757	1.1748	0.782272	1
SKAP1	1.03418	1.017512	1.039598	2.26119	1.1771	0.782272	1
CRYBA4	1.044054	1.027296	1.049502	1.81348	0.8588	0.782412	1
HOXA5	1.02277	1.038617	1.017618	0.45624	-1.1321	0.782425	1
C1orf229	1.052767	1.035994	1.058219	1.61744	0.6937	0.782444	1
ATG2B	1.157776	1.174418	1.152367	0.87357	-0.195	0.782464	1
RP11-634H22.1	1.060582	1.043859	1.066017	1.50523	0.59	0.78249	1
CLK3	1.410593	1.428538	1.40476	0.94451	-0.0824	0.782578	1
NR1H4	1.024035	1.039881	1.018884	0.4735	-1.0786	0.782592	1
RP11-176N18.2	1.035403	1.018775	1.040808	2.17355	1.1201	0.782621	1
MTRF1	1.114437	1.097409	1.119972	1.23164	0.3006	0.782723	1
RP11-115D19.2	1.050687	1.066616	1.045509	0.68315	-0.5497	0.782748	1
ZNF780A	1.119911	1.136232	1.114606	0.84125	-0.2494	0.782893	1
CTC-444N24.13	1.033693	1.017207	1.039052	2.2696	1.1824	0.782916	1
ZFP37	1.101941	1.085059	1.107428	1.26298	0.3368	0.782954	1
MMP24-AS1	3.201997	3.1696	3.212528	1.01979	0.0283	0.782955	1
RLIM	1.296576	1.31429	1.290818	0.92532	-0.112	0.783017	1
ATP5EP2	1.382441	1.362661	1.388871	1.07227	0.1007	0.783023	1
KDM3A	1.542892	1.562435	1.536539	0.95396	-0.068	0.783075	1
STX17-AS1	1.059726	1.043097	1.065131	1.51125	0.5957	0.783096	1
SLC25A48	1.018991	1.034772	1.013862	0.39865	-1.3268	0.783101	1
CHRFAM7A	1.048154	1.031514	1.053563	1.69964	0.7652	0.783119	1
STAG3	1.048203	1.031497	1.053634	1.70281	0.7679	0.783119	1
RP11-177G23.2	1.042555	1.026043	1.047922	1.84013	0.8798	0.783321	1
ABCA5	1.150207	1.132699	1.155899	1.17483	0.2325	0.783326	1
ARHGEF4	1.203142	1.185374	1.208917	1.12701	0.1725	0.783463	1
C10orf95	1.07166	1.054739	1.07716	1.40961	0.4953	0.783482	1
NAPG	1.264168	1.281378	1.258574	0.91896	-0.1219	0.783512	1
AL365181.1	1.058799	1.075008	1.05353	0.71365	-0.4867	0.783552	1
CTA-14H9.5	1.077203	1.093402	1.071937	0.77019	-0.3767	0.783669	1
CCBE1	1.042904	1.058639	1.037789	0.64444	-0.6339	0.783687	1
PANK2	1.437412	1.418082	1.443695	1.06126	0.0858	0.783926	1
SMG9	1.383724	1.365036	1.389799	1.06784	0.0947	0.784021	1
EFNA1	1.245018	1.261918	1.239525	0.9145	-0.1289	0.784094	1
CYSRT1	1.0488	1.032332	1.054154	1.6749	0.7441	0.784111	1
RP2	1.113299	1.09632	1.118818	1.23357	0.3028	0.784122	1
ELMO2	1.153368	1.136012	1.159009	1.16909	0.2254	0.784172	1
IFT74-AS1	1.028303	1.011999	1.033603	2.80043	1.4856	0.784226	1
AGR3	1.017105	1.032802	1.012002	0.3659	-1.4505	0.784372	1
RP11-767I20.1	1.017117	1.032853	1.012002	0.36534	-1.4527	0.784372	1
SPIN2A	1.051596	1.035044	1.056977	1.62584	0.7012	0.784406	1

NOP9	1.112735	1.129039	1.107435	0.83258	-0.2643	0.78442	1
RP11-637O19.2	1.067874	1.051163	1.073306	1.43278	0.5188	0.784556	1
DCAF5	1.429804	1.448075	1.423865	0.94597	-0.0801	0.784568	1
CFAP43	1.053469	1.036886	1.05886	1.59574	0.6742	0.784581	1
ANKAR	1.097916	1.114307	1.092588	0.80999	-0.304	0.784715	1
CUL4B	1.363085	1.380976	1.35727	0.93777	-0.0927	0.78472	1
PRPH2	1.040994	1.024686	1.046295	1.87534	0.9072	0.784911	1
DHX38	1.190192	1.172681	1.195884	1.13437	0.1819	0.784949	1
CYP26B1	1.032747	1.048585	1.027599	0.56806	-0.8159	0.785111	1
MECP2	1.766375	1.744972	1.773333	1.03807	0.0539	0.785268	1
HORMAD2	1.020947	1.036714	1.015821	0.43092	-1.2145	0.785307	1
KAT7	1.397353	1.378528	1.403473	1.0659	0.0921	0.785499	1
CTD-2501E16.2	1.031777	1.015495	1.03707	2.39235	1.2584	0.785509	1
SNX7	1.533392	1.513754	1.539775	1.05065	0.0713	0.785578	1
RMND1	1.280496	1.2621	1.286476	1.093	0.1283	0.785624	1
LINC01556	1.035753	1.019453	1.041051	2.11029	1.0774	0.785659	1
PBX2	1.827312	1.805312	1.834463	1.0362	0.0513	0.785675	1
FAM84A	1.414203	1.39567	1.420227	1.06206	0.0869	0.785717	1
TRMT61A	1.118748	1.134971	1.113475	0.84074	-0.2503	0.785827	1
RP4-565E6.1	1.034777	1.05045	1.029683	0.58837	-0.7652	0.785858	1
NPPC	1.034793	1.050537	1.029675	0.5872	-0.7681	0.785858	1
PGBD4	1.056896	1.040389	1.062261	1.54154	0.6244	0.785865	1
RP11-449J21.5	1.043039	1.026603	1.048381	1.81862	0.8628	0.785914	1
RP11-804H8.6	1.07578	1.09197	1.070518	0.76675	-0.3832	0.785947	1
SPAG7	2.611628	2.585469	2.620131	1.02186	0.0312	0.786004	1
CLN5	1.421267	1.438882	1.41554	0.94682	-0.0788	0.786106	1
MTMR6	1.191707	1.208612	1.186212	0.89262	-0.1639	0.786106	1
LINC01239	1.025762	1.041413	1.020674	0.49921	-1.0023	0.786302	1
RPRML	1.028111	1.043722	1.023036	0.52688	-0.9245	0.786328	1
POLR3G	1.116699	1.099874	1.122168	1.22322	0.2907	0.786412	1
RP11-154J22.1	1.045624	1.029312	1.050927	1.73742	0.7969	0.786428	1
NAT8L	1.203778	1.220219	1.198434	0.90107	-0.1503	0.786478	1
IFNAR1	1.491566	1.472488	1.497768	1.0535	0.0752	0.786605	1
RP11-803D5.4	1.051893	1.0676	1.046788	0.69212	-0.5309	0.786618	1
MIR155HG	1.098929	1.114998	1.093705	0.81484	-0.2954	0.78665	1
UCKL1-AS1	1.035807	1.019486	1.041113	2.1098	1.0771	0.786677	1
SNCA-AS1	1.034643	1.018353	1.039938	2.17611	1.1218	0.786715	1
NFKB1	1.142468	1.158891	1.13713	0.86304	-0.2125	0.786764	1
CARMIL3	1.035262	1.019043	1.040534	2.12856	1.0899	0.786789	1
CORO6	1.044016	1.027782	1.049293	1.77424	0.8272	0.78685	1
TM6SF2	1.044148	1.027748	1.049479	1.7832	0.8345	0.78685	1
GPR160	1.022367	1.038095	1.017255	0.45294	-1.1426	0.7869	1
RIIAD1	1.039193	1.022956	1.044471	1.93717	0.954	0.786939	1
RP11-227G15.8	1.039358	1.023009	1.044672	1.94154	0.9572	0.786939	1
CTD-2162K18.4	1.028362	1.012186	1.03362	2.75885	1.4641	0.787026	1

RP11-368I23.4	1.028365	1.012186	1.033624	2.75912	1.4642	0.787026	1
ZCCHC6	1.238977	1.256038	1.233431	0.9117	-0.1334	0.787088	1
SHBG	1.046455	1.030145	1.051757	1.71695	0.7798	0.78718	1
ENDOG	1.24097	1.257834	1.235488	0.91333	-0.1308	0.787199	1
CCDC175	1.031064	1.014881	1.036325	2.441	1.2875	0.787306	1
SUCLG2-AS1	1.057541	1.041243	1.062838	1.52362	0.6075	0.787315	1
SEC16B	1.057493	1.041169	1.062799	1.5254	0.6092	0.787315	1
C7orf31	1.095609	1.111429	1.090467	0.81188	-0.3007	0.787351	1
BRWD3	1.375082	1.356665	1.381068	1.06842	0.0955	0.787366	1
ZNF552	1.129229	1.112165	1.134776	1.20159	0.2649	0.787412	1
ZNF571	1.099288	1.082417	1.104772	1.27125	0.3462	0.787414	1
ISLR	1.023086	1.038708	1.018008	0.46522	-1.104	0.787483	1
CTD-2396E7.11	1.023066	1.038628	1.018007	0.46617	-1.1011	0.787483	1
SPATA12	1.066465	1.050116	1.07178	1.43228	0.5183	0.787537	1
ZNF311	1.057739	1.041396	1.063052	1.52316	0.6071	0.78756	1
AC108938.5	1.024474	1.039874	1.019469	0.48826	-1.0343	0.787637	1
TMEM235	1.024444	1.039917	1.019414	0.48635	-1.0399	0.787637	1
CTD-3035K23.7	1.049158	1.032905	1.054442	1.6545	0.7264	0.787689	1
RP11-385F7.1	1.080448	1.063908	1.085825	1.34295	0.4254	0.787833	1
CTH	1.080398	1.063924	1.085753	1.34147	0.4238	0.787833	1
LMO7-AS1	1.018745	1.034358	1.01367	0.39785	-1.3297	0.787849	1
NYX	1.021477	1.037015	1.016426	0.44378	-1.1721	0.787909	1
TKTL1	1.03798	1.021831	1.043229	1.98012	0.9856	0.787997	1
RP11-205M3.3	1.017841	1.033329	1.012806	0.38422	-1.38	0.78812	1
PARP8	1.139383	1.155247	1.134226	0.8646	-0.2099	0.788228	1
RP11-400F19.6	1.229957	1.212236	1.235717	1.11063	0.1514	0.788298	1
DLGAP1-AS3	1.017003	1.032561	1.011946	0.36689	-1.4466	0.788374	1
ADAMTS19	1.04993	1.033758	1.055187	1.63476	0.7091	0.788442	1
RBM27	1.194503	1.177134	1.200149	1.12993	0.1762	0.788515	1
LINC01424	1.034503	1.018394	1.039739	2.16046	1.1113	0.788597	1
AP2S1	5.422371	5.373762	5.438172	1.01473	0.0211	0.788649	1
JHDM1D-AS1	1.096365	1.079789	1.101753	1.27527	0.3508	0.788711	1
C1GALT1C1	1.189592	1.206154	1.184209	0.89355	-0.1624	0.788741	1
CAPNS2	1.017628	1.03323	1.012556	0.37785	-1.4041	0.788831	1
EFCAB10	1.052635	1.036432	1.057902	1.5893	0.6684	0.788945	1
HACD4	1.037989	1.053475	1.032955	0.61627	-0.6984	0.788951	1
NAP1L2	1.085157	1.100755	1.080086	0.79486	-0.3312	0.789016	1
ZC3H3	1.140805	1.123782	1.146338	1.18222	0.2415	0.789024	1
IFT46	1.237778	1.220665	1.24334	1.10276	0.1411	0.789077	1
CTB-58E17.5	1.04272	1.026522	1.047986	1.80926	0.8554	0.789236	1
C1orf220	1.042646	1.026525	1.047886	1.80532	0.8523	0.789236	1
RP11-705O1.8	1.030001	1.013947	1.03522	2.52534	1.3365	0.789252	1
NAPB	1.163106	1.145871	1.168708	1.15656	0.2098	0.789297	1
KLHL13	1.195328	1.178115	1.200924	1.12806	0.1738	0.78932	1
TYW5	1.261119	1.243032	1.266998	1.09861	0.1357	0.789347	1

RRN3	1.167336	1.150501	1.172809	1.14823	0.1994	0.789367	1
PCDH10	1.084393	1.099854	1.079367	0.79483	-0.3313	0.789406	1
RP11-568A7.4	1.031842	1.015826	1.037049	2.34098	1.2271	0.789425	1
RP11-314B1.2	1.024124	1.03959	1.019097	0.48238	-1.0518	0.789428	1
RP13-577H12.2	1.029245	1.013073	1.034502	2.6393	1.4002	0.789485	1
PLCB3	1.159661	1.142781	1.165148	1.15666	0.21	0.789485	1
GMPPB	1.280843	1.297638	1.275383	0.92523	-0.1121	0.789507	1
RP11-600F24.7	1.035213	1.019196	1.04042	2.10559	1.0742	0.789602	1
PPP1R3D	1.121924	1.138173	1.116642	0.84417	-0.2444	0.789667	1
SPRY3	1.053489	1.037368	1.05873	1.57164	0.6523	0.7897	1
LINC01301	1.053393	1.037162	1.058669	1.57876	0.6588	0.7897	1
RHPN1-AS1	1.064424	1.048288	1.069669	1.44278	0.5289	0.789753	1
ZNF622	1.28334	1.300204	1.277858	0.92556	-0.1116	0.789794	1
NALCN-AS1	1.029251	1.013274	1.034445	2.59486	1.3757	0.789835	1
XXbac-B444P24.14	1.029264	1.013274	1.034462	2.59616	1.3764	0.789835	1
ARMC5	1.112481	1.128518	1.107268	0.83465	-0.2608	0.789853	1
RUBCN	1.136238	1.119285	1.141748	1.18831	0.2489	0.789856	1
SNAPC4	1.102793	1.086219	1.10818	1.25472	0.3274	0.789894	1
SYNPO2	1.053411	1.069158	1.048293	0.6983	-0.5181	0.790007	1
MTFMT	1.286252	1.268485	1.292027	1.08769	0.1213	0.790008	1
AC144831.3	1.020695	1.036147	1.015672	0.43356	-1.2057	0.790023	1
VIT	1.020654	1.035983	1.015672	0.43554	-1.1991	0.790023	1
RHOXF1-AS1	1.020689	1.036125	1.015672	0.43382	-1.2048	0.790023	1
CARD11	1.020652	1.035973	1.015672	0.43565	-1.1988	0.790023	1
BCORL1	1.12913	1.14497	1.123981	0.85522	-0.2256	0.790025	1
LBX2-AS1	1.078226	1.094066	1.073077	0.77687	-0.3643	0.790208	1
DDX11-AS1	1.030713	1.014799	1.035886	2.42494	1.2779	0.790253	1
RP1-191J18.66	1.0308	1.014797	1.036002	2.43314	1.2828	0.790253	1
PSMC2	1.563951	1.582815	1.557819	0.95711	-0.0632	0.790597	1
C1orf132	1.035776	1.05119	1.030766	0.60103	-0.7345	0.790598	1
ATP6AP1L	1.192713	1.175703	1.198243	1.12828	0.1741	0.790641	1
LINC00106	1.032548	1.016579	1.037739	2.27636	1.1867	0.79078	1
UAP1	1.60247	1.583631	1.608594	1.04277	0.0604	0.790808	1
GALNS	1.137854	1.120993	1.143335	1.18465	0.2445	0.791032	1
GPR173	1.179111	1.195584	1.173756	0.8884	-0.1707	0.791055	1
AC007405.6	1.18734	1.203695	1.182024	0.89361	-0.1623	0.791082	1
RAB11FIP3	1.04933	1.03328	1.054547	1.63903	0.7128	0.791212	1
MUC4	1.024212	1.039545	1.019228	0.48623	-1.0403	0.791308	1
COL11A2	1.025315	1.040626	1.020338	0.50061	-0.9982	0.791346	1
SOX8	1.08726	1.103164	1.08209	0.79572	-0.3297	0.791372	1
ARVCF	1.275966	1.258378	1.281684	1.0902	0.1246	0.791383	1
DUSP16	1.173881	1.157226	1.179295	1.14036	0.1895	0.791385	1
ALG9	1.125128	1.1087	1.130468	1.20026	0.2634	0.791409	1
RNF111	1.280579	1.263143	1.286247	1.0878	0.1214	0.791437	1
ZNF205	1.244709	1.227468	1.250313	1.10043	0.1381	0.791499	1

RP11-334C17.5	1.090935	1.074484	1.096283	1.29266	0.3703	0.791552	1
DLEU7	1.049531	1.033554	1.054725	1.63097	0.7057	0.791779	1
RNF32	1.093882	1.077573	1.099183	1.27858	0.3545	0.791808	1
ABCA3	1.195858	1.17876	1.201417	1.12674	0.1722	0.791835	1
ATG16L1	1.118809	1.134592	1.113678	0.84462	-0.2436	0.791836	1
NAT16	1.036907	1.020949	1.042094	2.00939	1.0068	0.791837	1
AC009120.10	1.036869	1.020994	1.042029	2.0019	1.0014	0.791837	1
HELZ2	1.120526	1.10403	1.125888	1.21011	0.2751	0.791848	1
RP13-270P17.3	1.04899	1.033014	1.054184	1.64124	0.7148	0.791864	1
CLDN8	1.020741	1.035974	1.01579	0.43892	-1.188	0.791901	1
HCAR2	1.021893	1.03719	1.016921	0.45499	-1.1361	0.791939	1
CPEB1	1.083742	1.09938	1.078659	0.7915	-0.3373	0.791964	1
GRK5	1.124092	1.107499	1.129485	1.20452	0.2685	0.792	1
KDM4D	1.072286	1.05613	1.077537	1.38138	0.4661	0.792046	1
RP11-402D21.2	1.036	1.020114	1.041164	2.04652	1.0332	0.792179	1
MON1A	1.074175	1.05804	1.07942	1.36837	0.4525	0.792222	1
SMIM24	1.080979	1.096597	1.075902	0.78576	-0.3478	0.792252	1
C6orf226	1.211889	1.194814	1.21744	1.11614	0.1585	0.792278	1
ZNF763	1.03955	1.054892	1.034564	0.62967	-0.6673	0.79232	1
TRIM46	1.11282	1.096318	1.118185	1.22702	0.2952	0.792336	1
WDR53	1.088199	1.071941	1.093484	1.29945	0.3779	0.792502	1
C12orf60	1.080342	1.064145	1.085607	1.33459	0.4164	0.792564	1
EXO5	1.071474	1.055432	1.076689	1.38347	0.4683	0.792641	1
LINC00937	1.062997	1.04708	1.068171	1.448	0.5341	0.792689	1
ZNF417	1.062979	1.046999	1.068173	1.45052	0.5366	0.792689	1
BCL6	1.095106	1.110867	1.089982	0.81162	-0.3011	0.792757	1
CTD-3065J16.9	1.058643	1.04262	1.063852	1.49818	0.5832	0.792798	1
MYT1	1.037695	1.021814	1.042857	1.9647	0.9743	0.792828	1
RP11-157P1.4	1.036015	1.051214	1.031074	0.60675	-0.7208	0.792902	1
EXTL3-AS1	1.027473	1.011717	1.032594	2.78172	1.476	0.792975	1
SAP30	1.208524	1.191319	1.214116	1.11915	0.1624	0.792996	1
RP11-10L12.4	1.162837	1.146147	1.168262	1.15132	0.2033	0.7931	1
CNGA1	1.017975	1.033088	1.013062	0.39476	-1.341	0.793104	1
CSTA	1.017974	1.033084	1.013062	0.3948	-1.3408	0.793104	1
CTC-543D15.8	1.108231	1.091979	1.113514	1.23413	0.3035	0.793121	1
AK8	1.040316	1.024445	1.045475	1.86031	0.8955	0.793123	1
EDARADD	1.05253	1.036621	1.057702	1.57566	0.656	0.793123	1
HMP19	1.056625	1.040603	1.061833	1.52287	0.6068	0.793275	1
CBL	1.357857	1.339884	1.3637	1.07007	0.0977	0.793383	1
IMPDH1	1.290037	1.306757	1.284601	0.92777	-0.1082	0.793412	1
KCNJ15	1.023943	1.03902	1.019042	0.48799	-1.0351	0.793424	1
HOXC5	1.024045	1.039437	1.019042	0.48284	-1.0504	0.793424	1
GUCY2D	1.038629	1.053827	1.033688	0.62587	-0.6761	0.793457	1
C11orf1	1.469344	1.451169	1.475252	1.05338	0.075	0.793472	1
PRICKLE1	1.112117	1.12797	1.106964	0.83585	-0.2587	0.793494	1

TMEM178A	1.34336	1.324952	1.349343	1.07506	0.1044	0.793586	1
EFNB3	1.251585	1.234326	1.257195	1.09759	0.1343	0.793672	1
NUMBL	1.157329	1.173035	1.152223	0.87973	-0.1849	0.793872	1
SEC61A2	1.307615	1.324266	1.302202	0.93196	-0.1017	0.793887	1
SRRT	1.332055	1.314658	1.337709	1.07326	0.102	0.793925	1
CTC-444N24.11	1.374583	1.391677	1.369027	0.94217	-0.0859	0.793928	1
LCN12	1.085226	1.100733	1.080186	0.79603	-0.3291	0.793939	1
ANO9	1.035148	1.050204	1.030254	0.60262	-0.7307	0.794077	1
CD27	1.029396	1.013691	1.034501	2.52	1.3334	0.794253	1
JAK3	1.092199	1.0761	1.097432	1.28031	0.3565	0.794315	1
PPP5D1	1.100724	1.084526	1.10599	1.25392	0.3264	0.794333	1
DNAH6	1.04255	1.057786	1.037597	0.65063	-0.6201	0.794404	1
CTXN2	1.054087	1.069308	1.049139	0.709	-0.4961	0.794536	1
RNF43	1.056934	1.072185	1.051977	0.72005	-0.4738	0.794597	1
NPTX1	1.027105	1.042242	1.022185	0.5252	-0.9291	0.794615	1
ZNF667	1.081111	1.065074	1.086324	1.32654	0.4077	0.794736	1
PTTG2	1.031774	1.016045	1.036886	2.29893	1.201	0.794761	1
LHX5	1.033372	1.017512	1.038527	2.20007	1.1376	0.794933	1
ZNF790-AS1	1.03323	1.017512	1.038339	2.18932	1.1305	0.794933	1
CEBPA-AS1	1.051873	1.036027	1.057024	1.58284	0.6625	0.794985	1
CCDC47	1.576962	1.558163	1.583073	1.04463	0.063	0.795062	1
CXorf38	1.200749	1.183914	1.206221	1.12129	0.1652	0.795102	1
UPK1A-AS1	1.120707	1.136223	1.115664	0.84908	-0.236	0.795124	1
CX3CL1	1.063655	1.078805	1.058731	0.74527	-0.4242	0.795232	1
RP11-1055B8.3	1.022206	1.037287	1.017304	0.46408	-1.1076	0.795245	1
HLA-G	1.02373	1.039013	1.018762	0.4809	-1.0562	0.795247	1
RP11-342K6.1	1.065561	1.049528	1.070773	1.42893	0.5149	0.795301	1
SAFB2	1.436224	1.41773	1.442235	1.05866	0.0822	0.795348	1
RIMS1	1.049727	1.06475	1.044844	0.69257	-0.53	0.79542	1
PPP3R1	1.160945	1.177123	1.155686	0.87897	-0.1861	0.795495	1
CALCOCO2	1.382325	1.399102	1.376871	0.9443	-0.0827	0.795545	1
DAPK2	1.032812	1.017227	1.037878	2.19875	1.1367	0.795558	1
THPO	1.032789	1.01719	1.037859	2.20243	1.1391	0.795558	1
RP11-120D5.1	1.032812	1.017211	1.037883	2.20113	1.1382	0.795558	1
RP11-277P12.20	1.047148	1.062215	1.04225	0.6791	-0.5583	0.795755	1
AMPH	1.116036	1.100183	1.121189	1.20967	0.2746	0.795841	1
GIPR	1.046378	1.03062	1.051501	1.68193	0.7501	0.795918	1
IL1R1	1.021434	1.036363	1.016581	0.45598	-1.1329	0.795993	1
VIP	1.021415	1.036289	1.016581	0.45692	-1.13	0.795993	1
SMPD2	1.134136	1.117849	1.139429	1.18312	0.2426	0.79609	1
CECR1	1.330956	1.313343	1.336681	1.07448	0.1036	0.79613	1
NOL6	1.0781	1.093241	1.073179	0.78484	-0.3495	0.796166	1
MAP7D2	1.048642	1.032918	1.053753	1.63295	0.7075	0.796195	1
GBP2	1.117126	1.101271	1.12228	1.20745	0.272	0.796237	1
LFNG	1.084024	1.067947	1.089251	1.31354	0.3935	0.796283	1

ZBTB8B	1.116183	1.131646	1.111157	0.84436	-0.2441	0.796377	1
CTC1	1.06914	1.053343	1.074275	1.3924	0.4776	0.796547	1
PDE8B	1.072148	1.05635	1.077283	1.37149	0.4557	0.796573	1
GGCX	1.33634	1.31874	1.342061	1.07316	0.1019	0.796661	1
DUSP2	1.04592	1.060844	1.04107	0.675	-0.567	0.796753	1
GPATCH2L	1.790067	1.80892	1.783938	0.96912	-0.0453	0.796766	1
LINC00621	1.036284	1.020688	1.041354	1.99895	0.9992	0.796859	1
DEPTOR	1.036237	1.020714	1.041283	1.993	0.9949	0.796859	1
CCSER1	1.066647	1.081813	1.061717	0.75437	-0.4067	0.796868	1
RNGTT	1.183045	1.166496	1.188424	1.1317	0.1785	0.796869	1
HIST1H2AG	1.037783	1.02223	1.042839	1.9271	0.9464	0.796869	1
CCDC122	1.208489	1.191572	1.213989	1.11702	0.1596	0.79691	1
AC093388.3	1.050652	1.035047	1.055724	1.58996	0.669	0.796955	1
MED14OS	1.040255	1.055266	1.035376	0.64011	-0.6436	0.79698	1
ZNF845	1.049754	1.034243	1.054796	1.60024	0.6783	0.797191	1
RP11-413H22.2	1.05598	1.040243	1.061095	1.51816	0.6023	0.797225	1
CTC-559E9.1	1.123281	1.138784	1.118241	0.85198	-0.2311	0.797475	1
ZNF16	1.14355	1.127404	1.148799	1.16793	0.224	0.797506	1
THSD7B	1.031162	1.015676	1.036195	2.30904	1.2073	0.797512	1
CH507-9B2.9	1.038507	1.053457	1.033647	0.62943	-0.6679	0.797513	1
CTD-2371O3.2	1.040136	1.024562	1.045198	1.84019	0.8799	0.797542	1
RP11-98G7.1	1.04991	1.034395	1.054954	1.59775	0.676	0.797545	1
MEMO1	1.227148	1.210428	1.232583	1.10529	0.1444	0.797595	1
YPEL4	1.049981	1.065074	1.045074	0.69266	-0.5298	0.797643	1
TMEM94	1.191478	1.174875	1.196875	1.1258	0.171	0.797677	1
EIF4E3	1.136931	1.120797	1.142175	1.17697	0.2351	0.797844	1
DZIP1	1.846249	1.865039	1.840141	0.97122	-0.0421	0.797977	1
VASH1	1.317495	1.333856	1.312177	0.93507	-0.0969	0.797979	1
CLCC1	1.249984	1.266209	1.24471	0.91924	-0.1215	0.798001	1
PRPF18	1.262777	1.279019	1.257498	0.92287	-0.1158	0.798015	1
ADCY8	1.031413	1.015961	1.036435	2.28276	1.1908	0.798076	1
LEAP2	1.111179	1.094993	1.116441	1.22579	0.2937	0.798091	1
TEX19	1.030308	1.014833	1.035339	2.38251	1.2525	0.798114	1
SLC13A3	1.084769	1.100204	1.079752	0.79589	-0.3294	0.798119	1
GABRB2	1.03503	1.049873	1.030205	0.60564	-0.7235	0.798127	1
TMEM56-RWDD3	1.021752	1.036496	1.01696	0.4647	-1.1056	0.798142	1
MSR1	1.021772	1.036572	1.016962	0.46379	-1.1084	0.798142	1
ATP6V0E2-AS1	1.039769	1.024254	1.044812	1.84762	0.8857	0.798156	1
PTPN3	1.033344	1.048221	1.028508	0.5912	-0.7583	0.798212	1
HS1BP3	1.220319	1.236316	1.215119	0.9103	-0.1356	0.798265	1
CMC2	1.8647	1.845713	1.870872	1.02975	0.0423	0.798291	1
TCEA3	1.089606	1.073656	1.09479	1.28693	0.3639	0.798299	1
RAB40A	1.035022	1.01945	1.040084	2.06091	1.0433	0.798326	1
RP11-528I4.2	1.034935	1.01946	1.039966	2.05378	1.0383	0.798326	1
RP11-57G10.8	1.034961	1.019462	1.039998	2.0552	1.0393	0.798326	1

AKNA	1.151528	1.135013	1.156896	1.16208	0.2167	0.798337	1
TARS2	1.251151	1.234367	1.256607	1.09489	0.1308	0.79842	1
P3H2-AS1	1.075223	1.059369	1.080376	1.35384	0.4371	0.798423	1
TBL3	1.246226	1.22943	1.251686	1.09701	0.1336	0.798457	1
GRAMD1C	1.053321	1.03763	1.058422	1.55255	0.6346	0.798461	1
CSNK1G3	1.543401	1.560776	1.537753	0.95895	-0.0605	0.798494	1
CNNM4	1.056214	1.071414	1.051273	0.71797	-0.478	0.798667	1
RP1-90J20.8	1.056246	1.071095	1.05142	0.72326	-0.4674	0.798667	1
ARNT2	1.161497	1.145187	1.166799	1.14886	0.2002	0.798683	1
GPHN	1.217998	1.201235	1.223447	1.11038	0.1511	0.798706	1
ARHGDIG	1.031574	1.046306	1.026785	0.57843	-0.7898	0.798743	1
RP11-385D13.3	1.126196	1.110235	1.131384	1.19186	0.2532	0.798746	1
NDUFAF5	1.213884	1.23014	1.2086	0.9064	-0.1418	0.798765	1
CORO7	1.14593	1.129616	1.151233	1.16677	0.2225	0.798779	1
RP5-884G6.2	1.034707	1.019172	1.039757	2.07375	1.0522	0.798782	1
POLR1B	1.053334	1.037838	1.058371	1.54263	0.6254	0.798815	1
KCTD7	1.187565	1.171107	1.192915	1.12745	0.1731	0.798827	1
SLPI	1.032697	1.047516	1.02788	0.58675	-0.7692	0.798892	1
NDUFB9	3.963771	4.001376	3.951548	0.9834	-0.0242	0.798954	1
HMSD	1.034831	1.019501	1.039814	2.04164	1.0297	0.799347	1
SOSTDC1	1.204443	1.220239	1.199309	0.90497	-0.1441	0.799412	1
KCNN1	1.044737	1.029226	1.04978	1.70327	0.7683	0.799444	1
RP6-65G23.5	1.073838	1.058022	1.078979	1.36119	0.4449	0.799506	1
ZFPL1	1.413779	1.395771	1.419632	1.06029	0.0845	0.799557	1
DEUP1	1.027376	1.042177	1.022565	0.535	-0.9024	0.799686	1
MELTF	1.025558	1.040224	1.020791	0.51688	-0.9521	0.799724	1
RP11-11N5.3	1.115453	1.099504	1.120637	1.21239	0.2779	0.799769	1
NR2F6	1.878932	1.858108	1.885701	1.03216	0.0457	0.799774	1
GGN	1.038801	1.053667	1.033969	0.63296	-0.6598	0.799797	1
TBC1D19	1.221407	1.237231	1.216263	0.91161	-0.1335	0.799849	1
CIC	1.358559	1.374766	1.353291	0.9427	-0.0851	0.799951	1
RP11-425A6.5	1.023136	1.038036	1.018292	0.48092	-1.0561	0.799965	1
CTD-2515A14.1	1.030187	1.014881	1.035162	2.36285	1.2405	0.8	1
ZNF816-ZNF321P	1.038041	1.022706	1.043026	1.89491	0.9221	0.800048	1
AC002454.1	1.022975	1.037564	1.018232	0.48537	-1.0429	0.800073	1
RP11-589P10.5	1.056963	1.041526	1.06198	1.49257	0.5778	0.800081	1
HTR2A	1.025819	1.040652	1.020998	0.51653	-0.9531	0.800133	1
ATF1	1.418353	1.400509	1.424153	1.05903	0.0827	0.800208	1
USP19	1.127608	1.142848	1.122655	0.85864	-0.2199	0.800266	1
ZC3H11A	1.828981	1.808723	1.835566	1.03319	0.0471	0.800302	1
ZC2HC1A	1.570106	1.588184	1.56423	0.95927	-0.06	0.800448	1
DLST	1.368208	1.350908	1.373832	1.06533	0.0913	0.800563	1
PPP1R7	1.696136	1.714233	1.690254	0.96643	-0.0493	0.80057	1
MYL5	1.159604	1.174789	1.154669	0.88489	-0.1764	0.800643	1
NRG4	1.037852	1.022512	1.042839	1.90293	0.9282	0.800699	1

CXCL3	1.046596	1.031364	1.051547	1.64352	0.7168	0.800736	1
FAM155A	1.061089	1.045529	1.066148	1.45288	0.5389	0.800749	1
RPE65	1.022332	1.036979	1.017571	0.47518	-1.0735	0.800757	1
MSN	1.988043	1.966582	1.99502	1.02942	0.0418	0.800761	1
DNAJC6	1.07791	1.092592	1.073137	0.78989	-0.3403	0.800808	1
S100A8	1.020458	1.035097	1.015699	0.4473	-1.1607	0.80093	1
TRIM3	1.115952	1.131237	1.110984	0.84567	-0.2418	0.800945	1
HECW1	1.031897	1.046615	1.027112	0.58162	-0.7819	0.801025	1
WDTC1	1.332934	1.349018	1.327706	0.93894	-0.0909	0.801029	1
NDUFA11	7.06621	6.995968	7.089043	1.01552	0.0222	0.801043	1
LINC00052	1.021903	1.036565	1.017137	0.46867	-1.0933	0.801061	1
SRC	1.162445	1.177968	1.157399	0.88442	-0.1772	0.801081	1
CHADL	1.042379	1.056966	1.037638	0.6607	-0.5979	0.801088	1
EFHC1	2.383785	2.405913	2.376593	0.97915	-0.0304	0.801114	1
RP1-93H18.1	1.027219	1.011871	1.032208	2.71323	1.44	0.801199	1
KB-1125A3.12	1.027076	1.011871	1.032019	2.69728	1.4315	0.801199	1
GALR2	1.029172	1.043811	1.024413	0.55725	-0.8436	0.801308	1
RP11-219G17.4	1.095736	1.080061	1.100831	1.25943	0.3328	0.801376	1
EIF3G	3.519211	3.555247	3.507497	0.98131	-0.0272	0.801435	1
DHX37	1.085915	1.10081	1.081073	0.80422	-0.3143	0.801466	1
P2RY11	1.082966	1.067375	1.088034	1.30664	0.3859	0.801487	1
GHR	1.041743	1.026483	1.046704	1.76356	0.8185	0.801876	1
AC129492.6	1.040708	1.025349	1.0457	1.80281	0.8503	0.801914	1
FUT2	1.029144	1.013947	1.034084	2.44389	1.2892	0.801953	1
ARMC2	1.051575	1.036294	1.056543	1.5579	0.6396	0.802002	1
PRR19	1.050071	1.034779	1.055042	1.58262	0.6623	0.80202	1
TSGA10IP	1.031004	1.015826	1.035937	2.27076	1.1832	0.802127	1
ZNF862	1.066442	1.050997	1.071462	1.40129	0.4868	0.802182	1
PLIN5	1.028221	1.013102	1.033136	2.52914	1.3386	0.802295	1
CCDC9	1.063483	1.048133	1.068472	1.42257	0.5085	0.802353	1
RP11-301O19.1	1.03051	1.015371	1.035432	2.3051	1.2048	0.802477	1
ZNF33A	1.680194	1.661519	1.686265	1.03741	0.053	0.802482	1
COX20	3.436304	3.394096	3.450024	1.02336	0.0333	0.80249	1
CMTM8	1.340654	1.357114	1.335304	0.93893	-0.0909	0.802557	1
RP11-624L4.1	1.044994	1.029777	1.04994	1.67711	0.746	0.802566	1
NXNL2	1.040108	1.054629	1.035387	0.64778	-0.6264	0.802763	1
MIEF2	1.218061	1.233789	1.212948	0.91086	-0.1347	0.802988	1
CHST2	1.321054	1.33675	1.315952	0.93824	-0.092	0.803006	1
FAM166B	1.045295	1.03	1.050267	1.67558	0.7447	0.803134	1
DHX58	1.030784	1.015694	1.035689	2.2741	1.1853	0.803194	1
PRKCI	1.539239	1.52085	1.545216	1.04678	0.066	0.803204	1
LRRC7	1.044698	1.029559	1.049619	1.67864	0.7473	0.803207	1
UNC5A	1.032527	1.017464	1.037424	2.14292	1.0996	0.803232	1
FBXO28	1.226184	1.209732	1.231532	1.10394	0.1427	0.803244	1
DPY30	3.007853	2.980328	3.0168	1.01842	0.0263	0.803302	1

FAM167A	1.090271	1.074989	1.095239	1.27003	0.3449	0.803386	1
PABPC4L	1.069933	1.054465	1.07496	1.37632	0.4608	0.803435	1
SCAMP4	1.50124	1.518007	1.495789	0.95711	-0.0632	0.803493	1
HERC5	1.048709	1.063325	1.043958	0.69417	-0.5266	0.803562	1
PDE3A	1.06253	1.047387	1.067453	1.42343	0.5094	0.803586	1
RP11-307C12.11	1.066947	1.051679	1.07191	1.39146	0.4766	0.803606	1
CTD-2319I12.2	1.04058	1.02539	1.045518	1.79277	0.8422	0.803812	1
CCNG2	1.655099	1.636748	1.661065	1.03819	0.0541	0.803866	1
RP11-9G1.3	1.068801	1.053594	1.073745	1.376	0.4605	0.804206	1
MROH6	1.158528	1.142482	1.163744	1.14923	0.2007	0.804398	1
PEX12	1.094982	1.079568	1.099992	1.25669	0.3296	0.804478	1
KRTCAP2	2.831804	2.804042	2.840828	1.02039	0.0291	0.804487	1
RP11-318A15.2	1.036007	1.020962	1.040897	1.95104	0.9642	0.804507	1
EMC4	2.689664	2.713522	2.681909	0.98155	-0.0269	0.804539	1
NPAT	1.204667	1.188354	1.209969	1.11476	0.1567	0.804556	1
TIMMDC1	2.009725	2.029136	2.003415	0.97501	-0.0365	0.804707	1
PRRX2	1.021622	1.036062	1.016927	0.4694	-1.0911	0.804767	1
PLD4	1.02159	1.035937	1.016926	0.471	-1.0862	0.804767	1
CYP27C1	1.127786	1.112026	1.132908	1.18641	0.2466	0.804788	1
RP11-211N8.2	1.041218	1.026197	1.046101	1.75976	0.8154	0.804791	1
RP11-568N6.1	1.02279	1.03732	1.018066	0.48409	-1.0467	0.804805	1
RNASEH2B-AS1	1.035167	1.020114	1.04006	1.99168	0.994	0.80485	1
PARD3B	1.289651	1.272936	1.295085	1.08115	0.1126	0.804875	1
LETM2	1.101206	1.085621	1.106272	1.2412	0.3117	0.804893	1
RP11-1246C19.1	1.04641	1.060834	1.041722	0.68583	-0.5441	0.804907	1
SLC46A3	1.06963	1.084151	1.06491	0.77136	-0.3745	0.804919	1
ATXN10	2.657141	2.681786	2.64913	0.98058	-0.0283	0.804934	1
SUGP1	1.157273	1.141464	1.162412	1.14808	0.1992	0.805037	1
CBWD7	1.106069	1.090691	1.111068	1.22469	0.2924	0.805061	1
NME5	1.17799	1.162439	1.183044	1.12685	0.1723	0.80507	1
ZSCAN18	1.885867	1.86609	1.892296	1.03026	0.043	0.805077	1
URB1	1.095922	1.110667	1.09113	0.82346	-0.2802	0.805185	1
CTB-25B13.12	1.043055	1.057441	1.038379	0.66813	-0.5818	0.805235	1
ATP5H	3.589196	3.629001	3.576258	0.97994	-0.0292	0.805325	1
APBA1	1.057798	1.042767	1.062684	1.46573	0.5516	0.805372	1
INPP4A	1.217511	1.201664	1.222662	1.10412	0.1429	0.805511	1
POTEF	1.051005	1.036024	1.055875	1.55102	0.6332	0.805677	1
EYA4	1.028301	1.04267	1.02363	0.55379	-0.8526	0.805683	1
PCDHGA8	1.026603	1.011717	1.031442	2.6834	1.4241	0.80569	1
INTS1	1.208264	1.192069	1.213528	1.11172	0.1528	0.805692	1
GGT5	1.022763	1.037005	1.018134	0.49005	-1.029	0.805826	1
GATM-AS1	1.055729	1.040673	1.060623	1.49051	0.5758	0.80585	1
RP1-202O8.3	1.055763	1.040611	1.060688	1.49436	0.5795	0.80585	1
RP11-320M2.1	1.044776	1.029834	1.049633	1.66365	0.7344	0.806048	1
EFNB2	1.516419	1.533609	1.510832	0.95732	-0.0629	0.806052	1

TBC1D16	1.41731	1.433865	1.411929	0.94944	-0.0748	0.806086	1
RP11-188D8.1	1.038611	1.023639	1.043477	1.83923	0.8791	0.806122	1
EMCN	1.024849	1.039191	1.020187	0.51509	-0.9571	0.806298	1
SLC25A42	1.313613	1.297335	1.318904	1.07254	0.101	0.806418	1
ZNF200	1.13749	1.121519	1.142681	1.17415	0.2316	0.806521	1
POLR3B	1.061283	1.075685	1.056602	0.74786	-0.4192	0.806622	1
RXRG	1.040164	1.025323	1.044988	1.77657	0.8291	0.806747	1
RP11-525K10.3	1.036011	1.050215	1.031394	0.62519	-0.6776	0.806834	1
MTMR1	1.103375	1.118055	1.098603	0.83522	-0.2598	0.806838	1
LA16c-431H6.6	1.094639	1.079187	1.099662	1.25857	0.3318	0.806851	1
ZBTB3	1.055632	1.040633	1.060508	1.48914	0.5745	0.806884	1
RP11-156K23.3	1.113739	1.098281	1.118764	1.20841	0.2731	0.806888	1
RP11-620J15.2	1.028552	1.013691	1.033383	2.43834	1.2859	0.806974	1
DENND4C	1.19994	1.184138	1.205076	1.11371	0.1554	0.807046	1
SFXN2	1.115768	1.100647	1.120683	1.19907	0.2619	0.807069	1
ZFR	2.077478	2.056833	2.084188	1.02588	0.0369	0.807138	1
RP11-474G23.3	1.06639	1.051199	1.071328	1.39316	0.4784	0.807212	1
LINC00618	1.044828	1.029829	1.049703	1.66626	0.7366	0.807219	1
VCPKMT	1.137851	1.152757	1.133005	0.8707	-0.1998	0.807285	1
PACRG	1.094331	1.079046	1.0993	1.25622	0.3291	0.807322	1
SLC30A6	1.14824	1.132585	1.153329	1.15646	0.2097	0.807361	1
PPP2R1B	1.196724	1.212135	1.191714	0.90374	-0.146	0.807387	1
LURAP1L	1.125812	1.140705	1.120971	0.85975	-0.218	0.807392	1
SERP2	1.3885	1.371182	1.394129	1.06182	0.0865	0.807416	1
SPECC1L	1.221405	1.206067	1.22639	1.09863	0.1357	0.807437	1
XDH	1.086387	1.07112	1.09135	1.28446	0.3612	0.807448	1
TMEM52	1.054005	1.068404	1.049324	0.72107	-0.4718	0.807469	1
FMN2	1.129318	1.113894	1.134331	1.17944	0.2381	0.8076	1
PPP1R11	2.08498	2.104369	2.078678	0.97674	-0.034	0.807621	1
DKFZP434A062	1.04215	1.027282	1.046983	1.72214	0.7842	0.807634	1
SHQ1	1.140793	1.125345	1.145815	1.1633	0.2182	0.807696	1
LZTR1	1.152327	1.167267	1.14747	0.88165	-0.1817	0.807803	1
CCDC96	1.033526	1.018756	1.038327	2.04348	1.031	0.807991	1
CADM3	1.210173	1.193971	1.21544	1.11068	0.1514	0.808093	1
EPYC	1.024528	1.038641	1.01994	0.51604	-0.9544	0.808116	1
KCNQ1-AS1	1.024173	1.038308	1.019578	0.51108	-0.9684	0.808143	1
CFAP44	1.044582	1.029702	1.049419	1.66385	0.7345	0.808267	1
ZBTB39	1.145582	1.160595	1.140702	0.87613	-0.1908	0.808415	1
FBXO36	1.180101	1.164371	1.185215	1.12681	0.1722	0.808425	1
SPATA2L	1.1393	1.123692	1.144373	1.1672	0.2231	0.808444	1
BMT2	1.324232	1.307844	1.32956	1.07054	0.0983	0.808467	1
CCDC153	1.090584	1.10496	1.085911	0.81851	-0.2889	0.808481	1
MAP4K2	1.130862	1.145283	1.126174	0.86847	-0.2034	0.80851	1
ZNF471	1.083101	1.068048	1.087993	1.29311	0.3708	0.808683	1
ELF4	1.048834	1.063027	1.044221	0.70163	-0.5112	0.808695	1

PLCL2	1.047126	1.061452	1.042469	0.69109	-0.533	0.808734	1
CTA-212A2.4	1.034285	1.019535	1.03908	2.0005	1.0004	0.808744	1
NBEAL2	1.062375	1.047448	1.067227	1.41687	0.5027	0.808789	1
PIP5KL1	1.060421	1.07466	1.055792	0.74728	-0.4203	0.808791	1
BICDL2	1.020725	1.034767	1.01616	0.46482	-1.1053	0.808798	1
AP006216.10	1.020769	1.03499	1.016146	0.46145	-1.1158	0.808798	1
NRG2	1.054424	1.039491	1.059278	1.50105	0.586	0.808836	1
GPR107	1.227919	1.211928	1.233118	1.09998	0.1375	0.808935	1
CCDC134	1.035704	1.02101	1.04048	1.92675	0.9462	0.808947	1
ADNP-AS1	1.033016	1.047334	1.028362	0.5992	-0.7389	0.809075	1
PQLC1	1.293345	1.277076	1.298634	1.07781	0.1081	0.809109	1
LARS2	1.097303	1.082157	1.102227	1.24428	0.3153	0.809112	1
RP11-923I11.8	1.092116	1.106464	1.087453	0.82143	-0.2838	0.809166	1
SLC16A11	1.045529	1.059799	1.04089	0.6838	-0.5484	0.80933	1
JDP2	1.043652	1.057791	1.039056	0.67581	-0.5653	0.809369	1
MAP3K14-AS1	1.022278	1.036297	1.017721	0.48824	-1.0343	0.809428	1
ERCC4	1.265598	1.249463	1.270843	1.0857	0.1186	0.809446	1
CNNM2	1.101405	1.086329	1.106305	1.23139	0.3003	0.80974	1
KCNE2	1.030571	1.015934	1.035328	2.21713	1.1487	0.8098	1
MIR940	1.044279	1.02951	1.04908	1.66318	0.7339	0.809862	1
RC3H2	1.315139	1.330395	1.31018	0.93881	-0.0911	0.809907	1
APLN	1.049013	1.034269	1.053806	1.57009	0.6508	0.80991	1
MEIOB	1.048944	1.034098	1.053769	1.5769	0.6571	0.80991	1
C17orf100	1.288177	1.271482	1.293603	1.08148	0.113	0.809943	1
ZNF585B	1.037712	1.023048	1.042479	1.84304	0.8821	0.809988	1
CAMKV	1.037755	1.02306	1.042532	1.84444	0.8832	0.809988	1
TP53RK	1.443487	1.459818	1.438178	0.95294	-0.0695	0.810002	1
SBF2-AS1	1.133958	1.148681	1.129173	0.86879	-0.2029	0.810204	1
LL09NC01-251B2.3	1.030365	1.015676	1.035139	2.24168	1.1646	0.810258	1
CSTF3-AS1	1.030255	1.015676	1.034994	2.23242	1.1586	0.810258	1
MDM2	1.566019	1.547599	1.572007	1.04457	0.0629	0.810277	1
ZNF28	1.100791	1.085702	1.105695	1.23329	0.3025	0.810314	1
GRIK5	1.100858	1.085678	1.105793	1.23476	0.3042	0.810314	1
RP11-1072A3.4	1.04048	1.025761	1.045264	1.75706	0.8132	0.810472	1
TRAPPC4	1.736156	1.71769	1.742158	1.03409	0.0484	0.810481	1
PPM1H	1.158556	1.143156	1.163562	1.14255	0.1923	0.810661	1
ZNF551	1.123095	1.108059	1.127983	1.18438	0.2441	0.810757	1
TVP23A	1.1462	1.130766	1.151217	1.15639	0.2096	0.810798	1
LINC00648	1.040354	1.025714	1.045113	1.75444	0.811	0.810812	1
LEF1	1.186978	1.20171	1.182189	0.90322	-0.1468	0.810817	1
EMID1	1.109688	1.094558	1.114606	1.21202	0.2774	0.810894	1
FOLR1	1.034231	1.04816	1.029703	0.61676	-0.6972	0.8109	1
RP11-1114A5.4	1.276602	1.292096	1.271565	0.92971	-0.1051	0.810943	1
ADGB	1.034018	1.019476	1.038745	1.98937	0.9923	0.811053	1
EME2	1.090022	1.075366	1.094786	1.25767	0.3308	0.811132	1

GMIP	1.065236	1.07956	1.06058	0.76144	-0.3932	0.811175	1
LUZP2	1.035157	1.049252	1.030575	0.62078	-0.6879	0.811245	1
TERF2	1.147936	1.162519	1.143195	0.8811	-0.1826	0.811263	1
LYRM4	2.506919	2.4842	2.514304	1.02028	0.029	0.811282	1
FAM117A	1.079471	1.064514	1.084333	1.30721	0.3865	0.811307	1
LINC00926	1.061261	1.046526	1.06605	1.41965	0.5055	0.811323	1
PUSL1	1.252979	1.268189	1.248035	0.92485	-0.1127	0.811399	1
CDK20	1.162906	1.147592	1.167885	1.13749	0.1859	0.811474	1
RP11-299H21.1	1.033788	1.019172	1.038539	2.0102	1.0073	0.811512	1
IGFLR1	1.033714	1.019185	1.038437	2.00354	1.0026	0.811512	1
MYO1F	1.07515	1.060488	1.079916	1.32119	0.4018	0.811523	1
F8A1	1.105011	1.089843	1.109942	1.2237	0.2913	0.81153	1
GLCE	1.232869	1.217071	1.238005	1.09644	0.1328	0.81154	1
DUS1L	1.153737	1.138556	1.158672	1.14518	0.1956	0.811563	1
PRICKLE3	1.103667	1.08886	1.10848	1.22081	0.2878	0.811569	1
NR1H3	1.115652	1.100678	1.12052	1.19708	0.2595	0.811655	1
FSD1L	1.272929	1.25691	1.278135	1.08262	0.1145	0.811677	1
KIAA2026	1.437143	1.45306	1.431969	0.95345	-0.0688	0.811826	1
PROM2	1.027509	1.041409	1.02299	0.55519	-0.8489	0.811943	1
ELOVL3	1.033094	1.047101	1.028541	0.60594	-0.7228	0.812006	1
YWHAH	2.76888	2.737168	2.779189	1.02419	0.0345	0.81205	1
RP3-323P24.3	1.034025	1.019487	1.038751	1.98857	0.9917	0.812079	1
IFIT1	1.043806	1.029232	1.048543	1.66061	0.7317	0.812082	1
XXbac-BPG154L12.4	1.042388	1.027746	1.047148	1.69925	0.7649	0.812107	1
LINC01567	1.029055	1.043079	1.024497	0.56866	-0.8144	0.812184	1
RAB3C	1.058812	1.044074	1.063603	1.44308	0.5292	0.812243	1
ICE1	1.365297	1.380906	1.360222	0.9457	-0.0805	0.812266	1
NUDT16	1.379121	1.394272	1.374196	0.94908	-0.0754	0.812459	1
RP13-516M14.2	1.026573	1.012186	1.03125	2.56432	1.3586	0.812472	1
PAMR1	1.024051	1.037831	1.019571	0.51733	-0.9508	0.812589	1
RP11-517P14.2	1.029598	1.043478	1.025087	0.577	-0.7933	0.812647	1
EGFL8	1.029341	1.014881	1.034041	2.2875	1.1938	0.812754	1
RP11-692D12.1	1.029325	1.014881	1.03402	2.28613	1.1929	0.812755	1
FAM181A	1.037182	1.022703	1.041889	1.84507	0.8837	0.812762	1
RP11-359B12.2	1.107154	1.092219	1.112009	1.21459	0.2805	0.81277	1
IRF1	1.096748	1.110831	1.092171	0.83163	-0.266	0.81286	1
SIRT7	1.188692	1.173292	1.193698	1.11776	0.1606	0.812916	1
RAB11FIP4	1.176288	1.191288	1.171412	0.89609	-0.1583	0.812966	1
SLC46A1	1.106364	1.120627	1.101727	0.84332	-0.2458	0.813179	1
RP11-432I5.2	1.020615	1.034417	1.016128	0.46861	-1.0935	0.813237	1
RFPL1S	1.036977	1.022614	1.041645	1.84154	0.8809	0.813395	1
CDC27	1.459235	1.475691	1.453886	0.95416	-0.0677	0.813439	1
ESPNL	1.040993	1.026496	1.045705	1.72501	0.7866	0.813547	1
DGKE	1.117758	1.102768	1.12263	1.19327	0.2549	0.813563	1
MYL10	1.023191	1.036886	1.01874	0.50805	-0.977	0.81365	1

POTEI	1.05932	1.0448	1.06404	1.42946	0.5155	0.813661	1
RAD17	1.358461	1.342393	1.363685	1.06218	0.087	0.813664	1
ADAMTS12	1.158204	1.172766	1.15347	0.88831	-0.1709	0.813669	1
LYG1	1.051441	1.065356	1.046918	0.71788	-0.4782	0.81367	1
ADAMTS10	1.186345	1.171018	1.191327	1.11875	0.1619	0.813681	1
PCBP3	1.135623	1.150088	1.130921	0.8723	-0.1971	0.813749	1
RP1-78B3.1	1.047664	1.061731	1.043092	0.69806	-0.5186	0.813835	1
ZNF263	1.264238	1.279672	1.259221	0.92688	-0.1096	0.813999	1
C9orf153	1.040631	1.026229	1.045312	1.72755	0.7887	0.814007	1
USP43	1.096151	1.0814	1.100946	1.24013	0.3105	0.814043	1
CLN6	1.120411	1.105416	1.125285	1.18848	0.2491	0.814054	1
TMEM52B	1.018693	1.032423	1.014229	0.43886	-1.1882	0.814119	1
LINC00630	1.050805	1.036335	1.055509	1.52769	0.6113	0.814161	1
MCEE	1.199829	1.184294	1.204879	1.1117	0.1528	0.814227	1
RP4-625H18.2	1.075212	1.060626	1.079953	1.3188	0.3992	0.814311	1
NBPF26	1.075218	1.060621	1.079963	1.31906	0.3995	0.814311	1
PSME1	3.470142	3.439429	3.480125	1.01668	0.0239	0.814373	1
DHODH	1.125313	1.110644	1.130081	1.17567	0.2335	0.814405	1
RP11-438L19.1	1.025602	1.011304	1.030249	2.67588	1.42	0.81442	1
LINC00629	1.02564	1.011304	1.0303	2.68039	1.4224	0.81442	1
TPST1	1.22101	1.235758	1.216217	0.91711	-0.1248	0.814445	1
DYNLT1	4.995619	4.944806	5.012136	1.01707	0.0244	0.814539	1
DLX3	1.041072	1.026545	1.045794	1.72513	0.7867	0.814576	1
TAF4	1.084524	1.069873	1.089286	1.27783	0.3537	0.814627	1
PER1	1.128924	1.11387	1.133817	1.17517	0.2329	0.814647	1
AIP	1.654596	1.63622	1.660569	1.03827	0.0542	0.81467	1
CCDC102A	1.170276	1.155263	1.175156	1.12812	0.1739	0.814672	1
GHET1	1.030152	1.015826	1.034808	2.19942	1.1371	0.814889	1
RP11-620J15.4	1.033488	1.01921	1.03813	1.98491	0.9891	0.814985	1
LINC00290	1.027413	1.013102	1.032065	2.4474	1.2912	0.815058	1
GPR68	1.027369	1.013102	1.032007	2.44297	1.2886	0.815058	1
RGS11	1.040443	1.054245	1.035957	0.66286	-0.5932	0.81508	1
SP140L	1.062902	1.048292	1.067651	1.40087	0.4863	0.815151	1
HDX	1.126198	1.111313	1.131036	1.17719	0.2354	0.815193	1
ZNF502	1.064325	1.049875	1.069022	1.38391	0.4687	0.81549	1
NOL3	1.821848	1.798815	1.829336	1.03821	0.0541	0.815551	1
PREP	1.203025	1.187851	1.207958	1.10703	0.1467	0.815565	1
ZIK1	1.124772	1.110048	1.129558	1.17729	0.2355	0.815637	1
RP5-884C9.2	1.029091	1.014805	1.033735	2.2787	1.1882	0.815682	1
RP11-849H4.2	1.089444	1.075042	1.094126	1.25431	0.3269	0.815774	1
EGR2	1.044321	1.030012	1.048972	1.63171	0.7064	0.815819	1
RP11-46F15.2	1.044516	1.030064	1.049214	1.63698	0.711	0.815819	1
API5	1.505689	1.521679	1.500492	0.95939	-0.0598	0.815851	1
BRPF1	1.101567	1.087054	1.106285	1.22091	0.288	0.815916	1
NTN5	1.02986	1.015687	1.034467	2.19719	1.1357	0.815939	1

RRAGA	1.915205	1.896963	1.921134	1.02695	0.0384	0.816023	1
TP63	1.01967	1.033168	1.015282	0.46074	-1.118	0.816066	1
RP11-498C9.15	1.066201	1.051709	1.070912	1.37137	0.4556	0.816141	1
NAF1	1.167532	1.152732	1.172342	1.1284	0.1743	0.816166	1
RTCA-AS1	1.030765	1.016591	1.035372	2.13197	1.0922	0.816214	1
EPHA5-AS1	1.049255	1.06305	1.04477	0.71008	-0.494	0.816333	1
AGAP4	1.089083	1.074326	1.09388	1.26308	0.3369	0.816372	1
SLC25A27	1.141468	1.126565	1.146313	1.15603	0.2092	0.816477	1
CHERP	1.313028	1.297026	1.318229	1.07138	0.0995	0.816563	1
GHDC	1.082644	1.068108	1.087369	1.28281	0.3593	0.816703	1
SELENOI	1.19458	1.208412	1.190084	0.91206	-0.1328	0.816707	1
GSR	1.128174	1.113304	1.133007	1.1739	0.2313	0.816841	1
PPOX	1.359751	1.343775	1.364944	1.06158	0.0862	0.816922	1
SHROOM1	1.120966	1.135039	1.116392	0.86192	-0.2144	0.816978	1
RTN2	1.126742	1.112003	1.131533	1.17437	0.2319	0.816985	1
RP11-91J19.3	1.030273	1.043891	1.025847	0.58889	-0.7639	0.817003	1
SERPINB8	1.046732	1.032413	1.051387	1.58537	0.6648	0.817095	1
PNMA1	2.078826	2.059247	2.08519	1.02449	0.0349	0.817183	1
CCDC81	1.051353	1.03703	1.056009	1.51251	0.5969	0.817255	1
SETD1B	1.082632	1.096405	1.078156	0.8107	-0.3028	0.817263	1
CDYL	1.302376	1.317251	1.29754	0.93787	-0.0925	0.81743	1
ZNF732	1.040372	1.026271	1.044956	1.71127	0.7751	0.817481	1
NIPSNAP3A	1.321679	1.33673	1.316787	0.94077	-0.0881	0.817511	1
RP11-122M14.1	1.02965	1.015453	1.034265	2.21731	1.1488	0.81752	1
SKIV2L	1.166375	1.180537	1.161772	0.89606	-0.1583	0.817525	1
SH2B1	1.431115	1.446283	1.426184	0.95496	-0.0665	0.81757	1
COX8C	1.03423	1.020088	1.038828	1.93291	0.9508	0.817582	1
INO80B	1.109373	1.123184	1.104884	0.85144	-0.232	0.81761	1
ZNF432	1.144867	1.130048	1.149684	1.15099	0.2029	0.817659	1
GPAT3	1.022455	1.035918	1.018079	0.50335	-0.9904	0.817678	1
MCTS1	1.922248	1.902826	1.928562	1.02851	0.0406	0.81773	1
FAM206A	1.132744	1.146732	1.128197	0.87368	-0.1948	0.817789	1
MARK4	1.310504	1.32527	1.305705	0.93985	-0.0895	0.817795	1
RHBDL1	1.060023	1.045618	1.064705	1.41842	0.5043	0.817865	1
NEDD9	1.29713	1.31121	1.292553	0.94005	-0.0892	0.817899	1
FOXC1	1.032635	1.046143	1.028245	0.61211	-0.7081	0.817938	1
IKZF2	1.109531	1.094783	1.114325	1.20618	0.2704	0.817974	1
XIAP	1.497825	1.480689	1.503396	1.04724	0.0666	0.818043	1
SEZ6L	1.027187	1.040858	1.022743	0.55663	-0.8452	0.818101	1
WARS2	1.199068	1.213811	1.194276	0.90863	-0.1382	0.818224	1
MYO7A	1.023332	1.036756	1.018969	0.51607	-0.9544	0.818283	1
SLCO1A2	1.090297	1.104011	1.085839	0.82529	-0.277	0.818331	1
ENHO	1.110634	1.096083	1.115364	1.20066	0.2638	0.818387	1
ZNF517	1.038628	1.024532	1.04321	1.7614	0.8167	0.818492	1
COBL	1.03771	1.051208	1.033322	0.65072	-0.6199	0.818516	1

UBE2W	1.474687	1.490483	1.469553	0.95733	-0.0629	0.818518	1
C15orf39	1.065617	1.079265	1.061181	0.77186	-0.3736	0.818646	1
ACTN3	1.040564	1.026376	1.045176	1.71277	0.7763	0.818667	1
KYAT1	1.182832	1.16778	1.187725	1.11888	0.1621	0.818722	1
CCDC78	1.043924	1.029741	1.048534	1.6319	0.7066	0.818723	1
RP1-178F15.4	1.037675	1.02363	1.04224	1.7876	0.838	0.818728	1
ZNF625	1.096003	1.081452	1.100733	1.23672	0.3065	0.818773	1
MDN1	1.203751	1.18877	1.20862	1.10516	0.1443	0.818878	1
ZNF155	1.06352	1.049227	1.068166	1.38472	0.4696	0.819122	1
GAK	1.264257	1.27887	1.259507	0.93057	-0.1038	0.819256	1
C17orf80	1.163637	1.148863	1.168439	1.1315	0.1782	0.819268	1
SLN	1.055978	1.069303	1.051646	0.74522	-0.4243	0.81953	1
ZNF295-AS1	1.029223	1.01526	1.033762	2.21249	1.1457	0.819536	1
SLC29A1	1.257498	1.271977	1.252791	0.92946	-0.1055	0.819695	1
TCTEX1D1	1.058419	1.043994	1.063109	1.43449	0.5205	0.819707	1
IGDCC4	1.287065	1.301855	1.282257	0.93508	-0.0968	0.819709	1
CTD-2001C12.1	1.029197	1.015227	1.033738	2.21564	1.1477	0.819737	1
PHKG2	1.316886	1.300636	1.322168	1.07162	0.0998	0.819759	1
RP11-848P1.5	1.055883	1.069532	1.051447	0.7399	-0.4346	0.819779	1
ZBED1	1.322777	1.337724	1.317918	0.94135	-0.0872	0.819787	1
COG2	1.21696	1.202132	1.22178	1.0972	0.1338	0.819987	1
ERICH1	1.739426	1.756845	1.733764	0.9695	-0.0447	0.819988	1
MESTIT1	1.03652	1.022498	1.041078	1.82582	0.8685	0.820054	1
RP11-399K21.11	1.036634	1.022522	1.041222	1.83028	0.8721	0.820054	1
ZHX1-C8orf76	1.056686	1.070185	1.052298	0.74515	-0.4244	0.820128	1
ZSCAN16	1.095616	1.109259	1.091182	0.83454	-0.2609	0.820264	1
RP5-855D21.2	1.030071	1.016045	1.034631	2.15834	1.1099	0.82029	1
RP11-57H14.2	1.03002	1.016045	1.034563	2.15414	1.1071	0.82029	1
MAFK	1.094962	1.108517	1.090556	0.83449	-0.261	0.820307	1
RP11-110I1.12	1.041345	1.027295	1.045912	1.68207	0.7502	0.820334	1
MGRN1	1.511884	1.527667	1.506753	0.96037	-0.0583	0.820417	1
C11orf84	1.132214	1.117649	1.136948	1.16404	0.2191	0.820438	1
PRKAG2-AS1	1.104478	1.090134	1.10914	1.21087	0.276	0.820442	1
TRPC4AP	1.585884	1.569117	1.591334	1.03904	0.0552	0.820706	1
LRRC56	1.05761	1.043555	1.062179	1.4276	0.5136	0.820773	1
PLEKHH2	1.086187	1.072109	1.090763	1.25869	0.3319	0.820817	1
MYO18A	1.082966	1.068756	1.087585	1.27385	0.3492	0.820878	1
RP11-795F19.1	1.066468	1.052348	1.071058	1.35742	0.4409	0.820926	1
ZMYM6	1.206191	1.191194	1.211066	1.10394	0.1427	0.820966	1
GNB1L	1.143251	1.128762	1.147961	1.1491	0.2005	0.821017	1
TTN	1.023986	1.037417	1.01962	0.52435	-0.9314	0.821052	1
CMPK2	1.04743	1.033471	1.051967	1.55262	0.6347	0.821164	1
SHPK	1.080103	1.065963	1.084699	1.28404	0.3607	0.821303	1
MAST2	1.155055	1.168769	1.150597	0.89233	-0.1644	0.82134	1
RP11-356C4.3	1.053604	1.039582	1.058161	1.46938	0.5552	0.82148	1

SH3BP2	1.194687	1.208623	1.190157	0.91148	-0.1337	0.821538	1
CTD-3157E16.2	1.081602	1.067525	1.086177	1.27623	0.3519	0.821613	1
RDH5	1.18283	1.196845	1.178274	0.90566	-0.143	0.821638	1
KANSL1L	1.087998	1.101727	1.083535	0.82117	-0.2842	0.821639	1
GDF5OS	1.026568	1.039773	1.022276	0.56008	-0.8363	0.821649	1
ZC2HC1C	1.067332	1.053152	1.071941	1.3535	0.4367	0.82166	1
CDC14B	1.160611	1.174684	1.156036	0.89325	-0.1629	0.821677	1
LHX9	1.034897	1.021026	1.039406	1.87417	0.9062	0.821714	1
ADO	1.213131	1.227101	1.208591	0.9185	-0.1227	0.82196	1
CSTF2	1.100342	1.114089	1.095874	0.84034	-0.251	0.82196	1
NUP98	1.221329	1.206853	1.226034	1.09273	0.1279	0.821963	1
BARHL2	1.036251	1.02231	1.040783	1.82801	0.8703	0.821993	1
DACH2	1.060535	1.046583	1.06507	1.39688	0.4822	0.822007	1
FAIM	1.276393	1.26137	1.281277	1.07616	0.1059	0.822099	1
CACTIN	1.069861	1.055714	1.074459	1.33644	0.4184	0.822169	1
CTC-425F1.4	1.175292	1.160405	1.180131	1.12298	0.1673	0.822212	1
SULT2B1	1.023114	1.03628	1.018835	0.51915	-0.9458	0.822324	1
NOVA1-AS1	1.02315	1.036327	1.018866	0.51935	-0.9452	0.822324	1
FRK	1.01814	1.0313	1.013862	0.44286	-1.1751	0.822394	1
RP4-738P15.1	1.02574	1.011999	1.030207	2.51744	1.332	0.822452	1
RP4-550H1.7	1.02574	1.011999	1.030206	2.51736	1.3319	0.822452	1
CTA-373H7.7	1.048065	1.034113	1.052601	1.54198	0.6248	0.822468	1
TCAF2	1.048057	1.034185	1.052567	1.53771	0.6208	0.822468	1
C2orf91	1.029767	1.015934	1.034263	2.15027	1.1045	0.822603	1
TRIM68	1.09694	1.110404	1.092564	0.83841	-0.2543	0.82262	1
NPIPB6	1.038737	1.0519	1.034458	0.66393	-0.5909	0.822828	1
RP11-359E3.4	1.038339	1.024563	1.042817	1.74318	0.8017	0.822984	1
TAF6L	1.120842	1.134598	1.11637	0.86458	-0.2099	0.82303	1
RP11-932O9.8	1.029451	1.015676	1.033929	2.16447	1.114	0.823062	1
LINC01579	1.029408	1.015676	1.033872	2.16083	1.1116	0.823062	1
PTGES2-AS1	1.029459	1.015676	1.033939	2.16511	1.1144	0.823062	1
TFIP11	1.131049	1.116813	1.135676	1.16148	0.216	0.823064	1
C1QTNF6	1.224671	1.209804	1.229504	1.0939	0.1295	0.823071	1
GAREM1	1.038505	1.051698	1.034217	0.66186	-0.5954	0.823184	1
C1orf35	1.664231	1.647262	1.669747	1.03474	0.0493	0.823226	1
CYP3A5	1.103807	1.089681	1.108399	1.20872	0.2735	0.823312	1
RASA1	1.264665	1.278773	1.260079	0.93294	-0.1001	0.823324	1
LRP4	1.122577	1.108167	1.127262	1.17653	0.2345	0.823372	1
KCND1	1.03769	1.050813	1.033425	0.6578	-0.6043	0.82346	1
UQCRC2	2.558617	2.580467	2.551515	0.98168	-0.0267	0.823469	1
MICB	1.040761	1.053945	1.036476	0.67617	-0.5645	0.823525	1
AC011747.4	1.038013	1.024228	1.042494	1.75393	0.8106	0.823562	1
PRMT6	1.294018	1.279074	1.298875	1.07095	0.0989	0.823618	1
PPP2R2D	1.369218	1.38387	1.364455	0.94942	-0.0749	0.823657	1
ZNF133	1.078587	1.064457	1.08318	1.29046	0.3679	0.823821	1

TENM1	1.102999	1.116473	1.098619	0.84671	-0.2401	0.823875	1
ATP7A	1.235562	1.220415	1.240486	1.09106	0.1257	0.823966	1
SWSAP1	1.177218	1.162732	1.181927	1.11796	0.1609	0.824003	1
CFAP206	1.051746	1.037915	1.056242	1.48338	0.5689	0.824064	1
CACNA2D4	1.035977	1.049049	1.031728	0.64687	-0.6285	0.824085	1
ARRDC1	1.535572	1.519243	1.54088	1.04167	0.0589	0.824341	1
PNP	1.180357	1.194237	1.175845	0.90531	-0.1435	0.824385	1
NRSN2-AS1	1.06745	1.05354	1.071971	1.34426	0.4268	0.824439	1
MAPK3	1.463315	1.447231	1.468543	1.04765	0.0672	0.824511	1
TAF5	1.093124	1.079314	1.097613	1.23072	0.2995	0.824519	1
ARFRP1	1.385934	1.37026	1.391029	1.05609	0.0787	0.824622	1
ECD	1.187805	1.173105	1.192584	1.11253	0.1538	0.824626	1
RP11-351J23.1	1.020043	1.033029	1.015821	0.479	-1.0619	0.824626	1
PAXIP1-AS2	1.020031	1.032983	1.015821	0.47967	-1.0599	0.824626	1
MACC1	1.055729	1.069185	1.051356	0.7423	-0.4299	0.824744	1
MAIP1	1.282554	1.297047	1.277843	0.93535	-0.0964	0.824898	1
PSPC1	1.391877	1.406713	1.387054	0.95166	-0.0715	0.824959	1
ICAM5	1.028189	1.041211	1.023956	0.58131	-0.7826	0.825074	1
MBNL2	1.484152	1.499207	1.479258	0.96004	-0.0588	0.825141	1
CTB-58E17.1	1.091856	1.07795	1.096376	1.23637	0.3061	0.825239	1
FBXO45	1.152308	1.137766	1.157036	1.13988	0.1889	0.825266	1
CHST7	1.094719	1.108184	1.090342	0.83508	-0.26	0.825286	1
FOXH1	1.02915	1.042236	1.024896	0.58945	-0.7625	0.825419	1
CRYGN	1.024897	1.037798	1.020703	0.54773	-0.8685	0.825499	1
MLPH	1.03047	1.043397	1.026268	0.6053	-0.7243	0.825514	1
RP11-602N24.3	1.028446	1.014881	1.032855	2.20784	1.1426	0.825567	1
AC092198.1	1.028437	1.014881	1.032843	2.20701	1.1421	0.825567	1
CD14	1.028459	1.014881	1.032873	2.20904	1.1434	0.825567	1
TTC39A	1.115588	1.101536	1.120155	1.18337	0.2429	0.825633	1
RP11-10A14.5	1.024753	1.037713	1.02054	0.54463	-0.8766	0.825852	1
OSBPL5	1.116436	1.102209	1.121061	1.18444	0.2442	0.825884	1
PCAT1	1.037058	1.050008	1.032849	0.65688	-0.6063	0.826065	1
ACE	1.023856	1.036749	1.019665	0.53512	-0.9021	0.826127	1
SCGB1B2P	1.035437	1.021849	1.039854	1.82408	0.8672	0.826141	1
RP11-715J22.3	1.035484	1.02192	1.039893	1.81999	0.8639	0.826141	1
VWA5B2	1.021497	1.034409	1.0173	0.50276	-0.992	0.826171	1
WDR4	1.058254	1.044576	1.062701	1.40662	0.4922	0.826171	1
RP11-214O1.3	1.027024	1.039975	1.022814	0.57069	-0.8092	0.82618	1
MRPL3	2.088355	2.068849	2.094695	1.02418	0.0345	0.826211	1
CISD1	2.249058	2.268231	2.242826	0.97997	-0.0292	0.826266	1
GPAM	1.155881	1.141734	1.160479	1.13225	0.1792	0.826344	1
KIAA1147	1.247434	1.261491	1.242864	0.92877	-0.1066	0.826413	1
TP53I11	1.429628	1.413412	1.434898	1.05197	0.0731	0.826493	1
NUDT18	1.206871	1.192041	1.211691	1.10232	0.1405	0.826731	1
RXRB	1.355384	1.339854	1.360432	1.06055	0.0848	0.826737	1

LINC01018	1.025306	1.011871	1.029673	2.49964	1.3217	0.826775	1
AC007392.3	1.025323	1.011871	1.029695	2.50152	1.3228	0.826775	1
LMX1B	1.018003	1.030819	1.013838	0.44901	-1.1552	0.826846	1
LPAR5	1.01799	1.030765	1.013838	0.44979	-1.1527	0.826846	1
PCDHB14	1.15017	1.135782	1.154846	1.14041	0.1895	0.826854	1
CTA-276F8.1	1.06005	1.046391	1.06449	1.39014	0.4752	0.826856	1
ZSWIM6	1.24456	1.229634	1.249412	1.08613	0.1192	0.827009	1
LNPEP	1.507236	1.491044	1.5125	1.04369	0.0617	0.827009	1
GSDMB	1.061011	1.074353	1.056673	0.76222	-0.3917	0.827026	1
PPARG	1.019522	1.032291	1.015371	0.47602	-1.0709	0.827056	1
FBRSL1	1.152134	1.165626	1.147748	0.89206	-0.1648	0.82709	1
FOXJ2	1.08583	1.098872	1.08159	0.82521	-0.2772	0.827153	1
LINC00607	1.017819	1.030586	1.01367	0.44693	-1.1619	0.827198	1
LAMP3	1.017827	1.030616	1.01367	0.44649	-1.1633	0.827198	1
GALC	1.249232	1.234215	1.254113	1.08496	0.1176	0.827244	1
RP11-317P15.4	1.040024	1.026538	1.044407	1.67332	0.7427	0.827333	1
REM2	1.097469	1.083531	1.101999	1.2211	0.2882	0.827356	1
VSNL1	1.039484	1.026026	1.043858	1.68519	0.7529	0.827372	1
TAB3	1.128329	1.141831	1.12394	0.87386	-0.1945	0.827401	1
CFH	1.018742	1.031595	1.014564	0.46098	-1.1172	0.827434	1
RP11-165E7.1	1.027401	1.013947	1.031774	2.27827	1.1879	0.827534	1
AC005609.20	1.027462	1.013947	1.031855	2.28409	1.1916	0.827534	1
KCNE4	1.050862	1.064043	1.046578	0.72729	-0.4594	0.827568	1
LINC00491	1.070906	1.057319	1.075323	1.31409	0.3941	0.827588	1
ANAPC1	1.2404	1.25455	1.2358	0.92634	-0.1104	0.827659	1
RP4-761J14.9	1.016101	1.028882	1.011946	0.41362	-1.2736	0.82773	1
PTGER2	1.016092	1.028845	1.011946	0.41414	-1.2718	0.82773	1
GRIN2A	1.0161	1.028878	1.011946	0.41368	-1.2734	0.82773	1
NKIRAS1	1.231409	1.216806	1.236155	1.08925	0.1233	0.827809	1
TOMM40L	1.082424	1.095478	1.078181	0.81883	-0.2884	0.827814	1
EMG1	1.50322	1.487235	1.508417	1.04347	0.0614	0.827855	1
DUSP8	1.041472	1.05431	1.037299	0.68679	-0.5421	0.827932	1
CDK13	1.34518	1.359724	1.340453	0.94643	-0.0794	0.827946	1
RBFOX2	1.922236	1.903724	1.928254	1.02714	0.0386	0.82798	1
KIAA1462	1.026729	1.039518	1.022572	0.57117	-0.808	0.828059	1
NRP1	1.12872	1.141898	1.124436	0.87694	-0.1894	0.828085	1
FBXO6	1.146986	1.160474	1.142602	0.88863	-0.1703	0.828545	1
SPDYE3	1.043514	1.030057	1.047889	1.59329	0.672	0.828559	1
MFSD4A	1.038048	1.050972	1.033847	0.66403	-0.5907	0.828589	1
SNAPC5	1.526229	1.541296	1.521331	0.96312	-0.0542	0.828659	1
MAPKBP1	1.125131	1.138486	1.12079	0.87222	-0.1972	0.828716	1
SCML2	1.065267	1.051669	1.069687	1.34872	0.4316	0.82873	1
RELT	1.06533	1.051943	1.069682	1.34152	0.4239	0.82873	1
RP11-894P9.1	1.065345	1.051919	1.06971	1.34267	0.4251	0.82873	1
TBC1D4	1.089776	1.102879	1.085517	0.83124	-0.2667	0.828878	1

PNMA3	1.055309	1.068532	1.05101	0.74432	-0.426	0.828905	1
PCLO	1.242545	1.256358	1.238055	0.92861	-0.1069	0.828948	1
GPR150	1.032674	1.019315	1.037016	1.91642	0.9384	0.828956	1
KCNJ12	1.036076	1.022665	1.040435	1.78403	0.8351	0.828998	1
XXbac-BPG294E21.9	1.036155	1.022704	1.040527	1.78503	0.836	0.828998	1
AAED1	1.271067	1.285018	1.266532	0.93514	-0.0967	0.829102	1
PSMB6	3.580023	3.614481	3.568822	0.98254	-0.0254	0.829119	1
RP11-1143G9.4	1.029949	1.016593	1.03429	2.06648	1.0472	0.829126	1
PMM2	1.075562	1.088517	1.071351	0.80607	-0.311	0.829142	1
BBS5	1.107156	1.093387	1.111632	1.19537	0.2575	0.82932	1
TAF1A-AS1	1.08605	1.072595	1.090424	1.2456	0.3168	0.829353	1
RINL	1.113603	1.126611	1.109375	0.86387	-0.2111	0.829368	1
C11orf53	1.019845	1.032683	1.015672	0.4795	-1.0604	0.829398	1
WNT9B	1.019802	1.032508	1.015672	0.48208	-1.0527	0.829398	1
IL17RD	1.2033	1.216807	1.198909	0.91745	-0.1243	0.829403	1
SP1	1.282787	1.296898	1.278201	0.93702	-0.0938	0.829404	1
NDUFB8	5.307634	5.244869	5.328036	1.01959	0.028	0.829467	1
POMGNT1	1.523106	1.537643	1.518381	0.96417	-0.0526	0.829489	1
TSPYL1	1.710141	1.693032	1.715703	1.03271	0.0464	0.829499	1
ITGA4	1.069576	1.056133	1.073946	1.31732	0.3976	0.829596	1
PNPLA4	1.552917	1.537087	1.558063	1.03905	0.0553	0.829656	1
RP11-98D18.9	1.075031	1.061453	1.079444	1.29277	0.3705	0.829713	1
LINC01426	1.031576	1.018286	1.035895	1.96296	0.973	0.829726	1
IPO11	1.125362	1.138682	1.121032	0.87273	-0.1964	0.829731	1
RTN4RL2	1.046972	1.033601	1.051318	1.5273	0.611	0.829781	1
KDM5A	1.839246	1.821888	1.844889	1.02799	0.0398	0.829782	1
LINC01250	1.121521	1.107451	1.126095	1.17351	0.2308	0.829794	1
C18orf65	1.046474	1.033132	1.050811	1.53358	0.6169	0.829807	1
CTC-505O3.3	1.031201	1.044039	1.027028	0.61373	-0.7043	0.829909	1
PLA2G4C	1.031139	1.043976	1.026966	0.61318	-0.7056	0.829909	1
GLI2	1.178527	1.164137	1.183205	1.11617	0.1586	0.829919	1
GATAD1	1.670491	1.654514	1.675685	1.03235	0.0459	0.829997	1
CNTNAP5	1.027927	1.040602	1.023807	0.58635	-0.7702	0.830003	1
LCMT2	1.070979	1.057449	1.075377	1.31207	0.3918	0.830034	1
SLC25A23	1.633012	1.616221	1.638469	1.0361	0.0512	0.830195	1
RP11-521C20.5	1.036242	1.022839	1.040598	1.77761	0.8299	0.830199	1
RP11-771K4.1	1.039556	1.0262	1.043897	1.67547	0.7446	0.830227	1
OLFML3	1.164329	1.178131	1.159842	0.89733	-0.1563	0.830253	1
CH507-39O4.2	1.033423	1.020085	1.037759	1.87997	0.9107	0.830261	1
RABIF	1.332087	1.316767	1.337067	1.06408	0.0896	0.830361	1
GGNBP2	1.887552	1.904144	1.882159	0.97568	-0.0355	0.830407	1
STX1A	1.129683	1.11584	1.134182	1.15834	0.2121	0.830425	1
FPGT	1.084632	1.070991	1.089066	1.2546	0.3272	0.830449	1
LINC01405	1.023302	1.035833	1.019229	0.53664	-0.898	0.83063	1
RP11-201E8.1	1.023361	1.036083	1.019226	0.53283	-0.9083	0.83063	1

BCAN	1.02445	1.037037	1.020358	0.54967	-0.8634	0.830668	1
STXBP5-AS1	1.055684	1.068427	1.051542	0.75324	-0.4088	0.830684	1
CTD-3185P2.1	1.055501	1.068179	1.05138	0.75361	-0.4081	0.830684	1
KLHDC10	1.491739	1.475217	1.497109	1.04607	0.065	0.830688	1
C11orf24	1.261744	1.2758	1.257175	0.93247	-0.1009	0.830718	1
PODN	1.033491	1.0461	1.029392	0.63757	-0.6493	0.830848	1
SLC10A7	1.103184	1.08932	1.10769	1.20567	0.2698	0.830859	1
CSNK2A3	1.049827	1.036484	1.054164	1.48459	0.5701	0.831018	1
PAQR9	1.027987	1.040611	1.023884	0.58812	-0.7658	0.831033	1
SPTLC3	1.039732	1.05236	1.035627	0.68042	-0.5555	0.831171	1
RP11-219B17.3	1.024005	1.036638	1.019899	0.54311	-0.8807	0.831185	1
KCNK7	1.019884	1.032479	1.01579	0.48615	-1.0405	0.831298	1
COX7C	25.0572	25.18359	25.01612	0.99308	-0.01	0.831357	1
PCDHB3	1.038588	1.051153	1.034504	0.67452	-0.5681	0.831386	1
AL049794.1	1.039641	1.026356	1.043959	1.66789	0.738	0.831438	1
PAQR6	1.102697	1.115422	1.09856	0.85392	-0.2278	0.831445	1
MAGI2	1.223349	1.23669	1.219013	0.92531	-0.112	0.831503	1
ATP8B1	1.024497	1.037075	1.020408	0.55046	-0.8613	0.831697	1
DIP2B	1.282809	1.296813	1.278257	0.93748	-0.0931	0.831842	1
C4orf19	1.020538	1.033003	1.016487	0.49956	-1.0013	0.831849	1
C1QL1	1.036998	1.023791	1.041291	1.73555	0.7954	0.831855	1
EDIL3	1.107903	1.120753	1.103726	0.85899	-0.2193	0.831896	1
BATF	1.020767	1.033322	1.016686	0.50074	-0.9979	0.831904	1
LL22NC03-86G7.1	1.087476	1.100368	1.083285	0.8298	-0.2692	0.832123	1
MMP11	1.219632	1.232908	1.215316	0.92447	-0.1133	0.832133	1
ZNF395	1.380782	1.395491	1.376001	0.95072	-0.0729	0.832136	1
SV2C	1.026583	1.03919	1.022485	0.57375	-0.8015	0.832173	1
PP12613	1.038563	1.025286	1.042878	1.69573	0.7619	0.832197	1
PDZD8	1.315596	1.329169	1.311184	0.94536	-0.0811	0.83231	1
EVPL	1.021048	1.033535	1.016989	0.50661	-0.981	0.832363	1
SYT8	1.021043	1.033516	1.016989	0.5069	-0.9802	0.832363	1
MPP3	1.075703	1.062242	1.080079	1.28657	0.3635	0.832373	1
OSBP	1.226092	1.211998	1.230673	1.08809	0.1218	0.832379	1
APELA	1.028421	1.01526	1.0327	2.14288	1.0995	0.832384	1
RNF25	1.09863	1.085162	1.103008	1.20955	0.2745	0.832415	1
MIB2	1.472472	1.487069	1.467727	0.96029	-0.0585	0.832422	1
RP11-73E17.2	1.064557	1.051264	1.068878	1.34361	0.4261	0.832423	1
ZNF239	1.064666	1.051226	1.069035	1.34766	0.4305	0.832423	1
KCNJ6	1.017122	1.029613	1.013062	0.44109	-1.1809	0.832515	1
CA4	1.017109	1.029561	1.013062	0.44186	-1.1783	0.832515	1
CAD	1.265483	1.279347	1.260976	0.93424	-0.0981	0.832547	1
FBXL4	1.263842	1.249482	1.26851	1.07627	0.106	0.832563	1
RP11-797H7.5	1.032825	1.045374	1.028746	0.63354	-0.6585	0.832564	1
CLPSL2	1.026803	1.013691	1.031065	2.26906	1.1821	0.83259	1
C3orf18	1.18771	1.173552	1.192313	1.1081	0.1481	0.832667	1

ZNF454	1.043153	1.029906	1.047459	1.58693	0.6662	0.832672	1
RP11-449J10.1	1.046454	1.033204	1.05076	1.52872	0.6123	0.832674	1
VPS45	1.23785	1.25159	1.233383	0.92763	-0.1084	0.832722	1
PLCB2	1.040278	1.027108	1.044559	1.64375	0.717	0.832734	1
TEP1	1.045097	1.057565	1.041044	0.71299	-0.488	0.832743	1
RP11-302B13.5	1.0344	1.047031	1.030294	0.64413	-0.6346	0.832802	1
C5orf66-AS1	1.023131	1.03571	1.019042	0.53323	-0.9072	0.832838	1
SULT1E1	1.023136	1.035731	1.019042	0.53292	-0.908	0.832838	1
KLRC2	1.023144	1.035763	1.019042	0.53245	-0.9093	0.832838	1
ACSS1	1.067268	1.053976	1.071589	1.32632	0.4074	0.832843	1
LRRC8D	1.117445	1.10372	1.121907	1.17534	0.2331	0.83288	1
TTLL9	1.040591	1.027292	1.044914	1.64571	0.7187	0.83309	1
CLDN12	1.305286	1.291194	1.309867	1.06413	0.0897	0.833174	1
PCDH17	1.101012	1.113922	1.096815	0.84984	-0.2347	0.83318	1
IFNGR1	1.261821	1.275674	1.257317	0.93341	-0.0994	0.833181	1
PDZD7	1.084264	1.096933	1.080145	0.82681	-0.2744	0.833253	1
PTGER3	1.02826	1.04074	1.024204	0.59411	-0.7512	0.833357	1
RIMS2	1.167715	1.180901	1.163429	0.90342	-0.1465	0.83339	1
RP11-626G11.3	1.132467	1.145564	1.128209	0.88077	-0.1832	0.833431	1
ERN1	1.080433	1.093207	1.076281	0.81841	-0.2891	0.833438	1
KCNJ13	1.079133	1.065804	1.083465	1.26839	0.343	0.833468	1
RP11-315O6.1	1.042834	1.029736	1.047092	1.58366	0.6633	0.833687	1
NFS1	1.170464	1.156446	1.17502	1.11873	0.1619	0.833723	1
TSPEAR-AS1	1.044678	1.031464	1.048974	1.5565	0.6383	0.833726	1
LACC1	1.049779	1.062257	1.045723	0.73442	-0.4453	0.833875	1
AK7	1.0437	1.030627	1.04795	1.56558	0.6467	0.833964	1
RP11-776H12.1	1.026238	1.038661	1.0222	0.57422	-0.8003	0.833981	1
JSRP1	1.026238	1.038729	1.022178	0.57265	-0.8043	0.833981	1
SRGAP2C	1.340637	1.325728	1.345484	1.06065	0.0849	0.834018	1
RGMB	1.230964	1.244103	1.226694	0.92868	-0.1067	0.834081	1
C19orf84	1.027512	1.039993	1.023455	0.58648	-0.7698	0.834115	1
ADPGK	1.4135	1.427474	1.408957	0.95668	-0.0639	0.834133	1
CD302	1.062176	1.07475	1.058089	0.77711	-0.3638	0.834198	1
RP5-864K19.4	1.048842	1.061369	1.04477	0.72953	-0.455	0.834264	1
DUSP5	1.051963	1.064486	1.047892	0.74266	-0.4292	0.834317	1
ARMC4	1.043868	1.030846	1.0481	1.55939	0.641	0.834321	1
NR3C2	1.032671	1.019548	1.036937	1.88962	0.9181	0.834356	1
RP11-110I1.13	1.032647	1.019561	1.036901	1.88643	0.9157	0.834356	1
KIF5B	3.373044	3.400291	3.364187	0.98496	-0.0219	0.834416	1
CABYR	1.083512	1.070099	1.087872	1.25354	0.326	0.834427	1
GAL3ST4	1.093945	1.10656	1.089844	0.84313	-0.2462	0.834471	1
CHAC2	1.05927	1.071818	1.055191	0.76849	-0.3799	0.834487	1
KIF7	1.059293	1.071748	1.055244	0.76998	-0.3771	0.834487	1
RAB2B	1.254066	1.240015	1.258634	1.07757	0.1078	0.834497	1
LINC00493	3.415374	3.386217	3.424852	1.01619	0.0232	0.834531	1

SLC18A2	1.034136	1.021025	1.038397	1.82629	0.8689	0.834536	1
RP11-677M14.7	1.059685	1.046606	1.063937	1.37184	0.4561	0.834668	1
FAM157C	1.059689	1.046521	1.06397	1.37507	0.4595	0.834668	1
CCDC183	1.059741	1.046475	1.064053	1.37822	0.4628	0.834668	1
KRT15	1.022431	1.034767	1.01842	0.52982	-0.9164	0.834668	1
LINC00304	1.021314	1.033651	1.017304	0.51422	-0.9595	0.83468	1
RP11-74C3.1	1.022775	1.035135	1.018757	0.53386	-0.9055	0.834687	1
C7orf73	4.469687	4.425203	4.484147	1.01721	0.0246	0.83483	1
TESK1	1.149256	1.162048	1.145098	0.8954	-0.1594	0.834902	1
SESN2	1.218706	1.204205	1.223419	1.09409	0.1297	0.834981	1
SH3RF3	1.042973	1.055251	1.038982	0.70554	-0.5032	0.834994	1
MBNL1-AS1	1.042975	1.055381	1.038943	0.70318	-0.508	0.834994	1
GDAP2	1.174763	1.187714	1.170554	0.90858	-0.1383	0.835036	1
FAM20A	1.056028	1.068615	1.051936	0.75693	-0.4018	0.83516	1
VPS11	1.112211	1.125076	1.108029	0.86371	-0.2114	0.835173	1
LRIG2	1.226942	1.213071	1.231451	1.08626	0.1194	0.835174	1
RP11-656D10.3	1.047248	1.034275	1.051465	1.50151	0.5864	0.83519	1
TGFA	1.024015	1.036223	1.020047	0.55343	-0.8535	0.83526	1
BRI3BP	1.197422	1.183875	1.201825	1.09762	0.1344	0.835278	1
GRM7	1.024862	1.011999	1.029044	2.42047	1.2753	0.83531	1
HMGCS2	1.018989	1.031254	1.015001	0.47998	-1.059	0.835374	1
DYNLRB2	1.056106	1.042984	1.060372	1.40453	0.4901	0.835392	1
FLJ21408	1.028877	1.015934	1.033084	2.0763	1.054	0.835461	1
RP11-383I23.2	1.028867	1.015934	1.03307	2.07542	1.0534	0.835461	1
KB-226F1.2	1.028858	1.015934	1.033059	2.0747	1.0529	0.835461	1
INTS5	1.133491	1.146205	1.129358	0.88478	-0.1766	0.835475	1
C2orf27B	1.069923	1.056719	1.074215	1.30847	0.3879	0.835494	1
TMED8	1.298365	1.311833	1.293987	0.94277	-0.085	0.835612	1
RITA1	1.583816	1.598676	1.578986	0.96711	-0.0482	0.835662	1
UNC93B1	1.075826	1.088246	1.071788	0.8135	-0.2978	0.83572	1
ATP13A4	1.037534	1.02463	1.041729	1.69419	0.7606	0.83579	1
RP11-158M9.1	1.037552	1.024519	1.041789	1.70436	0.7692	0.83579	1
KLHL25	1.06363	1.050531	1.067887	1.34348	0.426	0.835824	1
FLT3LG	1.049001	1.035914	1.053255	1.48287	0.5684	0.835873	1
C2	1.039402	1.051743	1.035391	0.68397	-0.548	0.835928	1
STX5	1.55896	1.54285	1.564197	1.03932	0.0556	0.836	1
C16orf70	1.104874	1.117381	1.100809	0.85882	-0.2196	0.836069	1
AP001059.6	1.027858	1.040204	1.023845	0.5931	-0.7537	0.836213	1
WFIKKN2	1.040391	1.052665	1.036401	0.69119	-0.5329	0.836276	1
PLSCR4	1.038712	1.025809	1.042906	1.6624	0.7333	0.83629	1
MAPK8	1.369517	1.354837	1.374289	1.05482	0.077	0.836387	1
FBXL13	1.077714	1.064541	1.081996	1.27045	0.3453	0.836389	1
DNAH10	1.028764	1.015961	1.032926	2.06292	1.0447	0.836491	1
USHBP1	1.028807	1.015961	1.032983	2.06644	1.0471	0.836491	1
RP4-753F5.1	1.02832	1.015515	1.032483	2.09369	1.066	0.836529	1

ADAD2	1.0171	1.029269	1.013144	0.44906	-1.155	0.836665	1
RELL1	1.063124	1.075554	1.059083	0.782	-0.3548	0.836758	1
LINC01063	1.039519	1.026598	1.043719	1.64367	0.7169	0.836774	1
MYOZ3	1.067061	1.05411	1.071271	1.31714	0.3974	0.837015	1
VAMP3	1.985413	2.002273	1.979933	0.97771	-0.0325	0.837173	1
RECQL	1.447017	1.431629	1.452019	1.04724	0.0666	0.837177	1
RNF144A	1.213284	1.226278	1.20906	0.92391	-0.1142	0.837218	1
ALG1L2	1.127412	1.114008	1.13177	1.1558	0.2089	0.837351	1
SPAST	1.373216	1.358633	1.377957	1.05388	0.0757	0.837439	1
ARHGAP20	1.035309	1.047685	1.031286	0.6561	-0.608	0.837478	1
ARRDC3-AS1	1.035287	1.047581	1.031291	0.65764	-0.6046	0.837478	1
NDST2	1.055336	1.067647	1.051335	0.75886	-0.3981	0.837482	1
SHC4	1.042105	1.029246	1.046285	1.58262	0.6623	0.837528	1
SP2	1.13136	1.118092	1.135673	1.14887	0.2002	0.837576	1
PLSCR1	1.16094	1.173879	1.156734	0.90139	-0.1498	0.837684	1
PAQR9-AS1	1.031688	1.043855	1.027734	0.6324	-0.6611	0.837693	1
RP11-1109F11.5	1.029228	1.041339	1.025291	0.61179	-0.7089	0.837752	1
LINC00877	1.056354	1.043468	1.060543	1.3928	0.478	0.837832	1
PDK3	1.272498	1.258532	1.277038	1.07158	0.0997	0.837884	1
HIST1H3H	1.035741	1.022966	1.039894	1.73707	0.7967	0.837897	1
SNX12	1.474701	1.489059	1.470033	0.9611	-0.0572	0.837976	1
CTC-459F4.1	1.054266	1.04137	1.058458	1.41304	0.4988	0.83799	1
MED17	1.397031	1.382904	1.401623	1.04889	0.0689	0.837996	1
SPATA5	1.123648	1.110695	1.127859	1.15506	0.208	0.838	1
HIST1H2AC	1.09922	1.085981	1.103523	1.20402	0.2679	0.838041	1
PARP4	1.22162	1.234618	1.217394	0.92659	-0.11	0.838056	1
FAM131A	1.284465	1.297668	1.280174	0.94123	-0.0874	0.838061	1
PRRC1	1.444345	1.458178	1.439849	0.95999	-0.0589	0.838088	1
HIPK1-AS1	1.024864	1.012186	1.028984	2.37842	1.25	0.838151	1
RNF149	1.351473	1.365135	1.347031	0.95042	-0.0734	0.838167	1
NEFH	1.070533	1.057609	1.074734	1.29726	0.3755	0.838203	1
CTC-575N7.1	1.027623	1.014881	1.031765	2.13455	1.0939	0.838435	1
KATNA1	1.320984	1.306564	1.325671	1.06232	0.0872	0.838509	1
NDUFS1	1.471189	1.484995	1.466701	0.96228	-0.0555	0.838592	1
KIAA0040	1.025645	1.037849	1.021678	0.57276	-0.804	0.838693	1
RP11-126O1.5	1.060416	1.047555	1.064596	1.35834	0.4418	0.838782	1
SLC1A7	1.025577	1.037599	1.021669	0.57632	-0.7951	0.838802	1
SP110	1.057594	1.044686	1.061789	1.38276	0.4675	0.838845	1
PACSIN1	1.094394	1.106844	1.090347	0.8456	-0.242	0.838845	1
PUM3	1.357797	1.34344	1.362464	1.05539	0.0778	0.838858	1
MED29	1.548457	1.533115	1.553444	1.03813	0.054	0.838865	1
MAPT	1.134062	1.120663	1.138418	1.14715	0.1981	0.838916	1
RP11-621L6.3	1.037955	1.050033	1.034029	0.68013	-0.5561	0.838951	1
BCL11B	1.057518	1.044681	1.061691	1.38068	0.4654	0.838955	1
FCGR2A	1.034594	1.021867	1.038731	1.7712	0.8247	0.838969	1

ADARB2	1.034604	1.021852	1.03875	1.77328	0.8264	0.838969	1
NPAS1	1.133842	1.1464	1.12976	0.88634	-0.1741	0.839002	1
CTU2	1.12544	1.137861	1.121403	0.88062	-0.1834	0.839027	1
RASA3	1.149094	1.135295	1.15358	1.13515	0.1829	0.839036	1
ABL2	1.284869	1.270689	1.289479	1.06942	0.0968	0.839078	1
CTC-526N19.1	1.027865	1.039918	1.023947	0.59991	-0.7372	0.83911	1
AFP	1.022286	1.034275	1.018389	0.5365	-0.8983	0.839144	1
DNAJC11	1.162256	1.148875	1.166605	1.1191	0.1623	0.839197	1
LINC00574	1.028348	1.015668	1.03247	2.07239	1.0513	0.839376	1
PLEKHF1	1.119274	1.106318	1.123486	1.16148	0.216	0.839413	1
RP11-452F19.3	1.11955	1.106542	1.123778	1.16177	0.2163	0.839413	1
RNF223	1.022217	1.034376	1.018265	0.53134	-0.9123	0.839498	1
RBM19	1.254285	1.240483	1.258772	1.07605	0.1057	0.839517	1
AC108488.4	1.047882	1.035191	1.052007	1.47787	0.5635	0.839527	1
RP5-1024G6.5	1.038859	1.02623	1.042964	1.63799	0.7119	0.839571	1
HAAO	1.08287	1.0701	1.087021	1.24138	0.3119	0.83963	1
SLC25A34	1.024443	1.011871	1.028529	2.40329	1.265	0.839647	1
RP11-314N13.3	1.031055	1.018383	1.035175	1.91347	0.9362	0.839661	1
CYB561	1.158472	1.145165	1.162798	1.12147	0.1654	0.839712	1
LSG1	1.135959	1.122906	1.140202	1.14072	0.1899	0.839717	1
CLDN23	1.021266	1.0332	1.017387	0.52373	-0.9331	0.839774	1
EPN3	1.021277	1.03325	1.017385	0.52285	-0.9355	0.839774	1
DGKQ	1.061606	1.073819	1.057636	0.78077	-0.357	0.839779	1
KLK7	1.024473	1.036502	1.020562	0.56331	-0.828	0.839802	1
ZNF628	1.061838	1.049069	1.065989	1.34481	0.4274	0.839814	1
FAM85B	1.044786	1.031993	1.048945	1.52986	0.6134	0.839836	1
C3	1.018894	1.030939	1.014979	0.48414	-1.0465	0.839842	1
CTD-2555C10.3	1.01887	1.03083	1.014983	0.48599	-1.041	0.839842	1
YEATS2	1.381069	1.366767	1.385718	1.05167	0.0727	0.839887	1
DALRD3	1.726567	1.741167	1.721822	0.9739	-0.0382	0.839906	1
RPA4	1.031774	1.043767	1.027875	0.63689	-0.6509	0.839924	1
ZNF598	1.136721	1.149369	1.132609	0.8878	-0.1717	0.840049	1
PTCH2	1.061014	1.048302	1.065146	1.34873	0.4316	0.840055	1
RP11-148B18.1	1.018692	1.030585	1.014826	0.48475	-1.0447	0.840088	1
SGCZ	1.018797	1.030995	1.014832	0.47852	-1.0633	0.840088	1
RP11-513M16.8	1.039207	1.026477	1.043344	1.63705	0.7111	0.840144	1
RP11-368I23.3	1.038718	1.026044	1.042838	1.64487	0.718	0.840162	1
SH2B2	1.123085	1.109931	1.12736	1.15854	0.2123	0.840173	1
DUSP7	1.081866	1.094118	1.077883	0.82751	-0.2732	0.840179	1
RP1-124C6.1	1.018737	1.030824	1.014808	0.4804	-1.0577	0.840197	1
PSMC5	2.617644	2.597702	2.624126	1.01654	0.0237	0.840201	1
ACTR5	1.069914	1.057051	1.074095	1.29876	0.3771	0.840206	1
TAF6	1.771534	1.755589	1.776716	1.02796	0.0398	0.840218	1
PHF20L1	1.701628	1.716463	1.696806	0.97256	-0.0401	0.840285	1
RP11-715J22.2	1.024686	1.012187	1.028749	2.35907	1.2382	0.84037	1

ACTL6B	1.024779	1.012187	1.028873	2.36919	1.2444	0.84037	1
RP11-410N8.3	1.026514	1.013947	1.030599	2.19404	1.1336	0.840408	1
PCTP	1.195199	1.181472	1.199661	1.10023	0.1378	0.840471	1
CD2AP	1.516545	1.501679	1.521378	1.03927	0.0556	0.840565	1
DLGAP3	1.028399	1.015826	1.032485	2.05264	1.0375	0.840584	1
CALCB	1.031805	1.019283	1.035875	1.86047	0.8957	0.840597	1
PCDHA10	1.076949	1.064185	1.081098	1.26349	0.3374	0.840637	1
CTD-2116N20.1	1.025576	1.013073	1.02964	2.26736	1.181	0.840645	1
APLNR	1.025671	1.013073	1.029766	2.27702	1.1871	0.840645	1
RP11-196E1.3	1.016966	1.028898	1.013087	0.45287	-1.1428	0.84073	1
EML5	1.016996	1.029021	1.013087	0.45094	-1.149	0.84073	1
HOXA-AS2	1.016968	1.028922	1.013082	0.45234	-1.1445	0.84073	1
RP11-219D15.3	1.025601	1.013102	1.029664	2.26417	1.179	0.840754	1
RREB1	1.238858	1.251202	1.234846	0.93489	-0.0971	0.840757	1
ZNF850	1.042395	1.029705	1.04652	1.56608	0.6472	0.84078	1
RP11-387M24.5	1.027937	1.015369	1.032022	2.08349	1.059	0.840875	1
C17orf64	1.025847	1.013274	1.029934	2.25507	1.1732	0.841	1
ADAMTS4	1.059557	1.071849	1.055561	0.7733	-0.3709	0.841032	1
SHANK2	1.059496	1.071664	1.05554	0.77501	-0.3677	0.841032	1
WIPF2	1.397164	1.410352	1.392877	0.95741	-0.0628	0.841145	1
TRIML2	1.017546	1.029404	1.013692	0.46565	-1.1027	0.841194	1
PRR15L	1.01761	1.029645	1.013698	0.46207	-1.1138	0.841194	1
NKX3-1	1.01755	1.029412	1.013694	0.4656	-1.1028	0.841194	1
CTC-529P8.1	1.017574	1.0295	1.013697	0.46433	-1.1068	0.841194	1
AC009228.1	1.04268	1.030069	1.04678	1.55575	0.6376	0.841354	1
RP11-89C3.4	1.041464	1.028821	1.045574	1.58129	0.6611	0.841392	1
CCDC126	1.235473	1.248527	1.23123	0.9304	-0.1041	0.841401	1
PARN	1.208291	1.194606	1.21274	1.09318	0.1285	0.841405	1
ZNF513	1.12995	1.142538	1.125858	0.88298	-0.1795	0.84142	1
LRFN3	1.144453	1.15668	1.140479	0.8966	-0.1575	0.841483	1
PAUPAR	1.046235	1.033651	1.050326	1.49554	0.5807	0.841519	1
TNPO2	1.326973	1.339961	1.322752	0.94938	-0.0749	0.841531	1
ZMYND12	1.028213	1.015679	1.032288	2.05933	1.0422	0.841599	1
RP5-1112D6.8	1.064674	1.051903	1.068826	1.32604	0.4071	0.841622	1
NRXN1	1.031827	1.019331	1.035889	1.85654	0.8926	0.841814	1
RP11-48G14.3	1.035226	1.022705	1.039296	1.73071	0.7914	0.841814	1
AC025335.1	1.08039	1.06774	1.084502	1.24745	0.319	0.841819	1
LSINCT5	1.02906	1.016584	1.033115	1.99686	0.9977	0.841875	1
ZNRF3-AS1	1.051389	1.063603	1.047418	0.74553	-0.4237	0.841914	1
B4GALNT3	1.03133	1.018876	1.035379	1.87428	0.9063	0.842099	1
LINC01137	1.098769	1.085694	1.103019	1.20217	0.2656	0.842114	1
ADGRE5	1.074215	1.061169	1.078455	1.2826	0.3591	0.842299	1
ARHGAP32	1.204138	1.190504	1.20857	1.09483	0.1307	0.842323	1
NDUFA10	1.996423	2.012924	1.99106	0.97841	-0.0315	0.842345	1
EP400NL	1.138825	1.151332	1.13476	0.89049	-0.1673	0.842346	1

TMC5	1.020652	1.032451	1.016817	0.51823	-0.9483	0.842405	1
ADAM23	1.072965	1.084822	1.06911	0.81477	-0.2955	0.84247	1
RP11-635N19.1	1.225962	1.212683	1.230278	1.08273	0.1147	0.842531	1
RP13-638C3.4	1.030748	1.018312	1.03479	1.89985	0.9259	0.842565	1
LINC01088	1.049611	1.037044	1.053696	1.44953	0.5356	0.842718	1
DHDH	1.049645	1.03704	1.053743	1.45096	0.537	0.842718	1
ADCYAP1	1.031827	1.043701	1.027968	0.63998	-0.6439	0.842785	1
CELF1	2.261253	2.243462	2.267036	1.01896	0.0271	0.842786	1
ZNF524	1.197409	1.209752	1.193397	0.92202	-0.1171	0.842811	1
RP11-677M14.3	1.03169	1.019203	1.035749	1.86157	0.8965	0.842825	1
PVT1	1.129698	1.116335	1.134042	1.15221	0.2044	0.842863	1
WIPF1	1.315729	1.302326	1.320085	1.05874	0.0824	0.842992	1
ING1	1.257538	1.271048	1.253146	0.93396	-0.0986	0.843006	1
PDCD5	3.429918	3.454682	3.421868	0.98663	-0.0194	0.843013	1
PCDHB12	1.03533	1.022881	1.039377	1.72098	0.7832	0.84304	1
RP11-396F22.1	1.088498	1.075738	1.092645	1.22324	0.2907	0.843071	1
RP11-7F17.8	1.032501	1.020076	1.036539	1.82005	0.864	0.843101	1
LRP2BP	1.069496	1.056924	1.073582	1.29264	0.3703	0.843115	1
CDK16	3.313635	3.28284	3.323645	1.01787	0.0256	0.843227	1
ENO1-AS1	1.041385	1.028907	1.045442	1.572	0.6526	0.843313	1
HGF	1.041439	1.028954	1.045497	1.57134	0.652	0.843313	1
RP11-449P15.2	1.028303	1.040091	1.024471	0.61038	-0.7122	0.843469	1
AC137934.1	1.028347	1.040085	1.024532	0.612	-0.7084	0.843469	1
PPP4R1	1.329466	1.31541	1.334035	1.05905	0.0828	0.843512	1
AGPS	1.290419	1.276349	1.294993	1.06746	0.0942	0.843528	1
LRRC4	1.028596	1.040573	1.024703	0.60885	-0.7158	0.84354	1
RP11-295H24.4	1.049623	1.037077	1.053702	1.4484	0.5345	0.84376	1
CREBRF	1.376318	1.362156	1.380921	1.05181	0.0729	0.843762	1
PTGER1	1.034388	1.046328	1.030507	0.65851	-0.6027	0.843795	1
RP11-44F21.5	1.127346	1.114766	1.131435	1.14524	0.1957	0.843845	1
NBPF11	1.108339	1.120486	1.104391	0.86642	-0.2069	0.84389	1
ITGA2B	1.053086	1.040538	1.057164	1.41014	0.4958	0.843913	1
ADAMTS13	1.067287	1.079383	1.063355	0.79808	-0.3254	0.844065	1
ZNF575	1.036922	1.024469	1.040971	1.6744	0.7436	0.844085	1
MYBPC2	1.02485	1.036577	1.021039	0.57519	-0.7979	0.844155	1
SLX1B	1.025068	1.036832	1.021244	0.57678	-0.7939	0.844229	1
TSSC4	1.586141	1.599635	1.581754	0.97018	-0.0437	0.84423	1
RP11-76I14.1	1.038738	1.026409	1.042746	1.61861	0.6948	0.844262	1
MYCL	1.110149	1.097457	1.114274	1.17256	0.2297	0.844263	1
TCEB1	3.650917	3.621601	3.660447	1.01482	0.0212	0.844367	1
PRDM8	1.035988	1.023604	1.040013	1.69517	0.7614	0.844433	1
C5orf30	1.236159	1.249196	1.231921	0.93068	-0.1036	0.844462	1
RP11-16K12.1	1.031306	1.018985	1.035311	1.8599	0.8952	0.844467	1
STEAP1B	1.031315	1.018948	1.035335	1.86488	0.8991	0.844467	1
HGFAC	1.030885	1.042629	1.027067	0.63495	-0.6553	0.844481	1

CTU1	1.038259	1.02592	1.04227	1.63078	0.7056	0.844535	1
TFB2M	1.136088	1.123032	1.140332	1.14061	0.1898	0.844605	1
DNAJB5	1.14894	1.135925	1.153171	1.12687	0.1723	0.844692	1
SH3RF1	1.377429	1.363396	1.38199	1.05117	0.072	0.844744	1
ZNF14	1.077783	1.065026	1.08193	1.25996	0.3334	0.844792	1
ECHDC3	1.052448	1.040049	1.056479	1.41022	0.4959	0.84495	1
ACBD4	1.149242	1.136143	1.1535	1.12749	0.1731	0.844952	1
EPHA1-AS1	1.037621	1.025327	1.041618	1.64323	0.7165	0.845003	1
ZNF83	1.556258	1.541641	1.56101	1.03576	0.0507	0.845078	1
ASTE1	1.070251	1.057916	1.07426	1.28221	0.3586	0.845103	1
CHI3L1	1.027411	1.039067	1.023623	0.60468	-0.7258	0.845168	1
SMARCD2	1.28189	1.268074	1.286381	1.06829	0.0953	0.845219	1
RP11-173D3.1	1.027525	1.01526	1.031512	2.06503	1.0462	0.845286	1
MIS18A-AS1	1.040375	1.028092	1.044368	1.57936	0.6593	0.845302	1
RBM5	1.785554	1.768884	1.790973	1.02873	0.0409	0.845308	1
CXorf56	1.096587	1.108663	1.092662	0.85275	-0.2298	0.845356	1
RP11-459I19.1	1.021963	1.033751	1.018131	0.53721	-0.8964	0.845381	1
RP1-45N11.1	1.021941	1.033652	1.018134	0.53889	-0.8919	0.845381	1
RP11-932O9.10	1.02742	1.015222	1.031385	2.06188	1.044	0.845421	1
VKORC1	5.669821	5.710488	5.656601	0.98856	-0.0166	0.845521	1
LINC01483	1.01802	1.029773	1.0142	0.47694	-1.0681	0.845533	1
MMP9	1.045919	1.057744	1.042075	0.72864	-0.4567	0.845599	1
DIS3	1.372761	1.385866	1.368501	0.955	-0.0664	0.845606	1
DFNA5	1.155477	1.142518	1.15969	1.12048	0.1641	0.845655	1
LIMD1-AS1	1.028236	1.016045	1.032199	2.00681	1.0049	0.846045	1
CHGA	1.028319	1.016045	1.032309	2.01366	1.0098	0.846045	1
SYNRG	1.370855	1.383892	1.366617	0.955	-0.0664	0.846052	1
ATG9B	1.101244	1.088676	1.105329	1.1878	0.2483	0.846072	1
AMER2	1.055912	1.043595	1.059916	1.37438	0.4588	0.846137	1
ULBP2	1.029138	1.040763	1.02536	0.62213	-0.6847	0.846335	1
CHRNB1	1.430511	1.443668	1.426234	0.9607	-0.0578	0.84636	1
CRYGS	1.031803	1.043373	1.028042	0.64653	-0.6292	0.846414	1
BRINP1	1.031805	1.043387	1.02804	0.64627	-0.6298	0.846414	1
GPR39	1.03177	1.043274	1.028031	0.64774	-0.6265	0.846414	1
FBXL6	1.063671	1.075302	1.05989	0.79533	-0.3304	0.846553	1
MICU3	1.187066	1.17403	1.191303	1.09925	0.1365	0.846603	1
DHX29	1.58115	1.594653	1.576761	0.96991	-0.0441	0.846691	1
CTD-3025N20.3	1.045753	1.033586	1.049708	1.48005	0.5656	0.846693	1
HHIP-AS1	1.050669	1.062397	1.046857	0.75094	-0.4132	0.846805	1
ATG13	1.303365	1.289498	1.307872	1.06347	0.0888	0.846846	1
GABRA3	1.04286	1.030647	1.04683	1.52804	0.6117	0.846864	1
HTRA3	1.145004	1.15725	1.141024	0.89681	-0.1571	0.846907	1
FAM8A1	1.27203	1.284925	1.267839	0.94003	-0.0892	0.847041	1
AC093495.4	1.03166	1.019542	1.035599	1.82166	0.8653	0.847243	1
CASKIN2	1.078183	1.089744	1.074424	0.8293	-0.27	0.847249	1

ARFIP1	1.264158	1.277133	1.259941	0.93796	-0.0924	0.847364	1
RP11-74J13.8	1.058822	1.046695	1.062764	1.34413	0.4267	0.847379	1
RP11-764E7.1	1.028297	1.039895	1.024527	0.61479	-0.7018	0.847509	1
MLLT6	1.350954	1.3636	1.346843	0.95392	-0.0681	0.847602	1
RP11-166O4.6	1.088962	1.100905	1.08508	0.84316	-0.2461	0.847608	1
RP11-395D3.1	1.0233	1.034804	1.019561	0.56204	-0.8312	0.84766	1
IL10RB	1.272339	1.259228	1.276601	1.06702	0.0936	0.847684	1
ZNF587B	1.045514	1.033387	1.049456	1.4813	0.5669	0.847685	1
SNX2	1.865391	1.85002	1.870388	1.02396	0.0342	0.847719	1
NKRF	1.102483	1.090165	1.106486	1.18101	0.24	0.847722	1
CPB2-AS1	1.047234	1.034999	1.05121	1.46317	0.5491	0.847724	1
STAMBPL1	1.083286	1.070998	1.087281	1.22935	0.2979	0.847864	1
CHRNE	1.030074	1.017948	1.034015	1.89525	0.9224	0.847895	1
RP11-541G9.1	1.030055	1.017948	1.033991	1.8939	0.9214	0.847895	1
PRUNE1	1.145872	1.158085	1.141902	0.89763	-0.1558	0.847906	1
CAMKK2	1.19918	1.18624	1.203386	1.09207	0.1271	0.848041	1
NAA10	2.828487	2.850827	2.821225	0.98401	-0.0233	0.848067	1
RP11-484K9.4	1.046388	1.034087	1.050387	1.47817	0.5638	0.848074	1
RRP7A	1.459744	1.473015	1.455431	0.96283	-0.0547	0.848172	1
CNR1	1.043809	1.055276	1.040081	0.72511	-0.4637	0.848218	1
PPP1R16B	1.02403	1.011999	1.02794	2.32853	1.2194	0.84822	1
PTPN13	1.414912	1.427963	1.41067	0.95959	-0.0595	0.848225	1
HS6ST3	1.046469	1.034445	1.050377	1.46253	0.5485	0.848323	1
ESR2	1.027972	1.015934	1.031885	2.00103	1.0007	0.848372	1
AC104135.2	1.02799	1.015934	1.031909	2.00255	1.0018	0.848372	1
RASGRF1	1.095224	1.106905	1.091427	0.85522	-0.2256	0.848392	1
IMPA1	1.351003	1.337729	1.355318	1.05208	0.0732	0.84844	1
USP44	1.107612	1.119338	1.103801	0.8698	-0.2012	0.848524	1
CHCHD6	1.869382	1.853779	1.874454	1.02422	0.0345	0.848556	1
POFUT1	1.225174	1.211822	1.229515	1.08353	0.1157	0.848722	1
MAPRE3	1.25791	1.244278	1.262342	1.07395	0.1029	0.848726	1
AC113189.5	1.151977	1.139468	1.156043	1.11884	0.162	0.848828	1
AGAP2	1.027705	1.015676	1.031615	2.01682	1.0121	0.848834	1
TRIM14	1.037905	1.025799	1.04184	1.62174	0.6975	0.848862	1
AP5Z1	1.079325	1.091008	1.075528	0.8299	-0.269	0.848921	1
EBI3	1.021359	1.032654	1.017687	0.54165	-0.8846	0.848958	1
CTD-2090I13.1	1.021441	1.032909	1.017713	0.53824	-0.8937	0.848958	1
RP5-1024C24.1	1.021465	1.032901	1.017747	0.53943	-0.8905	0.848958	1
NEDD4	1.103264	1.114962	1.099462	0.86517	-0.2089	0.848973	1
TPST2	1.556798	1.542239	1.561531	1.03558	0.0504	0.849018	1
ZNF175	1.115808	1.103296	1.119876	1.1605	0.2147	0.849096	1
RNF169	1.154484	1.141708	1.158636	1.11945	0.1628	0.849138	1
CTD-2054N24.2	1.03633	1.02429	1.040244	1.6568	0.7284	0.849187	1
ANO7	1.127552	1.139266	1.123745	0.88855	-0.1705	0.849192	1
WIZ	1.315569	1.328072	1.311505	0.9495	-0.0748	0.849201	1

CLEC2A	1.517056	1.529736	1.512935	0.96828	-0.0465	0.849205	1
MAMLD1	1.045056	1.032976	1.048983	1.4854	0.5709	0.849367	1
RP11-1029J19.2	1.02794	1.015961	1.031834	1.99451	0.996	0.849405	1
AC012360.4	1.028009	1.015961	1.031925	2.00018	1.0001	0.849405	1
NAP1L6	1.02749	1.015521	1.031381	2.02182	1.0157	0.849422	1
CCDC93	1.287622	1.300091	1.283569	0.94494	-0.0817	0.849427	1
MRPS10	1.789741	1.773717	1.79495	1.02744	0.0391	0.849489	1
LINC01252	1.099799	1.111545	1.09598	0.86046	-0.2168	0.849558	1
CTC-523E23.11	1.031453	1.019441	1.035358	1.81875	0.8629	0.849575	1
ZNF415	1.158377	1.145849	1.162449	1.11382	0.1555	0.849611	1
MTMR9	1.417191	1.402989	1.421807	1.0467	0.0658	0.849639	1
RP11-393I2.4	1.066297	1.054208	1.070227	1.2955	0.3735	0.849674	1
PHEX	1.017937	1.029194	1.014278	0.4891	-1.0318	0.849686	1
DCAF4	1.081066	1.092663	1.077296	0.83416	-0.2616	0.849741	1
IRAK1	1.123964	1.135392	1.120249	0.88815	-0.1711	0.849872	1
RP11-488L18.8	1.025259	1.036699	1.02154	0.58693	-0.7687	0.849912	1
CWF19L2	1.513811	1.500044	1.518286	1.03648	0.0517	0.849941	1
LTBP4	2.191363	2.172819	2.197391	1.02095	0.0299	0.849993	1
INO80D	1.274206	1.286405	1.27024	0.94356	-0.0838	0.850014	1
DISC1	1.060226	1.071714	1.056492	0.78774	-0.3442	0.850152	1
SLC16A4	1.097956	1.109685	1.094143	0.85831	-0.2204	0.850227	1
AC005253.2	1.077744	1.089203	1.07402	0.82979	-0.2692	0.850418	1
FUOM	1.58775	1.601945	1.583136	0.96875	-0.0458	0.850508	1
MGAT5	1.158227	1.16987	1.154442	0.90918	-0.1374	0.850564	1
RP13-143G15.4	1.048553	1.036576	1.052446	1.4339	0.5199	0.850582	1
RP11-10C24.2	1.02179	1.033096	1.018115	0.54735	-0.8695	0.850639	1
TECPR2	1.207501	1.219326	1.203657	0.92856	-0.1069	0.850648	1
SLC9B1	1.053392	1.041472	1.057267	1.38085	0.4656	0.850731	1
CCNT2-AS1	1.043554	1.031568	1.047451	1.50313	0.588	0.851109	1
NUP188	1.147321	1.134845	1.151376	1.1226	0.1668	0.851132	1
GVQW1	1.034568	1.022692	1.038428	1.69348	0.76	0.851237	1
TRHDE	1.034567	1.022735	1.038414	1.68959	0.7567	0.851237	1
PCDH8	1.030815	1.042128	1.027138	0.64417	-0.6345	0.851308	1
SNTG1	1.026637	1.037858	1.02299	0.60726	-0.7196	0.851431	1
EGFR-AS1	1.02659	1.037765	1.022957	0.60789	-0.7181	0.851431	1
MATN1-AS1	1.040487	1.028554	1.044366	1.55374	0.6357	0.851487	1
RP11-48G14.2	1.044695	1.03271	1.048591	1.48551	0.571	0.851545	1
GLIDR	1.079563	1.067556	1.083466	1.23551	0.3051	0.851679	1
RFX1	1.274997	1.287108	1.27106	0.9441	-0.083	0.851776	1
RAG1	1.026474	1.03773	1.022815	0.60469	-0.7257	0.851787	1
SLC4A1AP	1.171064	1.158584	1.175121	1.10428	0.1431	0.851937	1
C2CD4C	1.279226	1.266215	1.283455	1.06476	0.0905	0.851962	1
AIM2	1.038314	1.026467	1.042166	1.59317	0.6719	0.851967	1
NMNAT3	1.154462	1.166284	1.15062	0.9058	-0.1427	0.852023	1
RP1-187B23.1	1.073809	1.061572	1.077786	1.26333	0.3372	0.852057	1

SPATA5L1	1.08336	1.071346	1.087265	1.22313	0.2906	0.852061	1
PARP6	1.281997	1.26843	1.286407	1.06697	0.0935	0.852156	1
ADTRP	1.046941	1.058254	1.043263	0.74266	-0.4292	0.85243	1
C17orf50	1.03808	1.026245	1.041927	1.5975	0.6758	0.852432	1
PKP2	1.18035	1.192146	1.176516	0.91866	-0.1224	0.852434	1
SLC7A6OS	1.346483	1.332791	1.350933	1.05452	0.0766	0.85248	1
MRGPRF	1.062067	1.073366	1.058394	0.79593	-0.3293	0.852538	1
UCKL1	1.248916	1.261008	1.244985	0.93861	-0.0914	0.852565	1
GPR156	1.023618	1.011871	1.027436	2.31124	1.2087	0.85257	1
SOHLH2	1.023594	1.011871	1.027405	2.30859	1.207	0.85257	1
PGBD1	1.149932	1.161628	1.14613	0.90411	-0.1454	0.852594	1
LINC00571	1.022113	1.033169	1.018519	0.55831	-0.8408	0.852784	1
SSUH2	1.022118	1.033239	1.018503	0.55666	-0.8451	0.852784	1
EXTL3	1.254235	1.241097	1.258506	1.07221	0.1006	0.852822	1
KBTBD2	1.211806	1.223554	1.207987	0.93037	-0.1041	0.852849	1
RP11-210M15.2	1.154884	1.142642	1.158863	1.11372	0.1554	0.852903	1
SRBD1	1.143953	1.131966	1.14785	1.12036	0.164	0.853024	1
OLIG2	1.023009	1.011304	1.026813	2.37195	1.2461	0.853036	1
RCOR1	1.193653	1.20551	1.189799	0.92355	-0.1147	0.853098	1
RAB11B	2.858787	2.836268	2.866107	1.01625	0.0233	0.853101	1
FIZ1	1.109414	1.120783	1.105718	0.87527	-0.1922	0.853163	1
AC011043.1	1.58318	1.569233	1.587713	1.03246	0.0461	0.853235	1
FUBP3	1.309305	1.296198	1.313566	1.05864	0.0822	0.853264	1
PRH1	1.1271	1.114744	1.131116	1.14268	0.1924	0.85328	1
LA16c-444G7.2	1.022333	1.033462	1.018715	0.5593	-0.8383	0.853291	1
SYCP2	1.022365	1.03356	1.018727	0.558	-0.8417	0.853291	1
CRIP3	1.025589	1.013947	1.029374	2.10617	1.0746	0.853334	1
APOBEC3D	1.02565	1.013947	1.029454	2.1119	1.0785	0.853334	1
LRRC34	1.070999	1.082502	1.06726	0.81525	-0.2947	0.85339	1
NUFIP2	1.865558	1.880323	1.860758	0.97778	-0.0324	0.85343	1
KCNA5	1.021867	1.032937	1.018268	0.55465	-0.8503	0.85351	1
RP5-984P4.6	1.024744	1.013073	1.028538	2.18307	1.1264	0.853571	1
AC009518.4	1.024728	1.013073	1.028517	2.18147	1.1253	0.853571	1
TMC7	1.05166	1.039795	1.055516	1.39506	0.4803	0.853574	1
ARHGAP25	1.051791	1.03988	1.055664	1.39579	0.4811	0.853574	1
RP11-817O13.8	1.051839	1.039906	1.055717	1.3962	0.4815	0.853574	1
RP11-290D2.6	1.041638	1.029742	1.045505	1.52998	0.6135	0.853623	1
LA16c-349E10.1	1.024804	1.013102	1.028608	2.18353	1.1267	0.85368	1
URB2	1.064469	1.052576	1.068335	1.29975	0.3782	0.853709	1
MTRNR2L4	1.017813	1.028856	1.014224	0.49291	-1.0206	0.853765	1
PDE1A	1.027253	1.015378	1.031113	2.02324	1.0167	0.853781	1
CPLX4	1.027109	1.015373	1.030924	2.0116	1.0083	0.853781	1
STAT5A	1.038857	1.050038	1.035223	0.70393	-0.5065	0.8538	1
LAT	1.047363	1.035655	1.051169	1.43514	0.5212	0.853861	1
PTPN21	1.091558	1.102887	1.087876	0.8541	-0.2275	0.854068	1

ZNF571-AS1	1.040667	1.028935	1.04448	1.53723	0.6203	0.854237	1
FHDC1	1.025732	1.036753	1.02215	0.60268	-0.7305	0.854296	1
ANKRD29	1.025773	1.036913	1.022152	0.60011	-0.7367	0.854296	1
RP11-707M3.3	1.027369	1.015676	1.031171	1.98846	0.9917	0.854507	1
CHTF8	1.630217	1.616073	1.634815	1.03042	0.0432	0.85451	1
ABHD18	1.119828	1.107801	1.123738	1.14784	0.1989	0.854533	1
ATP2A1	1.029106	1.017451	1.032894	1.88497	0.9145	0.854546	1
TSPAN3	5.442588	5.398954	5.456772	1.01314	0.0188	0.854575	1
SUMF2	2.161355	2.144127	2.166954	1.01995	0.0285	0.854609	1
SIRPA	1.075714	1.086882	1.072083	0.82967	-0.2694	0.854614	1
C21orf33	1.034434	1.022691	1.038251	1.68576	0.7534	0.854681	1
ATP1A3	1.100088	1.11135	1.096427	0.86599	-0.2076	0.854691	1
OXER1	1.030994	1.019367	1.034774	1.79551	0.8444	0.854722	1
MYH11	1.03102	1.019359	1.03481	1.79812	0.8465	0.854722	1
NOM1	1.180063	1.168018	1.183978	1.09499	0.1309	0.854746	1
RP11-239L20.6	1.028201	1.01659	1.031975	1.9274	0.9467	0.854784	1
YKT6	1.503268	1.515539	1.499279	0.96846	-0.0462	0.854961	1
RGS6	1.030428	1.018882	1.034182	1.81029	0.8562	0.854988	1
PCSK2	1.030555	1.018874	1.034352	1.82005	0.864	0.854988	1
UTP3	1.246678	1.258671	1.24278	0.93857	-0.0915	0.854991	1
FAM135B	1.022278	1.033264	1.018707	0.56237	-0.8304	0.855013	1
LINC00853	1.050806	1.039054	1.054627	1.39873	0.4841	0.855042	1
CCDC174	1.490862	1.477278	1.495278	1.03771	0.0534	0.855116	1
RPTOR	1.111995	1.099926	1.115918	1.16004	0.2142	0.855129	1
LLNLR-304G9.1	1.028375	1.016784	1.032143	1.91509	0.9374	0.85514	1
SPRY4-IT1	1.028337	1.016782	1.032093	1.91229	0.9353	0.85514	1
SGK3	1.089994	1.077925	1.093918	1.20524	0.2693	0.855156	1
FSIP1	1.054979	1.043241	1.058795	1.35971	0.4433	0.85517	1
CASP8	1.055016	1.043476	1.058767	1.35169	0.4348	0.85517	1
STRIP1	1.16244	1.174071	1.158659	0.91146	-0.1337	0.855321	1
DOK6	1.09507	1.106313	1.091415	0.85987	-0.2178	0.855347	1
AL592528.1	1.04522	1.033655	1.048979	1.45532	0.5413	0.855386	1
SLX1A	1.044213	1.032452	1.048036	1.48024	0.5658	0.855425	1
B3GALT1	1.113934	1.125229	1.110263	0.88048	-0.1836	0.855436	1
SAV1	1.491948	1.477707	1.496577	1.0395	0.0559	0.855439	1
RAP2C-AS1	1.25332	1.2405	1.257487	1.07063	0.0985	0.855456	1
ARMC3	1.048757	1.059718	1.045195	0.7568	-0.402	0.85551	1
HECTD1	1.995512	1.979835	2.000607	1.0212	0.0303	0.85554	1
RNF6	1.335882	1.323155	1.340019	1.05219	0.0734	0.855592	1
MOSPD3	1.816071	1.829593	1.811676	0.9784	-0.0315	0.855683	1
TBCEL	1.107884	1.119202	1.104205	0.87419	-0.194	0.855748	1
PELI3	1.081724	1.092874	1.078099	0.84091	-0.25	0.855769	1
ANKRD40	1.319746	1.331678	1.315867	0.95233	-0.0705	0.855819	1
AFAP1L1	1.032818	1.043786	1.029252	0.66807	-0.5819	0.855831	1
CWC22	1.346307	1.358878	1.342221	0.95359	-0.0686	0.855862	1

CTD-2047H16.4	1.037822	1.026198	1.041601	1.5879	0.6671	0.855876	1
OSMR-AS1	1.028601	1.039668	1.025003	0.63032	-0.6658	0.855929	1
RYR2	1.028499	1.039443	1.024941	0.63234	-0.6612	0.855929	1
CLDN2	1.034509	1.022941	1.03827	1.66818	0.7383	0.855931	1
ALOX12B	1.026943	1.015453	1.030678	1.98516	0.9893	0.856165	1
KIAA1614-AS1	1.02699	1.015453	1.03074	1.9892	0.9922	0.856165	1
TMEM138	1.377477	1.364197	1.381794	1.04832	0.0681	0.856227	1
ZNF34	1.113222	1.124545	1.109542	0.87954	-0.1852	0.856229	1
RP11-49I11.1	1.031875	1.020303	1.035636	1.75518	0.8116	0.856349	1
SPRY1	1.3993	1.386325	1.403517	1.0445	0.0628	0.856403	1
RP11-455O6.9	1.029209	1.040148	1.025654	0.63897	-0.6462	0.856449	1
AEN	1.223531	1.211271	1.227516	1.07689	0.1069	0.856509	1
EXOSC7	1.652746	1.639062	1.657195	1.02837	0.0404	0.85651	1
GABRE	1.048205	1.036532	1.051999	1.42338	0.5093	0.856549	1
TMC4	1.046444	1.057518	1.042844	0.74489	-0.4249	0.856603	1
SPAG6	1.025044	1.035917	1.021509	0.59885	-0.7397	0.856643	1
TRIM6	1.111521	1.099792	1.115334	1.15574	0.2088	0.856694	1
MRPL38	1.086455	1.074627	1.090301	1.21003	0.275	0.856752	1
CKMT1A	1.034184	1.022694	1.037919	1.6709	0.7406	0.856919	1
RASIP1	1.034151	1.022686	1.037877	1.66964	0.7395	0.856919	1
AGMAT	1.035989	1.024433	1.039746	1.62671	0.702	0.856958	1
RP3-486I3.7	1.03601	1.024503	1.03975	1.62223	0.698	0.856958	1
MIATNB	1.029699	1.04064	1.026143	0.64328	-0.6365	0.857006	1
PLS3-AS1	1.023195	1.011717	1.026926	2.29795	1.2003	0.85712	1
RP1-242N11.1	1.023115	1.011717	1.026819	2.28889	1.1946	0.85712	1
RYR3	1.02572	1.036519	1.022209	0.60815	-0.7175	0.857159	1
NIPAL1	1.025739	1.036547	1.022226	0.60815	-0.7175	0.857159	1
RP5-1148A21.3	1.02574	1.036603	1.022208	0.60675	-0.7208	0.857159	1
RCBTB1	1.188746	1.200702	1.18486	0.92107	-0.1186	0.857164	1
PPM1K	1.34625	1.333298	1.35046	1.05149	0.0724	0.857182	1
MOB3C	1.061003	1.049177	1.064848	1.31867	0.3991	0.85719	1
OAZ3	1.043019	1.053971	1.039459	0.73112	-0.4518	0.857301	1
NMNAT1	1.174374	1.162402	1.178265	1.09768	0.1345	0.857303	1
SNHG12	1.264749	1.252582	1.268704	1.06383	0.0893	0.857304	1
ZNF774	1.063531	1.074584	1.059938	0.80363	-0.3154	0.85731	1
PLEKHG2	1.16151	1.17315	1.157726	0.91092	-0.1346	0.857318	1
YLPM1	1.390532	1.376994	1.394932	1.04758	0.0671	0.857349	1
RP3-329A5.8	1.03042	1.018966	1.034143	1.80023	0.8482	0.857383	1
LINC01615	1.022716	1.033534	1.0192	0.57254	-0.8046	0.857398	1
ITCH	1.224952	1.2365	1.221199	0.9353	-0.0965	0.857401	1
BICC1	1.111941	1.100121	1.115783	1.15643	0.2097	0.85745	1
OPCML	1.149265	1.160773	1.145524	0.90515	-0.1438	0.857454	1
TCF20	1.197408	1.209055	1.193622	0.92618	-0.1106	0.857549	1
RP11-38C17.1	1.03531	1.023915	1.039015	1.63138	0.7061	0.857555	1
C3orf38	1.381424	1.368762	1.38554	1.0455	0.0642	0.857654	1

EDN3	1.026273	1.037118	1.022747	0.61284	-0.7064	0.857718	1
CYYR1-AS1	1.022297	1.033118	1.018779	0.56704	-0.8185	0.857871	1
TXNRD1	1.569373	1.581895	1.565303	0.97149	-0.0417	0.857897	1
SLITRK3	1.02247	1.033252	1.018965	0.57034	-0.8101	0.85797	1
CTA-292E10.6	1.151628	1.139554	1.155553	1.11464	0.1566	0.858046	1
RP11-706O15.3	1.058409	1.069277	1.054876	0.79212	-0.3362	0.858094	1
PHKA2	1.145721	1.157003	1.142053	0.90478	-0.1444	0.858144	1
FAM86C1	1.100117	1.111432	1.096439	0.86545	-0.2085	0.858181	1
TRAPPC6B	1.295509	1.28272	1.299666	1.05994	0.084	0.858199	1
NUDT17	1.122485	1.133637	1.11886	0.88942	-0.1691	0.8582	1
HIST1H2BN	1.044752	1.03333	1.048465	1.45408	0.5401	0.858255	1
SCG2	1.180763	1.19228	1.177019	0.92063	-0.1193	0.858297	1
KIAA1324	1.055345	1.043811	1.059094	1.34883	0.4317	0.858346	1
RP11-843A23.1	1.024975	1.013691	1.028643	2.09215	1.065	0.858419	1
TMPRSS13	1.022783	1.033572	1.019275	0.57416	-0.8005	0.858433	1
AC002076.10	1.022785	1.033531	1.019291	0.57533	-0.7975	0.858433	1
RNF167	2.060465	2.074615	2.055865	0.98255	-0.0254	0.858469	1
ATP5D	6.299041	6.244387	6.316806	1.01381	0.0198	0.858513	1
ZDHHC11	1.061309	1.049737	1.06507	1.30828	0.3877	0.858562	1
MAOA	1.040817	1.029377	1.044535	1.51599	0.6003	0.858583	1
GBP3	1.01882	1.029546	1.015334	0.51898	-0.9462	0.858585	1
SCT	1.033468	1.044286	1.029952	0.67633	-0.5642	0.85864	1
ASPRV1	1.036139	1.046929	1.032632	0.69535	-0.5242	0.858701	1
CD72	1.082285	1.093334	1.078693	0.84313	-0.2462	0.858779	1
CRHBP	1.024826	1.035579	1.021331	0.59954	-0.7381	0.858911	1
TTI2	1.164179	1.15262	1.167936	1.10036	0.138	0.858932	1
RP11-722G7.1	1.343025	1.330235	1.347182	1.05132	0.0722	0.85895	1
RP5-1021I20.1	1.054487	1.043018	1.058215	1.35327	0.4364	0.858966	1
OPRL1	1.054487	1.0431	1.058189	1.35011	0.4331	0.858966	1
RP11-794G24.1	1.027525	1.016045	1.031257	1.94805	0.962	0.858999	1
SCRT1	1.02749	1.016045	1.03121	1.94514	0.9599	0.858999	1
PADI2	1.02738	1.016045	1.031065	1.93609	0.9531	0.858999	1
RP11-276E15.4	1.028906	1.017512	1.03261	1.86217	0.897	0.85911	1
ZFP69B	1.07894	1.067367	1.082701	1.22763	0.2959	0.859289	1
LARP4B	1.298022	1.309475	1.294299	0.95096	-0.0725	0.859298	1
LSMEM1	1.041043	1.02977	1.044708	1.50176	0.5867	0.859314	1
RP11-817G13.3	1.030146	1.018773	1.033842	1.80276	0.8502	0.859416	1
RP11-1057B6.1	1.030167	1.018783	1.033867	1.80301	0.8504	0.859416	1
TRIM58	1.030115	1.018766	1.033805	1.80141	0.8491	0.859416	1
GAS8	1.053074	1.063854	1.04957	0.7763	-0.3653	0.859475	1
SLC27A3	1.150276	1.161673	1.146571	0.90659	-0.1415	0.859491	1
CDK17	1.20964	1.221212	1.205879	0.93068	-0.1036	0.859493	1
MGAT3	1.103059	1.113684	1.099605	0.87616	-0.1907	0.859542	1
RUFY2	1.428351	1.414972	1.432699	1.04272	0.0604	0.859549	1
NSUN4	1.125825	1.11401	1.129666	1.13732	0.1856	0.859684	1

AXIN2	1.063837	1.074668	1.060316	0.80779	-0.3079	0.859747	1
USP7	1.38056	1.368068	1.38462	1.04497	0.0635	0.859826	1
PYGB	1.309228	1.320553	1.305547	0.95319	-0.0692	0.85986	1
SLX4	1.080874	1.069261	1.084648	1.22217	0.2894	0.859952	1
ATP23	1.200556	1.211898	1.196869	0.92908	-0.1061	0.859965	1
RPUSD1	1.26719	1.25475	1.271234	1.06471	0.0905	0.860067	1
PRKCQ	1.029245	1.039944	1.025767	0.64506	-0.6325	0.86011	1
SMIM10L2A	1.140563	1.128643	1.144437	1.12278	0.1671	0.860214	1
MCM3AP	1.232327	1.2438	1.228598	0.93765	-0.0929	0.860218	1
OXSR1	1.536951	1.523466	1.541334	1.03413	0.0484	0.860229	1
RP11-317L10.1	1.032345	1.021009	1.036029	1.71495	0.7782	0.860334	1
TRAK2	1.107756	1.1184	1.104296	0.88088	-0.183	0.860431	1
BCAT1	2.802388	2.776204	2.810899	1.01953	0.0279	0.860602	1
ANKMY1	1.062355	1.051023	1.066038	1.29428	0.3721	0.860685	1
SARM1	1.160053	1.148153	1.163921	1.10643	0.1459	0.860725	1
RASD1	1.088778	1.099584	1.085266	0.85622	-0.2239	0.860737	1
C6orf223	1.024531	1.035154	1.021079	0.59961	-0.7379	0.860747	1
GCNT4	1.024507	1.035111	1.02106	0.59981	-0.7374	0.860747	1
RP1-102K2.6	1.023055	1.033748	1.019579	0.58015	-0.7855	0.860766	1
RP11-89K10.1	1.023038	1.03365	1.019588	0.58212	-0.7806	0.860766	1
U91319.1	1.033469	1.022319	1.037093	1.66191	0.7328	0.860766	1
RP11-326C3.12	1.025743	1.036276	1.022319	0.61526	-0.7007	0.860817	1
PPP1CB	3.527614	3.497631	3.53736	1.01591	0.0228	0.860853	1
LINC00237	1.029281	1.017948	1.032965	1.83673	0.8771	0.860854	1
OSTM1-AS1	1.02911	1.017948	1.032738	1.82411	0.8672	0.860854	1
RP11-158H5.8	1.047759	1.036425	1.051444	1.41233	0.4981	0.860959	1
STAC3	1.047786	1.036482	1.051461	1.41058	0.4963	0.860959	1
RP5-1098D14.1	1.023159	1.011999	1.026786	2.23233	1.1586	0.86118	1
RP11-665C14.2	1.023157	1.011999	1.026784	2.23216	1.1584	0.86118	1
ATRIP	1.023187	1.011999	1.026824	2.2355	1.1606	0.86118	1
RP11-109A6.3	1.023151	1.011999	1.026776	2.23149	1.158	0.86118	1
EXOC3L1	1.023126	1.011999	1.026743	2.22875	1.1562	0.86118	1
SSTR3	1.02318	1.011999	1.026815	2.2347	1.1601	0.86118	1
RP11-435I10.4	1.023164	1.011999	1.026793	2.23291	1.1589	0.86118	1
FRMD3	1.025699	1.036227	1.022277	0.61493	-0.7015	0.861238	1
USP36	1.202816	1.190848	1.206706	1.08309	0.1152	0.861383	1
POGK	1.286062	1.297472	1.282353	0.94918	-0.0753	0.861402	1
SLC6A6	1.322995	1.334461	1.319268	0.95457	-0.0671	0.861427	1
C4orf26	1.020737	1.03128	1.01731	0.55338	-0.8537	0.861438	1
ITPK1-AS1	1.022337	1.032946	1.018888	0.5733	-0.8026	0.861527	1
LINC01085	1.022323	1.03296	1.018866	0.57239	-0.8049	0.861527	1
WDR81	1.068357	1.079026	1.06489	0.82112	-0.2843	0.861611	1
PRRG2	1.041152	1.051744	1.037709	0.72876	-0.4565	0.861739	1
FCER1G	1.026851	1.015676	1.030483	1.94464	0.9595	0.861795	1
KHDC1L	1.026808	1.015676	1.030427	1.94107	0.9569	0.861795	1

BMI1	1.163405	1.151513	1.16727	1.104	0.1427	0.86184	1
PLD2	1.152321	1.163149	1.148801	0.91206	-0.1328	0.861885	1
TSC22D1-AS1	1.051812	1.04064	1.055444	1.36426	0.4481	0.861911	1
GRM3	1.03694	1.025726	1.040585	1.57758	0.6577	0.861974	1
PDHX	1.399739	1.387271	1.403792	1.04266	0.0603	0.861997	1
RP11-219B4.7	1.032721	1.021484	1.036374	1.69309	0.7597	0.862037	1
UHRF2	1.226519	1.237859	1.222832	0.93683	-0.0941	0.862055	1
RASGRF2-AS1	1.0354	1.024286	1.039013	1.60639	0.6838	0.862074	1
KCNK2	1.035418	1.024264	1.039043	1.60906	0.6862	0.862074	1
DHX8	1.188027	1.199104	1.184426	0.92628	-0.1105	0.862098	1
SEMA4G	1.08782	1.076453	1.091516	1.19702	0.2594	0.862168	1
LPO	1.017272	1.027763	1.013862	0.49929	-1.0021	0.862195	1
PQLC2L	1.017252	1.027682	1.013862	0.50074	-0.9979	0.862195	1
TNFSF9	1.017253	1.027684	1.013862	0.50071	-0.998	0.862195	1
RP11-351J23.2	1.017275	1.027775	1.013862	0.49907	-1.0027	0.862195	1
GATA5	1.017332	1.028006	1.013862	0.49495	-1.0146	0.862195	1
TRIP4	1.182813	1.170902	1.186685	1.09235	0.1274	0.862357	1
AC002398.11	1.02668	1.015515	1.03031	1.95362	0.9661	0.862364	1
SLC37A2	1.026575	1.015498	1.030175	1.94708	0.9613	0.862364	1
CTD-2126E3.4	1.025903	1.014833	1.029501	1.98893	0.992	0.862407	1
RP11-338K17.8	1.026022	1.014833	1.029659	1.99961	0.9997	0.862407	1
RP11-996F15.2	1.025962	1.014833	1.02958	1.99424	0.9958	0.862407	1
HPS6	1.143902	1.132046	1.147756	1.11897	0.1622	0.862421	1
VWDE	1.037687	1.048201	1.034269	0.71097	-0.4921	0.862475	1
FRS3	1.083402	1.071947	1.087126	1.21098	0.2762	0.862507	1
RP11-803B1.8	1.083235	1.071693	1.086987	1.21332	0.279	0.862507	1
HIST1H4I	1.030628	1.019445	1.034263	1.76204	0.8172	0.862517	1
FAM151B	1.037739	1.026593	1.041361	1.55532	0.6372	0.862526	1
AC009961.3	1.037781	1.026646	1.041401	1.55374	0.6357	0.862526	1
AC068492.1	1.037768	1.026651	1.041382	1.55271	0.6348	0.862526	1
NBPF12	1.148826	1.159903	1.145225	0.90821	-0.1389	0.862604	1
RP11-455F5.6	1.050007	1.060556	1.046578	0.76918	-0.3786	0.862636	1
TEAD1	1.491547	1.503669	1.487607	0.96811	-0.0468	0.862642	1
XYLT1	1.040529	1.051228	1.037051	0.72325	-0.4674	0.862647	1
ZFP90	1.620364	1.606668	1.624815	1.02991	0.0425	0.862731	1
GRAMD2	1.018847	1.029329	1.01544	0.52642	-0.9257	0.862739	1
TAF12	1.912478	1.898348	1.917071	1.02084	0.0298	0.862772	1
RP11-225B17.2	1.039984	1.050521	1.036559	0.72363	-0.4667	0.862863	1
CBWD1	1.537215	1.523807	1.541573	1.03392	0.0481	0.862889	1
TBX5	1.047346	1.058108	1.043848	0.7546	-0.4062	0.862926	1
RP11-326K13.4	1.04052	1.029382	1.044141	1.50231	0.5872	0.862927	1
ANKEF1	1.058516	1.047318	1.062156	1.31359	0.3935	0.862964	1
RP3-508I15.9	1.075097	1.063932	1.078727	1.23142	0.3003	0.8632	1
KIAA1161	1.034195	1.04458	1.03082	0.69133	-0.5325	0.863212	1
HLA-DMB	1.038879	1.027751	1.042496	1.53134	0.6148	0.863284	1

CYP4V2	1.091246	1.080097	1.09487	1.18445	0.2442	0.863326	1
MYBBP1A	1.100234	1.088787	1.103955	1.17083	0.2275	0.863332	1
CTD-2336O2.1	1.761397	1.775007	1.756973	0.97673	-0.034	0.86345	1
ATG2A	1.181436	1.192321	1.177898	0.92501	-0.1125	0.86348	1
MPP7	1.117158	1.105932	1.120807	1.14043	0.1896	0.863516	1
SIRT4	1.052505	1.041362	1.056128	1.35697	0.4404	0.863519	1
LPP-AS2	1.030048	1.018978	1.033647	1.77291	0.8261	0.863544	1
MEGF11	1.030098	1.019026	1.033697	1.77107	0.8246	0.863544	1
GPRIN1	1.030049	1.019008	1.033637	1.76968	0.8235	0.863544	1
DLAT	1.249757	1.260982	1.246108	0.94301	-0.0847	0.863561	1
DYNC1I2	3.855047	3.82754	3.863988	1.01289	0.0185	0.863618	1
ZNF284	1.086661	1.075272	1.090363	1.20048	0.2636	0.863678	1
GS1-72M22.1	1.034069	1.022942	1.037685	1.64264	0.716	0.863697	1
LINGO3	1.034054	1.022955	1.037661	1.64064	0.7143	0.863697	1
APOBEC3G	1.030933	1.041337	1.027551	0.66649	-0.5853	0.863704	1
NADSYN1	1.244163	1.255596	1.240446	0.94073	-0.0882	0.863774	1
ZNF841	1.05401	1.042798	1.057654	1.34711	0.4299	0.863793	1
ARMC12	1.032475	1.043071	1.029031	0.67403	-0.5691	0.863863	1
CRY2	1.127073	1.137855	1.123568	0.89636	-0.1579	0.863915	1
GID4	1.180771	1.191664	1.17723	0.92469	-0.113	0.864095	1
TFPT	1.245992	1.257248	1.242333	0.94202	-0.0862	0.86427	1
RP11-457M11.5	1.058756	1.047626	1.062374	1.30967	0.3892	0.864276	1
EPN2	1.48691	1.498336	1.483196	0.96962	-0.0445	0.864355	1
C9orf72	1.42843	1.415586	1.432605	1.04095	0.0579	0.864382	1
TTC25	1.064728	1.05347	1.068388	1.27899	0.355	0.864423	1
LINC00482	1.019185	1.029532	1.015821	0.53572	-0.9004	0.864448	1
IL18R1	1.019179	1.029507	1.015821	0.53618	-0.8992	0.864448	1
ENTPD3	1.019218	1.029668	1.015821	0.53327	-0.9071	0.864448	1
FHOD3	1.06893	1.079407	1.065525	0.82518	-0.2772	0.864471	1
TMLHE	1.151426	1.162757	1.147743	0.90775	-0.1396	0.864501	1
MALRD1	1.027391	1.037876	1.023982	0.63318	-0.6593	0.864694	1
LINC00235	1.03402	1.023045	1.037587	1.63108	0.7058	0.864737	1
PUS3	1.176433	1.187472	1.172845	0.92198	-0.1172	0.864757	1
MAP6D1	1.086943	1.097531	1.083502	0.85616	-0.2241	0.864771	1
ZNF430	1.298399	1.286482	1.302273	1.05512	0.0774	0.864884	1
MBTPS2	1.208284	1.219222	1.204728	0.93389	-0.0987	0.865079	1
ZSCAN22	1.046178	1.035306	1.049712	1.40804	0.4937	0.865187	1
SECISBP2L	1.676134	1.68825	1.672196	0.97667	-0.0341	0.865247	1
CTD-3138B18.5	1.206809	1.195266	1.210561	1.07833	0.1088	0.865289	1
SERINC5	1.324538	1.311959	1.328627	1.05343	0.0751	0.86531	1
EPM2A	1.056924	1.067521	1.053479	0.79203	-0.3364	0.865434	1
CTA-363E6.2	1.023873	1.034195	1.020518	0.60004	-0.7369	0.865439	1
RP11-46H11.3	1.029304	1.018372	1.032858	1.78849	0.8387	0.865513	1
IPCEF1	1.029357	1.018383	1.032925	1.79109	0.8408	0.865513	1
RP11-47A8.5	1.043097	1.032017	1.046699	1.45856	0.5445	0.865564	1

RNF123	1.107114	1.09578	1.110798	1.1568	0.2101	0.865615	1
RP11-181G12.2	1.045852	1.0348	1.049445	1.42082	0.5067	0.865691	1
AP1M1	1.535221	1.54768	1.531171	0.96986	-0.0442	0.865696	1
CYFIP2	1.471352	1.45879	1.475436	1.03628	0.0514	0.865732	1
EPHB1	1.023022	1.033302	1.01968	0.59097	-0.7588	0.865826	1
STK26	1.738581	1.751528	1.734373	0.97717	-0.0333	0.865829	1
RASL12	1.020634	1.031029	1.017255	0.55611	-0.8466	0.865938	1
RIT2	1.022138	1.011304	1.02566	2.26991	1.1826	0.866008	1
SHISA7	1.022138	1.011304	1.025659	2.26987	1.1826	0.866008	1
ROMO1	6.450147	6.493021	6.43621	0.98966	-0.015	0.866066	1
NMRK1	1.322762	1.333928	1.319132	0.95569	-0.0654	0.866089	1
OTUB2	1.084372	1.094983	1.080922	0.85196	-0.2311	0.866115	1
PHB	4.202707	4.1747	4.211811	1.01169	0.0168	0.866162	1
RP11-890B15.2	1.020452	1.030641	1.017139	0.55936	-0.8381	0.866185	1
POLR3A	1.163511	1.151978	1.16726	1.10055	0.1382	0.866204	1
POU3F1	1.023008	1.012187	1.026525	2.17654	1.122	0.866268	1
RP11-379K17.12	1.020421	1.030628	1.017104	0.55842	-0.8406	0.866295	1
ARHGAP27	1.020412	1.030647	1.017084	0.55745	-0.8431	0.866295	1
AKR7A3	1.024792	1.013947	1.028317	2.0304	1.0218	0.866307	1
ARHGAP19-SLIT1	1.024814	1.013947	1.028347	2.03251	1.0233	0.866307	1
XXbac-BPG27H4.8	1.024788	1.013947	1.028313	2.03007	1.0215	0.866307	1
ID2-AS1	1.024795	1.013947	1.028321	2.03068	1.022	0.866307	1
CES3	1.049719	1.03879	1.053271	1.37332	0.4577	0.86639	1
RP11-53I6.3	1.030052	1.019184	1.033585	1.75064	0.8079	0.866413	1
AC092835.2	1.063737	1.052684	1.06733	1.27798	0.3539	0.866444	1
RP11-21B23.2	1.021333	1.031604	1.017994	0.56937	-0.8126	0.866533	1
C12orf10	1.971396	1.98433	1.967191	0.98259	-0.0253	0.866535	1
GS1-24F4.2	1.023907	1.013073	1.027429	2.09819	1.0691	0.866545	1
RP11-787I22.3	1.023917	1.013102	1.027432	2.09382	1.0661	0.866655	1
PSMD6-AS2	1.023869	1.013102	1.027369	2.089	1.0628	0.866655	1
CNIH1	2.417598	2.434731	2.412029	0.98418	-0.023	0.866668	1
MUC15	1.017156	1.027362	1.013838	0.50573	-0.9836	0.866688	1
GADL1	1.017145	1.027321	1.013838	0.5065	-0.9814	0.866688	1
USP15	1.358433	1.345994	1.362477	1.04764	0.0671	0.866706	1
ABCB11	1.026192	1.015358	1.029714	1.93479	0.9522	0.866734	1
FASTKD2	1.23999	1.228183	1.243828	1.06857	0.0957	0.866762	1
ZNF586	1.118125	1.106895	1.121775	1.13921	0.188	0.866847	1
RBM12	1.236114	1.247512	1.232409	0.93898	-0.0908	0.866853	1
RP11-332H18.5	1.024075	1.013274	1.027586	2.07815	1.0553	0.866902	1
AC137932.5	1.024103	1.013274	1.027623	2.08096	1.0573	0.866902	1
RP11-1084I9.1	1.017019	1.027271	1.013687	0.50188	-0.9946	0.866934	1
HOXB7	1.029328	1.039569	1.025999	0.65706	-0.6059	0.867034	1
GZMA	1.016986	1.027189	1.01367	0.50276	-0.9921	0.867044	1
DNHD1	1.062862	1.051887	1.066429	1.28026	0.3564	0.86705	1
ADGRF4	1.019726	1.029878	1.016426	0.54977	-0.8631	0.867105	1

C1orf186	1.025561	1.014799	1.029059	1.96361	0.9735	0.867202	1
RP11-820I16.4	1.025567	1.014799	1.029067	1.96418	0.9739	0.867202	1
HPS3	1.166522	1.154845	1.170318	1.09992	0.1374	0.867206	1
ILKAP	1.43694	1.424537	1.440972	1.03871	0.0548	0.867211	1
TMEM30B	1.016104	1.026249	1.012806	0.48786	-1.0354	0.86732	1
GPR135	1.076538	1.086919	1.073163	0.84174	-0.2486	0.867336	1
DCUN1D2	1.103042	1.091889	1.106667	1.16082	0.2151	0.867366	1
PDE6A	1.026476	1.015669	1.029989	1.91387	0.9365	0.867462	1
CTC-304I17.6	1.028268	1.017469	1.031778	1.8191	0.8632	0.867501	1
AFF3	1.08307	1.071927	1.086692	1.20528	0.2694	0.867607	1
LIN7C	1.370873	1.382054	1.367239	0.96122	-0.0571	0.867646	1
GPR89B	1.105977	1.094932	1.109567	1.15416	0.2068	0.867677	1
RP13-131K19.2	1.03013	1.019331	1.03364	1.7402	0.7993	0.867678	1
CD226	1.027349	1.016592	1.030846	1.85904	0.8946	0.86774	1
RP11-15E18.1	1.027398	1.016571	1.030918	1.86578	0.8998	0.86774	1
GSG2	1.027295	1.016593	1.030774	1.8547	0.8912	0.86774	1
C12orf66	1.089044	1.077925	1.092659	1.18907	0.2498	0.867777	1
TRPV3	1.027391	1.01662	1.030892	1.85875	0.8943	0.867849	1
ZXDB	1.06618	1.055235	1.069738	1.26258	0.3364	0.867855	1
ABHD16A	1.036182	1.025397	1.039688	1.56271	0.6441	0.867882	1
TANGO6	1.05008	1.039075	1.053657	1.37319	0.4575	0.867895	1
DHDDS	1.219969	1.230798	1.216449	0.93783	-0.0926	0.867941	1
ATXN7L2	1.054147	1.043285	1.057677	1.33249	0.4141	0.867949	1
RP11-78L16.1	1.015845	1.025964	1.012556	0.48359	-1.0481	0.868046	1
CCL7	1.015834	1.025917	1.012556	0.48447	-1.0455	0.868046	1
RDH10-AS1	1.015847	1.025971	1.012556	0.48347	-1.0485	0.868046	1
RP11-131L12.2	1.015878	1.026099	1.012556	0.4811	-1.0556	0.868046	1
ZG16B	1.015827	1.025891	1.012556	0.48495	-1.0441	0.868046	1
RP3-395M20.8	1.015828	1.025894	1.012556	0.48491	-1.0442	0.868046	1
SUN1	1.774139	1.78644	1.770141	0.97927	-0.0302	0.868117	1
CCDC28B	2.349892	2.364803	2.345045	0.98552	-0.021	0.868148	1
CHGB	1.043257	1.032435	1.046775	1.44212	0.5282	0.868299	1
FAT4	1.096572	1.107078	1.093158	0.87	-0.2009	0.868528	1
KISS1R	1.29717	1.308272	1.293561	0.95228	-0.0705	0.868543	1
HYKK	1.086537	1.075532	1.090115	1.19307	0.2547	0.868747	1
CTD-2541J13.1	1.086611	1.075424	1.090247	1.19654	0.2589	0.868747	1
GXYLT2	1.086735	1.075553	1.090369	1.19611	0.2583	0.868747	1
TEF	1.113869	1.124308	1.110475	0.88872	-0.1702	0.86876	1
CFAP53	1.130793	1.140909	1.127504	0.90487	-0.1442	0.868803	1
TAC3	1.019688	1.0297	1.016433	0.55331	-0.8539	0.868804	1
LRRC46	1.030771	1.020105	1.034238	1.703	0.7681	0.86893	1
WI2-85898F10.1	1.030802	1.020108	1.034278	1.70469	0.7695	0.86893	1
WBSCR27	1.053404	1.042732	1.056872	1.3309	0.4124	0.869054	1
ZNF546	1.095688	1.084598	1.099292	1.1737	0.2311	0.869056	1
CHST6	1.039572	1.049632	1.036303	0.73144	-0.4512	0.869057	1

CXorf58	1.026082	1.015453	1.029537	1.91138	0.9346	0.869146	1
FBXO38	1.147312	1.13594	1.151008	1.11085	0.1517	0.869184	1
BDKRB2	1.018924	1.028929	1.015672	0.54174	-0.8843	0.869264	1
RP11-454P21.1	1.018925	1.028932	1.015672	0.54168	-0.8845	0.869264	1
HPSE2	1.030932	1.020304	1.034386	1.69361	0.7601	0.869288	1
HRH2	1.034123	1.044392	1.030785	0.69348	-0.5281	0.869306	1
ADIPOR2	1.405457	1.416864	1.401749	0.96374	-0.0533	0.869383	1
NUDT16L1	1.555486	1.5674	1.551613	0.97218	-0.0407	0.869419	1
DOCK9-AS2	1.046686	1.035995	1.050161	1.39353	0.4787	0.869466	1
RP11-356I2.4	1.047346	1.057496	1.044046	0.76607	-0.3845	0.869529	1
RP11-1038A11.3	1.051336	1.040524	1.054851	1.35354	0.4367	0.869539	1
RP11-90J7.2	1.016251	1.026254	1.013	0.49516	-1.014	0.869549	1
LINC00355	1.016263	1.026303	1.013	0.49423	-1.0168	0.869549	1
MKX	1.016264	1.026308	1.013	0.49415	-1.017	0.869549	1
LINC01564	1.016313	1.026506	1.013	0.49044	-1.0278	0.869549	1
RP11-403A3.3	1.016241	1.026211	1.013	0.49597	-1.0117	0.869549	1
KCNC4	1.030264	1.04022	1.027028	0.67199	-0.5735	0.869574	1
FES	1.047271	1.057469	1.043956	0.76486	-0.3867	0.86968	1
DVL2	1.59435	1.581192	1.598628	1.03	0.0426	0.869695	1
IL1RAPL1	1.036184	1.046322	1.032889	0.71001	-0.4941	0.869788	1
RP11-284M14.1	1.03336	1.022737	1.036813	1.61906	0.6952	0.869838	1
TLDC2	1.03331	1.022705	1.036757	1.61891	0.695	0.869838	1
ALG1	1.112873	1.101995	1.116409	1.14131	0.1907	0.869952	1
DAP	2.063506	2.047593	2.068679	1.02013	0.0288	0.869969	1
RP11-722E23.2	1.060106	1.049279	1.063625	1.29111	0.3686	0.869975	1
PAPOLB	1.022271	1.011717	1.025701	2.19344	1.1332	0.870103	1
RP11-215P8.3	1.034234	1.023597	1.037691	1.59729	0.6756	0.870117	1
DBNL	1.844613	1.830522	1.849194	1.02248	0.0321	0.870168	1
RP11-490O6.2	1.034235	1.023625	1.037684	1.59505	0.6736	0.870227	1
CARMIL2	1.026801	1.036789	1.023554	0.64025	-0.6433	0.870311	1
NF1	1.948864	1.934962	1.953382	1.0197	0.0281	0.870315	1
C20orf85	1.02955	1.01896	1.032993	1.74009	0.7992	0.870346	1
RP5-967N21.11	1.029475	1.018951	1.032895	1.73584	0.7956	0.870346	1
HIST1H2AJ	1.029488	1.01895	1.032913	1.73681	0.7964	0.870346	1
AARS	1.299575	1.310192	1.296124	0.95465	-0.067	0.870584	1
OXGR1	1.023147	1.033043	1.01993	0.60315	-0.7294	0.870929	1
PLPPR2	1.209388	1.197995	1.213091	1.07624	0.106	0.871039	1
AF127936.7	1.023335	1.03331	1.020092	0.60319	-0.7293	0.87105	1
NAIF1	1.077442	1.066636	1.080954	1.21487	0.2808	0.871093	1
RP11-563J2.3	1.063559	1.052942	1.067011	1.26574	0.34	0.871162	1
CTD-2008L17.1	1.054639	1.043807	1.05816	1.32765	0.4089	0.871166	1
ZNF764	1.141842	1.152154	1.13849	0.9102	-0.1358	0.871172	1
LARGE1	1.141881	1.152096	1.138561	0.91101	-0.1345	0.871172	1
GRHL2	1.019006	1.0289	1.01579	0.54636	-0.8721	0.87118	1
MARCO	1.019001	1.028879	1.01579	0.54674	-0.8711	0.87118	1

HCAR3	1.01905	1.02908	1.01579	0.54296	-0.8811	0.87118	1
RP1-121G13.2	1.01903	1.028998	1.01579	0.5445	-0.877	0.87118	1
RP11-342D14.1	1.020126	1.029984	1.016921	0.56433	-0.8254	0.871219	1
CLC	1.020162	1.030131	1.016921	0.56159	-0.8324	0.871219	1
SERPINB5	1.020144	1.030058	1.016921	0.56295	-0.8289	0.871219	1
HOXD-AS2	1.020182	1.030214	1.016921	0.56005	-0.8364	0.871219	1
CAMP	1.020194	1.030261	1.016921	0.55917	-0.8386	0.871219	1
RP11-65L3.4	1.025822	1.01526	1.029255	1.91713	0.9389	0.871236	1
RP11-367N14.2	1.037688	1.027228	1.041088	1.50902	0.5936	0.871299	1
USP51	1.09568	1.105975	1.092333	0.87127	-0.1988	0.871322	1
NRGN	1.076483	1.065846	1.07994	1.21404	0.2798	0.871338	1
AC010226.4	1.060509	1.049653	1.064038	1.28972	0.3671	0.871362	1
PRL	1.024177	1.013691	1.027586	2.0149	1.0107	0.871406	1
ATP6V1B1-AS1	1.024185	1.013691	1.027596	2.01569	1.0113	0.871406	1
RP11-195F19.9	1.03768	1.027103	1.041118	1.51712	0.6013	0.87141	1
RP11-278C7.4	1.037709	1.027123	1.04115	1.51716	0.6014	0.87141	1
CENPT	1.399879	1.387718	1.403833	1.04156	0.0587	0.871454	1
KLLN	1.039972	1.029596	1.043345	1.46454	0.5504	0.871465	1
RP11-718O11.1	1.023642	1.033561	1.020418	0.60838	-0.717	0.871514	1
LANCL3	1.023643	1.033593	1.020409	0.60753	-0.719	0.871514	1
FAM78B	1.033046	1.022519	1.036468	1.61944	0.6955	0.871541	1
MT3	1.037866	1.027281	1.041306	1.51409	0.5985	0.871659	1
TTLL11	1.037917	1.0275	1.041304	1.50196	0.5868	0.871659	1
COX10	1.154359	1.143724	1.157816	1.09805	0.1349	0.871671	1
ZNF600	1.07665	1.065886	1.080149	1.21648	0.2827	0.871704	1
GRHL3	1.019875	1.029686	1.016686	0.56208	-0.8312	0.871792	1
RP11-296I10.3	1.019916	1.029852	1.016686	0.55894	-0.8392	0.871792	1
COX6B2	1.058605	1.068579	1.055363	0.80729	-0.3088	0.871851	1
NDUFV2	3.516045	3.492673	3.523642	1.01242	0.0178	0.871878	1
KAZALD1	1.083382	1.093414	1.080121	0.8577	-0.2215	0.871971	1
RP11-572M11.4	1.026577	1.016045	1.03	1.86977	0.9029	0.871999	1
ANKRD42	1.124915	1.114099	1.12843	1.12561	0.1707	0.872077	1
METAP1	1.39297	1.38117	1.396806	1.04102	0.058	0.87209	1
SEC61G	10.2179	10.29358	10.1933	0.98921	-0.0157	0.872216	1
FTO	1.290568	1.301182	1.287117	0.9533	-0.069	0.872228	1
KLF2	1.020208	1.030109	1.016989	0.56425	-0.8256	0.872255	1
NFIA-AS2	1.020171	1.02996	1.016989	0.56705	-0.8184	0.872255	1
ADRB2	1.03226	1.042074	1.02907	0.69092	-0.5334	0.872267	1
USPL1	1.175203	1.164117	1.178806	1.08951	0.1237	0.872316	1
ZNF462	1.823425	1.809734	1.827875	1.0224	0.032	0.872343	1
GPBAR1	1.029267	1.018773	1.032678	1.74073	0.7997	0.872396	1
PAX3	1.016271	1.026145	1.013062	0.49959	-1.0012	0.872408	1
TSPAN12	1.26425	1.27512	1.260716	0.94765	-0.0776	0.872509	1
KCNMB2-AS1	1.043394	1.032921	1.046798	1.42151	0.5074	0.872637	1
KCNK15	1.041242	1.030803	1.044635	1.44908	0.5351	0.872838	1

ZFYVE28	1.028521	1.038351	1.025326	0.66037	-0.5987	0.872963	1
AC068282.3	1.028502	1.038245	1.025335	0.66244	-0.5941	0.872963	1
RP11-539G18.3	1.061745	1.051047	1.065222	1.27767	0.3535	0.872998	1
EDEM2	1.286886	1.297716	1.283365	0.9518	-0.0713	0.873048	1
ZNF174	1.191973	1.180568	1.19568	1.08369	0.1159	0.873233	1
FSIP2-AS1	1.100432	1.089599	1.103954	1.16021	0.2144	0.873272	1
NKAIN2	1.05176	1.061635	1.04855	0.78769	-0.3443	0.873352	1
OLFML2B	1.043707	1.033276	1.047098	1.41537	0.5012	0.873372	1
CTC-499B15.5	1.050733	1.040204	1.054155	1.347	0.4298	0.873443	1
TMEM255A	1.050659	1.040247	1.054043	1.34277	0.4252	0.873443	1
EFCAB12	1.047457	1.037008	1.050854	1.37412	0.4585	0.87359	1
KIAA1644	1.045558	1.055394	1.042361	0.76473	-0.387	0.873682	1
RP1-179N16.6	1.053507	1.042961	1.056935	1.32525	0.4063	0.873733	1
GRIP1	1.136018	1.146272	1.132685	0.90711	-0.1406	0.873738	1
TBC1D24	1.201913	1.212553	1.198454	0.93367	-0.099	0.873767	1
HSPBAP1	1.071321	1.060766	1.074752	1.23017	0.2989	0.873826	1
HTN3	1.023881	1.033623	1.020714	0.61607	-0.6988	0.873855	1
SUSD4	1.023875	1.033579	1.020721	0.61708	-0.6965	0.873855	1
CTD-2021H9.3	1.028326	1.017948	1.031699	1.76621	0.8207	0.873859	1
NAGS	1.161287	1.150389	1.164829	1.09601	0.1323	0.873883	1
RP11-712B9.2	1.026635	1.036459	1.023442	0.64297	-0.6372	0.873885	1
RP11-597D13.9	1.078522	1.088307	1.075341	0.85317	-0.2291	0.873919	1
KIAA1614	1.045415	1.055335	1.04219	0.76245	-0.3913	0.873933	1
AC012358.8	1.044902	1.034519	1.048277	1.39857	0.484	0.874013	1
STK17B	1.104958	1.115348	1.101581	0.88064	-0.1834	0.874093	1
AC145110.1	1.022283	1.011999	1.025626	2.13563	1.0947	0.874185	1
CCNE2	1.046214	1.035855	1.049582	1.38283	0.4676	0.874279	1
RP11-510M2.2	1.026241	1.015934	1.029591	1.85706	0.893	0.874339	1
HCG25	1.02635	1.015934	1.029735	1.86612	0.9	0.874339	1
NEURL1B	1.054942	1.064704	1.051769	0.80008	-0.3218	0.874343	1
TMEM65	1.201568	1.211569	1.198317	0.93737	-0.0933	0.874371	1
RNF7	5.449055	5.485429	5.437232	0.98925	-0.0156	0.874455	1
KDM4C	1.126629	1.116202	1.130018	1.1189	0.1621	0.874461	1
DOCK6	1.101826	1.111457	1.098695	0.8855	-0.1754	0.874463	1
RP11-231C14.7	1.020434	1.030061	1.017304	0.57564	-0.7968	0.874592	1
RP11-804N13.1	1.021942	1.031733	1.018759	0.59117	-0.7584	0.874604	1
SYN3	1.023158	1.032819	1.020017	0.60994	-0.7133	0.874618	1
RARS	1.848582	1.860315	1.844768	0.98193	-0.0263	0.874634	1
KCTD17	1.293643	1.282071	1.297404	1.05436	0.0764	0.874711	1
MTHFSD	1.146746	1.135674	1.150345	1.10813	0.1481	0.874747	1
RP5-1050D4.2	1.025977	1.015676	1.029326	1.8708	0.9037	0.874803	1
RP4-569M23.2	1.036028	1.025726	1.039377	1.53063	0.6141	0.874885	1
NXN	1.630979	1.618424	1.63506	1.0269	0.0383	0.875	1
RP11-319E16.2	1.03456	1.024273	1.037903	1.56152	0.643	0.875006	1
STON1-GTF2A1L	1.034534	1.024194	1.037896	1.56631	0.6474	0.875006	1

WNT16	1.023149	1.032907	1.019977	0.60709	-0.72	0.875046	1
RP11-533E19.5	1.023125	1.032789	1.019984	0.60945	-0.7144	0.875046	1
CH17-373J23.1	1.082987	1.09309	1.079702	0.85619	-0.224	0.875165	1
RP11-236L14.2	1.03041	1.040246	1.027213	0.67618	-0.5645	0.875234	1
NXPE3	1.284507	1.273058	1.288229	1.05556	0.078	0.875267	1
EVC2	1.068539	1.058022	1.071958	1.24019	0.3106	0.875284	1
DMRT2	1.018168	1.027928	1.014995	0.53692	-0.8972	0.875297	1
DSC1	1.018175	1.027938	1.015001	0.53695	-0.8971	0.875297	1
SLCO2A1	1.018121	1.027729	1.014998	0.54085	-0.8867	0.875297	1
ARSK	1.247814	1.23639	1.251528	1.06404	0.0896	0.875298	1
CLTCL1	1.040149	1.049816	1.037007	0.74289	-0.4288	0.875344	1
SLAMF9	1.019707	1.029325	1.016581	0.56541	-0.8226	0.875354	1
ELF5	1.019782	1.029629	1.016581	0.55961	-0.8375	0.875354	1
RP11-767C1.2	1.02622	1.015961	1.029555	1.85169	0.8888	0.875377	1
PCDHA7	1.026235	1.015961	1.029574	1.85291	0.8898	0.875377	1
SEL1L2	1.025049	1.014833	1.02837	1.91268	0.9356	0.875415	1
RP11-245J9.6	1.025081	1.014833	1.028412	1.91553	0.9377	0.875415	1
PKI55	1.13372	1.14379	1.130447	0.90721	-0.1405	0.875535	1
LINC01001	1.130165	1.119575	1.133608	1.11736	0.1601	0.87558	1
ZNRF1	1.2067	1.195622	1.210301	1.07504	0.1044	0.87566	1
RP11-294J22.6	1.230371	1.240923	1.226941	0.94197	-0.0863	0.875663	1
SH3BP5-AS1	1.052484	1.042063	1.055871	1.32827	0.4095	0.875748	1
PRADC1	1.361334	1.372209	1.3578	0.96129	-0.057	0.875806	1
CNTD1	1.019697	1.029376	1.016551	0.56341	-0.8277	0.875821	1
FAM150B	1.019672	1.029257	1.016557	0.56592	-0.8213	0.875821	1
MCF2L2	1.0972	1.107099	1.093983	0.87753	-0.1885	0.875866	1
MIER2	1.241371	1.230203	1.245001	1.06428	0.0899	0.87592	1
KIAA1468	1.12934	1.118772	1.132776	1.11791	0.1608	0.875946	1
CTC-360G5.9	1.029415	1.019213	1.032731	1.70358	0.7686	0.87597	1
ADPRH	1.026949	1.03662	1.023806	0.65008	-0.6213	0.876006	1
TEKT1	1.037909	1.027791	1.041198	1.48244	0.568	0.876198	1
NEDD8	7.370369	7.404911	7.359141	0.99285	-0.0103	0.876256	1
SHMT1	1.287957	1.276862	1.291563	1.0531	0.0746	0.876303	1
UBAP2	1.415867	1.426549	1.412395	0.96682	-0.0487	0.876467	1
LINC00924	1.029247	1.019055	1.03256	1.70877	0.773	0.876513	1
TDRD6	1.029632	1.019466	1.032936	1.69201	0.7587	0.876545	1
RP11-171I2.4	1.02976	1.019451	1.033111	1.70226	0.7675	0.876545	1
PANO1	1.073179	1.08289	1.070023	0.84477	-0.2434	0.876555	1
GJB7	1.037345	1.046876	1.034247	0.73058	-0.4529	0.876574	1
TNFRSF10A	1.044653	1.054191	1.041553	0.76679	-0.3831	0.876588	1
ATP2C2	1.016229	1.025722	1.013143	0.51095	-0.9687	0.8766	1
ZNF687	1.110686	1.100063	1.11414	1.14068	0.1899	0.876627	1
DMTN	1.058852	1.068418	1.055742	0.81473	-0.2956	0.87663	1
DPM1	1.694702	1.683042	1.698492	1.02262	0.0323	0.876988	1
THAP5	1.582065	1.593545	1.578333	0.97437	-0.0375	0.877016	1

SMIM18	1.022352	1.012186	1.025656	2.10529	1.074	0.87705	1
LINC00158	1.022294	1.012186	1.02558	2.09907	1.0697	0.87705	1
NECAB1	1.044168	1.053723	1.041062	0.76433	-0.3877	0.877051	1
EPHX3	1.04399	1.053763	1.040813	0.75912	-0.3976	0.877051	1
CAPN10	1.209167	1.219268	1.205883	0.93896	-0.0909	0.8771	1
RILPL2	1.294982	1.283738	1.298637	1.05251	0.0738	0.877144	1
HOXB-AS3	1.055128	1.044851	1.058469	1.30361	0.3825	0.877152	1
MIR92B	1.063883	1.053509	1.067255	1.25687	0.3298	0.877197	1
HMGN4	1.054884	1.044732	1.058184	1.3007	0.3793	0.877263	1
NAPSA	1.057304	1.046975	1.060662	1.29137	0.3689	0.877288	1
FMO5	1.024995	1.014881	1.028282	1.90053	0.9264	0.877336	1
RP11-193M21.1	1.025008	1.014881	1.028299	1.90167	0.9273	0.877336	1
PLEKHB2	1.575189	1.562718	1.579243	1.02937	0.0418	0.877461	1
TRMT2B	1.071976	1.061765	1.075296	1.21908	0.2858	0.877483	1
PLXDC2	1.653652	1.641365	1.657646	1.02539	0.0362	0.877524	1
RP11-923I11.3	1.02004	1.029489	1.016968	0.57541	-0.7973	0.877557	1
DDX49	1.349064	1.359691	1.34561	0.96085	-0.0576	0.877582	1
LINP1	1.029852	1.039334	1.026769	0.68056	-0.5552	0.877625	1
KIF24	1.032592	1.022501	1.035872	1.59423	0.6729	0.877671	1
RP11-46J23.1	1.043863	1.033645	1.047185	1.40242	0.4879	0.877768	1
LINC01550	1.031005	1.040552	1.027901	0.68804	-0.5394	0.877911	1
BBS1	1.059323	1.049084	1.062652	1.27643	0.3521	0.878068	1
FAM13A-AS1	1.02728	1.036758	1.024199	0.65834	-0.6031	0.878128	1
ZPR1	1.368116	1.379205	1.364512	0.96125	-0.057	0.878156	1
FARS2	1.206742	1.217016	1.203403	0.93727	-0.0935	0.878308	1
CEPT1	1.240263	1.250744	1.236856	0.94461	-0.0822	0.878335	1
NDST3	1.026466	1.035827	1.023423	0.65377	-0.6131	0.878389	1
FBXO27	1.127743	1.116966	1.131246	1.12209	0.1662	0.878404	1
GCLC	1.162643	1.152011	1.166098	1.09268	0.1279	0.878404	1
ZFYVE27	1.258072	1.246913	1.261699	1.05988	0.0839	0.878437	1
RP11-498P14.5	1.123918	1.11362	1.127265	1.12009	0.1636	0.878448	1
FZD2	1.41468	1.40336	1.418359	1.03718	0.0527	0.87845	1
CTD-2587H24.5	1.024717	1.034074	1.021676	0.63613	-0.6526	0.878507	1
CTB-131K11.1	1.028464	1.018382	1.031742	1.72684	0.7881	0.878508	1
ZNF182	1.086382	1.075908	1.089787	1.18285	0.2423	0.878523	1
NRSN1	1.021942	1.011871	1.025216	2.12421	1.0869	0.878558	1
PCCA-AS1	1.021948	1.011871	1.025224	2.12483	1.0873	0.878558	1
RP11-108M9.6	1.044939	1.03489	1.048206	1.38163	0.4664	0.878569	1
RP11-307B6.3	1.036116	1.026024	1.039396	1.51385	0.5982	0.878825	1
C5AR1	1.025639	1.035026	1.022587	0.64487	-0.6329	0.878857	1
ADCY9	1.03658	1.026455	1.039871	1.50711	0.5918	0.878869	1
ZMYND15	1.023855	1.033245	1.020802	0.62573	-0.6764	0.878896	1
RAB1B	1.482299	1.47061	1.486098	1.03291	0.0467	0.878929	1
IL21R-AS1	1.021293	1.011304	1.02454	2.17084	1.1183	0.879025	1
AF131216.1	1.02126	1.011304	1.024496	2.16698	1.1157	0.879025	1

EYA1	1.02126	1.011304	1.024496	2.167	1.1157	0.879025	1
GFRA3	1.022957	1.032355	1.019902	0.6151	-0.7011	0.879157	1
ZNF250	1.155742	1.145225	1.15916	1.09596	0.1322	0.879158	1
GOLT1A	1.021304	1.030678	1.018257	0.59512	-0.7487	0.879278	1
MTTP	1.022135	1.012187	1.025369	2.08167	1.0577	0.879286	1
CCDC106	1.604536	1.615363	1.601017	0.97669	-0.034	0.87932	1
RP11-482D24.2	1.023899	1.013947	1.027134	1.94557	0.9602	0.879325	1
MYT1L	1.023971	1.013947	1.027229	1.95236	0.9652	0.879325	1
DUOXA1	1.0213	1.030688	1.018248	0.59463	-0.7499	0.879388	1
GAS6	1.238428	1.227457	1.241994	1.06391	0.0894	0.879438	1
STARD5	1.039832	1.029744	1.043112	1.44945	0.5355	0.879447	1
TTC27	1.121414	1.110991	1.124802	1.12444	0.1692	0.879465	1
DDX25	1.061953	1.051781	1.065259	1.2603	0.3338	0.879466	1
RGMB-AS1	1.070892	1.060696	1.074206	1.22259	0.2899	0.879486	1
MEG9	1.025828	1.015826	1.029079	1.83743	0.8777	0.879502	1
RP11-417L19.4	1.0259	1.015826	1.029174	1.84341	0.8824	0.879502	1
FAM189A1	1.025787	1.015826	1.029025	1.83396	0.875	0.879502	1
RP11-883G14.1	1.023071	1.013073	1.026321	2.0135	1.0097	0.879564	1
RP11-50I19.2	1.022993	1.013073	1.026218	2.0056	1.004	0.879564	1
RP11-135A1.3	1.023026	1.013073	1.026261	2.00887	1.0064	0.879564	1
AF129408.17	1.020401	1.029723	1.017371	0.58442	-0.7749	0.879666	1
RP5-837J1.6	1.023042	1.013102	1.026273	2.00536	1.0039	0.879673	1
PRDM11	1.069328	1.078705	1.06628	0.84214	-0.2479	0.879701	1
USP27X-AS1	1.048418	1.038296	1.051708	1.35023	0.4332	0.879749	1
LNX2	1.096114	1.08591	1.099431	1.15739	0.2109	0.87976	1
FOXC2-AS1	1.017981	1.027225	1.014976	0.55006	-0.8623	0.879804	1
RP11-108H9.1	1.018013	1.027341	1.014981	0.54793	-0.8679	0.879804	1
DR1	1.690973	1.702553	1.687208	0.97816	-0.0319	0.879825	1
RP11-273B20.1	1.023183	1.013274	1.026405	1.98917	0.9922	0.879921	1
RP6-24A23.6	1.0232	1.013274	1.026426	1.99079	0.9933	0.879921	1
AC093627.9	1.039526	1.029495	1.042787	1.45064	0.5367	0.879975	1
SUPT7L	1.308602	1.318627	1.305343	0.95831	-0.0614	0.880055	1
FAXC	1.170513	1.180225	1.167356	0.9286	-0.1069	0.880149	1
ETV4	1.375593	1.364192	1.379299	1.04148	0.0586	0.880154	1
CKMT1B	1.024744	1.014817	1.027971	1.88776	0.9167	0.880201	1
RP11-373D23.3	1.020584	1.029847	1.017573	0.58876	-0.7643	0.880222	1
SRRD	1.255234	1.243822	1.258943	1.06202	0.0868	0.880343	1
SLC26A4	1.027424	1.036732	1.024398	0.66423	-0.5902	0.880374	1
RP11-307C18.1	1.020135	1.029411	1.01712	0.58211	-0.7806	0.880395	1
RP11-379F4.6	1.018728	1.028031	1.015704	0.56024	-0.8359	0.880399	1
ASB2	1.018748	1.028129	1.015699	0.55811	-0.8414	0.880399	1
NAALADL2	1.086909	1.0965	1.083792	0.86831	-0.2037	0.880418	1
DIRAS1	1.100786	1.11034	1.09768	0.88527	-0.1758	0.880426	1
CAPZA2	2.770692	2.752465	2.776616	1.01378	0.0197	0.880493	1
IL17RB	1.027352	1.017465	1.030566	1.75012	0.8075	0.880501	1

BCL2L2	1.133531	1.142861	1.130499	0.91346	-0.1306	0.880552	1
ZNF611	1.120196	1.109829	1.123566	1.12508	0.17	0.880592	1
AL022341.3	1.02922	1.019353	1.032428	1.67556	0.7446	0.880678	1
SLC17A8	1.016083	1.02531	1.013084	0.51692	-0.952	0.880698	1
ZNF750	1.016088	1.025318	1.013087	0.51691	-0.952	0.880698	1
HLA-DMA	1.067522	1.05744	1.0708	1.23259	0.3017	0.880735	1
LINC01320	1.026478	1.016593	1.029692	1.78945	0.8395	0.88074	1
ACBD7	1.026487	1.016573	1.02971	1.79268	0.8421	0.88074	1
GPN2	1.235038	1.245471	1.231647	0.94368	-0.0836	0.880825	1
NANOG	1.035356	1.025404	1.038591	1.51913	0.6032	0.88084	1
CCDC39	1.028768	1.018862	1.031988	1.69592	0.7621	0.880902	1
CNPY4	1.51231	1.52333	1.508728	0.9721	-0.0408	0.880917	1
B4GALNT1	1.060651	1.050662	1.063898	1.26126	0.3349	0.880961	1
RALGDS	1.417173	1.427012	1.413975	0.96947	-0.0447	0.880996	1
THAP6	1.294542	1.304925	1.291167	0.95488	-0.0666	0.881068	1
NR6A1	1.162966	1.152748	1.166288	1.08864	0.1225	0.881087	1
RP11-962G15.1	1.026664	1.016798	1.029871	1.77825	0.8305	0.881098	1
RP11-400N13.1	1.016711	1.025988	1.013695	0.52698	-0.9242	0.881166	1
LINC01291	1.016704	1.025946	1.0137	0.52803	-0.9213	0.881166	1
RP11-731C17.2	1.04359	1.033467	1.04688	1.40078	0.4862	0.881178	1
RP11-527D7.1	1.054341	1.04427	1.057614	1.30142	0.3801	0.881182	1
RASA4B	1.047115	1.037215	1.050333	1.35249	0.4356	0.881276	1
CLDND1	1.782537	1.769384	1.786813	1.02265	0.0323	0.881301	1
CLEC4A	1.028141	1.0183	1.031339	1.71251	0.7761	0.881372	1
FBXO46	1.14848	1.138044	1.151872	1.10017	0.1377	0.881404	1
EXD2	1.174569	1.184609	1.171305	0.92793	-0.1079	0.881533	1
ZNF296	1.03848	1.047965	1.035397	0.73798	-0.4384	0.881587	1
AC002456.2	1.029011	1.01918	1.032207	1.67919	0.7478	0.881633	1
C11orf88	1.034319	1.043571	1.031311	0.71863	-0.4767	0.881742	1
OTUD3	1.167074	1.176741	1.163931	0.92752	-0.1085	0.881758	1
LINC00504	1.029921	1.020073	1.033123	1.65011	0.7226	0.881912	1
DEGS2	1.029911	1.020097	1.033101	1.64709	0.7199	0.881912	1
FAM98C	1.270237	1.280422	1.266927	0.95187	-0.0712	0.881934	1
WDR45	1.916997	1.904227	1.921148	1.01871	0.0267	0.881951	1
TMEM81	1.030185	1.039356	1.027204	0.69123	-0.5328	0.882017	1
MGAM	1.029977	1.02011	1.033185	1.65015	0.7226	0.882022	1
CHRND	1.025215	1.015453	1.028388	1.83701	0.8774	0.88217	1
AP5B1	1.066985	1.076263	1.063969	0.83879	-0.2536	0.882256	1
SLC28A2	1.030108	1.020303	1.033296	1.6399	0.7136	0.882271	1
SEPSECS-AS1	1.057646	1.047822	1.06084	1.27222	0.3473	0.882312	1
KNDC1	1.079611	1.088943	1.076577	0.86098	-0.216	0.882375	1
RP5-1184F4.7	1.019844	1.029176	1.016811	0.57619	-0.7954	0.882387	1
TPD52L2	1.615046	1.602963	1.618974	1.02655	0.0378	0.882574	1
RCHY1	1.39939	1.388325	1.402986	1.03776	0.0535	0.882596	1
AKAP2	1.031112	1.040281	1.028131	0.69837	-0.5179	0.882633	1

HOXB9	1.017107	1.02624	1.014138	0.53882	-0.8921	0.882674	1
RHOBTB3	3.106635	3.083297	3.114221	1.01484	0.0213	0.88271	1
AC005932.1	1.043181	1.052406	1.040182	0.76676	-0.3832	0.882846	1
BCL7C	2.300116	2.313534	2.295754	0.98646	-0.0197	0.882868	1
WRAP73	1.238586	1.2487	1.235299	0.94612	-0.0799	0.882871	1
NCOA7	1.358146	1.367807	1.355006	0.9652	-0.0511	0.882895	1
ZNF621	1.224892	1.234896	1.22164	0.94357	-0.0838	0.882927	1
EIF1B	3.84949	3.87283	3.841903	0.98923	-0.0156	0.883022	1
AC078883.3	1.098993	1.089071	1.102218	1.1476	0.1986	0.88303	1
RP11-474P2.6	1.033364	1.023582	1.036544	1.54964	0.6319	0.88308	1
RP11-351I21.11	1.060262	1.050378	1.063475	1.25997	0.3334	0.883098	1
LIPC	1.021433	1.011717	1.024592	2.09876	1.0695	0.883129	1
RP11-989E6.10	1.021422	1.011717	1.024577	2.09749	1.0687	0.883129	1
SLC12A5	1.192183	1.181449	1.195673	1.07839	0.1089	0.883288	1
PCDHB4	1.061104	1.051378	1.064265	1.25083	0.3229	0.883439	1
RP11-342M1.3	1.033694	1.023791	1.036913	1.55151	0.6337	0.88344	1
SEC23B	1.202182	1.212235	1.198915	0.93724	-0.0935	0.883469	1
CTA-384D8.36	1.050459	1.040668	1.053642	1.31905	0.3995	0.88347	1
RP13-216E22.4	1.024494	1.033629	1.021525	0.64009	-0.6437	0.883582	1
FAM150A	1.024518	1.033816	1.021496	0.63567	-0.6537	0.883582	1
TNFRSF25	1.092631	1.082513	1.09592	1.16248	0.2172	0.883647	1
CD101	1.027981	1.037109	1.025014	0.67407	-0.569	0.88386	1
MOAP1	1.416675	1.40547	1.420317	1.03661	0.0519	0.883878	1
FAM173A	1.963626	1.951082	1.967704	1.01748	0.025	0.883912	1
SYTL3	1.035962	1.026239	1.039122	1.49097	0.5763	0.883964	1
NFIL3	1.078548	1.068626	1.081773	1.19158	0.2529	0.883984	1
FAM58A	1.352812	1.362681	1.349603	0.96394	-0.053	0.883984	1
MEF2C	1.194571	1.184047	1.197993	1.07577	0.1054	0.884059	1
WNT3A	1.024237	1.03324	1.021311	0.64112	-0.6413	0.884157	1
RP11-351I24.1	1.024974	1.01526	1.028132	1.84356	0.8825	0.884278	1
CD247	1.024918	1.01526	1.028058	1.83868	0.8787	0.884278	1
GRM2	1.02495	1.01526	1.0281	1.84144	0.8808	0.884278	1
RP4-561L24.3	1.024922	1.01526	1.028063	1.83902	0.8789	0.884278	1
UBALD1	1.233044	1.242702	1.229905	0.94727	-0.0781	0.884309	1
KRT16	1.019852	1.028858	1.016925	0.58647	-0.7699	0.884309	1
RP4-575N6.4	1.019871	1.028919	1.01693	0.58543	-0.7724	0.884309	1
CCER2	1.024905	1.015211	1.028056	1.8444	0.8832	0.884312	1
ADGRF3	1.021066	1.03028	1.018071	0.5968	-0.7447	0.884348	1
RHOH	1.021003	1.030012	1.018075	0.60225	-0.7316	0.884348	1
CTD-2034I21.2	1.023318	1.013691	1.026448	1.93179	0.9499	0.884435	1
RP11-673E11.2	1.023358	1.013691	1.0265	1.93564	0.9528	0.884435	1
LINC01030	1.023392	1.013691	1.026546	1.93896	0.9553	0.884435	1
ANKS6	1.128644	1.137989	1.125607	0.91026	-0.1356	0.884504	1
PKD1	1.299747	1.288868	1.303283	1.0499	0.0703	0.884504	1
CTD-2292M16.8	1.036969	1.027273	1.04012	1.47108	0.5569	0.884604	1

ABCB5	1.024554	1.033658	1.021595	0.64161	-0.6402	0.884622	1
RABEP1	1.782478	1.769997	1.786535	1.02148	0.0307	0.88472	1
CTB-78F1.1	1.020556	1.029589	1.017619	0.59548	-0.7479	0.884776	1
CTD-2396E7.10	1.020749	1.029756	1.017822	0.59894	-0.7395	0.884922	1
GACAT2	1.020748	1.029743	1.017824	0.59926	-0.7387	0.884922	1
TADA2B	1.066305	1.075269	1.063391	0.8422	-0.2478	0.884942	1
RCL1	1.14022	1.149791	1.137109	0.91533	-0.1276	0.884957	1
TMEM150C	1.185383	1.195054	1.182239	0.9343	-0.098	0.88502	1
RP11-473A10.2	1.02571	1.016045	1.028852	1.79817	0.8465	0.885043	1
GRM5	1.026568	1.035626	1.023623	0.6631	-0.5927	0.885104	1
PRR35	1.039348	1.02975	1.042468	1.42747	0.5135	0.885124	1
RP11-384K6.8	1.027125	1.017512	1.03025	1.7274	0.7886	0.885129	1
EMX2OS	1.03317	1.042055	1.030282	0.72005	-0.4738	0.885322	1
SSX1	1.02104	1.030004	1.018126	0.60411	-0.7271	0.885387	1
RP11-705O24.1	1.021039	1.029967	1.018137	0.60522	-0.7245	0.885387	1
NPNT	1.021041	1.030003	1.018127	0.60418	-0.727	0.885387	1
ELL	1.06702	1.057278	1.070186	1.22536	0.2932	0.88541	1
MVB12B	1.229582	1.239342	1.226409	0.94596	-0.0801	0.885432	1
ITGB1BP2	1.031687	1.040727	1.028748	0.70588	-0.5025	0.885455	1
USP12-AS2	1.028384	1.018735	1.031521	1.68243	0.7506	0.885491	1
NIP7	1.262226	1.251552	1.265696	1.05623	0.0789	0.885514	1
YES1	1.755481	1.766924	1.751761	0.98023	-0.0288	0.885546	1
BCL2	1.072559	1.081765	1.069567	0.85081	-0.2331	0.88586	1
HERC6	1.029342	1.038329	1.026421	0.68932	-0.5368	0.886019	1
DTX3L	1.121252	1.111364	1.124467	1.11766	0.1605	0.886043	1
RP4-594I10.3	1.029729	1.038699	1.026813	0.69287	-0.5293	0.886122	1
SCNN1A	1.028223	1.037217	1.025299	0.67977	-0.5569	0.886211	1
AC092669.3	1.042828	1.033206	1.045956	1.38395	0.4688	0.88628	1
AJAP1	1.030739	1.021089	1.033875	1.60632	0.6838	0.886316	1
RP4-798A10.2	1.044751	1.035082	1.047894	1.36519	0.4491	0.886319	1
SENP6	1.979746	1.967434	1.983747	1.01686	0.0241	0.886343	1
UBE2J2	1.732827	1.721561	1.736489	1.02069	0.0295	0.886385	1
IFT122	1.318394	1.30798	1.321779	1.04481	0.0632	0.886423	1
BEST3	1.046517	1.037055	1.049592	1.33832	0.4204	0.886498	1
AP000223.42	1.031883	1.022273	1.035007	1.57174	0.6524	0.886557	1
SLC25A25-AS1	1.031729	1.02222	1.034819	1.56703	0.648	0.886666	1
BLVRA	1.475982	1.486183	1.472666	0.9722	-0.0407	0.886706	1
ETFBKMT	1.069004	1.059391	1.072128	1.21447	0.2803	0.886746	1
RP11-546J1.1	1.030307	1.02069	1.033433	1.61587	0.6923	0.886802	1
AC015691.13	1.039119	1.029511	1.042243	1.43143	0.5175	0.886871	1
NCRNA00250	1.027485	1.017948	1.030585	1.70411	0.769	0.886906	1
ZNF670-ZNF695	1.027496	1.017948	1.0306	1.70496	0.7697	0.886906	1
AC061992.2	1.027428	1.017948	1.03051	1.69994	0.7655	0.886906	1
RIPPLY1	1.02749	1.017948	1.030592	1.70451	0.7694	0.886906	1
UQCRC1	2.673495	2.689368	2.668336	0.98755	-0.0181	0.886913	1

LINC01209	1.026276	1.035201	1.023375	0.66405	-0.5906	0.886925	1
RP1-34B20.21	1.026286	1.035228	1.02338	0.66366	-0.5915	0.886925	1
CMTM2	1.02483	1.033708	1.021945	0.65104	-0.6192	0.88697	1
FGD5-AS1	1.979057	1.966001	1.983301	1.01791	0.0256	0.886995	1
AP000473.8	1.021503	1.011999	1.024592	2.04946	1.0352	0.887234	1
RP11-363E6.4	1.02144	1.011999	1.024509	2.04255	1.0304	0.887234	1
RARRES3	1.047163	1.056041	1.044277	0.79007	-0.3399	0.887239	1
TNS2	1.048562	1.057455	1.045671	0.79491	-0.3311	0.887241	1
RP11-775C24.5	1.025367	1.015934	1.028433	1.7844	0.8354	0.887387	1
ZSCAN31	1.04633	1.036751	1.049444	1.34537	0.428	0.887432	1
NATD1	1.102029	1.092099	1.105257	1.14288	0.1927	0.88761	1
TSTD3	1.150156	1.140006	1.153455	1.09606	0.1323	0.887639	1
SRRM3	1.074042	1.064402	1.077175	1.19833	0.261	0.887707	1
FGF18	1.047144	1.037708	1.050212	1.3316	0.4132	0.887713	1
FGF10	1.022769	1.031592	1.019901	0.62995	-0.6667	0.88773	1
RP11-184I16.4	1.021328	1.030195	1.018446	0.61091	-0.711	0.887731	1
TMEM184A	1.024039	1.032864	1.02117	0.64417	-0.6345	0.887736	1
GNPDA2	1.342959	1.35335	1.339582	0.96103	-0.0573	0.887816	1
APOBEC3F	1.045655	1.054527	1.042771	0.7844	-0.3503	0.887839	1
NOD1	1.045699	1.05468	1.04278	0.78237	-0.3541	0.887839	1
RP11-20E24.1	1.025131	1.015676	1.028204	1.79927	0.8474	0.887852	1
GOLGA8S	1.025129	1.015676	1.028202	1.79911	0.8473	0.887852	1
LINC01513	1.025059	1.015676	1.02811	1.79323	0.8426	0.887852	1
PPM1N	1.075007	1.06517	1.078204	1.19999	0.263	0.887907	1
RNLS	1.079037	1.087997	1.076124	0.86508	-0.2091	0.887914	1
ZEB2-AS1	1.030894	1.021443	1.033967	1.58405	0.6636	0.888053	1
RP11-461M2.2	1.030934	1.021487	1.034005	1.58257	0.6623	0.888053	1
TTC21B	1.194029	1.184136	1.197245	1.07119	0.0992	0.888171	1
CPNE7	1.02485	1.015493	1.027892	1.80031	0.8482	0.888381	1
LY6G5B	1.024877	1.015504	1.027924	1.80114	0.8489	0.888381	1
GJA3	1.018976	1.027715	1.016136	0.5822	-0.7804	0.888425	1
RFX8	1.019047	1.0279	1.016169	0.57951	-0.7871	0.888425	1
CANT1	1.443989	1.454017	1.440729	0.97073	-0.0429	0.888431	1
SUPT5H	1.597566	1.607827	1.59423	0.97763	-0.0326	0.888439	1
SLC26A4-AS1	1.020573	1.029356	1.017718	0.60356	-0.7284	0.888495	1
CTD-2562J15.4	1.020595	1.02945	1.017717	0.60159	-0.7331	0.888495	1
RP11-1055B8.10	1.02057	1.02935	1.017717	0.60364	-0.7282	0.888495	1
PRSS12	1.020599	1.029423	1.017731	0.60261	-0.7307	0.888495	1
TMEM253	1.04977	1.040215	1.052876	1.31482	0.3949	0.88858	1
C10orf111	1.049759	1.040247	1.052851	1.31319	0.3931	0.88858	1
NFKBID	1.08024	1.089185	1.077333	0.8671	-0.2057	0.88866	1
RP11-930P14.2	1.037504	1.028006	1.040591	1.44938	0.5354	0.888678	1
LINC00605	1.040355	1.04913	1.037503	0.76335	-0.3896	0.8888	1
ERCC6	1.115015	1.105263	1.118185	1.12277	0.1671	0.888909	1
CERS6	1.402898	1.392005	1.406439	1.03682	0.0522	0.889067	1

TVP23C-CDRT4	1.037145	1.027767	1.040193	1.44751	0.5336	0.889153	1
ZNF852	1.037111	1.027656	1.040184	1.453	0.539	0.889153	1
RABEP2	1.213113	1.222207	1.210157	0.94577	-0.0804	0.889201	1
RP11-379F4.4	1.036006	1.044788	1.033151	0.74018	-0.434	0.889343	1
RNF166	1.234735	1.225197	1.237836	1.05612	0.0788	0.889398	1
C12orf56	1.028314	1.018989	1.031346	1.65074	0.7231	0.889524	1
RP1-90G24.11	1.028816	1.019451	1.03186	1.63795	0.7119	0.889577	1
ST8SIA5	1.028902	1.019457	1.031972	1.64317	0.7165	0.889577	1
AC026471.6	1.028821	1.019486	1.031856	1.63476	0.7091	0.889577	1
ADGRD1	1.027686	1.018364	1.030717	1.67262	0.7421	0.889616	1
APOM	1.070935	1.061682	1.073943	1.19877	0.2616	0.889669	1
RP11-109G23.3	1.032301	1.023063	1.035303	1.53073	0.6142	0.889678	1
LINC00535	1.032351	1.022933	1.035413	1.54418	0.6268	0.889678	1
FAM228A	1.032293	1.022977	1.035321	1.53726	0.6204	0.889678	1
RP11-452L6.7	1.056593	1.065427	1.053722	0.8211	-0.2844	0.889708	1
RP11-21A7A.4	1.017129	1.02584	1.014297	0.55327	-0.854	0.889731	1
RP11-817O13.9	1.095255	1.085592	1.098396	1.1496	0.2011	0.889764	1
NKILA	1.158615	1.148268	1.161978	1.09247	0.1276	0.889801	1
RP11-61J19.5	1.056929	1.047408	1.060024	1.26613	0.3404	0.889943	1
WDR11-AS1	1.04226	1.032883	1.045308	1.37787	0.4624	0.889946	1
CDK2AP2	2.176613	2.188571	2.172726	0.98667	-0.0194	0.890058	1
LRWD1	1.21683	1.226113	1.213812	0.9456	-0.0807	0.890118	1
CD3EAP	1.130133	1.139532	1.127078	0.91075	-0.1349	0.890128	1
C20orf196	1.115846	1.124946	1.112889	0.9035	-0.1464	0.890155	1
SETD1A	1.111402	1.120212	1.108538	0.90289	-0.1474	0.890266	1
KCTD14	1.034811	1.043489	1.03199	0.73558	-0.443	0.890359	1
DDX51	1.136989	1.145995	1.134062	0.91826	-0.123	0.890365	1
MTRNR2L10	1.024123	1.014881	1.027127	1.82294	0.8663	0.890391	1
LINC01481.1	1.024148	1.014881	1.02716	1.82513	0.868	0.890391	1
AC018890.6	1.024139	1.014881	1.027148	1.82435	0.8674	0.890391	1
RP11-1109F11.3	1.031732	1.040446	1.028899	0.71452	-0.485	0.89041	1
RNF215	1.055768	1.046483	1.058786	1.26469	0.3388	0.890599	1
ALDH1L1	1.020923	1.029624	1.018094	0.6108	-0.7112	0.890691	1
RAB27B	1.042982	1.033591	1.046034	1.37042	0.4546	0.890718	1
AC002116.7	1.032293	1.023012	1.035309	1.53438	0.6177	0.890722	1
EPOR	1.287137	1.296811	1.283992	0.95681	-0.0637	0.890773	1
CYTIP	1.08944	1.098286	1.086564	0.88074	-0.1832	0.890774	1
BCO1	1.029094	1.037751	1.02628	0.69613	-0.5226	0.890809	1
SLC27A6	1.029103	1.037699	1.026309	0.69788	-0.5189	0.890809	1
SIAE	1.112407	1.121139	1.109569	0.90449	-0.1448	0.890907	1
C8orf48	1.096249	1.08672	1.099346	1.1456	0.1961	0.890922	1
CPM	1.038669	1.047392	1.035833	0.75609	-0.4034	0.891102	1
RP11-132A1.4	1.048382	1.057142	1.045534	0.79686	-0.3276	0.891208	1
PCDHA3	1.024938	1.015688	1.027944	1.78123	0.8329	0.891257	1
RP11-1348G14.8	1.035479	1.026274	1.038471	1.46424	0.5502	0.891285	1

GAS6-AS1	1.045521	1.036213	1.048547	1.3406	0.4229	0.891326	1
CTD-3105H18.14	1.025755	1.034349	1.022961	0.66847	-0.5811	0.891352	1
ZNF236	1.209274	1.199603	1.212418	1.0642	0.0898	0.89136	1
USP45	1.204198	1.194487	1.207354	1.06616	0.0924	0.891432	1
EPS8L2	1.575929	1.565124	1.579441	1.02533	0.0361	0.891555	1
CKM	1.021088	1.011871	1.024084	2.02885	1.0207	0.891615	1
AC002310.7	1.021093	1.011871	1.024091	2.02941	1.0211	0.891615	1
EXOSC3	1.369962	1.380078	1.366674	0.96473	-0.0518	0.891651	1
SAMD9	1.025588	1.034092	1.022824	0.66949	-0.5789	0.891711	1
DTX3	1.621148	1.631103	1.617912	0.9791	-0.0305	0.891773	1
SRP68	1.459798	1.449453	1.46316	1.0305	0.0433	0.891787	1
ASH1L-AS1	1.108961	1.099357	1.112082	1.12808	0.1739	0.891816	1
RP11-299L17.3	1.035704	1.02651	1.038692	1.45956	0.5455	0.891864	1
AC105760.2	1.061808	1.070404	1.059014	0.83822	-0.2546	0.891897	1
ACADSB	1.137848	1.146741	1.134958	0.9197	-0.1208	0.891977	1
SLC11A2	1.383218	1.372985	1.386544	1.03635	0.0515	0.892215	1
PLGLB1	1.021274	1.012187	1.024228	1.98806	0.9914	0.892345	1
RAB40AL	1.023123	1.013947	1.026106	1.87185	0.9045	0.892384	1
KRT222	1.02306	1.013947	1.026023	1.86589	0.8999	0.892384	1
RP5-1157M23.2	1.02835	1.019216	1.031319	1.6298	0.7047	0.892405	1
SYNDIG1	1.045534	1.054128	1.04274	0.78961	-0.3408	0.892458	1
ZSCAN25	1.076841	1.067507	1.079875	1.1832	0.2427	0.892529	1
GS1-166A23.2	1.024964	1.015826	1.027934	1.76509	0.8197	0.892561	1
HKR1	1.215427	1.20567	1.218598	1.06285	0.0879	0.892585	1
HCFC2	1.091471	1.082029	1.094541	1.15253	0.2048	0.892621	1
HSPB9	1.022165	1.013073	1.025121	1.92166	0.9424	0.892623	1
MIGA2	1.113903	1.122804	1.111009	0.90395	-0.1457	0.892646	1
AP000442.1	1.031004	1.021909	1.033961	1.55011	0.6324	0.892693	1
AGBL5-AS1	1.031064	1.021903	1.034042	1.55419	0.6362	0.892693	1
ZNF37A	1.022165	1.013102	1.025111	1.91664	0.9386	0.892733	1
RP1-122P22.2	1.022172	1.013102	1.02512	1.91732	0.9391	0.892733	1
MPP4	1.023036	1.031471	1.020294	0.64485	-0.633	0.892745	1
TMOD3	1.654857	1.664899	1.651593	0.97999	-0.0292	0.892921	1
GJA4	1.018862	1.027311	1.016116	0.59009	-0.761	0.892944	1
LA16c-60D12.2	1.0223	1.013274	1.025234	1.901	0.9268	0.892981	1
CEBPA	1.02039	1.028881	1.01763	0.61042	-0.7121	0.893045	1
KL	1.018747	1.027294	1.015968	0.58504	-0.7734	0.893192	1
BTBD16	1.02389	1.014806	1.026843	1.81302	0.8584	0.89324	1
XKR5	1.023883	1.014801	1.026835	1.8131	0.8585	0.89324	1
TMEM221	1.051389	1.042143	1.054395	1.29072	0.3682	0.893353	1
FAM134A	1.798186	1.787048	1.801807	1.01875	0.0268	0.89339	1
PRR12	1.146452	1.136919	1.149551	1.09226	0.1273	0.893461	1
ABI3	1.024726	1.015685	1.027665	1.76379	0.8187	0.893502	1
RP11-312B8.1	1.024733	1.015706	1.027667	1.76153	0.8168	0.893502	1
RP11-475E11.9	1.024732	1.015696	1.027669	1.7628	0.8179	0.893502	1

LINC01460	1.024743	1.0157	1.027682	1.76319	0.8182	0.893502	1
BST1	1.026486	1.017461	1.02942	1.68494	0.7527	0.893541	1
GALNT9	1.026543	1.017454	1.029497	1.69001	0.757	0.893541	1
HSPA1A	1.019592	1.028066	1.016837	0.59992	-0.7372	0.893541	1
CECR6	1.03179	1.022688	1.034749	1.53157	0.615	0.89355	1
CTC-444N24.6	1.042414	1.033284	1.045381	1.36343	0.4472	0.893554	1
ANGPTL1	1.048268	1.056945	1.045448	0.7981	-0.3254	0.893561	1
WAS	1.03918	1.047741	1.036397	0.76239	-0.3914	0.893574	1
PPP4R1-AS1	1.017828	1.026258	1.015087	0.57458	-0.7994	0.89358	1
AC138969.4	1.028409	1.019333	1.03136	1.62213	0.6979	0.893718	1
LYPLA2	1.882805	1.870989	1.886645	1.01798	0.0257	0.893734	1
RP11-587P21.2	1.025638	1.016569	1.028586	1.72525	0.7868	0.89378	1
RP11-494H4.3	1.02566	1.016584	1.028611	1.72526	0.7868	0.89378	1
MOCOS	1.025599	1.016597	1.028525	1.71869	0.7813	0.89378	1
TMEM175	1.155318	1.164449	1.15235	0.92643	-0.1103	0.893783	1
RP11-546K22.1	1.025609	1.016627	1.028528	1.71574	0.7788	0.893891	1
RP11-375N15.2	1.027941	1.018869	1.030889	1.63705	0.7111	0.893922	1
RP11-413N10.3	1.02789	1.018943	1.030798	1.62581	0.7012	0.893922	1
RP11-1391J7.1	1.187162	1.196144	1.184242	0.93932	-0.0903	0.89394	1
HSPB8	1.220416	1.22969	1.217401	0.9465	-0.0793	0.894051	1
ADCY1	1.053357	1.044171	1.056342	1.27556	0.3511	0.894077	1
NR2E3	1.017538	1.02587	1.01483	0.57323	-0.8028	0.89431	1
CCDC88C	1.150464	1.159086	1.147662	0.92819	-0.1075	0.894491	1
U91328.19	1.045921	1.036802	1.048885	1.3283	0.4096	0.894683	1
DNAJC13	1.188026	1.178264	1.191199	1.07256	0.1011	0.894722	1
PSME3	1.408635	1.417716	1.405683	0.97119	-0.0422	0.894807	1
NLK	1.186675	1.195595	1.183776	0.93957	-0.0899	0.894879	1
CTC-529I10.1	1.029069	1.020083	1.03199	1.59287	0.6716	0.894934	1
XXbac-BPG157A10.21	1.029057	1.020174	1.031944	1.58342	0.663	0.894934	1
UGT8	1.021422	1.029759	1.018712	0.62878	-0.6694	0.895029	1
RP11-362K2.2	1.021393	1.029655	1.018707	0.63083	-0.6647	0.895029	1
IL18BP	1.029089	1.020136	1.031999	1.58912	0.6682	0.895044	1
TMEM268	1.067839	1.076445	1.065042	0.85083	-0.2331	0.895094	1
CDC42SE2	1.457639	1.44689	1.461133	1.03187	0.0453	0.895095	1
RP11-101E13.5	1.03107	1.03946	1.028343	0.71828	-0.4774	0.895098	1
B3GALT2	1.031161	1.039592	1.028421	0.71783	-0.4783	0.895098	1
FAM19A4	1.032292	1.040842	1.029513	0.72263	-0.4687	0.895137	1
AL356585.1	1.024499	1.015453	1.027439	1.77562	0.8283	0.895235	1
AP000221.1	1.024368	1.015453	1.027265	1.76436	0.8191	0.895235	1
NPL	1.046032	1.037126	1.048927	1.31786	0.3982	0.895265	1
RP11-791G15.2	1.046107	1.037025	1.04906	1.32506	0.4061	0.895265	1
HSD3B7	1.15846	1.148703	1.161632	1.08694	0.1203	0.895339	1
RAB13	4.876601	4.90492	4.867395	0.99039	-0.0139	0.895366	1
GIN1	1.144726	1.13549	1.147728	1.09033	0.1248	0.895389	1
ZNF836	1.218667	1.20905	1.221793	1.06095	0.0854	0.895451	1

CLUH	1.068729	1.077154	1.06599	0.8553	-0.2255	0.895451	1
TSPEAR-AS2	1.097223	1.087944	1.10024	1.13981	0.1888	0.895454	1
RP5-875H18.9	1.020635	1.028873	1.017958	0.62196	-0.6851	0.895535	1
ASIC2	1.031811	1.040193	1.029087	0.72367	-0.4666	0.895557	1
APOL6	1.07423	1.065044	1.077215	1.18713	0.2475	0.895609	1
PDE11A	1.088657	1.079505	1.091632	1.15253	0.2048	0.89581	1
AP003068.23	1.017968	1.026216	1.015286	0.5831	-0.7782	0.895822	1
RP11-327J17.9	1.03349	1.024455	1.036427	1.48952	0.5748	0.895843	1
MED27	1.096648	1.087588	1.099593	1.13706	0.1853	0.895862	1
PROC	1.027644	1.035924	1.024952	0.69457	-0.5258	0.895883	1
RP11-932O9.9	1.027642	1.03602	1.024919	0.69182	-0.5315	0.895883	1
INCA1	1.044472	1.052942	1.041719	0.78801	-0.3437	0.895942	1
NPIPB2	1.055247	1.046257	1.058169	1.2575	0.3306	0.895968	1
RP11-603J24.21	1.035298	1.026399	1.038191	1.44671	0.5328	0.896021	1
RP11-295I5.3	1.041468	1.032451	1.044398	1.36817	0.4522	0.896117	1
DLK2	1.041392	1.032434	1.044303	1.36597	0.4499	0.896117	1
GRM1	1.020545	1.011717	1.023415	1.99834	0.9988	0.896196	1
LINC01248	1.02057	1.011717	1.023448	2.00115	1.0008	0.896196	1
RP11-1299A16.3	1.020571	1.011717	1.023449	2.00127	1.0009	0.896196	1
CFHR3	1.020576	1.011717	1.023456	2.0018	1.0013	0.896196	1
EFCAB6	1.045596	1.053902	1.042896	0.79581	-0.3295	0.896224	1
ZBTB40	1.139534	1.130224	1.142561	1.09474	0.1306	0.896322	1
ITGA9	1.028334	1.03673	1.025605	0.6971	-0.5206	0.896338	1
DMXL1	1.277794	1.286684	1.274905	0.95891	-0.0605	0.896423	1
SEMA3D	1.19477	1.185437	1.197804	1.06669	0.0931	0.89657	1
MCMDC2	1.025318	1.033536	1.022646	0.67528	-0.5664	0.89671	1
BOLA2	1.025295	1.033569	1.022605	0.67339	-0.5705	0.89671	1
AC010524.4	1.083813	1.074797	1.086744	1.15973	0.2138	0.896729	1
PIGB	1.101421	1.092481	1.104327	1.1281	0.1739	0.896745	1
NEGR1	1.060991	1.069159	1.058336	0.84351	-0.2455	0.896785	1
FAM234A	1.891915	1.902737	1.888398	0.98412	-0.0231	0.896789	1
MORF4L2-AS1	1.0955	1.086289	1.098494	1.14145	0.1909	0.896928	1
STARD13	1.09551	1.086588	1.098411	1.13654	0.1847	0.896928	1
OAF	1.642771	1.652437	1.639629	0.98037	-0.0286	0.896958	1
JPH1	1.028807	1.037006	1.026141	0.70641	-0.5014	0.896968	1
PDE6D	1.371409	1.380326	1.36851	0.96893	-0.0455	0.896982	1
SLC16A13	1.036907	1.028036	1.039791	1.41929	0.5052	0.896988	1
ZNF697	1.070603	1.078954	1.067888	0.85985	-0.2178	0.897005	1
KLHL26	1.070413	1.078615	1.067747	0.86176	-0.2146	0.897005	1
KIF17	1.038742	1.029828	1.041639	1.39598	0.4813	0.897166	1
DCUN1D2-AS	1.036057	1.027264	1.038915	1.42736	0.5134	0.897229	1
NIPSNAP3B	1.035993	1.027121	1.038877	1.43344	0.5195	0.897339	1
RP11-439E19.10	1.024052	1.01526	1.02691	1.76345	0.8184	0.89736	1
CTD-2199O4.1	1.024065	1.01526	1.026927	1.76458	0.8193	0.89736	1
KHDC3L	1.023973	1.015208	1.026822	1.76365	0.8186	0.89736	1

AC007879.4	1.02401	1.015227	1.026865	1.76426	0.8191	0.89736	1
CYTH3	1.143024	1.134121	1.145917	1.08795	0.1216	0.897425	1
RNF19B	1.057544	1.065825	1.054853	0.83331	-0.2631	0.897466	1
NEUROG1	1.021889	1.030097	1.019221	0.63865	-0.6469	0.8975	1
RP11-158K1.3	1.022497	1.013691	1.02536	1.85231	0.8893	0.897504	1
LCTL	1.056557	1.047628	1.05946	1.24841	0.3201	0.897573	1
SCN2A	1.037515	1.045627	1.034879	0.76444	-0.3875	0.897577	1
FRAT1	1.036109	1.027305	1.03897	1.42722	0.5132	0.897589	1
NSF	1.196313	1.20508	1.193464	0.94336	-0.0841	0.897656	1
INSIG2	2.511175	2.525142	2.506635	0.98787	-0.0176	0.897679	1
CCAR2	1.239464	1.23029	1.242446	1.05278	0.0742	0.8977	1
NOXA1	1.104382	1.095194	1.107369	1.1279	0.1736	0.897706	1
GIP	1.067208	1.075434	1.064534	0.8555	-0.2252	0.897787	1
RP1-8B1.4	1.037576	1.028853	1.040411	1.40059	0.486	0.897826	1
CARD14	1.021428	1.029544	1.01879	0.63603	-0.6528	0.897908	1
TNXB	1.078313	1.086925	1.075514	0.86873	-0.203	0.89795	1
AC010287.1	1.021626	1.029768	1.018979	0.63757	-0.6493	0.898076	1
RP11-118K6.3	1.03854	1.029693	1.041416	1.39481	0.4801	0.898089	1
AC068057.1	1.024801	1.016045	1.027647	1.72311	0.785	0.898126	1
CTD-2587H19.3	1.024838	1.016045	1.027696	1.72612	0.7875	0.898126	1
RP11-379F4.9	1.02477	1.016045	1.027606	1.72053	0.7829	0.898126	1
RP1-159A19.4	1.024771	1.016045	1.027608	1.72065	0.783	0.898126	1
TMEM220	1.06615	1.057362	1.069007	1.20302	0.2667	0.8982	1
RP6-191P20.4	1.027432	1.035616	1.024772	0.69552	-0.5238	0.898236	1
RP1-20C7.6	1.027417	1.035562	1.02477	0.69653	-0.5218	0.898236	1
PARP3	1.119527	1.110551	1.122445	1.10759	0.1474	0.89824	1
HIST1H4J	1.039478	1.030565	1.042375	1.38641	0.4714	0.89837	1
CD36	1.033977	1.04208	1.031343	0.74485	-0.425	0.898399	1
QRICH2	1.056948	1.065258	1.054247	0.83127	-0.2666	0.898436	1
NLRP3	1.027513	1.01877	1.030355	1.61721	0.6935	0.898482	1
ANKRD23	1.027529	1.018788	1.03037	1.61641	0.6928	0.898482	1
RP11-313P13.5	1.027428	1.01871	1.030262	1.6174	0.6937	0.89852	1
PEAR1	1.021912	1.029981	1.019289	0.64338	-0.6363	0.898542	1
AC016700.5	1.104744	1.096154	1.107537	1.11838	0.1614	0.898606	1
CASKIN1	1.055271	1.046496	1.058124	1.25009	0.322	0.898671	1
AC007040.8	1.025896	1.017205	1.028721	1.66933	0.7393	0.898671	1
RP11-153I24.5	1.025906	1.01722	1.028729	1.66833	0.7384	0.898671	1
NDST4	1.073478	1.081848	1.070757	0.86449	-0.2101	0.898687	1
LINC00702	1.017962	1.025993	1.015351	0.5906	-0.7598	0.898696	1
RP11-431K24.1	1.01797	1.02606	1.015341	0.58866	-0.7645	0.898696	1
PATL2	1.034786	1.026066	1.03762	1.44328	0.5294	0.89871	1
RP11-111M22.4	1.034795	1.025965	1.037665	1.45063	0.5367	0.89871	1
ZNF267	1.109663	1.100605	1.112607	1.1193	0.1626	0.898826	1
LRP11	1.346246	1.336675	1.349357	1.03767	0.0533	0.898987	1
NTN1	1.023956	1.032048	1.021325	0.66543	-0.5876	0.899024	1

CCT6B	1.081698	1.072818	1.084584	1.16159	0.2161	0.899027	1
SELENOO	1.068656	1.059892	1.071505	1.1939	0.2557	0.899109	1
SDHAF2	1.43907	1.44815	1.436118	0.97315	-0.0393	0.89921	1
MRGPRG-AS1	1.03068	1.038994	1.027977	0.71747	-0.479	0.899223	1
SNAP91	1.053493	1.061713	1.050821	0.82351	-0.2801	0.899234	1
LINC00668	1.028301	1.01958	1.031135	1.59013	0.6691	0.89925	1
NLRP14	1.02824	1.019585	1.031053	1.58551	0.6649	0.89925	1
FAM214A	1.197289	1.187835	1.200362	1.06669	0.0931	0.899277	1
RTN4IP1	1.074305	1.082454	1.071656	0.86904	-0.2025	0.899318	1
KCNK5	1.029688	1.02107	1.032489	1.54196	0.6248	0.899368	1
SGK2	1.029724	1.021008	1.032557	1.54973	0.632	0.899368	1
OSBP2	1.069992	1.078156	1.067339	0.86159	-0.2149	0.899467	1
MSL1	1.817241	1.805626	1.821016	1.0191	0.0273	0.899472	1
CPOX	1.196459	1.20491	1.193712	0.94535	-0.0811	0.899492	1
MTRNR2L3	1.030876	1.022256	1.033679	1.51324	0.5976	0.899676	1
CRAT	1.356121	1.364933	1.353256	0.968	-0.0469	0.899769	1
SH3BP4	1.172013	1.180459	1.169268	0.93798	-0.0924	0.899792	1
PRSS27	1.084285	1.075435	1.087162	1.15545	0.2085	0.899851	1
RP11-367J11.3	1.038217	1.029455	1.041065	1.39415	0.4794	0.89986	1
NAP1L5	1.175564	1.166558	1.178491	1.07165	0.0998	0.899887	1
C17orf67	1.083354	1.091611	1.08067	0.88057	-0.1835	0.899903	1
SATB2	1.073443	1.064553	1.076332	1.18247	0.2418	0.899909	1
RP11-80H5.7	1.026522	1.017948	1.02931	1.63306	0.7076	0.899993	1
C1orf162	1.026573	1.017948	1.029377	1.63681	0.7109	0.899993	1
RRS1-AS1	1.026611	1.017948	1.029427	1.63961	0.7133	0.899993	1
MCEMP1	1.026614	1.017948	1.029431	1.63983	0.7135	0.899993	1
RP11-190C22.9	1.026623	1.017948	1.029444	1.64052	0.7142	0.899993	1
INO80E	1.49372	1.483869	1.496923	1.02698	0.0384	0.900013	1
DMRT3	1.027159	1.035295	1.024514	0.69456	-0.5258	0.900049	1
PRX	1.044522	1.035849	1.047341	1.32054	0.4011	0.900095	1
IRAK4	1.125447	1.116412	1.128384	1.10284	0.1412	0.900141	1
TEFM	1.155569	1.14659	1.158488	1.08117	0.1126	0.90015	1
RP11-998D10.4	1.020604	1.011999	1.023401	1.95025	0.9637	0.900321	1
SCRT2	1.020569	1.011999	1.023355	1.94634	0.9608	0.900321	1
SIX1	1.084192	1.092346	1.081541	0.88299	-0.1795	0.900357	1
CTD-2541J13.2	1.02452	1.015934	1.027311	1.71397	0.7773	0.900475	1
RP11-162D16.2	1.02453	1.015934	1.027324	1.71481	0.778	0.900475	1
DLX6	1.024518	1.015934	1.027308	1.71378	0.7772	0.900475	1
GATS	1.166482	1.15719	1.169503	1.07833	0.1088	0.900542	1
CAND2	1.055143	1.046476	1.057961	1.24712	0.3186	0.900628	1
RP11-418H16.1	1.046366	1.037653	1.049198	1.30662	0.3858	0.900641	1
LAIR1	1.034436	1.025755	1.037257	1.44662	0.5327	0.900715	1
MAP7D3	1.516431	1.525359	1.513529	0.97748	-0.0329	0.900719	1
OLFM1	1.067122	1.075203	1.064495	0.85761	-0.2216	0.900731	1
USP35	1.034348	1.025763	1.037139	1.44154	0.5276	0.900828	1

ZC3H11B	1.034344	1.025726	1.037145	1.44385	0.5299	0.900828	1
RIPK3	1.023631	1.031547	1.021058	0.6675	-0.5832	0.900879	1
RP5-916L7.2	1.023685	1.031769	1.021057	0.66281	-0.5933	0.900879	1
RP11-227H15.5	1.022206	1.030164	1.01962	0.65042	-0.6206	0.900892	1
RP11-745L13.2	1.024235	1.015676	1.027017	1.7235	0.7853	0.900941	1
SPAG8	1.077009	1.06818	1.079879	1.17159	0.2285	0.900998	1
RP11-48B3.5	1.041607	1.032989	1.044408	1.34614	0.4288	0.901007	1
CASC3	1.574489	1.584487	1.571239	0.97733	-0.0331	0.901053	1
P3H4	2.234663	2.222707	2.238549	1.01296	0.0186	0.901095	1
SLC1A1	1.040432	1.048332	1.037864	0.78341	-0.3522	0.901217	1
C3orf35	1.024851	1.032779	1.022274	0.67953	-0.5574	0.901235	1
RP11-160N1.10	1.06683	1.05796	1.069712	1.20276	0.2663	0.901376	1
SETDB1	1.31019	1.318852	1.307374	0.964	-0.0529	0.901425	1
RP11-329B9.3	1.024035	1.015531	1.026799	1.72549	0.787	0.901449	1
IL10RB-AS1	1.024059	1.015504	1.02684	1.73119	0.7918	0.901449	1
THSD1	1.035137	1.02656	1.037925	1.42787	0.5139	0.901485	1
PCDHB13	1.035172	1.026546	1.037976	1.4306	0.5166	0.901485	1
PCYT2	1.209929	1.218419	1.207169	0.94849	-0.0763	0.901488	1
HYAL1	1.02452	1.015961	1.027302	1.71051	0.7744	0.901517	1
TRMT2A	1.270815	1.261434	1.273864	1.04755	0.067	0.901518	1
RP5-991G20.6	1.023375	1.014833	1.026152	1.76317	0.8182	0.901556	1
RP11-1072C15.4	1.023326	1.014833	1.026086	1.75874	0.8145	0.901556	1
AC004878.2	1.02333	1.014833	1.026092	1.75909	0.8148	0.901556	1
IBA57-AS1	1.023324	1.014833	1.026084	1.75856	0.8144	0.901556	1
ADAM8	1.019881	1.027766	1.017318	0.62371	-0.6811	0.901575	1
FAR1	1.375731	1.384162	1.372991	0.97092	-0.0426	0.901603	1
PAPOLG	1.169827	1.160747	1.172779	1.07485	0.1041	0.901606	1
DMD	1.603493	1.59244	1.607087	1.02472	0.0352	0.901615	1
DOCK1	1.263713	1.254508	1.266706	1.04793	0.0675	0.901654	1
ACE2	1.021496	1.029524	1.018886	0.63968	-0.6446	0.901659	1
TNKS2-AS1	1.052568	1.043956	1.055368	1.25962	0.333	0.901709	1
STX11	1.020166	1.028004	1.017618	0.62914	-0.6686	0.901711	1
LINC01470	1.020194	1.028117	1.017618	0.62661	-0.6744	0.901711	1
ASB9	1.059639	1.067566	1.057063	0.84454	-0.2438	0.901819	1
RP11-30K9.6	1.037927	1.029311	1.040728	1.38952	0.4746	0.901826	1
OAS1	1.052088	1.043428	1.054903	1.26422	0.3382	0.901873	1
DSTYK	1.379064	1.369325	1.38223	1.03494	0.0496	0.901887	1
BLZF1	1.182221	1.173382	1.185095	1.06756	0.0943	0.901989	1
GABRB1	1.021391	1.029227	1.018844	0.64473	-0.6332	0.902058	1
RHBDL3	1.027749	1.019219	1.030522	1.58811	0.6673	0.90207	1
RP11-486A14.2	1.027666	1.019177	1.030425	1.58655	0.6659	0.90207	1
RP5-1159O4.2	1.027665	1.019189	1.03042	1.58529	0.6647	0.90207	1
KCNJ2-AS1	1.049178	1.057045	1.046621	0.81727	-0.2911	0.902237	1
FCRLB	1.060367	1.06834	1.057775	0.84541	-0.2423	0.902293	1
ZNF408	1.112778	1.104232	1.115556	1.10864	0.1488	0.902347	1

ASCL4	1.016424	1.024308	1.013862	0.57024	-0.8103	0.902406	1
RP11-62J1.4	1.016408	1.024241	1.013862	0.57183	-0.8064	0.902406	1
CLIC5	1.0164	1.024208	1.013862	0.57262	-0.8043	0.902406	1
RP11-408N14.1	1.016391	1.024173	1.013862	0.57344	-0.8023	0.902406	1
DACT3	1.092999	1.101049	1.090383	0.89445	-0.1609	0.90246	1
NCOA1	1.288058	1.29671	1.285245	0.96136	-0.0568	0.902474	1
ZGLP1	1.046513	1.054552	1.043899	0.80473	-0.3134	0.902529	1
WDR41	1.568049	1.577119	1.5651	0.97917	-0.0304	0.902538	1
RP11-192H23.4	1.059349	1.050738	1.062148	1.22488	0.2926	0.902568	1
MSS51	1.038614	1.030078	1.041389	1.37605	0.4605	0.902598	1
NOMO3	1.075993	1.067435	1.078775	1.16817	0.2242	0.902606	1
FAM69A	1.06641	1.07425	1.063861	0.86008	-0.2175	0.902628	1
RP11-258F1.1	1.027939	1.019469	1.030692	1.57649	0.6567	0.902647	1
RASD2	1.02791	1.019487	1.030648	1.57276	0.6533	0.902647	1
RBM5-AS1	1.026811	1.018336	1.029566	1.61244	0.6892	0.902686	1
RP11-295G20.2	1.151774	1.142954	1.154641	1.08176	0.1134	0.902697	1
SLC26A2	1.263087	1.271668	1.260298	0.95815	-0.0617	0.902698	1
GNAO1	1.031448	1.022979	1.034201	1.48835	0.5737	0.902727	1
LINC00900	1.056124	1.047401	1.05896	1.24384	0.3148	0.902835	1
CASC1	1.056131	1.047622	1.058896	1.23674	0.3065	0.902835	1
ZNF653	1.056041	1.047553	1.0588	1.23652	0.3063	0.902835	1
MATN4	1.056063	1.047597	1.058815	1.23569	0.3053	0.902835	1
RP11-290F5.2	1.040122	1.031544	1.04291	1.36032	0.4439	0.902847	1
CTD-2105E13.6	1.017973	1.025809	1.015426	0.59768	-0.7426	0.902885	1
TNNI2	1.017984	1.02587	1.01542	0.59607	-0.7464	0.902885	1
KRTDAP	1.035085	1.042934	1.032533	0.75776	-0.4002	0.902932	1
C6orf132	1.122755	1.13061	1.120202	0.92031	-0.1198	0.902967	1
RP11-23F23.2	1.033422	1.041349	1.030845	0.74598	-0.4228	0.903086	1
WBP1	1.073814	1.06516	1.076627	1.17599	0.2339	0.9031	1
CPT2	1.201669	1.210317	1.198858	0.94551	-0.0808	0.90318	1
XKR9	1.0311	1.022669	1.03384	1.49275	0.578	0.903195	1
PAX7	1.031212	1.022789	1.03395	1.48974	0.5751	0.903195	1
RP11-112L6.3	1.020554	1.012186	1.023274	1.90985	0.9335	0.903197	1
FUT8-AS1	1.033376	1.041178	1.03084	0.74895	-0.4171	0.903197	1
CA3-AS1	1.10211	1.110004	1.099544	0.90491	-0.1441	0.903203	1
ANKRD17	1.742845	1.75232	1.739765	0.98331	-0.0243	0.90327	1
SLC38A5	1.127801	1.135797	1.125201	0.92197	-0.1172	0.903399	1
RP11-725P16.2	1.02325	1.014881	1.02597	1.74516	0.8034	0.903485	1
GJA5	1.023289	1.014881	1.026023	1.74869	0.8063	0.903485	1
ZKSCAN4	1.090423	1.098417	1.087824	0.89236	-0.1643	0.903501	1
MRPL44	1.532458	1.542126	1.529315	0.97637	-0.0345	0.90364	1
ZFPM1	1.062726	1.054241	1.065483	1.20726	0.2717	0.903678	1
RP11-147L13.14	1.030881	1.022515	1.0336	1.49232	0.5776	0.903693	1
TMBIM4	2.111315	2.121811	2.107904	0.9876	-0.018	0.903707	1
CCL26	1.014523	1.022278	1.012002	0.53876	-0.8923	0.903713	1

GPR183	1.014522	1.022272	1.012002	0.53889	-0.8919	0.903713	1
WISP1	1.014522	1.022274	1.012002	0.53885	-0.892	0.903713	1
RP11-353N14.2	1.014513	1.022235	1.012002	0.5398	-0.8895	0.903713	1
ZNF252P-AS1	1.03141	1.022995	1.034145	1.4849	0.5704	0.903774	1
RP11-996F15.6	1.031425	1.023045	1.034149	1.48185	0.5674	0.903774	1
ART5	1.083452	1.074953	1.086215	1.15026	0.202	0.903824	1
SP6	1.021764	1.029485	1.019254	0.65301	-0.6148	0.903847	1
ACTR3C	1.021824	1.029721	1.019257	0.64792	-0.6261	0.903847	1
KBTBD11	1.137671	1.128938	1.140509	1.08974	0.124	0.903946	1
C1orf106	1.098706	1.106532	1.096163	0.90267	-0.1477	0.903988	1
ZNF25	1.200466	1.20872	1.197784	0.9476	-0.0776	0.904277	1
PI4K2B	1.109773	1.117634	1.107217	0.91145	-0.1338	0.904291	1
ADGRA1	1.034649	1.026285	1.037368	1.42166	0.5076	0.904316	1
STK11	1.207596	1.215963	1.204876	0.94866	-0.076	0.904406	1
AGBL2	1.052143	1.043715	1.054883	1.25549	0.3283	0.904422	1
RP11-250B2.5	1.043119	1.034776	1.045831	1.31787	0.3982	0.904444	1
TSTD2	1.250695	1.259138	1.247951	0.95683	-0.0637	0.904447	1
RP4-555D20.4	1.026667	1.034388	1.024157	0.70248	-0.5095	0.904465	1
ALG11	1.082646	1.090408	1.080123	0.88624	-0.1742	0.904478	1
GSG1L	1.040412	1.032013	1.043142	1.34764	0.4304	0.904479	1
CHIC1	1.115026	1.106393	1.117832	1.10752	0.1473	0.90455	1
RP11-489E7.4	1.100984	1.092338	1.103795	1.12407	0.1687	0.904591	1
GLUD2	1.026723	1.018388	1.029433	1.60068	0.6787	0.904618	1
ELMO1	1.049854	1.05745	1.047384	0.82479	-0.2779	0.904654	1
RP3-394A18.1	1.077062	1.068529	1.079836	1.165	0.2203	0.904672	1
LGSN	1.018321	1.026012	1.015821	0.60822	-0.7173	0.904674	1
RP11-865I6.2	1.018311	1.02597	1.015821	0.60921	-0.715	0.904674	1
RP11-1C8.4	1.01835	1.02613	1.015821	0.60547	-0.7239	0.904674	1
RP11-370A5.1	1.018328	1.026041	1.015821	0.60755	-0.7189	0.904674	1
RFX4	1.062185	1.069962	1.059657	0.85271	-0.2299	0.904696	1
RP11-1263C18.2	1.020211	1.011871	1.022922	1.93093	0.9493	0.904711	1
RP11-477D19.2	1.045513	1.037205	1.048214	1.29589	0.3739	0.90481	1
SH3GLB2	1.280747	1.288639	1.278182	0.96377	-0.0532	0.904866	1
SEPHS2	1.612511	1.621841	1.609478	0.98012	-0.029	0.904977	1
IKBKG	1.137497	1.145556	1.134878	0.92663	-0.1099	0.905101	1
AC000068.9	1.01956	1.011304	1.022243	1.96767	0.9765	0.90518	1
AC008074.3	1.019551	1.011304	1.022232	1.96664	0.9757	0.90518	1
AC003991.3	1.019564	1.011304	1.022249	1.96817	0.9769	0.90518	1
RP11-105C19.1	1.019563	1.011304	1.022248	1.96807	0.9768	0.90518	1
SMIM10L1	2.048712	2.057964	2.045705	0.98841	-0.0168	0.905182	1
SPATA6L	1.076121	1.067484	1.078929	1.16959	0.226	0.905303	1
LINC01607	1.071067	1.078788	1.068558	0.87015	-0.2007	0.905384	1
CHUK	1.103323	1.094703	1.106125	1.1206	0.1643	0.905391	1
SEMA6A-AS1	1.020394	1.012187	1.023062	1.89235	0.9202	0.905442	1
AC007386.2	1.020426	1.012187	1.023104	1.8958	0.9228	0.905442	1

RP11-338N10.1	1.022196	1.013947	1.024878	1.78378	0.8349	0.905481	1
TRPV6	1.022318	1.013947	1.025039	1.79534	0.8443	0.905481	1
FGFBP2	1.022171	1.013947	1.024845	1.78143	0.833	0.905481	1
AXDND1	1.022235	1.013947	1.024929	1.78748	0.8379	0.905481	1
DNAJC22	1.060219	1.051944	1.062908	1.21107	0.2763	0.905504	1
RP11-932O9.7	1.023009	1.030693	1.020511	0.66828	-0.5815	0.905533	1
ANO3	1.023027	1.03072	1.020526	0.66818	-0.5817	0.905533	1
PSD2	1.046746	1.054647	1.044177	0.80842	-0.3068	0.905612	1
RP4-694B14.8	1.024097	1.015826	1.026785	1.69248	0.7591	0.905659	1
ARHGEF7-AS2	1.024058	1.015826	1.026733	1.6892	0.7563	0.905659	1
CARD6	1.025805	1.033432	1.023326	0.69771	-0.5193	0.905706	1
DGKA	1.081962	1.089752	1.07943	0.88499	-0.1763	0.905712	1
ADAT3	1.021302	1.013073	1.023977	1.83415	0.8751	0.905721	1
RP11-468E2.5	1.03023	1.02191	1.032935	1.50318	0.588	0.905748	1
ST5	1.699067	1.688174	1.702608	1.02098	0.0299	0.905793	1
RP11-143K11.7	1.021361	1.013102	1.024046	1.83533	0.876	0.905831	1
RP13-650J16.1	1.021321	1.013102	1.023992	1.83125	0.8728	0.905831	1
RP11-334G22.1	1.025376	1.033183	1.022839	0.68827	-0.539	0.905839	1
CCDC110	1.080053	1.087789	1.077538	0.88324	-0.1791	0.905895	1
EXOC6B	1.160691	1.1519	1.163549	1.07669	0.1066	0.905895	1
PSMB9	1.0221	1.029675	1.019637	0.66175	-0.5956	0.905923	1
TIRAP	1.078092	1.085802	1.075586	0.88094	-0.1829	0.905935	1
SEC14L5	1.038325	1.030067	1.04101	1.36396	0.4478	0.906014	1
KCNK12	1.038323	1.030106	1.040994	1.36167	0.4454	0.906014	1
EVI5L	1.122922	1.114171	1.125766	1.10156	0.1395	0.906045	1
ZMYND19	1.090474	1.081981	1.093235	1.13727	0.1856	0.906049	1
RP13-753N3.1	1.021493	1.013274	1.024165	1.82045	0.8643	0.906079	1
RP11-500G22.4	1.021503	1.013274	1.024178	1.82147	0.8651	0.906079	1
RP11-3P17.4	1.021468	1.013274	1.024131	1.81788	0.8623	0.906079	1
TFAP2D	1.021527	1.013274	1.02421	1.82381	0.867	0.906079	1
PRCC	1.58632	1.594569	1.583639	0.98162	-0.0268	0.906176	1
ZBTB22	1.188029	1.17918	1.190905	1.06544	0.0914	0.906231	1
RP11-848P1.4	1.02298	1.014798	1.02564	1.73261	0.7929	0.906317	1
XXbac-BPGBPG55C20.3	1.02297	1.014791	1.025629	1.73278	0.7931	0.906317	1
C7orf43	1.055265	1.062779	1.052823	0.8414	-0.2491	0.906344	1
TXNDC11	1.164019	1.171803	1.161488	0.93996	-0.0893	0.906481	1
SPX	1.022314	1.029862	1.019861	0.6651	-0.5884	0.906526	1
LUCAT1	1.023866	1.015683	1.026526	1.69138	0.7582	0.906579	1
CTC-308K20.1	1.030944	1.0227	1.033624	1.48122	0.5668	0.906585	1
AC004951.6	1.126794	1.118105	1.129618	1.09748	0.1342	0.90662	1
AP001059.7	1.021885	1.029424	1.019434	0.66049	-0.5984	0.906655	1
AIMP2	1.298239	1.289345	1.30113	1.04073	0.0576	0.906695	1
TMEM198	1.155745	1.163801	1.153126	0.93483	-0.0972	0.906777	1
BORCS6	1.105678	1.097197	1.108435	1.11562	0.1578	0.906844	1
RP11-357H14.17	1.016308	1.023907	1.013838	0.57881	-0.7888	0.906929	1

LINC01611	1.016272	1.023761	1.013838	0.58237	-0.78	0.906929	1
ARAP1	1.249206	1.240305	1.2521	1.04909	0.0691	0.906962	1
CRH	1.024861	1.016598	1.027547	1.65972	0.7309	0.906969	1
RP11-109N23.4	1.027016	1.018882	1.029661	1.57088	0.6516	0.906979	1
ZC3HAV1	1.208605	1.216522	1.206031	0.95155	-0.0716	0.90699	1
ATP6V1G3	1.01782	1.025346	1.015374	0.60656	-0.7213	0.907004	1
FBXO24	1.017802	1.025289	1.015368	0.60772	-0.7185	0.907004	1
RP11-707A18.1	1.017827	1.025355	1.015381	0.60662	-0.7211	0.907004	1
KIRREL2	1.092972	1.08447	1.095736	1.13337	0.1806	0.90701	1
RP11-890B15.3	1.064197	1.071588	1.061795	0.8632	-0.2122	0.907016	1
L3MBTL3	1.208065	1.199051	1.210995	1.06	0.0841	0.90711	1
EMC3	1.499616	1.508438	1.496749	0.97701	-0.0336	0.907157	1
TMOD1	1.040531	1.032346	1.043192	1.3353	0.4172	0.907168	1
SCYL3	1.132492	1.124277	1.135162	1.08758	0.1211	0.907173	1
LINC01276	1.016129	1.02364	1.013687	0.57897	-0.7884	0.907177	1
AP003774.5	1.016132	1.023652	1.013687	0.57868	-0.7892	0.907177	1
PELP1	1.242328	1.2503	1.239736	0.95779	-0.0622	0.907196	1
RP11-323F24.3	1.02489	1.016773	1.027529	1.64122	0.7148	0.907218	1
USP22	2.528995	2.541292	2.524998	0.98943	-0.0153	0.907254	1
KCNH5	1.016108	1.02361	1.01367	0.57898	-0.7884	0.907287	1
HSPB3	1.016094	1.023553	1.01367	0.58038	-0.7849	0.907287	1
SAMSN1	1.016128	1.023692	1.01367	0.57696	-0.7934	0.907287	1
REN	1.016128	1.023692	1.01367	0.57696	-0.7934	0.907287	1
CSF3R	1.016117	1.023647	1.01367	0.57806	-0.7907	0.907287	1
LINC00857	1.016104	1.023594	1.01367	0.57937	-0.7874	0.907287	1
OLR1	1.018862	1.026354	1.016426	0.62329	-0.682	0.907349	1
SLC7A7	1.025705	1.033242	1.023255	0.69957	-0.5155	0.907359	1
FAM63B	1.942741	1.952365	1.939613	0.98661	-0.0194	0.907379	1
ATG14	1.238406	1.24686	1.235657	0.95462	-0.067	0.907434	1
LAMC3	1.026419	1.018289	1.029062	1.58909	0.6682	0.90745	1
SH3D21	1.087739	1.079256	1.090496	1.14182	0.1913	0.907455	1
ST8SIA6	1.018394	1.025876	1.015962	0.61686	-0.697	0.907474	1
FEV	1.017012	1.024541	1.014564	0.59348	-0.7527	0.907526	1
RP11-794M8.1	1.017043	1.024667	1.014564	0.59044	-0.7601	0.907526	1
RP6-99M1.3	1.017001	1.024497	1.014564	0.59454	-0.7502	0.907526	1
CTD-2377D24.6	1.017011	1.024536	1.014564	0.59359	-0.7525	0.907526	1
AC008074.1	1.017003	1.024504	1.014564	0.59436	-0.7506	0.907526	1
ARFGAP3	1.56637	1.575252	1.563483	0.97954	-0.0298	0.907547	1
ERC2	1.231868	1.222938	1.234771	1.05308	0.0746	0.907563	1
GRM8	1.015231	1.022691	1.012806	0.56437	-0.8253	0.907566	1
ROR1-AS1	1.015251	1.022771	1.012806	0.56237	-0.8304	0.907566	1
RP11-598F7.4	1.015239	1.022723	1.012806	0.56357	-0.8273	0.907566	1
MRPS36	2.535435	2.546177	2.531943	0.99079	-0.0133	0.907584	1
PLXNA2	1.050735	1.058275	1.048284	0.82854	-0.2714	0.907674	1
UST	1.053247	1.060841	1.050779	0.83462	-0.2608	0.907678	1

ZMIZ1-AS1	1.027281	1.019206	1.029906	1.55709	0.6388	0.907713	1
PKD1L3	1.014362	1.021796	1.011946	0.5481	-0.8675	0.907827	1
PRODH	1.014372	1.021834	1.011946	0.54712	-0.8701	0.907827	1
LINC00881	1.014373	1.021837	1.011946	0.54705	-0.8703	0.907827	1
RP11-434E6.4	1.014361	1.021789	1.011946	0.54826	-0.8671	0.907827	1
C16orf71	1.024968	1.032502	1.022519	0.69285	-0.5294	0.907885	1
CAPSL	1.024949	1.032413	1.022522	0.69486	-0.5252	0.907885	1
IL17D	1.068653	1.060398	1.071337	1.18111	0.2401	0.907919	1
BAALC-AS2	1.030977	1.022829	1.033625	1.47292	0.5587	0.90793	1
EED	1.239195	1.247241	1.23658	0.95688	-0.0636	0.907952	1
AP000253.1	1.037107	1.028899	1.039775	1.37633	0.4608	0.907996	1
LINC00910	1.036321	1.043819	1.033884	0.77327	-0.371	0.908013	1
SUCLA2	1.549036	1.557694	1.546222	0.97943	-0.03	0.908065	1
LINC00920	1.055934	1.047618	1.058637	1.23139	0.3003	0.908102	1
ADAMTS17	1.028255	1.020184	1.030879	1.52989	0.6134	0.908103	1
ABCA13	1.048766	1.05627	1.046327	0.8233	-0.2805	0.908131	1
PPP1R26-AS1	1.044753	1.036638	1.04739	1.29349	0.3713	0.908135	1
PINX1	1.151559	1.14303	1.154332	1.07901	0.1097	0.908217	1
ZNRF2	1.033092	1.040618	1.030646	0.75449	-0.4064	0.908238	1
ADAMTSL3	1.014985	1.022458	1.012556	0.55909	-0.8388	0.908296	1
LINC00501	1.01496	1.022357	1.012556	0.56161	-0.8324	0.908296	1
RP13-1032I1.11	1.023528	1.015453	1.026153	1.69237	0.759	0.908337	1
UNC13A	1.02835	1.0203	1.030967	1.52545	0.6092	0.908352	1
URB1-AS1	1.028387	1.020343	1.031001	1.52391	0.6078	0.908352	1
UFSP1	1.0284	1.020302	1.031033	1.52858	0.6122	0.908352	1
CTC-254B4.1	1.028438	1.020278	1.03109	1.53319	0.6165	0.908352	1
GOLGA8Q	1.028353	1.020302	1.03097	1.52547	0.6093	0.908352	1
STX6	1.320856	1.312064	1.323714	1.03733	0.0529	0.908362	1
CKMT2-AS1	1.095879	1.087583	1.098576	1.12551	0.1706	0.908513	1
GABARAPL2	4.595424	4.614861	4.589105	0.99287	-0.0103	0.908559	1
IP6K1	1.313153	1.304079	1.316102	1.03954	0.0559	0.908569	1
ZNF835	1.029893	1.021812	1.032519	1.49087	0.5762	0.908579	1
PMFBP1	1.029871	1.021825	1.032487	1.4885	0.5739	0.908579	1
RP11-573D15.2	1.0299	1.021825	1.032525	1.49028	0.5756	0.908579	1
ZSCAN2	1.143261	1.134681	1.14605	1.08442	0.1169	0.908589	1
GPD2	1.151754	1.143365	1.154481	1.07753	0.1077	0.908598	1
ZNF235	1.032602	1.040053	1.03018	0.75349	-0.4083	0.908638	1
RP11-10K16.1	1.032634	1.040113	1.030203	0.75295	-0.4094	0.908638	1
RP11-736N17.10	1.065334	1.07276	1.06292	0.86475	-0.2096	0.908762	1
GFRA2	1.058458	1.050382	1.061083	1.2124	0.2779	0.908785	1
RP1-80N2.3	1.03286	1.040287	1.030445	0.75571	-0.4041	0.908818	1
TAB1	1.236315	1.227639	1.239135	1.0505	0.0711	0.90889	1
MPZ	1.054393	1.046219	1.05705	1.23434	0.3037	0.908903	1
TMEM163	1.045292	1.052696	1.042886	0.81384	-0.2972	0.908968	1
TCTA	1.313065	1.321179	1.310428	0.96653	-0.0491	0.909047	1

TBC1D7	1.556876	1.565485	1.554078	0.97983	-0.0294	0.90918	1
C22orf46	1.13315	1.124929	1.135823	1.0872	0.1206	0.909228	1
RP11-356K23.1	1.031641	1.02363	1.034246	1.44923	0.5353	0.909233	1
NIPA1	1.174069	1.181581	1.171627	0.94518	-0.0813	0.909237	1
LINC00950	1.043908	1.051635	1.041396	0.80171	-0.3188	0.909259	1
RP11-397A16.3	1.019699	1.011717	1.022294	1.90262	0.928	0.9093	1
SERHL2	1.052259	1.04413	1.054902	1.24408	0.3151	0.909392	1
TMEM185B	1.114304	1.105816	1.117063	1.10629	0.1457	0.909413	1
PRKAR2B	1.114195	1.10612	1.11682	1.10083	0.1386	0.909413	1
RP11-458D21.1	1.027015	1.018966	1.029631	1.56231	0.6437	0.90948	1
LINC01546	1.026936	1.018953	1.029531	1.55809	0.6398	0.90948	1
RP4-563E14.1	1.026959	1.018951	1.029562	1.55992	0.6415	0.90948	1
IFNE	1.018052	1.025374	1.015672	0.61764	-0.6952	0.909522	1
ABHD3	1.120887	1.128705	1.118346	0.91951	-0.1211	0.909611	1
RP11-181E10.3	1.041352	1.048692	1.038967	0.80027	-0.3214	0.909672	1
RIMS3	1.07398	1.065718	1.076666	1.16659	0.2223	0.909689	1
KIF9-AS1	1.074024	1.065769	1.076708	1.16632	0.222	0.909689	1
C10orf90	1.015421	1.022869	1.013	0.56844	-0.8149	0.909809	1
ACVRL1	1.015375	1.022681	1.013	0.57315	-0.803	0.909809	1
RP11-92A5.2	1.01538	1.022703	1.013	0.5726	-0.8044	0.909809	1
DHH	1.015398	1.022774	1.013	0.57081	-0.8089	0.909809	1
CPEB3	1.036054	1.027988	1.038676	1.38187	0.4666	0.910007	1
HIST1H2BC	1.040318	1.047742	1.037905	0.79397	-0.3329	0.910055	1
RP11-166P13.4	1.060523	1.052497	1.063132	1.20259	0.2661	0.91012	1
KLHL7	1.595941	1.604593	1.593129	0.98104	-0.0276	0.91018	1
PGAP3	1.085907	1.093416	1.083466	0.89349	-0.1625	0.910259	1
ATG4D	1.389264	1.39727	1.386661	0.9733	-0.039	0.910317	1
TLK1	1.95089	1.940641	1.954221	1.01444	0.0207	0.910402	1
CXCL6	1.025935	1.033206	1.023571	0.70984	-0.4944	0.910437	1
LYSMD4	1.147911	1.13953	1.150636	1.0796	0.1105	0.91044	1
XXbac-BPG181B23.7	1.023131	1.015229	1.0257	1.68759	0.755	0.910444	1
RP11-142C4.6	1.023177	1.01526	1.02575	1.68747	0.7549	0.910478	1
DPP6	1.02317	1.01526	1.025741	1.68684	0.7543	0.910478	1
HBG2	1.035284	1.027322	1.037872	1.38611	0.471	0.91061	1
CTD-2515O10.5	1.021651	1.013691	1.024239	1.77043	0.8241	0.91061	1
RP1-278C19.8	1.021615	1.013691	1.024191	1.76696	0.8213	0.91061	1
RP11-2C24.9	1.02271	1.029964	1.020351	0.67919	-0.5581	0.910672	1
RP11-434D9.2	1.022771	1.030183	1.020362	0.67462	-0.5679	0.910672	1
KTI12	1.140394	1.132042	1.143109	1.08381	0.1161	0.91084	1
BEX4	6.524863	6.551933	6.516063	0.99354	-0.0094	0.910872	1
C16orf58	1.313682	1.321876	1.311019	0.96627	-0.0495	0.910879	1
RP1	1.091126	1.098208	1.088824	0.90445	-0.1449	0.91104	1
FOXJ1	1.082477	1.089677	1.080136	0.89361	-0.1623	0.911051	1
RABGGTA	1.310193	1.300866	1.313225	1.04108	0.0581	0.911135	1
RP11-483F11.7	1.046871	1.054545	1.044376	0.81358	-0.2976	0.911138	1

CLOCK	1.405958	1.397431	1.40873	1.02843	0.0404	0.911169	1
C19orf47	1.05489	1.062359	1.052462	0.84128	-0.2493	0.911209	1
TMEM27	1.063875	1.055892	1.066471	1.18928	0.2501	0.911244	1
RP3-466P17.2	1.023933	1.016045	1.026498	1.65146	0.7237	0.911246	1
RP1-244F24.1	1.023924	1.016045	1.026485	1.65068	0.7231	0.911246	1
RP11-90M2.5	1.022484	1.029756	1.02012	0.67618	-0.5645	0.91125	1
TF	1.022484	1.029762	1.020119	0.67599	-0.5649	0.91125	1
RP1-67K17.4	1.022518	1.029759	1.020164	0.67756	-0.5616	0.91125	1
WDR43	1.453789	1.461544	1.451268	0.97773	-0.0325	0.911269	1
CTC-366B18.2	1.025477	1.017512	1.028066	1.60269	0.6805	0.911307	1
TMEM61	1.025438	1.017512	1.028015	1.59977	0.6779	0.911307	1
MICU1	1.51702	1.50755	1.520098	1.02472	0.0352	0.911401	1
AGTR2	1.019295	1.026599	1.016921	0.63615	-0.6526	0.911489	1
FOXA1	1.019266	1.026479	1.016921	0.63904	-0.646	0.911489	1
EPHA1	1.019281	1.026542	1.016921	0.63753	-0.6494	0.911489	1
GJB3	1.019275	1.026517	1.016921	0.63812	-0.6481	0.911489	1
RP11-874J12.4	1.01928	1.026538	1.016921	0.63762	-0.6492	0.911489	1
RP4-568C11.4	1.019334	1.026755	1.016921	0.63245	-0.661	0.911489	1
FAM188B	1.089847	1.081953	1.092413	1.12763	0.1733	0.911645	1
LL0XNC01-7P3.1	1.022785	1.03002	1.020433	0.68065	-0.555	0.911717	1
TEX15	1.025025	1.01719	1.027572	1.60399	0.6817	0.911757	1
RP11-647K16.1	1.02505	1.017182	1.027608	1.6068	0.6842	0.911757	1
CTB-25B13.9	1.025017	1.017215	1.027553	1.6005	0.6785	0.911757	1
RP11-66B24.4	1.025018	1.017194	1.027562	1.60299	0.6808	0.911757	1
ZDHHC13	1.204167	1.195761	1.206899	1.0569	0.0798	0.911798	1
FAM20B	1.246437	1.237812	1.24924	1.04806	0.0677	0.911933	1
RP11-687M24.8	1.019062	1.026371	1.016686	0.63273	-0.6603	0.912065	1
NINJ2	1.019019	1.026196	1.016686	0.63696	-0.6507	0.912065	1
GPR37L1	1.019036	1.026267	1.016686	0.63523	-0.6546	0.912065	1
RP11-474O21.5	1.019042	1.026291	1.016686	0.63465	-0.656	0.912065	1
TFAP2C	1.024852	1.032063	1.022509	0.70201	-0.5104	0.9122	1
TOX3	1.10989	1.117363	1.10746	0.91563	-0.1272	0.9122	1
NEXN-AS1	1.042907	1.03505	1.045461	1.29703	0.3752	0.912248	1
CDC37L1	1.274569	1.281946	1.272172	0.96533	-0.0509	0.912249	1
HCCS	1.35647	1.364635	1.353816	0.97033	-0.0435	0.912406	1
TLR4	1.032668	1.039805	1.030348	0.76242	-0.3913	0.912426	1
GNE	1.069859	1.061729	1.072502	1.17453	0.2321	0.912439	1
C1orf105	1.029935	1.037145	1.027592	0.7428	-0.4289	0.912457	1
CTC-558O2.1	1.019332	1.026541	1.016989	0.6401	-0.6436	0.912532	1
PLA1A	1.019321	1.026494	1.016989	0.64124	-0.6411	0.912532	1
RP11-557H15.4	1.019324	1.026505	1.016989	0.64097	-0.6417	0.912532	1
AC022007.5	1.048219	1.055394	1.045887	0.82838	-0.2716	0.9126	1
CTB-13F3.1	1.030152	1.022293	1.032707	1.46718	0.5531	0.91268	1
KCNH1	1.015378	1.022505	1.013062	0.58038	-0.7849	0.912686	1
RP11-81H14.2	1.015386	1.022537	1.013062	0.57957	-0.7869	0.912686	1

RP11-445O16.3	1.015403	1.022607	1.013062	0.57778	-0.7914	0.912686	1
HSDL2	1.496999	1.488215	1.499855	1.02384	0.034	0.912698	1
UBXN1	3.65084	3.632265	3.656877	1.00935	0.0134	0.912762	1
RP11-410E4.1	1.042194	1.03437	1.044737	1.30165	0.3803	0.912852	1
TYW1B	1.042198	1.034326	1.044757	1.30386	0.3828	0.912852	1
FAM81A	1.037363	1.029595	1.039887	1.34778	0.4306	0.912885	1
PPIL4	1.540436	1.548754	1.537732	0.97991	-0.0293	0.912982	1
SLC12A3	1.021363	1.028504	1.019042	0.66803	-0.582	0.913014	1
RP11-437J19.1	1.021414	1.028713	1.019042	0.66318	-0.5925	0.913014	1
VPS13C	1.537827	1.545817	1.53523	0.9806	-0.0283	0.91303	1
MIER3	1.142613	1.134362	1.145296	1.08138	0.1129	0.913031	1
RP11-367N14.3	1.025688	1.017948	1.028204	1.57145	0.6521	0.913116	1
CYP4A22-AS1	1.025684	1.017948	1.028198	1.57113	0.6518	0.913116	1
RP11-396C23.4	1.025761	1.017948	1.0283	1.57683	0.657	0.913116	1
KLHL18	1.124873	1.132018	1.122551	0.92829	-0.1073	0.913165	1
TMEM100	1.100762	1.10828	1.098318	0.90799	-0.1392	0.913186	1
CTC-321K16.1	1.027986	1.035151	1.025658	0.72992	-0.4542	0.913192	1
KB-1732A1.1	1.027964	1.03507	1.025654	0.73152	-0.451	0.913192	1
SHROOM2	1.128952	1.120774	1.13161	1.08972	0.124	0.913198	1
RP11-639B1.1	1.026555	1.033692	1.024235	0.71932	-0.4753	0.913263	1
REV1	1.330266	1.321712	1.333046	1.03523	0.05	0.913345	1
MAP3K6	1.110689	1.102532	1.11334	1.10541	0.1446	0.913381	1
TNRC6C	1.38051	1.372038	1.383265	1.03018	0.0429	0.913393	1
AC078941.1	1.01971	1.011999	1.022216	1.85146	0.8887	0.913445	1
DLL4	1.019713	1.011999	1.022221	1.85184	0.889	0.913445	1
AC034243.1	1.019693	1.011999	1.022194	1.84963	0.8872	0.913445	1
TTLL10	1.019692	1.011999	1.022193	1.84954	0.8872	0.913445	1
HCG9	1.019696	1.011999	1.022198	1.85	0.8875	0.913445	1
AP001412.1	1.04839	1.040566	1.050933	1.25556	0.3283	0.913542	1
FAAH2	1.048295	1.04046	1.050842	1.25662	0.3295	0.913542	1
AC005954.4	1.023707	1.015934	1.026233	1.64635	0.7193	0.913599	1
SLC51A	1.023647	1.015934	1.026154	1.6414	0.7149	0.913599	1
XXbac-B135H6.15	1.045503	1.037662	1.048051	1.27584	0.3514	0.913605	1
STK32B	1.036393	1.043453	1.034098	0.7847	-0.3498	0.913643	1
MYD88	1.2142	1.205758	1.216945	1.05437	0.0764	0.913665	1
AC093818.1	1.088592	1.080699	1.091157	1.12959	0.1758	0.913708	1
ZNF425	1.063939	1.07117	1.061588	0.86537	-0.2086	0.913945	1
MAGEL2	1.065205	1.072503	1.062833	0.86664	-0.2065	0.913984	1
NDUFA6	4.237005	4.220638	4.242325	1.00673	0.0097	0.914025	1
RP11-806L2.2	1.025803	1.032961	1.023476	0.71224	-0.4896	0.914033	1
RP11-16P6.1	1.023387	1.015676	1.025893	1.65182	0.7241	0.914065	1
RP11-168E17.1	1.023348	1.015676	1.025842	1.64858	0.7212	0.914065	1
CTD-2619J13.3	1.023434	1.015676	1.025956	1.65581	0.7275	0.914065	1
CTD-3110H11.1	1.023353	1.015676	1.025848	1.64894	0.7215	0.914065	1
EML3	1.253368	1.244807	1.256151	1.04634	0.0653	0.91407	1

MPZL3	1.023038	1.03009	1.020746	0.68946	-0.5365	0.914072	1
KISS1	1.023093	1.030175	1.020791	0.68901	-0.5374	0.914072	1
FADS2	1.690561	1.698657	1.687929	0.98465	-0.0223	0.914086	1
PPP1R37	1.37938	1.370908	1.382135	1.03027	0.043	0.914093	1
STX17	1.382189	1.373827	1.384907	1.02964	0.0421	0.91419	1
CAPN12	1.041344	1.048377	1.039058	0.80737	-0.3087	0.914255	1
RP11-147L13.12	1.075859	1.067925	1.078438	1.15477	0.2076	0.914355	1
GS1-259H13.2	1.075622	1.067828	1.078156	1.15226	0.2045	0.914355	1
CTD-2145A24.5	1.025696	1.032814	1.023382	0.71256	-0.4889	0.914359	1
RAP1GDS1	1.396319	1.40391	1.393851	0.9751	-0.0364	0.914495	1
TRAF2	1.230075	1.237628	1.22762	0.95788	-0.0621	0.914508	1
FAM53B	1.129537	1.137004	1.12711	0.92778	-0.1081	0.914539	1
RP4-635E18.8	1.023135	1.0155	1.025617	1.65273	0.7249	0.914553	1
RP11-266K4.14	1.023167	1.015485	1.025664	1.65735	0.7289	0.914553	1
LRRC57	1.277596	1.269182	1.280331	1.04141	0.0585	0.914576	1
SMCR8	1.076742	1.068774	1.079332	1.15352	0.206	0.914585	1
FAM53A	1.023639	1.015961	1.026135	1.6374	0.7114	0.914643	1
MMP3	1.023646	1.015961	1.026144	1.638	0.7119	0.914643	1
AMER1	1.043969	1.051151	1.041634	0.81394	-0.297	0.914681	1
RP11-29P20.1	1.022473	1.014833	1.024957	1.68258	0.7507	0.914681	1
ACTA2-AS1	1.022482	1.014833	1.024969	1.6834	0.7514	0.914681	1
RP11-284F21.8	1.022522	1.014833	1.025021	1.68689	0.7544	0.914681	1
CTC-548K16.2	1.022485	1.014833	1.024973	1.68365	0.7516	0.914681	1
BSN-AS2	1.022448	1.014833	1.024924	1.68036	0.7488	0.914681	1
CTD-3076O17.2	1.022474	1.014833	1.024958	1.68265	0.7507	0.914681	1
C1QTNF9B-AS1	1.065424	1.057617	1.067962	1.17955	0.2382	0.914687	1
PDC	1.027132	1.019453	1.029627	1.52299	0.6069	0.914707	1
TM4SF19-AS1	1.027134	1.019424	1.02964	1.52594	0.6097	0.914707	1
MARCH11	1.0271	1.019436	1.029591	1.52252	0.6065	0.914707	1
RP11-734K2.4	1.090274	1.082345	1.092851	1.12759	0.1732	0.914753	1
CNPPD1	1.327677	1.335634	1.325091	0.96859	-0.046	0.91482	1
RP11-62C3.6	1.019603	1.026676	1.017304	0.64868	-0.6244	0.914883	1
CEMIP	1.019618	1.026735	1.017304	0.64725	-0.6276	0.914883	1
RP11-114G22.1	1.019604	1.02668	1.017304	0.64857	-0.6247	0.914883	1
RP11-161H23.9	1.021051	1.028108	1.018757	0.66732	-0.5836	0.914901	1
PTPN5	1.021071	1.02818	1.01876	0.66573	-0.587	0.914901	1
GPA33	1.021045	1.028049	1.018768	0.6691	-0.5797	0.914901	1
CD19	1.021058	1.028097	1.01877	0.66805	-0.582	0.914901	1
LINC00562	1.049165	1.041411	1.051685	1.24811	0.3197	0.91508	1
RP11-131L12.4	1.02682	1.019162	1.029309	1.52955	0.6131	0.915175	1
OGFR	1.394081	1.402275	1.391417	0.97301	-0.0395	0.915285	1
ANKRD20A3	1.032718	1.024957	1.035241	1.41208	0.4978	0.915327	1
CENPBD1	1.180068	1.187162	1.177763	0.94978	-0.0743	0.915513	1
PAQR8	1.177774	1.169987	1.180305	1.0607	0.085	0.915518	1
AZU1	1.06745	1.074416	1.065186	0.87598	-0.191	0.915544	1

ATP10B	1.017267	1.024228	1.015004	0.61926	-0.6914	0.915598	1
FBP1	1.017313	1.024434	1.014999	0.61386	-0.704	0.915598	1
HHIP	1.017258	1.024208	1.014998	0.61955	-0.6907	0.915598	1
STARD8	1.017281	1.02429	1.015003	0.61767	-0.6951	0.915598	1
RP11-76E17.4	1.017275	1.024262	1.015004	0.61839	-0.6934	0.915598	1
LAG3	1.037915	1.030265	1.040402	1.33495	0.4168	0.915639	1
CTD-2078B5.2	1.018888	1.025986	1.016581	0.63807	-0.6482	0.91565	1
RP11-366L20.2	1.02664	1.019014	1.02912	1.53152	0.615	0.915657	1
CD248	1.038559	1.045571	1.036279	0.7961	-0.329	0.915691	1
APOBEC3C	1.218229	1.209857	1.22095	1.05286	0.0743	0.915776	1
SMG8	1.02594	1.018377	1.028398	1.54529	0.6279	0.915792	1
RP11-248G5.9	1.025921	1.018352	1.028381	1.54645	0.629	0.915792	1
CTB-161M19.4	1.025932	1.018354	1.028395	1.54706	0.6295	0.915792	1
CTC-523E23.1	1.030602	1.022956	1.033088	1.4414	0.5275	0.915812	1
TBX19	1.061763	1.068944	1.059429	0.86199	-0.2143	0.915858	1
TUT1	1.15342	1.14579	1.1559	1.06935	0.0967	0.915878	1
NAPA	1.502983	1.494393	1.505775	1.02302	0.0328	0.916032	1
PCAT14	1.018867	1.025921	1.016574	0.63943	-0.6451	0.916056	1
FRY	1.034242	1.041211	1.031976	0.77591	-0.366	0.916171	1
RP11-217B1.2	1.026083	1.033052	1.023817	0.72059	-0.4727	0.916172	1
GBP4	1.052551	1.044969	1.055015	1.22341	0.2909	0.916187	1
ALDH1A2	1.030337	1.022725	1.032811	1.44378	0.5298	0.91628	1
LCN15	1.019757	1.012186	1.022218	1.8232	0.8665	0.916325	1
NR2C2	1.278455	1.285839	1.276055	0.96577	-0.0502	0.916422	1
RP6-65G23.3	1.208518	1.215581	1.206222	0.95658	-0.064	0.916506	1
RP11-342K6.4	1.035222	1.042124	1.032978	0.78288	-0.3531	0.916524	1
NUFIP1	1.18141	1.173715	1.183912	1.0587	0.0823	0.916551	1
AC079922.3	1.082498	1.074831	1.08499	1.13577	0.1837	0.916558	1
CTC-270D5.1	1.022492	1.014881	1.024966	1.6777	0.7465	0.916613	1
CTD-2283N19.1	1.02241	1.014881	1.024857	1.67037	0.7402	0.916613	1
CH17-340M24.3	1.264769	1.272179	1.26236	0.96392	-0.053	0.916652	1
RP11-368I7.4	1.030983	1.037995	1.028704	0.75547	-0.4046	0.916757	1
RP11-700J17.2	1.030489	1.022975	1.032931	1.43331	0.5194	0.916859	1
GRAMD4	1.146428	1.138596	1.148974	1.07488	0.1042	0.916862	1
HAUS3	1.14642	1.138623	1.148954	1.07452	0.1037	0.916862	1
MOV10L1	1.029497	1.021837	1.031988	1.46485	0.5508	0.916898	1
MARCH8	1.199028	1.191046	1.201622	1.05536	0.0777	0.916899	1
LINC00880	1.015375	1.022241	1.013144	0.59096	-0.7589	0.916909	1
RP11-764K9.1	1.015388	1.02231	1.013138	0.58887	-0.764	0.916909	1
IPO9-AS1	1.058087	1.050525	1.060545	1.19831	0.261	0.916996	1
AC083843.1	1.055842	1.048262	1.058306	1.2081	0.2727	0.917039	1
WDR77	1.394815	1.402149	1.392431	0.97584	-0.0353	0.917109	1
USP40	1.181482	1.173708	1.184009	1.0593	0.0831	0.917339	1
CFLAR	1.25682	1.248074	1.259662	1.04671	0.0659	0.917381	1
MIR296	1.023138	1.015675	1.025563	1.63078	0.7056	0.917438	1

KPNA3	1.944125	1.952866	1.941283	0.98784	-0.0176	0.917517	1
SLC33A1	1.304464	1.311961	1.302027	0.96816	-0.0467	0.917607	1
RP11-373N22.3	1.029053	1.036024	1.026787	0.74359	-0.4274	0.917663	1
TRIM35	1.060197	1.05269	1.062637	1.18879	0.2495	0.917782	1
DAGLA	1.060218	1.05255	1.062711	1.19334	0.255	0.917782	1
CTD-2311M21.3	1.044685	1.037135	1.047139	1.26937	0.3441	0.917813	1
CTD-2616J11.2	1.019282	1.011871	1.021691	1.82727	0.8697	0.917841	1
RP1-35C21.2	1.01927	1.011871	1.021675	1.82593	0.8686	0.917841	1
RP11-524C21.2	1.019291	1.011871	1.021703	1.82823	0.8704	0.917841	1
AP000350.5	1.019301	1.011871	1.021716	1.82935	0.8713	0.917841	1
RP11-1085N6.5	1.019174	1.025993	1.016957	0.65237	-0.6162	0.917871	1
TSNAX-DISC1	1.019199	1.026062	1.016968	0.65105	-0.6192	0.917871	1
AC005863.1	1.01918	1.025996	1.016964	0.65258	-0.6158	0.917871	1
BTD	1.118136	1.110302	1.120682	1.09411	0.1298	0.91794	1
RP5-1065J22.8	1.062158	1.054496	1.064648	1.18629	0.2465	0.917963	1
RP11-77K12.9	1.037525	1.029995	1.039972	1.33261	0.4143	0.918009	1
GCNT2	1.11058	1.102829	1.113099	1.09987	0.1373	0.918085	1
BRWD1	2.182933	2.170906	2.186843	1.01361	0.0195	0.918106	1
LRRC43	1.029679	1.036491	1.027465	0.75265	-0.41	0.918136	1
GPR27	1.117606	1.10979	1.120146	1.09433	0.13	0.918184	1
ADGRA1-AS1	1.01867	1.011304	1.021064	1.86336	0.8979	0.918311	1
RBP5	1.0187	1.011304	1.021104	1.86685	0.9006	0.918311	1
GLDN	1.018689	1.011304	1.02109	1.86562	0.8997	0.918311	1
ANKRD55	1.018666	1.011304	1.02106	1.86296	0.8976	0.918311	1
AC091729.8	1.018689	1.011304	1.02109	1.86563	0.8997	0.918311	1
LTB4R	1.059324	1.051854	1.061752	1.19088	0.252	0.918392	1
COX19	1.520774	1.512146	1.523578	1.02232	0.0319	0.918414	1
MITD1	1.567958	1.575799	1.565409	0.98196	-0.0263	0.918538	1
RP11-29B2.6	1.02663	1.019254	1.029028	1.50759	0.5922	0.918548	1
LINC00939	1.026548	1.019181	1.028942	1.50892	0.5935	0.918548	1
CTD-2049O4.1	1.019553	1.012187	1.021947	1.8009	0.8487	0.918573	1
LINC00922	1.019536	1.012187	1.021925	1.7991	0.8473	0.918573	1
SYT12	1.019572	1.012187	1.021973	1.80301	0.8504	0.918573	1
RP11-109A6.5	1.019558	1.012187	1.021954	1.80143	0.8491	0.918573	1
LINC00551	1.019548	1.012187	1.021941	1.80036	0.8483	0.918573	1
RP4-647C14.3	1.021308	1.013947	1.023701	1.69937	0.765	0.918612	1
RP11-386G11.8	1.021381	1.013947	1.023798	1.70634	0.7709	0.918612	1
LCMT1-AS1	1.023927	1.030665	1.021737	0.70886	-0.4964	0.918688	1
TMEM115	1.471779	1.47886	1.469477	0.98041	-0.0286	0.918718	1
PITPNM1	1.354105	1.345986	1.356745	1.0311	0.0442	0.918775	1
RP11-334J6.7	1.023241	1.015826	1.025651	1.62083	0.6967	0.91879	1
LAT2	1.023196	1.015826	1.025591	1.61701	0.6933	0.91879	1
PABPC1L2B	1.023203	1.015826	1.025601	1.61767	0.6939	0.91879	1
NPPA	1.023232	1.015826	1.02564	1.62008	0.6961	0.91879	1
MEF2C-AS1	1.029298	1.021943	1.031689	1.44418	0.5303	0.918837	1

HLA-F	1.03695	1.029507	1.039369	1.33424	0.416	0.918947	1
DND1	1.022727	1.015392	1.025111	1.63145	0.7062	0.918957	1
TMEM63C	1.022727	1.015384	1.025114	1.63243	0.707	0.918957	1
CTD-2031P19.5	1.020459	1.013102	1.02285	1.74408	0.8025	0.918963	1
RP11-365O16.6	1.020456	1.013102	1.022847	1.74386	0.8023	0.918963	1
RP11-498C9.3	1.020443	1.013102	1.02283	1.74251	0.8012	0.918963	1
LINC01307	1.020445	1.013102	1.022832	1.74271	0.8013	0.918963	1
AC004754.3	1.02617	1.033045	1.023935	0.72432	-0.4653	0.918973	1
ANO8	1.071652	1.064175	1.074083	1.15439	0.2071	0.919011	1
SCARF2	1.02479	1.031563	1.022589	0.71566	-0.4826	0.919039	1
ZBTB20-AS2	1.024794	1.031548	1.022599	0.71632	-0.4813	0.919039	1
RARA	1.508444	1.515932	1.50601	0.98077	-0.028	0.919056	1
ADCY5	1.023014	1.029781	1.020815	0.69895	-0.5167	0.919079	1
AC008746.12	1.036397	1.028927	1.038825	1.34215	0.4245	0.9191	1
AP000230.1	1.04112	1.033492	1.043599	1.30178	0.3805	0.919103	1
CDC42BPG	1.041047	1.033539	1.043488	1.29664	0.3748	0.919103	1
FOXG1-AS1	1.020584	1.013274	1.022959	1.72964	0.7905	0.919211	1
RP11-640N11.2	1.020643	1.013274	1.023039	1.73561	0.7954	0.919211	1
ANKRD13D	1.31732	1.324297	1.315052	0.97149	-0.0417	0.919253	1
RP11-1336O20.2	1.043814	1.05057	1.041618	0.82298	-0.2811	0.919268	1
LINC01204	1.020589	1.027283	1.018413	0.67491	-0.5672	0.919283	1
CPEB1-AS1	1.02213	1.014809	1.024509	1.65499	0.7268	0.919428	1
EPO	1.022139	1.014814	1.024519	1.65514	0.727	0.919428	1
ZNF256	1.050663	1.043289	1.05306	1.22571	0.2936	0.919461	1
LCN9	1.020444	1.027203	1.018247	0.67076	-0.5761	0.919533	1
SHISA3	1.020427	1.027165	1.018236	0.67133	-0.5749	0.919533	1
RP11-462L8.1	1.020433	1.02711	1.018263	0.67365	-0.5699	0.919533	1
STRN3	1.47974	1.486749	1.477462	0.98092	-0.0278	0.919553	1
RGS14	1.383723	1.375221	1.386486	1.03002	0.0427	0.919613	1
FLT4	1.03004	1.036891	1.027813	0.75393	-0.4075	0.919662	1
LEMD1-AS1	1.023115	1.015669	1.025536	1.62966	0.7046	0.919691	1
RP3-508I15.21	1.041016	1.047831	1.0388	0.81119	-0.3019	0.9197	1
MIR497HG	1.024775	1.017444	1.027159	1.55691	0.6387	0.91973	1
PSTPIP1	1.024767	1.017469	1.027139	1.55355	0.6356	0.91973	1
CTD-2600O9.1	1.026682	1.019341	1.029069	1.50294	0.5878	0.919908	1
TRIM63	1.019545	1.026237	1.01737	0.66206	-0.595	0.919923	1
CDYL2	1.019556	1.026225	1.017388	0.66303	-0.5929	0.919923	1
RBCK1	1.789755	1.797597	1.787206	0.98697	-0.0189	0.919943	1
TMEM201	1.130108	1.137066	1.127846	0.93273	-0.1005	0.919946	1
FAM212B-AS1	1.023889	1.016559	1.026272	1.58658	0.6659	0.91997	1
RP11-535A19.2	1.026162	1.018852	1.028538	1.5138	0.5982	0.92007	1
TERC	1.023932	1.016596	1.026317	1.58581	0.6652	0.920081	1
ESAM	1.017135	1.023781	1.014974	0.62967	-0.6673	0.920131	1
DAPP1	1.017127	1.023755	1.014972	0.63027	-0.666	0.920131	1
LINC01234	1.017162	1.023866	1.014983	0.6278	-0.6716	0.920131	1

SLC10A4	1.01714	1.023798	1.014976	0.62928	-0.6682	0.920131	1
RP11-474I16.8	1.017152	1.023833	1.01498	0.62855	-0.6699	0.920131	1
RP11-111H13.1	1.018695	1.025421	1.016508	0.6494	-0.6228	0.920185	1
MAK	1.044461	1.036995	1.046888	1.2674	0.3419	0.920192	1
ZNF354A	1.25161	1.243887	1.25412	1.04196	0.0593	0.920206	1
RP1-101D8.1	1.024053	1.016785	1.026416	1.57382	0.6543	0.92033	1
MGC16275	1.024096	1.016804	1.026466	1.57494	0.6553	0.92033	1
SFTPD-AS1	1.024111	1.016788	1.026492	1.578	0.6581	0.92033	1
RP11-521D12.5	1.016982	1.023611	1.014828	0.62798	-0.6712	0.92038	1
NEB	1.016966	1.023583	1.014815	0.62821	-0.6707	0.92049	1
FAM83F	1.016966	1.023594	1.014811	0.62774	-0.6718	0.92049	1
BAIAP2L2	1.026613	1.033358	1.02442	0.73207	-0.45	0.920497	1
ACKR4	1.017851	1.024447	1.015707	0.64248	-0.6383	0.92073	1
CYSLTR2	1.0161	1.022727	1.013946	0.61364	-0.7045	0.920769	1
ANPEP	1.016109	1.022774	1.013942	0.6122	-0.7079	0.920769	1
RP11-329J18.3	1.016104	1.022765	1.013939	0.61231	-0.7077	0.920769	1
C22orf23	1.052741	1.045519	1.055089	1.21023	0.2753	0.920794	1
LA16c-360H6.3	1.02823	1.021043	1.030567	1.45256	0.5386	0.920844	1
SPACA6	1.206396	1.213475	1.204095	0.95606	-0.0648	0.921024	1
ANKRD33	1.036274	1.028969	1.038648	1.33412	0.4159	0.921046	1
CC2D1B	1.079173	1.086089	1.076925	0.89356	-0.1624	0.92106	1
SYT3	1.027347	1.020119	1.029697	1.47606	0.5618	0.921085	1
KDELC2	1.272965	1.279743	1.270762	0.9679	-0.0471	0.921129	1
PTPRQ	1.029687	1.022488	1.032028	1.42422	0.5102	0.921178	1
TPRG1	1.029605	1.022365	1.031959	1.42896	0.515	0.921178	1
ZFP69	1.047798	1.040493	1.050173	1.23905	0.3092	0.921278	1
BTBD17	1.086739	1.079364	1.089136	1.12314	0.1675	0.921283	1
EBLN2	1.127863	1.120441	1.130276	1.08166	0.1132	0.921328	1
GPR61	1.023146	1.02975	1.021	0.70589	-0.5025	0.921334	1
RP11-615I2.2	1.072015	1.06462	1.074418	1.15163	0.2037	0.921352	1
KIAA0368	1.362476	1.354472	1.365078	1.02992	0.0425	0.921419	1
GORAB	1.114775	1.107259	1.117219	1.09285	0.1281	0.92142	1
C11orf72	1.022665	1.015453	1.02501	1.61839	0.6946	0.921472	1
RP11-557H15.3	1.022633	1.015453	1.024967	1.61562	0.6921	0.921472	1
AC093382.1	1.02266	1.015453	1.025003	1.61794	0.6942	0.921472	1
AC026202.3	1.022626	1.015453	1.024957	1.615	0.6915	0.921472	1
RP11-3N13.2	1.02269	1.015453	1.025042	1.62048	0.6964	0.921472	1
USH1G	1.015836	1.02241	1.013699	0.6113	-0.71	0.921501	1
CTC-431G16.2	1.01584	1.022428	1.013699	0.61078	-0.7113	0.921501	1
RP11-650L12.2	1.033527	1.040061	1.031404	0.7839	-0.3513	0.921735	1
ACOT2	1.064934	1.057691	1.067289	1.16637	0.222	0.921797	1
MYLPF	1.065072	1.057794	1.067437	1.16687	0.2226	0.921797	1
VPS33B	1.143463	1.136054	1.145872	1.07216	0.1005	0.921838	1
LAMA2	1.053429	1.046187	1.055783	1.20778	0.2724	0.92187	1
AC079305.10	1.05348	1.046122	1.055872	1.21139	0.2767	0.92187	1

RP11-557C18.4	1.022367	1.028969	1.020221	0.69802	-0.5187	0.921886	1
PLCG1-AS1	1.029912	1.022733	1.032245	1.41845	0.5043	0.921914	1
SLC14A2	1.02989	1.022696	1.032228	1.42	0.5059	0.921914	1
C20orf203	1.029904	1.022746	1.032231	1.41702	0.5029	0.921914	1
RNU12	1.03172	1.024515	1.034062	1.38943	0.4745	0.921954	1
TOMM5	1.656876	1.665289	1.654141	0.98324	-0.0244	0.92197	1
RP11-91I8.3	1.067165	1.073869	1.064986	0.87975	-0.1848	0.922017	1
FNDC11	1.067109	1.073996	1.06487	0.87666	-0.1899	0.922017	1
SIK1	1.039533	1.046233	1.037356	0.80798	-0.3076	0.922067	1
LGALS8-AS1	1.033597	1.026466	1.035915	1.35702	0.4404	0.922133	1
ATXN2L	1.571308	1.578642	1.568924	0.98321	-0.0244	0.922174	1
WNT2	1.019721	1.026273	1.017591	0.66956	-0.5787	0.922174	1
UBA7	1.03054	1.036999	1.02844	0.76868	-0.3796	0.922188	1
EIF2AK3	1.103625	1.096569	1.105918	1.0968	0.1333	0.922235	1
KCNMB1	1.033053	1.025837	1.035398	1.37009	0.4543	0.922283	1
RP11-506K6.4	1.01885	1.011717	1.021168	1.80656	0.8532	0.922436	1
LIN28B-AS1	1.018835	1.011717	1.021149	1.80491	0.8519	0.922436	1
KCNA4	1.118485	1.111235	1.120841	1.08636	0.1195	0.922482	1
NOP14-AS1	1.117479	1.1102	1.119844	1.08752	0.121	0.922546	1
LINC00637	1.026142	1.018962	1.028476	1.5017	0.5866	0.922595	1
LINC01549	1.026109	1.018973	1.028428	1.49829	0.5833	0.922595	1
MT1A	1.018978	1.025616	1.01682	0.65665	-0.6068	0.922729	1
HRAT92	1.018925	1.02542	1.016814	0.66143	-0.5963	0.922729	1
RP11-879F14.2	1.01891	1.025358	1.016813	0.66303	-0.5929	0.922729	1
GYS1	1.258215	1.264958	1.256023	0.96628	-0.0495	0.922826	1
SARDH	1.04268	1.049236	1.040548	0.82354	-0.2801	0.922873	1
NINL	1.369427	1.361138	1.372122	1.03041	0.0432	0.9229	1
SLAIN1	1.115366	1.122056	1.113192	0.92737	-0.1088	0.922954	1
TTC39C	1.358477	1.350506	1.361068	1.03013	0.0428	0.922965	1
SLC2A10	1.201057	1.207761	1.198878	0.95725	-0.063	0.922996	1
NODAL	1.016251	1.022748	1.014139	0.62157	-0.686	0.923017	1
ZDHHC18	1.139154	1.145859	1.136974	0.93909	-0.0907	0.923137	1
RP11-631N16.4	1.043828	1.050269	1.041734	0.83022	-0.2684	0.923187	1
GFOD1	1.030608	1.037097	1.028499	0.76822	-0.3804	0.923238	1
HIST1H4E	1.050851	1.043656	1.05319	1.21838	0.285	0.923282	1
SDCBP2	1.119772	1.112468	1.122146	1.08606	0.1191	0.923354	1
LINC01012	1.050198	1.042928	1.052561	1.2244	0.2921	0.923459	1
MMGT1	1.169042	1.17595	1.166796	0.94797	-0.0771	0.923501	1
TMEM205	2.426958	2.415252	2.430762	1.01096	0.0157	0.92351	1
LINC01317	1.022252	1.015215	1.02454	1.6129	0.6897	0.923561	1
PLD6	1.022344	1.015262	1.024646	1.61482	0.6914	0.923561	1
CTD-2249K22.1	1.022301	1.015211	1.024606	1.6176	0.6939	0.923561	1
AC005477.1	1.022277	1.015224	1.02457	1.61387	0.6905	0.923561	1
CIB4	1.022343	1.01526	1.024646	1.61507	0.6916	0.92363	1
PRSS57	1.022312	1.01526	1.024605	1.6124	0.6892	0.92363	1

RP11-177H2.2	1.022321	1.01526	1.024617	1.61319	0.6899	0.92363	1
SUSD5	1.022301	1.01526	1.02459	1.61143	0.6883	0.92363	1
SPI1	1.029584	1.022506	1.031885	1.41672	0.5026	0.923714	1
DNAAF5	1.206643	1.199074	1.209104	1.05038	0.0709	0.923715	1
CRKL	1.265254	1.257751	1.267692	1.03857	0.0546	0.923721	1
Z83844.1	1.020729	1.013691	1.023017	1.68121	0.7495	0.923749	1
RP5-963E22.6	1.020715	1.013691	1.022998	1.67984	0.7483	0.923749	1
AC003090.1	1.022448	1.028839	1.020371	0.70638	-0.5015	0.923821	1
UBE2R2	2.496073	2.482397	2.500518	1.01222	0.0175	0.923845	1
AC079586.1	1.023611	1.030111	1.021499	0.71398	-0.486	0.92386	1
SNURF	1.055935	1.062432	1.053823	0.86211	-0.2141	0.923918	1
FADD	1.462604	1.469858	1.460246	0.97954	-0.0298	0.923926	1
SDC3	1.322671	1.314543	1.325313	1.03424	0.0486	0.92395	1
CDK5R2	1.040376	1.046856	1.03827	0.81677	-0.292	0.924044	1
SDE2	1.11091	1.117465	1.10878	0.92606	-0.1108	0.924067	1
RP11-374M1.5	1.038623	1.031557	1.04092	1.29672	0.3749	0.924162	1
ASB1	1.246957	1.239383	1.249419	1.04192	0.0592	0.924226	1
RASL10A	1.02314	1.029488	1.021076	0.71474	-0.4845	0.924226	1
ZFR2	1.166611	1.173193	1.164472	0.94965	-0.0745	0.924328	1
QSOX2	1.143166	1.135571	1.145635	1.07423	0.1033	0.924328	1
ZNF324B	1.054243	1.047178	1.056539	1.19842	0.2611	0.924357	1
PKNOX2	1.148984	1.141616	1.151379	1.06894	0.0962	0.924395	1
RP11-2C24.7	1.023104	1.016045	1.025398	1.58293	0.6626	0.924398	1
ST8SIA1	1.123306	1.116126	1.12564	1.08193	0.1136	0.924409	1
TISP43	1.024567	1.017512	1.02686	1.53384	0.6171	0.924447	1
RP11-109D9.4	1.024519	1.017512	1.026797	1.5302	0.6137	0.924447	1
HSD17B13	1.024561	1.017512	1.026852	1.53338	0.6167	0.924447	1
PPM1M	1.095903	1.088825	1.098204	1.10559	0.1448	0.924504	1
SERPINB1	1.245904	1.252797	1.243663	0.96387	-0.0531	0.924529	1
C7orf33	1.05359	1.046506	1.055893	1.20184	0.2652	0.924539	1
RP11-158I9.5	1.019014	1.025414	1.016934	0.66632	-0.5857	0.924662	1
HTR1D	1.019021	1.025436	1.016936	0.66582	-0.5868	0.924662	1
COL4A3	1.019018	1.025452	1.016926	0.66504	-0.5885	0.924662	1
HOXB1	1.018999	1.025375	1.016926	0.66702	-0.5842	0.924662	1
CFAP157	1.02571	1.018721	1.027982	1.49469	0.5798	0.924682	1
RP4-800J21.3	1.020153	1.026558	1.018071	0.68044	-0.5555	0.9247	1
UPP2	1.020133	1.026513	1.018059	0.68114	-0.554	0.9247	1
RP11-77H9.8	1.020137	1.026521	1.018061	0.68103	-0.5542	0.9247	1
RP11-168O16.1	1.037001	1.043514	1.034884	0.80167	-0.3189	0.924729	1
RP11-538I12.3	1.037023	1.043447	1.034935	0.80408	-0.3146	0.924729	1
PRKAA1	1.33375	1.325768	1.336345	1.03247	0.0461	0.924819	1
CBLB	1.418715	1.410593	1.421355	1.02621	0.0373	0.92487	1
CHAC1	1.036882	1.043249	1.034812	0.80493	-0.3131	0.924903	1
RP11-61A14.4	1.023731	1.030212	1.021624	0.71575	-0.4825	0.924907	1
RP11-1191J2.2	1.044292	1.050576	1.042249	0.83535	-0.2596	0.925019	1

DGCR9	1.019703	1.026059	1.017637	0.67682	-0.5631	0.925061	1
DSCR8	1.019717	1.026164	1.017622	0.67351	-0.5702	0.925061	1
PIK3R4	1.097116	1.103722	1.094969	0.91561	-0.1272	0.925199	1
C6orf47	1.291525	1.28375	1.294052	1.03631	0.0515	0.925236	1
DNPEP	1.818477	1.809688	1.821334	1.01438	0.0206	0.92524	1
RP11-314C16.1	1.072928	1.079392	1.070827	0.89212	-0.1647	0.925257	1
ACADVL	3.459374	3.440704	3.465442	1.01014	0.0145	0.925273	1
RP11-524D16__A.3	1.019896	1.026272	1.017823	0.67841	-0.5598	0.925278	1
RP11-356N1.2	1.019874	1.02619	1.017821	0.68044	-0.5555	0.925278	1
TMEM204	1.019898	1.026244	1.017835	0.67959	-0.5573	0.925278	1
MAPK12	1.261677	1.253751	1.264254	1.04139	0.0585	0.925299	1
CTC-534A2.2	1.069068	1.061936	1.071386	1.15258	0.2049	0.925308	1
TRIM28	1.77057	1.762135	1.773312	1.01467	0.021	0.925348	1
HAS2-AS1	1.084417	1.077232	1.086753	1.12329	0.1677	0.925377	1
BANCR	1.026525	1.019615	1.028771	1.46674	0.5526	0.925482	1
ST3GAL6-AS1	1.026513	1.019566	1.028771	1.47046	0.5563	0.925482	1
SUSD3	1.027929	1.021061	1.030162	1.43214	0.5182	0.925575	1
STOML3	1.027963	1.021014	1.030222	1.43813	0.5242	0.925575	1
RP11-66H6.3	1.028023	1.021048	1.03029	1.43913	0.5252	0.925575	1
TAP2	1.058168	1.051124	1.060458	1.18259	0.242	0.925577	1
AKAP5	1.057146	1.050106	1.059434	1.18617	0.2463	0.925616	1
RP11-90L20.2	1.020196	1.026534	1.018136	0.68348	-0.549	0.925745	1
LINC01235	1.029335	1.022307	1.031619	1.41746	0.5033	0.925791	1
AC005592.1	1.029232	1.022297	1.031487	1.41212	0.4979	0.925791	1
STAC2	1.029245	1.02235	1.031486	1.40878	0.4944	0.925791	1
ZNF749	1.029175	1.02223	1.031433	1.41401	0.4998	0.925799	1
RP5-890E16.5	1.029219	1.022257	1.031482	1.41443	0.5002	0.925799	1
CHRD	1.029131	1.022263	1.031363	1.40878	0.4944	0.925799	1
TECPR1	1.152364	1.144938	1.154778	1.06789	0.0948	0.925866	1
AP003774.4	1.016257	1.022569	1.014205	0.62941	-0.6679	0.9259	1
LINC01013	1.016239	1.022524	1.014196	0.63024	-0.666	0.9259	1
MPC1	1.640779	1.632366	1.643514	1.01763	0.0252	0.925934	1
FAM83H-AS1	1.036428	1.029512	1.038676	1.31048	0.3901	0.925941	1
KIF25-AS1	1.036629	1.029601	1.038914	1.31462	0.3946	0.925941	1
GNG10	1.103129	1.10958	1.101032	0.92199	-0.1172	0.925989	1
RP11-582J16.5	1.027716	1.020694	1.029998	1.44958	0.5356	0.925999	1
GUCY1B3	1.234663	1.227196	1.23709	1.04355	0.0615	0.926027	1
CACNA1C-AS2	1.051808	1.05835	1.049681	0.85144	-0.232	0.926036	1
REPS1	1.286201	1.292881	1.28403	0.96978	-0.0443	0.926047	1
LINC00472	1.042796	1.035835	1.045059	1.25739	0.3304	0.926051	1
RELN	1.995063	1.985326	1.998228	1.01309	0.0188	0.92606	1
SLC5A12	1.240236	1.246833	1.238092	0.96459	-0.052	0.926168	1
KANSL2	1.210514	1.203262	1.212871	1.04728	0.0666	0.926197	1
AC116366.6	1.022272	1.02867	1.020193	0.70433	-0.5057	0.926229	1
AC092535.3	1.022241	1.028541	1.020194	0.70753	-0.4991	0.926229	1

IL1A	1.02223	1.028502	1.020192	0.70842	-0.4973	0.926229	1
SARNP	1.188042	1.194995	1.185781	0.95275	-0.0698	0.92623	1
NRDE2	1.34234	1.34924	1.340097	0.97382	-0.0383	0.926261	1
INSM2	1.024862	1.017948	1.027109	1.51047	0.595	0.926271	1
NXPH1	1.024847	1.017948	1.027089	1.50934	0.5939	0.926271	1
PCP2	1.02744	1.033906	1.025338	0.7473	-0.4202	0.926435	1
ALS2CL	1.027331	1.033587	1.025298	0.7532	-0.4089	0.926435	1
DHX35	1.095911	1.088721	1.098248	1.10738	0.1472	0.926506	1
NEU4	1.030212	1.023135	1.032513	1.40537	0.4909	0.926562	1
CTD-2378E12.1	1.029964	1.023047	1.032213	1.39773	0.4831	0.926562	1
ZBTB47	1.044632	1.037626	1.046909	1.24672	0.3181	0.926599	1
TPRG1-AS1	1.018836	1.011999	1.021059	1.75501	0.8115	0.9266	1
RP4-799P18.2	1.01884	1.011999	1.021064	1.75544	0.8118	0.9266	1
CTB-31O20.3	1.018826	1.011999	1.021045	1.75384	0.8105	0.9266	1
SCUBE2	1.018849	1.011999	1.021075	1.7564	0.8126	0.9266	1
RP11-173A6.3	1.01886	1.011999	1.02109	1.75762	0.8136	0.9266	1
RP11-428J1.5	1.075077	1.068134	1.077334	1.13503	0.1827	0.926629	1
LATS2-AS1	1.030001	1.036273	1.027962	0.77089	-0.3754	0.92663	1
PKNOX1	1.257996	1.250351	1.260481	1.04046	0.0572	0.926665	1
HCG23	1.045518	1.051786	1.043481	0.83963	-0.2522	0.926709	1
RP11-84G21.1	1.044609	1.037751	1.046838	1.24072	0.3112	0.926711	1
RP11-124N14.3	1.044575	1.037606	1.046841	1.24556	0.3168	0.926711	1
RP11-599B13.7	1.022791	1.015934	1.025019	1.57016	0.6509	0.926754	1
RP11-310E22.4	1.022788	1.015934	1.025016	1.56998	0.6508	0.926754	1
RDH12	1.022855	1.015934	1.025104	1.57549	0.6558	0.926754	1
TCEB3	1.289634	1.296892	1.287275	0.96761	-0.0475	0.926833	1
SLC30A10	1.03272	1.025848	1.034953	1.35228	0.4354	0.926866	1
RP11-95O2.5	1.044759	1.037852	1.047005	1.24182	0.3125	0.926963	1
WASL	1.802858	1.810118	1.800498	0.98813	-0.0172	0.926977	1
BRPF3	1.107187	1.100148	1.109476	1.09314	0.1285	0.927013	1
RP11-1000B6.8	1.039954	1.033088	1.042186	1.27495	0.3504	0.927049	1
ENO4	1.046378	1.052595	1.044358	0.84338	-0.2457	0.927064	1
AOC3	1.031088	1.024236	1.033316	1.37467	0.4591	0.927119	1
C11orf65	1.046265	1.039326	1.04852	1.23379	0.3031	0.927142	1
FBXL22	1.046279	1.039381	1.048521	1.23209	0.3011	0.927142	1
TMEM230	4.56288	4.542109	4.569632	1.00777	0.0112	0.927196	1
RP11-72M17.1	1.026634	1.032915	1.024593	0.74717	-0.4205	0.927206	1
AC092171.2	1.022544	1.015676	1.024777	1.58059	0.6605	0.927222	1
RP5-1068H6.6	1.022519	1.015676	1.024744	1.57849	0.6585	0.927222	1
RAB42	1.070796	1.063869	1.073048	1.14371	0.1937	0.927223	1
WWP1	1.287917	1.294223	1.285866	0.9716	-0.0416	0.927264	1
PTPRS	1.054236	1.047301	1.05649	1.19428	0.2561	0.92729	1
CTD-2227E11.1	1.028317	1.021439	1.030553	1.42511	0.5111	0.927358	1
CTD-2139B15.2	1.028292	1.021477	1.030508	1.42051	0.5064	0.927358	1
RP11-20I23.6	1.049005	1.042203	1.051216	1.21356	0.2792	0.927447	1

MAN2B1	1.06862	1.061699	1.07087	1.14865	0.1999	0.927717	1
RAP1GAP2	1.062471	1.068841	1.060401	0.87739	-0.1887	0.927742	1
GDPGP1	1.063998	1.057259	1.066189	1.15595	0.2091	0.927759	1
BRF2	1.202313	1.208799	1.200205	0.95884	-0.0606	0.927762	1
MARC2	1.09025	1.083224	1.092534	1.11187	0.153	0.927763	1
RP11-744I24.2	1.022744	1.015961	1.024948	1.56308	0.6444	0.9278	1
RP11-923I11.6	1.022729	1.015961	1.024929	1.56186	0.6433	0.9278	1
RP11-96L14.8	1.034984	1.028161	1.037202	1.32107	0.4017	0.92782	1
RP11-509J21.2	1.034917	1.028058	1.037147	1.32391	0.4048	0.92782	1
RBM44	1.021604	1.014833	1.023805	1.60491	0.6825	0.927839	1
LGALS4	1.021614	1.014833	1.023818	1.60582	0.6833	0.927839	1
AP003068.9	1.021605	1.014833	1.023806	1.60498	0.6826	0.927839	1
POU3F3	1.0216	1.014833	1.0238	1.60459	0.6822	0.927839	1
AP006285.2	1.026231	1.019444	1.028437	1.46255	0.5485	0.927843	1
RP11-710F7.3	1.026318	1.019449	1.028551	1.46801	0.5539	0.927843	1
RP11-110I1.11	1.021602	1.027761	1.019601	0.70605	-0.5022	0.927926	1
TMEM38A	1.118537	1.124754	1.116516	0.93396	-0.0986	0.92797	1
RP11-407N17.5	1.036068	1.029309	1.038265	1.30555	0.3847	0.928021	1
ADAMTS2	1.110948	1.103894	1.113241	1.08997	0.1243	0.928055	1
LINC01411	1.020431	1.026555	1.01844	0.69442	-0.5261	0.928101	1
GUCY2F	1.021891	1.028021	1.019898	0.71013	-0.4938	0.928107	1
PCBD1	4.176733	4.190923	4.17212	0.99411	-0.0085	0.928202	1
ZNF554	1.074245	1.067299	1.076502	1.13675	0.1849	0.928282	1
RP11-1148L6.9	1.025995	1.019172	1.028212	1.4715	0.5573	0.928311	1
RP11-285A1.1	1.025935	1.019196	1.028125	1.46515	0.5511	0.928311	1
ZNF578	1.025963	1.019171	1.028171	1.46949	0.5553	0.928311	1
ZBTB12	1.025982	1.019178	1.028194	1.47012	0.5559	0.928311	1
RP11-798G7.6	1.025938	1.019181	1.028134	1.46673	0.5526	0.928311	1
BARD1	1.305106	1.311625	1.302986	0.97228	-0.0406	0.928316	1
RP11-22N19.2	1.057701	1.050838	1.059932	1.17888	0.2374	0.928366	1
BMS1	1.395957	1.403291	1.393572	0.9759	-0.0352	0.928417	1
LMF1	1.120542	1.113352	1.122879	1.08404	0.1164	0.928423	1
CACNB2	1.031784	1.024968	1.034	1.36173	0.4454	0.928442	1
REL	1.414937	1.40774	1.417277	1.02339	0.0334	0.928443	1
LMOD3	1.050601	1.043682	1.05285	1.20988	0.2749	0.928495	1
RP11-171I2.3	1.036898	1.030185	1.03908	1.29467	0.3726	0.928712	1
RP11-20I23.13	1.054365	1.047461	1.056608	1.19272	0.2543	0.928716	1
CDK15	1.039466	1.045681	1.037446	0.81973	-0.2868	0.928768	1
RP11-783K16.14	1.0395	1.045689	1.037489	0.82051	-0.2854	0.928768	1
AC005224.2	1.025774	1.019011	1.027973	1.47142	0.5572	0.928772	1
RP11-425D10.10	1.025728	1.018994	1.027917	1.46978	0.5556	0.928772	1
BTC	1.025744	1.018997	1.027937	1.47062	0.5564	0.928772	1
DEPDC5	1.068592	1.061715	1.070827	1.14766	0.1987	0.928785	1
GALNT15	1.018128	1.024189	1.016158	0.668	-0.5821	0.928805	1
LINC01094	1.018138	1.024211	1.016164	0.66762	-0.5829	0.928805	1

KCNK15-AS1	1.067482	1.060682	1.069693	1.1485	0.1998	0.928825		1
OPA3	1.197903	1.204305	1.195822	0.95848	-0.0612	0.928848		1
TP53TG5	1.019707	1.025824	1.017718	0.68612	-0.5435	0.92887		1
ME2	1.479827	1.471944	1.48239	1.02213	0.0316	0.928883		1
RP11-746M1.1	1.026235	1.019454	1.028439	1.46188	0.5478	0.92889		1
HM13-AS1	1.026229	1.019461	1.028429	1.46077	0.5467	0.92889		1
STPG3-AS1	1.029693	1.022954	1.031883	1.38902	0.4741	0.928927		1
RP11-936I5.1	1.029782	1.022961	1.032	1.39365	0.4789	0.928927		1
TPH1	1.025134	1.018336	1.027344	1.49129	0.5766	0.928929		1
ZNF569	1.167864	1.1607	1.170192	1.05907	0.0828	0.92893		1
KCNIP1	1.038315	1.031584	1.040503	1.28237	0.3588	0.928937		1
SND1-IT1	1.093813	1.087015	1.096022	1.10352	0.1421	0.928942		1
C11orf95	1.275979	1.282214	1.273952	0.97073	-0.0429	0.928965		1
TRUB1	1.247264	1.240002	1.249625	1.0401	0.0567	0.92901		1
BAD	3.094223	3.078992	3.099174	1.00971	0.0139	0.929154		1
RP4-597N16.4	1.026952	1.033092	1.024956	0.75413	-0.4071	0.929336		1
RP11-73K9.3	1.026953	1.032977	1.024995	0.75794	-0.3999	0.929336		1
GBA	1.164423	1.170926	1.162309	0.94958	-0.0746	0.929358		1
RP5-1125N11.2	1.035119	1.041289	1.033113	0.80199	-0.3183	0.929382		1
CTB-179K24.3	1.035105	1.041356	1.033073	0.79973	-0.3224	0.929382		1
HIST1H2AM	1.029411	1.022756	1.031574	1.3875	0.4725	0.929396		1
RP11-961A15.3	1.037957	1.044105	1.035958	0.81527	-0.2946	0.929445		1
AP000708.1	1.037897	1.044085	1.035886	0.81402	-0.2969	0.929445		1
FSCN3	1.01886	1.012186	1.02103	1.72569	0.7872	0.929484		1
CTXN3	1.018831	1.012186	1.02099	1.72244	0.7845	0.929484		1
AF001548.5	1.018813	1.012186	1.020967	1.72053	0.7829	0.929484		1
SAXO1	1.018841	1.012186	1.021004	1.72354	0.7854	0.929484		1
RP5-1116H23.3	1.018841	1.012186	1.021004	1.72361	0.7854	0.929484		1
BATF3	1.035244	1.028564	1.037416	1.30992	0.3895	0.929522		1
LRRC37A3	1.096234	1.089321	1.098481	1.10255	0.1408	0.929599		1
MRVI1	1.106401	1.099312	1.108706	1.09459	0.1304	0.929667		1
ACKR3	1.063959	1.057213	1.066151	1.15622	0.2094	0.929733		1
CFAP161	1.021519	1.014881	1.023677	1.59105	0.67	0.929773		1
CTD-3065B20.3	1.021534	1.014881	1.023697	1.5924	0.6712	0.929773		1
LMNTD1	1.021522	1.014881	1.02368	1.59129	0.6702	0.929773		1
SHE	1.021541	1.014881	1.023706	1.59303	0.6718	0.929773		1
RP4-613B23.1	1.057226	1.050442	1.059431	1.1782	0.2366	0.929912		1
KIAA0196	1.160155	1.153142	1.162434	1.06068	0.085	0.929928		1
RP11-575H3.1	1.033196	1.026509	1.035369	1.33425	0.416	0.930008		1
AP006222.2	1.696065	1.68802	1.69868	1.01549	0.0222	0.930061		1
CTD-2021J15.1	1.016223	1.022224	1.014272	0.64219	-0.6389	0.930118		1
IRF5	1.016217	1.022203	1.014271	0.64276	-0.6376	0.930118		1
RP11-182J1.18	1.016238	1.02228	1.014273	0.64065	-0.6424	0.930118		1
ECSCR	1.016216	1.022181	1.014277	0.64367	-0.6356	0.930118		1
AC097662.2	1.031674	1.037823	1.029675	0.78456	-0.35	0.93013		1

PSMB8-AS1	1.03164	1.037641	1.029689	0.78874	-0.3424	0.93013	1
RP11-48B3.4	1.034436	1.040465	1.032476	0.80259	-0.3173	0.930304	1
TTC13	1.084287	1.077434	1.086515	1.11728	0.16	0.930309	1
RP11-510J16.5	1.022268	1.015675	1.024411	1.55732	0.6391	0.930578	1
GPRIN2	1.022266	1.01569	1.024404	1.55534	0.6372	0.930578	1
LRRK2	1.022319	1.015696	1.024472	1.55916	0.6408	0.930578	1
INTS12	1.160155	1.16641	1.158122	0.9502	-0.0737	0.93064	1
WNT5B	1.085276	1.091161	1.083363	0.91446	-0.129	0.930682	1
AC006116.17	1.024972	1.018374	1.027116	1.47577	0.5615	0.930866	1
VMAC	1.081107	1.087123	1.079152	0.90851	-0.1384	0.930907	1
ANKRD53	1.058318	1.051685	1.060474	1.17006	0.2266	0.930944	1
C10orf82	1.028195	1.034172	1.026253	0.76825	-0.3803	0.930992	1
DNASE1L3	1.018424	1.011871	1.020555	1.73151	0.792	0.931002	1
ZNF528	1.018426	1.011871	1.020557	1.73168	0.7922	0.931002	1
ANKRD34B	1.018421	1.011871	1.02055	1.73113	0.7917	0.931002	1
RP11-134K13.4	1.018438	1.011871	1.020572	1.733	0.7933	0.931002	1
RYR1	1.018449	1.011871	1.020587	1.73422	0.7943	0.931002	1
MYLK3	1.018422	1.011871	1.020551	1.73122	0.7918	0.931002	1
TTBK1	1.018414	1.011871	1.020541	1.73033	0.791	0.931002	1
VSTM2A	1.018427	1.011871	1.020558	1.73184	0.7923	0.931002	1
ZDHHC20	1.312229	1.304464	1.314753	1.03379	0.0479	0.93104	1
RP11-138P22.1	1.058547	1.051788	1.060744	1.17293	0.2301	0.931056	1
RP11-38G5.4	1.020046	1.025976	1.018119	0.69752	-0.5197	0.931083	1
LINC01376	1.031983	1.025382	1.034128	1.34461	0.4272	0.931098	1
PLA2G3	1.058484	1.052041	1.060579	1.16405	0.2192	0.93131	1
SIDT2	1.204857	1.197715	1.207179	1.04787	0.0675	0.931373	1
SALL4	1.048166	1.054092	1.04624	0.85485	-0.2263	0.93147	1
CFAP61	1.017811	1.011304	1.019927	1.76274	0.8178	0.931473	1
PPP1R13B	1.083142	1.089138	1.081193	0.91086	-0.1347	0.931479	1
BLCAP	1.532178	1.539305	1.529861	0.98249	-0.0255	0.931492	1
PCDHGC3	1.036341	1.029749	1.038483	1.29358	0.3714	0.931556	1
RP11-1152H14.1	1.036418	1.029847	1.038554	1.29175	0.3693	0.931556	1
RP11-81A22.5	1.068058	1.061286	1.07026	1.14643	0.1971	0.931573	1
LACTB2	1.315434	1.308369	1.317731	1.03036	0.0431	0.93159	1
PCDHAC1	1.024881	1.030805	1.022956	0.74522	-0.4243	0.931607	1
RP11-167P20.1	1.018722	1.012187	1.020847	1.71061	0.7745	0.931735	1
RP11-744I24.3	1.018679	1.012187	1.020789	1.70587	0.7705	0.931735	1
RP11-739B23.1	1.018737	1.012187	1.020866	1.71221	0.7759	0.931735	1
RP11-254F7.2	1.153113	1.159398	1.151069	0.94775	-0.0774	0.931735	1
RBL2	1.447935	1.440598	1.45032	1.02206	0.0315	0.931772	1
RP11-297P16.3	1.020483	1.013947	1.022607	1.62099	0.6969	0.931774	1
RP11-449D8.1	1.020446	1.013947	1.022559	1.61753	0.6938	0.931774	1
KCNF1	1.020457	1.013947	1.022573	1.61851	0.6947	0.931774	1
RNF40	1.345229	1.351686	1.34313	0.97567	-0.0355	0.931817	1
SHKBP1	1.607901	1.614989	1.605597	0.98473	-0.0222	0.931938	1

VWA3A	1.022342	1.015826	1.02446	1.54552	0.6281	0.931952	1
ODF3L2	1.02232	1.015826	1.024431	1.54373	0.6264	0.931952	1
RP11-108E14.1	1.022338	1.015826	1.024455	1.54522	0.6278	0.931952	1
PAH	1.022342	1.015826	1.02446	1.54553	0.6281	0.931952	1
RP11-126L15.4	1.022371	1.015826	1.024498	1.54794	0.6303	0.931952	1
PCDHA5	1.028455	1.021906	1.030584	1.39614	0.4814	0.931956	1
RP11-35G9.5	1.028477	1.021901	1.030615	1.39789	0.4833	0.931956	1
TNFAIP8L3	1.028444	1.021919	1.030565	1.39443	0.4797	0.931956	1
RP11-350N15.6	1.02471	1.0306	1.022796	0.74496	-0.4248	0.931968	1
ZNF232	1.240607	1.233584	1.24289	1.03984	0.0564	0.931976	1
CAMK2N2	1.019555	1.013073	1.021663	1.65713	0.7287	0.932015	1
CTB-43P18.1	1.021889	1.015389	1.024002	1.55972	0.6413	0.932098	1
RP11-282O18.3	1.021876	1.015374	1.02399	1.56041	0.6419	0.932098	1
NRG3	1.021849	1.015399	1.023946	1.55509	0.637	0.932098	1
FSCN2	1.021858	1.015363	1.02397	1.56023	0.6418	0.932098	1
ZSWIM2	1.019579	1.013102	1.021684	1.65509	0.7269	0.932125	1
USH2A	1.019587	1.013102	1.021695	1.65594	0.7277	0.932125	1
BMPER	1.019586	1.013102	1.021694	1.65582	0.7276	0.932125	1
RP11-403P17.3	1.03657	1.030032	1.038696	1.28848	0.3657	0.932138	1
NDUFB2-AS1	1.025633	1.031533	1.023715	0.75206	-0.4111	0.932209	1
PRPF40B	1.188945	1.182177	1.191145	1.04923	0.0693	0.932279	1
ARHGEF16	1.195184	1.201648	1.193083	0.95753	-0.0626	0.932298	1
RP11-480I12.10	1.019762	1.013274	1.021871	1.64764	0.7204	0.932374	1
RP11-47J17.2	1.019724	1.013274	1.021821	1.64389	0.7171	0.932374	1
AC007620.3	1.019754	1.013274	1.02186	1.6468	0.7197	0.932374	1
TSIX	1.02299	1.028827	1.021093	0.73171	-0.4507	0.932511	1
RP4-742C19.13	1.045008	1.050933	1.043081	0.84584	-0.2415	0.932522	1
GRB7	1.044955	1.050757	1.043069	0.84853	-0.237	0.932522	1
LINC00667	1.704288	1.711099	1.702073	0.98731	-0.0184	0.932623	1
THBD	1.050796	1.056742	1.048863	0.86114	-0.2157	0.932643	1
RP11-944C7.1	1.021295	1.027111	1.019404	0.71572	-0.4825	0.932725	1
HOXB-AS1	1.021322	1.027128	1.019435	0.71644	-0.4811	0.932725	1
RAPGEFL1	1.090565	1.083923	1.092724	1.10487	0.1439	0.932831	1
RP11-893F2.6	1.024061	1.029864	1.022175	0.74254	-0.4295	0.932898	1
CHMP4C	1.307571	1.299818	1.310091	1.03426	0.0486	0.932917	1
MRPL12	2.645867	2.636716	2.648842	1.00741	0.0106	0.932988	1
IGSF5	1.045351	1.051161	1.043462	0.84952	-0.2353	0.932996	1
USP3	1.569072	1.561792	1.571439	1.01717	0.0246	0.933027	1
RBPJ	2.969307	2.981419	2.96537	0.9919	-0.0117	0.933033	1
ZNF142	1.076742	1.082728	1.074797	0.90412	-0.1454	0.933037	1
NPIPA2	1.031928	1.025421	1.034043	1.33918	0.4213	0.933043	1
ADCY3	1.083366	1.089333	1.081427	0.9115	-0.1337	0.933046	1
RP11-629B11.5	1.039526	1.033054	1.04163	1.25945	0.3328	0.933072	1
ACTN2	1.022159	1.028019	1.020254	0.72287	-0.4682	0.933076	1
TRABD2B	1.02218	1.028017	1.020283	0.72395	-0.466	0.933076	1

AC004221.2	1.023026	1.016574	1.025123	1.51584	0.6001	0.933112	1
KCNQ3	1.020388	1.026202	1.018498	0.70596	-0.5023	0.933116	1
C11orf45	1.020405	1.026236	1.01851	0.70552	-0.5032	0.933116	1
CMP21-97G8.2	1.020415	1.026224	1.018527	0.70649	-0.5013	0.933116	1
BAIAP2L1	1.179006	1.184924	1.177083	0.95759	-0.0625	0.933118	1
PLA2G4A	1.04126	1.047159	1.039343	0.83426	-0.2614	0.933152	1
VSIG1	1.025294	1.018859	1.027386	1.4522	0.5382	0.93319	1
MYH7	1.025324	1.018859	1.027425	1.45419	0.5402	0.93319	1
RP11-171I2.5	1.040111	1.033614	1.042223	1.25611	0.329	0.933213	1
RNF112	1.023029	1.016602	1.025118	1.51298	0.5974	0.933223	1
CTC-498J12.3	1.023077	1.016627	1.025174	1.51402	0.5984	0.933223	1
AC079117.1	1.023026	1.016606	1.025113	1.51234	0.5968	0.933223	1
CXCL17	1.023037	1.01662	1.025123	1.51166	0.5961	0.933223	1
CPT1B	1.043587	1.037254	1.045645	1.22525	0.2931	0.933228	1
PTER	1.128831	1.13496	1.126838	0.93982	-0.0895	0.933301	1
HAND2-AS1	1.018011	1.023821	1.016122	0.67678	-0.5632	0.933346	1
RP11-281P23.1	1.018013	1.023828	1.016122	0.67662	-0.5636	0.933346	1
RP11-284F21.7	1.018044	1.023936	1.016128	0.6738	-0.5696	0.933346	1
SELPLG	1.018004	1.023819	1.016114	0.67653	-0.5638	0.933346	1
SNPH	1.030131	1.035893	1.028258	0.78727	-0.3451	0.933357	1
HDAC4	1.108729	1.114931	1.106714	0.9285	-0.107	0.933363	1
STX16-NPEPL1	1.019543	1.025348	1.017656	0.69653	-0.5217	0.933378	1
LDLRAD1	1.019561	1.025375	1.01767	0.69636	-0.5221	0.933378	1
RP11-360L9.4	1.019582	1.025497	1.017659	0.69258	-0.5299	0.933378	1
ESYT3	1.023165	1.016773	1.025243	1.50493	0.5897	0.933473	1
RNF31	1.02321	1.016778	1.025301	1.50802	0.5927	0.933473	1
TECTA	1.02322	1.016801	1.025306	1.50627	0.591	0.933473	1
RP11-33H15.1	1.023203	1.016768	1.025295	1.50856	0.5932	0.933473	1
TMEM110	1.038306	1.044129	1.036413	0.82515	-0.2773	0.933634	1
KCNIP4-IT1	1.027426	1.033158	1.025562	0.77092	-0.3754	0.933647	1
RP11-328K4.1	1.024718	1.018324	1.026796	1.46235	0.5483	0.933663	1
ZNHIT6	1.282293	1.288385	1.280313	0.97201	-0.041	0.93373	1
GPR3	1.020583	1.026358	1.018706	0.7097	-0.4947	0.933768	1
RP11-680G24.6	1.020597	1.026375	1.018718	0.70969	-0.4947	0.933768	1
RP11-1094H24.4	1.020571	1.026321	1.018702	0.71055	-0.493	0.933768	1
LGALSL	1.375524	1.381875	1.373459	0.97796	-0.0322	0.933789	1
USP6NL	1.253045	1.259186	1.251049	0.9686	-0.046	0.933828	1
PCF11-AS1	1.041852	1.047763	1.03993	0.836	-0.2584	0.933841	1
TMPRSS9	1.020128	1.025906	1.01825	0.70447	-0.5054	0.93385	1
COCH	1.127209	1.120485	1.129395	1.07394	0.1029	0.933866	1
KIAA0513	1.073399	1.079261	1.071493	0.902	-0.1488	0.933889	1
RP11-150C16.1	1.025636	1.019251	1.027712	1.43946	0.5255	0.933926	1
CAPN8	1.018707	1.024449	1.01684	0.68876	-0.5379	0.933946	1
RP11-266J6.2	1.027375	1.020975	1.029455	1.40432	0.4899	0.933966	1
RP11-341G23.4	1.016954	1.022688	1.01509	0.66513	-0.5883	0.933986	1

FAM41C	1.035414	1.028942	1.037518	1.2963	0.3744	0.934125	1
U47924.31	1.029213	1.022822	1.03129	1.37109	0.4553	0.934144	1
MYO15A	1.071083	1.06476	1.073139	1.12939	0.1755	0.934192	1
RP11-11N7.4	1.016078	1.021791	1.014221	0.65264	-0.6156	0.934248	1
UBXN10-AS1	1.056853	1.050499	1.058919	1.16673	0.2225	0.93429	1
RP11-429J17.2	1.055673	1.04941	1.057709	1.16796	0.224	0.934465	1
ATAD3A	1.266871	1.273383	1.264755	0.96844	-0.0463	0.9345	1
SLC7A4	1.023968	1.029668	1.022116	0.74544	-0.4238	0.934508	1
ATR	1.147294	1.153369	1.145319	0.94752	-0.0778	0.93451	1
SYNGR4	1.021765	1.015453	1.023817	1.54122	0.6241	0.934638	1
RP11-298I3.1	1.02183	1.015453	1.023902	1.54672	0.6292	0.934638	1
RP11-362K14.5	1.021802	1.015453	1.023866	1.54437	0.627	0.934638	1
PIH1D3	1.021769	1.015453	1.023822	1.54154	0.6244	0.934638	1
CTD-2005H7.1	1.021773	1.015453	1.023827	1.54186	0.6247	0.934638	1
CYBB	1.016699	1.022408	1.014843	0.66239	-0.5942	0.934719	1
GIT2	1.191428	1.184828	1.193574	1.04732	0.0667	0.934724	1
ZNF829	1.02818	1.021838	1.030242	1.3848	0.4697	0.934752	1
SNX1	1.348874	1.341837	1.351161	1.02728	0.0388	0.934792	1
DLGAP1-AS1	1.35376	1.3599	1.351764	0.97739	-0.033	0.93482	1
LIPT1	1.121836	1.127809	1.119894	0.93807	-0.0922	0.934964	1
PARP14	1.252865	1.259151	1.250822	0.96786	-0.0471	0.934987	1
RP11-346D14.1	1.029012	1.022683	1.031069	1.3697	0.4539	0.935016	1
RP11-1007O24.3	1.02902	1.022672	1.031083	1.37096	0.4552	0.935016	1
KCTD21	1.030803	1.024439	1.032871	1.34504	0.4277	0.935055	1
ABO	1.0346	1.040265	1.032759	0.81358	-0.2977	0.935069	1
SLC8A2	1.023303	1.028992	1.021454	0.74001	-0.4344	0.935082	1
RP11-10C24.1	1.023226	1.028959	1.021363	0.73768	-0.4389	0.935082	1
SLC9A8	1.075148	1.068766	1.077223	1.12298	0.1673	0.935167	1
WDR70	1.39207	1.398307	1.390042	0.97925	-0.0303	0.935221	1
PIWIL1	1.032726	1.026392	1.034785	1.31804	0.3984	0.935234	1
ABHD11-AS1	1.03276	1.026401	1.034827	1.31913	0.3996	0.935234	1
TBK1	1.19332	1.199288	1.191381	0.96032	-0.0584	0.935237	1
MFSD14B	1.265465	1.258649	1.267681	1.03492	0.0495	0.935282	1
CTD-2033C11.1	1.030243	1.035957	1.028385	0.78942	-0.3411	0.935302	1
SMIM11B	1.031392	1.037096	1.029538	0.79626	-0.3287	0.935342	1
CENPI	1.046899	1.040583	1.048952	1.20621	0.2705	0.935353	1
C6orf163	1.0322	1.025846	1.034265	1.32575	0.4068	0.935364	1
RIPPLY3	1.020549	1.026218	1.018707	0.71352	-0.487	0.935371	1
RP11-59H7.4	1.020554	1.026177	1.018726	0.71535	-0.4833	0.935371	1
RP11-631N16.2	1.087351	1.093012	1.085511	0.91936	-0.1213	0.935383	1
TMEM86B	1.02999	1.023676	1.032043	1.3534	0.4366	0.935408	1
LA16c-390H2.4	1.029902	1.023602	1.03195	1.35373	0.4369	0.935408	1
RP11-70D24.2	1.02993	1.023634	1.031976	1.35297	0.4361	0.935408	1
KCNIP2-AS1	1.029941	1.023597	1.032004	1.35628	0.4397	0.935408	1
C14orf79	1.123962	1.11736	1.126108	1.07454	0.1037	0.935596	1

RP5-991G20.2	1.017946	1.011717	1.019971	1.70438	0.7692	0.935603	1
CYP2D6	1.017954	1.011717	1.019981	1.70527	0.77	0.935603	1
C5orf22	1.116049	1.109689	1.118117	1.07683	0.1068	0.935616	1
ZNF407	1.08418	1.090108	1.082253	0.91283	-0.1316	0.935624	1
PPP1R3F	1.084275	1.089908	1.082444	0.91699	-0.125	0.935624	1
RP1-257A7.4	1.030922	1.03659	1.02908	0.79475	-0.3314	0.935695	1
RP11-96H19.1	1.074009	1.06745	1.076141	1.12884	0.1748	0.935737	1
FAM95B1	1.025255	1.018986	1.027292	1.43749	0.5236	0.935741	1
NSUN3	1.143985	1.137475	1.146102	1.06275	0.0878	0.935808	1
OGFOD3	1.352152	1.358188	1.35019	0.97767	-0.0326	0.93583	1
KCTD19	1.031624	1.025337	1.033667	1.32874	0.4101	0.935838	1
GOLGA8N	1.031575	1.025279	1.033621	1.32998	0.4114	0.935838	1
MAPK8IP3	1.205754	1.199022	1.207942	1.04482	0.0633	0.935843	1
FN3K	1.191773	1.185318	1.193871	1.04615	0.0651	0.935874	1
ASAP1	1.711279	1.702986	1.713975	1.01563	0.0224	0.935939	1
FANCC	1.10617	1.09966	1.108286	1.08656	0.1198	0.93594	1
IRF8	1.019845	1.025616	1.017969	0.70149	-0.5115	0.935949	1
RP11-626G11.1	1.0198	1.025454	1.017962	0.70564	-0.503	0.935949	1
PIK3IP1	1.188535	1.194397	1.186629	0.96004	-0.0588	0.936	1
OTUD6B	1.189652	1.195551	1.187735	0.96003	-0.0589	0.936042	1
PALM2	1.027949	1.033448	1.026161	0.78216	-0.3545	0.9362	1
SLC25A39	3.833646	3.819074	3.838383	1.00685	0.0098	0.936235	1
IGFBP7-AS1	1.017104	1.022713	1.015281	0.67276	-0.5718	0.936238	1
SEMA3B-AS1	1.036211	1.030032	1.03822	1.27264	0.3478	0.936321	1
ZDHHC19	1.036161	1.029947	1.038181	1.27495	0.3504	0.936321	1
FAM105A	1.036145	1.029944	1.03816	1.27437	0.3498	0.936321	1
NLGN1	1.237801	1.230998	1.240012	1.03902	0.0552	0.936395	1
P2RX2	1.075787	1.069632	1.077787	1.11712	0.1598	0.936407	1
MAMSTR	1.160321	1.153894	1.162411	1.05534	0.0777	0.936439	1
MRPL48	1.872451	1.865361	1.874756	1.01086	0.0156	0.936441	1
RP11-126K1.2	1.049185	1.042878	1.051235	1.19489	0.2569	0.936457	1
FAM83H	1.062397	1.068054	1.060558	0.88985	-0.1684	0.936572	1
ANAPC2	1.127499	1.121143	1.129565	1.06952	0.097	0.936623	1
TMEM86A	1.039666	1.045304	1.037833	0.83509	-0.26	0.936656	1
VPREB3	1.021418	1.015224	1.023431	1.53903	0.622	0.936707	1
HIST1H2BJ	1.021384	1.015213	1.023389	1.53743	0.6205	0.936707	1
RP11-96K19.5	1.02141	1.01521	1.023426	1.54011	0.623	0.936707	1
RP11-440L14.4	1.021405	1.015212	1.023419	1.53947	0.6224	0.936707	1
PCDHGA10	1.146507	1.152298	1.144624	0.94961	-0.0746	0.936749	1
FAM72A	1.027624	1.033211	1.025807	0.77706	-0.3639	0.93678	1
RP11-977P2.1	1.021445	1.01526	1.023456	1.53713	0.6202	0.936811	1
AP001468.58	1.021457	1.01526	1.023471	1.5381	0.6211	0.936811	1
RP11-584P21.2	1.021461	1.01526	1.023476	1.53844	0.6215	0.936811	1
AC068831.6	1.021459	1.01526	1.023474	1.5383	0.6213	0.936811	1
RHD	1.021431	1.01526	1.023437	1.53586	0.619	0.936811	1

RP11-108M9.3	1.021448	1.01526	1.02346	1.53737	0.6205	0.936811	1
SLC2A8	1.205884	1.199191	1.208059	1.04452	0.0628	0.936908	1
CTD-2240J17.1	1.019869	1.013691	1.021877	1.59797	0.6762	0.936917	1
TPBGL	1.019871	1.013691	1.02188	1.59813	0.6764	0.936917	1
ARPC4-TTLL3	1.019877	1.013691	1.021888	1.59874	0.6769	0.936917	1
RP11-27G24.1	1.019896	1.013691	1.021913	1.60058	0.6786	0.936917	1
AF131215.8	1.019846	1.013691	1.021847	1.59573	0.6742	0.936917	1
ZNF556	1.019843	1.013691	1.021842	1.5954	0.6739	0.936917	1
MYO15B	1.137561	1.131173	1.139637	1.06453	0.0902	0.936928	1
SRGAP3-AS4	1.023311	1.028931	1.021484	0.7426	-0.4293	0.937021	1
RP11-214O1.2	1.023327	1.02886	1.021528	0.74594	-0.4229	0.937021	1
RP11-977G19.12	1.080511	1.086221	1.078655	0.91225	-0.1325	0.937059	1
RP11-505K9.4	1.024506	1.03012	1.022681	0.75304	-0.4092	0.937061	1
RP11-90D4.3	1.038672	1.044148	1.036893	0.83566	-0.259	0.937068	1
TRAF3IP2	1.189921	1.19538	1.188146	0.96297	-0.0544	0.937171	1
MAST3	1.086379	1.092104	1.084518	0.91764	-0.124	0.937201	1
TRPS1	1.227422	1.233527	1.225438	0.96536	-0.0509	0.937202	1
MTMR12	1.121561	1.114774	1.123767	1.07836	0.1088	0.937221	1
ABCG4	1.028014	1.033663	1.026177	0.77762	-0.3629	0.93725	1
RBM38	1.183496	1.176938	1.185628	1.04912	0.0692	0.937267	1
DACT1	1.053311	1.047077	1.055338	1.17548	0.2332	0.937314	1
SERPINI1	1.059436	1.064976	1.057636	0.88702	-0.173	0.937322	1
ACVR1C	1.02401	1.02954	1.022212	0.75192	-0.4113	0.937405	1
BNIPL	1.023996	1.029572	1.022183	0.75015	-0.4148	0.937405	1
CD164L2	1.039129	1.032944	1.04114	1.24879	0.3205	0.937522	1
TMEM104	1.080765	1.086342	1.078952	0.91442	-0.1291	0.93754	1
RHEBL1	1.036226	1.041929	1.034372	0.81978	-0.2867	0.937543	1
AC007009.1	1.022213	1.016045	1.024217	1.50935	0.5939	0.93758	1
RP11-16C1.1	1.022171	1.016045	1.024162	1.5059	0.5906	0.93758	1
RP11-583F2.5	1.02225	1.016045	1.024267	1.51245	0.5969	0.93758	1
LYPLAL1-AS1	1.022182	1.016045	1.024178	1.50686	0.5915	0.93758	1
RP11-393I23.4	1.02216	1.016045	1.024148	1.505	0.5898	0.93758	1
CTA-212A2.2	1.022185	1.016045	1.024181	1.50707	0.5917	0.93758	1
RP11-1H15.2	1.022262	1.016045	1.024283	1.51344	0.5978	0.93758	1
RP11-70C1.1	1.023661	1.017512	1.02566	1.46528	0.5512	0.937616	1
ARHGEF18	1.02365	1.017512	1.025645	1.46443	0.5503	0.937616	1
CD160	1.023657	1.017512	1.025655	1.465	0.5509	0.937616	1
IRAK2	1.036625	1.042269	1.034791	0.82309	-0.2809	0.93773	1
FLVCR2	1.030046	1.035673	1.028217	0.79097	-0.3383	0.937735	1
RP11-517B11.4	1.029987	1.03554	1.028183	0.793	-0.3346	0.937735	1
ERVW-1	1.024834	1.018706	1.026826	1.43408	0.5201	0.937808	1
ADCK1	1.109351	1.102932	1.111438	1.08263	0.1145	0.937846	1
RP11-626G11.5	1.024896	1.018749	1.026895	1.43448	0.5205	0.937873	1
TNN	1.019881	1.025435	1.018075	0.71064	-0.4928	0.937885	1
HCFC1-AS1	1.019887	1.025473	1.018071	0.70942	-0.4953	0.937885	1

TNK1	1.035237	1.04094	1.033383	0.81542	-0.2944	0.937915	1
ZBTB49	1.057154	1.051003	1.059153	1.1598	0.2139	0.937947	1
MARS	1.340857	1.333817	1.343145	1.02794	0.0398	0.937948	1
RP11-540K16.1	1.0233	1.017201	1.025283	1.46986	0.5557	0.938024	1
AC007405.4	1.02339	1.017188	1.025406	1.47817	0.5638	0.938024	1
SLC34A2	1.023292	1.017212	1.025268	1.46803	0.5539	0.938024	1
RP11-83J21.3	1.062967	1.056964	1.064918	1.13964	0.1886	0.93803	1
ARHGEF37	1.103416	1.096863	1.105546	1.08964	0.1238	0.938168	1
PYGO2	1.262771	1.26862	1.260869	0.97115	-0.0422	0.938189	1
TNNI3K	1.020568	1.026071	1.018779	0.7203	-0.4733	0.938264	1
RP11-356J5.12	1.145459	1.138954	1.147574	1.06204	0.0868	0.9383	1
PRR29	1.041154	1.035033	1.043144	1.23153	0.3005	0.938303	1
FAM71D	1.041196	1.035121	1.04317	1.22919	0.2977	0.938303	1
CTA-204B4.2	1.085541	1.079299	1.08757	1.1043	0.1431	0.938345	1
CTB-55O6.12	1.070739	1.064532	1.072757	1.12745	0.1731	0.938455	1
PSCA	1.020753	1.026241	1.018969	0.72286	-0.4682	0.938503	1
RP11-115D19.4	1.020793	1.026384	1.018975	0.7192	-0.4755	0.938503	1
RP11-121C2.2	1.060884	1.05474	1.062881	1.14871	0.2	0.938523	1
ZNF18	1.082495	1.076156	1.084556	1.1103	0.151	0.938523	1
DDX58	1.082541	1.076155	1.084617	1.11111	0.152	0.938523	1
COPA	1.819449	1.811247	1.822115	1.0134	0.0192	0.938558	1
C7orf25	1.033212	1.038869	1.031374	0.80716	-0.3091	0.938624	1
UBA6	1.748313	1.74069	1.750791	1.01364	0.0195	0.938641	1
RUFY1	1.281596	1.274958	1.283754	1.03199	0.0454	0.938643	1
RP11-165J3.6	1.025663	1.019535	1.027655	1.41564	0.5015	0.938644	1
RP13-49I15.6	1.025672	1.019532	1.027667	1.41652	0.5023	0.938644	1
RP11-503P10.1	1.025658	1.01954	1.027647	1.41489	0.5007	0.938644	1
DNAJC28	1.051375	1.056883	1.049585	0.87169	-0.1981	0.938676	1
GCC2-AS1	1.027112	1.021048	1.029083	1.38172	0.4665	0.938724	1
STARD6	1.027086	1.021038	1.029051	1.38092	0.4656	0.938724	1
RP11-284F21.9	1.027088	1.021005	1.029066	1.38375	0.4686	0.938724	1
ZBTB37	1.21095	1.216585	1.209119	0.96553	-0.0506	0.938864	1
HIST3H2BB	1.034443	1.04004	1.032624	0.81478	-0.2955	0.938869	1
AC016831.7	1.028323	1.022277	1.030289	1.35963	0.4432	0.938905	1
CGREF1	1.040401	1.034295	1.042386	1.23593	0.3056	0.938909	1
ZNF79	1.040497	1.03434	1.042499	1.23761	0.3076	0.938909	1
LL09NC01-139C3.1	1.028433	1.022327	1.030417	1.36234	0.4461	0.938931	1
IL1RAP	1.09215	1.085824	1.094206	1.09766	0.1344	0.938935	1
DCAF17	1.144556	1.137913	1.146715	1.06383	0.0893	0.938954	1
BBS10	1.132228	1.137836	1.130405	0.94608	-0.08	0.939113	1
FLT3	1.017106	1.022517	1.015347	0.68158	-0.553	0.939125	1
RP11-245J9.5	1.017104	1.022528	1.015341	0.68098	-0.5543	0.939125	1
CTD-2653M23.3	1.017123	1.022542	1.015362	0.68149	-0.5532	0.939125	1
SPAG1	1.103371	1.097343	1.10533	1.08205	0.1138	0.939256	1
TVP23B	1.214325	1.207664	1.216491	1.0425	0.0601	0.939291	1

TEPSIN	1.102138	1.095804	1.104197	1.08761	0.1212	0.939296	1
KB-1466C5.1	1.02399	1.017948	1.025955	1.44612	0.5322	0.939455	1
STK32A	1.024001	1.017948	1.025969	1.44691	0.533	0.939455	1
TMEFF1	1.024016	1.017948	1.025988	1.44799	0.5341	0.939455	1
RP11-147L13.2	1.023972	1.017948	1.025931	1.44479	0.5309	0.939455	1
RP11-114O18.1	1.023985	1.017948	1.025948	1.44576	0.5318	0.939455	1
LIPH	1.023982	1.017948	1.025944	1.44551	0.5316	0.939455	1
PCDHGA12	1.024008	1.017948	1.025979	1.44746	0.5335	0.939455	1
CTD-2537I9.12	1.023973	1.017948	1.025931	1.44482	0.5309	0.939455	1
RP11-18B16.2	1.023108	1.028507	1.021353	0.74905	-0.4169	0.939455	1
RP11-2H3.6	1.023119	1.02867	1.021315	0.74346	-0.4277	0.939455	1
DEF6	1.029668	1.035057	1.027916	0.7963	-0.3286	0.93952	1
DENND1C	1.083452	1.089102	1.081615	0.91597	-0.1266	0.939536	1
RNF125	1.030973	1.036386	1.029214	0.80289	-0.3167	0.939539	1
PER2	1.064247	1.058277	1.066188	1.13575	0.1836	0.939571	1
CASC10	1.343781	1.349556	1.341903	0.97811	-0.0319	0.93967	1
RP11-455F5.3	2.402761	2.392382	2.406135	1.00988	0.0142	0.939714	1
RP11-666A8.8	1.017993	1.011999	1.019942	1.66194	0.7329	0.939784	1
RP11-685M7.3	1.017982	1.011999	1.019927	1.66066	0.7318	0.939784	1
RP11-370I10.12	1.017987	1.011999	1.019934	1.66124	0.7323	0.939784	1
RP11-230C9.2	1.021938	1.015934	1.02389	1.49926	0.5843	0.939939	1
KCNC2	1.021961	1.015934	1.023921	1.50121	0.5861	0.939939	1
KCNC1	1.021963	1.015934	1.023923	1.50137	0.5863	0.939939	1
CTD-2555O16.2	1.021984	1.015934	1.023951	1.50309	0.5879	0.939939	1
PUM1	1.979395	1.970908	1.982153	1.01158	0.0166	0.939951	1
ACTRT3	1.048519	1.053959	1.046751	0.86642	-0.2069	0.940021	1
TRMO	1.070324	1.075724	1.068569	0.90551	-0.1432	0.940071	1
SCUBE3	1.307147	1.300478	1.309316	1.02941	0.0418	0.940162	1
ANKRD28	1.283635	1.288726	1.28198	0.97664	-0.0341	0.94022	1
UBE4A	1.348637	1.354698	1.346667	0.97736	-0.033	0.940301	1
RP11-230C9.4	1.02162	1.015676	1.023553	1.50253	0.5874	0.940407	1
RP11-453A12.1	1.021627	1.015676	1.023561	1.50307	0.5879	0.940407	1
HCG15	1.021659	1.015676	1.023604	1.50577	0.5905	0.940407	1
DUSP13	1.021614	1.015676	1.023544	1.50195	0.5868	0.940407	1
LINC00957	1.046285	1.040382	1.048204	1.1937	0.2554	0.94041	1
FOXO4	1.044505	1.049791	1.042787	0.85933	-0.2187	0.940413	1
AF213884.2	1.027453	1.021441	1.029407	1.37153	0.4558	0.940522	1
ITPKA	1.027471	1.021485	1.029416	1.36912	0.4533	0.940522	1
DNAJB13	1.027438	1.021456	1.029382	1.36938	0.4535	0.940522	1
CTD-2007H13.3	1.027417	1.021441	1.029359	1.36933	0.4535	0.940522	1
ZNF527	1.072617	1.078008	1.070865	0.90843	-0.1386	0.940566	1
ACSS2	1.193859	1.187338	1.195978	1.04612	0.065	0.940578	1
PINK1-AS	1.042907	1.048504	1.041087	0.84709	-0.2394	0.940625	1
NPM2	1.027422	1.03272	1.0257	0.78544	-0.3484	0.940648	1
ZDHHC9	1.317438	1.310625	1.319653	1.02907	0.0413	0.940651	1

ZNF221	1.047111	1.041121	1.049058	1.19302	0.2546	0.940693	1
AC022182.1	1.021947	1.015961	1.023892	1.49692	0.582	0.940985	1
COL6A6	1.0219	1.015961	1.023831	1.49307	0.5783	0.940985	1
AGBL1	1.021916	1.015961	1.023852	1.49437	0.5795	0.940985	1
AP001469.7	1.02188	1.015961	1.023804	1.4914	0.5767	0.940985	1
MYPOP	1.1197	1.113541	1.121702	1.07188	0.1001	0.940987	1
FAM109A	1.161299	1.155001	1.163346	1.05384	0.0757	0.941003	1
CTB-133G6.2	1.025397	1.019427	1.027337	1.40715	0.4928	0.941006	1
PCAT29	1.02074	1.014833	1.022661	1.52778	0.6114	0.941024	1
AC004156.3	1.020735	1.014833	1.022653	1.52726	0.6109	0.941024	1
IGLL1	1.020738	1.014833	1.022658	1.52757	0.6112	0.941024	1
RP11-2B6.2	1.022426	1.027725	1.020703	0.74671	-0.4214	0.941121	1
GPR20	1.022453	1.02776	1.020728	0.74668	-0.4214	0.941121	1
RAD21-AS1	1.024051	1.02939	1.022315	0.75926	-0.3973	0.941244	1
RP11-318K12.2	1.024029	1.029298	1.022316	0.76167	-0.3928	0.941244	1
FAM210A	1.495893	1.502003	1.493906	0.98387	-0.0235	0.941252	1
RP11-1055B8.9	1.167126	1.160809	1.16918	1.05205	0.0732	0.941276	1
TMEM62	1.076571	1.081924	1.074831	0.91342	-0.1306	0.94128	1
RP11-884K10.7	1.063115	1.068371	1.061406	0.89813	-0.155	0.941293	1
RP11-276H19.2	1.110121	1.104147	1.112063	1.07601	0.1057	0.941309	1
DLD	1.508372	1.501915	1.510471	1.01705	0.0244	0.941366	1
AHRR	1.025103	1.019217	1.027017	1.40592	0.4915	0.941475	1
LINC00284	1.025092	1.019201	1.027007	1.40657	0.4922	0.941475	1
TMEM57	1.235996	1.241343	1.234258	0.97064	-0.043	0.941523	1
KCNJ5	1.031284	1.036512	1.029584	0.81025	-0.3036	0.941619	1
ARHGAP5-AS1	1.164308	1.169887	1.162495	0.95649	-0.0642	0.941696	1
CTC-241N9.1	1.212815	1.206547	1.214852	1.04021	0.0569	0.941712	1
PCDHB10	1.094233	1.099671	1.092466	0.92771	-0.1083	0.941716	1
RFLNA	1.041677	1.047025	1.039939	0.84932	-0.2356	0.941763	1
RP11-514O12.4	1.036015	1.030126	1.037929	1.259	0.3323	0.941812	1
GOLGA1	1.141326	1.146574	1.13962	0.95255	-0.0701	0.941862	1
RP5-858B6.3	1.050713	1.044728	1.052659	1.17732	0.2355	0.941874	1
VANGL2	1.292358	1.285679	1.29453	1.03098	0.044	0.941875	1
MKNK2	1.401011	1.406983	1.39907	0.98056	-0.0283	0.94202	1
LINC00488	1.01899	1.024264	1.017275	0.71196	-0.4901	0.942022	1
CBLC	1.018964	1.024184	1.017267	0.71398	-0.486	0.942022	1
CFHR1	1.025339	1.019504	1.027237	1.39649	0.4818	0.942055	1
RP11-227G15.11	1.025353	1.019466	1.027266	1.40073	0.4862	0.942055	1
VPS35	2.382577	2.371291	2.386245	1.01091	0.0156	0.942084	1
RP11-415F23.2	1.024198	1.018357	1.026097	1.42165	0.5076	0.942094	1
LIG4	1.112798	1.106726	1.114772	1.07539	0.1049	0.942094	1
AGO4	1.112756	1.106849	1.114675	1.07324	0.102	0.942094	1
IL18	1.020569	1.025828	1.018859	0.73017	-0.4537	0.942101	1
TOMM70	1.348306	1.353984	1.34646	0.97875	-0.031	0.942136	1
ZFYVE26	1.101235	1.106431	1.099547	0.93531	-0.0965	0.942142	1

ZNF304	1.059652	1.064899	1.057947	0.89288	-0.1635	0.942148	1
BANK1	1.038971	1.032859	1.040958	1.24645	0.3178	0.942173	1
RAB20	1.120932	1.126453	1.119137	0.94214	-0.086	0.942197	1
NRF1	1.076829	1.071049	1.078708	1.10781	0.1477	0.942199	1
LINC01583	1.019308	1.024507	1.017618	0.71891	-0.4761	0.942229	1
PAEP	1.019329	1.024592	1.017618	0.71643	-0.4811	0.942229	1
RP11-335O4.3	1.019328	1.024589	1.017618	0.71652	-0.4809	0.942229	1
SLC22A3	1.019311	1.024516	1.017618	0.71864	-0.4767	0.942229	1
LINC01040	1.019345	1.024655	1.017618	0.71461	-0.4848	0.942229	1
SLC4A10	1.019345	1.024655	1.017618	0.71461	-0.4848	0.942229	1
UCN2	1.019326	1.024577	1.017618	0.71686	-0.4802	0.942229	1
DGKK	1.019334	1.024613	1.017618	0.71582	-0.4823	0.942229	1
CTC-232P5.1	1.019333	1.024608	1.017618	0.71596	-0.4821	0.942229	1
SP140	1.019344	1.024653	1.017618	0.71465	-0.4847	0.942229	1
RP11-325L12.6	1.019344	1.024653	1.017618	0.71465	-0.4847	0.942229	1
SLC26A8	1.019324	1.024572	1.017618	0.71701	-0.4799	0.942229	1
KDELC1	1.223244	1.216838	1.225326	1.03914	0.0554	0.942247	1
RDH13	1.140766	1.134538	1.142791	1.06134	0.0859	0.942306	1
NARS2	1.424374	1.429979	1.422553	0.98273	-0.0251	0.942384	1
FAM134C	1.318969	1.324157	1.317283	0.9788	-0.0309	0.942505	1
CCDC65	1.028511	1.022684	1.030404	1.34032	0.4226	0.94254	1
PIWIL4	1.028601	1.022675	1.030527	1.34628	0.429	0.94254	1
TESK2	1.05501	1.049107	1.056929	1.15928	0.2132	0.942582	1
CTB-50L17.16	1.0344	1.028479	1.036324	1.27545	0.351	0.942645	1
RP11-661A12.5	1.034373	1.028467	1.036293	1.27492	0.3504	0.942645	1
GRIK2	1.034354	1.02849	1.03626	1.27275	0.348	0.942645	1
BTBD11	1.087523	1.092728	1.085831	0.92562	-0.1115	0.942671	1
RP11-902B17.1	1.017998	1.012186	1.019886	1.63187	0.7065	0.942672	1
CTA-243E7.4	1.017992	1.012186	1.019879	1.63121	0.7059	0.942672	1
ADAMTSL2	1.017962	1.012186	1.01984	1.62801	0.7031	0.942672	1
CTC-523E23.4	1.017975	1.012186	1.019857	1.62941	0.7044	0.942672	1
EXD1	1.017967	1.012186	1.019846	1.62855	0.7036	0.942672	1
PTK6	1.017997	1.012186	1.019886	1.63182	0.7065	0.942672	1
RP4-758J18.10	1.017983	1.012186	1.019868	1.63033	0.7052	0.942672	1
SLC25A6	15.01282	14.91266	15.04538	1.00954	0.0137	0.942776	1
RP11-147L13.15	1.139504	1.144793	1.137784	0.9516	-0.0716	0.942817	1
PCDHB5	1.054068	1.048121	1.056001	1.16376	0.2188	0.942826	1
RASAL3	1.039619	1.033725	1.041534	1.23154	0.3005	0.942861	1
NR5A2	1.015542	1.020709	1.013862	0.66935	-0.5792	0.942927	1
AC067959.1	1.015537	1.020692	1.013862	0.6699	-0.578	0.942927	1
CD200R1	1.015553	1.020756	1.013862	0.66785	-0.5824	0.942927	1
COL21A1	1.015546	1.020726	1.013862	0.66881	-0.5803	0.942927	1
LYZL4	1.015543	1.020717	1.013862	0.66911	-0.5797	0.942927	1
HYI-AS1	1.01554	1.020705	1.013862	0.66949	-0.5789	0.942927	1
U47924.27	1.015546	1.020729	1.013862	0.6687	-0.5806	0.942927	1

RASGEF1C	1.02067	1.014881	1.022552	1.51549	0.5998	0.94296	1
CHRNB2	1.020654	1.014881	1.02253	1.51399	0.5984	0.94296	1
RP11-185E8.1	1.020659	1.014881	1.022537	1.51443	0.5988	0.94296	1
RP11-93K22.6	1.020664	1.014881	1.022543	1.51489	0.5992	0.94296	1
RP4-728D4.2	1.020686	1.014881	1.022573	1.51689	0.6011	0.94296	1
LGALS7	1.020696	1.014881	1.022586	1.51776	0.6019	0.94296	1
ACSL5	1.020633	1.014881	1.022502	1.51214	0.5966	0.94296	1
ARL6	1.096538	1.090571	1.098477	1.08729	0.1207	0.942973	1
ERC1	1.383328	1.376548	1.385532	1.02386	0.034	0.943092	1
ITPR1-AS1	1.032386	1.026542	1.034286	1.29179	0.3694	0.943131	1
CDH15	1.027792	1.02183	1.02973	1.36187	0.4456	0.94316	1
TRANK1	1.145266	1.138902	1.147334	1.06071	0.085	0.943313	1
RP11-251G23.5	1.017105	1.02225	1.015432	0.69361	-0.5278	0.943338	1
CCL25	1.017097	1.022243	1.015425	0.69346	-0.5281	0.943338	1
ARL11	1.017099	1.022247	1.015425	0.69335	-0.5283	0.943338	1
ENTPD2	1.017098	1.022246	1.015425	0.69336	-0.5283	0.943338	1
AMDHD1	1.0171	1.022278	1.015417	0.69204	-0.5311	0.943338	1
RP11-57A19.7	1.017087	1.022197	1.015426	0.69498	-0.525	0.943338	1
CA5A	1.017102	1.022233	1.015434	0.69418	-0.5266	0.943338	1
METTL24	1.017096	1.022242	1.015424	0.69345	-0.5281	0.943338	1
IL20RB	1.017089	1.02225	1.015411	0.69261	-0.5299	0.943338	1
PABPC3	1.040661	1.035022	1.042494	1.21333	0.279	0.943501	1
MRPL43	2.771612	2.757669	2.776144	1.01051	0.0151	0.943567	1
CHST12	1.419739	1.424921	1.418055	0.98384	-0.0235	0.943612	1
TNFSF4	1.031621	1.036772	1.029946	0.81437	-0.2962	0.943656	1
CCDC114	1.037841	1.032029	1.03973	1.24046	0.3109	0.9437	1
CACNG4	1.126125	1.131477	1.124386	0.94607	-0.08	0.94373	1
CASC18	1.021488	1.015691	1.023372	1.48952	0.5748	0.943744	1
RP11-485G7.5	1.021416	1.015675	1.023282	1.48523	0.5707	0.943744	1
RP11-476H16.1	1.021447	1.015681	1.023322	1.4873	0.5727	0.943744	1
PXK	1.188797	1.182879	1.19072	1.04288	0.0606	0.943846	1
FAM193B	1.114038	1.119409	1.112292	0.94039	-0.0887	0.943934	1
SRP14-AS1	1.190037	1.183988	1.192004	1.04357	0.0615	0.944003	1
VWF	1.02409	1.018416	1.025934	1.40826	0.4939	0.944033	1
MAT1A	1.024118	1.018394	1.025979	1.41234	0.4981	0.944033	1
SLC38A9	1.168008	1.173523	1.166215	0.95788	-0.0621	0.944069	1
AP000704.5	1.048458	1.053749	1.046739	0.86957	-0.2016	0.944103	1
NUP205	1.187798	1.182158	1.189632	1.04103	0.058	0.944106	1
RGPD5	1.091461	1.096549	1.089807	0.93017	-0.1044	0.944146	1
C1orf168	1.029076	1.034153	1.027425	0.803	-0.3165	0.944148	1
RP11-315I20.1	1.032245	1.026509	1.034109	1.28669	0.3637	0.944183	1
RP11-57H12.3	1.032274	1.026554	1.034134	1.28544	0.3623	0.944183	1
HRK	1.032261	1.026499	1.034133	1.28809	0.3652	0.944183	1
VMO1	1.035799	1.030017	1.037678	1.25522	0.3279	0.944188	1
PGM5-AS1	1.017597	1.011871	1.019459	1.63921	0.713	0.944191	1

RP1-30M3.6	1.017599	1.011871	1.019461	1.63943	0.7132	0.944191	1
CTB-171A8.1	1.017542	1.011871	1.019385	1.63299	0.7075	0.944191	1
RP11-178C3.2	1.017561	1.011871	1.01941	1.63511	0.7094	0.944191	1
CTB-46B19.2	1.017561	1.011871	1.01941	1.63509	0.7094	0.944191	1
CMTM1	1.017551	1.011871	1.019398	1.63407	0.7085	0.944191	1
POMK	1.031129	1.02538	1.032997	1.30013	0.3787	0.944222	1
RP11-326A19.3	1.013659	1.018754	1.012002	0.63999	-0.6439	0.94424	1
ZAP70	1.013656	1.018742	1.012002	0.64041	-0.6429	0.94424	1
CILP	1.013653	1.01873	1.012002	0.6408	-0.6421	0.94424	1
KRT72	1.013648	1.018711	1.012002	0.64148	-0.6405	0.94424	1
ZPLD1	1.013654	1.018735	1.012002	0.64065	-0.6424	0.94424	1
CD3E	1.013665	1.018781	1.012002	0.63906	-0.646	0.94424	1
OR51E2	1.013663	1.01877	1.012002	0.63945	-0.6451	0.94424	1
LINC01117	1.013667	1.018787	1.012002	0.63886	-0.6464	0.94424	1
RP11-5C23.1	1.013648	1.018711	1.012002	0.64146	-0.6406	0.94424	1
GRM5-AS1	1.013655	1.01874	1.012002	0.64046	-0.6428	0.94424	1
POU3F4	1.029113	1.034085	1.027497	0.80672	-0.3099	0.944259	1
GLTSCR1	1.083956	1.089204	1.082251	0.92205	-0.1171	0.944291	1
NRTN	1.045492	1.039803	1.047341	1.18939	0.2502	0.944333	1
ZNF777	1.045538	1.039729	1.047427	1.19375	0.2555	0.944333	1
ENPP5	1.102383	1.096283	1.104366	1.08396	0.1163	0.944384	1
C11orf98	1.067118	1.061281	1.069015	1.12622	0.1715	0.944456	1
RP11-96B5.3	1.029989	1.035038	1.028347	0.80903	-0.3057	0.944501	1
RP11-294N21.3	1.016956	1.011304	1.018793	1.66249	0.7333	0.944662	1
CTD-2203K17.1	1.016956	1.011304	1.018793	1.66244	0.7333	0.944662	1
RP11-276M12.1	1.016982	1.011304	1.018827	1.66546	0.7359	0.944662	1
RP11-723O4.9	1.016961	1.011304	1.0188	1.6631	0.7339	0.944662	1
XXyac-YX155B6.7	1.016955	1.011304	1.018792	1.66241	0.7333	0.944662	1
RP1-76B20.11	1.016981	1.011304	1.018826	1.66537	0.7358	0.944662	1
RP11-770J1.4	1.016956	1.011304	1.018793	1.66249	0.7333	0.944662	1
ANKRD27	1.145745	1.139923	1.147638	1.05513	0.0774	0.944776	1
RP11-778D9.13	1.025758	1.030864	1.024098	0.78079	-0.357	0.944786	1
TEX22	1.062792	1.067998	1.061099	0.89854	-0.1543	0.944802	1
RP1-92O14.3	1.024884	1.019182	1.026738	1.39385	0.4791	0.944813	1
CTB-113D17.1	1.024916	1.019177	1.026782	1.39659	0.4819	0.944813	1
SMIM2-AS1	1.024832	1.019166	1.026674	1.39169	0.4768	0.944813	1
RP4-633O19__A.1	1.017839	1.012187	1.019676	1.61456	0.6911	0.944925	1
POU3F2	1.017816	1.012187	1.019646	1.61211	0.6889	0.944925	1
RP11-256L6.5	1.017808	1.012187	1.019635	1.61121	0.6881	0.944925	1
CTD-2013N17.7	1.017834	1.012187	1.019669	1.61399	0.6906	0.944925	1
SLC7A3	1.017818	1.012187	1.019649	1.61232	0.6891	0.944925	1
RP11-524O1.4	1.017817	1.012187	1.019648	1.61221	0.689	0.944925	1
RP11-46H11.12	1.046577	1.040792	1.048458	1.18793	0.2484	0.944949	1
SSSCA1	1.516067	1.509489	1.518206	1.01711	0.0245	0.944963	1
IRX6	1.019671	1.013947	1.021532	1.54389	0.6266	0.944964	1

CTB-193M12.1	1.019599	1.013947	1.021436	1.53698	0.6201	0.944964	1
RP11-713D19.1	1.019584	1.013947	1.021417	1.53563	0.6188	0.944964	1
KRTAP10-2	1.01968	1.013947	1.021544	1.54476	0.6274	0.944964	1
RP11-875O11.2	1.019606	1.013947	1.021446	1.53773	0.6208	0.944964	1
RP11-471M2.3	1.019617	1.013947	1.02146	1.53871	0.6217	0.944964	1
PARD6G-AS1	1.019613	1.013947	1.021454	1.53833	0.6214	0.944964	1
DHX36	2.493803	2.50129	2.491369	0.99339	-0.0096	0.945008	1
XYLB	1.046123	1.051256	1.044454	0.86729	-0.2054	0.945009	1
CTC-503J8.4	1.035309	1.040503	1.03362	0.83007	-0.2687	0.945048	1
TRAF6	1.112853	1.106973	1.114765	1.07285	0.1014	0.945114	1
RP11-328P23.4	1.021464	1.015826	1.023297	1.47205	0.5578	0.945142	1
ZBTB20-AS4	1.02148	1.015826	1.023318	1.47339	0.5591	0.945142	1
HPSE	1.021512	1.015826	1.02336	1.47602	0.5617	0.945142	1
CDC42BPA	1.615558	1.620388	1.613988	0.98968	-0.015	0.945145	1
CFB	1.025604	1.030602	1.02398	0.78359	-0.3518	0.945147	1
KLHL4	1.025588	1.030594	1.023961	0.7832	-0.3526	0.945147	1
RP11-408B11.2	1.025674	1.030857	1.02399	0.77746	-0.3632	0.945147	1
ADAMTS15	1.018696	1.013073	1.020524	1.56998	0.6507	0.945204	1
COL28A1	1.01871	1.013073	1.020542	1.5714	0.6521	0.945204	1
STEAP4	1.018707	1.013073	1.020539	1.57113	0.6518	0.945204	1
FOCAD-AS1	1.018697	1.013073	1.020526	1.57015	0.6509	0.945204	1
RP1-122K4.2	1.017483	1.022595	1.015821	0.7002	-0.5142	0.945205	1
CTD-3194G12.2	1.017493	1.022634	1.015821	0.69899	-0.5167	0.945205	1
ADGRG2	1.017451	1.022467	1.015821	0.70419	-0.506	0.945205	1
LINC01280	1.028522	1.033655	1.026853	0.7979	-0.3257	0.94521	1
CCDC60	1.039265	1.033579	1.041113	1.22438	0.292	0.945241	1
SLCO6A1	1.035758	1.030057	1.037611	1.25134	0.3235	0.945242	1
ONECUT3	1.021037	1.015359	1.022883	1.48991	0.5752	0.945266	1
EBF1	1.048128	1.042146	1.050073	1.18807	0.2486	0.945293	1
PCDHGA5	1.018733	1.013102	1.020563	1.56952	0.6503	0.945315	1
FAM90A1	1.018727	1.013102	1.020556	1.56898	0.6498	0.945315	1
HIST1H2AL	1.018702	1.013102	1.020523	1.56645	0.6475	0.945315	1
EEFSEC	1.183074	1.177182	1.184989	1.04406	0.0622	0.945338	1
MIRLET7BHG	1.02651	1.031565	1.024867	0.78781	-0.3441	0.945389	1
GARNL3	1.058356	1.06336	1.056729	0.89535	-0.1595	0.945423	1
UBQLN4	1.234792	1.240001	1.233098	0.97124	-0.0421	0.945455	1
ASAP3	1.131955	1.125894	1.133925	1.06379	0.0892	0.945484	1
RP11-382A20.7	1.01889	1.013274	1.020715	1.56057	0.6421	0.945564	1
RP11-78O7.2	1.018871	1.013274	1.02069	1.55865	0.6403	0.945564	1
RP11-182J1.5	1.018886	1.013274	1.020711	1.56023	0.6418	0.945564	1
RP11-61E11.2	1.018876	1.013274	1.020696	1.55914	0.6408	0.945564	1
CDX4	1.018865	1.013274	1.020683	1.55814	0.6398	0.945564	1
PRDM13	1.018896	1.013274	1.020723	1.56114	0.6426	0.945564	1
RP11-680A11.5	1.045875	1.050847	1.044259	0.87043	-0.2002	0.945595	1
ALPK1	1.168987	1.162887	1.17097	1.04963	0.0699	0.945604	1

ZNF879	1.080963	1.075294	1.082806	1.09977	0.1372	0.94563	1
RNF181	3.081671	3.071872	3.084857	1.00627	0.009	0.945788	1
ME1	1.073469	1.067959	1.075261	1.10745	0.1472	0.945795	1
LENG9	1.094218	1.099413	1.09253	0.93076	-0.1035	0.945853	1
RP11-736N17.11	1.028381	1.022742	1.030214	1.3286	0.4099	0.945879	1
ERLIN2	1.334442	1.328547	1.336359	1.02378	0.0339	0.94591	1
RP11-266L9.5	1.031785	1.036824	1.030147	0.81867	-0.2886	0.945926	1
LINC00592	1.022148	1.027116	1.020533	0.75723	-0.4012	0.945927	1
UBE2V1	1.168964	1.174306	1.167227	0.95939	-0.0598	0.945952	1
MCF2	1.021308	1.015709	1.023128	1.47227	0.558	0.946001	1
RP11-245G13.2	1.021276	1.015698	1.023089	1.47078	0.5566	0.946001	1
CACNA1E	1.023062	1.017468	1.02488	1.4243	0.5103	0.946041	1
UCP3	1.024953	1.019357	1.026772	1.38304	0.4678	0.946219	1
C5orf47	1.024941	1.019315	1.02677	1.38594	0.4709	0.946219	1
C2orf50	1.02495	1.019342	1.026773	1.38419	0.469	0.946219	1
RP11-697E2.6	1.024918	1.019333	1.026734	1.38287	0.4677	0.946219	1
RANBP3	1.307202	1.312728	1.305406	0.97659	-0.0342	0.94623	1
ZNF484	1.052615	1.047041	1.054427	1.15701	0.2104	0.94624	1
PEPD	1.61733	1.611019	1.619382	1.01369	0.0196	0.946244	1
LINC01220	1.023061	1.028053	1.021438	0.76422	-0.3879	0.946279	1
SNHG22	1.022157	1.016579	1.023971	1.44582	0.5319	0.946282	1
FRRS1	1.038081	1.032435	1.039916	1.23063	0.2994	0.946336	1
C1orf143	1.038031	1.032321	1.039886	1.23406	0.3034	0.946336	1
ZNF823	1.064636	1.059028	1.066459	1.12588	0.1711	0.946367	1
RP11-102G14.1	1.022179	1.016621	1.023986	1.44313	0.5292	0.946392	1
RP11-2E11.6	1.03101	1.035976	1.029395	0.81708	-0.2914	0.946517	1
C11orf96	1.020399	1.025376	1.018782	0.74014	-0.4341	0.946581	1
CTD-2291D10.4	1.020416	1.025416	1.018791	0.73932	-0.4357	0.946581	1
ZNF865	1.093143	1.08749	1.094981	1.08563	0.1185	0.946638	1
ZNF775	1.110218	1.104471	1.112086	1.07289	0.1015	0.946737	1
ZNF341	1.039114	1.044119	1.037487	0.84967	-0.235	0.946751	1
PPP2R5C	1.960167	1.953379	1.962373	1.00943	0.0135	0.946803	1
RNF217-AS1	1.023844	1.018291	1.025649	1.40227	0.4878	0.946811	1
RP13-753N3.3	1.023852	1.018339	1.025644	1.39839	0.4838	0.946811	1
GPR83	1.0187	1.023622	1.017101	0.72395	-0.466	0.946821	1
RP11-45M22.5	1.018714	1.023643	1.017111	0.72373	-0.4665	0.946821	1
TTC36	1.018717	1.023661	1.01711	0.72315	-0.4676	0.946821	1
RP11-64C12.8	1.018684	1.023568	1.017096	0.72537	-0.4632	0.946932	1
NDOR1	1.018685	1.023592	1.017091	0.72444	-0.4651	0.946932	1
RP11-472I20.3	1.018699	1.023622	1.017098	0.72383	-0.4663	0.946932	1
RP11-778D9.12	1.031796	1.026214	1.03361	1.28212	0.3585	0.94694	1
OSBPL10-AS1	1.021458	1.026368	1.019862	0.75325	-0.4088	0.946994	1
C20orf166-AS1	1.021019	1.025938	1.01942	0.74871	-0.4175	0.947054	1
LINC00867	1.02099	1.025932	1.019384	0.7475	-0.4199	0.947054	1
RBPMS-AS1	1.024739	1.019195	1.026542	1.38274	0.4675	0.947074	1

RP11-756H6.1	1.026567	1.021035	1.028365	1.34844	0.4313	0.947114	1
TMIE	1.026476	1.020957	1.02827	1.34896	0.4318	0.947114	1
ZDHHC8	1.176345	1.1814	1.174702	0.96308	-0.0543	0.947145	1
VTA1	1.351476	1.345706	1.353352	1.02212	0.0316	0.947169	1
INHBE	1.019577	1.024466	1.017988	0.73522	-0.4438	0.947172	1
SCGN	1.019594	1.024456	1.018013	0.73656	-0.4411	0.947172	1
RP11-582J16.4	1.019586	1.024485	1.017993	0.73487	-0.4444	0.947172	1
CABLES1	1.042095	1.036616	1.043877	1.19831	0.261	0.94718	1
RP11-403P17.4	1.017832	1.022788	1.016221	0.71184	-0.4904	0.947212	1
SYK	1.017827	1.022726	1.016234	0.71436	-0.4853	0.947212	1
CTD-2341M24.1	1.017813	1.022704	1.016223	0.71455	-0.4849	0.947212	1
SPIN2B	1.223428	1.217811	1.225253	1.03417	0.0485	0.947228	1
FXYD7	1.034467	1.028936	1.036265	1.25327	0.3257	0.947231	1
RNF14	1.312482	1.306158	1.314538	1.02737	0.039	0.94728	1
RP11-316O14.1	1.028401	1.022849	1.030205	1.32196	0.4027	0.947293	1
MR1	1.025607	1.020069	1.027407	1.36562	0.4496	0.947355	1
ZNF540	1.027623	1.032505	1.026036	0.80097	-0.3202	0.947401	1
RP11-262H14.3	1.027567	1.032459	1.025977	0.80031	-0.3214	0.947401	1
CTD-2012K14.7	1.027862	1.022369	1.029647	1.32532	0.4063	0.947406	1
AC005537.2	1.027907	1.022411	1.029693	1.32497	0.406	0.947406	1
ZXDC	1.09997	1.10504	1.098322	0.93605	-0.0953	0.94744	1
PTPRC	1.015419	1.020282	1.013838	0.68228	-0.5516	0.947469	1
RP11-622C24.2	1.015429	1.020326	1.013838	0.68081	-0.5547	0.947469	1
TMPRSS11E	1.015418	1.020279	1.013838	0.68238	-0.5514	0.947469	1
GJC3	1.015441	1.020371	1.013838	0.67929	-0.5579	0.947469	1
RP11-84D1.2	1.015417	1.020276	1.013838	0.68248	-0.5511	0.947469	1
ALOXE3	1.01542	1.020286	1.013838	0.68215	-0.5518	0.947469	1
AC013463.2	1.015423	1.020301	1.013838	0.68164	-0.5529	0.947469	1
SERPINA3	1.015425	1.020308	1.013838	0.68141	-0.5534	0.947469	1
CADM3-AS1	1.015424	1.020302	1.013838	0.6816	-0.553	0.947469	1
ITGB3	1.015411	1.020252	1.013838	0.68329	-0.5494	0.947469	1
ACTBL2	1.015411	1.02025	1.013838	0.68336	-0.5493	0.947469	1
IFNL2	1.015442	1.020377	1.013838	0.67908	-0.5584	0.947469	1
RP11-90C1.1	1.01543	1.020327	1.013838	0.68075	-0.5548	0.947469	1
MSTN	1.016984	1.021938	1.015374	0.70078	-0.513	0.947474	1
PRTN3	1.069712	1.063891	1.071605	1.12072	0.1644	0.947541	1
COG8.1	1.100728	1.105622	1.099137	0.9386	-0.0914	0.947597	1
AC005363.11	1.025861	1.02027	1.027679	1.36553	0.4495	0.947716	1
AJ006998.2	1.025793	1.02035	1.027562	1.35443	0.4377	0.947716	1
RTP4	1.015272	1.020147	1.013687	0.67937	-0.5577	0.947718	1
MICALCL	1.015261	1.020102	1.013687	0.68087	-0.5546	0.947718	1
CMKLR1	1.015257	1.020088	1.013687	0.68135	-0.5535	0.947718	1
HOXA1	1.015264	1.020114	1.013687	0.68046	-0.5554	0.947718	1
WNT11	1.015264	1.020114	1.013687	0.68046	-0.5554	0.947718	1
TCF4-AS2	1.015271	1.020144	1.013687	0.67947	-0.5575	0.947718	1

RGS9BP	1.015264	1.020117	1.013687	0.68038	-0.5556	0.947718	1
SSX5	1.015264	1.020115	1.013687	0.68045	-0.5554	0.947718	1
RP11-141J13.5	1.015266	1.020124	1.013687	0.68014	-0.5561	0.947718	1
CTD-2334D19.1	1.015276	1.020165	1.013687	0.67876	-0.559	0.947718	1
RP11-357N13.3	1.015276	1.020165	1.013687	0.67876	-0.559	0.947718	1
PPP1R26	1.180266	1.17438	1.182179	1.04473	0.0631	0.947731	1
RP11-319E12.1	1.015242	1.02008	1.01367	0.68075	-0.5548	0.947828	1
AC243756.1	1.015255	1.020133	1.01367	0.67897	-0.5586	0.947828	1
CARD16	1.015246	1.020094	1.01367	0.68027	-0.5558	0.947828	1
PRND	1.015255	1.020132	1.01367	0.67899	-0.5585	0.947828	1
RP11-946L16.1	1.015242	1.020078	1.01367	0.68081	-0.5547	0.947828	1
RFPL1	1.01525	1.020113	1.01367	0.67964	-0.5571	0.947828	1
INS	1.01525	1.020113	1.01367	0.67964	-0.5571	0.947828	1
RP11-268P4.4	1.015263	1.020166	1.01367	0.67785	-0.561	0.947828	1
P2RX7	1.015259	1.02015	1.01367	0.67839	-0.5598	0.947828	1
RP11-307P5.1	1.015246	1.020095	1.01367	0.68026	-0.5558	0.947828	1
GTSE1-AS1	1.020914	1.015453	1.022689	1.46821	0.5541	0.94783	1
RP13-131K19.6	1.02093	1.015453	1.022711	1.46961	0.5554	0.94783	1
GLIPR1L2	1.02095	1.015453	1.022737	1.47129	0.5571	0.94783	1
RP11-370I10.6	1.021018	1.015453	1.022827	1.47713	0.5628	0.94783	1
SIT1	1.020942	1.015453	1.022726	1.47063	0.5564	0.94783	1
GALP	1.020901	1.015453	1.022671	1.46706	0.5529	0.94783	1
RP11-689P11.2	1.020988	1.015453	1.022787	1.47454	0.5603	0.94783	1
RP1-267L14.6	1.02091	1.015453	1.022684	1.46789	0.5537	0.94783	1
RP11-582E3.6	1.113662	1.108043	1.115489	1.06892	0.0961	0.947878	1
ZSWIM3	1.051876	1.046169	1.053731	1.16379	0.2188	0.947888	1
PHOSPHO1	1.018003	1.022855	1.016426	0.7187	-0.4765	0.94789	1
RP11-758P17.3	1.018008	1.022875	1.016426	0.71809	-0.4778	0.94789	1
AP001626.1	1.018005	1.022863	1.016426	0.71846	-0.477	0.94789	1
AC007193.9	1.018015	1.022904	1.016426	0.71716	-0.4796	0.94789	1
AJ239322.1	1.017994	1.022819	1.016426	0.71985	-0.4742	0.94789	1
KRTAP2-3	1.017996	1.022826	1.016426	0.71963	-0.4747	0.94789	1
TMEM78	1.018016	1.022907	1.016426	0.71709	-0.4798	0.94789	1
CATSPERD	1.017998	1.022833	1.016426	0.7194	-0.4751	0.94789	1
SERPINE3	1.018004	1.02286	1.016426	0.71857	-0.4768	0.94789	1
SERPINB2	1.018021	1.022927	1.016426	0.71647	-0.481	0.94789	1
CASP10	1.018002	1.02285	1.016426	0.71888	-0.4762	0.94789	1
HAR1B	1.018027	1.02295	1.016426	0.71573	-0.4825	0.94789	1
AC093620.5	1.018013	1.022894	1.016426	0.7175	-0.479	0.94789	1
RGAG1	1.018013	1.022894	1.016426	0.7175	-0.479	0.94789	1
MISP	1.017544	1.022336	1.015986	0.71571	-0.4825	0.947946	1
SMPD3	1.017543	1.022384	1.015969	0.71339	-0.4872	0.947946	1
TLL2	1.017565	1.02243	1.015983	0.71258	-0.4889	0.947946	1
TTLL7	1.58604	1.579003	1.588327	1.01611	0.023	0.94797	1
CTA-363E6.7	1.016144	1.021002	1.014564	0.69348	-0.5281	0.948069	1

ZIM2-AS1	1.016144	1.021002	1.014564	0.69348	-0.5281	0.948069	1
RP11-840I19.5	1.016137	1.020974	1.014564	0.69442	-0.5261	0.948069	1
CKMT2	1.016137	1.020976	1.014564	0.69435	-0.5263	0.948069	1
CTAGE1	1.016137	1.020975	1.014564	0.69437	-0.5262	0.948069	1
LINC00494	1.016158	1.021059	1.014564	0.6916	-0.532	0.948069	1
PCDHA8	1.016142	1.020997	1.014564	0.69364	-0.5277	0.948069	1
AF067845.2	1.016142	1.020997	1.014564	0.69364	-0.5277	0.948069	1
IFNL1	1.016141	1.02099	1.014564	0.69386	-0.5273	0.948069	1
ATP8A2	1.064528	1.069278	1.062984	0.90914	-0.1374	0.948108	1
CIRBP-AS1	1.014371	1.019185	1.012806	0.66751	-0.5831	0.948108	1
AC104667.3	1.01438	1.019224	1.012806	0.66614	-0.5861	0.948108	1
KLKB1	1.014378	1.019214	1.012806	0.6665	-0.5853	0.948108	1
FAM83E	1.014383	1.019235	1.012806	0.66574	-0.587	0.948108	1
AC006273.5	1.014369	1.019176	1.012806	0.6678	-0.5825	0.948108	1
RP11-34P13.8	1.01439	1.019264	1.012806	0.66476	-0.5891	0.948108	1
RCVRN	1.028168	1.022708	1.029943	1.31862	0.399	0.948144	1
SLC26A1	1.029954	1.024467	1.031738	1.29717	0.3754	0.948183	1
RP11-500C11.3	1.045578	1.040187	1.04733	1.17774	0.236	0.948223	1
RP11-17M16.2	1.037929	1.032434	1.039716	1.22452	0.2922	0.948289	1
GSK3B	1.635359	1.628541	1.637575	1.01437	0.0206	0.948338	1
RP11-967K21.1	1.031884	1.026446	1.033652	1.27248	0.3476	0.948362	1
RP11-679C8.2	1.031861	1.026414	1.033632	1.27325	0.3485	0.948362	1
RP5-1096D14.6	1.03184	1.02632	1.033634	1.27788	0.3537	0.948362	1
DDX4	1.013518	1.018352	1.011946	0.65096	-0.6194	0.94837	1
ATP13A5	1.01352	1.018361	1.011946	0.65062	-0.6201	0.94837	1
APBB1IP	1.013505	1.018299	1.011946	0.65284	-0.6152	0.94837	1
MSC	1.013503	1.018293	1.011946	0.65304	-0.6147	0.94837	1
TNFSF15	1.013503	1.018291	1.011946	0.6531	-0.6146	0.94837	1
TBXA2R	1.013508	1.018312	1.011946	0.65236	-0.6162	0.94837	1
TM4SF19	1.013501	1.018285	1.011946	0.65334	-0.6141	0.94837	1
RP4-585I14.3	1.013524	1.01838	1.011946	0.64996	-0.6216	0.94837	1
S100Z	1.013504	1.018298	1.011946	0.65287	-0.6151	0.94837	1
PLEK2	1.013512	1.018329	1.011946	0.65178	-0.6175	0.94837	1
CLRN3	1.013502	1.01829	1.011946	0.65314	-0.6145	0.94837	1
MRM1	1.049045	1.053912	1.047463	0.88038	-0.1838	0.948379	1
RP11-347I19.7	1.029075	1.023657	1.030836	1.30343	0.3823	0.948425	1
TRAF1	1.044964	1.039482	1.046747	1.18401	0.2437	0.948436	1
CRELD1	1.861546	1.866309	1.859998	0.99272	-0.0105	0.948484	1
LRRC23	1.574776	1.579879	1.573118	0.98834	-0.0169	0.948516	1
SLC39A1	2.791498	2.782377	2.794464	1.00678	0.0098	0.948523	1
RP4-635E18.7	1.029147	1.023619	1.030944	1.31015	0.3897	0.948536	1
RP11-216B9.6	1.029018	1.023601	1.030779	1.30415	0.3831	0.948536	1
ATP9A	1.179195	1.173073	1.181185	1.04687	0.0661	0.948541	1
DIAPH1	1.348647	1.353844	1.346958	0.98054	-0.0284	0.948564	1
PARS2	1.119972	1.114432	1.121773	1.06415	0.0897	0.94872	1

RP11-545M17.1	1.017118	1.011717	1.018874	1.61079	0.6878	0.948796	1
RP11-286E11.1	1.017099	1.011717	1.018849	1.60863	0.6858	0.948796	1
CTD-2035E11.3	1.017108	1.011717	1.018861	1.60963	0.6867	0.948796	1
DLG3-AS1	1.017115	1.011717	1.018869	1.6104	0.6874	0.948796	1
RP11-755F10.1	1.017111	1.011717	1.018864	1.60994	0.687	0.948796	1
NFAM1	1.017116	1.011717	1.018871	1.61051	0.6875	0.948796	1
AC007966.1	1.017083	1.011717	1.018827	1.60681	0.6842	0.948796	1
RP11-503L19.1	1.017148	1.011717	1.018913	1.61409	0.6907	0.948796	1
FAM87A	1.014112	1.018898	1.012556	0.66442	-0.5898	0.948841	1
CTD-2358C21.5	1.014121	1.018936	1.012556	0.66309	-0.5927	0.948841	1
AC108004.3	1.014112	1.018897	1.012556	0.66445	-0.5898	0.948841	1
GNAI1	1.328633	1.322691	1.330565	1.0244	0.0348	0.9489	1
RP5-892K4.1	1.024392	1.018976	1.026153	1.37822	0.4628	0.948912	1
VSIG8	1.024447	1.019011	1.026214	1.37888	0.4635	0.948912	1
RP11-455J20.3	1.024384	1.018945	1.026152	1.38041	0.4651	0.948912	1
NSG1	1.1059	1.111097	1.10421	0.93801	-0.0923	0.948935	1
NAPA-AS1	1.077023	1.071566	1.078797	1.10103	0.1388	0.949138	1
FBXL19	1.189731	1.184191	1.191532	1.03985	0.0564	0.949159	1
LINC01372	1.020667	1.025441	1.019115	0.75132	-0.4125	0.949178	1
ZNF668	1.134355	1.13922	1.132774	0.9537	-0.0684	0.949234	1
CPTP	1.270663	1.264522	1.272659	1.03076	0.0437	0.949267	1
NELFB	1.371016	1.376115	1.369358	0.98204	-0.0262	0.94929	1
PARG	1.293177	1.28744	1.295041	1.02645	0.0377	0.949339	1
CTNNA3	1.027685	1.032339	1.026172	0.80931	-0.3052	0.949346	1
RP11-632L2.2	1.052925	1.05766	1.051386	0.8912	-0.1662	0.94935	1
WDR20	1.251758	1.245784	1.2537	1.03221	0.0457	0.949351	1
NFATC2IP	1.469139	1.463404	1.471003	1.0164	0.0235	0.949366	1
C9orf106	1.028858	1.03358	1.027323	0.81368	-0.2975	0.949385	1
RP5-907D15.4	1.028738	1.033494	1.027192	0.81185	-0.3007	0.949385	1
ZSCAN9	1.137947	1.132499	1.139717	1.05448	0.0765	0.949396	1
MAP3K5	1.017977	1.022757	1.016423	0.72169	-0.4705	0.949467	1
DOK3	1.017966	1.022702	1.016426	0.72357	-0.4668	0.949467	1
C15orf62	1.017975	1.02272	1.016433	0.7233	-0.4673	0.949467	1
CIART	1.204125	1.208999	1.202541	0.9691	-0.0453	0.949557	1
CTB-32O4.2	1.032534	1.027164	1.034279	1.26195	0.3357	0.949603	1
PHKA1	1.10887	1.103147	1.11073	1.07351	0.1023	0.949629	1
BATF2	1.032643	1.027263	1.034392	1.26148	0.3351	0.949854	1
RN7SL832P	1.032643	1.027283	1.034386	1.26034	0.3338	0.949854	1
KARS	1.991897	1.985366	1.99402	1.00878	0.0126	0.949994	1
JOSD2	1.707189	1.712639	1.705418	0.98987	-0.0147	0.950005	1
ZNFX1	1.119723	1.124529	1.11816	0.94886	-0.0757	0.950006	1
MED21	1.515847	1.509349	1.51796	1.01691	0.0242	0.950008	1
RP11-1081M5.2	1.020611	1.01526	1.02235	1.46465	0.5506	0.950018	1
LUADT1	1.020629	1.01526	1.022374	1.46622	0.5521	0.950018	1
RP11-863K10.2	1.020596	1.01526	1.022331	1.46341	0.5493	0.950018	1

OAS2	1.017218	1.021975	1.015672	0.71317	-0.4877	0.950071	1
LINC00309	1.017202	1.021909	1.015672	0.71533	-0.4833	0.950071	1
GJB5	1.017194	1.021877	1.015672	0.71637	-0.4812	0.950071	1
SOWAHA	1.017204	1.021919	1.015672	0.71499	-0.484	0.950071	1
RP11-96K19.2	1.017203	1.021912	1.015672	0.7152	-0.4836	0.950071	1
RP11-392P7.6	1.017196	1.021885	1.015672	0.71608	-0.4818	0.950071	1
RP11-452K12.7	1.017201	1.021905	1.015672	0.71546	-0.4831	0.950071	1
RP11-613M10.8	1.017221	1.021989	1.015672	0.71271	-0.4886	0.950071	1
RP11-589C21.5	1.017224	1.021999	1.015672	0.71239	-0.4893	0.950071	1
C10orf55	1.0172	1.0219	1.015672	0.71561	-0.4828	0.950071	1
ZP4	1.017208	1.021934	1.015672	0.71449	-0.485	0.950071	1
TSHR	1.017204	1.021918	1.015672	0.71502	-0.4839	0.950071	1
RP11-83N9.6	1.019002	1.013691	1.020728	1.51404	0.5984	0.950111	1
KB-431C1.5	1.01902	1.013691	1.020752	1.51575	0.6	0.950111	1
RP11-151A6.6	1.019027	1.013691	1.020762	1.51649	0.6007	0.950111	1
RP11-62H20.1	1.019015	1.013691	1.020745	1.51529	0.5996	0.950111	1
AC009473.1	1.019025	1.013691	1.020759	1.51625	0.6005	0.950111	1
TTC7A	1.081181	1.075675	1.082971	1.09641	0.1328	0.950117	1
UBA5	1.406594	1.401362	1.408294	1.01727	0.0247	0.950156	1
DOK1	1.217984	1.2123	1.219831	1.03548	0.0503	0.950166	1
LINC00324	1.080505	1.075205	1.082227	1.09337	0.1288	0.950174	1
RP1-142L7.8	1.024208	1.028849	1.0227	0.78685	-0.3458	0.95023	1
LARGE2	1.024203	1.028953	1.022659	0.78263	-0.3536	0.95023	1
CDKL5	1.092453	1.097069	1.090952	0.93698	-0.0939	0.950273	1
TMPRSS4	1.014525	1.019218	1.013	0.67644	-0.564	0.95036	1
AC068831.16	1.014525	1.019216	1.013	0.6765	-0.5638	0.95036	1
HR	1.014514	1.019173	1.013	0.67801	-0.5606	0.95036	1
APOD	1.014524	1.019214	1.013	0.6766	-0.5636	0.95036	1
MMP10	1.014532	1.019247	1.013	0.67542	-0.5661	0.95036	1
STX19	1.014521	1.0192	1.013	0.67708	-0.5626	0.95036	1
RP11-440G5.2	1.014518	1.019187	1.013	0.67753	-0.5616	0.95036	1
CDH26	1.014512	1.019164	1.013	0.67835	-0.5599	0.95036	1
AKAP10	1.343913	1.34918	1.342201	0.98001	-0.0291	0.950424	1
PEX26	1.292116	1.286315	1.294002	1.02685	0.0382	0.950563	1
SEMA4B	1.295539	1.300358	1.293972	0.97874	-0.031	0.950565	1
FAM168A	1.124877	1.119464	1.126636	1.06003	0.0841	0.950569	1
ACAA1	1.36663	1.37183	1.36494	0.98147	-0.027	0.950658	1
HOXB3	1.066595	1.071407	1.06503	0.9107	-0.135	0.950773	1
CAPN7	1.478062	1.472152	1.479983	1.01659	0.0237	0.950777	1
CTD-3064H18.2	1.021303	1.016045	1.023013	1.43425	0.5203	0.950788	1
RP11-179K3.2	1.021329	1.016045	1.023046	1.43635	0.5224	0.950788	1
PCDHGA9	1.021404	1.016045	1.023146	1.4426	0.5287	0.950788	1
RP11-536K7.3	1.021342	1.016045	1.023064	1.43747	0.5235	0.950788	1
DNASE2B	1.021325	1.016045	1.023041	1.43601	0.5221	0.950788	1
RP11-252I13.2	1.022834	1.017512	1.024563	1.40268	0.4882	0.95081	1

LINC01258	1.022805	1.017512	1.024526	1.40051	0.486	0.95081	1
RP11-1250I15.3	1.022812	1.017512	1.024535	1.40106	0.4865	0.95081	1
SPDEF	1.022792	1.017512	1.024508	1.39953	0.4849	0.95081	1
AC002550.6	1.022769	1.017512	1.024478	1.39782	0.4832	0.95081	1
MBOAT1	1.022773	1.017512	1.024483	1.39811	0.4835	0.95081	1
IGSF6	1.022794	1.017512	1.024511	1.39969	0.4851	0.95081	1
CTC-304I17.5	1.022778	1.017512	1.02449	1.3985	0.4839	0.95081	1
CNDP2	1.519146	1.513526	1.520973	1.0145	0.0208	0.95087	1
EPHA5	1.036264	1.030885	1.038013	1.23079	0.2996	0.950917	1
RP11-649A18.12	1.024007	1.018742	1.025718	1.37224	0.4565	0.95096	1
GLYCTK	1.062019	1.056689	1.063752	1.12458	0.1694	0.950993	1
TRIM62	1.064797	1.059443	1.066537	1.11933	0.1626	0.951016	1
RP11-80H5.2	1.024042	1.018799	1.025746	1.36957	0.4537	0.951059	1
TCEAL3-AS1	1.024063	1.018766	1.025785	1.37403	0.4584	0.951059	1
HULC	1.024037	1.018749	1.025756	1.37368	0.458	0.951059	1
ATP2A3	1.024059	1.018756	1.025783	1.37466	0.4591	0.951059	1
RP11-1069G10.1	1.02078	1.025434	1.019267	0.75753	-0.4006	0.951117	1
NEURL3	1.020731	1.025375	1.019221	0.7575	-0.4007	0.951117	1
SNX22	1.067075	1.061855	1.068772	1.11183	0.1529	0.951127	1
HYAL3	1.054056	1.05864	1.052566	0.89642	-0.1578	0.95113	1
PCYOX1L	1.144114	1.148683	1.142628	0.95928	-0.06	0.951139	1
NEK11	1.084609	1.079232	1.086357	1.08992	0.1242	0.951154	1
EGR3	1.021864	1.026481	1.020363	0.76899	-0.379	0.951156	1
MTCP1	1.021862	1.026509	1.020352	0.76773	-0.3813	0.951156	1
PIWIL2	1.021895	1.026583	1.020372	0.76634	-0.3839	0.951156	1
MAP1A	1.563417	1.569101	1.561569	0.98677	-0.0192	0.951177	1
MARCH4	1.056819	1.061404	1.055329	0.90107	-0.1503	0.951193	1
RP11-643M14.1	1.022432	1.017196	1.024134	1.4035	0.489	0.951197	1
BGLAP	1.022465	1.017196	1.024177	1.40602	0.4916	0.951197	1
RP11-85A1.3	1.022451	1.017203	1.024157	1.40428	0.4898	0.951197	1
NUP93	1.59083	1.595784	1.589219	0.98898	-0.016	0.951249	1
EMC1	1.148394	1.142676	1.150253	1.05311	0.0746	0.951266	1
DPP9	1.135404	1.140122	1.133871	0.95539	-0.0658	0.951284	1
HARS2	1.171836	1.166325	1.173627	1.0439	0.062	0.951318	1
ZNF26	1.172875	1.167297	1.174689	1.04418	0.0624	0.951349	1
COPS7B	1.50434	1.497956	1.506416	1.01699	0.0243	0.951382	1
MAP3K8	1.115722	1.120501	1.114169	0.94745	-0.0779	0.951417	1
PTPRJ	1.189785	1.184332	1.191557	1.0392	0.0555	0.951452	1
MYO9A	1.34956	1.343991	1.351371	1.02145	0.0306	0.951511	1
CFAP47	1.042307	1.037118	1.043994	1.18526	0.2452	0.95155	1
CLASP2	1.476748	1.470459	1.478792	1.01771	0.0253	0.951557	1
UPRT	1.155008	1.159728	1.153474	0.96084	-0.0576	0.951608	1
TMEM9B-AS1	1.039489	1.034182	1.041214	1.20572	0.2699	0.951613	1
RP11-679B19.1	1.068669	1.063241	1.070434	1.11375	0.1554	0.95173	1
MRAP2	1.021608	1.026224	1.020108	0.76677	-0.3831	0.951736	1

KB-1183D5.13	1.024762	1.019555	1.026455	1.35283	0.436	0.95183	1
RP11-774O3.3	1.035257	1.039771	1.03379	0.84961	-0.2351	0.951836	1
KCNA7	1.026246	1.020999	1.027952	1.33113	0.4126	0.951898	1
WEE2-AS1	1.026236	1.021039	1.027925	1.32731	0.4085	0.951898	1
HAPLN4	1.050617	1.055224	1.049119	0.88946	-0.169	0.951907	1
ELK1	1.241792	1.235856	1.243721	1.03334	0.0473	0.951935	1
ACRC	1.03966	1.034382	1.041376	1.20341	0.2671	0.951977	1
KLHL22	1.190952	1.195826	1.189367	0.96702	-0.0484	0.951978	1
MARS2	1.035196	1.039894	1.033669	0.84395	-0.2448	0.951981	1
RP11-949J7.8	1.017293	1.02192	1.01579	0.72034	-0.4733	0.952007	1
RP4-736L20.3	1.017291	1.021909	1.01579	0.7207	-0.4725	0.952007	1
RAMP3	1.018412	1.022997	1.016921	0.73579	-0.4426	0.952046	1
RP11-818F20.5	1.018408	1.022983	1.016921	0.73626	-0.4417	0.952046	1
CTB-107G13.1	1.018416	1.023015	1.016921	0.73522	-0.4437	0.952046	1
HAND2	1.018446	1.023138	1.016921	0.73132	-0.4514	0.952046	1
RP11-83A16.1	1.018423	1.023045	1.016921	0.73427	-0.4456	0.952046	1
EID2	1.224765	1.219046	1.226624	1.0346	0.0491	0.95209	1
SETSIP	1.02755	1.022261	1.029269	1.31483	0.3949	0.952097	1
PBX4	1.136793	1.131432	1.138536	1.05405	0.0759	0.952176	1
EIF2AK2	1.765498	1.770265	1.763948	0.9918	-0.0119	0.952177	1
RP11-394I13.2	1.021959	1.026541	1.02047	0.77123	-0.3748	0.952204	1
FREM1	1.017966	1.022551	1.016475	0.73055	-0.4529	0.952359	1
RP11-240G22.5	1.017986	1.022586	1.01649	0.73012	-0.4538	0.952359	1
ANO2	1.017978	1.022575	1.016483	0.73015	-0.4537	0.952359	1
MAP3K19	1.017973	1.022504	1.016501	0.73323	-0.4477	0.952359	1
GGA3	1.148045	1.142502	1.149847	1.05154	0.0725	0.952377	1
FOXN3-AS1	1.041994	1.036806	1.04368	1.18674	0.247	0.952385	1
PQLC2	1.282181	1.286865	1.280658	0.97836	-0.0316	0.952409	1
TMEM240	1.043804	1.038597	1.045496	1.17875	0.2373	0.952425	1
FICD	1.059668	1.064294	1.058164	0.90467	-0.1445	0.952532	1
EFNA5	3.404668	3.414883	3.401348	0.9944	-0.0081	0.952547	1
CTD-2023N9.3	1.018173	1.022751	1.016686	0.73342	-0.4473	0.952625	1
FRMPD3	1.018161	1.022699	1.016686	0.73508	-0.444	0.952625	1
RP11-169K16.4	1.018161	1.022701	1.016686	0.73503	-0.4441	0.952625	1
AKR1B15	1.018157	1.022682	1.016686	0.73562	-0.443	0.952625	1
ANTXRL	1.023098	1.017948	1.024773	1.38027	0.4649	0.952664	1
RSPH6A	1.023145	1.017948	1.024835	1.38372	0.4686	0.952664	1
AC009133.21	1.023126	1.017948	1.02481	1.38234	0.4671	0.952664	1
RP11-81H3.2	1.023125	1.017948	1.024808	1.38224	0.467	0.952664	1
FRMD7	1.023138	1.017948	1.024826	1.38323	0.468	0.952664	1
TMLHE-AS1	1.02312	1.017948	1.024802	1.38189	0.4666	0.952664	1
FKBP6	1.023105	1.017948	1.024781	1.38075	0.4655	0.952664	1
LRRC55	1.023133	1.017948	1.024819	1.38285	0.4676	0.952664	1
RP3-337H4.9	1.023087	1.017948	1.024758	1.37945	0.4641	0.952664	1
SFTA1P	1.023982	1.028654	1.022463	0.78396	-0.3511	0.952689	1

RP11-378A13.2	1.023936	1.028456	1.022466	0.78951	-0.341	0.952689	1
MRO	1.023975	1.028582	1.022478	0.78642	-0.3466	0.952689	1
CTD-3214H19.4	1.030577	1.035143	1.029093	0.82784	-0.2726	0.952698	1
AL133245.2	1.030591	1.035236	1.029081	0.82533	-0.2769	0.952698	1
AIM1L	1.031771	1.03646	1.030246	0.82957	-0.2696	0.952708	1
UGT2B7	1.031883	1.036353	1.03043	0.83706	-0.2566	0.952708	1
PHF8	1.151444	1.145771	1.153288	1.05157	0.0725	0.952749	1
KMT2B	1.141112	1.14559	1.139657	0.95924	-0.06	0.952824	1
LINC00886	1.028289	1.0232	1.029943	1.29064	0.3681	0.95287	1
GUCY1A3	1.211118	1.216075	1.209507	0.9696	-0.0445	0.952874	1
RP11-576D8.4	1.029702	1.024579	1.031367	1.27614	0.3518	0.952981	1
RP11-515O17.3	1.017127	1.011999	1.018794	1.56626	0.6473	0.952994	1
RP11-387H17.4	1.017116	1.011999	1.01878	1.56508	0.6462	0.952994	1
PPEF1	1.017114	1.011999	1.018776	1.56481	0.646	0.952994	1
LINC00852	1.017109	1.011999	1.01877	1.56426	0.6455	0.952994	1
LINC01198	1.017113	1.011999	1.018775	1.56467	0.6459	0.952994	1
RP1-92C4.2	1.017127	1.011999	1.018794	1.56631	0.6474	0.952994	1
IRX5	1.017137	1.011999	1.018808	1.5674	0.6484	0.952994	1
AC004878.8	1.017144	1.011999	1.018816	1.5681	0.649	0.952994	1
RP11-466F5.10	1.01712	1.011999	1.018785	1.56554	0.6467	0.952994	1
FAT3	1.530791	1.535749	1.529179	0.98774	-0.0178	0.952999	1
RP11-99L13.2	1.018458	1.022978	1.016989	0.73937	-0.4356	0.953093	1
LMF1-AS1	1.018474	1.02304	1.016989	0.73736	-0.4396	0.953093	1
CTC-510F12.2	1.018457	1.022971	1.016989	0.73958	-0.4352	0.953093	1
HNRNPCL1	1.018472	1.023034	1.016989	0.73756	-0.4392	0.953093	1
AC004012.1	1.01846	1.022987	1.016989	0.73908	-0.4362	0.953093	1
CTC-273B12.5	1.018471	1.023032	1.016989	0.73764	-0.439	0.953093	1
RP11-123K19.1	1.018453	1.022955	1.016989	0.74009	-0.4342	0.953093	1
C3orf70	1.030923	1.025812	1.032585	1.2624	0.3362	0.953109	1
RP11-230G5.2	1.021058	1.015934	1.022724	1.42609	0.5121	0.953148	1
HIST1H4F	1.021044	1.015934	1.022705	1.42494	0.5109	0.953148	1
RP4-666F24.3	1.021039	1.015934	1.022699	1.42452	0.5105	0.953148	1
AC009065.1	1.021055	1.015934	1.02272	1.42587	0.5118	0.953148	1
PMP2	1.021086	1.015934	1.022761	1.42841	0.5144	0.953148	1
SLC22A18AS	1.021061	1.015934	1.022728	1.42635	0.5123	0.953148	1
LRRTM3	1.021075	1.015934	1.022746	1.4275	0.5135	0.953148	1
CTC-325H20.4	1.021097	1.015934	1.022776	1.42937	0.5154	0.953148	1
AC073957.15	1.021045	1.015934	1.022707	1.42503	0.511	0.953148	1
SLC35G5	1.021065	1.015934	1.022732	1.42664	0.5126	0.953148	1
HBZ	1.021055	1.015934	1.02272	1.42585	0.5118	0.953148	1
RP11-41O4.1	1.021054	1.015934	1.022719	1.42578	0.5117	0.953148	1
NLRP12	1.021052	1.015934	1.022715	1.42556	0.5115	0.953148	1
ZBTB42	1.046272	1.050715	1.044828	0.88393	-0.178	0.953197	1
DAZL	1.014525	1.019027	1.013062	0.68647	-0.5427	0.953247	1
C1QL3	1.014519	1.019001	1.013062	0.68744	-0.5407	0.953247	1

RP11-968A15.2	1.014516	1.018991	1.013062	0.68779	-0.54	0.953247	1
IL1B	1.014517	1.018995	1.013062	0.68763	-0.5403	0.953247	1
SLC15A3	1.014543	1.019098	1.013062	0.68393	-0.5481	0.953247	1
SPANXD	1.014512	1.018975	1.013062	0.68838	-0.5387	0.953247	1
HRH4	1.014519	1.019002	1.013062	0.68739	-0.5408	0.953247	1
KCCAT211	1.014536	1.019073	1.013062	0.68484	-0.5462	0.953247	1
RP1-59D14.1	1.014524	1.019024	1.013062	0.6866	-0.5425	0.953247	1
RP11-249L12.1	1.014538	1.01908	1.013062	0.68458	-0.5467	0.953247	1
TMEM256-PLSCR3	1.029343	1.024245	1.031	1.27859	0.3545	0.953358	1
SALL3	1.020505	1.025005	1.019042	0.76152	-0.3931	0.953577	1
RP5-965G21.6	1.020515	1.025047	1.019042	0.76024	-0.3955	0.953577	1
AC016722.4	1.02051	1.025027	1.019042	0.76086	-0.3943	0.953577	1
RP11-179A10.1	1.020507	1.025016	1.019042	0.76117	-0.3937	0.953577	1
SLC2A14	1.020501	1.024988	1.019042	0.76202	-0.3921	0.953577	1
FLI1	1.020503	1.024997	1.019042	0.76176	-0.3926	0.953577	1
CTD-2639E6.9	1.020531	1.025111	1.019042	0.7583	-0.3992	0.953577	1
CCNI2	1.02051	1.025026	1.019042	0.76089	-0.3942	0.953577	1
CLCNKB	1.02835	1.032895	1.026873	0.81691	-0.2918	0.953582	1
RP11-356J5.13	1.028366	1.032903	1.026891	0.81728	-0.2911	0.953582	1
BRD1	1.135162	1.130056	1.136822	1.05202	0.0732	0.953598	1
RP11-723D22.3	1.020771	1.015676	1.022427	1.43069	0.5167	0.953617	1
CD300E	1.020774	1.015676	1.022432	1.43102	0.517	0.953617	1
LINC01598	1.020792	1.015676	1.022456	1.43254	0.5186	0.953617	1
TMEM72-AS1	1.020771	1.015676	1.022427	1.43072	0.5167	0.953617	1
RP11-431N15.2	1.020809	1.015676	1.022478	1.43396	0.52	0.953617	1
RP11-2B6.3	1.020775	1.015676	1.022433	1.43109	0.5171	0.953617	1
RIOK1	1.165838	1.160373	1.167615	1.04516	0.0637	0.953642	1
CNDP1	1.025653	1.030132	1.024198	0.80306	-0.3164	0.953687	1
ANGPTL6	1.026585	1.021456	1.028252	1.31672	0.397	0.95371	1
H3F3C	1.048585	1.043573	1.050214	1.15241	0.2047	0.953739	1
RP4-583P15.10	1.020584	1.015487	1.022241	1.43611	0.5222	0.95404	1
ELMOD1	1.020579	1.015535	1.022218	1.43019	0.5162	0.95404	1
EFNA2	1.020617	1.015501	1.02228	1.43739	0.5234	0.95404	1
RP11-7K24.3	1.020543	1.015499	1.022183	1.43129	0.5173	0.95404	1
RP11-371A19.2	1.02058	1.015514	1.022227	1.43273	0.5188	0.95404	1
SECTM1	1.020544	1.01552	1.022177	1.42898	0.515	0.95404	1
AC021224.1	1.034461	1.029424	1.036099	1.22685	0.295	0.954162	1
HIST2H2AB	1.024574	1.019449	1.02624	1.34921	0.4321	0.954195	1
RP11-589M4.1	1.021038	1.015961	1.022688	1.42148	0.5074	0.954196	1
RP11-707P17.2	1.021009	1.015961	1.02265	1.41909	0.505	0.954196	1
C11orf87	1.021022	1.015961	1.022667	1.42016	0.5061	0.954196	1
EFCC1	1.021013	1.015961	1.022655	1.41938	0.5053	0.954196	1
AC015849.19	1.021026	1.015961	1.022672	1.42046	0.5064	0.954196	1
VWA3B	1.021013	1.015961	1.022655	1.41939	0.5053	0.954196	1
NHEJ1	1.021004	1.015961	1.022643	1.41862	0.5045	0.954196	1

AKAP14	1.019881	1.014833	1.021522	1.45098	0.537	0.954235	1
KB-68A7.2	1.019889	1.014833	1.021533	1.45175	0.5378	0.954235	1
TFAP2E	1.01992	1.014833	1.021573	1.45447	0.5405	0.954235	1
KLHDC7A	1.019883	1.014833	1.021524	1.45117	0.5372	0.954235	1
RP11-131K5.1	1.019888	1.014833	1.021531	1.45162	0.5377	0.954235	1
CTB-55O6.13	1.019893	1.014833	1.021538	1.45206	0.5381	0.954235	1
AC007880.1	1.019879	1.014833	1.02152	1.45084	0.5369	0.954235	1
SOCS4	1.341382	1.346246	1.339801	0.98139	-0.0271	0.954251	1
FAM122A	1.421749	1.415786	1.423687	1.019	0.0272	0.954257	1
RP11-339B21.13	1.041645	1.036489	1.043321	1.18722	0.2476	0.954267	1
ABHD15	1.095771	1.100243	1.094317	0.94088	-0.0879	0.954361	1
RP11-1007O24.2	1.048918	1.043861	1.050562	1.15279	0.2051	0.954484	1
RP11-428J1.4	1.023645	1.028094	1.022199	0.79017	-0.3398	0.954547	1
SLC39A2	1.023638	1.028124	1.02218	0.78863	-0.3426	0.954547	1
AC141928.1	1.022188	1.026627	1.020745	0.77908	-0.3602	0.954568	1
IL27RA	1.165409	1.170041	1.163903	0.9639	-0.053	0.954572	1
DEPDC7	1.024197	1.019169	1.025831	1.34753	0.4303	0.954664	1
CYP19A1	1.024252	1.019189	1.025898	1.34965	0.4326	0.954664	1
SPACA6P-AS	1.024221	1.019174	1.025862	1.34881	0.4317	0.954664	1
RP11-536G4.2	1.024231	1.019208	1.025863	1.34649	0.4292	0.954664	1
AC072062.3	1.024225	1.019177	1.025867	1.34884	0.4317	0.954664	1
GDF9	1.024228	1.019204	1.025861	1.34659	0.4293	0.954664	1
RP11-140M13.1	1.024265	1.019188	1.025915	1.35059	0.4336	0.954664	1
PGBD5	1.040286	1.044695	1.038853	0.86931	-0.2021	0.954708	1
CDH10	1.024844	1.02926	1.023408	0.8	-0.3219	0.954722	1
BTN3A1	1.101702	1.096722	1.103321	1.06823	0.0952	0.954733	1
C2CD2L	1.064318	1.068716	1.062888	0.9152	-0.1278	0.95474	1
RP11-92G12.3	1.030008	1.024949	1.031653	1.2687	0.3434	0.954752	1
CASC4	1.909102	1.901888	1.911447	1.0106	0.0152	0.954854	1
CNNM1	1.049778	1.044744	1.051415	1.1491	0.2005	0.954881	1
ZNF154	1.112884	1.107598	1.114602	1.0651	0.091	0.954987	1
ACVR2A	1.143093	1.147556	1.141642	0.95992	-0.059	0.954995	1
SAMD10	1.108801	1.113188	1.107375	0.94864	-0.0761	0.955164	1
RP11-546K22.3	1.037854	1.032794	1.039499	1.20445	0.2684	0.955189	1
PCDHB6	1.028016	1.022987	1.02965	1.28989	0.3672	0.955238	1
LRRC25	1.028015	1.022961	1.029657	1.29162	0.3692	0.955238	1
RP3-412A9.16	1.024486	1.019477	1.026114	1.34075	0.423	0.955244	1
LINC01554	1.024467	1.019469	1.026091	1.34018	0.4224	0.955244	1
CTD-3076O17.1	1.024474	1.019459	1.026105	1.34151	0.4239	0.955244	1
RP11-20I23.8	1.024465	1.019457	1.026093	1.34102	0.4233	0.955244	1
FAM209B	1.019856	1.024279	1.018419	0.75865	-0.3985	0.955247	1
KCNE1	1.019861	1.024258	1.018432	0.75982	-0.3963	0.955247	1
CLIC2	1.023307	1.018328	1.024926	1.36002	0.4436	0.955283	1
OTUD6A	1.023365	1.018333	1.025001	1.36374	0.4476	0.955283	1
RP11-930O11.3	1.021466	1.02593	1.020015	0.7719	-0.3735	0.955339	1

RP11-21A7A.3	1.021435	1.025796	1.020017	0.77597	-0.3659	0.955339	1
CEP295NL	1.021468	1.025829	1.020051	0.77627	-0.3654	0.955339	1
INHA	1.091631	1.096057	1.090192	0.93895	-0.0909	0.955348	1
DGKH	1.263832	1.258265	1.265642	1.02857	0.0406	0.955408	1
HSPA12A	1.071632	1.07618	1.070153	0.92088	-0.1189	0.955416	1
FASTKD3	1.09546	1.090575	1.097048	1.07146	0.0996	0.955448	1
RP11-13J8.1	1.018746	1.023182	1.017304	0.74646	-0.4219	0.955452	1
RP11-96A15.1	1.018736	1.023141	1.017304	0.74776	-0.4194	0.955452	1
KRT6B	1.018719	1.02307	1.017304	0.75008	-0.4149	0.955452	1
RP11-543C4.1	1.018712	1.023041	1.017304	0.75101	-0.4131	0.955452	1
CECR3	1.01873	1.023117	1.017304	0.74853	-0.4179	0.955452	1
CTD-3064M3.4	1.018735	1.023136	1.017304	0.74794	-0.419	0.955452	1
RP3-402G11.28	1.018721	1.023079	1.017304	0.74977	-0.4155	0.955452	1
RP5-1142A6.8	1.020181	1.024545	1.018763	0.76441	-0.3876	0.955476	1
CCDC178	1.020194	1.024614	1.018757	0.76205	-0.392	0.955476	1
LINC01068	1.020195	1.024608	1.01876	0.76236	-0.3914	0.955476	1
AC073343.13	1.020187	1.024569	1.018762	0.76367	-0.389	0.955476	1
RP11-293M10.6	1.020178	1.024546	1.018758	0.76418	-0.388	0.955476	1
TFF1	1.020192	1.024569	1.018769	0.76391	-0.3885	0.955476	1
HIST1H2BL	1.02018	1.024546	1.018761	0.76431	-0.3878	0.955476	1
LGI4	1.052304	1.047295	1.053933	1.14036	0.1895	0.955528	1
CD2BP2	1.47396	1.46846	1.475748	1.01556	0.0223	0.955574	1
TTLL3	1.138715	1.142972	1.137331	0.96054	-0.0581	0.955604	1
AP1B1	1.31742	1.321629	1.316052	0.98266	-0.0252	0.95564	1
IFI30	1.028675	1.033012	1.027265	0.82591	-0.2759	0.955693	1
RP11-363J20.1	1.028681	1.033067	1.027255	0.82425	-0.2788	0.955693	1
GP1BA	1.027714	1.022727	1.029335	1.29078	0.3682	0.955708	1
AC005523.2	1.027729	1.022688	1.029368	1.2944	0.3723	0.955708	1
FUK	1.056025	1.051007	1.057656	1.13034	0.1768	0.95575	1
AHCYL2	1.168837	1.173297	1.167388	0.9659	-0.0501	0.955762	1
MAFTRR	1.037686	1.042007	1.036281	0.86369	-0.2114	0.955852	1
RP13-465B17.5	1.017106	1.012186	1.018705	1.53491	0.6181	0.955883	1
PRRT4	1.017106	1.012186	1.018705	1.53488	0.6181	0.955883	1
RP11-322E11.2	1.017111	1.012186	1.018712	1.53552	0.6187	0.955883	1
CTD-2587H24.10	1.017114	1.012186	1.018715	1.53577	0.619	0.955883	1
SCML4	1.017139	1.012186	1.018749	1.53854	0.6216	0.955883	1
AB015752.3	1.017166	1.012186	1.018785	1.54145	0.6243	0.955883	1
PCDHGA6	1.017114	1.012186	1.018716	1.53585	0.619	0.955883	1
AC008697.1	1.01717	1.012186	1.018789	1.54184	0.6247	0.955883	1
MAGED4	1.017098	1.012186	1.018694	1.53404	0.6173	0.955883	1
PLAA	1.226363	1.230832	1.22491	0.97434	-0.0375	0.955986	1
STK10	1.098011	1.09283	1.099696	1.07396	0.1029	0.956063	1
RP5-958B11.1	1.019787	1.014881	1.021382	1.43684	0.5229	0.956172	1
RP11-114N19.3	1.019799	1.014881	1.021398	1.43791	0.524	0.956172	1
RP11-650L12.1	1.019799	1.014881	1.021397	1.43787	0.5239	0.956172	1

GUCA1B	1.019773	1.014881	1.021363	1.4356	0.5217	0.956172	1
CORT	1.019825	1.014881	1.021432	1.4402	0.5263	0.956172	1
RP11-388M20.1	1.019787	1.014881	1.021382	1.43682	0.5229	0.956172	1
ITCH-AS1	1.019819	1.014881	1.021423	1.43963	0.5257	0.956172	1
SPINK6	1.019815	1.014881	1.021419	1.43932	0.5254	0.956172	1
RP11-102M11.2	1.01982	1.014881	1.021425	1.43976	0.5258	0.956172	1
RP11-103J8.1	1.019824	1.014881	1.02143	1.4401	0.5262	0.956172	1
FAM78A	1.019794	1.014881	1.021392	1.43749	0.5236	0.956172	1
TMPRSS7	1.016414	1.02074	1.015008	0.72362	-0.4667	0.956175	1
SLC2A9	1.016409	1.020752	1.014997	0.72269	-0.4685	0.956175	1
CTD-2589H19.6	1.016397	1.020706	1.014997	0.72426	-0.4654	0.956175	1
LINC01197	1.016398	1.0207	1.014999	0.72457	-0.4648	0.956175	1
CTC-498J12.1	1.016404	1.020704	1.015007	0.7248	-0.4643	0.956175	1
RP11-98I9.4	1.040173	1.035144	1.041808	1.18961	0.2505	0.95621	1
AC009404.2	1.041368	1.036414	1.042979	1.18027	0.2391	0.956212	1
MYRFL	1.017977	1.022271	1.016581	0.74449	-0.4257	0.956222	1
UBASH3A	1.017995	1.022344	1.016581	0.74205	-0.4304	0.956222	1
ICAM2	1.017972	1.022251	1.016581	0.74518	-0.4243	0.956222	1
RP11-1006G14.2	1.017986	1.022307	1.016581	0.74329	-0.428	0.956222	1
SPIB	1.017972	1.022254	1.016581	0.74508	-0.4245	0.956222	1
FCAR	1.01798	1.022283	1.016581	0.74411	-0.4264	0.956222	1
RP11-298E9.5	1.017977	1.022273	1.016581	0.74444	-0.4258	0.956222	1
CYP1A1	1.017984	1.022299	1.016581	0.74357	-0.4275	0.956222	1
RP11-706O15.1	1.578595	1.573083	1.580387	1.01275	0.0183	0.956265	1
ALDH3B1	1.031496	1.026464	1.033132	1.25194	0.3242	0.956278	1
FSTL5	1.027979	1.023001	1.029598	1.28679	0.3638	0.956289	1
RP11-297N6.4	1.041303	1.036367	1.042908	1.17985	0.2386	0.956304	1
MINPP1	1.217885	1.222646	1.216337	0.97167	-0.0415	0.956389	1
MERTK	1.065603	1.070006	1.064172	0.91666	-0.1255	0.956396	1
PSMD6	1.764335	1.758566	1.76621	1.01008	0.0145	0.956529	1
KCNN2	1.017971	1.022276	1.016571	0.74388	-0.4269	0.956564	1
SAMMSON	1.017975	1.022281	1.016575	0.74389	-0.4268	0.956564	1
C8orf34	1.025236	1.029585	1.023823	0.80522	-0.3125	0.956611	1
FGF1	1.025241	1.029599	1.023824	0.80488	-0.3131	0.956611	1
RP11-190A12.8	1.025243	1.029493	1.023861	0.80904	-0.3057	0.956611	1
ZNF547	1.032498	1.036791	1.031102	0.84538	-0.2423	0.956797	1
CASD1	1.259481	1.254209	1.261194	1.02748	0.0391	0.9568	1
CTD-3193K9.11	1.037028	1.032159	1.03861	1.20058	0.2637	0.956827	1
RP11-122G18.12	1.020564	1.015689	1.022149	1.41177	0.4975	0.956935	1
PGM3	1.486357	1.491296	1.484752	0.98668	-0.0193	0.956943	1
AC145124.2	1.263662	1.268172	1.262196	0.97772	-0.0325	0.957053	1
PRSS50	1.031719	1.035914	1.030356	0.84524	-0.2426	0.957061	1
FH	1.455152	1.449668	1.456934	1.01616	0.0231	0.957108	1
RP4-635E18.6	1.030923	1.026028	1.032514	1.2492	0.321	0.957159	1
CTD-2383M3.1	1.023273	1.01837	1.024866	1.35362	0.4368	0.957224	1

C1orf204	1.029908	1.034101	1.028545	0.83708	-0.2566	0.957311	1
IKBKB	1.282952	1.287427	1.281497	0.97937	-0.0301	0.957365	1
LILRB3	1.030291	1.025379	1.031887	1.25645	0.3294	0.95737	1
ADIRF	1.030296	1.025393	1.03189	1.25587	0.3287	0.95737	1
RP11-365P13.5	1.030292	1.025364	1.031894	1.25744	0.3305	0.95737	1
PCDHA11	1.030248	1.025331	1.031847	1.25725	0.3303	0.95737	1
FLVCR1-AS1	1.090673	1.08559	1.092325	1.07869	0.1093	0.957385	1
NACAD	1.09071	1.085474	1.092412	1.08116	0.1126	0.957385	1
RP5-1074L1.1	1.016691	1.011871	1.018258	1.53802	0.6211	0.957404	1
CTD-2619J13.19	1.016704	1.011871	1.018275	1.5395	0.6225	0.957404	1
LINC01143	1.016697	1.011871	1.018266	1.53875	0.6218	0.957404	1
DNAJC25-GNG10	1.016686	1.011871	1.018251	1.53745	0.6205	0.957404	1
AC005534.8	1.01668	1.011871	1.018243	1.53679	0.6199	0.957404	1
RP11-1277A3.3	1.016702	1.011871	1.018272	1.53921	0.6222	0.957404	1
IL36G	1.0167	1.011871	1.018269	1.53902	0.622	0.957404	1
TAF2	1.234829	1.229667	1.236506	1.02978	0.0423	0.957439	1
DTWD2	1.102527	1.097304	1.104224	1.07112	0.0991	0.957486	1
FAM138E	1.014507	1.018715	1.01314	0.70207	-0.5103	0.95749	1
RP11-157F20.3	1.014506	1.018714	1.013138	0.70207	-0.5103	0.95749	1
F13A1	1.014501	1.018699	1.013136	0.7025	-0.5094	0.95749	1
LINC00845	1.014507	1.018716	1.013138	0.70196	-0.5105	0.95749	1
CTB-119C2.1	1.014525	1.018751	1.013151	0.70137	-0.5117	0.95749	1
ZNF416	1.062514	1.066692	1.061156	0.917	-0.125	0.957508	1
HEATR4	1.021784	1.025939	1.020433	0.78773	-0.3442	0.957532	1
GDPD3	1.021806	1.026042	1.020428	0.78443	-0.3503	0.957532	1
SYTL4	1.021763	1.026038	1.020374	0.78245	-0.3539	0.957532	1
MS4A6E	1.021787	1.026015	1.020412	0.78464	-0.3499	0.957532	1
CUX1	1.918788	1.911872	1.921037	1.01005	0.0144	0.957612	1
FARP2	1.140448	1.135133	1.142176	1.05212	0.0733	0.957633	1
ZNF746	1.122436	1.117153	1.124153	1.05975	0.0837	0.957707	1
MPP5	1.312636	1.316694	1.311317	0.98302	-0.0247	0.957748	1
RAB33A	1.205183	1.209793	1.203684	0.97088	-0.0426	0.957835	1
RP11-770J1.5	1.01608	1.011304	1.017632	1.55974	0.6413	0.957875	1
GOLGA6L7P	1.016095	1.011304	1.017652	1.56156	0.643	0.957875	1
RP11-188P17.2	1.016102	1.011304	1.017662	1.56241	0.6438	0.957875	1
RP11-54C4.3	1.016113	1.011304	1.017677	1.56371	0.645	0.957875	1
SZT2-AS1	1.016086	1.011304	1.017641	1.56055	0.6421	0.957875	1
SRRM5	1.01613	1.011304	1.017699	1.56564	0.6468	0.957875	1
RP11-503C24.4	1.016124	1.011304	1.017691	1.56498	0.6461	0.957875	1
RP3-380B8.4	1.016075	1.011304	1.017626	1.55926	0.6409	0.957875	1
XX-FW83128A1.2	1.016101	1.011304	1.017661	1.5623	0.6437	0.957875	1
RP11-307L14.1	1.016106	1.011304	1.017666	1.5628	0.6441	0.957875	1
SCIMP	1.016081	1.011304	1.017633	1.55987	0.6414	0.957875	1
RP11-344N10.5	1.026627	1.030838	1.025258	0.81908	-0.2879	0.957971	1
B4GALT6	1.131256	1.12609	1.132935	1.05429	0.0763	0.958018	1

CCDC184	1.080052	1.084202	1.078703	0.9347	-0.0974	0.958084	1
GANC	1.104923	1.099943	1.106541	1.06602	0.0922	0.958093	1
LINC01219	1.016972	1.012187	1.018527	1.52028	0.6043	0.958138	1
RP11-122L9.1	1.016967	1.012187	1.018522	1.51981	0.6039	0.958138	1
RP11-158M2.3	1.016947	1.012187	1.018495	1.51762	0.6018	0.958138	1
CTB-129P6.4	1.016979	1.012187	1.018537	1.52109	0.6051	0.958138	1
RP11-326I11.4	1.01698	1.012187	1.018538	1.52114	0.6052	0.958138	1
PRLH	1.016961	1.012187	1.018513	1.51908	0.6032	0.958138	1
LINC01185	1.016982	1.012187	1.018541	1.52138	0.6054	0.958138	1
C6orf58	1.018723	1.013947	1.020276	1.45383	0.5399	0.958177	1
LINC00092	1.018775	1.013947	1.020345	1.45877	0.5447	0.958177	1
TMEM262	1.018732	1.013947	1.020288	1.45466	0.5407	0.958177	1
EOMES	1.018782	1.013947	1.020354	1.4594	0.5454	0.958177	1
GOLGA6L2	1.018784	1.013947	1.020357	1.45961	0.5456	0.958177	1
ZNF878	1.018723	1.013947	1.020275	1.45377	0.5398	0.958177	1
UCHL1	4.41994	4.427846	4.41737	0.99694	-0.0044	0.958333	1
CEACAM19	1.047099	1.042161	1.048704	1.15518	0.2081	0.958344	1
ESRRB	1.020599	1.015826	1.022151	1.39964	0.4851	0.958356	1
RP11-867G23.4	1.020607	1.015826	1.022161	1.40026	0.4857	0.958356	1
RP4-616B8.4	1.020597	1.015826	1.022148	1.39946	0.4849	0.958356	1
LINC00659	1.020612	1.015826	1.022168	1.40074	0.4862	0.958356	1
BHMT2	1.020634	1.015826	1.022197	1.40253	0.488	0.958356	1
RP11-186B7.7	1.020612	1.015826	1.022168	1.40073	0.4862	0.958356	1
SVEP1	1.034915	1.030167	1.036459	1.20856	0.2733	0.958369	1
PRKAR2A-AS1	1.034916	1.030163	1.036461	1.20879	0.2736	0.958369	1
CCDC61	1.061759	1.056982	1.063311	1.11108	0.152	0.958372	1
TIGD7	1.105797	1.100869	1.107399	1.06473	0.0905	0.958385	1
ARL17A	1.105875	1.100936	1.10748	1.06483	0.0906	0.958385	1
DIMT1	1.329528	1.334076	1.328049	0.98196	-0.0263	0.958392	1
RP11-268G13.1	1.017833	1.013073	1.01938	1.48253	0.5681	0.958418	1
RP11-230B22.1	1.017836	1.013073	1.019384	1.4828	0.5683	0.958418	1
CISTR	1.017827	1.013073	1.019373	1.48197	0.5675	0.958418	1
TEK	1.017815	1.013073	1.019357	1.48071	0.5663	0.958418	1
AC007403.2	1.017839	1.013073	1.019389	1.48319	0.5687	0.958418	1
RP4-616B8.5	1.017825	1.013073	1.019369	1.48168	0.5672	0.958418	1
RP11-497H16.9	1.046469	1.050707	1.045092	0.88926	-0.1693	0.958418	1
XXbac-BPG249D20.9	1.018315	1.022488	1.016958	0.75411	-0.4072	0.958456	1
RFPL2	1.01832	1.0225	1.016961	0.75381	-0.4077	0.958456	1
RP5-1061H20.4	1.018307	1.022446	1.016962	0.75567	-0.4042	0.958456	1
PHEX-AS1	1.01831	1.022454	1.016963	0.75544	-0.4046	0.958456	1
TSSK4	1.018339	1.022577	1.016962	0.75127	-0.4126	0.958456	1
CTC-297N7.1	1.018321	1.022518	1.016957	0.75304	-0.4092	0.958456	1
CCDC62	1.018326	1.022488	1.016973	0.75477	-0.4059	0.958456	1
RP1-178F10.1	1.018306	1.022424	1.016968	0.75669	-0.4022	0.958456	1
RP11-437B10.1	1.080101	1.075277	1.081669	1.08491	0.1176	0.958457	1

ABCC3	1.020133	1.015356	1.021686	1.41226	0.498	0.958458	1
LGALS7B	1.020184	1.015369	1.021749	1.41515	0.501	0.958458	1
RP3-388M5.9	1.020135	1.015376	1.021682	1.41012	0.4958	0.958458	1
TAPT1-AS1	1.020128	1.015365	1.021676	1.4107	0.4964	0.958458	1
RP11-963H4.3	1.020141	1.015374	1.02169	1.41084	0.4966	0.958458	1
RP5-875O13.1	1.020123	1.015367	1.021669	1.41012	0.4958	0.958458	1
TUBGCP6	1.10562	1.110038	1.104183	0.9468	-0.0789	0.958528	1
KLHL6	1.017856	1.013102	1.019402	1.48087	0.5664	0.958528	1
RP1-261G23.7	1.017858	1.013102	1.019404	1.48105	0.5666	0.958528	1
AC006273.4	1.017867	1.013102	1.019416	1.48197	0.5675	0.958528	1
LINC01516	1.017891	1.013102	1.019448	1.48439	0.5699	0.958528	1
FLJ42969	1.017891	1.013102	1.019448	1.48439	0.5699	0.958528	1
RP11-680F20.9	1.017858	1.013102	1.019403	1.48101	0.5666	0.958528	1
RP11-9N20.3	1.017852	1.013102	1.019396	1.48043	0.566	0.958528	1
AF011889.2	1.017849	1.013102	1.019392	1.48017	0.5658	0.958528	1
HPD	1.025616	1.029825	1.024247	0.813	-0.2987	0.958614	1
PSD3	1.553096	1.557941	1.551521	0.98849	-0.0167	0.95873	1
ZNF275	1.106091	1.101144	1.107699	1.0648	0.0906	0.95876	1
DCAF4L1	1.018024	1.013274	1.019568	1.47418	0.5599	0.958778	1
RP11-703H8.7	1.017999	1.013274	1.019535	1.47165	0.5574	0.958778	1
TUBA4B	1.018011	1.013274	1.019551	1.47284	0.5586	0.958778	1
MMP19	1.018011	1.013274	1.01955	1.4728	0.5586	0.958778	1
TRIM17	1.060329	1.064427	1.058997	0.91573	-0.127	0.95881	1
LINC01004	1.038069	1.033242	1.039638	1.1924	0.2539	0.958819	1
ARHGEF10L	1.107417	1.102366	1.109059	1.06539	0.0914	0.958829	1
RP11-415J8.5	1.019533	1.014799	1.021073	1.42395	0.5099	0.958931	1
RP11-496H1.2	1.019537	1.014812	1.021072	1.4227	0.5086	0.958931	1
HPCAL4	1.019519	1.014813	1.021048	1.42095	0.5069	0.958931	1
RP11-74E22.3	1.027475	1.022697	1.029028	1.27896	0.355	0.959028	1
ARHGAP15	1.027461	1.022711	1.029005	1.27713	0.3529	0.959028	1
RP5-933K21.3	1.027471	1.022777	1.028997	1.27305	0.3483	0.959028	1
HBS1L	1.430658	1.43536	1.42913	0.98569	-0.0208	0.95912	1
NRBF2	1.556721	1.550517	1.558737	1.01493	0.0214	0.959137	1
TMEM136	1.575397	1.579783	1.573972	0.98998	-0.0145	0.959179	1
MEI4	1.020403	1.015703	1.021931	1.39656	0.4819	0.959194	1
CCDC57	1.20075	1.195818	1.202354	1.03338	0.0474	0.959194	1
RP11-158H5.7	1.022183	1.017473	1.023714	1.35717	0.4406	0.959233	1
RP11-51J9.6	1.022215	1.01744	1.023767	1.36283	0.4466	0.959233	1
HCG22	1.022207	1.017446	1.023755	1.36168	0.4454	0.959233	1
SMPD5	1.023018	1.027171	1.021668	0.79745	-0.3265	0.959246	1
FSBP	1.022998	1.027168	1.021643	0.79663	-0.328	0.959246	1
RP4-671O14.7	1.023028	1.027101	1.021704	0.80088	-0.3203	0.959246	1
MCF2L-AS1	1.025803	1.029935	1.02446	0.81711	-0.2914	0.959309	1
SLC22A1	1.02587	1.030136	1.024483	0.8124	-0.2997	0.959309	1
CTD-2659N19.10	1.03021	1.025429	1.031763	1.24908	0.3209	0.959318	1

RP11-893F2.13	1.030193	1.025407	1.031748	1.24955	0.3214	0.959318	1
DMXL2	1.507908	1.50221	1.50976	1.01503	0.0215	0.95937	1
CNPY1	1.041867	1.037055	1.043431	1.17207	0.2291	0.959376	1
VGLL4	2.280625	2.285795	2.278945	0.99467	-0.0077	0.959384	1
CSMD3	1.02406	1.019335	1.025596	1.32382	0.4047	0.959412	1
LINC01251	1.024058	1.019332	1.025594	1.32395	0.4048	0.959412	1
RP11-455O6.8	1.0213	1.01659	1.022831	1.37621	0.4607	0.959474	1
TTBK2	1.386376	1.381057	1.388104	1.01849	0.0264	0.9595	1
RP4-593H12.1	1.02361	1.018872	1.02515	1.33267	0.4143	0.959509	1
SRCAP	1.043241	1.047553	1.041839	0.87983	-0.1847	0.959565	1
RP11-128A17.2	1.043346	1.047444	1.042014	0.88554	-0.1754	0.959565	1
RP11-5O24.1	1.021318	1.016596	1.022853	1.377	0.4615	0.959585	1
SPDYE1	1.021333	1.016615	1.022867	1.37631	0.4608	0.959585	1
PCDHA13	1.021319	1.01661	1.02285	1.37569	0.4602	0.959585	1
CHRNA10	1.021321	1.016599	1.022856	1.37693	0.4615	0.959585	1
LCN2	1.021291	1.016607	1.022813	1.37368	0.458	0.959585	1
RP11-736K20.6	1.031885	1.036103	1.030514	0.8452	-0.2426	0.959683	1
SMG6	1.424344	1.418808	1.426144	1.01752	0.0251	0.959764	1
RP5-994D16.3	1.021285	1.025431	1.019938	0.784	-0.3511	0.959791	1
DLGAP2	1.021243	1.025316	1.019919	0.78683	-0.3459	0.959791	1
NAALADL2-AS2	1.019705	1.023776	1.018382	0.77315	-0.3712	0.959802	1
S100A7	1.019756	1.023783	1.018447	0.77565	-0.3665	0.959802	1
KYNU	1.021464	1.016785	1.022984	1.36938	0.4535	0.959835	1
RP11-60A24.3	1.021516	1.016784	1.023055	1.3736	0.458	0.959835	1
LRRC8E	1.040022	1.043956	1.038743	0.88141	-0.1821	0.959874	1
TBC1D23	1.222057	1.226311	1.220674	0.97509	-0.0364	0.960006	1
ZNF821	1.426579	1.421351	1.428279	1.01644	0.0235	0.960008	1
OSTF1	1.203896	1.208087	1.202534	0.97331	-0.039	0.96003	1
MIR133A1HG	1.019574	1.023645	1.018251	0.77186	-0.3736	0.960052	1
C5orf66	1.019606	1.023791	1.018245	0.7669	-0.3829	0.960052	1
OSCAR	1.019575	1.02364	1.018253	0.77212	-0.3731	0.960052	1
NOCT	1.030957	1.026258	1.032484	1.23714	0.307	0.960068	1
KIAA1549L	1.030947	1.026432	1.032414	1.2263	0.2943	0.960068	1
CCDC88B	1.113959	1.109348	1.115458	1.05588	0.0784	0.960151	1
RP11-28H5.2	1.019568	1.02363	1.018247	0.77219	-0.373	0.960163	1
PTGFR	1.023854	1.019183	1.025373	1.32267	0.4035	0.960246	1
MRS2	1.68582	1.690777	1.684209	0.99049	-0.0138	0.960263	1
RP1-261D10.2	1.021854	1.025906	1.020537	0.79274	-0.3351	0.960264	1
CCNYL1	1.158974	1.163213	1.157597	0.96559	-0.0505	0.960268	1
RP11-486I11.2	1.025626	1.020959	1.027143	1.29504	0.373	0.960285	1
STPG2	1.025633	1.02099	1.027142	1.29313	0.3709	0.960285	1
NTNG1	1.025639	1.020945	1.027165	1.297	0.3752	0.960285	1
ALB	1.020429	1.024458	1.019119	0.78171	-0.3553	0.960404	1
PRR26	1.020449	1.024529	1.019123	0.77959	-0.3592	0.960404	1
MUC3A	1.018709	1.022709	1.017408	0.76658	-0.3835	0.960444	1

PWRN1	1.01869	1.022729	1.017377	0.76455	-0.3873	0.960444	1
CCL5	1.018659	1.022669	1.017356	0.76563	-0.3853	0.960444	1
TMEM132E	1.018669	1.022681	1.017365	0.76564	-0.3853	0.960444	1
GZF1	1.086934	1.082344	1.088426	1.07385	0.1028	0.960463	1
RP11-976B16.1	1.027502	1.022853	1.029013	1.26955	0.3443	0.960464	1
AC144450.2	1.027547	1.022926	1.029049	1.26711	0.3415	0.960464	1
RP1-199J3.7	1.027536	1.022878	1.02905	1.26977	0.3446	0.960464	1
TMEM151B	1.040651	1.035937	1.042183	1.17381	0.2312	0.960474	1
RMDN2	1.054946	1.050282	1.056462	1.12291	0.1672	0.960516	1
ACKR2	1.024789	1.020102	1.026312	1.3089	0.3884	0.960527	1
LINC00337	1.027094	1.022391	1.028623	1.27829	0.3542	0.960556	1
ADAMTSL4	1.071741	1.075903	1.070388	0.92734	-0.1088	0.960585	1
RP1-100J12.1	1.028464	1.032479	1.027158	0.83618	-0.2581	0.96059	1
LINC00959	1.091127	1.086554	1.092614	1.07001	0.0976	0.960623	1
RP3-439F8.1	1.024784	1.020094	1.026309	1.30927	0.3888	0.960638	1
RP1-234P15.4	1.024758	1.020151	1.026256	1.30294	0.3818	0.960638	1
DLX4	1.024848	1.02022	1.026353	1.30331	0.3822	0.960638	1
MAP1LC3B2	1.069542	1.064776	1.071091	1.09749	0.1342	0.960698	1
AL358852.1	1.017821	1.021821	1.016521	0.75709	-0.4015	0.960706	1
RP11-462B18.2	1.016281	1.020302	1.014974	0.73754	-0.4392	0.960723	1
RP11-568A7.3	1.016302	1.020347	1.014987	0.73657	-0.4411	0.960723	1
FGFBP1	1.016271	1.020271	1.014971	0.73855	-0.4372	0.960723	1
HUS1B	1.016301	1.020365	1.01498	0.73557	-0.4431	0.960723	1
RNASEK-C17orf49	1.024922	1.020278	1.026431	1.30344	0.3823	0.960888	1
RP11-416N2.4	1.024964	1.020371	1.026456	1.29872	0.3771	0.960888	1
MAP3K14	1.070483	1.065829	1.071996	1.09368	0.1292	0.960985	1
BAZ2A	1.628169	1.622471	1.630021	1.01213	0.0174	0.960987	1
FAM66D	1.026515	1.021873	1.028024	1.28122	0.3575	0.96103	1
C15orf59-AS1	1.026477	1.021806	1.027995	1.2838	0.3604	0.96103	1
SEC14L4	1.020042	1.015453	1.021534	1.39345	0.4787	0.961045	1
RP11-120C12.3	1.020089	1.015453	1.021596	1.39751	0.4829	0.961045	1
MIR548XHG	1.020067	1.015453	1.021567	1.3956	0.4809	0.961045	1
RP5-1116H23.6	1.020063	1.015453	1.021561	1.39524	0.4805	0.961045	1
TGM3	1.020052	1.015453	1.021547	1.39431	0.4796	0.961045	1
ZNF570	1.097529	1.101589	1.09621	0.94705	-0.0785	0.961075	1
FAM138B	1.016115	1.020119	1.014813	0.7363	-0.4416	0.961083	1
F2RL2	1.100639	1.095926	1.102171	1.06509	0.091	0.961111	1
NYAP1	1.108264	1.103383	1.10985	1.06255	0.0875	0.961124	1
CTD-2349P21.6	1.018863	1.022855	1.017565	0.76855	-0.3798	0.961145	1
RP11-687F6.5	1.018863	1.022855	1.017566	0.76857	-0.3797	0.961145	1
SOHLH1	1.018869	1.022874	1.017567	0.76798	-0.3809	0.961145	1
HP	1.018882	1.02293	1.017567	0.76609	-0.3844	0.961145	1
EGOT	1.018857	1.022823	1.017568	0.76976	-0.3775	0.961145	1
RP11-728K20.3	1.018413	1.022422	1.017109	0.76305	-0.3901	0.961179	1
ANXA9	1.018417	1.022365	1.017134	0.76611	-0.3844	0.961179	1

PLCL1	1.018411	1.022401	1.017114	0.76399	-0.3884	0.961179	1
RP3-461P17.10	1.018443	1.022424	1.017149	0.76475	-0.3869	0.961179	1
TMEM79	1.084803	1.088799	1.083504	0.94037	-0.0887	0.961265	1
RP1-239B22.5	1.058619	1.053937	1.060141	1.11503	0.1571	0.961279	1
WDR82	1.564616	1.559497	1.56628	1.01212	0.0174	0.961319	1
LINC01471	1.017021	1.021059	1.015709	0.74596	-0.4228	0.961324	1
RP5-1103G7.10	1.016995	1.020975	1.015701	0.74857	-0.4178	0.961324	1
PPT2-EGFL8	1.029097	1.024507	1.030589	1.24816	0.3198	0.961334	1
SLC27A2	1.029035	1.024452	1.030524	1.24836	0.32	0.961334	1
BID	1.818875	1.823818	1.817268	0.99205	-0.0115	0.961338	1
CHD5	1.015225	1.019179	1.01394	0.72684	-0.4603	0.961363	1
F10-AS1	1.015251	1.019284	1.01394	0.72288	-0.4682	0.961363	1
RP11-304L19.11	1.015238	1.01922	1.013943	0.72545	-0.4631	0.961363	1
CTD-2525I3.2	1.015227	1.019168	1.013946	0.72759	-0.4588	0.961363	1
RP4-680D5.2	1.015231	1.019204	1.01394	0.72587	-0.4622	0.961363	1
CPNE9	1.015246	1.019255	1.013943	0.72412	-0.4657	0.961363	1
RP11-711C17.2	1.015238	1.019213	1.013946	0.72587	-0.4622	0.961363	1
FAM179A	1.015235	1.019201	1.013945	0.7263	-0.4614	0.961363	1
LINC01465	1.015228	1.019194	1.013939	0.72623	-0.4615	0.961363	1
MYCT1	1.015231	1.019199	1.013941	0.72613	-0.4617	0.961363	1
HFE	1.048773	1.044128	1.050283	1.1395	0.1884	0.961424	1
PNMA6A	1.080056	1.07537	1.081579	1.08238	0.1142	0.961435	1
AC005592.2	1.057351	1.052777	1.058838	1.11485	0.1569	0.961518	1
NUP214	1.242212	1.237238	1.243828	1.02778	0.0395	0.961601	1
RP11-805J14.5	1.014373	1.018343	1.013082	0.71321	-0.4876	0.961625	1
PGM5P3-AS1	1.014365	1.018299	1.013087	0.71517	-0.4836	0.961625	1
RP5-1009E24.8	1.014361	1.01829	1.013084	0.71535	-0.4833	0.961625	1
SOX21-AS1	1.014368	1.018314	1.013085	0.71447	-0.4851	0.961625	1
RP11-12K11.2	1.014368	1.018308	1.013087	0.71479	-0.4844	0.961625	1
SEZ6	1.032013	1.036028	1.030708	0.85235	-0.2305	0.961633	1
KLHL11	1.028267	1.023752	1.029735	1.25191	0.3241	0.961687	1
CRTC3-AS1	1.028282	1.023627	1.029796	1.2611	0.3347	0.961687	1
MIDN	2.092026	2.085722	2.094074	1.00769	0.0111	0.961783	1
PLBD1-AS1	1.022387	1.026313	1.021111	0.80231	-0.3178	0.96179	1
KCND3	1.022283	1.026281	1.020983	0.79841	-0.3248	0.96179	1
BHLHB9	1.155896	1.160317	1.154459	0.96346	-0.0537	0.961807	1
FMNL1	1.03667	1.040542	1.035412	0.87346	-0.1952	0.961828	1
HIST1H2AE	1.048427	1.043865	1.049911	1.13783	0.1863	0.961899	1
C16orf95	1.085695	1.089696	1.084395	0.9409	-0.0879	0.961903	1
ACCS	1.076223	1.071465	1.07777	1.08823	0.122	0.961987	1
RP11-847H18.2	1.016266	1.011717	1.017744	1.51434	0.5987	0.962012	1
AC129492.1	1.016255	1.011717	1.01773	1.51311	0.5975	0.962012	1
NPIPA3	1.01624	1.011717	1.01771	1.51147	0.596	0.962012	1
TBR1	1.016271	1.011717	1.017751	1.51496	0.5993	0.962012	1
GABRG2	1.016226	1.011717	1.017691	1.50982	0.5944	0.962012	1

CTC-332L22.1	1.016242	1.011717	1.017712	1.51164	0.5961	0.962012	1
RP11-1081M5.1	1.016244	1.011717	1.017715	1.5119	0.5964	0.962012	1
AC008753.6	1.016243	1.011717	1.017714	1.51183	0.5963	0.962012	1
CTD-2240J17.4	1.016259	1.011717	1.017736	1.51363	0.598	0.962012	1
RP11-713P17.5	1.016239	1.011717	1.017708	1.5113	0.5958	0.962012	1
RP11-466F5.8	1.016232	1.011717	1.017699	1.51052	0.595	0.962012	1
TSSK6	1.016229	1.011717	1.017695	1.51018	0.5947	0.962012	1
AMIGO1	1.029874	1.025364	1.03134	1.23563	0.3052	0.962075	1
AC019186.1	1.014967	1.018891	1.013692	0.72477	-0.4644	0.962097	1
FAM46C	1.014969	1.018903	1.013691	0.72427	-0.4654	0.962097	1
DLG1-AS1	1.014962	1.018858	1.013696	0.72627	-0.4614	0.962097	1
TMPPE	1.014964	1.018866	1.013695	0.72592	-0.4621	0.962097	1
RAB37	1.014971	1.018877	1.013702	0.72585	-0.4623	0.962097	1
RP11-323J4.1	1.014967	1.018868	1.013698	0.72601	-0.4619	0.962097	1
RP11-580I16.2	1.023539	1.018979	1.025022	1.31837	0.3988	0.962106	1
RP5-1102E8.3	1.023502	1.018941	1.024984	1.31904	0.3995	0.962106	1
RFT1	1.173015	1.176931	1.171742	0.97067	-0.0429	0.962132	1
CACNB3	1.474765	1.469272	1.47655	1.01551	0.0222	0.962139	1
LPXN	1.082429	1.086457	1.081119	0.93827	-0.0919	0.962187	1
TMEM234	1.278141	1.273237	1.279735	1.02378	0.0339	0.962193	1
SLC26A10	1.046393	1.050254	1.045138	0.89821	-0.1549	0.962317	1
VN1R1	1.030762	1.02617	1.032255	1.23251	0.3016	0.962339	1
CEBPB-AS1	1.030733	1.026169	1.032217	1.23112	0.3	0.962339	1
C6orf89	1.409032	1.413278	1.407652	0.98638	-0.0198	0.962393	1
RP1-43E13.2	1.083431	1.078802	1.084936	1.07785	0.1082	0.962445	1
RP11-62F24.2	1.028524	1.032483	1.027237	0.83852	-0.2541	0.962537	1
HS3ST4	1.029649	1.033522	1.02839	0.84691	-0.2397	0.962576	1
CTA-228A9.4	1.033922	1.029438	1.035379	1.20179	0.2652	0.96264	1
KIAA0319	1.018813	1.022673	1.017558	0.77441	-0.3688	0.962702	1
MAP3K10	1.060022	1.064028	1.058719	0.91708	-0.1249	0.962879	1
RING1	1.557659	1.561776	1.556321	0.99029	-0.0141	0.962923	1
SYT16	1.057895	1.053309	1.059386	1.114	0.1558	0.962965	1
PACS2	1.339722	1.343623	1.338454	0.98496	-0.0219	0.963005	1
PAOX	1.130843	1.134929	1.129515	0.95987	-0.0591	0.963064	1
AC144449.1	1.019666	1.015217	1.021112	1.38742	0.4724	0.963074	1
LINC01431	1.097797	1.093102	1.099323	1.06682	0.0933	0.963103	1
PDE6G	1.02938	1.033239	1.028126	0.84618	-0.241	0.963159	1
RSAD2	1.026966	1.022478	1.028425	1.26457	0.3387	0.963163	1
AC011523.2	1.019737	1.01526	1.021193	1.38881	0.4738	0.963247	1
LINC01502	1.019724	1.01526	1.021175	1.38762	0.4726	0.963247	1
RP11-305L7.7	1.019748	1.01526	1.021207	1.38976	0.4748	0.963247	1
RP5-901A4.1	1.019706	1.01526	1.021151	1.38605	0.471	0.963247	1
DLX6-AS1	1.019722	1.01526	1.021173	1.38749	0.4725	0.963247	1
CH507-338C24.1	1.019718	1.01526	1.021167	1.38712	0.4721	0.963247	1
RP11-78O7.3	1.019718	1.01526	1.021167	1.38709	0.4721	0.963247	1

TMEM267	1.195702	1.190883	1.197269	1.03346	0.0475	0.963327	1
ZDHHC22	1.018142	1.013691	1.019589	1.4308	0.5168	0.963328	1
RP11-368I7.6	1.018123	1.013691	1.019564	1.42898	0.515	0.963328	1
AP000304.12	1.018152	1.013691	1.019602	1.43176	0.5178	0.963328	1
CTD-2366F13.2	1.018128	1.013691	1.01957	1.42946	0.5155	0.963328	1
TDRD9	1.018193	1.013691	1.019657	1.43576	0.5218	0.963328	1
LIN28A	1.018132	1.013691	1.019575	1.42982	0.5158	0.963328	1
LRRC2	1.018063	1.021907	1.016813	0.76747	-0.3818	0.963329	1
CTRC	1.018091	1.021993	1.016823	0.76492	-0.3866	0.963329	1
FTX	1.33318	1.33748	1.331783	0.98312	-0.0246	0.963407	1
CTC-246B18.10	1.036029	1.031577	1.037476	1.1868	0.2471	0.96342	1
PARK2	1.044017	1.039513	1.045481	1.15105	0.203	0.963463	1
AQP4	1.026184	1.030091	1.024914	0.82793	-0.2724	0.963483	1
PPIP5K1	1.057442	1.06128	1.056194	0.91701	-0.125	0.963517	1
ZNF419	1.123179	1.118526	1.124691	1.05202	0.0732	0.963523	1
PHF20	1.611219	1.616186	1.609604	0.98932	-0.0155	0.963548	1
RP11-699A5.2	1.01538	1.019204	1.014137	0.73615	-0.4419	0.963618	1
RP11-367H1.1	1.015386	1.019232	1.014136	0.73503	-0.4441	0.963618	1
IKZF1	1.015393	1.019267	1.014134	0.73362	-0.4469	0.963618	1
C9orf147	1.02974	1.033615	1.028481	0.84726	-0.2391	0.963629	1
GRIN3B	1.03511	1.030606	1.036575	1.195	0.257	0.963663	1
CERS1	1.07279	1.068233	1.074271	1.08849	0.1223	0.96368	1
NAGPA	1.108463	1.112328	1.107206	0.9544	-0.0673	0.963748	1
IDUA	1.083662	1.079153	1.085128	1.07549	0.105	0.963984	1
RP11-433J8.2	1.020472	1.016045	1.021911	1.36562	0.4496	0.964018	1
AC006004.1	1.020463	1.016045	1.021899	1.36486	0.4488	0.964018	1
BCL2L2-PABPN1	1.020454	1.016045	1.021887	1.36412	0.448	0.964018	1
AC012074.2	1.020458	1.016045	1.021892	1.36443	0.4483	0.964018	1
CNTNAP4	1.02045	1.016045	1.021882	1.36377	0.4476	0.964018	1
RP11-15B17.1	1.020437	1.016045	1.021864	1.36269	0.4465	0.964018	1
RP11-503C24.2	1.021916	1.017512	1.023347	1.33324	0.4149	0.964027	1
SNX15	1.021961	1.017512	1.023407	1.33664	0.4186	0.964027	1
FAM216B	1.021909	1.017512	1.023338	1.33272	0.4144	0.964027	1
AGRP	1.021918	1.017512	1.02335	1.33338	0.4151	0.964027	1
RP5-940J5.8	1.021958	1.017512	1.023403	1.33643	0.4184	0.964027	1
PPP1R17	1.021989	1.017512	1.023444	1.33877	0.4209	0.964027	1
AC079145.4	1.021944	1.017512	1.023384	1.33534	0.4172	0.964027	1
RP1-276E15.1	1.021938	1.017512	1.023376	1.33488	0.4167	0.964027	1
CAPRIN2	1.428851	1.423631	1.430547	1.01633	0.0234	0.964075	1
ZCWPW2	1.03965	1.043416	1.038426	0.88506	-0.1762	0.964096	1
KB-1410C5.3	1.036841	1.032448	1.038269	1.17939	0.238	0.964153	1
JMJD1C-AS1	1.039491	1.043291	1.038256	0.88368	-0.1784	0.964168	1
KB-1507C5.4	1.030475	1.026019	1.031923	1.2269	0.295	0.964217	1
SDHAF1	1.449266	1.453841	1.447778	0.98664	-0.0194	0.964248	1
MYLK4	1.02318	1.018784	1.024609	1.31013	0.3897	0.964267	1

MTUS2	1.023144	1.018795	1.024558	1.30666	0.3859	0.964267	1
RP11-399K21.14	1.0232	1.018768	1.024641	1.31291	0.3928	0.964267	1
SUSD2	1.023189	1.018756	1.02463	1.31318	0.3931	0.964267	1
RIMS4	1.021588	1.017191	1.023018	1.33893	0.4211	0.964393	1
CEL	1.02159	1.017195	1.023018	1.33863	0.4208	0.964393	1
NOXRED1	1.021598	1.01718	1.023035	1.34082	0.4231	0.964393	1
TSGA13	1.02162	1.017217	1.023051	1.33883	0.421	0.964393	1
KIFAP3	1.348464	1.343784	1.349985	1.01804	0.0258	0.964405	1
RUNX1T1	1.037702	1.033227	1.039157	1.17846	0.2369	0.964418	1
AC093901.1	1.026271	1.030114	1.025022	0.8309	-0.2673	0.964535	1
TSLP	1.022269	1.026016	1.021051	0.80915	-0.3055	0.96469	1
ZACN	1.022319	1.026193	1.02106	0.80401	-0.3147	0.96469	1
TMEM217	1.038506	1.034098	1.039938	1.17127	0.2281	0.964701	1
BRICD5	1.070872	1.074589	1.069664	0.93398	-0.0985	0.964811	1
ANKRD9	1.152297	1.156177	1.151036	0.96708	-0.0483	0.964905	1
RP11-665G4.1	1.022462	1.026195	1.021249	0.81118	-0.3019	0.964973	1
TAOK1	2.085611	2.079655	2.087548	1.00731	0.0105	0.964988	1
CTD-2168K21.2	1.023945	1.019535	1.025379	1.29913	0.3775	0.965039	1
RP11-700J17.1	1.023907	1.019548	1.025324	1.29552	0.3735	0.965039	1
RP11-22A3.1	1.023905	1.019564	1.025316	1.29401	0.3718	0.965039	1
USP2-AS1	1.0239	1.019535	1.025318	1.29603	0.3741	0.965039	1
NWD1	1.023916	1.019542	1.025338	1.29658	0.3747	0.965039	1
TMC3-AS1	1.023909	1.019545	1.025328	1.29587	0.3739	0.965039	1
C1orf56	1.477222	1.470762	1.479322	1.01818	0.026	0.965071	1
HIRA	1.110645	1.105946	1.112172	1.05877	0.0824	0.96508	1
SPATA41	1.0254	1.021029	1.02682	1.27541	0.351	0.965093	1
SEMA3G	1.025418	1.02101	1.026851	1.27801	0.3539	0.965093	1
CNIH3	1.156837	1.152107	1.158375	1.04121	0.0583	0.965158	1
RP11-693J15.5	1.053686	1.057502	1.052446	0.91206	-0.1328	0.965173	1
FAM160B2	1.233111	1.236572	1.231987	0.98062	-0.0282	0.96518	1
VTI1A	1.151549	1.15546	1.150277	0.96666	-0.0489	0.965184	1
TLN2	1.284928	1.280058	1.286511	1.02304	0.0329	0.965199	1
RARRES1	1.118412	1.114052	1.119828	1.05064	0.0713	0.965244	1
LMAN2L	1.290394	1.294511	1.289055	0.98147	-0.027	0.965255	1
CNTNAP2	1.397273	1.391915	1.399015	1.01811	0.0259	0.965257	1
EGFLAM	1.018148	1.021889	1.016932	0.77352	-0.3705	0.965267	1
RP11-557L19.1	1.018142	1.021868	1.016931	0.77423	-0.3692	0.965267	1
IAPP	1.033864	1.029594	1.035252	1.19117	0.2524	0.965267	1
RP11-849F2.9	1.033831	1.029537	1.035227	1.19264	0.2542	0.965267	1
OPHN1	1.121418	1.125132	1.120211	0.96067	-0.0579	0.965271	1
RP11-83M16.6	1.026645	1.022307	1.028055	1.25769	0.3308	0.965284	1
PHF24	1.019267	1.022981	1.018059	0.78585	-0.3477	0.965306	1
ZFPM2	1.019274	1.023008	1.01806	0.78494	-0.3493	0.965306	1
KB-1562D12.1	1.019285	1.02299	1.01808	0.78643	-0.3466	0.965306	1
NAPRT	1.17405	1.177655	1.172878	0.97311	-0.0393	0.965422	1

FGF22	1.025036	1.02069	1.026449	1.27837	0.3543	0.965454	1
SVOP	1.02507	1.020769	1.026468	1.27436	0.3498	0.965454	1
CFAP221	1.085957	1.08162	1.087366	1.0704	0.0981	0.965512	1
EIF3K	6.221348	6.232837	6.217613	0.99709	-0.0042	0.965567	1
OCM	1.018825	1.022496	1.017631	0.78376	-0.3515	0.965597	1
KCNK3	1.018809	1.0225	1.01761	0.78266	-0.3535	0.965597	1
CGB7	1.018821	1.02251	1.017622	0.78284	-0.3532	0.965597	1
RP11-500G22.5	1.018825	1.022526	1.017622	0.78227	-0.3543	0.965597	1
LRTM1	1.042	1.03764	1.043417	1.15348	0.206	0.965848	1
RP11-430B1.2	1.042045	1.03761	1.043486	1.15624	0.2094	0.965848	1
XPO4	1.185298	1.180999	1.186695	1.03147	0.0447	0.965855	1
QPCTL	1.032661	1.036381	1.031452	0.86451	-0.21	0.965881	1
POU5F1	1.019019	1.0227	1.017822	0.78509	-0.3491	0.965885	1
RP5-1125A11.7	1.019032	1.022717	1.017834	0.78505	-0.3491	0.965885	1
RP11-420N3.2	1.022229	1.017948	1.02362	1.31606	0.3962	0.965895	1
RBMXL2	1.022237	1.017948	1.023631	1.31667	0.3969	0.965895	1
GPR158-AS1	1.022246	1.017948	1.023643	1.31731	0.3976	0.965895	1
FAM151A	1.022274	1.017948	1.02368	1.31942	0.3999	0.965895	1
RP11-354E11.2	1.022237	1.017948	1.023631	1.31668	0.3969	0.965895	1
CTC-510F12.7	1.022228	1.017948	1.023619	1.31598	0.3961	0.965895	1
MYOM3	1.022234	1.017948	1.023628	1.31648	0.3967	0.965895	1
CCDC144NL	1.022249	1.017948	1.023647	1.31754	0.3979	0.965895	1
ADGRD2	1.024861	1.02871	1.02361	0.82238	-0.2821	0.965928	1
AC012507.3	1.024849	1.028535	1.023651	0.82886	-0.2708	0.965928	1
GHRLOS	1.030006	1.033697	1.028806	0.85483	-0.2263	0.966003	1
CFAP70	1.089992	1.085561	1.091432	1.06861	0.0957	0.966031	1
PLCE1	1.221421	1.21664	1.222975	1.02924	0.0416	0.966098	1
RP4-794H19.1	1.028852	1.024541	1.030253	1.23274	0.3019	0.966156	1
CTD-2377O17.1	1.028844	1.024542	1.030242	1.23224	0.3013	0.966156	1
CDKL1	1.028802	1.024502	1.030199	1.23255	0.3016	0.966156	1
RP11-408A13.4	1.028833	1.024521	1.030235	1.23301	0.3022	0.966156	1
GNG2	1.188608	1.183994	1.190107	1.03323	0.0472	0.966167	1
AC005481.5	1.016286	1.011999	1.01768	1.47343	0.5592	0.966225	1
TAS2R19	1.016246	1.011999	1.017626	1.46895	0.5548	0.966225	1
LHX4	1.016255	1.011999	1.017638	1.46994	0.5558	0.966225	1
RP11-473C18.3	1.016263	1.011999	1.017649	1.47089	0.5567	0.966225	1
PCAT5	1.016253	1.011999	1.017636	1.46974	0.5556	0.966225	1
AC011298.2	1.016254	1.011999	1.017637	1.46985	0.5557	0.966225	1
RP11-343H5.6	1.016254	1.011999	1.017637	1.46982	0.5556	0.966225	1
RP11-228B15.4	1.016267	1.011999	1.017654	1.47125	0.557	0.966225	1
LLNLR-284B4.2	1.016246	1.011999	1.017627	1.469	0.5548	0.966225	1
CTD-2256P15.4	1.016253	1.011999	1.017636	1.46979	0.5556	0.966225	1
ARHGAP22	1.01624	1.011999	1.017618	1.46829	0.5541	0.966225	1
CFLAR-AS1	1.016268	1.011999	1.017655	1.47136	0.5571	0.966225	1
AP001092.4	1.016237	1.011999	1.017614	1.46796	0.5538	0.966225	1

AC007906.1	1.01628	1.011999	1.017671	1.47271	0.5585	0.966225	1
RP3-323A16.1	1.016248	1.011999	1.01763	1.46925	0.5551	0.966225	1
GS1-590J6.3	1.016291	1.011999	1.017686	1.47393	0.5597	0.966225	1
CTD-2184C24.2	1.01623	1.011999	1.017605	1.46717	0.553	0.966225	1
C6orf99	1.016253	1.011999	1.017636	1.4698	0.5556	0.966225	1
CTC-428H11.2	1.030015	1.025786	1.031389	1.21729	0.2837	0.96624	1
RP11-38L15.3	1.030019	1.025764	1.031402	1.21886	0.2855	0.96624	1
CPA6	1.047204	1.050806	1.046033	0.90605	-0.1423	0.966252	1
GCN1	1.187943	1.191701	1.186721	0.97402	-0.038	0.96628	1
HEATR3	1.068289	1.071933	1.067104	0.93287	-0.1003	0.966338	1
SLC9A2	1.019312	1.022945	1.018132	0.79023	-0.3397	0.966354	1
RP11-278A23.1	1.019329	1.023018	1.018129	0.7876	-0.3445	0.966354	1
M1AP	1.019308	1.02295	1.018124	0.78974	-0.3406	0.966354	1
MYOG	1.019338	1.02304	1.018134	0.78707	-0.3454	0.966354	1
CTD-2026J24.1	1.019325	1.022998	1.018131	0.78838	-0.343	0.966354	1
ULBP3	1.020211	1.015934	1.021602	1.35569	0.439	0.966379	1
H2BFM	1.020196	1.015934	1.021581	1.35436	0.4376	0.966379	1
NPAS4	1.020198	1.015934	1.021583	1.35453	0.4378	0.966379	1
LINC01132	1.020183	1.015934	1.021564	1.35331	0.4365	0.966379	1
PPIAL4G	1.020209	1.015934	1.021599	1.3555	0.4388	0.966379	1
CTB-113P19.4	1.020231	1.015934	1.021628	1.35732	0.4408	0.966379	1
RP1-102G20.5	1.020206	1.015934	1.021595	1.35523	0.4385	0.966379	1
AC006994.3	1.020212	1.015934	1.021603	1.35576	0.4391	0.966379	1
AQP4-AS1	1.020213	1.015934	1.021604	1.35584	0.4392	0.966379	1
PPP1R27	1.020208	1.015934	1.021598	1.35543	0.4388	0.966379	1
RP11-541G9.2	1.020208	1.015934	1.021597	1.35537	0.4387	0.966379	1
RP11-43F13.4	1.020191	1.015934	1.021575	1.35398	0.4372	0.966379	1
ASB3	1.176356	1.180282	1.17508	0.97115	-0.0422	0.966445	1
LMLN	1.133385	1.129014	1.134805	1.04489	0.0634	0.966499	1
RP11-90P5.2	1.015384	1.019013	1.014205	0.74714	-0.4206	0.966508	1
LCMT1-AS2	1.015378	1.019007	1.014198	0.74699	-0.4208	0.966508	1
LINC00891	1.015391	1.019035	1.014206	0.74631	-0.4222	0.966508	1
AC015933.2	1.015394	1.019065	1.014201	0.74487	-0.4249	0.966508	1
NEURL4	1.062455	1.058077	1.063878	1.09988	0.1373	0.966548	1
RBBP8	1.244416	1.239858	1.245898	1.02518	0.0359	0.966554	1
FAM181B	1.027685	1.031231	1.026532	0.84954	-0.2352	0.966584	1
HIC1	1.027705	1.031283	1.026541	0.84841	-0.2372	0.966584	1
GATAD2A	1.317509	1.321105	1.316341	0.98516	-0.0216	0.966594	1
RP11-392E22.9	1.029232	1.032924	1.028032	0.85143	-0.232	0.966778	1
RP11-996F15.5	1.029193	1.032825	1.028012	0.85338	-0.2287	0.966778	1
RP11-61A14.1	1.027981	1.031608	1.026802	0.84795	-0.2379	0.966826	1
RP11-77K12.10	1.021356	1.024969	1.020182	0.80829	-0.3071	0.966838	1
BPI	1.021356	1.024987	1.020176	0.80746	-0.3085	0.966838	1
CIDEA	1.019912	1.015676	1.021289	1.35813	0.4416	0.966848	1
RP11-80H5.9	1.019936	1.015676	1.021321	1.36013	0.4437	0.966848	1

C5AR2	1.019887	1.015676	1.021256	1.35599	0.4393	0.966848	1
LRRC3-AS1	1.019901	1.015676	1.021275	1.35722	0.4407	0.966848	1
ROBO4	1.019905	1.015676	1.02128	1.35755	0.441	0.966848	1
LINC01010	1.019883	1.015676	1.02125	1.35564	0.439	0.966848	1
RP11-180C16.1	1.0199	1.015676	1.021273	1.3571	0.4405	0.966848	1
RP11-443O13.3	1.019948	1.015676	1.021337	1.36115	0.4448	0.966848	1
GHRHR	1.019882	1.015676	1.021249	1.35557	0.4389	0.966848	1
RP11-8L8.2	1.019904	1.015676	1.021278	1.35743	0.4409	0.966848	1
AC090587.5	1.019887	1.015676	1.021256	1.35598	0.4393	0.966848	1
RP4-781K5.2	1.019892	1.015676	1.021263	1.35644	0.4398	0.966848	1
GK-AS1	1.019916	1.015676	1.021294	1.35843	0.4419	0.966848	1
RP11-435J9.2	1.019898	1.015676	1.02127	1.35691	0.4403	0.966848	1
HPN	1.026491	1.030156	1.025299	0.83893	-0.2534	0.966905	1
RP1-122P22.4	1.025706	1.021449	1.02709	1.26299	0.3368	0.96692	1
EHHADH	1.025691	1.021515	1.027049	1.25723	0.3303	0.96692	1
SENP7	1.388466	1.39277	1.387067	0.98548	-0.0211	0.967043	1
DPH2	1.16221	1.157473	1.16375	1.03986	0.0564	0.967044	1
ZFP41	1.074288	1.069986	1.075686	1.08144	0.113	0.967054	1
SEPT4	1.222334	1.226491	1.220983	0.97568	-0.0355	0.967095	1
PPT2	1.134973	1.138724	1.133753	0.96417	-0.0526	0.967124	1
TCP11L1	1.079439	1.07506	1.080863	1.07731	0.1074	0.967249	1
AF146191.4	1.019699	1.015484	1.02107	1.36076	0.4444	0.96725	1
RP11-45P15.4	1.033515	1.029304	1.034884	1.19039	0.2514	0.967307	1
DSCAML1	1.023666	1.019468	1.025031	1.28579	0.3627	0.967405	1
RP11-575L7.8	1.02366	1.019477	1.025019	1.28456	0.3613	0.967405	1
NTSR1	1.020145	1.015961	1.021505	1.34734	0.4301	0.967427	1
RP11-569D9.5	1.020141	1.015961	1.021499	1.34699	0.4297	0.967427	1
RP11-410N8.4	1.020149	1.015961	1.021511	1.34771	0.4305	0.967427	1
MLN	1.020151	1.015961	1.021513	1.34784	0.4306	0.967427	1
PTCHD1	1.020147	1.015961	1.021508	1.3475	0.4303	0.967427	1
XXbac-BPG170G13.32	1.020152	1.015961	1.021515	1.34796	0.4308	0.967427	1
CHIT1	1.020165	1.015961	1.021531	1.34898	0.4319	0.967427	1
RP11-506H20.1	1.020119	1.015961	1.02147	1.34515	0.4278	0.967427	1
GBP7	1.020149	1.015961	1.02151	1.34764	0.4304	0.967427	1
LINC01358	1.020169	1.015961	1.021536	1.3493	0.4322	0.967427	1
HNF1B	1.02014	1.015961	1.021498	1.34691	0.4297	0.967427	1
CASQ1	1.020149	1.015961	1.021511	1.3477	0.4305	0.967427	1
RP11-297A16.2	1.020158	1.015961	1.021523	1.34846	0.4313	0.967427	1
GRIN1	1.020143	1.015961	1.021503	1.34722	0.43	0.967427	1
RP1-140A9.1	1.020146	1.015961	1.021507	1.34744	0.4302	0.967427	1
AC001226.7	1.020144	1.015961	1.021504	1.34727	0.43	0.967427	1
RP11-10K17.6	1.019013	1.014833	1.020372	1.37346	0.4578	0.967466	1
KRTAP11-1	1.019023	1.014833	1.020386	1.37439	0.4588	0.967466	1
TAF7L	1.019036	1.014833	1.020403	1.37554	0.46	0.967466	1
RAET1E-AS1	1.019	1.014833	1.020354	1.37228	0.4566	0.967466	1

LINC00854	1.01901	1.014833	1.020367	1.37316	0.4575	0.967466	1
AC008067.2	1.019012	1.014833	1.02037	1.37336	0.4577	0.967466	1
CTD-2256P15.1	1.019019	1.014833	1.02038	1.37402	0.4584	0.967466	1
RP11-261N11.8	1.019021	1.014833	1.020382	1.37414	0.4585	0.967466	1
SLC1A2	1.019024	1.014833	1.020387	1.37448	0.4589	0.967466	1
ZNF433	1.047902	1.043709	1.049265	1.12712	0.1726	0.967511	1
AGAP2-AS1	1.065916	1.061875	1.06723	1.08654	0.1197	0.967555	1
BCAP29	1.431249	1.426392	1.432828	1.01509	0.0216	0.967606	1
ARL15	1.139861	1.143624	1.138637	0.96528	-0.051	0.967742	1
PLEKHA6	1.109811	1.11332	1.10867	0.95896	-0.0605	0.967865	1
RP11-732A21.2	1.023365	1.019195	1.02472	1.28785	0.365	0.967874	1
SEMA3F-AS1	1.023377	1.019203	1.024734	1.28806	0.3652	0.967874	1
CHST8	1.023417	1.019175	1.024796	1.29315	0.3709	0.967874	1
RP6-74O6.2	1.023361	1.019207	1.024711	1.28656	0.3635	0.967874	1
SLFN11	1.023349	1.019177	1.024705	1.28831	0.3655	0.967874	1
RP11-63A1.1	1.023378	1.019204	1.024735	1.288	0.3651	0.967874	1
CTD-2033A16.3	1.023392	1.019228	1.024746	1.28698	0.364	0.967874	1
ZNF396	1.04888	1.044654	1.050254	1.12541	0.1704	0.967908	1
ZC3HAV1L	1.043349	1.046922	1.042187	0.89908	-0.1535	0.967914	1
RENBP	1.289598	1.293929	1.28819	0.98048	-0.0284	0.96793	1
FAM3D	1.029219	1.024988	1.030595	1.22439	0.2921	0.967941	1
RP11-25H12.1	1.029245	1.025061	1.030605	1.22124	0.2883	0.967941	1
MB	1.029147	1.025016	1.030489	1.2188	0.2855	0.967941	1
PEX5L	1.09324	1.09672	1.092109	0.95232	-0.0705	0.968041	1
BCAT2	1.619471	1.614175	1.621192	1.01142	0.0164	0.968066	1
COL4A6	1.188696	1.192197	1.187557	0.97586	-0.0353	0.96813	1
NAB2	1.38997	1.385903	1.391292	1.01396	0.02	0.968189	1
GPR176	1.09137	1.094955	1.090205	0.94998	-0.074	0.968226	1
RP11-102N12.3	1.050528	1.046414	1.051865	1.11744	0.1602	0.968268	1
HEYL	1.023177	1.018995	1.024536	1.29173	0.3693	0.968272	1
KIAA1755	1.023141	1.019016	1.024482	1.28745	0.3645	0.968272	1
HSD11B2	1.027165	1.023083	1.028492	1.23435	0.3038	0.968427	1
XXbac-B476C20.9	1.02709	1.022971	1.028429	1.23757	0.3075	0.968427	1
SLC13A5	1.023626	1.01946	1.024981	1.28369	0.3603	0.968455	1
GABRQ	1.022462	1.018332	1.023804	1.29853	0.3769	0.968494	1
RP11-355F16.1	1.022479	1.018356	1.023819	1.2976	0.3758	0.968494	1
PPP1R36	1.022438	1.018329	1.023773	1.29704	0.3752	0.968494	1
C1QTNF1	1.019579	1.023048	1.018451	0.80055	-0.3209	0.968716	1
OSR2	1.019582	1.023076	1.018446	0.79933	-0.3231	0.968716	1
KLK2	1.019593	1.023116	1.018448	0.79807	-0.3254	0.968716	1
LINC00626	1.01958	1.023051	1.018451	0.80044	-0.3211	0.968716	1
GLI1	1.019602	1.023134	1.018454	0.79771	-0.3261	0.968716	1
LINC00520	1.019584	1.023094	1.018443	0.7986	-0.3245	0.968716	1
RP11-626P14.2	1.021044	1.024534	1.019909	0.81147	-0.3014	0.968727	1
NRAS	1.622714	1.617648	1.624361	1.01087	0.0156	0.968871	1

L3MBTL4	1.029604	1.033099	1.028468	0.86008	-0.2175	0.968878	1
CTD-2002J20.1	1.026809	1.022727	1.028135	1.23797	0.308	0.968897	1
AANAT	1.052442	1.048244	1.053806	1.11529	0.1574	0.968932	1
NIPAL3	1.262709	1.257972	1.264249	1.02433	0.0347	0.968943	1
HOPX	1.03266	1.028448	1.034029	1.19616	0.2584	0.968959	1
RP11-814H16.2	1.032661	1.028496	1.034016	1.19371	0.2555	0.968959	1
PTGIS	1.072613	1.068584	1.073923	1.07785	0.1082	0.968965	1
SCN7A	1.036871	1.040554	1.035674	0.87967	-0.185	0.969013	1
CYS1	1.037713	1.033616	1.039045	1.16148	0.216	0.96909	1
YBX2	1.037734	1.033686	1.03905	1.15924	0.2132	0.96909	1
CPEB2-AS1	1.016256	1.012186	1.017579	1.44251	0.5286	0.969116	1
SLC23A3	1.016253	1.012186	1.017574	1.44213	0.5282	0.969116	1
BFSP2	1.016235	1.012186	1.017551	1.44026	0.5263	0.969116	1
RP4-564F22.5	1.016231	1.012186	1.017546	1.43978	0.5258	0.969116	1
RAD51AP2	1.016237	1.012186	1.017554	1.44045	0.5265	0.969116	1
RP11-638I2.4	1.016271	1.012186	1.017598	1.44411	0.5302	0.969116	1
RP11-316M1.3	1.01624	1.012186	1.017558	1.44077	0.5268	0.969116	1
GAL3ST1	1.016248	1.012186	1.017568	1.44159	0.5277	0.969116	1
CTC-420A11.2	1.01629	1.012186	1.017624	1.4462	0.5323	0.969116	1
CIITA	1.016262	1.012186	1.017586	1.44312	0.5292	0.969116	1
RP11-422J15.1	1.016242	1.012186	1.01756	1.44093	0.527	0.969116	1
FAM221B	1.016239	1.012186	1.017556	1.44065	0.5267	0.969116	1
C14orf105	1.016238	1.012186	1.017555	1.44056	0.5266	0.969116	1
ZUFSP	1.188472	1.192074	1.187301	0.97515	-0.0363	0.969186	1
RP11-33O4.1	1.026606	1.022493	1.027942	1.24229	0.313	0.96929	1
RP11-20J15.3	1.026597	1.022484	1.027934	1.24243	0.3132	0.96929	1
TTC23L	1.040404	1.036374	1.041714	1.14683	0.1976	0.969316	1
RP11-307N16.6	1.018926	1.014881	1.020241	1.36015	0.4438	0.969405	1
RP11-306O13.1	1.018924	1.014881	1.020239	1.36001	0.4436	0.969405	1
CTC-513N18.7	1.018959	1.014881	1.020284	1.36307	0.4469	0.969405	1
XXbac-BPG154L12.5	1.018945	1.014881	1.020265	1.36182	0.4455	0.969405	1
PRC1-AS1	1.018939	1.014881	1.020258	1.3613	0.445	0.969405	1
STMND1	1.018943	1.014881	1.020264	1.3617	0.4454	0.969405	1
CTD-2265O21.3	1.018948	1.014881	1.02027	1.36212	0.4459	0.969405	1
RP3-508I15.10	1.018916	1.014881	1.020227	1.35923	0.4428	0.969405	1
RP4-569D19.8	1.018958	1.014881	1.020284	1.36305	0.4468	0.969405	1
PXDNL	1.017258	1.020681	1.016145	0.78068	-0.3572	0.969427	1
CASC8	1.01727	1.020709	1.016152	0.77993	-0.3586	0.969427	1
RP11-333I13.1	1.017261	1.020716	1.016138	0.779	-0.3603	0.969427	1
CHST1	1.098331	1.094136	1.099695	1.05905	0.0828	0.969462	1
RP11-54O7.17	1.027059	1.022975	1.028387	1.23553	0.3051	0.969479	1
AKAP3	1.027046	1.022983	1.028366	1.23423	0.3036	0.969479	1
RASAL1	1.01884	1.022282	1.017721	0.79527	-0.3305	0.969487	1
RP11-290H9.5	1.018835	1.022251	1.017725	0.79661	-0.328	0.969487	1
SIM2	1.01884	1.02226	1.017728	0.79641	-0.3284	0.969487	1

RP11-511B23.1	1.018841	1.022274	1.017725	0.79576	-0.3296	0.969487	1
GPC5	1.018829	1.022249	1.017717	0.79631	-0.3286	0.969487	1
STX12	1.405855	1.40971	1.404602	0.98753	-0.0181	0.969495	1
AC009120.11	1.025997	1.021917	1.027323	1.24662	0.318	0.969518	1
RP11-1020A11.2	1.025887	1.021861	1.027195	1.244	0.315	0.969518	1
SEPT3	1.153516	1.149313	1.154882	1.0373	0.0528	0.96955	1
RP5-1180E21.5	1.054976	1.050937	1.056288	1.10507	0.1441	0.969555	1
CDKN2D	1.282488	1.285782	1.281417	0.98473	-0.0222	0.9696	1
AC144831.1	1.034229	1.037736	1.033089	0.87686	-0.1896	0.969658	1
STAT5B	1.183244	1.186729	1.182111	0.97527	-0.0361	0.969704	1
RP11-277A4.4	1.037029	1.040478	1.035908	0.88711	-0.1728	0.96972	1
BAIAP3	1.036519	1.039987	1.035392	0.88509	-0.1761	0.969756	1
TRIM32	1.100521	1.096418	1.101854	1.05638	0.0791	0.969794	1
BEAN1	1.018829	1.022278	1.017707	0.79482	-0.3313	0.969794	1
ALOX12-AS1	1.102437	1.098313	1.103777	1.05558	0.078	0.969835	1
TSSK3	1.030316	1.026235	1.031642	1.20611	0.2704	0.969917	1
HMGCLL1	1.055763	1.051657	1.057098	1.10533	0.1445	0.969954	1
CTD-2547L16.1	1.036115	1.032094	1.037422	1.166	0.2216	0.969973	1
MOB1A	2.009385	2.014343	2.007773	0.99352	-0.0094	0.969999	1
AC068831.10	1.041204	1.037128	1.04253	1.14549	0.196	0.970094	1
RP11-479O9.4	1.019735	1.015696	1.021048	1.34094	0.4232	0.970146	1
AC007193.10	1.01972	1.015691	1.021029	1.3402	0.4224	0.970146	1
RP11-93K22.13	1.019676	1.015693	1.020971	1.33637	0.4183	0.970146	1
AFDN	1.591799	1.58711	1.593323	1.01058	0.0152	0.97017	1
ZNF518B	1.364497	1.368053	1.363342	0.9872	-0.0186	0.970289	1
RP11-305K5.1	1.030063	1.02601	1.031381	1.20649	0.2708	0.970305	1
RPUSD2	1.147194	1.142982	1.148563	1.03904	0.0552	0.970307	1
F7	1.040398	1.043786	1.039297	0.89747	-0.1561	0.970373	1
FTCD	1.043861	1.039833	1.04517	1.13397	0.1814	0.970412	1
RP11-274H2.3	1.022393	1.018384	1.023696	1.28898	0.3662	0.970435	1
RP11-171I2.1	1.034019	1.03002	1.035319	1.17651	0.2345	0.970461	1
ADAP1	1.030791	1.034126	1.029707	0.87053	-0.2	0.970476	1
AP000692.10	1.030566	1.026451	1.031904	1.20616	0.2704	0.970499	1
BZW1	3.450492	3.443904	3.452633	1.00357	0.0051	0.970522	1
UBLCP1	1.358014	1.361479	1.356887	0.9873	-0.0184	0.970531	1
RP11-15H20.8	1.029372	1.025313	1.030692	1.21251	0.278	0.970538	1
ADGRA3	1.352148	1.347648	1.35361	1.01715	0.0245	0.970554	1
IGF1	1.068269	1.064348	1.069543	1.08073	0.112	0.970583	1
RP11-538D16.2	1.015862	1.011871	1.017159	1.44547	0.5315	0.970637	1
RP1-137D17.1	1.015838	1.011871	1.017127	1.44277	0.5288	0.970637	1
RP11-282A11.3	1.015836	1.011871	1.017125	1.44261	0.5287	0.970637	1
RP13-20L14.1	1.015829	1.011871	1.017115	1.44177	0.5278	0.970637	1
GNG13	1.015841	1.011871	1.017131	1.44314	0.5292	0.970637	1
NPIPB4	1.033101	1.036471	1.032006	0.87756	-0.1884	0.970681	1
SAMD1	1.492968	1.48819	1.494521	1.01297	0.0186	0.970681	1

CLCN6	1.148767	1.144742	1.150076	1.03685	0.0522	0.970704	1
ZNF623	1.114056	1.109959	1.115388	1.04937	0.0695	0.970711	1
RP11-147L13.11	1.121067	1.124266	1.120027	0.96589	-0.0501	0.970722	1
MRPL28	2.282789	2.276743	2.284755	1.00628	0.009	0.970733	1
LINC01389	1.015375	1.018741	1.014281	0.76204	-0.3921	0.970743	1
DNAJB7	1.015385	1.01874	1.014294	0.76275	-0.3907	0.970743	1
RP11-345P4.10	1.015369	1.018712	1.014282	0.76326	-0.3898	0.970743	1
RP13-514E23.2	1.015384	1.018775	1.014282	0.76069	-0.3946	0.970743	1
SLC4A1	1.015372	1.018741	1.014277	0.76181	-0.3925	0.970743	1
ACR	1.015396	1.01879	1.014292	0.76062	-0.3947	0.970743	1
RP11-116D17.1	1.015371	1.018746	1.014275	0.76149	-0.3931	0.970743	1
AC104654.2	1.022649	1.026083	1.021533	0.82555	-0.2766	0.970763	1
RHCG	1.022647	1.025958	1.021571	0.83101	-0.2671	0.970763	1
LSAMP-AS1	1.022633	1.025955	1.021553	0.83038	-0.2682	0.970763	1
TCF7	1.238756	1.234507	1.240137	1.02401	0.0342	0.970815	1
ZNF675	1.131412	1.127222	1.132775	1.04364	0.0616	0.970927	1
FAM175B	1.217104	1.212833	1.218493	1.02659	0.0379	0.970942	1
FLAD1	1.48598	1.489554	1.484818	0.99033	-0.014	0.970968	1
LBHD1	1.015235	1.011304	1.016513	1.46074	0.5467	0.971109	1
CDHR2	1.015245	1.011304	1.016526	1.46196	0.5479	0.971109	1
RP4-742J24.2	1.015266	1.011304	1.016554	1.46436	0.5503	0.971109	1
RP13-39P12.3	1.01528	1.011304	1.016573	1.46605	0.5519	0.971109	1
RP11-54A4.2	1.01528	1.011304	1.016573	1.46605	0.5519	0.971109	1
APOBEC2	1.015237	1.011304	1.016516	1.461	0.547	0.971109	1
SIGLEC10	1.015228	1.011304	1.016504	1.45997	0.5459	0.971109	1
CTC-1337H24.4	1.015233	1.011304	1.01651	1.46055	0.5465	0.971109	1
AC140912.1	1.015235	1.011304	1.016513	1.46074	0.5467	0.971109	1
RP11-211G23.2	1.015235	1.011304	1.016512	1.4607	0.5467	0.971109	1
RP11-384P7.5	1.015243	1.011304	1.016524	1.46172	0.5477	0.971109	1
RP11-879F14.1	1.015245	1.011304	1.016526	1.46189	0.5478	0.971109	1
FERMT3	1.015228	1.011304	1.016503	1.45992	0.5459	0.971109	1
AC007285.6	1.015229	1.011304	1.016504	1.45998	0.546	0.971109	1
TMPRSS2	1.015235	1.011304	1.016513	1.46076	0.5467	0.971109	1
CTD-2340E1.2	1.015231	1.011304	1.016507	1.46028	0.5462	0.971109	1
HAND1	1.01524	1.011304	1.016519	1.46133	0.5473	0.971109	1
ETV7	1.029048	1.032466	1.027937	0.8605	-0.2167	0.971134	1
CH17-360D5.2	1.028991	1.032345	1.027901	0.8626	-0.2132	0.971134	1
DSCAM	1.023134	1.019199	1.024413	1.2716	0.3466	0.971173	1
CTD-2342J14.6	1.023115	1.019196	1.024389	1.27052	0.3454	0.971173	1
HSPB2	1.023145	1.019189	1.024432	1.27322	0.3485	0.971173	1
RABL6	1.692217	1.687715	1.69368	1.00867	0.0125	0.971173	1
ACHE	1.15392	1.149907	1.155224	1.03547	0.0503	0.971215	1
CNOT6L	1.32608	1.322095	1.327376	1.01639	0.0235	0.971229	1
LCA5L	1.07668	1.080153	1.075551	0.9426	-0.0853	0.971241	1
AC007879.2	1.016096	1.012187	1.017366	1.425	0.511	0.971371	1

RP11-342I1.2	1.016099	1.012187	1.01737	1.42535	0.5113	0.971371	1
LINC01133	1.016093	1.012187	1.017363	1.42476	0.5107	0.971371	1
C10orf128	1.016102	1.012187	1.017375	1.42571	0.5117	0.971371	1
TEX12	1.016099	1.012187	1.017371	1.42543	0.5114	0.971371	1
SPATA22	1.016101	1.012187	1.017373	1.4256	0.5116	0.971371	1
RIPPLY2	1.016126	1.012187	1.017406	1.42828	0.5143	0.971371	1
CHST4	1.016113	1.012187	1.01739	1.42694	0.5129	0.971371	1
GCG	1.016087	1.012187	1.017355	1.42406	0.51	0.971371	1
RP11-423H2.3	1.02736	1.03063	1.026297	0.85854	-0.22	0.97141	1
RP11-404F10.2	1.017859	1.013947	1.019131	1.37175	0.456	0.971411	1
DRD1	1.017885	1.013947	1.019165	1.37416	0.4585	0.971411	1
PNMA5	1.017883	1.013947	1.019162	1.37397	0.4584	0.971411	1
RP11-30L15.6	1.017876	1.013947	1.019153	1.37332	0.4577	0.971411	1
GAS2L2	1.017874	1.013947	1.01915	1.37311	0.4575	0.971411	1
RP11-1228E12.1	1.017894	1.013947	1.019177	1.37502	0.4594	0.971411	1
AC003958.2	1.017876	1.013947	1.019153	1.3733	0.4576	0.971411	1
FRMPD2	1.017864	1.013947	1.019138	1.37222	0.4565	0.971411	1
CCDC177	1.017894	1.013947	1.019177	1.37504	0.4595	0.971411	1
MYO1H	1.017867	1.013947	1.019141	1.37246	0.4568	0.971411	1
Z83851.4	1.017862	1.013947	1.019135	1.37199	0.4563	0.971411	1
RP11-169E6.1	1.017861	1.013947	1.019133	1.37189	0.4562	0.971411	1
MIR137HG	1.017883	1.013947	1.019162	1.37399	0.4584	0.971411	1
AHSG	1.017888	1.013947	1.019169	1.37444	0.4588	0.971411	1
HIST1H2BM	1.017861	1.013947	1.019133	1.37185	0.4561	0.971411	1
NDST1	1.216129	1.219353	1.215081	0.98052	-0.0284	0.971421	1
ZNF843	1.060041	1.063427	1.05894	0.92925	-0.1059	0.971432	1
STK36	1.16039	1.156438	1.161675	1.03348	0.0475	0.971475	1
RP4-794I6.4	1.027349	1.030752	1.026243	0.85338	-0.2287	0.971521	1
RP11-114H21.2	1.019778	1.015826	1.021062	1.33087	0.4124	0.971589	1
RP11-231I16.1	1.019763	1.015826	1.021043	1.32963	0.411	0.971589	1
LINC00379	1.019731	1.015826	1.021	1.32694	0.4081	0.971589	1
LINC01254	1.019737	1.015826	1.021009	1.32746	0.4087	0.971589	1
TRPV4	1.019756	1.015826	1.021033	1.32901	0.4104	0.971589	1
RP11-676J12.4	1.019775	1.015826	1.021059	1.33064	0.4121	0.971589	1
REP15	1.019729	1.015826	1.020997	1.32676	0.4079	0.971589	1
AC002550.5	1.019742	1.015826	1.021016	1.3279	0.4091	0.971589	1
SCGB1D4	1.019752	1.015826	1.021028	1.32866	0.41	0.971589	1
WDR97	1.019733	1.015826	1.021003	1.32714	0.4083	0.971589	1
RP11-491F9.5	1.019737	1.015826	1.021009	1.32747	0.4087	0.971589	1
LINC00929	1.016997	1.013073	1.018273	1.3978	0.4832	0.971651	1
RP3-470B24.5	1.016976	1.013073	1.018245	1.39566	0.4809	0.971651	1
AC108004.2	1.016982	1.013073	1.018253	1.3963	0.4816	0.971651	1
RP5-956O18.3	1.016973	1.013073	1.018241	1.39536	0.4806	0.971651	1
PCDHGA1	1.016992	1.013073	1.018266	1.39728	0.4826	0.971651	1
CTD-2616J11.14	1.016982	1.013073	1.018253	1.39629	0.4816	0.971651	1

RP11-571I18.4	1.016961	1.013073	1.018226	1.39419	0.4794	0.971651	1
GSTA1	1.016971	1.013073	1.018238	1.39513	0.4804	0.971651	1
C8orf89	1.016994	1.013073	1.018268	1.39745	0.4828	0.971651	1
RP11-797D24.4	1.016961	1.013073	1.018225	1.39417	0.4794	0.971651	1
RP1-59D14.5	1.01701	1.013073	1.01829	1.3991	0.4845	0.971651	1
CTD-2189E23.1	1.019339	1.015372	1.020628	1.34197	0.4243	0.97167	1
AC062028.1	1.019302	1.015405	1.020569	1.33525	0.4171	0.97167	1
CD300A	1.079533	1.082785	1.078476	0.94795	-0.0771	0.971676	1
KDM6A	1.145299	1.148342	1.144309	0.97281	-0.0398	0.971679	1
RMDN3	1.291216	1.286938	1.292606	1.01975	0.0282	0.971707	1
KLK11	1.019167	1.022477	1.018091	0.80486	-0.3132	0.97171	1
RP4-604G5.1	1.019191	1.022498	1.018115	0.80519	-0.3126	0.97171	1
RP11-290M5.4	1.019218	1.022569	1.018128	0.80323	-0.3161	0.97171	1
HAPLN1	1.019182	1.0225	1.018103	0.80457	-0.3137	0.97171	1
SERPINI2	1.019173	1.022477	1.018099	0.80526	-0.3125	0.97171	1
TRDN	1.019186	1.022462	1.018121	0.80675	-0.3098	0.97171	1
LEMD3	1.199006	1.202454	1.197885	0.97743	-0.0329	0.971731	1
KCNN3	1.047744	1.050905	1.046716	0.91771	-0.1239	0.971756	1
RP5-1113E3.3	1.016991	1.013102	1.018255	1.39336	0.4786	0.971762	1
PLK5	1.016995	1.013102	1.018261	1.39378	0.479	0.971762	1
IDH3A	1.194272	1.197661	1.19317	0.97728	-0.0332	0.971917	1
AQP5	1.088927	1.085081	1.090178	1.0599	0.0839	0.971946	1
SPINK9	1.01715	1.013274	1.01841	1.3869	0.4719	0.972012	1
FGL2	1.017164	1.013274	1.018429	1.3883	0.4733	0.972012	1
RP1-137H15.2	1.017137	1.013274	1.018393	1.38562	0.4705	0.972012	1
RP11-560I19.1	1.017142	1.013274	1.018399	1.38607	0.471	0.972012	1
RP5-1116H23.5	1.017144	1.013274	1.018401	1.38625	0.4712	0.972012	1
FUT6	1.017168	1.013274	1.018434	1.38869	0.4737	0.972012	1
GRK1	1.017162	1.013274	1.018425	1.38805	0.4731	0.972012	1
DLEC1	1.017144	1.013274	1.018402	1.38634	0.4713	0.972012	1
HHLA2	1.017134	1.013274	1.018389	1.38529	0.4702	0.972012	1
CCDC182	1.017146	1.013274	1.018404	1.38648	0.4714	0.972012	1
RP11-603J24.17	1.01717	1.013274	1.018437	1.38894	0.474	0.972012	1
RP11-514P8.6	1.01716	1.013274	1.018422	1.38785	0.4728	0.972012	1
AC005616.2	1.017191	1.013274	1.018465	1.39102	0.4761	0.972012	1
CCR6	1.025586	1.028893	1.024511	0.84832	-0.2373	0.972067	1
CACNA1H	1.156294	1.152481	1.157533	1.03313	0.047	0.972137	1
CTD-2331H12.7	1.018704	1.01479	1.019977	1.35067	0.4337	0.972143	1
NOTUM	1.026566	1.022709	1.027819	1.22505	0.2928	0.972196	1
FBXL16	1.023861	1.027142	1.022795	0.83984	-0.2518	0.972346	1
NPPA-AS1	1.019528	1.015669	1.020783	1.32635	0.4075	0.972406	1
RGS7	1.019569	1.015685	1.020832	1.32814	0.4094	0.972406	1
AC093627.10	1.0213	1.017447	1.022552	1.29264	0.3703	0.972445	1
PCOLCE-AS1	1.041035	1.037158	1.042295	1.13826	0.1868	0.972481	1
RP4-671G15.2	1.029318	1.025427	1.030582	1.20273	0.2663	0.972487	1

BHLHE40-AS1	1.026717	1.030079	1.025625	0.85191	-0.2312	0.97252	1
RP11-318A15.8	1.026278	1.02969	1.025168	0.8477	-0.2384	0.972541	1
UBXN10	1.026154	1.029407	1.025097	0.85343	-0.2287	0.972541	1
CAPZA1	2.586768	2.589978	2.585725	0.99733	-0.0039	0.972581	1
MAGEE1	1.075172	1.071119	1.076489	1.0755	0.105	0.972588	1
PARP10	1.065281	1.061325	1.066567	1.08548	0.1183	0.972597	1
RP11-77H9.2	1.023234	1.019323	1.024505	1.26818	0.3428	0.972624	1
KLK6	1.282359	1.285787	1.281244	0.98411	-0.0231	0.972646	1
NR3C1	1.174674	1.178024	1.173585	0.97507	-0.0364	0.972685	1
AC079354.3	1.020429	1.01658	1.02168	1.30757	0.3869	0.972686	1
CACNG6	1.02475	1.027985	1.023699	0.84681	-0.2399	0.972699	1
TAT-AS1	1.022707	1.018871	1.023954	1.26935	0.3441	0.972699	1
CTBP1-AS	1.022756	1.018885	1.024015	1.27165	0.3467	0.972699	1
RP11-820I16.1	1.022762	1.018869	1.024027	1.27333	0.3486	0.972699	1
TEKT3	1.023015	1.026386	1.021919	0.83073	-0.2675	0.972739	1
LINC00858	1.020446	1.016597	1.021697	1.30731	0.3866	0.972797	1
KCNK9	1.020448	1.016633	1.021689	1.30399	0.3829	0.972797	1
AP001437.1	1.020445	1.016635	1.021683	1.30347	0.3824	0.972797	1
WDR38	1.020441	1.016599	1.02169	1.30671	0.3859	0.972797	1
NEFL	1.112056	1.107785	1.113445	1.05251	0.0738	0.972833	1
LATS1	1.258441	1.254118	1.259846	1.02254	0.0322	0.972844	1
EIF2S1	1.49024	1.49356	1.489161	0.99109	-0.0129	0.972869	1
PCDHB11	1.046532	1.042608	1.047807	1.12202	0.1661	0.972995	1
BRD3	2.173895	2.168908	2.175516	1.00565	0.0081	0.972998	1
RP11-188P20.3	1.022167	1.02543	1.021106	0.82997	-0.2689	0.973002	1
CNTD2	1.022114	1.025316	1.021073	0.8324	-0.2646	0.973002	1
RP11-161H23.5	1.020619	1.016782	1.021866	1.30295	0.3818	0.973047	1
ZNF639	1.296287	1.299999	1.29508	0.9836	-0.0239	0.973053	1
FBXL12	1.296324	1.299678	1.295234	0.98517	-0.0216	0.973053	1
ZNF418	1.071233	1.07442	1.070198	0.94327	-0.0843	0.973085	1
PFN4	1.051622	1.047803	1.052863	1.10586	0.1452	0.973086	1
KLHL17	1.052591	1.055908	1.051513	0.92139	-0.1181	0.973087	1
ARHGAP24	1.142571	1.14587	1.141499	0.97003	-0.0439	0.973211	1
WFDC3	1.030069	1.026189	1.03133	1.19632	0.2586	0.973216	1
METTL21B	1.157833	1.15399	1.159082	1.03307	0.0469	0.97322	1
SEL1L	1.358166	1.361157	1.357194	0.98903	-0.0159	0.973258	1
PDE1B	1.020443	1.023647	1.019401	0.82045	-0.2855	0.973286	1
C4orf22	1.020418	1.023597	1.019385	0.8215	-0.2837	0.973286	1
KIAA0825	1.054281	1.050522	1.055504	1.09861	0.1357	0.973293	1
AC023481.1	1.020409	1.023605	1.01937	0.82056	-0.2853	0.973397	1
SPPL3	1.462903	1.466303	1.461798	0.99034	-0.014	0.9734	1
RP11-413H22.3	1.022981	1.019215	1.024206	1.25972	0.3331	0.973436	1
CTD-2376I4.2	1.02299	1.019213	1.024218	1.26054	0.334	0.973436	1
CASC6	1.023196	1.026344	1.022173	0.84165	-0.2487	0.973459	1
RP11-686G8.5	1.023192	1.026408	1.022147	0.83866	-0.2538	0.973459	1

RP11-309M23.1	1.024786	1.021009	1.026014	1.23822	0.3083	0.973476	1
RP11-131L23.1	1.022731	1.025963	1.021681	0.83509	-0.26	0.973476	1
FUT1	1.022794	1.026108	1.021717	0.83179	-0.2657	0.973476	1
POU6F1	1.032743	1.029005	1.033958	1.17077	0.2275	0.973507	1
AF131217.1	1.040918	1.037073	1.042167	1.13743	0.1858	0.973539	1
RP11-16N11.2	1.040909	1.037088	1.042152	1.13655	0.1847	0.973539	1
MED20	1.192945	1.188744	1.194311	1.0295	0.0419	0.973544	1
FBXO8	1.2207	1.22391	1.219656	0.981	-0.0277	0.973574	1
RP1-266L20.2	1.021306	1.024464	1.02028	0.82895	-0.2706	0.973638	1
SH2D6	1.021316	1.024503	1.02028	0.82762	-0.273	0.973638	1
LRRC26	1.023859	1.02008	1.025087	1.24939	0.3212	0.973717	1
CTD-2320G14.2	1.026229	1.022417	1.027468	1.22531	0.2931	0.973725	1
RP11-950C14.3	1.026181	1.022373	1.027419	1.22555	0.2934	0.973725	1
RP11-329B9.5	1.029335	1.032523	1.028299	0.87011	-0.2007	0.97378	1
RP11-525G13.2	1.023969	1.020151	1.02521	1.25106	0.3231	0.973828	1
AC007292.6	1.023912	1.020136	1.025139	1.24846	0.3202	0.973828	1
CRHR1	1.023887	1.02015	1.025101	1.24573	0.317	0.973828	1
RP11-344B5.3	1.023915	1.020136	1.025143	1.24868	0.3204	0.973828	1
MAVS	1.482172	1.477759	1.483606	1.01224	0.0175	0.973876	1
SCN11A	1.036395	1.039684	1.035326	0.8902	-0.1678	0.97388	1
EEF1A2	1.078558	1.081674	1.077545	0.94945	-0.0748	0.973924	1
CTD-2129N1.1	1.018712	1.02201	1.01764	0.80145	-0.3193	0.973941	1
RP11-624J6.2	1.017153	1.020302	1.01613	0.7945	-0.3319	0.973979	1
LINC00843	1.017147	1.020301	1.016121	0.79409	-0.3326	0.973979	1
RP11-162A12.4	1.017135	1.020272	1.016116	0.79498	-0.331	0.973979	1
SMPX	1.017141	1.020275	1.016122	0.79519	-0.3306	0.973979	1
RP11-227D13.1	1.050006	1.046226	1.051234	1.10835	0.1484	0.973996	1
SMC2-AS1	1.050073	1.046253	1.051315	1.10943	0.1498	0.973996	1
RP11-468E2.4	1.067826	1.070978	1.066802	0.94117	-0.0875	0.974003	1
FAM155B	1.024026	1.020274	1.025246	1.24519	0.3164	0.974079	1
RP11-336K24.12	1.024048	1.020334	1.025255	1.24198	0.3126	0.974079	1
PYCARD-AS1	1.024022	1.02025	1.025248	1.24678	0.3182	0.974079	1
TAS2R31	1.025557	1.021841	1.026764	1.2254	0.2933	0.9742	1
OVOL1	1.016974	1.020093	1.01596	0.79428	-0.3323	0.974229	1
SPG20-AS1	1.016995	1.020132	1.015975	0.79353	-0.3336	0.974229	1
TUSC8	1.016984	1.020089	1.015975	0.79521	-0.3306	0.974229	1
CYP51A1-AS1	1.016987	1.020127	1.015966	0.79324	-0.3342	0.974229	1
RP11-471B22.3	1.017	1.020114	1.015988	0.79486	-0.3312	0.974229	1
RP11-613D13.5	1.016984	1.020122	1.015964	0.79338	-0.3339	0.974229	1
MKNK1	1.263139	1.26612	1.262171	0.98516	-0.0216	0.974229	1
GNL1	1.615862	1.611848	1.617167	1.00869	0.0125	0.974247	1
KCNJ11	1.019202	1.015453	1.02042	1.3214	0.4021	0.97428	1
CTD-2555O16.1	1.019177	1.015453	1.020387	1.31923	0.3997	0.97428	1
RP11-675F6.3	1.019186	1.015453	1.020399	1.32004	0.4006	0.97428	1
CCDC152	1.019176	1.015453	1.020385	1.31915	0.3996	0.97428	1

PIK3IP1-AS1	1.019168	1.015453	1.020375	1.31849	0.3989	0.97428	1
SALRNA2	1.019187	1.015453	1.020401	1.32014	0.4007	0.97428	1
RP11-136L23.2	1.019192	1.015453	1.020408	1.32059	0.4012	0.97428	1
RP11-549B18.1	1.019174	1.015453	1.020383	1.31901	0.3995	0.97428	1
RCSD1	1.016967	1.020067	1.015959	0.79527	-0.3305	0.97434	1
ASB16	1.019733	1.022839	1.018723	0.81979	-0.2867	0.974402	1
ERICH3	1.019722	1.022834	1.01871	0.8194	-0.2874	0.974402	1
RP11-354P11.8	1.019755	1.022927	1.018724	0.81667	-0.2922	0.974402	1
LINC00639	1.019767	1.022935	1.018737	0.81695	-0.2917	0.974402	1
S100P	1.019726	1.022859	1.018707	0.81835	-0.2892	0.974402	1
RP11-159F24.5	1.019756	1.022902	1.018734	0.81797	-0.2899	0.974402	1
GRIP2	1.019272	1.022364	1.018267	0.8168	-0.2919	0.974414	1
MTMR8	1.019299	1.022383	1.018297	0.81745	-0.2908	0.974414	1
CCDC163	1.050744	1.053838	1.049738	0.92385	-0.1143	0.974426	1
COLQ	1.037153	1.04026	1.036143	0.89775	-0.1556	0.974504	1
CTD-2541M15.3	1.036218	1.032459	1.037439	1.15343	0.2059	0.974524	1
SUPT4H1	2.634606	2.638888	2.633214	0.99654	-0.005	0.974552	1
AC006262.4	1.017847	1.020942	1.016841	0.80419	-0.3144	0.974581	1
KLHL33	1.017872	1.020988	1.016859	0.80326	-0.3161	0.974581	1
FAM124B	1.017857	1.020961	1.016848	0.8038	-0.3151	0.974581	1
HCG20	1.017852	1.020961	1.016841	0.80345	-0.3157	0.974581	1
AC092881.1	1.016109	1.019256	1.015086	0.78343	-0.3521	0.97462	1
KLHL34	1.016095	1.019196	1.015087	0.78594	-0.3475	0.97462	1
AC009410.1	1.016093	1.019204	1.015082	0.78535	-0.3486	0.97462	1
CD300C	1.016087	1.019174	1.015084	0.78668	-0.3462	0.97462	1
GGT6	1.016092	1.019181	1.015088	0.78658	-0.3463	0.97462	1
KCNIP3	1.048148	1.051213	1.047152	0.9207	-0.1192	0.974626	1
MAP2K3	1.264806	1.268232	1.263693	0.98308	-0.0246	0.974635	1
VCX	1.030149	1.026391	1.03137	1.18865	0.2493	0.974683	1
AC087294.2	1.027362	1.023599	1.028585	1.21128	0.2765	0.974745	1
ZNF233	1.027333	1.023573	1.028555	1.21132	0.2766	0.974745	1
SPN	1.029627	1.02591	1.030835	1.19008	0.2511	0.974748	1
KRT86	1.027347	1.023577	1.028573	1.21187	0.2772	0.974856	1
AC226118.1	1.027402	1.023637	1.028626	1.21106	0.2763	0.974856	1
FSTL4	1.02736	1.023617	1.028577	1.21003	0.275	0.974856	1
HRASLS2	1.015237	1.018319	1.014235	0.77705	-0.3639	0.974883	1
ASCL5	1.015221	1.018287	1.014224	0.7778	-0.3625	0.974883	1
RP11-421M1.8	1.037477	1.040584	1.036467	0.89855	-0.1543	0.974977	1
KIAA0391	1.045701	1.048707	1.044723	0.9182	-0.1231	0.97498	1
RP13-516M14.4	1.053526	1.049856	1.054719	1.09753	0.1343	0.974993	1
RBM14-RBM4	1.02315	1.026244	1.022144	0.84376	-0.2451	0.975003	1
GAB2	1.062688	1.065689	1.061712	0.93947	-0.0901	0.97518	1
RP11-420A6.2	1.029104	1.025417	1.030302	1.19221	0.2536	0.975223	1
RAB26	1.028974	1.025305	1.030167	1.19213	0.2535	0.975223	1
C2orf80	1.015374	1.011717	1.016562	1.41347	0.4992	0.975247	1

RP11-70D24.3	1.015374	1.011717	1.016563	1.41357	0.4993	0.975247	1
TDRD12	1.015375	1.011717	1.016565	1.41369	0.4995	0.975247	1
RP11-480D4.6	1.015374	1.011717	1.016563	1.41357	0.4993	0.975247	1
RP1-265C24.8	1.01541	1.011717	1.01661	1.41756	0.5034	0.975247	1
OGFR-AS1	1.015383	1.011717	1.016574	1.41453	0.5003	0.975247	1
BCL2L14	1.015385	1.011717	1.016577	1.41476	0.5006	0.975247	1
AC009264.1	1.0154	1.011717	1.016598	1.4165	0.5023	0.975247	1
HIST1H3F	1.015373	1.011717	1.016561	1.41337	0.4991	0.975247	1
RP11-927P21.2	1.015392	1.011717	1.016586	1.41551	0.5013	0.975247	1
AC137932.6	1.015378	1.011717	1.016567	1.41393	0.4997	0.975247	1
SYT17	1.416836	1.412711	1.418176	1.01324	0.019	0.975264	1
GOLGA8H	1.022642	1.018953	1.023842	1.25793	0.331	0.975319	1
CTD-2588E21.1	1.022632	1.018981	1.023819	1.25489	0.3276	0.975319	1
GALNTL6	1.022631	1.018966	1.023823	1.25611	0.329	0.975319	1
LINC01135	1.015826	1.018891	1.01483	0.78502	-0.3492	0.975355	1
RP4-798A10.4	1.015836	1.018913	1.014836	0.78442	-0.3503	0.975355	1
RP11-235C23.5	1.015837	1.018872	1.014851	0.78693	-0.3457	0.975355	1
ENOX1	1.047279	1.050378	1.046272	0.9185	-0.1226	0.975369	1
EID3	1.054566	1.057677	1.053555	0.92853	-0.107	0.97541	1
POMT1	1.219682	1.222984	1.218609	0.98038	-0.0286	0.975422	1
DOCK2	1.039487	1.04264	1.038462	0.90201	-0.1488	0.975466	1
C9orf50	1.031712	1.028009	1.032916	1.17517	0.2329	0.975527	1
CBLN3	1.03963	1.035997	1.04081	1.1337	0.181	0.975537	1
NCR3LG1	1.047736	1.044033	1.048939	1.11142	0.1524	0.975549	1
C10orf67	1.047746	1.044142	1.048918	1.1082	0.1482	0.975549	1
LGALS9	1.022393	1.025448	1.0214	0.84092	-0.25	0.975649	1
DRD5	1.022399	1.025472	1.0214	0.84013	-0.2513	0.975649	1
ZDHHC24	1.514537	1.51724	1.513658	0.99308	-0.01	0.975676	1
FAIM2	1.078712	1.07518	1.07986	1.06225	0.0871	0.975849	1
SPDYA	1.030848	1.027285	1.032005	1.17299	0.2302	0.975881	1
ZNF202	1.076507	1.07285	1.077695	1.0665	0.0929	0.975897	1
NTRK3	1.042275	1.045317	1.041286	0.91105	-0.1344	0.975931	1
SUSD6	1.138464	1.141417	1.137504	0.97233	-0.0405	0.975977	1
TEX26	1.056868	1.05315	1.058077	1.0927	0.1279	0.975987	1
CTD-2349P21.12	1.030979	1.027533	1.0321	1.16585	0.2214	0.976132	1
PCGF1	1.146152	1.149319	1.145122	0.97189	-0.0411	0.976155	1
SLC38A3	1.042799	1.045822	1.041817	0.91259	-0.132	0.976178	1
PELI1	1.187928	1.190792	1.186997	0.98011	-0.029	0.976198	1
RP11-435O5.4	1.018827	1.015229	1.019997	1.31308	0.3929	0.976288	1
NPAP1	1.018823	1.015209	1.019998	1.31488	0.3949	0.976288	1
TLL1	1.0439	1.046832	1.042946	0.91704	-0.1249	0.976315	1
RP11-218M22.1	1.06148	1.064406	1.060529	0.9398	-0.0896	0.976331	1
LACTB2-AS1	1.026093	1.022466	1.027272	1.21396	0.2797	0.976355	1
EXTL1	1.036099	1.039081	1.03513	0.89891	-0.1538	0.976387	1
RBM20	1.036108	1.039074	1.035144	0.89941	-0.1529	0.976387	1

KDM8	1.04119	1.044239	1.040199	0.90867	-0.1382	0.976446	1
CYP21A2	1.01886	1.01526	1.020031	1.31265	0.3925	0.976496	1
MED4-AS1	1.018848	1.01526	1.020015	1.31161	0.3913	0.976496	1
TIE1	1.018863	1.01526	1.020034	1.31287	0.3927	0.976496	1
IZUMO2	1.01885	1.01526	1.020017	1.31176	0.3915	0.976496	1
RP3-382I10.7	1.018858	1.01526	1.020028	1.31248	0.3923	0.976496	1
RP11-22C11.2	1.018847	1.01526	1.020013	1.31151	0.3912	0.976496	1
CTD-2310F14.1	1.018866	1.01526	1.020039	1.31318	0.3931	0.976496	1
AF131215.6	1.018877	1.01526	1.020053	1.31408	0.3941	0.976496	1
LINC01289	1.018846	1.01526	1.020012	1.31143	0.3911	0.976496	1
DEFB134	1.018842	1.01526	1.020006	1.31106	0.3907	0.976496	1
AC004637.1	1.018863	1.01526	1.020034	1.31286	0.3927	0.976496	1
CTD-2105E13.16	1.018856	1.01526	1.020025	1.31227	0.3921	0.976496	1
AC013275.2	1.018859	1.01526	1.020029	1.31253	0.3924	0.976496	1
EXOC3L2	1.01884	1.01526	1.020003	1.31086	0.3905	0.976496	1
RP11-386G21.2	1.018864	1.01526	1.020036	1.31298	0.3928	0.976496	1
VAC14-AS1	1.018839	1.01526	1.020002	1.31077	0.3904	0.976496	1
RP11-322E11.6	1.01885	1.01526	1.020017	1.31175	0.3915	0.976496	1
CTC-524C5.2	1.226096	1.222618	1.227226	1.0207	0.0296	0.976529	1
AC011997.1	1.017281	1.013691	1.018449	1.34752	0.4303	0.976563	1
RP11-338N10.3	1.017285	1.013691	1.018453	1.34787	0.4307	0.976563	1
CTD-2091N23.1	1.017263	1.013691	1.018424	1.34575	0.4284	0.976563	1
RP11-318C2.1	1.017279	1.013691	1.018446	1.34732	0.4301	0.976563	1
PRSS45	1.017263	1.013691	1.018425	1.34576	0.4284	0.976563	1
RP11-138I18.1	1.017271	1.013691	1.018435	1.34652	0.4292	0.976563	1
HS3ST5	1.017284	1.013691	1.018452	1.34775	0.4306	0.976563	1
KIF25	1.017266	1.013691	1.018428	1.346	0.4287	0.976563	1
NPY5R	1.017281	1.013691	1.018448	1.34746	0.4302	0.976563	1
ITFG1-AS1	1.017255	1.013691	1.018414	1.34498	0.4276	0.976563	1
SPESP1	1.017266	1.013691	1.018429	1.34607	0.4287	0.976563	1
CH507-145C22.1	1.017279	1.013691	1.018446	1.34732	0.4301	0.976563	1
IGFALS	1.017292	1.013691	1.018462	1.34851	0.4314	0.976563	1
CHRNA4	1.017263	1.013691	1.018424	1.34574	0.4284	0.976563	1
CTD-2337A12.1	1.017329	1.013691	1.018511	1.35211	0.4352	0.976563	1
CTA-212D2.2	1.017311	1.013691	1.018488	1.35041	0.4334	0.976563	1
RP11-114F3.4	1.018958	1.021991	1.017973	0.81729	-0.2911	0.976588	1
CSE1L-AS1	1.018941	1.021906	1.017978	0.82068	-0.2851	0.976588	1
C1orf158	1.018929	1.021882	1.01797	0.82119	-0.2842	0.976588	1
FKTN	1.192091	1.188289	1.193327	1.02676	0.0381	0.976606	1
RP3-467K16.4	1.025948	1.029	1.024955	0.86054	-0.2167	0.976659	1
KCNH3	1.025939	1.028886	1.024981	0.86482	-0.2095	0.976659	1
XPR1	1.477829	1.480561	1.476941	0.99247	-0.0109	0.976817	1
RP11-147L13.13	1.076406	1.079433	1.075422	0.9495	-0.0748	0.976869	1
RP11-265N7.1	1.016243	1.019197	1.015283	0.79609	-0.329	0.976877	1
U95743.1	1.016271	1.019272	1.015296	0.79369	-0.3334	0.976877	1

C21orf62	1.05446	1.050923	1.05561	1.09205	0.127	0.976956	1
GPR141	1.026677	1.029804	1.025661	0.86097	-0.216	0.976979	1
TPK1	1.089518	1.092577	1.088524	0.95622	-0.0646	0.97705	1
LINC00936	1.099834	1.096308	1.10098	1.04851	0.0683	0.977167	1
RNASE4	1.058562	1.06157	1.057584	0.93526	-0.0966	0.977215	1
SLC22A15	1.035913	1.032327	1.037079	1.14699	0.1979	0.977259	1
LINC01445	1.02111	1.017512	1.02228	1.2723	0.3474	0.977263	1
RP11-527J8.1	1.021105	1.017512	1.022273	1.2719	0.347	0.977263	1
KB-1592A4.15	1.021054	1.017512	1.022205	1.268	0.3426	0.977263	1
CTD-3154N5.2	1.021072	1.017512	1.022229	1.26939	0.3441	0.977263	1
RP11-324I22.2	1.021058	1.017512	1.022211	1.26835	0.343	0.977263	1
XXbac-BCX196D17.5	1.021089	1.017512	1.022252	1.27066	0.3456	0.977263	1
RP11-265N6.1	1.02108	1.017512	1.022239	1.26996	0.3448	0.977263	1
RP11-310N16.1	1.021053	1.017512	1.022204	1.26797	0.3425	0.977263	1
RP11-689C9.1	1.021044	1.017512	1.022192	1.26726	0.3417	0.977263	1
RP5-1116H23.4	1.02106	1.017512	1.022213	1.26846	0.3431	0.977263	1
ISL2	1.019593	1.016045	1.020747	1.29302	0.3707	0.977266	1
RP11-464F9.21	1.019584	1.016045	1.020734	1.29225	0.3699	0.977266	1
FITM1	1.019583	1.016045	1.020733	1.2922	0.3698	0.977266	1
RP3-483K16.4	1.019591	1.016045	1.020744	1.29283	0.3705	0.977266	1
TBPL2	1.019574	1.016045	1.020721	1.29142	0.369	0.977266	1
LILRB5	1.019602	1.016045	1.020758	1.29371	0.3715	0.977266	1
LINC00871	1.019594	1.016045	1.020747	1.29307	0.3708	0.977266	1
FOXR1	1.019581	1.016045	1.02073	1.29198	0.3696	0.977266	1
TBX5-AS1	1.019604	1.016045	1.020761	1.29392	0.3718	0.977266	1
TUSC5	1.019606	1.016045	1.020764	1.29411	0.372	0.977266	1
B4GALT1-AS1	1.026813	1.029748	1.02586	0.8693	-0.2021	0.97728	1
GDF6	1.032648	1.035419	1.031748	0.89636	-0.1579	0.97731	1
DAGLB	1.104156	1.100489	1.105348	1.04835	0.0681	0.977328	1
IFI35	1.14522	1.141487	1.146433	1.03496	0.0496	0.977383	1
CTD-2587M2.1	1.029548	1.025965	1.030712	1.18282	0.2422	0.977387	1
LRCOL1	1.022358	1.018766	1.023526	1.25362	0.3261	0.977494	1
C7orf57	1.022332	1.018768	1.02349	1.2516	0.3238	0.977494	1
RP11-358N2.2	1.022326	1.018782	1.023477	1.24999	0.3219	0.977494	1
TRAPPC12	1.264185	1.260484	1.265388	1.01883	0.0269	0.977495	1
RP11-444D3.1	1.038611	1.035207	1.039717	1.1281	0.1739	0.977564	1
PDE2A	1.022502	1.025438	1.021548	0.84707	-0.2394	0.977592	1
CTC-338M12.9	1.022506	1.025446	1.021551	0.84691	-0.2397	0.977592	1
FRGCA	1.022458	1.025375	1.021509	0.84764	-0.2385	0.977592	1
RP11-539L10.2	1.020731	1.017207	1.021877	1.27136	0.3464	0.977606	1
RP11-109E12.1	1.020716	1.017217	1.021853	1.26931	0.344	0.977606	1
TUNAR	1.020713	1.017212	1.02185	1.26951	0.3443	0.977606	1
SYN2	1.023591	1.026549	1.022629	0.85236	-0.2305	0.977631	1
PPP1R2P3	1.023631	1.026721	1.022627	0.84679	-0.2399	0.977631	1
IMPG2	1.023594	1.026494	1.022651	0.85496	-0.2261	0.977631	1

TIGD6	1.114075	1.117148	1.113076	0.96524	-0.051	0.977638	1
USP54	1.149717	1.146138	1.15088	1.03245	0.0461	0.977678	1
RP11-372K14.2	1.054044	1.056947	1.0531	0.93243	-0.1009	0.977705	1
RP11-231G3.1	1.027118	1.029972	1.026191	0.87382	-0.1946	0.977751	1
ZFP91	1.301747	1.305085	1.300662	0.9855	-0.0211	0.977755	1
ARIH2	1.676356	1.671911	1.6778	1.00876	0.0126	0.977784	1
RHCE	1.037687	1.034145	1.038839	1.13746	0.1858	0.977808	1
FZD9	1.035414	1.038349	1.03446	0.89861	-0.1542	0.977843	1
NUDCD3	1.404831	1.400981	1.406083	1.01272	0.0182	0.977884	1
RP3-475N16.1	1.023192	1.026076	1.022255	0.85347	-0.2286	0.977906	1
ULK4	1.150679	1.153646	1.149715	0.97442	-0.0374	0.977945	1
ZNF184	1.160143	1.163163	1.159162	0.97548	-0.0358	0.977981	1
NUDT9	1.297028	1.300247	1.295982	0.9858	-0.0206	0.978002	1
USP31	1.112232	1.10884	1.113335	1.0413	0.0584	0.978197	1
RP11-540E16.2	1.023331	1.026237	1.022386	0.85323	-0.229	0.978212	1
MYRIP	1.029185	1.032178	1.028212	0.87676	-0.1897	0.978237	1
AC145343.2	1.029143	1.032012	1.02821	0.88125	-0.1824	0.978237	1
RP11-572O6.1	1.023049	1.019541	1.02419	1.23788	0.3079	0.978266	1
RP1-292L20.3	1.023028	1.019542	1.024161	1.23633	0.3061	0.978266	1
FAM26D	1.023083	1.019535	1.024236	1.24061	0.3111	0.978266	1
RP4-756G23.5	1.023062	1.019568	1.024198	1.23658	0.3064	0.978266	1
NAT1	1.057673	1.05428	1.058776	1.08282	0.1148	0.978302	1
RP11-449H3.3	1.024557	1.021014	1.025709	1.22339	0.2909	0.978307	1
RP11-345J4.3	1.024572	1.021025	1.025726	1.22358	0.2911	0.978307	1
SLC31A1	1.418003	1.421086	1.417001	0.9903	-0.0141	0.978421	1
TCTEX1D4	1.025772	1.022256	1.026915	1.20931	0.2742	0.978489	1
CTD-2231E14.8	1.025783	1.022273	1.026924	1.20877	0.2735	0.978489	1
PDZRN3-AS1	1.019008	1.021885	1.018073	0.82582	-0.2761	0.978528	1
ATAD3C	1.040255	1.036705	1.041409	1.12813	0.1739	0.978538	1
AZGP1	1.020148	1.023013	1.019217	0.83504	-0.2601	0.978567	1
RP11-627D16.1	1.020169	1.023008	1.019246	0.83649	-0.2576	0.978567	1
SLC39A12	1.020139	1.022995	1.01921	0.83539	-0.2595	0.978567	1
PKN2-AS1	1.020133	1.022998	1.019202	0.83497	-0.2602	0.978567	1
GAS6-AS2	1.024167	1.020686	1.025298	1.22298	0.2904	0.978646	1
HAS3	1.023676	1.026534	1.022747	0.85727	-0.2222	0.978682	1
PSKH1	1.176146	1.178848	1.175267	0.97998	-0.0292	0.978786	1
LINC00856	1.019692	1.022522	1.018772	0.83349	-0.2628	0.978836	1
FAM69B	1.340663	1.343676	1.339683	0.98838	-0.0169	0.978838	1
RAVER2	1.121022	1.117619	1.122128	1.03833	0.0543	0.978846	1
FIS1	4.169918	4.163359	4.172051	1.00275	0.004	0.978891	1
ARHGAP31	1.041184	1.037675	1.042325	1.12341	0.1679	0.978934	1
FAM19A3	1.03236	1.035188	1.031441	0.89351	-0.1624	0.979063	1
PTGS1	1.021378	1.017948	1.022493	1.25327	0.3257	0.979144	1
RP11-129K12.1	1.021373	1.017948	1.022486	1.25285	0.3252	0.979144	1
CTD-2047H16.5	1.021392	1.017948	1.022511	1.25428	0.3269	0.979144	1

RP11-265N6.2	1.021371	1.017948	1.022484	1.25275	0.3251	0.979144	1
ATP5J2-PTCD1	1.021374	1.017948	1.022487	1.25294	0.3253	0.979144	1
CAPN9	1.021358	1.017948	1.022467	1.25181	0.324	0.979144	1
GPR50-AS1	1.021371	1.017948	1.022483	1.25272	0.3251	0.979144	1
RP11-381N20.1	1.0214	1.017948	1.022522	1.25486	0.3275	0.979144	1
RP11-40E6.2	1.021376	1.017948	1.022491	1.25312	0.3255	0.979144	1
RP11-305F18.1	1.021383	1.017948	1.0225	1.25364	0.3261	0.979144	1
CLEC12A	1.021392	1.017948	1.022511	1.25427	0.3268	0.979144	1
DCLK3	1.021369	1.017948	1.022482	1.25262	0.3249	0.979144	1
ZNF257	1.021391	1.017948	1.022511	1.25425	0.3268	0.979144	1
RP11-491F9.8	1.021404	1.017948	1.022527	1.25515	0.3279	0.979144	1
KRTAP5-10	1.021406	1.017948	1.02253	1.25533	0.3281	0.979144	1
CTD-2349P21.11	1.0214	1.017948	1.022522	1.25489	0.3276	0.979144	1
MSANTD1	1.019908	1.022725	1.018992	0.83571	-0.2589	0.979147	1
RP1-122O8.7	1.019896	1.022704	1.018984	0.83616	-0.2581	0.979147	1
FAM106A	1.01988	1.022693	1.018965	0.8357	-0.2589	0.979147	1
NEUROD2	1.025743	1.02867	1.024792	0.86475	-0.2096	0.979167	1
DNASE1L2	1.025689	1.028466	1.024786	0.87072	-0.1997	0.979167	1
CTD-2298J14.2	1.096431	1.092904	1.097578	1.05031	0.0708	0.979201	1
TMIGD2	1.026473	1.023047	1.027587	1.19699	0.2594	0.979262	1
RP11-700H13.1	1.026555	1.023033	1.027699	1.20257	0.2661	0.979262	1
RP11-307C12.12	1.061078	1.057675	1.062184	1.07818	0.1086	0.979286	1
SFMBT2	1.047968	1.050898	1.047015	0.92373	-0.1145	0.979307	1
KCNQ5	1.027986	1.024533	1.029109	1.18651	0.2467	0.979348	1
AP000569.9	1.029153	1.025738	1.030263	1.17582	0.2337	0.97939	1
CRAMP1	1.107184	1.109808	1.106331	0.96833	-0.0464	0.979449	1
RP11-347I19.8	1.015405	1.011999	1.016512	1.37606	0.4605	0.979474	1
RP11-661A12.12	1.015391	1.011999	1.016493	1.37454	0.4589	0.979474	1
AGTR1	1.015377	1.011999	1.016474	1.37296	0.4573	0.979474	1
CTD-2568A17.8	1.015384	1.011999	1.016484	1.37373	0.4581	0.979474	1
CA10	1.015375	1.011999	1.016473	1.3728	0.4571	0.979474	1
FGR	1.01538	1.011999	1.016479	1.37331	0.4577	0.979474	1
TCEAL6	1.015392	1.011999	1.016495	1.37467	0.4591	0.979474	1
CTD-2037L6.2	1.015392	1.011999	1.016495	1.37464	0.4591	0.979474	1
UBD	1.015378	1.011999	1.016477	1.37317	0.4575	0.979474	1
AP001604.3	1.015387	1.011999	1.016489	1.37416	0.4586	0.979474	1
RP11-442N1.1	1.01541	1.011999	1.016518	1.37662	0.4611	0.979474	1
WASIR1	1.01539	1.011999	1.016492	1.37439	0.4588	0.979474	1
RP1-37E16.12	1.015383	1.011999	1.016483	1.37367	0.458	0.979474	1
CTA-407F11.7	1.015396	1.011999	1.0165	1.37513	0.4596	0.979474	1
NDP-AS1	1.015396	1.011999	1.016501	1.37516	0.4596	0.979474	1
RP11-578F21.6	1.015382	1.011999	1.016482	1.37361	0.458	0.979474	1
CTD-3126B10.4	1.015397	1.011999	1.016501	1.37517	0.4596	0.979474	1
FAM157B	1.015387	1.011999	1.016488	1.37412	0.4585	0.979474	1
NGRN	1.281555	1.284377	1.280638	0.98685	-0.0191	0.979525	1

MBLAC1	1.074762	1.071362	1.075866	1.06311	0.0883	0.979597	1
RP11-770G2.5	1.020193	1.02295	1.019297	0.84082	-0.2501	0.979615	1
NUTM2E	1.020193	1.022951	1.019297	0.84076	-0.2502	0.979615	1
XXbac-BPG181M17.6	1.02018	1.022946	1.019281	0.84027	-0.2511	0.979615	1
AC005757.6	1.02019	1.022985	1.019282	0.8389	-0.2534	0.979615	1
RP11-83B20.1	1.020201	1.022976	1.019299	0.83996	-0.2516	0.979615	1
KCNS1	1.019347	1.015934	1.020456	1.28378	0.3604	0.979629	1
HIGD2B	1.019328	1.015934	1.020431	1.28222	0.3586	0.979629	1
RP11-429E11.3	1.01932	1.015934	1.020421	1.28159	0.3579	0.979629	1
NEUROG2	1.019344	1.015934	1.020453	1.28357	0.3602	0.979629	1
IGFL4	1.019331	1.015934	1.020436	1.28251	0.359	0.979629	1
FRMD6-AS2	1.019316	1.015934	1.020416	1.28124	0.3575	0.979629	1
RP1-286D6.5	1.019329	1.015934	1.020433	1.28233	0.3588	0.979629	1
AC010524.2	1.019362	1.015934	1.020476	1.28506	0.3618	0.979629	1
RP11-325L12.7	1.019329	1.015934	1.020433	1.28233	0.3588	0.979629	1
PCDHGA3	1.019324	1.015934	1.020426	1.28191	0.3583	0.979629	1
PTF1A	1.019334	1.015934	1.02044	1.28275	0.3592	0.979629	1
NOL8	1.337639	1.340574	1.336685	0.98858	-0.0166	0.979674	1
STXBP5L	1.027639	1.024194	1.028759	1.18873	0.2494	0.979683	1
AC012593.1	1.016255	1.019036	1.015351	0.80644	-0.3104	0.97977	1
RP3-333H23.8	1.016243	1.018997	1.015348	0.80791	-0.3077	0.97977	1
DOK7	1.016246	1.019024	1.015343	0.80648	-0.3103	0.97977	1
STT3A-AS1	1.016261	1.019082	1.015343	0.80406	-0.3146	0.97977	1
AC003092.1	1.016244	1.01898	1.015355	0.80901	-0.3058	0.97977	1
TRIM39	1.12442	1.120758	1.12561	1.04018	0.0568	0.979793	1
GPR137C	1.185032	1.181401	1.186213	1.02653	0.0378	0.979847	1
PET117	1.615822	1.618877	1.614829	0.99346	-0.0095	0.979895	1
RSPH4A	1.045553	1.048372	1.044637	0.92278	-0.1159	0.979962	1
RP4-545C24.1	1.028806	1.031529	1.027921	0.88556	-0.1753	0.980031	1
ANKRD44	1.028871	1.031676	1.027959	0.88268	-0.18	0.980031	1
RAB24	1.145599	1.142167	1.146715	1.03199	0.0454	0.980087	1
RP11-103H7.3	1.019039	1.015676	1.020133	1.28436	0.361	0.980097	1
RP11-85G18.6	1.019037	1.015676	1.02013	1.28418	0.3609	0.980097	1
RP11-332H18.7	1.019054	1.015676	1.020152	1.28559	0.3624	0.980097	1
AC005753.1	1.01904	1.015676	1.020133	1.28439	0.3611	0.980097	1
MSH5-SAPCD1	1.019028	1.015676	1.020118	1.28338	0.36	0.980097	1
CTD-3051D23.4	1.019065	1.015676	1.020167	1.28656	0.3635	0.980097	1
AC007395.3	1.019065	1.015676	1.020167	1.28656	0.3635	0.980097	1
TBX22	1.019031	1.015676	1.020122	1.28367	0.3603	0.980097	1
RP11-565A3.2	1.019037	1.015676	1.020129	1.28411	0.3608	0.980097	1
RP11-11N5.1	1.01905	1.015676	1.020147	1.28526	0.3621	0.980097	1
RP11-665J16.1	1.019045	1.015676	1.02014	1.28483	0.3616	0.980097	1
PFKFB1	1.019038	1.015676	1.020131	1.2842	0.3609	0.980097	1
C8orf49	1.019068	1.015676	1.020171	1.28677	0.3638	0.980097	1
AC079610.2	1.019046	1.015676	1.020141	1.2849	0.3617	0.980097	1

RP11-128P10.1	1.019047	1.015676	1.020142	1.28496	0.3617	0.980097	1
SPP2	1.019019	1.015676	1.020106	1.28265	0.3591	0.980097	1
CTD-2336O2.3	1.019034	1.015676	1.020125	1.28386	0.3605	0.980097	1
RP11-166B2.5	1.019032	1.015676	1.020123	1.28375	0.3604	0.980097	1
ZNF559-ZNF177	1.02222	1.02497	1.021326	0.85404	-0.2276	0.9801	1
TMEM72	1.022227	1.024987	1.021329	0.85361	-0.2284	0.9801	1
SLC6A11	1.022233	1.025012	1.02133	0.85278	-0.2298	0.9801	1
AC007038.7	1.022238	1.024994	1.021343	0.85391	-0.2278	0.9801	1
EPB41L4A-AS2	1.027394	1.030221	1.026474	0.87602	-0.191	0.980123	1
RP11-380B4.3	1.024836	1.021484	1.025926	1.20678	0.2712	0.980147	1
RP11-572M11.3	1.024884	1.021456	1.025998	1.21167	0.277	0.980147	1
PABPC1L2A	1.02486	1.021445	1.02597	1.21102	0.2762	0.980147	1
CYP2C8	1.029985	1.026567	1.031096	1.17047	0.2271	0.980255	1
SLC8A3	1.029971	1.026597	1.031068	1.1681	0.2242	0.980255	1
ANGPT4	1.030002	1.026573	1.031116	1.17098	0.2277	0.980255	1
RP11-121M22.1	1.030042	1.026662	1.031141	1.168	0.224	0.980255	1
RSPO2	1.030061	1.026593	1.031189	1.17281	0.23	0.980255	1
PIK3CD	1.06452	1.06106	1.065644	1.07509	0.1045	0.980268	1
ERCC2	1.212832	1.21593	1.211826	0.98099	-0.0277	0.980305	1
LAPTM5	1.031414	1.028057	1.032505	1.15852	0.2123	0.980386	1
AP001439.2	1.018841	1.0155	1.019927	1.28559	0.3624	0.980478	1
LINC01285	1.018836	1.015527	1.019911	1.28237	0.3588	0.980478	1
AC004069.2	1.047102	1.043762	1.048188	1.10114	0.139	0.980555	1
TBC1D10C	1.022796	1.01946	1.023881	1.22719	0.2954	0.980633	1
ULBP1	1.022767	1.019453	1.023845	1.22578	0.2937	0.980633	1
RBM8A	3.52813	3.520972	3.530457	1.00376	0.0054	0.980634	1
KCNMA1-AS3	1.019272	1.015961	1.020349	1.2749	0.3504	0.980677	1
TRIM60	1.019287	1.015961	1.020369	1.27615	0.3518	0.980677	1
RTP1	1.0193	1.015961	1.020386	1.27722	0.353	0.980677	1
CTD-2162K18.3	1.019284	1.015961	1.020364	1.27588	0.3515	0.980677	1
RP11-461L13.3	1.019271	1.015961	1.020347	1.27477	0.3502	0.980677	1
TNS4	1.019302	1.015961	1.020388	1.27733	0.3531	0.980677	1
CTC-336P14.1	1.019283	1.015961	1.020363	1.27581	0.3514	0.980677	1
LINC00884	1.019291	1.015961	1.020373	1.27645	0.3521	0.980677	1
RP13-192B19.2	1.019286	1.015961	1.020367	1.27605	0.3517	0.980677	1
GLIS2-AS1	1.019274	1.015961	1.020351	1.27502	0.3505	0.980677	1
DUOXA2	1.019317	1.015961	1.020408	1.27861	0.3546	0.980677	1
RP11-60H5.1	1.019283	1.015961	1.020363	1.2758	0.3514	0.980677	1
RP1-167A14.2	1.019273	1.015961	1.02035	1.27497	0.3505	0.980677	1
CTD-2369P2.8	1.019292	1.015961	1.020374	1.27651	0.3522	0.980677	1
C12orf50	1.019267	1.015961	1.020341	1.27442	0.3498	0.980677	1
RP11-798K3.4	1.01929	1.015961	1.020372	1.27634	0.352	0.980677	1
ZNF32-AS3	1.019265	1.015961	1.020339	1.27431	0.3497	0.980677	1
RP11-63L7.5	1.019269	1.015961	1.020345	1.27465	0.3501	0.980677	1
INE2	1.019313	1.015961	1.020402	1.27825	0.3542	0.980677	1

RP11-439C15.5	1.019276	1.015961	1.020353	1.27519	0.3507	0.980677	1
SLAMF7	1.01816	1.014833	1.019242	1.29726	0.3755	0.980716	1
RP11-495P10.3	1.018151	1.014833	1.019229	1.29642	0.3745	0.980716	1
RP11-245P10.8	1.018169	1.014833	1.019253	1.29804	0.3763	0.980716	1
CTD-2026K11.3	1.018165	1.014833	1.019248	1.29766	0.3759	0.980716	1
RP11-49K24.8	1.01815	1.014833	1.019229	1.29639	0.3745	0.980716	1
RP11-1136J12.1	1.018152	1.014833	1.019232	1.29659	0.3747	0.980716	1
TCP11X2	1.018176	1.014833	1.019263	1.29869	0.3771	0.980716	1
RP11-982M15.8	1.018158	1.014833	1.019238	1.29704	0.3752	0.980716	1
LBX1	1.018161	1.014833	1.019243	1.29735	0.3756	0.980716	1
CTD-3224I3.3	1.018148	1.014833	1.019226	1.29619	0.3743	0.980716	1
CTB-35F21.5	1.01814	1.014833	1.019215	1.29549	0.3735	0.980716	1
RAB7B	1.018142	1.014833	1.019218	1.29566	0.3737	0.980716	1
MIR1539	1.018164	1.014833	1.019246	1.29758	0.3758	0.980716	1
KB-1107E3.1	1.018168	1.014833	1.019252	1.29799	0.3763	0.980716	1
CT55	1.018152	1.014833	1.019231	1.29654	0.3747	0.980716	1
AC113167.1	1.018153	1.014833	1.019233	1.29667	0.3748	0.980716	1
PRLHR	1.018167	1.014833	1.01925	1.29786	0.3761	0.980716	1
GIMAP2	1.018167	1.014833	1.019251	1.29786	0.3761	0.980716	1
PAM16	1.025001	1.027709	1.02412	0.8705	-0.2001	0.980734	1
SLC35D1	1.091234	1.093863	1.09038	0.9629	-0.0545	0.980777	1
FECH	1.175535	1.178046	1.174719	0.98131	-0.0272	0.980817	1
PRDM15	1.047996	1.044751	1.04905	1.09607	0.1323	0.980839	1
MRPL49	1.506042	1.502543	1.50718	1.00923	0.0132	0.980863	1
EPHX4	1.093195	1.095897	1.092317	0.96267	-0.0549	0.980908	1
TRIM66	1.079554	1.076311	1.080608	1.0563	0.079	0.980921	1
SEC23A	2.049011	2.045398	2.050185	1.00458	0.0066	0.980968	1
XXbac-BPGBPG55C20.1	1.025386	1.028095	1.024505	0.87224	-0.1972	0.981003	1
SIRT6	1.297234	1.299986	1.29634	0.98784	-0.0176	0.981068	1
HDAC8	1.263542	1.266206	1.262676	0.98674	-0.0193	0.981076	1
STUM	1.022489	1.019189	1.023562	1.22791	0.2962	0.981102	1
RP13-467H17.1	1.022507	1.019189	1.023585	1.2291	0.2976	0.981102	1
RP1-251M9.2	1.022502	1.019175	1.023584	1.22996	0.2986	0.981102	1
RP11-11M20.4	1.028326	1.025022	1.0294	1.17496	0.2326	0.981147	1
SEPT7-AS1	1.041113	1.043893	1.040209	0.91606	-0.1265	0.981189	1
LINC01534	1.048221	1.04491	1.049297	1.09768	0.1345	0.981205	1
RPAP3	1.392245	1.395489	1.39119	0.98913	-0.0158	0.981275	1
ZSCAN23	1.036085	1.032787	1.037157	1.1333	0.1805	0.98144	1
RINT1	1.106282	1.103036	1.107337	1.04175	0.059	0.981449	1
RP11-861E21.2	1.059101	1.061732	1.058245	0.94352	-0.0839	0.981459	1
AGMO	1.022316	1.019082	1.023368	1.22464	0.2924	0.981478	1
SLC36A1	1.043401	1.040043	1.044492	1.1111	0.152	0.981482	1
RP11-166P13.3	1.081651	1.078259	1.082754	1.05743	0.0806	0.981602	1
Z69720.2	1.022734	1.019462	1.023797	1.22272	0.2901	0.981682	1
CCDC183-AS1	1.022778	1.019491	1.023846	1.22347	0.291	0.981682	1

HIST1H2AB	1.022709	1.019459	1.023765	1.22127	0.2884	0.981682	1
RP4-784A16.5	1.022753	1.019487	1.023814	1.22207	0.2893	0.981682	1
PPP1R12C	1.111939	1.114518	1.1111	0.97016	-0.0437	0.981703	1
RP13-616I3.1	1.021707	1.018354	1.022797	1.24207	0.3127	0.981721	1
STBD1	1.021613	1.018356	1.022672	1.23514	0.3047	0.981721	1
HIST1H3B	1.021577	1.018333	1.022631	1.23447	0.3039	0.981721	1
RP11-285F16.1	1.021629	1.018348	1.022695	1.23692	0.3068	0.981721	1
RP5-1158E12.3	1.023193	1.025753	1.02236	0.86826	-0.2038	0.981822	1
TCERG1L	1.097155	1.093874	1.098221	1.0463	0.0653	0.981836	1
ZNF2	1.077678	1.074419	1.078738	1.05804	0.0814	0.981882	1
RP5-968D22.3	1.021939	1.024597	1.021075	0.85681	-0.2229	0.981978	1
RP11-659E9.2	1.020438	1.023029	1.019596	0.85093	-0.2329	0.98198	1
LINC01415	1.02045	1.02309	1.019591	0.84846	-0.2371	0.98198	1
NCF1	1.023126	1.025727	1.022281	0.86607	-0.2074	0.982056	1
TLR2	1.023133	1.025844	1.022252	0.86101	-0.2159	0.982056	1
CCDC17	1.023158	1.025842	1.022285	0.86238	-0.2136	0.982056	1
RP11-303E16.5	1.030469	1.033023	1.029639	0.89755	-0.1559	0.982064	1
DNMBP	1.13381	1.136082	1.133071	0.97788	-0.0323	0.982077	1
RP11-381K20.2	1.039441	1.042145	1.038562	0.91497	-0.1282	0.982095	1
RP11-96O20.5	1.025951	1.022719	1.027001	1.1885	0.2491	0.982103	1
AC090616.2	1.025953	1.022725	1.027002	1.18822	0.2488	0.982103	1
HIST1H4H	1.036928	1.033731	1.037967	1.12558	0.1707	0.982231	1
TMEM169	1.262042	1.264552	1.261226	0.98743	-0.0183	0.982236	1
RP11-522I20.3	1.047423	1.05004	1.046573	0.9307	-0.1036	0.982242	1
CTD-2349P21.5	1.015378	1.012186	1.016415	1.34702	0.4298	0.982365	1
ZNF30-AS1	1.015376	1.012186	1.016413	1.34684	0.4296	0.982365	1
TP53AIP1	1.01538	1.012186	1.016419	1.3473	0.4301	0.982365	1
AC068535.2	1.015409	1.012186	1.016457	1.35045	0.4334	0.982365	1
CTD-2537I9.19	1.015417	1.012186	1.016467	1.35126	0.4343	0.982365	1
ZNF625-ZNF20	1.015386	1.012186	1.016426	1.34789	0.4307	0.982365	1
RP11-138H10.2	1.015391	1.012186	1.016432	1.34843	0.4313	0.982365	1
RP11-365N19.2	1.015373	1.012186	1.016409	1.34649	0.4292	0.982365	1
RP11-1070A24.2	1.015389	1.012186	1.01643	1.34821	0.431	0.982365	1
C9orf41-AS1	1.015415	1.012186	1.016464	1.35102	0.4341	0.982365	1
ERICH6B	1.015386	1.012186	1.016426	1.34791	0.4307	0.982365	1
NKG7	1.015383	1.012186	1.016422	1.34758	0.4304	0.982365	1
XKRX	1.015383	1.012186	1.016423	1.34762	0.4304	0.982365	1
LILRA2	1.015381	1.012186	1.01642	1.34739	0.4302	0.982365	1
RP11-214K3.25	1.015379	1.012186	1.016416	1.34711	0.4299	0.982365	1
PHYHIP	1.038423	1.035091	1.039506	1.12581	0.171	0.98245	1
FTCDNL1	1.025746	1.022475	1.026809	1.19284	0.2544	0.982475	1
HCG17	1.046844	1.043564	1.04791	1.09975	0.1372	0.982497	1
ERP27	1.04689	1.0436	1.04796	1.09999	0.1375	0.982497	1
ZNF223	1.067868	1.070479	1.067019	0.95091	-0.0726	0.982518	1
ASAH2B	1.092673	1.089336	1.093758	1.0495	0.0697	0.982592	1

PSMG3	1.420664	1.417387	1.421729	1.0104	0.0149	0.982612	1
RP11-873E20.1	1.029784	1.026476	1.03086	1.16558	0.221	0.98263	1
PCDHB1	1.018087	1.014881	1.01913	1.28549	0.3623	0.982654	1
LILRB1	1.018105	1.014881	1.019153	1.28705	0.3641	0.982654	1
SLCO1B1	1.018082	1.014881	1.019122	1.285	0.3618	0.982654	1
OR52H1	1.018065	1.014881	1.0191	1.28348	0.3601	0.982654	1
SCN4B	1.018073	1.014881	1.019111	1.28424	0.3609	0.982654	1
CTD-2199O4.6	1.018076	1.014881	1.019115	1.2845	0.3612	0.982654	1
RP11-69C13.1	1.018075	1.014881	1.019114	1.28442	0.3611	0.982654	1
RP11-174G6.1	1.018087	1.014881	1.019129	1.28544	0.3623	0.982654	1
AC073641.2	1.018084	1.014881	1.019125	1.28517	0.362	0.982654	1
RP11-304F15.3	1.018077	1.014881	1.019115	1.28454	0.3613	0.982654	1
ASB12	1.018055	1.014881	1.019087	1.28261	0.3591	0.982654	1
RP11-211G3.2	1.018098	1.014881	1.019144	1.28647	0.3634	0.982654	1
RP11-71E19.1	1.018068	1.014881	1.019103	1.28372	0.3603	0.982654	1
CNR2	1.018072	1.014881	1.019109	1.28409	0.3607	0.982654	1
SCN4A	1.018075	1.014881	1.019114	1.28441	0.3611	0.982654	1
RP11-234K24.6	1.018069	1.014881	1.019105	1.28382	0.3604	0.982654	1
AF131216.5	1.018069	1.014881	1.019105	1.28382	0.3604	0.982654	1
PLA2G7	1.018064	1.014881	1.019098	1.28338	0.36	0.982654	1
NLRC3	1.018063	1.014881	1.019097	1.2833	0.3599	0.982654	1
EFS	1.357832	1.354814	1.358812	1.01127	0.0162	0.982664	1
RP5-968P14.2	1.018154	1.020769	1.017304	0.83317	-0.2633	0.982678	1
GPR1	1.018154	1.020732	1.017316	0.83522	-0.2598	0.982678	1
TNF	1.018126	1.020675	1.017298	0.83669	-0.2572	0.982678	1
TMEM140	1.018143	1.020756	1.017294	0.83323	-0.2632	0.982678	1
RP3-496C20.1	1.01814	1.020747	1.017293	0.83353	-0.2627	0.982678	1
RP11-59C5.3	1.018145	1.020764	1.017293	0.83283	-0.2639	0.982678	1
P2RX5	1.018134	1.020696	1.017301	0.83594	-0.2585	0.982678	1
PLAC8L1	1.026196	1.02302	1.027229	1.18281	0.2422	0.982685	1
CD96	1.026229	1.023019	1.027273	1.18479	0.2446	0.982685	1
LONRF3	1.025103	1.021904	1.026142	1.1935	0.2552	0.982724	1
S1PR5	1.019724	1.022306	1.018884	0.84657	-0.2403	0.982751	1
LMTK2	1.054981	1.051709	1.056044	1.08383	0.1161	0.982843	1
DHRS12	1.120592	1.117289	1.121666	1.03731	0.0529	0.982922	1
UBTD2	1.328974	1.331316	1.328213	0.99063	-0.0136	0.982925	1
MYO5C	1.19174	1.193956	1.191019	0.98486	-0.022	0.98294	1
PDF	1.018465	1.02107	1.017618	0.83619	-0.2581	0.982956	1
RP11-308D13.3	1.018455	1.021029	1.017618	0.8378	-0.2553	0.982956	1
PLEKHD1	1.018459	1.021045	1.017618	0.83717	-0.2564	0.982956	1
IRX4	1.018456	1.021034	1.017618	0.83761	-0.2557	0.982956	1
ITGB2-AS1	1.018456	1.021034	1.017618	0.83761	-0.2557	0.982956	1
RP3-340B19.3	1.018456	1.021034	1.017618	0.83761	-0.2557	0.982956	1
RP11-644N4.1	1.018452	1.021016	1.017618	0.83832	-0.2544	0.982956	1
AL022344.7	1.018455	1.021029	1.017618	0.83782	-0.2553	0.982956	1

ANKS4B	1.018458	1.02104	1.017618	0.83738	-0.2561	0.982956	1
F8A3	1.018459	1.021043	1.017618	0.83726	-0.2562	0.982956	1
RP11-17A1.3	1.018451	1.021011	1.017618	0.83853	-0.2541	0.982956	1
AC007126.1	1.018471	1.021095	1.017618	0.8352	-0.2598	0.982956	1
KB-1043D8.8	1.018461	1.021054	1.017618	0.83681	-0.257	0.982956	1
DCDC2B	1.018461	1.021054	1.017618	0.83681	-0.257	0.982956	1
RP11-110I1.6	1.01846	1.021048	1.017618	0.83706	-0.2566	0.982956	1
RP11-498P14.3	1.018455	1.02103	1.017618	0.83779	-0.2553	0.982956	1
AP000442.4	1.018449	1.021005	1.017618	0.83878	-0.2536	0.982956	1
AC015849.16	1.01845	1.021007	1.017618	0.83868	-0.2538	0.982956	1
TMEM244	1.018451	1.021014	1.017618	0.83843	-0.2542	0.982956	1
IGF2BP2-AS1	1.018451	1.021013	1.017618	0.83845	-0.2542	0.982956	1
AC006277.2	1.018453	1.021022	1.017618	0.8381	-0.2548	0.982956	1
PGA5	1.018456	1.021031	1.017618	0.83776	-0.2554	0.982956	1
RDH16	1.019674	1.022235	1.018842	0.84743	-0.2388	0.983024	1
BARX1	1.019701	1.022336	1.018844	0.84369	-0.2452	0.983024	1
PROB1	1.035988	1.038582	1.035144	0.91089	-0.1347	0.983044	1
RP11-799D4.4	1.040334	1.037163	1.041364	1.11307	0.1545	0.983214	1
ZKSCAN7	1.101602	1.104109	1.100788	0.9681	-0.0468	0.983328	1
SPATA21	1.018826	1.015691	1.019845	1.26475	0.3388	0.983374	1
LLNLR-268E12.1	1.01886	1.015698	1.019888	1.26692	0.3413	0.983374	1
RP11-10N23.2	1.01885	1.015704	1.019873	1.26544	0.3396	0.983374	1
LRRN4CL	1.018827	1.015689	1.019847	1.26502	0.3392	0.983374	1
ADARB2-AS1	1.018826	1.015704	1.01984	1.26338	0.3373	0.983374	1
SCARF1	1.031832	1.034273	1.031038	0.90561	-0.143	0.98339	1
C1orf228	1.05571	1.058356	1.05485	0.93992	-0.0894	0.983453	1
KANK3	1.02919	1.026015	1.030222	1.1617	0.2162	0.983468	1
PAK1	1.566862	1.562925	1.568142	1.00927	0.0133	0.983477	1
SAP25	1.041854	1.038683	1.042885	1.10863	0.1488	0.983477	1
IGF2BP3	1.79348	1.789093	1.794906	1.00737	0.0106	0.983609	1
MFSD14A	1.611415	1.613782	1.610645	0.99489	-0.0074	0.983648	1
RP11-963H4.5	1.014687	1.017227	1.013862	0.80465	-0.3136	0.983655	1
AC004490.1	1.014693	1.01725	1.013862	0.80358	-0.3155	0.983655	1
RP11-6D1.3	1.014675	1.017179	1.013862	0.80691	-0.3095	0.983655	1
CCR9	1.014682	1.017205	1.013862	0.8057	-0.3117	0.983655	1
CTD-2313J17.6	1.01468	1.017197	1.013862	0.80605	-0.3111	0.983655	1
RP11-863P13.4	1.014679	1.017193	1.013862	0.80625	-0.3107	0.983655	1
RP11-50D16.4	1.014681	1.017201	1.013862	0.80587	-0.3114	0.983655	1
RP13-991F5.2	1.014683	1.017208	1.013862	0.80554	-0.312	0.983655	1
CTA-268H5.14	1.014684	1.017215	1.013862	0.80521	-0.3126	0.983655	1
GATA6-AS1	1.014683	1.017208	1.013862	0.80552	-0.312	0.983655	1
RP11-616M22.11	1.014683	1.017208	1.013862	0.80552	-0.312	0.983655	1
GSTM5	1.014678	1.01719	1.013862	0.80637	-0.3105	0.983655	1
RP11-554D15.3	1.014675	1.017176	1.013862	0.80706	-0.3092	0.983655	1
ADRA2A	1.014678	1.01719	1.013862	0.8064	-0.3104	0.983655	1

RP11-867G2.6	1.014675	1.017177	1.013862	0.80702	-0.3093	0.983655	1
RP11-521D12.1	1.014675	1.017177	1.013862	0.80702	-0.3093	0.983655	1
RXFP2	1.014679	1.017192	1.013862	0.8063	-0.3106	0.983655	1
RP11-866E20.3	1.014674	1.017173	1.013862	0.80717	-0.3091	0.983655	1
TMEM8C	1.014682	1.017203	1.013862	0.80575	-0.3116	0.983655	1
RP11-486L19.2	1.014677	1.017185	1.013862	0.80661	-0.3101	0.983655	1
RP11-240B13.2	1.014678	1.017189	1.013862	0.80645	-0.3103	0.983655	1
RP11-234K24.3	1.014682	1.017206	1.013862	0.80565	-0.3118	0.983655	1
CLEC2B	1.021513	1.018394	1.022526	1.22462	0.2923	0.983663	1
NR4A2	1.021532	1.018371	1.022559	1.22798	0.2963	0.983663	1
NALCN	1.021533	1.018382	1.022557	1.22715	0.2953	0.983663	1
SMO	1.40221	1.399017	1.403248	1.0106	0.0152	0.983679	1
DNAJC16	1.205226	1.201918	1.206301	1.0217	0.031	0.983709	1
RP11-38M8.1	1.034031	1.036687	1.033168	0.90407	-0.1455	0.983825	1
KPTN	1.149722	1.146447	1.150786	1.02963	0.0421	0.983854	1
RP11-114H23.2	1.014981	1.011871	1.015991	1.34712	0.4299	0.983887	1
LINC01600	1.014981	1.011871	1.015992	1.34719	0.43	0.983887	1
EYS	1.014969	1.011871	1.015975	1.34577	0.4284	0.983887	1
RP11-504P24.9	1.014974	1.011871	1.015983	1.34639	0.4291	0.983887	1
CCDC36	1.014978	1.011871	1.015988	1.3468	0.4295	0.983887	1
RP11-24N18.1	1.014967	1.011871	1.015974	1.34563	0.4283	0.983887	1
ACOT4	1.015024	1.011871	1.016049	1.35194	0.435	0.983887	1
RP11-78A19.3	1.014984	1.011871	1.015996	1.34754	0.4303	0.983887	1
RP11-45A12.1	1.014972	1.011871	1.01598	1.34615	0.4288	0.983887	1
DMRT1	1.014986	1.011871	1.015999	1.34772	0.4305	0.983887	1
SRGAP3-AS2	1.014983	1.011871	1.015994	1.34735	0.4301	0.983887	1
CTB-35F21.2	1.014963	1.011871	1.015968	1.34513	0.4277	0.983887	1
Z69666.2	1.014967	1.011871	1.015974	1.34562	0.4283	0.983887	1
SLC18A3	1.015013	1.011871	1.016035	1.35076	0.4338	0.983887	1
RP13-476E20.1	1.014977	1.011871	1.015986	1.34667	0.4294	0.983887	1
FOXD4	1.014966	1.011871	1.015972	1.3455	0.4281	0.983887	1
RP11-53B2.3	1.014985	1.011871	1.015997	1.34758	0.4304	0.983887	1
EXOC8	1.057753	1.060209	1.056955	0.94595	-0.0802	0.983946	1
AGER	1.058504	1.055263	1.059557	1.0777	0.108	0.983954	1
LY6G5C	1.058326	1.055195	1.059343	1.07515	0.1045	0.983954	1
ZNF804A	1.023522	1.025972	1.022725	0.87501	-0.1926	0.983994	1
TSPAN32	1.023529	1.026055	1.022708	0.87154	-0.1984	0.983994	1
GCKR	1.016249	1.018746	1.015437	0.82352	-0.2801	0.983995	1
P2RX5-TAX1BP3	1.016241	1.018709	1.015439	0.82522	-0.2772	0.983995	1
PIK3AP1	1.016241	1.018738	1.015429	0.82339	-0.2803	0.983995	1
CH507-42P11.8	1.016256	1.018799	1.015429	0.82077	-0.2849	0.983995	1
RP11-414H17.5	1.032642	1.035124	1.031835	0.90638	-0.1418	0.983996	1
SHROOM3	1.464383	1.4665	1.463694	0.99398	-0.0087	0.984018	1
RASGRP3	1.030714	1.033183	1.029911	0.9014	-0.1498	0.984035	1
SLC25A10	1.178661	1.181141	1.177855	0.98186	-0.0264	0.984138	1

RUNDC1	1.255395	1.257837	1.254601	0.98745	-0.0182	0.984162	1
ZNF671	1.06	1.05704	1.060962	1.06877	0.096	0.98428	1
R3HCC1	1.586623	1.583126	1.587759	1.00795	0.0114	0.984306	1
RP11-48B3.3	1.028358	1.030817	1.027558	0.89427	-0.1612	0.984346	1
SYNPO2L	1.028384	1.0308	1.027599	0.89606	-0.1583	0.984346	1
RSPH10B	1.014364	1.011304	1.015358	1.35863	0.4421	0.984359	1
CTD-2013N17.4	1.014375	1.011304	1.015373	1.35992	0.4435	0.984359	1
AC018755.17	1.014378	1.011304	1.015377	1.3603	0.4439	0.984359	1
RP11-697M17.2	1.014384	1.011304	1.015385	1.361	0.4447	0.984359	1
RP11-338N10.2	1.014359	1.011304	1.015352	1.3581	0.4416	0.984359	1
AC015922.6	1.014364	1.011304	1.015358	1.35863	0.4422	0.984359	1
ZNF32-AS2	1.014378	1.011304	1.015377	1.36026	0.4439	0.984359	1
LRRC37A	1.014379	1.011304	1.015379	1.36044	0.4441	0.984359	1
DENND5B-AS1	1.014374	1.011304	1.015372	1.35983	0.4434	0.984359	1
THCAT158	1.01437	1.011304	1.015366	1.35932	0.4429	0.984359	1
TGM4	1.014371	1.011304	1.015368	1.35944	0.443	0.984359	1
AC005609.18	1.01438	1.011304	1.01538	1.36054	0.4442	0.984359	1
CD34	1.01439	1.011304	1.015393	1.3617	0.4454	0.984359	1
PRPF6	1.728231	1.72483	1.729337	1.00622	0.0089	0.984377	1
AC092301.3	1.022324	1.019198	1.02334	1.21576	0.2819	0.984379	1
LINC01229	1.022274	1.019201	1.023273	1.2121	0.2775	0.984379	1
LINC01574	1.037807	1.040334	1.036986	0.91699	-0.125	0.984416	1
CTD-2506P8.6	1.032621	1.029556	1.033617	1.1374	0.1857	0.984458	1
POU5F2	1.06281	1.059751	1.063804	1.06783	0.0947	0.984588	1
DPH6-AS1	1.028222	1.030727	1.027408	0.892	-0.1649	0.984597	1
GLOD4	1.74841	1.74513	1.749476	1.00583	0.0084	0.984606	1
RP11-1C1.6	1.015238	1.012187	1.01623	1.33176	0.4133	0.984621	1
TP73	1.01523	1.012187	1.016219	1.33085	0.4124	0.984621	1
SSBP3-AS1	1.015236	1.012187	1.016227	1.33156	0.4131	0.984621	1
ACER2	1.015245	1.012187	1.01624	1.33257	0.4142	0.984621	1
RP11-195B17.1	1.01523	1.012187	1.016219	1.33091	0.4124	0.984621	1
UXT-AS1	1.01525	1.012187	1.016245	1.33303	0.4147	0.984621	1
RP11-439L18.1	1.01524	1.012187	1.016233	1.33198	0.4136	0.984621	1
RP11-244E17.1	1.015267	1.012187	1.016268	1.33487	0.4167	0.984621	1
AC007292.3	1.015232	1.012187	1.016221	1.33106	0.4126	0.984621	1
RP3-399L15.1	1.015253	1.012187	1.016249	1.33335	0.4151	0.984621	1
LINC00640	1.015228	1.012187	1.016217	1.33067	0.4122	0.984621	1
UBE2Q2L	1.015247	1.012187	1.016241	1.33271	0.4144	0.984621	1
ZNF648	1.015235	1.012187	1.016226	1.33144	0.413	0.984621	1
RP1-296L11.1	1.015248	1.012187	1.016243	1.33282	0.4145	0.984621	1
RP11-900F13.3	1.015248	1.012187	1.016242	1.3328	0.4145	0.984621	1
RP11-121C2.3	1.015264	1.012187	1.016264	1.33456	0.4164	0.984621	1
RP11-95I16.4	1.015252	1.012187	1.016249	1.33333	0.415	0.984621	1
RP11-98O2.1	1.015273	1.012187	1.016276	1.33556	0.4174	0.984621	1
RP11-96C23.15	1.015246	1.012187	1.01624	1.33258	0.4142	0.984621	1

LINC01207	1.015252	1.012187	1.016249	1.33334	0.415	0.984621	1
LINC00921	1.015234	1.012187	1.016225	1.33137	0.4129	0.984621	1
RP11-692C24.2	1.017007	1.013947	1.018001	1.29074	0.3682	0.984661	1
TAS2R46	1.017019	1.013947	1.018018	1.29194	0.3695	0.984661	1
LY86-AS1	1.017012	1.013947	1.018009	1.29126	0.3688	0.984661	1
KCNT1	1.016991	1.013947	1.017981	1.28929	0.3666	0.984661	1
RP5-864K19.6	1.017013	1.013947	1.01801	1.29133	0.3689	0.984661	1
RP11-23E10.3	1.016995	1.013947	1.017985	1.28958	0.3669	0.984661	1
RP11-900F13.2	1.017006	1.013947	1.018001	1.29067	0.3681	0.984661	1
RP11-21B23.3	1.017011	1.013947	1.018007	1.29117	0.3687	0.984661	1
HDC	1.017042	1.013947	1.018048	1.29409	0.3719	0.984661	1
RP11-439E19.7	1.017003	1.013947	1.017996	1.29035	0.3678	0.984661	1
CTA-398F10.2	1.016992	1.013947	1.017982	1.28935	0.3666	0.984661	1
DDTL	1.016992	1.013947	1.017982	1.28937	0.3667	0.984661	1
AC016629.3	1.017002	1.013947	1.017996	1.29032	0.3677	0.984661	1
RP11-402J6.1	1.017001	1.013947	1.017994	1.29019	0.3676	0.984661	1
LINC00987	1.032009	1.028908	1.033017	1.14214	0.1917	0.984718	1
NAV2-AS3	1.031982	1.028881	1.03299	1.14226	0.1919	0.984718	1
ADCY4	1.030983	1.033377	1.030205	0.90495	-0.1441	0.984771	1
RP11-138I1.4	1.030977	1.033436	1.030177	0.90252	-0.148	0.984771	1
RP11-269C23.5	1.030488	1.032942	1.02969	0.90128	-0.15	0.984776	1
CFL2	2.124493	2.120339	2.125843	1.00491	0.0071	0.984808	1
AC010975.1	1.018873	1.015826	1.019864	1.25513	0.3278	0.984839	1
RP11-94C24.13	1.018892	1.015826	1.019889	1.25672	0.3297	0.984839	1
CELSR3-AS1	1.018876	1.015826	1.019868	1.25539	0.3281	0.984839	1
RP11-635L1.3	1.018858	1.015826	1.019843	1.25383	0.3263	0.984839	1
AC067956.1	1.018872	1.015826	1.019863	1.25505	0.3277	0.984839	1
AP000265.1	1.018882	1.015826	1.019875	1.25582	0.3286	0.984839	1
LINC00836	1.018865	1.015826	1.019853	1.25443	0.327	0.984839	1
CTC-250I14.3	1.018871	1.015826	1.019861	1.25497	0.3277	0.984839	1
GAS1RR	1.018869	1.015826	1.019858	1.25474	0.3274	0.984839	1
ST8SIA3	1.018866	1.015826	1.019855	1.25454	0.3272	0.984839	1
CDC20B	1.018856	1.015826	1.019841	1.25371	0.3262	0.984839	1
LA16c-390E6.5	1.018874	1.015826	1.019865	1.2552	0.3279	0.984839	1
LONRF1	1.172211	1.17491	1.171333	0.97955	-0.0298	0.984849	1
CTB-50L17.14	1.018401	1.015363	1.019388	1.26198	0.3357	0.984898	1
HIST2H2BF	1.018419	1.015387	1.019405	1.26116	0.3348	0.984898	1
CYP3A43	1.016098	1.013073	1.017082	1.30672	0.386	0.984901	1
RP11-366L5.1	1.016099	1.013073	1.017082	1.30674	0.386	0.984901	1
RP5-921G16.1	1.016096	1.013073	1.017079	1.30651	0.3857	0.984901	1
CTC-529L17.1	1.016123	1.013073	1.017115	1.30922	0.3887	0.984901	1
AC005540.3	1.016113	1.013073	1.017102	1.30824	0.3876	0.984901	1
GRIK3	1.016108	1.013073	1.017095	1.30771	0.387	0.984901	1
RP11-227G15.12	1.016109	1.013073	1.017096	1.30775	0.3871	0.984901	1
LINC00636	1.016103	1.013073	1.017088	1.30718	0.3865	0.984901	1

CTD-2384B11.2	1.016116	1.013073	1.017105	1.3085	0.3879	0.984901	1
RP5-999L4.2	1.016111	1.013073	1.017099	1.30801	0.3874	0.984901	1
CTD-2616J11.9	1.016115	1.013073	1.017104	1.30836	0.3878	0.984901	1
AP000318.2	1.016115	1.013073	1.017104	1.30842	0.3878	0.984901	1
RP5-974N19.1	1.01614	1.013073	1.017138	1.31097	0.3906	0.984901	1
OLIG3	1.016136	1.013073	1.017132	1.31051	0.3901	0.984901	1
RP11-283I3.2	1.016113	1.013073	1.017101	1.30817	0.3876	0.984901	1
RP11-553P9.2	1.016114	1.013073	1.017102	1.30826	0.3876	0.984901	1
RP11-161M6.3	1.016123	1.013073	1.017114	1.30919	0.3887	0.984901	1
RP11-21M24.2	1.016112	1.013073	1.017101	1.30813	0.3875	0.984901	1
ATP13A5-AS1	1.016103	1.013073	1.017088	1.30715	0.3864	0.984901	1
RP11-439M11.1	1.020037	1.022438	1.019257	0.85826	-0.2205	0.984963	1
IL6R	1.020029	1.022448	1.019243	0.85721	-0.2223	0.984963	1
RP11-20I20.4	1.020041	1.022473	1.019251	0.85661	-0.2233	0.984963	1
RP11-138E2.1	1.020026	1.022466	1.019232	0.85607	-0.2242	0.984963	1
CTB-49A3.4	1.02004	1.02249	1.019243	0.85564	-0.2249	0.984963	1
RP11-318C24.2	1.020045	1.022525	1.019239	0.85411	-0.2275	0.984963	1
TBX1	1.012793	1.015224	1.012002	0.78837	-0.3431	0.98497	1
CTD-2008P7.9	1.012793	1.015224	1.012002	0.78837	-0.3431	0.98497	1
SPRR1B	1.012793	1.015224	1.012002	0.78837	-0.3431	0.98497	1
RP11-1060J15.9	1.012788	1.015205	1.012002	0.78935	-0.3413	0.98497	1
RP11-656E20.5	1.01279	1.015212	1.012002	0.789	-0.3419	0.98497	1
AC104076.3	1.012789	1.015211	1.012002	0.78907	-0.3418	0.98497	1
RP11-380F14.2	1.012797	1.015244	1.012002	0.78737	-0.3449	0.98497	1
DMP1	1.012789	1.015208	1.012002	0.78922	-0.3415	0.98497	1
C2CD4B	1.012793	1.015226	1.012002	0.7883	-0.3432	0.98497	1
IL36RN	1.01279	1.015211	1.012002	0.78905	-0.3418	0.98497	1
ITGBL1	1.01279	1.015211	1.012002	0.78905	-0.3418	0.98497	1
RP11-379F12.4	1.01279	1.015211	1.012002	0.78905	-0.3418	0.98497	1
RP11-352M15.1	1.012791	1.015218	1.012002	0.78871	-0.3424	0.98497	1
RP11-472G21.2	1.012795	1.015235	1.012002	0.78783	-0.344	0.98497	1
RP1-10C16.1	1.012788	1.015205	1.012002	0.78937	-0.3412	0.98497	1
RP11-412P11.1	1.012788	1.015204	1.012002	0.78943	-0.3411	0.98497	1
AC007381.3	1.01279	1.015213	1.012002	0.78894	-0.342	0.98497	1
HOXD4	1.012795	1.015231	1.012002	0.788	-0.3437	0.98497	1
LINC01597	1.012793	1.015227	1.012002	0.78823	-0.3433	0.98497	1
AC009495.2	1.012794	1.015231	1.012002	0.78804	-0.3437	0.98497	1
NAV2-AS2	1.016161	1.013102	1.017155	1.30939	0.3889	0.985012	1
RP4-678D15.1	1.016125	1.013102	1.017108	1.30583	0.385	0.985012	1
AC098820.4	1.016122	1.013102	1.017104	1.30549	0.3846	0.985012	1
RP11-160H22.3	1.016135	1.013102	1.017121	1.30678	0.386	0.985012	1
RP11-452C8.1	1.01613	1.013102	1.017115	1.30633	0.3855	0.985012	1
RP11-360L9.7	1.01612	1.013102	1.017101	1.30528	0.3844	0.985012	1
CTB-147C22.9	1.016135	1.013102	1.017121	1.30677	0.386	0.985012	1
HAGLROS	1.016131	1.013102	1.017116	1.30642	0.3856	0.985012	1

C2-AS1	1.016163	1.013102	1.017158	1.30965	0.3892	0.985012	1
RP11-73M7.9	1.016158	1.013102	1.017152	1.30917	0.3886	0.985012	1
RP11-281O15.4	1.01614	1.013102	1.017127	1.30726	0.3865	0.985012	1
RP11-80H18.3	1.016137	1.013102	1.017124	1.30699	0.3863	0.985012	1
AFAP1-AS1	1.01613	1.013102	1.017115	1.30633	0.3855	0.985012	1
RP4-650F12.2	1.016137	1.013102	1.017123	1.30695	0.3862	0.985012	1
FAM186B	1.016127	1.013102	1.017111	1.30601	0.3852	0.985012	1
GATA1	1.016144	1.013102	1.017133	1.30774	0.3871	0.985012	1
RAET1E	1.01613	1.013102	1.017114	1.30624	0.3854	0.985012	1
MSANTD3-TMEFF1	1.016121	1.013102	1.017102	1.30537	0.3845	0.985012	1
RP11-90B9.3	1.016165	1.013102	1.017161	1.30986	0.3894	0.985012	1
KCNT2	1.193575	1.196124	1.192747	0.98278	-0.0251	0.985081	1
RASSF1-AS1	1.093371	1.095791	1.092585	0.96653	-0.0491	0.985115	1
GRAMD3	1.15811	1.16036	1.157378	0.9814	-0.0271	0.98519	1
PCDHGA11	1.016287	1.013274	1.017266	1.30074	0.3793	0.985262	1
C5orf63	1.016305	1.013274	1.01729	1.30252	0.3813	0.985262	1
RP4-535B20.4	1.016296	1.013274	1.017278	1.30165	0.3803	0.985262	1
CH507-254M2.1	1.016295	1.013274	1.017277	1.30157	0.3803	0.985262	1
LINC00443	1.016272	1.013274	1.017247	1.29926	0.3777	0.985262	1
C4orf45	1.016289	1.013274	1.017269	1.30097	0.3796	0.985262	1
AC011247.3	1.01632	1.013274	1.01731	1.30403	0.383	0.985262	1
RP11-526F3.1	1.016334	1.013274	1.017329	1.30548	0.3846	0.985262	1
RP11-375I20.6	1.016292	1.013274	1.017273	1.30126	0.3799	0.985262	1
ADGRG3	1.016279	1.013274	1.017256	1.29999	0.3785	0.985262	1
ELL3	1.016281	1.013274	1.017258	1.30012	0.3786	0.985262	1
AE000662.93	1.016297	1.013274	1.01728	1.3018	0.3805	0.985262	1
AC005786.5	1.016282	1.013274	1.01726	1.30026	0.3788	0.985262	1
RP11-138B4.1	1.016285	1.013274	1.017263	1.30053	0.3791	0.985262	1
CTD-2545M3.8	1.024939	1.027323	1.024165	0.8844	-0.1772	0.985305	1
SMG5	1.219441	1.221665	1.218718	0.9867	-0.0193	0.985336	1
LGI2	1.017807	1.014823	1.018776	1.26673	0.3411	0.985371	1
RP11-459F6.3	1.01784	1.014821	1.018821	1.2699	0.3447	0.985371	1
AQP6	1.017835	1.014817	1.018816	1.26995	0.3448	0.985371	1
C14orf178	1.017819	1.014791	1.018803	1.27128	0.3463	0.985371	1
GRPR	1.025709	1.022716	1.026682	1.17462	0.2322	0.985381	1
FRMD8	1.157704	1.154805	1.158646	1.02481	0.0354	0.985382	1
SCN8A	1.036058	1.033032	1.037042	1.1214	0.1653	0.985445	1
MFSD10	2.221983	2.217469	2.22345	1.00491	0.0071	0.9855	1
RP13-20L14.6	1.024866	1.027314	1.024071	0.88126	-0.1824	0.985556	1
RP11-108M12.3	1.040077	1.037029	1.041068	1.10907	0.1493	0.985601	1
SEC61B	7.00606	7.008105	7.005395	0.99955	-0.0007	0.985626	1
RP11-475O6.1	1.018689	1.015687	1.019665	1.25354	0.326	0.985634	1
C15orf59	1.018674	1.015674	1.01965	1.25368	0.3262	0.985634	1
AC005618.6	1.018677	1.015684	1.01965	1.2529	0.3253	0.985634	1
IRF4	1.01868	1.015695	1.019651	1.25203	0.3243	0.985634	1

ELAC2	1.34	1.336617	1.3411	1.01332	0.0191	0.985661	1
NTN4	1.024742	1.027189	1.023946	0.88072	-0.1832	0.985667	1
CCDC154	1.028421	1.025404	1.029402	1.1574	0.2109	0.985672	1
SYCP3	1.02048	1.017473	1.021457	1.22799	0.2963	0.985673	1
ABHD12B	1.020549	1.017488	1.021544	1.23191	0.3009	0.985673	1
MIOX	1.020463	1.017477	1.021433	1.22636	0.2944	0.985673	1
LDHD	1.020453	1.017488	1.021417	1.22463	0.2923	0.985673	1
CPA5	1.0354	1.032393	1.036378	1.12303	0.1674	0.985711	1
CDK18	1.390401	1.387226	1.391433	1.01086	0.0156	0.985729	1
RP11-348P10.2	1.027495	1.029929	1.026704	0.89225	-0.1645	0.98573	1
CTD-2619J13.14	1.027523	1.029964	1.02673	0.89205	-0.1648	0.98573	1
RP13-977J11.2	1.044983	1.047258	1.044244	0.93621	-0.0951	0.985771	1
MED13	1.438466	1.435134	1.43955	1.01015	0.0146	0.985821	1
MMP1	1.022343	1.019345	1.023317	1.20532	0.2694	0.985852	1
TRAPPC12-AS1	1.022342	1.019331	1.023321	1.20641	0.2707	0.985852	1
RP1-153G14.4	1.021856	1.018931	1.022807	1.20479	0.2688	0.985906	1
GPR171	1.019577	1.016596	1.020546	1.23802	0.308	0.985914	1
CTC-487M23.8	1.0196	1.016578	1.020583	1.24159	0.3122	0.985914	1
LHFPL5	1.016608	1.019028	1.015821	0.83146	-0.2663	0.985936	1
VWA5B1	1.016587	1.018941	1.015821	0.83527	-0.2597	0.985936	1
RP11-149B9.2	1.016589	1.018949	1.015821	0.83491	-0.2603	0.985936	1
GJB6	1.016599	1.018993	1.015821	0.83298	-0.2636	0.985936	1
RP11-307O13.1	1.016589	1.018952	1.015821	0.83478	-0.2605	0.985936	1
RP11-123B3.2	1.016589	1.018952	1.015821	0.83478	-0.2605	0.985936	1
HOXC4	1.016597	1.018983	1.015821	0.83346	-0.2628	0.985936	1
RP11-659P15.1	1.016587	1.018942	1.015821	0.83526	-0.2597	0.985936	1
AP001046.6	1.016588	1.018948	1.015821	0.83498	-0.2602	0.985936	1
RP11-556I14.1	1.016602	1.019005	1.015821	0.83249	-0.2645	0.985936	1
NLRP7	1.016599	1.018993	1.015821	0.83298	-0.2637	0.985936	1
CLDN14	1.016599	1.018993	1.015821	0.83298	-0.2637	0.985936	1
RP11-442N1.2	1.016593	1.018969	1.015821	0.83404	-0.2618	0.985936	1
RP11-310E22.5	1.016591	1.01896	1.015821	0.83445	-0.2611	0.985936	1
RP11-707G14.1	1.016588	1.018947	1.015821	0.83504	-0.2601	0.985936	1
AC007292.4	1.016591	1.018959	1.015821	0.83449	-0.261	0.985936	1
DEFB136	1.016592	1.018966	1.015821	0.8342	-0.2615	0.985936	1
SPRN	1.023892	1.026279	1.023117	0.87967	-0.185	0.985949	1
RP4-669L17.10	1.023853	1.026238	1.023078	0.87957	-0.1851	0.985949	1
HIGD2A	3.882289	3.876429	3.884194	1.0027	0.0039	0.985971	1
CIPC	1.157299	1.159883	1.156458	0.97858	-0.0312	0.986012	1
APOBEC3B	1.019559	1.01661	1.020517	1.23527	0.3048	0.986025	1
LA16c-306E5.2	1.019615	1.016626	1.020586	1.23817	0.3082	0.986025	1
RP3-508I15.22	1.01958	1.0166	1.020548	1.23784	0.3078	0.986025	1
SFTA3	1.019593	1.016616	1.020561	1.23745	0.3074	0.986025	1
MATK	1.019577	1.016621	1.020537	1.23566	0.3053	0.986025	1
TMEM196	1.019574	1.016596	1.020543	1.23784	0.3078	0.986025	1

AC005614.3	1.019581	1.016608	1.020547	1.23714	0.307	0.986025	1
RP11-42O15.3	1.019626	1.016596	1.020612	1.24197	0.3126	0.986025	1
TMEM105	1.040583	1.042863	1.039842	0.92951	-0.1055	0.986084	1
FCHSD2	1.232733	1.229262	1.233861	1.02006	0.0287	0.986091	1
BCKDHA	1.04568	1.042684	1.046654	1.09301	0.1283	0.986094	1
MEIOC	1.045723	1.042653	1.046721	1.09538	0.1314	0.986094	1
POLN	1.050859	1.047622	1.051911	1.09006	0.1244	0.986144	1
RSPO4	1.045419	1.047687	1.044682	0.93698	-0.0939	0.986246	1
FAM162B	1.021516	1.023938	1.020728	0.86591	-0.2077	0.986268	1
LINC01118	1.021516	1.024014	1.020705	0.86219	-0.2139	0.986268	1
LINC01136	1.021487	1.023835	1.020724	0.86948	-0.2018	0.986268	1
CALML4	1.02147	1.023812	1.020709	0.86972	-0.2014	0.986268	1
RP11-981P6.1	1.019751	1.01679	1.020713	1.23363	0.3029	0.986276	1
RP11-165F24.3	1.019793	1.01677	1.020776	1.23887	0.309	0.986276	1
LRRC36	1.019714	1.016767	1.020672	1.23293	0.3021	0.986276	1
CHRNB3	1.019746	1.016781	1.02071	1.23411	0.3035	0.986276	1
HGSNAT	1.417578	1.413962	1.418754	1.01157	0.0166	0.986336	1
MMP17	1.021253	1.018287	1.022217	1.2149	0.2808	0.986379	1
RP11-352D13.6	1.021283	1.018383	1.022225	1.20903	0.2738	0.986379	1
RP11-1379J22.5	1.029169	1.026233	1.030123	1.14827	0.1995	0.98638	1
POLR2A	1.411428	1.413571	1.410731	0.99313	-0.0099	0.986394	1
RP11-71L14.4	1.021291	1.023627	1.020531	0.86896	-0.2026	0.986519	1
LRRC74B	1.021313	1.023665	1.020548	0.86831	-0.2037	0.986519	1
LRRC3B	1.060812	1.057868	1.061769	1.06742	0.0941	0.986523	1
LINGO1	1.067953	1.064918	1.06894	1.06196	0.0867	0.986525	1
CHODL	1.07143	1.073818	1.070654	0.95714	-0.0632	0.986618	1
ERFE	1.021294	1.023631	1.020535	0.86898	-0.2026	0.98663	1
WWTR1-AS1	1.021308	1.023627	1.020555	0.86998	-0.2009	0.98663	1
WWP2	1.140292	1.142412	1.139604	0.98028	-0.0287	0.986633	1
NR2C2AP	1.297561	1.294581	1.298529	1.0134	0.0192	0.986649	1
RPP14	1.253307	1.250216	1.254311	1.01637	0.0234	0.986663	1
DUSP10	1.10018	1.097143	1.101167	1.04142	0.0586	0.986665	1
ABTB2	1.031942	1.028907	1.032929	1.13913	0.1879	0.98667	1
RHBDL2	1.02395	1.020989	1.024912	1.1869	0.2472	0.986683	1
RP5-1142A6.2	1.024102	1.026478	1.02333	0.88112	-0.1826	0.986692	1
CD274	1.024078	1.026391	1.023326	0.88385	-0.1781	0.986692	1
RP11-473E2.4	1.024069	1.02641	1.023308	0.88255	-0.1802	0.986692	1
RP11-424N24.2	1.038916	1.036042	1.03985	1.10566	0.1449	0.9867	1
MAP3K3	1.095014	1.091938	1.096014	1.04434	0.0626	0.986707	1
WDR63	1.041536	1.043861	1.04078	0.92975	-0.1051	0.98671	1
AP000695.4	1.041615	1.04381	1.040902	0.93362	-0.0991	0.98671	1
RP1-151F17.2	1.025792	1.022882	1.026738	1.16851	0.2247	0.986862	1
CFAP57	1.025793	1.022854	1.026748	1.1704	0.227	0.986862	1
TBC1D29	1.025807	1.022946	1.026736	1.16518	0.2206	0.986862	1
CARD9	1.025784	1.02281	1.02675	1.17278	0.2299	0.986862	1

LINC00276	1.025823	1.022922	1.026766	1.16767	0.2236	0.986862	1
PCDHGB7	1.022161	1.024446	1.021418	0.87614	-0.1908	0.986871	1
RP11-799B12.4	1.020405	1.022766	1.019637	0.86255	-0.2133	0.98691	1
SZT2	1.124107	1.121068	1.125094	1.03325	0.0472	0.986913	1
SRXN1	1.023024	1.020096	1.023975	1.19304	0.2546	0.986924	1
TMEM220-AS1	1.023024	1.020097	1.023975	1.19297	0.2546	0.986924	1
ZNF674	1.03719	1.039458	1.036453	0.92385	-0.1143	0.987026	1
ANKRD13A	1.200629	1.203033	1.199848	0.98431	-0.0228	0.98703	1
AKT1S1	1.624689	1.621589	1.625696	1.00661	0.0095	0.987031	1
RP11-728F11.4	1.023025	1.020114	1.023972	1.1918	0.2531	0.987035	1
ASMTL-AS1	1.023081	1.020192	1.02402	1.18961	0.2505	0.987035	1
RP1-257A7.5	1.023036	1.020116	1.023985	1.19234	0.2538	0.987035	1
RP11-385F5.4	1.023021	1.020104	1.023969	1.19227	0.2537	0.987035	1
KCP	1.049181	1.046342	1.050104	1.08119	0.1126	0.987074	1
RP11-477N3.1	1.019523	1.021825	1.018775	0.86024	-0.2172	0.987174	1
RP11-360I2.1	1.019545	1.021851	1.018795	0.86015	-0.2173	0.987174	1
SLC44A4	1.019547	1.021876	1.018791	0.85898	-0.2193	0.987174	1
HBE1	1.01953	1.021808	1.01879	0.8616	-0.2149	0.987174	1
C1orf226	1.067168	1.069436	1.066431	0.95672	-0.0638	0.9872	1
ITGA10	1.018032	1.020298	1.017296	0.8521	-0.2309	0.987234	1
RP1-79C4.4	1.01803	1.020365	1.017271	0.84804	-0.2378	0.987234	1
PICK1	1.185142	1.187478	1.184382	0.98349	-0.024	0.987261	1
DKFZp779M0652	1.023181	1.020293	1.02412	1.1886	0.2493	0.987286	1
KRBA1	1.115249	1.117546	1.114502	0.97411	-0.0378	0.987333	1
RP11-497E19.2	1.024709	1.021875	1.02563	1.17169	0.2286	0.987385	1
RP11-1079K10.4	1.017844	1.020132	1.017101	0.84943	-0.2354	0.987484	1
RP11-1079K10.3	1.017854	1.02013	1.017114	0.85016	-0.2342	0.987484	1
RP3-500L14.2	1.017844	1.020109	1.017108	0.85073	-0.2332	0.987484	1
RP11-543D5.1	1.018348	1.015453	1.019288	1.24817	0.3198	0.98753	1
RP11-365H22.2	1.018312	1.015453	1.019241	1.24509	0.3163	0.98753	1
AC092295.4	1.018328	1.015453	1.019262	1.24645	0.3178	0.98753	1
ALDH1L1-AS2	1.018328	1.015453	1.019262	1.24646	0.3178	0.98753	1
SLC17A6	1.018367	1.015453	1.019314	1.24982	0.3217	0.98753	1
AC009299.2	1.018311	1.015453	1.01924	1.24506	0.3162	0.98753	1
MYLK-AS2	1.018326	1.015453	1.01926	1.24629	0.3176	0.98753	1
LINC01482	1.018369	1.015453	1.019317	1.25003	0.322	0.98753	1
RP11-390F4.10	1.018315	1.015453	1.019245	1.24537	0.3166	0.98753	1
RP11-386D6.1	1.018344	1.015453	1.019283	1.24782	0.3194	0.98753	1
RP11-95P2.3	1.018313	1.015453	1.019243	1.24523	0.3164	0.98753	1
RP11-728G15.1	1.018332	1.015453	1.019268	1.24685	0.3183	0.98753	1
RP11-341N2.1	1.018326	1.015453	1.01926	1.24633	0.3177	0.98753	1
TULP2	1.018366	1.015453	1.019312	1.2497	0.3216	0.98753	1
CTB-127C13.1	1.018338	1.015453	1.019276	1.24733	0.3188	0.98753	1
SNTN	1.018367	1.015453	1.019314	1.24979	0.3217	0.98753	1
VIPAS39	1.239324	1.236537	1.24023	1.01561	0.0223	0.98759	1

CH17-437K3.1	1.017824	1.020099	1.017085	0.85004	-0.2344	0.987595	1
ANKRD24	1.017841	1.020082	1.017113	0.85214	-0.2308	0.987595	1
CTC-457L16.2	1.017832	1.020053	1.01711	0.85326	-0.2289	0.987595	1
GNL3L	1.13791	1.134715	1.138948	1.03143	0.0446	0.987636	1
AC091878.1	1.020135	1.022384	1.019404	0.8669	-0.2061	0.987647	1
RP11-268G12.1	1.020604	1.022838	1.019878	0.8704	-0.2002	0.987657	1
RP5-994D16.11	1.035382	1.032552	1.036302	1.11518	0.1573	0.987666	1
CPSF2	1.469731	1.466775	1.470692	1.00839	0.0121	0.987686	1
GNMT	1.027389	1.024511	1.028324	1.15558	0.2086	0.987688	1
LINC00294	1.09636	1.098344	1.095715	0.97327	-0.0391	0.987755	1
SLC9C1	1.018754	1.021018	1.018018	0.85726	-0.2222	0.987836	1
SLC52A3	1.018733	1.020941	1.018016	0.86033	-0.217	0.987836	1
ARHGEF33	1.018711	1.020947	1.017984	0.85857	-0.22	0.987836	1
PRKCE	1.05524	1.052402	1.056162	1.07175	0.1	0.987848	1
SDR42E2	1.029258	1.026344	1.030205	1.14654	0.1973	0.987868	1
WFDC5	1.016989	1.019283	1.016243	0.84233	-0.2475	0.987875	1
MEOX1	1.016959	1.01923	1.01622	0.84351	-0.2455	0.987875	1
ACOT7	1.693266	1.695273	1.692613	0.99618	-0.0055	0.987939	1
FBXO44	1.449396	1.451345	1.448763	0.99428	-0.0083	0.988022	1
ZNF30	1.073133	1.075424	1.072388	0.95975	-0.0593	0.988039	1
RP11-7F18.2	1.046811	1.043904	1.047757	1.08775	0.1213	0.98804	1
TRAPPC2B	1.267948	1.265094	1.268876	1.01426	0.0204	0.988041	1
LINC01535	1.016074	1.018305	1.015349	0.83852	-0.2541	0.988138	1
CTD-2516F10.2	1.016109	1.018404	1.015363	0.83478	-0.2605	0.988138	1
RP1-69D17.3	1.014557	1.016768	1.013838	0.82526	-0.2771	0.988203	1
SLCO2B1	1.014556	1.016765	1.013838	0.82541	-0.2768	0.988203	1
DEFB1	1.014556	1.016765	1.013838	0.82541	-0.2768	0.988203	1
RP11-544L8__B.4	1.014565	1.016804	1.013838	0.82351	-0.2801	0.988203	1
CTC-281F24.5	1.014564	1.016797	1.013838	0.82381	-0.2796	0.988203	1
CIB3	1.014555	1.016762	1.013838	0.82553	-0.2766	0.988203	1
RP11-271M24.2	1.01456	1.016781	1.013838	0.82462	-0.2782	0.988203	1
RP11-260E18.1	1.014556	1.016767	1.013838	0.8253	-0.277	0.988203	1
RP11-715J22.4	1.014564	1.016797	1.013838	0.82381	-0.2796	0.988203	1
RP11-317N8.5	1.014556	1.016764	1.013838	0.82547	-0.2767	0.988203	1
DNAH8	1.014566	1.016804	1.013838	0.82348	-0.2802	0.988203	1
COL5A1-AS1	1.014558	1.016772	1.013838	0.82506	-0.2774	0.988203	1
RP13-820C6.4	1.014559	1.016776	1.013838	0.82487	-0.2778	0.988203	1
TRPC5OS	1.014557	1.016769	1.013838	0.8252	-0.2772	0.988203	1
SIAH3	1.014557	1.016769	1.013838	0.8252	-0.2772	0.988203	1
GABRA6	1.014563	1.016793	1.013838	0.82401	-0.2793	0.988203	1
IL15RA	1.014558	1.016772	1.013838	0.82504	-0.2775	0.988203	1
RP11-449J1.1	1.014555	1.016762	1.013838	0.82555	-0.2766	0.988203	1
CTC-218B8.3	1.014558	1.016773	1.013838	0.82498	-0.2776	0.988203	1
AC009133.17	1.014558	1.016773	1.013838	0.82498	-0.2776	0.988203	1
FAM138C	1.01456	1.016782	1.013838	0.82454	-0.2783	0.988203	1

RP11-161I2.1	1.01456	1.016782	1.013838	0.82454	-0.2783	0.988203	1
AC073316.1	1.01456	1.01678	1.013838	0.82468	-0.2781	0.988203	1
RP11-390K5.3	1.014572	1.016831	1.013838	0.82215	-0.2825	0.988203	1
ERMAP	1.111772	1.113778	1.11112	0.97664	-0.0341	0.988257	1
CARD8-AS1	1.026646	1.023827	1.027563	1.1568	0.2101	0.988293	1
CSNK2A1	2.072596	2.075376	2.071693	0.99657	-0.005	0.988366	1
PRR22	1.02813	1.025343	1.029035	1.14568	0.1962	0.988387	1
RP11-23P13.6	1.014401	1.016599	1.013687	0.82456	-0.2783	0.988453	1
LY6D	1.014419	1.016672	1.013687	0.82098	-0.2846	0.988453	1
DSG1-AS1	1.014404	1.01661	1.013687	0.824	-0.2793	0.988453	1
FAM109B	1.014404	1.01661	1.013687	0.824	-0.2793	0.988453	1
RP11-145M9.5	1.01441	1.016634	1.013687	0.82285	-0.2813	0.988453	1
CALHM3	1.014403	1.016606	1.013687	0.82424	-0.2789	0.988453	1
RP11-91A18.4	1.014402	1.0166	1.013687	0.82452	-0.2784	0.988453	1
CTD-2297D10.2	1.014412	1.016641	1.013687	0.82251	-0.2819	0.988453	1
VIPR1-AS1	1.014411	1.016636	1.013687	0.82271	-0.2815	0.988453	1
MOGAT3	1.014409	1.016629	1.013687	0.82309	-0.2809	0.988453	1
AICDA	1.01441	1.016635	1.013687	0.82279	-0.2814	0.988453	1
CTD-2545M3.2	1.014402	1.0166	1.013687	0.8245	-0.2784	0.988453	1
BDKRB1	1.014404	1.016608	1.013687	0.8241	-0.2791	0.988453	1
ART3	1.0144	1.016596	1.013687	0.82474	-0.278	0.988453	1
RP5-1028K7.2	1.014407	1.016622	1.013687	0.8234	-0.2803	0.988453	1
C17orf107	1.014405	1.016615	1.013687	0.82379	-0.2797	0.988453	1
RP11-264E20.1	1.014406	1.016618	1.013687	0.82361	-0.28	0.988453	1
FCMR	1.014399	1.016588	1.013687	0.82511	-0.2773	0.988453	1
AC093159.1	1.0144	1.016593	1.013687	0.82484	-0.2778	0.988453	1
SPANXA2-OT1	1.014407	1.016621	1.013687	0.82349	-0.2802	0.988453	1
RP11-498C9.16	1.0144	1.016592	1.013687	0.82494	-0.2776	0.988453	1
MGC15885	1.014402	1.016601	1.013687	0.82446	-0.2785	0.988453	1
MRPL58	1.329059	1.32571	1.330147	1.01362	0.0195	0.98849	1
AC073321.4	1.048285	1.050513	1.047561	0.94157	-0.0869	0.988491	1
ECT2L	1.014518	1.011717	1.015428	1.31672	0.3969	0.988497	1
KIR3DX1	1.014516	1.011717	1.015426	1.31652	0.3967	0.988497	1
AC074183.4	1.01453	1.011717	1.015444	1.31804	0.3984	0.988497	1
RP11-131N11.4	1.014518	1.011717	1.015429	1.31676	0.397	0.988497	1
AHSP	1.014509	1.011717	1.015417	1.31576	0.3959	0.988497	1
RP11-225H22.7	1.014531	1.011717	1.015446	1.31821	0.3986	0.988497	1
CTB-40H15.4	1.014517	1.011717	1.015428	1.31666	0.3969	0.988497	1
RP11-379L18.3	1.014521	1.011717	1.015432	1.31705	0.3973	0.988497	1
AC104134.2	1.014511	1.011717	1.015419	1.3159	0.3961	0.988497	1
C7orf55-LUC7L2	1.014517	1.011717	1.015428	1.31665	0.3969	0.988497	1
SLC34A3	1.01452	1.011717	1.015432	1.317	0.3973	0.988497	1
CNGB3	1.014512	1.011717	1.01542	1.31603	0.3962	0.988497	1
RP11-18C24.8	1.014516	1.011717	1.015425	1.31647	0.3967	0.988497	1
RP11-295K3.1	1.014527	1.011717	1.01544	1.31774	0.3981	0.988497	1

CPZ	1.014515	1.011717	1.015425	1.3164	0.3966	0.988497	1
RP11-881M11.1	1.014515	1.011717	1.015425	1.31642	0.3966	0.988497	1
RP11-326N17.2	1.014516	1.011717	1.015426	1.31655	0.3968	0.988497	1
AC025171.1	1.01452	1.011717	1.015431	1.31692	0.3972	0.988497	1
RP11-21L1.1	1.014514	1.011717	1.015424	1.31632	0.3965	0.988497	1
TMX2-CTNND1	1.014525	1.011717	1.015437	1.31748	0.3978	0.988497	1
AC078883.4	1.014547	1.011717	1.015467	1.31998	0.4005	0.988497	1
RP11-428O18.6	1.014516	1.011717	1.015426	1.31648	0.3967	0.988497	1
ASPG	1.014526	1.011717	1.015439	1.31767	0.398	0.988497	1
LA16c-380H5.6	1.014505	1.011717	1.015411	1.31521	0.3953	0.988497	1
RP11-585P4.5	1.014516	1.011717	1.015426	1.31652	0.3967	0.988497	1
RNU6ATAC35P	1.014521	1.011717	1.015433	1.31708	0.3973	0.988497	1
GSG1	1.02181	1.018991	1.022726	1.19667	0.259	0.988548	1
C1orf101	1.021754	1.01894	1.022669	1.19685	0.2592	0.988548	1
RP11-307L14.2	1.021816	1.018974	1.02274	1.19849	0.2612	0.988548	1
CTD-2296D1.2	1.021787	1.018973	1.022702	1.19651	0.2588	0.988548	1
ACRBP	1.059275	1.05644	1.060196	1.06655	0.093	0.988561	1
KCNH7	1.014382	1.016572	1.01367	0.82484	-0.2778	0.988563	1
RP11-11C20.3	1.014381	1.016571	1.01367	0.8249	-0.2777	0.988563	1
RP11-756H20.1	1.014382	1.016573	1.01367	0.82479	-0.2779	0.988563	1
LYL1	1.01438	1.016567	1.01367	0.8251	-0.2774	0.988563	1
PDZK1IP1	1.014378	1.016558	1.01367	0.82557	-0.2765	0.988563	1
SHISA8	1.014385	1.016586	1.01367	0.82418	-0.279	0.988563	1
VGLL2	1.014385	1.016586	1.01367	0.82418	-0.279	0.988563	1
LINC01416	1.014382	1.016573	1.01367	0.82483	-0.2778	0.988563	1
ENAM	1.01438	1.016564	1.01367	0.82524	-0.2771	0.988563	1
RP11-1057B8.2	1.01438	1.016564	1.01367	0.82524	-0.2771	0.988563	1
RP11-354K1.1	1.014379	1.016561	1.01367	0.82541	-0.2768	0.988563	1
ABRA	1.014392	1.016612	1.01367	0.82285	-0.2813	0.988563	1
IDI2	1.014384	1.01658	1.01367	0.82447	-0.2785	0.988563	1
AC004947.2	1.014384	1.01658	1.01367	0.82447	-0.2785	0.988563	1
TDRD15	1.014386	1.016591	1.01367	0.82393	-0.2794	0.988563	1
APOB	1.014382	1.016572	1.01367	0.82486	-0.2778	0.988563	1
PARVG	1.014389	1.016603	1.01367	0.8233	-0.2805	0.988563	1
AC005281.1	1.014387	1.016592	1.01367	0.82385	-0.2796	0.988563	1
LHFPL3	1.014385	1.016584	1.01367	0.82424	-0.2789	0.988563	1
H2BFS	1.014384	1.016584	1.01367	0.82429	-0.2788	0.988563	1
RP11-159F24.6	1.014388	1.016598	1.01367	0.82355	-0.2801	0.988563	1
LINC01284	1.014383	1.016579	1.01367	0.82449	-0.2784	0.988563	1
BAP1	1.327911	1.330236	1.327155	0.99067	-0.0135	0.988594	1
POR	1.327814	1.330071	1.327081	0.99094	-0.0131	0.988594	1
RP11-10C24.3	1.034574	1.036728	1.033873	0.92227	-0.1167	0.988595	1
RP5-1172N10.2	1.016699	1.018876	1.015992	0.84719	-0.2392	0.988611	1
RP11-547D24.3	1.01672	1.018941	1.015998	0.84465	-0.2436	0.988611	1
MTRNR2L11	1.016689	1.018868	1.01598	0.84693	-0.2397	0.988611	1

C7	1.017139	1.019332	1.016426	0.84968	-0.235	0.988626	1
CMB9-22P13.2	1.017141	1.01934	1.016426	0.84933	-0.2356	0.988626	1
RP11-886P16.6	1.01715	1.019377	1.016426	0.84771	-0.2384	0.988626	1
LINC01123	1.017146	1.019361	1.016426	0.84842	-0.2371	0.988626	1
CTD-2329K10.1	1.017146	1.019359	1.016426	0.84851	-0.237	0.988626	1
AC138517.6	1.017138	1.019329	1.016426	0.84984	-0.2347	0.988626	1
TSKS	1.017138	1.019329	1.016426	0.84984	-0.2347	0.988626	1
AC093110.3	1.017135	1.019317	1.016426	0.85036	-0.2339	0.988626	1
GFI1	1.017135	1.019317	1.016426	0.85036	-0.2339	0.988626	1
RP11-59E19.4	1.017136	1.019319	1.016426	0.85027	-0.234	0.988626	1
LBP	1.01714	1.019334	1.016426	0.8496	-0.2351	0.988626	1
UCHL1-AS1	1.017142	1.019342	1.016426	0.84924	-0.2358	0.988626	1
RP11-90D11.1	1.017142	1.019342	1.016426	0.84924	-0.2358	0.988626	1
IMPG1	1.017137	1.019324	1.016426	0.85004	-0.2344	0.988626	1
RP11-321N4.5	1.017146	1.019362	1.016426	0.84839	-0.2372	0.988626	1
RP11-70F11.11	1.017142	1.019344	1.016426	0.84915	-0.2359	0.988626	1
TEX11	1.017142	1.019344	1.016426	0.84915	-0.2359	0.988626	1
RP11-93I21.3	1.017147	1.019364	1.016426	0.84828	-0.2374	0.988626	1
RP11-692C24.1	1.017147	1.019364	1.016426	0.84828	-0.2374	0.988626	1
OXCT2	1.017139	1.019333	1.016426	0.84967	-0.235	0.988626	1
POU2AF1	1.017139	1.019333	1.016426	0.84967	-0.235	0.988626	1
GATA6	1.017137	1.019323	1.016426	0.85007	-0.2343	0.988626	1
RP1-45C12.1	1.017141	1.019341	1.016426	0.8493	-0.2357	0.988626	1
RP11-309L24.10	1.017148	1.019368	1.016426	0.84812	-0.2377	0.988626	1
VNN1	1.017139	1.019333	1.016426	0.84966	-0.235	0.988626	1
MAGEE2	1.017142	1.019346	1.016426	0.84908	-0.236	0.988626	1
RP11-347P5.1	1.029028	1.026333	1.029905	1.13563	0.1835	0.988651	1
IL11RA	1.329564	1.332252	1.32869	0.98928	-0.0156	0.988661	1
BLID	1.015271	1.017446	1.014564	0.83483	-0.2605	0.988804	1
RP11-405M12.3	1.015272	1.01745	1.014564	0.83466	-0.2607	0.988804	1
C1orf210	1.015276	1.017467	1.014564	0.83383	-0.2622	0.988804	1
FAM156A	1.015271	1.017446	1.014564	0.83484	-0.2604	0.988804	1
RP11-434H14.1	1.015271	1.017443	1.014564	0.83498	-0.2602	0.988804	1
ATP10A	1.015278	1.017471	1.014564	0.83362	-0.2625	0.988804	1
CTD-2023N9.1	1.015269	1.017437	1.014564	0.83527	-0.2597	0.988804	1
RP11-148K1.10	1.015272	1.01745	1.014564	0.83464	-0.2608	0.988804	1
RP11-362A1.1	1.015277	1.017468	1.014564	0.83378	-0.2623	0.988804	1
RP11-244F12.3	1.015269	1.017438	1.014564	0.83523	-0.2598	0.988804	1
CRHR2	1.015274	1.017456	1.014564	0.83434	-0.2613	0.988804	1
CLNK	1.015279	1.017476	1.014564	0.83337	-0.263	0.988804	1
UBL4B	1.015279	1.017476	1.014564	0.83337	-0.263	0.988804	1
HOXA9	1.015279	1.017476	1.014564	0.83337	-0.263	0.988804	1
RP11-385D13.1	1.015273	1.017454	1.014564	0.83447	-0.2611	0.988804	1
RP11-768G7.2	1.015274	1.017457	1.014564	0.83429	-0.2614	0.988804	1
FAM180B	1.01527	1.017442	1.014564	0.83503	-0.2601	0.988804	1

PI15	1.015269	1.017436	1.014564	0.83532	-0.2596	0.988804	1
RP11-107F6.3	1.015269	1.017436	1.014564	0.83532	-0.2596	0.988804	1
ARHGAP40	1.015273	1.017455	1.014564	0.83441	-0.2612	0.988804	1
FAM83C-AS1	1.015273	1.017452	1.014564	0.83453	-0.261	0.988804	1
RP11-508N12.2	1.015273	1.017454	1.014564	0.83446	-0.2611	0.988804	1
RP11-205K6.1	1.015279	1.017475	1.014564	0.83343	-0.2629	0.988804	1
AP000857.3	1.015278	1.017474	1.014564	0.8335	-0.2627	0.988804	1
CDC42EP5	1.079454	1.081645	1.078741	0.96443	-0.0522	0.988806	1
PGR	1.013518	1.015709	1.012806	0.81518	-0.2948	0.988843	1
XXyac-YR29IB3.1	1.013516	1.015699	1.012806	0.81569	-0.2939	0.988843	1
LINC00460	1.01351	1.015677	1.012806	0.81686	-0.2918	0.988843	1
HHEX	1.01351	1.015677	1.012806	0.81686	-0.2918	0.988843	1
RP11-429E11.2	1.013521	1.015719	1.012806	0.81467	-0.2957	0.988843	1
RP11-300E4.2	1.013515	1.015695	1.012806	0.81594	-0.2935	0.988843	1
AC147651.1	1.013516	1.015701	1.012806	0.81561	-0.294	0.988843	1
AC123023.1	1.01351	1.015678	1.012806	0.81681	-0.2919	0.988843	1
SLC6A13	1.013509	1.015672	1.012806	0.81709	-0.2914	0.988843	1
RP1-28H20.3	1.013508	1.015669	1.012806	0.81726	-0.2911	0.988843	1
AC062021.1	1.013511	1.01568	1.012806	0.81671	-0.2921	0.988843	1
RP5-947P14.1	1.013518	1.015707	1.012806	0.81527	-0.2946	0.988843	1
TTC6	1.013517	1.015703	1.012806	0.81549	-0.2943	0.988843	1
ESX1	1.013515	1.015697	1.012806	0.81583	-0.2937	0.988843	1
FARP1-AS1	1.013512	1.015683	1.012806	0.81654	-0.2924	0.988843	1
C22orf34	1.013515	1.015695	1.012806	0.81591	-0.2935	0.988843	1
LA16c-360A4.1	1.013512	1.015685	1.012806	0.81643	-0.2926	0.988843	1
AP006285.7	1.01352	1.015718	1.012806	0.81475	-0.2956	0.988843	1
OTOG	1.013513	1.015686	1.012806	0.81637	-0.2927	0.988843	1
IQCJ	1.013513	1.015686	1.012806	0.81637	-0.2927	0.988843	1
RP11-496D24.2	1.013512	1.015685	1.012806	0.81645	-0.2926	0.988843	1
OR5H15	1.013511	1.015681	1.012806	0.81666	-0.2922	0.988843	1
CTB-76P12.1	1.013511	1.015681	1.012806	0.81666	-0.2922	0.988843	1
RP11-6N13.1	1.013511	1.015681	1.012806	0.81666	-0.2922	0.988843	1
CYP4A22	1.013515	1.015696	1.012806	0.81589	-0.2936	0.988843	1
ZIM3	1.013515	1.015696	1.012806	0.81589	-0.2936	0.988843	1
SFTPD	1.032683	1.029849	1.033604	1.12582	0.171	0.98887	1
PTENP1-AS	1.032706	1.030066	1.033564	1.11634	0.1588	0.98887	1
AC007563.5	1.023242	1.025379	1.022548	0.88846	-0.1706	0.988883	1
CTC-340A15.2	1.023287	1.025475	1.022576	0.88621	-0.1743	0.988883	1
ANGPTL8	1.02326	1.025406	1.022563	0.88808	-0.1712	0.988883	1
AC093642.3	1.023258	1.025463	1.022541	0.88523	-0.1759	0.988883	1
INPP5B	1.075632	1.072977	1.076495	1.0482	0.0679	0.988892	1
MIR3911	1.031471	1.033617	1.030773	0.91542	-0.1275	0.98896	1
RP11-231E19.1	1.030007	1.027306	1.030886	1.13111	0.1777	0.989045	1
FRMPD4	1.012647	1.014805	1.011946	0.8069	-0.3095	0.989106	1
KLF8	1.012651	1.014821	1.011946	0.80603	-0.3111	0.989106	1

XACT	1.01265	1.014817	1.011946	0.80624	-0.3107	0.989106	1
HOXA10	1.01265	1.014817	1.011946	0.80624	-0.3107	0.989106	1
CLEC1A	1.01265	1.014814	1.011946	0.80643	-0.3104	0.989106	1
HLA-DQB2	1.012648	1.014809	1.011946	0.8067	-0.3099	0.989106	1
RP11-864I4.4	1.012647	1.014802	1.011946	0.80708	-0.3092	0.989106	1
RP11-681H18.2	1.012648	1.014806	1.011946	0.80682	-0.3097	0.989106	1
NETO1	1.012648	1.014806	1.011946	0.80682	-0.3097	0.989106	1
MYZAP	1.012645	1.014797	1.011946	0.80736	-0.3087	0.989106	1
RP11-33A14.1	1.012645	1.014797	1.011946	0.80736	-0.3087	0.989106	1
ZNF341-AS1	1.012648	1.014806	1.011946	0.80684	-0.3096	0.989106	1
RP11-173P15.3	1.012646	1.014798	1.011946	0.80727	-0.3089	0.989106	1
AF131216.6	1.012645	1.014794	1.011946	0.80752	-0.3084	0.989106	1
IL12A	1.012645	1.014795	1.011946	0.80743	-0.3086	0.989106	1
CTD-2515C13.2	1.01265	1.014816	1.011946	0.80631	-0.3106	0.989106	1
KLRF2	1.012653	1.014828	1.011946	0.80565	-0.3118	0.989106	1
DEFB135	1.012655	1.014834	1.011946	0.80532	-0.3124	0.989106	1
RP11-445K13.2	1.012645	1.014795	1.011946	0.80742	-0.3086	0.989106	1
AC005009.2	1.012649	1.014812	1.011946	0.80653	-0.3102	0.989106	1
AC073046.25	1.012644	1.014792	1.011946	0.8076	-0.3083	0.989106	1
LY6K	1.012644	1.014792	1.011946	0.8076	-0.3083	0.989106	1
RP11-6I2.3	1.012647	1.014805	1.011946	0.80692	-0.3095	0.989106	1
RP11-46C24.3	1.012644	1.014791	1.011946	0.80765	-0.3082	0.989106	1
RP11-295P9.12	1.012651	1.014818	1.011946	0.80622	-0.3108	0.989106	1
KERA	1.012653	1.014828	1.011946	0.80565	-0.3118	0.989106	1
AC003986.7	1.012653	1.014828	1.011946	0.80565	-0.3118	0.989106	1
XXyac-YX65C7_A.3	1.012653	1.014828	1.011946	0.80565	-0.3118	0.989106	1
RP11-757A13.1	1.012657	1.014845	1.011946	0.80472	-0.3134	0.989106	1
CTD-2589M5.4	1.012645	1.014797	1.011946	0.80735	-0.3087	0.989106	1
EPB41L1	1.346263	1.343859	1.347044	1.00926	0.0133	0.989149	1
RP11-1277A3.1	1.020608	1.022913	1.019858	0.86667	-0.2064	0.989173	1
DVL1	1.145426	1.142735	1.146301	1.02498	0.0356	0.989231	1
SSPN	1.505544	1.507634	1.504865	0.99455	-0.0079	0.989345	1
ANKRD34A	1.061012	1.063158	1.060314	0.95497	-0.0665	0.989455	1
DDC	1.017949	1.015211	1.018839	1.23853	0.3086	0.989516	1
DLEU7-AS1	1.01794	1.015218	1.018825	1.23704	0.3069	0.989516	1
DGUOK-AS1	1.017951	1.015211	1.018841	1.23865	0.3088	0.989516	1
NAMA	1.017964	1.015208	1.01886	1.24015	0.3105	0.989516	1
RP4-612B15.3	1.036962	1.039087	1.036272	0.92796	-0.1079	0.989556	1
HIST1H2BE	1.02522	1.022475	1.026112	1.16181	0.2164	0.989562	1
RP11-422P24.10	1.025274	1.022518	1.026169	1.16216	0.2168	0.989562	1
CTC-366B18.4	1.025236	1.022465	1.026137	1.16346	0.2184	0.989562	1
RP11-159K7.2	1.013245	1.015364	1.012556	0.81724	-0.2912	0.989578	1
LINC01322	1.013247	1.015371	1.012556	0.81687	-0.2918	0.989578	1
XIRP1	1.013262	1.015433	1.012556	0.81358	-0.2976	0.989578	1
RP11-173P15.5	1.01325	1.015385	1.012556	0.8161	-0.2932	0.989578	1

CTD-2376I20.1	1.013248	1.015378	1.012556	0.81648	-0.2925	0.989578	1
CTC-338M12.7	1.013248	1.015378	1.012556	0.81648	-0.2925	0.989578	1
DSG1	1.013251	1.015391	1.012556	0.81581	-0.2937	0.989578	1
ACAN	1.013254	1.015403	1.012556	0.81516	-0.2948	0.989578	1
AC006378.2	1.013245	1.015364	1.012556	0.81723	-0.2912	0.989578	1
RP11-404O13.5	1.013249	1.015382	1.012556	0.81626	-0.2929	0.989578	1
PLA2G4F	1.013245	1.015363	1.012556	0.81728	-0.2911	0.989578	1
TAS2R13	1.013245	1.015365	1.012556	0.81718	-0.2913	0.989578	1
C17orf99	1.013247	1.015372	1.012556	0.81683	-0.2919	0.989578	1
FGL1	1.013247	1.015373	1.012556	0.81674	-0.292	0.989578	1
SPRR2E	1.013247	1.015373	1.012556	0.81674	-0.292	0.989578	1
AC019330.1	1.013245	1.015365	1.012556	0.81716	-0.2913	0.989578	1
BIRC6-AS2	1.01325	1.015384	1.012556	0.81618	-0.293	0.989578	1
SHANK2-AS3	1.01325	1.015384	1.012556	0.81618	-0.293	0.989578	1
GBP5	1.013248	1.015376	1.012556	0.81662	-0.2923	0.989578	1
IGFL3	1.013245	1.015363	1.012556	0.81729	-0.2911	0.989578	1
FOXP3	1.013259	1.015422	1.012556	0.81418	-0.2966	0.989578	1
IL18RAP	1.013253	1.015398	1.012556	0.81545	-0.2943	0.989578	1
CA6	1.013253	1.015398	1.012556	0.81545	-0.2943	0.989578	1
AQP2	1.013247	1.015374	1.012556	0.8167	-0.2921	0.989578	1
CTD-2196E14.5	1.013248	1.015379	1.012556	0.81646	-0.2926	0.989578	1
GABRG1	1.013244	1.015361	1.012556	0.81739	-0.2909	0.989578	1
ALDH3B2	1.013244	1.01536	1.012556	0.81746	-0.2908	0.989578	1
UBQLNL	1.049313	1.046455	1.050242	1.08151	0.113	0.989646	1
C14orf169	1.105797	1.108059	1.105061	0.97225	-0.0406	0.989706	1
USP26	1.01799	1.01526	1.018878	1.23708	0.3069	0.989759	1
ANKRD20A4	1.017976	1.01526	1.018858	1.23583	0.3055	0.989759	1
AC009480.3	1.017996	1.01526	1.018885	1.23757	0.3075	0.989759	1
RP5-855F16.1	1.017978	1.01526	1.018861	1.23602	0.3057	0.989759	1
AC074363.1	1.017978	1.01526	1.018862	1.23608	0.3058	0.989759	1
CTD-3214H19.6	1.017978	1.01526	1.018862	1.23605	0.3057	0.989759	1
BCL2L15	1.017995	1.01526	1.018884	1.2375	0.3074	0.989759	1
RP11-455B3.1	1.017994	1.01526	1.018883	1.23743	0.3073	0.989759	1
RP11-335L23.5	1.017996	1.01526	1.018886	1.23761	0.3076	0.989759	1
RP11-863K10.7	1.017997	1.01526	1.018886	1.23766	0.3076	0.989759	1
NXF3	1.017997	1.01526	1.018886	1.23766	0.3076	0.989759	1
RP11-835E18.5	1.017997	1.01526	1.018886	1.23766	0.3076	0.989759	1
SCARA5	1.017993	1.01526	1.018881	1.23734	0.3072	0.989759	1
ARID3C	1.017992	1.01526	1.01888	1.23725	0.3071	0.989759	1
RP11-248M19.1	1.017979	1.01526	1.018863	1.23612	0.3058	0.989759	1
MIR194-2HG	1.017981	1.01526	1.018865	1.23626	0.306	0.989759	1
AF011889.5	1.017998	1.01526	1.018889	1.23781	0.3078	0.989759	1
RP11-497D6.3	1.017982	1.01526	1.018866	1.23635	0.3061	0.989759	1
RP11-480D4.1	1.017984	1.01526	1.01887	1.2366	0.3064	0.989759	1
LINC00484.1	1.018003	1.01526	1.018894	1.23819	0.3082	0.989759	1

RP5-1021I20.4	1.017983	1.01526	1.018868	1.23649	0.3062	0.989759	1
LINC01352	1.018003	1.01526	1.018895	1.23825	0.3083	0.989759	1
SIGLEC11	1.017979	1.01526	1.018863	1.23611	0.3058	0.989759	1
RP11-875O11.1	1.017994	1.01526	1.018882	1.23739	0.3073	0.989759	1
RP11-684B21.1	1.017986	1.01526	1.018872	1.23669	0.3065	0.989759	1
RP11-268P4.5	1.017995	1.01526	1.018884	1.23752	0.3075	0.989759	1
RP11-362K14.7	1.017976	1.01526	1.018859	1.23587	0.3055	0.989759	1
LZTS1-AS1	1.01799	1.01526	1.018877	1.23704	0.3069	0.989759	1
LINC01396	1.017975	1.01526	1.018857	1.23576	0.3054	0.989759	1
CYP4F8	1.017986	1.01526	1.018872	1.23674	0.3065	0.989759	1
RP11-1376P16.1	1.017986	1.01526	1.018872	1.23675	0.3066	0.989759	1
INAFM2	1.058357	1.060311	1.057721	0.95706	-0.0633	0.989763	1
SAR1A	2.198677	2.194207	2.200131	1.00496	0.0071	0.989771	1
P2RX3	1.016407	1.013691	1.01729	1.26286	0.3367	0.989813	1
RP11-95O2.1	1.016417	1.013691	1.017303	1.26386	0.3378	0.989813	1
AC100830.4	1.016407	1.013691	1.01729	1.26291	0.3367	0.989813	1
IGSF23	1.016417	1.013691	1.017303	1.26383	0.3378	0.989813	1
CTD-2600O9.2	1.016406	1.013691	1.017289	1.26282	0.3366	0.989813	1
RP11-242G20.1	1.016398	1.013691	1.017278	1.26203	0.3357	0.989813	1
LINC00167	1.016412	1.013691	1.017296	1.26334	0.3372	0.989813	1
SPINK2	1.016399	1.013691	1.017279	1.2621	0.3358	0.989813	1
TECTB	1.016426	1.013691	1.017315	1.26471	0.3388	0.989813	1
CTD-2619J13.27	1.016408	1.013691	1.017292	1.26301	0.3369	0.989813	1
LINC01241	1.016408	1.013691	1.017291	1.26294	0.3368	0.989813	1
LINC01268	1.016422	1.013691	1.01731	1.26438	0.3384	0.989813	1
RP11-166B2.8	1.016425	1.013691	1.017314	1.26466	0.3387	0.989813	1
RP11-757G1.6	1.016396	1.013691	1.017275	1.26178	0.3355	0.989813	1
CTD-3096M3.2	1.016405	1.013691	1.017287	1.26266	0.3365	0.989813	1
PCDHGA7	1.016412	1.013691	1.017297	1.26339	0.3373	0.989813	1
SMCR2	1.016408	1.013691	1.017291	1.26299	0.3368	0.989813	1
AC007365.3	1.016421	1.013691	1.017309	1.26429	0.3383	0.989813	1
SLC22A8	1.019791	1.021925	1.019098	0.87105	-0.1992	0.989845	1
MIA	1.019786	1.021896	1.0191	0.87229	-0.1971	0.989845	1
FGD3	1.019823	1.021945	1.019133	0.87183	-0.1979	0.989845	1
RP11-644K8.1	1.019794	1.021901	1.019109	0.8725	-0.1968	0.989845	1
LRP5L	1.062217	1.05948	1.063106	1.06097	0.0854	0.989856	1
RP11-540A21.2	1.026753	1.028845	1.026073	0.90389	-0.1458	0.989872	1
MICAL3	1.144254	1.14138	1.145188	1.02694	0.0384	0.989887	1
RP11-258F22.1	1.028	1.030197	1.027286	0.90359	-0.1463	0.989912	1
GS1-393G12.14	1.033397	1.030636	1.034294	1.11938	0.1627	0.990044	1
BRSK1	1.1375	1.134489	1.138479	1.02967	0.0422	0.990061	1
ABCD2	1.017101	1.019206	1.016417	0.85479	-0.2264	0.990134	1
RP11-649E7.5	1.027451	1.029482	1.026791	0.9087	-0.1381	0.99017	1
TCERG1L-AS1	1.027421	1.029496	1.026747	0.9068	-0.1411	0.99017	1
UPK1A	1.059038	1.061008	1.058398	0.95722	-0.0631	0.990246	1

ARMCX2	1.523848	1.52591	1.523177	0.9948	-0.0075	0.990271	1
ZHX3	1.12943	1.126332	1.130436	1.03249	0.0461	0.990283	1
PCED1B	1.035069	1.032451	1.03592	1.1069	0.1465	0.99038	1
FOXP4	1.502555	1.499822	1.503443	1.00725	0.0104	0.990469	1
ZNF101	1.06702	1.064302	1.067904	1.05602	0.0786	0.9905	1
BAALC-AS1	1.0335	1.035623	1.032809	0.92101	-0.1187	0.990502	1
WISP2	1.033518	1.035597	1.032843	0.92261	-0.1162	0.990502	1
PDPR	1.238784	1.240757	1.238143	0.98914	-0.0157	0.990511	1
GTF2A1L	1.020229	1.017512	1.021113	1.20562	0.2698	0.990513	1
RP11-19J5.2	1.020202	1.017512	1.021076	1.20352	0.2673	0.990513	1
MRLN	1.020172	1.017512	1.021037	1.20127	0.2646	0.990513	1
RP11-417F21.1	1.020188	1.017512	1.021058	1.2025	0.266	0.990513	1
RP13-890H12.2	1.020186	1.017512	1.021055	1.20232	0.2658	0.990513	1
RP11-239A17.1	1.020195	1.017512	1.021068	1.20305	0.2667	0.990513	1
RP13-941N14.1	1.020171	1.017512	1.021035	1.20119	0.2645	0.990513	1
SAP30L-AS1	1.020218	1.017512	1.021098	1.20478	0.2688	0.990513	1
RP11-368P15.3	1.020224	1.017512	1.021106	1.20525	0.2693	0.990513	1
AC026188.1	1.020201	1.017512	1.021075	1.20348	0.2672	0.990513	1
AC092684.1	1.020171	1.017512	1.021036	1.20123	0.2645	0.990513	1
MYO1G	1.02018	1.017512	1.021047	1.20188	0.2653	0.990513	1
SLFNL1	1.020187	1.017512	1.021056	1.20241	0.2659	0.990513	1
TUBB1	1.018744	1.016045	1.019621	1.22287	0.2903	0.99053	1
GABRD	1.01872	1.016045	1.019589	1.22088	0.2879	0.99053	1
RP3-431P23.5	1.018715	1.016045	1.019582	1.22047	0.2874	0.99053	1
RP11-295K2.3	1.018719	1.016045	1.019588	1.22083	0.2879	0.99053	1
RP11-235D19.4	1.01873	1.016045	1.019602	1.22171	0.2889	0.99053	1
RP11-411H5.1	1.018723	1.016045	1.019594	1.22116	0.2883	0.99053	1
AP000255.6	1.018728	1.016045	1.0196	1.22154	0.2887	0.99053	1
CTD-2058B24.3	1.018715	1.016045	1.019583	1.2205	0.2875	0.99053	1
RP4-584D14.6	1.018759	1.016045	1.019641	1.22414	0.2918	0.99053	1
ENPP6	1.018722	1.016045	1.019592	1.22109	0.2882	0.99053	1
RP11-794P6.6	1.018716	1.016045	1.019584	1.22058	0.2876	0.99053	1
RNU11	1.018714	1.016045	1.019581	1.22038	0.2873	0.99053	1
RP11-90L1.8	1.018722	1.016045	1.019592	1.22107	0.2881	0.99053	1
RP5-1029F21.3	1.018758	1.016045	1.01964	1.22408	0.2917	0.99053	1
DACT3-AS1	1.018758	1.016045	1.01964	1.22408	0.2917	0.99053	1
ERICH6	1.01871	1.016045	1.019576	1.22008	0.287	0.99053	1
RP11-207C16.4	1.01873	1.016045	1.019603	1.22175	0.2889	0.99053	1
RP1-206D15.6	1.01873	1.016045	1.019603	1.22172	0.2889	0.99053	1
NKX2-1	1.01873	1.016045	1.019603	1.22172	0.2889	0.99053	1
CTC-490E21.14	1.018723	1.016045	1.019593	1.22114	0.2882	0.99053	1
RP4-657D16.3	1.018714	1.016045	1.019581	1.22039	0.2873	0.99053	1
RNF219-AS1	1.018714	1.016045	1.019582	1.22045	0.2874	0.99053	1
SHOX	1.018713	1.016045	1.01958	1.22034	0.2873	0.99053	1
MPL	1.018727	1.016045	1.019599	1.22149	0.2886	0.99053	1

RP11-344E13.4	1.018733	1.016045	1.019606	1.22195	0.2892	0.99053	1
ACSM6	1.018726	1.016045	1.019598	1.22144	0.2886	0.99053	1
RP11-636O21.1	1.018736	1.016045	1.019611	1.22222	0.2895	0.99053	1
IRS4	1.021383	1.018701	1.022255	1.19004	0.251	0.990532	1
ZNF792	1.065701	1.067749	1.065035	0.95995	-0.059	0.990559	1
MAST1	1.111944	1.109276	1.112811	1.03235	0.0459	0.990623	1
AC072062.1	1.045729	1.043043	1.046602	1.08269	0.1146	0.990633	1
STX16	1.62496	1.6223	1.625824	1.00566	0.0081	0.990665	1
LINC01091	1.021437	1.018763	1.022306	1.18884	0.2496	0.990736	1
RP11-94B19.3	1.021452	1.018767	1.022325	1.18962	0.2505	0.990736	1
MS4A2	1.021494	1.01875	1.022386	1.19393	0.2557	0.990736	1
LINC00907	1.021412	1.018765	1.022272	1.18689	0.2472	0.990736	1
L3HYPDH	1.126615	1.128619	1.125963	0.97935	-0.0301	0.990743	1
CTD-2026D20.2	1.016341	1.018402	1.015672	0.85165	-0.2317	0.990809	1
ASPN	1.016335	1.018377	1.015672	0.85277	-0.2298	0.990809	1
LINC01592	1.016339	1.018394	1.015672	0.85202	-0.231	0.990809	1
RP11-567N4.3	1.016339	1.01839	1.015672	0.85219	-0.2308	0.990809	1
RP11-863H1.1	1.016336	1.018379	1.015672	0.85268	-0.2299	0.990809	1
HSPB7	1.016336	1.018379	1.015672	0.85268	-0.2299	0.990809	1
RP11-360O19.4	1.016337	1.018383	1.015672	0.85253	-0.2302	0.990809	1
KB-173C10.2	1.016336	1.018379	1.015672	0.85268	-0.2299	0.990809	1
CCDC125	1.016337	1.018382	1.015672	0.85257	-0.2301	0.990809	1
HOXC8	1.016346	1.018421	1.015672	0.85076	-0.2332	0.990809	1
RP11-1017G21.4	1.016336	1.018378	1.015672	0.85273	-0.2298	0.990809	1
NUTM2D	1.016337	1.018383	1.015672	0.8525	-0.2302	0.990809	1
RP11-168P13.1	1.016341	1.018401	1.015672	0.8517	-0.2316	0.990809	1
RP11-73M7.6	1.016342	1.018406	1.015672	0.85146	-0.232	0.990809	1
AC136352.2	1.016336	1.01838	1.015672	0.85265	-0.23	0.990809	1
MS4A8	1.016342	1.018403	1.015672	0.85159	-0.2318	0.990809	1
CTC-338M12.2	1.016342	1.018405	1.015672	0.85149	-0.2319	0.990809	1
RP11-166N17.1	1.016342	1.018405	1.015672	0.85149	-0.2319	0.990809	1
RP11-114H24.6	1.016341	1.0184	1.015672	0.85173	-0.2315	0.990809	1
RP11-59D5__B.2	1.016344	1.018413	1.015672	0.85111	-0.2326	0.990809	1
HOXA7	1.016344	1.018413	1.015672	0.85111	-0.2326	0.990809	1
RP11-61L19.2	1.016338	1.018386	1.015672	0.85239	-0.2304	0.990809	1
LINC01119	1.023345	1.025421	1.02267	0.89178	-0.1652	0.990827	1
RP11-266N13.2	1.023347	1.025351	1.022696	0.89527	-0.1596	0.990827	1
LINC00305	1.019896	1.017226	1.020763	1.20533	0.2694	0.990835	1
EVC	1.01986	1.017179	1.020732	1.20676	0.2711	0.990835	1
AF196972.9	1.019886	1.017203	1.020758	1.2066	0.2709	0.990835	1
CTD-2026K11.6	1.019863	1.017193	1.020731	1.20575	0.2699	0.990835	1
SCHLAP1	1.019855	1.017183	1.020724	1.2061	0.2703	0.990835	1
NCAN	1.019893	1.017175	1.020777	1.20971	0.2747	0.990835	1
AC104653.1	1.077396	1.079613	1.076676	0.9631	-0.0542	0.990854	1
STRC	1.039081	1.036453	1.039935	1.09553	0.1316	0.990894	1

DNAH5	1.057106	1.054309	1.058015	1.06822	0.0952	0.990964	1
UBE2QL1	1.027997	1.030041	1.027333	0.90987	-0.1363	0.990964	1
RP3-399L15.3	1.036272	1.038326	1.035605	0.92899	-0.1063	0.990979	1
LINC00539	1.013662	1.015698	1.013	0.82814	-0.272	0.991098	1
RP1-102E24.8	1.013662	1.015698	1.013	0.82814	-0.272	0.991098	1
ARL14EPL	1.013657	1.015678	1.013	0.82916	-0.2703	0.991098	1
RP4-539M6.14	1.01366	1.015692	1.013	0.82844	-0.2715	0.991098	1
CITF22-49E9.3	1.013655	1.01567	1.013	0.8296	-0.2695	0.991098	1
RP11-505P4.6	1.013657	1.015679	1.013	0.8291	-0.2704	0.991098	1
CH17-125A10.2	1.013659	1.015688	1.013	0.82864	-0.2712	0.991098	1
RP1-232L24.3	1.013659	1.015688	1.013	0.82864	-0.2712	0.991098	1
RP11-745C15.2	1.013662	1.0157	1.013	0.82801	-0.2723	0.991098	1
APOC2	1.013659	1.015686	1.013	0.82877	-0.271	0.991098	1
DNAH17	1.013659	1.015685	1.013	0.82878	-0.2709	0.991098	1
ACSM2B	1.013659	1.015685	1.013	0.82878	-0.2709	0.991098	1
FO538757.2	1.013657	1.015681	1.013	0.82903	-0.2705	0.991098	1
CSN1S1	1.013657	1.015681	1.013	0.82903	-0.2705	0.991098	1
THEGL	1.01366	1.015691	1.013	0.82849	-0.2714	0.991098	1
RP11-466I1.1	1.01366	1.015691	1.013	0.82849	-0.2714	0.991098	1
RP5-887A10.1	1.01366	1.015691	1.013	0.82849	-0.2714	0.991098	1
RP11-823E8.3	1.013658	1.015685	1.013	0.82882	-0.2709	0.991098	1
RP11-123O22.1	1.013656	1.015675	1.013	0.82932	-0.27	0.991098	1
AP000235.3	1.013656	1.015675	1.013	0.82932	-0.27	0.991098	1
BACH1-IT2	1.013656	1.015675	1.013	0.82932	-0.27	0.991098	1
RP11-894P9.2	1.01366	1.01569	1.013	0.82855	-0.2713	0.991098	1
RP11-486O13.2	1.013661	1.015696	1.013	0.82825	-0.2719	0.991098	1
MC2R	1.013657	1.015677	1.013	0.82923	-0.2702	0.991098	1
AC025442.3	1.013657	1.015677	1.013	0.82923	-0.2702	0.991098	1
DPEP1	1.013658	1.015682	1.013	0.82894	-0.2707	0.991098	1
LCMT1	2.112383	2.114945	2.11155	0.99696	-0.0044	0.991111	1
PRDM6	1.053104	1.055169	1.052432	0.95039	-0.0734	0.991116	1
TTC8	1.223115	1.220401	1.223997	1.01631	0.0233	0.991197	1
ZNF615	1.111633	1.113754	1.110944	0.9753	-0.0361	0.99124	1
MARCH9	1.177575	1.179233	1.177035	0.98774	-0.0178	0.991283	1
CYTH1	1.136451	1.13831	1.135847	0.98219	-0.0259	0.991339	1
USP20	1.118361	1.120306	1.117729	0.97858	-0.0312	0.991427	1
CTF1	1.035169	1.037174	1.034517	0.92853	-0.107	0.991449	1
TJP3	1.062206	1.06432	1.061519	0.95646	-0.0642	0.991505	1
KB-1980E6.3	1.023655	1.020999	1.024518	1.16758	0.2235	0.991536	1
UBE2D3	5.253132	5.247684	5.254903	1.0017	0.0024	0.991537	1
RP11-140I16.3	1.024824	1.022216	1.025671	1.15551	0.2085	0.991544	1
MRVI1-AS1	1.032152	1.029483	1.03302	1.11995	0.1634	0.99158	1
RP11-277B15.3	1.032147	1.029501	1.033007	1.11886	0.162	0.99158	1
RP11-45A17.2	1.032201	1.029485	1.033083	1.12203	0.1661	0.99158	1
CC2D2B	1.032146	1.029533	1.032995	1.11722	0.1599	0.99158	1

ZNF460	1.089089	1.086371	1.089973	1.0417	0.0589	0.991637	1
NPR3	1.051365	1.053393	1.050706	0.94968	-0.0745	0.991669	1
AC005944.2	1.024927	1.022274	1.025789	1.1578	0.2114	0.991709	1
AC007383.3	1.024937	1.022265	1.025806	1.15902	0.2129	0.991709	1
KANTR	1.019862	1.021882	1.019205	0.87767	-0.1882	0.991786	1
RP11-923I11.4	1.019893	1.021872	1.019249	0.88009	-0.1843	0.991786	1
LINC00487	1.019864	1.021845	1.01922	0.87984	-0.1847	0.991786	1
MCTP2	1.019856	1.021858	1.019206	0.87867	-0.1866	0.991786	1
AC015849.2	1.019859	1.021852	1.019211	0.87916	-0.1858	0.991786	1
LINC00997	1.059341	1.061417	1.058666	0.9552	-0.0661	0.9918	1
OPRD1	1.021055	1.023075	1.020398	0.88399	-0.1779	0.991825	1
PGAM4	1.021022	1.023041	1.020366	0.88391	-0.178	0.991825	1
RP11-324O2.3	1.021002	1.023002	1.020352	0.88481	-0.1766	0.991825	1
RP11-526I2.5	1.020998	1.02301	1.020344	0.88417	-0.1776	0.991825	1
RP11-218L14.4	1.020992	1.02298	1.020345	0.88534	-0.1757	0.991825	1
ZNF630	1.042498	1.039946	1.043328	1.08467	0.1173	0.991882	1
WDR5B	1.06646	1.063675	1.067366	1.05797	0.0813	0.991923	1
ZNF846	1.09993	1.101898	1.09929	0.97441	-0.0374	0.991942	1
SLC25A13	1.669044	1.671073	1.668384	0.99599	-0.0058	0.991952	1
PHF13	1.195944	1.197844	1.195326	0.98727	-0.0185	0.992065	1
NPIPB11	1.020568	1.022538	1.019928	0.8842	-0.1776	0.992073	1
CTD-2036P10.3	1.070044	1.071916	1.069436	0.96551	-0.0506	0.992239	1
PIGZ	1.060201	1.057558	1.061061	1.06086	0.0852	0.992261	1
ATAD3B	1.060234	1.057535	1.061111	1.06216	0.087	0.992261	1
FRMD6-AS1	1.146901	1.148611	1.146345	0.98475	-0.0222	0.992401	1
ADAMTSL1	1.026557	1.028477	1.025933	0.91067	-0.135	0.992404	1
SLCO4C1	1.020765	1.02276	1.020117	0.88387	-0.1781	0.992405	1
NOXO1	1.020757	1.022689	1.020129	0.88716	-0.1727	0.992405	1
AC079154.1	1.02052	1.017948	1.021356	1.18989	0.2508	0.992407	1
RP11-752G15.8	1.020499	1.017948	1.021328	1.18836	0.249	0.992407	1
AC004257.1	1.0205	1.017948	1.02133	1.18847	0.2491	0.992407	1
PTOV1-AS2	1.02052	1.017948	1.021356	1.1899	0.2508	0.992407	1
KLHL6-AS1	1.020502	1.017948	1.021333	1.18861	0.2493	0.992407	1
HIST1H2AI	1.020498	1.017948	1.021327	1.18828	0.2489	0.992407	1
RP1-197B17.4	1.0205	1.017948	1.021329	1.18841	0.249	0.992407	1
CXorf65	1.020519	1.017948	1.021355	1.18984	0.2508	0.992407	1
RP5-944M2.3	1.020515	1.017948	1.02135	1.18958	0.2505	0.992407	1
RP11-119H12.6	1.0205	1.017948	1.021329	1.1884	0.249	0.992407	1
KIZ-AS1	1.020529	1.017948	1.021369	1.19061	0.2517	0.992407	1
LINC00951	1.020501	1.017948	1.021331	1.18853	0.2492	0.992407	1
RP4-734G22.3	1.020507	1.017948	1.021339	1.18896	0.2497	0.992407	1
RP11-126K1.9	1.020491	1.017948	1.021317	1.18776	0.2482	0.992407	1
AC010987.6	1.020527	1.017948	1.021365	1.19042	0.2515	0.992407	1
RP11-326E22.1	1.020526	1.017948	1.021364	1.19035	0.2514	0.992407	1
ARAP1-AS1	1.020519	1.017948	1.021354	1.18981	0.2507	0.992407	1

AC096670.3	1.020508	1.017948	1.021341	1.18906	0.2498	0.992407	1
RP11-517C16.4	1.020524	1.017948	1.021361	1.19018	0.2512	0.992407	1
TCF23	1.020507	1.017948	1.021339	1.18894	0.2497	0.992407	1
RP11-70F11.8	1.020506	1.017948	1.021338	1.18891	0.2496	0.992407	1
ROPN1B	1.020497	1.017948	1.021326	1.18822	0.2488	0.992407	1
RP1-60O19.2	1.020526	1.017948	1.021364	1.19037	0.2514	0.992407	1
KIF4B	1.020498	1.017948	1.021327	1.18829	0.2489	0.992407	1
GSN-AS1	1.020498	1.017948	1.021327	1.18831	0.2489	0.992407	1
SYP-AS1	1.020518	1.017948	1.021354	1.18979	0.2507	0.992407	1
RP11-524G24.2	1.020514	1.017948	1.021348	1.18948	0.2503	0.992407	1
RP11-519G16.2	1.020514	1.017948	1.021348	1.18948	0.2503	0.992407	1
WNK4	1.025613	1.023048	1.026446	1.14745	0.1984	0.992482	1
FRY-AS1	1.025671	1.023057	1.026521	1.15022	0.2019	0.992482	1
RP11-468E2.10	1.025681	1.023081	1.026526	1.14924	0.2007	0.992482	1
MROH7	1.025647	1.023054	1.02649	1.14907	0.2005	0.992482	1
ENTPD8	1.025786	1.0232	1.026627	1.14771	0.1988	0.992482	1
NOMO2	1.025656	1.023071	1.026496	1.14847	0.1997	0.992482	1
RP11-78F17.1	1.027101	1.024557	1.027928	1.13728	0.1856	0.992555	1
RP4-660H19.1	1.02718	1.024573	1.028028	1.1406	0.1898	0.992555	1
SELENOV	1.027096	1.024562	1.02792	1.13673	0.1849	0.992555	1
GTF2A1	1.394938	1.391994	1.395895	1.00995	0.0143	0.992579	1
RIBC1	1.052548	1.049979	1.053383	1.0681	0.095	0.992594	1
SPRTN	1.19037	1.19245	1.189693	0.98567	-0.0208	0.992645	1
ABALON	1.028417	1.025865	1.029246	1.13074	0.1773	0.992679	1
RP5-1160K1.6	1.01453	1.011999	1.015353	1.27953	0.3556	0.992737	1
RP11-4B16.4	1.014529	1.011999	1.015351	1.27937	0.3554	0.992737	1
CTD-3193O13.14	1.014531	1.011999	1.015354	1.27962	0.3557	0.992737	1
RP11-335K5.2	1.014516	1.011999	1.015334	1.2779	0.3538	0.992737	1
SBSN	1.01453	1.011999	1.015352	1.27945	0.3555	0.992737	1
CPNE6	1.014521	1.011999	1.015341	1.27847	0.3544	0.992737	1
CTD-2649C14.3	1.014516	1.011999	1.015335	1.27798	0.3539	0.992737	1
RP11-244F12.2	1.01452	1.011999	1.01534	1.27841	0.3544	0.992737	1
LLNLR-246C6.1	1.014529	1.011999	1.015351	1.27934	0.3554	0.992737	1
ZNF492	1.01452	1.011999	1.01534	1.27839	0.3543	0.992737	1
AC006449.2	1.014538	1.011999	1.015364	1.28038	0.3566	0.992737	1
FETUB	1.014533	1.011999	1.015356	1.27975	0.3559	0.992737	1
CTD-2561B21.7	1.014526	1.011999	1.015348	1.27909	0.3551	0.992737	1
RP11-10O22.1	1.014533	1.011999	1.015356	1.27976	0.3559	0.992737	1
APOA5	1.014533	1.011999	1.015356	1.27976	0.3559	0.992737	1
RP11-196H14.3	1.014534	1.011999	1.015358	1.27993	0.3561	0.992737	1
RP11-218E20.5	1.014528	1.011999	1.01535	1.27923	0.3553	0.992737	1
PTGES3L-AARSD1	1.014514	1.011999	1.015332	1.27772	0.3536	0.992737	1
RP11-353N14.1	1.01452	1.011999	1.01534	1.2784	0.3543	0.992737	1
RP11-84A19.2	1.014517	1.011999	1.015335	1.27804	0.3539	0.992737	1
RP11-79O8.1	1.014526	1.011999	1.015347	1.27899	0.355	0.992737	1

UTF1	1.014517	1.011999	1.015335	1.27803	0.3539	0.992737	1
RP11-98D18.15	1.016416	1.018342	1.01579	0.86082	-0.2162	0.992747	1
RP11-809C18.3	1.01642	1.018359	1.01579	0.86003	-0.2175	0.992747	1
RP11-184E9.1	1.016415	1.018339	1.01579	0.86097	-0.216	0.992747	1
RP11-855A2.3	1.016411	1.018324	1.01579	0.86169	-0.2148	0.992747	1
TMPRSS15	1.016414	1.018336	1.01579	0.86113	-0.2157	0.992747	1
C2orf71	1.016419	1.018354	1.01579	0.86027	-0.2171	0.992747	1
SPINK8	1.016419	1.018354	1.01579	0.86027	-0.2171	0.992747	1
RP4-633I8.4	1.016412	1.018329	1.01579	0.86147	-0.2151	0.992747	1
MYH6	1.016416	1.018342	1.01579	0.86084	-0.2162	0.992747	1
RP3-413H6.2	1.016411	1.018324	1.01579	0.86171	-0.2147	0.992747	1
KCNK13	1.016412	1.018328	1.01579	0.86151	-0.2151	0.992747	1
RP13-57D9.3	1.016412	1.018326	1.01579	0.86161	-0.2149	0.992747	1
LINC01048	1.016424	1.018377	1.01579	0.85922	-0.2189	0.992747	1
ITGB2	1.016418	1.018352	1.01579	0.86037	-0.217	0.992747	1
MUC22	1.016418	1.018351	1.01579	0.86043	-0.2169	0.992747	1
GMNC	1.016415	1.01834	1.01579	0.86096	-0.216	0.992747	1
CEBPE	1.016415	1.01834	1.01579	0.86096	-0.216	0.992747	1
AMBN	1.016424	1.018376	1.01579	0.85925	-0.2189	0.992747	1
LINC00239	1.016417	1.018346	1.01579	0.86064	-0.2165	0.992747	1
C9orf92	1.016417	1.018347	1.01579	0.86061	-0.2166	0.992747	1
LINC01108	1.016419	1.018355	1.01579	0.86024	-0.2172	0.992747	1
RP11-713M15.2	1.016419	1.018355	1.01579	0.86024	-0.2172	0.992747	1
CCDC124	2.356145	2.352962	2.35718	1.00312	0.0045	0.992782	1
RP11-86A5.1	1.017543	1.019457	1.016921	0.86965	-0.2015	0.992786	1
RP11-25I15.2	1.017542	1.019454	1.016921	0.86982	-0.2012	0.992786	1
RP11-16E23.5	1.017542	1.019451	1.016921	0.86994	-0.201	0.992786	1
OSTN-AS1	1.017542	1.019451	1.016921	0.86994	-0.201	0.992786	1
RP11-281A20.2	1.017548	1.019475	1.016921	0.86888	-0.2028	0.992786	1
AL121656.5	1.017549	1.019481	1.016921	0.86858	-0.2033	0.992786	1
WWC2-AS1	1.017545	1.019463	1.016921	0.86939	-0.2019	0.992786	1
ITGAM	1.017549	1.019481	1.016921	0.8686	-0.2032	0.992786	1
RP11-342C20.2	1.017549	1.019481	1.016921	0.8686	-0.2032	0.992786	1
RP11-445O3.2	1.017544	1.01946	1.016921	0.86956	-0.2016	0.992786	1
RP11-881M11.2	1.017548	1.019474	1.016921	0.8689	-0.2027	0.992786	1
RP1-41C23.4	1.017542	1.01945	1.016921	0.86998	-0.2009	0.992786	1
LINC01603	1.017554	1.019501	1.016921	0.86771	-0.2047	0.992786	1
TNFSF18	1.017546	1.019469	1.016921	0.86911	-0.2024	0.992786	1
CFC1B	1.017546	1.019469	1.016921	0.86911	-0.2024	0.992786	1
RP11-733O18.1	1.017552	1.019491	1.016921	0.86817	-0.204	0.992786	1
RP11-103H7.1	1.017541	1.019448	1.016921	0.87007	-0.2008	0.992786	1
RNF222	1.01755	1.019486	1.016921	0.86839	-0.2036	0.992786	1
GDNF	1.017547	1.019471	1.016921	0.86903	-0.2025	0.992786	1
RP11-353N4.6	1.017541	1.019449	1.016921	0.87002	-0.2009	0.992786	1
HCN2	1.01755	1.019484	1.016921	0.86847	-0.2034	0.992786	1

PNLIPRP3	1.017545	1.019462	1.016921	0.86944	-0.2018	0.992786	1
CTA-989H11.1	1.01755	1.019486	1.016921	0.86836	-0.2036	0.992786	1
UGT2A3	1.01755	1.019486	1.016921	0.86836	-0.2036	0.992786	1
SCGB1D2	1.017554	1.019502	1.016921	0.86764	-0.2048	0.992786	1
CTD-2263F21.1	1.017542	1.019452	1.016921	0.86989	-0.2011	0.992786	1
CD3D	1.017542	1.019452	1.016921	0.86989	-0.2011	0.992786	1
TBL1XR1-AS1	1.017542	1.019452	1.016921	0.86989	-0.2011	0.992786	1
CTD-2349P21.9	1.046528	1.043935	1.047372	1.07823	0.1087	0.992847	1
RP4-761J14.10	1.021059	1.023019	1.020422	0.88714	-0.1728	0.992874	1
CYP17A1	1.018455	1.015934	1.019275	1.20964	0.2746	0.992892	1
RP11-442O1.4	1.018486	1.015934	1.019315	1.21216	0.2776	0.992892	1
CTD-3195I5.4	1.018475	1.015934	1.019301	1.21128	0.2765	0.992892	1
RP11-362F19.1	1.018475	1.015934	1.019301	1.21128	0.2765	0.992892	1
KCTD4	1.018481	1.015934	1.019309	1.21178	0.2771	0.992892	1
LA16c-316G12.2	1.018481	1.015934	1.019309	1.21178	0.2771	0.992892	1
RP11-573D15.8	1.018469	1.015934	1.019293	1.2108	0.276	0.992892	1
PASD1	1.018451	1.015934	1.01927	1.20932	0.2742	0.992892	1
AC116609.2	1.018452	1.015934	1.01927	1.20936	0.2742	0.992892	1
CASC16	1.018453	1.015934	1.019272	1.20945	0.2743	0.992892	1
LINC01305	1.018455	1.015934	1.019274	1.2096	0.2745	0.992892	1
OFCC1	1.018467	1.015934	1.019291	1.21065	0.2758	0.992892	1
RP11-379P15.1	1.018472	1.015934	1.019297	1.21105	0.2763	0.992892	1
RP11-109P14.10	1.018473	1.015934	1.019299	1.21114	0.2764	0.992892	1
AF064858.7	1.01845	1.015934	1.019268	1.20923	0.2741	0.992892	1
ZNF726	1.018452	1.015934	1.019271	1.20941	0.2743	0.992892	1
CTD-2542L18.1	1.018451	1.015934	1.019269	1.20928	0.2742	0.992892	1
RP11-134L10.1	1.018464	1.015934	1.019286	1.21034	0.2754	0.992892	1
RP11-256L11.3	1.018472	1.015934	1.019297	1.21105	0.2763	0.992892	1
RP11-173P15.10	1.018472	1.015934	1.019297	1.21105	0.2763	0.992892	1
CTC-499J9.1	1.018452	1.015934	1.01927	1.20935	0.2742	0.992892	1
RP11-667M19.2	1.01845	1.015934	1.019267	1.20919	0.274	0.992892	1
DISP2	1.02938	1.031244	1.028774	0.92093	-0.1188	0.992947	1
DBNDD1	1.191496	1.193239	1.190929	0.98805	-0.0173	0.993025	1
PF4V1	1.017103	1.019023	1.016479	0.86626	-0.2071	0.993029	1
KRT81	1.017098	1.019009	1.016477	0.86679	-0.2062	0.993029	1
POU2F3	1.017095	1.01899	1.01648	0.8678	-0.2046	0.993029	1
AP001626.2	1.017095	1.018995	1.016478	0.86751	-0.205	0.993029	1
ZNF627	1.160852	1.162855	1.160201	0.9837	-0.0237	0.993053	1
ST7	1.110316	1.107721	1.111159	1.03192	0.0453	0.993056	1
GRP	1.031005	1.032913	1.030385	0.92319	-0.1153	0.993168	1
FITM2	1.066658	1.068507	1.066057	0.96424	-0.0525	0.993177	1
KLHL42	1.356562	1.358356	1.355978	0.99336	-0.0096	0.993226	1
C10orf76	1.127025	1.128932	1.126405	0.9804	-0.0286	0.993228	1
RP11-467D6.1	1.02969	1.031589	1.029073	0.92033	-0.1198	0.993234	1
PUS7	1.140768	1.138389	1.141541	1.02278	0.0325	0.99325	1

PPARGC1B	1.038215	1.040071	1.037612	0.93862	-0.0914	0.993285	1
ZNF286B	1.130752	1.128388	1.131521	1.02441	0.0348	0.993307	1
ZNF157	1.028265	1.030138	1.027656	0.91766	-0.124	0.993338	1
RP11-927P21.1	1.028224	1.030146	1.0276	0.91555	-0.1273	0.993338	1
ZNF385B	1.028179	1.030051	1.027571	0.91749	-0.1242	0.993338	1
GRIK1-AS1	1.023115	1.025019	1.022496	0.89915	-0.1534	0.993359	1
LINC01032	1.01816	1.015676	1.018968	1.21005	0.2751	0.993361	1
PKHD1L1	1.018172	1.015676	1.018984	1.21107	0.2763	0.993361	1
RP11-98D18.16	1.018181	1.015676	1.018995	1.21177	0.2771	0.993361	1
RP11-80H18.4	1.018182	1.015676	1.018996	1.21184	0.2772	0.993361	1
TEKT4	1.018159	1.015676	1.018966	1.20993	0.2749	0.993361	1
LGALS9C	1.018164	1.015676	1.018973	1.21039	0.2755	0.993361	1
RP11-158D2.2	1.018177	1.015676	1.01899	1.21145	0.2767	0.993361	1
PABPN1L	1.018173	1.015676	1.018984	1.21108	0.2763	0.993361	1
RP4-665J23.2	1.018159	1.015676	1.018966	1.2099	0.2749	0.993361	1
CTD-2154I11.2	1.01817	1.015676	1.018981	1.21088	0.2761	0.993361	1
WI2-85898F10.2	1.018172	1.015676	1.018984	1.21105	0.2763	0.993361	1
CTD-2297D10.1	1.018178	1.015676	1.018992	1.21157	0.2769	0.993361	1
RP11-554E23.4	1.018171	1.015676	1.018982	1.21096	0.2761	0.993361	1
AY269186.1	1.018176	1.015676	1.018989	1.21138	0.2766	0.993361	1
AC026167.1	1.018183	1.015676	1.018998	1.21194	0.2773	0.993361	1
AC026150.8	1.018169	1.015676	1.018979	1.21077	0.2759	0.993361	1
CTD-2544H17.2	1.018161	1.015676	1.018969	1.21011	0.2751	0.993361	1
RP5-837I24.4	1.018161	1.015676	1.018969	1.21012	0.2752	0.993361	1
RP11-398G24.2	1.018158	1.015676	1.018965	1.20986	0.2748	0.993361	1
RP11-688I9.4	1.018161	1.015676	1.018968	1.21007	0.2751	0.993361	1
RP11-686D22.5	1.018164	1.015676	1.018973	1.21039	0.2755	0.993361	1
RP11-46E17.6	1.018177	1.015676	1.01899	1.21142	0.2767	0.993361	1
SLCO4A1-AS1	1.017302	1.019196	1.016686	0.86922	-0.2022	0.993365	1
PRAMEF12	1.017303	1.019203	1.016686	0.86891	-0.2027	0.993365	1
CTD-2035E11.4	1.017301	1.019192	1.016686	0.8694	-0.2019	0.993365	1
RP11-122K13.7	1.0173	1.019189	1.016686	0.86955	-0.2017	0.993365	1
C17orf98	1.017296	1.019175	1.016686	0.87018	-0.2006	0.993365	1
AC006116.21	1.017296	1.019175	1.016686	0.87018	-0.2006	0.993365	1
ENDOU	1.017307	1.019218	1.016686	0.86824	-0.2038	0.993365	1
RP1-111B22.3	1.017297	1.019176	1.016686	0.87012	-0.2007	0.993365	1
GS1-204I12.4	1.017297	1.019176	1.016686	0.87012	-0.2007	0.993365	1
RP11-301L8.2	1.017304	1.019206	1.016686	0.86877	-0.2029	0.993365	1
RP11-195B3.1	1.017297	1.019177	1.016686	0.87009	-0.2008	0.993365	1
PCDHB8	1.017296	1.019172	1.016686	0.87034	-0.2004	0.993365	1
RP11-179A16.2	1.017304	1.019208	1.016686	0.86868	-0.2031	0.993365	1
RP11-107I14.4	1.017297	1.019177	1.016686	0.87011	-0.2007	0.993365	1
RP11-502M1.2	1.017304	1.019207	1.016686	0.86873	-0.203	0.993365	1
RP11-335L23.4	1.017303	1.019203	1.016686	0.86889	-0.2028	0.993365	1
RP11-268G12.3	1.017302	1.019199	1.016686	0.86909	-0.2024	0.993365	1

AC020571.3	1.017298	1.019181	1.016686	0.86989	-0.2011	0.993365	1
IL12A-AS1	1.017296	1.019175	1.016686	0.8702	-0.2006	0.993365	1
RP5-1101C3.1	1.017299	1.019187	1.016686	0.86965	-0.2015	0.993365	1
SUCLA2-AS1	1.023969	1.021439	1.024792	1.15638	0.2096	0.993389	1
CTD-2571L23.8	1.023942	1.021449	1.024752	1.154	0.2066	0.993389	1
MAS1	1.023971	1.021476	1.024782	1.1539	0.2065	0.993389	1
TOR1AIP1	1.238127	1.239974	1.237527	0.9898	-0.0148	0.993502	1
RP11-191L17.1	1.056167	1.053736	1.056957	1.05993	0.084	0.99357	1
CHMP2A	3.924032	3.92353	3.924196	1.00023	0.0003	0.993577	1
ANKRD31	1.044515	1.04644	1.043889	0.94508	-0.0815	0.993587	1
METTL7B	1.031817	1.029364	1.032615	1.11071	0.1515	0.993646	1
MKL1	1.07649	1.078331	1.075892	0.96886	-0.0456	0.993668	1
MORN2	1.694998	1.692203	1.695906	1.00535	0.0077	0.99367	1
AC195454.1	1.017967	1.015513	1.018765	1.20967	0.2746	0.993719	1
AC007319.1	1.017981	1.015494	1.01879	1.21273	0.2783	0.993719	1
MATN1	1.017977	1.01554	1.018769	1.20776	0.2723	0.993719	1
DTX1	1.017961	1.015487	1.018766	1.21171	0.277	0.993719	1
RP11-65J3.2	1.017987	1.015511	1.018792	1.21154	0.2768	0.993719	1
VAX1	1.01797	1.015493	1.018775	1.21179	0.2771	0.993719	1
LINC01277	1.017982	1.015491	1.018792	1.21312	0.2787	0.993719	1
TPSD1	1.017593	1.01945	1.016989	0.8735	-0.1951	0.993834	1
SIX2	1.017601	1.019482	1.016989	0.87206	-0.1975	0.993834	1
XXbac-B444P24.8	1.017589	1.019434	1.016989	0.8742	-0.194	0.993834	1
GLT6D1	1.017594	1.019453	1.016989	0.87335	-0.1954	0.993834	1
AC114877.3	1.017595	1.01946	1.016989	0.87305	-0.1959	0.993834	1
CTB-191K22.6	1.017589	1.019435	1.016989	0.87413	-0.1941	0.993834	1
RP11-467C18.1	1.017594	1.019455	1.016989	0.87327	-0.1955	0.993834	1
CNTF	1.017594	1.019455	1.016989	0.87323	-0.1956	0.993834	1
CTA-992D9.7	1.01759	1.01944	1.016989	0.87393	-0.1944	0.993834	1
AP000487.4	1.017587	1.019427	1.016989	0.8745	-0.1935	0.993834	1
CTC-296K1.4	1.017587	1.019427	1.016989	0.8745	-0.1935	0.993834	1
LINC00326	1.017589	1.019436	1.016989	0.87411	-0.1941	0.993834	1
RP11-482D24.3	1.017595	1.019458	1.016989	0.87314	-0.1957	0.993834	1
RP11-867G23.12	1.017595	1.019458	1.016989	0.87312	-0.1958	0.993834	1
PLAGL2	1.257846	1.255372	1.25865	1.01283	0.0184	0.99386	1
NUP58	1.471624	1.473548	1.470998	0.99462	-0.0078	0.993866	1
RP11-245J9.4	1.021904	1.019431	1.022707	1.16857	0.2247	0.993874	1
BACH1-AS1	1.021935	1.019464	1.022738	1.16824	0.2243	0.993874	1
CDRT4	1.030242	1.027751	1.031052	1.11896	0.1622	0.993879	1
AC133644.2	1.025903	1.027669	1.025329	0.91544	-0.1275	0.993937	1
AC005498.3	1.025892	1.027737	1.025292	0.91185	-0.1331	0.993937	1
RP3-355L5.5	1.01843	1.015961	1.019233	1.20501	0.269	0.99394	1
RGSL1	1.01843	1.015961	1.019233	1.20501	0.269	0.99394	1
RP11-1143G9.5	1.018424	1.015961	1.019224	1.20443	0.2684	0.99394	1
C3orf80	1.018409	1.015961	1.019205	1.20322	0.2669	0.99394	1

RP11-583F2.2	1.018413	1.015961	1.019211	1.2036	0.2674	0.99394	1
RP11-700H6.1	1.018432	1.015961	1.019235	1.2051	0.2692	0.99394	1
AC004051.2	1.018419	1.015961	1.019218	1.20409	0.2679	0.99394	1
AC073133.1	1.018421	1.015961	1.019221	1.20426	0.2681	0.99394	1
FOXD1	1.018412	1.015961	1.019209	1.20347	0.2672	0.99394	1
RP1-166H4.2	1.018415	1.015961	1.019213	1.20374	0.2675	0.99394	1
CASC21	1.018421	1.015961	1.01922	1.20421	0.2681	0.99394	1
CST1	1.018417	1.015961	1.019215	1.20389	0.2677	0.99394	1
RP11-254F7.4	1.018412	1.015961	1.019209	1.20347	0.2672	0.99394	1
CTC-454I21.3	1.018418	1.015961	1.019216	1.20393	0.2678	0.99394	1
FGF17	1.018413	1.015961	1.019211	1.20359	0.2673	0.99394	1
RP5-1120P11.3	1.018425	1.015961	1.019225	1.20452	0.2685	0.99394	1
CTB-191K22.5	1.018424	1.015961	1.019224	1.20446	0.2684	0.99394	1
CTD-2154B17.4	1.018416	1.015961	1.019214	1.2038	0.2676	0.99394	1
CCDC146	1.081225	1.078881	1.081987	1.03939	0.0557	0.993965	1
RP11-10A14.4	1.017295	1.014833	1.018095	1.21995	0.2868	0.993979	1
AC092667.2	1.017272	1.014833	1.018064	1.2179	0.2844	0.993979	1
SEC62-AS1	1.017302	1.014833	1.018105	1.2206	0.2876	0.993979	1
SOX21	1.017282	1.014833	1.018078	1.21879	0.2855	0.993979	1
RP11-337N6.1	1.017294	1.014833	1.018094	1.21986	0.2867	0.993979	1
VWA7	1.017278	1.014833	1.018073	1.21844	0.285	0.993979	1
MOGAT1	1.017279	1.014833	1.018074	1.21857	0.2852	0.993979	1
CH17-140K24.5	1.017271	1.014833	1.018064	1.21788	0.2844	0.993979	1
CCR7	1.017275	1.014833	1.01807	1.21824	0.2848	0.993979	1
AJ003147.9	1.017284	1.014833	1.01808	1.21897	0.2857	0.993979	1
RP11-39E3.3	1.017282	1.014833	1.018078	1.21881	0.2855	0.993979	1
FAM47E-STBD1	1.017274	1.014833	1.018068	1.21811	0.2846	0.993979	1
RP11-403I13.7	1.017284	1.014833	1.018081	1.21898	0.2857	0.993979	1
AC006372.4	1.01728	1.014833	1.018076	1.21865	0.2853	0.993979	1
LINC01333	1.01729	1.014833	1.018089	1.21952	0.2863	0.993979	1
C1QTNF7	1.017271	1.014833	1.018063	1.21782	0.2843	0.993979	1
RP3-522J7.7	1.017287	1.014833	1.018085	1.21925	0.286	0.993979	1
CTD-2619J13.23	1.017284	1.014833	1.018081	1.21898	0.2857	0.993979	1
RP11-400N13.3	1.017273	1.014833	1.018066	1.218	0.2845	0.993979	1
NUP210L	1.0173	1.014833	1.018103	1.22047	0.2874	0.993979	1
RP11-44N11.3	1.017273	1.014833	1.018066	1.21801	0.2845	0.993979	1
RP11-31I22.2	1.017283	1.014833	1.01808	1.21895	0.2856	0.993979	1
SMARCA5-AS1	1.017285	1.014833	1.018083	1.21913	0.2859	0.993979	1
RP11-66D17.3	1.017273	1.014833	1.018066	1.218	0.2845	0.993979	1
LINC00330	1.017289	1.014833	1.018087	1.21942	0.2862	0.993979	1
LYPD5	1.017285	1.014833	1.018082	1.21906	0.2858	0.993979	1
SHANK2-AS1	1.01729	1.014833	1.018089	1.21954	0.2863	0.993979	1
TRPM5	1.017278	1.014833	1.018074	1.21851	0.2851	0.993979	1
AC079767.4	1.017285	1.014833	1.018083	1.21912	0.2858	0.993979	1
RP11-106M3.3	1.017283	1.014833	1.01808	1.21891	0.2856	0.993979	1

RP11-301G7.1	1.017275	1.014833	1.018068	1.21816	0.2847	0.993979	1
SLC36A2	1.013664	1.015518	1.013062	0.84172	-0.2486	0.993988	1
LINC01179	1.013658	1.015493	1.013062	0.84308	-0.2463	0.993988	1
RP5-902P8.12	1.013658	1.015493	1.013062	0.84308	-0.2463	0.993988	1
RP5-908M14.5	1.013661	1.015503	1.013062	0.84254	-0.2472	0.993988	1
GJC2	1.013659	1.015497	1.013062	0.84288	-0.2466	0.993988	1
RP13-895J2.2	1.013661	1.015504	1.013062	0.84249	-0.2473	0.993988	1
SLC24A3	1.013661	1.015504	1.013062	0.84249	-0.2473	0.993988	1
MNDA	1.013661	1.015504	1.013062	0.84249	-0.2473	0.993988	1
GRIN3A	1.013662	1.015509	1.013062	0.84221	-0.2478	0.993988	1
RP11-412H8.2	1.013658	1.015492	1.013062	0.84311	-0.2462	0.993988	1
RP11-316I3.1	1.013669	1.015537	1.013062	0.8407	-0.2503	0.993988	1
RP11-65D17.1	1.01366	1.015499	1.013062	0.84275	-0.2468	0.993988	1
KCNQ1	1.01366	1.015499	1.013062	0.84275	-0.2468	0.993988	1
RP3-416H24.1	1.01366	1.0155	1.013062	0.84271	-0.2469	0.993988	1
RP11-517I3.1	1.01366	1.015498	1.013062	0.84279	-0.2468	0.993988	1
RP11-290L1.5	1.013661	1.015503	1.013062	0.84252	-0.2472	0.993988	1
IL33	1.013669	1.015535	1.013062	0.84078	-0.2502	0.993988	1
RP11-3G20.2	1.013663	1.015514	1.013062	0.84195	-0.2482	0.993988	1
LINC00992	1.013659	1.015498	1.013062	0.84283	-0.2467	0.993988	1
CTD-2044J15.1	1.013659	1.015498	1.013062	0.84283	-0.2467	0.993988	1
FOXN1	1.013659	1.015498	1.013062	0.84283	-0.2467	0.993988	1
FMNL2	1.452011	1.449136	1.452946	1.00848	0.0122	0.994089	1
WNT7A	1.027566	1.029475	1.026945	0.91415	-0.1295	0.994112	1
RP11-348N5.9	1.02757	1.029411	1.026971	0.91702	-0.125	0.994112	1
ISL1	1.02752	1.029444	1.026894	0.9134	-0.1307	0.994112	1
NMU	1.06303	1.06491	1.062418	0.96161	-0.0565	0.994118	1
RGS19	1.2067	1.208389	1.206151	0.98926	-0.0156	0.9942	1
RP11-678G14.2	1.026231	1.028083	1.025629	0.91264	-0.1319	0.994227	1
GPR63	1.109235	1.110953	1.108676	0.97947	-0.0299	0.994231	1
KDM1B	1.166447	1.163802	1.167307	1.0214	0.0305	0.994282	1
COMMD3	1.622776	1.619898	1.623711	1.00615	0.0088	0.994312	1
RP11-17A4.2	1.019632	1.021447	1.019042	0.88786	-0.1716	0.994318	1
BFSP2-AS1	1.019636	1.021466	1.019042	0.88706	-0.1729	0.994318	1
CTD-2544N14.3	1.019632	1.021449	1.019042	0.88775	-0.1718	0.994318	1
SRGN	1.019639	1.021476	1.019042	0.88666	-0.1736	0.994318	1
RP11-432J24.2	1.019639	1.021476	1.019042	0.88666	-0.1736	0.994318	1
TSPAN8	1.019637	1.021466	1.019042	0.88705	-0.1729	0.994318	1
RP11-456O19.5	1.019637	1.021466	1.019042	0.88705	-0.1729	0.994318	1
C8B	1.01963	1.02144	1.019042	0.88812	-0.1712	0.994318	1
TRAF3IP3	1.019637	1.021467	1.019042	0.88701	-0.173	0.994318	1
CTD-2118P12.1	1.019632	1.021448	1.019042	0.88781	-0.1717	0.994318	1
RP11-1399P15.1	1.019634	1.021454	1.019042	0.88754	-0.1721	0.994318	1
LURAP1L-AS1	1.019631	1.021445	1.019042	0.88795	-0.1714	0.994318	1
LINC00348	1.019631	1.021445	1.019042	0.88794	-0.1715	0.994318	1

RP11-43F13.3	1.019639	1.021478	1.019042	0.88656	-0.1737	0.994318	1
RP11-327J17.2	1.019631	1.021444	1.019042	0.88799	-0.1714	0.994318	1
RP11-671P2.1	1.019631	1.021444	1.019042	0.88799	-0.1714	0.994318	1
RP3-331H24.5	1.019633	1.021452	1.019042	0.88763	-0.172	0.994318	1
CTD-2525I3.3	1.019631	1.021444	1.019042	0.88797	-0.1714	0.994318	1
RP11-350J20.12	1.019632	1.021446	1.019042	0.88789	-0.1716	0.994318	1
INSL4	1.019641	1.021483	1.019042	0.88636	-0.174	0.994318	1
GAST	1.019629	1.021438	1.019042	0.88824	-0.171	0.994318	1
RP11-34F13.2	1.019635	1.021461	1.019042	0.88725	-0.1726	0.994318	1
SPRR2A	1.019635	1.021461	1.019042	0.88725	-0.1726	0.994318	1
RP11-92C4.6	1.019632	1.021449	1.019042	0.88776	-0.1718	0.994318	1
RP11-90C4.1	1.019632	1.021449	1.019042	0.88776	-0.1718	0.994318	1
TVP23C	1.071198	1.068691	1.072013	1.04837	0.0681	0.994338	1
SNAI3	1.021648	1.019177	1.022451	1.17077	0.2275	0.994343	1
RP1-309F20.3	1.021628	1.019171	1.022427	1.16983	0.2263	0.994343	1
LTB4R2	1.02165	1.019208	1.022444	1.16845	0.2246	0.994343	1
RP11-531A24.5	1.021635	1.019172	1.022436	1.17028	0.2269	0.994343	1
ZSWIM4	1.097367	1.094852	1.098185	1.03513	0.0498	0.994348	1
RP5-827C21.6	1.027464	1.025042	1.028251	1.12815	0.174	0.994366	1
RMRP	1.027484	1.025035	1.02828	1.12964	0.1759	0.994366	1
IER5	1.11273	1.114706	1.112087	0.97717	-0.0333	0.994403	1
CUL9	1.112815	1.114823	1.112163	0.97684	-0.0338	0.994403	1
MRPS11	1.788826	1.790877	1.788159	0.99656	-0.005	0.994418	1
HMCN2	1.032589	1.030331	1.033323	1.09864	0.1357	0.994421	1
PITPNM3	1.05991	1.06174	1.059315	0.96072	-0.0578	0.994471	1
LMO2	1.099372	1.096985	1.100147	1.0326	0.0463	0.994533	1
DNAJC9-AS1	1.034111	1.031623	1.03492	1.10427	0.1431	0.994583	1
FAM120C	1.096461	1.093991	1.097264	1.03483	0.0494	0.994598	1
XX-C2158C12.2	1.049041	1.050842	1.048455	0.95304	-0.0694	0.994653	1
EFCAB13	1.021423	1.019028	1.022201	1.16675	0.2225	0.994698	1
SMIM11A	1.021454	1.019035	1.022241	1.16842	0.2246	0.994698	1
DDX20	1.096458	1.094281	1.097165	1.0306	0.0435	0.994713	1
ZNF468	1.07082	1.068412	1.071602	1.04663	0.0658	0.994819	1
SH2D5	1.02537	1.022987	1.026145	1.1374	0.1857	0.994853	1
IL17B	1.025368	1.022979	1.026145	1.13775	0.1862	0.994853	1
RP11-775D22.2	1.025426	1.022977	1.026221	1.1412	0.1906	0.994853	1
RP11-521O16.1	1.025368	1.023014	1.026133	1.13553	0.1834	0.994853	1
RP11-138A9.2	1.033728	1.031321	1.03451	1.10183	0.1399	0.994887	1
SYT15	1.033648	1.031194	1.034446	1.10425	0.1431	0.994887	1
FBLL1	1.021894	1.019452	1.022687	1.16631	0.2219	0.994924	1
RP11-129M16.4	1.021851	1.019457	1.02263	1.16303	0.2179	0.994924	1
GNG12-AS1	1.021858	1.019457	1.022639	1.1635	0.2185	0.994924	1
RFPL3S	1.021878	1.019487	1.022655	1.16261	0.2174	0.994924	1
HBB	1.021871	1.019487	1.022646	1.16214	0.2168	0.994924	1
CPA2	1.022429	1.024216	1.021848	0.90221	-0.1485	0.994929	1

ACSBG1	1.02076	1.018359	1.02154	1.17327	0.2305	0.994963	1
AQP7	1.020728	1.018346	1.021502	1.17198	0.2289	0.994963	1
MIR378D2	1.020747	1.018391	1.021513	1.16977	0.2262	0.994963	1
LLPH	1.680806	1.682861	1.680138	0.99601	-0.0058	0.995048	1
ACVR2B-AS1	1.024027	1.025779	1.023458	0.90997	-0.1361	0.99506	1
GRIA3	1.040313	1.042113	1.039728	0.94337	-0.0841	0.995212	1
SLFNL1-AS1	1.022816	1.024575	1.022245	0.90517	-0.1437	0.995225	1
CTD-2568A17.1	1.021328	1.023208	1.020717	0.89266	-0.1638	0.99524	1
IL23A	1.02132	1.023026	1.020765	0.90182	-0.1491	0.99524	1
TMC8	1.021319	1.023058	1.020753	0.90006	-0.1519	0.99524	1
MTAP	1.477326	1.479098	1.47675	0.9951	-0.0071	0.995301	1
HIST1H3A	1.025082	1.022749	1.02584	1.13584	0.1838	0.995323	1
KB-1208A12.3	1.036153	1.033862	1.036897	1.08963	0.1238	0.995386	1
CMC4	1.056426	1.058099	1.055883	0.96186	-0.0561	0.995419	1
AP3S1	3.323743	3.319987	3.324964	1.00215	0.0031	0.995468	1
ZNHIT2	1.154656	1.156222	1.154147	0.98672	-0.0193	0.995522	1
RP11-12M5.3	1.014537	1.012186	1.015301	1.25558	0.3284	0.995629	1
AC009060.2	1.014537	1.012186	1.015301	1.25558	0.3284	0.995629	1
RP1-278O22.1	1.014529	1.012186	1.01529	1.25469	0.3273	0.995629	1
ABCA10	1.014521	1.012186	1.015279	1.25381	0.3263	0.995629	1
AC010547.9	1.014547	1.012186	1.015315	1.25673	0.3297	0.995629	1
GNAT2	1.014538	1.012186	1.015303	1.25572	0.3285	0.995629	1
RP11-17E13.2	1.014514	1.012186	1.015271	1.2531	0.3255	0.995629	1
RAB6C	1.014516	1.012186	1.015273	1.2533	0.3257	0.995629	1
DENND6A-AS1	1.014527	1.012186	1.015288	1.25449	0.3271	0.995629	1
RP11-776A13.3	1.014538	1.012186	1.015303	1.25574	0.3285	0.995629	1
SULT1B1	1.014543	1.012186	1.015309	1.25627	0.3291	0.995629	1
ARSD-AS1	1.014523	1.012186	1.015283	1.2541	0.3266	0.995629	1
RP11-133F8.2	1.014514	1.012186	1.01527	1.25304	0.3254	0.995629	1
PSORS1C2	1.014538	1.012186	1.015303	1.25575	0.3286	0.995629	1
CTC-806A22.1	1.014519	1.012186	1.015277	1.25361	0.3261	0.995629	1
RP11-108K3.1	1.014526	1.012186	1.015287	1.25442	0.327	0.995629	1
RP11-1263C18.1	1.01453	1.012186	1.015292	1.25482	0.3275	0.995629	1
SLC35G6	1.01453	1.012186	1.015292	1.25482	0.3275	0.995629	1
RP11-367J11.2	1.01452	1.012186	1.015278	1.25373	0.3262	0.995629	1
ADAMTS19-AS1	1.014523	1.012186	1.015282	1.25404	0.3266	0.995629	1
RP3-467K16.7	1.014529	1.012186	1.01529	1.25467	0.3273	0.995629	1
GS1-304P7.3	1.014525	1.012186	1.015286	1.25432	0.3269	0.995629	1
RP11-31I22.3	1.014525	1.012186	1.015286	1.25432	0.3269	0.995629	1
RP11-229E13.4	1.014525	1.012186	1.015286	1.25432	0.3269	0.995629	1
LINC00538	1.014548	1.012186	1.015316	1.2568	0.3298	0.995629	1
RP11-304L19.12	1.014522	1.012186	1.015282	1.254	0.3265	0.995629	1
TPSG1	1.014519	1.012186	1.015277	1.2536	0.3261	0.995629	1
RP11-83M16.5	1.014532	1.012186	1.015295	1.25509	0.3278	0.995629	1
RP11-38L15.8	1.014532	1.012186	1.015295	1.25509	0.3278	0.995629	1

NR1I3	1.014532	1.012186	1.015295	1.25509	0.3278	0.995629	1
RP11-1103G16.1	1.014515	1.012186	1.015272	1.25319	0.3256	0.995629	1
RP4-536B24.4	1.014524	1.012186	1.015284	1.25421	0.3268	0.995629	1
LINC00462	1.014543	1.012186	1.015309	1.25625	0.3291	0.995629	1
RP11-447L10.1	1.014538	1.012186	1.015302	1.25566	0.3284	0.995629	1
CACNA1C-AS1	1.014533	1.012186	1.015295	1.25513	0.3278	0.995629	1
RP11-7F17.5	1.014523	1.012186	1.015283	1.25407	0.3266	0.995629	1
LINC00441	1.024882	1.022563	1.025636	1.13623	0.1843	0.995673	1
TTC39C-AS1	1.024857	1.022558	1.025605	1.13509	0.1828	0.995673	1
FAM168B	1.543749	1.541046	1.544627	1.00662	0.0095	0.995812	1
ZNF345	1.152033	1.153643	1.151509	0.98612	-0.0202	0.995814	1
BDNF-AS	1.140419	1.14184	1.139956	0.98672	-0.0193	0.995851	1
PCDHB15	1.054056	1.051649	1.054838	1.06174	0.0864	0.995859	1
RP11-421F16.3	1.017196	1.014881	1.017949	1.20612	0.2704	0.995918	1
RP11-802O23.3	1.017196	1.014881	1.017949	1.20614	0.2704	0.995918	1
AL022476.2	1.017195	1.014881	1.017948	1.20606	0.2703	0.995918	1
KB-1517D11.4	1.017208	1.014881	1.017965	1.2072	0.2717	0.995918	1
CTD-2006K23.1	1.017208	1.014881	1.017965	1.2072	0.2717	0.995918	1
HBA1	1.017197	1.014881	1.01795	1.20624	0.2705	0.995918	1
AF196970.3	1.017196	1.014881	1.017949	1.20615	0.2704	0.995918	1
RP11-282A11.4	1.017197	1.014881	1.017949	1.20618	0.2704	0.995918	1
MEIS1-AS3	1.017215	1.014881	1.017973	1.20777	0.2723	0.995918	1
RP11-252A24.5	1.0172	1.014881	1.017954	1.20651	0.2708	0.995918	1
RP11-977G19.10	1.01721	1.014881	1.017966	1.20732	0.2718	0.995918	1
ZNF860	1.017208	1.014881	1.017965	1.20721	0.2717	0.995918	1
ARTN	1.017202	1.014881	1.017956	1.20663	0.271	0.995918	1
PYHIN1	1.017199	1.014881	1.017952	1.20635	0.2706	0.995918	1
AC005330.2	1.017216	1.014881	1.017975	1.20792	0.2725	0.995918	1
FOXD3-AS1	1.017231	1.014881	1.017995	1.20923	0.2741	0.995918	1
ZNF177	1.017198	1.014881	1.017951	1.20631	0.2706	0.995918	1
RP11-485F13.1	1.017211	1.014881	1.017969	1.20748	0.272	0.995918	1
RP11-556N4.1	1.017196	1.014881	1.017948	1.2061	0.2704	0.995918	1
MNX1-AS1	1.017196	1.014881	1.017949	1.20613	0.2704	0.995918	1
RP11-348B17.1	1.017217	1.014881	1.017976	1.20797	0.2726	0.995918	1
RP11-385E5.5	1.017217	1.014881	1.017976	1.20797	0.2726	0.995918	1
RP1-136B1.1	1.017203	1.014881	1.017958	1.20674	0.2711	0.995918	1
RP11-54O7.16	1.017198	1.014881	1.017952	1.20633	0.2706	0.995918	1
RP5-1028K7.3	1.017197	1.014881	1.017949	1.20617	0.2704	0.995918	1
CALCA	1.017193	1.014881	1.017944	1.20583	0.27	0.995918	1
SLC5A4	1.018991	1.020735	1.018424	0.88855	-0.1705	0.995925	1
TCP11	1.019002	1.020691	1.018453	0.89185	-0.1651	0.995925	1
SNX31	1.019011	1.020747	1.018447	0.88916	-0.1695	0.995925	1
RP11-284N8.3	1.019011	1.020769	1.018439	0.8878	-0.1717	0.995925	1
FLNB-AS1	1.019001	1.020734	1.018438	0.88928	-0.1693	0.995925	1
KCNRG	1.024189	1.021869	1.024944	1.1406	0.1898	0.995943	1

RP11-809O17.1	1.05418	1.052026	1.054881	1.05486	0.0771	0.995972	1
KLRD1	1.038747	1.040368	1.03822	0.94679	-0.0789	0.996003	1
RP11-152N13.16	1.020568	1.02233	1.019995	0.89544	-0.1593	0.996011	1
PCDHA1	1.020589	1.022326	1.020025	0.89695	-0.1569	0.996011	1
TIMM23B	1.101055	1.102645	1.100539	0.97949	-0.0299	0.996046	1
DEDD2	1.450503	1.452361	1.449898	0.99456	-0.0079	0.996181	1
C9orf84	1.017856	1.019555	1.017304	0.8849	-0.1764	0.996195	1
RP1-155D22.2	1.017853	1.019541	1.017304	0.88553	-0.1754	0.996195	1
MZB1	1.017857	1.019559	1.017304	0.88473	-0.1767	0.996195	1
RP5-1050D4.4	1.01786	1.01957	1.017304	0.88422	-0.1775	0.996195	1
NHS-AS1	1.017854	1.019546	1.017304	0.88533	-0.1757	0.996195	1
C9orf64	1.017854	1.019546	1.017304	0.88533	-0.1757	0.996195	1
CTC-436P18.1	1.017853	1.019541	1.017304	0.88555	-0.1754	0.996195	1
ADGRF5	1.017852	1.019538	1.017304	0.88567	-0.1752	0.996195	1
TACR1	1.017859	1.019565	1.017304	0.88447	-0.1771	0.996195	1
GPX2	1.017855	1.019548	1.017304	0.88523	-0.1759	0.996195	1
SLC15A1	1.017855	1.019548	1.017304	0.88523	-0.1759	0.996195	1
RP11-407G23.1	1.017855	1.019548	1.017304	0.88523	-0.1759	0.996195	1
RP11-89M20.2	1.017859	1.019567	1.017304	0.88434	-0.1773	0.996195	1
RP11-673F18.1	1.017853	1.01954	1.017304	0.88559	-0.1753	0.996195	1
EPGN	1.017854	1.019546	1.017304	0.88532	-0.1757	0.996195	1
RP11-506M12.1	1.017857	1.019558	1.017304	0.88476	-0.1766	0.996195	1
TMED6	1.017864	1.019586	1.017304	0.8835	-0.1787	0.996195	1
ADPRM	1.207678	1.205106	1.208514	1.01662	0.0238	0.996198	1
EFCAB9	1.019322	1.021072	1.018753	0.88993	-0.1682	0.996225	1
LINC00470	1.019321	1.021044	1.018762	0.89156	-0.1656	0.996225	1
FP325317.1	1.019327	1.021067	1.018762	0.89058	-0.1672	0.996225	1
ITPKB-AS1	1.019322	1.021029	1.018767	0.89243	-0.1642	0.996225	1
FAM182B	1.019317	1.021012	1.018765	0.89306	-0.1632	0.996225	1
ADPRHL1	1.027844	1.02957	1.027283	0.92265	-0.1161	0.996231	1
PECAM1	1.028571	1.026227	1.029333	1.11841	0.1615	0.996299	1
MOCS1	1.095331	1.092951	1.096105	1.03394	0.0481	0.996363	1
DPP3	1.400819	1.402147	1.400387	0.99562	-0.0063	0.996376	1
PIGK	1.176421	1.178123	1.175868	0.98734	-0.0184	0.996399	1
CPSF1	1.337582	1.33557	1.338236	1.00795	0.0114	0.996475	1
SAPCD1-AS1	1.056549	1.058204	1.056011	0.96232	-0.0554	0.996486	1
SLC45A3	1.034175	1.035808	1.033644	0.93958	-0.0899	0.996488	1
VPS50	1.103302	1.100951	1.104066	1.03085	0.0438	0.996513	1
CDC37L1-AS1	1.128948	1.130746	1.128363	0.98178	-0.0265	0.996516	1
ECE2	1.197058	1.194915	1.197755	1.01457	0.0209	0.996516	1
RGS7BP	1.032745	1.03439	1.032211	0.93663	-0.0945	0.996551	1
AC007228.9	1.042042	1.039809	1.042768	1.07435	0.1035	0.996555	1
PHYH	1.413168	1.415295	1.412477	0.99321	-0.0098	0.996557	1
RP11-290L1.3	1.01806	1.015711	1.018824	1.19815	0.2608	0.996615	1
GLIPR1L1	1.017966	1.015687	1.018707	1.19252	0.254	0.996615	1

CTD-2033A16.1	1.018035	1.0157	1.018794	1.19708	0.2595	0.996615	1
ADAMTS16	1.052444	1.054026	1.05193	0.96122	-0.0571	0.996644	1
NXF1	1.177695	1.17925	1.17719	0.9885	-0.0167	0.996658	1
FAAP20	3.545335	3.546096	3.545087	0.9996	-0.0006	0.996729	1
PAPD5	1.207726	1.205414	1.208477	1.01491	0.0214	0.996788	1
CTD-3113P16.11	1.040563	1.038171	1.04134	1.08304	0.1151	0.996892	1
SLC8A1-AS1	1.020636	1.018382	1.021369	1.1625	0.2172	0.996905	1
TMEM110-MUSTN1	1.020709	1.018416	1.021454	1.16499	0.2203	0.996905	1
RP11-1212A22.4	1.020648	1.018382	1.021385	1.16334	0.2183	0.996905	1
USP50	1.01554	1.017186	1.015005	0.87307	-0.1958	0.996925	1
MSC-AS1	1.015541	1.017183	1.015007	0.87332	-0.1954	0.996925	1
RP11-640L9.1	1.01555	1.017215	1.015009	0.87183	-0.1979	0.996925	1
CTD-2655K5.1	1.015543	1.017217	1.014999	0.87118	-0.199	0.996925	1
RP11-489D6.2	1.015541	1.017212	1.014998	0.87138	-0.1986	0.996925	1
AP4B1-AS1	1.01554	1.017179	1.015008	0.87364	-0.1949	0.996925	1
STH	1.01554	1.017203	1.014999	0.87186	-0.1978	0.996925	1
RP11-572B2.1	1.015553	1.017243	1.015004	0.87015	-0.2007	0.996925	1
CTD-2530H12.4	1.01554	1.017204	1.014999	0.87183	-0.1979	0.996925	1
RP11-774D14.1	1.015534	1.017173	1.015001	0.87354	-0.1951	0.996925	1
LINC00868	1.017118	1.018772	1.016581	0.88325	-0.1791	0.996966	1
HEPHL1	1.017115	1.018759	1.016581	0.88391	-0.178	0.996966	1
RP11-213H15.1	1.017116	1.01876	1.016581	0.88382	-0.1782	0.996966	1
LINC01625	1.017117	1.018766	1.016581	0.88355	-0.1786	0.996966	1
FAM129C	1.017124	1.018796	1.016581	0.88214	-0.1809	0.996966	1
TAS2R43	1.017119	1.018776	1.016581	0.88308	-0.1794	0.996966	1
AC114730.7	1.017119	1.018776	1.016581	0.88308	-0.1794	0.996966	1
RP11-806K15.1	1.017119	1.018776	1.016581	0.88308	-0.1794	0.996966	1
CYTH4	1.017119	1.018776	1.016581	0.88308	-0.1794	0.996966	1
TMEM171	1.017112	1.018745	1.016581	0.88452	-0.177	0.996966	1
NLRP8	1.017126	1.018803	1.016581	0.8818	-0.1815	0.996966	1
ERVH48-1	1.017125	1.018799	1.016581	0.88201	-0.1811	0.996966	1
RP11-255P5.2	1.017123	1.018789	1.016581	0.88246	-0.1804	0.996966	1
BCL6B	1.01713	1.018819	1.016581	0.88109	-0.1826	0.996966	1
CACNG3	1.017121	1.018784	1.016581	0.88272	-0.18	0.996966	1
RP11-838N2.3	1.017113	1.018752	1.016581	0.88421	-0.1775	0.996966	1
LINC01527	1.017115	1.018759	1.016581	0.88388	-0.1781	0.996966	1
PRR5-ARHGAP8	1.017114	1.018754	1.016581	0.88413	-0.1777	0.996966	1
RP5-1057I20.5	1.017125	1.018797	1.016581	0.88208	-0.181	0.996966	1
AC098617.1	1.017118	1.01877	1.016581	0.88338	-0.1789	0.996966	1
TCF24	1.017114	1.018755	1.016581	0.88408	-0.1778	0.996966	1
RP11-1078H9.5	1.01712	1.018779	1.016581	0.88295	-0.1796	0.996966	1
LINC01350	1.017113	1.01875	1.016581	0.8843	-0.1774	0.996966	1
SLC39A11	1.07837	1.07983	1.077896	0.97578	-0.0354	0.997002	1
FAM120B	1.210803	1.208447	1.211569	1.01498	0.0215	0.997104	1
RP11-231N3.1	1.014108	1.011871	1.014835	1.24973	0.3216	0.99715	1

ADAM21	1.014101	1.011871	1.014826	1.24896	0.3207	0.99715	1
RP11-184A2.3	1.014107	1.011871	1.014834	1.2496	0.3215	0.99715	1
RP11-69I8.3	1.014106	1.011871	1.014833	1.24954	0.3214	0.99715	1
CES1	1.014115	1.011871	1.014845	1.25054	0.3226	0.99715	1
C19orf38	1.014108	1.011871	1.014835	1.24971	0.3216	0.99715	1
SLC7A14	1.014117	1.011871	1.014847	1.25074	0.3228	0.99715	1
CTA-254O6.1	1.014125	1.011871	1.014857	1.25157	0.3237	0.99715	1
RP11-264B17.2	1.014117	1.011871	1.014847	1.25069	0.3227	0.99715	1
RP11-288L9.1	1.014106	1.011871	1.014833	1.2495	0.3214	0.99715	1
SOWAHB	1.01411	1.011871	1.014839	1.24999	0.3219	0.99715	1
CTD-2550O8.5	1.01413	1.011871	1.014864	1.25211	0.3244	0.99715	1
FNDC9	1.014114	1.011871	1.014843	1.25036	0.3223	0.99715	1
AP001172.2	1.014118	1.011871	1.014848	1.25082	0.3229	0.99715	1
RP11-91P24.6	1.014118	1.011871	1.014849	1.25086	0.3229	0.99715	1
MIR7515HG	1.014118	1.011871	1.014849	1.25086	0.3229	0.99715	1
RP11-408O19.5	1.014116	1.011871	1.014846	1.25065	0.3227	0.99715	1
RP11-259O18.5	1.014105	1.011871	1.014831	1.24937	0.3212	0.99715	1
RP11-153K11.3	1.014106	1.011871	1.014833	1.24949	0.3213	0.99715	1
CTD-3051D23.1	1.014103	1.011871	1.014829	1.2492	0.321	0.99715	1
CTB-55O6.10	1.014113	1.011871	1.014841	1.25022	0.3222	0.99715	1
C16orf54	1.014127	1.011871	1.01486	1.25183	0.324	0.99715	1
KB-1043D8.6	1.017093	1.018746	1.016555	0.88314	-0.1793	0.997244	1
LINC00976	1.017124	1.018725	1.016603	0.88669	-0.1735	0.997244	1
TSPYL5	1.049571	1.051261	1.049022	0.95632	-0.0644	0.997325	1
IFIH1	1.030786	1.032412	1.030258	0.93355	-0.0992	0.997461	1
ZNF329	1.106812	1.104566	1.107542	1.02846	0.0405	0.997467	1
RP1-78O14.1	1.029225	1.030781	1.028719	0.93303	-0.1	0.997529	1
ZNF707	1.038736	1.040462	1.038175	0.94347	-0.084	0.997534	1
MT1M	1.021418	1.019186	1.022143	1.15413	0.2068	0.997599	1
FOXE3	1.021405	1.01919	1.022126	1.15299	0.2054	0.997599	1
RP11-342K6.3	1.013504	1.011304	1.014219	1.25782	0.3309	0.997622	1
RP3-428L16.1	1.013514	1.011304	1.014232	1.25896	0.3322	0.997622	1
SLC35F4	1.013512	1.011304	1.014229	1.25875	0.332	0.997622	1
GPC6-AS1	1.013512	1.011304	1.014229	1.25874	0.332	0.997622	1
RP11-15E1.5	1.01351	1.011304	1.014227	1.25851	0.3317	0.997622	1
RP11-856F16.2	1.013518	1.011304	1.014237	1.25942	0.3328	0.997622	1
RP11-15K19.2	1.013512	1.011304	1.01423	1.25879	0.332	0.997622	1
RP11-89N17.4	1.013513	1.011304	1.01423	1.25884	0.3321	0.997622	1
ANXA13	1.013511	1.011304	1.014228	1.25867	0.3319	0.997622	1
RP11-278H7.1	1.013504	1.011304	1.014219	1.25785	0.331	0.997622	1
RGPD1	1.01352	1.011304	1.01424	1.25967	0.333	0.997622	1
AC114765.1	1.013515	1.011304	1.014233	1.25909	0.3324	0.997622	1
RP11-6N17.10	1.013519	1.011304	1.014238	1.25955	0.3329	0.997622	1
HPDL	1.013513	1.011304	1.01423	1.25885	0.3321	0.997622	1
RP11-259O2.1	1.013515	1.011304	1.014234	1.25916	0.3325	0.997622	1

LINC01343	1.013515	1.011304	1.014234	1.25916	0.3325	0.997622	1
RP11-676J12.6	1.013536	1.011304	1.014261	1.26153	0.3352	0.997622	1
PABPC5-AS1	1.013511	1.011304	1.014228	1.25861	0.3318	0.997622	1
RP11-494O16.4	1.013521	1.011304	1.014241	1.25979	0.3332	0.997622	1
ABCB6	1.013508	1.011304	1.014224	1.2583	0.3315	0.997622	1
RP11-653J6.1	1.013516	1.011304	1.014235	1.25926	0.3326	0.997622	1
RP11-815J21.4	1.013512	1.011304	1.014229	1.25875	0.332	0.997622	1
DAW1	1.013507	1.011304	1.014223	1.25823	0.3314	0.997622	1
CTD-3224K15.3	1.013501	1.011304	1.014215	1.25747	0.3305	0.997622	1
PLCXD3	1.013516	1.011304	1.014235	1.25925	0.3326	0.997622	1
CTD-2521M24.6	1.013504	1.011304	1.014219	1.25787	0.331	0.997622	1
MID1IP1-AS1	1.013511	1.011304	1.014228	1.25865	0.3319	0.997622	1
SLC7A9	1.013507	1.011304	1.014223	1.25818	0.3313	0.997622	1
ZNF548	1.077314	1.078848	1.076815	0.97422	-0.0377	0.997662	1
SLC25A15	1.063842	1.061605	1.064569	1.04811	0.0678	0.997745	1
KLHL10	1.014394	1.012187	1.015111	1.23998	0.3103	0.997884	1
RP5-1177M21.1	1.014394	1.012187	1.015111	1.23998	0.3103	0.997884	1
RP11-264L1.4	1.014389	1.012187	1.015104	1.2394	0.3096	0.997884	1
AC068831.15	1.014373	1.012187	1.015083	1.23767	0.3076	0.997884	1
RP11-720L2.4	1.014376	1.012187	1.015088	1.23806	0.3081	0.997884	1
RP3-400B16.3	1.01438	1.012187	1.015093	1.23849	0.3086	0.997884	1
RP11-27K13.3	1.014377	1.012187	1.015089	1.23814	0.3082	0.997884	1
RP11-728E14.3	1.014388	1.012187	1.015104	1.23937	0.3096	0.997884	1
LINC01060	1.014373	1.012187	1.015084	1.23774	0.3077	0.997884	1
RP11-417L19.2	1.014378	1.012187	1.01509	1.23821	0.3083	0.997884	1
RP11-669C19.1	1.014366	1.012187	1.015075	1.23698	0.3068	0.997884	1
RP11-452K12.4	1.014374	1.012187	1.015085	1.23786	0.3078	0.997884	1
RP1-302G2.5	1.01437	1.012187	1.01508	1.23742	0.3073	0.997884	1
NCKAP1L	1.014389	1.012187	1.015105	1.23948	0.3097	0.997884	1
RP11-152H18.3	1.014383	1.012187	1.015097	1.23882	0.309	0.997884	1
ART4	1.014383	1.012187	1.015097	1.23882	0.309	0.997884	1
NAT2	1.014378	1.012187	1.01509	1.23824	0.3083	0.997884	1
RP11-521B24.5	1.014386	1.012187	1.015101	1.23916	0.3094	0.997884	1
NPSR1	1.014371	1.012187	1.015082	1.23754	0.3075	0.997884	1
CTD-2236F14.1	1.014373	1.012187	1.015083	1.23768	0.3076	0.997884	1
RP11-506F3.1	1.014372	1.012187	1.015082	1.23756	0.3075	0.997884	1
HIST1H2BI	1.014372	1.012187	1.015083	1.23764	0.3076	0.997884	1
RP11-35J1.2	1.014377	1.012187	1.015088	1.2381	0.3081	0.997884	1
ALDH1L1-AS1	1.014381	1.012187	1.015094	1.23859	0.3087	0.997884	1
SERINC4	1.014367	1.012187	1.015076	1.23706	0.3069	0.997884	1
RP4-777L9.2	1.014372	1.012187	1.015082	1.2376	0.3075	0.997884	1
RP11-304F15.6	1.014377	1.012187	1.015088	1.2381	0.3081	0.997884	1
CYP27B1	1.014377	1.012187	1.015088	1.2381	0.3081	0.997884	1
RP11-1008C21.1	1.014386	1.012187	1.015101	1.23917	0.3094	0.997884	1
RP11-443B7.1	1.014383	1.012187	1.015096	1.23876	0.3089	0.997884	1

GS1-259H13.13	1.014382	1.012187	1.015095	1.23866	0.3088	0.997884	1
RP1-150O5.3	1.024136	1.021945	1.024848	1.13227	0.1792	0.997888	1
CTC-548K16.1	1.024153	1.021932	1.024875	1.1342	0.1817	0.997888	1
ZNF410	1.029083	1.030691	1.028561	0.93059	-0.1038	0.997892	1
HAVCR2	1.016144	1.013947	1.016859	1.20881	0.2736	0.997924	1
MEG8	1.016147	1.013947	1.016862	1.20906	0.2739	0.997924	1
LDHAL6A	1.016142	1.013947	1.016856	1.2086	0.2733	0.997924	1
FAM9C	1.016132	1.013947	1.016842	1.20763	0.2722	0.997924	1
RP11-21C4.1	1.016149	1.013947	1.016864	1.2092	0.2741	0.997924	1
AC005220.3	1.016137	1.013947	1.016849	1.20809	0.2727	0.997924	1
CCDC13-AS1	1.016161	1.013947	1.016881	1.21042	0.2755	0.997924	1
XIRP2	1.016152	1.013947	1.016869	1.20955	0.2745	0.997924	1
TRIM74	1.016134	1.013947	1.016844	1.20778	0.2724	0.997924	1
AL133493.2	1.016126	1.013947	1.016834	1.20706	0.2715	0.997924	1
RP11-114B7.6	1.016141	1.013947	1.016854	1.20845	0.2732	0.997924	1
CTC-537E7.3	1.016126	1.013947	1.016835	1.20708	0.2715	0.997924	1
C11orf97	1.016136	1.013947	1.016848	1.208	0.2726	0.997924	1
RP3-324O17.8	1.016131	1.013947	1.016842	1.20758	0.2721	0.997924	1
ITIH1	1.016151	1.013947	1.016867	1.20941	0.2743	0.997924	1
APOBEC4	1.016137	1.013947	1.016849	1.20814	0.2728	0.997924	1
RP11-33B1.3	1.016145	1.013947	1.01686	1.20887	0.2737	0.997924	1
RP4-668E10.4	1.016134	1.013947	1.016846	1.20786	0.2725	0.997924	1
SOX1	1.016133	1.013947	1.016844	1.20774	0.2723	0.997924	1
AC079779.6	1.016136	1.013947	1.016848	1.20805	0.2727	0.997924	1
GOT2	1.666096	1.66412	1.666739	1.00394	0.0057	0.99793	1
RP11-53O19.3	1.119271	1.116943	1.120028	1.02638	0.0376	0.997952	1
KCTD8	1.031834	1.033438	1.031312	0.93643	-0.0948	0.997955	1
CCZ1	1.463748	1.46123	1.464566	1.00723	0.0104	0.997983	1
CTD-3234P18.6	1.018026	1.015826	1.018742	1.18422	0.2439	0.998102	1
CXorf36	1.018008	1.015826	1.018718	1.18272	0.2421	0.998102	1
RP11-164H13.1	1.018021	1.015826	1.018734	1.18377	0.2434	0.998102	1
LINC00243	1.018005	1.015826	1.018713	1.18242	0.2417	0.998102	1
RP11-387H17.6	1.018018	1.015826	1.01873	1.18351	0.2431	0.998102	1
RP11-120K19.4	1.018007	1.015826	1.018716	1.1826	0.242	0.998102	1
RP11-950C14.7	1.018008	1.015826	1.018717	1.18265	0.242	0.998102	1
CTD-2008A1.3	1.018004	1.015826	1.018712	1.18235	0.2417	0.998102	1
ADAM2	1.018004	1.015826	1.018712	1.18232	0.2416	0.998102	1
FLJ12825	1.018002	1.015826	1.018709	1.18216	0.2414	0.998102	1
RP11-624G17.3	1.018015	1.015826	1.018726	1.18325	0.2428	0.998102	1
LINC00534	1.018015	1.015826	1.018726	1.18325	0.2428	0.998102	1
RP11-296K13.4	1.018003	1.015826	1.01871	1.18222	0.2415	0.998102	1
LINC00403	1.018021	1.015826	1.018734	1.18376	0.2434	0.998102	1
CTC-338M12.6	1.018002	1.015826	1.018709	1.18216	0.2414	0.998102	1
RP11-661A12.7	1.017999	1.015826	1.018706	1.18197	0.2412	0.998102	1
AC016722.3	1.018015	1.015826	1.018727	1.18328	0.2428	0.998102	1

NFE4	1.017996	1.015826	1.018702	1.1817	0.2409	0.998102	1
RP3-380B4.1	1.018008	1.015826	1.018717	1.18264	0.242	0.998102	1
RP11-659E9.4	1.018004	1.015826	1.018711	1.18231	0.2416	0.998102	1
TAS2R5	1.018024	1.015826	1.018738	1.18399	0.2437	0.998102	1
RSPH10B2	1.018015	1.015826	1.018727	1.18327	0.2428	0.998102	1
CD163L1	1.018007	1.015826	1.018716	1.18261	0.242	0.998102	1
SCN1A	1.018	1.015826	1.018706	1.18199	0.2412	0.998102	1
RP1-40E16.11	1.017568	1.015377	1.018281	1.18882	0.2495	0.998139	1
TRIM67	1.017541	1.015376	1.018245	1.18666	0.2469	0.998139	1
SPATA13.1	1.015242	1.013073	1.015948	1.21995	0.2868	0.998164	1
CHRM1	1.015244	1.013073	1.015949	1.22008	0.287	0.998164	1
HLA-DRA	1.015249	1.013073	1.015956	1.22059	0.2876	0.998164	1
RP11-596C23.6	1.015252	1.013073	1.01596	1.22088	0.2879	0.998164	1
CTD-2193P3.2	1.015239	1.013073	1.015943	1.21956	0.2864	0.998164	1
RP11-65M17.3	1.01526	1.013073	1.015971	1.22174	0.2889	0.998164	1
RP11-556E13.1	1.015241	1.013073	1.015946	1.21979	0.2866	0.998164	1
RP11-511B23.2	1.01526	1.013073	1.015971	1.22172	0.2889	0.998164	1
RPH3A	1.015261	1.013073	1.015973	1.22186	0.2891	0.998164	1
RP11-703G6.1	1.015256	1.013073	1.015965	1.22128	0.2884	0.998164	1
CEMP1	1.015256	1.013073	1.015965	1.22128	0.2884	0.998164	1
RP11-317G6.1	1.015264	1.013073	1.015976	1.2221	0.2894	0.998164	1
RP11-474D1.2	1.015244	1.013073	1.01595	1.22011	0.287	0.998164	1
GCM2	1.015253	1.013073	1.015961	1.22099	0.288	0.998164	1
ABC12-49244600F4.4	1.015262	1.013073	1.015974	1.22198	0.2892	0.998164	1
LY75	1.015241	1.013073	1.015946	1.21981	0.2867	0.998164	1
GOLGA8M	1.015244	1.013073	1.015949	1.22006	0.287	0.998164	1
RP11-1348G14.5	1.015241	1.013073	1.015945	1.21976	0.2866	0.998164	1
RP11-557N21.1	1.015248	1.013073	1.015955	1.22052	0.2875	0.998164	1
CTB-43E15.1	1.015239	1.013073	1.015944	1.21963	0.2864	0.998164	1
HIST1H3C	1.015245	1.013073	1.015952	1.22024	0.2872	0.998164	1
ATP1A4	1.01524	1.013073	1.015944	1.21968	0.2865	0.998164	1
RP11-575L7.4	1.01526	1.013073	1.015971	1.22176	0.289	0.998164	1
BMP8B	1.015238	1.013073	1.015942	1.2195	0.2863	0.998164	1
CTD-2376I4.1	1.015243	1.013073	1.015949	1.22001	0.2869	0.998164	1
RP11-425A23.1	1.015237	1.013073	1.015941	1.21944	0.2862	0.998164	1
AC004158.3	1.015239	1.013073	1.015943	1.21956	0.2864	0.998164	1
RP11-624L4.2	1.015252	1.013073	1.01596	1.2209	0.2879	0.998164	1
KB-1836B5.4	1.015256	1.013073	1.015966	1.22133	0.2885	0.998164	1
SLC52A1	1.01524	1.013073	1.015944	1.21966	0.2865	0.998164	1
RP13-895J2.3	1.015246	1.013073	1.015953	1.22034	0.2873	0.998164	1
CTC-482H14.5	1.01525	1.013073	1.015958	1.22073	0.2877	0.998164	1
RP11-124N2.1	1.015252	1.013073	1.01596	1.22089	0.2879	0.998164	1
LA16c-321D4.2	1.015252	1.013073	1.01596	1.22089	0.2879	0.998164	1
ICAM4	1.020912	1.022476	1.020403	0.90777	-0.1396	0.998212	1
ZNF800	1.437104	1.43432	1.438009	1.00849	0.0122	0.998237	1

RP11-234G16.5	1.013656	1.015222	1.013147	0.86369	-0.2114	0.998241	1
CTC-518P12.6	1.013651	1.015217	1.013143	0.8637	-0.2114	0.998241	1
RP4-761J14.8	1.013653	1.015205	1.013148	0.86472	-0.2097	0.998241	1
ARHGAP30	1.013654	1.015229	1.013142	0.86295	-0.2126	0.998241	1
RP11-70L8.4	1.013653	1.015227	1.013142	0.86305	-0.2125	0.998241	1
CHI3L2	1.013646	1.01521	1.013137	0.86369	-0.2114	0.998241	1
HIST1H4A	1.013658	1.015235	1.013146	0.86288	-0.2128	0.998241	1
ATP2B3	1.013651	1.015217	1.013142	0.86368	-0.2114	0.998241	1
KLK13	1.01365	1.015212	1.013143	0.86397	-0.2109	0.998241	1
RORC	1.013651	1.015222	1.013141	0.86329	-0.2121	0.998241	1
SYNJ2BP-COX16	1.013651	1.01523	1.013138	0.86261	-0.2132	0.998241	1
RP5-940J5.6	1.013656	1.015235	1.013143	0.86265	-0.2132	0.998241	1
RP11-881M11.4	1.013654	1.015219	1.013145	0.86369	-0.2114	0.998241	1
MILR1	1.035481	1.033278	1.036197	1.0877	0.1213	0.998241	1
POU6F2-AS2	1.01527	1.013102	1.015975	1.2193	0.2861	0.998275	1
HIPK4	1.015264	1.013102	1.015967	1.2187	0.2853	0.998275	1
HLX	1.015274	1.013102	1.01598	1.21971	0.2865	0.998275	1
GPAT2	1.015258	1.013102	1.015959	1.21809	0.2846	0.998275	1
RP1-23E21.2	1.015261	1.013102	1.015963	1.21838	0.285	0.998275	1
DAB1-AS1	1.015276	1.013102	1.015982	1.21988	0.2867	0.998275	1
DNMBP-AS1	1.01528	1.013102	1.015989	1.22037	0.2873	0.998275	1
CTD-2561B21.5	1.015263	1.013102	1.015966	1.21866	0.2853	0.998275	1
TERT	1.015263	1.013102	1.015965	1.21859	0.2852	0.998275	1
AC108051.3	1.015273	1.013102	1.015978	1.21958	0.2864	0.998275	1
RP4-669H2.1	1.015273	1.013102	1.015978	1.21958	0.2864	0.998275	1
CSF2RB	1.015267	1.013102	1.015971	1.21903	0.2857	0.998275	1
MIXL1	1.015276	1.013102	1.015983	1.21997	0.2868	0.998275	1
NMUR2	1.015282	1.013102	1.015991	1.22052	0.2875	0.998275	1
ACOT12	1.01527	1.013102	1.015975	1.21929	0.286	0.998275	1
FOXB1	1.015283	1.013102	1.015993	1.22066	0.2877	0.998275	1
AC142119.1	1.015267	1.013102	1.01597	1.21897	0.2857	0.998275	1
RP1-144F13.3	1.015262	1.013102	1.015965	1.21855	0.2852	0.998275	1
C16orf96	1.015263	1.013102	1.015965	1.21859	0.2852	0.998275	1
RP11-304C12.5	1.015266	1.013102	1.01597	1.21892	0.2856	0.998275	1
RP11-287D1.3	1.01526	1.013102	1.015962	1.2183	0.2849	0.998275	1
AC006076.1	1.015273	1.013102	1.015979	1.2196	0.2864	0.998275	1
ZBED3	1.172915	1.174376	1.172441	0.9889	-0.0161	0.998299	1
PPP1R8	1.405061	1.406242	1.404677	0.99615	-0.0056	0.998424	1
HIST4H4	1.027311	1.028828	1.026818	0.93028	-0.1043	0.998438	1
TXNDC5	1.035164	1.036739	1.034653	0.94321	-0.0843	0.998501	1
RP4-621N11.2	1.015422	1.013274	1.01612	1.21437	0.2802	0.998525	1
RP5-837J1.4	1.01542	1.013274	1.016117	1.21419	0.28	0.998525	1
RP11-676J12.7	1.015449	1.013274	1.016155	1.21706	0.2834	0.998525	1
MYLK2	1.01542	1.013274	1.016118	1.21421	0.28	0.998525	1
HHIPL1	1.015427	1.013274	1.016127	1.21489	0.2808	0.998525	1

LINCR-0001	1.015423	1.013274	1.016121	1.21449	0.2803	0.998525	1
RP11-359D14.2	1.015425	1.013274	1.016124	1.21468	0.2806	0.998525	1
AC107218.3	1.015432	1.013274	1.016133	1.21538	0.2814	0.998525	1
TBX20	1.015417	1.013274	1.016113	1.21387	0.2796	0.998525	1
SP5	1.01542	1.013274	1.016118	1.21425	0.2801	0.998525	1
NKX6-3	1.015419	1.013274	1.016116	1.21412	0.2799	0.998525	1
CH17-408M7.1	1.015431	1.013274	1.016132	1.2153	0.2813	0.998525	1
LINC01126	1.015421	1.013274	1.016118	1.21426	0.2801	0.998525	1
FAM159B	1.01542	1.013274	1.016117	1.2142	0.28	0.998525	1
TMPRSS11D	1.015449	1.013274	1.016156	1.21714	0.2835	0.998525	1
ANKRD66	1.015423	1.013274	1.016121	1.21447	0.2803	0.998525	1
RP11-122K13.15	1.015419	1.013274	1.016116	1.21409	0.2799	0.998525	1
SCGB3A2	1.015419	1.013274	1.016116	1.21411	0.2799	0.998525	1
RP11-384F7.1	1.015435	1.013274	1.016137	1.21567	0.2818	0.998525	1
ZRSR1	1.01542	1.013274	1.016117	1.21419	0.28	0.998525	1
C20orf144	1.015422	1.013274	1.016121	1.21443	0.2803	0.998525	1
CTD-2540B15.6	1.015427	1.013274	1.016127	1.21494	0.2809	0.998525	1
LINC01102	1.015425	1.013274	1.016124	1.2147	0.2806	0.998525	1
RFPL3	1.015417	1.013274	1.016113	1.21386	0.2796	0.998525	1
RP5-1039K5.18	1.015454	1.013274	1.016162	1.21757	0.284	0.998525	1
CTC-529L17.2	1.015433	1.013274	1.016135	1.21552	0.2816	0.998525	1
MACC1-AS1	1.015433	1.013274	1.016135	1.21552	0.2816	0.998525	1
GPR132	1.015425	1.013274	1.016124	1.21467	0.2806	0.998525	1
RP11-103J8.2	1.015438	1.013274	1.016142	1.21603	0.2822	0.998525	1
RP1-69M21.2	1.015429	1.013274	1.01613	1.21514	0.2811	0.998525	1
RBP3	1.015413	1.013274	1.016108	1.21349	0.2792	0.998525	1
PIP4K2B	1.298894	1.296497	1.299673	1.01071	0.0154	0.998539	1
HSD17B3	1.016951	1.014806	1.017649	1.19205	0.2534	0.998612	1
GACAT3	1.016942	1.0148	1.017639	1.19178	0.2531	0.998612	1
RP11-171I2.2	1.016955	1.014824	1.017648	1.19046	0.2515	0.998612	1
JAK2	1.066674	1.064527	1.067373	1.0441	0.0623	0.998705	1
RP11-264B17.4	1.025619	1.027226	1.025097	0.92182	-0.1174	0.998761	1
OVOL1-AS1	1.034586	1.032511	1.03526	1.08454	0.1171	0.998865	1
CHRNA1	1.02757	1.025411	1.028272	1.1126	0.1539	0.998869	1
FAM231D	1.027569	1.025419	1.028268	1.11208	0.1533	0.998869	1
TRABD2A	1.025587	1.027096	1.025096	0.92618	-0.1106	0.998873	1
LINC01561	1.025553	1.027067	1.025062	0.92592	-0.111	0.998873	1
CTD-3116E22.8	1.017827	1.015719	1.018512	1.17765	0.2359	0.998875	1
KY	1.017814	1.015682	1.018507	1.18011	0.2389	0.998875	1
CD8A	1.017832	1.0157	1.018525	1.17999	0.2388	0.998875	1
SYCP2L	1.064557	1.062424	1.06525	1.04527	0.0639	0.998892	1
CYP51A1	1.05673	1.058152	1.056268	0.96759	-0.0475	0.998893	1
RP11-225H22.4	1.027961	1.02959	1.027432	0.92707	-0.1092	0.998913	1
B3GNT4	1.027932	1.029347	1.027472	0.93612	-0.0952	0.998913	1
VDR	1.019578	1.017474	1.020262	1.15957	0.2136	0.998914	1

RP11-20B24.4	1.019601	1.017444	1.020302	1.16383	0.2189	0.998914	1
RP11-642C5.1	1.019601	1.017452	1.020299	1.16319	0.2181	0.998914	1
TNMD	1.019627	1.017455	1.020333	1.1649	0.2202	0.998914	1
APOBEC3A	1.019613	1.017467	1.02031	1.1628	0.2176	0.998914	1
SELL	1.019592	1.017465	1.020283	1.16131	0.2158	0.998914	1
NMI	1.071467	1.072999	1.070969	0.97219	-0.0407	0.998921	1
CCDC192	1.028371	1.029859	1.027888	0.93399	-0.0985	0.998935	1
MEI1	1.021488	1.019333	1.022188	1.14771	0.1988	0.999093	1
ARHGAP31-AS1	1.021479	1.019341	1.022174	1.1465	0.1972	0.999093	1
CACNA1F	1.026534	1.028021	1.02605	0.92967	-0.1052	0.999115	1
RP3-465N24.6	1.02648	1.028024	1.025978	0.92698	-0.1094	0.999115	1
JAKMIP3	1.02652	1.02801	1.026035	0.92948	-0.1055	0.999115	1
CCM2L	1.021013	1.018859	1.021714	1.1514	0.2034	0.999125	1
RP11-105N14.1	1.064681	1.062547	1.065375	1.04521	0.0638	0.999147	1
RP11-111M22.5	1.024722	1.026208	1.024239	0.92485	-0.1127	0.999154	1
RP11-517A5.7	1.024743	1.02628	1.024244	0.92253	-0.1163	0.999154	1
LGR6	1.018683	1.016571	1.019369	1.1689	0.2252	0.999155	1
RP5-1021I20.8	1.018717	1.016595	1.019407	1.16944	0.2258	0.999155	1
RP11-266O8.1	1.018717	1.016611	1.019401	1.16794	0.224	0.999155	1
RP1-97J1.2	1.018715	1.01657	1.019412	1.17154	0.2284	0.999155	1
PCDHB7	1.018698	1.016572	1.019389	1.17004	0.2266	0.999155	1
NOSTRIN	1.018697	1.016564	1.01939	1.17059	0.2272	0.999155	1
KIF3B	1.246571	1.244007	1.247405	1.01392	0.02	0.999202	1
DENND2C	1.041488	1.043025	1.040988	0.95265	-0.07	0.999204	1
NOS3	1.044715	1.042538	1.045422	1.06778	0.0946	0.999206	1
USP2	1.044819	1.042663	1.04552	1.06696	0.0935	0.999206	1
GALR1	1.01746	1.018968	1.016969	0.8946	-0.1607	0.999207	1
RP11-613D13.10	1.017443	1.018936	1.016957	0.89548	-0.1593	0.999207	1
P2RY14	1.017453	1.018956	1.016964	0.89494	-0.1601	0.999207	1
RP11-395B7.2	1.017458	1.018968	1.016968	0.89451	-0.1608	0.999207	1
NKPD1	1.017451	1.018952	1.016963	0.89506	-0.1599	0.999207	1
RP3-510O8.4	1.017446	1.018942	1.01696	0.89534	-0.1595	0.999207	1
RP11-160E2.19	1.017448	1.01896	1.016957	0.89438	-0.161	0.999207	1
ADGRE2	1.017449	1.018942	1.016964	0.89555	-0.1592	0.999207	1
RP11-88G17.6	1.017451	1.018969	1.016958	0.89396	-0.1617	0.999207	1
CTD-2630F21.1	1.018738	1.016615	1.019429	1.16938	0.2257	0.999266	1
RP11-50D9.3	1.01873	1.016602	1.019421	1.16983	0.2263	0.999266	1
HSPA1L	1.0187	1.016602	1.019382	1.16744	0.2233	0.999266	1
AC003005.2	1.018721	1.016595	1.019412	1.16976	0.2262	0.999266	1
AC016738.3	1.018707	1.016605	1.01939	1.1677	0.2237	0.999266	1
PPP4C	3.217682	3.218445	3.217434	0.99954	-0.0007	0.999302	1
ZNF319	1.054867	1.0565	1.054336	0.9617	-0.0563	0.999413	1
B3GAT1	1.066985	1.064991	1.067634	1.04067	0.0575	0.999458	1
HEATR5A	1.137874	1.139092	1.137478	0.9884	-0.0168	0.999479	1
CBR3	1.022281	1.023741	1.021807	0.91851	-0.1226	0.999495	1

ALG10	1.055895	1.057542	1.05536	0.96208	-0.0558	0.999496	1
NCAM2	1.068723	1.066687	1.069384	1.04044	0.0572	0.999498	1
RP11-167N5.5	1.018867	1.016771	1.019549	1.16565	0.2211	0.999516	1
KRBOX1	1.018873	1.016773	1.019556	1.16591	0.2215	0.999516	1
C1QTNF2	1.018866	1.01676	1.019551	1.16652	0.2222	0.999516	1
DDN	1.020392	1.018291	1.021075	1.15222	0.2044	0.999598	1
OLAH	1.020377	1.018294	1.021054	1.15088	0.2027	0.999598	1
MUL1	1.215275	1.216899	1.214746	0.99007	-0.0144	0.999607	1
UGGT1	1.242471	1.243748	1.242056	0.99306	-0.01	0.999619	1
DOPEY2	1.102725	1.104212	1.102242	0.98109	-0.0275	0.999636	1
SCFD2	1.117012	1.118528	1.116519	0.98305	-0.0247	0.999691	1
WDR49	1.042822	1.040532	1.043567	1.07487	0.1042	0.999696	1
AFAP1L2	1.042687	1.040641	1.043353	1.06672	0.0932	0.999696	1
NFE2	1.022145	1.0236	1.021673	0.91834	-0.1229	0.999746	1
CFAP77	1.039126	1.036955	1.039831	1.07783	0.1081	0.999793	1
MAP3K15	1.038015	1.036037	1.038658	1.07273	0.1013	0.999832	1
SLC22A4	1.022131	1.023602	1.021653	0.91739	-0.1244	0.999857	1
MLKL	1.022144	1.023598	1.021671	0.91836	-0.1229	0.999857	1
BEND4	1.050916	1.048876	1.051579	1.0553	0.0777	0.999875	1
MTRNR2L1	1.024441	1.025973	1.023943	0.92184	-0.1174	0.999893	1
SIGLEC7	1.02307	1.020953	1.023759	1.1339	0.1813	0.999901	1
CTD-2020K17.1	1.02307	1.020973	1.023752	1.1325	0.1795	0.999901	1
ZNF490	1.024896	1.026363	1.024419	0.92623	-0.1106	0.99992	1
CTA-929C8.6	1.024929	1.0264	1.024451	0.92618	-0.1106	0.99992	1
RP11-305O4.3	1.014676	1.013691	1.014997	1.09539	0.1314	1	1
AGXT2	1.015544	1.013691	1.016146	1.17934	0.238	1	1
SEPT5	1.032817	1.031346	1.033295	1.06217	0.087	1	1
RP1-90G24.6	1.016387	1.017183	1.016128	0.93861	-0.0914	1	1
WASF3-AS1	1.015556	1.013691	1.016163	1.18055	0.2395	1	1
CTA-223H9.9	1.021572	1.020743	1.021841	1.05295	0.0744	1	1
SPRY4	1.061182	1.059272	1.061803	1.0427	0.0603	1	1
AC069155.1	1.01382	1.013691	1.013862	1.01249	0.0179	1	1
RP11-574O16.1	1.014677	1.013691	1.014997	1.09543	0.1315	1	1
SLC9A4	1.01382	1.013691	1.013862	1.01249	0.0179	1	1
LINC01260	1.01554	1.013691	1.016141	1.17898	0.2375	1	1
RP11-571L19.8	1.015542	1.013691	1.016143	1.17913	0.2377	1	1
KLK3	1.014677	1.013691	1.014997	1.09541	0.1315	1	1
AP003025.2	1.015551	1.013691	1.016155	1.18001	0.2388	1	1
RP5-1071N3.1	1.01382	1.013691	1.013862	1.01249	0.0179	1	1
LHFPL4	1.019859	1.020671	1.019595	0.94794	-0.0771	1	1
CTB-30L5.1	1.014682	1.013691	1.015004	1.09591	0.1321	1	1
RP11-244N9.4	1.014682	1.013691	1.015004	1.09592	0.1321	1	1
PCK1	1.015547	1.013691	1.016151	1.1797	0.2384	1	1
AC097721.2	1.018998	1.017191	1.019585	1.13926	0.1881	1	1
RP11-1223D19.4	1.015556	1.013691	1.016163	1.18055	0.2395	1	1

RP1-224A6.9	1.014684	1.013691	1.015006	1.0961	0.1324	1	1
SPATA45	1.016398	1.017212	1.016134	0.93736	-0.0933	1	1
ZNF251	1.10782	1.108535	1.107588	0.99127	-0.0126	1	1
RP11-47I22.1	1.015548	1.013691	1.016152	1.17975	0.2385	1	1
EMC9	1.768998	1.770731	1.768435	0.99702	-0.0043	1	1
RP11-473O4.5	1.014685	1.013691	1.015008	1.09622	0.1325	1	1
AC015987.1	1.017284	1.017219	1.017305	1.00497	0.0072	1	1
CYP2C9	1.01382	1.013691	1.013862	1.01249	0.0179	1	1
RP11-169K17.3	1.014678	1.013691	1.014999	1.09552	0.1316	1	1
UTAT33	1.01468	1.013691	1.015001	1.09571	0.1319	1	1
NLRP2	1.015555	1.013691	1.016161	1.18041	0.2393	1	1
RP11-507K2.3	1.014689	1.013691	1.015014	1.09666	0.1331	1	1
RP11-287D1.2	1.01815	1.017227	1.01845	1.07097	0.0989	1	1
LYN	1.228656	1.228177	1.228812	1.00278	0.004	1	1
LINC01114	1.015563	1.013691	1.016172	1.18124	0.2403	1	1
RP11-72I8.1	1.014684	1.013691	1.015006	1.09609	0.1324	1	1
CTD-2207P18.2	1.01382	1.013691	1.013862	1.01249	0.0179	1	1
RP11-529H2.1	1.014676	1.013691	1.014997	1.0954	0.1315	1	1
AC073342.12	1.014683	1.013691	1.015005	1.09602	0.1323	1	1
KRT6A	1.01382	1.013691	1.013862	1.01249	0.0179	1	1
WEE2	1.014677	1.013691	1.014998	1.0955	0.1316	1	1
RP11-458F8.4	1.036321	1.034841	1.036802	1.05627	0.079	1	1
AC024132.1	1.014677	1.013691	1.014997	1.09541	0.1315	1	1
RP13-228J13.1	1.015551	1.013691	1.016156	1.18007	0.2389	1	1
RP11-677I18.3	1.01727	1.017219	1.017287	1.00395	0.0057	1	1
BOLL	1.01382	1.013691	1.013862	1.01249	0.0179	1	1
RP5-1024G6.2	1.038017	1.038401	1.037892	0.98673	-0.0193	1	1
TRPM6	1.01382	1.013691	1.013862	1.01249	0.0179	1	1
LINGO1-AS1	1.016412	1.017197	1.016157	0.93952	-0.09	1	1
RP11-214K3.24	1.051	1.052347	1.050562	0.96589	-0.0501	1	1
ADRA2C	1.025938	1.02433	1.026461	1.08761	0.1212	1	1
RP11-121G22.3	1.014682	1.013691	1.015004	1.09589	0.1321	1	1
IYD	1.014682	1.013691	1.015004	1.09592	0.1321	1	1
TREM1	1.016401	1.017214	1.016136	0.93743	-0.0932	1	1
RP11-809N8.2	1.014677	1.013691	1.014997	1.09541	0.1315	1	1
CABP4	1.015542	1.013691	1.016144	1.1792	0.2378	1	1
LINC00964	1.01382	1.013691	1.013862	1.01249	0.0179	1	1
AC009299.3	1.016413	1.01719	1.016161	0.94016	-0.089	1	1
FIBP	2.428617	2.427716	2.428909	1.00084	0.0012	1	1
AC073254.1	1.015557	1.013691	1.016164	1.18065	0.2396	1	1
LRRD1	1.015543	1.013691	1.016145	1.17927	0.2379	1	1
RP11-425D17.1	1.019009	1.017196	1.019598	1.1397	0.1887	1	1
CHRM4	1.014683	1.013691	1.015005	1.096	0.1322	1	1
CLDN34	1.014677	1.013691	1.014997	1.0954	0.1315	1	1
LA16c-385E7.1	1.017255	1.01719	1.017276	1.00504	0.0072	1	1

RP11-159D12.11	1.01382	1.013691	1.013862	1.01249	0.0179	1	1
RP11-675F6.4	1.017274	1.017225	1.01729	1.00376	0.0054	1	1
LINC00930	1.015545	1.013691	1.016148	1.17951	0.2382	1	1
SCN2B	1.030205	1.031276	1.029857	0.95463	-0.067	1	1
MAGEA8-AS1	1.014677	1.013691	1.014998	1.09545	0.1315	1	1
RP11-611O2.5	1.01555	1.013691	1.016154	1.17995	0.2387	1	1
MSMP	1.047399	1.048797	1.046944	0.96204	-0.0558	1	1
AC010907.2	1.015554	1.013691	1.016159	1.18031	0.2392	1	1
CKLF-CMTM1	1.017259	1.017196	1.01728	1.00491	0.0071	1	1
DAND5	1.014686	1.013691	1.015009	1.09628	0.1326	1	1
PRR18	1.01898	1.017205	1.019556	1.13667	0.1848	1	1
RP11-768F21.1	1.016415	1.017198	1.016161	0.93968	-0.0898	1	1
AC093609.1	1.028535	1.02774	1.028793	1.03798	0.0538	1	1
RMND5A	1.423676	1.421471	1.424393	1.00693	0.01	1	1
CTD-2568P8.1	1.014678	1.013691	1.014999	1.09553	0.1316	1	1
TDRG1	1.01382	1.013691	1.013862	1.01249	0.0179	1	1
RP5-983L19.2	1.014677	1.013691	1.014997	1.09543	0.1315	1	1
LEKR1	1.04061	1.04188	1.040197	0.95981	-0.0592	1	1
INSRR	1.014678	1.013691	1.014999	1.09553	0.1316	1	1
CNEP1R1	1.183952	1.18243	1.184447	1.01106	0.0159	1	1
FCRL2	1.014685	1.013691	1.015009	1.09626	0.1326	1	1
RP11-190C22.8	1.023313	1.024232	1.023014	0.94974	-0.0744	1	1
LINC00463	1.014684	1.013691	1.015007	1.09616	0.1325	1	1
OTOGL	1.019843	1.020752	1.019548	0.94196	-0.0863	1	1
RP11-642D21.2	1.01382	1.013691	1.013862	1.01249	0.0179	1	1
CTD-2527I21.7	1.015541	1.013691	1.016142	1.17905	0.2376	1	1
RP11-158M2.2	1.01382	1.013691	1.013862	1.01249	0.0179	1	1
BTNL9	1.01382	1.013691	1.013862	1.01249	0.0179	1	1
CXCL13	1.014675	1.013691	1.014995	1.09526	0.1313	1	1
SPATA9	1.019821	1.020675	1.019544	0.94527	-0.0812	1	1
HIST1H4D	1.015545	1.013691	1.016148	1.17947	0.2381	1	1
RP3-326I13.1	1.018132	1.017173	1.018444	1.074	0.103	1	1
GRIFIN	1.01382	1.013691	1.013862	1.01249	0.0179	1	1
RP11-758N13.1	1.014682	1.013691	1.015004	1.09589	0.1321	1	1
KEL	1.014682	1.013691	1.015004	1.09592	0.1321	1	1
CTD-3075F15.1	1.014684	1.013691	1.015007	1.09611	0.1324	1	1
CD93	1.015536	1.013691	1.016136	1.17858	0.2371	1	1
AC010504.2	1.015557	1.013691	1.016164	1.18064	0.2396	1	1
AC124944.3	1.01555	1.013691	1.016154	1.17992	0.2387	1	1
ADAM33	1.026773	1.02779	1.026442	0.95152	-0.0717	1	1
SVOPL	1.017271	1.017177	1.017301	1.00725	0.0104	1	1
PDIA2	1.021673	1.020725	1.021981	1.06062	0.0849	1	1
SLC5A5	1.014681	1.013691	1.015002	1.0958	0.132	1	1
HERC3	1.088905	1.087624	1.089321	1.01938	0.0277	1	1
RP11-202D1.2	1.014675	1.013691	1.014995	1.09523	0.1312	1	1

LINC00525	1.021582	1.020679	1.021875	1.05788	0.0812	1	1
RP11-20G13.3	1.015535	1.013691	1.016135	1.17851	0.237	1	1
RP11-572M11.1	1.014683	1.013691	1.015006	1.09604	0.1323	1	1
RP11-154H23.5	1.014682	1.013691	1.015004	1.09591	0.1321	1	1
RP11-247L20.4	1.01727	1.017183	1.017299	1.00675	0.0097	1	1
RP11-526A4.1	1.015553	1.013691	1.016159	1.18025	0.2391	1	1
RP11-507M3.1	1.01639	1.017184	1.016132	0.93876	-0.0912	1	1
RP11-66N11.7	1.01555	1.013691	1.016154	1.17995	0.2387	1	1
TMEM71	1.01382	1.013691	1.013862	1.01249	0.0179	1	1
CLCNKA	1.025029	1.02425	1.025283	1.04259	0.0602	1	1
CTD-2538C1.2	1.055213	1.055823	1.055014	0.98551	-0.0211	1	1
ARL14	1.01382	1.013691	1.013862	1.01249	0.0179	1	1
CTD-3193O13.13	1.01555	1.013691	1.016155	1.17999	0.2388	1	1
RP3-406P24.3	1.014684	1.013691	1.015006	1.0961	0.1324	1	1
FMO4	1.055267	1.055916	1.055056	0.98461	-0.0224	1	1
LINC00838	1.01468	1.013691	1.015002	1.09576	0.1319	1	1
TMPRSS11B	1.014683	1.013691	1.015005	1.09599	0.1322	1	1
AC003956.1	1.014677	1.013691	1.014998	1.0955	0.1316	1	1
C1QTNF1-AS1	1.01555	1.013691	1.016154	1.17991	0.2387	1	1
TBX18	1.014679	1.013691	1.015001	1.09568	0.1318	1	1
RP11-674P19.2	1.016417	1.017204	1.016161	0.93938	-0.0902	1	1
AC006116.20	1.015539	1.013691	1.016139	1.17885	0.2374	1	1
ALKBH3-AS1	1.015541	1.013691	1.016142	1.17907	0.2377	1	1
CTC-242N15.1	1.015549	1.013691	1.016153	1.17988	0.2386	1	1
RP11-410K21.2	1.020732	1.020671	1.020751	1.00387	0.0056	1	1
FGD5	1.013543	1.013102	1.013687	1.04469	0.0631	1	1
RP11-115N4.1	1.013543	1.013102	1.013687	1.04469	0.0631	1	1
GAPDHS	1.014406	1.013102	1.01483	1.13192	0.1788	1	1
IFITM10	1.025598	1.023595	1.026249	1.11245	0.1537	1	1
RP11-819C21.1	1.063776	1.062433	1.064213	1.02851	0.0406	1	1
RP11-574F21.3	1.01526	1.016608	1.014821	0.89244	-0.1642	1	1
GFRA1	1.282811	1.283559	1.282568	0.99651	-0.005	1	1
SLC25A18	1.025621	1.023595	1.02628	1.11382	0.1555	1	1
TLR5	1.034335	1.034395	1.034315	0.99769	-0.0033	1	1
CTA-221G9.12	1.01871	1.020091	1.018261	0.90893	-0.1378	1	1
RP11-180P8.3	1.016121	1.016602	1.015965	0.96165	-0.0564	1	1
SH3RF3-AS1	1.013543	1.013102	1.013687	1.04469	0.0631	1	1
CST2	1.015267	1.016631	1.014824	0.89138	-0.1659	1	1
SLC25A16	1.132699	1.132719	1.132692	0.9998	-0.0003	1	1
RP11-798K3.3	1.013543	1.013102	1.013687	1.04469	0.0631	1	1
RP11-212D19.4	1.014405	1.013102	1.014829	1.13184	0.1787	1	1
RP11-335O13.7	1.014404	1.013102	1.014828	1.13174	0.1785	1	1
RP11-138J23.1	1.014404	1.013102	1.014828	1.13174	0.1785	1	1
GABRR2	1.013543	1.013102	1.013687	1.04469	0.0631	1	1
TMC2	1.014408	1.013102	1.014832	1.13211	0.179	1	1

UNC13C	1.015262	1.016602	1.014826	0.89304	-0.1632	1	1
ODF3L1	1.021304	1.020128	1.021685	1.07736	0.1075	1	1
RP11-36B15.1	1.013543	1.013102	1.013687	1.04469	0.0631	1	1
RP11-400N9.1	1.024775	1.023682	1.02513	1.06111	0.0856	1	1
AC005237.4	1.014401	1.013102	1.014823	1.13138	0.1781	1	1
AMER3	1.01614	1.016616	1.015985	0.96199	-0.0559	1	1
RP11-610P16.1	1.014401	1.013102	1.014823	1.13142	0.1781	1	1
RNF185-AS1	1.014402	1.013102	1.014824	1.13149	0.1782	1	1
RP11-429P3.3	1.014404	1.013102	1.014827	1.13174	0.1785	1	1
HOXC9	1.013543	1.013102	1.013687	1.04469	0.0631	1	1
ZYG11A	1.014403	1.013102	1.014826	1.13162	0.1784	1	1
CDX1	1.015259	1.016588	1.014826	0.89379	-0.162	1	1
SDHA	1.434365	1.434604	1.434288	0.99927	-0.001	1	1
KLHL41	1.014401	1.013102	1.014823	1.13143	0.1781	1	1
EREG	1.016118	1.016601	1.015961	0.96147	-0.0567	1	1
LINC01111	1.015273	1.016632	1.014831	0.8917	-0.1654	1	1
RP11-41O4.2	1.013543	1.013102	1.013687	1.04469	0.0631	1	1
RP4-647C14.2	1.014401	1.013102	1.014824	1.13145	0.1782	1	1
RP11-384C4.7	1.013543	1.013102	1.013687	1.04469	0.0631	1	1
PIK3R5	1.014402	1.013102	1.014825	1.13155	0.1783	1	1
ZMYM4-AS1	1.014403	1.013102	1.014826	1.13162	0.1784	1	1
LIN37	1.056645	1.055247	1.057099	1.03353	0.0476	1	1
TXK	1.015259	1.016599	1.014824	0.89305	-0.1632	1	1
AC131056.3	1.014402	1.013102	1.014824	1.13149	0.1782	1	1
CELSR3	1.077485	1.076421	1.077831	1.01844	0.0264	1	1
RP11-240G22.4	1.014405	1.013102	1.014829	1.13183	0.1787	1	1
RP11-482M8.3	1.025622	1.023623	1.026271	1.11209	0.1533	1	1
RP11-130L8.2	1.016114	1.01659	1.015959	0.96193	-0.056	1	1
DNAJC18	1.291595	1.291045	1.291773	1.0025	0.0036	1	1
STATH	1.013543	1.013102	1.013687	1.04469	0.0631	1	1
FCGBP	1.015261	1.016596	1.014828	0.89346	-0.1625	1	1
RP11-6O2.2	1.014406	1.013102	1.014831	1.13198	0.1788	1	1
RP11-45A17.4	1.020465	1.020127	1.020574	1.02225	0.0317	1	1
CCR1	1.019574	1.020094	1.019405	0.96573	-0.0503	1	1
LINC00620	1.014403	1.013102	1.014827	1.13167	0.1784	1	1
KB-1410C5.5	1.015274	1.016655	1.014825	0.89016	-0.1679	1	1
CTD-2544H17.1	1.014403	1.013102	1.014827	1.13168	0.1785	1	1
CFAP73	1.015265	1.016602	1.014831	0.89329	-0.1628	1	1
LMBRD2	1.120578	1.118452	1.121269	1.02378	0.0339	1	1
AC016907.3	1.025643	1.02372	1.026268	1.10744	0.1472	1	1
RP11-474G23.2	1.014402	1.013102	1.014825	1.13158	0.1783	1	1
RP11-284F21.11	1.014403	1.013102	1.014825	1.13158	0.1783	1	1
GS1-279B7.2	1.018729	1.020143	1.01827	0.90698	-0.1409	1	1
USP46-AS1	1.032533	1.03067	1.033138	1.08047	0.1117	1	1
PI4KB	1.304651	1.304518	1.304694	1.00058	0.0008	1	1

PNLDC1	1.014408	1.013102	1.014833	1.13214	0.1791	1	1
AC118754.4	1.013543	1.013102	1.013687	1.04469	0.0631	1	1
RP11-706P11.2	1.016125	1.016621	1.015964	0.96047	-0.0582	1	1
SATL1	1.014402	1.013102	1.014824	1.13149	0.1782	1	1
RANBP3L	1.02222	1.020113	1.022905	1.13882	0.1875	1	1
FAM163A	1.023053	1.02374	1.02283	0.96165	-0.0564	1	1
RP11-334E6.10	1.015263	1.0166	1.014829	0.8933	-0.1628	1	1
TEX13C	1.014406	1.013102	1.01483	1.13192	0.1788	1	1
LAMP5-AS1	1.014404	1.013102	1.014828	1.13175	0.1786	1	1
AC017002.2	1.016978	1.016595	1.017102	1.03059	0.0435	1	1
RP5-965G21.5	1.01441	1.013102	1.014835	1.13233	0.1793	1	1
RTEL1-TNFRSF6B	1.014402	1.013102	1.014825	1.13152	0.1783	1	1
RP3-508I15.18	1.013543	1.013102	1.013687	1.04469	0.0631	1	1
RP5-903G2.2	1.01785	1.01661	1.018253	1.0989	0.1361	1	1
CTB-60E11.9	1.014405	1.013102	1.014829	1.13186	0.1787	1	1
RP11-548H3.1	1.021293	1.020112	1.021677	1.07778	0.1081	1	1
CTD-2014B16.3	1.015259	1.016593	1.014825	0.89345	-0.1625	1	1
SLC4A11	1.01526	1.016604	1.014823	0.89275	-0.1637	1	1
CTB-178M22.1	1.013543	1.013102	1.013687	1.04469	0.0631	1	1
LINC00923	1.014402	1.013102	1.014825	1.13155	0.1783	1	1
RP1-228P16.8	1.015267	1.01662	1.014828	0.89217	-0.1646	1	1
RP11-484D2.2	1.014405	1.013102	1.014829	1.13183	0.1787	1	1
AF064860.7	1.014401	1.013102	1.014824	1.13146	0.1782	1	1
LA16c-60D12.1	1.014401	1.013102	1.014823	1.13139	0.1781	1	1
ACKR1	1.016989	1.016608	1.017113	1.03037	0.0432	1	1
CYSLTR1	1.017847	1.016602	1.018252	1.09941	0.1367	1	1
CD84	1.013543	1.013102	1.013687	1.04469	0.0631	1	1
CXCL5	1.017854	1.016636	1.01825	1.09697	0.1335	1	1
SART1	1.206686	1.206119	1.206871	1.00365	0.0053	1	1
CDHR5	1.015264	1.016627	1.014821	0.89138	-0.1659	1	1
AF127577.8	1.014405	1.013102	1.014829	1.13187	0.1787	1	1
TNFSF14	1.013543	1.013102	1.013687	1.04469	0.0631	1	1
YIPF7	1.02136	1.020141	1.021756	1.08023	0.1113	1	1
ZNF8	1.114573	1.115	1.114434	0.99508	-0.0071	1	1
RP11-159H22.2	1.013543	1.013102	1.013687	1.04469	0.0631	1	1
RP11-244H3.4	1.020467	1.020089	1.020589	1.02493	0.0355	1	1
RP11-342M21.2	1.014403	1.013102	1.014826	1.13166	0.1784	1	1
MGC45922	1.020476	1.02014	1.020585	1.0221	0.0315	1	1
SPHKAP	1.0187	1.020131	1.018235	0.90581	-0.1427	1	1
SMC1B	1.016125	1.016608	1.015968	0.96146	-0.0567	1	1
ILDR1	1.013543	1.013102	1.013687	1.04469	0.0631	1	1
RP11-738E22.3	1.017855	1.016599	1.018264	1.10031	0.1379	1	1
RP11-492E3.2	1.014402	1.013102	1.014825	1.13153	0.1783	1	1
RP1-142L7.9	1.014401	1.013102	1.014823	1.13139	0.1781	1	1
NLGN3	1.029932	1.030598	1.029716	0.97118	-0.0422	1	1

FBXW12	1.022151	1.020112	1.022814	1.13439	0.1819	1	1
SPATA3-AS1	1.014399	1.013102	1.014821	1.13122	0.1779	1	1
LINC00578	1.014402	1.013102	1.014825	1.13156	0.1783	1	1
PRB3	1.014399	1.013102	1.014821	1.13123	0.1779	1	1
RP11-122A21.2	1.014409	1.013102	1.014834	1.1322	0.1791	1	1
FIP1L1	1.612235	1.611025	1.612628	1.00262	0.0038	1	1
RP11-535M15.1	1.014402	1.013102	1.014824	1.13151	0.1782	1	1
AC004067.5	1.017001	1.016601	1.017132	1.03197	0.0454	1	1
ELOVL2-AS1	1.015274	1.016648	1.014827	0.89059	-0.1672	1	1
RP11-1017G21.6	1.021308	1.020124	1.021693	1.07797	0.1083	1	1
AC128709.3	1.014401	1.013102	1.014823	1.1314	0.1781	1	1
LL22NC03-2H8.4	1.0144	1.013102	1.014822	1.13135	0.178	1	1
KCNE5	1.036816	1.037665	1.03654	0.97015	-0.0437	1	1
RP11-9J18.1	1.014407	1.013102	1.014831	1.13201	0.1789	1	1
AC096669.1	1.013543	1.013102	1.013687	1.04469	0.0631	1	1
RP11-388C12.1	1.038614	1.037818	1.038872	1.02788	0.0397	1	1
CTB-176F20.3	1.014403	1.013102	1.014827	1.13167	0.1784	1	1
PCSK1	1.015259	1.016592	1.014825	0.89348	-0.1625	1	1
RP11-493L12.3	1.014402	1.013102	1.014825	1.13157	0.1783	1	1
RP11-409I10.2	1.014402	1.013102	1.014824	1.13151	0.1782	1	1
SLC38A11	1.013543	1.013102	1.013687	1.04469	0.0631	1	1
RP11-360L9.8	1.014399	1.013102	1.014821	1.13123	0.1779	1	1
AC002429.5	1.020447	1.02011	1.020557	1.02222	0.0317	1	1
C4orf32	1.018715	1.020146	1.01825	0.90589	-0.1426	1	1
RP11-996F15.4	1.013506	1.014805	1.013083	0.88371	-0.1784	1	1
RP11-472N13.2	1.013512	1.014828	1.013084	0.88241	-0.1805	1	1
IL26	1.012645	1.011304	1.013081	1.15719	0.2106	1	1
RP11-416N4.4	1.015237	1.014816	1.015373	1.03759	0.0532	1	1
IGSF9B	1.017815	1.018283	1.017663	0.96608	-0.0498	1	1
CTC-527H23.3	1.015223	1.014801	1.01536	1.03774	0.0534	1	1
RP11-162A12.2	1.022117	1.021779	1.022226	1.02055	0.0294	1	1
CCR4	1.01436	1.014791	1.01422	0.96138	-0.0568	1	1
AC007364.1	1.011789	1.011304	1.011946	1.05678	0.0797	1	1
EDRF1-AS1	1.013504	1.014798	1.013083	0.88414	-0.1777	1	1
AC104532.4	1.034192	1.032336	1.034796	1.07606	0.1058	1	1
RP5-893G23.1	1.011789	1.011304	1.011946	1.05678	0.0797	1	1
RP11-367G6.3	1.011789	1.011304	1.011946	1.05678	0.0797	1	1
KCNG4	1.012644	1.011304	1.01308	1.15707	0.2105	1	1
CTD-2526A2.2	1.015237	1.014813	1.015375	1.03794	0.0537	1	1
DCHS2	1.023015	1.021953	1.02336	1.06409	0.0896	1	1
RP11-265E18.1	1.01696	1.018323	1.016517	0.90143	-0.1497	1	1
HSPC324	1.012644	1.011304	1.01308	1.15707	0.2105	1	1
AP000473.5	1.013505	1.014798	1.013085	0.88422	-0.1775	1	1
PPP2R2C	1.013504	1.014798	1.013084	0.88417	-0.1776	1	1
ANGEL1	1.107467	1.105917	1.107971	1.01939	0.0277	1	1

QRICH1	1.290891	1.29203	1.290521	0.99483	-0.0075	1	1
ADAM32	1.017798	1.0183	1.017635	0.96366	-0.0534	1	1
LINC01304	1.013509	1.014798	1.01309	0.88459	-0.1769	1	1
E4F1	1.156597	1.155077	1.157091	1.01299	0.0186	1	1
SP9	1.012646	1.011304	1.013082	1.15722	0.2107	1	1
AC098823.3	1.011789	1.011304	1.011946	1.05678	0.0797	1	1
MCOLN2	1.017844	1.018361	1.017676	0.96267	-0.0549	1	1
RP11-321L2.1	1.011789	1.011304	1.011946	1.05678	0.0797	1	1
RP11-82L20.1	1.012649	1.011304	1.013085	1.15756	0.2111	1	1
GPRASP2	1.215154	1.214908	1.215233	1.00151	0.0022	1	1
HIST1H2AD	1.012647	1.011304	1.013083	1.15736	0.2108	1	1
RP11-436D10.3	1.012648	1.011304	1.013084	1.15746	0.211	1	1
ERGIC2	2.10411	2.102518	2.104628	1.00191	0.0028	1	1
RP11-18I14.11	1.015231	1.014788	1.015374	1.03965	0.0561	1	1
P4HA3	1.0135	1.014792	1.01308	0.88427	-0.1774	1	1
GSDMD	1.179818	1.179612	1.179884	1.00152	0.0022	1	1
RP3-460G2.2	1.012649	1.011304	1.013087	1.15767	0.2112	1	1
AAR2	1.244325	1.242918	1.244782	1.00767	0.011	1	1
RP11-745O10.4	1.012646	1.011304	1.013083	1.15731	0.2108	1	1
RP11-459O1.2	1.012646	1.011304	1.013082	1.15727	0.2107	1	1
U47924.32	1.012646	1.011304	1.013082	1.15723	0.2107	1	1
CCIN	1.011789	1.011304	1.011946	1.05678	0.0797	1	1
TUBA8	1.014374	1.014813	1.014232	0.96077	-0.0577	1	1
TH2LCRR	1.012647	1.011304	1.013083	1.15737	0.2109	1	1
CTA-228A9.3	1.02297	1.021787	1.023355	1.07197	0.1003	1	1
LINC01353	1.014374	1.014806	1.014233	0.96131	-0.0569	1	1
RP11-109D20.2	1.012645	1.011304	1.013081	1.1572	0.2106	1	1
RP11-439K3.3	1.015232	1.014799	1.015373	1.03879	0.0549	1	1
AC106786.1	1.01436	1.014795	1.014219	0.96109	-0.0573	1	1
ANKRD62	1.014371	1.014835	1.014221	0.9586	-0.061	1	1
RP11-247A12.7	1.031595	1.03244	1.03132	0.96549	-0.0507	1	1
TSACC	1.018654	1.018323	1.018761	1.02392	0.0341	1	1
DPP4	1.012651	1.011304	1.013089	1.15786	0.2115	1	1
RP11-135J2.3	1.012651	1.011304	1.013088	1.15783	0.2114	1	1
KCTD13	1.194505	1.193583	1.194805	1.00631	0.0091	1	1
RP11-400K9.4	1.012645	1.011304	1.013081	1.1572	0.2106	1	1
U91328.22	1.013503	1.014798	1.013082	0.88405	-0.1778	1	1
RP11-83N9.5	1.019547	1.018365	1.019931	1.08523	0.118	1	1
AC005740.6	1.015232	1.014816	1.015367	1.03722	0.0527	1	1
RP11-701H16.4	1.012648	1.011304	1.013085	1.1575	0.211	1	1
RP11-359E10.1	1.018666	1.018295	1.018787	1.02688	0.0383	1	1
CASS4	1.011789	1.011304	1.011946	1.05678	0.0797	1	1
KLHDC4	1.098727	1.098993	1.09864	0.99644	-0.0052	1	1
MESP1	1.334992	1.334404	1.335183	1.00233	0.0034	1	1
RP11-131L12.3	1.025562	1.025332	1.025637	1.01205	0.0173	1	1

RP11-348H3.8	1.011789	1.011304	1.011946	1.05678	0.0797	1	1
TDO2	1.037605	1.035866	1.03817	1.06422	0.0898	1	1
RP11-375A5.1	1.012645	1.011304	1.01308	1.15709	0.2105	1	1
BCL2L10	1.011789	1.011304	1.011946	1.05678	0.0797	1	1
AC012370.3	1.012644	1.011304	1.013079	1.15701	0.2104	1	1
FAM24B	1.011789	1.011304	1.011946	1.05678	0.0797	1	1
ERVV-1	1.012645	1.011304	1.01308	1.15709	0.2105	1	1
RP11-817J15.3	1.012652	1.011304	1.01309	1.15797	0.2116	1	1
GTSF1	1.012649	1.011304	1.013087	1.15766	0.2112	1	1
AC009227.2	1.012651	1.011304	1.013089	1.15786	0.2115	1	1
FKBP1C	1.023817	1.021796	1.024474	1.12289	0.1672	1	1
NXF2B	1.020433	1.021845	1.019973	0.9143	-0.1293	1	1
RP11-67L3.4	1.01265	1.011304	1.013088	1.15776	0.2113	1	1
CTD-2562J17.7	1.012644	1.011304	1.01308	1.15708	0.2105	1	1
RP11-497G19.2	1.012648	1.011304	1.013085	1.15753	0.211	1	1
RP11-370F5.4	1.011789	1.011304	1.011946	1.05678	0.0797	1	1
LENEP	1.016941	1.018303	1.016498	0.90141	-0.1497	1	1
CTD-3126B10.1	1.012647	1.011304	1.013083	1.15737	0.2109	1	1
KRTAP5-AS1	1.014361	1.014796	1.014219	0.96101	-0.0574	1	1
RP11-144G7.2	1.012645	1.011304	1.013081	1.1572	0.2106	1	1
ADGRG7	1.011789	1.011304	1.011946	1.05678	0.0797	1	1
RP11-369E15.3	1.014367	1.014806	1.014224	0.9607	-0.0578	1	1
RP11-276H7.3	1.014375	1.014792	1.014239	0.96259	-0.055	1	1
HIST1H3J	1.012646	1.011304	1.013082	1.15729	0.2107	1	1
LINC01563	1.014372	1.014821	1.014226	0.95983	-0.0592	1	1
RP5-1007H16.1	1.012649	1.011304	1.013087	1.15766	0.2112	1	1
RP11-131L23.2	1.012649	1.011304	1.013087	1.15767	0.2112	1	1
ATP2C1	1.581888	1.583207	1.581459	0.997	-0.0043	1	1
RP11-845M18.6	1.013508	1.014801	1.013088	0.88423	-0.1775	1	1
BMS1P14	1.026502	1.025321	1.026885	1.06177	0.0865	1	1
CTD-2036P10.6	1.018683	1.018325	1.018799	1.02588	0.0369	1	1
C15orf32	1.012649	1.011304	1.013085	1.15756	0.2111	1	1
RP11-815J21.2	1.012645	1.011304	1.013081	1.15719	0.2106	1	1
AC079354.5	1.020446	1.021931	1.019963	0.91023	-0.1357	1	1
HLA-DOB	1.012646	1.011304	1.013082	1.15729	0.2107	1	1
CCS	1.724355	1.723765	1.724547	1.00108	0.0016	1	1
FABP9	1.012645	1.011304	1.013081	1.15716	0.2106	1	1
RP11-221N13.3	1.01436	1.014801	1.014216	0.96052	-0.0581	1	1
ANXA2R	1.018668	1.018301	1.018788	1.0266	0.0379	1	1
NPHP3-ACAD11	1.011789	1.011304	1.011946	1.05678	0.0797	1	1
RP4-539M6.22	1.015237	1.014797	1.01538	1.03943	0.0558	1	1
BTN1A1	1.012652	1.011304	1.01309	1.15797	0.2116	1	1
WNT4	1.012647	1.011304	1.013084	1.15742	0.2109	1	1
PYDC1	1.013507	1.014798	1.013087	0.88437	-0.1773	1	1
RP11-345J4.5	1.064184	1.063793	1.064312	1.00813	0.0117	1	1

DLX5	1.018662	1.018316	1.018774	1.02502	0.0357	1	1
TEX38	1.012645	1.011304	1.013081	1.15716	0.2106	1	1
RP11-497H17.1	1.019553	1.018315	1.019955	1.08954	0.1237	1	1
PLCH2	1.025598	1.025428	1.025654	1.0089	0.0128	1	1
PLA2G4D	1.012647	1.011304	1.013083	1.15733	0.2108	1	1
RP11-542G1.1	1.012647	1.011304	1.013083	1.15736	0.2108	1	1
UPK3BL	1.012652	1.011304	1.013091	1.15801	0.2116	1	1
LINC01393	1.016096	1.014806	1.016516	1.11545	0.1576	1	1
RP11-292D4.3	1.012646	1.011304	1.013082	1.15722	0.2107	1	1
LINC01538	1.012651	1.011304	1.013088	1.15783	0.2114	1	1
RP11-431J17.1	1.012647	1.011304	1.013084	1.1574	0.2109	1	1
LINC01361	1.014366	1.014815	1.01422	0.95983	-0.0592	1	1
MIR29A	1.012645	1.011304	1.01308	1.15711	0.2105	1	1
RP11-214N1.1	1.013509	1.014812	1.013086	0.88349	-0.1787	1	1
CTD-2184D3.5	1.011789	1.011304	1.011946	1.05678	0.0797	1	1
LINC01545	1.023892	1.022004	1.024506	1.11366	0.1553	1	1
TNFRSF17	1.014381	1.014793	1.014247	0.96304	-0.0543	1	1
WFDC10B	1.011789	1.011304	1.011946	1.05678	0.0797	1	1
RP11-173M1.4	1.018671	1.018314	1.018787	1.02583	0.0368	1	1
RP5-942I16.1	1.022158	1.021829	1.022265	1.01994	0.0285	1	1
AC016912.3	1.016257	1.01526	1.016581	1.08657	0.1198	1	1
ZNF761	1.113183	1.113751	1.112998	0.99338	-0.0096	1	1
GS1-594A7.3	1.016257	1.01526	1.016581	1.08657	0.1198	1	1
RP11-291L22.4	1.016257	1.01526	1.016581	1.08657	0.1198	1	1
LINC01351	1.017125	1.01526	1.017732	1.162	0.2166	1	1
RP11-415J8.7	1.017117	1.01526	1.017721	1.1613	0.2157	1	1
RP11-593F23.1	1.016257	1.01526	1.016581	1.08657	0.1198	1	1
RP11-203B9.4	1.017116	1.01526	1.017719	1.16115	0.2156	1	1
KCNJ4	1.06904	1.068086	1.06935	1.01857	0.0265	1	1
RP11-426A6.5	1.017114	1.01526	1.017716	1.16099	0.2154	1	1
RP11-1C8.5	1.017119	1.01526	1.017723	1.16143	0.2159	1	1
SLC5A11	1.017119	1.01526	1.017723	1.16143	0.2159	1	1
RP11-709D24.5	1.018861	1.018799	1.018881	1.00437	0.0063	1	1
C11orf91	1.020563	1.01875	1.021152	1.12812	0.1739	1	1
RP3-453C12.15	1.024067	1.022301	1.024641	1.10494	0.144	1	1
RP11-429B14.4	1.017128	1.01526	1.017736	1.16226	0.2169	1	1
ABCG1	1.019727	1.018765	1.020039	1.0679	0.0948	1	1
RP11-7O11.3	1.019729	1.018789	1.020035	1.0663	0.0926	1	1
NPIPB7	1.017118	1.01526	1.017723	1.1614	0.2159	1	1
AC147651.4	1.017115	1.01526	1.017718	1.16111	0.2155	1	1
RP11-334A14.8	1.017114	1.01526	1.017717	1.16103	0.2154	1	1
AC008440.5	1.017114	1.01526	1.017717	1.16103	0.2154	1	1
C12orf49	1.543716	1.543479	1.543794	1.00058	0.0008	1	1
RP11-50B3.2	1.017116	1.01526	1.01772	1.16122	0.2156	1	1
MIRLET7DHG	1.017114	1.01526	1.017717	1.16103	0.2154	1	1

NR2F2-AS1	1.217986	1.216277	1.218542	1.01047	0.015	1	1
TRG-AS1	1.017121	1.01526	1.017726	1.16163	0.2161	1	1
RP11-645C24.5	1.017118	1.01526	1.017722	1.16134	0.2158	1	1
CLYBL-AS1	1.017118	1.01526	1.017723	1.1614	0.2159	1	1
LA16c-359F1.1	1.016257	1.01526	1.016581	1.08657	0.1198	1	1
OBP2B	1.017115	1.01526	1.017718	1.16111	0.2155	1	1
LINC01173	1.017983	1.018755	1.017733	0.94549	-0.0809	1	1
MRPL35	1.325759	1.324916	1.326033	1.00344	0.0049	1	1
RP11-252A24.3	1.026649	1.02586	1.026906	1.04044	0.0572	1	1
RP11-173P15.9	1.017117	1.01526	1.017721	1.16131	0.2158	1	1
RP5-1021I20.5	1.024073	1.022327	1.02464	1.10361	0.1422	1	1
RP11-154D17.1	1.017113	1.01526	1.017715	1.1609	0.2152	1	1
CTA-363E19.2	1.018841	1.018767	1.018865	1.00525	0.0076	1	1
KCNS2	1.017117	1.01526	1.017721	1.16131	0.2158	1	1
KIRREL3	1.025737	1.025761	1.025729	0.99878	-0.0018	1	1
PDCD1LG2	1.017123	1.01526	1.017729	1.16181	0.2164	1	1
PLN	1.017118	1.01526	1.017722	1.16135	0.2158	1	1
EIF3CL	1.024034	1.022264	1.02461	1.10535	0.1445	1	1
CARD19	1.642879	1.641898	1.643197	1.00202	0.0029	1	1
RP11-399E6.1	1.017119	1.01526	1.017723	1.16145	0.2159	1	1
CTD-2270F17.1	1.017121	1.01526	1.017726	1.16163	0.2161	1	1
POC1B-GALNT4	1.016257	1.01526	1.016581	1.08657	0.1198	1	1
RP11-328C8.4	1.016257	1.01526	1.016581	1.08657	0.1198	1	1
RP11-529E10.7	1.018848	1.018746	1.018881	1.00724	0.0104	1	1
RP11-552F3.9	1.016257	1.01526	1.016581	1.08657	0.1198	1	1
LINC01447	1.017114	1.01526	1.017717	1.16103	0.2154	1	1
RP11-796E10.1	1.023197	1.022322	1.023481	1.05193	0.073	1	1
RP11-830F9.5	1.016257	1.01526	1.016581	1.08657	0.1198	1	1
PCDHAC2	1.022321	1.022392	1.022298	0.9958	-0.0061	1	1
TRIM61	1.017117	1.01526	1.01772	1.16123	0.2157	1	1
RP4-669P10.20	1.016257	1.01526	1.016581	1.08657	0.1198	1	1
ST3GAL2	1.297382	1.296788	1.297575	1.00265	0.0038	1	1
RP11-13K12.1	1.020563	1.018772	1.021145	1.12637	0.1717	1	1
LINC01629	1.01798	1.018782	1.01772	0.94344	-0.084	1	1
ZNF280A	1.024035	1.022263	1.024611	1.10549	0.1447	1	1
GS1-166A23.1	1.017118	1.01526	1.017722	1.16139	0.2159	1	1
TIAF1	1.023196	1.022348	1.023471	1.05025	0.0707	1	1
RP11-356C4.5	1.017117	1.01526	1.01772	1.16124	0.2157	1	1
AC000403.4	1.017116	1.01526	1.017719	1.16119	0.2156	1	1
CTD-3037G24.5	1.016257	1.01526	1.016581	1.08657	0.1198	1	1
RP11-419I17.2	1.019701	1.018768	1.020004	1.06581	0.092	1	1
CCDC87	1.017979	1.018749	1.017729	0.94561	-0.0807	1	1
SLC6A2	1.016257	1.01526	1.016581	1.08657	0.1198	1	1
RP11-262A16.1	1.01884	1.018767	1.018864	1.00515	0.0074	1	1
RP11-44N12.5	1.016257	1.01526	1.016581	1.08657	0.1198	1	1

CTB-129P6.11	1.017116	1.01526	1.01772	1.1612	0.2156	1	1
USP30-AS1	1.017117	1.01526	1.017721	1.16127	0.2157	1	1
KLB	1.017117	1.01526	1.017721	1.16132	0.2158	1	1
CTD-3224K15.2	1.017117	1.01526	1.01772	1.16123	0.2157	1	1
C22orf42	1.017116	1.01526	1.01772	1.16121	0.2156	1	1
CST11	1.017971	1.018755	1.017717	0.94467	-0.0821	1	1
C1orf140	1.017114	1.01526	1.017717	1.16104	0.2154	1	1
AC016738.4	1.018837	1.018765	1.01886	1.00506	0.0073	1	1
RTL1	1.017121	1.01526	1.017726	1.16163	0.2162	1	1
THRIL	1.018852	1.01876	1.018881	1.00646	0.0093	1	1
RP11-434D9.1	1.017978	1.018769	1.017721	0.94419	-0.0829	1	1
CTC-564N23.2	1.017126	1.01526	1.017733	1.16205	0.2167	1	1
RP11-503E24.2	1.017122	1.01526	1.017728	1.16174	0.2163	1	1
RP11-616M22.1	1.016257	1.01526	1.016581	1.08657	0.1198	1	1
AC099342.1	1.017114	1.01526	1.017716	1.16099	0.2154	1	1
RP11-157E16.1	1.018859	1.018767	1.018888	1.00648	0.0093	1	1
CCR10	1.162562	1.163378	1.162297	0.99338	-0.0096	1	1
AL513523.2	1.017117	1.01526	1.017721	1.16126	0.2157	1	1
RP11-739L10.1	1.016257	1.01526	1.016581	1.08657	0.1198	1	1
RP11-665I14.1	1.017123	1.01526	1.017728	1.16176	0.2163	1	1
ARHGEF15	1.018864	1.018796	1.018886	1.00481	0.0069	1	1
P4HA2-AS1	1.017121	1.01526	1.017726	1.16163	0.2161	1	1
RP11-433J8.1	1.017115	1.01526	1.017719	1.16113	0.2155	1	1
RP13-516M14.10	1.017982	1.018795	1.017718	0.94274	-0.0851	1	1
RP11-143J12.3	1.016257	1.01526	1.016581	1.08657	0.1198	1	1
AP001055.6	1.01712	1.01526	1.017725	1.16157	0.2161	1	1
ANKRD30BL	1.017124	1.01526	1.01773	1.1619	0.2165	1	1
AC011525.2	1.016257	1.01526	1.016581	1.08657	0.1198	1	1
OAS3	1.02493	1.025797	1.024648	0.95546	-0.0657	1	1
PLLP	1.041263	1.039809	1.041736	1.04842	0.0682	1	1
SYNE1-AS1	1.017125	1.01526	1.017731	1.16193	0.2165	1	1
N4BP1	1.212603	1.21199	1.212803	1.00384	0.0055	1	1
CTB-180A7.3	1.017119	1.01526	1.017723	1.16143	0.2159	1	1
TEX40	1.018831	1.018754	1.018856	1.00547	0.0079	1	1
CMP21-97G8.1	1.019721	1.018755	1.020034	1.06822	0.0952	1	1
RP11-506O24.1	1.018857	1.01876	1.018889	1.00685	0.0098	1	1
LINC01054	1.017978	1.018773	1.01772	0.94391	-0.0833	1	1
TRIM7	1.031802	1.032817	1.031473	0.95904	-0.0603	1	1
YY2	1.081955	1.082025	1.081932	0.99885	-0.0017	1	1
RP11-274B21.9	1.017114	1.01526	1.017717	1.161	0.2154	1	1
RP11-495K9.5	1.017117	1.01526	1.017721	1.16129	0.2157	1	1
CTC-301O7.4	1.019698	1.018755	1.020005	1.06663	0.0931	1	1
GCSAM	1.017117	1.01526	1.017721	1.16127	0.2157	1	1
RP11-518L10.5	1.018844	1.018755	1.018872	1.00626	0.009	1	1
CDK11A	1.225546	1.226433	1.225257	0.99481	-0.0075	1	1

CTC-559E9.4	1.017117	1.01526	1.017721	1.16131	0.2158	1	1
RP3-508I15.14	1.022304	1.022295	1.022306	1.00048	0.0007	1	1
RP11-554E23.2	1.017983	1.018783	1.017723	0.94357	-0.0838	1	1
RP5-1125A11.6	1.017115	1.01526	1.017718	1.16107	0.2155	1	1
RP11-315E17.1	1.017126	1.01526	1.017733	1.16209	0.2167	1	1
RAPGEF3	1.031836	1.032917	1.031485	0.95649	-0.0642	1	1
AC073934.6	1.017114	1.01526	1.017717	1.161	0.2154	1	1
LINC00885	1.01712	1.01526	1.017725	1.16155	0.2161	1	1
RP11-1C8.6	1.01972	1.018767	1.020029	1.06724	0.0939	1	1
TBC1D30	1.057756	1.057467	1.05785	1.00668	0.0096	1	1
RP11-2H8.2	1.017121	1.01526	1.017726	1.16164	0.2162	1	1
DMRTA2	1.022291	1.022262	1.0223	1.00171	0.0025	1	1
TEX41	1.017002	1.017487	1.016844	0.96325	-0.054	1	1
FLJ27354	1.031686	1.031476	1.031755	1.00884	0.0127	1	1
CTD-2621I17.6	1.015271	1.013947	1.015702	1.12584	0.171	1	1
LINC00934	1.014413	1.013947	1.014564	1.0443	0.0625	1	1
LINC00911	1.014413	1.013947	1.014564	1.0443	0.0625	1	1
AC099850.1	1.030782	1.031446	1.030567	0.97205	-0.0409	1	1
AC009502.4	1.015278	1.013947	1.01571	1.12646	0.1718	1	1
CTD-2060L22.1	1.015272	1.013947	1.015702	1.12588	0.171	1	1
AC005775.2	1.016998	1.017447	1.016852	0.96593	-0.05	1	1
LINC01163	1.014413	1.013947	1.014564	1.0443	0.0625	1	1
SLC5A8	1.015271	1.013947	1.015701	1.12581	0.171	1	1
MYOC	1.014413	1.013947	1.014564	1.0443	0.0625	1	1
CTC-325J23.2	1.018725	1.017481	1.019129	1.09427	0.13	1	1
ANKRD7	1.014413	1.013947	1.014564	1.0443	0.0625	1	1
LINC01033	1.014413	1.013947	1.014564	1.0443	0.0625	1	1
KCNJ14	1.042045	1.042093	1.04203	0.99849	-0.0022	1	1
STK24-AS1	1.015274	1.013947	1.015706	1.12614	0.1714	1	1
LRRC66	1.018715	1.01744	1.01913	1.09691	0.1334	1	1
DLGAP2-AS1	1.015274	1.013947	1.015706	1.12614	0.1714	1	1
RP11-432J24.5	1.082791	1.080944	1.083392	1.03024	0.043	1	1
HSPA12B	1.018722	1.017449	1.019136	1.09669	0.1332	1	1
RP11-383M4.6	1.015278	1.013947	1.015711	1.12651	0.1719	1	1
CTD-2525I3.5	1.016134	1.017446	1.015708	0.90041	-0.1513	1	1
RP11-571I18.5	1.015273	1.013947	1.015704	1.12604	0.1713	1	1
CCDC63	1.015272	1.013947	1.015702	1.12589	0.1711	1	1
RP5-1056H1.2	1.015271	1.013947	1.015701	1.12578	0.1709	1	1
NYAP2	1.020481	1.021011	1.020309	0.96662	-0.049	1	1
ABCC11	1.015275	1.013947	1.015707	1.12625	0.1715	1	1
ARSF	1.014413	1.013947	1.014564	1.0443	0.0625	1	1
RP11-151A10.3	1.017873	1.017447	1.018011	1.03235	0.0459	1	1
HSPB6	1.016136	1.017454	1.015708	0.89998	-0.152	1	1
AC004449.6	1.015276	1.013947	1.015709	1.12634	0.1716	1	1
CTC-441N14.2	1.014413	1.013947	1.014564	1.0443	0.0625	1	1

APOC3	1.01527	1.013947	1.0157	1.12574	0.1709	1	1
RP11-576N17.5	1.016143	1.017473	1.01571	0.8991	-0.1534	1	1
GREM2	1.024824	1.02453	1.024919	1.01587	0.0227	1	1
CTB-147N14.6	1.024796	1.02449	1.024896	1.01659	0.0237	1	1
MNX1-AS2	1.014413	1.013947	1.014564	1.0443	0.0625	1	1
RP1-228P16.5	1.015273	1.013947	1.015704	1.126	0.1712	1	1
EDC4	1.017858	1.017467	1.017986	1.0297	0.0422	1	1
ANKRD30A	1.014413	1.013947	1.014564	1.0443	0.0625	1	1
TRERF1	1.142134	1.140383	1.142703	1.01652	0.0236	1	1
RP11-319E12.2	1.015271	1.013947	1.015701	1.12583	0.171	1	1
RP11-157I4.4	1.014413	1.013947	1.014564	1.0443	0.0625	1	1
GRPEL2-AS1	1.015275	1.013947	1.015707	1.12623	0.1715	1	1
BSND	1.015278	1.013947	1.015711	1.12651	0.1719	1	1
RECK	1.220039	1.217947	1.220719	1.01272	0.0182	1	1
MEOX2	1.015277	1.013947	1.015709	1.12636	0.1717	1	1
LINC00336	1.016987	1.017436	1.016841	0.96592	-0.05	1	1
GPR17	1.014413	1.013947	1.014564	1.0443	0.0625	1	1
RP1-20B21.4	1.014413	1.013947	1.014564	1.0443	0.0625	1	1
ZBTB11-AS1	1.056762	1.05605	1.056994	1.01685	0.0241	1	1
RP11-463J10.4	1.015278	1.013947	1.01571	1.12646	0.1718	1	1
GLRA1	1.015273	1.013947	1.015704	1.126	0.1712	1	1
BTN3A3	1.057716	1.056244	1.058195	1.0347	0.0492	1	1
DCAF10	1.539139	1.538224	1.539437	1.00225	0.0032	1	1
SRD5A2	1.015278	1.013947	1.015711	1.12649	0.1718	1	1
NRROS	1.024778	1.024427	1.024892	1.019	0.0272	1	1
TRIM72	1.016135	1.017465	1.015702	0.89905	-0.1535	1	1
CTB-113P19.5	1.015272	1.013947	1.015702	1.12588	0.171	1	1
AC007566.10	1.017	1.01745	1.016854	0.96585	-0.0501	1	1
FBXL18	1.055847	1.05612	1.055758	0.99355	-0.0093	1	1
RP11-154H23.4	1.016992	1.017468	1.016837	0.9639	-0.053	1	1
CTD-3064H18.4	1.016134	1.017442	1.015709	0.90065	-0.151	1	1
LVRN	1.01787	1.017446	1.018008	1.03218	0.0457	1	1
TNFSF13B	1.042893	1.042019	1.043177	1.02756	0.0392	1	1
RP11-456O19.3	1.014413	1.013947	1.014564	1.0443	0.0625	1	1
RP5-1186N24.3	1.030833	1.031558	1.030598	0.96956	-0.0446	1	1
NUAK1	1.301267	1.301924	1.301054	0.99712	-0.0042	1	1
RP11-196G11.6	1.028249	1.028158	1.028279	1.00429	0.0062	1	1
AF064858.8	1.015272	1.013947	1.015703	1.12593	0.1711	1	1
LINC01616	1.021328	1.021037	1.021423	1.01834	0.0262	1	1
FOXL1	1.024742	1.024453	1.024836	1.01567	0.0224	1	1
SPTBN5	1.015269	1.013947	1.015699	1.12568	0.1708	1	1
RP1-302D9.1	1.015275	1.013947	1.015707	1.1262	0.1715	1	1
ZNF613	1.061094	1.059751	1.061531	1.02979	0.0424	1	1
AC024592.12	1.015276	1.013947	1.015708	1.12627	0.1716	1	1
RP11-359J14.2	1.016143	1.017472	1.01571	0.89917	-0.1533	1	1

LINC00654	1.01527	1.013947	1.015701	1.12576	0.1709	1	1
HIST1H3I	1.015273	1.013947	1.015704	1.126	0.1712	1	1
DLGAP1-AS5	1.014413	1.013947	1.014564	1.0443	0.0625	1	1
EXOC1	1.46367	1.464461	1.463413	0.99774	-0.0033	1	1
FSD2	1.027376	1.028051	1.027156	0.9681	-0.0468	1	1
AC092415.1	1.015276	1.013947	1.015708	1.12629	0.1716	1	1
RP11-16E18.3	1.030819	1.031514	1.030593	0.9708	-0.0428	1	1
RP11-328J14.1	1.015272	1.013947	1.015702	1.12588	0.171	1	1
RP11-532M24.1	1.018753	1.017444	1.019178	1.09943	0.1368	1	1
ATXN2-AS	1.014413	1.013947	1.014564	1.0443	0.0625	1	1
RP11-84C10.4	1.017014	1.01749	1.016859	0.96396	-0.053	1	1
FOXS1	1.023049	1.024489	1.022581	0.9221	-0.117	1	1
CTD-3203P2.3	1.015274	1.013947	1.015706	1.12613	0.1714	1	1
CCDC91	1.476638	1.475176	1.477113	1.00408	0.0059	1	1
RP11-286N3.2	1.018723	1.01744	1.01914	1.09748	0.1342	1	1
IGBP1-AS2	1.014413	1.013947	1.014564	1.0443	0.0625	1	1
CPT1A	1.209756	1.211166	1.209298	0.99116	-0.0128	1	1
AC009495.3	1.016134	1.017447	1.015708	0.90031	-0.1515	1	1
LINC00598	1.028209	1.02802	1.028271	1.00894	0.0128	1	1
JAKMIP1	1.022161	1.020996	1.02254	1.07351	0.1023	1	1
RP11-24D15.1	1.014413	1.013947	1.014564	1.0443	0.0625	1	1
AC024560.2	1.018742	1.017454	1.019161	1.09779	0.1346	1	1
FAM19A1	1.017852	1.017464	1.017979	1.02948	0.0419	1	1
AC005253.4	1.014413	1.013947	1.014564	1.0443	0.0625	1	1
CD79A	1.019582	1.021009	1.019119	0.91001	-0.136	1	1
RP11-386I8.6	1.018741	1.017511	1.019141	1.0931	0.1284	1	1
LINC01337	1.015271	1.013947	1.015702	1.12586	0.171	1	1
RP11-347C12.11	1.016997	1.017454	1.016849	0.96534	-0.0509	1	1
FGFR4	1.0239	1.024485	1.023709	0.96831	-0.0465	1	1
RP11-314P15.2	1.014413	1.013947	1.014564	1.0443	0.0625	1	1
TMEM45B	1.017871	1.017457	1.018005	1.03138	0.0446	1	1
LINC00652	1.018725	1.017478	1.019131	1.09454	0.1303	1	1
XX-FW83563B9.5	1.037765	1.03867	1.037471	0.96898	-0.0455	1	1
SDSL	1.26886	1.266787	1.269534	1.0103	0.0148	1	1
CTC-260F20.3	1.015277	1.013947	1.015709	1.12638	0.1717	1	1
KBTBD8	1.018735	1.017469	1.019146	1.096	0.1322	1	1
KLF1	1.014413	1.013947	1.014564	1.0443	0.0625	1	1
TMEM156	1.015274	1.013947	1.015705	1.1261	0.1713	1	1
RP11-705C15.4	1.015274	1.013947	1.015705	1.1261	0.1713	1	1
SYPL2	1.015273	1.013947	1.015704	1.126	0.1712	1	1
MPDU1	1.523826	1.524064	1.523749	0.9994	-0.0009	1	1
ANGPT1	1.024791	1.024502	1.024885	1.01562	0.0224	1	1
MIR9-3HG	1.029042	1.027939	1.0294	1.05231	0.0736	1	1
OLIG1	1.015271	1.013947	1.015701	1.12582	0.171	1	1
AC007192.6	1.014413	1.013947	1.014564	1.0443	0.0625	1	1

ZBED4	1.252839	1.25301	1.252784	0.99911	-0.0013	1	1
PRR34-AS1	1.015276	1.013947	1.015708	1.12632	0.1716	1	1
RP11-13E5.2	1.017003	1.017446	1.016858	0.96634	-0.0494	1	1
MGAM2	1.015273	1.013947	1.015704	1.12604	0.1713	1	1
AC074212.6	1.016137	1.01745	1.01571	0.90029	-0.1515	1	1
SLC35G3	1.015271	1.013947	1.015701	1.12581	0.171	1	1
LINC00216	1.015277	1.013947	1.015709	1.12638	0.1717	1	1
RP11-417B4.2	1.015271	1.013947	1.015701	1.12578	0.1709	1	1
AC114730.5	1.014413	1.013947	1.014564	1.0443	0.0625	1	1
SMTN	1.136798	1.137046	1.136718	0.9976	-0.0035	1	1
AC140542.2	1.015276	1.013947	1.015708	1.12629	0.1716	1	1
CTD-2291D10.1	1.016141	1.017474	1.015707	0.89888	-0.1538	1	1
RP11-219B4.5	1.015273	1.013947	1.015704	1.12604	0.1713	1	1
FREM3	1.015269	1.013947	1.015699	1.12565	0.1708	1	1
SON	4.027011	4.025124	4.027624	1.00083	0.0012	1	1
SMCO2	1.015271	1.013947	1.015701	1.12582	0.171	1	1
PCDHA4	1.074108	1.073769	1.074218	1.00609	0.0088	1	1
TBC1D26	1.020184	1.018892	1.020604	1.0906	0.1251	1	1
TEX29	1.015826	1.015398	1.015965	1.03684	0.0522	1	1
RP11-213G2.2	1.016688	1.015364	1.017118	1.11418	0.156	1	1
TMEM74	1.022744	1.022565	1.022803	1.01055	0.0151	1	1
IZUMO1	1.012388	1.011871	1.012556	1.05772	0.081	1	1
AP001172.3	1.013248	1.011871	1.013696	1.15372	0.2063	1	1
SLC6A1	1.014103	1.015373	1.01369	0.89053	-0.1673	1	1
PPP1R1B	1.025348	1.025945	1.025155	0.96955	-0.0446	1	1
KIAA2012	1.022753	1.022401	1.022867	1.02081	0.0297	1	1
GOLGA7	2.402239	2.403735	2.401753	0.99859	-0.002	1	1
RP11-315D16.2	1.017547	1.018897	1.017108	0.9053	-0.1435	1	1
RP11-384O8.1	1.014961	1.015363	1.014831	0.96535	-0.0509	1	1
LINC01354	1.013252	1.011871	1.013701	1.15413	0.2068	1	1
RP4-777L9.3	1.013253	1.011871	1.013702	1.15427	0.207	1	1
CTD-2537I9.18	1.016712	1.015389	1.017143	1.11396	0.1557	1	1
AL354828.2	1.012388	1.011871	1.012556	1.05772	0.081	1	1
C3AR1	1.026137	1.025869	1.026224	1.0137	0.0196	1	1
RP6-201G10.2	1.01325	1.011871	1.013698	1.15389	0.2065	1	1
UBOX5	1.063223	1.06455	1.062792	0.97276	-0.0398	1	1
CMPK1	2.567274	2.567221	2.567291	1.00005	######	1	1
DUOX2	1.015845	1.015384	1.015995	1.03973	0.0562	1	1
TTC16	1.012388	1.011871	1.012556	1.05772	0.081	1	1
TRAPPC3L	1.012388	1.011871	1.012556	1.05772	0.081	1	1
RP11-170N16.3	1.013245	1.011871	1.013692	1.15342	0.2059	1	1
TFF3	1.014974	1.015364	1.014847	0.96632	-0.0494	1	1
CFAP58	1.02617	1.0259	1.026258	1.01383	0.0198	1	1
PROSER2-AS1	1.013247	1.011871	1.013694	1.15355	0.2061	1	1
GPHA2	1.015828	1.015364	1.015979	1.03999	0.0566	1	1

SLC24A2	1.013247	1.011871	1.013694	1.15361	0.2061	1	1
RP11-352G18.2	1.016723	1.015365	1.017165	1.11713	0.1598	1	1
ALOX15B	1.013245	1.011871	1.013692	1.15343	0.2059	1	1
SLC9C2	1.013248	1.011871	1.013696	1.15377	0.2064	1	1
RP11-535A19.1	1.012388	1.011871	1.012556	1.05772	0.081	1	1
OVCH2	1.013245	1.011871	1.013692	1.15339	0.2059	1	1
AP000431.2	1.012388	1.011871	1.012556	1.05772	0.081	1	1
SPATA25	1.012388	1.011871	1.012556	1.05772	0.081	1	1
C2orf72	1.032268	1.032935	1.032051	0.97318	-0.0392	1	1
LINC00222	1.015822	1.015361	1.015972	1.03979	0.0563	1	1
RP11-706O15.7	1.018452	1.018906	1.018305	0.96819	-0.0466	1	1
RP11-73M18.10	1.017535	1.018864	1.017102	0.9066	-0.1415	1	1
AC107081.5	1.013249	1.011871	1.013696	1.15379	0.2064	1	1
VWA8-AS1	1.014963	1.015369	1.014831	0.96496	-0.0515	1	1
RP11-646J21.4	1.01325	1.011871	1.013698	1.1539	0.2065	1	1
CTA-992D9.8	1.013249	1.011871	1.013697	1.15383	0.2064	1	1
RP11-187O7.3	1.013246	1.011871	1.013692	1.15344	0.2059	1	1
AC011893.3	1.013246	1.011871	1.013693	1.15352	0.206	1	1
CTD-2015H6.3	1.164139	1.163169	1.164454	1.00788	0.0113	1	1
RP11-327P2.5	1.028734	1.029369	1.028528	0.97136	-0.0419	1	1
CD81-AS1	1.022721	1.022416	1.022821	1.01806	0.0258	1	1
CTD-2081C10.7	1.019263	1.018858	1.019395	1.02846	0.0405	1	1
CNGB1	1.014982	1.015384	1.014851	0.96536	-0.0509	1	1
RP11-88E10.4	1.012388	1.011871	1.012556	1.05772	0.081	1	1
DTHD1	1.013247	1.011871	1.013695	1.15363	0.2062	1	1
LYZ	1.013251	1.011871	1.0137	1.15409	0.2068	1	1
LINC01176	1.014966	1.015364	1.014836	0.96566	-0.0504	1	1
RP11-374A4.1	1.013251	1.011871	1.013699	1.15402	0.2067	1	1
RP11-135A1.2	1.016695	1.015373	1.017125	1.11394	0.1557	1	1
DNASE1L1	1.343458	1.341636	1.34405	1.00707	0.0102	1	1
AC008522.1	1.014989	1.015363	1.014868	0.96776	-0.0473	1	1
ZNF534	1.013248	1.011871	1.013696	1.15377	0.2064	1	1
RP9	1.519331	1.517783	1.519835	1.00396	0.0057	1	1
NXPE2	1.013245	1.011871	1.013692	1.15337	0.2059	1	1
RP3-339A18.6	1.013246	1.011871	1.013693	1.15346	0.206	1	1
ITGA11	1.014974	1.015398	1.014836	0.96347	-0.0537	1	1
RP1-253P7.4	1.014111	1.015384	1.013698	0.89042	-0.1674	1	1
BOK-AS1	1.013244	1.011871	1.01369	1.15326	0.2057	1	1
LMAN1L	1.012388	1.011871	1.012556	1.05772	0.081	1	1
TCTE1	1.017614	1.018891	1.017199	0.91041	-0.1354	1	1
CHKB-CPT1B	1.01411	1.015363	1.013703	0.89192	-0.165	1	1
RP11-389G6.5	1.012388	1.011871	1.012556	1.05772	0.081	1	1
RP1-137D17.2	1.01583	1.015384	1.015975	1.03846	0.0544	1	1
AC110615.1	1.013245	1.011871	1.013692	1.15339	0.2059	1	1
RP1L1	1.013244	1.011871	1.013691	1.1533	0.2058	1	1

C1orf167	1.016683	1.015365	1.017112	1.1137	0.1554	1	1
JAKMIP2-AS1	1.013248	1.011871	1.013696	1.15373	0.2063	1	1
CTB-51A17.1	1.013246	1.011871	1.013693	1.15345	0.206	1	1
C11orf52	1.014967	1.015363	1.014838	0.96584	-0.0501	1	1
ALS2CR11	1.012388	1.011871	1.012556	1.05772	0.081	1	1
RP5-1077I2.3	1.013247	1.011871	1.013694	1.15362	0.2062	1	1
LRIT3	1.014106	1.015365	1.013697	0.89145	-0.1658	1	1
LA16c-380H5.5	1.017551	1.01887	1.017122	0.90735	-0.1403	1	1
C20orf141	1.012388	1.011871	1.012556	1.05772	0.081	1	1
LINC00518	1.012388	1.011871	1.012556	1.05772	0.081	1	1
LINC01449	1.014975	1.015371	1.014847	0.96588	-0.0501	1	1
RP11-676J12.9	1.014104	1.015363	1.013695	0.89144	-0.1658	1	1
RP3-406A7.7	1.02621	1.02611	1.026242	1.00507	0.0073	1	1
CACNA2D3-AS1	1.01325	1.011871	1.013698	1.15394	0.2066	1	1
CTD-2287O16.4	1.015822	1.015369	1.01597	1.03912	0.0554	1	1
TSPYL6	1.013248	1.011871	1.013695	1.15369	0.2062	1	1
RP11-180M15.6	1.01588	1.01536	1.016049	1.04484	0.0633	1	1
RPGR	1.17528	1.173333	1.175914	1.01489	0.0213	1	1
RP11-463O9.9	1.012388	1.011871	1.012556	1.05772	0.081	1	1
RP4-549L20.3	1.050363	1.05067	1.050262	0.99195	-0.0117	1	1
FAM107B	1.282193	1.282298	1.282158	0.9995	-0.0007	1	1
RP11-25O10.2	1.013249	1.011871	1.013697	1.15383	0.2064	1	1
TACR3	1.019281	1.018871	1.019415	1.02879	0.0409	1	1
HLA-DRB5	1.013243	1.011871	1.013689	1.15317	0.2056	1	1
CCDC70	1.013246	1.011871	1.013694	1.15354	0.2061	1	1
CAGE1	1.013248	1.011871	1.013696	1.15376	0.2063	1	1
CH25H	1.013245	1.011871	1.013691	1.15335	0.2058	1	1
ZBP1	1.013245	1.011871	1.013692	1.15343	0.2059	1	1
ST20-MTHFS	1.013251	1.011871	1.0137	1.15407	0.2067	1	1
TMEM51-AS1	1.014967	1.01537	1.014836	0.96526	-0.051	1	1
CTD-3222D19.11	1.012388	1.011871	1.012556	1.05772	0.081	1	1
SRRM2-AS1	1.091827	1.092866	1.091489	0.98517	-0.0216	1	1
RP4-656G21.1	1.012388	1.011871	1.012556	1.05772	0.081	1	1
SERAC1	1.241809	1.240318	1.242293	1.00822	0.0118	1	1
HIST1H2BK	1.016678	1.01537	1.017103	1.11277	0.1542	1	1
RP11-46A10.5	1.016687	1.015374	1.017114	1.1132	0.1547	1	1
CTD-2012J19.3	1.038239	1.036439	1.038824	1.06545	0.0915	1	1
RP11-80A15.1	1.023545	1.022378	1.023924	1.06908	0.0964	1	1
RP11-773H22.4	1.013247	1.011871	1.013694	1.15356	0.2061	1	1
C10orf91	1.013246	1.011871	1.013694	1.15355	0.2061	1	1
RP11-227D13.5	1.013244	1.011871	1.013691	1.1533	0.2058	1	1
CTD-2235C13.3	1.015831	1.015371	1.01598	1.03967	0.0561	1	1
ITM2A	1.070874	1.071544	1.070656	0.98759	-0.018	1	1
TRPC3	1.014961	1.015365	1.01483	0.96514	-0.0512	1	1
RP11-170M17.1	1.012388	1.011871	1.012556	1.05772	0.081	1	1

LINC01024	1.022734	1.022353	1.022858	1.02259	0.0322	1	1
RP11-423H2.4	1.015828	1.015363	1.015979	1.04004	0.0566	1	1
RP11-217B7.2	1.012388	1.011871	1.012556	1.05772	0.081	1	1
SYT2	1.012388	1.011871	1.012556	1.05772	0.081	1	1
TMED7-TICAM2	1.015823	1.015369	1.01597	1.03906	0.0553	1	1
RP11-302F12.3	1.012388	1.011871	1.012556	1.05772	0.081	1	1
SCARNA2	1.015847	1.015363	1.016004	1.04167	0.0589	1	1
VSTM1	1.013246	1.011871	1.013692	1.15344	0.2059	1	1
DNM1P35	1.020174	1.018863	1.0206	1.09209	0.1271	1	1
LRRC61	1.013247	1.011871	1.013695	1.15363	0.2062	1	1
OVOL2	1.013252	1.011871	1.0137	1.15412	0.2068	1	1
HP09025	1.013257	1.011871	1.013708	1.15478	0.2076	1	1
AC015969.3	1.012388	1.011871	1.012556	1.05772	0.081	1	1
RP11-435O5.5	1.021861	1.022414	1.021682	0.96733	-0.0479	1	1
RP11-544A12.4	1.016279	1.015826	1.016426	1.03792	0.0537	1	1
RP11-672A2.5	1.016279	1.015826	1.016426	1.03792	0.0537	1	1
ASB7	1.157304	1.156834	1.157456	1.00397	0.0057	1	1
IL1RAPL2	1.017146	1.015826	1.017575	1.11049	0.1512	1	1
NTN3	1.017138	1.015826	1.017564	1.1098	0.1503	1	1
ABCA9-AS1	1.016279	1.015826	1.016426	1.03792	0.0537	1	1
RP11-497G19.7	1.017996	1.019319	1.017566	0.90926	-0.1372	1	1
MIR2052HG	1.020594	1.019317	1.021009	1.08759	0.1211	1	1
GALNT4	1.017142	1.015826	1.017569	1.11014	0.1507	1	1
RP1-310O13.7	1.020628	1.019362	1.02104	1.08663	0.1199	1	1
RP11-944L7.4	1.017141	1.015826	1.017569	1.11012	0.1507	1	1
RNASE7	1.016279	1.015826	1.016426	1.03792	0.0537	1	1
CTD-3131K8.3	1.017143	1.015826	1.017571	1.11028	0.1509	1	1
RP11-680F8.3	1.022312	1.022839	1.02214	0.96938	-0.0449	1	1
RP11-266K4.9	1.024047	1.022874	1.024428	1.06793	0.0948	1	1
RP11-426C22.5	1.018868	1.019349	1.018711	0.96704	-0.0484	1	1
DHX34	1.131438	1.132118	1.131218	0.99319	-0.0099	1	1
RP11-737O24.5	1.016279	1.015826	1.016426	1.03792	0.0537	1	1
FBXL19-AS1	1.035248	1.033361	1.035861	1.07492	0.1042	1	1
RP11-646I6.5	1.025781	1.026368	1.02559	0.97048	-0.0432	1	1
IL2RG	1.017996	1.01933	1.017563	0.90857	-0.1383	1	1
NOX5	1.017139	1.015826	1.017566	1.10994	0.1505	1	1
RP11-173C1.1	1.018014	1.019377	1.017571	0.90681	-0.1411	1	1
CALN1	1.018004	1.019346	1.017568	0.90807	-0.1391	1	1
NTMT1	1.798208	1.797892	1.79831	1.00052	0.0008	1	1
KCNH8	1.021486	1.022874	1.021035	0.9196	-0.1209	1	1
RP11-144G16.1	1.017141	1.015826	1.017569	1.11013	0.1507	1	1
ZNF57	1.055249	1.054556	1.055474	1.01682	0.0241	1	1
SUMO4	1.069912	1.068722	1.070299	1.02295	0.0327	1	1
LINC00971	1.017138	1.015826	1.017565	1.10986	0.1504	1	1
MUC6	1.017145	1.015826	1.017574	1.11044	0.1511	1	1

CTD-2240J17.2	1.017143	1.015826	1.017571	1.11027	0.1509	1	1
HEPH	1.327879	1.325871	1.328532	1.00816	0.0117	1	1
TTC4	1.073309	1.072108	1.0737	1.02208	0.0315	1	1
RP11-724N1.1	1.016279	1.015826	1.016426	1.03792	0.0537	1	1
IL20RA	1.018001	1.019345	1.017564	0.90792	-0.1394	1	1
AC092839.3	1.017141	1.015826	1.017568	1.11005	0.1506	1	1
UGP2	2.073137	2.07347	2.073029	0.99959	-0.0006	1	1
TMOD4	1.017145	1.015826	1.017574	1.11046	0.1512	1	1
RP11-552D8.1	1.017146	1.015826	1.017576	1.11054	0.1513	1	1
PMF1-BGLAP	1.022332	1.02286	1.02216	0.96939	-0.0449	1	1
ABCC9	1.016279	1.015826	1.016426	1.03792	0.0537	1	1
RP4-789D17.5	1.020607	1.019356	1.021014	1.08562	0.1185	1	1
RP11-47L3.1	1.018877	1.019393	1.018709	0.96473	-0.0518	1	1
GAGE10	1.017139	1.015826	1.017566	1.10994	0.1505	1	1
LINC00623	1.056912	1.058044	1.056544	0.97415	-0.0378	1	1
RP4-790G17.7	1.02063	1.019371	1.021039	1.08609	0.1191	1	1
RP11-488C13.5	1.049116	1.047517	1.049635	1.04457	0.0629	1	1
CATSPER4	1.018877	1.019335	1.018728	0.96863	-0.046	1	1
RP11-498C9.2	1.020597	1.019319	1.021013	1.08767	0.1212	1	1
LINC01571	1.020601	1.019324	1.021016	1.08755	0.1211	1	1
CTA-384D8.33	1.017148	1.015826	1.017578	1.1107	0.1515	1	1
ENTPD5	1.125263	1.12501	1.125346	1.00268	0.0039	1	1
CTB-33G10.6	1.016279	1.015826	1.016426	1.03792	0.0537	1	1
WNT5A-AS1	1.035346	1.033435	1.035968	1.07577	0.1054	1	1
SAG	1.01714	1.015826	1.017567	1.10998	0.1505	1	1
RP11-220C2.1	1.017146	1.015826	1.017575	1.11053	0.1513	1	1
HRASLS5	1.017142	1.015826	1.01757	1.11017	0.1508	1	1
RP11-404E16.1	1.017146	1.015826	1.017576	1.11054	0.1513	1	1
RP11-523H24.3	1.017136	1.015826	1.017562	1.10966	0.1501	1	1
C1orf194	1.039636	1.040481	1.039362	0.97235	-0.0404	1	1
RHBDD1	1.11659	1.117966	1.116143	0.98455	-0.0225	1	1
AC105052.1	1.017143	1.015826	1.017571	1.11028	0.1509	1	1
PRH1-PRR4	1.017147	1.015826	1.017576	1.11055	0.1513	1	1
SPATA32	1.016279	1.015826	1.016426	1.03792	0.0537	1	1
C19orf18	1.026679	1.026468	1.026748	1.01058	0.0152	1	1
PALM3	1.032685	1.033429	1.032443	0.97051	-0.0432	1	1
CTD-3028N15.3	1.01714	1.015826	1.017567	1.10998	0.1505	1	1
RP11-173D14.3	1.017137	1.015826	1.017563	1.10975	0.1502	1	1
NUDCD1	1.290389	1.290786	1.29026	0.99819	-0.0026	1	1
RP11-117L5.4	1.017146	1.015826	1.017575	1.11049	0.1512	1	1
SPDYE16	1.020627	1.019345	1.021044	1.08784	0.1215	1	1
RP11-617D20.2	1.017139	1.015826	1.017566	1.10992	0.1505	1	1
FBXW10	1.017138	1.015826	1.017565	1.10985	0.1504	1	1
HLA-DOA	1.035295	1.033443	1.035897	1.07337	0.1021	1	1
RP11-127O4.3	1.016279	1.015826	1.016426	1.03792	0.0537	1	1

CACNA1G	1.026684	1.026457	1.026758	1.01135	0.0163	1	1
AC112715.2	1.016279	1.015826	1.016426	1.03792	0.0537	1	1
RP5-1142A6.9	1.016279	1.015826	1.016426	1.03792	0.0537	1	1
PROK2	1.017138	1.015826	1.017565	1.10986	0.1504	1	1
HIST1H4B	1.01888	1.019333	1.018733	0.96898	-0.0455	1	1
ZNF705A	1.01714	1.015826	1.017568	1.11004	0.1506	1	1
ALOX15	1.016279	1.015826	1.016426	1.03792	0.0537	1	1
RP11-650J17.1	1.017142	1.015826	1.01757	1.11017	0.1508	1	1
RP11-490B18.6	1.023231	1.022848	1.023355	1.02217	0.0316	1	1
RP11-435D7.3	1.017139	1.015826	1.017566	1.10993	0.1505	1	1
GLP2R	1.016279	1.015826	1.016426	1.03792	0.0537	1	1
CGB3	1.016279	1.015826	1.016426	1.03792	0.0537	1	1
RP1-269M15.3	1.017141	1.015826	1.017569	1.11012	0.1507	1	1
TTLL6	1.016279	1.015826	1.016426	1.03792	0.0537	1	1
RP11-541P9.3	1.018872	1.019339	1.01872	0.96801	-0.0469	1	1
BARHL1	1.017146	1.015826	1.017575	1.11052	0.1512	1	1
MESP2	1.025789	1.026374	1.025598	0.97057	-0.0431	1	1
TM6SF1	1.019739	1.019344	1.019867	1.02703	0.0385	1	1
LINC01324	1.016279	1.015826	1.016426	1.03792	0.0537	1	1
AC073657.1	1.017142	1.015826	1.017569	1.11015	0.1508	1	1
PCDHA6	1.017139	1.015826	1.017566	1.10994	0.1505	1	1
CABP2	1.017137	1.015826	1.017563	1.10972	0.1502	1	1
VAV3-AS1	1.016279	1.015826	1.016426	1.03792	0.0537	1	1
RP1-221C16.8	1.017141	1.015826	1.017569	1.11011	0.1507	1	1
AS3MT	1.017141	1.015826	1.017568	1.11006	0.1506	1	1
LINC00972	1.017142	1.015826	1.01757	1.11021	0.1508	1	1
RP5-821D11.7	1.046567	1.04764	1.046218	0.97016	-0.0437	1	1
OMG	1.017139	1.015826	1.017566	1.10991	0.1504	1	1
CTA-150C2.13	1.01801	1.019349	1.017575	0.90828	-0.1388	1	1
NCR2	1.016279	1.015826	1.016426	1.03792	0.0537	1	1
LINC01451	1.020593	1.019355	1.020995	1.08474	0.1174	1	1
RNASE6	1.016279	1.015826	1.016426	1.03792	0.0537	1	1
RP11-554A11.8	1.016279	1.015826	1.016426	1.03792	0.0537	1	1
CTD-2588C8.8	1.020605	1.019346	1.021014	1.08618	0.1193	1	1
RP11-850A17.1	1.040516	1.040548	1.040506	0.99895	-0.0015	1	1
C1QTNF3-AMACR	1.017141	1.015826	1.017568	1.11008	0.1507	1	1
RP13-554M15.8	1.024945	1.022899	1.02561	1.11841	0.1614	1	1
CCDC190	1.018859	1.019336	1.018704	0.96732	-0.0479	1	1
CTD-3037G24.3	1.016279	1.015826	1.016426	1.03792	0.0537	1	1
AC098973.1	1.020626	1.019362	1.021037	1.0865	0.1197	1	1
AC023469.1	1.016279	1.015826	1.016426	1.03792	0.0537	1	1
AC005152.3	1.019731	1.019315	1.019866	1.02854	0.0406	1	1
RP11-611O2.1	1.024913	1.022899	1.025567	1.11654	0.159	1	1
RP11-713P17.4	1.017137	1.015826	1.017563	1.10975	0.1502	1	1
ADRA1B	1.017143	1.015826	1.017571	1.11024	0.1509	1	1

PRMT5-AS1	1.017146	1.015826	1.017576	1.11054	0.1513	1	1
CTD-3179P9.1	1.017143	1.015826	1.017571	1.11028	0.1509	1	1
RP11-118H15.1	1.017139	1.015826	1.017566	1.10994	0.1505	1	1
CTA-212A2.3	1.023203	1.022899	1.023302	1.0176	0.0252	1	1
WAC	1.962293	1.961102	1.96268	1.00164	0.0024	1	1
RPGRIP1	1.017137	1.015826	1.017563	1.10972	0.1502	1	1
CATSPER1	1.019746	1.019355	1.019873	1.02676	0.0381	1	1
CTB-5E10.3	1.024108	1.022888	1.024505	1.07062	0.0985	1	1
RP1-151B14.6	1.017136	1.015826	1.017562	1.10966	0.1501	1	1
LA16c-358B7.4	1.020602	1.019315	1.02102	1.08829	0.1221	1	1
RP11-274H24.1	1.016279	1.015826	1.016426	1.03792	0.0537	1	1
RP4-583P15.15	1.017139	1.015826	1.017565	1.1099	0.1504	1	1
SLC22A16	1.018001	1.019341	1.017566	0.90822	-0.1389	1	1
RP11-307C19.1	1.026725	1.026395	1.026832	1.01656	0.0237	1	1
C12orf54	1.018863	1.019341	1.018707	0.96723	-0.0481	1	1
TLR7	1.017297	1.015676	1.017824	1.13707	0.1853	1	1
RAD9B	1.028583	1.029784	1.028193	0.94656	-0.0792	1	1
AF038458.5	1.019889	1.019181	1.020118	1.04886	0.0688	1	1
RP11-905K4.1	1.017301	1.015676	1.01783	1.13743	0.1858	1	1
CERS3	1.019019	1.019189	1.018964	0.98828	-0.017	1	1
RP11-589B3.6	1.018167	1.019199	1.017832	0.92877	-0.1066	1	1
ZBTB9	1.023348	1.022681	1.023565	1.03901	0.0552	1	1
SLC10A5	1.019017	1.019169	1.018968	0.98951	-0.0152	1	1
C7orf77	1.017295	1.015676	1.017821	1.13688	0.1851	1	1
DCAF12L2	1.057986	1.057903	1.058013	1.0019	0.0027	1	1
RP11-275I14.4	1.017302	1.015676	1.01783	1.13747	0.1858	1	1
GOLGA6L1	1.017304	1.015676	1.017833	1.13766	0.1861	1	1
CTB-152G17.6	1.068523	1.068719	1.06846	0.99623	-0.0055	1	1
RP11-993B23.3	1.017295	1.015676	1.017821	1.13685	0.185	1	1
LSMEM2	1.017311	1.015676	1.017842	1.13821	0.1868	1	1
RP11-501C14.6	1.017299	1.015676	1.017827	1.13727	0.1856	1	1
RP11-56G10.2	1.017306	1.015676	1.017836	1.13781	0.1863	1	1
RP11-798K3.2	1.019871	1.01917	1.020098	1.04842	0.0682	1	1
LINC01402	1.017303	1.015676	1.017832	1.13758	0.186	1	1
MMRN2	1.021632	1.022698	1.021286	0.93782	-0.0926	1	1
BCL2A1	1.01902	1.019175	1.018969	0.98928	-0.0155	1	1
CDHR4	1.019898	1.019174	1.020134	1.05009	0.0705	1	1
RP1-127D3.4	1.016438	1.015676	1.016686	1.06444	0.0901	1	1
CTD-2192J16.11	1.017311	1.015676	1.017842	1.13821	0.1868	1	1
AC026904.1	1.017301	1.015676	1.017829	1.1374	0.1857	1	1
RP4-751H13.7	1.017305	1.015676	1.017834	1.1377	0.1861	1	1
LINC00346	1.020768	1.019207	1.021276	1.10772	0.1476	1	1
RP1-309F20.4	1.018169	1.019205	1.017833	0.92855	-0.107	1	1
PHF23	1.541804	1.540569	1.542206	1.00303	0.0044	1	1
RP11-458J1.1	1.076046	1.075569	1.076201	1.00836	0.012	1	1

RP11-134G8.5	1.025084	1.026214	1.024717	0.94291	-0.0848	1	1
GAS8-AS1	1.017301	1.015676	1.01783	1.13742	0.1858	1	1
RP11-378J18.9	1.025093	1.02626	1.024713	0.94107	-0.0876	1	1
EBF2	1.019052	1.019205	1.019003	0.98947	-0.0153	1	1
RP11-223P11.3	1.017303	1.015676	1.017832	1.13754	0.1859	1	1
AC005082.12	1.016438	1.015676	1.016686	1.06444	0.0901	1	1
DLX2	1.038073	1.036818	1.038481	1.04516	0.0637	1	1
RP11-307C19.2	1.024222	1.022707	1.024714	1.08837	0.1222	1	1
TFDP3	1.017306	1.015676	1.017836	1.13781	0.1863	1	1
ZP2	1.017297	1.015676	1.017824	1.13704	0.1853	1	1
RP11-446E24.4	1.018159	1.019189	1.017824	0.92887	-0.1065	1	1
LINC01562	1.021618	1.022701	1.021266	0.9368	-0.0942	1	1
FUT3	1.016438	1.015676	1.016686	1.06444	0.0901	1	1
SLC6A12	1.021657	1.022813	1.021281	0.93284	-0.1003	1	1
SLC25A30-AS1	1.017294	1.015676	1.01782	1.13684	0.185	1	1
ALDH8A1	1.022488	1.022691	1.022423	0.98817	-0.0172	1	1
C9orf66	1.020783	1.019199	1.021298	1.10932	0.1497	1	1
RRP1	1.285524	1.283287	1.286251	1.01046	0.015	1	1
BVES-AS1	1.016438	1.015676	1.016686	1.06444	0.0901	1	1
AIRN	1.017303	1.015676	1.017832	1.13755	0.1859	1	1
AC013480.2	1.017307	1.015676	1.017838	1.13793	0.1864	1	1
RP4-764O22.1	1.018163	1.019201	1.017826	0.9284	-0.1072	1	1
RGPD6	1.017303	1.015676	1.017832	1.13757	0.186	1	1
RP11-457M11.7	1.017306	1.015676	1.017836	1.13781	0.1863	1	1
TM4SF19-TCTEX1D2	1.019016	1.019171	1.018966	0.98929	-0.0155	1	1
RP11-93G5.1	1.019887	1.019199	1.02011	1.04744	0.0669	1	1
AC133785.1	1.019895	1.019202	1.02012	1.0478	0.0674	1	1
AKNAD1	1.022492	1.022706	1.022423	0.98751	-0.0181	1	1
RP11-548P2.2	1.017295	1.015676	1.017821	1.13685	0.185	1	1
RP11-20I23.7	1.017304	1.015676	1.017833	1.13763	0.186	1	1
RP11-181G12.4	1.016438	1.015676	1.016686	1.06444	0.0901	1	1
AKR1E2	1.029402	1.029686	1.029309	0.98732	-0.0184	1	1
RP11-162D9.3	1.017296	1.015676	1.017823	1.13701	0.1852	1	1
RP11-116O18.3	1.017303	1.015676	1.017833	1.13761	0.186	1	1
CCDC105	1.0173	1.015676	1.017828	1.13729	0.1856	1	1
NUDT5	2.102204	2.101349	2.102483	1.00103	0.0015	1	1
RP11-142M10.2	1.017304	1.015676	1.017834	1.13769	0.1861	1	1
MFSD6L	1.016438	1.015676	1.016686	1.06444	0.0901	1	1
RP11-426C22.8	1.016438	1.015676	1.016686	1.06444	0.0901	1	1
FCHSD1	1.110789	1.11089	1.110756	0.99878	-0.0018	1	1
RP11-374M1.3	1.017302	1.015676	1.01783	1.13747	0.1858	1	1
RP11-727F15.12	1.049298	1.047439	1.049902	1.05192	0.073	1	1
AC068490.2	1.019033	1.019196	1.01898	0.98872	-0.0164	1	1
RP11-155O18.6	1.017303	1.015676	1.017833	1.13761	0.186	1	1
AC084809.2	1.016438	1.015676	1.016686	1.06444	0.0901	1	1

VSIG2	1.018161	1.019189	1.017827	0.92904	-0.1062	1	1
ADAM20	1.0268	1.02619	1.026998	1.03083	0.0438	1	1
CTDP1	1.100268	1.100013	1.100351	1.00338	0.0049	1	1
NMUR1	1.017296	1.015676	1.017823	1.137	0.1852	1	1
OXT	1.018164	1.019192	1.01783	0.92904	-0.1062	1	1
SLC22A11	1.01904	1.019189	1.018992	0.98973	-0.0149	1	1
RP1-16A9.1	1.017297	1.015676	1.017824	1.13705	0.1853	1	1
PSD4	1.123622	1.124791	1.123242	0.98759	-0.018	1	1
JMJD7-PLA2G4B	1.020762	1.019162	1.021282	1.11064	0.1514	1	1
RP11-454C18.1	1.017297	1.015676	1.017824	1.13708	0.1853	1	1
RP4-564M11.2	1.017303	1.015676	1.017832	1.13754	0.1859	1	1
RP5-1057J7.7	1.016438	1.015676	1.016686	1.06444	0.0901	1	1
NOS2	1.024247	1.022697	1.024751	1.09053	0.125	1	1
NPTX2	1.043217	1.043794	1.04303	0.98254	-0.0254	1	1
ZCCHC16	1.017298	1.015676	1.017825	1.13711	0.1854	1	1
LRFN2	1.019021	1.019189	1.018966	0.9884	-0.0168	1	1
LINC00299	1.017303	1.015676	1.017832	1.13754	0.1859	1	1
RP11-89C3.3	1.017303	1.015676	1.017833	1.13761	0.186	1	1
MGAT5B	1.040599	1.040292	1.040698	1.0101	0.0145	1	1
CHRM3-AS2	1.019881	1.019208	1.0201	1.04642	0.0655	1	1
CFP	1.019026	1.019177	1.018977	0.9896	-0.0151	1	1
FAM26E	1.016438	1.015676	1.016686	1.06444	0.0901	1	1
RP1-156L9.1	1.018176	1.019206	1.017841	0.92892	-0.1064	1	1
RP11-544D21.2	1.017301	1.015676	1.017829	1.13741	0.1857	1	1
RP11-12D24.10	1.017299	1.015676	1.017827	1.13725	0.1856	1	1
RP11-433A10.3	1.017297	1.015676	1.017824	1.13705	0.1853	1	1
RP1-142L7.5	1.016438	1.015676	1.016686	1.06444	0.0901	1	1
AVP	1.017298	1.015676	1.017826	1.13716	0.1854	1	1
RP11-127L20.3	1.017297	1.015676	1.017824	1.13708	0.1853	1	1
ALDH3A1	1.022469	1.022672	1.022403	0.98816	-0.0172	1	1
WDFY4	1.017303	1.015676	1.017833	1.13761	0.186	1	1
TMPRSS6	1.017295	1.015676	1.017822	1.13693	0.1851	1	1
NKX1-2	1.017297	1.015676	1.017824	1.13707	0.1853	1	1
RP13-188A5.1	1.017298	1.015676	1.017825	1.13711	0.1854	1	1
REC114	1.016438	1.015676	1.016686	1.06444	0.0901	1	1
LINC01290	1.018172	1.019186	1.017842	0.92998	-0.1047	1	1
CTD-2026K11.2	1.017301	1.015676	1.01783	1.13742	0.1858	1	1
NPY2R	1.017297	1.015676	1.017824	1.13708	0.1853	1	1
GOLGA8J	1.017307	1.015676	1.017838	1.13793	0.1864	1	1
RP11-119D9.1	1.019028	1.019194	1.018974	0.98857	-0.0166	1	1
AC091814.3	1.017301	1.015676	1.017829	1.13738	0.1857	1	1
RP11-434H6.6	1.017297	1.015676	1.017824	1.13705	0.1853	1	1
RP11-9M16.2	1.016438	1.015676	1.016686	1.06444	0.0901	1	1
RP11-27M24.1	1.019881	1.019176	1.020111	1.04872	0.0686	1	1
RP11-506M13.3	1.019016	1.01917	1.018965	0.98931	-0.0155	1	1

KB-1460A1.5	1.099511	1.100054	1.099335	0.99281	-0.0104	1	1
RP13-714J12.1	1.025948	1.026202	1.025865	0.98713	-0.0187	1	1
COL5A3	1.023369	1.022708	1.023583	1.03855	0.0546	1	1
SLC35G4	1.016438	1.015676	1.016686	1.06444	0.0901	1	1
ANKRD61	1.017301	1.015676	1.017829	1.13739	0.1857	1	1
IL12RB2	1.017302	1.015676	1.01783	1.13747	0.1858	1	1
XXyac-YX65C7_A.2	1.018152	1.019162	1.017824	0.93017	-0.1044	1	1
RP5-902P8.10	1.016247	1.015517	1.016484	1.06233	0.0872	1	1
RP11-233G1.4	1.013663	1.011999	1.014204	1.18371	0.2433	1	1
HDAC11-AS1	1.012801	1.011999	1.013062	1.08856	0.1224	1	1
LINC00624	1.014529	1.01553	1.014204	0.9146	-0.1288	1	1
RP5-1186P10.2	1.014526	1.01552	1.014202	0.91511	-0.128	1	1
RP1-154K9.2	1.01452	1.015514	1.014197	0.91511	-0.128	1	1
RP11-410D17.2	1.013666	1.011999	1.014208	1.18408	0.2438	1	1
GATA4	1.014516	1.015493	1.014199	0.91648	-0.1258	1	1
CTC-435M10.3	1.013661	1.011999	1.014201	1.1835	0.2431	1	1
RP11-426L16.10	1.013657	1.011999	1.014196	1.18308	0.2425	1	1
SH3GL2	1.017988	1.019037	1.017647	0.927	-0.1094	1	1
AC022154.7	1.017971	1.019012	1.017633	0.92749	-0.1086	1	1
RP4-673M15.1	1.016258	1.015509	1.016502	1.06402	0.0895	1	1
RP13-580F15.2	1.016242	1.01552	1.016476	1.06162	0.0863	1	1
LA16c-OS12.2	1.018811	1.019025	1.018742	0.98512	-0.0216	1	1
AC010761.8	1.034382	1.033035	1.034819	1.054	0.0759	1	1
RP11-849F2.7	1.016241	1.015514	1.016478	1.06211	0.0869	1	1
LRRC63	1.017118	1.015529	1.017635	1.13565	0.1835	1	1
SOD2	1.465575	1.46528	1.46567	1.00084	0.0012	1	1
NPIPB15	1.020567	1.018985	1.021081	1.11041	0.1511	1	1
RP11-15H20.7	1.014517	1.015501	1.014197	0.91592	-0.1267	1	1
STK31	1.019693	1.018994	1.01992	1.04871	0.0686	1	1
CLSTN2-AS1	1.012801	1.011999	1.013062	1.08856	0.1224	1	1
CTD-2373H9.5	1.013662	1.011999	1.014203	1.18362	0.2432	1	1
PLB1	1.019687	1.019017	1.019905	1.0467	0.0658	1	1
ATP6V0A4	1.012801	1.011999	1.013062	1.08856	0.1224	1	1
TMEM212	1.016232	1.015496	1.016471	1.06291	0.088	1	1
GPT	1.017948	1.018988	1.01761	0.92745	-0.1087	1	1
RP11-872D17.4	1.013661	1.011999	1.014201	1.1835	0.2431	1	1
PCDH9-AS4	1.012801	1.011999	1.013062	1.08856	0.1224	1	1
RP11-266K4.13	1.014521	1.015511	1.014199	0.91542	-0.1275	1	1
AC005042.4	1.016254	1.015498	1.0165	1.0647	0.0905	1	1
RP1-118J21.5	1.01452	1.015509	1.014199	0.91556	-0.1273	1	1
RP11-130C19.3	1.013662	1.011999	1.014203	1.18364	0.2432	1	1
LINC01140	1.016249	1.015518	1.016486	1.06242	0.0874	1	1
ANKFN1	1.017111	1.015498	1.017636	1.13796	0.1865	1	1
GNA15	1.01366	1.011999	1.0142	1.18343	0.243	1	1
RP11-402L1.4	1.013657	1.011999	1.014196	1.18308	0.2425	1	1

MTRNR2L6	1.012801	1.011999	1.013062	1.08856	0.1224	1	1
HRCT1	1.012801	1.011999	1.013062	1.08856	0.1224	1	1
INPP5J	1.046479	1.047234	1.046233	0.97881	-0.0309	1	1
MMD2	1.012801	1.011999	1.013062	1.08856	0.1224	1	1
SLFN14	1.012801	1.011999	1.013062	1.08856	0.1224	1	1
RP11-552M14.1	1.012801	1.011999	1.013062	1.08856	0.1224	1	1
CTC-428G20.6	1.017105	1.015499	1.017628	1.13738	0.1857	1	1
RP11-111A21.1	1.015375	1.01549	1.015338	0.99018	-0.0142	1	1
RGPD2	1.029166	1.029548	1.029042	0.98285	-0.025	1	1
DRICH1	1.012801	1.011999	1.013062	1.08856	0.1224	1	1
PWRN3	1.01366	1.011999	1.0142	1.1834	0.2429	1	1
CACNA1B	1.014518	1.015498	1.0142	0.91625	-0.1262	1	1
RP11-618L22.1	1.02056	1.019033	1.021057	1.1063	0.1457	1	1
TMEM182	1.062795	1.061333	1.063271	1.0316	0.0449	1	1
AC022201.5	1.013659	1.011999	1.014198	1.18327	0.2428	1	1
REG1A	1.012801	1.011999	1.013062	1.08856	0.1224	1	1
GRM4	1.01366	1.011999	1.0142	1.18339	0.2429	1	1
RP11-304M2.5	1.013658	1.011999	1.014197	1.1832	0.2427	1	1
AQP11	1.088623	1.089151	1.088452	0.99216	-0.0114	1	1
ADAM11	1.037776	1.036509	1.038188	1.04598	0.0649	1	1
KCNA1	1.02927	1.029773	1.029106	0.9776	-0.0327	1	1
MIR142	1.012801	1.011999	1.013062	1.08856	0.1224	1	1
RP5-1142A6.10	1.013662	1.011999	1.014203	1.18366	0.2433	1	1
UGT2B28	1.013663	1.011999	1.014203	1.18369	0.2433	1	1
HAPLN2	1.020573	1.019032	1.021074	1.10726	0.147	1	1
RP11-644F5.10	1.018838	1.019039	1.018773	0.98601	-0.0203	1	1
ZDHHC23	1.021399	1.022501	1.02104	0.93509	-0.0968	1	1
DNAI2	1.013662	1.011999	1.014202	1.18358	0.2432	1	1
TMIGD3	1.013658	1.011999	1.014197	1.18319	0.2427	1	1
RP11-305L7.1	1.017998	1.019033	1.017661	0.92791	-0.1079	1	1
RP11-491F9.1	1.01797	1.018988	1.017639	0.92897	-0.1063	1	1
CTD-2196P11.2	1.01366	1.011999	1.014199	1.18337	0.2429	1	1
RP11-86H7.6	1.014522	1.015514	1.0142	0.91528	-0.1277	1	1
CTD-2215E18.1	1.013663	1.011999	1.014203	1.18369	0.2433	1	1
CYP17A1-AS1	1.014525	1.015494	1.01421	0.91717	-0.1247	1	1
AIRE	1.013664	1.011999	1.014205	1.18382	0.2435	1	1
RP11-6E9.5	1.012801	1.011999	1.013062	1.08856	0.1224	1	1
AC012358.7	1.012801	1.011999	1.013062	1.08856	0.1224	1	1
TMEM53	1.32312	1.324499	1.322671	0.99437	-0.0082	1	1
FADS6	1.017978	1.019043	1.017631	0.92587	-0.1111	1	1
RP11-337L12.1	1.013661	1.011999	1.014202	1.18354	0.2431	1	1
GJA8	1.01538	1.015504	1.01534	0.98942	-0.0154	1	1
RP11-77H9.6	1.013661	1.011999	1.014201	1.18348	0.243	1	1
RP11-318M2.3	1.019706	1.01899	1.019939	1.04994	0.0703	1	1
AP003774.6	1.018835	1.019023	1.018774	0.98693	-0.019	1	1

RP11-686D22.4	1.020533	1.019025	1.021023	1.10502	0.1441	1	1
RP11-138C9.1	1.01366	1.011999	1.0142	1.1834	0.2429	1	1
MGAT4C	1.047305	1.047129	1.047363	1.00497	0.0072	1	1
RP5-1057I20.4	1.020563	1.019027	1.021063	1.10697	0.1466	1	1
RP11-16C18.3	1.012801	1.011999	1.013062	1.08856	0.1224	1	1
RP11-325L7.1	1.016248	1.015494	1.016493	1.06449	0.0902	1	1
CTD-2026K11.4	1.013659	1.011999	1.014199	1.18333	0.2428	1	1
SOWAHD	1.01711	1.015517	1.017628	1.13609	0.1841	1	1
ZNF662	1.013659	1.011999	1.014198	1.18326	0.2428	1	1
RP11-16L21.7	1.012801	1.011999	1.013062	1.08856	0.1224	1	1
RP11-415I12.3	1.015375	1.015501	1.015334	0.98926	-0.0156	1	1
RP11-282I1.1	1.012801	1.011999	1.013062	1.08856	0.1224	1	1
RP11-1078H9.1	1.013662	1.011999	1.014202	1.18359	0.2432	1	1
CTB-11I22.1	1.013662	1.011999	1.014202	1.18359	0.2432	1	1
RP11-290F5.1	1.013664	1.011999	1.014205	1.18385	0.2435	1	1
AC007557.1	1.013662	1.011999	1.014203	1.18363	0.2432	1	1
CTD-2574D22.7	1.015382	1.015504	1.015342	0.98955	-0.0152	1	1
FOXL2	1.013666	1.011999	1.014208	1.18408	0.2438	1	1
CTB-73N10.1	1.013662	1.011999	1.014203	1.18362	0.2432	1	1
HIST1H3E	1.023117	1.022513	1.023313	1.03552	0.0503	1	1
AC092718.1	1.01366	1.011999	1.0142	1.18346	0.243	1	1
WDR72	1.036989	1.036816	1.037046	1.00624	0.009	1	1
PCDHGA2	1.013658	1.011999	1.014197	1.18318	0.2427	1	1
ZFP3	1.013659	1.011999	1.014199	1.18331	0.2428	1	1
DUX4	1.013659	1.011999	1.014199	1.18333	0.2428	1	1
CSNK1A1L	1.013663	1.011999	1.014204	1.18372	0.2433	1	1
RP11-532L16.3	1.014521	1.015491	1.014205	0.91699	-0.125	1	1
RP11-196G11.2	1.015381	1.015504	1.015341	0.9895	-0.0152	1	1
HPR	1.018819	1.018981	1.018766	0.98868	-0.0164	1	1
RP11-629O1.2	1.047373	1.047084	1.047467	1.00815	0.0117	1	1
RP11-326G21.1	1.028337	1.029462	1.027971	0.94942	-0.0749	1	1
RP11-77H9.5	1.014525	1.015508	1.014206	0.91602	-0.1265	1	1
RP1-292B18.3	1.017595	1.015934	1.018135	1.13809	0.1866	1	1
SHANK1	1.017594	1.015934	1.018133	1.13798	0.1865	1	1
RP11-576I22.2	1.01673	1.015934	1.016989	1.0662	0.0925	1	1
RP3-454G6.2	1.01933	1.019451	1.019291	0.99175	-0.012	1	1
RP11-42O4.2	1.017595	1.015934	1.018135	1.1381	0.1866	1	1
RP11-19O2.2	1.017594	1.015934	1.018134	1.13804	0.1865	1	1
RP11-442N24__B.1	1.017592	1.015934	1.018131	1.13788	0.1863	1	1
RP11-864G5.3	1.01673	1.015934	1.016989	1.0662	0.0925	1	1
ERMN	1.019327	1.019445	1.019288	0.99194	-0.0117	1	1
LHX6	1.017598	1.015934	1.018139	1.13836	0.187	1	1
CTC-542B22.2	1.0176	1.015934	1.018142	1.13854	0.1872	1	1
AC092625.1	1.017592	1.015934	1.018131	1.13789	0.1864	1	1
CTC-250I14.6	1.019313	1.019449	1.019268	0.99072	-0.0134	1	1

BPNT1	1.271318	1.270201	1.271682	1.00548	0.0079	1	1
EXOC3L4	1.017593	1.015934	1.018132	1.13792	0.1864	1	1
EIF4E1B	1.017599	1.015934	1.01814	1.13845	0.1871	1	1
RNF224	1.01673	1.015934	1.016989	1.0662	0.0925	1	1
TEPP	1.0496	1.047708	1.050216	1.05257	0.0739	1	1
NOS1AP	1.175395	1.174658	1.175635	1.00559	0.008	1	1
RP11-452I5.2	1.017595	1.015934	1.018135	1.13809	0.1866	1	1
TYMP	1.268561	1.269226	1.268345	0.99673	-0.0047	1	1
NPIPB5	1.147635	1.146556	1.147986	1.00975	0.014	1	1
RP11-275N1.1	1.017593	1.015934	1.018132	1.1379	0.1864	1	1
ACTL10	1.022771	1.022949	1.022714	0.98974	-0.0149	1	1
MAGEB10	1.017591	1.015934	1.018129	1.13774	0.1862	1	1
RP11-525A16.4	1.017591	1.015934	1.018129	1.13774	0.1862	1	1
RP11-1105G2.3	1.017594	1.015934	1.018133	1.13801	0.1865	1	1
RP11-650P15.1	1.017588	1.015934	1.018126	1.13753	0.1859	1	1
NKX3-2	1.018466	1.019455	1.018145	0.93266	-0.1006	1	1
XXyac-YX155B6.5	1.017592	1.015934	1.018131	1.13787	0.1863	1	1
CTC-327F10.5	1.01673	1.015934	1.016989	1.0662	0.0925	1	1
ZFX-AS1	1.017588	1.015934	1.018126	1.13753	0.1859	1	1
LINC01031	1.01673	1.015934	1.016989	1.0662	0.0925	1	1
AL450992.2	1.02277	1.022941	1.022715	0.99015	-0.0143	1	1
SDR9C7	1.01845	1.019449	1.018125	0.93193	-0.1017	1	1
THSD4-AS1	1.01673	1.015934	1.016989	1.0662	0.0925	1	1
ARHGAP45	1.037472	1.037082	1.037599	1.01394	0.02	1	1
AP000654.4	1.01673	1.015934	1.016989	1.0662	0.0925	1	1
RP4-533D7.5	1.019318	1.019449	1.019276	0.99111	-0.0129	1	1
TTLL11-IT1	1.018456	1.019454	1.018132	0.93207	-0.1015	1	1
CELA1	1.017592	1.015934	1.01813	1.13782	0.1863	1	1
TMEM229A	1.01673	1.015934	1.016989	1.0662	0.0925	1	1
ST7-AS1	1.024489	1.022981	1.024979	1.08696	0.1203	1	1
LY6G6C	1.017603	1.015934	1.018145	1.13874	0.1874	1	1
KCNJ10	1.019321	1.019483	1.019268	0.98892	-0.0161	1	1
OVGP1	1.021085	1.019449	1.021616	1.11145	0.1524	1	1
FAM3B	1.017596	1.015934	1.018137	1.13822	0.1868	1	1
SLC32A1	1.017592	1.015934	1.018131	1.13787	0.1863	1	1
CTD-2503I6.1	1.017591	1.015934	1.01813	1.1378	0.1862	1	1
MMP13	1.01673	1.015934	1.016989	1.0662	0.0925	1	1
RP11-793H13.10	1.017589	1.015934	1.018127	1.13759	0.186	1	1
RP11-363E6.3	1.021911	1.022946	1.021574	0.94021	-0.0889	1	1
MYBPC3	1.017601	1.015934	1.018143	1.13862	0.1873	1	1
IRAK3	1.026215	1.026505	1.02612	0.98546	-0.0211	1	1
RP11-320G10.1	1.03406	1.033531	1.034233	1.02093	0.0299	1	1
AC093843.1	1.017589	1.015934	1.018127	1.1376	0.186	1	1
RP11-284F21.10	1.034854	1.033485	1.035299	1.05417	0.0761	1	1
RP11-109J4.1	1.01673	1.015934	1.016989	1.0662	0.0925	1	1

CTD-2020K17.3	1.039249	1.040629	1.0388	0.95498	-0.0665	1	1
RP11-769O8.1	1.017593	1.015934	1.018132	1.13792	0.1864	1	1
RP11-816J6.3	1.026248	1.026532	1.026156	0.98586	-0.0205	1	1
KLHL15	1.146705	1.146517	1.146767	1.0017	0.0025	1	1
GPRC6A	1.019312	1.019448	1.019268	0.99074	-0.0134	1	1
CTC-453G23.4	1.017591	1.015934	1.01813	1.13779	0.1862	1	1
LRTM2	1.017595	1.015934	1.018135	1.13809	0.1866	1	1
RP11-313A24.1	1.017589	1.015934	1.018127	1.13762	0.186	1	1
AC009487.4	1.017599	1.015934	1.018139	1.1384	0.187	1	1
RP11-13A1.1	1.017589	1.015934	1.018127	1.13764	0.186	1	1
AMY2B	1.040075	1.040564	1.039915	0.984	-0.0233	1	1
PON1	1.018461	1.019447	1.018141	0.93282	-0.1003	1	1
CTD-2033D15.3	1.017594	1.015934	1.018134	1.13806	0.1866	1	1
CTB-133P21.1	1.017595	1.015934	1.018135	1.1381	0.1866	1	1
XKR7	1.023643	1.022977	1.023859	1.03839	0.0544	1	1
RP11-982M15.2	1.021058	1.019476	1.021572	1.10763	0.1475	1	1
RP11-297J22.1	1.037477	1.037045	1.037618	1.01548	0.0222	1	1
RP11-802D6.1	1.025401	1.02659	1.025014	0.94074	-0.0881	1	1
CNBD1	1.018454	1.019448	1.018131	0.93227	-0.1012	1	1
RP11-175K6.1	1.01673	1.015934	1.016989	1.0662	0.0925	1	1
RALY-AS1	1.052134	1.051114	1.052466	1.02646	0.0377	1	1
KCNH6	1.020196	1.019453	1.020438	1.05059	0.0712	1	1
ELANE	1.017591	1.015934	1.01813	1.13779	0.1862	1	1
RP13-512J5.1	1.017587	1.015934	1.018124	1.13742	0.1858	1	1
MMP25	1.017591	1.015934	1.018129	1.13774	0.1862	1	1
RP11-346C20.4	1.01845	1.019428	1.018132	0.93328	-0.0996	1	1
CTD-2281M20.1	1.017593	1.015934	1.018132	1.1379	0.1864	1	1
RTBDN	1.023675	1.022972	1.023903	1.04051	0.0573	1	1
PLXNB3	1.017591	1.015934	1.018129	1.13776	0.1862	1	1
CTB-113P19.1	1.019335	1.019458	1.019295	0.99167	-0.0121	1	1
AC006129.2	1.01673	1.015934	1.016989	1.0662	0.0925	1	1
RP11-112J3.15	1.018449	1.019434	1.018129	0.93284	-0.1003	1	1
RP11-9N12.2	1.021062	1.019458	1.021584	1.10927	0.1496	1	1
RP11-379L18.1	1.017591	1.015934	1.018129	1.13774	0.1862	1	1
RP11-252E2.1	1.018462	1.019444	1.018143	0.93309	-0.0999	1	1
RP11-63M22.2	1.018454	1.01944	1.018133	0.9328	-0.1004	1	1
CD6	1.017598	1.015934	1.018139	1.13836	0.187	1	1
PLCE1-AS1	1.01673	1.015934	1.016989	1.0662	0.0925	1	1
LRRC71	1.019315	1.019447	1.019272	0.99098	-0.0131	1	1
RP11-293E1.2	1.017593	1.015934	1.018132	1.13794	0.1864	1	1
AP001058.3	1.01846	1.019441	1.018141	0.93312	-0.0999	1	1
SEC31B	1.134487	1.135756	1.134074	0.98761	-0.018	1	1
C1orf115	1.036645	1.037063	1.036509	0.98506	-0.0217	1	1
RP11-160H22.5	1.01673	1.015934	1.016989	1.0662	0.0925	1	1
RP11-546B15.1	1.017593	1.015934	1.018132	1.13791	0.1864	1	1

SLC25A31	1.017588	1.015934	1.018126	1.13753	0.1859	1	1
CTC-228N24.2	1.017587	1.015934	1.018124	1.13745	0.1858	1	1
DLGAP1-AS4	1.019331	1.019447	1.019293	0.99207	-0.0115	1	1
NR1I2	1.0176	1.015934	1.018141	1.13848	0.1871	1	1
CTC-490G23.6	1.018468	1.019469	1.018143	0.93189	-0.1018	1	1
PERM1	1.020192	1.019449	1.020433	1.0506	0.0712	1	1
RP11-68L18.1	1.028047	1.026542	1.028537	1.07516	0.1046	1	1
BPIFB1	1.019329	1.019433	1.019296	0.99293	-0.0102	1	1
AC010976.2	1.01673	1.015934	1.016989	1.0662	0.0925	1	1
PWRN4	1.017594	1.015934	1.018133	1.13801	0.1865	1	1
RP11-688G15.3	1.017593	1.015934	1.018133	1.13797	0.1865	1	1
CTD-3099C6.7	1.017589	1.015934	1.018127	1.13763	0.186	1	1
PDE6B	1.155428	1.153913	1.155921	1.01304	0.0187	1	1
RP11-10J21.6	1.019312	1.019428	1.019275	0.9921	-0.0114	1	1
RP11-292D4.1	1.01673	1.015934	1.016989	1.0662	0.0925	1	1
ZGPAT	1.017593	1.015934	1.018133	1.13797	0.1865	1	1
CDK11B	1.346907	1.346948	1.346893	0.99984	-0.0002	1	1
RP11-94I2.4	1.026263	1.026542	1.026172	0.98603	-0.0203	1	1
AC079776.1	1.019325	1.017512	1.019915	1.1372	0.1855	1	1
OCLM	1.019316	1.017512	1.019902	1.13649	0.1846	1	1
RP11-714M23.2	1.017592	1.017512	1.017618	1.00609	0.0088	1	1
CSGALNACT1	1.020201	1.021043	1.019927	0.94696	-0.0786	1	1
RP11-66B24.1	1.018452	1.017512	1.018758	1.07116	0.0992	1	1
UBXN7-AS1	1.01846	1.017512	1.018768	1.07172	0.0999	1	1
CTA-503F6.2	1.018465	1.017512	1.018775	1.07216	0.1005	1	1
DCDC2C	1.018456	1.017512	1.018763	1.07142	0.0995	1	1
AC130469.2	1.019345	1.017512	1.019941	1.13871	0.1874	1	1
ZBTB26	1.153696	1.151637	1.154365	1.01799	0.0257	1	1
POU4F1	1.019352	1.017512	1.01995	1.13924	0.1881	1	1
ZNF41	1.042683	1.04218	1.042847	1.01581	0.0226	1	1
RP11-461A8.5	1.01845	1.017512	1.018754	1.07095	0.0989	1	1
RP11-192H23.5	1.018452	1.017512	1.018758	1.07116	0.0992	1	1
AC092614.2	1.019323	1.017512	1.019911	1.13702	0.1853	1	1
LRP3	1.200107	1.200759	1.199895	0.9957	-0.0062	1	1
FGF14	1.048873	1.04918	1.048774	0.99175	-0.012	1	1
RP11-607P23.1	1.017592	1.017512	1.017618	1.00609	0.0088	1	1
RP11-574K11.29	1.019317	1.017512	1.019904	1.13663	0.1848	1	1
SLC51B	1.021033	1.021031	1.021034	1.00014	0.0002	1	1
KCNE1B	1.017592	1.017512	1.017618	1.00609	0.0088	1	1
C10orf62	1.019343	1.017512	1.019938	1.13855	0.1872	1	1
RP11-1100L3.8	1.018453	1.017512	1.018759	1.07124	0.0993	1	1
RP11-588H23.3	1.019322	1.017512	1.01991	1.13697	0.1852	1	1
CTD-2521M24.8	1.019313	1.017512	1.019898	1.13626	0.1843	1	1
RP11-1293J14.1	1.017592	1.017512	1.017618	1.00609	0.0088	1	1
NCK1-AS1	1.156316	1.155119	1.156705	1.01022	0.0147	1	1

GLS2	1.028872	1.028122	1.029116	1.03533	0.0501	1	1
CTC-453G23.8	1.018463	1.017512	1.018772	1.07198	0.1003	1	1
CYP2C18	1.017592	1.017512	1.017618	1.00609	0.0088	1	1
RP11-483L5.1	1.020167	1.021005	1.019895	0.94716	-0.0783	1	1
NOX1	1.017592	1.017512	1.017618	1.00609	0.0088	1	1
CTC-492K19.7	1.028025	1.028195	1.027969	0.99198	-0.0116	1	1
KB-1471A8.1	1.019312	1.017512	1.019897	1.13621	0.1842	1	1
RP11-670E13.6	1.064356	1.063559	1.064615	1.01661	0.0238	1	1
NAV2-AS4	1.019314	1.017512	1.0199	1.1364	0.1845	1	1
AP000302.58	1.017592	1.017512	1.017618	1.00609	0.0088	1	1
AC010884.1	1.027138	1.028101	1.026824	0.95458	-0.0671	1	1
AC005387.3	1.018451	1.017512	1.018756	1.07105	0.099	1	1
CLPSL1	1.017592	1.017512	1.017618	1.00609	0.0088	1	1
RP5-1182A14.5	1.018454	1.017512	1.018761	1.07131	0.0994	1	1
NICN1	1.018462	1.017512	1.018771	1.0719	0.1002	1	1
RP5-940J5.3	1.018455	1.017512	1.018762	1.07138	0.0995	1	1
LINC01347	1.017592	1.017512	1.017618	1.00609	0.0088	1	1
PTGER4P2-CDK2AP2P2	1.023635	1.024528	1.023345	0.95175	-0.0713	1	1
RP11-182J1.3	1.022762	1.021008	1.023333	1.11069	0.1514	1	1
KRT27	1.018451	1.017512	1.018756	1.07105	0.099	1	1
AC004854.4	1.022771	1.021029	1.023337	1.10976	0.1503	1	1
AF121898.3	1.018459	1.017512	1.018767	1.07168	0.0999	1	1
AC034110.1	1.018454	1.017512	1.018761	1.07131	0.0994	1	1
RP11-755B10.3	1.019323	1.017512	1.019912	1.13706	0.1853	1	1
GRID2IP	1.019335	1.017512	1.019928	1.13797	0.1865	1	1
SMTNL1	1.021991	1.021066	1.022292	1.05818	0.0816	1	1
RP1-45I4.3	1.021921	1.021025	1.022212	1.05648	0.0793	1	1
FLJ40288	1.019319	1.017512	1.019907	1.13675	0.1849	1	1
GPR88	1.019337	1.017512	1.01993	1.13807	0.1866	1	1
GPR35	1.021929	1.021013	1.022227	1.0578	0.0811	1	1
RP11-278A23.4	1.022798	1.021004	1.023381	1.11314	0.1546	1	1
RP11-351A20.1	1.019323	1.017512	1.019912	1.13705	0.1853	1	1
RP11-91K8.5	1.020166	1.021013	1.019891	0.9466	-0.0792	1	1
MIR3681HG	1.027958	1.028132	1.027901	0.99179	-0.0119	1	1
RP11-553A21.3	1.02452	1.024565	1.024505	0.99753	-0.0036	1	1
C8G	1.018449	1.017512	1.018753	1.07089	0.0988	1	1
AE000658.22	1.01934	1.017512	1.019934	1.13834	0.1869	1	1
IL2RB	1.017592	1.017512	1.017618	1.00609	0.0088	1	1
YEATS2-AS1	1.018454	1.017512	1.018761	1.07132	0.0994	1	1
RP11-295I5.4	1.018452	1.017512	1.018757	1.07112	0.0991	1	1
CTD-2302E22.4	1.02711	1.028094	1.02679	0.95361	-0.0685	1	1
DCST2	1.03055	1.031671	1.030186	0.95311	-0.0693	1	1
ADGRF1	1.018452	1.017512	1.018757	1.07111	0.0991	1	1
ECM2	1.021049	1.021014	1.021061	1.0022	0.0032	1	1
RP11-554A11.7	1.017592	1.017512	1.017618	1.00609	0.0088	1	1

RP11-147L13.8	1.028891	1.028202	1.029115	1.0324	0.046	1	1
SCAF8	1.28279	1.282248	1.282966	1.00254	0.0037	1	1
AC004895.4	1.02019	1.021012	1.019923	0.94817	-0.0768	1	1
RP11-146F11.5	1.01846	1.017512	1.018768	1.07172	0.0999	1	1
CTD-2616J11.11	1.019343	1.017512	1.019939	1.13859	0.1872	1	1
RP11-638L3.1	1.018452	1.017512	1.018757	1.07112	0.0991	1	1
RP11-513N24.1	1.019315	1.017512	1.019902	1.13646	0.1846	1	1
AC011738.4	1.018455	1.017512	1.018762	1.07138	0.0995	1	1
RP11-542B15.1	1.018456	1.017512	1.018762	1.07142	0.0995	1	1
ADCYAP1R1	1.02106	1.021046	1.021065	1.00089	0.0013	1	1
RASSF10	1.020181	1.021034	1.019904	0.94624	-0.0797	1	1
RP11-588G21.2	1.021052	1.021039	1.021057	1.00085	0.0012	1	1
LA16c-358B7.3	1.024519	1.024575	1.024501	0.99702	-0.0043	1	1
ADPGK-AS1	1.019315	1.017512	1.019901	1.13644	0.1845	1	1
RP11-561B11.2	1.019309	1.017512	1.019894	1.13602	0.184	1	1
RP1-118J21.25	1.017592	1.017512	1.017618	1.00609	0.0088	1	1
CTC-487M23.5	1.021038	1.021016	1.021045	1.0014	0.002	1	1
RP5-955M13.4	1.01845	1.017512	1.018755	1.07097	0.0989	1	1
SPTB	1.021918	1.021009	1.022213	1.0573	0.0804	1	1
RP11-717F1.2	1.028861	1.028136	1.029096	1.03413	0.0484	1	1
C17orf77	1.018459	1.017512	1.018767	1.0717	0.0999	1	1
CTD-2527I21.15	1.018458	1.017512	1.018765	1.07158	0.0997	1	1
OR7D2	1.019332	1.017512	1.019923	1.1377	0.1861	1	1
RP11-286E11.2	1.020201	1.021073	1.019918	0.94517	-0.0813	1	1
RP11-552F3.10	1.01845	1.017512	1.018754	1.07095	0.0989	1	1
RP1-151F17.1	1.018451	1.017512	1.018756	1.07103	0.099	1	1
KLHDC7B	1.017592	1.017512	1.017618	1.00609	0.0088	1	1
FGF4	1.018455	1.017512	1.018762	1.07137	0.0995	1	1
RP1-273N12.4	1.018449	1.017512	1.018753	1.07089	0.0988	1	1
IL16	1.017592	1.017512	1.017618	1.00609	0.0088	1	1
LINC00384	1.019339	1.017512	1.019933	1.13824	0.1868	1	1
SNRK	1.116336	1.116833	1.116174	0.99435	-0.0082	1	1
C6orf3	1.071211	1.070263	1.071518	1.01786	0.0255	1	1
PDZK1	1.022818	1.021046	1.023394	1.11156	0.1526	1	1
RP11-723O4.2	1.01845	1.017512	1.018755	1.07097	0.0989	1	1
AKR1D1	1.019323	1.017512	1.019911	1.13703	0.1853	1	1
CTD-3220F14.3	1.023655	1.02457	1.023358	0.95068	-0.073	1	1
IFFO1	1.124238	1.1235	1.124478	1.00791	0.0114	1	1
RP11-461L13.5	1.018452	1.017512	1.018758	1.07118	0.0992	1	1
MKRN3-AS1	1.019346	1.017512	1.019942	1.13879	0.1875	1	1
C8orf44-SGK3	1.023672	1.024575	1.023379	0.95131	-0.072	1	1
RP11-222G7.2	1.01931	1.017512	1.019894	1.13605	0.184	1	1
TLR3	1.023688	1.024611	1.023388	0.95032	-0.0735	1	1
ASCL2	1.031434	1.031559	1.031393	0.99476	-0.0076	1	1
SNAP25-AS1	1.022772	1.021006	1.023347	1.11145	0.1524	1	1

AOX1	1.018455	1.017512	1.018762	1.07139	0.0995	1	1
RP11-403A21.1	1.021035	1.021005	1.021045	1.00193	0.0028	1	1
RP11-178L8.3	1.017592	1.017512	1.017618	1.00609	0.0088	1	1
LINC01098	1.018452	1.017512	1.018757	1.07113	0.0991	1	1
RP11-392P7.7	1.018456	1.017512	1.018763	1.07143	0.0995	1	1
MAP1LC3C	1.01931	1.017512	1.019895	1.1361	0.1841	1	1
RP11-378A12.1	1.018451	1.017512	1.018756	1.07105	0.099	1	1
AC142293.3	1.017592	1.017512	1.017618	1.00609	0.0088	1	1
FAM95C	1.019344	1.017512	1.019939	1.13863	0.1873	1	1
ADRA1A	1.01845	1.017512	1.018754	1.07095	0.0989	1	1
MED26	1.100779	1.098839	1.10141	1.02602	0.0371	1	1
SRPRB	1.303401	1.303238	1.303454	1.00071	0.001	1	1
GRID1	1.022782	1.021071	1.023338	1.10756	0.1474	1	1
BEST2	1.01846	1.017512	1.018768	1.07175	0.1	1	1
RP11-829H16.3	1.017592	1.017512	1.017618	1.00609	0.0088	1	1
SDS	1.018454	1.017512	1.01876	1.07128	0.0993	1	1
OR13C3	1.018458	1.017512	1.018765	1.07157	0.0997	1	1
GOLGA7B	1.027145	1.02809	1.026837	0.95541	-0.0658	1	1
RGPD8	1.018463	1.017512	1.018772	1.07195	0.1002	1	1
RP11-762I7.5	1.018451	1.017512	1.018756	1.07105	0.099	1	1
RP4-680D5.9	1.018453	1.017512	1.018759	1.07124	0.0993	1	1
C8A	1.016417	1.014833	1.016932	1.14151	0.1909	1	1
AC106801.1	1.015555	1.014833	1.01579	1.06452	0.0902	1	1
RP11-665C16.6	1.016418	1.014833	1.016934	1.14168	0.1912	1	1
RP11-478K15.6	1.016422	1.014833	1.016938	1.14195	0.1915	1	1
LINC01314	1.017281	1.01834	1.016936	0.92349	-0.1148	1	1
SAA2	1.016414	1.014833	1.016928	1.14131	0.1907	1	1
RP11-93B14.4	1.01642	1.014833	1.016936	1.14182	0.1913	1	1
TAS1R3	1.019003	1.018351	1.019215	1.04709	0.0664	1	1
GAS5-AS1	1.016419	1.014833	1.016935	1.14176	0.1913	1	1
CCDC85A	1.03288	1.032404	1.033035	1.01946	0.0278	1	1
CD38	1.01727	1.018328	1.016926	0.92354	-0.1148	1	1
RP4-758J18.13	1.02161	1.021901	1.021516	0.98242	-0.0256	1	1
RP11-120J1.1	1.015555	1.014833	1.01579	1.06452	0.0902	1	1
ACTL8	1.016412	1.014833	1.016926	1.14114	0.1905	1	1
RP4-724E16.2	1.01641	1.014833	1.016923	1.14096	0.1902	1	1
NIM1K	1.025936	1.025387	1.026115	1.02868	0.0408	1	1
RP11-77I22.2	1.016417	1.014833	1.016932	1.14151	0.1909	1	1
RP5-842K16.2	1.015555	1.014833	1.01579	1.06452	0.0902	1	1
KCNE3	1.025971	1.025402	1.026156	1.02969	0.0422	1	1
RNF39	1.015555	1.014833	1.01579	1.06452	0.0902	1	1
RP11-260M19.2	1.021617	1.021921	1.021519	0.98166	-0.0267	1	1
RP11-29H23.4	1.015555	1.014833	1.01579	1.06452	0.0902	1	1
LINC00645	1.019024	1.018339	1.019247	1.04949	0.0697	1	1
GGT1	1.023347	1.021876	1.023826	1.0891	0.1231	1	1

RP11-280H21.1	1.01642	1.014833	1.016936	1.14184	0.1914	1	1
RP11-763F8.1	1.019022	1.018342	1.019243	1.0491	0.0691	1	1
SLC7A1	1.184849	1.183697	1.185223	1.00831	0.0119	1	1
CTD-2245F17.6	1.019878	1.018319	1.020384	1.11273	0.1541	1	1
RP11-463D19.2	1.017284	1.018369	1.016931	0.92171	-0.1176	1	1
AC005329.7	1.016418	1.014833	1.016933	1.14162	0.1911	1	1
AC241377.1	1.015555	1.014833	1.01579	1.06452	0.0902	1	1
TMPRSS3	1.015555	1.014833	1.01579	1.06452	0.0902	1	1
USP29	1.016415	1.014833	1.016929	1.14134	0.1907	1	1
RP11-21K12.3	1.017274	1.01835	1.016924	0.9223	-0.1167	1	1
CTD-2024P10.2	1.028568	1.028837	1.028481	0.98766	-0.0179	1	1
RP11-834C11.6	1.016415	1.014833	1.016929	1.14138	0.1908	1	1
RP11-930P14.1	1.016417	1.014833	1.016932	1.14152	0.191	1	1
PCNX3	1.034548	1.035942	1.034096	0.94863	-0.0761	1	1
PCDHGB2	1.01642	1.014833	1.016936	1.1418	0.1913	1	1
AC131097.4	1.016411	1.014833	1.016924	1.14101	0.1903	1	1
GLRA3	1.01642	1.014833	1.016936	1.1418	0.1913	1	1
PADI1	1.015555	1.014833	1.01579	1.06452	0.0902	1	1
RP11-756G20.1	1.016413	1.014833	1.016927	1.14118	0.1905	1	1
ZNF350-AS1	1.016426	1.014833	1.016944	1.14235	0.192	1	1
RP11-51J9.4	1.016415	1.014833	1.01693	1.14138	0.1908	1	1
IKZF3	1.021582	1.021849	1.021495	0.98377	-0.0236	1	1
SERF1B	1.023317	1.021869	1.023788	1.08776	0.1214	1	1
RP5-1180C18.1	1.017278	1.018338	1.016933	0.92341	-0.115	1	1
FAM153B	1.016418	1.014833	1.016933	1.14162	0.1911	1	1
RP11-849H4.4	1.015555	1.014833	1.01579	1.06452	0.0902	1	1
RP11-296A18.6	1.01642	1.014833	1.016936	1.14183	0.1914	1	1
RP11-212I21.4	1.024206	1.02538	1.023825	0.93871	-0.0913	1	1
CST5	1.015555	1.014833	1.01579	1.06452	0.0902	1	1
SLC22A2	1.016412	1.014833	1.016926	1.14114	0.1905	1	1
CRYAA	1.015555	1.014833	1.01579	1.06452	0.0902	1	1
CTD-2192J16.26	1.017279	1.018356	1.016929	0.92223	-0.1168	1	1
ADGRL4	1.044053	1.042851	1.044444	1.03715	0.0526	1	1
RP11-374M1.4	1.01729	1.018391	1.016932	0.92068	-0.1192	1	1
CTD-3193O13.1	1.016413	1.014833	1.016927	1.14118	0.1905	1	1
C9orf139	1.019	1.018339	1.019215	1.04773	0.0673	1	1
RP11-74E22.8	1.022473	1.021874	1.022668	1.03629	0.0514	1	1
AL354828.1	1.017278	1.018355	1.016928	0.92226	-0.1168	1	1
RP11-319F12.2	1.018161	1.018391	1.018085	0.98337	-0.0242	1	1
RP11-231E6.1	1.016419	1.014833	1.016934	1.14171	0.1912	1	1
SERPINC1	1.017282	1.018354	1.016934	0.9226	-0.1162	1	1
TEX14	1.019857	1.01834	1.02035	1.1096	0.15	1	1
RP11-849I19.1	1.016417	1.014833	1.016932	1.14152	0.191	1	1
TCF15	1.01989	1.018329	1.020398	1.11289	0.1543	1	1
AP000769.7	1.019015	1.018329	1.019239	1.04964	0.0699	1	1

CTD-3088G3.8	1.015555	1.014833	1.01579	1.06452	0.0902	1	1
NCMAP	1.019863	1.018329	1.020361	1.11086	0.1517	1	1
AC022182.3	1.017277	1.018336	1.016933	0.92349	-0.1148	1	1
KPNA7	1.015555	1.014833	1.01579	1.06452	0.0902	1	1
TNK2-AS1	1.015555	1.014833	1.01579	1.06452	0.0902	1	1
C11orf16	1.017273	1.018322	1.016932	0.92414	-0.1138	1	1
AGXT	1.016416	1.014833	1.016931	1.1415	0.1909	1	1
RP11-384F7.2	1.018131	1.01832	1.01807	0.98631	-0.0199	1	1
DYSF	1.015555	1.014833	1.01579	1.06452	0.0902	1	1
CTD-2013N24.2	1.01642	1.014833	1.016936	1.14179	0.1913	1	1
RP13-349O20.2	1.021602	1.021827	1.021529	0.98637	-0.0198	1	1
RP4-677H15.4	1.016411	1.014833	1.016924	1.14103	0.1903	1	1
AKT1	1.505333	1.504327	1.50566	1.00264	0.0038	1	1
FRMPD1	1.016412	1.014833	1.016926	1.14114	0.1905	1	1
RP4-622L5.7	1.039823	1.039587	1.039899	1.00788	0.0113	1	1
RP11-649A16.1	1.016417	1.014833	1.016932	1.14152	0.191	1	1
LINC00244	1.016422	1.014833	1.016939	1.14201	0.1916	1	1
AGFG2	1.065708	1.064109	1.066228	1.03306	0.0469	1	1
RP11-111J6.2	1.019883	1.01834	1.020385	1.11151	0.1525	1	1
ZNF618	1.374361	1.374016	1.374474	1.00122	0.0018	1	1
CTD-2562J15.6	1.016412	1.014833	1.016926	1.14114	0.1905	1	1
AP001065.15	1.015555	1.014833	1.01579	1.06452	0.0902	1	1
FASTKD5	1.092507	1.092419	1.092535	1.00125	0.0018	1	1
ERICD	1.01642	1.014833	1.016936	1.14184	0.1914	1	1
RP11-253M7.4	1.023357	1.021863	1.023843	1.09057	0.1251	1	1
RP11-385F5.5	1.017269	1.01833	1.016924	0.92332	-0.1151	1	1
DCST1	1.016416	1.014833	1.01693	1.14142	0.1908	1	1
CTD-2325A15.5	1.016414	1.014833	1.016927	1.14124	0.1906	1	1
RP11-13P5.2	1.016421	1.014833	1.016938	1.14193	0.1915	1	1
AC092431.3	1.016412	1.014833	1.016926	1.14112	0.1904	1	1
HCG16	1.016419	1.014833	1.016934	1.14171	0.1912	1	1
ARHGEF38	1.017295	1.018393	1.016938	0.92086	-0.1189	1	1
RP5-971N18.3	1.016419	1.014833	1.016935	1.14176	0.1913	1	1
RP5-867C24.5	1.016419	1.014833	1.016934	1.14171	0.1912	1	1
C3orf49	1.016418	1.014833	1.016933	1.14163	0.1911	1	1
CLCA2	1.015555	1.014833	1.01579	1.06452	0.0902	1	1
AC079807.2	1.029375	1.028923	1.029522	1.02069	0.0295	1	1
RP11-697E22.1	1.016418	1.014833	1.016934	1.14166	0.1911	1	1
CLYBL-AS2	1.016422	1.014833	1.016939	1.142	0.1916	1	1
EPS15L1	1.379755	1.377789	1.380394	1.0069	0.0099	1	1
AC006160.5	1.023319	1.021829	1.023803	1.09044	0.1249	1	1
CIAPIN1	1.644722	1.645458	1.644482	0.99849	-0.0022	1	1
AP000462.3	1.01642	1.014833	1.016936	1.14184	0.1914	1	1
AF186192.1	1.0251	1.025482	1.024976	0.98014	-0.0289	1	1
GMFG	1.037167	1.035964	1.037558	1.04433	0.0626	1	1

WBSCR28	1.022475	1.021901	1.022662	1.03471	0.0492	1	1
RP11-561B11.6	1.015555	1.014833	1.01579	1.06452	0.0902	1	1
RP11-130F10.1	1.074456	1.074593	1.074411	0.99756	-0.0035	1	1
AC012368.1	1.015555	1.014833	1.01579	1.06452	0.0902	1	1
CTD-2246P4.1	1.01728	1.01834	1.016935	0.92338	-0.115	1	1
XPNPEP2	1.01642	1.014833	1.016936	1.14182	0.1913	1	1
RP11-443B20.1	1.018142	1.018355	1.018072	0.98463	-0.0223	1	1
CTD-2194D22.3	1.017276	1.018352	1.016926	0.92228	-0.1167	1	1
DNAJB5-AS1	1.015555	1.014833	1.01579	1.06452	0.0902	1	1
PCDH11Y	1.030339	1.029076	1.030749	1.05752	0.0807	1	1
UBXN2B	1.340637	1.339156	1.341118	1.00578	0.0083	1	1
RP11-247I13.11	1.020733	1.021854	1.020369	0.93203	-0.1015	1	1
C6orf222	1.016421	1.014833	1.016937	1.14191	0.1915	1	1
BMP8A	1.014564	1.013274	1.014983	1.12874	0.1747	1	1
RP11-473E2.2	1.014554	1.013274	1.01497	1.12777	0.1735	1	1
GET4	1.01715	1.016797	1.017265	1.02788	0.0397	1	1
RP3-337H4.10	1.015417	1.016772	1.014977	0.89295	-0.1633	1	1
AC100830.5	1.016285	1.016771	1.016128	0.96165	-0.0564	1	1
TXNRD3	1.063624	1.062251	1.064071	1.02923	0.0416	1	1
RP11-115H18.1	1.020593	1.020291	1.020691	1.0197	0.0281	1	1
MVB12A	2.176907	2.177325	2.17677	0.99953	-0.0007	1	1
GIT1	1.264837	1.262775	1.265507	1.0104	0.0149	1	1
RP11-88E10.5	1.019728	1.020276	1.01955	0.96422	-0.0526	1	1
FBXO40	1.0137	1.013274	1.013838	1.04247	0.06	1	1
DDR1-AS1	1.018029	1.016781	1.018435	1.09853	0.1356	1	1
KB-1440D3.14	1.014555	1.013274	1.014972	1.1279	0.1736	1	1
C10orf53	1.016276	1.016782	1.016111	0.96006	-0.0588	1	1
ESM1	1.015433	1.016828	1.014979	0.89015	-0.1679	1	1
YY1AP1	1.236228	1.234367	1.236833	1.01052	0.0151	1	1
ACP7	1.014555	1.013274	1.014972	1.12787	0.1736	1	1
AC084082.3	1.014555	1.013274	1.014972	1.1279	0.1736	1	1
RP11-253E3.3	1.020587	1.020299	1.020681	1.01879	0.0269	1	1
RP11-108O10.2	1.014558	1.013274	1.014975	1.12816	0.174	1	1
RP5-966M1.6	1.016293	1.0168	1.016128	0.96004	-0.0588	1	1
TMEM92-AS1	1.01456	1.013274	1.014978	1.12834	0.1742	1	1
PRRT3	1.223432	1.224415	1.223112	0.99419	-0.0084	1	1
RP11-91P24.7	1.0137	1.013274	1.013838	1.04247	0.06	1	1
RP11-622A1.2	1.014561	1.013274	1.014979	1.12847	0.1744	1	1
RP11-309L24.4	1.0137	1.013274	1.013838	1.04247	0.06	1	1
GPR62	1.018858	1.0203	1.018389	0.90588	-0.1426	1	1
COL20A1	1.014556	1.013274	1.014972	1.12792	0.1737	1	1
ST3GAL6	1.142429	1.143527	1.142072	0.98987	-0.0147	1	1
BX470102.3	1.016278	1.016778	1.016115	0.9605	-0.0581	1	1
CEACAM8	1.014564	1.013274	1.014984	1.12878	0.1748	1	1
ACOT1	1.033485	1.03434	1.033207	0.96701	-0.0484	1	1

LDLRAD2	1.0137	1.013274	1.013838	1.04247	0.06	1	1
CTD-2540B15.9	1.018875	1.020347	1.018397	0.90416	-0.1454	1	1
COMP	1.016288	1.016788	1.016126	0.96054	-0.0581	1	1
RP3-325F22.5	1.014562	1.013274	1.014981	1.12858	0.1745	1	1
AC007383.4	1.021494	1.020427	1.021841	1.06922	0.0966	1	1
RP11-214D15.2	1.014558	1.013274	1.014975	1.12811	0.1739	1	1
CTD-2288F12.1	1.01456	1.013274	1.014978	1.12837	0.1742	1	1
CPVL	1.116122	1.115124	1.116447	1.01149	0.0165	1	1
VSTM5	1.018005	1.016773	1.018406	1.09732	0.134	1	1
LINC01392	1.014561	1.013274	1.014979	1.12847	0.1744	1	1
RP4-777O23.1	1.014564	1.013274	1.014983	1.12875	0.1747	1	1
LINC01138	1.182935	1.182164	1.183186	1.00561	0.0081	1	1
NKX6-1	1.014561	1.013274	1.014979	1.12847	0.1744	1	1
RP11-757F18.5	1.017143	1.016791	1.017257	1.02777	0.0395	1	1
SLC5A10	1.014556	1.013274	1.014973	1.128	0.1738	1	1
AF165138.7	1.014555	1.013274	1.014971	1.12782	0.1735	1	1
RP11-805I24.1	1.016287	1.0168	1.01612	0.95957	-0.0595	1	1
LINC00345	1.01456	1.013274	1.014978	1.12836	0.1742	1	1
GPS2	1.238076	1.238304	1.238002	0.99873	-0.0018	1	1
ASPHD2	1.06713	1.065915	1.067525	1.02442	0.0348	1	1
RPEL1	1.014559	1.013274	1.014977	1.12829	0.1741	1	1
AIPL1	1.021452	1.020282	1.021832	1.07645	0.1063	1	1
RP4-564F22.7	1.0137	1.013274	1.013838	1.04247	0.06	1	1
DHRS11	1.195708	1.195884	1.195651	0.99881	-0.0017	1	1
AC007204.2	1.0137	1.013274	1.013838	1.04247	0.06	1	1
RP1-149A16.3	1.014557	1.013274	1.014974	1.12806	0.1738	1	1
LINC00519	1.0137	1.013274	1.013838	1.04247	0.06	1	1
RP11-618K13.2	1.014565	1.013274	1.014985	1.12885	0.1749	1	1
HIST1H3G	1.032685	1.030914	1.03326	1.07588	0.1055	1	1
ARMCX3-AS1	1.017155	1.016819	1.017264	1.02649	0.0377	1	1
RP11-87C12.2	1.01801	1.016781	1.01841	1.09704	0.1336	1	1
AC005256.1	1.014556	1.013274	1.014973	1.12799	0.1738	1	1
CFAP100	1.017152	1.01678	1.017273	1.02939	0.0418	1	1
RP11-92F20.1	1.014555	1.013274	1.014971	1.12785	0.1736	1	1
RP11-382A20.4	1.014561	1.013274	1.014979	1.12841	0.1743	1	1
CAPN14	1.018873	1.020269	1.018419	0.90875	-0.1381	1	1
MAB21L3	1.014557	1.013274	1.014974	1.12802	0.1738	1	1
C9orf170	1.0137	1.013274	1.013838	1.04247	0.06	1	1
RP11-79N23.1	1.020591	1.02027	1.020695	1.02098	0.03	1	1
UCP1	1.014556	1.013274	1.014973	1.128	0.1738	1	1
RP11-575F12.3	1.0137	1.013274	1.013838	1.04247	0.06	1	1
AC012442.6	1.014556	1.013274	1.014972	1.12792	0.1737	1	1
RP5-965G21.3	1.020578	1.020276	1.020676	1.01974	0.0282	1	1
RP11-108K3.2	1.019738	1.020278	1.019562	0.9647	-0.0518	1	1
ZC3H12D	1.01456	1.013274	1.014978	1.12836	0.1742	1	1

RAX2	1.014571	1.013274	1.014993	1.12949	0.1757	1	1
RP11-507K2.2	1.018868	1.020328	1.018393	0.90481	-0.1443	1	1
AC011718.2	1.015426	1.016796	1.014981	0.89192	-0.165	1	1
F2RL3	1.01889	1.020291	1.018434	0.90849	-0.1385	1	1
FGF23	1.024893	1.023764	1.02526	1.06293	0.088	1	1
HTR5A-AS1	1.0137	1.013274	1.013838	1.04247	0.06	1	1
USE1	1.555889	1.554972	1.556187	1.00219	0.0032	1	1
LINC01270	1.01715	1.016777	1.017271	1.02943	0.0418	1	1
LINC01182	1.0137	1.013274	1.013838	1.04247	0.06	1	1
SERPINB9P1	1.016284	1.016765	1.016128	0.96201	-0.0559	1	1
RP11-269M20.3	1.017153	1.016769	1.017278	1.03033	0.0431	1	1
GRIN2C	1.021503	1.020365	1.021873	1.07405	0.1031	1	1
RP11-255M2.3	1.060362	1.059157	1.060753	1.02697	0.0384	1	1
C1orf234	1.018873	1.02026	1.018422	0.90929	-0.1372	1	1
FLT1	1.017139	1.016765	1.01726	1.02956	0.042	1	1
RP11-573G6.8	1.014559	1.013274	1.014977	1.12826	0.1741	1	1
RP11-758M4.4	1.03183	1.030729	1.032188	1.04748	0.0669	1	1
RP11-123I22.1	1.0137	1.013274	1.013838	1.04247	0.06	1	1
RP11-505E24.2	1.01802	1.016788	1.01842	1.09721	0.1338	1	1
SOX30	1.0137	1.013274	1.013838	1.04247	0.06	1	1
LINC01489	1.014557	1.013274	1.014975	1.12811	0.1739	1	1
NCBP2-AS1	1.01456	1.013274	1.014978	1.12836	0.1742	1	1
GNGT2	1.024092	1.023786	1.024191	1.01705	0.0244	1	1
WT1	1.014555	1.013274	1.014972	1.12787	0.1736	1	1
RP11-435O5.7	1.014561	1.013274	1.014979	1.12844	0.1743	1	1
AC006159.4	1.014557	1.013274	1.014974	1.12806	0.1738	1	1
PLSCR2	1.014554	1.013274	1.014971	1.1278	0.1735	1	1
RP11-559M23.1	1.025809	1.023869	1.02644	1.10767	0.1475	1	1
UROC1	1.014562	1.013274	1.01498	1.12852	0.1744	1	1
STYK1	1.014559	1.013274	1.014977	1.1283	0.1741	1	1
FAM131B	1.107785	1.108133	1.107671	0.99573	-0.0062	1	1
LINC00437	1.014558	1.013274	1.014975	1.12813	0.1739	1	1
WWC2-AS2	1.040577	1.041345	1.040327	0.97538	-0.036	1	1
LINC00426	1.0137	1.013274	1.013838	1.04247	0.06	1	1
CTD-3035K23.6	1.015418	1.016791	1.014972	0.89169	-0.1654	1	1
FLJ13224	1.018862	1.020272	1.018404	0.90788	-0.1394	1	1
AC009505.2	1.014557	1.013274	1.014974	1.12807	0.1739	1	1
RP11-701H24.4	1.01542	1.016782	1.014977	0.89241	-0.1642	1	1
ZNF317	1.148644	1.147097	1.149146	1.01393	0.02	1	1
EPN2-AS1	1.014557	1.013274	1.014974	1.12805	0.1738	1	1
RP5-1051H14.2	1.0137	1.013274	1.013838	1.04247	0.06	1	1
RP11-182J1.13	1.0137	1.013274	1.013838	1.04247	0.06	1	1
AP003419.16	1.018865	1.020321	1.018392	0.90506	-0.1439	1	1
HIST1H2BG	1.039648	1.037906	1.040214	1.06087	0.0852	1	1
RP11-408H20.3	1.014559	1.013274	1.014976	1.12821	0.174	1	1

FAM90A26	1.0137	1.013274	1.013838	1.04247	0.06	1	1
KLRG2	1.0137	1.013274	1.013838	1.04247	0.06	1	1
DPAGT1	1.307823	1.308328	1.307659	0.99783	-0.0031	1	1
RP11-863P13.1	1.014563	1.013274	1.014982	1.12864	0.1746	1	1
CTB-60B18.12	1.0137	1.013274	1.013838	1.04247	0.06	1	1
RP11-462P6.1	1.021548	1.021948	1.021418	0.97587	-0.0352	1	1
TRPM8	1.01893	1.018377	1.01911	1.0399	0.0564	1	1
CFAP58-AS1	1.018081	1.018382	1.017983	0.97831	-0.0316	1	1
TNFRSF11A	1.016333	1.014881	1.016805	1.12931	0.1754	1	1
CD1D	1.018064	1.018384	1.01796	0.97697	-0.0336	1	1
CCDC141	1.021522	1.02189	1.021402	0.97771	-0.0325	1	1
CTD-2089N3.1	1.016337	1.014881	1.01681	1.1296	0.1758	1	1
SLC4A9	1.015478	1.014881	1.015672	1.05313	0.0747	1	1
ENPP1	1.04316	1.043005	1.043211	1.00479	0.0069	1	1
RP11-398A8.5	1.016337	1.014881	1.016811	1.12967	0.1759	1	1
RP11-434P11.2	1.021524	1.021889	1.021406	0.97793	-0.0322	1	1
HYPK	1.075322	1.074912	1.075455	1.00724	0.0104	1	1
RP11-357N13.2	1.016335	1.014881	1.016808	1.12946	0.1756	1	1
GRAPL	1.016337	1.014881	1.01681	1.12963	0.1759	1	1
YRDC	1.102852	1.102968	1.102814	0.99851	-0.0022	1	1
EEF1E1-BLOC1S5	1.016345	1.014881	1.016821	1.13035	0.1768	1	1
RP11-353N14.4	1.01634	1.014881	1.016814	1.12986	0.1761	1	1
XAF1	1.016346	1.014881	1.016822	1.13045	0.1769	1	1
RP11-469L4.1	1.016337	1.014881	1.01681	1.12959	0.1758	1	1
RP11-407G23.7	1.016345	1.014881	1.01682	1.13032	0.1767	1	1
AC006547.15	1.015478	1.014881	1.015672	1.05313	0.0747	1	1
RP11-297K8.2	1.021525	1.021892	1.021405	0.97777	-0.0324	1	1
ARHGAP6	1.043058	1.042915	1.043105	1.00442	0.0064	1	1
RP11-17J14.2	1.018926	1.01837	1.019107	1.0401	0.0567	1	1
PTH2R	1.019798	1.018392	1.020255	1.10131	0.1392	1	1
LA16c-352F7.1	1.016336	1.014881	1.016809	1.12957	0.1758	1	1
LDB3	1.025006	1.02552	1.024839	0.97332	-0.039	1	1
RP11-408A13.3	1.019787	1.018381	1.020244	1.10135	0.1393	1	1
CTD-2269F5.1	1.031083	1.032438	1.030643	0.94465	-0.0822	1	1
ANKUB1	1.017202	1.018394	1.016815	0.91412	-0.1295	1	1
RP11-546O6.4	1.019804	1.01838	1.020266	1.10263	0.141	1	1
LINC01510	1.015478	1.014881	1.015672	1.05313	0.0747	1	1
RP5-937E21.8	1.016342	1.014881	1.016817	1.13005	0.1764	1	1
RP11-876N24.4	1.018953	1.018433	1.019122	1.03734	0.0529	1	1
WFDC10A	1.01634	1.014881	1.016814	1.12988	0.1762	1	1
AC006000.5	1.016339	1.014881	1.016813	1.1298	0.1761	1	1
PITRM1-AS1	1.021536	1.021907	1.021415	0.97753	-0.0328	1	1
ASPDH	1.017203	1.018386	1.016818	0.91474	-0.1286	1	1
RP11-638I2.8	1.015478	1.014881	1.015672	1.05313	0.0747	1	1
RP11-599B13.3	1.016342	1.014881	1.016817	1.13009	0.1764	1	1

NDUFV2-AS1	1.064823	1.06431	1.06499	1.01058	0.0152	1	1
RP11-101E5.1	1.01634	1.014881	1.016815	1.12993	0.1762	1	1
ATP4A	1.019807	1.018401	1.020264	1.10129	0.1392	1	1
POLR3C	1.17813	1.177062	1.178476	1.00799	0.0115	1	1
C10orf142	1.018928	1.018386	1.019104	1.03907	0.0553	1	1
RP1-30M3.5	1.022396	1.021962	1.022537	1.02617	0.0373	1	1
RP11-1101H11.1	1.015478	1.014881	1.015672	1.05313	0.0747	1	1
CTA-126B4.7	1.016337	1.014881	1.016811	1.12966	0.1759	1	1
RP11-54G14.1	1.016341	1.014881	1.016816	1.13001	0.1763	1	1
AP001257.1	1.015478	1.014881	1.015672	1.05313	0.0747	1	1
RP4-530I15.9	1.016338	1.014881	1.016811	1.12971	0.176	1	1
SLC28A1	1.016344	1.014881	1.01682	1.13029	0.1767	1	1
RP11-106M3.5	1.016343	1.014881	1.016818	1.13013	0.1765	1	1
IL3RA	1.018076	1.018382	1.017976	0.97789	-0.0323	1	1
INHBA-AS1	1.0172	1.0184	1.01681	0.9136	-0.1304	1	1
RP11-24F11.5	1.016341	1.014881	1.016815	1.12996	0.1763	1	1
RP11-375O18.2	1.016337	1.014881	1.01681	1.12963	0.1759	1	1
AK3	2.193342	2.191516	2.193935	1.00203	0.0029	1	1
NCR3	1.016342	1.014881	1.016817	1.13009	0.1764	1	1
HCN4	1.018076	1.018389	1.017975	0.97745	-0.0329	1	1
RP11-753C18.12	1.016338	1.014881	1.016812	1.12972	0.176	1	1
BBOX1	1.018934	1.018413	1.019104	1.0375	0.0531	1	1
WNT8B	1.01634	1.014881	1.016814	1.12988	0.1762	1	1
CTC-350I8.1	1.019819	1.018405	1.020279	1.10184	0.1399	1	1
TBC1D3D	1.015478	1.014881	1.015672	1.05313	0.0747	1	1
RP11-1260E13.2	1.022387	1.02189	1.022548	1.03008	0.0428	1	1
PRKG2	1.020649	1.021885	1.020247	0.92514	-0.1123	1	1
DOCK3	1.079652	1.078374	1.080068	1.0216	0.0308	1	1
LINC00613	1.015478	1.014881	1.015672	1.05313	0.0747	1	1
RAB40C	1.203219	1.201635	1.203734	1.01041	0.0149	1	1
AC109642.1	1.018063	1.018389	1.017958	0.97652	-0.0343	1	1
LL22NC03-N95F10.1	1.016336	1.014881	1.016809	1.12954	0.1757	1	1
CTD-2292P10.4	1.018068	1.018397	1.017961	0.9763	-0.0346	1	1
RP11-531F16.4	1.015478	1.014881	1.015672	1.05313	0.0747	1	1
RP11-250B2.6	1.02499	1.025431	1.024847	0.97701	-0.0335	1	1
CYP2E1	1.02669	1.025428	1.027101	1.0658	0.0919	1	1
RP11-569A11.1	1.018959	1.018421	1.019134	1.03873	0.0548	1	1
TEN1-CDK3	1.016346	1.014881	1.016822	1.13045	0.1769	1	1
RP11-646I6.6	1.018933	1.018441	1.019093	1.03537	0.0501	1	1
LKAAEAR1	1.033649	1.032455	1.034036	1.04872	0.0686	1	1
RP11-306G20.1	1.04228	1.043065	1.042025	0.97585	-0.0353	1	1
AC083843.2	1.016337	1.014881	1.01681	1.12963	0.1758	1	1
PTPRH	1.057806	1.057203	1.058002	1.01397	0.02	1	1
AC097724.3	1.024123	1.025438	1.023696	0.93149	-0.1024	1	1
VCAM1	1.030183	1.028954	1.030582	1.05623	0.0789	1	1

RBMXL3	1.016336	1.014881	1.016809	1.12958	0.1758	1	1
RP11-1070N10.4	1.01634	1.014881	1.016814	1.12988	0.1762	1	1
ZNF442	1.042287	1.04314	1.04201	0.97381	-0.0383	1	1
RP11-31K23.2	1.016341	1.014881	1.016815	1.12996	0.1763	1	1
AC005487.2	1.016337	1.014881	1.01681	1.12959	0.1758	1	1
TMEM249	1.017195	1.018381	1.016809	0.91452	-0.1289	1	1
RP11-6O2.3	1.017205	1.018417	1.016811	0.91284	-0.1316	1	1
RP11-573D15.9	1.023236	1.021907	1.023667	1.08037	0.1115	1	1
RP11-99J16__A.2	1.015478	1.014881	1.015672	1.05313	0.0747	1	1
CTC-591M7.1	1.015478	1.014881	1.015672	1.05313	0.0747	1	1
HAMP	1.016337	1.014881	1.01681	1.12963	0.1759	1	1
RP3-406P24.4	1.018083	1.018382	1.017986	0.97842	-0.0315	1	1
RP11-540O11.1	1.015478	1.014881	1.015672	1.05313	0.0747	1	1
CABP1	1.06046	1.060575	1.060423	0.99748	-0.0036	1	1
AC079630.4	1.017196	1.018382	1.016811	0.9145	-0.1289	1	1
C11orf63	1.084573	1.085258	1.08435	0.98934	-0.0155	1	1
RP11-415F23.4	1.019822	1.018395	1.020285	1.10274	0.1411	1	1
CFAP45	1.027544	1.028906	1.027101	0.93756	-0.093	1	1
ATP6V0C	1.036261	1.036087	1.036318	1.00642	0.0092	1	1
AC005624.2	1.016342	1.014881	1.016817	1.13009	0.1764	1	1
RP11-3D4.3	1.024977	1.025397	1.02484	0.97806	-0.032	1	1
TAF9B	1.161524	1.162728	1.161132	0.99019	-0.0142	1	1
PRSS53	1.021519	1.021907	1.021392	0.97651	-0.0343	1	1
ABCA12	1.015478	1.014881	1.015672	1.05313	0.0747	1	1
RP11-65J3.3	1.023289	1.021871	1.02375	1.08589	0.1189	1	1
CTC-523E23.14	1.018058	1.018392	1.017949	0.97594	-0.0351	1	1
KCNAB1-AS1	1.016333	1.014881	1.016805	1.12931	0.1754	1	1
SORCS3	1.030186	1.028956	1.030585	1.05625	0.079	1	1
CATSPER3	1.01979	1.01838	1.020248	1.10164	0.1397	1	1
RP11-46O21.2	1.015478	1.014881	1.015672	1.05313	0.0747	1	1
RP11-427M20.1	1.016338	1.014881	1.016811	1.12971	0.176	1	1
NME1-NME2	1.015478	1.014881	1.015672	1.05313	0.0747	1	1
ARL5C	1.016337	1.014881	1.016811	1.12966	0.1759	1	1
SNCB	1.06324	1.064308	1.062893	0.97799	-0.0321	1	1
RP11-452L6.5	1.076024	1.074786	1.076427	1.02194	0.0313	1	1
UBE2F-SCLY	1.016344	1.014881	1.016819	1.13023	0.1766	1	1
AC002306.1	1.016343	1.014881	1.016818	1.13013	0.1765	1	1
SFTA2	1.022405	1.021885	1.022575	1.03151	0.0448	1	1
CTB-102L5.4	1.016344	1.014881	1.016819	1.13025	0.1766	1	1
DNM3	1.045653	1.04644	1.045397	0.97755	-0.0328	1	1
RP11-379B18.6	1.0172	1.018397	1.01681	0.91373	-0.1302	1	1
CTC-756D1.2	1.014385	1.013073	1.014812	1.13304	0.1802	1	1
SERPINB7	1.029044	1.02719	1.029646	1.09032	0.1247	1	1
RP4-613B23.5	1.013523	1.013073	1.01367	1.04568	0.0644	1	1
KLF15	1.020437	1.020101	1.020546	1.0221	0.0315	1	1

RP11-958F21.1	1.017837	1.01659	1.018242	1.09963	0.137	1	1
SERPINB11	1.014381	1.013073	1.014806	1.13261	0.1796	1	1
ZNF208	1.014381	1.013073	1.014806	1.13262	0.1797	1	1
RP11-227D13.4	1.014384	1.013073	1.014811	1.13297	0.1801	1	1
RP11-955H22.1	1.014382	1.013073	1.014808	1.13277	0.1799	1	1
AC005592.3	1.017839	1.016591	1.018245	1.09971	0.1371	1	1
RP11-325N19.3	1.01956	1.020117	1.019379	0.96332	-0.0539	1	1
MYL2	1.013523	1.013073	1.01367	1.04568	0.0644	1	1
FAM86B2	1.014386	1.013073	1.014812	1.1331	0.1803	1	1
IFNL3	1.013523	1.013073	1.01367	1.04568	0.0644	1	1
RP11-446H18.5	1.014382	1.013073	1.014808	1.13273	0.1798	1	1
AP001059.5	1.02042	1.020119	1.020518	1.0198	0.0283	1	1
SPDYE5	1.01524	1.016573	1.014807	0.89348	-0.1625	1	1
EWSAT1	1.016116	1.016598	1.015959	0.9615	-0.0566	1	1
RP11-325K19.1	1.014381	1.013073	1.014807	1.13268	0.1797	1	1
AC005009.1	1.016968	1.016571	1.017097	1.03175	0.0451	1	1
TARID	1.014382	1.013073	1.014807	1.13272	0.1798	1	1
SLC15A2	1.035976	1.034216	1.036549	1.06819	0.0952	1	1
LRMP	1.013523	1.013073	1.01367	1.04568	0.0644	1	1
IL7	1.015245	1.016561	1.014817	0.89469	-0.1605	1	1
CTD-2532N20.1	1.019566	1.0201	1.019393	0.96481	-0.0517	1	1
LGALS12	1.013523	1.013073	1.01367	1.04568	0.0644	1	1
NPIPB3	1.013523	1.013073	1.01367	1.04568	0.0644	1	1
RP5-958B11.2	1.016983	1.016555	1.017121	1.0342	0.0485	1	1
RP11-82O19.1	1.014382	1.013073	1.014808	1.13277	0.1799	1	1
AC012360.6	1.014382	1.013073	1.014808	1.13274	0.1798	1	1
RP11-545G3.1	1.013523	1.013073	1.01367	1.04568	0.0644	1	1
EBF3	1.014385	1.013073	1.014812	1.13304	0.1802	1	1
SEPT12	1.016104	1.016572	1.015952	0.96257	-0.055	1	1
LINC00704	1.013523	1.013073	1.01367	1.04568	0.0644	1	1
RP11-168L7.1	1.016103	1.016565	1.015952	0.96299	-0.0544	1	1
RP13-726E6.1	1.014386	1.013073	1.014813	1.13318	0.1804	1	1
RP11-573J24.1	1.016117	1.016595	1.015961	0.96181	-0.0562	1	1
ZNF699	1.073177	1.073033	1.073224	1.00261	0.0038	1	1
PCDHGB5	1.014385	1.013073	1.014812	1.13306	0.1802	1	1
RP4-537K23.4	1.019549	1.020085	1.019374	0.96462	-0.052	1	1
RP11-446H18.6	1.017833	1.016558	1.018247	1.10205	0.1402	1	1
DPPA3	1.014382	1.013073	1.014807	1.13271	0.1798	1	1
RP11-192P3.4	1.014383	1.013073	1.014809	1.13283	0.1799	1	1
RP11-449L23.2	1.01438	1.013073	1.014805	1.13251	0.1795	1	1
XXbac-BPG308J9.3	1.014384	1.013073	1.01481	1.13292	0.1801	1	1
RP4-601P9.2	1.014387	1.013073	1.014815	1.13328	0.1805	1	1
RP3-402G11.25	1.015257	1.016593	1.014823	0.89335	-0.1627	1	1
GNA14	1.015243	1.016584	1.014807	0.89281	-0.1636	1	1
AC008269.2	1.017835	1.016595	1.018238	1.099	0.1362	1	1

MIR670HG	1.016973	1.016572	1.017103	1.03203	0.0455	1	1
SLA2	1.014384	1.013073	1.01481	1.13292	0.1801	1	1
LA16c-312E8.2	1.029903	1.03065	1.02966	0.9677	-0.0474	1	1
C2CD4D	1.049741	1.048269	1.050219	1.04042	0.0572	1	1
CLEC14A	1.017821	1.016584	1.018223	1.09884	0.136	1	1
CGB2	1.013523	1.013073	1.01367	1.04568	0.0644	1	1
RP11-431J24.2	1.014383	1.013073	1.014809	1.13287	0.18	1	1
SLC22A14	1.014381	1.013073	1.014806	1.13261	0.1796	1	1
ABI3BP	1.013523	1.013073	1.01367	1.04568	0.0644	1	1
RP11-22L13.1	1.014384	1.013073	1.01481	1.1329	0.18	1	1
CHRNA9	1.022144	1.020063	1.02282	1.13745	0.1858	1	1
RERGL	1.015242	1.016564	1.014812	0.89427	-0.1612	1	1
CD86	1.014388	1.013073	1.014816	1.13335	0.1806	1	1
RP5-1024G6.7	1.022143	1.02008	1.022813	1.13614	0.1841	1	1
CSAG1	1.014383	1.013073	1.014809	1.13283	0.1799	1	1
LRIT1	1.013523	1.013073	1.01367	1.04568	0.0644	1	1
RP5-994D16.12	1.01438	1.013073	1.014805	1.13251	0.1795	1	1
HTR7	1.015243	1.016561	1.014815	0.89456	-0.1607	1	1
PNPLA7	1.030824	1.030703	1.030863	1.00519	0.0075	1	1
TAPBPL	1.016965	1.016575	1.017092	1.03122	0.0444	1	1
CTC-203F4.2	1.018697	1.020104	1.01824	0.90727	-0.1404	1	1
C7orf61	1.023851	1.023607	1.02393	1.01369	0.0196	1	1
RP11-370I10.10	1.0161	1.016579	1.015944	0.96169	-0.0564	1	1
RP11-90E5.1	1.015246	1.016586	1.014811	0.89297	-0.1633	1	1
DENND2D	1.013523	1.013073	1.01367	1.04568	0.0644	1	1
RP11-406D1.2	1.014383	1.013073	1.014809	1.1328	0.1799	1	1
RP11-747D18.1	1.014382	1.013073	1.014808	1.13278	0.1799	1	1
OPN4	1.014383	1.013073	1.014809	1.13283	0.1799	1	1
PDGFRL	1.016099	1.016578	1.015943	0.9617	-0.0563	1	1
BAK1	1.336394	1.336289	1.336429	1.00042	0.0006	1	1
AC004014.3	1.014385	1.013073	1.014812	1.13303	0.1802	1	1
RP1-12G14.7	1.016111	1.016561	1.015964	0.96396	-0.053	1	1
RP11-43N5.1	1.013523	1.013073	1.01367	1.04568	0.0644	1	1
RP11-3D4.2	1.013523	1.013073	1.01367	1.04568	0.0644	1	1
BOLA2B	1.162067	1.160615	1.162539	1.01198	0.0172	1	1
RP1-140K8.5	1.013523	1.013073	1.01367	1.04568	0.0644	1	1
PLIN1	1.020488	1.020133	1.020604	1.02336	0.0333	1	1
RP3-393E18.2	1.013523	1.013073	1.01367	1.04568	0.0644	1	1
RP11-256I23.1	1.014388	1.013073	1.014815	1.13333	0.1806	1	1
ZNF805	1.035052	1.034075	1.035369	1.038	0.0538	1	1
RP11-503G7.1	1.017849	1.016598	1.018255	1.09982	0.1373	1	1
AC004160.4	1.013523	1.013073	1.01367	1.04568	0.0644	1	1
RP11-445P19.3	1.013523	1.013073	1.01367	1.04568	0.0644	1	1
OPN1SW	1.014382	1.013073	1.014808	1.13274	0.1798	1	1
RP11-256I23.3	1.016111	1.016573	1.015961	0.9631	-0.0542	1	1

RP11-479J7.2	1.015249	1.016598	1.014811	0.89231	-0.1644	1	1
RP11-553L6.2	1.013523	1.013073	1.01367	1.04568	0.0644	1	1
CTC-508F8.1	1.014381	1.013073	1.014807	1.13266	0.1797	1	1
LRRIQ4	1.013523	1.013073	1.01367	1.04568	0.0644	1	1
WTH3DI	1.014379	1.013073	1.014804	1.13246	0.1795	1	1
RP11-118E18.2	1.014382	1.013073	1.014807	1.13271	0.1798	1	1
CTD-2302E22.6	1.020412	1.020086	1.020517	1.02148	0.0307	1	1
MC5R	1.014385	1.013073	1.014811	1.13301	0.1802	1	1
LINC00266-1	1.014379	1.013073	1.014804	1.13246	0.1795	1	1
RAB44	1.013523	1.013073	1.01367	1.04568	0.0644	1	1
XCL1	1.023031	1.023698	1.022814	0.96273	-0.0548	1	1
CTD-2260A17.2	1.036841	1.037669	1.036572	0.97088	-0.0426	1	1
DNLZ	1.020435	1.020123	1.020537	1.02054	0.0293	1	1
RP11-344B5.4	1.014381	1.013073	1.014806	1.13261	0.1796	1	1
STEAP3-AS1	1.014385	1.013073	1.014811	1.13301	0.1802	1	1
RP11-269F20.1	1.01438	1.013073	1.014804	1.13249	0.1795	1	1
RP11-354P11.4	1.013523	1.013073	1.01367	1.04568	0.0644	1	1
OGFRP1	1.016107	1.016559	1.01596	0.96378	-0.0532	1	1
NPEPL1	1.296571	1.297711	1.2962	0.99492	-0.0073	1	1
SMAD5-AS1	1.021265	1.020074	1.021652	1.0786	0.1092	1	1
C16orf89	1.013523	1.013073	1.01367	1.04568	0.0644	1	1
RP4-816N1.7	1.013523	1.013073	1.01367	1.04568	0.0644	1	1
BHMG1	1.014387	1.013073	1.014815	1.13327	0.1805	1	1
RP11-326C3.15	1.019558	1.020059	1.019396	0.96692	-0.0485	1	1
RP1-56K13.5	1.018696	1.02015	1.018223	0.90435	-0.145	1	1
IL4I1	1.017822	1.016567	1.01823	1.1004	0.138	1	1
RP11-347C18.5	1.02569	1.023788	1.026309	1.10596	0.1453	1	1
RP1-261G23.5	1.014385	1.013073	1.014811	1.13301	0.1802	1	1
CTB-22K21.2	1.016118	1.016565	1.015973	0.96423	-0.0526	1	1
SPINK5	1.079215	1.079915	1.078988	0.9884	-0.0168	1	1
C10orf126	1.02304	1.023585	1.022863	0.96937	-0.0449	1	1
ADGRG5	1.014386	1.013073	1.014812	1.13309	0.1803	1	1
AC003088.1	1.014383	1.013073	1.014809	1.13283	0.1799	1	1
GNAT3	1.013523	1.013073	1.01367	1.04568	0.0644	1	1
RP11-624D11.2	1.029061	1.027102	1.029697	1.09578	0.132	1	1
RP11-444E17.6	1.015251	1.016598	1.014813	0.89242	-0.1642	1	1
LINC00354	1.018773	1.017948	1.019042	1.06096	0.0854	1	1
ZCCHC24	1.121146	1.120291	1.121424	1.00941	0.0135	1	1
RP11-4B16.3	1.021369	1.021435	1.021347	0.99587	-0.006	1	1
XAGE3	1.019642	1.017948	1.020192	1.12508	0.17	1	1
MYO16	1.023101	1.021478	1.023629	1.10014	0.1377	1	1
RP11-123K3.9	1.01963	1.017948	1.020177	1.12419	0.1689	1	1
RP11-519M16.1	1.018773	1.017948	1.019042	1.06096	0.0854	1	1
RP11-338I21.1	1.018773	1.017948	1.019042	1.06096	0.0854	1	1
FGGY	1.13152	1.131354	1.131574	1.00167	0.0024	1	1

DNAH2	1.019633	1.017948	1.020181	1.12445	0.1692	1	1
TEX35	1.019632	1.017948	1.02018	1.12437	0.1691	1	1
HMGA1P4	1.028296	1.028515	1.028225	0.98983	-0.0147	1	1
ZNF23	1.044812	1.046171	1.04437	0.961	-0.0574	1	1
RP11-350G8.5	1.021381	1.021483	1.021348	0.99374	-0.0091	1	1
NRIP2	1.023117	1.021445	1.02366	1.10331	0.1418	1	1
RP11-15I11.3	1.020496	1.021449	1.020186	0.94113	-0.0875	1	1
AC016745.3	1.019633	1.017948	1.020181	1.12445	0.1692	1	1
GNAT1	1.019643	1.017948	1.020195	1.1252	0.1702	1	1
RP1-50J22.4	1.021355	1.021456	1.021322	0.99375	-0.0091	1	1
RP11-536C5.7	1.023121	1.021476	1.023655	1.10148	0.1394	1	1
RP11-17A19.2	1.040491	1.039074	1.040952	1.04807	0.0677	1	1
RP11-344F13.1	1.018773	1.017948	1.019042	1.06096	0.0854	1	1
RP1-266L20.4	1.019639	1.017948	1.020188	1.12484	0.1697	1	1
ZFP92	1.018773	1.017948	1.019042	1.06096	0.0854	1	1
MCCC1-AS1	1.019633	1.017948	1.020181	1.12443	0.1692	1	1
RP11-177F15.1	1.020509	1.021478	1.020194	0.94021	-0.0889	1	1
AP000350.10	1.019642	1.017948	1.020193	1.12511	0.1701	1	1
RP11-227H15.4	1.02225	1.021473	1.022502	1.04792	0.0675	1	1
HIST2H4B	1.02225	1.021475	1.022502	1.04785	0.0674	1	1
MIR3179-3	1.022229	1.021446	1.022484	1.04841	0.0682	1	1
RP11-456N14.4	1.018773	1.017948	1.019042	1.06096	0.0854	1	1
OTOF	1.019644	1.017948	1.020195	1.12523	0.1702	1	1
RP11-354M20.3	1.019639	1.017948	1.020189	1.12486	0.1697	1	1
EN2	1.019639	1.017948	1.020189	1.12486	0.1697	1	1
SFTPA2	1.019641	1.017948	1.020191	1.12499	0.1699	1	1
SLC47A2	1.02136	1.021481	1.021321	0.99259	-0.0107	1	1
CATSPERG	1.057872	1.056875	1.058196	1.02322	0.0331	1	1
CMYA5	1.076981	1.078369	1.076531	0.97655	-0.0342	1	1
RP11-567J20.2	1.022236	1.021439	1.022495	1.04924	0.0693	1	1
AC009014.3	1.019632	1.017948	1.020179	1.12435	0.1691	1	1
NADK2-AS1	1.018773	1.017948	1.019042	1.06096	0.0854	1	1
GNLY	1.019632	1.017948	1.020179	1.12432	0.1691	1	1
VPS37B	1.165418	1.166348	1.165116	0.9926	-0.0107	1	1
AC006126.3	1.019634	1.017948	1.020182	1.12451	0.1693	1	1
PSMG3-AS1	1.066415	1.067187	1.066165	0.98479	-0.0221	1	1
PLPP7	1.045637	1.046288	1.045425	0.98136	-0.0271	1	1
RP11-572C15.5	1.01963	1.017948	1.020177	1.12421	0.1689	1	1
CTD-2589M5.5	1.019641	1.017948	1.020191	1.12499	0.1699	1	1
CSMD1	1.019637	1.017948	1.020186	1.12471	0.1696	1	1
DDX39B-AS1	1.018773	1.017948	1.019042	1.06096	0.0854	1	1
RP11-15J22.8	1.021379	1.021453	1.021355	0.99544	-0.0066	1	1
POTEE	1.042245	1.042808	1.042062	0.98258	-0.0254	1	1
RP11-498C9.13	1.019629	1.017948	1.020175	1.12413	0.1688	1	1
ADAT1	1.159073	1.158966	1.159107	1.00089	0.0013	1	1

ANXA8	1.022234	1.021471	1.022482	1.04709	0.0664	1	1
RP11-307C12.13	1.027463	1.028595	1.027095	0.94751	-0.0778	1	1
FGD2	1.019631	1.017948	1.020178	1.12429	0.169	1	1
EHF	1.018773	1.017948	1.019042	1.06096	0.0854	1	1
RP11-156K13.1	1.019631	1.017948	1.020179	1.1243	0.169	1	1
RP11-143A12.3	1.019633	1.017948	1.020181	1.12441	0.1692	1	1
SLC3A1	1.019631	1.017948	1.020179	1.1243	0.169	1	1
MTMR2	1.391616	1.392163	1.391439	0.99815	-0.0027	1	1
CTD-2308B18.3	1.019641	1.017948	1.020191	1.12498	0.1699	1	1
MOG	1.019641	1.017948	1.020191	1.12499	0.1699	1	1
RP11-14N9.2	1.019642	1.017948	1.020192	1.12506	0.17	1	1
RP11-306I1.2	1.023123	1.021504	1.023649	1.09976	0.1372	1	1
RP11-596D21.1	1.024853	1.024935	1.024826	0.99563	-0.0063	1	1
KBTBD12	1.019634	1.017948	1.020182	1.12451	0.1693	1	1
CTC-297N7.9	1.01963	1.017948	1.020177	1.12419	0.1689	1	1
TUBE1	1.238195	1.236958	1.238597	1.00692	0.0099	1	1
CTD-2213F21.2	1.020491	1.021452	1.020178	0.94062	-0.0883	1	1
TRMT6	1.157449	1.1555	1.158083	1.01661	0.0238	1	1
CFTR	1.018773	1.017948	1.019042	1.06096	0.0854	1	1
SHOX2	1.027491	1.028617	1.027125	0.94785	-0.0773	1	1
RP5-1039K5.17	1.018773	1.017948	1.019042	1.06096	0.0854	1	1
RP11-569G13.2	1.019634	1.017948	1.020183	1.12453	0.1693	1	1
LINC01618	1.01964	1.017948	1.02019	1.12491	0.1698	1	1
QPCT	1.019642	1.017948	1.020192	1.12506	0.17	1	1
AGAP9	1.114393	1.113477	1.114691	1.0107	0.0154	1	1
CTD-2026G6.3	1.019631	1.017948	1.020179	1.1243	0.169	1	1
CYP2J2	1.019636	1.017948	1.020185	1.12468	0.1695	1	1
ATXN7L1	1.088179	1.088396	1.088108	0.99674	-0.0047	1	1
RP11-307E17.8	1.023091	1.021469	1.023618	1.10013	0.1377	1	1
RP11-309M7.1	1.019633	1.017948	1.020181	1.12443	0.1692	1	1
NXNL1	1.019643	1.017948	1.020194	1.12514	0.1701	1	1
SAMD12-AS1	1.01963	1.017948	1.020177	1.12421	0.1689	1	1
RP11-7F17.3	1.02136	1.021456	1.021329	0.99406	-0.0086	1	1
AC010642.1	1.02481	1.024979	1.024755	0.99102	-0.013	1	1
RP11-758P17.2	1.018773	1.017948	1.019042	1.06096	0.0854	1	1
DPH1	1.030006	1.028485	1.0305	1.07071	0.0986	1	1
PANX2	1.061229	1.060269	1.06154	1.02109	0.0301	1	1
DHRS7C	1.021365	1.021438	1.021342	0.99554	-0.0064	1	1
RP11-131K5.2	1.019631	1.017948	1.020179	1.1243	0.169	1	1
RP11-220I1.5	1.0231	1.021469	1.023631	1.10071	0.1384	1	1
ZIC4-AS1	1.019642	1.017948	1.020193	1.12509	0.17	1	1
RP11-545D19.1	1.019637	1.017948	1.020185	1.12469	0.1695	1	1
UTS2B	1.020502	1.021452	1.020192	0.94127	-0.0873	1	1
MFSD3	1.56367	1.562405	1.564082	1.00298	0.0043	1	1
PRSS48	1.019631	1.017948	1.020178	1.12426	0.169	1	1

FAM71F1	1.019636	1.017948	1.020185	1.12466	0.1695	1	1
ST8SIA6-AS1	1.020498	1.021458	1.020185	0.94071	-0.0882	1	1
RP4-778K6.3	1.019635	1.017948	1.020184	1.12458	0.1694	1	1
TXNDC2	1.018773	1.017948	1.019042	1.06096	0.0854	1	1
RP11-767C1.1	1.018773	1.017948	1.019042	1.06096	0.0854	1	1
CDH9	1.01964	1.017948	1.02019	1.12492	0.1698	1	1
RP11-257P3.3	1.02223	1.021447	1.022485	1.04841	0.0682	1	1
RP11-346C20.3	1.030866	1.032082	1.03047	0.94975	-0.0744	1	1
RP11-164N3.2	1.022243	1.021504	1.022484	1.04554	0.0642	1	1
RP11-679B19.2	1.023995	1.025062	1.023648	0.94356	-0.0838	1	1
SIGLEC14	1.019637	1.017948	1.020186	1.12474	0.1696	1	1
AOAH	1.022228	1.021484	1.02247	1.04588	0.0647	1	1
CTC-436K13.1	1.019633	1.017948	1.020181	1.12441	0.1692	1	1
RP11-432J22.2	1.021346	1.021438	1.021316	0.99432	-0.0082	1	1
PRR29-AS1	1.028349	1.028615	1.028262	0.98767	-0.0179	1	1
CTD-3193O13.11	1.023094	1.021444	1.02363	1.10194	0.14	1	1
IGSF22	1.019629	1.017948	1.020176	1.12416	0.1689	1	1
RP11-206L10.4	1.021378	1.021445	1.021356	0.99585	-0.006	1	1
RP11-197K3.1	1.019639	1.017948	1.020189	1.12486	0.1697	1	1
PILRA	1.028343	1.028587	1.028263	0.98866	-0.0165	1	1
TBC1D3I	1.018773	1.017948	1.019042	1.06096	0.0854	1	1
RP11-182N22.8	1.019638	1.017948	1.020187	1.12477	0.1696	1	1
RP4-760C5.3	1.019632	1.017948	1.02018	1.12437	0.1691	1	1
RP11-981G7.6	1.028358	1.028565	1.02829	0.99036	-0.014	1	1
CXorf21	1.018773	1.017948	1.019042	1.06096	0.0854	1	1
MKRN2OS	1.02137	1.021445	1.021346	0.9954	-0.0067	1	1
RP11-394I13.1	1.023116	1.02149	1.023645	1.10028	0.1379	1	1
ITGB7	1.020491	1.021456	1.020177	0.94042	-0.0886	1	1
OPLAH	1.072631	1.071014	1.073157	1.03018	0.0429	1	1
HCLS1	1.020494	1.021461	1.02018	0.94028	-0.0888	1	1
CLDN16	1.049992	1.049665	1.050098	1.00872	0.0125	1	1
PSMF1	1.883487	1.88316	1.883593	1.00049	0.0007	1	1
PHOX2A	1.020487	1.021444	1.020175	0.94084	-0.088	1	1
AJ239322.3	1.019634	1.017948	1.020182	1.12449	0.1693	1	1
RP11-428G5.5	1.017457	1.015453	1.018108	1.17175	0.2287	1	1
GP6	1.017458	1.015453	1.018109	1.17185	0.2288	1	1
RP11-903H12.3	1.01659	1.015453	1.01696	1.09747	0.1342	1	1
ZNF710	1.080608	1.078818	1.08119	1.03009	0.0428	1	1
CPO	1.016597	1.015453	1.016968	1.09802	0.1349	1	1
RIMBP3C	1.016593	1.015453	1.016963	1.0977	0.1345	1	1
RP5-894D12.5	1.016589	1.015453	1.016958	1.09739	0.1341	1	1
CITF22-1A6.3	1.022676	1.022504	1.022732	1.01013	0.0145	1	1
GORASP1	1.149568	1.148954	1.149767	1.00546	0.0079	1	1
MPPED1	1.023521	1.022589	1.023823	1.05462	0.0767	1	1
RP5-1050D4.5	1.023531	1.022443	1.023885	1.06425	0.0898	1	1

RP11-285E23.2	1.017474	1.015453	1.01813	1.17322	0.2305	1	1
RP11-1090M7.1	1.017481	1.015453	1.01814	1.17385	0.2312	1	1
AC067968.3	1.017475	1.015453	1.018132	1.17331	0.2306	1	1
RP11-164P12.3	1.016592	1.015453	1.016962	1.0976	0.1343	1	1
CLPS	1.016594	1.015453	1.016965	1.09783	0.1347	1	1
SCGB1A1	1.016594	1.015453	1.016965	1.09782	0.1346	1	1
GBX2	1.016597	1.015453	1.016969	1.09809	0.135	1	1
METTL4	1.266435	1.26558	1.266714	1.00427	0.0061	1	1
VIPR1	1.016593	1.015453	1.016964	1.09773	0.1345	1	1
RP11-466P24.6	1.016592	1.015453	1.016963	1.09767	0.1344	1	1
RP11-475I24.3	1.026098	1.026047	1.026115	1.00259	0.0037	1	1
TSPEAR	1.016588	1.015453	1.016957	1.0973	0.134	1	1
RP4-693M11.3	1.01659	1.015453	1.016959	1.09742	0.1341	1	1
LY6G6E	1.017461	1.015453	1.018114	1.17217	0.2292	1	1
RP11-48G14.1	1.016593	1.015453	1.016963	1.09767	0.1344	1	1
CXCR6	1.016597	1.015453	1.016969	1.09807	0.135	1	1
SHANK3	1.019176	1.018941	1.019252	1.01643	0.0235	1	1
RBM34	1.117709	1.117389	1.117813	1.00361	0.0052	1	1
RP11-122K13.12	1.016592	1.015453	1.016962	1.09762	0.1344	1	1
FAM198A	1.018321	1.018986	1.018104	0.95355	-0.0686	1	1
CTD-2126E3.3	1.017468	1.015453	1.018123	1.17272	0.2299	1	1
RP11-629N8.3	1.015731	1.015453	1.015821	1.02379	0.0339	1	1
PDE4A	1.060729	1.061514	1.060473	0.98309	-0.0246	1	1
PCDH20	1.016589	1.015453	1.016957	1.09733	0.134	1	1
RP3-400B16.1	1.016593	1.015453	1.016964	1.09773	0.1345	1	1
RP11-871F6.3	1.017456	1.015453	1.018107	1.17172	0.2286	1	1
RP11-326C3.7	1.020958	1.018945	1.021613	1.14084	0.1901	1	1
LINC00602	1.016599	1.015453	1.016971	1.09819	0.1351	1	1
AC012314.8	1.017466	1.015453	1.01812	1.17255	0.2296	1	1
RP11-192H23.7	1.015731	1.015453	1.015821	1.02379	0.0339	1	1
MTMR7	1.051989	1.050682	1.052413	1.03415	0.0484	1	1
RP11-186N15.3	1.020943	1.01896	1.021588	1.13858	0.1872	1	1
RHOXF1	1.027867	1.025984	1.02848	1.09603	0.1323	1	1
IL37	1.016588	1.015453	1.016956	1.09726	0.1339	1	1
AC084149.1	1.015731	1.015453	1.015821	1.02379	0.0339	1	1
LINC00887	1.031304	1.029557	1.031872	1.07832	0.1088	1	1
CTD-2033D15.1	1.016597	1.015453	1.016968	1.09803	0.1349	1	1
RP11-401P9.5	1.016595	1.015453	1.016966	1.09785	0.1347	1	1
LINC01082	1.016588	1.015453	1.016957	1.09729	0.1339	1	1
RP11-475C16.2	1.016596	1.015453	1.016968	1.09801	0.1349	1	1
RP11-789C17.3	1.016589	1.015453	1.016957	1.09733	0.134	1	1
FEZ1	2.138358	2.135182	2.139391	1.00371	0.0053	1	1
CLEC4G	1.017443	1.015453	1.01809	1.17061	0.2273	1	1
MRGPRF-AS1	1.01745	1.015453	1.018099	1.17119	0.228	1	1
SUN3	1.01659	1.015453	1.016959	1.09745	0.1342	1	1

RP11-15L13.4	1.020046	1.018966	1.020397	1.07546	0.1049	1	1
MLIP-AS1	1.015731	1.015453	1.015821	1.02379	0.0339	1	1
AMZ1	1.01659	1.015453	1.016959	1.09746	0.1342	1	1
MOGAT2	1.015731	1.015453	1.015821	1.02379	0.0339	1	1
CLCN1	1.016593	1.015453	1.016964	1.09772	0.1345	1	1
SSTR1	1.01659	1.015453	1.01696	1.09747	0.1342	1	1
RP11-298A10.1	1.016599	1.015453	1.016972	1.09824	0.1352	1	1
AP000866.1	1.026928	1.025982	1.027236	1.04829	0.068	1	1
AC105760.3	1.021791	1.02253	1.021551	0.95655	-0.0641	1	1
RP11-392O17.1	1.015731	1.015453	1.015821	1.02379	0.0339	1	1
HDAC10	1.283158	1.283602	1.283014	0.99793	-0.003	1	1
RP11-379K17.4	1.019194	1.018949	1.019274	1.01713	0.0245	1	1
CYP26C1	1.015731	1.015453	1.015821	1.02379	0.0339	1	1
RP11-62I21.1	1.016588	1.015453	1.016956	1.09725	0.1339	1	1
AC109333.10	1.015731	1.015453	1.015821	1.02379	0.0339	1	1
IL25	1.016599	1.015453	1.016972	1.09826	0.1352	1	1
ANKK1	1.018304	1.018942	1.018096	0.95538	-0.0659	1	1
QRFP	1.017472	1.015453	1.018128	1.17304	0.2303	1	1
AP002954.3	1.016589	1.015453	1.016958	1.09735	0.134	1	1
LINC00269	1.017473	1.015453	1.01813	1.17319	0.2304	1	1
RP11-120K24.3	1.018355	1.018971	1.018155	0.95696	-0.0635	1	1
NAALADL1	1.033871	1.032934	1.034175	1.0377	0.0534	1	1
SNED1	1.020042	1.01895	1.020396	1.07634	0.1061	1	1
AC105393.1	1.015731	1.015453	1.015821	1.02379	0.0339	1	1
CPA3	1.015731	1.015453	1.015821	1.02379	0.0339	1	1
PCAT4	1.020048	1.018967	1.020399	1.0755	0.105	1	1
LINC01214	1.016587	1.015453	1.016955	1.09716	0.1338	1	1
RP11-464F9.22	1.022668	1.022456	1.022736	1.01248	0.0179	1	1
RP11-818O24.3	1.017466	1.015453	1.01812	1.17258	0.2297	1	1
C4orf51	1.017466	1.015453	1.018121	1.17259	0.2297	1	1
DCTN5	1.671504	1.670383	1.671869	1.00222	0.0032	1	1
SLC25A47	1.015731	1.015453	1.015821	1.02379	0.0339	1	1
AC009312.1	1.018331	1.018976	1.018121	0.95494	-0.0665	1	1
CFAP99	1.01659	1.015453	1.016959	1.09746	0.1342	1	1
NAV2-AS5	1.016597	1.015453	1.016969	1.09809	0.135	1	1
AP004372.1	1.017464	1.015453	1.018117	1.17238	0.2294	1	1
RP11-90K6.1	1.02007	1.018972	1.020427	1.07668	0.1066	1	1
RP11-888D10.3	1.01747	1.015453	1.018126	1.17291	0.2301	1	1
CLK2	1.150648	1.149171	1.151128	1.01312	0.0188	1	1
AL035610.2	1.017464	1.015453	1.018117	1.17238	0.2294	1	1
ITGAX	1.018317	1.018956	1.01811	0.95537	-0.0659	1	1
CTD-2531D15.5	1.017467	1.015453	1.018122	1.17268	0.2298	1	1
RP11-91I20.3	1.017447	1.015453	1.018096	1.17097	0.2277	1	1
CTD-2017F17.1	1.016591	1.015453	1.016961	1.09753	0.1343	1	1
OLFM4	1.017466	1.015453	1.018121	1.17261	0.2297	1	1

RP1-225E12.2	1.016596	1.015453	1.016968	1.09798	0.1349	1	1
RP11-470P21.2	1.015731	1.015453	1.015821	1.02379	0.0339	1	1
FOXF1	1.020044	1.019005	1.020382	1.07248	0.101	1	1
RP11-334J6.6	1.015731	1.015453	1.015821	1.02379	0.0339	1	1
AP001628.7	1.015731	1.015453	1.015821	1.02379	0.0339	1	1
GLIS3-AS1	1.016591	1.015453	1.016961	1.09755	0.1343	1	1
ANKRD34C	1.018313	1.01899	1.018093	0.95274	-0.0698	1	1
RP11-731D1.4	1.017452	1.015453	1.018101	1.17134	0.2282	1	1
RP11-452H21.1	1.017475	1.015453	1.018133	1.17337	0.2307	1	1
SLC22A9	1.017457	1.015453	1.018108	1.1718	0.2287	1	1
RP3-468B3.2	1.017446	1.015453	1.018094	1.17085	0.2276	1	1
TAS2R10	1.01833	1.018969	1.018123	0.95537	-0.0659	1	1
KRTAP10-4	1.0166	1.015453	1.016973	1.09835	0.1353	1	1
ALDH4A1	1.110829	1.110405	1.110967	1.00509	0.0073	1	1
RP11-587P21.3	1.015731	1.015453	1.015821	1.02379	0.0339	1	1
KRT79	1.016592	1.015453	1.016962	1.09762	0.1344	1	1
RP3-395M20.9	1.016592	1.015453	1.016962	1.0976	0.1344	1	1
TBC1D26.1	1.01659	1.015453	1.01696	1.09746	0.1342	1	1
AATK-AS1	1.023512	1.022496	1.023843	1.05987	0.0839	1	1
RP11-332K15.1	1.018321	1.01896	1.018113	0.95535	-0.0659	1	1
RP11-428K3.1	1.018318	1.018985	1.018101	0.95342	-0.0688	1	1
RP11-403A3.1	1.015731	1.015453	1.015821	1.02379	0.0339	1	1
TTYH2	1.066771	1.064945	1.067365	1.03726	0.0528	1	1
UPB1	1.024386	1.022456	1.025013	1.11388	0.1556	1	1
LINC00589	1.015731	1.015453	1.015821	1.02379	0.0339	1	1
HEPACAM2	1.017464	1.015453	1.018118	1.17243	0.2295	1	1
RP11-431K24.3	1.015731	1.015453	1.015821	1.02379	0.0339	1	1
AC074366.3	1.015731	1.015453	1.015821	1.02379	0.0339	1	1
UST-AS1	1.016591	1.015453	1.01696	1.09751	0.1342	1	1
RP11-118G23.1	1.017453	1.015453	1.018103	1.17147	0.2283	1	1
RP11-119J18.1	1.020088	1.018972	1.020451	1.07799	0.1083	1	1
ALS2CR12	1.016599	1.015453	1.016971	1.09819	0.1351	1	1
RP11-104H15.8	1.016592	1.015453	1.016962	1.0976	0.1343	1	1
LINC00434	1.017461	1.015453	1.018114	1.17217	0.2292	1	1
RP5-1070A16.1	1.019196	1.018963	1.019271	1.01623	0.0232	1	1
KLRC1	1.017468	1.015453	1.018122	1.17271	0.2298	1	1
RP11-1L9.1	1.018313	1.018986	1.018095	0.95305	-0.0694	1	1
LINC01332	1.015731	1.015453	1.015821	1.02379	0.0339	1	1
RP11-116D17.5	1.016596	1.015453	1.016968	1.09801	0.1349	1	1
RP4-655J12.4	1.016597	1.015453	1.016968	1.09802	0.1349	1	1
RP11-522B15.3	1.035605	1.03653	1.035304	0.96645	-0.0492	1	1
RP11-473M20.5	1.019205	1.018958	1.019285	1.01723	0.0246	1	1
AL121578.2	1.017443	1.015453	1.01809	1.17061	0.2273	1	1
RP11-462G2.1	1.016597	1.015453	1.016969	1.09809	0.135	1	1
PRDM12	1.019177	1.018945	1.019253	1.01627	0.0233	1	1

SYBU	1.054572	1.05414	1.054713	1.01059	0.0152	1	1
MDFIC	1.299305	1.297552	1.299875	1.00781	0.0112	1	1
CTD-2017C7.1	1.017459	1.015453	1.018111	1.17198	0.2289	1	1
RP11-2E11.5	1.019192	1.018948	1.019271	1.01703	0.0244	1	1
LINC01228	1.017463	1.015453	1.018117	1.17233	0.2294	1	1
GUCA2A	1.015731	1.015453	1.015821	1.02379	0.0339	1	1
OR4D10	1.016595	1.015453	1.016966	1.09785	0.1347	1	1
TBC1D15	1.111022	1.111259	1.110945	0.99718	-0.0041	1	1
TMEM256-PLSCR3.1	1.017555	1.015961	1.018074	1.13236	0.1793	1	1
RP11-864J10.4	1.017547	1.015961	1.018062	1.13162	0.1784	1	1
PXT1	1.018412	1.019491	1.018061	0.92666	-0.1099	1	1
HEXA-AS1	1.024488	1.023012	1.024968	1.08497	0.1176	1	1
LINC01539	1.017547	1.015961	1.018062	1.13162	0.1784	1	1
RP11-89H19.2	1.016686	1.015961	1.016921	1.06016	0.0843	1	1
RP11-195C7.1	1.01755	1.015961	1.018067	1.13192	0.1788	1	1
GON4L	1.447261	1.445834	1.447725	1.00424	0.0061	1	1
MMEL1	1.019281	1.019479	1.019217	0.98653	-0.0196	1	1
RP11-254F7.3	1.016686	1.015961	1.016921	1.06016	0.0843	1	1
RP11-34P13.7	1.022768	1.023045	1.022678	0.98405	-0.0232	1	1
SLA	1.017547	1.015961	1.018062	1.13165	0.1784	1	1
RNF212B	1.021874	1.022986	1.021513	0.93589	-0.0956	1	1
RP11-118B22.4	1.017546	1.015961	1.018062	1.13161	0.1784	1	1
RP11-253M7.1	1.034022	1.033681	1.034133	1.01342	0.0192	1	1
SMIM17	1.019278	1.019451	1.019221	0.9882	-0.0171	1	1
AC007743.1	1.02104	1.019491	1.021543	1.10526	0.1444	1	1
RP11-118G23.3	1.017551	1.015961	1.018067	1.13196	0.1788	1	1
RP11-347C12.3	1.021008	1.019449	1.021515	1.10623	0.1457	1	1
RP11-262K1.1	1.016686	1.015961	1.016921	1.06016	0.0843	1	1
SYNE3	1.027924	1.026542	1.028373	1.06899	0.0962	1	1
FA2H	1.017549	1.015961	1.018065	1.13184	0.1787	1	1
TNNC2	1.036572	1.037132	1.03639	0.98003	-0.0291	1	1
RP11-176H8.1	1.045215	1.044109	1.045574	1.03321	0.0471	1	1
AC005339.2	1.017548	1.015961	1.018064	1.13177	0.1786	1	1
PRR27	1.016686	1.015961	1.016921	1.06016	0.0843	1	1
GIPC2	1.018412	1.019501	1.018058	0.92599	-0.1109	1	1
RP11-799M12.2	1.019286	1.019448	1.019234	0.98897	-0.016	1	1
CTC-265N9.1	1.016686	1.015961	1.016921	1.06016	0.0843	1	1
RP4-591N18.2	1.017554	1.015961	1.018071	1.13221	0.1791	1	1
KCNB1	1.02445	1.022994	1.024923	1.08388	0.1162	1	1
LYRM9	1.38153	1.379322	1.382248	1.00771	0.0111	1	1
CCDC168	1.027931	1.026513	1.028392	1.07088	0.0988	1	1
LINC00174	1.032248	1.033523	1.031834	0.94961	-0.0746	1	1
RP11-495O11.1	1.017548	1.015961	1.018063	1.13171	0.1785	1	1
RP11-235E17.2	1.020144	1.019497	1.020355	1.04398	0.0621	1	1
CTD-2547L24.4	1.01755	1.015961	1.018066	1.13191	0.1788	1	1

RP11-434B12.1	1.020999	1.019495	1.021488	1.10219	0.1404	1	1
RP11-218I7.2	1.01755	1.015961	1.018066	1.1319	0.1787	1	1
THOC5	1.233102	1.231198	1.233721	1.01091	0.0157	1	1
RP11-370I10.11	1.020991	1.019457	1.02149	1.10446	0.1433	1	1
RP11-242J7.1	1.019273	1.019487	1.019203	0.98541	-0.0212	1	1
RP11-367G18.1	1.020983	1.019452	1.021481	1.10428	0.1431	1	1
CD5	1.0184	1.01946	1.018056	0.92784	-0.108	1	1
PLA2G2C	1.017546	1.015961	1.018061	1.13156	0.1783	1	1
ZSCAN5B	1.017544	1.015961	1.018058	1.13138	0.1781	1	1
RP11-1C8.7	1.021863	1.022972	1.021502	0.93603	-0.0954	1	1
RTEL1	1.017553	1.015961	1.018071	1.13217	0.1791	1	1
RP11-75C23.1	1.016686	1.015961	1.016921	1.06016	0.0843	1	1
ZAR1L	1.019266	1.019484	1.019195	0.98517	-0.0216	1	1
CCDC144A	1.051386	1.051287	1.051418	1.00255	0.0037	1	1
SLC6A4	1.023663	1.023065	1.023857	1.03433	0.0487	1	1
PLA2G1B	1.016686	1.015961	1.016921	1.06016	0.0843	1	1
AC012456.4	1.018413	1.019477	1.018067	0.92762	-0.1084	1	1
AC241585.1	1.016686	1.015961	1.016921	1.06016	0.0843	1	1
PRIMA1	1.016686	1.015961	1.016921	1.06016	0.0843	1	1
SAPCD1	1.018402	1.019462	1.018057	0.9278	-0.1081	1	1
ADH1C	1.016686	1.015961	1.016921	1.06016	0.0843	1	1
OTUD1	1.06346	1.061735	1.064021	1.03704	0.0525	1	1
NPC1L1	1.017549	1.015961	1.018066	1.13187	0.1787	1	1
RP11-166N6.2	1.017543	1.015961	1.018057	1.13133	0.178	1	1
CYP4F26P	1.017545	1.015961	1.01806	1.13153	0.1783	1	1
RP11-290O12.2	1.066792	1.065115	1.067337	1.03414	0.0484	1	1
AMBP	1.017547	1.015961	1.018062	1.13162	0.1784	1	1
CTB-70G10.1	1.016686	1.015961	1.016921	1.06016	0.0843	1	1
RP11-510M2.1	1.017544	1.015961	1.018059	1.13142	0.1781	1	1
GDNF-AS1	1.016686	1.015961	1.016921	1.06016	0.0843	1	1
ACADL	1.026219	1.026472	1.026137	0.98735	-0.0184	1	1
RP5-851M4.1	1.018408	1.019455	1.018067	0.92866	-0.1068	1	1
LINC00458	1.021006	1.019469	1.021505	1.10462	0.1435	1	1
ACOT6	1.017543	1.015961	1.018057	1.13134	0.178	1	1
HNF4A	1.016686	1.015961	1.016921	1.06016	0.0843	1	1
RP1-56K13.2	1.018415	1.019488	1.018066	0.92705	-0.1093	1	1
ZNF366	1.01755	1.015961	1.018066	1.13191	0.1788	1	1
PTAFR	1.017549	1.015961	1.018066	1.13187	0.1787	1	1
AC005013.5	1.017553	1.015961	1.01807	1.13214	0.179	1	1
RP11-100M12.3	1.01755	1.015961	1.018067	1.13195	0.1788	1	1
RP11-452H21.4	1.024462	1.022986	1.024942	1.08509	0.1178	1	1
ACTN1-AS1	1.016686	1.015961	1.016921	1.06016	0.0843	1	1
ACSL6	1.021008	1.019495	1.0215	1.10282	0.1412	1	1
LINC00540	1.018405	1.019456	1.018063	0.9284	-0.1072	1	1
CTRL	1.019266	1.019457	1.019204	0.98698	-0.0189	1	1

AC114765.2	1.017554	1.015961	1.018072	1.13228	0.1792	1	1
RP11-235E17.6	1.017542	1.015961	1.018056	1.13123	0.1779	1	1
RP11-85O21.5	1.019272	1.019474	1.019207	0.98629	-0.0199	1	1
RP11-407N8.6	1.017545	1.015961	1.018059	1.13146	0.1782	1	1
RP11-810M2.2	1.016686	1.015961	1.016921	1.06016	0.0843	1	1
RUBCNL	1.01927	1.019491	1.019198	0.98498	-0.0218	1	1
NAAA	1.082422	1.082596	1.082366	0.99722	-0.004	1	1
RP5-1056L3.1	1.017546	1.015961	1.018061	1.13159	0.1784	1	1
RP11-80H5.6	1.01755	1.015961	1.018067	1.13192	0.1788	1	1
RP1-28O10.1	1.019285	1.019457	1.019229	0.98825	-0.0171	1	1
RP11-737O24.2	1.021027	1.01946	1.021537	1.10674	0.1463	1	1
RP11-352D3.2	1.017545	1.015961	1.018059	1.13146	0.1782	1	1
HNRNPCL2	1.016686	1.015961	1.016921	1.06016	0.0843	1	1
GLYAT	1.016686	1.015961	1.016921	1.06016	0.0843	1	1
RP11-160E2.11	1.017545	1.015961	1.01806	1.1315	0.1782	1	1
RNF148	1.017551	1.015961	1.018068	1.13199	0.1789	1	1
PLIN4	1.016686	1.015961	1.016921	1.06016	0.0843	1	1
VAV1	1.016686	1.015961	1.016921	1.06016	0.0843	1	1
ANKDD1B	1.016686	1.015961	1.016921	1.06016	0.0843	1	1
SLC6A14	1.016686	1.015961	1.016921	1.06016	0.0843	1	1
CMB9-55A18.1	1.017551	1.015961	1.018067	1.13197	0.1788	1	1
PARP12	1.02361	1.022995	1.02381	1.03541	0.0502	1	1
KCNV2	1.020148	1.019481	1.020365	1.04536	0.064	1	1
RP11-111K18.2	1.021012	1.019481	1.02151	1.10413	0.1429	1	1
RP11-218C14.5	1.017549	1.015961	1.018065	1.13184	0.1787	1	1
RP11-17E3.1	1.018409	1.019457	1.018069	0.92862	-0.1068	1	1
ANKRD20A1	1.017545	1.015961	1.01806	1.1315	0.1782	1	1
CTC-487M23.7	1.01755	1.015961	1.018067	1.13195	0.1788	1	1
SRL	1.016686	1.015961	1.016921	1.06016	0.0843	1	1
CTD-2192J16.22	1.017853	1.016045	1.01844	1.14929	0.2007	1	1
MAGEA10	1.017859	1.016045	1.018448	1.14977	0.2013	1	1
PCDHA2	1.02045	1.019558	1.02074	1.06043	0.0846	1	1
ELFN2	1.020499	1.01957	1.020801	1.06291	0.088	1	1
KB-68A7.1	1.023062	1.02305	1.023065	1.00066	0.0009	1	1
PLAC1	1.017861	1.016045	1.018451	1.14997	0.2016	1	1
AC007950.2	1.02047	1.01962	1.020746	1.05737	0.0805	1	1
RP11-800A3.7	1.028253	1.026559	1.028804	1.08456	0.1171	1	1
ITK	1.016995	1.016045	1.017304	1.07848	0.109	1	1
TBX3	1.162739	1.161136	1.163261	1.01319	0.0189	1	1
FYCO1	1.10447	1.103774	1.104696	1.00888	0.0128	1	1
RP11-45M22.3	1.017853	1.016045	1.018441	1.14935	0.2008	1	1
RP11-89G4.1	1.017861	1.016045	1.018451	1.14997	0.2016	1	1
RP11-67L2.2	1.074062	1.072333	1.074624	1.03168	0.045	1	1
TDRD10	1.018725	1.01956	1.018453	0.9434	-0.0841	1	1
RP11-12L8.1	1.017852	1.016045	1.01844	1.14924	0.2007	1	1

LDHC	1.017852	1.016045	1.018439	1.14923	0.2007	1	1
RP11-463C8.7	1.017855	1.016045	1.018443	1.14944	0.2009	1	1
RP11-65L3.2	1.023044	1.023112	1.023022	0.99608	-0.0057	1	1
AC023115.2	1.025701	1.026676	1.025384	0.95157	-0.0716	1	1
LLNLR-307A6.1	1.017857	1.016045	1.018446	1.14964	0.2012	1	1
RP11-547D23.1	1.016995	1.016045	1.017304	1.07848	0.109	1	1
ZNF474	1.023939	1.023092	1.024215	1.04862	0.0685	1	1
RP11-25G10.2	1.017856	1.016045	1.018444	1.14953	0.201	1	1
KCNU1	1.017855	1.016045	1.018444	1.1495	0.201	1	1
KB-1568E2.1	1.017858	1.016045	1.018447	1.1497	0.2013	1	1
DHRS9	1.019587	1.019559	1.019596	1.00189	0.0027	1	1
RP11-571M6.17	1.041251	1.0407	1.041431	1.01796	0.0257	1	1
RP3-329E20.2	1.017858	1.016045	1.018448	1.14974	0.2013	1	1
DDX47	1.036028	1.037192	1.03565	0.95853	-0.0611	1	1
AC107057.1	1.017857	1.016045	1.018446	1.14966	0.2012	1	1
CTD-2245E15.3	1.017853	1.016045	1.018441	1.1493	0.2008	1	1
SERPINA3.1	1.016995	1.016045	1.017304	1.07848	0.109	1	1
RP11-499A10.3	1.017854	1.016045	1.018442	1.14939	0.2009	1	1
RP11-276H7.2	1.019585	1.019565	1.019591	1.00131	0.0019	1	1
WARS2-IT1	1.017857	1.016045	1.018446	1.14964	0.2012	1	1
ACRV1	1.016995	1.016045	1.017304	1.07848	0.109	1	1
VRTN	1.017855	1.016045	1.018443	1.14944	0.2009	1	1
NADK	1.37019	1.367801	1.370966	1.00861	0.0124	1	1
RP3-395M20.2	1.020453	1.019551	1.020746	1.06113	0.0856	1	1
DNAH12	1.017857	1.016045	1.018446	1.14966	0.2012	1	1
PLCD4	1.024809	1.023072	1.025373	1.09976	0.1372	1	1
LINC00642	1.01786	1.016045	1.01845	1.14989	0.2015	1	1
ERVK-28	1.017851	1.016045	1.018439	1.14919	0.2006	1	1
ANKHD1-EIF4EBP3	1.025651	1.026601	1.025342	0.9527	-0.0699	1	1
HIST1H2BB	1.017859	1.016045	1.018448	1.14977	0.2013	1	1
CTD-2410N18.3	1.019585	1.019566	1.019591	1.00128	0.0018	1	1
RP11-141O15.1	1.016995	1.016045	1.017304	1.07848	0.109	1	1
GOLGA8K	1.017856	1.016045	1.018445	1.14959	0.2011	1	1
RP11-465B22.8	1.016995	1.016045	1.017304	1.07848	0.109	1	1
PLPPR4	1.018739	1.019571	1.018468	0.94367	-0.0836	1	1
RP11-158M2.5	1.017858	1.016045	1.018447	1.14971	0.2013	1	1
RP3-523C21.2	1.016995	1.016045	1.017304	1.07848	0.109	1	1
RP11-1228E12.2	1.02477	1.023025	1.025337	1.10038	0.138	1	1
AP000679.2	1.016995	1.016045	1.017304	1.07848	0.109	1	1
RP11-863P13.3	1.017852	1.016045	1.01844	1.14924	0.2007	1	1
MPND	1.274864	1.273397	1.275341	1.00711	0.0102	1	1
RP11-463J10.3	1.016995	1.016045	1.017304	1.07848	0.109	1	1
MADD	1.179115	1.178326	1.179372	1.00586	0.0084	1	1
RP1-293L6.1	1.017854	1.016045	1.018441	1.14936	0.2008	1	1
CTD-2233C11.2	1.017858	1.016045	1.018447	1.14971	0.2013	1	1

ADM2	1.029131	1.030204	1.028782	0.95292	-0.0696	1	1
AL023806.1	1.022151	1.02303	1.021865	0.94942	-0.0749	1	1
PLG	1.017856	1.016045	1.018444	1.14953	0.201	1	1
FBXL14	1.023022	1.023095	1.022999	0.99584	-0.006	1	1
SYNDIG1L	1.020484	1.019558	1.020785	1.06276	0.0878	1	1
RP5-965G21.4	1.016995	1.016045	1.017304	1.07848	0.109	1	1
GDA	1.022217	1.023086	1.021935	0.95014	-0.0738	1	1
HBA2	1.018723	1.019564	1.018449	0.94302	-0.0846	1	1
ARMS2	1.017853	1.016045	1.018441	1.14933	0.2008	1	1
RP11-424D14.1	1.017854	1.016045	1.018442	1.14937	0.2008	1	1
RP11-568J23.4	1.017853	1.016045	1.018441	1.14932	0.2008	1	1
LHB	1.016995	1.016045	1.017304	1.07848	0.109	1	1
TPO	1.023887	1.02309	1.024146	1.04577	0.0646	1	1
RP11-37N22.1	1.024822	1.023041	1.025401	1.10245	0.1407	1	1
AC016999.2	1.017855	1.016045	1.018443	1.14945	0.2009	1	1
SCHIP1	1.016995	1.016045	1.017304	1.07848	0.109	1	1
NPHP3-AS1	1.016995	1.016045	1.017304	1.07848	0.109	1	1
NUDC	2.649654	2.650272	2.649453	0.9995	-0.0007	1	1
RS1	1.016995	1.016045	1.017304	1.07848	0.109	1	1
TACR2	1.018719	1.019555	1.018447	0.94332	-0.0842	1	1
CTD-2396E7.9	1.017864	1.016045	1.018455	1.15023	0.2019	1	1
AC016751.3	1.017854	1.016045	1.018441	1.14936	0.2008	1	1
PLVAP	1.017853	1.016045	1.018441	1.1493	0.2008	1	1
RP11-81H14.1	1.018719	1.019558	1.018446	0.94314	-0.0845	1	1
AC135178.7	1.017867	1.016045	1.01846	1.15051	0.2023	1	1
C1orf141	1.018717	1.019547	1.018447	0.9437	-0.0836	1	1
VCX3B	1.033451	1.033692	1.033372	0.99051	-0.0138	1	1
RP11-575I8.1	1.017853	1.016045	1.01844	1.14929	0.2007	1	1
AC007040.6	1.017857	1.016045	1.018447	1.14968	0.2012	1	1
SLC39A5	1.019592	1.019552	1.019605	1.00275	0.004	1	1
TRPM2	1.016995	1.016045	1.017304	1.07848	0.109	1	1
RP11-524H19.2	1.016995	1.016045	1.017304	1.07848	0.109	1	1
RP11-96B5.4	1.016995	1.016045	1.017304	1.07848	0.109	1	1
RP11-85L21.4	1.017857	1.016045	1.018446	1.14963	0.2012	1	1
GSTA3	1.016995	1.016045	1.017304	1.07848	0.109	1	1
CTD-2516F10.4	1.016995	1.016045	1.017304	1.07848	0.109	1	1
RP11-220I1.2	1.017857	1.016045	1.018446	1.14962	0.2012	1	1
RP11-193H5.1	1.017855	1.016045	1.018444	1.1495	0.201	1	1
RP11-243A14.1	1.019571	1.019546	1.019579	1.00171	0.0025	1	1
FAM189A2	1.019577	1.019565	1.01958	1.00075	0.0011	1	1
D2HGDH	1.331352	1.32993	1.331815	1.00571	0.0082	1	1
MS4A7	1.016995	1.016045	1.017304	1.07848	0.109	1	1
NOS1	1.017859	1.016045	1.018448	1.14977	0.2013	1	1
RP11-334C17.6	1.023091	1.023059	1.023101	1.00183	0.0026	1	1
AOC2	1.031742	1.030193	1.032246	1.068	0.0949	1	1

NRARP	1.035187	1.033694	1.035673	1.05872	0.0823	1	1
RP11-343L5.2	1.020452	1.019539	1.020749	1.06191	0.0867	1	1
CCNB3	1.024758	1.023076	1.025304	1.09657	0.133	1	1
RP11-411K7.1	1.018723	1.019546	1.018455	0.94421	-0.0828	1	1
HMX2	1.019576	1.019545	1.019585	1.00206	0.003	1	1
RP11-260M19.1	1.017855	1.016045	1.018443	1.14944	0.2009	1	1
RP3-461P17.9	1.017858	1.016045	1.018447	1.14973	0.2013	1	1
AC008753.4	1.016995	1.016045	1.017304	1.07848	0.109	1	1
RP11-164O23.8	1.017855	1.016045	1.018443	1.14948	0.201	1	1
CACNA1I	1.017853	1.016045	1.018441	1.14935	0.2008	1	1
RP11-320M16.2	1.016995	1.016045	1.017304	1.07848	0.109	1	1
SLC19A3	1.023042	1.023141	1.02301	0.99433	-0.0082	1	1
AF127577.12	1.017858	1.016045	1.018447	1.1497	0.2013	1	1
PLA2G4B	1.017866	1.016045	1.018458	1.1504	0.2021	1	1
ELDR	1.020435	1.019546	1.020725	1.06032	0.0845	1	1
RP11-574F21.2	1.020467	1.019585	1.020753	1.05963	0.0836	1	1
RP13-631K18.2	1.022226	1.023208	1.021907	0.94392	-0.0833	1	1
LINC00968	1.016995	1.016045	1.017304	1.07848	0.109	1	1
SLC25A41	1.018717	1.01956	1.018443	0.94286	-0.0849	1	1
CD37	1.027439	1.026606	1.027709	1.04146	0.0586	1	1
RP11-320N7.2	1.017854	1.016045	1.018441	1.14936	0.2008	1	1
FAM160A1	1.039523	1.04068	1.039147	0.9623	-0.0554	1	1
RP11-65B7.2	1.017861	1.016045	1.018451	1.14997	0.2016	1	1
FPR1	1.020449	1.01956	1.020739	1.06029	0.0845	1	1
ZBTB1	1.286397	1.287432	1.28606	0.99523	-0.0069	1	1
DNAH1	1.03526	1.033774	1.035744	1.05833	0.0818	1	1
SLCO1C1	1.017863	1.016045	1.018454	1.15013	0.2018	1	1
NPIPB13	1.022197	1.023113	1.0219	0.94752	-0.0778	1	1
RP5-858L17.1	1.028258	1.026661	1.028778	1.07937	0.1102	1	1
MASP1	1.025652	1.026617	1.025338	0.95195	-0.071	1	1
SHROOM4	1.154641	1.153622	1.154972	1.00879	0.0126	1	1
RP5-823G15.5	1.016995	1.016045	1.017304	1.07848	0.109	1	1
RP11-667K14.4	1.017857	1.016045	1.018446	1.14963	0.2012	1	1
RP11-143I21.1	1.015382	1.015693	1.015281	0.97379	-0.0383	1	1
U52111.14	1.0128	1.012186	1.013	1.06675	0.0932	1	1
RP4-809F18.1	1.015376	1.015678	1.015278	0.97444	-0.0374	1	1
RP11-727A23.10	1.01452	1.015682	1.014143	0.90185	-0.149	1	1
RP11-286N22.10	1.013662	1.012186	1.014142	1.16048	0.2147	1	1
CCL18	1.014528	1.015709	1.014144	0.90034	-0.1515	1	1
KLRB1	1.013662	1.012186	1.014141	1.1604	0.2146	1	1
LINC01433	1.0128	1.012186	1.013	1.06675	0.0932	1	1
MYOZ2	1.0128	1.012186	1.013	1.06675	0.0932	1	1
SPEM1	1.015385	1.015706	1.01528	0.9729	-0.0396	1	1
ANKFY1	1.317078	1.317718	1.31687	0.99733	-0.0039	1	1
MAGIX	1.085198	1.085819	1.084996	0.99041	-0.0139	1	1

AC004893.11	1.045594	1.043901	1.046144	1.0511	0.0719	1	1
HHIPL2	1.0128	1.012186	1.013	1.06675	0.0932	1	1
AC093702.1	1.013665	1.012186	1.014145	1.16076	0.2151	1	1
CTB-111F10.1	1.01366	1.012186	1.014139	1.16021	0.2144	1	1
TNFAIP8L2	1.013659	1.012186	1.014138	1.16012	0.2143	1	1
GLTPD2	1.019715	1.019272	1.019859	1.03044	0.0433	1	1
EFCAB6-AS1	1.013658	1.012186	1.014136	1.16001	0.2141	1	1
RP11-10A14.3	1.01366	1.012186	1.014139	1.16024	0.2144	1	1
RP11-849F2.4	1.01366	1.012186	1.014139	1.16027	0.2145	1	1
AC025165.8	1.01452	1.015696	1.014137	0.90072	-0.1508	1	1
PRKG1-AS1	1.01366	1.012186	1.014139	1.16026	0.2144	1	1
NAV2-AS1	1.013661	1.012186	1.014141	1.16039	0.2146	1	1
HSPA2	1.028297	1.029675	1.027849	0.93845	-0.0917	1	1
ATP2B2	1.013662	1.012186	1.014142	1.16046	0.2147	1	1
RP11-566J3.2	1.015386	1.015717	1.015279	0.97214	-0.0408	1	1
LINC00265	1.018831	1.019206	1.018709	0.97415	-0.0378	1	1
PKD1L1	1.014523	1.015687	1.014144	0.90165	-0.1494	1	1
ATOH1	1.013662	1.012186	1.014142	1.16048	0.2147	1	1
DNAH10OS	1.028303	1.029762	1.027829	0.93503	-0.0969	1	1
XXbac-B33L19.4	1.013661	1.012186	1.01414	1.16032	0.2145	1	1
CCNT1	1.26813	1.268529	1.268	0.99803	-0.0028	1	1
GTF3C2-AS1	1.013655	1.012186	1.014132	1.1597	0.2137	1	1
RP11-407N8.5	1.013657	1.012186	1.014136	1.15995	0.2141	1	1
CACNG2	1.013662	1.012186	1.014141	1.16042	0.2147	1	1
RP5-857K21.4	1.01366	1.012186	1.014139	1.16026	0.2144	1	1
RP11-1M18.1	1.0128	1.012186	1.013	1.06675	0.0932	1	1
RP4-784A16.2	1.0128	1.012186	1.013	1.06675	0.0932	1	1
RP11-329N22.1	1.013658	1.012186	1.014137	1.16007	0.2142	1	1
RP11-388P9.2	1.013662	1.012186	1.014142	1.16047	0.2147	1	1
CTB-167B5.1	1.014517	1.015694	1.014134	0.90058	-0.1511	1	1
RP11-567C2.1	1.015385	1.0157	1.015283	0.97347	-0.0388	1	1
SBK2	1.015394	1.015698	1.015295	0.97432	-0.0375	1	1
RP11-797D24.3	1.0128	1.012186	1.013	1.06675	0.0932	1	1
RP11-313P22.1	1.013666	1.012186	1.014147	1.16086	0.2152	1	1
RP11-706O15.5	1.024011	1.022698	1.024437	1.07661	0.1065	1	1
STPG3	1.01366	1.012186	1.014139	1.1602	0.2144	1	1
GFAP	1.016242	1.015681	1.016424	1.04742	0.0668	1	1
LINC00242	1.016241	1.015692	1.016419	1.04635	0.0654	1	1
SLC6A7	1.013657	1.012186	1.014135	1.1599	0.214	1	1
TCN1	1.0128	1.012186	1.013	1.06675	0.0932	1	1
LINC00407	1.013655	1.012186	1.014133	1.15973	0.2138	1	1
RP13-379L11.2	1.013659	1.012186	1.014138	1.16012	0.2143	1	1
PTCHD1-AS	1.013662	1.012186	1.014141	1.16042	0.2146	1	1
DCAF4L2	1.013662	1.012186	1.014141	1.16043	0.2147	1	1
SATB2-AS1	1.013655	1.012186	1.014133	1.15973	0.2138	1	1

TUBB8	1.015379	1.015689	1.015279	0.97385	-0.0382	1	1
STX1B	1.06025	1.061374	1.059885	0.97575	-0.0354	1	1
GATAD2B	1.406922	1.405965	1.407233	1.00312	0.0045	1	1
BICD1	1.645839	1.643639	1.646554	1.00453	0.0065	1	1
HIST1H2AK	1.015378	1.015678	1.015281	0.97465	-0.037	1	1
XX-C00717C00720L.1	1.01366	1.012186	1.014139	1.16021	0.2144	1	1
AC007292.7	1.0128	1.012186	1.013	1.06675	0.0932	1	1
RP11-317N12.1	1.015396	1.015694	1.015299	0.97479	-0.0368	1	1
ZSWIM1	1.03351	1.033235	1.033599	1.01097	0.0157	1	1
USH1C	1.014528	1.015711	1.014144	0.90026	-0.1516	1	1
RP11-353K11.1	1.013663	1.012186	1.014143	1.16053	0.2148	1	1
RP11-290F24.6	1.030048	1.029799	1.030128	1.01105	0.0159	1	1
RP11-166N6.3	1.013663	1.012186	1.014143	1.1606	0.2149	1	1
ATP8B2	1.096434	1.096566	1.096391	0.99819	-0.0026	1	1
CTD-2193G5.1	1.013666	1.012186	1.014147	1.16092	0.2153	1	1
MANEAL	1.026561	1.026235	1.026666	1.01644	0.0235	1	1
XIST	1.015381	1.015691	1.01528	0.97378	-0.0383	1	1
RP11-46D6.1	1.017118	1.015682	1.017585	1.12132	0.1652	1	1
PUM2	1.650397	1.648357	1.65106	1.00417	0.006	1	1
IDO1	1.0128	1.012186	1.013	1.06675	0.0932	1	1
CHRNA6	1.014522	1.015681	1.014146	0.9021	-0.1486	1	1
MRAP	1.01366	1.012186	1.014139	1.16026	0.2144	1	1
RP11-105N14.2	1.013663	1.012186	1.014143	1.16053	0.2148	1	1
RP11-69E11.4	1.046432	1.047374	1.046125	0.97365	-0.0385	1	1
ARHGEF11	1.098108	1.09657	1.098608	1.0211	0.0301	1	1
LRRC38	1.016245	1.015698	1.016422	1.04617	0.0651	1	1
RP11-281O15.8	1.013663	1.012186	1.014143	1.16053	0.2148	1	1
AC034228.2	1.013662	1.012186	1.014141	1.16042	0.2147	1	1
RP4-635E18.9	1.013662	1.012186	1.014141	1.1604	0.2146	1	1
NPFFR1	1.01366	1.012186	1.01414	1.16028	0.2145	1	1
UTS2	1.01366	1.012186	1.014139	1.16026	0.2144	1	1
ATP4B	1.015377	1.015672	1.015281	0.97503	-0.0365	1	1
FAM196B	1.0128	1.012186	1.013	1.06675	0.0932	1	1
RP11-1100L3.7	1.013659	1.012186	1.014138	1.16013	0.2143	1	1
CTD-2201E9.1	1.01366	1.012186	1.014139	1.1602	0.2144	1	1
RP11-103H7.2	1.0128	1.012186	1.013	1.06675	0.0932	1	1
RP11-227G15.10	1.024872	1.026274	1.024416	0.92927	-0.1058	1	1
RP5-978I12.1	1.013661	1.012186	1.01414	1.16036	0.2146	1	1
C9orf129	1.017106	1.015669	1.017573	1.12148	0.1654	1	1
ADCY10	1.013658	1.012186	1.014136	1.15999	0.2141	1	1
RP11-534C12.1	1.016244	1.015696	1.016422	1.04622	0.0652	1	1
PSAPL1	1.013657	1.012186	1.014135	1.15987	0.214	1	1
PYM1	1.396457	1.394912	1.396958	1.00518	0.0075	1	1
FBXL20	1.360443	1.359558	1.360731	1.00326	0.0047	1	1
RP11-555G19.1	1.0128	1.012186	1.013	1.06675	0.0932	1	1

AP001891.1	1.0128	1.012186	1.013	1.06675	0.0932	1	1
FYB	1.0128	1.012186	1.013	1.06675	0.0932	1	1
KCNG3	1.017099	1.015682	1.01756	1.11971	0.1631	1	1
RP11-10L7.1	1.0128	1.012186	1.013	1.06675	0.0932	1	1
SAMD3	1.015376	1.015673	1.01528	0.9749	-0.0367	1	1
KLF16	1.115373	1.114143	1.115773	1.01428	0.0205	1	1
ADORA2A-AS1	1.017956	1.019196	1.017553	0.91444	-0.129	1	1
AC113608.1	1.0128	1.012186	1.013	1.06675	0.0932	1	1
CH507-42P11.7	1.013655	1.012186	1.014132	1.15968	0.2137	1	1
PDYN	1.01366	1.012186	1.014139	1.1602	0.2144	1	1
RP11-166B2.7	1.01366	1.012186	1.014139	1.1602	0.2144	1	1
ZBTB45	1.165574	1.166928	1.165134	0.98925	-0.0156	1	1
EIF2B5-AS1	1.015372	1.015691	1.015268	0.97305	-0.0394	1	1
LINC01247	1.013665	1.012186	1.014146	1.16077	0.2151	1	1
CYP39A1	1.097343	1.096618	1.097579	1.00995	0.0143	1	1
RP11-336A10.2	1.013663	1.012186	1.014143	1.1606	0.2149	1	1
PRAM1	1.019703	1.019271	1.019844	1.02977	0.0423	1	1
C7orf65	1.013659	1.012186	1.014138	1.16016	0.2143	1	1
CTD-2631K10.1	1.013657	1.012186	1.014136	1.15996	0.2141	1	1
HCG27	1.02484	1.026222	1.024391	0.93018	-0.1044	1	1
CTC-232P5.3	1.013662	1.012186	1.014141	1.16042	0.2147	1	1
AWAT2	1.0128	1.012186	1.013	1.06675	0.0932	1	1
GGT2	1.0128	1.012186	1.013	1.06675	0.0932	1	1
AC084809.3	1.014514	1.015672	1.014138	0.90209	-0.1487	1	1
RP11-271F18.1	1.013661	1.012186	1.014141	1.16038	0.2146	1	1
PKHD1	1.0128	1.012186	1.013	1.06675	0.0932	1	1
DSG3	1.0128	1.012186	1.013	1.06675	0.0932	1	1
TOMM20L	1.013663	1.012186	1.014143	1.16053	0.2148	1	1
RP11-130L8.1	1.028333	1.029802	1.027856	0.93469	-0.0974	1	1
RP4-710M16.2	1.013651	1.011717	1.01428	1.21868	0.2853	1	1
CTB-35F21.1	1.012791	1.011717	1.01314	1.12141	0.1653	1	1
RP11-637O19.3	1.012791	1.011717	1.01314	1.12141	0.1653	1	1
CTD-2083E4.6	1.018808	1.018723	1.018836	1.00603	0.0087	1	1
RP11-1038A11.1	1.013658	1.011717	1.014289	1.21949	0.2863	1	1
RWDD2B	1.12826	1.127483	1.128513	1.00808	0.0116	1	1
PLA2G4E-AS1	1.012794	1.011717	1.013144	1.12175	0.1658	1	1
EIF4EBP3	1.025714	1.025731	1.025708	0.99908	-0.0013	1	1
NPR1	1.011932	1.011717	1.012002	1.02433	0.0347	1	1
LRP1-AS	1.013659	1.011717	1.01429	1.21961	0.2864	1	1
LINC00678	1.012793	1.011717	1.013142	1.12162	0.1656	1	1
CTC-367F4.1	1.012791	1.011717	1.01314	1.12141	0.1653	1	1
CTD-2293H3.2	1.01279	1.011717	1.013138	1.12126	0.1651	1	1
FAM71F2	1.013668	1.011717	1.014302	1.22055	0.2875	1	1
RP11-191L9.4	1.013661	1.011717	1.014292	1.21977	0.2866	1	1
FAM66B	1.028316	1.029242	1.028015	0.95804	-0.0618	1	1

RP11-250B2.3	1.028321	1.029311	1.027999	0.95522	-0.0661	1	1
CTD-2201E18.5	1.013666	1.011717	1.0143	1.22039	0.2873	1	1
ACER1	1.013657	1.011717	1.014288	1.21936	0.2861	1	1
LINC01039	1.012791	1.011717	1.01314	1.12144	0.1654	1	1
RP11-1149O23.4	1.011932	1.011717	1.012002	1.02433	0.0347	1	1
HIST1H2BF	1.017096	1.015208	1.01771	1.1645	0.2197	1	1
PRCAT47	1.016235	1.01522	1.016565	1.08838	0.1222	1	1
RP11-159D12.10	1.015374	1.015208	1.015428	1.01444	0.0207	1	1
NTF3	1.033442	1.032694	1.033685	1.03033	0.0431	1	1
RP11-527F13.1	1.012793	1.011717	1.013143	1.12166	0.1656	1	1
CTC-251D13.1	1.014506	1.015208	1.014278	0.93882	-0.0911	1	1
NRN1L	1.012789	1.011717	1.013138	1.12125	0.1651	1	1
RP11-303E16.7	1.013667	1.011717	1.014301	1.22046	0.2874	1	1
RP13-270P17.1	1.015368	1.015224	1.015414	1.01247	0.0179	1	1
UBE2L5P	1.012795	1.011717	1.013146	1.12189	0.1659	1	1
RP11-111A22.1	1.01624	1.01521	1.016575	1.08968	0.1239	1	1
C6orf47-AS1	1.013655	1.011717	1.014285	1.21911	0.2858	1	1
RESP18	1.012795	1.011717	1.013145	1.12185	0.1659	1	1
TUBA3D	1.013672	1.011717	1.014308	1.22106	0.2881	1	1
CTD-2651B20.3	1.059347	1.057243	1.060031	1.04871	0.0686	1	1
RP11-803P9.1	1.011932	1.011717	1.012002	1.02433	0.0347	1	1
CTB-58E17.9	1.017961	1.018733	1.01771	0.94538	-0.081	1	1
RP11-65N13.8	1.012791	1.011717	1.01314	1.12144	0.1654	1	1
TPTE2	1.013661	1.011717	1.014293	1.21986	0.2867	1	1
CTD-2555A7.3	1.012793	1.011717	1.013143	1.12167	0.1657	1	1
RP11-381P6.1	1.01451	1.015226	1.014278	0.9377	-0.0928	1	1
KLHL1	1.012789	1.011717	1.013138	1.12123	0.1651	1	1
MAFA	1.034348	1.0328	1.034851	1.06255	0.0875	1	1
FEM1C	1.159276	1.158962	1.159378	1.00262	0.0038	1	1
RP11-706C16.7	1.012794	1.011717	1.013144	1.12173	0.1657	1	1
RP11-469N6.1	1.013657	1.011717	1.014288	1.21938	0.2861	1	1
RP11-98D18.17	1.014523	1.015228	1.014293	0.93861	-0.0914	1	1
PZP	1.012789	1.011717	1.013138	1.12121	0.1651	1	1
HYLS1	1.087702	1.089093	1.08725	0.97932	-0.0302	1	1
RP11-13P5.1	1.014513	1.015236	1.014278	0.93716	-0.0936	1	1
CXCL11	1.012792	1.011717	1.013141	1.12149	0.1654	1	1
RD3L	1.021407	1.022249	1.021133	0.94986	-0.0742	1	1
CTD-2008P7.1	1.011932	1.011717	1.012002	1.02433	0.0347	1	1
Z83001.1	1.012792	1.011717	1.013141	1.12149	0.1654	1	1
AC006946.15	1.013658	1.011717	1.014289	1.21949	0.2863	1	1
TFAP2A-AS1	1.01625	1.015215	1.016587	1.09012	0.1245	1	1
LRG1	1.011932	1.011717	1.012002	1.02433	0.0347	1	1
RP4-545L17.11	1.018815	1.018725	1.018845	1.00638	0.0092	1	1
RBMS3-AS2	1.016246	1.015224	1.016578	1.08894	0.1229	1	1
CD79B	1.014505	1.01521	1.014276	0.93858	-0.0914	1	1

RP5-894D12.4	1.011932	1.011717	1.012002	1.02433	0.0347	1	1
RP11-651P23.5	1.014505	1.015211	1.014275	0.93845	-0.0916	1	1
METTL1	1.164459	1.162887	1.164969	1.01279	0.0183	1	1
TMC3	1.014516	1.015208	1.014292	0.93975	-0.0896	1	1
RP11-407N17.4	1.015371	1.015219	1.015421	1.01325	0.019	1	1
DMGDH	1.023087	1.022192	1.023377	1.0534	0.0751	1	1
RP11-571M6.18	1.017117	1.015207	1.017738	1.16639	0.2221	1	1
SLC14A2-AS1	1.015365	1.015218	1.015413	1.0128	0.0184	1	1
TAS2R30	1.029185	1.029203	1.029179	0.99918	-0.0012	1	1
RP11-403P17.6	1.01453	1.015239	1.0143	0.93836	-0.0918	1	1
SLC35D2	1.234931	1.2362	1.234518	0.99288	-0.0103	1	1
AC007228.5	1.012793	1.011717	1.013143	1.12167	0.1657	1	1
SLC25A19	1.120394	1.120409	1.120389	0.99984	-0.0002	1	1
LINC00847	1.139556	1.138259	1.139977	1.01243	0.0178	1	1
RP11-379B8.1	1.013647	1.011717	1.014275	1.21826	0.2848	1	1
AMY1C	1.011932	1.011717	1.012002	1.02433	0.0347	1	1
ZNF555	1.114579	1.113462	1.114941	1.01304	0.0187	1	1
RP4-697K14.15	1.013649	1.011717	1.014277	1.2185	0.2851	1	1
AC007182.6	1.011932	1.011717	1.012002	1.02433	0.0347	1	1
RP11-508N22.12	1.013663	1.011717	1.014295	1.21998	0.2869	1	1
LINC00176	1.018828	1.018799	1.018838	1.00208	0.003	1	1
RP11-226E21.4	1.023986	1.022209	1.024564	1.10606	0.1454	1	1
RHBG	1.011932	1.011717	1.012002	1.02433	0.0347	1	1
RP11-239H6.2	1.01279	1.011717	1.013138	1.12126	0.1651	1	1
RP11-616M22.12	1.020554	1.01873	1.021147	1.12909	0.1752	1	1
RP11-481J13.1	1.01796	1.018747	1.017704	0.94437	-0.0826	1	1
AC009120.4	1.013652	1.011717	1.014281	1.21881	0.2855	1	1
RP11-6F2.5	1.013652	1.011717	1.014281	1.21877	0.2854	1	1
DBH-AS1	1.022327	1.022232	1.022358	1.00567	0.0082	1	1
STAU2-AS1	1.013661	1.011717	1.014293	1.21986	0.2867	1	1
C10orf105	1.015365	1.015211	1.015415	1.0134	0.0192	1	1
RP11-77E14.2	1.013657	1.011717	1.014287	1.21931	0.2861	1	1
CFAP52	1.022242	1.022209	1.022252	1.00197	0.0028	1	1
MFRP	1.011932	1.011717	1.012002	1.02433	0.0347	1	1
SERTM1	1.015371	1.015208	1.015423	1.01413	0.0202	1	1
RP11-380O24.1	1.013657	1.011717	1.014287	1.21931	0.2861	1	1
RP5-1069C8.2	1.012791	1.011717	1.01314	1.12141	0.1653	1	1
RP11-196H14.2	1.012788	1.011717	1.013136	1.12106	0.1649	1	1
CTD-2619J13.9	1.012789	1.011717	1.013138	1.12121	0.1651	1	1
RP11-96B2.1	1.012791	1.011717	1.01314	1.12141	0.1653	1	1
RP11-277P12.10	1.012793	1.011717	1.013142	1.12162	0.1656	1	1
SUCNR1	1.011932	1.011717	1.012002	1.02433	0.0347	1	1
ZFP57	1.011932	1.011717	1.012002	1.02433	0.0347	1	1
LMNTD2	1.065368	1.064325	1.065707	1.02149	0.0307	1	1
RP11-1099M24.7	1.013658	1.011717	1.014289	1.21951	0.2863	1	1

CRACR2A	1.012793	1.011717	1.013143	1.12167	0.1657	1	1
SFRP4	1.014521	1.01521	1.014297	0.93994	-0.0894	1	1
RP11-388M20.6	1.012789	1.011717	1.013137	1.12115	0.165	1	1
HIST2H2AA4	1.01279	1.011717	1.013139	1.12135	0.1652	1	1
GRTP1-AS1	1.013656	1.011717	1.014286	1.2192	0.2859	1	1
HIGD1B	1.011932	1.011717	1.012002	1.02433	0.0347	1	1
RP11-119D18.1	1.012791	1.011717	1.013139	1.12138	0.1653	1	1
C6	1.01366	1.011717	1.014292	1.21973	0.2866	1	1
LINC01180	1.012793	1.011717	1.013142	1.12162	0.1656	1	1
EPHA10	1.02749	1.025789	1.028043	1.08738	0.1209	1	1
CTC-498M16.4	1.012795	1.011717	1.013145	1.12185	0.1659	1	1
RP11-457I16.2	1.011932	1.011717	1.012002	1.02433	0.0347	1	1
RP4-613A2.1	1.012794	1.011717	1.013144	1.12175	0.1657	1	1
LINC01419	1.01366	1.011717	1.014291	1.21966	0.2865	1	1
AC004231.2	1.021405	1.022201	1.021146	0.95249	-0.0702	1	1
UNC79	1.040353	1.039816	1.040527	1.01785	0.0255	1	1
LINC01293	1.011932	1.011717	1.012002	1.02433	0.0347	1	1
PRM1	1.01279	1.011717	1.013139	1.12135	0.1652	1	1
RP11-483I13.5	1.052492	1.053919	1.052029	0.96495	-0.0515	1	1
RP11-84A19.4	1.01538	1.01521	1.015435	1.01475	0.0211	1	1
LINC00896	1.013666	1.011717	1.0143	1.22039	0.2873	1	1
AC138035.2	1.011932	1.011717	1.012002	1.02433	0.0347	1	1
CXCR5	1.012789	1.011717	1.013138	1.12125	0.1651	1	1
LINC01422	1.013644	1.011717	1.01427	1.21789	0.2844	1	1
RP5-921G16.2	1.011932	1.011717	1.012002	1.02433	0.0347	1	1
RP11-398H6.1	1.012793	1.011717	1.013142	1.12162	0.1656	1	1
RP11-542A14.2	1.011932	1.011717	1.012002	1.02433	0.0347	1	1
SRPK3	1.013653	1.011717	1.014283	1.21893	0.2856	1	1
RP11-524F11.2	1.013657	1.011717	1.014287	1.21933	0.2861	1	1
TSNARE1	1.037815	1.036255	1.038322	1.057	0.08	1	1
GFI1B	1.014521	1.015226	1.014292	0.9387	-0.0913	1	1
AC007255.8	1.014525	1.015232	1.014295	0.93846	-0.0916	1	1
RP11-956E11.1	1.012793	1.011717	1.013142	1.12162	0.1656	1	1
RP11-717K11.2	1.013517	1.012187	1.013949	1.14459	0.1948	1	1
RP11-755F10.3	1.014367	1.015685	1.013939	0.88869	-0.1703	1	1
AC010761.6	1.013511	1.012187	1.013942	1.14401	0.1941	1	1
AC011995.1	1.013513	1.012187	1.013945	1.14424	0.1944	1	1
CLEC4C	1.017811	1.01919	1.017362	0.90476	-0.1444	1	1
RP1-101G11.3	1.013515	1.012187	1.013947	1.14446	0.1947	1	1
RP11-110I1.14	1.016107	1.015701	1.016239	1.03425	0.0486	1	1
TBC1D3L	1.013515	1.012187	1.013946	1.14438	0.1946	1	1
AP000688.29	1.013516	1.012187	1.013948	1.14453	0.1948	1	1
ZNF75D	1.085174	1.085962	1.084918	0.98786	-0.0176	1	1
RP11-568A7.2	1.013518	1.012187	1.013951	1.14473	0.195	1	1
RP5-1185I7.1	1.012654	1.012187	1.012806	1.0508	0.0715	1	1

RP11-444A22.1	1.013519	1.012187	1.013953	1.14489	0.1952	1	1
RP11-781A6.1	1.013512	1.012187	1.013943	1.14412	0.1942	1	1
SLC30A3	1.014376	1.015711	1.013943	0.88742	-0.1723	1	1
AC007796.1	1.012654	1.012187	1.012806	1.0508	0.0715	1	1
RP11-177J6.1	1.015243	1.015719	1.015088	0.95984	-0.0591	1	1
RP1-117B12.4	1.012654	1.012187	1.012806	1.0508	0.0715	1	1
RP11-253I19.3	1.013516	1.012187	1.013948	1.14455	0.1948	1	1
CCDC39-AS1	1.013519	1.012187	1.013952	1.14485	0.1952	1	1
MYL1	1.014369	1.015685	1.013942	0.88886	-0.17	1	1
RP11-680F20.10	1.015233	1.015694	1.015083	0.96111	-0.0572	1	1
RP11-153F10.2	1.013513	1.012187	1.013944	1.14423	0.1944	1	1
PRMT8	1.01788	1.019264	1.01743	0.9048	-0.1443	1	1
RNF103-CHMP3	1.017828	1.019193	1.017385	0.90578	-0.1428	1	1
WI2-1959D15.1	1.016106	1.015694	1.016239	1.03471	0.0492	1	1
TEC	1.031598	1.029688	1.032218	1.08521	0.118	1	1
ZNF596	1.029034	1.029822	1.028779	0.96503	-0.0514	1	1
CTD-2027I19.3	1.018684	1.019192	1.018519	0.96495	-0.0515	1	1
AC000032.2	1.013514	1.012187	1.013945	1.14431	0.1945	1	1
BORCS8-MEF2B	1.014369	1.015687	1.01394	0.88866	-0.1703	1	1
RP4-813D12.3	1.029946	1.029727	1.030017	1.00973	0.014	1	1
RP11-356O9.1	1.014372	1.015684	1.013946	0.8892	-0.1694	1	1
RP11-3B12.2	1.013513	1.012187	1.013944	1.14421	0.1943	1	1
SLC18A1	1.012654	1.012187	1.012806	1.0508	0.0715	1	1
RP11-830F9.6	1.016955	1.01568	1.017369	1.10775	0.1476	1	1
AC092580.4	1.014367	1.015684	1.013939	0.88876	-0.1701	1	1
NOD2	1.012654	1.012187	1.012806	1.0508	0.0715	1	1
ADGRL3	1.272187	1.271906	1.272279	1.00137	0.002	1	1
RP11-257O5.4	1.012654	1.012187	1.012806	1.0508	0.0715	1	1
ASAP2	1.443753	1.443667	1.443781	1.00026	0.0004	1	1
RP11-455F5.4	1.013515	1.012187	1.013946	1.14438	0.1946	1	1
C12orf80	1.014371	1.015688	1.013943	0.88875	-0.1702	1	1
RP11-760H22.2	1.013509	1.012187	1.013939	1.14381	0.1938	1	1
TYROBP	1.029881	1.029644	1.029958	1.01058	0.0152	1	1
AURKC	1.080709	1.078881	1.081303	1.0307	0.0436	1	1
RAPSN	1.013512	1.012187	1.013942	1.14405	0.1942	1	1
RP11-561O23.8	1.013516	1.012187	1.013948	1.14452	0.1947	1	1
METTL11B	1.012654	1.012187	1.012806	1.0508	0.0715	1	1
HNMT	1.155726	1.156013	1.155633	0.99757	-0.0035	1	1
LRRC19	1.013518	1.012187	1.01395	1.14471	0.195	1	1
HLF	1.045427	1.043828	1.045946	1.04834	0.0681	1	1
INHBB	1.016093	1.015683	1.016226	1.03466	0.0492	1	1
CTD-2012K14.6	1.033368	1.033241	1.03341	1.00509	0.0073	1	1
LRGUK	1.016961	1.015676	1.017379	1.10868	0.1488	1	1
TPSB2	1.016102	1.015696	1.016234	1.03428	0.0486	1	1
AC000068.10	1.015241	1.015708	1.015089	0.96057	-0.058	1	1

RP4-569M23.5	1.01611	1.015694	1.016245	1.03505	0.0497	1	1
RP11-626K17.3	1.013517	1.012187	1.013949	1.14462	0.1949	1	1
RP11-66N24.6	1.013521	1.012187	1.013955	1.1451	0.1955	1	1
FFAR4	1.013512	1.012187	1.013943	1.14412	0.1942	1	1
LINC00670	1.014375	1.015707	1.013943	0.88768	-0.1719	1	1
HTR3A	1.013515	1.012187	1.013947	1.14441	0.1946	1	1
NKX2-5	1.01351	1.012187	1.01394	1.14389	0.1939	1	1
HCN1	1.012654	1.012187	1.012806	1.0508	0.0715	1	1
PALM2-AKAP2	1.016956	1.015695	1.017366	1.10645	0.1459	1	1
CTC-345K18.2	1.013517	1.012187	1.013949	1.14462	0.1949	1	1
CDH22	1.015242	1.015693	1.015095	0.9619	-0.056	1	1
TNFRSF8	1.01437	1.015672	1.013947	0.88988	-0.1683	1	1
ATP7B	1.083323	1.082464	1.083602	1.0138	0.0198	1	1
CSF2RA	1.026449	1.026293	1.0265	1.0079	0.0114	1	1
EQTN	1.015233	1.015695	1.015083	0.96102	-0.0574	1	1
CTA-339C12.1	1.012654	1.012187	1.012806	1.0508	0.0715	1	1
OR2B6	1.013512	1.012187	1.013943	1.14408	0.1942	1	1
RP11-430G17.3	1.078309	1.079131	1.078042	0.98624	-0.02	1	1
HEPN1	1.016096	1.015688	1.016228	1.03441	0.0488	1	1
RP5-1039K5.16	1.013513	1.012187	1.013944	1.14416	0.1943	1	1
RP4-594A5.1	1.013515	1.012187	1.013947	1.1444	0.1946	1	1
ADRA2B	1.015233	1.015684	1.015087	0.96191	-0.056	1	1
ALK	1.013511	1.012187	1.013941	1.14395	0.194	1	1
ABCG2	1.013515	1.012187	1.013946	1.14437	0.1946	1	1
RASL11A	1.013513	1.012187	1.013945	1.14424	0.1944	1	1
RP11-362K14.6	1.023903	1.022751	1.024277	1.06707	0.0937	1	1
RP11-260O18.1	1.013518	1.012187	1.01395	1.14471	0.195	1	1
CTA-984G1.5	1.018691	1.019222	1.018518	0.96339	-0.0538	1	1
KCNA3	1.012654	1.012187	1.012806	1.0508	0.0715	1	1
SAMD9L	1.023017	1.022772	1.023096	1.01423	0.0204	1	1
RP11-497G19.1	1.013516	1.012187	1.013948	1.1445	0.1947	1	1
RP11-861L17.3	1.013515	1.012187	1.013946	1.14438	0.1946	1	1
LINC01505	1.015233	1.015701	1.01508	0.96045	-0.0582	1	1
LINC00310	1.016084	1.015681	1.016215	1.03405	0.0483	1	1
ITIH4	1.016941	1.015676	1.017353	1.10697	0.1466	1	1
RP11-460B17.3	1.013518	1.012187	1.01395	1.14472	0.195	1	1
SLC7A10	1.016998	1.015684	1.017425	1.11101	0.1519	1	1
H2BFWT	1.013515	1.012187	1.013947	1.14446	0.1947	1	1
ARR3	1.013509	1.012187	1.013939	1.14378	0.1938	1	1
C15orf41	1.19806	1.198514	1.197913	0.99697	-0.0044	1	1
TLR6	1.022995	1.022701	1.02309	1.01715	0.0245	1	1
IMMP2L	1.500515	1.500045	1.500667	1.00124	0.0018	1	1
RAB39A	1.020416	1.019202	1.02081	1.08374	0.116	1	1
PGAM5	1.095269	1.096326	1.094926	0.98546	-0.0211	1	1
RP11-135L13.4	1.03165	1.029703	1.032283	1.08683	0.1201	1	1

AP000355.2	1.014372	1.015685	1.013946	0.88909	-0.1696	1	1
RP5-1139B12.2	1.016966	1.015683	1.017384	1.10846	0.1486	1	1
CTD-2528L19.6	1.03334	1.033288	1.033357	1.00205	0.003	1	1
GTF2H4	1.021287	1.022792	1.020798	0.9125	-0.1321	1	1
RP1-125I3.2	1.013517	1.012187	1.01395	1.14467	0.1949	1	1
AC002398.9	1.013515	1.012187	1.013947	1.14445	0.1947	1	1
RP11-783K16.13	1.018682	1.019212	1.018509	0.9634	-0.0538	1	1
AC004953.1	1.013512	1.012187	1.013943	1.14414	0.1943	1	1
CTD-2314G24.2	1.014385	1.015709	1.013955	0.88833	-0.1708	1	1
TTLL2	1.013517	1.012187	1.013949	1.1446	0.1948	1	1
LRP5	1.316175	1.314474	1.316727	1.00716	0.0103	1	1
RP11-329B9.1	1.013512	1.012187	1.013943	1.14412	0.1942	1	1
HNF4G	1.013518	1.012187	1.013951	1.14473	0.195	1	1
LINC00445	1.012654	1.012187	1.012806	1.0508	0.0715	1	1
SPAG11B	1.013512	1.012187	1.013943	1.14412	0.1942	1	1
ALG1L	1.016107	1.015695	1.016241	1.03484	0.0494	1	1
OR10C1	1.013512	1.012187	1.013942	1.14405	0.1942	1	1
AC005037.3	1.016965	1.015703	1.017375	1.10646	0.146	1	1
METTL21C	1.01351	1.012187	1.01394	1.14389	0.1939	1	1
RP11-485G7.6	1.013509	1.012187	1.013939	1.14376	0.1938	1	1
PKD2L2	1.016967	1.015696	1.01738	1.10731	0.1471	1	1
C10orf113	1.016965	1.015703	1.017375	1.10645	0.1459	1	1
AC034187.2	1.013515	1.012187	1.013946	1.14439	0.1946	1	1
FAT2	1.012654	1.012187	1.012806	1.0508	0.0715	1	1
SPINK4	1.012654	1.012187	1.012806	1.0508	0.0715	1	1
RP11-74C13.4	1.013512	1.012187	1.013943	1.14411	0.1942	1	1
AC007131.1	1.013512	1.012187	1.013942	1.14406	0.1942	1	1
RP11-793H13.3	1.018683	1.019155	1.01853	0.96735	-0.0479	1	1
RP11-572C21.1	1.013518	1.012187	1.01395	1.1447	0.195	1	1
RP11-693J15.6	1.013515	1.012187	1.013947	1.14447	0.1947	1	1
NRIR	1.012654	1.012187	1.012806	1.0508	0.0715	1	1
DQX1	1.016089	1.015695	1.016218	1.03329	0.0472	1	1
RP11-104O19.4	1.015259	1.015733	1.015105	0.96011	-0.0587	1	1
