# Supplementary material for: Single cell RNA sequencing of stem cell-derived retinal ganglion cells
Source: Sci Data. 2018 Feb 13;5:180013. doi: 10.1038/sdata.2018.13 (PMC5810423; doi:10.1038/sdata.2018.13)
Supplement: Supplementary Tables [file sdata201813-s2.zip › Supplementary tables/table s4.pdf]

ReactomePathway	RatioOfProteinInPathway	NumberOfProteinInPathway	ProteinFromGeneSet	P-value	FDR	HitGenes
mRNA Splicing - Major Pathway	0.0179	127	61	1.11E-16	2.28E-14	SRSF2,SRSF3,SRSF4,SRSF5,SRSF7,SRSF9,BCAS2,SRSF1,HNRNPA1,HNRNPA0,HNRNPA3,CDC5L,SNRPD2,SNRPD1,SNRPD3,HNRNPA2B1,PPIH,HNRNPR,HNRNPM,HNRNPK,HNRNPF,HNRNPD,HNRNPC,SNRPG,SNRPE,SNRPF,SNRPA,SNRPB,DNAJC8,POLR2C,POLR2E,POLR2G,POLR2H,SNRNP40,MAGOH,RNPS1,HNRNPH1,HNRNPH2,CWC15,YBX1,
Respiratory electron transport, ATP synthesis by chemiosmotic coupling, and heat production by uncoupling proteins.	0.0165	117	64	1.11E-16	2.28E-14	ATP5C1,TMEM126B,ATP5A1,UQCRB,UQCRH,ETFA,ETFB,NDUFAF4,NDUFAF2,COX7C,COX8A,COX5B,COX5A,COX6C,NDUFAB1,COX6A1,SDHC,SDHD,SDHB,COX6B1,NDUFB10,NDUFB11,NDUFA13,NDUFA11,NDUFA12,ATP5J2,ATP5G3,ATP5G2,ATP5F1,UQCRC1,UQCRC2,ECSIT,COX4I1,NDUFC2,NDUFC1,NDUFB9,NDUFB8,NDUFB6,NDUFB5,NDUFB4,NDUFB3,NDUFA8,NDUFA7,NDUFA6,NDUFA5,NDUFA4,NDU
Respiratory electron transport	0.0136	97	53	1.11E-16	2.28E-14	TMEM126B,UQCRB,UQCRH,ETFA,ETFB,NDUFAF4,NDUFAF2,COX7C,COX8A,COX5B,COX5A,COX6C,NDUFAB1,COX6A1,SDHC,SDHD,SDHB,COX6B1,NDUFB10,NDUFB11,NDUFA13,NDUFA11,NDUFA12,UQCRC1,UQCRC2,ECSIT,COX4I1,NDUFC2,NDUFC1,NDUFB9,NDUFB8,NDUFB6,NDUFB5,NDUFB4,NDUFB3,NDUFA8,NDUFA7,NDUFA6,NDUF
The citric acid (TCA) cycle and respiratory electron transport	0.0224	159	81	1.11E-16	2.28E-14	ATP5C1,TMEM126B,ATP5A1,UQCRB,UQCRH,ACO2,ETFA,ETFB,SUCLA2,BSG,NDUFAF4,NDUFAF2,COX7C,COX8A,COX5B,COX5A,COX6C,NDUFAB1,COX6A1,SDHC,SDHD,SDHB,COX6B1,NDUFB10,NDUFB11,PDHA1,LDHB,LDHA,MPC2,IDH3B,PDHB,IDH3G,SUCLG2,SUCLG1,NDUFA13,NDUFA11,NDUFA12,ATP5J2,IDH2,ATP5G3,ATP5G2,ATP5F1,UQCRC1,UQCRC2,ECSIT,FH,SLC16A1,MDH2,COX4I1,NDUFC2,NDUFC1,NDUFB9,NDUFB8,NDUFB6,NDUFB5,NDUFB4,NDUFB3,NDUFA8,
mRNA Splicing	0.0188	134	62	1.11E-16	2.28E-14	ZCRB1,SRSF2,SRSF3,SRSF4,SRSF5,SRSF7,SRSF9,BCAS2,SRSF1,HNRNPA1,HNRNPA0,HNRNPA3,CDC5L,SNRPD2,SNRPD1,SNRPD3,HNRNPA2B1,PPIH,HNRNPR,HNRNPM,HNRNPK,HNRNPF,HNRNPD,HNRNPC,SNRPG,SNRPE,SNRPF,SNRPA,SNRPB,DNAJC8,POLR2C,POLR2E,POLR2G,POLR2H,SNRNP40,MAGOH,RNPS1,HNRNPH1,HNRNPH2,CWC15,YBX1,RBMX,PCBP1,PCBP2,RBM8A,NCBP2,SNU13,UPF3B,SF3B5,S

Processing of Capped Intron-Containing Pre-mRNA	0.0238	169	67	4.44E-16	7.11E-14	ZCRB1,SRSF2,SRSF3,SRSF4,SRSF5,SRSF7,SRSF9,BCAS2,SRSF1,HNRNPA1,EIF4E,HNRNPA0,HNRNPA3,CDC5L,SNRPD2,SNRPD1,SNRPD3,RAE1,HNRNPA2B1,PPIH,HNRNPR,NUP93,HNRNPM,HNRNPK,HNRNPF,HNRNPD,HNRNPC,SNRPG,SNRPE,SNRPF,SNRPA,SNRPB,DNAJC8,POLR2C,POLR2E,POLR2G,POLR2H,SNRNP40,MAGOH,RNPS1,HNRNPH1,HNRNPH2,CWC15,YBX1,RBMX,PCBP1,PCBP2,RBM8A,NCBP2,N
APC/C-mediated degradation of cell cycle proteins	0.0115	82	45	5.55E-16	7.11E-14	NEK2,CDC20,UBE2C,ANAPC15,ANAPC16,PLK1,UBB,BUB3,PSMD8,PSMD6,PSMD7,PSMD4,PSMD3,PSME1,PSME2,PSMF1,PSMA5,PSMA3,PSMA4,PSMA1,PSMA2,PSMA7,PSMB6,PSMB7,PSMB5,PSMB2,PSMB3,PSMB1,PSMC5,PSMC6,PSMC3,PSMC1,UBE2E1,MAD2L1,PSM
Regulation of mitotic cell cycle	0.0115	82	45	5.55E-16	7.11E-14	NEK2,CDC20,UBE2C,ANAPC15,ANAPC16,PLK1,UBB,BUB3,PSMD8,PSMD6,PSMD7,PSMD4,PSMD3,PSME1,PSME2,PSMF1,PSMA5,PSMA3,PSMA4,PSMA1,PSMA2,PSMA7,PSMB6,PSMB7,PSMB5,PSMB2,PSMB3,PSMB1,PSMC5,PSMC6,PSMC3,PSMC1,UBE2E1,MAD2L1,PSM
Cell Cycle, Mitotic	0.0561	399	113	9.99E-16	1.13E-13	OPTN,SET,CDCA8,CENPA,CENPE,CENPF,CENPH,APEX1,CENPM,CENPN,GMNN,NEK2,CDC25B,CDC20,CSNK2B,DYNC1I2,PCNA,RAE1,UBE2C,POLD2,SMC3,SMC4,SMC2,NUP93,ANAPC15,ANAPC16,CEP70,PLK1,CDKN1A,CDKN2B,TUBB4B,CEP41,PPP1CC,VRK1,MCM7,RFC5,TFDP2,RAB1A,PPP2R1A,TOP2A,RAB8A,TUBB,TYMS,PPP2CA,PMF1,UBB,BUB3,PSMD8,PSMD6,PSMD7,PSMD4,PSMD3,PSME1,PSME2,PSMF1,TUBA1A,DYNLL1,PSMA5,PSMA3,PSMA4,PSMA1,PSMA2,PSMA7,PSMB6,PSMB7,PSMB5,PSMB2,PSMB3,PSMB1,PSMC5,PSMC6,PSMC3,PSMC1,UBE2E1,HSP90AA1,DBF4,BIRC5,KIF20A,CETN2,KIF2C,
Activation of APC/C and APC/C:Cdc20 mediated degradation of mitotic proteins	0.0103	73	42	1.11E-15	1.13E-13	NEK2,CDC20,UBE2C,ANAPC15,ANAPC16,PLK1,UBB,BUB3,PSMD8,PSMD6,PSMD7,PSMD4,PSMD3,PSME1,PSME2,PSMF1,PSMA5,PSMA3,PSMA4,PSMA1,PSMA2,PSMA7,PSMB6,PSMB7,PSMB5,PSMB2,PSMB3,PSMB1,PSMC5,PSMC6,PSMC3,PSMC1,UBE2E1,MAD2L1,PSM
APC/C:Cdc20 mediated degradation of mitotic proteins	0.0101	72	41	3.22E-15	2.99E-13	NEK2,CDC20,UBE2C,ANAPC15,ANAPC16,UBB,BUB3,PSMD8,PSMD6,PSMD7,PSMD4,PSMD3,PSME1,PSME2,PSMF1,PSMA5,PSMA3,PSMA4,PSMA1,PSMA2,PSMA7,PSMB6,PSMB7,PSMB5,PSMB2,PSMB3,PSMB1,PSMC5,PSMC6,PSMC3,PSMC1,UBE2E1,MAD2L1,PSMD10,PS
Mitotic Anaphase	0.0228	162	63	7.55E-15	6.42E-13	CDCA8,CENPA,CENPE,CENPF,CENPH,CENPM,CENPN,CDC20,UBE2C,SMC3,ANAPC15,ANAPC16,PLK1,PPP1CC,VRK1,PPP2R1A,PPP2CA,PMF1,UBB,BUB3,PSMD8,PSMD6,PSMD7,PSMD4,PSMD3,PSME1,PSME2,PSMF1,PSMA5,PSMA3,PSMA4,PSMA1,PSMA2,PSMA7,PSMB6,PSMB7,PSMB5,PSMB2,PSMB3,PSMB1,PSMC5,PSMC6,PSMC3,PSM

Mitotic Metaphase and Anaphase	0.0229	163	63	9.88E-15	7.71E-13	CDCA8,CENPA,CENPE,CENPF,CENPH,CENPM,CENPN,CDC20,UBE2C,SMC3,ANAPC15,ANAPC16,PLK1,PPP1CC,VRK1,PPP2R1A,PPP2CA,PMF1,UBB,BUB3,PSMD8,PSMD6,PSMD7,PSMD4,PSMD3,PSME1,PSME2,PSMF1,PSMA5,PSMA3,PSMA4,PSMA1,PSMA2,PSMA7,PSMB6,PSMB7,PSMB5,PSMB2,PSMB3,PSMB1,PSMC5,PSMC6,PSMC3,PSM
Cell Cycle Checkpoints	0.021	149	59	2.62E-14	1.91E-12	BRE,CDC20,TP53,UBE2C,UBE2N,ANAPC15,ANAPC16,CDKN1A,MCM7,RFC5,UBB,H2AFX,BUB3,PSMD8,PSMD6,PSMD7,PSMD4,PSMD3,PSME1,PSME2,PSMF1,PSMA5,PSMA3,PSMA4,PSMA1,PSMA2,PSMA7,PSMB6,PSMB7,PSMB5,PSMB2,PSMB3,PSMB1,PSMC5,PSMC6,PSMC3,PSMC1,UBE2E1,SUMO1,DBF4,BABAM1,RPA2,RPA3,MAD2L1,
Regulation of APC/C activators between G1/S and early anaphase	0.0108	77	41	2.86E-14	1.95E-12	CDC20,UBE2C,ANAPC15,ANAPC16,PLK1,UBB,BUB3,PSMD8,PSMD6,PSMD7,PSMD4,PSMD3,PSME1,PSME2,PSMF1,PSMA5,PSMA3,PSMA4,PSMA1,PSMA2,PSMA7,PSMB6,PSMB7,PSMB5,PSMB2,PSMB3,PSMB1,PSMC5,PSMC6,PSMC3,PSMC1,UBE2E1,MAD2L1,PSMD10,PS
APC:Cdc20 mediated degradation of cell cycle proteins prior to satisfation of the cell cycle checkpoint	0.0098	70	39	3.04E-14	1.95E-12	NEK2,CDC20,UBE2C,ANAPC15,ANAPC16,UBB,BUB3,PSMD8,PSMD6,PSMD7,PSMD4,PSMD3,PSME1,PSME2,PSMF1,PSMA5,PSMA3,PSMA4,PSMA1,PSMA2,PSMA7,PSMB6,PSMB7,PSMB5,PSMB2,PSMB3,PSMB1,PSMC5,PSMC6,PSMC3,PSMC1,UBE2E1,MAD2L1,PSMD10,PS
M Phase	0.0316	225	75	4.64E-14	2.68E-12	SET,CDCA8,CENPA,CENPE,CENPF,CENPH,CENPM,CENPN,CDC20,CSNK2B,RAE1,UBE2C,SMC3,SMC4,SMC2,NUP93,ANAPC15,ANAPC16,PLK1,PPP1CC,VRK1,RAB1A,PPP2R1A,PPP2CA,PMF1,UBB,BUB3,PSMD8,PSMD6,PSMD7,PSMD4,PSMD3,PSME1,PSME2,PSMF1,PSMA5,PSMA3,PSMA4,PSMA1,PSMA2,PSMA7,PSMB6,PSMB7,PSMB5,PSMB2,PSMB3,PSMB1,PSMC5,PSMC6,PSMC3,PSMC1,UBE2E1,BIRC5,KIF
APC/C:Cdh1 mediated degradation of Cdc20 and other APC/C:Cdh1 targeted proteins in late mitosis/early G1	0.01	71	39	4.71E-14	2.68E-12	CDC20,UBE2C,ANAPC15,ANAPC16,PLK1,UBB,PSMD8,PSMD6,PSMD7,PSMD4,PSMD3,PSME1,PSME2,PSMF1,PSMA5,PSMA3,PSMA4,PSMA1,PSMA2,PSMA7,PSMB6,PSMB7,PSMB5,PSMB2,PSMB3,PSMB1,PSMC5,PSMC6,PSMC3,PSMC1,UBE2E1,PSMD10,PSMD11,PSMD14
SCF(Skp2)-mediated degradation of p27/p21	0.0073	52	33	9.15E-14	4.70E-12	CDKN1A,UBB,PSMD8,PSMD6,PSMD7,PSMD4,PSMD3,PSME1,PSME2,PSMF1,PSMA5,PSMA3,PSMA4,PSMA1,PSMA2,PSMA7,PSMB6,PSMB7,PSMB5,PSMB2,PSMB3,PSMB1,PSMC5,PSMC6,PSMC3,PSMC1,
Cdc20:Phospho-APC/C mediated degradation of Cyclin A	0.0097	69	38	9.21E-14	4.70E-12	CDC20,UBE2C,ANAPC15,ANAPC16,UBB,BUB3,PSMD8,PSMD6,PSMD7,PSMD4,PSMD3,PSME1,PSME2,PSMF1,PSMA5,PSMA3,PSMA4,PSMA1,PSMA2,PSMA7,PSMB6,PSMB7,PSMB5,PSMB2,PSMB3,PSMB1,PSMC5,PSMC6,PSMC3,PSMC1,UBE2E1,MAD2L1,PSMD10,PSMD1

Separation of Sister Chromatids	0.0217	154	59	1.03E-13	4.93E-12	CDCA8,CENPA,CENPE,CENPF,CENPH,CENPM,CENPN,CDC20,UBE2C,SMC3,ANAPC15,ANAPC16,PLK1,PPP1CC,PMF1,UBB,BUB3,PSMD8,PSMD6,PSMD7,PSMD4,PSMD3,PSME1,PSME2,PSMF1,PSMA5,PSMA3,PSMA4,PSMA1,PSMA2,PSMA7,PSMB6,PSMB7,PSMB5,PSMB2,PSMB3,PSMB1,PSMC5,PSMC6,PSMC3,PSMC1,UBE2E1,BIRC5,KIF2C,M
AUF1 (hnRNP D0) binds and destabilizes mRNA	0.0075	53	33	1.53E-13	7.03E-12	PABPC1,HNRNPD,UBB,PSMD8,PSMD6,PSMD7,PSMD4,PSMD3,PSME1,PSME2,PSMF1,PSMA5,PSMA3,PSMA4,PSMA1,PSMA2,PSMA7,PSMB6,PSMB7,PSMB5,PSMB2,PSMB3,PSMB1,PSMC5,PSMC6,PSMC
APC/C:Cdc20 mediated degradation of Securin	0.0093	66	36	5.42E-13	2.39E-11	CDC20,UBE2C,ANAPC15,ANAPC16,UBB,PSMD8,PSMD6,PSMD7,PSMD4,PSMD3,PSME1,PSME2,PSMF1,PSMA5,PSMA3,PSMA4,PSMA1,PSMA2,PSMA7,PSMB6,PSMB7,PSMB5,PSMB2,PSMB3,PSMB1,PSM
Ubiquitin-dependent degradation of Cyclin D	0.007	50	31	9.24E-13	3.79E-11	UBB,PSMD8,PSMD6,PSMD7,PSMD4,PSMD3,PSME1,PSME2,PSMF1,PSMA5,PSMA3,PSMA4,PSMA1,PSMA2,PSMA7,PSMB6,PSMB7,PSMB5,PSMB2,PSMB3,PSMB1,PSMC5,PSMC6,PSMC3,PSMC1,PSMD10,
Ubiquitin-dependent degradation of Cyclin D1	0.007	50	31	9.24E-13	3.79E-11	UBB,PSMD8,PSMD6,PSMD7,PSMD4,PSMD3,PSME1,PSME2,PSMF1,PSMA5,PSMA3,PSMA4,PSMA1,PSMA2,PSMA7,PSMB6,PSMB7,PSMB5,PSMB2,PSMB3,PSMB1,PSMC5,PSMC6,PSMC3,PSMC1,PSMD10,
ER-Phagosome pathway	0.009	64	35	1.06E-12	4.14E-11	CALR,SEC61G,SEC61B,UBB,PSMD8,PSMD6,PSMD7,PSMD4,PSMD3,PSME1,PSME2,PSMF1,PSMA5,PSMA3,PSMA4,PSMA1,PSMA2,PSMA7,PSMB6,PSMB7,PSMB5,PSMB2,PSMB3,PSMB1,PSMC5,PSMC6,P
SCF-beta-TrCP mediated degradation of Emi1	0.0076	54	32	1.28E-12	4.86E-11	CDC20,UBB,PSMD8,PSMD6,PSMD7,PSMD4,PSMD3,PSME1,PSME2,PSMF1,PSMA5,PSMA3,PSMA4,PSMA1,PSMA2,PSMA7,PSMB6,PSMB7,PSMB5,PSMB2,PSMB3,PSMB1,PSMC5,PSMC6,PSMC3,PSMC1,P
Autodegradation of the E3 ubiquitin ligase COP1	0.0072	51	31	1.52E-12	5.47E-11	TP53,UBB,PSMD8,PSMD6,PSMD7,PSMD4,PSMD3,PSME1,PSME2,PSMF1,PSMA5,PSMA3,PSMA4,PSMA1,PSMA2,PSMA7,PSMB6,PSMB7,PSMB5,PSMB2,PSMB3,PSMB1,PSMC5,PSMC6,PSMC3,PSMC1,PS
p53-Dependent G1/S DNA damage checkpoint	0.0077	55	32	2.05E-12	6.63E-11	TP53,CDKN1A,UBB,PSMD8,PSMD6,PSMD7,PSMD4,PSMD3,PSME1,PSME2,PSMF1,PSMA5,PSMA3,PSMA4,PSMA1,PSMA2,PSMA7,PSMB6,PSMB7,PSMB5,PSMB2,PSMB3,PSMB1,PSMC5,PSMC6,PSMC3,P
Complex I biogenesis	0.0077	55	32	2.05E-12	6.63E-11	TMEM126B,NDUFAF4,NDUFAF2,NDUFAB1,NDUFB10,NDUFB11,NDUFA13,NDUFA11,NDUFA12,ECSIT,NDUFC2,NDUFC1,NDUFB9,NDUFB8,NDUFB6,NDUFB5,NDUFB4,NDUFB3,NDUFA8,NDUFA7,NDUFA6,NDUFA5,NDUFA2,NDUFV2,NDUFV1,NDUFS8,NDUFS7,NDUFS6,ND
p53-Dependent G1 DNA Damage Response	0.0077	55	32	2.05E-12	6.63E-11	TP53,CDKN1A,UBB,PSMD8,PSMD6,PSMD7,PSMD4,PSMD3,PSME1,PSME2,PSMF1,PSMA5,PSMA3,PSMA4,PSMA1,PSMA2,PSMA7,PSMB6,PSMB7,PSMB5,PSMB2,PSMB3,PSMB1,PSMC5,PSMC6,PSMC3,P

Autodegradation of Cdh1 by Cdh1:APC/C	0.0087	62	34	2.07E-12	6.63E-11	UBE2C,ANAPC15,ANAPC16,UBB,PSMD8,PSMD6,PSMD7,PSMD4,PSMD3,PSME1,PSME2,PSMF1,PSMA5,PSMA3,PSMA4,PSMA1,PSMA2,PSMA7,PSMB6,PSMB7,PSMB5,PSMB2,PSMB3,PSMB1,PSMC5,PSM
Stabilization of p53	0.0073	52	31	2.47E-12	7.65E-11	TP53,UBB,PSMD8,PSMD6,PSMD7,PSMD4,PSMD3,PSME1,PSME2,PSMF1,PSMA5,PSMA3,PSMA4,PSMA1,PSMA2,PSMA7,PSMB6,PSMB7,PSMB5,PSMB2,PSMB3,PSMB1,PSMC5,PSMC6,PSMC3,PSMC1,PS
Regulation of activated PAK-2p34 by proteasome mediated degradation	0.0069	49	30	2.92E-12	8.75E-11	UBB,PSMD8,PSMD6,PSMD7,PSMD4,PSMD3,PSME1,PSME2,PSMF1,PSMA5,PSMA3,PSMA4,PSMA1,PSMA2,PSMA7,PSMB6,PSMB7,PSMB5,PSMB2,PSMB3,PSMB1,PSMC5,PSMC6,PSMC3,PSMC1,PSMD10,
Antigen processing: Ubiquitination & Proteasome degradation	0.0075	53	31	3.97E-12	1.15E-10	UBB,BLMH,PSMD8,PSMD6,PSMD7,PSMD4,PSMD3,PSME1,PSME2,PSMF1,PSMA5,PSMA3,PSMA4,PSMA1,PSMA2,PSMA7,PSMB6,PSMB7,PSMB5,PSMB2,PSMB3,PSMB1,PSMC5,PSMC6,PSMC3,PSMC1,P
Regulation of Apoptosis	0.007	50	30	4.76E-12	1.28E-10	UBB,PSMD8,PSMD6,PSMD7,PSMD4,PSMD3,PSME1,PSME2,PSMF1,PSMA5,PSMA3,PSMA4,PSMA1,PSMA2,PSMA7,PSMB6,PSMB7,PSMB5,PSMB2,PSMB3,PSMB1,PSMC5,PSMC6,PSMC3,PSMC1,PSMD10,
Regulation of ornithine decarboxylase (ODC)	0.007	50	30	4.76E-12	1.28E-10	ODC1,PSMD8,PSMD6,PSMD7,PSMD4,PSMD3,PSME1,PSME2,OAZ2,PSMF1,PSMA5,PSMA3,PSMA4,PSMA1,PSMA2,PSMA7,PSMB6,PSMB7,PSMB5,PSMB2,PSMB3,PSMB1,PSMC5,PSMC6,PSMC3,PSMC1,P
CDK-mediated phosphorylation and removal of Cdc6	0.007	50	30	4.76E-12	1.28E-10	UBB,PSMD8,PSMD6,PSMD7,PSMD4,PSMD3,PSME1,PSME2,PSMF1,PSMA5,PSMA3,PSMA4,PSMA1,PSMA2,PSMA7,PSMB6,PSMB7,PSMB5,PSMB2,PSMB3,PSMB1,PSMC5,PSMC6,PSMC3,PSMC1,PSMD10,
Cyclin A:Cdk2-associated events at S phase entry	0.0086	61	33	6.21E-12	1.62E-10	CDKN1A,UBB,PSMD8,PSMD6,PSMD7,PSMD4,PSMD3,PSME1,PSME2,PSMF1,PSMA5,PSMA3,PSMA4,PSMA1,PSMA2,PSMA7,PSMB6,PSMB7,PSMB5,PSMB2,PSMB3,PSMB1,PSMC5,PSMC6,PSMC3,PSMC1,
G1/S DNA Damage Checkpoints	0.0082	58	32	7.90E-12	1.97E-10	TP53,CDKN1A,UBB,PSMD8,PSMD6,PSMD7,PSMD4,PSMD3,PSME1,PSME2,PSMF1,PSMA5,PSMA3,PSMA4,PSMA1,PSMA2,PSMA7,PSMB6,PSMB7,PSMB5,PSMB2,PSMB3,PSMB1,PSMC5,PSMC6,PSMC3,P
Regulation of mRNA stability by proteins that bind AU-rich elements	0.012	85	39	1.03E-11	2.57E-10	SET,PABPC1,HNRNPD,UBB,PSMD8,PSMD6,PSMD7,PSMD4,PSMD3,PSME1,PSME2,PSMF1,PSMA5,PSMA3,PSMA4,PSMA1,PSMA2,PSMA7,PSMB6,PSMB7,PSMB5,PSMB2,PSMB3,PSMB1,PSMC5,PSMC6,PSMC3,PSMC1,EXOSC7,EXOSC8,PSMD10,PSMD11,PSMD14,PSMD13
Degradation of GLI1 by the proteasome	0.0083	59	32	1.21E-11	2.57E-10	UBB,PSMD8,PSMD6,PSMD7,PSMD4,PSMD3,PSME1,PSME2,PSMF1,PSMA5,PSMA3,PSMA4,PSMA1,PSMA2,PSMA7,PSMB6,PSMB7,PSMB5,PSMB2,PSMB3,PSMB1,PSMC5,PSMC6,PSMC3,PSMC1,RBX1,PS

Degradation of GLI2 by the proteasome	0.0083	59	32	1.21E-11	2.57E-10	UBB,PSMD8,PSMD6,PSMD7,PSMD4,PSMD3,PSME1,PSME2,PSMF1,PSMA5,PSMA3,PSMA4,PSMA1,PSMA2,PSMA7,PSMB6,PSMB7,PSMB5,PSMB2,PSMB3,PSMB1,PSMC5,PSMC6,PSMC3,PSMC1,RBX1,PS
GLI3 is processed to GLI3R by the proteasome	0.0083	59	32	1.21E-11	2.57E-10	UBB,PSMD8,PSMD6,PSMD7,PSMD4,PSMD3,PSME1,PSME2,PSMF1,PSMA5,PSMA3,PSMA4,PSMA1,PSMA2,PSMA7,PSMB6,PSMB7,PSMB5,PSMB2,PSMB3,PSMB1,PSMC5,PSMC6,PSMC3,PSMC1,RBX1,PS
Ubiquitin Mediated Degradation of Phosphorylated Cdc25A	0.0073	52	30	1.22E-11	2.57E-10	UBB,PSMD8,PSMD6,PSMD7,PSMD4,PSMD3,PSME1,PSME2,PSMF1,PSMA5,PSMA3,PSMA4,PSMA1,PSMA2,PSMA7,PSMB6,PSMB7,PSMB5,PSMB2,PSMB3,PSMB1,PSMC5,PSMC6,PSMC3,PSMC1,PSMD10,
p53-Independent G1/S DNA damage checkpoint	0.0073	52	30	1.22E-11	2.57E-10	UBB,PSMD8,PSMD6,PSMD7,PSMD4,PSMD3,PSME1,PSME2,PSMF1,PSMA5,PSMA3,PSMA4,PSMA1,PSMA2,PSMA7,PSMB6,PSMB7,PSMB5,PSMB2,PSMB3,PSMB1,PSMC5,PSMC6,PSMC3,PSMC1,PSMD10,
p53-Independent DNA Damage Response	0.0073	52	30	1.22E-11	2.57E-10	UBB,PSMD8,PSMD6,PSMD7,PSMD4,PSMD3,PSME1,PSME2,PSMF1,PSMA5,PSMA3,PSMA4,PSMA1,PSMA2,PSMA7,PSMB6,PSMB7,PSMB5,PSMB2,PSMB3,PSMB1,PSMC5,PSMC6,PSMC3,PSMC1,PSMD10,
Cyclin E associated events during G1/S transition	0.0089	63	33	1.43E-11	3.00E-10	CDKN1A,UBB,PSMD8,PSMD6,PSMD7,PSMD4,PSMD3,PSME1,PSME2,PSMF1,PSMA5,PSMA3,PSMA4,PSMA1,PSMA2,PSMA7,PSMB6,PSMB7,PSMB5,PSMB2,PSMB3,PSMB1,PSMC5,PSMC6,PSMC3,PSMC1,
Degradation of DVL	0.0079	56	31	1.54E-11	3.08E-10	UBB,PSMD8,PSMD6,PSMD7,PSMD4,PSMD3,PSME1,PSME2,PSMF1,PSMA5,PSMA3,PSMA4,PSMA1,PSMA2,PSMA7,PSMB6,PSMB7,PSMB5,PSMB2,PSMB3,PSMB1,PSMC5,PSMC6,PSMC3,PSMC1,RBX1,PS
Cross-presentation of soluble exogenous antigens (endosomes)	0.0065	46	28	1.76E-11	3.52E-10	PSMD8,PSMD6,PSMD7,PSMD4,PSMD3,PSME1,PSME2,PSMF1,PSMA5,PSMA3,PSMA4,PSMA1,PSMA2,PSMA7,PSMB6,PSMB7,PSMB5,PSMB2,PSMB3,PSMB1,PSMC5,PSMC6,PSMC3,PSMC1,PSMD10,PSM
Degradation of beta-catenin by the destruction complex	0.0096	68	34	2.38E-11	4.76E-10	TLE4,UBB,PSMD8,PSMD6,PSMD7,PSMD4,PSMD3,PSME1,PSME2,PSMF1,PSMA5,PSMA3,PSMA4,PSMA1,PSMA2,PSMA7,PSMB6,PSMB7,PSMB5,PSMB2,PSMB3,PSMB1,PSMC5,PSMC6,PSMC3,PSMC1,RB
Antigen processing-Cross presentation	0.0107	76	36	2.71E-11	5.15E-10	ITGAV,CALR,SEC61G,SEC61B,UBB,PSMD8,PSMD6,PSMD7,PSMD4,PSMD3,PSME1,PSME2,PSMF1,PSMA5,PSMA3,PSMA4,PSMA1,PSMA2,PSMA7,PSMB6,PSMB7,PSMB5,PSMB2,PSMB3,PSMB1,PSMC5,PS
Degradation of AXIN	0.0076	54	30	3.00E-11	5.71E-10	UBB,PSMD8,PSMD6,PSMD7,PSMD4,PSMD3,PSME1,PSME2,PSMF1,PSMA5,PSMA3,PSMA4,PSMA1,PSMA2,PSMA7,PSMB6,PSMB7,PSMB5,PSMB2,PSMB3,PSMB1,PSMC5,PSMC6,PSMC3,PSMC1,PSMD10,
CDT1 association with the CDC6:ORC:origin complex	0.0082	58	31	3.62E-11	6.52E-10	GMNN,UBB,PSMD8,PSMD6,PSMD7,PSMD4,PSMD3,PSME1,PSME2,PSMF1,PSMA5,PSMA3,PSMA4,PSMA1,PSMA2,PSMA7,PSMB6,PSMB7,PSMB5,PSMB2,PSMB3,PSMB1,PSMC5,PSMC6,PSMC3,PSMC1,P

Asymmetric localization of PCP proteins	0.0082	58	31	3.62E-11	6.52E-10	UBB,PSMD8,PSMD6,PSMD7,PSMD4,PSMD3,PSME1,PSME2,PSMF1,PSMA5,PSMA3,PSMA4,PSMA1,PSMA2,PSMA7,PSMB6,PSMB7,PSMB5,PSMB2,PSMB3,PSMB1,PSMC5,PSMC6,PSMC3,PSMC1,PSMD10,
NIK-->noncanonical NF-kB signaling	0.0082	58	31	3.62E-11	6.52E-10	UBB,PSMD8,PSMD6,PSMD7,PSMD4,PSMD3,PSME1,PSME2,PSMF1,PSMA5,PSMA3,PSMA4,PSMA1,PSMA2,PSMA7,PSMB6,PSMB7,PSMB5,PSMB2,PSMB3,PSMB1,PSMC5,PSMC6,PSMC3,PSMC1,PSMD10,
Metabolism of polyamines	0.0103	73	35	3.71E-11	6.57E-10	ENOPH1,ODC1,ADI1,CKB,PSMD8,PSMD6,PSMD7,PSMD4,PSMD3,PSME1,PSME2,OAZ2,PSMF1,PSMA5,PSMA3,PSMA4,PSMA1,PSMA2,PSMA7,PSMB6,PSMB7,PSMB5,PSMB2,PSMB3,PSMB1,PSMC5,PSM
PCP/CE pathway	0.0108	77	36	3.86E-11	6.57E-10	RAC1,UBB,PSMD8,PSMD6,PSMD7,PSMD4,PSMD3,PSME1,PSME2,PSMF1,PSMA5,PSMA3,PSMA4,PSMA1,PSMA2,PSMA7,PSMB6,PSMB7,PSMB5,PSMB2,PSMB3,PSMB1,AP2S1,PSMC5,PSMC6,PSMC3,PS
Dectin-1 mediated noncanonical NF-kB signaling	0.0083	59	31	5.48E-11	9.31E-10	UBB,PSMD8,PSMD6,PSMD7,PSMD4,PSMD3,PSME1,PSME2,PSMF1,PSMA5,PSMA3,PSMA4,PSMA1,PSMA2,PSMA7,PSMB6,PSMB7,PSMB5,PSMB2,PSMB3,PSMB1,PSMC5,PSMC6,PSMC3,PSMC1,PSMD10,
Mitotic G1-G1/S phases	0.0177	126	47	8.29E-11	1.41E-09	PCNA,CDKN1A,CDKN2B,MCM7,TFDP2,TOP2A,TYMS,UBB,PSMD8,PSMD6,PSMD7,PSMD4,PSMD3,PSME1,PSME2,PSMF1,PSMA5,PSMA3,PSMA4,PSMA1,PSMA2,PSMA7,PSMB6,PSMB7,PSMB5,PSMB2,PSMB3,PSMB1,PSMC5,PSMC6,PSMC3,PSMC1,DBF4,RPA2,RPA3,PSM
Hedgehog ligand biogenesis	0.0087	62	31	1.80E-10	2.87E-09	UBB,PSMD8,PSMD6,PSMD7,PSMD4,PSMD3,PSME1,PSME2,PSMF1,PSMA5,PSMA3,PSMA4,PSMA1,PSMA2,PSMA7,PSMB6,PSMB7,PSMB5,PSMB2,PSMB3,PSMB1,PSMC5,PSMC6,PSMC3,PSMC1,P4HB,PS
Removal of licensing factors from origins	0.0098	70	33	2.04E-10	3.27E-09	GMNN,CDKN1A,MCM7,UBB,PSMD8,PSMD6,PSMD7,PSMD4,PSMD3,PSME1,PSME2,PSMF1,PSMA5,PSMA3,PSMA4,PSMA1,PSMA2,PSMA7,PSMB6,PSMB7,PSMB5,PSMB2,PSMB3,PSMB1,PSMC5,PSMC6,
G1/S Transition	0.0145	103	41	2.09E-10	3.34E-09	PCNA,CDKN1A,MCM7,TYMS,UBB,PSMD8,PSMD6,PSMD7,PSMD4,PSMD3,PSME1,PSME2,PSMF1,PSMA5,PSMA3,PSMA4,PSMA1,PSMA2,PSMA7,PSMB6,PSMB7,PSMB5,PSMB2,PSMB3,PSMB1,PSMC5,PSMC6,PSMC3,PSMC1,DBF4,RPA2,RPA3,PSMD10,PSMD11,PSMD14,P
Assembly of the pre-replicative complex	0.0094	67	32	2.80E-10	4.47E-09	GMNN,MCM7,UBB,PSMD8,PSMD6,PSMD7,PSMD4,PSMD3,PSME1,PSME2,PSMF1,PSMA5,PSMA3,PSMA4,PSMA1,PSMA2,PSMA7,PSMB6,PSMB7,PSMB5,PSMB2,PSMB3,PSMB1,PSMC5,PSMC6,PSMC3,P
Orc1 removal from chromatin	0.0096	68	32	4.00E-10	6.00E-09	CDKN1A,MCM7,UBB,PSMD8,PSMD6,PSMD7,PSMD4,PSMD3,PSME1,PSME2,PSMF1,PSMA5,PSMA3,PSMA4,PSMA1,PSMA2,PSMA7,PSMB6,PSMB7,PSMB5,PSMB2,PSMB3,PSMB1,PSMC5,PSMC6,PSMC3,

Switching of origins to a post-replicative state	0.0096	68	32	4.00E-10	6.00E-09	CDKN1A,MCM7,UBB,PSMD8,PSMD6,PSMD7,PSMD4,PSMD3,PSME1,PSME2,PSMF1,PSMA5,PSMA3,PSMA4,PSMA1,PSMA2,PSMA7,PSMB6,PSMB7,PSMB5,PSMB2,PSMB3,PSMB1,PSMC5,PSMC6,PSMC3,
Regulation of RAS by GAPs	0.0091	65	31	5.48E-10	8.22E-09	UBB,PSMD8,PSMD6,PSMD7,PSMD4,PSMD3,PSME1,PSME2,PSMF1,PSMA5,PSMA3,PSMA4,PSMA1,PSMA2,PSMA7,PSMB6,PSMB7,PSMB5,PSMB2,PSMB3,PSMB1,PSMC5,PSMC6,PSMC3,PSMC1,RBX1,PS
Regulation of DNA replication	0.0103	73	33	5.75E-10	8.62E-09	GMNN,CDKN1A,MCM7,UBB,PSMD8,PSMD6,PSMD7,PSMD4,PSMD3,PSME1,PSME2,PSMF1,PSMA5,PSMA3,PSMA4,PSMA1,PSMA2,PSMA7,PSMB6,PSMB7,PSMB5,PSMB2,PSMB3,PSMB1,PSMC5,PSMC6,
M/G1 Transition	0.0115	82	35	7.60E-10	1.06E-08	GMNN,MCM7,UBB,PSMD8,PSMD6,PSMD7,PSMD4,PSMD3,PSME1,PSME2,PSMF1,PSMA5,PSMA3,PSMA4,PSMA1,PSMA2,PSMA7,PSMB6,PSMB7,PSMB5,PSMB2,PSMB3,PSMB1,PSMC5,PSMC6,PSMC3,P
DNA Replication Pre-Initiation	0.0115	82	35	7.60E-10	1.06E-08	GMNN,MCM7,UBB,PSMD8,PSMD6,PSMD7,PSMD4,PSMD3,PSME1,PSME2,PSMF1,PSMA5,PSMA3,PSMA4,PSMA1,PSMA2,PSMA7,PSMB6,PSMB7,PSMB5,PSMB2,PSMB3,PSMB1,PSMC5,PSMC6,PSMC3,P
Activation of NF-kappaB in B cells	0.0093	66	31	7.83E-10	1.10E-08	UBB,PSMD8,PSMD6,PSMD7,PSMD4,PSMD3,PSME1,PSME2,PSMF1,PSMA5,PSMA3,PSMA4,PSMA1,PSMA2,PSMA7,PSMB6,PSMB7,PSMB5,PSMB2,PSMB3,PSMB1,PSMC5,PSMC6,PSMC3,PSMC1,PSMD10,
Class I MHC mediated antigen processing & presentation	0.0128	91	37	9.10E-10	1.27E-08	ITGAV,CALR,SEC61G,SEC61B,UBB,BLMH,PSMD8,PSMD6,PSMD7,PSMD4,PSMD3,PSME1,PSME2,PSMF1,PSMA5,PSMA3,PSMA4,PSMA1,PSMA2,PSMA7,PSMB6,PSMB7,PSMB5,PSMB2,PSMB3,PSMB1,PSM
Synthesis of DNA	0.0135	96	38	1.11E-09	1.56E-08	APEX1,PCNA,POLD2,CDKN1A,MCM7,RFC5,UBB,PSMD8,PSMD6,PSMD7,PSMD4,PSMD3,PSME1,PSME2,PSMF1,PSMA5,PSMA3,PSMA4,PSMA1,PSMA2,PSMA7,PSMB6,PSMB7,PSMB5,PSMB2,PSMB3,PSM
Protein folding	0.0053	38	23	1.22E-09	1.59E-08	SPHK1,ACTB,TUBB2B,TUBB2A,TCP1,TUBB4B,PFDN2,PFDN4,PFDN5,TUBA1C,TUBA1B,TUBA1A,ARL2,VBP1,CCT3,CCT2,CCT8,CCT7,CCT5,
S Phase	0.0169	120	43	1.72E-09	2.23E-08	APEX1,PCNA,POLD2,SMC3,CDKN1A,MCM7,RFC5,UBB,PSMD8,PSMD6,PSMD7,PSMD4,PSMD3,PSME1,PSME2,PSMF1,PSMA5,PSMA3,PSMA4,PSMA1,PSMA2,PSMA7,PSMB6,PSMB7,PSMB5,PSMB2,PSMB3,PSMB1,PSMC5,PSMC6,PSMC3,PSMC1,RPA2,RPA3,PSMD10,PSM
Apoptosis	0.0212	151	49	3.05E-09	3.97E-08	FNTA,TP53,SPTAN1,LMNB1,GSN,UBB,CASP7,CASP6,PSMD8,PSMD6,PSMD7,PSMD4,PSMD3,PSME1,PSME2,PSMF1,DYNLL1,PSMA5,PSMA3,PSMA4,PSMA1,PSMA2,PSMA7,PSMB6,PSMB7,PSMB5,PSMB2,PSMB3,PSMB1,PSMC5,PSMC6,PSMC3,PSMC1,DSP,DSG2,DBNL,BC
Chaperonin-mediated protein folding	0.0045	32	20	8.58E-09	1.12E-07	SPHK1,ACTB,TUBB2B,TUBB2A,TCP1,TUBB4B,PFDN2,PFDN4,PFDN5,TUBA1C,TUBA1B,TUBA1A,VBP1,CCT3,CCT2,CCT8,CCT7,CCT5,CCT4,

Prefoldin mediated transfer of substrate to CCT/TriC	0.0037	26	18	1.03E-08	1.34E-07	ACTB,TUBB2B,TUBB2A,TCP1,TUBB4B,PFDN2,PFDN4,PFDN5,TUBA1C,TUBA1A,VBP1,CCT3,CCT2,CCT8,CCT7,CCT5,CCT4,CCT6A
MAPK6/MAPK4 signaling	0.0124	88	34	1.48E-08	1.78E-07	JUN,RAC1,TNRC6B,UBB,PSMD8,PSMD6,PSMD7,PSMD4,PSMD3,PSME1,PSME2,PSMF1,PSMA5,PSMA3,PSMA4,PSMA1,PSMA2,PSMA7,PSMB6,PSMB7,PSMB5,PSMB2,PSMB3,PSMB1,PSMC5,PSMC6,PSM
Mitochondrial translation elongation	0.0118	84	33	1.64E-08	1.96E-07	MRPS26,MRPS27,MRPS23,MRPS35,MRPS36,MRPS34,TUFM,MRPL16,MRPL15,MRPL12,MRPL13,MRPL10,MRPL11,MRPL28,MRPL23,MRPL21,MRPL22,MRPL42,MRPL47,MRPL48,MRPL45,MRPL43,MRP
Beta-catenin independent WNT signaling	0.0156	111	39	1.64E-08	1.97E-07	RAC1,TNRC6B,UBB,PSMD8,PSMD6,PSMD7,PSMD4,PSMD3,PSME1,PSME2,PSMF1,PSMA5,PSMA3,PSMA4,PSMA1,PSMA2,PSMA7,PSMB6,PSMB7,PSMB5,PSMB2,PSMB3,PSMB1,AP2S1,PSMC5,PSMC6,PSMC3,PSMC1,AP2M1,CALM1,PSMD10,PSMD11,PSMD14,PSMD13,U
Hedgehog 'off' state	0.0125	89	34	1.94E-08	2.32E-07	IFT52,IFT57,UBB,PSMD8,PSMD6,PSMD7,PSMD4,PSMD3,PSME1,PSME2,PSMF1,PSMA5,PSMA3,PSMA4,PSMA1,PSMA2,PSMA7,PSMB6,PSMB7,PSMB5,PSMB2,PSMB3,PSMB1,PSMC5,PSMC6,PSMC3,PSM
Hedgehog 'on' state	0.0114	81	32	2.37E-08	2.85E-07	SPOP,UBB,PSMD8,PSMD6,PSMD7,PSMD4,PSMD3,PSME1,PSME2,PSMF1,PSMA5,PSMA3,PSMA4,PSMA1,PSMA2,PSMA7,PSMB6,PSMB7,PSMB5,PSMB2,PSMB3,PSMB1,PSMC5,PSMC6,PSMC3,PSMC1,RB
Cooperation of Prefoldin and TriC/CCT in actin and tubulin	0.0044	31	19	2.72E-08	3.26E-07	ACTB,TUBB2B,TUBB2A,TCP1,TUBB4B,PFDN2,PFDN4,PFDN5,TUBA1C,TUBA1B,TUBA1A,VBP1,CCT3,CCT2,CCT8,CCT7,CCT5,CCT4,CCT6A
Mitochondrial translation initiation	0.0117	83	32	4.10E-08	4.92E-07	MRPS26,MRPS27,MRPS23,MRPS35,MRPS36,MRPS34,MRPL16,MRPL15,MRPL12,MRPL13,MRPL10,MRPL11,MRPL28,MRPL23,MRPL21,MRPL22,MRPL42,MRPL47,MRPL48,MRPL45,MRPL43,MRPL50,MR
Mitochondrial translation termination	0.0118	84	32	5.36E-08	5.89E-07	MRPS26,MRPS27,MRPS23,MRPS35,MRPS36,MRPS34,MRPL16,MRPL15,MRPL12,MRPL13,MRPL10,MRPL11,MRPL28,MRPL23,MRPL21,MRPL22,MRPL42,MRPL47,MRPL48,MRPL45,MRPL43,MRPL50,MR
Mitochondrial translation	0.0125	89	33	6.16E-08	6.78E-07	MRPS26,MRPS27,MRPS23,MRPS35,MRPS36,MRPS34,TUFM,MRPL16,MRPL15,MRPL12,MRPL13,MRPL10,MRPL11,MRPL28,MRPL23,MRPL21,MRPL22,MRPL42,MRPL47,MRPL48,MRPL45,MRPL43,MRP
mRNA Splicing - Minor Pathway	0.007	50	23	1.68E-07	1.85E-06	ZCRB1,SRSF2,SRSF7,SRSF1,SNRPD2,SNRPD1,SNRPD3,SNRPG,SNRPE,SNRPF,SNRPB,POLR2C,POLR2E,POLR2G,POLR2H,SNRNP40,YBX1,N
CLEC7A (Dectin-1) signaling	0.0135	96	33	3.32E-07	3.65E-06	UBE2N,UBB,PSMD8,PSMD6,PSMD7,PSMD4,PSMD3,PSME1,PSME2,PSMF1,PSMA5,PSMA3,PSMA4,PSMA1,PSMA2,PSMA7,PSMB6,PSMB7,PSMB5,PSMB2,PSMB3,PSMB1,PSMC5,PSMC6,PSMC3,PSMC1,C

TNFR2 non-canonical NF-kB pathway	0.0135	96	32	9.50E-07	1.05E-05	UBB,PSMD8,PSMD6,PSMD7,PSMD4,PSMD3,PSME1,PSME2,PSMF1,PSMA5,PSMA3,PSMA4,PSMA1,PSMA2,PSMA7,PSMB6,PSMB7,PSMB5,PSMB2,PSMB3,PSMB1,PSMC5,PSMC6,PSMC3,PSMC1,TNFRSF12
Nucleotide Excision Repair	0.0143	102	33	1.22E-06	1.34E-05	ACTB,PCNA,TCEA1,PPIE,UBE2I,UBE2N,POLD2,ERCC1,HMGN1,COPS4,COPS6,COPS5,COPS8,RFC5,POLR2C,POLR2E,POLR2G,POLR2H,PARP1,UBB,ACTL6A,RUVBL1,RBX1,SUMO1,SUMO2,RAD23B,CETN2,RP
Signaling by Wnt	0.0322	229	56	2.36E-06	2.59E-05	WLS,CSNK2B,SMARCA4,RAC1,VPS29,VPS35,TLE4,TNRC6B,UBB,WNT2B,PSMD8,PSMD6,PSMD7,PSMD4,PSMD3,PSME1,PSME2,PSMF1,PSMA5,PSMA3,PSMA4,PSMA1,PSMA2,PSMA7,PSMB6,PSMB7,PSMB5,PSMB2,PSMB3,PSMB1,AP2S1,PSMC5,PSMC6,PSMC3,PSMC1,RUVBL1,AP2M1,RBX1,SFRP2,SNX3,CALM1,PSMD10,PSMD11,PSMD14
Pyruvate metabolism and Citric Acid (TCA) cycle	0.0065	46	20	2.43E-06	2.67E-05	ACO2,SUCLA2,BSG,SDHC,SDHD,SDHB,PDHA1,LDHB,LDHA,MPC2,IDH3B,PDHB,IDH3G,SUCLG2,SUCLG1,IDH2,FH,SLC16A1,MDH2,GLO1
Folding of actin by CCT/TriC	0.0013	9	9	2.77E-06	2.77E-05	ACTB,TCP1,CCT3,CCT2,CCT8,CCT7,CCT5,CCT4,CCT6A
Association of TriC/CCT with	0.0013	9	9	2.77E-06	2.77E-05	SPHK1,TCP1,CCT3,CCT2,CCT8,CCT7,CCT5,CCT4,CCT6A
Formation of tubulin folding intermediates by CCT/TriC	0.0034	24	14	3.12E-06	3.12E-05	TUBB2B,TUBB2A,TCP1,TUBB4B,TUBA1C,TUBA1B,TUBA1A,CCT3,CCT2,CCT8,CCT7,CCT5,CCT4,CCT6A
Signaling by Hedgehog	0.0172	122	36	3.19E-06	3.19E-05	SPOP,IFT52,IFT57,UBB,PSMD8,PSMD6,PSMD7,PSMD4,PSMD3,PSME1,PSME2,PSMF1,PSMA5,PSMA3,PSMA4,PSMA1,PSMA2,PSMA7,PSMB6,PSMB7,PSMB5,PSMB2,PSMB3,PSMB1,PSMC5,PSMC6,PSMC
Downstream TCR signaling	0.0143	102	32	3.29E-06	3.29E-05	UBE2N,UBB,PSMD8,PSMD6,PSMD7,PSMD4,PSMD3,PSME1,PSME2,PSMF1,PSMA5,PSMA3,PSMA4,PSMA1,PSMA2,PSMA7,PSMB6,PSMB7,PSMB5,PSMB2,PSMB3,PSMB1,PSMC5,PSMC6,PSMC3,PSMC1,P
Citric acid cycle (TCA cycle)	0.0025	18	12	4.16E-06	4.16E-05	ACO2,SUCLA2,SDHC,SDHD,SDHB,IDH3B,IDH3G,SUCLG2,SUCLG1,ID
Detoxification of Reactive Oxygen	0.0037	26	14	7.62E-06	7.62E-05	GSTP1,TXN2,CAT,TXN,ERO1A,P4HB,SOD3,SOD1,PRDX3,PRDX2,PRD
C-type lectin receptors (CLRs)	0.0167	119	34	1.15E-05	1.15E-04	RAF1,UBE2N,UBB,PSMD8,PSMD6,PSMD7,PSMD4,PSMD3,PSME1,PSME2,PSMF1,PSMA5,PSMA3,PSMA4,PSMA1,PSMA2,PSMA7,PSMB6,PSMB7,PSMB5,PSMB2,PSMB3,PSMB1,PSMC5,PSMC6,PSMC3,PS
Mitotic Prometaphase	0.0139	99	30	1.26E-05	1.26E-04	CDCA8,CENPA,CENPE,CENPF,CENPH,CENPM,CENPN,CDC20,CSNK2B,SMC3,SMC4,SMC2,PLK1,PPP1CC,PMF1,BUB3,BIRC5,KIF2C,MAD2L1,CCNB2,CCNB1,AURKB,NUP37,SKA2,RAD21,NUF2,NUDC,MAD1L
TCF dependent signaling in response to WNT	0.0222	158	41	1.42E-05	1.28E-04	CSNK2B,SMARCA4,TLE4,UBB,PSMD8,PSMD6,PSMD7,PSMD4,PSMD3,PSME1,PSME2,PSMF1,PSMA5,PSMA3,PSMA4,PSMA1,PSMA2,PSMA7,PSMB6,PSMB7,PSMB5,PSMB2,PSMB3,PSMB1,PSMC5,PSMC6,PSMC3,PSMC1,RUVBL1,RBX1,SFRP2,PSMD10,PSMD11,PSMD14,PS

Global Genome Nucleotide Excision Repair (GG-NER)	0.0107	76	25	1.76E-05	1.59E-04	ACTB,PCNA,UBE2I,UBE2N,POLD2,ERCC1,COPS4,COPS6,COPS5,COPS8,RFC5,PARP1,UBB,ACTL6A,RUVBL1,RBX1,SUMO1,SUMO2,RAD23
Formation of ATP by	0.0025	18	11	2.28E-05	2.06E-04	ATP5C1,ATP5A1,ATP5J2,ATP5G3,ATP5G2,ATP5F1,ATP5J,ATP5H,AT
Chromosome Maintenance	0.009	64	22	2.91E-05	2.62E-04	CENPA,CENPH,APEX1,CENPM,CENPN,SMARCA5,PCNA,MIS18A,POLD2,OIP5,RFC5,TERF2IP,RUVBL2,RUVBL1,NPM1,RPA2,RPA3,DKC1,N
Regulation of PLK1 Activity at G2/M Transition	0.0113	80	25	4.01E-05	3.61E-04	OPTN,NEK2,DYNC1I2,CEP70,PLK1,TUBB4B,CEP41,PPP2R1A,RAB8A,TUBB,UBB,TUBA1A,DYNLL1,HSP90AA1,CETN2,CCNB2,CCNB1,YWH
Resolution of Sister Chromatid Cohesion	0.0128	91	27	4.78E-05	4.30E-04	CDCA8,CENPA,CENPE,CENPF,CENPH,CENPM,CENPN,CDC20,SMC3,PLK1,PPP1CC,PMF1,BUB3,BIRC5,KIF2C,MAD2L1,CCNB2,CCNB1,AU
Glycolysis	0.0038	27	13	4.95E-05	4.45E-04	PGK1,GAPDH,GPI,TPI1,PKM,HK2,PGAM1,ENO1,ENO3,PFKL,PFKM,A
Cleavage of Growing Transcript in the Termination Region	0.0062	44	17	5.74E-05	5.17E-04	SRSF2,SRSF3,SRSF4,SRSF5,SRSF7,SRSF9,SRSF1,SNRPD3,SNRPG,SNRPE,SNRPF,SNRPB,MAGOH,RNPS1,RBM8A,NCBP2,UPF3B
RNA Polymerase II Transcription Termination	0.0062	44	17	5.74E-05	5.17E-04	SRSF2,SRSF3,SRSF4,SRSF5,SRSF7,SRSF9,SRSF1,SNRPD3,SNRPG,SNRPE,SNRPF,SNRPB,MAGOH,RNPS1,RBM8A,NCBP2,UPF3B
TCR signaling	0.0169	120	32	7.38E-05	6.64E-04	UBE2N,UBB,PSMD8,PSMD6,PSMD7,PSMD4,PSMD3,PSME1,PSME2,PSMF1,PSMA5,PSMA3,PSMA4,PSMA1,PSMA2,PSMA7,PSMB6,PSMB7,PSMB5,PSMB2,PSMB3,PSMB1,PSMC5,PSMC6,PSMC3,PSMC1,P
RHO GTPases Activate Formins	0.0142	101	28	1.08E-04	9.76E-04	CDCA8,CENPA,CENPE,CENPF,CENPH,CENPM,CENPN,ITGB1,ACTB,CDC20,RAC1,ACTG1,PLK1,PPP1CC,PMF1,BUB3,BIRC5,KIF2C,MAD2L1,AURKB,NUP37,SKA2,NUF2,NUDC,MAD1L1,EVL,RHOA,ITGB3BP
Metabolism of amino acids and derivatives	0.0374	266	56	1.38E-04	1.21E-03	QARS,ENOPH1,ODC1,AIMP1,HIBADH,PHGDH,EEF1E1,NDUFAB1,PDHA1,ADI1,CKB,PDHB,PYCR1,PYCR2,DARS,GRHPR,DCT,PSMD8,PSMD6,PSMD7,PSMD4,PSMD3,PSME1,PSME2,OAZ2,PSMF1,PSMA5,PSMA3,PSMA4,PSMA1,PSMA2,PSMA7,PSMB6,PSMB7,PSMB5,PSMB2,PSMB3,PSMB1,PSMC5,PSMC6,PSMC3,PSMC1,SARS,GOT1,HSD17B
Metabolism of nucleotides	0.0087	62	20	1.52E-04	1.21E-03	ATIC,NUDT15,DCTPP1,NUDT1,NUDT5,APRT,CAT,ITPA,ADSL,TXN,DTYMK,TYMS,UPP1,NT5C3A,PAICS,HPRT1,AK2,IMPDH2,NME1,GMPR
TP53 Regulates Metabolic Genes	0.0101	72	22	1.55E-04	1.24E-03	TP53,COX7C,COX8A,COX5B,COX5A,COX6C,COX6A1,COX6B1,GPI,TXN,COX4I1,NDUFA4,YWHAE,YWHAB,YWHAQ,YWHAH,PRDX2,PRDX5
Transcriptional Regulation by TP53	0.0101	72	22	1.55E-04	1.24E-03	TP53,COX7C,COX8A,COX5B,COX5A,COX6C,COX6A1,COX6B1,GPI,TXN,COX4I1,NDUFA4,YWHAE,YWHAB,YWHAQ,YWHAH,PRDX2,PRDX5
Formation of Incision Complex in GG-NER	0.0055	39	15	1.61E-04	1.29E-03	UBE2I,UBE2N,ERCC1,PARP1,UBB,RBX1,SUMO1,SUMO2,RAD23B,CETN2,RPA2,RPA3,UBE2V2,UBA52,GTF2H5
G2/M Transition	0.0153	109	29	1.63E-04	1.31E-03	OPTN,CENPF,NEK2,CDC25B,DYNC1I2,CEP70,PLK1,TUBB4B,CEP41,PPP2R1A,RAB8A,TUBB,UBB,TUBA1A,DYNLL1,HSP90AA1,CETN2,CCNB2,CCNB1,YWHAE,CCNA2,DCTN2,DCTN3,AURKA,UBA52,CDK1,RBB

HSF1 activation	0.0017	12	8	1.64E-04	1.31E-03	EEF1A1,PTGES3,HSP90AB1,HSP90AA1,RPA2,RPA3,YWHAE,HSBP1
Translation	0.0203	144	35	2.02E-04	1.62E-03	EIF1AX,EIF4H,EIF4E,EIF4B,EIF3M,EIF3K,EIF3L,EIF3I,EIF3G,EIF3H,EIF3E,EIF3F,EIF3D,PABPC1,SRP14,EEF1B2,EEF1A1,EIF2B1,SEC11A,SEC61G,SEC61B,SPCS2,SPCS1,EIF2S3,EIF5,SRP9,EEF2,DDOST,EIF4A2,EI
Mitotic G2-G2/M phases	0.0156	111	29	2.20E-04	1.76E-03	OPTN,CENPF,NEK2,CDC25B,DYNC1I2,CEP70,PLK1,TUBB4B,CEP41,PPP2R1A,RAB8A,TUBB,UBB,TUBA1A,DYNLL1,HSP90AA1,CETN2,CCNB2,CCNB1,YWHAE,CCNA2,DCTN2,DCTN3,AURKA,UBA52,CDK1,RBB
Transcription-Coupled Nucleotide Excision Repair (TC-NER)	0.0104	74	22	2.25E-04	1.80E-03	PCNA,TCEA1,PPIE,POLD2,ERCC1,HMGN1,COPS4,COPS6,COPS5,COPS8,RFC5,POLR2C,POLR2E,POLR2G,POLR2H,UBB,RBX1,RPA2,RPA3,U
Glucose metabolism	0.0098	70	21	2.75E-04	2.20E-03	PGK1,PGM1,GAPDH,GPI,TPI1,PKM,UBB,HK2,PGAM1,MDH1,MDH2,GOT1,CALM1,ENO1,ENO3,UBA52,GYG1,PFKL,PFKM,ALDOC,ALDOA
RNA Polymerase II Transcription	0.0152	108	28	3.15E-04	2.52E-03	SRSF2,SRSF3,SRSF4,SRSF5,SRSF7,SRSF9,SRSF1,SNRPD3,TCEB1,TCEA1,SSRP1,SUPT16H,SNRPG,SNRPE,SNRPF,SNRPB,POLR2C,POLR2E,POLR2G,POLR2H,MAGOH,RNPS1,NELFE,RBM8A,NCBP2,UPF3B,GTF2
Deposition of new CENPA-	0.0041	29	12	3.72E-04	2.84E-03	CENPA,CENPH,CENPM,CENPN,SMARCA5,MIS18A,OIP5,RUVBL1,NP
Nucleosome assembly	0.0041	29	12	3.72E-04	2.84E-03	CENPA,CENPH,CENPM,CENPN,SMARCA5,MIS18A,OIP5,RUVBL1,NP
Ribosomal scanning and start codon recognition	0.008	57	18	4.06E-04	2.84E-03	EIF1AX,EIF4H,EIF4E,EIF4B,EIF3M,EIF3K,EIF3L,EIF3I,EIF3G,EIF3H,EIF3E,EIF3F,EIF3D,EIF2S3,EIF5,EIF4A2,EIF4A1,FAU
Translation initiation complex formation	0.008	57	18	4.06E-04	2.84E-03	EIF1AX,EIF4H,EIF4E,EIF4B,EIF3M,EIF3K,EIF3L,EIF3I,EIF3G,EIF3H,EIF3E,EIF3F,EIF3D,PABPC1,EIF2S3,EIF4A2,EIF4A1,FAU
NCAM signaling for neurite out-growth	0.0314	223	47	4.53E-04	3.17E-03	DUSP6,AGRN,SHC1,SPTAN1,PEBP1,RAF1,FGF19,PHB,UBB,COL6A1,PSMD8,PSMD6,PSMD7,PSMD4,PSMD3,PSME1,PSME2,PSMF1,PSMA5,PSMA3,PSMA4,PSMA1,PSMA2,PSMA7,PSMB6,PSMB7,PSMB5,PSMB2,PSMB3,PSMB1,PSMC5,PSMC6,PSMC3,PSMC1,RBX1,COL9A1,
Activation of the mRNA upon binding of the cap-binding	0.0082	58	18	4.95E-04	3.47E-03	EIF1AX,EIF4H,EIF4E,EIF4B,EIF3M,EIF3K,EIF3L,EIF3I,EIF3G,EIF3H,EIF3E,EIF3F,EIF3D,PABPC1,EIF2S3,EIF4A2,EIF4A1,FAU
Gluconeogenesis	0.0044	31	12	6.64E-04	4.65E-03	PGK1,GAPDH,GPI,TPI1,PGAM1,MDH1,MDH2,GOT1,ENO1,ENO3,AL
DNA Damage Recognition in GG-	0.0051	36	13	7.60E-04	5.32E-03	ACTB,COPS4,COPS6,COPS5,COPS8,PARP1,UBB,ACTL6A,RUVBL1,RB
Dual incision in TC-NER	0.0086	61	18	8.73E-04	6.11E-03	PCNA,TCEA1,PPIE,POLD2,ERCC1,HMGN1,RFC5,POLR2C,POLR2E,POLR2G,POLR2H,UBB,RBX1,RPA2,RPA3,UBA52,GTF2H5,PRPF19
Collagen biosynthesis and modifying enzymes	0.0086	61	18	8.73E-04	6.11E-03	COL3A1,PPIB,P3H2,COL18A1,COLGALT2,COL6A1,COL1A1,P4HB,COL9A1,COL9A3,PCOLCE,COL5A1,COL5A2,COL8A2,COL8A1,COL4A2,C
MAPK1/MAPK3 signaling	0.0271	193	41	8.82E-04	6.17E-03	DUSP6,SHC1,SPTAN1,PEBP1,RAF1,FGF19,PHB,UBB,PSMD8,PSMD6,PSMD7,PSMD4,PSMD3,PSME1,PSME2,PSMF1,PSMA5,PSMA3,PSMA4,PSMA1,PSMA2,PSMA7,PSMB6,PSMB7,PSMB5,PSMB2,PSMB3,PSMB1,PSMC5,PSMC6,PSMC3,PSMC1,RBX1,CALM1,PSMD10,PSMD

SOS-mediated signalling	0.0264	188	40	9.85E-04	6.51E-03	DUSP6,SHC1,SPTAN1,PEBP1,RAF1,FGF19,PHB,UBB,PSMD8,PSMD6,PSMD7,PSMD4,PSMD3,PSME1,PSME2,PSMF1,PSMA5,PSMA3,PSMA4,PSMA1,PSMA2,PSMA7,PSMB6,PSMB7,PSMB5,PSMB2,PSMB3,PSMB1,PSMC5,PSMC6,PSMC3,PSMC1,RBX1,CALM1,PSMD10,PSMD
GRB2 events in EGFR signaling	0.0264	188	40	9.85E-04	6.51E-03	DUSP6,SHC1,SPTAN1,PEBP1,RAF1,FGF19,PHB,UBB,PSMD8,PSMD6,PSMD7,PSMD4,PSMD3,PSME1,PSME2,PSMF1,PSMA5,PSMA3,PSMA4,PSMA1,PSMA2,PSMA7,PSMB6,PSMB7,PSMB5,PSMB2,PSMB3,PSMB1,PSMC5,PSMC6,PSMC3,PSMC1,RBX1,CALM1,PSMD10,PSMD
GRB2 events in ERBB2 signaling	0.0264	188	40	9.85E-04	6.51E-03	DUSP6,SHC1,SPTAN1,PEBP1,RAF1,FGF19,PHB,UBB,PSMD8,PSMD6,PSMD7,PSMD4,PSMD3,PSME1,PSME2,PSMF1,PSMA5,PSMA3,PSMA4,PSMA1,PSMA2,PSMA7,PSMB6,PSMB7,PSMB5,PSMB2,PSMB3,PSMB1,PSMC5,PSMC6,PSMC3,PSMC1,RBX1,CALM1,PSMD10,PSMD
SHC1 events in ERBB4 signaling	0.0264	188	40	9.85E-04	6.51E-03	DUSP6,SHC1,SPTAN1,PEBP1,RAF1,FGF19,PHB,UBB,PSMD8,PSMD6,PSMD7,PSMD4,PSMD3,PSME1,PSME2,PSMF1,PSMA5,PSMA3,PSMA4,PSMA1,PSMA2,PSMA7,PSMB6,PSMB7,PSMB5,PSMB2,PSMB3,PSMB1,PSMC5,PSMC6,PSMC3,PSMC1,RBX1,CALM1,PSMD10,PSMD
SHC1 events in ERBB2 signaling	0.0264	188	40	9.85E-04	6.51E-03	DUSP6,SHC1,SPTAN1,PEBP1,RAF1,FGF19,PHB,UBB,PSMD8,PSMD6,PSMD7,PSMD4,PSMD3,PSME1,PSME2,PSMF1,PSMA5,PSMA3,PSMA4,PSMA1,PSMA2,PSMA7,PSMB6,PSMB7,PSMB5,PSMB2,PSMB3,PSMB1,PSMC5,PSMC6,PSMC3,PSMC1,RBX1,CALM1,PSMD10,PSMD
SHC1 events in EGFR signaling	0.0264	188	40	9.85E-04	6.51E-03	DUSP6,SHC1,SPTAN1,PEBP1,RAF1,FGF19,PHB,UBB,PSMD8,PSMD6,PSMD7,PSMD4,PSMD3,PSME1,PSME2,PSMF1,PSMA5,PSMA3,PSMA4,PSMA1,PSMA2,PSMA7,PSMB6,PSMB7,PSMB5,PSMB2,PSMB3,PSMB1,PSMC5,PSMC6,PSMC3,PSMC1,RBX1,CALM1,PSMD10,PSMD
RAF/MAP kinase cascade	0.0264	188	40	9.85E-04	6.51E-03	DUSP6,SHC1,SPTAN1,PEBP1,RAF1,FGF19,PHB,UBB,PSMD8,PSMD6,PSMD7,PSMD4,PSMD3,PSME1,PSME2,PSMF1,PSMA5,PSMA3,PSMA4,PSMA1,PSMA2,PSMA7,PSMB6,PSMB7,PSMB5,PSMB2,PSMB3,PSMB1,PSMC5,PSMC6,PSMC3,PSMC1,RBX1,CALM1,PSMD10,PSMD
SUMO is transferred from E1 to E2	0.0008	6	5	1.05E-03	6.51E-03	UBE2I,UBA2,SAE1,SUMO1,SUMO2
Senescence-Associated Secretory Phenotype (SASP)	0.0073	52	16	1.08E-03	6.51E-03	JUN,FOS,UBE2C,ANAPC15,ANAPC16,CDKN1A,CDKN2B,CEBPB,UBB,STAT3,UBE2E1,UBA52,H3F3A,IGFBP7,CDK6,CDK4
FRS-mediated FGFR2 signaling	0.0266	189	40	1.08E-03	6.51E-03	DUSP6,SHC1,SPTAN1,PEBP1,RAF1,FGF19,PHB,UBB,PSMD8,PSMD6,PSMD7,PSMD4,PSMD3,PSME1,PSME2,PSMF1,PSMA5,PSMA3,PSMA4,PSMA1,PSMA2,PSMA7,PSMB6,PSMB7,PSMB5,PSMB2,PSMB3,PSMB1,PSMC5,PSMC6,PSMC3,PSMC1,RBX1,CALM1,PSMD10,PSMD

FRS-mediated FGFR3 signaling	0.0266	189	40	1.08E-03	6.51E-03	DUSP6,SHC1,SPTAN1,PEBP1,RAF1,FGF19,PHB,UBB,PSMD8,PSMD6,PSMD7,PSMD4,PSMD3,PSME1,PSME2,PSMF1,PSMA5,PSMA3,PSMA4,PSMA1,PSMA2,PSMA7,PSMB6,PSMB7,PSMB5,PSMB2,PSMB3,PSMB1,PSMC5,PSMC6,PSMC3,PSMC1,RBX1,CALM1,PSMD10,PSMD
FRS-mediated FGFR4 signaling	0.0266	189	40	1.08E-03	6.51E-03	DUSP6,SHC1,SPTAN1,PEBP1,RAF1,FGF19,PHB,UBB,PSMD8,PSMD6,PSMD7,PSMD4,PSMD3,PSME1,PSME2,PSMF1,PSMA5,PSMA3,PSMA4,PSMA1,PSMA2,PSMA7,PSMB6,PSMB7,PSMB5,PSMB2,PSMB3,PSMB1,PSMC5,PSMC6,PSMC3,PSMC1,RBX1,CALM1,PSMD10,PSMD
FRS-mediated FGFR1 signaling	0.0266	189	40	1.08E-03	6.51E-03	DUSP6,SHC1,SPTAN1,PEBP1,RAF1,FGF19,PHB,UBB,PSMD8,PSMD6,PSMD7,PSMD4,PSMD3,PSME1,PSME2,PSMF1,PSMA5,PSMA3,PSMA4,PSMA1,PSMA2,PSMA7,PSMB6,PSMB7,PSMB5,PSMB2,PSMB3,PSMB1,PSMC5,PSMC6,PSMC3,PSMC1,RBX1,CALM1,PSMD10,PSMD
VEGFR2 mediated cell proliferation	0.0284	202	42	1.15E-03	6.89E-03	SPHK1,DUSP6,SHC1,SPTAN1,VEGFA,PEBP1,RAF1,FGF19,PHB,UBB,PSMD8,PSMD6,PSMD7,PSMD4,PSMD3,PSME1,PSME2,PSMF1,PSMA5,PSMA3,PSMA4,PSMA1,PSMA2,PSMA7,PSMB6,PSMB7,PSMB5,PSMB2,PSMB3,PSMB1,PSMC5,PSMC6,PSMC3,PSMC1,RBX1,CALM1,P
Signaling by Leptin	0.0276	196	41	1.17E-03	7.03E-03	DUSP6,SHC1,SPTAN1,PEBP1,RAF1,FGF19,PHB,UBB,PSMD8,PSMD6,PSMD7,PSMD4,PSMD3,PSME1,PSME2,PSMF1,PSMA5,PSMA3,PSMA4,PSMA1,PSMA2,PSMA7,PSMB6,PSMB7,PSMB5,PSMB2,PSMB3,PSMB1,STAT3,PSMC5,PSMC6,PSMC3,PSMC1,RBX1,CALM1,PSMD10,
Formation of TC-NER Pre-Incision Complex	0.0075	53	16	1.31E-03	7.86E-03	TCEA1,PPIE,HMGN1,COPS4,COPS6,COPS5,COPS8,POLR2C,POLR2E,POLR2G,POLR2H,UBB,RBX1,UBA52,GTF2H5,PRPF19
Transport of Mature mRNA derived from an Intron-Containing	0.0075	53	16	1.31E-03	7.86E-03	SRSF2,SRSF3,SRSF4,SRSF5,SRSF7,SRSF9,SRSF1,EIF4E,RAE1,NUP93,MAGOH,RNPS1,RBM8A,NCBP2,NUP37,UPF3B
RHO GTPase Effectors	0.0295	210	43	1.35E-03	8.07E-03	CDCA8,CENPA,CENPE,CENPF,CENPH,CENPM,CENPN,ARPC4,ARPC3,ITGB1,ACTB,CDC20,RAC1,ACTG1,PLK1,PPP1CC,KDM1A,PMF1,MYH9,BUB3,MYL6,FLNA,BIRC5,PRC1,CALM1,KIF2C,MAD2L1,YWHAE,YWHAB,YWHAQ,YWHAH,AURKB,NUP37,SKA2,H3F3A,NUF2,NUDC,MA
APC-Cdc20 mediated degradation	0.0035	25	10	1.43E-03	8.57E-03	NEK2,CDC20,UBE2C,ANAPC15,ANAPC16,UBB,BUB3,UBE2E1,MAD2
Signalling to p38 via RIT and RIN	0.027	192	40	1.44E-03	8.62E-03	DUSP6,SHC1,SPTAN1,PEBP1,RAF1,FGF19,PHB,UBB,PSMD8,PSMD6,PSMD7,PSMD4,PSMD3,PSME1,PSME2,PSMF1,PSMA5,PSMA3,PSMA4,PSMA1,PSMA2,PSMA7,PSMB6,PSMB7,PSMB5,PSMB2,PSMB3,PSMB1,PSMC5,PSMC6,PSMC3,PSMC1,RBX1,CALM1,PSMD10,PSMD

ARMS-mediated activation	0.0271	193	40	1.58E-03	9.45E-03	DUSP6,SHC1,SPTAN1,PEBP1,RAF1,FGF19,PHB,UBB,PSMD8,PSMD6,PSMD7,PSMD4,PSMD3,PSME1,PSME2,PSMF1,PSMA5,PSMA3,PSMA4,PSMA1,PSMA2,PSMA7,PSMB6,PSMB7,PSMB5,PSMB2,PSMB3,PSMB1,PSMC5,PSMC6,PSMC3,PSMC1,RBX1,CALM1,PSMD10,PSMD
DNA Double Strand Break Response	0.0062	44	14	1.59E-03	9.54E-03	BRE,TP53,SMARCA5,UBE2I,UBE2N,APBB1,UBB,H2AFX,KPNA2,SUMO1,BABAM1,UBE2V2,FAM175A,UBA52
Deadenylation-dependent mRNA decay	0.0069	49	15	1.60E-03	9.60E-03	EIF4E,EIF4B,PABPC1,PAIP1,EIF4A2,EIF4A1,EIF4A3,EXOSC7,EXOSC8,LSM5,LSM4,LSM3,LSM2,LSM7,LSM6
Frs2-mediated activation	0.0273	194	40	1.72E-03	0.0103	DUSP6,SHC1,SPTAN1,PEBP1,RAF1,FGF19,PHB,UBB,PSMD8,PSMD6,PSMD7,PSMD4,PSMD3,PSME1,PSME2,PSMF1,PSMA5,PSMA3,PSMA4,PSMA1,PSMA2,PSMA7,PSMB6,PSMB7,PSMB5,PSMB2,PSMB3,PSMB1,PSMC5,PSMC6,PSMC3,PSMC1,RBX1,CALM1,PSMD10,PSMD
Attenuation phase	0.0014	10	6	1.80E-03	0.0108	FKBP4,PTGES3,HSP90AB1,HSP90AA1,HSBP1,HSPA8
SLBP independent Processing of	0.0014	10	6	1.80E-03	0.0108	SNRPD3,SNRPG,SNRPE,SNRPF,SNRPB,NCBP2
Gap-filling DNA repair synthesis and ligation in TC-NER	0.0084	60	17	1.83E-03	0.011	PCNA,TCEA1,PPIE,POLD2,HMGN1,RFC5,POLR2C,POLR2E,POLR2G,POLR2H,UBB,RBX1,RPA2,RPA3,UBA52,GTF2H5,PRPF19
mRNA 3'-end processing	0.0049	35	12	1.83E-03	0.011	SRSF2,SRSF3,SRSF4,SRSF5,SRSF7,SRSF9,SRSF1,MAGOH,RNPS1,RBM
G2/M DNA damage checkpoint	0.0056	40	13	1.92E-03	0.0115	BRE,UBE2N,H2AFX,SUMO1,BABAM1,UBE2V2,CCNB1,FAM175A,YWHAE,YWHAB,YWHAQ,YWHAH,CDK1
Interleukin receptor SHC signaling	0.0276	196	40	2.06E-03	0.0124	DUSP6,SHC1,SPTAN1,PEBP1,RAF1,FGF19,PHB,UBB,PSMD8,PSMD6,PSMD7,PSMD4,PSMD3,PSME1,PSME2,PSMF1,PSMA5,PSMA3,PSMA4,PSMA1,PSMA2,PSMA7,PSMB6,PSMB7,PSMB5,PSMB2,PSMB3,PSMB1,PSMC5,PSMC6,PSMC3,PSMC1,RBX1,CALM1,PSMD10,PSMD
Prolonged ERK activation events	0.0276	196	40	2.06E-03	0.0124	DUSP6,SHC1,SPTAN1,PEBP1,RAF1,FGF19,PHB,UBB,PSMD8,PSMD6,PSMD7,PSMD4,PSMD3,PSME1,PSME2,PSMF1,PSMA5,PSMA3,PSMA4,PSMA1,PSMA2,PSMA7,PSMB6,PSMB7,PSMB5,PSMB2,PSMB3,PSMB1,PSMC5,PSMC6,PSMC3,PSMC1,RBX1,CALM1,PSMD10,PSMD
Post-chaperonin tubulin folding	0.0031	22	9	2.09E-03	0.0125	TUBB2B,TUBB2A,TUBB4B,TUBA1C,TUBA1B,TUBA1A,ARL2,TBCB,TB
Mitochondrial Fatty Acid Beta-	0.002	14	7	2.15E-03	0.0127	ECHS1,PCCA,ECI1,DECR1,HADHB,HADH,ACADM
Axon guidance	0.0631	449	78	2.31E-03	0.0127	DPYSL4,UNC5D,DUSP6,AGRN,SHC1,ARPC4,ARPC3,ITGB1,ITGAV,ITGA1,ACTB,CSNK2B,RRAS,NRP2,RAC1,SPTAN1,VEGFA,PEBP1,ACTG1,RAF1,FGF19,ALCAM,PLXND1,CFL1,PHB,PITPNA,UBB,COL6A1,DCX,MYH9,PSMD8,PSMD6,PSMD7,PSMD4,PSMD3,MYL6,PSME1,PSME2,PSMF1,PSMA5,PSMA3,PSMA4,PSMA1,PSMA2,PSMA7,PSMB6,PSMB7,PSMB5,PSMB2,PSMB3,PSMB1,AP2S1,PSMC5,PSMC6,PSMC3,PS

Loss of proteins required for interphase microtubule	0.0087	62	17	2.54E-03	0.0127	NEK2,DYNC1I2,CEP70,PLK1,TUBB4B,CEP41,PPP2R1A,TUBB,TUBA1A,DYNLL1,HSP90AA1,CETN2,YWHAE,DCTN2,DCTN3,CDK1,TUBG1
Loss of Nlp from mitotic centrosomes	0.0087	62	17	2.54E-03	0.0127	NEK2,DYNC1I2,CEP70,PLK1,TUBB4B,CEP41,PPP2R1A,TUBB,TUBA1A,DYNLL1,HSP90AA1,CETN2,YWHAE,DCTN2,DCTN3,CDK1,TUBG1
APC/C:Cdc20 mediated	0.0032	23	9	2.80E-03	0.014	CDC20,UBE2C,ANAPC15,ANAPC16,UBB,UBE2E1,CCNB1,UBA52,CD
Condensation of Prometaphase	0.0015	11	6	2.87E-03	0.0144	CSNK2B,SMC4,SMC2,CCNB2,CCNB1,CDK1
SLBP Dependent Processing of	0.0015	11	6	2.87E-03	0.0144	SNRPD3,SNRPG,SNRPE,SNRPF,SNRPB,NCBP2
Signalling to RAS	0.0281	200	40	2.91E-03	0.0145	DUSP6,SHC1,SPTAN1,PEBP1,RAF1,FGF19,PHB,UBB,PSMD8,PSMD6,PSMD7,PSMD4,PSMD3,PSME1,PSME2,PSMF1,PSMA5,PSMA3,PSMA4,PSMA1,PSMA2,PSMA7,PSMB6,PSMB7,PSMB5,PSMB2,PSMB3,PSMB1,PSMC5,PSMC6,PSMC3,PSMC1,RBX1,CALM1,PSMD10,PSMD
Scavenging by Class A Receptors	0.0027	19	8	3.05E-03	0.0152	COL3A1,HSP90B1,APOE,CALR,FTH1,COL1A1,COL4A2,COL4A1
Transport of Mature Transcript to Cytoplasm	0.0082	58	16	3.16E-03	0.0158	SRSF2,SRSF3,SRSF4,SRSF5,SRSF7,SRSF9,SRSF1,EIF4E,RAE1,NUP93,MAGOH,RNPS1,RBM8A,NCBP2,NUP37,UPF3B
Interleukin-2 signaling	0.0284	202	40	3.43E-03	0.0172	DUSP6,SHC1,SPTAN1,PEBP1,RAF1,FGF19,PHB,UBB,PSMD8,PSMD6,PSMD7,PSMD4,PSMD3,PSME1,PSME2,PSMF1,PSMA5,PSMA3,PSMA4,PSMA1,PSMA2,PSMA7,PSMB6,PSMB7,PSMB5,PSMB2,PSMB3,PSMB1,PSMC5,PSMC6,PSMC3,PSMC1,RBX1,CALM1,PSMD10,PSMD
G2/M Checkpoints	0.0105	75	19	3.47E-03	0.0174	BRE,UBE2N,MCM7,RFC5,H2AFX,SUMO1,DBF4,BABAM1,RPA2,RPA3,UBE2V2,CCNB2,CCNB1,FAM175A,YWHAE,YWHAB,YWHAQ,YWH
Recruitment and ATM-mediated phosphorylation of repair and	0.006	43	13	3.52E-03	0.0176	BRE,TP53,SMARCA5,UBE2I,UBE2N,APBB1,UBB,H2AFX,SUMO1,BABAM1,UBE2V2,FAM175A,UBA52
Downstream signaling events of B Cell Receptor (BCR)	0.0232	165	34	3.71E-03	0.0185	FGF19,CDKN1A,TNRC6B,UBB,PSMD8,PSMD6,PSMD7,PSMD4,PSMD3,PSME1,PSME2,PSMF1,PSMA5,PSMA3,PSMA4,PSMA1,PSMA2,PSMA7,PSMB6,PSMB7,PSMB5,PSMB2,PSMB3,PSMB1,PSMC5,PSMC6,
Centrosome maturation	0.0098	70	18	3.76E-03	0.0188	NEK2,DYNC1I2,CEP70,PLK1,TUBB4B,CEP41,PPP2R1A,TUBB,TUBA1A,DYNLL1,HSP90AA1,CETN2,CCNB1,YWHAE,DCTN2,DCTN3,CDK1,T
Recruitment of mitotic centrosome proteins and	0.0098	70	18	3.76E-03	0.0188	NEK2,DYNC1I2,CEP70,PLK1,TUBB4B,CEP41,PPP2R1A,TUBB,TUBA1A,DYNLL1,HSP90AA1,CETN2,CCNB1,YWHAE,DCTN2,DCTN3,CDK1,T
SUMO is conjugated to E1	0.0007	5	4	3.83E-03	0.0192	UBA2,SAE1,SUMO1,SUMO2
Beta oxidation of octanoyl-CoA to	0.0007	5	4	3.83E-03	0.0192	ECHS1,HADHB,HADH,ACADM
Catabolism of glucuronate to	0.0007	5	4	3.83E-03	0.0192	CRYL1,AKR1A1,SORD,DCXR
Beta oxidation of decanoyl-CoA to	0.0007	5	4	3.83E-03	0.0192	ECHS1,HADHB,HADH,ACADM
Extension of Telomeres	0.0041	29	10	4.07E-03	0.0204	APEX1,PCNA,POLD2,RFC5,RUVBL2,RUVBL1,RPA2,RPA3,DKC1,NHP2

IRS-mediated signalling	0.0333	237	45	4.24E-03	0.0212	DUSP6,SHC1,EIF4E,EIF4B,SPTAN1,PEBP1,RAF1,FGF19,PHB,UBB,PSMD8,PSMD6,PSMD7,PSMD4,PSMD3,PSME1,PSME2,PSMF1,PSMA5,PSMA3,PSMA4,PSMA1,PSMA2,PSMA7,PSMB6,PSMB7,PSMB5,PSMB2,PSMB3,PSMB1,PSMC5,PSMC6,PSMC3,PSMC1,RBX1,CALM1,P
Chk1/Chk2(Cds1) mediated	0.0017	12	6	4.37E-03	0.0219	CCNB1,YWHAE,YWHAB,YWHAQ,YWHAH,CDK1
Translesion synthesis by REV1	0.0023	16	7	4.43E-03	0.0221	PCNA,RFC5,UBB,RPA2,MAD2L2,RPA3,UBA52
SUMOylation	0.0141	100	23	4.50E-03	0.0225	CDCA8,PCNA,RAE1,UBE2I,UBA2,SMC3,NUP93,HNRNPK,HNRNPC,TOP2A,TOP2B,PARP1,SAE1,SUMO1,SUMO2,BIRC5,NSMCE1,CETN2,
MAPK family signaling cascades	0.0326	232	44	4.75E-03	0.0235	JUN,DUSP6,SHC1,RAC1,SPTAN1,PEBP1,RAF1,FGF19,PHB,TNRC6B,UBB,PSMD8,PSMD6,PSMD7,PSMD4,PSMD3,PSME1,PSME2,PSMF1,PSMA5,PSMA3,PSMA4,PSMA1,PSMA2,PSMA7,PSMB6,PSMB7,PSMB5,PSMB2,PSMB3,PSMB1,PSMC5,PSMC6,PSMC3,PSMC1,RBX1,CAL
Basigin interactions	0.0035	25	9	4.78E-03	0.0235	SLC7A6,SLC7A8,ITGB1,BSG,PPIA,SLC3A2,ATP1B3,ATP1B1,SLC16A1
Collagen formation	0.0101	72	18	4.98E-03	0.0235	COL3A1,PPIB,P3H2,COL18A1,COLGALT2,COL6A1,COL1A1,P4HB,COL9A1,COL9A3,PCOLCE,COL5A1,COL5A2,COL8A2,COL8A1,COL4A2,C
SUMOylation of DNA replication	0.0063	45	13	5.09E-03	0.0235	CDCA8,PCNA,RAE1,UBE2I,NUP93,TOP2A,TOP2B,SUMO1,SUMO2,BI
Platelet degranulation	0.011	78	19	5.21E-03	0.0235	FN1,ACTN1,ACTN4,VEGFA,PPIA,CD9,TIMP1,CFL1,FLNA,LEFTY2,CALM1,VCL,SOD1,SERPINE1,SERPING1,SPARC,ALDOA,SCG3,TGFB2
Insulin receptor signalling cascade	0.0338	240	45	5.28E-03	0.0235	DUSP6,SHC1,EIF4E,EIF4B,SPTAN1,PEBP1,RAF1,FGF19,PHB,UBB,PSMD8,PSMD6,PSMD7,PSMD4,PSMD3,PSME1,PSME2,PSMF1,PSMA5,PSMA3,PSMA4,PSMA1,PSMA2,PSMA7,PSMB6,PSMB7,PSMB5,PSMB2,PSMB3,PSMB1,PSMC5,PSMC6,PSMC3,PSMC1,RBX1,CALM1,P
Mitotic Spindle Checkpoint	0.003	21	8	5.49E-03	0.0235	CDC20,UBE2C,ANAPC15,ANAPC16,BUB3,UBE2E1,MAD2L1,MAD1L
Signalling to ERKs	0.0293	208	40	5.53E-03	0.0235	DUSP6,SHC1,SPTAN1,PEBP1,RAF1,FGF19,PHB,UBB,PSMD8,PSMD6,PSMD7,PSMD4,PSMD3,PSME1,PSME2,PSMF1,PSMA5,PSMA3,PSMA4,PSMA1,PSMA2,PSMA7,PSMB6,PSMB7,PSMB5,PSMB2,PSMB3,PSMB1,PSMC5,PSMC6,PSMC3,PSMC1,RBX1,CALM1,PSMD10,PSMD
Interleukin-3, 5 and GM-CSF signaling	0.0293	208	40	5.53E-03	0.0235	DUSP6,SHC1,SPTAN1,PEBP1,RAF1,FGF19,PHB,UBB,PSMD8,PSMD6,PSMD7,PSMD4,PSMD3,PSME1,PSME2,PSMF1,PSMA5,PSMA3,PSMA4,PSMA1,PSMA2,PSMA7,PSMB6,PSMB7,PSMB5,PSMB2,PSMB3,PSMB1,PSMC5,PSMC6,PSMC3,PSMC1,RBX1,CALM1,PSMD10,PSMD
IRS-related events triggered by IGF1R	0.0339	241	45	5.67E-03	0.0235	DUSP6,SHC1,EIF4E,EIF4B,SPTAN1,PEBP1,RAF1,FGF19,PHB,UBB,PSMD8,PSMD6,PSMD7,PSMD4,PSMD3,PSME1,PSME2,PSMF1,PSMA5,PSMA3,PSMA4,PSMA1,PSMA2,PSMA7,PSMB6,PSMB7,PSMB5,PSMB2,PSMB3,PSMB1,PSMC5,PSMC6,PSMC3,PSMC1,RBX1,CALM1,P

Signaling by Type 1 Insulin-like Growth Factor 1 Receptor (IGF1R)	0.0339	241	45	5.67E-03	0.0235	DUSP6,SHC1,EIF4E,EIF4B,SPTAN1,PEBP1,RAF1,FGF19,PHB,UBB,PSMD8,PSMD6,PSMD7,PSMD4,PSMD3,PSME1,PSME2,PSMF1,PSMA5,PSMA3,PSMA4,PSMA1,PSMA2,PSMA7,PSMB6,PSMB7,PSMB5,PSMB2,PSMB3,PSMB1,PSMC5,PSMC6,PSMC3,PSMC1,RBX1,CALM1,P
IGF1R signaling cascade	0.0339	241	45	5.67E-03	0.0235	DUSP6,SHC1,EIF4E,EIF4B,SPTAN1,PEBP1,RAF1,FGF19,PHB,UBB,PSMD8,PSMD6,PSMD7,PSMD4,PSMD3,PSME1,PSME2,PSMF1,PSMA5,PSMA3,PSMA4,PSMA1,PSMA2,PSMA7,PSMB6,PSMB7,PSMB5,PSMB2,PSMB3,PSMB1,PSMC5,PSMC6,PSMC3,PSMC1,RBX1,CALM1,P
Processing and activation of	0.0013	9	5	5.87E-03	0.0235	UBE2I,UBA2,SAE1,SUMO1,SUMO2
Translesion synthesis by POLK	0.0024	17	7	6.10E-03	0.0244	PCNA,RFC5,UBB,RPA2,MAD2L2,RPA3,UBA52
Translesion synthesis by POLI	0.0024	17	7	6.10E-03	0.0244	PCNA,RFC5,UBB,RPA2,MAD2L2,RPA3,UBA52
Dual Incision in GG-NER	0.0051	36	11	6.43E-03	0.0257	PCNA,POLD2,ERCC1,RFC5,PARP1,UBB,RBX1,RPA2,RPA3,UBA52,GT
Apoptotic execution phase	0.0066	47	13	7.19E-03	0.0287	FNTA,SPTAN1,LMNB1,GSN,CASP7,CASP6,DSP,DSG2,DBNL,BCAP31,
Phosphorylation of Emi1	0.0008	6	4	7.21E-03	0.0288	CDC20,PLK1,CCNB1,CDK1
mitochondrial fatty acid beta-	0.0008	6	4	7.21E-03	0.0288	ECI1,DECR1,HADHB,ACADM
Activation of NIMA Kinases NEK9,	0.0008	6	4	7.21E-03	0.0288	PLK1,CCNB2,CCNB1,CDK1
Telomere Extension By	0.0008	6	4	7.21E-03	0.0288	RUVBL2,RUVBL1,DKC1,NHP2
Telomere Maintenance	0.0052	37	11	7.79E-03	0.0312	APEX1,PCNA,POLD2,RFC5,TERF2IP,RUVBL2,RUVBL1,RPA2,RPA3,DK
Condensation of Prophase	0.002	14	6	8.97E-03	0.0359	SET,SMC4,SMC2,PLK1,CCNB1,CDK1
Cellular Senescence	0.0176	125	26	9.18E-03	0.0367	JUN,FOS,TP53,UBE2C,LMNB1,ANAPC15,ANAPC16,CDKN1A,CDKN2B,CEBPB,TNRC6B,TXN,UBB,TERF2IP,STAT3,UBE2E1,ID1,HMGA1,UB
Purine metabolism	0.0032	23	8	9.19E-03	0.0368	ATIC,APRT,CAT,ADSL,PAICS,HPRT1,IMPDH2,GMPR2
Apoptotic cleavage of cellular	0.0053	38	11	9.37E-03	0.0375	FNTA,SPTAN1,LMNB1,GSN,CASP7,CASP6,DSP,DSG2,DBNL,BCAP31,
Response to elevated platelet cytosolic Ca2+	0.0117	83	19	9.66E-03	0.0386	FN1,ACTN1,ACTN4,VEGFA,PPIA,CD9,TIMP1,CFL1,FLNA,LEFTY2,CALM1,VCL,SOD1,SERPINE1,SERPING1,SPARC,ALDOA,SCG3,TGFB2
SUMO E3 ligases SUMOylate target proteins	0.0134	95	21	9.74E-03	0.039	CDCA8,PCNA,RAE1,UBE2I,SMC3,NUP93,HNRNPK,HNRNPC,TOP2A,TOP2B,PARP1,SUMO1,SUMO2,BIRC5,NSMCE1,CETN2,AURKB,AURK
DNA Double-Strand Break Repair	0.016	114	24	0.0105	0.0419	BRE,TP53,SMARCA5,UBE2I,UBE2N,ERCC1,PRKDC,RFC5,PARP1,APBB1,UBB,H2AFX,KPNA2,SUMO1,SUMO2,XRCC6,XRCC5,BABAM1,RPA
Signaling by Insulin receptor	0.0371	264	47	0.0108	0.043	DUSP6,SHC1,EIF4E,EIF4B,ATP6V1C2,SPTAN1,PEBP1,RAF1,FGF19,ATP6V1B1,PHB,UBB,PSMD8,PSMD6,PSMD7,PSMD4,PSMD3,PSME1,PSME2,PSMF1,PSMA5,PSMA3,PSMA4,PSMA1,PSMA2,PSMA7,PSMB6,PSMB7,PSMB5,PSMB2,PSMB3,PSMB1,PSMC5,PSMC6,PSMC3,PS
Resolution of AP sites via the	0.0027	19	7	0.0108	0.0431	APEX1,PCNA,POLD2,RFC5,PARP1,RPA2,RPA3
Phosphorylation of the APC/C	0.0027	19	7	0.0108	0.0431	UBE2C,ANAPC15,ANAPC16,PLK1,UBE2E1,CCNB1,CDK1

Anchoring of the basal body to the plasma membrane	0.0127	90	20	0.0109	0.0435	NEK2,DYNC1I2,CEP70,PLK1,TUBB4B,CEP41,PPP2R1A,RAB8A,TUBB, RAB11A,TUBA1A,DYNLL1,HSP90AA1,CETN2,YWHAE,DCTN2,DCTN3
Assembly of collagen fibrils and	0.0055	39	11	0.0112	0.0447	COL3A1,COL18A1,COL6A1,COL1A1,COL5A1,COL5A2,COL8A2,COL8
Assembly of the primary cilium	0.0242	172	33	0.0114	0.0454	NEK2,DYNC1I2,IFT52,IFT57,CEP70,PLK1,WDR34,TCP1,TUBB4B,CEP 41,PPP2R1A,RAB8A,TUBB,RAB11A,DYNLRB1,ARF4,HSPB11,TUBA1 A,DYNLL1,ARL3,HSP90AA1,CETN2,CCT3,CCT2,CCT8,CCT5,CCT4,YW
Metabolism of carbohydrates	0.0336	239	43	0.0121	0.0486	BGN,AGRN,PGK1,G6PC3,PGLS,PGM1,GAPDH,PRPS1,TALDO1,RAE1, GALK1,NUP93,GPI,TPI1,TKT,HMMR,PKM,UBB,CRYL1,HK2,PGAM1, MDH1,MDH2,GOT1,CALM1,AKR1B1,AKR1A1,GPC1,VCAN,NUP37,E
VEGFA-VEGFR2 Pathway	0.0374	266	47	0.0122	0.0486	SPHK1,DUSP6,SHC1,ITGAV,RAC1,SPTAN1,VEGFA,PEBP1,RAF1,FGF1 9,PHB,UBB,PSMD8,PSMD6,PSMD7,PSMD4,PSMD3,PSME1,PSME2, PSMF1,PSMA5,PSMA3,PSMA4,PSMA1,PSMA2,PSMA7,PSMB6,PSM B7,PSMB5,PSMB2,PSMB3,PSMB1,PSMC5,PSMC6,PSMC3,PSMC1,R
mRNA decay by 5' to 3'	0.0021	15	6	0.0122	0.049	LSM5,LSM4,LSM3,LSM2,LSM7,LSM6
Signaling by VEGF	0.0385	274	48	0.0131	0.0523	SPHK1,DUSP6,SHC1,ITGAV,NRP2,RAC1,SPTAN1,VEGFA,PEBP1,RAF1 ,FGF19,PHB,UBB,PSMD8,PSMD6,PSMD7,PSMD4,PSMD3,PSME1,PS ME2,PSMF1,PSMA5,PSMA3,PSMA4,PSMA1,PSMA2,PSMA7,PSMB6 ,PSMB7,PSMB5,PSMB2,PSMB3,PSMB1,PSMC5,PSMC6,PSMC3,PSM
SeMet incorporation into proteins	0.0015	11	5	0.0131	0.0524	QARS,AIMP1,EEF1E1,DARS,LARS
Golgi Cisternae Pericentriolar	0.0015	11	5	0.0131	0.0524	PLK1,RAB1A,CCNB2,CCNB1,CDK1
Inactivation of APC/C via direct	0.0028	20	7	0.0139	0.0556	CDC20,UBE2C,ANAPC15,ANAPC16,BUB3,UBE2E1,MAD2L1
Inhibition of the proteolytic activity of APC/C required for the	0.0028	20	7	0.0139	0.0556	CDC20,UBE2C,ANAPC15,ANAPC16,BUB3,UBE2E1,MAD2L1
Mitochondrial protein import	0.0049	35	10	0.014	0.0558	MTX1,MTX2,PMPCB,SAMM50,TIMM17B,TIMM10,TOMM20,CHCH
Cyclin D associated events in G1	0.0042	30	9	0.0144	0.0577	CDKN1A,CDKN2B,TFDP2,UBB,UBA52,CDK6,CDK4,SKP1,CKS1B
G1 Phase	0.0042	30	9	0.0144	0.0577	CDKN1A,CDKN2B,TFDP2,UBB,UBA52,CDK6,CDK4,SKP1,CKS1B
Nonhomologous End-Joining	0.0051	36	10	0.0166	0.0664	BRE,UBE2N,PRKDC,H2AFX,SUMO1,XRCC6,XRCC5,BABAM1,UBE2V2
Gap-filling DNA repair synthesis	0.003	21	7	0.0176	0.0705	PCNA,POLD2,RFC5,UBB,RPA2,RPA3,UBA52
HSF1-dependent transactivation	0.003	21	7	0.0176	0.0705	CRYAB,FKBP4,PTGES3,HSP90AB1,HSP90AA1,HSBP1,HSPA8
Pyruvate metabolism	0.0037	26	8	0.0179	0.0714	BSG,PDHA1,LDHB,LDHA,MPC2,PDHB,SLC16A1,GLO1
Recognition of DNA damage by	0.0037	26	8	0.0179	0.0714	PCNA,POLD2,RFC5,UBB,RBX1,RPA2,RPA3,UBA52
Processive synthesis on the C-	0.0017	12	5	0.0183	0.0733	APEX1,PCNA,POLD2,RPA2,RPA3
Caspase-mediated cleavage of	0.0017	12	5	0.0183	0.0733	SPTAN1,GSN,CASP7,CASP6,DBNL
Processing of DNA double-strand break ends	0.0091	65	15	0.0187	0.0747	BRE,UBE2I,UBE2N,RFC5,UBB,H2AFX,SUMO1,SUMO2,BABAM1,RPA 2,RPA3,UBE2V2,FAM175A,UBA52,PPP4C
Nuclear Envelope Reassembly	0.0011	8	4	0.0188	0.0752	VRK1,PPP2R1A,PPP2CA,BANF1

mitochondrial fatty acid beta-	0.0011	8	4	0.0188	0.0752	ECHS1,HADHB,HADH,ACADM
eNOS activation	0.0011	8	4	0.0188	0.0752	DDAH2,LYPLA1,HSP90AA1,CALM1
Initiation of Nuclear Envelope	0.0011	8	4	0.0188	0.0752	VRK1,PPP2R1A,PPP2CA,BANF1
Cellular response to heat stress	0.0108	77	17	0.0189	0.0754	CRYAB,RAE1,EEF1A1,NUP93,FKBP4,DNAJC7,PTGES3,HSP90AB1,HSP90AA1,RPA2,RPA3,YWHAE,NUP37,ST13,HSBP1,BAG1,HSPA8
PCNA-Dependent Long Patch Base	0.0024	17	6	0.0212	0.0764	APEX1,PCNA,POLD2,RFC5,RPA2,RPA3
Membrane Trafficking	0.034	242	42	0.0221	0.0764	RAC1,SPTAN1,DYNC1I2,GJA1,TFG,AP1S2,AP1S1,VPS25,VPS28,COPZ1,TRAPPC1,TRAPPC4,DAB2,CNIH1,RAB1A,FTH1,RAB8A,RAB11A,UBB,CHMP2A,COPE,TRAPPC6A,ARF4,ARF1,DYNLL1,VAMP8,AP2M1,KDELR1,CALM1,YWHAE,YWHAB,YWHAQ,YWHAH,DCTN6,DCTN2,DC
RNA Polymerase II Transcription	0.0062	44	11	0.0245	0.0764	TCEB1,TCEA1,SSRP1,SUPT16H,POLR2C,POLR2E,POLR2G,POLR2H,N
Formation of RNA Pol II	0.0062	44	11	0.0245	0.0764	TCEB1,TCEA1,SSRP1,SUPT16H,POLR2C,POLR2E,POLR2G,POLR2H,N
Cyclin B2 mediated events	0.0007	5	3	0.0255	0.0764	CDC25B,CCNB2,CDK1
G2/M DNA replication checkpoint	0.0007	5	3	0.0255	0.0764	CCNB2,CCNB1,CDK1
Beta oxidation of lauroyl-CoA to	0.0007	5	3	0.0255	0.0764	ECHS1,HADHB,HADH
Phosphate bond hydrolysis by	0.0007	5	3	0.0255	0.0764	NUDT15,NUDT1,NUDT5
Beta oxidation of hexanoyl-CoA to	0.0007	5	3	0.0255	0.0764	ECHS1,HADHB,HADH
Formation of the ternary complex,	0.007	50	12	0.0255	0.0766	EIF1AX,EIF3M,EIF3K,EIF3L,EIF3I,EIF3G,EIF3H,EIF3E,EIF3F,EIF3D,EIF2
Termination of translesion DNA	0.0039	28	8	0.0262	0.0786	PCNA,POLD2,RFC5,UBB,UBE2L6,RPA2,RPA3,UBA52
Metabolism of nitric oxide	0.0025	18	6	0.027	0.0809	GCHFR,NOSIP,DDAH2,LYPLA1,HSP90AA1,CALM1
eNOS activation and regulation	0.0025	18	6	0.027	0.0809	GCHFR,NOSIP,DDAH2,LYPLA1,HSP90AA1,CALM1
Deadenylation of mRNA	0.0032	23	7	0.0271	0.0813	EIF4E,EIF4B,PABPC1,PAIP1,EIF4A2,EIF4A1,EIF4A3
Purine ribonucleoside	0.0013	9	4	0.0274	0.0821	ATIC,ADSL,PAICS,IMPDH2
Signaling by PDGF	0.0425	302	50	0.0283	0.0849	PDGFC,DUSP6,SHC1,SPTAN1,PEBP1,RAF1,FGF19,CDKN1A,PHB,TNRC6B,UBB,COL6A1,PSMD8,PSMD6,PSMD7,PSMD4,PSMD3,PSME1,PSME2,PSMF1,PSMA5,PSMA3,PSMA4,PSMA1,PSMA2,PSMA7,PSMB6,PSMB7,PSMB5,PSMB2,PSMB3,PSMB1,STAT3,PSMC5,PSMC6,PS
snRNP Assembly	0.0072	51	12	0.029	0.0871	SNRPD2,SNRPD1,SNRPD3,RAE1,NUP93,SNRPG,SNRPE,SNRPF,SNRP
Metabolism of non-coding RNA	0.0072	51	12	0.029	0.0871	SNRPD2,SNRPD1,SNRPD3,RAE1,NUP93,SNRPG,SNRPE,SNRPF,SNRP
Mitotic Telophase/Cytokinesis	0.002	14	5	0.0325	0.0975	SMC3,PLK1,KIF20A,RAD21,NUDC
Cytosolic tRNA aminoacylation	0.0034	24	7	0.0329	0.0988	QARS,PPA1,AIMP1,EEF1E1,DARS,SARS,LARS
RMTs methylate histone arginines	0.0027	19	6	0.0337	0.1011	SMARCB1,SMARCC1,SMARCA4,CDK4,RBBP7,PRMT1
Translesion Synthesis by POLH	0.0027	19	6	0.0337	0.1011	PCNA,RFC5,UBB,RPA2,RPA3,UBA52
RHO GTPases activate PKNs	0.0049	35	9	0.034	0.102	KDM1A,MYH9,MYL6,YWHAE,YWHAB,YWHAQ,YWHAH,H3F3A,RHO
Translesion synthesis by Y family	0.0049	35	9	0.034	0.102	PCNA,POLD2,RFC5,UBB,UBE2L6,RPA2,MAD2L2,RPA3,UBA52

Signaling by SCF-KIT	0.0373	265	44	0.0364	0.1091	DUSP6,SHC1,RAC1,SPTAN1,PEBP1,RAF1,FGF19,CDKN1A,PHB,TNRC6B,UBB,PSMD8,PSMD6,PSMD7,PSMD4,PSMD3,PSME1,PSME2,PSMF1,PSMA5,PSMA3,PSMA4,PSMA1,PSMA2,PSMA7,PSMB6,PSMB7,PSMB5,PSMB2,PSMB3,PSMB1,STAT3,PSMC5,PSMC6,PSMC3,PSM
Oncogene Induced Senescence	0.0042	30	8	0.0369	0.1107	TP53,CDKN2B,TNRC6B,UBB,ID1,UBA52,CDK6,CDK4
Removal of the Flap Intermediate	0.0014	10	4	0.0379	0.1137	PCNA,POLD2,RPA2,RPA3
Elastic fibre formation	0.0051	36	9	0.0394	0.1182	ITGB1,ITGAV,FBN2,FBN1,FBLN1,BMP4,ELN,LTBP3,TGFB2
Lysosome Vesicle Biogenesis	0.0035	25	7	0.0395	0.1186	AP1S2,AP1S1,CHMP2A,ARF1,CLTC,CLTA,HSPA8
MASTL Facilitates Mitotic	0.0008	6	3	0.0402	0.1207	CCNB1,ENSA,CDK1
Methionine salvage pathway	0.0008	6	3	0.0402	0.1207	ENOPH1,ADI1,GOT1
Mitotic Prophase	0.0093	66	14	0.0408	0.1225	SET,RAE1,SMC4,SMC2,NUP93,PLK1,VRK1,RAB1A,BANF1,CCNB2,CC
MicroRNA (miRNA) biogenesis	0.0028	20	6	0.0414	0.1243	POLR2C,POLR2E,POLR2G,POLR2H,PRKRA,RAN
Amino acid synthesis and	0.0021	15	5	0.0416	0.1248	PHGDH,PYCR1,PYCR2,GOT1,GLUL
Base Excision Repair	0.0044	31	8	0.0432	0.1297	APEX1,PCNA,POLD2,NTHL1,RFC5,PARP1,RPA2,RPA3
Resolution of Abasic Sites (AP	0.0044	31	8	0.0432	0.1297	APEX1,PCNA,POLD2,NTHL1,RFC5,PARP1,RPA2,RPA3
ISG15 antiviral mechanism	0.0094	67	14	0.0452	0.1356	EIF4E,RAE1,UBE2N,NUP93,UBB,FLNB,KPNA2,UBE2E1,EIF4A2,EIF4A1,EIF4A3,UBE2L6,NUP37,UBA52
Antiviral mechanism by IFN-stimulated genes	0.0094	67	14	0.0452	0.1356	EIF4E,RAE1,UBE2N,NUP93,UBB,FLNB,KPNA2,UBE2E1,EIF4A2,EIF4A1,EIF4A3,UBE2L6,NUP37,UBA52
Lagging Strand Synthesis	0.003	21	6	0.0502	0.1507	APEX1,PCNA,POLD2,RFC5,RPA2,RPA3
BBSome-mediated cargo-targeting	0.003	21	6	0.0502	0.1507	TCP1,CCT3,CCT2,CCT8,CCT5,CCT4
SUMOylation of transcription	0.0015	11	4	0.0505	0.1515	UBE2I,SUMO1,SUMO2,MITF
Formation of Senescence-	0.0015	11	4	0.0505	0.1515	TP53,LMNB1,HMGA1,ASF1A
Polo-like kinase mediated events	0.0023	16	5	0.0521	0.1562	CENPF,PLK1,CCNB2,CCNB1,RBBP4
Processive synthesis on the	0.0023	16	5	0.0521	0.1562	APEX1,PCNA,POLD2,RPA2,RPA3
Synthesis and interconversion of	0.0023	16	5	0.0521	0.1562	DCTPP1,TXN,DTYMK,AK2,NME1
DNA Damage Bypass	0.0062	44	10	0.0526	0.1579	PCNA,POLD2,RFC5,UBB,RBX1,UBE2L6,RPA2,MAD2L2,RPA3,UBA52
Eukaryotic Translation Initiation	0.016	114	21	0.053	0.1591	EIF1AX,EIF4H,EIF4E,EIF4B,EIF3M,EIF3K,EIF3L,EIF3I,EIF3G,EIF3H,EIF3E,EIF3F,EIF3D,PABPC1,EIF2B1,EIF2S3,EIF5,EIF4A2,EIF4A1,UBA52,F
Cap-dependent Translation Initiation	0.016	114	21	0.053	0.1591	EIF1AX,EIF4H,EIF4E,EIF4B,EIF3M,EIF3K,EIF3L,EIF3I,EIF3G,EIF3H,EIF3E,EIF3F,EIF3D,PABPC1,EIF2B1,EIF2S3,EIF5,EIF4A2,EIF4A1,UBA52,F
Beta oxidation of butanoyl-CoA to	0.0004	3	2	0.0547	0.1618	ECHS1,HADH
Phosphorylation of proteins involved in the G2/M transition by	0.0004	3	2	0.0547	0.1618	CCNA2,CDK1
Gap junction degradation	0.0004	3	2	0.0547	0.1618	DAB2,AP2M1
Synthesis of dolichyl-phosphate	0.0004	3	2	0.0547	0.1618	DPM1,DPM3

Clearance of Nuclear Envelope	0.0004	3	2	0.0547	0.1618	VRK1,BANF1
Breakdown of the nuclear lamina	0.0004	3	2	0.0547	0.1618	LMNB1,CASP6
Golgi Associated Vesicle	0.0038	27	7	0.0552	0.1618	AP1S2,AP1S1,FTH1,ARF1,CLTC,CLTA,HSPA8
Smooth Muscle Contraction	0.0046	33	8	0.0579	0.1618	ITGA1,ACTG2,TPM4,TPM1,MYL6,CALM1,VCL,ANXA6
Pentose phosphate pathway	0.001	7	3	0.0584	0.1618	PGLS,TALDO1,TKT
Gap junction trafficking	0.001	7	3	0.0584	0.1618	GJA1,DAB2,AP2M1
MHC class II antigen presentation	0.0125	89	17	0.0586	0.1618	CENPE,DYNC1I2,AP1S2,AP1S1,RAB7A,ARF1,DYNLL1,AP2S1,AP2M1,CTSL,KIF2C,CD74,DCTN6,DCTN2,DCTN3,CLTC,CLTA
Signaling by FGFR3	0.0387	275	44	0.0587	0.1618	DUSP6,SHC1,SPTAN1,PEBP1,RAF1,FGF19,CDKN1A,PHB,TNRC6B,PPP2R1A,PPP2CA,UBB,PSMD8,PSMD6,PSMD7,PSMD4,PSMD3,PSME1,PSME2,PSMF1,PSMA5,PSMA3,PSMA4,PSMA1,PSMA2,PSMA7,PSMB6,PSMB7,PSMB5,PSMB2,PSMB3,PSMB1,PSMC5,PSMC6,PSMC3,P
Signaling by FGFR4	0.0387	275	44	0.0587	0.1618	DUSP6,SHC1,SPTAN1,PEBP1,RAF1,FGF19,CDKN1A,PHB,TNRC6B,PPP2R1A,PPP2CA,UBB,PSMD8,PSMD6,PSMD7,PSMD4,PSMD3,PSME1,PSME2,PSMF1,PSMA5,PSMA3,PSMA4,PSMA1,PSMA2,PSMA7,PSMB6,PSMB7,PSMB5,PSMB2,PSMB3,PSMB1,PSMC5,PSMC6,PSMC3,P
mTORC1-mediated signalling	0.0031	22	6	0.0601	0.1618	EIF4E,EIF4B,YWHAB,LAMTOR5,RHEB,RRAGA
Cholesterol biosynthesis	0.0031	22	6	0.0601	0.1618	FDFT1,TM7SF2,LBR,PMVK,FDPS,DHCR24
Signaling by FGFR1	0.0388	276	44	0.0614	0.1618	DUSP6,SHC1,SPTAN1,PEBP1,RAF1,FGF19,CDKN1A,PHB,TNRC6B,PPP2R1A,PPP2CA,UBB,PSMD8,PSMD6,PSMD7,PSMD4,PSMD3,PSME1,PSME2,PSMF1,PSMA5,PSMA3,PSMA4,PSMA1,PSMA2,PSMA7,PSMB6,PSMB7,PSMB5,PSMB2,PSMB3,PSMB1,PSMC5,PSMC6,PSMC3,P
Signaling by FGFR2	0.0388	276	44	0.0614	0.1618	DUSP6,SHC1,SPTAN1,PEBP1,RAF1,FGF19,CDKN1A,PHB,TNRC6B,PPP2R1A,PPP2CA,UBB,PSMD8,PSMD6,PSMD7,PSMD4,PSMD3,PSME1,PSME2,PSMF1,PSMA5,PSMA3,PSMA4,PSMA1,PSMA2,PSMA7,PSMB6,PSMB7,PSMB5,PSMB2,PSMB3,PSMB1,PSMC5,PSMC6,PSMC3,P
Signaling by ERBB4	0.0378	269	43	0.0618	0.1618	DUSP6,SHC1,SPTAN1,PEBP1,RAF1,FGF19,CDKN1A,PHB,TNRC6B,UBB,PSMD8,PSMD6,PSMD7,PSMD4,PSMD3,PSME1,PSME2,PSMF1,PSMA5,PSMA3,PSMA4,PSMA1,PSMA2,PSMA7,PSMB6,PSMB7,PSMB5,PSMB2,PSMB3,PSMB1,PSMC5,PSMC6,PSMC3,PSMC1,RBX1,CALM
Oxidative Stress Induced	0.009	64	13	0.0621	0.1618	JUN,FOS,TP53,CDKN2B,TNRC6B,TXN,UBB,UBA52,H3F3A,CDK6,CDK
Homology Directed Repair	0.0127	90	17	0.0634	0.1618	BRE,UBE2I,UBE2N,ERCC1,RFC5,PARP1,UBB,H2AFX,SUMO1,SUMO2,BABAM1,RPA2,RPA3,UBE2V2,FAM175A,UBA52,PPP4C
Cyclin A/B1 associated events	0.0024	17	5	0.064	0.1618	CDC25B,CCNB2,CCNB1,CCNA2,CDK1
Regulation of Insulin-like Growth Factor (IGF) transport and uptake	0.0024	17	5	0.064	0.1618	PAPPA2,IGFBP5,IGFBP3,IGFBP6,PAPPA

Signaling by FGFR	0.039	277	44	0.0642	0.1618	DUSP6,SHC1,SPTAN1,PEBP1,RAF1,FGF19,CDKN1A,PHB,TNRC6B,PPP2R1A,PPP2CA,UBB,PSMD8,PSMD6,PSMD7,PSMD4,PSMD3,PSME1,PSME2,PSMF1,PSMA5,PSMA3,PSMA4,PSMA1,PSMA2,PSMA7,PSMB6,PSMB7,PSMB5,PSMB2,PSMB3,PSMB1,PSMC5,PSMC6,PSMC3,P
IRF3-mediated induction of type I	0.0017	12	4	0.0651	0.1618	PRKDC,IFI16,XRCC6,XRCC5
Synthesis, secretion, and inactivation of Glucose-dependent	0.0017	12	4	0.0651	0.1618	SEC11A,SPCS2,SPCS1,PAX6
The NLRP3 inflammasome	0.0017	12	4	0.0651	0.1618	TXNIP,SUGT1,TXN,HSP90AB1
NOTCH1 Intracellular Domain	0.0056	40	9	0.0669	0.1618	TLE4,HDAC3,UBB,HES1,RBX1,CCNC,SNW1,UBA52,SKP1
Signaling by ERBB2	0.0391	278	44	0.0671	0.1618	DUSP6,SHC1,CDC37,SPTAN1,PEBP1,RAF1,FGF19,CDKN1A,PHB,TNRC6B,UBB,PSMD8,PSMD6,PSMD7,PSMD4,PSMD3,PSME1,PSME2,PSMF1,PSMA5,PSMA3,PSMA4,PSMA1,PSMA2,PSMA7,PSMB6,PSMB7,PSMB5,PSMB2,PSMB3,PSMB1,PSMC5,PSMC6,PSMC3,PSMC1,RBX
Integrin cell surface interactions	0.0091	65	13	0.0682	0.1618	FN1,AGRN,ITGB1,ITGAV,ITGA1,BSG,FBN1,TNC,COL18A1,COL9A1,C
Signaling by NOTCH1	0.0083	59	12	0.0705	0.1618	JAG1,TLE4,HDAC3,DNER,UBB,HES1,RBX1,CCNC,SNW1,UBA52,SKP1
Activation of BH3-only proteins	0.0032	23	6	0.071	0.1618	TP53,DYNLL1,YWHAE,YWHAB,YWHAQ,YWHAH
Telomere C-strand (Lagging	0.0032	23	6	0.071	0.1618	APEX1,PCNA,POLD2,RFC5,RPA2,RPA3
HDR through Homologous Recombination (HR) or Single	0.012	85	16	0.0717	0.1618	BRE,UBE2I,UBE2N,ERCC1,RFC5,UBB,H2AFX,SUMO1,SUMO2,BABAM1,RPA2,RPA3,UBE2V2,FAM175A,UBA52,PPP4C
Signaling by NOTCH	0.0129	92	17	0.074	0.1618	JUN,JAG1,TP53,TLE4,TFDP2,HDAC3,TNRC6B,DNER,UBB,HES1,RBX1,MDK,CCNC,SNW1,UBA52,SKP1,DLK1
Amyloid fiber formation	0.0058	41	9	0.0752	0.1618	MFGE8,GSN,TTR,SNCA,UBE2L6,H3F3A,B2M,ITM2B,TGFBI
Conversion from APC/C:Cdc20 to	0.0025	18	5	0.0772	0.1618	CDC20,UBE2C,ANAPC15,ANAPC16,UBE2E1
Syndecan interactions	0.0025	18	5	0.0772	0.1618	ITGB1,ITGAV,ACTN1,TNC,TRAPPC4
L13a-mediated translational silencing of Ceruloplasmin	0.0149	106	19	0.0775	0.1618	EIF1AX,EIF4H,EIF4E,EIF4B,EIF3M,EIF3K,EIF3L,EIF3I,EIF3G,EIF3H,EIF3E,EIF3F,EIF3D,PABPC1,EIF2S3,EIF4A2,EIF4A1,UBA52,FAU
Sema4D mediated inhibition of	0.0011	8	3	0.0798	0.1618	RRAS,RAC1,RHOA
HuR (ELAVL1) binds and stabilizes	0.0011	8	3	0.0798	0.1618	SET,ANP32A,ELAVL1
Purine salvage	0.0011	8	3	0.0798	0.1618	APRT,HPRT1,GMPR2
Downstream signaling of activated FGFR2	0.0377	268	42	0.0809	0.1618	DUSP6,SHC1,SPTAN1,PEBP1,RAF1,FGF19,CDKN1A,PHB,TNRC6B,UBB,PSMD8,PSMD6,PSMD7,PSMD4,PSMD3,PSME1,PSME2,PSMF1,PSMA5,PSMA3,PSMA4,PSMA1,PSMA2,PSMA7,PSMB6,PSMB7,PSMB5,PSMB2,PSMB3,PSMB1,PSMC5,PSMC6,PSMC3,PSMC1,RBX1,CALM

Downstream signaling of activated FGFR1	0.0377	268	42	0.0809	0.1618	DUSP6,SHC1,SPTAN1,PEBP1,RAF1,FGF19,CDKN1A,PHB,TNRC6B,UBB,PSMD8,PSMD6,PSMD7,PSMD4,PSMD3,PSME1,PSME2,PSMF1,PSMA5,PSMA3,PSMA4,PSMA1,PSMA2,PSMA7,PSMB6,PSMB7,PSMB5,PSMB2,PSMB3,PSMB1,PSMC5,PSMC6,PSMC3,PSMC1,RBX1,CALM
Downstream signaling of activated FGFR4	0.0377	268	42	0.0809	0.1618	DUSP6,SHC1,SPTAN1,PEBP1,RAF1,FGF19,CDKN1A,PHB,TNRC6B,UBB,PSMD8,PSMD6,PSMD7,PSMD4,PSMD3,PSME1,PSME2,PSMF1,PSMA5,PSMA3,PSMA4,PSMA1,PSMA2,PSMA7,PSMB6,PSMB7,PSMB5,PSMB2,PSMB3,PSMB1,PSMC5,PSMC6,PSMC3,PSMC1,RBX1,CALM
Downstream signaling of activated FGFR3	0.0377	268	42	0.0809	0.1618	DUSP6,SHC1,SPTAN1,PEBP1,RAF1,FGF19,CDKN1A,PHB,TNRC6B,UBB,PSMD8,PSMD6,PSMD7,PSMD4,PSMD3,PSME1,PSME2,PSMF1,PSMA5,PSMA3,PSMA4,PSMA1,PSMA2,PSMA7,PSMB6,PSMB7,PSMB5,PSMB2,PSMB3,PSMB1,PSMC5,PSMC6,PSMC3,PSMC1,RBX1,CALM
Advanced glycosylation	0.0018	13	4	0.0816	0.1633	CAPZA2,DDOST,PRKCSH,HMGB1
Synthesis of Prostaglandins (PG)	0.0018	13	4	0.0816	0.1633	CBR1,PTGES3,PTGR1,PTGDS
Activation of BAD and	0.0018	13	4	0.0816	0.1633	YWHAE,YWHAB,YWHAQ,YWHAH
Cargo trafficking to the periciliary	0.0068	48	10	0.0824	0.1647	TCP1,RAB8A,RAB11A,ARF4,ARL3,CCT3,CCT2,CCT8,CCT5,CCT4
DAP12 signaling	0.0398	283	44	0.0829	0.1657	DUSP6,SHC1,RAC1,SPTAN1,PEBP1,RAF1,FGF19,CDKN1A,PHB,TNRC6B,UBB,PSMD8,PSMD6,PSMD7,PSMD4,PSMD3,PSME1,PSME2,PSMF1,PSMA5,PSMA3,PSMA4,PSMA1,PSMA2,PSMA7,PSMB6,PSMB7,PSMB5,PSMB2,PSMB3,PSMB1,PSMC5,PSMC6,PSMC3,PSMC1,RBX
Processing of Capped Intronless	0.0034	24	6	0.0829	0.1659	SNRPD3,SNRPG,SNRPE,SNRPF,SNRPB,NCBP2
GTP hydrolysis and joining of the 60S ribosomal subunit	0.015	107	19	0.0829	0.1659	EIF1AX,EIF4H,EIF4E,EIF4B,EIF3M,EIF3K,EIF3L,EIF3I,EIF3G,EIF3H,EIF3E,EIF3F,EIF3D,EIF2S3,EIF5,EIF4A2,EIF4A1,UBA52,FAU
Glutathione conjugation	0.0042	30	7	0.0851	0.1703	GSTM3,GSTO1,GSTP1,GSTA4,AKR1A1,ESD,MGST3
SUMOylation of chromatin	0.0006	4	2	0.0898	0.1797	UBE2I,SUMO2
Fructose metabolism	0.0006	4	2	0.0898	0.1797	AKR1B1,SORD
tRNA processing in the	0.0006	4	2	0.0898	0.1797	TRMT10C,HSD17B10
Antagonism of Activin by	0.0006	4	2	0.0898	0.1797	FSTL3,INHBA
Hypusine synthesis from eIF5A-	0.0006	4	2	0.0898	0.1797	EIF5A,DHPS
Clathrin derived vesicle budding	0.006	43	9	0.0938	0.1876	AP1S2,AP1S1,FTH1,CHMP2A,ARF1,VAMP8,CLTC,CLTA,HSPA8
trans-Golgi Network Vesicle	0.006	43	9	0.0938	0.1876	AP1S2,AP1S1,FTH1,CHMP2A,ARF1,VAMP8,CLTC,CLTA,HSPA8
Non-integrin membrane-ECM	0.0052	37	8	0.0958	0.1917	AGRN,ITGB1,ITGAV,ACTN1,TNC,TRAPPC4,TTR,DMD
Negative regulation of MAPK	0.0035	25	6	0.096	0.1919	DUSP6,PEBP1,RAF1,UBB,YWHAB,UBA52

Signaling by Interleukins	0.0394	280	43	0.0983	0.1965	DUSP6,SHC1,SPTAN1,PEBP1,RAF1,FGF19,UBE2N,PHB,UBB,PSMD8,PSMD6,PSMD7,PSMD4,PSMD3,PSME1,PSME2,PSMF1,PSMA5,PSMA3,PSMA4,PSMA1,PSMA2,PSMA7,PSMB6,PSMB7,PSMB5,PSMB2,PSMB3,PSMB1,STAT3,PSMC5,PSMC6,PSMC3,PSMC1,RBX1,CALM1,P
Removal of the Flap Intermediate	0.002	14	4	0.1	0.2001	PCNA,POLD2,RPA2,RPA3
Mismatch repair (MMR) directed	0.002	14	4	0.1	0.2001	PCNA,POLD2,RPA2,RPA3
Mismatch repair (MMR) directed	0.002	14	4	0.1	0.2001	PCNA,POLD2,RPA2,RPA3
N-glycan trimming in the ER and	0.002	14	4	0.1	0.2001	CALR,MLEC,PRKCSH,PDIA3
Downstream signal transduction	0.0395	281	43	0.1022	0.2043	DUSP6,SHC1,SPTAN1,PEBP1,RAF1,FGF19,CDKN1A,PHB,TNRC6B,UBB,PSMD8,PSMD6,PSMD7,PSMD4,PSMD3,PSME1,PSME2,PSMF1,PSMA5,PSMA3,PSMA4,PSMA1,PSMA2,PSMA7,PSMB6,PSMB7,PSMB5,PSMB2,PSMB3,PSMB1,STAT3,PSMC5,PSMC6,PSMC3,PSMC1,RBX1
Signaling by EGFR	0.0416	296	45	0.1038	0.2075	DUSP6,SHC1,SPTAN1,PEBP1,RAF1,FGF19,CDKN1A,PHB,TNRC6B,UBB,PSMD8,PSMD6,PSMD7,PSMD4,PSMD3,PSME1,PSME2,PSMF1,PSMA5,PSMA3,PSMA4,PSMA1,PSMA2,PSMA7,PSMB6,PSMB7,PSMB5,PSMB2,PSMB3,PSMB1,AP2S1,PSMC5,PSMC6,PSMC3,PSMC1,AP2
Nuclear Envelope Breakdown	0.0062	44	9	0.104	0.208	RAE1,NUP93,PLK1,VRK1,BANF1,CCNB2,CCNB1,NUP37,CDK1
Gap junction trafficking and	0.0013	9	3	0.1041	0.2081	GJA1,DAB2,AP2M1
CHL1 interactions	0.0013	9	3	0.1041	0.2081	ITGB1,ITGA1,HSPA8
SMAD2/SMAD3:SMAD4	0.0045	32	7	0.1093	0.2187	CDKN2B,TFDP2,UBB,CCNC,SNW1,SERPINE1,UBA52
RNA Polymerase II Pre-	0.009	64	12	0.1097	0.2195	TCEB1,TCEA1,SSRP1,SUPT16H,POLR2C,POLR2E,POLR2G,POLR2H,N
Cellular response to hypoxia	0.0037	26	6	0.11	0.22	TCEB1,VEGFA,CA9,UBB,RBX1,UBA52
WNT ligand biogenesis and	0.0037	26	6	0.11	0.22	WLS,VPS29,VPS35,WNT2B,SNX3,VPS26A
Regulation of Hypoxia-inducible	0.0037	26	6	0.11	0.22	TCEB1,VEGFA,CA9,UBB,RBX1,UBA52
NGF signalling via TRKA from the plasma membrane	0.0442	314	47	0.1171	0.2343	DUSP6,SHC1,SPTAN1,PEBP1,RAF1,FGF19,CDKN1A,PHB,TNRC6B,UBB,PSMD8,PSMD6,PSMD7,PSMD4,PSMD3,PSME1,PSME2,PSMF1,PSMA5,PSMA3,PSMA4,PSMA1,PSMA2,PSMA7,PSMB6,PSMB7,PSMB5,PSMB2,PSMB3,PSMB1,AP2S1,STAT3,PSMC5,PSMC6,PSMC3,PSMC
Semaphorin interactions	0.0091	65	12	0.1189	0.2377	DPYSL4,ITGB1,ITGA1,RRAS,RAC1,PLXND1,CFL1,MYH9,MYL6,HSP90
STING mediated induction of host	0.0021	15	4	0.1202	0.2404	PRKDC,IFI16,XRCC6,XRCC5
Glycogen synthesis	0.0021	15	4	0.1202	0.2404	PGM1,UBB,UBA52,GYG1
Mismatch Repair	0.0021	15	4	0.1202	0.2404	PCNA,POLD2,RPA2,RPA3
RHO GTPases activate CIT	0.0021	15	4	0.1202	0.2404	MYH9,MYL6,PRC1,RHOA
RNA Polymerase I Transcription	0.0073	52	10	0.1207	0.2413	ZNRD1,PTRF,HDAC2,POLR2E,POLR2H,CBX3,H3F3A,GTF2H5,RBBP4,
DNA strand elongation	0.0046	33	7	0.1227	0.2453	APEX1,PCNA,POLD2,MCM7,RFC5,RPA2,RPA3
Formation of the Early Elongation	0.0046	33	7	0.1227	0.2453	POLR2C,POLR2E,POLR2G,POLR2H,NELFE,NCBP2,GTF2H5

NCAM1 interactions	0.0046	33	7	0.1227	0.2453	AGRN,COL6A1,COL9A1,COL9A3,COL4A2,COL4A1,COL4A5
L1CAM interactions	0.0111	79	14	0.1239	0.2478	ITGB1,ITGAV,ITGA1,CSNK2B,NRP2,RAC1,SPTAN1,ALCAM,DCX,AP2S
Signaling by Rho GTPases	0.0454	323	48	0.1239	0.2478	ITGB1,ACTB,CDC20,RAC1,ACTG1,ECT2,PLK1,PPP1CC,KDM1A,ARHGDIB,PMF1,MYH9,BUB3,MYL6,FLNA,BIRC5,PRC1,CALM1,KIF2C,MAD2L1,YWHAE,YWHAB,YWHAQ,YWHAH,AURKB,NUP37,SKA2,ABR,H3F3A,NUF2,NUDC,MAD1L1,BRK1,EVL,GDI2,RHOA,CTNNB1,ARHGAP
