# Supplementary material for: Single cell RNA sequencing of stem cell-derived retinal ganglion cells
Source: Sci Data. 2018 Feb 13;5:180013. doi: 10.1038/sdata.2018.13 (PMC5810423; doi:10.1038/sdata.2018.13)
Supplement: Supplementary Tables [file sdata201813-s2.zip › Supplementary tables/table s5.pdf]

ReactomePathway	RatioOfProteinInPathway	NumberOfProteinInPathway	ProteinFromGeneSet	P-value	FDR	HitGenes
Regulation of Apoptosis	0.007	50	36	1.11E-16	6.11E-15	UBB,UBC,PSMD8,PSMD6,PSMD7,PSMD4,PSMD2,PSMD3,PSMD1,PSME1,PSME2,PSMF1,PSMA5,PSMA3,PSMA4,PSMA1,PSMA2,PSMA7,PSMB6,PSMB7,PSMB5,PSMB2,PSMB3,PSMB1,PSMC5,PSMC6,PSMC3,PSMC4,PSMC1,PSMC2,PSMD10,PSMD12,PSMD11,PSMD14,PSMD13,UBA52
Respiratory electron transport, ATP synthesis by chemiosmotic coupling, and heat production by uncoupling proteins.	0.0165	117	71	1.11E-16	6.11E-15	ATP5C1,ATP5A1,UQCRB,UQCRH,UQCRQ,ETFA,ETFB,NDUFAF3,COX7B,COX7C,COX8A,COX5B,COX5A,COX6C,NDUFAB1,COX6A1,SDHC,SDHB,COX6B1,NDUFB10,NDUFB11,NDUFA13,NDUFA11,NDUFA12,NDUFA10,ATP5J2,ATP5G3,ATP5F1,UQCRC1,UQCRC2,COX4I1,NDUFC2,NDUFC1,NDUFB9,NDUFB8,NDUFB7,NDUFB6,NDUFB5,NDUFB4,NDUFB3,NDUFB2,NDUFB1,NDUFA9,NDUFA8,NDUFA6,NDUFA5,NDUFA4,NDUFA3,NDUFA2,NDUFA1,ATP5J,ATP5I,ATP5H,ATP5O,ATP5L,ATP5B,ATP5E,ATP5D,CYC1,NDUFV2,NDUFV1,CYCS,UQCRFS1,NDUFS8,NDUFS7,NDUFS6,NDUFS5,NDUFS4,NDUFS3,TIMMDC1,UQCR11
Antigen processing-Cross presentation	0.0107	76	47	1.11E-16	6.11E-15	ITGAV,CALR,HLA-B,HLA-C,HLA-A,HLA-E,SEC61G,SEC61B,UBB,UBC,PSMD8,PSMD6,PSMD7,PSMD4,PSMD2,PSMD3,PSMD1,PSME1,PSME2,PSMF1,PSMA5,PSMA3,PSMA4,PSMA1,PSMA2,PSMA7,PSMB6,PSMB7,PSMB5,PSMB2,PSMB3,PSMB1,PSMC5,PSMC6,PSMC3,PSMC4,PSMC1,PSMC2,CYBA,PSMD10,PSMD12,PSMD11,PSMD14,PSMD13,UBA52,B2M,PDIA3
Respiratory electron transport	0.0136	97	58	1.11E-16	6.11E-15	UQCRB,UQCRH,UQCRQ,ETFA,ETFB,NDUFAF3,COX7B,COX7C,COX8A,COX5B,COX5A,COX6C,NDUFAB1,COX6A1,SDHC,SDHB,COX6B1,NDUFB10,NDUFB11,NDUFA13,NDUFA11,NDUFA12,NDUFA10,UQCRC1,UQCRC2,COX4I1,NDUFC2,NDUFC1,NDUFB9,NDUFB8,NDUFB7,NDUFB6,NDUFB5,NDUFB4,NDUFB3,NDUFB2,NDUFB1,NDUFA9,NDUFA8,NDUFA6,NDUFA5,NDUFA4,NDUFA3,NDUFA2,NDUFA1,CYC1,NDUFV2,NDUFV1,CYCS,UQCRFS1,NDUFS8,NDUFS7,NDUFS6,NDUFS5,NDUFS4,NDUFS3,TIMMDC1,UQCR11

Ubiquitin-dependent degradation of Cyclin D	0.007	50	38	1.11E-16	6.11E-15	UBB,UBC,PSMD8,PSMD6,PSMD7,PSMD4,PSMD2,PSMD3,PSMD1,PSME1,PSME2,PSMF1,PSMA5,PSMA3,PSMA4,PSMA1,PSMA2,PSMA7,PSMB6,PSMB7,PSMB5,PSMB2,PSMB3,PSMB1,PSMC5,PSMC6,PSMC3,PSMC4,PSMC1,PSMC2,CCND1,PSMD10,PSMD12,PSMD11,PSMD14,PSMD13,UBA52,CDK4
Class I MHC mediated antigen processing & presentation	0.0128	91	49	1.11E-16	6.11E-15	ITGAV,CALR,CANX,HLA-B,HLA-C,HLA-A,HLA-E,SEC61G,SEC61B,UBB,UBC,PSMD8,PSMD6,PSMD7,PSMD4,PSMD2,PSMD3,PSMD1,PSME1,PSME2,PSMF1,PSMA5,PSMA3,PSMA4,PSMA1,PSMA2,PSMA7,PSMB6,PSMB7,PSMB5,PSMB2,PSMB3,PSMB1,PSMC5,PSMC6,PSMC3,PSMC4,PSMC1,PSMC2,SEC13,CYBA,PSMD10,PSMD12,PSMD11,PSMD14,PSMD13,UBA52,B2M,PDIA3
Ubiquitin Mediated Degradation of Phosphorylated Cdc25A	0.0073	52	37	1.11E-16	6.11E-15	UBB,UBC,PSMD8,PSMD6,PSMD7,PSMD4,PSMD2,PSMD3,PSMD1,PSME1,PSME2,PSMF1,PSMA5,PSMA3,PSMA4,PSMA1,PSMA2,PSMA7,PSMB6,PSMB7,PSMB5,PSMB2,PSMB3,PSMB1,PSMC5,PSMC6,PSMC3,PSMC4,PSMC1,PSMC2,PSMD10,PSMD12,PSMD11,PSMD14,PSMD13,UBA52,CHEK1
Regulation of ornithine decarboxylase (ODC)	0.007	50	36	1.11E-16	6.11E-15	ODC1,PSMD8,PSMD6,PSMD7,PSMD4,PSMD2,PSMD3,PSMD1,PSME1,PSME2,OAZ1,PSMF1,PSMA5,PSMA3,PSMA4,PSMA1,PSMA2,PSMA7,PSMB6,PSMB7,PSMB5,PSMB2,PSMB3,PSMB1,PSMC5,PSMC6,PSMC3,PSMC4,PSMC1,PSMC2,PSMD10,PSMD12,PSMD11,PSMD14,PSMD13,NQO1
Ubiquitin-dependent degradation of Cyclin D1	0.007	50	38	1.11E-16	6.11E-15	UBB,UBC,PSMD8,PSMD6,PSMD7,PSMD4,PSMD2,PSMD3,PSMD1,PSME1,PSME2,PSMF1,PSMA5,PSMA3,PSMA4,PSMA1,PSMA2,PSMA7,PSMB6,PSMB7,PSMB5,PSMB2,PSMB3,PSMB1,PSMC5,PSMC6,PSMC3,PSMC4,PSMC1,PSMC2,CCND1,PSMD10,PSMD12,PSMD11,PSMD14,PSMD13,UBA52,CDK4
AUF1 (hnRNP D0) binds and destabilizes mRNA	0.0075	53	38	1.11E-16	6.11E-15	UBB,UBC,PSMD8,PSMD6,PSMD7,PSMD4,PSMD2,PSMD3,PSMD1,PSME1,PSME2,PSMF1,PSMA5,PSMA3,PSMA4,PSMA1,PSMA2,PSMA7,PSMB6,PSMB7,PSMB5,PSMB2,PSMB3,PSMB1,PSMC5,PSMC6,PSMC3,PSMC4,PSMC1,PSMC2,PSMD10,PSMD12,PSMD11,PSMD14,PSMD13,UBA52,HSPA8,HSPB1
Regulation of activated PAK-2p34 by proteasome mediated degradation	0.0069	49	36	1.11E-16	6.11E-15	UBB,UBC,PSMD8,PSMD6,PSMD7,PSMD4,PSMD2,PSMD3,PSMD1,PSME1,PSME2,PSMF1,PSMA5,PSMA3,PSMA4,PSMA1,PSMA2,PSMA7,PSMB6,PSMB7,PSMB5,PSMB2,PSMB3,PSMB1,PSMC5,PSMC6,PSMC3,PSMC4,PSMC1,PSMC2,PSMD10,PSMD12,PSMD11,PSMD14,PSMD13,UBA52

The citric acid (TCA) cycle and respiratory electron transport	0.0224	159	85	1.11E-16	6.11E-15	ATP5C1,ATP5A1,UQCRB,UQCRH,UQCRQ,ACO2,ETFA,ETFB,SUCLA2,BSG,NDUFAF3,COX7B,COX7C,COX8A,COX5B,COX5A,COX6C,NDUFAB1,COX6A1,SDHC,SDHB,COX6B1,NDUFB10,NDUFB11,PDHA1,LDHB,LDHA,IDH3B,PDHB,IDH3G,SUCLG1,NDUFA13,NDUFA11,NDUFA12,NDUFA10,ATP5J2,ATP5G3,ATP5F1,UQCRC1,UQCRC2,FH,MDH2,SLC16A3,COX4I1,NDUFC2,NDUFC1,NDUFB9,NDUFB8,NDUFB7,NDUFB6,NDUFB5,NDUFB4,NDUFB3,NDUFB2,NDUFB1,NDUFA9,NDUFA8,NDUFA6,NDUFA5,NDUFA4,NDUFA3,NDUFA2,NDUFA1,ATP5J,ATP5I,ATP5H,ATP5O,ATP5L,ATP5B,ATP5E,ATP5D,CYC1,NDUFV2,NDUFV1,CYCS,UQCRFS1,NDUFS8,NDUFS7,NDUFS6,NDUFS5,NDUFS4,NDUFS3,TIMMDC1,GLO1,UQCR11
CDK-mediated phosphorylation and removal of Cdc6	0.007	50	36	1.11E-16	6.11E-15	UBB,UBC,PSMD8,PSMD6,PSMD7,PSMD4,PSMD2,PSMD3,PSMD1,PSME1,PSME2,PSMF1,PSMA5,PSMA3,PSMA4,PSMA1,PSMA2,PSMA7,PSMB6,PSMB7,PSMB5,PSMB2,PSMB3,PSMB1,PSMC5,PSMC6,PSMC3,PSMC4,PSMC1,PSMC2,PSMD10,PSMD12,PSMD11,PSMD14,PSMD13,UBA52
ER-Phagosome pathway	0.009	64	45	1.11E-16	6.11E-15	CALR,HLA-B,HLA-C,HLA-A,HLA-E,SEC61G,SEC61B,UBB,UBC,PSMD8,PSMD6,PSMD7,PSMD4,PSMD2,PSMD3,PSMD1,PSME1,PSME2,PSMF1,PSMA5,PSMA3,PSMA4,PSMA1,PSMA2,PSMA7,PSMB6,PSMB7,PSMB5,PSMB2,PSMB3,PSMB1,PSMC5,PSMC6,PSMC3,PSMC4,PSMC1,PSMC2,PSMD10,PSMD12,PSMD11,PSMD14,PSMD13,UBA52,B2M,PDIA3
Autodegradation of the E3 ubiquitin ligase COP1	0.0072	51	36	1.11E-16	6.11E-15	UBB,UBC,PSMD8,PSMD6,PSMD7,PSMD4,PSMD2,PSMD3,PSMD1,PSME1,PSME2,PSMF1,PSMA5,PSMA3,PSMA4,PSMA1,PSMA2,PSMA7,PSMB6,PSMB7,PSMB5,PSMB2,PSMB3,PSMB1,PSMC5,PSMC6,PSMC3,PSMC4,PSMC1,PSMC2,PSMD10,PSMD12,PSMD11,PSMD14,PSMD13,UBA52
SCF-beta-TrCP mediated degradation of Emi1	0.0076	54	38	1.11E-16	6.11E-15	CDC20,UBB,UBC,PSMD8,PSMD6,PSMD7,PSMD4,PSMD2,PSMD3,PSMD1,PSME1,PSME2,PSMF1,PSMA5,PSMA3,PSMA4,PSMA1,PSMA2,PSMA7,PSMB6,PSMB7,PSMB5,PSMB2,PSMB3,PSMB1,PSMC5,PSMC6,PSMC3,PSMC4,PSMC1,PSMC2,PSMD10,PSMD12,PSMD11,PSMD14,PSMD13,UBA52,SKP1

p53-Independent G1/S DNA damage checkpoint	0.0073	52	37	1.11E-16	6.11E-15	UBB,UBC,PSMD8,PSMD6,PSMD7,PSMD4,PSMD2,PSMD3,PSMD1,PSME1,PSME2,PSMF1,PSMA5,PSMA3,PSMA4,PSMA1,PSMA2,PSMA7,PSMB6,PSMB7,PSMB5,PSMB2,PSMB3,PSMB1,PSMC5,PSMC6,PSMC3,PSMC4,PSMC1,PSMC2,PSMD10,PSMD12,PSMD11,PSMD14,PSMD13,UBA52,CHEK1
p53-Independent DNA Damage Response	0.0073	52	37	1.11E-16	6.11E-15	UBB,UBC,PSMD8,PSMD6,PSMD7,PSMD4,PSMD2,PSMD3,PSMD1,PSME1,PSME2,PSMF1,PSMA5,PSMA3,PSMA4,PSMA1,PSMA2,PSMA7,PSMB6,PSMB7,PSMB5,PSMB2,PSMB3,PSMB1,PSMC5,PSMC6,PSMC3,PSMC4,PSMC1,PSMC2,PSMD10,PSMD12,PSMD11,PSMD14,PSMD13,UBA52,CHEK1
SCF(Skp2)-mediated degradation of p27/p21	0.0073	52	38	1.11E-16	6.11E-15	CDKN1A,UBB,UBC,PSMD8,PSMD6,PSMD7,PSMD4,PSMD2,PSMD3,PSMD1,PSME1,PSME2,PSMF1,PSMA5,PSMA3,PSMA4,PSMA1,PSMA2,PSMA7,PSMB6,PSMB7,PSMB5,PSMB2,PSMB3,PSMB1,PSMC5,PSMC6,PSMC3,PSMC4,PSMC1,PSMC2,PSMD10,PSMD12,PSMD11,PSMD14,PSMD13,UBA52,SKP1
CDT1 association with the CDC6:ORC:origin complex	0.0082	58	38	2.22E-16	9.10E-15	ORC6,UBB,UBC,PSMD8,PSMD6,PSMD7,PSMD4,PSMD2,PSMD3,PSMD1,PSME1,PSME2,PSMF1,PSMA5,PSMA3,PSMA4,PSMA1,PSMA2,PSMA7,PSMB6,PSMB7,PSMB5,PSMB2,PSMB3,PSMB1,PSMC5,PSMC6,PSMC3,PSMC4,PSMC1,PSMC2,PSMD10,PSMD12,PSMD11,PSMD14,PSMD13,UBA52,CDT1
p53-Dependent G1/S DNA damage checkpoint	0.0077	55	37	2.22E-16	9.10E-15	CDKN1A,UBB,UBC,PSMD8,PSMD6,PSMD7,PSMD4,PSMD2,PSMD3,PSMD1,PSME1,PSME2,PSMF1,PSMA5,PSMA3,PSMA4,PSMA1,PSMA2,PSMA7,PSMB6,PSMB7,PSMB5,PSMB2,PSMB3,PSMB1,PSMC5,PSMC6,PSMC3,PSMC4,PSMC1,PSMC2,PSMD10,PSMD12,PSMD11,PSMD14,PSMD13,UBA52
G1/S DNA Damage Checkpoints	0.0082	58	38	2.22E-16	9.10E-15	CDKN1A,UBB,UBC,PSMD8,PSMD6,PSMD7,PSMD4,PSMD2,PSMD3,PSMD1,PSME1,PSME2,PSMF1,PSMA5,PSMA3,PSMA4,PSMA1,PSMA2,PSMA7,PSMB6,PSMB7,PSMB5,PSMB2,PSMB3,PSMB1,PSMC5,PSMC6,PSMC3,PSMC4,PSMC1,PSMC2,PSMD10,PSMD12,PSMD11,PSMD14,PSMD13,UBA52,CHEK1
Cyclin A:Cdk2-associated events at S phase entry	0.0086	61	39	2.22E-16	9.10E-15	CDKN1A,UBB,UBC,WEE1,PSMD8,PSMD6,PSMD7,PSMD4,PSMD2,PSMD3,PSMD1,PSME1,PSME2,PSMF1,PSMA5,PSMA3,PSMA4,PSMA1,PSMA2,PSMA7,PSMB6,PSMB7,PSMB5,PSMB2,PSMB3,PSMB1,PSMC5,PSMC6,PSMC3,PSMC4,PSMC1,PSMC2,PSMD10,PSMD12,PSMD11,PSMD14,PSMD13,UBA52,SKP1

Stabilization of p53	0.0073	52	36	2.22E-16	9.10E-15	UBB,UBC,PSMD8,PSMD6,PSMD7,PSMD4,PSMD2,PSMD3,PSMD1,PSME1,PSME2,PSMF1,PSMA5,PSMA3,PSMA4,PSMA1,PSMA2,PSMA7,PSMB6,PSMB7,PSMB5,PSMB2,PSMB3,PSMB1,PSMC5,PSMC6,PSMC3,PSMC4,PSMC1,PSMC2,PSMD10,PSMD12,PSMD11,PSMD14,PSMD13,UBA52
p53-Dependent G1 DNA Damage Response	0.0077	55	37	2.22E-16	9.10E-15	CDKN1A,UBB,UBC,PSMD8,PSMD6,PSMD7,PSMD4,PSMD2,PSMD3,PSMD1,PSME1,PSME2,PSMF1,PSMA5,PSMA3,PSMA4,PSMA1,PSMA2,PSMA7,PSMB6,PSMB7,PSMB5,PSMB2,PSMB3,PSMB1,PSMC5,PSMC6,PSMC3,PSMC4,PSMC1,PSMC2,PSMD10,PSMD12,PSMD11,PSMD14,PSMD13,UBA52
Degradation of GLI1 by the proteasome	0.0083	59	38	3.33E-16	1.20E-14	UBB,UBC,PSMD8,PSMD6,PSMD7,PSMD4,PSMD2,PSMD3,PSMD1,PSME1,PSME2,PSMF1,PSMA5,PSMA3,PSMA4,PSMA1,PSMA2,PSMA7,PSMB6,PSMB7,PSMB5,PSMB2,PSMB3,PSMB1,PSMC5,PSMC6,PSMC3,PSMC4,PSMC1,PSMC2,RBX1,PSMD10,PSMD12,PSMD11,PSMD14,PSMD13,UBA52,SKP1
Degradation of GLI2 by the proteasome	0.0083	59	38	3.33E-16	1.20E-14	UBB,UBC,PSMD8,PSMD6,PSMD7,PSMD4,PSMD2,PSMD3,PSMD1,PSME1,PSME2,PSMF1,PSMA5,PSMA3,PSMA4,PSMA1,PSMA2,PSMA7,PSMB6,PSMB7,PSMB5,PSMB2,PSMB3,PSMB1,PSMC5,PSMC6,PSMC3,PSMC4,PSMC1,PSMC2,RBX1,PSMD10,PSMD12,PSMD11,PSMD14,PSMD13,UBA52,SKP1
GLI3 is processed to GLI3R by the proteasome	0.0083	59	38	3.33E-16	1.20E-14	UBB,UBC,PSMD8,PSMD6,PSMD7,PSMD4,PSMD2,PSMD3,PSMD1,PSME1,PSME2,PSMF1,PSMA5,PSMA3,PSMA4,PSMA1,PSMA2,PSMA7,PSMB6,PSMB7,PSMB5,PSMB2,PSMB3,PSMB1,PSMC5,PSMC6,PSMC3,PSMC4,PSMC1,PSMC2,RBX1,PSMD10,PSMD12,PSMD11,PSMD14,PSMD13,UBA52,SKP1
APC/C:Cdc20 mediated degradation of mitotic proteins	0.0101	72	42	3.33E-16	1.20E-14	CDC20,UBB,UBC,PSMD8,PSMD6,PSMD7,PSMD4,PSMD2,PSMD3,PSMD1,PSME1,PSME2,PSMF1,PSMA5,PSMA3,PSMA4,PSMA1,PSMA2,PSMA7,PSMB6,PSMB7,PSMB5,PSMB2,PSMB3,PSMB1,PSMC5,PSMC6,PSMC3,PSMC4,PSMC1,PSMC2,UBE2D1,UBE2E1,MAD2L1,PSMD10,PSMD12,PSMD11,PSMD14,PSMD13,CCNB1,UBA52,PTTG1
Degradation of DVL	0.0079	56	37	4.44E-16	1.42E-14	UBB,UBC,PSMD8,PSMD6,PSMD7,PSMD4,PSMD2,PSMD3,PSMD1,PSME1,PSME2,PSMF1,PSMA5,PSMA3,PSMA4,PSMA1,PSMA2,PSMA7,PSMB6,PSMB7,PSMB5,PSMB2,PSMB3,PSMB1,PSMC5,PSMC6,PSMC3,PSMC4,PSMC1,PSMC2,RBX1,PSMD10,PSMD12,PSMD11,PSMD14,PSMD13,UBA52

Antigen processing: Ubiquitination & Proteasome degradation	0.0075	53	36	4.44E-16	1.42E-14	UBB,UBC,PSMD8,PSMD6,PSMD7,PSMD4,PSMD2,PSMD3,PSMD1,PSME1,PSME2,PSMF1,PSMA5,PSMA3,PSMA4,PSMA1,PSMA2,PSMA7,PSMB6,PSMB7,PSMB5,PSMB2,PSMB3,PSMB1,PSMC5,PSMC6,PSMC3,PSMC4,PSMC1,PSMC2,PSMD10,PSMD12,PSMD11,PSMD14,PSMD13,UBA52
APC/C:Cdc20 mediated degradation of Securin	0.0093	66	40	4.44E-16	1.42E-14	CDC20,UBB,UBC,PSMD8,PSMD6,PSMD7,PSMD4,PSMD2,PSMD3,PSMD1,PSME1,PSME2,PSMF1,PSMA5,PSMA3,PSMA4,PSMA1,PSMA2,PSMA7,PSMB6,PSMB7,PSMB5,PSMB2,PSMB3,PSMB1,PSMC5,PSMC6,PSMC3,PSMC4,PSMC1,PSMC2,UBE2D1,UBE2E1,PSMD10,PSMD12,PSMD11,PSMD14,PSMD13,UBA52,PTTG1
Activation of APC/C and APC/C:Cdc20 mediated degradation of mitotic proteins	0.0103	73	42	5.55E-16	1.67E-14	CDC20,UBB,UBC,PSMD8,PSMD6,PSMD7,PSMD4,PSMD2,PSMD3,PSMD1,PSME1,PSME2,PSMF1,PSMA5,PSMA3,PSMA4,PSMA1,PSMA2,PSMA7,PSMB6,PSMB7,PSMB5,PSMB2,PSMB3,PSMB1,PSMC5,PSMC6,PSMC3,PSMC4,PSMC1,PSMC2,UBE2D1,UBE2E1,MAD2L1,PSMD10,PSMD12,PSMD11,PSMD14,PSMD13,CCNB1,UBA52,PTTG1
Cyclin E associated events during G1/S transition	0.0089	63	39	5.55E-16	1.67E-14	CDKN1A,UBB,UBC,WEE1,PSMD8,PSMD6,PSMD7,PSMD4,PSMD2,PSMD3,PSMD1,PSME1,PSME2,PSMF1,PSMA5,PSMA3,PSMA4,PSMA1,PSMA2,PSMA7,PSMB6,PSMB7,PSMB5,PSMB2,PSMB3,PSMB1,PSMC5,PSMC6,PSMC3,PSMC4,PSMC1,PSMC2,PSMD10,PSMD12,PSMD11,PSMD14,PSMD13,UBA52,SKP1
Removal of licensing factors from origins	0.0098	70	41	6.66E-16	1.93E-14	CDKN1A,ORC6,MCM4,UBB,UBC,PSMD8,PSMD6,PSMD7,PSMD4,PSMD2,PSMD3,PSMD1,PSME1,PSME2,PSMF1,PSMA5,PSMA3,PSMA4,PSMA1,PSMA2,PSMA7,PSMB6,PSMB7,PSMB5,PSMB2,PSMB3,PSMB1,PSMC5,PSMC6,PSMC3,PSMC4,PSMC1,PSMC2,MCM10,PSMD10,PSMD12,PSMD11,PSMD14,PSMD13,UBA52,CDT1
Degradation of AXIN	0.0076	54	36	7.77E-16	2.25E-14	UBB,UBC,PSMD8,PSMD6,PSMD7,PSMD4,PSMD2,PSMD3,PSMD1,PSME1,PSME2,PSMF1,PSMA5,PSMA3,PSMA4,PSMA1,PSMA2,PSMA7,PSMB6,PSMB7,PSMB5,PSMB2,PSMB3,PSMB1,PSMC5,PSMC6,PSMC3,PSMC4,PSMC1,PSMC2,PSMD10,PSMD12,PSMD11,PSMD14,PSMD13,UBA52
Regulation of mRNA stability by proteins that bind AU-rich elements	0.012	85	45	8.88E-16	2.49E-14	SET,UBB,UBC,PSMD8,PSMD6,PSMD7,PSMD4,PSMD2,PSMD3,PSMD1,PSME1,PSME2,PSMF1,PSMA5,PSMA3,PSMA4,PSMA1,PSMA2,PSMA7,PSMB6,PSMB7,PSMB5,PSMB2,PSMB3,PSMB1,PSMC5,PSMC6,PSMC3,PSMC4,PSMC1,PSMC2,EXOSC7,PSMD10,PSMD12,PSMD11,PSMD14,PSMD13,YWHAB,YWHAZ,UBA52,ANP32A,ZFP36L1,HSPA8,HSPB1,ELAVL1

Asymmetric localization of PCP proteins	0.0082	58	37	1.22E-15	2.93E-14	UBB,UBC,PSMD8,PSMD6,PSMD7,PSMD4,PSMD2,PSMD3,PSMD1,PSME1,PSME2,PSMF1,PSMA5,PSMA3,PSMA4,PSMA1,PSMA2,PSMA7,PSMB6,PSMB7,PSMB5,PSMB2,PSMB3,PSMB1,PSMC5,PSMC6,PSMC3,PSMC4,PSMC1,PSMC2,PSMD10,PSMD12,PSMD11,PSMD14,PSMD13,UBA52,FZD5
Orc1 removal from chromatin	0.0096	68	40	1.22E-15	2.93E-14	CDKN1A,ORC6,MCM4,UBB,UBC,PSMD8,PSMD6,PSMD7,PSMD4,PSMD2,PSMD3,PSMD1,PSME1,PSME2,PSMF1,PSMA5,PSMA3,PSMA4,PSMA1,PSMA2,PSMA7,PSMB6,PSMB7,PSMB5,PSMB2,PSMB3,PSMB1,PSMC5,PSMC6,PSMC3,PSMC4,PSMC1,PSMC2,PSMD10,PSMD12,PSMD11,PSMD14,PSMD13,UBA52,CDT1
Degradation of beta-catenin by the destruction complex	0.0096	68	40	1.22E-15	2.93E-14	TLE4,UBB,UBC,PSMD8,PSMD6,PSMD7,PSMD4,PSMD2,PSMD3,PSMD1,PSME1,PSME2,PSMF1,PSMA5,PSMA3,PSMA4,PSMA1,PSMA2,PSMA7,PSMB6,PSMB7,PSMB5,PSMB2,PSMB3,PSMB1,PSMC5,PSMC6,PSMC3,PSMC4,PSMC1,PSMC2,RBX1,PSMD10,PSMD12,PSMD11,PSMD14,PSMD13,UBA52,SKP1,CTNNB1
NIK-->noncanonical NF-kB signaling	0.0082	58	37	1.22E-15	2.93E-14	UBB,UBC,PSMD8,PSMD6,PSMD7,PSMD4,PSMD2,PSMD3,PSMD1,PSME1,PSME2,PSMF1,PSMA5,PSMA3,PSMA4,PSMA1,PSMA2,PSMA7,PSMB6,PSMB7,PSMB5,PSMB2,PSMB3,PSMB1,PSMC5,PSMC6,PSMC3,PSMC4,PSMC1,PSMC2,PSMD10,PSMD12,PSMD11,PSMD14,PSMD13,UBA52,SKP1
Switching of origins to a post-replicative state	0.0096	68	40	1.22E-15	2.93E-14	CDKN1A,ORC6,MCM4,UBB,UBC,PSMD8,PSMD6,PSMD7,PSMD4,PSMD2,PSMD3,PSMD1,PSME1,PSME2,PSMF1,PSMA5,PSMA3,PSMA4,PSMA1,PSMA2,PSMA7,PSMB6,PSMB7,PSMB5,PSMB2,PSMB3,PSMB1,PSMC5,PSMC6,PSMC3,PSMC4,PSMC1,PSMC2,PSMD10,PSMD12,PSMD11,PSMD14,PSMD13,UBA52,CDT1
Hedgehog ligand biogenesis	0.0087	62	38	1.78E-15	4.09E-14	UBB,UBC,PSMD8,PSMD6,PSMD7,PSMD4,PSMD2,PSMD3,PSMD1,PSME1,PSME2,PSMF1,PSMA5,PSMA3,PSMA4,PSMA1,PSMA2,PSMA7,PSMB6,PSMB7,PSMB5,PSMB2,PSMB3,PSMB1,PSMC5,PSMC6,PSMC3,PSMC4,PSMC1,PSMC2,P4HB,PSMD10,PSMD12,VCP,PSMD11,PSMD14,PSMD13,UBA52
Cross-presentation of soluble exogenous antigens (endosomes)	0.0065	46	33	1.78E-15	4.09E-14	PSMD8,PSMD6,PSMD7,PSMD4,PSMD2,PSMD3,PSMD1,PSME1,PSME2,PSMF1,PSMA5,PSMA3,PSMA4,PSMA1,PSMA2,PSMA7,PSMB6,PSMB7,PSMB5,PSMB2,PSMB3,PSMB1,PSMC5,PSMC6,PSMC3,PSMC4,PSMC1,PSMC2,PSMD10,PSMD12,PSMD11,PSMD14,PSMD13

Autodegradation of Cdh1 by Cdh1:APC/C	0.0087	62	38	1.78E-15	4.09E-14	UBB,UBC,PSMD8,PSMD6,PSMD7,PSMD4,PSMD2,PSMD3,PSMD1,PSME1,PSME2,PSMF1,PSMA5,PSMA3,PSMA4,PSMA1,PSMA2,PSMA7,PSMB6,PSMB7,PSMB5,PSMB2,PSMB3,PSMB1,PSMC5,PSMC6,PSMC3,PSMC4,PSMC1,PSMC2,UBE2D1,UBE2E1,PSMD10,PSMD12,PSMD11,PSMD14,PSMD13,UBA52
Cdc20:Phospho-APC/C mediated degradation of Cyclin A	0.0097	69	40	2.00E-15	4.40E-14	CDC20,UBB,UBC,PSMD8,PSMD6,PSMD7,PSMD4,PSMD2,PSMD3,PSMD1,PSME1,PSME2,PSMF1,PSMA5,PSMA3,PSMA4,PSMA1,PSMA2,PSMA7,PSMB6,PSMB7,PSMB5,PSMB2,PSMB3,PSMB1,PSMC5,PSMC6,PSMC3,PSMC4,PSMC1,PSMC2,UBE2D1,UBE2E1,MAD2L1,PSMD10,PSMD12,PSMD11,PSMD14,PSMD13,UBA52
Dectin-1 mediated noncanonical NF-kB signaling	0.0083	59	37	2.11E-15	4.64E-14	UBB,UBC,PSMD8,PSMD6,PSMD7,PSMD4,PSMD2,PSMD3,PSMD1,PSME1,PSME2,PSMF1,PSMA5,PSMA3,PSMA4,PSMA1,PSMA2,PSMA7,PSMB6,PSMB7,PSMB5,PSMB2,PSMB3,PSMB1,PSMC5,PSMC6,PSMC3,PSMC4,PSMC1,PSMC2,PSMD10,PSMD12,PSMD11,PSMD14,PSMD13,UBA52,SKP1
Regulation of DNA replication	0.0103	73	41	2.44E-15	5.13E-14	CDKN1A,ORC6,MCM4,UBB,UBC,PSMD8,PSMD6,PSMD7,PSMD4,PSMD2,PSMD3,PSMD1,PSME1,PSME2,PSMF1,PSMA5,PSMA3,PSMA4,PSMA1,PSMA2,PSMA7,PSMB6,PSMB7,PSMB5,PSMB2,PSMB3,PSMB1,PSMC5,PSMC6,PSMC3,PSMC4,PSMC1,PSMC2,MCM10,PSMD10,PSMD12,PSMD11,PSMD14,PSMD13,UBA52,CDT1
Metabolism of polyamines	0.0103	73	41	2.44E-15	5.13E-14	ENOPH1,ODC1,SMS,CKB,PSMD8,PSMD6,PSMD7,PSMD4,PSMD2,PSMD3,PSMD1,PSME1,PSME2,OAZ1,PSMF1,PSMA5,PSMA3,PSMA4,PSMA1,PSMA2,PSMA7,PSMB6,PSMB7,PSMB5,PSMB2,PSMB3,PSMB1,PSMC5,PSMC6,PSMC3,PSMC4,PSMC1,PSMC2,SAT1,GOT1,PSMD10,PSMD12,PSMD11,PSMD14,PSMD13,NQO1
PCP/CE pathway	0.0108	77	42	3.00E-15	6.00E-14	RAC1,UBB,UBC,PSMD8,PSMD6,PSMD7,PSMD4,PSMD2,PSMD3,PSMD1,PSME1,PSME2,PSMF1,PSMA5,PSMA3,PSMA4,PSMA1,PSMA2,PSMA7,PSMB6,PSMB7,PSMB5,PSMB2,PSMB3,PSMB1,AP2S1,PSMC5,PSMC6,PSMC3,PSMC4,PSMC1,PSMC2,AP2M1,PSMD10,PSMD12,PSMD11,PSMD14,PSMD13,UBA52,FZD5,PFN1,RHOA
Regulation of APC/C activators between G1/S and early anaphase	0.0108	77	42	3.00E-15	6.00E-14	CDC20,UBB,UBC,PSMD8,PSMD6,PSMD7,PSMD4,PSMD2,PSMD3,PSMD1,PSME1,PSME2,PSMF1,PSMA5,PSMA3,PSMA4,PSMA1,PSMA2,PSMA7,PSMB6,PSMB7,PSMB5,PSMB2,PSMB3,PSMB1,PSMC5,PSMC6,PSMC3,PSMC4,PSMC1,PSMC2,UBE2D1,UBE2E1,MAD2L1,PSMD10,PSMD12,PSMD11,PSMD14,PSMD13,CCNB1,UBA52,SKP1

APC:Cdc20 mediated degradation of cell cycle proteins prior to satisfation of the cell cycle checkpoint	0.0098	70	40	3.11E-15	6.22E-14	CDC20,UBB,UBC,PSMD8,PSMD6,PSMD7,PSMD4,PSMD2,PSMD3,PSMD1,PSME1,PSME2,PSMF1,PSMA5,PSMA3,PSMA4,PSMA1,PSMA2,PSMA7,PSMB6,PSMB7,PSMB5,PSMB2,PSMB3,PSMB1,PSMC5,PSMC6,PSMC3,PSMC4,PSMC1,PSMC2,UBE2D1,UBE2E1,MAD2L1,PSMD10,PSMD12,PSMD11,PSMD14,PSMD13,UBA52
Assembly of the pre-replicative complex	0.0094	67	39	3.89E-15	7.38E-14	ORC6,MCM4,UBB,UBC,PSMD8,PSMD6,PSMD7,PSMD4,PSMD2,PSMD3,PSMD1,PSME1,PSME2,PSMF1,PSMA5,PSMA3,PSMA4,PSMA1,PSMA2,PSMA7,PSMB6,PSMB7,PSMB5,PSMB2,PSMB3,PSMB1,PSMC5,PSMC6,PSMC3,PSMC4,PSMC1,PSMC2,PSMD10,PSMD12,PSMD11,PSMD14,PSMD13,UBA52,CDT1
APC/C:Cdh1 mediated degradation of Cdc20 and other APC/C:Cdh1 targeted proteins in late mitosis/early G1	0.01	71	40	5.00E-15	9.49E-14	CDC20,UBB,UBC,PSMD8,PSMD6,PSMD7,PSMD4,PSMD2,PSMD3,PSMD1,PSME1,PSME2,PSMF1,PSMA5,PSMA3,PSMA4,PSMA1,PSMA2,PSMA7,PSMB6,PSMB7,PSMB5,PSMB2,PSMB3,PSMB1,PSMC5,PSMC6,PSMC3,PSMC4,PSMC1,PSMC2,UBE2D1,UBE2E1,PSMD10,PSMD12,PSMD11,PSMD14,PSMD13,UBA52,PTTG1
APC/C-mediated degradation of cell cycle proteins	0.0115	82	43	5.33E-15	9.59E-14	CDC20,UBB,UBC,PSMD8,PSMD6,PSMD7,PSMD4,PSMD2,PSMD3,PSMD1,PSME1,PSME2,PSMF1,PSMA5,PSMA3,PSMA4,PSMA1,PSMA2,PSMA7,PSMB6,PSMB7,PSMB5,PSMB2,PSMB3,PSMB1,PSMC5,PSMC6,PSMC3,PSMC4,PSMC1,PSMC2,UBE2D1,UBE2E1,MAD2L1,PSMD10,PSMD12,PSMD11,PSMD14,PSMD13,CCNB1,UBA52,SKP1,PTTG1
Regulation of mitotic cell cycle	0.0115	82	43	5.33E-15	9.59E-14	CDC20,UBB,UBC,PSMD8,PSMD6,PSMD7,PSMD4,PSMD2,PSMD3,PSMD1,PSME1,PSME2,PSMF1,PSMA5,PSMA3,PSMA4,PSMA1,PSMA2,PSMA7,PSMB6,PSMB7,PSMB5,PSMB2,PSMB3,PSMB1,PSMC5,PSMC6,PSMC3,PSMC4,PSMC1,PSMC2,UBE2D1,UBE2E1,MAD2L1,PSMD10,PSMD12,PSMD11,PSMD14,PSMD13,CCNB1,UBA52,SKP1,PTTG1
Regulation of RAS by GAPs	0.0091	65	38	7.77E-15	1.40E-13	KRAS,UBB,UBC,PSMD8,PSMD6,PSMD7,PSMD4,PSMD2,PSMD3,PSMD1,PSME1,PSME2,PSMF1,PSMA5,PSMA3,PSMA4,PSMA1,PSMA2,PSMA7,PSMB6,PSMB7,PSMB5,PSMB2,PSMB3,PSMB1,PSMC5,PSMC6,PSMC3,PSMC4,PSMC1,PSMC2,RBX1,PSMD10,PSMD12,PSMD11,PSMD14,PSMD13,UBA52

Complex I biogenesis	0.0077	55	35	7.99E-15	1.44E-13	NDUFAF3,NDUFAB1,NDUFB10,NDUFB11,NDUFA13,NDUFA11,NDUFA12,NDUFA10,NDUFC2,NDUFC1,NDUFB9,NDUFB8,NDUFB7,NDUFB6,NDUFB5,NDUFB4,NDUFB3,NDUFB2,NDUFB1,NDUFA9,NDUFA8,NDUFA6,NDUFA5,NDUFA3,NDUFA2,NDUFA1,NDUFV2,NDUFV1,NDUFS8,NDUFS7,NDUFS6,NDUFS5,NDUFS4,NDUFS3,TIMMDC1
Cell Cycle Checkpoints	0.021	149	59	1.03E-14	1.76E-13	BRE,CDC20,CDKN1A,ORC6,RFC4,MCM4,UBB,UBC,WEE1,PSMD8,PSMD6,PSMD7,PSMD4,PSMD2,PSMD3,PSMD1,PSME1,PSME2,PSMF1,PSMA5,PSMA3,PSMA4,PSMA1,PSMA2,PSMA7,PSMB6,PSMB7,PSMB5,PSMB2,PSMB3,PSMB1,PSMC5,PSMC6,PSMC3,PSMC4,PSMC1,PSMC2,UBE2D1,UBE2E1,MCM10,BRCA1,BABAM1,MAD2L1,UBE2V2,PSMD10,PSMD12,PSMD11,PSMD14,PSMD13,CCNB2,CCNB1,YWHAE,YWHAB,YWHAQ,YWHAH,YWHAZ,UBA52,RAD9A,CHEK1
Activation of NF-kappaB in B cells	0.0093	66	38	1.23E-14	2.10E-13	UBB,UBC,PSMD8,PSMD6,PSMD7,PSMD4,PSMD2,PSMD3,PSMD1,PSME1,PSME2,PSMF1,PSMA5,PSMA3,PSMA4,PSMA1,PSMA2,PSMA7,PSMB6,PSMB7,PSMB5,PSMB2,PSMB3,PSMB1,PSMC5,PSMC6,PSMC3,PSMC4,PSMC1,PSMC2,PSMD10,PSMD12,PSMD11,PSMD14,PSMD13,UBA52,SKP1,NFKBIA
G1/S Transition	0.0145	103	46	1.68E-13	2.86E-12	DHFR,CDKN1A,ORC6,TFDP1,MCM4,UBB,UBC,WEE1,PSMD8,PSMD6,PSMD7,PSMD4,PSMD2,PSMD3,PSMD1,PSME1,PSME2,PSMF1,PSMA5,PSMA3,PSMA4,PSMA1,PSMA2,PSMA7,PSMB6,PSMB7,PSMB5,PSMB2,PSMB3,PSMB1,PSMC5,PSMC6,PSMC3,PSMC4,PSMC1,PSMC2,MCM10,PSMD10,PSMD12,PSMD11,PSMD14,PSMD13,CCNB1,UBA52,SKP1,CDT1
M/G1 Transition	0.0115	82	40	4.50E-13	7.20E-12	ORC6,MCM4,UBB,UBC,PSMD8,PSMD6,PSMD7,PSMD4,PSMD2,PSMD3,PSMD1,PSME1,PSME2,PSMF1,PSMA5,PSMA3,PSMA4,PSMA1,PSMA2,PSMA7,PSMB6,PSMB7,PSMB5,PSMB2,PSMB3,PSMB1,PSMC5,PSMC6,PSMC3,PSMC4,PSMC1,PSMC2,MCM10,PSMD10,PSMD12,PSMD11,PSMD14,PSMD13,UBA52,CDT1
DNA Replication Pre-Initiation	0.0115	82	40	4.50E-13	7.20E-12	ORC6,MCM4,UBB,UBC,PSMD8,PSMD6,PSMD7,PSMD4,PSMD2,PSMD3,PSMD1,PSME1,PSME2,PSMF1,PSMA5,PSMA3,PSMA4,PSMA1,PSMA2,PSMA7,PSMB6,PSMB7,PSMB5,PSMB2,PSMB3,PSMB1,PSMC5,PSMC6,PSMC3,PSMC4,PSMC1,PSMC2,MCM10,PSMD10,PSMD12,PSMD11,PSMD14,PSMD13,UBA52,CDT1

Synthesis of DNA	0.0135	96	43	9.51E-13	1.52E-11	APEX1,CDKN1A,ORC6,RFC4,MCM4,GINS2,UBB,UBC,PSMD8,PSMD6,PSMD7,PSMD4,PSMD2,PSMD3,PSMD1,PSME1,PSME2,PSMF1,PSMA5,PSMA3,PSMA4,PSMA1,PSMA2,PSMA7,PSMB6,PSMB7,PSMB5,PSMB2,PSMB3,PSMB1,PSMC5,PSMC6,PSMC3,PSMC4,PSMC1,PSMC2,PSMD10,PSMD12,PSMD11,PSMD14,PSMD13,UBA52,CDT1
S Phase	0.0169	120	48	2.36E-12	3.77E-11	APEX1,CDKN1A,ORC6,RFC4,MCM4,GINS2,UBB,UBC,WEE1,PSMD8,PSMD6,PSMD7,PSMD4,PSMD2,PSMD3,PSMD1,PSME1,PSME2,PSMF1,PSMA5,PSMA3,PSMA4,PSMA1,PSMA2,PSMA7,PSMB6,PSMB7,PSMB5,PSMB2,PSMB3,PSMB1,PSMC5,PSMC6,PSMC3,PSMC4,PSMC1,PSMC2,CCND1,PSMD10,PSMD12,PSMD11,PSMD14,PSMD13,UBA52,RAD21,CDK4,SKP1,CDT1
Mitotic G1-G1/S phases	0.0177	126	49	3.71E-12	5.56E-11	DHFR,CDKN1A,CDKN2A,ORC6,TFDP1,MCM4,UBB,UBC,WEE1,PSMD8,PSMD6,PSMD7,PSMD4,PSMD2,PSMD3,PSMD1,PSME1,PSME2,PSMF1,PSMA5,PSMA3,PSMA4,PSMA1,PSMA2,PSMA7,PSMB6,PSMB7,PSMB5,PSMB2,PSMB3,PSMB1,PSMC5,PSMC6,PSMC3,PSMC4,PSMC1,PSMC2,MCM10,CCND1,PSMD10,PSMD12,PSMD11,PSMD14,PSMD13,CCNB1,UBA52,CDK4,SKP1,CDT1
MAPK6/MAPK4 signaling	0.0124	88	40	3.82E-12	5.74E-11	CDC42,RAC1,TNRC6B,UBB,UBC,PSMD8,PSMD6,PSMD7,PSMD4,PSMD2,PSMD3,PSMD1,PSME1,PSME2,PSMF1,PSMA5,PSMA3,PSMA4,PSMA1,PSMA2,PSMA7,PSMB6,PSMB7,PSMB5,PSMB2,PSMB3,PSMB1,PSMC5,PSMC6,PSMC3,PSMC4,PSMC1,PSMC2,PSMD10,PSMD12,PSMD11,PSMD14,PSMD13,UBA52,HSPB1
Hedgehog 'on' state	0.0114	81	38	5.36E-12	8.03E-11	UBB,UBC,PSMD8,PSMD6,PSMD7,PSMD4,PSMD2,PSMD3,PSMD1,PSME1,PSME2,PSMF1,PSMA5,PSMA3,PSMA4,PSMA1,PSMA2,PSMA7,PSMB6,PSMB7,PSMB5,PSMB2,PSMB3,PSMB1,PSMC5,PSMC6,PSMC3,PSMC4,PSMC1,PSMC2,RBX1,PSMD10,PSMD12,PSMD11,PSMD14,PSMD13,UBA52,CDON
Apoptosis	0.0212	151	54	6.68E-12	1.00E-10	GSN,TFDP1,MAGED1,LMNA,UBB,UBC,PSMD8,PSMD6,PSMD7,PSMD4,PSMD2,PSMD3,PSMD1,PSME1,PSME2,PSMF1,DYNLL1,PSMA5,PSMA3,PSMA4,PSMA1,PSMA2,PSMA7,PSMB6,PSMB7,PSMB5,PSMB2,PSMB3,PSMB1,PSMC5,PSMC6,PSMC3,PSMC4,PSMC1,PSMC2,DSP,DSG2,DBNL,BCAP31,PSMD10,PSMD12,PSMD11,PSMD14,PSMD13,CYCS,YWHAE,YWHAB,HMGB1,YWHAQ,YWHAH,YWHAZ,VIM,UBA52,CTNNB1

Beta-catenin independent WNT signaling	0.0156	111	45	7.49E-12	1.05E-10	RAC1,TNRC6B,UBB,UBC,PSMD8,PSMD6,PSMD7,PSMD4,PSMD2,PSMD3,PSMD1,PSME1,PSME2,PSMF1,PSMA5,PSMA3,PSMA4,PSMA1,PSMA2,PSMA7,PSMB6,PSMB7,PSMB5,PSMB2,PSMB3,PSMB1,AP2S1,PSMC5,PSMC6,PSMC3,PSMC4,PSMC1,PSMC2,AP2M1,CALM1,PSMD10,PSMD12,PSMD11,PSMD14,PSMD13,UBA52,FZD5,PFN1,RHOA,CTNNB1
CLEC7A (Dectin-1) signaling	0.0135	96	41	1.37E-11	1.91E-10	UBB,UBC,PSMD8,PSMD6,PSMD7,PSMD4,PSMD2,PSMD3,PSMD1,PSME1,PSME2,PSMF1,PSMA5,PSMA3,PSMA4,PSMA1,PSMA2,PSMA7,PSMB6,PSMB7,PSMB5,PSMB2,PSMB3,PSMB1,PSMC5,PSMC6,PSMC3,PSMC4,PSMC1,PSMC2,UBE2D2,UBE2D1,CALM1,PSMD10,PSMD12,PSMD11,PSMD14,PSMD13,UBA52,SKP1,NFKBIA
Hedgehog 'off' state	0.0125	89	39	2.06E-11	2.88E-10	GNAS,UBB,UBC,PSMD8,PSMD6,PSMD7,PSMD4,PSMD2,PSMD3,PSMD1,PSME1,PSME2,PSMF1,PSMA5,PSMA3,PSMA4,PSMA1,PSMA2,PSMA7,PSMB6,PSMB7,PSMB5,PSMB2,PSMB3,PSMB1,PSMC5,PSMC6,PSMC3,PSMC4,PSMC1,PSMC2,RBX1,PSMD10,PSMD12,PSMD11,PSMD14,PSMD13,UBA52,SKP1
Prefoldin mediated transfer of substrate to CCT/TriC	0.0037	26	20	1.86E-10	2.60E-09	ACTB,TUBB2B,TUBB2A,TCP1,TUBB4B,PFDN1,PFDN2,PFDN4,PFDN5,TUBA1C,TUBA1A,VBP1,CCT3,CCT2,CCT8,CCT7,CCT5,CCT4,TUBB6,CCT6A
Mitotic Anaphase	0.0228	162	53	2.44E-10	3.42E-09	NUP107,CENPU,CENPK,CDC20,PPP2R1A,PPP2CA,LMNA,UBB,UBC,PSMD8,PSMD6,PSMD7,PSMD4,PSMD2,PSMD3,PSMD1,PSME1,PSME2,PSMF1,PSMA5,PSMA3,PSMA4,PSMA1,PSMA2,PSMA7,PSMB6,PSMB7,PSMB5,PSMB2,PSMB3,PSMB1,PSMC5,PSMC6,PSMC3,PSMC4,PSMC1,PSMC2,UBE2D1,UBE2E1,SEC13,MAD2L1,BANF1,PSMD10,PSMD12,PSMD11,PSMD14,PSMD13,UBA52,RAD21,NUDC,ZWINT,MAPRE1,PTTG1
Downstream TCR signaling	0.0143	102	40	2.84E-10	3.69E-09	UBB,UBC,PSMD8,PSMD6,PSMD7,PSMD4,PSMD2,PSMD3,PSMD1,PSME1,PSME2,PSMF1,PSMA5,PSMA3,PSMA4,PSMA1,PSMA2,PSMA7,PSMB6,PSMB7,PSMB5,PSMB2,PSMB3,PSMB1,PSMC5,PSMC6,PSMC3,PSMC4,PSMC1,PSMC2,UBE2D2,UBE2D1,PSMD10,PSMD12,PSMD11,PSMD14,PSMD13,UBA52,SKP1,NFKBIA

Mitotic Metaphase and Anaphase	0.0229	163	53	3.02E-10	3.93E-09	NUP107,CENPU,CENPK,CDC20,PPP2R1A,PPP2CA,LMNA,UBB,UBC,PSMD8,PSMD6,PSMD7,PSMD4,PSMD2,PSMD3,PSMD1,PSME1,PSME2,PSMF1,PSMA5,PSMA3,PSMA4,PSMA1,PSMA2,PSMA7,PSMB6,PSMB7,PSMB5,PSMB2,PSMB3,PSMB1,PSMC5,PSMC6,PSMC3,PSMC4,PSMC1,PSMC2,UBE2D1,UBE2E1,SEC13,MAD2L1,BANF1,PSMD10,PSMD12,PSMD11,PSMD14,PSMD13,UBA52,RAD21,NUDC,ZWINT,MAPRE1,PTTG1
TNFR2 non-canonical NF-kB pathway	0.0135	96	38	6.16E-10	8.01E-09	UBB,UBC,PSMD8,PSMD6,PSMD7,PSMD4,PSMD2,PSMD3,PSMD1,PSME1,PSME2,PSMF1,PSMA5,PSMA3,PSMA4,PSMA1,PSMA2,PSMA7,PSMB6,PSMB7,PSMB5,PSMB2,PSMB3,PSMB1,PSMC5,PSMC6,PSMC3,PSMC4,PSMC1,PSMC2,TNFRSF12A,PSMD10,PSMD12,PSMD11,PSMD14,PSMD13,UBA52,SKP1
Cooperation of Prefoldin and TriC/CCT in actin and tubulin folding	0.0044	31	21	6.24E-10	8.11E-09	ACTB,TUBB2B,TUBB2A,TCP1,TUBB4B,PFDN1,PFDN2,PFDN4,PFDN5,TUBA1C,TUBA1B,TUBA1A,VBP1,CCT3,CCT2,CCT8,CCT7,CCT5,CCT4,TUBB6,CCT6A
Cell Cycle, Mitotic	0.0561	399	95	7.54E-10	9.80E-09	NUP107,OPTN,SET,DHFR,CENPU,APEX1,CENPK,CDC20,CSNK2B,ACTR1A,DYNC1I2,SSNA1,DYNC1H1,NUP93,CDKN1A,CDKN2A,TUBB4B,RAB2A,CEP135,ORC6,RFC4,TFDP1,MCM4,RAB1A,CEP152,PPP2R1A,TUBB,PPP2CA,LMNA,GINS2,UBB,UBC,WEE1,PSMD8,PSMD6,PSMD7,PSMD4,PSMD2,PSMD3,PSMD1,PSME1,PSME2,PSMF1,TUBA1A,DYNLL1,PSMA5,PSMA3,PSMA4,PSMA1,PSMA2,PSMA7,PSMB6,PSMB7,PSMB5,PSMB2,PSMB3,PSMB1,PSMC5,PSMC6,PSMC3,PSMC4,PSMC1,PSMC2,UBE2D1,UBE2E1,MCM10,HSP90AA1,PPP1R12A,CETN2,SEC13,SFI1,MAD2L1,CCND1,BANF1,PSMD10,PSMD12,PSMD11,PSMD14,PSMD13,CCNB2,CCNB1,YWHAE,GORASP2,DCTN2,DCTN3,UBA52,RAD21,NUDC,CDK4,SKP1,CDT1,ZWINT,TUBG1,MAPRE1,PTTG1
Protein folding	0.0053	38	23	8.13E-10	1.06E-08	ACTB,TUBB2B,TUBB2A,TCP1,TUBB4B,PFDN1,PFDN2,PFDN4,PFDN5,TUBA1C,TUBA1B,TUBA1A,VBP1,CCT3,CCT2,CCT8,CCT7,CCT5,CCT4,TUBB6,CCT6A,TBCB,TBCA
Chaperonin-mediated protein folding	0.0045	32	21	1.08E-09	1.30E-08	ACTB,TUBB2B,TUBB2A,TCP1,TUBB4B,PFDN1,PFDN2,PFDN4,PFDN5,TUBA1C,TUBA1B,TUBA1A,VBP1,CCT3,CCT2,CCT8,CCT7,CCT5,CCT4,TUBB6,CCT6A

C-type lectin receptors (CLRs)	0.0167	119	42	2.19E-09	2.62E-08	KRAS,UBB,UBC,PSMD8,PSMD6,PSMD7,PSMD4,PSMD2,PSMD3,PSMD1,PSME1,PSME2,PSMF1,PSMA5,PSMA3,PSMA4,PSMA1,PSMA2,PSMA7,PSMB6,PSMB7,PSMB5,PSMB2,PSMB3,PSMB1,PSMC5,PSMC6,PSMC3,PSMC4,PSMC1,PSMC2,UBE2D2,UBE2D1,CALM1,PSMD10,PSMD12,PSMD11,PSMD14,PSMD13,UBA52,SKP1,NFKBIA
Separation of Sister Chromatids	0.0217	154	49	2.84E-09	3.40E-08	NUP107,CENPU,CENPK,CDC20,UBB,UBC,PSMD8,PSMD6,PSMD7,PSMD4,PSMD2,PSMD3,PSMD1,PSME1,PSME2,PSMF1,PSMA5,PSMA3,PSMA4,PSMA1,PSMA2,PSMA7,PSMB6,PSMB7,PSMB5,PSMB2,PSMB3,PSMB1,PSMC5,PSMC6,PSMC3,PSMC4,PSMC1,PSMC2,UBE2D1,UBE2E1,SEC13,MAD2L1,PSMD10,PSMD12,PSMD11,PSMD14,PSMD13,UBA52,RAD21,NUDC,ZWINT,MAPRE1,PTTG1
Signaling by Wnt	0.0322	229	63	4.11E-09	4.93E-08	WLS,LGR4,CSNK2B,RAC1,CAV1,VPS29,VPS35,TLE4,TNRC6B,UBB,UBC,WNT2B,PSMD8,PSMD6,PSMD7,PSMD4,PSMD2,PSMD3,PSMD1,PSME1,PSME2,PSMF1,PSMA5,PSMA3,PSMA4,PSMA1,PSMA2,PSMA7,PSMB6,PSMB7,PSMB5,PSMB2,PSMB3,PSMB1,AP2S1,PSMC5,PSMC6,PSMC3,PSMC4,PSMC1,PSMC2,RUVBL1,AP2M1,RBX1,SFRP1,SFRP2,SNX3,CALM1,PSMD10,PSMD12,PSMD11,PSMD14,PSMD13,YWHAZ,UBA52,VPS26A,FZD5,SOX2,SOX4,SKP1,PFN1,RHOA,CTNNB1
Signaling by Hedgehog	0.0172	122	42	4.42E-09	5.30E-08	GNAS,UBB,UBC,PSMD8,PSMD6,PSMD7,PSMD4,PSMD2,PSMD3,PSMD1,PSME1,PSME2,PSMF1,PSMA5,PSMA3,PSMA4,PSMA1,PSMA2,PSMA7,PSMB6,PSMB7,PSMB5,PSMB2,PSMB3,PSMB1,PSMC5,PSMC6,PSMC3,PSMC4,PSMC1,PSMC2,RBX1,P4HB,PSMD10,PSMD12,VCP,PSMD11,PSMD14,PSMD13,UBA52,CDON,SKP1
TCF dependent signaling in response to WNT	0.0222	158	49	6.31E-09	7.58E-08	LGR4,CSNK2B,CAV1,TLE4,UBB,UBC,PSMD8,PSMD6,PSMD7,PSMD4,PSMD2,PSMD3,PSMD1,PSME1,PSME2,PSMF1,PSMA5,PSMA3,PSMA4,PSMA1,PSMA2,PSMA7,PSMB6,PSMB7,PSMB5,PSMB2,PSMB3,PSMB1,PSMC5,PSMC6,PSMC3,PSMC4,PSMC1,PSMC2,RUVBL1,RBX1,SFRP1,SFRP2,PSMD10,PSMD12,PSMD11,PSMD14,PSMD13,YWHAZ,UBA52,FZD5,SOX2,SOX4,CTNNB1
TCR signaling	0.0169	120	41	8.30E-09	9.96E-08	VASP,UBB,UBC,PSMD8,PSMD6,PSMD7,PSMD4,PSMD2,PSMD3,PSMD1,PSME1,PSME2,PSMF1,PSMA5,PSMA3,PSMA4,PSMA1,PSMA2,PSMA7,PSMB6,PSMB7,PSMB5,PSMB2,PSMB3,PSMB1,PSMC5,PSMC6,PSMC3,PSMC4,PSMC1,PSMC2,UBE2D2,UBE2D1,PSMD10,PSMD12,PSMD11,PSMD14,PSMD13,UBA52,SKP1,NFKBIA

M Phase	0.0316	225	61	1.23E-08	1.35E-07	NUP107,SET,CENPU,CENPK,CDC20,CSNK2B,NUP93,RAB2A,RAB1A,PPP2R1A,PPP2CA,LMNA,UBB,UBC,PSMD8,PSMD6,PSMD7,PSMD4,PSMD2,PSMD3,PSMD1,PSME1,PSME2,PSMF1,PSMA5,PSMA3,PSMA4,PSMA1,PSMA2,PSMA7,PSMB6,PSMB7,PSMB5,PSMB2,PSMB3,PSMB1,PSMC5,PSMC6,PSMC3,PSMC4,PSMC1,PSMC2,UBE2D1,UBE2E1,SEC13,MAD2L1,BANF1,PSMD10,PSMD12,PSMD11,PSMD14,PSMD13,CCNB2,CCNB1,GORASP2,UBA52,RAD21,NUDC,ZWINT,MAPRE1,PTTG1
Axon guidance	0.0631	449	98	2.92E-08	3.21E-07	DPYSL3,JAK1,DUSP6,ARPC4,ARPC5,ARPC2,ARPC3,ITGB1,ITGAV,ACTR3,ACTR2,CDC42,ACTB,CSNK2B,SDC2,RRAS,NRP2,RAC1,CAP1,CAP2,PEBP1,ACTG1,KRAS,ALCAM,ARPC1B,CFL1,MAP2K1,PHB,MYL12A,MYL12B,SPTBN1,VASP,NRCAM,UBB,UBC,DNM1,COL6A2,COL6A1,MYH9,PSMD8,PSMD6,PSMD7,PSMD4,PSMD2,PSMD3,PSMD1,MYL6,PSME1,PSME2,MYL9,PSMF1,PSMA5,PSMA3,PSMA4,PSMA1,PSMA2,PSMA7,PSMB6,PSMB7,PSMB5,PSMB2,PSMB3,PSMB1,AP2S1,PSMC5,PSMC6,PSMC3,PSMC4,PSMC1,PSMC2,AP2M1,RBX1,HSP90AB1,HSP90AA1,COL9A1,COL9A3,CALM1,PSMD10,PSMD12,PSMD11,PSMD14,PSMD13,PRNP,YWHAB,UBA52,CLTA,LAMTOR2,SEMA5A,SEMA6A,CDK5,EZR,RHOC,RHOA,COL4A2,COL4A1,SDCBP,MYH14,HSPA8
Formation of tubulin folding intermediates by CCT/TriC	0.0034	24	15	4.63E-07	5.09E-06	TUBB2B,TUBB2A,TCP1,TUBB4B,TUBA1C,TUBA1B,TUBA1A,CCT3,CCT2,CCT8,CCT7,CCT5,CCT4,TUBB6,CCT6A
TP53 Regulates Metabolic Genes	0.0101	72	27	5.14E-07	5.66E-06	DDIT4,COX7B,COX7C,COX8A,COX5B,COX5A,COX6C,COX6A1,COX6B1,TXN,COX4I1,NDUFA4,G6PD,CYCS,YWHAE,YWHAB,YWHAQ,YWHAH,YWHAZ,PRDX2,PRDX5,PRDX1,LAMTOR2,LAMTOR4,LAMTOR5,RHEB,RRAGA
Transcriptional Regulation by TP53	0.0101	72	27	5.14E-07	5.66E-06	DDIT4,COX7B,COX7C,COX8A,COX5B,COX5A,COX6C,COX6A1,COX6B1,TXN,COX4I1,NDUFA4,G6PD,CYCS,YWHAE,YWHAB,YWHAQ,YWHAH,YWHAZ,PRDX2,PRDX5,PRDX1,LAMTOR2,LAMTOR4,LAMTOR5,RHEB,RRAGA
Formation of ATP by chemiosmotic coupling	0.0025	18	13	5.52E-07	6.07E-06	ATP5C1,ATP5A1,ATP5J2,ATP5G3,ATP5F1,ATP5J,ATP5I,ATP5H,ATP5O,ATP5L,ATP5B,ATP5E,ATP5D

Signaling by Insulin receptor	0.0371	264	63	5.93E-07	6.53E-06	JAK1,DUSP6,EIF4E,ATP6V1E1,ATP6V1G1,ATP6V1C2,PEBP1,ATP6AP1,KRAS,MAP2K1,PHB,ATP6V0E1,ATP6V0D1,SPTBN1,UBB,UBC,PSMD8,PSMD6,PSMD7,PSMD4,PSMD2,PSMD3,PSMD1,PSME1,PSME2,PSMF1,PSMA5,PSMA3,PSMA4,PSMA1,PSMA2,PSMA7,PSMB6,PSMB7,PSMB5,PSMB2,PSMB3,PSMB1,PSMC5,PSMC6,PSMC3,PSMC4,PSMC1,PSMC2,RBX1,CALM1,PSMD10,PSMD12,PSMD11,PSMD14,PSMD13,YWHAB,UBA52,LAMTOR2,LAMTOR4,LAMTOR5,GRB10,RHEB,RRAGA,ATP6V0B,ATP6V1H,ATP6V1D,ATP6V1F
Membrane Trafficking	0.034	242	58	1.47E-06	1.62E-05	RAC1,NAPA,DYNC1I2,DYNC1H1,GJA1,TFG,AP1S2,VPS25,VPS28,COPZ1,TRAPPC3,RAB4A,TRAPPC1,TRAPPC4,CNIH1,RAB1A,RAB14,RAB13,FTH1,SPTBN1,TMED3,TMED2,TMED7,TMED9,RAB11A,TRAPPC2L,CHMP4B,COPB2,UBB,UBC,CHMP2A,COPE,SNF8,ARF4,ARF1,DYNLL1,VAMP8,AP2M1,VPS37A,KDELR1,CALM1,CTSC,SEC13,CD59,TMED10,YWHAE,YWHAB,YWHAQ,YWHAH,YWHAZ,DCTN6,DCTN2,DCTN3,UBA52,CLTA,APP,HSPA8,CHMP5
Folding of actin by CCT/TriC	0.0013	9	9	2.33E-06	2.33E-05	ACTB,TCP1,CCT3,CCT2,CCT8,CCT7,CCT5,CCT4,CCT6A
NCAM signaling for neurite out-growth	0.0314	223	54	2.53E-06	2.53E-05	JAK1,DUSP6,PEBP1,KRAS,MAP2K1,PHB,SPTBN1,UBB,UBC,COL6A2,COL6A1,PSMD8,PSMD6,PSMD7,PSMD4,PSMD2,PSMD3,PSMD1,PSME1,PSME2,PSMF1,PSMA5,PSMA3,PSMA4,PSMA1,PSMA2,PSMA7,PSMB6,PSMB7,PSMB5,PSMB2,PSMB3,PSMB1,PSMC5,PSMC6,PSMC3,PSMC4,PSMC1,PSMC2,RBX1,COL9A1,COL9A3,CALM1,PSMD10,PSMD12,PSMD11,PSMD14,PSMD13,PRNP,YWHAB,UBA52,LAMTOR2,COL4A2,COL4A1
Regulation of PLK1 Activity at G2/M Transition	0.0113	80	27	3.57E-06	3.57E-05	OPTN,ACTR1A,DYNC1I2,SSNA1,DYNC1H1,TUBB4B,CEP135,CEP152,PPP2R1A,TUBB,UBB,UBC,TUBA1A,DYNLL1,HSP90AA1,PPP1R12A,CETN2,SFI1,CCNB2,CCNB1,YWHAE,DCTN2,DCTN3,UBA52,SKP1,TUBG1,MAPRE1
Signalling to p38 via RIT and RIN	0.027	192	48	4.01E-06	4.01E-05	JAK1,DUSP6,RIT1,PEBP1,KRAS,MAP2K1,PHB,SPTBN1,UBB,UBC,PSMD8,PSMD6,PSMD7,PSMD4,PSMD2,PSMD3,PSMD1,PSME1,PSME2,PSMF1,PSMA5,PSMA3,PSMA4,PSMA1,PSMA2,PSMA7,PSMB6,PSMB7,PSMB5,PSMB2,PSMB3,PSMB1,PSMC5,PSMC6,PSMC3,PSMC4,PSMC1,PSMC2,RBX1,CALM1,PSMD10,PSMD12,PSMD11,PSMD14,PSMD13,YWHAB,UBA52,LAMTOR2

SOS-mediated signalling	0.0264	188	47	5.05E-06	4.54E-05	JAK1,DUSP6,PEBP1,KRAS,MAP2K1,PHB,SPTBN1,UBB,UBC,PSMD8,PSMD6,PSMD7,PSMD4,PSMD2,PSMD3,PSMD1,PSME1,PSME2,PSMF1,PSMA5,PSMA3,PSMA4,PSMA1,PSMA2,PSMA7,PSMB6,PSMB7,PSMB5,PSMB2,PSMB3,PSMB1,PSMC5,PSMC6,PSMC3,PSMC4,PSMC1,PSMC2,RBX1,CALM1,PSMD10,PSMD12,PSMD11,PSMD14,PSMD13,YWHAB,UBA52,LAMTOR2
GRB2 events in EGFR signaling	0.0264	188	47	5.05E-06	4.54E-05	JAK1,DUSP6,PEBP1,KRAS,MAP2K1,PHB,SPTBN1,UBB,UBC,PSMD8,PSMD6,PSMD7,PSMD4,PSMD2,PSMD3,PSMD1,PSME1,PSME2,PSMF1,PSMA5,PSMA3,PSMA4,PSMA1,PSMA2,PSMA7,PSMB6,PSMB7,PSMB5,PSMB2,PSMB3,PSMB1,PSMC5,PSMC6,PSMC3,PSMC4,PSMC1,PSMC2,RBX1,CALM1,PSMD10,PSMD12,PSMD11,PSMD14,PSMD13,YWHAB,UBA52,LAMTOR2
GRB2 events in ERBB2 signaling	0.0264	188	47	5.05E-06	4.54E-05	JAK1,DUSP6,PEBP1,KRAS,MAP2K1,PHB,SPTBN1,UBB,UBC,PSMD8,PSMD6,PSMD7,PSMD4,PSMD2,PSMD3,PSMD1,PSME1,PSME2,PSMF1,PSMA5,PSMA3,PSMA4,PSMA1,PSMA2,PSMA7,PSMB6,PSMB7,PSMB5,PSMB2,PSMB3,PSMB1,PSMC5,PSMC6,PSMC3,PSMC4,PSMC1,PSMC2,RBX1,CALM1,PSMD10,PSMD12,PSMD11,PSMD14,PSMD13,YWHAB,UBA52,LAMTOR2
SHC1 events in ERBB4 signaling	0.0264	188	47	5.05E-06	4.54E-05	JAK1,DUSP6,PEBP1,KRAS,MAP2K1,PHB,SPTBN1,UBB,UBC,PSMD8,PSMD6,PSMD7,PSMD4,PSMD2,PSMD3,PSMD1,PSME1,PSME2,PSMF1,PSMA5,PSMA3,PSMA4,PSMA1,PSMA2,PSMA7,PSMB6,PSMB7,PSMB5,PSMB2,PSMB3,PSMB1,PSMC5,PSMC6,PSMC3,PSMC4,PSMC1,PSMC2,RBX1,CALM1,PSMD10,PSMD12,PSMD11,PSMD14,PSMD13,YWHAB,UBA52,LAMTOR2
SHC1 events in ERBB2 signaling	0.0264	188	47	5.05E-06	4.54E-05	JAK1,DUSP6,PEBP1,KRAS,MAP2K1,PHB,SPTBN1,UBB,UBC,PSMD8,PSMD6,PSMD7,PSMD4,PSMD2,PSMD3,PSMD1,PSME1,PSME2,PSMF1,PSMA5,PSMA3,PSMA4,PSMA1,PSMA2,PSMA7,PSMB6,PSMB7,PSMB5,PSMB2,PSMB3,PSMB1,PSMC5,PSMC6,PSMC3,PSMC4,PSMC1,PSMC2,RBX1,CALM1,PSMD10,PSMD12,PSMD11,PSMD14,PSMD13,YWHAB,UBA52,LAMTOR2
SHC1 events in EGFR signaling	0.0264	188	47	5.05E-06	4.54E-05	JAK1,DUSP6,PEBP1,KRAS,MAP2K1,PHB,SPTBN1,UBB,UBC,PSMD8,PSMD6,PSMD7,PSMD4,PSMD2,PSMD3,PSMD1,PSME1,PSME2,PSMF1,PSMA5,PSMA3,PSMA4,PSMA1,PSMA2,PSMA7,PSMB6,PSMB7,PSMB5,PSMB2,PSMB3,PSMB1,PSMC5,PSMC6,PSMC3,PSMC4,PSMC1,PSMC2,RBX1,CALM1,PSMD10,PSMD12,PSMD11,PSMD14,PSMD13,YWHAB,UBA52,LAMTOR2

RAF/MAP kinase cascade	0.0264	188	47	5.05E-06	4.54E-05	JAK1,DUSP6,PEBP1,KRAS,MAP2K1,PHB,SPTBN1,UBB,UBC,PSMD8,PSMD6,PSMD7,PSMD4,PSMD2,PSMD3,PSMD1,PSME1,PSME2,PSMF1,PSMA5,PSMA3,PSMA4,PSMA1,PSMA2,PSMA7,PSMB6,PSMB7,PSMB5,PSMB2,PSMB3,PSMB1,PSMC5,PSMC6,PSMC3,PSMC4,PSMC1,PSMC2,RBX1,CALM1,PSMD10,PSMD12,PSMD11,PSMD14,PSMD13,YWHAB,UBA52,LAMTOR2
FRS-mediated FGFR2 signaling	0.0266	189	47	5.78E-06	5.20E-05	JAK1,DUSP6,PEBP1,KRAS,MAP2K1,PHB,SPTBN1,UBB,UBC,PSMD8,PSMD6,PSMD7,PSMD4,PSMD2,PSMD3,PSMD1,PSME1,PSME2,PSMF1,PSMA5,PSMA3,PSMA4,PSMA1,PSMA2,PSMA7,PSMB6,PSMB7,PSMB5,PSMB2,PSMB3,PSMB1,PSMC5,PSMC6,PSMC3,PSMC4,PSMC1,PSMC2,RBX1,CALM1,PSMD10,PSMD12,PSMD11,PSMD14,PSMD13,YWHAB,UBA52,LAMTOR2
FRS-mediated FGFR3 signaling	0.0266	189	47	5.78E-06	5.20E-05	JAK1,DUSP6,PEBP1,KRAS,MAP2K1,PHB,SPTBN1,UBB,UBC,PSMD8,PSMD6,PSMD7,PSMD4,PSMD2,PSMD3,PSMD1,PSME1,PSME2,PSMF1,PSMA5,PSMA3,PSMA4,PSMA1,PSMA2,PSMA7,PSMB6,PSMB7,PSMB5,PSMB2,PSMB3,PSMB1,PSMC5,PSMC6,PSMC3,PSMC4,PSMC1,PSMC2,RBX1,CALM1,PSMD10,PSMD12,PSMD11,PSMD14,PSMD13,YWHAB,UBA52,LAMTOR2
FRS-mediated FGFR4 signaling	0.0266	189	47	5.78E-06	5.20E-05	JAK1,DUSP6,PEBP1,KRAS,MAP2K1,PHB,SPTBN1,UBB,UBC,PSMD8,PSMD6,PSMD7,PSMD4,PSMD2,PSMD3,PSMD1,PSME1,PSME2,PSMF1,PSMA5,PSMA3,PSMA4,PSMA1,PSMA2,PSMA7,PSMB6,PSMB7,PSMB5,PSMB2,PSMB3,PSMB1,PSMC5,PSMC6,PSMC3,PSMC4,PSMC1,PSMC2,RBX1,CALM1,PSMD10,PSMD12,PSMD11,PSMD14,PSMD13,YWHAB,UBA52,LAMTOR2
FRS-mediated FGFR1 signaling	0.0266	189	47	5.78E-06	5.20E-05	JAK1,DUSP6,PEBP1,KRAS,MAP2K1,PHB,SPTBN1,UBB,UBC,PSMD8,PSMD6,PSMD7,PSMD4,PSMD2,PSMD3,PSMD1,PSME1,PSME2,PSMF1,PSMA5,PSMA3,PSMA4,PSMA1,PSMA2,PSMA7,PSMB6,PSMB7,PSMB5,PSMB2,PSMB3,PSMB1,PSMC5,PSMC6,PSMC3,PSMC4,PSMC1,PSMC2,RBX1,CALM1,PSMD10,PSMD12,PSMD11,PSMD14,PSMD13,YWHAB,UBA52,LAMTOR2
ARMS-mediated activation	0.0271	193	47	9.79E-06	8.82E-05	JAK1,DUSP6,PEBP1,KRAS,MAP2K1,PHB,SPTBN1,UBB,UBC,PSMD8,PSMD6,PSMD7,PSMD4,PSMD2,PSMD3,PSMD1,PSME1,PSME2,PSMF1,PSMA5,PSMA3,PSMA4,PSMA1,PSMA2,PSMA7,PSMB6,PSMB7,PSMB5,PSMB2,PSMB3,PSMB1,PSMC5,PSMC6,PSMC3,PSMC4,PSMC1,PSMC2,RBX1,CALM1,PSMD10,PSMD12,PSMD11,PSMD14,PSMD13,YWHAB,UBA52,LAMTOR2

MAPK1/MAPK3 signaling	0.0271	193	47	9.79E-06	8.82E-05	JAK1,DUSP6,PEBP1,KRAS,MAP2K1,PHB,SPTBN1,UBB,UBC,PSMD8,PSMD6,PSMD7,PSMD4,PSMD2,PSMD3,PSMD1,PSME1,PSME2,PSMF1,PSMA5,PSMA3,PSMA4,PSMA1,PSMA2,PSMA7,PSMB6,PSMB7,PSMB5,PSMB2,PSMB3,PSMB1,PSMC5,PSMC6,PSMC3,PSMC4,PSMC1,PSMC2,RBX1,CALM1,PSMD10,PSMD12,PSMD11,PSMD14,PSMD13,YWHAB,UBA52,LAMTOR2
Frs2-mediated activation	0.0273	194	47	1.11E-05	1.00E-04	JAK1,DUSP6,PEBP1,KRAS,MAP2K1,PHB,SPTBN1,UBB,UBC,PSMD8,PSMD6,PSMD7,PSMD4,PSMD2,PSMD3,PSMD1,PSME1,PSME2,PSMF1,PSMA5,PSMA3,PSMA4,PSMA1,PSMA2,PSMA7,PSMB6,PSMB7,PSMB5,PSMB2,PSMB3,PSMB1,PSMC5,PSMC6,PSMC3,PSMC4,PSMC1,PSMC2,RBX1,CALM1,PSMD10,PSMD12,PSMD11,PSMD14,PSMD13,YWHAB,UBA52,LAMTOR2
Metabolism of amino acids and derivatives	0.0374	266	59	1.20E-05	1.08E-04	ENOPH1,OAT,ODC1,SMS,AIMP1,PHGDH,EEF1E1,NDUFAB1,PDHA1,PAPSS1,CKB,PDHB,PSAT1,DARS,GRHPR,DCT,PSMD8,PSMD6,PSMD7,PSMD4,PSMD2,PSMD3,PSMD1,PSME1,PSME2,OAZ1,PSMF1,PSMA5,PSMA3,PSMA4,PSMA1,PSMA2,PSMA7,PSMB6,PSMB7,PSMB5,PSMB2,PSMB3,PSMB1,PSMC5,PSMC6,PSMC3,PSMC4,PSMC1,PSMC2,SAT1,SARS,GOT1,HSD17B10,PSMD10,PSMD12,PSMD11,PSMD14,PSMD13,UBA52,CDO1,NQO1,FAH,FAU
Interleukin receptor SHC signaling	0.0276	196	47	1.44E-05	1.15E-04	JAK1,DUSP6,PEBP1,KRAS,MAP2K1,PHB,SPTBN1,UBB,UBC,PSMD8,PSMD6,PSMD7,PSMD4,PSMD2,PSMD3,PSMD1,PSME1,PSME2,PSMF1,PSMA5,PSMA3,PSMA4,PSMA1,PSMA2,PSMA7,PSMB6,PSMB7,PSMB5,PSMB2,PSMB3,PSMB1,PSMC5,PSMC6,PSMC3,PSMC4,PSMC1,PSMC2,RBX1,CALM1,PSMD10,PSMD12,PSMD11,PSMD14,PSMD13,YWHAB,UBA52,LAMTOR2
Prolonged ERK activation events	0.0276	196	47	1.44E-05	1.15E-04	JAK1,DUSP6,PEBP1,KRAS,MAP2K1,PHB,SPTBN1,UBB,UBC,PSMD8,PSMD6,PSMD7,PSMD4,PSMD2,PSMD3,PSMD1,PSME1,PSME2,PSMF1,PSMA5,PSMA3,PSMA4,PSMA1,PSMA2,PSMA7,PSMB6,PSMB7,PSMB5,PSMB2,PSMB3,PSMB1,PSMC5,PSMC6,PSMC3,PSMC4,PSMC1,PSMC2,RBX1,CALM1,PSMD10,PSMD12,PSMD11,PSMD14,PSMD13,YWHAB,UBA52,LAMTOR2

Signaling by Leptin	0.0276	196	47	1.44E-05	1.15E-04	JAK1,DUSP6,PEBP1,KRAS,MAP2K1,PHB,SPTBN1,UBB,UBC,PSMD8,PSMD6,PSMD7,PSMD4,PSMD2,PSMD3,PSMD1,PSME1,PSME2,PSMF1,PSMA5,PSMA3,PSMA4,PSMA1,PSMA2,PSMA7,PSMB6,PSMB7,PSMB5,PSMB2,PSMB3,PSMB1,PSMC5,PSMC6,PSMC3,PSMC4,PSMC1,PSMC2,RBX1,CALM1,PSMD10,PSMD12,PSMD11,PSMD14,PSMD13,YWHAB,UBA52,LAMTOR2
Oxygen-dependent proline hydroxylation of Hypoxia-inducible Factor Alpha	0.0025	18	11	1.88E-05	1.50E-04	EGLN3,TCEB2,TCEB1,UBB,UBC,UBE2D2,UBE2D3,UBE2D1,RBX1,UBA52,HIF1A
Association of TriC/CCT with target proteins during biosynthesis	0.0013	9	8	1.95E-05	1.56E-04	TCP1,CCT3,CCT2,CCT8,CCT7,CCT5,CCT4,CCT6A
Downstream signaling events of B Cell Receptor (BCR)	0.0232	165	41	2.29E-05	1.83E-04	KRAS,CDKN1A,TNRC6B,UBB,UBC,PSMD8,PSMD6,PSMD7,PSMD4,PSMD2,PSMD3,PSMD1,PSME1,PSME2,PSMF1,PSMA5,PSMA3,PSMA4,PSMA1,PSMA2,PSMA7,PSMB6,PSMB7,PSMB5,PSMB2,PSMB3,PSMB1,PSMC5,PSMC6,PSMC3,PSMC4,PSMC1,PSMC2,PSMD10,PSMD12,PSMD11,PSMD14,PSMD13,UBA52,SKP1,NFKBIA
Signalling to RAS	0.0281	200	47	2.35E-05	1.88E-04	JAK1,DUSP6,PEBP1,KRAS,MAP2K1,PHB,SPTBN1,UBB,UBC,PSMD8,PSMD6,PSMD7,PSMD4,PSMD2,PSMD3,PSMD1,PSME1,PSME2,PSMF1,PSMA5,PSMA3,PSMA4,PSMA1,PSMA2,PSMA7,PSMB6,PSMB7,PSMB5,PSMB2,PSMB3,PSMB1,PSMC5,PSMC6,PSMC3,PSMC4,PSMC1,PSMC2,RBX1,CALM1,PSMD10,PSMD12,PSMD11,PSMD14,PSMD13,YWHAB,UBA52,LAMTOR2
Detoxification of Reactive Oxygen Species	0.0037	26	13	2.72E-05	2.18E-04	GSTP1,TXN,ERO1A,ATOX1,P4HB,CYCS,SOD1,PRDX3,PRDX2,PRDX5,PRDX1,PRDX6,GPX1
VEGFR2 mediated cell proliferation	0.0284	202	47	2.99E-05	2.39E-04	JAK1,DUSP6,PEBP1,KRAS,MAP2K1,PHB,SPTBN1,UBB,UBC,PSMD8,PSMD6,PSMD7,PSMD4,PSMD2,PSMD3,PSMD1,PSME1,PSME2,PSMF1,PSMA5,PSMA3,PSMA4,PSMA1,PSMA2,PSMA7,PSMB6,PSMB7,PSMB5,PSMB2,PSMB3,PSMB1,PSMC5,PSMC6,PSMC3,PSMC4,PSMC1,PSMC2,RBX1,CALM1,PSMD10,PSMD12,PSMD11,PSMD14,PSMD13,YWHAB,UBA52,LAMTOR2

Interleukin-2 signaling	0.0284	202	47	2.99E-05	2.39E-04	JAK1,DUSP6,PEBP1,KRAS,MAP2K1,PHB,SPTBN1,UBB,UBC,PSMD8,PSMD6,PSMD7,PSMD4,PSMD2,PSMD3,PSMD1,PSME1,PSME2,PSMF1,PSMA5,PSMA3,PSMA4,PSMA1,PSMA2,PSMA7,PSMB6,PSMB7,PSMB5,PSMB2,PSMB3,PSMB1,PSMC5,PSMC6,PSMC3,PSMC4,PSMC1,PSMC2,RBX1,CALM1,PSMD10,PSMD12,PSMD11,PSMD14,PSMD13,YWHAB,UBA52,LAMTOR2
Signalling to ERKs	0.0293	208	48	3.01E-05	2.41E-04	JAK1,DUSP6,RIT1,PEBP1,KRAS,MAP2K1,PHB,SPTBN1,UBB,UBC,PSMD8,PSMD6,PSMD7,PSMD4,PSMD2,PSMD3,PSMD1,PSME1,PSME2,PSMF1,PSMA5,PSMA3,PSMA4,PSMA1,PSMA2,PSMA7,PSMB6,PSMB7,PSMB5,PSMB2,PSMB3,PSMB1,PSMC5,PSMC6,PSMC3,PSMC4,PSMC1,PSMC2,RBX1,CALM1,PSMD10,PSMD12,PSMD11,PSMD14,PSMD13,YWHAB,UBA52,LAMTOR2
Interleukin-3, 5 and GM-CSF signaling	0.0293	208	48	3.01E-05	2.41E-04	JAK1,DUSP6,PEBP1,KRAS,MAP2K1,PHB,SPTBN1,UBB,UBC,PSMD8,PSMD6,PSMD7,PSMD4,PSMD2,PSMD3,PSMD1,PSME1,PSME2,PSMF1,PSMA5,PSMA3,PSMA4,PSMA1,PSMA2,PSMA7,PSMB6,PSMB7,PSMB5,PSMB2,PSMB3,PSMB1,PSMC5,PSMC6,PSMC3,PSMC4,PSMC1,PSMC2,RBX1,CALM1,PSMD10,PSMD12,PSMD11,PSMD14,PSMD13,YWHAB,YWHAZ,UBA52,LAMTOR2
Insulin receptor signalling cascade	0.0338	240	53	3.71E-05	2.89E-04	JAK1,DUSP6,EIF4E,PEBP1,KRAS,MAP2K1,PHB,SPTBN1,UBB,UBC,PSMD8,PSMD6,PSMD7,PSMD4,PSMD2,PSMD3,PSMD1,PSME1,PSME2,PSMF1,PSMA5,PSMA3,PSMA4,PSMA1,PSMA2,PSMA7,PSMB6,PSMB7,PSMB5,PSMB2,PSMB3,PSMB1,PSMC5,PSMC6,PSMC3,PSMC4,PSMC1,PSMC2,RBX1,CALM1,PSMD10,PSMD12,PSMD11,PSMD14,PSMD13,YWHAB,UBA52,LAMTOR2,LAMTOR4,LAMTOR5,GRB10,RHEB,RRAGA
RHO GTPases activate PKNs	0.0049	35	15	3.91E-05	2.89E-04	PKN2,MYL12B,MYH9,MYL6,MYL9,PPP1R12A,YWHAE,YWHAB,YWHAQ,YWHAH,YWHAZ,H3F3A,RHOC,RHOA,MYH14
IRS-related events triggered by IGF1R	0.0339	241	53	4.13E-05	2.89E-04	JAK1,DUSP6,EIF4E,IGF2,PEBP1,KRAS,MAP2K1,PHB,SPTBN1,UBB,UBC,PSMD8,PSMD6,PSMD7,PSMD4,PSMD2,PSMD3,PSMD1,PSME1,PSME2,PSMF1,PSMA5,PSMA3,PSMA4,PSMA1,PSMA2,PSMA7,PSMB6,PSMB7,PSMB5,PSMB2,PSMB3,PSMB1,PSMC5,PSMC6,PSMC3,PSMC4,PSMC1,PSMC2,RBX1,CALM1,PSMD10,PSMD12,PSMD11,PSMD14,PSMD13,YWHAB,UBA52,LAMTOR2,LAMTOR4,LAMTOR5,RHEB,RRAGA

Signaling by Type 1 Insulin-like Growth Factor 1 Receptor (IGF1R)	0.0339	241	53	4.13E-05	2.89E-04	JAK1,DUSP6,EIF4E,IGF2,PEBP1,KRAS,MAP2K1,PHB,SPTBN1,UBB,UBC,PSMD8,PSMD6,PSMD7,PSMD4,PSMD2,PSMD3,PSMD1,PSME1,PSME2,PSMF1,PSMA5,PSMA3,PSMA4,PSMA1,PSMA2,PSMA7,PSMB6,PSMB7,PSMB5,PSMB2,PSMB3,PSMB1,PSMC5,PSMC6,PSMC3,PSMC4,PSMC1,PSMC2,RBX1,CALM1,PSMD10,PSMD12,PSMD11,PSMD14,PSMD13,YWHAB,UBA52,LAMTOR2,LAMTOR4,LAMTOR5,RHEB,RRAGA
IGF1R signaling cascade	0.0339	241	53	4.13E-05	2.89E-04	JAK1,DUSP6,EIF4E,IGF2,PEBP1,KRAS,MAP2K1,PHB,SPTBN1,UBB,UBC,PSMD8,PSMD6,PSMD7,PSMD4,PSMD2,PSMD3,PSMD1,PSME1,PSME2,PSMF1,PSMA5,PSMA3,PSMA4,PSMA1,PSMA2,PSMA7,PSMB6,PSMB7,PSMB5,PSMB2,PSMB3,PSMB1,PSMC5,PSMC6,PSMC3,PSMC4,PSMC1,PSMC2,RBX1,CALM1,PSMD10,PSMD12,PSMD11,PSMD14,PSMD13,YWHAB,UBA52,LAMTOR2,LAMTOR4,LAMTOR5,RHEB,RRAGA
VEGFA-VEGFR2 Pathway	0.0374	266	57	4.29E-05	3.00E-04	JAK1,DUSP6,ITGAV,CDC42,RAC1,PEBP1,CAV1,KRAS,MAP2K1,PHB,SPTBN1,UBB,UBC,PSMD8,PSMD6,PSMD7,PSMD4,PSMD2,PSMD3,PSMD1,PSME1,PSME2,PSMF1,PSMA5,PSMA3,PSMA4,PSMA1,PSMA2,PSMA7,PSMB6,PSMB7,PSMB5,PSMB2,PSMB3,PSMB1,PSMC5,PSMC6,PSMC3,PSMC4,PSMC1,PSMC2,RBX1,HSP90AA1,CALM1,CYBA,PSMD10,PSMD12,PSMD11,PSMD14,PSMD13,YWHAB,UBA52,LAMTOR2,BRK1,RHOA,CTNNA1,HSPB1
Signaling by VEGF	0.0385	274	58	5.11E-05	3.58E-04	JAK1,DUSP6,ITGAV,CDC42,NRP2,RAC1,PEBP1,CAV1,KRAS,MAP2K1,PHB,SPTBN1,UBB,UBC,PSMD8,PSMD6,PSMD7,PSMD4,PSMD2,PSMD3,PSMD1,PSME1,PSME2,PSMF1,PSMA5,PSMA3,PSMA4,PSMA1,PSMA2,PSMA7,PSMB6,PSMB7,PSMB5,PSMB2,PSMB3,PSMB1,PSMC5,PSMC6,PSMC3,PSMC4,PSMC1,PSMC2,RBX1,HSP90AA1,CALM1,CYBA,PSMD10,PSMD12,PSMD11,PSMD14,PSMD13,YWHAB,UBA52,LAMTOR2,BRK1,RHOA,CTNNA1,HSPB1
IRS-mediated signalling	0.0333	237	52	5.16E-05	3.61E-04	JAK1,DUSP6,EIF4E,PEBP1,KRAS,MAP2K1,PHB,SPTBN1,UBB,UBC,PSMD8,PSMD6,PSMD7,PSMD4,PSMD2,PSMD3,PSMD1,PSME1,PSME2,PSMF1,PSMA5,PSMA3,PSMA4,PSMA1,PSMA2,PSMA7,PSMB6,PSMB7,PSMB5,PSMB2,PSMB3,PSMB1,PSMC5,PSMC6,PSMC3,PSMC4,PSMC1,PSMC2,RBX1,CALM1,PSMD10,PSMD12,PSMD11,PSMD14,PSMD13,YWHAB,UBA52,LAMTOR2,LAMTOR4,LAMTOR5,RHEB,RRAGA

DNA Damage Recognition in GG-NER	0.0051	36	15	5.35E-05	3.74E-04	DDB2,ACTB,COPS4,COPS6,COPS5,COPS8,UBB,UBC,ACTL6A,RUVBL1,RBX1,RAD23B,CETN2,UBA52,GPS1
MAPK family signaling cascades	0.0326	232	51	5.80E-05	4.06E-04	JAK1,DUSP6,CDC42,RAC1,PEBP1,KRAS,MAP2K1,PHB,TNRC6B,SPTBN1,UBB,UBC,PSMD8,PSMD6,PSMD7,PSMD4,PSMD2,PSMD3,PSMD1,PSME1,PSME2,PSMF1,PSMA5,PSMA3,PSMA4,PSMA1,PSMA2,PSMA7,PSMB6,PSMB7,PSMB5,PSMB2,PSMB3,PSMB1,PSMC5,PSMC6,PSMC3,PSMC4,PSMC1,PSMC2,RBX1,CALM1,PSMD10,PSMD12,PSMD11,PSMD14,PSMD13,YWHAB,UBA52,LAMTOR2,HSPB1
Smooth Muscle Contraction	0.0046	33	14	7.78E-05	5.44E-04	ACTA2,TPM4,TPM3,TPM2,TPM1,MYL12A,MYL12B,MYL6,MYL9,CALM1,CALD1,ANXA6,ANXA1,ANXA2
Cellular response to hypoxia	0.0037	26	12	1.15E-04	8.05E-04	EGLN3,TCEB2,TCEB1,CITED2,UBB,UBC,UBE2D2,UBE2D3,UBE2D1,RBX1,UBA52,HIF1A
Regulation of Hypoxia-inducible Factor (HIF) by oxygen	0.0037	26	12	1.15E-04	8.05E-04	EGLN3,TCEB2,TCEB1,CITED2,UBB,UBC,UBE2D2,UBE2D3,UBE2D1,RBX1,UBA52,HIF1A
Platelet degranulation	0.011	78	23	1.35E-04	9.45E-04	FN1,ACTN1,ACTN4,IGF2,CAP1,PPIA,CD9,TIMP1,CLU,CFL1,LAMP2,WDR1,CALM1,CD63,SOD1,SERPINE1,TMSB4X,PSAP,APP,SPARC,PFN1,ALDOA,HSPA5
Chk1/Chk2(Cds1) mediated inactivation of Cyclin B:Cdk1 complex	0.0017	12	8	1.42E-04	9.91E-04	WEE1,CCNB1,YWHAE,YWHAB,YWHAQ,YWHAH,YWHAZ,CHEK1
EPH-Ephrin signaling	0.0125	89	25	1.48E-04	1.03E-03	ARPC4,ARPC5,ARPC2,ARPC3,ACTR3,ACTR2,CDC42,ACTB,SDC2,RAC1,ACTG1,ARPC1B,CFL1,MYL12A,MYL12B,DNM1,MYH9,MYL6,MYL9,AP2S1,AP2M1,HSP90AA1,RHOA,SDCBP,MYH14
Mitochondrial translation elongation	0.0118	84	24	1.55E-04	1.09E-03	GADD45GIP1,MRPS15,MRPS23,MRPS35,MRPS36,TUFM,MRPL17,MRPL12,MRPL28,MRPL21,MRPL22,MRPL34,MRPL32,MRPL33,MRPL40,MRPL47,MRPL48,MRPL43,MRPL52,MRPL51,MRPL3,MRPL9,MRPS6,AURKAIP1
Cell-extracellular matrix interactions	0.0023	16	9	1.95E-04	1.36E-03	LIMS1,ITGB1,ACTN1,VASP,PARVA,PARVB,RSU1,FBLIM1,ILK
Glucose metabolism	0.0098	70	21	2.07E-04	1.45E-03	PGK1,PGM1,GAPDH,TPI1,PKM,UBB,UBC,HK1,PGAM1,MDH1,MDH2,GOT1,SLC25A11,CALM1,ENO1,ENO2,UBA52,GYG1,PFKL,PFKP,ALDOA
Pyruvate metabolism and Citric Acid (TCA) cycle	0.0065	46	16	2.36E-04	1.65E-03	ACO2,SUCLA2,BSG,SDHC,SDHB,PDHA1,LDHB,LDHA,IDH3B,PDHB,IDH3G,SUCLG1,FH,MDH2,SLC16A3,GLO1

G2/M Transition	0.0153	109	28	2.59E-04	1.81E-03	OPTN,ACTR1A,DYNC1I2,SSNA1,DYNC1H1,TUBB4B,CEP135,CEP152,PPP2R1A,TUBB,UBB,UBC,WEE1,TUBA1A,DYNLL1,HSP90AA1,PPP1R12A,CETN2,SFI1,CCNB2,CCNB1,YWHAE,DCTN2,DCTN3,UBA52,SKP1,TUBG1,MAPRE1
Loss of proteins required for interphase microtubule organizationÃ,Â from the centrosome	0.0087	62	19	3.14E-04	1.93E-03	ACTR1A,DYNC1I2,SSNA1,DYNC1H1,TUBB4B,CEP135,CEP152,PPP2R1A,TUBB,TUBA1A,DYNLL1,HSP90AA1,CETN2,SFI1,YWHAE,DCTN2,DCTN3,TUBG1,MAPRE1
Loss of Nlp from mitotic centrosomes	0.0087	62	19	3.14E-04	1.93E-03	ACTR1A,DYNC1I2,SSNA1,DYNC1H1,TUBB4B,CEP135,CEP152,PPP2R1A,TUBB,TUBA1A,DYNLL1,HSP90AA1,CETN2,SFI1,YWHAE,DCTN2,DCTN3,TUBG1,MAPRE1
Response to elevated platelet cytosolic Ca2+	0.0117	83	23	3.21E-04	1.93E-03	FN1,ACTN1,ACTN4,IGF2,CAP1,PPIA,CD9,TIMP1,CLU,CFL1,LAMP2,WDR1,CALM1,CD63,SOD1,SERPINE1,TMSB4X,PSAP,APP,SPARC,PFN1,ALDOA,HSPA5
Mitochondrial translation initiation	0.0117	83	23	3.21E-04	1.93E-03	GADD45GIP1,MRPS15,MRPS23,MRPS35,MRPS36,MRPL17,MRPL12,MRPL28,MRPL21,MRPL22,MRPL34,MRPL32,MRPL33,MRPL40,MRPL47,MRPL48,MRPL43,MRPL52,MRPL51,MRPL3,MRPL9,MRPS6,AURKAIP1
Mitotic G2-G2/M phases	0.0156	111	28	3.44E-04	2.06E-03	OPTN,ACTR1A,DYNC1I2,SSNA1,DYNC1H1,TUBB4B,CEP135,CEP152,PPP2R1A,TUBB,UBB,UBC,WEE1,TUBA1A,DYNLL1,HSP90AA1,PPP1R12A,CETN2,SFI1,CCNB2,CCNB1,YWHAE,DCTN2,DCTN3,UBA52,SKP1,TUBG1,MAPRE1
Mitochondrial translation	0.0125	89	24	3.54E-04	2.12E-03	GADD45GIP1,MRPS15,MRPS23,MRPS35,MRPS36,TUFM,MRPL17,MRPL12,MRPL28,MRPL21,MRPL22,MRPL34,MRPL32,MRPL33,MRPL40,MRPL47,MRPL48,MRPL43,MRPL52,MRPL51,MRPL3,MRPL9,MRPS6,AURKAIP1
Mitochondrial translation termination	0.0118	84	23	3.78E-04	2.27E-03	GADD45GIP1,MRPS15,MRPS23,MRPS35,MRPS36,MRPL17,MRPL12,MRPL28,MRPL21,MRPL22,MRPL34,MRPL32,MRPL33,MRPL40,MRPL47,MRPL48,MRPL43,MRPL52,MRPL51,MRPL3,MRPL9,MRPS6,AURKAIP1
Citric acid cycle (TCA cycle)	0.0025	18	9	4.54E-04	2.73E-03	ACO2,SUCLA2,SDHC,SDHB,IDH3B,IDH3G,SUCLG1,FH,MDH2
Centrosome maturation	0.0098	70	20	5.29E-04	3.17E-03	ACTR1A,DYNC1I2,SSNA1,DYNC1H1,TUBB4B,CEP135,CEP152,PPP2R1A,TUBB,TUBA1A,DYNLL1,HSP90AA1,CETN2,SFI1,CCNB1,YWHAE,DCTN2,DCTN3,TUBG1,MAPRE1

Recruitment of mitotic centrosome proteins and complexes	0.0098	70	20	5.29E-04	3.17E-03	ACTR1A,DYNC1I2,SSNA1,DYNC1H1,TUBB4B,CEP135,CEP152,PPP2R1A,TUBB,TUBA1A,DYNLL1,HSP90AA1,CETN2,SFI1,CCNB1,YWHAE,DCTN2,DCTN3,TUBG1,MAPRE1
Glycolysis	0.0038	27	11	6.12E-04	3.67E-03	PGK1,GAPDH,TPI1,PKM,HK1,PGAM1,ENO1,ENO2,PFKL,PFKP,ALDOA
COPI-mediated anterograde transport	0.01	71	20	6.29E-04	3.77E-03	NAPA,DYNC1I2,DYNC1H1,COPZ1,RAB1A,SPTBN1,TMED3,TMED2,TMED7,TMED9,COPB2,COPE,ARF4,DYNLL1,KDELR1,CD59,TMED10,DCTN6,DCTN2,DCTN3
EPHB-mediated forward signaling	0.0058	41	14	6.74E-04	4.04E-03	ARPC4,ARPC5,ARPC2,ARPC3,ACTR3,ACTR2,CDC42,ACTB,SDC2,RAC1,ACTG1,ARPC1B,CFL1,RHOA
Collagen biosynthesis and modifying enzymes	0.0086	61	18	6.85E-04	4.11E-03	COL3A1,PPIB,COL2A1,P3H2,COL18A1,COLGALT2,COL6A2,COL6A1,COL1A1,COL11A1,P4HB,COL9A1,COL9A3,COL5A1,SERPINH1,COL8A1,COL4A2,COL4A1
Signaling by SCF-KIT	0.0373	265	52	7.03E-04	4.22E-03	JAK1,DUSP6,RAC1,PEBP1,KRAS,CDKN1A,MAP2K1,PHB,TNRC6B,SPTBN1,UBB,UBC,PSMD8,PSMD6,PSMD7,PSMD4,PSMD2,PSMD3,PSMD1,PSME1,PSME2,PSMF1,PSMA5,PSMA3,PSMA4,PSMA1,PSMA2,PSMA7,PSMB6,PSMB7,PSMB5,PSMB2,PSMB3,PSMB1,PSMC5,PSMC6,PSMC3,PSMC4,PSMC1,PSMC2,RBX1,CALM1,PSMD10,PSMD12,PSMD11,PSMD14,PSMD13,YWHAB,UBA52,LAMTOR2,GRB10,CHEK1
RHO GTPases Activate WASPs and WAVEs	0.0045	32	12	7.23E-04	4.34E-03	ARPC4,ARPC5,ARPC2,ARPC3,ACTR3,ACTR2,CDC42,ACTB,RAC1,ACTG1,ARPC1B,BRK1
Collagen formation	0.0101	72	20	7.44E-04	4.46E-03	COL3A1,LOXL1,PPIB,COL2A1,P3H2,COL18A1,COLGALT2,COL6A2,COL6A1,COL1A1,COL11A1,P4HB,COL9A1,COL9A3,COL5A1,SERPINH1,CD151,COL8A1,COL4A2,COL4A1
ER to Golgi Anterograde Transport	0.0165	117	28	7.63E-04	4.58E-03	NAPA,DYNC1I2,DYNC1H1,TFG,COPZ1,TRAPPC3,TRAPPC1,TRAPPC4,CNIH1,RAB1A,SPTBN1,TMED3,TMED2,TMED7,TMED9,TRAPPC2L,COPB2,COPE,ARF4,DYNLL1,KDELR1,CTSC,SEC13,CD59,TMED10,DCTN6,DCTN2,DCTN3
Asparagine N-linked glycosylation	0.0267	190	40	8.01E-04	4.81E-03	NEU1,CALR,NANS,CANX,NAPA,DYNC1I2,DYNC1H1,TFG,COPZ1,TRAPPC3,TRAPPC1,TRAPPC4,CNIH1,DAD1,RAB1A,SPTBN1,TMED3,TMED2,TMED7,TMED9,TRAPPC2L,COPB2,COPE,ARF4,DYNLL1,DDOST,KDELR1,CTSC,SEC13,CD59,TMED10,PRKCSH,DCTN6,DCTN2,DCTN3,RPN2,TUSC3,CMAS,PDIA3,DPM1

RHO GTPase Effectors	0.0295	210	43	8.91E-04	5.35E-03	NUP107,CENPU,CENPK,PKN2,ARPC4,ARPC5,ARPC2,ARPC3,ITGB1,ACTR3,ACTR2,CDC42,ACTB,CDC20,RAC1,TAX1BP3,ACTG1,ARPC1B,MYL12B,MYH9,MYL6,MYL9,PPP1R12A,CALM1,SEC13,MAD2L1,CYBA,YWHAE,YWHAB,YWHAQ,YWHAH,YWHAZ,H3F3A,NUDC,BRK1,ZWINT,RHOC,RHOD,RHOA,MAPRE1,CTNNA1,CTNNB1,MYH14
RHO GTPases Activate ROCKs	0.0023	16	8	9.27E-04	5.56E-03	MYL12B,MYH9,MYL6,MYL9,PPP1R12A,RHOC,RHOA,MYH14
Assembly of collagen fibrils and other multimeric structures	0.0055	39	13	1.28E-03	7.65E-03	COL3A1,LOXL1,COL2A1,COL18A1,COL6A2,COL6A1,COL1A1,COL11A1,COL5A1,CD151,COL8A1,COL4A2,COL4A1
RHO GTPases activate PAKs	0.003	21	9	1.32E-03	7.94E-03	CDC42,RAC1,MYL12B,MYH9,MYL6,MYL9,PPP1R12A,CALM1,MYH14
Generic Transcription Pathway	0.0311	221	44	1.34E-03	8.03E-03	DDIT4,NR2F1,COX7B,COX7C,COX8A,COX5B,COX5A,COX6C,COX6A1,COX6B1,TGIF1,NR1H2,TFDP1,TXN,UBB,UBC,UBE2D3,UBE2D1,CTGF,COX4I1,NDUFA4,SNW1,G6PD,CYCS,YWHAE,CHD9,YWHAB,YWHAQ,YWHAH,YWHAZ,MAML2,SERPINE1,JUNB,MED4,UBA52,PRDX2,PRDX5,PRDX1,LAMTOR2,LAMTOR4,LAMTOR5,RHEB,RRAGA,SMAD2
Signaling by PDGF	0.0425	302	56	1.59E-03	9.04E-03	JAK1,DUSP6,SPP1,PEBP1,KRAS,CDKN1A,MAP2K1,PHB,TNRC6B,SPTBN1,UBB,UBC,COL6A2,COL6A1,PSMD8,PSMD6,PSMD7,PSMD4,PSMD2,PSMD3,PSMD1,PSME1,PSME2,PSMF1,PSMA5,PSMA3,PSMA4,PSMA1,PSMA2,PSMA7,PSMB6,PSMB7,PSMB5,PSMB2,PSMB3,PSMB1,PSMC5,PSMC6,PSMC3,PSMC4,PSMC1,PSMC2,RBX1,COL9A1,COL9A3,CALM1,PSMD10,PSMD12,PSMD11,PSMD14,PSMD13,YWHAB,UBA52,LAMTOR2,COL4A2,COL4A1
Attenuation phase	0.0014	10	6	1.62E-03	9.04E-03	FKBP4,PTGES3,HSP90AB1,HSP90AA1,HSBP1,HSPA8
Insulin receptor recycling	0.0037	26	10	1.62E-03	9.04E-03	ATP6V1E1,ATP6V1G1,ATP6V1C2,ATP6AP1,ATP6V0E1,ATP6V0D1,ATP6V0B,ATP6V1H,ATP6V1D,ATP6V1F
Post-chaperonin tubulin folding pathway	0.0031	22	9	1.81E-03	9.04E-03	TUBB2B,TUBB2A,TUBB4B,TUBA1C,TUBA1B,TUBA1A,TUBB6,TBCB,TBCA
Gluconeogenesis	0.0044	31	11	1.82E-03	9.09E-03	PGK1,GAPDH,TPI1,PGAM1,MDH1,MDH2,GOT1,SLC25A11,ENO1,ENO2,ALDOA
Transferrin endocytosis and recycling	0.0038	27	10	2.12E-03	0.0106	ATP6V1E1,ATP6V1G1,ATP6V1C2,ATP6AP1,ATP6V0E1,ATP6V0D1,ATP6V0B,ATP6V1H,ATP6V1D,ATP6V1F
Antigen Presentation: Folding, assembly and peptide loading of class I MHC	0.0032	23	9	2.43E-03	0.0121	CALR,CANX,HLA-B,HLA-C,HLA-A,HLA-E,SEC13,B2M,PDIA3

mRNA Splicing - Major Pathway	0.0179	127	28	2.46E-03	0.0123	SRSF4,SRSF5,FUS,BCAS2,HNRNPA0,HNRNPA3,SNRPD3,HNRNPA2B1,PABPN1,HNRNPM,HNRNPK,HNRNPF,SNRPG,SNRPB,DNAJC8,POLR2E,POLR2G,POLR2L,MAGOH,HNRNPH2,CTNNBL1,PCBP1,RBM8A,SNU13,SF3B5,SF3B6,SNRPB2,HSPA8
Signaling by FGFR3	0.0387	275	51	2.51E-03	0.0126	JAK1,DUSP6,PEBP1,KRAS,CDKN1A,MAP2K1,PHB,TNRC6B,PPP2R1A,SPTBN1,PPP2CA,UBB,UBC,PSMD8,PSMD6,PSMD7,PSMD4,PSMD2,PSMD3,PSMD1,PSME1,PSME2,PSMF1,PSMA5,PSMA3,PSMA4,PSMA1,PSMA2,PSMA7,PSMB6,PSMB7,PSMB5,PSMB2,PSMB3,PSMB1,PSMC5,PSMC6,PSMC3,PSMC4,PSMC1,PSMC2,RBX1,CALM1,PSMD10,PSMD12,PSMD11,PSMD14,PSMD13,YWHAB,UBA52,LAMTOR2
Signaling by FGFR4	0.0387	275	51	2.51E-03	0.0126	JAK1,DUSP6,PEBP1,KRAS,CDKN1A,MAP2K1,PHB,TNRC6B,PPP2R1A,SPTBN1,PPP2CA,UBB,UBC,PSMD8,PSMD6,PSMD7,PSMD4,PSMD2,PSMD3,PSMD1,PSME1,PSME2,PSMF1,PSMA5,PSMA3,PSMA4,PSMA1,PSMA2,PSMA7,PSMB6,PSMB7,PSMB5,PSMB2,PSMB3,PSMB1,PSMC5,PSMC6,PSMC3,PSMC4,PSMC1,PSMC2,RBX1,CALM1,PSMD10,PSMD12,PSMD11,PSMD14,PSMD13,YWHAB,UBA52,LAMTOR2
Signaling by ERBB4	0.0378	269	50	2.64E-03	0.0132	JAK1,DUSP6,PEBP1,KRAS,CDKN1A,MAP2K1,PHB,TNRC6B,SPTBN1,UBB,UBC,PSMD8,PSMD6,PSMD7,PSMD4,PSMD2,PSMD3,PSMD1,PSME1,PSME2,PSMF1,PSMA5,PSMA3,PSMA4,PSMA1,PSMA2,PSMA7,PSMB6,PSMB7,PSMB5,PSMB2,PSMB3,PSMB1,PSMC5,PSMC6,PSMC3,PSMC4,PSMC1,PSMC2,RBX1,CALM1,PSMD10,PSMD12,PSMD11,PSMD14,PSMD13,YWHAB,UBA52,LAMTOR2,SKP1
Scavenging by Class A Receptors	0.0027	19	8	2.68E-03	0.0134	COL3A1,HSP90B1,APOE,CALR,FTH1,COL1A1,COL4A2,COL4A1
Signaling by FGFR1	0.0388	276	51	2.70E-03	0.0135	JAK1,DUSP6,PEBP1,KRAS,CDKN1A,MAP2K1,PHB,TNRC6B,PPP2R1A,SPTBN1,PPP2CA,UBB,UBC,PSMD8,PSMD6,PSMD7,PSMD4,PSMD2,PSMD3,PSMD1,PSME1,PSME2,PSMF1,PSMA5,PSMA3,PSMA4,PSMA1,PSMA2,PSMA7,PSMB6,PSMB7,PSMB5,PSMB2,PSMB3,PSMB1,PSMC5,PSMC6,PSMC3,PSMC4,PSMC1,PSMC2,RBX1,CALM1,PSMD10,PSMD12,PSMD11,PSMD14,PSMD13,YWHAB,UBA52,LAMTOR2
Signaling by FGFR2	0.0388	276	51	2.70E-03	0.0135	JAK1,DUSP6,PEBP1,KRAS,CDKN1A,MAP2K1,PHB,TNRC6B,PPP2R1A,SPTBN1,PPP2CA,UBB,UBC,PSMD8,PSMD6,PSMD7,PSMD4,PSMD2,PSMD3,PSMD1,PSME1,PSME2,PSMF1,PSMA5,PSMA3,PSMA4,PSMA1,PSMA2,PSMA7,PSMB6,PSMB7,PSMB5,PSMB2,PSMB3,PSMB1,PSMC5,PSMC6,PSMC3,PSMC4,PSMC1,PSMC2,RBX1,CALM1,PSMD10,PSMD12,PSMD11,PSMD14,PSMD13,YWHAB,UBA52,LAMTOR2

mRNA Splicing	0.0188	134	29	2.70E-03	0.0135	ZCRB1,SRSF4,SRSF5,FUS,BCAS2,HNRNPA0,HNRNPA3,SNRPD3,HNRNPA2B1,PABPN1,HNRNPM,HNRNPK,HNRNPF,SNRPG,SNRPB,DNAJC8,POLR2E,POLR2G,POLR2L,MAGOH,HNRNPH2,CTNNBL1,PCBP1,RBM8A,SNU13,SF3B5,SF3B6,SNRPB2,HSPA8
Endosomal Sorting Complex Required For Transport (ESCRT)	0.0039	28	10	2.75E-03	0.0137	VPS25,VPS28,CHMP4B,UBB,UBC,CHMP2A,SNF8,VPS37A,UBA52,CHMP5
DAP12 signaling	0.0398	283	52	2.75E-03	0.0138	JAK1,DUSP6,RAC1,PEBP1,KRAS,CDKN1A,MAP2K1,PHB,TNRC6B,HLA-E,SPTBN1,UBB,UBC,PSMD8,PSMD6,PSMD7,PSMD4,PSMD2,PSMD3,PSMD1,PSME1,PSME2,PSMF1,PSMA5,PSMA3,PSMA4,PSMA1,PSMA2,PSMA7,PSMB6,PSMB7,PSMB5,PSMB2,PSMB3,PSMB1,PSMC5,PSMC6,PSMC3,PSMC4,PSMC1,PSMC2,RBX1,CALM1,PSMD10,PSMD12,PSMD11,PSMD14,PSMD13,YWHAB,UBA52,LAMTOR2,B2M
RHO GTPases activate CIT	0.0021	15	7	2.78E-03	0.0139	MYL12B,MYH9,MYL6,MYL9,RHOC,RHOA,MYH14
Signaling by FGFR	0.039	277	51	2.89E-03	0.0145	JAK1,DUSP6,PEBP1,KRAS,CDKN1A,MAP2K1,PHB,TNRC6B,PPP2R1A,SPTBN1,PPP2CA,UBB,UBC,PSMD8,PSMD6,PSMD7,PSMD4,PSMD2,PSMD3,PSMD1,PSME1,PSME2,PSMF1,PSMA5,PSMA3,PSMA4,PSMA1,PSMA2,PSMA7,PSMB6,PSMB7,PSMB5,PSMB2,PSMB3,PSMB1,PSMC5,PSMC6,PSMC3,PSMC4,PSMC1,PSMC2,RBX1,CALM1,PSMD10,PSMD12,PSMD11,PSMD14,PSMD13,YWHAB,UBA52,LAMTOR2
DAP12 interactions	0.0419	298	54	3.06E-03	0.0153	JAK1,DUSP6,RAC1,PEBP1,KRAS,CDKN1A,MAP2K1,PHB,TNRC6B,HLA-B,HLA-C,HLA-E,SPTBN1,UBB,UBC,PSMD8,PSMD6,PSMD7,PSMD4,PSMD2,PSMD3,PSMD1,PSME1,PSME2,PSMF1,PSMA5,PSMA3,PSMA4,PSMA1,PSMA2,PSMA7,PSMB6,PSMB7,PSMB5,PSMB2,PSMB3,PSMB1,PSMC5,PSMC6,PSMC3,PSMC4,PSMC1,PSMC2,RBX1,CALM1,PSMD10,PSMD12,PSMD11,PSMD14,PSMD13,YWHAB,UBA52,LAMTOR2,B2M
Signaling by ERBB2	0.0391	278	51	3.10E-03	0.0155	JAK1,DUSP6,CDC37,PEBP1,KRAS,CDKN1A,MAP2K1,PHB,TNRC6B,SPTBN1,UBB,UBC,PSMD8,PSMD6,PSMD7,PSMD4,PSMD2,PSMD3,PSMD1,PSME1,PSME2,PSMF1,PSMA5,PSMA3,PSMA4,PSMA1,PSMA2,PSMA7,PSMB6,PSMB7,PSMB5,PSMB2,PSMB3,PSMB1,PSMC5,PSMC6,PSMC3,PSMC4,PSMC1,PSMC2,RBX1,HSP90AA1,CALM1,PSMD10,PSMD12,PSMD11,PSMD14,PSMD13,YWHAB,UBA52,LAMTOR2
Semaphorin interactions	0.0091	65	17	3.28E-03	0.0164	DPYSL3,ITGB1,RRAS,RAC1,CFL1,MYL12B,MYH9,MYL6,MYL9,HSP90AB1,HSP90AA1,SEMA5A,SEMA6A,CDK5,RHOC,RHOA,MYH14

Regulation of cytoskeletal remodeling and cell spreading by IPP complex components	0.0011	8	5	3.29E-03	0.0164	LIMS1,ACTN1,PARVA,PARVB,RSU1
HSF1 activation	0.0017	12	6	3.94E-03	0.0197	PTGES3,HSP90AB1,HSP90AA1,VCP,YWHAE,HSBP1
Downstream signaling of activated FGFR2	0.0377	268	49	3.94E-03	0.0197	JAK1,DUSP6,PEBP1,KRAS,CDKN1A,MAP2K1,PHB,TNRC6B,SPTBN1,UBB,UBC,PSMD8,PSMD6,PSMD7,PSMD4,PSMD2,PSMD3,PSMD1,PSME1,PSME2,PSMF1,PSMA5,PSMA3,PSMA4,PSMA1,PSMA2,PSMA7,PSMB6,PSMB7,PSMB5,PSMB2,PSMB3,PSMB1,PSMC5,PSMC6,PSMC3,PSMC4,PSMC1,PSMC2,RBX1,CALM1,PSMD10,PSMD12,PSMD11,PSMD14,PSMD13,YWHAB,UBA52,LAMTOR2
Downstream signaling of activated FGFR1	0.0377	268	49	3.94E-03	0.0197	JAK1,DUSP6,PEBP1,KRAS,CDKN1A,MAP2K1,PHB,TNRC6B,SPTBN1,UBB,UBC,PSMD8,PSMD6,PSMD7,PSMD4,PSMD2,PSMD3,PSMD1,PSME1,PSME2,PSMF1,PSMA5,PSMA3,PSMA4,PSMA1,PSMA2,PSMA7,PSMB6,PSMB7,PSMB5,PSMB2,PSMB3,PSMB1,PSMC5,PSMC6,PSMC3,PSMC4,PSMC1,PSMC2,RBX1,CALM1,PSMD10,PSMD12,PSMD11,PSMD14,PSMD13,YWHAB,UBA52,LAMTOR2
Downstream signaling of activated FGFR4	0.0377	268	49	3.94E-03	0.0197	JAK1,DUSP6,PEBP1,KRAS,CDKN1A,MAP2K1,PHB,TNRC6B,SPTBN1,UBB,UBC,PSMD8,PSMD6,PSMD7,PSMD4,PSMD2,PSMD3,PSMD1,PSME1,PSME2,PSMF1,PSMA5,PSMA3,PSMA4,PSMA1,PSMA2,PSMA7,PSMB6,PSMB7,PSMB5,PSMB2,PSMB3,PSMB1,PSMC5,PSMC6,PSMC3,PSMC4,PSMC1,PSMC2,RBX1,CALM1,PSMD10,PSMD12,PSMD11,PSMD14,PSMD13,YWHAB,UBA52,LAMTOR2
Downstream signaling of activated FGFR3	0.0377	268	49	3.94E-03	0.0197	JAK1,DUSP6,PEBP1,KRAS,CDKN1A,MAP2K1,PHB,TNRC6B,SPTBN1,UBB,UBC,PSMD8,PSMD6,PSMD7,PSMD4,PSMD2,PSMD3,PSMD1,PSME1,PSME2,PSMF1,PSMA5,PSMA3,PSMA4,PSMA1,PSMA2,PSMA7,PSMB6,PSMB7,PSMB5,PSMB2,PSMB3,PSMB1,PSMC5,PSMC6,PSMC3,PSMC4,PSMC1,PSMC2,RBX1,CALM1,PSMD10,PSMD12,PSMD11,PSMD14,PSMD13,YWHAB,UBA52,LAMTOR2
Sema4D in semaphorin signaling	0.0035	25	9	4.16E-03	0.0208	RRAS,RAC1,MYL12B,MYH9,MYL6,MYL9,RHOC,RHOA,MYH14
Basigin interactions	0.0035	25	9	4.16E-03	0.0208	SLC7A8,ITGB1,BSG,PPIA,CAV1,SLC3A2,ATP1B3,ATP1B1,SLC16A3

Signaling by EGFR	0.0416	296	53	4.20E-03	0.021	JAK1,DUSP6,CDC42,PEBP1,KRAS,CDKN1A,MAP2K1,PHB,TNRC6B,SPTBN1,UBB,UBC,PSMD8,PSMD6,PSMD7,PSMD4,PSMD2,PSMD3,PSMD1,PSME1,PSME2,PSMF1,PSMA5,PSMA3,PSMA4,PSMA1,PSMA2,PSMA7,PSMB6,PSMB7,PSMB5,PSMB2,PSMB3,PSMB1,AP2S1,PSMC5,PSMC6,PSMC3,PSMC4,PSMC1,PSMC2,AP2M1,RBX1,CALM1,PSMD10,PSMD12,PSMD11,PSMD14,PSMD13,YWHAB,UBA52,CLTA,LAMTOR2
Anchoring of the basal body to the plasma membrane	0.0127	90	21	4.35E-03	0.0217	ACTR1A,DYNC1I2,SSNA1,DYNC1H1,AHI1,TUBB4B,CEP135,CEP152,PPP2R1A,TUBB,RAB11A,TUBA1A,DYNLL1,HSP90AA1,CETN2,SFI1,YWHAE,DCTN2,DCTN3,TUBG1,MAPRE1
G2/M DNA damage checkpoint	0.0056	40	12	4.43E-03	0.0221	BRE,WEE1,BRCA1,BABAM1,UBE2V2,CCNB1,YWHAE,YWHAB,YWHAQ,YWHAH,YWHAZ,CHEK1
Transport to the Golgi and subsequent modification	0.0188	134	28	5.04E-03	0.023	NAPA,DYNC1I2,DYNC1H1,TFG,COPZ1,TRAPPC3,TRAPPC1,TRAPPC4,CNIH1,RAB1A,SPTBN1,TMED3,TMED2,TMED7,TMED9,TRAPPC2L,COPB2,COPE,ARF4,DYNLL1,KDELR1,CTSC,SEC13,CD59,TMED10,DCTN6,DCTN2,DCTN3
Endosomal/Vacuolar pathway	0.0013	9	5	5.37E-03	0.023	HLA-B,HLA-C,HLA-A,HLA-E,B2M
NGF signalling via TRKA from the plasma membrane	0.0442	314	55	5.51E-03	0.023	JAK1,DUSP4,DUSP6,RIT1,PEBP1,KRAS,CDKN1A,MAP2K1,PHB,TNRC6B,SPTBN1,UBB,UBC,PSMD8,PSMD6,PSMD7,PSMD4,PSMD2,PSMD3,PSMD1,PSME1,PSME2,PSMF1,PSMA5,PSMA3,PSMA4,PSMA1,PSMA2,PSMA7,PSMB6,PSMB7,PSMB5,PSMB2,PSMB3,PSMB1,AP2S1,PSMC5,PSMC6,PSMC3,PSMC4,PSMC1,PSMC2,AP2M1,RBX1,CALM1,PSMD10,PSMD12,PSMD11,PSMD14,PSMD13,YWHAB,UBA52,CLTA,LAMTOR2,RHOA
Signaling by Interleukins	0.0394	280	50	5.60E-03	0.023	JAK1,DUSP6,PEBP1,KRAS,MAP2K1,PHB,SPTBN1,UBB,UBC,PSMD8,PSMD6,PSMD7,PSMD4,PSMD2,PSMD3,PSMD1,PSME1,PSME2,PSMF1,PSMA5,PSMA3,PSMA4,PSMA1,PSMA2,PSMA7,PSMB6,PSMB7,PSMB5,PSMB2,PSMB3,PSMB1,PSMC5,PSMC6,PSMC3,PSMC4,PSMC1,PSMC2,RBX1,SQSTM1,CALM1,PSMD10,PSMD12,PSMD11,PSMD14,PSMD13,YWHAB,YWHAZ,UBA52,LAMTOR2,SKP1
Advanced glycosylation endproduct receptor signaling	0.0018	13	6	5.76E-03	0.023	LGALS3,CAPZA2,DDOST,PRKCSH,HMGB1,APP
NF-kB is activated and signals survival	0.0018	13	6	5.76E-03	0.023	UBB,UBC,NGFR,SQSTM1,UBA52,NFKBIA

G2/M Checkpoints	0.0105	75	18	6.01E-03	0.024	BRE,ORC6,RFC4,MCM4,WEE1,MCM10,BRCA1,BABAM1,UBE2V2,CCNB2,CCNB1,YWHAE,YWHAB,YWHAQ,YWHAH,YWHAZ,RAD9A,CHEK1
Sema4D induced cell migration and growth-cone collapse	0.0031	22	8	6.33E-03	0.0253	RAC1,MYL12B,MYH9,MYL6,MYL9,RHOC,RHOA,MYH14
Formation of TC-NER Pre-Incision Complex	0.0075	53	14	6.62E-03	0.0265	TCEA1,COPS4,COPS6,COPS5,COPS8,POLR2E,POLR2G,POLR2L,UBB,UBC,RBX1,UBA52,GPS1,GTF2H5
Processing of Capped Intron-Containing Pre-mRNA	0.0238	169	33	6.64E-03	0.0266	NUP107,ZCRB1,SRSF4,SRSF5,FUS,BCAS2,EIF4E,HNRNPA0,HNRNPA3,SNRPD3,HNRNPA2B1,PABPN1,NUP93,HNRNPM,HNRNPK,HNRNPF,SNRPG,SNRPB,DNAJC8,POLR2E,POLR2G,POLR2L,MAGOH,HNRNPH2,CTNNBL1,PCBP1,RBM8A,SNU13,SF3B5,SF3B6,SNRPB2,HSPA8,WTAP
Global Genome Nucleotide Excision Repair (GG-NER)	0.0107	76	18	6.83E-03	0.0273	DDB2,ACTB,COPS4,COPS6,COPS5,COPS8,RFC4,UBB,UBC,ACTL6A,RUVBL1,RBX1,RAD23B,CETN2,UBE2V2,UBA52,GPS1,GTF2H5
MHC class II antigen presentation	0.0125	89	20	7.82E-03	0.0313	LGMN,OSBPL1A,ACTR1A,CANX,DYNC1I2,DYNC1H1,AP1S2,RAB7A,ARF1,DYNLL1,AP2S1,AP2M1,CTSL,CTSD,SEC13,CD74,DCTN6,DCTN2,DCTN3,CLTA
Nucleotide Excision Repair	0.0143	102	22	8.55E-03	0.0342	DDB2,ACTB,TCEA1,COPS4,COPS6,COPS5,COPS8,RFC4,POLR2E,POLR2G,POLR2L,UBB,UBC,ACTL6A,RUVBL1,RBX1,RAD23B,CETN2,UBE2V2,UBA52,GPS1,GTF2H5
Downstream signal transduction	0.0395	281	49	9.17E-03	0.0367	JAK1,DUSP6,PEBP1,KRAS,CDKN1A,MAP2K1,PHB,TNRC6B,SPTBN1,UBB,UBC,PSMD8,PSMD6,PSMD7,PSMD4,PSMD2,PSMD3,PSMD1,PSME1,PSME2,PSMF1,PSMA5,PSMA3,PSMA4,PSMA1,PSMA2,PSMA7,PSMB6,PSMB7,PSMB5,PSMB2,PSMB3,PSMB1,PSMC5,PSMC6,PSMC3,PSMC4,PSMC1,PSMC2,RBX1,CALM1,PSMD10,PSMD12,PSMD11,PSMD14,PSMD13,YWHAB,UBA52,LAMTOR2
Iron uptake and transport	0.0055	39	11	9.65E-03	0.0386	ATP6V1E1,ATP6V1G1,ATP6V1C2,ATP6AP1,FTH1,ATP6V0E1,ATP6V0D1,ATP6V0B,ATP6V1H,ATP6V1D,ATP6V1F
Signaling by TGF-beta Receptor Complex	0.0103	73	17	9.79E-03	0.0392	TGIF1,TFDP1,UBB,UBC,UBE2D3,UBE2D1,PPP1R15A,SNW1,NEDD8,PPP1CA,FKBP1A,SERPINE1,JUNB,UBA52,RHOA,STRAP,SMAD2
ROS, RNS production in response to bacteria	0.0048	34	10	0.0101	0.0405	ATP6V1E1,ATP6V1G1,ATP6V1C2,ATP6V0E1,ATP6V0D1,CYBA,ATP6V0B,ATP6V1H,ATP6V1D,ATP6V1F

Signalling by NGF	0.0548	390	64	0.0113	0.0451	JAK1,DUSP4,DUSP6,RAC1,RIT1,PEBP1,KRAS,CDKN1A,MAP2K1,PHB,HDAC2,TNRC6B,MAGED1,SPTBN1,ARHGDIA,UBB,UBC,PSMD8,PSMD6,PSMD7,PSMD4,PSMD2,PSMD3,PSMD1,PSME1,PSME2,PSMF1,NGFR,PSMA5,PSMA3,PSMA4,PSMA1,PSMA2,PSMA7,PSMB6,PSMB7,PSMB5,PSMB2,PSMB3,PSMB1,AP2S1,PSMC5,PSMC6,PSMC3,PSMC4,PSMC1,PSMC2,AP2M1,RBX1,SQSTM1,CALM1,PSMD10,PSMD12,PSMD11,PSMD14,PSMD13,RTN4,YWHAE,YWHAB,UBA52,CLTA,LAMTOR2,RHOA,NFKBIA
RHO GTPases activate IQGAPs	0.0015	11	5	0.012	0.0482	CDC42,RAC1,CALM1,CTNNA1,CTNNB1
Golgi Cisternae Pericentriolar Stack Reorganization	0.0015	11	5	0.012	0.0482	RAB2A,RAB1A,CCNB2,CCNB1,GORASP2
Cyclin D associated events in G1	0.0042	30	9	0.0127	0.0509	CDKN1A,CDKN2A,TFDP1,UBB,UBC,CCND1,UBA52,CDK4,SKP1
Glutathione conjugation	0.0042	30	9	0.0127	0.0509	GSTM3,GSTO1,GSTP1,GSTA4,GGCT,ESD,MGST3,MGST1,MGST2
G1 Phase	0.0042	30	9	0.0127	0.0509	CDKN1A,CDKN2A,TFDP1,UBB,UBC,CCND1,UBA52,CDK4,SKP1
Negative regulation of MAPK pathway	0.0035	25	8	0.0129	0.0516	DUSP6,PEBP1,KRAS,MAP2K1,UBB,UBC,YWHAB,UBA52
IRE1alpha activates chaperones	0.0073	52	13	0.0132	0.0526	YIF1A,DNAJB11,DDX11,DNAJC3,ATP6V0D1,SEC61G,SEC61B,LMNA,SEC62,MYDGF,ASNA1,PDIA6,HSPA5
Localization of the PINCH-ILK-PARVIN complex to focal adhesions	0.0006	4	3	0.0135	0.0539	ITGB1,PARVA,ILK
Axonal growth stimulation	0.0006	4	3	0.0135	0.0539	ARHGDIA,NGFR,RHOA
p75NTR signals via NF-kB	0.0023	16	6	0.0148	0.0593	UBB,UBC,NGFR,SQSTM1,UBA52,NFKBIA
Unfolded Protein Response (UPR)	0.0117	83	18	0.0155	0.0619	HSP90B1,CALR,HERPUD1,YIF1A,DNAJB11,DDX11,DNAJC3,ATP6V0D1,SEC61G,SEC61B,LMNA,CCL2,SEC62,MYDGF,EXOSC7,ASNA1,PDIA6,HSPA5
HSF1-dependent transactivation	0.003	21	7	0.0159	0.0635	CRYAB,FKBP4,PTGES3,HSP90AB1,HSP90AA1,HSBP1,HSPA8
FCERI mediated NF-kB activation	0.0321	228	40	0.0159	0.0637	UBB,UBC,PSMD8,PSMD6,PSMD7,PSMD4,PSMD2,PSMD3,PSMD1,PSME1,PSME2,PSMF1,PSMA5,PSMA3,PSMA4,PSMA1,PSMA2,PSMA7,PSMB6,PSMB7,PSMB5,PSMB2,PSMB3,PSMB1,PSMC5,PSMC6,PSMC3,PSMC4,PSMC1,PSMC2,UBE2D2,UBE2D1,PSMD10,PSMD12,PSMD11,PSMD14,PSMD13,UBA52,SKP1,NFKBIA
Synthesis, secretion, and inactivation of Glucose-dependent Insulinotropic Polypeptide (GIP)	0.0017	12	5	0.0169	0.0675	SEC11A,SEC11C,SPCS2,SPCS1,PAX6

Nuclear Envelope Reassembly	0.0011	8	4	0.0175	0.0701	PPP2R1A,PPP2CA,LMNA,BANF1
Axonal growth inhibition (RHOA activation)	0.0011	8	4	0.0175	0.0701	ARHGDIA,NGFR,RTN4,RHOA
eNOS activation	0.0011	8	4	0.0175	0.0701	CAV1,DDAH2,HSP90AA1,CALM1
Initiation of Nuclear Envelope Reformation	0.0011	8	4	0.0175	0.0701	PPP2R1A,PPP2CA,LMNA,BANF1
SMAD2/SMAD3:SMAD4 heterotrimer regulates transcription	0.0045	32	9	0.0184	0.0738	TGIF1,TFDP1,UBB,UBC,SNW1,SERPINE1,JUNB,UBA52,SMAD2
TGF-beta receptor signaling activates SMADs	0.0045	32	9	0.0184	0.0738	UBB,UBC,PPP1R15A,NEDD8,PPP1CA,FKBP1A,UBA52,STRAP,SMAD2
Regulation of Insulin-like Growth Factor (IGF) transport and uptake by Insulin-like Growth Factor Binding Proteins (IGFBPs)	0.0024	17	6	0.0193	0.0772	IGF2,IGFBP5,IGFBP4,IGFBP3,IGFBP2,IGFBP6
Pink/Parkin Mediated Mitophagy	0.0031	22	7	0.0199	0.0795	MAP1LC3B,UBB,UBC,SQSTM1,UBA52,VDAC1,TOMM7
mTORC1-mediated signalling	0.0031	22	7	0.0199	0.0795	EIF4E,YWHAB,LAMTOR2,LAMTOR4,LAMTOR5,RHEB,RRAGA
Downregulation of SMAD2/3:SMAD4 transcriptional activity	0.0031	22	7	0.0199	0.0795	TGIF1,UBB,UBC,UBE2D3,UBE2D1,UBA52,SMAD2
Translation	0.0203	144	27	0.0208	0.0832	EIF5B,EIF4H,EIF4E,EIF3M,EIF3K,EIF3I,EIF3J,EIF3G,SRP19,SRP14,SEC11A,SEC11C,SEC61G,SEC61B,SPCS2,SPCS1,EIF2S2,EIF5,DDOST,EIF4A2,EIF4A1,UBA52,RPN2,SSR4,SSR2,SSR3,FAU
Transcriptional activity of SMAD2/SMAD3:SMAD4 heterotrimer	0.0062	44	11	0.0214	0.0856	TGIF1,TFDP1,UBB,UBC,UBE2D3,UBE2D1,SNW1,SERPINE1,JUNB,UBA52,SMAD2
XBP1(S) activates chaperone genes	0.007	50	12	0.0221	0.0884	YIF1A,DNAJB11,DDX11,DNAJC3,ATP6V0D1,SEC61G,SEC61B,LMNA,SEC62,MYDGF,ASNA1,PDIA6
p75NTR recruits signalling complexes	0.0018	13	5	0.0228	0.0914	UBB,UBC,NGFR,SQSTM1,UBA52
Synthesis of Prostaglandins (PG) and Thromboxanes (TX)	0.0018	13	5	0.0228	0.0914	CBR1,PTGES3,PTGR1,PTGDS,PTGES
Activation of BAD and translocation to mitochondria	0.0018	13	5	0.0228	0.0914	YWHAE,YWHAB,YWHAQ,YWHAH,YWHAZ
G2/M DNA replication checkpoint	0.0007	5	3	0.0241	0.0964	WEE1,CCNB2,CCNB1
Galactose catabolism	0.0007	5	3	0.0241	0.0964	PGM1,GALK1,GALE

Activation of BH3-only proteins	0.0032	23	7	0.0245	0.098	TFDP1,DYNLL1,YWHAE,YWHAB,YWHAQ,YWHAH,YWHAZ
APC/C:Cdc20 mediated degradation of Cyclin B	0.0032	23	7	0.0245	0.098	CDC20,UBB,UBC,UBE2D1,UBE2E1,CCNB1,UBA52
Synthesis, secretion, and inactivation of Glucagon-like Peptide-1 (GLP-1)	0.0025	18	6	0.0246	0.0984	GNB3,SEC11A,SEC11C,SPCS2,SPCS1,PAX6
Serine biosynthesis	0.0003	2	2	0.0253	0.0995	PHGDH,PSAT1
p75NTR regulates axonogenesis	0.0013	9	4	0.0255	0.0995	ARHGDIA,NGFR,RTN4,RHOA
COPII (Coat Protein 2) Mediated Vesicle Transport	0.0082	58	13	0.0286	0.0995	NAPA,TFG,TRAPPC3,TRAPPC1,TRAPPC4,CNIH1,RAB1A,TMED2,TRAPPC2L,CTSC,SEC13,CD59,TMED10
Metabolism of carbohydrates	0.0336	239	40	0.0303	0.0995	NUP107,B4GAT1,BGN,PGK1,G6PC3,PGM1,GAPDH,PRPS1,TALDO1,SDC2,GALK1,GALE,NUP93,TPI1,TKT,PAPSS1,PKM,SLC35B2,UBB,UBC,HK1,HEXB,PGAM1,MDH1,MDH2,GOT1,SLC25A11,CALM1,AKR1B1,HYAL2,G6PD,ENO1,ENO2,UBA52,CHPF,GYG1,PFKL,PFKP,ALDOA,SLC2A1
Oncogene Induced Senescence	0.0042	30	8	0.0332	0.0995	CDKN2A,TFDP1,TNRC6B,UBB,UBC,ID1,UBA52,CDK4
Assembly of the primary cilium	0.0242	172	30	0.0355	0.1065	ACTR1A,DYNC1I2,SSNA1,DYNC1H1,AHI1,TCP1,TUBB4B,CEP135,CEP152,PPP2R1A,TUBB,RAB11A,DYNLRB1,ARF4,TUBA1A,DYNLL1,ARL3,HSP90AA1,CETN2,CCT3,CCT2,SFI1,CCT8,CCT5,CCT4,YWHAE,DCTN2,DCTN3,TUBG1,MAPRE1
APC-Cdc20 mediated degradation of Nek2A	0.0035	25	7	0.0359	0.1077	CDC20,UBB,UBC,UBE2D1,UBE2E1,MAD2L1,UBA52
Scavenging by Class F Receptors	0.0008	6	3	0.0381	0.1144	CALR,HSPH1,HSP90AA1
NRIF signals cell death from the nucleus	0.0021	15	5	0.0385	0.1155	UBB,UBC,NGFR,SQSTM1,UBA52
Glycogen synthesis	0.0021	15	5	0.0385	0.1155	PGM1,UBB,UBC,UBA52,GYG1
Transcription-Coupled Nucleotide Excision Repair (TC-NER)	0.0104	74	15	0.0417	0.1251	TCEA1,COPS4,COPS6,COPS5,COPS8,RFC4,POLR2E,POLR2G,POLR2L,UBB,UBC,RBX1,UBA52,GPS1,GTF2H5
Pyruvate metabolism	0.0037	26	7	0.0427	0.1282	BSG,PDHA1,LDHB,LDHA,PDHB,SLC16A3,GLO1
Downregulation of TGF-beta receptor signaling	0.0037	26	7	0.0427	0.1282	UBB,UBC,PPP1R15A,PPP1CA,UBA52,STRAP,SMAD2
BBSome-mediated cargo-targeting to cilium	0.003	21	6	0.0461	0.1384	TCP1,CCT3,CCT2,CCT8,CCT5,CCT4
IKK complex recruitment mediated by RIP1	0.003	21	6	0.0461	0.1384	UBB,UBC,UBE2D2,UBE2D3,UBE2D1,UBA52

Incretin synthesis, secretion, and inactivation	0.003	21	6	0.0461	0.1384	GNB3,SEC11A,SEC11C,SPCS2,SPCS1,PAX6
Golgi to ER Retrograde Transport	0.0015	11	4	0.0473	0.142	COPZ1,COPB2,COPE,ARF1
COPI Mediated Transport	0.0015	11	4	0.0473	0.142	COPZ1,COPB2,COPE,ARF1
MAP3K8 (TPL2)-dependent MAPK1/3 activation	0.0023	16	5	0.0483	0.1449	MAP2K1,UBB,UBC,UBA52,SKP1
Spry regulation of FGF signaling	0.0023	16	5	0.0483	0.1449	PPP2R1A,PPP2CA,UBB,UBC,UBA52
TGF-beta receptor signaling in EMT (epithelial to mesenchymal transition)	0.0023	16	5	0.0483	0.1449	UBB,UBC,FKBP1A,UBA52,RHOA
EGFR downregulation	0.0038	27	7	0.0503	0.151	CDC42,UBB,UBC,AP2S1,AP2M1,UBA52,CLTA
Ribosomal scanning and start codon recognition	0.008	57	12	0.0507	0.1522	EIF4H,EIF4E,EIF3M,EIF3K,EIF3I,EIF3J,EIF3G,EIF2S2,EIF5,EIF4A2,EIF4A1,FAU
Cell junction organization	0.0098	70	14	0.0523	0.1569	LIMS1,ITGB1,ACTN1,VASP,PARVA,PARVB,RSU1,CADM1,FBLIM1,PARD6B,ILK,CDH6,CD151,CTNNA1
Formation of Incision Complex in GG-NER	0.0055	39	9	0.0531	0.1592	DDB2,UBB,UBC,RBX1,RAD23B,CETN2,UBE2V2,UBA52,GTF2H5
Pentose phosphate pathway (hexose monophosphate shunt)	0.001	7	3	0.0555	0.1664	TALDO1,TKT,G6PD
Senescence-Associated Secretory Phenotype (SASP)	0.0073	52	11	0.058	0.174	FOS,CDKN1A,CDKN2A,UBB,UBC,UBE2D1,UBE2E1,UBA52,H3F3A,IGFBP7,CDK4
Translocation of GLUT4 to the plasma membrane	0.0073	52	11	0.058	0.174	RAC1,RAB4A,RAB14,RAB13,RAB11A,CALM1,YWHAE,YWHAB,YWHAQ,YWHAH,YWHAZ
MAP2K and MAPK activation	0.0024	17	5	0.0595	0.1784	PEBP1,KRAS,MAP2K1,YWHAB,LAMTOR2
Processing of DNA double-strand break ends	0.0091	65	13	0.0598	0.1794	RMI2,BRE,BRIP1,RFC4,UBB,UBC,BRCA1,BABAM1,UBE2V2,UBA52,PPP4C,RAD9A,CHEK1
NOTCH1 Intracellular Domain Regulates Transcription	0.0056	40	9	0.0602	0.1805	TLE4,UBB,UBC,RBX1,SNW1,MAML2,UBA52,HIF1A,SKP1
Trafficking and processing of endosomal TLR	0.0017	12	4	0.0611	0.1834	LGMN,HSP90B1,CTSL,CTSB
The NLRP3 inflammasome	0.0017	12	4	0.0611	0.1834	SUGT1,TXN,HSP90AB1,APP
Regulation of innate immune responses to cytosolic DNA	0.0017	12	4	0.0611	0.1834	UBB,UBC,UBA52,DTX4
HDR through Homologous Recombination (HR) or Single Strand Annealing (SSA)	0.012	85	16	0.0619	0.1856	RMI2,BRE,BRIP1,RFC4,UBB,UBC,XRCC2,BRCA1,BRCA2,BABAM1,UBE2V2,UBA52,PPP4C,RAD51AP1,RAD9A,CHEK1

L1CAM interactions	0.0111	79	15	0.0651	0.1953	ITGB1,ITGAV,CSNK2B,NRP2,RAC1,ALCAM,MAP2K1,SPTBN1,NRCAM,AP2S1,AP2M1,CLTA,EZR,SDCBP,HSPA8
Amyloid fiber formation	0.0058	41	9	0.0678	0.2035	MFGE8,GSN,CST3,SNCA,H3F3A,APP,B2M,ITM2B,TGFBI
Mitochondrial protein import	0.0049	35	8	0.0685	0.2056	MTX1,MTX2,SAMM50,TIMM17A,TIMM17B,TOMM7,CHCHD2,HSPA9
DNA Double-Strand Break Repair	0.016	114	20	0.0715	0.2144	RMI2,BRE,BRIP1,RFC4,UBB,UBC,KPNA2,XRCC6,XRCC5,XRCC2,BRCA1,BRCA2,BABAM1,DCLRE1C,UBE2V2,UBA52,PPP4C,RAD51AP1,RAD9A,CHEK1
ISG15 antiviral mechanism	0.0094	67	13	0.0718	0.2154	NUP107,JAK1,EIF4E,NUP93,UBB,UBC,KPNA2,UBE2E1,EIF4A2,EIF4A1,EIF4A3,UBA52,EIF4G2
Phase II conjugation	0.0094	67	13	0.0718	0.2154	GSTM3,GSTO1,GSTP1,PAPSS1,SLC35B2,GSTA4,COMT,GGCT,MAT2A,ESD,MGST3,MGST1,MGST2
Antiviral mechanism by IFN-stimulated genes	0.0094	67	13	0.0718	0.2154	NUP107,JAK1,EIF4E,NUP93,UBB,UBC,KPNA2,UBE2E1,EIF4A2,EIF4A1,EIF4A3,UBA52,EIF4G2
Metabolism of nitric oxide	0.0025	18	5	0.0719	0.2158	DHFR,CAV1,DDAH2,HSP90AA1,CALM1
Syndecan interactions	0.0025	18	5	0.0719	0.2158	ITGB1,ITGAV,ACTN1,SDC2,TRAPPC4
eNOS activation and regulation	0.0025	18	5	0.0719	0.2158	DHFR,CAV1,DDAH2,HSP90AA1,CALM1
RNA Polymerase II Transcription	0.0152	108	19	0.0758	0.2274	SRSF4,SRSF5,SNRPD3,TCEB2,TCEB1,TCEA1,PABPN1,SNRPG,SNRPB,POLR2E,POLR2G,POLR2L,MAGOH,NELFE,SUPT4H1,RBM8A,TAF9,GTF2H5,GTF2A2
Sema4D mediated inhibition of cell attachment and migration	0.0011	8	3	0.0759	0.2278	RRAS,RAC1,RHOA
HuR (ELAVL1) binds and stabilizes mRNA	0.0011	8	3	0.0759	0.2278	SET,ANP32A,ELAVL1
Downregulation of ERBB4 signaling	0.0011	8	3	0.0759	0.2278	UBB,UBC,UBA52
RHO GTPases activate KTN1	0.0011	8	3	0.0759	0.2278	CDC42,RAC1,RHOA
Homologous DNA Pairing and Strand Exchange	0.0059	42	9	0.0761	0.2282	RMI2,BRIP1,RFC4,XRCC2,BRCA1,BRCA2,RAD51AP1,RAD9A,CHEK1
Cytosolic tRNA aminoacylation	0.0034	24	6	0.0766	0.2299	PPA1,AIMP1,EEF1E1,GARS,DARS,SARS
Aflatoxin activation and detoxification	0.0018	13	4	0.0768	0.2305	AKR7A2,MGST3,MGST1,MGST2
Calnexin/calreticulin cycle	0.0018	13	4	0.0768	0.2305	CALR,CANX,PRKCSH,PDIA3
Elastic fibre formation	0.0051	36	8	0.0776	0.2329	EFEMP2,ITGB1,ITGAV,LOXL1,MFAP2,FBLN1,BMP4,ELN
Intrinsic Pathway for Apoptosis	0.0051	36	8	0.0776	0.2329	TFDP1,DYNLL1,CYCS,YWHAE,YWHAB,YWHAQ,YWHAH,YWHAZ
Clathrin derived vesicle budding	0.006	43	9	0.0849	0.2498	NAPA,AP1S2,FTH1,CHMP2A,ARF1,VAMP8,CLTA,APP,HSPA8

trans-Golgi Network Vesicle Budding	0.006	43	9	0.0849	0.2498	NAPA,AP1S2,FTH1,CHMP2A,ARF1,VAMP8,CLTA,APP,HSPA8
RMTs methylate histone arginines	0.0027	19	5	0.0857	0.2498	SMARCB1,CCND1,CDK4,RBBP7,PRMT1
Translesion Synthesis by POLH	0.0027	19	5	0.0857	0.2498	RFC4,UBB,UBC,VCP,UBA52
Lysosome Vesicle Biogenesis	0.0035	25	6	0.0889	0.2498	AP1S2,CHMP2A,ARF1,CLTA,APP,HSPA8
mRNA Splicing - Minor Pathway	0.007	50	10	0.0905	0.2498	ZCRB1,SNRPD3,SNRPG,SNRPB,POLR2E,POLR2G,POLR2L,SNU13,SF3B5,SF3B6
Homology Directed Repair	0.0127	90	16	0.0905	0.2498	RMI2,BRE,BRIP1,RFC4,UBB,UBC,XRCC2,BRCA1,BRCA2,BABAM1,UBE2V2,UBA52,PPP4C,RAD51AP1,RAD9A,CHEK1
N-glycan trimming in the ER and Calnexin/Calreticulin cycle	0.002	14	4	0.0943	0.2498	CALR,CANX,PRKCSH,PDIA3
RNA Polymerase II Transcription Elongation	0.0062	44	9	0.0944	0.2498	TCEB2,TCEB1,TCEA1,POLR2E,POLR2G,POLR2L,NELFE,SUPT4H1,GTF2H5
Formation of RNA Pol II elongation complex	0.0062	44	9	0.0944	0.2498	TCEB2,TCEB1,TCEA1,POLR2E,POLR2G,POLR2L,NELFE,SUPT4H1,GTF2H5
Cellular response to heat stress	0.0108	77	14	0.0946	0.2498	NUP107,CRYAB,NUP93,FKBP4,DNAJB6,PTGES3,HSPH1,HSP90AB1,HSP90AA1,VCP,YWHAE,ST13,HSBP1,HSPA8
Translation initiation complex formation	0.008	57	11	0.0947	0.2498	EIF4H,EIF4E,EIF3M,EIF3K,EIF3I,EIF3J,EIF3G,EIF2S2,EIF4A2,EIF4A1,FAU
Signaling by Rho GTPases	0.0454	323	48	0.0967	0.2498	NUP107,CENPU,CENPK,PKN2,ARPC4,ARPC5,ARPC2,ARPC3,ITGB1,ACTR3,ACTR2,CDC42,ACTB,CDC20,RAC1,TAX1BP3,ACTG1,ARPC1B,SRGAP3,MYL12B,ARHGDIA,ARHGDIB,MYH9,MYL6,MYL9,ARHGAP11B,PPP1R12A,CALM1,SEC13,MAD2L1,CYBA,YWHAE,YWHAB,YWHAQ,YWHAH,YWHAZ,H3F3A,NUDC,BRK1,ZWINT,GDI2,RHOC,RHOD,RHOA,MAPRE1,CTNNA1,CTNNB1,MYH14
Apoptotic cleavage of cellular proteins	0.0053	38	8	0.0979	0.2498	GSN,LMNA,DSP,DSG2,DBNL,BCAP31,VIM,CTNNB1
Formyl peptide receptors bind formyl peptides and many other ligands	0.0013	9	3	0.0992	0.2498	ANXA1,APP,HEBP1
RHO GTPases Activate Rhotekin and Rhophilins	0.0013	9	3	0.0992	0.2498	TAX1BP3,RHOC,RHOA
MicroRNA (miRNA) biogenesis	0.0028	20	5	0.1008	0.2498	POLR2E,POLR2G,POLR2L,PRKRA,RAN
WNT ligand biogenesis and trafficking	0.0037	26	6	0.1021	0.2498	WLS,VPS29,VPS35,WNT2B,SNX3,VPS26A

Recognition of DNA damage by PCNA-containing replication complex	0.0037	26	6	0.1021	0.2498	UBE2B,RFC4,UBB,UBC,RBX1,UBA52
Resolution of D-loop Structures through Synthesis-Dependent Strand Annealing (SDSA)	0.0037	26	6	0.1021	0.2498	RMI2,BRIP1,XRCC2,BRCA1,BRCA2,RAD51AP1
Activation of the mRNA upon binding of the cap-binding complex and eIFs, and subsequent binding to 43S	0.0082	58	11	0.1034	0.2498	EIF4H,EIF4E,EIF3M,EIF3K,EIF3I,EIF3J,EIF3G,EIF2S2,EIF4A2,EIF4A1,FAU
Presynaptic phase of homologous DNA pairing and strand exchange	0.0055	39	8	0.109	0.2498	RMI2,BRIP1,RFC4,XRCC2,BRCA1,BRCA2,RAD9A,CHEK1
Arachidonic acid metabolism	0.0055	39	8	0.109	0.2498	CBR1,PTGES3,PTGR1,CYP1B1,GPX1,GPX4,PTGDS,PTGES
NCAM1 interactions	0.0046	33	7	0.1131	0.2498	COL6A2,COL6A1,COL9A1,COL9A3,PRNP,COL4A2,COL4A1
Association of licensing factors with the pre-replicative complex	0.0021	15	4	0.1135	0.2498	UBB,UBC,UBA52,CDT1
Amino acid synthesis and interconversion (transamination)	0.0021	15	4	0.1135	0.2498	OAT,PHGDH,PSAT1,GOT1
Cytosolic sensors of pathogen-associated DNA	0.0093	66	12	0.115	0.2498	POLR2E,POLR2L,UBB,UBC,XRCC6,XRCC5,HMGB1,UBA52,APP,CTNNB1,DTX4,NFKBIA
Golgi Associated Vesicle Biogenesis	0.0038	27	6	0.1162	0.2498	NAPA,AP1S2,FTH1,ARF1,CLTA,HSPA8
Signal transduction by L1	0.003	21	5	0.1172	0.2498	ITGB1,ITGAV,CSNK2B,RAC1,MAP2K1
RHO GTPases Activate Formins	0.0142	101	17	0.1185	0.2498	NUP107,CENPU,CENPK,ITGB1,CDC42,ACTB,CDC20,RAC1,ACTG1,SEC13,MAD2L1,NUDC,ZWINT,RHOC,RHOD,RHOA,MAPRE1
Deactivation of the beta-catenin transactivating complex	0.0056	40	8	0.1208	0.2498	TLE4,UBB,UBC,YWHAZ,UBA52,SOX2,SOX4,CTNNB1
Macroautophagy	0.0084	60	11	0.1222	0.2498	MAP1LC3B,RB1CC1,CHMP4B,CHMP2A,GABARAPL2,DYNLL1,LAMTOR2,LAMTOR4,LAMTOR5,RHEB,RRAGA
ATF6-alpha activates chaperone genes	0.0014	10	3	0.1249	0.2498	HSP90B1,CALR,HSPA5
WNT5A-dependent internalization of FZD2, FZD5 and ROR2	0.0014	10	3	0.1249	0.2498	AP2S1,AP2M1,FZD5
IRAK2 mediated activation of TAK1 complex	0.0014	10	3	0.1249	0.2498	UBB,UBC,UBA52

Tetrahydrobiopterin (BH4) synthesis, recycling, salvage and regulation	0.0014	10	3	0.1249	0.2498	DHFR,HSP90AA1,CALM1
IRAK2 mediated activation of TAK1 complex upon TLR7/8 or 9 stimulation	0.0014	10	3	0.1249	0.2498	UBB,UBC,UBA52
SLBP independent Processing of Histone Pre-mRNAs	0.0014	10	3	0.1249	0.2498	SNRPD3,SNRPG,SNRPB
Dissolution of Fibrin Clot	0.0014	10	3	0.1249	0.2498	SERPINE1,S100A10,ANXA2
Retrograde neurotrophin signalling	0.0014	10	3	0.1249	0.2498	AP2S1,AP2M1,CLTA
