# Supplementary material for: Single cell RNA sequencing of stem cell-derived retinal ganglion cells
Source: Sci Data. 2018 Feb 13;5:180013. doi: 10.1038/sdata.2018.13 (PMC5810423; doi:10.1038/sdata.2018.13)
Supplement: Supplementary Tables [file sdata201813-s2.zip › Supplementary tables/table s6.pdf]

ReactomePathway	RatioOfProteinInPathway	NumberOfProteinInPathway	ProteinFromGeneSet	P-value	FDR	HitGenes
Cell Cycle, Mitotic	0.0561	399	113	2.55E-13	2.80E-10	NUP107,OPTN,SET,CDCA5,CDCA8,DHFR,CENPA,CENPU,CENPE,CENPF,CENPH,APEX1,CENPK,CENPM,CENPN,GMNN,NEK2,CDC25B,ODF2,NDC80,CEP290,CDC20,FBXO5,CSNK1D,PCM1,CNTRL,PCNA,SSNA1,UBE2C,MYBL2,POLD2,RRM2,SMC3,SMC4,SMC2,ANAPC15,ANAPC16,CEP70,PLK1,CEP57,CDKN1A,CDKN2B,CDKN2A,CEP41,PPP1CC,RAB2A,CEP135,VRK1,ORC6,MCM7,RFC5,RFC4,RFC1,SMC1A,TFDP2,MCM3,MCM4,RAB1A,TOP2A,TUBB,TYMS,LMNA,GINS2,GINS4,LIG1,UBC,BUB3,PSMD2,NCAPG,TUBA1A,OFD1,PSMA4,PSMB8,FOXM1,DBF4,BIRC5,KIF20A,CETN2,KIF23,RPA2,RPA3,SPC24,MAD2L1,SPC25,CCND1,BANF1,CCNB2,CCNB1,CCNA2,DCTN2,AURKB,AURKA,NUP37,UBA52,SKA2,RAD21,ENSA,NUF2,MAD1L1,CDK6,CDK4,CDK1,RBBP4,GOLGA2,CDT1,ZWINT,STAG2,FEN1,PRIM1,PTTG1,KIF18A,CKS1B,ITGB3BP
mRNA Splicing - Major Pathway	0.0179	127	53	1.79E-12	9.84E-10	SRSF2,SRSF3,SRSF5,SRSF6,SRSF7,SRSF9,FUS,SRSF1,HNRNPA1,HNRNPA0,HNRNPA3,SNRPD2,SNRPD1,SNRPD3,HNRNPA2B1,PABPN1,HNRNPU,HNRNPR,HNRNPM,HNRNPK,HNRNPD,HNRNPC,SNRPG,SNRPE,SNRPF,SNRPA,SNRPB,SNRNP70,SMC1A,POLR2G,POLR2H,POLR2I,POLR2L,SNRNP40,SRRM1,CSTF3,RNPS1,HNRNPH1,YBX1,RBMX,PCBP2,HNRNPUL1,NCBP2,UPF3B,LSM5,LSM4,LSM3,LSM2,LSM8,LSM7,LSM6,SNRPA1,HSPA8
mRNA Splicing	0.0188	134	53	1.26E-11	4.63E-09	SRSF2,SRSF3,SRSF5,SRSF6,SRSF7,SRSF9,FUS,SRSF1,HNRNPA1,HNRNPA0,HNRNPA3,SNRPD2,SNRPD1,SNRPD3,HNRNPA2B1,PABPN1,HNRNPU,HNRNPR,HNRNPM,HNRNPK,HNRNPD,HNRNPC,SNRPG,SNRPE,SNRPF,SNRPA,SNRPB,SNRNP70,SMC1A,POLR2G,POLR2H,POLR2I,POLR2L,SNRNP40,SRRM1,CSTF3,RNPS1,HNRNPH1,YBX1,RBMX,PCBP2,HNRNPUL1,NCBP2,UPF3B,LSM5,LSM4,LSM3,LSM2,LSM8,LSM7,LSM6,SNRPA1,HSPA8

Processing of Capped Intron-Containing Pre-mRNA	0.0238	169	55	5.25E-09	1.44E-06	NUP107,SRSF2,SRSF3,SRSF5,SRSF6,SRSF7,SRSF9,FUS,SRSF1,HNRNPA1,HNRNPA0,HNRNPA3,SNRPD2,SNRPD1,SNRPD3,HNRNPA2B1,PABPN1,HNRNPU,HNRNPR,HNRNPM,HNRNPK,HNRNPD,HNRNPC,SNRPG,SNRPE,SNRPF,SNRPA,SNRPB,SNRNP70,SMC1A,POLR2G,POLR2H,POLR2I,POLR2L,SNRNP40,SRRM1,CSTF3,RNPS1,HNRNPH1,YBX1,RBMX,PCBP2,HNRNPUL1,NCBP2,NUP37,UPF3B,LSM5,LSM4,LSM3,LSM2,LSM8,LSM7,LSM6,SNRPA1,HSPA8
Mitotic Prometaphase	0.0139	99	38	2.19E-08	4.83E-06	NUP107,CDCA5,CDCA8,CENPA,CENPU,CENPE,CENPF,CENPH,CENPK,CENPM,CENPN,NDC80,CDC20,SMC3,SMC4,SMC2,PLK1,PPP1CC,SMC1A,BUB3,NCAPG,BIRC5,SPC24,MAD2L1,SPC25,CCNB2,CCNB1,AURKB,NUP37,SKA2,RAD21,NUF2,MAD1L1,CDK1,ZWINT,STAG2,KIF18A,ITGB3BP
Chromosome Maintenance	0.009	64	29	3.48E-08	6.37E-06	CENPA,CENPU,CENPW,CENPH,APEX1,CENPK,CENPM,CENPN,SMARCA5,MIS18BP1,PCNA,MIS18A,POLD2,OIP5,RFC5,RFC4,RFC1,LIG1,NPM1,RPA2,RPA3,DKC1,HJURP,NHP2,RBBP4,RBBP7,FEN1,PRIM1,ITGB3BP
Resolution of Sister Chromatid Cohesion	0.0128	91	35	7.46E-08	1.17E-05	NUP107,CDCA5,CDCA8,CENPA,CENPU,CENPE,CENPF,CENPH,CENPK,CENPM,CENPN,NDC80,CDC20,SMC3,PLK1,PPP1CC,SMC1A,BUB3,BIRC5,SPC24,MAD2L1,SPC25,CCNB2,CCNB1,AURKB,NUP37,SKA2,RAD21,NUF2,MAD1L1,CDK1,ZWINT,STAG2,KIF18A,ITGB3BP
RHO GTPase Effectors	0.0295	210	59	2.21E-07	3.02E-05	NUP107,CDCA8,SFN,CENPA,CENPU,CENPE,CENPF,CENPH,CENPK,CENPM,PKN2,CENPN,ARPC5,NDC80,ARPC2,ITGB1,ACTR3,ACTR2,ACTB,CDC20,TAX1BP3,NCKAP1,PLK1,ARPC1B,PPP1CC,MYL12B,MYH9,BUB3,MYL6,MYL9,IQGAP1,FLNA,BIRC5,PRC1,CALM1,KIF14,CTTN,SPC24,MAD2L1,SPC25,KIF5A,CYBA,YWHAQ,YWHAZ,AURKB,NUP37,SKA2,H3F3A,NUF2,KTN1,MAD1L1,EVL,ZWINT,RHOC,RHOD,CTNNA1,MYH14,KIF18A,ITGB3BP
Assembly of collagen fibrils and other multimeric structures	0.0055	39	20	7.11E-07	8.68E-05	COL3A1,ITGA6,LOXL1,PLEC,COL2A1,COL27A1,COL18A1,LOX,COL6A2,COL6A1,COL1A1,BMP1,COL5A1,COL5A2,LAMC2,CD151,COL8A2,COL8A1,COL4A2,COL4A1
Platelet degranulation	0.011	78	29	1.84E-06	2.02E-04	FN1,ACTN1,ACTN4,IGF2,CAP1,VEGFA,PPIA,CLU,LAMP2,WDR1,TLN1,FLNA,LEFTY2,A2M,CALM1,CD63,VCL,SERPINE1,SERPING1,TMSB4X,PSAP,APP,SPARC,PFN1,ALDOA,SCG3,TGFB2,TGFB1,HSPA5

Collagen formation	0.0101	72	27	3.53E-06	2.83E-04	COL3A1,PLOD3,ITGA6,LOXL1,CRTAP,PLEC,COL2A1,COL27A1,P3H2,COL18A1,LOX,COLGALT2,COL6A2,COL6A1,COL1A1,COL9A1,COL9A3,BMP1,COL5A1,COL5A2,LAMC2,SERPINH1,CD151,COL8A2,COL8A1,COL4A2,COL4A1
Elastic fibre formation	0.0051	36	18	3.54E-06	2.83E-04	EFEMP2,EFEMP1,ITGB1,ITGAV,ITGB6,LOXL1,FBN2,FBN1,MFAP2,LOX,FBLN1,FURIN,BMP7,BMP4,ELN,LTBP1,TGFB2,TGFB1
Deposition of new CENPA-containing nucleosomes at the centromere	0.0041	29	16	3.63E-06	2.83E-04	CENPA,CENPU,CENPW,CENPH,CENPK,CENPM,CENPN,SMARCA5,MIS18BP1,MIS18A,OIP5,NPM1,HJURP,RBBP4,RBBP7,ITGB3BP
Nucleosome assembly	0.0041	29	16	3.63E-06	2.83E-04	CENPA,CENPU,CENPW,CENPH,CENPK,CENPM,CENPN,SMARCA5,MIS18BP1,MIS18A,OIP5,NPM1,HJURP,RBBP4,RBBP7,ITGB3BP
M Phase	0.0316	225	58	3.90E-06	2.85E-04	NUP107,SET,CDCA5,CDCA8,CENPA,CENPU,CENPE,CENPF,CENPH,CENPK,CENPM,CENPN,NDC80,CDC20,FBXO5,UBE2C,SMC3,SMC4,SMC2,ANAPC15,ANAPC16,PLK1,PPP1CC,RAB2A,VRK1,SMC1A,RAB1A,LMNA,UBC,BUB3,PSMD2,NCAPG,PSMA4,PSMB8,BIRC5,KIF20A,KIF23,SPC24,MAD2L1,SPC25,BANF1,CCNB2,CCNB1,AURKB,NUP37,UBA52,SKA2,RAD21,ENSA,NUF2,MAD1L1,CDK1,GOLGA2,ZWINT,STAG2,PTTG1,KIF18A,ITGB3BP
Smooth Muscle Contraction	0.0046	33	17	4.46E-06	3.03E-04	ITGA1,ACTG2,ACTA2,TPM4,TPM3,TPM2,TPM1,TLN1,MYL12A,MYL12B,MYL6,MYL9,CALM1,VCL,CALD1,ANXA1,ANXA2
Response to elevated platelet cytosolic Ca2+	0.0117	83	29	5.93E-06	3.80E-04	FN1,ACTN1,ACTN4,IGF2,CAP1,VEGFA,PPIA,CLU,LAMP2,WDR1,TLN1,FLNA,LEFTY2,A2M,CALM1,CD63,VCL,SERPINE1,SERPING1,TMSB4X,PSAP,APP,SPARC,PFN1,ALDOA,SCG3,TGFB2,TGFB1,HSPA5
Mitotic Metaphase and Anaphase	0.0229	163	45	9.24E-06	5.64E-04	NUP107,CDCA5,CDCA8,CENPA,CENPU,CENPE,CENPF,CENPH,CENPK,CENPM,CENPN,NDC80,CDC20,FBXO5,UBE2C,SMC3,ANAPC15,ANAPC16,PLK1,PPP1CC,VRK1,SMC1A,LMNA,UBC,BUB3,PSMD2,PSMA4,PSMB8,BIRC5,SPC24,MAD2L1,SPC25,BANF1,AURKB,NUP37,UBA52,SKA2,RAD21,NUF2,MAD1L1,ZWINT,STAG2,PTTG1,KIF18A,ITGB3BP
RHO GTPases Activate Formins	0.0142	101	32	1.39E-05	7.66E-04	NUP107,CDCA8,CENPA,CENPU,CENPE,CENPF,CENPH,CENPK,CENPM,CENPN,NDC80,ITGB1,ACTB,CDC20,PLK1,PPP1CC,BUB3,BIRC5,SPC24,MAD2L1,SPC25,AURKB,NUP37,SKA2,NUF2,MAD1L1,EVL,ZWINT,RHOC,RHOD,KIF18A,ITGB3BP
Cleavage of Growing Transcript in the Termination Region	0.0062	44	19	1.47E-05	7.66E-04	SRSF2,SRSF3,SRSF5,SRSF6,SRSF7,SRSF9,SRSF1,SNRPD3,PABPN1,SNRPG,SNRPE,SNRPF,SNRPB,SRRM1,CSTF3,RNPS1,LSM10,NCBP2,UPF3B

RNA Polymerase II Transcription Termination	0.0062	44	19	1.47E-05	7.66E-04	SRSF2,SRSF3,SRSF5,SRSF6,SRSF7,SRSF9,SRSF1,SNRPD3,PABPN1,SNRPG,SNRPE,SNRPF,SNRPB,SRRM1,CSTF3,RNPS1,LSM10,NCBP2,UPF3B
Mitotic Anaphase	0.0228	162	44	1.70E-05	8.23E-04	NUP107,CDCA5,CDCA8,CENPA,CENPU,CENPE,CENPF,CENPH,CENPK,CENPM,CENPN,NDC80,CDC20,UBE2C,SMC3,ANAPC15,ANAPC16,PLK1,PPP1CC,VRK1,SMC1A,LMNA,UBC,BUB3,PSMD2,PSMA4,PSMB8,BIRC5,SPC24,MAD2L1,SPC25,BANF1,AURKB,NUP37,UBA52,SKA2,RAD21,NUF2,MAD1L1,ZWINT,STAG2,PTTG1,KIF18A,ITGB3BP
DNA strand elongation	0.0046	33	16	1.75E-05	8.23E-04	APEX1,PCNA,POLD2,MCM7,RFC5,RFC4,RFC1,MCM3,MCM4,GINS2,GINS4,LIG1,RPA2,RPA3,FEN1,PRIM1
Signaling by Rho GTPases	0.0454	323	73	2.04E-05	9.19E-04	NUP107,CDCA8,SFN,CENPA,CENPU,CENPE,CENPF,CENPH,CENPK,CENPM,PKN2,CENPN,ARPC5,NDC80,ARPC2,ITGB1,ACTR3,ACTR2,ACTB,CDC20,NET1,RAC3,TAX1BP3,NCKAP1,DEPDC1B,PLK1,ARPC1B,PPP1CC,SRGAP3,MYL12B,VAV3,ARHGDIB,MYH9,BUB3,MYL6,MYL9,IQGAP1,FLNA,ARHGAP11A,ARHGAP11B,BIRC5,PRC1,A2M,CALM1,KIF14,CTTN,SPC24,MAD2L1,SPC25,KIF5A,CYBA,YWHAQ,YWHAZ,AURKB,NUP37,TRIO,SKA2,ABR,H3F3A,NUF2,KTN1,MAD1L1,EVL,ZWINT,RHOF,RHOC,RHOD,CTNNA1,DLC1,MYH14,ARHGAP29,KIF18A,ITGB3BP
Laminin interactions	0.0032	23	13	2.26E-05	9.94E-04	ITGB1,ITGAV,ITGA3,ITGA2,ITGA1,ITGA6,COL18A1,HSPG2,LAMC2,LAMC1,LAMA5,LAMB2,LAMB1
Separation of Sister Chromatids	0.0217	154	41	5.03E-05	2.11E-03	NUP107,CDCA5,CDCA8,CENPA,CENPU,CENPE,CENPF,CENPH,CENPK,CENPM,CENPN,NDC80,CDC20,UBE2C,SMC3,ANAPC15,ANAPC16,PLK1,PPP1CC,SMC1A,UBC,BUB3,PSMD2,PSMA4,PSMB8,BIRC5,SPC24,MAD2L1,SPC25,AURKB,NUP37,UBA52,SKA2,RAD21,NUF2,MAD1L1,ZWINT,STAG2,PTTG1,KIF18A,ITGB3BP
Resolution of AP sites via the multiple-nucleotide patch replacement pathway	0.0027	19	11	7.66E-05	3.06E-03	APEX1,PCNA,POLD2,RFC5,RFC4,RFC1,PARP1,LIG1,RPA2,RPA3,FEN1
mRNA Splicing - Minor Pathway	0.007	50	19	7.92E-05	3.09E-03	SRSF2,SRSF6,SRSF7,SRSF1,SNRPD2,SNRPD1,SNRPD3,SNRPG,SNRPE,SNRPF,SNRPB,POLR2G,POLR2H,POLR2I,POLR2L,SNRNP40,YBX1,NCBP2,LSM2
Cell-extracellular matrix interactions	0.0023	16	10	8.67E-05	3.21E-03	LIMS1,ITGB1,ACTN1,VASP,PARVB,RSU1,FERMT2,FLNA,FLNC,FBLIM1

Collagen biosynthesis and modifying enzymes	0.0086	61	21	1.33E-04	4.79E-03	COL3A1,PLOD3,CRTAP,COL2A1,COL27A1,P3H2,COL18A1,COLGALT2,COL6A2,COL6A1,COL1A1,COL9A1,COL9A3,BMP1,COL5A1,COL5A2,SERPINH1,COL8A2,COL8A1,COL4A2,COL4A1
PCNA-Dependent Long Patch Base Excision Repair	0.0024	17	10	1.41E-04	4.94E-03	APEX1,PCNA,POLD2,RFC5,RFC4,RFC1,LIG1,RPA2,RPA3,FEN1
Gap-filling DNA repair synthesis and ligation in GG-NER	0.003	21	11	1.81E-04	5.97E-03	PCNA,POLD2,RFC5,RFC4,RFC1,LIG1,UBC,XRCC1,RPA2,RPA3,UBA52
Lagging Strand Synthesis	0.003	21	11	1.81E-04	5.97E-03	APEX1,PCNA,POLD2,RFC5,RFC4,RFC1,LIG1,RPA2,RPA3,FEN1,PRIM1
Basigin interactions	0.0035	25	12	2.08E-04	6.64E-03	SLC7A5,SLC7A6,SLC7A8,ITGB1,ITGA3,ITGA6,PPIA,CAV1,SLC3A2,ATP1B1,SLC16A1,SLC16A3
Non-integrin membrane-ECM interactions	0.0052	37	15	2.20E-04	6.64E-03	AGRN,ITGB1,ITGAV,ITGA2,ACTN1,SDC4,SDC2,TNC,TTR,HSPG2,LAMC1,LAMA5,LAMB2,LAMB1,TGFB1
Extension of Telomeres	0.0041	29	13	2.21E-04	6.64E-03	APEX1,PCNA,POLD2,RFC5,RFC4,RFC1,LIG1,RPA2,RPA3,DKC1,NHP2,FEN1,PRIM1
G2/M Transition	0.0153	109	30	2.87E-04	8.32E-03	OPTN,CENPF,NEK2,CDC25B,ODF2,CEP290,CSNK1D,PCM1,CNTRL,SSNA1,MYBL2,CEP70,PLK1,CEP57,CEP41,CEP135,TUBB,UBC,TUBA1A,OFD1,FOXM1,CETN2,CCNB2,CCNB1,CCNA2,DCTN2,AURKA,UBA52,CDK1,RBBP4
Integrin cell surface interactions	0.0091	65	21	3.07E-04	8.60E-03	FN1,SPP1,AGRN,ITGB1,ITGAV,ITGB6,ITGA3,ITGA2,ITGA1,ITGA6,FBN1,TNC,COL18A1,LUM,HSPG2,COL9A1,COL9A3,CD47,CD44,COL4A2,COL4A1
S Phase	0.0169	120	32	3.15E-04	8.81E-03	CDCA5,APEX1,PCNA,POLD2,SMC3,CDKN1A,ORC6,MCM7,RFC5,RFC4,RFC1,SMC1A,MCM3,MCM4,GINS2,GINS4,LIG1,UBC,PSMD2,PSMA4,PSMB8,RPA2,RPA3,CCND1,UBA52,RAD21,CDK4,CDT1,STAG2,FEN1,PRIM1,CKS1B
Regulation of PLK1 Activity at G2/M Transition	0.0113	80	24	3.44E-04	9.29E-03	OPTN,NEK2,ODF2,CEP290,CSNK1D,PCM1,CNTRL,SSNA1,CEP70,PLK1,CEP57,CEP41,CEP135,TUBB,UBC,TUBA1A,OFD1,CETN2,CCNB2,CCNB1,DCTN2,AURKA,UBA52,CDK1
Mitotic G2-G2/M phases	0.0156	111	30	3.85E-04	9.46E-03	OPTN,CENPF,NEK2,CDC25B,ODF2,CEP290,CSNK1D,PCM1,CNTRL,SSNA1,MYBL2,CEP70,PLK1,CEP57,CEP41,CEP135,TUBB,UBC,TUBA1A,OFD1,FOXM1,CETN2,CCNB2,CCNB1,CCNA2,DCTN2,AURKA,UBA52,CDK1,RBBP4
Telomere C-strand (Lagging Strand) Synthesis	0.0032	23	11	3.87E-04	9.46E-03	APEX1,PCNA,POLD2,RFC5,RFC4,RFC1,LIG1,RPA2,RPA3,FEN1,PRIM1

ECM proteoglycans	0.0068	48	17	4.08E-04	9.46E-03	BGN,AGRN,ITGB1,ITGAV,ITGB6,ITGA2,TNC,DCN,LAMC1,LAMA5,LAMB2,LAMB1,SERPINE1,SPARC,TGFB2,TGFB1,NCAM1
Glycolysis	0.0038	27	12	4.10E-04	9.46E-03	PGK1,GAPDH,GPI,TPI1,PKM,HK2,PGAM1,ENO1,ENO2,PFKM,PFKP,ALDOA
Translesion synthesis by REV1	0.0023	16	9	4.13E-04	9.46E-03	PCNA,RFC5,RFC4,RFC1,UBC,RPA2,MAD2L2,RPA3,UBA52
Base Excision Repair	0.0044	31	13	4.13E-04	9.46E-03	APEX1,PCNA,POLD2,RFC5,RFC4,RFC1,PARP1,LIG1,UNG,XRCC1,RPA2,RPA3,FEN1
Resolution of Abasic Sites (AP sites)	0.0044	31	13	4.13E-04	9.46E-03	APEX1,PCNA,POLD2,RFC5,RFC4,RFC1,PARP1,LIG1,UNG,XRCC1,RPA2,RPA3,FEN1
Metabolism of nucleotides	0.0087	62	20	4.30E-04	9.46E-03	NUDT15,RRM2B,DCTPP1,NUDT1,APRT,RRM1,RRM2,TK1,ADSL,DTYMK,TYMS,UPP1,NT5C3A,DUT,PAICS,HPRT1,GPX1,IMPDH2,NME4,NME1
SLBP independent Processing of Histone Pre-mRNAs	0.0014	10	7	5.06E-04	0.0111	SNRPD3,SNRPG,SNRPE,SNRPF,SNRPB,LSM10,NCBP2
Termination of translesion DNA synthesis	0.0039	28	12	5.63E-04	0.0123	PCNA,POLD2,RFC5,RFC4,RFC1,KIAA0101,UBC,UBE2L6,RPA2,RPA3,ISG15,UBA52
DNA Double-Strand Break Repair	0.016	114	30	5.88E-04	0.0123	RMI2,TP53,SMARCA5,UBE2I,WHSC1,RHNO1,PRKDC,RFC5,RFC4,PARP1,TIMELESS,UBC,H2AFX,KPNA2,SUMO1,SUMO2,XRCC6,XRCC2,XRCC1,BRCA1,BRCA2,RPA2,RPA3,DCLRE1C,FAM175A,UBA52,RAD51AP1,RAD9A,CHEK1,FEN1
Translesion synthesis by POLK	0.0024	17	9	6.33E-04	0.0127	PCNA,RFC5,RFC4,RFC1,UBC,RPA2,MAD2L2,RPA3,UBA52
Translesion synthesis by POLI	0.0024	17	9	6.33E-04	0.0127	PCNA,RFC5,RFC4,RFC1,UBC,RPA2,MAD2L2,RPA3,UBA52
Mitotic G1-G1/S phases	0.0177	126	32	7.05E-04	0.0141	DHFR,FBXO5,PCNA,MYBL2,RRM2,CDKN1A,CDKN2B,CDKN2A,ORC6,MCM7,TFDP2,MCM3,MCM4,TOP2A,TYMS,UBC,PSMD2,PSMA4,PSMB8,DBF4,RPA2,RPA3,CCND1,CCNB1,UBA52,CDK6,CDK4,CDK1,RBBP4,CDT1,PRIM1,CKS1B
Molecules associated with elastic fibres	0.0041	29	12	7.60E-04	0.0151	EFEMP2,EFEMP1,ITGB1,ITGAV,ITGB6,MFAP2,FBLN1,BMP7,BMP4,LTBP1,TGFB2,TGFB1
G2/M Checkpoints	0.0105	75	22	7.93E-04	0.0151	SFN,WHSC1,ORC6,MCM7,RFC5,RFC4,MCM3,MCM4,H2AFX,SUMO1,DBF4,BRCA1,RPA2,RPA3,CCNB2,CCNB1,FAM175A,YWHAQ,YWHAZ,CDK1,RAD9A,CHEK1
Cell Cycle Checkpoints	0.021	149	36	8.18E-04	0.0155	SFN,CDC20,TP53,UBE2C,WHSC1,ANAPC15,ANAPC16,CDKN1A,ORC6,MCM7,RFC5,RFC4,MCM3,MCM4,UBC,H2AFX,BUB3,PSMD2,PSMA4,PSMB8,SUMO1,DBF4,BRCA1,RPA2,RPA3,MAD2L1,CCNB2,CCNB1,FAM175A,YWHAQ,YWHAZ,UBA52,MAD1L1,CDK1,RAD9A,CHEK1

SLBP Dependent Processing of Replication-Dependent Histone Pre-mRNAs	0.0015	11	7	8.78E-04	0.0158	SNRPD3,SNRPG,SNRPE,SNRPF,SNRPB,LSM10,NCBP2
Golgi Cisternae Pericentriolar Stack Reorganization	0.0015	11	7	8.78E-04	0.0158	PLK1,RAB2A,RAB1A,CCNB2,CCNB1,CDK1,GOLGA2
Senescence-Associated Secretory Phenotype (SASP)	0.0073	52	17	9.71E-04	0.0175	FOS,UBE2C,ANAPC15,ANAPC16,CDKN1A,CDKN2B,CDKN2A,CEBPB,UBC,EHMT2,EHMT1,STAT3,UBA52,H3F3A,IGFBP7,CDK6,CDK4
RHO GTPases activate CIT	0.0021	15	8	1.19E-03	0.0209	MYL12B,MYH9,MYL6,MYL9,PRC1,KIF14,RHOC,MYH14
mRNA 3'-end processing	0.0049	35	13	1.23E-03	0.0209	SRSF2,SRSF3,SRSF5,SRSF6,SRSF7,SRSF9,SRSF1,PABPN1,SRRM1,CSTF3,RNPS1,NCBP2,UPF3B
Translesion synthesis by Y family DNA polymerases bypasses lesions on DNA template	0.0049	35	13	1.23E-03	0.0209	PCNA,POLD2,RFC5,RFC4,RFC1,KIAA0101,UBC,UBE2L6,RPA2,MAD2L2,RPA3,ISG15,UBA52
Processive synthesis on the C-strand of the telomere	0.0017	12	7	1.44E-03	0.024	APEX1,PCNA,POLD2,LIG1,RPA2,RPA3,FEN1
Phosphorylation of Emi1	0.0008	6	5	1.50E-03	0.024	CDC20,FBXO5,PLK1,CCNB1,CDK1
Anchoring of the basal body to the plasma membrane	0.0127	90	24	1.67E-03	0.0267	NEK2,ODF2,CEP290,CSNK1D,PCM1,CNTRL,SSNA1,AHI1,CEP70,PLK1,CEP83,CEP57,CEP41,CEP135,TUBB,RAB11A,TUBA1A,OFD1,TMEM67,CETN2,DCTN2,RAB3IP,CDK1,IQCB1
Homology Directed Repair	0.0127	90	24	1.67E-03	0.0267	RMI2,UBE2I,WHSC1,RHNO1,RFC5,RFC4,PARP1,TIMELESS,UBC,H2AFX,SUMO1,SUMO2,XRCC2,XRCC1,BRCA1,BRCA2,RPA2,RPA3,FAM175A,UBA52,RAD51AP1,RAD9A,CHEK1,FEN1
Processive synthesis on the lagging strand	0.0023	16	8	1.77E-03	0.0272	APEX1,PCNA,POLD2,LIG1,RPA2,RPA3,FEN1,PRIM1
Cell junction organization	0.0098	70	20	1.81E-03	0.0272	JUP,LIMS1,ITGB1,ITGA6,ACTN1,PLEC,VASP,PARVB,RSU1,CADM1,FERMT2,FLNA,FLNC,FBLIM1,PARD6B,LAMC2,CDH6,CDH2,CD151,CTNNA1
Semaphorin interactions	0.0091	65	19	1.81E-03	0.0272	DPYSL4,DPYSL2,DPYSL3,ITGB1,ITGA1,RRAS,PLXND1,TLN1,MYL12B,MYH9,MYL6,MYL9,HSP90AB1,MET,SEMA5A,SEMA6A,SEMA3E,RHOC,MYH14
Telomere Maintenance	0.0052	37	13	1.99E-03	0.0299	APEX1,PCNA,POLD2,RFC5,RFC4,RFC1,LIG1,RPA2,RPA3,DKC1,NHP2,FEN1,PRIM1

Axon guidance	0.0631	449	84	2.03E-03	0.0304	DPYSL4,DPYSL2,DPYSL3,UNC5B,UNC5D,JAK1,DUSP6,AGRN,ARPC5,ARPC2,ITGB1,ITGAV,ACTR3,ACTR2,ITGA2,ITGA1,MYO10,ACTB,SDC2,RRAS,NRP2,SPTAN1,CAP1,CAP2,VEGFA,PEBP1,FGF19,ALCAM,ARPC1B,PEA15,PLXND1,FGF9,PITPNA,TLN1,MYL12A,MYL12B,SPTBN1,VASP,ROBO1,HRAS,VAV3,NRCAM,UBC,FGFR3,FGFR2,FGFR1,COL6A2,COL6A1,DCX,MYH9,PSMD2,MYL6,MYL9,PSMA4,PSMB8,PTPRA,HSP90AB1,MET,ST8SIA2,COL9A1,COL9A3,CALM1,CD24,GPC1,PRNP,LAMC1,MMP2,LAMB1,TRIO,UBA52,SEMA5A,IRS2,SEMA6A,EVL,SEMA3E,EZR,RHOC,COL4A2,COL4A1,SDCBP,MYH14,HSPA8,DLG1,NCAM1
Degradation of the extracellular matrix	0.0114	81	22	2.05E-03	0.0307	CAPNS1,MMP14,SPP1,TIMP2,COL18A1,DCN,HSPG2,FURIN,A2M,COL9A1,COL9A3,CTSV,CTSD,CTSB,CD44,BMP1,LAMC2,LAMC1,MMP2,LAMA5,LAMB1,ADAM9
Loss of proteins required for interphase microtubule organizationÃ,Â from the centrosome	0.0087	62	18	2.53E-03	0.0354	NEK2,ODF2,CEP290,CSNK1D,PCM1,CNTRL,SSNA1,CEP70,PLK1,CEP57,CEP41,CEP135,TUBB,TUBA1A,OFD1,CETN2,DCTN2,CDK1
Loss of Nlp from mitotic centrosomes	0.0087	62	18	2.53E-03	0.0354	NEK2,ODF2,CEP290,CSNK1D,PCM1,CNTRL,SSNA1,CEP70,PLK1,CEP57,CEP41,CEP135,TUBB,TUBA1A,OFD1,CETN2,DCTN2,CDK1
Regulation of Insulin-like Growth Factor (IGF) transport and uptake by Insulin-like Growth Factor Binding Proteins (IGFBPs)	0.0024	17	8	2.57E-03	0.0359	IGF2,PAPPA2,MMP2,IGFBP5,IGFBP3,IGFBP2,IGFBP6,PAPPA
Iron uptake and transport	0.0055	39	13	3.10E-03	0.0435	FTL,ATP6V1E1,ATP6V1G1,ATP6V1C2,ATP6AP1,ATP6V1B1,TCIRG1,FTH1,ATP6V0E1,CP,ATP6V0B,ATP6V1D,ATP6V1F
DNA Damage Bypass	0.0062	44	14	3.31E-03	0.0438	PCNA,POLD2,USP1,RFC5,RFC4,RFC1,KIAA0101,UBC,UBE2L6,RPA2,MAD2L2,RPA3,ISG15,UBA52
Mitotic Telophase/Cytokinesis	0.002	14	7	3.37E-03	0.0438	SMC3,PLK1,SMC1A,KIF20A,KIF23,RAD21,STAG2
Recognition of DNA damage by PCNA-containing replication complex	0.0037	26	10	3.39E-03	0.044	PCNA,POLD2,USP1,RFC5,RFC4,RFC1,UBC,RPA2,RPA3,UBA52
Detoxification of Reactive Oxygen Species	0.0037	26	10	3.39E-03	0.044	GSTP1,TXN2,ERO1A,ATOX1,CYCS,SOD3,PRDX2,PRDX1,GPX1,GPX3
Insulin receptor recycling	0.0037	26	10	3.39E-03	0.044	ATP6V1E1,ATP6V1G1,ATP6V1C2,ATP6AP1,ATP6V1B1,TCIRG1,ATP6V0E1,ATP6V0B,ATP6V1D,ATP6V1F
Syndecan interactions	0.0025	18	8	3.61E-03	0.0469	ITGB1,ITGAV,ITGA2,ACTN1,SDC4,SDC2,TNC,TGFB1

Synthesis of DNA	0.0135	96	24	3.73E-03	0.048	APEX1,PCNA,POLD2,CDKN1A,ORC6,MCM7,RFC5,RFC4,RFC1,MCM3,MCM4,GINS2,GINS4,LIG1,UBC,PSMD2,PSMA4,PSMB8,RPA2,RPA3,UBA52,CDT1,FEN1,PRIM1
Nucleotide Excision Repair	0.0143	102	25	4.00E-03	0.048	DDB2,ACTB,PCNA,UBE2I,POLD2,HMGN1,RFC5,RFC4,RFC1,POLR2G,POLR2H,POLR2I,POLR2L,PARP1,LIG1,UBC,ACTL6A,SUMO1,SUMO2,XRCC1,CETN2,RPA2,RPA3,UBA52,GTF2H2
Centrosome maturation	0.0098	70	19	4.00E-03	0.048	NEK2,ODF2,CEP290,CSNK1D,PCM1,CNTRL,SSNA1,CEP70,PLK1,CEP57,CEP41,CEP135,TUBB,TUBA1A,OFD1,CETN2,CCNB1,DCTN2,CDK1
Recruitment of mitotic centrosome proteins and complexes	0.0098	70	19	4.00E-03	0.048	NEK2,ODF2,CEP290,CSNK1D,PCM1,CNTRL,SSNA1,CEP70,PLK1,CEP57,CEP41,CEP135,TUBB,TUBA1A,OFD1,CETN2,CCNB1,DCTN2,CDK1
Processing of DNA double-strand break ends	0.0091	65	18	4.09E-03	0.0483	RMI2,UBE2I,WHSC1,RHNO1,RFC5,RFC4,TIMELESS,UBC,H2AFX,SUMO1,SUMO2,BRCA1,RPA2,RPA3,FAM175A,UBA52,RAD9A,CHEK1
Gap-filling DNA repair synthesis and ligation in TC-NER	0.0084	60	17	4.15E-03	0.0483	PCNA,POLD2,HMGN1,RFC5,RFC4,RFC1,POLR2G,POLR2H,POLR2I,POLR2L,LIG1,UBC,XRCC1,RPA2,RPA3,UBA52,GTF2H2
RNA Polymerase II Transcription	0.0152	108	26	4.24E-03	0.0483	SRSF2,SRSF3,SRSF5,SRSF6,SRSF7,SRSF9,SRSF1,SNRPD3,PABPN1,SSRP1,SUPT16H,SNRPG,SNRPE,SNRPF,SNRPB,POLR2G,POLR2H,POLR2I,POLR2L,SRRM1,CSTF3,RNPS1,LSM10,NCBP2,UPF3B,GTF2H2
Condensation of Prometaphase Chromosomes	0.0015	11	6	4.27E-03	0.0483	SMC4,SMC2,NCAPG,CCNB2,CCNB1,CDK1
Dual Incision in GG-NER	0.0051	36	12	4.39E-03	0.0483	DDB2,PCNA,POLD2,RFC5,RFC4,RFC1,PARP1,UBC,RPA2,RPA3,UBA52,GTF2H2
Transferrin endocytosis and recycling	0.0038	27	10	4.39E-03	0.0483	ATP6V1E1,ATP6V1G1,ATP6V1C2,ATP6AP1,ATP6V1B1,TCIRG1,ATP6V0E1,ATP6V0B,ATP6V1D,ATP6V1F
Global Genome Nucleotide Excision Repair (GG-NER)	0.0107	76	20	4.48E-03	0.0492	DDB2,ACTB,PCNA,UBE2I,POLD2,RFC5,RFC4,RFC1,PARP1,LIG1,UBC,ACTL6A,SUMO1,SUMO2,XRCC1,CETN2,RPA2,RPA3,UBA52,GTF2H2
mRNA decay by 5' to 3' exoribonuclease	0.0021	15	7	4.87E-03	0.0536	LSM1,LSM5,LSM4,LSM3,LSM2,LSM7,LSM6
G1/S-Specific Transcription	0.0021	15	7	4.87E-03	0.0536	DHFR,FBXO5,PCNA,RRM2,TYMS,CDK1,CDT1
Translesion Synthesis by POLH	0.0027	19	8	4.96E-03	0.0545	PCNA,RFC5,RFC4,RFC1,UBC,RPA2,RPA3,UBA52
Chaperonin-mediated protein folding	0.0045	32	11	5.00E-03	0.0549	SPHK1,ACTB,TUBB2B,TUBB2A,TUBA1B,TUBA1A,CCT3,CCT8,CCT7,CCT4,TUBB6
E2F mediated regulation of DNA replication	0.0039	28	10	5.61E-03	0.0605	DHFR,FBXO5,PCNA,RRM2,ORC6,TYMS,CCNB1,CDK1,CDT1,PRIM1
Metabolism of fat-soluble vitamins	0.0059	42	13	5.67E-03	0.0605	RDH11,LGMN,AGRN,SDC4,SDC2,APOE,TTR,HSPG2,RBP1,LRP2,GPC1,GPC4,CUBN

SUMOylation	0.0141	100	24	6.05E-03	0.0605	NUP107,CDCA8,PCNA,UBE2I,UBA2,SMC3,HNRNPK,HNRNPC,CDKN2A,SMC1A,TOP2A,TOP2B,PARP1,SUMO1,SUMO2,BIRC5,CETN2,BRCA1,AURKB,AURKA,NUP37,RAD21,MITF,STAG2
Formation of tubulin folding intermediates by CCT/TriC	0.0034	24	9	6.20E-03	0.062	TUBB2B,TUBB2A,TUBA1B,TUBA1A,CCT3,CCT8,CCT7,CCT4,TUBB6
Processing of Capped Intronless Pre-mRNA	0.0034	24	9	6.20E-03	0.062	SNRPD3,PABPN1,SNRPG,SNRPE,SNRPF,SNRPB,CSTF3,LSM10,NCBP2
SUMO E3 ligases SUMOylate target proteins	0.0134	95	23	6.44E-03	0.0644	NUP107,CDCA8,PCNA,UBE2I,SMC3,HNRNPK,HNRNPC,CDKN2A,SMC1A,TOP2A,TOP2B,PARP1,SUMO1,SUMO2,BIRC5,CETN2,BRCA1,AURKB,AURKA,NUP37,RAD21,MITF,STAG2
Chk1/Chk2(Cds1) mediated inactivation of Cyclin B:Cdk1 complex	0.0017	12	6	6.44E-03	0.0644	SFN,CCNB1,YWHAQ,YWHAZ,CDK1,CHEK1
Protein folding	0.0053	38	12	6.62E-03	0.0661	SPHK1,ACTB,TUBB2B,TUBB2A,TUBA1B,TUBA1A,ARL2,CCT3,CCT8,CCT7,CCT4,TUBB6
L1CAM interactions	0.0111	79	20	6.72E-03	0.0672	DPYSL2,ITGB1,ITGAV,ITGA2,ITGA1,NRP2,SPTAN1,ALCAM,SPTBN1,NRCAM,FGFR1,DCX,CD24,LAMC1,LAMB1,EZR,SDCBP,HSPA8,DLG1,NCAM1
Polo-like kinase mediated events	0.0023	16	7	6.83E-03	0.0683	CENPF,MYBL2,PLK1,FOXM1,CCNB2,CCNB1,RBBP4
Synthesis and interconversion of nucleotide di- and triphosphates	0.0023	16	7	6.83E-03	0.0683	RRM2B,DCTPP1,RRM1,RRM2,DTYMK,NME4,NME1
HDR through Homologous Recombination (HR) or Single Strand Annealing (SSA)	0.012	85	21	7.23E-03	0.0707	RMI2,UBE2I,WHSC1,RHNO1,RFC5,RFC4,TIMELESS,UBC,H2AFX,SUMO1,SUMO2,XRCC2,BRCA1,BRCA2,RPA2,RPA3,FAM175A,UBA52,RAD51AP1,RAD9A,CHEK1
Cellular Senescence	0.0176	125	28	7.86E-03	0.0707	AGO3,AGO1,FOS,TP53,UBE2C,LMNB1,ANAPC15,ANAPC16,CDKN1A,CDKN2B,CDKN2A,CEBPB,TNRC6B,UBC,EHMT2,EHMT1,EZH2,STAT3,HMGA1,H1F0,MAPK10,UBA52,H3F3A,IGFBP7,CDK6,CDK4,RBBP4,RBBP7
A tetrasaccharide linker sequence is required for GAG synthesis	0.0035	25	9	7.96E-03	0.0717	BGN,AGRN,SDC4,SDC2,DCN,HSPG2,GPC1,GPC4,B4GALT7
APC-Cdc20 mediated degradation of Nek2A	0.0035	25	9	7.96E-03	0.0717	NEK2,CDC20,UBE2C,ANAPC15,ANAPC16,UBC,BUB3,MAD2L1,UBA52
Folding of actin by CCT/TriC	0.0013	9	5	8.20E-03	0.0738	ACTB,CCT3,CCT8,CCT7,CCT4
Endosomal/Vacuolar pathway	0.0013	9	5	8.20E-03	0.0738	HLA-B,HLA-C,HLA-A,HLA-E,B2M

Association of TriC/CCT with target proteins during biosynthesis	0.0013	9	5	8.20E-03	0.0738	SPHK1,CCT3,CCT8,CCT7,CCT4
RHO GTPases activate PAKs	0.003	21	8	8.76E-03	0.0763	MYL12B,MYH9,MYL6,MYL9,FLNA,CALM1,CTTN,MYH14
Cyclin D associated events in G1	0.0042	30	10	8.85E-03	0.0763	CDKN1A,CDKN2B,CDKN2A,TFDP2,UBC,CCND1,UBA52,CDK6,CDK4,CKS1B
G1 Phase	0.0042	30	10	8.85E-03	0.0763	CDKN1A,CDKN2B,CDKN2A,TFDP2,UBC,CCND1,UBA52,CDK6,CDK4,CKS1B
Oncogene Induced Senescence	0.0042	30	10	8.85E-03	0.0763	AGO3,AGO1,TP53,CDKN2B,CDKN2A,TNRC6B,UBC,UBA52,CDK6,CDK4
RHO GTPases activate PKNs	0.0049	35	11	9.38E-03	0.0763	SFN,PKN2,MYL12B,MYH9,MYL6,MYL9,YWHAQ,YWHAZ,H3F3A,RHOC,MYH14
SUMO is transferred from E1 to E2 (UBE2I, UBC9)	0.0008	6	4	9.53E-03	0.0763	UBE2I,UBA2,SUMO1,SUMO2
Activation of NIMA Kinases NEK9, NEK6, NEK7	0.0008	6	4	9.53E-03	0.0763	PLK1,CCNB2,CCNB1,CDK1
POLB-Dependent Long Patch Base Excision Repair	0.0008	6	4	9.53E-03	0.0763	APEX1,PARP1,LIG1,FEN1
Prefoldin mediated transfer of substrate to CCT/TriC	0.0037	26	9	0.0101	0.0807	ACTB,TUBB2B,TUBB2A,TUBA1A,CCT3,CCT8,CCT7,CCT4,TUBB6
Cooperation of Prefoldin and TriC/CCT in actin and tubulin folding	0.0044	31	10	0.0109	0.0874	ACTB,TUBB2B,TUBB2A,TUBA1B,TUBA1A,CCT3,CCT8,CCT7,CCT4,TUBB6
Downregulation of SMAD2/3:SMAD4 transcriptional activity	0.0031	22	8	0.0113	0.0907	TGIF1,TGIF2,PARP1,UBC,UBA52,NEDD4L,SMAD2,SMAD3
Amyloid fiber formation	0.0058	41	12	0.0115	0.0922	MFGE8,TTR,CST3,SNCA,HSPG2,FURIN,UBE2L6,H3F3A,APP,B2M,ITM2B,TGFBI
Removal of the Flap Intermediate from the C-strand	0.0014	10	5	0.0125	0.0934	PCNA,POLD2,RPA2,RPA3,FEN1
Regulation of gene expression by Hypoxia-inducible Factor	0.0014	10	5	0.0125	0.0934	VEGFA,CA9,CITED2,HIF1A,HIF3A

Metabolism of carbohydrates	0.0336	239	46	0.0125	0.0934	NUP107,B4GAT1,BGN,AGRN,PGK1,PGLS,GAPDH,SDC4,SDC2,GALE,GNS,ST3GAL1,GPI,TPI1,TKT,PGD,PKM,UBC,HK2,DCN,LUM,HSPG2,HEXB,PGAM1,SLC25A11,CALM1,AKR1B1,AKR1A1,CD44,GPC1,GPC4,NUP37,ENO1,ENO2,UBA52,CHST11,CHPF,GYG1,PFKM,PFKP,ALDOA,SLC2A1,SLC2A3,DCXR,B4GALT7,PRELP
Polymerase switching	0.002	14	6	0.013	0.0934	PCNA,POLD2,RFC5,RFC4,RFC1,PRIM1
Leading Strand Synthesis	0.002	14	6	0.013	0.0934	PCNA,POLD2,RFC5,RFC4,RFC1,PRIM1
Polymerase switching on the C-strand of the telomere	0.002	14	6	0.013	0.0934	PCNA,POLD2,RFC5,RFC4,RFC1,PRIM1
Condensation of Prophase Chromosomes	0.002	14	6	0.013	0.0934	SET,SMC4,SMC2,PLK1,CCNB1,CDK1
Removal of the Flap Intermediate	0.002	14	6	0.013	0.0934	PCNA,POLD2,RPA2,RPA3,FEN1,PRIM1
Mismatch repair (MMR) directed by MSH2:MSH6 (MutSalpha)	0.002	14	6	0.013	0.0934	MSH6,PCNA,POLD2,LIG1,RPA2,RPA3
SMAD2/SMAD3:SMAD4 heterotrimer regulates transcription	0.0045	32	10	0.0133	0.0934	TGIF1,TGIF2,CDKN2B,TFDP2,UBC,SERPINE1,JUNB,UBA52,SMAD2,SMAD3
Apoptotic execution phase	0.0066	47	13	0.0135	0.0942	SPTAN1,LMNB1,PLEC,LMNA,CASP7,KPNB1,DSP,DSG2,BCAP31,H1F0,HMGB2,HMGB1,VIM
Retinoid metabolism and transport	0.0052	37	11	0.0137	0.0956	RDH11,AGRN,SDC4,SDC2,APOE,TTR,HSPG2,RBP1,LRP2,GPC1,GPC4
Activation of ATR in response to replication stress	0.0052	37	11	0.0137	0.0956	ORC6,MCM7,RFC5,RFC4,MCM3,MCM4,DBF4,RPA2,RPA3,RAD9A,CHEK1
Homologous DNA Pairing and Strand Exchange	0.0059	42	12	0.0137	0.0956	RMI2,RHNO1,RFC5,RFC4,XRCC2,BRCA1,BRCA2,RPA2,RPA3,RAD51AP1,RAD9A,CHEK1
Meiosis	0.0089	63	16	0.0139	0.0971	UBE2I,LMNB1,SMC3,SMC1A,PSMC3IP,LMNA,H2AFX,BRCA1,BRCA2,RPA2,RPA3,RAD21,H3F3A,CDK4,MND1,STAG2
APC/C:Cdc20 mediated degradation of Cyclin B	0.0032	23	8	0.0144	0.1009	CDC20,UBE2C,ANAPC15,ANAPC16,UBC,CCNB1,UBA52,CDK1
Metabolism of vitamins and cofactors	0.016	114	25	0.0146	0.1021	SHMT2,RDH11,DHFR,LGMN,AGRN,ACP5,PCCA,SDC4,SDC2,APOE,TCN2,TTR,PDXK,HSPG2,RBP1,CYB5R3,LRP2,MMAB,GPC1,GPC4,AASDHPPT,CUBN,RFK,SLC2A1,SLC2A3
G1/S Transition	0.0145	103	23	0.0154	0.1079	DHFR,FBXO5,PCNA,RRM2,CDKN1A,ORC6,MCM7,MCM3,MCM4,TYMS,UBC,PSMD2,PSMA4,PSMB8,DBF4,RPA2,RPA3,CCNB1,UBA52,CDK1,CDT1,PRIM1,CKS1B

Oxidative Stress Induced Senescence	0.009	64	16	0.0158	0.1107	AGO3,AGO1,FOS,TP53,CDKN2B,CDKN2A,TNRC6B,UBC,EZH2,MAPK10,UBA52,H3F3A,CDK6,CDK4,RBBP4,RBBP7
NCAM1 interactions	0.0046	33	10	0.0161	0.113	AGRN,COL6A2,COL6A1,ST8SIA2,COL9A1,COL9A3,PRNP,COL4A2,COL4A1,NCAM1
Scavenging by Class A Receptors	0.0027	19	7	0.0162	0.1133	COL3A1,FTL,APOE,FTH1,COL1A1,COL4A2,COL4A1
Glucose metabolism	0.0098	70	17	0.0169	0.1155	PGK1,GAPDH,GPI,TPI1,PKM,UBC,HK2,PGAM1,SLC25A11,CALM1,ENO1,ENO2,UBA52,GYG1,PFKM,PFKP,ALDOA
Mismatch Repair	0.0021	15	6	0.0176	0.1155	MSH6,PCNA,POLD2,LIG1,RPA2,RPA3
Crosslinking of collagen fibrils	0.0006	4	3	0.0177	0.1155	LOXL1,LOX,BMP1
Establishment of Sister Chromatid Cohesion	0.0015	11	5	0.018	0.1155	CDCA5,SMC3,SMC1A,RAD21,STAG2
APC/C-mediated degradation of cell cycle proteins	0.0115	82	19	0.0186	0.1155	NEK2,CDC20,FBXO5,UBE2C,ANAPC15,ANAPC16,PLK1,UBC,BUB3,PSMD2,PSMA4,PSMB8,MAD2L1,CCNB1,AURKB,AURKA,UBA52,CDK1,PTTG1
Regulation of mitotic cell cycle	0.0115	82	19	0.0186	0.1155	NEK2,CDC20,FBXO5,UBE2C,ANAPC15,ANAPC16,PLK1,UBC,BUB3,PSMD2,PSMA4,PSMB8,MAD2L1,CCNB1,AURKB,AURKA,UBA52,CDK1,PTTG1
Transcriptional activity of SMAD2/SMAD3:SMAD4 heterotrimer	0.0062	44	12	0.0188	0.1155	TGIF1,TGIF2,CDKN2B,TFDP2,PARP1,UBC,SERPINE1,JUNB,UBA52,NEDD4L,SMAD2,SMAD3
Presynaptic phase of homologous DNA pairing and strand exchange	0.0055	39	11	0.0193	0.1155	RMI2,RHNO1,RFC5,RFC4,XRCC2,BRCA1,BRCA2,RPA2,RPA3,RAD9A,CHEK1
Formation of Incision Complex in GG-NER	0.0055	39	11	0.0193	0.1155	DDB2,UBE2I,PARP1,UBC,SUMO1,SUMO2,CETN2,RPA2,RPA3,UBA52,GTF2H2
ROS, RNS production in response to bacteria	0.0048	34	10	0.0194	0.1162	ATP6V1E1,ATP6V1G1,ATP6V1C2,ATP6V1B1,TCIRG1,ATP6V0E1,CYBA,ATP6V0B,ATP6V1D,ATP6V1F
Mitotic Prophase	0.0093	66	16	0.0203	0.122	NUP107,SET,SMC4,SMC2,PLK1,RAB2A,VRK1,RAB1A,LMNA,BANF1,CCNB2,CCNB1,NUP37,ENSA,CDK1,GOLGA2
Dual incision in TC-NER	0.0086	61	15	0.0216	0.1297	PCNA,POLD2,HMGN1,RFC5,RFC4,RFC1,POLR2G,POLR2H,POLR2I,POLR2L,UBC,RPA2,RPA3,UBA52,GTF2H2
SUMOylation of DNA replication proteins	0.0063	45	12	0.0219	0.1315	NUP107,CDCA8,PCNA,UBE2I,TOP2A,TOP2B,SUMO1,SUMO2,BIRC5,AURKB,AURKA,NUP37
Sema4D in semaphorin signaling	0.0035	25	8	0.0224	0.1342	RRAS,MYL12B,MYH9,MYL6,MYL9,MET,RHOC,MYH14
G2/M DNA damage checkpoint	0.0056	40	11	0.0226	0.1357	SFN,WHSC1,H2AFX,SUMO1,BRCA1,CCNB1,FAM175A,YWHAQ,YWHAZ,CDK1,CHEK1

Activation of the pre-replicative complex	0.0042	30	9	0.023	0.1382	ORC6,MCM7,MCM3,MCM4,DBF4,RPA2,RPA3,CDT1,PRIM1
Transcriptional regulation of pluripotent stem cells	0.0049	35	10	0.023	0.1382	PBX1,ZIC3,POLR2G,POLR2H,POLR2I,POLR2L,STAT3,HIF3A,SOX2,SMAD2
RHO GTPases Activate ROCKs	0.0023	16	6	0.0232	0.1393	MYL12B,MYH9,MYL6,MYL9,RHOC,MYH14
Regulation of cytoskeletal remodeling and cell spreading by IPP complex components	0.0011	8	4	0.0245	0.1467	LIMS1,ACTN1,PARVB,RSU1
Activation of DNA fragmentation factor	0.0011	8	4	0.0245	0.1467	KPNB1,H1F0,HMGB2,HMGB1
Apoptosis induced DNA fragmentation	0.0011	8	4	0.0245	0.1467	KPNB1,H1F0,HMGB2,HMGB1
Type I hemidesmosome assembly	0.0011	8	4	0.0245	0.1467	ITGA6,PLEC,LAMC2,CD151
Unwinding of DNA	0.0017	12	5	0.025	0.1499	MCM7,MCM3,MCM4,GINS2,GINS4
IRF3-mediated induction of type I IFN	0.0017	12	5	0.025	0.1499	PRKDC,IFI16,XRCC6,IRF3,DTX4
HSF1 activation	0.0017	12	5	0.025	0.1499	EEF1A1,PTGES3,HSP90AB1,RPA2,RPA3
Chondroitin sulfate/dermatan sulfate metabolism	0.0065	46	12	0.0254	0.1521	BGN,AGRN,SDC4,SDC2,DCN,HSPG2,HEXB,GPC1,GPC4,CHST11,CHPF,B4GALT7
Mitotic Spindle Checkpoint	0.003	21	7	0.026	0.1533	CDC20,UBE2C,ANAPC15,ANAPC16,BUB3,MAD2L1,MAD1L1
Transcription-Coupled Nucleotide Excision Repair (TC-NER)	0.0104	74	17	0.0268	0.1533	PCNA,POLD2,HMGN1,RFC5,RFC4,RFC1,POLR2G,POLR2H,POLR2I,POLR2L,LIG1,UBC,XRCC1,RPA2,RPA3,UBA52,GTF2H2
Cellular response to hypoxia	0.0037	26	8	0.0273	0.1533	EGLN3,VEGFA,CA9,CITED2,UBC,UBA52,HIF1A,HIF3A
Downregulation of TGF-beta receptor signaling	0.0037	26	8	0.0273	0.1533	PPP1CC,UBC,BAMBI,UBA52,NEDD4L,SMAD2,SMAD3,TGFB1
Regulation of Hypoxia-inducible Factor (HIF) by oxygen	0.0037	26	8	0.0273	0.1533	EGLN3,VEGFA,CA9,CITED2,UBC,UBA52,HIF1A,HIF3A
Gluconeogenesis	0.0044	31	9	0.0276	0.1533	PGK1,GAPDH,GPI,TPI1,PGAM1,SLC25A11,ENO1,ENO2,ALDOA
PRC2 methylates histones and DNA	0.0024	17	6	0.0299	0.1533	MTF2,EZH2,H3F3A,DNMT1,RBBP4,RBBP7
Cyclin A/B1 associated events during G2/M transition	0.0024	17	6	0.0299	0.1533	CDC25B,FOXM1,CCNB2,CCNB1,CCNA2,CDK1
Serine biosynthesis	0.0003	2	2	0.0307	0.1533	PHGDH,PSAT1
Cyclin B2 mediated events	0.0007	5	3	0.0314	0.1568	CDC25B,CCNB2,CDK1
SUMO is conjugated to E1 (UBA2:SAE1)	0.0007	5	3	0.0314	0.1568	UBA2,SUMO1,SUMO2

G2/M DNA replication checkpoint	0.0007	5	3	0.0314	0.1568	CCNB2,CCNB1,CDK1
Transport of Mature mRNA derived from an Intron-Containing Transcript	0.0075	53	13	0.0314	0.1572	NUP107,SRSF2,SRSF3,SRSF5,SRSF6,SRSF7,SRSF9,SRSF1,SRRM1,RNPS1,NCBP2,NUP37,UPF3B
Membrane Trafficking	0.034	242	44	0.0326	0.1629	SFN,PRKAG2,ARCN1,FTL,MCFD2,PRKAA2,MYO1C,CSNK1D,SPTAN1,GJA1,AP1S1,VPS28,TRAPPC3,DAB2,RAB1A,FTH1,SPTBN1,TMED3,TMED2,TMED7,TMED9,RAB11A,TRAPPC2L,CHMP4B,UBC,TRAPPC6A,SNF8,VAMP8,VPS37A,ANK3,CALM1,CTSZ,CTSC,CD59,TMED10,YWHAQ,YWHAZ,DCTN2,UBA52,CLTC,LMAN1,APP,GOLGA2,HSPA8
Meiotic recombination	0.0045	32	9	0.0327	0.1635	PSMC3IP,H2AFX,BRCA1,BRCA2,RPA2,RPA3,H3F3A,CDK4,MND1
TGF-beta receptor signaling activates SMADs	0.0045	32	9	0.0327	0.1635	PPP1CC,UBC,BAMBI,FURIN,UBA52,NEDD4L,SMAD2,SMAD3,TGFB1
Signaling by Activin	0.0018	13	5	0.0335	0.1674	FSTL3,INHBA,ACVR2B,SMAD2,SMAD3
NF-kB is activated and signals survival	0.0018	13	5	0.0335	0.1674	UBC,NGFR,SQSTM1,UBA52,NFKBIA
Role of Abl in Robo-Slit signaling	0.0013	9	4	0.0353	0.1766	CAP1,CAP2,ROBO1,GPC1
CHL1 interactions	0.0013	9	4	0.0353	0.1766	ITGB1,ITGA2,ITGA1,HSPA8
Processing and activation of SUMO	0.0013	9	4	0.0353	0.1766	UBE2I,UBA2,SUMO1,SUMO2
HS-GAG degradation	0.0025	18	6	0.0378	0.1889	AGRN,SDC4,SDC2,HSPG2,GPC1,GPC4
HDR through Homologous Recombination (HRR)	0.0069	49	12	0.038	0.1901	RMI2,RHNO1,RFC5,RFC4,XRCC2,BRCA1,BRCA2,RPA2,RPA3,RAD51AP1,RAD9A,CHEK1
Deadenylation-dependent mRNA decay	0.0069	49	12	0.038	0.1901	EIF4B,PABPC1,CNOT10,EIF4A2,EXOSC8,LSM1,LSM5,LSM4,LSM3,LSM2,LSM7,LSM6
DNA Double Strand Break Response	0.0062	44	11	0.0404	0.2022	TP53,SMARCA5,UBE2I,WHSC1,UBC,H2AFX,KPNA2,SUMO1,BRCA1,FAM175A,UBA52
Mismatch repair (MMR) directed by MSH2:MSH3 (MutSbeta)	0.002	14	5	0.0436	0.2179	PCNA,POLD2,LIG1,RPA2,RPA3
Activation of APC/C and APC/C:Cdc20 mediated degradation of mitotic proteins	0.0103	73	16	0.044	0.2202	NEK2,CDC20,UBE2C,ANAPC15,ANAPC16,PLK1,UBC,BUB3,PSMD2,PSMA4,PSMB8,MAD2L1,CCNB1,UBA52,CDK1,PTTG1
Signaling by TGF-beta Receptor Complex	0.0103	73	16	0.044	0.2202	TGIF1,TGIF2,CDKN2B,PPP1CC,TFDP2,PARP1,UBC,BAMBI,FURIN,SERPINE1,JUNB,UBA52,NEDD4L,SMAD2,SMAD3,TGFB1

Platelet activation, signaling and aggregation	0.0285	203	37	0.0455	0.2273	FN1,JAK1,BCAR1,ACTN1,ACTN4,IGF2,CAP1,VEGFA,PPIA,GNB1,CLU,LAMP2,WDR1,TLN1,VAV3,PDPN,FLNA,LEFTY2,ARRB2,A2M,CALM1,CD63,VCL,YWHAZ,SERPINE1,SERPING1,TMSB4X,F2R,PSAP,APP,SPARC,PFN1,ALDOA,SCG3,TGFB2,TGFB1,HSPA5
Other semaphorin interactions	0.0027	19	6	0.0468	0.2286	ITGB1,ITGA1,PLXND1,SEMA5A,SEMA6A,SEMA3E
Phosphorylation of the APC/C	0.0027	19	6	0.0468	0.2286	UBE2C,ANAPC15,ANAPC16,PLK1,CCNB1,CDK1
Amino acid transport across the plasma membrane	0.0027	19	6	0.0468	0.2286	SLC7A5,SLC7A6,SLC7A8,SLC7A2,SLC3A2,SLC38A2
RMTs methylate histone arginines	0.0027	19	6	0.0468	0.2286	SMARCB1,SMARCC1,CCND1,CDK4,RBBP7,PRMT1
Assembly of the primary cilium	0.0242	172	32	0.0478	0.2286	NEK2,ODF2,CEP290,CSNK1D,PCM1,CNTRL,BBS4,SSNA1,AHI1,CEP70,PLK1,CEP83,CEP57,WDR34,CEP41,CEP135,TUBB,RAB11A,DYNLRB1,HSPB11,TUBA1A,OFD1,TMEM67,CETN2,CCT3,CCT8,CCT4,DCTN2,IFT81,RAB3IP,CDK1,IQCB1
Attenuation phase	0.0014	10	4	0.0486	0.2286	DNAJB1,PTGES3,HSP90AB1,HSPA8
Tetrahydrobiopterin (BH4) synthesis, recycling, salvage and regulation	0.0014	10	4	0.0486	0.2286	DHFR,GCHFR,PTS,CALM1
Cohesin Loading onto Chromatin	0.0014	10	4	0.0486	0.2286	SMC3,SMC1A,RAD21,STAG2
Dissolution of Fibrin Clot	0.0014	10	4	0.0486	0.2286	PLAU,SERPINE1,S100A10,ANXA2
Vitamin D (calciferol) metabolism	0.0008	6	3	0.0492	0.2286	LGMN,LRP2,CUBN
MASTL Facilitates Mitotic Progression	0.0008	6	3	0.0492	0.2286	CCNB1,ENSA,CDK1
Meiotic synapsis	0.0049	35	9	0.052	0.2286	UBE2I,LMNB1,SMC3,SMC1A,LMNA,H2AFX,BRCA1,RAD21,STAG2
NRIF signals cell death from the nucleus	0.0021	15	5	0.0553	0.2286	UBC,NGFR,SQSTM1,UBA52,ITGB3BP
STING mediated induction of host immune responses	0.0021	15	5	0.0553	0.2286	PRKDC,IFI16,XRCC6,IRF3,DTX4
COPII (Coat Protein 2) Mediated Vesicle Transport	0.0082	58	13	0.0563	0.2286	MCFD2,CSNK1D,TRAPPC3,RAB1A,TMED2,TRAPPC2L,TRAPPC6A,CTSZ,CTSC,CD59,TMED10,LMAN1,GOLGA2
Transport of Mature Transcript to Cytoplasm	0.0082	58	13	0.0563	0.2286	NUP107,SRSF2,SRSF3,SRSF5,SRSF6,SRSF7,SRSF9,SRSF1,SRRM1,RNPS1,NCBP2,NUP37,UPF3B
Inactivation of APC/C via direct inhibition of the APC/C complex	0.0028	20	6	0.0572	0.2286	CDC20,UBE2C,ANAPC15,ANAPC16,BUB3,MAD2L1

Inhibition of the proteolytic activity of APC/C required for the onset of anaphase by mitotic spindle checkpoint components	0.0028	20	6	0.0572	0.2286	CDC20,UBE2C,ANAPC15,ANAPC16,BUB3,MAD2L1
MicroRNA (miRNA) biogenesis	0.0028	20	6	0.0572	0.2286	POLR2G,POLR2H,POLR2I,POLR2L,DICER1,RAN
Cell surface interactions at the vascular wall	0.0141	100	20	0.058	0.232	CXADR,FN1,SLC7A5,SLC7A6,SLC7A8,ITGB1,ITGAV,ITGA3,ITGA6,PPIA,CAV1,SLC3A2,HRAS,ATP1B1,SLC16A1,SLC16A3,MIF,CD47,CD44,CD74
Intrinsic Pathway for Apoptosis	0.0051	36	9	0.0598	0.2391	SFN,BMF,TP53,BCL2L1,PMAIP1,CASP7,CYCS,YWHAQ,YWHAZ
Fanconi Anemia Pathway	0.0051	36	9	0.0598	0.2391	UBE2T,USP1,FANCL,FANCB,FANCD2,UBC,RPA2,RPA3,UBA52
