# Supplementary material for: Single cell RNA sequencing of stem cell-derived retinal ganglion cells
Source: Sci Data. 2018 Feb 13;5:180013. doi: 10.1038/sdata.2018.13 (PMC5810423; doi:10.1038/sdata.2018.13)
Supplement: Supplementary Tables [file Table s1.pdf › Supplementary tables/Table s1.pdf]

id	baseMean	baseMeanA	baseMeanB	foldChange	log2FoldChange	pval
FN1	3.034981	1.3508185	4.42579027	9.7651371	3.2876403	6.60E-97
CTGF	2.798627	1.3553231	3.99053041	8.4163684	3.07319786	2.56E-85
ELN	2.712724	1.3487157	3.83914508	8.1417196	3.02533354	4.74E-83
IGFBP7	5.601269	2.1096845	8.48467685	6.7448692	2.75379048	1.54E-76
DCT	8.904439	1.8469355	14.7326415	16.214506	4.01921319	6.40E-75
PGK1	11.07602	15.718509	7.24216779	0.4241033	-1.2375125	1.79E-74
STMN1	13.95965	19.24108	9.59815366	0.4713621	-1.0850923	9.27E-65
PKM	19.56027	25.354428	14.7753673	0.5656206	-0.8220933	4.29E-61
MGP	1.90409	1.0187539	2.63521546	87.193583	6.44615006	3.15E-59
KRT19	3.617095	1.9042152	5.03161891	4.4586938	2.15662113	8.16E-59
SFRP2	35.90345	51.907822	22.6867816	0.426001	-1.2310714	2.52E-55
CRABP1	26.44976	38.420962	16.5637472	0.4159099	-1.2656572	3.90E-54
DAPL1	13.743	19.080439	9.33525909	0.4610098	-1.1171308	4.57E-54
ENO1	32.92379	45.735605	22.3435877	0.4771052	-1.0676208	2.53E-52
RAN	10.07196	13.077013	7.59033175	0.5456922	-0.8738407	5.51E-52
TUBB	23.84702	29.934864	18.8195696	0.6158512	-0.6993464	1.32E-48
TPI1	28.62179	37.465475	21.3185261	0.557199	-0.8437354	1.41E-48
HSP90AA1	18.59744	24.262395	13.9192262	0.5553696	-0.84848	4.69E-47
PRDX1	10.03516	13.064239	7.53369446	0.5415753	-0.884766	3.96E-46
CPE	3.766224	5.1254924	2.64371688	0.3984293	-1.3276045	2.13E-45
SERPINF1	4.116021	2.5168768	5.43662106	2.9248395	1.54835744	4.77E-45
COL1A1	4.025737	2.3699233	5.39313479	3.2068472	1.6811556	5.64E-43
H3F3A	35.95869	43.996151	29.3212167	0.6586919	-0.6023243	6.20E-43
PPIA	17.7728	21.982197	14.2966188	0.6337096	-0.6581063	7.34E-41
SIX6	4.677512	6.1892322	3.42910807	0.4681055	-1.0950944	1.74E-38
PRDX2	14.45734	19.155334	10.5776562	0.5275395	-0.9226489	2.89E-38
PTTG1	4.621397	6.5897296	2.99591534	0.3570683	-1.485728	3.82E-38
TUBA1A	15.99203	22.986361	10.2159903	0.4191685	-1.2543977	6.32E-38
LDHA	26.03806	33.602872	19.7909176	0.5763577	-0.7949636	9.66E-38
S100A9	1.70409	1.0321431	2.25899505	39.16844	5.29161976	2.02E-37
PSMA4	4.37448	5.6806946	3.29578614	0.4904798	-1.0277344	2.96E-37
KRT7	1.970263	1.2534364	2.56222982	6.1641896	2.62391123	5.30E-37
ATP5B	5.376131	6.9740102	4.05657526	0.5116455	-0.9667836	6.12E-37
LDHB	14.75122	18.279054	11.8378773	0.6272263	-0.672942	1.21E-36
PEBP1	10.10392	12.615444	8.0298706	0.6052176	-0.7244742	2.24E-36
NPM1	26.23858	31.607081	21.8051796	0.6797505	-0.5569227	3.53E-36
HSPD1	10.56728	12.964776	8.58739344	0.6341442	-0.6571171	1.78E-35
TYRP1	1.703217	1.0823969	2.21589982	14.756625	3.88329094	6.53E-35
ALDOA	31.22104	39.206251	24.6267217	0.6183994	-0.6933892	1.69E-34
CKB	9.249314	13.921887	5.39062687	0.3397822	-1.5573179	7.78E-34
HNRNPA1	49.03652	60.007436	39.976554	0.6605363	-0.5982902	1.23E-33
CFL1	24.01326	28.844022	20.0239346	0.6832323	-0.549552	1.65E-33
HSP90AB1	24.82443	29.722519	20.7795144	0.6886414	-0.5381753	6.46E-32

PMEL	5.187152	3.0646179	6.93997597	2.877034	1.52458228	8.08E-32
CCNB1	1.798501	2.4346749	1.27313874	0.1903837	-2.393018	1.08E-30
PRMT1	3.437837	4.3604705	2.67591154	0.4987134	-1.0037172	1.33E-30
RBP1	23.77806	28.372493	19.9839058	0.6935395	-0.52795	4.10E-30
IGFBP3	1.745576	1.1537964	2.2342771	8.0253988	3.00457308	5.81E-30
KRT17	1.754374	1.1516602	2.25210561	8.2559944	3.045442	7.17E-30
TRPM1	1.715868	1.1581101	2.17647316	7.4408472	2.89546689	7.56E-29
COL3A1	1.602309	1.0388456	2.06762573	27.483809	4.78051005	1.21E-28
HMGB1	19.63298	23.770711	16.2159818	0.668226	-0.5815919	1.53E-28
FOS	3.926178	5.3789483	2.72645601	0.3942627	-1.3427708	2.00E-28
HNRNPK	7.831877	9.6190068	6.35603593	0.6214215	-0.686356	2.11E-28
ERH	7.973533	9.7379362	6.51646049	0.631323	-0.6635497	2.23E-28
TMEM98	5.535272	3.9179205	6.87090675	2.0120174	1.00864279	1.31E-27
TUBA1B	21.56051	28.736329	15.6346013	0.527633	-0.9223934	1.95E-27
MAB21L1	5.345335	6.7870295	4.15475966	0.5451432	-0.8752929	1.54E-26
SSB	3.704228	4.6184172	2.94927483	0.5387092	-0.8924213	2.28E-26
PTMA	80.67723	96.419019	67.6773945	0.6987852	-0.5170791	8.17E-26
EID1	6.408729	7.8771663	5.19606835	0.610145	-0.7127761	2.45E-25
FGF19	2.213944	2.8409946	1.69611549	0.3781192	-1.4030868	3.76E-25
AKR1B1	2.707497	3.3608353	2.16795898	0.4947228	-1.0153078	5.08E-25
TUBB4B	3.14963	4.0626176	2.39566919	0.4557112	-1.1338082	5.36E-25
ATP6V1C2	1.804722	1.1999193	2.30417777	6.5235196	2.70565053	5.63E-25
UCHL1	4.41994	5.4597668	3.56123386	0.5742977	-0.8001293	6.64E-25
PDIA6	5.418215	6.6855656	4.37161573	0.5930132	-0.7538638	7.40E-25
HMGB3	4.578773	5.8635852	3.51775462	0.5176746	-0.9498825	1.14E-24
ARL6IP1	4.009789	5.2150827	3.01443719	0.4779117	-1.0651841	3.10E-24
SKP1	9.167784	11.601471	7.15800505	0.5808632	-0.7837295	8.65E-24
YWHAE	10.96609	13.096852	9.20647332	0.6783974	-0.5597974	9.21E-24
ATP6V1B1	2.607975	3.4322007	1.92731496	0.3812658	-1.391131	1.11E-23
PTGDS	3.572607	1.9039217	4.95063487	4.3705501	2.12781487	3.56E-23
TNFRSF12A	2.108731	1.5363504	2.58141256	2.9484689	1.559966	3.89E-23
MAD2L1	2.000503	2.5274827	1.56531418	0.3700953	-1.4340312	4.68E-23
RCN1	5.081459	6.2025159	4.1556725	0.6065666	-0.721262	6.08E-23
FAM162A	7.947525	9.9863977	6.26379055	0.5857509	-0.7716408	7.66E-23
PDIA3	3.911934	4.9108007	3.08705386	0.5336641	-0.9059962	8.16E-23
TGM2	1.70045	1.2110348	2.1046167	5.2342872	2.38799307	9.49E-23
IGFBP5	13.50654	7.3778595	18.567699	2.754482	1.46178102	9.62E-23
CLNS1A	3.646119	4.4326499	2.99658969	0.5816468	-0.7817848	1.19E-22
ILF2	3.395307	4.1433387	2.77756954	0.5655037	-0.8223917	1.50E-22
FBLN1	2.61102	3.2794991	2.05897952	0.4645668	-1.1060421	2.10E-22
TTR	4.275116	1.4040487	6.64608992	13.973784	3.80465087	2.27E-22
CCT7	3.248322	4.0450988	2.59033067	0.5222591	-0.9371622	2.64E-22
GJA1	3.472938	2.369241	4.3843885	2.471726	1.30551883	5.05E-22
TECR	3.616705	4.4749281	2.90796988	0.5490674	-0.8649447	5.57E-22
GGH	2.171969	2.658681	1.77003342	0.4642444	-1.1070435	5.71E-22

PRDX4	5.950437	7.2218069	4.9005187	0.6269109	-0.6736676	6.08E-22
ETFB	3.865822	4.785236	3.10655502	0.5565188	-0.8454976	9.02E-22
NME1	5.443532	6.7351339	4.37690619	0.5888103	-0.7641251	1.50E-21
MORF4L2	10.05405	11.935367	8.50042551	0.685887	-0.5439573	1.53E-21
CCT8	4.288367	5.1866522	3.54654867	0.6082542	-0.7172538	2.49E-21
FKBP3	3.038267	3.81345	2.39810745	0.496937	-1.0088651	2.70E-21
YWHAQ	7.949211	9.8642608	6.36773044	0.6055474	-0.7236881	3.12E-21
UBB	8.035811	9.7055397	6.65692097	0.649807	-0.6219167	4.28E-21
POLR2G	3.063017	3.7619417	2.48583307	0.5379669	-0.8944108	4.81E-21
BIRC5	2.363441	3.0879881	1.76509842	0.3664285	-1.4483962	5.32E-21
GAPDH	128.8507	148.79199	112.382922	0.7536465	-0.40804	5.36E-21
GAP43	2.823529	3.5613751	2.2142041	0.4740438	-1.0769076	6.25E-21
HNRNPA2B1	10.68478	13.476358	8.37944458	0.5914743	-0.7576127	6.32E-21
PTMS	12.3391	14.377531	10.6557291	0.7217871	-0.4703548	7.66E-21
XRCC6	3.086588	3.7578099	2.5322815	0.5556153	-0.8478417	8.47E-21
WLS	1.531049	1.1230638	1.86797023	7.0530093	2.81823894	8.52E-21
SLC7A8	1.810348	1.3576556	2.18418818	3.310973	1.72725522	1.51E-20
SMARCB1	3.34389	4.0721651	2.74246883	0.5671794	-0.8181229	1.62E-20
EGR1	2.181158	2.7664957	1.69777682	0.3950062	-1.3400527	2.09E-20
BNIP3	13.52632	16.235966	11.2886544	0.6752873	-0.5664267	2.19E-20
HNRNPA0	6.200884	7.3837689	5.22403785	0.661684	-0.5957857	2.78E-20
PSIP1	4.425719	5.5073307	3.53250609	0.5618638	-0.8317076	7.25E-20
HMGB2	6.181981	8.4279317	4.32723822	0.447936	-1.1586354	1.05E-19
SRI	4.203528	5.1535406	3.41899105	0.5823925	-0.7799362	1.62E-19
TYMS	3.177232	3.8869721	2.59111715	0.551137	-0.8595171	2.10E-19
ECHS1	2.937223	3.5525099	2.42910873	0.5598837	-0.8368009	2.81E-19
PHGDH	2.241102	2.7648385	1.80859242	0.4581679	-1.1260516	2.95E-19
IL32	1.69624	1.2395036	2.07342014	4.481854	2.16409567	3.07E-19
NIPSNAP1	2.931533	3.5529201	2.41838173	0.5555919	-0.8479025	3.31E-19
SPATS2L	1.72846	1.324886	2.06173893	3.2680357	1.70842376	3.53E-19
CSRP2	3.335001	4.0431357	2.75021186	0.5751344	-0.7980291	4.14E-19
DCTN2	3.032264	3.6287072	2.53971203	0.5857298	-0.7716929	5.28E-19
NACA	32.7749	37.032023	29.2592951	0.7842828	-0.3505542	7.38E-19
TPM1	8.67694	5.2905603	11.473469	2.4410492	1.2875014	8.02E-19
PARP1	3.614042	4.3936676	2.97021478	0.5805562	-0.7844924	1.27E-18
ANP32A	2.69245	3.2277281	2.2504082	0.561293	-0.833174	1.51E-18
HDAC2	4.12933	4.9791707	3.42751795	0.6100562	-0.7129858	2.13E-18
ATP5O	7.041199	8.5075298	5.83027947	0.6433913	-0.6362316	2.20E-18
PSMB1	5.028892	5.9541253	4.26481911	0.6590102	-0.6016273	2.42E-18
PPP2R1A	3.611977	4.3866658	2.9722271	0.5823507	-0.7800399	3.05E-18
EIF3I	3.62181	4.358985	3.01303872	0.5992997	-0.7386504	3.65E-18
MORF4L1	8.235658	9.931211	6.83544237	0.6533764	-0.6140138	3.83E-18
SPCS2	2.999517	3.6050833	2.4994295	0.5755783	-0.7969158	4.20E-18
MDH1	2.579366	3.0972349	2.15170099	0.5491521	-0.8647223	4.61E-18
YBX1	37.12853	41.360221	33.6339368	0.8085669	-0.306561	4.83E-18

UBE2C	2.623051	3.4914938	1.90587719	0.363588	-1.4596236	4.88E-18
CALM2	13.08121	15.48504	11.0960843	0.6970008	-0.5207678	5.50E-18
GHITM	3.204231	3.8148696	2.69995492	0.6039196	-0.7275716	5.80E-18
TUBB2B	10.95661	12.941227	9.31768442	0.6965519	-0.5216972	6.79E-18
WNT2B	1.56493	1.1542518	1.90407575	5.8610396	2.55115659	8.59E-18
PLD3	5.784213	6.8526659	4.90186729	0.666682	-0.5849292	9.31E-18
RPA3	2.413192	2.8768586	2.03028795	0.5489428	-0.8652723	1.06E-17
CCT5	2.537791	3.0730904	2.0957316	0.5285498	-0.9198886	1.15E-17
CETN2	2.724434	3.2515824	2.28910659	0.5725336	-0.8045677	1.16E-17
NEAT1	53.20754	42.346763	62.1765436	1.4795969	0.56520423	1.27E-17
ATP1B1	3.003385	2.2807287	3.60016599	2.0302238	1.0216388	1.30E-17
PRDX6	10.11144	12.108733	8.46204913	0.6717282	-0.5740505	1.40E-17
MARCKSL1	16.77148	19.470638	14.5424765	0.7331894	-0.4477421	1.66E-17
SPARC	8.037737	6.5343626	9.27924858	1.4959715	0.58108267	1.69E-17
H2AFZ	18.06459	21.430719	15.2847779	0.6991814	-0.5162613	2.08E-17
TXN	10.91859	12.834494	9.33640036	0.7044155	-0.5055015	2.53E-17
PAIP2	5.806315	6.9707037	4.84474424	0.6439349	-0.6350133	3.89E-17
DARS	3.214924	3.8944842	2.65373202	0.5713391	-0.8075808	6.31E-17
APEX1	4.752964	5.6808748	3.98667928	0.6380601	-0.6482358	6.45E-17
BNIP3L	4.633546	5.5353736	3.88880137	0.6369489	-0.6507504	6.48E-17
UQCRC1	2.673495	3.207934	2.2321472	0.5580544	-0.8415224	1.30E-16
NUSAP1	1.915444	2.3720734	1.53835155	0.3923635	-1.3497372	1.36E-16
HN1	6.096708	7.3544452	5.05804859	0.6386157	-0.64698	1.74E-16
SLC25A3	11.52922	13.364316	10.0137717	0.729015	-0.4559796	1.84E-16
CCT3	4.098611	4.9144469	3.42488	0.6194694	-0.6908951	1.89E-16
RBBP7	3.752143	4.4699873	3.15933528	0.6222891	-0.6843431	1.90E-16
SNRPF	6.082596	7.1905129	5.16765989	0.6732334	-0.5708214	1.91E-16
ACTG1	66.84634	75.136368	60.0002951	0.7958347	-0.3294593	2.34E-16
COL4A2	2.292283	1.8295368	2.67442658	2.0185079	1.01328921	3.38E-16
PSMB3	4.077223	4.8990719	3.39852726	0.6151534	-0.7009819	3.44E-16
RPN2	2.816896	3.3720635	2.35842967	0.5726785	-0.8042027	3.67E-16
ST13	8.886361	10.347168	7.68000206	0.7146552	-0.4846808	4.03E-16
BMP4	1.591418	1.2216355	1.89679041	4.0462396	2.01658174	4.82E-16
TFPI2	1.483107	1.0939037	1.80451787	8.5674784	3.09887065	5.32E-16
LAPTM4A	4.575201	5.4868958	3.82230925	0.6290115	-0.6688416	8.42E-16
CALR	6.811507	8.1694141	5.69012488	0.6541852	-0.6122289	9.73E-16
CBX1	4.55697	5.4485092	3.8207226	0.6340827	-0.6572572	1.04E-15
SRP9	10.65359	12.624211	9.02622219	0.6904746	-0.5343398	1.11E-15
RAB11A	3.832299	4.5476166	3.24157807	0.6318547	-0.6623354	1.22E-15
ATP5A1	4.644255	5.4829306	3.9516628	0.6584226	-0.6029143	1.33E-15
CDC20	1.498246	1.8817041	1.18158063	0.2059428	-2.2796843	1.35E-15
CRTAC1	1.901695	2.2753475	1.59312637	0.4650704	-1.1044789	1.55E-15
CKS2	4.991044	6.7853485	3.50927699	0.4337296	-1.2051321	1.56E-15
GPNMB	1.51841	1.1531834	1.82001928	5.3531861	2.42039781	1.75E-15
DCTN3	2.63756	3.1130913	2.244859	0.5891175	-0.7633728	2.02E-15

SNRPD2	10.59507	12.103291	9.34956056	0.7519897	-0.4112151	2.11E-15
GSTA4	2.12305	2.5194217	1.79571967	0.523699	-0.9331901	2.25E-15
ANXA5	4.049167	4.8569267	3.38210529	0.6176175	-0.6952145	2.50E-15
PTOV1	3.069067	3.6380014	2.59923081	0.6062282	-0.7220672	2.70E-15
BTF3	17.67848	20.197719	15.5980471	0.7604053	-0.3951596	2.73E-15
ATP6AP2	2.390915	2.8242527	2.0330568	0.5662904	-0.820386	3.38E-15
PSMD8	3.606953	4.2747938	3.05543841	0.6276543	-0.6719579	3.40E-15
NUDCD2	2.951927	3.4931669	2.50496282	0.603635	-0.7282516	4.14E-15
ARPC3	5.361442	6.323155	4.56724406	0.6701372	-0.5774717	4.27E-15
TBCB	3.690375	4.3479416	3.14734617	0.641393	-0.6407196	4.35E-15
IER3	1.639523	1.2789463	1.93729309	3.3601206	1.748513	4.36E-15
RBMX	7.858935	9.2042863	6.74792201	0.7005999	-0.5133373	4.49E-15
NRP2	1.513812	1.1831932	1.78684335	4.2951569	2.10271082	4.55E-15
ISYNA1	3.933747	4.699661	3.30124288	0.6220145	-0.6849798	4.77E-15
PTGR1	2.070553	2.4594809	1.74937072	0.5134502	-0.9617038	5.05E-15
CCNB2	1.59947	1.9854724	1.28070223	0.2848403	-1.8117749	6.18E-15
C14orf166	4.883802	5.6876637	4.21996046	0.6869009	-0.5418261	7.37E-15
BSG	10.01403	11.697109	8.6241123	0.7127264	-0.4885797	8.69E-15
IMPDH2	6.191982	7.2473588	5.32043419	0.6915617	-0.5320701	9.38E-15
PTGES3	7.260695	8.9441102	5.87050336	0.6130962	-0.7058147	1.29E-14
ACTB	60.32267	71.314362	51.2455573	0.7145846	-0.4848234	1.39E-14
TKT	13.53211	15.901539	11.5754021	0.7096852	-0.4947488	1.42E-14
SNRPB2	3.337788	3.9657902	2.81917278	0.6133855	-0.705134	1.42E-14
PSMB6	3.580023	4.2560747	3.0217279	0.6209096	-0.6875449	1.55E-14
FOSB	1.699968	2.043939	1.4159103	0.3984048	-1.3276931	1.71E-14
RACK1	58.99207	65.654015	53.490523	0.811868	-0.300683	2.04E-14
APOC1	1.9502	1.5373168	2.29116543	2.4029871	1.26482892	2.05E-14
KRT18	5.236058	3.591211	6.59440064	2.1589908	1.1103571	2.14E-14
VBP1	2.232483	2.6110568	1.91985071	0.570961	-0.8085358	2.65E-14
SEPT7	4.973153	5.9954966	4.12888464	0.6263411	-0.6749796	2.75E-14
CCT2	3.407703	3.9815497	2.93381009	0.6485923	-0.6246163	2.87E-14
FSCN1	3.477899	4.1192842	2.9482328	0.6245769	-0.6790489	2.93E-14
ACTN4	2.452079	1.9961178	2.82861892	1.8357456	0.87636612	3.10E-14
MGARP	4.148376	5.0285484	3.42151453	0.6010886	-0.7343504	3.14E-14
MDH2	3.584333	4.2110776	3.06675609	0.6436332	-0.6356894	3.49E-14
PSME2	2.43125	2.8498005	2.08560337	0.5868759	-0.7688726	3.70E-14
ANP32E	3.172959	3.7319521	2.71133309	0.626414	-0.6748116	3.95E-14
CACYBP	2.777308	3.3290752	2.3216487	0.5674564	-0.8174184	4.03E-14
RCN2	6.027166	7.2003105	5.05836461	0.6545422	-0.611442	4.86E-14
MGST3	4.852608	5.7540598	4.10817393	0.6537936	-0.6130928	5.34E-14
CCNA2	1.632289	2.0004226	1.32827859	0.3281399	-1.607617	6.88E-14
PHYHIPL	1.80772	2.1524972	1.52299724	0.4537948	-1.1398879	7.27E-14
CTSH	1.900304	1.51005	2.22258227	2.3969851	1.26122096	7.56E-14
SOD1	9.447725	11.374659	7.85643077	0.6608825	-0.5975343	7.65E-14
XRCC5	3.720235	4.3946443	3.16329646	0.6372675	-0.650029	7.72E-14

BANF1	6.082936	7.0853625	5.25511542	0.6992378	-0.5161449	8.70E-14
ITGAV	2.218818	1.8070439	2.55886861	1.9315784	0.94978021	8.77E-14
MIF	15.15873	17.045663	13.6004619	0.7852877	-0.3487068	9.42E-14
ATP5G3	9.708028	11.416511	8.29713429	0.7005354	-0.5134702	9.97E-14
CALB1	3.610691	4.8064109	2.62324648	0.4264507	-1.2295492	1.00E-13
ESD	4.272627	5.0027998	3.66963783	0.6669426	-0.5843654	1.08E-13
KRT8	5.610781	4.3091741	6.68567028	1.7181539	0.78085923	1.09E-13
C12orf75	2.137209	1.7131128	2.48743416	2.0858329	1.06062362	1.11E-13
SNRPE	8.603666	10.029333	7.4263273	0.7117167	-0.4906249	1.14E-13
HSP90B1	7.401954	8.9434032	6.12900056	0.6456931	-0.6310795	1.16E-13
CYP1B1	11.93356	14.40711	9.8908532	0.6631446	-0.5926045	1.18E-13
LY6E	2.664418	2.1326404	3.1035689	1.8572258	0.89314922	1.36E-13
TIMP3	1.752726	1.3864644	2.05519081	2.7303703	1.44909664	1.37E-13
FABP7	4.374693	5.7207053	3.26313313	0.4794057	-1.060681	1.38E-13
ATPIF1	5.95366	6.8700197	5.19691425	0.7149745	-0.4840364	1.58E-13
TALDO1	2.952659	3.4615368	2.53242019	0.6225461	-0.6837474	1.64E-13
PSMA7	8.077288	9.5895774	6.828414	0.6785449	-0.5594838	2.06E-13
NUDC	2.649654	3.0892401	2.28663575	0.6158391	-0.6993746	2.46E-13
NDUFB4	4.999487	5.9752846	4.19365763	0.6419045	-0.6395694	2.76E-13
VDAC3	2.738103	3.2275049	2.33394669	0.5988524	-0.7397276	2.98E-13
SERBP1	8.038561	9.411013	6.90516743	0.7020756	-0.5103016	3.16E-13
CENPF	3.628055	4.7984713	2.66150655	0.4374145	-1.1929269	3.47E-13
VPS28	3.247567	3.8072624	2.78536115	0.6359794	-0.652948	3.49E-13
DNAJC8	2.831061	3.3068803	2.43812126	0.6234052	-0.6817578	3.79E-13
MZT1	2.227133	2.6473855	1.88008208	0.5342296	-0.9044682	3.79E-13
EBPL	3.364111	3.9444681	2.88484316	0.6401303	-0.6435626	3.88E-13
IFITM1	1.723443	2.0644618	1.44182418	0.4150681	-1.2685799	4.56E-13
SET	7.917051	9.0826922	6.95444554	0.7366909	-0.4408687	4.79E-13
KHDRBS1	4.422453	5.3075467	3.69152748	0.62484	-0.6784413	5.41E-13
SSBP1	3.366208	3.959369	2.87636556	0.6340424	-0.6573487	5.72E-13
CKS1B	4.373549	5.2495799	3.65010796	0.6236165	-0.6812691	6.02E-13
PRDX3	2.918615	3.3800283	2.53757263	0.6460312	-0.6303242	7.55E-13
AASDHPPT	2.257907	2.6702063	1.91742283	0.5492871	-0.8643676	7.55E-13
HNRNPH3	3.815229	4.6141174	3.15549359	0.5964094	-0.7456251	7.66E-13
RHOA	5.999425	7.039717	5.1403346	0.685518	-0.5447336	8.20E-13
CDKN3	1.713916	2.0776568	1.41353285	0.3837334	-1.3818239	8.25E-13
SUB1	6.606048	7.696478	5.70555316	0.7026908	-0.5090382	8.78E-13
LMNB1	1.599804	1.8953265	1.35575578	0.3973475	-1.3315268	1.01E-12
PPA1	5.038831	6.1404235	4.12911861	0.6087278	-0.7161309	1.03E-12
FUNDC2	2.852902	3.3275464	2.46093255	0.6276707	-0.6719203	1.03E-12
ATP5J2	7.38927	8.8097454	6.21621809	0.6679114	-0.5822713	1.15E-12
EIF3E	10.31344	11.884461	9.01606896	0.736469	-0.4413032	1.15E-12
ATXN10	2.657141	3.0943766	2.29606486	0.6188309	-0.692383	1.33E-12
EIF4A1	4.007888	4.6749043	3.45705457	0.6686037	-0.5807768	1.37E-12
SELENOP	2.456326	2.9038103	2.08678689	0.5708483	-0.8088207	1.54E-12

ARHGDIB	1.90801	1.543905	2.20869383	2.2222519	1.15202238	1.60E-12
PLIN2	3.505191	2.7456692	4.13241737	1.7943935	0.84349628	1.74E-12
H2AFY	5.108722	6.0111467	4.36348555	0.6712008	-0.5751837	1.77E-12
GPM6A	2.178381	2.5943572	1.83486172	0.5236353	-0.9333657	1.84E-12
COPE	4.114494	4.8309881	3.52280138	0.658525	-0.6026899	1.86E-12
EEF1B2	19.90031	22.066764	18.1112188	0.8122377	-0.3000262	1.91E-12
TMEM97	2.40484	2.7917407	2.08533055	0.6057408	-0.7232274	2.08E-12
HAUS1	2.17678	2.5237863	1.89021643	0.5842134	-0.7754326	2.27E-12
CENPA	1.530336	1.8619072	1.25651923	0.2976182	-1.7484656	2.38E-12
RBX1	6.51164	7.5049236	5.69137072	0.721203	-0.4715226	2.46E-12
NDUFV1	2.272662	2.6539871	1.95775739	0.5790598	-0.7882158	2.62E-12
GYG1	2.917474	3.489816	2.44482408	0.5802935	-0.7851453	2.84E-12
TIMP2	1.947842	1.6227358	2.21632039	1.9531885	0.9658312	2.89E-12
HACD3	3.177081	3.7142311	2.73349411	0.6386686	-0.6468606	3.28E-12
BZW2	2.220689	2.5795185	1.92436182	0.5852175	-0.7729553	3.35E-12
AIMP1	2.37925	2.7418488	2.07981008	0.6199218	-0.6898418	3.50E-12
NASP	3.171457	3.7362366	2.70505215	0.6231377	-0.6823771	3.58E-12
NDUFC2	4.761906	5.5795413	4.08668978	0.6740172	-0.5691426	3.60E-12
PAX6	10.13785	11.676571	8.86714627	0.7368607	-0.4405361	3.71E-12
CCT6A	3.547614	4.0916759	3.09831917	0.6786996	-0.559155	3.72E-12
LY6H	1.953057	1.5823782	2.25916904	2.1621158	1.1124438	4.01E-12
NDN	3.09269	3.5905199	2.68157312	0.6491257	-0.6234302	4.64E-12
MAP1B	7.777296	9.6793718	6.20653072	0.5998741	-0.7372682	4.86E-12
CCNG1	6.456437	7.5078679	5.58814874	0.7050157	-0.5042728	4.87E-12
MYH9	2.450935	2.0247699	2.80286852	1.759291	0.81499417	4.99E-12
PSME1	3.470142	4.0302157	3.00762373	0.6625349	-0.5939316	4.99E-12
SNRPD1	4.79455	5.5395053	4.17935414	0.7003746	-0.5138014	5.11E-12
DNPH1	3.312885	3.8239244	2.89086005	0.6695859	-0.5786589	5.38E-12
COPS6	3.43167	3.9860225	2.97387729	0.661039	-0.5971927	5.65E-12
RSL1D1	3.38083	3.9445842	2.91527234	0.650439	-0.6205144	6.19E-12
COL4A1	1.791397	1.4654175	2.060597	2.2788077	1.18827919	6.34E-12
PSMB2	3.324043	3.8526815	2.8874839	0.6616525	-0.5958543	6.69E-12
HPRT1	2.322619	2.683473	2.02461935	0.6086343	-0.7163526	7.08E-12
RTN4	7.320447	8.452098	6.38591043	0.7227375	-0.4684564	7.10E-12
NDUFS4	3.373559	3.8825579	2.95321855	0.6775991	-0.5614962	9.84E-12
CD9	2.465179	2.9917002	2.0303689	0.5173313	-0.9508396	1.02E-11
OSTC	5.981285	6.8802539	5.23890087	0.7208704	-0.4721882	1.11E-11
ATP1B3	3.142438	3.6140252	2.7529932	0.6706107	-0.5764527	1.25E-11
VDAC1	5.745015	6.9026269	4.78903971	0.6419243	-0.6395249	1.33E-11
HADH	1.913585	2.2255726	1.65594053	0.5352115	-0.9018191	1.33E-11
SAP18	4.616109	5.2838382	4.06468784	0.715407	-0.4831638	1.39E-11
NDUFS3	2.401126	2.7630974	2.10220494	0.6251526	-0.6777197	1.63E-11
STC2	2.366687	1.8698591	2.77697482	2.0428307	1.03056964	1.66E-11
KPNA2	2.415188	2.9006893	2.01425228	0.5336234	-0.9061061	1.67E-11
LSM5	4.939297	5.7114644	4.30162822	0.7007648	-0.5129979	1.69E-11

LMAN1	4.046216	4.6668981	3.53364663	0.6909509	-0.5333449	1.71E-11
AKR7A2	2.591061	3.0159146	2.24020987	0.6152095	-0.7008503	1.86E-11
CCT4	4.55623	5.2725966	3.96464202	0.6938736	-0.5272552	1.97E-11
GSTO1	2.782516	3.2181211	2.42278519	0.6414371	-0.6406203	2.13E-11
RANBP1	7.121175	8.3431112	6.11207981	0.6961735	-0.5224811	2.14E-11
ODC1	4.136707	4.8218064	3.57094056	0.672703	-0.5719584	2.24E-11
DTD1	2.319231	2.6971657	2.00712683	0.5934169	-0.752882	2.35E-11
UBE2S	2.17356	2.5918718	1.82811035	0.5202117	-0.9428292	2.41E-11
SLC6A15	1.565421	1.2805583	1.80066613	2.8538315	1.51290014	2.56E-11
SNRPD3	2.602533	2.9845232	2.28707952	0.6485586	-0.6246912	2.56E-11
WDR18	1.6707	1.9499828	1.44006336	0.463233	-1.1101902	2.99E-11
SCCPDH	2.216344	2.5577101	1.93443886	0.5998798	-0.7372545	3.37E-11
C9orf135	1.998444	2.3333422	1.72187872	0.5414054	-0.8852187	3.63E-11
COL8A1	1.363926	1.0984227	1.58318283	5.9252849	2.56688452	3.66E-11
WDR54	4.240111	4.8445699	3.74093946	0.7129379	-0.4881518	3.66E-11
TMSB15A	4.694445	5.4581835	4.06373634	0.6872163	-0.5411639	3.78E-11
MAGED2	3.136155	3.7134358	2.65942666	0.6115592	-0.7094359	3.97E-11
TRH	3.442689	4.2539349	2.77274878	0.5448016	-0.8761973	4.27E-11
TPPP3	2.657507	3.2407825	2.17582825	0.52474	-0.9303252	5.13E-11
NDUFV2	3.516045	4.0732673	3.05588139	0.6689563	-0.5800162	5.26E-11
DHRS3	1.526305	1.2364399	1.76568029	3.2383719	1.69526869	5.49E-11
ACTL6A	1.610028	1.8796568	1.38736471	0.4403589	-1.1832483	5.80E-11
VEGFA	1.981229	1.6570809	2.24891535	1.9007027	0.92653287	6.09E-11
NHP2	5.0487	5.8074484	4.42211254	0.7118355	-0.4903842	6.26E-11
TSPAN10	1.543126	1.2650056	1.77280158	2.9161706	1.54407513	7.08E-11
DBI	4.75042	5.4079478	4.20742254	0.7276453	-0.4586927	7.25E-11
PDHB	2.087648	2.4009671	1.8289033	0.5916651	-0.7571473	7.26E-11
RSL24D1	3.73136	4.3044069	3.25812859	0.6833688	-0.5492637	7.43E-11
HSPE1	10.25988	11.80266	8.98583438	0.739247	-0.4358715	7.50E-11
ID3	10.87907	13.624089	8.61219693	0.6029898	-0.7297944	7.50E-11
ACTN1	2.000631	1.6575293	2.28396975	1.9527186	0.96548406	7.76E-11
AHSA1	1.992242	2.3042531	1.7345786	0.5632178	-0.8282351	8.29E-11
STOML2	4.211603	4.8914114	3.65020658	0.6810399	-0.5541887	8.33E-11
CCNI	12.57524	14.135667	11.2866183	0.7831059	-0.3527207	8.34E-11
NCL	5.564756	6.4022073	4.87317574	0.7169617	-0.480032	8.56E-11
COL5A1	1.496884	1.2249693	1.72143526	3.2068162	1.68114168	8.66E-11
ATP5F1	3.564048	4.1203337	3.10465888	0.674498	-0.5681139	9.06E-11
IER2	2.437344	2.844442	2.10115649	0.5970134	-0.7441649	9.52E-11
MYL6	25.95646	28.983878	23.4563612	0.8024749	-0.3174717	9.55E-11
H2AFY2	2.821903	3.26714	2.45421835	0.641433	-0.6406296	9.71E-11
RNF5	3.036697	3.4963588	2.65709984	0.6638068	-0.5911647	1.01E-10
FDCSP	1.366775	1.05629	1.62317789	11.070841	3.46869289	1.07E-10
NUCKS1	7.492754	8.8620257	6.36198626	0.6820108	-0.5521336	1.10E-10
HPF1	2.075651	2.3785415	1.82551966	0.5988355	-0.7397682	1.17E-10
PPIB	5.771615	6.803494	4.91947152	0.6753641	-0.5662626	1.20E-10

ARL3	3.411533	3.9627791	2.95630495	0.6602939	-0.5988198	1.48E-10
TAF7	4.011038	4.6143802	3.51278816	0.6952197	-0.5244592	1.51E-10
COX5B	9.033483	10.193401	8.07560359	0.7696394	-0.3777455	1.59E-10
PLK2	2.339952	1.935219	2.67418681	1.7901547	0.8400843	1.62E-10
EIF3H	6.172016	7.0248809	5.46770649	0.7415427	-0.4313983	1.68E-10
PSMA3	2.717471	3.1103082	2.39305897	0.6601211	-0.5991974	1.71E-10
PPT1	1.970468	2.2861542	1.70976903	0.5518538	-0.8576421	1.78E-10
TMBIM6	4.88415	5.5823085	4.30759993	0.7218196	-0.4702898	2.00E-10
SEZ6L2	1.575655	1.3172867	1.78901915	2.4867706	1.31427344	2.00E-10
HNRNPM	3.418147	3.9661027	2.96563547	0.6626997	-0.5935728	2.02E-10
EIF5	5.116108	5.9178782	4.45399357	0.7023341	-0.5097706	2.06E-10
PSMA2	3.907508	4.6270923	3.31326284	0.6377734	-0.6488843	2.12E-10
PARK7	7.271528	8.4952741	6.26093798	0.7019007	-0.5106612	2.12E-10
FLNA	3.192801	2.6844458	3.61260876	1.5510198	0.63321711	2.15E-10
ALCAM	1.589105	1.3314568	1.80187496	2.4192445	1.27455661	2.23E-10
PTH2	7.974922	9.6938692	6.55538748	0.6390006	-0.6461109	2.27E-10
CP	1.939714	2.2953087	1.64605789	0.4987675	-1.0035606	2.52E-10
FSTL1	2.520187	2.1063535	2.86193837	1.6829507	0.75099291	2.70E-10
GSTM3	2.183721	2.5157267	1.90954558	0.6000723	-0.7367918	2.76E-10
TUFM	3.831956	4.4251951	3.34205004	0.6837713	-0.5484143	2.79E-10
SMARCE1	3.346911	3.8482838	2.93286979	0.6786086	-0.5593485	3.26E-10
PDIA4	2.780734	3.1792214	2.45165674	0.6661355	-0.5861124	3.36E-10
PFN2	7.260481	8.3785708	6.33714379	0.7233303	-0.4672735	3.46E-10
PA2G4	3.574406	4.0806861	3.15631134	0.6999452	-0.5146862	3.54E-10
SHMT2	2.109456	2.4297294	1.84496953	0.5909996	-0.7587709	3.61E-10
CDK4	4.316041	4.9592024	3.78490772	0.7034012	-0.5075803	3.61E-10
BHLHE40	1.836021	1.5184168	2.09830392	2.1185731	1.08309288	3.66E-10
SSRP1	2.706623	3.1326046	2.35484024	0.6352984	-0.6544938	3.87E-10
ADH5	2.20749	2.5152934	1.95330096	0.6291197	-0.6685935	3.92E-10
NDUFS5	10.82147	12.748386	9.23019574	0.7005384	-0.5134639	4.39E-10
NDUFC1	6.110148	6.952244	5.41473195	0.741692	-0.4311078	4.46E-10
MED4	2.246003	2.5615603	1.98540978	0.6310418	-0.6641926	5.07E-10
UBE2T	1.831501	2.1262761	1.58807048	0.522137	-0.9374996	6.43E-10
SRSF9	7.143102	8.2630237	6.21825221	0.7184683	-0.4770036	6.75E-10
OLA1	4.090658	4.684774	3.60002791	0.7056139	-0.503049	6.94E-10
UBE2L6	2.180564	2.5012978	1.91569765	0.6099374	-0.7132669	7.33E-10
ATP5C1	3.850749	4.5332878	3.28709655	0.6472998	-0.627494	7.95E-10
GPM6B	3.070292	3.5765478	2.65221811	0.6412526	-0.6410352	8.16E-10
FAM60A	3.028619	3.5202397	2.62263023	0.6438396	-0.6352267	8.24E-10
ETFA	2.126653	2.4212799	1.88334553	0.6215141	-0.6861409	8.34E-10
DPM3	2.701043	3.0664195	2.39930925	0.6771661	-0.5624183	8.42E-10
ANP32B	4.413054	5.0600926	3.87871803	0.7090277	-0.4960862	8.44E-10
ECH1	2.638728	3.03038	2.31529449	0.6478071	-0.6263639	8.62E-10
HNRNPA3	5.870529	6.8121209	5.09294629	0.7042087	-0.505925	9.71E-10
TGFBI	1.43323	1.1649461	1.65478408	3.9696844	1.98902431	1.00E-09

IMMP1L	2.864314	3.3142078	2.49278466	0.6450521	-0.6325124	1.07E-09
COX5A	6.378946	7.3144503	5.60639191	0.7295001	-0.45502	1.07E-09
EXOSC8	1.980166	2.25873	1.75012302	0.5959364	-0.7467698	1.13E-09
GLO1	3.617357	4.252492	3.09285188	0.643461	-0.6360753	1.16E-09
RTN3	3.222727	3.6915658	2.83555241	0.6819645	-0.5522314	1.17E-09
FTH1	111.8545	98.317008	123.033973	1.253984	0.32651897	1.19E-09
TMEM256	6.005283	6.8707368	5.29057644	0.7308412	-0.4523701	1.25E-09
UFC1	3.452923	3.9681938	3.02740357	0.6830429	-0.549952	1.30E-09
CENPV	6.188917	7.1480443	5.39685474	0.7151631	-0.4836557	1.42E-09
HMX1	2.62705	3.0552623	2.27342583	0.6195929	-0.6906076	1.55E-09
HSPA9	2.100807	2.4159213	1.84058075	0.5936635	-0.7522828	1.66E-09
ENY2	4.655667	5.3142564	4.11179379	0.7212816	-0.4713654	1.66E-09
TMEM258	9.349573	10.335993	8.53497097	0.8070883	-0.3092016	1.69E-09
TNC	1.530344	1.2588096	1.75458095	2.9155835	1.54378466	1.71E-09
EEF1A1	200.3231	217.54412	186.101662	0.8547988	-0.2263432	1.72E-09
MEST	9.043304	10.686476	7.68634629	0.6902765	-0.5347538	1.77E-09
MFGE8	3.020437	2.5672866	3.3946555	1.5278989	0.61154911	1.82E-09
SSBP2	2.549475	2.9005719	2.25953251	0.6627124	-0.5935453	1.82E-09
CDK1	1.737449	2.0385457	1.48879873	0.4706569	-1.0872523	1.91E-09
EIF4A2	11.48243	12.994337	10.2338817	0.7698535	-0.3773442	2.09E-09
GTSE1	1.672459	1.9590537	1.43578454	0.4543901	-1.1379966	2.27E-09
WDR61	1.888623	2.1556081	1.66814226	0.5781737	-0.7904251	2.27E-09
PCBP2	7.146865	8.01519	6.42978804	0.7740044	-0.3695863	2.30E-09
TAGLN3	1.408161	1.6974223	1.16928426	0.2427285	-2.0425847	2.34E-09
TPX2	1.790015	2.1038729	1.53082557	0.4808756	-1.0562643	2.59E-09
ITM2B	6.542578	7.4620678	5.78324856	0.740204	-0.4340051	2.61E-09
VSX2	1.910883	2.188695	1.68146073	0.5732848	-0.8026761	2.67E-09
HSD17B10	2.380272	2.7436641	2.08017635	0.6194865	-0.6908553	2.77E-09
CDKN1C	1.775385	1.4729651	2.02512897	2.1674517	1.11599984	2.81E-09
HNRNPR	4.013233	4.6489303	3.48826355	0.6819159	-0.5523343	2.81E-09
BDH2	2.117218	2.408511	1.87666353	0.6224045	-0.6840757	3.22E-09
NDUFB6	2.392937	2.73449	2.11087699	0.6404632	-0.6428124	3.35E-09
SRSF3	5.468587	6.3063066	4.7767835	0.7117537	-0.49055	3.53E-09
FKBP7	1.746773	2.0004621	1.53727234	0.5370242	-0.896941	3.54E-09
RTFDC1	2.184114	2.4952479	1.92717473	0.6200809	-0.6894716	3.60E-09
ANKRD1	1.315537	1.041743	1.54164004	12.97559	3.69772819	3.69E-09
TIMP1	6.665133	7.4936993	5.98088962	0.7670342	-0.3826371	4.04E-09
NDUFA8	2.277427	2.5880577	2.02090241	0.6428623	-0.6374184	4.20E-09
RBMS1	2.236527	1.9300082	2.4896549	1.6017653	0.67966279	4.25E-09
EAPP	1.738913	1.9907664	1.53092909	0.5358772	-0.9000258	4.35E-09
RARRES2	3.494702	4.0058379	3.07259817	0.6895243	-0.5363268	4.46E-09
SERPING1	1.700552	1.9496511	1.49484163	0.5210773	-0.9404306	4.81E-09
CRIM1	1.649116	1.3939603	1.85982755	2.1825233	1.12599706	4.95E-09
ATP5G2	16.90747	19.685879	14.6130047	0.7285183	-0.4569629	5.35E-09
DNAJA1	3.494536	3.9878664	3.08713633	0.6985374	-0.5175908	5.40E-09

SNRPB	5.176596	5.8644121	4.60858648	0.741834	-0.4308316	5.42E-09
PLPP3	2.242824	2.5629903	1.97842449	0.6259952	-0.6757764	5.46E-09
ZNF503	1.57127	1.3287266	1.77156574	2.3471353	1.23090099	5.65E-09
MRPL9	2.089683	2.3739045	1.85496893	0.6222914	-0.6843378	5.79E-09
FAM132A	1.674954	1.4216476	1.88413942	2.096868	1.06823606	6.08E-09
SLC2A3	2.440414	2.0667194	2.74901633	1.6396217	0.713363	6.18E-09
HNRNPC	5.743711	6.4315687	5.17566732	0.7687774	-0.3793622	6.32E-09
TSPAN6	2.183546	2.468703	1.94805793	0.6455069	-0.6314955	6.71E-09
MRPL47	2.099378	2.3746358	1.87206607	0.6343979	-0.6565401	6.80E-09
SDHC	3.152502	3.6139742	2.77141127	0.6776698	-0.5613457	7.17E-09
C1QBP	3.517283	3.9949673	3.12280274	0.70879	-0.4965699	7.32E-09
NDUFB3	3.37979	3.8806569	2.96616547	0.6825407	-0.5510131	7.38E-09
TFDP2	3.034577	3.4523838	2.68954546	0.6889401	-0.5375496	7.42E-09
DECR1	2.149056	2.4512106	1.89953242	0.6198497	-0.6900097	7.57E-09
UQCRFS1	3.270928	3.755911	2.87042111	0.6786943	-0.5591662	8.02E-09
COL9A3	1.559625	1.319545	1.75788634	2.3717674	1.24596251	9.04E-09
RGS16	1.425251	1.7030921	1.1958052	0.2784916	-1.8442945	9.20E-09
NDUFB11	6.599954	7.4935156	5.86203557	0.7487524	-0.4174393	9.67E-09
RAD21	2.633109	2.9744549	2.35121976	0.6843508	-0.5471921	9.78E-09
SLC2A1	11.06684	9.4842585	12.3737639	1.3405725	0.42284928	1.08E-08
SMC4	2.497638	2.9183129	2.15023774	0.599609	-0.737906	1.08E-08
GNPDA1	2.144234	2.4396878	1.90024416	0.6253051	-0.6773677	1.10E-08
HINT1	22.90186	25.278629	20.9390768	0.8212604	-0.2840883	1.28E-08
ECI2	2.177228	2.4546695	1.94811178	0.6517713	-0.6175623	1.28E-08
CHCHD2	14.29653	16.321634	12.6241672	0.7586767	-0.3984428	1.29E-08
SARS	2.142023	2.4496478	1.88798058	0.6125492	-0.7071024	1.32E-08
UBE2V2	2.841083	3.2152636	2.53207914	0.6916013	-0.5319875	1.33E-08
UXT	3.787604	4.2897043	3.37296162	0.7213298	-0.4712692	1.33E-08
FAM64A	1.598556	1.8523313	1.38898481	0.4563775	-1.1317006	1.33E-08
ITGB1	4.581598	3.9517597	5.10172881	1.3895876	0.47465681	1.36E-08
MRFAP1	4.997397	5.7057557	4.41242221	0.7251592	-0.4636303	1.44E-08
PSMA1	4.470262	5.0979078	3.95194196	0.7203534	-0.4732232	1.46E-08
LSM6	2.566905	2.9694349	2.23448868	0.6268238	-0.6738681	1.47E-08
GNAI3	2.172562	2.4476155	1.94541842	0.6530867	-0.6146536	1.51E-08
DSG2	1.610702	1.3759125	1.80459412	2.140376	1.09786425	1.52E-08
PSMD13	1.838307	2.0877257	1.63233308	0.5813351	-0.7825582	1.57E-08
DSP	1.697247	1.4355797	1.91333607	2.096829	1.06820923	1.61E-08
LGALS1	4.354816	2.8154355	5.62606167	2.5481828	1.3494688	1.63E-08
FKBP4	2.173581	2.5007975	1.90336024	0.6019201	-0.732356	1.65E-08
BEX1	4.801705	5.5626987	4.17326306	0.6954794	-0.5239203	1.80E-08
PPP1CC	4.194172	4.7776994	3.71228458	0.7179726	-0.4779993	1.81E-08
EIF5A	7.393059	8.6294579	6.37202071	0.7041157	-0.5061157	1.91E-08
NUF2	1.43409	1.6601966	1.2473669	0.3746867	-1.4162433	1.96E-08
NDFIP1	3.024407	3.5024928	2.62959497	0.6511887	-0.6188525	2.02E-08
SSR2	4.821033	5.4548423	4.29762257	0.7402333	-0.4339481	2.02E-08

MLLT11	5.634186	6.3880209	5.01165702	0.7445511	-0.4255572	2.12E-08
PAFAH1B3	4.605192	5.3825021	3.96327602	0.6761608	-0.5645618	2.15E-08
NELFE	2.352485	2.6738236	2.08711849	0.6494821	-0.6226383	2.21E-08
NDUFA13	5.649591	6.3583774	5.06426435	0.7584879	-0.3988019	2.22E-08
DIO3	3.154498	3.8838163	2.55221408	0.53825	-0.8936517	2.35E-08
APOE	6.574608	5.3970237	7.54707724	1.4889793	0.57432369	2.37E-08
HNRNPDL	11.76466	13.509595	10.3236629	0.7453209	-0.4240663	2.38E-08
OTX2	1.611874	1.3582725	1.82130303	2.2923979	1.19685749	2.38E-08
PPID	1.703664	1.9406549	1.50795345	0.5399998	-0.8889692	2.44E-08
SERPINB6	2.785165	3.1630124	2.47313243	0.6810559	-0.5541548	2.45E-08
SNRPA1	1.937954	2.1894721	1.73024579	0.6139243	-0.7038674	2.55E-08
SH3BGRL	3.228754	3.6302415	2.89719939	0.7213023	-0.471324	2.56E-08
HNRNPF	2.835193	3.1831022	2.54788454	0.7090298	-0.4960818	2.57E-08
NAE1	2.51687	2.837665	2.25195165	0.6812731	-0.5536949	2.60E-08
PSMC5	2.617644	2.9391919	2.35210446	0.6972515	-0.520249	2.61E-08
CUEDC2	2.933891	3.3341068	2.60338669	0.686938	-0.5417482	2.74E-08
TMEFF2	1.832765	1.4988018	2.10855652	2.2224388	1.15214371	2.78E-08
EIF4E	2.214102	2.4875941	1.98824711	0.6643258	-0.5900372	2.94E-08
SNRPG	8.486478	9.3914574	7.7391309	0.8030942	-0.3163588	2.99E-08
CAPZA2	2.770692	3.1381958	2.46720074	0.6861863	-0.5433277	3.10E-08
SMC3	2.586593	2.9488718	2.28741654	0.6605958	-0.5981603	3.16E-08
FDPS	2.769104	3.126858	2.47366451	0.6928834	-0.5293156	3.25E-08
SUMO2	16.926	19.458808	14.8343704	0.7494726	-0.4160524	3.29E-08
SOX2	3.662195	4.2367331	3.1877317	0.6759074	-0.5651026	3.29E-08
CAPZB	4.372254	4.9217321	3.91848646	0.744183	-0.4262706	3.43E-08
PRSS33	1.298261	1.0844339	1.47484311	5.6238462	2.49155714	3.47E-08
FBL	3.658373	4.1026657	3.29146953	0.7385486	-0.4372352	3.47E-08
MT1G	1.396873	1.1292144	1.61790962	4.7820476	2.2576285	3.58E-08
SNRPC	3.255684	3.6943394	2.89343522	0.7027456	-0.5089255	3.66E-08
MIR4435-2HG	2.189419	1.8051611	2.5067461	1.8713598	0.90408698	3.69E-08
PSMA5	2.237015	2.5326354	1.99288709	0.6478299	-0.626313	3.74E-08
SEPT2	4.423072	4.9752328	3.96708905	0.7463938	-0.4219911	3.75E-08
MRPL13	2.099753	2.3899674	1.86008978	0.6187841	-0.6924919	3.83E-08
STRAP	4.298981	4.9018137	3.8011521	0.7179103	-0.4781246	3.84E-08
CYTOR	2.62822	2.201279	2.98079471	1.6489049	0.72150816	3.85E-08
HSPA8	11.21542	12.941174	9.79026983	0.7361311	-0.4419653	3.85E-08
CPXM1	1.719852	1.9540862	1.52641786	0.5517508	-0.8579112	4.02E-08
DDOST	2.549522	2.8672765	2.28711432	0.6893003	-0.5367954	4.26E-08
CDC123	1.756579	1.9924029	1.56183121	0.5661321	-0.8207893	4.34E-08
ACP1	4.489601	5.0973255	3.9877317	0.7291907	-0.4556319	4.54E-08
TRPM3	4.545056	3.7079804	5.23632624	1.5643858	0.64559638	4.69E-08
ITM2C	4.098314	3.5420301	4.557703	1.3995519	0.48496499	4.69E-08
COPZ1	2.563556	2.9485794	2.24559661	0.6392332	-0.6455858	5.28E-08
ALDH7A1	1.949781	2.1992419	1.74377153	0.6202014	-0.6891913	5.60E-08
CBX3	5.439129	6.1431108	4.85777012	0.750085	-0.414874	6.02E-08

IDH3B	1.765966	2.0006721	1.57214125	0.571757	-0.806526	6.03E-08
RNASEH2A	1.822968	2.059702	1.62746994	0.5921192	-0.7560404	6.51E-08
CIRBP	11.70404	12.726927	10.8593292	0.8407428	-0.2502636	6.55E-08
PABPC1	13.70969	15.312472	12.3860803	0.7955356	-0.3300016	6.73E-08
PCMT1	2.364811	2.6711878	2.11179971	0.6652752	-0.5879769	7.51E-08
SRP14	20.42887	22.250831	18.9242729	0.8434622	-0.2456047	7.74E-08
PSMC3	2.446369	2.8065441	2.14892977	0.6359821	-0.6529419	8.09E-08
GABARAPL2	4.595424	5.144889	4.14166614	0.7579615	-0.3998036	8.12E-08
RRAGA	1.915205	2.1567339	1.71574575	0.6187644	-0.6925379	8.29E-08
FRG1	1.891468	2.1308321	1.69379679	0.6135277	-0.7047997	8.35E-08
AKR1A1	3.350424	3.7521256	3.01869237	0.733503	-0.4471253	8.37E-08
CECR5	2.247021	2.5113831	2.02870722	0.6806396	-0.555037	8.54E-08
PAICS	2.847208	3.2311749	2.5301224	0.6857922	-0.5441566	9.14E-08
TMEM123	3.370602	3.8705696	2.95771978	0.681997	-0.5521628	9.20E-08
COPS8	2.35404	2.6606464	2.10083921	0.662898	-0.5931412	9.60E-08
PNRC2	2.734181	3.0828394	2.44625235	0.6943658	-0.5262323	9.90E-08
MAD2L2	2.777674	3.1381167	2.48001528	0.6922051	-0.5307285	1.01E-07
KIF5B	3.373044	3.7807609	3.03634419	0.7322975	-0.4494983	1.02E-07
ERP29	4.199982	4.742314	3.75211502	0.7354046	-0.4433899	1.04E-07
MAGOH	1.961101	2.2033176	1.76107478	0.6324804	-0.6609074	1.05E-07
TXNL1	2.939144	3.3191012	2.62536853	0.7008614	-0.5127989	1.07E-07
PRC1	1.526897	1.7523292	1.34073123	0.4529018	-1.1427299	1.07E-07
FAM192A	2.012342	2.2581294	1.80936729	0.64331	-0.6364139	1.12E-07
DKK3	5.900725	6.6289556	5.29933937	0.7637899	-0.3887523	1.16E-07
ARL2BP	2.47997	2.7701984	2.2402946	0.7006529	-0.5132283	1.21E-07
NAP1L1	18.43816	21.050288	16.2810296	0.7621352	-0.3918812	1.21E-07
ATP5L	13.16998	14.485699	12.0834399	0.8218662	-0.2830246	1.22E-07
CYC1	3.157269	3.5540225	2.82962284	0.7163691	-0.4812249	1.23E-07
APOO	1.921124	2.1600922	1.72378002	0.6238987	-0.6806162	1.29E-07
COX6C	11.07139	12.726383	9.70467688	0.7423156	-0.4298955	1.31E-07
RUVBL1	1.681178	1.9035352	1.49755134	0.5506718	-0.8607354	1.35E-07
TERF2IP	2.487376	2.8165438	2.21554335	0.6691517	-0.5795948	1.35E-07
CMBL	1.842866	2.0776331	1.648991	0.6022375	-0.7315956	1.36E-07
SAE1	1.778266	2.0063395	1.58991896	0.5862027	-0.7705284	1.39E-07
TRAPPC6A	1.803347	2.0336457	1.61316235	0.5932036	-0.7534007	1.42E-07
EIF6	2.36048	2.6420995	2.12791422	0.6868732	-0.5418842	1.47E-07
GRHPR	2.467483	2.785008	2.2052654	0.6752157	-0.5665797	1.49E-07
S100A6	1.35904	1.1022143	1.57113026	5.5875764	2.48222264	1.49E-07
EEF1E1	1.938249	2.1806632	1.7380591	0.6251225	-0.6777892	1.50E-07
ERI3	2.349434	2.6231127	2.12342632	0.6921431	-0.5308577	1.56E-07
NPM3	2.99813	3.4346485	2.63764609	0.6726417	-0.5720899	1.59E-07
DPCD	2.773387	3.1271206	2.48126767	0.6963722	-0.5220694	1.59E-07
SCOC	3.854059	4.3277788	3.46285335	0.7400893	-0.4342288	1.63E-07
HINT2	2.142643	2.3980436	1.93172832	0.6664516	-0.5854281	1.67E-07
CDC25B	1.586639	1.8058059	1.40564817	0.5034068	-0.9902035	1.69E-07

CD24	9.089113	10.609336	7.83368664	0.7111507	-0.4917727	1.74E-07
UCP2	2.210117	2.5251883	1.94992565	0.6228251	-0.6831009	1.75E-07
MZT2B	9.53733	10.526034	8.72084135	0.8104991	-0.3031176	1.84E-07
DHRS7	1.697439	1.9187793	1.51465201	0.5601476	-0.836121	1.92E-07
TSC22D1	11.26575	13.265724	9.61414099	0.7022937	-0.5098535	2.12E-07
CALM3	4.924643	5.6191675	4.35109239	0.7254754	-0.4630014	2.19E-07
GMNN	1.781924	2.006774	1.59623846	0.5922267	-0.7557785	2.23E-07
ELAVL1	2.466204	2.7818719	2.20551971	0.6765468	-0.5637384	2.23E-07
IFITM2	5.607067	6.3868591	4.96310254	0.7356982	-0.442814	2.31E-07
TXNDC17	4.634894	5.1970753	4.17063572	0.7554393	-0.4046122	2.39E-07
CKAP2	1.872616	2.1203695	1.66801771	0.5962477	-0.7460164	2.41E-07
FAM84B	1.477474	1.2668391	1.65141929	2.4412442	1.28761663	2.51E-07
HMGN2	12.48244	14.076829	11.1657591	0.7773872	-0.3632948	2.54E-07
LPP	2.213564	1.9438162	2.43632686	1.521829	0.60580627	2.57E-07
PSMB5	4.335779	4.8588767	3.90379626	0.7524978	-0.4102407	2.62E-07
FLNB	1.483463	1.2812346	1.65046679	2.3128976	1.20970138	2.62E-07
CPNE3	2.505986	2.8213412	2.24556135	0.6838704	-0.5482051	2.66E-07
UBA52	27.79234	30.473631	25.5780942	0.8339011	-0.2620518	2.66E-07
CENPH	2.020205	2.2988293	1.79011176	0.6083261	-0.7170832	2.76E-07
CCDC25	2.425123	2.7270044	2.1758252	0.6808467	-0.5545981	2.78E-07
TSPAN3	5.442588	6.1040996	4.89630122	0.763367	-0.3895513	2.79E-07
EIF3M	3.276719	3.6507827	2.9678103	0.7423507	-0.4298273	2.83E-07
CNBP	5.553707	6.4022782	4.85294315	0.7132071	-0.487607	3.06E-07
PDHA1	1.777955	2.004408	1.59094682	0.5883533	-0.7652453	3.10E-07
LAYN	1.417084	1.2207173	1.57924752	2.6243867	1.3919803	3.15E-07
EIF4A3	1.606566	1.8174759	1.43239226	0.5289358	-0.9188355	3.15E-07
ARF1	4.174752	4.6852565	3.75316979	0.7470768	-0.4206715	3.15E-07
DNER	1.498543	1.7104491	1.32354769	0.4554129	-1.1347529	3.23E-07
LHX2	2.483289	2.8329963	2.19449462	0.6516623	-0.6178035	3.33E-07
PBK	1.513301	1.7351113	1.330126	0.449083	-1.154946	3.39E-07
SLC25A5	8.349455	9.4287032	7.4581932	0.7662143	-0.3841801	3.48E-07
NDUFB5	3.290784	3.7216154	2.93499684	0.7109736	-0.492132	3.55E-07
KDELR1	6.043914	6.8187537	5.40403858	0.7568697	-0.4018831	3.57E-07
NDUFA5	3.236678	3.6298251	2.91201104	0.7270487	-0.459876	3.75E-07
PGRMC1	4.523668	5.2103895	3.95656272	0.7022065	-0.5100328	3.89E-07
STXBP6	1.438478	1.2416996	1.60098124	2.4864805	1.31410511	3.98E-07
AHNAK2	1.780864	1.5540218	1.96819377	1.7475735	0.80535313	4.10E-07
PFKL	2.260226	2.5517429	2.01948589	0.6569941	-0.6060478	4.14E-07
PHPT1	6.418453	7.0873873	5.86603589	0.7993636	-0.3230762	4.22E-07
IFITM3	9.241149	10.516132	8.18824761	0.7553749	-0.4047352	4.25E-07
NEIL2	1.988513	2.2256415	1.7926891	0.6467545	-0.62871	4.36E-07
C8orf33	2.673311	2.9804227	2.41969349	0.7168639	-0.4802289	4.47E-07
YWHAB	4.559303	5.0785681	4.13048597	0.7675454	-0.3816761	4.68E-07
PDLIM1	1.964572	1.7013205	2.18196927	1.6853483	0.7530468	4.90E-07
CHD7	2.837945	3.1860894	2.55044235	0.709231	-0.4956725	4.95E-07

SNX17	2.135677	2.3786327	1.93504067	0.6782377	-0.5601371	5.15E-07
C12orf57	4.292719	4.8055957	3.86917616	0.7539361	-0.4074858	5.15E-07
HOXB5	1.232585	1.4782626	1.02970039	0.0621006	-4.0092493	5.20E-07
NCCRP1	1.307597	1.0985782	1.4802076	4.8713355	2.28431735	5.23E-07
BCAS2	1.694695	1.9078202	1.5186929	0.5713609	-0.8075259	5.25E-07
UBE2E3	4.896173	5.5031549	4.39491668	0.7538974	-0.4075599	5.35E-07
PSMD4	2.91748	3.3218076	2.58357957	0.682046	-0.5520591	5.42E-07
MINOS1	4.515827	5.0769758	4.05242146	0.7486975	-0.4175452	5.45E-07
TSEN34	2.800301	3.1161854	2.53943866	0.7274593	-0.4590616	5.56E-07
HMGN1	15.27596	17.425096	13.5011702	0.7611018	-0.3938387	5.59E-07
G6PC3	2.340231	2.6304002	2.1006042	0.6750515	-0.5669304	5.84E-07
PPP4C	3.217682	3.5871388	2.91257909	0.7392642	-0.4358381	5.86E-07
POLR2E	3.362812	3.7523253	3.04114486	0.7416074	-0.4312724	6.01E-07
SNRNP40	1.62501	1.8315826	1.45441856	0.5464503	-0.8718378	6.07E-07
RAB31	3.140863	3.5202087	2.82759322	0.7251754	-0.4635982	6.18E-07
CHCHD3	2.273345	2.5513647	2.04375257	0.6727964	-0.5717581	6.24E-07
ADSL	2.133707	2.3695721	1.93892499	0.6855609	-0.5446434	6.31E-07
SF3B6	5.203602	5.8719417	4.65167633	0.749532	-0.415938	6.40E-07
DLGAP5	1.491421	1.7046902	1.3153	0.4474306	-1.1602641	6.42E-07
EIF3K	6.221348	7.0021201	5.57657302	0.7624927	-0.3912045	6.49E-07
DNAJC1	4.423465	4.9359137	4.00027604	0.762282	-0.3916033	6.70E-07
MYRF	1.311811	1.1276952	1.46385697	3.6325335	1.86097609	6.78E-07
CCDC80	1.795553	1.5613753	1.98894149	1.7616405	0.81691951	6.88E-07
SYNCRIP	2.226257	2.4694378	2.02543415	0.6978411	-0.5190295	7.02E-07
HNRNPA1L2	1.770485	1.9928505	1.5868511	0.591077	-0.758582	7.07E-07
JUN	2.967549	3.4732871	2.54990158	0.6266566	-0.6742531	7.28E-07
FXR1	2.997144	3.3627129	2.6952519	0.7175023	-0.4789447	7.31E-07
PCP4	1.377327	1.1588564	1.55774322	3.51099	1.81187787	7.45E-07
PSMD7	2.415383	2.7071271	2.17445506	0.6879717	-0.539579	7.72E-07
LEFTY2	3.851251	5.3989696	2.57312027	0.3576111	-1.4835367	7.74E-07
PCNA	1.887108	2.1182174	1.69625359	0.622646	-0.6835159	7.77E-07
NDUFAB1	3.558776	4.0639236	3.1416164	0.6989784	-0.5166802	7.86E-07
LBR	1.709338	1.9187505	1.53640219	0.5838388	-0.776358	7.91E-07
VPS25	2.330685	2.5910056	2.11570825	0.7012598	-0.5119791	8.26E-07
ATP5H	3.589196	4.0987192	3.16842406	0.6997808	-0.5150251	8.40E-07
HTATSF1	1.852217	2.0706597	1.67182428	0.6274863	-0.6723442	8.41E-07
PGLS	4.013166	4.5048322	3.60713996	0.7438701	-0.4268773	8.45E-07
ACAA2	2.722035	3.0349661	2.46361214	0.7192317	-0.4754715	8.62E-07
MCM7	1.903737	2.1283383	1.71825733	0.6365621	-0.6516269	8.71E-07
H2AFV	3.718963	4.1444147	3.36761791	0.7529598	-0.4093552	8.72E-07
AP1S1	2.965445	3.3012035	2.68817005	0.7336031	-0.4469283	8.85E-07
GDI2	4.413321	5.0607231	3.8786857	0.7089096	-0.4963264	8.97E-07
VCL	1.79507	1.581782	1.97120585	1.6693639	0.73929848	9.02E-07
NUDT5	2.102204	2.3370955	1.90822745	0.679254	-0.557977	9.14E-07
GSN	2.171598	2.4877618	1.91050399	0.6119958	-0.7084063	9.31E-07

SLC5A3	1.956265	1.7268686	2.14570408	1.5762189	0.65646789	9.44E-07
PRKRA	2.262881	2.5105506	2.05835157	0.7006396	-0.5132556	9.69E-07
TIMM17B	4.386251	4.9384294	3.93025339	0.7440157	-0.426595	9.73E-07
ASS1	2.00792	2.2560172	1.80303799	0.6393527	-0.6453161	1.00E-06
THOC3	2.075248	2.3204259	1.87277651	0.6609811	-0.5973191	1.03E-06
AK2	2.125529	2.3591819	1.93257377	0.6861288	-0.5434488	1.06E-06
SCHIP1.1	2.296332	2.5885528	2.05501162	0.6641338	-0.5904542	1.07E-06
SERPINB9	1.405556	1.2080073	1.56869418	2.7340103	1.45101868	1.08E-06
IDH2	3.368513	3.8054186	3.00770985	0.7156543	-0.4826652	1.09E-06
PGM1	2.152552	2.392707	1.95422882	0.6851612	-0.5454846	1.11E-06
GOLM1	2.61914	2.9149341	2.37486753	0.7179712	-0.4780021	1.18E-06
RIDA	1.778698	1.9896358	1.6045021	0.6108329	-0.7111503	1.19E-06
RUVBL2	1.992277	2.2220251	1.80254743	0.6567357	-0.6066153	1.20E-06
SCP2	3.277163	3.6545258	2.9655311	0.7404453	-0.433535	1.38E-06
CMSS1	2.313352	2.5753294	2.09700712	0.6963668	-0.5220806	1.40E-06
GCSH	4.4906	5.0739978	4.00881968	0.7385423	-0.4372476	1.46E-06
CCNB1IP1	3.69386	4.1334303	3.33085522	0.7438669	-0.4268835	1.47E-06
EIF3L	7.626006	8.5630009	6.85221952	0.773796	-0.3699749	1.49E-06
ARL2	3.915234	4.3550184	3.55205344	0.7606675	-0.3946621	1.51E-06
LSM7	5.654382	6.3186091	5.10585239	0.7719786	-0.3733672	1.53E-06
SPAG4	2.490754	2.8289454	2.2114703	0.6623874	-0.5942529	1.56E-06
DCX	1.277608	1.4967648	1.09662468	0.1945079	-2.3620993	1.57E-06
NOB1	1.936739	2.1580519	1.75397575	0.6510725	-0.6191099	1.57E-06
H1FX	3.019838	3.3728397	2.72832354	0.7283777	-0.4572413	1.61E-06
ZFAS1	16.45838	17.85799	15.3025566	0.8484141	-0.2371595	1.61E-06
BEX4	6.524863	7.2051921	5.9630352	0.7998198	-0.3222532	1.65E-06
DCTPP1	2.298929	2.5441416	2.09642777	0.7100565	-0.4939943	1.68E-06
DDAH2	5.399123	6.0038161	4.89975795	0.7793568	-0.3596442	1.70E-06
CDKN1A	2.289847	1.9066777	2.60627405	1.7716041	0.82505627	1.70E-06
NDUFA12	3.325772	3.7342226	2.98846692	0.7272513	-0.4594742	1.71E-06
MKI67	2.28797	2.7307817	1.92228787	0.5328736	-0.9081348	1.73E-06
ARPC4	2.694339	3.0091928	2.43432785	0.7138826	-0.4862412	1.76E-06
FDFT1	2.322532	2.5896761	2.10192012	0.6931727	-0.5287132	1.82E-06
CDCA8	1.333311	1.5121994	1.18558264	0.362325	-1.4646439	1.87E-06
FMC1	2.329922	2.6058451	2.10206071	0.6862808	-0.543129	1.88E-06
UBXN1	3.65084	4.069699	3.3049385	0.7508679	-0.4133689	1.90E-06
EML1	1.817557	2.0354842	1.6375889	0.6157399	-0.6996071	1.90E-06
RLBP1	1.292469	1.1103893	1.442834	4.0115653	2.00416529	2.00E-06
MRPL21	2.135784	2.3679606	1.94404944	0.6901145	-0.5350923	2.05E-06
POLR2H	2.545305	2.8105176	2.32628843	0.7325466	-0.4490076	2.14E-06
PGAM1	9.942629	11.208546	8.8972145	0.7735886	-0.3703616	2.19E-06
HNRNPH1	3.942196	4.5942592	3.40371228	0.6687643	-0.5804302	2.25E-06
CHMP5	2.636721	2.9201959	2.40262295	0.7304583	-0.4531262	2.28E-06
GBAS	2.300298	2.5643281	2.0822572	0.6918352	-0.5314997	2.37E-06
IK	2.14346	2.3917035	1.93845609	0.6743218	-0.5684908	2.39E-06

DKC1	1.675483	1.8736785	1.51180959	0.58581	-0.7714953	2.49E-06
ITIH5	1.20885	1.0414495	1.34709125	8.3738388	3.06588914	2.55E-06
RTCB	1.685696	1.8846581	1.52138953	0.5893684	-0.7627583	2.55E-06
BASP1	3.530742	3.9438685	3.18957447	0.7437746	-0.4270627	2.58E-06
EIF4B	4.695964	5.1670975	4.30689403	0.7935725	-0.333566	2.63E-06
RBPMS	1.606969	1.4146129	1.76581912	1.8470701	0.8852386	2.63E-06
DCAF13	1.777459	1.9810608	1.60932078	0.6210836	-0.6871407	2.64E-06
DYNLL1	8.916629	9.9135232	8.09337648	0.7957994	-0.3295233	2.66E-06
SLIRP	6.29966	6.9465652	5.76543548	0.8013761	-0.3194485	2.66E-06
RPA2	1.733482	1.9345003	1.56747695	0.6072518	-0.7196333	2.68E-06
C1orf54	1.843329	2.0524809	1.67060746	0.6371683	-0.6502536	2.72E-06
CCDC34	2.883315	3.2067227	2.6162388	0.7324159	-0.449265	2.75E-06
PSMB7	3.722549	4.1528119	3.36723045	0.7508315	-0.4134389	2.75E-06
BZW1	3.450492	3.8063734	3.15659844	0.7684645	-0.3799494	2.76E-06
POLD2	3.651505	4.1360027	3.25139911	0.71792	-0.478105	2.78E-06
PDGFC	1.308451	1.1351676	1.4515518	3.3406816	1.74014249	2.82E-06
ARMCX1	1.697469	1.9023449	1.52827863	0.5854509	-0.7723798	2.88E-06
DGUOK	3.921523	4.3638863	3.55621203	0.7598985	-0.3961214	2.92E-06
MGAT4B	1.596792	1.4057689	1.75454116	1.8595344	0.89494147	2.93E-06
LDOC1	1.741678	1.9426116	1.57574358	0.6107962	-0.711237	2.94E-06
PFKM	1.827623	2.0336065	1.65751767	0.6361392	-0.6525855	2.99E-06
BOD1	2.944657	3.2658716	2.67939266	0.7411685	-0.4321265	3.00E-06
NDUFB8	5.307634	5.9647384	4.76498625	0.7583453	-0.3990731	3.18E-06
STAT3	1.830293	2.0389984	1.65794003	0.6332445	-0.6591654	3.22E-06
FAU	31.1044	34.136686	28.6002955	0.8329226	-0.2637456	3.28E-06
SNW1	1.881871	2.0890188	1.71080496	0.6527022	-0.6155033	3.42E-06
MTCH2	3.060723	3.4559038	2.73437573	0.7062067	-0.5018376	3.51E-06
LINC01508	1.615445	1.8105053	1.45436021	0.5605888	-0.8349852	3.53E-06
LHFP	1.408692	1.2310938	1.55535557	2.4031613	1.26493348	3.56E-06
CCDC12	1.968184	2.1802626	1.79304612	0.6719235	-0.5736312	3.57E-06
MAGEF1	2.421392	2.6995335	2.1916977	0.7011911	-0.5121205	3.60E-06
HSBP1	11.05428	12.343178	9.98988857	0.792537	-0.3354498	3.65E-06
TCEAL4	7.468307	8.2376907	6.83293708	0.8059113	-0.3113069	3.77E-06
SKA2	2.406622	2.6919217	2.17101617	0.692122	-0.5309018	3.79E-06
TTYH1	2.973845	3.4163562	2.60841118	0.665635	-0.5871969	3.80E-06
DYNC1I2	3.855047	4.2703088	3.51211683	0.7681589	-0.3805233	3.88E-06
PTRF	1.418434	1.2402101	1.56561346	2.354661	1.23551935	3.89E-06
DHPS	1.970703	2.1814937	1.79662879	0.6742556	-0.5686324	3.96E-06
PTRHD1	2.40146	2.7053892	2.15047027	0.6746086	-0.5678773	4.06E-06
AHNAK	1.386863	1.211666	1.531543	2.5112344	1.32839667	4.10E-06
AKAP12	5.401181	4.5610914	6.09494111	1.4307246	0.516746	4.29E-06
ARL6IP5	3.495057	3.8873929	3.17106022	0.7519102	-0.4113677	4.36E-06
THOC7	2.492589	2.7718793	2.26194613	0.7122077	-0.48963	4.56E-06
IFT57	2.52244	2.8224549	2.27468315	0.6994319	-0.5157445	4.63E-06
FIGN	2.916925	3.2695587	2.62571428	0.716313	-0.4813381	4.63E-06

PSMD10	1.806938	2.007377	1.64141254	0.6367155	-0.6512792	4.66E-06
SPTAN1	1.667519	1.4761207	1.82557958	1.7339713	0.79407998	4.67E-06
SMARCC1	2.851934	3.1399852	2.614056	0.754237	-0.4069102	4.69E-06
TOP2A	3.1728	3.8469352	2.61608778	0.5676588	-0.8169041	4.77E-06
LTA4H	2.829402	3.1471363	2.56701181	0.7298148	-0.4543977	4.82E-06
CEBPB	1.466854	1.2872254	1.61519338	2.1418491	1.09885683	4.83E-06
PHB	4.202707	4.6614574	3.8238632	0.7712402	-0.3747478	4.85E-06
SNX2	1.865391	2.068384	1.69775668	0.6530954	-0.6146344	5.02E-06
EFHD2	2.130193	2.3589848	1.94125235	0.6926143	-0.5298759	5.03E-06
CPLX3	1.231325	1.0581719	1.37431756	6.4346747	2.68586722	5.07E-06
LINC01551	1.444852	1.6257481	1.29546586	0.4721802	-1.0825905	5.20E-06
ASPM	1.959857	2.2690102	1.70455392	0.5551996	-0.8489216	5.26E-06
NDUFS8	4.434524	4.8689176	4.07579405	0.7950012	-0.3309711	5.26E-06
NME7	1.687369	1.8798471	1.52841817	0.6005796	-0.7355727	5.27E-06
ACTR10	1.942071	2.1485379	1.77156749	0.6717823	-0.5739342	5.31E-06
SYNPR	1.962733	2.1706554	1.79102679	0.6757128	-0.565518	5.37E-06
SUGT1	2.108625	2.3358535	1.92097676	0.6894294	-0.5365252	5.45E-06
SMARCA4	2.668131	2.9489595	2.43621844	0.7369155	-0.4404289	5.62E-06
MRPS34	3.025631	3.3579044	2.75123367	0.7427077	-0.4291336	5.76E-06
SRSF7	3.970273	4.4019825	3.61376044	0.7683051	-0.3802488	5.87E-06
EIF2S3	4.912311	5.4165857	4.49587184	0.7915327	-0.3372792	5.91E-06
BAG1	2.541222	2.8051047	2.3233035	0.7330896	-0.4479385	6.06E-06
MARCH3	1.838413	2.0425079	1.66986731	0.6425537	-0.638111	6.06E-06
PALLD	3.327494	3.7081777	3.01311869	0.7433481	-0.4278901	6.08E-06
IMP3	2.034081	2.2494115	1.85625769	0.6853288	-0.5451318	6.11E-06
AURKB	1.595783	1.7990479	1.42792414	0.5355425	-0.900927	6.17E-06
GLRX3	2.482861	2.7396788	2.27077729	0.7304666	-0.4531098	6.34E-06
FAM216A	1.811081	2.008814	1.64778988	0.6421302	-0.6390623	6.42E-06
BCHE	1.654297	1.8436312	1.49794182	0.5902364	-0.7606352	6.50E-06
SMC2	1.792271	1.9944551	1.62530462	0.6287912	-0.6693471	6.50E-06
MRPL45	1.855225	2.0551898	1.6900916	0.6539976	-0.6126427	6.63E-06
RBM3	3.838693	4.2687762	3.48352354	0.7597717	-0.3963622	6.87E-06
GTF2H5	3.480143	3.926561	3.1114841	0.7214899	-0.470949	6.89E-06
CEP70	1.445403	1.6217646	1.29976132	0.4821138	-1.0525543	6.92E-06
SF3B5	4.405333	4.8758418	4.01677922	0.7783546	-0.3615005	6.99E-06
COX7C	25.0572	26.738433	23.6688084	0.8807377	-0.1832157	7.05E-06
TXNDC12	2.611989	2.8938294	2.37924044	0.7282813	-0.4574324	7.43E-06
SERPINE1	1.309646	1.1064092	1.47748142	4.4872204	2.16582205	7.43E-06
NUP37	1.495002	1.6731208	1.3479081	0.5168584	-0.9521591	7.73E-06
MEAF6	2.99377	3.309765	2.73281686	0.7502135	-0.4146269	7.78E-06
SHISA2	1.980777	2.2275982	1.77694887	0.6329016	-0.6599468	7.81E-06
H3F3B	18.10379	19.589844	16.8765868	0.8540463	-0.2276139	7.87E-06
RNH1	2.452902	2.6998473	2.24897078	0.7347547	-0.4446655	7.96E-06
TRAP1	1.888376	2.0877833	1.72370193	0.6652997	-0.5879236	7.97E-06
C4orf3	10.7017	11.760929	9.82697424	0.8202799	-0.2858118	8.24E-06

OARD1	2.255681	2.4976335	2.05587245	0.7050273	-0.504249	8.40E-06
BUB3	2.192442	2.4152833	2.00841627	0.712519	-0.4889995	8.93E-06
CCNDBP1	1.850342	2.0496394	1.68575833	0.6533275	-0.6141217	9.27E-06
PNRC1	4.313799	4.7413134	3.96074976	0.7913664	-0.3375823	9.29E-06
COL18A1	2.731936	2.4407996	2.97236171	1.3689355	0.45305451	9.33E-06
PLK1	1.346401	1.5290528	1.19556457	0.3696504	-1.4357666	9.43E-06
ZNHIT3	2.545765	2.8575173	2.28831388	0.6935676	-0.5278916	9.73E-06
NUDT15	1.62283	1.8064922	1.47115898	0.5842078	-0.7754466	9.93E-06
FAM103A1	2.699871	3.0040002	2.44871532	0.7229118	-0.4681085	1.01E-05
TINAGL1	1.233479	1.0419406	1.39165525	9.3383217	3.2231633	1.04E-05
LSM2	4.531945	5.0490995	4.10486956	0.766805	-0.3830684	1.07E-05
MRPL50	1.821295	2.0153046	1.66107879	0.6511138	-0.6190184	1.08E-05
LSM4	7.620229	8.5262743	6.87200227	0.7802004	-0.3580833	1.10E-05
ACOT13	1.861268	2.0585174	1.69837705	0.6597691	-0.5999669	1.13E-05
RB1CC1	2.176645	2.4084662	1.98520254	0.6994861	-0.5156327	1.13E-05
HMGN5	1.876735	2.0719906	1.71548918	0.6674398	-0.5832905	1.14E-05
GLTSCR2	5.338254	5.8590547	4.90816828	0.8043063	-0.3141831	1.16E-05
PYCR1	2.10286	2.3140331	1.92846943	0.7065799	-0.5010753	1.17E-05
DRAM2	1.857087	2.059381	1.69002848	0.6513507	-0.6184937	1.19E-05
AMZ2	2.327402	2.5589407	2.13619357	0.7288241	-0.4563575	1.19E-05
SNX6	2.360056	2.5946653	2.16631147	0.7313832	-0.4513005	1.19E-05
ERO1A	2.200959	1.9591898	2.40061644	1.4602078	0.54617372	1.22E-05
NUDT1	1.787169	1.9774928	1.62999657	0.6445025	-0.6337422	1.22E-05
PSMC6	2.149293	2.3574397	1.97740151	0.7200331	-0.4738648	1.24E-05
UNC50	1.651969	1.8346534	1.50110499	0.6003749	-0.7360644	1.25E-05
LARS	2.433082	2.6897331	2.22113497	0.7226792	-0.4685727	1.26E-05
KRTCAP2	2.831804	3.1133065	2.59933441	0.7567924	-0.4020304	1.27E-05
P4HB	4.82351	5.3138621	4.41856982	0.7924615	-0.3355872	1.28E-05
SPAG16	2.352773	2.6334925	2.12095084	0.6862296	-0.5432368	1.32E-05
STMN2	2.256205	2.8992526	1.72516504	0.381816	-1.3890505	1.32E-05
FRZB	1.431648	1.2488291	1.58262316	2.3414589	1.22740772	1.33E-05
TROAP	1.400623	1.5683118	1.26214361	0.4612672	-1.1163253	1.34E-05
CXCL14	2.466849	1.9938446	2.85746408	1.8689684	0.90224217	1.37E-05
SDHAF3	2.103362	2.3103659	1.93241455	0.7115681	-0.4909263	1.38E-05
CSTB	6.645434	5.9763778	7.1979523	1.2454746	0.31669564	1.39E-05
C14orf2	11.77064	13.092388	10.6791196	0.8004308	-0.3211514	1.41E-05
TBX2-AS1	2.506593	2.8118441	2.25451179	0.692395	-0.5303328	1.41E-05
CASP7	1.881232	2.0861058	1.71204358	0.6555932	-0.6091272	1.41E-05
CA9	1.49093	1.6737671	1.33993968	0.5045359	-0.9869711	1.44E-05
TMEM18	1.749793	1.936558	1.59555853	0.6359014	-0.653125	1.50E-05
HEBP1	2.337568	2.5733314	2.14287002	0.7264013	-0.4611613	1.55E-05
STX8	1.804023	1.9933232	1.64769546	0.6520491	-0.6169476	1.55E-05
TPRKB	2.058478	2.2700019	1.88379752	0.6959025	-0.5230429	1.56E-05
SCARA3	1.303271	1.1436509	1.43508744	3.0287833	1.59873837	1.56E-05
FLRT3	2.065463	2.3470654	1.83291082	0.6183151	-0.6935859	1.57E-05

SEC11A	5.865233	6.4304947	5.39843028	0.8099502	-0.3040949	1.58E-05
KNSTRN	1.386502	1.5515923	1.2501684	0.4535386	-1.1407028	1.60E-05
RP11-247C2.2	1.689044	1.8877732	1.52493017	0.5912886	-0.7580656	1.64E-05
ACTC1	1.836642	1.4610782	2.14678938	2.4871908	1.3145172	1.71E-05
MRPL11	2.810237	3.104123	2.56754083	0.7449854	-0.424716	1.71E-05
EVA1A	1.386924	1.2232993	1.52204773	2.3378834	1.225203	1.73E-05
PRPF40A	2.69363	3.0081511	2.43389412	0.714037	-0.4859293	1.73E-05
PCCA	2.032427	2.2567794	1.84715332	0.6740668	-0.5690365	1.75E-05
ATP5J	7.419622	8.3397257	6.65978492	0.7711167	-0.3749789	1.76E-05
ANXA3	1.326048	1.1646961	1.45929584	2.7887474	1.47961725	1.76E-05
PEPD	1.61733	1.7951819	1.47045747	0.5916351	-0.7572205	1.78E-05
C8orf4	1.916871	1.6950157	2.10008226	1.5828165	0.66249397	1.81E-05
NDUFA11	7.06621	7.6824068	6.55734491	0.8316382	-0.265972	1.82E-05
MARCKS	6.850932	7.7061665	6.14466604	0.7671545	-0.3824109	1.83E-05
PCNP	3.817413	4.20195	3.49985576	0.7807292	-0.3571059	1.83E-05
AURKA	1.377597	1.5410157	1.24264259	0.4484945	-1.1568378	1.83E-05
TPGS1	1.720526	1.9034307	1.56947978	0.6303525	-0.6657694	1.89E-05
SUMO1	6.215932	6.7965054	5.73648448	0.8171276	-0.2913667	2.00E-05
B2M	10.84644	9.7713878	11.7342295	1.2237778	0.29134161	2.01E-05
PQBP1	3.037094	3.3335467	2.79227774	0.7680488	-0.3807301	2.04E-05
S100A10	6.710315	5.46648	7.7374936	1.5084571	0.59307369	2.04E-05
IVNS1ABP	2.407102	2.1266227	2.63872642	1.4545476	0.54057054	2.08E-05
HTRA1	1.869609	1.6631564	2.04010038	1.5684089	0.64930174	2.10E-05
PSMC1	2.44782	2.7001001	2.23948309	0.7290648	-0.4558811	2.11E-05
MRPS18B	1.89584	2.0864451	1.73843551	0.6796805	-0.5570714	2.13E-05
SLC16A1	2.289068	2.5113087	2.10553777	0.7315102	-0.4510501	2.13E-05
EIF1	20.146	22.229094	18.4257542	0.8208431	-0.2848217	2.13E-05
PRPH	1.295159	1.1335803	1.42859405	3.2085126	1.68190463	2.14E-05
MRPL51	4.954141	5.4460235	4.54793667	0.7980022	-0.3255353	2.14E-05
ING4	1.94427	2.1378559	1.78440412	0.6893704	-0.5366488	2.15E-05
ITPA	1.767466	1.9516681	1.6153488	0.6466002	-0.6290541	2.16E-05
METAP2	3.505581	3.8547311	3.21724799	0.7766924	-0.3645847	2.16E-05
GLRX5	2.48861	2.7231329	2.29493718	0.7515016	-0.4121518	2.20E-05
AAMP	1.786359	1.9708985	1.63396408	0.6529664	-0.6149194	2.22E-05
TTC19	2.364301	2.6212914	2.15207337	0.7105899	-0.4929108	2.23E-05
SAMD11	2.192177	2.4706414	1.96221627	0.6542834	-0.6120124	2.24E-05
FUNDC1	1.601905	1.7761568	1.45800564	0.5900942	-0.7609828	2.25E-05
MACROD1	1.604064	1.7794512	1.45922578	0.5891656	-0.763255	2.27E-05
C5orf46	1.180656	1.0321176	1.3033218	9.4441045	3.23941401	2.32E-05
TGFB2	2.001451	1.7850883	2.1801267	1.5031771	0.58801496	2.36E-05
C20orf27	2.587569	2.8417794	2.3776371	0.7479924	-0.4189044	2.37E-05
PUF60	2.170643	2.3793789	1.9982663	0.7237071	-0.4665222	2.40E-05
CIAPIN1	1.644722	1.8210922	1.49907222	0.6078151	-0.7182957	2.43E-05
FIS1	4.169918	4.5521569	3.85425949	0.8035286	-0.3155788	2.47E-05
RORB	1.518059	1.7051636	1.36354565	0.5155479	-0.9558215	2.53E-05

SGO2	1.474727	1.6471143	1.33236684	0.5136138	-0.9612441	2.53E-05
SRSF5	6.560237	7.2123787	6.02168755	0.8083357	-0.3069735	2.55E-05
ZNF22	2.327875	2.5642222	2.13269594	0.7241273	-0.4656848	2.56E-05
LYPLAL1	1.719498	1.8994841	1.57086333	0.6346564	-0.6559524	2.59E-05
B9D1	1.66435	1.8413079	1.51821461	0.6159631	-0.6990842	2.59E-05
ACBD6	2.045305	2.2494734	1.87669971	0.7016554	-0.5111655	2.60E-05
PPIL1	1.766956	1.9484602	1.61706723	0.650599	-0.6201596	2.62E-05
C19orf33	1.27723	1.1079441	1.41702966	3.8633854	1.94986562	2.68E-05
SLTM	2.347494	2.5716431	2.16238813	0.7396006	-0.4351818	2.71E-05
NEK2	1.26442	1.4321955	1.12586809	0.2912296	-1.7797713	2.72E-05
CBR4	2.033467	2.2384094	1.86422235	0.6978487	-0.5190138	2.73E-05
MRPL22	2.145293	2.3486934	1.97732117	0.724643	-0.4646577	2.75E-05
CD81	1.850362	1.6659587	2.00264541	1.5055669	0.59030679	2.81E-05
CASP6	1.620911	1.7941802	1.47782172	0.601654	-0.732994	2.83E-05
SNU13	4.712378	5.1738161	4.33131471	0.798146	-0.3252754	2.85E-05
TMEM50A	2.699364	2.9560011	2.48742935	0.760444	-0.3950861	2.89E-05
UQCRB	10.5922	11.403485	9.92222682	0.857619	-0.2215912	2.90E-05
CHMP2A	3.924032	4.331936	3.58717864	0.7764791	-0.364981	2.90E-05
NDUFS2	2.422075	2.6776046	2.21105461	0.7218951	-0.4701388	2.99E-05
BRE	1.46322	1.6282043	1.32697246	0.5204875	-0.9420646	3.03E-05
C21orf59	1.965982	2.1716281	1.79615692	0.6795304	-0.55739	3.07E-05
YEATS4	2.073815	2.2689971	1.91262966	0.719174	-0.4755873	3.09E-05
GNL3	2.022093	2.2380437	1.84375734	0.6815247	-0.5531622	3.14E-05
ADPRHL2	1.644728	1.819439	1.50044829	0.6107206	-0.7114155	3.27E-05
JAGN1	1.925633	2.1137514	1.77028093	0.6916094	-0.5319707	3.31E-05
THYN1	2.373745	2.6304758	2.16173271	0.7125115	-0.4890149	3.35E-05
CETN3	1.82213	2.0043531	1.67164664	0.6687355	-0.5804923	3.36E-05
TCP1	3.039474	3.333131	2.79696717	0.7701956	-0.3767033	3.36E-05
UBA2	2.401038	2.6739104	2.17569571	0.7023648	-0.5097076	3.38E-05
BIN1	2.384843	2.6101373	2.19879083	0.7445271	-0.4256038	3.48E-05
CCDC88A	1.879062	2.0643015	1.72608883	0.682221	-0.5516889	3.54E-05
GLUL	2.92248	3.2604973	2.64333922	0.7269813	-0.4600099	3.65E-05
DIXDC1	1.785084	1.9649143	1.63657727	0.6597241	-0.6000652	3.74E-05
ALDOC	1.564234	1.7320798	1.42562426	0.5813905	-0.7824206	3.79E-05
FHL1	2.296566	2.5390488	2.09632004	0.7123361	-0.4893699	3.81E-05
UBE2N	2.73943	2.9941815	2.52905288	0.7667571	-0.3831584	4.03E-05
CRYL1	1.735392	1.9153252	1.58680064	0.6410844	-0.6414139	4.05E-05
SHFM1	6.321734	6.9755303	5.78181885	0.8002334	-0.3215073	4.08E-05
ATXN7L3B	2.383606	2.6117955	2.19516308	0.7415104	-0.4314613	4.14E-05
LMAN2	2.602785	2.8633602	2.38759846	0.7446754	-0.4253165	4.17E-05
C16orf45	2.811332	3.0944602	2.57752041	0.7531871	-0.4089198	4.17E-05
HNRNPD	3.215822	3.5268276	2.958989	0.7752761	-0.3672179	4.20E-05
AP2M1	5.998841	6.6528187	5.4587754	0.7887703	-0.3423229	4.20E-05
PRPSAP1	2.398805	2.6193725	2.2166566	0.7513136	-0.4125129	4.21E-05
ASCL1	1.62593	1.8127448	1.47165504	0.5803237	-0.7850703	4.21E-05

UPF3B	1.736867	1.9128707	1.59151977	0.6479776	-0.6259842	4.24E-05
HIBADH	1.813839	1.9991442	1.66081078	0.6613768	-0.5964557	4.27E-05
PFDN2	2.688126	2.9456776	2.47543512	0.7583143	-0.3991322	4.31E-05
AAMDC	2.154514	2.3537912	1.98994706	0.7312406	-0.451582	4.36E-05
PDCL3	1.485198	1.6475312	1.35114086	0.5422764	-0.8828997	4.38E-05
MRPL3	2.088355	2.2909802	1.92102375	0.7134298	-0.4871567	4.41E-05
RFXANK	1.872064	2.0546473	1.72128282	0.683909	-0.5481237	4.47E-05
NOSIP	2.322149	2.5338041	2.14736091	0.7480492	-0.418795	4.52E-05
SPHK1	2.985112	3.3345962	2.69650285	0.7266793	-0.4606092	4.55E-05
GTF2A2	3.238744	3.5784368	2.95822042	0.7594603	-0.3969535	4.59E-05
APMAP	1.905661	2.0888775	1.7543587	0.6927857	-0.529519	4.60E-05
GCHFR	4.840785	5.31641	4.44800541	0.7988132	-0.3240699	4.65E-05
UBAC2	2.077328	2.2728886	1.91583008	0.7194896	-0.4749543	4.66E-05
NONO	5.267737	5.8484465	4.78817747	0.7813178	-0.3560186	4.67E-05
EXOSC7	1.652746	1.8228947	1.51223501	0.6224795	-0.6839019	4.77E-05
SHISA5	2.485579	2.2126884	2.71093737	1.4108632	0.49657807	4.78E-05
MRPL28	2.282789	2.5113194	2.0940652	0.723914	-0.4661098	4.80E-05
SVIL	1.512033	1.3503502	1.64555317	1.8425938	0.88173809	4.85E-05
PPP1CA	2.813943	3.081288	2.59316436	0.7654704	-0.3855815	4.86E-05
PCBP1	4.171824	4.5951912	3.82220036	0.7849931	-0.3492481	4.88E-05
SEC61G	10.2179	11.309637	9.31632175	0.8066551	-0.3099761	4.92E-05
MRPL48	1.872451	2.0542562	1.72231355	0.6851404	-0.5455284	4.92E-05
ADI1	2.023107	2.2181814	1.86201098	0.7076212	-0.4989508	4.96E-05
S100A4	10.18826	7.7728513	12.1829467	1.6511431	0.72346517	5.02E-05
MDK	26.78933	28.65767	25.2464182	0.8766616	-0.189908	5.03E-05
ARF5	2.498244	2.7373573	2.30078081	0.7487123	-0.4175166	5.09E-05
SRSF4	2.408602	2.6474028	2.2113959	0.7353368	-0.4435229	5.14E-05
SEPT11	2.359057	2.593105	2.16577724	0.7317642	-0.4505492	5.14E-05
COPS5	1.848587	2.0282774	1.70019633	0.6809411	-0.5543981	5.24E-05
CFAP97	2.132738	2.3276026	1.97181508	0.7320075	-0.4500696	5.34E-05
PLXNB2	1.680471	1.5108638	1.82053606	1.6061739	0.68362812	5.35E-05
CRIPT	1.770446	1.9471977	1.62448117	0.6592934	-0.6010074	5.40E-05
GSTP1	29.32064	31.664134	27.3853356	0.8604624	-0.2168159	5.46E-05
DNAJC3	1.876171	1.6776644	2.04010031	1.5348309	0.61807976	5.47E-05
PLIN3	1.77127	1.9463646	1.62667453	0.6621914	-0.5946798	5.47E-05
DLK1	5.839202	7.1432914	4.76226303	0.6124181	-0.7074111	5.49E-05
TOMM20	4.54362	4.9593405	4.20031113	0.808294	-0.307048	5.51E-05
NCBP2	3.096237	3.4442863	2.80881247	0.7400166	-0.4343704	5.51E-05
ZBTB16	1.380866	1.5353012	1.25333085	0.4732492	-1.0793281	5.56E-05
NDUFS7	2.961477	3.2530305	2.72070649	0.7637298	-0.3888658	5.62E-05
DCXR	2.676463	2.9239444	2.47208968	0.7651415	-0.3862015	5.73E-05
GNG11	1.741724	1.5478706	1.90181145	1.6460301	0.71899067	5.73E-05
NUTF2	2.976199	3.3087571	2.70156721	0.7370057	-0.4402522	5.77E-05
BGN	1.284162	1.1228997	1.41733562	3.3957405	1.7637262	5.80E-05
SCG3	1.215523	1.3768414	1.0823037	0.2184041	-2.1949282	5.82E-05

RAMP2	2.159287	2.3717591	1.98382402	0.7171988	-0.4795549	5.84E-05
SPSB1	1.262486	1.1163349	1.38317912	3.2937602	1.71973553	5.92E-05
SNHG7	5.014655	5.4645509	4.64312399	0.8160113	-0.293339	5.93E-05
CLIC1	6.982772	7.6062886	6.46786224	0.8276754	-0.2728631	5.98E-05
TUBB2A	2.186002	2.4447317	1.97233869	0.6730237	-0.5712708	6.00E-05
UBE2L3	3.207497	3.5705527	2.9076802	0.7421284	-0.4302593	6.07E-05
KIAA1143	1.949038	2.1322913	1.7977049	0.704505	-0.5053182	6.34E-05
SNRPA	2.142953	2.347825	1.97376609	0.7224722	-0.468986	6.51E-05
TOP2B	2.364573	2.5788226	2.18764203	0.7522327	-0.4107491	6.72E-05
PPCS	1.95703	2.1421147	1.80418405	0.7041185	-0.5061099	6.86E-05
SYF2	2.583093	2.8354415	2.37469951	0.7489749	-0.4170108	7.08E-05
SULT1A1	1.522188	1.6816628	1.39049035	0.5728497	-0.8037713	7.13E-05
KCNQ1OT1	7.25591	6.3889227	7.97188164	1.2937431	0.37155119	7.14E-05
VPS72	1.807277	1.9811318	1.66370418	0.6764679	-0.5639066	7.25E-05
VPS29	2.313565	2.5220104	2.14142691	0.7499469	-0.4151397	7.29E-05
IFT52	1.838822	2.0143272	1.69388633	0.6840853	-0.5477519	7.37E-05
EMC4	2.689664	2.9241216	2.49604549	0.7775213	-0.363046	7.42E-05
QARS	3.112939	3.4028639	2.87351342	0.7797002	-0.3590086	7.42E-05
CMC1	1.874302	2.0516439	1.72784961	0.6921066	-0.5309339	7.53E-05
LSM3	3.510974	3.8454852	3.23472865	0.7853594	-0.348575	7.57E-05
MPST	2.865235	3.1821449	2.60352529	0.7348391	-0.4444998	7.62E-05
HSPA4	1.899223	2.1025839	1.7312851	0.6632467	-0.5923825	7.62E-05
ADAM28	1.172712	1.0341412	1.2871467	8.4105603	3.07220191	7.63E-05
PHF6	3.116827	3.389243	2.89186161	0.7918247	-0.336747	7.64E-05
SDHB	1.84073	2.015716	1.69622366	0.6854511	-0.5448744	7.68E-05
DNAJC12	1.448655	1.6036214	1.32068083	0.5312615	-0.912506	7.68E-05
UQCRC2	2.558617	2.788757	2.36856389	0.7650921	-0.3862946	7.73E-05
MRPL42	2.951632	3.2247671	2.72607298	0.7758444	-0.3661608	7.75E-05
PARD6B	1.536136	1.3777979	1.66689376	1.7652129	0.81984219	7.75E-05
HDAC3	2.135106	2.3255697	1.97781728	0.7376581	-0.4389758	7.83E-05
FNDC3B	1.388282	1.2377294	1.51261098	2.1562792	1.10854402	7.86E-05
PHB2	3.698093	4.0572212	3.40151872	0.7855234	-0.3482739	7.86E-05
FKBP8	4.240001	4.6258653	3.92134807	0.8056968	-0.3116911	7.92E-05
BRD8	1.531041	1.6906018	1.39927374	0.5781533	-0.7904759	8.00E-05
PNMA1	2.078826	2.2645787	1.92542809	0.7318074	-0.450464	8.06E-05
NKAIN4	3.848864	4.1962203	3.56201062	0.8015751	-0.3190904	8.06E-05
TROVE2	2.494097	2.7112739	2.31474865	0.7682865	-0.3802837	8.16E-05
MPHOSPH6	1.71717	1.8853055	1.57832024	0.6532437	-0.6143068	8.30E-05
RAD23B	2.381616	2.5920836	2.20780883	0.758634	-0.398524	8.36E-05
SPCS1	4.673907	5.1089994	4.31460079	0.8066686	-0.309952	8.43E-05
NAP1L4	2.356524	2.5817346	2.17054231	0.7400371	-0.4343304	8.43E-05
UBE2I	4.800841	5.3673381	4.33301819	0.7631693	-0.389925	8.62E-05
NDUFA2	4.650002	5.0831608	4.29229247	0.8063098	-0.3105939	8.85E-05
NUDT4	5.136292	5.6594592	4.70425139	0.794996	-0.3309805	9.01E-05
EIF3D	3.444233	3.7558133	3.18692406	0.7935676	-0.333575	9.02E-05

PIH1D1	2.80989	3.1162424	2.55689909	0.7356903	-0.4428295	9.18E-05
PIN4	2.831021	3.1006914	2.60832279	0.7656159	-0.3853072	9.23E-05
ARL4C	1.954951	1.7655829	2.11133361	1.4516175	0.53766139	9.35E-05
RBM8A	3.52813	3.9138918	3.20956123	0.7582853	-0.3991874	9.38E-05
HSPB11	2.439027	2.6707201	2.24769129	0.7467985	-0.4212091	9.39E-05
FABP5	11.8644	13.444673	10.5593809	0.7681504	-0.3805392	9.44E-05
CRYAB	4.116092	2.5212538	5.43313635	2.9141333	1.54306686	9.46E-05
DHX40	1.775769	1.9463617	1.63489122	0.6708759	-0.5758822	9.50E-05
PBDC1	1.539148	1.6965889	1.40913029	0.5873339	-0.7677472	9.69E-05
INHBA	1.187713	1.0463847	1.30442421	6.5630282	2.71436162	9.72E-05
OAZ2	4.997891	5.4282328	4.64250726	0.8225645	-0.2817992	9.72E-05
PDCD5	3.429918	3.7420061	3.17218974	0.79219	-0.3360816	9.82E-05
CREB3L4	1.405488	1.5565873	1.28070809	0.5043379	-0.9875374	9.87E-05
EVL	1.863999	1.6914354	2.00650536	1.4556753	0.54168856	9.94E-05
UBA1	2.590451	2.8191071	2.40162221	0.7705001	-0.3761329	0.0001
GPAA1	2.248554	2.4408924	2.08971864	0.7562803	-0.403007	0.0001
PRPSAP2	1.79386	1.9630988	1.65410002	0.6791619	-0.5581725	0.0001
SLC12A8	1.201614	1.063997	1.31526009	4.9261697	2.30046632	0.0001
ID2	4.157657	4.7052849	3.70541681	0.7301508	-0.4537336	0.00011
COLGALT2	1.812824	1.6368665	1.95813308	1.5044489	0.58923515	0.00011
COPS4	1.627661	1.7892098	1.49425117	0.6262608	-0.6751645	0.00011
TCEB1	3.650917	3.9934994	3.36800756	0.7910499	-0.3381593	0.00011
PPP1R7	1.696136	1.8602492	1.56060942	0.6516826	-0.6177587	0.00011
MRPL23	2.470661	2.7096993	2.27325849	0.7447265	-0.4252173	0.00011
CTNNB1	3.354718	3.7765381	3.00637161	0.7226163	-0.4686983	0.00011
MAF1	3.025231	3.3392228	2.76593099	0.7549221	-0.4056003	0.00011
RTF1	3.618234	4.0210456	3.28558516	0.7565543	-0.4024844	0.00011
PYURF	3.721541	4.0765012	3.42840887	0.7893411	-0.3412792	0.00011
CENPM	1.503167	1.6572048	1.37596023	0.5720595	-0.8057629	0.00011
CDO1	1.562363	1.7196403	1.43248056	0.6009676	-0.7346408	0.00011
IGFBP6	1.704212	1.5227018	1.8541053	1.6340201	0.70842572	0.00011
CENPN	1.48177	1.6351907	1.35507259	0.5590016	-0.8390758	0.00012
NDUFB10	4.365887	4.7696434	4.03245763	0.8044415	-0.3139406	0.00012
CCDC28B	2.349892	2.5514656	2.18342933	0.7627815	-0.3906582	0.00012
ERCC1	2.639344	2.8860832	2.43558317	0.7611452	-0.3937564	0.00012
CSNK2B	3.77687	4.117499	3.49557306	0.8005048	-0.321018	0.00012
SPAG7	2.611628	2.8391107	2.4237687	0.7741615	-0.3692935	0.00012
MEIS1	2.08378	2.2844968	1.91802537	0.7146965	-0.4845974	0.00012
ADAMTS18	1.519184	1.3660343	1.64565723	1.7639255	0.81878962	0.00012
CLRN1	1.781711	1.9497491	1.64294296	0.6769608	-0.5628557	0.00012
TRAPPC1	4.634038	5.0861515	4.26067499	0.7979819	-0.325572	0.00012
BTF3L4	3.362971	3.6763317	3.10419331	0.7862229	-0.3469897	0.00013
SPOP	1.691324	1.8542651	1.55676528	0.6517477	-0.6176146	0.00013
HSD17B2	2.57913	2.2280136	2.86908729	1.5220412	0.60600746	0.00013
LAMTOR5	6.418801	6.9791696	5.95603987	0.8288843	-0.2707573	0.00013

IDH3G	1.877546	2.0479927	1.73678843	0.7030473	-0.5083064	0.00013
ALDH1A1	4.683427	5.2975199	4.1762988	0.7391004	-0.4361577	0.00013
CLTC	2.020728	1.8404914	2.16957122	1.3915327	0.47667477	0.00013
PRCD	1.395009	1.2446601	1.51916984	2.1220047	1.08542786	0.00013
TM7SF2	2.714571	2.9849862	2.49125752	0.7512685	-0.4125996	0.00014
SLC35G1	1.497411	1.3460142	1.62243661	1.798876	0.84709577	0.00014
AP2S1	5.422371	5.8531185	5.06665262	0.8379463	-0.2550703	0.00014
COQ5	1.589539	1.7456136	1.46064965	0.6178128	-0.6947583	0.00014
MKRN1	2.394714	2.6458723	2.18730373	0.7213826	-0.4711634	0.00014
APRT	3.455898	3.7673559	3.19869134	0.7945098	-0.3318631	0.00014
SOX11	3.338599	3.7464489	3.00179001	0.7288648	-0.4562768	0.00014
SDHD	1.669688	1.829656	1.53758314	0.6479591	-0.6260253	0.00014
MRPS35	1.707509	1.869831	1.57346016	0.6592777	-0.6010419	0.00014
LYPD1	1.669277	1.4921871	1.81552163	1.6569343	0.72851643	0.00014
PRKDC	3.214442	3.5036255	2.97562952	0.7891074	-0.3417064	0.00014
HOMER2	3.286635	3.586788	3.03876397	0.788145	-0.3434671	0.00014
PPIE	1.633291	1.7913568	1.50275842	0.6353119	-0.6544631	0.00014
PAPPA2	1.316795	1.1706072	1.4375187	2.5644791	1.3586658	0.00014
NDUFA6	4.237005	4.5847905	3.94979723	0.8228646	-0.281273	0.00015
RNF26	1.779654	1.9441938	1.64377443	0.6818245	-0.5525277	0.00015
RP11-390P2.4	1.597536	1.7545674	1.46785746	0.620034	-0.6895807	0.00015
IFI16	1.997328	2.1815455	1.84519754	0.7153322	-0.4833148	0.00015
HDDC2	4.392007	4.8017528	4.05363276	0.8032171	-0.3161381	0.00015
CENPE	1.405603	1.5532572	1.28366698	0.5127217	-0.9637522	0.00016
TMEM45A	2.160178	2.3719651	1.98528058	0.7181528	-0.4776373	0.00016
CWC27	1.708386	1.8695302	1.57531102	0.6616343	-0.5958941	0.00016
TMEM55A	1.669065	1.8287943	1.53715728	0.648119	-0.6256695	0.00016
MRPS6	5.203506	5.7994586	4.71135892	0.773287	-0.3709241	0.00016
ATF4	2.757621	2.993132	2.5631325	0.7842594	-0.3505972	0.00017
VDAC2	4.978289	5.3854479	4.64205057	0.8304854	-0.2679732	0.00017
SDK2	1.973635	1.8003606	2.11672776	1.3952808	0.48055554	0.00017
TMX1	1.676461	1.8347088	1.54577765	0.6538539	-0.6129597	0.00018
MRPS5	2.060641	2.2396587	1.91280582	0.7363364	-0.4415631	0.00018
MLF2	5.210762	5.6756063	4.82688558	0.818479	-0.2889827	0.00018
PDLIM3	1.581767	1.4269552	1.70961367	1.6620329	0.73294896	0.00018
NUDT14	1.589107	1.4361217	1.71544481	1.6404707	0.71410983	0.00018
ENOPH1	1.928156	2.1023623	1.78429409	0.7114667	-0.4911318	0.00018
CUTA	7.002492	7.6033794	6.50626993	0.8338564	-0.2621292	0.00018
RAC1	7.150249	7.760539	6.64626056	0.835179	-0.2598426	0.00019
CFAP36	2.364214	2.5849818	2.18190019	0.7456869	-0.4233581	0.00019
NARF	2.841846	3.0976988	2.63055894	0.7773084	-0.363441	0.00019
HDHD2	1.632562	1.7883498	1.50390973	0.6391956	-0.6456706	0.00019
DPYSL4	1.621618	1.7769294	1.49335966	0.6350123	-0.6551437	0.00019
FAM175A	1.820194	1.9866676	1.68271774	0.691943	-0.5312749	0.00019
DNTTIP1	1.461689	1.608813	1.34019126	0.5587779	-0.8396531	0.00019

RNPEP	1.696403	1.8546301	1.56573613	0.6619661	-0.5951708	0.00019
DCTN6	1.744347	1.9042972	1.61225784	0.6770538	-0.5626576	0.0002
SLC37A4	1.777814	1.9390815	1.64463661	0.6864544	-0.5427643	0.0002
HDGF	2.49239	2.7253673	2.29999399	0.7534593	-0.4083986	0.0002
POLR2C	1.968965	2.1395515	1.82809212	0.7266825	-0.460603	0.0002
HES1	5.633512	6.5425078	4.88284773	0.7005579	-0.5134238	0.0002
BCAP31	2.693011	2.9247414	2.50164397	0.7801796	-0.3581218	0.00021
NDUFS6	4.745947	5.2237599	4.35136064	0.7934543	-0.3337809	0.00021
CCDC115	1.852412	2.0164608	1.71693732	0.7053271	-0.5036357	0.00021
MIS18A	1.514514	1.662797	1.39206015	0.5915237	-0.757492	0.00022
EIF2B1	1.438912	1.5839445	1.31914145	0.546527	-0.8716353	0.00022
NOP56	2.206117	2.4012392	2.04498272	0.7457561	-0.4232242	0.00022
TMBIM4	2.111315	2.2872032	1.9660641	0.750514	-0.414049	0.00022
SLC39A6	2.271008	2.4770512	2.10085374	0.7453051	-0.424097	0.00022
HNRNPH2	1.733244	1.8910877	1.60289391	0.676582	-0.5636633	0.00022
ZCRB1	3.249854	3.5176354	3.02871561	0.805802	-0.3115028	0.00023
RP4-614C10.2	2.474832	2.2647303	2.64833749	1.3033115	0.38218189	0.00023
ROMO1	6.450147	7.055342	5.95036635	0.8175205	-0.2906732	0.00023
DAD1	4.197203	4.5787048	3.88215229	0.8053618	-0.312291	0.00023
TMEM30A	1.652148	1.4985325	1.77900566	1.5625976	0.64394627	0.00023
GOLIM4	2.252491	2.4571207	2.08350504	0.7435932	-0.4274145	0.00023
ARL6IP6	2.017496	2.1881365	1.87657885	0.7377762	-0.4387449	0.00023
EIF4H	3.776547	4.1200551	3.49287186	0.7989833	-0.3237628	0.00023
CHPF	2.736312	2.4915589	2.9384331	1.2996021	0.37806999	0.00023
SLC7A6	1.337062	1.1992184	1.45089517	2.2633213	1.17844139	0.00024
YIPF3	2.484343	2.7081244	2.29954039	0.7607996	-0.3944116	0.00024
METTL5	2.190163	2.3916798	2.02374638	0.7356192	-0.442969	0.00025
TLE4	3.372192	3.0319173	3.65319567	1.3057597	0.38488939	0.00025
CRB1	1.966446	2.1606156	1.80609772	0.6945433	-0.5258636	0.00025
COX4I1	13.08624	14.428101	11.9781137	0.8175478	-0.2906251	0.00025
COX6B1	12.2573	13.115421	11.5486444	0.8706791	-0.199787	0.00025
TXNIP	4.071495	4.4870194	3.7283485	0.78243	-0.3539665	0.00026
TMEM126A	1.718577	1.8749418	1.58944812	0.6736998	-0.5698223	0.00026
PFDN5	14.17556	15.299552	13.247344	0.8564844	-0.2235011	0.00026
TMED1	1.932068	2.100822	1.79270903	0.7201064	-0.4737179	0.00026
ACAT2	1.558731	1.7073735	1.43598	0.6163363	-0.6982103	0.00026
NT5C	2.395609	2.5920996	2.23334425	0.7746653	-0.368355	0.00026
RP11-71N10.1	2.11902	2.3068065	1.96394304	0.7376326	-0.4390258	0.00026
FCGRT	1.343656	1.2064599	1.45695559	2.2132902	1.1461926	0.00026
CFL2	2.124493	2.3036398	1.97655062	0.7490955	-0.4167785	0.00026
GPI	4.085113	4.4256856	3.80386298	0.8184823	-0.2889769	0.00026
GNG3	1.456114	1.6003483	1.33700328	0.5613463	-0.8330371	0.00026
FLOT1	2.564978	2.7802538	2.38720036	0.7792149	-0.3599068	0.00027
FBXO21	1.739363	1.8961417	1.60989316	0.6805767	-0.5551703	0.00027
TTYH3	1.938503	1.7743404	2.07407072	1.3870783	0.47204925	0.00027

LINC00493	3.415374	3.7123575	3.17012105	0.8000867	-0.3217718	0.00028
PJA1	1.692466	1.8466281	1.56515563	0.6675371	-0.5830801	0.00028
PLGRKT	1.656623	1.8097917	1.5301346	0.6546555	-0.6111921	0.00029
TPRN	1.249305	1.1178915	1.35782883	3.0352394	1.60181033	0.00029
PEF1	1.891209	2.0536759	1.75704043	0.7184756	-0.4769889	0.00029
TXN2	2.975295	3.2138686	2.77827718	0.8032442	-0.3160893	0.00029
POMP	6.202022	6.6954713	5.79452365	0.8418133	-0.2484277	0.00029
SHC1	2.205522	2.0287592	2.35149631	1.3137149	0.39365227	0.0003
DCAF7	2.533055	2.751254	2.35286241	0.7725107	-0.3723732	0.0003
VRK1	1.428999	1.569795	1.31272815	0.5488433	-0.8655339	0.0003
ANXA6	1.75408	1.9098711	1.62542457	0.6873771	-0.5408263	0.00031
DTYMK	2.411335	2.6062049	2.25040782	0.7784859	-0.3612572	0.00031
CPNE1	2.19291	2.3935524	2.02721709	0.7371213	-0.4400261	0.00031
COL5A2	1.406024	1.2640082	1.52330371	1.9821497	0.9870659	0.00031
LCA5	1.661844	1.8135367	1.53657458	0.659558	-0.6004286	0.00031
ATIC	1.670396	1.8220902	1.54512408	0.6630952	-0.592712	0.00031
BAALC	1.71005	1.5550392	1.83806	1.5099114	0.59446386	0.00031
CBR1	1.79738	1.9544077	1.66770422	0.6996006	-0.5153966	0.00032
ILVBL	1.519669	1.6644337	1.40011997	0.602197	-0.7316925	0.00032
AGRN	1.482189	1.340586	1.5991263	1.7591043	0.81484106	0.00033
CTHRC1	1.27353	1.1418854	1.3822449	2.6940399	1.42977124	0.00033
COX6A1	7.857874	8.4236863	7.39061646	0.8608414	-0.2161806	0.00033
EIF3F	8.056569	8.7476184	7.48588887	0.8371461	-0.2564486	0.00033
PSMD3	1.875417	2.0349675	1.74365701	0.7185318	-0.4768761	0.00034
P3H2	1.819361	1.6628533	1.94860757	1.4310972	0.51712169	0.00034
ANAPC16	5.984398	6.4584602	5.59291002	0.8414296	-0.2490855	0.00034
PRCP	1.591053	1.7382776	1.46947284	0.6359029	-0.6531217	0.00034
COL9A1	1.794176	1.9518151	1.66399484	0.697609	-0.5195094	0.00034
FAM198B	1.550095	1.4052135	1.6697407	1.6528094	0.72492032	0.00034
TOX	1.699274	1.8519314	1.5732075	0.672833	-0.5716797	0.00035
PFDN4	2.811729	3.044695	2.6193416	0.7919722	-0.3364783	0.00035
DDX1	1.951041	2.1248141	1.80753607	0.7179285	-0.478088	0.00035
DPY30	3.007853	3.2453339	2.81173665	0.8068896	-0.3095567	0.00036
CFDP1	4.10431	4.4695791	3.80266494	0.8077824	-0.3079614	0.00037
FAM118A	1.771979	1.6178631	1.89925138	1.4554218	0.54143734	0.00037
ACO2	1.587034	1.7333945	1.4661667	0.6356289	-0.6537434	0.00037
MRPS27	1.601488	1.74869	1.47992703	0.6410224	-0.6415534	0.00037
SMIM14	1.817733	1.9753668	1.68755602	0.7049205	-0.5044676	0.00038
PITPNA	1.402919	1.2663705	1.51568271	1.9359603	0.95304934	0.00038
COL8A2	1.154548	1.0300775	1.25733728	8.5558082	3.09690414	0.00038
C1orf43	3.700012	4.0510333	3.41013359	0.7899401	-0.3401848	0.00038
EEF2	28.75527	31.160486	26.7690057	0.8543962	-0.2270228	0.00038
ECSIT	1.673961	1.8240298	1.55003193	0.6674903	-0.5831812	0.00038
TCEA1	3.786594	4.145005	3.49061154	0.7919261	-0.3365623	0.00039
RAB11B	2.858787	3.1051516	2.65533479	0.7863257	-0.3468011	0.00039

C14orf1	2.438245	2.6482479	2.26482175	0.7673735	-0.3819992	0.00039
NSA2	3.405516	3.689947	3.17062864	0.806941	-0.3094648	0.00039
ANAPC15	2.014982	2.1793098	1.87927834	0.7455873	-0.4235509	0.00039
ZNF428	5.220665	5.6783519	4.84270004	0.821379	-0.28388	0.0004
MRPL10	2.105085	2.282332	1.9587107	0.7476306	-0.4196024	0.0004
NBEAL1	3.976397	4.325793	3.6878602	0.8081862	-0.3072403	0.0004
CCDC167	3.07782	3.3594443	2.84524942	0.7820695	-0.3546313	0.0004
HADHB	2.02638	2.1933111	1.88852578	0.7445885	-0.4254847	0.0004
GAD2	2.039781	2.2708603	1.84895268	0.6680142	-0.5820494	0.0004
TSEN15	1.707705	1.8584853	1.58318808	0.6793222	-0.5578322	0.00041
S100A16	4.312459	3.8209876	4.71832434	1.3180931	0.39845229	0.00041
PMF1	1.943217	2.1041299	1.81033254	0.7339105	-0.446324	0.00041
TMEM261	2.967233	3.2465551	2.73656458	0.77299	-0.3714784	0.00042
CDC37	2.963311	3.2078957	2.7613284	0.7977408	-0.3260081	0.00042
GNB2	3.899769	4.21649	3.63821545	0.8202157	-0.2859248	0.00042
BLMH	1.470896	1.6099811	1.35603651	0.5836845	-0.7767393	0.00043
NUP93	1.59083	1.7361657	1.47080899	0.6395421	-0.6448887	0.00043
TRIOBP	2.080723	1.9156521	2.21704113	1.3291523	0.41050646	0.00043
ID1	5.38396	6.1067047	4.78710529	0.7415947	-0.4312971	0.00043
TNNI3	1.522591	1.6642791	1.40558211	0.6105598	-0.7117954	0.00044
ISCA2	2.19592	2.3796107	2.04422549	0.7568987	-0.4018279	0.00044
MSANTD4	1.908852	2.07263	1.77360214	0.7212199	-0.4714889	0.00044
WTAP	2.021016	2.1960733	1.87645153	0.7327741	-0.4485596	0.00044
YTHDC1	2.091608	2.2654912	1.94801321	0.7491266	-0.4167185	0.00044
HMMR	1.231286	1.3583187	1.12638038	0.3527038	-1.5034709	0.00044
GABPB1	2.331288	2.5174664	2.177538	0.7759895	-0.3658909	0.00045
HMGN3	9.309426	10.137421	8.62565417	0.8345521	-0.2609259	0.00045
FBXO7	2.062864	2.2338853	1.92163238	0.7469352	-0.420945	0.00046
MSI1	2.250879	2.466276	2.07300014	0.7317859	-0.4505065	0.00047
PSMD14	2.124635	2.294447	1.98440194	0.7604807	-0.3950165	0.00047
RAB1A	3.053213	3.3499153	2.80819178	0.769471	-0.3780611	0.00047
NSMCE1	1.416238	1.5523715	1.30381641	0.5500218	-0.8624392	0.00048
GPX3	1.696475	1.8629452	1.5590006	0.6477823	-0.6264191	0.00048
KIF20A	1.20382	1.328788	1.10061892	0.3060298	-1.7082559	0.00049
ABR	1.638717	1.493593	1.7585635	1.5368197	0.61994796	0.00049
C12orf10	1.971396	2.1313106	1.83933513	0.741914	-0.4306762	0.00049
MOB4	1.777773	1.9294283	1.65253294	0.7020799	-0.5102929	0.00049
STIP1	1.765505	1.9158392	1.64135611	0.7002933	-0.5139688	0.0005
PWP1	1.687775	1.8357385	1.56558433	0.6767479	-0.5633095	0.0005
MFSD10	2.221983	2.398968	2.07582576	0.7690139	-0.3789185	0.0005
ABHD2	1.369738	1.2378211	1.47867717	2.0127611	1.00917597	0.0005
SLC39A1	2.791498	3.040491	2.58587637	0.7772033	-0.363636	0.0005
TCTEX1D2	2.285204	2.4763739	2.12733199	0.7635816	-0.3891457	0.00051
DACH1	2.496889	2.7119189	2.31931371	0.7706637	-0.3758267	0.00051
TRAPPC4	1.736156	1.8852091	1.61306485	0.692565	-0.5299785	0.00052

EIF1AX	3.489951	3.7792604	3.25103457	0.8099401	-0.3041128	0.00052
ENO3	1.870531	2.0274495	1.74094541	0.7211502	-0.4716284	0.00053
ARHGAP29	1.591349	1.426123	1.72779487	1.7079455	0.77226196	0.00053
NIFK	1.723698	1.8721196	1.60112814	0.6892726	-0.5368535	0.00053
TAGLN	12.32001	10.063518	14.1834614	1.4545634	0.54058613	0.00054
NTPCR	2.32518	2.521831	2.162783	0.7640684	-0.3882262	0.00054
H2AFX	2.087542	2.2836089	1.92562596	0.7211122	-0.4717044	0.00054
FDX1	2.368515	2.5593183	2.21094697	0.7765874	-0.3647798	0.00054
NKAP	1.834614	1.98733	1.70849844	0.7175903	-0.4787677	0.00055
NTHL1	1.508467	1.6470896	1.39398999	0.6088647	-0.7158065	0.00055
GAR1	1.624377	1.7683281	1.50550008	0.6579221	-0.6040112	0.00055
AFF4	2.180616	2.0022684	2.32789881	1.3248935	0.40587636	0.00055
NPRL2	1.491579	1.6299149	1.37733824	0.5990305	-0.7392987	0.00055
CDC5L	1.738091	1.8867321	1.61534047	0.6939418	-0.5271134	0.00055
RFC5	1.44406	1.5789007	1.33270578	0.5747199	-0.799069	0.00056
CYB5B	2.00338	2.1679628	1.8674652	0.7427164	-0.4291166	0.00056
PAPPA	1.179973	1.0509471	1.28652442	5.6239616	2.49158675	0.00056
CHURC1	2.92271	3.1675887	2.72048549	0.7937324	-0.3332753	0.00056
PMPCB	2.17282	2.3539449	2.02324395	0.7557501	-0.4040189	0.00056
FHL2	1.439373	1.2948578	1.55871635	1.894867	0.92209663	0.00056
NDUFA7	2.281615	2.466971	2.12854435	0.7693024	-0.3783772	0.00056
THOC6	1.437713	1.572568	1.3263469	0.5699706	-0.8110407	0.00056
OTUB1	2.242688	2.4311055	2.08709059	0.759616	-0.3966578	0.00057
DUSP1	1.763289	1.9283829	1.62695091	0.675315	-0.5663674	0.00057
PRPF19	1.464175	1.6001632	1.3518728	0.5862951	-0.770301	0.00057
MRPL15	1.707807	1.854651	1.58654101	0.6862931	-0.5431033	0.00057
TNRC6B	4.259657	4.6322449	3.95196751	0.8127116	-0.2991847	0.00058
CNIH4	2.481771	2.7009073	2.30080406	0.7647707	-0.3869008	0.00059
TSPAN31	1.737335	1.8854381	1.61502942	0.6946047	-0.525736	0.00059
PSMF1	1.883487	2.0381508	1.7557632	0.7279898	-0.4580099	0.00059
TMEM107	2.083184	2.2704417	1.92854405	0.7308828	-0.452288	0.00059
TULP4	1.724762	1.5784692	1.84557216	1.461741	0.54768774	0.0006
TRMT10C	1.550876	1.6903225	1.43571937	0.6311823	-0.6638713	0.0006
RBM22	1.520121	1.657991	1.40626577	0.6174336	-0.695644	0.00061
ZC3H15	2.865083	3.1131965	2.66018633	0.785628	-0.3480818	0.00061
CALM1	4.239642	4.5951974	3.94601821	0.8194316	-0.2873046	0.00062
GPN3	1.485495	1.6212571	1.37338086	0.6010086	-0.7345424	0.00062
DEPDC1	1.291801	1.4184617	1.18720213	0.4473578	-1.1604988	0.00062
SOX4	10.1746	10.981546	9.50821266	0.8523943	-0.2304072	0.00062
PMVK	2.127276	2.290701	1.99231715	0.7688203	-0.3792816	0.00062
COL4A5	1.652577	1.5098447	1.77044771	1.5111418	0.59563909	0.00063
SUCLG2	1.618258	1.7598153	1.50135787	0.6598418	-0.5998079	0.00065
DNAJC19	2.817115	3.0417635	2.63159637	0.7991114	-0.3235315	0.00065
RNF19A	1.458893	1.3255271	1.56902812	1.7480207	0.80572228	0.00066
BLVRA	1.475982	1.6112375	1.36428546	0.5959802	-0.7466637	0.00066

ILF3	3.57655	3.8510223	3.34988549	0.8242256	-0.2788889	0.00067
GLOD4	1.74841	1.8949748	1.62737434	0.7009966	-0.5125206	0.00069
VCAN	2.005364	1.8255277	2.15387642	1.397744	0.48310017	0.0007
CAPN2	2.811475	2.5749577	3.00679557	1.2741902	0.34958062	0.0007
LGALS3BP	2.054216	2.2150277	1.92141427	0.7583484	-0.3990674	0.00071
AQP1	1.179215	1.0598008	1.277829	4.6459045	2.21595949	0.00071
SRSF1	3.031828	3.2700612	2.83509078	0.8083883	-0.3068797	0.00071
SAMM50	1.825674	1.9746419	1.70265424	0.7209358	-0.4720573	0.00072
SDR39U1	1.97492	2.1305197	1.8464237	0.7487032	-0.4175342	0.00072
OIP5	1.289661	1.4147399	1.18636816	0.4493615	-1.1540516	0.00072
FAM57B	1.207496	1.3290233	1.10713596	0.3256181	-1.6187471	0.00073
FUBP1	2.115472	2.2766916	1.98233436	0.7694374	-0.3781241	0.00073
SLC25A37	2.11434	1.945333	2.2539089	1.3264204	0.40753805	0.00074
PYCR2	1.660481	1.80175	1.54381846	0.6782893	-0.5600274	0.00075
ZFR	2.077478	2.236003	1.94656455	0.765827	-0.3849095	0.00075
DDT	3.805983	4.1228159	3.54433743	0.8147574	-0.2955575	0.00075
MANF	3.32957	3.5846322	3.11893568	0.819821	-0.2866192	0.00075
NR2F2	1.849467	1.6954095	1.97669104	1.4044832	0.49003938	0.00076
VAPA	3.800333	4.0806937	3.56880636	0.8338402	-0.2621571	0.00076
CMTM6	2.071314	2.2287346	1.94131258	0.7660829	-0.3844276	0.00076
PRSS35	1.30829	1.4339547	1.20451365	0.4712788	-1.0853472	0.00077
XXYLT1	1.787761	1.9347346	1.66638833	0.7129171	-0.4881937	0.00078
C11orf54	1.426405	1.5570898	1.31848271	0.5716901	-0.8066948	0.00079
MRPL43	2.771612	3.0173717	2.56865906	0.7775756	-0.3629451	0.00079
CLTA	5.831089	6.3753129	5.38166047	0.8151452	-0.294871	0.0008
ENSA	3.263176	3.5402921	3.03432982	0.8008252	-0.3204408	0.00081
ATRAID	3.749209	4.1034473	3.45667281	0.7915948	-0.3371659	0.00081
TMEM80	1.464863	1.5972615	1.3555255	0.5952594	-0.7484096	0.00082
MRPL4	2.088908	2.2650227	1.94347015	0.7458128	-0.4231145	0.00084
WDR34	1.838453	1.9861859	1.71645353	0.7264893	-0.4609865	0.00086
SOX9	1.791315	1.6396356	1.91657352	1.432962	0.51900038	0.00086
FNTA	1.847646	1.9950982	1.72587722	0.7294529	-0.4551134	0.00087
MITF	1.470666	1.3398805	1.57867105	1.7025722	0.76771602	0.00088
PUDP	1.532211	1.6663664	1.42142368	0.6324203	-0.6610444	0.0009
CDCA3	1.337006	1.4619358	1.23383741	0.5062119	-0.9821866	0.0009
NAA20	1.853363	2.0006865	1.7317006	0.7311986	-0.4516647	0.0009
PCOLCE	1.692683	1.8365071	1.57391146	0.6860808	-0.5435497	0.00091
PAIP1	2.757728	2.9655947	2.58606874	0.8069155	-0.3095105	0.00092
PPP2CA	2.027318	2.1819197	1.89964526	0.7611729	-0.3937038	0.00093
UQCR10	8.603195	9.4493223	7.90444974	0.8171602	-0.2913092	0.00093
AC004556.1	1.648532	1.7875661	1.53371596	0.6776777	-0.5613288	0.00094
SF3B2	3.005421	3.2487349	2.8044888	0.8024462	-0.3175234	0.00094
ARSI	1.398745	1.2722895	1.50317394	1.8479372	0.88591572	0.00095
PRDX5	8.046229	8.6772617	7.52511149	0.8499269	-0.2345893	0.00096
TM9SF3	2.137063	1.9806124	2.26626325	1.2912984	0.36882239	0.00096

NUCB2	2.379147	2.5640737	2.22643219	0.7841269	-0.350841	0.00098
DPM1	1.694702	1.834266	1.57944734	0.6945595	-0.5258298	0.00098
NEUROD1	1.195761	1.347062	1.07081438	0.2040396	-2.2930792	0.00099
DKK1	1.306359	1.4640251	1.17615562	0.3796252	-1.3973523	0.00099
ECT2	1.346719	1.4711998	1.24392134	0.5176601	-0.9499231	0.00102
CTSL	1.731061	1.87159	1.61500965	0.7056181	-0.5030406	0.00103
GPC1	1.838186	1.693983	1.95727051	1.3793862	0.46402643	0.00103
NOL7	2.167683	2.3387479	2.02641563	0.7666982	-0.3832693	0.00103
UCHL3	1.498764	1.6386833	1.38321561	0.6000088	-0.7369444	0.00105
TMA16	1.993913	2.160617	1.85624624	0.7377509	-0.4387943	0.00105
FBN1	1.518355	1.3877791	1.62618655	1.6148024	0.69135763	0.00106
HMGA1	15.20249	16.249627	14.3377481	0.8746278	-0.1932589	0.00106
ABAT	1.806737	1.9503544	1.68813514	0.7240827	-0.4657736	0.00106
MAD1L1	1.416752	1.5442078	1.31149613	0.5723845	-0.8049435	0.00107
PDCD6	2.698109	2.937316	2.50056855	0.7745605	-0.3685501	0.00109
MPI	1.504214	1.635104	1.39612251	0.6237128	-0.6810462	0.0011
PSMG2	2.506254	2.7066307	2.34077915	0.7856294	-0.3480793	0.00111
PON2	2.20185	2.3685038	2.06422535	0.7776561	-0.3627957	0.00112
CDCA7L	2.29119	2.4709093	2.14277475	0.7769172	-0.3641672	0.00112
SLF1	1.86136	2.0087154	1.73967111	0.7332803	-0.4475633	0.00112
SELENOM	2.665874	2.4548707	2.84012352	1.2648021	0.33891166	0.00113
VPS35	2.382577	2.5815351	2.21827369	0.7703109	-0.3764873	0.00113
UBL5	14.08244	14.925613	13.3861375	0.8894501	-0.1690144	0.00114
ALDH2	1.553295	1.6854428	1.44416482	0.6479969	-0.6259411	0.00114
FABP3	1.365883	1.2430144	1.46734993	1.9231369	0.94346148	0.00115
KDM1A	1.85205	1.9961693	1.73303399	0.7358528	-0.4425108	0.00115
ACYP1	1.95057	2.0998709	1.8272751	0.7521566	-0.4108951	0.00116
TCN2	1.562697	1.6945567	1.45380464	0.653373	-0.6140212	0.00117
MRPS9	1.585691	1.7189917	1.4756099	0.6614957	-0.5961963	0.00117
NDUFAF2	2.077652	2.2415485	1.94230288	0.7589739	-0.3978779	0.00117
MRPS36	2.535435	2.7223581	2.38107065	0.8018487	-0.318598	0.00117
ZMAT2	2.067089	2.2195246	1.94120539	0.7717806	-0.3737374	0.00117
ITGA1	1.203775	1.0887018	1.29880343	3.3686268	1.75216063	0.00119
DUSP6	1.261276	1.1432191	1.35876994	2.5050417	1.32483461	0.0012
TSN	1.736073	1.8753788	1.62103146	0.7094431	-0.495241	0.0012
TIMM10	2.226472	2.3954916	2.08689345	0.7788606	-0.3605629	0.0012
DBNL	1.844613	1.9897838	1.72472903	0.7322094	-0.4496717	0.00121
ALKBH7	3.487665	3.8092562	3.22209034	0.7909889	-0.3382707	0.00121
TMEM161B-AS1	1.832016	1.9795542	1.71017668	0.7249998	-0.4639474	0.00122
PERP	2.626423	2.379539	2.83030358	1.3267502	0.40789672	0.00122
FAM13C	1.621797	1.7556975	1.51122043	0.6764882	-0.5638634	0.00123
ZNF436-AS1	2.03872	2.2127655	1.89499006	0.7379745	-0.4383571	0.00123
ZNRD1	1.563035	1.694833	1.4541946	0.6536744	-0.6133558	0.00123
DMKN	1.522175	1.3933181	1.62858713	1.5981649	0.67641631	0.00124
OPTN	1.857825	1.714184	1.9764468	1.3672202	0.45124563	0.00125

PSMD6	1.764335	1.9037956	1.64916626	0.7182667	-0.4774086	0.00125
TUBG1	1.882306	2.0269962	1.7628176	0.7427658	-0.4290207	0.00126
ASF1A	1.618071	1.7518986	1.50755326	0.6750289	-0.5669789	0.00126
PRKCSH	2.479028	2.6806807	2.3125007	0.780934	-0.3567275	0.00127
TCEAL5	2.050056	2.2092445	1.91859518	0.7596439	-0.3966048	0.00127
PRPF31	1.978098	2.1261456	1.85583721	0.7599703	-0.3959851	0.00127
CDK6	1.533815	1.4041245	1.64091544	1.5859357	0.66533432	0.00128
GPN1	1.41466	1.5398222	1.31129906	0.5766696	-0.7941832	0.00128
DCAKD	1.591045	1.7239329	1.48130347	0.6648454	-0.5889091	0.00128
MLEC	3.196878	3.42657	3.00719414	0.8271734	-0.2737383	0.0013
GTF3C6	3.072538	3.3146725	2.87257996	0.8090043	-0.3057808	0.00131
TBCA	9.280246	10.035043	8.65692172	0.8474693	-0.238767	0.00131
NDUFB9	3.963771	4.2794129	3.70310952	0.8242663	-0.2788176	0.00131
IDS	2.35157	2.1710246	2.50066756	1.2814996	0.35783306	0.00132
RNF181	3.081671	3.3145377	2.88936668	0.8163041	-0.2928213	0.00133
ACADM	1.724441	1.8613343	1.61139295	0.7098207	-0.4944734	0.00133
ARF4	3.075775	3.3645619	2.8372897	0.7770106	-0.3639938	0.00133
TCEAL8	5.294607	5.7150367	4.9474094	0.8371959	-0.2563629	0.00134
ABRACL	3.048432	3.2784108	2.85851187	0.8157053	-0.29388	0.00139
MRPS7	2.354628	2.5495323	2.19367341	0.7703443	-0.3764246	0.00139
TOMM34	1.613425	1.745718	1.50417508	0.6760935	-0.5647053	0.0014
TRIM59	1.38304	1.5054718	1.28193431	0.5577647	-0.8422714	0.00142
TADA3	2.679914	2.8874219	2.50855064	0.7992652	-0.3232538	0.00142
LRP2	1.796866	1.9421248	1.67690951	0.7184924	-0.4769552	0.00142
DYNLRB1	4.094591	4.4196405	3.82616051	0.8264496	-0.2750013	0.00143
DAB2	1.244688	1.1291062	1.34013758	2.6345569	1.39756033	0.00144
GOT1	1.275752	1.3931966	1.17876502	0.4546454	-1.1371864	0.00145
DMD	1.603493	1.4717958	1.71225151	1.5096605	0.59422419	0.00145
FH	1.455152	1.5803109	1.35179304	0.6062147	-0.7220992	0.00145
MRPS23	1.737058	1.8737619	1.62416612	0.7143435	-0.4853101	0.00147
CDKN2B	1.137471	1.0274986	1.22828742	8.3017709	3.05341912	0.00147
SNCA	3.027411	2.7729716	3.23753112	1.2620231	0.33573833	0.00148
SMAP1	2.432287	2.6215032	2.27602937	0.7869423	-0.3456703	0.00148
FANCL	1.652272	1.7854246	1.54231193	0.6904697	-0.5343499	0.00149
MTX1	1.769659	1.907467	1.65585529	0.7227319	-0.4684676	0.0015
SRPK2	1.946902	2.0956394	1.82407204	0.752138	-0.4109307	0.0015
TATDN1	1.918426	2.0622853	1.7996244	0.7527398	-0.4097768	0.00151
SSFA2	1.564243	1.4348535	1.67109417	1.5432648	0.62598566	0.00151
COL6A1	2.572856	2.3841132	2.72872269	1.2489749	0.32074453	0.00151
DBF4	1.597323	1.727763	1.4896042	0.6727523	-0.5718527	0.00152
CSN3	1.203992	1.0619388	1.32130197	5.1874112	2.37501472	0.00152
RBBP4	3.518093	3.776311	3.30485176	0.830185	-0.2684952	0.00153
RCC1L	1.52122	1.6490001	1.41569646	0.6405183	-0.6426883	0.00154
SLC3A2	2.399024	2.1924597	2.56960878	1.3162782	0.39646446	0.00155
KCTD12	1.216835	1.0933736	1.31879197	3.4141536	1.77152797	0.00158

EIF3G	3.519211	3.7806955	3.30327205	0.8283079	-0.271761	0.00159
MTX2	1.890987	2.032656	1.77399511	0.7495188	-0.4159634	0.00161
RHEB	5.018643	5.4145313	4.6917126	0.8362638	-0.25797	0.00161
TEKT2	1.302268	1.4200733	1.20498168	0.4879664	-1.0351461	0.00162
NFU1	1.840026	1.9799974	1.72443601	0.7392224	-0.4359196	0.00163
EIF1B	3.84949	4.1333164	3.61510099	0.8346112	-0.2608238	0.00164
FAT1	1.676674	1.5441721	1.78609582	1.444572	0.5306421	0.00164
FSTL3	1.356467	1.2379927	1.45430526	1.908904	0.93274457	0.00167
TOM1L1	1.539794	1.6680166	1.43390496	0.6495422	-0.6225049	0.00167
FAM127B	2.858672	3.0572856	2.69465301	0.8237325	-0.2797522	0.00168
PRPS1	1.759575	1.8955272	1.64730425	0.7228192	-0.4682933	0.0017
LTBP3	1.570242	1.4425702	1.67567478	1.5267063	0.61042255	0.00171
ITGB3BP	2.141788	2.2927967	2.01708326	0.786731	-0.3460576	0.00171
JAG1	1.284434	1.1694198	1.3794139	2.2394897	1.16317006	0.00173
CD74	1.702132	1.56954	1.81162783	1.4250585	0.51102116	0.00173
RAB8A	1.49704	1.6217883	1.3940208	0.6336897	-0.6581516	0.00176
LBH	1.424617	1.3037132	1.52446173	1.7268322	0.78812793	0.00176
SRSF2	4.557276	4.8869034	4.28506396	0.8451622	-0.2426998	0.00179
C4orf48	7.36429	6.9225972	7.72904733	1.136165	0.18417231	0.0018
SEPT6	1.727829	1.8621386	1.61691422	0.7155627	-0.4828499	0.00181
MRPS26	3.166903	3.4078462	2.96792853	0.8172983	-0.2910654	0.00183
MCTS1	1.922248	2.0638643	1.80529951	0.7569569	-0.4017168	0.00183
PPIH	1.517595	1.6428003	1.41419919	0.6443668	-0.6340459	0.00184
RAB7A	2.892361	3.1028458	2.71853847	0.8172442	-0.2911609	0.00187
PHF14	3.525403	3.7904951	3.30648483	0.8265504	-0.2748253	0.00188
C12orf29	1.415316	1.5361827	1.31550155	0.5884217	-0.7650776	0.0019
UROD	1.732317	1.8652876	1.62250755	0.7194227	-0.4750884	0.00194
FAHD2A	1.755629	1.8897485	1.64487008	0.7247779	-0.4643891	0.00194
DBN1	2.101183	2.2721299	1.96001209	0.7546494	-0.4061215	0.00197
NFIB	1.209011	1.0981607	1.30055322	3.0618493	1.61440328	0.00198
TMED4	2.915398	3.1190788	2.74719503	0.8245069	-0.2783966	0.00198
GADD45A	2.182699	2.4838348	1.93401555	0.6294606	-0.667812	0.00198
EFHC1	2.383785	2.2034336	2.53272299	1.2736248	0.34894038	0.00198
CGNL1	1.219431	1.1084548	1.3110763	2.8682583	1.52017496	0.00199
KLHL7	1.595941	1.7241233	1.49008637	0.6767996	-0.5631994	0.00199
SUCLG1	1.950793	2.0940531	1.83248704	0.7609201	-0.3941831	0.00199
SNX3	5.522124	5.8842724	5.22305572	0.8646233	-0.2098564	0.002
FAM92A	2.486443	2.6650923	2.33891055	0.8041059	-0.3145426	0.00201
DHCR24	1.317781	1.20279	1.41274171	2.035316	1.02525281	0.00203
DYNLT1	4.995619	5.4061668	4.65658167	0.8298782	-0.2690285	0.00204
UQCRH	12.47436	13.330734	11.767153	0.8731964	-0.1956218	0.00204
MICU2	1.584277	1.711108	1.47953712	0.6743521	-0.5684261	0.00205
AP3S1	3.323743	3.5680159	3.12201893	0.8263262	-0.2752166	0.00205
TP53	1.535733	1.6609621	1.43231626	0.6540712	-0.6124804	0.00206
FAM136A	2.562321	2.7466944	2.4100619	0.8072745	-0.3088687	0.00206

HLTF	2.04772	2.2106422	1.91317694	0.7542913	-0.4068062	0.00206
PIR	1.627874	1.7559123	1.5221379	0.6907388	-0.5337879	0.00207
DCBLD2	8.724311	8.0426302	9.28725511	1.1767273	0.23478001	0.00207
TXLNG	1.417736	1.5375041	1.31882921	0.5931661	-0.753492	0.00208
ASUN	1.360334	1.4777233	1.26339175	0.551348	-0.8589649	0.00209
SEC61B	7.00606	7.4423799	6.64573908	0.8763437	-0.1904313	0.00209
CLDN3	1.841559	1.9818213	1.72572859	0.7391657	-0.4360303	0.00209
PLAU	1.190857	1.0810316	1.28155207	3.4745966	1.79684547	0.00209
MIAT	7.384079	8.1237386	6.77325529	0.8104249	-0.3032496	0.00212
S100A14	1.141332	1.0188405	1.24248754	12.870581	3.6860053	0.00213
UBE2E1	2.230196	2.3855725	2.10188371	0.7952552	-0.3305102	0.00214
PET100	3.431595	3.7302179	3.18498666	0.8002975	-0.3213916	0.00215
SYAP1	2.906878	3.1037336	2.74431058	0.8291499	-0.2702951	0.00215
HIKESHI	2.511503	2.7106836	2.34701713	0.7874145	-0.3448048	0.00217
RRAS	1.529827	1.406984	1.63127358	1.5511016	0.63329319	0.00218
ARMC1	1.755428	1.8883593	1.64565068	0.72679	-0.4603895	0.00219
FAM204A	2.440923	2.6158766	2.29644425	0.8023163	-0.3177569	0.00219
MPC2	2.746949	2.9330784	2.59323995	0.8241983	-0.2789366	0.00219
TPGS2	3.03853	3.2787621	2.84014177	0.8075181	-0.3084334	0.00222
LGI1	1.153379	1.0464184	1.24170908	5.2071787	2.38050192	0.00222
GMPR2	1.900644	2.0385988	1.78671787	0.7574801	-0.4007202	0.00222
NT5C3A	1.730945	1.8620557	1.62267207	0.7223107	-0.4693085	0.00223
RAE1	1.391508	1.5094234	1.2941316	0.5773814	-0.7924036	0.00223
SOD3	1.307292	1.1941451	1.40073077	2.0640783	1.0454977	0.00225
MAP2	2.217133	2.3897239	2.07460517	0.7732508	-0.3709916	0.00226
TPM4	6.243764	5.7936386	6.61548549	1.1714453	0.22828961	0.00226
REEP6	1.626861	1.7542101	1.52169448	0.6917098	-0.5317613	0.00226
XRN2	2.993068	3.2169124	2.8082138	0.8156451	-0.2939865	0.00227
HIGD2A	3.882289	4.1684385	3.64598151	0.8351058	-0.2599691	0.00227
PARL	1.760272	1.8927763	1.65084802	0.7290158	-0.4559781	0.00227
THRAP3	2.389938	2.5799883	2.2329912	0.78038	-0.3577513	0.00228
ELP6	1.809469	1.9437796	1.69855323	0.7401657	-0.4340799	0.00229
SCD	2.315088	2.1388885	2.46059565	1.2824747	0.35893033	0.00229
MMADHC	2.284844	2.4743444	2.12835148	0.7653242	-0.385857	0.00229
INTS10	1.68534	1.8158688	1.57754708	0.7078921	-0.4983986	0.00231
HK2	1.772459	1.6189901	1.89919662	1.4526834	0.53872027	0.00232
CAT	1.486779	1.6082022	1.38650596	0.6354893	-0.6540603	0.00232
SNAPIN	2.18217	2.3342383	2.05659026	0.7919052	-0.3366004	0.00233
SPON2	1.145874	1.0399037	1.23338569	5.8487292	2.54812318	0.00234
CEP41	1.517303	1.6393725	1.41649664	0.6514146	-0.6183519	0.00234
TFG	2.269544	2.4354266	2.13255592	0.789003	-0.3418973	0.00235
VAMP8	1.333742	1.2046677	1.44033321	2.1514544	1.10531227	0.00235
SLC25A38	1.427084	1.5460649	1.3288276	0.6021767	-0.7317411	0.00235
LINC01474	1.408805	1.2333993	1.55365863	2.372152	1.24619645	0.00236
SUPT16H	2.196914	2.35049	2.07008835	0.7923705	-0.335753	0.00236

SMARCA5	2.69942	2.8885872	2.54320268	0.8171202	-0.2913798	0.00236
COX8A	8.610922	9.2221597	8.10615231	0.8642683	-0.2104488	0.00237
MRPL16	1.559179	1.6831185	1.45682748	0.6687383	-0.5804864	0.00237
ELAVL2	1.29545	1.4153871	1.19640433	0.4728224	-1.0806297	0.00238
VPS26A	1.7124	1.8414056	1.60586558	0.7200637	-0.4738036	0.00245
CNIH1	2.417598	2.5974624	2.26906349	0.7944247	-0.3320177	0.00245
FBXO2	1.402781	1.2866167	1.49871204	1.7399964	0.79908431	0.00248
PAF1	1.403836	1.5206708	1.30735274	0.5903014	-0.7604762	0.00248
RNPS1	4.042944	4.3293946	3.8063879	0.8429124	-0.2465454	0.00249
PSRC1	1.418449	1.5354384	1.32183761	0.6010731	-0.7343876	0.0025
GALK1	1.62242	1.7482063	1.51854394	0.6930494	-0.5289699	0.0025
BRK1	8.34729	8.948557	7.85075423	0.8618865	-0.2144301	0.00252
RNF175	1.57705	1.7007052	1.47493373	0.6777939	-0.5610815	0.00252
APBB1	1.505044	1.6259827	1.40517068	0.6472554	-0.6275929	0.00253
PCSK1N	3.091968	2.8376392	3.30199688	1.2526925	0.32503237	0.00257
DNAAF2	1.631016	1.7573253	1.52670828	0.6954848	-0.523909	0.00258
NDUFA4	17.73083	18.789536	16.8565297	0.8913402	-0.1659518	0.00258
SORD	1.468961	1.5880371	1.37062544	0.6302756	-0.6659454	0.0026
STK25	2.3452	2.5099887	2.20911399	0.8007437	-0.3205875	0.00262
NAT14	2.295856	2.4545501	2.16480468	0.8008007	-0.3204849	0.00266
FZD3	2.396044	2.5777946	2.24595202	0.7896795	-0.3406609	0.00266
PSMD11	1.914639	2.049903	1.80293538	0.764771	-0.3869002	0.00267
C8orf76	1.448544	1.5669377	1.35077319	0.6187156	-0.6926516	0.00267
MTCH1	4.485632	4.8345983	4.19744935	0.8338421	-0.262154	0.00268
TUBA1C	2.881456	3.1097927	2.69289099	0.8023969	-0.3176122	0.00268
LYPLA1	3.061983	3.3186175	2.8500499	0.7979108	-0.3257007	0.0027
VPS51	1.654013	1.7805595	1.54950828	0.7039928	-0.5063674	0.0027
ARL14EP	1.696679	1.8243482	1.59124701	0.7172297	-0.4794929	0.00272
RAB5C	2.953223	3.1539322	2.78747453	0.8298657	-0.2690501	0.00272
ECI1	1.443772	1.5611122	1.34687003	0.618183	-0.6938941	0.00275
WDR45	1.916997	2.0527776	1.80486749	0.764518	-0.3873776	0.00275
MRPL2	1.632534	1.7575735	1.52927517	0.6986453	-0.5173679	0.00277
SSR4	7.108806	7.5357603	6.75622058	0.880727	-0.1832332	0.00277
VKORC1	5.669821	6.0273647	5.37455483	0.8701487	-0.2006661	0.00279
RPF1	1.411446	1.5271599	1.31588784	0.5992259	-0.7388282	0.0028
FBXO17	1.756545	1.8859897	1.64964747	0.733245	-0.4476328	0.0028
MED10	2.29157	2.4445481	2.16523846	0.8066456	-0.309993	0.00282
BABAM1	2.291469	2.4507864	2.15990144	0.7994984	-0.3228329	0.00282
ACTG2	1.153105	1.0320743	1.25305409	7.8896204	2.97995588	0.00283
NAA10	2.828487	3.0345448	2.65832116	0.8150822	-0.2949826	0.00285
CAV2	1.253839	1.1455899	1.34323204	2.3575258	1.23727354	0.00289
POLR3GL	2.391725	2.5598428	2.25289109	0.8032163	-0.3161396	0.00291
ADAM9	1.317255	1.2059183	1.40919781	1.9871854	0.99072649	0.00292
UBXN4	3.521015	3.7741787	3.31194747	0.8333809	-0.2629521	0.00293
SUCLA2	1.549036	1.6701497	1.4490188	0.6700276	-0.5777075	0.00294

PPP1R10	1.59923	1.7223585	1.49754838	0.6887832	-0.5378781	0.00295
LIMD2	2.747531	2.9466041	2.58313291	0.8132793	-0.2981771	0.00296
CWC15	2.776727	2.9774058	2.61100371	0.8147057	-0.2956491	0.00297
KIF2C	1.219488	1.326612	1.13102326	0.4011588	-1.3177547	0.00297
C9orf116	1.631867	1.7557944	1.52952637	0.7006223	-0.5132912	0.00298
SMIM7	2.280762	2.4442088	2.14578425	0.7933647	-0.3339439	0.00298
AP1S2	5.74977	6.1431797	5.42488596	0.8603405	-0.2170203	0.00298
FAM3C	2.676482	2.4937555	2.82738129	1.223347	0.29083369	0.00299
CLCN3	2.361575	2.202289	2.49311658	1.2418949	0.31254304	0.00299
ISOC1	1.406296	1.5212832	1.31133696	0.5972511	-0.7435905	0.00301
VGF	1.489832	1.6127697	1.38830718	0.6336919	-0.6581465	0.00303
ESPN	1.502205	1.6244461	1.40125605	0.6425791	-0.638054	0.00303
PRPF38A	1.826707	1.9568897	1.7191991	0.7516008	-0.4119615	0.00306
NRN1	1.380185	1.4939215	1.28625997	0.5795658	-0.7869557	0.00307
PDRG1	1.59712	1.7190267	1.49644676	0.6904427	-0.5344064	0.00307
TBRG4	1.427757	1.5432979	1.33234188	0.6117121	-0.7090753	0.00309
SPA17	1.272505	1.3814611	1.18252698	0.4784943	-1.0634264	0.0031
ICMT	1.451316	1.3348329	1.54751055	1.6351754	0.70944537	0.00311
FBN2	1.880539	1.7431116	1.99402935	1.3376582	0.41970949	0.00311
UNC5D	1.164157	1.0605695	1.24970154	4.1225602	2.04354057	0.00313
ELK3	1.464241	1.3481377	1.56012118	1.6089069	0.68608084	0.00313
RAB38	1.445337	1.3294266	1.54105687	1.64242	0.71582306	0.00315
TMEM126B	1.994157	2.1347937	1.87801665	0.7737236	-0.3701099	0.00316
MRPL12	2.645867	2.8180337	2.5036893	0.8270965	-0.2738724	0.00316
BEX3	23.39936	24.477374	22.509116	0.9161636	-0.1263228	0.00316
RAF1	1.681833	1.8072204	1.57828632	0.7163921	-0.4811787	0.00316
NDUFAF4	1.431745	1.547338	1.33628701	0.6144047	-0.702739	0.00317
IARS2	1.908145	2.0414822	1.79803331	0.7662477	-0.3841172	0.00318
CCNC	1.932401	2.0667306	1.82146853	0.7700806	-0.3769187	0.00318
MCRIP1	4.132633	4.4347749	3.88311984	0.8393912	-0.2525848	0.0032
MAGOHB	1.543476	1.6628674	1.44488008	0.6711449	-0.5753037	0.00321
SGO1	1.428688	1.5435345	1.33384559	0.6142123	-0.7031907	0.00322
MT1E	1.309428	1.172266	1.42269802	2.4537514	1.2949891	0.00322
YWHAH	2.76888	2.9683129	2.60418567	0.8150054	-0.2951185	0.00328
ZFAND6	2.687791	2.8820434	2.52737461	0.8115512	-0.3012459	0.00329
UPP1	1.57286	1.4533034	1.67159209	1.4815511	0.5671084	0.00331
REPIN1	2.131953	2.2746787	2.01408759	0.7955633	-0.3299514	0.00332
PLXND1	1.272373	1.164568	1.36139975	2.1960505	1.13491126	0.00334
DNAJC7	2.270637	2.4312074	2.13803552	0.7951576	-0.3306872	0.00334
NABP2	1.792907	1.921635	1.68660171	0.7449823	-0.424722	0.00334
BTG3	2.607483	2.8005092	2.44807851	0.8042605	-0.3142651	0.00335
RALYL	1.344665	1.4562655	1.25250344	0.5534134	-0.8535706	0.00336
BMPR1B	1.729946	1.8661558	1.61746185	0.7128762	-0.4882765	0.00337
DEPDC1B	1.257376	1.364397	1.16899687	0.4637713	-1.1085147	0.0034
MRPS33	2.564065	2.7372943	2.42100989	0.8179443	-0.2899255	0.0034

TRA2A	2.101117	2.2609688	1.96910861	0.7685429	-0.3798024	0.0034
CAPRIN1	2.433974	2.6007712	2.29623008	0.8097535	-0.3044453	0.00345
ZNF385A	1.5481	1.6671993	1.44974625	0.6740809	-0.5690064	0.00346
ALKBH2	1.635165	1.7574151	1.53420905	0.7053055	-0.5036797	0.00349
ZNF106	1.945472	1.8131681	2.0547314	1.2970645	0.37525021	0.0035
HYAL2	2.222267	2.3673825	2.10242861	0.8062328	-0.3107316	0.00351
FILIP1L	1.289575	1.1793407	1.38060868	2.1222655	1.08560518	0.00351
RALA	1.748065	1.8742266	1.64387862	0.7365122	-0.4412186	0.00351
BTG2	1.933661	2.0664491	1.82400218	0.7726597	-0.372095	0.00353
TRMT112	5.94395	6.3275296	5.62718421	0.8685422	-0.2033322	0.00353
MED28	2.888064	3.0847479	2.72563986	0.8277451	-0.2727415	0.00358
MRPS21	5.17252	5.6304636	4.79434225	0.8194303	-0.2873068	0.00358
SNHG15	2.033201	2.1766748	1.91471766	0.7773751	-0.3633173	0.00359
BLOC1S1	3.605073	3.8633069	3.3918198	0.8353348	-0.2595736	0.0036
HAT1	1.599952	1.719255	1.50142926	0.6971509	-0.5204572	0.00364
DDX17	5.476655	5.0690145	5.81329037	1.1829131	0.24234405	0.00365
BEX2	5.119862	5.4634828	4.83609391	0.8594396	-0.2185318	0.00365
MORN2	1.694998	1.8190075	1.59258877	0.723545	-0.4668454	0.00365
TSSC1	1.46843	1.583759	1.37318891	0.639286	-0.6454667	0.00366
PPDPF	11.75344	12.344989	11.2649271	0.9047983	-0.1443318	0.00368
ATP5I	11.20039	11.791064	10.7126006	0.9000596	-0.1519075	0.00371
STMN4	1.318666	1.4406008	1.21797069	0.4947124	-1.0153381	0.00373
NDUFB1	5.076983	5.4275378	4.78748915	0.8554391	-0.2252629	0.00375
ZC3H13	2.098684	2.2477268	1.97560281	0.7819042	-0.3549363	0.00376
NAXE	2.729985	2.9360989	2.55977268	0.8056265	-0.3118169	0.00377
ORC4	1.557316	1.6753031	1.45988051	0.6809987	-0.5542761	0.00379
RRM2	1.340267	1.4495082	1.25005316	0.5562817	-0.8461125	0.0038
KIF2A	1.875101	2.0070472	1.76613753	0.7607762	-0.394456	0.00381
CA3	2.078619	2.2726846	1.91835695	0.7215903	-0.4707481	0.00384
RBM7	1.829006	1.9614076	1.71966674	0.7485553	-0.4178193	0.00385
ABHD8	1.505739	1.6217112	1.40996698	0.6594171	-0.6007367	0.00386
NME6	1.416639	1.5291309	1.32374132	0.611836	-0.7087831	0.00387
PTP4A3	1.728824	1.5960462	1.83847326	1.4067254	0.49234071	0.00387
GGPS1	2.024562	2.1645835	1.90892971	0.7804762	-0.3575734	0.00387
TCTN3	1.61202	1.7313256	1.51349593	0.7021441	-0.510161	0.0039
CHCHD1	2.483409	2.6516347	2.34448584	0.8140334	-0.29684	0.0039
AK6	2.003557	2.1403466	1.89059477	0.7809861	-0.3566313	0.00394
ATP5G1	4.238653	4.5157284	4.00983992	0.8561071	-0.2241369	0.00397
SRP72	2.208639	2.3654381	2.07915205	0.7903339	-0.3394658	0.00397
AGGF1	1.972935	2.1050633	1.86382171	0.7816943	-0.3553236	0.00398
RSRC2	3.363428	3.5895934	3.17665619	0.8405398	-0.250612	0.00399
CLTB	2.494747	2.3137412	2.64422369	1.2515583	0.32372544	0.00399
CDC42	5.387422	5.8054333	5.04222048	0.8411771	-0.2495185	0.00401
PPP2R5E	1.885063	2.0141453	1.7784639	0.7676059	-0.3815624	0.00403
RNF114	1.761544	1.8859152	1.65883683	0.7436794	-0.4272473	0.00405

RHOD	1.268562	1.1640322	1.35488485	2.1635069	1.11337169	0.0041
MAPRE1	2.123327	2.264175	2.00701188	0.7965763	-0.3281155	0.00413
TTC9C	1.582739	1.7005842	1.48542015	0.6928791	-0.5293244	0.00414
TMEM141	2.558497	2.7209338	2.42435442	0.8276637	-0.2728834	0.00415
TSR2	1.569697	1.6868958	1.47291272	0.6884781	-0.5385173	0.00415
PRKCDBP	2.422531	2.598101	2.27754265	0.799413	-0.3229871	0.00416
CEP55	1.202051	1.3046688	1.11730813	0.3850349	-1.3769389	0.00417
LARP7	1.99187	2.1368226	1.87216625	0.7671964	-0.3823321	0.0042
GSPT1	1.947526	2.0786996	1.83920035	0.7779741	-0.362206	0.0042
TBC1D9	1.443328	1.3312331	1.53589716	1.6178852	0.69410923	0.00421
GPR37	1.496782	1.6114	1.40212895	0.6577182	-0.6044584	0.00421
TIMM8B	4.480095	4.8037907	4.21278115	0.8446262	-0.2436152	0.00422
CKAP4	2.407288	2.2488105	2.53816052	1.2317005	0.30065153	0.00422
NCAM1	1.668907	1.790355	1.56861231	0.7194391	-0.4750555	0.00424
XAGE2	1.153118	1.0296632	1.25506948	8.5988558	3.1041447	0.00424
REEP4	1.305796	1.4122325	1.21789827	0.528581	-0.9198034	0.00425
UPF3A	2.630473	2.8386655	2.45854397	0.7932623	-0.3341301	0.00425
NDUFA10	1.996423	2.1315754	1.88481238	0.7819297	-0.3548892	0.00426
KIF22	1.496782	1.6108519	1.40258205	0.6590501	-0.6015399	0.00426
ACAT1	1.525368	1.6407179	1.43011083	0.6712952	-0.5749808	0.00429
RPP25L	1.70906	1.83071	1.60859964	0.7326259	-0.4488514	0.00429
ATP6V0D1	1.615618	1.7340211	1.51783954	0.7054832	-0.5033163	0.00434
CMAS	1.931342	2.0617293	1.82366534	0.7757771	-0.3662858	0.00434
ANAPC11	6.974481	7.5069034	6.53479776	0.850604	-0.2334405	0.00435
PVRL3-AS1	1.52062	1.4061886	1.6151198	1.51437	0.59871772	0.00437
VAX2	1.372983	1.4822025	1.28278774	0.5864502	-0.7699194	0.00439
GRPEL1	1.557878	1.6740872	1.46191053	0.6852385	-0.5453218	0.00441
UBE2D3	5.253132	5.6216885	4.94877166	0.8544002	-0.2270161	0.00441
MYH14	1.328479	1.222254	1.41620167	1.8726395	0.9050732	0.00449
PAAF1	1.52929	1.6437678	1.43475189	0.6753241	-0.5663481	0.00451
COX7B	7.710076	8.2214769	7.28775225	0.8707017	-0.1997495	0.00455
NDRG1	2.029376	1.8728113	2.15866934	1.3275141	0.40872715	0.00456
N4BP2	2.054718	2.1908353	1.94231038	0.791302	-0.3376997	0.00456
CA8	1.245176	1.3486244	1.15974616	0.4582186	-1.1258922	0.00458
MESDC2	2.286687	2.4425139	2.15800229	0.8027668	-0.3169471	0.00461
AK4	2.22479	2.3727246	2.10262313	0.803237	-0.3161024	0.00464
PPP2R2A	2.024789	2.1621953	1.91131667	0.7841338	-0.3508282	0.00465
EIF2A	1.798766	1.9228314	1.69631042	0.7545369	-0.4063365	0.00468
DRG2	1.374986	1.4834176	1.28544185	0.5904664	-0.7600732	0.0047
LYRM1	1.785478	1.9098481	1.68277051	0.7504225	-0.4142249	0.00471
EXTL2	1.76967	1.8918861	1.66874277	0.7498074	-0.4154081	0.00471
DNAJB6	3.146316	3.3637124	2.96678556	0.8320748	-0.2652148	0.00472
COX11	1.887813	2.0180338	1.78027384	0.7664518	-0.383733	0.00472
PGD	2.793185	2.9857733	2.63414161	0.8229245	-0.2811679	0.00476
VCP	2.449604	2.6126909	2.31492433	0.8153604	-0.2944902	0.00479

TMEM35A	1.265954	1.370189	1.17987444	0.4858989	-1.041272	0.00481
TMEM245	1.750497	1.6289816	1.85084691	1.3527374	0.43588183	0.00481
PIF1	1.285722	1.3932696	1.19690795	0.5006946	-0.9979972	0.00481
SCAND1	3.521036	3.7649024	3.3196465	0.8389614	-0.2533236	0.00481
GABRP	1.135325	1.0169971	1.23304247	13.710703	3.77723059	0.00482
SPATC1L	2.0267	2.1629669	1.91416852	0.7860658	-0.3472779	0.00482
LRRC41	1.767858	1.8901425	1.66687341	0.749176	-0.4166234	0.00483
RSPH9	1.798749	1.9219283	1.69702509	0.7560513	-0.4034439	0.00484
DICER1	2.053794	1.9225652	2.16216538	1.2597109	0.33309267	0.00486
VWA9	1.36103	1.46831	1.27243626	0.5817435	-0.781545	0.00487
ABT1	1.812782	1.9407166	1.70713095	0.7516939	-0.4117828	0.00487
UROS	2.596984	2.7777765	2.44768329	0.8143225	-0.2963279	0.00489
EEF1D	11.43391	12.141284	10.8497421	0.8840761	-0.1777576	0.00496
IAH1	2.059224	2.2039584	1.93970013	0.7805088	-0.3575132	0.00498
TTC3	9.171796	9.6889661	8.74470907	0.8913269	-0.1659735	0.00499
LRRC39	1.13442	1.036841	1.21500162	5.8359387	2.54496474	0.005
PITPNA-AS1	1.646171	1.7640388	1.5488344	0.7183331	-0.477275	0.005
METTL23	1.794282	1.9198303	1.69060306	0.750794	-0.4135109	0.005
KIFC1	1.30547	1.4098292	1.21928853	0.535073	-0.9021924	0.00502
MRPS15	2.488138	2.6429621	2.36028083	0.8279441	-0.2723947	0.00504
ETFRF1	1.880984	2.0067141	1.77715422	0.7719712	-0.3733811	0.00504
CENPW	1.818257	1.9510905	1.7085615	0.7449991	-0.4246895	0.00505
NET1	1.399184	1.291396	1.48819619	1.6753702	0.74447991	0.00506
UBE2D1	1.791574	1.9147186	1.68987848	0.7541975	-0.4069857	0.00507
KREMEN1	1.384885	1.2777793	1.47333494	1.7039963	0.7689222	0.00509
PLEKHA4	1.777642	1.899761	1.67679478	0.7521939	-0.4108234	0.0051
EIF3J	2.263971	2.4260451	2.13012737	0.7924906	-0.3355342	0.00517
HSPB1	8.912559	8.2340501	9.47288369	1.1712503	0.22804947	0.00519
FEN1	1.342694	1.4485163	1.25530484	0.5692209	-0.8129394	0.00519
CLDN6	1.667152	1.5300227	1.78039523	1.4723808	0.55815088	0.00521
DUSP23	2.079318	2.2207989	1.96248081	0.7884024	-0.3429958	0.00527
SMAD2	2.815741	3.0152359	2.65099478	0.8192563	-0.2876132	0.00528
CREB1	1.842164	1.9660049	1.73989377	0.7659317	-0.3847124	0.00529
DHRS4-AS1	1.772153	1.8934228	1.67200643	0.7521707	-0.410868	0.00531
SUPT4H1	2.634606	2.8131539	2.48715813	0.8202051	-0.2859433	0.00538
LRRC17	3.125108	2.8430518	3.35803389	1.2794181	0.35548783	0.00538
BTG1	10.59957	11.24578	10.0659212	0.8848444	-0.1765042	0.00541
TIMM9	2.153952	2.3050402	2.02918126	0.7886203	-0.3425972	0.00541
COMMD9	1.469553	1.5799556	1.3783807	0.6524305	-0.6161039	0.00543
TM2D2	1.530796	1.643564	1.43766968	0.6800718	-0.5562411	0.00546
NAPA	1.502983	1.6140487	1.41126272	0.6697559	-0.5782927	0.00547
NLGN2	1.448841	1.3398446	1.53885177	1.5855829	0.66501329	0.00548
PTPRF	2.036441	1.8960887	2.15234707	1.2859744	0.36286189	0.00548
YARS	1.395712	1.5028683	1.30722001	0.6109353	-0.7109086	0.00551
GRIA4	1.903722	2.0314451	1.79824567	0.77391	-0.3697623	0.00555

MSN	1.988043	2.1203746	1.87876201	0.7843466	-0.3504367	0.00555
PRPS2	1.531589	1.6436631	1.43903568	0.6820892	-0.5519676	0.00555
NT5C3B	2.75382	2.955544	2.58723207	0.8116575	-0.3010569	0.00556
MMP14	1.383782	1.2779355	1.47119156	1.6953267	0.76156333	0.00556
HSD17B8	1.285974	1.3888924	1.20098217	0.5168066	-0.9523035	0.00557
ASF1B	1.347979	1.4529104	1.26132403	0.5769883	-0.7933859	0.00557
SH3BGRL3	14.17534	15.17766	13.3476014	0.8709196	-0.1993886	0.00562
IRS2	1.348233	1.2437638	1.43450561	1.782486	0.83389078	0.00562
PSMC2	1.563951	1.6769508	1.47063336	0.6952253	-0.5244475	0.00563
DEF8	1.921951	1.7976136	2.02463004	1.2846196	0.36134117	0.00565
COPS2	2.09132	2.2378656	1.97030024	0.7838494	-0.3513516	0.00566
WDR25	1.667119	1.7918616	1.56410458	0.7123778	-0.4892856	0.00567
PPP2R5C	1.960167	2.0866745	1.85569495	0.7874437	-0.3447513	0.00573
DLG1	1.695167	1.5784946	1.79151769	1.368237	0.45231819	0.00577
CDON	2.362134	2.535697	2.21880285	0.793648	-0.3334289	0.00579
CHTOP	2.154867	2.2883632	2.04462279	0.8108139	-0.3025572	0.00579
MRPL41	3.351681	3.1388313	3.52745657	1.1816998	0.24086362	0.00582
C9orf3	1.287282	1.1855786	1.37127092	2.0006131	1.00044222	0.00583
DCUN1D5	1.936795	2.0630032	1.83257071	0.783225	-0.3525012	0.00583
ATP5D	6.299041	6.7401401	5.93477396	0.8596957	-0.2181019	0.00586
REEP2	1.97145	2.1015772	1.86398935	0.7843203	-0.3504851	0.00589
IMMT	1.75384	1.8727835	1.655615	0.7511771	-0.412775	0.00589
AIP	1.654596	1.7710318	1.55844111	0.7242776	-0.4653853	0.0059
PLOD2	2.226018	2.3681867	2.10861248	0.8102787	-0.3035099	0.00591
NDUFA1	5.384534	5.8281461	5.01819134	0.8322431	-0.2649231	0.00599
STMN3	1.846139	1.9681697	1.74536446	0.7698697	-0.3773139	0.00599
HNRNPU	5.166722	5.5148334	4.87924675	0.8592226	-0.2188962	0.006
PFDN6	1.921706	2.0492798	1.81635325	0.7780129	-0.3621339	0.006
PAK2	2.678371	2.8580998	2.52994735	0.8233935	-0.280346	0.00601
SYNE2	2.108083	1.970174	2.22196974	1.2595367	0.33289311	0.00602
CYR61	1.979583	1.7583283	2.16229885	1.5327119	0.61608657	0.00603
LAMP5	1.632262	1.7482305	1.53649375	0.7170167	-0.4799214	0.00604
FAM96B	3.767885	3.9957914	3.57967637	0.8611001	-0.2157471	0.00608
CCL2	2.946343	2.3204698	3.46319869	1.8653957	0.89948171	0.00614
SARAF	4.830788	5.1916216	4.53280596	0.8428256	-0.246694	0.00615
MZT2A	6.128639	6.6271519	5.71695854	0.8382497	-0.254548	0.00615
XRCC1	1.591891	1.704645	1.498777	0.7078415	-0.4985017	0.00623
SPDL1	1.466373	1.5742195	1.37731086	0.6570847	-0.6058488	0.00626
HSPG2	1.262659	1.1631267	1.34485529	2.1140339	1.07999852	0.00629
GADD45B	1.513121	1.3851957	1.61876312	1.6063604	0.68379564	0.00629
PES1	1.399444	1.5052375	1.31207737	0.6176845	-0.6950579	0.0063
CEP63	1.505878	1.615387	1.4154429	0.6750921	-0.5668437	0.00631
C11orf58	5.863684	6.3402589	5.47012055	0.8370606	-0.2565959	0.00634
ECHDC1	1.991229	2.1164856	1.88779044	0.7951652	-0.3306735	0.00634
TCF25	3.340792	3.583775	3.14013297	0.828297	-0.27178	0.00638

PDAP1	3.943361	4.2118893	3.7216062	0.8473537	-0.2389638	0.0064
CD44	1.218522	1.120792	1.29922897	2.4772249	1.30872486	0.00642
CXCL12	1.156083	1.0606055	1.23492904	3.8763651	1.95470445	0.00645
PCDH19	1.230481	1.1324	1.31147831	2.3525554	1.23422869	0.00646
RP11-14N7.2	1.533377	1.4243045	1.62345053	1.469347	0.55517511	0.0065
CNTRL	1.355006	1.4586318	1.2694299	0.5874645	-0.7674265	0.0066
CCK	1.395146	1.5056539	1.30388684	0.600978	-0.734616	0.00665
CPAMD8	1.994116	2.1228062	1.88784197	0.7907348	-0.3387342	0.00667
C9orf78	1.812851	1.9319799	1.71447189	0.7666173	-0.3834215	0.00672
BPHL	1.662647	1.7766634	1.5684912	0.731966	-0.4501515	0.00674
WBP11	2.308046	2.4521675	2.18902782	0.8187953	-0.2884254	0.00675
MSI2	6.483071	6.864513	6.16806945	0.8812444	-0.1823859	0.00675
MRPL39	1.406142	1.5108273	1.31969052	0.625829	-0.6761596	0.00677
ZNF883	1.641065	1.7543802	1.54748762	0.725745	-0.4624654	0.00678
RSRC1	1.722271	1.8386033	1.62620229	0.7467205	-0.4213597	0.0068
ZCCHC9	1.3474	1.4504714	1.26228222	0.5822394	-0.7803156	0.0068
CHMP6	1.377578	1.4815086	1.29174998	0.6059081	-0.722829	0.00681
NLRP1	2.829113	3.0264774	2.66612644	0.8221786	-0.2824762	0.00683
ONECUT2	1.128344	1.2261825	1.0475465	0.210213	-2.250076	0.00684
KIF20B	1.557483	1.6686613	1.46567086	0.6964226	-0.5219651	0.00685
FGF13	1.444929	1.5511494	1.35720973	0.6481177	-0.6256722	0.00688
AKAP8L	1.596607	1.708338	1.50433834	0.7120024	-0.490046	0.00688
FAM50A	2.285254	2.4229808	2.17151742	0.8232841	-0.2805378	0.0069
BMF	1.171072	1.0760563	1.24953786	3.2809632	1.71411943	0.00692
CHCHD5	2.95535	3.166701	2.78081208	0.8219003	-0.2829648	0.00696
FAM83D	1.148176	1.2425159	1.07026905	0.2897503	-1.7871179	0.00698
GAS2L3	1.3564	1.4588096	1.27182801	0.5924636	-0.7552016	0.00699
HADHA	2.647541	2.8230468	2.50260621	0.824228	-0.2788847	0.007
PDZD11	1.892323	2.0139559	1.79187733	0.7809781	-0.356646	0.00701
RP11-553A10.1	1.766477	1.6504337	1.86230705	1.3257417	0.40679973	0.00702
CASP3	1.549126	1.658756	1.45859197	0.6961485	-0.5225331	0.00708
NAA50	1.815313	1.9347539	1.71667732	0.7667016	-0.383263	0.00711
ZIC1	1.400318	1.5040606	1.31464572	0.6242219	-0.679869	0.00713
HES6	2.491911	3.0955316	1.99343181	0.4740715	-1.0768234	0.00714
CAST	2.759774	2.5560746	2.92799233	1.2390102	0.30918807	0.00719
VAMP2	3.744818	3.9947515	3.53841955	0.8476228	-0.2385058	0.00721
UBE2B	2.542562	2.7094059	2.40477973	0.8217941	-0.2831511	0.00723
AAAS	1.435555	1.5402954	1.34905838	0.646051	-0.63028	0.00723
SMDT1	4.211835	4.499952	3.97390269	0.8496981	-0.2349777	0.00724
PLTP	1.870476	1.9898607	1.77188544	0.779792	-0.3588387	0.0073
SCRN2	1.766404	1.8830815	1.67005053	0.7587641	-0.3982767	0.00731
CD47	2.483392	2.6505404	2.34535721	0.815101	-0.2949492	0.00731
SH3BP5	1.418527	1.3148271	1.50416417	1.6014003	0.67933401	0.00736
PIGP	2.041254	2.1661147	1.93814232	0.8045026	-0.313831	0.00737
USO1	1.936447	2.0583541	1.83577327	0.7896915	-0.3406389	0.00738

RBM10	1.390169	1.493441	1.3048844	0.6178741	-0.6946152	0.00738
SUCO	2.024127	1.9008348	2.12594394	1.2498895	0.32180051	0.00743
RP11-620J15.3	2.353174	2.4975578	2.23393909	0.8239676	-0.2793405	0.00743
GPSM2	1.265566	1.3637766	1.18446141	0.5070733	-0.9797338	0.00747
LSM14A	3.106437	3.3022674	2.94471769	0.8446967	-0.2434947	0.00749
AIG1	1.791842	1.9086539	1.69537664	0.7652822	-0.3859363	0.00751
TCEAL7	4.081497	4.3672484	3.8455194	0.8450578	-0.2428782	0.00753
KLHDC8B	2.550171	2.7386775	2.39449877	0.8020457	-0.3182437	0.00754
NAP1L3	1.440002	1.5447283	1.35351823	0.6489809	-0.6237521	0.00755
GLT8D1	1.87521	1.9947005	1.77653187	0.780669	-0.3572171	0.00756
PPP2R3C	1.898413	2.0189217	1.79889539	0.7840596	-0.3509647	0.00757
HACD1	1.605025	1.4949654	1.69591478	1.4059868	0.49158305	0.00758
GPC2	1.477647	1.5837734	1.39000662	0.6680787	-0.58191	0.00765
DERA	1.624071	1.7351923	1.53230435	0.7240342	-0.4658703	0.00766
LIPA	1.396942	1.5004139	1.31149268	0.6224701	-0.6839235	0.00767
TWF2	1.541874	1.649986	1.45259247	0.6963111	-0.5221961	0.00768
PLAGL1	1.426972	1.3230497	1.51279348	1.5873518	0.66662192	0.00774
MTHFD2	1.71892	1.8324052	1.62520232	0.7510793	-0.4129629	0.00774
RPIA	1.590346	1.7006005	1.49929563	0.7126681	-0.4886978	0.00776
CIB1	2.495588	2.656197	2.36295459	0.8229423	-0.2811368	0.00777
G6PD	1.822913	1.940009	1.72621391	0.7725606	-0.37228	0.00779
LINC00998	4.188672	4.4426335	3.97894599	0.8653102	-0.2087107	0.0078
ATXN2	1.833872	1.7163105	1.93095693	1.2996556	0.37812938	0.00784
CISD1	2.249058	2.410504	2.11573329	0.7910175	-0.3382185	0.00785
APEH	1.487447	1.5933961	1.39995272	0.6740063	-0.569166	0.00788
MRPL57	4.883751	5.1924469	4.62882463	0.8655625	-0.2082902	0.0079
SFT2D1	1.957192	2.0784084	1.85708875	0.794772	-0.3313871	0.00795
COPB2	1.68907	1.801984	1.5958246	0.7429383	-0.4286858	0.00798
PDXK	1.722873	1.6094276	1.81655857	1.3398778	0.42210145	0.00798
WDR83OS	4.396195	4.6901299	4.1534592	0.8545659	-0.2267363	0.00798
PLS3	1.888191	1.7685197	1.98701675	1.2843089	0.36099228	0.00799
NIT2	1.648417	1.759929	1.55632913	0.7320804	-0.449926	0.00801
EIF2S2	3.388769	3.5976682	3.21625689	0.8531717	-0.229092	0.00801
PRNP	1.655218	1.5443314	1.74678942	1.3719389	0.45621621	0.00801
LETMD1	1.754588	1.8702509	1.65907138	0.7573349	-0.4009966	0.00802
ZNF511	1.890687	2.0162421	1.78700203	0.7744238	-0.3688048	0.00808
NARS	2.354873	2.5016465	2.23366506	0.8215416	-0.2835945	0.00812
PSMD2	2.260883	2.4065456	2.14059285	0.8109178	-0.3023725	0.00814
CFAP20	1.737079	1.8505603	1.64336483	0.7564012	-0.4027765	0.00815
NUCB1	2.209447	2.3383849	2.10296791	0.8241036	-0.2791023	0.00822
SLC38A2	2.018529	1.8925531	2.12256127	1.257697	0.33078435	0.00824
RASL11B	1.262534	1.1661038	1.34216748	2.0599622	1.04261784	0.00824
HM13	2.050126	2.1753324	1.94672791	0.805498	-0.312047	0.00825
NEFM	1.203244	1.3316605	1.09719477	0.293055	-1.7707565	0.00826
BPGM	1.519297	1.6260407	1.43114716	0.6886887	-0.5380761	0.00829

TBCC	1.299119	1.397455	1.21791212	0.5482687	-0.867045	0.00832
ADK	1.399082	1.5007823	1.31509543	0.6292064	-0.6683948	0.00833
ASPH	2.734532	2.5571695	2.88100111	1.2079617	0.27257466	0.00838
PCBP4	2.076168	2.2342014	1.94566126	0.7662131	-0.3841824	0.00841
AHCY	3.577483	3.8100708	3.38540738	0.848878	-0.2363708	0.00842
ACTR3	2.458414	2.6038821	2.33828497	0.8344036	-0.2611827	0.00846
MIS18BP1	2.12854	2.274549	2.00796365	0.7908395	-0.3385432	0.00847
SIGIRR	2.0612	2.188138	1.95637214	0.8049335	-0.3130584	0.00851
MRNIP	1.660444	1.7715897	1.56865868	0.7369962	-0.4402708	0.00852
EFNA4	1.73466	1.84774	1.64127675	0.7564545	-0.4026747	0.00854
ANKLE2	1.663165	1.5526109	1.75446313	1.3652701	0.44918635	0.00855
CHCHD6	1.869382	1.9873591	1.77195499	0.7818381	-0.3550581	0.00856
DZIP3	1.731331	1.8448759	1.63756439	0.7546249	-0.4061684	0.0086
INA	1.140224	1.2325112	1.06401191	0.2753068	-1.8608876	0.00862
ITGA3	1.357955	1.2585672	1.44003142	1.7018071	0.76706752	0.00862
EPC1	2.02098	2.1429577	1.92024874	0.8051468	-0.3126762	0.00863
PLEKHJ1	2.754111	2.9144118	2.6217313	0.8471173	-0.2393664	0.00863
APPL1	2.221215	2.3649092	2.10254927	0.8077821	-0.3079619	0.0087
SELENOF	2.979602	3.187755	2.80770625	0.8262837	-0.2752909	0.00878
PINLYP	1.313517	1.4118252	1.23233268	0.5641536	-0.82584	0.00878
C1QTNF4	1.392767	1.4938925	1.30925616	0.6261609	-0.6753947	0.0088
SNAPC1	1.752365	1.8647207	1.65957931	0.7627657	-0.3906881	0.0088
ITFG1	1.771691	1.8858588	1.67741015	0.7646931	-0.3870472	0.00885
DEK	4.995175	5.2964825	4.74635103	0.8719577	-0.1976699	0.00887
LAMC1	1.604719	1.4966651	1.69395235	1.3972239	0.48256319	0.00893
COMMD4	1.768794	1.8840765	1.67359255	0.7619166	-0.392295	0.00893
OPTC	1.171182	1.2635182	1.09492901	0.3602371	-1.4729814	0.00893
GRB2	1.909696	2.027651	1.81228606	0.7904298	-0.3392907	0.00894
TMEM259	1.639855	1.530768	1.72994089	1.3752542	0.45969827	0.00903
CSNK1E	2.263948	2.1218832	2.38126671	1.2312036	0.30006939	0.00905
ALKBH5	2.76873	2.9310343	2.63469734	0.8465398	-0.2403502	0.00907
POLE3	1.86588	1.9876514	1.76531935	0.7748881	-0.3679401	0.00916
DDX18	2.221384	2.3689251	2.09954247	0.8032159	-0.3161402	0.00921
ANXA1	4.420578	4.0079199	4.76135831	1.2504849	0.32248761	0.00922
RAB27A	1.268131	1.1724818	1.34712011	2.0125031	1.00899101	0.00928
UQCC2	4.797853	5.1374767	4.51738644	0.8501284	-0.2342474	0.0093
CDCA2	1.196604	1.2893667	1.11999919	0.4146959	-1.2698743	0.00931
SIGMAR1	2.215453	2.3575859	2.0980778	0.8088459	-0.3060632	0.00933
HSD17B11	1.424588	1.5258039	1.34100281	0.6485361	-0.6247411	0.00939
PDCD10	1.721087	1.8324668	1.62910778	0.7557152	-0.4040855	0.00946
S100A11	10.67896	9.7282814	11.4640422	1.1988663	0.26167074	0.00948
ARID5B	1.370293	1.2718816	1.45156307	1.6608815	0.73194914	0.0095
MRPL54	2.102645	2.2330385	1.99496395	0.8069204	-0.3095017	0.00951
NEDD8	7.370369	7.7504579	7.05648505	0.8971962	-0.1565046	0.00951
SNRPN	5.432729	5.752839	5.16837682	0.8770288	-0.1893038	0.00951

RFLNB	1.258294	1.1633472	1.33670277	2.06127	1.04353348	0.00953
RPF2	1.553697	1.6594643	1.46635294	0.7071694	-0.4998723	0.00954
ILK	2.230947	2.3777646	2.10970308	0.8054374	-0.3121557	0.00956
CTNNBIP1	2.259132	2.4020742	2.14108734	0.8138566	-0.2971535	0.00963
ERGIC1	1.987574	2.1074965	1.88853921	0.8022953	-0.3177948	0.00966
EIF5AL1	1.305043	1.4019346	1.22502799	0.5598622	-0.8368562	0.00967
NGDN	1.496753	1.5998275	1.41163216	0.6862509	-0.543192	0.00974
SCLT1	1.277794	1.3730894	1.19909765	0.5336459	-0.9060453	0.00978
FARSA	1.501197	1.6045379	1.41585639	0.6878913	-0.5397475	0.00978
LINC00665	1.943243	2.0692606	1.83917621	0.7848191	-0.3495679	0.00978
RAB14	1.875465	1.9911412	1.77993832	0.7869094	-0.3457305	0.00985
MYL9	7.480745	6.8636893	7.99031956	1.1921367	0.25354974	0.00986
SOCS6	1.329222	1.2322159	1.40933149	1.7627196	0.81780299	0.00989
TOMM22	2.888459	3.0693532	2.73907297	0.8403944	-0.2508615	0.00992
FAM110B	1.423231	1.5239776	1.34003201	0.6489438	-0.6238345	0.00993
PABPC4	2.771694	2.9683076	2.60932777	0.81762	-0.2904975	0.00994
IPO5	2.448538	2.5949133	2.32765945	0.8324336	-0.2645929	0.00994
ERGIC3	4.045893	4.2743444	3.85723393	0.8726125	-0.1965869	0.00994
ZYX	1.605668	1.4999325	1.69298665	1.3861605	0.47109431	0.00996
CSDE1	5.057829	5.3952057	4.77921789	0.8598501	-0.217843	0.01
NELFCD	2.218319	2.3447622	2.11389941	0.8283245	-0.2717321	0.01002
CTSZ	1.29275	1.1978662	1.37110716	1.8755464	0.90731098	0.01003
WRB	2.161929	2.2990274	2.04871107	0.8073048	-0.3088146	0.01005
CD320	2.428423	2.5793972	2.30374562	0.8254704	-0.2767117	0.01008
NIPBL	2.196962	2.3257914	2.0905717	0.8225817	-0.2817691	0.0101
SMAD9	1.785555	1.9083244	1.68416937	0.7532214	-0.4088541	0.01011
RNF167	2.060465	2.1811852	1.96077133	0.813396	-0.2979702	0.0102
RTN1	1.202784	1.315901	1.10937088	0.3462189	-1.5302437	0.01028
DCTD	2.085732	2.2141356	1.97969371	0.8069063	-0.3095269	0.01032
HMGCL	1.51056	1.6139399	1.42518732	0.6925552	-0.529999	0.01032
CTNNBL1	1.483992	1.5861022	1.39966764	0.6819078	-0.5523514	0.01037
RAB13	4.876601	5.2525924	4.56610031	0.8385709	-0.2539953	0.01047
C15orf57	1.393683	1.492389	1.31217003	0.6339906	-0.6574666	0.01047
CCDC58	1.336536	1.4338804	1.25614771	0.5903648	-0.7603214	0.01049
COA1	3.678926	3.8858409	3.50805145	0.8690886	-0.2024248	0.01052
BOP1	1.307993	1.4037416	1.22892215	0.5670017	-0.8185751	0.01053
POP4	1.432515	1.5328406	1.34966436	0.6562269	-0.6077334	0.01056
MTERF2	1.527362	1.6310563	1.44173034	0.6999857	-0.5146027	0.01058
LRRC42	1.536718	1.6401472	1.45130373	0.705	-0.5043049	0.01059
RPUSD3	1.829298	1.9420669	1.73617083	0.7814422	-0.3557889	0.0106
WSB2	1.51871	1.4166493	1.60299267	1.4472427	0.53330692	0.01073
SNCG	1.731032	1.8949566	1.59566019	0.6655744	-0.5873282	0.01076
CREG1	1.765893	1.8767428	1.67435229	0.7691564	-0.3786512	0.01079
RRBP1	1.812724	1.7005126	1.9053895	1.2924671	0.37012756	0.01084
C5orf15	1.804649	1.9162198	1.71251293	0.777666	-0.3627775	0.01084

SMAD3	1.393503	1.2958805	1.47412142	1.6024085	0.68024202	0.01087
MRPL19	1.841536	1.9538614	1.7487754	0.7849939	-0.3492466	0.0109
TBCD	1.371949	1.4695696	1.29133301	0.6204256	-0.68867	0.0109
NUBP1	1.309678	1.4050838	1.23089079	0.5699828	-0.8110096	0.01095
GADD45GIP1	4.811562	5.0974006	4.57551125	0.8726292	-0.1965594	0.01096
ASNA1	1.817613	1.9306006	1.7243056	0.7783206	-0.3615635	0.01099
GTF2F2	1.728268	1.8378902	1.63774002	0.761126	-0.3937927	0.01105
FBLIM1	1.411537	1.3136808	1.49234788	1.5695826	0.65038099	0.01105
HES4	2.782923	2.5865546	2.94508636	1.2259813	0.29393702	0.01106
ECE1	1.435885	1.3369702	1.5175706	1.5359538	0.6191348	0.01106
NBL1	1.887359	2.0077548	1.78793364	0.7818704	-0.3549986	0.01108
PFDN1	2.463318	2.6016643	2.34906952	0.8422923	-0.2476071	0.01112
PPP1R1A	1.24149	1.3348935	1.1643562	0.4907715	-1.0268766	0.01113
MRPS22	1.36126	1.4579665	1.28139738	0.6144497	-0.7026333	0.01115
RASSF4	1.835025	1.9520691	1.73836821	0.7755405	-0.3667259	0.01118
RP11-195F19.5	1.493407	1.5949182	1.40957805	0.6884612	-0.5385528	0.01118
TXNL4A	2.497123	2.6591757	2.36329651	0.821671	-0.2833673	0.01124
ATF7IP	1.990302	2.107147	1.89380863	0.807308	-0.3088089	0.01125
ODF2	1.471853	1.5722719	1.38892474	0.6796153	-0.5572098	0.01133
C11orf74	1.897789	2.0180687	1.79846018	0.7842891	-0.3505426	0.01133
TIMMDC1	2.009725	2.1280825	1.91198329	0.8084367	-0.3067933	0.01141
USP11	2.577551	2.7275252	2.4536995	0.8414925	-0.2489777	0.01142
SFN	1.106073	1.0175675	1.17916161	10.198494	3.35028427	0.01142
DSC2	1.699924	1.5929651	1.78825172	1.3293391	0.41070912	0.01148
DYNC1LI1	1.480642	1.5815475	1.39731243	0.6831986	-0.5496232	0.01148
KLHDC9	1.476958	1.5774178	1.39399729	0.6823435	-0.5514299	0.01151
FBXO22	1.73329	1.8423161	1.64325463	0.7636736	-0.3889719	0.01165
DUT	5.15201	5.4871144	4.87527493	0.8636452	-0.2114893	0.01167
GRID2	1.114237	1.0272531	1.18607053	6.8274916	2.77135563	0.01172
FAM32A	3.164951	3.3600814	3.00380845	0.8490421	-0.236092	0.01178
ELP5	2.039475	2.1638567	1.93675813	0.8048741	-0.3131649	0.01181
GOT2	1.666096	1.7724104	1.57830046	0.7486958	-0.4175484	0.01183
CCDC160	1.395175	1.4921726	1.31507276	0.6401672	-0.6434793	0.01194
CUTC	1.372693	1.4689748	1.29318259	0.6251563	-0.6777111	0.01197
CASC15	1.961007	1.8449914	2.05681463	1.2506809	0.32271372	0.01202
TUSC3	3.633895	3.8579943	3.44883029	0.8568353	-0.2229102	0.01205
JAM2	1.447205	1.5460103	1.36560909	0.6696011	-0.5786263	0.01209
RNF11	2.02144	1.9040254	2.11840332	1.2371371	0.30700534	0.01209
MRPS28	1.658549	1.7644488	1.57109574	0.7470686	-0.4206873	0.01212
TPM3	3.166427	3.3471967	3.01714473	0.8593846	-0.2186242	0.01216
NCOR1	2.655038	2.8165779	2.52163553	0.8376385	-0.2556004	0.01217
CRMP1	1.299862	1.3974	1.21931335	0.5518705	-0.8575983	0.01218
NCAPD2	1.336154	1.4309853	1.25784134	0.5982602	-0.7411551	0.01221
AAGAB	1.425153	1.5230333	1.34432212	0.6583178	-0.6031439	0.01222
MSRB2	2.265483	2.3980092	2.15604055	0.8269192	-0.2741818	0.01222

PDCD2	2.182037	2.3070985	2.07875866	0.8253078	-0.2769957	0.01232
NHLH1	1.117531	1.2169371	1.03543897	0.1633606	-2.6138684	0.01233
WIPI2	2.163653	2.2860907	2.06254216	0.8261798	-0.2754723	0.01233
EBAG9	1.552421	1.6547376	1.46792701	0.7146787	-0.4846333	0.01236
LAMB1	1.201358	1.1121745	1.27500798	2.4516076	1.29372811	0.01237
TRUB2	1.394913	1.4913311	1.31528899	0.6417037	-0.6400208	0.01242
CLSTN2	1.448675	1.3509979	1.52933767	1.5080937	0.59272611	0.01242
SVBP	2.657038	2.8200759	2.52239948	0.8364483	-0.2576516	0.01245
TWISTNB	1.339759	1.4344115	1.26159265	0.6021771	-0.7317402	0.01248
RNASEH2B	1.734642	1.8424863	1.64558282	0.7662828	-0.3840511	0.01248
CLDN7	1.112284	1.0195686	1.18885074	9.6507085	3.27063486	0.01249
TMEM91	1.490655	1.391441	1.57258671	1.4627662	0.54869918	0.01255
FBXO5	1.468133	1.5669943	1.38649232	0.6816512	-0.5528945	0.01256
LTBP4	2.191363	2.0670215	2.29404607	1.2127648	0.27829979	0.01261
PGAM2	1.319814	1.4341237	1.22541465	0.5192406	-0.9455249	0.01264
ANGPTL4	1.614638	1.4852834	1.72146075	1.4866794	0.57209352	0.0127
TMEM151A	1.123343	1.0369605	1.1946785	5.2672023	2.39703686	0.01271
RAC3	2.302427	2.4332243	2.19441275	0.8333746	-0.2629629	0.01274
SERPINE2	1.609614	1.7233705	1.51567161	0.7128734	-0.4882822	0.01275
PRPF4	1.358739	1.4538424	1.28020117	0.6173975	-0.6957286	0.01281
BHLHE41	1.218756	1.1289032	1.29295856	2.2727015	1.18440818	0.01283
FNBP1L	1.946357	2.0599412	1.85255807	0.8043447	-0.3141142	0.01284
HSD17B12	2.233341	2.3572054	2.13105178	0.8333682	-0.2629741	0.01289
AIMP2	1.298239	1.3906872	1.22189347	0.5679569	-0.8161466	0.01299
FGFR2	1.141749	1.0552174	1.21320782	3.8612419	1.94906494	0.01301
C8orf59	4.005912	4.2405593	3.8121365	0.8677936	-0.2045762	0.01303
LTBP1	1.299329	1.2069859	1.37558697	1.8145533	0.85961444	0.01311
SLC16A3	3.179108	3.3732002	3.01882257	0.8506752	-0.2333197	0.01317
MYL6B	6.577476	6.9030588	6.30860489	0.8992973	-0.1531299	0.01318
GALNT10	1.20471	1.1158965	1.27805316	2.3991509	1.2625239	0.01321
KLHDC2	2.299317	2.4252254	2.19534057	0.8387028	-0.2537683	0.01321
GMPR	1.141214	1.0551165	1.21231516	3.8521155	1.94565094	0.01323
CEBPZOS	1.952837	2.0749485	1.85199457	0.792591	-0.3353514	0.01324
SCPEP1	1.482466	1.5810528	1.40105179	0.6902157	-0.5348808	0.01335
IGBP1	2.13432	2.253703	2.03573064	0.8261372	-0.2755468	0.01336
TPP2	1.383456	1.4791171	1.3044571	0.6354545	-0.6541393	0.01343
MTERF3	1.248895	1.3391663	1.17434747	0.5140471	-0.9600275	0.01344
PRKAG1	1.765316	1.8729256	1.6764493	0.774922	-0.367877	0.01349
MARVELD1	1.46669	1.3695455	1.54691357	1.4799625	0.56556065	0.0135
PTGFRN	1.379577	1.2847724	1.4578689	1.6078414	0.68512507	0.01351
POLR2J	3.743153	4.0066126	3.52558428	0.8400099	-0.2515218	0.01355
WDR12	1.355768	1.4496904	1.27820465	0.6186582	-0.6927854	0.01358
ZCCHC7	1.803833	1.9128106	1.71383675	0.7820206	-0.3547214	0.01358
HAX1	2.195682	2.3408373	2.07580969	0.8023417	-0.3177113	0.01358
HEY1	1.416921	1.513042	1.33754351	0.6579257	-0.6040034	0.01361

GEMIN8	1.475376	1.5733119	1.39449852	0.6881046	-0.5393002	0.01369
ITGB6	1.172043	1.0835132	1.24515288	2.9354976	1.55360507	0.01385
NSFL1C	1.728473	1.8345609	1.64086373	0.7679053	-0.3809997	0.01387
CXXC5	3.280851	3.0933838	3.43566363	1.1635055	0.21847807	0.01388
PDE4D	1.269405	1.1791117	1.34397091	1.9204264	0.94142665	0.01397
RP11-352M15.2	1.796813	1.90513	1.70736292	0.7815042	-0.3556744	0.01404
BAMBI	1.465206	1.3685101	1.5450588	1.4790879	0.56470782	0.01407
ZNF512	1.509021	1.6078472	1.42740954	0.7031529	-0.5080897	0.01416
RALBP1	2.130704	2.2633072	2.02119876	0.8083535	-0.3069418	0.01422
MACF1	2.193697	2.0652762	2.29974907	1.2201052	0.28700553	0.01423
BCO2	1.416944	1.512264	1.33822684	0.6602589	-0.5988963	0.01424
SPNS2	1.119562	1.0347722	1.18958264	5.4521396	2.44682249	0.0143
GLIPR1	1.149466	1.0639768	1.22006476	3.4397571	1.78230671	0.01441
MAP1LC3B	2.892078	3.0541728	2.75821765	0.8559249	-0.2244439	0.01442
BCKDK	2.671158	2.8293944	2.54048433	0.8420734	-0.2479822	0.01454
NSMCE4A	1.528483	1.6270857	1.44705571	0.7129101	-0.488208	0.01457
IFRD1	2.518001	2.6683924	2.3938057	0.8354184	-0.2594291	0.01458
PSMC4	1.671338	1.7742466	1.58635503	0.7573233	-0.4010187	0.01462
KLHL23	1.839893	1.9481914	1.75045842	0.791463	-0.3374062	0.01465
KLHDC3	2.538234	2.6805192	2.42073225	0.8454127	-0.2422723	0.01467
SDF4	2.237728	2.1066245	2.34599616	1.2163079	0.28250852	0.01468
TAF9	2.010254	2.1247751	1.91568105	0.8141015	-0.2967195	0.01474
MKKS	2.374783	2.533724	2.24352605	0.8107887	-0.3026021	0.01484
FAHD2B	1.372645	1.4662014	1.29538495	0.6335995	-0.6583569	0.01485
GOLGA8A	1.795103	1.6888641	1.88283595	1.2815821	0.35792593	0.01491
ATP5SL	1.747931	1.8537183	1.66057021	0.7737567	-0.3700482	0.01495
BEND5	1.508907	1.6067012	1.42814697	0.7056966	-0.50288	0.01496
HIBCH	2.038005	2.153144	1.942921	0.8176958	-0.2903639	0.015
ABCE1	1.753743	1.8589847	1.66683241	0.7763031	-0.3653081	0.015
RP1-39G22.7	1.460593	1.557364	1.38067711	0.6829955	-0.5500521	0.01504
BACE2	1.228245	1.1404183	1.30077398	2.1419854	1.09894865	0.01506
THRA	1.724947	1.8298346	1.63832817	0.7692234	-0.3785255	0.01507
MTFR1	1.882463	1.9914897	1.79242663	0.7992283	-0.3233204	0.0151
STK17A	1.340846	1.246702	1.41859079	1.6967468	0.76277132	0.01517
ATP1A1	2.135365	2.0107016	2.23831386	1.2252022	0.2930199	0.01518
GNPAT	1.844119	1.9524638	1.75464526	0.7923086	-0.3358657	0.01524
CCNL2	1.71891	1.6151644	1.80458417	1.3079174	0.38727144	0.01525
CNDP2	1.519146	1.6168624	1.43845071	0.7107755	-0.4925341	0.01528
TWF1	1.809952	1.9173974	1.72122094	0.7861598	-0.3471056	0.0153
NDC80	1.230363	1.3180685	1.15793382	0.4965402	-1.0100176	0.01535
TMEM9	2.737185	2.8837599	2.61614189	0.8579341	-0.2210613	0.01538
PHF19	1.371638	1.4642275	1.29517552	0.6358424	-0.653259	0.01541
HLA-A	3.550085	3.7973136	3.34592025	0.8386333	-0.253888	0.01541
ZNF146	2.167596	2.2897084	2.06675339	0.8271276	-0.2738182	0.01546
NUDT16L1	1.555486	1.6544052	1.47379689	0.7240115	-0.4659155	0.0155

MPPED2	1.339352	1.4313307	1.26339388	0.6106541	-0.7115726	0.01552
DUSP4	1.394821	1.2923559	1.47943828	1.6399134	0.71361962	0.01557
METTL10	1.330433	1.4220484	1.25477481	0.6036626	-0.7281856	0.0156
RSBN1	1.60612	1.706433	1.52328063	0.7407363	-0.432968	0.01564
MID1IP1	1.821555	1.9292535	1.73261599	0.788392	-0.343015	0.01568
PIGU	1.321507	1.4121598	1.24664407	0.5984186	-0.7407731	0.0157
GDF15	1.178194	1.0825865	1.25714784	3.1136782	1.63861984	0.01571
MFAP3L	1.114649	1.0311958	1.18356666	5.8843298	2.55687811	0.01572
TMEM59	3.278787	3.5028666	3.09373755	0.8365358	-0.2575008	0.01573
SHC2	1.216343	1.1295766	1.28799531	2.2225877	1.15224037	0.01575
PAFAH1B1	2.331037	2.2059942	2.43430009	1.1893092	0.2501239	0.01579
PIN1	3.274081	3.490444	3.09540435	0.8413778	-0.2491743	0.01582
HSBP1L1	1.417435	1.3239603	1.49462886	1.5268194	0.61052942	0.01588
ADGRG6	1.121508	1.0385836	1.18998888	4.924078	2.2998536	0.01592
TMX2	1.473168	1.5685123	1.39443028	0.6937938	-0.5274212	0.01595
NDUFAF3	3.476137	3.6777674	3.3096273	0.8625198	-0.2133706	0.01595
LSAMP	2.539899	2.6936351	2.41294123	0.8342654	-0.2614216	0.01599
TMEM39B	1.338222	1.4292639	1.26303771	0.6127645	-0.7065953	0.01602
RP11-96L14.7	2.42836	2.2688192	2.56011107	1.2295772	0.29816228	0.01606
MAP3K2	1.604876	1.505714	1.68676477	1.3580101	0.4414942	0.01611
SEMA3C	1.166463	1.0817489	1.23642145	2.892046	1.5320905	0.01616
SEPHS1	1.498563	1.594946	1.41896862	0.7042128	-0.5059166	0.01623
OXR1	1.819342	1.9293461	1.72849884	0.7838832	-0.3512894	0.01624
GTF2B	1.267769	1.3564913	1.19450047	0.5455967	-0.8740931	0.01624
DHX32	1.557843	1.6554826	1.4772105	0.7280293	-0.4579317	0.01631
APIP	1.52708	1.6238931	1.44712927	0.7166761	-0.4806069	0.01632
SREK1IP1	2.657062	2.8227313	2.52024884	0.8340499	-0.2617944	0.01633
ACOT7	1.693266	1.7956871	1.60868472	0.76498	-0.386506	0.01641
PSAT1	2.218917	2.3591769	2.10308845	0.8115856	-0.3011848	0.01643
PTBP3	1.382258	1.2901451	1.45832695	1.5796476	0.65960271	0.0165
TPBG	1.368099	1.2765989	1.44366152	1.6039885	0.6816638	0.0165
NUBP2	2.193876	2.31944	2.0901825	0.8262464	-0.275356	0.01655
MBIP	1.441028	1.5353812	1.36310889	0.678225	-0.5601642	0.01657
CYTH2	1.913474	2.022538	1.82340629	0.8052574	-0.3124781	0.01657
DHRS4L2	1.74186	1.8452477	1.65648085	0.7766727	-0.3646213	0.0166
SLC4A2	1.320632	1.2308393	1.39478496	1.7102153	0.77417795	0.01665
MRPL24	1.593576	1.6922777	1.51206561	0.7396823	-0.4350223	0.01666
ERP44	1.658998	1.7595903	1.57592778	0.7582084	-0.3993336	0.01667
GADD45G	1.235648	1.3237031	1.16293038	0.5033327	-0.9904157	0.01669
DNAJB1	1.757721	1.6507287	1.84607719	1.3001996	0.37873312	0.01675
FAM96A	2.486249	2.6323498	2.36559688	0.8365835	-0.2574186	0.01683
SNRNP70	3.510892	3.7082358	3.34792156	0.8669561	-0.2059691	0.017
HDAC1	1.443572	1.5370082	1.366411	0.6823192	-0.5514812	0.01715
CNTN2	1.10831	1.1972387	1.0348707	0.1767944	-2.4998557	0.01719
MET	1.148509	1.0652985	1.21722647	3.3266696	1.73407858	0.01719

GPX4	13.90558	14.401846	13.4957627	0.9323912	-0.1009927	0.01722
EBP	1.660983	1.7613137	1.57812846	0.7593827	-0.397101	0.01727
EGFL6	1.12902	1.0429625	1.20008763	4.6572613	2.21948182	0.01733
ZSWIM7	2.055167	2.1693413	1.96087975	0.8217274	-0.2832683	0.01733
CAV1	1.677154	1.562172	1.77210787	1.373437	0.45779077	0.01733
LAMTOR2	3.112562	3.3191352	2.94197095	0.8373686	-0.2560653	0.01736
CD46	1.93128	1.8231087	2.02060884	1.2399441	0.3102751	0.01739
KNL1	1.240928	1.3273985	1.16951986	0.5177784	-0.9495933	0.01742
SSR3	3.230778	3.4082166	3.08424692	0.8654732	-0.2084389	0.01744
MS4A6A	1.111797	1.024371	1.18399498	7.5497498	2.91642884	0.01753
NMRK2	1.16116	1.07768	1.23009894	2.9621369	1.5666383	0.01759
KPNA3	1.944125	2.052285	1.85480379	0.812331	-0.2998603	0.0176
SF3B1	2.384608	2.5116956	2.27965619	0.8465039	-0.2404114	0.01764
TIMM13	5.544234	5.904007	5.24712715	0.8660524	-0.2074737	0.01764
CNN3	5.206491	5.5136661	4.95282027	0.8757449	-0.1914173	0.01765
CNOT7	3.081388	3.2704889	2.925226	0.8479346	-0.2379752	0.01774
MAPK3	1.463315	1.5572864	1.38571234	0.6921259	-0.5308936	0.01779
ANTXR1	1.489409	1.395169	1.56723408	1.4354216	0.52147457	0.0178
STK16	1.766585	1.8698219	1.68132986	0.7832981	-0.3523667	0.01797
RHOBTB3	3.106635	3.3131632	2.93608028	0.8369839	-0.2567283	0.01799
CTTN	2.49379	2.3491671	2.61322163	1.1957167	0.2578756	0.01801
GNB3	1.334591	1.2451328	1.40846722	1.6663099	0.7366567	0.01802
CD164	2.957817	2.7973971	3.09029521	1.1629568	0.21779756	0.0181
COPS7A	1.684699	1.7848946	1.60195629	0.7669263	-0.3828402	0.01815
AZI2	1.919234	2.0273599	1.8299424	0.80784	-0.3078585	0.01816
ARMC6	1.301447	1.3895672	1.22867557	0.586999	-0.7685701	0.01823
MED31	1.674625	1.7747534	1.59193736	0.7640333	-0.3882926	0.01828
BORCS7	1.998337	2.1073651	1.90829974	0.8202351	-0.2858906	0.01829
DDX39A	1.501011	1.5949447	1.42343813	0.7117268	-0.4906045	0.01842
CLPP	2.286668	2.4200585	2.17651255	0.8284958	-0.2714337	0.01842
COL1A2	4.209665	3.9034151	4.46257201	1.1925859	0.25409323	0.01843
RARS	1.848582	1.9541194	1.76142778	0.7980425	-0.3254625	0.0185
INAFM1	1.950767	2.0674338	1.8544218	0.8004448	-0.3211262	0.01853
HNRNPUL1	2.665095	2.814963	2.54133228	0.8492362	-0.2357622	0.01854
VPS4A	1.728275	1.8294186	1.64474869	0.7773502	-0.3633634	0.01854
PIFO	1.256958	1.1705663	1.32830191	1.924776	0.94469059	0.01855
TPR	3.09982	3.2772064	2.95333083	0.857775	-0.2213288	0.01856
TMEM181	1.320977	1.2322882	1.39421769	1.6971056	0.76307637	0.01856
STAMBP	1.582643	1.6796835	1.50250518	0.7393223	-0.4357247	0.0187
HDAC7	1.542221	1.4469813	1.62087171	1.3890328	0.47408069	0.0187
SH3BGR	1.261898	1.3483827	1.19047813	0.5467497	-0.8710475	0.01875
ELAC2	1.34	1.4294564	1.26612607	0.6196812	-0.6904018	0.01876
TIPRL	1.756147	1.8578356	1.67217082	0.783566	-0.3518733	0.01886
KIF5C	2.259284	2.123865	2.37111492	1.2199997	0.28688079	0.01904
GNG12	1.450088	1.3584869	1.52573358	1.4665348	0.55241129	0.01906

MFAP1	1.668413	1.7670857	1.58692704	0.7651388	-0.3862067	0.01906
TM2D3	1.700293	1.8000761	1.61789007	0.7722891	-0.372787	0.0191
NDUFV3	2.541688	2.679501	2.42788031	0.8501813	-0.2341576	0.01921
SPC25	1.250142	1.3355681	1.17959522	0.5351976	-0.9018566	0.0193
PPHLN1	2.009442	2.1201203	1.91804224	0.8195926	-0.2870212	0.01933
EMC3	1.499616	1.5934231	1.42214928	0.71138	-0.4913078	0.01934
GABARAP	1.844919	1.9503316	1.75786729	0.7974766	-0.3264858	0.01934
YTHDF2	2.340425	2.4680363	2.23504108	0.8412878	-0.2493286	0.01938
COA6	1.638605	1.7367359	1.55756667	0.7568067	-0.4020032	0.01938
RAB34	3.702993	3.9052598	3.53595799	0.8728851	-0.1961363	0.0194
YIPF4	1.927209	2.0337717	1.83920856	0.8117929	-0.3008164	0.01943
LAP3	1.439616	1.5313471	1.36386279	0.684793	-0.54626	0.01947
PAPSS1	1.567383	1.6627957	1.4885897	0.7371649	-0.4399407	0.01967
MPLKIP	2.581039	2.7268166	2.46065395	0.8458651	-0.2415005	0.01968
SLC7A2	1.15485	1.0729931	1.22244811	3.0475217	1.60763652	0.01972
FOXP2	1.673179	1.7714771	1.5920031	0.7673632	-0.3820185	0.01981
RPAIN	3.321529	3.5104285	3.16553263	0.8626147	-0.2132117	0.01983
COIL	1.515858	1.6094089	1.43860134	0.719716	-0.4745003	0.01993
TBX2	1.452547	1.5484562	1.37334287	0.6807159	-0.5548754	0.01993
SRSF11	3.680924	3.9287154	3.4762939	0.8455222	-0.2420854	0.01995
BIRC7	2.130752	1.9848962	2.2512029	1.2703907	0.34527225	0.01995
GMPS	1.885255	1.9894208	1.79923279	0.8077784	-0.3079685	0.01999
MRPS14	2.012846	2.1245573	1.92059301	0.818627	-0.2887218	0.02004
MMP2	1.157143	1.0754387	1.22461516	2.9774547	1.57407955	0.02008
TRIAP1	2.086975	2.1991238	1.994361	0.8292397	-0.270139	0.02015
MED30	1.538654	1.6325617	1.46110367	0.7289465	-0.4561151	0.02025
UNC45A	1.469373	1.5615995	1.39321134	0.7001632	-0.5142368	0.02026
AMOTL2	1.291303	1.2055331	1.36213367	1.7619242	0.81715186	0.02031
ICE2	1.717746	1.8171001	1.63569786	0.7779926	-0.3621716	0.02037
AMD1	2.205942	2.325693	2.10705016	0.8350728	-0.2600261	0.02047
SPG21	3.098783	3.299868	2.93272368	0.8403629	-0.2509157	0.02047
PIGF	1.936637	2.0418548	1.84974615	0.815609	-0.2940503	0.02049
C14orf119	2.033178	2.1450579	1.94078634	0.8216059	-0.2834815	0.02055
FBXW11	1.668267	1.5710889	1.74851846	1.3106865	0.39032265	0.02067
CHCHD7	1.889351	1.9931806	1.8036063	0.809124	-0.3055672	0.0208
PRCC	1.58632	1.6821361	1.50719379	0.7435376	-0.4275225	0.02089
IER5L	1.362155	1.2742794	1.43472326	1.5849649	0.66445088	0.02097
TES	1.755974	1.6563262	1.83826474	1.2772075	0.35299296	0.02102
MAPKAPK5-AS1	1.734799	1.8343421	1.6525942	0.7821662	-0.3544529	0.02103
SCNM1	1.829966	1.9324911	1.74529976	0.7992567	-0.3232692	0.02105
MRPL27	2.11496	2.2258498	2.02338453	0.8348368	-0.2604339	0.02115
LAS1L	1.250787	1.3352614	1.18102751	0.5399593	-0.8890775	0.02132
RLF	1.559538	1.6538111	1.48168618	0.7367361	-0.4407802	0.02133
FGFBP3	1.702344	1.8007038	1.62111763	0.7757146	-0.3664021	0.02136
SRP54	1.67339	1.7707077	1.59302389	0.7694537	-0.3780935	0.02138

COMMD1	1.668018	1.7650506	1.58788764	0.7684297	-0.3800148	0.02139
UGP2	2.073137	2.1849049	1.98083685	0.8277768	-0.2726862	0.02147
RNF130	2.114635	2.2275042	2.02142555	0.8321157	-0.265144	0.02149
PTN	1.555278	1.6628207	1.46646757	0.7037613	-0.5068419	0.02156
HELLS	2.056469	1.9339261	2.15766761	1.2395709	0.30984079	0.02159
BLVRB	2.145095	2.2645856	2.04641714	0.8274783	-0.2732067	0.0216
CCDC181	1.393339	1.4819192	1.3201879	0.6644017	-0.5898724	0.02163
PLEKHB1	1.716399	1.618658	1.79711555	1.2884592	0.36564683	0.02163
ARHGAP11A	1.308727	1.3947194	1.23771309	0.6022331	-0.7316062	0.02171
CYB5D2	1.51637	1.6359336	1.41763259	0.6567236	-0.6066418	0.02192
FOSL2	1.268633	1.1846721	1.33797005	1.8301091	0.87192962	0.02192
FLNC	1.358905	1.2718694	1.43078044	1.5845122	0.66403879	0.02207
GREM1	1.179771	1.0990177	1.24645875	2.4890365	1.31558741	0.02209
ASCC1	1.394039	1.4825728	1.32092655	0.6650325	-0.5885033	0.02214
PTK7	1.569789	1.4763294	1.64696911	1.3582389	0.44173724	0.02221
ADM	2.346304	2.122528	2.53110129	1.363976	0.44781825	0.02228
MTFP1	1.733935	1.8326808	1.65238837	0.7834795	-0.3520325	0.02232
ASAH1	2.851179	2.693454	2.98143151	1.1700533	0.22657426	0.02234
FOXP1	2.810923	2.9621849	2.68600877	0.8592507	-0.218849	0.02238
C1orf122	3.119689	2.9502015	3.25965513	1.1586778	0.21247942	0.02239
CTB-50L17.10	1.609949	1.7046544	1.53174038	0.7546116	-0.4061937	0.02241
LGALS3	2.89546	3.1076518	2.72022918	0.8161828	-0.2930358	0.02251
ZNF207	2.855362	3.0232147	2.71674671	0.8485242	-0.2369722	0.02255
SNAP47	1.559799	1.6529469	1.48287598	0.7395333	-0.435313	0.0226
TRIM16L	1.268287	1.3527328	1.19855076	0.5628928	-0.8290679	0.02273
SMC1A	1.818753	1.9188601	1.7360834	0.8010832	-0.3199759	0.02282
SMARCA1	2.071156	2.1804208	1.98092351	0.8309947	-0.2670887	0.02288
TAX1BP1	2.624756	2.7602932	2.51282766	0.859418	-0.2185681	0.0229
RHOT1	1.681032	1.777948	1.60099741	0.7725418	-0.372315	0.02293
UBTD1	1.383735	1.2963871	1.45586905	1.5380865	0.62113668	0.02294
AMOTL1	1.369438	1.2824904	1.44124057	1.5619669	0.64336386	0.02294
TMEM205	2.426958	2.5785245	2.30179104	0.8246885	-0.2780788	0.02295
PDCL	1.398433	1.4862673	1.32589811	0.6702036	-0.5773287	0.02302
HEG1	1.139806	1.0605984	1.20521768	3.3865221	1.75980443	0.02304
KTN1	4.483953	4.2246038	4.69812724	1.146847	0.19767297	0.02306
FUZ	1.4893	1.5801835	1.4142477	0.7139943	-0.4860156	0.02307
DPYSL3	1.330957	1.2460213	1.40109847	1.6303402	0.70517303	0.0231
CCNH	1.437118	1.5260928	1.36364066	0.6912101	-0.5328039	0.0231
DVL3	1.475574	1.3858536	1.54966679	1.4245475	0.51050376	0.02311
PSMA6	1.450735	1.5403467	1.3767322	0.6972046	-0.5203461	0.02311
ORMDL1	2.726039	2.8773973	2.60104453	0.8528001	-0.2297205	0.02315
RP11-490M8.1	1.664878	1.5683686	1.7445771	1.310025	0.3895943	0.02317
OAT	1.563474	1.6560968	1.48698496	0.7422456	-0.4300314	0.02318
CIAO1	1.482398	1.5727893	1.40775111	0.7118693	-0.4903156	0.02321
ZNF581	2.250775	2.3814719	2.14284395	0.8272654	-0.2735778	0.02324

ZCCHC10	1.614438	1.7089033	1.53642727	0.7567002	-0.4022062	0.02333
PLP2	2.58237	2.437043	2.70238392	1.1846437	0.24445315	0.02349
ANKRD46	1.387587	1.4753187	1.31513614	0.6629997	-0.5929198	0.02349
ARID1B	2.460461	2.3338944	2.56498248	1.1732432	0.23050204	0.02352
PPM1G	2.871909	3.0241877	2.74615482	0.8626447	-0.2131616	0.02354
ROBO1	1.300003	1.21563	1.36968006	1.7144183	0.77771912	0.02354
NDUFA4L2	3.245663	3.6135206	2.94187994	0.7430131	-0.4285405	0.02356
RP11-89K21.1	2.231777	2.358128	2.12743359	0.8301379	-0.268577	0.0236
LHFPL2	1.164099	1.0842982	1.22999952	2.728405	1.44805783	0.02363
DPH5	1.745728	1.8433895	1.66507704	0.7885764	-0.3426776	0.02373
RAB25	1.092404	1.0143261	1.15688128	10.950737	3.45295608	0.02373
S100A3	1.23831	1.1565544	1.30582542	1.9534773	0.96604447	0.02382
COMMD8	1.556816	1.6488247	1.4808328	0.7410828	-0.4322934	0.02403
ARF3	1.912306	2.0139561	1.82836178	0.8169602	-0.2916623	0.02405
PAWR	2.001097	1.8958199	2.08803735	1.2145715	0.28044739	0.02418
ISG15	1.474337	1.3748452	1.55649833	1.4846086	0.57008262	0.02418
CCDC90B	2.523804	2.6599232	2.41139521	0.8502774	-0.2339944	0.02426
HMCES	1.307519	1.3918089	1.23791146	0.6072129	-0.7197256	0.0243
TJP1	1.982127	1.8784517	2.06774366	1.2154836	0.28153043	0.02432
KLHL20	1.950533	1.8481382	2.03509262	1.2204292	0.28738866	0.02435
TMPO	2.046743	2.1554392	1.9569794	0.8282387	-0.2718815	0.02439
GPX7	2.466856	2.5941165	2.36176194	0.8542424	-0.2272826	0.02439
CBX5	4.708633	4.9967456	4.47070463	0.8683827	-0.2035971	0.0244
P3H4	2.234663	2.3562053	2.13429091	0.8363711	-0.2577849	0.02457
CNN2	3.423677	3.5987599	3.2790901	0.8769914	-0.1893654	0.02459
LINC00973	1.261206	1.1790338	1.32906594	1.8380106	0.87814509	0.02465
ALKBH4	1.413019	1.5003758	1.34087863	0.6812452	-0.5537539	0.02465
ANAPC13	3.268037	3.4488802	3.11869453	0.8651687	-0.2089466	0.02469
DYNC2LI1	1.601717	1.69474	1.52489628	0.7555291	-0.4044408	0.02472
RAB3IP	1.603132	1.5102783	1.67981297	1.3322395	0.41385346	0.02475
TLN1	1.946783	1.8444107	2.03132407	1.2213536	0.28848095	0.02479
DAAM2	1.121149	1.0432716	1.18546129	4.2859839	2.09962644	0.0248
ZNF644	2.086683	2.1940009	1.99805756	0.8358934	-0.258609	0.02489
WDR1	2.473345	2.3345778	2.58794203	1.189846	0.25077487	0.02503
SLC24A5	1.12541	1.0474136	1.18982028	4.0035016	2.00126239	0.02506
DUSP12	1.609815	1.7029829	1.53287462	0.7580194	-0.3996934	0.02507
MIF4GD	1.696637	1.7923657	1.617583	0.7794167	-0.3595333	0.02508
C1D	1.8341	1.9334536	1.75205227	0.8056665	-0.3117453	0.0251
OCIAD1	2.942106	3.0954227	2.81549401	0.8664094	-0.2068791	0.0251
OXTR	1.124816	1.0472765	1.18884915	3.9945685	1.99803966	0.02519
NDUFA9	1.598268	1.6906386	1.52198745	0.755804	-0.4039159	0.02524
MYL12B	4.956481	5.2268556	4.73320146	0.8832101	-0.1791715	0.0253
TIMELESS	1.443889	1.5314162	1.3716071	0.699277	-0.516064	0.02536
NARS2	1.424374	1.512092	1.35193546	0.6872504	-0.5410922	0.02537
PITHD1	1.583436	1.6757515	1.5072009	0.7505732	-0.4139354	0.02539

RIN2	1.594642	1.6943215	1.51232534	0.7378791	-0.4385436	0.02541
MRPL46	1.294622	1.3780651	1.22571298	0.5970214	-0.7441454	0.02542
COMMD10	1.439832	1.5275142	1.36742313	0.696518	-0.5217675	0.02557
BTBD3	1.618937	1.7117618	1.54228089	0.7618853	-0.3923542	0.02558
SDHAF4	1.31106	1.3951728	1.24159802	0.6113732	-0.7098748	0.02559
GTF3C5	1.485998	1.5752007	1.41233373	0.7168519	-0.4802531	0.02566
HIST3H2A	1.630468	1.7236405	1.55352494	0.764917	-0.3866248	0.02573
SIRT3	1.342351	1.4271186	1.27234797	0.6376401	-0.6491857	0.02576
MRPL14	3.95516	4.1733451	3.77497902	0.8744649	-0.1935276	0.0258
ATP6V0E1	4.060226	3.8373053	4.24431763	1.1434503	0.19339365	0.02597
GCFC2	1.361245	1.4465512	1.29079723	0.6512069	-0.6188122	0.026
SOX6	1.164454	1.0858892	1.22933351	2.6701076	1.41689791	0.02601
SLC7A5	1.197416	1.1176915	1.26325468	2.2368199	1.16144911	0.02614
TPD52L1	4.060137	3.8193467	4.25898605	1.1559366	0.20906227	0.02616
PXN-AS1	1.390822	1.4765699	1.32000957	0.671485	-0.5745729	0.02617
SUMF2	2.161355	2.287664	2.05704615	0.8209022	-0.2847178	0.02619
SNN	1.466789	1.5552436	1.39374083	0.7091317	-0.4958746	0.02624
EMP3	3.725889	3.9204268	3.56523591	0.8783771	-0.1870877	0.02628
RAP1B	1.712332	1.8076651	1.63360411	0.7844886	-0.3501755	0.02637
GCC2	2.051218	1.9425958	2.1409195	1.2104017	0.27548591	0.02638
KCTD6	1.582623	1.6736525	1.50744992	0.7532814	-0.4087392	0.02638
ELF3	1.138103	1.0611261	1.20167118	3.2992643	1.72214434	0.02684
TPP1	1.400515	1.3148184	1.47128381	1.4970019	0.58207604	0.02687
RORA	1.36097	1.276923	1.43037708	1.5541396	0.63611612	0.02688
FOPNL	1.508199	1.5973339	1.43459046	0.7275503	-0.4588811	0.02693
CDK5	1.487184	1.5756218	1.4141499	0.7194827	-0.4749681	0.02695
ANK3	1.458626	1.3713348	1.53071221	1.4292014	0.51520924	0.02699
UFL1	1.488715	1.5765972	1.4161409	0.7217186	-0.4704917	0.02704
C14orf39	1.277479	1.3591467	1.21003731	0.5848231	-0.7739278	0.02713
GPS1	1.761254	1.856921	1.68225139	0.796166	-0.3288588	0.02715
ITGAE	2.390417	2.5360738	2.27013201	0.8268691	-0.2742691	0.02719
PLEKHA5	1.471609	1.3841946	1.54379735	1.4154218	0.50123202	0.02723
LMNA	2.086757	1.964253	2.18792234	1.2319612	0.30095682	0.02724
TSHZ2	1.266568	1.1854977	1.33351688	1.7979566	0.84635823	0.02727
PSMB4	1.965884	2.0671344	1.88226912	0.8267648	-0.2744512	0.02736
PIAS2	2.172705	2.0632918	2.26306075	1.1878778	0.24838642	0.02736
SYPL1	1.873806	1.9769147	1.78865756	0.8072942	-0.3088336	0.02747
FADS1	3.089223	3.2396572	2.96499148	0.8773626	-0.1887548	0.02747
RRAGD	1.325887	1.2429477	1.39437957	1.6233104	0.69893892	0.02751
NKIRAS2	1.76248	1.8586445	1.68306569	0.7955163	-0.3300367	0.02754
SLC12A2	1.432109	1.3463123	1.50296092	1.4523333	0.5383726	0.02763
C8orf46	1.158866	1.2560789	1.07858676	0.306885	-1.7042301	0.02767
CCNL1	2.271522	2.3849473	2.17785378	0.8504683	-0.2336706	0.02773
HIST1H4C	12.79828	14.424112	11.4556331	0.7788696	-0.3605463	0.02779
SPOCD1	1.201377	1.1228431	1.26623121	2.1672451	1.1158623	0.02785

ATP6V0E2	2.163373	2.2737841	2.07219338	0.8417387	-0.2485556	0.02797
ELP4	1.594043	1.5030423	1.66919285	1.3302913	0.41174221	0.02799
PYGL	1.269814	1.3511309	1.20266091	0.5771663	-0.792941	0.028
YPEL5	2.362747	2.4925677	2.25553813	0.8411934	-0.2494905	0.02804
NRCAM	1.424514	1.5101946	1.35375833	0.6933792	-0.5282836	0.02808
ZIC2	1.681418	1.7741601	1.60482953	0.7812719	-0.3561034	0.0281
ZNF827	1.528764	1.4404172	1.6017216	1.3662536	0.45022525	0.0281
EPCAM	1.229632	1.1375968	1.30563612	2.221244	1.15136787	0.02817
MITD1	1.567958	1.6579493	1.49364163	0.750273	-0.4145124	0.0283
THTPA	1.455142	1.5419159	1.38348201	0.7076411	-0.4989102	0.02832
FEZ2	1.952915	2.0539122	1.8695095	0.8250303	-0.2774811	0.02835
WASF1	1.450766	1.5375872	1.37906791	0.7051283	-0.5040424	0.02835
STAU2	1.609141	1.7004315	1.53375195	0.762033	-0.3920746	0.02837
PCCB	1.222251	1.3016767	1.1566595	0.519296	-0.9453711	0.02839
SLC45A2	1.097295	1.0223623	1.15917563	7.1180483	2.83148172	0.02866
REXO2	2.043604	2.1516635	1.95436739	0.8286859	-0.2711026	0.02868
QPRT	4.249864	4.4806589	4.0592691	0.8789339	-0.1861734	0.02878
ENC1	1.3998	1.3154059	1.46949375	1.4885381	0.57389618	0.02885
EIF2B3	1.245404	1.3254666	1.17928665	0.5508604	-0.8602413	0.02887
SSPN	1.505544	1.5933525	1.43303091	0.7298039	-0.4544193	0.02892
LINC00632	2.298795	2.4286919	2.19152394	0.8339964	-0.2618869	0.02908
LYPLA2	1.882805	1.9808358	1.80184886	0.8175159	-0.2906814	0.02914
UBN1	1.588766	1.4986694	1.66317004	1.3298791	0.41129507	0.02915
MED19	1.514521	1.6025694	1.44180857	0.7332077	-0.4477061	0.02919
FEZ1	2.138358	2.248427	2.04746159	0.8390251	-0.2532141	0.0292
GULP1	2.864364	3.0215669	2.73454362	0.8580194	-0.2209178	0.02921
TRIO	1.4732	1.3870336	1.54435743	1.4064863	0.49209551	0.02924
NOG	1.102033	1.0270345	1.16396834	6.0651543	2.60054434	0.02924
ANOS1	1.332669	1.2504497	1.40056697	1.5993907	0.67752243	0.02926
TRAM2	1.187737	1.1102053	1.25176307	2.284492	1.19187341	0.02927
SNHG19	1.566096	1.6555483	1.49222497	0.7508599	-0.4133843	0.02928
C6orf203	1.40187	1.4861949	1.33223306	0.6833331	-0.549339	0.02935
EPN1	2.223359	2.3468426	2.12138405	0.8326022	-0.2643008	0.0294
PGP	1.950885	1.8487883	2.03519874	1.2196195	0.28643116	0.02945
SEPT10	1.657239	1.7496781	1.58090125	0.7748676	-0.3679783	0.02952
SGF29	1.227585	1.3066986	1.16225259	0.5290295	-0.9185799	0.02954
TSPYL1	1.710141	1.8036466	1.63292329	0.7875642	-0.3445305	0.02967
PRR3	1.444234	1.5299714	1.37343138	0.7046256	-0.5050713	0.02977
TSFM	1.315145	1.3966108	1.24786983	0.6249699	-0.6781414	0.02978
HBP1	1.57847	1.667955	1.50457227	0.7553986	-0.4046901	0.02987
CHRNA3	1.417036	1.3313198	1.4878217	1.4723592	0.55812964	0.02988
SMIM20	2.690181	2.8328222	2.57238488	0.8579037	-0.2211124	0.02997
MRPS16	2.670311	2.8068833	2.55752705	0.8619965	-0.2142461	0.02999
PKIG	2.285768	2.4039073	2.18820749	0.8463575	-0.2406609	0.03002
SF3A3	1.603481	1.6928325	1.52969256	0.7645319	-0.3873513	0.03004

PEX19	1.533368	1.6210168	1.46098537	0.7423074	-0.4299114	0.03005
CENPK	1.734396	1.8273556	1.65762861	0.7948561	-0.3312345	0.03015
PLRG1	1.484476	1.5710855	1.41295252	0.7231011	-0.4677307	0.03039
CAAP1	1.507701	1.5946377	1.43590764	0.7330643	-0.4479883	0.03042
LDLRAD3	1.333065	1.4150982	1.26532112	0.6391768	-0.6457131	0.03042
PLEKHA2	1.180155	1.1033655	1.2435686	2.356383	1.23657406	0.0305
RP11-1260E13.1	1.565271	1.6635489	1.48411106	0.7295785	-0.4548648	0.0305
LINC01315	1.245053	1.3243138	1.17959725	0.5537762	-0.852625	0.03057
MXRA7	1.681578	1.5905732	1.75673174	1.2813513	0.35766607	0.03061
WDR45B	2.780264	2.9302321	2.65641751	0.8581442	-0.220708	0.03066
ZNF516	1.83359	1.9381866	1.74721213	0.796443	-0.328357	0.0308
SQSTM1	2.339225	2.216989	2.44016931	1.1833873	0.24292229	0.03085
EFCAB2	1.2126	1.2908777	1.14795656	0.5086556	-0.9752389	0.03093
GAMT	2.636264	2.7656014	2.52945464	0.8662514	-0.2071424	0.03099
BLOC1S4	1.83293	1.9281465	1.75429903	0.8126939	-0.299216	0.03104
DIS3L	1.277938	1.3579051	1.21189916	0.592054	-0.7561994	0.03104
FAR2	1.300924	1.3815665	1.23432733	0.6141193	-0.7034092	0.03105
PIGBOS1	1.640759	1.7308215	1.56638331	0.7749954	-0.3677403	0.03112
CCDC77	1.267964	1.3475048	1.2022777	0.582086	-0.7806958	0.03115
TATDN3	1.225519	1.3040479	1.16066778	0.5284291	-0.9202182	0.03117
CHD1L	1.359029	1.4416282	1.2908164	0.6585095	-0.6027237	0.0312
NMD3	1.477504	1.5634233	1.40655114	0.7215731	-0.4707825	0.03124
ATP6V1G1	7.566079	7.9079231	7.28377937	0.9096481	-0.1366195	0.03129
ALG3	1.636624	1.7268872	1.56208265	0.7732736	-0.3709492	0.03133
OSBPL9	1.831047	1.9263435	1.75235031	0.8121721	-0.3001427	0.03139
UBL7	1.692218	1.7837339	1.61664278	0.7868012	-0.345929	0.03144
GOPC	1.582045	1.6706529	1.50887092	0.7587694	-0.3982666	0.03151
MALT1	1.42892	1.3450553	1.49817621	1.4437578	0.52982873	0.03157
HNRNPLL	1.637446	1.7272899	1.56325074	0.7744515	-0.3687532	0.03157
GNG5	10.18754	10.806128	9.67670055	0.8848243	-0.1765371	0.03167
ALDH3A2	1.401909	1.4852159	1.33311242	0.6865241	-0.5426178	0.03176
ITGA6	1.336159	1.2550023	1.40317976	1.5810827	0.66091287	0.03176
CCDC47	1.576962	1.6650636	1.50420671	0.758133	-0.3994771	0.03182
TNFRSF19	1.141329	1.0664709	1.20314842	3.0562011	1.61173948	0.03184
NHSL1	1.537612	1.6248834	1.46554105	0.7450047	-0.4246785	0.03188
MAMDC2	1.151839	1.0767334	1.21386178	2.7870758	1.47875226	0.03189
B4GALT7	1.725302	1.633311	1.80127005	1.2652079	0.33937449	0.03192
WFDC2	2.349603	2.4834991	2.23902846	0.8352067	-0.2597948	0.03196
RBFOX2	1.922236	2.02005	1.84145987	0.8249202	-0.2776735	0.0321
NAA38	3.946237	4.1512433	3.77693913	0.8812202	-0.1824256	0.03216
C19orf43	4.287968	4.5578262	4.06511556	0.8615136	-0.2150545	0.03216
DUSP26	1.255514	1.3345142	1.19027484	0.5688094	-0.8139828	0.03217
URI1	1.715237	1.8092918	1.63756555	0.7878068	-0.3440862	0.03225
EHMT2	2.015306	2.1147818	1.9331577	0.8370766	-0.2565685	0.03227
NRDC	2.157582	2.26304	2.07049256	0.8475524	-0.2386256	0.03232

CCNA1	1.125386	1.1996944	1.06402174	0.3205986	-1.64116	0.03232
POLR1D	3.731719	3.9448143	3.5557422	0.8678789	-0.2044343	0.03236
CHST11	1.194294	1.1181792	1.2571516	2.175947	1.12164342	0.03237
URM1	1.986601	2.0874717	1.9033009	0.8306431	-0.2676993	0.03245
MXRA5	1.189494	1.1132817	1.25243221	2.228358	1.155981	0.03251
PAPD4	1.388205	1.4711186	1.31973322	0.6786683	-0.5592215	0.03255
NFYB	1.596534	1.6853669	1.52317465	0.7633498	-0.3895838	0.03269
MRPS10	1.789741	1.8835147	1.71230094	0.8062128	-0.3107673	0.03271
HSPBP1	1.539937	1.6267762	1.46822383	0.7470351	-0.420752	0.03276
HMG20B	2.613126	2.7414276	2.50717217	0.8654808	-0.2084262	0.03277
TCF12	1.962612	2.0620494	1.88049504	0.8290528	-0.2704641	0.03279
FZD5	2.066686	2.1915868	1.96354097	0.8086201	-0.3064661	0.03296
NANS	1.465432	1.5497467	1.3958032	0.7199738	-0.4739837	0.03301
GRSF1	2.097486	2.2020259	2.01115546	0.8412094	-0.2494631	0.03301
FAH	1.44648	1.5302122	1.37733184	0.7116619	-0.4907361	0.03301
DDX46	2.797283	2.9292511	2.68830237	0.8751076	-0.1924676	0.03302
KIN	1.321434	1.4016914	1.25515698	0.6352065	-0.6547024	0.03303
ELOVL4	1.262316	1.3409832	1.19735145	0.5787717	-0.7889337	0.03305
RAB4A	2.963553	3.1425139	2.81576332	0.847492	-0.2387284	0.03315
KAT8	1.39961	1.4820629	1.33151916	0.6877093	-0.5401292	0.03323
CDKN2A	1.190412	1.1027555	1.26279957	2.5575237	1.35474759	0.03324
ZNF638	2.641208	2.7719028	2.53327852	0.8653288	-0.2086797	0.03325
EXD3	1.38905	1.4713106	1.32111734	0.6813286	-0.5535774	0.03331
UBXN11	1.43678	1.5204082	1.36771863	0.7065966	-0.5010414	0.03334
LRRC4C	1.356223	1.4373297	1.28924415	0.661387	-0.5964334	0.0334
VWA5A	1.268025	1.3466274	1.20311402	0.5859722	-0.7710959	0.03352
RP3-525N10.2	1.530928	1.6177364	1.4592397	0.7434234	-0.4277441	0.03359
PROX1	1.304989	1.2260805	1.37015374	1.6372655	0.71128826	0.03359
H1F0	4.040642	4.2634704	3.85662583	0.8753338	-0.1920949	0.0336
NDE1	1.215256	1.2917052	1.15212315	0.5214962	-0.9392712	0.03364
LYPD6B	1.368872	1.2880709	1.43559864	1.512123	0.59657549	0.03369
PTK2	1.954342	2.054377	1.87173174	0.8267743	-0.2744346	0.03372
ARRB2	1.51434	1.599963	1.44363066	0.73943	-0.4355144	0.03381
TRIM37	1.465327	1.3812521	1.53475654	1.4026325	0.48813702	0.03397
SLC35D3	1.095811	1.0232104	1.155765	6.7110015	2.74652808	0.03401
YARS2	1.298947	1.378223	1.2334799	0.6173075	-0.6959388	0.03401
KIF14	1.218938	1.295262	1.15590759	0.5280314	-0.9213045	0.03411
RBMXL1	1.485183	1.5703393	1.41485893	0.7273896	-0.4591997	0.03418
PHACTR2	1.48517	1.4011901	1.55452284	1.3821946	0.46696075	0.03423
MRPS31	1.558985	1.6455231	1.48752026	0.7552329	-0.4050065	0.03427
WDR48	1.405569	1.4879318	1.33755323	0.6918042	-0.5315644	0.03435
RWDD4	1.668373	1.7586144	1.59384947	0.7828081	-0.3532695	0.03435
APH1A	2.676854	2.8149936	2.56277524	0.8610362	-0.2158542	0.03448
SGTA	1.541196	1.6270767	1.47027368	0.749946	-0.4151414	0.0346
DDX56	1.406324	1.4880837	1.33880597	0.6941555	-0.5266691	0.03462

COPG2	1.336071	1.4163029	1.26981387	0.6481191	-0.6256691	0.03462
RAB2A	3.287106	3.4526954	3.15035905	0.876733	-0.1897905	0.03462
GS1-600G8.5	1.131404	1.0427126	1.20464698	4.7912587	2.26040471	0.03467
C17orf58	1.517514	1.6026278	1.44722547	0.7421255	-0.4302649	0.03469
CCS	1.724355	1.8150791	1.64943377	0.7967739	-0.3277578	0.0347
PIGT	1.684738	1.7741798	1.61087463	0.7890604	-0.3417923	0.03471
DNM1	1.741014	1.6503864	1.81585594	1.2544173	0.32701741	0.03477
TK1	1.366357	1.4469109	1.29983377	0.6709028	-0.5758243	0.03482
ITGA7	1.224466	1.1481799	1.2874647	1.9399705	0.95603472	0.03484
ZCCHC17	2.47493	2.590624	2.37938742	0.8671989	-0.2055651	0.03488
PLPPR1	1.128181	1.2015138	1.06762232	0.3355716	-1.5753076	0.03518
MSX1	1.13613	1.0628195	1.19667142	3.1307404	1.64650387	0.03521
RP11-1338A24.1	1.332583	1.4121269	1.26689375	0.647601	-0.6268229	0.03522
PIGX	2.150071	2.2587891	2.06028941	0.8423091	-0.2475784	0.03526
DRG1	1.612341	1.6995808	1.54029627	0.7723143	-0.3727399	0.03526
NBEA	2.120436	2.2300129	2.02994474	0.8373447	-0.2561065	0.03527
NCOR2	1.486837	1.402779	1.55625303	1.3810377	0.46575268	0.03533
PTGES	1.547894	1.4552967	1.62436264	1.3713312	0.45557709	0.03537
PSME4	1.351301	1.271385	1.41729649	1.5376547	0.62073157	0.03542
ARG2	1.327417	1.406417	1.26217762	0.645095	-0.6324163	0.03546
TRAK1	1.298892	1.2206697	1.36348929	1.6472101	0.72002459	0.03557
ARL16	2.530303	2.6668605	2.41753103	0.8504197	-0.233753	0.03558
HP1BP3	3.070319	3.237211	2.93249705	0.8637974	-0.2112351	0.03564
HSPA5	4.136696	4.4063397	3.91402024	0.8554697	-0.2252114	0.03571
OSGEP	1.566849	1.6528646	1.49581521	0.7594457	-0.3969814	0.03597
TMED10	3.087645	3.2385586	2.96301824	0.8769117	-0.1894965	0.03618
KIF3A	1.557761	1.6434949	1.4869611	0.7567444	-0.4021221	0.03645
TMEM160	3.468377	3.6324073	3.33291863	0.8862301	-0.1742468	0.0365
CALU	3.409631	3.2436707	3.5466845	1.1350527	0.18275927	0.03661
SLC20A2	1.235551	1.1596895	1.29819952	1.8673708	0.9010084	0.03672
ATP6V1H	1.703387	1.7926818	1.62964535	0.794323	-0.3322024	0.03672
SSNA1	3.413045	3.2453059	3.55156679	1.1364005	0.18447141	0.03677
LRRC75A	1.350308	1.4310821	1.2836039	0.6578883	-0.6040854	0.0371
RAD50	2.241761	2.3543591	2.14877529	0.8482058	-0.2375137	0.03711
CCDC171	1.409019	1.4908751	1.34142092	0.6955352	-0.5238045	0.03716
CPEB4	1.462155	1.3799453	1.53004481	1.3950558	0.48032278	0.03721
SHKBP1	1.607901	1.6943942	1.53647275	0.7725767	-0.37225	0.03739
CALD1	8.316254	7.9205825	8.64300624	1.1043877	0.14324674	0.03739
CDC26	2.348342	2.468192	2.24936866	0.8509573	-0.2328414	0.03743
FUT11	1.571037	1.4845735	1.64244024	1.3257849	0.40684671	0.03752
DKK2	1.096059	1.0157693	1.16236388	10.296176	3.36403666	0.03753
RHOC	4.903801	4.6606511	5.10459818	1.1212754	0.16514071	0.03765
MYO5A	1.240478	1.1649832	1.30282297	1.8354775	0.87615544	0.03777
BCCIP	1.586314	1.6720468	1.51551433	0.767081	-0.3825492	0.03787
ACOT11	1.229696	1.1544348	1.29184742	1.889777	0.918216	0.03792

CLDN4	1.316083	1.2262776	1.39024505	1.7246293	0.78628632	0.03803
ERAL1	1.463983	1.5463882	1.39593074	0.7246326	-0.4646783	0.03811
EPPK1	1.128416	1.05683	1.18753333	3.299903	1.7224236	0.03815
NDP	1.304676	1.382216	1.2406425	0.6295983	-0.6674965	0.03816
CCDC124	2.356145	2.4678569	2.26389183	0.8610457	-0.2158384	0.0382
USMG5	9.879455	10.311873	9.52235622	0.9152139	-0.1278191	0.03822
CTNNA1	2.357969	2.4854633	2.25268277	0.8432943	-0.2458919	0.03827
KHDC1	1.397091	1.4779365	1.33032731	0.6911531	-0.5329227	0.03842
LSM8	2.737985	2.8755953	2.62434487	0.8660423	-0.2074906	0.03856
NUDT22	1.896505	1.9903368	1.81901799	0.8270095	-0.2740242	0.03862
UTRN	2.077576	2.1826915	1.99076998	0.8377248	-0.2554517	0.0387
SAT2	5.522136	5.8023855	5.2907014	0.8934521	-0.1625377	0.03885
MRPL1	1.626937	1.7131538	1.55573801	0.7792681	-0.3598083	0.03886
C11orf1	1.469344	1.5514117	1.40157168	0.728261	-0.4574725	0.03886
MED7	1.282805	1.3594095	1.21954409	0.6108467	-0.7111177	0.03891
SETX	1.435017	1.3539302	1.50198022	1.4183029	0.50416567	0.03895
TMEM101	1.671473	1.7588131	1.59934564	0.7898462	-0.3403564	0.03897
TMX4	1.809886	1.9002447	1.73526662	0.8167408	-0.2920497	0.03913
BUB1	1.180228	1.2533109	1.11987577	0.4732358	-1.0793689	0.03921
MRPS17	1.369691	1.4486375	1.30449543	0.6787114	-0.5591298	0.03923
TRMT1	1.521566	1.6050578	1.4526168	0.7480554	-0.4187829	0.03928
VMP1	2.474316	2.3371895	2.58755782	1.1872348	0.24760526	0.03929
TCEB2	13.62546	14.372667	13.0083977	0.8979808	-0.1552436	0.03934
MAD2L1BP	1.273882	1.3502034	1.21085379	0.6020895	-0.73195	0.03942
TMEM128	1.601979	1.6872019	1.53160015	0.773572	-0.3703925	0.03945
DDA1	1.643556	1.5567921	1.7152075	1.2845146	0.36122326	0.03949
RNF213	1.482688	1.401214	1.5499707	1.3707666	0.45498298	0.03957
MAPK1IP1L	2.292298	2.4006359	2.20283025	0.8587744	-0.2196489	0.0397
HOXB6	1.105393	1.1893567	1.03605474	0.1904064	-2.3928458	0.03982
ECM1	1.248348	1.323259	1.1864851	0.5768908	-0.79363	0.03989
TGIF2	1.658207	1.7444977	1.58694733	0.7883803	-0.3430364	0.04001
PITRM1	1.248314	1.1730774	1.31044477	1.7936756	0.84291896	0.04004
SLC35F3	1.267389	1.3431319	1.20483943	0.59697	-0.7442696	0.04011
ACADS	1.251479	1.3267334	1.18933189	0.579469	-0.7871966	0.04015
AKIRIN2	1.550837	1.634328	1.48188954	0.7596851	-0.3965266	0.04033
S100A13	6.826461	7.1670519	6.54519616	0.8991648	-0.1533425	0.04041
CAPZA1	2.586768	2.7269616	2.4709944	0.8517818	-0.2314442	0.04047
CD63	12.23492	12.814135	11.756599	0.9104855	-0.135292	0.04054
MYO1C	1.205699	1.1322958	1.26631737	2.0130448	1.00937926	0.04059
TEAD2	2.128317	2.2446616	2.03223706	0.8293315	-0.2699793	0.0407
CLMP	1.115384	1.0444925	1.17392688	3.9091314	1.96684807	0.04072
DHRS4	1.263684	1.3392054	1.20131774	0.593498	-0.752685	0.0408
DGCR2	1.447253	1.3667269	1.51375246	1.4009131	0.48636747	0.04101
INF2	1.24358	1.1691749	1.30502582	1.803021	0.85041617	0.04106
EXOSC5	1.358721	1.4364883	1.29450019	0.6747035	-0.5676744	0.0411

MRPL49	1.506042	1.588022	1.43834233	0.7454523	-0.4238121	0.0411
KCNG1	1.11787	1.0473647	1.17609484	3.7178533	1.89446984	0.04112
GPC4	1.462432	1.5432468	1.39569441	0.728388	-0.457221	0.04113
BTBD10	1.383494	1.4622179	1.31848172	0.6890294	-0.5373626	0.04119
ARPC1B	2.273823	2.1608659	2.36710446	1.1776592	0.23592213	0.0412
SPINT1	1.256622	1.181896	1.31833284	1.7500817	0.80742225	0.0412
C1orf52	2.003736	2.1029137	1.92183321	0.8358162	-0.2587423	0.04155
POLE4	2.696899	2.8426191	2.57656086	0.8556087	-0.224977	0.04156
PPA2	1.919286	2.0116224	1.84303251	0.833347	-0.2630108	0.04159
TMEM14C	5.677445	5.9605598	5.44364434	0.8957949	-0.1587596	0.04165
UNC5B	1.266966	1.191843	1.32900303	1.7149595	0.77817452	0.04176
NXT2	1.352999	1.4308839	1.28867957	0.6699706	-0.5778303	0.0418
METTL21A	1.768596	1.8571549	1.69546203	0.811361	-0.3015841	0.04193
ZFYVE16	1.555158	1.4725338	1.62339029	1.3192501	0.3997181	0.04196
UTP18	1.432301	1.5121106	1.36639332	0.7154574	-0.4830622	0.04205
TTC14	2.424213	2.3132434	2.51585339	1.1542821	0.20699586	0.04205
GLUD1	1.714353	1.8011664	1.64266094	0.8021566	-0.3180441	0.04211
SELENOK	4.211137	4.4070367	4.04936062	0.8950184	-0.1600107	0.04227
FRMD6	1.170673	1.0987207	1.23009167	2.3307344	1.22078464	0.04228
NHLRC2	1.408457	1.4880223	1.34275072	0.702326	-0.5097874	0.04234
EEF1AKMT1	1.338932	1.4158111	1.27544486	0.6624279	-0.5941646	0.04265
DST	2.562507	2.436398	2.66664997	1.1602981	0.21449555	0.04276
AKAP13	1.789509	1.7013441	1.86231765	1.2295215	0.29809691	0.0429
ARPC5	3.360381	3.5239185	3.22532953	0.8816963	-0.1816463	0.04311
VAMP7	1.372981	1.4509445	1.30859747	0.6843357	-0.5472238	0.04333
VRK3	1.519898	1.6017271	1.45232246	0.751707	-0.4117576	0.04336
TGFB1I1	1.558326	1.4763975	1.62598356	1.3139942	0.39395893	0.04336
RASSF8	1.342807	1.2664771	1.40584125	1.5229875	0.60690407	0.04338
BAG2	1.386204	1.464431	1.32160309	0.692467	-0.5301829	0.04341
NSL1	1.6543	1.7393492	1.58406551	0.7899725	-0.3401257	0.04343
CERK	1.798666	1.8878732	1.72499646	0.8165541	-0.2923797	0.04345
PTP4A1	2.059905	1.962488	2.14035448	1.1847987	0.24464194	0.04363
ADAM12	1.182939	1.1110952	1.24226897	2.1807339	1.12481375	0.04369
UFSP2	1.685678	1.7713908	1.61489505	0.7971252	-0.3271217	0.04376
YBX3	3.10026	3.2407213	2.98426427	0.8855471	-0.175359	0.04379
PRELID1	5.212362	5.4345734	5.02885666	0.9085105	-0.1384249	0.04394
CRYZL1	1.997618	2.0939766	1.91804384	0.8391805	-0.252947	0.04399
BMPR2	1.558763	1.4767648	1.62647914	1.3140213	0.39398868	0.04401
FYN	1.601546	1.6855334	1.53218697	0.7763108	-0.3652938	0.04413
SYNJ2BP	1.719343	1.8090442	1.64526713	0.7975672	-0.326322	0.04415
ABCC5	1.272199	1.1980379	1.33344243	1.6837307	0.75166143	0.0442
ACVR2B	1.592288	1.6759897	1.52316654	0.7739268	-0.3697309	0.04436
DNMT1	2.019501	2.1138979	1.94154715	0.8452724	-0.2425117	0.04438
BBS4	1.441769	1.5206446	1.37663306	0.7233976	-0.4671394	0.0444
MPP1	1.280324	1.3547291	1.21887963	0.6170333	-0.6965798	0.04452

FRMD4B	1.85642	1.9461434	1.78232407	0.8268557	-0.2742926	0.04453
PTPRM	1.180373	1.1090981	1.23923241	2.19282	1.13278739	0.04459
FGFR3	1.142739	1.0723094	1.20090031	2.7783435	1.47422497	0.04468
AUH	1.266749	1.3407551	1.20563271	0.6034618	-0.7286657	0.04476
FUT8	1.240657	1.1673874	1.30116434	1.7992054	0.84735993	0.04477
LIMA1	1.646303	1.562289	1.71568399	1.2728044	0.34801076	0.04506
MRPS2	1.802732	1.8904752	1.7302723	0.8200928	-0.2861409	0.04526
NIF3L1	1.22985	1.3026653	1.16971808	0.5607451	-0.834583	0.04532
GLIPR2	1.432192	1.3542365	1.49656901	1.4018009	0.48728142	0.04556
ATL1	1.200434	1.2722859	1.1410976	0.5181964	-0.948429	0.04558
HHLA3	1.472643	1.5522882	1.40687117	0.7367009	-0.4408492	0.04567
DNASE2	1.541694	1.6227018	1.47479631	0.7624778	-0.3912327	0.04593
C19orf81	2.057795	2.1532461	1.97896929	0.8488815	-0.2363649	0.04593
EPB41L4A-AS1	2.955241	3.1200619	2.81912917	0.8580547	-0.2208584	0.04595
KARS	1.991897	2.0841662	1.91569966	0.8446119	-0.2436396	0.04606
AGBL5	1.347183	1.4233925	1.28424844	0.6713591	-0.5748434	0.04614
CRLF1	1.180521	1.1098777	1.23885981	2.173869	1.12026501	0.0463
ANKRD37	1.595173	1.4962168	1.67689221	1.3641059	0.44795567	0.04635
KIAA0232	1.588062	1.5057963	1.65599864	1.2969622	0.37513639	0.04635
BUD31	2.926935	3.0619776	2.81541519	0.8804243	-0.1837291	0.04642
SHMT1	1.287957	1.3622093	1.22663787	0.6257097	-0.6764346	0.04643
C12orf65	1.504372	1.5841181	1.43851638	0.7507323	-0.4136294	0.04647
GTF2IRD1	1.270767	1.1971573	1.33155539	1.6816795	0.74990278	0.04655
MED21	1.515847	1.596492	1.44924949	0.7531525	-0.408986	0.04666
R3HCC1	1.586623	1.6687758	1.51877953	0.7757152	-0.366401	0.04667
STRIP2	1.137112	1.067634	1.19448894	2.8756092	1.52386764	0.04677
OXA1L	2.356655	2.4656694	2.26662835	0.8641979	-0.2105665	0.04689
C19orf70	3.028902	3.1744961	2.90866758	0.8777517	-0.1881152	0.04717
RBM39	4.928397	5.1717117	4.72746321	0.8935093	-0.1624453	0.04723
LINC00467	1.876758	1.9742811	1.79622095	0.8172394	-0.2911693	0.04725
WDR13	1.897773	1.9880653	1.82320764	0.833151	-0.26335	0.04729
ADA	1.61734	1.7007096	1.54849204	0.7827666	-0.3533459	0.04732
MED8	1.42769	1.5053864	1.36352699	0.719305	-0.4753245	0.04737
SERINC1	1.835676	1.7479841	1.90809264	1.2140535	0.27983194	0.04748
CHMP1B	1.966179	2.0575527	1.89072183	0.8422482	-0.2476827	0.04755
TMEM159	1.462595	1.5412528	1.39763855	0.7346633	-0.4448448	0.04778
AP1M1	1.535221	1.6156718	1.46878389	0.7614185	-0.3932384	0.04779
LMNB2	1.423826	1.5014591	1.35971552	0.7173377	-0.4792756	0.04785
DPYSL2	3.183122	3.3355755	3.05722308	0.8808206	-0.1830798	0.048
ETV4	1.375593	1.451724	1.31272208	0.6922857	-0.5305606	0.04802
TANC1	1.326024	1.2514057	1.38764493	1.5419099	0.62471849	0.04809
DGCR6L	2.953814	3.119143	2.81728305	0.8575557	-0.2216978	0.04811
SLC39A13	1.437118	1.3596863	1.50106269	1.3930548	0.47825203	0.04814
BAX	3.713376	3.8944672	3.5638282	0.8857686	-0.1749982	0.04836
IFT43	1.683719	1.767665	1.61439582	0.8003437	-0.3213085	0.04843

TSG101	1.875276	1.9638304	1.8021465	0.8322486	-0.2649136	0.04846
SKIDA1	1.266163	1.3395633	1.20554721	0.6053281	-0.7242109	0.04853
VAMP3	1.985413	2.0799572	1.907337	0.8401601	-0.2512638	0.04857
DUSP14	1.401233	1.4778908	1.33792861	0.7071252	-0.4999624	0.04863
NDUFB2	6.897221	7.2955184	6.56830004	0.8844863	-0.1770882	0.04865
CBX6	1.970264	1.8692101	2.05371625	1.2122688	0.27770965	0.0488
CRACR2B	2.529183	2.6541812	2.42595788	0.8620325	-0.2141859	0.04889
KLF6	1.75416	1.6650221	1.82777194	1.2447284	0.31583101	0.04893
QTRT1	2.006263	2.099605	1.92917967	0.8450122	-0.2429559	0.049
PPP1R13L	1.299141	1.2257893	1.35971545	1.5931463	0.67187877	0.04923
MXD3	1.344383	1.4185632	1.28312444	0.6764198	-0.5640092	0.04936
HTRA2	1.498396	1.5771416	1.43336581	0.750883	-0.41334	0.04947
RASGEF1B	1.376128	1.4532908	1.31240633	0.6891963	-0.5370132	0.04972
ANKRD45	4.163238	3.9279066	4.35757785	1.1467503	0.19755129	0.04973
PIGH	1.335262	1.4096552	1.27382656	0.6684318	-0.5811478	0.04982
MAP2K7	1.515076	1.5945344	1.44945713	0.7559818	-0.4035767	0.04994
ZNF423	1.322515	1.3964289	1.26147561	0.6595775	-0.6003859	0.04998
MANBAL	2.028021	2.1205175	1.95163581	0.8492824	-0.2356837	0.0501
SLC20A1	1.425015	1.3485326	1.48817458	1.4006568	0.48610345	0.05015
DCN	1.081021	1.0141592	1.13623676	9.6218064	3.26630777	0.05016
IFT81	1.923186	2.0120554	1.84979642	0.8396738	-0.2520991	0.05019
TOP1MT	1.292543	1.3655004	1.2322943	0.6355514	-0.6539192	0.05034
LINC00685	2.237471	2.3514515	2.14334334	0.8460113	-0.2412511	0.05039
CTNNAL1	2.053686	2.1472718	1.97640099	0.8510633	-0.2326616	0.05048
TRIM8	1.289689	1.2173824	1.34940088	1.6073101	0.68464832	0.05049
COQ8A	1.177238	1.2467842	1.11980564	0.4854672	-1.0425543	0.05059
GSK3B	1.635359	1.7176804	1.56737671	0.7905701	-0.3390346	0.05061
MED11	1.511058	1.5898648	1.44597864	0.7560693	-0.4034097	0.05066
IBTK	1.894951	1.8070283	1.96755818	1.1989149	0.26172923	0.05087
TYW3	1.326527	1.4005074	1.26543321	0.6627424	-0.5934799	0.051
DRAP1	4.077901	4.2754921	3.91472756	0.8898594	-0.1683506	0.05103
TFAM	1.643137	1.7243758	1.57604907	0.7952351	-0.3305466	0.05108
CSNK2A1	2.072596	2.1708183	1.99148288	0.846829	-0.2398575	0.05115
AHDC1	1.367923	1.2933814	1.42948048	1.4638979	0.54981497	0.05148
IMPACT	1.496516	1.5746072	1.43202662	0.7518643	-0.4114557	0.05151
RRP1B	1.584493	1.66484	1.51814078	0.7793465	-0.3596631	0.05162
LYAR	1.311531	1.3844925	1.25127767	0.6535307	-0.613673	0.05175
SSSCA1	1.516067	1.5946729	1.45115368	0.7586585	-0.3984775	0.05176
RNF13	1.837324	1.9237693	1.76593551	0.8291416	-0.2703096	0.05177
IDE	1.269735	1.1980208	1.32895815	1.6612303	0.73225207	0.05177
DNALI1	1.567479	1.647418	1.50146371	0.7745594	-0.3685523	0.05179
KIF23	1.248643	1.3191708	1.1904003	0.5965467	-0.7452929	0.05222
PCBD1	4.176733	4.3790973	4.00961728	0.8906572	-0.1670579	0.05227
AGPAT2	2.024084	1.9257009	2.10532987	1.1940464	0.25585893	0.05234
ELAVL3	1.151296	1.2200266	1.09453749	0.4296639	-1.2187197	0.05246

RNF20	1.370059	1.4447186	1.30840364	0.6934805	-0.5280728	0.05254
IP6K2	2.720942	2.8542009	2.61089495	0.8687812	-0.2029351	0.05259
KLF5	1.149641	1.0818676	1.20560912	2.5114834	1.32853976	0.0526
LTV1	1.197253	1.2669242	1.1397165	0.5234313	-0.9339279	0.05268
AGTRAP	2.013761	2.1098003	1.9344508	0.8419991	-0.2481095	0.05287
MTHFD1	1.347568	1.4211851	1.28677418	0.6808744	-0.5545393	0.05291
CELSR2	1.307646	1.2353665	1.36733631	1.5606993	0.64219259	0.05294
LRRN1	1.414563	1.4899577	1.35230141	0.7190446	-0.4758469	0.05295
PLCD3	1.305012	1.2329219	1.36454539	1.5650974	0.64625244	0.05317
MNAT1	1.774616	1.858988	1.70493985	0.8206632	-0.2851379	0.0532
ITGA2	1.253615	1.1831317	1.31182059	1.7027123	0.76783472	0.05333
CTSV	1.218072	1.1486722	1.27538301	1.8522831	0.88930461	0.05345
RBM23	2.157095	2.2510291	2.07952179	0.862907	-0.212723	0.0535
MYCN	1.330042	1.40289	1.26988305	0.6698678	-0.5780516	0.0535
CEP57L1	1.353315	1.426935	1.29251764	0.6851573	-0.5454928	0.05351
HNRNPAB	2.612607	2.7350225	2.51151409	0.8711784	-0.1989599	0.05352
SYTL2	1.405208	1.4802248	1.34325719	0.7147844	-0.4844199	0.05369
METRN	2.899901	3.0283835	2.79379815	0.8843486	-0.1773129	0.05371
SPATA7	1.485737	1.5629089	1.42200774	0.749691	-0.4156321	0.05374
CKLF	1.814897	1.8996357	1.74491927	0.8280232	-0.2722568	0.05391
MTIF2	1.496594	1.573803	1.43283434	0.7543257	-0.4067406	0.05394
MCUB	1.697157	1.7797549	1.62894686	0.8065956	-0.3100826	0.05408
CTSA	1.427059	1.5022611	1.36495653	0.7266271	-0.4607129	0.05417
CLDND1	1.782537	1.8669285	1.71284556	0.8222657	-0.2823234	0.05422
SRP68	1.459798	1.5356555	1.39715303	0.7414337	-0.4316104	0.05432
C11orf49	1.641959	1.7226325	1.57533727	0.7961686	-0.3288542	0.05442
SBNO1	1.843517	1.929751	1.77230349	0.8306563	-0.2676765	0.05459
EMC2	1.495221	1.572126	1.43171225	0.7545754	-0.406263	0.05461
LCORL	1.54235	1.6204311	1.47786937	0.7702214	-0.3766548	0.05461
MOSPD3	1.816071	1.9009371	1.74598768	0.8280131	-0.2722746	0.05473
TAF6	1.771534	1.8562985	1.70153319	0.8192624	-0.2876024	0.05478
SNAPC3	1.977819	2.0664441	1.90463159	0.8482691	-0.237406	0.05495
PTPRD-AS1	1.200561	1.2697596	1.14341473	0.531639	-0.9114812	0.05509
PBX1	3.410634	3.564039	3.28395021	0.8907627	-0.166887	0.05511
DNAJC4	2.313195	2.4147578	2.22932283	0.8689281	-0.2026913	0.05518
WSB1	9.281884	8.8289795	9.65590006	1.105623	0.14485959	0.05519
SGCE	1.274832	1.3457279	1.2162856	0.6255949	-0.6766993	0.05527
FBXW9	1.270379	1.3414087	1.21172221	0.620143	-0.6893271	0.05532
B4GALT5	1.305829	1.2343069	1.36489335	1.5573308	0.63907544	0.05535
PXDN	2.198098	2.0946653	2.28351355	1.1725169	0.22960875	0.05538
HOXB2	1.153649	1.2370063	1.08481151	0.357845	-1.4825933	0.05543
IFNGR1	1.261821	1.3321995	1.2037006	0.6131876	-0.7055996	0.05545
CDK5RAP3	2.171695	2.2727204	2.08826743	0.8550719	-0.2258824	0.05549
ZCCHC11	2.918271	3.0462168	2.81261148	0.8858355	-0.1748893	0.0555
RPP30	1.430868	1.5059335	1.36887763	0.7291029	-0.4558056	0.05579

CDK16	3.313635	3.4663554	3.18751573	0.8869426	-0.1730873	0.05591
EEF1G	1.553489	1.6325067	1.48823507	0.771905	-0.3735047	0.05599
GDF11	1.549534	1.4717866	1.61373985	1.3008844	0.37949277	0.05599
GPRC5A	1.120085	1.0538204	1.17480754	3.2479773	1.69954153	0.05611
YAF2	1.915065	2.0021501	1.84314836	0.8413394	-0.2492403	0.05617
GIPC1	1.938052	1.8515392	2.00949601	1.1854957	0.24549043	0.05623
NEDD1	1.374306	1.4479526	1.31348743	0.6998228	-0.5149383	0.05636
PCNX1	1.395126	1.3213031	1.45608994	1.4195006	0.50538342	0.05642
PSPH	1.498346	1.5748752	1.43514712	0.7569418	-0.4017456	0.05651
DOK5	1.194967	1.2677136	1.13489085	0.5038626	-0.9888978	0.05653
WASF3	1.718269	1.8006398	1.65024579	0.8121577	-0.3001682	0.0568
GTF2F1	1.834142	1.9186687	1.76433885	0.8320071	-0.2653323	0.05695
TRIP6	1.639038	1.7194324	1.57264765	0.7959715	-0.3292113	0.05695
FKBP1A	6.172622	6.4922141	5.90869746	0.8937557	-0.1620476	0.05701
TLE6	1.231209	1.3006057	1.17389998	0.5784987	-0.7896143	0.05708
FURIN	1.260142	1.1903692	1.31776127	1.6691841	0.7391431	0.05709
ACTR1A	1.743816	1.8261587	1.67581652	0.8180226	-0.2897874	0.05715
ACTR1B	2.216865	2.3121783	2.13815328	0.867377	-0.2052689	0.0572
DDX24	2.691705	2.8048014	2.59830897	0.8855872	-0.1752938	0.05723
PLCB4	1.254333	1.1846072	1.31191439	1.6896113	0.75669143	0.05729
FAM199X	1.541515	1.4643316	1.60525414	1.3034955	0.38238563	0.05746
XRCC4	1.281919	1.3522293	1.22385599	0.6355405	-0.653944	0.05752
CLUAP1	1.542699	1.6199012	1.47894505	0.7726151	-0.3721781	0.05758
SCRN3	1.338376	1.4106401	1.27869924	0.6786947	-0.5591654	0.05763
CEP126	1.411189	1.4856909	1.34966404	0.7199312	-0.474069	0.05779
ZNF462	1.823425	1.9079084	1.7536565	0.8301019	-0.2686397	0.05785
GLG1	1.976288	1.8881475	2.04907555	1.1811952	0.24024738	0.05787
PPP3CA	1.755301	1.6732005	1.82310162	1.2226693	0.29003429	0.05805
TRMT11	1.479458	1.5551079	1.41698429	0.7511771	-0.4127751	0.05815
FGF9	1.369727	1.4423034	1.30979183	0.7004057	-0.5137372	0.05833
STK26	1.738581	1.820249	1.67113906	0.8182138	-0.2894502	0.05852
CHD1	1.893629	1.9788059	1.82328877	0.8411154	-0.2496243	0.05859
RNF145	2.237821	2.341568	2.15214459	0.8588045	-0.2195984	0.05865
TACC3	1.612477	1.6910854	1.54756037	0.7923195	-0.3358459	0.05871
LRRFIP2	1.424004	1.350404	1.48478473	1.3835023	0.46832503	0.05873
SLC15A4	1.262616	1.1932168	1.3199269	1.655792	0.72752144	0.05888
MRPL44	1.532458	1.6088422	1.46937894	0.770937	-0.3753152	0.05906
IQCK	1.478394	1.5536486	1.41624728	0.7518258	-0.4115297	0.05913
HCCS	1.35647	1.4285474	1.29694773	0.692917	-0.5292456	0.05916
MRPL32	1.929149	2.0153038	1.85800185	0.8450691	-0.2428588	0.05924
UNG	1.472023	1.5470028	1.41010307	0.7497276	-0.4155616	0.05931
SSBP4	3.078613	3.211609	2.9687832	0.890204	-0.1677921	0.05932
ZC3H8	1.67446	1.7547574	1.60814965	0.8057552	-0.3115866	0.05934
EIF3A	3.297822	3.4478909	3.17389206	0.8880674	-0.1712589	0.05952
SFR1	1.283246	1.353146	1.22552173	0.6386076	-0.6469985	0.05955

MRTO4	1.487059	1.5620772	1.42510854	0.7563169	-0.4029372	0.05956
C1orf27	1.379827	1.4523063	1.31997194	0.7074232	-0.4993546	0.05957
LMBRD1	1.406858	1.4801496	1.34633198	0.7213002	-0.4713283	0.05969
DAZAP2	2.318647	2.4261596	2.22986076	0.8623584	-0.2136404	0.05973
RP11-25K19.1	1.498667	1.5742803	1.43622387	0.7596009	-0.3966864	0.05976
MALSU1	1.688664	1.7695938	1.62183097	0.807999	-0.3075747	0.05986
KEAP1	1.593789	1.6714829	1.5296284	0.7887445	-0.3423701	0.05992
PAPOLA	4.03504	4.215073	3.88636632	0.8977608	-0.1555971	0.05993
EPRS	1.83521	1.9187255	1.76624227	0.8340274	-0.2618332	0.05999
TAF11	2.316341	2.4153468	2.23457949	0.8722805	-0.1971359	0.06006
ISCA1	2.227744	2.321466	2.15034699	0.8705082	-0.2000703	0.06018
C6orf1	1.636608	1.5582432	1.70132334	1.2563042	0.32918589	0.06052
COQ8B	1.452093	1.5263359	1.39078205	0.7424576	-0.4296195	0.06054
MICA	1.296146	1.2262794	1.35384323	1.5637447	0.64500499	0.06058
ASNSD1	1.54587	1.6224619	1.48261942	0.7753397	-0.3670996	0.06067
B4GAT1	1.636351	1.7146254	1.57171144	0.8000155	-0.3219001	0.06071
MALAT1	648.8266	626.11486	667.582253	1.0663356	0.09266161	0.06072
SUDS3	1.869389	1.9540038	1.79951326	0.8380609	-0.2548731	0.06075
LOXL2	1.435262	1.3616099	1.49608544	1.3718801	0.45615445	0.06093
SIX3-AS1	1.826167	1.9148145	1.75295967	0.8230736	-0.2809066	0.06108
GPATCH2L	1.790067	1.7081519	1.85771325	1.2111995	0.27643647	0.06121
SEPHS2	1.612511	1.6904509	1.54814664	0.7938966	-0.3329769	0.06122
RCHY1	1.39939	1.471994	1.33943163	0.719144	-0.4756474	0.06125
FAM49B	1.970827	2.0565462	1.9000393	0.8518693	-0.231296	0.06134
RP11-245D16.4	1.172479	1.2390678	1.11748902	0.4914464	-1.0248939	0.06135
NDUFB7	5.111195	5.3589261	4.90661507	0.8962334	-0.1580536	0.06166
AK1	2.270426	2.3689961	2.18902437	0.8685375	-0.20334	0.06167
BCL2L12	1.597378	1.6743936	1.5337777	0.7914929	-0.3373518	0.0617
SEC24D	1.293904	1.2243455	1.35134631	1.5660946	0.64717137	0.06178
S100A2	2.328821	2.0770905	2.5367039	1.4267175	0.51269973	0.06178
GNS	1.463602	1.3897312	1.52460587	1.3460711	0.42875459	0.06205
ADRM1	1.924504	2.0096353	1.85420177	0.8460498	-0.2411854	0.06227
HDAC5	1.470301	1.5442383	1.40924211	0.7519538	-0.411284	0.06269
ITPR2	1.159092	1.0933518	1.21338173	2.285781	1.19268718	0.06275
BCLAF1	2.924645	3.0495809	2.82147111	0.8887042	-0.1702248	0.06282
CRTAP	2.77695	2.6513425	2.88067872	1.1388787	0.18761406	0.06283
CNOT10	1.456637	1.5300424	1.39601755	0.7471432	-0.4205433	0.06294
ODF2L	2.305652	2.410353	2.21918905	0.8644566	-0.2101345	0.06294
RB1	1.504336	1.4300262	1.56570194	1.3155058	0.39561765	0.06299
CYB5R3	2.746757	2.8713772	2.64384376	0.8784139	-0.1870272	0.06305
SEPT8	1.358808	1.2880448	1.41724492	1.4485418	0.53460127	0.06308
EIF4EBP1	3.233456	3.4025624	3.09380566	0.8714886	-0.1984464	0.0631
LINC00969	2.253513	2.159684	2.33099801	1.1477247	0.19877666	0.06313
PIP5K1A	1.414218	1.3417476	1.47406455	1.3871774	0.47215228	0.06315
GNL1	1.615862	1.6933782	1.55184772	0.7958827	-0.3293723	0.06315

NUDT11	1.606554	1.6838613	1.54271217	0.7935997	-0.3335166	0.06324
HYOU1	1.371945	1.300594	1.43086697	1.4333849	0.5194261	0.0633
POLR2I	4.879417	5.1130945	4.68644147	0.8962696	-0.1579954	0.0634
MIIP	1.425486	1.4985228	1.36517033	0.7325047	-0.44909	0.06358
YPEL1	1.549367	1.6255241	1.48647431	0.7777067	-0.362702	0.06363
PDCD4	1.720311	1.800209	1.65433079	0.8176999	-0.2903567	0.06392
IFT74	1.369387	1.4404528	1.3107002	0.7054108	-0.5034643	0.06392
TSPAN14	1.692075	1.7713096	1.62664191	0.8124389	-0.2996688	0.06393
SMURF2	1.360379	1.2899849	1.41851218	1.4432205	0.52929178	0.06393
HINT3	1.496582	1.571007	1.43512025	0.7620226	-0.3920944	0.06407
THUMPD3	1.585451	1.6619278	1.52229611	0.789053	-0.3418058	0.06416
JUNB	2.408357	2.5593001	2.28370625	0.823258	-0.2805835	0.06418
CD53	1.101758	1.0380931	1.15433269	4.0514584	2.01844132	0.06421
NUFIP2	1.865558	1.7828518	1.93385731	1.1928916	0.2544629	0.06435
DUSP11	1.324191	1.3940397	1.26650916	0.6763511	-0.5641557	0.06445
ACTR6	1.294832	1.3635588	1.23807663	0.6548505	-0.6107626	0.06445
DDX5	6.118116	5.8054072	6.37635584	1.1188138	0.16196994	0.06486
PDE4DIP	1.345921	1.2759535	1.40370097	1.4629309	0.54886164	0.06496
XPNPEP1	1.799716	1.8805176	1.73298938	0.8324528	-0.2645596	0.06496
CAPG	1.470533	1.3973868	1.53093823	1.3360741	0.41799998	0.06503
DPP8	1.291097	1.2222814	1.34792521	1.5652467	0.64639009	0.0653
ARMC10	2.272875	2.3744118	2.18902387	0.8651147	-0.2090366	0.06545
NISCH	1.378734	1.308211	1.43697395	1.4177752	0.50362882	0.06564
STN1	1.482435	1.5561015	1.42160008	0.7581352	-0.399473	0.06568
MIR210HG	1.972132	2.0587317	1.90061691	0.8506564	-0.2333516	0.06574
AASDH	1.459993	1.5330996	1.39962108	0.7496181	-0.4157723	0.06575
NOL4	1.115476	1.1799402	1.06224058	0.3458959	-1.5315901	0.06576
PCAT6	1.778562	1.8680484	1.70466187	0.8117772	-0.3008443	0.06581
TM9SF2	1.737729	1.8175745	1.6717917	0.8216886	-0.2833363	0.06583
RAB11FIP1	1.404545	1.3331083	1.46353936	1.3915576	0.47670066	0.06587
ASRGL1	1.489405	1.5630991	1.42854726	0.7610513	-0.3939344	0.06595
HMBS	1.470112	1.5437644	1.4092884	0.7526944	-0.4098639	0.06598
TXNDC9	1.402751	1.4743688	1.34360866	0.7243491	-0.4652429	0.06608
DLC1	1.103554	1.0405129	1.1556143	3.8411063	1.94152189	0.06625
ARID1A	2.179683	2.2767406	2.09953133	0.8612018	-0.2155767	0.06625
VGLL4	2.280625	2.3734648	2.20395714	0.8765839	-0.1900359	0.0665
LDAH	1.370852	1.4413288	1.31265142	0.7084319	-0.4972988	0.06652
DYM	1.640432	1.7177667	1.57656723	0.8032794	-0.3160262	0.06655
TBC1D7	1.556876	1.6318922	1.49492644	0.783245	-0.3524644	0.06656
SRSF6	2.017126	2.1023391	1.9467558	0.8588607	-0.2195039	0.06667
BAZ1A	1.919573	2.0026519	1.85096469	0.848714	-0.2366497	0.06675
HRAS	1.454488	1.3822637	1.51413232	1.3449677	0.42757149	0.0668
COA3	2.944399	3.0756845	2.83598125	0.8845185	-0.1770359	0.06681
KRAS	1.803112	1.8842584	1.7360993	0.8324482	-0.2645676	0.06682
MAFB	1.43131	1.509539	1.36670651	0.7196829	-0.4745667	0.06728

TNFRSF9	1.15653	1.0922013	1.20965377	2.2738691	1.1851492	0.06756
ASH1L	2.148736	2.0565423	2.22487178	1.1593211	0.21328025	0.06762
MLLT1	1.388331	1.3179675	1.44643788	1.4040363	0.48958023	0.06768
CDKN2AIPNL	1.885897	1.9725312	1.81435381	0.8373549	-0.2560888	0.06784
RARS2	1.712163	1.7911689	1.6469183	0.8176741	-0.2904022	0.0679
RAX	1.574917	1.6495877	1.51325182	0.7901193	-0.3398576	0.068
PLEC	1.423432	1.352495	1.48201235	1.3674302	0.45146723	0.06803
LAMA5	1.405283	1.3347942	1.46349459	1.3844162	0.46927777	0.06807
PRELP	1.268638	1.3359105	1.21308318	0.6343452	-0.65666	0.06815
PGF	1.206376	1.1408695	1.26047195	1.8490303	0.88676884	0.06825
C19orf60	3.100717	3.2435778	2.98274094	0.8837407	-0.178305	0.06828
SLC25A6	15.01282	15.761236	14.3947717	0.9074288	-0.1401436	0.06828
COMMD3	1.622776	1.698941	1.55987685	0.8010359	-0.3200611	0.06849
CBFB	1.469405	1.5421953	1.40929392	0.7548828	-0.4056755	0.0685
DDX19B	1.403774	1.4745941	1.34528935	0.7275466	-0.4588884	0.06872
TXNDC15	1.508665	1.5821971	1.44794033	0.7693964	-0.378201	0.06873
EFHD1	1.239836	1.1734987	1.29461754	1.6980967	0.76391861	0.06887
LAMB2	1.340444	1.2716622	1.39724507	1.4622759	0.54821552	0.06888
SULF1	1.261262	1.1945627	1.31634354	1.625921	0.70125716	0.06936
CDH20	1.353693	1.4229568	1.29649293	0.7010005	-0.5125126	0.06942
RPA1	1.57244	1.6466431	1.51116111	0.7904841	-0.3391917	0.06958
SFPQ	2.932511	3.0571718	2.82956394	0.8893589	-0.1691624	0.06961
SAT1	6.43535	6.0485008	6.75481572	1.1399059	0.18891471	0.06965
OBSCN	1.19039	1.1254953	1.24398111	1.9441461	0.95913662	0.06975
IMP4	1.777993	1.8573427	1.71246405	0.8310143	-0.2670548	0.07007
PCSK7	1.511373	1.4386465	1.571432	1.3027165	0.38152321	0.07023
TMEM11	1.686199	1.7631905	1.62261755	0.8158088	-0.293697	0.07029
TACO1	1.32961	1.3981574	1.27300254	0.6856648	-0.5444246	0.07029
DMAP1	1.476133	1.5485179	1.41635715	0.7590585	-0.397717	0.07033
PDLIM4	2.499644	2.3763879	2.60143067	1.1635025	0.21847427	0.07034
ZFP36L2	5.408747	5.7154089	5.15550012	0.8812598	-0.1823608	0.07042
CXCL16	1.105925	1.043591	1.15740231	3.6108866	1.85235312	0.07043
THAP2	1.57551	1.650255	1.51378407	0.790127	-0.3398434	0.07044
TRAPPC2	1.284868	1.3526263	1.2289125	0.6491645	-0.623344	0.07045
C9orf16	6.606089	6.3287696	6.83510393	1.095019	0.1309559	0.07046
SEMA3E	1.640602	1.7180045	1.57668163	0.8031727	-0.3162178	0.07062
CTDSP2	1.969345	2.055319	1.89834653	0.8512559	-0.2323351	0.07068
KDSR	1.598315	1.673213	1.53646321	0.7968699	-0.3275839	0.0708
CADPS	1.191232	1.1267467	1.24448523	1.9289274	0.94779885	0.07081
DLD	1.508372	1.5808793	1.44849442	0.7720957	-0.3731483	0.07088
D2HGDH	1.331352	1.2625969	1.3881319	1.4780519	0.56369693	0.07099
TEX2	1.164665	1.1009694	1.21726626	2.1518037	1.10554649	0.07105
PHYH	1.413168	1.4833811	1.35518538	0.7347937	-0.4445889	0.07106
GNB1	2.449082	2.3506279	2.53038761	1.1330935	0.18026685	0.07109
RP11-128P17.4	1.225998	1.2913591	1.1720223	0.5904134	-0.7602025	0.07111

C6orf52	1.151497	1.2151577	1.09892502	0.4597791	-1.1209871	0.07114
DDIT4	12.81026	13.708541	12.0684452	0.8709454	-0.1993458	0.07118
RABEPK	1.356812	1.4260563	1.29962904	0.7032616	-0.5078666	0.07118
TH	1.241324	1.3094404	1.18507303	0.5980895	-0.7415667	0.07128
DCPS	1.244971	1.3107707	1.19063328	0.6134209	-0.7050507	0.07142
TOB1	1.377242	1.4466748	1.31990315	0.716188	-0.4815897	0.07142
HTATIP2	1.088846	1.0271913	1.13976124	5.1399211	2.36174622	0.07147
GATA3	1.142384	1.0796036	1.19422904	2.4399544	1.28685416	0.07168
TRNT1	1.340135	1.408775	1.28345064	0.6934148	-0.5282096	0.07179
FAM161A	1.639811	1.7151085	1.57762948	0.8077508	-0.3080178	0.07195
FAM177A1	2.04532	1.9540492	2.1206935	1.1746705	0.23225615	0.07195
NKTR	3.430805	3.2813277	3.55424573	1.1196312	0.16302362	0.07197
CDC40	1.481432	1.5530838	1.42226031	0.7634653	-0.3893656	0.07199
ARGLU1	4.920725	5.1279988	4.74955447	0.9083226	-0.1387234	0.07202
TMEM216	1.378403	1.4477939	1.32109826	0.7170671	-0.4798199	0.07204
PUM2	1.650397	1.574651	1.71294851	1.2406634	0.31111181	0.07205
CXXC4	1.598108	1.6725611	1.5366227	0.7978794	-0.3257573	0.07228
MPV17	2.164195	2.2522906	2.09144349	0.8715577	-0.1983319	0.07229
CASK	1.443943	1.373035	1.5025007	1.3470606	0.42981471	0.07237
CTA-29F11.1	1.338541	1.406761	1.28220337	0.6937817	-0.5274462	0.07238
PCM1	3.163194	3.2977608	3.05206705	0.8930725	-0.1631508	0.07245
MPHOSPH10	1.421984	1.4921457	1.36404377	0.7397073	-0.4349737	0.07246
TMEM132A	1.463572	1.3922047	1.52250762	1.332232	0.41384536	0.07247
IFI6	1.830305	1.9422828	1.73783095	0.7830249	-0.3528699	0.07285
ZNF605	1.459641	1.531053	1.40066864	0.7544796	-0.4064461	0.07291
MAPRE2	1.420271	1.4908034	1.36202415	0.7376154	-0.4390593	0.07293
SPIDR	1.377969	1.4471765	1.32081622	0.7174264	-0.4790973	0.07296
SLC44A1	1.438507	1.367783	1.4969114	1.3510993	0.43413372	0.07298
SLITRK4	1.189574	1.1255969	1.24240751	1.9300443	0.94863399	0.07302
TOP1	2.249522	2.3391393	2.17551511	0.8778139	-0.188013	0.0732
PSMD12	1.689316	1.7658209	1.62613748	0.817603	-0.2905275	0.07328
FBXW7	1.460721	1.5318879	1.40195106	0.7557063	-0.4041025	0.07333
CDK18	1.390401	1.3211147	1.44761892	1.3939535	0.47918246	0.07336
AKAP9	3.016513	2.8678384	3.13929022	1.1453294	0.19576258	0.07343
RYK	1.517866	1.4457728	1.57740089	1.2952806	0.37326466	0.07357
NOTCH3	1.473168	1.4020073	1.53193427	1.3231955	0.40402625	0.07357
ALDH9A1	1.535854	1.608417	1.47593109	0.7822449	-0.3543078	0.07364
CSTF3	2.010688	2.0963552	1.93994333	0.8573347	-0.2220696	0.07368
TNIP1	2.050092	2.1348342	1.98011048	0.8636596	-0.2114652	0.07375
KCTD3	1.430266	1.5004939	1.3722707	0.7438066	-0.4270005	0.0738
C20orf96	1.727151	1.8045436	1.66323811	0.8243656	-0.2786437	0.074
SEC11C	2.374484	2.4778575	2.28911708	0.8722878	-0.1971239	0.07401
MRPL40	1.935118	2.0169818	1.86751351	0.8530276	-0.2293357	0.07403
LRRC69	1.26297	1.3290513	1.2083986	0.6333316	-0.6589669	0.07406
LUC7L2	1.859459	1.9400413	1.79291188	0.8434862	-0.2455636	0.07406

TLN2	1.284928	1.2184396	1.33983491	1.5557383	0.63759937	0.07407
FOXM1	1.24903	1.3144073	1.19504052	0.6203435	-0.6888607	0.07411
NDUFA3	4.79466	4.9860845	4.63657886	0.9123186	-0.1323904	0.07419
HDDC3	1.573301	1.646864	1.51255162	0.7923638	-0.3357651	0.07423
KCNK1	1.248606	1.3139815	1.19461767	0.619838	-0.6900368	0.07433
STOM	1.206846	1.1427255	1.25979749	1.8202596	0.86414419	0.07443
PPP1R14C	1.234199	1.1694553	1.28766533	1.6975886	0.76348689	0.07461
TRMT1L	1.342072	1.4098259	1.28612061	0.6981516	-0.5183878	0.07474
PLP1	1.265424	1.3342834	1.20855824	0.6238965	-0.6806214	0.07474
SF3A2	1.742932	1.8203553	1.67899521	0.8276843	-0.2728475	0.07482
GOSR1	1.421201	1.4908908	1.36364977	0.7407957	-0.4328524	0.07483
MECOM	1.221143	1.2857397	1.16779719	0.5872378	-0.7679832	0.07486
PEX2	2.180032	2.2732234	2.1030726	0.8663621	-0.2069579	0.07496
IDH1	1.762025	1.8394821	1.69805889	0.8315352	-0.2661508	0.07497
KNOP1	2.208131	2.3002472	2.13206065	0.8706503	-0.1998347	0.07498
PMEPA1	1.48655	1.4160776	1.544748	1.309246	0.38873621	0.07508
PANK3	1.499235	1.428278	1.55783162	1.3024989	0.38128219	0.07514
WNK2	1.326867	1.2597187	1.38231981	1.4720535	0.55783006	0.07518
C12orf45	1.612979	1.6872574	1.55163859	0.8026667	-0.3171271	0.07524
TPM2	7.765399	8.1469994	7.4502678	0.9025141	-0.1479786	0.07549
PGM2	1.229706	1.2943801	1.17629758	0.5988774	-0.7396674	0.07562
ZNF330	1.715813	1.7924971	1.65248687	0.8233303	-0.2804567	0.07572
ELFN1	1.376197	1.4444443	1.31983633	0.7196319	-0.4746689	0.07582
PTK2B	1.114553	1.053033	1.16535808	3.1180196	1.64063001	0.07582
AAK1	1.743187	1.6668305	1.80624297	1.2090673	0.27389454	0.07602
OSBPL8	2.468509	2.5681361	2.38623482	0.8840016	-0.1778791	0.07606
TTC28	1.639388	1.5650223	1.70080009	1.2403052	0.31069515	0.07634
KIF18A	1.217595	1.2814656	1.16485003	0.5856845	-0.7718043	0.07647
TAGLN2	4.416222	4.662458	4.21287612	0.8772459	-0.1889468	0.07673
MRPS18C	2.229317	2.3300865	2.14610005	0.8616733	-0.2147871	0.07673
RNF2	1.443635	1.5136435	1.38582108	0.7511456	-0.4128355	0.0768
SCFD1	1.78629	1.863938	1.72216608	0.8359003	-0.2585972	0.07703
CADM2	1.606168	1.6817433	1.54375628	0.7975968	-0.3262685	0.07704
TPMT	1.330983	1.3982067	1.27546932	0.6917747	-0.5316258	0.07707
ATG3	1.575514	1.6483596	1.51535711	0.7948631	-0.3312217	0.07724
ME3	1.35891	1.4264685	1.3031195	0.7107664	-0.4925527	0.0773
MIDN	2.092026	2.1845229	2.01563979	0.8574252	-0.2219172	0.07739
LRRC28	1.43787	1.5078427	1.38008475	0.74843	-0.4180607	0.07742
TMEM223	1.610272	1.6835229	1.54977975	0.8043326	-0.3141359	0.07765
RHNO1	1.86661	1.9456477	1.80133938	0.8473974	-0.2388894	0.07795
SMU1	1.446897	1.5165624	1.38936579	0.7537634	-0.4078164	0.07805
ISY1	1.350895	1.4181222	1.29537751	0.7064382	-0.5013648	0.07816
MIR7-3HG	1.084546	1.145043	1.03458574	0.2384516	-2.0682315	0.07817
RAD9A	1.507721	1.43657	1.56647791	1.2975648	0.37580655	0.07824
RP11-736K20.5	1.104299	1.0435522	1.15446409	3.5466405	1.8264531	0.07825

DDR1	2.287704	2.3870192	2.20568854	0.869266	-0.2021305	0.0783
METTL26	3.398405	3.5681375	3.25823682	0.8793286	-0.1855257	0.07833
ARFGAP3	1.56637	1.6385856	1.50673348	0.7935247	-0.333653	0.07834
TSNAX	1.49825	1.5687004	1.44007044	0.7738177	-0.3699343	0.07838
SERGEF	1.699429	1.7749226	1.63708511	0.8221274	-0.282566	0.07845
MAT2B	1.389139	1.4570078	1.33309269	0.7288556	-0.456295	0.07855
HOXB8	1.083488	1.1526845	1.02634397	0.1725386	-2.5350087	0.07869
NCOA4	1.591372	1.6641829	1.53124441	0.7998466	-0.3222048	0.0787
HAUS6	1.473083	1.5429837	1.41535765	0.7649542	-0.3865548	0.07879
RPAP3	1.392245	1.4602296	1.33610165	0.7302912	-0.4534562	0.07881
EBNA1BP2	1.942022	2.0223138	1.87571511	0.8566011	-0.2233046	0.07883
MAL2	1.113643	1.0528671	1.16383196	3.0989372	1.63177353	0.07906
PSMD1	1.67564	1.7502531	1.61402419	0.8184227	-0.2890819	0.07944
AVL9	1.404061	1.4723113	1.34769908	0.7361651	-0.4418987	0.0795
TANC2	1.301075	1.2358549	1.35493575	1.5048906	0.58965861	0.0795
RGS5	1.247183	1.3113051	1.19422957	0.6239203	-0.6805663	0.07954
GINS2	1.900334	1.9863282	1.82931883	0.8408143	-0.2501409	0.07962
SCAMP1-AS1	1.305811	1.3717696	1.25134207	0.6760695	-0.5647566	0.07971
STK32C	1.458375	1.5278234	1.40102342	0.7597681	-0.3963689	0.07988
UBR4	1.366531	1.2995704	1.42182735	1.4081074	0.49375736	0.07988
S1PR3	1.65414	1.7284459	1.59277719	0.8137559	-0.2973319	0.07994
GALT	1.544904	1.6167002	1.48561362	0.7874387	-0.3447604	0.07994
HAGH	1.994899	2.0763098	1.92766903	0.8618978	-0.2144113	0.07996
TTC1	1.65465	1.7286697	1.59352279	0.8145292	-0.2959616	0.07997
CLDN1	2.127397	2.2219967	2.04927574	0.8586568	-0.2198465	0.08
CKAP2L	1.245297	1.3091235	1.1925874	0.6230112	-0.6826701	0.08004
EXOC5	1.68429	1.6095791	1.74598696	1.2237738	0.2913369	0.08026
MFNG	1.098295	1.1590479	1.04812423	0.3025769	-1.7246261	0.08029
KIAA1147	1.247434	1.1832236	1.3004592	1.6398497	0.71356358	0.08031
EDF1	7.086414	7.4065186	6.8220671	0.9087724	-0.1380091	0.08037
CDH7	1.377205	1.4444634	1.32166155	0.7237075	-0.4665213	0.08054
NRBP2	1.209523	1.146563	1.26151641	1.7843273	0.83538031	0.08056
RP11-61K9.3	1.422196	1.4910548	1.36533045	0.7439708	-0.4266821	0.08057
MCM3AP-AS1	1.43649	1.5053086	1.37965819	0.7513393	-0.4124635	0.08077
USP14	1.895316	1.9734947	1.83075502	0.853374	-0.2287499	0.0808
ADGRL1	1.460208	1.3911551	1.5172338	1.322324	0.40307574	0.08082
PACS1	1.348701	1.2823733	1.40347478	1.4288702	0.51487483	0.08091
DPM2	2.208408	2.3129573	2.12207	0.8546127	-0.2266573	0.08103
NTM	1.14852	1.0874368	1.19896387	2.2755154	1.18619336	0.08104
KIF4A	1.198113	1.2606973	1.14642897	0.5616819	-0.8321749	0.0812
KLF7	1.292813	1.3581001	1.23889755	0.667125	-0.5839711	0.08124
THNSL2	1.301823	1.3672128	1.24782335	0.6748767	-0.5673042	0.08136
KCTD20	1.616807	1.6899258	1.55642515	0.8065	-0.3102536	0.08141
PMM1	1.519525	1.5900096	1.46131724	0.781881	-0.3549791	0.08146
NFIC	1.421165	1.3532196	1.47727609	1.3512163	0.43425867	0.08148

UBE2J2	1.732827	1.8077693	1.67093795	0.8306059	-0.2677639	0.08174
ZC4H2	1.288929	1.3539234	1.2352549	0.6647057	-0.5892124	0.08177
CNOT8	1.446824	1.5158217	1.38984408	0.7557729	-0.4039753	0.08187
FAM104B	1.558401	1.6295174	1.49967162	0.7937376	-0.333266	0.08199
PARM1	1.172039	1.1101481	1.22315035	2.0259128	1.01857206	0.08202
RAB5A	1.815315	1.8923077	1.75173325	0.8424597	-0.2473204	0.08205
ALDH18A1	1.358874	1.4255822	1.30378541	0.7138113	-0.4863853	0.0821
HILPDA	6.656417	6.2240887	7.01344012	1.1510984	0.20301116	0.08211
AURKAIP1	3.875694	4.0657533	3.71874059	0.88681	-0.1733031	0.08222
ALDH16A1	1.153154	1.2146467	1.10237287	0.4769367	-1.0681304	0.08225
TBC1D14	1.40556	1.3380001	1.46135284	1.3649488	0.4488468	0.08234
SNRNP27	2.185749	2.282985	2.1054503	0.8616237	-0.2148701	0.08249
ZC3H11A	1.828981	1.75195	1.89259512	1.1870405	0.24736913	0.08251
GSTZ1	1.304305	1.369477	1.25048439	0.6779431	-0.5607638	0.08257
NPRL3	1.647295	1.7207321	1.58664893	0.8139625	-0.2969657	0.08264
STX10	1.783587	1.8598214	1.72063132	0.8381174	-0.2547757	0.08274
SYP	1.228227	1.2915058	1.17597003	0.6036588	-0.7281947	0.08277
C12orf76	2.512187	2.6225345	2.42105979	0.8758272	-0.1912819	0.08287
ALOX5AP	1.093316	1.0342606	1.1420849	4.1471807	2.0521309	0.08292
SPSB3	1.760744	1.8361073	1.69850706	0.8354275	-0.2594134	0.08297
TMEM161A	1.291127	1.3557868	1.23772946	0.6681796	-0.5816922	0.08297
ORMDL2	1.471988	1.5411383	1.41488336	0.7666864	-0.3832915	0.08311
STRADB	2.057487	2.1410201	1.98850353	0.8663331	-0.2070062	0.08329
COQ7	1.390752	1.4579583	1.33525226	0.7320584	-0.4499693	0.08335
ILKAP	1.43694	1.5051425	1.38061786	0.7534862	-0.408347	0.08351
EHD2	1.153127	1.0925001	1.20319297	2.1966796	1.13532443	0.08364
WDR74	1.3543	1.4204031	1.2997101	0.7129112	-0.4882057	0.08382
CHMP4B	2.423896	2.5305707	2.33580163	0.8727474	-0.1963639	0.0839
USP16	1.855341	1.9324982	1.79162318	0.8489273	-0.2362871	0.08395
PRKAA1	1.33375	1.2682817	1.38781531	1.4455524	0.53162092	0.08399
GRK6	1.28478	1.3492786	1.23151608	0.6628407	-0.5932659	0.08403
ANXA7	1.56762	1.6386397	1.5089712	0.7969614	-0.3274182	0.08405
SRR	1.211915	1.2745585	1.16018214	0.5834172	-0.7774001	0.08405
LITAF	2.456475	2.5555225	2.37468002	0.8837417	-0.1783034	0.08406
SPOCK2	1.238195	1.3014224	1.18598103	0.6170113	-0.6966312	0.08421
LAMC2	1.096001	1.0369195	1.14479168	3.9218232	1.9715245	0.08427
GABPB1-AS1	3.679346	3.8290203	3.55574334	0.9034023	-0.1465596	0.08441
ARPC2	6.248985	5.9994939	6.4550194	1.0911143	0.12580227	0.08445
SAMD4A	1.125525	1.0654692	1.17512088	2.6748608	1.41946379	0.08448
LCNL1	1.086281	1.0271059	1.13514824	4.9859434	2.31786651	0.08458
FAM208B	1.30207	1.2371782	1.35565783	1.4995383	0.58451837	0.08461
TOR2A	1.334389	1.3997253	1.28043232	0.7015626	-0.5113562	0.08464
COL27A1	1.310101	1.245464	1.36347969	1.4807863	0.56636344	0.08473
DDX10	1.328017	1.3933933	1.2740277	0.6965743	-0.5216508	0.08492
LRPPRC	1.623908	1.6962039	1.56420411	0.8104006	-0.3032928	0.08525

DCAF11	1.559602	1.6299219	1.50153153	0.7961805	-0.3288325	0.08536
FXN	1.396311	1.4630685	1.34118222	0.7367857	-0.4406831	0.08546
ZNF608	1.805346	1.8835233	1.74078616	0.8384455	-0.2542111	0.08551
RHOF	1.34164	1.276697	1.39527117	1.4285344	0.51453582	0.08577
ZNF664	1.487563	1.4191355	1.54407196	1.2980813	0.37638069	0.08589
NDRG3	1.428262	1.4958324	1.37246166	0.7511845	-0.4127607	0.086
FNBP4	1.897897	1.9769198	1.83263806	0.8523095	-0.2305507	0.08605
TRIM33	1.660681	1.5882203	1.72051995	1.2249151	0.29268179	0.08626
FYTTD1	1.986839	2.0664336	1.92110919	0.8637286	-0.2113501	0.08633
AC090498.1	4.232224	4.412235	4.08356715	0.9036796	-0.1461167	0.08639
NECTIN2	2.280433	2.3664946	2.20936192	0.8850104	-0.1762337	0.08641
FN3KRP	1.369993	1.4359734	1.31550583	0.7236813	-0.4665736	0.08657
FGFRL1	1.140723	1.0805205	1.19043953	2.3651076	1.24190579	0.08659
DALRD3	1.726567	1.8007679	1.66529137	0.8308167	-0.2673978	0.08672
ARSB	1.18799	1.1267176	1.23859057	1.8828522	0.91291973	0.08692
BCAP29	1.431249	1.4982896	1.37588606	0.7543527	-0.4066889	0.08711
RNF10	1.9754	2.0556853	1.90909901	0.8611458	-0.2156705	0.08715
C8orf82	1.508187	1.5770476	1.45132067	0.7821203	-0.3545376	0.08718
TSTD1	2.637827	2.7547301	2.54128576	0.8783606	-0.1871147	0.08731
MPHOSPH8	2.163984	2.2511906	2.09196673	0.8727421	-0.1963727	0.08741
LRRC47	1.473515	1.5414707	1.41739623	0.7708565	-0.3754657	0.08743
PLAC9	1.083871	1.0252913	1.13224738	5.2289589	2.38652373	0.08745
EML2	1.386826	1.4531424	1.33206074	0.7327955	-0.4485174	0.08752
BRI3	2.675325	2.5715231	2.7610465	1.1205985	0.16426951	0.08758
HLA-C	2.560704	2.4396341	2.66068498	1.1535465	0.20607621	0.08759
YAE1D1	1.288916	1.3525354	1.23637896	0.6705113	-0.5766665	0.08764
SURF2	1.441402	1.5095961	1.38508575	0.7556686	-0.4041745	0.08797
BRD3	2.173895	2.2607339	2.10218137	0.8742379	-0.1939022	0.08804
SYT4	1.089048	1.1479019	1.04044572	0.2734631	-1.8705819	0.08807
OSTM1	1.240107	1.1779935	1.2914016	1.6371475	0.71118431	0.08811
DNAJA2	1.610114	1.6810316	1.55154938	0.8098734	-0.3042317	0.08817
MOAP1	1.416675	1.4834973	1.36149145	0.7476598	-0.4195462	0.08826
SPECC1	1.396985	1.3313003	1.45122906	1.3619943	0.44572063	0.08832
POP5	1.773913	1.8494294	1.71155019	0.8376802	-0.2555286	0.08841
GTPBP8	1.283123	1.3464457	1.2308293	0.6662784	-0.585803	0.0885
SSU72	3.7697	3.9183121	3.64697369	0.9070221	-0.1407903	0.08853
C18orf21	1.458603	1.5261441	1.40282555	0.7656182	-0.3853029	0.0886
JUP	1.503083	1.4348656	1.55941848	1.2864168	0.36335821	0.08861
SDCBP	4.436821	4.6454609	4.26452341	0.8955036	-0.1592288	0.08873
COMMD6	12.53081	12.997079	12.1457594	0.9290394	-0.1061882	0.08882
FKBP2	4.407445	4.58899	4.25752235	0.9076432	-0.1398028	0.0889
HIST1H1D	1.601776	1.6805085	1.53675755	0.7887595	-0.3423426	0.08893
RP11-792A8.4	1.706763	1.7799163	1.64635108	0.8287442	-0.2710013	0.08905
NUAK2	1.175741	1.1152228	1.22571737	1.958964	0.97009089	0.08905
AP3D1	2.122172	2.203373	2.05511431	0.8767974	-0.1896846	0.08915

ITSN1	1.72357	1.7972845	1.66269568	0.831191	-0.2667481	0.0892
MXRA8	2.177172	2.2642573	2.10525541	0.874233	-0.1939103	0.08936
TSPAN9	1.191763	1.1309153	1.24201148	1.8486116	0.88644215	0.0895
RNF24	1.594313	1.6645906	1.53627712	0.8069285	-0.3094872	0.08987
EIF2B2	1.384556	1.4501183	1.33041348	0.7340592	-0.4460317	0.08998
ANAPC7	1.266114	1.3288818	1.21428001	0.651541	-0.6180721	0.09001
SMIM19	2.113632	2.1997411	2.04252223	0.868956	-0.202645	0.09002
GOLGA8B	1.659054	1.5877575	1.717931	1.2214748	0.28862409	0.09003
ADCY6	1.191097	1.1303523	1.24126072	1.8508354	0.88817658	0.09003
STARD7	2.264515	2.3629623	2.18321613	0.8681209	-0.2040321	0.09008
SHISA4	1.48899	1.4216182	1.54462634	1.2917525	0.3693297	0.09009
PXDC1	1.126528	1.0676405	1.17515905	2.5895602	1.3727071	0.09029
VAPB	1.470322	1.5377888	1.41460667	0.770947	-0.3752964	0.09036
PRRX1	1.075718	1.0178619	1.12349601	6.9139195	2.7895038	0.09037
PIK3CD-AS2	1.434523	1.5012892	1.37938655	0.7568217	-0.4019746	0.0904
JAM3	1.571883	1.6414164	1.514462	0.8020718	-0.3181968	0.09054
NIT1	1.616482	1.686929	1.5583054	0.8127556	-0.2991065	0.09058
MIR762HG	1.281038	1.3442963	1.22879799	0.6645381	-0.5895763	0.0906
UBE3A	2.296708	2.3853672	2.22349207	0.8831536	-0.1792637	0.09061
SLC50A1	1.637123	1.7084731	1.57820131	0.8161231	-0.2931413	0.09064
EMG1	1.50322	1.5714182	1.44690173	0.7820923	-0.3545893	0.09067
MFSD12	1.270767	1.2081828	1.32245059	1.5488823	0.63122756	0.09078
ISOC2	2.250362	2.3390185	2.17714807	0.8791126	-0.1858801	0.09096
TMEM176A	2.429905	2.2993458	2.53772309	1.1834595	0.2430103	0.09098
DNPEP	1.818477	1.8930395	1.75690227	0.8475574	-0.238617	0.09106
B3GAT3	2.088856	2.1696227	2.02215843	0.8739215	-0.1944244	0.09107
C4orf47	1.350288	1.4148643	1.29695938	0.7157989	-0.4823738	0.09111
LPIN2	1.595777	1.5260951	1.65332172	1.241832	0.31246999	0.09112
NOP10	5.442678	5.6487808	5.27247423	0.9190526	-0.1217806	0.09112
PROSC	1.586723	1.6563235	1.5292453	0.8063787	-0.3104705	0.09124
SPTBN1	2.001108	1.9211274	2.06715738	1.158534	0.21230042	0.09134
FADD	1.462604	1.5295816	1.40729324	0.7690849	-0.3787852	0.09151
CNN1	1.319785	1.2565388	1.37201441	1.4501291	0.53618134	0.09157
KMT5A	1.286114	1.3491038	1.23409688	0.6705653	-0.5765503	0.09169
RALY	3.228408	3.3578856	3.12148297	0.8997396	-0.1524206	0.09171
CDC42EP1	1.298493	1.235772	1.35028897	1.4857107	0.57115322	0.09197
IQGAP1	1.623973	1.5538486	1.68188315	1.2311725	0.30003294	0.09199
LTB	1.080093	1.0225241	1.12763432	5.6665681	2.50247524	0.09219
FAM229B	2.764787	2.8748392	2.67390391	0.8928253	-0.1635501	0.0923
NMB	1.771203	1.6962645	1.83308868	1.1965118	0.2588346	0.09232
LACTB2	1.315434	1.378888	1.26303341	0.6942246	-0.5265255	0.09234
EZH2	1.71871	1.7908467	1.65913766	0.8334582	-0.2628183	0.09271
SEC22A	1.186692	1.2469758	1.13690899	0.5543417	-0.8511525	0.09287
PRKAG2	1.224443	1.1634822	1.27478458	1.6808225	0.74916736	0.09297
SEPT4	1.222334	1.2949144	1.16239644	0.5506562	-0.8607761	0.09311

NORAD	2.990777	2.8789632	3.083115	1.1086513	0.14880568	0.0933
UBE2D2	4.878666	5.0996199	4.6961994	0.9015956	-0.1494476	0.09332
HK1	1.723582	1.7959962	1.66378075	0.8338994	-0.2620548	0.09332
RBMX2	1.397847	1.4630758	1.34398027	0.7428163	-0.4289226	0.09341
PIPOX	1.131975	1.1908696	1.08333954	0.4366308	-1.1955143	0.09354
TMEM2	1.697263	1.6254076	1.75660225	1.2097746	0.27473832	0.0937
DES	1.142845	1.2017052	1.09423795	0.4672062	-1.0978686	0.0939
VMA21	1.923299	2.0013952	1.85880509	0.8576085	-0.2216088	0.0939
NAA15	1.601368	1.670772	1.54405283	0.8110846	-0.3020757	0.09405
HIST2H2BE	1.132464	1.1912223	1.08394014	0.4389662	-1.1878183	0.09419
M6PR	1.782459	1.8554244	1.72220342	0.8442633	-0.244235	0.09426
CYB5R4	1.349694	1.4141485	1.29646705	0.7158472	-0.4822764	0.09429
DDX41	1.620306	1.6906907	1.56218053	0.8139396	-0.2970063	0.09431
RHOQ	1.403831	1.3387896	1.45754386	1.350525	0.43352034	0.09438
RSF1	2.574629	2.6721405	2.49410298	0.8935272	-0.1624165	0.09447
ATP5E	24.80436	25.353195	24.351122	0.9588525	-0.0606192	0.09463
SLC29A4	1.340373	1.2770495	1.39266731	1.4173181	0.50316358	0.09464
C14orf159	1.438699	1.5047755	1.38413195	0.7609957	-0.3940398	0.09492
RBMS3	1.154766	1.0960286	1.20327154	2.1167803	1.08187155	0.09508
SLITRK2	1.157185	1.2162975	1.10836808	0.5010141	-0.9970769	0.0953
TNFRSF1A	2.152879	2.2385775	2.08210791	0.8736699	-0.1948397	0.09548
RP5-940J5.9	1.2588	1.3204497	1.20788922	0.6487422	-0.6242828	0.09574
RBP7	1.141021	1.0829586	1.18896961	2.2778782	1.18769061	0.0958
HIPK3	1.265673	1.20425	1.31639752	1.5490696	0.63140197	0.09582
F3	1.103478	1.0461844	1.15079181	3.2649965	1.70708147	0.09598
C22orf39	2.069432	2.1511668	2.00193435	0.8703642	-0.2003089	0.0961
CCDC59	1.87531	1.9496206	1.81394373	0.8571252	-0.2224222	0.09625
COX7A2	10.67762	11.276829	10.1827911	0.8935432	-0.1623906	0.09658
IPO7	2.439307	2.533845	2.36123666	0.8874669	-0.1722348	0.09684
PTPRE	1.188377	1.1291865	1.23725751	1.83655	0.87699814	0.09689
LUC7L3	4.427223	4.5944967	4.28908542	0.9150336	-0.1281033	0.09726
RPUSD4	1.265323	1.3268907	1.21447919	0.6561189	-0.6079708	0.09732
MTA3	1.608921	1.6779613	1.55190622	0.8140674	-0.2967799	0.09746
SGSM2	1.18857	1.1293773	1.23745271	1.8353502	0.87605538	0.0975
DGKZ	1.312944	1.2505405	1.36447795	1.4547665	0.54078762	0.09763
SYT7	1.642677	1.7122449	1.58522693	0.8216653	-0.2833772	0.09765
PTBP1	2.502358	2.6026357	2.41954758	0.8857581	-0.1750153	0.09783
SLC18B1	1.279316	1.3407229	1.22860549	0.6709425	-0.5757389	0.09787
LRP1	1.569099	1.5013089	1.62508127	1.2468985	0.31834403	0.09788
THUMPD3-AS1	2.484411	2.5935684	2.39426745	0.8749342	-0.1927536	0.09801
CNTFR	1.631633	1.700853	1.57447022	0.8196729	-0.2868798	0.09804
LXN	1.176797	1.2354498	1.12836002	0.5451693	-0.8752237	0.09811
MMAB	1.85867	1.932225	1.79792771	0.855939	-0.2244202	0.09828
UBQLN1	1.724872	1.6540088	1.7833912	1.1978298	0.26042295	0.09829
ANKRD17	1.742845	1.6709994	1.80217621	1.1954947	0.25760771	0.09837

SLC6A6	1.322995	1.260681	1.37445477	1.4364481	0.52250591	0.09838
SNHG12	1.264749	1.2035949	1.31525058	1.5484207	0.63079752	0.09841
VPS13B	1.206587	1.1466837	1.25605662	1.7456374	0.80375388	0.09843
TRA2B	2.749407	2.8545711	2.66256113	0.8964667	-0.1576782	0.09847
RAB29	1.243499	1.1827968	1.29362785	1.6063077	0.68374828	0.09848
QTRT2	1.184009	1.2431896	1.13513708	0.5556861	-0.847658	0.09848
C3orf33	1.18378	1.2428793	1.13497523	0.5557297	-0.8475448	0.09857
STARD10	1.765832	1.6939737	1.82517435	1.1890571	0.24981803	0.09895
COASY	1.667836	1.7380179	1.60987793	0.8263728	-0.2751354	0.099
ABHD14B	1.429371	1.4943121	1.37574189	0.7601309	-0.3956803	0.09913
TNIK	1.337227	1.2749253	1.38867719	1.4137558	0.49953293	0.09924
FBXO3	1.378289	1.44227	1.32545327	0.7358701	-0.4424769	0.09932
BMP7	1.353556	1.2908773	1.40531708	1.3934299	0.47864045	0.09934
CARHSP1	3.224647	3.3449886	3.1252664	0.9063014	-0.1419372	0.09944
PRMT5	1.341991	1.4044385	1.29042071	0.7180838	-0.4777759	0.0995
BLOC1S5	1.321657	1.3840012	1.27017135	0.7035691	-0.5072359	0.09959
PMAIP1	1.580414	1.5131612	1.63595191	1.239283	0.3095057	0.09965
NYNRIN	1.298334	1.2367043	1.34922837	1.4753784	0.56108499	0.09994
TRPT1	2.330107	2.4362653	2.24243926	0.8650486	-0.2091469	0.09994
PAIP2B	1.268589	1.3295466	1.21824839	0.6622686	-0.5945116	0.1
NME4	6.159684	6.4468411	5.9225457	0.9037432	-0.1460152	0.10011
MGAT1	1.616774	1.6851918	1.5602727	0.8176874	-0.2903787	0.10012
CRYM	1.100415	1.1569645	1.05371474	0.3422095	-1.5470482	0.10013
TMEM68	1.542059	1.6096317	1.48625681	0.7976239	-0.3262195	0.10027
C16orf72	1.533459	1.4664948	1.58875916	1.2620915	0.33581655	0.1003
SAYSD1	1.283644	1.3446342	1.23327686	0.6768825	-0.5630227	0.10035
STAU1	2.703842	2.8133789	2.61338442	0.8897117	-0.1685902	0.10042
MRPL52	4.004252	4.148582	3.88506272	0.9163054	-0.1260996	0.10052
ZMAT3	2.277763	2.1901896	2.35008345	1.1343432	0.18185715	0.10052
PPIL4	1.540436	1.6072456	1.48526326	0.7991219	-0.3235125	0.10056
NDUFS1	1.471189	1.5367512	1.41704573	0.7769814	-0.364048	0.10066
TSTA3	1.760091	1.8316577	1.7009901	0.8428829	-0.2465958	0.10072
GSKIP	1.374851	1.4383104	1.32244444	0.7356532	-0.4429022	0.10081
ASNS	1.369368	1.4326675	1.31709374	0.7328808	-0.4483495	0.10093
UTP11	1.640254	1.7094656	1.58309832	0.8218839	-0.2829935	0.10102
SLC35A5	1.35494	1.417791	1.30303608	0.7253293	-0.4632919	0.10124
CUL1	1.679803	1.7491062	1.6225716	0.8310859	-0.2669305	0.10141
ANAPC10	1.419584	1.4836465	1.36668074	0.7581585	-0.3994286	0.10142
THAP3	1.39333	1.456869	1.34085906	0.7460761	-0.4226052	0.10166
HIGD1A	2.672477	2.7869224	2.57796672	0.8830639	-0.1794102	0.10173
LIAS	1.337305	1.3995826	1.28587503	0.7154341	-0.4831091	0.10185
MGME1	1.542301	1.608747	1.48742896	0.8007086	-0.3206508	0.10208
TMEM42	1.755285	1.8271183	1.69596309	0.8414311	-0.249083	0.1022
RRAGB	1.310202	1.3723314	1.25889483	0.6953344	-0.524221	0.10222
RP13-942N8.1	1.565176	1.6327626	1.50936248	0.804982	-0.3129717	0.10225

USF1	1.311061	1.3727162	1.26014488	0.6979704	-0.5187623	0.10227
ILF3-AS1	1.861335	1.9352499	1.80029548	0.8557023	-0.2248191	0.1023
RPE	1.365479	1.427981	1.3138635	0.7333584	-0.4474096	0.1025
TARSL2	1.539243	1.6057387	1.48433018	0.7995694	-0.3227048	0.10267
MAF	1.804616	1.7327432	1.86396982	1.1790895	0.23767329	0.10283
VAV3	1.128276	1.0715969	1.17508336	2.4454048	1.29007331	0.10287
ATP5EP2	1.382441	1.4463965	1.32962591	0.7384151	-0.437496	0.10292
ITGB1BP1	3.641994	3.7838061	3.52488326	0.9069896	-0.140842	0.10294
RNF115	1.731394	1.802257	1.67287466	0.8387271	-0.2537267	0.10307
TFE3	1.457667	1.3932634	1.51085246	1.2990083	0.37741067	0.10309
WNT6	1.070198	1.0148661	1.11589281	7.7957706	2.96269164	0.1035
PPP6R1	1.320833	1.2595917	1.37140689	1.430735	0.51675645	0.10352
UFD1L	1.882677	1.9562332	1.82193396	0.8595539	-0.2183401	0.10374
NTS	1.15631	1.2261757	1.09861374	0.4360049	-1.1975836	0.10383
RACGAP1	1.235181	1.2943507	1.18631741	0.6329775	-0.6597738	0.10408
U2SURP	2.390681	2.4884008	2.30998315	0.8801279	-0.1842148	0.1042
PALMD	1.375702	1.4384256	1.32390458	0.7387903	-0.4367631	0.10426
ASXL2	1.239888	1.1804524	1.28897151	1.6013721	0.67930857	0.10439
GOLGA4	3.174037	3.0553371	3.2720613	1.1054446	0.14462674	0.10446
SSC4D	1.322196	1.261438	1.37237116	1.4243192	0.51027252	0.1045
C1orf123	1.749781	1.8201806	1.69164296	0.8432813	-0.2459142	0.10452
GPX1	8.146334	7.8960199	8.3530471	1.0662741	0.0925783	0.10452
RNF113A	1.322517	1.3839293	1.27180215	0.7079485	-0.4982837	0.10457
CKAP5	1.503572	1.5686882	1.44979758	0.7909389	-0.3383619	0.10469
ACAP1	1.367876	1.3059531	1.41901245	1.3695319	0.45368282	0.1047
HCG11	1.234086	1.293725	1.18483503	0.6292792	-0.6682279	0.10491
DICER1-AS1	1.502227	1.567584	1.44825463	0.7897591	-0.3405155	0.10492
EIF2AK4	2.385717	2.4836353	2.30485354	0.8794975	-0.1852486	0.10497
OS9	1.634806	1.702598	1.57882197	0.8238309	-0.2795799	0.10498
CRNDE	1.602564	1.6702437	1.54667375	0.8156343	-0.2940057	0.105
PPP6C	1.443207	1.5074983	1.39011473	0.7687015	-0.3795046	0.10502
TMA7	16.02746	15.548687	16.4228306	1.060084	0.08417863	0.10515
SRPK1	2.01204	2.088505	1.94889304	0.8717397	-0.1980307	0.10519
AFAP1	1.363384	1.3015143	1.41447759	1.374653	0.45906749	0.10534
PTPRN2	1.376708	1.3142198	1.42831122	1.3630942	0.44688527	0.10538
SEC13	1.751748	1.8217971	1.69390117	0.8443704	-0.2440521	0.10546
PSMD9	1.40712	1.470425	1.35484239	0.7543017	-0.4067865	0.10548
COPS3	1.658379	1.7267773	1.60189411	0.8281686	-0.2720036	0.10551
TMEM87A	1.522657	1.58833	1.46842282	0.7961906	-0.3288142	0.10581
TSKU	1.299405	1.2390843	1.34921875	1.4606512	0.54661173	0.10582
VARS2	1.139161	1.195962	1.09225352	0.4707724	-1.0868983	0.10584
RSRP1	2.596852	2.4913976	2.6839373	1.1291002	0.17517351	0.10595
LAMA1	1.139987	1.0834132	1.18670737	2.2383439	1.16243172	0.10597
SEMA3B	1.281112	1.2210527	1.33070978	1.4960673	0.58117509	0.10607
PLPPR3	1.397915	1.4610928	1.34574094	0.7498293	-0.4153658	0.10613

NAT6	1.283568	1.343955	1.23369949	0.6794478	-0.5575654	0.10646
SLFN5	1.240548	1.1815785	1.28924525	1.5929486	0.6716997	0.10653
TMEM60	1.777815	1.8480979	1.71977353	0.8486915	-0.2366878	0.10653
RNF126	1.71749	1.7873804	1.6597735	0.8379349	-0.2550899	0.1066
FBXL15	1.755458	1.8259312	1.69726073	0.8442117	-0.2443233	0.10678
PTCD3	1.519124	1.5844993	1.46513659	0.7957864	-0.3295468	0.10684
GOLGA6L10	1.274057	1.2140268	1.32363151	1.5121075	0.59656074	0.10691
SH3PXD2A	1.267248	1.2078687	1.31628364	1.5215549	0.6055464	0.10693
METTL13	1.193372	1.251371	1.14547579	0.5787294	-0.7890393	0.10734
TMEM92	1.118373	1.0626794	1.1643657	2.6223238	1.39084584	0.10753
BCL2L13	1.608633	1.5417213	1.6638896	1.2255188	0.29339261	0.1077
RDH11	1.616997	1.5501853	1.67217081	1.221717	0.28891019	0.10779
LINC00662	1.589895	1.6565658	1.53483627	0.8145966	-0.2958423	0.10787
LINC01184	1.600999	1.6680618	1.54561755	0.8167171	-0.2920916	0.10798
RP11-182L21.6	1.459684	1.5236381	1.40687019	0.7770065	-0.3640014	0.10826
AC011043.1	1.58318	1.5166546	1.63811771	1.2350954	0.30462244	0.1083
DDX52	1.63568	1.7030634	1.58003457	0.8250103	-0.277516	0.10832
RFC3	1.332479	1.3933252	1.28223067	0.7175505	-0.4788478	0.10833
RNFT1	1.27378	1.3333388	1.22459546	0.6737754	-0.5696604	0.10842
SERP1	3.59723	3.7387296	3.48037781	0.9056673	-0.1429469	0.10845
ASPHD1	1.134824	1.0787563	1.18112501	2.299815	1.20151782	0.10848
SNRNP35	1.343949	1.4050059	1.29352784	0.7247496	-0.4644454	0.10848
BAZ2B	2.809489	2.9245681	2.71445534	0.890826	-0.1667844	0.10859
OC90	1.078749	1.0237109	1.12420089	5.2381377	2.38905399	0.10865
APP	3.519465	3.3810833	3.63374354	1.1061114	0.14549676	0.10876
PHLDA2	1.541803	1.4568142	1.61198865	1.3396884	0.4218975	0.10884
CDKL3	1.204195	1.2621807	1.15631019	0.5961926	-0.7461497	0.10887
ME2	1.479827	1.5442022	1.42666534	0.7840199	-0.3510378	0.10911
AGTPBP1	1.524649	1.5898516	1.47080438	0.7981743	-0.3252243	0.10914
PECR	1.208515	1.266332	1.16076787	0.603637	-0.7282469	0.10918
PCID2	1.594112	1.6600503	1.53965983	0.8176041	-0.2905257	0.10925
PEAK1	1.30664	1.2467509	1.35609776	1.4431465	0.52921776	0.1093
GCDH	1.228084	1.2865138	1.17983254	0.6276575	-0.6719506	0.10933
TNPO1	2.03897	2.1144119	1.97666832	0.8763979	-0.190342	0.10938
HJURP	1.200526	1.2580769	1.15299977	0.5928456	-0.7542716	0.10944
CASZ1	1.273671	1.3329543	1.22471431	0.6749104	-0.5672322	0.10958
CHST12	1.419739	1.4828551	1.36761684	0.7613398	-0.3933875	0.10964
LECT1	1.111331	1.0558656	1.15713477	2.8127261	1.49196909	0.10967
PIK3R1	1.943234	2.0179929	1.88149735	0.865917	-0.2076993	0.10967
IMPAD1	1.419187	1.3564772	1.47097349	1.3211883	0.40183607	0.10974
RHOG	1.451995	1.3887776	1.50420024	1.296886	0.37505165	0.10979
TMCO1	2.771731	2.873416	2.68775766	0.9008985	-0.1505635	0.10983
FAT3	1.530791	1.4656931	1.58454948	1.2552247	0.3279457	0.10998
ASGR1	1.398409	1.4608163	1.34687127	0.7527322	-0.4097914	0.11021
CDC73	1.455587	1.5188282	1.40336103	0.7774462	-0.3631853	0.11025

ZNF765	1.201853	1.2598506	1.15395731	0.592484	-0.7551519	0.11032
KATNB1	1.398168	1.4603289	1.34683474	0.7534498	-0.4084167	0.11046
CREB3L2	1.372329	1.3108614	1.42308989	1.3610243	0.4446928	0.1105
LPGAT1	1.658628	1.5910116	1.71446651	1.2088875	0.27368	0.11057
SRSF8	2.066591	2.1426987	2.00373914	0.8783936	-0.1870606	0.11065
TMEM69	1.464897	1.5286098	1.41228207	0.7799365	-0.3585715	0.11072
ZNF506	1.260266	1.3195326	1.21132333	0.6613514	-0.596511	0.11096
FAM114A1	1.53205	1.4676263	1.58525277	1.2515394	0.32370376	0.11096
RPAP2	1.465609	1.5289092	1.41333506	0.7814859	-0.3557083	0.11125
LUM	1.117572	1.0577574	1.16696703	2.8908358	1.53148666	0.11126
TARBP1	1.303576	1.2435742	1.35312654	1.4497696	0.53582367	0.11137
PARPBP	1.291086	1.3503836	1.24211736	0.6910066	-0.5332287	0.11141
TUG1	2.02309	1.9405602	2.09124368	1.1602061	0.2143811	0.11164
EIF2S1	1.49024	1.5543093	1.43733076	0.7889652	-0.3419664	0.11171
MFHAS1	1.183491	1.1267413	1.23035577	1.8175268	0.86197662	0.11185
AIDA	1.903486	1.9760353	1.84357267	0.864285	-0.210421	0.11198
POU2F1	1.566609	1.6322128	1.51243246	0.810538	-0.3030483	0.11202
LHX5-AS1	1.073838	1.1343107	1.0238988	0.1779367	-2.4905642	0.1121
FXYD6	3.080511	3.2139878	2.9702833	0.8899251	-0.1682442	0.11213
HSDL2	1.496999	1.5607131	1.44438312	0.792532	-0.3354589	0.11214
PEG10	2.531679	2.4330096	2.61316168	1.1257159	0.17084273	0.11222
TXNRD1	1.569373	1.6349885	1.51518706	0.8113329	-0.3016341	0.11228
SNHG18	2.444095	2.5387082	2.36596154	0.8877326	-0.1718028	0.11238
OLFM2	1.530547	1.466613	1.58334436	1.2501675	0.32212139	0.11244
NEDD9	1.29713	1.2381058	1.34587237	1.4525996	0.53863706	0.11251
DYNC1LI2	1.997345	2.0730183	1.93485321	0.8712369	-0.198863	0.11262
XPO1	2.16938	2.2531675	2.10018726	0.8779251	-0.1878302	0.11282
MPDU1	1.523826	1.5877837	1.47100876	0.80133	-0.3195316	0.11318
CTDNEP1	1.971063	1.8981758	2.03125472	1.1481658	0.19933093	0.1132
TNFRSF1B	1.170162	1.2263786	1.12373796	0.5465975	-0.8714492	0.11325
POGZ	2.259089	2.1755791	2.32805259	1.1297007	0.17594065	0.11329
MAB21L2	1.572687	1.6382376	1.51855462	0.812479	-0.2995976	0.11354
CEP57	2.221574	2.3110372	2.14769393	0.8754092	-0.1919706	0.11361
ZNF90	1.590043	1.6553935	1.53607539	0.8179443	-0.2899255	0.11378
LEFTY1	1.088593	1.1430345	1.04363344	0.3050553	-1.7128572	0.1139
HOXB4	1.123704	1.1873011	1.07118482	0.3800554	-1.3957182	0.11393
PODXL2	1.487394	1.5506243	1.4351765	0.7903329	-0.3394676	0.11412
SOCS2	1.496306	1.433364	1.54828514	1.2651839	0.33934712	0.11425
SENP7	1.388466	1.3270597	1.43917626	1.3428016	0.42524621	0.11426
CRBN	1.62099	1.6868768	1.5665803	0.8248645	-0.2777709	0.11437
PDIA5	1.192699	1.249322	1.14593966	0.5853461	-0.7726382	0.1144
SH3PXD2B	1.219061	1.1619767	1.26620167	1.6434569	0.71673366	0.11445
DDRGK1	2.046384	2.1207125	1.98500313	0.8789079	-0.1862161	0.11458
IGF1R	2.030561	1.9574476	2.09093863	1.1394239	0.18830452	0.11473
DKKL1	1.148336	1.2042279	1.10217888	0.500318	-0.9990828	0.11491

LINC01089	1.638573	1.7051614	1.58358324	0.8275882	-0.2730151	0.11497
CRYBB2	1.097762	1.0293152	1.15428612	5.2629992	2.39588518	0.11499
BCS1L	1.24819	1.3062724	1.20022513	0.6537486	-0.6131921	0.11505
LNPEP	1.507236	1.4436741	1.55972667	1.2615716	0.33522211	0.11511
ATG4B	1.570715	1.6358919	1.51689153	0.8128607	-0.29892	0.11515
COL6A2	1.917944	1.8437934	1.97917906	1.1604488	0.21468291	0.11517
TBC1D5	1.596948	1.6631184	1.54230293	0.8178071	-0.2901674	0.11523
LSM10	2.299874	2.2082292	2.37555657	1.1384897	0.18712125	0.1153
POU4F2	1.07147	1.125523	1.02683144	0.2137572	-2.2259552	0.11535
TCERG1	1.884495	1.9551978	1.82610807	0.8648555	-0.209469	0.11539
PRR11	1.210571	1.2675589	1.16350958	0.6111162	-0.7104814	0.1155
MRPL58	1.329059	1.389202	1.27939178	0.717858	-0.4782296	0.11561
CREBBP	1.448365	1.3855017	1.50027909	1.2977352	0.37599603	0.11587
SERINC3	1.577977	1.6433207	1.52401575	0.8145482	-0.295928	0.11591
GON7	1.402533	1.4633762	1.35228763	0.7602627	-0.3954301	0.11599
RAB10	2.249308	2.3332459	2.17999	0.8850505	-0.1761683	0.11602
EFNA5	3.404668	3.5543647	3.28104578	0.8929993	-0.1632691	0.11615
UBAC1	1.743133	1.8112686	1.68686626	0.846657	-0.2401505	0.11623
EXOSC9	1.315598	1.3749146	1.2666126	0.7111289	-0.4918171	0.11624
PLPP4	1.177703	1.121672	1.22397367	1.8407984	0.88033161	0.11636
MSX2	1.099635	1.0457391	1.14414297	3.1514178	1.65600102	0.11656
KANK4	1.071393	1.0180913	1.11541125	6.3793717	2.67341433	0.11664
ATAD5	1.491777	1.4284397	1.54408226	1.2699157	0.34473273	0.11667
ACSL4	1.575838	1.6404986	1.52243965	0.8156765	-0.293931	0.11674
AUTS2	1.310003	1.2511816	1.3585779	1.4275643	0.51355574	0.11678
ACTR3B	1.684223	1.7510383	1.6290458	0.8375682	-0.2557214	0.11682
BNIP1	1.198643	1.2554513	1.15173057	0.5939706	-0.7515365	0.11683
TMEM177	1.244796	1.302389	1.19723498	0.6522557	-0.6164903	0.11686
TYSND1	1.257829	1.3157457	1.2100013	0.6650964	-0.5883647	0.11711
CCDC151	2.092635	2.1737821	2.02562272	0.8737761	-0.1946644	0.1172
DDIT3	1.549479	1.4793696	1.60737667	1.2670322	0.34145315	0.11727
CRX	1.140872	1.0856981	1.18643544	2.1754919	1.12134167	0.11733
HLA-B	2.69565	2.5780396	2.79277463	1.1360771	0.18406069	0.11733
COG4	1.290917	1.349313	1.24269302	0.6947723	-0.5253878	0.11771
CABP7	1.069968	1.1235065	1.02575481	0.2085301	-2.2616727	0.11771
LINC01420	5.15917	5.3689232	4.98595315	0.9123422	-0.132353	0.11788
SCG5	2.16196	2.0753317	2.23349883	1.1470868	0.1979746	0.11791
MINCR	1.842759	1.9122505	1.78537215	0.8609172	-0.2160537	0.11791
ZC3H7B	1.314917	1.2561062	1.36348467	1.4192732	0.50515232	0.11792
REEP3	1.735074	1.6673677	1.79098675	1.1852339	0.24517174	0.11802
CTC-378H22.2	1.068826	1.1222521	1.02470634	0.2020935	-2.3069055	0.11809
UQCR11	10.13926	10.477139	9.86023811	0.9349064	-0.0971061	0.11813
GLRX	1.513593	1.5769837	1.4612448	0.799407	-0.3229979	0.11823
RAD23A	3.449462	3.5859716	3.33673035	0.903618	-0.1462152	0.11863
KLHL13	1.195328	1.2518227	1.14867463	0.5903941	-0.7602499	0.1187

TMEM187	1.321969	1.3809919	1.27322619	0.7171444	-0.4796645	0.11872
FUS	5.546448	5.7400897	5.38653468	0.9254117	-0.1118327	0.11875
WBP1L	1.303327	1.2448571	1.35161311	1.4359932	0.52204891	0.11881
GEM	1.34384	1.2769141	1.39910931	1.4412749	0.52734553	0.11909
SMIM15	1.974882	2.0466485	1.91561577	0.8748073	-0.1929628	0.1193
C21orf58	1.525732	1.4624152	1.57801962	1.2500014	0.32192966	0.11938
GDF7	1.065997	1.0132655	1.10954308	8.2577602	3.04575052	0.11949
BACE1	1.395859	1.3352717	1.44589248	1.3299438	0.41136528	0.11972
CCDC91	1.476638	1.5390133	1.42512733	0.788714	-0.3424259	0.1198
MMP23B	1.302031	1.2441881	1.34979809	1.4324944	0.5185295	0.12002
THY1	1.147803	1.0932071	1.1928888	2.0694644	1.04925745	0.12003
ZNF677	1.764765	1.8329443	1.70846202	0.8505515	-0.2335296	0.12057
TBL2	1.365762	1.4251145	1.3167474	0.7450873	-0.4245187	0.12078
XAB2	1.252046	1.3092693	1.20479052	0.6621754	-0.5947147	0.12088
NME2	1.203398	1.2596324	1.15695788	0.604539	-0.7260927	0.12091
TAF15	1.747451	1.8146359	1.69196829	0.8494204	-0.2354494	0.12112
FAM107A	1.116615	1.0627913	1.16106375	2.5650639	1.35899475	0.12122
SESN3	3.381602	3.2416388	3.49718601	1.1140002	0.15574944	0.12181
NAA35	1.308347	1.3667395	1.26012588	0.7092934	-0.4955456	0.12181
NFYC	1.500808	1.5631726	1.44930607	0.7978124	-0.3258786	0.12184
TAX1BP3	2.918448	2.8098846	3.0081017	1.1095192	0.14993464	0.12186
ADAMTS9-AS1	1.259874	1.3169898	1.2127076	0.6710235	-0.5755649	0.12204
CSRP1	1.361628	1.302692	1.4102978	1.3554961	0.43882098	0.12205
ZNF830	1.342925	1.4016528	1.29442643	0.7330372	-0.4480417	0.12214
RNF168	1.421375	1.3608616	1.47134861	1.3061755	0.38534873	0.12216
USP39	1.426335	1.4872607	1.37602194	0.7717059	-0.373877	0.1222
IGSF3	1.202858	1.1472875	1.24874937	1.6888699	0.75605821	0.12233
ZNF706	4.608057	4.7781259	4.4676111	0.9178125	-0.1237287	0.12234
IQCB1	1.453891	1.5153164	1.40316552	0.782365	-0.3540863	0.12241
ZFAND5	2.655581	2.5563151	2.73755706	1.1164558	0.15892617	0.1225
DTWD1	2.049724	2.1280862	1.98501112	0.8731701	-0.1956654	0.12257
ZNF397	1.929593	2.0027764	1.86915627	0.8667498	-0.2063124	0.12259
HEMK1	1.407836	1.4681707	1.35801125	0.7647023	-0.3870299	0.12274
SLC35B2	1.644015	1.7090403	1.59031576	0.832556	-0.2643807	0.12276
C6orf48	5.368718	5.5645249	5.20701688	0.9216768	-0.1176671	0.12278
ALG8	1.5891	1.6532259	1.53614447	0.8207643	-0.2849601	0.12279
TNKS1BP1	1.21823	1.1623503	1.26437661	1.6284329	0.70348429	0.12285
TTC32	1.256965	1.3139896	1.20987348	0.668409	-0.581197	0.12288
CD151	4.344201	4.5101926	4.20712332	0.9136602	-0.1302703	0.12299
PHLDB2	1.107345	1.0541792	1.15125	2.7916601	1.48112329	0.12299
PLEKHB2	1.575189	1.6391933	1.52233342	0.817176	-0.2912813	0.12299
GOLGA7	2.402239	2.486928	2.33230168	0.8960095	-0.158414	0.12301
EWSR1	2.07036	2.1539383	2.00134021	0.8677589	-0.2046338	0.12304
DPAGT1	1.307823	1.3660391	1.2597473	0.7096162	-0.4948892	0.12311
MGMT	1.790144	1.857316	1.73467276	0.8569452	-0.2227252	0.12325

AGK	1.213421	1.2695068	1.16710442	0.6200378	-0.689572	0.12331
SRD5A1	1.452113	1.5132369	1.4016357	0.7825543	-0.3537372	0.12338
PMPCA	1.452078	1.5131624	1.40163302	0.7826627	-0.3535374	0.12344
AC009005.2	1.341613	1.4003951	1.29306885	0.7319491	-0.4501848	0.1235
NOLC1	1.419287	1.4792516	1.36976722	0.7715513	-0.374166	0.12375
GUF1	1.383263	1.4427291	1.33415499	0.7547617	-0.4059068	0.12379
HIC2	1.249573	1.1928913	1.29638124	1.5365191	0.61966567	0.12383
SELENOH	7.73354	8.0146891	7.50136209	0.9268211	-0.1096371	0.12387
BAG4	1.313408	1.3714413	1.26548295	0.7147372	-0.4845152	0.12391
MRPS18A	1.618928	1.6829893	1.5660256	0.8287474	-0.2709957	0.12393
TMTC4	1.667043	1.7324054	1.61306498	0.8370569	-0.2566024	0.12395
F2R	1.643306	1.5789322	1.6964665	1.2030191	0.26665959	0.124
PRMT9	1.334878	1.2763782	1.38318796	1.3864623	0.47140842	0.12436
SMIM10	1.259473	1.3162227	1.21260736	0.6723342	-0.5727495	0.12439
PPAT	1.34806	1.4064273	1.29985895	0.7377923	-0.4387134	0.12444
MLST8	2.193617	2.269367	2.1310617	0.8910439	-0.1664316	0.1245
NUPL2	1.304073	1.3617309	1.25645755	0.7089733	-0.4961968	0.12455
RNF207	1.27625	1.2195442	1.3230795	1.4715923	0.557378	0.12458
GCA	1.607895	1.6714784	1.55538664	0.8271102	-0.2738484	0.12464
TERF1	1.97049	2.0406702	1.91253484	0.8768723	-0.1895613	0.12468
SLC35A1	1.242529	1.2987425	1.19610764	0.6564437	-0.6072568	0.1247
KANK2	1.198866	1.1436275	1.24448283	1.7022004	0.76740092	0.12472
RAB3B	1.27602	1.2195721	1.3226351	1.4693808	0.55520836	0.12495
SF3B4	1.434832	1.4951792	1.38499626	0.7774887	-0.3631063	0.12512
FAM210B	2.381963	2.4714128	2.30809398	0.8890055	-0.1697358	0.12513
STRA6	1.9778	2.0522816	1.91629143	0.8707664	-0.1996424	0.12604
UNC119	2.460812	2.5545162	2.38343013	0.8899426	-0.1682158	0.1262
CTCF	1.624091	1.6877116	1.57155112	0.8310912	-0.2669212	0.12638
CDKAL1	1.337921	1.3962534	1.28974996	0.7312238	-0.451615	0.12648
ECHDC2	1.301982	1.244542	1.34941668	1.4288615	0.51486606	0.12649
SFRP5	1.068719	1.0166975	1.11168001	6.6884094	2.74166316	0.12657
NUB1	1.426064	1.486325	1.37630008	0.7737625	-0.3700372	0.12661
ZC3H4	1.320184	1.3783598	1.27214222	0.7192683	-0.4753982	0.12691
HNRNPL	1.625406	1.6894385	1.57252717	0.8304252	-0.2680778	0.12735
POLR2K	2.887234	2.9866223	2.80515664	0.9086562	-0.1381936	0.12748
DPP3	1.400819	1.4603101	1.35169	0.7640284	-0.3883018	0.12763
ZNF672	1.466096	1.5270947	1.4157231	0.7887067	-0.3424393	0.12763
ZNF451	1.609959	1.5469243	1.66201358	1.21043	0.27551969	0.12809
FZD7	1.413526	1.353873	1.46278759	1.3077787	0.38711846	0.12811
B4GALT3	1.491586	1.5529731	1.44089128	0.7973105	-0.3267864	0.12811
TMEM246	1.615446	1.6789199	1.56302858	0.8293004	-0.2700333	0.12819
SELENON	1.515102	1.4537873	1.56573709	1.2467011	0.31811556	0.12833
PAQR4	1.318206	1.2606112	1.36576834	1.4035019	0.48903105	0.12853
ZMAT1	1.399453	1.3403239	1.44828253	1.3172233	0.39749998	0.12854
LRRFIP1	1.673087	1.6089135	1.7260832	1.1924242	0.25389752	0.12866

FIBP	2.428617	2.5117494	2.35996412	0.8995963	-0.1526504	0.12893
BET1	1.855613	1.9265557	1.79702697	0.8602041	-0.2172492	0.1292
C7orf73	4.469687	4.6417666	4.32758162	0.9137273	-0.1301644	0.12924
INO80C	1.554301	1.6168441	1.50265106	0.8148754	-0.2953486	0.12934
MFSD14A	1.611415	1.6749911	1.55891232	0.8280292	-0.2722464	0.12944
IGF2BP2	3.471828	3.6193532	3.34999983	0.897168	-0.15655	0.12956
RBM42	1.465527	1.5255999	1.41591728	0.7913192	-0.3376683	0.12958
ANKFY1	1.317078	1.2597651	1.36440718	1.4028337	0.48834397	0.12959
TTLL7	1.58604	1.5235543	1.63764144	1.2179089	0.28440625	0.12964
ATXN1	1.256698	1.2010188	1.30267964	1.5057281	0.59046126	0.12998
LPAR6	1.431797	1.4912194	1.38272423	0.7791309	-0.3600624	0.13014
NPS	1.143294	1.2100415	1.08817268	0.419787	-1.2522706	0.13028
NPHP3	1.319819	1.2626645	1.36701764	1.397287	0.48262839	0.1304
ZNF83	1.556258	1.4943522	1.60738171	1.2286417	0.29706425	0.13049
SNHG8	3.195954	3.3164874	3.09641506	0.9049974	-0.1440144	0.13076
NCOA1	1.288058	1.2315673	1.33470854	1.4454048	0.53147356	0.13083
SLC8A1	1.235646	1.1805751	1.28112454	1.5568287	0.63861021	0.13084
FEZF2	1.134458	1.1875197	1.09063847	0.4833543	-1.0488469	0.13102
TCIRG1	1.153488	1.1000613	1.19760927	1.9748813	0.98176595	0.13103
BNC2	1.138417	1.0854496	1.1821591	2.131773	1.09205383	0.13115
DEAF1	2.099681	2.1843861	2.0297302	0.869421	-0.2018731	0.13118
SNAI2	1.118487	1.0658502	1.16195476	2.4594424	1.29833129	0.13126
PPIF	1.463443	1.5233347	1.41398275	0.7910478	-0.3381632	0.13127
ITPKB	1.151304	1.0980899	1.19524867	1.9905079	0.99313661	0.1314
PEX11A	1.255881	1.3116097	1.20986027	0.6734717	-0.5703108	0.13142
PBX3	1.314232	1.2576751	1.3609368	1.4007437	0.48619302	0.13172
OXLD1	1.994849	1.9232083	2.05401028	1.141682	0.19116088	0.13203
MRE11	1.370503	1.4285983	1.32252616	0.7525138	-0.41021	0.13227
TMEM41A	1.421479	1.4805286	1.37271559	0.7756367	-0.3665471	0.13241
CCDC6	1.618711	1.5559741	1.6705208	1.2060287	0.2702643	0.13243
NOTCH2	1.432318	1.3730418	1.4812685	1.2901196	0.36750485	0.13245
EIF2AK1	2.413818	2.5019723	2.34101918	0.8928388	-0.1635283	0.13259
ZNF32	2.206091	2.2806929	2.14448381	0.8936442	-0.1622276	0.13276
FOXK1	1.44262	1.38318	1.49170605	1.2832247	0.35977381	0.13283
DHRS1	1.527636	1.5889215	1.47702501	0.8099976	-0.3040105	0.13287
MOK	1.266245	1.3218402	1.22033349	0.6846052	-0.5466558	0.13313
SH3RF1	1.377429	1.435625	1.32936943	0.7560848	-0.40338	0.13315
JARID2	1.436501	1.3772754	1.485411	1.2866223	0.36358865	0.1332
AP3M2	1.399811	1.4580444	1.3517218	0.7678771	-0.3810528	0.13323
COA4	2.584357	2.6746588	2.50978426	0.9015474	-0.1495248	0.13338
DIABLO	1.215342	1.2699091	1.1702789	0.630875	-0.664574	0.13342
UBL7-AS1	1.391089	1.4494628	1.34288204	0.7628707	-0.3904895	0.13342
POR	1.327814	1.3845865	1.28093077	0.7304748	-0.4530936	0.1337
KLHL5	1.367069	1.3096627	1.4144759	1.3384752	0.42059038	0.1339
PHLDA1	2.041155	1.9545604	2.11266604	1.1656319	0.22111227	0.13395

SIPA1L2	1.174776	1.1215238	1.21875199	1.8000752	0.84805718	0.13403
PARVA	1.613591	1.6764818	1.56165472	0.8302584	-0.2683677	0.13408
NUDT21	1.742034	1.8065432	1.6887614	0.8539671	-0.2277476	0.13429
R3HDM4	1.346607	1.4039631	1.29924078	0.7407626	-0.4329168	0.13451
ARHGEF39	1.152013	1.2048568	1.10837299	0.5290183	-0.9186104	0.13463
GORASP2	1.629995	1.6927343	1.57818399	0.8346403	-0.2607735	0.13486
CTTNBP2NL	1.479987	1.4202164	1.52934661	1.2597	0.33308015	0.13487
GNAI1	1.328633	1.3853467	1.28179837	0.7312852	-0.4514939	0.13488
TMEM191B	1.143216	1.0907117	1.18657472	2.0567888	1.04039363	0.13506
TSPAN7	1.102681	1.1545675	1.05983172	0.3870913	-1.369254	0.13509
MVB12A	2.176907	2.2638436	2.10511256	0.8744061	-0.1936246	0.1353
DAG1	1.411449	1.3532023	1.4595506	1.3010974	0.37972899	0.13533
CDCP1	1.078924	1.028036	1.12094857	4.3140415	2.10904004	0.13541
ERCC4	1.265598	1.3209776	1.21986457	0.6849842	-0.5458575	0.13545
HAUS4	1.212879	1.2670879	1.16811302	0.6294296	-0.667883	0.13558
C19orf24	2.077621	2.0071212	2.13583998	1.1278086	0.17352228	0.13563
MRC2	1.327128	1.2705602	1.37384289	1.3817362	0.46648222	0.13564
ARL1	1.957908	2.0257532	1.90188015	0.879237	-0.185676	0.13571
MAPKAPK3	1.880673	1.9511965	1.82243371	0.8646307	-0.209844	0.13578
PEX13	1.508649	1.5696077	1.45830773	0.8046024	-0.3136521	0.13582
ZNF536	1.210012	1.1561496	1.25449338	1.6298051	0.70469949	0.13582
PLAUR	1.298083	1.2421445	1.34427757	1.4217858	0.50770411	0.13615
CHORDC1	1.565842	1.6272676	1.51511522	0.8212049	-0.2841858	0.13664
CHID1	2.70798	2.8076125	2.62570185	0.8993641	-0.1530228	0.13667
QSER1	1.631852	1.6947991	1.5798698	0.8345862	-0.260867	0.13668
CCDC117	1.364808	1.4221244	1.31747523	0.7520893	-0.4110242	0.13673
TP53INP1	1.311711	1.256037	1.3576868	1.397012	0.48234441	0.13696
ZDHHC3	1.556712	1.4963678	1.60654448	1.2219657	0.28920383	0.13734
WDR77	1.394815	1.4527834	1.34694386	0.7662469	-0.3841187	0.13747
GNG8	1.100202	1.1541623	1.05563988	0.3609175	-1.4702589	0.1376
PEMT	2.160253	2.2321587	2.10087229	0.8934501	-0.162541	0.13773
CAP1	2.487019	2.5772588	2.41249676	0.895539	-0.1591719	0.13807
RAB28	1.497	1.5568731	1.44755494	0.8036928	-0.3152839	0.13816
ZPR1	1.368116	1.4249402	1.32118966	0.7558467	-0.4038345	0.13827
SESN1	1.327023	1.3833472	1.28050924	0.7317369	-0.4506032	0.13836
FAM129A	1.112357	1.0609146	1.1548386	2.5418973	1.34590575	0.13841
AFF1	1.2518	1.197316	1.29679358	1.5041534	0.58895173	0.13847
DCP1A	1.536944	1.5974232	1.48699846	0.8151649	-0.2948362	0.13863
LNP1	1.188561	1.2416554	1.14471411	0.5988451	-0.7397453	0.13864
NKAIN3	1.197426	1.2509238	1.15324645	0.6107291	-0.7113954	0.13868
NT5DC2	1.920665	1.9881032	1.86497344	0.8753877	-0.192006	0.1387
ZBTB41	1.524848	1.5852071	1.47500302	0.8116836	-0.3010106	0.13878
SS18L2	1.886207	1.953278	1.83081855	0.8715386	-0.1983635	0.13894
GPR108	1.573839	1.6351654	1.52319451	0.8237137	-0.279785	0.13904
C11orf71	1.367642	1.4248933	1.32036368	0.7539862	-0.4073901	0.13951

LENG1	1.252295	1.3068315	1.20725723	0.6754757	-0.5660243	0.13978
UGDH	1.643782	1.7060533	1.59235775	0.8389703	-0.2533083	0.13998
SFXN1	2.349156	2.4349236	2.27832703	0.8908676	-0.166717	0.14004
LRCH2	1.301223	1.2463541	1.34653556	1.4066566	0.49227015	0.14009
RP11-146F11.1	1.335008	1.3913741	1.28846088	0.7370465	-0.4401724	0.1401
FAM234A	1.891915	1.9583771	1.83703004	0.8733827	-0.1953141	0.14018
CCKBR	1.223881	1.2775768	1.1795374	0.6468025	-0.6286028	0.14019
TMEM54	1.103139	1.0521849	1.14521728	2.7827437	1.47650806	0.14042
TAMM41	1.173936	1.2266419	1.13041007	0.5754014	-0.7973593	0.14045
RRM1	1.61696	1.678279	1.56632156	0.834939	-0.2602573	0.1406
APLP1	2.28167	2.1908783	2.35664705	1.1391987	0.18801944	0.14114
MFAP4	1.329297	1.384959	1.28333111	0.7360034	-0.4422156	0.14126
TNFAIP2	1.100238	1.0495065	1.14213377	2.8710126	1.52155967	0.1413
BYSL	1.196621	1.2496904	1.15279605	0.611942	-0.7085332	0.14137
CENPX	3.038699	3.1544071	2.94314592	0.90194	-0.1488966	0.14164
TUBB6	1.997393	2.0717857	1.93595846	0.8732702	-0.1955001	0.14178
C5orf24	2.668095	2.7552854	2.59609123	0.9093058	-0.1371625	0.14186
TFB1M	1.27113	1.3257917	1.22598858	0.6936596	-0.5277001	0.1419
PCYOX1	1.793162	1.7286471	1.84644032	1.1616601	0.21618802	0.1422
MAP2K6	1.214638	1.2678832	1.17066722	0.6370957	-0.650418	0.14222
POLR3D	1.465942	1.5244044	1.41766293	0.796452	-0.3283407	0.14235
YY1	2.699127	2.7963324	2.61885283	0.9011989	-0.1500825	0.14244
RNF217	1.386052	1.3296424	1.43263635	1.3124413	0.39225293	0.14269
ST3GAL1	1.112333	1.0615083	1.15430524	2.50869	1.32693418	0.14277
FNDC10	1.194506	1.1417048	1.23811069	1.6803293	0.74874397	0.14279
DAZAP1	1.683518	1.7460413	1.63188492	0.8469838	-0.2395936	0.14293
IARS	1.616011	1.6773135	1.56538643	0.8347485	-0.2605865	0.1431
CNTROB	1.21215	1.2657765	1.16786471	0.631601	-0.6629145	0.14322
EVI5	1.587722	1.5269995	1.6378685	1.2103779	0.27545753	0.14329
KCNMA1	1.300568	1.2455416	1.34601054	1.4091729	0.49484865	0.14372
NECAB2	1.231946	1.285425	1.18778219	0.6579038	-0.6040514	0.14383
TP53RK	1.443487	1.5011935	1.39583184	0.7897785	-0.34048	0.14408
POLR2J3	4.079815	3.9169616	4.21430177	1.1019349	0.14003901	0.14472
NOC3L	1.380084	1.4366267	1.33339073	0.7635602	-0.3891862	0.14479
GLI3	1.37036	1.3142622	1.41668599	1.3259183	0.40699193	0.14488
SEC31A	1.884494	1.8156157	1.94137529	1.1541898	0.20688046	0.14508
PPIL3	1.707065	1.7699142	1.65516305	0.8509559	-0.2328437	0.14529
ABCB7	1.288524	1.3433085	1.24328293	0.7086424	-0.4968703	0.14536
PCDHB2	1.32235	1.3776952	1.27664568	0.7324574	-0.4491831	0.14567
MAP1A	1.563417	1.6234411	1.51384755	0.8242119	-0.2789128	0.14568
ING3	1.331407	1.3866702	1.28576908	0.7390511	-0.4362539	0.14574
GSTM4	1.448838	1.506303	1.4013817	0.7927697	-0.3350263	0.14584
LLPH	1.680806	1.7427715	1.62963391	0.8476819	-0.2384051	0.14585
NRL	1.134409	1.0834692	1.17647601	2.114264	1.08015552	0.14586
JAZF1	1.143342	1.092038	1.18571057	2.0177603	1.01275477	0.14587

DDX39B	1.434439	1.3767805	1.48205425	1.2794033	0.3554711	0.14589
KPNA4	1.925111	1.995767	1.86676263	0.8704473	-0.2001712	0.14597
HBEGF	1.082526	1.03285	1.12354935	3.7610181	1.91112326	0.14607
RWDD2A	1.172596	1.2245511	1.12969146	0.5775587	-0.7919604	0.14607
KPNB1	4.024924	4.1698052	3.90527819	0.9165479	-0.1257179	0.14621
RCOR2	1.309121	1.3638523	1.26392305	0.7253576	-0.4632356	0.14643
KMT5C	1.557438	1.6176293	1.50773021	0.8220631	-0.282679	0.14649
PLAG1	1.581715	1.5218647	1.63113997	1.209394	0.27428428	0.14653
SIAH1	1.465874	1.5235803	1.41821871	0.7987671	-0.3241532	0.14661
CEP83	1.388406	1.4447064	1.34191259	0.7688501	-0.3792258	0.14676
GOLPH3L	1.266051	1.3196439	1.22179224	0.6938729	-0.5272567	0.14717
INPP1	1.28691	1.2328889	1.33152178	1.4235192	0.50946195	0.14756
CXCR4	2.007123	2.1655103	1.87632504	0.751881	-0.4114238	0.14772
IRAK1BP1	1.20839	1.2607963	1.16511199	0.633107	-0.6594787	0.14777
AMFR	1.343004	1.3989493	1.29680327	0.7439625	-0.4266983	0.14798
ERGIC2	2.10411	2.1728127	2.04737486	0.8930453	-0.1631947	0.14889
ERRFI1	1.296532	1.2428401	1.34087097	1.4036848	0.48921901	0.14912
ZSCAN1	1.243956	1.2971856	1.19999841	0.6729748	-0.5713756	0.14939
NOL10	1.245323	1.2982985	1.20157587	0.6757523	-0.5654336	0.14964
UQCRHL	1.268088	1.3218094	1.22372411	0.695207	-0.5244855	0.14982
CENPS	1.360727	1.4160364	1.31505074	0.7572672	-0.4011256	0.14987
GATC	1.349928	1.4054204	1.30410074	0.7500874	-0.4148694	0.14987
MTPN	2.504316	2.586262	2.43664435	0.9056791	-0.1429281	0.14993
NR2F1	5.499608	5.7281406	5.31088283	0.9117501	-0.1332896	0.14994
NBN	1.303728	1.3577127	1.25914688	0.7244554	-0.4650312	0.15004
PISD	1.293874	1.2400218	1.33834635	1.4096482	0.49533517	0.15029
TCF7L2	1.599398	1.5396431	1.64874378	1.2021719	0.26564317	0.15037
SLC6A8	1.279353	1.2259558	1.32344945	1.431472	0.51749942	0.15061
HSCB	1.727252	1.789332	1.6759845	0.8564007	-0.2236421	0.15096
PHTF1	1.832983	1.8969045	1.7801961	0.8698764	-0.2011177	0.15116
CPSF4	1.415184	1.4713868	1.3687717	0.7823124	-0.3541833	0.15131
CSRNP1	1.129455	1.0792657	1.17090265	2.1560725	1.10840568	0.15143
COMMD2	1.6535	1.7143946	1.60321168	0.8443676	-0.2440569	0.15148
U2AF2	1.599892	1.659098	1.550999	0.8359895	-0.2584432	0.15224
ZMAT5	1.656972	1.7177237	1.60680151	0.8454528	-0.2422039	0.15224
RP11-486G15.2	1.572879	1.6322352	1.52386235	0.8285878	-0.2712735	0.15241
RP11-115C21.2	1.479322	1.5367958	1.43185918	0.804513	-0.3138124	0.15263
CENPC	1.434551	1.4907292	1.38815812	0.7909823	-0.3382826	0.15263
CANX	4.92489	5.1053524	4.77586079	0.919741	-0.1207005	0.15265
CNPY4	1.51231	1.5701714	1.46452669	0.8147141	-0.2956342	0.15285
GPR180	1.279679	1.2267583	1.32338187	1.4261084	0.51208362	0.1531
VHL	1.299524	1.3536045	1.2548631	0.7207575	-0.4724141	0.15326
RNF34	1.377365	1.4327211	1.33165134	0.7664322	-0.38377	0.1533
BBS12	1.169417	1.220596	1.12715311	0.5764073	-0.7948396	0.15337
GOLT1B	1.335198	1.2808362	1.38009005	1.3534223	0.43661208	0.15342

C1orf53	1.354897	1.4096431	1.3096861	0.7559901	-0.4035607	0.15343
ECE2	1.197058	1.2484532	1.1546153	0.6223115	-0.6842912	0.15386
CAB39	1.350728	1.2964995	1.3955111	1.333935	0.41568834	0.15395
MIA3	1.503578	1.5613408	1.45587718	0.812122	-0.3002317	0.15409
RRP1	1.285524	1.3389751	1.24138335	0.7120975	-0.4898533	0.1542
SRSF10	3.212603	3.3199133	3.12398413	0.9155446	-0.1272979	0.15435
PPP1R14B	2.11212	2.0398248	2.17182268	1.1269424	0.17241376	0.15461
LRP10	1.709925	1.6493821	1.7599231	1.1702249	0.2267858	0.15482
PPFIA3	1.446602	1.5032313	1.39983643	0.7945381	-0.3318117	0.15485
COX20	3.436304	3.5766054	3.32044092	0.9005806	-0.1510727	0.15488
RGS3	1.496822	1.4397112	1.54398563	1.2371429	0.30701218	0.15511
RSPO3	1.078999	1.0305009	1.11904878	3.9031262	1.96463012	0.15517
PSENEN	2.56593	2.6600992	2.48816326	0.8964303	-0.1577366	0.15523
VPS39	1.177427	1.1266987	1.21931848	1.7310242	0.79162587	0.15524
TRAM1L1	1.147863	1.1981862	1.10630458	0.5363875	-0.8986524	0.15525
CSNK1G2	1.335456	1.2812872	1.38018903	1.3516042	0.43467276	0.15554
RAB18	1.846507	1.9099443	1.79411917	0.8727119	-0.1964227	0.1557
ADAM19	1.09995	1.0510585	1.14032598	2.7483399	1.45856042	0.15574
SYNE1	1.350958	1.2965683	1.39587408	1.3348495	0.4166771	0.15578
BCL10	1.288486	1.3416476	1.24458344	0.7158939	-0.4821823	0.15585
PHF5A	1.862835	1.9264528	1.81029929	0.8746256	-0.1932626	0.1559
PIGC	1.63061	1.6909673	1.58076577	0.8405113	-0.250661	0.15598
MAP3K7	1.677961	1.7388486	1.62767915	0.8495369	-0.2352514	0.15616
B4GALT1	1.382764	1.32834	1.42770792	1.3026373	0.38143546	0.15616
SUMO3	3.168321	3.2963611	3.06258371	0.8981966	-0.1548968	0.15628
NEK1	1.473533	1.5305977	1.42640886	0.8036387	-0.3153811	0.15632
FOXJ3	1.334369	1.2803845	1.37895075	1.3515397	0.43460385	0.15634
CD200	1.488615	1.5463774	1.44091437	0.8069776	-0.3093994	0.15638
MPPE1	1.231899	1.1801715	1.27461714	1.5241982	0.60805052	0.15643
HS3ST1	1.0904	1.0417625	1.13056642	3.126403	1.64450377	0.15656
SMYD3	1.524111	1.5817605	1.476504	0.8190725	-0.287937	0.15667
DUSP3	1.393753	1.3389493	1.43901006	1.2952086	0.37318451	0.15697
BECN1	1.421709	1.4777155	1.37545822	0.7859452	-0.3474994	0.15702
NUP35	1.165976	1.2165872	1.12418066	0.5733517	-0.8025078	0.15715
SVIP	2.40086	2.3147383	2.47198122	1.1196002	0.16298364	0.15719
PIK3C2A	1.546759	1.4891444	1.59433833	1.215057	0.28102401	0.1572
TBPL1	1.670801	1.7314576	1.62070933	0.8485923	-0.2368565	0.15763
TCEAL1	2.156233	2.2245007	2.09985675	0.8982083	-0.154878	0.15764
MDP1	1.24803	1.3001822	1.20496127	0.6827895	-0.5504873	0.15774
MIR124-2HG	1.296216	1.3492298	1.25243647	0.7228377	-0.4682564	0.15776
ITGA5	1.149786	1.0997989	1.19106695	1.9145195	0.93698238	0.15788
NRM	1.371276	1.4253234	1.32664347	0.7679885	-0.3808435	0.158
SLC9A7	1.220602	1.1692943	1.26297349	1.5533509	0.6353838	0.15809
PLEKHG3	1.136856	1.0874572	1.17765119	2.0312921	1.02239768	0.15809
CDC42EP3	1.80594	1.7441251	1.85698845	1.1516725	0.20373049	0.15811

ZNF3	1.541322	1.5994615	1.49330869	0.8229197	-0.2811764	0.15814
FANCF	1.376139	1.4305983	1.3311663	0.7690841	-0.3787867	0.1583
UBL3	1.695982	1.6362212	1.74533259	1.1714991	0.22835585	0.15852
CCDC14	2.457452	2.3733854	2.52687612	1.1117608	0.15284648	0.15855
SENP6	1.979746	2.0447947	1.92602693	0.8863243	-0.1740934	0.15878
WWC2	1.302613	1.249634	1.34636366	1.3874858	0.47247297	0.15879
HLA-DPB1	1.574178	1.632888	1.52569517	0.8306291	-0.2677237	0.15884
RPP38	1.39074	1.4458479	1.34523098	0.7743245	-0.3689898	0.1589
PGRMC2	1.815407	1.877483	1.76414423	0.8708365	-0.1995262	0.15912
ZNF444	1.579282	1.6376215	1.53110476	0.8329467	-0.2637039	0.15934
CRLS1	1.748184	1.8096685	1.6974083	0.8613504	-0.2153278	0.15958
BOLA1	1.500229	1.5572545	1.45313665	0.8131593	-0.2983901	0.15958
MECR	1.379309	1.4336746	1.33441243	0.7711138	-0.3749844	0.15963
ARPC1A	3.095397	3.1923789	3.01530819	0.9192335	-0.1214966	0.15969
FUOM	1.58775	1.6457086	1.53988744	0.8361162	-0.2582246	0.15977
IGF2R	1.279995	1.2277184	1.32316588	1.419147	0.50502408	0.15993
XPO6	1.253351	1.2015349	1.29614074	1.4694262	0.55525295	0.16017
COQ9	1.699853	1.7606578	1.64963887	0.8540488	-0.2276096	0.16019
RAPGEF1	1.154203	1.104561	1.19519851	1.8668384	0.90059702	0.16041
SYMPK	2.051924	2.1189148	1.99660214	0.8906864	-0.1670106	0.16044
TJP2	1.198146	1.1477487	1.23976526	1.6227913	0.69847748	0.16054
HEXA	1.689073	1.6287865	1.73885886	1.1750553	0.23272863	0.16064
HECTD1	1.995512	2.0629428	1.93982565	0.8841733	-0.1775989	0.16081
FIP1L1	1.612235	1.6703682	1.56422674	0.8416669	-0.2486787	0.16087
ROCK1	2.008063	2.0734041	1.95410398	0.8888581	-0.1699749	0.16091
MYO1E	1.221296	1.1706092	1.26315392	1.5424367	0.62521131	0.16109
GCAT	1.346631	1.3999809	1.30257292	0.7564685	-0.4026481	0.16122
CCDC53	1.55346	1.6109513	1.50598267	0.8281882	-0.2719695	0.16123
MAEA	1.490382	1.5463444	1.4441669	0.8129797	-0.2987087	0.16135
KLHL9	1.510541	1.5670074	1.46390923	0.8181714	-0.289525	0.16136
COLGALT1	1.292212	1.2401234	1.33522796	1.3960651	0.48136618	0.16167
YLPM1	1.390532	1.3365265	1.43513047	1.293005	0.37072786	0.16213
ZCCHC12	1.170901	1.2206988	1.1297766	0.588026	-0.7660483	0.1624
SPC24	1.251418	1.303215	1.20864341	0.6881039	-0.5393016	0.16255
NIP7	1.262226	1.3142211	1.21928783	0.6978775	-0.5189543	0.1626
LOXL1	2.654407	2.7421936	2.58191149	0.9079998	-0.139236	0.16272
ASIC4	1.092962	1.1412788	1.05306089	0.3755757	-1.4128244	0.16294
RP11-85O21.2	1.34441	1.3977022	1.30039984	0.7553387	-0.4048044	0.16298
SSC5D	1.139751	1.0906503	1.18029881	1.9889497	0.99200681	0.1632
THRB	1.240217	1.2924283	1.19709944	0.6740095	-0.5691593	0.16333
RP11-54H7.4	1.062924	1.0156039	1.10200102	6.536892	2.70860485	0.16358
PEX16	1.341901	1.3954874	1.29764888	0.7526128	-0.4100203	0.1638
ARRDC3	2.025553	1.9438915	2.09299014	1.1579617	0.21158748	0.16383
OAZ1	12.8179	13.088725	12.5942494	0.9590961	-0.0602527	0.16387
TMEM176B	2.331433	2.240503	2.40652508	1.1338345	0.1812101	0.16401

SLC39A7	1.549858	1.6070059	1.50266502	0.8281056	-0.2721133	0.16402
SUN2	1.228395	1.2791898	1.18644759	0.6678167	-0.5824759	0.16408
NCALD	1.405696	1.4602985	1.36060403	0.7834134	-0.3521543	0.16417
AC009506.1	1.158653	1.2084271	1.11754816	0.5639773	-0.8262911	0.16417
PRAF2	2.199368	2.1312985	2.25558028	1.1098576	0.15037459	0.16418
MKRN2	1.476426	1.5324063	1.43019577	0.8080216	-0.3075342	0.16425
ACBD3	1.327787	1.2748856	1.37147404	1.3513767	0.43442991	0.16426
TIMM44	1.666308	1.7255546	1.61738195	0.8509104	-0.2329209	0.16432
FAM133B	2.358233	2.4315199	2.2977107	0.9065265	-0.1415789	0.16464
RNF212	1.184337	1.1344789	1.22551124	1.6769262	0.74581918	0.16478
FTX	1.33318	1.2800813	1.37703027	1.3461458	0.42883473	0.16486
CCDC28A	1.270055	1.3220472	1.22711962	0.7052371	-0.5038197	0.16486
SMG7	1.352103	1.4059194	1.30766125	0.7579367	-0.3998507	0.16496
CDKN2D	1.282488	1.3346819	1.23938556	0.7152629	-0.4834545	0.165
IPO4	1.214162	1.2646544	1.172465	0.6516613	-0.6178059	0.1652
TTLL5	1.224911	1.1742492	1.26674818	1.5308428	0.61432615	0.16536
TMED3	2.806828	2.7170904	2.88093507	1.0954199	0.13148405	0.16546
PPIC	1.858911	1.7966721	1.9103097	1.1426403	0.19237137	0.16546
BRIX1	1.448511	1.503134	1.40340184	0.8017782	-0.318725	0.16559
ATP2A2	1.735399	1.6755018	1.78486239	1.1618954	0.21648017	0.1659
SLC25A24	1.528421	1.5852616	1.48148044	0.8226756	-0.2816045	0.16597
POSTN	1.059572	1.0124282	1.09850327	7.9258171	2.98655968	0.16625
TRRAP	1.203833	1.1536551	1.24527065	1.5962414	0.67467889	0.16644
NUDT3	2.027856	2.0971557	1.97062638	0.8846752	-0.1767802	0.16648
DDX50	1.755162	1.8152048	1.70557777	0.8655221	-0.2083575	0.1665
COMMD7	2.041307	2.1074232	1.98670706	0.8909937	-0.1665129	0.16655
SALL1	1.19752	1.2475049	1.15624085	0.6312638	-0.6636852	0.16674
IDH3A	1.194272	1.2441929	1.15304649	0.6267443	-0.6740511	0.16683
UCHL5	1.380079	1.4334605	1.33599621	0.7751484	-0.3674555	0.16725
TOMM40	1.976046	2.0398012	1.9233953	0.8880498	-0.1712875	0.1675
CYSTM1	3.090095	2.9877961	3.17457465	1.0939626	0.12956346	0.1676
DLGAP1	1.166168	1.1172911	1.20653187	1.760848	0.81627038	0.16762
IL10RB	1.272339	1.3235444	1.23005305	0.71104	-0.4919973	0.16794
DLL3	1.520191	1.5768869	1.47337072	0.8205607	-0.285318	0.16804
CTA-392E5.1	1.075303	1.0284388	1.11400335	4.0087206	2.00314186	0.16833
SYNJ2	1.083279	1.0360792	1.12225724	3.3885841	1.76068259	0.16859
MCAT	1.242754	1.2934894	1.20085613	0.6843726	-0.5471461	0.16864
AFMID	1.39983	1.453578	1.35544425	0.7836453	-0.3517273	0.16879
DNAJC24	1.438663	1.4929903	1.39379874	0.7987961	-0.3241008	0.16907
EPAS1	1.108335	1.0607324	1.14764539	2.4310814	1.28159818	0.16945
MAP4K3	1.254452	1.2037118	1.29635403	1.4547709	0.540792	0.16948
TMEM158	1.079149	1.0320617	1.11803407	3.681465	1.88027999	0.16951
FAM215B	1.551547	1.6080185	1.50491207	0.8304222	-0.268083	0.16958
MLLT6	1.350954	1.2983962	1.39435678	1.3215879	0.40227235	0.16958
VAV2	1.182936	1.1338354	1.22348432	1.6698442	0.73971353	0.16985

LRRC73	1.207307	1.2574748	1.16587702	0.6442458	-0.634317	0.16986
ST8SIA2	1.1938	1.144261	1.23470937	1.626977	0.70219389	0.16991
PTH1R	1.081312	1.0341604	1.12025027	3.5201643	1.81564279	0.16992
RHBDF1	1.138765	1.0904975	1.17862426	1.9738037	0.9809785	0.17013
ZNF576	1.309791	1.3617371	1.26689338	0.7378104	-0.4386779	0.17014
TUBGCP2	1.6954	1.7542953	1.64676365	0.8574409	-0.2218909	0.17023
ATAT1	1.61164	1.668966	1.56430011	0.8435408	-0.2454703	0.17026
TMEM38B	1.518096	1.5732927	1.47251338	0.8242096	-0.2789168	0.17046
HTN1	1.455179	1.2813825	1.59870358	2.1277218	1.08930951	0.17046
PUS7L	1.447061	1.5013423	1.40223539	0.8023168	-0.317756	0.17058
CLGN	1.318493	1.3702117	1.27578287	0.7449329	-0.4248177	0.17097
SAAL1	1.328959	1.3809402	1.28603294	0.7508605	-0.4133831	0.17107
ERICH5	2.611489	2.5213796	2.6859035	1.1081413	0.14814183	0.17135
CCDC43	1.454696	1.5089705	1.4098757	0.8053035	-0.3123955	0.1714
MCM3	1.582215	1.638249	1.53594087	0.839705	-0.2520455	0.17141
KAT5	1.296325	1.3474511	1.25410413	0.7313378	-0.4513901	0.17145
AC002310.12	1.23393	1.2843622	1.1922822	0.6761876	-0.5645046	0.17169
PPFIBP1	1.645959	1.5887207	1.69322711	1.1775144	0.23574473	0.1717
LOX	1.414737	1.3615438	1.45866502	1.2686291	0.34327033	0.17178
ECEL1	1.153884	1.2023602	1.11385202	0.5626206	-0.8297658	0.17182
FAM98B	1.345938	1.3982747	1.30271722	0.7600715	-0.395793	0.17201
SRPRA	1.30699	1.2556875	1.34935716	1.3663445	0.45032125	0.17209
SPRED1	1.498009	1.4429818	1.54345096	1.2268021	0.29490252	0.17213
CDH23	1.216586	1.2662934	1.17553717	0.659187	-0.6012403	0.17231
DHTKD1	1.278429	1.3294039	1.23633376	0.7174588	-0.479032	0.17237
DYX1C1	1.219024	1.2690348	1.1777237	0.6605975	-0.5981567	0.17246
SEC61A1	1.837443	1.7769178	1.88742498	1.1422379	0.19186319	0.17266
ABCA1	1.086286	1.0394785	1.12493963	3.1647471	1.66209023	0.17272
TRIP12	1.734874	1.6759572	1.78352831	1.1591389	0.21305349	0.17274
IRS1	1.240677	1.1904228	1.28217728	1.4818461	0.56739566	0.17274
SART3	1.216554	1.2662861	1.17548521	0.6590101	-0.6016275	0.17293
PDHX	1.399739	1.4526827	1.35601712	0.7864607	-0.3465535	0.17302
USF2	1.707807	1.6495847	1.75588866	1.1636491	0.21865604	0.17302
EMILIN2	1.0879	1.0410574	1.12658268	3.0830628	1.62436427	0.17333
BIRC2	1.589133	1.6457506	1.54237801	0.8399187	-0.2516784	0.17341
STEAP1	1.130005	1.1775731	1.09072305	0.5109054	-0.9688719	0.17344
CACUL1	1.942528	2.0048072	1.89109609	0.8868329	-0.1732659	0.17349
MTMR14	1.235771	1.2857675	1.19448279	0.6805629	-0.5551996	0.17362
GPR143	1.33411	1.2823918	1.37681953	1.3343857	0.4161757	0.17371
BCAR1	1.627779	1.5709905	1.67467579	1.1815884	0.2407276	0.17377
CPVL	1.116122	1.1633884	1.07708907	0.471815	-1.0837069	0.17378
PTPRA	2.802174	2.8898158	2.72979736	0.9153259	-0.1276426	0.17401
ERC1	1.383328	1.4362482	1.33962584	0.7785152	-0.3612029	0.17402
SORBS3	1.767393	1.7079325	1.81649603	1.153353	0.20583411	0.17412
RIOK2	1.239963	1.2898267	1.19878448	0.6858736	-0.5439854	0.1742

NECAB3	1.361051	1.4135959	1.31765908	0.7680421	-0.3807427	0.17422
BCL7A	1.703344	1.7620664	1.65485043	0.8593089	-0.2187513	0.17422
SAPCD2	1.155692	1.2036689	1.11607281	0.5699094	-0.8111954	0.17461
KPNA6	1.571913	1.5160952	1.61800871	1.1974704	0.25998994	0.17464
FOXG1	1.257822	1.3078235	1.21652979	0.703422	-0.5075376	0.17478
PLEKHO1	1.899467	1.9613481	1.84836487	0.8824742	-0.180374	0.17493
SNX5	1.877722	1.9385792	1.82746587	0.8816154	-0.1817786	0.17501
UIMC1	1.327103	1.3792945	1.28400154	0.7487626	-0.4174197	0.17519
BCL11A	1.554065	1.6099548	1.50790975	0.8327006	-0.2641303	0.17525
EMC7	1.607474	1.6636706	1.56106653	0.8453991	-0.2422954	0.1753
NUP210	1.210982	1.1616032	1.25176049	1.557893	0.63959612	0.17542
MMACHC	1.096111	1.1432777	1.05716081	0.3989512	-1.3257159	0.17607
FRAS1	1.235586	1.1862604	1.27631992	1.483514	0.56901856	0.17633
WBSCR22	1.886594	1.9473011	1.83646074	0.8829935	-0.1795253	0.17637
SEC22C	1.793462	1.8537752	1.74365472	0.8710194	-0.1992233	0.17648
P3H3	1.348297	1.2964893	1.39108058	1.3190378	0.39948588	0.17653
ERV3-1	1.758609	1.8175546	1.70993022	0.8683582	-0.2036378	0.17666
GTF3A	3.188403	3.2898746	3.10460588	0.9190922	-0.1217185	0.1768
GOLGA2	1.458978	1.4052006	1.50338745	1.2423167	0.31303302	0.17712
SDHA	1.434365	1.4874479	1.39052865	0.80117	-0.3198197	0.17713
GPD1	1.082979	1.0366769	1.12121528	3.3049502	1.72462852	0.17725
LTN1	1.445954	1.4996118	1.4016423	0.8039088	-0.3148963	0.17748
HINFP	1.265254	1.3156427	1.2236419	0.7085285	-0.4971021	0.17748
HDAC10	1.283158	1.2325838	1.32492298	1.3970149	0.48234736	0.1775
SLF2	1.630159	1.5733677	1.67705912	1.1808464	0.23982126	0.17763
TAB2	1.425905	1.3731926	1.46943661	1.2578936	0.33100989	0.17779
NFE2L1	1.708205	1.6507418	1.75565824	1.1612259	0.21564867	0.17796
RAB30	1.220776	1.2700145	1.18011458	0.6670551	-0.5841222	0.17816
LINC01503	1.216556	1.1677938	1.25682422	1.530594	0.61409162	0.17821
C15orf40	1.852106	1.9122661	1.80242437	0.8795946	-0.1850893	0.17833
SOCS3	1.8946	1.9655914	1.83597455	0.8657643	-0.2079538	0.17834
FLJ37453	1.539097	1.5939248	1.49381887	0.8314502	-0.2662983	0.17847
EPHB4	1.244003	1.1945979	1.2848028	1.4635451	0.54946721	0.1786
NUDT9	1.297028	1.3474078	1.25542343	0.7352265	-0.4437394	0.17868
NRBF2	1.556721	1.6122981	1.51082385	0.8342731	-0.2614084	0.17869
BICD2	1.263705	1.2138841	1.30484817	1.4252959	0.51126147	0.17885
SUPT3H	1.305879	1.3570904	1.26358838	0.7381559	-0.4380026	0.17893
PHF1	1.380599	1.328821	1.42335741	1.2875012	0.3645738	0.17954
COX7A2L	6.743587	6.9440661	6.57802873	0.9384197	-0.0916948	0.17999
NFS1	1.170464	1.2183829	1.13089121	0.5993656	-0.7384918	0.18022
NEFL	1.112056	1.1641676	1.06902224	0.4204375	-1.2500367	0.1803
MINPP1	1.217885	1.2666075	1.17764845	0.6663296	-0.5856922	0.1803
SNHG21	1.344406	1.3958239	1.30194518	0.7628271	-0.3905719	0.18031
C1orf50	1.184936	1.2335093	1.14482299	0.6202023	-0.6891892	0.18047
LRRC37B	1.315427	1.3661079	1.27357449	0.7472511	-0.420335	0.18052

FUCA2	1.502959	1.5566827	1.45859249	0.8237952	-0.2796424	0.1807
BTN3A2	1.239814	1.2891212	1.19909558	0.6886232	-0.5382133	0.18073
CACNA1D	1.255248	1.3046775	1.21442836	0.7037879	-0.5067874	0.18075
ZNF618	1.374361	1.3224097	1.41726405	1.2942043	0.37206538	0.18083
ESYT2	1.281058	1.2312701	1.32217354	1.3930617	0.47825915	0.1809
CENPQ	1.253974	1.3034461	1.21311971	0.7023314	-0.5097762	0.18106
LIF	1.079006	1.0330827	1.11692973	3.534468	1.8214931	0.18112
SMARCAD1	1.393977	1.341796	1.43706908	1.2787424	0.3547257	0.18136
IGSF8	1.303875	1.35403	1.262457	0.7413411	-0.4317907	0.1814
FAM169A	1.377096	1.4288553	1.334353	0.7796405	-0.359119	0.18167
ZC3H12B	1.111584	1.1582743	1.07302721	0.4613966	-1.1159207	0.18182
EIF4G2	7.387281	7.19985	7.54206457	1.0551972	0.07751268	0.18208
MXD4	1.58821	1.6438278	1.54228035	0.8422755	-0.247636	0.1821
SNX7	1.533392	1.5876874	1.48855399	0.8313161	-0.2665309	0.18214
PPP1R11	2.08498	2.1500536	2.03124165	0.8966901	-0.1573187	0.18215
ZBTB38	1.925492	1.9871935	1.87453721	0.8858822	-0.1748132	0.18242
SH3GL1	1.476322	1.5295974	1.43232666	0.8163307	-0.2927743	0.18278
SQLE	2.145314	2.2124103	2.08990501	0.8989572	-0.1536756	0.18279
VEZT	1.934621	1.99606	1.88388359	0.8873799	-0.1723762	0.18294
TMEM67	1.514245	1.5679135	1.46992392	0.8274569	-0.2732439	0.18305
ABCF1	1.87596	1.9357109	1.82661721	0.8834109	-0.1788434	0.18312
ZDBF2	1.210001	1.1617602	1.24983852	1.5444996	0.62713945	0.18316
EMC9	1.768998	1.8266424	1.72139444	0.8726801	-0.1964751	0.18352
STARD4-AS1	1.43246	1.4853358	1.38879504	0.8010847	-0.3199733	0.18387
BMP1	1.218633	1.1706036	1.25829559	1.5140104	0.59837511	0.18396
RAI1	1.182553	1.1349169	1.22189132	1.6446515	0.71778195	0.18398
NTMT1	1.798208	1.8571099	1.74956518	0.8745264	-0.1934262	0.18412
IGF2BP3	1.79348	1.852292	1.74491244	0.8740109	-0.1942769	0.18412
PRPF4B	2.167043	2.0999451	2.2224529	1.1113763	0.15234736	0.18415
ZSCAN16-AS1	2.126527	2.1964863	2.06875301	0.893243	-0.1628755	0.18431
CEBPZ	1.828979	1.8879721	1.78026126	0.8787002	-0.186557	0.1844
SELENOW	6.770529	6.5316251	6.96781979	1.0788547	0.1095006	0.1845
USP48	1.589255	1.6440581	1.54399753	0.8446405	-0.2435906	0.18466
PPP2R5D	1.208942	1.2572222	1.1690713	0.6572968	-0.6053832	0.18492
NIPSNAP3A	1.321679	1.3719864	1.28013449	0.7530773	-0.4091302	0.18497
HIPK1	1.357178	1.3065349	1.39900062	1.3016484	0.38033983	0.18498
CEP350	1.736692	1.6792227	1.78415115	1.1544831	0.20724709	0.18502
AGO3	1.715911	1.7734977	1.66835558	0.8640692	-0.2107812	0.18509
METTL16	1.378994	1.4300894	1.33679802	0.7830883	-0.3527531	0.18531
RILPL1	1.189836	1.1420497	1.2292983	1.6142123	0.69083029	0.18533
MRPL18	2.311714	2.3855056	2.2507763	0.902758	-0.1475887	0.18533
SNX4	1.502736	1.556007	1.45874381	0.8250684	-0.2774144	0.18536
MAX	1.56218	1.507441	1.60738459	1.1969561	0.25937023	0.1854
C1orf56	1.477222	1.5309372	1.43286285	0.8152807	-0.2946313	0.18546
HSPA4L	1.260453	1.3092277	1.22017331	0.7120103	-0.49003	0.18563

LIMK1	1.365101	1.3143259	1.4070317	1.2949353	0.37288004	0.18582
CDV3	1.784415	1.7271619	1.8316956	1.1437557	0.19377894	0.18599
MATN2	1.129246	1.0832412	1.16723708	2.0090664	1.00652525	0.18633
ZNF430	1.298399	1.2486701	1.33946617	1.3651266	0.4490347	0.18636
CREB5	1.305741	1.2558048	1.34697963	1.3564236	0.43980775	0.18642
CBLN2	1.138139	1.1846993	1.09968862	0.5397348	-0.8896775	0.18654
DCAF8	1.48524	1.5386489	1.4411334	0.8189628	-0.2881301	0.18666
SEC23B	1.202182	1.2498496	1.16281822	0.6516649	-0.6177977	0.18673
KBTBD7	1.242329	1.2909556	1.2021717	0.6948542	-0.5252178	0.18676
SPATA6	1.433586	1.4860977	1.39022017	0.8027607	-0.3169581	0.1868
FAM129B	1.189828	1.1424641	1.22894235	1.6070174	0.68438559	0.18688
FKBP11	1.267895	1.2188966	1.30835791	1.4086919	0.49435609	0.18693
GSX2	1.0708	1.0256585	1.10807768	4.212153	2.07455783	0.18714
OSER1-AS1	1.354717	1.4057194	1.31259815	0.7704787	-0.376173	0.1874
A2M	1.39853	1.4497987	1.35619113	0.7918901	-0.3366278	0.18768
KBTBD3	1.346077	1.3968224	1.30416982	0.7665137	-0.3836165	0.18788
CPNE8	1.381211	1.4321786	1.33912182	0.7846798	-0.3498241	0.18793
CNPPD1	1.327677	1.3776515	1.28640737	0.7583907	-0.3989869	0.18825
POLR1E	1.216316	1.2641861	1.17678439	0.6691662	-0.5795635	0.18832
GLMN	1.372903	1.4239099	1.33078148	0.7803108	-0.3578792	0.18843
EZR	1.647201	1.5911085	1.69352273	1.173258	0.23052025	0.18849
FAM174B	1.105346	1.059767	1.14298585	2.3923881	1.25845142	0.18849
MPC1	1.640779	1.6964069	1.59484081	0.8541569	-0.2274269	0.18857
GART	1.338842	1.3894793	1.29702412	0.7626185	-0.3909665	0.18874
PNMA2	1.146735	1.1931293	1.10842255	0.5613988	-0.8329021	0.18889
C1orf174	1.624591	1.679608	1.57915711	0.8521929	-0.2307481	0.18896
TNNT1	1.509181	1.4563776	1.55278694	1.2112492	0.27649569	0.18898
MAP1S	1.192546	1.2399724	1.15338138	0.6391627	-0.6457449	0.18909
C10orf35	1.497278	1.5504739	1.45334862	0.8235605	-0.2800534	0.1891
SLITRK6	1.153478	1.2000886	1.11498679	0.5746793	-0.7991711	0.18921
VPS13D	1.180313	1.133204	1.21921703	1.6457245	0.71872288	0.18952
FOXO3	1.808569	1.8677656	1.75968262	0.8754468	-0.1919086	0.18965
CALCOCO2	1.382325	1.4331741	1.34033233	0.785671	-0.3480027	0.18969
RND2	1.514034	1.5673796	1.46998019	0.8283347	-0.2717142	0.18971
DSC3	1.101043	1.0556863	1.13849935	2.4871361	1.31448545	0.18983
VAT1L	1.183203	1.1361422	1.22206712	1.6311412	0.70588166	0.19001
BNIP2	1.473673	1.4210734	1.5171101	1.2280758	0.29639963	0.19026
NOTCH1	1.281563	1.2323892	1.32217063	1.3863408	0.47128194	0.19029
RAI14	1.940249	1.880705	1.98942187	1.123443	0.1679269	0.19035
ERBIN	1.769476	1.826807	1.72213168	0.8733982	-0.1952886	0.19043
CDCA7	1.427445	1.3754563	1.470379	1.2528196	0.32517866	0.19046
RMDN1	1.446156	1.4985447	1.4028928	0.8081378	-0.3073267	0.19048
UCK1	1.554655	1.5006848	1.5992237	1.1968083	0.25919211	0.19053
DDHD2	1.484357	1.5369549	1.44092057	0.8211501	-0.2842822	0.19064
RAP1A	1.786907	1.844509	1.73933916	0.8754663	-0.1918764	0.19068

EXOC3-AS1	1.330746	1.3810026	1.28924302	0.7591629	-0.3975186	0.19091
KMO	1.11973	1.0742572	1.15728197	2.1180695	1.08274996	0.19098
MUT	1.278426	1.3273308	1.23803908	0.7272126	-0.4595509	0.19105
NNT	1.466702	1.5188533	1.42363423	0.8164818	-0.2925074	0.19115
KIF1C	1.394101	1.343764	1.43567063	1.267354	0.34181957	0.1912
LGR4	2.462395	2.5346223	2.40274792	0.9140672	-0.1296278	0.19142
MINK1	1.18744	1.1404908	1.22621075	1.6101468	0.68719225	0.19146
CH17-189H20.1	1.514459	1.4618758	1.5578834	1.2078645	0.27245864	0.19171
TRNAU1AP	1.606531	1.6610088	1.56154263	0.8495237	-0.2352739	0.19181
CHMP4A	2.03318	2.0941177	1.98285726	0.8983103	-0.1547142	0.19199
SLC25A39	3.833646	3.9566451	3.73207146	0.9240444	-0.1139659	0.19215
RASL10B	1.133198	1.0874674	1.17096371	1.9546	0.96687343	0.19224
SEC24B	1.212973	1.1656	1.25209497	1.5223123	0.60626436	0.1925
SRFBP1	1.355452	1.4054988	1.31412164	0.7746549	-0.3683743	0.19255
ATOH7	1.088439	1.1339928	1.0508202	0.3792757	-1.3986813	0.19256
GEMIN2	1.268871	1.3174023	1.22879247	0.720828	-0.4722731	0.19262
AKAP17A	1.51327	1.4607714	1.55662455	1.2080275	0.27265329	0.19293
VKORC1L1	1.306758	1.3561007	1.26601019	0.7470083	-0.4208039	0.19309
PKN2	2.149509	2.0854209	2.20243413	1.1078045	0.14770331	0.1931
KXD1	2.575232	2.6557486	2.50874091	0.9112137	-0.1341386	0.1932
ATOH8	1.156664	1.2028905	1.11848982	0.5840087	-0.7759383	0.19323
RP11-538P18.2	1.199343	1.2469367	1.16003964	0.6480999	-0.6257119	0.1933
C1GALT1	1.563882	1.509848	1.60850479	1.1935024	0.25520151	0.1933
SDCCAG3	1.165553	1.1192272	1.2038088	1.7094149	0.77350259	0.19337
USP10	1.701712	1.7572219	1.65587119	0.8661546	-0.2073036	0.1935
SPP1	2.06648	1.9693321	2.14670597	1.1829857	0.24243259	0.19356
PTPN4	1.446275	1.4981963	1.40339678	0.8097145	-0.3045149	0.19375
PHC1	1.292429	1.3412476	1.25211401	0.7388008	-0.4367428	0.19376
MSMO1	1.868707	1.8024142	1.92345293	1.1508433	0.20269138	0.19381
CEP152	1.230166	1.1822824	1.26970967	1.4796251	0.56523173	0.19393
IPO9	1.919097	1.8602995	1.9676532	1.1247865	0.16965113	0.19399
ENDOD1	1.175705	1.1292563	1.21406371	1.6561185	0.7278059	0.19407
DDX42	1.540613	1.5937133	1.49676229	0.836704	-0.2572107	0.19412
MIR4500HG	1.090434	1.1352573	1.05341738	0.3949317	-1.3403248	0.19417
RNF208	1.214763	1.1674299	1.25385164	1.5161665	0.60042816	0.19419
VPS26B	1.483731	1.5364096	1.44022824	0.8206942	-0.2850833	0.1943
CDCA5	1.315786	1.3646581	1.27542584	0.7552988	-0.4048806	0.19431
COL6A3	1.066936	1.0224556	1.10366783	4.6165753	2.20682303	0.19453
SFT2D3	1.184917	1.2315463	1.14640887	0.6323092	-0.6612978	0.19453
RP11-115D19.1	3.244376	3.3784592	3.13364771	0.8970714	-0.1567053	0.19466
MIR325HG	1.309855	1.3589439	1.26931729	0.7503048	-0.4144514	0.19467
E2F4	1.431979	1.3807533	1.47428126	1.2456392	0.31688628	0.19475
CEP44	1.275241	1.3237158	1.23520925	0.7265918	-0.460783	0.19482
TARDBP	1.966696	2.0265048	1.91730551	0.8936203	-0.1622662	0.19494
BHMT	1.061761	1.1118589	1.02038891	0.1822735	-2.4558233	0.19503

LEPROTL1	1.723697	1.7801443	1.67708257	0.867894	-0.2044092	0.1953
ZIC3	1.281353	1.3297546	1.24138187	0.7320047	-0.4500752	0.1954
STOX2	1.419135	1.4699802	1.37714534	0.8024706	-0.3174795	0.19543
AP1S3	1.1248	1.0797545	1.16199927	2.0312243	1.02234954	0.19559
TMEM254	1.407874	1.4589633	1.36568414	0.7967611	-0.3277809	0.19563
PEX5	1.233109	1.2807575	1.19376025	0.6901339	-0.5350519	0.19564
DIMT1	1.329528	1.3786275	1.28898048	0.7632317	-0.389807	0.19569
CPSF3L	1.504631	1.5569049	1.46146327	0.8286214	-0.2712151	0.19569
PAG1	1.190248	1.1439456	1.22848503	1.5873012	0.66657592	0.19578
KDELC1	1.223244	1.2706086	1.18412903	0.6804256	-0.5554906	0.19593
ZNF75A	1.494116	1.5465492	1.45081643	0.8248415	-0.2778112	0.19594
VTA1	1.351476	1.4009176	1.31064655	0.7748389	-0.3680317	0.19605
TIMM50	1.541344	1.5941693	1.49772064	0.8376748	-0.2555377	0.19616
MED25	1.387084	1.4370059	1.34585711	0.7914244	-0.3374765	0.19617
LDLRAP1	1.236752	1.1893247	1.2759181	1.4573805	0.54337758	0.19619
KYAT3	1.296512	1.3448789	1.25656932	0.7439402	-0.4267413	0.1962
CENPT	1.399879	1.3494599	1.44151686	1.2634266	0.33734181	0.19633
ARID3A	2.100075	2.1682587	2.04376716	0.8934384	-0.1625599	0.19653
BCAS1	1.059553	1.0156039	1.09584726	6.1425187	2.61883034	0.19665
GATB	1.183857	1.2302329	1.14555976	0.6322284	-0.6614823	0.19697
VTCN1	1.07534	1.0309049	1.11203509	3.6251561	1.8580431	0.197
ZNF426	1.356238	1.4060954	1.31506565	0.7758415	-0.3661662	0.19704
PHKG2	1.316886	1.3663285	1.27605597	0.753575	-0.4081771	0.19707
KAT14	1.1796	1.2259747	1.14130278	0.6253036	-0.6773712	0.19724
FILIP1	1.125026	1.0798093	1.16236613	2.0344263	1.02462202	0.19739
MYCBP2	1.645399	1.5905525	1.69069194	1.1695691	0.22597713	0.19762
TMEM87B	1.184422	1.138014	1.22274555	1.6139339	0.69058153	0.19767
CCM2	1.456993	1.4056884	1.49936054	1.2308968	0.29970978	0.19796
MBLAC2	1.146969	1.1925784	1.10930345	0.567579	-0.8171068	0.19812
METTL17	1.497959	1.446511	1.54044471	1.2103726	0.27545118	0.19813
RP11-390E23.6	1.401735	1.451987	1.36023603	0.7970053	-0.3273388	0.19813
KMT2D	1.153766	1.1081239	1.19145817	1.7707299	0.82434414	0.19814
NSRP1	1.88498	1.9433355	1.83678926	0.8870537	-0.1729067	0.19826
ATXN2L	1.571308	1.5183495	1.6150415	1.1865382	0.24675855	0.19844
AXL	1.960878	1.8892916	2.01999589	1.1469758	0.19783495	0.19844
TAF1B	1.311216	1.3598902	1.27102084	0.7530653	-0.4091532	0.19851
ARMCX2	1.523848	1.5760038	1.48077642	0.8346758	-0.2607122	0.19866
GNPTAB	1.184041	1.137869	1.22217074	1.6114622	0.68837032	0.19878
ZNF566	1.281589	1.3300078	1.24160382	0.7321154	-0.449857	0.19893
ULK3	1.303013	1.3513075	1.26313122	0.7490054	-0.4169519	0.19983
OGG1	1.437162	1.4877931	1.39535089	0.8104889	-0.3031357	0.19995
CXADR	2.401675	2.3238107	2.4659772	1.1073919	0.14716585	0.20032
COL25A1	1.201169	1.2472977	1.16307537	0.6594293	-0.60071	0.20047
NT5C2	1.349669	1.3990163	1.30891641	0.774195	-0.3692311	0.20052
LINC01003	1.598319	1.6517669	1.55418053	0.8502741	-0.2340002	0.2006

EPB41L4A	1.117849	1.0733653	1.15458422	2.1070494	1.07522416	0.20076
LL22NC03-2H8.5	1.395511	1.3456029	1.43672594	1.2636639	0.33761276	0.20082
MEA1	2.744499	2.8278042	2.67570341	0.916785	-0.1253447	0.20102
BBS9	1.255406	1.3029994	1.21610271	0.7132116	-0.487598	0.20107
ANKS1B	1.193838	1.2399391	1.15576681	0.649193	-0.6232806	0.20118
MYLK-AS1	1.308079	1.3564386	1.26814319	0.7522844	-0.4106499	0.20135
MRPL53	1.226625	1.2741448	1.1873828	0.6835176	-0.5489496	0.20141
C1GALT1C1	1.189592	1.2354672	1.15170785	0.6442844	-0.6342303	0.20142
STAT1	1.372459	1.3231161	1.41320645	1.2788171	0.35480997	0.20145
CTSD	2.243967	2.1662284	2.30816478	1.1217055	0.16569397	0.2015
HELB	1.210215	1.2567691	1.17176933	0.6689641	-0.5799994	0.20158
PCDHB16	1.194511	1.1483276	1.23264934	1.5684835	0.64937034	0.20163
EML4	1.732937	1.7884628	1.68708254	0.8714203	-0.1985593	0.20169
ARL6IP4	5.987798	6.1491326	5.85456519	0.9427928	-0.0849873	0.20178
SWI5	1.859563	1.9171574	1.81200051	0.8853448	-0.1756887	0.20179
ROM1	1.136351	1.1816123	1.09897377	0.5449729	-0.8757435	0.20187
RYBP	1.551813	1.4990952	1.59534771	1.1928539	0.2544174	0.20196
IFT20	1.888261	1.9468715	1.83985916	0.8869833	-0.1730212	0.20213
EMC6	2.846796	2.7604507	2.91810158	1.0895514	0.12373427	0.20218
BOLA2B	1.162067	1.2078196	1.12428409	0.5980384	-0.7416899	0.20224
SMIM4	1.771278	1.8272293	1.72507214	0.8765069	-0.1901627	0.2023
CCDC15	1.130916	1.0861152	1.16791346	1.9498705	0.96337831	0.20259
STAT6	1.276545	1.2286722	1.31607858	1.3822344	0.46700231	0.20267
ATP6V1G2	1.265539	1.3130496	1.22630303	0.7228982	-0.4681356	0.20271
JAKMIP2	1.453058	1.5038048	1.41115008	0.81609	-0.2931998	0.20292
SMIM10L1	2.048712	2.1093793	1.99861239	0.9001542	-0.151756	0.20308
DNMT3A	1.814825	1.8746656	1.76540821	0.8750867	-0.1925021	0.20342
SFRP1	4.773819	4.595207	4.92131977	1.0907077	0.12526447	0.20364
SOCS1	1.081506	1.0376722	1.11770378	3.1244189	1.64358788	0.2037
NAIP	1.253936	1.3007707	1.2152591	0.7156918	-0.4825896	0.20378
ZDHHC4	1.803606	1.8613168	1.75594719	0.8776645	-0.1882586	0.20381
C12orf43	1.308822	1.3566159	1.26935325	0.7553035	-0.4048716	0.20391
NINJ1	2.228183	2.1654808	2.2799639	1.0982282	0.13517789	0.20402
COA5	1.829614	1.8862214	1.78286692	0.8833762	-0.1789001	0.20429
RP11-445F12.1	1.094859	1.0511444	1.13095913	2.5605763	1.35646854	0.20435
CUBN	1.140021	1.0955823	1.17671851	1.8488629	0.88663826	0.2047
RANGAP1	1.329905	1.3780416	1.29015288	0.7675157	-0.3817319	0.20474
ARL5B	1.488246	1.5392955	1.44608821	0.8271684	-0.273747	0.20485
ABHD11	1.58096	1.6338279	1.53730136	0.8477086	-0.2383597	0.20489
ANKMY2	1.227834	1.2744411	1.18934492	0.6899291	-0.5354799	0.20493
SMKR1	1.222351	1.2685106	1.18423155	0.6861238	-0.5434591	0.20526
ATE1	1.335606	1.2873781	1.37543312	1.3064083	0.38560589	0.20539
ARHGAP42	1.113685	1.0695346	1.15014523	2.1592883	1.11055587	0.20541
C2orf76	1.296313	1.343816	1.25708382	0.7477367	-0.4193978	0.20545
HSDL1	1.501658	1.5527407	1.45947258	0.8312624	-0.2666241	0.20558

SOAT1	1.175782	1.1305434	1.2131407	1.6327188	0.70727632	0.2056
LASP1	1.798952	1.7437514	1.84453794	1.1355111	0.18334178	0.20565
FGF2	1.137017	1.0923368	1.1739152	1.883487	0.91340609	0.2057
CD276	1.893405	1.8354338	1.94127839	1.1266942	0.17209601	0.20611
ARV1	1.248676	1.2951732	1.21027832	0.7123896	-0.4892617	0.20614
E2F5	1.28657	1.3339261	1.2474628	0.7410705	-0.4323173	0.20614
AHCYL1	2.055888	2.1188392	2.00390184	0.8972709	-0.1563844	0.20616
C10orf88	1.164947	1.2104509	1.12736901	0.6052196	-0.7244694	0.20629
MOSPD2	1.643148	1.6963553	1.59920877	0.8604929	-0.2167648	0.20639
TAZ	1.281745	1.3290103	1.24271259	0.737705	-0.438884	0.20641
FARSB	1.303049	1.3505636	1.2638114	0.752535	-0.4101695	0.20677
SAP30L	1.217607	1.1716957	1.25552062	1.4882184	0.57358623	0.2068
DSCR3	1.479691	1.5304715	1.4377559	0.8252205	-0.2771485	0.20747
CLIP1	1.472425	1.4221553	1.51393883	1.2174164	0.28382266	0.20757
PTPRD	1.728221	1.7833584	1.68268815	0.8714889	-0.1984457	0.20768
ROCK2	1.417362	1.4669894	1.37637812	0.8059672	-0.311207	0.20775
PPP1CB	3.527614	3.6584627	3.41955728	0.910134	-0.1358491	0.2078
PDLIM2	2.186307	2.1251368	2.23682259	1.0992642	0.13653817	0.20786
ZNF48	1.283469	1.3306123	1.24453641	0.7396471	-0.435091	0.20788
GNAQ	1.559844	1.5083414	1.60237507	1.1849814	0.24486441	0.20791
NR1H2	1.603189	1.6563096	1.55932069	0.8522208	-0.2307008	0.20799
CDC42BPB	1.460079	1.4101463	1.50131385	1.2222806	0.28957556	0.20806
DTNA	2.044505	2.1106203	1.98990678	0.8913098	-0.1660011	0.20834
GUCD1	1.342213	1.390389	1.30242768	0.7746829	-0.3683221	0.20836
ZNF593	1.885744	1.9425214	1.83885662	0.8900133	-0.1681012	0.20837
YES1	1.755481	1.8103128	1.71020016	0.8764519	-0.1902532	0.20874
IVD	1.444268	1.4939934	1.40320414	0.8162136	-0.2929814	0.20886
RMND5B	1.421182	1.4707446	1.38025224	0.8077675	-0.3079879	0.20907
RP11-197K6.1	1.147438	1.1923696	1.11033239	0.5735438	-0.8020244	0.2091
ABCD4	2.741299	2.8335244	2.6651373	0.9081621	-0.1389783	0.2094
CABIN1	1.565039	1.6167721	1.52231619	0.8468545	-0.239814	0.2095
SNX24	1.422049	1.4717118	1.38103706	0.807775	-0.3079745	0.20959
LCMT1	2.112383	2.1765918	2.05935843	0.9003619	-0.151423	0.20971
NAT9	1.640558	1.6941557	1.59629668	0.8590244	-0.219229	0.20985
LEO1	1.289802	1.3364721	1.25126061	0.7467503	-0.4213022	0.21022
BBIP1	1.953931	2.011475	1.90641116	0.8961281	-0.1582231	0.21065
RNF7	5.449055	5.6268225	5.30225214	0.9298503	-0.1049297	0.21065
ELL2	1.329828	1.3778652	1.29015866	0.7678894	-0.3810296	0.21084
CCZ1	1.463748	1.5138456	1.42237614	0.8219904	-0.2828066	0.21087
KIFAP3	1.348464	1.3964064	1.30887207	0.7791803	-0.3599709	0.21087
YEATS2	1.381069	1.332381	1.42127621	1.2674496	0.34192833	0.21101
C11orf57	1.584033	1.635919	1.54118446	0.8510273	-0.2327227	0.21118
MFAP2	2.336246	2.2656058	2.39458156	1.1019083	0.1400042	0.21126
RANBP9	1.277786	1.3246866	1.23905533	0.7362649	-0.4417032	0.21175
TMCC1	1.302146	1.2551909	1.34092185	1.3359482	0.41786411	0.21176

RAB3A	1.423593	1.4729564	1.38282832	0.8094367	-0.3050097	0.21178
ADAMTS6	1.62607	1.6788337	1.58249673	0.8580845	-0.2208083	0.21182
OSGEPL1	1.224039	1.2695542	1.18645142	0.6917028	-0.5317757	0.21196
USP42	1.266886	1.3135842	1.22832129	0.728102	-0.4577876	0.212
LZTS2	1.570468	1.5186799	1.61323614	1.1823017	0.24159824	0.21215
SPOCK3	1.091275	1.1343625	1.05569202	0.4144909	-1.2705877	0.21215
KATNA1	1.320984	1.3683704	1.28185071	0.7651286	-0.3862258	0.2122
CGRRF1	1.299077	1.345979	1.26034488	0.7524875	-0.4102605	0.2126
ATG2A	1.181436	1.1364947	1.21855009	1.6011621	0.6791194	0.2128
PLXNA1	1.292892	1.2459667	1.33164283	1.3483243	0.43116754	0.21284
YTHDF3	1.313051	1.2659245	1.3519691	1.3235676	0.4044319	0.2129
PCF11	1.670241	1.7236851	1.62610655	0.8651643	-0.2089539	0.21291
ANO6	1.4202	1.3713354	1.46055402	1.2402642	0.31064749	0.21305
CNOT2	1.876313	1.9317925	1.8304976	0.8912903	-0.1660327	0.21305
ANKRA2	1.696755	1.7502193	1.65260343	0.8698835	-0.2011059	0.21345
WNK3	1.40756	1.456438	1.36719518	0.8044799	-0.3138718	0.2136
PTRH1	1.395823	1.4442591	1.3558233	0.8009365	-0.3202403	0.21366
RFWD2	1.382175	1.430474	1.34228847	0.7951432	-0.3307134	0.21368
NEIL1	1.352247	1.3043198	1.39182602	1.2875468	0.36462489	0.21376
USP37	1.258706	1.2125065	1.29685889	1.39694	0.48227007	0.21413
ANAPC1	1.2404	1.1945628	1.27825267	1.4301429	0.51615927	0.21427
MRS2	1.68582	1.7391276	1.64179829	0.8683187	-0.2037035	0.21436
N6AMT1	1.150158	1.1941575	1.11382157	0.5862331	-0.7704537	0.2148
PIEZO1	1.159299	1.1153341	1.19560584	1.6959934	0.76213054	0.21493
TMEM37	1.107631	1.064352	1.14337133	2.2279239	1.15569993	0.21508
SMCHD1	1.930809	1.9885619	1.88311638	0.8933344	-0.1627277	0.21524
LGMN	1.577834	1.6292344	1.53538593	0.850853	-0.2330183	0.21533
SERPINH1	2.733437	2.8268166	2.65632264	0.9066716	-0.141348	0.21549
STX7	1.704237	1.7573724	1.6603578	0.8719063	-0.1977549	0.2157
NQO1	1.991329	2.0622158	1.93278895	0.8781539	-0.1874542	0.21608
BAG3	1.287141	1.333405	1.24893465	0.7466435	-0.4215086	0.21612
PEX10	1.7138	1.7669911	1.66987358	0.8733786	-0.195321	0.21619
ANKIB1	1.478539	1.4290046	1.51944579	1.2108163	0.27597999	0.21643
ARFGAP2	1.545014	1.5952703	1.50351119	0.845853	-0.2415211	0.21646
BICD1	1.645839	1.5938666	1.6887589	1.1597873	0.21386024	0.21655
ESYT1	1.513907	1.4636278	1.55542918	1.1980066	0.26063591	0.21672
BRCA2	1.393582	1.3450863	1.43363087	1.2565866	0.32951013	0.21673
TOB2	1.641716	1.6944229	1.59818976	0.8614199	-0.2152114	0.21687
BRCC3	1.248849	1.2943263	1.21129226	0.7178843	-0.4781767	0.2169
CNP	1.702266	1.7552123	1.65854288	0.8719971	-0.1976048	0.21706
SAMD15	1.129808	1.1733384	1.09385902	0.5414784	-0.8850242	0.21724
CMPK1	2.567274	2.6406658	2.50666594	0.9183259	-0.1229218	0.21727
IKZF4	1.331314	1.284232	1.37019424	1.3024368	0.38121336	0.21735
CLASP2	1.476748	1.4276814	1.51726717	1.2094686	0.27437333	0.21782
XRCC2	1.371502	1.3235114	1.41113421	1.2708491	0.34579279	0.2179

FCHO2	1.197436	1.242132	1.16052459	0.6629631	-0.5929996	0.21798
LZTFL1	1.335861	1.3828962	1.29701928	0.7757174	-0.3663969	0.21812
ZNF720	1.552901	1.6038594	1.51081875	0.8459234	-0.2414011	0.21817
COL13A1	1.240395	1.2854078	1.20322279	0.7120436	-0.4899626	0.21839
SAR1B	1.364069	1.4119902	1.32449465	0.7876271	-0.3444153	0.21862
MLANA	1.079617	1.0372364	1.11461563	3.0780523	1.62201773	0.21884
DDX55	1.225442	1.2702835	1.18841079	0.6970857	-0.520592	0.21938
YPEL2	1.170619	1.1268527	1.20676153	1.6299345	0.70481402	0.21962
SPEG	1.232838	1.1879777	1.26988378	1.4357222	0.52177664	0.21969
VAMP5	1.997756	1.9393767	2.04596666	1.1134689	0.1550612	0.21996
AC004540.4	1.32768	1.3749787	1.28862042	0.7696982	-0.3776351	0.22013
SPARCL1	1.079245	1.0371941	1.11397123	3.0642287	1.615524	0.22031
C2orf69	1.387174	1.4343472	1.34821814	0.8017046	-0.3188573	0.22044
HS3ST3B1	1.085802	1.0437833	1.12050236	2.7522422	1.46060745	0.22052
RAB30-AS1	1.654873	1.7068162	1.61197706	0.8658221	-0.2078575	0.22066
RAB39B	1.201199	1.2455885	1.16454117	0.6699874	-0.5777942	0.22099
ZBTB17	1.224843	1.2695912	1.18788917	0.696941	-0.5208916	0.22107
PTRH2	1.190784	1.2348508	1.15439337	0.6574105	-0.6051336	0.22119
C11orf70	1.190967	1.2350941	1.15452632	0.6572956	-0.6053857	0.22119
GSPT2	1.256339	1.3013502	1.2191678	0.7272862	-0.459405	0.22124
BBS2	1.269564	1.3156424	1.2315122	0.7334635	-0.4472029	0.22126
CAD	1.265483	1.2200525	1.30300036	1.3769456	0.46147152	0.2213
RP11-66N11.8	1.185494	1.2296037	1.14906701	0.6492361	-0.623185	0.22142
HGS	1.470107	1.4213911	1.51033659	1.2110759	0.27628932	0.22153
PIP5K1C	1.258183	1.2130274	1.29547373	1.3870224	0.47199111	0.22168
MYOF	1.257342	1.2125772	1.29430951	1.384483	0.46934735	0.22173
ARHGEF26	1.255739	1.3009861	1.21837334	0.7255263	-0.4629002	0.22174
FAM228B	1.492235	1.5414766	1.45157131	0.8339627	-0.2619452	0.22184
UBE2R2	2.496073	2.5714006	2.43386539	0.9124761	-0.1321414	0.2224
TMEM206	1.248769	1.2937931	1.21158827	0.7201949	-0.4735408	0.22258
KIAA0141	1.280963	1.2353208	1.31865448	1.354128	0.43736412	0.2228
ATP13A3	1.302037	1.2560242	1.34003514	1.3281367	0.40940362	0.2228
LAMP1	1.919795	1.975062	1.87415538	0.8965126	-0.1576042	0.22288
RASSF2	1.132139	1.0892358	1.16756891	1.8778208	0.90905936	0.22305
TMEM88	1.162134	1.1188634	1.19786778	1.6646647	0.73523162	0.22315
TSR1	1.29766	1.3434175	1.25987251	0.7567247	-0.4021596	0.22316
C19orf48	2.467931	2.5374822	2.41049425	0.9174052	-0.1243689	0.22345
JOSD1	1.314243	1.2680101	1.35242325	1.3149625	0.39502168	0.22348
EDNRA	1.080839	1.0388642	1.11550301	2.9719638	1.57141655	0.22367
CTNND2	1.318214	1.3644077	1.28006591	0.768551	-0.3797871	0.22371
HIF3A	1.619862	1.5692693	1.66164263	1.1622664	0.2169408	0.22385
ALPK2	1.106823	1.0645144	1.14176246	2.1973779	1.13578297	0.22393
EGLN3	1.581748	1.522599	1.6305938	1.2066495	0.27100671	0.22398
CLPTM1L	1.282094	1.2367944	1.31950235	1.3492819	0.43219184	0.22407
RELL2	1.174184	1.1304156	1.21032931	1.6127621	0.68953362	0.22413

MANEA	1.270586	1.3160601	1.2330326	0.7373046	-0.4396673	0.22414
PROS1	1.631948	1.683063	1.58973648	0.8633706	-0.2119482	0.22414
DHRS13	1.329338	1.3753023	1.29137963	0.7763864	-0.3651532	0.22415
STXBP3	1.470645	1.5190987	1.43063089	0.8295743	-0.2695569	0.22425
KIAA1109	1.619346	1.5684491	1.66137698	1.1634761	0.21844163	0.22435
SEC14L1	1.915868	1.8607855	1.96135573	1.1168354	0.15941652	0.22443
NEK3	1.210007	1.2540566	1.17362925	0.6834273	-0.5491401	0.22452
KITLG	1.337427	1.290883	1.37586352	1.2921467	0.36976988	0.22452
MED24	1.393319	1.4403058	1.35451648	0.8051597	-0.3126532	0.22462
CHP1	1.739246	1.7973015	1.69130252	0.8670528	-0.2058082	0.22467
PDLIM7	4.614632	4.4746735	4.73021221	1.0735432	0.10238028	0.22484
FERMT2	1.423317	1.3755276	1.46278169	1.2323506	0.30141273	0.22498
DNAJC17	1.23019	1.2746074	1.19350917	0.7046759	-0.5049683	0.225
CBWD3	1.222938	1.2677588	1.18592501	0.694375	-0.526213	0.22525
FLJ16779	1.235839	1.1914232	1.27251768	1.4236396	0.50958393	0.22525
INSIG1	1.588453	1.5317426	1.63528455	1.194722	0.25667495	0.22538
ZNF302	1.862415	1.9170135	1.81732634	0.8912915	-0.1660308	0.2257
FDXR	1.231565	1.2760267	1.19484747	0.7059007	-0.5024628	0.2263
FAM160B2	1.233111	1.1888549	1.26965908	1.427864	0.51385861	0.2263
CLK1	1.877211	1.8209471	1.92367441	1.1251326	0.17009507	0.22632
NFATC2IP	1.469139	1.4205986	1.50922483	1.2107146	0.27585879	0.22643
RTCA	1.583741	1.5335636	1.62517889	1.1717046	0.22860888	0.22651
SAMD5	1.069469	1.0281523	1.10358857	3.6795716	1.87953782	0.22667
RPP21	1.337774	1.3840594	1.29955022	0.779958	-0.3585317	0.22669
TARBP2	1.412911	1.4602608	1.37380862	0.812167	-0.3001516	0.22672
PHF23	1.541804	1.5915005	1.50076418	0.8465998	-0.2402479	0.22733
ATF1	1.418353	1.4657318	1.37922619	0.8142588	-0.2964407	0.2274
BPNT1	1.271318	1.3164079	1.23408264	0.739813	-0.4347675	0.2274
PREP	1.203025	1.2467753	1.1668959	0.6763072	-0.5642493	0.22761
EXOSC2	1.252407	1.2968018	1.21574574	0.7269017	-0.4601679	0.22763
C17orf49	1.185384	1.2289359	1.14941846	0.6526651	-0.6155852	0.22766
CWC25	1.399019	1.4463623	1.35992284	0.8063469	-0.3105274	0.22771
ATP2C1	1.581888	1.6318528	1.54062661	0.8556211	-0.2249561	0.2278
CTBP1	1.60728	1.5567866	1.64897869	1.1655788	0.2210465	0.22781
GPRC5C	1.247889	1.2036751	1.2844019	1.3963511	0.48166178	0.22799
CRY1	1.185862	1.1425512	1.22162865	1.5547303	0.63666435	0.2282
ATPAF2	1.300519	1.3458317	1.26309938	0.7607729	-0.3944622	0.22831
ARHGEF10	1.388217	1.3415255	1.42677626	1.2496175	0.3214866	0.22831
HIST1H2BD	1.151978	1.1949871	1.11646082	0.5972746	-0.7435338	0.22843
SNTB2	1.200834	1.1571462	1.23691175	1.5075878	0.592242	0.22863
TMEM190	1.133108	1.0907755	1.16806637	1.8514516	0.88865687	0.22877
NTAN1	1.675208	1.6239844	1.71750896	1.1498829	0.20148694	0.22885
SETD9	1.379071	1.4259484	1.34035842	0.7990602	-0.3236239	0.2289
CPXM2	1.211151	1.1675853	1.24712907	1.4746462	0.56036888	0.22891
TRIT1	1.450143	1.4979929	1.41062754	0.8245651	-0.2782948	0.22892

ATM	1.882393	1.9369744	1.83731786	0.89364	-0.1622343	0.22902
RNF146	1.636445	1.6869563	1.59473238	0.8657499	-0.2079778	0.22913
PBXIP1	1.366423	1.3199072	1.40483732	1.2654835	0.33968867	0.22928
AES	2.902092	2.994789	2.82554176	0.9151553	-0.1279115	0.22945
GAS7	1.204974	1.1615278	1.24085332	1.4910948	0.57637198	0.22962
HERPUD2	1.402899	1.4500126	1.36399246	0.8088495	-0.3060569	0.22963
ELOVL1	1.400531	1.3538273	1.43909926	1.2409989	0.31150186	0.22976
CYB561D2	1.292565	1.3375304	1.25543148	0.7567659	-0.4020811	0.22992
AK3	2.193342	2.2564873	2.14119492	0.9082423	-0.1388509	0.23002
GPR153	1.16057	1.1178179	1.19587546	1.6625271	0.73337782	0.23015
CTD-2256P15.2	1.136152	1.1786026	1.10109564	0.5660369	-0.821032	0.23021
TMOD3	1.654857	1.705992	1.61262975	0.8677574	-0.2046364	0.23026
RASGRP1	1.132589	1.090485	1.16736002	1.8495879	0.88720384	0.23038
ICA1	1.230214	1.2741612	1.19392178	0.7073275	-0.4995497	0.23042
CNFN	1.201834	1.2455401	1.16574076	0.675005	-0.5670299	0.23049
LINC01164	1.125679	1.0838692	1.16020675	1.9101972	0.93372161	0.23051
PDK1	2.225525	2.2930541	2.16975847	0.9046477	-0.144572	0.23051
PRSS8	1.13133	1.0891031	1.16620236	1.8652821	0.89939381	0.23053
WDR20	1.251758	1.2961277	1.21511721	0.7264339	-0.4610965	0.23098
GTF3C1	1.248958	1.20491	1.2853333	1.3924813	0.4776579	0.23112
HIST1H1C	1.396742	1.4429479	1.35858473	0.8095415	-0.3048231	0.23133
TNFAIP1	1.511258	1.4631077	1.55102121	1.1898339	0.25076024	0.23156
COPS7B	1.50434	1.5529733	1.4641786	0.8394232	-0.2525298	0.2316
DCK	1.504123	1.5526171	1.46407515	0.839777	-0.2519218	0.2316
HEPH	1.327879	1.3737329	1.29001213	0.7759877	-0.3658942	0.23169
LLGL2	1.096338	1.0548234	1.13062102	2.3825766	1.25252262	0.23171
KIF9	1.429618	1.4770741	1.39042855	0.8183813	-0.2891549	0.23172
RBM15B	1.612115	1.6625817	1.57043846	0.8609331	-0.216027	0.23183
EPB41	1.482622	1.5308447	1.44279981	0.834142	-0.2616351	0.23185
NR2F2-AS1	1.217986	1.1742203	1.25412935	1.4586668	0.54465035	0.23223
ZDHHC24	1.514537	1.5630551	1.47446927	0.8426693	-0.2469615	0.23228
ZADH2	1.431476	1.4791232	1.39212738	0.8184271	-0.2890742	0.23242
RAB1B	1.482299	1.5304189	1.44256048	0.8343604	-0.2612574	0.23246
SST	1.072211	1.0257826	1.11055309	4.2878936	2.10026912	0.23281
AGAP3	1.25365	1.2095479	1.29007037	1.3842678	0.46912305	0.23303
GSTK1	2.068151	2.1309587	2.01628424	0.8986042	-0.1542423	0.23311
HMOX2	1.546477	1.59562	1.50589343	0.849356	-0.2355588	0.23312
SLC36A4	2.010273	2.067537	1.9629832	0.9020607	-0.1487036	0.23339
LPCAT2	1.111026	1.069489	1.14532778	2.0913787	1.06445434	0.23348
MSL3	1.922164	1.9768688	1.87698809	0.8977542	-0.1556075	0.23354
MBOAT2	1.699802	1.6483538	1.74228936	1.1448832	0.19520044	0.23367
TGFBR3L	1.207046	1.1638778	1.24269514	1.4809518	0.56652472	0.23382
FNIP2	1.387682	1.3413922	1.42590822	1.2475627	0.31911236	0.23392
ZC3HC1	1.207393	1.2505618	1.17174273	0.6854305	-0.5449178	0.23394
CEP68	1.342909	1.3885167	1.30524554	0.785669	-0.3480064	0.23394

CPQ	1.165985	1.1237422	1.20086913	1.6232878	0.69891882	0.23445
UTP14C	1.211726	1.1683986	1.24750609	1.4697635	0.55558401	0.2346
AFG3L2	1.530692	1.5791636	1.49066277	0.8471921	-0.239239	0.23466
RIF1	1.877193	1.8210471	1.9235594	1.1248556	0.16973979	0.23473
SRSF12	1.260803	1.3048856	1.22439918	0.7360111	-0.4422005	0.23483
PLCH1	1.274898	1.3192189	1.23829648	0.7464987	-0.4217883	0.23491
NPTN	1.164032	1.1216814	1.19900565	1.6354644	0.7097004	0.23491
PELO	1.424919	1.4716018	1.38636676	0.8192649	-0.2875982	0.23514
ZNF514	1.356664	1.4026082	1.31872264	0.7916448	-0.3370749	0.23533
USP47	1.915922	1.9700511	1.87122127	0.898119	-0.1550216	0.23547
KANSL3	1.32082	1.365754	1.2837123	0.7756917	-0.3664448	0.2355
CADM4	1.489888	1.4421083	1.52934434	1.1973182	0.2598066	0.23559
STUB1	2.979148	2.8936606	3.04974473	1.0824245	0.11426644	0.23573
FPGS	1.320184	1.3657947	1.2825178	0.7723397	-0.3726925	0.23575
ATP2B1	1.984689	2.0420715	1.93730085	0.8994593	-0.1528701	0.23581
RPH3AL	1.445368	1.4923246	1.40659018	0.825858	-0.2760344	0.23595
CCDC106	1.604536	1.654545	1.56323817	0.8605034	-0.2167473	0.23605
GARS	1.57955	1.6291533	1.5385869	0.8560503	-0.2242325	0.23606
RANGRF	2.032287	2.0896914	1.98488169	0.9038171	-0.1458973	0.23609
TMEM237	1.543129	1.5919202	1.50283567	0.8494991	-0.2353156	0.23625
DFFA	1.906755	1.9606884	1.86221548	0.8974975	-0.1560202	0.23631
LRIF1	1.376348	1.422287	1.33841118	0.8013772	-0.3194466	0.23637
PPP1R21	1.218242	1.2614121	1.18259083	0.698479	-0.5177113	0.23645
PIEZO2	1.287025	1.3312677	1.25048846	0.7561512	-0.4032533	0.23662
SFXN4	1.34817	1.393902	1.31040454	0.7880248	-0.343687	0.23679
RBM45	1.126871	1.1684311	1.09255015	0.5494838	-0.8638513	0.23742
MEN1	1.342547	1.3878855	1.30510657	0.7865893	-0.3463175	0.23757
CENPB	1.175271	1.1327779	1.21036238	1.584318	0.66386193	0.23765
RASSF1	1.252138	1.2958153	1.21606869	0.7304177	-0.4532064	0.23767
EFEMP1	1.198578	1.1560789	1.23367521	1.4971606	0.58222903	0.23768
HDAC6	1.380046	1.4257851	1.34227345	0.8038643	-0.314976	0.23797
SSH1	1.184314	1.1419379	1.21930807	1.5450991	0.62769934	0.23804
MND1	1.36346	1.4086513	1.32614091	0.798091	-0.3253749	0.23813
MSH3	1.369054	1.4146057	1.33143645	0.7994016	-0.3230077	0.23822
GSS	1.397521	1.4434807	1.35956641	0.8107826	-0.302613	0.23825
CPSF3	1.468996	1.5162819	1.42994656	0.8327748	-0.2640017	0.23827
MAGI3	1.538604	1.5869288	1.49869688	0.8496719	-0.2350223	0.23827
LTBP2	1.130061	1.0885199	1.16436682	1.856834	0.89284486	0.2384
RBM24	1.145885	1.1042775	1.18024541	1.7285166	0.78953442	0.23859
TMEM167A	2.288279	2.2289924	2.33723936	1.0880778	0.12178173	0.23864
REPS1	1.286201	1.330677	1.24947282	0.7544305	-0.40654	0.23874
RP11-722G7.1	1.343025	1.2978227	1.3803536	1.277114	0.35288734	0.23882
CD2BP2	1.47396	1.5210936	1.43503626	0.8348524	-0.2604069	0.23902
VGLL3	1.070457	1.0300787	1.10380235	3.451026	1.78702535	0.23914
HERC2	1.862218	1.9154249	1.81827891	0.8938788	-0.1618488	0.2392

RP11-136K7.2	1.118179	1.1594928	1.08406197	0.527058	-0.9239665	0.2394
OTX2-AS1	1.072683	1.0323426	1.10599685	3.277314	1.71251391	0.23945
TRAF3IP2-AS1	1.334996	1.3801407	1.29771556	0.783172	-0.352599	0.23959
ASB8	1.288759	1.3329173	1.25229178	0.7578211	-0.4000707	0.23965
EPB41L3	1.625299	1.5760113	1.66600142	1.1562298	0.20942813	0.2397
KHSRP	1.808844	1.8608238	1.76591911	0.8897514	-0.1685259	0.23973
NOVA1	2.730548	2.8193888	2.6571815	0.9108452	-0.1347222	0.23976
GPAT4	1.36685	1.4127197	1.32897045	0.7970795	-0.3272044	0.23978
NSDHL	1.180405	1.22266	1.14551102	0.6535123	-0.6137138	0.23981
RAP1GDS1	1.396319	1.4423369	1.35831632	0.810053	-0.3039117	0.23985
PIK3CB	1.127512	1.086163	1.16165851	1.876193	0.90780828	0.2399
RARG	1.097463	1.0567268	1.1311042	2.3111509	1.20861146	0.23999
CLVS1	1.233218	1.2763614	1.19759019	0.7149704	-0.4840446	0.24043
ZNF224	1.389671	1.3440238	1.42736759	1.2422617	0.31296918	0.24045
VWA1	1.402805	1.4485717	1.36500929	0.8137145	-0.2974055	0.24059
TRIM56	1.839489	1.787386	1.88251578	1.1208172	0.16455104	0.24076
INSR	1.543246	1.4957473	1.58247088	1.1749351	0.23258111	0.24094
LMO4	2.381497	2.4486706	2.32602473	0.915339	-0.1276219	0.24113
RNF170	1.598622	1.6475843	1.55818727	0.861953	-0.2143189	0.24117
NDRG4	1.465667	1.5125912	1.42691687	0.8328604	-0.2638534	0.2414
SENP5	1.563621	1.5152488	1.60356746	1.1714098	0.2282459	0.24156
NAGK	1.64327	1.6928526	1.60232391	0.8693392	-0.2020088	0.24159
UBE3D	1.266892	1.3105815	1.23081339	0.7431653	-0.4282449	0.24186
FAIM	1.276393	1.3199309	1.24043879	0.7515334	-0.4120908	0.24205
KIAA2022	1.152487	1.1941101	1.11811442	0.6084919	-0.7166901	0.2422
GNPDA2	1.342959	1.3872813	1.30635777	0.7910472	-0.3381643	0.24235
SRD5A3	1.201238	1.2437864	1.16610172	0.6813413	-0.5535504	0.24241
KIAA2026	1.437143	1.3908062	1.47540783	1.2164797	0.2827123	0.24249
DENND3	1.180166	1.1382701	1.21476362	1.5532178	0.63526016	0.24316
PRR14L	1.555023	1.6032433	1.51520231	0.8540539	-0.2276009	0.2435
ISG20L2	1.539043	1.5867868	1.49961472	0.8514416	-0.2320204	0.24358
SGCD	1.095056	1.0545056	1.12854252	2.3583379	1.23777043	0.24368
MAP7D3	1.516431	1.5642478	1.47694343	0.845273	-0.2425107	0.2438
ANXA2	12.46781	12.981131	12.043893	0.9217738	-0.1175153	0.24387
CELF3	1.066678	1.1068263	1.03352236	0.3138024	-1.6720718	0.24389
SARS2	1.192168	1.2342288	1.15743262	0.6721317	-0.5731841	0.24392
IL13RA1	1.735729	1.786066	1.69416005	0.8830811	-0.1793821	0.24392
MT1H	1.140489	1.0845239	1.18670532	2.208905	1.14333136	0.24404
UBE2M	1.313663	1.2698675	1.34982986	1.2963024	0.3744023	0.24434
LIX1L	1.640357	1.6899758	1.59938068	0.8686982	-0.2030731	0.24446
ALAD	1.259203	1.2159783	1.29489815	1.3654062	0.44933025	0.24466
CDC42EP4	1.318238	1.3626001	1.28160225	0.7766193	-0.3647205	0.24493
GPR107	1.227919	1.1852146	1.26318586	1.4209779	0.50688409	0.2452
SMIM3	1.365779	1.3209135	1.40282923	1.2552581	0.32798407	0.24525
RP11-455F5.3	2.402761	2.4678206	2.34903391	0.9190728	-0.121749	0.24539

UTP6	1.265886	1.3089833	1.23029486	0.7453311	-0.4240467	0.2454
RMDN3	1.291216	1.3348643	1.2551696	0.7620089	-0.3921203	0.2456
MED6	1.6364	1.6851826	1.59611505	0.870009	-0.2008977	0.24573
TCF7	1.238756	1.1959699	1.27408901	1.3986278	0.48401207	0.24579
KCTD17	1.293643	1.3373623	1.25753815	0.7633875	-0.3895126	0.24634
PRRC2C	3.674404	3.7773314	3.58940464	0.9323355	-0.1010789	0.24638
ORC3	1.349236	1.3937445	1.31247943	0.7936097	-0.3334984	0.2464
TSHZ1	1.26268	1.3061862	1.22675116	0.7405662	-0.4332994	0.2464
CDH2	3.315289	3.2215963	3.39266186	1.0770012	0.10701985	0.24653
ZNF140	1.181047	1.2227533	1.14660435	0.6581466	-0.6035192	0.24653
PFN1	21.55027	22.117066	21.0821954	0.9509936	-0.0724924	0.24658
FGFR1	1.839264	1.787637	1.88189836	1.1196762	0.16308154	0.24669
SDAD1	1.57121	1.6190024	1.53174177	0.8590303	-0.2192191	0.24693
LIG3	1.38918	1.434159	1.35203566	0.810845	-0.302502	0.24701
WDR59	1.380539	1.4256325	1.34330014	0.8065647	-0.3101378	0.24734
GPBP1	3.976388	4.0829457	3.88839021	0.936893	-0.0940438	0.24737
CNKSR3	1.161011	1.1197546	1.19508135	1.6290097	0.70399522	0.24759
RP11-617F23.1	1.142035	1.1830873	1.10813291	0.5906085	-0.7597261	0.24764
NPC1	1.113708	1.07322	1.14714423	2.0096171	1.00692061	0.24765
BBX	3.214287	3.2968557	3.14610015	0.9343644	-0.0979428	0.24767
RP11-539L10.3	1.296934	1.3409433	1.26059016	0.764321	-0.3877494	0.24768
PSMA3-AS1	1.970971	2.0249218	1.92641679	0.9038903	-0.1457805	0.24786
FIGNL1	1.207832	1.1654155	1.2428603	1.4681834	0.55403223	0.24792
CLN8	1.415659	1.3706365	1.45283844	1.2217858	0.28899133	0.24801
TNFRSF10D	1.183607	1.1422103	1.21779307	1.5314854	0.61493165	0.24805
MEF2A	1.480965	1.4345859	1.5192655	1.1948513	0.25683111	0.24807
EFNB2	1.516419	1.4695349	1.55513755	1.1823137	0.24161284	0.24819
UTP14A	1.120584	1.161034	1.08717969	0.5413746	-0.885301	0.24841
CTC-524C5.2	1.226096	1.2683167	1.19122904	0.7126991	-0.4886351	0.24858
REXO1	1.108516	1.0683205	1.141711	2.0742089	1.0525612	0.24863
GUSB	1.302781	1.3461411	1.26697296	0.7712835	-0.3746668	0.2487
SMUG1	1.55067	1.5982757	1.51135662	0.8547174	-0.2264806	0.2488
UNC13D	1.090204	1.05014	1.12329031	2.458921	1.29802538	0.24884
AP4M1	1.515793	1.5629803	1.47682527	0.8469662	-0.2396237	0.24908
MAN1B1-AS1	1.196804	1.238471	1.16239432	0.6809815	-0.5543125	0.24916
SFXN5	1.327804	1.2838104	1.36413427	1.2830196	0.35954316	0.24918
RMND1	1.280496	1.3238495	1.24469356	0.7555781	-0.4043472	0.2494
PCIF1	1.324056	1.367819	1.28791524	0.7827633	-0.353352	0.24942
TMEM169	1.262042	1.3050929	1.22648916	0.7423612	-0.4298068	0.24957
SYT5	1.242926	1.2854066	1.20784408	0.7282386	-0.4575169	0.2496
FAM200A	1.238767	1.2810599	1.20384114	0.7252587	-0.4634325	0.24963
ST6GAL2	1.118468	1.078249	1.15168073	1.9384362	0.95489325	0.24973
FAM114A2	1.207518	1.249507	1.17284303	0.6927383	-0.5296176	0.24981
FAM63B	1.942741	1.9959248	1.89882086	0.9024987	-0.1480032	0.24993
SLX4IP	1.272526	1.3159404	1.23667297	0.7491063	-0.4167576	0.25015

NSMCE2	1.536943	1.5840094	1.49807498	0.8528544	-0.2296287	0.25037
SEC23A	2.049011	2.1033898	2.00410423	0.9100177	-0.1360335	0.25043
TMEM41B	1.569567	1.5217804	1.60903055	1.1672163	0.22307194	0.25043
ARMCX3	2.383265	2.4443774	2.33279736	0.9227487	-0.1159903	0.25045
LINC00545	1.069526	1.030297	1.10192161	3.3640822	1.75021295	0.25051
WDYHV1	1.515612	1.5625218	1.47687309	0.8477416	-0.2383036	0.25056
EPN2	1.48691	1.4410613	1.52477242	1.1897947	0.25071266	0.25081
KIFC3	1.260523	1.2178077	1.29579822	1.3580703	0.44155812	0.2509
LRP8	1.149055	1.1083725	1.18265196	1.6854082	0.75309805	0.25092
COX16	1.386732	1.4312494	1.34996937	0.8115242	-0.3012939	0.25107
SNHG9	1.764889	1.8151984	1.72334273	0.8873211	-0.1724718	0.25117
SCAMP2	1.406889	1.4518445	1.3697641	0.8183436	-0.2892213	0.25144
RBFA	1.534058	1.5805354	1.49567607	0.8538258	-0.2279863	0.25183
CCDC102A	1.170276	1.2112684	1.13642371	0.6457365	-0.6309826	0.25193
DDAH1	1.514263	1.4678534	1.55258915	1.1811161	0.24015075	0.25218
CCDC127	1.320868	1.3644029	1.28491549	0.7818694	-0.3550005	0.25219
UBALD2	1.229046	1.271277	1.1941709	0.7157661	-0.4824398	0.2523
TMEM129	1.796879	1.847588	1.75500219	0.8907655	-0.1668823	0.2523
ZCWPW1	1.157443	1.1985777	1.1234735	0.6217893	-0.6855022	0.25239
SDC4	1.43369	1.3874296	1.4718933	1.2180103	0.28452634	0.25257
DESI2	1.790622	1.7402448	1.83222422	1.1242553	0.16896974	0.25271
ATP13A2	1.415569	1.3708905	1.45246488	1.2199419	0.28681243	0.25301
CTD-2547G23.4	1.281564	1.3245329	1.24607879	0.7582553	-0.3992445	0.25306
LINC00908	1.493196	1.5398259	1.45468814	0.8422866	-0.2476169	0.25309
WFS1	1.158347	1.1175225	1.1920606	1.6342447	0.70862403	0.25336
B9D2	1.218142	1.2599055	1.18365325	0.7066155	-0.5010026	0.25336
RBM4B	1.492483	1.5387791	1.45424997	0.8431098	-0.2462076	0.25342
CINP	1.386816	1.4313316	1.35005342	0.8115646	-0.3012222	0.25342
MYOM2	1.119822	1.1599795	1.08665888	0.5416874	-0.8844677	0.25351
KMT2A	1.951717	2.006279	1.90665902	0.9010016	-0.1503984	0.25354
PRUNE2	1.093917	1.0543473	1.1265942	2.3293573	1.21993195	0.25361
RCOR3	1.338797	1.295	1.37496589	1.2710707	0.34604423	0.25366
PGPEP1	1.292618	1.2497114	1.32805106	1.3137209	0.3936588	0.25368
PCDH18	1.119357	1.0792097	1.1525113	1.9254124	0.94516747	0.25372
PABPC5	1.20967	1.2516721	1.17498372	0.6952845	-0.5243246	0.25393
PPP2CB	1.553668	1.5071428	1.59208975	1.167501	0.22342377	0.25396
FOXN3	1.503288	1.4568719	1.5416184	1.1854928	0.24548693	0.25411
PTPN14	1.140993	1.1006413	1.17431667	1.7320584	0.79248754	0.2545
SEC24A	1.161151	1.1205705	1.1946622	1.6145093	0.69109573	0.25476
GTF3C3	1.303715	1.3466337	1.26827297	0.773938	-0.36971	0.25497
CPSF1	1.337582	1.2939996	1.37357357	1.2706601	0.3455782	0.25526
TMEM47	2.111795	2.0557923	2.15804305	1.0968474	0.13336288	0.25527
DDX11	1.315528	1.272226	1.3512883	1.2904287	0.36785044	0.25542
RP11-12G12.7	1.479297	1.5249362	1.44160653	0.8412576	-0.2493805	0.25554
REEP5	2.236981	2.3019549	2.18332512	0.9088833	-0.137833	0.25589

STT3B	1.922857	1.8701253	1.96640293	1.110648	0.15140171	0.25593
ADAM17	1.716916	1.7665824	1.67590022	0.8817059	-0.1816306	0.25617
TMEM155	1.19036	1.2316829	1.15623532	0.6743498	-0.568431	0.25626
YTHDF1	1.245071	1.2031801	1.27966468	1.3764372	0.46093876	0.25692
SLC25A22	1.401319	1.4453113	1.36498921	0.8196271	-0.2869604	0.25706
GCLM	1.283973	1.3264845	1.24886559	0.7622584	-0.3916479	0.25714
CHST10	1.153798	1.1945339	1.12015815	0.6176721	-0.695087	0.25723
DDX19A	1.326134	1.3693756	1.29042489	0.7862589	-0.3469236	0.25735
ELAC1	1.212192	1.2534217	1.17814366	0.7029534	-0.508499	0.25747
DNAJA3	1.421732	1.4661568	1.38504514	0.8259991	-0.2757878	0.25762
CD70	1.112963	1.0735405	1.14551888	1.9787587	0.98459571	0.25799
ST14	1.069751	1.03101	1.10174313	3.2809813	1.71412737	0.25807
SCAPER	1.455362	1.5007822	1.41785349	0.8344016	-0.2611862	0.25816
KLC4	1.272231	1.3145478	1.23728416	0.7543659	-0.4066637	0.2582
TRIM44	2.182334	2.2416943	2.1333129	0.9127149	-0.1317638	0.2582
FABP4	1.097863	1.0588781	1.1300582	2.2089411	1.14335492	0.2583
SMAD7	1.151602	1.1115119	1.18470922	1.6564085	0.72805851	0.25851
CTC-338M12.5	1.411797	1.4561053	1.37520686	0.8226322	-0.2816806	0.25863
TMEM260	1.338714	1.3820231	1.30294857	0.7930112	-0.3345869	0.25878
ZBTB1	1.286397	1.2438853	1.32150336	1.3182567	0.39863128	0.25878
OXSM	1.165761	1.2062694	1.13230857	0.6414359	-0.6406229	0.25879
SOX5	1.219309	1.2609546	1.18491665	0.7086161	-0.4969238	0.25892
MAP3K13	1.88487	1.937141	1.84170311	0.8981606	-0.1549547	0.25899
TMEM33	1.643176	1.6914539	1.60330757	0.8725204	-0.1967393	0.25902
EFS	1.357832	1.3141423	1.39391109	1.2539258	0.32645193	0.25913
C9orf69	1.269784	1.2277623	1.30448556	1.3368569	0.41884503	0.25941
ZFHX4	1.984313	1.9305139	2.0287413	1.1055626	0.14478069	0.25941
CACNA2D3	1.262923	1.304915	1.22824474	0.7485521	-0.4178254	0.25962
THAP8	1.416016	1.4604386	1.37933053	0.823846	-0.2795534	0.25982
PCAT7	1.474754	1.5202229	1.43720537	0.8404193	-0.2508188	0.25986
NELL2	2.041175	2.0959266	1.9959608	0.9087842	-0.1379904	0.26005
DBP	1.632479	1.5848073	1.67184762	1.1488359	0.20017268	0.26006
ARL8B	1.350279	1.307121	1.38592005	1.2565733	0.32949481	0.26007
CCDC22	1.409443	1.453785	1.37282464	0.8215888	-0.2835116	0.26015
ZNF268	1.334571	1.3775603	1.29906988	0.7921116	-0.3362243	0.26051
DLX1	1.050224	1.0888069	1.01836134	0.2067559	-2.2739998	0.26061
TMEM99	1.462368	1.5075928	1.42502155	0.8373278	-0.2561355	0.26077
NR2E1	1.166957	1.207059	1.13384032	0.6463874	-0.629529	0.2609
PFKFB4	1.609697	1.6567935	1.57080472	0.8690779	-0.2024426	0.26097
SREK1	2.151076	2.2090308	2.10321545	0.9124792	-0.1321365	0.26121
RAD51C	1.579398	1.6262219	1.54073025	0.8634803	-0.2117648	0.26122
ADARB1	1.614881	1.6622449	1.57576796	0.8694184	-0.2018774	0.26165
ATOX1	5.268991	5.13031	5.3835169	1.0613046	0.08583874	0.26191
NSG1	1.1059	1.1451957	1.07344819	0.5058567	-0.9831994	0.26193
TMEM184B	1.44687	1.4024729	1.48353305	1.2014053	0.26472289	0.26275

CCDC112	1.441608	1.4859634	1.40497794	0.8333506	-0.2630045	0.26286
PSTK	1.310091	1.3524823	1.27508342	0.7804177	-0.3576817	0.26338
AARS	1.299575	1.3418974	1.26462487	0.7739891	-0.3696148	0.2634
C17orf75	1.447391	1.4916975	1.41080182	0.8354767	-0.2593285	0.26341
GBP1	1.057523	1.0194662	1.08895086	4.5695031	2.1920373	0.26353
PITPNB	1.497259	1.5426653	1.45976145	0.8472284	-0.2391772	0.26361
DRAIC	1.145168	1.1849413	1.11232299	0.607344	-0.7194143	0.26378
RBM26	1.820245	1.7709201	1.86097779	1.1168185	0.15939474	0.26407
CELF2	2.065153	2.0096787	2.11096551	1.1003159	0.13791778	0.26426
SAP30BP	1.520784	1.5664383	1.48308187	0.8528411	-0.2296511	0.26432
XPOT	1.674442	1.7224401	1.63480387	0.8786942	-0.186567	0.26448
RP11-1275H24.1	1.204278	1.2449004	1.17073117	0.6971453	-0.5204687	0.26458
MALL	1.071812	1.0334181	1.10351838	3.0976694	1.63118316	0.26473
RNF138	1.386382	1.4295117	1.35076515	0.8166604	-0.2921919	0.26536
DEGS1	1.617483	1.6642164	1.57888894	0.8715366	-0.1983668	0.26541
PCMTD1	2.190113	2.2592233	2.13304059	0.8997932	-0.1523346	0.26576
STARD9	1.211955	1.2527931	1.17823079	0.705046	-0.5042106	0.26576
MIF-AS1	1.170425	1.210681	1.13718123	0.6511323	-0.6189774	0.2658
ZNF362	1.224774	1.1841772	1.25829952	1.4024511	0.48795049	0.26589
TBC1D20	1.361349	1.4045992	1.32563232	0.8048268	-0.3132497	0.26596
PAM	1.965755	2.0173604	1.92313852	0.907386	-0.1402118	0.26622
BORA	1.110369	1.1492067	1.07829563	0.5247459	-0.930309	0.26625
MGRN1	1.511884	1.4666461	1.54924166	1.1769982	0.23511213	0.26633
MRRF	1.467546	1.5122244	1.43065055	0.8407458	-0.2502584	0.26662
AC245100.1	1.127933	1.0888984	1.16016792	1.8016958	0.84935544	0.26679
UGGT2	1.394714	1.4382232	1.35878387	0.818724	-0.2885509	0.2668
EIF4E2	2.170065	2.2285388	2.12177718	0.9130987	-0.1311573	0.26685
ANKRD36B	1.339943	1.2973767	1.37509486	1.2613457	0.33496378	0.2669
ACAA1	1.36663	1.4093987	1.33131093	0.8092623	-0.3053208	0.26698
OCEL1	1.551357	1.5969487	1.51370678	0.8605543	-0.2166619	0.26709
PEX3	1.204247	1.2449933	1.17059808	0.6963378	-0.5221408	0.26722
TMED5	1.520554	1.5658071	1.48318363	0.8539724	-0.2277387	0.26802
BRD4	1.774195	1.7257988	1.81416047	1.121744	0.16574347	0.2681
CELF5	1.317501	1.3597978	1.28257102	0.7853607	-0.3485728	0.26813
ANTXR2	1.243668	1.2844967	1.20995025	0.7379708	-0.4383645	0.26832
PHAX	1.528796	1.5737959	1.49163493	0.8568115	-0.2229502	0.26854
WDR43	1.453789	1.4981255	1.41717487	0.8374896	-0.2558569	0.26856
BDNF	1.118156	1.0794707	1.15010363	1.8887911	0.91746315	0.26885
VSIG10L	1.218417	1.2592411	1.18470291	0.7124755	-0.4890876	0.26896
AGFG1	1.269597	1.2285508	1.30349375	1.3279052	0.40915212	0.26914
MYD88	1.2142	1.2546304	1.1808127	0.7100986	-0.4939087	0.26919
MEIS2	3.223919	3.1248287	3.30574862	1.0851457	0.11788872	0.26929
ARID3B	1.203169	1.2434533	1.16990192	0.697883	-0.5189429	0.26969
MIR99AHG	1.58565	1.539706	1.62359177	1.1554286	0.20842814	0.26979
ALDH6A1	1.333187	1.3750748	1.29859577	0.7960966	-0.3289845	0.27003

EHMT1	1.75543	1.8035518	1.71569072	0.8906591	-0.1670547	0.27009
SUPT20H	1.920514	1.971281	1.87859008	0.9045684	-0.1446985	0.27025
CLCN5	1.462286	1.4184151	1.49851501	1.1914365	0.25270203	0.2703
FXYD5	1.642258	1.5960221	1.68043949	1.1416347	0.19110113	0.27031
PPP2R2B	1.182185	1.1425614	1.21490618	1.5074641	0.59212364	0.27031
AACS	1.319371	1.3615973	1.28450039	0.7867879	-0.3459534	0.27046
AGAP1	1.389465	1.3468197	1.42468225	1.2245045	0.29219803	0.27048
PSAP	4.183466	4.0666319	4.27994895	1.0695607	0.09701833	0.27063
SMAD6	1.083294	1.0452047	1.11474957	2.5384428	1.34394376	0.27083
NCAPH2	1.255055	1.2958039	1.22140458	0.7484844	-0.4179559	0.27092
TOMM70	1.348306	1.3905565	1.31341502	0.8024831	-0.317457	0.27113
PUM1	1.979395	1.9282076	2.02166573	1.1006867	0.1384039	0.27158
SCN5A	1.178908	1.139133	1.21175444	1.5219569	0.6059275	0.27172
SSBP3	1.774351	1.8227652	1.73436927	0.8925624	-0.163975	0.27179
GRWD1	1.212928	1.2527857	1.18001344	0.7121188	-0.4898101	0.272
SLC16A10	1.082545	1.0446282	1.11385714	2.5512358	1.35119627	0.27209
OGFRL1	1.45535	1.4122946	1.49090517	1.1906659	0.2517687	0.27293
CCNF	1.224531	1.2644541	1.19156234	0.724369	-0.4652033	0.27296
GORASP1	1.149568	1.1884889	1.11742609	0.6229869	-0.6827264	0.27336
ACSF3	1.196435	1.2361479	1.16363982	0.6929549	-0.5291666	0.27346
TBK1	1.19332	1.1535278	1.22618188	1.4732311	0.55898377	0.27348
FCHO1	1.193636	1.2333027	1.16087792	0.6895675	-0.5362364	0.27377
RAB5B	1.717494	1.6703971	1.75638664	1.1282665	0.17410791	0.27383
SCAMP3	1.363099	1.3208406	1.39799709	1.2404824	0.31090131	0.27389
PPP1R12A	1.992801	2.0450364	1.94966377	0.9087375	-0.1380645	0.27391
MICU1	1.51702	1.5616937	1.4801273	0.8547849	-0.2263666	0.27416
RNF6	1.335882	1.3779118	1.30117349	0.7969412	-0.3274549	0.2742
PAXIP1-AS1	1.765406	1.8135137	1.7256778	0.892029	-0.1648376	0.27426
RP11-758H9.2	1.220226	1.2604248	1.18702906	0.7181691	-0.4776045	0.27435
GEN1	1.138059	1.0993407	1.17003284	1.7116123	0.77535596	0.27435
ZSCAN5A	1.139448	1.1781806	1.10746274	0.6031115	-0.7295034	0.27443
CDC16	1.578091	1.6235383	1.54055964	0.8669229	-0.2060245	0.2748
PLA2R1	1.115426	1.0773511	1.14686934	1.8987351	0.92503861	0.27485
PI4KA	1.324127	1.2826453	1.3583826	1.2679588	0.34250789	0.27503
ZHX1	1.66039	1.7069653	1.62192796	0.8797149	-0.184892	0.27509
AIF1	1.161168	1.2003816	1.12878565	0.6427021	-0.6377779	0.27509
SKIL	2.498936	2.5696604	2.44052981	0.9177334	-0.123853	0.27575
ST18	1.04756	1.084812	1.01679584	0.1980361	-2.3361649	0.27601
SMS	5.821849	5.9804	5.6909154	0.9418752	-0.0863921	0.27603
TMUB2	1.536297	1.5807522	1.49958597	0.8602395	-0.2171897	0.27621
SERTAD4-AS1	1.086638	1.0490149	1.1177071	2.4014564	1.26390964	0.27631
PPP4R3B	1.69052	1.7375945	1.65164579	0.8834743	-0.1787399	0.27643
UNC5B-AS1	1.104634	1.0668637	1.13582544	2.0313766	1.02245774	0.27645
GLIS3	1.257179	1.2169197	1.290425	1.3388593	0.42100431	0.27658
LHX1	1.080824	1.0434311	1.11170317	2.5719613	1.36286896	0.27658

WWC3	1.206697	1.1673076	1.23922581	1.4298565	0.51587036	0.27674
TP53I13	1.846544	1.7959872	1.88829425	1.1159655	0.1582924	0.27674
ARHGAP23	1.13111	1.0928396	1.1627141	1.7526372	0.8095274	0.27675
KIAA1324L	1.243262	1.28339	1.2101234	0.7414636	-0.4315521	0.27679
KIDINS220	1.853214	1.8041171	1.89375901	1.1114787	0.15248031	0.27694
DCC	1.103102	1.1414913	1.07139971	0.5046227	-0.9867231	0.27717
LMTK3	1.109149	1.0714181	1.14030731	1.9645916	0.97422941	0.27752
PRKCI	1.539239	1.5838897	1.50236562	0.8603777	-0.216958	0.27755
DMWD	1.243193	1.2029168	1.27645378	1.3623994	0.44614971	0.27759
CSE1L	1.460429	1.5034143	1.42493145	0.8440989	-0.2445161	0.27761
FAM127C	1.32776	1.36887	1.2938108	0.7965159	-0.328225	0.27768
LINC01224	1.260081	1.3003503	1.22682615	0.7552052	-0.4050593	0.27769
FANCA	1.219491	1.1796811	1.25236668	1.4045253	0.49008262	0.27788
NIN	1.489293	1.5328192	1.45334908	0.8508497	-0.2330238	0.2779
AKAP6	1.677143	1.7268698	1.63607818	0.8750923	-0.1924929	0.27795
LAGE3	3.521272	3.6141433	3.44457673	0.9351349	-0.0967535	0.2781
ARMT1	1.364883	1.4069169	1.33017162	0.8113982	-0.3015179	0.27827
ICK	1.24581	1.2858637	1.21273231	0.7441738	-0.4262885	0.27828
CDC37L1	1.274569	1.3153836	1.24086445	0.7637189	-0.3888864	0.27836
OSBPL1A	2.404319	2.3379698	2.45911056	1.0905407	0.12504362	0.27856
CEP76	1.134945	1.1732423	1.10331812	0.5963792	-0.7456981	0.27866
PORCN	1.150563	1.1892769	1.11859314	0.626559	-0.6744776	0.27878
YKT6	1.503268	1.547582	1.46667258	0.8522423	-0.2306644	0.27902
C16orf87	1.909563	1.9588871	1.86883069	0.9060824	-0.1422859	0.27908
C16orf86	1.182589	1.2217916	1.15021573	0.6772832	-0.5621688	0.27913
UBQLN2	1.455043	1.4985043	1.41915187	0.840819	-0.2501329	0.27917
MCF2L	1.191651	1.1522961	1.22415178	1.4718157	0.55759704	0.27938
JOSD2	1.707189	1.7539787	1.6685495	0.8866955	-0.1734893	0.27995
TULP1	1.110053	1.0722195	1.14129739	1.9564994	0.96827465	0.27995
RALGPS1	1.11826	1.0805106	1.14943401	1.8560776	0.89225703	0.28014
ATP6V1B2	1.546339	1.5906734	1.50972697	0.8629591	-0.2126359	0.28034
C17orf62	1.75463	1.8016194	1.71582453	0.8929731	-0.1633114	0.28069
PRR7	2.13292	2.1951636	2.08151894	0.9049129	-0.1441492	0.28086
KIAA1958	1.387135	1.4294823	1.35216375	0.8199727	-0.2863523	0.28092
DENND4B	1.181447	1.142513	1.2135995	1.4988072	0.58381478	0.28099
MAML2	1.56094	1.5165411	1.59760454	1.1569352	0.21030807	0.28116
NQO2	1.962175	2.0128382	1.92033609	0.9086704	-0.138171	0.28116
MRPL34	2.93755	3.0081971	2.87920886	0.9357691	-0.0957754	0.28139
PSME3	1.408635	1.451099	1.37356752	0.8281277	-0.2720749	0.28146
HRC	1.088194	1.1255872	1.05731459	0.456373	-1.1317147	0.2816
PGM5	1.166953	1.1286199	1.19860936	1.5441575	0.62681993	0.28174
EEFSEC	1.183074	1.221914	1.15099842	0.6804367	-0.5554672	0.28187
PARVB	1.316863	1.2765344	1.35016638	1.2662672	0.34058186	0.28195
CYBA	3.073842	3.1575407	3.00472224	0.9291701	-0.1059854	0.28201
RAB32	1.673637	1.6281501	1.71120145	1.1322158	0.17914891	0.28201

MARC1	1.193251	1.2320274	1.16122842	0.6948679	-0.5251894	0.28202
MGST1	1.691378	1.6422796	1.731924	1.1395722	0.18849228	0.28238
SRRM1	3.783274	3.8781271	3.70494219	0.9398272	-0.0895325	0.28244
TKFC	1.240773	1.2805587	1.20791805	0.7410858	-0.4322876	0.28249
KDM2B	1.292292	1.332793	1.25884635	0.7777998	-0.3625293	0.28256
PPWD1	1.335484	1.3766375	1.3014986	0.8005007	-0.3210253	0.28271
PEX7	1.249818	1.2099957	1.2827046	1.3462402	0.4289358	0.28293
POLR3K	1.51767	1.5613583	1.48159214	0.8579051	-0.2211101	0.28301
LANCL1	1.448763	1.4917598	1.41325491	0.8403593	-0.2509218	0.28312
ELOVL6	1.43242	1.4750045	1.39725271	0.8363136	-0.2578841	0.28326
TSPAN13	1.595944	1.6408018	1.55889889	0.8721868	-0.1972909	0.2834
E2F3	1.36492	1.4068278	1.33031195	0.8119207	-0.3005893	0.28355
ZNF419	1.123179	1.085437	1.15434638	1.8065524	0.85323907	0.28383
CDR2	1.136533	1.0986354	1.16782882	1.7015076	0.76681362	0.28395
ZSCAN32	1.205181	1.2448107	1.1724533	0.7044353	-0.5054609	0.28401
SAMD8	1.246471	1.2068438	1.27919582	1.3497908	0.43273587	0.28403
VEGFD	1.127119	1.1647361	1.09605402	0.5830781	-0.7782389	0.28449
YIPF2	1.580547	1.6251198	1.54373832	0.8698146	-0.2012202	0.2848
CAPN7	1.478062	1.4349141	1.51369454	1.1811402	0.24018021	0.28481
FBRS	1.25577	1.2160983	1.28853077	1.3351832	0.4170377	0.28484
MCM5	1.303398	1.3438078	1.27002616	0.7853987	-0.3485029	0.28504
FNDC4	1.628952	1.6743737	1.59144271	0.8770252	-0.1893098	0.28509
MAPK13	1.114449	1.0770004	1.14537499	1.8879766	0.91684088	0.28509
CAMK4	1.548561	1.5929978	1.51186481	0.8631816	-0.212264	0.28519
PPARD	1.145794	1.1080077	1.17699829	1.6387565	0.71260146	0.28546
GPX8	1.913834	1.9629564	1.87326801	0.9068614	-0.141046	0.28559
TMEM63A	1.361175	1.40274	1.32684991	0.8115656	-0.3012205	0.28587
E2F7	1.158836	1.1207555	1.19028359	1.5757756	0.65606208	0.28589
HAUS7	1.12736	1.1649089	1.09635226	0.5842756	-0.7752791	0.286
PLOD1	2.151129	2.2070944	2.10491171	0.9153482	-0.1276074	0.28613
GOLPH3	1.495399	1.4522014	1.53107305	1.1744171	0.23194488	0.28626
ZMYM1	1.234816	1.274142	1.20233932	0.7380821	-0.4381468	0.28645
ERI2	1.207155	1.2461645	1.17494102	0.7106672	-0.4927539	0.28647
NACA2	1.162317	1.2006121	1.13069208	0.6514665	-0.618237	0.28651
LRRC23	1.574776	1.6198077	1.53758885	0.8673479	-0.2053174	0.28659
BIVM	1.384744	1.426158	1.35054308	0.822566	-0.2817967	0.28668
RP11-983P16.4	1.164087	1.2022198	1.13259603	0.6557025	-0.6088867	0.28683
TOPORS-AS1	1.669172	1.7150605	1.63127727	0.8828306	-0.1797915	0.28687
ZNF84	1.531257	1.575061	1.49508318	0.8609229	-0.2160441	0.28706
MPP6	1.364792	1.4062505	1.33055413	0.8136708	-0.2974829	0.28748
MZF1-AS1	1.212708	1.2517625	1.18045635	0.7167723	-0.4804132	0.2875
SGSM3	1.562652	1.6065662	1.52638782	0.8678159	-0.204539	0.28751
YIPF5	1.623413	1.6682535	1.58638291	0.8774857	-0.1885525	0.28776
TRIM26	1.132782	1.1703996	1.1017161	0.596927	-0.7443735	0.2878
ABCD3	1.298042	1.3382023	1.26487656	0.7831896	-0.3525665	0.28801

USP1	1.964621	2.0145426	1.92339566	0.9101596	-0.1358086	0.28813
FAM172A	1.754902	1.8013916	1.7165096	0.8940817	-0.1615214	0.28838
TTC38	1.170413	1.2083908	1.13905052	0.6672584	-0.5836825	0.28844
MGA	1.426535	1.4685448	1.39184277	0.8362973	-0.2579122	0.28858
SP1	1.282787	1.2429939	1.31564944	1.2990014	0.37740297	0.28879
TET1	2.306926	2.3647836	2.25914597	0.9225975	-0.1162266	0.28886
RLN2	1.103362	1.1404439	1.07273956	0.517926	-0.9491821	0.28891
DNM1L	1.683989	1.7294006	1.64648719	0.8863267	-0.1740896	0.28892
ABHD17B	1.31825	1.35817	1.28528378	0.7965039	-0.3282467	0.289
ABHD6	1.084713	1.0480928	1.11495504	2.3902757	1.25717701	0.28916
RP11-430H10.1	1.181108	1.2195255	1.14938153	0.6804747	-0.5553866	0.28922
ALG14	1.211232	1.2502668	1.17899614	0.7152212	-0.4835387	0.2893
EMD	1.603549	1.5595117	1.63991609	1.1437046	0.19371453	0.28957
SYS1	1.476365	1.5190756	1.44109467	0.8497696	-0.2348564	0.28965
CDK12	1.690961	1.7371788	1.65279346	0.8855293	-0.175388	0.28965
CISD3	1.424375	1.3828788	1.45864361	1.1978819	0.26048569	0.28967
CDIP1	1.651957	1.6970071	1.61475434	0.8819914	-0.1811634	0.28967
C21orf91	1.235429	1.2747365	1.20296878	0.7387761	-0.4367908	0.28982
NKILA	1.158615	1.1970135	1.12690457	0.6441416	-0.6345503	0.29033
ACVR1	1.115594	1.0785226	1.14620811	1.8619877	0.89684352	0.2904
KCNIP4	1.113914	1.0767725	1.14458589	1.8833036	0.9132656	0.29058
MAL	1.411857	1.4592503	1.37271892	0.8115811	-0.3011927	0.29059
MCM10	1.164339	1.1262586	1.19578657	1.5506784	0.63289955	0.29066
CNPY2	5.614358	5.4849656	5.72121235	1.0526753	0.07406047	0.2907
KRR1	1.5416	1.5850455	1.5057215	0.8644139	-0.2102058	0.29079
GNAS	6.258693	6.4158682	6.12889501	0.9470125	-0.0785446	0.29115
CYB5R1	1.403303	1.3620781	1.43734712	1.2078806	0.27247791	0.29116
PLK3	1.186293	1.1482628	1.21769946	1.4683348	0.55418096	0.29127
ZFPL1	1.413779	1.4555324	1.37929748	0.8326465	-0.264224	0.29132
BCAS3	1.210359	1.2490128	1.17843816	0.7165823	-0.4807958	0.29141
MBP	1.197231	1.1591154	1.22870671	1.437364	0.52342544	0.29143
F2RL1	1.078991	1.0427241	1.10894081	2.5498665	1.35042169	0.29146
PDE6D	1.371409	1.412754	1.33726571	0.8171106	-0.2913966	0.29187
SHARPIN	1.977146	2.0275119	1.93555328	0.9105036	-0.1352633	0.29235
SIVA1	3.636409	3.7451254	3.54662877	0.9276912	-0.1082834	0.29248
TNKS2	1.493647	1.4511676	1.52872713	1.1719083	0.22885974	0.29251
PIBF1	1.383807	1.4249086	1.34986447	0.8233877	-0.2803563	0.29256
TRDMT1	1.218193	1.2566899	1.18640166	0.7261745	-0.4616119	0.29263
ZNF263	1.264238	1.2247696	1.29683176	1.3206048	0.40119885	0.29271
C1orf21	2.047337	1.99787	2.08818728	1.09051	0.12500304	0.29294
TOR1AIP2	1.869098	1.921679	1.82567643	0.8958394	-0.1586879	0.29299
CORO1B	1.768076	1.814296	1.72990673	0.8963654	-0.1578412	0.29311
CALCRL	1.109001	1.145793	1.07861843	0.539247	-0.8909819	0.29314
MKNK2	1.401011	1.3596146	1.43519666	1.2101751	0.27521577	0.29322
BCAS4	1.452277	1.4110314	1.48633866	1.1832153	0.24271266	0.29329

KDM5B	2.204978	2.261502	2.15829964	0.9181909	-0.123134	0.29341
SRM	1.918276	1.8705049	1.95772699	1.1001971	0.13776203	0.29377
VEPH1	1.112604	1.0759541	1.1428697	1.8810003	0.91150004	0.29384
CPT2	1.201669	1.2396672	1.17028929	0.7105239	-0.493045	0.29433
CSNK1G1	1.250969	1.2121198	1.2830517	1.3343955	0.41618629	0.2947
CPTP	1.270663	1.3099921	1.23818418	0.7683555	-0.3801541	0.29478
ZNF738	1.464802	1.5068604	1.43006908	0.8484961	-0.23702	0.29485
PTAR1	1.895666	1.8474928	1.93544887	1.1037839	0.14245775	0.29491
LMF2	1.383216	1.3423492	1.41696536	1.2179533	0.28445884	0.29502
ACER3	1.242893	1.2041807	1.27486235	1.3461718	0.42886259	0.29503
COG3	1.168808	1.1312722	1.19980642	1.5220767	0.6060411	0.29508
RP11-660L16.2	1.428203	1.470051	1.39364342	0.8374482	-0.2559281	0.29516
OFD1	1.763799	1.8096959	1.7258966	0.8965052	-0.1576161	0.29517
ZNF214	1.142994	1.1801859	1.11228037	0.6231363	-0.6823804	0.29526
CNOT3	1.378509	1.4192145	1.34489347	0.8227137	-0.2815376	0.29544
TMEM134	2.775203	2.7042432	2.83380243	1.0760216	0.10570702	0.29547
NXPH4	1.12314	1.1599426	1.09274826	0.5798845	-0.7861625	0.29551
PYROXD1	1.130007	1.1669907	1.09946461	0.5956297	-0.7475125	0.29552
KHNYN	1.294714	1.2550906	1.3274364	1.283608	0.36020468	0.2959
ZFP82	1.290623	1.3299336	1.25815965	0.7824594	-0.3539122	0.29599
PLEKHM2	1.216939	1.1787583	1.24846901	1.3899715	0.47505532	0.29628
PIGL	1.258167	1.2969722	1.22612113	0.761422	-0.3932318	0.29629
TBC1D22A	1.250941	1.2898856	1.21877979	0.7547108	-0.4060043	0.29636
GPATCH11	1.1898	1.2276437	1.15854856	0.6964767	-0.5218529	0.29639
NUMA1	1.647428	1.6034596	1.68373752	1.1330294	0.18018533	0.29662
RBAK-RBAKDN	1.24729	1.2860284	1.215299	0.7527189	-0.4098169	0.2967
RNF25	1.09863	1.1351087	1.06850618	0.5070451	-0.9798142	0.29719
USP5	1.385341	1.4258919	1.35185357	0.826157	-0.2755121	0.2972
PCNT	1.228038	1.2662717	1.1964632	0.7378299	-0.4386398	0.29733
XPO4	1.185298	1.1473921	1.2166008	1.4695545	0.55537887	0.29733
LNPK	1.943518	1.9918547	1.90360127	0.9110218	-0.1344425	0.29742
ALS2	1.230104	1.1915716	1.26192483	1.3672426	0.45126924	0.29759
TNKS	1.497781	1.4556054	1.53261027	1.1690167	0.22529553	0.2977
SRRM4	1.085271	1.1216625	1.05521884	0.4538689	-1.1396525	0.29773
PLEKHF2	1.157687	1.1206178	1.18829939	1.561125	0.64258605	0.29788
TRIP13	1.193481	1.2311075	1.16240786	0.7027373	-0.5089427	0.2981
TDP2	1.333264	1.3732946	1.30020615	0.804207	-0.3143611	0.29816
SLC41A3	1.566775	1.6101033	1.53099419	0.8703349	-0.2003574	0.29821
PDK2	1.487954	1.5298506	1.45335468	0.8556274	-0.2249454	0.29835
ZNF480	1.344216	1.3843698	1.31105576	0.8092618	-0.3053216	0.2985
C8orf88	1.269447	1.3082688	1.23738801	0.7700683	-0.3769416	0.29856
MOCS2	1.910553	1.9581996	1.87120623	0.9092116	-0.137312	0.2986
INTS8	1.406724	1.447416	1.37312021	0.8339448	-0.2619763	0.29864
NFE2L3	1.161886	1.124871	1.19245294	1.5412137	0.62406692	0.29893
PLPP5	1.414778	1.4554605	1.38118182	0.8369153	-0.2568465	0.29908

EFCAB7	1.157169	1.1942728	1.12652775	0.6512891	-0.6186301	0.2991
NUDT2	1.740484	1.7856867	1.7031551	0.894956	-0.1601113	0.29913
SPRR2F	1.051359	1.0159741	1.08058068	5.044452	2.33469754	0.29921
PPARA	1.146644	1.1099584	1.17693984	1.6091532	0.68630165	0.29952
DDX3X	2.121037	2.0702538	2.16297382	1.0866337	0.11986565	0.29996
MRM2	1.436708	1.4777037	1.40285254	0.8433105	-0.2458641	0.30008
REXO4	1.711144	1.7561566	1.67397259	0.8913134	-0.1659952	0.30014
PTGR2	1.282952	1.3218183	1.25085527	0.7794936	-0.3593909	0.30017
MTA2	1.134949	1.1720379	1.10431984	0.6063771	-0.7217128	0.30028
TNFRSF18	1.14127	1.104457	1.17167049	1.6434561	0.71673292	0.30038
OGFOD2	1.225492	1.2635556	1.19405809	0.7363079	-0.4416189	0.30045
TNFAIP3	1.084144	1.0484217	1.11364452	2.3469767	1.23080354	0.30057
NF2	1.337159	1.2973889	1.37000159	1.2441675	0.31518074	0.30072
DSEL	1.655219	1.6994384	1.61870209	0.8845698	-0.1769521	0.30082
MAP2K3	1.264806	1.3032404	1.2330664	0.7685862	-0.379721	0.3009
ZNHIT1	4.149742	4.2792482	4.0427928	0.9278934	-0.107969	0.30096
WDFY3	1.261585	1.2232803	1.29321738	1.3132254	0.39311461	0.30112
HMBOX1	1.410889	1.4516102	1.37726007	0.8353666	-0.2595186	0.30126
CMIP	1.385541	1.3454686	1.41863357	1.2117849	0.27713361	0.3013
ARFIP1	1.264158	1.3026163	1.23239861	0.7679646	-0.3808882	0.30147
TMED9	4.119555	4.2232642	4.0339095	0.9412537	-0.0873444	0.30151
MIOS	1.205927	1.243423	1.17496226	0.7187582	-0.4764217	0.30175
CUX1	1.918788	1.8704536	1.95870406	1.1013844	0.13931812	0.30181
CDK19	1.608779	1.6520296	1.57306173	0.8788892	-0.1862468	0.30204
MAP1LC3A	1.71012	1.6652649	1.74716196	1.1231045	0.16749216	0.30282
RP11-1094M14.11	1.579256	1.6220438	1.54392036	0.8744084	-0.1936208	0.30286
RP11-314A20.2	1.095554	1.1313986	1.06595223	0.5019248	-0.9944568	0.30298
YAP1	1.837876	1.7923304	1.87548898	1.1049545	0.1439869	0.30322
DHRS2	1.067897	1.0327271	1.09694	2.9620709	1.56660617	0.30327
PJA2	2.289802	2.2330253	2.33668889	1.0840725	0.11646128	0.30339
SKI	1.316435	1.277291	1.34876117	1.2577444	0.33083882	0.3035
TYR	1.053734	1.018682	1.08267995	4.4256459	2.14588804	0.30353
CHRDL1	1.176379	1.2136123	1.14563086	0.6817531	-0.5526787	0.30358
RP11-629G13.1	1.095511	1.1312529	1.06599477	0.5028061	-0.991926	0.30359
TFDP1	1.67288	1.6287279	1.70934136	1.1282167	0.1740442	0.30369
SLC37A1	1.162422	1.1992874	1.13197852	0.6622523	-0.5945472	0.30388
ELMOD2	1.483494	1.52509	1.44914393	0.8553656	-0.2253869	0.30394
AKAP11	1.381636	1.3416109	1.41468916	1.2139225	0.27967634	0.30411
NSUN5	1.624554	1.6685599	1.588213	0.8798209	-0.1847183	0.3042
RABAC1	4.03873	4.1375246	3.9571445	0.9425088	-0.085422	0.30427
EDEM3	1.227991	1.1901856	1.25921205	1.3629424	0.44672456	0.30436
ZMYND11	1.623547	1.6671032	1.58757735	0.8807893	-0.1831312	0.30459
CHRAC1	1.664962	1.708877	1.6286956	0.8868895	-0.1731737	0.30462
EPG5	1.189675	1.1523882	1.22046786	1.4467517	0.53281736	0.30469
NOA1	1.463229	1.5048555	1.42885306	0.849457	-0.2353872	0.30477

C3orf38	1.381424	1.4211878	1.34858713	0.8276288	-0.2729443	0.30498
GRAMD3	1.15811	1.121367	1.18845239	1.5527476	0.63482333	0.30504
STX6	1.320856	1.3599217	1.28859507	0.8018273	-0.3186366	0.30508
PRIM2	1.198225	1.2356141	1.16734882	0.7102664	-0.4935678	0.30516
GLE1	1.166368	1.2031597	1.13598557	0.6693531	-0.5791606	0.30526
GLA	1.253184	1.2913379	1.22167559	0.7608883	-0.3942434	0.30527
GSTO2	1.40871	1.4490302	1.37541367	0.8360544	-0.2583312	0.30529
KIAA1551	1.27733	1.238852	1.30910607	1.2941323	0.37198515	0.30562
NENF	3.549336	3.4693064	3.61542582	1.0591743	0.08293997	0.30579
METTL22	1.316203	1.3554523	1.28379076	0.7983933	-0.3248285	0.30584
RP5-908M14.9	1.125781	1.162034	1.09584277	0.5914979	-0.7575551	0.3059
EYA3	1.376439	1.4165995	1.34327368	0.8239897	-0.2793018	0.30607
LIN52	1.204387	1.2415682	1.17368252	0.7189793	-0.4759778	0.30618
PAQR5	1.163439	1.2004117	1.1329069	0.6631693	-0.5925508	0.30631
IFT27	2.046155	2.0967586	2.0043663	0.9157588	-0.1269604	0.30641
CPLX2	1.045057	1.080074	1.01613888	0.2015495	-2.3107937	0.30656
ETNK1	1.743902	1.6997121	1.78039488	1.1153085	0.15744279	0.30661
MYEF2	2.686777	2.7482545	2.63600853	0.9357954	-0.0957349	0.30669
CEP85L	2.230358	2.2924026	2.17912036	0.9123476	-0.1323446	0.3069
MTF2	2.623765	2.6877569	2.57091983	0.9307738	-0.1034975	0.30698
RP11-1114A5.4	1.276602	1.2384566	1.30810289	1.2920711	0.36968543	0.307
KDM3B	1.467866	1.5089094	1.4339724	0.8527498	-0.2298055	0.30714
PRR5L	1.131332	1.0951965	1.16117375	1.6930641	0.75963661	0.30716
CSPP1	1.260184	1.2980886	1.22888153	0.7678306	-0.38114	0.3072
DCUN1D3	1.072289	1.0371877	1.10127648	2.7233889	1.44540302	0.30723
NELFB	1.371016	1.4107052	1.33823992	0.8235588	-0.2800564	0.30727
MCMBP	1.482349	1.5233195	1.44851515	0.857058	-0.2225352	0.30732
DYRK1A	1.343642	1.3042366	1.37618405	1.2364852	0.30624494	0.30734
SYTL1	1.241535	1.2039762	1.27255158	1.336193	0.41812844	0.30744
MMP24-AS1	3.201997	3.129146	3.26215837	1.0624722	0.08742507	0.30746
NEK9	1.285723	1.2473394	1.31742051	1.2833396	0.359903	0.3076
PDE12	1.186962	1.1501127	1.21739231	1.4481938	0.53425472	0.30762
CARS	1.31168	1.3505434	1.27958507	0.7975762	-0.3263057	0.30766
VARS	1.417426	1.4577717	1.38410835	0.8390827	-0.2531151	0.30786
TCTN1	1.758851	1.8032691	1.72216994	0.8990387	-0.1535449	0.30809
IFT122	1.318394	1.2795355	1.35048318	1.2538054	0.32631348	0.30809
SLC23A2	1.100292	1.0647049	1.12967975	2.0041723	1.00300657	0.30811
BARD1	1.305106	1.2663948	1.33707351	1.2653158	0.33949756	0.30818
MAGI2	1.223349	1.1859417	1.25424127	1.3673168	0.4513476	0.30819
BORCS5	1.157568	1.1937866	1.12765847	0.6587579	-0.6021797	0.3086
DENND4C	1.19994	1.2371991	1.16917024	0.7131994	-0.4876227	0.30873
FAM221A	1.356428	1.3958746	1.32385172	0.8180665	-0.2897099	0.30873
SLC39A9	1.237059	1.2751452	1.20560736	0.7472685	-0.4203014	0.3088
PDPR	1.238784	1.2013054	1.2697343	1.3399261	0.4221534	0.30888
ATG4C	1.274129	1.3123104	1.24259773	0.7767839	-0.3644147	0.30893

MCCC1	1.239097	1.2771211	1.20769556	0.7494757	-0.4160464	0.30897
FAHD1	1.379182	1.4187191	1.34653146	0.827599	-0.2729962	0.30907
SLC2A13	1.194982	1.1580589	1.22547311	1.4265132	0.51249315	0.3093
ADAMTS7	1.256842	1.2193052	1.28784087	1.3125127	0.39233135	0.30954
ITPR3	1.059167	1.0246097	1.08770559	3.5638638	1.8334422	0.31006
FUCA1	1.279143	1.3168546	1.24800074	0.7826958	-0.3534765	0.3102
CBX7	1.134649	1.0985586	1.16445384	1.6685901	0.73862959	0.31021
MAN2A2	1.276616	1.2384326	1.30814834	1.2923919	0.37004365	0.31023
STK38L	1.430894	1.3908459	1.46396684	1.1870837	0.2474217	0.31037
PCDHB14	1.15017	1.1866747	1.12002315	0.6429536	-0.6372135	0.31038
SSTR2	1.074486	1.1095775	1.0455074	0.4152989	-1.267778	0.31042
MRPS30	1.572814	1.6149242	1.53803935	0.8749686	-0.1926969	0.31059
CEP250	1.434718	1.4748911	1.40154193	0.8455454	-0.242046	0.31064
LLNLR-245B6.1	2.569512	2.6348365	2.51556637	0.9270446	-0.1092894	0.31068
RNF121	1.215343	1.2524582	1.18469202	0.7315747	-0.4509229	0.31081
STX17	1.382189	1.4216445	1.34960575	0.8291481	-0.2702982	0.31108
PNPLA4	1.552917	1.5946912	1.51842003	0.8717466	-0.1980193	0.31149
SF1	2.473863	2.5303377	2.42722487	0.9326208	-0.1006374	0.31158
CDK8	1.6087	1.6513667	1.57346504	0.8804027	-0.1837645	0.31175
PARD6A	1.31096	1.349846	1.27884734	0.7970573	-0.3272446	0.31185
USP32	1.257674	1.2203364	1.28850795	1.3093975	0.38890312	0.31199
CHD4	3.568759	3.6579161	3.49513123	0.9387547	-0.0911799	0.31204
MSH2	1.38239	1.4215741	1.35003079	0.8302948	-0.2683044	0.31207
KIF21B	1.066878	1.0321004	1.09559746	2.9780815	1.57438322	0.31222
LOXL1-AS1	1.314433	1.2761861	1.34601722	1.2528407	0.32520301	0.31234
SLC2A1-AS1	1.460404	1.4195798	1.49411713	1.1776476	0.23590783	0.31235
SLC26A6	1.180964	1.2178894	1.15047116	0.6905852	-0.5341088	0.31238
PTPRG	1.593322	1.5506377	1.62857141	1.1415336	0.19097337	0.31256
C2orf49	1.511634	1.5528843	1.47756918	0.8637779	-0.2112678	0.31258
DPY19L2	1.306632	1.2681222	1.33843451	1.2622398	0.33598597	0.31275
TNFRSF14	1.075743	1.0408951	1.10452103	2.5558344	1.35379434	0.31277
IRF2BP2	1.684419	1.6410987	1.72019383	1.1233744	0.16783886	0.313
TMEM130	1.067571	1.0329249	1.09618286	2.9212829	1.54660207	0.31323
TFRC	1.357832	1.3970962	1.32540629	0.8194646	-0.2872464	0.31325
MCC	1.162854	1.1268759	1.19256609	1.5177518	0.60193589	0.3133
APTX	1.559469	1.6014059	1.52483663	0.8726829	-0.1964706	0.31333
ETNK2	1.147127	1.1115024	1.17654619	1.5833402	0.66297129	0.31343
GALNT6	1.050855	1.0165706	1.07916714	4.7775693	2.2562768	0.31371
ATP11A	1.155231	1.1190898	1.18507704	1.554096	0.63607565	0.31372
RRM2B	1.410918	1.371808	1.4432161	1.1920564	0.25345254	0.3142
MPG	2.686551	2.7535128	2.63125359	0.9302776	-0.1042668	0.31422
MDFI	1.948991	1.9020373	1.98776674	1.0950398	0.13098327	0.31434
MIR181A2HG	1.182926	1.2194047	1.15280107	0.6964348	-0.5219398	0.31435
ARHGEF1	1.267075	1.2294518	1.29814561	1.2993825	0.37782618	0.31438
UAP1	1.60247	1.6446308	1.56765261	0.8805856	-0.1834648	0.31442

ZNF185	1.089647	1.054867	1.11836875	2.1573766	1.10927807	0.3149
PAFAH1B2	2.094348	2.1435166	2.05374375	0.921494	-0.1179533	0.31501
MARCH1	1.108386	1.0735158	1.13718163	1.8660153	0.8999608	0.31518
ITPRIPL2	1.252399	1.215183	1.28313289	1.3157772	0.39591519	0.31537
THNSL1	1.169864	1.206046	1.1399837	0.6793809	-0.5577074	0.31539
AVPI1	1.24443	1.2073055	1.27508748	1.3269667	0.40813214	0.31545
MUTYH	1.304597	1.3424627	1.27332741	0.7981232	-0.3253167	0.31571
AP006222.2	1.696065	1.7394299	1.66025342	0.8929222	-0.1633936	0.31578
PRDM4	1.305538	1.3438545	1.27389497	0.7965432	-0.3281755	0.31582
RNF41	1.426491	1.4666249	1.39334738	0.8429627	-0.2464593	0.316
RP11-294J22.6	1.230371	1.2675777	1.19964476	0.7461188	-0.4225228	0.31601
GUK1	7.313712	7.4727356	7.18238736	0.9551429	-0.0662115	0.31604
AGPAT5	1.31823	1.3564305	1.28668317	0.8043171	-0.3141636	0.31633
NPLOC4	1.231174	1.1944544	1.26149686	1.3447721	0.42736168	0.31637
ZNF770	1.797306	1.84193	1.76045486	0.9032281	-0.1468378	0.3164
RRP15	1.533128	1.5745038	1.49895849	0.8685033	-0.2033967	0.31642
UPF1	1.165056	1.1291423	1.19471425	1.5077498	0.59239702	0.31643
ZMIZ1	1.389501	1.3506817	1.4215584	1.2021111	0.26557019	0.31659
RNPC3	1.404288	1.4440295	1.37146857	0.8365854	-0.2574153	0.31671
LIN7C	1.370873	1.4098494	1.33868543	0.8263655	-0.275148	0.31683
ASMTL	1.238422	1.2755172	1.20778876	0.754177	-0.4070249	0.31701
CRYBB3	1.106644	1.1465312	1.0737045	0.5029951	-0.9913837	0.31729
IQGAP3	1.110921	1.1461803	1.08180347	0.5596068	-0.8375147	0.31743
EIF2B4	1.477456	1.517775	1.44416007	0.8578244	-0.2212457	0.31745
PRPF38B	2.100613	2.1494748	2.06026238	0.9223885	-0.1165535	0.31749
MBNL1	1.486648	1.4463345	1.51993936	1.1649096	0.22021801	0.31774
PEA15	1.93191	1.8866985	1.96924578	1.0930951	0.12841892	0.31786
KIF16B	1.186877	1.2232359	1.15685073	0.7026233	-0.5091767	0.31802
LRP3	1.200107	1.1637129	1.23016236	1.4058902	0.49148391	0.31822
PNMAL1	1.35861	1.3970216	1.32688944	0.8233542	-0.2804149	0.31838
LIMS2	1.078011	1.0436722	1.10636819	2.4356045	1.2842799	0.31841
DTD2	1.19217	1.2285295	1.16214362	0.7095086	-0.4951079	0.31842
SECISBP2	2.079499	2.1271574	2.04014138	0.9228005	-0.1159094	0.31844
THSD4	1.058659	1.0245909	1.08679315	3.5294776	1.81945465	0.31849
RABEP1	1.782478	1.8266233	1.74602256	0.902494	-0.1480108	0.31852
MAFF	1.147688	1.1121729	1.1770174	1.5780765	0.65816711	0.31855
WFIKKN1	1.111525	1.1466106	1.08255087	0.5630619	-0.8286346	0.31877
MED16	1.301577	1.3391944	1.27051184	0.7975127	-0.3264205	0.31882
JMJD4	1.231996	1.2691085	1.20134706	0.7482003	-0.4185035	0.31886
MAPKAPK2	1.278136	1.2408545	1.30892421	1.2826173	0.35909075	0.31897
FNDC3A	2.373699	2.4288862	2.32812359	0.9294817	-0.1055017	0.31912
ZNF91	1.511264	1.5516293	1.47793036	0.8663978	-0.2068986	0.31912
OGFOD3	1.352152	1.3904821	1.32049871	0.8207769	-0.284938	0.31928
SLC39A10	2.713068	2.7766439	2.66056558	0.9346643	-0.0974798	0.31932
RAP2C-AS1	1.25332	1.2161241	1.28403727	1.3142323	0.39422029	0.31955

THUMPD2	1.3684	1.4070358	1.33649315	0.8266918	-0.2745785	0.31957
CARD8	1.373043	1.4118403	1.34100446	0.8280018	-0.2722943	0.31961
ATP1A3	1.100088	1.1352433	1.07105634	0.5253965	-0.9285214	0.31962
SACM1L	1.300555	1.2629917	1.33157472	1.2607801	0.33431663	0.31968
TACC1	1.278472	1.2410865	1.30934523	1.2831294	0.35966667	0.31973
FXYD3	1.055587	1.0215767	1.0836728	3.8779217	1.95528368	0.31975
SMG1	1.709964	1.6670809	1.74537671	1.1173708	0.16010797	0.31989
PRKG1	1.103011	1.0682769	1.13169448	1.9288284	0.94772483	0.31999
THOP1	1.343501	1.38153	1.31209615	0.8180121	-0.2898059	0.32001
ING2	1.799865	1.8441794	1.76326913	0.9041551	-0.1453578	0.32018
ZEB1	1.414311	1.4534477	1.3819909	0.8424144	-0.2473979	0.3202
KRIT1	1.674573	1.7172846	1.63930055	0.8912787	-0.1660514	0.32031
SLC25A13	1.669044	1.7115012	1.63398189	0.8910482	-0.1664245	0.32081
GDE1	1.898463	1.8530257	1.93598541	1.0972534	0.13389677	0.32092
AC104655.3	1.141105	1.1765033	1.11187174	0.6338225	-0.6578492	0.32097
CNTN3	1.155698	1.1202745	1.18495098	1.53774	0.62081161	0.32107
PPP3CB	1.425264	1.4644137	1.3929327	0.8460834	-0.2411282	0.32117
UMPS	1.20944	1.2454285	1.17971923	0.7322672	-0.4495579	0.3212
SBF1	1.219871	1.1834916	1.2499143	1.3619932	0.44571953	0.32125
WDCP	1.109491	1.1443218	1.08072692	0.5593535	-0.8381677	0.32125
CST3	7.067367	6.869721	7.23058539	1.061479	0.08607578	0.32133
AK9	1.194871	1.2310722	1.16497528	0.7139555	-0.4860939	0.32145
ATPAF1	1.541164	1.5816318	1.50774554	0.8729673	-0.1960005	0.32152
SLC30A4	1.112246	1.0774407	1.14098912	1.8206064	0.86441908	0.32164
HEXIM2	1.193962	1.2301239	1.16409864	0.7130882	-0.4878476	0.32171
GGA2	1.779818	1.7360525	1.81596088	1.1085634	0.1486913	0.32191
CHAMP1	1.362133	1.4004716	1.33047266	0.8252087	-0.277169	0.32198
TOR1A	1.335724	1.3737708	1.30430419	0.8141465	-0.2966397	0.32206
SIRT2	1.434275	1.3946599	1.46699015	1.1832725	0.24278231	0.32211
RP11-798M19.6	2.326875	2.3816861	2.28161046	0.9275699	-0.1084721	0.32215
PNKP	1.732191	1.7750969	1.69675865	0.898931	-0.1537177	0.32219
CLASP1	1.350496	1.3122071	1.38211645	1.2239198	0.29150904	0.32246
CA11	1.823864	1.8687766	1.78677365	0.905611	-0.1430366	0.32247
RND1	1.062155	1.0960611	1.03415548	0.3555599	-1.4918355	0.32252
MTAP	1.477326	1.516972	1.44458552	0.8599799	-0.2176251	0.32256
SFSWAP	1.572378	1.6131604	1.53869896	0.8785612	-0.1867853	0.32257
CREB3L1	1.15511	1.1199423	1.18415143	1.5353329	0.61855151	0.32262
TGFB1	1.58781	1.5465634	1.62187124	1.1377842	0.18622693	0.32273
UBE2V1	1.168964	1.1333845	1.19834523	1.4870181	0.5724222	0.32306
CSPG5	1.147024	1.1823507	1.11785121	0.6462888	-0.6297491	0.32315
CBFA2T2	1.4248	1.3860117	1.45683208	1.183467	0.24301944	0.32324
RP11-532F12.5	1.097135	1.0625403	1.1257034	2.0099594	1.00716635	0.3233
EMILIN1	1.078562	1.0444184	1.10675876	2.4034779	1.26512353	0.32342
RP1-102K2.8	1.064888	1.0310019	1.09287094	2.9956552	1.58287159	0.32352
METAP1	1.39297	1.4313718	1.36125789	0.8374629	-0.2559028	0.32356

QSOX1	1.382964	1.3442976	1.41489558	1.2050495	0.26909236	0.32363
ELOVL2	1.167765	1.2033661	1.13836535	0.6803755	-0.5555968	0.32374
SCOC-AS1	1.112195	1.1469147	1.0835225	0.5685103	-0.8147416	0.32396
LIPG	1.060086	1.0263433	1.08795205	3.338694	1.73928386	0.32397
TIPIN	1.280338	1.317454	1.24968617	0.7865271	-0.3464317	0.32412
COPS9	4.789348	4.6800826	4.87958067	1.0542102	0.07616257	0.32417
ACP6	1.598698	1.6399108	1.56466353	0.8824098	-0.1804793	0.32426
PSPC1	1.391877	1.4304741	1.36000245	0.8362929	-0.2579198	0.32434
LHPP	1.246478	1.2831078	1.21622838	0.7637668	-0.3887958	0.32436
PTPRU	1.408345	1.3696855	1.44027005	1.1909313	0.25209018	0.3245
ST3GAL3	1.28778	1.3250213	1.25702598	0.7907972	-0.3386203	0.3245
NTRK1	1.06031	1.0941654	1.03235187	0.3435643	-1.5413478	0.32454
ANXA11	1.674584	1.6324857	1.70934918	1.121526	0.16546306	0.32477
IFT22	2.460278	2.5198635	2.41107204	0.9284203	-0.1071501	0.32487
SCARB2	3.43872	3.3606717	3.5031734	1.0603649	0.08456083	0.32505
UPF2	1.55598	1.5965097	1.52251063	0.8759466	-0.1910852	0.32521
OLFML2A	1.05824	1.0247285	1.08591449	3.4743164	1.79672914	0.32529
EMX2	1.112686	1.1532781	1.07916499	0.5164794	-0.9532173	0.32536
UGCG	1.490686	1.4515038	1.52304245	1.1584454	0.21219003	0.32546
CCNJ	1.26044	1.2974109	1.22990942	0.7730364	-0.3713917	0.3255
IFI27L2	3.721267	3.6316023	3.79531359	1.0622097	0.08706867	0.32565
SLC30A7	1.372511	1.3345861	1.40382982	1.2069535	0.27137011	0.32579
ABHD4	1.199394	1.2351599	1.16985761	0.722307	-0.469316	0.32582
AKAP7	1.142306	1.1772583	1.11344107	0.6399761	-0.64391	0.32605
ZNF195	1.438624	1.4775021	1.40651812	0.8513431	-0.2321874	0.32612
HAS1	1.102774	1.0685164	1.13106375	1.9128819	0.93574778	0.32621
EXT1	1.223706	1.1876236	1.2535031	1.3511256	0.43416179	0.32625
AC004540.5	1.275517	1.2390375	1.3056422	1.2786371	0.35460689	0.32627
ST3GAL4	1.242945	1.2064741	1.27306273	1.3225038	0.40327185	0.3264
RP11-11N9.4	2.209455	2.2651968	2.16342204	0.9195582	-0.1209872	0.3264
MAN1C1	1.177952	1.1426468	1.20710807	1.4518939	0.53793599	0.3266
WDR33	1.836359	1.7922495	1.87278585	1.1016553	0.13967283	0.32674
PIP4K2A	1.172258	1.137142	1.20125646	1.4675037	0.55336417	0.32681
IGF2	5.961897	5.6699156	6.20301914	1.114157	0.15595252	0.3269
H6PD	1.257219	1.2938739	1.22694857	0.7722652	-0.3728317	0.32691
PIAS4	1.206998	1.2428262	1.17741068	0.7306075	-0.4528315	0.32695
PLOD3	1.320722	1.2833452	1.35158833	1.2408482	0.31132664	0.32695
CREBRF	1.376318	1.3381446	1.40784164	1.2061161	0.27036873	0.32706
DBX2	1.115213	1.1496207	1.08679836	0.5801226	-0.7855703	0.32707
CTNNA2	1.200171	1.2359706	1.17060718	0.7230018	-0.4679289	0.3272
ASAP2	1.443753	1.4047599	1.47595491	1.1758945	0.23375862	0.32726
CLDND2	1.348304	1.3859176	1.31724166	0.8220452	-0.2827104	0.32732
PRADC1	1.361334	1.3233518	1.39270121	1.2144703	0.28032721	0.32734
PPP1R8	1.405061	1.4435782	1.37325284	0.8414589	-0.2490352	0.32756
FASTKD2	1.23999	1.2761749	1.21010846	0.7607805	-0.3944477	0.3276

NUDT8	1.410014	1.4484485	1.37827424	0.8435177	-0.2455098	0.32775
ZNF71	1.331977	1.3694327	1.30104466	0.8148837	-0.295334	0.32778
BAD	3.094223	3.169695	3.031897	0.9364897	-0.094665	0.32786
NOL12	1.305431	1.2681063	1.33625511	1.2541857	0.32675102	0.32787
ATP6V0A2	1.137157	1.1025551	1.16573189	1.6160274	0.69245167	0.32807
SNX19	1.166551	1.1315064	1.19549181	1.4865574	0.57197514	0.32812
TMEM115	1.471779	1.5114272	1.43903699	0.8584544	-0.2201865	0.32822
CDK11B	1.346907	1.3843861	1.31595535	0.8219739	-0.2828355	0.32825
CADPS2	1.284357	1.3211047	1.25400931	0.7910482	-0.3381625	0.32826
RP11-110G21.1	1.181768	1.2171441	1.15255342	0.7025445	-0.5093386	0.32865
NUMB	1.613394	1.5721931	1.64741792	1.1314676	0.17819531	0.32869
RC3H1	1.422706	1.4611315	1.39097448	0.847859	-0.2381038	0.32874
SHROOM3	1.464383	1.425334	1.49662978	1.1676231	0.22357462	0.32875
MRPL37	2.077562	2.1267366	2.0369532	0.9203155	-0.1197995	0.32899
LRRC4B	1.296532	1.2595381	1.3270821	1.260247	0.33370656	0.329
GBF1	1.233658	1.1977102	1.26334437	1.3319714	0.41356307	0.32903
FLCN	1.340258	1.3031426	1.37090763	1.2235418	0.29106336	0.32911
TMEM116	1.224171	1.1880612	1.25399115	1.3505769	0.43357579	0.32914
SLIT3	1.112805	1.0786335	1.14102359	1.793429	0.84272062	0.32918
LRSAM1	1.138509	1.1730424	1.1099916	0.6356338	-0.6537321	0.32921
SH3BP1	1.180657	1.2162078	1.15129822	0.6997815	-0.5150235	0.32922
MAP2K1	1.790223	1.833308	1.75464358	0.9055998	-0.1430545	0.32929
ZNF493	1.439376	1.4782996	1.40723183	0.8514157	-0.2320644	0.3293
ZBTB4	1.243443	1.2075593	1.27307555	1.315651	0.39577689	0.32937
NBPF19	1.235723	1.1996615	1.26550385	1.3297697	0.41117637	0.32952
TTC8	1.223115	1.2589568	1.19351573	0.7472897	-0.4202605	0.32955
ZNF791	1.573923	1.6145613	1.54036321	0.8792665	-0.1856276	0.32966
ABCA5	1.150207	1.1852383	1.12127828	0.6547147	-0.6110616	0.32967
PQLC3	1.25427	1.2907787	1.22412101	0.7707614	-0.3756438	0.32969
SEC62	3.506095	3.5850798	3.44086799	0.9442138	-0.0828146	0.3297
CDIPT	2.010671	1.9647773	2.04857161	1.0868535	0.12015752	0.32972
CCDC137	1.470471	1.5096939	1.43807972	0.8594958	-0.2184376	0.32993
TMEM184C	1.276259	1.3131817	1.24576804	0.7847459	-0.3497026	0.33012
CARNMT1	1.314441	1.3513983	1.28392138	0.807976	-0.3076156	0.33015
GDPD1	1.187862	1.2231456	1.15872397	0.7113022	-0.4914654	0.33023
FTL	103.3532	101.00744	105.290348	1.0428259	0.06049832	0.33037
EXOC3	1.157813	1.1228724	1.18666669	1.5191917	0.60330394	0.33044
SLC38A6	1.270566	1.2341333	1.300652	1.2841061	0.36076437	0.3306
JRKL	1.194546	1.1591498	1.22377713	1.4060788	0.49167748	0.33064
METTL9	4.169223	4.2656614	4.08958338	0.946082	-0.0799629	0.33075
ZFP36L1	9.161817	8.9710289	9.31937341	1.0437013	0.06170892	0.3308
GPALPP1	1.411134	1.4498666	1.37914744	0.8427997	-0.2467383	0.33088
CCNO	1.076831	1.0432498	1.10456269	2.4176432	1.27360132	0.33142
MED9	1.215686	1.2512891	1.18628521	0.7413184	-0.4318348	0.33142
UFM1	2.217851	2.2678961	2.17652228	0.9279328	-0.1079078	0.33155

TMSB10	80.68803	79.201316	81.9157745	1.0347112	0.04922811	0.33156
MSL1	1.817241	1.7745456	1.85249923	1.1006443	0.13834831	0.33162
PHKG1	1.216753	1.2544173	1.18564886	0.7297022	-0.4546202	0.33166
DOCK7	1.574386	1.5339803	1.60775335	1.1381569	0.18669948	0.33168
PIGS	1.448637	1.4097506	1.48074963	1.1732738	0.23053971	0.33169
WDR35	1.172083	1.2072042	1.14308024	0.6905277	-0.5342287	0.33171
COQ6	1.172129	1.2071876	1.14317666	0.6910484	-0.5331414	0.33171
DNAJB2	2.186188	2.2403891	2.14142839	0.920218	-0.1199524	0.33187
TRIM73	1.235923	1.2000148	1.26557673	1.3277854	0.40902196	0.33188
FAM122A	1.421749	1.460294	1.38991765	0.8471057	-0.2393862	0.33206
SAMD13	1.186986	1.2220729	1.15801002	0.7115232	-0.4910174	0.33215
THG1L	1.300318	1.3371811	1.26987534	0.8003868	-0.3212307	0.33216
RUNDC3B	1.068951	1.1024744	1.04126702	0.4027056	-1.3122025	0.33221
PPP1R15A	1.663097	1.7044075	1.62898229	0.8929238	-0.163391	0.33239
AC073283.4	1.21792	1.2538268	1.18826749	0.7417162	-0.4310608	0.33239
GDAP1	1.22205	1.2578717	1.19246728	0.7463683	-0.4220403	0.33245
LRR1	1.265398	1.3013149	1.23573814	0.7823647	-0.3540868	0.33253
CHD8	1.402181	1.3640485	1.43367056	1.1912438	0.25246875	0.33256
PLEKHG5	1.126229	1.0919968	1.1544992	1.6793983	0.74794441	0.3326
KIF3C	1.175568	1.1408846	1.20420979	1.4494828	0.5355382	0.33266
FRA10AC1	2.113145	2.1609293	2.07368348	0.9248483	-0.1127113	0.33267
ABHD12	1.579269	1.538845	1.61265173	1.1369721	0.18519691	0.33271
YIPF6	1.564262	1.604332	1.53117137	0.8789397	-0.1861639	0.3328
RFNG	1.298196	1.2615663	1.32844578	1.2556886	0.32847873	0.33291
RSAD1	1.469171	1.5078325	1.43724353	0.8609995	-0.2159157	0.33343
PDP1	1.11378	1.0798327	1.14181496	1.7764018	0.82895792	0.33361
HSD17B4	1.466861	1.5057352	1.43475883	0.8596571	-0.2181668	0.33368
AREL1	1.15923	1.1246457	1.1877897	1.5065878	0.59128478	0.33392
FGFR1OP2	1.678804	1.7203832	1.6444668	0.8946167	-0.1606585	0.33411
SCYL1	1.36206	1.3992702	1.3313309	0.8298413	-0.2690927	0.33413
CYB5R2	1.142591	1.1084717	1.17076797	1.5743093	0.65471899	0.33427
KDM2A	1.303394	1.2666836	1.33370972	1.2513319	0.32346448	0.33435
DSCC1	1.132392	1.1663955	1.10431049	0.626883	-0.6737319	0.33436
VPS41	1.382802	1.3456437	1.41348888	1.1962866	0.25856309	0.33441
TSC22D2	1.371059	1.3336625	1.40194126	1.2046341	0.26859498	0.33446
STX3	1.449218	1.4108069	1.48093925	1.1707184	0.22739415	0.33451
ZDHHC2	1.226021	1.1906459	1.25523452	1.3387881	0.42092764	0.33452
NFIA	1.198743	1.1636164	1.22775184	1.3919866	0.47714527	0.33459
ACAD9	1.236808	1.2723855	1.20742728	0.761521	-0.3930442	0.33468
ZRANB3	1.171056	1.2059486	1.14224047	0.6906601	-0.5339522	0.33477
SLC40A1	1.095219	1.061666	1.12292851	1.9934581	0.99527327	0.33478
LRWD1	1.21683	1.252123	1.18768399	0.7444143	-0.4258223	0.33481
DAB1	1.208089	1.172641	1.23736329	1.3748948	0.45932127	0.33484
RGS20	1.425311	1.4637552	1.39356341	0.8486447	-0.2367673	0.33492
NAA60	1.471222	1.5102739	1.438973	0.8602694	-0.2171396	0.33521

DNAAF3	1.121428	1.1552691	1.09348088	0.602057	-0.732028	0.33523
ACADVL	3.459374	3.5540195	3.38121354	0.9323396	-0.1010726	0.33525
ZZEF1	1.14388	1.1094886	1.17228066	1.5735028	0.65397975	0.33533
ENKUR	1.141035	1.175073	1.11292516	0.6450176	-0.6325895	0.33559
PHACTR4	1.683942	1.7250986	1.64995413	0.8963666	-0.1578392	0.3359
BMS1	1.395957	1.4337356	1.36475818	0.840969	-0.2498755	0.33595
MMS22L	1.429893	1.3915492	1.46155754	1.1787984	0.237317	0.33597
NCK1	1.500105	1.538849	1.46810879	0.8687197	-0.2030373	0.33643
ARHGAP12	1.296509	1.333212	1.26619865	0.7988867	-0.3239371	0.33643
CMTM8	1.340654	1.3777792	1.30999598	0.8205745	-0.2852937	0.33644
GGCX	1.33634	1.3732719	1.30584064	0.8193509	-0.2874467	0.33648
CBY1	1.855567	1.8987095	1.81993844	0.9123509	-0.1323392	0.33649
TPD52L2	1.615046	1.6557817	1.58140616	0.886585	-0.1736692	0.3366
CRYGD	1.129204	1.1630111	1.10128624	0.6213456	-0.6865321	0.33678
KSR2	1.105164	1.0714435	1.13301032	1.8617546	0.8966629	0.33684
SEC16A	1.198312	1.1634301	1.22711724	1.3896906	0.47476376	0.33697
FNBP1	1.584894	1.5446339	1.61814179	1.1349676	0.18265114	0.33697
AMPD2	1.322505	1.2858395	1.3527832	1.2342005	0.30357679	0.33724
TMEM231	1.331822	1.3687147	1.30135624	0.8173156	-0.2910348	0.33745
PPP1R16A	1.525437	1.4862316	1.55781267	1.1472159	0.19813695	0.33767
RABL3	1.117914	1.1516393	1.09006227	0.5939243	-0.751649	0.33773
NES	1.921732	1.8764659	1.95911427	1.0942974	0.13000482	0.33778
TMEM248	1.714329	1.6730458	1.74842146	1.1119919	0.15314629	0.33789
HIST1H2AC	1.09922	1.132794	1.07149411	0.5383835	-0.8932938	0.33793
BCAT2	1.619471	1.6602405	1.58580233	0.8872559	-0.1725778	0.33795
KIF5A	1.245444	1.2813943	1.21575624	0.76674	-0.3831907	0.33819
VEGFB	1.534749	1.4955319	1.56713452	1.1444964	0.19471292	0.33837
BUB1B	1.158921	1.1929717	1.130801	0.6778247	-0.561016	0.33859
RP11-344P13.6	1.086811	1.1201047	1.0593156	0.4938656	-1.0178095	0.33866
C10orf11	1.076072	1.042826	1.10352697	2.4173851	1.27344729	0.33879
GFER	1.585339	1.6252	1.55242075	0.8835904	-0.1785503	0.33888
CHKB	1.242115	1.2777344	1.21269914	0.7658364	-0.3848919	0.33892
XKR4	1.58935	1.5486755	1.62294047	1.1353532	0.18314119	0.33896
SCUBE1	1.050737	1.0181221	1.07767108	4.2859901	2.09962852	0.33915
ATP6V1D	2.180772	2.1300608	2.22264961	1.0819326	0.11361068	0.33919
PAX2	1.12245	1.0886201	1.15038758	1.6969919	0.76297967	0.33919
MDFIC	1.299305	1.2628499	1.32941041	1.2532264	0.32564711	0.33928
DFNA5	1.155477	1.1896284	1.12727459	0.6711791	-0.5752304	0.33934
RASD1	1.088778	1.1220926	1.06126689	0.5018068	-0.9947962	0.33935
DANT2	1.267965	1.2323317	1.29739088	1.2800274	0.35617469	0.33951
JPH4	1.464318	1.5029129	1.43244612	0.8598827	-0.2177882	0.33953
DPYSL5	1.4201	1.4579097	1.38887617	0.849242	-0.2357523	0.33987
DSTN	8.219998	8.3821439	8.08609543	0.9598967	-0.059049	0.33992
UBAP1L	1.083527	1.0504592	1.11083436	2.1965135	1.13521534	0.34002
KIF15	1.16161	1.1955803	1.13355671	0.6828742	-0.5503082	0.34013

PTCHD4	1.147333	1.113513	1.17526188	1.5439806	0.62665459	0.34014
MCM6	1.284693	1.3204492	1.25516457	0.7962716	-0.3286676	0.34039
MBNL2	1.484152	1.4456822	1.51592063	1.1575975	0.21113373	0.34054
ARRDC1-AS1	1.419443	1.4571922	1.38826879	0.8492463	-0.235745	0.34058
PAK3	1.347723	1.3847078	1.31718089	0.8244722	-0.2784572	0.34059
DHRSX	1.560865	1.5212926	1.5935453	1.1386029	0.18726465	0.34065
LINC01473	1.368686	1.4057689	1.33806277	0.8331412	-0.2633671	0.34066
ZDHHC8	1.176345	1.1419089	1.2047829	1.4430587	0.52913001	0.34067
RRAGC	1.392769	1.4306448	1.36149	0.8394158	-0.2525425	0.34068
ATP13A1	1.213833	1.1788414	1.24273031	1.3572381	0.44067379	0.34077
PPARGC1A	1.083104	1.0500998	1.11035971	2.2027984	1.13933749	0.34079
RAPGEF2	1.202972	1.1681154	1.23175673	1.3785571	0.463159	0.34111
STX5	1.55896	1.5988378	1.52602904	0.8784165	-0.1870229	0.34121
RFFL	1.223995	1.2590726	1.19502808	0.752793	-0.4096748	0.34133
TMEM230	4.56288	4.6712969	4.47334773	0.946082	-0.0799629	0.34169
STK38	1.265408	1.2299333	1.29470269	1.2816877	0.35804481	0.34174
NDUFAF1	1.181195	1.2154608	1.15289726	0.709629	-0.4948631	0.34174
ZFP62	1.262022	1.2974921	1.232731	0.7823098	-0.3541881	0.3418
SMC5	1.794224	1.7520131	1.82908202	1.1024835	0.14075707	0.34197
AKT2	1.553839	1.5935637	1.52103286	0.8778044	-0.1880286	0.34218
UBR5-AS1	1.109999	1.1435607	1.08228309	0.573159	-0.8029927	0.34226
DHX33	1.259784	1.2951935	1.23054301	0.7809896	-0.3566248	0.34231
EIF2D	1.597073	1.6372934	1.56385777	0.8847695	-0.1766264	0.34244
PHLDA3	2.064009	2.0165033	2.10324	1.0853285	0.1181318	0.34256
WNT5A	1.497107	1.4586945	1.52882816	1.1528985	0.20526548	0.34264
PPOX	1.359751	1.3964933	1.32940907	0.8308062	-0.267416	0.34279
TULP3	1.296223	1.3321232	1.26657618	0.8026425	-0.3171706	0.34284
DENND5B	1.327835	1.2912957	1.35801005	1.2290261	0.2975155	0.34284
SS18L1	1.238943	1.2038609	1.26791491	1.3142043	0.39418953	0.34309
STAG2	2.174861	2.2292	2.12998701	0.9192865	-0.1214135	0.34314
CNOT6	1.423474	1.4610759	1.39242084	0.8510981	-0.2326026	0.3432
CACNB4	1.178512	1.2127507	1.15023632	0.7061612	-0.5019305	0.34337
ZFP64	1.230365	1.2656528	1.20122366	0.7574687	-0.4007418	0.34354
TMEM218	1.520221	1.5591418	1.48807921	0.8729077	-0.196099	0.34361
SPATA33	1.738095	1.6968684	1.77214098	1.1080155	0.14797803	0.34361
CYP20A1	1.229504	1.2642652	1.20079787	0.7598346	-0.3962427	0.34371
KATNAL2	1.229567	1.2645132	1.20070843	0.7587841	-0.3982387	0.34384
SLC7A1	1.184849	1.1504948	1.21321912	1.416787	0.50262287	0.34393
DIDO1	1.473021	1.5112377	1.44146049	0.8635132	-0.2117099	0.34397
DNAJC9	1.706807	1.7469832	1.67362831	0.9017985	-0.149123	0.34405
COL12A1	1.092768	1.0599097	1.11990369	2.0014054	1.00101344	0.34414
COL23A1	1.227528	1.2622574	1.19884743	0.7582146	-0.3993218	0.34429
NAGA	1.276532	1.3119904	1.24724974	0.7924915	-0.3355326	0.34434
DPP7	2.143296	2.1901761	2.10458213	0.9280829	-0.1076743	0.34453
PPP2R5B	1.239732	1.274967	1.21063405	0.7660339	-0.3845198	0.34479

G3BP1	2.038463	2.0828491	2.00180745	0.9251589	-0.1122269	0.34493
ALG12	1.338641	1.3748268	1.30875862	0.8237367	-0.2797448	0.34494
PDE8A	1.140951	1.1073688	1.1686831	1.5710621	0.65174022	0.34501
RIMKLB	1.455845	1.4937189	1.42456887	0.8599404	-0.2176914	0.34505
PAPOLG	1.169827	1.2039635	1.14163685	0.6944224	-0.5261145	0.34506
CDT1	1.613219	1.5732568	1.6462203	1.127279	0.17284463	0.34527
HUWE1	1.764312	1.7232658	1.79820871	1.1036173	0.14224001	0.34532
CC2D1A	1.309679	1.2740152	1.33913134	1.2376371	0.30758839	0.34535
MAGED1	3.030954	2.9584575	3.09082202	1.0675861	0.09435246	0.34544
GRN	1.971334	2.0150517	1.93523145	0.9213633	-0.1181579	0.3455
LAMTOR4	4.754227	4.8616105	4.66554858	0.9492279	-0.0751735	0.34554
LYRM2	2.154733	2.2039347	2.11410096	0.9253832	-0.1118771	0.34556
MED22	1.255952	1.2912708	1.22678564	0.7786076	-0.3610316	0.34556
CD99L2	1.671828	1.7123929	1.63832835	0.8960341	-0.1583745	0.34573
CDH6	4.011964	4.1308059	3.91382238	0.930694	-0.1036211	0.34579
ZSCAN18	1.885867	1.9287641	1.8504421	0.9156708	-0.1270991	0.34588
TMEM251	1.498179	1.5365226	1.46651465	0.8695153	-0.2017166	0.34611
ABCC1	1.190601	1.1564796	1.21877883	1.3981299	0.48349842	0.34614
CDC42SE1	1.52098	1.5625894	1.48661868	0.8649625	-0.2092906	0.34619
CRELD2	1.858923	1.9035984	1.8220299	0.9097293	-0.1364908	0.34629
VPS33B	1.143463	1.1770709	1.11570969	0.6534652	-0.6138177	0.34636
AC018816.3	1.094466	1.0618249	1.12142126	1.9639537	0.97376094	0.34642
BTBD19	1.08379	1.0511886	1.11071339	2.1628507	1.11293411	0.34651
LMAN2L	1.290394	1.3258854	1.26108437	0.8011538	-0.3198488	0.34664
EPB41L5	1.217526	1.1831632	1.24590257	1.3425323	0.42495678	0.34667
SNX10	1.405323	1.4424867	1.37463337	0.8466545	-0.2401547	0.3467
DDX59	1.191923	1.226117	1.16368474	0.7238942	-0.4661493	0.34697
ARFIP2	1.596586	1.6360164	1.56402358	0.8868067	-0.1733085	0.34702
ADGRB2	1.191958	1.1577559	1.22020319	1.3958477	0.48114155	0.34702
RBBP6	1.997369	1.9526894	2.03426605	1.0856277	0.11852948	0.34725
NFATC3	1.432698	1.3954919	1.46342357	1.171765	0.22868327	0.34726
DGKH	1.263832	1.2287575	1.29279788	1.2799489	0.35608617	0.34751
DOCK9	1.142986	1.1094853	1.17065069	1.5586635	0.64030952	0.34774
TRPM4	1.153808	1.1875093	1.12597669	0.6718425	-0.5738051	0.3478
HPS4	1.827723	1.8700098	1.79280141	0.9112557	-0.1340721	0.34784
SCN9A	1.153621	1.1200997	1.18130357	1.5096083	0.59417423	0.34802
RP11-32K4.1	1.130766	1.1639578	1.10335536	0.6303777	-0.6657115	0.34828
CDC34	1.692286	1.6522527	1.72534538	1.1120619	0.15323715	0.34853
TIPARP-AS1	1.060976	1.0931264	1.03442629	0.3696729	-1.4356789	0.34891
MOV10	1.309127	1.3446067	1.27982686	0.8120179	-0.3004165	0.34895
RP11-499E18.1	1.216341	1.2508444	1.18784792	0.7488623	-0.4172276	0.34895
HMP19	1.056625	1.0888863	1.02998303	0.337319	-1.5678146	0.34898
SGMS2	1.058464	1.0264981	1.08486195	3.2025621	1.67922652	0.34903
STK19	1.391596	1.4279843	1.36154681	0.8447666	-0.2433753	0.34906
EMC10	3.193737	3.2791623	3.12319106	0.9315664	-0.1022695	0.34909

SEPT9	2.15259	2.2014899	2.11220805	0.9256908	-0.1113978	0.34921
AC137932.4	1.084751	1.1173524	1.05782853	0.4927768	-1.0209938	0.34948
SLC16A14	1.112308	1.0792597	1.13959955	1.7612931	0.81663499	0.34982
SLC44A2	1.387209	1.3504233	1.41758697	1.1916643	0.25297793	0.34986
ADD1	1.76652	1.7254481	1.80043761	1.10337	0.1419166	0.34998
ZNF703	1.30096	1.3366239	1.27150904	0.8065649	-0.3101375	0.35024
GOLGA5	1.254071	1.2890388	1.22519362	0.7791121	-0.3600971	0.35032
SPTSSA	2.353182	2.4044887	2.31081262	0.9333024	-0.0995835	0.35038
ENOSF1	1.507931	1.4696824	1.53951677	1.1486842	0.19998216	0.35041
STAM-AS1	1.106307	1.1391046	1.07922179	0.5695124	-0.8122009	0.35056
ABCD1	1.151294	1.1846593	1.12373956	0.6700964	-0.5775594	0.35078
RBM47	1.144824	1.1117329	1.17215056	1.5407335	0.62361732	0.35081
ACD	1.472645	1.5101576	1.44166597	0.8657442	-0.2079873	0.35085
ABI2	2.954016	3.0196192	2.89983917	0.9406918	-0.088206	0.3511
PHOSPHO2	1.149714	1.1832803	1.12199518	0.6656209	-0.5872274	0.35112
DDX28	1.086639	1.118982	1.05993047	0.5036936	-0.9893818	0.35115
CLK3	1.410593	1.4474964	1.38011729	0.8494309	-0.2354315	0.35115
CLPX	1.441762	1.4787017	1.41125592	0.8591069	-0.2190904	0.35135
ADCY7	1.12007	1.0871348	1.14726868	1.6901242	0.75712926	0.35147
EMB	1.080364	1.0483566	1.10679619	2.2085142	1.1430761	0.35159
THBS2	1.080556	1.0481617	1.10730789	2.2280766	1.15579882	0.35159
TGDS	1.233958	1.2682774	1.20561698	0.7664343	-0.3837661	0.35183
RAD54L2	1.254772	1.2894716	1.22611573	0.7811328	-0.3563603	0.35201
BCKDHB	1.275324	1.310378	1.24637673	0.7937957	-0.3331603	0.35225
TP53I3	1.441926	1.4788961	1.41139541	0.8590495	-0.2191869	0.35225
TST	1.222423	1.2566456	1.19416074	0.7565326	-0.4025259	0.35253
RHBDF2	1.113433	1.0809593	1.14024967	1.732348	0.79272876	0.35265
SCUBE3	1.307147	1.2721947	1.33601207	1.2344547	0.3038739	0.353
TGS1	1.353503	1.3892017	1.32402179	0.8325292	-0.2644271	0.35304
ANKS1A	1.194824	1.1611721	1.22261517	1.3812263	0.46594974	0.35308
INCENP	1.072348	1.1042778	1.04597924	0.4409305	-1.1813769	0.35323
HIVEP1	1.210256	1.1762909	1.23830482	1.3517706	0.43485039	0.35332
GLB1L	1.106541	1.1392052	1.07956551	0.5715699	-0.806998	0.35352
HUNK	1.098233	1.0657225	1.12508096	1.9031673	0.92840239	0.35356
SHD	1.091304	1.1237241	1.06453059	0.5215684	-0.9390717	0.35372
RALGAPA1	1.541789	1.5035721	1.57334902	1.1385639	0.18721528	0.35372
TDRP	1.265266	1.2306528	1.29385034	1.273994	0.34935852	0.35394
DLST	1.368208	1.4042491	1.3384451	0.8372192	-0.2563226	0.35412
NDUFAF7	1.262746	1.2972548	1.23424776	0.788037	-0.3436647	0.35417
MFSD14C	1.253309	1.2881952	1.2244986	0.7789811	-0.3603398	0.35426
CYP24A1	1.046582	1.0150885	1.07258944	4.8109264	2.26631472	0.3544
ZDHHC7	1.352773	1.3171552	1.38218711	1.2050476	0.26909008	0.35459
CXCL1	1.049163	1.0175016	1.07531042	4.3030672	2.10536538	0.35465
TBC1D23	1.222057	1.2560365	1.19399548	0.7576869	-0.4003263	0.35476
MAP6	1.485007	1.5230269	1.45360974	0.867278	-0.2054336	0.355

MEGF6	1.123218	1.0905125	1.15022755	1.6597429	0.73095975	0.35504
CALCOCO1	1.645614	1.6060325	1.67830033	1.1192475	0.16252916	0.35548
GTF2H3	1.284012	1.319144	1.25499907	0.7990096	-0.3237153	0.35556
ZFAND2B	1.482929	1.5204109	1.45197655	0.8684994	-0.2034032	0.35558
CPSF2	1.469731	1.506765	1.43914791	0.8665712	-0.2066099	0.35587
NFIX	1.0542	1.0226329	1.08026854	3.5465405	1.82641241	0.35587
OTUD5	1.55778	1.5196209	1.58929151	1.1340796	0.1815219	0.35592
TRAF2	1.230075	1.2640823	1.20199147	0.7648808	-0.3866931	0.3563
FKBP1B	1.728003	1.7689667	1.69417454	0.9027368	-0.1476227	0.35638
EHD3	1.107644	1.0753272	1.13433135	1.7833053	0.83455368	0.35639
TRAFD1	1.199874	1.2333816	1.17220349	0.7378624	-0.4385762	0.35641
ARMCX5	1.251281	1.2854196	1.22308842	0.7816158	-0.3554685	0.35668
RNASET2	1.782125	1.8229108	1.74844274	0.9095065	-0.1368441	0.35675
SNUPN	1.293119	1.3277989	1.26447931	0.806834	-0.3096563	0.35678
NPAT	1.204667	1.1707551	1.23267158	1.3626038	0.44636614	0.3568
FAM185A	1.154764	1.1877956	1.12748598	0.6788551	-0.5588243	0.35703
ATP11C	1.491576	1.4545344	1.52216552	1.1487922	0.20011787	0.35715
ZNF85	1.155666	1.1888099	1.12829506	0.6794933	-0.5574689	0.35719
ENDOG	1.24097	1.2750645	1.21281424	0.7736886	-0.370175	0.3572
OTX1	1.102394	1.0702726	1.12891971	1.834565	0.87543806	0.35722
UQCC3	2.385663	2.4382855	2.3422066	0.933199	-0.0997433	0.35724
TRPM7	1.32706	1.291993	1.35601914	1.219273	0.28602113	0.35748
ITSN2	1.310764	1.2758607	1.339587	1.231009	0.29984126	0.35751
SPTY2D1-AS1	1.097421	1.1294111	1.07100301	0.5486623	-0.8660096	0.35789
ACOX2	1.113572	1.1457636	1.08698792	0.5967741	-0.7447432	0.35794
RIC8A	1.686083	1.7257792	1.65330045	0.9001367	-0.151784	0.35798
DPH6	1.323828	1.359038	1.29475062	0.8209455	-0.2846417	0.35804
REC8	1.97833	2.0250611	1.93973779	0.9167627	-0.1253797	0.35804
PARN	1.208291	1.2421295	1.18034728	0.7448382	-0.425001	0.35805
RBPJ	2.969307	3.0416686	2.90954926	0.9352886	-0.0965166	0.35824
MIR22HG	1.059542	1.0281501	1.08546658	3.0361003	1.60221945	0.35831
PPM1L	1.221886	1.1878926	1.24995808	1.3303242	0.41177789	0.35845
TMEM185B	1.114304	1.1467001	1.08755009	0.5967966	-0.7446889	0.35854
KRT4	1.045168	1.0139027	1.07098757	5.1060224	2.35219988	0.35902
ZBTB8OS	1.660386	1.7001359	1.6275604	0.8963409	-0.1578806	0.35913
TACSTD2	1.045543	1.0143306	1.07131835	4.9766548	2.31517633	0.3592
ERBB4	1.151434	1.1843159	1.12427938	0.6742739	-0.5685933	0.3593
FAM175B	1.217104	1.2509133	1.18918441	0.7539831	-0.4073958	0.35942
DNAJB4	1.395384	1.4317043	1.36539076	0.8463913	-0.2406033	0.35947
SLC35F2	1.166223	1.1993273	1.13888437	0.6967655	-0.5212548	0.35961
HMGXB3	1.32043	1.2855211	1.349259	1.2232336	0.2907	0.35983
LLGL1	1.300128	1.3348121	1.27148528	0.8108586	-0.3024778	0.36015
RBM17	2.559346	2.6150177	2.51337117	0.9370617	-0.0937841	0.36015
DTX4	1.404358	1.4401933	1.37476421	0.8513627	-0.2321541	0.36034
RP11-430G17.3	1.078309	1.046567	1.10452175	2.2445436	1.16642213	0.36034

PHTF2	1.301435	1.2666246	1.33018254	1.2383799	0.30845401	0.36042
PPIG	3.091858	3.1608072	3.03491874	0.9417401	-0.0865991	0.36043
IL17RC	1.236885	1.2707906	1.20888471	0.7713883	-0.3744708	0.36078
PRRT2	2.448345	2.3868279	2.49914697	1.0809899	0.1123531	0.36089
METTL15	1.326336	1.3614762	1.297316	0.822505	-0.2819036	0.3609
CLSTN1	1.76353	1.7233466	1.79671457	1.1014285	0.13937581	0.36098
KIAA0895	1.174425	1.2073897	1.14720304	0.7097896	-0.4945367	0.36121
AKTIP	1.330471	1.3657654	1.30132405	0.8238177	-0.2796029	0.3614
PARP11	1.21673	1.2501595	1.18912339	0.7560112	-0.4035204	0.36142
C12orf73	1.83942	1.8809504	1.8051236	0.9139262	-0.1298505	0.36143
INSM1	1.056297	1.0878117	1.03027105	0.3447267	-1.536475	0.36144
UACA	1.760351	1.8001292	1.72750165	0.9092303	-0.1372824	0.36157
CTB-12O2.1	1.046293	1.015165	1.07199913	4.7477191	2.24723457	0.36166
ADAMTS12	1.158204	1.1254543	1.18524849	1.4766211	0.56229965	0.36171
RASSF6	1.121238	1.0893965	1.1475336	1.6503293	0.72275394	0.3619
ADAMTS1	1.121585	1.0895288	1.14805721	1.6537391	0.72573164	0.3619
SLCO5A1	1.143674	1.1761699	1.11683879	0.6632164	-0.5924484	0.36221
POLE2	1.111661	1.143461	1.08540077	0.5952891	-0.7483377	0.36298
ALAS1	1.296525	1.3312954	1.26781084	0.8083749	-0.3069037	0.36314
KIRREL	1.199768	1.1666566	1.2271115	1.3627514	0.4465224	0.36342
NCBP3	1.554818	1.5923947	1.52378618	0.8841845	-0.1775806	0.3635
SLC35E3	1.658126	1.6971074	1.62593528	0.8979037	-0.1553673	0.36362
CCAR1	1.873878	1.9148064	1.84007808	0.9183124	-0.1229431	0.36362
UBE2F	1.507084	1.4702872	1.53747052	1.142856	0.19264363	0.36385
RP11-395A13.2	1.233267	1.1995813	1.26108539	1.3081655	0.38754504	0.3639
NCK2	1.232258	1.1988656	1.25983365	1.3065793	0.38579468	0.364
NCKAP1	1.951859	1.91088	1.98570038	1.0821408	0.11388822	0.36408
WASF2	2.799958	2.8676244	2.74407737	0.933848	-0.0987403	0.36412
SDC3	1.322671	1.2879993	1.35130355	1.219807	0.28665289	0.36436
C10orf10	1.056675	1.0257187	1.08223975	3.1976644	1.67701851	0.36437
ERCC6L2	1.353124	1.3883752	1.32401301	0.8342784	-0.2613993	0.36439
SCAMP5	1.161159	1.128541	1.18809627	1.4633178	0.54924312	0.36467
ARPP19	3.173662	3.2391334	3.11959549	0.9466142	-0.0791515	0.36467
CSTF1	1.298179	1.3322295	1.27005972	0.812871	-0.2989017	0.36479
SLC39A3	1.592983	1.5550982	1.62426913	1.1246103	0.16942512	0.36491
RP11-841O20.2	1.463522	1.4272603	1.49346788	1.1549583	0.20784078	0.36497
LSG1	1.135959	1.1680501	1.10945753	0.6513386	-0.6185203	0.36499
SACS	1.583667	1.545893	1.61486145	1.1263407	0.17164328	0.3651
GPC6	2.698449	2.642946	2.74428358	1.0616804	0.08634952	0.36567
KLHL28	1.390122	1.4257002	1.3607417	0.8474078	-0.2388717	0.36608
CLOCK	1.405958	1.3700491	1.43561207	1.1771736	0.23532707	0.3663
KANK1	1.202169	1.1691171	1.22946365	1.3568327	0.44024283	0.36645
SYCE1L	1.949035	1.9072547	1.98353823	1.0840817	0.11647351	0.36646
TCAIM	1.429923	1.4659282	1.40019016	0.8589096	-0.2194218	0.36678
APH1B	1.145585	1.1134777	1.1720992	1.5165904	0.60083148	0.36699

HDHD3	1.430556	1.3949704	1.45994314	1.1645002	0.21971095	0.36702
KCTD2	1.140642	1.108441	1.16723348	1.5421611	0.62495353	0.36723
ANAPC5	2.213389	2.2643597	2.17129627	0.9263948	-0.1103009	0.36727
PARP16	1.210111	1.2435758	1.18247448	0.7491488	-0.4166759	0.36728
RAP2B	1.469675	1.4339697	1.49916184	1.1502227	0.20191327	0.36733
UBLCP1	1.358014	1.3931081	1.32903231	0.8370021	-0.2566968	0.3674
ZNF44	1.161646	1.1293807	1.18829169	1.4553302	0.54134651	0.36794
ANLN	1.211131	1.2441041	1.18390148	0.7533732	-0.4085635	0.36847
SCARB1	1.293221	1.2588458	1.32160782	1.2424685	0.3132093	0.3691
COMT	1.306437	1.3405212	1.27828926	0.817245	-0.2911594	0.36923
ARHGAP21	1.712097	1.673233	1.7441907	1.1053984	0.14456647	0.36933
HABP4	1.400101	1.4356789	1.37072077	0.8509037	-0.2329322	0.36939
TRABD	1.654125	1.6923105	1.62259166	0.8992954	-0.153133	0.36949
CDH1	1.043002	1.0124282	1.06825095	5.4916403	2.45723714	0.36954
ISPD	1.080233	1.0491282	1.10591921	2.155976	1.1083411	0.36984
RARB	1.170345	1.1381221	1.19695547	1.4259514	0.51192479	0.37017
RP11-120I21.2	1.241503	1.274768	1.21403145	0.7789532	-0.3603914	0.37018
TNK2	1.147113	1.1150748	1.17356983	1.5083221	0.59294453	0.37034
RASSF8-AS1	1.513631	1.4769757	1.54390171	1.1403133	0.18943021	0.37049
DNMT3B	1.147847	1.18002	1.12127826	0.6736933	-0.5698362	0.3705
NPPB	1.045615	1.0149471	1.07094075	4.7461337	2.24675275	0.37061
CRB3	1.048279	1.0176743	1.07355199	4.1615244	2.0571121	0.37062
CLMN	1.066959	1.0360874	1.09245337	2.561928	1.35722991	0.37064
UBE2H	2.002422	1.9606305	2.03693337	1.07943	0.11026974	0.37069
KCNC3	1.229084	1.1961428	1.25628786	1.3066393	0.38586093	0.37085
N4BP2L2	4.479138	4.3793162	4.56157181	1.0539327	0.07578271	0.37091
CYB5RL	1.112965	1.0814658	1.13897688	1.7059534	0.77057825	0.37094
CHN2	1.060272	1.0295081	1.08567671	2.9034971	1.53779158	0.37096
GTPBP10	1.17033	1.2027207	1.14358193	0.7082747	-0.4976192	0.37109
AFDN-AS1	1.26678	1.3003949	1.23902035	0.7956872	-0.3297267	0.37123
QRSL1	1.250207	1.2835691	1.22265571	0.7851902	-0.348886	0.37143
CHTF8	1.630217	1.6681266	1.59891053	0.8964028	-0.1577809	0.37146
SUPT6H	1.315802	1.2818627	1.34382878	1.2198449	0.28669776	0.37158
CCDC138	1.229878	1.2628897	1.20261557	0.7707248	-0.3757124	0.37159
ZDHHC12	2.174661	2.1308504	2.21084042	1.0707344	0.0986006	0.37182
AP000769.1	1.156333	1.1885139	1.1297581	0.688321	-0.5388466	0.37183
AZIN2	1.156371	1.1884817	1.12985304	0.6889426	-0.5375444	0.37183
NOC2L	1.645109	1.682943	1.6138645	0.8988517	-0.153845	0.37185
G3BP2	2.04008	2.0827347	2.00485599	0.9280723	-0.107691	0.37212
SETDB2	1.233478	1.2004097	1.26078605	1.3012644	0.37991409	0.37213
IWS1	1.516153	1.5529007	1.48580568	0.8786491	-0.186641	0.3722
KYAT1	1.182832	1.2154632	1.15588505	0.7234879	-0.4669592	0.37231
FST	1.197214	1.1648219	1.22396337	1.3588207	0.44235505	0.37247
NUP88	1.203356	1.235648	1.17668825	0.7497975	-0.415427	0.37248
JUND	2.084279	2.0398534	2.12096695	1.0780048	0.10836354	0.37249

ATP5S	1.494045	1.5303142	1.4640933	0.875129	-0.1924324	0.37254
ZNF786	1.123842	1.1554479	1.09774184	0.6287756	-0.6693828	0.37259
RBFOX3	1.043955	1.0744325	1.01878617	0.2523918	-1.9862629	0.37282
ARMC7	1.130144	1.1619681	1.10386351	0.6412589	-0.6410212	0.37294
SFT2D2	1.373725	1.3389174	1.40246988	1.1875161	0.24794705	0.37317
ZEB1-AS1	1.309091	1.343059	1.28103918	0.8192154	-0.2876853	0.37331
VASN	1.085075	1.0540376	1.11070575	2.0486807	1.03469516	0.37335
SPATS2	2.390794	2.4498474	2.34202634	0.9256328	-0.1114881	0.37337
MED23	1.237065	1.2702546	1.20965705	0.7757761	-0.3662878	0.37339
PTPRJ	1.189785	1.1573976	1.21653088	1.3756939	0.46015951	0.37349
N4BP1	1.212603	1.1801577	1.23939723	1.3288203	0.41014599	0.37356
RFTN1	1.064824	1.0342664	1.09005952	2.6282154	1.39408351	0.37368
B3GNTL1	1.134079	1.1655404	1.10809702	0.6529949	-0.6148564	0.37375
DENND2A	1.154778	1.1228206	1.18116989	1.4750779	0.56079115	0.37379
SLC4A5	1.077384	1.0465904	1.10281345	2.2067512	1.14192397	0.37384
INPPL1	1.385855	1.3510103	1.41463009	1.1812478	0.2403116	0.37387
SMARCAL1	1.135158	1.166658	1.10914503	0.6549041	-0.6106444	0.37389
TCEA2	2.48433	2.5399693	2.43838291	0.9340335	-0.0984538	0.37423
MELK	1.202837	1.2351885	1.17612009	0.7488464	-0.4172582	0.37423
DOT1L	1.335082	1.3008255	1.36337092	1.2079125	0.27251597	0.37433
NAA25	1.337246	1.3032023	1.36535914	1.2050013	0.26903469	0.37448
EDN1	1.080041	1.0494341	1.10531597	2.1304314	1.0911456	0.37454
PREPL	1.735759	1.6968462	1.76789354	1.1019555	0.14006598	0.37455
DKFZP434L187	1.191151	1.2234564	1.16447273	0.7360394	-0.442145	0.37465
PRRC1	1.444345	1.4800689	1.41484407	0.8641344	-0.2106724	0.37484
C11orf80	1.250243	1.2832115	1.22301706	0.7874576	-0.3447259	0.37503
ZNF253	1.250692	1.2836355	1.22348591	0.7879335	-0.3438542	0.37503
C20orf24	2.266311	2.3148532	2.22622476	0.9325944	-0.1006783	0.37522
CEP135	1.368482	1.3339696	1.39698235	1.188678	0.24935792	0.37525
SYT11	1.678823	1.6407572	1.71025909	1.1084683	0.14856751	0.37558
BAP1	1.327911	1.3618865	1.29985343	0.8285842	-0.2712797	0.37561
HSD17B14	1.467334	1.4319692	1.49653856	1.1494767	0.20097719	0.37561
VASP	1.700822	1.6627901	1.73222987	1.1047689	0.14374458	0.37565
RAB33A	1.205183	1.2377094	1.17832124	0.7501647	-0.4147206	0.37594
DAP3	1.758807	1.7976382	1.7267404	0.9111153	-0.1342944	0.376
NDUFAF8	3.685231	3.7629827	3.62102291	0.9486208	-0.0760966	0.37601
TBC1D24	1.201913	1.1694961	1.22868362	1.3491968	0.43210081	0.37609
HAUS2	1.207855	1.2403609	1.18101064	0.7530786	-0.4091276	0.37616
AKAP8	1.201013	1.2333336	1.17432271	0.7470964	-0.4206337	0.3766
DNAJC21	1.523335	1.5597403	1.49327119	0.8812501	-0.1823765	0.37669
RP5-827C21.4	1.097436	1.0665441	1.12294785	1.8476146	0.88566382	0.37678
RBM38	1.183496	1.1515838	1.20985017	1.3843843	0.46924445	0.37717
LARP4	1.513398	1.47721	1.54328285	1.1384566	0.18707929	0.37718
L3MBTL2	1.167916	1.1360094	1.19426453	1.4283171	0.51431631	0.37722
MIR222HG	1.143336	1.111811	1.16937031	1.5147909	0.59911869	0.37723

RP11-434H6.7	1.121377	1.1528159	1.09541424	0.6243737	-0.6795182	0.37728
NOP16	1.297432	1.3307994	1.26987654	0.8158315	-0.2936569	0.3773
KBTBD4	1.194149	1.2262312	1.16765523	0.7410792	-0.4323004	0.37742
SEC23IP	1.30472	1.2709468	1.33261086	1.2275872	0.29582549	0.37747
UBTD2	1.328974	1.3629619	1.30090668	0.829031	-0.270502	0.3775
NFYA	1.169017	1.200938	1.14265583	0.7099496	-0.4942114	0.37762
ZNF382	1.166246	1.1979706	1.1400476	0.707416	-0.4993693	0.37773
HUS1	1.162745	1.1947335	1.13632797	0.7000745	-0.5144196	0.37785
CHPT1	1.47193	1.5073532	1.44267681	0.8725219	-0.1967368	0.37791
OSCP1	1.17194	1.2038303	1.14560372	0.714338	-0.4853213	0.37803
RALGAPB	1.297564	1.2639803	1.32529784	1.2322806	0.30133086	0.37803
MAN1A2	1.974659	2.0164526	1.94014483	0.9249274	-0.112588	0.37804
TMX3	1.410527	1.3754631	1.43948293	1.1705091	0.2271361	0.37819
COMTD1	1.159916	1.1283708	1.18596653	1.4486673	0.53472631	0.37834
GPKOW	1.203833	1.2359977	1.17727105	0.7511559	-0.4128157	0.37838
FAM45A	1.781359	1.8203312	1.74917454	0.9132586	-0.1309047	0.37878
ZMAT4	1.206237	1.2384223	1.17965749	0.7535265	-0.4082699	0.37879
LRRIQ1	1.353879	1.3879125	1.32577443	0.8398141	-0.2518581	0.37891
ZNF670	1.130141	1.1613522	1.1043655	0.6468178	-0.6285686	0.37912
GMCL1	1.637231	1.6746645	1.60631854	0.8986963	-0.1540944	0.37913
MARCH7	1.843592	1.8829552	1.81108581	0.9186036	-0.1224857	0.37919
DERL3	1.172563	1.2040845	1.14653241	0.7179987	-0.4779469	0.37922
CDC14B	1.160611	1.1288029	1.1868781	1.450884	0.53693214	0.37925
STPG1	1.131623	1.1628516	1.1058339	0.6498794	-0.6217562	0.37932
EPHA7	1.086316	1.0556795	1.11161678	2.0046303	1.00333617	0.37941
DCAF10	1.539139	1.57588	1.50879834	0.8835145	-0.1786742	0.37985
RHBDD3	1.430377	1.4655082	1.40136553	0.8622094	-0.2138898	0.38004
MAZ	2.133137	2.1764637	2.09735672	0.9327587	-0.1004242	0.38007
C3orf14	1.612387	1.6494548	1.58177628	0.8957919	-0.1587646	0.38015
STXBP2	1.378249	1.4126593	1.34983187	0.8477498	-0.2382896	0.38019
RFC2	1.434748	1.4693602	1.40616502	0.8653588	-0.2086297	0.3802
DLG5	1.267071	1.2340734	1.29432018	1.2573839	0.3304252	0.38022
BBC3	1.535958	1.572489	1.50579042	0.8834938	-0.1787082	0.38023
PREB	1.59235	1.6292471	1.56188031	0.8929407	-0.1633637	0.38026
COPZ2	1.197414	1.1655628	1.2237178	1.3512566	0.43430164	0.3805
BAZ1B	2.045091	2.0866043	2.0108095	0.9302462	-0.1043155	0.38095
MDM1	1.226766	1.259091	1.20007068	0.7722024	-0.3729491	0.38111
ARAP3	1.119991	1.0891961	1.1454224	1.630368	0.7051976	0.38112
ARHGEF3	1.217843	1.1856341	1.24444125	1.3167907	0.39702608	0.38114
ZNF24	1.773816	1.8127994	1.74162258	0.91243	-0.1322142	0.38126
COQ4	2.020051	2.0617405	1.98562369	0.9283094	-0.1073223	0.38149
SIN3B	1.210558	1.1784583	1.23706559	1.328409	0.40969939	0.38157
SH3BP2	1.194687	1.1630253	1.22083332	1.3545953	0.43786185	0.38182
POC1A	1.196239	1.2279017	1.17009212	0.7463398	-0.4220955	0.38213
AFDN	1.591799	1.5548366	1.6223223	1.1216316	0.1655989	0.38216

A2M-AS1	1.243301	1.2757147	1.2165328	0.7853509	-0.3485907	0.38257
MAN2A1	1.330089	1.2965146	1.35781612	1.2067402	0.27111507	0.38278
METTL2B	1.349651	1.3833921	1.32178696	0.8393156	-0.2527146	0.38286
ARHGAP4	1.180148	1.21169	1.15410022	0.7279525	-0.4580838	0.38296
DNAH9	1.076318	1.1063627	1.05150741	0.4842621	-1.0461401	0.38306
MTFR1L	1.807468	1.8466814	1.77508503	0.9154388	-0.1274646	0.38314
ITFG2	1.26264	1.2952992	1.23566914	0.7980689	-0.3254148	0.3834
C18orf32	1.536555	1.5725936	1.5067929	0.885083	-0.1761153	0.38371
RP11-83A24.2	1.634124	1.6710877	1.60359846	0.899433	-0.1529123	0.38376
POLL	1.367938	1.402061	1.3397584	0.845042	-0.242905	0.38378
EIF4EBP2	2.038692	2.0805412	2.00413294	0.929287	-0.1058038	0.38388
ERCC8	1.172335	1.203689	1.14644266	0.7189522	-0.4760322	0.38392
GRIA1	1.098373	1.0679909	1.12346238	1.8158652	0.86065711	0.38396
PLCB1	1.301845	1.3349359	1.27451879	0.819616	-0.28698	0.38399
TRIQK	1.539243	1.5751005	1.5096319	0.8861615	-0.1743585	0.384
GPR162	1.589931	1.6265356	1.55970194	0.8933282	-0.1627378	0.38413
ONECUT1	1.052822	1.0827883	1.02807543	0.3391232	-1.5601186	0.38414
CLP1	1.107502	1.138043	1.08228051	0.59605	-0.7464946	0.38421
RABGEF1	1.114246	1.0838346	1.13936024	1.6623239	0.73320148	0.38448
C16orf46	1.174061	1.2054525	1.14813694	0.7210278	-0.4718731	0.3845
RPP25	1.202943	1.1713104	1.22906528	1.3371359	0.41914606	0.38453
FAM111B	1.155819	1.1244941	1.18168761	1.4594072	0.54538252	0.38472
FAM19A5	1.230943	1.2633906	1.20414669	0.775072	-0.3675978	0.38487
TSPYL2	1.230639	1.2628578	1.20403161	0.7762053	-0.3654897	0.38487
CYFIP1	1.390629	1.4244016	1.3627385	0.8547057	-0.2265003	0.38488
MYO10	1.504001	1.4685627	1.53326639	1.1380897	0.1866143	0.38498
RHOU	1.448438	1.4135467	1.47725204	1.1540462	0.20670095	0.38498
AP2A1	1.78236	1.8211071	1.75036177	0.9138415	-0.1299841	0.38505
MTPAP	1.267375	1.2998398	1.24056453	0.8023103	-0.3177678	0.38507
APCDD1	1.17932	1.2108822	1.1532559	0.7267369	-0.460495	0.38519
NFAT5	1.572669	1.5370376	1.60209458	1.1211406	0.16496716	0.38524
ALKBH3	1.19935	1.2309917	1.17322035	0.7498984	-0.4152329	0.38536
KIAA1217	1.47652	1.5116168	1.44753666	0.8747498	-0.1930577	0.38553
ADAMTS9	1.188753	1.157532	1.21453631	1.361859	0.4455773	0.38556
GLIS2	1.258241	1.2259983	1.28486813	1.2604877	0.33398207	0.38608
CEND1	1.108624	1.1392552	1.08332741	0.598379	-0.7408686	0.38612
HGSNAT	1.417578	1.383521	1.44570331	1.1621355	0.21677826	0.38621
CLEC2A	1.517056	1.4824209	1.54565887	1.1310847	0.17770695	0.38628
MYADM	1.556908	1.5926955	1.52735323	0.8897541	-0.1685214	0.38629
AKT1S1	1.624689	1.661509	1.59428176	0.898373	-0.1546136	0.38638
MTCL1	1.130351	1.0995602	1.155779	1.5646711	0.64585946	0.38638
COPB1	1.68381	1.7209912	1.65310487	0.905843	-0.1426671	0.38644
SYVN1	1.128355	1.0977327	1.15364413	1.5720847	0.65267899	0.3866
NPAS2	1.081868	1.0518026	1.10669628	2.0596723	1.04241481	0.38661
TRIM13	1.581649	1.6177883	1.55180478	0.893194	-0.1629545	0.38681

POLDIP3	1.558067	1.5939991	1.5283941	0.8895537	-0.1688463	0.38696
FHAD1	1.170839	1.2019401	1.14515439	0.7187994	-0.4763389	0.38719
GS1-124K5.4	1.352752	1.3863102	1.32503957	0.8413953	-0.2491443	0.38742
EZH1	1.304032	1.2711782	1.33116379	1.2212038	0.28830394	0.3876
RGS14	1.383723	1.4177984	1.35558239	0.851086	-0.2326232	0.3877
OMA1	1.144406	1.1753808	1.11882582	0.6775305	-0.5616421	0.38774
MIGA2	1.113903	1.0834779	1.13902802	1.6654464	0.73590891	0.38775
CCAR2	1.239464	1.2712953	1.21317674	0.7857739	-0.3478139	0.38792
UBA6	1.748313	1.7104818	1.77955449	1.0972196	0.13385226	0.38799
MYDGF	3.016572	3.0787615	2.96521491	0.9453778	-0.0810372	0.38819
LAPTM4B	3.245219	3.3129776	3.18926351	0.9465131	-0.0793057	0.38836
MGLL	1.165474	1.1342024	1.19129884	1.4254507	0.51141817	0.38866
C10orf54	1.051895	1.022544	1.07613341	3.3771036	1.75578642	0.38866
BRCA1	1.432125	1.397429	1.46077813	1.1593973	0.213375	0.38868
ZZZ3	1.458368	1.4235857	1.48709255	1.1499267	0.20154196	0.38881
HAS2	1.315563	1.2823083	1.34302581	1.2150752	0.2810456	0.38912
IQSEC1	1.368571	1.3354888	1.39589009	1.1800398	0.23883551	0.38914
C14orf80	1.145116	1.1758675	1.11972083	0.6807446	-0.5548145	0.38914
ATP2A1-AS1	1.419315	1.4533764	1.39118649	0.8628295	-0.2128526	0.38916
SOS1	1.370381	1.3366799	1.39821183	1.182761	0.24215853	0.38928
MARCH5	1.88726	1.927431	1.85408658	0.9209166	-0.1188576	0.38943
SGIP1	1.333156	1.3003	1.36028991	1.1997667	0.26275393	0.38953
RAMP1	1.482631	1.4481323	1.51112137	1.140559	0.18974113	0.38958
EIF5B	3.247933	3.3167118	3.19113494	0.9457952	-0.0804003	0.38971
MBD4	1.699671	1.7366561	1.66912747	0.9083309	-0.1387102	0.38982
FAN1	1.252869	1.2853307	1.22606158	0.7922792	-0.3359191	0.39033
EVA1C	1.043931	1.0146773	1.06808852	4.6390304	2.21382329	0.39037
NLN	1.396502	1.4301978	1.36867562	0.856991	-0.222648	0.39053
DTX3	1.621148	1.584979	1.6510164	1.1128886	0.15430914	0.39061
UBR2	1.305599	1.2727748	1.33270551	1.2197075	0.28653524	0.39061
FAM43A	1.16315	1.1322308	1.18868294	1.4269212	0.51290571	0.39062
UNC119B	1.362638	1.3960285	1.33506272	0.846057	-0.2411732	0.39076
NDFIP2	1.369393	1.3360073	1.39696417	1.1814155	0.24051639	0.39079
BOD1L1	1.448929	1.414476	1.47738159	1.1517715	0.20385454	0.39104
RP11-149I23.3	1.093346	1.0634728	1.11801588	1.8593128	0.89476947	0.39122
PPM1K	1.34625	1.3795146	1.31877992	0.8399674	-0.2515947	0.39138
COX15	1.297381	1.3296153	1.27076125	0.8214463	-0.2837618	0.39156
PTPN2	1.596713	1.6330497	1.5667062	0.8952002	-0.1597178	0.39162
ACOT8	1.311199	1.3442725	1.28388567	0.8245958	-0.278241	0.39171
NCAPD3	1.204649	1.2360139	1.17874814	0.7573626	-0.4009438	0.39187
MEGF8	1.271888	1.239676	1.29848847	1.2453832	0.31658971	0.39195
LINC00599	1.04837	1.0780105	1.02389268	0.3062752	-1.7070994	0.39199
MEF2D	1.100996	1.0709175	1.12583575	1.7743953	0.82732741	0.3921
CHMP3	1.75888	1.7963502	1.72793595	0.9140902	-0.1295915	0.39215
ZDHHC21	1.507949	1.5430921	1.47892767	0.8818534	-0.1813892	0.39221

LRRC59	1.737246	1.7749013	1.7061493	0.9112764	-0.1340394	0.39232
CELF1	2.261253	2.3091216	2.2217229	0.9332387	-0.099682	0.39248
STAT5B	1.183244	1.1521104	1.20895388	1.3736992	0.45806613	0.3927
PMP22	1.15899	1.1284122	1.18424164	1.4347678	0.52081726	0.39274
MRI1	1.297218	1.2649308	1.3238819	1.2225152	0.2898524	0.39303
ZC3H7A	1.561194	1.5965912	1.5319617	0.8916687	-0.1654204	0.39309
VEGFC	1.054942	1.0257446	1.07905407	3.0707024	1.61856869	0.39317
ARFGAP1	1.265869	1.2337931	1.29235701	1.2504944	0.32249855	0.39327
ZNF579	1.235701	1.2044349	1.26152152	1.2792408	0.35528789	0.3933
SLC25A1	1.940505	1.9809575	1.90709795	0.9247067	-0.1129323	0.39358
ZNF45	1.091163	1.1208725	1.06662917	0.551235	-0.8592605	0.3937
PDXDC1	1.592008	1.5564133	1.62140205	1.1167993	0.15937	0.39372
GJC1	1.258227	1.2261879	1.28468564	1.2586246	0.33184805	0.39381
ANK2	1.267929	1.3005967	1.24095177	0.8015784	-0.3190845	0.39438
DHFR2	1.133406	1.1636139	1.1084598	0.6629007	-0.5931353	0.39446
NEDD4L	1.323412	1.291015	1.35016671	1.20326	0.26694837	0.39451
SLC30A9	1.727421	1.7648437	1.69651675	0.9106654	-0.135007	0.39454
MDM4	2.007553	2.0533567	1.96972681	0.9206063	-0.1193438	0.39459
MIEN1	2.812419	2.8669222	2.76741027	0.9466973	-0.0790248	0.39481
DHX29	1.58115	1.617077	1.55148106	0.893699	-0.1621391	0.39486
TIA1	2.20196	2.159551	2.23698206	1.0667768	0.09325831	0.395
BCL2L1	1.438272	1.4047582	1.46594768	1.1511754	0.20310766	0.395
CREM	1.990713	2.031048	1.95740338	0.928573	-0.1069127	0.39505
HEATR6	1.151627	1.1820589	1.12649575	0.6948067	-0.5253165	0.39527
ULK1	1.201322	1.1703844	1.22687034	1.3315207	0.41307489	0.39535
PABPC1L	1.19274	1.1615689	1.21848094	1.3522465	0.43535816	0.39535
PNP	1.180357	1.2111027	1.15496653	0.7340812	-0.4459884	0.39548
TBKBP1	1.133983	1.1037273	1.15896888	1.5325649	0.61594814	0.39551
TRIM36	1.245222	1.2139248	1.271067	1.2671133	0.34154555	0.39572
GRAMD1B	1.120546	1.0905738	1.14529737	1.6041877	0.68184292	0.39578
TENM2	1.082511	1.0530257	1.10686018	2.0152538	1.01096154	0.39585
TLE1	1.539619	1.5747358	1.51061845	0.8884404	-0.1706531	0.39598
SLC25A26	1.728798	1.7663485	1.69778754	0.9105356	-0.1352127	0.39603
CHMP4C	1.307571	1.340254	1.28058027	0.8246202	-0.2781984	0.39652
NPW	3.930624	3.8328128	4.01139747	1.0630415	0.08819787	0.39665
TBCE	1.159731	1.1900271	1.13471239	0.7089115	-0.4963225	0.39684
MESP1	1.334992	1.36765	1.3080224	0.8378143	-0.2552975	0.39685
TTC9	1.080597	1.0512626	1.10482135	2.0447936	1.03195521	0.39696
PANK4	1.13188	1.1620954	1.10692731	0.6596565	-0.6002132	0.39702
DTL	1.20984	1.1785668	1.23566505	1.3197587	0.40027416	0.39716
TMEM120A	1.252949	1.2844692	1.22691968	0.7976951	-0.3260907	0.39724
JRK	1.221689	1.1904756	1.24746532	1.2991969	0.37762014	0.39726
PDS5B	1.568715	1.6040774	1.5395119	0.8931171	-0.1630787	0.39733
KCTD18	1.238827	1.2701602	1.21295116	0.7882402	-0.3432927	0.39733
ETNPPL	1.048965	1.0779159	1.02505668	0.3215864	-1.6367219	0.3974

PSMD5	1.240013	1.2714163	1.21408032	0.7887525	-0.3423554	0.39758
POLRMT	1.205663	1.2366156	1.18010239	0.7611602	-0.3937281	0.39803
XPA	1.445962	1.4796074	1.418177	0.8719152	-0.1977403	0.39818
ANKRD35	1.169187	1.2000718	1.14368193	0.7181518	-0.4776393	0.39832
ELAVL4	1.043296	1.0723239	1.01932418	0.2671895	-1.9040648	0.39835
TMEM147-AS1	1.239374	1.2709362	1.21331027	0.7873082	-0.3449996	0.39845
SLC19A1	1.173387	1.2039072	1.14818311	0.7267182	-0.460532	0.39849
ABHD17A	1.540073	1.5057595	1.56841008	1.1238743	0.16848062	0.39855
BRIP1	1.165402	1.1349416	1.19055707	1.4121446	0.49788779	0.3987
BTBD1	1.249826	1.2815365	1.22363879	0.794351	-0.3321515	0.39874
ETV3	1.144234	1.1139695	1.16922609	1.4848369	0.5703045	0.39892
RFC1	2.677913	2.7308945	2.63416063	0.9441134	-0.082968	0.39901
RP5-1172A22.1	1.087223	1.0578509	1.11147901	1.9270048	0.94636018	0.39924
SIL1	1.374994	1.4077948	1.34790604	0.8531399	-0.2291458	0.39926
PDGFRA	1.807261	1.7671842	1.84035635	1.0953775	0.13142817	0.3994
TRMT10A	1.222886	1.2540024	1.19719027	0.7763324	-0.3652536	0.39974
TENM4	1.242297	1.2110552	1.26809744	1.2702716	0.34513703	0.39989
MAFG	1.187966	1.1573608	1.21324037	1.3551045	0.43840406	0.40006
EGLN1	1.995315	2.034779	1.96272528	0.930368	-0.1041266	0.40017
LYSMD1	1.114334	1.1444067	1.08950028	0.6197792	-0.6901738	0.40032
DYRK3	1.10947	1.139247	1.0848794	0.6095598	-0.7141604	0.40034
PRKCB	1.071672	1.0425386	1.09573044	2.2504394	1.17020672	0.40047
EMP2	1.432008	1.3985946	1.45960108	1.153054	0.20546006	0.40057
CROT	1.332842	1.3655031	1.30587011	0.8368469	-0.2569644	0.40067
GALNT1	1.853921	1.8161385	1.88512178	1.084524	0.11706203	0.40077
NREP	4.645134	4.753586	4.55557262	0.9472469	-0.0781876	0.40079
ZNF440	1.089899	1.0605305	1.11415157	1.8858532	0.91521734	0.40083
CDK5RAP1	1.167212	1.1975491	1.14215952	0.7196162	-0.4747005	0.40086
SYT6	1.118623	1.1479932	1.09436838	0.6376533	-0.6491558	0.40088
EDN2	1.098786	1.0694012	1.1230533	1.7730724	0.82625147	0.40095
ID4	1.487775	1.5217319	1.4597328	0.8811667	-0.1825132	0.40111
MAP4	1.951687	1.9132244	1.98344953	1.076898	0.10688163	0.40119
ZNF609	1.41604	1.3828964	1.44341047	1.158043	0.21168884	0.40122
SLC25A51	1.174789	1.1443036	1.19996441	1.3857199	0.47063564	0.40158
MRPL30	1.373848	1.406701	1.34671668	0.8525099	-0.2302115	0.40159
YME1L1	1.844616	1.8825845	1.81326114	0.9214541	-0.1180158	0.40181
RP4-621F18.2	1.090814	1.061551	1.11497982	1.8680429	0.90152758	0.40195
DIP2A	1.44449	1.4111775	1.47200059	1.1479242	0.19902741	0.40204
SMCR5	1.38036	1.3473698	1.40760344	1.1733992	0.23069386	0.40209
LST1	1.095487	1.1247937	1.07128566	0.5712282	-0.8078608	0.40231
FAM171B	1.527189	1.5617726	1.4986301	0.8876013	-0.1720163	0.40234
CACNA1H	1.156294	1.1261723	1.18116817	1.4358794	0.52193457	0.40245
POLI	1.311103	1.3430786	1.28469615	0.8298277	-0.2691163	0.40261
CCDC33	1.416781	1.4501643	1.38921318	0.8646025	-0.209891	0.40266
DUSP18	1.270763	1.3023487	1.2446792	0.8092617	-0.3053217	0.40283

CRNKL1	1.290635	1.3225233	1.26430018	0.8194761	-0.2872261	0.40284
OSER1	1.391946	1.4247516	1.36485509	0.8589846	-0.2192958	0.40284
FKBP10	2.868798	2.8123686	2.91539847	1.0568482	0.07976813	0.40291
UNK	1.364883	1.397996	1.33753737	0.8480924	-0.2377067	0.40299
WDR82	1.564616	1.5297717	1.59339136	1.1200888	0.16361308	0.40302
ZNF614	1.148604	1.1187073	1.17329366	1.4598401	0.54581034	0.4031
TMEM242	1.48596	1.5197227	1.45807852	0.8813902	-0.1821472	0.40341
ADD2	1.235774	1.2668122	1.21014177	0.7876019	-0.3444615	0.40342
TANGO2	1.090629	1.0613317	1.11482367	1.8721763	0.90471631	0.40346
RAB6B	1.329146	1.2969994	1.35569391	1.1976249	0.26017612	0.40355
TOR1AIP1	1.238127	1.2691409	1.2125155	0.7896068	-0.3407938	0.40358
RP11-16E12.1	1.132129	1.1618812	1.10755875	0.66443	-0.5898109	0.40358
FAM46A	1.331418	1.2993644	1.35788897	1.1954963	0.25760963	0.40365
ZNF771	1.351695	1.3838753	1.32512088	0.846944	-0.2396614	0.40387
APBA2	1.442633	1.4759623	1.41510888	0.8721465	-0.1973576	0.40414
YIF1A	2.398477	2.4512861	2.35486604	0.9335623	-0.0991818	0.40414
KMT2C	1.952951	1.9915842	1.92104752	0.9288647	-0.1064597	0.40418
RDH14	1.519415	1.5533576	1.49138436	0.8880051	-0.1713601	0.40443
CTC-425F1.4	1.175292	1.2057528	1.15013718	0.7296968	-0.454631	0.40447
ZNF649	1.170415	1.200467	1.14559839	0.726296	-0.4613705	0.40459
SLC35A2	1.345961	1.3783374	1.31922403	0.8437549	-0.2451042	0.40459
ACP2	1.329757	1.3616841	1.30339051	0.8388273	-0.2535543	0.40463
RP11-469A15.2	1.13618	1.1658655	1.1116652	0.6732275	-0.570834	0.40478
LRRC32	1.052159	1.0236165	1.07573027	3.2066636	1.68107302	0.40491
CDR1-AS	1.26851	1.2999633	1.24253493	0.8085485	-0.3065937	0.40513
C6orf106	1.280917	1.2496977	1.30669897	1.2282814	0.29664108	0.40515
SCAF11	3.00349	3.0653558	2.9523993	0.9453089	-0.0811422	0.40542
HRASLS	1.355961	1.3884571	1.32912478	0.8472616	-0.2391206	0.40563
GFRA1	1.282811	1.2514962	1.30867193	1.2273422	0.29553756	0.40577
IKBKB	1.282952	1.2514124	1.30899703	1.2290443	0.29753692	0.40577
ZNF100	1.198198	1.2286909	1.17301736	0.7565555	-0.4024823	0.40577
OCRL	1.161384	1.131595	1.18598344	1.4133013	0.49906905	0.40592
SP100	1.085292	1.0562517	1.10927365	1.9425857	0.95797821	0.406
RP11-872J21.3	1.094731	1.1237835	1.07073961	0.5714785	-0.8072288	0.4061
STEAP2	1.143752	1.173462	1.11921613	0.6872753	-0.54104	0.40649
POU2F2	1.224119	1.1935219	1.24938585	1.2886696	0.3658824	0.4065
CHMP2B	1.982218	2.0214867	1.94978943	0.9298108	-0.1049908	0.40671
TMEM44	1.130655	1.1010136	1.1551336	1.5357694	0.61896163	0.40688
CHD9	3.062611	3.0037424	3.11122586	1.0536414	0.07538389	0.40711
PPIL6	1.19429	1.2246851	1.16918981	0.7530086	-0.4092618	0.40717
MPHOSPH9	1.500054	1.4663116	1.52791935	1.1321172	0.17902327	0.4073
CUL4B	1.363085	1.330873	1.3896865	1.1777526	0.2360365	0.40733
RHOJ	1.110533	1.0813581	1.13462544	1.6547269	0.72659316	0.40734
KCTD13	1.194505	1.2249162	1.16939101	0.7531294	-0.4090303	0.40734
CNTN1	1.069198	1.0404815	1.09291197	2.2951726	1.19860266	0.40738

UBR7	1.173305	1.2034905	1.14837648	0.7291568	-0.455699	0.4074
TMEM8A	1.256844	1.2259498	1.28235681	1.2496441	0.32151726	0.40741
ATP1A2	1.157076	1.1868019	1.13252839	0.7094594	-0.495208	0.40752
ANKRD52	1.166773	1.1371478	1.19123799	1.3943934	0.47963762	0.40764
TCTN2	1.183907	1.2143504	1.15876664	0.7406874	-0.4330633	0.40812
MREG	1.10434	1.1335085	1.08025204	0.6011004	-0.734322	0.40824
CMTM3	1.823491	1.8609409	1.79256387	0.9205787	-0.119387	0.40828
GPSM1	1.21909	1.1885398	1.24431957	1.2958515	0.37390035	0.40834
EPHX2	1.608661	1.6441703	1.57933627	0.8993526	-0.1530412	0.40866
PDE7B	1.122181	1.1514209	1.09803506	0.6474341	-0.6271949	0.40882
FAM92B	1.095059	1.123882	1.07125606	0.5751932	-0.7978816	0.40883
RUFY2	1.428351	1.4615505	1.40093342	0.8686664	-0.2031258	0.40899
FHIT	1.254767	1.2858357	1.22911028	0.8015455	-0.3191437	0.40899
ZNF891	1.299503	1.3309662	1.27352102	0.8264318	-0.2750324	0.40912
NSMAF	1.326268	1.3579643	1.30009304	0.8383323	-0.254406	0.40928
DDX54	1.591168	1.6258064	1.56256252	0.8989402	-0.153703	0.40938
DNAJC15	1.697619	1.6622141	1.72685746	1.097617	0.13437467	0.40949
ARL5A	1.735276	1.6990404	1.76519949	1.0946428	0.13046016	0.40956
RBM6	2.438495	2.3852157	2.48249356	1.0702258	0.09791518	0.41022
RNF40	1.345229	1.3134503	1.37147212	1.185107	0.24501735	0.41028
ZNF655	1.430312	1.3975582	1.45736049	1.150424	0.20216568	0.41034
F11R	1.152802	1.1232124	1.17723713	1.4384688	0.52453395	0.41049
MAIP1	1.282554	1.3134114	1.2570712	0.8202358	-0.2858894	0.41071
PLD1	1.106744	1.0778301	1.13062232	1.6783016	0.74700199	0.41073
CARNS1	1.047156	1.0189474	1.07045121	3.7182555	1.89462589	0.4108
PSTPIP2	1.149582	1.1792576	1.12507486	0.6977381	-0.5192424	0.41087
PLAT	1.402914	1.3700484	1.43005491	1.1621585	0.2168069	0.41088
RP5-1074L1.4	1.165429	1.1357482	1.18993979	1.3992064	0.48460884	0.41088
AMT	1.105547	1.1345038	1.08163397	0.606927	-0.7204051	0.41089
FAM217B	1.541403	1.5074238	1.5694628	1.1222627	0.16641042	0.41093
BRAF	1.230848	1.2005027	1.25590697	1.2763266	0.35199756	0.41163
ERI1	1.252474	1.2829193	1.22733163	0.8035212	-0.315592	0.41173
IFT46	1.237778	1.2682164	1.21264094	0.7927961	-0.3349783	0.41174
CCNT1	1.26813	1.2372773	1.29360877	1.2374076	0.30732078	0.41186
RPP40	1.135377	1.1646381	1.11121308	0.6755003	-0.5659718	0.41187
TUBG2	1.260846	1.2917301	1.23534224	0.8067122	-0.309874	0.41192
ARFRP1	1.385934	1.4182264	1.35926709	0.8590254	-0.2192273	0.41197
C15orf65	1.170423	1.2000577	1.14594994	0.7295392	-0.4549425	0.41202
RP11-867G23.8	1.135774	1.1651952	1.11147764	0.6748237	-0.5674174	0.41207
GRIA2	1.053683	1.082024	1.03027885	0.3691462	-1.4377356	0.41254
S100A1	1.068776	1.0404814	1.09214217	2.27616	1.186602	0.41257
AP1G1	1.345943	1.3143695	1.37201714	1.1833755	0.24290787	0.41258
GNA13	1.258987	1.2285663	1.28410941	1.2430067	0.31383409	0.41277
CDK2	1.357894	1.3896102	1.33170242	0.8513701	-0.2321417	0.41317
DSE	1.23785	1.2075655	1.26285954	1.2663931	0.34072525	0.41331

NHLH2	1.087572	1.1161144	1.064002	0.5511978	-0.8593579	0.41339
MED13L	1.434923	1.4022986	1.46186469	1.1480643	0.19920344	0.41343
AGO1	1.711457	1.7475829	1.68162418	0.9117706	-0.1332571	0.41356
TAF10	1.893776	1.8566421	1.92444156	1.0791457	0.10988962	0.41388
GSDMD	1.179818	1.209553	1.15526148	0.7409174	-0.4326154	0.41392
COQ3	1.116856	1.1456552	1.09307384	0.6390011	-0.6461096	0.41393
SEMA3A	1.674146	1.6388049	1.70333208	1.1010123	0.13883064	0.41402
DESI1	1.384388	1.3526474	1.41059984	1.1643353	0.21950657	0.41404
DIAPH2	1.578502	1.6124292	1.55048431	0.8988538	-0.1538417	0.41424
SRP14-AS1	1.190037	1.2198896	1.16538504	0.7521274	-0.410951	0.41434
ETV2	1.133601	1.1626236	1.10963427	0.6741599	-0.5688374	0.41451
DNAJB14	1.692298	1.6570915	1.72137177	1.0978255	0.13464878	0.41479
HIP1	1.515452	1.4819879	1.54308765	1.1267663	0.17218826	0.41484
TRNP1	1.075545	1.0472656	1.09889853	2.0924018	1.0651599	0.41494
ARHGEF17	1.212106	1.1822816	1.23673631	1.2987398	0.37711237	0.41515
SAMD1	1.492968	1.4600991	1.52011159	1.130434	0.17687671	0.4155
DNTTIP2	1.366559	1.397898	1.34067851	0.8561957	-0.2239875	0.41558
CHEK2	1.148884	1.1777804	1.12502152	0.7032357	-0.5079198	0.41576
EML3	1.253368	1.2839311	1.22812823	0.8034634	-0.3156958	0.41578
RP11-418J17.1	1.073595	1.1019051	1.05021612	0.4927732	-1.0210042	0.41641
TRIB2	1.597746	1.5635788	1.62596233	1.1106919	0.15145862	0.41658
WDR6	1.881497	1.8441086	1.91237333	1.080872	0.11219565	0.4167
STS	1.084727	1.0562549	1.10824044	1.9241073	0.94418925	0.41684
CCDC94	1.147237	1.1761654	1.12334817	0.700184	-0.5141941	0.4173
FAM69C	1.147557	1.1765823	1.12358785	0.6998881	-0.5148039	0.4173
SH2B1	1.431115	1.4637477	1.4041662	0.8715218	-0.1983913	0.41744
KCNB2	1.146062	1.1751197	1.12206624	0.6970447	-0.5206768	0.41761
FAM134B	1.22125	1.1915532	1.2457747	1.2830623	0.35959119	0.41763
PLA2G12A	2.154796	2.1083096	2.19318607	1.0765819	0.10645812	0.41764
SRPRB	1.303401	1.3343963	1.2778041	0.8307632	-0.2674909	0.41769
OSBPL11	1.310126	1.3411864	1.2844751	0.833782	-0.2622579	0.41787
KLHL18	1.124873	1.1538584	1.10093699	0.6560383	-0.6081481	0.4179
ATRX	3.587767	3.6526853	3.53415611	0.9553173	-0.0659481	0.41797
OSGIN1	1.05788	1.0299222	1.0809677	2.7059451	1.43613257	0.41811
SUGP2	2.005418	1.9675269	2.03670834	1.0715034	0.09963643	0.41819
RING1	1.557659	1.5917657	1.52949267	0.8947674	-0.1604153	0.41833
SCAF1	1.208715	1.1789699	1.23327862	1.3034519	0.38233735	0.41871
AC093673.5	1.344967	1.3139438	1.3705867	1.1804238	0.23930494	0.41875
BHLHE22	1.060537	1.0885905	1.03736925	0.42182	-1.2453007	0.41898
TTPAL	1.183856	1.2133162	1.15952694	0.7478424	-0.4191938	0.41903
CCDC85B	3.097855	3.0277347	3.15576089	1.0631375	0.08832823	0.41925
C2orf42	1.092766	1.1209604	1.06948281	0.5744263	-0.7998064	0.41933
TTC30B	1.099047	1.1273915	1.07563914	0.5937536	-0.7520638	0.41936
CSRNP3	1.414573	1.4467943	1.38796437	0.8683288	-0.2036867	0.41945
RP11-332H14.2	1.373562	1.4049216	1.34766426	0.8585966	-0.2199477	0.41961

ARSJ	1.051275	1.023592	1.07413685	3.1424554	1.65189226	0.41962
LYPD2	1.05121	1.0235478	1.07405448	3.1448612	1.65299634	0.41976
CDKL5	1.092453	1.0640657	1.11589513	1.8090047	0.85519617	0.41976
DENND1A	1.263265	1.2328617	1.28837227	1.2383842	0.30845893	0.4199
CELSR1	1.171038	1.1419437	1.19506512	1.374243	0.45863713	0.42
RILPL2	1.294982	1.3255652	1.26972576	0.8284847	-0.2714531	0.42005
CAMSAP2	1.504429	1.4712751	1.53180731	1.1284434	0.17433408	0.42006
OLFML3	1.164329	1.1932528	1.14044304	0.7267323	-0.460504	0.42009
FAM222A	1.091657	1.119984	1.06826331	0.5689369	-0.8136595	0.42028
FAM89B	1.586449	1.5526391	1.6143691	1.1117004	0.15276802	0.4203
TMEM234	1.278141	1.3086608	1.25293674	0.819465	-0.2872458	0.42031
THBS3	1.288564	1.319287	1.26319185	0.8243112	-0.2787389	0.42041
PIDD1	1.403832	1.3721889	1.42996336	1.1552287	0.20817852	0.42042
PCK2	1.167393	1.1964958	1.14336022	0.7295843	-0.4548534	0.42043
EEA1	1.640056	1.6057636	1.6683754	1.1033602	0.14190383	0.42068
KLHL35	1.08984	1.1181331	1.06647517	0.5627141	-0.829526	0.42081
NCKAP5L	1.256259	1.2261226	1.28114592	1.2433342	0.31421412	0.42092
PHACTR1	1.287334	1.3177552	1.2622122	0.825202	-0.2771808	0.42101
WDR11	1.663741	1.6295048	1.69201426	1.0992994	0.13658443	0.42116
TYK2	1.290146	1.3207637	1.26486102	0.82572	-0.2762755	0.42125
ST20-AS1	1.167149	1.138331	1.19094765	1.3803676	0.46505249	0.42145
SEC63	2.271953	2.3164357	2.23521845	0.9383052	-0.0918708	0.42168
ACO1	1.317277	1.347844	1.29203468	0.8395564	-0.2523008	0.42169
TLCD2	1.123469	1.0950173	1.14696491	1.5467174	0.62920959	0.42191
CDKN2AIP	1.246078	1.2758467	1.22149449	0.8029621	-0.3165962	0.42193
SIX5	1.320616	1.3514794	1.29512817	0.8396742	-0.2520985	0.42193
FLJ20021	1.231139	1.2610418	1.20644459	0.7908486	-0.3385265	0.42199
FOXN4	1.268748	1.2987296	1.24398915	0.8167558	-0.2920234	0.4225
ZNF740	1.149438	1.178276	1.12562272	0.7046531	-0.505015	0.42252
MORC2-AS1	1.16442	1.1934953	1.14040989	0.7256501	-0.462654	0.42254
SPRY1	1.3993	1.4310098	1.3731129	0.8656715	-0.2081084	0.42256
SMARCA2	1.244772	1.2147873	1.26953413	1.2548883	0.32755901	0.42289
RP11-421L21.3	1.310548	1.3409318	1.28545692	0.8372846	-0.25621	0.42299
SLC43A2	1.102408	1.1304706	1.0792334	0.6072893	-0.7195442	0.42316
CASC9	1.307348	1.3383055	1.28178253	0.8329232	-0.2637446	0.42318
SLC47A1	1.053396	1.0257591	1.07621951	2.9589316	1.56507633	0.42348
KIFC2	1.141809	1.1132123	1.16542536	1.4611962	0.54714988	0.42367
FSD1	1.489731	1.5224645	1.46269846	0.8856075	-0.1752606	0.42367
TMBIM1	1.290717	1.2603338	1.31580812	1.2130891	0.27868547	0.42381
LINC00294	1.09636	1.0683512	1.11948968	1.7481725	0.80584754	0.42382
AC012123.1	1.117564	1.1461161	1.09398584	0.643227	-0.6366002	0.42396
DPY19L4	1.341168	1.3101423	1.36678992	1.1826505	0.24202378	0.42399
TMEM214	1.225044	1.1956332	1.24933198	1.2744869	0.34991659	0.42414
AR	1.194788	1.2242691	1.17044113	0.7599848	-0.3959574	0.42425
METTL2A	1.306163	1.3366127	1.28101656	0.8348364	-0.2604345	0.42443

CYP27A1	1.263806	1.2339016	1.28850128	1.2334301	0.30267593	0.42463
PATZ1	1.418869	1.4509627	1.39236578	0.8700626	-0.2008088	0.42464
PWWP2B	1.101963	1.0739384	1.12510574	1.692026	0.75875176	0.42479
ASB7	1.157304	1.1283596	1.18120599	1.4117054	0.49743906	0.42486
POT1	1.182533	1.2117134	1.15843477	0.7483454	-0.4182237	0.42498
GLTP	1.671314	1.7067207	1.64207417	0.9085261	-0.1384001	0.425
LRP11	1.346246	1.3771608	1.32071591	0.8503427	-0.2338838	0.42502
GDPD5	1.052636	1.0251789	1.07531133	2.991051	1.58065253	0.42504
CDK5R1	1.084732	1.112662	1.06166646	0.5473583	-0.8694426	0.4251
DHX57	1.213803	1.2431282	1.18958617	0.7797785	-0.3588637	0.42534
HSF1	1.882606	1.9213826	1.85058396	0.9231604	-0.1153467	0.42543
RP11-119B16.2	1.278827	1.2484902	1.30387917	1.2229019	0.2903087	0.42545
ADGRB1	1.046252	1.0189739	1.06877905	3.6249215	1.85794973	0.42563
ING5	1.435428	1.4672343	1.40916132	0.875709	-0.1914765	0.42563
TMEM51	1.152209	1.1235482	1.17587827	1.4235599	0.50950325	0.42564
AHR	1.191858	1.1628769	1.21579064	1.3248694	0.40585016	0.42569
KCNAB2	1.095514	1.0676987	1.11848443	1.7501731	0.80749762	0.42576
LSM12	1.701626	1.7382179	1.67140792	0.9094983	-0.1368572	0.42577
IQCD	1.100646	1.1285127	1.07763392	0.6040953	-0.7271521	0.42589
SAFB	1.607888	1.6414087	1.58020561	0.9045802	-0.1446797	0.42601
SSH2	1.340466	1.371214	1.31507452	0.848768	-0.2365579	0.42607
CTB-193M12.5	1.06003	1.0874898	1.03735269	0.4269379	-1.2279019	0.42613
FAM110A	1.119833	1.1480187	1.09655731	0.652332	-0.6163217	0.42613
PTHLH	1.065305	1.0378447	1.08798262	2.3248333	1.21712725	0.42618
CMTR1	1.216708	1.2463312	1.19224447	0.780431	-0.3576571	0.42622
ORAI3	1.788506	1.7531717	1.81768622	1.0856572	0.11856861	0.42632
SDC2	2.356022	2.3073889	2.39618463	1.0679183	0.09480134	0.42653
IPP	1.163511	1.1919931	1.13999008	0.7291413	-0.4557297	0.42686
N4BP3	1.143581	1.1152226	1.16700005	1.4493691	0.53542505	0.42707
GTF2H1	1.319122	1.3496483	1.29391257	0.8405949	-0.2505173	0.42733
ZSWIM8	1.156046	1.1276084	1.17953064	1.4068874	0.49250683	0.42741
SMCO4	1.466908	1.4350128	1.4932481	1.1338702	0.18125555	0.42745
NUFIP1	1.18141	1.2101983	1.15763646	0.7499415	-0.41515	0.4275
RP11-386I14.4	1.20325	1.2325102	1.17908685	0.7702323	-0.3766345	0.42777
ZYG11B	1.436045	1.4675506	1.41002642	0.876967	-0.1894056	0.42784
LANCL2	1.14628	1.1748581	1.12268057	0.7016009	-0.5112775	0.42789
SPACA9	1.25215	1.281795	1.22766892	0.807924	-0.3077085	0.42801
MZF1	1.291483	1.3219632	1.26631282	0.827153	-0.2737738	0.42803
CTDSPL2	1.497581	1.4649374	1.52453886	1.1281925	0.17401321	0.42811
ZFAND2A	1.237	1.2075094	1.2613547	1.2594834	0.33283216	0.42827
C6orf226	1.211889	1.2411028	1.18776443	0.7787734	-0.3607244	0.4285
LGR5	1.14149	1.169679	1.11821032	0.6966702	-0.5214523	0.42858
PIM2	1.310592	1.3406222	1.28579189	0.8390289	-0.2532076	0.42861
PKN3	1.117856	1.1459489	1.09465604	0.6485562	-0.6246964	0.42871
KDM5C	1.555516	1.5223393	1.58291443	1.1159688	0.15829675	0.42904

PQLC2	1.282181	1.3121175	1.25745825	0.8248761	-0.2777506	0.42906
IQSEC2	1.312582	1.3430331	1.28743434	0.8379202	-0.2551152	0.42927
POMC	1.078087	1.1053768	1.0555499	0.5271547	-0.9237017	0.4294
HOOK1	1.289814	1.2594742	1.31486859	1.2134869	0.27915854	0.42947
ORMDL3	1.261642	1.2912834	1.23716378	0.8142029	-0.2965398	0.42947
CLCC1	1.249984	1.2203707	1.27443894	1.2453511	0.31655253	0.42947
MLYCD	1.256636	1.2268766	1.28121157	1.2394913	0.30974815	0.42949
USP34	2.286849	2.2421054	2.32379823	1.0657697	0.09189566	0.42957
RP11-260M2.1	1.093336	1.1213066	1.07023654	0.579	-0.7883647	0.4296
CTSS	1.064139	1.0369205	1.08661594	2.3460098	1.23020903	0.42964
C19orf68	1.171648	1.2003214	1.14796929	0.7386594	-0.4370188	0.42964
SLC38A5	1.127801	1.1560079	1.10450702	0.669883	-0.578019	0.42979
GPR82	1.143629	1.1151704	1.16713122	1.4511652	0.5372118	0.4298
RC3H2	1.315139	1.2850377	1.33999704	1.1928143	0.25436946	0.42983
POP1	1.156129	1.1844881	1.13270912	0.7193371	-0.4752601	0.42984
ARL4A	3.512835	3.4213035	3.58842276	1.0690204	0.09628934	0.42984
TSPAN5	1.433767	1.4022375	1.45980478	1.1431176	0.1929738	0.42985
USP12	1.203795	1.1746683	1.22784863	1.3044644	0.38345753	0.42986
HEXIM1	1.397368	1.4288449	1.37137465	0.8659882	-0.2075807	0.42997
C19orf66	1.506723	1.539522	1.47963734	0.8890042	-0.1697379	0.42999
MINA	1.199763	1.2285027	1.17603012	0.7703634	-0.376389	0.43002
AAED1	1.271067	1.3007564	1.24654885	0.8197626	-0.286722	0.43009
ANKRD36	1.385966	1.3550814	1.41147025	1.1588054	0.21263835	0.43031
DHX9	2.15087	2.192589	2.11641691	0.9361288	-0.095221	0.43034
HECTD2	1.251225	1.2810597	1.22658638	0.8061859	-0.3108156	0.43053
ADAM23	1.072965	1.0455488	1.09560525	2.0989637	1.06967723	0.43055
FMR1	1.443722	1.4120764	1.4698561	1.1402159	0.18930698	0.43058
ZNF487	1.250214	1.2796176	1.22593259	0.8080057	-0.3075625	0.43058
DAPK3	1.455564	1.4236357	1.48193103	1.1376072	0.18600255	0.43064
FAM91A1	1.322776	1.292874	1.34746896	1.1864111	0.24660399	0.43077
GDPD2	1.089433	1.0618243	1.11223322	1.8153565	0.86025288	0.43081
BIRC6	1.671852	1.6379018	1.69988937	1.0971741	0.13379244	0.43082
PTX3	1.11303	1.0852271	1.13599026	1.5956227	0.67411954	0.43092
ACTR2	2.456409	2.5089098	2.41305363	0.9364732	-0.0946904	0.43094
IFI27L1	1.947712	1.9110239	1.9780093	1.0735277	0.10235936	0.43101
ZNF586	1.118125	1.1460722	1.09504538	0.6506741	-0.6199929	0.43109
RRP12	1.155098	1.1267154	1.17853664	1.4089579	0.49462853	0.4311
KIAA1586	1.344958	1.37541	1.31981108	0.8518982	-0.2312471	0.43113
RERG	1.122205	1.1499971	1.09925462	0.6617101	-0.5957287	0.43137
ZNF169	1.122306	1.1502419	1.09923592	0.6605078	-0.5983526	0.43137
FRMD6-AS1	1.146901	1.118596	1.17027556	1.4357613	0.52181595	0.4314
LRRC14	1.268971	1.2393349	1.29344491	1.2260847	0.29405863	0.43143
ATF6	1.460943	1.4290221	1.48730459	1.1358495	0.18377168	0.43179
ZNF281	1.443097	1.4116977	1.4690279	1.139253	0.18808817	0.43212
NUDT7	1.147578	1.175632	1.1244104	0.7083586	-0.4974482	0.43213

PARP9	1.17169	1.2002748	1.14808453	0.7394068	-0.4355597	0.43228
CCPG1	1.486515	1.5184758	1.46012062	0.8874487	-0.1722644	0.43231
MPRIP	1.352818	1.3223894	1.37794653	1.1723294	0.22937795	0.43252
NCAPH	1.104329	1.1317469	1.08168744	0.6200331	-0.6895829	0.4327
TMEM64	1.251654	1.2222495	1.27593668	1.2415629	0.31215734	0.43273
DTNB	1.235558	1.2649666	1.21127109	0.79735	-0.3267149	0.43281
MSANTD2	1.177964	1.2064002	1.15448051	0.7484513	-0.4180197	0.43285
SAFB2	1.436224	1.4677344	1.41020188	0.8769974	-0.1893555	0.43287
CTD-2517O10.6	1.147118	1.1753079	1.12383861	0.7064062	-0.5014302	0.43287
CCDC66	2.391932	2.4352398	2.35616682	0.9449061	-0.0817571	0.43301
ABHD10	1.201813	1.2305722	1.17806323	0.7722668	-0.3728288	0.43302
UHRF1	1.2799	1.3093197	1.25560522	0.8263463	-0.2751816	0.43324
MEIS1-AS2	1.109759	1.1375263	1.08682882	0.6313615	-0.6634618	0.43324
MPZL1	1.993253	1.9559927	2.02402227	1.0711612	0.09917556	0.43333
UBAP1	1.478541	1.5106197	1.45204931	0.8852955	-0.175769	0.43337
RCBTB2	1.216252	1.2449696	1.19253709	0.7859631	-0.3474666	0.43346
RP11-179B2.2	1.137112	1.1652081	1.11390933	0.68949	-0.5363984	0.43347
CDC25C	1.124145	1.1517321	1.10136246	0.6680359	-0.5820024	0.43368
GTDC1	1.220392	1.2492791	1.19653682	0.7884209	-0.3429622	0.43374
EPHB2	1.574778	1.6080719	1.54728257	0.9000294	-0.151956	0.43374
BTBD7	1.542024	1.5742304	1.51542779	0.8975976	-0.1558593	0.43383
TMEM8B	1.302519	1.3325668	1.27770522	0.835036	-0.2600896	0.43399
TMEM185A	1.314703	1.344682	1.28994647	0.8411999	-0.2494793	0.43419
FAM212A	1.058016	1.0309009	1.08040889	2.6021548	1.37970677	0.4342
ACTA1	1.05899	1.0312521	1.08189598	2.620499	1.38984154	0.43445
MID1	2.321982	2.2771985	2.35896519	1.0640204	0.08952578	0.4345
TMEM132D	1.075466	1.0483735	1.09783909	2.0225763	1.0161941	0.43453
RP11-148L24.1	1.075254	1.1026238	1.05265222	0.5130603	-0.9627996	0.43456
DTNBP1	1.391027	1.4217819	1.36562914	0.8668677	-0.2061162	0.4349
PPP4R2	2.338871	2.3840673	2.30154662	0.9403782	-0.0886871	0.435
TACC2	1.22553	1.1966648	1.24936796	1.267985	0.34253764	0.43502
PGM3	1.486357	1.5179146	1.46029653	0.8887498	-0.1701508	0.43505
ANKRD28	1.283635	1.2543768	1.30779713	1.2100047	0.27501266	0.43537
KIF11	1.230051	1.2587523	1.20634883	0.7974763	-0.3264865	0.43546
GATM	1.268649	1.2981735	1.24426804	0.8192144	-0.287687	0.43547
RUNX1	1.133342	1.1055074	1.15632752	1.4816738	0.56722791	0.43549
FASN	1.579022	1.5463269	1.60602222	1.1092667	0.14960631	0.43566
MESDC1	1.479183	1.447639	1.50523292	1.1286614	0.17461275	0.43574
BEX5	1.404619	1.4355393	1.37908433	0.8703792	-0.200284	0.43583
ZNF131	1.603805	1.6369497	1.57643313	0.90499	-0.1440262	0.43585
ZNF343	1.09076	1.0633121	1.11342738	1.7915589	0.84121549	0.43611
CAMK1D	1.33206	1.361844	1.30746476	0.8497164	-0.2349466	0.43615
DPH2	1.16221	1.190332	1.13898623	0.7302305	-0.4535762	0.43646
ZNF212	1.147202	1.1749807	1.12426105	0.7101415	-0.4938216	0.43647
PIGZ	1.060201	1.0333333	1.08238947	2.4716893	1.3054974	0.43655

CEP131	1.22741	1.256137	1.20368696	0.7952267	-0.3305619	0.43658
ACP5	1.198833	1.1701585	1.22251348	1.3076834	0.38701325	0.4368
ZFC3H1	1.412527	1.4434968	1.3869523	0.872503	-0.1967681	0.43684
TEX264	1.742523	1.7764868	1.7144747	0.9201375	-0.1200786	0.43684
RGS2	1.199387	1.170768	1.22302123	1.3059893	0.38514302	0.43717
SERP2	1.3885	1.4194763	1.36291847	0.8651704	-0.2089439	0.43721
WDR86	1.324186	1.2946551	1.34857245	1.1829845	0.24243115	0.43732
NABP1	1.146542	1.1187734	1.16947441	1.4268723	0.51285619	0.43757
SPINT2	4.84487	4.7584262	4.91625651	1.0419937	0.0593466	0.43758
B3GALNT1	1.458245	1.4894053	1.43251199	0.8837502	-0.1782895	0.43759
PENK	1.041757	1.0148895	1.06394395	4.2945701	2.10251374	0.43773
GNB1L	1.143251	1.1711732	1.12019258	0.7021692	-0.5101093	0.43773
COPRS	2.143661	2.1842809	2.11011567	0.9373753	-0.0933013	0.438
PRELID3B	1.27929	1.3086358	1.25505577	0.8263972	-0.2750927	0.43805
TMEM40	1.037805	1.0113227	1.05967527	5.2703945	2.39791094	0.43812
ZNF414	1.387184	1.4175487	1.36210894	0.8672257	-0.2055206	0.4382
ARL8A	1.31468	1.2850142	1.33917871	1.1900414	0.25101179	0.43824
NCBP2-AS2	2.318709	2.363711	2.28154471	0.939748	-0.0896541	0.43842
GPR89A	1.201948	1.2301599	1.17865004	0.7761997	-0.3655003	0.43848
RCAN3	1.174504	1.1464428	1.19767804	1.3498651	0.43281527	0.43848
ZMIZ2	1.207322	1.1787535	1.23091473	1.2918054	0.36938877	0.43856
SLC9A3R1	1.319057	1.2894812	1.34348033	1.1865377	0.24675791	0.43858
RBFOX1	1.10254	1.0752313	1.12509244	1.6627703	0.73358891	0.43866
HGH1	1.153761	1.181739	1.13065591	0.7189205	-0.4760959	0.43874
TRMT2A	1.270815	1.3000925	1.24663668	0.8218689	-0.2830198	0.43881
ANKZF1	1.590114	1.6226295	1.56326135	0.9046493	-0.1445696	0.43882
APLP2	7.279165	7.439338	7.14689185	0.9545844	-0.0670553	0.43909
ZNF681	1.190282	1.2184949	1.16698392	0.7642462	-0.3878906	0.4391
PI4K2A	1.123529	1.0959247	1.14632546	1.5254205	0.60920695	0.4391
PIGA	1.147996	1.1759918	1.12487717	0.7095624	-0.4949985	0.43919
PCYT1B	1.156848	1.184642	1.13389513	0.7251608	-0.4636271	0.43926
SDHAF1	1.449266	1.4181681	1.47494672	1.1357795	0.18368275	0.43934
C2orf40	1.25554	1.2266667	1.27938351	1.2325742	0.30167453	0.43937
GLI4	1.376757	1.4070614	1.3517312	0.864074	-0.2107733	0.43961
CPEB2	1.079951	1.0531155	1.10211298	1.9224716	0.94296227	0.43961
FAM184B	1.244205	1.2733344	1.2201494	0.8054216	-0.312184	0.43973
CHN1	1.60868	1.6417805	1.58134567	0.9058326	-0.1426836	0.43981
TUSC2	2.09379	2.137488	2.0577041	0.9298596	-0.1049152	0.4399
PLXNB1	1.280488	1.2514391	1.3044776	1.2109398	0.27612719	0.43993
CTBP2	1.69733	1.7310068	1.66951935	0.9158867	-0.126759	0.44001
CHRNB4	1.069429	1.0962315	1.04729498	0.4914708	-1.0248225	0.44019
RAP1GAP	1.060316	1.0336954	1.08230043	2.4424794	1.28834637	0.44022
PPP5C	1.338595	1.3684466	1.31394257	0.8520707	-0.2309549	0.44024
PROCA1	1.213907	1.2423975	1.19037908	0.7854003	-0.3485	0.4405
PGAP2	1.369044	1.3989903	1.34431426	0.862964	-0.2126278	0.44062

PDDC1	1.583416	1.6156438	1.55680257	0.9044233	-0.1449299	0.44079
CMTR2	1.506272	1.5380664	1.48001544	0.8921118	-0.1647035	0.44106
AGA	1.341578	1.3119372	1.36605508	1.1734897	0.23080522	0.44106
PRRT1	1.243991	1.2730753	1.21997249	0.8055379	-0.3119757	0.44116
SETD4	1.341766	1.3715675	1.31715492	0.8535595	-0.2284364	0.44133
RP4-639F20.1	1.271957	1.2431159	1.29577451	1.2165989	0.28285357	0.44142
ARHGDIA	2.213847	2.2524417	2.18197454	0.9437362	-0.0835445	0.44173
CYTL1	1.056166	1.0295873	1.07811469	2.6401428	1.40061596	0.442
CPNE5	1.092445	1.0656151	1.11460155	1.7465741	0.80452789	0.44237
NCAPG	1.255219	1.2836008	1.23178118	0.8172797	-0.2910982	0.44273
KMT2E	3.528086	3.6060076	3.46373638	0.9454065	-0.0809934	0.44288
ATP8B2	1.096434	1.0694415	1.118725	1.7097132	0.7737543	0.443
AC010729.2	1.06322	1.0898511	1.041227	0.458837	-1.1239462	0.44348
RP11-400F19.6	1.229957	1.2586545	1.2062581	0.797427	-0.3265757	0.44367
CEP290	2.161605	2.2029179	2.1274879	0.9372941	-0.0934263	0.44385
NUDT10	1.153264	1.1807032	1.13060418	0.7227551	-0.4684211	0.44391
ARIH2OS	1.104167	1.1309817	1.08202261	0.6262144	-0.6752714	0.44398
INPP5E	1.161899	1.1343797	1.18462514	1.3739065	0.45828387	0.444
PPP1R18	1.499393	1.4683501	1.5250293	1.1210188	0.16481048	0.44405
NOL11	1.251963	1.2804088	1.22847231	0.814783	-0.2955123	0.44405
MOB1B	1.260891	1.2320365	1.28471866	1.2270425	0.29518518	0.44412
GTF3C4	1.292654	1.321981	1.26843612	0.8337017	-0.2623969	0.44415
CCDC8	1.623613	1.5909999	1.65054482	1.1007528	0.13849054	0.44416
LRRC27	1.374289	1.4042194	1.34957135	0.864806	-0.2095515	0.44427
FASTK	1.723895	1.7577266	1.69595702	0.9184804	-0.1226791	0.44455
DCDC1	1.604928	1.5725278	1.6316837	1.103324	0.14185654	0.44466
PLEKHA3	1.735853	1.7025946	1.76331898	1.0864287	0.11959349	0.44473
THAP6	1.294542	1.2655146	1.31851313	1.1996067	0.26256149	0.44475
MTHFS	1.41747	1.3868313	1.44277185	1.1446121	0.19485879	0.44476
SPATA6L	1.076121	1.0494516	1.09814507	1.984669	0.98889844	0.44478
SPR	1.088675	1.0620449	1.11066585	1.7836413	0.83482547	0.44493
SLC16A5	1.096603	1.0697241	1.11880092	1.7038717	0.76881668	0.44512
SC5D	1.369144	1.3389583	1.39407173	1.1625966	0.21735056	0.44522
NT5DC3	1.080742	1.0541798	1.10267758	1.8951262	0.92229389	0.44523
MAPKAP1	1.685356	1.6526031	1.71240362	1.0916338	0.12648902	0.44532
PITX1	1.041351	1.015126	1.06300714	4.1654829	2.05848374	0.4454
CHRNA5	1.179371	1.2071135	1.15646118	0.7554368	-0.404617	0.44542
MARCH6	2.621815	2.6784353	2.57505784	0.9384084	-0.0917121	0.44542
NNT-AS1	1.788893	1.7548915	1.81697167	1.0822372	0.11401668	0.44547
MPZL2	1.055729	1.0292903	1.07756333	2.6480924	1.40495346	0.44549
BMP2K	1.228529	1.2000591	1.25204035	1.2598292	0.33322821	0.44557
ZSWIM6	1.24456	1.2161024	1.26806153	1.2404375	0.31084909	0.44563
IFNGR2	2.331703	2.373507	2.29717999	0.9444291	-0.0824856	0.44598
ENTPD3-AS1	1.158669	1.1861447	1.13597868	0.7304998	-0.4530441	0.44598
SZRD1	1.803633	1.8378499	1.77537693	0.9254366	-0.111794	0.4461

GALNT7	1.719983	1.7529614	1.69274826	0.9200316	-0.1202446	0.44611
CTSB	4.335317	4.2380129	4.41567226	1.0548668	0.0770608	0.4462
MAPK10	1.443274	1.473816	1.41805213	0.8823091	-0.180644	0.44621
MAP4K5	1.594148	1.6267226	1.56724684	0.9051004	-0.1438503	0.44624
SNAP29	1.575444	1.6074162	1.5490403	0.9038947	-0.1457734	0.44645
FAM229A	1.179525	1.1520023	1.20225446	1.3306012	0.41207827	0.44658
RABGAP1L	1.179191	1.1517273	1.20187032	1.3304816	0.41194851	0.44681
PWWP2A	1.360291	1.3304686	1.38491885	1.1647668	0.22004112	0.44688
SEMA5A	1.847723	1.8833773	1.8182795	0.9263081	-0.110436	0.44689
LRRCC1	1.612603	1.6445763	1.58619863	0.9094325	-0.1369615	0.44694
C9orf72	1.42843	1.3980645	1.45350667	1.1392795	0.18812169	0.44701
SURF1	1.988021	2.0239108	1.95838334	0.9360028	-0.0954153	0.44708
MPP5	1.312636	1.283639	1.3365817	1.1866553	0.24690095	0.44728
HN1L	1.949643	1.9853275	1.9201749	0.9338772	-0.0986952	0.44736
RPP14	1.253307	1.224924	1.27674526	1.2303947	0.29912118	0.44761
EID2B	1.392496	1.4223428	1.3678474	0.8709687	-0.1993072	0.44773
PIM1	1.220171	1.1918733	1.24353986	1.2692744	0.34400395	0.44777
POLB	1.283912	1.3128249	1.26003586	0.8312506	-0.2666446	0.44784
DDX6	2.021331	2.0593698	1.98991849	0.934441	-0.0978246	0.44794
MCRS1	1.652815	1.6851829	1.626085	0.9137488	-0.1301305	0.44807
COA7	1.181979	1.2094706	1.15927659	0.7603767	-0.3952139	0.44815
RP11-436D23.1	1.073411	1.0998878	1.05154675	0.5160465	-0.9544269	0.44824
AP001469.9	1.071913	1.0984651	1.04998668	0.5076587	-0.9780693	0.44839
PIK3CA	1.253573	1.2252278	1.27698041	1.2297787	0.29839874	0.4484
RABGGTA	1.310193	1.3393873	1.28608372	0.8429418	-0.246495	0.4488
MECP2	1.766375	1.8007346	1.73800097	0.9216549	-0.1177014	0.44884
THBS1	1.102326	1.0757873	1.12424187	1.639349	0.71312303	0.44897
POT1-AS1	1.144369	1.1716548	1.12183563	0.7097713	-0.4945738	0.44899
HSD3B7	1.15846	1.1860221	1.1356987	0.7294762	-0.4550672	0.44908
RAB8B	1.353607	1.3242157	1.37787795	1.165514	0.22096636	0.44917
ARAP1	1.249206	1.2777861	1.22560471	0.8121527	-0.3001772	0.44918
RGS9	1.279295	1.2505359	1.30304509	1.2095873	0.27451494	0.44924
KPTN	1.149722	1.1768722	1.12730016	0.7197297	-0.474473	0.44946
EFCAB11	1.223014	1.2508418	1.20003357	0.7974491	-0.3265356	0.44957
PLEKHO2	1.090039	1.0635427	1.11192022	1.761339	0.81667257	0.44959
TTLL1	1.281875	1.3104681	1.25826254	0.8318488	-0.2656068	0.4496
OGFOD1	1.240918	1.2689077	1.21780307	0.8099547	-0.3040869	0.44965
XPO7	1.303626	1.2749479	1.32730869	1.190439	0.25149369	0.44966
UBAP2	1.415867	1.3859179	1.44059936	1.1416921	0.19117362	0.44985
AMMECR1	1.309402	1.3384562	1.28540856	0.8432659	-0.2459404	0.45022
MEIS3	1.739591	1.7065432	1.76688156	1.0853993	0.11822593	0.45027
RFK	1.594762	1.5635491	1.62053891	1.1011266	0.13898033	0.4504
ANKS3	1.400146	1.4300421	1.37545718	0.8730708	-0.1958295	0.45115
ZNHIT2	1.154656	1.181817	1.13222625	0.727249	-0.4594786	0.45115
LIMD1	1.132161	1.1052818	1.15435809	1.4661418	0.55202463	0.45117

PRKD2	1.142963	1.1159384	1.1652804	1.4255879	0.51155697	0.45138
NBPF12	1.148826	1.1217455	1.17118927	1.4061237	0.49172349	0.45138
ZNF512B	1.165643	1.1385205	1.18804118	1.3574975	0.44094954	0.45183
PGM2L1	1.445397	1.4157014	1.46992055	1.1304282	0.17686933	0.45234
TCOF1	1.397395	1.3678783	1.42177107	1.146496	0.19723138	0.45243
AARSD1	1.220171	1.2480339	1.19716176	0.7948986	-0.3311573	0.45247
MT1F	1.210202	1.2403666	1.18529235	0.770874	-0.375433	0.4525
INIP	1.427851	1.4578311	1.40309229	0.8804389	-0.1837053	0.45263
ADGRV1	1.357463	1.3869701	1.33309595	0.8607796	-0.2162842	0.45264
PAPD5	1.207726	1.1798795	1.23072141	1.2826442	0.35912106	0.45273
RUFY3	2.127355	2.1653794	2.09595371	0.9404265	-0.0886129	0.45302
GRINA	2.510134	2.4634868	2.54865596	1.0581961	0.08160696	0.45307
RABGGTB	2.089247	2.0483862	2.12299133	1.0711618	0.09917647	0.4531
AC007040.11	1.136936	1.1636793	1.11485092	0.7016828	-0.5111092	0.4531
RCAN1	1.106106	1.0795995	1.12799465	1.6079836	0.68525273	0.45317
MYL4	1.09161	1.0652656	1.11336492	1.7369774	0.79657896	0.45318
FLOT2	1.465047	1.4345812	1.49020626	1.127997	0.17376322	0.45322
CDK7	1.293201	1.3215937	1.26975402	0.8388037	-0.2535949	0.45323
MLH1	1.347597	1.318238	1.3718421	1.1684403	0.22458402	0.45331
WDR75	1.268719	1.296919	1.24543051	0.8265907	-0.2747549	0.45338
TMCC1-AS1	1.256772	1.2848724	1.23356644	0.8198984	-0.2864829	0.45342
ZNF776	1.218886	1.1911342	1.24180456	1.2651034	0.33925527	0.45355
NMNAT3	1.154462	1.1814237	1.13219731	0.7286663	-0.4566698	0.45372
ITGB5	1.119135	1.0926756	1.14098572	1.5212821	0.60528772	0.4538
CD68	1.117154	1.0904583	1.1391997	1.5388274	0.62183139	0.45381
HELQ	1.187817	1.2152543	1.16515959	0.7672768	-0.382181	0.4539
LIN7B	1.221816	1.1942592	1.24457309	1.2590038	0.33228261	0.45393
CASC4	1.909102	1.8751445	1.93714447	1.0708454	0.09875017	0.45401
IGSF10	1.324074	1.295166	1.34794687	1.1788177	0.23734065	0.4543
YDJC	1.720251	1.752824	1.69335247	0.9210021	-0.1187237	0.45454
TRMU	1.522161	1.5533898	1.49637192	0.8969661	-0.1568746	0.4547
DHX16	1.185821	1.2129732	1.16339807	0.7672235	-0.3822812	0.45483
C4orf46	1.346038	1.3746797	1.32238472	0.8604275	-0.2168745	0.4549
TDP1	1.257552	1.28556	1.23442214	0.8209209	-0.2846849	0.45506
ARHGAP33	1.164292	1.191141	1.14212043	0.7435372	-0.4275231	0.45513
GLRX2	1.214154	1.2417615	1.19135565	0.791506	-0.3373278	0.45517
SLC35F5	1.307803	1.279261	1.33137268	1.1866057	0.24684063	0.45517
ARHGEF40	1.465006	1.434626	1.49009349	1.1276212	0.17328255	0.45531
RP11-299J3.8	1.099327	1.1257349	1.07751864	0.6165242	-0.6977705	0.45539
CCDC84	1.216171	1.1886698	1.23888281	1.2661425	0.34043982	0.45547
UNC5C	1.19374	1.2211401	1.17111277	0.7737755	-0.3700131	0.45551
MYO19	1.242673	1.2147747	1.26571248	1.2371682	0.30704168	0.45557
TSPAN11	1.085101	1.0589685	1.1066826	1.809147	0.85530965	0.45559
LL21NC02-21A1.1	1.063739	1.0378261	1.08513843	2.2507843	1.17042779	0.45574
TGOLN2	1.447974	1.4181504	1.47260236	1.1302211	0.17660497	0.45593

MTG2	1.210116	1.2377114	1.18732649	0.7880416	-0.3436563	0.45602
MPND	1.274864	1.3031323	1.25151936	0.8297345	-0.2692783	0.45604
TBC1D8B	1.151375	1.124526	1.17354772	1.3936667	0.47888558	0.45613
RBL2	1.447935	1.4176232	1.47296665	1.13252	0.1795365	0.45628
BOLA3-AS1	1.172502	1.1996428	1.15008868	0.7517861	-0.4116059	0.45647
FAM126A	1.381577	1.4107826	1.35745855	0.8701892	-0.200599	0.4565
DCAF16	1.411717	1.4410565	1.38748787	0.8785447	-0.1868123	0.45656
ACACB	1.126902	1.1003841	1.14880137	1.4823201	0.567857	0.45663
TMEM70	1.419478	1.448688	1.39535616	0.8811383	-0.1825596	0.457
TIAM1	1.354609	1.383632	1.3306412	0.8618707	-0.2144567	0.45719
GRPEL2	1.118903	1.0924095	1.14078193	1.5234572	0.60734894	0.45721
SPATA24	1.119115	1.1453701	1.09743308	0.6702414	-0.5772474	0.45725
PHF20L1	1.701628	1.7341939	1.67473531	0.9190151	-0.1218395	0.4573
MYLK	1.118403	1.0921415	1.1400893	1.5203706	0.60442307	0.45759
SYNE4	1.099101	1.0730745	1.12059457	1.650297	0.72272567	0.45773
RP11-544A12.8	1.182495	1.2095678	1.16013835	0.7641362	-0.3880983	0.45773
SPEN	1.495401	1.5261092	1.47004111	0.8934287	-0.1625755	0.45791
ZIC5	1.237804	1.2655305	1.21490709	0.8093501	-0.3051643	0.45826
CREBL2	1.305506	1.2771375	1.32893247	1.1868926	0.24718934	0.45826
FAM3A	1.586388	1.5548052	1.61246888	1.1039349	0.14265514	0.45829
LATS2	1.179544	1.1526787	1.20173024	1.3212728	0.40192838	0.45836
GTF2H2	1.844063	1.8777923	1.81620935	0.9298434	-0.1049403	0.45837
HOTAIRM1	1.06045	1.0347107	1.08170542	2.3539006	1.23505339	0.45839
SEPSECS	1.121474	1.1477578	1.09976761	0.6752105	-0.5665908	0.45866
ZFYVE19	1.272674	1.3007175	1.24951493	0.829732	-0.2692826	0.4587
SLC26A11	1.293213	1.2649331	1.31656647	1.1948921	0.25688039	0.45875
PREX2	1.140686	1.1673577	1.11865955	0.7090178	-0.4961063	0.4588
SCN3A	1.210311	1.2374319	1.18791465	0.7914467	-0.3374359	0.45894
PIGK	1.176421	1.2033559	1.15417861	0.7581713	-0.3994042	0.45897
CYP27C1	1.127786	1.1542552	1.10592658	0.686697	-0.5422545	0.45905
IPO13	1.119344	1.0931439	1.14097964	1.5135675	0.59795304	0.45924
TTC33	1.201486	1.2285919	1.17910144	0.7834988	-0.3519971	0.45939
EPS8L2	1.575929	1.6068009	1.55043389	0.907108	-0.1406538	0.45961
SPAG5	1.15766	1.1839907	1.1359162	0.7387124	-0.4369154	0.45974
CBWD2	1.413596	1.4429978	1.38931559	0.8788206	-0.1863593	0.45986
MAVS	1.482172	1.4519206	1.50715367	1.1222185	0.16635354	0.45988
CTH	1.080398	1.1062961	1.05901101	0.5551569	-0.8490326	0.46001
SNAP23	1.513572	1.5435444	1.48881983	0.899319	-0.1530951	0.46003
UBXN7	1.739311	1.7063851	1.76650101	1.0851036	0.11783275	0.46007
CPSF6	1.787063	1.8201499	1.75973944	0.9263422	-0.1103829	0.46009
MTMR4	1.704773	1.7371602	1.67802633	0.9197815	-0.1206369	0.46025
THAP9-AS1	1.814664	1.8479507	1.78717501	0.9283263	-0.107296	0.46038
ORC5	1.133561	1.1600036	1.11172432	0.6982614	-0.5181609	0.46046
PPP1R3B	1.211176	1.1841214	1.23351847	1.2682855	0.34287957	0.46074
KLHL21	1.112469	1.0862762	1.13409884	1.5542969	0.63626213	0.4609

CHCHD4	1.241201	1.26888	1.21834256	0.8120446	-0.3003691	0.4611
THAP1	1.281864	1.3096121	1.25894888	0.8363656	-0.2577944	0.46114
TSPAN2	1.223994	1.1968625	1.24639891	1.2516295	0.32380752	0.46116
BACH2	1.09025	1.0644701	1.11153919	1.7300916	0.79084839	0.46117
SRRM2	3.834879	3.7693238	3.88901598	1.0432207	0.06104442	0.46124
TRAPPC10	1.10177	1.0757926	1.1232227	1.6257887	0.70113974	0.46125
GALK2	1.335726	1.3643827	1.31206081	0.8564096	-0.2236272	0.4615
LRRC1	1.217382	1.190213	1.23981838	1.2607888	0.33432661	0.46167
LMBR1L	1.293679	1.3217995	1.27045712	0.8404523	-0.2507621	0.46169
UBXN6	1.502505	1.4725544	1.52723931	1.1157218	0.15797739	0.46191
MSRB3	1.299982	1.2721143	1.32299512	1.1869831	0.24729941	0.46199
LINC00461	1.048434	1.0739951	1.02732602	0.3692948	-1.437155	0.46205
RBBP5	1.251444	1.2795871	1.22820231	0.8162119	-0.2929844	0.46213
GABPA	1.36245	1.3909286	1.33893151	0.8669909	-0.2059112	0.46216
EP300	1.368893	1.3400833	1.39268519	1.1546736	0.20748514	0.46223
PPFIA2	1.085469	1.1113437	1.0641005	0.5756996	-0.796612	0.4623
HSF2BP	1.076228	1.101844	1.05507377	0.5407657	-0.8869244	0.4627
PDGFB	1.07415	1.0486711	1.09519064	1.9557954	0.96775544	0.46296
PPP1R9A	1.530613	1.561098	1.50543763	0.900801	-0.1507196	0.4631
ZSCAN26	1.240514	1.2680316	1.21778892	0.8125495	-0.2994725	0.46336
ENTPD6	1.282556	1.3104544	1.25951772	0.8359286	-0.2585483	0.46343
CLK4	1.330962	1.3591876	1.30765296	0.8565245	-0.2234336	0.46357
RNF14	1.312482	1.3407529	1.28913583	0.8485206	-0.2369784	0.46357
DLL1	1.123183	1.1491548	1.10173433	0.6820723	-0.5520034	0.46391
FAM222B	1.367544	1.3392956	1.39087215	1.1520107	0.20415412	0.46405
SALL2	1.329999	1.3582732	1.30664981	0.8559105	-0.2244682	0.46406
MAPK12	1.261677	1.2342367	1.28433855	1.2138942	0.27964264	0.46408
ZNF273	1.257915	1.2852131	1.23537157	0.825248	-0.2771003	0.4641
TMEM168	1.219711	1.2468177	1.19732589	0.7994803	-0.3228655	0.46416
C2orf68	1.542399	1.5729485	1.51717079	0.9026479	-0.1477647	0.46426
GNA11	1.215832	1.1890118	1.23798033	1.2590765	0.33236589	0.46429
LATS1	1.258441	1.286144	1.23556318	0.8232329	-0.2806275	0.46444
FBXO4	1.122308	1.1482406	1.10089161	0.6805937	-0.5551343	0.46465
CCP110	1.504735	1.4746752	1.52955904	1.115624	0.15785094	0.46469
RP5-882C2.2	1.135548	1.1092537	1.15726163	1.4394173	0.52548488	0.46472
ZNF219	1.543496	1.5740085	1.5182976	0.9029441	-0.1472914	0.46473
PRTFDC1	1.252607	1.2800813	1.22991812	0.8208977	-0.2847257	0.46475
IGF2BP1	2.02846	1.993804	2.05707886	1.0636694	0.08904977	0.46477
MBTD1	1.299838	1.3279024	1.2766619	0.8437326	-0.2451423	0.46478
WDR60	2.059219	2.0955381	2.02922559	0.9394704	-0.0900804	0.46491
MAP4K4	1.811299	1.844811	1.78362351	0.9275726	-0.1084679	0.46501
RASA4	1.428843	1.4582751	1.40453815	0.8827408	-0.1799383	0.46503
SPNS1	1.579565	1.610127	1.55432658	0.9085429	-0.1383734	0.46503
MCCC2	1.344966	1.3733623	1.32151638	0.8611378	-0.2156839	0.46512
ANKAR	1.097916	1.0720271	1.11929508	1.6562523	0.72792244	0.46512

DLGAP4	1.759591	1.7921931	1.73266806	0.9248605	-0.1126924	0.46515
MCAM	1.106192	1.0804957	1.12741187	1.5828411	0.66251639	0.4654
KDM4B	1.552857	1.5831545	1.5278367	0.9051404	-0.1437865	0.46546
B3GLCT	1.126961	1.1010243	1.14837948	1.46875	0.55458887	0.46552
FBXO42	1.288876	1.2609938	1.3119014	1.1950531	0.25707471	0.46571
TIAM2	1.172613	1.1461934	1.1944302	1.3299517	0.41137384	0.46574
ZBTB37	1.21095	1.1840989	1.23312472	1.2663012	0.34062062	0.46578
CSNK1D	1.967912	1.933579	1.99626536	1.0671463	0.09375799	0.46596
SUN1	1.774139	1.741942	1.80072798	1.0792326	0.11000584	0.46631
ZDHHC18	1.139154	1.1129641	1.16078225	1.4233046	0.50924444	0.46635
WWC1	1.148108	1.1220216	1.16965001	1.3903277	0.47542495	0.46637
GPER1	1.092069	1.0664605	1.11321734	1.7035279	0.76852553	0.46646
DDR2	1.092152	1.0665437	1.11329896	1.7026258	0.76776137	0.46646
ZNF789	1.145176	1.1712899	1.12361025	0.7216434	-0.4706421	0.46652
B3GALT6	1.109214	1.0833826	1.1305459	1.5656256	0.64673923	0.46656
CDK11A	1.225546	1.252472	1.2033095	0.8052754	-0.3124458	0.46658
AKT3	1.565227	1.5350802	1.59012328	1.1028689	0.14126135	0.46662
PDZD8	1.315596	1.2875773	1.33873429	1.1778897	0.23620442	0.46664
TUBB4A	1.271635	1.2992775	1.24880717	0.8313595	-0.2664556	0.46669
NRAP	1.070628	1.0960384	1.04964351	0.5169132	-0.952006	0.46671
ROGDI	1.641721	1.673018	1.61587489	0.9150942	-0.1280078	0.46674
KCNAB1	1.040738	1.0157004	1.06141527	3.9116957	1.96779416	0.46697
RP11-111M22.2	1.22581	1.1989031	1.24802968	1.2469875	0.31844699	0.46714
LRTOMT	1.204726	1.2315383	1.18258378	0.7885685	-0.3426921	0.4673
LDLR	1.167275	1.1410569	1.18892556	1.3393575	0.42154108	0.4673
ATG10	1.580737	1.6113823	1.5554303	0.9084827	-0.138469	0.46748
RP11-53O19.1	1.165353	1.191912	1.14341961	0.7473197	-0.4202026	0.46752
MUM1L1	1.149177	1.1756057	1.12735246	0.7252182	-0.4635131	0.46754
CNBD2	1.264127	1.2919199	1.24117455	0.8261669	-0.2754948	0.46755
MTMR2	1.391616	1.4205979	1.36768301	0.8741913	-0.1939791	0.46761
APBB3	1.19763	1.2244043	1.17551964	0.782158	-0.354468	0.4684
ZKSCAN5	1.137591	1.1635282	1.11617175	0.7104082	-0.4932799	0.46847
SLC12A4	1.128157	1.10241	1.14941978	1.4590344	0.54501392	0.46851
TCEAL2	1.529217	1.5592158	1.50444406	0.9020561	-0.1487109	0.46855
FECH	1.175535	1.2021305	1.15357253	0.7597692	-0.3963669	0.46865
ZBTB44	1.731764	1.6999866	1.75800587	1.0828862	0.11488169	0.46882
ATP1B2	1.433112	1.4043908	1.45683041	1.1296756	0.17590854	0.46887
OST4	14.1061	14.270417	13.9704093	0.9773928	-0.0329897	0.469
KIAA1524	1.212845	1.2391197	1.19114683	0.7993773	-0.3230515	0.46918
RPAP1	1.205274	1.2319567	1.18323928	0.7899718	-0.3401269	0.46926
ASCC2	1.246306	1.2732473	1.22405674	0.8199779	-0.286343	0.46929
CRELD1	1.861546	1.89484	1.83405138	0.9320676	-0.1014935	0.46934
TMEM57	1.235996	1.263189	1.21354008	0.8113565	-0.3015921	0.46938
RAPGEF6	1.13415	1.1600644	1.11274882	0.7043967	-0.5055399	0.46953
CHST2	1.321054	1.2932566	1.3440093	1.1730659	0.23028407	0.46963

ARFGEF3	1.256761	1.2297505	1.27906641	1.21465	0.28054064	0.46965
PPCDC	1.171124	1.1974692	1.14936838	0.7564136	-0.4027528	0.46968
ZNF718	1.132899	1.1069265	1.15434844	1.4435008	0.52957192	0.46981
CDC42SE2	1.457639	1.4287452	1.48149935	1.1230432	0.16741339	0.46983
PROM1	1.276552	1.3038421	1.25401604	0.8360134	-0.2584021	0.4699
ZNF69	1.085093	1.1105241	1.06409125	0.5798849	-0.7861616	0.46992
ASL	1.195539	1.2219938	1.17369149	0.7824159	-0.3539924	0.47002
RIT1	1.485306	1.5153441	1.46049982	0.8935774	-0.1623353	0.47003
ZNF385C	1.203204	1.2297675	1.181268	0.7889192	-0.3420505	0.47011
ATXN7L3	1.232806	1.206097	1.25486306	1.2366172	0.30639897	0.47015
STOML1	1.137784	1.1638124	1.11628911	0.7098919	-0.4943288	0.47019
RSPO1	1.042706	1.0178705	1.06321481	3.5373888	1.82268478	0.47034
GATAD2B	1.406922	1.378084	1.43073693	1.1392626	0.18810032	0.47041
UG0898H09	1.179319	1.153082	1.20098636	1.3129327	0.39279292	0.47045
SCGB3A1	1.112961	1.1385081	1.09186347	0.6632356	-0.5924066	0.47048
RP13-1032I1.7	1.146251	1.120181	1.16777982	1.3960594	0.48136028	0.47077
REV3L	1.878232	1.8455577	1.90521428	1.070553	0.09835617	0.4708
CCDC150	1.204523	1.1778397	1.22655791	1.2739447	0.34930263	0.47095
ZNF280D	1.131347	1.1572058	1.10999154	0.6996658	-0.5152622	0.47108
DDX23	1.347831	1.3197314	1.37103584	1.160461	0.21469806	0.4711
HIST1H1A	1.231618	1.2581274	1.20972625	0.8124912	-0.2995758	0.47127
AMOT	1.233429	1.2065154	1.25565523	1.2379476	0.30795024	0.47132
ZNF292	3.230459	3.2866541	3.18405213	0.9551301	-0.0662309	0.47149
JPX	2.349684	2.3973458	2.31032403	0.9377235	-0.0927655	0.47169
SOD2	1.465575	1.4947446	1.44148537	0.89235	-0.1643184	0.47172
CELF4	1.345769	1.3744433	1.32209014	0.8601841	-0.2172827	0.47175
RP13-436F16.1	1.039447	1.0147517	1.05984148	4.0565826	2.02026487	0.47184
RICTOR	1.647016	1.6165102	1.67220904	1.0903454	0.12478516	0.47198
C6orf141	1.078335	1.0532403	1.09905827	1.8605897	0.89575995	0.47203
CENPJ	1.238041	1.2648611	1.21589182	0.8151133	-0.2949274	0.47207
C17orf100	1.288177	1.3162539	1.26498975	0.8379019	-0.2551468	0.47217
NT5DC1	1.294491	1.3218142	1.27192793	0.8449842	-0.2430037	0.47221
KALRN	1.153382	1.1275345	1.17472705	1.3700373	0.45421516	0.47223
AHSA2	1.696581	1.6651828	1.7225097	1.0861821	0.11926598	0.47233
NPHP1	1.264255	1.2914425	1.24180267	0.8296755	-0.269381	0.47236
RAB11FIP2	1.424694	1.4534458	1.4009497	0.8842285	-0.1775089	0.4724
TESMIN	1.152676	1.1784576	1.13138453	0.7362225	-0.4417863	0.47252
NUP214	1.242212	1.2154013	1.26435216	1.2272544	0.29543438	0.47253
NKAIN1	1.094615	1.1198396	1.07378432	0.6156923	-0.6997185	0.47265
BRMS1L	1.218859	1.2454364	1.19691105	0.8022896	-0.3178049	0.47269
GPR85	1.302447	1.3299878	1.27970316	0.8476168	-0.238516	0.47272
ATP10D	1.193045	1.1666964	1.21480441	1.2885966	0.36580072	0.47274
PIK3C3	1.464292	1.4934641	1.44020096	0.8920628	-0.1647828	0.47286
ZRANB2	2.648332	2.6951334	2.60968271	0.9495906	-0.0746225	0.4731
MFSD9	1.127189	1.1529049	1.10595242	0.6929304	-0.5292177	0.4732

ZC3H12C	1.161992	1.1878866	1.14060714	0.7483618	-0.4181921	0.47323
SV2A	1.342465	1.3144469	1.36560209	1.1626833	0.21745813	0.47331
PHF3	2.48899	2.5348208	2.45114253	0.9454801	-0.080881	0.47336
PRPF18	1.262777	1.2898319	1.2404349	0.8295667	-0.26957	0.47369
FHOD1	1.159084	1.1331771	1.18047816	1.3551747	0.4384788	0.47374
TMEM170A	1.776091	1.8081589	1.74960858	0.9275509	-0.1085016	0.47383
SLC29A3	1.089879	1.1148755	1.06923728	0.6027157	-0.7304505	0.47396
NGF	1.044336	1.0197789	1.06461577	3.2669067	1.70792525	0.47409
PPP1R15B	1.26387	1.2368932	1.28614876	1.2079233	0.27252886	0.47416
SLC2A4	1.117717	1.0920337	1.13892646	1.5095179	0.59408787	0.47457
RP11-763B22.4	1.072427	1.0976844	1.05156901	0.5279147	-0.9216233	0.4746
CORO1C	1.583596	1.5533423	1.60858045	1.0998264	0.13727586	0.47474
C19orf54	1.202297	1.2285757	1.18059497	0.7900881	-0.3399145	0.47488
RALGDS	1.417173	1.3892462	1.44023633	1.1309972	0.17759532	0.47504
SUFU	1.131733	1.1571592	1.11073514	0.7046049	-0.5051136	0.47531
RITA1	1.583816	1.5539854	1.60845135	1.0983165	0.13529391	0.47538
FAM149B1	1.131719	1.1573031	1.11059205	0.7030508	-0.5082991	0.47586
C6orf136	1.255179	1.2819529	1.23306868	0.8266227	-0.2746991	0.47587
MTRR	1.132947	1.1586203	1.11174542	0.7044836	-0.5053621	0.47592
SESTD1	1.554101	1.5840705	1.52935178	0.9063149	-0.1419157	0.47609
DAXX	1.393484	1.4216689	1.37020761	0.8779581	-0.187776	0.47633
FBXL20	1.360443	1.3327175	1.38333977	1.152148	0.204326	0.47643
SLC27A1	1.226615	1.2531395	1.20471034	0.8086859	-0.3063486	0.47651
NOC4L	1.201057	1.2270963	1.17955356	0.7906495	-0.3388899	0.47692
TMEM167B	1.425484	1.4543009	1.40168685	0.8841868	-0.1775768	0.47696
NTRK2	1.580653	1.5505642	1.60550084	1.0997824	0.13721812	0.47704
MYL7	1.067003	1.0422967	1.08740516	2.0664777	1.0471738	0.47711
ALG6	1.171213	1.1969452	1.14996369	0.7614487	-0.3931813	0.47712
DEPDC4	1.090792	1.1159305	1.07003293	0.604094	-0.727155	0.47716
MCL1	1.946354	1.9792689	1.91917225	0.9386311	-0.0913698	0.47727
ESRRG	1.301894	1.3289125	1.27958261	0.8500213	-0.2344291	0.47751
VLDLR	1.280494	1.2533205	1.30293462	1.1958552	0.2580427	0.47758
GNPTG	1.197389	1.1712651	1.21896277	1.278502	0.35445438	0.47771
DRAXIN	1.070019	1.0948793	1.04948887	0.521598	-0.9389898	0.47775
EIF2AK2	1.765498	1.7341811	1.79135945	1.0778804	0.10819715	0.47775
BANP	1.240928	1.2673551	1.21910491	0.8195276	-0.2871355	0.4779
FBXO36	1.180101	1.2061849	1.1585612	0.7690242	-0.3788991	0.4783
SLC38A1	2.25785	2.2214884	2.28787889	1.0543521	0.07635679	0.47843
CAMK2G	1.245651	1.2725248	1.22345853	0.8199568	-0.2863802	0.47848
PTPN9	1.308673	1.2815799	1.33104647	1.175675	0.23348925	0.47859
MYOCD	1.13111	1.156455	1.11018022	0.7042293	-0.5058829	0.47884
GRIK1	1.044018	1.0195919	1.06419011	3.2763601	1.71209395	0.47886
JADE3	1.088451	1.0635955	1.1089779	1.7136104	0.77703916	0.47887
RSPH3	1.303344	1.3308304	1.28064466	0.8483036	-0.2373473	0.47921
API5	1.505689	1.5348026	1.48164678	0.9006067	-0.1510309	0.47941

RP11-521B24.3	1.196854	1.222946	1.17530724	0.7863216	-0.3468086	0.47942
OTUD7B	1.323692	1.3511426	1.30102225	0.857265	-0.2221868	0.47945
RP11-159D12.2	1.166898	1.141192	1.18812674	1.3324174	0.41404605	0.4795
KIF27	1.219358	1.2456103	1.19767909	0.8048487	-0.3132105	0.47981
HPS1	1.843779	1.8119979	1.87002492	1.0714621	0.09958079	0.47988
RNF8	1.595809	1.6257125	1.57111492	0.9127434	-0.1317188	0.47993
CPB1	1.041034	1.0166975	1.06113206	3.661141	1.87229333	0.47998
LINC00327	1.041049	1.0166975	1.06115812	3.6627016	1.87290818	0.47998
MAP7D1	1.57267	1.602319	1.548185	0.910124	-0.135865	0.48014
MAP7	1.312908	1.3402367	1.29033915	0.8533445	-0.2287998	0.48014
ZBTB43	1.287334	1.2602863	1.30967063	1.189731	0.25063539	0.48015
SYNGR1	1.727531	1.6967562	1.75294562	1.0806442	0.11189166	0.48022
CCDC136	1.280215	1.3075218	1.25766455	0.837874	-0.2551948	0.48025
RP11-178L8.7	1.199033	1.2252223	1.17740593	0.7876927	-0.3442951	0.4804
ZNF215	1.239828	1.2661267	1.21811023	0.8195729	-0.2870558	0.48049
ZNRF1	1.2067	1.1807972	1.2280908	1.2615837	0.33523589	0.48062
BRD7	1.741518	1.7102699	1.76732322	1.0803263	0.11146708	0.48065
NUP62	1.493136	1.5217348	1.46951931	0.8999194	-0.1521323	0.48065
ZNF561	1.493787	1.5229284	1.46972212	0.8982533	-0.1548058	0.48065
RANBP17	1.279413	1.2526485	1.30151476	1.1934158	0.25509676	0.48121
ZNF580	3.381559	3.4392107	3.33394837	0.9568457	-0.0636418	0.48125
CLIC4	1.730803	1.6998608	1.75635516	1.0807222	0.11199575	0.48129
RP11-1275H24.3	1.080269	1.1051864	1.05969128	0.5674812	-0.8173554	0.48139
ZNF853	1.080704	1.0560906	1.1010298	1.8011891	0.84894967	0.4814
CASP2	1.28486	1.311704	1.26269088	0.8427575	-0.2468105	0.48147
GAS6	1.238428	1.2651267	1.21637946	0.816136	-0.2931186	0.48181
PRDM1	1.063931	1.0885346	1.04361308	0.4926106	-1.0214805	0.48189
MICAL2	1.048032	1.0238822	1.06797485	2.8462605	1.50906771	0.48197
EBF4	1.107995	1.083063	1.12858414	1.5480317	0.63043506	0.48198
RP11-499F3.2	1.601439	1.631554	1.57656972	0.912938	-0.1314111	0.48218
FAM214A	1.197289	1.1716928	1.21842697	1.2721967	0.34732177	0.48232
ZBED5-AS1	1.217969	1.2441272	1.19636736	0.8043651	-0.3140776	0.48244
PKNOX1	1.257996	1.2845565	1.2360618	0.8295778	-0.2695507	0.48255
USP31	1.112232	1.0872349	1.13287488	1.523185	0.60709116	0.48299
UBE2Z	1.518173	1.4891736	1.54212141	1.1082393	0.14826941	0.4831
RGP1	1.531746	1.5027737	1.55567224	1.1052134	0.14432494	0.48313
SF3A1	1.408478	1.4363042	1.38549907	0.8835556	-0.1786072	0.48315
TRAF5	1.181063	1.1554053	1.20225127	1.3014437	0.38011289	0.48318
SIAH2	1.314381	1.2874728	1.33660265	1.1709025	0.22762092	0.48324
GSK3A	1.371749	1.4000018	1.34841761	0.8710401	-0.1991889	0.48328
EFCAB1	1.070668	1.0462783	1.09080892	1.9622353	0.97249803	0.48342
MEGF9	1.3298	1.3027619	1.35212811	1.1630529	0.21791669	0.48345
P4HA1	3.234907	3.180976	3.27944439	1.0451488	0.06370833	0.48347
MTA1	1.645122	1.614899	1.67008135	1.0897421	0.12398671	0.48355
PICALM	1.446555	1.418654	1.46959692	1.1216827	0.16566464	0.48357

TRIM5	1.411737	1.439965	1.38842635	0.8828575	-0.1797475	0.48404
NUP85	1.277375	1.3040125	1.2553766	0.84002	-0.2515044	0.48409
WDR92	1.194443	1.2201355	1.17322524	0.7869029	-0.3457425	0.48436
CTSO	1.175729	1.1501285	1.19687013	1.3113444	0.39104663	0.48439
RPUSD1	1.26719	1.2937254	1.24527744	0.835057	-0.2600535	0.48441
JKAMP	1.620135	1.6496681	1.59574656	0.9170014	-0.1250042	0.48447
ATAD3A	1.266871	1.2930839	1.24522481	0.8367052	-0.2572087	0.4845
ATG4A	1.173425	1.1479581	1.19445676	1.3142687	0.3942603	0.48461
MAN1B1	1.387624	1.415299	1.36477034	0.8783318	-0.187162	0.48461
LRRC20	1.26685	1.2932271	1.24506818	0.8357623	-0.2588355	0.48472
PAK1IP1	1.32697	1.3536761	1.30491588	0.8621331	-0.2140175	0.48486
TEFM	1.155569	1.1807148	1.13480338	0.7459453	-0.4228583	0.48486
ARHGAP10	1.165195	1.1399758	1.18602234	1.3289608	0.41029854	0.48499
ANKRD26	1.829816	1.8621508	1.80311356	0.9315233	-0.1023362	0.48504
CXCL10	1.041651	1.0177401	1.06139623	3.4608811	1.7911394	0.48506
ACYP2	1.675689	1.7064027	1.65032442	0.9206143	-0.1193312	0.48511
MTX3	1.291354	1.2646855	1.31337818	1.1839644	0.24362565	0.48519
PYCRL	1.173663	1.1990246	1.1527183	0.7673337	-0.382074	0.48521
NEK4	1.259465	1.2857117	1.23778953	0.8322708	-0.264875	0.48522
TMEM117	1.180915	1.2066619	1.15965211	0.7725281	-0.3723407	0.4853
PLXNA3	1.204838	1.1793047	1.2259238	1.2599995	0.33342313	0.48549
ICAM3	1.165152	1.1904937	1.14422432	0.7571079	-0.4014292	0.48561
VAT1	2.368092	2.3313011	2.39847465	1.0504571	0.07101721	0.48561
ADIPOR1	1.98974	1.9566199	2.01709067	1.063213	0.08843065	0.48573
GALNT16	1.136646	1.1614179	1.1161898	0.7198075	-0.474317	0.48584
ZBED9	1.089525	1.1140817	1.06924625	0.6069882	-0.7202595	0.48588
NGLY1	1.547456	1.5182946	1.5715388	1.1027297	0.14107919	0.48607
POLD1	1.225474	1.2511507	1.20426891	0.8133319	-0.2980839	0.48611
IFI44L	1.076862	1.0524946	1.09698533	1.8475307	0.88559837	0.48616
KBTBD2	1.211806	1.237841	1.19030609	0.8001401	-0.3216754	0.48621
KLHL29	1.09181	1.0673523	1.11200775	1.6630121	0.73379867	0.48627
TEX30	1.376487	1.4036634	1.3540447	0.8770789	-0.1892214	0.4863
IQCG	1.17177	1.1465257	1.1926166	1.3145588	0.39457871	0.4863
RASSF9	1.085215	1.1096783	1.06501367	0.5927667	-0.7544638	0.48638
RP11-696N14.1	1.076715	1.1011266	1.05655477	0.5592472	-0.8384419	0.48641
ADGRB3	1.195202	1.220768	1.17408843	0.7885583	-0.3427107	0.48652
SRRM2-AS1	1.091827	1.0672449	1.11212641	1.6674343	0.73762991	0.48659
MCM3AP	1.232327	1.2062601	1.25385376	1.2307459	0.2995329	0.48679
ZNF217	1.221798	1.1961497	1.2429784	1.2387399	0.30887332	0.4868
ATF5	1.199737	1.2254493	1.17850247	0.7917632	-0.3368592	0.48726
DHRS7B	1.358826	1.385816	1.33653677	0.8722727	-0.1971489	0.48733
UBR3	1.223666	1.1978767	1.24496394	1.2379623	0.3079674	0.48734
LINC01515	1.167302	1.1419586	1.1882301	1.3259503	0.40702673	0.48736
POLR2L	11.97873	12.148319	11.8386801	0.9722255	-0.0406372	0.48745
SOSTDC1	1.204443	1.2300057	1.18333343	0.7970821	-0.3271999	0.48754

FAM27C	1.111433	1.1360905	1.09106955	0.6691839	-0.5795254	0.48763
ZNF554	1.074245	1.098566	1.05415976	0.5494769	-0.8638693	0.488
FAM207A	1.516632	1.4879313	1.540334	1.1073978	0.14717357	0.48811
ZBTB18	1.175211	1.1500507	1.19598901	1.3061522	0.38532297	0.48817
PLPPR2	1.209388	1.2352298	1.18804688	0.7994178	-0.3229784	0.48822
THEM4	1.466695	1.4951651	1.44318471	0.8950241	-0.1600016	0.48824
FHL3	1.364092	1.3367186	1.38669728	1.1484288	0.19966136	0.48831
CRB2	2.786899	2.8434562	2.74019287	0.9439838	-0.0831659	0.48834
FANK1	1.258455	1.2325163	1.27987639	1.203685	0.2674579	0.48854
GTF2E2	1.728868	1.6982801	1.75412726	1.0799781	0.11100206	0.48863
ENAH	2.879299	2.9304711	2.83704036	0.9516021	-0.0715697	0.48867
CDPF1	1.294539	1.3208303	1.27282684	0.8503775	-0.2338247	0.48886
RP11-806H10.4	1.179356	1.2046116	1.15849955	0.774636	-0.3684095	0.48889
NID2	1.053468	1.0295839	1.07319217	2.4740522	1.30687593	0.48933
MAML1	1.192413	1.1669762	1.21341952	1.2781434	0.35404971	0.48934
YIPF1	1.315946	1.3423924	1.29410589	0.8589732	-0.2193149	0.48994
ZC3H10	1.176831	1.2018793	1.15614648	0.7734646	-0.3705928	0.48998
MLXIP	1.243026	1.2171975	1.26435631	1.217124	0.28347614	0.49001
LINC00649	1.241106	1.2671219	1.21962133	0.8221763	-0.2824803	0.49018
PQLC1	1.293345	1.2670786	1.31503633	1.179564	0.23825369	0.49026
SRBD1	1.143953	1.1686646	1.12354584	0.732494	-0.4491111	0.49036
SNRNP48	1.349578	1.3762883	1.32752087	0.8703987	-0.2002517	0.49039
NPEPL1	1.296571	1.270149	1.31839078	1.1785748	0.23704337	0.49049
PCSK4	1.20984	1.1843983	1.23085079	1.251914	0.32413544	0.49069
PRELID2	1.451714	1.4238182	1.47475067	1.1201752	0.16372444	0.49074
RP11-1391J7.1	1.187162	1.1619159	1.20801058	1.2846825	0.3614119	0.4908
RND3	2.046009	2.0015366	2.08273421	1.081073	0.11246396	0.49093
ZNF582-AS1	1.190043	1.2154369	1.16907311	0.7847919	-0.349618	0.49094
MYCBP	1.272323	1.2982702	1.25089485	0.8411665	-0.2495368	0.49105
RP4-773N10.4	1.048351	1.0245884	1.06797501	2.7645165	1.46702716	0.49106
EFR3B	1.420211	1.3930765	1.44261997	1.1260403	0.17125847	0.49111
PRKCH	1.050859	1.0270642	1.07050852	2.6052343	1.38141312	0.49122
BROX	1.742946	1.7733238	1.71785977	0.9282784	-0.1073706	0.49146
ZNF394	1.179226	1.1540604	1.20000873	1.2982492	0.37656729	0.49152
ZNF730	1.192755	1.1674026	1.21369179	1.2765139	0.35220923	0.49154
MMAA	1.084499	1.1088456	1.06439317	0.5916008	-0.7573041	0.49158
ZNF592	1.118825	1.0944415	1.13896207	1.471409	0.55719829	0.49178
KCMF1	1.426532	1.3991323	1.44915908	1.1253387	0.17035929	0.4918
PUM3	1.357797	1.3846594	1.33561438	0.8724975	-0.1967771	0.49206
RGCC	1.040617	1.0170009	1.06011969	3.5362608	1.82222469	0.49209
AC074289.1	1.15418	1.1789816	1.13369867	0.7469967	-0.4208261	0.4921
TEX10	1.172266	1.1971871	1.15168561	0.7692471	-0.378481	0.49212
TM7SF3	1.672726	1.7024544	1.64817551	0.9227297	-0.11602	0.49214
COQ2	1.222854	1.2482172	1.20190845	0.8134345	-0.2979019	0.49224
EHBP1L1	1.077827	1.0539609	1.09753552	1.8075235	0.85401444	0.49236

FANCI	1.305016	1.3312106	1.28338452	0.8556022	-0.2249879	0.49244
USP30	1.152542	1.1278803	1.17290851	1.352112	0.43521467	0.49244
COPA	1.819449	1.7887251	1.8448216	1.071123	0.09912422	0.49246
ZNF483	1.099381	1.123638	1.07934988	0.6417921	-0.639822	0.49248
RCL1	1.14022	1.1649652	1.11978508	0.7261234	-0.4617133	0.49261
GUSBP1	1.067309	1.0911927	1.04758473	0.521804	-0.9384202	0.49263
TOE1	1.144735	1.1691737	1.12455222	0.7362385	-0.4417548	0.49284
RP11-658F2.8	1.157528	1.1826435	1.13678653	0.7489263	-0.4171044	0.49295
CEP170B	1.102895	1.0787972	1.12279609	1.5583821	0.64004903	0.49299
GLS	1.376613	1.3496072	1.3989149	1.1410374	0.19034608	0.49307
ZNF695	1.069225	1.0930889	1.04951781	0.5319413	-0.9106612	0.49335
RRAD	1.070031	1.0462699	1.0896535	1.9376221	0.95428722	0.49339
GPR87	1.037753	1.0141505	1.05724422	4.0453782	2.01627459	0.4934
EBLN3P	1.995669	2.0282737	1.96874281	0.942106	-0.0860387	0.49348
MAPK8	1.369517	1.3962314	1.34745657	0.8769031	-0.1895107	0.49355
GTPBP2	1.141659	1.1170644	1.16196888	1.3835876	0.46841404	0.4936
KIAA0101	3.400487	3.4794772	3.33525617	0.9418341	-0.0864551	0.49368
SEH1L	1.244763	1.2703935	1.22359675	0.826931	-0.2741612	0.49376
SLC4A8	1.302036	1.3280954	1.28051597	0.8549829	-0.2260325	0.4938
GDAP1L1	1.076872	1.1008627	1.05705958	0.5657153	-0.8218519	0.4938
RP5-1136G13.2	1.149991	1.1748782	1.12943863	0.7401644	-0.4340823	0.49407
EFHC2	1.068652	1.0925832	1.04888871	0.5280517	-0.921249	0.4941
PAOX	1.130843	1.1552694	1.11067098	0.7127673	-0.4884969	0.49411
GBA2	1.211272	1.1857944	1.23231114	1.2503665	0.32235105	0.49421
MTMR1	1.103375	1.0791823	1.12335321	1.5578384	0.63954554	0.49433
SMC6	1.437235	1.4094657	1.46016728	1.1238238	0.16841589	0.4944
LMO1	1.313064	1.2849278	1.33629869	1.1802944	0.23914678	0.49448
FIG4	1.254346	1.2800251	1.2331396	0.8325668	-0.2643621	0.49467
SMYD4	1.33771	1.3112209	1.3595859	1.1554042	0.20839765	0.4948
STXBP5	1.163457	1.1388714	1.18376005	1.3232385	0.40407315	0.4955
CNTNAP1	1.10507	1.08102	1.12493099	1.5419771	0.62478137	0.49573
NFX1	1.228583	1.203248	1.24950509	1.2275892	0.29582792	0.49574
DOLPP1	1.101537	1.1255633	1.08169603	0.650636	-0.6200774	0.49581
TNFAIP8	1.194991	1.1698791	1.21572965	1.2699009	0.34471597	0.49591
TIMM17A	1.92655	1.9602173	1.89874694	0.9359829	-0.0954459	0.49617
RP11-631N16.2	1.087351	1.063376	1.10715	1.6907039	0.757624	0.49655
NOL3	1.821848	1.8540299	1.79527254	0.9311998	-0.1028373	0.49666
RABGAP1	1.489429	1.4615981	1.51241133	1.1100811	0.15066511	0.49676
NETO2	1.581304	1.6097468	1.55781496	0.9148304	-0.1284237	0.49681
DNAJC3-AS1	1.295304	1.2692292	1.31683747	1.1768316	0.2349079	0.49691
NDRG2	1.161976	1.1864946	1.14172864	0.7599613	-0.3960021	0.49693
HERPUD1	2.095535	2.049581	2.1334855	1.079941	0.11095246	0.49698
CYCS	3.618518	3.6813475	3.56663199	0.9572172	-0.0630817	0.4973
LIG1	1.327437	1.3537352	1.3057186	0.8642583	-0.2104655	0.49731
FKBP9	1.34563	1.3194093	1.36728285	1.1498814	0.20148505	0.4974

ZNF774	1.063531	1.0399271	1.08302308	2.0793658	1.05614359	0.49745
MYT1	1.037695	1.0610422	1.01841414	0.3016623	-1.7289938	0.49774
NUTM2A-AS1	1.30352	1.2773599	1.32512381	1.1722092	0.22923004	0.49782
TPD52	1.816911	1.8478666	1.7913476	0.9333397	-0.0995258	0.49806
ZNF404	1.100044	1.0761611	1.11976648	1.5725416	0.65309814	0.49809
FOXC2	1.047201	1.0238871	1.06645334	2.7819803	1.47611222	0.49825
FBXW5	1.79738	1.7672887	1.82222908	1.0716032	0.09977082	0.49856
THAP4	1.321328	1.3477667	1.29949525	0.8611959	-0.2155866	0.4986
SLC25A21-AS1	1.266077	1.2915638	1.24502954	0.8403975	-0.2508562	0.49873
OPA3	1.197903	1.2227625	1.17737354	0.796245	-0.3287158	0.49894
R3HDM2	1.716925	1.6872924	1.74139602	1.07872	0.10932041	0.49911
PABPN1	4.173098	4.2391416	4.11855743	0.9627728	-0.0547327	0.49911
LPCAT3	1.200331	1.2254299	1.17960382	0.7967168	-0.3278611	0.49917
SMARCD1	1.813536	1.8439099	1.7884529	0.9342857	-0.0980644	0.49917
ADD3	1.68905	1.6594198	1.71351878	1.0820402	0.11375412	0.49926
DDX27	1.354651	1.38121	1.33271877	0.8727965	-0.1962827	0.49933
ALMS1	1.298551	1.3246743	1.27697822	0.8530956	-0.2292206	0.4994
RP11-727F15.9	1.088109	1.1119335	1.06843361	0.6113776	-0.7098644	0.49946
HIF1A	2.519207	2.5607805	2.48487482	0.9513668	-0.0719264	0.49979
SNAPC4	1.102793	1.1267648	1.08299628	0.6547268	-0.6110351	0.49983
AZIN1	1.991701	1.9594543	2.01833164	1.0613655	0.08592155	0.49988
FTO	1.290568	1.3165044	1.26914849	0.8503784	-0.2338231	0.50007
RANBP2	1.491355	1.4638054	1.51410521	1.1084502	0.14854398	0.50034
ASB1	1.246957	1.2722336	1.22608362	0.8304764	-0.2679889	0.50051
COG2	1.21696	1.241732	1.19650294	0.8128959	-0.2988574	0.50052
IFT88	1.387902	1.4146692	1.3657981	0.8821444	-0.1809133	0.50066
TTN-AS1	1.304158	1.2783306	1.32548657	1.1694243	0.22579852	0.50073
RRP9	1.216764	1.2413677	1.19644549	0.8138849	-0.2971034	0.50074
DCAF12	1.660872	1.6899615	1.63684952	0.9230218	-0.1155634	0.50077
DEC1	1.042005	1.0188971	1.06108775	3.2326597	1.69272163	0.50079
PLPP2	1.066292	1.0429625	1.08555855	1.9914709	0.99383437	0.50108
RP11-496I9.1	1.180867	1.1563842	1.20108581	1.2858448	0.36271651	0.50121
ZNF165	1.045943	1.0690635	1.02685	0.3887724	-1.3630022	0.50122
MYL5	1.159604	1.1352947	1.17967987	1.3280631	0.40932368	0.50144
ZNF354A	1.25161	1.2768977	1.2307265	0.8332554	-0.2631693	0.50146
ABCA2	1.170906	1.1463082	1.19121962	1.3069646	0.3862201	0.50175
VASH2	1.137713	1.1621114	1.11756374	0.7252032	-0.4635428	0.50185
ISCU	2.774598	2.8275807	2.7308446	0.9470688	-0.0784589	0.50194
AMER2	1.055912	1.0791763	1.03669983	0.4635204	-1.1092953	0.50209
SMARCD3	1.640105	1.6689327	1.61629921	0.9213172	-0.1182302	0.50211
ARHGEF35	1.079368	1.0558488	1.09879087	1.7688996	0.82285218	0.50225
GRAMD1A	2.05462	2.0885754	2.0265787	0.9430478	-0.0845971	0.50232
LRRC24	1.120614	1.1444451	1.10093437	0.6987733	-0.5171035	0.50235
ST3GAL2	1.297382	1.2718569	1.31846057	1.1714273	0.22826742	0.50237
EIF3J-AS1	2.015174	2.0504351	1.98605459	0.9387106	-0.0912476	0.50246

GNG4	1.388646	1.3618915	1.41074063	1.1349828	0.18267041	0.50246
NUDT6	1.221564	1.2462133	1.20120736	0.8172077	-0.2912254	0.5025
ANKRD13C	1.245144	1.2200237	1.26588956	1.2084586	0.27316802	0.50256
HAP1	1.144655	1.1689539	1.12458779	0.7374071	-0.4394669	0.50264
IFT172	1.266615	1.2925471	1.24520073	0.8381581	-0.2547057	0.5027
CCDC71L	1.138178	1.1140767	1.15808065	1.3857401	0.47065666	0.50273
ZBTB8B	1.116183	1.092394	1.13582865	1.4701021	0.55591637	0.50274
SLC35C2	1.635739	1.6644807	1.61200363	0.9210254	-0.1186871	0.50299
IRAK1	1.123964	1.1002171	1.14357471	1.4326365	0.51867256	0.503
ZNF138	1.268897	1.2942029	1.24799934	0.8429534	-0.2464752	0.50302
EMC8	1.640456	1.6694285	1.61653091	0.920981	-0.1187566	0.50302
AC007163.3	1.05496	1.0782266	1.03574577	0.4569513	-1.1298876	0.50309
PRR14	1.495672	1.4681606	1.51839161	1.1072943	0.1470387	0.50326
STX2	1.291903	1.266311	1.31303817	1.1754611	0.23322675	0.5033
ETV5	1.378214	1.4044927	1.35651201	0.8813805	-0.1821632	0.50359
ZNF701	1.128336	1.1522026	1.10862659	0.7136973	-0.4866158	0.50383
SERPINA1	1.038711	1.0157693	1.05765596	3.6562068	1.87034767	0.50389
HOMER3	1.835148	1.8054037	1.85971207	1.06743	0.09414143	0.50402
TAOK3	1.531639	1.5597335	1.50843886	0.9083589	-0.1386657	0.50407
PRDM2	1.525168	1.4974513	1.54805714	1.1017302	0.13977097	0.50409
TMEM144	1.330263	1.3046166	1.35144289	1.1537222	0.2062959	0.50443
DIO3OS	1.81082	1.8413323	1.78562293	0.9337844	-0.0988387	0.50451
PXMP4	1.141554	1.1656896	1.12162151	0.7340322	-0.4460847	0.50455
ZNF563	1.119794	1.1435185	1.10020273	0.698187	-0.5183145	0.50462
TRAF3IP1	1.254557	1.2796457	1.23383794	0.8361936	-0.2580911	0.50469
C1orf35	1.664231	1.692856	1.64059182	0.9245671	-0.1131501	0.50476
UBR1	1.218935	1.1941589	1.23939544	1.232987	0.30215757	0.50482
SPOCK1	2.502646	2.5448526	2.46779129	0.9501174	-0.0738223	0.50482
PDSS1	1.100581	1.0769329	1.12010995	1.5612298	0.64268287	0.50493
GPATCH2	1.40221	1.4287898	1.38025925	0.8868198	-0.1732871	0.50504
SGSH	1.108548	1.0847045	1.12823852	1.5139512	0.59831868	0.50513
PNPT1	1.357917	1.3840809	1.33630985	0.8756225	-0.191619	0.50526
ZNF766	1.364581	1.3909897	1.34277291	0.8766801	-0.1898777	0.50529
CASP9	1.144473	1.168512	1.12462082	0.7395367	-0.4353064	0.50535
EXOG	1.314357	1.3400521	1.29313803	0.8620385	-0.2141759	0.50543
RIOK3	1.597984	1.5701316	1.62098491	1.0891957	0.12326314	0.50549
HDAC4	1.108729	1.0850727	1.12826565	1.507718	0.59236664	0.5055
CISH	1.113133	1.1367264	1.09364992	0.6849438	-0.5459425	0.50559
CIZ1	1.593585	1.6216429	1.57041363	0.9175905	-0.1240776	0.50561
STARD4	1.575568	1.5479595	1.59836743	1.0919921	0.12696247	0.50574
INO80D	1.274206	1.2490522	1.29497754	1.1844004	0.24415692	0.50582
AGPAT3	1.25868	1.2335846	1.27940485	1.1961613	0.25841189	0.50612
MRGBP	1.437825	1.4644368	1.41584908	0.8953837	-0.1594221	0.50615
ITPR1	1.066552	1.0433916	1.08567768	1.9745212	0.98150284	0.50624
HSD11B1L	1.915268	1.9463052	1.88963683	0.9401162	-0.089089	0.50625

FAM72D	1.061308	1.0843507	1.04227836	0.5012213	-0.9964804	0.50626
EXOSC6	1.273867	1.2992297	1.2529226	0.8452456	-0.2425574	0.50648
TBC1D2B	1.165855	1.1418615	1.1856696	1.3088089	0.38825446	0.50661
FKBPL	1.139112	1.1628718	1.11949129	0.7336524	-0.4468314	0.50697
STX18	1.201969	1.2266024	1.18162687	0.8015222	-0.3191856	0.50697
P2RX2	1.075787	1.0990213	1.05659932	0.5715873	-0.8069542	0.50697
SDSL	1.26886	1.2939027	1.24818005	0.8444292	-0.2439516	0.50698
INTS6	1.53745	1.5099363	1.56017041	1.0985105	0.13554861	0.50722
POLR3F	1.209226	1.233819	1.18891655	0.8079607	-0.3076429	0.50758
MED20	1.192945	1.2175855	1.17259722	0.7932388	-0.3341729	0.50766
TBX3	1.162739	1.1869039	1.14278374	0.7639419	-0.3884652	0.50771
TMEM135	1.176024	1.2003522	1.15593419	0.7783004	-0.361601	0.5078
RAB21	1.46035	1.4336651	1.48238763	1.1123505	0.15361141	0.50782
GFOD2	1.412576	1.4390212	1.39073709	0.8900187	-0.1680925	0.50789
PPP1R3C	1.185109	1.2094269	1.16502693	0.787993	-0.3437452	0.50791
KIAA0586	1.29264	1.3174921	1.27211741	0.857084	-0.2224914	0.50797
TYMP	1.268561	1.2435138	1.28924573	1.1878	0.24829191	0.50818
STK33	1.422044	1.4483115	1.40035139	0.8930205	-0.1632348	0.50831
CAPN1	1.592463	1.6205146	1.56929782	0.9174609	-0.1242815	0.50833
REST	2.057922	2.0912672	2.03038464	0.9442093	-0.0828214	0.50842
LYPD6	1.137799	1.1140281	1.15742854	1.380612	0.46530792	0.50848
PCDH1	1.045556	1.022763	1.06437874	2.8282148	1.49989168	0.50849
RXRB	1.355384	1.3811937	1.33407012	0.8763789	-0.1903733	0.50851
ACAD10	1.213075	1.237583	1.19283536	0.8116545	-0.3010623	0.50852
FRMD8	1.157704	1.1816306	1.13794474	0.7594797	-0.3969166	0.50866
TNFSF12	1.064402	1.0413892	1.08340575	2.0151554	1.01089107	0.5087
LRRC45	1.171946	1.1961696	1.15194102	0.7745391	-0.36859	0.5087
SPRYD4	1.251345	1.2760532	1.23093975	0.8365771	-0.2574297	0.50871
PRNCR1	1.070504	1.047478	1.08951856	1.8854739	0.91492715	0.50892
JMJD8	1.38604	1.3596735	1.40781313	1.1338427	0.18122048	0.50902
WWTR1	1.546949	1.5199256	1.56926605	1.0948992	0.13079801	0.5091
AP006621.5	1.310417	1.2849188	1.33147418	1.1633987	0.21834565	0.50913
OTUD6B-AS1	2.507309	2.546949	2.4745738	0.9532143	-0.0691276	0.50923
SEMA3F	1.135265	1.1114793	1.15490672	1.3895554	0.4746234	0.50925
CA5B	1.182631	1.2068619	1.1626204	0.7861302	-0.3471598	0.50941
ZNF626	1.339891	1.3138332	1.36141047	1.1516003	0.20364012	0.50961
ZNF680	1.206842	1.2315099	1.18647022	0.8054525	-0.3121286	0.50966
SULT1A2	1.064557	1.087514	1.04559962	0.5210551	-0.9404923	0.5099
METTL8	1.31187	1.3375265	1.29068165	0.8612114	-0.2155607	0.50992
KTN1-AS1	1.090313	1.1135904	1.07109014	0.6258464	-0.6761195	0.50997
TIMM23	1.280852	1.3058855	1.26017861	0.8505751	-0.2334895	0.50998
BAIAP2-AS1	1.252287	1.2274059	1.27283493	1.1997706	0.26275864	0.51016
RP11-88I18.2	1.119561	1.0960481	1.13897828	1.4469657	0.53303073	0.51019
CDK9	1.3521	1.3779835	1.33072564	0.8749738	-0.1926883	0.51019
PLPPR5	1.081866	1.0586595	1.10103104	1.7223303	0.78436188	0.5102

PI4KB	1.304651	1.330301	1.28346895	0.8582141	-0.2205904	0.51028
PTBP2	1.392001	1.4183524	1.37023898	0.8849932	-0.1762618	0.51044
TARS2	1.251151	1.2758262	1.23077366	0.8366633	-0.257281	0.51067
PRPF39	1.26668	1.2918303	1.24590979	0.8426467	-0.2470003	0.5107
CEP83-AS1	1.071779	1.0948753	1.05270605	0.5555296	-0.8480644	0.51075
TNIP3	1.049479	1.0721772	1.03073436	0.4258181	-1.231691	0.51082
GAS1	2.112591	2.0756248	2.14311882	1.0627486	0.08780041	0.51085
PMS2	1.373577	1.3478166	1.3948501	1.135225	0.18297821	0.51115
ITPK1	1.155358	1.1312349	1.17527884	1.335611	0.41749989	0.51119
HOOK2	1.933845	1.9034174	1.95897319	1.0614951	0.08609774	0.51146
ABCB10	1.078064	1.0551084	1.09702061	1.7605407	0.81601862	0.51158
PTTG1IP	3.445178	3.50067	3.39935204	0.9594837	-0.0596698	0.51168
ALG5	1.6797	1.708128	1.65622296	0.9267011	-0.109824	0.51178
WDR41	1.568049	1.5956569	1.54524924	0.9153747	-0.1275657	0.5118
CCDC85C	1.48058	1.5076411	1.45823176	0.9026688	-0.1477314	0.51181
RDH5	1.18283	1.1588158	1.2026609	1.276075	0.35171313	0.51181
HBQ1	1.109145	1.1324734	1.08987947	0.6784718	-0.5596393	0.51195
JTB	4.201916	4.2729867	4.14322385	0.9603534	-0.0583627	0.51198
SELENOS	2.160915	2.1956476	2.13223244	0.9469617	-0.0786221	0.51235
CAMK2D	1.574679	1.6023772	1.55180618	0.9160476	-0.1265056	0.51249
NKD2	1.089113	1.0659602	1.10823305	1.6408831	0.71447243	0.51255
FAM168A	1.124877	1.1014419	1.1442292	1.4217915	0.50770988	0.51267
JMY	1.204164	1.1800095	1.22411147	1.244998	0.31614341	0.51279
ABHD14A	1.865285	1.8949379	1.84079798	0.9395043	-0.0900284	0.51292
TSSC4	1.586141	1.5585436	1.60893103	1.0902121	0.12460887	0.51294
SEC24B-AS1	1.121184	1.1445435	1.10189286	0.7049287	-0.5044508	0.513
UQCRQ	11.58827	11.83779	11.3822142	0.9579641	-0.0619565	0.51312
EPOR	1.287137	1.2619366	1.30794766	1.1756574	0.23346771	0.51316
ZNF510	1.098766	1.121995	1.07958301	0.6523464	-0.6162898	0.51328
CLPTM1	1.46254	1.4362955	1.48421324	1.1098288	0.15033709	0.51346
FBXO30	1.129667	1.1532308	1.11020803	0.7192289	-0.4754771	0.51355
SLC6A9	1.104089	1.1272147	1.0849923	0.6681013	-0.5818613	0.51358
ZRANB1	1.354182	1.328742	1.3751912	1.1412938	0.19067019	0.51359
IDH1-AS1	1.067542	1.0905538	1.04853921	0.5360261	-0.8996248	0.51365
FUBP3	1.309305	1.3344706	1.28852279	0.8626253	-0.213194	0.51377
PML	1.254224	1.2788215	1.23391143	0.8389289	-0.2533796	0.51398
UBA5	1.406594	1.4320716	1.38555381	0.8923377	-0.1643382	0.5141
WDR78	1.158982	1.1827847	1.13932619	0.7622422	-0.3916787	0.51414
AC062017.1	1.224235	1.2485719	1.20413652	0.8212374	-0.2841288	0.51439
SLC25A4	1.983608	2.0152672	1.95746313	0.9430652	-0.0845706	0.51443
FLRT1	1.04625	1.0236389	1.06492315	2.7464501	1.45756807	0.51447
PARP8	1.139383	1.1161199	1.15859419	1.36578	0.44972509	0.51466
LIN7A	1.512868	1.5394425	1.49092284	0.910056	-0.1359728	0.5147
ARF6	1.977407	1.9457452	2.00355458	1.0611258	0.08559564	0.51499
SCAI	1.409605	1.4357747	1.38799363	0.8903537	-0.1675496	0.51518

SERPINB1	1.245904	1.2701287	1.22589864	0.8362631	-0.2579712	0.5152
MTHFSD	1.146746	1.1232307	1.16616452	1.3484019	0.43125061	0.51529
TSEN54	1.268618	1.2933195	1.24821931	0.8462421	-0.2408577	0.51536
COMMD5	1.392241	1.3665797	1.41343238	1.1278102	0.17352435	0.51539
ARMCX6	1.756151	1.7850016	1.73232486	0.932896	-0.1002118	0.51553
MMP7	1.050231	1.0264499	1.06986888	2.6415523	1.40138595	0.51584
DMTF1	1.515219	1.4880961	1.53761826	1.1014598	0.1394169	0.51593
AC008079.10	1.058894	1.0817497	1.0400203	0.4895467	-1.0304815	0.51595
RP11-262H14.4	1.172925	1.1965336	1.15342913	0.7806763	-0.3572036	0.51597
RAD17	1.358461	1.3838165	1.33752263	0.8793853	-0.1854327	0.51597
ZSCAN21	1.121596	1.1447706	1.10245729	0.7077214	-0.4987465	0.51609
SEMA6B	1.105733	1.08269	1.12476278	1.5088015	0.59340303	0.51623
JAK1	1.834135	1.805316	1.85793396	1.0653382	0.09131151	0.51654
OCLN	1.214456	1.1903591	1.23435558	1.2311234	0.29997539	0.51656
RP11-166O4.6	1.088962	1.0660728	1.10786411	1.6325036	0.70708621	0.51657
SEL1L	1.358166	1.384192	1.33667304	0.8763145	-0.1904793	0.51658
MED29	1.548457	1.575415	1.52619539	0.9144624	-0.1290042	0.51661
MEPCE	1.18721	1.2110222	1.16754597	0.7939733	-0.3328377	0.51665
AIFM1	1.31546	1.3405006	1.29478068	0.8657273	-0.2080155	0.51719
GYG2	1.245054	1.2207577	1.26511845	1.2009476	0.26417315	0.51721
GTPBP1	1.233791	1.2095597	1.25380201	1.2111203	0.27634221	0.5174
MME	1.06532	1.0427573	1.08395314	1.9634831	0.97341519	0.51752
NFASC	1.051623	1.0740709	1.0330853	0.4466708	-1.1627161	0.51768
PKP3	1.037858	1.0156039	1.05623517	3.6039173	1.84956592	0.51774
MX1	1.066229	1.0436264	1.08489416	1.9459334	0.96046232	0.51774
IPO8	1.256181	1.2317423	1.27636321	1.1925454	0.25404418	0.51801
CTA-293F17.1	1.140617	1.1639466	1.12135178	0.7401909	-0.4340307	0.51803
ARPP21	1.155012	1.1785831	1.13554645	0.7590103	-0.3978086	0.51804
LYRM4	2.506919	2.543772	2.47648487	0.9564138	-0.0642931	0.51827
CCDC107	2.119633	2.0856483	2.1476987	1.0571551	0.0801871	0.51854
PINX1	1.151559	1.1749551	1.13223896	0.755845	-0.4038377	0.51857
FAM57A	1.311362	1.2865773	1.33182916	1.1579046	0.21151642	0.51865
DYRK1B	1.361531	1.3870184	1.34048321	0.8797598	-0.1848183	0.51879
NRAV	1.168817	1.1923126	1.14941388	0.7769323	-0.3641392	0.51902
APOLD1	1.232312	1.2562027	1.21258251	0.8297435	-0.2692627	0.51913
CBLB	1.418715	1.4449594	1.3970421	0.8923108	-0.1643818	0.51918
IFNAR2	1.386349	1.4119481	1.36520925	0.8865419	-0.1737393	0.51929
COG1	1.224135	1.2000719	1.24400699	1.2195967	0.28640413	0.51931
TEX261	1.280986	1.3057364	1.26054654	0.8521935	-0.2307471	0.51935
ASXL1	1.30433	1.2797305	1.32464488	1.160563	0.21482487	0.51936
RSPRY1	1.545554	1.5724666	1.52332987	0.9141667	-0.1294709	0.51937
ACOT2	1.064934	1.0874213	1.04636436	0.5303555	-0.9149683	0.51941
PPL	1.152575	1.1293351	1.1717662	1.3280709	0.40933213	0.51945
MLF1	1.503647	1.530157	1.48175406	0.9087008	-0.1381227	0.51963
MT1X	2.324063	2.4240076	2.24152772	0.8718547	-0.1978404	0.5197

EPB41L2	1.515612	1.5417167	1.4940545	0.9120164	-0.1328683	0.5197
UGT3A2	1.064244	1.0869221	1.04551685	0.5236509	-0.9333228	0.51998
KAT6B	1.478845	1.5054439	1.45687998	0.9039183	-0.1457357	0.52001
TRMT6	1.157449	1.1807232	1.13822876	0.7648646	-0.3867237	0.52009
ERVK3-1	1.276015	1.3002925	1.25596654	0.8523906	-0.2304133	0.52018
GNGT1	1.064874	1.0424855	1.08336229	1.9621336	0.97242327	0.52022
RBPMS2	1.231764	1.2079419	1.25143706	1.2091695	0.27401655	0.52031
ARHGAP5	2.324972	2.3624475	2.29402368	0.9497788	-0.0743366	0.52036
GAPVD1	1.335852	1.3108859	1.35646937	1.1466245	0.19739304	0.52046
GPHN	1.217998	1.2421659	1.19804053	0.8177886	-0.2902001	0.52049
PLEKHG4	1.078268	1.100743	1.05970861	0.5926828	-0.754668	0.52058
SCO1	1.32086	1.3457774	1.30028315	0.8684289	-0.2035203	0.52059
SNTA1	1.13216	1.1091544	1.1511576	1.3848052	0.46968303	0.52059
RAD51D	1.21861	1.1946888	1.23836458	1.2243363	0.29199992	0.52073
HMGXB4	1.997136	2.027952	1.97168826	0.9452662	-0.0812074	0.52088
ETS1	1.057328	1.0351307	1.0756595	2.153655	1.10678715	0.52104
PRKX	1.577638	1.6049728	1.5550637	0.9175019	-0.124217	0.52109
FBXW8	1.122807	1.0997589	1.14184007	1.4218281	0.50774708	0.52109
CHD6	1.731722	1.7033192	1.75517685	1.0737328	0.10263496	0.52122
TNS1	1.087955	1.0653738	1.10660341	1.6306742	0.70546861	0.5213
KCNJ3	1.084773	1.0621581	1.10344934	1.6642936	0.73490994	0.52138
SLC30A1	1.109643	1.0868642	1.12845345	1.4787853	0.56441262	0.52152
VCPIP1	1.20475	1.228573	1.18507599	0.809702	-0.3045371	0.52156
SLC35E1	1.310398	1.2858698	1.33065379	1.1566588	0.20996336	0.52162
ANKRD18B	1.136174	1.1593008	1.11707474	0.7349286	-0.4443239	0.52166
ZGRF1	1.15818	1.1349247	1.17738527	1.3146986	0.39473213	0.52185
KLHL42	1.356562	1.3314228	1.3773216	1.1384901	0.1871217	0.52186
AAR2	1.244325	1.2683814	1.22445799	0.8363395	-0.2578393	0.52188
RMND5A	1.423676	1.3979812	1.44489612	1.1178823	0.16076835	0.52191
RIC3	1.892804	1.9227704	1.86805702	0.9407075	-0.0881819	0.52219
TICAM1	1.091951	1.0693663	1.11060163	1.5944566	0.67306487	0.52236
CTD-3184A7.4	1.452349	1.4782678	1.43094559	0.9010549	-0.150313	0.52257
SNX27	1.516692	1.5433778	1.49465507	0.9103337	-0.1355326	0.52267
TTC37	2.092218	2.1250884	2.06507375	0.9466578	-0.079085	0.52278
NXT1	1.738749	1.7670135	1.71540819	0.9327192	-0.1004853	0.52279
ZNF143	1.213998	1.2377793	1.19435869	0.8173912	-0.2909014	0.5228
MUC16	1.034248	1.0122555	1.05240925	4.2763937	2.09639469	0.52282
GRB10	1.652408	1.6793075	1.63019448	0.9277014	-0.1082676	0.52291
ALDH1B1	1.170104	1.19333	1.15092272	0.7806484	-0.3572552	0.52295
NOTCH4	1.100917	1.1235644	1.08221442	0.6653569	-0.5877997	0.52295
RP11-567M16.6	1.126107	1.1490282	1.10717855	0.7191831	-0.475569	0.52326
NRDE2	1.34234	1.3174386	1.36290393	1.1432255	0.19311003	0.52336
THAP7	1.741818	1.7708161	1.71787076	0.9313126	-0.1026626	0.5234
WSCD2	1.09747	1.1200629	1.07881167	0.6564199	-0.6073092	0.52348
DYNC1H1	2.406818	2.4462042	2.37429136	0.9502748	-0.0735834	0.5235

MIR4453	1.118543	1.1413588	1.09970129	0.7053066	-0.5036776	0.52364
EXOC7	1.883234	1.912837	1.85878753	0.9407896	-0.088056	0.5238
FAM13A	1.697201	1.6678464	1.72144203	1.0802514	0.11136708	0.52387
FBXL12	1.296324	1.3205047	1.27635518	0.8622501	-0.2138216	0.52392
TMEM53	1.32312	1.3478205	1.30272105	0.8703369	-0.2003542	0.52393
KIAA0355	1.551187	1.5245754	1.57316393	1.0926244	0.12779757	0.52417
HBD	1.046657	1.0246172	1.06485786	2.6346579	1.39761564	0.52421
SEMA3D	1.19477	1.1711324	1.21429047	1.252191	0.32445461	0.52435
SLITRK5	1.582998	1.6096812	1.56096245	0.9200915	-0.1201508	0.52459
APTR	1.402459	1.4277053	1.38161111	0.8922292	-0.1645137	0.52461
RALGPS2	1.537005	1.5635731	1.51506411	0.913926	-0.1298508	0.52461
COX4I2	1.08235	1.1047186	1.06387771	0.6099938	-0.7131335	0.52475
FSD1L	1.272929	1.2487401	1.29290369	1.177549	0.23578712	0.52476
RER1	1.901183	1.9304679	1.87699828	0.9425347	-0.0853823	0.52499
TMCC3	1.097414	1.0750201	1.11590721	1.5450163	0.6276221	0.52501
IMMP2L	1.500515	1.5266322	1.47894612	0.9094509	-0.1369323	0.5251
RP11-1042B17.3	1.160712	1.1836865	1.14173883	0.7716343	-0.3740108	0.52512
KMT2E-AS1	1.161712	1.1850287	1.14245599	0.769913	-0.3772327	0.5252
TCEAL9	8.609863	8.490605	8.70834852	1.0290689	0.04133956	0.52527
SHTN1	1.351167	1.326541	1.37150366	1.1376936	0.18611207	0.52533
GMPPA	1.268723	1.2928714	1.24878167	0.849457	-0.2353871	0.52538
AP3B2	1.043814	1.065663	1.02577023	0.3924619	-1.3493756	0.52553
DCAF15	1.19875	1.2222563	1.17933833	0.8068986	-0.3095408	0.52559
MYPOP	1.1197	1.0970868	1.13837446	1.4252648	0.51123003	0.52575
STK11IP	1.151879	1.1749113	1.13285857	0.7595767	-0.3967325	0.5264
ST6GALNAC4	1.199349	1.2227948	1.17998779	0.8078634	-0.3078167	0.5265
LENG8	2.001466	1.970116	2.02735565	1.0590029	0.08270654	0.52652
UBE2G2	1.534196	1.5607414	1.51227495	0.9135673	-0.1304171	0.52667
MARVELD2	1.086097	1.0638684	1.10445424	1.6354592	0.70969579	0.52672
DMXL1	1.277794	1.2535903	1.29778236	1.1742656	0.2317588	0.52675
SIRT5	1.284409	1.308601	1.26443067	0.8568691	-0.2228532	0.52695
PIAS3	1.29372	1.2693879	1.31381355	1.1649133	0.22022256	0.52711
SNX8	1.157785	1.1347047	1.17684503	1.3128348	0.39268543	0.52732
CCDC61	1.061759	1.0837536	1.04359483	0.5205131	-0.9419936	0.52738
CLIP4	1.169385	1.1465866	1.18821225	1.2839664	0.36060742	0.528
ZNF800	1.437104	1.4115645	1.4581946	1.1132997	0.15484196	0.52815
PPP6R2	1.41334	1.3882135	1.43409009	1.1181736	0.16114416	0.52833
BRD9	1.465379	1.4912558	1.44400864	0.9038238	-0.1458866	0.52843
UBTF	1.517611	1.5432849	1.49640946	0.9137185	-0.1301783	0.52862
NAV3	1.052893	1.0310466	1.07093438	2.2847682	1.19204779	0.52863
SELENOI	1.19458	1.1715786	1.21357471	1.244763	0.31587104	0.5288
SRPX	1.37901	1.4035087	1.35877939	0.8891491	-0.1695027	0.52882
SS18	1.56948	1.5960197	1.54756277	0.9186992	-0.1223356	0.52887
DNAJC2	1.564013	1.5906797	1.54199053	0.917571	-0.1241083	0.52912
MCM8	1.203202	1.1799582	1.22239779	1.2358302	0.30548059	0.52915

PTPRR	1.090206	1.0679885	1.10855425	1.5966565	0.67505399	0.5293
HCFC1R1	3.632341	3.5664121	3.68678655	1.0469038	0.06612884	0.5295
EDIL3	1.107903	1.1303681	1.08935091	0.6853742	-0.5450362	0.52972
PRRC2A	1.999116	2.0300387	1.97357983	0.9451876	-0.0813273	0.52985
RP11-563J2.3	1.063559	1.0415575	1.08172901	1.9666492	0.97573962	0.52988
CBARP	1.063577	1.0414792	1.08182531	1.9726831	0.98015918	0.52988
PTGES2	1.791465	1.7633755	1.81466249	1.0671844	0.0938095	0.52997
INTS9	1.076775	1.0988353	1.05855691	0.5924693	-0.7551877	0.53037
AC006262.5	1.036632	1.0148661	1.05460693	3.6732485	1.87705651	0.53041
PPP1R3G	1.134042	1.1112796	1.15283876	1.3734666	0.4578218	0.53044
SYT13	1.073351	1.0513687	1.0915043	1.7813256	0.83295123	0.53053
PRRT3	1.223432	1.1997131	1.24301919	1.2168414	0.28314112	0.5306
AKR1C2	1.117649	1.1398772	1.09929288	0.7098573	-0.4943991	0.53069
ZBTB25	1.317117	1.3417964	1.29673681	0.8681683	-0.2039533	0.5307
ANGPTL7	1.03757	1.0160668	1.05532741	3.4435795	1.78390898	0.53071
EFL1	1.129908	1.1524528	1.11129057	0.7300001	-0.4540315	0.53083
MMP11	1.219632	1.2429843	1.20034643	0.8245242	-0.2783662	0.53119
DDHD1	1.608179	1.635054	1.58598563	0.9227336	-0.1160139	0.53121
KIAA1211	1.114628	1.1372125	1.09597746	0.6994805	-0.5156443	0.53122
CUL5	1.857874	1.886287	1.83440915	0.941466	-0.087019	0.53132
MGAT2	1.429529	1.4544569	1.4089439	0.8998519	-0.1522406	0.53135
NACC2	1.133381	1.1108557	1.15198226	1.3709916	0.45521977	0.53141
TMEM59L	1.312785	1.2890637	1.3323749	1.1498328	0.20142404	0.53151
GINS3	1.094552	1.1165493	1.07638692	0.6554044	-0.6095428	0.53165
RAD51AP1	1.513723	1.4875545	1.53533387	1.0979979	0.13487531	0.53171
C1QTNF3	1.103944	1.0818086	1.12222294	1.4940101	0.5791899	0.53177
CSNK2A3	1.049827	1.0714572	1.03196358	0.447311	-1.1606499	0.53182
ZNF274	1.271474	1.2953026	1.25179599	0.852671	-0.229939	0.53211
TMEM192	1.383054	1.4078577	1.36257141	0.8889654	-0.1698009	0.53239
SPRR3	1.037749	1.0159741	1.05573188	3.4888859	1.8027664	0.53262
ESRP1	1.037515	1.0159741	1.05530362	3.4620764	1.79163758	0.53262
CACNA2D1	1.411157	1.435839	1.39077413	0.896602	-0.1574604	0.53262
CHGB	1.043257	1.0648883	1.0253931	0.3913358	-1.3535209	0.53275
CHRM3	1.16542	1.1424452	1.18439357	1.2944878	0.3723814	0.53275
ETV1	1.317871	1.2936688	1.33785712	1.1504698	0.20222312	0.53299
RP11-437B10.1	1.080101	1.0580721	1.09829245	1.6925932	0.75923527	0.53302
CD99	3.925405	3.8552882	3.98330815	1.0448361	0.06327661	0.53307
ZNF724	1.10376	1.081467	1.1221691	1.499615	0.58459218	0.53313
PKP4	1.618919	1.6456865	1.59681425	0.9243097	-0.1135518	0.53344
IRF3	1.6051	1.5784987	1.62706694	1.0839557	0.11630577	0.53346
PCMTD2	1.546553	1.5725847	1.52505596	0.9169927	-0.1250179	0.53353
AP1M2	1.040153	1.0185978	1.05795283	3.1161084	1.63974542	0.53376
ARSK	1.247814	1.2713973	1.22833928	0.8413469	-0.2492273	0.53377
XPNPEP3	1.358021	1.3825061	1.33780173	0.8831278	-0.1793059	0.53389
SNTB1	1.0604	1.0387013	1.07831842	2.0236654	1.01697079	0.53391

RBM5	1.785554	1.7584995	1.80789689	1.0651252	0.09102303	0.53395
DEXI	1.56549	1.5397065	1.58678186	1.087224	0.12064922	0.53396
CNTN6	1.054827	1.0331724	1.07270936	2.1918657	1.13215939	0.534
FBXO25	1.207938	1.1851899	1.22672303	1.2242729	0.29192515	0.53405
REV1	1.330266	1.3058845	1.35040025	1.1455313	0.19601682	0.5344
GABRB3	1.1145	1.0924706	1.13269263	1.4349713	0.52102189	0.53454
LMCD1	1.197111	1.1743523	1.21590643	1.2383345	0.30840106	0.53463
KLHDC10	1.491739	1.4666752	1.51243716	1.0980596	0.13495636	0.53463
ALX3	1.037044	1.0156039	1.05475016	3.508748	1.81095633	0.53473
RNF4	1.481827	1.5071689	1.46089943	0.9087692	-0.1380142	0.53485
ZNF420	1.222497	1.2455035	1.20349842	0.8289023	-0.2707261	0.5349
ATP2B4	1.939372	1.9682929	1.91548883	0.9454668	-0.0809013	0.5349
VIPAS39	1.239324	1.2625208	1.2201674	0.8386665	-0.2538309	0.53503
FAM118B	1.179046	1.2019158	1.16015966	0.7932004	-0.3342427	0.5352
CDC42BPA	1.615558	1.5895514	1.63703546	1.0805427	0.11175606	0.5353
P4HTM	1.779199	1.8064316	1.7567107	0.9383445	-0.0918103	0.53531
MCFD2	2.117469	2.0867525	2.14283499	1.0516056	0.07259377	0.53536
TAOK2	1.207442	1.1845658	1.22633272	1.2262984	0.29431004	0.53547
HMGCS1	1.757526	1.726365	1.78325966	1.078328	0.10879606	0.53552
NRGN	1.076483	1.0547153	1.09445827	1.7263583	0.7877319	0.53569
SOBP	1.142237	1.1198892	1.1606931	1.3403468	0.42260628	0.5358
GLMP	1.783377	1.8114676	1.76017934	0.9367956	-0.0941937	0.53597
EFCAB14	1.547516	1.5220131	1.56857635	1.0891994	0.12326814	0.53599
APCDD1L	1.202432	1.1796795	1.22122198	1.2312034	0.30006915	0.53609
PRDM16	1.076849	1.0551066	1.09480471	1.7203872	0.78273328	0.53618
KLHL36	1.310362	1.2867754	1.3298411	1.1501722	0.20184993	0.53621
DCAF5	1.429804	1.4547786	1.40917967	0.8997338	-0.1524299	0.53641
LINC00877	1.056354	1.0348247	1.07413363	2.128766	1.0900174	0.53648
SLC1A5	1.370994	1.3951552	1.35104184	0.8883644	-0.1707765	0.53657
LRBA	1.621549	1.6481578	1.59957566	0.9250458	-0.1124033	0.5369
SLC35B1	1.817233	1.8452806	1.79407126	0.9394174	-0.0901618	0.53699
FOSL1	1.04769	1.026271	1.06537743	2.488576	1.31532044	0.53701
TNRC6C	1.38051	1.3558761	1.40085385	1.1263858	0.1717011	0.53701
COBLL1	1.049808	1.028432	1.06746073	2.3727046	1.24653249	0.53701
FZD6	1.222833	1.1996584	1.24197135	1.2119264	0.27730212	0.53725
RP3-467N11.1	1.172891	1.1503431	1.19151205	1.2738329	0.34917608	0.53728
ANAPC2	1.127499	1.1495826	1.10926148	0.7304422	-0.453158	0.53733
PXN	1.474739	1.4495708	1.49552384	1.1022154	0.14040615	0.53734
ARHGEF2	1.25864	1.2820418	1.23931428	0.8485065	-0.2370024	0.53734
RP11-398K22.12	1.316253	1.3407215	1.29604683	0.8688822	-0.2027675	0.53743
ATG13	1.303365	1.3274456	1.28347817	0.8657259	-0.2080177	0.53766
TRAIP	1.080709	1.1021967	1.0629635	0.6161012	-0.6987608	0.53786
PLEKHA1	2.914503	2.9598338	2.87706842	0.9577692	-0.0622501	0.53795
RPRD1B	1.286934	1.2633231	1.30643306	1.1637152	0.21873807	0.53801
ZNF780B	1.136001	1.1582449	1.11763169	0.7433523	-0.427882	0.53802

LRP5	1.316175	1.2925073	1.3357195	1.1477303	0.19878366	0.53802
RNF182	1.260401	1.2835407	1.2412912	0.8509932	-0.2327805	0.53814
DLEU1	1.339637	1.3634623	1.31996084	0.8803137	-0.1839104	0.53823
UBN2	1.372972	1.3487208	1.39299969	1.1269752	0.17245573	0.53828
CRYBA1	1.044078	1.0653143	1.02654102	0.4063585	-1.2991749	0.53828
C14orf37	1.248202	1.2710972	1.22929501	0.8458037	-0.2416053	0.53838
FAM76B	1.295197	1.3189209	1.27560594	0.8641826	-0.2105918	0.53879
SETD1A	1.111402	1.0894322	1.12954513	1.4485295	0.53458902	0.53888
MORC3	1.254851	1.2783129	1.23547522	0.846081	-0.2411323	0.53894
LINC00667	1.704288	1.6774435	1.7264558	1.0723489	0.10077436	0.53908
RP5-1092A3.4	1.16271	1.1852265	1.14411508	0.7780479	-0.3620691	0.53914
CHST3	1.098893	1.0772492	1.11676615	1.5115508	0.59602945	0.53919
NEU1	1.592108	1.6178076	1.57088541	0.9240505	-0.1139564	0.53922
PHLDB1	1.241214	1.2183627	1.26008462	1.1910671	0.2522547	0.53931
RP11-635N19.1	1.225962	1.2489462	1.20698106	0.831429	-0.266335	0.53938
ABLIM1	1.593848	1.5681742	1.6150504	1.0825032	0.11437128	0.53956
UQCC1	1.411825	1.4363195	1.39159788	0.8975026	-0.156012	0.53957
SNAPC5	1.526229	1.5008414	1.54719422	1.0925498	0.12769907	0.53959
CEBPD	1.431394	1.4067486	1.45174669	1.1106287	0.15137655	0.5397
BAG6	2.028187	2.058696	2.00299258	0.9473848	-0.0779775	0.53991
ZFP1	1.234159	1.257078	1.21523274	0.8372274	-0.2563085	0.53995
INPP5D	1.244959	1.2685869	1.22544653	0.8393804	-0.2526034	0.54018
IRGQ	1.222611	1.1997692	1.24147468	1.208768	0.27353738	0.54024
TUBGCP4	1.545939	1.5713504	1.52495418	0.9187955	-0.1221844	0.5406
PDE5A	1.10808	1.0862931	1.12607176	1.4609722	0.54692877	0.54064
DENR	2.627243	2.6653774	2.59575076	0.9581917	-0.0616138	0.54075
SMN2	1.080322	1.1018421	1.06254968	0.6141827	-0.7032603	0.54079
CTD-2147F2.1	1.148524	1.1263415	1.16684267	1.3205689	0.40115953	0.54117
ZCCHC3	1.395146	1.4197389	1.37483676	0.8930236	-0.1632298	0.54124
TNFRSF4	1.073395	1.0522141	1.090887	1.7406599	0.79963437	0.5414
SIPA1L3	1.095856	1.0741882	1.11374889	1.5332486	0.61659164	0.54142
STKLD1	1.065379	1.0867323	1.04774581	0.5504963	-0.8611952	0.54149
TMED7	1.750538	1.7237558	1.77265477	1.0675627	0.09432086	0.54159
THRA1/BTR	1.034221	1.0552478	1.0168571	0.305118	-1.7125607	0.54171
ZHX3	1.12943	1.1517192	1.11102254	0.7317635	-0.4505506	0.54179
ZNF460	1.089089	1.0674945	1.10692199	1.5841581	0.66371634	0.54183
ANO5	1.133362	1.1114369	1.15146898	1.3592358	0.44279581	0.54198
MIER2	1.241371	1.2644594	1.22230357	0.8405962	-0.2505152	0.54205
RUNDC3A	1.059632	1.081013	1.0419753	0.5181306	-0.9486123	0.54213
TSPOAP1-AS1	1.082174	1.1036445	1.0644433	0.6217722	-0.6855419	0.54234
WDR4	1.058254	1.0794099	1.04078399	0.5135883	-0.9613158	0.54237
DBR1	1.152095	1.1740453	1.1339673	0.7697266	-0.377582	0.54252
RP3-473L9.4	1.068987	1.0903802	1.05132051	0.5678288	-0.816472	0.54258
LAMTOR3	1.761727	1.7890799	1.73913854	0.9367094	-0.0943266	0.54259
KIAA1522	1.220951	1.1987753	1.23926449	1.2036932	0.26746768	0.54277

ATG9A	1.272447	1.249335	1.29153373	1.1692449	0.22557716	0.54291
XKR8	1.294072	1.270782	1.31330588	1.157041	0.21043999	0.54306
SCAP	1.33451	1.3582962	1.31486677	0.8787889	-0.1864114	0.54312
RP5-832C2.5	1.077901	1.099369	1.06017276	0.6055485	-0.7236856	0.54317
SND1	2.281678	2.3163297	2.25306116	0.9519356	-0.0710641	0.54321
INTS6L	1.100301	1.0786373	1.11819126	1.5029919	0.5878372	0.54345
PAK5	1.064441	1.0858713	1.04674349	0.5443433	-0.8774113	0.54362
P3H1	1.500885	1.4755977	1.52176675	1.0970758	0.13366322	0.54411
PHF12	1.251187	1.2281632	1.2702006	1.1842428	0.24396492	0.54412
HS2ST1	1.571149	1.5967891	1.54997539	0.9215573	-0.1178542	0.54435
TXNRD2	1.09836	1.0769167	1.11606865	1.5090185	0.59361047	0.54442
SUPV3L1	1.174324	1.1966623	1.15587661	0.7926106	-0.3353158	0.5446
LINC00511	1.110082	1.0884881	1.12791418	1.4455524	0.53162092	0.54472
G2E3	1.504959	1.5294937	1.48469867	0.9154003	-0.1275253	0.54499
TTK	1.141098	1.1627409	1.12322466	0.757183	-0.401286	0.54503
CLDN5	1.041035	1.0620325	1.02369424	0.381965	-1.3884878	0.54527
ANKRD9	1.152297	1.1303464	1.17042477	1.3074762	0.38678467	0.54534
PTPN13	1.414912	1.3908678	1.43476815	1.112315	0.15356537	0.54545
SYNC	1.613546	1.641819	1.59019827	0.9195712	-0.1209668	0.54559
LINC01273	1.100313	1.1219708	1.08242724	0.6757947	-0.565343	0.54561
CMTM4	1.240273	1.2176655	1.25894239	1.1896345	0.2505184	0.54572
PLEKHH1	1.20951	1.2322467	1.19073297	0.8212516	-0.2841039	0.54579
THUMPD1	1.60164	1.6270895	1.58062273	0.925901	-0.1110702	0.54597
NGFR	1.429787	1.4537686	1.40998247	0.9035056	-0.1463946	0.54601
DCLK1	1.522913	1.5511133	1.49962436	0.9065729	-0.1415051	0.54622
DIP2B	1.282809	1.2597167	1.30187924	1.1623405	0.21703271	0.54641
TNS3	1.336951	1.313516	1.35630462	1.1364797	0.18457195	0.54658
MTMR3	1.158183	1.136306	1.17624881	1.2930375	0.3707641	0.54676
ZNF470	1.153004	1.1309088	1.17125042	1.3081663	0.38754593	0.54686
BEGAIN	1.066418	1.0453353	1.08382925	1.8490962	0.88682027	0.54689
MAPK14	1.316397	1.2929247	1.33577996	1.1463013	0.19698631	0.5469
CDNF	1.040445	1.0195519	1.05769951	2.9510945	1.5612501	0.54692
WDR31	1.100558	1.122064	1.08279888	0.6783233	-0.559955	0.54699
SLFN12	1.171402	1.1934512	1.15319377	0.7918987	-0.3366122	0.54716
DLEU2	1.132717	1.1542631	1.11492348	0.7449837	-0.4247192	0.54726
VPS16	1.169963	1.1920953	1.15168495	0.7896337	-0.3407446	0.54746
KAT2B	1.087799	1.0666233	1.10528537	1.5803081	0.66020582	0.54747
ARHGAP28	1.185807	1.1634742	1.20425016	1.2494333	0.32127383	0.5476
E2F6	1.457788	1.4825232	1.43736174	0.9064057	-0.1417712	0.54767
GNA12	1.214619	1.1921711	1.23315707	1.2132785	0.27891073	0.54774
TMEM5	1.605713	1.631847	1.58413092	0.9244816	-0.1132835	0.54792
LAMP2	1.60707	1.5814442	1.62823301	1.0804699	0.1116589	0.54795
LCMT2	1.070979	1.0920784	1.05355533	0.5816275	-0.7818326	0.54797
CDKN2C	1.101547	1.1228138	1.08398458	0.6838366	-0.5482765	0.54839
ACTA2	2.435814	2.4960961	2.38603195	0.9264325	-0.1102423	0.54877

MAPK8IP3	1.205754	1.2280884	1.18730974	0.8212155	-0.2841672	0.54879
PPP4R4	1.069206	1.0482563	1.08650687	1.7926533	0.84209652	0.54882
PIP5KL1	1.060421	1.039432	1.07775341	1.9718332	0.97953754	0.54886
HOXB-AS3	1.055128	1.0761071	1.03780319	0.4967101	-1.0095239	0.54895
RASA3	1.149094	1.1710524	1.13096061	0.7656168	-0.3853056	0.5491
GGNBP2	1.887552	1.859627	1.9106134	1.0593122	0.0831279	0.5491
SLC2A4RG	1.363214	1.3873455	1.34328591	0.8862526	-0.1742102	0.54912
WRNIP1	1.256895	1.2342152	1.27562393	1.1767979	0.23486654	0.54939
SMPDL3A	1.254443	1.2767928	1.2359859	0.8525724	-0.2301057	0.54942
LINC00869	1.412342	1.4364379	1.39244312	0.8991957	-0.1532929	0.54946
ARHGAP18	1.199558	1.2217184	1.18125845	0.8175164	-0.2906805	0.5495
SEC14L6	1.074683	1.0536806	1.09202662	1.7143381	0.7776517	0.5495
ZNF93	1.212013	1.2342689	1.19363388	0.8265454	-0.2748341	0.54958
STK24	1.459686	1.4863686	1.43765061	0.8998331	-0.1522706	0.54965
FAM173B	1.228783	1.2514441	1.21006917	0.8354509	-0.259373	0.54972
KIAA0319L	1.473429	1.4490593	1.49355317	1.0990824	0.13629956	0.54975
HECTD4	1.272038	1.2492589	1.29084968	1.1668576	0.22262847	0.54978
RP11-247A12.8	1.102804	1.0815443	1.12036141	1.476024	0.56171622	0.54985
RP11-390F4.6	1.217903	1.2401232	1.1995528	0.8310433	-0.2670044	0.5499
SLC35D1	1.091234	1.0700153	1.10875743	1.5533385	0.63537224	0.54993
FAM109A	1.161299	1.1831912	1.14321993	0.7818057	-0.355118	0.55
LARGE1	1.141881	1.1202942	1.15970792	1.3276439	0.40886821	0.55029
C6orf47	1.291525	1.314556	1.2725048	0.8663157	-0.2070352	0.55031
HLA-DPA1	1.07682	1.0979818	1.0593448	0.605672	-0.7233914	0.55034
FAM171A1	1.41003	1.4340828	1.39016623	0.8988291	-0.1538813	0.55055
BIRC3	1.046287	1.0255686	1.063396	2.4794515	1.31002103	0.55059
LYRM7	1.406302	1.4301339	1.38662211	0.8988414	-0.1538616	0.55081
SLC26A2	1.263087	1.2403702	1.28184729	1.172555	0.22965562	0.55092
SPON1	1.079067	1.0579647	1.09649417	1.6647048	0.73526639	0.55098
ZNF234	1.15728	1.1788644	1.13945494	0.7796685	-0.3590672	0.5511
GRIN2D	1.053918	1.0331789	1.07104467	2.1412591	1.09845936	0.55113
C1orf109	1.317083	1.3398229	1.29830477	0.8778241	-0.1879962	0.55118
FOXN2	1.142244	1.1637445	1.12448841	0.7602599	-0.3954354	0.5512
NECTIN1	1.137558	1.1159775	1.15537927	1.3397363	0.42194906	0.55125
SLC25A44	1.099164	1.0780693	1.11658375	1.4933375	0.57854025	0.55126
ZNF446	1.197053	1.2188733	1.17903362	0.8179783	-0.2898656	0.55136
RP11-636O21.2	1.079269	1.0583588	1.09653704	1.654198	0.72613196	0.55136
AP4S1	1.089467	1.06845	1.1068228	1.5605967	0.64209776	0.55136
THAP5	1.582065	1.5571021	1.6026798	1.0818121	0.11344999	0.55138
RNMT	3.349726	3.397616	3.31017704	0.9635309	-0.0535972	0.55145
DLG4	1.309396	1.2862357	1.32852194	1.1477322	0.19878602	0.55146
WWOX	1.376617	1.3998584	1.35742376	0.8938759	-0.1618535	0.55148
ORC2	1.188124	1.2100998	1.16997622	0.8090262	-0.3057417	0.55151
H2AFJ	2.437831	2.4031721	2.46645203	1.0450978	0.06363795	0.55154
ELMO2	1.153368	1.1752106	1.13532948	0.772382	-0.3726135	0.5516

IGDCC3	1.373072	1.3967065	1.35355437	0.891224	-0.16614	0.55163
PYM1	1.396457	1.4202295	1.3768244	0.896711	-0.157285	0.55164
GRK2	1.134452	1.1131403	1.15205172	1.3439222	0.42644959	0.55169
ULK4	1.150679	1.1289791	1.1685994	1.3071837	0.38646194	0.55197
SGK223	1.085384	1.0644373	1.10268179	1.5935144	0.67221201	0.55199
GATS	1.166482	1.1448232	1.18436852	1.2730589	0.34829912	0.55202
NOMO1	1.123885	1.1025229	1.14152551	1.3804285	0.46511613	0.55204
OPHN1	1.121418	1.100117	1.1390091	1.3884671	0.473493	0.55207
ROR2	1.084289	1.0632651	1.10165009	1.6067319	0.68412925	0.55208
EPC2	1.237396	1.2597699	1.21891965	0.8427446	-0.2468325	0.55209
FAM219B	2.010876	1.9826493	2.03418551	1.0524462	0.07374647	0.55215
C1orf131	1.424424	1.4482713	1.404731	0.9028707	-0.1474087	0.55223
MTBP	1.088285	1.0672308	1.10567237	1.571786	0.65240483	0.55226
GNPNAT1	1.378628	1.4020217	1.35930905	0.8937553	-0.1620482	0.55227
PFKFB2	1.076775	1.0558358	1.09406732	1.6847142	0.75250383	0.55228
ROBO2	1.10833	1.129571	1.09078931	0.7006917	-0.5131483	0.55231
MAP4K2	1.130862	1.1094499	1.14854397	1.3571875	0.44062003	0.55238
FAM188A	1.189902	1.2118519	1.17177626	0.8108317	-0.3025257	0.55262
FRMD4A	1.834032	1.8612332	1.81156965	0.9423344	-0.085689	0.55263
DENND5A	1.369801	1.3463218	1.38919092	1.123784	0.16836475	0.55277
NFKB2	1.142898	1.1214343	1.16062325	1.3227168	0.40350417	0.55333
CLEC19A	1.212286	1.2342158	1.19417547	0.829045	-0.2704777	0.55336
APOL2	1.279329	1.3022913	1.26036547	0.8613066	-0.2154011	0.55341
NME8	1.140541	1.1620958	1.12274106	0.7572132	-0.4012286	0.55346
HSF4	1.194327	1.1720933	1.21268822	1.2358893	0.30554952	0.5535
SPOPL	1.171038	1.149204	1.18906809	1.2671788	0.34162011	0.55357
CTC-559E9.1	1.123281	1.1018859	1.1409489	1.3833988	0.46821716	0.55371
PIGV	1.128987	1.1505946	1.1111435	0.7380311	-0.4382465	0.55374
POU6F2	1.177141	1.1989759	1.15911016	0.7996453	-0.3225679	0.55391
CNKSR2	1.115793	1.1371251	1.09817667	0.715964	-0.482041	0.55394
PLXDC2	1.653652	1.6281944	1.67467557	1.0739916	0.10298275	0.55411
CYP11A1	1.100743	1.12177	1.08337871	0.684723	-0.5464076	0.55421
RP11-345P4.9	1.416169	1.4397525	1.39669392	0.9020844	-0.1486656	0.55425
ANO4	1.038633	1.0181568	1.05554309	3.0590776	1.61309672	0.55425
MIR217HG	1.098773	1.1198366	1.08137889	0.6790823	-0.5583416	0.55426
SOGA1	1.268358	1.2458386	1.28695444	1.167247	0.22310994	0.55428
SPIN2B	1.223428	1.2454709	1.20522371	0.8360409	-0.2583546	0.55446
SMIM13	1.097878	1.0768996	1.11520169	1.4980791	0.58311384	0.55451
NFATC2	1.043176	1.0227073	1.06007887	2.6457952	1.40370139	0.55452
MFSD6	1.043152	1.0226923	1.06004726	2.6461568	1.40389855	0.55452
OAF	1.642771	1.6167697	1.66424334	1.0769715	0.10698002	0.55454
RP11-378J18.8	1.084623	1.1055328	1.06735523	0.63824	-0.6478291	0.55456
MTHFD2L	1.71406	1.7402986	1.69239253	0.9352882	-0.0965171	0.55464
ST6GALNAC3	1.28173	1.3047033	1.26275832	0.8623415	-0.2136688	0.55468
RAB11FIP5	1.069911	1.0493132	1.0869216	1.7626426	0.81773999	0.55476

ZNF181	1.17654	1.1982902	1.15857873	0.7997306	-0.322414	0.55477
CTA-445C9.14	1.178671	1.2005876	1.16057209	0.8005084	-0.3210116	0.55486
HECW2	1.08083	1.0600141	1.09802037	1.6332901	0.7077811	0.55491
SLMAP	1.498438	1.4742957	1.51837573	1.0929378	0.12821133	0.55532
PRR13	2.954721	2.9100994	2.99157048	1.0426528	0.06025879	0.55535
FAM104A	1.540571	1.5161694	1.56072198	1.0863138	0.1194409	0.55541
RIPK4	1.035142	1.0147414	1.05198985	3.5267821	1.81835243	0.55551
SARNP	1.188042	1.2098778	1.17000914	0.8100389	-0.3039369	0.55568
FAM84A	1.414203	1.4377664	1.39474323	0.9017211	-0.1492468	0.55573
YWHAZ	5.641624	5.7173401	5.57909592	0.9706945	-0.0429108	0.55584
MYO6	1.811819	1.7854569	1.83358975	1.06128	0.08580539	0.55588
ADPGK	1.4135	1.3894427	1.43336614	1.1127854	0.15417534	0.55589
GPATCH4	1.282074	1.3042201	1.26378535	0.8670872	-0.2057509	0.556
PCLO	1.242545	1.2647056	1.22424489	0.8471483	-0.2393136	0.55603
TTC7B	1.098011	1.0771084	1.11527351	1.4949533	0.58010041	0.55605
ELOF1	2.284299	2.2508864	2.31189144	1.0487695	0.06869758	0.55612
C1R	1.098031	1.0769628	1.11542896	1.4998019	0.58477196	0.55622
ZNF620	1.245568	1.2678979	1.22712832	0.8478168	-0.2381756	0.55625
OTUD4	1.248712	1.2266851	1.26690288	1.1774168	0.23562508	0.55631
ISG20	1.07294	1.0523844	1.08991513	1.7164471	0.77942537	0.55646
EIF4G1	1.892588	1.8656365	1.91484468	1.0568462	0.0797655	0.5565
BANK1	1.038971	1.0596361	1.02190553	0.3673199	-1.4448911	0.55655
RP11-254F7.2	1.153113	1.1315243	1.17094036	1.2996862	0.37816331	0.55658
ZSCAN2	1.143261	1.1645451	1.1256838	0.763826	-0.3886841	0.5566
PCTP	1.195199	1.2172737	1.17696913	0.8144987	-0.2960157	0.55666
PHLPP2	1.081531	1.0608769	1.09858832	1.6194705	0.6955222	0.55669
NOL8	1.337639	1.3605665	1.31870568	0.8839025	-0.1780408	0.55669
TSPAN1	1.052209	1.0318206	1.06904671	2.1698723	1.11761013	0.55676
L1CAM	1.199723	1.1782338	1.21746846	1.2201304	0.28703537	0.5568
VPS13A	1.493554	1.4694522	1.51345825	1.0937392	0.12926875	0.55681
RP11-451G4.2	1.03357	1.0132213	1.05037446	3.8100908	1.92982537	0.55691
CEP120	1.150888	1.1723492	1.13316547	0.7726492	-0.3721145	0.55716
C22orf29	1.23047	1.2526428	1.2121599	0.8397623	-0.251947	0.55717
ZEB2	1.719841	1.7454095	1.69872663	0.9373728	-0.0933052	0.55729
TTC5	1.320054	1.342734	1.30132488	0.87918	-0.1857695	0.55737
RALGAPA2	1.261845	1.2842917	1.24330773	0.8558384	-0.2245897	0.55756
BTN2A2	1.240872	1.2189629	1.25896513	1.1826898	0.2420717	0.55765
PFKP	2.011903	1.9838669	2.03505584	1.0520284	0.07317361	0.55766
FAM21C	1.255326	1.2778502	1.23672468	0.8519867	-0.2310972	0.55767
CHD3	1.481384	1.5058148	1.46120832	0.9118126	-0.1331907	0.55803
TBC1D13	1.188968	1.1673026	1.20686026	1.2364439	0.30619677	0.55813
ALDH5A1	1.174733	1.1963668	1.15686757	0.7988497	-0.3240039	0.55819
SLC12A7	1.141097	1.1202382	1.15832275	1.3167423	0.396973	0.55826
LYPD3	1.135282	1.114151	1.15273255	1.3379872	0.42006429	0.55838
AC093323.3	1.332688	1.3556005	1.3137666	0.882357	-0.1805656	0.55846

FAM227B	1.278641	1.300859	1.26029363	0.8651681	-0.2089476	0.55859
EID2	1.224765	1.246661	1.20668264	0.8379217	-0.2551126	0.55867
FOXJ1	1.082477	1.0618496	1.09951097	1.608918	0.68609084	0.55885
MYC	1.074602	1.0539663	1.09164251	1.6981425	0.7639575	0.55886
APOA1	1.176362	1.1976103	1.15881453	0.8036756	-0.3153149	0.55893
PDGFRB	1.569547	1.5948529	1.54864948	0.922328	-0.1166483	0.55897
FAM20B	1.246437	1.2244508	1.264593	1.1788462	0.23737552	0.55914
UBE2E2	1.721205	1.6958013	1.742183	1.0666594	0.09309951	0.55922
SHB	1.185064	1.1636715	1.20273064	1.2386438	0.3087614	0.55927
TTBK2	1.386376	1.3631681	1.40554069	1.1166749	0.15920928	0.55942
RP11-1000B6.3	1.205467	1.1837207	1.22342518	1.216113	0.28227727	0.55956
PIK3IP1	1.188535	1.2100054	1.17080417	0.8133322	-0.2980833	0.55969
KCTD10	1.273415	1.2963109	1.2545074	0.8589202	-0.219404	0.5597
MCUR1	1.892435	1.8659024	1.9143453	1.0559449	0.0785346	0.55971
PSEN1	1.350019	1.3729577	1.33107623	0.8877046	-0.1718485	0.55973
IL17RE	1.141733	1.1206351	1.15915634	1.3193203	0.39979489	0.55975
CREB3	1.744151	1.7184604	1.76536731	1.0652881	0.09124369	0.5598
MIPEP	1.164465	1.1856993	1.14692927	0.7912216	-0.3378463	0.55987
NECTIN4	1.037188	1.0170009	1.05385932	3.1680236	1.66358308	0.5599
RFESD	1.127676	1.1487796	1.1102491	0.741023	-0.4324098	0.56003
ZC3H18	1.371158	1.3940809	1.35222704	0.8937936	-0.1619863	0.56006
PCGF2	2.013538	1.9862877	2.03604092	1.050445	0.07100059	0.56016
VOPP1	1.69631	1.6709445	1.71725753	1.0690266	0.09629772	0.56016
ARAP2	1.080357	1.0597866	1.09734501	1.6282088	0.70328572	0.56018
CAMK1G	1.076662	1.0971911	1.05970956	0.6143524	-0.7028617	0.56026
AMHR2	1.038892	1.0187101	1.0555579	2.969408	1.57017534	0.5604
CCND1	3.656554	3.7242129	3.60068096	0.9546541	-0.0669501	0.56051
FXR2	1.201427	1.1798562	1.21924019	1.218975	0.28566859	0.56053
ZSCAN16	1.095616	1.1163743	1.07847401	0.674324	-0.5684861	0.56065
MTMR6	1.191707	1.2131353	1.17401101	0.8164346	-0.2925907	0.5608
LARP6	1.548927	1.5247988	1.56885304	1.0839449	0.11629144	0.56085
SH3YL1	1.625887	1.6507296	1.60537086	0.9302955	-0.104239	0.56094
ZNF286A	1.172357	1.1938531	1.15460504	0.7975372	-0.3263763	0.56095
XYLT2	1.173615	1.1524411	1.19110073	1.2536038	0.32608151	0.56096
CAMK2B	1.09296	1.1136043	1.07591161	0.6682106	-0.5816253	0.56098
SPATA20	1.454887	1.4788705	1.43508103	0.9085568	-0.1383514	0.56116
ZNF467	1.25621	1.2342824	1.27431884	1.1708894	0.22760483	0.56139
TAF9B	1.161524	1.182633	1.14409098	0.7889647	-0.3419673	0.56152
HAGHL	1.379302	1.3562117	1.39837014	1.1183521	0.16137451	0.56168
WNT10A	1.04738	1.0272619	1.06399337	2.3473594	1.23103876	0.56169
DCTN1	1.463936	1.4877866	1.44423891	0.9107238	-0.1349145	0.56173
LAMA4	1.121769	1.1009463	1.138964	1.3766132	0.46112327	0.56203
HIST1H2BH	1.074725	1.0949842	1.05799417	0.6105664	-0.7117798	0.56207
RP11-479G22.8	1.066474	1.0461744	1.08323764	1.8026791	0.8501426	0.56208
TFIP11	1.131049	1.1518344	1.11388336	0.7500499	-0.4149415	0.5622

FERMT1	1.050267	1.0301381	1.06688902	2.2194193	1.15018228	0.56223
LRIG3	1.074073	1.0537547	1.09085134	1.6901092	0.75711643	0.56246
RNF185	1.231738	1.2534152	1.21383641	0.8438183	-0.2449957	0.56252
MAST3	1.086379	1.0658751	1.10331069	1.5682806	0.64918373	0.56294
CCDC121	1.082053	1.1026765	1.06502267	0.6332772	-0.659091	0.56299
SPIN1	1.998368	1.9710663	2.02091397	1.0513329	0.0722196	0.56303
NANOS1	1.059099	1.0387917	1.07586973	1.9558248	0.96777712	0.56309
DNAJC27-AS1	1.168387	1.1472092	1.18587545	1.2626616	0.33646806	0.56329
GOLGA6L9	1.229313	1.2075747	1.24726563	1.1912128	0.25243113	0.56347
PHF8	1.151444	1.1305744	1.16867873	1.2918204	0.36940554	0.56347
DCDC2	1.042862	1.0227912	1.05943637	2.6078689	1.38287132	0.56349
FRYL	1.565228	1.5899475	1.54481495	0.9234974	-0.1148202	0.56357
VSX1	1.050277	1.0299325	1.06707791	2.2409749	1.16412647	0.5636
DYNLT3	1.785976	1.8122008	1.76431839	0.9410461	-0.0876627	0.56363
TMEM143	1.132246	1.153078	1.11504324	0.7515336	-0.4120905	0.56367
NHLRC3	2.04134	2.0135161	2.0643168	1.0501232	0.07055858	0.56367
MYBPHL	1.042436	1.0224912	1.05890678	2.6191044	1.38907358	0.56367
CUL4A	1.412705	1.3892702	1.43205874	1.1099199	0.15045554	0.56369
ADGRA3	1.352148	1.3294146	1.37092074	1.1259997	0.17120643	0.56373
RAPGEFL1	1.090565	1.0701156	1.10745177	1.5324944	0.61588182	0.56374
AMBRA1	1.090689	1.0701598	1.10764322	1.5342572	0.61754034	0.56374
GPR161	1.304335	1.2821299	1.32267227	1.1437009	0.19370982	0.56377
FARP1	2.040248	2.0116111	2.06389671	1.0516855	0.07270332	0.56385
CFAP126	1.038844	1.0189092	1.05530715	2.924886	1.54838038	0.56394
ANKRD40	1.319746	1.3424454	1.30099979	0.8789715	-0.1861117	0.56416
WEE1	1.660476	1.6349315	1.68157127	1.0734564	0.10226363	0.56424
RANBP3	1.307202	1.329257	1.28898945	0.8777017	-0.1881975	0.56424
NVL	1.212268	1.233991	1.19432922	0.8304985	-0.2679505	0.56425
NR3C1	1.174674	1.1533961	1.1922453	1.253261	0.3256869	0.56432
ZNF574	1.131002	1.1518341	1.11379857	0.7494927	-0.4160137	0.56432
PITX3	1.08471	1.0644137	1.10147021	1.5752898	0.65561726	0.56441
IRF2BPL	1.906435	1.9344431	1.88330525	0.9452745	-0.0811947	0.56446
RANBP6	1.177154	1.1558472	1.19474993	1.2496207	0.3214903	0.56484
LYRM9	1.38153	1.3585692	1.40049126	1.1169147	0.15951903	0.56491
TNNI1	1.38748	1.410848	1.3681819	0.8961511	-0.1581862	0.56523
COL4A6	1.188696	1.1675691	1.20614227	1.2301928	0.29888443	0.5653
AGPAT1	1.739056	1.71408	1.75968231	1.0638616	0.08931046	0.56547
SON	4.027011	4.0820666	3.98154527	0.9673851	-0.0478378	0.56551
MEX3D	1.192433	1.1713704	1.20982753	1.2244091	0.29208564	0.56561
ZFAND4	1.113035	1.1338449	1.0958503	0.7161295	-0.4817076	0.56576
RRP7A	1.459744	1.4830029	1.4405372	0.9120798	-0.1327681	0.56583
CSF1R	1.032771	1.0129919	1.04910412	3.7795891	1.91822941	0.56587
DOC2B	1.458173	1.481732	1.43871673	0.910707	-0.1349412	0.56601
WNK1	2.09105	2.0626358	2.11451413	1.0488204	0.06876764	0.56611
TUBGCP5	1.235969	1.2141412	1.25399391	1.1861047	0.24623133	0.56618

IGFBP4	1.719117	1.7442476	1.69836401	0.938349	-0.0918035	0.56633
NFATC4	1.417286	1.4401095	1.39843816	0.905316	-0.1435067	0.5664
SPATA17	1.09371	1.1139728	1.07697648	0.6753936	-0.5661995	0.56645
LRRC8C	1.053195	1.0331809	1.06972233	2.10128	1.07126842	0.56662
ABCC4	1.199535	1.2209569	1.18184496	0.8229883	-0.2810561	0.56677
DHX36	2.493803	2.5335384	2.46098856	0.9526912	-0.0699194	0.56696
BCL2L11	1.208802	1.1873661	1.22650494	1.2088895	0.27368232	0.56701
EARS2	1.148564	1.1692234	1.13150358	0.7771006	-0.3638268	0.56713
C9orf142	2.535859	2.569403	2.50815757	0.9609753	-0.0574287	0.56727
FAM76A	1.251242	1.2729345	1.2333277	0.8548852	-0.2261974	0.56737
NEO1	1.408022	1.3853647	1.42673192	1.1073457	0.14710565	0.56757
FOXK2	1.196057	1.1750725	1.21338606	1.2188437	0.28551317	0.56765
ARHGEF37	1.103416	1.0830302	1.12025085	1.4482793	0.53433985	0.56778
ERAP1	1.105899	1.0852682	1.1229358	1.4417547	0.52782572	0.5678
ABCB4	1.136716	1.1158828	1.1539209	1.3282463	0.40952271	0.56796
FAM227A	1.527752	1.5042137	1.54718956	1.0852334	0.11800538	0.568
CNTN5	1.365571	1.3426394	1.38450862	1.1221962	0.16632488	0.56802
ANKRD13B	1.140287	1.1609569	1.12321732	0.76553	-0.3854691	0.56805
INVS	1.101738	1.1221289	1.08489953	0.695163	-0.5245768	0.56816
USP38	1.153875	1.1331897	1.1709572	1.2835613	0.36015221	0.56829
NPTXR	1.080348	1.0601597	1.09702033	1.612714	0.68949065	0.56834
TRIB3	1.151367	1.1305171	1.16858604	1.2916781	0.36924655	0.56852
SLC35A4	1.428624	1.4514891	1.40974093	0.9075323	-0.1399791	0.5686
MRAS	1.31049	1.2885961	1.32857061	1.1385138	0.18715176	0.56875
STON2	1.505273	1.4813868	1.52499941	1.0905979	0.12511932	0.5689
COG6	1.245537	1.2671954	1.22765059	0.8520005	-0.2310738	0.56921
OPRK1	1.10865	1.1289777	1.09186291	0.7122386	-0.4895674	0.56935
NLGN1	1.237801	1.2594514	1.21992209	0.8476428	-0.2384717	0.56953
PRICKLE3	1.103667	1.1238826	1.08697295	0.7020592	-0.5103354	0.56968
KANSL1	1.486741	1.4635226	1.50591515	1.0914573	0.12625571	0.56969
RP11-672L10.6	1.220987	1.2423832	1.20331739	0.8388263	-0.253556	0.56987
FAM206A	1.132744	1.1121375	1.14976038	1.335507	0.41738756	0.57039
NMRK1	1.322762	1.3008678	1.34084191	1.1328626	0.17997283	0.57058
CERCAM	1.419063	1.3962916	1.43786771	1.1049129	0.14393265	0.57064
USP6	1.073055	1.0529491	1.0896596	1.6933177	0.7598527	0.57067
KLHDC8A	1.209563	1.2311185	1.19176194	0.8297127	-0.2693163	0.57073
TCEAL3	2.761597	2.7187745	2.79696096	1.0454897	0.06417882	0.57073
WBP4	1.899192	1.9250623	1.87782789	0.9489392	-0.0756124	0.57102
HECA	1.129896	1.1094506	1.14677961	1.3410582	0.42337181	0.57122
RGS4	1.066972	1.0471259	1.08336184	1.7689171	0.82286642	0.57132
PALB2	1.114033	1.1342996	1.0972973	0.7244795	-0.4649831	0.57139
CYLD	1.18508	1.164168	1.20235018	1.23258	0.3016813	0.57142
ZBTB2	1.074201	1.0940909	1.05777591	0.6140434	-0.7035874	0.57144
MCF2L2	1.0972	1.0770643	1.11382922	1.4770688	0.56273705	0.57157
SMIM1	1.862597	1.8370965	1.88365484	1.0556188	0.07808895	0.57172

ABLIM2	1.114846	1.1352148	1.09802541	0.7249606	-0.4640255	0.57179
SAR1A	2.198677	2.232226	2.17097249	0.9502903	-0.0735598	0.5718
PUS10	1.129875	1.1501455	1.1131357	0.753507	-0.4083072	0.57191
PLAA	1.226363	1.2476771	1.20876055	0.8428738	-0.2466115	0.57196
NXPE3	1.284507	1.2627	1.30251539	1.1515623	0.20359249	0.57198
ABCA7	1.069033	1.0491792	1.08542821	1.7370785	0.79666291	0.57207
LINC00475	1.123715	1.1442669	1.10674265	0.7398969	-0.4346039	0.57218
FOCAD	1.234539	1.2133897	1.25200438	1.1809584	0.23995819	0.57219
RBM15	1.178691	1.1577938	1.19594748	1.2417948	0.31242682	0.57229
COL11A1	2.225535	2.193616	2.25189495	1.0488256	0.06877478	0.57246
RP11-2E11.9	1.146449	1.167095	1.12939889	0.774403	-0.3688436	0.5725
KLHL15	1.146705	1.1674174	1.12960098	0.7741189	-0.369373	0.57267
NAP1L5	1.175564	1.1964254	1.15833554	0.8060847	-0.3109967	0.57278
RAB22A	1.390233	1.3675888	1.40893383	1.1124763	0.15377457	0.5728
PTPMT1	2.104574	2.1339381	2.0803254	0.9527199	-0.069876	0.57281
CLIP3	1.531901	1.5558952	1.51208599	0.9211916	-0.1184269	0.57302
C20orf196	1.115846	1.1360304	1.09917826	0.729089	-0.4558331	0.57308
ABCA3	1.195858	1.1750018	1.21308224	1.2176	0.28404025	0.57312
FOXRED2	1.182083	1.2028897	1.16490076	0.8127608	-0.2990974	0.57317
MSL2	1.181989	1.2028271	1.16478116	0.812422	-0.2996989	0.57317
IKBKAP	1.191164	1.2119123	1.17402919	0.8212322	-0.2841379	0.57321
PNMAL2	1.086669	1.1067293	1.07010236	0.6568236	-0.606422	0.57328
BUD13	1.166089	1.1865978	1.14915256	0.7993266	-0.3231429	0.57345
LDOC1L	1.289799	1.3115514	1.27183585	0.8725234	-0.1967343	0.5735
POLQ	1.142006	1.121525	1.15892004	1.3077143	0.38704743	0.57379
SLC17A7	1.047645	1.0670735	1.03160062	0.471134	-1.0857907	0.57389
IL11	1.071716	1.0519404	1.08804725	1.6951586	0.76142028	0.57405
LINC01134	1.032644	1.0521725	1.01651672	0.3165788	-1.6593634	0.57414
FBXL2	1.304445	1.3262868	1.28640803	0.8777799	-0.1880688	0.57435
SFXN3	1.411469	1.3890583	1.42997564	1.1051702	0.14426861	0.57446
HACE1	1.149358	1.1698635	1.13242491	0.7795959	-0.3592015	0.57449
CLCN4	1.249794	1.2285325	1.26735227	1.1698656	0.22634285	0.57457
PACRGL	1.217173	1.2380924	1.19989761	0.8395799	-0.2522604	0.57465
GINS1	1.190073	1.1691277	1.20737079	1.2261195	0.29409959	0.57478
PRIM1	1.313209	1.3347997	1.29537856	0.8822544	-0.1807333	0.57497
PKIA	1.451913	1.4293914	1.47051191	1.0957646	0.13193796	0.575
RPN1	1.487906	1.4650833	1.50675272	1.0895956	0.12379285	0.5754
NUP62CL	1.090759	1.110552	1.07441441	0.673117	-0.5710708	0.57556
TJAP1	1.191032	1.170302	1.20815078	1.222245	0.28953346	0.57566
AC069277.2	1.307637	1.2857734	1.32569172	1.1396852	0.1886354	0.57596
SKA3	1.137541	1.1577269	1.12087173	0.7663356	-0.3839518	0.57598
COX14	3.037723	2.9980648	3.07047313	1.0362392	0.05135713	0.576
PSMG4	1.522721	1.4990748	1.54224912	1.0865086	0.11969966	0.57603
ANKRD36C	1.606609	1.5827583	1.62630478	1.0747247	0.10396717	0.57617
FBXO8	1.2207	1.241674	1.20337888	0.8415423	-0.2488923	0.57622

ICOSLG	1.046505	1.0270616	1.06256199	2.3118357	1.20903888	0.57624
WBSCR17	1.181291	1.1604734	1.19848162	1.2368507	0.30667134	0.57627
P4HA2	1.530362	1.5069017	1.54973543	1.0845011	0.11703157	0.57659
UPK1B	1.03433	1.0149397	1.05034314	3.3697601	1.75264589	0.57668
EPHA4	1.22156	1.2004158	1.23902194	1.1926304	0.25414697	0.57676
ANAPC4	1.204169	1.2253041	1.18671549	0.8287264	-0.2710322	0.57688
PKDCC	1.343497	1.365899	1.3249964	0.8882134	-0.1710218	0.5769
TRAPPC6B	1.295509	1.3172212	1.27757929	0.8750339	-0.1925891	0.57705
AHCYL2	1.168837	1.1483064	1.18579205	1.2527583	0.32510806	0.57705
RBM19	1.254285	1.2753583	1.23688299	0.8602717	-0.2171357	0.57706
AC016549.1	1.098891	1.0789834	1.11533051	1.4601865	0.54615267	0.57713
DBT	1.297677	1.3192668	1.27984725	0.8765311	-0.1901229	0.57722
PTDSS2	1.366542	1.3887153	1.34823091	0.8958508	-0.1586696	0.57723
RP11-77P16.4	1.084597	1.1045052	1.06815564	0.6521748	-0.6166695	0.57725
ATG9B	1.101244	1.0811365	1.11784853	1.4524717	0.53851001	0.57729
RP11-1055B8.9	1.167126	1.1465492	1.18411917	1.2563646	0.32925524	0.57729
STX12	1.405855	1.4280359	1.38753785	0.9053863	-0.1433946	0.5773
ARL13B	1.331574	1.3530619	1.31382914	0.8888785	-0.1699419	0.57755
TMEM132C	1.036418	1.0169854	1.05246663	3.0889312	1.62710775	0.57756
C8orf58	1.217988	1.1971954	1.23515843	1.1925146	0.25400698	0.57782
AC108142.1	1.17367	1.1940255	1.15686007	0.8084507	-0.3067683	0.57798
RP11-527D7.1	1.054341	1.073969	1.03813127	0.5155034	-0.9559461	0.57803
SLC25A11	2.199684	2.2296028	2.17497611	0.9555737	-0.0655609	0.57808
FAF1	1.202081	1.2226601	1.18508565	0.8312475	-0.2666499	0.57828
ZNF438	1.109272	1.1290367	1.09294965	0.7203347	-0.4732606	0.57842
GDI1	1.82797	1.8535679	1.80683158	0.945246	-0.0812383	0.57842
ZNF652	1.442599	1.4654543	1.4237245	0.9103461	-0.135513	0.57851
C5orf51	1.222656	1.2432929	1.20561399	0.8451293	-0.242756	0.57855
ARL4D	1.433638	1.4117118	1.45174538	1.0972368	0.13387494	0.57876
SEPT1	1.528877	1.5524696	1.50939442	0.9220316	-0.1171119	0.57876
ZNF768	1.25783	1.2789637	1.24037762	0.8616807	-0.2147748	0.57877
ERF	1.220768	1.200173	1.23777622	1.1878534	0.24835683	0.57879
CBWD5	1.464701	1.4874034	1.44595305	0.9149568	-0.1282244	0.5788
TRIP4	1.182813	1.2032559	1.16593102	0.816365	-0.2927137	0.57885
PDCD7	1.580413	1.5562512	1.600366	1.0793073	0.11010574	0.57889
USP25	1.149462	1.1292657	1.16614109	1.2852683	0.36206957	0.5789
IL17RD	1.2033	1.1827114	1.22030187	1.2057369	0.26991518	0.57894
CTD-2336O2.1	1.761397	1.7366625	1.78182255	1.0613035	0.08583734	0.57896
CCDC189	1.191971	1.1715095	1.20886828	1.2178234	0.28430495	0.57903
NOTCH2NL	1.105439	1.1252437	1.08908438	0.7112884	-0.4914935	0.57916
PNPLA6	1.218055	1.1975106	1.23502077	1.1899149	0.25085845	0.57917
PLXDC1	1.076436	1.0569887	1.09249557	1.6230501	0.69870751	0.5793
AP3B1	1.439503	1.4621271	1.4208195	0.9106141	-0.1350882	0.57968
SUV39H1	1.075054	1.0946149	1.05889983	0.6225215	-0.6838043	0.58
FAM50B	1.112743	1.1325505	1.09638604	0.7271649	-0.4596455	0.58014

FER1L5	1.049008	1.0683509	1.03303482	0.4833122	-1.0489727	0.58015
SLC30A5	1.390849	1.4128625	1.37266938	0.9026477	-0.1477651	0.58029
STK36	1.16039	1.1402468	1.1770244	1.2622352	0.33598076	0.58033
RP11-1017G21.5	1.115045	1.0952456	1.13139617	1.3795516	0.46419943	0.5804
RP11-126K1.6	1.182865	1.20329	1.16599751	0.8165551	-0.2923778	0.58078
ZNF486	1.098174	1.1179013	1.08188301	0.694505	-0.5259431	0.58095
TAP1	1.159932	1.1398386	1.17652627	1.2623572	0.3361202	0.58105
PARD3	1.388389	1.3663928	1.40655401	1.1096125	0.15005595	0.58106
OSTF1	1.203896	1.2243737	1.18698585	0.833368	-0.2629745	0.58108
DLG3	1.185193	1.1647902	1.20204252	1.2260593	0.29402879	0.58115
ZNF793	1.438814	1.4165032	1.45723924	1.0978049	0.13462172	0.58123
MYOZ1	1.100732	1.0809387	1.11707799	1.4465015	0.53256777	0.58125
VPS54	1.185383	1.1650562	1.20216945	1.2248521	0.29260761	0.58153
BAG5	1.374281	1.3526229	1.39216597	1.1121399	0.15333828	0.58159
PRMT3	1.11801	1.137685	1.10176174	0.739091	-0.4361762	0.58172
ZNF526	1.13183	1.1518843	1.11526798	0.7589198	-0.3979808	0.58175
SERF2	25.02224	25.379812	24.7269568	0.9732215	-0.0391599	0.58185
ELMSAN1	1.237788	1.2169899	1.25496276	1.1749983	0.23265866	0.58197
RBBP8	1.244416	1.2235438	1.26165308	1.1704778	0.22709752	0.58232
TXNDC16	1.279981	1.3011303	1.26251573	0.8717678	-0.1979842	0.58239
WHAMM	1.358476	1.3800251	1.34067997	0.896467	-0.1576777	0.58263
LLNLF-187D8.1	1.19326	1.2135847	1.17647462	0.8262512	-0.2753476	0.58285
TENM3	1.697623	1.6729993	1.71795766	1.0668031	0.09329387	0.58286
MEF2C	1.194571	1.1742798	1.21132865	1.2125824	0.27808274	0.58288
ANKRD10	2.027526	2.0009549	2.04946799	1.0484668	0.06828123	0.58295
CLSTN3	1.232712	1.212223	1.24963201	1.176272	0.2342217	0.58303
FAM122C	1.111468	1.1309873	1.09534933	0.7279279	-0.4581325	0.58309
TCTE3	1.151025	1.1710668	1.13447381	0.7860894	-0.3472347	0.58313
TNRC6A	1.788808	1.8138381	1.76813692	0.9438449	-0.0833783	0.58339
NLE1	1.16456	1.1845677	1.14803816	0.8020806	-0.3181809	0.58347
DNAJB11	1.806662	1.8318021	1.7859001	0.9448162	-0.0818944	0.5836
WDR89	1.19267	1.2128471	1.17600827	0.8269233	-0.2741745	0.58362
TOP3A	1.180474	1.1601302	1.19727468	1.231964	0.30096008	0.58372
IRF2	1.242051	1.2629214	1.22481524	0.8550664	-0.2258917	0.58375
FGF18	1.047144	1.0280823	1.06288615	2.2393495	1.16307971	0.58384
KLHL30	1.04241	1.0233542	1.05814715	2.4897965	1.31602785	0.58409
CCDC57	1.20075	1.1802344	1.21769269	1.2078309	0.27241852	0.5842
DUOX1	1.200708	1.1802785	1.21757942	1.2069069	0.27131444	0.5842
SPINK1	1.033536	1.0146773	1.04910958	3.3459506	1.74241614	0.58428
CHPF2	1.135759	1.1159605	1.15210829	1.3117253	0.39146562	0.58437
EFNB1	2.033216	2.0065631	2.05522684	1.0483465	0.06811561	0.58454
RP11-386G11.10	1.245679	1.2662767	1.22866996	0.8587682	-0.2196593	0.58456
TUFT1	1.129863	1.1099316	1.14632287	1.3310353	0.41254881	0.58457
ZNF781	1.198131	1.2182782	1.18149277	0.8314747	-0.2662557	0.5846
OLFM3	1.180788	1.2008999	1.16417992	0.8172226	-0.2911989	0.58466

HEBP2	2.53869	2.5723728	2.51087473	0.9608884	-0.0575593	0.58472
CNRIP1	1.219645	1.2401396	1.20271995	0.8441756	-0.244385	0.58472
HIPK2	1.935927	1.9104322	1.95698057	1.0511277	0.07193799	0.58475
APC	1.42251	1.4006434	1.44056764	1.0996504	0.13704496	0.5848
RP11-9G1.3	1.068801	1.0495666	1.08468555	1.7085194	0.77274666	0.58483
RP11-73K9.2	1.09507	1.1146231	1.07892295	0.6885432	-0.5383809	0.58494
ATF2	1.739436	1.7637884	1.71932614	0.9417871	-0.0865271	0.58518
NPDC1	2.625747	2.6598667	2.59756959	0.9624686	-0.0551886	0.58528
SLC45A4	1.039418	1.0204506	1.05508137	2.6933875	1.42942179	0.58535
USB1	1.572021	1.5952232	1.55285995	0.928828	-0.1065167	0.58536
RGAG4	1.139354	1.1591989	1.12296486	0.7723975	-0.3725846	0.58541
MON2	1.238467	1.2591407	1.22139517	0.8543436	-0.2271117	0.58576
NDEL1	1.17909	1.1591264	1.19557685	1.2290659	0.29756226	0.58581
UBE2Q1	1.246289	1.2256266	1.26335277	1.1672063	0.22305956	0.58603
SF3B3	1.650229	1.6737505	1.63080493	0.936259	-0.0950205	0.58607
VANGL2	1.292358	1.2716752	1.30943903	1.1390037	0.18777248	0.58614
NPR2	1.06814	1.0489843	1.08395901	1.7139968	0.77736445	0.58624
LEPROT	2.537786	2.5031825	2.5663624	1.0420308	0.05939789	0.58646
WDHD1	1.238079	1.2584245	1.22127776	0.8562569	-0.2238844	0.5865
AP001347.6	1.248846	1.2690308	1.23217673	0.8630118	-0.2125477	0.58658
MGAT3	1.103059	1.0837631	1.11899393	1.4206011	0.50650155	0.58662
RP11-432J24.5	1.082791	1.0634516	1.09876182	1.55649	0.63829629	0.58665
ZNF783	1.188144	1.1679729	1.2048024	1.2192585	0.28600407	0.58667
CRYBB1	1.035867	1.0170541	1.05140267	3.0140859	1.59172054	0.5867
ZMPSTE24	1.242674	1.2631128	1.22579601	0.8581719	-0.2206614	0.58679
TOMM5	1.656876	1.6803912	1.63745687	0.9368976	-0.0940367	0.58682
AATK	1.09501	1.0757726	1.11089657	1.4635439	0.54946602	0.58697
CDADC1	1.27673	1.2978777	1.25926502	0.8703741	-0.2002925	0.58699
CHST9	1.102424	1.1218254	1.08640243	0.7092318	-0.4956708	0.58699
ZNF485	1.058701	1.077741	1.04297718	0.5528248	-0.8551059	0.58707
USP21	1.130108	1.1496432	1.11397549	0.7616481	-0.3928035	0.58728
RFX7	1.266593	1.2459724	1.28362209	1.1530646	0.2054734	0.5873
DOCK11	1.077973	1.0588236	1.09378636	1.5943673	0.67298405	0.5873
SLC2A6	1.127911	1.1083418	1.14407134	1.3297854	0.4111934	0.58735
IRX2	1.050048	1.031013	1.06576673	2.1206181	1.08448482	0.58746
KIF1B	1.956465	1.9831897	1.9343945	0.9503705	-0.073438	0.58797
CHD2	1.738673	1.7146201	1.75853599	1.0614535	0.08604122	0.58807
XPR1	1.477829	1.4558629	1.49596945	1.0879794	0.12165119	0.58845
RP11-196G18.23	1.088438	1.1076572	1.07256685	0.6740549	-0.569062	0.58845
ABHD14A-ACY1	1.104242	1.1237776	1.08810959	0.7118377	-0.4903798	0.58848
CCZ1B	1.360644	1.3817622	1.34320487	0.8990018	-0.1536041	0.5885
RASEF	1.047027	1.0282113	1.06256567	2.217751	1.14909736	0.58888
FAM210A	1.495893	1.5180511	1.4775936	0.9219044	-0.117311	0.58895
CLDN11	1.166051	1.1856661	1.14985244	0.8071071	-0.309168	0.58903
LINC00115	1.178131	1.1581615	1.19462252	1.2305307	0.2992806	0.58903

KCTD7	1.187565	1.207619	1.1710043	0.8236447	-0.279906	0.58912
GMEB1	1.2567	1.2773693	1.2396316	0.8639442	-0.2109899	0.58923
DGKB	1.041174	1.0223802	1.05669391	2.5332146	1.3409693	0.58945
LRP4-AS1	1.108861	1.1280538	1.09301106	0.7263434	-0.4612764	0.58948
PALM	1.559395	1.5370264	1.57786652	1.0760487	0.10574338	0.58959
SYCE3	1.04478	1.0637503	1.02911342	0.4566791	-1.1307473	0.58969
RP4-798A10.2	1.044751	1.0636526	1.02914128	0.4578176	-1.1271551	0.58969
C16orf74	1.06714	1.048113	1.08285288	1.7220481	0.78412543	0.58971
FCF1	1.222883	1.2431714	1.20612875	0.8476685	-0.238428	0.58973
UVSSA	1.149357	1.1296737	1.16561261	1.2771485	0.35292632	0.58993
GIGYF1	1.369586	1.391138	1.35178772	0.8993954	-0.1529726	0.58997
ZMYM4	1.691276	1.7146366	1.67198385	0.9403155	-0.0887832	0.59031
RP11-497H16.9	1.046469	1.0276989	1.06197	2.2372729	1.16174124	0.59038
RAD1	1.38261	1.4041636	1.36480995	0.9026294	-0.1477944	0.59049
PURA	1.344118	1.3653012	1.32662465	0.8941242	-0.1614529	0.59069
ARMCX4	1.209538	1.2299322	1.19269589	0.8380553	-0.2548826	0.59086
MUS81	1.429867	1.4517193	1.41182187	0.9116764	-0.1334062	0.5909
RPRM	1.547256	1.5693438	1.52901642	0.9291687	-0.1059876	0.59095
DCUN1D1	1.488744	1.5106767	1.47063122	0.9215834	-0.1178133	0.591
GID4	1.180771	1.1609352	1.19715131	1.2250353	0.29282336	0.59113
RBMS2	1.180879	1.1612788	1.19706573	1.2218946	0.28911989	0.59113
COQ10A	1.274633	1.295209	1.25764076	0.8727401	-0.1963759	0.5913
PAQR6	1.102697	1.0835328	1.11852265	1.4188754	0.50474785	0.59138
FZD2	1.41468	1.4359416	1.39712117	0.9109505	-0.1345555	0.59139
GXYLT2	1.086735	1.1060174	1.0708106	0.6679148	-0.5822639	0.59161
ELOVL5	1.65133	1.6283935	1.67027121	1.0666425	0.09307669	0.59162
MYF6	1.096954	1.1160623	1.08117481	0.6994075	-0.5157948	0.59182
PYCARD	1.062025	1.0432172	1.07755606	1.794563	0.8436326	0.59185
CASC3	1.574489	1.5514617	1.5935052	1.0762401	0.10600003	0.59186
LDB1	1.190243	1.1704629	1.20657699	1.2118589	0.27722176	0.59198
SLC2A10	1.201057	1.1809981	1.21762234	1.2023457	0.26585173	0.59204
C3orf67	1.232472	1.2123732	1.24907079	1.1727974	0.22995385	0.59209
NIPAL4	1.030853	1.0123699	1.04611606	3.7280926	1.89843771	0.59213
RAVER2	1.121022	1.1016003	1.13706007	1.3490119	0.43190305	0.59218
ARHGEF11	1.098108	1.0788438	1.11401715	1.4461151	0.53218243	0.59219
MYCL	1.110149	1.1293639	1.09428038	0.7288	-0.4564051	0.59225
ZFX	1.508528	1.5306781	1.49023699	0.9237935	-0.1143576	0.59265
CLINT1	1.544775	1.5225631	1.56311776	1.0776073	0.10783151	0.59265
STK35	1.172983	1.1531554	1.18935738	1.2363741	0.30611536	0.59268
CA14	1.361782	1.3829604	1.34429262	0.8990292	-0.1535601	0.59292
EMC1	1.148394	1.1287686	1.16460104	1.2782705	0.35419317	0.59304
NADSYN1	1.244163	1.2643729	1.22747256	0.8604231	-0.2168818	0.59312
CTD-2034I4.2	1.958958	1.9854849	1.93705106	0.9508527	-0.0727062	0.59315
RFX2	1.2858	1.3063043	1.26886677	0.8777768	-0.188074	0.59317
GEMIN6	1.387409	1.4085324	1.36996484	0.905595	-0.1430622	0.59317

CAMK1	1.355007	1.3338261	1.37249787	1.1158441	0.15813546	0.59337
TSC22D4	1.680352	1.6572356	1.69944113	1.0642168	0.08979209	0.5934
HSD17B1	1.115858	1.1350451	1.10001353	0.7405936	-0.433246	0.59341
RP11-793H13.11	1.067454	1.0861793	1.05199051	0.6032831	-0.729093	0.59342
ZDHHC20	1.312229	1.2917377	1.3291508	1.1282422	0.1740768	0.59342
USP33	1.512231	1.4899181	1.53065778	1.0831561	0.11524121	0.59359
SH3KBP1	1.435134	1.4137912	1.45275848	1.0941714	0.12983872	0.59365
GOLGB1	2.353398	2.3220734	2.3792655	1.0432594	0.06109789	0.59383
ZNF431	1.428754	1.4504107	1.41086988	0.9122116	-0.1325595	0.59391
RGL1	1.11124	1.0920982	1.12704786	1.3794821	0.46412671	0.594
CENPU	1.606087	1.6284053	1.58765611	0.9351546	-0.0967232	0.59402
MAPK8IP1	1.197399	1.2174827	1.18081366	0.8313933	-0.266397	0.59406
WAC	1.962293	1.9876447	1.9413572	0.9531335	-0.0692498	0.59407
SASS6	1.236853	1.2569624	1.22024711	0.8571182	-0.2224339	0.5942
CYP26A1	1.107385	1.1291835	1.08938437	0.691918	-0.531327	0.59421
SPRY2	1.173619	1.1541314	1.18971224	1.2308477	0.29965228	0.59446
RNASEH2C	2.907709	2.9427033	2.87881015	0.9671112	-0.0482463	0.59448
SNX12	1.474701	1.496629	1.45659198	0.9193825	-0.121263	0.59455
MXD1	1.108386	1.0894046	1.12406137	1.3876399	0.47263324	0.59467
LINC00920	1.055934	1.0372637	1.07135154	1.9147717	0.93717236	0.59475
AHCTF1	1.321831	1.3426487	1.30463881	0.8890703	-0.1696306	0.59481
NAPRT	1.17405	1.1937833	1.15775328	0.8140704	-0.2967745	0.59496
FBLN5	1.041328	1.0227778	1.05664665	2.4869289	1.31436523	0.595
CCND2-AS1	1.078608	1.0598858	1.09406828	1.5707939	0.65149394	0.59508
PTEN	1.508232	1.486474	1.52619942	1.08166	0.1132471	0.59516
EBF1	1.048128	1.0670104	1.03253489	0.4855199	-1.0423977	0.59516
EGLN2	1.857449	1.8328526	1.87776106	1.0539212	0.07576705	0.5954
NF1	1.948864	1.9242506	1.9691895	1.048622	0.06849474	0.59541
COG8.1	1.100728	1.0815497	1.11656524	1.4293767	0.51538621	0.5955
CADM3	1.210173	1.1904438	1.22646569	1.1891469	0.24992701	0.59551
NAB2	1.38997	1.4108221	1.37274945	0.9073258	-0.1403074	0.59557
VAMP1	1.137197	1.1564929	1.12126246	0.7748749	-0.3679647	0.59562
SART1	1.206686	1.2265152	1.19031116	0.8401694	-0.2512478	0.59562
LUZP1	1.565677	1.5434115	1.58406427	1.0748102	0.10408191	0.59588
TMEM186	1.262779	1.2422569	1.27972574	1.1546657	0.20747526	0.59603
KMT2B	1.141112	1.1218013	1.15705938	1.2894719	0.36678036	0.59607
HVCN1	1.06874	1.0874089	1.05332348	0.6100465	-0.713009	0.59614
PPAN	1.361548	1.3823751	1.34434885	0.9005524	-0.1511178	0.59623
FAM98A	1.293498	1.2729296	1.31048326	1.1375947	0.1859867	0.59645
ZNF20	1.054957	1.0736275	1.03953912	0.5370155	-0.8969645	0.59649
ZBTB20	2.269874	2.2408841	2.2938142	1.0426552	0.06026214	0.59659
NAB1	1.321533	1.3009087	1.3385651	1.1251424	0.17010765	0.59659
RABEP2	1.213113	1.2330568	1.19664339	0.8437574	-0.2450998	0.59659
TLE2	1.132273	1.1514512	1.11643448	0.7687922	-0.3793345	0.5969
SLC27A5	1.664589	1.6879826	1.64527087	0.9379175	-0.0924671	0.59692

EMILIN3	1.113164	1.0941881	1.12883489	1.3678462	0.45190606	0.59693
MAFG-AS1	1.40194	1.4229411	1.38459699	0.9093393	-0.1371094	0.597
PSEN2	1.179765	1.1993448	1.1635956	0.8206664	-0.2851323	0.59701
KREMEN2	1.092858	1.0739694	1.10845606	1.4662292	0.55211067	0.5971
CTAGE5	1.150082	1.1309199	1.16590666	1.2672376	0.34168703	0.59712
ZNF692	1.389501	1.4107744	1.37193395	0.9054458	-0.1432998	0.59714
HMCN1	1.042942	1.0244339	1.05822702	2.3830448	1.25280607	0.59718
OXSR1	1.536951	1.5591032	1.51865711	0.927659	-0.1083334	0.59732
SPHK2	1.311775	1.3324784	1.29467864	0.8863091	-0.1741182	0.59741
EXOSC1	1.479237	1.4574275	1.49724776	1.0870525	0.12042165	0.5977
ETV6	1.163334	1.1826185	1.1474086	0.8071943	-0.3090121	0.59774
LINC01569	1.066259	1.0847892	1.05095624	0.6009758	-0.7346213	0.59792
ERLEC1	2.097456	2.0720171	2.11846419	1.0433268	0.06119117	0.59802
PDZRN4	1.067928	1.0493774	1.08324724	1.6859374	0.75355097	0.59846
B4GALT6	1.131256	1.1122628	1.14694092	1.3089015	0.38835648	0.59848
CXCL3	1.046596	1.0282297	1.06176254	2.1878531	1.12951588	0.59848
SCX	1.105408	1.0864115	1.12109598	1.4013879	0.48685634	0.59848
C4orf33	1.182843	1.2022485	1.16681839	0.8248189	-0.2778508	0.59859
SMAD4	1.447372	1.4687961	1.42968029	0.9165612	-0.1256969	0.5986
PPP2R5A	1.189892	1.1704777	1.20592536	1.2079315	0.2725387	0.59867
SOX3	1.083373	1.102072	1.06793105	0.665521	-0.5874439	0.59872
KIAA1033	1.396215	1.3753977	1.41340581	1.1012477	0.13913896	0.59874
LINC01001	1.130165	1.1111115	1.14590006	1.3130957	0.39297203	0.59879
ZC2HC1A	1.570106	1.5922898	1.55178668	0.9316161	-0.1021926	0.59886
NCS1	1.175276	1.1560215	1.19117707	1.2253255	0.29316503	0.59895
B3GAT1	1.066985	1.0483815	1.08234884	1.7020725	0.7672925	0.599
NAT10	1.261352	1.2814527	1.24475285	0.8696056	-0.2015669	0.59914
PABPC4L	1.069933	1.0885619	1.05454804	0.6159313	-0.6991586	0.5996
RP11-307C12.11	1.066947	1.0854178	1.05169339	0.6051831	-0.7245565	0.59961
CXorf40A	1.170987	1.1902795	1.15505544	0.8148826	-0.2953359	0.59971
NPIPB2	1.055247	1.0736973	1.04000984	0.5428939	-0.8812578	0.59982
FGFR1OP	1.277792	1.2576623	1.29441534	1.1426405	0.19237162	0.59985
UBXN8	1.201951	1.2216809	1.18565725	0.8374976	-0.255843	0.59997
CTB-113I20.2	1.186801	1.2063227	1.17068001	0.8272478	-0.2736085	0.6
C9orf24	1.097529	1.078797	1.11299887	1.4340509	0.52009619	0.60004
RP13-577H12.2	1.029245	1.0476064	1.01408228	0.2958067	-1.7572735	0.60016
ZNF277	1.545615	1.5676585	1.52741139	0.9290998	-0.1060945	0.60017
SAC3D1	1.686511	1.6635693	1.70545734	1.0631254	0.08831178	0.60017
TMEM179B	1.98086	1.9555835	2.00173323	1.0482948	0.06804452	0.60018
LRRC75B	1.101651	1.0828626	1.11716599	1.4139796	0.49976131	0.60019
FZD1	1.302393	1.3226963	1.28562537	0.8851212	-0.176053	0.60025
WAC-AS1	1.681755	1.7049655	1.6625865	0.939885	-0.0894438	0.60027
BRMS1	1.454501	1.4758449	1.43687565	0.9181052	-0.1232687	0.60053
PRKAB1	1.203376	1.2229289	1.18722932	0.839861	-0.2517776	0.60056
ARHGEF5	1.072427	1.0540403	1.08761029	1.621202	0.69706388	0.60069

PIGQ	1.392314	1.4130359	1.37520245	0.9084016	-0.1385978	0.60074
SOS2	1.199546	1.1800541	1.21564186	1.1976504	0.26020688	0.60096
WDSUB1	1.189322	1.208686	1.17333054	0.8305805	-0.2678081	0.60098
INTS2	1.161927	1.1426844	1.17781828	1.2462352	0.31757637	0.60101
KRCC1	1.938755	1.914496	1.95878842	1.0484337	0.06823563	0.60116
RAB36	1.196694	1.2160502	1.18070927	0.8364228	-0.2576958	0.60133
PAQR7	1.181611	1.2012276	1.16541156	0.8220123	-0.2827682	0.60141
NUBPL	1.328228	1.3483533	1.31160883	0.8945196	-0.160815	0.60165
FBXO10	1.094109	1.0755916	1.10940076	1.4472609	0.53332504	0.60174
FASTKD3	1.09546	1.1139014	1.08023028	0.7043838	-0.5055664	0.60177
NFKBIL1	1.710874	1.7341614	1.69164204	0.9420844	-0.0860718	0.60178
RFX5	1.387941	1.4085936	1.37088649	0.9077149	-0.1396889	0.60183
AC007228.11	1.072931	1.0913642	1.05770918	0.631639	-0.6628279	0.60192
CGA	1.03285	1.0146773	1.04785765	3.260654	1.70516138	0.60234
CCDC169	1.068008	1.0864325	1.05279255	0.6107949	-0.7112401	0.60249
RGMB-AS1	1.070892	1.0893592	1.05564138	0.6226709	-0.6834583	0.60252
ANGPTL2	1.101536	1.0827817	1.11702429	1.41365	0.49942501	0.60256
TCEA3	1.089606	1.0710877	1.10489828	1.4756171	0.56131843	0.60272
GDF5	1.176741	1.157561	1.1925801	1.2222576	0.28954833	0.60281
LINC00472	1.042796	1.0246313	1.05779733	2.3465031	1.23051238	0.60316
SERTAD1	1.276632	1.2968953	1.25989814	0.8753864	-0.1920081	0.60317
PLEKHH2	1.086187	1.0677145	1.10144178	1.4980798	0.58311451	0.6033
RP11-544I20.2	1.086957	1.0684989	1.10219956	1.491988	0.57723596	0.60339
CDC23	1.278349	1.2981356	1.26200906	0.8788253	-0.1863517	0.60341
IP6K1	1.313153	1.2929397	1.32984517	1.1259832	0.17118531	0.60345
METTL18	1.160688	1.1795585	1.14510468	0.8081192	-0.3073601	0.60354
MAPK7	1.250927	1.2310658	1.26732861	1.156937	0.21031033	0.60364
GUCA1A	1.620696	1.6437642	1.60164553	0.9345744	-0.0976185	0.60371
GALNT13	1.056955	1.0386527	1.07207021	1.8645604	0.89883552	0.60385
NUP98	1.221329	1.2015975	1.23762289	1.1786995	0.23719599	0.60389
DARS2	1.070982	1.0892245	1.05591735	0.6267039	-0.6741441	0.60395
DHFR	1.954104	1.9285898	1.97517408	1.0501667	0.07061839	0.60398
MTIF3	2.386354	2.417614	2.36053894	0.9597387	-0.0592865	0.6042
RBM14	1.493013	1.4714609	1.51081122	1.0834648	0.11565223	0.60435
WDR27	1.348958	1.3287342	1.36565952	1.1123257	0.15357923	0.60442
RCC2	1.640184	1.6176866	1.6587621	1.066499	0.09288255	0.60454
TMEM183A	2.50008	2.5316382	2.47401875	0.9623805	-0.0553207	0.60457
NEU3	1.073016	1.0914376	1.05780263	0.6321541	-0.6616518	0.6046
ADGRL3	1.272187	1.252414	1.28851605	1.143027	0.1928595	0.6047
ZSWIM5	1.080777	1.0994745	1.06533669	0.6568184	-0.6064336	0.60473
RIPK1	1.103236	1.1218753	1.08784408	0.7207701	-0.4723889	0.60474
ZNF124	1.327574	1.3479812	1.31072064	0.8929236	-0.1633913	0.60478
LHX5	1.033372	1.0515849	1.01833122	0.3553604	-1.4926452	0.60483
C20orf194	1.229891	1.210407	1.24598142	1.1690744	0.22536669	0.60492
UBA3	1.460933	1.4817522	1.44374018	0.9210964	-0.118576	0.60492

POLK	1.518531	1.5402428	1.50060059	0.9266216	-0.1099478	0.60516
CMTM5	1.059562	1.0777299	1.04455948	0.5732607	-0.8027367	0.60522
FCHSD1	1.110789	1.0920544	1.12625969	1.3715777	0.45583634	0.60537
SLC30A6	1.14824	1.1294414	1.16376392	1.2651592	0.33931888	0.60549
EIF1AD	1.215673	1.1962566	1.231707	1.1806332	0.23956079	0.60564
ZNF322	1.849846	1.8740147	1.82988768	0.9495123	-0.0747415	0.60575
PCED1A	1.433239	1.4543507	1.41580522	0.9151636	-0.1278984	0.60578
GLB1	1.43262	1.4535358	1.41534785	0.9157996	-0.1268962	0.60578
PITX2	1.031739	1.0137737	1.04657529	3.3814717	1.75765129	0.60579
C18orf25	1.158119	1.1392018	1.17374068	1.2481211	0.31975787	0.60585
ATXN7L1	1.088179	1.0697315	1.10341317	1.4830196	0.56853768	0.60596
AMN	1.043577	1.0255476	1.05846587	2.2885094	1.19440821	0.60596
SEMA4C	1.230209	1.2107347	1.24629102	1.1687257	0.22493642	0.60611
HNRNPUL2	1.408434	1.387633	1.4256125	1.0979779	0.13484898	0.60624
FAM131C	1.139022	1.1579602	1.12338254	0.7810989	-0.3564229	0.6063
ELOVL7	1.048379	1.0304238	1.06320623	2.0775251	1.05486592	0.60634
SLC22A17	1.52976	1.5083411	1.54744889	1.0769321	0.10692734	0.60636
TCEB3	1.289634	1.3092435	1.27344009	0.8842225	-0.1775187	0.60636
PHKA2	1.145721	1.1269502	1.16122179	1.269961	0.34478421	0.60655
ZNF606	1.29675	1.3169748	1.2800481	0.8835026	-0.1786937	0.60679
NRSN2	1.48767	1.5091516	1.46993077	0.9229682	-0.1156472	0.60696
COLEC12	1.383374	1.3628384	1.40033328	1.1033378	0.14187461	0.60699
NAA30	1.324496	1.3044574	1.34104487	1.1201725	0.16372093	0.6071
CHST1	1.098331	1.0799916	1.11347635	1.4186032	0.50447112	0.60711
NDC1	1.286039	1.3056443	1.26984906	0.882886	-0.1797009	0.60713
GTF2E1	1.150957	1.1699367	1.13528357	0.7960822	-0.3290107	0.60717
MFSD3	1.56367	1.5852856	1.5458201	0.9325706	-0.1007151	0.60718
VPS37B	1.165418	1.14637	1.18114879	1.2376085	0.30755497	0.60721
CFLAR	1.25682	1.2770101	1.24014594	0.8669213	-0.2060271	0.60729
CEBPA-AS1	1.051873	1.0338027	1.06679651	1.9760717	0.98263532	0.60732
MED12L	1.213456	1.1941201	1.22942358	1.1818644	0.24106449	0.60749
UBE2J1	1.822084	1.8464008	1.80200249	0.9475446	-0.0777342	0.60764
PITPNM1	1.354105	1.3744528	1.33730221	0.9007871	-0.1507419	0.60792
GPATCH8	1.779242	1.8022842	1.76021368	0.9475616	-0.0777083	0.60822
LINC00339	1.200138	1.1807414	1.21615575	1.1959395	0.25814435	0.60863
RNF128	1.053249	1.0353612	1.06802059	1.9235946	0.94380479	0.60867
PDE6B	1.155428	1.1364626	1.17109041	1.2537535	0.3262537	0.60867
KIZ	1.318018	1.3383978	1.30118844	0.8900426	-0.1680538	0.60872
RP11-147L13.11	1.121067	1.1025585	1.1363521	1.3295062	0.41089046	0.60883
SNAPC2	1.49939	1.5206033	1.48187173	0.9256025	-0.1115353	0.60898
FBXO28	1.226184	1.2456327	1.21012281	0.8554349	-0.2252701	0.60901
ETAA1	1.395046	1.4154549	1.37819146	0.9103068	-0.1355753	0.6093
EXOSC4	1.60543	1.6272445	1.58741562	0.9365018	-0.0946463	0.60941
GCK	2.117925	2.1679188	2.07663993	0.9218449	-0.1174041	0.60945
TRAK2	1.107756	1.0894417	1.12288092	1.3738658	0.45824107	0.60952

ETV3L	1.032832	1.0150885	1.04748574	3.1471577	1.65404945	0.60954
TUBE1	1.238195	1.2575071	1.22224638	0.863069	-0.2124522	0.60963
AKR1C1	1.08995	1.1082321	1.07485233	0.6915911	-0.5320087	0.60986
C15orf48	1.037754	1.0200564	1.05236862	2.6110684	1.38464028	0.60998
CNOT9	1.494558	1.5165657	1.47638442	0.9222145	-0.1168257	0.61025
LL22NC03-86G7.1	1.087476	1.0692433	1.10253301	1.4807648	0.56634251	0.61028
NUDCD1	1.290389	1.3097803	1.27437471	0.8857074	-0.1750979	0.61041
WDR70	1.39207	1.4125612	1.37514733	0.9093131	-0.1371509	0.61046
RIC1	1.149798	1.1311195	1.16522215	1.2600878	0.33352427	0.61047
NEURL1	1.052584	1.0346237	1.06741581	1.9471015	0.96132806	0.61054
UBE3B	1.326208	1.3062357	1.34270193	1.119079	0.16231191	0.61075
ZNF280C	1.111148	1.1294499	1.0960338	0.7418608	-0.4307796	0.61099
RNF169	1.154484	1.1358378	1.16988144	1.2506195	0.32264293	0.61105
CARMIL1	1.1547	1.1359701	1.17016781	1.2515093	0.32366902	0.61105
S100B	1.234131	1.2150193	1.24991324	1.1622831	0.21696147	0.61129
PRSS56	1.031639	1.0492303	1.01711196	0.3475902	-1.5245406	0.61148
MARCH2	1.227142	1.208199	1.24278473	1.1661187	0.22171466	0.61161
TIGD4	1.120615	1.1389293	1.10549042	0.7593102	-0.3972387	0.61165
RIOK1	1.165838	1.1846313	1.15031852	0.8141553	-0.296624	0.61168
TAF2	1.234829	1.2539258	1.21905781	0.8626844	-0.2130952	0.61174
ZNF629	1.312949	1.332642	1.29668685	0.8919103	-0.1650294	0.61176
CTDSPL	1.184323	1.1657981	1.19962113	1.2040013	0.26783692	0.61181
ARMC9	1.266016	1.2853822	1.25002334	0.8761	-0.1908325	0.61182
PDGFA	1.07219	1.05429	1.08697297	1.6020073	0.6798807	0.61184
COTL1	5.287091	5.2111807	5.34977819	1.0329118	0.04671705	0.61194
ABCF3	1.139814	1.12131	1.1550949	1.2785005	0.35445268	0.61195
EI24	3.63997	3.6826544	3.60472062	0.970949	-0.0425326	0.61199
GPS2	1.238076	1.2572474	1.22224464	0.8639335	-0.2110078	0.61207
KIAA1429	1.273765	1.2933557	1.25758589	0.8780667	-0.1875975	0.61211
CITF22-92A6.1	1.090408	1.1085087	1.07546028	0.695431	-0.5240207	0.61216
DCHS1	1.259091	1.239661	1.2751366	1.1480242	0.19915301	0.6123
RP4-816N1.6	1.118029	1.1362442	1.10298679	0.7558987	-0.4037351	0.61236
MED15	1.416097	1.3956917	1.43294816	1.0941552	0.12981737	0.61238
VPS36	1.547484	1.5684276	1.53018752	0.9327265	-0.100474	0.61266
CLVS2	1.125765	1.1441053	1.11061837	0.7676217	-0.3815327	0.61266
NPIPA5	1.054382	1.0722017	1.03966639	0.5493834	-0.8641147	0.61288
ANKRD65	1.139908	1.1584029	1.12463516	0.7868237	-0.3458876	0.61292
TRIM38	1.156684	1.1382147	1.17193648	1.2439811	0.31496456	0.61304
ZCCHC14	1.220413	1.2394269	1.20471143	0.8550059	-0.2259937	0.61324
EMX1	1.086089	1.1040368	1.07126777	0.6850249	-0.5457716	0.61333
EPHA6	1.086184	1.1041733	1.07132782	0.6847032	-0.5464493	0.6135
IP6K3	1.049758	1.0320918	1.06434724	2.0050973	1.00367225	0.61369
PXYLP1	1.239277	1.2200417	1.25516198	1.1596076	0.21363669	0.61371
FAM126B	1.268052	1.2487902	1.28395793	1.1413549	0.19074743	0.61372
PRR36	1.171205	1.1897578	1.15588423	0.8214906	-0.2836841	0.61374

NBPF20	1.204149	1.2228955	1.18866817	0.8464421	-0.2405167	0.61382
FBXW4	1.270361	1.290006	1.25413834	0.8763211	-0.1904685	0.61386
ADAMTS3	1.106787	1.1248813	1.09184444	0.7354541	-0.4432928	0.61386
RP11-315D16.4	1.037688	1.0552915	1.02315082	0.4187048	-1.2559946	0.61389
TTC21B	1.194029	1.1752733	1.2095185	1.195382	0.25747171	0.61396
XRN1	1.428083	1.4488065	1.41096977	0.9156949	-0.1270612	0.61397
SLC25A14	1.260987	1.2415865	1.27700872	1.1466233	0.19739154	0.614
XK	1.047051	1.0293617	1.06165836	2.0999609	1.07036246	0.61407
FN3K	1.191773	1.1727212	1.20750614	1.2013934	0.26470859	0.61421
RP11-977B10.2	1.035427	1.0530632	1.0208622	0.3931573	-1.3468215	0.61424
WTIP	1.126735	1.1087296	1.14160351	1.3023455	0.38111222	0.61427
PCDH7	3.280485	3.3227504	3.24558127	0.9667768	-0.0487452	0.61436
USP43	1.096151	1.1141423	1.08129422	0.7122184	-0.4896085	0.61437
ATRNL1	1.115135	1.097022	1.13009322	1.3408635	0.42316235	0.61449
EDEM2	1.286886	1.3060995	1.27101853	0.8853937	-0.175609	0.61451
C1orf226	1.067168	1.0493919	1.08184819	1.6571163	0.72867484	0.61454
CYP39A1	1.097343	1.1153144	1.08250189	0.7154518	-0.4830734	0.61456
MOB2	1.360084	1.3799309	1.3436948	0.9046244	-0.1446092	0.61465
POPDC3	1.030793	1.0132661	1.04526735	3.4122624	1.7707286	0.61467
PDK3	1.272498	1.2917825	1.25657305	0.8793297	-0.1855239	0.61473
CSAD	1.578612	1.5567674	1.59665251	1.071637	0.09981634	0.61481
SGCB	1.52746	1.548483	1.51009857	0.9300171	-0.1046709	0.61484
RP11-395B7.4	1.075296	1.0930798	1.06060993	0.6511607	-0.6189146	0.61518
MGAT5	1.158227	1.139792	1.17345008	1.2407722	0.31123825	0.61524
PAX8	1.065152	1.0827976	1.05057919	0.6108772	-0.7110457	0.61538
SERTAD3	1.31887	1.3383465	1.30278646	0.8949006	-0.1602007	0.61543
KSR1	1.091331	1.073439	1.10610598	1.4448186	0.53088841	0.61545
PPP1R37	1.37938	1.3992919	1.36293712	0.9089519	-0.1377241	0.61548
TELO2	1.173497	1.1920211	1.15820006	0.823868	-0.2795149	0.61569
FEM1B	1.438587	1.4183867	1.45526966	1.0881553	0.12188449	0.61571
ENDOV	1.72041	1.742592	1.70209224	0.9454616	-0.0809093	0.61573
KIF13A	1.193082	1.174588	1.20835538	1.1934119	0.25509207	0.61588
INPP4A	1.217511	1.1985218	1.23319317	1.1746479	0.23222837	0.61605
SLC38A7	1.145548	1.1271091	1.16077547	1.2648625	0.33898051	0.61605
C1S	1.104564	1.0866463	1.11936161	1.3775731	0.46212885	0.61615
TRAPPC9	1.109255	1.0911614	1.12419654	1.3623805	0.44612969	0.61624
NSUN4	1.125825	1.1440539	1.11077215	0.7689631	-0.3790138	0.61642
USP36	1.202816	1.2215141	1.18737434	0.8458801	-0.2414749	0.61642
OGDH	1.200678	1.1819809	1.21611887	1.187591	0.24803801	0.61645
APLN	1.049013	1.0666169	1.03447574	0.5175222	-0.9503073	0.61647
USP15	1.358433	1.3385066	1.37488915	1.1074795	0.14727998	0.61648
TTC13	1.084287	1.0663177	1.09912644	1.4947197	0.579875	0.61657
MMRN1	1.237457	1.2566314	1.22162306	0.8635851	-0.2115897	0.61663
PER3	1.091238	1.0733395	1.10601827	1.445582	0.53165045	0.61677
AASS	1.252203	1.2710878	1.23660819	0.87281	-0.1962604	0.61683

KCNK6	1.066134	1.0484735	1.08071863	1.6652101	0.73570421	0.61695
SMIM5	1.034267	1.016834	1.04866398	2.8908182	1.53147789	0.61698
FAM214B	1.390087	1.3702782	1.40644565	1.0976765	0.13445293	0.61701
MAP2K5	1.280571	1.2997816	1.26470602	0.8829963	-0.1795207	0.61709
NR2C1	1.362775	1.3826124	1.34639354	0.905338	-0.1434715	0.6171
TBL3	1.246226	1.2650944	1.23064484	0.8700479	-0.2008332	0.61715
SH3RF2	1.042095	1.0246794	1.05647738	2.2884435	1.19436665	0.61727
RP11-46F15.2	1.044516	1.0621757	1.02993254	0.4814189	-1.0546354	0.61745
CORO2B	1.099496	1.0817055	1.11418848	1.3975624	0.48291269	0.61788
CCDC184	1.080052	1.0978839	1.06532611	0.6673836	-0.5834118	0.61791
RAB20	1.120932	1.1390415	1.10597691	0.762196	-0.391766	0.61793
OGT	1.45675	1.4771401	1.43991105	0.9219745	-0.1172012	0.61802
C22orf46	1.13315	1.1148803	1.14823829	1.2903722	0.36778729	0.61802
LYN	1.228656	1.2474392	1.21314447	0.8614013	-0.2152426	0.6181
PDE3B	1.172647	1.1912153	1.15731375	0.8227046	-0.2815536	0.61818
RP11-1E4.1	1.032795	1.050259	1.01837353	0.3655769	-1.451753	0.61818
PAK6	1.037993	1.0206429	1.05232043	2.5345529	1.3417313	0.61832
KIAA0430	1.347705	1.3279665	1.36400454	1.1098834	0.1504081	0.61839
POLR2M	1.466246	1.4864165	1.44958826	0.9242867	-0.1135877	0.61842
CYBRD1	1.227452	1.2088327	1.24282839	1.1627889	0.21758915	0.61854
FBXO16	1.173018	1.1913194	1.15790512	0.825348	-0.2769255	0.61855
MKS1	1.171991	1.1901801	1.15697004	0.8253759	-0.2768768	0.61863
AK8	1.040316	1.0577389	1.02592705	0.4490396	-1.1550854	0.61864
ADAMTSL4	1.071741	1.0539829	1.08640604	1.6006183	0.67862933	0.61866
ZNF641	1.104962	1.1228744	1.09016887	0.7338298	-0.4464825	0.61867
DGCR14	1.164922	1.183192	1.14983397	0.8179067	-0.2899917	0.61867
FKRP	1.142682	1.1245216	1.15767863	1.2662751	0.34059088	0.61883
LYSMD2	1.112795	1.130856	1.09787936	0.7479927	-0.4189039	0.61888
MLLT3	1.308582	1.2892438	1.3245509	1.1220669	0.16615872	0.6191
PIK3C2B	1.065958	1.048304	1.08053757	1.6673076	0.73752026	0.61912
LINC00909	1.289687	1.3089652	1.27376629	0.8860749	-0.1744995	0.61923
SERTAD2	1.372038	1.3527332	1.38797952	1.0999235	0.13740323	0.61928
RFT1	1.173015	1.1913458	1.15787711	0.8250879	-0.2773803	0.6193
BRICD5	1.070872	1.0532704	1.08540825	1.6032958	0.68104061	0.61958
RSG1	1.074726	1.0923852	1.06014257	0.6509982	-0.6192746	0.61959
KDM4A	1.268743	1.2877565	1.25304089	0.8793575	-0.1854782	0.61966
CLIC6	1.233018	1.214554	1.24826642	1.1571281	0.21054864	0.61979
ZNF784	1.108531	1.1263755	1.09379534	0.7421957	-0.4301284	0.6199
GPR27	1.117606	1.1357176	1.10264895	0.7563424	-0.4028886	0.62008
TIMM21	1.299371	1.2797663	1.31556132	1.1279461	0.17369816	0.62012
GINM1	1.961393	1.936608	1.98186062	1.0483154	0.06807288	0.62012
FAM122B	1.338755	1.3191454	1.35494815	1.1121831	0.15339437	0.6202
COL14A1	1.036109	1.0188445	1.05036548	2.672691	1.41829303	0.62021
VTI1A	1.151549	1.1333305	1.16659339	1.249477	0.32132438	0.62027
FLVCR1-AS1	1.090673	1.0729825	1.1052823	1.4425682	0.52863956	0.62028

SLC13A4	1.041184	1.0238889	1.0554663	2.3218409	1.21526912	0.62031
C14orf169	1.105797	1.123482	1.09119154	0.7385007	-0.4373288	0.62041
MAT2A	2.218007	2.190243	2.24093418	1.0425889	0.06017043	0.62057
SLU7	1.414553	1.4345127	1.3980693	0.9161281	-0.1263788	0.62069
VRK2	1.272614	1.2914964	1.25702033	0.8817272	-0.1815957	0.62069
MMP25-AS1	1.14421	1.1624045	1.12918509	0.7954528	-0.3301518	0.62077
POLR1C	1.296825	1.3156638	1.28126716	0.8910338	-0.1664479	0.62088
UXS1	1.410307	1.3902541	1.42686721	1.0938187	0.12937362	0.62093
NMRAL1	1.735198	1.7568675	1.71730221	0.9477249	-0.0774597	0.62094
SLC39A4	1.110114	1.0923032	1.1248231	1.3523158	0.43543213	0.62099
SGTB	1.237791	1.2567658	1.22212213	0.8650767	-0.2091	0.62106
ESCO1	1.636807	1.6581967	1.61914294	0.9406656	-0.0882462	0.62109
HEXB	1.629344	1.6504832	1.61188749	0.9406661	-0.0882454	0.62121
ZC3H6	1.828893	1.8514787	1.81024142	0.9515698	-0.0716185	0.62126
POMZP3	1.143597	1.1254496	1.15858275	1.264115	0.33812777	0.62134
CLIC3	1.049161	1.0318991	1.06341623	1.9880262	0.9913368	0.6214
ENTPD4	1.538538	1.5181238	1.55539693	1.0719387	0.10022237	0.62143
ITGB8	1.429721	1.4097235	1.44623606	1.0891152	0.12315663	0.62156
TONSL	1.144779	1.1266185	1.15977667	1.2618749	0.33556884	0.6217
KIF1BP	1.204653	1.2231756	1.18935608	0.8484625	-0.2370772	0.62173
CD40	1.135007	1.1171825	1.14972621	1.277718	0.35356949	0.62174
RP5-991G20.1	1.080423	1.0628373	1.09494596	1.5109815	0.595486	0.62203
RBM12B	1.552165	1.5730997	1.53487589	0.9333033	-0.0995821	0.62209
S1PR1	1.097244	1.0796609	1.11176391	1.4029958	0.4885107	0.62209
MPP2	1.180372	1.1985787	1.16533568	0.8325951	-0.2643131	0.62211
IGSF9	1.308819	1.2894039	1.32485152	1.122485	0.16669616	0.62219
SETMAR	1.190426	1.2086537	1.17537395	0.8405025	-0.250676	0.62236
TBP	1.246014	1.2274752	1.26132395	1.148802	0.20013017	0.62266
MRPL36	2.0753	2.1005913	2.05441461	0.9580437	-0.0618366	0.62269
PDCD2L	1.172067	1.1904314	1.15690213	0.8239298	-0.2794068	0.62271
PLA2G15	1.172079	1.1537063	1.18725189	1.2182448	0.28480408	0.62286
RP11-386G11.5	1.123149	1.1409714	1.1084312	0.7691714	-0.378623	0.62287
CRAT	1.356121	1.3757545	1.33990733	0.9045995	-0.144649	0.62293
L3MBTL1	1.229377	1.2108814	1.24465113	1.1601359	0.21429382	0.62296
NR0B1	1.099232	1.1168366	1.08469453	0.7248973	-0.4641514	0.62303
WHRN	1.250759	1.2321192	1.26615188	1.1466172	0.19738385	0.62317
ATP6V1E1	1.673345	1.6953423	1.6551788	0.9422393	-0.0858346	0.62321
USP18	1.137703	1.1198019	1.15248595	1.2728174	0.34802551	0.62323
DISC1	1.060226	1.0429059	1.07453011	1.7370594	0.79664711	0.62332
ZFYVE26	1.101235	1.0836855	1.11572854	1.3828986	0.46769538	0.62338
DCAF1	1.130417	1.1482733	1.11567172	0.7801251	-0.3582226	0.62342
PLEKHG4B	1.297341	1.3165252	1.28149925	0.8893424	-0.1691892	0.62342
SLC18A2	1.034136	1.0513682	1.01990468	0.3874904	-1.3677677	0.62347
TRAM2-AS1	1.179802	1.1982922	1.16453304	0.8297504	-0.2692506	0.62348
SMYD2	1.520665	1.5412797	1.50364089	0.9304633	-0.1039789	0.62353

HKDC1	1.048169	1.0310323	1.06232025	2.0082389	1.00593093	0.62366
DENND6B	1.204445	1.1858788	1.21977795	1.1823725	0.24168457	0.62371
DAAM1	2.512813	2.5458373	2.48554078	0.9609943	-0.0574002	0.62382
LIPT2	1.107002	1.1244296	1.09261035	0.7442791	-0.4260843	0.62389
LCN12	1.085226	1.0677659	1.09964559	1.470438	0.55624595	0.62391
PPME1	1.519629	1.5399448	1.50285196	0.9313025	-0.1026783	0.62396
MTFMT	1.286252	1.3053904	1.27044726	0.8855788	-0.1753075	0.62398
DROSHA	1.241492	1.2228141	1.25691695	1.153055	0.20546138	0.62406
ZBTB33	1.188921	1.2072177	1.17381057	0.8387823	-0.2536317	0.62407
PWAR6	1.795166	1.7734491	1.81310016	1.0512652	0.07212667	0.62409
CST6	1.030777	1.0137737	1.0448194	3.25399	1.70220981	0.62416
SCYL2	1.415825	1.3960395	1.4321634	1.0912128	0.12593248	0.62447
MICALL2	1.135655	1.1179759	1.15025547	1.2736119	0.3489257	0.62454
DLX3	1.041072	1.0583794	1.02677888	0.4587044	-1.1243633	0.62461
NADK2	1.19881	1.2171663	1.18365165	0.845673	-0.2418282	0.62465
IL17D	1.068653	1.0513444	1.08294763	1.6155155	0.69199462	0.62467
TSGA10	1.311637	1.3306548	1.29593175	0.8949869	-0.1600616	0.62477
L2HGDH	1.151866	1.1699952	1.136895	0.8052876	-0.3124239	0.62484
RP5-1159O4.1	1.122552	1.1048547	1.13716649	1.3081574	0.38753614	0.62491
RP11-563N4.1	1.068235	1.0856092	1.05388731	0.629457	-0.6678203	0.62496
KB-1125A3.11	1.100121	1.117685	1.08561706	0.7275103	-0.4589605	0.62496
MOGS	1.213571	1.1952365	1.22871232	1.1714627	0.228311	0.62497
ZER1	1.119628	1.1020281	1.13416278	1.3149588	0.39501757	0.62515
BRWD1	2.182933	2.1567592	2.20454842	1.041313	0.05840375	0.62516
ZNF658	1.078731	1.0962523	1.06426167	0.6676375	-0.5828632	0.62524
RP11-710C12.1	1.085562	1.1028523	1.07128352	0.6930667	-0.528934	0.62525
RNF166	1.234735	1.2529183	1.21971916	0.8687358	-0.2030106	0.62529
FANCG	1.2863	1.3048204	1.27100604	0.8890678	-0.1696347	0.62557
ASB13	1.196229	1.2146317	1.18103196	0.8434538	-0.245619	0.62557
RP11-796E2.4	1.196947	1.1784867	1.21219158	1.188837	0.24955098	0.62559
MAU2	1.262487	1.2813548	1.24690633	0.8775622	-0.1884266	0.62562
PAN2	1.120317	1.1379446	1.10576063	0.7666893	-0.3832861	0.62573
RHOB	1.527702	1.5075184	1.54436957	1.0726105	0.10112623	0.62573
HPCAL1	1.377391	1.3584203	1.39305643	1.0966354	0.13308394	0.62592
COL26A1	1.105025	1.0875436	1.11946206	1.3646006	0.44847875	0.62597
PKD2	1.216346	1.1977393	1.23171175	1.1718041	0.22873136	0.62605
ARRB1	1.061701	1.0788243	1.04755993	0.603366	-0.7288946	0.62607
KRT23	1.030049	1.0131484	1.04400571	3.3468488	1.74280337	0.62609
GRASP	1.054906	1.0377293	1.06909061	1.8312185	0.87280398	0.62613
OR2A1-AS1	1.068165	1.0508805	1.08243899	1.6202482	0.6962148	0.62621
A1BG-AS1	1.082547	1.0652169	1.0968582	1.4851705	0.57062854	0.62634
TIMM10B	1.403578	1.3840421	1.41971117	1.0928779	0.12813228	0.62653
CENPBD1	1.180068	1.1983094	1.16500464	0.8320564	-0.2652469	0.62671
NFKBIE	1.175635	1.1576248	1.190508	1.2086166	0.27335661	0.62675
FAM127A	2.507674	2.5381678	2.48249259	0.9638042	-0.053188	0.62686

C15orf41	1.19806	1.2160726	1.18318578	0.8477975	-0.2382084	0.62689
TNFRSF21	1.255696	1.2744005	1.24024923	0.8755421	-0.1917515	0.62695
MED27	1.096648	1.1140612	1.08226802	0.7212624	-0.4714039	0.62701
KRBA2	1.13778	1.1199621	1.15249428	1.2711867	0.34617599	0.62703
RP11-107N15.1	1.079631	1.0971618	1.06515434	0.6705756	-0.5765281	0.62716
RP3-522D1.1	1.070341	1.0875455	1.05613315	0.6411885	-0.6411795	0.6272
TSTD2	1.250695	1.2321271	1.2660292	1.1460498	0.19666972	0.62725
DTWD2	1.102527	1.1201274	1.08799144	0.7324845	-0.4491298	0.62741
SPACA6	1.206396	1.1883697	1.22128254	1.1747247	0.23232266	0.62757
MFSD14B	1.265465	1.2466257	1.28102308	1.139472	0.1883655	0.62761
CH17-353B19.1	1.03162	1.048481	1.01769524	0.3649931	-1.454059	0.62777
ZFHX4-AS1	1.054171	1.0370876	1.06827912	1.8410234	0.88050793	0.62784
HELZ	1.559268	1.5386541	1.57629201	1.069874	0.09744096	0.62786
CYTH1	1.136451	1.1188721	1.15096851	1.2700083	0.34483796	0.62793
CABP1	1.06046	1.0432974	1.07463336	1.7237364	0.78553918	0.62795
LINC00526	1.25879	1.2775933	1.24326202	0.8763251	-0.1904619	0.62797
CCDC3	1.182943	1.2011899	1.16787413	0.8344064	-0.2611778	0.62812
PDE8B	1.072148	1.0548997	1.0863916	1.5736255	0.6540922	0.62822
TPST2	1.556798	1.5363282	1.57370227	1.069685	0.09718601	0.62826
SIX3	3.297278	3.3340894	3.26687778	0.9712043	-0.0421532	0.62834
KCTD1	1.172264	1.1902462	1.15741457	0.8274255	-0.2732987	0.62844
HIST1H1B	1.138525	1.1208537	1.15311813	1.266971	0.3413835	0.6287
ZNF148	1.801342	1.7792581	1.81957985	1.0517438	0.07278331	0.62874
GTPBP4	1.473466	1.4935462	1.45688337	0.9257156	-0.1113591	0.62878
RBM28	1.396721	1.3768082	1.41316539	1.0964871	0.13288889	0.62883
TRPC4AP	1.585884	1.6067099	1.56868495	0.937326	-0.0933772	0.62897
ACOX1	1.264052	1.2455154	1.2793592	1.1378479	0.18630776	0.62915
CAMLG	4.047523	4.0964273	4.00713631	0.9711632	-0.0422143	0.62921
TYW5	1.261119	1.2426855	1.27634117	1.1386801	0.18736244	0.62927
FLJ46066	1.071346	1.0541904	1.08551405	1.5780295	0.6581242	0.62931
IKZF2	1.109531	1.0921864	1.12385501	1.3435273	0.42602568	0.62933
MTM1	1.109816	1.1273656	1.09532289	0.7484196	-0.4180808	0.62936
CCDC157	1.217718	1.2356579	1.20290372	0.8610095	-0.215899	0.62952
HENMT1	1.107489	1.1248114	1.09318456	0.7466031	-0.4215865	0.62953
GALNT12	1.078903	1.0617275	1.09308627	1.5080191	0.59265471	0.62956
BCL9L	1.113681	1.0964359	1.12792273	1.3265052	0.40763031	0.62969
FAM66C	1.081278	1.0642025	1.09537865	1.4855903	0.57103626	0.62972
LINC01021	3.038041	3.088427	2.9964309	0.9559496	-0.0649936	0.62976
APPL2	1.229876	1.2481011	1.21482483	0.8658763	-0.2077671	0.62994
SLC35A3	1.252831	1.2345294	1.26794427	1.1424763	0.19216424	0.63
MED12	1.186364	1.2042851	1.1715646	0.8398292	-0.2518322	0.63001
MARK2	1.233036	1.2148779	1.24803093	1.1542879	0.20700304	0.63014
TYW1	1.142059	1.1594874	1.12766655	0.8004802	-0.3210623	0.63015
METAP1D	1.23496	1.2532086	1.21989023	0.8684153	-0.203543	0.6302
RP1-197B17.3	1.067707	1.084756	1.05362716	0.6327243	-0.6603512	0.63045

CDC6	1.177312	1.1594417	1.19207038	1.2046437	0.26860647	0.63067
POU5F1B	1.042047	1.0252669	1.0559036	2.2125233	1.14569267	0.63082
QKI	2.253922	2.2269568	2.27619049	1.0401266	0.05675919	0.63108
ZNF627	1.160852	1.1428362	1.17572931	1.2302855	0.29899318	0.63112
B3GALT1	1.113934	1.0966449	1.12821188	1.3266287	0.40776462	0.63114
NKD1	1.079715	1.0626201	1.09383186	1.4984301	0.58345177	0.63122
ITGA8	1.034552	1.0176654	1.04849746	2.7453416	1.45698566	0.63126
AEBP1	2.07693	2.0520381	2.09748621	1.0432	0.06101581	0.63136
ATP5L2	1.13618	1.1538019	1.12162758	0.7908065	-0.3386033	0.63138
CLPB	1.070593	1.0876986	1.05646765	0.6438834	-0.6351286	0.6314
HOXB3	1.066595	1.0836147	1.05253917	0.6283486	-0.670363	0.63143
GALNT3	1.060532	1.043492	1.07460357	1.7153396	0.7784942	0.63145
AGAP4	1.089083	1.1065012	1.07469948	0.7013958	-0.5116994	0.63161
BBS7	1.388776	1.4081278	1.37279445	0.9134257	-0.1306407	0.63161
SLC9A3R2	1.165332	1.1478094	1.17980184	1.2164441	0.28267006	0.63164
BBOF1	1.148305	1.1659047	1.13377024	0.8063077	-0.3105976	0.63172
PIK3R4	1.097116	1.0797918	1.11142347	1.3964283	0.4817415	0.63177
TCF7L1	1.409185	1.3901856	1.42487544	1.0889061	0.12287953	0.63185
HS6ST2	1.207466	1.2257439	1.19237186	0.8521687	-0.230789	0.63186
NPAS3	1.275779	1.2572574	1.29107496	1.1314543	0.17817825	0.63191
ST6GAL1	1.157704	1.1400702	1.17226559	1.2298517	0.29848436	0.63194
DACT3	1.092999	1.0757446	1.10724819	1.4159184	0.50173817	0.63194
PLCG2	1.196092	1.1779625	1.21106367	1.1860008	0.246105	0.63203
LRFN4	1.12297	1.1056829	1.13724623	1.2986602	0.37702394	0.63205
ABLIM3	1.033628	1.0169852	1.04737209	2.7890277	1.47976228	0.63217
C22orf15	1.056737	1.0398803	1.07065827	1.7717576	0.82518123	0.63218
SNX18	1.215183	1.1973315	1.22992422	1.1651674	0.22053729	0.6322
GPATCH1	1.097979	1.0808001	1.11216571	1.3881879	0.47320281	0.63244
PSMG3	1.420664	1.4399866	1.40470705	0.9198168	-0.1205815	0.63272
SGCG	1.041288	1.0247053	1.0549818	2.2255071	1.15413407	0.63277
TSPAN17	1.454947	1.4748336	1.43852473	0.9235334	-0.1147639	0.63283
CTC-444N24.11	1.374583	1.3555198	1.39032601	1.0979023	0.13474972	0.63286
ZBTB8A	1.253245	1.2714188	1.23823753	0.8777487	-0.1881201	0.63303
GPSM3	1.160282	1.1427478	1.1747623	1.2242732	0.29192556	0.63309
SNX9	1.450609	1.4704955	1.43418689	0.922829	-0.1158648	0.63345
CPLX1	1.042418	1.0591537	1.02859693	0.4834341	-1.0486089	0.63352
SNRNP200	1.875864	1.8984075	1.85724766	0.9541857	-0.067658	0.63369
STRN4	1.284053	1.2658	1.29912648	1.1253818	0.17041457	0.63381
ADAMTS9-AS2	1.125351	1.1424776	1.11120678	0.7805214	-0.3574899	0.63385
HIVEP3	1.302946	1.2843276	1.31832085	1.1195565	0.16292737	0.63423
TM6SF2	1.044148	1.0609303	1.03028927	0.4971132	-1.0083538	0.6343
ENPP2	1.087264	1.0703009	1.10127303	1.440566	0.52663577	0.63436
CDKN1B	1.412497	1.4314799	1.39682091	0.9196742	-0.1208052	0.63444
SIDT2	1.204857	1.1870429	1.21956892	1.1738958	0.23130434	0.63445
MTERF4	1.401313	1.4204572	1.38550404	0.9168687	-0.1252129	0.63462

ZNF611	1.120196	1.1374517	1.10594622	0.770789	-0.3755922	0.63463
SORT1	1.257956	1.2397417	1.2729979	1.1387167	0.18740882	0.63464
PCYT1A	1.188582	1.2065252	1.17376428	0.8413709	-0.2491861	0.63478
PHF11	1.271887	1.2535294	1.28704748	1.1322058	0.17913626	0.63486
GPANK1	1.679323	1.7001531	1.66212125	0.9456807	-0.0805749	0.6349
AP000487.5	1.076451	1.093291	1.06254369	0.670415	-0.5768736	0.63493
GLRA2	1.064857	1.081786	1.050877	0.6220744	-0.684841	0.63501
GANAB	1.787354	1.7662129	1.80481259	1.0503773	0.07090761	0.63505
SNTG2	1.04047	1.0570772	1.02675563	0.4687623	-1.0930715	0.6354
NSMCE3	1.417235	1.4365853	1.40125439	0.9190746	-0.1217462	0.63541
TNFRSF11B	1.036153	1.0195869	1.04983313	2.544203	1.34721378	0.63548
RFX4	1.062185	1.0453423	1.0760937	1.678206	0.74691983	0.63559
TRAPPC11	1.222082	1.2400086	1.20727797	0.8636271	-0.2115195	0.63561
PBRM1	1.824947	1.8464694	1.80717289	0.9535759	-0.0685802	0.63582
RBKS	1.094662	1.1117702	1.08053329	0.7205255	-0.4728786	0.63595
SLC35C1	1.184219	1.1667206	1.19866951	1.1916316	0.25293827	0.63615
C15orf52	1.057638	1.0410051	1.07137342	1.7405968	0.79958209	0.63616
PPM1D	1.163196	1.1807746	1.14867937	0.8224573	-0.2819872	0.63617
ALPL	1.090146	1.0731715	1.10416397	1.42356	0.50950331	0.63618
ZNF691	1.176951	1.1942945	1.1626286	0.837021	-0.2566642	0.63629
MBTPS1	1.611361	1.6317301	1.59454069	0.9411308	-0.0875328	0.63637
EIF2S3L	1.083011	1.0998791	1.06908197	0.6916561	-0.5318732	0.63654
GLT1D1	1.056306	1.0397911	1.06994466	1.7577984	0.81376965	0.63658
RBCK1	1.789755	1.8114841	1.77181053	0.9511099	-0.0723161	0.6366
CANT1	1.443989	1.4635025	1.42787384	0.9231318	-0.1153915	0.63673
CCNG2	1.655099	1.6755973	1.63817172	0.9446036	-0.082219	0.63674
ZNF667	1.081111	1.0979918	1.06717009	0.6854663	-0.5448424	0.63686
TRPC4	1.05748	1.0408543	1.07121049	1.7430372	0.80160333	0.63687
ARRDC2	1.115916	1.133098	1.10172674	0.7642998	-0.3877895	0.63701
ELF2	1.762422	1.7836547	1.74488719	0.9505299	-0.0731961	0.63703
PNMA3	1.055309	1.0384898	1.06919803	1.7978256	0.84625305	0.63715
MBOAT7	1.708837	1.6876851	1.72630446	1.0561584	0.07882627	0.63724
ZCCHC8	1.219723	1.2375587	1.20499321	0.8629162	-0.2127077	0.63728
ARHGAP11B	1.288454	1.3064944	1.27355532	0.8925296	-0.1640281	0.63756
RDM1	1.122967	1.1398744	1.10900454	0.7793029	-0.3597438	0.63777
MRPL55	2.635882	2.6074282	2.65937989	1.0323198	0.04588991	0.6378
MAN2C1	1.237059	1.2191325	1.25186327	1.1493654	0.20083748	0.63781
ANKRD42	1.124915	1.1420175	1.1107908	0.7801208	-0.3582306	0.63786
ZNF674-AS1	1.132423	1.1495293	1.11829622	0.7911242	-0.3380238	0.63791
AKIP1	1.582507	1.6030597	1.56553347	0.9377736	-0.0926885	0.63794
ZW10	1.135311	1.1524632	1.12114738	0.794601	-0.3316975	0.63806
MTMR11	1.319357	1.30121	1.33434331	1.1100007	0.15056063	0.63809
EFEMP2	1.998129	1.9760961	2.01632439	1.0412134	0.05826582	0.63811
SMURF1	1.110135	1.093199	1.12412012	1.3317748	0.41335018	0.63812
NFXL1	1.218395	1.2360293	1.20383272	0.8635906	-0.2115806	0.63814

WDR3	1.184235	1.2016871	1.16982205	0.8420077	-0.2480947	0.63817
METTL21B	1.157833	1.1753226	1.1433894	0.8178601	-0.290074	0.6382
ATP6V1C1	1.44166	1.4225031	1.45747938	1.0827835	0.11474486	0.63844
ELL	1.06702	1.0836679	1.05327136	0.6366999	-0.6513146	0.63856
TLK2	1.347801	1.3664856	1.33237145	0.9069153	-0.1409602	0.63866
KAZALD1	1.083382	1.1002578	1.06944547	0.692669	-0.529762	0.63889
IRAK4	1.125447	1.1083688	1.13955017	1.2877338	0.36483441	0.63896
BLOC1S2	2.058912	2.0363552	2.07753908	1.0397391	0.05622158	0.63904
CTD-2008L17.2	1.279297	1.2974378	1.26431532	0.8886407	-0.1703279	0.63913
ZNF282	1.104963	1.0880975	1.11889037	1.3495319	0.43245903	0.63919
ITGA4	1.069576	1.0860752	1.05595076	0.6500215	-0.6214406	0.63924
LAD1	1.031044	1.0146773	1.04455911	3.0359166	1.60213216	0.63925
SLC16A6	1.057645	1.0742574	1.04392629	0.5915411	-0.7574497	0.63929
PDIK1L	1.151772	1.1690422	1.13750963	0.8134631	-0.2978511	0.63931
HIST1H1E	1.264533	1.2822302	1.24991817	0.8855118	-0.1754166	0.63933
NME3	2.371339	2.398871	2.34860283	0.9640652	-0.0527974	0.63947
CTD-2020K17.4	1.064628	1.0480084	1.0783536	1.6320826	0.70671408	0.63954
RAP2C	1.723546	1.7442927	1.70641238	0.9491056	-0.0753595	0.63957
RP3-510D11.2	1.222833	1.2407592	1.20802851	0.864052	-0.2108099	0.63971
TGFBRAP1	1.122308	1.105302	1.13635263	1.2948718	0.37280932	0.63972
CNPY3	1.962697	1.9852915	1.94403778	0.9581304	-0.061706	0.63976
RNF219	1.323505	1.3050346	1.33875839	1.1105572	0.15128371	0.63976
DYDC2	1.043474	1.0598313	1.02996573	0.5008372	-0.9975864	0.63976
DGAT1	1.21939	1.2019012	1.23383301	1.1581559	0.21182944	0.63986
EPS8	1.202138	1.1847279	1.21651623	1.1720819	0.22907343	0.63991
NKRF	1.102483	1.1193246	1.08857421	0.742296	-0.4299335	0.64011
YY2	1.081955	1.0653236	1.09568866	1.4648401	0.55074323	0.64013
SLC4A1AP	1.171064	1.188368	1.1567747	0.832279	-0.2648608	0.64014
GM2A	1.20627	1.1885215	1.22092692	1.1718925	0.22884023	0.6402
C18orf54	1.158063	1.1406177	1.17246929	1.2265123	0.29456165	0.64025
ZNF793-AS1	1.229707	1.2474032	1.21509318	0.8694033	-0.2019025	0.64027
PTPN11	1.676292	1.697324	1.65892384	0.9449321	-0.0817174	0.64032
AC093818.1	1.088592	1.1052821	1.0748084	0.7105519	-0.4929881	0.64043
TOP3B	1.341091	1.3230409	1.35599656	1.1020168	0.14014624	0.6405
RP11-351I21.11	1.060262	1.0436976	1.07394193	1.6921278	0.75883851	0.6405
GABRA5	1.041641	1.025327	1.05511339	2.1760707	1.12172545	0.6407
KCNMB3	1.090683	1.1075831	1.07672735	0.7131916	-0.4876384	0.64071
WRAP53	1.190205	1.2074962	1.17592548	0.847849	-0.2381207	0.6408
TRIP10	1.390686	1.371958	1.40615173	1.0919289	0.12687898	0.64086
MARVELD3	1.038906	1.0225171	1.05244006	2.3289019	1.21964989	0.64101
ACOX3	1.109663	1.0926137	1.12374217	1.3361112	0.41804013	0.64102
OGFR	1.394081	1.4128294	1.37859748	0.9170798	-0.1248809	0.6412
MAP3K11	1.427423	1.4083927	1.44313936	1.0850816	0.11780355	0.64127
BARX2	1.035114	1.0186051	1.04874804	2.6201461	1.38964728	0.64138
ZKSCAN2	1.118453	1.10143	1.13251034	1.3064215	0.38562041	0.64158

RCE1	1.212893	1.2301816	1.19861602	0.8628665	-0.2127908	0.64165
PDCD6IP	1.785561	1.8068397	1.76798839	0.9518476	-0.0711976	0.64184
RAB40B	1.587015	1.6071296	1.57040375	0.939509	-0.090021	0.64189
SRGAP3	2.355519	2.3821996	2.33348648	0.9647568	-0.0517628	0.64193
RAB15	1.084129	1.067549	1.09782132	1.4481542	0.53421525	0.64208
TTL	1.292254	1.2743254	1.30706037	1.119329	0.16263411	0.6421
ABCB9	1.107905	1.1247177	1.09402149	0.7538743	-0.4076041	0.64211
LINC00926	1.061261	1.0776671	1.04771194	0.6143133	-0.7029534	0.64221
DNAL1	1.270519	1.2883928	1.25575776	0.8868383	-0.1732571	0.64224
DOCK5	1.050552	1.0341944	1.06406123	1.8734424	0.90569162	0.64228
SIRT7	1.188692	1.1713287	1.20303151	1.1850406	0.24493644	0.64229
ADGRE5	1.074215	1.0909716	1.06037693	0.6636898	-0.5914191	0.64231
MEGF10	1.081618	1.0980527	1.06804667	0.6939807	-0.5270326	0.64247
BEST1	1.282089	1.3001518	1.26717212	0.8901233	-0.1679229	0.64257
RNF139-AS1	1.117756	1.1344881	1.10393845	0.772845	-0.3717489	0.64264
DNAL4	1.466172	1.4854893	1.4502196	0.9273523	-0.1088105	0.64264
PSMB10	1.243421	1.2256902	1.25806393	1.1434433	0.19338484	0.64271
CPD	1.487532	1.4684561	1.50328442	1.0743471	0.10346013	0.64275
CTTNBP2	1.061841	1.0455123	1.07532481	1.6550443	0.72686982	0.64278
TAL2	1.034295	1.0505556	1.02086703	0.412754	-1.2766461	0.64298
TMEM200B	1.041467	1.0252132	1.05488926	2.1770057	1.12234521	0.64305
RP11-666O2.2	1.077212	1.0940366	1.06331841	0.6733382	-0.5705969	0.64311
PCDHGA10	1.146507	1.1295305	1.16052604	1.239291	0.30951502	0.64316
RUBCN	1.136238	1.1534229	1.12204566	0.7954853	-0.3300927	0.64322
ZFP36	1.173451	1.1561869	1.18770822	1.2018179	0.26521835	0.64323
TRIM6	1.111521	1.1281319	1.09780391	0.7633066	-0.3896655	0.64332
MGAT4A	1.118429	1.1014641	1.13243841	1.3052731	0.38435171	0.64332
ENHO	1.110634	1.0939696	1.12439537	1.3237829	0.40466659	0.64332
ALG9	1.125128	1.1082152	1.13909501	1.2853555	0.36216744	0.64343
KCNJ8	1.053344	1.0696115	1.03990984	0.5733228	-0.8025804	0.64351
PROSER3	1.298965	1.3171258	1.28396785	0.8954424	-0.1593275	0.64366
GAK	1.264257	1.2467211	1.27873829	1.1297706	0.17602984	0.64381
TRMT61B	1.291681	1.309582	1.27689892	0.8944284	-0.1609621	0.64395
MON1B	1.271591	1.2894206	1.25686656	0.8875198	-0.1721487	0.64402
AC105760.2	1.061808	1.0454027	1.07535597	1.6597227	0.73094221	0.64425
EPB41L4B	1.061772	1.0453698	1.07531696	1.6600694	0.73124355	0.64425
DNAH6	1.04255	1.0263029	1.05596734	2.1278018	1.08936376	0.64441
MARCH4	1.056819	1.0405524	1.07025234	1.7323846	0.79275922	0.64456
DLAT	1.249757	1.2676799	1.23495511	0.8777466	-0.1881237	0.64461
THOC2	1.91672	1.8948817	1.93475503	1.0445571	0.06289132	0.64469
GS1-124K5.11	1.086163	1.0696449	1.09980317	1.4330297	0.51906855	0.64472
FAAH	1.15815	1.1410056	1.17230878	1.2219993	0.28924343	0.64476
SETD5	2.537238	2.5698877	2.51027551	0.9620277	-0.0558496	0.64482
RNFT2	1.266339	1.2840646	1.25170016	0.8860667	-0.1745128	0.64485
NCLN	1.205136	1.1877874	1.21946287	1.1686771	0.22487641	0.64485

DHX8	1.188027	1.2053238	1.17374276	0.8461891	-0.240948	0.6449
C11orf63	1.084573	1.0679878	1.09826853	1.4453846	0.53145342	0.64503
ARRDC1	1.535572	1.515872	1.55184032	1.0697234	0.09723774	0.64513
TET2	1.195521	1.1783378	1.20971126	1.1759219	0.2337922	0.6452
TRIM65	1.175544	1.1582939	1.18978885	1.198965	0.26178952	0.64524
ZNF275	1.106091	1.0893881	1.11988405	1.3411631	0.42348467	0.64529
NOP2	1.097508	1.1140327	1.0838611	0.7354129	-0.4433737	0.64533
GCH1	1.058539	1.0423071	1.07194415	1.7005212	0.76597698	0.64551
FAM81B	1.036591	1.0205148	1.04986776	2.4308198	1.28144295	0.64561
CLDN10	1.081593	1.0979658	1.06807257	0.6948605	-0.5252048	0.64573
DRD4	1.061084	1.0447874	1.07454284	1.6643718	0.73497777	0.64584
SCN3B	1.078199	1.0618837	1.09167244	1.4813661	0.56692819	0.64586
DYRK4	1.87647	1.8976364	1.85898987	0.9569464	-0.06349	0.64595
CUL3	1.433247	1.4523173	1.41749886	0.9230221	-0.1155629	0.64598
ADSS	1.856929	1.8784172	1.83918386	0.9553363	-0.0659194	0.64601
ATP6V1A	1.44915	1.4682028	1.43341542	0.9257002	-0.1113831	0.64614
MRPL20	3.333842	3.3728054	3.30166552	0.9700187	-0.0439156	0.64624
ZBTB21	1.113656	1.0970879	1.12733904	1.311585	0.39131134	0.64625
C2orf88	1.034746	1.0186638	1.04802613	2.5732235	1.36357679	0.64627
PPP1R26	1.180266	1.1632816	1.19429161	1.1899175	0.25086161	0.64629
CAPN3	1.078392	1.0621169	1.09183212	1.4783762	0.56401343	0.64647
COL16A1	1.093257	1.0768813	1.10678042	1.3889005	0.4739433	0.64657
C17orf96	1.07863	1.0950611	1.06506008	0.6844029	-0.5470822	0.64706
C6orf132	1.122755	1.1060818	1.13652377	1.286967	0.36397504	0.6471
PCGF1	1.146152	1.1629462	1.13228246	0.8118166	-0.3007742	0.64755
IMPA1	1.351003	1.3688756	1.33624348	0.9115362	-0.1336281	0.64764
TRIP11	1.81685	1.7956935	1.8343207	1.0485453	0.0683892	0.64766
LRRC58	1.60694	1.5865913	1.62374504	1.0633384	0.08860078	0.64773
LINC01088	1.049611	1.0657188	1.0363095	0.5524984	-0.8559579	0.64787
MBD6	1.209596	1.1925238	1.22369412	1.1619039	0.21649072	0.64799
IL4R	1.100895	1.084401	1.1145156	1.3568044	0.44021271	0.64808
DOCK4	1.129125	1.1458069	1.11534822	0.7911025	-0.3380635	0.6482
SEC22B	1.649731	1.6697992	1.63315894	0.9452967	-0.0811609	0.64822
RELA	1.389493	1.4079232	1.37427385	0.9175105	-0.1242035	0.64867
PIGG	1.14778	1.1646462	1.13385241	0.8129701	-0.2987257	0.64875
C16orf70	1.104874	1.0884295	1.1184545	1.3395362	0.42173354	0.6488
GANC	1.104923	1.0884789	1.11850192	1.3393234	0.42150431	0.6488
FANCC	1.10617	1.1226804	1.09253518	0.7542785	-0.4068307	0.64899
LRRC37A2	1.100273	1.0838435	1.11384149	1.3577847	0.44125478	0.64903
ACY1	1.082828	1.0992409	1.0692743	0.6980416	-0.5186151	0.64919
AC010894.3	1.122697	1.1393211	1.10896767	0.7821335	-0.3545133	0.64929
RP11-159H10.3	1.080425	1.0968432	1.06686716	0.6904683	-0.534353	0.64951
ZNF439	1.36286	1.381005	1.34787599	0.9130482	-0.131237	0.64951
TESK2	1.05501	1.0710831	1.04173717	0.5871599	-0.7681748	0.64952
PPP2R2D	1.369218	1.3511003	1.38417902	1.0942146	0.12989572	0.64952

PHC2	1.447197	1.4663671	1.43136583	0.9249491	-0.1125542	0.64959
SYNPO2	1.053411	1.0373435	1.06668015	1.7855906	0.83640133	0.6496
EPSTI1	1.06034	1.0442172	1.0736551	1.6657565	0.73617751	0.6497
TRIM71	1.099005	1.1152036	1.08562727	0.7432688	-0.428044	0.64971
PEX11G	1.099092	1.1155236	1.0855223	0.7403017	-0.4338148	0.64971
GLCCI1	1.358333	1.3404258	1.37312124	1.0960429	0.13230427	0.65006
B4GALT4	1.423062	1.4413116	1.407992	0.9244988	-0.1132567	0.65008
HDAC9	1.293017	1.3109317	1.27822366	0.8948064	-0.1603525	0.65015
GNAL	1.273468	1.291138	1.25887495	0.8891828	-0.169448	0.65026
CYB561	1.158472	1.1752801	1.14459145	0.8249163	-0.2776803	0.65032
SLC16A8	1.050903	1.0348427	1.06416636	1.8416028	0.88096195	0.65056
ELP3	1.1756	1.1922137	1.16188097	0.8421926	-0.2477778	0.65061
SLC5A6	1.351091	1.3330291	1.36600766	1.0990262	0.13622578	0.65067
RBM12	1.236114	1.2532591	1.2219547	0.8763939	-0.1903486	0.65073
AC007773.2	1.116977	1.133353	1.10345257	0.77578	-0.3662805	0.65088
RP11-127B20.3	1.175747	1.1927458	1.16170997	0.8389807	-0.2532904	0.65099
RP11-774O3.3	1.035257	1.0194099	1.04834367	2.4906695	1.3165336	0.65099
SGSM1	1.078646	1.0623656	1.09209091	1.4766301	0.56230846	0.651
DENND1B	1.326298	1.3441866	1.31152514	0.9051053	-0.1438424	0.65103
OPN3	1.688645	1.6692302	1.70467865	1.0529691	0.07446306	0.65104
CWF19L2	1.513811	1.4946689	1.52961939	1.0706544	0.09849281	0.65121
RWDD1	3.553946	3.5946519	3.52033039	0.9713559	-0.0419281	0.65131
HES7	1.10668	1.1228389	1.09333642	0.7598282	-0.3962548	0.65138
C1orf198	1.409899	1.391578	1.4250288	1.0854255	0.11826072	0.65147
RNF149	1.351473	1.3335327	1.36628761	1.098206	0.13514875	0.65148
HRH1	1.044917	1.0290721	1.05800255	1.9951259	0.99647977	0.65149
PTGER4	1.050062	1.0341532	1.06319886	1.8504493	0.88787564	0.65149
POPDC2	1.050078	1.0341818	1.06320523	1.8490915	0.88681659	0.65149
SCN1B	1.07235	1.0564956	1.08544313	1.5123854	0.59682583	0.65179
TLDC1	1.295088	1.3128875	1.28038817	0.8961309	-0.1582186	0.6521
ARIH2	1.676356	1.6964497	1.65976165	0.9473214	-0.0780742	0.65217
NOV	1.047909	1.0320675	1.06099174	1.9019774	0.92750011	0.65217
ETFDH	1.228485	1.2110807	1.24285695	1.1505407	0.202312	0.65223
GGA1	1.800567	1.7796822	1.81781458	1.0489076	0.06888759	0.65224
NMI	1.071467	1.0874818	1.05824205	0.6657616	-0.5869225	0.65228
MICAL3	1.144254	1.127657	1.15796001	1.2373789	0.30728729	0.65231
ALG2	1.196947	1.2140067	1.18285948	0.8544569	-0.2269203	0.65234
ZNF454	1.043153	1.0273094	1.05623683	2.0592491	1.04211836	0.65238
CTD-2012K14.8	1.080185	1.0639483	1.09359374	1.4635856	0.54950715	0.65241
RP1-179N16.6	1.053507	1.0375361	1.06669566	1.776839	0.82931296	0.65242
DCLRE1B	1.125711	1.1422284	1.11206988	0.7879572	-0.3438108	0.65246
HACD2	1.248949	1.2662277	1.23467962	0.8814995	-0.1819684	0.65248
TSPAN19	1.146465	1.1628937	1.13289735	0.815853	-0.2936189	0.65265
ACBD4	1.149242	1.1658553	1.1355232	0.8171172	-0.291385	0.65271
RP11-54O7.3	1.112137	1.1284726	1.0986461	0.7678374	-0.3811272	0.6528

RSPH1	1.118874	1.1025043	1.1323918	1.2915732	0.36912943	0.65282
MADCAM1	1.11469	1.0982915	1.12823154	1.3046049	0.38361297	0.65283
RNASEL	1.064566	1.0485162	1.07782103	1.6040201	0.68169222	0.65287
SYNM	1.157392	1.140669	1.17120295	1.2170628	0.28340359	0.65316
SMO	1.40221	1.3838972	1.41733338	1.0870966	0.12048009	0.65318
RP11-479O16.1	1.037723	1.0219452	1.05075286	2.3127075	1.20958284	0.65332
TCP11L2	1.081721	1.0655271	1.09509426	1.4512211	0.53726731	0.65343
ZNF174	1.191973	1.2090115	1.1779016	0.8511571	-0.2325026	0.65345
FNDC5	1.087872	1.1040905	1.07447798	0.715512	-0.4829521	0.65345
SMOC1	1.088244	1.1043116	1.07497506	0.7187603	-0.4764173	0.6537
APEX2	1.156228	1.1727431	1.14258941	0.8254419	-0.2767615	0.65383
NEBL	1.373976	1.3918395	1.35922381	0.9167627	-0.1253798	0.65387
BLOC1S3	1.095195	1.1113709	1.08183672	0.7348127	-0.4445516	0.65397
RAB6A	2.18036	2.1577765	2.19900967	1.0356141	0.05048649	0.65406
VEZF1	1.88979	1.9109119	1.87234677	0.9576632	-0.0624098	0.65407
ASCC3	1.79196	1.7719224	1.80850809	1.0473956	0.06680641	0.65427
MYSM1	1.265086	1.2825748	1.25064427	0.8870016	-0.1729914	0.65433
GMFB	1.504353	1.5230377	1.48892338	0.9347765	-0.0973066	0.65447
SLIT2	1.457775	1.4393922	1.4729557	1.0763862	0.10619579	0.6545
TCEB3-AS1	1.241695	1.2587445	1.22761565	0.8796928	-0.1849283	0.65456
DDX25	1.061953	1.0779388	1.04875094	0.6255026	-0.6769123	0.65459
PTPN18	1.399535	1.3813931	1.41451712	1.0868501	0.12015296	0.65464
USP9X	1.761398	1.7411703	1.77810198	1.0498289	0.0701542	0.65466
PLXNA4	1.098799	1.08266	1.11212645	1.356478	0.43986567	0.65469
FBF1	1.158125	1.1416775	1.17170785	1.211963	0.27734571	0.65472
PTPRZ1	1.194061	1.2109964	1.18007469	0.8534493	-0.2286226	0.65474
RP11-326C3.11	1.140994	1.1573268	1.12750646	0.8104562	-0.3031939	0.65475
DUSP22	1.355718	1.3735892	1.34095917	0.912658	-0.1318538	0.65476
NEK6	1.584576	1.6036053	1.56886175	0.94244	-0.0855274	0.65491
ITPRIP	1.173506	1.1901666	1.15974687	0.8400364	-0.2514763	0.65499
MYBL1	1.114124	1.1303037	1.10076308	0.7732939	-0.3709113	0.65505
FAM102A	1.123974	1.1077102	1.13740509	1.2756929	0.35128106	0.65505
GLI2	1.178527	1.1620133	1.19216461	1.1861041	0.24623066	0.65511
LDB2	1.065929	1.0817926	1.05282838	0.6458821	-0.6306572	0.65519
WDR5	1.255891	1.2730348	1.24173416	0.8853603	-0.1756634	0.65523
AXIN1	1.141335	1.1248341	1.15496151	1.2413391	0.31189731	0.6554
RP11-342K6.4	1.035222	1.019513	1.04819454	2.4698651	1.30443226	0.65551
RP11-803D5.4	1.051893	1.0360804	1.06495181	1.8001985	0.84815602	0.65571
INPP5K	1.15888	1.1423301	1.17254714	1.2123026	0.2777498	0.65573
USF3	1.262021	1.2449876	1.27608668	1.1269413	0.1724124	0.65581
RP11-348J24.2	1.18533	1.2020073	1.17155783	0.8492654	-0.2357127	0.6559
DMRTA1	1.166914	1.1504815	1.18048339	1.1993729	0.26228029	0.65601
NBPF15	1.180101	1.1633554	1.19392935	1.1871625	0.24751743	0.65608
SLC48A1	1.331375	1.3139424	1.3457709	1.1013832	0.13931649	0.65608
RP11-44N21.1	1.067095	1.0830787	1.05389478	0.6487199	-0.6243325	0.65612

KCNQ4	1.037992	1.0223186	1.05093536	2.2821885	1.19041795	0.65618
IRF6	1.032597	1.0170195	1.04546064	2.6710987	1.4174333	0.65619
TAB1	1.236315	1.2531922	1.22237776	0.8782964	-0.1872202	0.65623
PLEKHG2	1.16151	1.145102	1.17505974	1.2064601	0.27078022	0.65645
RP11-545I5.3	1.153788	1.1374075	1.16731476	1.2176536	0.28410379	0.65655
PRKCZ	1.377957	1.3958806	1.36315454	0.9173334	-0.1244819	0.65666
PIK3R3	1.214398	1.2310928	1.20061194	0.8681011	-0.204065	0.65675
SAP130	1.193076	1.1763339	1.20690117	1.1733488	0.23063192	0.65677
ZNF837	1.11051	1.1266155	1.09720959	0.7677543	-0.3812833	0.65692
DIS3	1.372761	1.3905217	1.35809437	0.9169641	-0.1250629	0.65697
CACNB1	1.075426	1.091197	1.06240151	0.6842498	-0.5474049	0.65713
PDE6H	1.039302	1.0237147	1.05217339	2.2000417	1.13753088	0.65716
SH2D2A	1.095142	1.1111848	1.08189431	0.7365601	-0.4411248	0.65718
ACIN1	1.653709	1.6733173	1.63751674	0.9468296	-0.0788233	0.65732
INPP5F	1.673742	1.6932895	1.6575993	0.9485204	-0.0762492	0.65732
MUM1	3.256348	3.2941481	3.22513251	0.9699167	-0.0440673	0.65736
FAM188B	1.089847	1.0737913	1.10310596	1.3972647	0.48260537	0.65741
PDE1A	1.027253	1.0429681	1.01427482	0.3322187	-1.5897948	0.65743
AP1B1	1.31742	1.3351436	1.30278422	0.9034461	-0.1464895	0.65749
NEGR1	1.060991	1.0452253	1.07401078	1.63649	0.71060481	0.65751
SAPCD1-AS1	1.056549	1.0407882	1.06956416	1.7054975	0.77019263	0.65761
P2RY6	1.046411	1.0308658	1.05924809	1.9195383	0.94075935	0.65761
RP11-572O17.1	1.079522	1.06357	1.09269561	1.4581654	0.54415434	0.65765
RP9	1.519331	1.5378234	1.50406054	0.9372232	-0.0935355	0.65776
PCOLCE2	1.150858	1.1346575	1.16423731	1.2196673	0.2864877	0.65776
SCAF8	1.28279	1.2659953	1.2966588	1.1152786	0.15740409	0.65777
SEMA6D	1.125432	1.109148	1.13888017	1.2724021	0.3475547	0.65784
TSEN2	1.287896	1.3051229	1.27367056	0.896919	-0.1569504	0.65791
FAXC	1.170513	1.1539734	1.18417156	1.196126	0.25836941	0.65805
ICE1	1.365297	1.347643	1.37987517	1.0927164	0.12791907	0.65816
TNFAIP8L1	1.174219	1.1904336	1.16082791	0.8445352	-0.2437706	0.65828
BEST4	1.086914	1.1027694	1.07382089	0.7183158	-0.4773099	0.65828
KLF13	1.168424	1.1519802	1.18200352	1.1975474	0.26008281	0.6583
ZBTB26	1.153696	1.1373327	1.1672089	1.2175464	0.2839767	0.65833
VPS4B	1.434114	1.4521715	1.41920228	0.9270869	-0.1092236	0.65836
GNG7	1.141763	1.125494	1.15519741	1.2366915	0.30648561	0.65841
MYO3B	1.034269	1.0187441	1.04708917	2.5122133	1.32895893	0.65846
RP11-111M22.3	1.435027	1.4530552	1.42013989	0.9273481	-0.108817	0.65846
CTR9	1.339518	1.3570773	1.32501657	0.9102135	-0.135723	0.65853
REPS2	1.093326	1.077285	1.10657249	1.378955	0.46357534	0.65858
SLC4A7	1.882265	1.902894	1.86523005	0.9582853	-0.0614729	0.6587
ARNTL	1.126681	1.142749	1.11341158	0.7944823	-0.331913	0.65887
HCRT	1.128373	1.1121103	1.14180277	1.2648501	0.33896645	0.65899
FAP	1.031402	1.0159741	1.04414209	2.7633505	1.46641857	0.65905
PHF21B	1.09849	1.1144897	1.08527747	0.7448486	-0.4249808	0.6591

MARK1	1.125781	1.1097665	1.13900554	1.2663747	0.34070432	0.6593
MYH15	1.037815	1.05332	1.02501038	0.4690619	-1.0921497	0.65932
FAM183A	1.220353	1.2370983	1.20652507	0.8710524	-0.1991685	0.65933
BRINP2	1.07475	1.0589514	1.08779596	1.4892933	0.57462786	0.65961
CBL	1.357857	1.3756059	1.34320043	0.9137248	-0.1301684	0.65995
C8orf48	1.096249	1.112101	1.08315746	0.7418086	-0.4308811	0.66006
B4GALNT4	1.324495	1.3075923	1.33845326	1.1003307	0.13793717	0.66013
RAB3IL1	1.061105	1.0454689	1.07401671	1.6278542	0.70297152	0.66036
APOOL	1.326783	1.3441745	1.31242162	0.907742	-0.1396458	0.66037
RP3-508I15.9	1.075097	1.0907438	1.06217615	0.6851838	-0.5454371	0.66042
NLK	1.186675	1.2031366	1.17308119	0.8520433	-0.2310013	0.66043
CACNG4	1.126125	1.1100888	1.13936851	1.2659642	0.34023658	0.66065
SH3BP5L	1.126274	1.1102425	1.13951274	1.2655079	0.33971655	0.66065
TMEM102	1.082777	1.0985177	1.06977842	0.7082833	-0.4976016	0.66071
RP11-96H19.1	1.074009	1.0898284	1.06094509	0.6784616	-0.559661	0.66078
RWDD3	1.090654	1.0747803	1.10376282	1.3875688	0.47255934	0.6608
RP11-817O13.8	1.051839	1.0674729	1.03892777	0.5769391	-0.7935091	0.66082
PC	1.058699	1.0742121	1.04588801	0.6183362	-0.6935366	0.66082
SLC44A5	1.126387	1.1104201	1.13957331	1.2640208	0.33802018	0.66101
MIER1	1.790873	1.8112666	1.77403209	0.9541032	-0.0677828	0.66113
IRF9	1.22966	1.213135	1.24330737	1.1415648	0.19101273	0.66117
MC1R	1.067919	1.0523057	1.08081256	1.545006	0.62761241	0.66125
RP11-219G17.4	1.095736	1.0799321	1.10878681	1.3609901	0.44465661	0.66126
SRGAP1	1.613042	1.5942322	1.62857506	1.0577938	0.08105836	0.66131
NEMP1	1.208115	1.2244676	1.19461017	0.8669857	-0.2059198	0.66136
PNKD	2.853542	2.825224	2.87692748	1.0283272	0.04029934	0.66138
CCDC134	1.035704	1.0510257	1.02305108	0.4517542	-1.1463902	0.66142
HTT	1.301728	1.2847557	1.3157433	1.1088218	0.1490275	0.66154
OGDHL	1.043001	1.0275358	1.05577249	2.025454	1.01824529	0.66157
ADAM15	1.333413	1.3158944	1.34788074	1.1012563	0.13915021	0.66159
NR2F1-AS1	1.783175	1.8032433	1.76660209	0.9543834	-0.0673591	0.6616
PGM5P4-AS1	1.029121	1.0137737	1.04179567	3.0344603	1.60143995	0.66165
CDHR3	1.060532	1.076227	1.04757136	0.6240752	-0.6802082	0.66169
ENPEP	1.06269	1.0471108	1.07555584	1.603791	0.68148619	0.6617
PLEKHG1	1.374239	1.3916045	1.35989806	0.9190345	-0.121809	0.6618
ARHGAP8	1.04829	1.032824	1.06106182	1.8602781	0.89551831	0.66184
CHDH	1.137375	1.153371	1.12416475	0.809571	-0.3047705	0.66187
ACVR1B	1.179895	1.1963082	1.16634059	0.847344	-0.2389803	0.66188
AJUBA	1.304383	1.2874051	1.3184032	1.1078551	0.14776921	0.66202
AC098973.2	1.252441	1.2693075	1.23851225	0.8856504	-0.1751908	0.66204
CHRNB1	1.430511	1.4481889	1.41591242	0.9279846	-0.1078272	0.66207
WDR76	1.191281	1.1748911	1.20481555	1.1711033	0.22786828	0.66215
PCSK5	1.077647	1.0621386	1.09045334	1.455671	0.54168432	0.66236
CTB-58E17.5	1.04272	1.0581732	1.02995927	0.5150016	-0.9573512	0.66243
CXorf40B	1.207024	1.1903546	1.22079041	1.15989	0.213988	0.66251

MMP28	1.034244	1.0495637	1.02159195	0.4356405	-1.1987901	0.66254
KCTD9	1.187357	1.1710286	1.20084161	1.1743157	0.2318203	0.66254
ZNF713	1.078683	1.0944027	1.06570184	0.6959744	-0.5228938	0.66274
SUPT7L	1.308602	1.3257175	1.29446765	0.9040585	-0.145512	0.6628
NAMPT	1.792819	1.812817	1.77630512	0.9550798	-0.0663068	0.66292
PODXL	1.10012	1.0844443	1.11306454	1.3389244	0.42107449	0.66306
C16orf52	1.22479	1.2085023	1.23823988	1.1426245	0.19235133	0.66307
ZSWIM4	1.097367	1.1130711	1.08439836	0.7464182	-0.4219439	0.6631
OSMR	1.037656	1.0223731	1.05027611	2.247172	1.16811054	0.66317
TMEM164	1.307238	1.290275	1.32124577	1.1066946	0.14625719	0.66338
ZNF225	1.063538	1.0789168	1.05083877	0.6442074	-0.6344029	0.66346
HTRA3	1.145004	1.1288344	1.15835741	1.2291551	0.29766694	0.66348
KDM3A	1.542892	1.5242311	1.55830171	1.0649916	0.09084202	0.6635
KLF3	1.874644	1.8541743	1.89154854	1.0437548	0.06178286	0.66356
XPC	1.648952	1.6679528	1.63326112	0.9480627	-0.0769457	0.66367
ZFP42	1.033477	1.0181575	1.04612745	2.5404124	1.34506269	0.66372
LINC01481	1.611941	1.6310406	1.59616802	0.944738	-0.0820139	0.66378
ZNF468	1.07082	1.0862999	1.05803587	0.6724905	-0.5724142	0.66381
SH2B2	1.123085	1.1389469	1.10998532	0.7915637	-0.3372227	0.66381
SORBS2	1.611354	1.5930042	1.62650691	1.0564965	0.07928799	0.66404
GPR137C	1.185032	1.1688162	1.19842407	1.1753854	0.23313392	0.6641
KIAA1644	1.045558	1.0302623	1.05818972	1.922845	0.94324244	0.66411
ARL10	1.316266	1.2991867	1.33037078	1.1042293	0.14303981	0.66413
ZNF582	1.118332	1.1340796	1.10532711	0.7855568	-0.3482124	0.66435
EIF4E3	1.136931	1.1210914	1.15001095	1.238824	0.30897121	0.66443
SRCIN1	1.060251	1.044791	1.07301845	1.6302053	0.70505364	0.66444
PPM1J	1.091587	1.1070828	1.07879057	0.7357913	-0.4426315	0.66444
ARL17A	1.105875	1.1216281	1.09286539	0.763519	-0.389264	0.66446
LTBR	1.067497	1.052067	1.08023865	1.5410647	0.62392739	0.66455
SLC25A53	1.188792	1.2049775	1.17542609	0.8558308	-0.2246025	0.66455
MGST2	1.367135	1.3845751	1.35273354	0.9172032	-0.1246867	0.66461
KRTCAP3	1.041454	1.0261164	1.05412083	2.0722927	1.05122778	0.66467
ZNF329	1.106812	1.091005	1.1198657	1.3171327	0.39740069	0.66472
POLR2B	1.84671	1.8665115	1.83035844	0.9582775	-0.0614846	0.66477
RP11-783K16.5	1.102331	1.0868177	1.11514196	1.3262498	0.40735251	0.66478
WFDC1	1.213511	1.1971586	1.22701531	1.1514349	0.20343282	0.66487
KDM4A-AS1	1.101213	1.1169125	1.08824893	0.7548286	-0.405779	0.66487
SWAP70	1.19825	1.2147178	1.1846508	0.8599696	-0.2176425	0.6649
IRX3	1.053335	1.0379138	1.06607047	1.7426492	0.80128216	0.66513
BOC	1.466213	1.4839541	1.45156126	0.9330663	-0.0999485	0.66515
RTN2	1.126742	1.1424254	1.11379042	0.7989475	-0.3238273	0.66537
GALNT11	1.716136	1.7355336	1.70011708	0.9518492	-0.0711951	0.66539
CBX8	1.135662	1.1514407	1.12263116	0.8097638	-0.304427	0.66545
TRMT5	1.187851	1.2041831	1.17436446	0.8539611	-0.2277578	0.66551
MKLN1	1.662816	1.6438095	1.67851186	1.0539015	0.07574006	0.66563

RD3	1.032736	1.0174967	1.04532175	2.5903013	1.3731199	0.66565
RECQL5	1.111023	1.126773	1.09801648	0.7731651	-0.3711516	0.66566
MATR3.1	1.110606	1.0948565	1.12361289	1.3031574	0.38201136	0.66598
OAS1	1.052088	1.0367361	1.06476569	1.7630008	0.81803312	0.66604
DUSP5	1.051963	1.0366967	1.06456943	1.7595445	0.81520204	0.66604
SHROOM4	1.154641	1.1385976	1.16788938	1.2113441	0.27660876	0.66605
SLC29A1	1.257498	1.240867	1.27123188	1.1260651	0.17129018	0.66606
KANSL2	1.210514	1.2268255	1.19704345	0.8687007	-0.2030688	0.66614
ZNF496	1.18977	1.2062476	1.17616263	0.8541318	-0.2274694	0.66622
RP11-497E19.1	1.053134	1.0684596	1.04047831	0.5912729	-0.758104	0.6663
CD59	2.669167	2.6398448	2.693381	1.0326471	0.04634737	0.6664
SOX13	1.055073	1.0397665	1.06771305	1.7027652	0.76787954	0.66645
BCOR	1.240436	1.223822	1.2541556	1.1355257	0.18336038	0.66647
CABLES2	1.077123	1.0616838	1.08987305	1.4569949	0.54299584	0.66655
BACE1-AS	1.155603	1.1716059	1.14238706	0.8297328	-0.2692813	0.6668
CNKSR1	1.044738	1.0295437	1.0572856	1.9390124	0.95532201	0.66683
ZNF335	1.096738	1.0811123	1.10964286	1.3517416	0.43481939	0.66686
OTOS	1.205678	1.1859017	1.22200995	1.1942331	0.25608445	0.66692
PPP1R12C	1.111939	1.0963121	1.12484337	1.2962374	0.37432991	0.66699
RP11-181C3.1	1.060402	1.0758984	1.04760514	0.6272218	-0.6729524	0.667
LETM1	1.335996	1.3532837	1.32171962	0.9106553	-0.1350231	0.66706
TMEM219	3.378408	3.3443888	3.40650142	1.0264941	0.0377254	0.66707
ANKDD1A	1.114131	1.098586	1.12696876	1.2878988	0.36501919	0.66716
STARD3	1.630645	1.6494964	1.61507709	0.9470061	-0.0785544	0.66737
LRRC8A	1.231415	1.2150747	1.2449083	1.1387125	0.18740354	0.6674
SESN2	1.218706	1.2352562	1.20503843	0.871554	-0.1983381	0.66756
SH2B3	1.060549	1.0453098	1.07313404	1.6140871	0.69071845	0.66776
PDE4A	1.060729	1.0453119	1.07345997	1.6212052	0.69706671	0.66776
INSIG2	2.511175	2.4832096	2.53426979	1.0344254	0.04882966	0.66778
PANX1	1.146826	1.1307036	1.16014076	1.2252203	0.29304117	0.66794
POLD4	1.208292	1.1921742	1.22160193	1.1531304	0.20555568	0.66796
ARHGAP19	1.106055	1.1215544	1.09325627	0.767198	-0.3823292	0.66798
NOP14	1.228003	1.2441402	1.21467644	0.8793161	-0.1855463	0.66798
KLK6	1.282359	1.2988808	1.26871432	0.8990686	-0.1534969	0.66801
EFNA1	1.245018	1.2616938	1.23124698	0.8836549	-0.1784451	0.66808
LZIC	1.678011	1.6969077	1.66240511	0.9504918	-0.0732538	0.66809
RASGRF1	1.095224	1.0798086	1.10795485	1.352672	0.43581208	0.66832
RABL2A	1.407714	1.4251789	1.39329044	0.9249998	-0.112475	0.66839
ADAMTS4	1.059557	1.0441655	1.07226694	1.6362747	0.71041496	0.66839
JAG2	1.057026	1.0417812	1.06961473	1.6661751	0.73654006	0.66839
WDR53	1.088199	1.1035482	1.07552324	0.7293533	-0.4553103	0.66849
DUSP19	1.088296	1.1036917	1.07558174	0.7289086	-0.4561902	0.66849
ARMC8	1.418903	1.4011925	1.43352867	1.0806	0.11183264	0.6686
PAQR3	1.250554	1.2341841	1.2640723	1.1276267	0.17328957	0.66861
PROCR	1.061842	1.0465786	1.074446	1.5982889	0.67652818	0.66866

MFF	2.417341	2.4417883	2.39715159	0.9690407	-0.0453708	0.66873
SLC6A17	1.029891	1.0149309	1.04224618	2.8294484	1.50052082	0.66888
MN1	1.189203	1.1731383	1.20246993	1.1694119	0.22578312	0.66898
CEP128	1.123028	1.1386017	1.11016638	0.7948413	-0.3312613	0.669
ZNF865	1.093143	1.0777119	1.10588717	1.3625609	0.44632069	0.66915
SUSD1	1.049918	1.0347672	1.06242922	1.7956344	0.84449363	0.66935
TPPP	1.036867	1.0217993	1.04931069	2.262027	1.17761616	0.66944
RASAL3	1.039619	1.0547801	1.02709812	0.4946704	-1.0154606	0.66966
SCD5	1.439762	1.4568499	1.42565038	0.9317074	-0.1020512	0.66998
KDM4C	1.126629	1.1108447	1.13966325	1.2599906	0.33341297	0.67
TPK1	1.089518	1.0740961	1.10225389	1.380017	0.46468601	0.67008
POLR2A	1.411428	1.39411	1.42572894	1.0802286	0.11133671	0.67024
UBQLNL	1.049313	1.0342444	1.0617566	1.8034081	0.85072593	0.67029
KHK	1.214041	1.2300507	1.20082071	0.8729412	-0.1960436	0.67029
ZNF33B	1.388748	1.4058495	1.37462481	0.9230634	-0.1154984	0.67033
DUS4L	1.371485	1.3885211	1.35741627	0.9199404	-0.1203877	0.67036
TCEANC	1.050573	1.0656199	1.03814614	0.5813195	-0.7825968	0.67043
MIR155HG	1.098929	1.0834341	1.11172423	1.3390713	0.42123273	0.67054
PLD5	1.049871	1.0350189	1.06213662	1.7743743	0.82731038	0.6706
ADAL	1.513106	1.5308193	1.49847804	0.9390729	-0.0906909	0.67069
HYI	1.596367	1.578228	1.6113461	1.0572752	0.0803509	0.6707
RP11-498P14.5	1.123918	1.1083163	1.13680202	1.2629865	0.3368392	0.67071
MRPS25	1.79127	1.7716199	1.80749826	1.0464974	0.06556876	0.67076
SFI1	1.367023	1.3499258	1.38114295	1.0892109	0.12328334	0.67107
C5orf34	1.113579	1.0980242	1.1264244	1.289726	0.36706455	0.6711
DCLRE1C	1.624279	1.6425927	1.60915468	0.9479639	-0.077096	0.67114
LMLN	1.133385	1.1177652	1.14628354	1.242163	0.31285453	0.67121
KIAA0368	1.362476	1.3796191	1.34831921	0.9175493	-0.1241424	0.67122
FASTKD1	1.159734	1.1754195	1.14678113	0.8367437	-0.2571423	0.67127
RNASEH1	1.433049	1.4157084	1.44736839	1.0761592	0.10589146	0.67141
SLX1A	1.044213	1.0593335	1.03172618	0.5347098	-0.9031718	0.6715
MAPK15	1.101739	1.0863612	1.11443869	1.325117	0.40611969	0.67162
PPFIBP2	1.045057	1.030048	1.05745213	1.9120136	0.93509279	0.67169
XXbac-BPG252P9.10	1.155686	1.1712986	1.142793	0.8335913	-0.2625879	0.67175
CHMP7	1.303392	1.3198183	1.28982756	0.9062256	-0.1420579	0.67178
RP11-1008C21.2	1.068611	1.0836406	1.05619997	0.671922	-0.5736343	0.67183
GLIDR	1.079563	1.0946672	1.06709035	0.7086966	-0.49676	0.67195
C7orf33	1.05359	1.0686198	1.04117816	0.6000913	-0.736746	0.67195
TMEM175	1.155318	1.1708956	1.1424538	0.8335723	-0.2626208	0.67199
RPRD1A	1.717391	1.7364076	1.70168691	0.9528513	-0.0696771	0.6721
MTFR2	1.08628	1.1012985	1.07387744	0.7293048	-0.4554063	0.67216
LRPAP1	2.009812	2.0301826	1.99299023	0.9638973	-0.0530487	0.67245
SNX33	1.096943	1.0816937	1.10953577	1.3408111	0.42310603	0.67246
DGCR8	1.189996	1.1740222	1.2031875	1.1675952	0.22354013	0.67255
MYCNOS	1.087132	1.1023884	1.07453359	0.7279495	-0.4580898	0.67261

TUSC1	1.225861	1.2419199	1.21259858	0.8787974	-0.1863976	0.67273
PTPRB	1.05926	1.0442861	1.07162646	1.6173583	0.69363933	0.6729
RASGEF1A	1.071614	1.0565007	1.0840943	1.488376	0.57373906	0.67291
TFCP2	1.130934	1.1462478	1.1182871	0.8088128	-0.3061222	0.67304
CAPN10	1.209167	1.1930879	1.22244504	1.15204	0.20419084	0.6731
CTB-186G2.1	1.034433	1.0195825	1.04669592	2.3845799	1.25373512	0.67318
GMPPB	1.280843	1.2974918	1.26709392	0.8978194	-0.1555029	0.67325
TMEM56	1.272859	1.289047	1.2594911	0.897747	-0.1556192	0.67343
PRLR	1.057398	1.0723602	1.0450428	0.6224801	-0.6839004	0.67359
RP11-164P12.4	1.054839	1.0399373	1.0671445	1.6812464	0.74953119	0.67363
ENG	1.033309	1.0185516	1.04549503	2.4523495	1.29416461	0.67364
HSPA6	1.033409	1.0185436	1.04568591	2.4636959	1.30082422	0.67364
BFAR	1.937145	1.9576128	1.92024232	0.9609754	-0.0574286	0.67367
NR1D1	1.163376	1.1478012	1.17623874	1.1924036	0.25387264	0.67368
FAM168B	1.543749	1.526194	1.55824572	1.0609123	0.08530541	0.67372
DONSON	1.32152	1.3047904	1.33533473	1.1002143	0.13778455	0.67373
SERAC1	1.241809	1.2578184	1.2285877	0.886623	-0.1736073	0.67373
TTC4	1.073309	1.0883796	1.06086395	0.688665	-0.5381258	0.6738
SMIM12	1.58842	1.6066176	1.57339218	0.9452283	-0.0812652	0.6738
WWP2	1.140292	1.124797	1.15308877	1.226702	0.29478477	0.67395
SLC25A17	1.226569	1.2104268	1.23989903	1.1400594	0.18910898	0.67401
TTYH2	1.066771	1.081718	1.05442748	0.6660403	-0.5863185	0.67411
ADGRA2	1.38047	1.3635036	1.3944814	1.0852202	0.11798777	0.67419
XYLB	1.046123	1.0311692	1.05847165	1.875942	0.90761524	0.67434
RP11-655M14.13	1.182489	1.1982876	1.16944161	0.8545245	-0.2268062	0.67449
ARHGEF19	1.110576	1.1258704	1.09794516	0.7781429	-0.361893	0.6745
PXK	1.188797	1.2044963	1.17583152	0.8598276	-0.2178807	0.67454
FAAP100	1.231841	1.247852	1.21861901	0.8820545	-0.1810603	0.67458
HLA-DMA	1.067522	1.0825598	1.05510386	0.6674419	-0.5832858	0.67464
FLJ31104	1.047998	1.0628438	1.03573851	0.5686875	-0.8142919	0.67469
NFE2L2	1.982687	2.0031001	1.96582877	0.9628439	-0.0546262	0.6749
RELL1	1.063124	1.0481317	1.07550476	1.5687115	0.64958007	0.6749
TEX9	1.425677	1.4428531	1.41149351	0.9291874	-0.1059585	0.67491
TMEM145	1.111381	1.1267061	1.0987255	0.7791695	-0.3599909	0.67492
CHFR	1.201454	1.1855663	1.21457514	1.1563261	0.20954836	0.67495
WIPI1	1.279211	1.263257	1.29238697	1.1106522	0.15140716	0.67497
KRI1	1.146653	1.1620924	1.13390328	0.8260922	-0.2756252	0.67503
TMEM131	1.252086	1.2358769	1.26547166	1.1254668	0.17052344	0.67504
ARHGEF6	1.100131	1.085045	1.11258953	1.3238826	0.40477515	0.67516
CLUH	1.068729	1.0537239	1.08111955	1.5099334	0.59448488	0.67517
ZNF782	1.068876	1.0539586	1.08119479	1.5047595	0.58953296	0.67517
TRAF3	1.175723	1.1601663	1.18857037	1.1773411	0.23553232	0.67524
ARMC3	1.048757	1.0339093	1.06101922	1.799486	0.84758491	0.67527
NKIRAS1	1.231409	1.2475635	1.21806776	0.8808559	-0.183022	0.67536
DGKE	1.117758	1.1023519	1.13047988	1.2748169	0.35029008	0.67539

PRRG2	1.041152	1.0263752	1.05335469	2.0229142	1.01643515	0.67552
RNF216	1.439134	1.4563113	1.42494887	0.9312696	-0.1027293	0.67555
PRSS22	1.029642	1.0148661	1.04184435	2.8147472	1.49300536	0.67567
EEPD1	1.122589	1.1072767	1.13523334	1.2606031	0.33411409	0.67572
C17orf67	1.083354	1.0682755	1.09580549	1.4032191	0.4887403	0.67572
ERP27	1.04689	1.0319774	1.05920539	1.8514742	0.88867446	0.67594
CCBE1	1.042904	1.0282458	1.05500892	1.947509	0.96163002	0.67595
SFMBT2	1.047968	1.0331737	1.06018495	1.8142378	0.8593636	0.67607
ZDHHC9	1.317438	1.3341545	1.30363361	0.9086624	-0.1381838	0.67613
EPB42	1.068968	1.0840128	1.05654328	0.6730319	-0.5712532	0.67614
CAPS2	1.162985	1.178345	1.15030031	0.8427505	-0.2468226	0.67617
RP11-10L12.4	1.162837	1.1783029	1.15006538	0.8416319	-0.2487387	0.67617
WDR91	1.060135	1.0452341	1.07244024	1.6014515	0.67938011	0.67626
C1orf216	1.159146	1.1436515	1.17194144	1.1969348	0.25934462	0.67628
SENP8	1.085892	1.1008809	1.07351402	0.7287208	-0.4565619	0.67635
C1orf228	1.05571	1.040816	1.06801025	1.6662626	0.73661577	0.67643
MIGA1	1.326892	1.3435015	1.31317496	0.9117134	-0.1333476	0.67645
SNHG25	1.720024	1.7388419	1.70448296	0.9534962	-0.0687009	0.67667
MFSD7	1.044923	1.0302636	1.05702874	1.8844016	0.91410644	0.67676
AKNA	1.151528	1.1672846	1.13851534	0.8280223	-0.2722585	0.67686
NEK7	1.288566	1.2724164	1.30190204	1.1082372	0.14826676	0.67691
NFKBIB	1.181976	1.1976658	1.16901933	0.8550765	-0.2258746	0.67697
LINC00958	1.090302	1.0751326	1.10282852	1.368628	0.45273035	0.67713
SPATA2	1.08028	1.0653261	1.09262943	1.4179539	0.50381064	0.67713
SULF2	1.324966	1.3085939	1.33848586	1.0968652	0.13338619	0.67723
MAPT	1.134062	1.149462	1.12134469	0.8118765	-0.3006677	0.67729
POLA1	1.288346	1.3042967	1.2751742	0.9042956	-0.1451337	0.67744
SDK1	1.148244	1.1635603	1.1355954	0.8290242	-0.2705139	0.67746
MCTP1	1.082619	1.0676374	1.09499012	1.4044015	0.48995547	0.67749
TMEM25	1.403517	1.4204026	1.38957295	0.9266664	-0.109878	0.67755
PRKCA	1.063859	1.0490985	1.07604766	1.548878	0.63122353	0.67768
ZNF773	1.064816	1.0795516	1.05264729	0.6618008	-0.5955311	0.67782
EIF5A2	1.157627	1.17317	1.14479105	0.8361211	-0.2582162	0.67785
RP5-1125N11.2	1.035119	1.0204446	1.04723749	2.3105169	1.20821566	0.67792
MANSC1	1.220285	1.2357986	1.20747303	0.8798737	-0.1846316	0.67804
LINC01122	1.132655	1.1479643	1.12001271	0.8110921	-0.3020623	0.67805
C1orf106	1.098706	1.0837347	1.11107024	1.3264541	0.40757477	0.67812
HDAC8	1.263542	1.2474258	1.27685013	1.1189217	0.16210908	0.67831
CPSF7	1.442982	1.4599513	1.42896786	0.9326376	-0.1006116	0.67833
DIRAS2	1.156284	1.1717336	1.1435261	0.8357483	-0.2588595	0.67842
KIF13B	1.085154	1.0700706	1.09761002	1.3930232	0.47821929	0.67852
STPG2-AS1	1.030896	1.0162589	1.04298326	2.6436804	1.40254776	0.67853
HARS2	1.171836	1.1872051	1.15914343	0.8501018	-0.2342925	0.67863
CBX2	1.22209	1.2377178	1.20918525	0.8799731	-0.1844686	0.67871
FAM120AOS	1.566478	1.5846004	1.55151249	0.9434009	-0.0840571	0.67878

PANK2	1.437412	1.4545397	1.42326682	0.9311989	-0.1028388	0.67878
RP1-78B3.1	1.047664	1.0328517	1.05989671	1.8232434	0.86650718	0.67886
GGCT	2.553646	2.5272035	2.57548276	1.0316128	0.04490162	0.67891
AC129492.6	1.040708	1.0554024	1.02857256	0.5157277	-0.9553186	0.67902
TM9SF1	1.135132	1.1501812	1.12270421	0.8170411	-0.2915195	0.67906
RP11-355B11.2	1.073587	1.0884069	1.0613491	0.69394	-0.5271171	0.67907
PRPF8	1.677968	1.6597233	1.69303472	1.0504931	0.07106663	0.67925
ZKSCAN3	1.094775	1.1098044	1.08236269	0.7500857	-0.4148727	0.67925
SYT10	1.03348	1.018922	1.04550258	2.4047465	1.26588483	0.67945
PHLDB3	1.164017	1.1793366	1.15136517	0.8440281	-0.2446371	0.67949
CCHCR1	1.258309	1.2743054	1.24509826	0.8935232	-0.1624229	0.67968
NHSL2	1.237384	1.2217216	1.25031822	1.1289754	0.17501408	0.67971
FBXL7	1.180446	1.1958909	1.16769112	0.8560436	-0.2242439	0.67989
SHF	1.156466	1.1718756	1.14374049	0.8363054	-0.2578982	0.67997
RP11-456O19.2	1.18043	1.1957782	1.16775448	0.8568598	-0.222869	0.68008
FIZ1	1.109414	1.09436	1.12184573	1.2912862	0.36880876	0.6801
VPS8	1.368139	1.3849331	1.35427094	0.9203442	-0.1197546	0.68013
CXCL2	1.044631	1.0300607	1.05666365	1.8849733	0.9145441	0.68021
BTN2A1	1.359303	1.3427422	1.37297889	1.08822	0.12197026	0.68022
TMEM62	1.076571	1.0913448	1.06437047	0.7046977	-0.5049236	0.68029
MDGA2	1.032198	1.0177118	1.04416157	2.4933432	1.3180815	0.68029
C9orf163	1.05091	1.0655514	1.03881935	0.592197	-0.7558508	0.68039
RP11-60L3.6	1.09286	1.1076677	1.08063234	0.7488998	-0.4171554	0.68043
KLC3	1.040061	1.025456	1.05212213	2.0475367	1.03388932	0.68059
CPNE2	1.291434	1.3078156	1.27790548	0.902831	-0.1474722	0.68063
RP11-267M23.4	1.0362	1.0507554	1.02417952	0.4763934	-1.0697747	0.68065
OVOS2	1.039883	1.0252382	1.05197755	2.0594797	1.04227987	0.68085
PXMP2	1.964738	1.9454149	1.9806953	1.0373173	0.05285731	0.68091
TRMT10B	1.289698	1.3059398	1.27628592	0.9030728	-0.1470858	0.68093
SHISA9	1.15416	1.1694509	1.14153249	0.8352419	-0.2597339	0.68097
FAM35A	1.199557	1.215222	1.18662089	0.8671088	-0.205715	0.68126
SYTL3	1.035962	1.0504479	1.02399864	0.475711	-1.0718428	0.68127
OSBPL6	1.16376	1.1790204	1.15115803	0.8443623	-0.244066	0.68131
GLTSCR1	1.083956	1.0691045	1.09622131	1.3924024	0.47757619	0.68143
IRF7	1.20899	1.2247003	1.19601554	0.872342	-0.1970342	0.68143
G0S2	1.079966	1.0653492	1.09203725	1.4083914	0.49404835	0.68162
MAG	1.045633	1.0602279	1.03357988	0.557547	-0.8428346	0.68167
RIN1	1.121355	1.1363495	1.10897284	0.799217	-0.3233408	0.68172
TRIM69	1.338988	1.3553355	1.32548742	0.9160002	-0.1265801	0.68184
PRH1	1.1271	1.1119216	1.13963426	1.2476076	0.3191642	0.6819
RP11-192H23.4	1.059349	1.0447357	1.07141752	1.596431	0.67485021	0.68191
LINC00982	1.077357	1.0625836	1.08955627	1.4309857	0.51700922	0.68191
ZNF555	1.114579	1.1297305	1.1020659	0.7867531	-0.3460172	0.68192
PVALB	1.126427	1.1112913	1.13892579	1.2483081	0.31997402	0.68194
GNL2	1.448493	1.4649253	1.43492237	0.9354673	-0.0962409	0.682

CFAP74	1.089093	1.1039146	1.07685273	0.7395759	-0.4352298	0.68203
CTD-2366F13.1	1.116711	1.131588	1.10442522	0.7935773	-0.3335574	0.68219
ERICH1	1.739426	1.7576108	1.72440901	0.9561757	-0.0646524	0.68225
ZNF365	1.032329	1.0178739	1.04426647	2.4766038	1.30836306	0.68242
TMC6	1.117377	1.1023717	1.12976947	1.2676299	0.34213354	0.68243
UMAD1	1.266147	1.2818196	1.25320417	0.8984619	-0.1544708	0.68243
ZNF532	1.475093	1.49216	1.46099818	0.9366835	-0.0943664	0.68248
CCDC51	1.351897	1.335691	1.36527959	1.0881425	0.12186747	0.68257
RP11-372K14.2	1.054044	1.0685833	1.04203639	0.6129245	-0.7062186	0.68261
SPTY2D1	1.270663	1.254859	1.28371494	1.113223	0.15474266	0.68264
SNF8	3.095103	3.1246267	3.07072157	0.9746284	-0.0370758	0.68264
TCERG1L	1.097155	1.1118582	1.08501254	0.7600028	-0.3959233	0.6827
ARIH1	1.56927	1.5520913	1.58345607	1.0568109	0.0797172	0.68271
SLC6A16	1.105276	1.1203468	1.09282977	0.7713525	-0.3745378	0.68281
SGK1	1.17993	1.1952076	1.16731346	0.8571053	-0.2224556	0.68289
HIRIP3	1.265905	1.2814069	1.25310404	0.8994238	-0.152927	0.68293
FBXL8	1.108639	1.1235227	1.09634722	0.7799958	-0.3584617	0.68301
ATXN1L	1.187284	1.1719752	1.19992714	1.1625347	0.21727384	0.68306
RP11-727F15.12	1.049298	1.0347621	1.06130135	1.763455	0.81840477	0.68312
LMBR1	1.276024	1.2920055	1.26282652	0.9000739	-0.1518847	0.68327
TAF1C	1.16364	1.1483817	1.17624133	1.1877564	0.24823904	0.68333
ARNTL2	1.066467	1.0519324	1.07846998	1.5110013	0.59550493	0.68335
BMP2	1.062009	1.0474181	1.07405783	1.5618051	0.64321444	0.68338
LETM2	1.101206	1.115973	1.08901186	0.7675224	-0.3817192	0.68341
PHF2	1.141772	1.1266525	1.15425878	1.2179689	0.28447733	0.68346
WNT3	1.034914	1.0205123	1.04680762	2.2819298	1.19025443	0.68358
GRIP1	1.136018	1.1208405	1.14855192	1.2293222	0.29786304	0.6836
NCF2	1.02729	1.013034	1.03906326	2.9970337	1.5835353	0.68373
PHKA1	1.10887	1.0940537	1.12110483	1.2876133	0.36469935	0.68375
MBTPS2	1.208284	1.2237327	1.19552594	0.8739266	-0.194416	0.68375
RP11-37B2.1	1.174975	1.1596449	1.18763536	1.1753292	0.23306489	0.68377
ZNF334	1.278537	1.2942793	1.26553677	0.9023291	-0.1482744	0.68377
PRICKLE2-AS1	1.058568	1.0732635	1.04643227	0.6337711	-0.6579663	0.68385
ALDH4A1	1.110829	1.125773	1.0984885	0.7830657	-0.3527948	0.68386
CACNA1C	1.06966	1.0549968	1.08176926	1.4868005	0.57221109	0.68394
TMEM39A	1.187638	1.2028763	1.17505466	0.8628642	-0.2127947	0.68397
BCR	1.170812	1.1556052	1.18337069	1.1784359	0.23687326	0.68401
RPPH1	1.067453	1.082053	1.05539668	0.6751326	-0.5667572	0.68401
PIM3	1.412216	1.395702	1.4258541	1.0761991	0.10594507	0.68404
SLC25A21	1.091309	1.1059545	1.07921461	0.7476286	-0.4196063	0.68412
RP11-473I1.5	1.049246	1.0636501	1.03735033	0.5868071	-0.7690418	0.68416
TMTC1	1.19032	1.2057083	1.17761139	0.8634136	-0.2118762	0.68427
ADAM22	1.131083	1.1460312	1.11873915	0.8131081	-0.2984808	0.68429
ANGPT2	1.109631	1.0949139	1.12178453	1.2831053	0.3596396	0.68442
LINC01152	1.059577	1.0740792	1.04760059	0.6425635	-0.638089	0.68445

WDR55	1.276982	1.2608938	1.29026824	1.1125916	0.15392412	0.6846
ACTL6B	1.024779	1.0390638	1.01298307	0.3323558	-1.5891996	0.68466
MAML3	1.207223	1.222863	1.1943064	0.8718646	-0.197824	0.68467
ERG	1.029421	1.0150885	1.04125635	2.7342994	1.45117122	0.68468
ACSM3	1.049084	1.0346267	1.06102297	1.7623083	0.81746629	0.68505
FAAP20	3.545335	3.5825391	3.51461069	0.973697	-0.0384551	0.68508
KCNMB2-AS1	1.043394	1.057698	1.03158084	0.547347	-0.8694722	0.68509
STT3A	1.287168	1.3030901	1.27402018	0.9040882	-0.1454645	0.68511
CXXC1	1.33287	1.3487688	1.31974123	0.9167713	-0.1253662	0.68524
FKBP5	1.23691	1.2522178	1.2242681	0.8891844	-0.1694454	0.68535
TMUB1	2.05212	2.07242	2.03535669	0.9654396	-0.0507421	0.68538
CTD-2165H16.4	1.052974	1.067299	1.04114346	0.6113536	-0.709921	0.68548
RP1-40E16.9	1.152882	1.1680357	1.14036768	0.8353443	-0.2595571	0.68561
PLXNC1	1.226288	1.2417371	1.21352952	0.883313	-0.1790034	0.68563
C21orf33	1.034434	1.0487678	1.02259678	0.4633544	-1.1098119	0.68579
CHAF1A	1.271445	1.2871337	1.25848888	0.9002386	-0.1516207	0.68579
ZNF687	1.110686	1.0959499	1.12285592	1.2804168	0.35661356	0.6859
PCDHB4	1.061104	1.0754298	1.04927258	0.653224	-0.6143504	0.68611
ZNF135	1.168753	1.1839016	1.15624223	0.8495969	-0.2351496	0.68617
TPCN1	1.13104	1.116217	1.14328165	1.2328803	0.3020327	0.6864
EIF3B	1.516455	1.5335802	1.50231218	0.9413995	-0.087121	0.68649
MTRNR2L12	1.388621	1.3724614	1.4019663	1.0792161	0.10998381	0.68649
TFEC	1.079107	1.0646426	1.09105148	1.4085372	0.49419764	0.68651
GMDS	1.254417	1.2699536	1.24158673	0.8949194	-0.1601704	0.68659
TMSB4X	411.5709	415.34319	408.455592	0.9833771	-0.0241834	0.68673
PART1	1.026597	1.0124282	1.03829787	3.0815417	1.62365229	0.68681
TCHP	1.205496	1.190124	1.21818972	1.1476179	0.19864237	0.68687
MMD	1.800204	1.8185966	1.78501493	0.9589765	-0.0604326	0.68711
CPB2-AS1	1.047234	1.0614727	1.03547464	0.5770796	-0.7931577	0.68718
DMPK	1.163713	1.1486385	1.17616121	1.1851655	0.24508853	0.68721
TRIM32	1.100521	1.1150141	1.08855191	0.7699223	-0.3772152	0.68762
ZFYVE27	1.258072	1.2425233	1.27091229	1.1170566	0.15970233	0.68765
KLF12	1.1748	1.1598711	1.1871281	1.1704933	0.22711662	0.68787
DNAJC14	1.174853	1.1595952	1.18745291	1.1745526	0.23211135	0.68787
ISLR2	1.13359	1.1484308	1.12133478	0.8174503	-0.290797	0.68792
SMYD5	1.118518	1.1332129	1.10638209	0.7985867	-0.324479	0.68794
FBXO32	1.397346	1.3808411	1.41097669	1.0791291	0.1098675	0.68798
FLVCR1	1.328417	1.3125007	1.34156064	1.0929917	0.12828242	0.68801
CDK20	1.162906	1.1778721	1.15054763	0.8463815	-0.2406201	0.68808
CACHD1	1.138162	1.1236416	1.15015342	1.2144242	0.2802725	0.68812
HAPLN3	1.076823	1.0623326	1.08878964	1.4244505	0.51040549	0.68831
ZNF621	1.224892	1.2094268	1.23766281	1.1348254	0.1824704	0.68841
MYL12A	4.028619	4.0754249	3.98996612	0.9722124	-0.0406566	0.68844
CEP164	1.466938	1.4833248	1.45340543	0.9380968	-0.0921913	0.68846
B3GALT5-AS1	1.035922	1.0218141	1.04757281	2.1808285	1.12487631	0.68853

POLH	1.148167	1.1629949	1.13592125	0.833899	-0.2620555	0.68854
SLC25A28	1.088579	1.0742472	1.10041387	1.3524259	0.43554952	0.68871
ZBED3	1.172915	1.1879951	1.1604624	0.8535457	-0.2284598	0.6888
AIFM2	1.120027	1.1347006	1.10790942	0.8011056	-0.3199357	0.68886
UBE2E1-AS1	1.032498	1.0466171	1.02083832	0.44701	-1.1616209	0.68887
WNT7B	1.086593	1.0723565	1.09835033	1.3592459	0.44280651	0.68896
LRRTM1	1.036122	1.0219467	1.04782825	2.17929	1.12385817	0.68915
BHLHB9	1.155896	1.1407741	1.16838445	1.1961327	0.25837741	0.68922
RAB11FIP3	1.04933	1.0635755	1.03756516	0.5908751	-0.759075	0.6894
IER5	1.11273	1.0980447	1.12485678	1.2734682	0.34876292	0.68959
LMF1	1.120542	1.135337	1.1083232	0.8003958	-0.3212146	0.6896
GUCY1A2	1.048916	1.0347515	1.06061405	1.7442163	0.80257898	0.68961
CCDC142	1.130196	1.1154654	1.14236108	1.2329331	0.30209453	0.68969
AKAP1	1.146124	1.1608554	1.13395786	0.8327843	-0.2639853	0.68971
UBE4B	1.35896	1.3428631	1.37225377	1.0857212	0.11865373	0.68972
ATR	1.147294	1.1324644	1.15954045	1.2044022	0.26831721	0.68977
TFPT	1.245992	1.2613982	1.23326953	0.8923917	-0.1642511	0.68993
CDYL	1.302376	1.2867248	1.3153006	1.099663	0.13706141	0.69008
UBE2W	1.474687	1.491841	1.46052175	0.9363224	-0.0949228	0.69021
KHDRBS2	1.1562	1.1709446	1.14402287	0.8425117	-0.2472314	0.69035
NSMF	1.468542	1.4520219	1.48218394	1.0667269	0.09319087	0.69054
ST3GAL6	1.142429	1.1277124	1.1545825	1.2103952	0.27547817	0.69059
NCBP1	1.214103	1.2294241	1.20145002	0.8780681	-0.1875952	0.69069
RP11-495P10.1	1.078693	1.0932932	1.06663502	0.7142535	-0.4854919	0.69071
S100PBP	1.433931	1.4177781	1.44726975	1.0705917	0.09840839	0.69085
RP11-732A21.3	1.10868	1.1232114	1.09668016	0.7846687	-0.3498445	0.691
C1orf132	1.035776	1.0216948	1.04740533	2.185102	1.12770065	0.69103
ZNF697	1.070603	1.0848723	1.05881849	0.6930233	-0.5290242	0.69107
SYNGR2	1.483846	1.4672602	1.49754327	1.0648098	0.0905958	0.69115
NOS1AP	1.175395	1.1906712	1.16278059	0.8537241	-0.2281581	0.69124
BTD	1.118136	1.1327818	1.10604124	0.7986125	-0.3244324	0.69131
PCYOX1L	1.144114	1.1588934	1.13190825	0.8301682	-0.2685245	0.69131
DARS-AS1	1.087892	1.1024265	1.07588905	0.7409121	-0.4326256	0.69136
APOBEC3G	1.030933	1.0169433	1.04248552	2.507518	1.32626005	0.69138
ANKRD33B	1.052959	1.0387781	1.06466909	1.6676701	0.73783388	0.69139
PNMA6A	1.080056	1.0942135	1.06836382	0.7256267	-0.4627006	0.69141
LINC01521	1.080084	1.0944303	1.06823599	0.7226072	-0.4687165	0.69141
LEMD1	1.411914	1.4279544	1.3986682	0.9315669	-0.1022687	0.69142
ENOX2	1.26024	1.2757472	1.24743468	0.8973244	-0.1562985	0.69142
PSMC3IP	1.34209	1.3259303	1.3554355	1.0905262	0.12502443	0.6915
HCG18	1.584321	1.5674611	1.59824419	1.054247	0.07621289	0.69151
INTS6-AS1	1.130588	1.1159637	1.14266439	1.2302508	0.29895248	0.69155
CDH3	1.060437	1.0461276	1.07225428	1.5663997	0.64745242	0.69156
SGK3	1.089994	1.0756178	1.10186679	1.3471279	0.42988681	0.69167
TFPI	1.498472	1.4820398	1.5120418	1.0622396	0.08710924	0.6918

SEMA6C	1.194308	1.209441	1.18181128	0.8680788	-0.2041021	0.69191
RBSN	1.281148	1.2967923	1.2682285	0.9037582	-0.1459913	0.69198
RCC1	1.472233	1.4887028	1.45863176	0.9384677	-0.091621	0.69214
SH3GLB2	1.280747	1.265523	1.29331909	1.1046843	0.14363419	0.69214
TOR3A	1.290193	1.305597	1.27747163	0.9079659	-0.13929	0.69218
AP4B1	1.083032	1.0973829	1.07117997	0.7309289	-0.452197	0.69224
SNX14	1.459248	1.4758715	1.44552051	0.9362202	-0.0950802	0.69234
MORN4	1.3292	1.345126	1.31604733	0.9157448	-0.1269824	0.69247
MIS12	1.341448	1.3571821	1.32845519	0.9195735	-0.1209631	0.69253
FASTKD5	1.092507	1.1067552	1.08073986	0.7563085	-0.4029533	0.69258
ZFAND3	1.866148	1.8848538	1.85069969	0.9614015	-0.0567891	0.69266
PGAP1	1.587921	1.5705901	1.60223294	1.0554563	0.07786683	0.69275
MIEF2	1.218061	1.2029277	1.23055794	1.136158	0.18416343	0.69286
RSU1	1.540676	1.5578135	1.52652277	0.9439047	-0.0832868	0.6929
KCND2	1.03739	1.0234205	1.04892646	2.089047	1.06284494	0.69321
TAF4	1.084524	1.098769	1.07275945	0.7366629	-0.4409234	0.69324
IGSF5	1.045351	1.031343	1.05691861	1.8159925	0.86075823	0.69326
NSUN2	1.140342	1.1258459	1.15231337	1.2103168	0.27538469	0.69327
ALOX12-AS1	1.102437	1.1166992	1.09065822	0.7768542	-0.3642843	0.69329
GATAD2A	1.317509	1.3020902	1.3302428	1.0931926	0.12854763	0.69339
SQRDL	1.045164	1.0311973	1.05669783	1.8173958	0.86187264	0.69344
PLEKHG6	1.033418	1.0194993	1.04491148	2.303232	1.20365972	0.69368
RAD18	1.151599	1.1368697	1.1637625	1.196485	0.25880233	0.69374
LIMS1	1.905728	1.8878512	1.92049141	1.0367631	0.05208631	0.69379
PARP14	1.252865	1.237425	1.26561616	1.1187373	0.16187135	0.69382
NUP205	1.187798	1.1728056	1.2001798	1.1584104	0.21214649	0.69383
FAM98C	1.270237	1.2854715	1.25765698	0.9025664	-0.147895	0.69412
POLR2J2	1.189982	1.1750404	1.20232144	1.1558554	0.20896092	0.69412
RP11-554J4.1	1.085246	1.0709949	1.09701396	1.3664927	0.45047774	0.69422
C5	1.07912	1.0934095	1.06731994	0.7206965	-0.4725363	0.69435
DDX60	1.066967	1.0810376	1.05534744	0.6829846	-0.5500751	0.69447
SLC9A1	1.106705	1.0923854	1.11853053	1.2830011	0.35952237	0.69451
SMAGP	1.152052	1.1378072	1.16381526	1.1887283	0.24941902	0.6946
LEPR	1.134478	1.1199389	1.14648485	1.221329	0.28845191	0.69461
RDH13	1.140766	1.1263895	1.15263914	1.2076888	0.27224869	0.69463
BARHL2	1.036251	1.0502771	1.0246684	0.4906486	-1.0272381	0.69465
C16orf95	1.085695	1.0999775	1.07390101	0.7391763	-0.4360096	0.69482
PCGF6	1.344302	1.3598112	1.3314941	0.9213002	-0.1182568	0.69485
TGFBR2	1.063582	1.0496217	1.07511022	1.5136559	0.59803723	0.69486
HACL1	1.142556	1.1570168	1.13061471	0.831852	-0.2656012	0.69496
C11orf24	1.261744	1.2465074	1.27432663	1.1128534	0.15426351	0.69502
ST5	1.699067	1.716993	1.68426353	0.9543518	-0.067407	0.69502
KIAA2013	1.313606	1.2982963	1.3262483	1.0937055	0.12922433	0.69512
MYH10	2.170963	2.1915453	2.15396555	0.9684614	-0.0462336	0.6952
ADAMTS8	1.064406	1.0503677	1.07599926	1.5088882	0.59348596	0.6954

ELMOD3	1.320284	1.3046097	1.33322838	1.0939519	0.12954926	0.69543
ZNF792	1.065701	1.0798838	1.05398856	0.6758385	-0.5652495	0.69549
SPAST	1.373216	1.3891971	1.36001917	0.9250304	-0.1124273	0.69557
ERBB3	1.042333	1.0284554	1.05379391	1.8904671	0.91874277	0.69561
DNAJC18	1.291595	1.3070931	1.27879563	0.9078538	-0.1394681	0.69566
ESF1	2.523692	2.5494796	2.50239628	0.9696135	-0.0445184	0.69568
COL17A1	1.026746	1.0129919	1.03810451	2.9329395	1.5523473	0.69576
ZNF524	1.197409	1.1827297	1.20953123	1.146673	0.19745398	0.69584
LPIN3	1.158738	1.144084	1.17083911	1.1856909	0.245728	0.6959
SYNGAP1	1.460843	1.4773022	1.44725127	0.9370399	-0.0938175	0.69593
TBXAS1	1.079468	1.0652185	1.09123491	1.3989112	0.48430441	0.69596
CCDC186	1.55633	1.5398344	1.56995319	1.0557927	0.07832663	0.69599
AP3M1	1.423065	1.4389621	1.40993625	0.9338762	-0.0986968	0.69605
NGEF	1.095853	1.0817716	1.10748187	1.3144155	0.39442136	0.69611
CTD-2561B21.11	1.074304	1.0883958	1.06266618	0.7089267	-0.4962916	0.69621
OSBPL5	1.116436	1.1307613	1.10460626	0.7999785	-0.3219668	0.69623
RAPH1	1.210718	1.1959618	1.22290405	1.1374874	0.1858506	0.69633
CROCC	1.193759	1.1789181	1.20601396	1.1514427	0.20344268	0.69634
CPED1	1.028503	1.0146773	1.03992118	2.7199235	1.44356607	0.69642
TSC22D3	2.000968	2.0216195	1.98391403	0.9630925	-0.0542538	0.69659
GIGYF2	1.490099	1.5069947	1.4761461	0.939154	-0.0905663	0.69692
INTS5	1.133491	1.147845	1.12163743	0.822736	-0.2814984	0.69693
POC1B	1.242675	1.2574334	1.230487	0.8953268	-0.1595138	0.69693
NEUROD4	1.036651	1.0504819	1.02522838	0.4997511	-1.0007183	0.69702
SOX12	1.522651	1.5062971	1.53615641	1.0589759	0.0826698	0.69716
DPF3	1.107335	1.0931125	1.11908101	1.2788935	0.35489615	0.69736
ST6GALNAC2	1.039137	1.0252904	1.05057126	1.9996205	0.99972619	0.69752
ZNF597	1.03911	1.0253106	1.05050506	1.9954088	0.99668433	0.69752
VGLL1	1.029696	1.0159362	1.04105972	2.5764988	1.3654119	0.6976
SMARCC2	1.763241	1.7816987	1.74799836	0.9568883	-0.0635775	0.69765
CALB2	1.039592	1.0257813	1.05099713	1.9780662	0.98409073	0.69777
SERINC5	1.324538	1.3092968	1.33712405	1.0899696	0.12428785	0.69777
ESRRA	1.206764	1.1920835	1.21888799	1.1395459	0.18845901	0.69783
TRIM22	1.156373	1.1420297	1.16821874	1.1843916	0.24414616	0.69783
RP3-399L15.3	1.036272	1.0225154	1.04763286	2.115564	1.08104234	0.69798
COX17	3.202064	3.1724188	3.22654571	1.0249155	0.03550496	0.69798
ZC2HC1C	1.067332	1.0813424	1.05576105	0.6855099	-0.5447506	0.69808
SLC11A2	1.383218	1.3674941	1.39620322	1.0781212	0.10851942	0.69819
NMNAT1	1.174374	1.188647	1.16258675	0.8618569	-0.2144798	0.6982
CDC27	1.459235	1.4751743	1.44607295	0.9387565	-0.091177	0.69822
SLC5A12	1.240236	1.2253088	1.25256379	1.1209672	0.16474401	0.69825
ZNF473	1.095247	1.1094223	1.08354072	0.7634711	-0.3893546	0.6984
ZWINT	1.424659	1.4400391	1.41195777	0.9361844	-0.0951353	0.69844
SULT1A3	1.03178	1.018068	1.04310368	2.3856372	1.25437467	0.69845
MID2	1.031742	1.0180812	1.04302417	2.3794962	1.25065615	0.69845

SYNJ1	1.076952	1.0910222	1.06533266	0.7177662	-0.4784141	0.69847
SMOC2	1.032432	1.0186038	1.04385201	2.3571506	1.23704391	0.69859
ADNP2	1.225146	1.2102688	1.23743174	1.1291821	0.17527812	0.69874
MTOR	1.207796	1.1933229	1.21974848	1.1366915	0.1848407	0.69876
GUCY1A3	1.211118	1.2257567	1.1990296	0.881611	-0.1817858	0.69899
PIWIL1	1.032726	1.0464026	1.02143224	0.4618759	-1.114423	0.69901
C8orf44	1.267902	1.2831519	1.25530811	0.9016648	-0.1493368	0.69902
SH3GL3	1.065264	1.0513691	1.07673936	1.4938804	0.57906466	0.69912
USP13	1.194705	1.1801113	1.20675637	1.1479367	0.19904314	0.69935
KIAA1191	1.982166	1.9639373	1.99722035	1.0345282	0.04897296	0.69953
YBEY	1.703332	1.7208373	1.68887503	0.9556596	-0.0654313	0.69961
FAM71E1	1.264984	1.2800147	1.25257136	0.9019933	-0.1488114	0.69964
CAPN15	1.130027	1.1157737	1.14179739	1.2247805	0.29252326	0.6998
ELP2	1.614225	1.5976257	1.62793252	1.050712	0.07136731	0.6998
ZBTB6	1.091052	1.0770171	1.10264166	1.3327133	0.41436642	0.69989
CITED2	2.004406	1.9844115	2.02091755	1.0370841	0.05253289	0.69998
MYO3A	1.248111	1.2628188	1.23596493	0.8978237	-0.1554959	0.69999
RP1-93H18.1	1.027219	1.0409812	1.01585459	0.3868751	-1.3700603	0.70002
CCDC110	1.080053	1.0939885	1.06854461	0.7292868	-0.4554418	0.70005
KIAA0391	1.045701	1.0320266	1.05699284	1.7795483	0.83151112	0.70017
MAMDC4	1.123019	1.1371698	1.11133284	0.8116425	-0.3010837	0.70017
PHF10	1.53477	1.5511452	1.52124641	0.9457516	-0.0804668	0.7003
RP11-706O15.1	1.578595	1.5953469	1.5647617	0.9486262	-0.0760884	0.70035
IQGAP2	1.108249	1.0941957	1.11985533	1.2724076	0.34756089	0.7004
NCSTN	1.38327	1.3676285	1.39618683	1.0776827	0.10793249	0.70044
FAM58A	1.352812	1.368298	1.3400227	0.923227	-0.1152426	0.70049
METTL6	1.208295	1.2229325	1.19620739	0.8801204	-0.1842273	0.70074
FAM20C	1.148142	1.133771	1.16001027	1.1961503	0.25839864	0.70074
MUL1	1.215275	1.229728	1.20333864	0.885128	-0.1760421	0.70089
PCDH17	1.101012	1.1150139	1.08944845	0.7777188	-0.3626795	0.70102
KIAA0196	1.160155	1.17454	1.14827522	0.8495202	-0.2352799	0.70128
SLC38A4	1.055766	1.069459	1.04445868	0.6400706	-0.643697	0.70135
TAPBP	1.736323	1.719177	1.75048165	1.0435285	0.06146997	0.70139
GPR158	1.036931	1.0504229	1.02578982	0.5114702	-0.9672779	0.70145
CTD-3131K8.2	1.162748	1.1483096	1.17467093	1.1777453	0.23602759	0.70149
PARP10	1.065281	1.0790409	1.05391835	0.6821574	-0.5518234	0.70166
C6orf89	1.409032	1.4249369	1.39589736	0.9316615	-0.1021223	0.70168
MDM2	1.566019	1.5498357	1.57938423	1.0537406	0.07551981	0.70168
FAM133A	1.146075	1.1603014	1.13432631	0.8379608	-0.2550454	0.70176
ZNF607	1.093772	1.1075356	1.08240521	0.7663064	-0.3840068	0.70188
TEX19	1.030308	1.0438526	1.01912296	0.436074	-1.197355	0.70192
CASD1	1.259481	1.2446328	1.27174256	1.1108183	0.15162286	0.70202
LINC00271	1.066057	1.0522714	1.0774411	1.4815194	0.56707753	0.7021
TRIM11	1.256136	1.2413568	1.26834076	1.111801	0.15289855	0.70211
SELENBP1	1.264421	1.2789888	1.25238998	0.90466	-0.1445524	0.70214

PPP2R3B	1.179119	1.1934238	1.16730556	0.8649688	-0.2092801	0.70217
PRRC2B	2.464845	2.4429996	2.4828861	1.0276414	0.0393369	0.70219
WDR47	1.120181	1.1061364	1.13177979	1.2416083	0.31221005	0.7022
RP11-499P20.2	1.082253	1.0961193	1.07080193	0.7366045	-0.4410379	0.70232
MMP15	1.12734	1.1131795	1.13903321	1.2284315	0.29681745	0.70234
ALG1L2	1.127412	1.1133579	1.13901871	1.2263701	0.29439437	0.70234
ZNF654	1.274158	1.2890097	1.2618934	0.906175	-0.1421384	0.70237
CBWD7	1.106069	1.1199936	1.09456965	0.7881224	-0.3435084	0.70268
RP11-296O14.3	1.066282	1.0800734	1.05489262	0.6855291	-0.5447101	0.70277
GJB2	1.033463	1.0200492	1.04454066	2.2215734	1.15158181	0.70278
FAM171A2	1.147519	1.1332827	1.15927471	1.1950144	0.25702804	0.70287
JAK3	1.092199	1.1061567	1.08067239	0.7599369	-0.3960484	0.70294
TAF1	1.213387	1.1987744	1.22545434	1.1342221	0.18170321	0.70314
ARHGAP35	1.403256	1.3875552	1.41622122	1.0739662	0.10294861	0.70315
GPC3	1.359367	1.375646	1.3459234	0.9208762	-0.1189209	0.70327
HIST1H4J	1.039478	1.0530604	1.02826139	0.5326272	-0.9088019	0.70327
ZKSCAN7	1.101602	1.1156432	1.09000722	0.7783182	-0.3615681	0.70329
NUP58	1.471624	1.4875602	1.45846323	0.9403213	-0.0887743	0.70342
ZNF398	1.10327	1.1171448	1.09181174	0.7837461	-0.3515418	0.70355
CRK	1.64728	1.6641643	1.63333648	0.953584	-0.068568	0.70357
NR2F6	1.878932	1.8979003	1.86326838	0.9614301	-0.0567461	0.70357
GAD1	1.118795	1.1046573	1.13046954	1.2466355	0.31803975	0.70358
PSORS1C1	1.045372	1.0319481	1.05645773	1.7671679	0.82143914	0.70366
CCDC174	1.490862	1.5068654	1.47764639	0.9423536	-0.0856596	0.7037
RP11-38L15.2	1.087335	1.1011492	1.07592623	0.7506359	-0.4138147	0.70382
SERPINI1	1.059436	1.0730478	1.04819584	0.659785	-0.5999321	0.70387
C21orf2	1.409571	1.4256452	1.39629739	0.931051	-0.103068	0.70394
TTLL4	1.216461	1.2019493	1.22844467	1.1311979	0.17785139	0.70401
SLC30A2	1.031104	1.0175088	1.042332	2.4177526	1.27366665	0.70413
DZIP1	1.846249	1.8289473	1.86053615	1.0381072	0.05395541	0.70418
CTD-2035E11.5	1.038041	1.0516739	1.02678336	0.5183147	-0.9480997	0.70422
S100A5	1.045702	1.032207	1.05684693	1.7650464	0.8197061	0.70423
SCYL3	1.132492	1.1463437	1.12105229	0.827178	-0.2737303	0.7043
PLAC8	1.041306	1.0276922	1.05254786	1.8975665	0.92415044	0.70437
TBC1D19	1.221407	1.2067294	1.23352836	1.1296333	0.17585452	0.70439
KATNBL1	2.218885	2.1979291	2.23619088	1.0319399	0.04535899	0.70447
ZNF43	1.43885	1.4545131	1.42591437	0.9370783	-0.0937584	0.70448
HKR1	1.215427	1.2299652	1.20342026	0.8845695	-0.1769525	0.70448
UAP1L1	1.075724	1.0620758	1.08699415	1.4014173	0.48688664	0.70449
SLC38A8	1.035199	1.0485762	1.02415195	0.4971974	-1.0081094	0.70454
CDCA4	1.648919	1.665024	1.63561855	0.9557829	-0.0652451	0.70464
VPS9D1-AS1	1.063687	1.0772908	1.05245304	0.678645	-0.559271	0.70483
TM4SF1	1.025706	1.0123699	1.03671913	2.9684303	1.56970024	0.70484
RP11-112J3.16	1.170561	1.1564028	1.18225356	1.1652834	0.22068082	0.70497
C11orf68	1.188139	1.1738491	1.19994063	1.1500813	0.2017358	0.70507

BICDL1	1.060978	1.0473749	1.07221106	1.5242468	0.60809651	0.70508
TNFRSF10B	1.792997	1.8112646	1.77791143	0.9588874	-0.0605667	0.70517
CPEB1	1.083742	1.0699423	1.09513887	1.3602482	0.44386994	0.70517
OLMALINC	1.119401	1.1334067	1.10783436	0.808313	-0.3070141	0.70519
TM9SF4	1.362429	1.3473936	1.37484459	1.0790198	0.10972137	0.7054
TRIM28	1.77057	1.7879695	1.75620103	0.9596831	-0.0593699	0.70546
MROH1	1.12868	1.1427696	1.11704495	0.8198173	-0.2866257	0.70552
RGMA	1.129204	1.1152128	1.14075753	1.2217183	0.28891162	0.70571
LYNX1	1.076209	1.0627051	1.08736147	1.3932115	0.47841431	0.70576
HSPA12A	1.071632	1.0852333	1.06039941	0.7086363	-0.4968828	0.70577
ZNF385D	1.48064	1.496874	1.46723384	0.9403467	-0.0887354	0.70578
ST6GALNAC6	1.504974	1.5209686	1.49176616	0.9439458	-0.0832241	0.70579
IST1	1.873725	1.8914166	1.85911568	0.9637645	-0.0532474	0.7059
CCDC18	1.206717	1.2210931	1.19484516	0.881281	-0.182326	0.70592
NIPA1	1.174069	1.1598598	1.18580343	1.16229	0.21697002	0.70601
DCTN4	1.44538	1.4611873	1.43232547	0.9374184	-0.0932351	0.70605
ACSS3	1.334706	1.3499519	1.32211507	0.9204554	-0.1195803	0.70613
TBC1D12	1.188929	1.1745165	1.2008305	1.1507821	0.20261466	0.70615
CNIH3	1.156837	1.1428147	1.16841762	1.1792739	0.23789889	0.70628
CTD-2270L9.4	1.142093	1.156044	1.13057228	0.8367657	-0.2571044	0.70645
VILL	1.073324	1.0869667	1.06205729	0.7135752	-0.4868626	0.70649
PCGF5	1.317512	1.332721	1.30495271	0.9165417	-0.1257276	0.70653
CAB39L	1.108877	1.0952869	1.1201004	1.2604083	0.33389117	0.70654
TMEM254-AS1	1.080747	1.0943574	1.06950696	0.7366347	-0.4409787	0.70654
GRIPAP1	1.590286	1.573898	1.6038202	1.0521385	0.07332461	0.70655
ZNF200	1.13749	1.1237152	1.14886502	1.203288	0.26698199	0.70658
ZFP41	1.074288	1.0607234	1.08548929	1.4078464	0.49348995	0.7066
FZD4	1.055854	1.0423192	1.0670304	1.5839243	0.66350337	0.70672
ATP6V0E2-AS1	1.039769	1.053152	1.02871658	0.5402732	-0.8882391	0.70677
POLD3	1.336262	1.3511169	1.32399462	0.9227543	-0.1159815	0.70689
KIF17	1.038742	1.0521175	1.02769549	0.5314047	-0.9121171	0.70706
RP11-402D21.2	1.036	1.0493521	1.02497433	0.5060441	-0.9826651	0.70707
BRI3BP	1.197422	1.1832095	1.20915836	1.1416349	0.19110137	0.7071
CAMSAP3	1.087691	1.0740277	1.09897493	1.3369991	0.41899851	0.70716
ACTR5	1.069914	1.0834944	1.05869919	0.703031	-0.5083397	0.70721
CLCF1	1.035987	1.0227185	1.04694345	2.0663097	1.0470565	0.70732
RIPK2	1.273473	1.2588815	1.28552217	1.102907	0.14131109	0.7074
ZNF721	1.636746	1.6534377	1.6229615	0.9533603	-0.0689066	0.70755
RP11-165A20.3	1.041641	1.0550087	1.03060156	0.556304	-0.8460546	0.7076
TET3	1.104095	1.0902377	1.1155395	1.2803906	0.35658396	0.70765
JADE2	1.085782	1.0722402	1.09696433	1.3422491	0.42465243	0.70776
CERS2	1.593498	1.5774543	1.60674759	1.0507283	0.07138968	0.70777
KLHL24	1.75067	1.7675293	1.73674817	0.9598959	-0.0590502	0.7078
SKAP1	1.03418	1.0475106	1.02317063	0.4876944	-1.0359506	0.70788
TTC9B	1.034132	1.0474913	1.02309907	0.4863848	-1.03983	0.70788

ATP6AP1	1.607516	1.5910451	1.62111788	1.0508807	0.07159886	0.70794
UBE2K	2.031046	2.0129216	2.04601277	1.0326691	0.04637802	0.70799
SLC43A3	1.137597	1.15131	1.1262722	0.8345262	-0.2609708	0.70801
EPHA5	1.036264	1.04964	1.02521849	0.508028	-0.97702	0.70807
LIN54	1.154743	1.1406985	1.16634184	1.1822575	0.24154429	0.70808
PTTG2	1.031774	1.0451256	1.02074742	0.4597708	-1.1210133	0.70815
OSGEPL1-AS1	1.043484	1.0567301	1.03254508	0.5736824	-0.8016757	0.70828
RIMS3	1.07398	1.0875958	1.06273649	0.7162046	-0.4815564	0.7083
LA16c-431H6.6	1.094639	1.0810166	1.10588893	1.3070029	0.38626239	0.7083
B4GALNT1	1.060651	1.0740747	1.04956515	0.6691241	-0.5796543	0.70836
RARA-AS1	1.230835	1.2452227	1.21895311	0.8928745	-0.1634707	0.70845
CTD-2540F13.2	1.047506	1.0341446	1.05853976	1.714466	0.77775927	0.70854
KHDRBS3	1.147308	1.1612128	1.13582523	0.8425216	-0.2472145	0.70858
DUS1L	1.153737	1.1397595	1.16527999	1.1826033	0.2419662	0.70862
RNF150	1.259377	1.2448401	1.27138224	1.1084059	0.14848629	0.70867
SLIT1	1.053311	1.0669135	1.04207735	0.6288317	-0.6692541	0.70867
RAB27B	1.042982	1.0295626	1.05406317	1.8287692	0.87087305	0.70868
NUP133	1.253207	1.2675277	1.24138118	0.9022663	-0.1483748	0.7091
KLK10	1.027444	1.0142685	1.03832492	2.6859855	1.42545151	0.7092
MX2	1.137389	1.123481	1.14887419	1.2056442	0.26980421	0.70921
RP11-160E2.6	1.114901	1.1286122	1.10357812	0.805352	-0.3123085	0.70939
GRIK4	1.03362	1.0203973	1.04453908	2.183579	1.12669474	0.70941
TMEM154	1.028284	1.0151109	1.03916215	2.591641	1.37386588	0.70944
CSPG4	1.028284	1.0151186	1.03915568	2.589899	1.37289585	0.70944
AP5M1	1.425038	1.4098775	1.43755811	1.0675339	0.09428191	0.70949
TTC39A	1.115588	1.129296	1.10426764	0.8064259	-0.3103862	0.7096
NIPAL3	1.262709	1.2484237	1.2745066	1.1049937	0.1440381	0.70961
CLIP2	1.195386	1.2099245	1.18338038	0.873554	-0.1950312	0.7097
VWC2	1.043923	1.0572735	1.03289789	0.5744002	-0.7998718	0.70974
C19orf47	1.05489	1.0415134	1.06593615	1.5883084	0.66749106	0.70978
AURKC	1.080709	1.094173	1.0695903	0.7389619	-0.4364281	0.7098
MLLT10	1.55087	1.5665988	1.53788179	0.9493169	-0.0750384	0.70983
ZNF449	1.089576	1.0759653	1.10081602	1.3271331	0.40831305	0.70995
ZNF529-AS1	1.17875	1.164764	1.19029928	1.1549809	0.20786894	0.71007
SRGAP2C	1.340637	1.3257763	1.35290973	1.0832884	0.11541737	0.71007
RP11-422P24.11	1.079036	1.0924984	1.06791848	0.7342669	-0.4456236	0.71014
POC5	1.164931	1.1788017	1.15347624	0.8583599	-0.2203454	0.71041
LY96	1.034876	1.0217291	1.0457329	2.1046854	1.0736046	0.71041
KIAA0895L	1.341633	1.3563737	1.32945963	0.924478	-0.1132891	0.71044
BBOX1-AS1	1.27553	1.2904583	1.26320286	0.9061641	-0.1421558	0.71048
AC011747.4	1.038013	1.0512302	1.02709765	0.5289386	-0.9188279	0.71061
DYNC1I1	1.052124	1.0388687	1.06307134	1.6226757	0.6983747	0.71061
CAPS	1.093316	1.0799613	1.10434382	1.3049293	0.38397163	0.71078
ZNF530	1.097893	1.1113786	1.08675685	0.7789365	-0.3604224	0.71079
RP11-181E10.3	1.041352	1.0282123	1.0522036	1.8503861	0.88782634	0.7109

MVP	1.143445	1.1297811	1.15472855	1.1922275	0.25365953	0.71108
LAMTOR1	3.825054	3.7902968	3.85375663	1.022743	0.03244372	0.71111
SIK3	1.193601	1.2077837	1.18188838	0.8753735	-0.1920293	0.71123
USP24	1.271889	1.2867587	1.25960965	0.9053245	-0.1434931	0.71128
RSBN1L	1.855365	1.8725214	1.8411977	0.9640997	-0.0527457	0.71164
LPCAT4	1.530877	1.5153381	1.5437099	1.0550546	0.0773177	0.71168
THEM6	1.228644	1.2428071	1.21694824	0.8935003	-0.1624598	0.71177
CSGALNACT2	1.169165	1.1830729	1.15767986	0.8612957	-0.2154194	0.71189
RHPN1-AS1	1.064424	1.077653	1.05349897	0.688949	-0.5375308	0.71196
GAA	1.277316	1.2628593	1.28925458	1.100416	0.13804904	0.71202
RP11-22P6.2	1.075251	1.0887448	1.06410716	0.7223762	-0.4691778	0.71209
RP11-147L13.15	1.139504	1.1259344	1.15070948	1.1967304	0.25909816	0.71215
SH3TC1	1.036821	1.0236777	1.04767575	2.0135273	1.009725	0.71218
ORAOV1	1.188041	1.2020273	1.17649169	0.8736031	-0.1949501	0.71233
RINT1	1.106282	1.1197457	1.09516381	0.794716	-0.3314886	0.71233
KIAA0556	1.121992	1.1356557	1.11070831	0.8160976	-0.2931864	0.71238
SEMA4A	1.036542	1.0234413	1.04736039	2.0203829	1.0146287	0.71255
CAMTA1	4.293947	4.328731	4.2652219	0.9809209	-0.0277912	0.71264
MGEA5	2.299374	2.3188705	2.28327314	0.9730092	-0.0394746	0.71271
MIR193BHG	1.04924	1.0361223	1.06007312	1.663046	0.73382806	0.71285
SLC9A5	1.058067	1.0712242	1.0472008	0.6627075	-0.5935559	0.71302
FNDC11	1.067109	1.0537612	1.07813139	1.4533037	0.53933621	0.71316
FAIM2	1.078712	1.0919007	1.0678212	0.7379835	-0.4383396	0.71322
PREX1	1.113829	1.1272607	1.10273711	0.8072961	-0.3088301	0.71333
EXOSC10	1.278995	1.2645345	1.29093653	1.0998054	0.1372483	0.71334
POLDIP2	1.888979	1.9063069	1.87467018	0.9650928	-0.0512605	0.7135
CTD-2553L13.10	1.077697	1.0910794	1.06664496	0.7317238	-0.450629	0.7137
SCEL	1.029836	1.0166975	1.04068548	2.4366145	1.28487803	0.71374
ATRN	1.213914	1.1998654	1.2255149	1.1283337	0.17419386	0.71376
HPS5	1.152961	1.1666694	1.14164027	0.8498276	-0.2347579	0.71388
PAXBP1-AS1	1.051638	1.0647271	1.04082793	0.63077	-0.6648141	0.7139
PRR7-AS1	1.086503	1.0997131	1.07559454	0.7581201	-0.3995017	0.71398
RP11-522I20.3	1.047423	1.0605249	1.03660354	0.6047677	-0.7255469	0.71415
WDFY1	1.394041	1.4090435	1.38165235	0.933036	-0.0999953	0.71426
ATP8A1	1.126155	1.1396285	1.11502837	0.8238174	-0.2796036	0.71433
NEXN	1.174921	1.1613352	1.18613973	1.1537453	0.20632472	0.71434
AP001059.6	1.027858	1.0148661	1.03858637	2.5955921	1.37606368	0.71443
PRKAB2	1.315346	1.3008957	1.32727884	1.087682	0.12125687	0.71451
PTDSS1	1.393452	1.4086733	1.38088142	0.9319948	-0.1016062	0.71456
SNX30	1.155045	1.168744	1.1437314	0.8517717	-0.2314613	0.71464
RNPEPL1	1.158059	1.1444239	1.16931896	1.1723747	0.22943376	0.71471
IFFO2	1.0539	1.0408154	1.06470548	1.5853203	0.66477439	0.71473
MARCH9	1.177575	1.1639672	1.18881162	1.1515205	0.20354009	0.7148
CYTH3	1.143024	1.1292377	1.15440828	1.1947621	0.25672343	0.71516
ZFR2	1.166611	1.153017	1.17783793	1.1622099	0.2168707	0.71517

KIF18B	1.082032	1.0952122	1.07114796	0.7472571	-0.4203235	0.71518
LINC00562	1.049165	1.0360607	1.05998586	1.6634685	0.73419457	0.71518
PCDHA9	1.073287	1.0865984	1.06229405	0.7193444	-0.4752455	0.71519
IFI35	1.14522	1.131432	1.15660577	1.1915348	0.25282103	0.7152
UPRT	1.155008	1.1687888	1.14362746	0.8509298	-0.2328879	0.7153
SLC7A11	1.157966	1.1717066	1.14661891	0.8538923	-0.227874	0.71534
SUPT5H	1.597566	1.5817594	1.61061915	1.0496078	0.06985033	0.71539
MRPL17	2.127575	2.1466925	2.11178731	0.9695601	-0.0445978	0.71549
RRN3	1.167336	1.1808445	1.15618129	0.8636221	-0.2115279	0.71555
RP11-680F20.4	1.03006	1.0429672	1.01940041	0.4515163	-1.14715	0.71556
FAM26F	1.136207	1.1499436	1.12486264	0.8327309	-0.2640777	0.71569
EPS15L1	1.379755	1.3647646	1.39213382	1.0750325	0.10438021	0.71575
VSNL1	1.039484	1.0523497	1.02885858	0.5512656	-0.8591806	0.71582
CHGA	1.028319	1.0412954	1.01760343	0.4262801	-1.2301265	0.71591
POLR3E	1.24058	1.2544708	1.2291096	0.9003375	-0.1514621	0.71606
TTC23	1.220951	1.2349774	1.20936842	0.891015	-0.1664784	0.71607
RP11-356K23.1	1.031641	1.0187509	1.0422867	2.2551859	1.17324634	0.71612
DHRS11	1.195708	1.209604	1.18423305	0.8789577	-0.1861344	0.71616
H1FX-AS1	1.162626	1.1761731	1.15143908	0.859604	-0.2182559	0.71619
NKAIN2	1.05176	1.03868	1.06256138	1.6174086	0.69368418	0.7162
ASB16-AS1	1.257848	1.272367	1.24585846	0.9026734	-0.1477241	0.71622
CES3	1.049719	1.0628137	1.03890493	0.61937	-0.6911265	0.71635
C5orf49	1.132515	1.1192256	1.143489	1.2035081	0.2672459	0.71651
MFSD11	1.265634	1.2514773	1.27732432	1.1027809	0.14114619	0.71651
GALNT14	1.033398	1.0205477	1.04401027	2.1418611	1.09886492	0.71657
CRYZ	1.162882	1.1765627	1.15158463	0.8585316	-0.2200569	0.71667
ODAM	1.028809	1.0159362	1.03943989	2.4748545	1.3073437	0.71711
ASPRV1	1.036139	1.0232231	1.04680583	2.015482	1.01112492	0.71715
SLC26A7	1.053495	1.0404447	1.06427253	1.5891443	0.66825015	0.71715
DISP3	1.03089	1.0436589	1.02034466	0.4659907	-1.1016268	0.71716
ZNF775	1.110218	1.12361	1.09915899	0.8021926	-0.3179795	0.71719
PANK1	1.176065	1.1624261	1.18732823	1.1533136	0.20578482	0.71719
ERN1	1.080433	1.0672379	1.0913304	1.3583168	0.44181996	0.71725
SIPA1L1	1.210957	1.1971689	1.22234401	1.1276827	0.17336114	0.7174
LINC00342	1.124887	1.1114843	1.13595522	1.2195006	0.28629042	0.71748
MRVI1	1.106401	1.0932151	1.11729069	1.2582804	0.33145348	0.71772
SCRN1	1.768608	1.7523563	1.78202974	1.0394407	0.0558074	0.71777
RP11-327J17.9	1.03349	1.0464382	1.0227974	0.4909189	-1.0264434	0.71786
GEMIN4	1.114198	1.1272456	1.10342361	0.8127875	-0.2990498	0.71793
RGL2	1.464126	1.4489935	1.47662317	1.0615369	0.08615447	0.71805
F2RL2	1.100639	1.087443	1.11153602	1.2755279	0.35109441	0.71806
GRIN2B	1.094976	1.1080136	1.08420925	0.7796174	-0.3591619	0.71809
SETD1B	1.082632	1.0695771	1.09341382	1.3425947	0.42502382	0.7181
MSLN	1.029877	1.0170724	1.04045056	2.3693599	1.24449733	0.71811
BID	1.818875	1.8358177	1.80488305	0.9629887	-0.0544092	0.71819

UVRAG	1.373134	1.3881121	1.36076462	0.9295372	-0.1054155	0.71823
NDUFAF6	1.297055	1.3112261	1.28535263	0.9168661	-0.125217	0.71831
SULT1C2	1.13324	1.1197159	1.14440809	1.206257	0.27053737	0.71834
FYCO1	1.10447	1.1179345	1.09335055	0.7915456	-0.3372556	0.71837
DNAH11	1.113293	1.0999013	1.12435297	1.2447585	0.31586589	0.71839
LRP12	1.242047	1.2563216	1.2302586	0.8983193	-0.1546998	0.71841
RAB3GAP2	1.505264	1.4905071	1.5174512	1.0549311	0.07714877	0.71849
AUP1	1.950675	1.9680771	1.93630496	0.9671802	-0.0481435	0.71858
ASH2L	1.253143	1.2672147	1.24152329	0.9038549	-0.1458369	0.7186
RP11-483F11.7	1.046871	1.0338525	1.05762129	1.7021286	0.76734005	0.71869
B3GNT7	1.060157	1.0474278	1.07066809	1.4900141	0.57532601	0.7187
NFYC-AS1	1.070907	1.0837797	1.06027739	0.719475	-0.4749835	0.71876
B3GALNT2	1.366994	1.3814679	1.35504042	0.9307215	-0.1035785	0.71878
HLA-DRB1	1.104233	1.1172513	1.09348159	0.7972756	-0.3268496	0.7188
TGFBR1	1.406684	1.3921939	1.41864945	1.0674553	0.0941757	0.71889
ZNF230	1.123431	1.1366625	1.11250438	0.823228	-0.280636	0.71891
MLPH	1.03047	1.0177175	1.04100148	2.3141854	1.21050444	0.71901
DRC3	1.115502	1.1285359	1.10473921	0.8148633	-0.29537	0.71902
RP11-214N9.1	1.124788	1.1114362	1.13581356	1.2187562	0.28540954	0.71911
MXI1	1.591367	1.606766	1.57865013	0.9536627	-0.068449	0.71913
CIT	1.132674	1.145694	1.12192202	0.8368365	-0.2569824	0.71917
PROSER2	1.057718	1.044805	1.06838155	1.5262041	0.60994795	0.7192
SGPL1	1.290718	1.2769187	1.30211427	1.0909855	0.12563188	0.7193
EIF4G3	1.78038	1.7638666	1.79401615	1.0394697	0.05584764	0.71933
LOXL4	1.077657	1.0648269	1.0882517	1.3613429	0.44503046	0.71938
TUBB3	1.141972	1.1550848	1.13114298	0.8456208	-0.2419171	0.71939
GNB4	1.500769	1.5160915	1.48811538	0.9457924	-0.0804046	0.72015
CSRNP2	1.173153	1.1597152	1.1842506	1.15362	0.20616808	0.72015
TMEM119	1.02405	1.0113227	1.03456059	3.0523188	1.60990563	0.7203
RUNX1T1	1.037702	1.0504492	1.02717517	0.5386645	-0.8925411	0.72034
RP11-18H7.1	1.056586	1.0436953	1.06723127	1.538639	0.62165479	0.72038
MKL2	1.264349	1.250261	1.27598314	1.1027814	0.14114685	0.72041
ZDHHC19	1.036161	1.0233817	1.04671413	1.9978951	0.99848084	0.72049
SBF2	3.127027	3.0904195	3.15725886	1.0319741	0.04540681	0.72051
SEC31B	1.134487	1.1211129	1.14553113	1.2016151	0.26497483	0.72065
RP11-295G20.2	1.151774	1.1384875	1.16274617	1.1751685	0.23286759	0.72069
NUTM2B-AS1	2.414122	2.3947932	2.4300833	1.0253013	0.03604797	0.72089
DDIT4L	1.074827	1.0617358	1.0856382	1.387172	0.47214672	0.72096
FGD5-AS1	1.979057	1.9972189	1.96405872	0.9667474	-0.0487892	0.72103
CECR5-AS1	1.128936	1.1422677	1.11792588	0.8289014	-0.2707276	0.72108
KIAA0907	1.588771	1.5731205	1.60169556	1.0498587	0.07019514	0.72109
SYNRG	1.370855	1.3854247	1.35882266	0.9309799	-0.1031781	0.72121
MIR24-2	1.106996	1.0939036	1.11780798	1.2545635	0.32718548	0.72125
RTN4RL1	1.030611	1.0178779	1.04112636	2.3004071	1.20188919	0.72128
EFNB3	1.251585	1.2378615	1.26291757	1.1053387	0.14448854	0.72132

PLSCR4	1.038712	1.0514465	1.02819519	0.5480492	-0.8676227	0.72134
GTF2A1	1.394938	1.3801975	1.40711063	1.0707873	0.09867197	0.72144
CPA5	1.0354	1.0227077	1.04588235	2.0205673	1.0147604	0.72145
C22orf31	1.0305	1.0178371	1.0409577	2.2962102	1.19925471	0.72146
RXRA	1.220779	1.2345677	1.20939128	0.892669	-0.1638028	0.72148
DOCK10	1.057496	1.0447183	1.06804728	1.5216869	0.60567152	0.72155
ZDHHC15	1.139285	1.152549	1.12833167	0.8412488	-0.2493956	0.72163
KCNIP3	1.048148	1.0352955	1.05876173	1.6648527	0.7353945	0.72164
KCNC4	1.030264	1.0175943	1.04072658	2.3147538	1.21085878	0.72173
RNU12	1.03172	1.0443935	1.02125348	0.4787523	-1.0626488	0.72175
HS1BP3	1.220319	1.2339571	1.20905671	0.8935686	-0.1623496	0.72177
VPS18	1.122759	1.1361404	1.11170763	0.8205325	-0.2853676	0.72182
LDLRAD4	1.212948	1.1990695	1.22440821	1.1272858	0.17285328	0.72185
TMEM105	1.040583	1.0533208	1.03006386	0.5638295	-0.826669	0.7219
SNX25	1.154129	1.1408682	1.16507926	1.1718702	0.22881281	0.72195
AC000068.5	1.075925	1.0888869	1.0652202	0.7337441	-0.4466511	0.72199
CDAN1	1.084197	1.0972135	1.07344833	0.7555364	-0.4044268	0.72199
NLRX1	1.102655	1.1155617	1.09199643	0.796081	-0.329013	0.72215
RP11-356C4.3	1.053604	1.0663906	1.04304377	0.6483413	-0.6251745	0.72219
LINC00159	1.032621	1.0200174	1.04302989	2.1496265	1.104086	0.72226
GPBP1L1	1.725777	1.7419136	1.71245173	0.9602894	-0.0584589	0.72226
TMEM178A	1.34336	1.3291003	1.35513509	1.079109	0.10984056	0.72236
CEP192	1.195911	1.1823276	1.20712917	1.1360273	0.1839975	0.72251
IFT80	1.275229	1.2890202	1.26384063	0.9128797	-0.1315033	0.72257
DUS2	1.121025	1.1340621	1.11025857	0.8224439	-0.2820108	0.72263
TANK	1.413845	1.4284241	1.40180461	0.9378665	-0.0925455	0.72267
TCF19	1.168483	1.1550999	1.17953587	1.15755	0.21107454	0.72288
SPINK5	1.079215	1.0664605	1.0897482	1.350399	0.43338572	0.7229
CORO2A	1.044572	1.0320159	1.05494081	1.7160503	0.77909187	0.72305
AC007325.4	1.314198	1.3000102	1.32591495	1.0863463	0.11948408	0.72341
TCFL5	1.101241	1.0881765	1.11202992	1.2705192	0.3454182	0.72348
ZNF337-AS1	1.101357	1.0885784	1.11190899	1.2633888	0.33729865	0.72348
FANCD2	1.245947	1.2320387	1.25743354	1.1094422	0.14983449	0.72349
GFY	1.124071	1.1370409	1.11336103	0.8272061	-0.2736813	0.72354
ZWILCH	1.315992	1.3300476	1.30438397	0.9222427	-0.1167816	0.72354
TAF1D	2.654429	2.6302118	2.67442765	1.0271228	0.03860866	0.7236
LUC7L	1.696545	1.7123556	1.68348886	0.9594771	-0.0596797	0.72362
INSL3	1.047446	1.0348707	1.05783167	1.658461	0.72984508	0.72364
ABCB11	1.026192	1.0387343	1.01583466	0.4088016	-1.2905273	0.72364
UGGT1	1.242471	1.2286486	1.25388659	1.110379	0.15105214	0.72368
HIF1A-AS2	1.074525	1.0616464	1.08515983	1.3814234	0.46615558	0.72368
ZBTB7B	1.087111	1.0743758	1.09762816	1.3126343	0.39246508	0.72373
ARX	1.040422	1.0277882	1.05085464	1.83008	0.87190674	0.72384
SULT4A1	1.046643	1.0339861	1.05709478	1.679943	0.74841227	0.72384
STXBP4	1.197849	1.211298	1.1867424	0.8837871	-0.1782293	0.72401

RPGRIP1L	1.322407	1.3364808	1.31078456	0.9236325	-0.1146092	0.72403
CHTF18	1.181405	1.1948155	1.1703301	0.8743151	-0.1937748	0.72423
PCP4L1	1.05468	1.0674288	1.04415256	0.6548028	-0.6108676	0.7243
ZNF843	1.060041	1.0473344	1.07053337	1.4901065	0.57541545	0.72439
RGS10	1.197284	1.2106153	1.18627426	0.8844288	-0.1771821	0.7244
FAM89A	1.305015	1.2910547	1.31654337	1.0875736	0.12111306	0.72448
ARHGAP5-AS1	1.164308	1.1776095	1.15332391	0.8632643	-0.2121257	0.72451
ZNF41	1.042683	1.0300072	1.05315177	1.7713029	0.82481094	0.72454
CDH8	1.173157	1.1600224	1.18400404	1.149864	0.20146319	0.72458
ATF7	1.227612	1.2413797	1.21624296	0.8958621	-0.1586515	0.72461
TSPAN18	1.245171	1.2588668	1.23386146	0.9034045	-0.1465559	0.72461
CASP4	1.031091	1.0184888	1.04149852	2.2445182	1.16640581	0.72463
SPRED3	1.092599	1.0799073	1.10307989	1.2899935	0.36736383	0.72473
TDG	1.835822	1.8523925	1.82213808	0.9645065	-0.0521371	0.7248
DSN1	1.235834	1.2493628	1.22466225	0.9009455	-0.1504883	0.72481
TP53I11	1.429628	1.4447071	1.41717468	0.9380886	-0.0922039	0.72494
TP53BP1	1.634668	1.6196493	1.64707161	1.0442546	0.06247353	0.72494
BCL2L2	1.133531	1.1468212	1.12255656	0.8347335	-0.2606125	0.72502
TRIM52	1.15338	1.1400461	1.1643918	1.1738407	0.23123658	0.72508
RECQL	1.447017	1.4324741	1.45902636	1.0613961	0.08596311	0.72524
GALM	1.284426	1.2981323	1.27310711	0.91606	-0.126486	0.72524
PATL1	1.244792	1.2586751	1.23332703	0.9020082	-0.1487876	0.72532
NCK1-AS1	1.156316	1.1430492	1.16727119	1.1693265	0.22567777	0.72533
ITCH	1.224952	1.2115606	1.23601142	1.1155734	0.15778545	0.72533
LIX1	1.303354	1.3170694	1.29202726	0.92102	-0.1186956	0.72542
P2RX4	1.183642	1.1970419	1.17257527	0.8758304	-0.1912766	0.72546
ZNF407	1.08418	1.0713128	1.09480642	1.3294442	0.41082324	0.72547
PPP1R3F	1.084275	1.0714414	1.09487384	1.3279961	0.40925096	0.72547
WIF1	1.024535	1.0120052	1.03488247	2.9056248	1.53884844	0.72553
AC108938.5	1.024474	1.0120052	1.03477169	2.8963964	1.53425906	0.72553
RP11-290O12.2	1.066792	1.0540847	1.07728595	1.4289793	0.51498497	0.7256
ACACA	1.273478	1.2872642	1.26209329	0.9123773	-0.1322976	0.72581
FAM19A4	1.032292	1.0197349	1.04266243	2.1617718	1.11221421	0.72582
FAM173A	1.963626	1.9814056	1.9489437	0.966923	-0.0485271	0.72585
LSM1	2.142594	2.1235231	2.1583424	1.0309912	0.04403197	0.72587
C16orf91	1.406297	1.4206919	1.39440887	0.9375243	-0.093072	0.72587
ENTPD7	1.04986	1.0372032	1.06031171	1.6211448	0.69701296	0.72596
SGPP2	1.038881	1.0263896	1.04919582	1.8642106	0.89856487	0.72607
RP11-20I23.6	1.049005	1.0615515	1.03864368	0.627827	-0.671561	0.72619
TIGAR	1.214439	1.2011669	1.22539992	1.120462	0.16409374	0.72634
RP11-276H19.2	1.110121	1.122936	1.09953838	0.8096764	-0.3045828	0.72643
EXOC1	1.46367	1.4490692	1.47572727	1.059363	0.08319705	0.72644
SIX1	1.084192	1.0968928	1.07370275	0.7606631	-0.3946705	0.72645
PLEKHN1	1.040541	1.0280318	1.05087161	1.8147829	0.85979698	0.72674
BMT2	1.324232	1.338153	1.31273639	0.924837	-0.112729	0.72685

RECQL4	1.130658	1.1435204	1.12003517	0.836363	-0.2577989	0.7269
FGD1	1.145575	1.1325227	1.15635425	1.1798299	0.23857887	0.72696
MAATS1	1.170147	1.1567993	1.18117061	1.1554301	0.20843001	0.72697
PRDM15	1.047996	1.0604635	1.03769934	0.6235054	-0.681526	0.72707
SOCS7	1.202309	1.1888207	1.21344721	1.1304226	0.17686218	0.72719
CBS	1.04252	1.054932	1.03227068	0.5874654	-0.7674243	0.72728
RP11-177G23.2	1.042555	1.0550449	1.03223979	0.5856995	-0.7717675	0.72728
RP11-141B14.1	1.13889	1.1517742	1.12825071	0.84501	-0.2429597	0.72731
CTD-2325P2.4	1.068748	1.0560659	1.07922086	1.4129953	0.49875664	0.72737
KCNMB4	1.358556	1.3446761	1.37001774	1.0735231	0.10235321	0.72747
RGS19	1.2067	1.1933792	1.21770047	1.1257696	0.17091166	0.72747
SLC25A33	1.358475	1.3724268	1.34695292	0.9316002	-0.1022172	0.72772
RNF103	1.123236	1.1103654	1.13386382	1.2129143	0.27847761	0.72775
LRRC40	1.205759	1.2189034	1.1949032	0.8903615	-0.1675369	0.72781
ATP6V1F	5.967991	5.9218439	6.00610098	1.017119	0.02448851	0.72787
HCN3	1.076723	1.0639808	1.08724486	1.3636093	0.44743029	0.72791
SCRIB	1.247471	1.2612433	1.2360971	0.9037442	-0.1460135	0.72791
WIZ	1.315569	1.3018444	1.32690307	1.0830184	0.1150577	0.72794
FAM83G	1.065614	1.0528654	1.07614119	1.440284	0.52635335	0.72799
FRMD5	1.08613	1.0734635	1.09658984	1.3148001	0.39484344	0.728
UHRF1BP1L	1.198608	1.211815	1.18770214	0.8861606	-0.1743599	0.72834
RP11-109G23.3	1.032301	1.0446289	1.02211971	0.4956371	-1.012644	0.72836
PER1	1.128924	1.1161534	1.13947001	1.2007402	0.26392401	0.72849
PACSIN3	1.485613	1.5000124	1.47372167	0.9474198	-0.0779243	0.72856
ARHGEF9	1.306149	1.2922409	1.31763373	1.0868899	0.12020586	0.72861
PHKB	1.584076	1.5993853	1.57143406	0.9533668	-0.0688968	0.72863
MTRF1	1.114437	1.1271724	1.10391973	0.817156	-0.2913166	0.72873
CDC37L1-AS1	1.128948	1.116019	1.13962477	1.2034642	0.26719328	0.72875
ZNF287	1.134878	1.1477381	1.12425752	0.8410662	-0.2497087	0.72877
GRIK5	1.100858	1.1135722	1.09035887	0.7956074	-0.3298714	0.72878
RP11-455F5.6	1.050007	1.0375027	1.06033306	1.608765	0.68595364	0.72884
KCNH2	1.172179	1.1589	1.18314428	1.152576	0.20486194	0.72896
TSR3	1.520723	1.5357202	1.50833833	0.9488878	-0.0756906	0.72902
IL15	1.027397	1.0151161	1.03753816	2.4833234	1.31227213	0.72903
RP11-210M15.2	1.154884	1.1418254	1.16566773	1.1681108	0.22417714	0.72919
PAPD7	1.184262	1.1975937	1.17325262	0.8768124	-0.1896599	0.72944
RP4-591C20.9	1.089605	1.1021773	1.07922287	0.7753468	-0.3670864	0.72951
AUNIP	1.054089	1.0664239	1.04390206	0.6609376	-0.597414	0.72961
MPDZ	1.70347	1.7188083	1.69080295	0.9610393	-0.0573327	0.72975
SLC35F1	1.236537	1.2227615	1.2479132	1.1129088	0.15433534	0.72976
ALKBH1	1.129062	1.1418428	1.1185071	0.8354817	-0.2593198	0.72988
TMEM31	1.046667	1.0590711	1.03642354	0.6166051	-0.6975813	0.72993
SMIM10L2B	1.123365	1.1106377	1.1338762	1.2100414	0.27505644	0.72998
RP6-65G23.3	1.208518	1.1953957	1.21935405	1.1226143	0.16686239	0.73001
ANKS6	1.128644	1.1158426	1.13921599	1.2017681	0.26515854	0.73007

CNNM4	1.056214	1.0436538	1.06658617	1.5253226	0.60911441	0.73012
RP11-406H21.2	1.039767	1.0522065	1.02949347	0.5649382	-0.8238351	0.73013
AC009404.2	1.041368	1.0289349	1.05163605	1.7845601	0.83556849	0.73018
ATAD2B	1.186064	1.1729844	1.19686448	1.1380478	0.18656119	0.7302
SPAG17	1.108518	1.1212556	1.09799891	0.808201	-0.307214	0.73022
PHF7	1.108418	1.1209985	1.09802812	0.8101601	-0.303721	0.73041
SLC16A12	1.033642	1.0214511	1.04370978	2.037645	1.02690274	0.73045
ASXL3	1.094101	1.0815167	1.10449252	1.2818545	0.35823253	0.73051
EPS8L1	1.084743	1.0975415	1.07417465	0.7604421	-0.3950898	0.7307
FPGT	1.084632	1.0971751	1.07427346	0.7643263	-0.3877394	0.7307
GEMIN7	1.725942	1.7415739	1.71303323	0.9615134	-0.0566211	0.73076
SPATA2L	1.1393	1.152132	1.12870286	0.8459945	-0.2412797	0.73088
FICD	1.059668	1.0472426	1.06992916	1.4802139	0.56580567	0.73091
CACFD1	1.288795	1.3025431	1.27744142	0.9170311	-0.1249574	0.73097
SLX4	1.080874	1.0682758	1.09127712	1.3368886	0.41887929	0.73117
RP5-1098D14.1	1.023159	1.0353279	1.01310914	0.371071	-1.4302329	0.73126
CCDC9	1.063483	1.0758776	1.05324686	0.7017471	-0.510977	0.73132
BIK	1.046396	1.034063	1.05658059	1.6610583	0.73210274	0.73133
YY1AP1	1.236228	1.249611	1.22517664	0.9021102	-0.1486243	0.73139
MKNK1	1.263139	1.2498294	1.27413092	1.0972724	0.1339217	0.73143
ARCN1	1.513908	1.4995174	1.5257919	1.0525997	0.07395695	0.73145
ZNF383	1.113854	1.1265078	1.10340465	0.8173777	-0.2909252	0.7316
ZNF18	1.082495	1.0950161	1.07215497	0.759397	-0.3970737	0.7317
ST8SIA1	1.123306	1.1360491	1.11278303	0.8289874	-0.2705779	0.73179
EHBP1	1.487474	1.5023422	1.47519516	0.945959	-0.0801505	0.7318
ZHX2	1.186927	1.1999978	1.1761334	0.8806765	-0.1833159	0.73184
SREBF1	1.092051	1.1046794	1.081622	0.7797332	-0.3589475	0.73188
SEMA5B	1.078163	1.0657274	1.08843197	1.3454358	0.4280736	0.73194
EXOC6B	1.160691	1.1736709	1.14997225	0.863543	-0.2116602	0.73194
MTHFD1L	1.28667	1.2731438	1.29783929	1.0904122	0.12487356	0.73196
RP11-259K5.2	1.035626	1.0233886	1.04573253	1.9553322	0.96741371	0.73209
ARHGAP39	1.093714	1.1062661	1.0833488	0.7843401	-0.3504488	0.7321
NEFH	1.070533	1.0828971	1.06032205	0.727674	-0.4586358	0.73215
ZC3HAV1	1.208605	1.1955846	1.21935717	1.1215463	0.16548919	0.7322
N4BP2L1	1.197131	1.1841228	1.20787432	1.1289984	0.1750435	0.7323
GPLD1	1.073319	1.0608369	1.08362662	1.3746044	0.45901646	0.73233
MDN1	1.203751	1.2169083	1.19288501	0.8892467	-0.1693443	0.73237
ZNF236	1.209274	1.1962178	1.22005646	1.1214908	0.16541773	0.73246
NDST1	1.216129	1.2031807	1.22682177	1.1163546	0.15879541	0.73247
MKRN3	1.073483	1.086127	1.06304148	0.7319593	-0.4501648	0.73254
ZSWIM3	1.051876	1.0643705	1.04155702	0.6455911	-0.6313075	0.73264
DAPK2	1.032812	1.0205959	1.04289965	2.0829227	1.05860932	0.73267
RAB33B	1.08913	1.1015746	1.07885377	0.7763137	-0.3652884	0.73272
LSM11	1.082659	1.0951192	1.07236823	0.7608159	-0.3943807	0.73273
S1PR2	1.144023	1.1313837	1.15445985	1.1756389	0.23344505	0.73282

ZNF543	1.10797	1.1204057	1.09769996	0.8114231	-0.3014738	0.73301
ASPSCR1	1.777211	1.7923615	1.76469926	0.9650889	-0.0512663	0.73302
NTF3	1.033442	1.0456014	1.02340101	0.5131647	-0.9625062	0.73306
LL0XNC01-237H1.2	1.078585	1.0910909	1.06825793	0.7493388	-0.41631	0.73311
LCLAT1	1.131577	1.1188554	1.14208294	1.1954267	0.25752562	0.73348
ZFP69B	1.07894	1.0913073	1.06872608	0.7526902	-0.409872	0.7335
ATMIN	1.27804	1.2645283	1.28919797	1.0932592	0.12863554	0.73355
APBB2	1.222241	1.2092279	1.2329882	1.1135616	0.15518138	0.73371
RP11-274B21.10	1.062048	1.0744625	1.05179642	0.6956041	-0.5236617	0.73371
MTURN	1.105014	1.0925196	1.11533205	1.2465683	0.31796196	0.73375
PRICKLE1	1.112117	1.0995459	1.12249805	1.2305681	0.29932448	0.73385
ERCC2	1.212832	1.2257826	1.20213798	0.895277	-0.159594	0.7339
KANSL1-AS1	1.116915	1.1295496	1.10648158	0.8219365	-0.2829012	0.73409
LGALSL	1.375524	1.3894244	1.36404411	0.9348262	-0.0972299	0.73411
FBXL13	1.077714	1.0653309	1.08794036	1.3460768	0.4287607	0.73422
APOL1	1.027886	1.0157961	1.03786995	2.3974252	1.26148583	0.73429
PHYKPL	1.868643	1.8852124	1.85495907	0.9658237	-0.0501683	0.73429
LARS2	1.097303	1.0848292	1.10760451	1.2684845	0.34310584	0.73434
GK5	1.295219	1.3086496	1.28412778	0.9205513	-0.1194299	0.73439
ALG11	1.082646	1.0702487	1.09288465	1.3222259	0.40296868	0.7344
MRM3	1.145216	1.1578317	1.13479689	0.8540546	-0.2275997	0.73447
WRN	1.278768	1.2919863	1.26785149	0.9173426	-0.1244675	0.73464
RP11-157P1.4	1.036015	1.0239347	1.04599042	1.9214983	0.94223169	0.73478
KISS1R	1.29717	1.2835851	1.30838781	1.0874613	0.12096407	0.7348
LINS1	1.156798	1.1695155	1.1462952	0.8630196	-0.2125348	0.73501
R3HDM1	1.547746	1.5624474	1.53560543	0.9522765	-0.0705476	0.73511
GATAD1	1.670491	1.6552123	1.68310874	1.0425761	0.06015276	0.7352
LINC00205	1.149162	1.136461	1.15965154	1.1699427	0.22643784	0.73522
CCDC159	1.209918	1.1968267	1.22072875	1.1214373	0.16534894	0.73524
INTU	1.517248	1.5024845	1.52943977	1.0536439	0.07538739	0.73531
ZKSCAN1	2.953865	2.9786638	2.93338491	0.9771164	-0.0333976	0.73531
TESC	1.048716	1.0610204	1.03855559	0.6318475	-0.6623517	0.73536
MVK	1.191915	1.2048064	1.18126933	0.8850764	-0.176126	0.73539
ZNF839	1.149639	1.1368941	1.16016397	1.1699848	0.22648973	0.73546
CDH13	1.038293	1.0261855	1.04829135	1.8442017	0.88299648	0.73549
CACNA2D2	1.084618	1.0968913	1.07448163	0.7687136	-0.3794819	0.73551
ZBTB48	1.084614	1.0969074	1.07446243	0.7683878	-0.3800936	0.73551
RP11-736N17.10	1.065334	1.0531051	1.07543247	1.420438	0.50633583	0.73556
SPANXB1	1.026787	1.0146773	1.03678794	2.5064487	1.32564473	0.73562
ANKRD18A	1.089456	1.0770068	1.09973639	1.2951634	0.37313416	0.73585
MFSD2A	1.067606	1.0799481	1.05741411	0.7181423	-0.4776583	0.73588
DLX2	1.038073	1.050153	1.02809703	0.5602265	-0.8359179	0.73602
RP5-1180E21.5	1.054976	1.0427829	1.06504435	1.5203353	0.60438951	0.73602
PPP1R42	1.050436	1.0624673	1.04050115	0.6483579	-0.6251377	0.73605
TLK1	1.95089	1.9677019	1.93700581	0.9682794	-0.0465047	0.73606

USP45	1.204198	1.2170499	1.19358409	0.8918875	-0.1650664	0.73614
UCN	1.062113	1.0743337	1.05202051	0.6998243	-0.5149353	0.73622
FAM160A2	1.306966	1.2934624	1.3181169	1.0840126	0.11638154	0.73629
C19orf53	5.835395	5.8819764	5.79692728	0.9825789	-0.0253548	0.73629
TCF3	1.710218	1.6953017	1.72253621	1.0391694	0.0554308	0.73639
SORCS1	1.449922	1.4635282	1.43868664	0.9464076	-0.0794664	0.73641
VMAC	1.081107	1.0935217	1.07085552	0.7576376	-0.4004202	0.73658
COL4A4	1.027963	1.0159741	1.03786317	2.3702819	1.24505868	0.73664
ZNF311	1.057739	1.0455121	1.06783675	1.4905202	0.57581595	0.73676
EPHB6	1.160956	1.1482425	1.17145512	1.1565852	0.20987153	0.73677
HIST1H2AH	1.051767	1.0396045	1.06181064	1.5606966	0.64219011	0.73679
ZNF79	1.040497	1.0283321	1.05054351	1.7839676	0.8350894	0.73688
BRF2	1.202313	1.1892144	1.21312992	1.1263937	0.17171113	0.73695
CORO1A	1.07666	1.0888712	1.06657667	0.749137	-0.4166985	0.73697
GCNT1	1.028256	1.0162589	1.03816269	2.3471923	1.23093603	0.73705
CFAP46	1.134721	1.1471521	1.12445518	0.8457591	-0.2416814	0.73716
WWP1	1.287917	1.3013938	1.27678669	0.9183555	-0.1228754	0.73722
LIM2	1.150504	1.1370679	1.16159917	1.1789718	0.23752917	0.73724
CHAC2	1.05927	1.0713129	1.04932434	0.691661	-0.531863	0.73727
MTDH	3.537494	3.5647259	3.51500456	0.9806134	-0.0282437	0.73736
DNAJC13	1.188026	1.1752878	1.19854525	1.1326816	0.17974233	0.7374
NRIP3	1.097894	1.0856369	1.1080166	1.2613323	0.33494839	0.73741
LMNTD2	1.065368	1.0532434	1.07537991	1.4157616	0.50157835	0.73746
PAN3	1.373269	1.3871231	1.36182824	0.9346595	-0.0974873	0.73754
LINC01567	1.029055	1.0170606	1.03896088	2.2836742	1.19135682	0.73777
CSNK2A2	1.402467	1.4166116	1.39078647	0.9380115	-0.0923226	0.73778
PTPRN	1.095524	1.1079475	1.08526376	0.7898636	-0.3403246	0.73782
RNF111	1.280579	1.2938076	1.26965509	0.9177948	-0.1237565	0.73789
LRFN1	1.081598	1.0694311	1.09164583	1.3199535	0.40048711	0.73804
ZNF552	1.129229	1.1167776	1.13951105	1.1946726	0.25661534	0.73812
KAT6A	1.49807	1.4839931	1.50969489	1.0531037	0.07464749	0.73816
PP7080	1.341309	1.3549128	1.33007413	0.9300148	-0.1046744	0.73819
TM4SF18	1.024689	1.012846	1.03446962	2.6832889	1.42400242	0.73847
GNAI2	2.503413	2.4837035	2.51968996	1.0242545	0.03457419	0.73862
TMEM27	1.063875	1.0759355	1.05391609	0.7100245	-0.4940594	0.7389
ESCO2	1.156254	1.1436424	1.16666929	1.1603074	0.21450707	0.73894
IFI44	1.027552	1.0156039	1.0374188	2.3980411	1.2618564	0.73905
C3orf52	1.037162	1.0252324	1.04701429	1.8632539	0.89782425	0.73918
LIFR-AS1	1.061855	1.0738604	1.0519411	0.7032334	-0.5079244	0.7392
PYROXD2	1.155037	1.1675449	1.14470697	0.8636906	-0.2114135	0.73921
LMX1A	1.025614	1.0137737	1.03539152	2.5695048	1.36149036	0.73934
NUP54	1.376728	1.3903048	1.36551621	0.9364892	-0.0946657	0.73944
GFPT2	1.104494	1.0922771	1.11458314	1.2417284	0.31234961	0.73954
NEDD4	1.103264	1.0911091	1.11330207	1.2435863	0.31450668	0.73968
C6orf62	2.214116	2.2329807	2.19853741	0.972065	-0.0408752	0.73973

SCRT1	1.02749	1.039461	1.01760343	0.4460964	-1.1645727	0.73976
SUZ12	1.717074	1.7021199	1.72942424	1.0388884	0.05504071	0.73992
ZMYM2	2.486903	2.4646615	2.50527112	1.0277263	0.03945611	0.73995
CERS1	1.07279	1.0605991	1.08285684	1.3672957	0.45132524	0.73998
FUT9	1.075489	1.0876886	1.06541434	0.7459844	-0.4227826	0.73999
LYSMD3	1.18775	1.1750509	1.19823705	1.1324539	0.17945227	0.74
PEBP4	1.032541	1.0206384	1.04237085	2.0530091	1.03774	0.74014
ZNF660	1.29611	1.3092301	1.28527534	0.9225343	-0.1163255	0.74017
JAK2	1.066674	1.0787199	1.05672701	0.7206185	-0.4726925	0.74018
APC2	1.095595	1.1076685	1.08562491	0.795264	-0.3304942	0.74027
ADAM10	1.745102	1.7605853	1.73231587	0.9628321	-0.0546439	0.74036
ARSA	1.121063	1.1089935	1.13102983	1.2021802	0.26565312	0.74036
ZBED8	1.130267	1.1425955	1.1200856	0.8421415	-0.2478654	0.74036
SYNPO	1.039417	1.0274909	1.04926489	1.7920427	0.84160505	0.74045
LSP1	1.034178	1.0223758	1.04392439	1.9630318	0.97308354	0.74056
AP3S2	1.205384	1.2182099	1.19479291	0.8926861	-0.1637751	0.74057
CCSAP	1.194745	1.1819279	1.20532952	1.1286311	0.17457403	0.74059
FLRT2	1.443092	1.457074	1.43154587	0.9441489	-0.0829137	0.74067
RP11-396C23.2	1.055844	1.0678576	1.04592212	0.6767427	-0.5633206	0.74067
FAM161B	1.086579	1.0743572	1.09667211	1.3001039	0.37862696	0.7407
CTD-2325M2.1	1.147258	1.1596868	1.13699437	0.857894	-0.2211286	0.74074
NEPRO	1.388094	1.3740977	1.39965148	1.0683078	0.09532731	0.74077
DHCR7	1.367965	1.3543371	1.37921936	1.0702221	0.09791022	0.74079
NADK	1.37019	1.3568943	1.3811698	1.0680189	0.09493712	0.74088
LCA5L	1.07668	1.0646202	1.0866395	1.3407493	0.42303952	0.74089
NCOA3	1.234369	1.221565	1.24494282	1.1055123	0.14471509	0.74093
ADTRP	1.046941	1.0350373	1.05677111	1.6203055	0.69626589	0.74097
PCDHB9	1.104988	1.1172718	1.09484319	0.8087466	-0.3062403	0.74103
IQCH	1.06966	1.057587	1.07963013	1.3827803	0.46757192	0.74104
RPUSD2	1.147194	1.1347709	1.15745353	1.1683051	0.22441702	0.74109
NRF1	1.076829	1.0888282	1.06692062	0.7533715	-0.4085665	0.7411
TCAP	1.034611	1.0227	1.0444481	1.9580632	0.96942734	0.74112
MAPKAPK5	1.720269	1.7355238	1.70767139	0.9621325	-0.0556925	0.7412
ZNF276	1.178095	1.190672	1.16770866	0.8795663	-0.1851357	0.74122
CDK14	1.177797	1.1902684	1.16749782	0.880324	-0.1838935	0.74122
TBC1D4	1.089776	1.0776338	1.09980336	1.2855655	0.36240307	0.74124
AKT1	1.505333	1.4916369	1.51664337	1.0508637	0.07157559	0.74138
ANKRD27	1.145745	1.1579362	1.13567786	0.8590673	-0.2191569	0.7414
SIK2	1.169581	1.1571936	1.17981002	1.1438765	0.19393128	0.74142
TASP1	1.1428	1.1551226	1.13262298	0.8549562	-0.2260776	0.74142
ZNF213-AS1	1.282222	1.295454	1.27129526	0.9182317	-0.1230699	0.74145
RP11-298I3.4	1.058446	1.0703461	1.04861934	0.6911444	-0.5329409	0.7415
DENND6A	1.20889	1.2215317	1.19845093	0.8958126	-0.1587311	0.74151
SIAE	1.112407	1.1246634	1.10228602	0.8204974	-0.2854294	0.74155
PAPLN	1.099706	1.0874486	1.10982813	1.2559168	0.32874084	0.7416

CLYBL	1.103408	1.1154628	1.09345245	0.8093729	-0.3051235	0.74184
BEND6	1.054268	1.0661122	1.04448613	0.6728881	-0.5715615	0.74188
PDP2	1.095427	1.0833024	1.10543967	1.2657459	0.33998782	0.74192
RP11-266L9.5	1.031785	1.019966	1.04154547	2.0808088	1.05714443	0.74194
CTIF	1.180127	1.1928578	1.1696132	0.8794728	-0.1852891	0.74212
FAM19A3	1.03236	1.0205314	1.04212816	2.0518875	1.03695165	0.74217
LINC00618	1.044828	1.0330212	1.05457769	1.6528092	0.72492023	0.74226
CARS2	1.38944	1.4028894	1.37833399	0.9390517	-0.0907236	0.74229
SP4	1.152611	1.1647696	1.14256979	0.8652675	-0.2087819	0.7423
ZNRF3	1.161055	1.1488381	1.1711439	1.1498664	0.20146621	0.74248
BDP1	2.030047	2.0475446	2.01559743	0.9695029	-0.044683	0.74248
UPK1A-AS1	1.120707	1.1085088	1.13078126	1.2052598	0.26934417	0.74248
CYTIP	1.08944	1.1013659	1.07959142	0.7851895	-0.3488872	0.74272
FBXL19	1.189731	1.1773145	1.19998447	1.1278517	0.17357743	0.74278
PRSS23	3.012743	3.0449408	2.98615376	0.9712525	-0.0420818	0.74279
LAMA3	1.026436	1.0147423	1.03609344	2.4482918	1.29177549	0.74285
NELFA	1.407442	1.4207887	1.39642073	0.9420897	-0.0860636	0.74289
STX16	1.62496	1.6106075	1.63681191	1.0429153	0.06062204	0.74297
RBM27	1.194503	1.1819664	1.20485654	1.125793	0.17094153	0.743
ADAMTSL5	1.031279	1.0195935	1.04092902	2.0889094	1.06274992	0.74306
LNX1	1.137695	1.1255409	1.14773138	1.1767593	0.23481927	0.74331
ASIC2	1.031811	1.0200468	1.04152651	2.0714807	1.05066235	0.74332
LINC01186	1.029208	1.0175063	1.03887069	2.2203857	1.15081031	0.74344
COLCA2	1.046469	1.0582204	1.03676463	0.6314732	-0.6632065	0.74358
STRBP	1.590214	1.5760354	1.60192273	1.0449405	0.06342085	0.74367
KCNA4	1.118485	1.1306009	1.1084788	0.8306127	-0.2677522	0.74378
EMID1	1.109688	1.1219008	1.09960248	0.8170783	-0.2914538	0.74379
UBR5	1.846043	1.8614803	1.83329464	0.9672823	-0.0479911	0.74394
RUSC1-AS1	1.058594	1.0704189	1.04882855	0.6934014	-0.5282374	0.74403
PPFIA4	1.384038	1.370639	1.39510287	1.0660046	0.09221371	0.74404
ATP9A	1.179195	1.1668811	1.18936464	1.1347276	0.18234599	0.74404
SECISBP2L	1.676134	1.691041	1.66382399	0.9606144	-0.0579706	0.74415
NCEH1	1.100679	1.0885091	1.11072896	1.2510455	0.3231343	0.74426
CLEC11A	1.9941	2.012115	1.97922356	0.9675023	-0.047663	0.74436
APAF1	1.312521	1.3254492	1.301844	0.9274688	-0.1086293	0.74437
EGR2	1.044321	1.0560233	1.03465659	0.6186103	-0.6928973	0.74438
TBC1D32	1.203054	1.1905922	1.21334585	1.119384	0.16270502	0.74444
MORC2	1.181856	1.1942127	1.17165135	0.8838319	-0.1781561	0.74446
NUDT19	1.183915	1.1717251	1.19398184	1.1296066	0.17582041	0.74457
CEP19	1.351028	1.3639215	1.34037953	0.9353104	-0.0964829	0.74464
PTGIS	1.072613	1.0843729	1.0629016	0.7455191	-0.4236829	0.74469
CLN6	1.120411	1.1083096	1.13040462	1.2039991	0.26783427	0.74478
RP11-373I8.1	1.065549	1.077267	1.05587207	0.7231038	-0.4677254	0.74479
DIRAS3	1.041088	1.02951	1.0506485	1.7163168	0.77931587	0.74482
PRKAA2	1.745284	1.7601821	1.73298046	0.9642169	-0.0525704	0.74484

WRAP73	1.238586	1.2511315	1.22822648	0.9087928	-0.1379767	0.74486
AREG	1.023842	1.0122555	1.03340982	2.7261135	1.44684563	0.74494
CTD-2017D11.1	1.226791	1.2393626	1.21640892	0.9041049	-0.1454379	0.74498
LINC00672	1.273976	1.2866436	1.26351407	0.9193091	-0.1213781	0.74508
SLC39A11	1.07837	1.0665064	1.08816795	1.3257056	0.40676046	0.74517
TBC1D2	1.040813	1.0290856	1.05049764	1.7361747	0.79591209	0.74519
PBX4	1.136793	1.1245424	1.1469099	1.1795979	0.2382951	0.7452
FLII	1.37281	1.3863849	1.36159964	0.9358534	-0.0956455	0.74523
ENOX1	1.047279	1.0589841	1.03761361	0.6376909	-0.6490709	0.74524
FAM218A	1.047242	1.0588783	1.03763341	0.6391729	-0.6457218	0.74524
SPRED2	1.150354	1.1381283	1.16045095	1.1616077	0.21612289	0.74527
FBXO9	1.625249	1.6399391	1.61311702	0.9580865	-0.0617722	0.74538
SH2D4A	1.061062	1.0493299	1.07075022	1.4342272	0.52027358	0.74539
CD82	1.13157	1.1195323	1.14151117	1.1838743	0.24351594	0.74555
KCTD21	1.030803	1.0424124	1.02121538	0.5002158	-0.9993775	0.7456
NSD1	2.319947	2.3393428	2.30392929	0.973559	-0.0386596	0.74565
CRIP2	1.697423	1.6837344	1.70872664	1.0365526	0.05179326	0.74576
SNTG1	1.026637	1.0150407	1.03621322	2.4076742	1.26764019	0.74579
RP11-126K1.2	1.049185	1.0608382	1.0395618	0.6502794	-0.6208685	0.7458
HIF1AN	1.349859	1.3630509	1.33896496	0.933657	-0.0990355	0.7458
MIEF1	1.349725	1.362889	1.3388531	0.9337652	-0.0988682	0.7458
TMEM232	1.122531	1.1346711	1.11250638	0.8354162	-0.2594329	0.74583
C2orf81	1.178147	1.1904889	1.16795491	0.8817043	-0.1816331	0.74603
MRPS12	2.087561	2.0712829	2.10100435	1.0277438	0.03948073	0.74611
CD109	1.056918	1.0451765	1.06661425	1.474532	0.56025715	0.74612
CLCN2	1.127998	1.1156473	1.13819819	1.1949976	0.25700767	0.74631
HMG20A	1.282169	1.294824	1.27171816	0.9216284	-0.1177429	0.7464
GPRIN3	1.063923	1.0521393	1.07365427	1.4126435	0.49839742	0.74643
NEK8	1.110396	1.0981288	1.12052602	1.228243	0.29659602	0.74665
PTPN1	1.292854	1.3058291	1.28213903	0.9225381	-0.1163195	0.74665
PRAP1	1.02649	1.0149608	1.03601027	2.4069743	1.26722073	0.7468
PGGHG	1.28516	1.2721257	1.29592352	1.0874515	0.12095104	0.74691
CDK13	1.34518	1.3320768	1.35600178	1.0720467	0.1003677	0.74697
MYO1D	1.092486	1.0807819	1.10215159	1.2645358	0.33860792	0.747
FO538757.1	1.425336	1.4122863	1.43611246	1.0577903	0.08105362	0.74702
RP11-284M14.1	1.03336	1.0217559	1.04294319	1.9738596	0.9810194	0.74707
DNAJC10	2.016642	2.0009244	2.02962202	1.0286712	0.04078187	0.74709
ERMARD	1.135336	1.1474233	1.12535463	0.8503043	-0.2339488	0.74723
PRKD3	1.534235	1.5203502	1.54570099	1.0487188	0.06862789	0.74726
SPIN4	1.144963	1.1571548	1.13489422	0.8583525	-0.2203578	0.74735
IGLON5	1.197213	1.1850965	1.20721824	1.1195148	0.16287364	0.7474
FOXP4-AS1	1.094814	1.1066448	1.08504484	0.7974591	-0.3265176	0.74741
RP11-127B20.2	1.086962	1.0988627	1.0771335	0.7802081	-0.358069	0.74748
GUCY1B3	1.234663	1.2471701	1.22433375	0.9076088	-0.1398575	0.74749
TRUB1	1.247264	1.2599512	1.23678718	0.9108907	-0.1346501	0.74751

CDS1	1.193652	1.1814854	1.20369953	1.1224015	0.16658884	0.74767
NME9	1.063728	1.0753363	1.05414084	0.7186553	-0.4766281	0.74773
CXCL8	1.028537	1.0169863	1.03807555	2.2415395	1.1644899	0.74777
ENO4	1.046378	1.0347391	1.05599034	1.6117372	0.68861654	0.74787
C16orf58	1.313682	1.3265126	1.30308704	0.9282552	-0.1074066	0.74793
STAG1	1.513829	1.4998204	1.52539807	1.0511737	0.07200114	0.74809
FOXH1	1.02915	1.0175759	1.03870767	2.2023138	1.13902006	0.74812
LINC00890	1.033132	1.0446317	1.0236348	0.5295524	-0.9171547	0.74814
AEN	1.223531	1.2109788	1.23389629	1.1086248	0.14877119	0.7482
PRUNE1	1.145872	1.1579224	1.13591992	0.8606754	-0.2164588	0.74824
USP7	1.38056	1.3936657	1.36973712	0.939216	-0.0904711	0.74828
RANBP10	1.100533	1.1124289	1.09070865	0.8068093	-0.3097004	0.74831
CACNG7	1.149233	1.1370159	1.15932148	1.1627953	0.21759713	0.74834
PPP4R3A	1.628334	1.6426758	1.61649053	0.9592559	-0.0600123	0.74837
RHOT2	1.612695	1.6268969	1.60096663	0.9586371	-0.0609433	0.74841
ERMAP	1.111772	1.099945	1.12153873	1.2160561	0.28220983	0.74841
PRDM5	1.094789	1.1065369	1.08508723	0.7986645	-0.3243385	0.74845
SMIM22	1.025823	1.0142717	1.03536194	2.4777752	1.30904528	0.7486
RP1-315G1.3	1.1204	1.132255	1.11061059	0.8363436	-0.2578324	0.74887
NIPSNAP3B	1.035993	1.0244593	1.04551804	1.8609718	0.8960562	0.7489
TBL1X	1.572493	1.5584467	1.58409286	1.0459241	0.06477812	0.7489
DOPEY2	1.102725	1.0908298	1.11254817	1.2391108	0.30930514	0.74893
PACSIN1	1.094394	1.0826646	1.10408061	1.2590712	0.33235984	0.749
MAPK4	1.054943	1.0434015	1.0644738	1.4855186	0.57096668	0.74902
USP44	1.107612	1.0957757	1.11738709	1.2256463	0.29354274	0.74903
PRKD1	1.082873	1.0946543	1.07314326	0.7727412	-0.3719428	0.74904
CTD-3157E16.2	1.081602	1.0932288	1.07199975	0.7722906	-0.3727844	0.74911
PTPRO	1.148223	1.1603953	1.13817026	0.8614361	-0.2151844	0.74913
RP11-637O19.2	1.067874	1.0560848	1.07760911	1.3837816	0.46861627	0.74916
EPHA2	1.268717	1.256494	1.27881133	1.0870092	0.12036415	0.74917
PPP1R35	1.403578	1.4165727	1.39284735	0.9430463	-0.0845994	0.74917
ZNF160	1.433007	1.4463918	1.42195413	0.9452551	-0.0812244	0.74919
ZNF316	1.085244	1.0736459	1.09482099	1.2875249	0.36460038	0.74926
ADGRG1	1.216189	1.2041007	1.22617131	1.1081358	0.14813473	0.74935
BCL2	1.072559	1.0608311	1.08224496	1.3520222	0.43511881	0.74947
CLUL1	1.137542	1.1256328	1.14737657	1.1730743	0.23029445	0.74959
RP11-527L4.2	1.130261	1.1422378	1.12036977	0.8462575	-0.2408314	0.74972
FRRS1	1.038081	1.0265335	1.04761663	1.7945882	0.8436528	0.74988
CHRFAM7A	1.048154	1.0597019	1.03861747	0.6468382	-0.6285231	0.74989
ZBTB11	1.155701	1.1435484	1.16573722	1.1545735	0.20736005	0.7499
PLXNA2	1.050735	1.0392286	1.06023673	1.5355303	0.61873696	0.7499
ZSCAN9	1.137947	1.1498298	1.12813313	0.8551914	-0.2256808	0.7499
AGO2	1.56764	1.5534175	1.57938466	1.0469214	0.06615314	0.75004
PIGO	1.103145	1.0913098	1.11291938	1.2366625	0.30645181	0.75008
LINC00094	1.782597	1.7972787	1.77047274	0.9663782	-0.0493402	0.75014

TMEM191C	1.146248	1.1344431	1.15599607	1.1603129	0.21451385	0.75016
VCX3B	1.033451	1.0219557	1.04294358	1.9559232	0.9678497	0.7502
KLHL2	1.097226	1.0855161	1.1068966	1.2500167	0.32194735	0.75024
FAM160B1	1.208079	1.1959935	1.21805969	1.1125862	0.15391709	0.75032
RP11-452L6.1	1.104902	1.1169461	1.09495663	0.8119691	-0.3005033	0.75035
TMEM106C	3.178577	3.2075692	3.15463543	0.9760217	-0.0350149	0.75038
GRK4	1.188382	1.2007648	1.17815561	0.8873846	-0.1723685	0.75043
PID1	1.035213	1.0237647	1.04466658	1.8795352	0.91037592	0.75071
CCT6B	1.081698	1.0699905	1.09136558	1.3053998	0.38449168	0.75072
NPR3	1.051365	1.0398579	1.06086805	1.5271281	0.61082107	0.75072
FAM13B	1.216611	1.2291533	1.20625403	0.9000702	-0.1518906	0.75078
SOCS5	1.179007	1.1668674	1.18903218	1.1328288	0.17992981	0.75102
SUOX	1.121393	1.1094747	1.13123582	1.1987771	0.26156344	0.75106
PCSK6	1.06627	1.0778737	1.0566877	0.7279437	-0.4581012	0.75108
RP11-256I23.2	1.077415	1.0656947	1.08709385	1.3257366	0.40679412	0.75114
ZNF778	1.083614	1.0954863	1.07380883	0.7729784	-0.3714999	0.75118
RP11-277P12.20	1.047148	1.0357036	1.05659876	1.5852408	0.66470199	0.75134
APITD1-CORT	1.042975	1.0543482	1.03358321	0.6179266	-0.6944926	0.75148
ECHDC3	1.052448	1.0638494	1.04303296	0.6739764	-0.56923	0.75157
CXorf57	1.133822	1.1456159	1.12408309	0.8521262	-0.230861	0.7516
TWIST1	1.029967	1.0185674	1.03938056	2.1209488	1.08470979	0.75163
KRT80	1.029889	1.0185692	1.03923648	2.1129866	1.0792836	0.75163
FRAT1	1.036109	1.0474779	1.02671999	0.562788	-0.8293364	0.75163
ESPL1	1.070099	1.0816625	1.06055044	0.7414718	-0.4315362	0.75164
GLYCTK	1.062019	1.0504643	1.07156151	1.4180617	0.50392033	0.75165
RP11-108M9.4	2.078163	2.0620738	2.091449	1.0276583	0.03936067	0.75173
ZNF573	1.153052	1.1650468	1.14314714	0.8673126	-0.2053761	0.75174
TFAP2A	1.162325	1.1741353	1.15257129	0.8761653	-0.190725	0.7518
GNRH1	1.052488	1.0410146	1.06196325	1.510761	0.59527543	0.75191
RNF152	1.042634	1.0312929	1.05199979	1.6617147	0.73267271	0.752
AC023590.1	1.071042	1.0825573	1.0615322	0.7453275	-0.4240535	0.752
RP11-105N14.1	1.064681	1.0761908	1.05517682	0.7241931	-0.4655536	0.752
C15orf61	1.505463	1.4919409	1.51662992	1.0501869	0.07064614	0.75201
RP11-571M6.17	1.041251	1.0527001	1.03179692	0.603356	-0.7289186	0.75205
CTD-2298J14.2	1.096431	1.0847823	1.10605111	1.2508634	0.32292423	0.75211
RGMB	1.230964	1.2436296	1.22050532	0.9050841	-0.1438762	0.75214
TMEM263	1.861731	1.8469374	1.87394771	1.0318918	0.04529165	0.75217
CLRN1-AS1	1.050199	1.0616358	1.04075506	0.6612241	-0.5967889	0.75218
CCDC92	1.192662	1.1805881	1.20263354	1.122076	0.1661704	0.75219
PIP5K1B	1.308765	1.3213368	1.29838335	0.9285689	-0.1069191	0.75221
GNAO1	1.031448	1.0427872	1.02208416	0.516139	-0.9541685	0.7523
RP11-47A8.5	1.043097	1.0545373	1.03364921	0.6169947	-0.6966699	0.7523
IPPK	1.072127	1.0837453	1.06253202	0.7466931	-0.4214127	0.75235
CDC25A	1.095224	1.08348	1.10492312	1.2568646	0.32982926	0.75242
CWC22	1.346307	1.3333338	1.35702126	1.0710623	0.0990424	0.75244

CUL2	1.297284	1.3097298	1.28700517	0.9266308	-0.1099335	0.75245
AC009501.4	1.127923	1.116393	1.13744556	1.1808747	0.23985593	0.75246
CRCP	1.270082	1.2825191	1.25981201	0.9196264	-0.1208802	0.75258
YIF1B	1.716152	1.7304307	1.70436055	0.9643085	-0.0524333	0.75267
TRIM66	1.079554	1.0912649	1.069883	0.7657162	-0.3851182	0.75268
TMEM266	1.033557	1.0449088	1.02418216	0.538473	-0.893054	0.75278
GALNS	1.137854	1.1498364	1.12795912	0.8539925	-0.2277047	0.7528
DHX30	2.02097	2.038606	2.00640504	0.9689959	-0.0454375	0.75283
AMN1	1.317836	1.3302656	1.30757084	0.9312833	-0.102708	0.7529
NARFL	1.261026	1.2733666	1.25083533	0.9175786	-0.1240963	0.75301
RHPN1	1.345933	1.3587773	1.3353259	0.9346351	-0.0975248	0.75301
ZMYND10	1.088349	1.0997982	1.07889417	0.7905374	-0.3390944	0.75307
FAM66B	1.028316	1.0170375	1.03762969	2.2086347	1.14315484	0.75321
LRRC49	1.332054	1.3447409	1.3215766	0.9328066	-0.1003501	0.75322
C9orf47	1.108994	1.0971955	1.11873762	1.2216371	0.28881582	0.75324
TMCO6	1.175034	1.1869505	1.16519309	0.8836193	-0.1785032	0.75329
RP11-234B24.2	1.02798	1.0166975	1.03729678	2.2336686	1.15941518	0.7533
HDAC11	1.183215	1.1712956	1.193058	1.127046	0.17254637	0.75344
RP11-93H24.3	1.155629	1.167462	1.14585706	0.8709858	-0.1992788	0.75345
THAP7-AS1	1.139037	1.1508289	1.12929961	0.8572602	-0.222195	0.7535
RP11-192H23.6	1.194118	1.2064924	1.1838991	0.8905853	-0.1671743	0.75352
AC005609.20	1.027462	1.0387985	1.0181003	0.4665207	-1.099987	0.75356
VCPKMT	1.137851	1.1496527	1.12810472	0.8560133	-0.2242949	0.75358
RP11-166P13.3	1.081651	1.0931323	1.07216974	0.7749168	-0.3678867	0.75362
THADA	1.15491	1.1668428	1.1450553	0.8694129	-0.2018865	0.75363
FANCE	1.074846	1.0634587	1.08425065	1.3276447	0.40886913	0.75392
NOL4L	1.493286	1.4802725	1.50403352	1.049474	0.06966644	0.75401
APBA3	1.119588	1.1313636	1.1098633	0.8363297	-0.2578562	0.75402
RNASEK	1.164925	1.1769105	1.15502759	0.8763054	-0.1904944	0.75421
LINGO2	1.039691	1.0283916	1.04902167	1.7266254	0.78795512	0.75426
VWA8	1.11249	1.1241092	1.10289531	0.8290706	-0.2704331	0.75428
SVEP1	1.034915	1.0235555	1.044296	1.8804925	0.91111059	0.75435
NXN	1.630979	1.6448668	1.61951052	0.9606798	-0.0578725	0.75438
BRWD1-AS2	1.067333	1.0787511	1.05790442	0.7352835	-0.4436276	0.7544
CRH	1.024861	1.0361605	1.0155299	0.4294709	-1.2193677	0.75448
CTA-392C11.1	1.03451	1.023312	1.04375726	1.8770275	0.90844979	0.75462
SLC38A9	1.168008	1.1562096	1.17775121	1.1379016	0.18637583	0.75481
SORCS2	1.136237	1.1243256	1.14607323	1.1749252	0.23256887	0.75482
ZNF319	1.054867	1.0434194	1.06432044	1.4813743	0.56693618	0.75488
ANKRD49	1.472498	1.4590889	1.48357115	1.0533279	0.07495457	0.7549
GTF2I	4.463764	4.4311115	4.49072848	1.0173754	0.02485213	0.75503
ADAMTS13	1.067287	1.0557704	1.07679705	1.3770209	0.4615505	0.75507
OPA1	1.439799	1.4266734	1.45063793	1.056166	0.07883654	0.75534
TCEANC2	1.198369	1.2101719	1.18862153	0.897463	-0.1560756	0.75534
RP11-158I9.8	1.108718	1.097067	1.11833877	1.2191453	0.28587009	0.7554

SETBP1	1.121735	1.1099843	1.13143908	1.1950711	0.2570964	0.75548
MPV17L2	1.182118	1.1941901	1.17214841	0.8864945	-0.1738164	0.7555
PHIP	2.708184	2.6874097	2.72533982	1.0224783	0.03207023	0.75557
SCML1	1.265456	1.277673	1.25536622	0.9196652	-0.1208194	0.75583
TDRKH	1.053398	1.0645861	1.04415909	0.6837246	-0.5485128	0.75583
SGCA	1.036821	1.0256556	1.0460409	1.7945762	0.84364315	0.75584
BST2	1.678164	1.6932368	1.66571588	0.9603009	-0.0584416	0.75595
KCNJ4	1.06904	1.0576179	1.07847284	1.3619523	0.44567613	0.75595
RSPH14	1.078703	1.0901476	1.06925178	0.768204	-0.3804386	0.75598
KCNMB2	1.088111	1.0993755	1.07880926	0.7930449	-0.3345255	0.75603
LSMEM1	1.041043	1.0521097	1.03190429	0.6122529	-0.7078005	0.7561
CACNB2	1.031784	1.0429824	1.02253628	0.5243144	-0.9314961	0.75611
HIST1H2AE	1.048427	1.0596136	1.03918969	0.6573947	-0.6051684	0.75614
EGFL7	1.792163	1.8070497	1.77986907	0.966321	-0.0494256	0.75619
LINC01504	1.036578	1.0253846	1.04582259	1.8051334	0.85210547	0.75622
PARP4	1.22162	1.2095044	1.23162449	1.1055832	0.14480758	0.75622
WDR36	1.180203	1.1921368	1.17034759	0.8865953	-0.1736524	0.75623
MAP1LC3B2	1.069542	1.0808419	1.06021034	0.7447915	-0.4250915	0.75631
DUSP28	1.184315	1.1725148	1.1940591	1.1248835	0.16977562	0.75632
SOX8	1.08726	1.0757123	1.09679551	1.2784644	0.35441198	0.75632
MEX3C	1.272959	1.2607582	1.28303441	1.0854286	0.11826482	0.75632
BVES	1.046511	1.0352254	1.05583093	1.5849633	0.66444945	0.75635
RP11-214K3.24	1.051	1.0397489	1.06029072	1.5167898	0.60102119	0.75644
RP11-227G15.8	1.039358	1.0506345	1.03004526	0.5933754	-0.752983	0.75644
RP1-191J18.66	1.0308	1.0419327	1.02160663	0.515269	-0.9566024	0.75656
KLF11	1.14076	1.1291301	1.15036355	1.1644347	0.21962968	0.75656
PPT2	1.134973	1.1465713	1.12539455	0.8555192	-0.2251278	0.75657
BMP6	1.031955	1.0208378	1.04113574	1.9740936	0.98119042	0.75658
IGFL2	1.044666	1.0334874	1.05389737	1.6094842	0.68659843	0.7566
ZDHHC13	1.204167	1.1920692	1.21415733	1.1150011	0.15704509	0.7566
SOX7	1.044156	1.032986	1.05338054	1.6182762	0.69445782	0.75663
TRIM68	1.09694	1.0854982	1.10638923	1.2443443	0.31538572	0.75666
SMIM18	1.022352	1.0334869	1.01315569	0.3928602	-1.3479119	0.75668
MAP3K7CL	1.029193	1.0180243	1.03841714	2.1314113	1.09180904	0.75671
CALCB	1.031805	1.0429106	1.02263338	0.5274542	-0.9228824	0.75677
TRPS1	1.227422	1.2151582	1.23755011	1.104072	0.14283427	0.75679
SYN1	1.077165	1.0884987	1.06780508	0.7661703	-0.3842631	0.75683
CX3CL1	1.063655	1.0524004	1.07294951	1.3921553	0.47732013	0.7569
BTBD17	1.086739	1.0981735	1.07729617	0.7873425	-0.3449368	0.75696
RGR	1.026773	1.0157004	1.03591751	2.2876783	1.19388417	0.75699
MSANTD3	1.373444	1.3865468	1.36262333	0.9381099	-0.0921712	0.75704
ZNF142	1.076742	1.0654234	1.08608979	1.315886	0.39603447	0.75709
ZGLP1	1.046513	1.0352027	1.05585233	1.5865923	0.66593143	0.75716
RP11-326I11.3	1.051605	1.0628206	1.04234295	0.6740294	-0.5691166	0.75721
SMPD2	1.134136	1.1458595	1.12445369	0.8532434	-0.2289707	0.75723

SDF2L1	2.257742	2.2401925	2.27223491	1.0258366	0.03680097	0.75724
EXT2	1.285143	1.2727395	1.29538541	1.0830314	0.11507514	0.75727
RAB24	1.145599	1.1572143	1.13600744	0.8651085	-0.2090471	0.75743
ZNF599	1.240572	1.2527921	1.23048079	0.9117403	-0.1333052	0.75749
TBCCD1	1.136758	1.1482986	1.12722739	0.8579136	-0.2210957	0.75753
MAFK	1.094962	1.0835033	1.10442544	1.2505542	0.32256758	0.75753
CRIP3	1.025589	1.0365832	1.01651055	0.4513151	-1.147793	0.75756
SNCA-AS1	1.034643	1.0457789	1.02544678	0.555863	-0.8471988	0.7576
C2	1.039402	1.0282788	1.04858822	1.7181835	0.78088416	0.75766
DTX2	1.108467	1.097074	1.11787594	1.2142891	0.28011192	0.75783
TNNC1	1.140854	1.1293239	1.1503756	1.1627831	0.21758198	0.75793
INTS4	1.155804	1.1673738	1.14625005	0.873793	-0.1946366	0.75811
ZNF197	1.258063	1.2702412	1.24800653	0.9177228	-0.1238696	0.75812
NEIL3	1.073052	1.0842096	1.06383737	0.7580768	-0.3995841	0.75814
RP11-346D14.1	1.029012	1.0179348	1.0381597	2.1276865	1.08928562	0.75815
GGT7	1.258764	1.2705421	1.24903734	0.9205123	-0.1194912	0.75815
CRTC2	1.152304	1.1407159	1.16187359	1.1503576	0.20208238	0.75816
RP1	1.091126	1.0798434	1.1004437	1.2580085	0.33114172	0.7583
LINC00683	1.045751	1.0568629	1.0365744	0.6432036	-0.6366526	0.75832
CTD-2350C19.2	1.065697	1.0770002	1.05636186	0.7319707	-0.4501421	0.75834
SWSAP1	1.177218	1.1889226	1.16755278	0.8868858	-0.1731797	0.75837
WDR26	1.527556	1.5144036	1.53841759	1.0466831	0.06582468	0.75838
TNFRSF13C	1.053665	1.0424295	1.06294418	1.4835016	0.56900644	0.75863
AK7	1.0437	1.0547674	1.03456081	0.6310474	-0.6641797	0.7587
KCNA1	1.02927	1.0181252	1.03847336	2.1226459	1.08586371	0.75879
NEBL-AS1	1.082931	1.0942925	1.07354831	0.7800017	-0.3584508	0.75898
WDR46	1.327601	1.3397226	1.3175914	0.9348552	-0.0971852	0.75898
ZNF787	1.083384	1.0720999	1.09270239	1.2857485	0.36260851	0.75907
AGPAT4	1.367656	1.3798736	1.35756728	0.9412796	-0.0873047	0.75924
MYO9A	1.34956	1.3621992	1.33912323	0.9362893	-0.0949737	0.75925
FARS2	1.206742	1.2184442	1.19707824	0.9021901	-0.1484966	0.75946
FMN1	1.051695	1.0407194	1.06075944	1.4921493	0.5773919	0.7597
CXorf38	1.200749	1.2124891	1.19105355	0.8991216	-0.1534118	0.75974
VPS9D1	1.102827	1.0915838	1.11211194	1.2241466	0.29177636	0.75976
CFAP206	1.051746	1.0628565	1.04257103	0.6772733	-0.56219	0.75979
CFAP70	1.089992	1.0787887	1.09924298	1.2596091	0.33297606	0.75979
ATN1	1.545639	1.5328043	1.55623797	1.0439818	0.06209654	0.7598
FAM111A	1.427088	1.4398413	1.4165563	0.9470604	-0.0784716	0.75987
PTPN23	1.146396	1.1348372	1.15594056	1.1565098	0.20977743	0.75994
MTHFR	1.18891	1.1771231	1.19864456	1.121506	0.16543731	0.75997
BTBD2	1.352108	1.364835	1.34159737	0.9363065	-0.0949473	0.76006
AC068499.10	1.039499	1.0505963	1.03033472	0.5995439	-0.7380626	0.76009
SLC46A1	1.106364	1.0949141	1.11581896	1.2202497	0.28717646	0.76014
RP11-977G19.12	1.080511	1.0692252	1.08983187	1.2976761	0.37593033	0.76019
BAZ2A	1.628169	1.6421752	1.61660256	0.9601781	-0.058626	0.76019

SPATA5	1.123648	1.1348719	1.11437901	0.848057	-0.2377669	0.76027
RP11-1275H24.2	1.063903	1.0528025	1.07306955	1.383827	0.46866359	0.76056
RP5-884C9.2	1.029091	1.040098	1.02000144	0.4988146	-1.0034243	0.76057
SPATA18	1.152366	1.1639706	1.14278188	0.8707772	-0.1996244	0.7606
RP11-399K21.13	1.053497	1.0424232	1.06264148	1.4765863	0.5622657	0.76063
LCTL	1.056557	1.0677073	1.0473494	0.6993249	-0.5159653	0.76069
PEX12	1.094982	1.106263	1.0856658	0.8061679	-0.3108478	0.76072
CAMTA2	1.134073	1.1226431	1.14351182	1.1701584	0.22670388	0.76075
RNF220	1.311546	1.3239967	1.30126405	0.9298369	-0.1049504	0.76075
ALPK1	1.168987	1.157438	1.17852465	1.1339363	0.18133955	0.76076
ZNF816	1.076378	1.0876091	1.06710294	0.765936	-0.3847043	0.76084
LIPC	1.021433	1.0323288	1.01243594	0.3846713	-1.3783019	0.76087
USP46	1.562878	1.5501636	1.57337786	1.0421953	0.05962561	0.76092
TFAP2B	1.02887	1.0178761	1.03794899	2.1228919	1.08603092	0.76094
KLK4	1.041321	1.0302206	1.05048757	1.6706359	0.74039737	0.76101
BOK	1.267393	1.2554261	1.27727628	1.0855441	0.11841831	0.76107
NUP50-AS1	1.081481	1.0702345	1.09076932	1.2923756	0.37002539	0.76114
SLC11A1	1.049863	1.0608636	1.04077819	0.6699931	-0.5777819	0.76117
ABCC10	1.161956	1.1503321	1.17155558	1.1411775	0.19052325	0.76119
LSM14B	1.212643	1.2008812	1.22235588	1.1069026	0.14652824	0.76121
KDM6B	1.560011	1.5736527	1.54874618	0.9565826	-0.0640385	0.76123
AC113189.5	1.151977	1.1634709	1.14248534	0.8716251	-0.1982203	0.76124
SBDS	2.135753	2.1516713	2.12260673	0.9747631	-0.0368764	0.76126
TBC1D31	1.142096	1.1533651	1.13278996	0.8658423	-0.2078238	0.76142
BRAT1	1.273203	1.2608933	1.28336823	1.0861463	0.11921841	0.76144
POLR3B	1.061283	1.0501616	1.07046802	1.4048202	0.49038547	0.76172
OTUD6B	1.189652	1.2014071	1.17994499	0.893439	-0.1625589	0.76176
KLHL12	1.275119	1.2873098	1.26505101	0.9225268	-0.1163373	0.76181
ABL1	1.369124	1.356709	1.37937693	1.0635473	0.08888423	0.76182
RP11-890B15.3	1.064197	1.0531214	1.07334411	1.3806877	0.46538698	0.76186
SH3BP5-AS1	1.052484	1.0635865	1.04331493	0.6811966	-0.5538569	0.76189
RP11-176N18.2	1.035403	1.0464681	1.02626565	0.5652411	-0.8230616	0.76198
IFRD2	1.508258	1.4955693	1.51873595	1.0467476	0.06591366	0.76198
RP11-843A23.1	1.024975	1.0357524	1.01607509	0.449623	-1.1532121	0.762
TPCN2	1.109012	1.1203037	1.09968656	0.8286242	-0.2712102	0.76202
ARHGAP31	1.041184	1.0302504	1.05021314	1.6599152	0.73110956	0.76203
DXO	1.242401	1.254266	1.23260289	0.9148013	-0.1284697	0.76208
AC010136.2	1.034577	1.0454846	1.02556981	0.5621639	-0.8309372	0.76214
IPO11	1.125362	1.1139219	1.13480857	1.1833425	0.24286772	0.76228
CEP97	1.265931	1.2777101	1.25620358	0.9225578	-0.1162888	0.76229
MAP2K2	1.461827	1.4492018	1.47225363	1.0513173	0.07219812	0.76246
SETDB1	1.31019	1.2981935	1.32009612	1.073451	0.10225632	0.76249
INTS7	1.121733	1.1330626	1.11237696	0.8445423	-0.2437585	0.76252
BTAF1	1.299537	1.2874883	1.30948674	1.0765195	0.1063744	0.76256
LGALS8-AS1	1.033597	1.0444608	1.02462519	0.5538634	-0.852398	0.76258

LINC00937	1.062997	1.0518096	1.07223607	1.3942607	0.47950039	0.76261
CITED1	1.057748	1.0466607	1.06690442	1.4338498	0.51989392	0.76269
MRGPRF	1.062067	1.0510315	1.07118016	1.394827	0.4800862	0.76272
SURF6	1.444889	1.4572345	1.43469338	0.9507012	-0.0729362	0.76278
NYAP1	1.108264	1.0970659	1.11751117	1.2106335	0.27576212	0.76278
RP4-555D20.2	1.025559	1.0147414	1.0344918	2.3397851	1.22637602	0.76279
FAM174A	1.407619	1.4201761	1.39724872	0.9454338	-0.0809517	0.76284
SRGAP2B	1.312927	1.3250344	1.30292844	0.9319889	-0.1016152	0.76288
RP11-141M1.3	1.03653	1.0474141	1.02754206	0.5808838	-0.7836786	0.76299
FNTB	1.187318	1.1987525	1.17787468	0.8949558	-0.1601117	0.76308
RNF157	1.195312	1.2068968	1.18574462	0.8977647	-0.1555907	0.76319
RP1-257A7.4	1.030922	1.0200217	1.03992411	1.9940423	0.99569601	0.7632
USP8	1.784134	1.770448	1.79543599	1.0324331	0.04604829	0.7632
PGS1	1.236855	1.2484994	1.22723914	0.9144455	-0.1290309	0.76332
SLC16A7	1.10933	1.0979539	1.11872483	1.2120475	0.27744629	0.76335
MSS51	1.038614	1.0277204	1.04761088	1.7175391	0.7803429	0.76336
ACKR3	1.063959	1.0527695	1.07319911	1.3871484	0.47212218	0.76342
MIR92B	1.063883	1.0528864	1.07296354	1.379628	0.46427932	0.76342
MYLPF	1.065072	1.0761287	1.05594058	0.7348155	-0.4445461	0.76342
RP11-966I7.2	1.056823	1.0677426	1.04780457	0.7056796	-0.5029147	0.76351
CYYR1	1.397484	1.4096857	1.38740713	0.9456204	-0.0806669	0.76356
SMN1	1.116804	1.1055072	1.12613353	1.1954972	0.25761078	0.76356
HPSE2	1.030932	1.0417138	1.02202741	0.52806	-0.9212264	0.76361
DFFB	1.058418	1.0474395	1.06748431	1.4225336	0.50846275	0.76363
FAM181B	1.027685	1.0168057	1.03666897	2.1819386	1.12561051	0.76363
RP11-98G7.1	1.04991	1.0607699	1.0409425	0.6737298	-0.569758	0.76376
LL0XNC01-116E7.2	1.059451	1.0704368	1.05037821	0.7152256	-0.4835297	0.76383
AC108488.4	1.047882	1.0587482	1.03890877	0.6622976	-0.5944485	0.76384
RP5-1050D4.2	1.025977	1.0367909	1.01704689	0.4633455	-1.1098397	0.76385
DUSP10	1.10018	1.0889278	1.10947171	1.2310174	0.29985112	0.76399
REM2	1.097469	1.1086609	1.08822613	0.8119398	-0.3005553	0.76405
HAPLN4	1.050617	1.039738	1.05960029	1.4998302	0.58479918	0.76406
P2RY2	1.022181	1.0113663	1.03111184	2.7371966	1.45269909	0.76436
HIST1H3H	1.035741	1.0465438	1.02682046	0.5762411	-0.7952554	0.76443
ORC6	1.635253	1.6484875	1.62432441	0.9627393	-0.0547829	0.76454
BCL7C	2.300116	2.3171757	2.28602753	0.9763523	-0.0345263	0.76457
RIN3	1.065061	1.0541181	1.07409824	1.369194	0.45332689	0.76465
LHCGR	1.054844	1.0657397	1.04584562	0.6973808	-0.5199815	0.76466
LRRC3	1.11161	1.1229589	1.10223721	0.8314749	-0.2662553	0.76475
CTD-2186M15.3	1.141031	1.1521462	1.13185115	0.866608	-0.2065485	0.76484
IFFO1	1.124238	1.1354905	1.11494524	0.848364	-0.2372447	0.76484
PIP4K2C	1.101523	1.0903841	1.11072095	1.2250049	0.29278757	0.76487
ZBED6	1.495924	1.4829519	1.50663721	1.0490427	0.06907341	0.7649
ZNF749	1.029175	1.0399283	1.02029543	0.5082969	-0.9762567	0.76498
EPS15	1.406039	1.3935507	1.41635169	1.0579365	0.08125309	0.76498

ETHE1	2.071022	2.0861557	2.05852465	0.9745607	-0.0371761	0.76502
RP11-115D19.3	1.047879	1.0370296	1.05683771	1.5349253	0.61816842	0.76509
LINC01250	1.121521	1.1327421	1.11225433	0.8456572	-0.2418551	0.7651
IMPA2	1.685928	1.6992681	1.67491225	0.9651695	-0.0511457	0.76513
CRTC3	1.26946	1.2576169	1.27924085	1.0839385	0.11628289	0.76517
JPH3	1.040321	1.0295088	1.04924999	1.668996	0.73898048	0.76526
ST6GALNAC1	1.022774	1.0120052	1.03166721	2.6378008	1.39933563	0.76528
TTC27	1.121414	1.1326247	1.11215643	0.8456678	-0.2418371	0.7653
FMO5	1.024995	1.0357183	1.01613888	0.4518372	-1.1461251	0.76533
LINC01355	1.285071	1.2730941	1.29496231	1.0800757	0.11113249	0.76534
TGFB3	1.047631	1.0367335	1.056631	1.5416726	0.6244964	0.76537
MBD1	1.330039	1.3179658	1.34001013	1.0693293	0.09670626	0.76537
DPY19L3	1.284	1.2964811	1.27369233	0.9231358	-0.1153852	0.76548
RP11-814H16.2	1.032661	1.0218473	1.04159188	1.9037569	0.92884928	0.76551
ANKRD11	2.258906	2.2430144	2.27202882	1.023342	0.03328833	0.76577
TAOK1	2.085611	2.1012083	2.07273118	0.9741401	-0.0377988	0.7659
LINC01030	1.023392	1.0341559	1.01450349	0.4246258	-1.2357362	0.76601
CALHM2	1.145622	1.1343354	1.15494175	1.1533944	0.2058859	0.76605
C2CD3	1.079164	1.0901342	1.07010484	0.7777825	-0.3625613	0.76608
BTBD8	1.129154	1.1180315	1.13833969	1.1720574	0.22904327	0.76623
YPEL3	1.581124	1.5941547	1.57036301	0.9599571	-0.0589582	0.76624
IGIP	1.038834	1.0280146	1.0477692	1.7051536	0.76990167	0.76628
IGFBPL1	1.081131	1.0919982	1.07215701	0.7843304	-0.3504667	0.76638
FOXA3	1.045663	1.0348627	1.05458112	1.5656	0.64671567	0.76642
LRRC46	1.030771	1.0414455	1.02195626	0.5297619	-0.9165841	0.76658
MAP6D1	1.086943	1.0759129	1.09605221	1.2652942	0.33947285	0.76659
RP1-17K7.2	1.038633	1.0494263	1.02971916	0.6012827	-0.7338847	0.76667
ZNF845	1.049754	1.0604655	1.04090883	0.6765651	-0.5636994	0.76676
ARHGAP9	1.02839	1.0176665	1.03724511	2.1082357	1.07603615	0.76677
AP5B1	1.066985	1.0778395	1.05802076	0.7453898	-0.423933	0.76681
MTSS1L	1.111011	1.0999952	1.12010745	1.2011323	0.26439512	0.76682
ASIC1	1.059332	1.0701533	1.05039476	0.718352	-0.4772372	0.76684
USP53	1.366758	1.354723	1.37669681	1.0619463	0.0867108	0.767
ZNF711	1.385158	1.3726733	1.39546894	1.061168	0.08565307	0.76704
STK4	1.637086	1.6237749	1.64807892	1.0389629	0.05514411	0.76718
POU5F2	1.06281	1.0519013	1.07181819	1.3837447	0.46857777	0.76725
AMY2B	1.040075	1.0293691	1.04891545	1.6655418	0.73599152	0.76732
RP13-131K19.7	1.044994	1.0342088	1.05390038	1.5756285	0.65592737	0.76741
KIAA0922	1.045574	1.0561738	1.03682063	0.655477	-0.6093829	0.76748
ASAH2B	1.092673	1.0818902	1.10157729	1.2404085	0.31081535	0.76755
TRIM21	1.086755	1.0758925	1.09572477	1.2613207	0.33493512	0.76764
TNNT2	1.0301	1.0193891	1.0389445	2.0085729	1.0061708	0.76779
SLC37A3	1.339716	1.3518291	1.32971215	0.9371372	-0.0936678	0.7678
AC002451.3	1.045486	1.0562233	1.03661972	0.651326	-0.6185484	0.76812
ACTR8	1.14867	1.1599318	1.13937029	0.8714358	-0.1985338	0.7682

KCNS3	1.048293	1.0376439	1.05708696	1.5164995	0.60074501	0.76823
RP3-323P24.3	1.034025	1.0447127	1.02519912	0.5635786	-0.8273112	0.76825
ZNF415	1.158377	1.1695229	1.14917256	0.8799552	-0.1844979	0.76827
ZNF598	1.136721	1.1255348	1.14595841	1.1626932	0.21747042	0.76829
DDX51	1.136989	1.1258659	1.14617467	1.1613523	0.21580566	0.76829
RP1-267D11.6	1.172363	1.1609225	1.18181091	1.1298044	0.176073	0.76832
FGF8	1.081342	1.0921388	1.07242581	0.7860509	-0.3473053	0.76842
RP11-171I2.1	1.034019	1.0446514	1.02523808	0.5652249	-0.8231031	0.76844
MICU3	1.187066	1.1985553	1.17757721	0.8943462	-0.1610947	0.76845
SLC24A4	1.024897	1.0142701	1.03367257	2.3596655	1.23858235	0.76858
STRIP1	1.16244	1.151094	1.1718092	1.1371011	0.18536052	0.76858
PDS5A	1.6614	1.6485719	1.67199338	1.0361123	0.05118044	0.76859
PI4K2B	1.109773	1.1208646	1.1006127	0.8324417	-0.2645789	0.76863
TP53BP2	1.354754	1.3428203	1.36460986	1.0635598	0.0889012	0.76865
ACCS	1.076223	1.065346	1.08520598	1.3039196	0.38285486	0.7687
TBC1D16	1.41731	1.4051387	1.42736208	1.0548539	0.07704319	0.76872
ACAD8	1.2126	1.2009762	1.22219908	1.1055992	0.1448285	0.76878
CTPS2	1.395926	1.4081863	1.38580114	0.9451595	-0.0813703	0.76882
SMAD5	1.997955	2.0128442	1.98565903	0.9731596	-0.0392517	0.76891
MUC12	1.19642	1.2082008	1.18669182	0.896691	-0.1573172	0.76903
PM20D2	1.460928	1.4734344	1.45059936	0.9517673	-0.0713192	0.7691
NR2C2AP	1.297561	1.2858385	1.30724133	1.0748773	0.10417193	0.76913
PTGES3L	1.103384	1.0923111	1.11252726	1.2189998	0.28569785	0.76913
MYNN	1.192121	1.1806859	1.20156459	1.1155521	0.15775789	0.76924
FITM2	1.066658	1.0557682	1.07565034	1.3565142	0.43990412	0.76925
MED17	1.397031	1.3846084	1.40728987	1.0589729	0.08266567	0.76928
CABS1	1.024743	1.0141637	1.03347991	2.3637792	1.2410953	0.76933
RP4-605O3.4	1.195962	1.2073026	1.1865967	0.9001175	-0.1518147	0.76949
PCDH11X	1.281966	1.2935934	1.27236343	0.9276891	-0.1082866	0.76957
FAM72C	1.04223	1.0527922	1.03350681	0.6346926	-0.65587	0.76957
ACSL1	1.075066	1.0643146	1.08394492	1.3052233	0.3842967	0.76965
PDE1C	1.08498	1.0742536	1.09383738	1.2637421	0.33770204	0.76973
EOGT	1.049351	1.0386146	1.05821725	1.5076501	0.59230163	0.76973
LINC00535	1.032351	1.0216951	1.04115121	1.8968	0.92356758	0.76975
CERKL	1.039459	1.0501179	1.03065686	0.6116945	-0.7091167	0.76978
SETD7	1.164385	1.1533341	1.17351181	1.131593	0.17835519	0.7698
RFWD3	1.242977	1.2316674	1.25231628	1.0891317	0.12317841	0.76982
ABHD17C	1.068772	1.0580008	1.07766671	1.3390617	0.42122248	0.76989
C12orf60	1.080342	1.0912011	1.07137375	0.7825971	-0.3536584	0.76994
RP11-468E2.4	1.067826	1.0571011	1.07668302	1.3429342	0.42538861	0.7701
VASH1	1.317495	1.3058343	1.32712529	1.069616	0.09709298	0.77013
SRF	1.120466	1.1095424	1.12948664	1.1820684	0.24131355	0.77019
PRR19	1.050071	1.0606699	1.0413191	0.6810481	-0.5541713	0.77037
CH507-254M2.3	1.155382	1.1442566	1.1645703	1.1408165	0.19006678	0.77044
RP11-53O19.3	1.119271	1.1083309	1.1283059	1.1843886	0.24414256	0.77045

HSF2	1.384244	1.3961342	1.37442407	0.9451951	-0.0813159	0.77063
TIGD5	1.093438	1.0825599	1.10242042	1.2405583	0.31098958	0.77066
ZMYM5	1.609945	1.5969661	1.62066285	1.0396954	0.0561609	0.7707
CCR10	1.162562	1.151373	1.17180168	1.1349561	0.1826365	0.7707
TSPAN4	2.35945	2.3783873	2.34381117	0.9749156	-0.0366508	0.77083
TOPORS	1.440213	1.4276728	1.45056914	1.053537	0.07524094	0.77093
BACH1	1.391482	1.3794802	1.4013936	1.0577457	0.08099287	0.77099
SLC17A9	1.068355	1.0576718	1.07717728	1.3382157	0.42031071	0.77104
PUS3	1.176433	1.187438	1.16734523	0.8928033	-0.1635857	0.77108
SATB1	1.240103	1.2515296	1.23066607	0.9170534	-0.1249223	0.77146
TRAF7	1.843637	1.8576057	1.83210196	0.9702617	-0.0435541	0.77147
RP11-615I2.2	1.072015	1.082777	1.06312682	0.7626134	-0.3909762	0.77173
RP11-357K6.1	1.044536	1.055205	1.03572548	0.6471424	-0.627845	0.77182
ARHGEF7	1.310593	1.2988788	1.32026757	1.0715634	0.09971726	0.77197
F12	1.351848	1.3398415	1.36176302	1.0645051	0.09018287	0.77198
C11orf88	1.034319	1.0238225	1.04298671	1.8044594	0.85156668	0.77201
PARP3	1.119527	1.1303627	1.11057956	0.8482454	-0.2374464	0.77202
LURAP1L	1.125812	1.1147859	1.13491756	1.1753841	0.23313223	0.77202
C1orf145	1.042009	1.0315167	1.05067319	1.607819	0.68510499	0.77213
MARCH10	1.053114	1.0636283	1.04443058	0.6982828	-0.5181166	0.77213
IFIT2	1.041439	1.0309687	1.05008531	1.6172861	0.69357488	0.77216
WDR63	1.041536	1.0310054	1.05023169	1.6200934	0.69607695	0.77216
DGKQ	1.061606	1.0509155	1.07043494	1.3833696	0.46818664	0.77224
MON1A	1.074175	1.0635247	1.08297044	1.3061123	0.38527892	0.77237
RP11-397A16.1	1.043924	1.0545026	1.03518829	0.6456258	-0.6312298	0.77237
MCEE	1.199829	1.1887112	1.209011	1.1075709	0.14739911	0.7724
DIAPH1	1.348647	1.3368651	1.35837753	1.0638605	0.08930906	0.77241
TTC29	1.053434	1.0640086	1.04470099	0.6983588	-0.5179596	0.77245
RET	1.030114	1.0194771	1.03889868	1.9971473	0.99794071	0.77249
RP11-83J21.3	1.062967	1.0734575	1.05430296	0.739243	-0.4358794	0.77252
SDC1	1.262547	1.2511166	1.2719861	1.083107	0.11517572	0.77261
TBC1D22B	1.099801	1.1107004	1.09080073	0.8202383	-0.285885	0.77263
BTBD16	1.02389	1.03432	1.01527752	0.4451496	-1.167638	0.77266
XKR5	1.023883	1.0343054	1.01527591	0.4452913	-1.1671788	0.77266
CEP162	1.377641	1.3891435	1.36814147	0.9460302	-0.0800419	0.77269
ZBTB40	1.139534	1.1503818	1.13057644	0.8682996	-0.2037351	0.7727
CCDC144NL-AS1	2.833727	2.8559542	2.81537089	0.9781335	-0.0318968	0.77272
ZNF81	1.044662	1.0341175	1.05336921	1.5642758	0.64549493	0.77276
NIPAL2	1.088222	1.0772736	1.09726328	1.2586872	0.33191984	0.77277
RNF43	1.056934	1.046348	1.06567676	1.417036	0.50287643	0.77279
SIRT1	1.168236	1.1791459	1.15922566	0.8888042	-0.1700624	0.77287
AP000344.3	1.090796	1.080125	1.09960816	1.2431597	0.3140117	0.77295
BMP5	1.026633	1.0161994	1.035249	2.1759509	1.12164598	0.773
MT2A	4.513595	4.4261901	4.58577472	1.0465778	0.06567963	0.77308
PYY	1.137133	1.1478456	1.12828575	0.8677006	-0.2047307	0.77311

LONP1	1.371168	1.3833647	1.36109535	0.9419109	-0.0863375	0.77316
RAB2B	1.254066	1.2653576	1.24474146	0.9223082	-0.1166791	0.77318
KCNE5	1.036816	1.026326	1.04547934	1.727543	0.78872161	0.77319
NECAP1	1.280107	1.2915761	1.27063649	0.9281848	-0.107516	0.77321
ARFGEF1	1.549263	1.5619458	1.53878942	0.9587926	-0.0607094	0.77323
MED1	1.269759	1.2581775	1.27932392	1.0819065	0.1135758	0.77325
ZSCAN30	1.218119	1.2292771	1.20890533	0.9111478	-0.134243	0.77327
UBQLN4	1.234792	1.2234918	1.24412372	1.0923163	0.12739067	0.7733
DEPDC5	1.068592	1.0578499	1.07746271	1.3390284	0.42118655	0.7733
UTP23	1.348622	1.3600602	1.33917644	0.9419993	-0.0862021	0.77335
HOXA3	1.027113	1.0166975	1.03571357	2.1388516	1.09683639	0.77335
USP6NL	1.253045	1.2417211	1.26239609	1.0855323	0.11840261	0.77355
GAREM1	1.038505	1.0280268	1.04715887	1.682633	0.75072055	0.77357
RBM48	1.151371	1.1623448	1.14230871	0.876583	-0.1900375	0.77359
LYG1	1.051441	1.040954	1.06010161	1.4675396	0.55339945	0.77364
UBE2Q2	1.57107	1.5842504	1.56018532	0.9588103	-0.0606827	0.77369
TXNRD3	1.063624	1.0530731	1.07233772	1.362984	0.44676865	0.7737
ABL2	1.284869	1.296416	1.27533356	0.9288753	-0.1064431	0.77374
SLC33A1	1.304464	1.3162452	1.29473516	0.9319831	-0.1016243	0.77375
PIGM	1.301022	1.3124938	1.29154756	0.9329707	-0.1000963	0.77376
FGF14-AS2	1.42051	1.408244	1.43063924	1.0548575	0.07704813	0.7738
SETD2	1.59065	1.6030941	1.58037264	0.9623251	-0.0554037	0.77384
DCP2	1.665268	1.6521769	1.67607824	1.0366485	0.05192684	0.77388
SLC25A32	1.112748	1.1019756	1.12164379	1.192872	0.25443925	0.77391
DIRC3	1.135007	1.1241038	1.14401045	1.1604029	0.21462578	0.77397
GPRASP1	1.099967	1.0892729	1.10879872	1.2187203	0.28536711	0.77399
PKD1	1.299747	1.288203	1.30927998	1.0731325	0.10182816	0.77402
RP11-190A12.8	1.025243	1.0148661	1.03381156	2.2744049	1.18548914	0.77405
BLOC1S6	1.878689	1.8647618	1.89018959	1.0294044	0.04180989	0.7741
ZNF154	1.112884	1.1021071	1.12178324	1.1927012	0.25423269	0.77411
MEDAG	1.03397	1.0235978	1.04253492	1.8024936	0.84999415	0.77415
TMEM173	1.041278	1.0308401	1.04989812	1.6179644	0.69417986	0.77417
RP11-70P17.1	1.068044	1.0785604	1.05935891	0.7555829	-0.404338	0.77423
LINC01560	1.279183	1.2906078	1.26974864	0.9282224	-0.1074575	0.77426
NINL	1.369427	1.3815324	1.35943079	0.9420715	-0.0860915	0.77426
AC005253.2	1.077744	1.0884642	1.06889158	0.7787508	-0.3607664	0.77427
USP27X-AS1	1.048418	1.0589503	1.03971984	0.6737852	-0.5696393	0.77436
C5orf30	1.236159	1.2247852	1.245551	1.0923806	0.12747557	0.77442
MORF4L2-AS1	1.0955	1.0847989	1.10433708	1.2304053	0.29913367	0.77446
CTA-212A2.4	1.034285	1.0238624	1.04289303	1.7975121	0.84600151	0.77457
BTBD11	1.087523	1.0768711	1.09631924	1.2529968	0.32538279	0.77458
DNAJB5	1.14894	1.1598352	1.13994269	0.8755434	-0.1917494	0.77463
RAB9A	1.794067	1.8077212	1.78279129	0.9691355	-0.0452297	0.77469
RP11-219D15.3	1.025601	1.0358732	1.01711814	0.477185	-1.0673795	0.77469
AP001258.4	1.094975	1.1055812	1.08621643	0.8165888	-0.2923183	0.77473

RP11-819C21.1	1.063776	1.0744498	1.05496229	0.7382467	-0.437825	0.77475
FAM134C	1.318969	1.3075659	1.32838654	1.067695	0.09449952	0.77476
ERCC6	1.115015	1.1259884	1.10595359	0.8409789	-0.2498586	0.77483
RASSF3	1.133797	1.1447408	1.12475979	0.8619531	-0.2143187	0.77492
SPSB4	1.103264	1.1138457	1.09452494	0.8302902	-0.2683124	0.77496
NOP58	1.539859	1.5522847	1.52959702	0.9589202	-0.0605173	0.77504
MYB	1.042266	1.0318327	1.05088281	1.5984442	0.67666841	0.77505
NCAPG2	1.171531	1.1824388	1.16252357	0.890839	-0.1667633	0.77505
RP11-484K9.4	1.046388	1.0359892	1.05497562	1.5275589	0.61122799	0.77517
RP11-47I22.2	1.035007	1.0246762	1.04353927	1.7644269	0.81919969	0.77523
IRAK2	1.036625	1.0262066	1.04522882	1.7258581	0.78731382	0.77527
DOPEY1	1.146089	1.1570118	1.13706962	0.8729892	-0.1959643	0.77535
VAC14	1.244482	1.2331221	1.25386302	1.08897	0.12296416	0.77535
ABHD1	1.051596	1.0619383	1.04305446	0.6951188	-0.5246685	0.77548
BRAP	1.184165	1.194973	1.17524023	0.8987925	-0.15394	0.7755
GPD2	1.151754	1.1409007	1.16071719	1.1406418	0.18984586	0.77555
SLC27A6	1.029103	1.0187673	1.03763895	2.0055554	1.0040018	0.7756
ABI1	1.367226	1.3555494	1.37686792	1.0599595	0.08400914	0.7757
SAMD12	1.126505	1.1373526	1.11754724	0.8558062	-0.224644	0.77576
DUS3L	1.127179	1.1377138	1.11847839	0.8603234	-0.217049	0.77586
MAP4K1	1.066269	1.0558506	1.07487303	1.3405951	0.42287362	0.77591
BFSP1	1.052226	1.0626166	1.04364441	0.6970104	-0.5207479	0.77593
LNX2	1.096114	1.0854652	1.10490752	1.2274885	0.29570952	0.77608
PACSIN2	1.334586	1.3456802	1.32542386	0.9414014	-0.0871181	0.77609
CA13	1.080947	1.0705587	1.08952539	1.2688072	0.34347291	0.77614
RP11-57A19.2	1.041971	1.052246	1.03348569	0.640924	-0.6417749	0.77615
PUSL1	1.252979	1.2416325	1.26234907	1.0857358	0.11867308	0.77619
METTL3	1.402645	1.3904703	1.4126991	1.0569283	0.07987746	0.7763
CTC-534A2.2	1.069068	1.0795655	1.06039858	0.7591054	-0.3976278	0.7763
DLEU7	1.049531	1.0598899	1.04097681	0.6842018	-0.5475061	0.77644
BAHD1	1.104974	1.0943856	1.11371878	1.2048313	0.26883114	0.77651
AC091729.9	1.146614	1.1574628	1.13765399	0.8741998	-0.193965	0.77655
RP11-385F7.1	1.080448	1.0909193	1.0718008	0.7897201	-0.3405868	0.7766
MOB3A	1.230986	1.2194973	1.24047388	1.0955665	0.13167709	0.77673
TC2N	1.02618	1.0159362	1.03463895	2.1735949	1.12008309	0.7768
ZNF10	1.089589	1.1000778	1.08092711	0.8086423	-0.3064264	0.7768
SLC25A29	1.362193	1.3505725	1.37179013	1.0605228	0.08477562	0.77681
SEMA4F	1.204386	1.215573	1.19514812	0.9052532	-0.1436067	0.77682
RP11-686O6.2	1.051645	1.061921	1.04315813	0.6969874	-0.5207956	0.7769
DNASE1L1	1.343458	1.3553382	1.33364678	0.9389556	-0.0908712	0.77709
TOLLIP	1.498911	1.4868472	1.50887422	1.0452442	0.06384011	0.77713
ZSCAN22	1.046178	1.0356785	1.05484931	1.5373226	0.62041993	0.77717
ATG16L1	1.118809	1.1295154	1.10996696	0.8490647	-0.2360536	0.77721
TMEM106A	1.092566	1.0820586	1.10124385	1.2337995	0.30310801	0.77724
ENO2	3.114946	3.1382629	3.09569141	0.9800906	-0.0290129	0.77725

CFAP65	1.062687	1.0524061	1.07117781	1.358196	0.44169172	0.77736
SOX10	1.028342	1.0180979	1.03680191	2.0334947	1.0239612	0.77742
RP11-736N17.11	1.028381	1.01807	1.03689627	2.0418503	1.02987712	0.77742
DUBR	1.140217	1.1512073	1.13114015	0.8672874	-0.205418	0.77748
TOX4	1.716146	1.7031715	1.72685971	1.0336877	0.04780042	0.77753
IPMK	1.13804	1.1272057	1.14698765	1.1555119	0.20853207	0.77755
FAM83H	1.062397	1.0519945	1.07098669	1.3652719	0.44918827	0.77774
NOM1	1.180063	1.1691026	1.18911373	1.118337	0.16135503	0.77777
RHOBTB1	1.542553	1.5547881	1.53244928	0.9597346	-0.0592926	0.77785
RP11-1191J2.2	1.044292	1.0339279	1.05285028	1.5577232	0.63943888	0.77788
GNAZ	1.143851	1.1331085	1.15272258	1.1473545	0.1983112	0.7779
AC005754.8	1.064671	1.0750851	1.0560705	0.7467596	-0.4212841	0.77791
ZNF610	1.093	1.1034017	1.08440991	0.8163297	-0.2927762	0.77795
RP11-115C10.1	1.10245	1.1128263	1.09388057	0.8320807	-0.2652047	0.77796
PRPF3	1.223426	1.2349158	1.21393753	0.9106986	-0.1349544	0.77801
AC084219.4	1.134103	1.1448049	1.1252653	0.8650628	-0.2091232	0.77805
ZNF136	1.196039	1.1849911	1.20516228	1.1090387	0.14930965	0.77808
TOPBP1	1.449187	1.436606	1.45957658	1.0526118	0.07397341	0.77815
SFMBT1	1.075828	1.086133	1.06731734	0.7815515	-0.3555872	0.77823
RNGTT	1.183045	1.1719035	1.19224565	1.1183346	0.16135193	0.7784
TSPOAP1	1.156778	1.1677058	1.14775397	0.8810306	-0.1827359	0.77843
DHX34	1.131438	1.142156	1.1225875	0.862345	-0.213663	0.77855
AC005537.2	1.027907	1.0177323	1.03630893	2.0476104	1.03394125	0.77856
RP11-225H22.4	1.027961	1.0177308	1.03640988	2.0534801	1.03807094	0.77856
TBRG1	1.521305	1.5090076	1.53146058	1.0441113	0.06227553	0.77862
IDUA	1.083662	1.09407	1.07506739	0.7979948	-0.3255488	0.77869
SLC2A8	1.205884	1.2168488	1.19682868	0.9076771	-0.1397489	0.77872
SLC4A4	1.208436	1.2195086	1.19929285	0.9079043	-0.1393878	0.77886
FMNL3	1.099266	1.0886727	1.10801362	1.2181157	0.28465119	0.77891
ERLIN2	1.334442	1.3229624	1.34392241	1.0648992	0.09071686	0.77903
HIST1H2AJ	1.029488	1.0395579	1.02117202	0.5352161	-0.9018066	0.77907
NUP153	1.266779	1.2556458	1.27597308	1.0795134	0.11038113	0.77919
FKBP15	1.191956	1.2027563	1.18303631	0.9027404	-0.147617	0.77921
ABHD13	1.266266	1.2549589	1.27560368	1.0809732	0.11233071	0.77924
AZIN1-AS1	1.202927	1.1920802	1.21188515	1.1031078	0.14157379	0.77924
LINC01574	1.037807	1.0275568	1.04627171	1.6791389	0.74772153	0.77942
CTXN1	1.977896	1.9923512	1.96595885	0.9734042	-0.0388891	0.77963
LENG8-AS1	1.071911	1.061533	1.08048151	1.307941	0.38729748	0.7797
CDK10	1.423765	1.435738	1.41387684	0.9498296	-0.0742594	0.77971
SIRT4	1.052505	1.0626639	1.04411648	0.7040169	-0.5063181	0.77978
PCSK2	1.030555	1.0407542	1.02213302	0.5430852	-0.8807495	0.77984
DNAH7	1.072364	1.062023	1.08090286	1.3044004	0.38338675	0.77997
CLEC16A	1.112289	1.1016503	1.12107472	1.1910906	0.25228322	0.78001
FBXW2	1.395138	1.4070878	1.38526909	0.9464029	-0.0794737	0.78001
ATP8B3	1.078172	1.088313	1.06979801	0.7903481	-0.3394399	0.78002

GAL	2.571026	2.6146451	2.53500441	0.950676	-0.0729743	0.78006
PELI1	1.187928	1.1771299	1.19684538	1.1113054	0.15225534	0.78006
CTD-2090I13.1	1.021441	1.0113227	1.02979707	2.6316148	1.39594834	0.78007
RNLS	1.079037	1.089344	1.07052541	0.7893697	-0.341227	0.78013
TMEM253	1.04977	1.0600569	1.04127485	0.6872629	-0.541066	0.78015
INO80	1.21883	1.229748	1.20981384	0.9132346	-0.1309426	0.78022
RP11-160N1.10	1.06683	1.0772982	1.05818425	0.7527241	-0.409807	0.78025
FAM65A	1.185877	1.175444	1.19449254	1.1085733	0.14870415	0.78033
RP11-461M2.2	1.030934	1.0410702	1.0225637	0.5493939	-0.8640873	0.78037
ZNF184	1.160143	1.1495163	1.16891951	1.1297728	0.17603273	0.7804
RAB3D	1.248031	1.2594524	1.2385994	0.9196269	-0.1208795	0.78042
TANGO6	1.05008	1.0603553	1.04159437	0.6891584	-0.5370925	0.78046
CTD-2132N18.2	1.086832	1.0971478	1.07831331	0.8061255	-0.3109236	0.78053
IFIT3	1.07652	1.0867921	1.06803779	0.7839165	-0.3512282	0.78055
RBAK	1.257822	1.26927	1.24836756	0.9223737	-0.1165768	0.78059
VTI1B	2.78403	2.8056069	2.76621222	0.978182	-0.0318251	0.78063
ZFP69	1.047798	1.0578929	1.03946157	0.6816309	-0.5529374	0.78064
BCAT1	2.802388	2.824451	2.78416765	0.9779203	-0.0322112	0.78068
IER3IP1	2.674696	2.6943001	2.65850701	0.9788744	-0.0308044	0.78069
PEX14	1.21442	1.2251692	1.20554322	0.912839	-0.1315676	0.78075
KNTC1	1.17559	1.1648606	1.1844512	1.1188313	0.1619925	0.78076
TAF1A-AS1	1.08605	1.0963161	1.07757187	0.8053884	-0.3122434	0.78077
ZNF500	1.190799	1.2017385	1.18176504	0.9009933	-0.1504117	0.78079
ZC3H12A	1.044909	1.0347291	1.05331521	1.5351739	0.6184021	0.78093
ZNF14	1.077783	1.0675317	1.08624951	1.2771699	0.35295041	0.78101
ALX1	1.028008	1.0178764	1.03637556	2.034842	1.02491676	0.78104
SUGP1	1.157273	1.1678764	1.14851684	0.8846799	-0.1767726	0.78121
RP11-258F22.1	1.028	1.0178326	1.03639693	2.0410329	1.02929942	0.78122
RBMS3-AS3	1.034509	1.0243779	1.04287589	1.7588031	0.81459398	0.78122
KDM7A	1.139149	1.1285709	1.14788449	1.1502175	0.20190673	0.78135
IKBIP	1.988191	2.0031771	1.9758153	0.9727248	-0.0398964	0.78145
POLG2	1.195659	1.2061988	1.1869546	0.9066716	-0.141348	0.78157
PDE10A	1.089216	1.0788165	1.09780466	1.2409163	0.31140577	0.78165
ADCK1	1.109351	1.0989408	1.11794821	1.192109	0.25351619	0.78175
FBXO44	1.449396	1.4379746	1.45882833	1.047614	0.06710724	0.78179
RP11-596C23.2	1.079422	1.0690427	1.08799391	1.2744846	0.34991389	0.78184
PSTPIP1	1.024767	1.0347555	1.01651847	0.4752766	-1.0731607	0.78185
ARHGAP19-SLIT1	1.024814	1.0348548	1.01652224	0.47403	-1.0769498	0.78185
C16orf59	1.284911	1.2960446	1.27571684	0.9313355	-0.1026271	0.78187
ZNF491	1.06276	1.0729014	1.05438426	0.7459973	-0.4227577	0.7819
JPH2	1.030879	1.0208746	1.03914076	1.8750378	0.9069197	0.78202
ETS2	1.10746	1.0969461	1.11614242	1.1980102	0.26064023	0.78212
MMP9	1.045919	1.0357223	1.05433914	1.5211545	0.60516665	0.78225
DR1	1.690973	1.7036111	1.68053531	0.9672037	-0.0481083	0.78244
FAM189B	1.66696	1.6546105	1.67715907	1.0344458	0.04885812	0.78245

CSMD2	1.02583	1.0157693	1.03413851	2.1648666	1.11427812	0.78248
RREB1	1.238858	1.2281123	1.24773202	1.0860089	0.11903595	0.78249
RP11-677M14.7	1.059685	1.0494725	1.06811929	1.3769117	0.46143599	0.7825
TEX26	1.056868	1.0466741	1.06528627	1.3987683	0.48415696	0.78254
PAXIP1	1.143057	1.1535761	1.1343701	0.8749417	-0.1927413	0.78279
MARC2	1.09025	1.1004655	1.08181432	0.8143525	-0.2962746	0.78281
RP5-862P8.2	1.054565	1.0444739	1.06289893	1.4142893	0.50007726	0.78283
PIAS1	1.881054	1.8677742	1.89202115	1.0279416	0.03975826	0.78285
RP5-984P4.6	1.024744	1.0147414	1.03300475	2.2389097	1.16279633	0.7829
MED13	1.438466	1.4268337	1.44807301	1.0497603	0.07005988	0.78308
PCBD2	1.193127	1.2038263	1.18429219	0.9041629	-0.1453453	0.78309
CHST6	1.039572	1.0295869	1.04781867	1.6162093	0.69261404	0.78313
SIK1	1.039533	1.0294616	1.04785092	1.6241809	0.69971234	0.78313
RIMS2	1.167715	1.1572563	1.17635228	1.1214325	0.16534279	0.78313
TBC1D10A	1.118814	1.1083364	1.12746624	1.1765782	0.23459722	0.78321
HCG17	1.046844	1.0569275	1.03851611	0.6765815	-0.5636644	0.78324
ZBED5	1.560829	1.5488828	1.57069413	1.0397376	0.05621952	0.78337
CERS5	1.390657	1.4021365	1.38117736	0.9478804	-0.077223	0.78341
LTC4S	1.084823	1.0950792	1.07635238	0.8030398	-0.3164567	0.78341
SPAG6	1.025044	1.0150885	1.03326515	2.204676	1.14056665	0.78346
NAT1	1.057673	1.0676966	1.04939492	0.7296515	-0.4547206	0.78347
PYGO1	1.265969	1.2551881	1.27487182	1.0771342	0.10719801	0.78348
ST3GAL5	1.169781	1.1593284	1.1784128	1.1197803	0.16321567	0.78371
DNM3	1.045653	1.0356319	1.05392827	1.5134814	0.59787097	0.78381
CPA1	1.031787	1.041672	1.02362334	0.5668871	-0.8188668	0.78383
METTL14	1.296476	1.3077015	1.28720613	0.933392	-0.0994449	0.78385
SETD3	1.353157	1.3417157	1.36260453	1.0611293	0.08560046	0.78391
PPP1R14A	1.6061	1.5943456	1.61580623	1.0361081	0.0511745	0.78396
CDK5R2	1.040376	1.0303378	1.04866636	1.6041488	0.68180801	0.78396
TGM1	1.03847	1.0284993	1.04670355	1.638762	0.71260638	0.78403
CH507-9B2.9	1.038507	1.0285021	1.04676932	1.6409053	0.71449201	0.78403
GPR146	1.135006	1.124528	1.14365969	1.1536341	0.20618573	0.78405
CSNK1A1	3.795342	3.8219434	3.77337498	0.982789	-0.0250463	0.78409
SEMA4B	1.295539	1.2844447	1.3047001	1.0712102	0.09924158	0.7841
RP11-545E17.3	1.147676	1.1372736	1.15626589	1.1383531	0.18694814	0.78421
SHBG	1.046455	1.0565195	1.0381438	0.6748788	-0.5672997	0.78424
ORAI1	1.178008	1.1675645	1.18663174	1.1137902	0.15547747	0.78424
SAMHD1	1.075015	1.0851713	1.06662688	0.7822693	-0.3542627	0.78428
ATG16L2	1.209376	1.1983869	1.2184508	1.101135	0.13899134	0.78433
ZNF583	1.201078	1.2118792	1.19215791	0.906922	-0.1409497	0.78435
SPG20	1.706699	1.7186873	1.69679898	0.9695441	-0.0446216	0.78442
ARHGAP20	1.035309	1.025249	1.04361597	1.7274317	0.78862868	0.78444
FLJ22447	1.035249	1.0253345	1.04343627	1.7145126	0.77779854	0.78444
HSPH1	1.925474	1.9121742	1.93645758	1.0266214	0.03790422	0.78445
C1orf61	1.046071	1.0559831	1.03788504	0.6767232	-0.5633623	0.78448

AIM1	1.021241	1.0113663	1.02939535	2.586181	1.37082326	0.7845
AGR2	1.021327	1.0113663	1.0295525	2.6000067	1.37851535	0.7845
NPY1R	1.065247	1.0754621	1.0568103	0.752832	-0.4096002	0.78452
NBPF3	1.105398	1.1156797	1.09690771	0.8377241	-0.2554529	0.78455
ZNF625	1.096003	1.1063636	1.08744794	0.8221605	-0.282508	0.78455
PHF13	1.195944	1.1853572	1.20468668	1.1042823	0.14310904	0.78469
ZNF391	1.084407	1.074305	1.09274891	1.2482192	0.31987125	0.78474
GCLC	1.162643	1.1731702	1.15394857	0.8890016	-0.169742	0.78476
HSPA14	1.270001	1.2807858	1.26109391	0.9298686	-0.1049013	0.78477
SLC45A1	1.03425	1.0242483	1.04250881	1.7530616	0.80987666	0.78483
FGF13-AS1	1.0519	1.0418745	1.06017914	1.43713	0.52319053	0.78493
CCDC180	1.095579	1.0854652	1.10393155	1.2160687	0.28222471	0.78494
STRC	1.039081	1.0291356	1.04729433	1.623248	0.69888345	0.78496
HIRA	1.110645	1.1004907	1.11902986	1.1844867	0.24426205	0.78501
KRT5	1.022131	1.0122555	1.03028707	2.4713086	1.30527515	0.78504
CTB-58E17.3	1.0584	1.068323	1.05020477	0.7348152	-0.4445467	0.78518
RP11-38L15.3	1.030019	1.0398917	1.02186629	0.5481416	-0.8673794	0.78521
RP11-156K23.3	1.113739	1.1033741	1.12229913	1.1830733	0.24253944	0.78526
COCH	1.127209	1.1375001	1.11871056	0.863349	-0.2119843	0.7853
RWDD2B	1.12826	1.1179649	1.13676199	1.1593448	0.21330975	0.7854
LINC00052	1.021903	1.0120052	1.03007649	2.505298	1.3249822	0.78542
KMT5B	1.667225	1.6794556	1.65712469	0.9671341	-0.0482121	0.78548
CNTLN	1.473955	1.4854844	1.46443307	0.9566385	-0.0639543	0.78556
RP11-458F8.4	1.036321	1.0263235	1.04457667	1.6934179	0.75993808	0.78562
HS3ST4	1.029649	1.019744	1.03782808	1.9159266	0.9380423	0.78564
MORN5	1.070282	1.0806436	1.0617247	0.7654008	-0.3857127	0.78569
DACH2	1.060535	1.0705746	1.05224423	0.7402691	-0.4338782	0.78574
ZNF615	1.111633	1.1218108	1.10322848	0.8474494	-0.2388009	0.78581
ARL9	1.08957	1.0995947	1.08129168	0.8162249	-0.2929613	0.78584
NUMBL	1.157329	1.146976	1.16587833	1.1286079	0.1745444	0.78602
UBE3C	1.24626	1.2356758	1.25500048	1.0819967	0.11369611	0.78612
WARS	1.277026	1.2661415	1.28601536	1.074674	0.10389904	0.78616
PBX2	1.827312	1.841	1.8160074	0.9702823	-0.0435236	0.78619
GRHL1	1.031808	1.0218608	1.04002166	1.8307502	0.87243496	0.78627
RP11-17M16.2	1.037929	1.0279957	1.04613245	1.6478422	0.72057805	0.78632
PRL	1.024177	1.0339804	1.01608117	0.4732482	-1.079331	0.78633
RP11-42I10.1	1.042047	1.0321385	1.0502292	1.5628973	0.64422301	0.78638
RP11-430B1.2	1.042045	1.0321224	1.05023907	1.5639888	0.64523019	0.78638
CTBP1-AS2	1.210436	1.2211657	1.20157482	0.9114201	-0.133812	0.78671
SYT14	1.066288	1.0563829	1.07446835	1.3207602	0.40136853	0.78671
RP11-393I2.4	1.066297	1.0562251	1.07461486	1.3270738	0.40824857	0.78671
PCDH15	1.043751	1.0338282	1.05194543	1.5355642	0.6187688	0.78674
PELI2	1.150405	1.1608308	1.14179481	0.8816397	-0.181739	0.78683
ZNF704	1.245659	1.2563506	1.23682942	0.9238498	-0.1142698	0.78689
RP11-452L6.7	1.056593	1.0464862	1.06493982	1.3969694	0.48230043	0.78692

MAP7D2	1.048642	1.0387144	1.05683971	1.4681807	0.55402954	0.78693
RP11-1109F11.3	1.031732	1.0218349	1.03990456	1.8275562	0.86991576	0.78703
LIPT1	1.121836	1.1318923	1.11353064	0.8607827	-0.2162789	0.78714
LINC00116	1.882282	1.8955781	1.87130168	0.972893	-0.0396469	0.78729
RP11-303E16.5	1.030469	1.0205721	1.03864263	1.8783987	0.90950335	0.7873
MFSD1	1.187815	1.1981992	1.17923866	0.904336	-0.1450692	0.78735
HERC1	1.320817	1.3096283	1.33005597	1.0659747	0.0921732	0.78737
SEMA7A	1.044067	1.0341557	1.05225109	1.5297917	0.61333519	0.78738
GXYLT1	1.436418	1.4477132	1.42708982	0.9539362	-0.0680353	0.78741
AC009133.12	1.125135	1.1352783	1.11675788	0.8630939	-0.2124105	0.78741
WAS	1.03918	1.0292448	1.04738423	1.6202612	0.69622645	0.78754
TMED8	1.298365	1.2876214	1.30723706	1.0681996	0.09518123	0.78762
SULT1C4	1.474168	1.4627492	1.48359858	1.0450555	0.06357953	0.78773
POMT2	1.180159	1.1697132	1.18878502	1.112377	0.15364587	0.78773
RP11-161M6.2	1.253971	1.2648236	1.24500842	0.9251761	-0.1122001	0.78777
UCK2	1.215236	1.2046735	1.22395913	1.0942265	0.12991146	0.78781
TMEM132B	1.092447	1.0823012	1.10082591	1.225084	0.29288067	0.78785
LHFPL1	1.036589	1.0464286	1.02846339	0.613057	-0.7059068	0.78791
AMMECR1L	1.131724	1.1215429	1.14013081	1.1529325	0.20530808	0.78793
MAP3K14	1.070483	1.0604618	1.07875912	1.3026267	0.38142375	0.78801
SNX21	1.215809	1.2264602	1.20701389	0.9141294	-0.1295297	0.78802
RP11-392O17.2	1.123386	1.1337796	1.11480265	0.8581478	-0.220702	0.78815
UBASH3B	1.047433	1.0375639	1.05558229	1.4796727	0.56527808	0.78819
DEUP1	1.027376	1.0175728	1.03547119	2.0185241	1.01330079	0.78822
GVQW1	1.034568	1.044334	1.02650253	0.5977929	-0.7422824	0.78823
NMU	1.06303	1.0729072	1.05487269	0.7526379	-0.4099721	0.78824
PCDHB13	1.035172	1.045024	1.02703653	0.6004913	-0.7357848	0.78827
C1orf74	1.073175	1.0829528	1.06510035	0.7847875	-0.349626	0.78827
ERMP1	1.088623	1.0785691	1.09692554	1.2336336	0.30291393	0.7883
OPCML	1.149265	1.1595997	1.14073004	0.8817687	-0.1815278	0.78834
CARD10	1.040181	1.0303982	1.04825985	1.5875897	0.66683815	0.78844
NANP	1.10163	1.1115604	1.09342888	0.8374737	-0.2558842	0.78848
BLACAT1	1.103741	1.1137007	1.0955164	0.8400685	-0.2514212	0.78849
SFXN2	1.115768	1.1054442	1.12429264	1.1787522	0.2372605	0.78851
PAK4	1.489922	1.5013853	1.48045622	0.9582575	-0.0615147	0.78853
RP11-299G20.2	1.046616	1.0565264	1.03843164	0.6798888	-0.5566293	0.78853
POMGNT1	1.523106	1.53522	1.5131018	0.9586745	-0.0608871	0.78858
ANO7	1.127552	1.1173498	1.13597765	1.1587382	0.21255461	0.7886
RP11-57G10.8	1.034961	1.0447723	1.02685796	0.5998795	-0.7372553	0.78869
CYP2W1	1.024009	1.0142605	1.03206036	2.2482005	1.16877068	0.78873
COX18	1.270725	1.2813788	1.26192679	0.930869	-0.10335	0.78878
POFUT2	1.222229	1.2115228	1.23107046	1.0924138	0.12751943	0.78885
PCDH9	4.199958	4.2337518	4.1720508	0.9809197	-0.0277931	0.78885
PGGT1B	1.271848	1.2824739	1.26307358	0.93132	-0.1026512	0.78892
DET1	1.253441	1.264132	1.24461135	0.9260951	-0.1107678	0.78892

MYOM1	1.024853	1.0151206	1.03288955	2.1751522	1.12111634	0.78893
CTA-228A9.3	1.02297	1.0132688	1.03098141	2.3349002	1.22336088	0.78894
STAT4	1.022992	1.013265	1.03102562	2.3389072	1.22583461	0.78894
DIP2C	1.277082	1.2663346	1.28595649	1.0736737	0.10255567	0.78907
GPR157	1.058514	1.048589	1.0667095	1.3729347	0.45726299	0.78914
FOXO6	1.039881	1.0300822	1.04797251	1.5947154	0.67329897	0.78916
FAM65C	1.061185	1.0511354	1.06948364	1.3588172	0.44235138	0.78917
FUT4	1.055959	1.0461465	1.06406276	1.3882486	0.47326599	0.78922
SNCAIP	1.048803	1.0388937	1.05698682	1.4651949	0.55109263	0.78923
SH3TC2	1.036003	1.0262547	1.04405367	1.6779316	0.74668391	0.78923
CHMP1A	1.609852	1.621716	1.60005506	0.9651594	-0.0511608	0.78925
STK11	1.207596	1.197058	1.2162984	1.0976381	0.13440249	0.78931
RP11-586K2.1	1.053638	1.0436726	1.06186797	1.4166321	0.50246514	0.78932
MICB	1.040761	1.0309624	1.04885369	1.5778404	0.65795127	0.78937
ZNF667-AS1	1.53214	1.5204562	1.54178856	1.0409879	0.05795328	0.78946
RBM33	1.4401	1.4288341	1.44940352	1.047966	0.06759186	0.78948
CD69	1.023857	1.0141478	1.0318757	2.2530463	1.17187697	0.78949
C5orf56	1.077794	1.0876915	1.06962101	0.7939314	-0.3329137	0.78962
MIPOL1	1.238997	1.2496736	1.23017928	0.9219207	-0.1172855	0.7897
FIBIN	1.043964	1.0342393	1.05199574	1.518599	0.60274099	0.78974
CFAP69	1.071652	1.0815444	1.0634833	0.7785119	-0.361209	0.78976
GSTT2B	1.099204	1.0891731	1.10748685	1.205373	0.26947964	0.78977
EFR3A	1.309457	1.2987149	1.31832738	1.0656561	0.0917419	0.78985
MIR3911	1.031471	1.0216708	1.03956365	1.8256656	0.86842252	0.79006
CTC-499B15.5	1.050733	1.0409423	1.05881742	1.4365932	0.52265161	0.79016
TMEM14A	2.267827	2.2847621	2.25384248	0.9759336	-0.0351451	0.79019
ZNRF3-AS1	1.051389	1.041499	1.05955549	1.4351055	0.52115676	0.7903
MIR181A1HG	1.16503	1.1753728	1.1564893	0.8923236	-0.1643611	0.7903
ERO1B	1.153298	1.1635576	1.14482496	0.8854674	-0.1754889	0.79037
MAOB	1.10709	1.1170731	1.09884594	0.8443095	-0.2441562	0.7904
ACSL3	1.471093	1.4598169	1.48040417	1.0447728	0.06318926	0.79041
CACTIN	1.069861	1.0797304	1.06171049	0.7739897	-0.3696138	0.79047
JHDM1D-AS1	1.096365	1.1063401	1.08812735	0.8287315	-0.2710233	0.79068
CTB-25B13.5	1.037746	1.0473696	1.0297987	0.6290674	-0.6687135	0.79089
NAGS	1.161287	1.1714331	1.15290746	0.8919366	-0.1649869	0.79097
TRIM9	1.22436	1.2346409	1.21587034	0.920003	-0.1202895	0.79098
CD36	1.033977	1.0243168	1.04195516	1.7253536	0.78689207	0.79101
LIMCH1	1.498291	1.4866163	1.50793134	1.0438026	0.06184887	0.79105
RSPH4A	1.045553	1.0358056	1.05360332	1.4970654	0.58213721	0.79112
NAALADL1	1.033871	1.0435827	1.02585036	0.593134	-0.7535699	0.79128
CSTF2	1.100342	1.1101571	1.09223735	0.8373254	-0.2561397	0.79134
KATNAL1	1.258032	1.2476346	1.26661871	1.0766618	0.10656517	0.79136
AC092835.2	1.063737	1.0538514	1.07190057	1.3351658	0.41701896	0.7914
CFI	1.32359	1.3341791	1.31484453	0.942143	-0.0859821	0.79145
SRGAP2	1.167938	1.1781014	1.15954441	0.8958065	-0.158741	0.79167

AC073321.4	1.048285	1.0386022	1.05628149	1.4579868	0.5439777	0.79172
CAHM	1.225939	1.2363813	1.21731533	0.9193421	-0.1213262	0.79178
RP11-589P10.5	1.056963	1.0666685	1.04894724	0.7341881	-0.4457784	0.79182
ADCK2	1.22343	1.2339575	1.21473535	0.917839	-0.123687	0.79191
GAL3ST3	1.213919	1.2243007	1.20534636	0.915496	-0.1273746	0.79198
TRAPPC13	1.146488	1.136411	1.15481008	1.1348802	0.18253996	0.79211
KIAA0513	1.073399	1.0636246	1.08147044	1.2804869	0.3566925	0.79211
CHSY3	1.109613	1.1193817	1.10154634	0.850602	-0.2334439	0.79214
ZFP91	1.301747	1.2907794	1.31080439	1.0688665	0.09608167	0.79214
HOMER1	1.251872	1.2416117	1.26034566	1.0775375	0.10773811	0.79216
CCDC13	1.051835	1.0615195	1.04383766	0.7125811	-0.4888738	0.79228
RP11-802E16.3	1.030935	1.0213055	1.03888672	1.8252004	0.8680549	0.79235
KIAA1211L	1.030958	1.0213435	1.03889766	1.8224585	0.86588592	0.79235
FEM1C	1.159276	1.1694813	1.15084798	0.8900569	-0.1680305	0.79244
HARBI1	1.061728	1.0714873	1.0536678	0.750732	-0.4136301	0.79254
RNF141	1.469394	1.480507	1.46021714	0.9577741	-0.0622427	0.79254
MPZ	1.054393	1.0641545	1.04633242	0.7222002	-0.4695292	0.79257
MSRB1	1.483288	1.4947335	1.47383624	0.9577606	-0.0622631	0.79259
RP11-242D8.1	1.479154	1.4901485	1.47007536	0.9590467	-0.060327	0.79263
RP11-539G18.3	1.061745	1.0715427	1.05365343	0.7499498	-0.415134	0.79272
ATL3	1.242929	1.2323081	1.25169922	1.0834716	0.11566129	0.79288
TNFRSF10A	1.044653	1.0543	1.0366868	0.675632	-0.5656905	0.79304
SPATA5L1	1.08336	1.0931312	1.07529115	0.8084417	-0.3067844	0.79305
SRRM3	1.074042	1.0838637	1.06593044	0.7861619	-0.3471017	0.79311
RP11-48G14.3	1.035226	1.0448178	1.02730512	0.6092477	-0.7148992	0.79312
CASP8AP2	1.481971	1.4934099	1.47252384	0.9576699	-0.0623996	0.79315
ANKRD29	1.025773	1.0161878	1.03368867	2.0811087	1.05735236	0.79317
SBF2-AS1	1.133958	1.1238882	1.14227418	1.148408	0.19963527	0.79319
FSIP1	1.054979	1.0646483	1.04699473	0.7269292	-0.4601132	0.79322
SIGLEC15	1.031845	1.0223022	1.03972548	1.781235	0.83287789	0.79326
MRPS11	1.788826	1.8011084	1.77868324	0.9720074	-0.0409608	0.79333
PCDHGA8	1.026603	1.036106	1.01875591	0.519468	-0.9448932	0.79336
KAT7	1.397353	1.3862491	1.40652336	1.0524903	0.07380687	0.79352
CFAP221	1.085957	1.0761804	1.09402988	1.234306	0.30370009	0.79362
RP11-367N14.2	1.037688	1.0280687	1.04563136	1.6257036	0.70106422	0.79364
GATA2	1.037781	1.0282311	1.04566769	1.6176352	0.69388626	0.79364
DERL2	1.542225	1.5536908	1.53275608	0.9621906	-0.0556054	0.79375
CGN	1.236752	1.2262756	1.24540424	1.0845371	0.11707935	0.79383
FBXL18	1.055847	1.0462351	1.06378458	1.37957	0.4642187	0.79389
BTN3A3	1.057716	1.0674677	1.04966373	0.7361111	-0.4420045	0.79394
LRFN3	1.144453	1.1345734	1.15261264	1.134048	0.18148164	0.79395
TKTL1	1.03798	1.0474811	1.03013361	0.6346444	-0.6559796	0.79396
SPPL3	1.462903	1.4738896	1.4538307	0.9576719	-0.0623966	0.79397
FXYD1	1.38006	1.3691159	1.38909716	1.0541327	0.07605643	0.7941
TTC39B	1.106825	1.0969642	1.11496865	1.185682	0.24571707	0.79413

C1QL2	1.02446	1.0148661	1.03238263	2.1782851	1.1231928	0.79428
B3GNT2	1.10409	1.0942269	1.11223446	1.1911087	0.25230504	0.79428
KRT13	1.025322	1.0158097	1.0331771	2.0985264	1.06937664	0.79431
ATG7	1.197705	1.2080852	1.1891336	0.9089238	-0.1377687	0.79438
ZUFSP	1.188472	1.1781922	1.19696047	1.1053261	0.14447204	0.79442
RP11-637A17.2	1.087166	1.0969797	1.07906205	0.815243	-0.2946979	0.79443
COG7	1.188052	1.1981645	1.17970158	0.9068302	-0.1410957	0.7945
RP11-546J1.1	1.030307	1.0398722	1.0224073	0.5619787	-0.8314127	0.7945
RP11-444D3.1	1.038611	1.0289787	1.04656476	1.6068606	0.68424476	0.79454
SUGCT	1.104159	1.1139736	1.09605385	0.8427728	-0.2467843	0.79458
C15orf39	1.065617	1.0558904	1.07364979	1.3177529	0.39807988	0.79459
TAF12	1.912478	1.9247208	1.90236769	0.9758271	-0.0353025	0.79461
UST	1.053247	1.0436276	1.06119169	1.4025918	0.48809516	0.79462
TMCO4	1.028379	1.0188473	1.03625106	1.9234086	0.94366525	0.79474
CHST15	1.320061	1.3307376	1.31124449	0.9410617	-0.0876389	0.79479
CHKB-AS1	1.056647	1.0662454	1.0487199	0.7354462	-0.4433082	0.79485
RHCE	1.037687	1.0472227	1.02981243	0.6313158	-0.6635663	0.79493
DNAJC9-AS1.1	1.141486	1.1315366	1.14970229	1.1381034	0.1866316	0.79511
SDPR	1.035145	1.025657	1.04298011	1.6751824	0.74431821	0.79511
IKBKG	1.137497	1.1473304	1.12937698	0.8781416	-0.1874744	0.79512
FADS3	1.191544	1.1813649	1.19995063	1.1024768	0.14074832	0.79524
RBM34	1.117709	1.1275262	1.10960155	0.8594437	-0.218525	0.79524
ZNF350	1.117744	1.1275992	1.10960619	0.8589879	-0.2192903	0.79524
RP11-525K10.3	1.036011	1.0265282	1.043842	1.6526547	0.72478535	0.79525
LIN28B	1.303737	1.3142448	1.29505953	0.9389479	-0.090883	0.79545
RASGRP2	1.059873	1.0502828	1.06779199	1.3482133	0.43104879	0.79545
ITM2A	1.070874	1.0803629	1.06303775	0.7844136	-0.3503135	0.79561
C2orf73	1.042976	1.0524815	1.03512539	0.6692904	-0.5792957	0.79569
SLC27A4	1.065279	1.055624	1.07325193	1.3169126	0.39715965	0.79575
ADAMTS17	1.028255	1.01876	1.03609671	1.9241338	0.94420913	0.79577
USP27X	1.164784	1.1748121	1.15650322	0.8952654	-0.1596127	0.79595
POLR3H	1.528038	1.540085	1.51808956	0.9592741	-0.059985	0.79595
XXbac-BPG308K3.5	1.042572	1.0520641	1.03473392	0.6671373	-0.5839444	0.79595
MYO9B	1.430956	1.4199705	1.44002783	1.0477589	0.06730681	0.79599
XXbac-BPG252P9.9	1.107374	1.1170118	1.09941422	0.8496082	-0.2351304	0.79605
BRPF3	1.107187	1.1169333	1.09913923	0.8478272	-0.2381579	0.79605
ZNF416	1.062514	1.0720433	1.05464481	0.7584991	-0.3987807	0.79607
LINC01431	1.097797	1.1075852	1.08971408	0.8338885	-0.2620736	0.7961
TMEM44-AS1	1.119026	1.1289	1.11087099	0.8601316	-0.2173707	0.79618
ZSCAN12	1.126695	1.1364911	1.11860491	0.8689572	-0.202643	0.79622
EML6	1.108911	1.0991586	1.11696532	1.1795777	0.23827048	0.79623
RP4-565E6.1	1.034777	1.0253433	1.0425681	1.6796603	0.7481695	0.79625
GGA3	1.148045	1.1380392	1.15630772	1.132343	0.1793111	0.7963
ALOX5	1.042573	1.033092	1.05040284	1.5231115	0.60702158	0.79634
BTBD6	1.048072	1.038524	1.05595615	1.4525021	0.53854027	0.79641

ZNF213	1.126795	1.1366881	1.11862575	0.8678573	-0.2044702	0.79641
AC007246.3	1.479032	1.4679743	1.48816275	1.04314	0.06093277	0.79641
NUP50	1.440629	1.4516571	1.43152165	0.9554187	-0.065795	0.79645
ALDH1A3	1.373358	1.3844744	1.36417805	0.9472101	-0.0782436	0.79648
SERF1A	1.079015	1.0887727	1.07095639	0.7993041	-0.3231836	0.79648
MCOLN3	1.042936	1.0334596	1.05076191	1.5171125	0.60132807	0.79659
RP3-403A15.5	1.040991	1.0505174	1.03312306	0.6556756	-0.6089459	0.79663
CDKL2	1.22931	1.2395551	1.22084867	0.921912	-0.1172991	0.79664
C19orf44	1.079061	1.0887223	1.07108304	0.8011853	-0.3197921	0.79667
MIR1302-9	1.150641	1.1606284	1.14239275	0.886473	-0.1738514	0.79668
HERC4	1.613221	1.62517	1.60335242	0.9651014	-0.0512476	0.79689
SHOX2	1.027491	1.0180243	1.0353084	1.9589366	0.9700707	0.79703
RP6-191P20.4	1.027432	1.0180243	1.03520149	1.9530048	0.96569547	0.79703
PCDH8	1.030815	1.0213721	1.03861366	1.8067335	0.85338371	0.79707
GALNT8	1.043114	1.0525495	1.03532262	0.6721778	-0.5730853	0.7971
RP11-1348G14.4	1.045259	1.0358144	1.05305825	1.4814773	0.56703648	0.79724
ZNF226	1.607304	1.6188748	1.59774865	0.9658636	-0.0501086	0.79727
GLTSCR1L	1.075933	1.0663997	1.08380561	1.2621387	0.33587044	0.7973
ACBD5	1.389856	1.4008468	1.38077979	0.9499384	-0.0740942	0.79745
STAP2	1.333852	1.344571	1.32499945	0.9432003	-0.0843639	0.79756
RP11-74E22.3	1.027475	1.0180706	1.03524146	1.9502076	0.96362772	0.79761
CTD-2308L22.1	1.040543	1.0310482	1.04838453	1.5583663	0.6400344	0.79769
CCDC86	1.19938	1.2093604	1.19113857	0.9129642	-0.1313698	0.79771
CTD-2515A14.1	1.030187	1.0395698	1.02243818	0.5670531	-0.8184442	0.79789
TNIP2	1.378489	1.3679264	1.38721185	1.0524167	0.073706	0.79791
SHANK2	1.059496	1.0499509	1.06737824	1.3488906	0.43177335	0.79796
DNM3OS	1.028063	1.0185931	1.03588431	1.929985	0.94858964	0.79801
RP11-329B9.4	1.036385	1.0458196	1.02859352	0.6240458	-0.6802761	0.79805
CTC-241F20.4	1.036317	1.0457219	1.02854962	0.6244193	-0.6794131	0.79805
TIAL1	2.178306	2.1927045	2.16641595	0.9779589	-0.0321543	0.79806
PDLIM5	2.234533	2.2217037	2.24512815	1.0191736	0.02739979	0.79807
ZNF232	1.240607	1.250788	1.23219938	0.925879	-0.1111044	0.79809
NAT16	1.036907	1.0463383	1.02911834	0.6283857	-0.6702777	0.79811
NCKAP5	1.076802	1.0864638	1.06882238	0.7959676	-0.3292183	0.79815
TBL1XR1	2.202691	2.1896693	2.2134442	1.0199844	0.02854713	0.79817
CTPS1	1.277145	1.2873732	1.26869867	0.9350165	-0.0969363	0.79832
ERLIN1	1.296025	1.3064038	1.2874546	0.938156	-0.0921002	0.79836
LINC01235	1.029335	1.0387818	1.02153367	0.5552524	-0.8487844	0.79846
ATCAY	1.131638	1.1219302	1.13965562	1.1453731	0.19581766	0.79851
R3HCC1L	1.190908	1.1807417	1.1993036	1.1026986	0.14103858	0.79853
SMIM10L2A	1.140563	1.130882	1.14855724	1.1350471	0.18275222	0.79857
RP11-620J15.2	1.028552	1.0378707	1.02085653	0.5507294	-0.8605844	0.79858
SPCS3	1.862526	1.8749591	1.85225912	0.974056	-0.0379234	0.79863
NEMF	1.887203	1.8744037	1.89777327	1.0267263	0.03805165	0.79864
SLC9A8	1.075148	1.0846489	1.06730256	0.7950793	-0.3308293	0.79864

TIMM23B	1.101055	1.0914695	1.1089716	1.1913437	0.25258966	0.79868
TMEM165	2.226089	2.2125681	2.23725491	1.0203591	0.02907704	0.79872
CLDN9	1.022143	1.012846	1.02982065	2.3213891	1.21498837	0.79873
RNF223	1.022217	1.012846	1.02995618	2.3319399	1.22153061	0.79873
OBSL1	3.361607	3.3812018	3.34542528	0.9849755	-0.0218403	0.79875
HOXA2	1.027179	1.0178144	1.03491253	1.9597973	0.97070446	0.79875
SYT9	1.073992	1.0835155	1.06612676	0.7917906	-0.3368092	0.79876
PIP4K2B	1.298894	1.2887619	1.30726159	1.0640657	0.0895872	0.79898
NUAK1	1.301267	1.3119209	1.29246963	0.9376402	-0.0928936	0.79899
PCGF3	1.435658	1.4248257	1.44460436	1.0465571	0.06565108	0.79917
ADGRA1	1.034649	1.0252691	1.0423951	1.677747	0.74652515	0.79918
DDB2	2.073833	2.0600848	2.08518613	1.0236786	0.03376281	0.79923
CPA4	1.083771	1.074157	1.0917107	1.2367101	0.30650736	0.79932
OAS3	1.02493	1.0156039	1.03263122	2.091222	1.06434624	0.79933
ARHGAP1	1.22855	1.2189843	1.23644943	1.0797552	0.11070423	0.79933
DLG2	1.163416	1.1733144	1.15524173	0.8957231	-0.1588753	0.79955
CNTNAP2	1.397273	1.3868975	1.40584145	1.0489636	0.06896466	0.79956
FBXO11	1.474926	1.4639978	1.48395125	1.0430033	0.06074369	0.7996
TTF2	1.507091	1.5176303	1.49838801	0.9628262	-0.0546527	0.79961
STK10	1.098011	1.1075983	1.09009428	0.8373209	-0.2561475	0.79961
HIST1H4E	1.050851	1.0602486	1.04309057	0.7152128	-0.4835556	0.79963
PLS1	1.074065	1.0645404	1.08193064	1.2694473	0.3442005	0.79973
SLC22A15	1.035913	1.026633	1.04357691	1.6362	0.71034914	0.79974
GBGT1	1.128463	1.1187725	1.13646523	1.1489634	0.20033288	0.79975
TNPO2	1.326973	1.3376453	1.31816014	0.942291	-0.0857554	0.79978
EAF1	1.214044	1.204063	1.22228609	1.0893014	0.12340323	0.7998
TBX19	1.061763	1.0523158	1.06956475	1.3297082	0.41110974	0.79982
CRYBA4	1.044054	1.0535198	1.03623769	0.6770896	-0.5625813	0.79999
AMIGO2	1.141275	1.131319	1.14949645	1.1384218	0.18703524	0.80005
PAK1	1.566862	1.5781122	1.55757115	0.9644688	-0.0521936	0.80011
RP11-91G21.1	1.125006	1.1345336	1.11713854	0.8707008	-0.1997511	0.80014
RP5-1068E13.7	1.11824	1.1085459	1.12624475	1.1630536	0.21791755	0.80018
REEP1	1.077115	1.0867238	1.06917929	0.7976972	-0.3260869	0.8002
C1orf204	1.029908	1.0206219	1.03757688	1.8221868	0.86567083	0.80021
PNISR	7.209827	7.1622549	7.24911319	1.0140952	0.02019312	0.80025
TP53INP2	1.105659	1.096148	1.1135131	1.1806078	0.23952978	0.80029
DCAF12L2	1.057986	1.067383	1.05022619	0.7453839	-0.4239445	0.80032
C16orf62	1.289012	1.299045	1.28072666	0.938744	-0.0911963	0.80045
RP11-677M14.3	1.03169	1.0409652	1.02403018	0.5866	-0.7695511	0.80054
CD83	1.106122	1.0967417	1.11386916	1.1770435	0.23516769	0.80054
ADSSL1	1.220608	1.2106645	1.22882019	1.0861828	0.11926699	0.8007
DNAAF5	1.206643	1.2166056	1.19841627	0.9160254	-0.1265405	0.80073
ADGRL2	1.50708	1.5181813	1.49791292	0.9608855	-0.0575636	0.80074
KIF9-AS1	1.074024	1.0645255	1.08186843	1.2687756	0.34343698	0.80078
DRD2	1.058333	1.0678156	1.05050279	0.7447073	-0.4252545	0.80079

CCND3	1.351945	1.3623171	1.34337938	0.9477317	-0.0774494	0.8008
RIIAD1	1.039193	1.0484415	1.03155526	0.6514098	-0.6183626	0.80089
LRCH4	1.260298	1.2703702	1.25198069	0.931984	-0.101623	0.80093
RXRG	1.040164	1.0494179	1.0325217	0.6580948	-0.6036326	0.80097
SIRPA	1.075714	1.0663971	1.08340755	1.2561918	0.32905672	0.801
TTC30A	1.139698	1.1493191	1.13175254	0.8823555	-0.1805681	0.80116
MOB1A	2.009385	1.9967358	2.01983018	1.02317	0.03304591	0.80118
XBP1	1.792654	1.7806831	1.8025389	1.0279957	0.03983421	0.80122
C6orf118	1.135305	1.125517	1.14338886	1.1423857	0.19204977	0.80124
NCOA2	1.270669	1.2605715	1.27900825	1.0707551	0.0986285	0.80144
DPP10	1.137808	1.1283316	1.14563387	1.1348247	0.1824695	0.80149
RP11-467J12.4	1.032504	1.0232925	1.04011071	1.7220453	0.7841231	0.8015
PRPF40B	1.188945	1.1790847	1.19708849	1.1005325	0.13820177	0.80154
PLS3-AS1	1.023195	1.0324131	1.01558225	0.4807395	-1.0566727	0.80156
ZNF567	1.172282	1.1818169	1.16440853	0.9042534	-0.145201	0.80157
ZNF497	1.072661	1.0820079	1.06494125	0.7918898	-0.3366284	0.80166
TEAD1	1.491547	1.5025799	1.48243608	0.9599191	-0.0590153	0.80168
TMEM120B	1.182183	1.1724148	1.19024903	1.1034382	0.14200584	0.80171
RP11-452L6.5	1.076024	1.085427	1.06825886	0.7990314	-0.3236758	0.80174
METTL4	1.266435	1.2767297	1.25793437	0.9320806	-0.1014733	0.80178
ZNF772	1.103591	1.1131554	1.09569262	0.8456742	-0.2418261	0.80188
RP11-1379J22.5	1.029169	1.0199548	1.03677752	1.8430421	0.88208905	0.80205
CHSY1	1.20348	1.1937004	1.21155703	1.0921868	0.12721961	0.80205
C10orf95	1.07166	1.0624448	1.07926995	1.2694412	0.34419361	0.80205
FCGR2A	1.034594	1.0438007	1.02699137	0.6162313	-0.6984562	0.80214
ZBTB42	1.046272	1.0370617	1.05387858	1.4537553	0.53978441	0.80219
APOL4	1.030042	1.0208113	1.0376647	1.8098182	0.85584481	0.80221
SMIM8	1.742348	1.7535471	1.73309927	0.9728645	-0.0396892	0.80229
ZNF283	1.079454	1.0700836	1.08719247	1.2441202	0.31512585	0.80233
NPHS1	1.029763	1.0205224	1.03739334	1.8220709	0.86557907	0.80243
SGK2	1.029724	1.0205155	1.03732762	1.819481	0.86352695	0.80243
HES2	1.041095	1.0318418	1.04873584	1.530561	0.61406052	0.80245
RP11-55K13.1	1.041164	1.0319384	1.04878312	1.5274153	0.61109238	0.80245
PINK1	1.309002	1.319533	1.30030529	0.9398256	-0.089535	0.80249
RP11-181G12.2	1.045852	1.055088	1.0382252	0.6938938	-0.5272131	0.8026
SLC12A9	1.151477	1.1418637	1.15941505	1.1237197	0.16828216	0.80265
YWHAG	2.23656	2.2514949	2.22422633	0.9782112	-0.0317821	0.80265
GAPLINC	1.024922	1.0157693	1.03248033	2.0597139	1.04244398	0.80276
ATG14	1.238406	1.228338	1.24671973	1.0805023	0.11170217	0.80276
RP11-259N19.1	1.044803	1.0539811	1.03722308	0.6895571	-0.5362582	0.80302
DENND2C	1.041488	1.0322749	1.04909591	1.5211811	0.60519195	0.8031
CDK2AP2	2.176613	2.1896667	2.16583339	0.9799664	-0.0291958	0.80315
MOCS1	1.095331	1.1047467	1.08755589	0.8358817	-0.2586293	0.80322
RP11-417L19.4	1.0259	1.0351397	1.01826902	0.5198972	-0.9437016	0.80324
PHF21A	1.506494	1.4956516	1.51544878	1.0399418	0.0565028	0.80326

GATA3-AS1	1.042113	1.0330383	1.0496063	1.5014772	0.5863826	0.80335
SH2D3A	1.042062	1.0328542	1.04966684	1.5117345	0.5962048	0.80335
MICALL1	1.080611	1.0714325	1.08819137	1.2346109	0.30405647	0.8034
TRO	1.235704	1.2256079	1.24404175	1.0817075	0.11331048	0.80348
ARHGAP17	1.235546	1.2254423	1.24389007	1.0818291	0.11347257	0.80348
TMEM108	1.249915	1.239939	1.25815407	1.0759156	0.10556493	0.8035
ADAP2	1.036285	1.0271621	1.04381831	1.6132161	0.68993967	0.8036
SHPK	1.080103	1.0893809	1.0724404	0.8104687	-0.3031716	0.80362
LCOR	1.660808	1.6496277	1.67004147	1.0314238	0.04463728	0.80363
ADO	1.213131	1.2032957	1.22125401	1.088336	0.12212399	0.80368
KCNRG	1.024189	1.0150885	1.03170493	2.1012714	1.07126249	0.80375
MUC4	1.024212	1.0150885	1.03174714	2.1040686	1.07318175	0.80375
LINC01106	1.026593	1.0175091	1.03409553	1.9473037	0.96147789	0.80377
CES2	1.66174	1.6507643	1.67080415	1.0307944	0.04375658	0.80382
PAN3-AS1	1.081157	1.0716688	1.08899234	1.2417166	0.3123359	0.80384
MFSD4B	1.275342	1.2654717	1.28349352	1.067886	0.09475769	0.8039
SLC35B3	1.255205	1.2649583	1.24715033	0.9327894	-0.1003766	0.80395
TMEM201	1.130108	1.1204881	1.13805159	1.1457699	0.19631729	0.80397
RP11-44N11.2	1.04853	1.0576269	1.04101741	0.7117758	-0.4905052	0.80397
SLC16A9	1.178152	1.168488	1.18613237	1.1047217	0.14368303	0.80413
C2CD5	1.204644	1.2143921	1.19659347	0.9169809	-0.1250365	0.80414
SCRT2	1.020569	1.0296021	1.01310914	0.4428457	-1.1751241	0.80414
SNX32	1.113545	1.1229836	1.10575004	0.8598713	-0.2178073	0.80416
GAS2	1.039246	1.0300619	1.04682977	1.5577788	0.6394904	0.80424
PPP6R3	1.532464	1.5218782	1.54120605	1.0370353	0.05246494	0.80428
PDPK1	1.276282	1.2663291	1.2845019	1.0682344	0.09522828	0.80432
ZNF112	1.083465	1.074056	1.09123492	1.2319714	0.30096874	0.80433
IQCE	1.247979	1.238017	1.25620553	1.0764169	0.10623692	0.80442
ZNF780A	1.119911	1.1105434	1.12764654	1.1547184	0.20754102	0.80443
SPTBN2	1.17374	1.1641638	1.18164728	1.1065001	0.14600354	0.80443
EGF	1.075509	1.0847408	1.06788532	0.8010939	-0.3199568	0.8045
RHBDL3	1.027749	1.0368713	1.02021647	0.5482975	-0.8669692	0.80458
ZNF74	1.242723	1.2330063	1.25074643	1.0761356	0.10585992	0.80466
GNG2	1.188608	1.1985918	1.18036255	0.9082075	-0.1389061	0.80468
RTN4RL2	1.046972	1.0561706	1.03937564	0.7010013	-0.512511	0.80473
TTLL3	1.138715	1.1294493	1.14636643	1.1306857	0.17719801	0.80475
PLA2G3	1.058484	1.0492277	1.06612851	1.343318	0.42580089	0.80477
CEBPA	1.02039	1.0113663	1.02784204	2.4495217	1.2925001	0.8048
CHRNE	1.030074	1.0391799	1.02255347	0.5756385	-0.796765	0.80487
RP11-541G9.1	1.030055	1.0390767	1.02260549	0.5784909	-0.7896339	0.80487
ZBTB14	1.169458	1.1789699	1.16160234	0.9029583	-0.1472687	0.80489
USP20	1.118361	1.1278865	1.11049492	0.8640077	-0.2108839	0.80495
RP3-402G11.26	1.176449	1.1861069	1.16847323	0.9052496	-0.1436125	0.80504
TXLNA	1.428997	1.4182993	1.437832	1.0466954	0.06584172	0.80511
CPS1	1.235902	1.2457807	1.2277449	0.9266184	-0.1099528	0.80537

LRCH3	1.385558	1.3753772	1.39396492	1.0495175	0.06972618	0.80538
C10orf107	1.04582	1.036678	1.05336982	1.4550901	0.54110844	0.80542
LINC00648	1.040354	1.049426	1.03286259	0.6648846	-0.588824	0.80547
YRDC	1.102852	1.1120625	1.09524549	0.8499318	-0.2345809	0.80553
MTRNR2L3	1.030876	1.0398495	1.02346619	0.5888704	-0.7639779	0.80557
SLC5A2	1.036214	1.0270651	1.04376872	1.6171627	0.69346487	0.80562
VPREB3	1.021418	1.0303915	1.01400677	0.4608781	-1.1175428	0.80567
FZD9	1.035414	1.0263374	1.04291003	1.6292429	0.70420169	0.80581
AC100830.3	1.058432	1.0492368	1.06602603	1.3409892	0.42329758	0.80582
COL22A1	1.10824	1.0990244	1.11585102	1.1699241	0.22641499	0.80594
HERC5	1.048709	1.0395665	1.05625984	1.4219066	0.50782672	0.80602
PAMR1	1.024051	1.0150455	1.03148757	2.092816	1.06544546	0.80617
CALY	1.254832	1.2648143	1.24658864	0.9311756	-0.1028749	0.80627
C5orf38	1.072736	1.0635366	1.08033245	1.2643482	0.33839386	0.80643
COX10	1.154359	1.1636821	1.1466598	0.8960038	-0.1584232	0.80647
KIAA1549L	1.030947	1.0218712	1.03844134	1.7576222	0.81362498	0.80648
AP000253.1	1.037107	1.0280464	1.04458986	1.5898611	0.66890071	0.8065
MAPK9	1.196161	1.1867427	1.2039388	1.0920846	0.12708461	0.80656
TTC7A	1.081181	1.0903565	1.07360413	0.8145971	-0.2958414	0.80657
GPR137B	1.356815	1.3467444	1.36513152	1.053028	0.07454379	0.80657
CEP72	1.047999	1.0388557	1.05554944	1.4296344	0.51564625	0.80659
ZNF417	1.062979	1.0537343	1.07061307	1.3141165	0.3940932	0.80669
RP11-319E16.2	1.03456	1.0435437	1.02714067	0.6232972	-0.6820079	0.80672
TSPYL4	1.320377	1.3102919	1.32870467	1.0593402	0.08316604	0.80682
KIAA1024L	1.059304	1.0683745	1.05181284	0.7577799	-0.4001492	0.80685
RP11-398C13.6	1.059247	1.0682529	1.05180887	0.7590716	-0.3976921	0.80685
CATSPERG	1.057872	1.0487389	1.0654137	1.3421237	0.42451767	0.80693
TNKS2-AS1	1.052568	1.0615879	1.0451198	0.7326079	-0.4488868	0.80698
LACC1	1.049779	1.0588413	1.04229516	0.7188011	-0.4763355	0.80699
CYP2R1	1.28214	1.2919324	1.27405361	0.9387572	-0.091176	0.80704
LINC00894	1.056075	1.0468899	1.06365928	1.3576322	0.44109272	0.80719
TERC	1.023932	1.014929	1.03136751	2.1011095	1.07115137	0.8072
COL15A1	1.026678	1.0176696	1.03411705	1.9308317	0.94922239	0.80721
LINC00473	1.026649	1.0176698	1.03406397	1.927802	0.94695685	0.80721
VN1R1	1.030762	1.0217476	1.03820652	1.7568131	0.81296071	0.80725
NDNF	1.030761	1.0217439	1.03820664	1.7571194	0.81321226	0.80725
DLGAP1-AS2	1.129169	1.119922	1.13680516	1.1407844	0.19002614	0.80726
KB-1460A1.5	1.099511	1.0903861	1.10704691	1.1843287	0.24406958	0.80738
B3GNT5	1.188375	1.1786136	1.19643675	1.0997864	0.13722333	0.80743
CENPL	1.168723	1.1781006	1.16097801	0.9038601	-0.1458286	0.80744
INHA	1.091631	1.0823688	1.09927994	1.20531	0.26940421	0.8075
KIF25-AS1	1.036629	1.045751	1.02909652	0.6359754	-0.6529572	0.80759
KIAA1614-AS1	1.02699	1.0359206	1.01961496	0.5460645	-0.8728566	0.80764
RP4-563E14.1	1.026959	1.0358832	1.01958849	0.5458958	-0.8733026	0.80764
CHAF1B	1.133485	1.1426016	1.12595721	0.8832804	-0.1790565	0.80764

AFTPH	1.306254	1.2964871	1.31431912	1.0601443	0.08426064	0.80766
TMEM138	1.377477	1.3671112	1.38603782	1.0515554	0.0725249	0.8077
CAMSAP1	1.34689	1.3367347	1.35527725	1.0550657	0.0773328	0.80778
FAM86B1	1.173796	1.1832732	1.16596966	0.9055861	-0.1430764	0.80785
CFAP54	1.056333	1.0652572	1.04896351	0.7503153	-0.4144311	0.80789
NRG4	1.037852	1.0468196	1.03044726	0.6503108	-0.6207986	0.80795
PCDHB3	1.038588	1.0295852	1.04602353	1.5556269	0.63749609	0.80798
GNG10	1.103129	1.1123417	1.09552122	0.8502738	-0.2340005	0.80799
KTI12	1.140394	1.1496738	1.13273057	0.8867992	-0.1733206	0.80799
MFSD4A	1.038048	1.0290276	1.0454965	1.5673552	0.6483322	0.80802
C12orf56	1.028314	1.0371961	1.02097953	0.564025	-0.8261689	0.80812
AQP3	1.082288	1.0732397	1.08975946	1.2255578	0.29343847	0.80819
MLXIPL	1.046643	1.0375507	1.05415121	1.4420825	0.52815369	0.8083
RP11-73E17.2	1.064557	1.0736134	1.05707866	0.7753841	-0.367017	0.80838
SGPP1	1.090746	1.081653	1.09825591	1.2033343	0.2670375	0.80839
RP11-3P17.4	1.021468	1.0303228	1.01415473	0.4668012	-1.0991199	0.80841
TFAP2D	1.021527	1.0304541	1.01415473	0.4647883	-1.1053542	0.80841
UNC93B1	1.075826	1.0848948	1.0683361	0.80495	-0.313029	0.80854
NOMO3	1.075993	1.0849683	1.06858177	0.8071454	-0.3090994	0.80854
WDR5B	1.06646	1.0755872	1.05892343	0.7795423	-0.3593008	0.80864
PRKAR1B	1.155233	1.1646687	1.14744118	0.8953805	-0.1594272	0.8087
RCVRN	1.028168	1.0370326	1.02084819	0.5629681	-0.828875	0.80877
GPR63	1.109235	1.1000317	1.11683474	1.1679767	0.22401151	0.80883
PLPP1	1.249514	1.2400982	1.25728903	1.0715993	0.09976558	0.80892
TK2	1.243619	1.2533079	1.2356181	0.9301648	-0.1044418	0.80893
FRS2	1.24343	1.252871	1.23563391	0.9318343	-0.1018547	0.80893
GDF10	1.064802	1.0558742	1.07217504	1.2917421	0.36931811	0.80901
RP11-23F23.2	1.033422	1.0245075	1.04078345	1.664121	0.73476037	0.80907
ZNF267	1.109663	1.1002412	1.11744353	1.1716098	0.22849219	0.80908
SDCBP2-AS1	1.139148	1.1486482	1.13130238	0.8833094	-0.1790092	0.80909
EAF1-AS1	1.047417	1.0563846	1.04001227	0.7096311	-0.4948589	0.80921
TGFA	1.024015	1.0151338	1.03134997	2.0715228	1.0506917	0.80923
DCAF6	1.281762	1.2918796	1.27340634	0.9367094	-0.0943266	0.80928
HIVEP2	1.088369	1.0976645	1.08069247	0.8262211	-0.2754002	0.80942
GRIN3B	1.03511	1.0261582	1.04250323	1.624851	0.70030746	0.80944
ZNF223	1.067868	1.0587385	1.0754067	1.283769	0.36038562	0.80953
C3orf18	1.18771	1.1973027	1.17978889	0.911234	-0.1341065	0.8096
ZDHHC14	1.079038	1.0700335	1.08647397	1.2347516	0.30422082	0.80965
CDH18	1.047535	1.0386805	1.05484798	1.4179756	0.50383272	0.80982
SENP2	1.392383	1.3821772	1.40081079	1.0487564	0.06867955	0.80992
ZNF8	1.114573	1.1237464	1.10699729	0.8646494	-0.2098129	0.80995
LFNG	1.084024	1.0749427	1.09152437	1.2212583	0.28836832	0.81
DRAM1	1.08394	1.0749043	1.09140239	1.2202552	0.28718289	0.81
AP001372.2	1.212713	1.2031934	1.22057362	1.0855356	0.11840705	0.81012
KIAA1257	1.105031	1.1142559	1.097413	0.852586	-0.2300827	0.81012

STRADA	1.050761	1.0597605	1.04332931	0.7250489	-0.4638498	0.81017
KIAA2012	1.022753	1.0139097	1.03005611	2.160797	1.11156355	0.81018
RP11-521O16.2	1.071776	1.0808678	1.06426876	0.794739	-0.3314469	0.81023
NOX4	1.081915	1.0727341	1.08949591	1.2304539	0.29919057	0.81026
SLCO3A1	1.084287	1.0752387	1.09175858	1.2195658	0.28636754	0.81028
GABRG3	1.050691	1.0417162	1.05810308	1.3928167	0.47800542	0.81029
HMGN3-AS1	1.050803	1.0419157	1.05814185	1.3871134	0.47208575	0.81029
TXLNB	1.074672	1.0655365	1.0822162	1.2545101	0.32712407	0.8103
RP11-172H24.4	1.066965	1.0580058	1.07436315	1.281995	0.35839064	0.8103
DNAJB9	1.710575	1.7217854	1.70131643	0.9716412	-0.0415044	0.81032
CACNA2D4	1.035977	1.0271348	1.04327902	1.5949633	0.67352321	0.81034
HOPX	1.03266	1.0237856	1.03998805	1.681184	0.74947761	0.81034
FOXC1	1.032635	1.0237999	1.03993186	1.6778147	0.74658337	0.81034
UBA7	1.03054	1.0216997	1.03783978	1.7437931	0.80222891	0.81036
MCM9	1.107202	1.0979671	1.11482863	1.1721142	0.2291132	0.81051
FGFBP2	1.022171	1.0309273	1.01494071	0.4830909	-1.0496335	0.81052
TRPV6	1.022318	1.0312509	1.01494071	0.4780883	-1.0646509	0.81052
CBX4	1.080036	1.071067	1.08744258	1.2304242	0.29915573	0.81057
IL34	1.0258	1.0169707	1.03309054	1.9498609	0.96337123	0.81062
C14orf28	1.196135	1.2054167	1.1884693	0.9174976	-0.1242237	0.81064
DPH7	1.331062	1.321147	1.33924968	1.0563687	0.07911345	0.81071
NT5M	1.139294	1.1485659	1.13163689	0.8860507	-0.1745389	0.81072
RP11-673E11.2	1.023358	1.0321746	1.01607711	0.4996838	-1.0009127	0.8109
APOBEC3F	1.045655	1.0367842	1.05298112	1.4403234	0.52639279	0.81093
RP5-1085F17.3	1.15189	1.1426178	1.15954681	1.1187022	0.16182604	0.81099
AP5S1	1.167397	1.1766817	1.15973032	0.9040571	-0.1455142	0.81106
TMEM200A	1.05729	1.0484655	1.06457798	1.3324519	0.41408345	0.81113
WNT9A	1.031561	1.0227396	1.03884527	1.7082655	0.77253218	0.81113
CCDC155	1.038674	1.0475656	1.03133096	0.6586897	-0.6023291	0.81123
SEC61A2	1.307615	1.2977511	1.31576008	1.0604834	0.08472198	0.81159
ZBTB49	1.057154	1.0482293	1.06452379	1.3378545	0.41992119	0.8116
ZNF211	1.083412	1.0923857	1.07600064	0.8226448	-0.2816585	0.81179
EDNRB	1.065605	1.0567327	1.07293116	1.2855233	0.36235573	0.81183
C17orf97	1.103304	1.1122586	1.09590933	0.8543605	-0.2270831	0.81185
FGGY	1.13152	1.1404511	1.12414503	0.8839021	-0.1780415	0.81186
C7orf50	3.52566	3.5461852	3.50870922	0.9852815	-0.0213921	0.81192
RNF32	1.093882	1.1030765	1.08628835	0.8371295	-0.2564772	0.81193
GIT1	1.264837	1.2746676	1.25671882	0.9346528	-0.0974975	0.81194
ARL6	1.096538	1.0874109	1.10407481	1.1906394	0.2517366	0.81196
KIF7	1.059293	1.0504817	1.0665693	1.3186829	0.39909771	0.81206
EXOSC3	1.369962	1.3795985	1.36200366	0.9536489	-0.0684698	0.8121
PARD6G	1.174817	1.1655404	1.18247775	1.1023158	0.14053757	0.81215
POLR1A	1.137427	1.12822	1.14503098	1.1311107	0.17774016	0.81215
BMI1	1.163405	1.1542062	1.17100102	1.1089115	0.14914417	0.81216
PET117	1.615822	1.6051346	1.62464779	1.0322461	0.0457869	0.81221

ZNF117	1.280099	1.2705693	1.28796845	1.0643058	0.08991277	0.81224
RHBDD1	1.11659	1.1074382	1.12414848	1.1555336	0.20855924	0.81227
CYP2S1	1.032575	1.0238197	1.03980569	1.6711255	0.74082007	0.81234
KIAA1671	1.180599	1.1714922	1.18812035	1.0969617	0.13351315	0.81247
RGS8	1.052874	1.0617565	1.04553886	0.7373935	-0.4394935	0.81251
CNOT11	1.303183	1.3128779	1.29517748	0.9434272	-0.084017	0.81251
ST20	1.719843	1.7308406	1.71076057	0.9725247	-0.0401932	0.81256
CTB-13F3.1	1.030152	1.0213092	1.03745521	1.757701	0.81368971	0.81265
MEMO1	1.227148	1.2366902	1.21926842	0.926394	-0.1103022	0.81274
CD27	1.029396	1.020585	1.03667184	1.7814822	0.83307807	0.81286
IQCH-AS1	1.11831	1.1092218	1.12581527	1.1519249	0.2040467	0.81293
CTC-304I17.6	1.028268	1.0195103	1.03550027	1.8195684	0.8635963	0.81295
CNIH2	1.157361	1.1479851	1.16510388	1.1156793	0.15792245	0.81302
PITPNM2	1.042675	1.0338666	1.04994917	1.4748778	0.56059545	0.81305
MCM2	1.289099	1.2986024	1.28125079	0.9418907	-0.0863684	0.81309
MCEMP1	1.026614	1.0353596	1.0193919	0.5484196	-0.8666479	0.81312
TMEM55B	1.421007	1.4107577	1.42947065	1.0455572	0.06427203	0.81313
ADHFE1	1.098254	1.0892054	1.10572587	1.1851962	0.2451259	0.81314
ARAF	1.270857	1.2804531	1.26293252	0.9375276	-0.093067	0.81316
GS1-72M22.1	1.034069	1.0428662	1.02680348	0.6252819	-0.6774214	0.81319
RP11-320G10.1	1.03406	1.0428358	1.02681361	0.6259627	-0.6758515	0.81319
ERBB2	1.366027	1.3562398	1.37410987	1.0501631	0.07061335	0.81325
DLX6	1.024518	1.0331911	1.01735489	0.5228771	-0.9354563	0.81329
SMOX	1.272399	1.2629626	1.28019222	1.0655212	0.09155928	0.81338
GPR89B	1.105977	1.1149145	1.09859649	0.8579988	-0.2209524	0.81338
AATF	1.335838	1.3455318	1.32783222	0.9487758	-0.0758609	0.8134
RP11-438L19.1	1.025602	1.034306	1.01841338	0.5367386	-0.8977084	0.81345
XXbac-BPG294E21.9	1.036155	1.0449563	1.02888596	0.6425343	-0.6381546	0.81348
INPP4B	1.059313	1.0504741	1.06661242	1.3197338	0.400247	0.8135
AC005076.5	1.117456	1.1264027	1.11006843	0.8707756	-0.1996271	0.81354
MAGEE1	1.075172	1.0663054	1.08249359	1.2441452	0.31515485	0.81357
FRMD3	1.025699	1.0170105	1.03287472	1.9326141	0.95055357	0.81367
BSN	1.080368	1.089275	1.07301178	0.8178304	-0.2901265	0.81372
FLAD1	1.48598	1.4963025	1.47745568	0.9620256	-0.0558528	0.81373
KCNK15-AS1	1.067482	1.0763639	1.06014787	0.7876475	-0.3443781	0.8138
LINC00491	1.070906	1.0797097	1.06363595	0.7983463	-0.3249134	0.81383
NAIF1	1.077442	1.0863491	1.07008575	0.8116562	-0.3010593	0.81383
SOX15	1.069572	1.0607859	1.07682786	1.2639086	0.33789218	0.81387
VPS37A	1.518866	1.508727	1.52723806	1.036387	0.05156289	0.81388
SLC19A2	1.115764	1.1248541	1.10825706	0.8670686	-0.2057819	0.81392
TSPYL5	1.049571	1.0407755	1.05683474	1.3938457	0.47907083	0.81394
TMEM200C	1.080756	1.0718985	1.08807125	1.2249382	0.29270897	0.81397
RHBDD2	2.267251	2.2813403	2.25561592	0.9799239	-0.0292585	0.81421
TRIM55	1.024709	1.0160357	1.03187229	1.9875831	0.99101518	0.81426
LIMS3	1.024722	1.0160691	1.03186814	1.9831955	0.98782688	0.81426

RP11-95O2.5	1.044759	1.035979	1.05201031	1.4455731	0.53164154	0.81429
STK17B	1.104958	1.1137173	1.09772455	0.8593639	-0.2186589	0.81432
SMTNL2	1.148154	1.1570419	1.14081485	0.8966705	-0.1573502	0.81433
AMACR	1.061817	1.0529822	1.06911247	1.3044458	0.38343698	0.81456
LRIG1	1.120528	1.111661	1.12785092	1.1449918	0.19533726	0.81459
ITPR1-AS1	1.032386	1.0236502	1.03960095	1.6744459	0.74368378	0.8146
ZNF28	1.100791	1.1097121	1.09342306	0.8515296	-0.2318715	0.81473
RAVER1	1.050089	1.0587663	1.04292237	0.7303913	-0.4532585	0.81474
PTGER1	1.034388	1.0256105	1.04163691	1.6257781	0.70113038	0.81478
LINC00174	1.032248	1.0235476	1.0394329	1.6746054	0.74382122	0.81479
GKAP1	1.53174	1.5419028	1.52334777	0.9657595	-0.0502641	0.81489
DDX49	1.349064	1.3586314	1.34116351	0.951293	-0.0720383	0.815
SP110	1.057594	1.06631	1.05039539	0.7599974	-0.3959336	0.81503
CTD-2515O10.5	1.021651	1.0303063	1.01450349	0.4785639	-1.0632165	0.81503
USPL1	1.175203	1.1845801	1.1674586	0.9072409	-0.1404423	0.81505
GTF2IRD2	1.287097	1.2773159	1.29517392	1.064396	0.09003497	0.81507
SMNDC1	1.520129	1.5304525	1.51160278	0.9644648	-0.0521995	0.81517
F10	1.177395	1.1864083	1.16995247	0.9117213	-0.1333352	0.8152
TMEM217	1.038506	1.0471195	1.03139245	0.6662304	-0.585907	0.81529
EFCAB12	1.047457	1.038731	1.05466308	1.4113516	0.49707742	0.81534
NPIPB5	1.147635	1.1386624	1.15504515	1.1181483	0.16111159	0.81537
CAMK2A	1.066481	1.057824	1.07363025	1.2733514	0.34863062	0.81542
CLCN7	1.244446	1.2350776	1.25218311	1.0727652	0.10133435	0.81546
RP1-193H18.2	1.127355	1.1363302	1.11994358	0.879802	-0.1847493	0.8155
GPR68	1.027369	1.0359091	1.02031625	0.5657688	-0.8217154	0.81551
TUBGCP6	1.10562	1.0966278	1.11304512	1.1699023	0.2263881	0.81565
ZDHHC11	1.061309	1.0526215	1.06848324	1.3014314	0.38009931	0.81568
THOC1	1.505612	1.5158638	1.49714569	0.963715	-0.0533215	0.81569
RP11-861E21.2	1.059101	1.0503334	1.06634081	1.3180266	0.3983795	0.8157
LMO3	1.059025	1.0503249	1.06620979	1.3156472	0.39577262	0.8157
EGFR	1.617815	1.6076988	1.62616826	1.0303924	0.04319389	0.81573
DACT1	1.053311	1.0619901	1.0461439	0.7443758	-0.4258969	0.81578
DNAJC30	1.34502	1.3544789	1.33720807	0.9512782	-0.0720608	0.81588
USP3-AS1	1.043887	1.0525983	1.03669378	0.6976226	-0.5194814	0.81599
UBE2G1	1.602254	1.5916268	1.61102926	1.0327951	0.04655413	0.81603
TBC1D10B	1.218237	1.2089169	1.22593296	1.0814487	0.11296529	0.8161
GATA2-AS1	1.030115	1.021499	1.03722947	1.7316828	0.7921747	0.81611
HOOK3	1.828614	1.8399343	1.81926562	0.9753925	-0.0359453	0.81613
AC010761.8	1.034382	1.0430123	1.02725444	0.6336423	-0.6582594	0.81619
RHOV	1.033016	1.0243885	1.04014069	1.6458882	0.71886637	0.81643
TRIM16	1.546805	1.5368639	1.55501364	1.033807	0.04796689	0.81649
LRRC10B	1.085942	1.077127	1.0932214	1.2086746	0.27342586	0.81655
GHR	1.041743	1.0331136	1.04887005	1.4758286	0.56152522	0.81657
SIRT6	1.297234	1.2876792	1.30512482	1.0606424	0.08493839	0.81657
TMEM61	1.025438	1.0340443	1.01833122	0.5384513	-0.8931123	0.8166

MB21D2	1.081761	1.0904455	1.07458853	0.8246791	-0.2780953	0.81661
VPS33A	1.184177	1.1931568	1.17676168	0.9151202	-0.1279668	0.81662
TMEM170B	1.162493	1.1534044	1.16999881	1.1081743	0.14818477	0.81663
PPFIA1	1.524214	1.5143498	1.53236014	1.0350158	0.04965285	0.81663
GPR176	1.09137	1.0825796	1.09863005	1.1943637	0.25624228	0.81664
ZC3H14	1.624461	1.6346828	1.61601892	0.9705934	-0.0430611	0.81667
AP000695.4	1.041615	1.0503746	1.03438136	0.682514	-0.5510694	0.81673
RP11-923I11.8	1.092116	1.100983	1.0847941	0.8396867	-0.252077	0.8168
RASIP1	1.034151	1.0426485	1.02713316	0.6362036	-0.6524396	0.81684
RP11-390F4.3	1.05137	1.0600203	1.04422563	0.7368442	-0.4405686	0.81688
NR3C2	1.032671	1.0412859	1.02555751	0.6190368	-0.691903	0.81688
FAM65B	1.036991	1.0283641	1.0441153	1.5553213	0.63721268	0.81689
SERTAD4	1.037033	1.0284373	1.04413215	1.5519112	0.63404605	0.81689
RP11-440L14.1	1.181359	1.1903858	1.17390461	0.9134327	-0.1306296	0.81696
NUDT13	1.062758	1.0713752	1.05564226	0.7795745	-0.3592411	0.81702
UEVLD	1.132708	1.1237219	1.14012835	1.1326077	0.17964823	0.81702
TADA2A	1.12325	1.1322249	1.11583791	0.8760671	-0.1908867	0.81704
GPR39	1.03177	1.0232192	1.0388316	1.6723905	0.74191173	0.81709
P3H2-AS1	1.075223	1.0665576	1.08237872	1.2377054	0.30766792	0.81719
ADAT1	1.159073	1.1499845	1.166578	1.1106347	0.15138431	0.8172
OSTN	1.024504	1.0159741	1.03154891	1.9750017	0.98185388	0.81726
LCP1	1.024484	1.0159741	1.03151199	1.9726903	0.98016451	0.81726
CERS4	1.582531	1.5928571	1.57400279	0.9681975	-0.0466268	0.8174
ZNF746	1.122436	1.1137053	1.12964529	1.1401871	0.18927064	0.81741
PTPRT	1.024492	1.0159362	1.03155823	1.9802797	0.98570421	0.81744
WDR66	1.115903	1.1248773	1.10849167	0.8687861	-0.202927	0.81752
TMTC2	1.249686	1.2588198	1.24214355	0.935568	-0.0960856	0.81753
LINC01456	1.088139	1.0968344	1.08095744	0.8360403	-0.2583556	0.81762
KCNN1	1.044737	1.0533273	1.03764368	0.7058989	-0.5024665	0.81763
RASSF7	1.513427	1.5038504	1.52133467	1.0347013	0.04921436	0.81783
PKN1	1.409457	1.4193177	1.40131395	0.9570642	-0.0633125	0.81786
AL049794.1	1.039641	1.0311016	1.04669263	1.5012911	0.58620369	0.81794
RP1-239B22.5	1.058619	1.0499943	1.06574153	1.3149818	0.39504283	0.81803
CAMKK1	1.051307	1.0427219	1.05839676	1.366906	0.45091403	0.81814
AF003626.1	1.025601	1.0171526	1.03257818	1.8993112	0.92547634	0.81816
TBC1D9B	1.527196	1.5174277	1.53526318	1.0344694	0.048891	0.81824
FKTN	1.192091	1.2014774	1.18434024	0.9149425	-0.1282471	0.81829
LAMA2	1.053429	1.044775	1.06057561	1.3528903	0.43604484	0.81832
AGBL5-AS1	1.031064	1.0395897	1.02402304	0.6067998	-0.7207075	0.81836
MAP3K4	1.317799	1.3083119	1.32563296	1.0561803	0.07885613	0.81838
ARL15	1.139861	1.1487127	1.13255062	0.89132	-0.1659846	0.8184
FAAP24	1.182336	1.191215	1.17500421	0.9152224	-0.1278057	0.81846
SLC35B4	1.363239	1.3728303	1.35531846	0.9530299	-0.0694066	0.81847
ZCCHC4	1.09373	1.1022792	1.08666942	0.8473806	-0.238918	0.81847
TRPV1	1.085722	1.0944817	1.07848805	0.8307218	-0.2675627	0.8185

COL2A1	2.715604	2.6979772	2.73016082	1.0189541	0.0270891	0.81862
SLC22A5	1.142186	1.1333039	1.14952081	1.1216534	0.16562689	0.81865
NFKB1	1.142468	1.1335885	1.14980131	1.1213639	0.16525452	0.81865
LRCH1	1.122238	1.1132944	1.12962315	1.1441268	0.19424695	0.81884
ZNF141	1.386051	1.3957114	1.37807303	0.9554262	-0.0657837	0.81884
HPS3	1.166522	1.1754797	1.15912523	0.9068014	-0.1411415	0.81893
RP11-424N24.2	1.038916	1.0303578	1.04598309	1.5147023	0.59903431	0.81894
B3GALT4	1.06649	1.0579189	1.07356818	1.2701931	0.34504785	0.81894
CASKIN2	1.078183	1.0694821	1.08536747	1.2286252	0.29704487	0.81897
MYO1B	1.51556	1.5260031	1.50693622	0.9637514	-0.0532671	0.81899
JADE1	1.373117	1.3826855	1.36521492	0.9543475	-0.0674134	0.81902
HRH2	1.034123	1.0255466	1.04120627	1.6129834	0.68973157	0.81903
GRAMD4	1.146428	1.1554113	1.1390093	0.8944609	-0.1609097	0.81907
PGBD4	1.056896	1.0654344	1.04984443	0.7617469	-0.3926163	0.81908
RP11-488L18.8	1.025259	1.0167613	1.03227584	1.9256112	0.94531646	0.81911
RP11-783K16.14	1.0395	1.0310448	1.04648309	1.4972916	0.58235523	0.81918
CMPK2	1.04743	1.0559646	1.04038156	0.721556	-0.4708167	0.81927
RP11-84G21.1	1.044609	1.0530961	1.03759952	0.7081404	-0.4978926	0.81928
VAMP4	1.158424	1.1495472	1.16575419	1.108374	0.14844478	0.81939
FARP2	1.140448	1.1318258	1.14756803	1.1194172	0.16274786	0.81939
RNASEH2B-AS1	1.035167	1.043684	1.02813292	0.6440101	-0.6348448	0.81947
RP11-465N4.4	1.033733	1.0252253	1.04075908	1.6157997	0.69224836	0.8195
RP11-1109F11.5	1.029228	1.0207783	1.03620578	1.7424835	0.80114495	0.81951
RP11-333E1.1	1.059912	1.0684987	1.05282181	0.771136	-0.3749427	0.81959
TRAPPC2B	1.267948	1.277125	1.26036931	0.9395376	-0.0899772	0.81973
ZBBX	1.046345	1.0378577	1.05335332	1.409313	0.49499205	0.81974
NR1H4	1.024035	1.0156039	1.03099699	1.9864896	0.99022122	0.81974
RP11-488C13.5	1.049116	1.0405761	1.05616786	1.3842598	0.46911475	0.81975
AGBL2	1.052143	1.043622	1.05918068	1.3566712	0.44007108	0.8199
STEAP1B	1.031315	1.0397511	1.02434867	0.6125274	-0.7071536	0.81992
PTCD1	1.118466	1.1273321	1.1111448	0.872873	-0.1961563	0.81993
UGDH-AS1	1.097613	1.1061672	1.09054931	0.8528933	-0.2295629	0.81998
EPHB3	1.149416	1.140433	1.15683505	1.1167965	0.15936632	0.82002
MYL3	1.022188	1.0137737	1.02913663	2.1153854	1.08092055	0.82004
MMGT1	1.169042	1.1778707	1.16175031	0.9093702	-0.1370603	0.82004
CTA-373H7.7	1.048065	1.03952	1.05512242	1.3947989	0.48005717	0.82005
LINC00893	1.110426	1.1017113	1.11762252	1.1564355	0.20968479	0.82006
MCRIP2	1.852645	1.8639177	1.84333627	0.9761766	-0.0347859	0.82008
SZT2	1.124107	1.1152838	1.13139245	1.13973	0.18869207	0.82018
ADORA2B	1.105306	1.0963786	1.11267916	1.1691309	0.22543644	0.8202
ILDR2	1.323825	1.3142078	1.33176624	1.0558816	0.07844811	0.82022
RBBP9	1.205924	1.2149031	1.19850857	0.9237118	-0.1144852	0.82022
AGBL3	1.104748	1.0960199	1.11195489	1.1659548	0.22151191	0.82024
CACNG8	1.15181	1.1427251	1.1593132	1.1162239	0.1586264	0.82026
PARG	1.293177	1.2838069	1.30091418	1.0602779	0.08444247	0.82047

RP11-38C17.1	1.03531	1.0437306	1.02835694	0.6484461	-0.6249414	0.82051
DMTN	1.058852	1.0674285	1.05176875	0.7677581	-0.3812763	0.82055
RPRD2	1.322677	1.3320882	1.31490469	0.9482562	-0.0766511	0.82055
DMBX1	1.031876	1.0234202	1.03885955	1.6592311	0.73051481	0.82056
MIB2	1.472472	1.4625952	1.48062857	1.0389831	0.05517214	0.82063
C17orf64	1.025847	1.034283	1.01888078	0.5507333	-0.8605744	0.82069
CTNND1	1.634606	1.6246541	1.6428253	1.02909	0.04136913	0.8207
PCNX4	1.799846	1.8107445	1.79084619	0.9754568	-0.0358502	0.82074
MAN2B1	1.06862	1.0771551	1.06157156	0.7980229	-0.3254979	0.82088
RP11-799D4.4	1.040334	1.0487613	1.0333739	0.6844341	-0.5470165	0.82088
MEIOC	1.045723	1.0372467	1.05272246	1.415493	0.50130459	0.82097
PRRT3-AS1	1.032519	1.0409515	1.0255547	0.6240243	-0.680326	0.82098
HCG14	1.032489	1.0408247	1.02560501	0.6271948	-0.6730146	0.82098
NEK5	1.136718	1.1280176	1.14390217	1.1240813	0.16874641	0.82101
RP11-380N8.7	1.061061	1.0695391	1.0540605	0.7774113	-0.3632501	0.8211
RAB9B	1.252074	1.2428233	1.25971408	1.0695599	0.0970173	0.82117
LEMD1-AS1	1.023115	1.0315902	1.01611633	0.5101685	-0.9709543	0.8212
AMPD3	1.102499	1.1111455	1.09535922	0.8579675	-0.2210051	0.82138
PPP3R1	1.160945	1.1519454	1.16837709	1.1081418	0.14814248	0.82138
CD2AP	1.516545	1.5066307	1.52473312	1.035731	0.05064931	0.82144
SP2	1.13136	1.1400366	1.12419456	0.886872	-0.1732021	0.82146
MYCBPAP	1.051879	1.0434208	1.05886366	1.355655	0.43899005	0.82147
RP11-1246C19.1	1.04641	1.0380601	1.05330592	1.4005706	0.48601475	0.82169
RP11-797H7.5	1.032825	1.0244351	1.03975366	1.6269102	0.7021346	0.82171
SEPT5	1.032817	1.0244124	1.0397569	1.628554	0.70359152	0.82171
NBPF26	1.075218	1.0666417	1.08230058	1.2349717	0.30447793	0.82173
LINC00664	1.032682	1.0410731	1.02575291	0.6270021	-0.6734578	0.82175
FLJ27354	1.031686	1.0233168	1.03859797	1.6553709	0.72715452	0.82183
NACC1	1.282333	1.2732834	1.28980632	1.0604606	0.08469101	0.82184
RP11-11M20.4	1.028326	1.0199251	1.03526361	1.7698077	0.82359263	0.82188
PCDHA7	1.026235	1.0178455	1.03316281	1.8583253	0.89400309	0.8219
RASA2	1.237712	1.2285981	1.24523761	1.0727896	0.10136715	0.82192
FCHSD2	1.232733	1.2239436	1.23999064	1.0716566	0.09984262	0.82194
DSCAM	1.023134	1.0314134	1.01629649	0.5187752	-0.9468185	0.82196
PPM1F	1.187992	1.1792935	1.19517459	1.088576	0.12244209	0.82204
TRAPPC12	1.264185	1.2551074	1.27168138	1.0649685	0.09081072	0.82206
LINC01252	1.099799	1.1082655	1.09280628	0.8572098	-0.2222798	0.82213
RP11-639B1.1	1.026555	1.0181595	1.03348816	1.8441133	0.88292726	0.82214
OPRL1	1.054487	1.0628924	1.04754654	0.7559988	-0.4035441	0.82217
DOK4	1.282544	1.2738639	1.28971222	1.0578693	0.08116144	0.82227
TRIM17	1.060329	1.0518239	1.06735331	1.2996573	0.37813129	0.82241
SPATA4	1.03452	1.0261276	1.04145062	1.5864701	0.66582036	0.82241
TCAF1	2.518499	2.5317968	2.50751776	0.9841499	-0.02305	0.82242
GOLGA8R	1.045604	1.0540175	1.03865558	0.7156118	-0.482751	0.82246
IPCEF1	1.029357	1.0377213	1.02245049	0.5951677	-0.7486318	0.8225

ZNF557	1.146725	1.1380168	1.15391581	1.1151958	0.1572971	0.82251
UBXN10-AS1	1.056853	1.0652268	1.04993803	0.7656061	-0.3853258	0.82259
AGMAT	1.035989	1.0443172	1.02911221	0.6569058	-0.6062416	0.82264
NATD1	1.102029	1.0934405	1.10912226	1.1678263	0.22382566	0.82266
FREM2	1.040298	1.0319485	1.04719353	1.4771768	0.56284255	0.8227
RP11-338K17.8	1.026022	1.0343973	1.01910571	0.5554417	-0.8482925	0.82281
RP11-996F15.2	1.025962	1.0342706	1.01910046	0.5573427	-0.8433635	0.82281
GALNT9	1.026543	1.0348693	1.01966706	0.564022	-0.8261766	0.82281
LOH12CR2	1.096975	1.0882547	1.10417559	1.1803971	0.23927223	0.82285
LZTR1	1.152327	1.1609421	1.14521227	0.902264	-0.1483785	0.82289
RP11-347C12.10	1.064697	1.0731171	1.05774366	0.7897426	-0.3405455	0.82297
ZNF852	1.037111	1.0454715	1.03020585	0.6642806	-0.5901354	0.82307
LINC01534	1.048221	1.0398166	1.05516113	1.385381	0.47028276	0.8231
FAM8A1	1.27203	1.2627983	1.279654	1.0641392	0.08968685	0.82318
PTGS2	1.021284	1.013034	1.02809742	2.1557063	1.10816067	0.82318
AANAT	1.052442	1.0609263	1.04543492	0.7457354	-0.4232642	0.82319
GABRA3	1.04286	1.0511381	1.03602408	0.7044473	-0.5054363	0.8234
PINK1-AS	1.042907	1.0511195	1.03612458	0.70667	-0.5008915	0.8234
RP11-563K23.1	1.090462	1.0989685	1.08343631	0.8430589	-0.2462946	0.82341
CTD-2623N2.11	1.035874	1.0274483	1.04283244	1.5604782	0.64198817	0.82346
ZNF571	1.099288	1.0909284	1.10619184	1.1678625	0.22387044	0.82354
PRRG4	1.026992	1.0186556	1.03387595	1.8158642	0.86065633	0.82355
DNAJC27	1.143175	1.1518655	1.13599872	0.8955208	-0.1592011	0.82357
RP4-671O14.7	1.023028	1.0147451	1.02986834	2.025646	1.01838207	0.8236
C10orf126	1.02304	1.014755	1.02988195	2.0252037	1.01806699	0.8236
AC002454.1	1.022975	1.0147414	1.02977404	2.0197512	1.01417756	0.8236
RP11-521C20.5	1.036242	1.0445495	1.02938081	0.6595099	-0.6005339	0.8236
UCKL1-AS1	1.035807	1.0273802	1.04276656	1.5619533	0.64335128	0.82365
SLCO6A1	1.035758	1.027379	1.04267733	1.5587613	0.64040002	0.82365
ARFGEF2	1.157346	1.1663494	1.14991143	0.9011842	-0.1501061	0.82366
RP11-494H4.3	1.02566	1.0339742	1.01879483	0.5532094	-0.8541024	0.82371
DMXL2	1.507908	1.4981136	1.51599685	1.0359019	0.05088736	0.82374
RP11-122M14.1	1.02965	1.0379933	1.02276044	0.5990642	-0.7392174	0.82406
CTD-2231E14.8	1.025783	1.0175054	1.03261837	1.8633324	0.89788504	0.8242
PODNL1	1.10902	1.1004806	1.11607168	1.1551654	0.20809942	0.82422
ABCC6	1.097519	1.1062497	1.09030822	0.8499624	-0.234529	0.82423
IGSF1	1.174246	1.1829783	1.16703435	0.912864	-0.1315281	0.82424
MPV17L	1.053911	1.0455353	1.06082705	1.3358227	0.41772851	0.82428
ZNF587	1.292855	1.2835273	1.3005582	1.0600679	0.08415666	0.82435
SOWAHC	1.125652	1.1171219	1.13269651	1.1329779	0.18011974	0.82443
RAP1GAP2	1.062471	1.0540869	1.06939491	1.2830273	0.35955183	0.82444
TMEM255B	1.067815	1.0594516	1.07472195	1.2568541	0.32981717	0.82445
TMEM199	1.277107	1.2858739	1.26986739	0.9440084	-0.0831284	0.82446
RP11-563J2.2	1.040089	1.0483357	1.03327953	0.6885081	-0.5384544	0.82451
AC007405.6	1.18734	1.1961432	1.18007045	0.9180561	-0.1233458	0.82453

RP11-342M1.3	1.033694	1.0421251	1.02673126	0.634568	-0.6561534	0.82465
ZNF320	1.171428	1.1802046	1.16417993	0.9110751	-0.1343582	0.82465
HCST	1.062302	1.0539106	1.06923198	1.2841991	0.36086886	0.82473
NRK	1.062256	1.053962	1.0691059	1.2806407	0.35686579	0.82473
INO80B	1.109373	1.1010047	1.11628358	1.1512687	0.20322462	0.82487
ADAP1	1.030791	1.0225369	1.03760781	1.668719	0.73874105	0.82499
AARD	1.033621	1.0253539	1.04044747	1.5953155	0.67384178	0.825
AK5	1.020609	1.0123699	1.02741333	2.2161357	1.14804622	0.82503
EME1	1.074682	1.0662073	1.08167986	1.2336988	0.30299025	0.82506
EP400NL	1.138825	1.1301154	1.14601836	1.1222218	0.16635781	0.82508
KCNAB3	1.131472	1.139927	1.12448895	0.8896708	-0.1686565	0.82509
SAP30	1.208524	1.2172375	1.20132748	0.926762	-0.1097292	0.8251
FLT3LG	1.049001	1.057384	1.04207793	0.7332697	-0.4475841	0.82521
GPR135	1.076538	1.0681057	1.08350141	1.2260562	0.29402505	0.82525
MESTIT1	1.03652	1.0282388	1.04335905	1.5354414	0.61865343	0.82527
PRR15	1.032494	1.0242885	1.03926971	1.6168009	0.69314199	0.82536
AL592528.1	1.04522	1.0369024	1.05208862	1.4115232	0.49725285	0.82539
ST8SIA5	1.028902	1.0371885	1.02205876	0.5931614	-0.7535033	0.82541
DNAH10	1.028764	1.0369214	1.0220283	0.5966265	-0.7451	0.82541
ZEB2-AS1	1.030894	1.039097	1.02412038	0.616937	-0.6968049	0.82542
FAM110D	1.049857	1.0580106	1.0431231	0.7433665	-0.4278544	0.82543
KBTBD11	1.137671	1.1461434	1.13067364	0.8941469	-0.1614162	0.82552
RP4-742C19.13	1.045008	1.0367719	1.05180883	1.4089257	0.4945955	0.82567
GBP2	1.117126	1.1255023	1.11020906	0.8781438	-0.1874708	0.82568
RP11-573D15.2	1.0299	1.0380943	1.02313382	0.6072777	-0.7195717	0.82568
ATL2	1.220247	1.2288685	1.21312792	0.9312242	-0.1027995	0.82572
ROPN1L	1.079468	1.0711858	1.08630701	1.2124182	0.27788744	0.82586
TMCC2	1.063234	1.0549071	1.07011125	1.2769074	0.35265389	0.82597
KDM4D	1.072286	1.0805681	1.06544578	0.8123042	-0.2999079	0.826
DOCK1	1.263713	1.2728204	1.25619268	0.9390526	-0.0907222	0.82603
CNTN4	1.208287	1.2168662	1.20120248	0.9277723	-0.1081573	0.82611
PAQR9	1.027987	1.019807	1.03474271	1.7540652	0.81070236	0.82616
PRSS16	1.077514	1.0691658	1.08440813	1.2203743	0.2873237	0.82617
FMOD	1.020138	1.0120052	1.02685355	2.2368351	1.16145893	0.82617
CDH11	1.587815	1.5779208	1.59598573	1.0312585	0.04440604	0.82631
PALM2	1.027949	1.0197524	1.03471764	1.7576381	0.81363801	0.82634
QSOX2	1.143166	1.1349055	1.14998757	1.1117977	0.15289434	0.82635
SLC25A45	1.064697	1.0729457	1.05788546	0.7935418	-0.3336219	0.8265
ZNF7	1.329808	1.3391067	1.32212924	0.9499349	-0.0740995	0.82652
LINC00668	1.028301	1.0200776	1.03509136	1.7477856	0.80552823	0.82659
PTGER3	1.02826	1.0201224	1.034981	1.7384144	0.79777204	0.82659
FLYWCH1	1.235145	1.2441872	1.22767845	0.932393	-0.1009899	0.82664
COLEC11	1.033795	1.0420372	1.02698796	0.6420027	-0.6393488	0.82675
ANKRD33	1.036274	1.0280804	1.04303996	1.5327428	0.6161156	0.82684
CCDC183	1.059741	1.0513472	1.06667195	1.2984529	0.37679363	0.82694

TMEM17	1.117987	1.109383	1.12509149	1.1436098	0.19359486	0.82698
CH17-360D5.2	1.028991	1.0208763	1.03569256	1.7097149	0.77375579	0.82706
VSTM4	1.028963	1.020871	1.03564621	1.7079264	0.77224579	0.82706
DAGLA	1.060218	1.0520305	1.06697969	1.2873163	0.36436656	0.82709
UNC13B	1.172242	1.1634891	1.17947006	1.0977493	0.13454865	0.82715
SP8	1.072923	1.0646137	1.07978509	1.2348013	0.30427889	0.82717
DHX15	1.761018	1.7712237	1.75258924	0.9758378	-0.0352867	0.82718
CCND2	2.607634	2.5944598	2.61851286	1.0150854	0.02160115	0.82719
HCFC1	1.569956	1.5602297	1.57798772	1.0316977	0.04502024	0.82723
RNASEH1-AS1	1.457776	1.4675377	1.44971489	0.9618794	-0.056072	0.82724
USP22	2.528995	2.5148881	2.54064527	1.0170027	0.02432347	0.82733
RP11-1398P2.1	1.029859	1.0217859	1.03652675	1.6766219	0.7455574	0.82761
ZNF714	1.674157	1.6639168	1.68261386	1.0281617	0.04006719	0.82762
MESP2	1.025789	1.017671	1.03249223	1.8387346	0.87871322	0.82766
HTR2A	1.025819	1.0176749	1.03254504	1.8413135	0.88073524	0.82766
CARD6	1.025805	1.0176744	1.03251969	1.839933	0.87965324	0.82766
IREB2	1.880132	1.8910327	1.87112971	0.977663	-0.0325909	0.82784
WNT10B	1.037554	1.0292764	1.04438937	1.516218	0.60047722	0.82789
RP11-359I18.5	1.046875	1.0550024	1.04016271	0.7301997	-0.453637	0.82791
AP000251.3	1.086887	1.0950655	1.08013387	0.8429333	-0.2465096	0.82803
AC009403.2	1.294937	1.286049	1.30227709	1.0567318	0.07960931	0.82809
RAD51B	1.16149	1.1529331	1.16855708	1.1021619	0.14033622	0.82816
RP11-501C14.5	1.077278	1.0854333	1.070544	0.8257199	-0.2762755	0.82818
EXOC6	1.245389	1.236282	1.25291	1.0703734	0.09811422	0.82822
ATG101	1.408317	1.3990839	1.415942	1.0422421	0.05969038	0.82823
FBXL5	1.536436	1.5460045	1.52853343	0.968002	-0.046918	0.82824
PEX11B	1.302156	1.2931754	1.30957219	1.0559283	0.07851192	0.82828
MCHR1	1.146422	1.1547411	1.13955185	0.9018408	-0.1490554	0.82831
COLQ	1.037153	1.0290256	1.04386448	1.5112342	0.59572726	0.82836
RP11-285J16.1	1.032757	1.0245863	1.03950504	1.6067891	0.68418059	0.82839
TSNAXIP1	1.074055	1.0657692	1.0808971	1.2300141	0.29867491	0.8284
TAB3	1.128329	1.1199121	1.1352802	1.1281612	0.17397319	0.82841
PPIP5K1	1.057442	1.0655819	1.05071958	0.7733779	-0.3707545	0.82852
GS1-124K5.3	1.05753	1.0656475	1.05082651	0.7742334	-0.3691596	0.82852
EFCAB3	1.039525	1.0313769	1.04625412	1.4741454	0.55987881	0.82865
C19orf84	1.027512	1.0194114	1.03420232	1.761972	0.81719099	0.82865
STXBP5-AS1	1.055684	1.047501	1.06244237	1.3145474	0.3945662	0.82866
LURAP1	1.076979	1.0853577	1.07006048	0.8207873	-0.2849197	0.82867
CMYA5	1.076981	1.0851304	1.07025194	0.8252275	-0.2771362	0.82867
TMEM94	1.191478	1.1829119	1.1985519	1.0855054	0.11836686	0.82873
LIPE	1.191515	1.1829303	1.19860356	1.0856791	0.11859775	0.82873
KIAA0753	1.181918	1.173398	1.18895465	1.0897167	0.12395309	0.82874
FAM131A	1.284465	1.2935224	1.27698575	0.9436613	-0.083659	0.82875
SCARF1	1.031832	1.0399377	1.02513772	0.6294229	-0.6678985	0.8288
SLC46A3	1.06963	1.0614203	1.07641024	1.2440559	0.31505131	0.82881

GPN2	1.235038	1.2435918	1.22797462	0.9358879	-0.0955924	0.82883
PNPO	1.067282	1.0753466	1.06062161	0.8045696	-0.3137109	0.82886
C5AR1	1.025639	1.017591	1.03228492	1.8353093	0.87602323	0.82895
FAM213B	1.419212	1.4099918	1.42682584	1.0410595	0.05805256	0.82896
TAF13	1.118101	1.1264828	1.11117833	0.8789995	-0.1860657	0.82898
TMEM127	1.184682	1.1763145	1.19159154	1.0866462	0.11988235	0.82898
ZNF239	1.064666	1.0564298	1.0714679	1.2664932	0.34083929	0.82906
FAM135A	1.321278	1.3123378	1.3286606	1.0522601	0.07349131	0.82908
GHRH	1.036388	1.044394	1.02977644	0.6707307	-0.5761944	0.82911
NPL	1.046032	1.0540435	1.03941598	0.7293378	-0.4553409	0.82921
RP1-202O8.3	1.055763	1.0638977	1.04904433	0.7675442	-0.3816783	0.82922
RNF144A	1.213284	1.2048021	1.22028827	1.0756152	0.1051621	0.82927
RP11-347P5.1	1.029028	1.0369731	1.02246761	0.6076748	-0.7186286	0.82929
PPP1R12B	1.18422	1.1757714	1.1911962	1.0877546	0.12135313	0.82932
AVEN	1.138044	1.1463393	1.13119429	0.8965076	-0.1576122	0.82936
BPTF	2.649745	2.635332	2.66164806	1.0160922	0.0230313	0.82943
CTB-32O4.2	1.032534	1.024464	1.03919771	1.6022598	0.68010813	0.82943
AC046143.3	1.054381	1.0463241	1.06103383	1.3175381	0.39784466	0.82945
PSD	1.140126	1.1318381	1.14697071	1.1147816	0.15676105	0.82946
ZNF836	1.218667	1.2273405	1.2115039	0.9303395	-0.1041708	0.82955
RP11-35G9.5	1.028477	1.020383	1.0351614	1.7250343	0.78662506	0.82955
NUP107	1.514136	1.5235359	1.50637418	0.9672197	-0.0480845	0.82959
TRIM41	1.174234	1.1656151	1.18135251	1.0950239	0.1309624	0.82966
RP11-533E19.5	1.023125	1.0151141	1.02974056	1.9677311	0.97653307	0.82967
STIL	1.101064	1.1093586	1.09421466	0.8615201	-0.2150436	0.82973
FAM19A2	1.082983	1.0911402	1.07624644	0.8365841	-0.2574174	0.82974
UNKL	1.208424	1.2170417	1.20130681	0.9275027	-0.1085766	0.82976
ME1	1.073469	1.081497	1.06684	0.8201526	-0.2860357	0.82992
E2F1	1.117971	1.1097516	1.12475862	1.1367362	0.18489753	0.82995
JMJD1C-AS1	1.039491	1.0475346	1.03284874	0.6910492	-0.5331397	0.82999
CUEDC1	1.267721	1.2588424	1.27505311	1.0626278	0.08763633	0.83006
CCDC146	1.081225	1.0730361	1.08798779	1.2047158	0.26869283	0.8302
PIANP	1.290694	1.2817576	1.29807423	1.0579101	0.08121708	0.83022
GPT2	1.347432	1.3563056	1.34010353	0.9545277	-0.0671411	0.83024
SLC35F6	1.309071	1.3001407	1.31644632	1.0543266	0.07632188	0.83025
ANKRD34A	1.061012	1.0690736	1.05435471	0.7869106	-0.3457284	0.83029
LBX2	1.098064	1.106314	1.09125054	0.8583113	-0.2204271	0.83035
CTD-3138B18.5	1.206809	1.1981446	1.21396435	1.0798397	0.11081717	0.83037
LINC01248	1.02057	1.0285204	1.01400499	0.4910523	-1.0260513	0.83041
ZNF286B	1.130752	1.1391733	1.12379833	0.8895266	-0.1688904	0.83045
CTB-58E17.1	1.091856	1.0836721	1.09861369	1.1785734	0.23704164	0.83064
IGDCC4	1.287065	1.2783501	1.29426113	1.057162	0.08019643	0.83079
KPNA1	1.400552	1.4099761	1.39276882	0.9580286	-0.0618593	0.83082
AC159540.1	1.198329	1.1898083	1.20536602	1.0819656	0.11365467	0.83087
GHRLOS	1.030006	1.0219609	1.03664907	1.6688307	0.73883764	0.83104

SLC8A3	1.029971	1.0219497	1.03659508	1.6672255	0.73744924	0.83104
RSPO2	1.030061	1.0220401	1.03668526	1.6644805	0.73507199	0.83104
CRYGN	1.024897	1.0169687	1.03144445	1.853088	0.88993143	0.83107
PCDHAC1	1.024881	1.0169536	1.0314283	1.8537791	0.89046934	0.83107
CIART	1.204125	1.2124962	1.197212	0.9280732	-0.1076895	0.83112
RP5-864K19.4	1.048842	1.0408532	1.05543929	1.3570369	0.4404599	0.8312
RP11-1007O24.2	1.048918	1.0408024	1.05562079	1.3631758	0.44697161	0.8312
PCDH9-AS1	1.063139	1.0550775	1.06979624	1.2672374	0.34168678	0.83121
PCDHGB6	1.183734	1.1751957	1.19078463	1.0889798	0.12297719	0.83126
FMO1	1.031817	1.0238384	1.03840589	1.6110914	0.68803837	0.83129
RP11-138A9.2	1.033728	1.0417132	1.02713294	0.6504643	-0.6204582	0.83134
FRS3	1.083402	1.0916368	1.07660223	0.8359327	-0.2585413	0.83135
OTULIN	1.28517	1.2939459	1.27792306	0.9454905	-0.0808651	0.83137
PTCH1	1.13262	1.1411236	1.12559699	0.8899785	-0.1681576	0.8315
RRAS2	1.500299	1.5100157	1.4922744	0.9652143	-0.0510789	0.83152
TNS2	1.048562	1.0405832	1.05515055	1.3589516	0.44249408	0.83159
HSD17B6	1.039413	1.047322	1.03288176	0.6948521	-0.5252222	0.83167
ZNF19	1.147272	1.1389365	1.15415586	1.1095419	0.14996409	0.83168
RP11-441O15.3	1.065497	1.0735001	1.0588888	0.8012073	-0.3197525	0.83169
CLDN12	1.305286	1.3138125	1.29824501	0.9503924	-0.0734047	0.8319
RP11-1143G9.4	1.029949	1.0378984	1.02338395	0.617017	-0.6966178	0.83192
LINC00449	1.045143	1.0531839	1.03850294	0.7239587	-0.4660208	0.83204
RP11-315A16.1	1.071799	1.0636109	1.07856015	1.235011	0.30452385	0.83204
ZNF790	1.270007	1.2610055	1.27744136	1.0629714	0.08810273	0.83208
NPPA	1.023232	1.0311423	1.0166999	0.5362452	-0.8990353	0.83215
STRN	1.154153	1.1457608	1.16108291	1.1051181	0.14420053	0.83215
TRAF4	1.821516	1.8123244	1.82910581	1.0206586	0.02950032	0.83226
CNEP1R1	1.183952	1.1924767	1.17691296	0.9191396	-0.1216441	0.83228
KLHL7-AS1	1.082903	1.0910263	1.07619463	0.837062	-0.2565936	0.83228
NPHP4	1.093833	1.1018809	1.08718694	0.855773	-0.2246999	0.83231
LRP4	1.122577	1.114318	1.12939807	1.1319133	0.17876345	0.83247
ZNF684	1.073136	1.0810751	1.0665805	0.8212205	-0.2841585	0.83249
RFPL1S	1.036977	1.0448695	1.03045835	0.6788204	-0.5588982	0.83253
FZD8	1.101408	1.093196	1.10818933	1.1608797	0.21521852	0.83255
ACAD11	1.063751	1.0718392	1.05707224	0.7944447	-0.3319813	0.83269
AGT	1.039539	1.0315479	1.04613811	1.462476	0.54841293	0.83275
ZNF763	1.03955	1.031605	1.04611182	1.4590036	0.54498345	0.83275
ZSCAN31	1.04633	1.0542662	1.03977684	0.7329944	-0.4481259	0.83291
BISPR	1.068318	1.0601759	1.07504117	1.2470301	0.31849624	0.83317
RP11-147L13.12	1.075859	1.0840488	1.06909565	0.82209	-0.2826317	0.83319
AC093609.1	1.028535	1.0206316	1.03506103	1.6993882	0.76501548	0.8333
GNMT	1.027389	1.0194628	1.0339344	1.7435545	0.80203148	0.8334
ZMIZ1-AS1	1.027281	1.0350834	1.02083836	0.5939671	-0.751545	0.83346
RP11-165J3.6	1.025663	1.0335296	1.0191667	0.5716349	-0.806834	0.8335
MAK	1.044461	1.0524828	1.03783711	0.7209434	-0.472042	0.83353

HMSD	1.034831	1.0426672	1.02835961	0.6646697	-0.5892906	0.83354
ZNF205	1.244709	1.2530716	1.23780278	0.9396661	-0.0897799	0.83366
INO80E	1.49372	1.5027626	1.48625319	0.9671626	-0.0481696	0.83373
RIPPLY1	1.02749	1.0353823	1.02097258	0.5927428	-0.7545219	0.83375
RPTOR	1.111995	1.1037021	1.11884281	1.1460021	0.19660971	0.83383
FOLR1	1.034231	1.026419	1.04068196	1.5398752	0.62281342	0.83388
RCN3	1.115254	1.1232543	1.10864804	0.8814946	-0.1819764	0.83392
STOX1	1.107096	1.115082	1.10050046	0.8732941	-0.1954605	0.83395
VCAM1	1.030183	1.0222929	1.03669825	1.6461844	0.71912598	0.834
RP1-111C20.4	1.030163	1.0222886	1.03666628	1.645068	0.71814724	0.834
ECD	1.187805	1.1962088	1.18086551	0.9218014	-0.1174721	0.8341
TNFRSF25	1.092631	1.0845815	1.09927841	1.1737606	0.23113813	0.8341
CCER2	1.024905	1.0170425	1.0313975	1.8423059	0.88151262	0.83412
FAM110C	1.054209	1.0462896	1.06074856	1.3123583	0.39216163	0.83414
RP11-770J1.3	1.068913	1.0768448	1.06236322	0.8115475	-0.3012525	0.83416
KCNE4	1.050862	1.0428496	1.05747962	1.3414283	0.42376993	0.83419
BBS1	1.059323	1.0672105	1.05280999	0.7857407	-0.3478748	0.8344
C1orf229	1.052767	1.0606863	1.04622628	0.7617249	-0.392658	0.8344
FMN2	1.129318	1.1212109	1.13601265	1.1221157	0.16622141	0.83441
RP11-345J4.3	1.024572	1.0166975	1.03107557	1.8610862	0.89614484	0.83447
PLA2G4A	1.04126	1.03334	1.04780059	1.4337291	0.51977248	0.8345
ITPRIPL1	1.086802	1.094686	1.08029079	0.8479688	-0.2379169	0.83462
HECTD3	1.119361	1.1275497	1.11259852	0.8827816	-0.1798715	0.83462
RASGRF2	1.113853	1.1221131	1.10703079	0.8764891	-0.190192	0.83478
NRIP1	2.320308	2.3323272	2.3103817	0.9835284	-0.0239613	0.83484
MIS18A-AS1	1.040375	1.0481759	1.03393364	0.7043698	-0.505595	0.83486
ARHGAP25	1.051791	1.0597525	1.04521714	0.7567406	-0.4021293	0.83493
TMC7	1.05166	1.0595404	1.04515138	0.7583315	-0.3990995	0.83493
RAB40C	1.203219	1.2117128	1.19620475	0.9267495	-0.1097486	0.83496
ADIRF	1.030296	1.038129	1.02382745	0.6249163	-0.6782652	0.83508
WDR11-AS1	1.04226	1.0500928	1.03579122	0.714498	-0.4849981	0.83516
RP3-512B11.3	1.155762	1.147519	1.1625686	1.1020183	0.14014824	0.83517
RP11-464F9.22	1.022668	1.0148661	1.02911041	1.9581722	0.96950761	0.83519
IKBKE	1.093369	1.0855157	1.09985416	1.1676705	0.2236332	0.83524
NR4A3	1.031364	1.0235675	1.0378022	1.6039977	0.68167206	0.83524
KCTD16	1.31752	1.3091367	1.32444269	1.0495122	0.06971897	0.8353
ANKRD53	1.058318	1.0662001	1.05180874	0.782608	-0.3536382	0.83531
RP11-386G11.8	1.021381	1.02918	1.01494071	0.5120196	-0.9657292	0.83532
CLSPN	1.246547	1.2376548	1.25389048	1.0683162	0.09533871	0.83537
NTNG2	1.08165	1.0895799	1.07510068	0.8383651	-0.2543495	0.83541
ZFYVE9	1.269987	1.2783132	1.26311167	0.9453799	-0.0810339	0.83545
ASB6	1.128384	1.1365354	1.1216518	0.8909909	-0.1665174	0.83547
AOC2	1.031742	1.0239085	1.03821158	1.5982436	0.67648734	0.83548
FAM83B	1.031115	1.0233464	1.03753089	1.6075694	0.68488102	0.83551
CTC-505O3.3	1.031201	1.0233784	1.03766115	1.610937	0.68790008	0.83551

FADS2	1.690561	1.6812013	1.69828965	1.0250856	0.03574444	0.83554
DPP9	1.135404	1.12733	1.14207254	1.1157822	0.15805546	0.83556
DNM2	1.303398	1.3119243	1.29635602	0.9500896	-0.0738644	0.83563
DNAAF1	1.04658	1.0387087	1.0530801	1.3712701	0.45551276	0.83563
WARS2	1.199068	1.2072525	1.19230874	0.9278957	-0.1079654	0.83564
SIMC1	1.415418	1.4244768	1.40793803	0.9610374	-0.0573356	0.8357
OCIAD2	5.094956	5.0711877	5.11458493	1.0106596	0.01529718	0.83575
SNX16	1.172933	1.1644781	1.1799159	1.0938592	0.12942708	0.83579
RP11-278C7.4	1.037709	1.0454671	1.03130192	0.6884528	-0.5385704	0.83583
ITGB4	1.058324	1.0506857	1.06463222	1.2751572	0.3506751	0.83589
PLBD1	1.189444	1.197542	1.18275628	0.9251515	-0.1122384	0.83589
XXbac-B135H6.15	1.045503	1.0376743	1.05196733	1.3793825	0.4640226	0.83593
CEP170	1.840931	1.8313263	1.84886266	1.0210944	0.03011628	0.83595
RP11-293A21.1	1.052975	1.0607481	1.04655666	0.7663886	-0.3838519	0.83598
C4orf26	1.020737	1.0129919	1.02713259	2.0884205	1.06241225	0.83605
WDR73	1.692266	1.7020236	1.68420746	0.9746217	-0.0370858	0.83606
FAM120A	1.492407	1.5016772	1.48475151	0.9662617	-0.0495141	0.83607
UBOX5	1.063223	1.0710585	1.0567522	0.7986681	-0.324332	0.83608
MYH11	1.03102	1.0387753	1.02461553	0.6348245	-0.6555704	0.83608
CRTC3-AS1	1.028282	1.0360978	1.02182845	0.6047028	-0.7257019	0.83609
C10orf25	1.066394	1.0741734	1.05996931	0.808502	-0.3066768	0.8361
LRRTM4	1.117579	1.1094834	1.12426383	1.1350013	0.18269391	0.83618
ATXN7	1.284674	1.2935396	1.27735248	0.9448553	-0.0818347	0.83624
NUS1	1.428479	1.4374009	1.42111077	0.9627571	-0.0547563	0.8363
RP11-977G19.11	1.07379	1.08154	1.06738928	0.8264566	-0.2749891	0.83633
AC002467.7	1.092412	1.1003007	1.08589698	0.8563947	-0.2236523	0.83639
TMEM121	1.297618	1.2889825	1.30474991	1.054562	0.07664391	0.83652
RP5-1050D4.3	1.064394	1.0720786	1.05804847	0.8053501	-0.3123121	0.83652
USP28	1.490672	1.4995527	1.48333851	0.9675425	-0.047603	0.83653
APOM	1.070935	1.0786971	1.0645251	0.8199168	-0.2864506	0.83654
KIAA1841	1.355164	1.3639542	1.34790517	0.9559038	-0.0650627	0.83662
MSMP	1.047399	1.039594	1.05384413	1.3599057	0.44350664	0.83665
KDELR2	3.270999	3.254705	3.28445455	1.0131944	0.01891107	0.83665
ERN2	1.054893	1.0627085	1.04843814	0.7724334	-0.3725176	0.8367
RP11-1260E13.2	1.022387	1.0146773	1.02875331	1.95903	0.9701395	0.83675
HOXA4	1.022409	1.0146773	1.02879336	1.9617592	0.97214799	0.83675
PAXBP1	1.562922	1.5536163	1.570607	1.0306904	0.04361099	0.83678
ZIC4	1.135695	1.1438006	1.12900117	0.8970834	-0.156686	0.83679
TMEM198	1.155745	1.147565	1.16249983	1.1012083	0.13908734	0.83684
RP11-16N11.2	1.040909	1.0330963	1.04736157	1.4310217	0.51704557	0.83693
RP11-239L20.6	1.028201	1.0204767	1.03457913	1.6887088	0.75592057	0.83695
P2RY1	1.114681	1.1225917	1.10814799	0.8821805	-0.1808542	0.83699
RP4-550H1.7	1.02574	1.033391	1.01942133	0.5816343	-0.7818158	0.83699
ZNF589	1.126318	1.1343666	1.11967123	0.890632	-0.1670987	0.83705
NPC2	7.861868	7.8174589	7.89854201	1.0118935	0.0170574	0.83709

SPRY4-IT1	1.028337	1.0359548	1.0220462	0.6131649	-0.7056531	0.83713
TAF4B	1.063747	1.0715978	1.05726343	0.7997927	-0.3223021	0.83725
POLR3G	1.116699	1.1247308	1.11006575	0.8824266	-0.1804518	0.8373
ZNF513	1.12995	1.1219056	1.13659289	1.120481	0.16411823	0.83731
GLRB	1.146942	1.1549791	1.14030421	0.9053106	-0.1435152	0.83734
NR4A1	1.056056	1.0482613	1.06249219	1.2948728	0.37281033	0.83736
DYNLL2	1.324898	1.3334856	1.31780583	0.9529823	-0.0694787	0.83738
CBFA2T3	1.067759	1.0599467	1.07421027	1.2379383	0.30793944	0.83755
CTD-2024P10.2	1.028568	1.0208792	1.03491736	1.67235	0.74187679	0.83757
TIMM22	1.466426	1.4753774	1.45903304	0.9656182	-0.0504753	0.83759
HIST2H2AC	1.324959	1.3335827	1.31783794	0.9528011	-0.069753	0.83761
INMT	1.043758	1.0515566	1.03731749	0.7238166	-0.4663039	0.83762
HIST1H2BN	1.044752	1.052436	1.0384069	0.7324535	-0.4491909	0.83783
ROR1	1.177563	1.1693276	1.1843643	1.0888026	0.12274242	0.83797
RP11-396C23.4	1.025761	1.0180243	1.03214965	1.7836866	0.83486216	0.83797
SP140L	1.062902	1.0551803	1.06927863	1.2554959	0.32825729	0.838
NBR1	1.518813	1.5279202	1.51129263	0.9685036	-0.0461707	0.83804
PRKAR2A	1.803384	1.7935322	1.81151895	1.0226667	0.03233602	0.83815
ZDHHC5	1.174388	1.1664558	1.18093867	1.0870075	0.12036192	0.83815
SOCS4	1.341382	1.3326603	1.34858522	1.0478714	0.06746172	0.83816
CXorf56	1.096587	1.1044459	1.09009726	0.8626216	-0.2132002	0.83816
RP11-328K4.1	1.024718	1.0323542	1.01841134	0.5690555	-0.8133587	0.8382
GPD1L	1.057824	1.0499482	1.06432775	1.2878881	0.36500721	0.83823
RP13-463N16.6	1.023348	1.0157004	1.02966259	1.8892865	0.91784152	0.83826
BNC1	1.023377	1.0157004	1.02971688	1.892744	0.92047931	0.83826
FAM46B	1.035919	1.0282432	1.04225775	1.4962111	0.58131377	0.83831
DMC1	1.070372	1.062464	1.07690278	1.2311526	0.30000963	0.83832
NRP1	1.12872	1.1208821	1.13519185	1.1183777	0.16140755	0.83844
PTOV1-AS1	1.069179	1.0770073	1.06271486	0.8144013	-0.2961883	0.83857
TSPEAR-AS2	1.097223	1.089393	1.10369003	1.1599343	0.21404311	0.8388
RETSAT	1.109737	1.1018091	1.11628332	1.1421708	0.19177837	0.8388
PRKCG	1.068316	1.0760322	1.06194448	0.814714	-0.2956345	0.83881
RP11-35G9.3	1.076842	1.0846194	1.07041882	0.8321833	-0.2650268	0.83885
NPIPA2	1.031928	1.0395904	1.02560082	0.6466425	-0.6289597	0.83895
RP11-467P9.1	1.038033	1.0456319	1.03175789	0.6959582	-0.5229275	0.83898
AL365181.1	1.058799	1.0510128	1.06522846	1.2786679	0.35464163	0.83901
MPRIP-AS1	1.058877	1.0667424	1.05238166	0.7848329	-0.3495425	0.83902
KCTD21-AS1	1.067259	1.0749604	1.06089849	0.8124085	-0.2997228	0.83913
CTC-270D5.1	1.022492	1.0301854	1.01613888	0.5346576	-0.9033128	0.83916
CMTM7	1.49397	1.4850605	1.50132776	1.0335365	0.04758932	0.83918
TNRC18	1.197197	1.18907	1.2039088	1.0784833	0.10900388	0.83919
EPHA5-AS1	1.049255	1.0569376	1.04290976	0.7536285	-0.4080746	0.83923
LRRC6	1.089295	1.0970833	1.08286259	0.8535206	-0.2285021	0.83927
HACD4	1.037989	1.0303475	1.04429899	1.4597259	0.54569746	0.83933
ZBTB7A	1.157528	1.1495968	1.16407717	1.096796	0.13329523	0.83937

HMGN4	1.054884	1.0625233	1.04857517	0.7769131	-0.3641748	0.83943
RP11-533E19.7	1.079743	1.0875115	1.07332719	0.837915	-0.2551242	0.83948
TRMT61A	1.118748	1.1266135	1.11225286	0.886579	-0.1736789	0.8395
VPS13C	1.537827	1.5288019	1.54527984	1.0311608	0.04426934	0.83954
CD248	1.038559	1.0309082	1.04487671	1.4519336	0.53797549	0.83956
CECR2	1.270887	1.2796129	1.26368119	0.9430224	-0.0846361	0.83963
LINC01127	1.024354	1.0167647	1.03062054	1.8264882	0.86907245	0.83964
AKIRIN1	2.364523	2.377353	2.35392722	0.9829921	-0.0247482	0.83968
RP11-1069G10.2	1.020399	1.012846	1.02663617	2.0734933	1.05206335	0.83969
NRXN3	1.048269	1.0559942	1.04188998	0.7481123	-0.4186732	0.8397
FAAH2	1.048295	1.0559094	1.04200734	0.7513462	-0.4124504	0.8397
PAGR1	1.237703	1.2293847	1.24457235	1.0662104	0.09249217	0.83973
NLRC5	1.025794	1.0181781	1.03208305	1.7649299	0.8196109	0.83985
RYR3	1.02572	1.0181186	1.03199657	1.7659531	0.82044707	0.83985
CYP4V2	1.091246	1.0833574	1.09776055	1.1727873	0.22994144	0.83989
PTPRK	1.3557	1.3471404	1.36276891	1.0450208	0.06353161	0.83991
LRP6	1.447609	1.4391112	1.45462585	1.0353319	0.05009328	0.83991
RP11-76I14.1	1.038738	1.0462943	1.03249803	0.7019874	-0.5104829	0.83998
FER	1.37063	1.3618818	1.37785511	1.0441395	0.0623145	0.84003
AP001412.1	1.04839	1.0407072	1.05473508	1.344603	0.42718028	0.84005
KDELC2	1.272965	1.2644336	1.2800113	1.0589098	0.08257975	0.84007
FAM234B	1.194079	1.1859189	1.200818	1.0801379	0.11121547	0.84011
TOMM7	10.73549	10.695716	10.7683314	1.0074895	0.01076474	0.84017
CTC-503J8.4	1.035309	1.0275834	1.04168854	1.5113645	0.59585169	0.84018
ATP8A2	1.064528	1.0721996	1.05819275	0.8059983	-0.3111513	0.84021
TCP10L	1.06002	1.0523443	1.06635789	1.2677199	0.34223604	0.84027
MISP3	1.164553	1.1726782	1.15784312	0.9140885	-0.1295943	0.84036
SLC45A3	1.034175	1.0265635	1.04046096	1.5231796	0.60708611	0.84039
KRT15	1.022431	1.0148929	1.0286554	1.9241041	0.94418688	0.84051
KAZN	1.398039	1.3894071	1.40516776	1.0404735	0.05724021	0.84052
ZNF304	1.059652	1.0519421	1.06601912	1.2710145	0.34598047	0.84055
DPPA4	1.045722	1.0533848	1.03939433	0.7379319	-0.4384405	0.84076
LRRC9	1.069589	1.0773784	1.06315703	0.8162104	-0.292987	0.84093
SMG5	1.219441	1.2114053	1.22607654	1.0693986	0.09679964	0.84093
MAP3K8	1.115722	1.1235959	1.10921981	0.8836846	-0.1783965	0.84101
C10orf82	1.028195	1.0206592	1.03441858	1.6660156	0.7364019	0.84102
MATN3	1.030907	1.0233619	1.03713818	1.5896933	0.66874849	0.84103
LINC01550	1.031005	1.0234154	1.03727196	1.5917739	0.6706354	0.84103
ADCY4	1.030983	1.023427	1.0372231	1.5889005	0.66802878	0.84103
CATSPER2	1.271925	1.2803248	1.26498835	0.9452903	-0.0811707	0.84109
DFNB59	1.062235	1.0700585	1.0557745	0.7961136	-0.3289537	0.84118
RIMKLA	1.255242	1.2472457	1.26184607	1.0590523	0.08277378	0.84119
RNF44	1.505318	1.4963028	1.51276344	1.0331664	0.0470727	0.84122
RP11-31F15.2	1.042881	1.0503706	1.03669505	0.7285006	-0.456998	0.84122
HEXDC	1.241487	1.2495451	1.23483194	0.9410401	-0.0876719	0.84129

PCAT14	1.018867	1.0113227	1.02509762	2.216569	1.14832828	0.84131
CEP78	1.767301	1.776632	1.75959571	0.9780639	-0.0319994	0.84134
C1orf220	1.042646	1.0350563	1.04891298	1.3952711	0.48054544	0.84135
GPR156	1.023618	1.0311078	1.01743276	0.5603991	-0.8354734	0.84146
CCSER2	1.58972	1.5995196	1.58162755	0.970156	-0.0437114	0.84148
RP11-849H4.2	1.089444	1.0816095	1.09591461	1.1752869	0.23301303	0.84157
DGKG	1.050437	1.0579876	1.04420117	0.7622517	-0.3916607	0.8416
THSD7B	1.031162	1.0386747	1.02495703	0.6453071	-0.6319421	0.8416
NUDT16	1.379121	1.3877412	1.37200286	0.9594103	-0.0597802	0.84161
ZNF317	1.148644	1.1564102	1.14222994	0.9093395	-0.1371091	0.84163
RP3-508I15.21	1.041016	1.0486527	1.03470874	0.7133984	-0.4872201	0.84164
ZNF26	1.172875	1.1648126	1.17953392	1.0893214	0.12342966	0.84167
TOX2	1.12139	1.129125	1.11500239	0.8906283	-0.1671046	0.84168
SIPA1	1.291001	1.2827813	1.2977887	1.0530706	0.07460219	0.84169
CDC42BPG	1.041047	1.0487088	1.03472033	0.7128146	-0.4884013	0.84183
AP000230.1	1.04112	1.0488392	1.03474522	0.7114203	-0.4912259	0.84183
PPP3CC	1.248166	1.2566179	1.24118635	0.9398658	-0.0894733	0.84188
TLR4	1.032668	1.02515	1.03887703	1.5458075	0.62836071	0.842
TMEM81	1.030185	1.0227168	1.03635225	1.6002382	0.6782867	0.84203
PCDH11Y	1.030339	1.0227497	1.03660561	1.6090563	0.6862148	0.84203
SLC25A12	1.103677	1.1114702	1.09724141	0.8723536	-0.1970151	0.84204
RP11-218M22.1	1.06148	1.0539002	1.06773943	1.2567575	0.32970633	0.84213
FAM21A	1.163297	1.1716534	1.15639623	0.9111166	-0.1342924	0.84216
FTCD	1.043861	1.0514245	1.03761443	0.7314498	-0.4511693	0.84218
ERICH6-AS1	1.095028	1.1028394	1.08857634	0.8613072	-0.2154002	0.84221
AC012354.6	1.132487	1.1247107	1.13890857	1.1138463	0.15555014	0.84221
RUSC2	1.096353	1.0887362	1.10264368	1.1567281	0.21004974	0.84224
DGCR6	1.438684	1.4301757	1.44571005	1.0361117	0.05117959	0.84228
AC099850.1	1.030782	1.023281	1.03697675	1.5882772	0.66746275	0.8423
CD4	1.065424	1.073053	1.05912298	0.8093166	-0.3052239	0.84237
ZNF823	1.064636	1.056891	1.07103154	1.2485543	0.32025852	0.84238
MAP10	1.081704	1.0740423	1.088031	1.188928	0.2496614	0.84244
ZC3H11B	1.034344	1.0418061	1.02818085	0.6740842	-0.5689994	0.84245
KLHL8	1.297636	1.3058287	1.29086997	0.9510879	-0.0723494	0.84264
WNK4	1.025613	1.0181535	1.03177263	1.7502251	0.80754052	0.84269
CTB-47B11.3	1.025703	1.0181557	1.03193641	1.7590302	0.81478025	0.84269
RP11-432I5.2	1.020615	1.0131484	1.02678098	2.0368237	1.02632109	0.84271
AHRR	1.025103	1.0176056	1.03129505	1.7775616	0.82989952	0.84272
DRC7	1.025127	1.0176004	1.03134322	1.7808271	0.83254747	0.84272
SREBF2	1.512964	1.50444	1.5200025	1.030851	0.04383575	0.84281
RP5-933K21.3	1.027471	1.01999	1.03364879	1.6832769	0.7512725	0.84289
RP11-764E7.1	1.028297	1.0208294	1.03446425	1.654597	0.72647986	0.84302
AQP11	1.088623	1.0962911	1.08229146	0.8546112	-0.22666	0.84305
MIR34A	1.128029	1.12026	1.13444469	1.11795	0.16085564	0.84313
ZNF575	1.036922	1.0444571	1.03070023	0.6905583	-0.5341648	0.84318

CIPC	1.157299	1.1494017	1.16381983	1.0965059	0.13291359	0.84319
PDE7A	1.458412	1.4497778	1.46554301	1.0350512	0.04970215	0.84323
GAB2	1.062688	1.070288	1.05641146	0.8025759	-0.3172902	0.84326
SEC24C	1.256967	1.2651273	1.25022799	0.9438031	-0.0834422	0.84329
SMPDL3B	1.044684	1.0371888	1.05087397	1.3679903	0.45205795	0.84335
TECPR1	1.152364	1.1445963	1.15877896	1.0980845	0.13498912	0.84336
RP11-290L1.2	1.047516	1.0550439	1.04129863	0.7502848	-0.4144898	0.84337
CTC-321K16.1	1.027986	1.0205005	1.03416839	1.6667138	0.73700641	0.84338
CTC-492K19.7	1.028025	1.0205002	1.03423849	1.6701577	0.73998436	0.84338
STOML3	1.027963	1.0204914	1.03413307	1.6657286	0.73615339	0.84338
PNPLA8	1.522758	1.5313879	1.51563091	0.9703475	-0.0434266	0.84342
SYCP2L	1.064557	1.05701	1.07078949	1.2417019	0.31231888	0.84344
DEDD	1.197548	1.2054121	1.19105309	0.9300966	-0.1045475	0.84346
TEKT1	1.037909	1.0452875	1.03181545	0.7025214	-0.5093859	0.8436
UCKL1	1.248916	1.2570144	1.24222806	0.9424689	-0.085483	0.84367
USP54	1.149717	1.1417891	1.15626365	1.1020851	0.1402356	0.84367
RP11-505K9.1	1.127404	1.1350878	1.12105945	0.8961541	-0.1581812	0.8437
RP11-651L5.3	1.115315	1.1228744	1.10907148	0.8876664	-0.1719106	0.84374
PPP1R1C	1.113045	1.1056	1.11919394	1.1287302	0.17470067	0.84375
CORIN	1.023208	1.0157693	1.02935019	1.861219	0.89624782	0.84376
GPR155	1.278092	1.2863966	1.27123347	0.9470556	-0.078479	0.84378
PARP6	1.281997	1.2739044	1.28868009	1.0539446	0.07579899	0.84383
NEMP2	1.054787	1.0471684	1.06107877	1.2949077	0.37284928	0.84384
RP11-284F21.10	1.034854	1.027406	1.04100456	1.4961877	0.58129113	0.84393
PRKAR2A-AS1	1.034916	1.0274035	1.04111962	1.5005224	0.58546486	0.84412
COG5	1.209627	1.217972	1.20273512	0.9300969	-0.1045471	0.84414
MCU	1.132452	1.124559	1.13897059	1.1157005	0.15794982	0.84429
USP40	1.181482	1.1891462	1.17515307	0.9260193	-0.1108859	0.84431
TNK1	1.035237	1.0276797	1.04147799	1.4984999	0.58351894	0.84436
POGLUT1	1.23093	1.2393941	1.22393983	0.9354442	-0.0962764	0.84442
KCNJ2	1.030078	1.0226323	1.0362272	1.6006847	0.67868919	0.84443
LA16c-349E10.1	1.024804	1.0322272	1.01867321	0.5794244	-0.7873076	0.84447
RP11-1000B6.8	1.039954	1.0473562	1.03384104	0.7146057	-0.4847806	0.84447
AC116614.1	1.133144	1.140815	1.12680897	0.9005361	-0.1511439	0.84452
KRT10	4.290364	4.2697251	4.30740826	1.0115249	0.0165318	0.84456
C2orf48	1.05284	1.0452511	1.05910653	1.3061887	0.3853633	0.84457
RP11-1191J2.5	1.078176	1.0857844	1.07189225	0.8380575	-0.2548789	0.84462
ATP6AP1L	1.192713	1.1849113	1.19915664	1.0770385	0.10706977	0.84472
MYO15B	1.137561	1.1299102	1.14387927	1.1075284	0.14734374	0.84475
AC007383.3	1.024937	1.01752	1.03106213	1.7729531	0.8261544	0.84476
BMP3	1.022453	1.0150885	1.02853471	1.8911622	0.91927308	0.84476
CTC-338M12.9	1.022506	1.0150885	1.02863224	1.8976259	0.92419563	0.84476
SMCO3	1.022435	1.0150885	1.02850104	1.8889306	0.9175697	0.84476
RP11-343J3.2	1.068025	1.0754961	1.06185577	0.8193244	-0.2874933	0.84479
PPIP5K2	1.621926	1.6129843	1.62931057	1.0266341	0.03792209	0.84484

KCNQ1-AS1	1.024173	1.0168034	1.03025891	1.8007645	0.84860956	0.84496
FAM167B	1.058135	1.065805	1.05180146	0.7871968	-0.3452038	0.84501
SCN2A	1.037515	1.0301397	1.04360655	1.4468148	0.53288024	0.84501
COL9A2	2.435903	2.4506085	2.42375954	0.9814912	-0.0269527	0.84503
HPGD	1.023011	1.0156722	1.0290707	1.8549246	0.89136056	0.84506
EDRF1	1.244788	1.2529639	1.23803532	0.9409853	-0.087756	0.84511
GLYR1	1.377456	1.3858428	1.37053035	0.9603143	-0.0584214	0.84524
C1RL	1.330536	1.338622	1.32385886	0.9564024	-0.0643103	0.84544
SH3BP4	1.172013	1.179716	1.16565245	0.9217455	-0.1175597	0.84548
LINC00324	1.080505	1.0729208	1.08676778	1.1898909	0.25082935	0.84553
GLTPD2	1.019715	1.0123699	1.02578027	2.0841164	1.05943582	0.84561
WNT2	1.019721	1.0123699	1.02579148	2.0850229	1.06006321	0.84561
PRRG3	1.119692	1.1273176	1.11339479	0.8906447	-0.167078	0.8457
RP11-522B15.3	1.035605	1.0282226	1.04170128	1.4775846	0.56324072	0.84573
TATDN2	1.100646	1.0931189	1.1068623	1.1475901	0.19860737	0.84574
RP11-273G15.2	1.020571	1.0132186	1.02664352	2.0156051	1.011213	0.84578
NAA40	1.25784	1.249826	1.26445804	1.0585689	0.08211511	0.8458
NEU4	1.030212	1.0377811	1.02396162	0.634223	-0.6569378	0.84582
RP11-57H14.2	1.03002	1.0374071	1.02392032	0.6394588	-0.6450767	0.84582
ERAP2	1.044624	1.037208	1.050749	1.3639288	0.44776831	0.84602
SGMS1-AS1	1.076136	1.0835798	1.06998888	0.8373894	-0.2560294	0.84605
TINCR	1.056803	1.0493725	1.06293966	1.274791	0.35026078	0.84615
LINC01125	1.056609	1.049235	1.06269794	1.2734428	0.34873418	0.84615
PDE3A	1.06253	1.0550131	1.06873832	1.2494901	0.32133949	0.84617
TGIF1	1.993898	1.9843646	2.0017717	1.0176836	0.02528907	0.84626
SHC4	1.042105	1.0494775	1.03601719	0.7279507	-0.4580873	0.84634
DCP1B	1.293135	1.2849785	1.29986991	1.0522544	0.07348351	0.84639
SLC44A3	1.105318	1.097707	1.11160337	1.1422244	0.19184613	0.84644
POLR3A	1.163511	1.1712644	1.15710781	0.9173405	-0.1244708	0.84647
SLC31A1	1.418003	1.409186	1.42528413	1.0393419	0.05567032	0.84647
CTD-3222D19.8	1.071052	1.063532	1.07726187	1.2161099	0.28227363	0.84656
ZNF639	1.296287	1.2879496	1.30317172	1.0528639	0.07431899	0.84663
ZNF345	1.152033	1.1597371	1.14567022	0.9119375	-0.1329932	0.84665
HSPA13	1.498507	1.4891665	1.50622033	1.034863	0.04943975	0.84678
SPTBN4	1.065537	1.0729297	1.05943239	0.8149269	-0.2952574	0.8468
DMGDH	1.023087	1.0303023	1.01712767	0.5652267	-0.8230986	0.84692
NPIPA1	1.222638	1.214819	1.2290953	1.0664573	0.09282627	0.84695
DGCR9	1.019703	1.0124282	1.02571094	2.068766	1.04877049	0.84703
ANKRD22	1.019739	1.0124282	1.0257773	2.0741052	1.05248908	0.84703
TWIST2	1.027458	1.0200498	1.03357614	1.6746365	0.74384799	0.84715
CARMIL2	1.026801	1.0195164	1.03281609	1.6814623	0.74971641	0.84718
GPR160	1.022367	1.0150488	1.02841118	1.8879396	0.91681259	0.84718
C2orf54	1.022353	1.0150395	1.02839233	1.8878463	0.91674133	0.84718
EVI5L	1.122922	1.1307101	1.11649008	0.8912097	-0.1661632	0.8472
ENTPD1-AS1	1.166642	1.1742124	1.160391	0.9206638	-0.1192537	0.84724

RP11-602N24.3	1.028446	1.0357032	1.02245285	0.6288754	-0.6691538	0.84731
CD14	1.028459	1.0357092	1.0224724	0.6293172	-0.6681408	0.84731
CTB-131K11.1	1.028464	1.0357155	1.02247632	0.6293148	-0.6681461	0.84731
PCNX3	1.034548	1.0418217	1.02854207	0.682471	-0.5511603	0.84735
HSD17B7	1.313905	1.3219172	1.30728863	0.954558	-0.0670952	0.84735
RNF144B	1.056183	1.0489648	1.06214453	1.2691679	0.3438829	0.8474
CCDC74B	1.222306	1.2144369	1.22880387	1.0669983	0.09355794	0.84746
SLC8B1	1.090335	1.0828665	1.09650338	1.1645652	0.21979136	0.84748
SGK494	1.113873	1.121181	1.10783864	0.8898969	-0.1682899	0.84752
ZNF829	1.02818	1.0208432	1.03423921	1.6427002	0.71606924	0.84756
FALEC	1.168799	1.1766549	1.16231082	0.9188017	-0.1221745	0.84756
TSC1	1.252674	1.2449552	1.25904881	1.0575353	0.08070586	0.84761
LONRF3	1.025103	1.0323628	1.01910729	0.59041	-0.7602109	0.84767
ICAM1	1.127588	1.120239	1.13365652	1.1115905	0.15262544	0.84774
NAPA-AS1	1.077023	1.0844487	1.07089063	0.8394522	-0.2524799	0.84776
C18orf65	1.046474	1.0391405	1.05253107	1.3421169	0.42451033	0.84778
RP11-576D8.4	1.029702	1.0223998	1.03573172	1.5951783	0.67371768	0.84783
FAM193B	1.114038	1.1065981	1.12018199	1.1274306	0.17303857	0.84786
GINS4	1.266171	1.2738573	1.25982416	0.9487574	-0.0758888	0.84791
ZNF594	1.150402	1.1580975	1.14404691	0.9111271	-0.1342758	0.84797
RHOBTB2	1.154442	1.1468033	1.1607494	1.0949984	0.1309287	0.84804
CEACAM1	1.070615	1.0779124	1.06458802	0.8289821	-0.2705872	0.84814
FTSJ3	1.17877	1.1710014	1.18518612	1.0829509	0.11496782	0.84822
MTERF1	1.14085	1.1482669	1.1347254	0.9086683	-0.1381744	0.84824
SLC25A30	1.05488	1.0475861	1.06090307	1.279851	0.35597589	0.84825
TCF20	1.197408	1.1896755	1.20379388	1.0744341	0.10357702	0.84835
USP46-AS1	1.032533	1.0397386	1.02658234	0.66893	-0.5800729	0.84836
C1orf115	1.036645	1.0293405	1.04267646	1.454524	0.54054709	0.84837
SIX4	1.112331	1.1048221	1.11853264	1.1307979	0.17734107	0.84842
IPO9-AS1	1.058087	1.065393	1.05205416	0.796021	-0.3291217	0.84847
NECTIN3	1.196174	1.1885578	1.20246383	1.0737497	0.10265769	0.84859
IGHMBP2	1.115898	1.1084098	1.12208094	1.1261057	0.17134225	0.84859
CCL28	1.029215	1.0219877	1.03518275	1.60011	0.67817109	0.84877
SAMD10	1.108801	1.1162066	1.10268564	0.8836473	-0.1784574	0.84879
ABCB1	1.085109	1.0923121	1.07916111	0.8575375	-0.2217284	0.8488
ACTRT3	1.048519	1.0556965	1.04259232	0.7647222	-0.3869922	0.84882
USE1	1.555889	1.5647155	1.54860044	0.9714635	-0.0417684	0.84893
SPRTN	1.19037	1.1825454	1.19683108	1.0782581	0.10870252	0.84893
GMEB2	1.142722	1.1351084	1.1490101	1.1028932	0.14129307	0.84895
HYLS1	1.087702	1.094957	1.08171064	0.8605019	-0.2167498	0.84896
PLSCR1	1.16094	1.1684594	1.15473027	0.9185018	-0.1226456	0.84897
TYW1B	1.042198	1.0494894	1.03617699	0.731005	-0.4520469	0.84909
SORL1	1.110137	1.1028593	1.11614695	1.1291826	0.17527875	0.8491
HHIP-AS1	1.050669	1.0432951	1.0567584	1.3109666	0.39063095	0.84915
TTF1	1.526278	1.5347174	1.51930894	0.971184	-0.0421835	0.84918

ATP6V0D2	1.026592	1.0194004	1.03253001	1.6767676	0.74568274	0.84922
ATAD2	1.408375	1.3998329	1.41542833	1.039005	0.05520254	0.84924
RP5-894A10.2	1.068028	1.0752624	1.06205346	0.8244946	-0.278418	0.8493
ANO8	1.071652	1.0642792	1.0777411	1.2094282	0.27432513	0.84937
TRIM25	1.217367	1.2095637	1.22381138	1.0679876	0.09489484	0.84939
F8	1.039149	1.0319497	1.04509482	1.4114315	0.4971591	0.84944
FGF12	1.039284	1.0320039	1.04529538	1.41531	0.50111806	0.84944
RP11-385D13.3	1.126196	1.1186655	1.1324145	1.1158634	0.15816037	0.84948
ZBTB20-AS2	1.024794	1.0175993	1.03073615	1.7464463	0.80442228	0.84952
CTC-543D15.8	1.108231	1.1008025	1.11436558	1.1345515	0.18212208	0.84953
AJAP1	1.030739	1.0380647	1.02468843	0.6485907	-0.6246197	0.8496
EFNA2	1.020617	1.0278097	1.01467742	0.5277806	-0.9219899	0.84962
SECTM1	1.020544	1.027638	1.01468601	0.5313695	-0.9122128	0.84962
RP11-998D10.4	1.020604	1.0277716	1.01468539	0.5287909	-0.9192307	0.84962
SNAP25	1.151044	1.1588818	1.14457159	0.9099319	-0.1361695	0.84963
FAM184A	1.132415	1.1399713	1.12617442	0.9014305	-0.1497119	0.84966
CENPI	1.046899	1.054117	1.040938	0.7564723	-0.4026409	0.84971
CTD-2371O3.3	1.150333	1.1426759	1.15665651	1.0979886	0.13486312	0.84972
RP11-33H15.1	1.023203	1.0303531	1.01729912	0.5699283	-0.8111477	0.84972
ARL17B	1.117945	1.1253378	1.11183951	0.8923049	-0.1643914	0.84973
ZNF585B	1.037712	1.0448523	1.03181577	0.7093459	-0.4954387	0.84974
NUDT17	1.122485	1.1149672	1.12869345	1.1193929	0.16271656	0.84992
PSMB8-AS1	1.03164	1.0244688	1.03756121	1.5350654	0.61830016	0.84993
TRPV2	1.034394	1.0272644	1.04028153	1.477443	0.56310243	0.84994
RP11-48B3.4	1.034436	1.0272747	1.04034971	1.4793837	0.5649963	0.84994
AC005523.2	1.027729	1.0349507	1.0217657	0.6227541	-0.6832654	0.84999
PHC3	1.402213	1.4104665	1.39539629	0.9632852	-0.0539651	0.85004
TAS2R4	1.171478	1.1789122	1.16533791	0.9241286	-0.1138345	0.85005
PP12613	1.038563	1.0458336	1.03255795	0.7103512	-0.4933956	0.85013
RP5-1120P11.1	1.131687	1.1390852	1.12557828	0.9028875	-0.1473818	0.85017
KCNIP2	1.048306	1.0410823	1.05427141	1.3210425	0.40167685	0.85018
RP1-101K10.6	1.097366	1.1048314	1.09120031	0.8699717	-0.2009596	0.85018
ARHGAP45	1.037472	1.0446043	1.0315826	0.7080624	-0.4980515	0.85018
RP11-421M1.8	1.037477	1.0446563	1.03154849	0.7064733	-0.5012931	0.85018
AP5Z1	1.079325	1.0719656	1.08540292	1.1867194	0.24697881	0.85023
RP11-706O15.3	1.058409	1.0512126	1.06435176	1.2565615	0.32948123	0.85054
MYO1F	1.07515	1.0677453	1.08126551	1.1995745	0.2625227	0.85062
TAPT1	1.112554	1.1198766	1.1065062	0.8884655	-0.1706123	0.8507
CLHC1	1.235474	1.2432483	1.22905347	0.9416448	-0.0867451	0.85075
CLDN23	1.021266	1.0141633	1.02713232	1.915684	0.93785962	0.85085
LINC01139	1.230367	1.2227407	1.23666432	1.0625104	0.08747693	0.85091
DERL1	1.480496	1.4719527	1.48755198	1.0330526	0.04691374	0.85091
PIGW	1.0799	1.0871117	1.07394498	0.8488529	-0.2364136	0.85094
RASGRF2-AS1	1.0354	1.0282578	1.04129816	1.4614756	0.54742577	0.85103
RAB4B	1.206603	1.2141314	1.2003852	0.9358046	-0.0957208	0.85104

JPH1	1.028807	1.0216628	1.0347064	1.6021237	0.67998551	0.85117
UBE2D4	1.405593	1.4139436	1.39869623	0.9631655	-0.0541444	0.85118
CTC-273B12.10	1.042436	1.0495596	1.03655249	0.7375464	-0.4391942	0.8512
L3HYPDH	1.126615	1.1192859	1.13266659	1.1121729	0.15338104	0.85123
CRISPLD2	1.028766	1.0216484	1.03464435	1.6003191	0.67835958	0.85136
RP5-1042K10.10	1.112629	1.1198609	1.10665739	0.8898433	-0.1683769	0.85138
KDM6A	1.145299	1.1528679	1.13904755	0.9095927	-0.1367075	0.85141
E2F8	1.062051	1.0547667	1.06806731	1.242859	0.3136626	0.85143
RFXAP	1.303201	1.3108665	1.29687155	0.9549807	-0.0664565	0.85144
AVIL	1.06219	1.0693759	1.05625557	0.8108811	-0.3024377	0.85153
POTEI	1.05932	1.0665123	1.05338076	0.8025696	-0.3173016	0.85154
SMC5-AS1	1.075843	1.0831222	1.0698315	0.8401068	-0.2513553	0.8516
SHOC2	1.769659	1.7609357	1.77686279	1.020931	0.0298853	0.85161
AMER1	1.043969	1.0368191	1.04987335	1.3545498	0.4378134	0.85162
C19orf12	1.572965	1.5816097	1.56582631	0.9728626	-0.039692	0.85163
GPRASP2	1.215154	1.2074862	1.22148537	1.0674701	0.09419571	0.85165
C10orf76	1.127025	1.1195321	1.13321249	1.1144498	0.15633163	0.85169
ZNF324	1.213476	1.2058982	1.21973469	1.0672005	0.09383131	0.85174
PAQR8	1.177774	1.1850971	1.17172583	0.9277608	-0.1081752	0.85176
HERC3	1.088905	1.0816165	1.09492387	1.1630471	0.21790958	0.85179
GLB1L2	1.114167	1.1213114	1.10826647	0.8924676	-0.1641283	0.85183
NPEPPS	2.554724	2.5674396	2.54422283	0.9851881	-0.0215289	0.85186
RP1-120G22.11	1.052621	1.0597384	1.04674248	0.7824532	-0.3539236	0.85187
CHCHD10	2.500482	2.4896907	2.50939377	1.0132263	0.01895637	0.85204
NRXN2	1.242022	1.2344614	1.24826557	1.058876	0.08253368	0.85211
HAUS8	1.156072	1.1632567	1.15013803	0.9196442	-0.1208524	0.8524
AFF3	1.08307	1.0903869	1.07702744	0.852197	-0.2307412	0.85242
WSCD1	1.058362	1.0512017	1.06427593	1.2553469	0.32808614	0.85244
HMGA2	3.626969	3.6484174	3.60925725	0.9852137	-0.0214914	0.85246
RP11-154J22.1	1.045624	1.0384159	1.051577	1.3425934	0.42502242	0.85253
EFCAB6	1.045596	1.0384845	1.05146811	1.3373709	0.41939962	0.85253
LINC00954	1.268826	1.2612814	1.27505585	1.0527189	0.07412027	0.85254
CEBPB-AS1	1.030733	1.0236832	1.03655514	1.5435031	0.62620839	0.85257
GARNL3	1.058356	1.0512269	1.06424243	1.2540748	0.32662346	0.85262
ZBTB10	1.524058	1.5325923	1.51700983	0.9707422	-0.0428399	0.85264
SCMH1	1.358485	1.3506151	1.36498334	1.04098	0.05794238	0.85271
RP11-359B12.2	1.107154	1.1144187	1.10115517	0.8840788	-0.1777532	0.85285
SLC9A6	1.11745	1.1101377	1.1234891	1.1212249	0.16507571	0.85285
MTUS1	1.085267	1.0780699	1.09121062	1.1683196	0.22443497	0.85292
PELP1	1.242328	1.2498904	1.23608192	0.9447418	-0.0820081	0.85295
SUSD6	1.138464	1.1312327	1.14443607	1.1006103	0.13830376	0.85295
MATN1-AS1	1.040487	1.0333718	1.04636263	1.3892752	0.47433242	0.85298
SOGA3	1.040536	1.0333557	1.04646491	1.3930146	0.47821035	0.85298
C20orf85	1.02955	1.0225453	1.03533491	1.5672859	0.64826839	0.85299
TRABD2A	1.025587	1.0185596	1.03139036	1.6913294	0.75815768	0.85307

CTD-2116N20.1	1.025576	1.0185483	1.03137921	1.6917531	0.758519	0.85307
ELF1	1.396854	1.389013	1.40332834	1.0367991	0.05213634	0.85314
CAMK2N1	1.295566	1.2876725	1.30208459	1.0500991	0.07052545	0.85315
ULK2	1.26584	1.2582623	1.27209841	1.0535737	0.07529119	0.85325
TTLL9	1.040591	1.0478138	1.03462629	0.7241907	-0.4655585	0.85327
MURC	1.162891	1.1554676	1.16902197	1.0871849	0.12059726	0.85359
AC009120.11	1.025997	1.0189149	1.03184473	1.6835821	0.75153408	0.85371
KLHL31	1.039435	1.0324463	1.04520634	1.3932656	0.47847035	0.85371
ZNF624	1.100426	1.1076616	1.09445066	0.877292	-0.188871	0.85378
SNAI1	1.027625	1.0205786	1.03344338	1.6251508	0.70057361	0.85386
HIC1	1.027705	1.0206167	1.03355803	1.627709	0.70284282	0.85386
TICRR	1.057531	1.0503882	1.06342931	1.2588137	0.33206477	0.85388
ZBTB46	1.057631	1.0505374	1.06348845	1.2562667	0.3291428	0.85388
PPM1E	1.139345	1.1464555	1.1334735	0.9113589	-0.1339088	0.85389
BORCS8	1.269651	1.2622013	1.27580222	1.0518722	0.07295947	0.85389
DNMBP	1.13381	1.141412	1.1275316	0.9018443	-0.1490497	0.85394
RP11-60A24.3	1.021516	1.0285269	1.01572679	0.5512974	-0.8590974	0.85394
ZFP90	1.620364	1.6290279	1.61320847	0.974851	-0.0367464	0.85397
JAKMIP3	1.02652	1.0194804	1.03233288	1.6597624	0.73097676	0.85398
IDI1	1.991882	1.9814579	2.00049003	1.0193917	0.02770854	0.85407
DNASE1L3	1.018424	1.0253511	1.01270407	0.5011255	-0.996756	0.85412
RYR1	1.018449	1.0254047	1.01270407	0.500067	-0.9998066	0.85412
RP3-467K16.2	1.03791	1.0449588	1.03208863	0.7137349	-0.4865399	0.85419
RP11-572M11.4	1.026577	1.0336335	1.02074964	0.6169345	-0.6968108	0.85423
LGALS8	1.365112	1.3729572	1.35863363	0.9615946	-0.0564993	0.85425
KLC1	1.719498	1.7281789	1.71232949	0.9782342	-0.0317482	0.85431
LINC01556	1.035753	1.0427244	1.02999536	0.702067	-0.5103195	0.85437
SEMA3B-AS1	1.036211	1.0291801	1.04201805	1.4399565	0.52602519	0.85439
RP11-767C1.2	1.02622	1.0331869	1.02046691	0.6167168	-0.6973199	0.85447
RP11-739B23.1	1.018737	1.0257046	1.01298307	0.5050878	-0.9853938	0.8545
GPR61	1.023146	1.0161807	1.02889877	1.7860005	0.83673248	0.85456
SNCB	1.06324	1.0705437	1.05720794	0.810957	-0.3023027	0.85456
RP4-671G15.2	1.029318	1.0223262	1.03509136	1.5717569	0.65237813	0.85456
PGM5-AS1.1	1.114657	1.1219433	1.10863927	0.8909001	-0.1666645	0.85464
HCG25	1.02635	1.0334135	1.02051617	0.6140077	-0.7036713	0.85466
RP11-510M2.2	1.026241	1.0331619	1.02052501	0.6189338	-0.692143	0.85466
ATP6V1E2	1.139057	1.1463267	1.1330537	0.909292	-0.1371844	0.85467
CYB561D2.1	1.139343	1.1466601	1.13330104	0.9089113	-0.1377887	0.85467
TMEM150C	1.185383	1.1928089	1.17925084	0.9296814	-0.1051916	0.8547
BICC1	1.111941	1.1190727	1.1060513	0.8906436	-0.1670799	0.85485
ZNF251	1.10782	1.1006263	1.11376048	1.1305239	0.17699148	0.85487
ACAP2	1.741715	1.7503168	1.73461132	0.9790682	-0.0305187	0.85495
SH3RF3	1.042973	1.0359425	1.04877862	1.3571297	0.44055864	0.85499
CTD-2001C12.1	1.029197	1.0361253	1.02347576	0.649842	-0.6218391	0.85502
SKIV2L2	1.529568	1.5380514	1.52256253	0.9712131	-0.0421403	0.85508

HLA-G	1.02373	1.0166975	1.02953708	1.7689476	0.82289133	0.85513
RP5-916L7.2	1.023685	1.0166975	1.02945524	1.7640463	0.81888847	0.85513
CDA	1.023719	1.0166975	1.02951816	1.7678143	0.82196672	0.85513
YPEL4	1.049981	1.0430085	1.05573832	1.2959826	0.37404639	0.85517
CMC2	1.8647	1.8734175	1.85750082	0.9817766	-0.0265333	0.85519
ZNF256	1.050663	1.0436275	1.05647238	1.2944204	0.37230621	0.8552
PPM1N	1.075007	1.0680387	1.08076078	1.1869833	0.2472997	0.85525
FSCN3	1.01886	1.0257684	1.01315569	0.5105356	-0.9699167	0.85527
PRELID3A	1.120156	1.12742	1.11415644	0.8959064	-0.1585801	0.8553
TRIM14	1.037905	1.0308637	1.04372004	1.4165528	0.50238441	0.85532
TLR6	1.022995	1.0160435	1.02873555	1.7910992	0.84084523	0.85533
RP11-397A16.3	1.019699	1.0265912	1.01400739	0.5267684	-0.9247592	0.85535
TIPARP	1.231973	1.2394825	1.22577141	0.9427469	-0.0850576	0.85535
LINC01424	1.034503	1.0413642	1.02883609	0.6971264	-0.5205078	0.85543
RP11-467L13.7	1.08986	1.0827622	1.09572191	1.1565902	0.20987782	0.85546
RUNDC1	1.255395	1.2478436	1.26163145	1.0556311	0.07810577	0.85552
PPP1R13B	1.083142	1.0902491	1.07727249	0.8562131	-0.2239581	0.85562
GNL3L	1.13791	1.1307353	1.14383435	1.1001951	0.13775935	0.85562
GSAP	1.059341	1.0663965	1.053514	0.8059762	-0.3111908	0.85569
JMJD6	1.167068	1.174408	1.16100736	0.9231652	-0.1153393	0.85582
MSR1	1.021772	1.0148661	1.0274757	1.8482102	0.88612888	0.85584
GDPD3	1.021806	1.0148661	1.02753645	1.8522967	0.8893152	0.85584
MAP3K9	1.128722	1.1214835	1.13470017	1.1087943	0.14899169	0.85594
BSPRY	1.023114	1.0162291	1.02880059	1.7746294	0.82751778	0.85598
ATRIP	1.023187	1.0162198	1.02894137	1.7843287	0.83538143	0.85598
FZD10	1.023154	1.0162239	1.02887753	1.7799337	0.83182348	0.85598
RASL10A	1.02314	1.0162435	1.02883521	1.7751844	0.82796889	0.85598
UBL4A	1.518714	1.510561	1.52544691	1.0291561	0.04146177	0.85601
KDM5A	1.839246	1.8485015	1.83160317	0.9800845	-0.0290219	0.85601
GNAS-AS1	1.057432	1.0503217	1.06330466	1.2580001	0.33113199	0.85601
ZNF600	1.07665	1.0837048	1.07082458	0.846123	-0.2410607	0.85605
RP11-66N24.3	1.093965	1.1011615	1.0880225	0.8701188	-0.2007157	0.85605
CTD-3025N20.3	1.045753	1.038702	1.05157597	1.3326428	0.41429013	0.85605
LPAR4	1.288835	1.2812907	1.29506521	1.0489689	0.06897191	0.85609
ADCY2	1.03025	1.0233232	1.03597	1.5422398	0.62502714	0.85609
ACHE	1.15392	1.1611135	1.14797859	0.9184739	-0.1226894	0.8561
RP11-559M23.1	1.025809	1.0188528	1.0315536	1.6736811	0.74302466	0.85611
VSTM2B	1.06457	1.0715046	1.05884358	0.8229347	-0.2811502	0.85612
SYT17	1.416836	1.4087017	1.42355286	1.0363373	0.05149369	0.85613
RP11-384P7.7	1.038896	1.0319576	1.04462527	1.3963916	0.48170356	0.85617
THAP9	1.092124	1.099231	1.0862545	0.8692297	-0.2021907	0.8562
SPSB2	1.155665	1.1484891	1.16159136	1.0882372	0.121993	0.85624
PTPDC1	1.182144	1.1748626	1.18815641	1.0760242	0.10571053	0.85626
AC007405.4	1.02339	1.0303491	1.01764321	0.5813411	-0.7825432	0.85642
RP11-247A12.7	1.031595	1.0246741	1.03731036	1.5121294	0.59658161	0.85646

GOLGA8N	1.031575	1.0246724	1.03727467	1.5107859	0.59529921	0.85646
EVC	1.01986	1.0129919	1.02553204	1.9652247	0.9746943	0.85671
GSC	1.019915	1.0129919	1.02563187	1.972909	0.98032441	0.85671
NRXN1	1.031827	1.0387067	1.0261461	0.6754932	-0.5659868	0.85676
RP11-568A7.4	1.031842	1.0386977	1.02618113	0.6765553	-0.5637202	0.85676
SETD6	1.199641	1.1922603	1.2057353	1.0700872	0.09772843	0.85683
RP11-356I2.4	1.047346	1.0404066	1.05307615	1.3135509	0.39347215	0.85695
RP11-722E23.2	1.060106	1.0530627	1.06592159	1.2423343	0.31305349	0.857
ZSWIM2	1.019579	1.0263778	1.01396402	0.5293852	-0.9176102	0.85713
AC016907.3	1.025643	1.0187597	1.03132758	1.6699395	0.73979588	0.85716
ASH1L-AS1	1.108961	1.1160828	1.10307888	0.8879769	-0.1714059	0.85716
ACVR2A	1.143093	1.1358708	1.14905685	1.0970487	0.13362758	0.85725
RP11-560J1.2	1.045477	1.0384808	1.05125446	1.3319504	0.41354031	0.85725
CHRND	1.025215	1.0319914	1.01961906	0.6132609	-0.7054271	0.85729
EVA1B	1.654726	1.6463497	1.66164372	1.0236621	0.0337396	0.85733
POTEE	1.042245	1.0353581	1.04793244	1.3556275	0.43896076	0.85741
SLC6A20	1.02152	1.0146773	1.02717164	1.8512676	0.88851344	0.85741
C2orf72	1.032268	1.0253588	1.03797382	1.4974596	0.58251706	0.85741
LINC00864	1.038892	1.0458075	1.03318186	0.7243765	-0.4651883	0.85746
CARM1	1.396547	1.3885753	1.40313068	1.0374584	0.05305345	0.85747
RAD52	1.099442	1.1065631	1.09356176	0.8779941	-0.1877168	0.85747
ZNF266	1.155626	1.1483471	1.16163767	1.0895907	0.12378629	0.85748
TMOD2	1.113842	1.1067552	1.11969367	1.1211977	0.16504068	0.85749
ZNF568	1.108186	1.1152686	1.10233778	0.8878204	-0.1716603	0.85752
CADM1	2.153275	2.1641896	2.14426199	0.9828828	-0.0249086	0.85755
ZNF785	1.391316	1.3993868	1.38465012	0.9631016	-0.0542401	0.85765
ZNF235	1.032602	1.0257249	1.03828146	1.4881097	0.57348091	0.85768
MRPL38	1.086455	1.093449	1.08068009	0.8633593	-0.211967	0.85768
NPTX2	1.043217	1.0501037	1.03753028	0.7490526	-0.4168611	0.85778
PHLPP1	1.114465	1.1214443	1.10870165	0.8950741	-0.159921	0.85781
PSMB8	1.354791	1.346856	1.36134306	1.0417667	0.05903228	0.85785
ACLY	1.540097	1.5485267	1.53313562	0.9719411	-0.0410592	0.85792
CTXN2	1.054087	1.0472089	1.05976662	1.2660022	0.34027995	0.85794
RP11-731C17.2	1.04359	1.0506299	1.03777544	0.746109	-0.4225416	0.85807
PLPP7	1.045637	1.0524433	1.04001559	0.7630256	-0.3901966	0.85808
RP11-299H21.1	1.033788	1.0407071	1.0280735	0.6896465	-0.536071	0.8581
RP11-1057B6.1	1.030167	1.0232773	1.03585585	1.5403784	0.62328478	0.85814
AC007620.3	1.019754	1.0265336	1.01415473	0.5334634	-0.9065388	0.85817
DGKA	1.081962	1.0749003	1.08779409	1.1721457	0.22915189	0.85819
IKZF5	1.120723	1.1134642	1.12671667	1.1167983	0.15936863	0.85822
DNASE1	1.384261	1.3921247	1.37776719	0.9633852	-0.0538153	0.85823
ARPC5L	1.942629	1.9336988	1.95000377	1.0174628	0.02497604	0.85824
RP11-380L11.4	1.062071	1.0552661	1.06769112	1.2248219	0.29257195	0.85825
HERC6	1.029342	1.0225005	1.0349924	1.5551805	0.637082	0.85831
LA16c-313D11.9	1.029344	1.0224826	1.03500951	1.5571824	0.63893792	0.85831

TRIM2	1.566386	1.574669	1.55954547	0.9736831	-0.0384757	0.85832
ZFYVE21	1.639445	1.6314466	1.64604948	1.0231262	0.03298404	0.85832
TESK1	1.149256	1.1566218	1.14317274	0.9141305	-0.1295279	0.85835
CDK17	1.20964	1.2168562	1.2036811	0.9392451	-0.0904265	0.85843
GGT5	1.022763	1.0159741	1.0283701	1.7760042	0.82863499	0.85846
LINC00862	1.022756	1.0159741	1.02835649	1.7751519	0.82794252	0.85846
NIFK-AS1	1.569815	1.5782701	1.56283329	0.9733052	-0.0390358	0.85849
SBNO2	1.085625	1.0786665	1.09137163	1.1615057	0.21599622	0.85856
ADGB	1.034018	1.0407969	1.02841933	0.6966058	-0.5215855	0.85857
NMT1	1.953724	1.96287	1.9461716	0.9826576	-0.0252392	0.85859
PSPN	1.056058	1.0491664	1.06174897	1.2559185	0.3287428	0.85862
ZNF518A	2.340951	2.3304219	2.34964639	1.0144499	0.02069766	0.85863
CRHBP	1.024826	1.0180243	1.03044302	1.6890015	0.75617062	0.85864
EMCN	1.024849	1.0180243	1.03048529	1.6913468	0.7581725	0.85864
RP11-568N6.1	1.02279	1.0159362	1.02844953	1.7852086	0.8360927	0.85865
CTD-3076O17.1	1.024474	1.0312593	1.01887133	0.6037024	-0.7280906	0.85869
RP11-190C22.9	1.026623	1.0334589	1.02097858	0.6269958	-0.6734722	0.85871
TEPP	1.0496	1.0425813	1.0553969	1.3009672	0.37958458	0.85883
RP11-961A15.3	1.037957	1.031096	1.04362234	1.4028299	0.4883401	0.85883
AGAP5	1.055038	1.0481288	1.06074427	1.2621176	0.33584637	0.85886
PLCH2	1.025598	1.0323433	1.0200283	0.6192409	-0.6914273	0.85891
RAC2	1.022468	1.0157004	1.02805738	1.7870464	0.83757713	0.85893
RP11-436K8.1	1.022511	1.0157004	1.0281346	1.7919649	0.84154238	0.85893
SLITRK3	1.02247	1.0157004	1.02805974	1.7871967	0.83769841	0.85893
RP11-90M2.5	1.022484	1.0157004	1.02808553	1.7888391	0.83902361	0.85893
GTF2IRD2B	1.184671	1.191778	1.17880231	0.9323402	-0.1010716	0.85896
ASTN1	1.182153	1.17492	1.18812555	1.0754949	0.10500063	0.85907
ANK1	1.069076	1.075999	1.06335841	0.8336737	-0.2624452	0.85912
MANEAL	1.026561	1.0332748	1.0210158	0.6315829	-0.662956	0.85912
NCOA7	1.358146	1.3661865	1.35150566	0.9599087	-0.0590309	0.85914
CTD-2145A24.5	1.025696	1.0188993	1.03130819	1.6565793	0.72820725	0.85917
TDRD3	1.412804	1.4200741	1.40680033	0.9684014	-0.046323	0.85923
MAPK1	1.608381	1.6001521	1.6151769	1.0250351	0.03567325	0.85924
FKBP1C	1.023817	1.0170619	1.02939616	1.7229101	0.78484746	0.85924
MRFAP1L1	1.530141	1.5380413	1.52361717	0.9731913	-0.0392046	0.85929
FBXO41	1.064717	1.0578552	1.07038338	1.2165432	0.28278757	0.85933
LINC01006	1.267543	1.2751003	1.26130211	0.9498432	-0.0742388	0.85939
TSC2	1.432678	1.424839	1.43915099	1.033688	0.04780087	0.85941
ARSG	1.201207	1.1939067	1.2072362	1.0687419	0.09591352	0.85943
ARHGAP32	1.204138	1.1970322	1.21000669	1.0658495	0.09200372	0.85946
CEP104	1.373698	1.3815616	1.36720326	0.9623696	-0.055337	0.85958
MTRNR2L8	1.052474	1.0455519	1.05818949	1.2774321	0.35324657	0.8596
RP11-458J1.1	1.076046	1.0690941	1.08178777	1.1837151	0.24332194	0.85964
SPAG1	1.103371	1.0962585	1.10924367	1.1348985	0.18256327	0.85965
MTMR9	1.417191	1.4251733	1.41059875	0.9657209	-0.0503218	0.85968

RP11-129M6.1	1.071148	1.0780689	1.06543273	0.838141	-0.2547352	0.86
SDF2	1.762613	1.7712521	1.75547928	0.979549	-0.0298104	0.86001
LEKR1	1.04061	1.0473291	1.03506091	0.7407901	-0.4328633	0.86003
XXbac-BPGBPG55C20.2	1.342912	1.3356961	1.34887034	1.0392447	0.05553532	0.8601
ZNF121	1.242315	1.2494448	1.23642618	0.9478094	-0.0773311	0.86012
KCTD15	1.159183	1.152225	1.16492819	1.0834501	0.11563266	0.86016
TMEM235	1.024444	1.0177366	1.02998247	1.6904277	0.75738835	0.86019
RABIF	1.332087	1.3398999	1.32563483	0.9580316	-0.0618549	0.86022
RP11-315I20.1	1.032245	1.0255048	1.03781056	1.4824888	0.56802123	0.86026
RP11-351D16.3	1.098504	1.0915751	1.10422536	1.1381405	0.18667864	0.86028
RP11-580I16.2	1.023539	1.0167697	1.02913014	1.7370732	0.79665857	0.8603
RP11-449D8.1	1.020446	1.0271131	1.01494071	0.5510513	-0.8597414	0.86031
AMTN	1.019534	1.012846	1.02505704	1.950566	0.96389285	0.86037
LINC00441	1.024882	1.018125	1.03046268	1.6806979	0.74906041	0.86046
E2F2	1.064479	1.0576302	1.07013511	1.2169848	0.2833111	0.86046
SRRD	1.255234	1.2479847	1.26122012	1.0533721	0.07501509	0.86046
RP11-124N14.3	1.044575	1.037727	1.05023059	1.3314221	0.41296805	0.86047
TSPAN15	1.188921	1.1818966	1.19472148	1.0705066	0.09829375	0.86055
TFEB	1.089349	1.0963335	1.08358139	0.8676256	-0.2048555	0.8606
CIB4	1.022343	1.0290603	1.01679584	0.5779644	-0.7909476	0.86061
KIAA1328	1.210748	1.2037931	1.21649214	1.0623132	0.0872092	0.86061
CTC-529I10.1	1.029069	1.0357873	1.02352078	0.6572382	-0.6055118	0.86087
DHODH	1.125313	1.1320894	1.11971654	0.9063296	-0.1418924	0.86088
UHRF2	1.226519	1.2338581	1.22045784	0.9426994	-0.0851303	0.8609
LINC01085	1.022323	1.0156039	1.02787251	1.7862522	0.83693582	0.86097
CDKN2B-AS1	1.02234	1.0156039	1.02790286	1.7881975	0.83850605	0.86097
ITPK1-AS1	1.022337	1.0156039	1.02789691	1.7878165	0.83819864	0.86097
AC226118.1	1.027402	1.0341501	1.02182909	0.6392108	-0.6456364	0.86099
LINC00290	1.027413	1.0341862	1.02181901	0.6382403	-0.6478283	0.86099
FAM78B	1.033046	1.0397554	1.02750563	0.6918723	-0.5314223	0.86121
LINC00457	1.020456	1.0137737	1.02597509	1.8858503	0.91521518	0.86127
C1orf116	1.020438	1.0137737	1.02594155	1.8834154	0.91335123	0.86127
UNC80	1.060613	1.067302	1.05508893	0.8185338	-0.288886	0.86131
C5orf42	1.529061	1.5211815	1.5355688	1.0276052	0.03928613	0.86152
ZNF469	1.047167	1.040446	1.05271791	1.3034138	0.38229518	0.86167
ZFPM1	1.062726	1.0695036	1.0571282	0.8219455	-0.2828854	0.86173
UHMK1	1.398872	1.3915417	1.40492492	1.0341808	0.04848837	0.86179
ANGPTL8	1.02326	1.0165851	1.028773	1.7348665	0.79482463	0.86187
NDST4	1.073478	1.0665284	1.0792168	1.1907209	0.25183528	0.86197
FAM47E	1.154105	1.1611129	1.14831849	0.9205872	-0.1193737	0.86197
DDC	1.017949	1.0113227	1.02342103	2.0684958	1.04858204	0.862
WNT5B	1.085276	1.0922357	1.07952868	0.8622334	-0.2138497	0.862
MORN1	1.101576	1.1084256	1.09591904	0.8846531	-0.1768162	0.86206
COL24A1	1.042139	1.0353779	1.04772264	1.3489379	0.43182392	0.8621
QRICH2	1.056948	1.0502773	1.06245726	1.2422563	0.31296284	0.8621

LINC00623	1.056912	1.0501568	1.06248992	1.2458902	0.31717696	0.8621
LMO7	1.059819	1.0530705	1.06539143	1.2321616	0.30119152	0.86212
MRPS24	1.059837	1.0530056	1.06547831	1.2353099	0.30487297	0.86212
RTKN	1.350321	1.3578152	1.34413196	0.9617589	-0.0562528	0.86219
CEP89	1.44693	1.4392061	1.45330897	1.0321098	0.04559645	0.86224
RP11-48G14.2	1.044695	1.0379979	1.05022509	1.3217863	0.40248899	0.86241
PRKCD	1.091959	1.0986653	1.08642006	0.8758908	-0.191177	0.86245
AC114803.3	1.336615	1.3441334	1.33040697	0.9601132	-0.0587236	0.86251
RP11-715J22.2	1.024686	1.0312526	1.01926371	0.6163879	-0.6980897	0.86258
RP5-1068H6.6	1.022519	1.0291454	1.01704689	0.5848914	-0.7737593	0.86265
SLC26A1	1.029954	1.023273	1.03547159	1.5241524	0.60800716	0.86289
NXPH1	1.024847	1.0314519	1.0193919	0.616558	-0.6976914	0.86294
SENP1	1.121899	1.1151884	1.12744002	1.1063618	0.14582324	0.86297
HDX	1.126198	1.1330667	1.12052535	0.9057514	-0.142813	0.86305
ZBTB11-AS1	1.056762	1.0634926	1.05120448	0.8064635	-0.3103188	0.86309
NNMT	1.159685	1.1666294	1.15395091	0.9239118	-0.114173	0.86311
FAM150A	1.024518	1.0178342	1.03003806	1.6842968	0.75214638	0.86312
SLC25A43	1.135284	1.1422114	1.12956284	0.9110577	-0.1343856	0.86316
HBS1L	1.430658	1.4382108	1.42442074	0.968531	-0.0461298	0.86321
XKR6	1.202635	1.1953546	1.20864784	1.0680466	0.09497453	0.86328
NLGN4X	1.236853	1.2296367	1.2428115	1.0573725	0.08048372	0.86334
RP1-92O14.3	1.024884	1.0314606	1.0194532	0.6183359	-0.6935374	0.86336
CDHR1	1.024815	1.0181646	1.03030751	1.6684891	0.73854223	0.86336
CMTM2	1.02483	1.0181568	1.03034167	1.6710906	0.74078997	0.86336
ZNF550	1.152035	1.1451956	1.15768282	1.086003	0.11902811	0.86337
CHST14	1.228562	1.2355332	1.22280459	0.9459583	-0.0801515	0.86339
WNT3A	1.024237	1.0176051	1.02971394	1.6878076	0.75515041	0.8634
RP11-480I12.10	1.019762	1.0131484	1.02522374	1.9183882	0.93989472	0.8634
FAM196A	1.053475	1.0467607	1.05901928	1.2621545	0.33588857	0.86341
COL7A1	1.11141	1.1046703	1.1169757	1.1175639	0.16035738	0.86342
RP11-15A1.3	1.140077	1.1468466	1.13448571	0.9158243	-0.1268573	0.86345
RARRES3	1.047163	1.0537673	1.04170891	0.7757304	-0.3663727	0.86345
NOTUM	1.026566	1.0199486	1.03202992	1.6056242	0.6831343	0.8635
RNF183	1.02659	1.0199894	1.03204128	1.6029108	0.68069413	0.8635
EMSY	1.259203	1.2521276	1.26504532	1.0512348	0.07208495	0.86357
TBX6	1.083599	1.0768648	1.08915955	1.1599528	0.21406607	0.8636
PRDM11	1.069328	1.0626192	1.07486859	1.1956164	0.25775454	0.86364
PFN4	1.051622	1.0449066	1.05716788	1.2730397	0.34827735	0.86367
MRM1	1.049045	1.0557099	1.0435408	0.7815637	-0.3555646	0.86376
CTD-3035K23.7	1.049158	1.055754	1.04371147	0.7840055	-0.3510644	0.86376
C17orf80	1.163637	1.1705871	1.15789746	0.925612	-0.1115205	0.86377
CTD-2269F5.1	1.031083	1.0377566	1.02557226	0.6772926	-0.5621489	0.86384
RP11-140K17.3	1.354557	1.361951	1.34845029	0.9627	-0.0548418	0.86386
ZNF521	1.355972	1.3486606	1.36200946	1.038286	0.0542039	0.86397
NPTX1	1.027105	1.0205028	1.03255813	1.5879862	0.66719837	0.86404

PLA2G16	2.068409	2.0596278	2.07566002	1.0151301	0.02166461	0.86408
RAD54B	1.150473	1.1574927	1.14467521	0.9186153	-0.1224673	0.86411
PHYHD1	1.238708	1.2316045	1.24457423	1.0559996	0.07860929	0.86412
FEZF1-AS1	1.167592	1.1607921	1.17320826	1.0772186	0.1073111	0.86421
CDH15	1.027792	1.0345395	1.02221956	0.6433085	-0.6364173	0.86421
GFPT1	1.409167	1.4017088	1.41532541	1.0338968	0.04809221	0.86422
FAM212B	1.090259	1.0968211	1.08483966	0.8762516	-0.1905829	0.86424
CRIP1	1.053923	1.0473558	1.05934575	1.2531879	0.32560269	0.86424
VSTM2L	1.120976	1.1277557	1.11537778	0.9031129	-0.1470217	0.86428
KDF1	1.019608	1.013034	1.02503689	1.920894	0.94177789	0.86445
RPE65	1.022332	1.0157693	1.02775197	1.7598687	0.81546783	0.86446
SYCP2	1.022365	1.0157693	1.02781256	1.7637115	0.8186146	0.86446
PIK3C2G	1.02236	1.0157693	1.0278018	1.7630287	0.81805599	0.86446
FAM193A	1.205132	1.2122733	1.19923456	0.9385758	-0.0914548	0.86453
KLHL22	1.190952	1.1978779	1.18523165	0.9360908	-0.0952796	0.86456
ATP6V0B	3.064419	3.0775438	3.05358055	0.9884656	-0.0167374	0.86463
RP11-950C14.3	1.026181	1.0196309	1.03159017	1.609207	0.68634988	0.86465
TLX2	1.026184	1.0196936	1.03154388	1.6017301	0.67963108	0.86465
RP11-253M7.1	1.034022	1.0274086	1.03948286	1.4405308	0.52660051	0.86471
IFIT5	1.227362	1.2343571	1.22158559	0.945504	-0.0808445	0.86475
RRP8	1.275346	1.2822496	1.26964516	0.9553431	-0.0659092	0.86476
RP11-502I4.3	1.050357	1.0569521	1.0449099	0.7885551	-0.3427166	0.86478
AF131215.8	1.019846	1.0263153	1.01450349	0.5511425	-0.8595026	0.86487
GPR141	1.026677	1.0200676	1.03213526	1.6013537	0.67929196	0.86493
TSPO	2.158839	2.1491559	2.16683486	1.0153843	0.02202583	0.86493
CYB561A3	1.237744	1.2447073	1.23199312	0.9480433	-0.0769751	0.86494
PRR16	1.034349	1.0277191	1.03982453	1.4367164	0.52277531	0.86495
RP11-306G20.1	1.04228	1.0356494	1.04775554	1.3395896	0.42179105	0.86502
EPHA3	1.088126	1.0814985	1.0935996	1.1484819	0.19972816	0.86504
AC087294.2	1.027362	1.0338814	1.02197799	0.6486741	-0.6244342	0.86509
RP11-15E18.1	1.027398	1.0339634	1.02197674	0.6470706	-0.628005	0.86509
ZNF233	1.027333	1.033853	1.02194812	0.6483368	-0.6251846	0.86509
CTC-459F4.1	1.054266	1.0608866	1.0487981	0.8014586	-0.3193001	0.8651
TMEM125	1.036961	1.0304448	1.04234312	1.3908178	0.47593343	0.86523
ETF1	1.497737	1.5051469	1.49161719	0.9732164	-0.0391675	0.86538
SNURF	1.055935	1.049342	1.06137937	1.2439583	0.31493812	0.86543
KLHL26	1.070413	1.0770295	1.06494918	0.8431728	-0.2460997	0.86548
DHX35	1.095911	1.1026159	1.09037435	0.8807049	-0.1832694	0.86565
MAP3K10	1.060022	1.0666134	1.0545783	0.8193292	-0.2874848	0.86568
HSPA1B	1.101589	1.0947121	1.1072689	1.1325783	0.17961077	0.86573
ZFP2	1.071428	1.0779963	1.06600331	0.8462364	-0.2408673	0.86576
CTSK	1.071383	1.0779568	1.0659536	0.8460272	-0.241224	0.86576
ST7L	1.239779	1.2325401	1.24575662	1.0568353	0.07975058	0.86579
ADAMTS19	1.04993	1.05638	1.04460366	0.7911255	-0.3380215	0.86586
FAM167A	1.090271	1.0835046	1.09585966	1.147956	0.19906731	0.8659

EDEM1	1.14149	1.1345382	1.14723143	1.0943467	0.1300699	0.86591
BCO1	1.029094	1.0225518	1.03449609	1.5296353	0.6131877	0.86609
RP11-295H24.4	1.049623	1.0561219	1.04425635	0.7885751	-0.3426799	0.86611
CABYR	1.083512	1.0901768	1.07800858	0.8650629	-0.2091231	0.86612
NRG1	1.051784	1.0584277	1.04629708	0.7923818	-0.3357323	0.86612
LINC00960	1.25568	1.2627966	1.24980322	0.9505574	-0.0731544	0.86616
RP11-665C14.2	1.023157	1.0295806	1.01785255	0.6035218	-0.7285222	0.86617
RP11-109A6.3	1.023151	1.0295855	1.01783741	0.6029106	-0.7299839	0.86617
KIAA1755	1.023141	1.0295827	1.01782161	0.602434	-0.731125	0.86617
HEYL	1.023177	1.0296571	1.01782506	0.6010397	-0.7344679	0.86617
ZNF326	2.245973	2.2549118	2.23859205	0.9869953	-0.0188849	0.86619
CWF19L1	1.125594	1.1321957	1.1201429	0.9088262	-0.1379237	0.86621
CLEC7A	1.018833	1.0123699	1.02417087	1.9540103	0.96643807	0.86631
RP11-111M22.4	1.034795	1.0282519	1.04019848	1.422857	0.50879072	0.86631
CAPSL	1.024949	1.0184658	1.03030253	1.6410101	0.71458412	0.86634
CREG2	1.030839	1.0243036	1.03623593	1.4909672	0.57624854	0.86643
FAM150B	1.019672	1.0132122	1.02500725	1.8927405	0.9204766	0.86647
RP11-286N22.8	1.042297	1.0488308	1.03690069	0.7556853	-0.4041426	0.86648
HMGCLL1	1.055763	1.0491695	1.06120867	1.2448497	0.31597157	0.8665
PCDHA4	1.074108	1.0807906	1.0685898	0.8489823	-0.2361936	0.8665
RP11-707A18.1	1.017827	1.0113663	1.02316308	2.0378704	1.02706233	0.86653
TAF5	1.093124	1.0995838	1.08778957	0.8815651	-0.1818609	0.86655
CARMN	1.051784	1.0451389	1.05727089	1.2687701	0.34343066	0.86657
TBCK	1.302531	1.3096232	1.29667377	0.9581769	-0.0616361	0.86657
SCN8A	1.036058	1.0295392	1.04144188	1.4029438	0.48845724	0.86658
NPIPB6	1.038737	1.0322693	1.04407841	1.365954	0.44990889	0.86665
LINC00575	1.040144	1.046673	1.03475194	0.7445828	-0.4254958	0.86665
LINC01011	1.133983	1.1407419	1.12840149	0.9123186	-0.1323904	0.86674
PPP4R1	1.329466	1.322239	1.33543458	1.0409497	0.05790032	0.86675
DDI2	1.226744	1.2199562	1.23234945	1.0563441	0.07907987	0.86683
PLEKHH3	1.17035	1.1773476	1.1645719	0.9279626	-0.1078614	0.86687
ANKRD50	1.236557	1.2438301	1.23055103	0.9455398	-0.0807899	0.8669
KCTD19	1.031624	1.0380987	1.02627624	0.6896884	-0.5359833	0.86696
TSPAN33	1.044537	1.0380636	1.04988359	1.3105338	0.39015456	0.86699
PRX	1.044522	1.0380134	1.0498964	1.3126015	0.39242895	0.86699
CTD-2095E4.5	1.169844	1.1770604	1.16388472	0.9255866	-0.1115601	0.86703
RAB17	1.033586	1.0270092	1.0390176	1.4446045	0.53067462	0.86704
VPS52	1.195507	1.1886834	1.20114187	1.0660283	0.09224568	0.86706
VTN	1.062806	1.056249	1.06822026	1.2128253	0.27837171	0.86706
CCL20	1.018659	1.0122555	1.02394683	1.9539696	0.96640805	0.86708
PWRN1	1.01869	1.0122555	1.02400413	1.9586446	0.96985564	0.86708
CTA-984G1.5	1.018691	1.0122555	1.02400486	1.9587042	0.96989951	0.86708
RP11-793H13.3	1.018683	1.0122555	1.0239909	1.9575658	0.96906084	0.86708
AMPH	1.116036	1.109291	1.1216055	1.1126764	0.15403408	0.86724
WDTC1	1.332934	1.3404312	1.32674315	0.9597919	-0.0592065	0.86726

RP4-798A10.7	1.071472	1.0780316	1.06605414	0.8465055	-0.2404087	0.86739
ARSE	1.026263	1.0197874	1.03161069	1.5975132	0.67582787	0.86739
RP11-816J6.3	1.026248	1.0197666	1.03160132	1.5987233	0.67692026	0.86739
C14orf132	1.581993	1.5900309	1.57535496	0.9751268	-0.0363383	0.86744
RP3-461P17.10	1.018443	1.0120052	1.02375911	1.9790755	0.98482663	0.86747
FDX2	1.488537	1.4965072	1.48195437	0.9706895	-0.0429182	0.86748
HMGA1P4	1.028296	1.0218202	1.03364356	1.5418567	0.62466864	0.86757
PRR29-AS1	1.028349	1.0218732	1.0336965	1.5405381	0.62343433	0.86757
EYA4	1.028301	1.0218417	1.03363467	1.5399321	0.62286676	0.86757
AQP4	1.026184	1.0197494	1.03149768	1.5948709	0.67343964	0.86758
CD96	1.026229	1.019764	1.03156851	1.597272	0.67561004	0.86758
ABCB8	1.151209	1.1579364	1.14565299	0.9222258	-0.1168081	0.86759
ZNF284	1.086661	1.0932215	1.08124305	0.8715052	-0.1984188	0.86761
AC007879.4	1.02401	1.0304194	1.01871703	0.6152983	-0.7006421	0.86769
RP11-188D8.1	1.038611	1.0321486	1.04394703	1.3669985	0.45101163	0.86771
UPK3B	1.018845	1.0124282	1.02414355	1.9426495	0.95802564	0.86774
ST3GAL6-AS1	1.026513	1.0200654	1.03183799	1.5867092	0.66603777	0.86783
CYGB	1.114057	1.1073986	1.11955637	1.1132023	0.15471575	0.86788
MED14OS	1.040255	1.0338014	1.04558498	1.3486135	0.43147696	0.86801
EGFL8	1.029341	1.035716	1.02407572	0.674088	-0.5689911	0.86806
LINC01301	1.053393	1.0469788	1.05869019	1.2492907	0.32110927	0.86808
RELT	1.06533	1.0587266	1.07078329	1.2053026	0.26939538	0.86809
EIF3C	1.079556	1.0729786	1.08498855	1.1645689	0.21979596	0.86834
RP11-480C16.1	1.071901	1.065324	1.07733255	1.1838312	0.24346335	0.86839
SUMF1	1.204943	1.1977991	1.21084218	1.0659411	0.09212777	0.86844
EXO5	1.071474	1.0779171	1.06615324	0.8490205	-0.2361287	0.86846
FAM219A	1.303529	1.2966966	1.30917182	1.0420472	0.05942064	0.86853
ZBTB47	1.044632	1.0510675	1.0393167	0.7698967	-0.3772632	0.86857
SEMA6A	1.592892	1.5852466	1.59920518	1.0238507	0.03400542	0.86858
C9orf172	1.131643	1.1384673	1.12600786	0.9100187	-0.1360319	0.86859
RP11-395G23.3	1.908233	1.9174554	1.90061709	0.9816467	-0.0267242	0.86859
ZNF92	1.21412	1.2071924	1.21984076	1.0610463	0.08548764	0.86859
CTB-25B13.12	1.043055	1.0366953	1.04830684	1.3164307	0.3966316	0.86862
OAZ3	1.043019	1.0365551	1.04835742	1.3228635	0.40366417	0.86862
RLN1	1.042983	1.0494833	1.03761513	0.760158	-0.3956288	0.86863
AC079922.3	1.082498	1.0759308	1.08792153	1.1579165	0.21153123	0.86868
TMEM151B	1.040651	1.0342256	1.04595682	1.3427617	0.42520334	0.86873
NTRK3	1.042275	1.0358442	1.04758549	1.3275663	0.40878387	0.86873
NT5E	1.042187	1.0357984	1.04746325	1.3258503	0.40691794	0.86873
FGD4	1.525479	1.533194	1.5191087	0.9735832	-0.0386239	0.86874
PLEKHA6	1.109811	1.1165518	1.10424377	0.8943984	-0.1610105	0.86877
PELI3	1.081724	1.0751915	1.08711793	1.1586147	0.2124009	0.8688
OSBP	1.226092	1.2328865	1.22048035	0.9467288	-0.0789768	0.86884
HOXB-AS1	1.021322	1.0149316	1.02659997	1.7814535	0.83305484	0.86893
TMEM139	1.136192	1.1297971	1.141473	1.0899547	0.12426823	0.86893

RP11-473E2.4	1.024069	1.0176765	1.0293475	1.6602595	0.73140879	0.86894
RP13-143G15.4	1.048553	1.0549337	1.04328307	0.7879149	-0.3438882	0.86896
ZCCHC6	1.238977	1.2456836	1.23343802	0.950157	-0.0737622	0.869
NECAP2	1.532212	1.5396567	1.52606344	0.9748112	-0.0368052	0.86906
RP11-375N15.2	1.027941	1.021484	1.0332727	1.5487203	0.63107666	0.86912
AF003625.3	1.027885	1.0214932	1.03316352	1.5429804	0.62571978	0.86912
CTC-526N19.1	1.027865	1.0214842	1.0331338	1.5422398	0.62502712	0.86912
RSAD2	1.026966	1.0205595	1.03225728	1.5689723	0.64981993	0.86922
RP11-458D21.1	1.027015	1.0205823	1.03232737	1.5706386	0.65135121	0.86922
TMEM220-AS1	1.023024	1.0166552	1.02828321	1.6981606	0.76397287	0.8693
ABCA4	1.023035	1.0166847	1.02827858	1.6948791	0.76118237	0.8693
XCL1	1.023031	1.0166418	1.02830735	1.7009767	0.76636341	0.8693
SNX22	1.067075	1.0734521	1.06180847	0.8414804	-0.2489985	0.86931
RP11-347C18.5	1.02569	1.0320268	1.0204577	0.6387686	-0.6466348	0.86932
ZNF814	1.348963	1.3412505	1.35533247	1.0412657	0.05833827	0.86937
RP11-449P15.2	1.028303	1.0219059	1.03358586	1.5331902	0.61653667	0.86939
KB-1208A12.3	1.036153	1.0298386	1.04136679	1.386353	0.47129466	0.8694
RP11-787I22.3	1.023917	1.0302272	1.01870538	0.6188254	-0.6923958	0.86947
TMLHE	1.151426	1.157861	1.14611267	0.925578	-0.1115735	0.86951
RGS12	1.529569	1.5373432	1.52314904	0.9735845	-0.0386218	0.86963
RP11-326C3.12	1.025743	1.0194146	1.03096845	1.5951148	0.67366025	0.86991
RP13-131K19.1	1.059608	1.0660964	1.05425004	0.8207715	-0.2849475	0.86995
RP11-318E3.9	1.038437	1.0320111	1.04374307	1.3664968	0.45048203	0.87003
VPS50	1.103302	1.096644	1.10879967	1.125778	0.17092238	0.87003
ACOT1	1.033485	1.0397627	1.0283003	0.7117307	-0.4905967	0.87011
CRTC1	1.188782	1.1820124	1.19437333	1.0679124	0.09479331	0.87013
LIFR	1.143252	1.1368416	1.14854507	1.0855256	0.11839377	0.87013
EFCAB10	1.052635	1.0461868	1.05796023	1.2549104	0.32758434	0.87019
SERPINF2	1.023921	1.017598	1.02914261	1.6560175	0.72771795	0.87021
MEX3B	1.285427	1.2784777	1.29116492	1.0455592	0.06427468	0.87026
ZNF519	1.312251	1.3050792	1.31817403	1.0429228	0.06063236	0.87026
PRICKLE2	1.170649	1.1641511	1.17601421	1.0722692	0.10066721	0.87026
INPP5A	1.081714	1.0753073	1.08700527	1.1553361	0.20831264	0.87027
CLDN16	1.049992	1.0563933	1.04470531	0.7927416	-0.3350775	0.87032
ZNF816-ZNF321P	1.038041	1.0443433	1.03283639	0.7405037	-0.4334212	0.87039
SMIM11A	1.021454	1.0277439	1.01626046	0.586092	-0.770801	0.87042
STK40	1.232144	1.2253027	1.23779427	1.0554436	0.07784954	0.87049
MGC16275	1.024096	1.0304077	1.01888282	0.6209872	-0.6873645	0.87052
RP11-17A19.2	1.040491	1.0469071	1.0351926	0.7502622	-0.4145333	0.87069
CKM	1.021088	1.0274268	1.01585339	0.5780246	-0.7907972	0.8707
STK32B	1.036393	1.0301074	1.04158317	1.3811612	0.46588166	0.87075
CATIP	1.0374	1.0310164	1.04267238	1.3758004	0.46027114	0.87075
KIAA1462	1.026729	1.0203842	1.0319684	1.5682944	0.64919643	0.87079
RRNAD1	1.319185	1.3264602	1.31317736	0.9593125	-0.0599272	0.87081
LONRF1	1.172211	1.1786105	1.16692535	0.9345774	-0.0976139	0.87085

RP11-7F17.8	1.032501	1.0261735	1.03772589	1.4413795	0.52745024	0.87088
HOMEZ	1.228086	1.221273	1.23371289	1.0562196	0.07890976	0.87091
EBI3	1.021359	1.015118	1.02651238	1.7536997	0.81040176	0.87094
RP13-977J11.2	1.044983	1.0386272	1.05023219	1.3004365	0.37899593	0.87098
SLK	1.449514	1.4420975	1.45563873	1.0306296	0.04352586	0.87102
MCF2	1.021308	1.0275718	1.01613572	0.585225	-0.7729366	0.87108
AMIGO1	1.029874	1.0361132	1.02472173	0.684563	-0.5467448	0.87119
TBX5	1.047346	1.0409377	1.05263875	1.2858246	0.36269385	0.8712
TLE3	1.415772	1.4233704	1.40949678	0.9672306	-0.0480682	0.8714
OCA2	1.046263	1.0398837	1.05153059	1.2920216	0.36963023	0.87142
CCNE2	1.046214	1.0398808	1.05144494	1.2899684	0.3673357	0.87142
STC1	1.557092	1.5653342	1.55028548	0.9733809	-0.0389237	0.87149
AF129408.17	1.020401	1.0141599	1.02555489	1.8047398	0.85179083	0.87156
GPR173	1.179111	1.1724691	1.1845963	1.0703155	0.0980361	0.87172
ARRDC4	1.230184	1.2370682	1.22449835	0.946978	-0.0785972	0.87177
CNOT6L	1.32608	1.3330849	1.32029593	0.9616044	-0.0564845	0.87179
RP11-473M20.16	1.043298	1.04963	1.03806879	0.7670517	-0.3826043	0.87182
CASC18	1.021488	1.0277753	1.01629556	0.5866933	-0.7693217	0.87185
MT1M	1.021418	1.0276263	1.01629093	0.5896892	-0.7619732	0.87185
AC016723.4	1.027964	1.0216962	1.03313944	1.5274311	0.6111073	0.87185
RP5-1198O20.4	1.103106	1.1094853	1.097838	0.8936181	-0.1622697	0.87189
RP11-727F15.11	1.10373	1.1103479	1.09826459	0.8904979	-0.1673159	0.87193
FLVCR2	1.030046	1.0237492	1.03524568	1.4840774	0.5695663	0.87203
TRIM35	1.060197	1.0538621	1.06542854	1.2147423	0.28065025	0.87204
CCDC168	1.027931	1.0216422	1.03312467	1.5305616	0.61406107	0.87204
SYNE3	1.027924	1.0216391	1.03311433	1.5302976	0.61381226	0.87204
A1BG	2.768744	2.7809654	2.75865198	0.9874712	-0.0181895	0.87207
LRRN2	1.154941	1.1483855	1.16035424	1.0806601	0.11191277	0.87213
CAPN6	1.043136	1.0368558	1.04832185	1.3111067	0.39078506	0.87215
DRP2	1.045763	1.0521449	1.04049244	0.7765367	-0.364874	0.87216
A4GALT	1.027686	1.0214339	1.03284929	1.5325862	0.6159682	0.87232
GRB14	1.06717	1.0734523	1.06198192	0.8438387	-0.2449608	0.87234
OLFM1	1.067122	1.073432	1.06191052	0.8430996	-0.2462251	0.87234
ANG	1.237845	1.2311007	1.24341459	1.0532835	0.07489384	0.87234
DKFZp779M0652	1.023181	1.0169456	1.02833106	1.6718863	0.74147675	0.87235
NGRN	1.281555	1.2888397	1.27553937	0.9539525	-0.0680107	0.87238
RP11-53I6.3	1.030052	1.0237835	1.03522854	1.4812171	0.56678314	0.87242
TDRD7	1.030854	1.0246615	1.03596853	1.4584878	0.5444733	0.87252
RP11-373N22.3	1.029053	1.0227707	1.03424082	1.5037211	0.58853704	0.87263
GABBR2	1.028988	1.0228018	1.03409633	1.4953339	0.58046766	0.87263
CDH24	1.137478	1.1309389	1.14287864	1.0911858	0.12589679	0.87264
RP11-629O1.2	1.047373	1.0410601	1.05258623	1.2807123	0.35694642	0.87265
XXbac-BPG27H4.8	1.024788	1.0309862	1.01967012	0.6348027	-0.6556199	0.87266
GREM2	1.024824	1.0310511	1.01968093	0.6338244	-0.657845	0.87266
TAF6L	1.120842	1.1272546	1.11554557	0.9079874	-0.1392558	0.87272

SND1-IT1	1.093813	1.0872797	1.09920766	1.136663	0.1848046	0.87276
SPG7	1.737969	1.7300305	1.74452472	1.0198542	0.02836293	0.87282
PDE9A	1.19762	1.1911413	1.20296981	1.0618835	0.08662548	0.87285
CTD-3064M3.7	1.03061	1.0243404	1.03578822	1.4703222	0.55613235	0.87295
DCST2	1.03055	1.0243048	1.03570723	1.4691406	0.55497249	0.87295
AC007040.8	1.025896	1.0320725	1.0207948	0.6483684	-0.6251143	0.873
RP11-410L14.2	1.218788	1.2120742	1.22433233	1.057801	0.08106827	0.87312
RP1-43E13.2	1.083431	1.0771102	1.08865172	1.1496756	0.20122685	0.87314
ADCY3	1.083366	1.0769528	1.08866303	1.1521744	0.20435908	0.87314
SNIP1	1.083482	1.0770053	1.08882999	1.1535571	0.20608936	0.87314
LA16c-325D7.2	1.042371	1.0361126	1.04753847	1.3163966	0.3965942	0.87316
FXYD2	1.077017	1.0833795	1.07176217	0.8606689	-0.2164698	0.87336
CTC-498J12.3	1.023077	1.0168623	1.02821005	1.6729638	0.7424062	0.8734
LINC00276	1.025823	1.0195795	1.03097852	1.582191	0.66192374	0.87341
ZNF429	1.17049	1.1770572	1.16506645	0.9322774	-0.1011687	0.87348
FAM81A	1.037363	1.0435621	1.03224283	0.7401574	-0.4340959	0.87352
C2CD2L	1.064318	1.0579694	1.06956081	1.1999576	0.26298343	0.87356
FAM212B-AS1	1.023889	1.0300573	1.01879576	0.6253302	-0.67731	0.87357
TPRG1	1.029605	1.0234057	1.03472518	1.4836179	0.56911957	0.87358
RDX	3.722855	3.7387681	3.70971342	0.9893913	-0.0153868	0.87359
MROH6	1.158528	1.1651594	1.15305139	0.926689	-0.1098429	0.8736
FAM69A	1.06641	1.0601307	1.07159491	1.1906558	0.25175637	0.87374
RP11-368I23.3	1.038718	1.0449185	1.03359809	0.7479788	-0.4189308	0.87375
PRKACA	1.304591	1.2976953	1.31028575	1.0422929	0.05976078	0.87379
RBM41	1.758279	1.7510137	1.76427911	1.0176634	0.02526047	0.87381
RP11-305K5.1	1.030063	1.0239088	1.03514546	1.4699826	0.55579911	0.87385
NSF	1.196313	1.2028321	1.19092972	0.9413191	-0.0872442	0.87386
TMEM241	1.144823	1.1383698	1.1501519	1.0851494	0.11789372	0.87389
CAPN12	1.041344	1.0476143	1.03616639	0.7595699	-0.3967453	0.87391
CYP26B1	1.032747	1.0264985	1.0379071	1.4305394	0.51655922	0.87394
KIF19	1.045992	1.0523838	1.04071431	0.7772312	-0.3635844	0.87423
U91328.19	1.045921	1.0521378	1.04078681	0.7822882	-0.3542278	0.87423
TVP23B	1.214325	1.2208928	1.20890173	0.9457156	-0.0805217	0.87424
RNF180	1.436594	1.4436087	1.43080173	0.97113	-0.0422636	0.87431
LINC-PINT	1.067096	1.0608744	1.07223366	1.1866025	0.24683673	0.87432
MARS	1.340857	1.3478153	1.33511073	0.9634732	-0.0536836	0.87435
RP11-98I9.4	1.040173	1.0339963	1.04527354	1.3317193	0.41328998	0.87446
GFRA2	1.058458	1.0646806	1.053319	0.8243432	-0.2786829	0.87448
DPYD	1.098183	1.1045061	1.09296203	0.8895368	-0.1688738	0.8745
RP11-791G15.2	1.046107	1.0523278	1.04097062	0.7829607	-0.3529881	0.87451
ADAM33	1.026773	1.0206216	1.03185246	1.5446195	0.62725151	0.87454
CCDC153	1.090584	1.0968556	1.08540402	0.8817661	-0.1815321	0.87459
KIAA0825	1.054281	1.0480181	1.05945393	1.2381574	0.30819476	0.87459
THOC5	1.233102	1.2397013	1.22765246	0.9497339	-0.0744048	0.87465
RP11-171I2.3	1.036898	1.0430049	1.03185434	0.7407148	-0.4330099	0.87465

CCNY	1.32108	1.3144035	1.32659389	1.0387732	0.05488069	0.87468
DLL4	1.019713	1.0257951	1.01469051	0.569508	-0.8122119	0.87469
HCG9	1.019696	1.0257567	1.01469176	0.570405	-0.8099414	0.87469
PAUPAR	1.046235	1.0524551	1.0410991	0.7835101	-0.3519762	0.8747
IL11RA	1.329564	1.3361416	1.32413158	0.9642709	-0.0524895	0.87473
FAM208A	1.864148	1.8564681	1.87049011	1.0163719	0.0234284	0.87474
YJEFN3	1.1298	1.1362563	1.12446845	0.9134877	-0.1305428	0.87479
CD3EAP	1.130133	1.1362012	1.12512225	0.9186573	-0.1224013	0.87483
NUP160	1.431722	1.438672	1.42598237	0.9710727	-0.0423488	0.87486
ELMO3	1.04713	1.0409349	1.05224626	1.2763253	0.35199611	0.87493
AP2B1	2.903953	2.9159364	2.89405728	0.9885805	-0.0165697	0.87495
EPHA1-AS1	1.037621	1.0437262	1.03257943	0.7450778	-0.424537	0.87497
RP11-794G24.1	1.027525	1.0337763	1.02236245	0.6620752	-0.5949329	0.87505
EXOC2	1.073718	1.0674566	1.07888837	1.1694677	0.225852	0.87506
BLM	1.129883	1.1361606	1.12469885	0.9158218	-0.1268612	0.87509
BBS10	1.132228	1.1384619	1.12707946	0.9177936	-0.1237584	0.8751
KCP	1.049181	1.0429341	1.05433997	1.2656609	0.33989093	0.8751
PPARGC1B	1.038215	1.032027	1.04332511	1.3527681	0.43591456	0.87518
CD164L2	1.039129	1.0329471	1.0442349	1.3426055	0.42503545	0.8752
CCDC154	1.028421	1.022306	1.03347146	1.5005618	0.58550277	0.87525
USP19	1.127608	1.1213003	1.13281765	1.094949	0.13086365	0.87525
CTD-2049O4.1	1.019553	1.0255984	1.01456007	0.5687883	-0.8140362	0.87535
FLJ37035	1.093317	1.0995697	1.08815278	0.8853374	-0.1757008	0.87536
C6orf120	1.422555	1.4156988	1.42821772	1.0301152	0.04280576	0.8754
XXbac-BPG181B23.7	1.023131	1.0170284	1.02817116	1.6543587	0.7262721	0.87541
NOVA1-AS1	1.02315	1.0170649	1.02817495	1.651048	0.72338207	0.87541
PVT1	1.129698	1.1234749	1.1348375	1.0920235	0.12700384	0.87544
IL21R-AS1	1.021293	1.0151549	1.02636197	1.7395066	0.79867817	0.87549
RP11-410E4.1	1.042194	1.0359406	1.04735837	1.317684	0.3980044	0.87551
PLCXD1	1.494611	1.4873537	1.50060494	1.0271903	0.03870347	0.87554
TRIM46	1.11282	1.119132	1.10760815	0.9032681	-0.1467738	0.87561
XX-C2158C12.2	1.049041	1.042848	1.05415487	1.2638821	0.33786185	0.87562
FSIP2-AS1	1.100432	1.094174	1.10560034	1.121332	0.16521355	0.87565
CTDP1	1.100268	1.0939757	1.10546479	1.1222559	0.16640168	0.87565
TSPAN12	1.26425	1.257459	1.26985745	1.0481571	0.067855	0.87571
TRMT13	1.394934	1.4019515	1.38913967	0.968126	-0.0467332	0.87579
NOXA1	1.104382	1.1106369	1.09921731	0.8967834	-0.1571686	0.87586
HOXA5	1.02277	1.0166975	1.02778421	1.6639699	0.73462936	0.87589
LINC00571	1.022113	1.0160429	1.02712518	1.6907932	0.7577002	0.87604
HDLBP	2.764397	2.776045	2.75477862	0.988026	-0.0173791	0.87608
SYCE2	1.061181	1.0673131	1.05611688	0.833669	-0.2624535	0.87616
RP11-15A1.7	1.05846	1.0645942	1.05339395	0.8266055	-0.2747292	0.87617
TBC1D1	1.447408	1.4547118	1.44137604	0.9706721	-0.0429441	0.8762
C7orf26	1.223307	1.216826	1.22865974	1.0545772	0.07666476	0.8762
PRSS27	1.084285	1.0904578	1.07918713	0.8754046	-0.1919781	0.87623

LONP2	1.61345	1.6207418	1.6074288	0.9785531	-0.0312779	0.87624
RP11-936I5.1	1.029782	1.0236622	1.03483651	1.4722455	0.55801827	0.87631
MIATNB	1.029699	1.0235963	1.03473931	1.4722348	0.55800778	0.87631
CORO7	1.14593	1.1523333	1.14064166	0.9232497	-0.1152072	0.87636
PSD3	1.553096	1.5605021	1.54698011	0.9758752	-0.0352315	0.87641
ZNF395	1.380782	1.3876932	1.37507531	0.9674538	-0.0477353	0.87648
RP11-171I2.4	1.02976	1.0235985	1.03484766	1.4766915	0.56236847	0.8765
TRAM1	2.204994	2.2141168	2.19746042	0.9862811	-0.0199292	0.87654
FBXO46	1.14848	1.1548129	1.14324945	0.9253071	-0.1119958	0.87655
LRTM1	1.042	1.0358756	1.04705764	1.3116904	0.3914272	0.87657
HORMAD2	1.020947	1.0148661	1.02596788	1.7467831	0.80470047	0.87661
MEIOB	1.048944	1.055098	1.04386133	0.7960602	-0.3290505	0.87662
SLC25A16	1.132699	1.1390501	1.12745379	0.9166036	-0.1256302	0.87665
B3GNT8	1.09177	1.0979961	1.08662912	0.8840062	-0.1778717	0.87669
TSLP	1.022269	1.0162299	1.02725678	1.6794139	0.74795781	0.8767
RP11-621L6.3	1.037955	1.0319482	1.04291616	1.343306	0.42578799	0.87677
AC021224.1	1.034461	1.0404899	1.0294829	0.7281544	-0.4576838	0.87679
STAMBPL1	1.083286	1.0894032	1.07823491	0.8750796	-0.1925139	0.87681
AKAP10	1.343913	1.3370623	1.34956991	1.0371078	0.05256589	0.87683
NALT1	1.168089	1.174379	1.16289392	0.934137	-0.0982939	0.87683
ZNF461	1.068752	1.0625407	1.07388196	1.1813419	0.24042651	0.87684
NDUFV2-AS1	1.064823	1.0586988	1.06988056	1.1904938	0.25156015	0.87685
WHSC1L1	2.91362	2.925292	2.90398095	0.988931	-0.0160582	0.87688
CACNA1E	1.023062	1.0290769	1.01809491	0.6223115	-0.6842911	0.87691
RAB40AL	1.023123	1.0291974	1.01810696	0.6201573	-0.6892939	0.87691
PTPN21	1.091558	1.0853978	1.09664553	1.1317102	0.17850462	0.87692
RRS1	1.166076	1.1594999	1.17150682	1.0752788	0.10471081	0.87692
RP11-284F21.8	1.022522	1.0285798	1.01751851	0.6129693	-0.7061132	0.87697
PPP1R36	1.022438	1.0284094	1.01750589	0.616201	-0.698527	0.87697
NAAA	1.082422	1.0886502	1.07727947	0.8717353	-0.198038	0.8771
ZNF880	1.084708	1.0909192	1.07957847	0.8752656	-0.1922073	0.87711
ARHGAP26	1.075252	1.0690849	1.08034449	1.1629817	0.21782842	0.87713
DVL2	1.59435	1.6018524	1.58815521	0.9772417	-0.0332127	0.87718
CYFIP2	1.471352	1.4642667	1.47720335	1.0278646	0.03965022	0.87721
GRM2	1.02495	1.0309772	1.01997259	0.6447505	-0.6331871	0.87722
NR2C2	1.278455	1.2851825	1.27289942	0.9569292	-0.063516	0.8773
SLC52A2	1.444572	1.4380515	1.44995751	1.0271794	0.03868818	0.87739
ADRB2	1.03226	1.0261878	1.03727395	1.4233346	0.50927482	0.8774
SMPD4	1.252674	1.2460696	1.25812872	1.0490068	0.06902407	0.8774
FAM178B	1.046598	1.0405307	1.05160822	1.2733107	0.34858454	0.87747
SLC25A48	1.018991	1.0129919	1.02394584	1.8431333	0.88216045	0.87748
KB-431C1.5	1.01902	1.0129919	1.02399749	1.8471086	0.88526872	0.87748
HMGCS2	1.018989	1.0129919	1.02394077	1.8427428	0.88185474	0.87748
PTENP1-AS	1.032706	1.0386397	1.02780496	0.719596	-0.4747409	0.87752
GPR150	1.032674	1.0386477	1.02774006	0.7177667	-0.478413	0.87752

AP001062.7	1.045723	1.0396451	1.05074145	1.279891	0.356021	0.87756
RP4-548D19.3	1.045729	1.0396652	1.05073684	1.2791257	0.35515801	0.87756
SATB2	1.073443	1.0673672	1.07846003	1.1646628	0.21991231	0.87763
PAQR9-AS1	1.031688	1.0257142	1.03662181	1.4241852	0.51013678	0.87765
KDM1B	1.166447	1.1729849	1.16104855	0.9309979	-0.1031502	0.87769
ZNF354B	1.12875	1.1225485	1.13387147	1.0923956	0.1274954	0.87771
ZNF808	1.112086	1.1058904	1.11720236	1.1068268	0.1464295	0.87775
RP11-345J4.5	1.064184	1.0701727	1.05923904	0.8441894	-0.2443614	0.87776
FAM213A	3.874119	3.8936803	3.85796528	0.9876576	-0.0179171	0.8778
CTD-2020K17.3	1.039249	1.0331927	1.04424959	1.3331103	0.41479618	0.87783
CCDC60	1.039265	1.0331659	1.0443009	1.3357365	0.41763541	0.87783
CDK5RAP2	1.371463	1.3781059	1.3659773	0.9679228	-0.0470362	0.87786
AC144450.2	1.027547	1.0214887	1.0325498	1.5147378	0.59906807	0.87788
MICAL1	1.126891	1.120659	1.1320366	1.0942958	0.13000274	0.8779
RP11-406A9.2	1.063457	1.0694321	1.0585227	0.8428771	-0.2466057	0.87791
RP11-77H9.2	1.023234	1.02925	1.01826632	0.6244903	-0.6792489	0.87798
RP11-513M16.8	1.039207	1.0331544	1.04420458	1.3332941	0.41499502	0.87802
POLE	1.146542	1.1402283	1.15175559	1.0822035	0.11397185	0.87811
ZNF799	1.068274	1.0621353	1.07334421	1.1803948	0.23926948	0.87811
CXorf58	1.026082	1.0320525	1.02115215	0.6599224	-0.5996318	0.87812
HIST1H2BG	1.039648	1.0456968	1.03465222	0.7583074	-0.3991453	0.87813
ERCC3	1.18335	1.1897937	1.17802928	0.9380148	-0.0923175	0.87814
ZNF709	1.046299	1.0522661	1.04137195	0.7915641	-0.3372219	0.87816
CARD11	1.020652	1.0146773	1.02558598	1.743233	0.80176539	0.87819
TMC5	1.020652	1.0146773	1.02558629	1.7432536	0.80178249	0.87819
AC144831.3	1.020695	1.0146773	1.02566369	1.748527	0.80614005	0.87819
RHOXF1-AS1	1.020689	1.0146773	1.02565416	1.7478782	0.80560469	0.87819
VIT	1.020654	1.0146773	1.02559017	1.7435181	0.80200138	0.87819
MAP3K3	1.095014	1.0888883	1.1000733	1.1258318	0.17099129	0.8782
TMEM220	1.06615	1.0721992	1.06115527	0.8470357	-0.2395053	0.87823
TAF5L	1.066297	1.0724422	1.06122188	0.845113	-0.2427838	0.87823
LINC01063	1.039519	1.0334053	1.04456749	1.3341435	0.41591388	0.87827
REL	1.414937	1.4079504	1.42070724	1.0312705	0.04442282	0.87828
PICK1	1.185142	1.191444	1.17993753	0.9398962	-0.0894266	0.87831
POLR2F	3.69601	3.7099764	3.68447574	0.9905901	-0.0136399	0.87833
MAP2K4	1.506781	1.5002592	1.51216666	1.0238026	0.03393751	0.87846
PLEKHM1	1.093202	1.086994	1.09832803	1.1302853	0.17668699	0.87846
ATP13A4	1.037534	1.0435239	1.03258802	0.7487386	-0.417466	0.87847
CHM	1.218795	1.2122328	1.22421484	1.056457	0.0792341	0.87859
TMEM50B	2.060562	2.0684447	2.05405268	0.98653	-0.0195652	0.87862
RP11-54O7.2	1.046555	1.0525027	1.04164358	0.7931699	-0.3342981	0.87874
RP11-1055B8.4	1.140443	1.1466813	1.13529138	0.9223491	-0.1166151	0.87874
RP11-392E22.9	1.029232	1.0232245	1.03419326	1.4722946	0.5580664	0.87884
SELENOO	1.068656	1.0625375	1.07370952	1.1786445	0.23712864	0.87888
FBXO34	1.307782	1.3011017	1.31329845	1.0405071	0.05728676	0.87899

RP11-489E7.4	1.100984	1.1071443	1.09589758	0.8950322	-0.1599885	0.87899
PKNOX2	1.148984	1.142771	1.15411454	1.0794529	0.11030027	0.87909
DOHH	1.193383	1.1997555	1.18812138	0.9417582	-0.0865714	0.87913
IL17RB	1.027352	1.0213662	1.03229595	1.5115413	0.5960204	0.87914
GREB1L	1.121724	1.1280428	1.11650496	0.9098906	-0.136235	0.87919
RP11-701H24.9	1.258681	1.2518836	1.26429402	1.0492704	0.0693865	0.8792
SEPT7-AS1	1.041113	1.035111	1.04606926	1.3121047	0.3918828	0.87924
RP1-45N11.1	1.021941	1.0159741	1.02686862	1.6820094	0.75018577	0.87924
PEAR1	1.021912	1.0159741	1.02681555	1.678687	0.74733329	0.87924
RP11-20E24.1	1.025131	1.0310774	1.02022015	0.6506383	-0.6200723	0.87927
TMEM38A	1.118537	1.1247839	1.1133779	0.9085939	-0.1382925	0.87927
ENTPD8	1.025786	1.0318143	1.02080815	0.6540506	-0.6125258	0.8793
RP11-528I4.2	1.034935	1.0408054	1.03008749	0.7373417	-0.4395947	0.87933
TRPC1	1.194655	1.2009854	1.18942745	0.9424938	-0.085445	0.87936
CNTNAP3B	1.291633	1.2984121	1.28603386	0.9585198	-0.0611199	0.87938
HOXC5	1.024045	1.0180243	1.02901678	1.6098728	0.68694674	0.87942
KCNJ15	1.023943	1.0180243	1.02883043	1.5995335	0.67765121	0.87942
HCAR2	1.021893	1.0159362	1.02681299	1.6825156	0.75061989	0.87943
NCRNA00250	1.027485	1.0334094	1.02259188	0.6762134	-0.5644495	0.87956
IBA57	1.112182	1.1184305	1.10702171	0.9036665	-0.1461376	0.87958
USP49	1.184007	1.1901723	1.17891566	0.940808	-0.0880278	0.87962
POU3F2	1.017816	1.0236691	1.01298307	0.5485238	-0.8663739	0.87962
RP11-227H15.5	1.022206	1.0162589	1.02711807	1.6678937	0.73802731	0.87968
RP11-1055B8.3	1.022206	1.0162589	1.02711771	1.667872	0.73800859	0.87968
CDC45	1.108926	1.1148665	1.10402084	0.90558	-0.143086	0.87969
RP11-468E2.5	1.03023	1.0242361	1.03518038	1.4515666	0.5376108	0.8797
GHDC	1.082644	1.0887116	1.07763266	0.8751125	-0.1924596	0.87971
AC010287.1	1.021626	1.0157004	1.02651948	1.6890935	0.75624921	0.87971
LINC01562	1.021618	1.0157004	1.02650489	1.6881642	0.75545522	0.87971
MMRN2	1.021632	1.0157004	1.02653118	1.6898389	0.75688569	0.87971
TAC1	1.024017	1.0181035	1.02890132	1.5964476	0.67486517	0.87974
COX19	1.520774	1.5279009	1.51488803	0.9753497	-0.0360085	0.87975
RP11-379F4.4	1.036006	1.0299954	1.04096942	1.3658577	0.44980715	0.87976
TTI2	1.164179	1.1577371	1.16949812	1.0745607	0.10374701	0.87979
ZBTB22	1.188029	1.1818747	1.19311118	1.0617814	0.08648679	0.8798
PNMT	1.048588	1.0425327	1.05358847	1.2599345	0.33334874	0.87982
C3orf35	1.024851	1.0189188	1.02975012	1.5725125	0.65307152	0.87987
RP11-250B2.3	1.028321	1.0342051	1.02346159	0.6859082	-0.5439127	0.88
MTRF1L	1.567723	1.5607507	1.57348076	1.0227019	0.03238564	0.88003
ATP7A	1.235562	1.2420123	1.23023584	0.9513396	-0.0719677	0.88011
SLC10A3	1.169723	1.1635648	1.17480774	1.0687369	0.09590675	0.88018
RP1-76B20.11	1.016981	1.0228456	1.0121375	0.5312843	-0.9124441	0.88018
TMEM267	1.195702	1.2020797	1.19043577	0.9423797	-0.0856196	0.88023
RP11-553A21.3	1.02452	1.0186106	1.02939928	1.5797081	0.659658	0.88036
LA16c-358B7.3	1.024519	1.0185977	1.02940971	1.5813605	0.66116627	0.88036

PTK6	1.017997	1.0238598	1.01315569	0.5513751	-0.858894	0.8804
RP4-758J18.10	1.017983	1.0238295	1.01315569	0.5520757	-0.8570621	0.8804
AP001462.6	1.099944	1.1061779	1.09479611	0.8928043	-0.1635841	0.8804
ZFP37	1.101941	1.1079569	1.09697257	0.898253	-0.1548063	0.88041
RP11-506K6.4	1.01885	1.0247187	1.01400275	0.5664831	-0.8198952	0.88045
LINC00882	1.090967	1.0968987	1.08606911	0.8882377	-0.1709822	0.88047
ACE	1.023856	1.017967	1.02871886	1.5984182	0.67664494	0.88052
MACROD2	1.092483	1.0863649	1.09753528	1.1293398	0.1754797	0.88062
HAUS5	1.267973	1.2747217	1.26240055	0.9551505	-0.0662	0.88072
RP11-16E18.3	1.030819	1.0366913	1.02597002	0.7077973	-0.4985918	0.88074
VWF	1.02409	1.0299032	1.01928886	0.6450432	-0.6325323	0.8808
FJX1	1.348564	1.3418404	1.35411664	1.0359121	0.05090153	0.88082
EPHX1	1.25831	1.2645229	1.2531798	0.9571188	-0.0632301	0.88088
RP11-513G11.4	1.080522	1.0863637	1.07569858	0.8765094	-0.1901585	0.88096
SPECC1L	1.221405	1.227587	1.21629906	0.9504016	-0.0733909	0.88098
LMO7-AS1	1.018745	1.012846	1.02361627	1.8384092	0.8784579	0.88115
ACVR1C	1.02401	1.0181327	1.02886322	1.591778	0.67063912	0.88118
RP11-329B9.3	1.024035	1.0181233	1.02891682	1.5955586	0.67406162	0.88118
RP11-368I23.2	1.053008	1.0589956	1.0480631	0.8146898	-0.2956773	0.88118
UPK3A	1.031683	1.0258688	1.03648478	1.4103756	0.49607941	0.8812
SYDE2	1.147016	1.1408364	1.15211995	1.0801178	0.11118864	0.88121
KLHDC1	1.070488	1.0644884	1.0754422	1.1698574	0.2263327	0.88121
RP11-382D12.2	1.05423	1.0601051	1.04937761	0.8215207	-0.2836311	0.88121
CTDSP1	1.597009	1.6037434	1.59144752	0.9796338	-0.0296855	0.88121
SPATA12	1.066465	1.0604364	1.07144391	1.1821336	0.24139308	0.88123
AC004156.3	1.020735	1.0265406	1.01593993	0.6005868	-0.7355554	0.88125
SLC1A4	1.082897	1.0886889	1.07811441	0.8807684	-0.1831653	0.88126
SMAP2	1.195059	1.1888313	1.20020147	1.0602133	0.08435458	0.8814
IQUB	1.05685	1.062724	1.05199848	0.8290052	-0.270547	0.88143
ZBTB34	1.076913	1.0708881	1.08188863	1.1551808	0.20811862	0.88149
RP11-158K1.3	1.022497	1.0283651	1.01765111	0.6222819	-0.6843599	0.8815
STAM2	1.127107	1.1210625	1.13209899	1.0911639	0.12586776	0.88157
BAIAP2L1	1.179006	1.1728923	1.18405529	1.0645663	0.09026582	0.88158
STX1B	1.06025	1.0543169	1.06515008	1.1994439	0.26236572	0.88159
NCKIPSD	1.19703	1.1909018	1.2020911	1.0586131	0.08217539	0.88162
ZNF30	1.073133	1.0789902	1.06829567	0.8646098	-0.2098789	0.88167
NAV1	1.766514	1.7743826	1.76001672	0.9814486	-0.0270154	0.88169
PPP1R26-AS1	1.044753	1.0506162	1.03991024	0.7884873	-0.3428405	0.88172
LRRC7	1.044698	1.0505858	1.03983536	0.7874809	-0.3446831	0.88172
ACE2	1.021496	1.0156039	1.02636122	1.6893994	0.75651044	0.88176
IL1R1	1.021434	1.0156039	1.02624792	1.6821379	0.750296	0.88176
VIP	1.021415	1.0156039	1.02621467	1.680007	0.74846727	0.88176
BAALC-AS2	1.030977	1.0368225	1.02614947	0.7101499	-0.4938046	0.88178
GSDMB	1.061011	1.0550174	1.06595985	1.1988912	0.26170078	0.88181
NAALADL2	1.086909	1.0808779	1.09189006	1.1361585	0.18416414	0.88182

DNAJC9-AS1	1.034111	1.0283466	1.03887106	1.3712764	0.45551936	0.88184
GNB5	1.397867	1.4048797	1.39207612	0.9683769	-0.0463594	0.88194
TAF3	1.260311	1.2665977	1.25511874	0.9569428	-0.0634955	0.88195
IRX6	1.019671	1.0137737	1.02454158	1.7817746	0.83331487	0.88206
APOBEC3A	1.019613	1.0137737	1.02443489	1.7740289	0.8270295	0.88206
NHS	1.127245	1.1330662	1.12243791	0.9201279	-0.1200936	0.88211
BDNF-AS	1.140419	1.1344567	1.14534191	1.0809572	0.11230943	0.88223
HPSE	1.021512	1.0273382	1.0166999	0.6108632	-0.7110789	0.88226
TMEM163	1.045292	1.0512545	1.04036899	0.787619	-0.3444302	0.88231
TMEM19	1.2669	1.2730514	1.26182089	0.9588703	-0.0605924	0.88237
ZNF846	1.09993	1.0939003	1.1049086	1.1172339	0.1599313	0.8824
C2orf27B	1.069923	1.0639948	1.07481856	1.1691347	0.22544122	0.88249
CLEC2D	1.309104	1.3023567	1.31467678	1.040747	0.05761942	0.8825
RP3-428L16.2	1.123774	1.1178303	1.12868262	1.0921017	0.12710716	0.88252
PRKRIP1	1.419905	1.4264561	1.41449582	0.9719543	-0.0410396	0.88254
RP11-277B15.3	1.032147	1.0263232	1.03695621	1.4039423	0.48948369	0.88256
ZNF264	1.201546	1.2079523	1.19625526	0.9437513	-0.0835214	0.88259
C3orf58	1.318029	1.3116149	1.32332674	1.0375843	0.05322857	0.8826
ADAMTS5	1.02238	1.0165756	1.02717303	1.6393406	0.71311564	0.88265
CTC-444N24.6	1.042414	1.0482796	1.03756933	0.7781609	-0.3618596	0.88267
KCNJ2-AS1	1.049178	1.0433758	1.05396999	1.2442412	0.31526615	0.88268
ARC	1.028319	1.0225053	1.03311951	1.4716348	0.55741974	0.88268
AC062029.1	1.076114	1.0700826	1.08109498	1.1571343	0.21055636	0.88268
TMEM9B	1.540425	1.5335984	1.54606177	1.0233572	0.03330987	0.8827
RP11-449J21.5	1.043039	1.0489056	1.03819338	0.7809608	-0.3566779	0.8827
EAF2	1.094684	1.1004879	1.08989182	0.8945533	-0.1607606	0.88274
AC005224.2	1.025774	1.0315685	1.02098902	0.6648726	-0.5888501	0.88274
LMTK2	1.054981	1.0491044	1.05983359	1.2184966	0.2851022	0.88278
RP11-767I20.1	1.017117	1.0113227	1.02190251	1.9343834	0.95187379	0.88279
AGR3	1.017105	1.0113227	1.02187979	1.9323771	0.95037666	0.88279
RP11-21A7A.4	1.017129	1.0113227	1.02192305	1.9361975	0.9532261	0.88279
LINC00936	1.099834	1.1057756	1.09492715	0.8974388	-0.1561146	0.88284
UNC13A	1.02835	1.0341327	1.02357512	0.6906908	-0.533888	0.88284
UFSP1	1.0284	1.0341669	1.02363845	0.6918515	-0.5314657	0.88284
LLNLR-304G9.1	1.028375	1.0341679	1.0235913	0.6904525	-0.5343859	0.88284
RAPGEF4	1.055039	1.0608822	1.05021381	0.8247697	-0.2779367	0.88287
CASP8	1.055016	1.0607565	1.0502748	0.8274803	-0.2732031	0.88287
PPP1R1B	1.025348	1.0311134	1.02058759	0.661696	-0.5957595	0.88301
LINC01018	1.025306	1.0310503	1.02056195	0.6622149	-0.5946286	0.88301
DNAJC11	1.162256	1.1682613	1.15729608	0.9348324	-0.0972204	0.88302
RFTN2	1.162765	1.1688317	1.15775447	0.9343888	-0.0979051	0.88302
KCNA7	1.026246	1.0320437	1.02145869	0.6696691	-0.5784797	0.8831
SP2-AS1	1.033441	1.0391305	1.02874251	0.73453	-0.4451066	0.88313
AFAP1L2	1.042687	1.0485057	1.03788244	0.7809888	-0.3566262	0.88314
ALDH1L2	1.04472	1.0505388	1.03991562	0.7898018	-0.3404374	0.88317

MOSPD1	1.260166	1.2537428	1.26547011	1.0462174	0.06518267	0.88322
ZNF507	1.213345	1.2196867	1.20810813	0.947295	-0.0781144	0.88324
CECR1	1.330956	1.3378443	1.32526711	0.9627724	-0.0547333	0.88329
TMEM133	1.056925	1.0510781	1.06175425	1.2090153	0.27383247	0.88343
CTA-246H3.12	1.030273	1.024443	1.035087	1.435463	0.52151618	0.88344
CTD-2619J13.3	1.023434	1.0292524	1.01862865	0.6368237	-0.6510342	0.88353
CHST8	1.023417	1.0292333	1.01861432	0.6367508	-0.6511993	0.88353
RP11-168E17.1	1.023348	1.0290626	1.01862919	0.6410011	-0.6416013	0.88353
RP3-466P17.2	1.023933	1.0296814	1.01918675	0.6464235	-0.6294484	0.88357
RP11-22A3.1	1.023905	1.0296263	1.01917944	0.6473786	-0.6273184	0.88357
CTD-2168K21.2	1.023945	1.0297319	1.0191667	0.644651	-0.6334098	0.88357
CASKIN1	1.055271	1.0493874	1.06013011	1.2175195	0.28394488	0.88357
ZNF346	1.25038	1.2439882	1.25565831	1.0478305	0.06740537	0.8836
YOD1	1.165077	1.1590349	1.17006732	1.0693708	0.0967622	0.88361
NBPF14	1.276958	1.2704266	1.28235137	1.0440963	0.06225476	0.88363
TXNDC5	1.035164	1.0408741	1.03044908	0.7449475	-0.4247893	0.88367
RP11-61A14.4	1.023731	1.0178847	1.02855908	1.5968407	0.67522041	0.88371
PDCD4-AS1	1.119614	1.1254529	1.11479209	0.9150217	-0.1281222	0.88377
MMP3	1.023646	1.0294114	1.01888504	0.6420985	-0.6391334	0.88381
RP11-367N14.3	1.025688	1.031388	1.02098068	0.66843	-0.5811516	0.88383
FAM53A	1.023639	1.0178402	1.02842757	1.5934594	0.67216227	0.8839
AC003075.4	1.041669	1.0358969	1.0464361	1.2935972	0.37138841	0.88403
VLDLR-AS1	1.112509	1.106637	1.11735825	1.1005397	0.13821114	0.88409
ASTN2	1.098749	1.1045935	1.0939226	0.8979773	-0.1552491	0.8841
SEMA3F-AS1	1.023377	1.0176058	1.02814358	1.5985358	0.67675101	0.88419
MYO7A	1.023332	1.0175952	1.02807002	1.5953197	0.67384553	0.88419
RP11-16P6.1	1.023387	1.0176	1.02816521	1.6002921	0.67833529	0.88419
POLN	1.050859	1.0452281	1.0555082	1.2272951	0.29548222	0.88419
RP11-167N5.5	1.018867	1.0131484	1.02359007	1.7941399	0.84329236	0.8842
CTD-2555C10.3	1.01887	1.0131484	1.02359577	1.7945732	0.84364075	0.8842
FHDC1	1.025732	1.0199791	1.03048307	1.5257516	0.6095201	0.88421
SLC7A7	1.025705	1.0199563	1.03045221	1.5259478	0.60970557	0.88421
PRKAG2-AS1	1.104478	1.0985289	1.10939006	1.1102327	0.15086215	0.88422
CDR2L	1.130676	1.1247889	1.13553814	1.0861391	0.11920892	0.88425
GDF5OS	1.026568	1.0207949	1.03133641	1.5069288	0.59161126	0.88433
PLEKHM3	1.174017	1.1800259	1.16905409	0.9390544	-0.0907193	0.88433
LCAT	1.042931	1.0371824	1.04767799	1.2822746	0.35870519	0.88434
RP11-374M1.5	1.038623	1.0443382	1.03390317	0.7646499	-0.3871287	0.88453
TMPRSS5	1.038022	1.0436942	1.03333711	0.7629636	-0.3903138	0.88453
MYO15A	1.071083	1.0768561	1.06631557	0.8628536	-0.2128122	0.88466
NFAM1	1.017116	1.0227831	1.01243594	0.5458415	-0.873446	0.88473
DLG3-AS1	1.017115	1.0227808	1.01243594	0.5458962	-0.8733013	0.88473
RP11-545M17.1	1.017118	1.0227885	1.01243594	0.5457114	-0.8737898	0.88473
LINC01209	1.026276	1.0205093	1.03103895	1.513409	0.59780197	0.88482
RUNDC3A-AS1	1.026265	1.0205249	1.0310061	1.5106616	0.59518048	0.88482

RP11-776H12.1	1.026238	1.0205082	1.03097054	1.5101543	0.59469597	0.88482
RP1-34B20.21	1.026286	1.0205041	1.03106133	1.5148841	0.59920738	0.88482
RP11-354P11.3	1.093313	1.0874552	1.09814997	1.1222885	0.16644353	0.88482
RP11-1072A3.4	1.04048	1.0462449	1.03571881	0.7723843	-0.3726093	0.88484
TTC23L	1.040404	1.0460608	1.03573316	0.7757829	-0.3662751	0.88484
GAREM2	1.078163	1.0722948	1.08300923	1.1482041	0.19937912	0.88487
IZUMO4	1.061974	1.0676803	1.05726174	0.8460626	-0.2411637	0.88491
PDE11A	1.088657	1.0944619	1.08386265	0.8877936	-0.1717038	0.88496
TP53TG1	2.615933	2.6072369	2.62311446	1.0098788	0.01418218	0.88496
LRRC3B	1.060812	1.0665289	1.05609107	0.8431081	-0.2462104	0.88498
MELTF	1.025558	1.0198552	1.03026773	1.5244229	0.6082632	0.88499
HPD	1.025616	1.0198914	1.0303426	1.5254111	0.60919809	0.88499
SLC27A2	1.029035	1.0346886	1.02436567	0.7024122	-0.5096101	0.88499
NSUN7	1.209863	1.2158227	1.20494162	0.9495834	-0.0746334	0.885
MAN1A1	1.946786	1.9386609	1.95349678	1.0158054	0.02262404	0.88501
CTD-3193O13.12	1.048315	1.0540154	1.0436075	0.8073168	-0.3087931	0.88505
HCG23	1.045518	1.0512929	1.04074928	0.7944429	-0.3319846	0.88506
CTD-2630F21.1	1.018738	1.013034	1.02344933	1.7990927	0.84726952	0.88525
TTC36	1.018717	1.013034	1.02341044	1.7961091	0.84487502	0.88525
SGCZ	1.018797	1.013034	1.02355623	1.8072943	0.85383149	0.88525
RP11-148B18.1	1.018692	1.013034	1.02336489	1.7926139	0.84206481	0.88525
NYX	1.021477	1.0157693	1.02619023	1.6608325	0.73190662	0.88526
HPCA	1.116031	1.1101056	1.12092374	1.098252	0.13520909	0.88538
MEX3A	2.155563	2.1477538	2.1620123	1.0124229	0.0178121	0.8854
PNPLA3	1.086946	1.0812003	1.09169035	1.1291875	0.17528512	0.88541
KRTAP10-2	1.01968	1.0254197	1.01494071	0.5877614	-0.7666974	0.88546
UPB1	1.024386	1.018672	1.02910398	1.5586969	0.6403404	0.88554
KLRD1	1.038747	1.044469	1.0340214	0.7650585	-0.3863579	0.88555
TMCO3	1.894456	1.9020799	1.88816074	0.9845699	-0.0224345	0.88556
RP11-795F19.5	1.069224	1.0749518	1.06449392	0.8604721	-0.2167996	0.88556
ZNF569	1.167864	1.1619846	1.17271912	1.0662686	0.09257091	0.88556
WDR44	1.166965	1.1611657	1.17175427	1.0657002	0.09180168	0.8856
TMEM229B	1.020474	1.0147486	1.02520195	1.708774	0.77296162	0.88563
RP11-379K17.12	1.020421	1.0147444	1.02510952	1.7029825	0.7680636	0.88563
AC079354.3	1.020429	1.014744	1.02512362	1.7039923	0.7689188	0.88563
SPACA3	1.055174	1.0608294	1.05050306	0.8302417	-0.2683968	0.88564
SLC9B2	1.246906	1.2530439	1.24183657	0.9557099	-0.0653554	0.88566
CTD-2541J13.1	1.086611	1.0809082	1.09132022	1.1286888	0.1746478	0.8857
RP11-108M9.3	1.021448	1.0270819	1.01679584	0.6201881	-0.6892222	0.88576
RP11-339B21.13	1.041645	1.0473685	1.03691802	0.7793797	-0.3596017	0.88581
ZNF445	1.3989	1.3925391	1.40415273	1.0295859	0.04206414	0.88582
CH17-340M24.3	1.264769	1.2710663	1.25956797	0.9575809	-0.0625336	0.88596
RP11-244M2.1	1.159174	1.1532859	1.16403615	1.0701322	0.09778905	0.88596
STX1A	1.129683	1.123815	1.13452812	1.0865255	0.11972209	0.88614
RAB12	1.061005	1.0669342	1.05610802	0.8382567	-0.2545361	0.88615

PLBD2	1.116569	1.1223548	1.11179098	0.9136622	-0.1302672	0.8862
SYN3	1.023158	1.0175144	1.02781823	1.5883101	0.66749261	0.88624
TRIM29	1.023167	1.0175001	1.02784674	1.5912365	0.67014827	0.88624
RP11-796E10.1	1.023197	1.0175028	1.02789899	1.5939718	0.67262613	0.88624
CLDN8	1.020741	1.0150885	1.02540922	1.6840174	0.75190702	0.88627
RP11-1069G10.1	1.02078	1.0150885	1.02548007	1.6887131	0.75592423	0.88627
PITPNM3	1.05991	1.0655554	1.05524758	0.8427616	-0.2468034	0.88629
EIF2AK3	1.103625	1.0977274	1.10849442	1.1101739	0.1507857	0.88639
HPS6	1.143902	1.1496942	1.13911865	0.9293522	-0.1057026	0.8864
OTUB2	1.084372	1.0900955	1.07964484	0.8840048	-0.177874	0.8864
RP11-2B6.2	1.022426	1.0167889	1.02708054	1.6130051	0.68975097	0.88641
CCNT2-AS1	1.043554	1.0492911	1.03881683	0.7875015	-0.3446455	0.88653
MTR	1.481836	1.4749626	1.4875127	1.0264232	0.03762575	0.88664
KCNMB1	1.033053	1.0386939	1.02839419	0.7338159	-0.44651	0.88676
KLHL11	1.028267	1.0225415	1.03299535	1.4637582	0.54967723	0.8868
OXER1	1.030994	1.0252933	1.03570209	1.411521	0.4972506	0.88681
USP2	1.044819	1.0390754	1.0495625	1.2683823	0.34298961	0.88687
AC009228.1	1.04268	1.0369254	1.04743297	1.2845618	0.36127634	0.88689
NPPC	1.034793	1.0291176	1.03947897	1.3558454	0.43919268	0.88695
RP11-486O12.2	1.077836	1.0720482	1.08261625	1.1466802	0.19746305	0.88703
CRLF3	1.123463	1.1177738	1.12816134	1.0881992	0.12194272	0.88706
RNF187	1.373887	1.3679057	1.3788263	1.0296832	0.04220052	0.88707
NEK11	1.084609	1.0788154	1.0893932	1.1342099	0.18168762	0.88708
RP11-801F7.1	1.084703	1.0789174	1.08948156	1.1338639	0.18124749	0.88708
AP003068.23	1.017968	1.0123699	1.02259025	1.8262306	0.86886894	0.88712
IGF1	1.068269	1.0739803	1.06355216	0.8590418	-0.2191998	0.88713
B4GALT2	1.301251	1.2951188	1.3063144	1.0379361	0.05371759	0.88718
RP13-516M14.1	1.041018	1.0467961	1.03624615	0.7745557	-0.3685591	0.88719
CCDC68	1.035222	1.0296015	1.03986399	1.3466869	0.42941443	0.88722
SYTL5	1.035201	1.0295445	1.03987231	1.3495666	0.43249618	0.88722
BEAN1	1.018829	1.0132316	1.02345087	1.7723332	0.82564989	0.88727
NPAP1	1.018823	1.0132224	1.02344885	1.7734119	0.82652762	0.88727
GRAMD2	1.018847	1.0132144	1.02349844	1.778242	0.83045168	0.88727
SOHLH2	1.023594	1.029136	1.01901776	0.6527236	-0.615456	0.88729
EML5	1.016996	1.0113663	1.02164497	1.9043087	0.9292674	0.88734
DLGAP1-AS3	1.017003	1.0113663	1.02165833	1.9054835	0.93015709	0.88734
HOXA-AS2	1.016968	1.0113663	1.02159397	1.8998219	0.92586418	0.88734
TMEM100	1.100762	1.1064391	1.09607335	0.9026137	-0.1478194	0.88736
RP5-1112D6.8	1.064674	1.0588842	1.06945609	1.1795374	0.23822113	0.88738
RP11-456H18.2	1.209171	1.2152648	1.20413891	0.9483152	-0.0765614	0.88745
AC078883.3	1.098993	1.093196	1.10378045	1.1135719	0.15519472	0.88746
GRK3	1.175162	1.1810544	1.17029637	0.9405812	-0.0883756	0.88747
NR1H3	1.115652	1.1214753	1.11084331	0.9124764	-0.1321409	0.88749
ZNF227	1.180571	1.1864234	1.17573857	0.9426853	-0.0851519	0.88758
SPEF2	1.171764	1.1655274	1.17691482	1.068795	0.09598521	0.88762

ACSS1	1.067268	1.0729026	1.06261548	0.8588927	-0.2194501	0.88765
HLA-E	1.777905	1.7709106	1.78368118	1.0165656	0.02370337	0.88769
LPAR2	1.225454	1.2312561	1.22066344	0.9541952	-0.0676437	0.88771
SLC30A10	1.03272	1.0270505	1.0374011	1.3826394	0.46742492	0.88775
STX18-AS1	1.058761	1.053059	1.06346908	1.1961993	0.25845774	0.88781
MYLIP	1.113179	1.1073475	1.11799437	1.0991815	0.13642969	0.88782
RP11-1H15.2	1.022262	1.0279036	1.01760343	0.6308663	-0.6645938	0.88784
CTD-3193K9.4	1.146543	1.1522681	1.14181558	0.9313547	-0.1025973	0.88785
RABL6	1.692217	1.6991062	1.68652759	0.9820076	-0.0261939	0.88787
AP000254.8	1.031893	1.0262926	1.03651789	1.3889024	0.47394518	0.88788
RP13-582O9.7	1.087499	1.0931481	1.08283329	0.8892649	-0.1693149	0.88788
RP11-205M3.3	1.017841	1.0122555	1.02245282	1.8320641	0.87346995	0.8879
ADAT2	1.141786	1.1474913	1.13707409	0.9293705	-0.1056743	0.88798
THEMIS2	1.049249	1.0549198	1.04456553	0.8114662	-0.3013971	0.88801
TSTD3	1.150156	1.1561198	1.14523102	0.9302535	-0.1043043	0.88803
USP35	1.034348	1.039895	1.02976757	0.746147	-0.4224682	0.88804
DBNDD1	1.191496	1.1857291	1.19625839	1.0566918	0.07955471	0.88808
DNAJC16	1.205226	1.1992998	1.21011924	1.054287	0.07626772	0.88808
AC022182.1	1.021947	1.0275681	1.01730453	0.6277018	-0.6718486	0.88809
DECR2	1.171272	1.1769802	1.16655789	0.9411101	-0.0875645	0.88809
LIPH	1.023982	1.0295403	1.0193919	0.6564553	-0.6072313	0.8881
ZNF436	1.217281	1.2231461	1.21243738	0.9520101	-0.0709512	0.88816
RTTN	1.105788	1.1115109	1.10106134	0.9062915	-0.141953	0.8882
OLIG2	1.023009	1.0285351	1.01844497	0.6463951	-0.6295119	0.88824
KCNC2	1.021961	1.0275396	1.01735489	0.6301793	-0.6661656	0.88828
CTD-2555O16.2	1.021984	1.0275895	1.01735489	0.6290385	-0.6687797	0.88828
MISP	1.017544	1.0120052	1.0221176	1.8423416	0.88154062	0.88828
PRR15L	1.01761	1.0120052	1.02223925	1.852475	0.8894541	0.88828
TCTE1	1.017614	1.0120052	1.02224584	1.853024	0.88988159	0.88828
NKX3-1	1.01755	1.0120052	1.02212922	1.8433097	0.88229847	0.88828
CAPNS2	1.017628	1.0120052	1.02227086	1.8551085	0.89150355	0.88828
TRIML2	1.017546	1.0120052	1.02212206	1.8427139	0.8818321	0.88828
CTC-529P8.1	1.017574	1.0120052	1.02217287	1.8469461	0.88514177	0.88828
RIMBP2	1.052192	1.0465818	1.05682508	1.2198998	0.28676269	0.88835
CTD-2007H13.3	1.027417	1.0218386	1.03202351	1.4663689	0.55224808	0.88836
RP5-827C21.6	1.027464	1.0218384	1.03210947	1.4703236	0.55613375	0.88836
COL11A2	1.025315	1.0197281	1.02992867	1.5170566	0.60127495	0.88837
SLC25A46	1.398576	1.4049119	1.39334334	0.9714293	-0.041819	0.88844
SLC36A1	1.043401	1.0376793	1.04812535	1.2772347	0.3530237	0.88845
GLDC	1.097974	1.0921575	1.10277743	1.1152365	0.15734965	0.88846
MAP3K19	1.017973	1.0124282	1.02255282	1.8146559	0.85969602	0.88856
CNGA1	1.017975	1.0124282	1.022555	1.814831	0.85983522	0.88856
LINC00702	1.017962	1.0124282	1.02253119	1.8129155	0.85831165	0.88856
CSTA	1.017974	1.0124282	1.02255318	1.8146849	0.85971908	0.88856
RP11-491F9.1	1.01797	1.0124282	1.02254626	1.8141278	0.85927607	0.88856

RP11-305L7.1	1.017998	1.0124282	1.02259682	1.8181961	0.86250778	0.88856
LSR	1.515063	1.5084445	1.52052901	1.0237677	0.0338884	0.88857
RP11-473A10.2	1.02571	1.0200706	1.03036712	1.5130133	0.59742469	0.88862
CNDP1	1.025653	1.0200533	1.030278	1.5098796	0.59443354	0.88862
RP11-503P10.1	1.025658	1.0201042	1.03024415	1.5043691	0.58915856	0.88862
HEY2	1.025684	1.0200615	1.03032751	1.5117287	0.59619929	0.88862
ABHD3	1.120887	1.114992	1.12575525	1.0936	0.12908517	0.88865
SLX1B	1.025068	1.0195038	1.02966331	1.5209012	0.60492641	0.88865
RP11-219A15.1	1.020586	1.0150463	1.02516074	1.6722165	0.74176163	0.88868
PRMT7	1.306383	1.3003924	1.31132952	1.0364096	0.05159431	0.88874
GOSR2	1.483534	1.4901735	1.47805165	0.9752702	-0.0361261	0.88875
AC009120.10	1.036869	1.0423565	1.03233688	0.7634464	-0.3894013	0.88878
MATN4	1.056063	1.0616139	1.05147892	0.8355079	-0.2592746	0.88878
SLC25A23	1.633012	1.6266891	1.63823275	1.0184201	0.02633278	0.88879
CCNT2	1.476674	1.4699673	1.4822126	1.0260557	0.03710908	0.88887
IQSEC3	1.026417	1.0208665	1.03100086	1.485673	0.5711166	0.8889
ZNF623	1.114056	1.1198024	1.10931068	0.912425	-0.1322222	0.88905
TIGD6	1.114075	1.1198132	1.10933605	0.9125546	-0.1320172	0.88905
AQP5	1.088927	1.0945183	1.08431025	0.891999	-0.164886	0.88913
FOXI3	1.05479	1.0490353	1.05954294	1.2142861	0.28010834	0.88915
LINC01410	1.069623	1.0751697	1.06504185	0.8652665	-0.2087835	0.88925
KB-1732A1.1	1.027964	1.0224575	1.03251145	1.4476869	0.53374958	0.88928
RP4-622L5.7	1.039823	1.0342182	1.0444509	1.299042	0.37744807	0.88936
TMED2	4.23287	4.2503977	4.21839448	0.9901541	-0.0142751	0.8894
ARID5A	1.2266	1.2326345	1.22161705	0.9526404	-0.0699963	0.88945
SARM1	1.160053	1.1542532	1.16484264	1.0686499	0.0957893	0.88956
TMEM52	1.054005	1.0484257	1.05861234	1.210357	0.2754326	0.88964
RP11-271F18.4	1.054036	1.048393	1.05869571	1.2128956	0.27845535	0.88964
EP400	1.475528	1.4690435	1.48088208	1.0252397	0.0359613	0.88965
RP11-244H3.4	1.020467	1.0149371	1.02503299	1.6758982	0.74493452	0.88973
MGC45922	1.020476	1.0149297	1.02505598	1.6782668	0.7469721	0.88973
RP11-890B15.2	1.020452	1.0149403	1.02500279	1.673515	0.74288144	0.88973
ELSPBP1	1.023184	1.0176665	1.02774121	1.5702731	0.65101548	0.88974
RP11-490B18.6	1.023231	1.0176782	1.02781589	1.573458	0.65393866	0.88974
MFN2	1.235298	1.2413902	1.23026723	0.9539211	-0.0680582	0.8898
RP11-214O1.3	1.027024	1.0215099	1.03157676	1.4680093	0.55386108	0.88985
PIH1D2	1.108485	1.1140042	1.10392803	0.9116158	-0.1335022	0.88989
MAPK6	2.036109	2.0439083	2.0296679	0.9863585	-0.0198159	0.8899
PRIMPOL	1.228223	1.2340605	1.22340147	0.9544605	-0.0672426	0.89001
ANKRD54	1.393836	1.3875204	1.39905111	1.0297552	0.04230142	0.89001
MIR503HG	1.103799	1.0981244	1.10848559	1.1055923	0.14481948	0.89003
ARHGAP6	1.043058	1.0375411	1.04761412	1.2683208	0.34291967	0.89005
ENPP1	1.04316	1.0376626	1.04769991	1.266507	0.3408551	0.89005
TCERG1L-AS1	1.027421	1.0219506	1.03193916	1.4550478	0.54106658	0.89012
FAM69B	1.340663	1.3466261	1.33573779	0.9685877	-0.0460454	0.89014

CTD-3074O7.5	1.1247	1.1303631	1.12002406	0.9206903	-0.1192122	0.89017
ACBD7	1.026487	1.0319856	1.021946	0.6861217	-0.5434637	0.89017
LINC01320	1.026478	1.0319163	1.02198765	0.6889158	-0.5376004	0.89017
RP11-87C12.5	1.036998	1.031397	1.04162302	1.3257015	0.40675592	0.89022
AGAP6	1.070482	1.0760531	1.06588059	0.8662441	-0.2071545	0.8903
LMOD3	1.050601	1.0560921	1.046067	0.8212746	-0.2840635	0.89033
RP13-20L14.6	1.024866	1.0303598	1.0203298	0.6696283	-0.5785676	0.89033
RP1-234P15.4	1.024758	1.0301919	1.02027065	0.6713945	-0.5747673	0.89033
TMEM221	1.051389	1.0568916	1.04684527	0.8234127	-0.2803125	0.89034
IGFBP2	8.085889	8.0614259	8.10609177	1.0063253	0.00909679	0.8904
MAOA	1.040817	1.0462892	1.03629738	0.7841439	-0.3508096	0.89048
AC010226.4	1.060509	1.0661868	1.05582043	0.8433766	-0.2457511	0.89051
FBXL4	1.263842	1.2577918	1.26883798	1.0428492	0.06053061	0.8906
ZNF133	1.078587	1.0730153	1.08318765	1.1393183	0.18817081	0.89071
CRYGC	1.05155	1.0460687	1.05607679	1.2172439	0.28361825	0.89072
ING1	1.257538	1.2633757	1.25271692	0.95953	-0.0596002	0.89085
WBP2	1.689551	1.6829452	1.69500576	1.0176596	0.02525508	0.89086
SLC13A3	1.084769	1.0902517	1.08024108	0.8890807	-0.1696137	0.89087
RP11-370I10.6	1.021018	1.0265553	1.01644507	0.6192767	-0.6913439	0.89092
RNF122	1.072734	1.0783614	1.06808766	0.868893	-0.2027495	0.89092
LINC00853	1.050806	1.0562988	1.04627078	0.8218791	-0.2830019	0.89098
SCN7A	1.036871	1.0313341	1.04144428	1.3226559	0.40343779	0.891
CHUK	1.103323	1.0976545	1.10800419	1.1059829	0.14532913	0.891
ISLR	1.023086	1.0176013	1.02761514	1.5689297	0.6497807	0.89101
RP5-1024G6.5	1.038859	1.0442995	1.03436527	0.7757486	-0.3663389	0.89115
NR1D2	1.492911	1.4866014	1.49812178	1.0236752	0.03375797	0.8912
PDGFD	1.106199	1.1006157	1.11080937	1.101313	0.13922457	0.89125
NLGN3	1.029932	1.0244424	1.03446593	1.4100894	0.49578666	0.89126
SLPI	1.032697	1.0272369	1.03720568	1.3660034	0.44996107	0.89127
RP11-428J1.5	1.075077	1.0805258	1.07057815	0.876466	-0.1902299	0.89136
GTPBP6	1.386313	1.3925781	1.38113951	0.970863	-0.0426604	0.89136
KIF26B	1.07948	1.0738884	1.08409814	1.1381778	0.1867259	0.89138
RP11-416N2.4	1.024964	1.0303717	1.02049752	0.6748879	-0.5672801	0.89139
CFAP77	1.039126	1.0445912	1.03461191	0.7762055	-0.3654895	0.89143
TNNT3	1.035631	1.0300883	1.04020759	1.336318	0.41826341	0.89144
SNRK	1.116336	1.1217813	1.11183851	0.9183554	-0.1228756	0.89145
RP11-448A19.1	1.24778	1.2417967	1.25272103	1.04518	0.06375137	0.89151
WDFY2	1.307083	1.3010811	1.31203907	1.0363954	0.05157447	0.89153
PCCA-AS1	1.021948	1.0273835	1.01745913	0.6375788	-0.6493245	0.89158
NRSN1	1.021942	1.0274107	1.01742666	0.635761	-0.6534436	0.89158
LINC00941	1.127566	1.1216354	1.13246286	1.0890159	0.12302497	0.89159
CAMKMT	1.224698	1.2304207	1.21997277	0.9546573	-0.0669451	0.89163
HLA-F	1.03695	1.0314169	1.04151889	1.321548	0.40222888	0.89167
ANKH	1.199833	1.2056203	1.19505425	0.9486139	-0.0761071	0.89167
GGACT	1.140738	1.1350769	1.14541245	1.0765164	0.10637023	0.89173

RP11-616M22.12	1.020554	1.0151567	1.02501167	1.6502018	0.72264247	0.89174
RP11-111H13.1	1.018695	1.0132641	1.02317947	1.7475335	0.80532009	0.89182
CTD-2129N1.1	1.018712	1.0132573	1.02321678	1.7512449	0.80838081	0.89182
SLC7A6OS	1.346483	1.3528789	1.34120091	0.9669066	-0.0485516	0.89188
TDRD6	1.029632	1.0349913	1.02520563	0.7203408	-0.4732485	0.8919
MDGA1	1.089679	1.0952176	1.08510502	0.8937951	-0.1619839	0.89201
SSPO	1.086092	1.0918189	1.08136295	0.8861246	-0.1744186	0.89203
SEL1L3	1.346265	1.3525728	1.34105568	0.967334	-0.047914	0.89209
PRKAR1A	2.756056	2.7458321	2.76449854	1.010692	0.01534342	0.89211
RECK	1.220039	1.2143836	1.2247096	1.048166	0.0678672	0.89212
RP11-778D9.12	1.031796	1.0371916	1.02733974	0.7351058	-0.4439762	0.8922
C14orf93	1.128361	1.1338728	1.1238092	0.9248273	-0.1127442	0.89222
UTP4	1.215309	1.2094077	1.22018256	1.0514541	0.07238592	0.89228
SEZ6	1.032013	1.0265751	1.03650404	1.3736207	0.45798364	0.89228
CTC-444N24.13	1.033693	1.0282285	1.03820572	1.3534453	0.43663661	0.89232
RP11-74J13.8	1.058822	1.0532364	1.0634355	1.191581	0.25287702	0.89238
TPT1	53.40383	53.521905	53.3063138	0.9958952	-0.0059342	0.89244
RP11-414H17.5	1.032642	1.027267	1.03708085	1.3599146	0.44351609	0.8925
EYA1	1.02126	1.0266095	1.0168427	0.6329576	-0.6598193	0.89253
LINC01137	1.098769	1.0933715	1.10322554	1.1055354	0.14474516	0.89253
LINC01229	1.022274	1.0276101	1.01786798	0.6471548	-0.6278172	0.89274
VPS37D	1.253362	1.2594305	1.24835139	0.9572944	-0.0629655	0.89276
CTD-2081C10.7	1.019263	1.0138992	1.02369248	1.7045959	0.7694298	0.89277
RP1-242N11.1	1.023115	1.0284236	1.0187305	0.6589769	-0.6017001	0.8928
MYRIP	1.029185	1.0237468	1.03367569	1.4181149	0.50397447	0.89282
RP11-498C9.15	1.066201	1.0608251	1.07064038	1.1613688	0.21582615	0.89283
PRDM6	1.053104	1.0584555	1.04868443	0.8328464	-0.2638777	0.89296
CLK2	1.150648	1.1565367	1.14578452	0.9313123	-0.1026631	0.89299
KIF21A	1.820629	1.8277156	1.81477751	0.984369	-0.0227289	0.893
RP5-994D16.11	1.035382	1.0299336	1.03988141	1.3323283	0.4139496	0.89303
NANOG	1.035356	1.029911	1.03985293	1.3323818	0.41400754	0.89303
PLB1	1.019687	1.0143227	1.02411732	1.6838488	0.75176258	0.89304
CILP2	1.041146	1.0357121	1.04563367	1.2778201	0.35368475	0.89305
PDZD2	1.027355	1.0219806	1.03179379	1.4464492	0.53251561	0.89308
RP11-131L12.4	1.02682	1.0214342	1.03126718	1.4587544	0.54473699	0.89311
B4GALT1-AS1	1.026813	1.0214318	1.03125757	1.4584653	0.54445107	0.89311
AFP	1.022286	1.016941	1.0266995	1.5760269	0.65629215	0.89316
RHEBL1	1.036226	1.0415047	1.031867	0.7677925	-0.3812115	0.89341
ZNF747	1.15167	1.1572487	1.14706286	0.9352246	-0.0966153	0.89343
LINGO1	1.067953	1.0734562	1.06340928	0.8632258	-0.2121902	0.89345
PIKFYVE	1.269873	1.2639083	1.27479927	1.0412679	0.05834127	0.89356
NSUN6	1.340837	1.3467645	1.33594131	0.9687879	-0.0457472	0.89365
RP11-250B2.5	1.043119	1.0376772	1.04761268	1.2636994	0.33765334	0.89375
PTS	1.762373	1.7558872	1.76772941	1.0156667	0.02242698	0.89376
GALE	1.61626	1.6095338	1.62181429	1.0201474	0.02877761	0.89406

FBXL19-AS1	1.035248	1.0405575	1.03086251	0.7609567	-0.3941137	0.89407
TRIB1	1.368576	1.3635466	1.37272876	1.0252571	0.03598567	0.89412
EMP1	1.299364	1.2932363	1.30442393	1.0381521	0.05401781	0.8942
FAM102B	1.11769	1.1123433	1.12210599	1.0869008	0.12022022	0.8942
RP13-554M15.8	1.024945	1.0195751	1.02938012	1.5008925	0.58582067	0.89422
ZNF490	1.024896	1.0195713	1.02929241	1.4966996	0.58178465	0.89422
C5orf47	1.024941	1.0195801	1.02936801	1.4998916	0.58485823	0.89422
SHQ1	1.140793	1.1461199	1.13639415	0.93344	-0.0993708	0.89425
KLHL14	1.209221	1.2034619	1.21397752	1.0516835	0.07270059	0.89427
TSC22D1-AS1	1.051812	1.0463702	1.05630611	1.2142736	0.28009354	0.89428
TNNC2	1.036572	1.0312452	1.04097089	1.3112717	0.3909667	0.89429
ACKR2	1.024789	1.0301351	1.02037368	0.6760783	-0.5647379	0.89445
AC009518.4	1.024728	1.0299801	1.02039162	0.6801725	-0.5560274	0.89445
PRTG	1.518723	1.5126075	1.52377347	1.0217827	0.03108835	0.89447
ARF4-AS1	1.068789	1.0741023	1.06440178	0.869093	-0.2024176	0.89449
RP11-196G18.24	1.050651	1.0453048	1.05506603	1.215456	0.28149767	0.89451
ZNF501	1.092405	1.0976601	1.08806475	0.9017477	-0.1492043	0.89454
HIST1H4H	1.036928	1.0315269	1.04138765	1.3127714	0.39261573	0.89454
ZNF114	1.08042	1.0857656	1.07600561	0.8862013	-0.1742936	0.89457
C19orf45	1.057506	1.0628706	1.0530761	0.844212	-0.2443227	0.89458
RP11-455O6.9	1.029209	1.0238456	1.03363872	1.410688	0.49639897	0.89458
LINC00924	1.029247	1.0238527	1.03370131	1.4128959	0.49865517	0.89458
RGPD2	1.029166	1.0238171	1.033583	1.4100368	0.4957328	0.89458
SLC25A36	2.854523	2.844443	2.86284684	1.009978	0.01432382	0.89459
MEG9	1.025828	1.0311274	1.02145204	0.6891688	-0.5370707	0.89461
FAM189A1	1.025787	1.0310676	1.02142577	0.6896499	-0.5360639	0.89461
TOM1	1.242134	1.2477805	1.23747131	0.958394	-0.0613092	0.89461
DKFZP434A062	1.04215	1.0474218	1.03779584	0.7970146	-0.327322	0.89461
ZNF707	1.038736	1.0333451	1.04318765	1.2951719	0.37314359	0.89466
CMC4	1.056426	1.0618546	1.05194335	0.8397649	-0.2519427	0.89472
MTG1	1.196368	1.2019835	1.19173069	0.9492392	-0.0751564	0.89472
ANO1	1.058525	1.0638158	1.05415639	0.8486355	-0.2367831	0.8948
MCM4	1.369111	1.3748679	1.36435749	0.9719624	-0.0410276	0.89486
TDO2	1.037605	1.0322905	1.04199298	1.3004742	0.37903777	0.89488
SLC35E2	1.130904	1.1363423	1.12641391	0.9271807	-0.1090775	0.895
GIN1	1.144726	1.1391347	1.1493429	1.0733692	0.10214644	0.89502
LPP-AS2	1.030048	1.0353286	1.02568765	0.7271064	-0.4597616	0.89503
MEGF11	1.030098	1.0353979	1.0257216	0.7266416	-0.4606842	0.89503
CH17-373J23.1	1.082987	1.0883328	1.0785718	0.8894979	-0.1689369	0.89504
NFKBID	1.08024	1.0748576	1.08468507	1.1312829	0.17795975	0.89506
RN7SL832P	1.032643	1.0378639	1.02833208	0.7482609	-0.4183868	0.89514
DGAT2	1.039254	1.0338329	1.04373167	1.2925766	0.37024974	0.89514
PNOC	1.026626	1.0213203	1.03100826	1.4543994	0.54042351	0.89517
RP11-712B9.2	1.026635	1.0213074	1.0310347	1.4565244	0.54252985	0.89517
ADRA2C	1.025938	1.0206791	1.03028134	1.4643418	0.55025232	0.89533

RP11-153I24.5	1.025906	1.0205821	1.03030187	1.4722417	0.55801459	0.89533
CBWD1	1.537215	1.5438016	1.53177575	0.9778856	-0.0322624	0.89538
EDC3	1.185003	1.1793046	1.189708	1.0580206	0.08136769	0.8954
FAM151B	1.037739	1.043011	1.03338452	0.7761864	-0.365525	0.89544
AC068492.1	1.037768	1.0429644	1.03347695	0.7791796	-0.3599722	0.89544
RP11-118K6.3	1.03854	1.0332202	1.04293302	1.2923753	0.37002512	0.89544
AL133245.2	1.030591	1.0358238	1.02626976	0.7333056	-0.4475135	0.89547
RP11-96D1.11	1.024755	1.0194803	1.02911142	1.4944008	0.57956709	0.89548
SCARF2	1.02479	1.0194805	1.02917519	1.4976636	0.58271363	0.89548
CACNG6	1.02475	1.0194782	1.02910381	1.4941712	0.57934543	0.89548
ERICH2	1.046063	1.0408038	1.0504065	1.235338	0.3049058	0.89554
PTPA	1.883371	1.8772517	1.88842524	1.012737	0.01825955	0.89554
GALC	1.249232	1.2435328	1.25393848	1.0427282	0.06036321	0.89556
RP11-686D22.4	1.020533	1.0257117	1.01625602	0.6322412	-0.661453	0.8956
ZNF689	1.220543	1.2260808	1.2159692	0.9552743	-0.066013	0.89566
RP11-273B20.1	1.023183	1.0283928	1.0188816	0.6650145	-0.5885422	0.89567
AC097662.2	1.031674	1.0263444	1.03607459	1.3693438	0.45348475	0.89571
RP11-138P22.1	1.058547	1.0533048	1.06287642	1.179564	0.2382537	0.89575
CES4A	1.178686	1.1843843	1.17397975	0.9435712	-0.0837967	0.89582
CTU1	1.038259	1.0329963	1.04260539	1.2912168	0.36873124	0.89584
ARPIN	1.223838	1.2181082	1.22857008	1.0479663	0.06759231	0.89587
DNM1P35	1.020174	1.0254273	1.01583589	0.6227912	-0.6831795	0.89588
LGALS7B	1.020184	1.0254426	1.01584105	0.6226187	-0.6835793	0.89588
RP5-875O13.1	1.020123	1.0252981	1.01584989	0.6265242	-0.6745579	0.89588
GP1BA	1.027714	1.032977	1.0233677	0.7086066	-0.4969432	0.8959
WDR93	1.039557	1.0447588	1.03526048	0.7877883	-0.34412	0.89591
LIMD1-AS1	1.028236	1.033446	1.02393433	0.7156121	-0.4827502	0.89593
LINC00886	1.028289	1.0335072	1.02397916	0.7156425	-0.482689	0.89593
RP11-800A3.7	1.028253	1.0335391	1.02388856	0.7122599	-0.4895242	0.89593
MLX	1.387191	1.3814927	1.39189636	1.0272709	0.03881666	0.89595
RP11-46H11.12	1.046577	1.0413252	1.05091396	1.2320331	0.30104105	0.89598
PTPN3	1.033344	1.0281053	1.03767065	1.3403395	0.42259852	0.89603
RGN	1.102712	1.0974575	1.10705097	1.0984373	0.13545252	0.89608
TRAF3IP2	1.189921	1.184519	1.19438124	1.0534481	0.07511927	0.8961
RP11-258F1.1	1.027939	1.0331588	1.02362812	0.7125749	-0.4888865	0.89618
DPH1	1.030006	1.0352264	1.02569416	0.7294014	-0.4552152	0.89619
RP11-92G12.3	1.030008	1.0352495	1.02568015	0.7285254	-0.4569488	0.89619
MAP3K14-AS1	1.022278	1.0170649	1.02658346	1.5577874	0.63949834	0.89621
AC079354.5	1.020446	1.0151589	1.02481157	1.6367691	0.71085078	0.8963
CTD-2291D10.4	1.020416	1.0151937	1.02472871	1.6275632	0.70271353	0.8963
ZBTB5	1.176937	1.1823417	1.17247374	0.945882	-0.0802678	0.89634
RP11-68L18.1	1.028047	1.0333199	1.02369346	0.7110909	-0.4918941	0.89637
FGF14	1.048873	1.0436075	1.0532219	1.2204758	0.28744367	0.89655
ZNF66	1.054306	1.0594742	1.0500381	0.8413407	-0.249238	0.89657
SLC25A27	1.141468	1.135894	1.14607165	1.0748941	0.10419449	0.89659

FANCB	1.274878	1.2689528	1.27977182	1.0402266	0.05689782	0.89661
EFNA3	1.567611	1.5609007	1.57315207	1.0218423	0.03117253	0.89663
RELB	1.102282	1.1077952	1.0977282	0.9066098	-0.1414464	0.89666
MED18	1.0925	1.0977548	1.08816003	0.9018481	-0.1490436	0.89666
PROSER1	1.158197	1.1636433	1.15369998	0.9392376	-0.0904379	0.8967
GPR35	1.021929	1.0166975	1.02624988	1.5720805	0.65267514	0.89676
FAM216B	1.021909	1.0166975	1.02621284	1.5698621	0.6506378	0.89676
SPTB	1.021918	1.0166975	1.0262284	1.5707941	0.65149413	0.89676
SMTNL1	1.021991	1.0166975	1.02636271	1.5788378	0.65886293	0.89676
RP5-940J5.8	1.021958	1.0166975	1.02630226	1.5752173	0.6555509	0.89676
RP11-493L12.4	1.04241	1.0476974	1.03804277	0.7975855	-0.3262889	0.89678
RCAN2	1.167523	1.1620125	1.17207426	1.0621047	0.08692593	0.8968
AC003991.3	1.019564	1.024747	1.01528375	0.6175997	-0.695256	0.89682
EPN3	1.021277	1.0160611	1.02558384	1.5929072	0.67166221	0.89686
ZNF613	1.061094	1.055752	1.0655059	1.1749506	0.23260007	0.89688
CTD-2541M15.3	1.036218	1.04145	1.03189675	0.769524	-0.3779619	0.89695
RTKN2	1.281673	1.2871383	1.27716046	0.9652507	-0.0510245	0.89702
HIST1H2BJ	1.021384	1.0265212	1.01714109	0.6463159	-0.6296885	0.89709
EXOC8	1.057753	1.052434	1.06214593	1.1852216	0.24515683	0.89714
MCOLN1	1.19991	1.2054325	1.19534999	0.9509207	-0.072603	0.89723
FAM120C	1.096461	1.0911738	1.10082781	1.1058857	0.14520223	0.89726
HGFAC	1.030885	1.0256769	1.03518565	1.3703243	0.45451739	0.89728
C9orf106	1.028858	1.0236369	1.03316978	1.4033052	0.48882885	0.89729
RP5-907D15.4	1.028738	1.0235432	1.03302866	1.4028952	0.48840726	0.89729
EMG1.1	1.076646	1.0820216	1.07220738	0.8803461	-0.1838573	0.8974
RMI1	1.256763	1.262328	1.25216757	0.9612683	-0.0569889	0.89743
RP11-351J23.1	1.020043	1.0148661	1.02431729	1.6357531	0.70995501	0.89748
CTB-49A3.4	1.02004	1.0148661	1.02431252	1.6354318	0.70967159	0.89748
RP11-38G5.4	1.020046	1.0148661	1.02432384	1.6361935	0.71034341	0.89748
RP11-923I11.3	1.02004	1.0148661	1.02431202	1.6353984	0.70964211	0.89748
RP11-439M11.1	1.020037	1.0148661	1.02430806	1.6351321	0.70940719	0.89748
PAXIP1-AS2	1.020031	1.0148661	1.02429678	1.634373	0.70873729	0.89748
RP11-363E6.4	1.02144	1.0162286	1.02574389	1.5863287	0.66569176	0.89752
ULBP2	1.029138	1.0238431	1.03351126	1.4054935	0.4910768	0.89754
ADM2	1.029131	1.0238917	1.03345734	1.4003781	0.48581636	0.89754
LRRC4	1.028596	1.0232887	1.03297969	1.416125	0.50194858	0.89757
GABRE	1.048205	1.0429739	1.05252465	1.2222455	0.2895341	0.89762
RP11-332H18.5	1.024075	1.0188492	1.02839033	1.5061851	0.5908991	0.89762
SFTPD-AS1	1.024111	1.0188752	1.02843566	1.5065069	0.59120726	0.89762
BCAR3	1.152955	1.1475094	1.15745267	1.0674079	0.09411159	0.89763
GSE1	1.515581	1.5216618	1.51055876	0.978716	-0.0310379	0.89776
MYT1L	1.023971	1.0291517	1.01969187	0.6754962	-0.5659805	0.89782
OTUD6A	1.023365	1.0285572	1.0190777	0.6680525	-0.5819666	0.8979
DHX38	1.190192	1.1846972	1.19472966	1.0543183	0.07631047	0.89793
STX4	1.452994	1.4591213	1.44793332	0.9756317	-0.0355914	0.89805

CTC-459F4.3	1.150767	1.156117	1.14634942	0.9374343	-0.0932106	0.89805
HESX1	1.107972	1.1132939	1.10357708	0.9142335	-0.1293653	0.89814
TEX15	1.025025	1.0301419	1.0207999	0.6900667	-0.5351923	0.89815
ASCL2	1.031434	1.0262593	1.03570682	1.3597784	0.44337161	0.89818
ZCCHC24	1.121146	1.115948	1.12543865	1.0818527	0.1135041	0.8982
ARID2	1.565874	1.5594508	1.5711789	1.0209636	0.02993146	0.8982
AP000345.1	1.182961	1.1882127	1.17862408	0.9490544	-0.0754374	0.89823
SCG2	1.180763	1.1860859	1.17636635	0.9477684	-0.0773935	0.89825
RP11-356J5.12	1.145459	1.1402519	1.14975964	1.0677905	0.09462859	0.89832
CLN5	1.421267	1.4153553	1.42614806	1.0259843	0.03700863	0.89832
GALNT15	1.018128	1.0129919	1.02236997	1.7218375	0.78394903	0.89836
GPR1	1.018154	1.0129919	1.02241705	1.7254611	0.78698194	0.89836
TMEM140	1.018143	1.0129919	1.0223976	1.723964	0.78572963	0.89836
TDRD9	1.018193	1.0129919	1.02248834	1.7309486	0.79156288	0.89836
FRK	1.01814	1.0129919	1.0223908	1.7234403	0.78529134	0.89836
CC2D2A	1.472983	1.4673748	1.47761489	1.0219098	0.03126788	0.89838
ZNF296	1.03848	1.03329	1.04276585	1.2846467	0.36137164	0.89853
RP11-399K21.11	1.036634	1.0418928	1.03229194	0.7708229	-0.3755286	0.89854
FAM83H-AS1	1.036428	1.0414863	1.03225023	0.7773698	-0.3633271	0.89854
AZU1	1.06745	1.0623229	1.07168491	1.150217	0.20190601	0.89855
BOLA3	2.284391	2.2933308	2.27700919	0.9873802	-0.0183224	0.89865
CTD-2008L17.1	1.054639	1.0600303	1.05018688	0.836026	-0.2583803	0.89866
CELSR3	1.077485	1.0722198	1.08183306	1.1331103	0.18028836	0.89866
HLA-DQB1	1.073521	1.068333	1.07780573	1.1386267	0.18729478	0.89868
DIAPH3	1.240418	1.234705	1.24513495	1.0444384	0.06272736	0.8988
CUL7	1.204174	1.2100012	1.19936254	0.9493398	-0.0750035	0.89884
RP4-613B23.1	1.057226	1.0520129	1.06153032	1.1829813	0.24242728	0.89884
CGREF1	1.040401	1.0455276	1.03616812	0.7944214	-0.3320236	0.89887
L3MBTL4	1.029604	1.0243966	1.03390369	1.389688	0.47476098	0.8989
YIPF7	1.02136	1.0264983	1.01711686	0.64596	-0.6304833	0.89892
CFB	1.025604	1.0204776	1.02983814	1.4571101	0.54310994	0.89902
RP11-626G11.1	1.0198	1.0146773	1.02402986	1.6372109	0.71124016	0.89906
RP11-415F23.4	1.019822	1.0146773	1.02406971	1.639926	0.71363071	0.89906
WNT9B	1.019802	1.0146773	1.02403415	1.6375032	0.71149775	0.89906
RP11-103J8.1	1.019824	1.0146773	1.02407386	1.6402085	0.71387925	0.89906
C11orf53	1.019845	1.0146773	1.02411248	1.6428396	0.71619159	0.89906
IRF8	1.019845	1.0146773	1.02411245	1.6428374	0.71618966	0.89906
MIA	1.019786	1.0146773	1.02400467	1.6354947	0.70972705	0.89906
ZNF732	1.040372	1.0351766	1.0446628	1.2696723	0.34445617	0.8991
TRAPPC3	2.401201	2.3936306	2.40745218	1.0099177	0.01423772	0.89917
PLCD1	1.121461	1.1266259	1.11719578	0.9255278	-0.1116518	0.89919
STAC	1.088935	1.0941599	1.08462041	0.8986882	-0.1541074	0.89924
FAXDC2	1.26931	1.2752534	1.26440109	0.9605734	-0.0580323	0.89926
ZNF529	1.222612	1.2171854	1.22709324	1.0456194	0.06435788	0.89928
RP4-753F5.1	1.02832	1.033385	1.0241381	0.7230227	-0.4678871	0.89932

RP11-46C24.7	1.087649	1.0928774	1.0833307	0.8972123	-0.1564788	0.89933
C11orf65	1.046265	1.0514241	1.042004	0.8168159	-0.2919172	0.89938
RP11-176H8.1	1.045215	1.0503496	1.04097419	0.8137933	-0.2972657	0.89945
CTC-471J1.11	1.080353	1.0854802	1.07611896	0.8904865	-0.1673344	0.89946
B3GNT4	1.027932	1.0330806	1.02368005	0.7158282	-0.4823147	0.89959
FANCM	1.155761	1.1502619	1.16030238	1.0668199	0.09331658	0.89962
TUBGCP3	1.215447	1.2207268	1.21108701	0.9563272	-0.0644238	0.89962
CPOX	1.196459	1.2020142	1.19187136	0.9497914	-0.0743174	0.89962
MCPH1	1.196483	1.2016627	1.19220544	0.9531034	-0.0692954	0.89962
RP11-248G5.9	1.025921	1.020794	1.03015412	1.4501325	0.53618469	0.8997
CDC42EP5	1.079454	1.074243	1.08375704	1.1281469	0.17395493	0.89971
MNT	1.258534	1.2641248	1.25391766	0.9613548	-0.0568591	0.89972
AP001439.2	1.018841	1.0238827	1.01467723	0.6145551	-0.7023858	0.89991
RP3-465N24.6	1.02648	1.0214023	1.03067251	1.4331441	0.51918371	0.89994
GRIN2C	1.021503	1.0265552	1.01733066	0.6526268	-0.6156699	0.89997
RP11-500G22.4	1.021503	1.0265803	1.01731093	0.6512682	-0.6186764	0.89997
AC004076.5	1.101226	1.0960457	1.10550396	1.0984762	0.13550365	0.90006
SSX1	1.02104	1.0159741	1.0252227	1.578973	0.65898649	0.90013
HBZ	1.021055	1.0159741	1.02525125	1.58076	0.66061839	0.90013
EVPL	1.021048	1.0159741	1.02523812	1.5799381	0.65986808	0.90013
OVGP1	1.021085	1.0159741	1.02530496	1.5841224	0.6636838	0.90013
RP4-666F24.3	1.021039	1.0159741	1.02522216	1.5789393	0.65895574	0.90013
SYT8	1.021043	1.0159741	1.02522965	1.5794081	0.65938396	0.90013
PHRF1	1.143973	1.1387706	1.14826974	1.0684525	0.09552277	0.90016
GS1-393G12.14	1.033397	1.0282889	1.03761462	1.3296598	0.41105716	0.90016
ZNF578	1.025963	1.0310339	1.02177598	0.7016829	-0.511109	0.90019
RP11-700H13.1	1.026555	1.031695	1.02230959	0.7038845	-0.5065893	0.90022
MSH5	1.062086	1.0671087	1.0579381	0.8633474	-0.2119869	0.90028
ARVCF	1.275966	1.2816124	1.27130371	0.9633939	-0.0538023	0.90028
KLRC2	1.023144	1.0180243	1.0273713	1.5185802	0.60272306	0.9003
RP11-536C5.7	1.023121	1.0180243	1.02732919	1.5162438	0.60050175	0.9003
SULT1E1	1.023136	1.0180243	1.02735698	1.5177856	0.601968	0.9003
RP11-18B16.2	1.023108	1.0180243	1.02730617	1.5149665	0.59928587	0.9003
GRIK1-AS1	1.023115	1.0180243	1.02731934	1.5156976	0.59998196	0.9003
C5orf66-AS1	1.023131	1.0180243	1.0273476	1.5172653	0.60147338	0.9003
AC007743.1	1.02104	1.0159362	1.02525455	1.5847238	0.66423143	0.90032
OPRD1	1.021055	1.0159362	1.02528237	1.5864697	0.66581993	0.90032
RP11-737O24.2	1.021027	1.0159362	1.02523121	1.5832589	0.66289716	0.90032
RHOH	1.021003	1.0159362	1.02518797	1.5805456	0.6604227	0.90032
RP11-526I2.5	1.020998	1.0159362	1.02517853	1.5799534	0.65988204	0.90032
KCNN3	1.047744	1.0427956	1.05182961	1.2110968	0.27631418	0.90043
SV2B	1.023142	1.0180681	1.0273327	1.5127595	0.59718262	0.90056
LINC00304	1.021314	1.0162589	1.02548934	1.5677191	0.64866705	0.90056
TMC8	1.021319	1.0162589	1.02549703	1.5681917	0.64910192	0.90056
C15orf59	1.018674	1.023675	1.01454488	0.6143566	-0.7028518	0.90057

BATF	1.020767	1.0157004	1.02495071	1.5891748	0.66827781	0.9006
GACAT2	1.020748	1.0157004	1.02491658	1.5870008	0.66630288	0.9006
MYOZ3	1.067061	1.0618956	1.07132683	1.1523737	0.20460864	0.9006
DGKI	1.077281	1.0722447	1.08144084	1.1272923	0.1728617	0.90067
RP11-649A18.12	1.024007	1.0189297	1.02819964	1.4897041	0.5750258	0.90067
NECAB1	1.044168	1.0493999	1.03984778	0.8066365	-0.3100094	0.90072
LPIN1	1.310025	1.3155083	1.30549747	0.9682709	-0.0465173	0.90072
DNAH10OS	1.028303	1.033307	1.02417089	0.7257007	-0.4625535	0.90076
BEND7	1.12899	1.1237868	1.13328724	1.0767483	0.10668111	0.90078
RMI2	1.416763	1.4109731	1.42154416	1.0257221	0.03663987	0.90084
AP000569.9	1.029153	1.0341558	1.02502075	0.7325474	-0.4490061	0.90084
RP11-26J3.1	1.029216	1.0342498	1.02505972	0.7316742	-0.4507268	0.90084
RP13-766D20.4	1.111054	1.1058273	1.11537116	1.0901836	0.12457108	0.90093
FAM71D	1.041196	1.0462163	1.03704946	0.8016527	-0.3189507	0.90104
TMOD1	1.040531	1.0455382	1.0363961	0.799243	-0.3232939	0.90105
ZNF571-AS1	1.040667	1.0457284	1.03648705	0.7979082	-0.3257053	0.90105
CHRNA7	1.084396	1.0896535	1.08005408	0.8929273	-0.1633854	0.90111
WDR7	1.113327	1.1082327	1.11753452	1.0859423	0.1189475	0.90112
SCGB1B2P	1.035437	1.0304069	1.03959165	1.302062	0.38079815	0.90119
PIK3CD	1.06452	1.0696493	1.06028365	0.8655311	-0.2083424	0.9012
RP5-1116H23.3	1.018841	1.0238227	1.01472744	0.6182095	-0.6938323	0.90135
RIMS1	1.049727	1.0446982	1.05388043	1.2054272	0.26954456	0.90137
MAMDC2-AS1	1.075422	1.070316	1.07963845	1.132579	0.17961172	0.90141
UBC	6.367864	6.3439621	6.38760183	1.0081662	0.01173346	0.90142
C2CD4D	1.049741	1.054724	1.0456257	0.8337414	-0.2623281	0.90143
DDX20	1.096458	1.0911608	1.10083197	1.1060888	0.14546726	0.90152
NOD1	1.045699	1.0405639	1.04993929	1.231125	0.29997725	0.90164
RINL	1.113603	1.108523	1.11779873	1.0854722	0.11832274	0.9017
DND1	1.022727	1.0177058	1.02687314	1.5177552	0.60193915	0.90173
CD81-AS1	1.022721	1.0176939	1.02687285	1.5187639	0.60289764	0.90173
GDF6	1.032648	1.0275957	1.03682111	1.3343085	0.4160923	0.90173
LIN9	1.12672	1.1215503	1.13098997	1.0776607	0.10790302	0.90176
PPM1M	1.095903	1.1009966	1.09169751	0.9079272	-0.1393515	0.9018
DNAH5	1.057106	1.0520816	1.06125451	1.1761259	0.23404245	0.90184
RP11-120D5.1	1.032812	1.0377658	1.02872083	0.760498	-0.3949837	0.90193
SP6	1.021764	1.0167757	1.0258835	1.5429201	0.62566331	0.90195
AC105760.3	1.021791	1.0167793	1.02592928	1.5453101	0.62789641	0.90195
TMEM56-RWDD3	1.021752	1.0167656	1.02586989	1.5430304	0.62576648	0.90195
PGBD5	1.040286	1.0453058	1.03614108	0.7977147	-0.3260553	0.90196
NUPR1	1.030928	1.0259608	1.03502915	1.3493115	0.4322234	0.90199
RP11-435I10.4	1.023164	1.0181662	1.02729114	1.502306	0.58717871	0.902
CTC-457L16.2	1.017832	1.012846	1.02195	1.7086986	0.77289792	0.90204
LINC00607	1.017819	1.012846	1.02192648	1.7068673	0.77135089	0.90204
LAMP3	1.017827	1.012846	1.02193987	1.7079099	0.77223183	0.90204
ANKRD24	1.017841	1.012846	1.02196586	1.7099332	0.77393997	0.90204

ZNF331	1.214601	1.2091009	1.21914367	1.0480282	0.06767753	0.90208
LINC01239	1.025762	1.020727	1.0299193	1.4434958	0.52956692	0.90209
RP11-212P7.2	1.148921	1.1540176	1.14471264	0.9395852	-0.0899041	0.90211
RP11-51J9.6	1.022215	1.0272341	1.01807032	0.6635173	-0.5917941	0.90212
AXDND1	1.022235	1.027242	1.01810032	0.6644278	-0.5898156	0.90212
DIO2	1.033497	1.0285872	1.03755221	1.3136036	0.39352996	0.90212
RP13-616I3.1	1.021707	1.0267813	1.01751734	0.6540877	-0.6124439	0.90221
XIAP	1.497825	1.4918057	1.50279651	1.022348	0.03188632	0.90225
RP11-839D17.3	1.072011	1.0769814	1.06790649	0.8821153	-0.1809608	0.90234
MBLAC1	1.074762	1.0797658	1.07062891	0.8854538	-0.175511	0.90235
RCCD1	1.440282	1.4341263	1.44536559	1.0258894	0.0368752	0.90237
ZNF280A	1.024035	1.0290026	1.01993243	0.6872626	-0.5410666	0.90242
RP11-540K16.1	1.0233	1.0282396	1.0192217	0.6806649	-0.5549833	0.90243
EED	1.239195	1.2445502	1.23477319	0.9600204	-0.058863	0.90245
C1RL-AS1	1.0717	1.0666128	1.07590082	1.1394321	0.18831496	0.90263
SLC26A4-AS1	1.020573	1.0156039	1.02467725	1.5814792	0.66127456	0.90265
TRAF1	1.044964	1.0399086	1.04913952	1.2313026	0.3001854	0.90266
TMEM243	1.38114	1.3755652	1.38574463	1.0271042	0.03858254	0.90272
LMOD1	1.134231	1.1289177	1.13861801	1.0752441	0.10466419	0.90273
FOXO1	1.120916	1.1161627	1.12484147	1.0747124	0.10395068	0.90274
SLC16A11	1.045529	1.0405252	1.04966104	1.2254368	0.29329613	0.90282
TBC1D8	1.062587	1.0676298	1.05842283	0.8638623	-0.2111268	0.90285
SLC52A3	1.018733	1.0137737	1.02282903	1.6574393	0.72895601	0.90295
RP11-379F4.6	1.018728	1.0137737	1.02281956	1.6567517	0.72835737	0.90295
CFH	1.018742	1.0137737	1.02284529	1.6586202	0.72998353	0.90295
SLC9C1	1.018754	1.0137737	1.02286726	1.6602149	0.73137001	0.90295
PCDHA10	1.076949	1.0819616	1.07280894	0.8883298	-0.1708328	0.90296
ZSCAN25	1.076841	1.081857	1.07269862	0.8881175	-0.1711776	0.90296
RP11-455O6.8	1.0213	1.026225	1.01723322	0.6571306	-0.605748	0.90303
FAM43B	1.053935	1.0588787	1.04985302	0.8467071	-0.2400651	0.90304
IL6ST	1.780008	1.7735499	1.78534209	1.0152443	0.0218269	0.90311
SLC25A18	1.025621	1.0206456	1.02973014	1.4400195	0.52608836	0.90315
AC138969.4	1.028409	1.0233989	1.03254719	1.3909727	0.47609414	0.90316
MMP1	1.022343	1.0272671	1.01827607	0.6702602	-0.5772068	0.90322
TMEM196	1.019574	1.024473	1.01552898	0.6345352	-0.6562278	0.90323
BMPER	1.019586	1.0244992	1.01552859	0.6338409	-0.6578073	0.90323
AC005614.3	1.019581	1.0244802	1.01553476	0.6345835	-0.6561182	0.90323
RP11-42O15.3	1.019626	1.024588	1.01552913	0.6315742	-0.6629759	0.90323
GLYATL2	1.120783	1.1259021	1.11655513	0.9257598	-0.1112902	0.90331
RNF139	1.341573	1.3471332	1.33698095	0.9707541	-0.0428222	0.90336
KAT2A	1.411164	1.4167797	1.40652657	0.9753992	-0.0359353	0.90339
HIST1H2BC	1.040318	1.0352891	1.04447179	1.2602132	0.3336678	0.90341
BCL3	1.219705	1.225203	1.21516451	0.9554248	-0.0657857	0.90342
TRERF1	1.142134	1.1472206	1.13793258	0.9369107	-0.0940165	0.90342
PRSS53	1.021519	1.0165764	1.02559991	1.5443579	0.62700714	0.90353

SAXO2	1.109136	1.1142414	1.10491932	0.9183998	-0.1228058	0.90367
CAP2	1.366255	1.3609755	1.37061419	1.0267018	0.0380172	0.90367
ECSCR	1.016216	1.0113227	1.02025644	1.7890059	0.83915812	0.90368
RBMS3-AS2	1.016246	1.0113227	1.02031143	1.7938627	0.84306947	0.90368
CTD-2240J17.4	1.016259	1.0113227	1.02033588	1.796022	0.84480504	0.90368
ATP2C2	1.016229	1.0113227	1.02028042	1.7911242	0.84086538	0.90368
CCDC149	1.17056	1.1653729	1.17484365	1.0572691	0.08034256	0.90384
NAGPA	1.108463	1.1033052	1.11272223	1.0911571	0.12585887	0.90388
DNAI1	1.043717	1.0487774	1.03953789	0.810578	-0.302977	0.9039
SSH3	1.195707	1.1904857	1.20001857	1.0500451	0.07045129	0.90394
ANKRD13D	1.31732	1.3230139	1.3126176	0.9678148	-0.047197	0.90397
UHRF1BP1	1.211201	1.2059308	1.21555397	1.0467302	0.0658896	0.90402
GAS2L1	1.130245	1.1252867	1.1343393	1.0722548	0.10064776	0.90408
ITIH3	1.030089	1.0252078	1.03412022	1.3535589	0.43675763	0.90409
CNOT1	1.532379	1.526437	1.53728596	1.0206083	0.02942925	0.9041
PPP1R9B	1.205463	1.2003826	1.2096586	1.0462914	0.06528477	0.90411
RBP4	1.027653	1.0227567	1.03169675	1.3928518	0.47804178	0.90417
HNMT	1.155726	1.160881	1.15146904	0.9414973	-0.0869712	0.9042
ZNF813	1.122787	1.1278688	1.11859113	0.9274438	-0.1086682	0.90421
LINC01198	1.017113	1.0219604	1.01310914	0.5969451	-0.7443297	0.90423
CHRNB3	1.019746	1.0246153	1.01572566	0.6388559	-0.6464375	0.90428
NIPA2	1.543267	1.5374413	1.54807748	1.0197904	0.02827273	0.90444
DEPDC7	1.024197	1.0290615	1.02018013	0.6943935	-0.5261746	0.90448
RBM11	1.099325	1.1042724	1.09523862	0.913364	-0.1307382	0.9045
RP11-579D7.4	1.090376	1.0854056	1.09448083	1.1062605	0.1456911	0.9045
CTD-2587H19.3	1.024838	1.0297445	1.02078524	0.6987922	-0.5170645	0.90452
GRM3	1.03694	1.0418766	1.03286263	0.7847499	-0.3496952	0.90457
LL0XNC01-7P3.1	1.022785	1.0178815	1.02683393	1.5006518	0.58558925	0.9046
TMPRSS13	1.022783	1.017878	1.02683278	1.5008825	0.58581107	0.9046
AL450992.2	1.02277	1.0178705	1.02681653	1.5006056	0.58554488	0.9046
AP000708.1	1.037897	1.0329383	1.04199178	1.2748611	0.35034013	0.90462
RFLNA	1.041677	1.0367371	1.04575699	1.2455254	0.31675442	0.90471
CERS6	1.402898	1.3973329	1.4074933	1.0255715	0.03642812	0.90474
ADAMTS20	1.048959	1.0538167	1.0449479	0.8352031	-0.259801	0.90475
KCNB1	1.02445	1.0292879	1.02045477	0.6984025	-0.5178693	0.90476
RRS1-AS1	1.026611	1.0315144	1.02256167	0.7159158	-0.4821382	0.90478
RP11-2C24.9	1.02271	1.0178302	1.02673919	1.4996593	0.58463478	0.90479
LINC01185	1.016982	1.0218243	1.01298307	0.5948911	-0.7493026	0.90489
PRLH	1.016961	1.0217775	1.01298307	0.5961702	-0.7462038	0.90489
RP11-2E11.6	1.03101	1.0261055	1.03505982	1.3430052	0.42546486	0.90493
RP11-162D16.2	1.02453	1.0293685	1.02053432	0.6991948	-0.5162336	0.90495
LAMB3	1.042036	1.037116	1.04609941	1.2420369	0.31270809	0.90498
AC012358.8	1.044902	1.0398014	1.04911356	1.2339647	0.30330115	0.90499
HCG27	1.02484	1.019972	1.02886044	1.4450429	0.53111229	0.90503
RP11-89K10.1	1.023038	1.0181635	1.0270634	1.4899845	0.57529736	0.90503

SLC19A3	1.023042	1.0181646	1.02707066	1.4902988	0.57560163	0.90503
AC009948.5	1.144807	1.1397214	1.14900638	1.0664532	0.0928207	0.90504
LINC01607	1.071067	1.0759641	1.06702361	0.8823067	-0.1806479	0.90505
LINC00939	1.026548	1.031351	1.02258114	0.7202688	-0.4733926	0.90506
ALDH8A1	1.022488	1.017615	1.02651305	1.5051439	0.58990142	0.90507
RP13-467H17.1	1.022507	1.017595	1.02656251	1.5096621	0.59422566	0.90507
LMX1B	1.018003	1.0131484	1.02201285	1.6741848	0.74345879	0.9051
LPAR5	1.01799	1.0131484	1.02198893	1.6723651	0.74188982	0.9051
RP11-108H9.1	1.018013	1.0131484	1.02203021	1.6755049	0.74459588	0.9051
RP11-284F21.7	1.018044	1.0131484	1.02208648	1.6797843	0.74827598	0.9051
CFAP157	1.02571	1.020829	1.02974089	1.4278595	0.51385398	0.90513
RP11-313P13.5	1.027428	1.032258	1.02344005	0.7266431	-0.4606812	0.90513
RAB3C	1.058812	1.0637243	1.05475569	0.8592596	-0.2188341	0.90516
AC083843.1	1.055842	1.0607507	1.05178854	0.8524766	-0.2302678	0.90517
CUL9	1.112815	1.1077589	1.11699082	1.0856723	0.11858865	0.90522
ZNF408	1.112778	1.1076433	1.11701809	1.0870916	0.12047357	0.90522
MERTK	1.065603	1.06064	1.06970176	1.1494349	0.20092481	0.90531
NTF4	1.066474	1.0615509	1.0705399	1.146042	0.1966599	0.90533
FOXP4	1.502555	1.5082799	1.49782699	0.9794348	-0.0299786	0.90538
TMEM182	1.062795	1.0675861	1.05883902	0.8705789	-0.199953	0.90543
RP11-503C24.4	1.016124	1.0209519	1.0121375	0.5793024	-0.7876114	0.90545
RP11-54C4.3	1.016113	1.0209281	1.0121375	0.579961	-0.7859723	0.90545
PPRC1	1.079601	1.0845951	1.07547644	0.8922078	-0.1645484	0.90553
LRRIQ3	1.061593	1.066517	1.05752687	0.8648448	-0.2094868	0.90555
CTA-292E10.6	1.151628	1.1465829	1.15579421	1.0628401	0.08792461	0.90556
RAB3GAP1	1.386541	1.3811236	1.3910144	1.0259517	0.03696275	0.90561
SCML4	1.017139	1.021963	1.01315569	0.5989942	-0.739386	0.90567
AC008697.1	1.01717	1.0220301	1.01315569	0.5971683	-0.7437905	0.90567
MAGED4	1.017098	1.0218715	1.01315569	0.6014997	-0.733364	0.90567
AB015752.3	1.017166	1.0220222	1.01315569	0.5973837	-0.7432701	0.90567
AMH	1.113259	1.1082007	1.11743709	1.0853634	0.11817813	0.90567
TCAF2	1.048057	1.0529098	1.04404997	0.8325489	-0.2643931	0.90568
C21orf62-AS1	1.08599	1.0811028	1.09002659	1.1100303	0.15059901	0.90568
SCIN	1.025408	1.0205008	1.02945974	1.4370056	0.52306571	0.90569
SPATA41	1.0254	1.020551	1.02940363	1.4307622	0.51678389	0.90569
AC142472.6	1.104977	1.09994	1.10913646	1.0920197	0.1269989	0.90569
RP5-991G20.2	1.017946	1.0227115	1.01401049	0.6168895	-0.6969159	0.9057
ARMC5	1.112481	1.1074869	1.11660558	1.0848356	0.11747638	0.90571
RBM18	1.326858	1.32154	1.33125011	1.0301986	0.04292252	0.90572
RBPMS-AS1	1.024739	1.0198562	1.02877176	1.4490031	0.53506072	0.90581
XX-FW83563B9.5	1.037765	1.0328722	1.04180563	1.2717631	0.34682995	0.90582
MOV10L1	1.029497	1.0344431	1.02541306	0.7378266	-0.4386463	0.90592
KLRG1	1.106341	1.1112323	1.1023011	0.9197064	-0.1207547	0.90594
APOL6	1.07423	1.0694018	1.07821651	1.1270102	0.17250058	0.90596
VPS37C	1.158034	1.1527314	1.16241222	1.0633843	0.08866306	0.90596

PDE4C	1.219641	1.2249901	1.21522407	0.9565933	-0.0640223	0.906
BIN3	1.299938	1.2943338	1.30456536	1.0347619	0.04929883	0.90601
CTD-2383M3.1	1.023273	1.0281182	1.01927115	0.685362	-0.5450618	0.90602
CTD-2036P10.3	1.070044	1.0749908	1.0659588	0.879558	-0.1851493	0.90606
RP11-495P10.6	1.071251	1.0760983	1.06724867	0.8837075	-0.1783592	0.90607
ZNF418	1.071233	1.0761636	1.06716212	0.8818139	-0.1814539	0.90607
SLC41A1	1.2569	1.2516344	1.26124795	1.0382046	0.05409084	0.9061
MOB3B	1.495278	1.5009576	1.49058735	0.9792992	-0.0301784	0.9061
RP5-903G2.2	1.01785	1.013034	1.02182691	1.6746162	0.74383053	0.90615
BHMT2	1.020634	1.0157693	1.02465104	1.5632262	0.64452652	0.90616
RP11-373D23.3	1.020584	1.0157693	1.02455988	1.5574452	0.63918143	0.90616
RP11-680G24.6	1.020597	1.0157693	1.02458328	1.5589292	0.64055542	0.90616
LINC01571	1.020601	1.0157693	1.02459068	1.5593981	0.64098928	0.90616
SPDYE16	1.020627	1.0157693	1.02463943	1.56249	0.64384696	0.90616
UGT2B7	1.031883	1.0367915	1.02782889	0.7563947	-0.4027888	0.90617
MTRNR2L1	1.024441	1.0195825	1.02845365	1.453018	0.53905262	0.9062
DNAJB12	1.273135	1.2675956	1.27771035	1.0377987	0.05352666	0.90629
CTD-2319I12.2	1.04058	1.0356668	1.04463756	1.2515174	0.32367832	0.90632
RP1-138B7.7	1.039714	1.0445297	1.03573734	0.8025505	-0.317336	0.90634
ZNF527	1.072617	1.0776405	1.06846901	0.8818721	-0.1813587	0.90636
TSHZ3	1.105087	1.1001291	1.10918133	1.0904059	0.12486524	0.90638
FAM149A	1.078364	1.0734443	1.08242653	1.1223005	0.16645906	0.90642
ABHD12B	1.020549	1.0254197	1.01652706	0.650168	-0.6211156	0.90643
TRAPPC8	1.169383	1.1744511	1.16519742	0.9469556	-0.0786313	0.90644
PCBP3	1.135623	1.1407752	1.13136861	0.9331804	-0.0997721	0.90649
RP11-28H5.2	1.019568	1.0147494	1.02354693	1.5964618	0.67487799	0.90651
CTC-487M23.8	1.0196	1.0147444	1.02361069	1.6013282	0.679269	0.90651
KB-68A7.2	1.019889	1.0246717	1.01593993	0.6460812	-0.6302126	0.90653
PRRG1	1.378086	1.3730248	1.38226591	1.0247734	0.03530499	0.90656
GYPC	2.353188	2.3602013	2.34739713	0.9905866	-0.013645	0.90659
EXOC4	1.511086	1.5053982	1.51578307	1.0205479	0.02934396	0.90663
RP11-279O9.4	1.075073	1.0701544	1.07913445	1.1280043	0.17377262	0.90666
PDK4	1.127003	1.1218489	1.13125869	1.0772253	0.10731997	0.90677
POU3F4	1.029113	1.0340022	1.02507537	0.7374622	-0.4393589	0.90678
STARD13	1.09551	1.0904448	1.09969357	1.1022588	0.14046293	0.90684
ADORA1	1.062807	1.0675652	1.05887823	0.8714286	-0.1985457	0.9069
DENND4A	1.339948	1.3453564	1.33548118	0.9714058	-0.041854	0.90691
CCDC40	1.128628	1.133662	1.12447039	0.9312327	-0.1027864	0.90692
ZC3HAV1L	1.043349	1.0481034	1.03942202	0.8195269	-0.2871368	0.90695
RP11-379H18.1	1.125625	1.1307688	1.12137678	0.9281785	-0.1075257	0.90699
NAALAD2	1.248354	1.2431979	1.25261109	1.0387058	0.05478713	0.907
GABBR1	1.159799	1.1647115	1.15574236	0.9455465	-0.0807797	0.90702
AP2A2	1.657059	1.6511527	1.66193734	1.0165624	0.02369881	0.90704
RP11-96L14.8	1.034984	1.0301285	1.03899393	1.2942524	0.37211896	0.90706
COG8	1.200203	1.2053575	1.19594682	0.954174	-0.0676757	0.90706

L1TD1	1.046757	1.0419468	1.05072997	1.2093894	0.27427881	0.90709
EEF1A2	1.078558	1.0833955	1.07456367	0.8940974	-0.1614961	0.9071
KCNK7	1.019884	1.0150885	1.02384385	1.5802717	0.66017259	0.90717
CTD-2245F17.6	1.019878	1.0150885	1.02383288	1.579544	0.65950814	0.90717
AC007880.1	1.019879	1.0150885	1.02383536	1.5797086	0.65965846	0.90717
TFAP2E	1.01992	1.0150885	1.02390966	1.5846333	0.66414898	0.90717
RAP2A	1.325077	1.3304814	1.32061389	0.970142	-0.0437321	0.9072
GBE1	1.801971	1.7952521	1.8075192	1.0154254	0.02208423	0.9072
LINC00525	1.021582	1.0167947	1.02553492	1.5204127	0.60446294	0.90729
RAB42	1.070796	1.0658944	1.07484462	1.1358263	0.1837422	0.9073
ANKRD16	1.127913	1.1329107	1.1237852	0.931341	-0.1026186	0.9073
STARD3NL	2.048857	2.0564873	2.04255587	0.9868134	-0.0191507	0.90737
SPICE1	1.321046	1.3263514	1.31666425	0.9703167	-0.0434723	0.90739
BLNK	1.041908	1.0370807	1.0458952	1.2377121	0.3076758	0.90739
PCF11-AS1	1.041852	1.0369403	1.04590744	1.2427471	0.31353274	0.90739
CTD-2292M16.8	1.036969	1.0417119	1.03305169	0.7923803	-0.3357351	0.90742
PUS7	1.140768	1.1356156	1.14502228	1.069363	0.0967517	0.90742
S100A8	1.020458	1.0156669	1.0244141	1.5583188	0.63999042	0.90743
CCDC144A	1.051386	1.0560905	1.04750092	0.8468619	-0.2398013	0.90743
TAF1A	1.115843	1.120611	1.11190572	0.9278232	-0.1080781	0.90753
RP11-680F20.9	1.017858	1.0225722	1.01396402	0.6186366	-0.692836	0.90754
LINC01516	1.017891	1.0226462	1.01396402	0.616616	-0.6975557	0.90754
FLJ42969	1.017891	1.0226462	1.01396402	0.616616	-0.6975557	0.90754
FHOD3	1.06893	1.0641934	1.07284239	1.1347325	0.18235225	0.90757
RP11-38M8.1	1.034031	1.0387639	1.03012235	0.777073	-0.3638779	0.90757
HEATR3	1.068289	1.0634804	1.07225997	1.1383035	0.18688524	0.9076
AATK-AS1	1.023512	1.0282509	1.01959942	0.6937632	-0.5274847	0.9076
CCDC81	1.051353	1.0562108	1.04734154	0.8422141	-0.247741	0.90762
CTA-228A9.4	1.033922	1.0291457	1.0378655	1.2991809	0.37760231	0.90768
EXTL3	1.254235	1.2491953	1.25839755	1.0369278	0.05231542	0.90768
RP11-290D2.6	1.041638	1.0465633	1.03757139	0.8068884	-0.309559	0.90769
ADNP-AS1	1.033016	1.0281885	1.03700336	1.3127095	0.39254765	0.90778
RP11-128I7.1	1.118629	1.1234851	1.114619	0.928201	-0.1074909	0.90784
SH2D3C	1.042811	1.0475443	1.03890155	0.8182169	-0.2894448	0.90791
RP11-65J3.3	1.023289	1.0185034	1.02724091	1.4722139	0.55798726	0.908
C9orf129	1.017106	1.0123699	1.02101677	1.6990277	0.76470935	0.90803
IGFBP7-AS1	1.017104	1.0123699	1.02101375	1.6987839	0.76450234	0.90803
RGL3	1.21632	1.2218992	1.21171326	0.9540964	-0.067793	0.90814
CTD-2105E13.6	1.017973	1.0132184	1.02189937	1.6567297	0.72833825	0.90816
KCNN2	1.017971	1.0132256	1.02188909	1.6550512	0.72687586	0.90816
PHEX	1.017937	1.0132207	1.02183237	1.6513814	0.72367337	0.90816
RP11-442H21.2	1.068116	1.0727946	1.06425292	0.8826608	-0.180069	0.90818
ZNF559	1.162862	1.1678854	1.15871327	0.9453669	-0.0810537	0.90819
KIF1A	2.135806	2.1303148	2.14034005	1.0088694	0.01273943	0.90819
GRIN2A	1.0161	1.0113663	1.02000854	1.7603368	0.81585149	0.90824

RP4-761J14.9	1.016101	1.0113663	1.0200104	1.7605002	0.81598537	0.90824
ZNF750	1.016088	1.0113663	1.01998642	1.7583905	0.81425547	0.90824
PTGER2	1.016092	1.0113663	1.01999411	1.7590674	0.81481076	0.90824
CTC-366B18.2	1.025477	1.0303107	1.02148512	0.7088286	-0.4964913	0.90833
F8A1	1.105011	1.1001152	1.10905468	1.0892923	0.12339114	0.90837
RP1-90J20.8	1.056246	1.061143	1.05220282	0.8537819	-0.2280605	0.90839
NOL6	1.0781	1.0733494	1.08202393	1.1182636	0.16126025	0.90847
CTB-50L17.16	1.0344	1.0391773	1.03045412	0.777341	-0.3633805	0.90853
ABI3	1.024726	1.0294154	1.02085421	0.7089549	-0.4962343	0.90859
RP11-475E11.9	1.024732	1.0293917	1.02088414	0.7105451	-0.4930019	0.90859
FBXO48	1.078085	1.0732436	1.08208358	1.1206926	0.16439055	0.90867
FAM228A	1.032293	1.0369748	1.02842684	0.7688174	-0.3792871	0.90871
JDP2	1.043652	1.0389199	1.04755954	1.2219867	0.28922858	0.90875
AC092171.2	1.022544	1.0272816	1.01863153	0.6829338	-0.5501824	0.90879
FAM26D	1.023083	1.0278243	1.0191667	0.6888468	-0.5377449	0.90882
RP11-705O1.8	1.030001	1.0252082	1.03395959	1.347167	0.4299287	0.90885
FBXO31	1.097067	1.1017615	1.09318989	0.9157677	-0.1269464	0.90891
ABCB5	1.024554	1.0198252	1.02845968	1.4355287	0.52158219	0.90906
HIST2H2AB	1.024574	1.0198163	1.02850362	1.4383921	0.52445704	0.90906
C1orf194	1.039636	1.0348874	1.04355793	1.2485286	0.32022888	0.90906
RP11-352G18.2	1.016723	1.0120052	1.02061938	1.7175436	0.78034676	0.90919
RP11-547D24.3	1.01672	1.0120052	1.02061389	1.7170868	0.77996296	0.90919
CTD-2537I9.18	1.016712	1.0120052	1.02059969	1.7159035	0.77896842	0.90919
P2RX6	1.087999	1.0926543	1.08415489	0.9082673	-0.1388112	0.90921
ANGPTL6	1.026585	1.0218203	1.03051997	1.3986937	0.48408004	0.90924
SV2C	1.026583	1.0218524	1.03048979	1.3952588	0.4805327	0.90924
GRM5	1.026568	1.0218179	1.0304903	1.3974894	0.48283733	0.90924
RP13-216E22.4	1.024494	1.0197964	1.02837404	1.4332935	0.51933409	0.90925
RDH12	1.022855	1.0276084	1.01892889	0.6856197	-0.5445196	0.90926
MOCS3	1.089482	1.094334	1.08547431	0.9060815	-0.1422872	0.90927
ARHGEF25	1.183567	1.1885056	1.17948914	0.9521689	-0.0707107	0.9093
RP11-245J9.5	1.017104	1.0124282	1.02096548	1.6869345	0.75440399	0.90947
KCNJ6	1.017122	1.0124282	1.02099823	1.6895694	0.75665557	0.90947
CTD-2653M23.3	1.017123	1.0124282	1.02100078	1.6897749	0.75683107	0.90947
CA4	1.017109	1.0124282	1.02097499	1.6877	0.7550585	0.90947
PALD1	1.080884	1.085663	1.07693785	0.8981459	-0.1549783	0.90947
KB-1183D5.13	1.024762	1.0200598	1.02864517	1.4279864	0.51398219	0.9095
AC068057.1	1.024801	1.0200705	1.02870772	1.4303473	0.51636546	0.9095
ZFP14	1.528815	1.5236263	1.53310076	1.0180939	0.0258706	0.90954
TEPSIN	1.102138	1.1071	1.09804081	0.9154139	-0.1275039	0.90954
MAPRE3	1.25791	1.252842	1.2620959	1.0365995	0.05185858	0.90956
RP11-932O9.10	1.02742	1.0226961	1.03132091	1.3800118	0.46468056	0.90958
EXTL3-AS1	1.027473	1.0227848	1.03134413	1.3756584	0.46012223	0.90958
DCLK2	1.155194	1.1504032	1.15914948	1.0581522	0.08154719	0.90959
GEMIN5	1.105316	1.1099819	1.10146363	0.9225485	-0.1163034	0.90967

LINC00629	1.02564	1.0209783	1.02948989	1.4057301	0.49131958	0.90971
CLASRP	1.292154	1.2974145	1.28781026	0.9677077	-0.0473568	0.90977
C2orf74	1.411914	1.4171677	1.40757492	0.9770051	-0.0335621	0.90984
PYGB	1.309228	1.3145829	1.3048057	0.9689203	-0.0455502	0.90991
RP11-418H16.1	1.046366	1.0511371	1.04242622	0.8296557	-0.2694153	0.90993
DISP1	1.112424	1.1174312	1.10828962	0.922154	-0.1169204	0.90999
AC008753.6	1.016243	1.0208536	1.01243594	0.596344	-0.7457833	0.91001
TBR1	1.016271	1.0209149	1.01243594	0.5945976	-0.7500144	0.91001
SDCCAG8	1.782922	1.7891594	1.77777138	0.9855694	-0.0209706	0.91013
RP11-121C2.2	1.060884	1.0561159	1.06482115	1.1551299	0.20805511	0.91014
SWT1	1.099825	1.0951127	1.10371699	1.0904643	0.1249426	0.91014
GCC2-AS1	1.027112	1.0224164	1.03098899	1.3824268	0.46720314	0.91014
LA16c-360H6.3	1.02823	1.0328517	1.0244139	0.7431553	-0.4282644	0.91016
DYNC2H1	1.529606	1.5241587	1.53410472	1.0189752	0.02711901	0.91017
AC006942.4	1.168897	1.1640548	1.17289503	1.0538857	0.07571843	0.91017
ZNF549	1.095996	1.1007798	1.09204467	0.9133243	-0.1308008	0.91021
PRTN3	1.069712	1.074611	1.06566716	0.8801267	-0.1842169	0.91029
RP11-390P24.1	1.051557	1.05621	1.04771366	0.8488461	-0.236425	0.91031
COQ10B	1.27255	1.2778217	1.26819659	0.965355	-0.0508686	0.91031
RP11-128P17.2	1.073116	1.068263	1.07712347	1.1297992	0.17606633	0.91032
CFAP161	1.021519	1.0261199	1.01771955	0.6783928	-0.5598073	0.91033
SHE	1.021541	1.0261773	1.01771268	0.676642	-0.5635353	0.91033
CTD-3065B20.3	1.021534	1.0261658	1.01770935	0.6768118	-0.5631734	0.91033
PDE4B	1.115795	1.1204697	1.11193384	0.9291453	-0.1060238	0.91035
MFSD13A	1.074719	1.0699767	1.07863544	1.1237371	0.16830457	0.91045
L3MBTL3	1.208065	1.2130269	1.20396726	0.9574721	-0.0626977	0.9105
RBM26-AS1	1.09996	1.1046231	1.09610931	0.918624	-0.1224536	0.9106
RP11-634H22.1	1.060582	1.055885	1.0644601	1.1534415	0.20594482	0.91062
USH2A	1.019587	1.0149336	1.0234301	1.5689524	0.6498016	0.91063
LA16c-306E5.2	1.019615	1.014931	1.02348263	1.5727449	0.65328465	0.91063
OSCAR	1.019575	1.0149315	1.02340944	1.5677938	0.64873582	0.91063
PAH	1.022342	1.0176743	1.02619632	1.4821707	0.56771162	0.91064
SPX	1.022314	1.0176711	1.02614856	1.4797354	0.56533919	0.91064
CH507-154B10.1	1.10683	1.1020159	1.11080533	1.0861576	0.11923352	0.91068
LINC00092	1.018775	1.0234186	1.01494071	0.6379833	-0.6484094	0.91075
GOLGA6L2	1.018784	1.0234382	1.01494071	0.6374517	-0.6496121	0.91075
EOMES	1.018782	1.0234333	1.01494071	0.6375833	-0.6493142	0.91075
C8orf34	1.025236	1.0205673	1.02909182	1.4144682	0.50025976	0.91087
CTA-276F8.1	1.06005	1.0646777	1.05622767	0.8693516	-0.2019883	0.91092
CACNB3	1.474765	1.4802191	1.47026007	0.9792616	-0.0302338	0.91093
RP11-626G11.3	1.132467	1.1278438	1.13628451	1.0660239	0.09223978	0.91098
PPM1A	1.799637	1.8055374	1.79476351	0.9866252	-0.0194259	0.91099
MYH7B	1.076925	1.0721354	1.08088056	1.1212331	0.16508627	0.911
RP1-78O14.1	1.029225	1.0245612	1.03307649	1.3466947	0.42942285	0.91101
NCAN	1.019893	1.0245297	1.01606403	0.6548804	-0.6106966	0.91104

TPBGL	1.019871	1.0244603	1.01608063	0.6574173	-0.6051187	0.91104
CCDC7	1.061115	1.0658257	1.05722418	0.8693285	-0.2020266	0.91114
RAD51	1.13803	1.1425133	1.13432703	0.9425577	-0.0853471	0.91123
DLGAP3	1.028399	1.0330029	1.02459624	0.7452762	-0.4241529	0.91124
RP11-264B17.4	1.025619	1.0301645	1.02186598	0.7248902	-0.4641656	0.91125
ZNF821	1.426579	1.4212406	1.43098839	1.0231407	0.03300459	0.91133
RP11-16K12.1	1.031306	1.0359197	1.02749636	0.7654957	-0.3855339	0.91137
KPNA5	1.458569	1.4637668	1.45427631	0.979536	-0.0298296	0.91142
CDC7	1.191606	1.1962145	1.18779947	0.9571133	-0.0632384	0.91143
ARHGEF16	1.195184	1.1901643	1.1993297	1.0481975	0.06791063	0.91147
RILP	1.169265	1.1642718	1.17338846	1.0554974	0.07792304	0.91151
RP11-132A1.4	1.048382	1.0436657	1.05227611	1.1971883	0.25965007	0.91151
RP11-318K12.2	1.024029	1.0194051	1.02784693	1.4350321	0.52108302	0.91159
TMEM184A	1.024039	1.0194105	1.02786086	1.4353471	0.52139969	0.91159
ACVR2B-AS1	1.024027	1.0194041	1.02784466	1.4349851	0.52103577	0.91159
PRRX2	1.021622	1.016987	1.02544871	1.4981243	0.58315728	0.91165
RP11-260M19.2	1.021617	1.0169894	1.02543886	1.4973348	0.58239685	0.91165
PLD4	1.02159	1.016985	1.02539271	1.4950056	0.58015092	0.91165
TRIL	1.070343	1.0656716	1.07420121	1.1298824	0.17617266	0.91168
DDX58	1.082541	1.0779036	1.08637105	1.1086915	0.14885795	0.91171
SCAMP4	1.50124	1.4958947	1.50565345	1.0196791	0.02811515	0.91176
HOGA1	1.051572	1.0561179	1.04781707	0.8520821	-0.2309357	0.91177
CEP95	1.385497	1.3904888	1.38137417	0.9766585	-0.0340739	0.91183
DHDH	1.049645	1.0542087	1.04587682	0.8463007	-0.2407578	0.91188
PTPN7	1.05582	1.0511682	1.05966183	1.1659938	0.22156007	0.91189
RP11-848P1.5	1.055883	1.0512159	1.05973752	1.166387	0.22204659	0.91189
RP11-158H5.7	1.022183	1.0175649	1.02599716	1.4800642	0.5656598	0.9119
RP11-307L14.2	1.021816	1.0264112	1.018022	0.6823613	-0.5513922	0.91191
PIH1D3	1.021769	1.0263144	1.01801558	0.6846281	-0.5466075	0.91191
ADCK5	1.140963	1.1457975	1.1369699	0.939453	-0.0901071	0.91191
EIF4ENIF1	1.182124	1.1870067	1.17809168	0.9523278	-0.0704698	0.91195
CYP46A1	1.147511	1.1523045	1.14355241	0.9425356	-0.0853809	0.91199
BCKDHA	1.04568	1.0411043	1.04945863	1.203247	0.26693288	0.912
RP3-395M20.12	1.097128	1.1017566	1.09330528	0.9169458	-0.1250917	0.91202
RP1-168L15.5	1.062389	1.0576909	1.06626961	1.1487004	0.20000251	0.91202
C1orf168	1.029076	1.0244632	1.0328845	1.3442445	0.42679561	0.91207
SRPX2	1.029075	1.0244269	1.03291435	1.3474606	0.43024304	0.91207
KCTD8	1.031834	1.0271733	1.03568206	1.3131279	0.39300748	0.91208
ZFHX2	1.184163	1.1890195	1.1801532	0.953093	-0.0693111	0.91223
EXOC3L1	1.023126	1.0276391	1.01939936	0.7018811	-0.5107015	0.91225
SSTR3	1.02318	1.0277519	1.0194047	0.6992211	-0.5161794	0.91225
ATXN3	1.564043	1.569215	1.55977258	0.9834115	-0.0241329	0.91225
LINC01023	1.143054	1.1477104	1.13920809	0.9424394	-0.0855283	0.91227
RP11-128A17.2	1.043346	1.0386444	1.04722884	1.2221386	0.28940791	0.91229
PNPLA7	1.030824	1.0262334	1.03461407	1.3194649	0.39995293	0.91238

SLC26A10	1.046393	1.0418382	1.05015473	1.1987782	0.26156481	0.91239
LPL	1.066271	1.0708489	1.06249076	0.8820285	-0.1811028	0.9124
RP11-3D4.3	1.024977	1.0203911	1.02876354	1.4105898	0.49629852	0.91246
CD55	1.071143	1.0665252	1.07495704	1.1267464	0.17216283	0.91247
CTBP1-AS	1.022756	1.0273318	1.01897772	0.6943461	-0.5262731	0.91252
TMEM63C	1.022727	1.027258	1.0189851	0.6964974	-0.5218101	0.91252
ARID4B	2.657436	2.649473	2.66401132	1.0088139	0.01266004	0.91253
NPY	1.019676	1.0151072	1.02344854	1.5521393	0.63425804	0.91263
RP5-1021I20.1	1.054487	1.0590538	1.0507161	0.8588125	-0.2195849	0.91264
GCN1	1.187943	1.183361	1.19172647	1.0456231	0.06436288	0.91265
MTO1	1.122576	1.1272806	1.11869116	0.9325157	-0.1008001	0.91272
MTRNR2L4	1.017813	1.0132644	1.02156967	1.626128	0.70144079	0.91273
RP11-459F6.3	1.01784	1.0132712	1.02161283	1.6285539	0.70359145	0.91273
AL358852.1	1.017821	1.0132614	1.02158646	1.6277712	0.70289797	0.91273
PDPN	1.834958	1.8410457	1.82992985	0.9867833	-0.0191948	0.91276
DUSP9	1.119681	1.1242603	1.11589958	0.9327163	-0.1004898	0.91276
BCL6	1.095106	1.0996849	1.09132387	0.9161257	-0.1263826	0.91279
CTD-2376I4.2	1.02299	1.0275041	1.01926268	0.700356	-0.5138396	0.91291
UBA6-AS1	1.188803	1.1838807	1.1928677	1.048874	0.06884141	0.91292
ANKLE1	1.057884	1.0532068	1.06174731	1.1605152	0.2147654	0.91293
HIST1H4I	1.030628	1.0352126	1.02684201	0.7622854	-0.3915969	0.91301
PPY	1.079915	1.0752338	1.08378036	1.1135997	0.15523078	0.91302
RP11-101E13.5	1.03107	1.0265074	1.03483877	1.3143038	0.39429875	0.91309
LINC01598	1.020792	1.025328	1.01704689	0.6730454	-0.5712242	0.91311
CD300E	1.020774	1.0252883	1.01704689	0.6741022	-0.5689608	0.91311
CLEC4F	1.045534	1.0409199	1.0493436	1.2058578	0.27005981	0.91317
ITGA2B	1.053086	1.048416	1.0569421	1.176101	0.23401196	0.91317
AC005618.6	1.018677	1.0141483	1.02241762	1.5844758	0.66400565	0.91329
RP11-167P20.1	1.018722	1.0141585	1.02249128	1.5885404	0.66770175	0.91329
TIRAP	1.078092	1.0827242	1.07426687	0.897765	-0.1555902	0.9133
RAB35	1.185041	1.1897864	1.18112248	0.9543488	-0.0674114	0.91331
C11orf87	1.021022	1.0255239	1.01730453	0.6779727	-0.5607008	0.91339
ZNF182	1.086382	1.0911004	1.08248609	0.9054415	-0.1433067	0.9134
TTC39C	1.358477	1.3638745	1.35401976	0.9729173	-0.0396109	0.9134
RSPH6A	1.023145	1.0276899	1.0193919	0.7003236	-0.5139064	0.9134
RP11-81H3.2	1.023125	1.0276456	1.0193919	0.701447	-0.5115941	0.9134
FKBP6	1.023105	1.0276009	1.0193919	0.7025818	-0.5092618	0.9134
EPB41L1	1.346263	1.3513805	1.34203642	0.9734074	-0.0388843	0.91342
SHISA7	1.022138	1.0266324	1.01842611	0.6918671	-0.5314331	0.91347
C12orf4	1.150589	1.155214	1.14676938	0.9455934	-0.0807081	0.91352
ARHGEF28	1.124166	1.1289261	1.12023491	0.9325876	-0.1006889	0.91356
RP11-41O4.1	1.021054	1.0255341	1.01735489	0.6796756	-0.5570818	0.91358
RP11-728K20.3	1.018413	1.0138969	1.02214156	1.593269	0.67198985	0.91368
CTD-2021H9.3	1.028326	1.0237381	1.0321146	1.3528741	0.43602764	0.9137
CTD-2139B15.2	1.028292	1.0237083	1.03207801	1.353029	0.43619273	0.9137

PLCG1	1.248439	1.2530961	1.24459318	0.9664044	-0.0493011	0.9137
PTGIR	1.026213	1.0216884	1.02994911	1.3808822	0.46559022	0.91371
RP11-226E21.4	1.023986	1.0284604	1.02029145	0.7129715	-0.4880837	0.91374
ALG10	1.055895	1.051263	1.05972076	1.1649882	0.22031537	0.91377
NEURL1B	1.054942	1.0504056	1.05868826	1.1643206	0.21948836	0.91381
POLA2	1.150536	1.1552269	1.14666193	0.9448232	-0.0818837	0.91382
RP3-486I3.7	1.03601	1.0404804	1.03231827	0.7983676	-0.3248749	0.91387
CTC-246B18.10	1.036029	1.0404782	1.03235484	0.7993157	-0.3231626	0.91387
DPH3	1.665602	1.660352	1.66993744	1.0145157	0.02079114	0.9139
TTLL12	1.307841	1.3026656	1.31211446	1.0312187	0.04435038	0.91395
OCM	1.018825	1.0143212	1.02254388	1.5741619	0.65458392	0.91395
SCUBE2	1.018849	1.0143306	1.02258006	1.5756558	0.65595241	0.91395
SEC14L2	1.292065	1.297086	1.28791805	0.9691403	-0.0452226	0.91395
RP11-368I23.4	1.028365	1.0237884	1.03214393	1.3512412	0.43428518	0.91396
RP11-285A1.1	1.025935	1.0214112	1.02967063	1.3857535	0.47067063	0.914
RP13-753N3.1	1.021493	1.0169431	1.02525076	1.4903252	0.57562718	0.91405
ZNF250	1.155742	1.1604766	1.15183187	0.9461307	-0.0798886	0.91408
RASA1	1.264665	1.2599589	1.26855149	1.0330537	0.04691526	0.91409
RP11-30K9.6	1.037927	1.0424414	1.03419914	0.8057971	-0.3115115	0.91418
RP11-147L13.13	1.076406	1.0810054	1.0726085	0.896342	-0.1578788	0.91422
TVP23C-CDRT4	1.037145	1.0416212	1.03344865	0.8036441	-0.3153713	0.91423
RP11-597D13.9	1.078522	1.0830959	1.07474395	0.8994899	-0.1528209	0.91423
NAF1	1.167532	1.1721811	1.16369189	0.9506961	-0.0729438	0.91424
LEMD2	1.35918	1.354223	1.36327399	1.0255515	0.0364	0.91436
OSGIN2	1.131993	1.1273099	1.13586085	1.0671661	0.09378468	0.91447
CXorf23	1.163111	1.1680422	1.15903852	0.94642	-0.0794475	0.91455
CTB-161M19.4	1.025932	1.0303618	1.02227399	0.7336199	-0.4468954	0.91455
RP11-147L13.8	1.028891	1.0243348	1.03265409	1.3418705	0.42424546	0.9146
RP11-276E15.4	1.028906	1.0244353	1.0325984	1.3340717	0.41583617	0.9146
RP11-388M20.1	1.019787	1.0242046	1.01613888	0.6667702	-0.5847385	0.91464
GBP4	1.052551	1.0570126	1.04886571	0.8571044	-0.2224572	0.91475
ABALON	1.028417	1.0329307	1.02468893	0.7497246	-0.4155673	0.91477
STXBP5L	1.027639	1.0321395	1.02392314	0.7443532	-0.4259408	0.91479
RP11-582J16.5	1.027716	1.032222	1.02399421	0.7446537	-0.4253585	0.91479
ZNF202	1.076507	1.0719223	1.08029243	1.1163781	0.15882569	0.9148
SASH1	1.296933	1.2925765	1.3005308	1.0271872	0.03869908	0.9148
RENBP	1.289598	1.2941933	1.28580346	0.9714817	-0.0417412	0.91482
NEURL2	1.105079	1.109417	1.10149687	0.9276155	-0.1084011	0.91486
RP2	1.113299	1.1179046	1.10949479	0.9286726	-0.1067581	0.91493
STIM1	1.166277	1.1618004	1.16997394	1.050516	0.0710982	0.91506
RP11-299L17.3	1.035704	1.0312083	1.03941627	1.2630047	0.33686	0.91508
USP4	1.178022	1.1826771	1.17417711	0.95347	-0.0687405	0.9151
RP11-944C7.1	1.021295	1.0168351	1.02497726	1.4836398	0.56914089	0.91511
RP11-1017G21.6	1.021308	1.0168422	1.02499614	1.4841398	0.56962697	0.91511
AC015691.13	1.039119	1.0435432	1.03546604	0.8145031	-0.2960079	0.91511

RP11-893F2.6	1.024061	1.0196055	1.02774127	1.4149723	0.50077385	0.91512
CTB-5E10.3	1.024108	1.0196075	1.02782482	1.4190918	0.50496792	0.91512
SEPSECS-AS1	1.057646	1.0621196	1.05395235	0.8685236	-0.2033631	0.91512
RP11-387M24.5	1.027937	1.0234419	1.03164923	1.3501159	0.43308331	0.91513
FAM124A	1.049697	1.0542317	1.04595284	0.8473428	-0.2389823	0.91525
SLC25A25	1.090206	1.0857285	1.09390336	1.0953573	0.13140154	0.91525
RP11-73K9.3	1.026953	1.0225572	1.03058269	1.3557839	0.43912719	0.91532
GON4L	1.447261	1.4524957	1.44293761	0.9788769	-0.0308006	0.91536
CTBS	1.457595	1.4524747	1.46182345	1.0206613	0.02950418	0.91536
MILR1	1.035481	1.030972	1.03920406	1.2657899	0.34003797	0.91536
RP11-587P21.2	1.025638	1.0300788	1.02197122	0.7304547	-0.4531333	0.91539
RP11-408B11.2	1.025674	1.0300657	1.02204785	0.7333214	-0.4474825	0.91539
RP11-326G21.1	1.028337	1.0238969	1.03200367	1.3392416	0.42141624	0.9154
TRAPPC5	1.139552	1.1350128	1.1432999	1.0613802	0.08594161	0.91542
GUCY2D	1.038629	1.0341686	1.04231198	1.23833	0.30839588	0.91542
RP11-368I7.4	1.030983	1.0265061	1.03468018	1.3083839	0.38778595	0.91546
TEAD4	1.103719	1.0992677	1.10739573	1.0818803	0.11354082	0.91551
RP11-307C19.1	1.026725	1.0312145	1.02301779	0.7374063	-0.4394684	0.91556
RP5-967N21.11	1.029475	1.0338186	1.02588726	0.7654729	-0.3855769	0.91567
HAAO	1.08287	1.0873664	1.07915664	0.9060312	-0.1423674	0.91568
ZNF484	1.052615	1.0481603	1.05629321	1.1688724	0.22511751	0.91574
CENPO	1.112719	1.1079619	1.11664676	1.0804432	0.11162325	0.91578
KDELR3	1.470877	1.4656151	1.47522158	1.0206319	0.02946265	0.91579
TOM1L2	1.252266	1.2472331	1.2564221	1.0371673	0.05264868	0.91587
DNA2	1.162178	1.1575147	1.16602939	1.0540563	0.0759519	0.91592
C2CD4C	1.279226	1.2745233	1.28310957	1.0312771	0.04443203	0.91593
NFATC1	1.050024	1.0454888	1.05377003	1.182051	0.24129226	0.91593
NCR3LG1	1.047736	1.0521302	1.0441067	0.8460866	-0.2411227	0.91595
POM121C	1.454386	1.4494466	1.45846539	1.0200664	0.02866306	0.91599
AC006994.2	1.033591	1.0379242	1.03001342	0.7914046	-0.3375127	0.916
TCTEX1D4	1.025772	1.0213156	1.0294522	1.3817214	0.46646674	0.91606
KCNN4	1.025743	1.0213415	1.0293776	1.3765482	0.46105512	0.91606
RP11-806L2.2	1.025803	1.0213554	1.02947537	1.3802298	0.46490852	0.91606
SP3	1.555537	1.5501667	1.55997167	1.0178218	0.02548498	0.91608
IBA57-AS1	1.023324	1.0188795	1.02699393	1.4298003	0.51581369	0.91613
RP11-1072C15.4	1.023326	1.0188807	1.02699646	1.4298412	0.51585489	0.91613
RP11-201E8.1	1.023361	1.0189101	1.02703753	1.4297934	0.51580674	0.91613
RP5-991G20.6	1.023375	1.0189029	1.02706882	1.4319912	0.51802263	0.91613
LINC01405	1.023302	1.0189108	1.02692895	1.4240008	0.50994998	0.91613
KCND1	1.03769	1.0333063	1.04131046	1.2403195	0.31071183	0.91617
RGS6	1.030428	1.034791	1.02682574	0.7710536	-0.375097	0.91618
CCDC163	1.050744	1.0551902	1.04707171	0.8528999	-0.2295517	0.91619
CLCNKA	1.025029	1.0206035	1.02868426	1.3922049	0.47737151	0.9162
MAST4	1.433875	1.4289087	1.43797554	1.0211393	0.03017972	0.91624
RP11-90D4.3	1.038672	1.0432	1.03493347	0.8086445	-0.3064226	0.91629

ENPP4	1.135226	1.1306968	1.13896632	1.0632725	0.08851135	0.91634
LAPTM5	1.031414	1.0357546	1.02782881	0.7783273	-0.3615511	0.9164
SURF4	1.97826	1.971804	1.98359194	1.0121299	0.01739449	0.91642
SPDYA	1.030848	1.0263547	1.03455785	1.3112603	0.39095406	0.91652
AP000473.8	1.021503	1.0259028	1.01786906	0.6898515	-0.5356423	0.91657
UBIAD1	1.139963	1.1353205	1.14379663	1.0626377	0.08764987	0.91662
GNGT2	1.024092	1.0285174	1.02043668	0.7166381	-0.4806833	0.91663
RP1-101D8.1	1.024053	1.0283841	1.02047655	0.7214081	-0.4711125	0.91663
CTD-2270P14.5	1.036471	1.0319836	1.04017666	1.2561644	0.32902526	0.91665
TUBD1	1.189672	1.1944371	1.18573711	0.9552554	-0.0660417	0.91673
CCDC88B	1.113959	1.1094632	1.11767259	1.0749969	0.10433246	0.91673
ATP9B	1.189835	1.1852152	1.19364949	1.045538	0.06424554	0.91678
RP11-389G6.3	1.03256	1.028053	1.03628221	1.2933432	0.37110515	0.91682
LINC00106	1.032548	1.0281164	1.0362081	1.2877948	0.36490274	0.91682
ONECUT3	1.021037	1.0253927	1.01744032	0.6868241	-0.5419873	0.91685
CCM2L	1.021013	1.0253828	1.01740504	0.6857028	-0.5443447	0.91685
SLC1A1	1.040432	1.0359963	1.04409449	1.2249716	0.29274832	0.91685
SNX15	1.021961	1.0263558	1.01833122	0.6955277	-0.52382	0.91695
RP11-503C24.2	1.021916	1.0262567	1.01833122	0.6981538	-0.5183832	0.91695
SNX13	1.361783	1.366665	1.35775195	0.9756917	-0.0355027	0.91699
PIK3R2	1.094075	1.098522	1.09040304	0.9175925	-0.1240745	0.91701
POLR2D	1.559862	1.5543462	1.56441728	1.0181675	0.02597489	0.91709
RP5-1024C24.1	1.021465	1.0170419	1.02511721	1.4738497	0.55958939	0.9171
AC026471.6	1.028821	1.0332062	1.02520018	0.7589	-0.3980183	0.91712
SLC38A10	1.303185	1.307838	1.29934295	0.9724042	-0.040372	0.91718
SLC44A4	1.019547	1.0151598	1.02317091	1.5284409	0.61206077	0.9172
RP11-83N9.5	1.019547	1.0151653	1.02316501	1.5274983	0.61117077	0.9172
RP11-360L9.4	1.019582	1.0151772	1.02321921	1.5298729	0.61341179	0.9172
RP11-295P9.13	1.019546	1.015183	1.02314968	1.5247132	0.60853794	0.9172
UBE2A	1.776398	1.781767	1.77196392	0.9874603	-0.0182053	0.91723
MVD	1.283398	1.2880702	1.27953969	0.9703875	-0.0433672	0.91728
VIM-AS1	1.226082	1.2307594	1.22222003	0.9629943	-0.0544008	0.91733
DPP10-AS1	1.060129	1.0557759	1.06372415	1.1425033	0.19219828	0.91741
AP4E1	1.096787	1.0923046	1.100488	1.0886564	0.12254874	0.91742
C9orf43	1.034563	1.0301898	1.03817406	1.2644683	0.33853083	0.91753
CRADD	1.241581	1.2461673	1.23779323	0.9659821	-0.0499316	0.91755
PGBD1	1.149932	1.1453327	1.1537293	1.0577749	0.08103263	0.91767
CD19	1.021058	1.0166975	1.02465895	1.4768008	0.56247524	0.91772
RP11-337C18.8	1.069833	1.0741242	1.06628861	0.8942908	-0.1611841	0.91775
EPM2A	1.056924	1.0524131	1.0606487	1.1571292	0.21054997	0.91775
XRCC3	1.110192	1.1055912	1.11399105	1.0795502	0.11043034	0.91776
CMP21-97G8.2	1.020415	1.016097	1.02398096	1.4897778	0.5750972	0.91777
RAB39A	1.020416	1.0160431	1.02402698	1.4976558	0.58270609	0.91777
RP11-799B12.4	1.020405	1.0160505	1.02400029	1.4952972	0.58043226	0.91777
FBLN2	1.084209	1.0797961	1.08785414	1.1009829	0.13879204	0.91783

SCFD2	1.117012	1.1213728	1.11340993	0.9343934	-0.097898	0.91785
TMEM257	1.052382	1.0566709	1.04884051	0.8618264	-0.2145308	0.91787
HEATR5B	1.10082	1.0964474	1.10443126	1.0827789	0.11473875	0.9179
ESR2	1.027972	1.0235727	1.03160484	1.3407416	0.42303118	0.91798
LRRC25	1.028015	1.0236287	1.03163666	1.3389079	0.42105674	0.91798
FOXE3	1.021405	1.0257068	1.01785328	0.6944966	-0.5259604	0.91802
CFAP52	1.022242	1.0265238	1.01870551	0.7052358	-0.5038224	0.91805
PMM2	1.075562	1.0799155	1.07196702	0.9005395	-0.1511385	0.91809
TMPRSS9	1.020128	1.0157987	1.02370377	1.5003579	0.58530667	0.91816
RP11-307C18.1	1.020135	1.0158194	1.02369956	1.4981341	0.58316672	0.91816
LRRC56	1.05761	1.0619321	1.05404117	0.8725868	-0.1966294	0.91818
ZNF518B	1.364497	1.3596583	1.36849371	1.024566	0.03501293	0.9182
TOMM40L	1.082424	1.0780693	1.08602045	1.1018476	0.1399247	0.91821
TM2D1	2.025183	2.0190918	2.0302138	1.0109137	0.01565979	0.91822
CEP85	1.136618	1.132061	1.14038082	1.0629995	0.08814093	0.91835
SKP2	1.390043	1.3947472	1.38615755	0.97824	-0.0317396	0.91837
CHRNA1	1.02757	1.0319007	1.02399346	0.7521306	-0.4109449	0.91838
FLT4	1.03004	1.0256695	1.033649	1.3108555	0.39050865	0.91841
SCNN1A	1.028223	1.023853	1.03183092	1.334463	0.41625931	0.91842
RP5-858L17.1	1.028258	1.0238847	1.0318704	1.3343423	0.41612877	0.91842
RNF215	1.055768	1.051366	1.05940281	1.1564607	0.20971619	0.91842
ENTPD3	1.019218	1.0148661	1.02281184	1.5344861	0.61775555	0.91844
TSNAX-DISC1	1.019199	1.0148661	1.02277634	1.5320981	0.61550864	0.91844
IL18R1	1.019179	1.0148661	1.02273981	1.5296406	0.6131927	0.91844
RP4-604G5.1	1.019191	1.0148661	1.02276186	1.531124	0.61459115	0.91844
RP5-1070A16.1	1.019196	1.0148661	1.0227713	1.5317591	0.61518939	0.91844
SERPINI2	1.019173	1.0148661	1.02273004	1.528983	0.61257237	0.91844
RP11-290M5.4	1.019218	1.0148661	1.02281097	1.534427	0.61770005	0.91844
RP11-1085N6.5	1.019174	1.0148661	1.02273075	1.5290307	0.61261741	0.91844
TRDN	1.019186	1.0148661	1.02275385	1.5305847	0.61408292	0.91844
LINC00482	1.019185	1.0148661	1.022751	1.5303929	0.61390211	0.91844
PPP1R3E	1.513162	1.5080113	1.51741618	1.0185132	0.02646464	0.91848
TECTA	1.02322	1.0188705	1.02681155	1.4208206	0.50672443	0.91852
KIAA0100	1.374048	1.368985	1.37822886	1.0250522	0.03569731	0.91864
WDPCP	1.136687	1.1322141	1.14038143	1.0617734	0.08647585	0.91876
FOXL1	1.024742	1.0290162	1.0212131	0.7310785	-0.4519018	0.91879
ID2-AS1	1.024795	1.0290743	1.02126083	0.7312583	-0.451547	0.91879
CTB-147N14.6	1.024796	1.0291044	1.02123856	0.7297368	-0.4545518	0.91879
ANGPT1	1.024791	1.0290908	1.02124011	0.7301322	-0.4537704	0.91879
DCAF17	1.144556	1.1491342	1.14077511	0.9439494	-0.0832185	0.91881
LMO2	1.099372	1.1037988	1.09571566	0.922127	-0.1169626	0.91894
GIP	1.067208	1.0715835	1.06359398	0.8883884	-0.1707375	0.91898
ZNF565	1.065191	1.0694672	1.0616596	0.8876074	-0.1720064	0.91903
SKAP2	1.076523	1.072072	1.08019862	1.1127573	0.15413891	0.91903
GALNT5	1.030567	1.0263096	1.03408222	1.2954308	0.37343197	0.91904

FUT8-AS1	1.033376	1.0376784	1.02982343	0.7915258	-0.3372918	0.91909
TNFSF13B	1.042893	1.038525	1.0465002	1.2070136	0.27144192	0.9191
RP11-158H5.8	1.047759	1.0520836	1.04418822	0.8484091	-0.237168	0.91913
SLC25A40	1.192988	1.1883219	1.19684157	1.0452401	0.06383444	0.91916
GMIP	1.065236	1.0695085	1.06170762	0.8877702	-0.1717418	0.91923
STARD8	1.017281	1.0129919	1.02082352	1.6028057	0.68059952	0.91932
RP11-351J23.2	1.017275	1.0129919	1.02081198	1.6019175	0.67979984	0.91932
LPO	1.017272	1.0129919	1.02080646	1.6014926	0.67941717	0.91932
TNFSF9	1.017253	1.0129919	1.02077125	1.5987822	0.67697339	0.91932
HHIP	1.017258	1.0129919	1.02078035	1.5994824	0.67760516	0.91932
GATA5	1.017332	1.0129919	1.02091532	1.6098718	0.68694581	0.91932
PQLC2L	1.017252	1.0129919	1.02077029	1.5987084	0.67690685	0.91932
RP11-76E17.4	1.017275	1.0129919	1.020812	1.6019186	0.67980085	0.91932
RP11-77K12.9	1.037525	1.0332439	1.04105958	1.2350996	0.30462735	0.91932
RP11-138A9.1	1.037472	1.0331145	1.04107089	1.2402709	0.31065528	0.91932
APOBEC3C	1.218229	1.2228125	1.21444304	0.9624373	-0.0552355	0.91939
ZNF33A	1.680194	1.6854173	1.67588106	0.9860869	-0.0202133	0.91948
B3GALT5	1.039489	1.0352013	1.04303049	1.2224129	0.28973163	0.9195
SLFN13	1.132123	1.1275747	1.13587944	1.0650971	0.09098503	0.91954
FAM163A	1.023053	1.0187432	1.0266122	1.4198299	0.50571806	0.91958
RP1-257A7.5	1.023036	1.0187669	1.02656112	1.4153171	0.50112535	0.91958
SLC22A1	1.02587	1.0215249	1.02945754	1.3685346	0.45263189	0.91959
RP11-646I6.5	1.025781	1.0214725	1.02933893	1.366352	0.45032917	0.91959
MCF2L-AS1	1.025803	1.0215279	1.02933396	1.3626025	0.44636477	0.91959
RP11-22N19.2	1.057701	1.0620174	1.05413725	0.8729362	-0.1960519	0.91964
CTA-243E7.1	1.105948	1.1015974	1.10954072	1.0781842	0.1086037	0.91971
YBX2	1.037734	1.0334128	1.041303	1.2361442	0.3058471	0.91976
THBS4	1.08111	1.076816	1.08465642	1.1020682	0.14021354	0.91977
RP11-44F21.5	1.127346	1.1227878	1.13111009	1.0677778	0.09461144	0.91981
HIP1R	1.195853	1.1915486	1.19940822	1.0410318	0.05801407	0.91981
GATM-AS1	1.055729	1.0599889	1.0522107	0.8703397	-0.2003495	0.91982
GPRIN1	1.030049	1.025696	1.03364297	1.3092708	0.38876356	0.91986
RP11-644F5.11	1.171094	1.1663303	1.17502725	1.0522871	0.07352836	0.91989
C2orf50	1.02495	1.029227	1.02141845	0.7328306	-0.4484484	0.91989
PKIB	2.635598	2.6452168	2.62765479	0.9893254	-0.0154829	0.91993
LINC00844	1.046018	1.0504582	1.04235062	0.8393209	-0.2527057	0.91994
XXbac-B135H6.18	1.100619	1.0962369	1.10423757	1.0831353	0.1152135	0.91996
EPHX4	1.093195	1.097489	1.08964985	0.9195897	-0.1209378	0.91998
C1orf158	1.018929	1.0146773	1.02244088	1.5289494	0.6125407	0.92003
RP11-876N24.4	1.018953	1.0146773	1.02248378	1.5318724	0.61529615	0.92003
RP11-454P21.1	1.018925	1.0146773	1.02243224	1.5283613	0.61198564	0.92003
CSE1L-AS1	1.018941	1.0146773	1.02246241	1.5304166	0.61392439	0.92003
XXbac-BPG154L12.5	1.018945	1.0146773	1.02246862	1.5308397	0.61432325	0.92003
BDKRB2	1.018924	1.0146773	1.02243074	1.528259	0.6118891	0.92003
TRPM8	1.01893	1.0146773	1.02244265	1.5290705	0.6126549	0.92003

MT1A	1.018978	1.0146773	1.02252962	1.5349955	0.61823444	0.92003
HRAT92	1.018925	1.0146773	1.02243324	1.5284288	0.61204937	0.92003
RP11-306O13.1	1.018924	1.0146773	1.02243153	1.5283126	0.61193968	0.92003
ANGPTL1	1.048268	1.0524991	1.0447744	0.8528596	-0.2296198	0.92024
APLF	1.077269	1.0816448	1.07365581	0.9021499	-0.148561	0.92025
TBC1D15	1.111022	1.1152631	1.10752012	0.9328234	-0.1003241	0.92025
VSIG10	1.319444	1.3146478	1.32340436	1.0278299	0.03960146	0.92028
GSTCD	1.234385	1.2298263	1.23815008	1.0362178	0.05132733	0.9203
RP11-290F5.2	1.040122	1.0359007	1.04360838	1.2146946	0.28059358	0.92035
RUSC1	1.469684	1.4648849	1.47364773	1.0188495	0.02694095	0.9204
ZNF347	1.125848	1.1302813	1.12218765	0.9378757	-0.0925314	0.92041
BTBD9	1.071729	1.0760704	1.0681445	0.8958079	-0.1587387	0.92045
CCDC39	1.028768	1.0330147	1.02526099	0.7651437	-0.3861974	0.9205
RP11-348N5.9	1.02757	1.0233552	1.03104977	1.3294605	0.41084092	0.92052
RP11-67L2.2	1.074062	1.0696923	1.07767117	1.1144869	0.15637969	0.92065
ANKRD12	2.609038	2.6159934	2.60329477	0.9921419	-0.0113816	0.92077
NTNG1	1.025639	1.0214434	1.02910455	1.3572716	0.44070947	0.92082
TIFA	1.249471	1.2538439	1.24585933	0.9685454	-0.0461083	0.92084
ZNF788	1.09298	1.0884579	1.09671403	1.0933339	0.12873412	0.92084
MAFA-AS1	1.104	1.1083818	1.10038149	0.9261839	-0.1106294	0.92086
ZNF596	1.029034	1.0331926	1.02560053	0.7712716	-0.3746891	0.92089
TMEM268	1.067839	1.0720342	1.06437468	0.8936685	-0.1621884	0.9209
NRG2	1.054424	1.0586841	1.0509061	0.8674595	-0.2051317	0.9209
RP11-318M2.3	1.019706	1.0239029	1.01624052	0.6794383	-0.5575856	0.92091
ZFHX3	1.61713	1.6222887	1.61286964	0.9848638	-0.0220039	0.92094
SSX2IP	1.633625	1.6288349	1.63758018	1.0139072	0.01992559	0.92095
HLA-DOA	1.035295	1.0310356	1.03881278	1.2505896	0.3226084	0.92098
RP11-603J24.21	1.035298	1.031058	1.03880037	1.2492894	0.32110767	0.92098
PCDHB12	1.03533	1.0310613	1.03885575	1.2509362	0.32300825	0.92098
CARF	1.302114	1.3067455	1.2982888	0.9724309	-0.0403323	0.92103
RP11-770G2.5	1.020193	1.0159741	1.02367651	1.4821793	0.56772003	0.92109
NUTM2E	1.020193	1.0159741	1.02367735	1.4822321	0.5677714	0.92109
RP11-83B20.1	1.020201	1.0159741	1.02369151	1.4831186	0.56863395	0.92109
NFIA-AS2	1.020171	1.0159741	1.02363719	1.4797182	0.56532241	0.92109
KLF2	1.020208	1.0159741	1.02370397	1.4838983	0.56939222	0.92109
PERM1	1.020192	1.0159741	1.02367471	1.4820665	0.56761017	0.92109
CTB-113P19.4	1.020231	1.0159741	1.02374662	1.4865682	0.57198561	0.92109
FCER1G	1.026851	1.031043	1.02338863	0.7534278	-0.4084589	0.92116
RP11-524C21.2	1.019291	1.0234592	1.01584836	0.6755707	-0.5658214	0.92119
SPIN2A	1.051596	1.0557942	1.04812967	0.8626282	-0.2131893	0.9212
AC144831.1	1.034229	1.0299658	1.03774963	1.2597578	0.33314635	0.92126
AC007038.7	1.022238	1.0180243	1.02571842	1.4268773	0.51286128	0.92127
AC116366.6	1.022272	1.0180243	1.0257804	1.4303156	0.51633355	0.92127
SLC6A11	1.022233	1.0180243	1.02570828	1.4263147	0.51229231	0.92127
ANXA8	1.022234	1.0180243	1.02571087	1.4264583	0.51243757	0.92127

HIST2H4B	1.02225	1.0180243	1.02573988	1.4280678	0.51406443	0.92127
RP11-77H9.8	1.020137	1.0159362	1.0236053	1.4812332	0.56679879	0.92128
SERPINB5	1.020144	1.0159362	1.02361861	1.4820685	0.56761217	0.92128
CLC	1.020162	1.0159362	1.02365117	1.4841118	0.56959979	0.92128
RP11-342D14.1	1.020126	1.0159362	1.0235856	1.4799973	0.56559456	0.92128
CAMP	1.020194	1.0159362	1.02370951	1.4877724	0.5731538	0.92128
UPP2	1.020133	1.0159362	1.02359921	1.480851	0.56642645	0.92128
RP4-800J21.3	1.020153	1.0159362	1.02363545	1.483125	0.56864016	0.92128
HOXD-AS2	1.020182	1.0159362	1.02368833	1.4864433	0.57186441	0.92128
RP11-410N8.4	1.020149	1.0159362	1.02362864	1.4826977	0.56822453	0.92128
TNFAIP8L3	1.028444	1.0242166	1.03193469	1.3187105	0.39912789	0.92136
RP1-100J12.1	1.028464	1.0242783	1.03191999	1.3147547	0.39479364	0.92136
HS3ST3A1	1.088493	1.0842081	1.09203078	1.0928973	0.12815778	0.9214
ENTPD5	1.125263	1.1209321	1.12883999	1.0653908	0.09138277	0.92141
PRR22	1.02813	1.0322534	1.02472422	0.766561	-0.3835275	0.92145
LINC00237	1.029281	1.0336011	1.02571341	0.7652549	-0.3859877	0.92145
OSTM1-AS1	1.02911	1.0332221	1.02571425	0.7740104	-0.3695752	0.92145
AC092301.3	1.022324	1.0180838	1.02582519	1.428081	0.51407777	0.92146
LINC00158	1.022294	1.0180691	1.02578354	1.4269389	0.5129236	0.92146
FAM135B	1.022278	1.0180623	1.02575905	1.4261204	0.51209575	0.92146
TCF4	2.262596	2.2695766	2.25683119	0.9899609	-0.0145565	0.92148
RP11-231C14.7	1.020434	1.0162589	1.02388116	1.4688083	0.55464613	0.92153
RP11-574F21.2	1.020467	1.0162589	1.02394173	1.4725332	0.55830021	0.92153
SULT2B1	1.023114	1.0189195	1.02657841	1.4048158	0.490381	0.92156
TLR2	1.023133	1.0189178	1.02661384	1.4068112	0.49242872	0.92156
MSANTD1	1.019908	1.0157004	1.02338239	1.4892842	0.57461913	0.92157
GK-AS1	1.019916	1.0157004	1.02339689	1.4902077	0.57551347	0.92157
RP1-122O8.7	1.019896	1.0157004	1.02336147	1.4879521	0.57332806	0.92157
LRRC3-AS1	1.019901	1.0157004	1.0233708	1.4885458	0.5739036	0.92157
RP11-296I10.3	1.019916	1.0157004	1.02339675	1.4901991	0.57550513	0.92157
CDHR4	1.019898	1.0157004	1.02336523	1.4881916	0.57356023	0.92157
GRHL3	1.019875	1.0157004	1.02332204	1.4854405	0.57089083	0.92157
RP11-443O13.3	1.019948	1.0157004	1.0234555	1.4939411	0.57912325	0.92157
LINC00922	1.019536	1.0236607	1.01612997	0.6817193	-0.5527502	0.92158
RGS7	1.019569	1.0237293	1.01613422	0.6799276	-0.556547	0.92158
CTC-241N9.1	1.212815	1.217429	1.2090046	0.9612547	-0.0570094	0.9216
C22orf23	1.052741	1.0569097	1.04929865	0.8662604	-0.2071273	0.92162
PDC	1.027132	1.0313269	1.02366713	0.7554899	-0.4045156	0.92163
DACT2	1.083377	1.0875917	1.07989687	0.9121509	-0.1326556	0.92164
ZNF564	1.083342	1.0875299	1.07988362	0.9126442	-0.1318756	0.92164
PTER	1.128831	1.1242327	1.13262764	1.0675746	0.09433683	0.92167
RP11-258C19.7	1.18211	1.186341	1.17861534	0.95854	-0.0610895	0.92174
GPR137	1.547272	1.5426209	1.55111354	1.0156511	0.02240495	0.92184
AC013271.5	1.03702	1.0327692	1.04052995	1.2368317	0.30664923	0.92187
RP11-168O16.1	1.037001	1.0327655	1.04049896	1.236024	0.30570679	0.92187

OVOL1-AS1	1.034586	1.0303619	1.03807395	1.2540022	0.32653992	0.922
RP11-297D21.4	1.137738	1.1331793	1.14150276	1.0624984	0.08746064	0.922
TTC31	1.168928	1.1732857	1.16532963	0.954087	-0.0678073	0.92203
FAM41C	1.035414	1.0395902	1.0319659	0.8074189	-0.3086107	0.9221
CTD-2331H12.7	1.018704	1.0228627	1.01527044	0.6679204	-0.5822518	0.92214
DLX5	1.018662	1.022759	1.01527789	0.6712902	-0.5749915	0.92214
HES5	1.043964	1.0481029	1.04054695	0.8429219	-0.2465291	0.92214
LUZP2	1.035157	1.030968	1.03861567	1.2469534	0.3184075	0.92218
TTC25	1.064728	1.0605312	1.06819398	1.1265917	0.17196473	0.92218
LINC01258	1.022805	1.0186034	1.02627464	1.4123537	0.49810148	0.92218
RP11-74C3.1	1.022775	1.0185978	1.0262247	1.4100951	0.49579244	0.92218
ST3GAL4-AS1	1.140576	1.1451232	1.13682101	0.942792	-0.0849886	0.92225
SH3GLB1	2.015241	2.0102016	2.01940208	1.0091076	0.01308001	0.92235
ACOXL	1.042026	1.0377845	1.04552958	1.2049816	0.26901116	0.92235
GRM1	1.020545	1.024651	1.017155	0.6959153	-0.5230164	0.92238
H3F3C	1.048585	1.0443308	1.05209779	1.1752053	0.23291283	0.9224
SPNS3	1.029691	1.0255874	1.03308019	1.2928324	0.37053527	0.92243
EID3	1.054566	1.0587687	1.05109492	0.869424	-0.2018682	0.92252
NAV2-AS3	1.031982	1.0361027	1.02857891	0.7916008	-0.337155	0.92253
PASK	1.110695	1.1063175	1.11430955	1.0751713	0.10456659	0.92255
ANKEF1	1.058516	1.0541996	1.0620802	1.1453986	0.19584981	0.9226
BEND3	1.066127	1.0703591	1.06263196	0.8901754	-0.1678385	0.92267
GALNT18	1.134653	1.1303266	1.13822646	1.0606162	0.08490271	0.92272
TMEM147	4.66388	4.6748636	4.65480923	0.9945428	-0.0078946	0.92277
RPA4	1.031774	1.0275814	1.03523579	1.2775207	0.35334668	0.92286
KIT	1.039476	1.0436031	1.03606762	0.8271804	-0.273726	0.92289
AC092652.1	1.022328	1.0181901	1.02574599	1.4153883	0.50119791	0.9229
CYYR1-AS1	1.022297	1.0181511	1.02572056	1.4170243	0.50286452	0.9229
RP11-932O9.8	1.029451	1.0252595	1.03291302	1.3029982	0.38183514	0.9229
LINC01579	1.029408	1.0252618	1.03283224	1.2996773	0.37815349	0.9229
GLIPR1L2	1.02095	1.0167623	1.0244081	1.4561326	0.54214172	0.92291
ACRBP	1.059275	1.0550903	1.06272991	1.1386747	0.18735567	0.92292
CTD-2545M3.8	1.024939	1.0207927	1.0283638	1.3641248	0.44797566	0.92298
NEB	1.016966	1.012846	1.02036886	1.5856148	0.66504235	0.92301
RCSD1	1.016967	1.012846	1.02036941	1.5856572	0.66508095	0.92301
C8orf89	1.016994	1.012846	1.02041864	1.5894898	0.66856374	0.92301
AC005009.1	1.016968	1.012846	1.02037235	1.5858859	0.66528899	0.92301
GZMA	1.016986	1.012846	1.02040517	1.5884411	0.66761162	0.92301
FAM83F	1.016966	1.012846	1.0203681	1.5855554	0.66498827	0.92301
KRT222	1.02306	1.0271555	1.01967862	0.7246644	-0.464615	0.92311
RP11-713P17.3	1.046414	1.050519	1.04302323	0.8516251	-0.2317095	0.92314
RP11-29P20.1	1.022473	1.0265861	1.01907676	0.7175459	-0.478857	0.92321
OTUD1	1.06346	1.0676746	1.05998014	0.886302	-0.1741297	0.92328
ADGRL4	1.044053	1.0398957	1.0474861	1.1902559	0.25127173	0.92338
CTC-444N24.8	1.077683	1.0817549	1.07431986	0.9090571	-0.1375573	0.92343

AGAP2-AS1	1.065916	1.0616167	1.06946639	1.1273962	0.17299458	0.92346
ASAH2	1.07434	1.0701944	1.07776419	1.1078402	0.14774981	0.92346
RP11-317L10.1	1.032345	1.0281814	1.03578289	1.269735	0.34452742	0.92348
SNHG23	1.085099	1.0810361	1.08845434	1.0915423	0.12636801	0.92353
RP11-231E19.1	1.030007	1.0340904	1.02663563	0.7813243	-0.3560066	0.92356
GDAP2	1.174763	1.1704784	1.17830164	1.04589	0.06473107	0.92361
CH507-338C24.1	1.019718	1.0156039	1.02311525	1.4813763	0.56693817	0.92362
S1PR5	1.019724	1.0156039	1.02312556	1.4820368	0.56758131	0.92362
SLAMF9	1.019707	1.0156039	1.02309571	1.4801238	0.56571781	0.92362
ELF5	1.019782	1.0156039	1.02323186	1.4888496	0.57419799	0.92362
TCTA	1.313065	1.3176393	1.309288	0.9737083	-0.0384384	0.92369
CH507-154B10.2	1.054358	1.0501762	1.05781217	1.1521833	0.20437019	0.92372
ZNF675	1.131412	1.1272335	1.13486354	1.0599692	0.08402234	0.92374
DDIAS	1.065663	1.061442	1.06914934	1.1254401	0.17048926	0.92375
SNX31	1.019011	1.0148897	1.02241465	1.5053786	0.59012641	0.92378
GJA3	1.018976	1.0148892	1.02235104	1.5011578	0.5860756	0.92378
RP11-425D17.1	1.019009	1.0148887	1.02241085	1.5052216	0.58997589	0.92378
RP11-151A6.6	1.019027	1.0148842	1.02244867	1.5082244	0.59285112	0.92378
RP11-413H22.2	1.05598	1.0601355	1.0525481	0.8738283	-0.1945782	0.92391
HIST1H2BM	1.017861	1.0137737	1.02123548	1.5417443	0.62456349	0.92393
TMEM45B	1.017871	1.0137737	1.02125411	1.5430966	0.62582834	0.92393
DRD1	1.017885	1.0137737	1.02127974	1.5449575	0.62756716	0.92393
RP11-151A10.3	1.017873	1.0137737	1.02125774	1.5433601	0.62607474	0.92393
AC006262.4	1.017847	1.0137737	1.02121125	1.5399852	0.62291645	0.92393
PNMA5	1.017883	1.0137737	1.02127617	1.5446985	0.62732532	0.92393
FRMPD2	1.017864	1.0137737	1.02124259	1.54226	0.62504603	0.92393
HMOX1	1.039208	1.0350884	1.04260991	1.2143598	0.2801959	0.92394
SRRT	1.332055	1.3366797	1.32823502	0.9749178	-0.0366476	0.92402
RP11-400N9.1	1.024775	1.0206725	1.02816216	1.3623016	0.44604611	0.92404
LRAT	1.024795	1.0206532	1.02821618	1.3661908	0.45015901	0.92404
PCSK9	1.027529	1.0233963	1.03094249	1.3225359	0.40330691	0.92405
SLC9A9	1.053969	1.0580359	1.05061006	0.872047	-0.1975223	0.92409
NCAM2	1.068723	1.0728085	1.06534844	0.8975384	-0.1559544	0.92419
FAM160A1	1.039523	1.0354651	1.04287381	1.2089025	0.2736979	0.92419
KB-1507C5.4	1.030475	1.0264027	1.03383758	1.2815933	0.3579385	0.92421
KCNK9	1.020448	1.0244649	1.01713135	0.7002428	-0.5140728	0.92424
LINC01307	1.020445	1.024466	1.01712444	0.6999272	-0.5147233	0.92424
AC016700.5	1.104744	1.1004028	1.10832928	1.0789463	0.10962312	0.92424
METRNL	1.131856	1.1277648	1.13523457	1.0584646	0.08197307	0.92425
GALNT2	1.610424	1.6060037	1.61407346	1.0133164	0.01908469	0.92434
ATG4D	1.389264	1.3847981	1.39295121	1.0211881	0.03024864	0.92434
RAG1	1.026474	1.0223688	1.0298642	1.3350857	0.41693236	0.92436
LRRC8D	1.117445	1.11325	1.12090962	1.0676345	0.09441786	0.92437
KCNK10	1.045339	1.0493612	1.04201825	0.8512406	-0.2323611	0.92438
INTS3	1.377321	1.3819712	1.37348128	0.9777735	-0.0324278	0.92441

ACAP3	1.362939	1.3675926	1.35909546	0.9768842	-0.0337405	0.92446
CNNM3	1.077465	1.0815459	1.07409519	0.9086319	-0.1382322	0.92449
LGALS7	1.020696	1.0165789	1.02409578	1.4534003	0.53943215	0.92449
LINC01372	1.020667	1.0165808	1.02404078	1.4499205	0.53597381	0.92449
PCDHB11	1.046532	1.0506169	1.04315867	0.8526528	-0.2299698	0.92453
RP11-111F5.3	1.046603	1.050623	1.04328334	0.8550132	-0.2259814	0.92453
FMO4	1.055267	1.0511306	1.05868227	1.1476937	0.19873767	0.92455
NAP1L6	1.02749	1.0314934	1.02418425	0.767916	-0.3809797	0.92455
TJP3	1.062206	1.0663524	1.05878243	0.8859127	-0.1747635	0.92456
CD302	1.062176	1.0663143	1.05875863	0.8860629	-0.174519	0.92456
RP11-69E11.4	1.046432	1.0505044	1.04306831	0.8527642	-0.2297813	0.9246
LINC01389	1.015375	1.0113227	1.0187218	1.6534702	0.72549708	0.92466
BCL2L14	1.015385	1.0113227	1.01873952	1.6550355	0.72686212	0.92466
RP11-480D4.6	1.015374	1.0113227	1.01872027	1.6533351	0.72537914	0.92466
SLC4A1	1.015372	1.0113227	1.01871594	1.6529528	0.72504552	0.92466
LINC00880	1.015375	1.0113227	1.01872218	1.6535038	0.72552636	0.92466
FES	1.047271	1.0513336	1.04391556	0.8554941	-0.2251702	0.92473
ZNF2	1.077678	1.073465	1.08115767	1.1047114	0.14366949	0.92474
LINC00337	1.027094	1.0312015	1.02370242	0.7596571	-0.3965797	0.92482
CPLX4	1.027109	1.0311538	1.02376888	0.7629531	-0.3903338	0.92482
RP11-109N23.4	1.027016	1.0310349	1.02369799	0.7635925	-0.3891252	0.92482
RP5-884G6.2	1.034707	1.0388833	1.03125825	0.8038981	-0.3149155	0.92485
RP11-10C24.3	1.034574	1.0386334	1.03122124	0.8081406	-0.3073218	0.92485
PCDHB10	1.094233	1.0901214	1.09762875	1.0833023	0.11543591	0.92488
VANGL1	1.121342	1.1171535	1.12480137	1.0652805	0.09123333	0.92489
EPDR1	1.177399	1.1733993	1.18070177	1.0421135	0.05951248	0.92491
CTNS	1.114066	1.1182238	1.11063211	0.9357856	-0.0957501	0.92492
AC005592.1	1.029232	1.0251131	1.03263413	1.299486	0.37794112	0.92497
GPBAR1	1.029267	1.0251654	1.03265374	1.2975671	0.3758091	0.92497
RP11-61A14.1	1.027981	1.0320204	1.02464482	0.7696594	-0.3777079	0.92502
SUSD3	1.027929	1.0319175	1.02463545	0.7718466	-0.3736139	0.92502
HFE	1.048773	1.0446488	1.05217927	1.1686599	0.22485513	0.92506
AC068282.3	1.028502	1.0245032	1.03180359	1.297936	0.37621922	0.92509
UBXN2A	1.995095	1.9898794	1.99940262	1.0096206	0.01381324	0.92513
AJ011932.1	1.0527	1.0566957	1.04939994	0.8713177	-0.1987293	0.92517
TENM1	1.102999	1.0988055	1.1064619	1.0774894	0.10767371	0.92523
RP11-666A8.8	1.017993	1.0219938	1.01468982	0.667906	-0.5822831	0.92526
AC007319.1	1.017981	1.0219855	1.01467437	0.6674577	-0.5832517	0.92526
RP11-165E7.1	1.027401	1.0233372	1.03075659	1.3179233	0.39826646	0.92528
RP11-79N23.1	1.020591	1.0245902	1.01728813	0.7030495	-0.5083017	0.92529
XXbac-BPG299F13.17	1.073209	1.0772228	1.06989478	0.9051051	-0.1438428	0.9253
TSNARE1	1.037815	1.041855	1.03447837	0.823758	-0.2797075	0.92539
TLR5	1.034335	1.030228	1.03772651	1.2480631	0.31969093	0.92541
RP11-490O6.2	1.034235	1.0301819	1.03758192	1.2451826	0.31635727	0.92541
RGS17	1.236874	1.2410841	1.23339749	0.9681165	-0.0467474	0.92542

SYNGR3	1.176488	1.1807091	1.17300295	0.9573563	-0.0628722	0.92543
GOLGA8S	1.025129	1.0291725	1.02178991	0.7469342	-0.4209469	0.92548
PCDHA12	1.116529	1.1207	1.11308464	0.936907	-0.0940222	0.9255
ZDHHC6	1.292507	1.2883696	1.29592361	1.0261955	0.03730561	0.92553
RP1-30M3.6	1.017599	1.021629	1.01427165	0.6598385	-0.5998151	0.92553
RP1-40E16.11	1.017568	1.0215571	1.01427455	0.6621755	-0.5947145	0.92553
PGM5-AS1	1.017597	1.0216218	1.01427397	0.660165	-0.5991015	0.92553
RP11-459I19.1	1.021963	1.0178798	1.02533494	1.4169614	0.50280048	0.92556
SNX29	1.157239	1.15308	1.1606736	1.0496055	0.06984714	0.92562
TMEM255A	1.050659	1.0465455	1.05405525	1.1613424	0.21579338	0.92563
SLC15A2	1.035976	1.0319358	1.03931302	1.2310027	0.2998339	0.92569
RP11-147L13.2	1.023972	1.0199216	1.02731737	1.3712467	0.45548815	0.92574
PCDHGA12	1.024008	1.019968	1.0273451	1.369445	0.45359136	0.92574
MTCP1	1.021862	1.0178461	1.02517872	1.410882	0.49659732	0.92575
PIWIL2	1.021895	1.0178326	1.02525019	1.4159563	0.50177669	0.92575
RP11-1C8.7	1.021863	1.0178302	1.02519288	1.4129349	0.49869503	0.92575
GNG12-AS1	1.021858	1.0178418	1.02517531	1.4110322	0.49675092	0.92575
RNF212B	1.021874	1.0178415	1.02520458	1.412696	0.49845109	0.92575
RFPL3S	1.021878	1.0178591	1.02519693	1.4108742	0.49658937	0.92575
BMS1P14	1.026502	1.0305749	1.02313787	0.7567597	-0.4020929	0.92578
C15orf59-AS1	1.026477	1.0304665	1.02318243	0.7609155	-0.3941918	0.92578
FAM72B	1.043067	1.0470367	1.03978908	0.8459151	-0.2414153	0.92578
STXBP1	1.404826	1.4003354	1.40853381	1.0204788	0.02924621	0.9258
RP11-324I22.4	1.064702	1.0606399	1.06805575	1.1222931	0.16644955	0.92582
RP11-247A12.2	1.085013	1.0810193	1.08831046	1.0899931	0.12431896	0.92583
RP11-256L6.5	1.017808	1.0217543	1.01454934	0.6688039	-0.5803449	0.92592
ARHGAP15	1.027461	1.0314235	1.02418828	0.7697523	-0.3775337	0.92599
KCNIP4-IT1	1.027426	1.0314266	1.02412171	0.767558	-0.3816523	0.92599
LYPLAL1-AS1	1.022182	1.0181535	1.02550974	1.4052282	0.49080445	0.926
AC141928.1	1.022188	1.0181546	1.02551857	1.4056281	0.49121498	0.926
RP13-631K18.2	1.022226	1.0181732	1.02557262	1.4071591	0.49278549	0.926
RP11-583F2.5	1.02225	1.0181546	1.02563246	1.411901	0.49763895	0.926
TMEM209	1.179761	1.1754558	1.18331613	1.0447997	0.06322639	0.92601
CAND1	2.337394	2.3441371	2.33182459	0.9908398	-0.0132762	0.92604
AC016831.7	1.028323	1.0323074	1.02503347	0.7748528	-0.3680059	0.92605
RP11-474I16.8	1.017152	1.0131484	1.02045797	1.5559284	0.63777565	0.92608
LINC01234	1.017162	1.0131484	1.02047683	1.5573623	0.63910458	0.92608
ARMCX3-AS1	1.017155	1.0131484	1.02046337	1.5563387	0.63815611	0.92608
MUC15	1.017156	1.0131484	1.02046471	1.5564406	0.63825051	0.92608
RP5-1116H23.5	1.017144	1.0131484	1.02044286	1.5547791	0.63670958	0.92608
FLT1	1.017139	1.0131484	1.02043426	1.5541249	0.63610244	0.92608
GADL1	1.017145	1.0131484	1.02044622	1.5550347	0.63694674	0.92608
XXbac-BPGBPG55C20.3	1.02297	1.0189659	1.02627739	1.3855056	0.47041252	0.92614
P2RY11	1.082966	1.0870672	1.07957969	0.9140035	-0.1297284	0.92628
LMCD1-AS1	1.083144	1.0872017	1.07979301	0.9150399	-0.1280935	0.92628

TCTEX1D1	1.058419	1.0545822	1.06158836	1.1283607	0.17422836	0.92635
ART5	1.083452	1.0874327	1.08016531	0.91688	-0.1251952	0.92636
RP5-1024G6.2	1.038017	1.0339463	1.04137792	1.2189224	0.28560626	0.92638
GABPB2	1.135904	1.1401087	1.13243206	0.9452096	-0.0812938	0.92641
AC008746.12	1.036397	1.0324507	1.03965512	1.2220117	0.28925807	0.92644
PEX1	1.313611	1.3178695	1.31009519	0.9755425	-0.0357234	0.92644
RP11-294N21.3	1.016956	1.0208893	1.01370805	0.656224	-0.6077398	0.92648
NHLRC4	1.041396	1.0453434	1.03813657	0.8410617	-0.2497165	0.9265
CTD-2574D22.2	1.058309	1.0542337	1.06167404	1.1371901	0.1854735	0.92655
SPIRE1	1.214046	1.2095693	1.21774287	1.0390015	0.05519776	0.92658
RP11-488L18.10	1.174006	1.1781725	1.17056459	0.9573005	-0.0629563	0.92663
GCNT4	1.024507	1.0205264	1.02779437	1.3540762	0.43730896	0.92664
EPYC	1.024528	1.0205716	1.0277949	1.3511269	0.43416318	0.92664
RP11-449H3.3	1.024557	1.0204991	1.02790866	1.3614595	0.44515409	0.92664
CEPT1	1.240263	1.2357812	1.24396408	1.0347053	0.04921991	0.9267
RP11-290L1.3	1.01806	1.0220879	1.01473434	0.6670781	-0.5840724	0.9267
CTC-523E23.4	1.017975	1.0219075	1.01472744	0.6722567	-0.5729159	0.9267
CTA-243E7.4	1.017992	1.0219357	1.01473434	0.6717051	-0.5741001	0.9267
LIN28B-AS1	1.018835	1.0227756	1.01558063	0.6840925	-0.5477367	0.92672
RP5-821D11.7	1.046567	1.0425122	1.04991584	1.1741539	0.23162155	0.92678
RP4-549L20.3	1.050363	1.0462604	1.05375019	1.1619049	0.21649196	0.92681
CHEK1	1.406281	1.4017492	1.41002331	1.0205953	0.02941094	0.92685
FOXJ2	1.08583	1.0818634	1.08910496	1.0884591	0.12228716	0.92688
RP11-670E13.6	1.064356	1.0682986	1.06109962	0.8945957	-0.1606923	0.92692
BSDC1	1.777991	1.7834663	1.77346951	0.9872403	-0.0185269	0.92694
AC018890.6	1.024139	1.028111	1.02085903	0.7420234	-0.4304633	0.92702
WISP2	1.033518	1.0294937	1.03684199	1.2491476	0.32094396	0.92705
RP11-350N15.5	1.039448	1.0434171	1.03617023	0.8330876	-0.2634598	0.92706
ADCY8	1.031413	1.0274256	1.03470521	1.2654323	0.33963029	0.92707
CAPN10-AS1	1.19415	1.1984258	1.19061927	0.9606578	-0.0579055	0.92709
TUSC8	1.016984	1.013034	1.02024683	1.553389	0.63541915	0.92714
AC004067.5	1.017001	1.013034	1.02027781	1.5557657	0.63762481	0.92714
RP11-521D12.5	1.016982	1.013034	1.020243	1.5530948	0.63514594	0.92714
RP11-471B22.3	1.017	1.013034	1.0202752	1.5555656	0.63743927	0.92714
RP11-1084I9.1	1.017019	1.013034	1.0203107	1.5582887	0.63996258	0.92714
ASB16	1.019733	1.0157693	1.02300603	1.4589092	0.54489009	0.92714
ADGRF4	1.019726	1.0157693	1.02299387	1.4581377	0.54412695	0.92714
RP11-159F24.5	1.019756	1.0157693	1.02304865	1.4616114	0.54755982	0.92714
RP11-114H21.2	1.019778	1.0157693	1.02308827	1.4641243	0.55003803	0.92714
AKR1C3	1.061474	1.0654378	1.05820107	0.8894102	-0.1690791	0.92724
AC093495.4	1.03166	1.0276557	1.03496754	1.2643881	0.33843941	0.92731
ITGB1BP2	1.031687	1.0277022	1.0349774	1.2626206	0.33642124	0.92731
PLA2G4C	1.031139	1.0271137	1.03446236	1.2710305	0.34599865	0.92735
RP11-20D14.6	1.034366	1.0303873	1.03765236	1.2390817	0.30927134	0.92735
GALNTL6	1.022631	1.0186568	1.02591345	1.388958	0.47400293	0.92737

GOLGA8H	1.022642	1.0186535	1.02593652	1.3904363	0.47553763	0.92737
IL10RB-AS1	1.024059	1.0201023	1.02732631	1.3593655	0.44293338	0.92737
CHST7	1.094719	1.0988469	1.09130925	0.9237439	-0.1144352	0.92742
SLC12A6	1.151433	1.1556711	1.1479336	0.9502956	-0.0735518	0.92742
FAR1	1.375731	1.3804178	1.37186122	0.9775073	-0.0328206	0.92742
EGFR-AS1	1.02659	1.022664	1.02983176	1.3162607	0.3964453	0.92744
RP4-647C14.3	1.021308	1.0251888	1.01810275	0.7186837	-0.4765712	0.92745
AC093627.10	1.0213	1.0251998	1.01807942	0.7174424	-0.4790651	0.92745
PDZD4	1.073103	1.0690635	1.07643954	1.1068001	0.14639467	0.92747
RP1-124C6.1	1.018737	1.0147451	1.02203298	1.4942583	0.5794296	0.92748
RP11-64C12.8	1.018684	1.0147424	1.02193864	1.4881332	0.5735037	0.92748
CTC-203F4.2	1.018697	1.014739	1.02196556	1.4902967	0.57559955	0.92748
RP11-472I20.3	1.018699	1.0147443	1.02196426	1.489679	0.57500146	0.92748
NOSTRIN	1.018697	1.0147402	1.02196453	1.4901157	0.57542439	0.92748
TMEM106B	2.144306	2.1502084	2.13943177	0.9906307	-0.0135808	0.92754
CUX2	1.047344	1.0512786	1.04409497	0.85991	-0.2177423	0.92758
EEF2KMT	1.184077	1.1882589	1.18062341	0.9594417	-0.0597329	0.9277
TUB	1.131676	1.1359686	1.12813101	0.9423576	-0.0856535	0.92771
AP001505.10	1.080032	1.075909	1.08343617	1.0991607	0.13640229	0.92773
DPP6	1.02317	1.0270771	1.01994258	0.7365101	-0.4412228	0.92775
RP11-142C4.6	1.023177	1.0270617	1.01996872	0.7378966	-0.4385094	0.92775
GSTM2	1.076122	1.0719256	1.07958669	1.1065136	0.14602117	0.92781
RP11-138I1.4	1.030977	1.0348998	1.02773683	0.7947561	-0.331416	0.92788
ATP23	1.200556	1.2046002	1.19721607	0.9639094	-0.0530305	0.92788
PLIN5	1.028221	1.0320593	1.02505111	0.7813991	-0.3558686	0.92789
PLEKHF1	1.119274	1.1232256	1.11601086	0.9414509	-0.0870422	0.92797
CTD-2012K14.6	1.033368	1.0294482	1.03660562	1.2430531	0.31388789	0.92809
RP11-21A7A.3	1.021435	1.0175076	1.02467783	1.4095459	0.49523042	0.9281
CASC1	1.056131	1.0521247	1.05943866	1.1403175	0.18943554	0.92812
SMG9	1.383724	1.3794633	1.38724295	1.0205018	0.02927871	0.92813
HCAR3	1.01905	1.0150885	1.02232152	1.4793777	0.56499045	0.92816
HTR1D	1.019021	1.0150885	1.02226916	1.4759077	0.5616025	0.92816
RP1-121G13.2	1.01903	1.0150885	1.02228481	1.476945	0.5626161	0.92816
GRHL2	1.019006	1.0150885	1.0222406	1.4740144	0.55975066	0.92816
RP11-763F8.1	1.019022	1.0150885	1.0222697	1.4759433	0.56163729	0.92816
MARCO	1.019001	1.0150885	1.02223148	1.4734102	0.55915915	0.92816
HOXB1	1.018999	1.0150885	1.02222749	1.4731456	0.5589	0.92816
ZNF25	1.200466	1.2046493	1.19701205	0.9626811	-0.0548702	0.92818
PTPRS	1.054236	1.0581635	1.05099246	0.8767096	-0.189829	0.92818
AP006216.10	1.020769	1.0168039	1.0240436	1.4308342	0.51685649	0.92824
BICDL2	1.020725	1.016827	1.02394344	1.422915	0.50884951	0.92824
TUNAR	1.020713	1.0167909	1.02395107	1.4264333	0.51241231	0.92824
RMDN2	1.054946	1.0588235	1.05174402	0.8796487	-0.1850006	0.92835
DAGLB	1.104156	1.1001209	1.10748807	1.0735825	0.10243303	0.92835
RALY-AS1	1.052134	1.056001	1.04894146	0.8739393	-0.194395	0.92836

DNHD1	1.062862	1.0589156	1.06612081	1.1222974	0.16645503	0.92837
TRIM23	1.152785	1.1488161	1.15606303	1.0486975	0.06859856	0.92837
SCGN	1.019594	1.0156993	1.02280984	1.4529247	0.53895996	0.9284
CTB-193M12.1	1.019599	1.0156689	1.02284368	1.4578986	0.54389033	0.9284
RP11-20B24.4	1.019601	1.0156807	1.02283785	1.4564258	0.54243223	0.9284
RP11-642C5.1	1.019601	1.0156858	1.0228338	1.4556948	0.5417079	0.9284
NAPG	1.264168	1.2683526	1.26071272	0.9715305	-0.0416689	0.9284
RP11-91I8.3	1.067165	1.0711759	1.06385329	0.897119	-0.1566287	0.92843
XRRA1	1.760141	1.765236	1.75593414	0.9878445	-0.0176442	0.92845
ATAD3B	1.060234	1.0562853	1.063495	1.1280923	0.17388512	0.92845
STAT2	1.238562	1.2342784	1.2420992	1.0333826	0.04737443	0.9285
U47924.31	1.029213	1.0253014	1.03244305	1.2822645	0.35869392	0.92851
HSPA1L	1.0187	1.0225359	1.01553183	0.6892044	-0.5369962	0.92858
AL356585.1	1.024499	1.0284029	1.02127518	0.7490491	-0.4168678	0.92861
RP11-455J20.3	1.024384	1.0282473	1.02119428	0.7503109	-0.4144395	0.92861
CC2D2B	1.032146	1.0281699	1.03542952	1.2577097	0.33079896	0.92865
MGAT5B	1.040599	1.0366073	1.04389488	1.1990748	0.26192162	0.92865
LINC01588	1.043648	1.0474742	1.04048869	0.8528565	-0.2296251	0.92868
IFIT1	1.043806	1.0398704	1.04705564	1.1802137	0.23904805	0.92868
TPRA1	1.427404	1.4231669	1.43090264	1.0182805	0.02613504	0.9287
MED26	1.100779	1.1048081	1.0974524	0.9298174	-0.1049806	0.92873
RP11-650L12.2	1.033527	1.0296238	1.03675114	1.2405962	0.31103365	0.92876
KIAA1468	1.12934	1.1332662	1.12609855	0.9462159	-0.0797587	0.92878
FAM85B	1.044786	1.0486803	1.04157033	0.8539459	-0.2277834	0.92882
RP4-738P15.1	1.02574	1.0295974	1.02255534	0.7620709	-0.3920029	0.92888
RP11-320M2.1	1.044776	1.0486553	1.04157268	0.8544336	-0.2269597	0.92889
ARID4A	1.551651	1.5559558	1.54809671	0.9858639	-0.0205396	0.92892
TXNL4B	1.165148	1.1691364	1.16185445	0.9569461	-0.0634904	0.92895
URB1-AS1	1.028387	1.0322099	1.02522935	0.783279	-0.3524019	0.92895
CTC-254B4.1	1.028438	1.0324047	1.02516144	0.7764746	-0.3649894	0.92895
SNRNP25	2.412038	2.4063799	2.4167096	1.0073449	0.01055766	0.92896
TRIM39	1.12442	1.12844	1.12110025	0.9428547	-0.0848926	0.92901
RP11-90J7.2	1.016251	1.0123699	1.01945659	1.5729004	0.65342731	0.92902
LINC00355	1.016263	1.0123699	1.01947868	1.574686	0.65506421	0.92902
MKX	1.016264	1.0123699	1.01948058	1.5748396	0.65520492	0.92902
RP11-403A3.3	1.016241	1.0123699	1.01943737	1.5713466	0.65200146	0.92902
LINC01564	1.016313	1.0123699	1.01956956	1.5820329	0.66177964	0.92902
ALG13	1.401848	1.3974715	1.40546254	1.0201046	0.02871707	0.92905
RFX3-AS1	1.057148	1.0610322	1.05394083	0.8838088	-0.1781939	0.92906
PNN	3.555664	3.5644936	3.54837224	0.9937136	-0.0090979	0.92906
SLC1A3	1.283891	1.2881588	1.28036637	0.972958	-0.0395506	0.92908
ADAD2	1.0171	1.0132269	1.02029759	1.534564	0.61782886	0.92913
CA5A	1.017102	1.0132213	1.02030621	1.5358679	0.6190541	0.92913
ARL11	1.017099	1.0132157	1.02030503	1.5364289	0.61958096	0.92913
NDST2	1.055336	1.0513117	1.05865963	1.143201	0.19307904	0.92915

AC007392.3	1.025323	1.0291654	1.02214928	0.7594373	-0.3969972	0.92916
CDHR2	1.015245	1.0113663	1.01844879	1.6231113	0.69876192	0.92923
RP4-539M6.22	1.015237	1.0113663	1.01843323	1.6217416	0.69754396	0.92923
HRASLS2	1.015237	1.0113663	1.01843291	1.6217136	0.69751908	0.92923
RP13-39P12.3	1.01528	1.0113663	1.01851254	1.6287194	0.70373808	0.92923
RP11-54A4.2	1.01528	1.0113663	1.01851254	1.6287194	0.70373808	0.92923
CYB5A	1.846807	1.8514536	1.84296998	0.9900363	-0.0144467	0.92936
CA2	1.826424	1.8318833	1.8219165	0.988019	-0.0173893	0.92937
AC114271.2	1.066107	1.0699849	1.06290463	0.8988315	-0.1538774	0.92937
AC104532.4	1.034192	1.0380138	1.03103637	0.8164492	-0.292565	0.92939
RNF125	1.030973	1.0270969	1.03417447	1.2611969	0.33479354	0.92942
RGS5.1	1.05087	1.054778	1.04764313	0.8697488	-0.2013293	0.92944
BATF3	1.035244	1.0390863	1.03207159	0.820533	-0.2853668	0.92946
RP11-473C18.3	1.016263	1.0200828	1.01310914	0.6527547	-0.6153872	0.92962
AC005481.5	1.016286	1.0201338	1.01310914	0.6511016	-0.6190454	0.92962
CTD-2184C24.2	1.01623	1.0200084	1.01310914	0.6551811	-0.6100344	0.92962
CFLAR-AS1	1.016268	1.0200923	1.01310914	0.6524466	-0.6160684	0.92962
MTMR12	1.121561	1.1256701	1.11816748	0.9402988	-0.0888088	0.92965
CTA-384D8.36	1.050459	1.0464976	1.05373119	1.1555693	0.2086038	0.92966
RP4-569M23.2	1.036028	1.0398393	1.03288128	0.8253476	-0.2769262	0.9297
TEAD3	1.184761	1.1807974	1.18803436	1.040028	0.05662244	0.9297
PPT2-EGFL8	1.029097	1.0252229	1.03229681	1.2804578	0.35665975	0.92973
ATP2A1	1.029106	1.0252443	1.03229479	1.2792891	0.3553423	0.92973
TMEM30B	1.016104	1.0122555	1.01928149	1.5732954	0.65378961	0.92979
ANPEP	1.016109	1.0122555	1.01929102	1.574073	0.65450248	0.92979
RP6-74O6.2	1.023361	1.0271857	1.02020198	0.7431103	-0.4283518	0.92982
RP1-244F24.1	1.023924	1.0277289	1.0207819	0.7494661	-0.416065	0.92985
FAM107B	1.282193	1.2862998	1.27880085	0.9738074	-0.0382916	0.92988
RP11-282A11.3	1.015836	1.0196287	1.01270407	0.6472189	-0.6276743	0.92989
GNG13	1.015841	1.0196392	1.01270407	0.6468724	-0.6284469	0.92989
QDPR	1.617022	1.6215782	1.61326003	0.9866177	-0.019437	0.92992
RP11-718O11.1	1.023642	1.0197734	1.0268368	1.3572165	0.44065083	0.93002
RP11-575L7.8	1.02366	1.0197836	1.02686036	1.3577054	0.44117043	0.93002
DSCAML1	1.023666	1.0197753	1.02687984	1.3592615	0.44282308	0.93002
DLK2	1.041392	1.0452063	1.03824119	0.8459264	-0.241396	0.93008
GHRL	1.079891	1.083781	1.07667942	0.9152369	-0.1277829	0.93008
EHHADH	1.025691	1.0294909	1.02255382	0.7647716	-0.3868992	0.93011
FAM53B	1.129537	1.1254779	1.13288882	1.0590615	0.08278636	0.93017
GPHA2	1.015828	1.0120052	1.01898482	1.5813894	0.66119266	0.93018
ZG16B	1.015827	1.0120052	1.01898378	1.5813027	0.6611136	0.93018
RP11-78L16.1	1.015845	1.0120052	1.01901651	1.5840286	0.66359841	0.93018
RP4-798A10.4	1.015836	1.0120052	1.01899939	1.5826024	0.66229887	0.93018
USH1G	1.015836	1.0120052	1.01899928	1.5825934	0.66229065	0.93018
RP3-395M20.8	1.015828	1.0120052	1.01898483	1.5813901	0.66119334	0.93018
CCL7	1.015834	1.0120052	1.01899541	1.5822712	0.66199694	0.93018

RP11-235C23.5	1.015837	1.0120052	1.01900185	1.5828073	0.66248565	0.93018
RP11-180M15.6	1.01588	1.0120052	1.01907935	1.5892629	0.66835779	0.93018
RP11-131L12.2	1.015878	1.0120052	1.01907668	1.5890411	0.66815645	0.93018
RDH10-AS1	1.015847	1.0120052	1.01901931	1.5842622	0.66381111	0.93018
DNASE1L2	1.025689	1.0218476	1.02886093	1.3210102	0.40164157	0.9302
TVP23A	1.1462	1.1503085	1.14280743	0.9500957	-0.0738553	0.93021
AC079586.1	1.023611	1.0197488	1.02680097	1.357093	0.44051963	0.93021
GCG	1.016087	1.0198454	1.01298307	0.6542114	-0.6121713	0.93028
RP11-342I1.2	1.016099	1.0198715	1.01298307	0.6533522	-0.6140672	0.93028
CHST4	1.016113	1.0199039	1.01298307	0.6522879	-0.6164193	0.93028
AC005954.4	1.023707	1.0275893	1.02050068	0.7430672	-0.4284355	0.93029
XKR7	1.023643	1.0274371	1.02050936	0.7475047	-0.4198455	0.93029
GRPR	1.025709	1.0294723	1.02260178	0.7668833	-0.382921	0.93033
C12orf66	1.089044	1.0849823	1.09239903	1.0872745	0.12071621	0.93034
FSIP2	1.366897	1.3629238	1.37017812	1.0199885	0.02855289	0.93035
GS1-590J6.3	1.016291	1.0124282	1.01948074	1.5674688	0.64843671	0.93046
PAX3	1.016271	1.0124282	1.0194451	1.5646013	0.64579511	0.93046
RP3-323A16.1	1.016248	1.0124282	1.01940338	1.5612444	0.64269638	0.93046
AP003774.4	1.016257	1.0124282	1.0194187	1.5624767	0.64383464	0.93046
AC012593.1	1.016255	1.0124282	1.01941583	1.5622458	0.64362148	0.93046
AC003092.1	1.016244	1.0124282	1.01939582	1.5606358	0.64213391	0.93046
LLNLR-284B4.2	1.016246	1.0124282	1.01939927	1.5609132	0.64239027	0.93046
RP3-333H23.8	1.016243	1.0124282	1.01939408	1.5604957	0.64200441	0.93046
AC005042.4	1.016254	1.0124282	1.01941421	1.5621156	0.64350118	0.93046
HTN3	1.023881	1.0200523	1.02704215	1.3485811	0.43144223	0.93046
SUSD4	1.023875	1.0200493	1.02703517	1.3484332	0.43128402	0.93046
TTN	1.023986	1.0201224	1.02717586	1.3505304	0.43352608	0.93046
TMEM63B	1.291777	1.2876634	1.29517465	1.0261112	0.03718709	0.93047
SLFN11	1.023349	1.0194993	1.02652843	1.3604802	0.44411596	0.9305
RP11-540E16.2	1.023331	1.0195095	1.02648612	1.3575983	0.44105665	0.9305
CTD-2033A16.3	1.023392	1.0195088	1.02659907	1.3634382	0.44724932	0.9305
DNAJC6	1.07791	1.0742132	1.08096218	1.09094	0.12557181	0.93052
C1orf234	1.018873	1.0150523	1.02202873	1.4634758	0.54939885	0.93055
GJA4	1.018862	1.0150505	1.02201032	1.4624272	0.54836479	0.93055
C3	1.018894	1.015052	1.02206664	1.4660225	0.55190721	0.93055
HYKK	1.086537	1.0826088	1.08978172	1.0868296	0.12012573	0.93065
ABTB2	1.031942	1.0357819	1.02877167	0.8040851	-0.31458	0.93072
RP11-326K13.4	1.04052	1.0366373	1.0437268	1.1935046	0.25520408	0.93073
RP11-35O15.1	1.04045	1.0443037	1.03726795	0.8411923	-0.2494924	0.93074
SPTLC3	1.039732	1.0359049	1.04289159	1.1945891	0.25651446	0.9308
ADM5	1.039719	1.0357837	1.04296964	1.2008149	0.26401379	0.9308
TTC26	1.214532	1.2183036	1.21141677	0.968453	-0.0462461	0.9308
RP11-384P7.5	1.015243	1.0190043	1.0121375	0.6386702	-0.646857	0.93084
RP4-742J24.2	1.015266	1.0190542	1.0121375	0.6369998	-0.6506352	0.93084
SLC39A8	1.272343	1.2763871	1.26900337	0.9732846	-0.0390663	0.93088

ZFAT	1.061818	1.0656465	1.05865569	0.8935079	-0.1624476	0.93095
EBLN2	1.127863	1.1238436	1.13118242	1.0592583	0.08305449	0.93099
EDARADD	1.05253	1.0563612	1.04936697	0.8759034	-0.1911562	0.931
CTU2	1.12544	1.1214697	1.12871928	1.0596818	0.0836311	0.93101
RP11-638I2.4	1.016271	1.0200429	1.01315569	0.6563764	-0.6074048	0.93106
RP11-286E11.1	1.017099	1.0208443	1.01400662	0.6719638	-0.5735447	0.93106
TYRO3	1.194448	1.1984436	1.19114897	0.9632405	-0.054032	0.93107
JSRP1	1.026238	1.0223888	1.02941698	1.3139178	0.39387502	0.93109
AF127936.9	1.087187	1.0909894	1.08404744	0.9237054	-0.1144953	0.93115
RP11-422P24.12	1.071771	1.0756676	1.06855378	0.9059864	-0.1424386	0.93116
ADPRM	1.207678	1.2118819	1.20420685	0.9637769	-0.0532289	0.93122
JMJD1C	2.431834	2.4390848	2.42584652	0.9908009	-0.0133329	0.93125
RP11-575H3.1	1.033196	1.0293555	1.03636693	1.238846	0.30899688	0.93131
ADAMTS10	1.186345	1.182303	1.18968325	1.0404835	0.05725413	0.93136
SFTA2	1.022405	1.026195	1.01927588	0.73586	-0.4424969	0.93136
CDRT4	1.030242	1.0340194	1.02712286	0.7972769	-0.3268473	0.93143
ZNRF2	1.033092	1.0292857	1.03623606	1.2373285	0.30722855	0.93151
SPAG8	1.077009	1.0731475	1.08019819	1.0963905	0.13276176	0.93151
KBTBD11-OT1	1.041453	1.037538	1.04468617	1.1904248	0.25147652	0.93152
ESRP2	1.025297	1.0215041	1.02842877	1.3220179	0.40274169	0.93156
FAM90A1	1.018727	1.0149319	1.02186154	1.4640817	0.54999609	0.93161
KL	1.018747	1.0149371	1.02189246	1.4656471	0.55153776	0.93161
RP11-50D9.3	1.01873	1.0149371	1.02186172	1.4635893	0.54951078	0.93161
PCDHGA5	1.018733	1.0149371	1.02186719	1.4639552	0.54987143	0.93161
GS1-279B7.2	1.018729	1.0149371	1.02186086	1.4635317	0.54945398	0.93161
RP11-97C16.1	1.086344	1.0900834	1.08325663	0.9242173	-0.113696	0.93165
SLC27A3	1.150276	1.1540482	1.14716041	0.9552882	-0.065992	0.93165
SYDE1	1.173095	1.1691109	1.17638542	1.043016	0.06076135	0.93172
RP13-401N8.1	1.043019	1.0467642	1.03992632	0.8537791	-0.2280652	0.93174
RP1-80N2.3	1.03286	1.0291109	1.03595554	1.2351217	0.30465323	0.93179
TNMD	1.019627	1.023399	1.01651203	0.7056734	-0.5029274	0.9318
RP11-471M2.3	1.019617	1.023357	1.01652797	0.7076227	-0.4989477	0.9318
RP5-892K4.1	1.024392	1.0205787	1.0275417	1.338357	0.42046304	0.93182
RP11-425D10.10	1.025728	1.0219123	1.02887888	1.3179332	0.3982772	0.93184
RP5-1148A21.3	1.02574	1.0219532	1.02886636	1.3149029	0.39495625	0.93184
RP11-48B3.3	1.028358	1.0245772	1.03147965	1.2808488	0.35710021	0.93189
RP11-130F10.1	1.074456	1.0783881	1.07120821	0.9084055	-0.1385916	0.93191
TAF7L	1.019036	1.0227858	1.01593993	0.6995563	-0.515488	0.93192
RP11-261N11.8	1.019021	1.0227512	1.01593993	0.7006197	-0.5132965	0.93192
GREB1	1.238905	1.2428588	1.23564078	0.970279	-0.0435284	0.93193
PTPN6	1.06272	1.0665462	1.05955935	0.8950069	-0.1600292	0.93194
AP001059.5	1.02042	1.0166482	1.02353447	1.4136362	0.49941085	0.93195
ARHGAP27	1.020412	1.0166501	1.02351784	1.4124702	0.49822039	0.93195
SLC29A2	1.203168	1.2070908	1.19992793	0.9654118	-0.0507837	0.93196
RP5-963E22.6	1.020715	1.024406	1.01766692	0.7238757	-0.4661861	0.93207

RP11-539L10.2	1.020731	1.0244629	1.01764986	0.7214947	-0.4709392	0.93207
RP11-410K21.2	1.020732	1.0244876	1.0176301	0.71996	-0.4740114	0.93207
RP11-109E12.1	1.020716	1.0244128	1.01766263	0.7234987	-0.4669378	0.93207
NRAS	1.622714	1.6270536	1.61913009	0.987364	-0.0183461	0.93216
ZNF70	1.061847	1.0579754	1.06504437	1.1219305	0.16598334	0.93216
CYP7B1	1.050737	1.0543591	1.04774567	0.8783383	-0.1871514	0.9322
AL022341.3	1.02922	1.032916	1.0261686	0.795011	-0.3309533	0.93221
CCDC50	1.867141	1.8723044	1.86287761	0.9891933	-0.0156757	0.93224
TIGD2	1.113125	1.1092444	1.11632949	1.0648555	0.09065773	0.93224
LINC00471	1.051414	1.0550725	1.0483925	0.8787054	-0.1865486	0.93225
ROBO3	1.14088	1.1448933	1.13756619	0.949431	-0.0748649	0.93227
PRPF6	1.728231	1.7235509	1.73209605	1.01181	0.01693844	0.93229
RP1-313I6.12	1.176392	1.1722219	1.17983572	1.0442093	0.06241087	0.93238
AC092669.3	1.042828	1.0465681	1.03973948	0.853362	-0.2287702	0.93241
DIAPH2-AS1	1.068147	1.0719763	1.0649852	0.9028691	-0.1474112	0.93243
AQR	1.349341	1.3533009	1.34606998	0.9795332	-0.0298337	0.93258
RP11-247I13.11	1.020733	1.0169821	1.02383047	1.4032702	0.48879286	0.93263
AQP7	1.020728	1.0169845	1.02381857	1.4023703	0.48786732	0.93263
ACSBG1	1.02076	1.0169934	1.02387056	1.4046922	0.49025406	0.93263
BGLAP	1.022465	1.0187061	1.02556863	1.3668642	0.45086991	0.9327
GPR20	1.022453	1.0187083	1.02554559	1.3654712	0.4493989	0.9327
RP11-85A1.3	1.022451	1.0187034	1.02554605	1.3658519	0.44980102	0.9327
ADAMTS15	1.018696	1.0223796	1.01565341	0.6994495	-0.5157082	0.93271
RP5-1021I20.8	1.018717	1.0224185	1.01565988	0.6985247	-0.517617	0.93271
STEAP4	1.018707	1.0224034	1.01565461	0.6987608	-0.5171294	0.93271
RP11-266O8.1	1.018717	1.0224092	1.0156673	0.6991459	-0.5163345	0.93271
RP1-97J1.2	1.018715	1.0224277	1.0156487	0.6977392	-0.5192403	0.93271
DLGAP1-AS1	1.35376	1.3498305	1.3570041	1.0205058	0.0292844	0.93275
KCNQ2	1.105891	1.1098151	1.10264996	0.9347529	-0.097343	0.93276
FAM66A	1.033164	1.0368993	1.03007902	0.8151651	-0.2948357	0.93276
HEATR1	1.234917	1.2308038	1.23831364	1.0325377	0.04619439	0.93277
ZNF23	1.044812	1.0410244	1.04794	1.1685725	0.22474729	0.93277
C9orf153	1.040631	1.0442897	1.03760948	0.8491693	-0.2358758	0.93282
AC068831.10	1.041204	1.0449296	1.03812795	0.8486152	-0.2368176	0.93286
RP11-21B23.2	1.021333	1.0175788	1.02443316	1.3899219	0.47500382	0.93286
GCC1	1.072097	1.0683519	1.07518902	1.1000281	0.13754034	0.9329
GK	1.099035	1.1026786	1.09602601	0.9352094	-0.0966387	0.93292
KCTD14	1.034811	1.031027	1.03793612	1.2226806	0.29004759	0.93293
RP11-676J12.4	1.019775	1.0234993	1.0166999	0.7106564	-0.492776	0.93293
WDR97	1.019733	1.0234068	1.0166999	0.7134651	-0.4870853	0.93293
PLK5	1.016995	1.0206654	1.01396402	0.6757182	-0.5655063	0.93294
RP11-557H15.3	1.022633	1.0263035	1.01960208	0.7452263	-0.4242495	0.93295
RP11-3N13.2	1.02269	1.0263988	1.01962685	0.7434737	-0.4276463	0.93295
C11orf72	1.022665	1.0263944	1.01958588	0.7420456	-0.4304202	0.93295
ICAM5	1.028189	1.0244524	1.03127492	1.2790122	0.35502998	0.93295

MYEOV	1.030907	1.0271817	1.03398298	1.2502136	0.32217455	0.93296
ZNF595	1.161304	1.1651484	1.15812926	0.9574982	-0.0626583	0.93301
NRTN	1.045492	1.0417146	1.0486106	1.1653134	0.22071799	0.93309
HAS2-AS1	1.084417	1.0806614	1.08751924	1.0850196	0.11772107	0.93309
TLDC2	1.03331	1.0369672	1.03028979	0.8193695	-0.2874139	0.93315
CARMIL3	1.035262	1.0315316	1.03834295	1.2160159	0.28216213	0.9332
SAMD4B	1.555523	1.5600586	1.55177832	0.9852153	-0.021489	0.93324
LA16c-312E8.2	1.029903	1.0261572	1.03299591	1.2614481	0.33508085	0.93325
RP11-344N10.5	1.026627	1.0302915	1.02360096	0.7791276	-0.3600684	0.93328
PCOLCE-AS1	1.041035	1.0447428	1.03797244	0.8486821	-0.2367038	0.9333
DNAH14	1.641455	1.6457251	1.63792796	0.987925	-0.0175266	0.93336
RP1-150O5.3	1.024136	1.0203912	1.02722764	1.3352661	0.41712728	0.93341
RP11-314B1.2	1.024124	1.0203928	1.02720593	1.3340928	0.41585902	0.93341
MAP3K5	1.017977	1.0142772	1.02103213	1.4731219	0.55887682	0.93349
CCDC102B	1.173129	1.176791	1.17010406	0.9621759	-0.0556275	0.93355
AKAP5	1.057146	1.0534041	1.06023566	1.1279214	0.17366655	0.93355
LINC00176	1.018828	1.0151092	1.02189941	1.4494097	0.53546545	0.9336
RP11-265E18.1	1.01696	1.0132758	1.02000284	1.5067166	0.59140805	0.93371
RP11-196E1.3	1.016966	1.0132703	1.02001784	1.5084659	0.59308211	0.93371
ZNF696	1.078953	1.0826254	1.07591971	0.9188426	-0.1221104	0.93374
RP11-996F15.6	1.031425	1.0350845	1.02840314	0.8095647	-0.3047818	0.93378
MANEA-AS1	1.210407	1.2066206	1.21353351	1.0334571	0.04747856	0.93386
CSNK1G3	1.543401	1.5390455	1.54699815	1.0147533	0.02112897	0.93388
RP11-365P13.5	1.030292	1.0265611	1.0333734	1.2564754	0.32938244	0.93398
RP11-603J24.17	1.01717	1.0208224	1.01415473	0.6797852	-0.5568491	0.934
HHLA2	1.017134	1.0207415	1.01415473	0.6824348	-0.551237	0.934
RP1-137H15.2	1.017137	1.0207488	1.01415473	0.6821963	-0.5517411	0.934
LONRF2	1.142084	1.1458884	1.13894216	0.9523867	-0.0703806	0.934
MAFA	1.034348	1.0379854	1.03134378	0.8251526	-0.2772671	0.93401
CCDC120	1.086743	1.0904726	1.08366225	0.9247243	-0.1129047	0.93407
SNAI3	1.021648	1.0253207	1.01861505	0.7351714	-0.4438475	0.93416
HCG15	1.021659	1.0253483	1.01861201	0.7342512	-0.4456544	0.93416
FAM159A	1.07146	1.0677488	1.07452549	1.1000261	0.13753773	0.93417
AC007009.1	1.022213	1.0258895	1.01917625	0.7406973	-0.4330441	0.9342
RP11-403P17.4	1.017832	1.0141472	1.02087577	1.4756148	0.56131619	0.93427
PPP4R1-AS1	1.017828	1.014159	1.02085742	1.4730824	0.55883816	0.93427
MADD	1.179115	1.1752909	1.1822733	1.039833	0.05635188	0.93428
XYLT1	1.040529	1.0367777	1.04362651	1.1862213	0.24637313	0.93429
ACTN3	1.040564	1.0368914	1.04359712	1.1817701	0.24094936	0.93429
XXbac-BPG32J3.19	1.040549	1.036826	1.04362309	1.1845715	0.24436533	0.93429
TIGD3	1.040496	1.0367562	1.04358356	1.1857468	0.24579598	0.93429
CBLN3	1.03963	1.0432522	1.03663799	0.8470789	-0.2394317	0.93438
CTD-2571L23.8	1.023942	1.0275527	1.02096018	0.7607308	-0.3945421	0.93446
DCHS2	1.023015	1.0266051	1.02005046	0.7536336	-0.4080648	0.93446
RP11-848P1.4	1.02298	1.0265925	1.01999711	0.7519837	-0.4112267	0.93446

TSIX	1.02299	1.0266284	1.01998601	0.7505513	-0.4139775	0.93446
MSTO1	1.156939	1.152932	1.16024772	1.0478365	0.06741365	0.93446
IL17B	1.025368	1.0216746	1.02841838	1.3111366	0.39081804	0.93448
RP11-802D6.1	1.025401	1.0216882	1.02846662	1.3125413	0.39236282	0.93448
HARS	1.491992	1.4957999	1.48884757	0.9859775	-0.0203733	0.9345
ANKRD39	1.474015	1.4781877	1.47056995	0.9840696	-0.0231677	0.93454
FCRLB	1.060367	1.0641304	1.05725889	0.8928507	-0.1635092	0.93454
CCNYL1	1.158974	1.1627375	1.15586652	0.9577788	-0.0622356	0.9346
RAD54L	1.100541	1.0967239	1.10369313	1.0720528	0.10037594	0.93464
GAS8	1.053074	1.0493402	1.05615829	1.1381861	0.1867365	0.93465
ABCF2	1.264511	1.2685661	1.261163	0.9724348	-0.0403266	0.93466
CHI3L1	1.027411	1.0237488	1.03043592	1.2815766	0.3579197	0.93466
RP1-20C7.6	1.027417	1.0237555	1.03044126	1.2814421	0.35776832	0.93466
CMTM1	1.017551	1.0139016	1.0205654	1.4793555	0.56496882	0.93466
NR2E3	1.017538	1.013898	1.02054409	1.4782087	0.56384995	0.93466
RP11-73M18.10	1.017535	1.013898	1.02053779	1.4777552	0.5634073	0.93466
LA16c-380H5.5	1.017551	1.0139007	1.02056526	1.4794368	0.5650481	0.93466
RP11-334C17.5	1.090935	1.0871265	1.09408062	1.0798162	0.11078583	0.93467
MCMDC2	1.025318	1.0216533	1.02834428	1.3090069	0.38847274	0.93467
RP11-227G15.11	1.025353	1.0216463	1.02841347	1.3126245	0.39245424	0.93467
RP11-140I16.3	1.024824	1.0284161	1.02185702	0.7691764	-0.3786137	0.93471
LSS	1.261933	1.2658709	1.25868091	0.9729569	-0.0395523	0.93487
ANKHD1-EIF4EBP3	1.025651	1.0220169	1.028652	1.3013653	0.38002601	0.93492
NOMO2	1.025656	1.0220161	1.02866193	1.301861	0.38057548	0.93492
RP11-468E2.10	1.025681	1.0220334	1.02869284	1.302245	0.38100095	0.93492
AC023115.2	1.025701	1.0220045	1.02875337	1.3067053	0.38593385	0.93492
RP11-268J15.5	1.163748	1.16007	1.16678601	1.041957	0.05929573	0.93493
LINC01483	1.01802	1.0143272	1.02107009	1.4706344	0.55643859	0.93493
RP11-431K24.1	1.01797	1.0143257	1.02097988	1.4644898	0.55039816	0.93493
RP11-378J18.9	1.025093	1.0214523	1.02809867	1.3098183	0.38936669	0.93495
LINC01204	1.020589	1.0169689	1.02357886	1.3895329	0.47460001	0.93502
RP11-253E3.3	1.020587	1.0169336	1.02360442	1.3939381	0.47916647	0.93502
RP11-161H23.5	1.020619	1.0169551	1.02364476	1.3945495	0.47979917	0.93502
RP11-115H18.1	1.020593	1.0169626	1.02359014	1.3907117	0.47582337	0.93502
CEBPG	1.382143	1.3780723	1.38550382	1.0196563	0.02808293	0.93504
CTD-2659N19.10	1.03021	1.033837	1.02721391	0.8042653	-0.3142567	0.93504
MFAP5	1.026433	1.0227782	1.02945058	1.2929272	0.37064109	0.93506
FBN3	1.067532	1.071255	1.06445784	0.904608	-0.1446353	0.93511
SYT1	4.092151	4.1020217	4.08400047	0.9941905	-0.0084058	0.93515
GAL3ST4	1.093945	1.0976407	1.09089259	0.9308882	-0.1033202	0.93519
TAS2R14	1.050747	1.0543562	1.04776604	0.8787599	-0.1864591	0.9353
LINC01356	1.03977	1.0361139	1.04278976	1.184857	0.24471296	0.93531
SEMA4D	1.168638	1.1647213	1.17187172	1.043409	0.06130478	0.93534
ATG12	2.206349	2.2113275	2.20223714	0.9924956	-0.0108674	0.93536
RP1-265C24.8	1.01541	1.0190107	1.01243594	0.6541533	-0.6122993	0.93542

OGFR-AS1	1.015383	1.0189515	1.01243594	0.6561982	-0.6077965	0.93542
GRIA3	1.040313	1.0366436	1.04334382	1.182847	0.24226344	0.93546
DCLRE1A	1.125705	1.129194	1.1228241	0.9506952	-0.0729451	0.93553
KCNQ5	1.027986	1.0243826	1.03096187	1.2698334	0.34463919	0.93554
ZC3H3	1.140805	1.1446146	1.13765909	0.951903	-0.0711135	0.93563
WASIR2	1.064469	1.0608083	1.06749243	1.1099213	0.15045739	0.93566
RP11-185E8.1	1.020659	1.0242225	1.01771539	0.7313603	-0.4513458	0.9357
ATAD1	1.65051	1.64568	1.65449893	1.0136583	0.01957141	0.93574
SPRYD3	1.12765	1.1241984	1.13049951	1.050734	0.07139753	0.93577
CTD-3222D19.12	1.058623	1.0622023	1.0556679	0.894949	-0.1601227	0.93581
FRY	1.034242	1.0378306	1.03127786	0.8267863	-0.2744137	0.93583
COX8C	1.03423	1.0377926	1.03128868	0.8279048	-0.2724632	0.93583
NFRKB	1.19603	1.1996159	1.19306895	0.9672023	-0.0481104	0.93585
FBRSL1	1.152134	1.1484259	1.15519634	1.0456152	0.06435198	0.93593
CCDC170	1.050585	1.0541749	1.04762097	0.8790221	-0.1860287	0.93597
RP11-420A23.1	1.058282	1.061905	1.05528934	0.8931315	-0.1630554	0.93607
TAP2	1.058168	1.0617397	1.05521897	0.8943836	-0.1610344	0.93607
RP11-209D14.2	1.051456	1.05509	1.04845516	0.8795634	-0.1851405	0.93607
WDR38	1.020441	1.0168745	1.02338673	1.3859187	0.47084261	0.93608
C4orf22	1.020418	1.0168367	1.02337559	1.3883693	0.47339135	0.93608
RP11-462L8.1	1.020433	1.0168533	1.02338971	1.3878441	0.47284549	0.93608
LCN9	1.020444	1.0168269	1.02343096	1.3924743	0.47765073	0.93608
CASC6	1.023196	1.0196026	1.02616361	1.3347039	0.4165197	0.93609
MYL10	1.023191	1.0196295	1.02613252	1.331285	0.41281946	0.93609
RP11-686G8.5	1.023192	1.0196181	1.02614423	1.3326605	0.41430927	0.93609
RP11-734K2.4	1.090274	1.0866046	1.09330435	1.0773599	0.10750023	0.93612
CCDC177	1.017894	1.0214703	1.01494071	0.6958781	-0.5230935	0.93615
GAS2L2	1.017874	1.0214254	1.01494071	0.6973349	-0.5200765	0.93615
RP11-1228E12.1	1.017894	1.0214697	1.01494071	0.6958959	-0.5230565	0.93615
PRKACB	1.42661	1.422917	1.42966069	1.0159458	0.02282339	0.93616
NRARP	1.035187	1.0315747	1.0381707	1.2089013	0.2736965	0.93619
DNAH1	1.03526	1.0317373	1.03816989	1.202681	0.26625409	0.93619
LACTB2-AS1	1.026093	1.0224769	1.02907977	1.2937651	0.37157565	0.93627
RP11-475I24.3	1.026098	1.0224776	1.02908798	1.2940874	0.37193501	0.93627
ALKBH8	1.116063	1.1199433	1.11285934	0.9409391	-0.0878267	0.93629
LINC00323	1.027482	1.0239209	1.03042281	1.2718074	0.34688024	0.93629
MLKL	1.022144	1.0185669	1.02509778	1.3517455	0.43482351	0.93641
HSPB9	1.022165	1.0185734	1.02513142	1.3530882	0.43625592	0.93641
RP11-62H20.1	1.019015	1.0225678	1.01608078	0.7125535	-0.4889297	0.93641
DLX6-AS1	1.019722	1.0232658	1.01679584	0.7219115	-0.4701061	0.93645
DOCK3	1.079652	1.0761898	1.08251149	1.0829728	0.11499697	0.93649
KNDC1	1.079611	1.0760296	1.08256823	1.0860014	0.1190259	0.93649
SLC35G2	1.197134	1.2009655	1.19397053	0.9651933	-0.0511103	0.93664
YTHDF3-AS1	1.150797	1.1544497	1.1477805	0.9568198	-0.0636808	0.93668
AHI1	2.126855	2.1317083	2.1228463	0.9921693	-0.0113417	0.93671

SAP25	1.041854	1.0454129	1.03891518	0.8569199	-0.2227678	0.93671
RP11-966I7.4	1.049892	1.0461444	1.05298625	1.1482695	0.19946133	0.93674
THBD	1.050796	1.0543597	1.04785323	0.8803074	-0.1839207	0.93676
RP11-930P14.2	1.037504	1.0339205	1.04046318	1.1928821	0.25445146	0.93677
RP11-158M9.1	1.037552	1.033921	1.04055062	1.1954416	0.25754362	0.93677
ADCYAP1	1.031827	1.0353808	1.02889304	0.8166298	-0.2922459	0.93682
CTD-2501E16.2	1.031777	1.0353225	1.02884972	0.8167523	-0.2920295	0.93682
KLHDC4	1.098727	1.0950543	1.1017594	1.0705396	0.09833818	0.93682
ARHGEF12	1.729467	1.725522	1.73272505	1.009928	0.01425248	0.93683
ASIC3	1.036818	1.0331848	1.0398183	1.1998959	0.26290922	0.93697
RP4-561L24.3	1.024922	1.0213329	1.02788615	1.3071904	0.38646926	0.93702
BAHCC1	1.136899	1.133219	1.13993811	1.0504364	0.07098882	0.93702
SIKE1	1.710432	1.7060115	1.71408239	1.0114317	0.01639886	0.93703
RP3-470B24.5	1.016976	1.0204799	1.01408228	0.6876138	-0.5403297	0.93707
AC108004.2	1.016982	1.0204939	1.01408228	0.6871464	-0.5413106	0.93707
RP11-319G6.1	1.095493	1.0989224	1.09266019	0.9366959	-0.0943474	0.93707
B4GALNT3	1.03133	1.0348434	1.02842907	0.8159093	-0.2935193	0.93709
FRGCA	1.022458	1.018896	1.02539869	1.3441312	0.42667397	0.9371
RP11-355F16.1	1.022479	1.0189117	1.02542483	1.3443936	0.42695557	0.9371
CTC-548K16.2	1.022485	1.0188996	1.02544627	1.3463902	0.4290966	0.9371
GPAT3	1.022455	1.0189088	1.02538413	1.3424471	0.4248652	0.9371
ATP6V1B1-AS1	1.024185	1.0206213	1.02712821	1.3155461	0.39566182	0.93715
GAS6-AS2	1.024167	1.0205802	1.02712819	1.3181682	0.39853445	0.93715
PEX6	1.207234	1.2034986	1.21031884	1.0335149	0.04755914	0.93729
RP11-326C3.7	1.020958	1.0245298	1.01800872	0.7341581	-0.4458373	0.93729
LINC01220	1.023061	1.019513	1.02599108	1.3319893	0.41358254	0.93732
FOXS1	1.023049	1.0194828	1.02599477	1.3342411	0.41601939	0.93732
SIGLEC7	1.02307	1.0195281	1.02599579	1.3311961	0.41272314	0.93732
CTD-2396E7.11	1.023066	1.019518	1.0259954	1.3318673	0.41345039	0.93732
KCNIP2-AS1	1.029941	1.0263346	1.03291959	1.25005	0.32198583	0.9374
WDR24	1.152615	1.1560608	1.14977019	0.9596913	-0.0593577	0.93745
CTD-2311M21.3	1.044685	1.041189	1.04757164	1.1549605	0.20784348	0.93745
DRC1	1.035604	1.0320479	1.03853995	1.202574	0.26612572	0.9375
CASC2	1.077076	1.0807263	1.07406168	0.9174421	-0.1243109	0.93758
AC145110.1	1.022283	1.0257949	1.01938285	0.7514222	-0.4123044	0.93759
AGMO	1.022316	1.0258102	1.01943122	0.7528518	-0.4095621	0.93759
AIFM3	1.042079	1.0456093	1.03916448	0.8586951	-0.2197822	0.93762
FGF7	1.171795	1.1755774	1.1686706	0.9606623	-0.0578987	0.93762
RNASEK-C17orf49	1.024922	1.0284403	1.02201635	0.774125	-0.3693615	0.93763
USP3	1.569072	1.5646019	1.57276395	1.0144563	0.02070673	0.93766
RP11-166P13.4	1.060523	1.0568692	1.06354085	1.1173163	0.16003765	0.93767
PDE6G	1.02938	1.0328792	1.02649057	0.8056942	-0.3116958	0.93782
AKR1E2	1.029402	1.0329365	1.02648269	0.8040538	-0.314636	0.93782
RP5-855D21.2	1.030071	1.0336564	1.02711063	0.805512	-0.3120219	0.93786
SLC25A15	1.063842	1.0673181	1.06097126	0.9057181	-0.142866	0.93793

FBLN7	1.131477	1.1348937	1.12865489	0.9537499	-0.0683171	0.93795
TAC3	1.019688	1.0161837	1.02258183	1.3953413	0.4806181	0.93797
TP63	1.01967	1.0161779	1.02255347	1.394089	0.47932267	0.93797
SNAP25-AS1	1.022772	1.0262553	1.01989607	0.7577919	-0.4001263	0.93802
AC002550.6	1.022769	1.0262306	1.01991088	0.7590695	-0.397696	0.93802
SPDEF	1.022792	1.0262971	1.01989737	0.7566363	-0.4023282	0.93802
GRID1	1.022782	1.0262402	1.01992544	0.7593467	-0.3971694	0.93802
MBOAT1	1.022773	1.026247	1.01990444	0.7583508	-0.3990627	0.93802
DDX11-AS1	1.030713	1.0341726	1.027856	0.8151563	-0.2948515	0.93805
RP13-638C3.4	1.030748	1.034229	1.02787279	0.8143031	-0.2963623	0.93805
RP11-1299A16.3	1.020571	1.0170839	1.02345125	1.3727081	0.45702491	0.93806
CFHR3	1.020576	1.0170239	1.02350938	1.3809602	0.4656717	0.93806
POLG	1.286784	1.2829896	1.28991671	1.0244782	0.03488929	0.93818
ANXA2R	1.018668	1.015165	1.02156129	1.421784	0.50770235	0.93818
TMEM52B	1.018693	1.015176	1.02159673	1.4230803	0.50901707	0.93818
C2orf91	1.029767	1.0332415	1.0268969	0.8091362	-0.3055454	0.9383
RP11-302B13.5	1.0344	1.0308047	1.03736892	1.2130899	0.27868647	0.9383
LAIR1	1.034436	1.0308716	1.03737902	1.2107901	0.27594876	0.9383
KIAA1683	1.044711	1.0482801	1.0417643	0.8650409	-0.2091598	0.93836
SYT15	1.033648	1.0300955	1.03658236	1.2155444	0.28160255	0.93839
RP11-435J9.2	1.019898	1.0233501	1.01704689	0.7300572	-0.4539186	0.93852
RP11-15B17.1	1.020437	1.0238677	1.01760343	0.7375426	-0.4392017	0.93855
CNTNAP4	1.02045	1.0238968	1.01760343	0.7366428	-0.440963	0.93855
AP001596.6	1.032555	1.0291002	1.03540769	1.216752	0.28303509	0.93861
UNC5A	1.032527	1.0289756	1.03545997	1.2237854	0.29135056	0.93861
SPIN3	1.219018	1.2226715	1.21600071	0.9700422	-0.0438806	0.93864
ATF3	1.133628	1.1372711	1.13061997	0.9515476	-0.0716523	0.93872
UPK2	1.045164	1.0417015	1.04802264	1.15158	0.2036147	0.93873
TEP1	1.045097	1.0415726	1.04800692	1.1547744	0.207611	0.93873
RP11-109A6.5	1.019558	1.0160514	1.0224531	1.3988283	0.48421892	0.93875
SYT12	1.019572	1.0160982	1.02244099	1.3940097	0.47924061	0.93875
TRIM63	1.019545	1.0160525	1.02243003	1.3972935	0.48263513	0.93875
CDYL2	1.019556	1.0160655	1.02243828	1.3966763	0.48199764	0.93875
STX11	1.020166	1.0166975	1.02303079	1.3792922	0.46392808	0.93876
HIST1H2BL	1.02018	1.0166975	1.02305624	1.3808165	0.46552159	0.93876
RP11-417F21.1	1.020188	1.0166975	1.02307045	1.3816674	0.46641036	0.93876
RP5-1142A6.8	1.020181	1.0166975	1.02305813	1.3809297	0.46563988	0.93876
RP11-286E11.2	1.020201	1.0166975	1.02309461	1.3831141	0.46792018	0.93876
LINC01470	1.020194	1.0166975	1.02308144	1.3823253	0.46709715	0.93876
TFF1	1.020192	1.0166975	1.0230772	1.3820714	0.46683219	0.93876
AC026188.1	1.020201	1.0166975	1.02309408	1.3830828	0.46788749	0.93876
NTSR1	1.020145	1.0235844	1.01730453	0.7337276	-0.4466836	0.9388
AC001226.7	1.020144	1.0235827	1.01730453	0.733782	-0.4465766	0.9388
CASQ1	1.020149	1.0235941	1.01730453	0.7334269	-0.4472749	0.9388
CHIT1	1.020165	1.023628	1.01730453	0.7323727	-0.4493501	0.9388

RP11-354E11.2	1.022237	1.0256822	1.0193919	0.7550706	-0.4053166	0.93881
FAM151A	1.022274	1.0257643	1.0193919	0.752667	-0.4099164	0.93881
GPR158-AS1	1.022246	1.0257012	1.0193919	0.7545127	-0.406383	0.93881
ZNF665	1.119934	1.1164053	1.12284811	1.0553478	0.07771858	0.93885
RP1-102G20.5	1.020206	1.0236584	1.01735489	0.7335603	-0.4470126	0.93899
PPP1R27	1.020208	1.0236639	1.01735489	0.7333915	-0.4473446	0.93899
H2BFM	1.020196	1.0236354	1.01735489	0.7342745	-0.4456086	0.93899
RP11-43F13.4	1.020191	1.0236253	1.01735489	0.7345885	-0.4449918	0.93899
DOC2A	1.090292	1.0867412	1.0932252	1.0747512	0.10400268	0.939
GPRIN2	1.022266	1.0256627	1.01946109	0.7583421	-0.3990792	0.93903
LRRK2	1.022319	1.0257173	1.01951235	0.7587237	-0.3983534	0.93903
RP11-679B19.1	1.068669	1.072154	1.06579174	0.911824	-0.1331727	0.93905
CCDC17	1.023158	1.0265765	1.02033442	0.7651269	-0.3862291	0.93905
LARP1	1.986958	1.9914856	1.98321955	0.9916629	-0.0120783	0.93912
MTMR8	1.019299	1.0157935	1.02219409	1.4052632	0.4908404	0.93913
AC062028.1	1.019302	1.0158119	1.0221849	1.4030479	0.48856426	0.93913
B3GNT9	1.118695	1.1152075	1.12157566	1.055276	0.07762034	0.93914
STK39	1.214335	1.2181301	1.21120165	0.9682372	-0.0465676	0.93921
ABTB1	1.104112	1.1075824	1.10124594	0.9411016	-0.0875777	0.93925
CHL1	1.112798	1.116417	1.1098086	0.9432354	-0.0843102	0.93925
LINC01144	1.029221	1.0257339	1.03210143	1.2474396	0.31896993	0.9393
CTC-428H11.2	1.030015	1.0265534	1.03287348	1.2380162	0.30803025	0.93935
PCP2	1.02744	1.0239181	1.03034847	1.2688518	0.34352361	0.93937
ALS2CL	1.027331	1.0238817	1.03017989	1.2637263	0.337684	0.93937
AC092198.1	1.028437	1.031819	1.02564331	0.8059107	-0.3113081	0.93937
CTD-2002J20.1	1.026809	1.0233043	1.02970247	1.2745469	0.34998449	0.9394
DEPTOR	1.036237	1.039629	1.03343554	0.8437142	-0.2451737	0.93941
CTD-2054N24.2	1.03633	1.039792	1.03347177	0.8411689	-0.2495326	0.93941
LINC00856	1.019692	1.0162259	1.02255425	1.390019	0.47510458	0.93941
HLCS	1.17179	1.1681337	1.17480893	1.0397016	0.0561696	0.93943
RP11-386D6.1	1.018344	1.0148661	1.02121554	1.4271076	0.51309408	0.93948
SNTN	1.018367	1.0148661	1.02125735	1.4299196	0.51593404	0.93948
TAS2R10	1.01833	1.0148661	1.02119132	1.4254779	0.51144567	0.93948
CCDC62	1.018326	1.0148661	1.02118349	1.4249512	0.5109125	0.93948
RP11-370A5.1	1.018328	1.0148661	1.0211871	1.4251942	0.5111585	0.93948
LINC01482	1.018369	1.0148661	1.0212626	1.430273	0.51629054	0.93948
LGSN	1.018321	1.0148661	1.02117437	1.4243379	0.51029147	0.93948
RP11-341N2.1	1.018326	1.0148661	1.02118374	1.4249682	0.51092968	0.93948
RP11-865I6.2	1.018311	1.0148661	1.02115529	1.4230545	0.50899092	0.93948
RP11-1C8.4	1.01835	1.0148661	1.02122732	1.4278998	0.51389474	0.93948
WASL	1.802858	1.8072903	1.79919726	0.9899751	-0.0145359	0.93953
RP13-270P17.3	1.04899	1.0524238	1.04615478	0.8804158	-0.1837431	0.9396
KIRREL2	1.092972	1.089373	1.09594486	1.0735328	0.10236628	0.93962
NDUFB2-AS1	1.025633	1.0290415	1.02281777	0.7856941	-0.3479604	0.93979
KCNJ14	1.042045	1.0385828	1.04490438	1.1638443	0.21889803	0.93988

TPH1	1.025134	1.0286167	1.02225875	0.7778252	-0.3624821	0.9399
SLC22A18	1.243761	1.2475631	1.24062162	0.9719606	-0.0410303	0.93998
KCNK5	1.029688	1.0261955	1.03257152	1.2434035	0.31429455	0.93998
ZNF396	1.04888	1.0453877	1.05176379	1.1404812	0.18964264	0.94001
RP11-307N16.6	1.018926	1.0223009	1.01613888	0.7236887	-0.4665588	0.94006
STMND1	1.018943	1.0223393	1.01613888	0.722442	-0.4690464	0.94006
ANKRD23	1.027529	1.030931	1.02471923	0.7991742	-0.3234181	0.9401
DOK1	1.217984	1.2141977	1.22111046	1.0322727	0.04582416	0.9401
LINC00240	1.08303	1.0865311	1.08013839	0.9261223	-0.1107254	0.9401
LRRC57	1.277596	1.2814054	1.2744496	0.9752818	-0.036109	0.94022
RP11-367J11.3	1.038217	1.0416232	1.03540404	0.8505852	-0.2334724	0.94029
ZNF502	1.064325	1.0608805	1.06716959	1.1033031	0.14182913	0.94034
RP11-166B2.8	1.016425	1.0129919	1.01926066	1.482511	0.5680428	0.94036
SLC2A9	1.016409	1.0129919	1.01923078	1.4802108	0.56580265	0.94036
IGSF23	1.016417	1.0129919	1.0192451	1.4813128	0.56687634	0.94036
CTC-498J12.1	1.016404	1.0129919	1.01922237	1.4795634	0.5651715	0.94036
RP11-408N14.1	1.016391	1.0129919	1.01919838	1.4777169	0.56336994	0.94036
RP11-95O2.1	1.016417	1.0129919	1.01924555	1.4813481	0.56691067	0.94036
ASCL4	1.016424	1.0129919	1.01925915	1.4823943	0.56792926	0.94036
RP11-62J1.4	1.016408	1.0129919	1.01922903	1.4800761	0.56567139	0.94036
CLIC5	1.0164	1.0129919	1.01921393	1.4789143	0.56453845	0.94036
RP11-768F21.1	1.016415	1.0129919	1.01924233	1.4810997	0.56666877	0.94036
CTD-3065J16.9	1.058643	1.0621037	1.05578579	0.8982683	-0.1547816	0.94036
RP11-89F3.2	1.049673	1.0531027	1.04684112	0.8820856	-0.1810095	0.94036
RP11-102G14.1	1.022179	1.018735	1.02502313	1.3356338	0.41752449	0.94054
RANBP3L	1.02222	1.0187717	1.02506781	1.3354022	0.41727434	0.94054
GS1-166A23.2	1.024964	1.0214884	1.02783437	1.2953196	0.37330808	0.94055
CTA-929C8.6	1.024929	1.0214826	1.02777575	1.2929415	0.37065703	0.94055
ZNF254	1.424605	1.4209401	1.42763133	1.015896	0.02275266	0.94056
CPM	1.038669	1.0352146	1.04152087	1.1790801	0.23766171	0.94061
THAP12	1.449745	1.4459388	1.4528879	1.0155831	0.02230827	0.94065
RP11-1152H14.1	1.036418	1.0329374	1.03929271	1.1929494	0.2545328	0.94066
IL17RA	1.072983	1.0695999	1.07577702	1.0887516	0.12267482	0.94071
LRIG2	1.226942	1.2234575	1.22981926	1.0284695	0.04049897	0.94071
NAP1L2	1.085157	1.0818298	1.08790419	1.0742318	0.10330531	0.94074
CTA-212D2.2	1.017311	1.0207114	1.01450349	0.7002665	-0.514024	0.94077
CTD-2337A12.1	1.017329	1.0207502	1.01450349	0.6989558	-0.5167268	0.94077
NPY5R	1.017281	1.0206438	1.01450349	0.7025576	-0.5093115	0.94077
RP11-138I18.1	1.017271	1.0206225	1.01450349	0.7032859	-0.5078167	0.94077
AC011997.1	1.017281	1.0206453	1.01450349	0.7025091	-0.5094111	0.94077
RP11-338N10.3	1.017285	1.0206533	1.01450349	0.702236	-0.5099721	0.94077
RP11-318C2.1	1.017279	1.0206408	1.01450349	0.7026627	-0.5090957	0.94077
LEMD3	1.199006	1.1954295	1.20195948	1.0334134	0.04741747	0.94077
KLHL17	1.052591	1.0491313	1.05544861	1.1285812	0.17451022	0.94083
EEF2K	1.24433	1.2406825	1.2473413	1.0276662	0.03937177	0.94089

RP1-151F17.2	1.025792	1.0291505	1.02301935	0.7896731	-0.3406725	0.94091
RNF112	1.023029	1.0263891	1.02025435	0.7675262	-0.3817121	0.94092
CLCN6	1.148767	1.1522378	1.14590121	0.9583768	-0.0613352	0.94092
NXF1	1.177695	1.1741212	1.18064666	1.0374763	0.05307843	0.94101
CTRC	1.018091	1.0146773	1.02091026	1.4246649	0.51062262	0.94107
SLCO1B1	1.018082	1.0146773	1.02089354	1.4235258	0.50946862	0.94107
CTD-2199O4.6	1.018076	1.0146773	1.02088318	1.4228198	0.50875298	0.94107
IFNE	1.018052	1.0146773	1.02083842	1.4197702	0.50565742	0.94107
CFAP58-AS1	1.018081	1.0146773	1.02089226	1.4234386	0.50938022	0.94107
CD1D	1.018064	1.0146773	1.02086103	1.4213105	0.50722176	0.94107
SCN4B	1.018073	1.0146773	1.02087793	1.4224623	0.50839041	0.94107
RP3-406P24.4	1.018083	1.0146773	1.02089533	1.423648	0.50959247	0.94107
DPY19L1	1.398836	1.3948757	1.40210614	1.0183107	0.02617787	0.94111
FAS	1.091923	1.0884403	1.09479965	1.0719052	0.10017734	0.94112
ZP1	1.064085	1.0606883	1.06688962	1.1021823	0.14036288	0.94114
ZNF616	1.149996	1.1533913	1.14719284	0.9595904	-0.0595094	0.94115
TIGD1	1.23566	1.2319866	1.23869334	1.02891	0.04111676	0.9412
ZNF628	1.061838	1.0651853	1.05907374	0.9062437	-0.142029	0.94122
HIST1H3G	1.032685	1.0360239	1.02992698	0.8307539	-0.267507	0.94123
FNIP1	1.801106	1.7965584	1.80486169	1.0104239	0.0149607	0.94125
LY6G5C	1.058326	1.0548907	1.06116253	1.1142611	0.15608738	0.94129
EXD2	1.174569	1.177862	1.17184873	0.9661915	-0.0496189	0.94139
RP11-324E6.10	1.038522	1.0351377	1.04131728	1.1758664	0.23372412	0.9414
RP11-98D18.9	1.075031	1.0716317	1.07783755	1.0866351	0.11986753	0.94141
ZNF75D	1.085174	1.0887723	1.08220275	0.9259959	-0.1109222	0.94146
CENPP	1.085053	1.0883151	1.08235929	0.9325617	-0.1007289	0.94146
RP11-72M17.1	1.026634	1.0232262	1.02944848	1.2678969	0.34243741	0.94147
RP11-382A20.3	2.316405	2.3116824	2.32030423	1.0065731	0.00945198	0.94149
LAG3	1.037915	1.0413054	1.03511573	0.850149	-0.2342124	0.94149
RP11-415F23.2	1.024198	1.0207938	1.02700977	1.2989355	0.37732983	0.94157
RP1-142L7.8	1.024208	1.0208221	1.02700485	1.2969297	0.37510028	0.94157
AC005498.3	1.025892	1.0225154	1.02868018	1.2738024	0.34914149	0.94159
RP11-420L9.5	1.065328	1.0618982	1.06815961	1.1011561	0.13901897	0.9416
GPR19	1.046098	1.0426482	1.04894682	1.1476868	0.198729	0.94161
SALRNA2	1.019187	1.0225076	1.01644507	0.7306466	-0.4527544	0.94165
OSBPL7	1.063026	1.0664076	1.06023393	0.9070342	-0.1407711	0.94167
NRROS	1.024778	1.021412	1.02755721	1.2869984	0.36401025	0.94178
RBM4	1.880556	1.8762295	1.88412821	1.0090144	0.01294681	0.94182
C20orf203	1.029904	1.0332359	1.02715252	0.8169637	-0.2916561	0.94183
PLCG1-AS1	1.029912	1.0332589	1.02714776	0.8162555	-0.2929072	0.94183
NAT8L	1.203778	1.2074862	1.20071601	0.9673704	-0.0478597	0.94187
CLEC18C	1.048991	1.0455956	1.05179586	1.135983	0.18394129	0.94189
RP11-680A11.5	1.045875	1.0424845	1.04867532	1.1457202	0.19625479	0.9419
AC142528.1	1.082598	1.0791412	1.08545349	1.0797591	0.11070951	0.94192
RP11-618L22.1	1.02056	1.0238595	1.01783567	0.7475294	-0.4197978	0.94195

RP5-1057I20.4	1.020563	1.0238757	1.01782787	0.7466948	-0.4214095	0.94195
RP4-583P15.10	1.020584	1.023938	1.0178145	0.7441925	-0.4262523	0.94195
NPIPB15	1.020567	1.0239137	1.01780313	0.7444749	-0.4257048	0.94195
CCDC88C	1.150464	1.1469899	1.15333346	1.0431566	0.06095574	0.94195
POMGNT2	1.289261	1.2856673	1.2922289	1.0229693	0.03276292	0.94206
RP11-110I1.12	1.041345	1.0379906	1.04411564	1.161224	0.21564635	0.94206
ERMN	1.019327	1.0159741	1.02209537	1.383198	0.46800768	0.94213
SLC9A2	1.019312	1.0159741	1.02206909	1.3815527	0.46629061	0.94213
CTC-558O2.1	1.019332	1.0159741	1.02210577	1.383849	0.46868658	0.94213
RP11-557H15.4	1.019324	1.0159741	1.02208969	1.3828426	0.46763691	0.94213
RP11-278A23.1	1.019329	1.0159741	1.02209864	1.3834029	0.46822139	0.94213
PCDHGA3	1.019324	1.0159741	1.02209092	1.3829193	0.46771702	0.94213
BPIFB1	1.019329	1.0159741	1.0221004	1.383513	0.46833615	0.94213
MYOG	1.019338	1.0159741	1.02211587	1.3844816	0.46934592	0.94213
PLA1A	1.019321	1.0159741	1.02208479	1.3825356	0.46731668	0.94213
CTB-113P19.1	1.019335	1.0159741	1.02211079	1.3841634	0.46901424	0.94213
CTC-250I14.6	1.019313	1.0159741	1.02206968	1.3815895	0.46632907	0.94213
HIGD2B	1.019328	1.0159741	1.02209766	1.3833414	0.46815728	0.94213
PTCH2	1.061014	1.0643507	1.05825815	0.9053232	-0.1434952	0.94216
RP11-927P21.1	1.028224	1.0315274	1.02549674	0.808718	-0.3062915	0.9422
MAGIX	1.085198	1.0885695	1.082413	0.9304893	-0.1039385	0.94227
ENTPD1	1.14859	1.1453305	1.15128254	1.0409555	0.05790835	0.94229
ZNF540	1.027623	1.0242754	1.03038755	1.2517825	0.32398387	0.9423
FAM231D	1.027569	1.0241818	1.03036708	1.2557839	0.32858823	0.9423
DOCK8	1.027553	1.0242459	1.03028391	1.2490326	0.32081119	0.9423
KRTAP5-10	1.021406	1.0180243	1.02419877	1.3425656	0.42499258	0.94231
MKRN2OS	1.02137	1.0180243	1.02413379	1.3389609	0.42111382	0.94231
RP11-491F9.8	1.021404	1.0180243	1.02419427	1.3423161	0.4247245	0.94231
SLC12A3	1.021363	1.0180243	1.02412021	1.3382071	0.42030141	0.94231
RP11-40E6.2	1.021376	1.0180243	1.02414421	1.3395389	0.42173648	0.94231
RP11-7F17.3	1.02136	1.0180243	1.02411513	1.3379254	0.41999766	0.94231
RP11-350G8.5	1.021381	1.0180243	1.02415386	1.3400743	0.42231304	0.94231
RP11-206L10.4	1.021378	1.0180243	1.02414702	1.3396948	0.42190434	0.94231
CLEC12A	1.021392	1.0180243	1.02417256	1.3411116	0.42342924	0.94231
PTGS1	1.021378	1.0180243	1.02414786	1.3397415	0.42195469	0.94231
RP11-4B16.3	1.021369	1.0180243	1.02413056	1.3387813	0.42092036	0.94231
RP11-437J19.1	1.021414	1.0180243	1.02421363	1.3433902	0.42587839	0.94231
RP11-874J12.4	1.01928	1.0159362	1.02204198	1.3831351	0.46794203	0.94232
CTD-2162K18.3	1.019284	1.0159362	1.0220489	1.3835691	0.46839467	0.94232
FOXA1	1.019266	1.0159362	1.02201565	1.3814825	0.46621732	0.94232
RP1-28O10.1	1.019285	1.0159362	1.02205021	1.3836512	0.46848028	0.94232
RP13-192B19.2	1.019286	1.0159362	1.02205257	1.3837992	0.4686346	0.94232
GLIS2-AS1	1.019274	1.0159362	1.02202993	1.3823788	0.467153	0.94232
GJB3	1.019275	1.0159362	1.02203266	1.3825503	0.46733199	0.94232
KB-1562D12.1	1.019285	1.0159362	1.02204986	1.3836295	0.46845772	0.94232

EPHA1	1.019281	1.0159362	1.02204367	1.3832409	0.46805242	0.94232
RP4-568C11.4	1.019334	1.0159362	1.02213915	1.3892324	0.47428799	0.94232
SMIM17	1.019278	1.0159362	1.02203724	1.3828375	0.46763168	0.94232
AGTR2	1.019295	1.0159362	1.02206937	1.3848535	0.46973336	0.94232
RP11-85O21.5	1.019272	1.0159362	1.02202752	1.3822272	0.46699481	0.94232
SLC16A2	1.514065	1.5100594	1.5173732	1.014339	0.02053992	0.94235
LINC01445	1.02111	1.0244759	1.01833122	0.7489489	-0.4170609	0.94237
RP11-527J8.1	1.021105	1.0244645	1.01833122	0.7493002	-0.4163842	0.94237
RP11-265N6.1	1.02108	1.0244077	1.01833122	0.7510411	-0.4130361	0.94237
RP11-310N16.1	1.021053	1.0243495	1.01833122	0.7528392	-0.4095864	0.94237
CTD-2015H6.3	1.164139	1.1605811	1.16707761	1.0404564	0.05721649	0.94237
RP11-362K2.2	1.021393	1.0180802	1.02412817	1.3345045	0.41630419	0.94243
MSH6	1.618007	1.6218407	1.61484047	0.9887427	-0.016333	0.94256
PLEKHA7	1.064984	1.0683704	1.06218835	0.9095798	-0.1367278	0.94257
LILRB5	1.019602	1.0162589	1.02236196	1.3753701	0.45981989	0.94257
RP11-62C3.6	1.019603	1.0162589	1.02236504	1.3755594	0.46001841	0.94257
GLI1	1.019602	1.0162589	1.02236257	1.3754072	0.45985878	0.94257
CEMIP	1.019618	1.0162589	1.02239137	1.3771787	0.46171574	0.94257
KLK2	1.019593	1.0162589	1.02234647	1.3744171	0.45881988	0.94257
RP11-114G22.1	1.019604	1.0162589	1.02236698	1.3756784	0.46014324	0.94257
AL132709.1	1.121106	1.1175528	1.12404023	1.0551872	0.07749903	0.94257
CTD-3051D23.4	1.019065	1.0157004	1.02184442	1.391327	0.47646149	0.94261
AC007395.3	1.019065	1.0157004	1.02184442	1.391327	0.47646149	0.94261
RP11-474O21.5	1.019042	1.0157004	1.02180162	1.3886007	0.4736318	0.94261
RP11-687M24.8	1.019062	1.0157004	1.02183743	1.3908817	0.47599973	0.94261
GPR37L1	1.019036	1.0157004	1.02179084	1.3879142	0.47291835	0.94261
BCL2A1	1.01902	1.0157004	1.02176063	1.3859903	0.47091714	0.94261
NINJ2	1.019019	1.0157004	1.02175899	1.3858858	0.47080843	0.94261
CTD-2336O2.3	1.019034	1.0157004	1.02178618	1.3876175	0.47260994	0.94261
RP11-11N5.1	1.01905	1.0157004	1.02181636	1.38954	0.47460733	0.94261
RP5-1125A11.7	1.019032	1.0157004	1.02178299	1.3874141	0.47239841	0.94261
MSH5-SAPCD1	1.019028	1.0157004	1.02177585	1.3869598	0.47192593	0.94261
SLITRK1	1.074537	1.0710347	1.07742977	1.0900269	0.1243638	0.94261
PCDHB6	1.028016	1.0313426	1.02526805	0.8061895	-0.3108091	0.94264
C3orf70	1.030923	1.0342507	1.02817516	0.8226146	-0.2817115	0.94266
C16orf82	1.042938	1.0462393	1.0402125	0.8696599	-0.2014768	0.94267
C9orf135-AS1	1.042341	1.0456627	1.03959866	0.8671988	-0.2055653	0.94271
KCNK2	1.035418	1.0320844	1.03817026	1.1896831	0.25057728	0.94281
CH507-9B2.5	1.07501	1.0716342	1.07779763	1.0860408	0.11907833	0.94289
TTPA	1.0448	1.0479839	1.04217142	0.8788667	-0.1862838	0.94298
EFCAB5	1.044892	1.0482291	1.04213585	0.8736611	-0.1948543	0.94298
ATP6V0C	1.036261	1.0395504	1.03354525	0.8481645	-0.237584	0.94301
ABHD16A	1.036182	1.0394362	1.03349542	0.8493579	-0.2355554	0.94301
KBTBD6	1.199127	1.2025202	1.19632449	0.9694069	-0.0448257	0.94302
CAMKK2	1.19918	1.2027895	1.19619887	0.9675002	-0.0476661	0.94302

CORO6	1.044016	1.0472903	1.04131191	0.8735804	-0.1949877	0.94304
POM121	1.36574	1.3622605	1.36861428	1.0175393	0.02508448	0.94306
TPST1	1.22101	1.2245812	1.21806139	0.9709691	-0.0425027	0.94309
RNF38	1.406426	1.4102231	1.40329073	0.983101	-0.0245885	0.94313
RP11-496H1.2	1.019537	1.0227784	1.01685941	0.740148	-0.4341144	0.94318
HPCAL4	1.019519	1.0227599	1.01684174	0.7399728	-0.4344558	0.94318
RP11-804N13.1	1.021942	1.018601	1.02470073	1.3279226	0.40917104	0.94321
GUCY2F	1.021891	1.0185937	1.02461407	1.3237847	0.40466847	0.94321
RP1-45I4.3	1.021921	1.0185944	1.02466834	1.3266571	0.40779557	0.94321
GTF2H4	1.021287	1.0179643	1.02403169	1.3377498	0.41980833	0.94321
ALPK3	1.104254	1.1011399	1.10682482	1.056208	0.07889398	0.94323
CTC-367J11.1	1.071776	1.0684018	1.07456275	1.0900699	0.12442062	0.94323
MKL1	1.07649	1.0796904	1.07384748	0.9266799	-0.109857	0.94328
ATP11B	1.201478	1.2050752	1.19850815	0.9679773	-0.0469548	0.94335
KB-226F1.2	1.028858	1.0255653	1.03157694	1.2351466	0.30468232	0.94337
FAM179B	1.465076	1.4690501	1.46179459	0.9845315	-0.0224907	0.9434
PCAT1	1.037058	1.0338052	1.03974512	1.1757111	0.23353359	0.94353
RP11-346C20.3	1.030866	1.0275232	1.03362589	1.2217295	0.28892493	0.94355
RP1-90G24.11	1.028816	1.0254814	1.03156894	1.2389016	0.30906161	0.94357
USHBP1	1.028807	1.0254917	1.03154468	1.2374489	0.30736893	0.94357
C20orf166-AS1	1.021019	1.0177182	1.02374416	1.3400977	0.42233818	0.9436
OSBP2	1.069992	1.0666513	1.07275142	1.0915223	0.12634165	0.9436
CASC10	1.343781	1.3476223	1.34060853	0.9798237	-0.029406	0.94369
GLUD2	1.026723	1.0299843	1.02403039	0.8014324	-0.3193473	0.94371
SLC25A25-AS1	1.031729	1.0284427	1.03444201	1.2109284	0.27611356	0.94378
PIWIL4	1.028601	1.0252759	1.03134703	1.2401921	0.31056363	0.94385
ABHD5	1.14341	1.1400003	1.14622491	1.0444617	0.06275953	0.94388
ZDHHC23	1.021399	1.0181279	1.02410001	1.3294462	0.41082538	0.94388
AL356053.1	1.047345	1.0506276	1.04463496	0.881632	-0.1817514	0.94391
MGAT4C	1.047305	1.0505377	1.04463627	0.8832278	-0.1791425	0.94391
PCAT4	1.020048	1.0168007	1.02272976	1.3529031	0.43605847	0.94394
MIR548XHG	1.020067	1.0167623	1.02279638	1.3599808	0.44358624	0.94394
RP11-119J18.1	1.020088	1.0167623	1.02283513	1.3622929	0.44603695	0.94394
PSMG3-AS1	1.066415	1.0698052	1.06361623	0.91134	-0.1339388	0.94394
FAM155B	1.024026	1.0207485	1.02673272	1.2884195	0.36560242	0.94394
PMFBP1	1.029871	1.0265648	1.03260149	1.2272428	0.29542069	0.94396
TUBA4A	1.049895	1.0532477	1.04712647	0.885042	-0.1761822	0.94405
KCNH5	1.016108	1.012846	1.01880186	1.4636313	0.54955219	0.94406
CTC-529L17.1	1.016123	1.012846	1.01882947	1.4657803	0.55166885	0.94406
FAM138B	1.016115	1.012846	1.01881442	1.4646091	0.55051568	0.94406
RP11-168L7.1	1.016103	1.012846	1.01879211	1.4628723	0.54880381	0.94406
SAMSN1	1.016128	1.012846	1.01883891	1.4665154	0.55239221	0.94406
REN	1.016128	1.012846	1.01883891	1.4665154	0.55239221	0.94406
RP11-161M6.3	1.016123	1.012846	1.01882896	1.4657407	0.55162992	0.94406
RP11-573J24.1	1.016117	1.012846	1.01881732	1.4648345	0.55073765	0.94406

LINC00857	1.016104	1.012846	1.01879483	1.4630839	0.54901248	0.94406
OGFRP1	1.016107	1.012846	1.01879968	1.463462	0.54938529	0.94406
CSF3R	1.016117	1.012846	1.0188188	1.4649502	0.55085166	0.94406
HSPB3	1.016094	1.012846	1.01877644	1.4616526	0.54760044	0.94406
EWSAT1	1.016116	1.012846	1.01881677	1.4647918	0.55069564	0.94406
FUT10	1.175097	1.178561	1.17223675	0.9645821	-0.0520241	0.94411
TMEM109	1.432159	1.4282356	1.43539869	1.0167269	0.02393224	0.94418
PPP1R32	1.111954	1.1152353	1.1092447	0.9480142	-0.0770195	0.94418
AC006160.5	1.023319	1.0265519	1.02064946	0.777702	-0.3627106	0.94426
SERF1B	1.023317	1.0265263	1.02066723	0.7791212	-0.3600803	0.94426
RFX3	1.552036	1.5482387	1.5551711	1.0126448	0.01812821	0.94437
EFTUD2	1.29269	1.2891995	1.29557173	1.0220339	0.03144307	0.94442
CFAP43	1.053469	1.0502141	1.05615782	1.1183667	0.16139337	0.94442
CTB-25B13.9	1.025017	1.0282275	1.02236536	0.7923237	-0.3358382	0.94443
RP11-647K16.1	1.02505	1.0282831	1.02238028	0.7912949	-0.3377127	0.94443
SVOP	1.02507	1.028297	1.02240453	0.7917646	-0.3368565	0.94443
WIPF1	1.315729	1.3119649	1.3188371	1.0220288	0.03143585	0.94443
LACE1	1.067115	1.0637729	1.06987427	1.0956734	0.13181782	0.94449
RP11-255M2.3	1.060362	1.057023	1.06311876	1.1068992	0.14652384	0.94459
ST7	1.110316	1.1136309	1.10757789	0.9467311	-0.0789733	0.94463
ARHGEF15	1.018864	1.0156039	1.02155683	1.3815024	0.46623804	0.94467
RP11-529E10.7	1.018848	1.0156039	1.02152725	1.379607	0.46425738	0.94467
RP11-386G21.2	1.018864	1.0156039	1.02155634	1.3814713	0.46620556	0.94467
SIM2	1.01884	1.0156039	1.02151272	1.3786753	0.46328277	0.94467
CTD-2078B5.2	1.018888	1.0156039	1.02160009	1.3842747	0.46913027	0.94467
RP11-506O24.1	1.018857	1.0156039	1.02154381	1.380668	0.46536642	0.94467
AC013275.2	1.018859	1.0156039	1.02154698	1.3808714	0.46557898	0.94467
RP11-680F8.1	1.093737	1.0905213	1.09639316	1.0648675	0.09067399	0.94475
AARS2	1.102009	1.0987149	1.10472872	1.0609211	0.08531741	0.94476
MOXD1	1.40424	1.4006561	1.40719883	1.0163301	0.02336912	0.94479
NUDCD3	1.404831	1.4012695	1.40777244	1.016206	0.02319288	0.94479
SSR1	2.432476	2.4276745	2.43644143	1.0061407	0.00883203	0.9448
DMRT2	1.018168	1.0148827	1.02088024	1.4029849	0.48849946	0.94481
DSC1	1.018175	1.0148936	1.02088498	1.4022741	0.48776838	0.94481
CTD-2366F13.2	1.018128	1.0148936	1.02079901	1.396502	0.48181761	0.94481
SLCO2A1	1.018121	1.0148871	1.02079139	1.396608	0.4819271	0.94481
FEM1A	1.133343	1.1366205	1.1306367	0.9562015	-0.0646134	0.94486
RP11-269C23.5	1.030488	1.0271881	1.03321254	1.2215839	0.288753	0.94492
MIER3	1.142613	1.1393716	1.14529066	1.0424694	0.06000504	0.94495
RP6-99M1.3	1.017001	1.0137737	1.01966624	1.4278139	0.51380794	0.94497
FEV	1.017012	1.0137737	1.01968572	1.4292278	0.5152359	0.94497
TAS2R46	1.017019	1.0137737	1.01969977	1.4302482	0.51626548	0.94497
RP11-900F13.2	1.017006	1.0137737	1.01967539	1.4284781	0.51447888	0.94497
RP11-692C24.2	1.017007	1.0137737	1.01967662	1.4285675	0.51456921	0.94497
HDC	1.017042	1.0137737	1.01974114	1.433252	0.51929226	0.94497

LINC01471	1.017021	1.0137737	1.01970351	1.4305193	0.51653896	0.94497
AC005775.2	1.016998	1.0137737	1.01966114	1.4274437	0.51343389	0.94497
RP11-794M8.1	1.017043	1.0137737	1.01974246	1.4333476	0.51938854	0.94497
AC008074.1	1.017003	1.0137737	1.01966946	1.4280473	0.51404375	0.94497
RP5-864K19.6	1.017013	1.0137737	1.01968804	1.4293963	0.51540598	0.94497
CTD-2377D24.6	1.017011	1.0137737	1.01968377	1.4290863	0.51509301	0.94497
RP11-84C10.4	1.017014	1.0137737	1.01968961	1.4295101	0.51552084	0.94497
CTA-363E6.2	1.023873	1.0206223	1.02655822	1.2878412	0.36495471	0.945
RP11-344B5.3	1.023915	1.020647	1.02661329	1.2889665	0.36621474	0.945
CARD19	1.642879	1.6472485	1.63926988	0.987673	-0.0178947	0.94501
GOLGA1	1.141326	1.1446932	1.13854529	0.9575107	-0.0626395	0.94502
RP11-50I19.2	1.022993	1.0261623	1.02037646	0.7788477	-0.3605869	0.94506
PILRB	1.27404	1.2707538	1.27675439	1.0221627	0.03162479	0.94507
MFAP3	1.071643	1.0749052	1.06894977	0.9204941	-0.1195196	0.94507
DNAJC25	1.265994	1.2624115	1.26895326	1.0249295	0.03552464	0.94507
CTD-2587M2.1	1.029548	1.0263345	1.03220101	1.2227669	0.29014943	0.94514
FBXO43	1.082821	1.0793679	1.08567347	1.0794478	0.11029343	0.94515
RP11-147L13.14	1.030881	1.0276252	1.03356965	1.2151812	0.28117145	0.94519
CITED4	1.054414	1.051218	1.05705368	1.1139371	0.15566777	0.94521
NDUFAF5	1.213884	1.2171884	1.21115462	0.9722189	-0.0406469	0.94523
CTD-2006H14.2	1.070893	1.0742288	1.06813807	0.9179461	-0.1235186	0.94524
RP11-143K11.7	1.021361	1.0245887	1.01869533	0.7603236	-0.3953146	0.94528
RP11-5O24.1	1.021318	1.0245176	1.01867643	0.7617574	-0.3925965	0.94528
ODF3L1	1.021304	1.0244738	1.01868541	0.7634862	-0.389326	0.94528
RP11-71L14.4	1.021291	1.0244538	1.01867847	0.7638257	-0.3886846	0.94528
RP13-650J16.1	1.021321	1.0244904	1.01870281	0.7636803	-0.3889593	0.94528
ZNF471	1.083101	1.0862347	1.08051234	0.9336424	-0.099058	0.9453
KLHL4	1.025588	1.0223602	1.02825428	1.2635996	0.33753939	0.94531
LINC01561	1.025553	1.0223633	1.02818799	1.2604596	0.33394988	0.94531
SAMD9	1.025588	1.0223684	1.02824709	1.2628118	0.33663961	0.94531
LINC01546	1.026936	1.0301148	1.02431147	0.8072941	-0.3088338	0.94531
ZNF700	1.100601	1.1038817	1.09789112	0.9423327	-0.0856917	0.94534
APOPT1	2.030505	2.0351725	2.02665132	0.9917683	-0.0119249	0.94535
LAT	1.047363	1.0504799	1.04478942	0.8872729	-0.1725502	0.94537
NPB	1.41236	1.40874	1.41534962	1.0161708	0.02314297	0.94545
ITGA9	1.028334	1.031447	1.02576315	0.819257	-0.2876121	0.94551
RP11-644K8.1	1.019794	1.0165698	1.02245641	1.3552646	0.43857457	0.94553
RP5-1184F4.7	1.019844	1.016578	1.02254146	1.3597172	0.44330664	0.94553
ATP4A	1.019807	1.0165705	1.02247996	1.356629	0.44002624	0.94553
FAM220A	1.189335	1.1857179	1.192322	1.0355599	0.05041107	0.94566
GFI1B	1.014521	1.0113227	1.01716283	1.5157848	0.60006498	0.94571
CCL26	1.014523	1.0113227	1.01716594	1.5160597	0.6003266	0.94571
AC025171.1	1.01452	1.0113227	1.01715997	1.5155323	0.59982458	0.94571
AC007255.8	1.014525	1.0113227	1.01716918	1.5163461	0.60059909	0.94571
CTB-119C2.1	1.014525	1.0113227	1.01716935	1.5163608	0.60061306	0.94571

RP11-353N14.2	1.014513	1.0113227	1.01714675	1.5143654	0.59871338	0.94571
RP11-651P23.5	1.014505	1.0113227	1.01713245	1.5131018	0.59750903	0.94571
C7orf55-LUC7L2	1.014517	1.0113227	1.01715555	1.5151425	0.59945346	0.94571
SFRP4	1.014521	1.0113227	1.01716174	1.5156893	0.59997404	0.94571
WISP1	1.014522	1.0113227	1.01716431	1.5159156	0.60018944	0.94571
ECT2L	1.014518	1.0113227	1.01715664	1.5152386	0.59954501	0.94571
RP11-403P17.6	1.01453	1.0113227	1.01717873	1.5171896	0.60140141	0.94571
TMC3	1.014516	1.0113227	1.01715371	1.5149793	0.59929811	0.94571
ASPG	1.014526	1.0113227	1.01717197	1.5165926	0.6008336	0.94571
AC078883.4	1.014547	1.0113227	1.01720935	1.5198937	0.60397038	0.94571
GPR183	1.014522	1.0113227	1.01716352	1.5158461	0.60012332	0.94571
SUMO4	1.069912	1.0731372	1.06724942	0.9194961	-0.1210846	0.94573
ATAD3C	1.040255	1.0370502	1.04290117	1.1579205	0.21153614	0.94583
VPS45	1.23785	1.2343454	1.24074363	1.0273023	0.03886084	0.94585
CLCNKB	1.02835	1.0251484	1.03099424	1.2324556	0.30153571	0.94592
DYNLRB2	1.056106	1.0529192	1.05873856	1.1099669	0.15051662	0.94594
ADAMTS2	1.110948	1.1078163	1.11353398	1.0530318	0.07454899	0.94597
SMC2-AS1	1.050073	1.0468708	1.05271802	1.1247521	0.16960701	0.946
C10orf67	1.047746	1.0444817	1.05044209	1.1339975	0.1814174	0.94601
KCNH3	1.025939	1.0227529	1.02857082	1.2557017	0.32849379	0.94603
SMG8	1.02594	1.0227129	1.02860499	1.2594176	0.33275675	0.94603
CDKL1	1.028802	1.0319754	1.02618074	0.8187786	-0.2884547	0.94604
CS	1.687666	1.6920004	1.68408674	0.9885641	-0.0165936	0.94606
EDA2R	1.21381	1.2172252	1.21098884	0.9712907	-0.042025	0.94612
CYB5D1	1.113667	1.1103444	1.11641046	1.054974	0.07720751	0.94613
TMTC3	1.318387	1.3148646	1.32129651	1.0204275	0.02917372	0.94615
NOL9	1.177769	1.1811824	1.174951	0.9656071	-0.0504919	0.94617
ZMYND12	1.028213	1.0314289	1.02555803	0.8132006	-0.2983169	0.94618
RP11-225B17.2	1.039984	1.0368021	1.04261141	1.1578524	0.21145139	0.94622
TMIE	1.026476	1.0232745	1.02912015	1.2511628	0.32326948	0.94623
RP11-756H6.1	1.026567	1.023379	1.02919955	1.2489624	0.32073006	0.94623
FBXO27	1.127743	1.1244408	1.13046997	1.0484501	0.06825826	0.94623
LINC00959	1.091127	1.0942899	1.08851595	0.9387641	-0.0911654	0.94624
PDZD7	1.084264	1.0811235	1.08685701	1.0706763	0.09852237	0.94625
PMS1	1.417895	1.4142417	1.42091143	1.0161012	0.02304403	0.94625
KIF6	1.048123	1.0449197	1.05076765	1.1301869	0.17656143	0.94626
VMO1	1.035799	1.0389747	1.03317564	0.8512106	-0.232412	0.94627
FAM105A	1.036145	1.0328999	1.03882417	1.1800697	0.23887213	0.94629
UTP20	1.098064	1.0947387	1.10080984	1.0640831	0.08961083	0.9463
RCBTB1	1.188746	1.1851879	1.19168455	1.0350812	0.0497439	0.94631
RP11-173A6.3	1.01886	1.0220104	1.01625811	0.7386574	-0.4370226	0.94632
HPR	1.018819	1.0219457	1.01623618	0.7398351	-0.4347244	0.94632
LYSMD4	1.147911	1.1447886	1.15049009	1.0393781	0.05572053	0.94634
KYNU	1.021464	1.0246074	1.01886728	0.7667333	-0.3832033	0.94634
RP11-126O1.5	1.060416	1.0636406	1.05775246	0.9074782	-0.1400651	0.9465

PPM1H	1.158556	1.161816	1.15586377	0.9632163	-0.0540683	0.94652
LINC00847	1.139556	1.1428161	1.13686338	0.958319	-0.0614222	0.94653
LRRTM3	1.021075	1.0178803	1.0237133	1.3262247	0.40732518	0.94659
RP4-761J14.10	1.021059	1.0178734	1.02368944	1.325402	0.40643002	0.94659
SLC22A18AS	1.021061	1.0178804	1.0236879	1.3247957	0.40576985	0.94659
RP11-705O24.1	1.021039	1.0178893	1.02364036	1.3214825	0.40215729	0.94659
RP11-230G5.2	1.021058	1.0178763	1.02368577	1.3249852	0.40597621	0.94659
NPNT	1.021041	1.0178732	1.02365639	1.3235691	0.4044335	0.94659
SLC35G5	1.021065	1.017878	1.02369639	1.3254501	0.40648231	0.94659
MYLK3	1.018422	1.0215271	1.01585723	0.7366179	-0.4410116	0.9466
RP11-46H11.3	1.029304	1.0261042	1.03194693	1.2238209	0.2913924	0.94674
DZIP1L	1.134354	1.1310557	1.13707753	1.0459486	0.06481194	0.94676
RP11-307E17.8	1.023091	1.0199209	1.02570869	1.2905388	0.36797353	0.94677
TMLHE-AS1	1.02312	1.0199274	1.02575697	1.2925433	0.37021264	0.94677
ADGRF3	1.021066	1.017855	1.0237181	1.3283701	0.40965713	0.94678
RP11-370I10.11	1.020991	1.0178371	1.02359623	1.3228744	0.40367613	0.94678
RP11-324O2.3	1.021002	1.0178412	1.02361295	1.3235075	0.40436637	0.94678
ZBED4	1.252839	1.2563326	1.24995407	0.9751163	-0.0363538	0.94678
HM13-AS1	1.026229	1.0294032	1.02360742	0.8028874	-0.3167304	0.9468
CTD-2227E11.1	1.028317	1.0314579	1.02572358	0.8177154	-0.2903292	0.94682
HELZ2	1.120526	1.1172004	1.12327147	1.0518009	0.07286166	0.94684
MIR296	1.023138	1.0199638	1.0257584	1.290255	0.36765625	0.94689
MELTF-AS1	1.229283	1.2259853	1.2320066	1.0266447	0.037937	0.94694
ZNF764	1.141842	1.1451499	1.13911053	0.9583919	-0.0613123	0.94695
HSPA2	1.028297	1.0314375	1.02570341	0.8176036	-0.2905266	0.94696
CTD-2162K18.4	1.028362	1.0314819	1.02578603	0.8190739	-0.2879344	0.94696
PRR29	1.041154	1.0442915	1.03856323	0.8706681	-0.1998052	0.94699
AP006285.2	1.026231	1.0293502	1.0236549	0.8059528	-0.3112327	0.94699
RP11-710F7.3	1.026318	1.0295073	1.02368422	0.8026557	-0.3171469	0.94699
CHRD	1.029131	1.032213	1.0265854	0.8252992	-0.2770109	0.94701
RP11-184I16.4	1.021328	1.0181646	1.02394112	1.3180065	0.39835748	0.94703
CTD-2396E7.10	1.020749	1.0175961	1.02335345	1.3271933	0.40837854	0.94707
PSCA	1.020753	1.0175943	1.02336104	1.327758	0.40899225	0.94707
RP11-723D22.3	1.020771	1.0176051	1.02338485	1.3283034	0.40958473	0.94707
GATSL3	1.259032	1.2559011	1.26161776	1.0223395	0.03187431	0.94711
RIT2	1.022138	1.0189699	1.02475459	1.3049403	0.38398378	0.94711
EPO	1.022139	1.0189883	1.02474012	1.302915	0.38174301	0.94711
C10orf111	1.049759	1.0464899	1.0524591	1.1283978	0.17427581	0.94711
RP11-568A7.3	1.016302	1.0131484	1.0189063	1.437916	0.52397944	0.94713
LINC01611	1.016272	1.0131484	1.01885187	1.4337766	0.51982024	0.94713
CH507-254M2.1	1.016295	1.0131484	1.01889392	1.4369743	0.52303421	0.94713
RP4-535B20.4	1.016296	1.0131484	1.01889547	1.4370925	0.52315292	0.94713
COMP	1.016288	1.0131484	1.01888149	1.4360289	0.52208478	0.94713
PCDHGA11	1.016287	1.0131484	1.01887882	1.4358259	0.5218808	0.94713
RP11-357H14.17	1.016308	1.0131484	1.01891733	1.4387547	0.52482065	0.94713

RP11-526F3.1	1.016334	1.0131484	1.0189655	1.4424187	0.52848998	0.94713
RADIL	1.042287	1.0453582	1.03975	0.8763573	-0.1904088	0.94735
ZNF570	1.097529	1.1006896	1.09491959	0.9426952	-0.0851368	0.94738
CA3-AS1	1.10211	1.0990661	1.10462451	1.0561079	0.07875718	0.94748
STIM2	1.360685	1.3644309	1.3575914	0.9812324	-0.0273332	0.9475
MCOLN2	1.017844	1.0209262	1.01529822	0.7310572	-0.4519437	0.94755
NUDT18	1.206871	1.2035767	1.20959115	1.029544	0.04200555	0.94758
ZNFX1	1.119723	1.1229357	1.11706903	0.9522783	-0.0705448	0.94759
EXPH5	1.093769	1.0906265	1.09636348	1.0633037	0.0885537	0.94762
ZNF844	1.112501	1.1092436	1.11519136	1.0544445	0.07648321	0.94765
CTD-3220F14.3	1.023655	1.0205008	1.02625997	1.2809249	0.35718588	0.94766
RP11-428J1.4	1.023645	1.0205195	1.02622644	1.2781236	0.35402737	0.94766
IRF1	1.096748	1.1000507	1.09402111	0.9397344	-0.089675	0.94767
SAMD9L	1.023017	1.0198762	1.02561029	1.2884906	0.36568197	0.94767
EPHB1	1.023022	1.0198915	1.02560697	1.2873318	0.364384	0.94767
PACS2	1.339722	1.3434254	1.33666338	0.9803102	-0.0286898	0.94771
WASH1	1.565312	1.5618491	1.56817126	1.0112524	0.01614313	0.94772
BRD1	1.135162	1.1319076	1.13784987	1.0450484	0.06356971	0.94772
RPGR	1.17528	1.1720227	1.17797083	1.0345778	0.04904212	0.94775
CTXN3	1.018831	1.0218946	1.01630016	0.7444843	-0.4256867	0.94777
AF001548.5	1.018813	1.0218583	1.01629812	0.7456274	-0.4234732	0.94777
AC144449.1	1.019666	1.0227149	1.01714758	0.754905	-0.405633	0.94777
CTC-523E23.1	1.030602	1.0275074	1.03315825	1.20543	0.2695479	0.94781
PCYT2	1.209929	1.2132642	1.20717498	0.9714473	-0.0417923	0.94784
NBEAL2	1.062375	1.0654973	1.05979635	0.9129584	-0.1313789	0.94792
RP11-219B4.7	1.032721	1.0295871	1.03530868	1.193379	0.25505228	0.94799
CCDC96	1.033526	1.0366074	1.0309812	0.846309	-0.2407436	0.94805
RP11-250B2.6	1.02499	1.0280861	1.02243298	0.7987209	-0.3242367	0.94806
TAT-AS1	1.022707	1.0195895	1.02528133	1.2905542	0.36799068	0.94807
RP11-388C12.1	1.038614	1.041638	1.03611625	0.8673863	-0.2052534	0.94807
TRANK1	1.145266	1.1422469	1.14775911	1.0387514	0.05485038	0.94809
CTB-147C22.9	1.016135	1.013034	1.01869533	1.4343537	0.5204008	0.94819
GATA1	1.016144	1.013034	1.01871284	1.4356969	0.52175116	0.94819
AP003774.5	1.016132	1.013034	1.01868964	1.4339172	0.5199617	0.94819
LINC01276	1.016129	1.013034	1.01868425	1.4335039	0.51954581	0.94819
RP11-73M7.9	1.016158	1.013034	1.01873859	1.4376728	0.52373537	0.94819
RP11-80H18.3	1.016137	1.013034	1.01869936	1.434663	0.52071194	0.94819
EGOT	1.018857	1.0157693	1.02140751	1.3575404	0.44099517	0.94819
HIST1H4B	1.01888	1.0157693	1.0214488	1.3601584	0.44377472	0.94819
RP11-687F6.5	1.018863	1.0157693	1.02141839	1.3582299	0.4417277	0.94819
RP11-541P9.3	1.018872	1.0157693	1.02143374	1.3592035	0.44276144	0.94819
AP000265.1	1.018882	1.0157693	1.02145172	1.3603437	0.44397116	0.94819
GAS1RR	1.018869	1.0157693	1.0214281	1.3588459	0.44238191	0.94819
CTD-2349P21.6	1.018863	1.0157693	1.0214176	1.35818	0.44167468	0.94819
RP11-47L3.1	1.018877	1.0157693	1.02144372	1.3598361	0.44343275	0.94819

OLR1	1.018862	1.0157693	1.02141529	1.3580332	0.44151875	0.94819
ZNF443	1.062494	1.0655576	1.05996417	0.9146788	-0.1286629	0.94821
ZCCHC18	1.284905	1.2816965	1.28755456	1.0207958	0.0296943	0.94822
ZNF584	1.209989	1.2067171	1.21269189	1.0289033	0.04110745	0.94825
DBF4B	1.15847	1.1553009	1.1610875	1.0372603	0.05277802	0.94828
RP11-91J19.3	1.030273	1.0271817	1.03282675	1.2076786	0.27223655	0.94829
RP11-266K4.14	1.023167	1.0200392	1.02574976	1.2849721	0.36173707	0.94834
ZNF585A	1.157629	1.1543666	1.16032258	1.0385835	0.05461724	0.94836
GSG1	1.02181	1.0186979	1.02437984	1.3038781	0.38280903	0.94839
RP11-10C24.2	1.02179	1.0186943	1.02434737	1.3023979	0.38117028	0.94839
CTD-2005H7.1	1.021773	1.0186524	1.0243498	1.3054524	0.38454987	0.94839
CTD-2296D1.2	1.021787	1.0186862	1.02434831	1.3030092	0.38184723	0.94839
AJ006998.2	1.025793	1.0226287	1.02840634	1.2553245	0.32806032	0.94839
PRSS50	1.031719	1.0286388	1.03426289	1.1963811	0.25867704	0.9484
LINC01426	1.031576	1.0285025	1.03411348	1.1968589	0.25925305	0.9484
HCG22	1.022207	1.0252972	1.01965593	0.7770005	-0.3640126	0.9485
RP11-446H18.6	1.017833	1.0147515	1.02037777	1.3814078	0.4661393	0.94852
AC007403.2	1.017839	1.0147444	1.02039534	1.3832563	0.46806849	0.94852
SLC1A6	1.221766	1.2249521	1.21913498	0.9741406	-0.0377981	0.94854
TMF1	1.839268	1.8428509	1.83630957	0.9922391	-0.0112403	0.94856
TCP11L1	1.079439	1.0826407	1.07679554	0.9292707	-0.1058292	0.94859
KIF3B	1.246571	1.2436372	1.24899426	1.021988	0.03137831	0.94863
RP13-349O20.2	1.021602	1.0246654	1.01907271	0.773256	-0.3709819	0.94863
LGALS4	1.021614	1.0246568	1.01910111	0.7746784	-0.3683307	0.94863
RBM44	1.021604	1.0246259	1.01910821	0.775939	-0.3659849	0.94863
AP003068.9	1.021605	1.0246275	1.01910821	0.7758904	-0.3660753	0.94863
ADCY1	1.053357	1.0563496	1.0508848	0.9030196	-0.1471708	0.94868
BCDIN3D	1.133776	1.1367957	1.13128254	0.9596975	-0.0593483	0.94877
CTD-2034I21.2	1.023318	1.0263885	1.02078283	0.7875719	-0.3445164	0.94878
RP11-190C22.8	1.023313	1.0263531	1.02080199	0.7893565	-0.341251	0.94878
UPK1A	1.059038	1.0560657	1.06149328	1.096807	0.13330972	0.94878
TCEAL3-AS1	1.024063	1.0271542	1.02151061	0.7921661	-0.3361251	0.94881
RP11-439E19.10	1.024052	1.0270812	1.02155022	0.7957645	-0.3295865	0.94881
HULC	1.024037	1.0270889	1.02151656	0.7942942	-0.3322547	0.94881
CTD-2199O4.1	1.024065	1.0271084	1.02155141	0.7950081	-0.3309585	0.94881
TARS	1.70737	1.703488	1.71057537	1.0100746	0.0144618	0.94881
SMAD1	1.1826	1.1857576	1.17999303	0.9689672	-0.0454803	0.94883
MAP3K6	1.110689	1.1137394	1.10816947	0.9510293	-0.0724384	0.94884
ZNF790-AS1	1.03323	1.0301289	1.03579049	1.1879108	0.2484265	0.94884
C7orf25	1.033212	1.0300864	1.03579395	1.1897051	0.250604	0.94884
RP11-967K21.1	1.031884	1.0349425	1.0293583	0.8401896	-0.2512131	0.94885
HLF	1.045427	1.0484518	1.04292832	0.8860009	-0.17462	0.9489
PRR4	1.869992	1.8740209	1.86666491	0.9915837	-0.0121935	0.94893
ZNF547	1.032498	1.029456	1.03500984	1.188546	0.2491977	0.94897
AC053503.4	1.040157	1.0369935	1.04276946	1.1561357	0.20931071	0.94902

CTD-3001H11.2	1.108593	1.111727	1.10600512	0.9487867	-0.0758442	0.94902
PGAM5	1.095269	1.092123	1.09786728	1.0623541	0.08726476	0.94906
ELK1	1.241792	1.2385481	1.24446997	1.0248245	0.03537688	0.94908
MAPKBP1	1.125131	1.128212	1.12258704	0.9561276	-0.064725	0.94909
IFNAR1	1.491566	1.4879269	1.49457177	1.0136185	0.01951474	0.94913
IL18	1.020569	1.0175039	1.02309945	1.3196746	0.40018226	0.94913
RP11-1055B8.10	1.02057	1.0174989	1.0231067	1.3204625	0.40104329	0.94913
RP11-152N13.16	1.020568	1.0175018	1.02310034	1.3198807	0.40040752	0.94913
CTD-2562J15.4	1.020595	1.0174994	1.02315169	1.3229984	0.40381128	0.94913
PRSS12	1.020599	1.0175226	1.02313974	1.320562	0.40115207	0.94913
AC113167.1	1.018153	1.0150885	1.0206846	1.3708892	0.45511195	0.94921
RP11-49K24.8	1.01815	1.0150885	1.02067888	1.3705103	0.45471317	0.94921
RP11-317P15.4	1.040024	1.0368923	1.04260959	1.1549725	0.20785854	0.94921
OTOGL	1.019843	1.0167748	1.02237673	1.3339458	0.41570009	0.94927
C6orf163	1.0322	1.0291776	1.03469579	1.1891253	0.24990079	0.94936
SRCAP	1.043241	1.0462457	1.0407593	0.8813643	-0.1821896	0.94939
PLCE1	1.221421	1.2248388	1.21859881	0.9722468	-0.0406055	0.94939
ADAT3	1.021302	1.0243168	1.01881219	0.7736306	-0.3702833	0.94942
ASB2	1.018748	1.0156669	1.02129303	1.3591097	0.44266192	0.94944
LINC00652	1.018725	1.0156724	1.0212466	1.3556729	0.43900914	0.94944
KBTBD8	1.018735	1.0156721	1.02126409	1.3568137	0.44022262	0.94944
CAPN8	1.018707	1.0156705	1.02121369	1.3537345	0.43694486	0.94944
HSPA12B	1.018722	1.015676	1.021238	1.3548095	0.43809002	0.94944
KIF26A	1.189659	1.1863662	1.19237766	1.032256	0.04580079	0.94944
KIAA0040	1.025645	1.0225595	1.02819344	1.2497383	0.32162607	0.94946
ANKRD31	1.044515	1.0475362	1.04202042	0.8839677	-0.1779345	0.94947
FBXO18	1.306849	1.303482	1.30963047	1.0202599	0.02893668	0.94948
KCNJ5	1.031284	1.0282745	1.03376837	1.1943036	0.25616966	0.94957
RP11-793J2.1	1.142405	1.1392026	1.14505	1.0420063	0.05936397	0.94958
RP11-680F8.3	1.022312	1.025303	1.01984112	0.7841396	-0.3508175	0.94965
TRAPPC12-AS1	1.022342	1.0253711	1.01984011	0.7819965	-0.3547659	0.94965
APOBEC3B	1.019559	1.0225279	1.01710694	0.759366	-0.3971327	0.94965
AC008088.4	1.036669	1.0396827	1.03418082	0.8613539	-0.215322	0.94967
ZNHIT6	1.282293	1.2855756	1.27958187	0.9790119	-0.0306018	0.94972
SLC2A12	1.095627	1.0924634	1.09823934	1.0624675	0.08741876	0.94975
BET1L	1.218905	1.2220834	1.21628068	0.9738716	-0.0381965	0.94975
SLC39A14	1.230773	1.2339341	1.22816163	0.9753242	-0.0360463	0.94981
SMCR8	1.076742	1.0734742	1.07944119	1.0812124	0.11264991	0.94985
RP11-366L20.2	1.02664	1.0296409	1.02416258	0.8151773	-0.2948142	0.94988
RP11-20J15.3	1.026597	1.0295976	1.02411944	0.8149116	-0.2952844	0.94988
XXbac-B444P24.14	1.029264	1.0322534	1.02679554	0.8307824	-0.2674574	0.94992
NALCN-AS1	1.029251	1.0322547	1.02677076	0.8299788	-0.2688535	0.94992
RP11-159D12.5	1.081644	1.0847083	1.0791128	0.9339435	-0.0985928	0.94995
RGPD5	1.091461	1.0945234	1.08893133	0.9408395	-0.0879795	0.94996
NR4A2	1.021532	1.0184875	1.02404597	1.3006581	0.37924176	0.94998

PAX5	1.042711	1.0396964	1.04519969	1.138635	0.18730541	0.95001
PRSS36	1.061907	1.058806	1.06446752	1.0962742	0.13260864	0.95004
ACVRL1	1.015375	1.0123699	1.01785643	1.4435414	0.52961245	0.95007
DHH	1.015398	1.0123699	1.01789806	1.4469069	0.53297212	0.95007
XIST	1.015381	1.0123699	1.01786677	1.4443773	0.53044768	0.95007
RP11-143I21.1	1.015382	1.0123699	1.0178701	1.4446463	0.53071627	0.95007
SBK2	1.015394	1.0123699	1.01789104	1.4463394	0.53240614	0.95007
RP11-1070A24.2	1.015389	1.0123699	1.01788179	1.4455913	0.53165977	0.95007
RP11-92A5.2	1.01538	1.0123699	1.01786616	1.4443279	0.53039831	0.95007
CTD-2537I9.19	1.015417	1.0123699	1.01793308	1.4497378	0.53579203	0.95007
C10orf90	1.015421	1.0123699	1.01794059	1.4503449	0.53639602	0.95007
UBE4A	1.348637	1.3450603	1.35159113	1.0189265	0.02705003	0.95008
UBXN10	1.026154	1.029101	1.02372059	0.815112	-0.2949298	0.95015
RP11-182J1.18	1.016238	1.013216	1.01873274	1.4174285	0.50327594	0.95017
P2RX5-TAX1BP3	1.016241	1.0132327	1.01872498	1.4150582	0.50086136	0.95017
TSSK6	1.016229	1.0132144	1.01871793	1.4164775	0.50230764	0.95017
CH507-42P11.8	1.016256	1.0132159	1.01876659	1.4200008	0.50589177	0.95017
RP11-713P17.5	1.016239	1.0132213	1.01873041	1.4166815	0.50251542	0.95017
MANBA	1.312393	1.3094694	1.31480813	1.0172512	0.02467594	0.95023
TTC12	1.112469	1.1091552	1.11520503	1.0554241	0.07782288	0.95024
PKD1L3	1.014362	1.0113663	1.0168366	1.4812719	0.56683653	0.95028
RP11-12K11.2	1.014368	1.0113663	1.01684597	1.4820959	0.56763883	0.95028
PRODH	1.014372	1.0113663	1.01685394	1.482797	0.56832106	0.95028
RP11-697M17.2	1.014384	1.0113663	1.01687623	1.4847586	0.5702284	0.95028
RP11-434E6.4	1.014361	1.0113663	1.01683358	1.4810061	0.56657758	0.95028
LINC01353	1.014374	1.0113663	1.01685733	1.4830954	0.56861143	0.95028
RP11-276H7.3	1.014375	1.0113663	1.01685873	1.4832185	0.56873111	0.95028
SOX21-AS1	1.014368	1.0113663	1.0168463	1.4821247	0.56766687	0.95028
LRRC37A	1.014379	1.0113663	1.01686746	1.4839868	0.56947829	0.95028
LINC00881	1.014373	1.0113663	1.01685522	1.4829101	0.56843112	0.95028
PGM5P3-AS1	1.014365	1.0113663	1.01684167	1.4817178	0.56727074	0.95028
THCAT158	1.01437	1.0113663	1.01685002	1.4824526	0.56798594	0.95028
TUBA8	1.014374	1.0113663	1.01685823	1.4831745	0.56868836	0.95028
PCED1B	1.035069	1.0380196	1.03263201	0.8582952	-0.2204542	0.95029
DDX60L	1.091576	1.0945433	1.08912521	0.9426923	-0.0851411	0.95029
TINF2	1.544509	1.5408538	1.54752692	1.0123381	0.01769114	0.9503
PTPRH	1.057806	1.0546865	1.06038237	1.1041543	0.14294186	0.95031
CIB2	1.457993	1.4612775	1.45528077	0.9869996	-0.0188785	0.95032
RRP36	1.193306	1.190357	1.19574118	1.0282844	0.04023935	0.95032
GAB1	1.153914	1.1508882	1.15641303	1.0366155	0.05188093	0.95036
ZNF384	1.153885	1.1506032	1.15659452	1.0397819	0.05628089	0.95036
RTN4R	1.091183	1.0880463	1.09377312	1.0650431	0.09091181	0.95037
ICA1L	1.248494	1.2451487	1.25125728	1.0249178	0.03550818	0.95038
GOLGA7B	1.027145	1.0301813	1.02463694	0.8162976	-0.2928329	0.95039
RP11-284F21.9	1.027088	1.0300465	1.02464508	0.8202322	-0.2858956	0.95039

STARD6	1.027086	1.0300138	1.02466734	0.8218658	-0.2830252	0.95039
EXTL1	1.036099	1.0391084	1.03361411	0.8595108	-0.2184123	0.95043
PPTC7	1.112611	1.1156698	1.11008538	0.9517207	-0.0713899	0.95047
NOP9	1.112735	1.1157659	1.11023156	0.9521937	-0.0706729	0.95047
STK3	1.419211	1.4155197	1.42225998	1.0162214	0.02321468	0.95049
KCNJ12	1.036076	1.0390546	1.03361657	0.8607593	-0.2163182	0.95051
TADA1	1.134945	1.138171	1.13228024	0.9573659	-0.0628577	0.95053
PDE6A	1.026476	1.0294602	1.02401175	0.8150585	-0.2950245	0.95054
NOP14-AS1	1.117479	1.1205086	1.11497627	0.9540919	-0.0677999	0.95054
PGAP3	1.085907	1.0829236	1.08837061	1.0656869	0.09178362	0.95057
CHAC1	1.036882	1.0398231	1.03445307	0.8651529	-0.2089729	0.95058
LINGO3	1.034054	1.0370701	1.03156278	0.8514354	-0.2320311	0.95061
RP11-515O17.3	1.017127	1.0200799	1.01468854	0.7315058	-0.4510588	0.95071
RP11-15A1.2	1.035118	1.0320923	1.03761667	1.1721397	0.22914451	0.95071
RP11-981P6.1	1.019751	1.022717	1.01730141	0.7616065	-0.3928823	0.95072
RP11-47J17.2	1.019724	1.0226605	1.0172999	0.7634386	-0.389416	0.95072
FBXL3	1.226988	1.224162	1.22932127	1.0230159	0.0328285	0.95081
ACER2	1.015245	1.0122555	1.01771461	1.4454446	0.53151327	0.95085
RP11-98O2.1	1.015273	1.0122555	1.01776476	1.4495365	0.53559163	0.95085
UXT-AS1	1.01525	1.0122555	1.01772227	1.4460692	0.53213662	0.95085
RP11-95I16.4	1.015252	1.0122555	1.01772731	1.4464804	0.53254674	0.95085
RP11-598F7.4	1.015239	1.0122555	1.01770221	1.4444328	0.53050305	0.95085
RP11-121C2.3	1.015264	1.0122555	1.01774803	1.4481711	0.53423209	0.95085
RP11-104O19.4	1.015259	1.0122555	1.01773976	1.4474963	0.5335597	0.95085
RP1-296L11.1	1.015248	1.0122555	1.01771882	1.4457878	0.53185577	0.95085
RP11-711C17.2	1.015238	1.0122555	1.01770155	1.4443787	0.53044906	0.95085
GRM8	1.015231	1.0122555	1.01768772	1.4432503	0.52932155	0.95085
ROR1-AS1	1.015251	1.0122555	1.01772397	1.4462077	0.5322748	0.95085
LENG9	1.094218	1.0972091	1.09174881	0.9438298	-0.0834014	0.95085
RP11-745L13.2	1.024235	1.0272008	1.02178506	0.8008976	-0.3203103	0.95088
RP11-37N22.1	1.024822	1.0278607	1.02231307	0.80088	-0.320342	0.95091
CCNB3	1.024758	1.0276975	1.02233004	0.8062117	-0.3107695	0.95091
RP11-102N12.3	1.050528	1.0475025	1.05302583	1.1162752	0.15869275	0.95094
TIMP4	1.036773	1.0337967	1.03923029	1.1607743	0.21508746	0.95097
RP5-1074L1.1	1.016691	1.0196239	1.01426865	0.7271054	-0.4597635	0.95098
RP11-1277A3.3	1.016702	1.0196372	1.01427722	0.7270501	-0.4598733	0.95098
ULBP1	1.022767	1.0197862	1.0252295	1.2751088	0.35062039	0.95105
AC002076.10	1.022785	1.0198156	1.02523644	1.2735637	0.3488711	0.95105
RP11-80H5.7	1.026522	1.0294048	1.02414194	0.8210193	-0.2845119	0.95118
RP11-572M11.3	1.024884	1.0218351	1.02740171	1.2549368	0.32761475	0.95123
RP11-380B4.3	1.024836	1.0218141	1.02733158	1.2529326	0.32530882	0.95123
LINC00501	1.01496	1.0120052	1.01740086	1.4494493	0.53550484	0.95124
RAB37	1.014971	1.0120052	1.01742059	1.4510927	0.53713968	0.95124
C11orf52	1.014967	1.0120052	1.01741274	1.4504384	0.53648905	0.95124
VWA8-AS1	1.014963	1.0120052	1.0174057	1.4498522	0.53590584	0.95124

DMRT1	1.014986	1.0120052	1.01744767	1.4533482	0.53938039	0.95124
ACOT4	1.015024	1.0120052	1.01751662	1.4590913	0.54507018	0.95124
TMEM51-AS1	1.014967	1.0120052	1.01741329	1.4504847	0.53653505	0.95124
SLC18A3	1.015013	1.0120052	1.01749733	1.4574852	0.54348124	0.95124
ADAMTSL3	1.014985	1.0120052	1.01744603	1.4532114	0.53924455	0.95124
AC008522.1	1.014989	1.0120052	1.01745318	1.4538075	0.53983625	0.95124
RP11-434D9.2	1.022771	1.0197598	1.0252585	1.278277	0.35420054	0.95124
LINC01615	1.022716	1.0197377	1.02517551	1.275501	0.35106401	0.95124
HIST1H2AB	1.022709	1.0197558	1.02514714	1.2728961	0.3481147	0.95124
RP11-744I24.2	1.022744	1.019774	1.02519601	1.2741986	0.34959018	0.95124
TMEM189	1.430211	1.4268178	1.43301312	1.0145152	0.02079049	0.95124
CDK15	1.039466	1.0424138	1.03703222	0.8731169	-0.1957532	0.95132
AC069513.4	1.172703	1.169755	1.17513824	1.0317118	0.04504005	0.95133
CTB-113D17.1	1.024916	1.0218761	1.02742711	1.2537478	0.32624716	0.95135
RP11-227G15.10	1.024872	1.0218901	1.0273338	1.2486826	0.32040677	0.95135
ST7-AS1	1.024489	1.0273905	1.0220925	0.8065763	-0.3101171	0.95135
SLC7A10	1.016998	1.019963	1.0145495	0.7288242	-0.4563572	0.95137
CTB-129P6.4	1.016979	1.0198989	1.01456819	0.7321093	-0.449869	0.95137
BCL7B	1.532164	1.5355774	1.52934556	0.9883643	-0.0168852	0.95139
IDNK	1.147367	1.1505205	1.1447622	0.9617441	-0.056275	0.9514
FBXO38	1.147312	1.1504591	1.1447128	0.9618082	-0.0561789	0.9514
EIF4EBP3	1.025714	1.0227364	1.02817236	1.2390889	0.30927968	0.95141
GSR	1.128174	1.1311878	1.12568477	0.9580523	-0.0618236	0.95141
RP1-102K2.6	1.023055	1.0200548	1.02553272	1.2731506	0.34840303	0.95149
KCNH1	1.015378	1.0124282	1.0178149	1.4334311	0.51947256	0.95151
RP11-81H14.2	1.015386	1.0124282	1.01782902	1.4345669	0.52061527	0.95151
FGR	1.01538	1.0124282	1.01781714	1.4336114	0.51965403	0.95151
LINC00891	1.015391	1.0124282	1.01783714	1.4352204	0.52127231	0.95151
RP11-445O16.3	1.015403	1.0124282	1.01786037	1.4370899	0.52315034	0.95151
FAM157B	1.015387	1.0124282	1.01783057	1.4346919	0.52074099	0.95151
TF	1.022484	1.0195164	1.02493515	1.2776543	0.35349753	0.95152
ZIK1	1.124772	1.1217507	1.12726688	1.0453069	0.06392654	0.95156
LINC01545	1.023892	1.0209037	1.0263598	1.2610111	0.33458095	0.95156
RP13-753N3.3	1.023852	1.0208668	1.02631767	1.2612211	0.33482117	0.95156
KCTD11	1.238535	1.2354784	1.24105868	1.0236976	0.03378959	0.95157
RASSF5	1.078344	1.0752086	1.08093316	1.076115	0.10583231	0.95158
COPG1	1.284831	1.2880644	1.28215994	0.9795031	-0.029878	0.95159
RP11-281P23.1	1.018013	1.0150452	1.02046304	1.3601023	0.44371514	0.95159
RP1-79C4.4	1.01803	1.0150542	1.02048703	1.3608808	0.44454067	0.95159
HAND2-AS1	1.018011	1.015052	1.02045382	1.358873	0.44241065	0.95159
FOXC2-AS1	1.017981	1.0150469	1.0204035	1.3559931	0.43934982	0.95159
PABPC3	1.040661	1.0435153	1.03830365	0.880234	-0.184041	0.95166
POU6F1	1.032743	1.0356472	1.03034499	0.851258	-0.2323317	0.95168
ZNF77	1.085236	1.088124	1.08285107	0.9401652	-0.0890138	0.95177
DCBLD1	1.107597	1.1106404	1.1050845	0.9497845	-0.0743279	0.95179

RP11-307L14.1	1.016106	1.0190084	1.01370863	0.7211871	-0.4715544	0.95193
SRRM5	1.01613	1.019066	1.01370531	0.7188359	-0.4762657	0.95193
RP3-380B8.4	1.016075	1.0189401	1.01370986	0.7238552	-0.4662269	0.95193
RP11-770J1.5	1.01608	1.0189431	1.01371489	0.7240063	-0.4659258	0.95193
LINC01393	1.016096	1.018991	1.01370606	0.7217118	-0.4705053	0.95193
C7orf49	1.588606	1.5853605	1.59128675	1.0101241	0.0145326	0.95198
FKBP14	1.330978	1.3339814	1.3284983	0.9835828	-0.0238816	0.95201
PSMG1	1.526378	1.5295484	1.52376066	0.9890704	-0.0158548	0.95207
CACNA1A	1.429566	1.4327026	1.4269763	0.9867661	-0.01922	0.95208
CYP2D6	1.017954	1.020823	1.01558456	0.7484315	-0.4180578	0.95214
DGUOK-AS1	1.017951	1.0208422	1.01556309	0.7467095	-0.4213809	0.95214
PRRT4	1.017106	1.0199765	1.01473466	0.7375984	-0.4390926	0.95215
RP11-322E11.2	1.017111	1.0200014	1.01472489	0.7361923	-0.4418454	0.95215
GIPR	1.046378	1.0434075	1.04883177	1.124962	0.16987633	0.95217
RP11-798L4.1	1.051644	1.0486796	1.05409282	1.1112018	0.15212088	0.95219
TTC21A	1.136738	1.133701	1.13924641	1.0414764	0.0586302	0.9522
SAMD14	1.352968	1.3561484	1.35034211	0.9836969	-0.0237142	0.95222
RAB11FIP4	1.176288	1.173118	1.1789059	1.0334332	0.04744508	0.95227
LINC01547	1.03971	1.0426104	1.03731464	0.8757174	-0.1914626	0.9524
RAPGEF3	1.031836	1.0347223	1.02945244	0.8482284	-0.2374753	0.9524
CRYGS	1.031803	1.0347272	1.0293877	0.8462455	-0.2408518	0.9524
RP11-573D15.9	1.023236	1.0261197	1.02085378	0.7983928	-0.3248294	0.95242
HRK	1.032261	1.0293834	1.0346366	1.1787816	0.23729647	0.95244
RP4-625H18.2	1.075212	1.0781321	1.07280032	0.9317589	-0.1019714	0.95245
METTL1	1.164459	1.1674601	1.16197987	0.9672744	-0.0480029	0.95252
NEK10	1.06367	1.0665342	1.06130403	0.921391	-0.1181146	0.95256
RP11-1336O20.2	1.043814	1.0467097	1.04142252	0.8868075	-0.1733071	0.95256
PURB	1.365072	1.3685391	1.36220853	0.9828226	-0.0249971	0.95258
RP11-738E22.3	1.017855	1.0149316	1.02026954	1.3574903	0.44094194	0.95265
RP3-500L14.2	1.017844	1.0149347	1.02024631	1.3556577	0.43899291	0.95265
RP11-1079K10.3	1.017854	1.0149349	1.02026424	1.356838	0.44024845	0.95265
MBD5	1.418923	1.4221192	1.41628393	0.9861762	-0.0200827	0.95266
RP4-790G17.7	1.02063	1.0176758	1.02306899	1.3051173	0.38417945	0.95266
MIR2052HG	1.020594	1.0176698	1.02300816	1.302114	0.38085575	0.95266
HMCN2	1.032589	1.0296071	1.03505074	1.1838609	0.24349962	0.95269
CTA-204B4.2	1.085541	1.0885496	1.08305668	0.9379682	-0.092389	0.95269
CTD-2287O16.5	1.107191	1.1103505	1.1045818	0.9477242	-0.0774609	0.95277
PCNX2	1.183205	1.1802097	1.18567801	1.0303444	0.04312667	0.95278
NCOA5	1.135319	1.138424	1.13275488	0.9590453	-0.0603292	0.95281
POGK	1.286062	1.2892521	1.28342759	0.9798636	-0.0293472	0.95282
MPPED1	1.023521	1.0205765	1.02595183	1.2612341	0.33483605	0.95283
TSPAN32	1.023529	1.0206251	1.02592673	1.2570458	0.33003721	0.95283
NCOA6	1.282668	1.2798132	1.2850249	1.0186255	0.02662375	0.95295
DCUN1D4	1.320739	1.3237949	1.31821573	0.9827693	-0.0250753	0.95299
PARS2	1.119972	1.1167973	1.12259414	1.0496313	0.06988264	0.953

RP11-111J6.2	1.019883	1.0227554	1.01751074	0.7695187	-0.3779717	0.95301
XXbac-BPG157A10.21	1.029057	1.0318728	1.02673137	0.8386889	-0.2537923	0.95302
SERPINB7	1.029044	1.0318471	1.02672846	0.8392743	-0.2527856	0.95302
RP11-347I19.7	1.029075	1.0318948	1.02674591	0.8385653	-0.2540049	0.95302
LSINCT5	1.02906	1.0319132	1.02670317	0.8367431	-0.2571433	0.95302
PRR12	1.146452	1.1435237	1.14887085	1.0372564	0.05277255	0.95309
MBD2	1.146592	1.1436909	1.14898755	1.0368611	0.05222258	0.95309
NOXRED1	1.021598	1.024461	1.01923445	0.7863327	-0.3467883	0.95314
PDIA2	1.021673	1.0246241	1.01923631	0.7812002	-0.3562358	0.95314
SPRYD7	1.255604	1.2586855	1.25305858	0.9782482	-0.0317275	0.95314
RP11-358N2.2	1.022326	1.0252155	1.01993903	0.7907451	-0.3387153	0.95318
DMRTA2	1.022291	1.0251471	1.01993228	0.7926258	-0.3352882	0.95318
LRCOL1	1.022358	1.0252763	1.01994831	0.7892091	-0.3415206	0.95318
SUSD5	1.022301	1.0251557	1.01994379	0.7928135	-0.3349466	0.95318
RP11-177H2.2	1.022321	1.0251667	1.0199716	0.7935723	-0.3335665	0.95318
RP11-809O17.1	1.05418	1.0512326	1.05661476	1.1050535	0.14411626	0.95319
RP13-131K19.2	1.03013	1.033007	1.02775405	0.8408546	-0.2500718	0.95321
FSTL4	1.02736	1.03025	1.02497313	0.8255572	-0.2765599	0.95322
TRPV3	1.027391	1.0302814	1.02500393	0.8257179	-0.2762791	0.95322
AC079305.10	1.05348	1.0563369	1.05112125	0.9074209	-0.1401562	0.95326
AGFG2	1.065708	1.0686096	1.06331271	0.9227973	-0.1159143	0.95326
ATXN7L2	1.054147	1.0570186	1.05177505	0.9080377	-0.1391758	0.95334
ZNF222	1.069523	1.0665077	1.07201348	1.0827835	0.11474478	0.95343
LRP2BP	1.069496	1.0665451	1.07193212	1.0809534	0.11230428	0.95343
C1QL1	1.036998	1.0398295	1.03465974	0.8702027	-0.2005767	0.95348
AC240274.1	1.097475	1.1003503	1.0951011	0.9476916	-0.0775104	0.95351
RP3-508I15.14	1.022304	1.0194085	1.02469432	1.2723428	0.34748747	0.95359
AC015849.2	1.019859	1.0169921	1.02222647	1.3080441	0.3874112	0.95368
RP4-575N6.4	1.019871	1.0169912	1.02224924	1.3094558	0.38896741	0.95368
HCFC1-AS1	1.019887	1.0169819	1.02228606	1.3123405	0.39214207	0.95368
KRT16	1.019852	1.0169822	1.02222216	1.3085529	0.38797227	0.95368
AKAP14	1.019881	1.0169844	1.02227268	1.3113629	0.39106702	0.95368
RP11-131K5.1	1.019888	1.0169987	1.02227394	1.3103315	0.38993183	0.95368
KLHDC7A	1.019883	1.0169938	1.02226864	1.3103954	0.39000224	0.95368
SPTLC2	1.311234	1.314403	1.30861694	0.9815966	-0.0267978	0.95373
XDH	1.086387	1.0834679	1.08879829	1.0638609	0.08930955	0.95378
GPR171	1.019577	1.0224118	1.01723566	0.7690447	-0.3788607	0.95379
RP11-325N19.3	1.01956	1.0223724	1.01723749	0.7704799	-0.3761708	0.95379
LDHD	1.020453	1.0175789	1.02282655	1.2985189	0.37686705	0.95389
NYAP2	1.020481	1.01762	1.02284419	1.2964943	0.37461583	0.95389
KRT86	1.027347	1.024468	1.02972483	1.2148433	0.28077026	0.9539
SOST	1.027404	1.0246032	1.02971705	1.2078526	0.27244436	0.9539
VCX	1.030149	1.0272928	1.03250691	1.1910448	0.25222769	0.95391
DUSP8	1.041472	1.0386144	1.04383242	1.1351307	0.18285842	0.95391
PLPP6	1.13336	1.1303353	1.13585751	1.0423694	0.05986665	0.95393

RP11-509J21.2	1.034917	1.0378078	1.03253052	0.8604189	-0.2168888	0.95401
ZNF804A	1.023522	1.0263863	1.02115618	0.801786	-0.3187109	0.95402
LA16c-358B7.4	1.020602	1.023435	1.01826263	0.7792895	-0.3597687	0.95403
LINC00659	1.020612	1.0234338	1.01828244	0.7801731	-0.3581338	0.95403
RP11-498C9.2	1.020597	1.023422	1.01826432	0.7797932	-0.3588366	0.95403
AC098973.1	1.020626	1.0234628	1.01828377	0.7792664	-0.3598114	0.95403
ARHGAP44	1.145474	1.1484696	1.143001	0.963167	-0.0541421	0.95404
CTC-529I10.2	1.034286	1.0370644	1.03199232	0.8631551	-0.2123082	0.95407
CKMT1A	1.034184	1.0369959	1.03186124	0.861211	-0.2155613	0.95407
CNPY1	1.041867	1.0447744	1.0394657	0.8814343	-0.182075	0.95419
ZNF367	1.069307	1.0665381	1.07159327	1.0759749	0.10564439	0.9542
NOVA2	1.30288	1.3059626	1.30033442	0.9816052	-0.0267853	0.95423
CPNE7	1.02485	1.0276526	1.02253613	0.8149719	-0.2951779	0.95425
ZHX1-C8orf76	1.056686	1.0537558	1.05910648	1.0995362	0.13689504	0.9543
ZNF862	1.066442	1.063652	1.06874529	1.0800175	0.11105472	0.95442
ZMYND19	1.090474	1.0934452	1.08802046	0.9419473	-0.0862817	0.95448
RP11-110I1.13	1.032647	1.0355632	1.03023926	0.8502957	-0.2339635	0.95451
SLC25A34	1.024443	1.0272077	1.02215927	0.8144497	-0.2961024	0.95452
PRICKLE4	1.112413	1.1152524	1.11006885	0.9550244	-0.0663905	0.95453
PCDHGA6	1.017114	1.0142633	1.01946899	1.3649668	0.44886591	0.95454
ABCD2	1.017101	1.0142691	1.01943992	1.3623758	0.44612474	0.95454
KCNG3	1.017099	1.0142667	1.01943841	1.362502	0.44625832	0.95454
HOXB9	1.017107	1.0142696	1.01945014	1.3630477	0.4468361	0.95454
RP11-1038A11.3	1.051336	1.0484512	1.0537184	1.1087125	0.14888526	0.95461
TNNI2	1.017984	1.015126	1.02034369	1.3449484	0.42755085	0.95463
CTB-58E17.9	1.017961	1.0151437	1.02028769	1.3396757	0.42188382	0.95463
NAMA	1.017964	1.0151179	1.02031451	1.3437422	0.42625636	0.95463
STAM	1.249282	1.2523059	1.24678519	0.9781189	-0.0319183	0.95469
SH3D21	1.087739	1.0907233	1.0852745	0.9399402	-0.0893591	0.95471
C9orf85	1.265573	1.2624772	1.26812979	1.0215356	0.03073943	0.95475
SLC17A8	1.016083	1.0132641	1.01841083	1.388019	0.47302731	0.95476
CTD-2516F10.2	1.016109	1.0132691	1.01845398	1.3907528	0.47586601	0.95476
RP11-11N7.4	1.016078	1.0132703	1.01839726	1.3863454	0.47128677	0.95476
AC002116.7	1.032293	1.035113	1.02996327	0.8533379	-0.228811	0.95476
RP11-57H12.3	1.032274	1.0350168	1.03000925	0.8569952	-0.2226411	0.95476
GRIK2	1.034354	1.037192	1.03201071	0.860688	-0.2164377	0.95478
RP11-661A12.5	1.034373	1.0372184	1.032024	0.8604337	-0.2168641	0.95478
RP13-516M14.4	1.053526	1.0506231	1.05592297	1.1046918	0.14364391	0.95479
BRINP1	1.031805	1.0289811	1.0341362	1.1778781	0.23619021	0.9548
TRIM7	1.031802	1.0289917	1.03412347	1.1770084	0.23512465	0.9548
ARNT2	1.161497	1.1645603	1.15896725	0.9660124	-0.0498864	0.95481
CD22	1.031121	1.0282876	1.03346178	1.1829149	0.24234628	0.95488
DOCK9-AS2	1.046686	1.0494995	1.04436184	0.8962071	-0.1580959	0.9549
RP11-15H20.8	1.029372	1.0265196	1.03172798	1.1963966	0.25869573	0.95493
FBXO15	1.077894	1.080644	1.07562374	0.9377481	-0.0927276	0.95497

METTL25	1.08007	1.0828662	1.07776141	0.9383974	-0.0917291	0.95501
RP11-505E24.2	1.01802	1.0207944	1.01572873	0.7563916	-0.4027948	0.9551
TUBA4B	1.018011	1.0207758	1.01572772	0.7570228	-0.4015913	0.9551
DDR1-AS1	1.018029	1.0208189	1.01572566	0.7553551	-0.4047731	0.9551
RP1-37E16.12	1.015383	1.0181365	1.01310914	0.7228057	-0.4683203	0.9551
CTA-407F11.7	1.015396	1.0181656	1.01310914	0.7216479	-0.4706329	0.9551
RP11-442N1.1	1.01541	1.0181954	1.01310914	0.7204651	-0.4729995	0.9551
CTD-3126B10.4	1.015397	1.0181665	1.01310914	0.7216105	-0.4707079	0.9551
RP11-347I19.8	1.015405	1.0181842	1.01310914	0.7209065	-0.472116	0.9551
NDP-AS1	1.015396	1.0181662	1.01310914	0.721621	-0.4706867	0.9551
RP11-661A12.12	1.015391	1.0181538	1.01310914	0.7221154	-0.4696986	0.9551
RP11-61J19.5	1.056929	1.0598027	1.0545564	0.9122725	-0.1324632	0.95511
RP11-96B5.3	1.029989	1.0271526	1.03233077	1.1907045	0.25181538	0.95511
GRK5	1.124092	1.1212987	1.12639827	1.0420419	0.05941322	0.95514
RP4-756G23.5	1.023062	1.0258453	1.02076369	0.8033833	-0.3158397	0.95528
HS6ST3	1.046469	1.0436611	1.0487874	1.117411	0.16015998	0.95529
AC005037.3	1.016965	1.0141489	1.01929057	1.3633934	0.44720193	0.95532
LINC01219	1.016972	1.0141518	1.01930058	1.3638218	0.44765518	0.95532
LRGUK	1.016961	1.0141739	1.01926342	1.3590782	0.44262849	0.95532
RP11-830F9.6	1.016955	1.0141514	1.01927003	1.3617082	0.44541757	0.95532
C10orf113	1.016965	1.0141633	1.01927789	1.3611204	0.44479467	0.95532
FOXD4	1.014966	1.0177054	1.01270407	0.7175273	-0.4788944	0.95537
Z69666.2	1.014967	1.0177076	1.01270407	0.7174348	-0.4790804	0.95537
RP11-504P24.9	1.014974	1.017723	1.01270407	0.7168138	-0.4803298	0.95537
RP11-53B2.3	1.014985	1.0177464	1.01270407	0.715868	-0.4822346	0.95537
LINC01600	1.014981	1.0177388	1.01270407	0.7161743	-0.4816173	0.95537
ATP8B1	1.024497	1.0216786	1.02682364	1.2373341	0.30723508	0.9555
CCDC183-AS1	1.022778	1.0255758	1.02046732	0.8002623	-0.3214551	0.95553
RP11-34P13.7	1.022768	1.0255411	1.02047756	0.8017483	-0.3187788	0.95553
INSM2	1.024862	1.0276516	1.02255802	0.8157943	-0.2937227	0.95554
SPATA13	1.160446	1.1575825	1.16281141	1.0331821	0.04709453	0.95558
SLC12A1	1.032852	1.0301382	1.03509286	1.1643985	0.2195849	0.9556
MORN3	1.073117	1.0702355	1.0754974	1.0749172	0.1042255	0.95562
ADAMTSL1	1.026557	1.0237702	1.02885903	1.2140836	0.27986774	0.95567
C1orf162	1.026573	1.0237372	1.02891522	1.2181414	0.28468164	0.95567
RP11-452H21.4	1.024462	1.0216989	1.02674374	1.2324935	0.30157998	0.95569
BCAN	1.02445	1.0216709	1.02674438	1.2341177	0.30348	0.95569
ZMYM6	1.206191	1.2034484	1.20845587	1.0246127	0.03507874	0.9557
CTD-2619J13.19	1.016704	1.0139009	1.01901919	1.3682026	0.4522819	0.95571
RP11-400N13.1	1.016711	1.0139057	1.01902703	1.3682932	0.45237745	0.95571
RP11-46A10.5	1.016687	1.0138991	1.01899018	1.36629	0.45026377	0.95571
LINC01291	1.016704	1.0139143	1.01900821	1.3660925	0.45005514	0.95571
TBC1D10C	1.022796	1.0255521	1.02052007	0.8030673	-0.3164072	0.95572
ACTL10	1.022771	1.0255256	1.0204968	0.8029902	-0.3165457	0.95572
WDR81	1.068357	1.0711198	1.06607629	0.9290837	-0.1061195	0.95573

LINC01207	1.015252	1.0180005	1.01298307	0.7212608	-0.4714071	0.95576
RP11-244E17.1	1.015267	1.0180317	1.01298307	0.7200132	-0.4739046	0.95576
LINC00921	1.015234	1.0179605	1.01298307	0.722867	-0.4681979	0.95576
RP3-399L15.1	1.015253	1.0180007	1.01298307	0.7212525	-0.4714237	0.95576
LINC00640	1.015228	1.0179464	1.01298307	0.7234355	-0.4670636	0.95576
RP11-195B17.1	1.01523	1.0179513	1.01298307	0.7232389	-0.4674557	0.95576
OSBPL2	1.333256	1.3303111	1.33568716	1.0162758	0.02329191	0.9558
BAIAP2L2	1.026613	1.0238191	1.02891965	1.2141369	0.27993115	0.9558
EHD1	1.317476	1.3205553	1.31493391	0.9824635	-0.0255243	0.95592
PLCD4	1.024809	1.0219704	1.02715295	1.2358893	0.30554954	0.95594
MUC20	1.063012	1.0602262	1.06531235	1.0844504	0.11696402	0.95595
CRIPAK	1.1021	1.0993273	1.10439042	1.0509745	0.07172769	0.95596
CTC-428G20.6	1.017105	1.014329	1.0193981	1.3537657	0.43697804	0.95598
RP11-387H17.4	1.017116	1.0143303	1.01941699	1.3549562	0.43824625	0.95598
PPEF1	1.017114	1.0143217	1.01941972	1.3559647	0.43931965	0.95598
LRRC63	1.017118	1.0143264	1.0194241	1.3558236	0.43916953	0.95598
RP11-466F5.10	1.01712	1.0143272	1.01942723	1.3559668	0.43932182	0.95598
SOWAHD	1.01711	1.0143316	1.01940483	1.3539896	0.43721661	0.95598
TAS2R31	1.025557	1.0227731	1.02785543	1.2231729	0.29062835	0.95601
CH17-264L24.1	1.117415	1.1203875	1.11496039	0.95492	-0.0665483	0.95603
AP000442.1	1.031004	1.0336717	1.02880119	0.8553541	-0.2254063	0.95603
RP11-108K3.2	1.019738	1.0169524	1.02203765	1.29997	0.37847829	0.95605
PAFAH2	1.119184	1.1166135	1.12130752	1.0402531	0.05693455	0.95614
APPBP2	1.592178	1.5953884	1.58952674	0.9901549	-0.0142739	0.95621
EVC2	1.068539	1.071377	1.06619593	0.9274127	-0.1087167	0.95622
MTMR7	1.051989	1.049181	1.05430735	1.1042344	0.14304651	0.95629
BRD2	2.356839	2.3525892	2.36034862	1.0057367	0.0082527	0.95629
SCLY	1.132177	1.1350122	1.1298361	0.9616617	-0.0563986	0.95631
RP11-338N10.2	1.014359	1.0170499	1.0121375	0.7118819	-0.4902901	0.95632
CD34	1.01439	1.0171177	1.0121375	0.7090621	-0.496016	0.95632
AC005609.18	1.01438	1.0170959	1.0121375	0.7099636	-0.4941831	0.95632
CCDC24	1.139776	1.1425757	1.13746422	0.9641493	-0.0526715	0.95633
RP11-466F5.8	1.016232	1.0189364	1.01399813	0.7392172	-0.4359298	0.95652
TFAP2A-AS1	1.01625	1.0189717	1.01400275	0.7380874	-0.4381364	0.95652
RP11-365N19.2	1.015373	1.0180579	1.01315569	0.7285278	-0.4569442	0.95654
RP11-138H10.2	1.015391	1.0180974	1.01315569	0.7269374	-0.460097	0.95654
RP11-214K3.25	1.015379	1.0180705	1.01315569	0.7280183	-0.4579534	0.95654
CTD-2349P21.5	1.015378	1.0180688	1.01315569	0.7280904	-0.4578105	0.95654
C9orf41-AS1	1.015415	1.0181501	1.01315569	0.7248271	-0.4642912	0.95654
ZNF625-ZNF20	1.015386	1.0180864	1.01315569	0.727382	-0.4592148	0.95654
AC068535.2	1.015409	1.0181385	1.01315569	0.7252924	-0.4633654	0.95654
C3orf62	1.15151	1.1545434	1.14900536	0.9641652	-0.0526478	0.95654
RP11-384K6.8	1.027125	1.0243445	1.02942141	1.2085448	0.27327096	0.95654
DMRT3	1.027159	1.0243791	1.02945447	1.2081862	0.27284277	0.95654
RP11-78F17.1	1.027101	1.0243491	1.02937444	1.206388	0.27069395	0.95654

CTD-2302E22.4	1.02711	1.024307	1.02942497	1.2105575	0.27567162	0.95654
UBP1	1.262073	1.2650235	1.25963555	0.9796698	-0.0296326	0.95663
ANO10	1.305576	1.3028152	1.30785581	1.0166457	0.023817	0.95668
BCAM	1.550222	1.5473503	1.55259333	1.009579	0.01375382	0.95669
CCDC74A	1.745855	1.7489245	1.74331945	0.9925158	-0.010838	0.9567
NPAS1	1.133842	1.1310926	1.13611241	1.0382922	0.05421251	0.95672
RP11-139K4.2	1.035124	1.0377972	1.03291635	0.870867	-0.1994756	0.95676
ZNF442	1.042287	1.0394557	1.0446254	1.1310249	0.17763066	0.95676
POLR3C	1.17813	1.1809087	1.17583454	0.9719517	-0.0410435	0.95677
RP11-434P11.2	1.021524	1.024245	1.01927763	0.7951177	-0.3307596	0.95679
CLEC2B	1.021513	1.0242156	1.01928064	0.7962092	-0.3287806	0.95679
ZBED1	1.322777	1.3197397	1.32528511	1.0173435	0.02480685	0.95681
RBM20	1.036108	1.0333093	1.0384195	1.1534176	0.20591489	0.95685
AC004381.6	1.089161	1.0864645	1.09138773	1.0569396	0.07989295	0.95686
LINC00235	1.03402	1.0311847	1.03636116	1.1659918	0.22155766	0.95687
CABLES1	1.042095	1.0447802	1.03987839	0.8905357	-0.1672546	0.9569
TRMO	1.070324	1.0674687	1.07268199	1.0772698	0.10737955	0.95692
VIPR2	1.082595	1.0852808	1.08037771	0.9425061	-0.0854261	0.95697
RP11-535A19.2	1.026162	1.0234195	1.02842622	1.2137821	0.27950948	0.95697
AC004754.3	1.02617	1.0233826	1.0284712	1.2176227	0.28406719	0.95697
B3GAT2	1.410895	1.4138344	1.40846839	0.9870333	-0.0188293	0.957
CCDC93	1.287622	1.2847667	1.28998054	1.018309	0.0261754	0.95703
MIR133A1HG	1.019574	1.0168339	1.02183731	1.2972259	0.37542975	0.95712
LA16c-444G7.2	1.022333	1.0195886	1.02459895	1.2557812	0.32858512	0.95712
PMF1-BGLAP	1.022332	1.0195969	1.02459061	1.2548198	0.32748018	0.95712
IFI27	1.107556	1.1103331	1.10526345	0.954051	-0.0678617	0.95713
LINC00630	1.050805	1.0480501	1.05308018	1.1046846	0.1436345	0.95719
ZFP30	1.22784	1.2250705	1.23012731	1.0224675	0.03205501	0.95723
AC002398.11	1.02668	1.0239076	1.02897013	1.2117554	0.27709856	0.95725
TMEM262	1.018732	1.0214111	1.01651963	0.7715461	-0.3741758	0.95725
C6orf58	1.018723	1.0214014	1.01651168	0.7715241	-0.3742169	0.95725
MARK4	1.310504	1.3135573	1.3079832	0.9822229	-0.0258777	0.95726
CTC-366B18.4	1.025236	1.0224987	1.02749625	1.2221266	0.28939374	0.95727
HIST1H2BE	1.02522	1.0224485	1.0275085	1.2254071	0.29326115	0.95727
EHD4	1.208269	1.2113014	1.20576477	0.9737976	-0.0383062	0.95729
MIR1539	1.018164	1.0208564	1.01593993	0.764272	-0.3878419	0.9574
TCP11X2	1.018176	1.0208837	1.01593993	0.7632715	-0.3897317	0.9574
CTD-2026K11.3	1.018165	1.0208583	1.01593993	0.7642013	-0.3879754	0.9574
RP11-245P10.8	1.018169	1.0208676	1.01593993	0.7638585	-0.3886228	0.9574
RP11-982M15.8	1.018158	1.020843	1.01593993	0.7647628	-0.3869157	0.9574
GIMAP2	1.018167	1.0208633	1.01593993	0.7640187	-0.3883201	0.9574
LBX1	1.018161	1.0208507	1.01593993	0.7644807	-0.387448	0.9574
DUOXA1	1.0213	1.0185793	1.02354691	1.26737	0.34183774	0.95743
ELF4	1.048834	1.0461083	1.05108577	1.1079518	0.14789518	0.95749
RP11-27G24.1	1.019896	1.0225484	1.01770578	0.7852337	-0.348806	0.9575

PSKH1	1.176146	1.1790097	1.17378023	0.9707865	-0.042774	0.95754
RP11-13K12.1	1.020563	1.0232191	1.01836919	0.7911237	-0.3380248	0.95756
LUADT1	1.020629	1.0233552	1.01837729	0.786862	-0.3458174	0.95756
SLC38A3	1.042799	1.0455414	1.04053457	0.8900606	-0.1680246	0.95761
ZNF285	1.052417	1.0550505	1.05024285	0.9126692	-0.1318361	0.95764
RP11-483I13.5	1.052492	1.0552187	1.05024085	0.9098513	-0.1362973	0.95764
CTD-2506P8.6	1.032621	1.0352722	1.03043093	0.8627456	-0.2129929	0.95776
KIF24	1.032592	1.0352888	1.03036448	0.8604564	-0.2168261	0.95776
HBG2	1.035284	1.0380012	1.03303958	0.8694358	-0.2018485	0.95783
CPT1B	1.043587	1.0408169	1.04587376	1.1238902	0.16850114	0.95784
ARHGAP24	1.142571	1.1397296	1.14491755	1.0371282	0.05259422	0.95786
RP11-834C11.4	1.08498	1.0821165	1.0873439	1.0636589	0.08903558	0.95787
LRRN3	1.091892	1.0891061	1.09419217	1.0570783	0.08008223	0.95801
NSUN3	1.143985	1.1466561	1.14177996	0.9667512	-0.0487835	0.95803
RP3-453C12.15	1.024067	1.0213129	1.02634178	1.2359553	0.30562652	0.95804
RAD21-AS1	1.024051	1.0213293	1.02629774	1.2329409	0.30210364	0.95804
EIF3CL	1.024034	1.0213486	1.0262524	1.2297006	0.2983071	0.95804
STARD5	1.039832	1.0425367	1.03759911	0.8839215	-0.1780098	0.95805
DOCK6	1.101826	1.0992844	1.10392458	1.0467359	0.06589755	0.95808
STBD1	1.021613	1.0189003	1.02385294	1.2620386	0.33575602	0.95814
RP4-758J18.13	1.02161	1.0188888	1.02385758	1.2630531	0.33691526	0.95814
RP11-395D3.1	1.0233	1.0205954	1.02553434	1.2398065	0.31011494	0.95815
RP11-958F21.1	1.017837	1.0204761	1.01565758	0.7646761	-0.3870792	0.95817
TEK	1.017815	1.0204285	1.01565673	0.7664146	-0.3838031	0.95817
RP11-503G7.1	1.017849	1.0204975	1.01566155	0.7640712	-0.3882209	0.95817
CLEC14A	1.017821	1.0204431	1.01565488	0.7657773	-0.3850032	0.95817
NME5	1.17799	1.1806984	1.17575272	0.9726303	-0.0400365	0.9583
RP4-635E18.7	1.029147	1.02656	1.03128356	1.1778438	0.23614821	0.95834
RP11-338N10.1	1.022196	1.0195159	1.02440943	1.2507437	0.32278617	0.95835
JAKMIP1	1.022161	1.0194857	1.02437009	1.2506627	0.32269278	0.95835
SYNGR4	1.021765	1.0243811	1.01960533	0.8041205	-0.3145165	0.95838
RP11-298I3.1	1.02183	1.0245218	1.01960619	0.7995417	-0.3227549	0.95838
AC010975.1	1.018873	1.0215051	1.0166999	0.7765547	-0.3648405	0.95842
RP11-94C24.13	1.018892	1.0215473	1.0166999	0.7750353	-0.3676661	0.95842
CTC-250I14.3	1.018871	1.0215011	1.0166999	0.7767011	-0.3645686	0.95842
CELSR3-AS1	1.018876	1.0215121	1.0166999	0.7763027	-0.3653088	0.95842
AC067956.1	1.018872	1.021503	1.0166999	0.7766297	-0.3647013	0.95842
FAM186B	1.016127	1.0187467	1.01396402	0.7448794	-0.4249211	0.95843
C2-AS1	1.016163	1.0188262	1.01396402	0.7417349	-0.4310245	0.95843
RP11-90B9.3	1.016165	1.0188308	1.01396402	0.741552	-0.4313803	0.95843
RP11-452C8.1	1.01613	1.0187537	1.01396402	0.7445991	-0.4254642	0.95843
HAGLROS	1.016131	1.0187556	1.01396402	0.7445245	-0.4256088	0.95843
NAV2-AS2	1.016161	1.0188206	1.01396402	0.7419546	-0.4305971	0.95843
RP4-678D15.1	1.016125	1.0187428	1.01396402	0.7450337	-0.4246224	0.95843
ACADSB	1.137848	1.135105	1.14011379	1.0370732	0.05251777	0.95844

CKMT2-AS1	1.095879	1.0931965	1.09809472	1.0525585	0.07390041	0.95847
FOXO4	1.044505	1.041818	1.04672474	1.1173363	0.16006343	0.95849
RP11-307B6.3	1.036116	1.0334007	1.03835823	1.1484241	0.19965556	0.9585
RP11-108M12.3	1.040077	1.0427542	1.03786585	0.8856632	-0.1751699	0.95853
FABP6	1.040086	1.0427253	1.03790626	0.8872089	-0.1726542	0.95853
MAGT1	1.490842	1.4936182	1.48854847	0.9897295	-0.0148939	0.95859
RTCA-AS1	1.030765	1.0280723	1.032989	1.175144	0.23283753	0.95861
SERINC2	2.223447	2.2199466	2.22633684	1.0052381	0.0075373	0.95862
RP4-635E18.8	1.023135	1.0257478	1.02097724	0.8147192	-0.2956252	0.95862
INTS12	1.160155	1.1628601	1.15792177	0.9696774	-0.0444232	0.95863
RP11-778D9.13	1.025758	1.0283609	1.02360798	0.8324125	-0.2646294	0.95865
SORBS1	1.100679	1.10329	1.09852321	0.9538507	-0.0681646	0.95869
LINC01099	1.056156	1.0588015	1.05397176	0.9178632	-0.123649	0.9588
ALDH1A2	1.030337	1.0329646	1.02816628	0.854441	-0.2269472	0.95883
LL09NC01-251B2.3	1.030365	1.0329685	1.02821434	0.8557961	-0.2246609	0.95883
AC006116.17	1.024972	1.0222934	1.02718346	1.2193509	0.28611341	0.95887
C16orf71	1.024968	1.0222801	1.02718743	1.2202551	0.28718283	0.95887
LDB3	1.025006	1.0223143	1.02722897	1.2202503	0.28717707	0.95887
TMEM74	1.022744	1.0252931	1.02063982	0.8160264	-0.2933123	0.9589
LINC01024	1.022734	1.0253903	1.02054072	0.8089975	-0.3057929	0.9589
FUT1	1.022794	1.02539	1.0206501	0.8133156	-0.2981128	0.9589
LLNLR-268E12.1	1.01886	1.0161828	1.02107039	1.3020237	0.38075571	0.959
SLC41A2	1.072295	1.0696412	1.07448565	1.0695623	0.09702052	0.95904
MAST2	1.155055	1.1523938	1.15725212	1.0318799	0.04527508	0.95904
CNTD1	1.019697	1.0170078	1.02191838	1.2887255	0.36594495	0.95908
RP11-70C1.1	1.023661	1.0263092	1.02147396	0.8162135	-0.2929815	0.95911
ARHGEF18	1.02365	1.0262714	1.02148469	0.8177983	-0.2901831	0.95911
KB-1980E6.3	1.023655	1.026312	1.02146075	0.815627	-0.2940185	0.95911
TLR3	1.023688	1.0263242	1.0215108	0.8171478	-0.291331	0.95911
AP000692.10	1.030566	1.0332386	1.02835956	0.8532113	-0.2290251	0.95912
BRPF1	1.101567	1.0988284	1.10382937	1.0506028	0.07121739	0.95918
FBXO24	1.017802	1.0151718	1.01997414	1.3165287	0.39673901	0.95922
RP11-45P15.4	1.033515	1.0307908	1.03576453	1.1615312	0.21602789	0.95923
U91319.1	1.033469	1.0360579	1.03133062	0.868898	-0.2027412	0.95926
C8orf37	1.108203	1.1054481	1.11047777	1.0476976	0.06722238	0.95927
TNPO3	1.473335	1.4764322	1.47077791	0.9881319	-0.0172244	0.95927
C14orf79	1.123962	1.1266168	1.12176885	0.9617119	-0.0563234	0.95932
HYAL3	1.054056	1.0567443	1.05183566	0.9134952	-0.130531	0.95932
RP11-401F2.3	1.09088	1.0934609	1.08874854	0.9495796	-0.0746392	0.95933
SMG6	1.424344	1.4273767	1.42183963	0.987044	-0.0188138	0.95935
ZNF57	1.055249	1.0578463	1.05310409	0.9180201	-0.1234024	0.95942
BMPR1A	1.379062	1.3822062	1.37646485	0.9849783	-0.0218362	0.95944
ELL3	1.016281	1.018855	1.01415473	0.7507149	-0.4136629	0.95948
AE000662.93	1.016297	1.0188922	1.01415473	0.7492361	-0.4165077	0.95948
AC011247.3	1.01632	1.0189416	1.01415473	0.7472832	-0.420273	0.95948

RP11-375I20.6	1.016292	1.0188804	1.01415473	0.7497044	-0.4156061	0.95948
C4orf45	1.016289	1.0188738	1.01415473	0.7499656	-0.4151036	0.95948
RP11-138B4.1	1.016285	1.0188641	1.01415473	0.750354	-0.4143567	0.95948
C9orf50	1.031712	1.0290758	1.03388944	1.1655531	0.22101477	0.95954
ZNF189	1.147486	1.144842	1.14967023	1.0333345	0.0473073	0.95958
RP11-2B6.3	1.020775	1.0233743	1.0186291	0.7969919	-0.327363	0.95963
JMJD7-PLA2G4B	1.020762	1.0233694	1.01860843	0.7962725	-0.3286659	0.95963
PCDHGA9	1.021404	1.0240982	1.01917983	0.7959045	-0.3293328	0.95966
CTD-3064H18.2	1.021303	1.023875	1.01917967	0.8033386	-0.3159199	0.95966
RP11-179K3.2	1.021329	1.023934	1.0191772	0.8012523	-0.3196715	0.95966
SMTN	1.136798	1.1393749	1.13467079	0.9662487	-0.0495335	0.95973
NBPF10	1.092109	1.0894984	1.09426433	1.0532512	0.07484954	0.95973
RP11-475O6.1	1.018689	1.0160653	1.02085558	1.2981763	0.37648627	0.95978
CCL5	1.018659	1.0160417	1.02082076	1.297911	0.37619141	0.95978
CTD-2027I19.3	1.018684	1.016048	1.02086134	1.299934	0.37843838	0.95978
RP11-335O4.3	1.019328	1.0166975	1.02150114	1.287683	0.36477742	0.95985
GRID2IP	1.019335	1.0166975	1.02151349	1.2884225	0.36560573	0.95985
C10orf62	1.019343	1.0166975	1.02152738	1.2892544	0.36653699	0.95985
SLC26A8	1.019324	1.0166975	1.02149353	1.2872268	0.36426631	0.95985
DGKK	1.019334	1.0166975	1.02151177	1.2883195	0.36549047	0.95985
RP11-574K11.29	1.019317	1.0166975	1.02148105	1.2864796	0.36342857	0.95985
LINC01040	1.019345	1.0166975	1.02153052	1.2894421	0.36674703	0.95985
SLC4A10	1.019345	1.0166975	1.02153052	1.2894421	0.36674703	0.95985
AC130469.2	1.019345	1.0166975	1.02153137	1.2894934	0.36680443	0.95985
RP11-351A20.1	1.019323	1.0166975	1.02149124	1.2870897	0.36411256	0.95985
PAEP	1.019329	1.0166975	1.02150251	1.2877647	0.36486896	0.95985
MKRN3-AS1	1.019346	1.0166975	1.02153312	1.289598	0.36692143	0.95985
FAM95C	1.019344	1.0166975	1.02152932	1.2893708	0.36666721	0.95985
SP140	1.019344	1.0166975	1.02152995	1.2894081	0.36670898	0.95985
RP11-325L12.6	1.019344	1.0166975	1.02152995	1.2894081	0.36670898	0.95985
LINC00384	1.019339	1.0166975	1.02151987	1.2888046	0.36603352	0.95985
FP325317.1	1.019327	1.0166975	1.0214991	1.2875609	0.36464072	0.95985
UCN2	1.019326	1.0166975	1.02149586	1.2873666	0.36442297	0.95985
SLC22A3	1.019311	1.0166975	1.02146854	1.2857305	0.36258832	0.95985
LINC01583	1.019308	1.0166975	1.02146446	1.2854859	0.36231377	0.95985
RP11-755B10.3	1.019323	1.0166975	1.02149146	1.2871031	0.36412767	0.95985
CTC-232P5.1	1.019333	1.0166975	1.0215097	1.2881955	0.36535161	0.95985
AE000658.22	1.01934	1.0166975	1.02152249	1.2889615	0.36620916	0.95985
RP11-188P20.3	1.022167	1.0247461	1.020037	0.8097016	-0.3045378	0.95986
RP11-434B12.1	1.020999	1.0235556	1.0188876	0.8018308	-0.3186302	0.95991
RP11-707P17.2	1.021009	1.023573	1.01889198	0.801423	-0.3193642	0.95991
LRRC55	1.023133	1.0257464	1.02097538	0.8146904	-0.2956762	0.95992
RP3-337H4.9	1.023087	1.0256421	1.02097725	0.8180775	-0.2896906	0.95992
NRIP2	1.023117	1.0257307	1.02095833	0.8145268	-0.295966	0.95992
CCNK	1.443531	1.4409963	1.44562449	1.0104948	0.01506189	0.96005

NBPF1	1.287118	1.2897797	1.28492033	0.9832309	-0.0243978	0.96009
RP11-982M15.2	1.021058	1.0236216	1.01894136	0.8018659	-0.3185671	0.9601
RFC4	1.540517	1.5367355	1.54363937	1.0128627	0.01843866	0.9601
RP11-981G7.6	1.028358	1.0257272	1.03052998	1.1866811	0.24693231	0.96012
PILRA	1.028343	1.0257244	1.03050482	1.1858306	0.24589795	0.96012
PLAC8L1	1.026196	1.0235818	1.02835512	1.2024151	0.26593503	0.96013
ACADL	1.026219	1.0235731	1.02840475	1.2049641	0.26899014	0.96013
VSTM2A	1.018427	1.0158069	1.02059127	1.3026779	0.38148045	0.96017
KLC2	1.191259	1.1940659	1.18894093	0.9735917	-0.0386112	0.96018
LINC00574	1.028348	1.0256623	1.03056662	1.1911101	0.25230675	0.96024
MLIP	1.028271	1.0256616	1.03042539	1.185637	0.24566233	0.96024
RP11-130L8.1	1.028333	1.0256979	1.03050911	1.1872238	0.24759192	0.96024
RP5-1157M23.2	1.02835	1.0257187	1.03052296	1.1867983	0.24707477	0.96024
CRAMP1	1.107184	1.1045105	1.10939184	1.0467063	0.06585662	0.96031
HPN	1.026491	1.0238551	1.02866708	1.2017193	0.26509996	0.96038
RP11-329B9.5	1.029335	1.0319249	1.02719617	0.85188	-0.231278	0.96039
CXCL6	1.025935	1.0233201	1.0280937	1.2047012	0.26867533	0.96041
ZBTB12	1.025982	1.023393	1.02812021	1.2020791	0.2655318	0.96041
RP13-714J12.1	1.025948	1.0233411	1.02810019	1.2038939	0.26770827	0.96041
AC090616.2	1.025953	1.0233188	1.02812796	1.2062367	0.27051309	0.96041
GBP3	1.01882	1.016219	1.0209683	1.2928232	0.37052502	0.96045
RP11-644F5.10	1.018838	1.0162321	1.02099023	1.2931331	0.37087074	0.96045
RP11-500G22.5	1.018825	1.0162193	1.02097637	1.2932989	0.37105575	0.96045
KCNK3	1.018809	1.0162253	1.02094296	1.2907561	0.36821643	0.96045
TGFB2-AS1	1.048599	1.051233	1.04642371	0.9061282	-0.142213	0.96045
FAM162B	1.021516	1.0189027	1.02367335	1.2523775	0.32466947	0.96051
VWA5B2	1.021497	1.0189245	1.02362103	1.2481746	0.31981973	0.96051
CALML4	1.02147	1.018868	1.0236194	1.2518229	0.32403043	0.96051
LINC01118	1.021516	1.018873	1.02369949	1.2557325	0.32852917	0.96051
AC007383.4	1.021494	1.0188533	1.02367482	1.2557405	0.32853837	0.96051
CSDC2	1.179514	1.176926	1.18165126	1.0267073	0.038025	0.96054
RP11-452H21.1	1.017475	1.0148661	1.01963024	1.3204691	0.40105049	0.96057
RP11-731D1.4	1.017452	1.0148661	1.01958696	1.3175574	0.39786577	0.96057
RP1-122K4.2	1.017483	1.0148661	1.01964383	1.3213831	0.40204884	0.96057
AL035610.2	1.017464	1.0148661	1.01960907	1.3190445	0.39949319	0.96057
RP11-285E23.2	1.017474	1.0148661	1.01962685	1.3202405	0.40080081	0.96057
ADGRG2	1.017451	1.0148661	1.01958646	1.3175238	0.39782899	0.96057
GALR1	1.01746	1.0148661	1.01960145	1.3185324	0.398933	0.96057
P2RY14	1.017453	1.0148661	1.01958936	1.317719	0.39804272	0.96057
RP11-395B7.2	1.017458	1.0148661	1.0195992	1.3183807	0.39876708	0.96057
AC067968.3	1.017475	1.0148661	1.0196289	1.3203787	0.4009518	0.96057
RP11-888D10.3	1.01747	1.0148661	1.01962044	1.3198094	0.40032964	0.96057
RP3-510O8.4	1.017446	1.0148661	1.019577	1.3168877	0.39713229	0.96057
RP11-1090M7.1	1.017481	1.0148661	1.01964034	1.3211485	0.40179264	0.96057
HEPACAM2	1.017464	1.0148661	1.01961016	1.319118	0.3995736	0.96057

ADGRE2	1.017449	1.0148661	1.01958257	1.3172623	0.39754261	0.96057
CTD-3194G12.2	1.017493	1.0148661	1.01966141	1.3225659	0.40333958	0.96057
CTD-2531D15.5	1.017467	1.0148661	1.01961555	1.3194806	0.39997017	0.96057
KLRC1	1.017468	1.0148661	1.01961605	1.3195146	0.40000727	0.96057
OLFM4	1.017466	1.0148661	1.0196139	1.3193695	0.39984869	0.96057
RASAL2	1.611287	1.614389	1.60872481	0.9907808	-0.0133622	0.96059
ANKMY1	1.062355	1.0596727	1.06456958	1.0820622	0.11378339	0.96062
LPAR1	1.171784	1.168977	1.17410246	1.0303322	0.04310959	0.96062
FAM117A	1.079471	1.0769114	1.08158559	1.0607735	0.08511667	0.96068
PLEKHA8	1.474683	1.4718711	1.47700464	1.0108792	0.01561058	0.9607
DCTN5	1.671504	1.669051	1.6735304	1.0066952	0.00962694	0.96081
CACNA1F	1.026534	1.0291472	1.02437516	0.8362778	-0.2579458	0.96082
BST1	1.026486	1.028993	1.02441581	0.8421264	-0.2478913	0.96082
NAPB	1.163106	1.1605414	1.16522353	1.0291646	0.04147369	0.96087
RP11-225H22.7	1.014531	1.0170683	1.01243594	0.7285992	-0.4568028	0.96091
RP11-18C24.8	1.014516	1.0170342	1.01243594	0.7300555	-0.4539219	0.96091
KIR3DX1	1.014516	1.0170353	1.01243594	0.7300111	-0.4540097	0.96091
AC074183.4	1.01453	1.0170649	1.01243594	0.7287446	-0.4565147	0.96091
RP11-131N11.4	1.014518	1.0170399	1.01243594	0.7298123	-0.4544026	0.96091
AC104134.2	1.014511	1.0170232	1.01243594	0.7305285	-0.4529875	0.96091
CTD-2126E3.4	1.025903	1.0284079	1.02383352	0.8389753	-0.2532998	0.96094
OSBPL10	1.049753	1.0471497	1.05190295	1.1008126	0.1385689	0.96095
CTD-2377O17.1	1.028844	1.0262397	1.03099448	1.1812051	0.24025945	0.96098
ANKRD44	1.028871	1.026269	1.03101955	1.1808431	0.23981731	0.96098
PFKFB3	1.567637	1.5706189	1.56517438	0.9904585	-0.0138316	0.96103
LRRK1	1.036268	1.038781	1.03419312	0.8816986	-0.1816426	0.9611
ORAI2	1.862883	1.8663717	1.86000201	0.9926478	-0.0106461	0.96111
EPHX3	1.04399	1.0413152	1.0461983	1.1181912	0.16116686	0.96116
PARK2	1.044017	1.0413831	1.04619247	1.116217	0.1586175	0.96116
CORT	1.019825	1.022371	1.01772236	0.792202	-0.3360597	0.96117
PTH2R	1.019798	1.0223254	1.0177114	0.7933307	-0.3340057	0.96117
CTC-350I8.1	1.019819	1.0223646	1.01771698	0.7921901	-0.3360814	0.96117
RP11-650L12.1	1.019799	1.0223275	1.0177105	0.7932141	-0.3342177	0.96117
RP11-408A13.3	1.019787	1.0223057	1.01770631	0.7938001	-0.3331523	0.96117
INTS1	1.208264	1.2054369	1.2105984	1.0251247	0.03579946	0.96124
CCDC114	1.037841	1.035213	1.0400108	1.13625	0.18428028	0.96127
CTD-2012K14.7	1.027862	1.0252809	1.02999267	1.1863757	0.2465609	0.96142
ZNF35	1.108503	1.1059423	1.11061759	1.0441302	0.06230164	0.96142
CD93	1.015536	1.0129919	1.01763681	1.3575213	0.44097483	0.96145
AC010504.2	1.015557	1.0129919	1.01767571	1.3605156	0.44415348	0.96145
HYI-AS1	1.01554	1.0129919	1.01764509	1.3581589	0.44165225	0.96145
RP11-489D6.2	1.015541	1.0129919	1.01764669	1.3582821	0.44178319	0.96145
AGXT2	1.015544	1.0129919	1.01765118	1.3586273	0.44214972	0.96145
HIST1H4D	1.015545	1.0129919	1.01765362	1.3588157	0.44234978	0.96145
LYZL4	1.015543	1.0129919	1.01765038	1.3585662	0.44208485	0.96145

AC067959.1	1.015537	1.0129919	1.01763949	1.3577282	0.44119466	0.96145
AP4B1-AS1	1.01554	1.0129919	1.01764496	1.3581489	0.44164165	0.96145
MSC-AS1	1.015541	1.0129919	1.01764525	1.358171	0.44166513	0.96145
WASF3-AS1	1.015556	1.0129919	1.01767393	1.3603786	0.44400826	0.96145
CTC-242N15.1	1.015549	1.0129919	1.01766126	1.3594032	0.44297345	0.96145
U47924.27	1.015546	1.0129919	1.01765606	1.3590036	0.44254926	0.96145
COL21A1	1.015546	1.0129919	1.0176546	1.3588906	0.44242931	0.96145
RP11-640L9.1	1.01555	1.0129919	1.01766235	1.3594872	0.44306253	0.96145
CD200R1	1.015553	1.0129919	1.01766788	1.3599127	0.44351404	0.96145
RP11-47I22.1	1.015548	1.0129919	1.01765885	1.3592178	0.44277667	0.96145
AC124944.3	1.01555	1.0129919	1.01766205	1.3594641	0.44303803	0.96145
RP13-228J13.1	1.015551	1.0129919	1.01766496	1.3596884	0.44327606	0.96145
CTD-2530H12.4	1.01554	1.0129919	1.01764364	1.3580476	0.44153402	0.96145
NR5A2	1.015542	1.0129919	1.01764712	1.3583152	0.44181834	0.96145
RP11-611O2.5	1.01555	1.0129919	1.01766266	1.3595115	0.44308831	0.96145
RP11-66N11.7	1.01555	1.0129919	1.01766266	1.3595115	0.44308831	0.96145
FBXO33	1.150052	1.1476358	1.15204681	1.0298774	0.04247261	0.96148
OSBPL3	1.231428	1.2340854	1.2292329	0.9792703	-0.030221	0.96152
KLLN	1.039972	1.0424338	1.03793916	0.8940781	-0.1615273	0.96152
DDX47	1.036028	1.0333915	1.03820597	1.144181	0.19431528	0.96154
AP1G2	1.3191	1.3218248	1.31685022	0.9845425	-0.0224746	0.96156
PCDHA13	1.021319	1.0187726	1.02342189	1.2476648	0.3192304	0.96157
GOLT1A	1.021304	1.0187645	1.02340179	1.2471325	0.31861473	0.96157
CSMD3	1.02406	1.0215166	1.02616	1.215808	0.28191538	0.96158
CTA-398F10.2	1.016992	1.0194761	1.01494071	0.7671299	-0.3824572	0.96164
RP11-439E19.7	1.017003	1.0194995	1.01494071	0.7662081	-0.3841918	0.96164
IFT74-AS1	1.028303	1.0257346	1.03042426	1.1822317	0.24151275	0.96169
AC137934.1	1.028347	1.0258209	1.03043371	1.1786453	0.23712967	0.96169
ALOX12B	1.026943	1.0243792	1.02906002	1.1919995	0.25338368	0.96171
ADPRH	1.026949	1.0243706	1.02907874	1.193189	0.25482255	0.96171
PYGO2	1.262771	1.2601839	1.26490662	1.0181514	0.02595216	0.96171
MOCOS	1.025599	1.0281215	1.0235164	0.8362431	-0.2580058	0.96174
ZFYVE1	1.147089	1.1444529	1.14926645	1.0333225	0.04729062	0.96175
GCGR	1.089014	1.0863328	1.09122752	1.0566958	0.07956013	0.96182
MSRA	1.371549	1.3688564	1.37377198	1.0133267	0.01909935	0.96185
SRXN1	1.023024	1.0204696	1.02513296	1.2278206	0.29609981	0.96187
RP3-326I13.1	1.018132	1.0206377	1.0160633	0.7783461	-0.3615164	0.96188
LIN28A	1.018132	1.020613	1.01608269	0.7802214	-0.3580445	0.96188
RP6-24A23.3	1.049994	1.0524889	1.04793303	0.9132032	-0.1309922	0.9619
RP11-108L7.15	1.064011	1.0613637	1.06619753	1.0787732	0.10939155	0.9619
DEFB134	1.018842	1.0213197	1.01679584	0.7878105	-0.3440795	0.96195
LINC01289	1.018846	1.0213291	1.01679584	0.787461	-0.3447196	0.96195
AF131215.6	1.018877	1.0213966	1.01679584	0.7849761	-0.3492794	0.96195
RNF19B	1.057544	1.0549627	1.05967622	1.085759	0.11870391	0.96195
C19orf18	1.026679	1.0291707	1.02462173	0.8440572	-0.2445874	0.96196

PSMD6-AS2	1.023869	1.026331	1.02183581	0.8292821	-0.2700651	0.96197
FBXL16	1.023861	1.0263254	1.02182627	0.8290948	-0.270391	0.96197
RP11-525G13.2	1.023969	1.0265456	1.02184114	0.8227779	-0.2814251	0.96197
SPI1	1.029584	1.0320902	1.02751494	0.8574262	-0.2219155	0.96198
EYA2	1.111622	1.1142515	1.10945127	0.9579857	-0.061924	0.96199
ASPHD2	1.06713	1.0646394	1.0691871	1.0703554	0.09808995	0.96205
KCNIP1	1.038315	1.0357964	1.0403952	1.128471	0.17436933	0.9621
CTD-2369P2.10	1.118377	1.1208965	1.11629722	0.9619571	-0.0559556	0.96212
ZNF544	1.477965	1.4807819	1.47563848	0.989302	-0.0155171	0.96215
OAS2	1.017218	1.0146773	1.01931611	1.316052	0.39621655	0.96216
C10orf55	1.0172	1.0146773	1.01928255	1.3137654	0.3937077	0.96216
LINC00309	1.017202	1.0146773	1.01928642	1.3140289	0.39399704	0.96216
ZP4	1.017208	1.0146773	1.01929795	1.3148143	0.39485908	0.96216
AF196970.3	1.017196	1.0146773	1.01927672	1.3133682	0.39327138	0.96216
HBA1	1.017197	1.0146773	1.0192785	1.3134893	0.39340445	0.96216
GJB5	1.017194	1.0146773	1.01927211	1.3130537	0.39292595	0.96216
RP11-96K19.2	1.017203	1.0146773	1.01928819	1.3141496	0.39412954	0.96216
RP11-452K12.7	1.017201	1.0146773	1.01928465	1.3139085	0.39386485	0.96216
TSHR	1.017204	1.0146773	1.0192906	1.3143134	0.39430932	0.96216
ANKUB1	1.017202	1.0146773	1.0192874	1.3140959	0.39407062	0.96216
AC005330.2	1.017216	1.0146773	1.01931293	1.3158354	0.395979	0.96216
RP11-589C21.5	1.017224	1.0146773	1.01932686	1.3167842	0.39701894	0.96216
RP11-977G19.10	1.01721	1.0146773	1.01930072	1.315003	0.39506606	0.96216
KB-1517D11.4	1.017208	1.0146773	1.01929821	1.3148319	0.39487838	0.96216
CTD-2006K23.1	1.017208	1.0146773	1.01929821	1.3148319	0.39487838	0.96216
RP11-392P7.6	1.017196	1.0146773	1.01927607	1.3133234	0.39322226	0.96216
RP11-613M10.8	1.017221	1.0146773	1.01932248	1.3164856	0.39669174	0.96216
RP11-485F13.1	1.017211	1.0146773	1.01930397	1.3152243	0.39530889	0.96216
SOWAHA	1.017204	1.0146773	1.01929098	1.3143397	0.3943382	0.96216
HSPB8	1.220416	1.2176585	1.22269282	1.0231295	0.03298874	0.96218
IGSF11	1.033615	1.0360912	1.03157066	0.8747465	-0.1930631	0.96218
ZNF688	1.481219	1.4837425	1.47913505	0.9904754	-0.0138069	0.96219
TRMT12	1.095759	1.0983631	1.09360795	0.9516573	-0.071486	0.96224
UBAC2-AS1	1.154535	1.1571658	1.15236178	0.9694334	-0.0447863	0.9623
RP11-191L17.1	1.056167	1.0586682	1.05410074	0.9221473	-0.116931	0.96237
RP11-301O19.1	1.03051	1.0329636	1.02848453	0.8641206	-0.2106955	0.9624
RP11-65L3.4	1.025822	1.0233023	1.02790216	1.1973999	0.259905	0.96249
KIRREL3	1.025737	1.0231981	1.02783362	1.1998213	0.26281956	0.96249
AGPS	1.290419	1.2879157	1.29248628	1.0158749	0.02272272	0.96249
LINC01170	1.109398	1.1068796	1.111478	1.043024	0.06077231	0.96254
MAK16	1.141959	1.144465	1.13989003	0.9683314	-0.0464272	0.96255
OLIG3	1.016136	1.0186225	1.01408228	0.756195	-0.4031698	0.96256
AP000318.2	1.016115	1.018577	1.01408228	0.7580485	-0.399638	0.96256
ATP13A5-AS1	1.016103	1.0185494	1.01408228	0.759179	-0.397488	0.96256
GRIK3	1.016108	1.0185616	1.01408228	0.758677	-0.3984423	0.96256

CTD-2616J11.9	1.016115	1.0185757	1.01408228	0.758103	-0.3995342	0.96256
RP5-974N19.1	1.01614	1.0186326	1.01408228	0.755787	-0.4039485	0.96256
RP5-921G16.1	1.016096	1.0185355	1.01408228	0.759748	-0.3964072	0.96256
CTD-2384B11.2	1.016116	1.0185788	1.01408228	0.7579754	-0.3997771	0.96256
RP11-227G15.12	1.016109	1.0185625	1.01408228	0.7586424	-0.3985081	0.96256
LARP1B	1.37332	1.3759792	1.37112435	0.9870874	-0.0187502	0.96257
FGF22	1.025036	1.0224967	1.02713345	1.2061064	0.27035717	0.96259
RP11-66B24.4	1.025018	1.0224789	1.02711516	1.2062481	0.27052665	0.96259
PAM16	1.025001	1.0224753	1.02708602	1.2051486	0.26921099	0.96259
RP11-214O1.2	1.023327	1.0208356	1.02538408	1.2183036	0.28487374	0.9626
SRGAP3-AS4	1.023311	1.0207883	1.02539487	1.2215919	0.28876236	0.9626
RP11-253M7.4	1.023357	1.0208696	1.02541118	1.2176145	0.28405739	0.9626
TMPO-AS1	1.16786	1.1703581	1.16579747	0.9732291	-0.0391486	0.96262
DIRAS1	1.100786	1.0982482	1.10288142	1.0471579	0.06647905	0.96265
KCNK12	1.038323	1.0408146	1.03626572	0.8885476	-0.170479	0.96268
SEC14L5	1.038325	1.0407917	1.03628886	0.889614	-0.1687486	0.96268
F7	1.040398	1.0428641	1.03836228	0.8949751	-0.1600805	0.9627
RP11-120C12.3	1.020089	1.0225954	1.01801984	0.797499	-0.3264454	0.96276
RP11-297N6.4	1.041303	1.0438106	1.03923222	0.8954956	-0.1592417	0.96278
DHX58	1.030784	1.0331974	1.02879122	0.8672744	-0.2054395	0.96279
LIG4	1.112798	1.1152561	1.11076852	0.9610642	-0.0572953	0.96285
FBXL22	1.046279	1.0436933	1.04841362	1.1080339	0.14800202	0.96289
ADAM11	1.037776	1.0352834	1.03983515	1.129006	0.17505319	0.96292
BORCS6	1.105678	1.1081212	1.10366004	0.9587389	-0.0607901	0.96296
ASB9	1.059639	1.0571541	1.06169191	1.0793964	0.11022476	0.963
PYCARD-AS1	1.024022	1.0264404	1.02202439	0.8329838	-0.2636397	0.96303
AC137932.5	1.024103	1.0265326	1.02209661	0.8328088	-0.2639428	0.96303
RP11-336K24.12	1.024048	1.0264926	1.02202858	0.8314985	-0.2662145	0.96303
PECAM1	1.028571	1.0310415	1.02653081	0.8546887	-0.226529	0.9632
RP11-99L13.2	1.018458	1.0159741	1.02050978	1.2839383	0.36057592	0.96323
CTC-490G23.6	1.018468	1.0159741	1.020528	1.2850785	0.36185648	0.96323
KCTD4	1.018481	1.0159741	1.02055105	1.2865216	0.36347566	0.96323
LA16c-316G12.2	1.018481	1.0159741	1.02055105	1.2865216	0.36347566	0.96323
RP11-346C20.4	1.01845	1.0159741	1.02049447	1.28298	0.35949866	0.96323
RP11-252E2.1	1.018462	1.0159741	1.02051682	1.2843786	0.36107053	0.96323
LINC01305	1.018455	1.0159741	1.02050339	1.2835378	0.36012581	0.96323
RP11-123K19.1	1.018453	1.0159741	1.02049968	1.2833059	0.35986506	0.96323
CTC-273B12.5	1.018471	1.0159741	1.02053386	1.2854457	0.36226863	0.96323
RP11-442O1.4	1.018486	1.0159741	1.02055946	1.2870483	0.36406617	0.96323
AP001058.3	1.01846	1.0159741	1.02051246	1.2841061	0.36076438	0.96323
RP11-134L10.1	1.018464	1.0159741	1.02051959	1.284552	0.36126535	0.96323
NKX3-2	1.018466	1.0159741	1.02052448	1.2848582	0.36160915	0.96323
RP11-256L11.3	1.018472	1.0159741	1.02053503	1.2855189	0.36235085	0.96323
HNRNPCL1	1.018472	1.0159741	1.02053503	1.2855189	0.36235085	0.96323
RP11-173P15.10	1.018472	1.0159741	1.02053503	1.2855189	0.36235085	0.96323

AC004012.1	1.01846	1.0159741	1.02051379	1.2841892	0.36085772	0.96323
LMF1-AS1	1.018474	1.0159741	1.02053778	1.2856908	0.36254372	0.96323
CTC-510F12.2	1.018457	1.0159741	1.02050687	1.2837558	0.36037084	0.96323
CTD-3195I5.4	1.018475	1.0159741	1.0205401	1.2858364	0.36270706	0.96323
RP11-362F19.1	1.018475	1.0159741	1.0205401	1.2858364	0.36270706	0.96323
LINP1	1.029852	1.0323003	1.02782962	0.8615901	-0.2149265	0.96336
ZNF835	1.029893	1.0323048	1.02790056	0.863665	-0.2114563	0.96336
FAF2	1.411504	1.408667	1.41384755	1.0126766	0.0181735	0.96339
UTS2B	1.020502	1.0180243	1.02254731	1.2509419	0.32301474	0.96341
CTD-2639E6.9	1.020531	1.0180243	1.02260033	1.2538834	0.32640315	0.96341
AC016722.4	1.02051	1.0180243	1.0225626	1.25179	0.32399257	0.96341
RP11-326E22.1	1.020526	1.0180243	1.02259188	1.2534145	0.32586354	0.96341
RP11-177F15.1	1.020509	1.0180243	1.0225601	1.2516513	0.32383272	0.96341
ITGB7	1.020491	1.0180243	1.02252769	1.2498532	0.3217587	0.96341
RP11-179A10.1	1.020507	1.0180243	1.02255793	1.2515306	0.32369353	0.96341
RP5-944M2.3	1.020515	1.0180243	1.02257276	1.2523533	0.32464165	0.96341
RP11-519G16.2	1.020514	1.0180243	1.02257042	1.2522235	0.32449206	0.96341
ST8SIA6-AS1	1.020498	1.0180243	1.02254006	1.2505395	0.32255057	0.96341
HIST1H2AI	1.020498	1.0180243	1.02254068	1.2505735	0.3225899	0.96341
RP5-965G21.6	1.020515	1.0180243	1.02257166	1.2522924	0.32457145	0.96341
KIZ-AS1	1.020529	1.0180243	1.02259819	1.2537647	0.32626663	0.96341
PTOV1-AS2	1.02052	1.0180243	1.0225806	1.2527883	0.3251427	0.96341
SLC2A14	1.020501	1.0180243	1.02254549	1.2508405	0.32289783	0.96341
CCNI2	1.02051	1.0180243	1.02256214	1.2517646	0.32396325	0.96341
AC096670.3	1.020508	1.0180243	1.02255995	1.2516431	0.32382325	0.96341
RP1-60O19.2	1.020526	1.0180243	1.02259223	1.253434	0.325886	0.96341
FLI1	1.020503	1.0180243	1.02254932	1.2510534	0.32314334	0.96341
AC079154.1	1.02052	1.0180243	1.02258045	1.2527805	0.32513365	0.96341
TCF23	1.020507	1.0180243	1.02255691	1.251474	0.32362835	0.96341
SALL3	1.020505	1.0180243	1.02255288	1.2512506	0.32337073	0.96341
RP5-851M4.1	1.018408	1.0159362	1.020449	1.2831752	0.35971817	0.96342
RP11-83A16.1	1.018423	1.0159362	1.02047741	1.2849584	0.3617216	0.96342
FGF17	1.018413	1.0159362	1.020459	1.2838028	0.36042359	0.96342
RP5-1120P11.3	1.018425	1.0159362	1.02047943	1.2850848	0.36186355	0.96342
RGSL1	1.01843	1.0159362	1.02049024	1.2857629	0.36262458	0.96342
RP11-700H6.1	1.018432	1.0159362	1.0204923	1.2858921	0.3627696	0.96342
CTB-107G13.1	1.018416	1.0159362	1.02046401	1.2841173	0.360777	0.96342
CASC21	1.018421	1.0159362	1.02047277	1.2846669	0.36139429	0.96342
HAND2	1.018446	1.0159362	1.02051904	1.2875705	0.36465142	0.96342
SAPCD1	1.018402	1.0159362	1.0204383	1.282504	0.35896329	0.96342
AC004051.2	1.018419	1.0159362	1.02047	1.2844933	0.36119935	0.96342
RP11-818F20.5	1.018408	1.0159362	1.02044954	1.2832093	0.35975655	0.96342
RAMP3	1.018412	1.0159362	1.02045606	1.2836186	0.36021658	0.96342
CTC-454I21.3	1.018418	1.0159362	1.02046656	1.2842773	0.36095673	0.96342
CTD-2154B17.4	1.018416	1.0159362	1.02046362	1.284093	0.36074971	0.96342

RP11-1143G9.5	1.018424	1.0159362	1.02047756	1.2849672	0.36173153	0.96342
LINC00540	1.018405	1.0159362	1.02044364	1.2828389	0.35934002	0.96342
RP11-122G18.12	1.020564	1.018045	1.02264508	1.2549255	0.32760177	0.96346
RIPPLY3	1.020549	1.0180617	1.02260352	1.2514648	0.3236177	0.96346
CPA6	1.047204	1.0447548	1.04922649	1.0999158	0.13739314	0.96347
PPP1R17	1.021989	1.0244894	1.01992408	0.8135811	-0.297642	0.96349
RP1-276E15.1	1.021938	1.0243966	1.01990688	0.8159706	-0.293411	0.96349
AGRP	1.021918	1.0243594	1.01990131	0.8169873	-0.2916145	0.96349
ZNF577	1.091162	1.0885868	1.09328846	1.0530739	0.07460673	0.9635
RP11-989E6.10	1.021422	1.0189561	1.02345883	1.2375372	0.30747192	0.96353
RP11-96K19.5	1.02141	1.0189138	1.02347215	1.2410084	0.31151292	0.96353
TOX3	1.10989	1.1074532	1.11190167	1.0413995	0.05852367	0.96354
NOCT	1.030957	1.0333955	1.02894309	0.866677	-0.2064336	0.96358
CECR3	1.01873	1.0162589	1.02077116	1.2775278	0.35335467	0.96367
RP11-344E13.4	1.018733	1.0162589	1.02077533	1.2777843	0.35364436	0.96367
RP11-13J8.1	1.018746	1.0162589	1.02079998	1.2793004	0.35535503	0.96367
RP11-96A15.1	1.018736	1.0162589	1.0207819	1.2781883	0.35410036	0.96367
RP3-402G11.28	1.018721	1.0162589	1.02075407	1.2764766	0.35216709	0.96367
RP11-543C4.1	1.018712	1.0162589	1.02073697	1.2754253	0.35097838	0.96367
AP000255.6	1.018728	1.0162589	1.02076633	1.2772307	0.35301909	0.96367
RP11-207C16.4	1.01873	1.0162589	1.02077091	1.2775127	0.35333759	0.96367
C1orf141	1.018717	1.0162589	1.02074668	1.2760224	0.35165364	0.96367
RP4-584D14.6	1.018759	1.0162589	1.02082382	1.2807666	0.35700763	0.96367
KRT6B	1.018719	1.0162589	1.02074983	1.2762158	0.35187227	0.96367
RP11-411K7.1	1.018723	1.0162589	1.02075727	1.2766735	0.35238963	0.96367
GABRD	1.01872	1.0162589	1.02075174	1.2763334	0.35200525	0.96367
RP11-235D19.4	1.01873	1.0162589	1.02077006	1.2774605	0.35327869	0.96367
CTD-3064M3.4	1.018735	1.0162589	1.02077943	1.2780363	0.35392883	0.96367
NKX2-1	1.01873	1.0162589	1.02077038	1.27748	0.35330076	0.96367
LDLRAD1	1.019561	1.0170989	1.02159349	1.2628576	0.336692	0.96368
PPARG	1.019522	1.0170813	1.02153762	1.2608908	0.33444339	0.96368
STX16-NPEPL1	1.019543	1.0170997	1.0215605	1.2608701	0.33441962	0.96368
HBE1	1.01953	1.0170713	1.02156108	1.2630037	0.33685886	0.96368
RP11-360I2.1	1.019545	1.0170711	1.02158771	1.264577	0.33865487	0.96368
RP11-497H17.1	1.019553	1.0170674	1.02160524	1.2658738	0.34013364	0.96368
PLGLB1	1.021274	1.0236857	1.01928256	0.8141018	-0.296719	0.96368
RP11-245G13.2	1.021276	1.0236742	1.01929557	0.815046	-0.2950466	0.96368
LINC01572	1.09334	1.0906137	1.09559207	1.0549407	0.07716188	0.96368
RNF165	1.433053	1.4354324	1.43108794	0.9900227	-0.0144665	0.96368
ATF6B	2.231259	2.2362744	2.22711735	0.992593	-0.0107258	0.96369
AY269186.1	1.018176	1.0157004	1.02022056	1.2878992	0.36501963	0.9637
RP11-589B3.6	1.018167	1.0157004	1.02020451	1.2868772	0.36387436	0.9637
AC026167.1	1.018183	1.0157004	1.02023272	1.2886735	0.36588676	0.9637
RP11-46E17.6	1.018177	1.0157004	1.02022141	1.2879533	0.36508026	0.9637
PKHD1L1	1.018172	1.0157004	1.02021389	1.2874742	0.36454352	0.9637

CTD-2154I11.2	1.01817	1.0157004	1.02020975	1.2872107	0.36424825	0.9637
RP1-156L9.1	1.018176	1.0157004	1.02022009	1.2878694	0.36498626	0.9637
FRMPD3	1.018161	1.0157004	1.02019284	1.2861336	0.36304053	0.9637
CTD-2023N9.3	1.018173	1.0157004	1.02021579	1.2875954	0.36467929	0.9637
WI2-85898F10.2	1.018172	1.0157004	1.02021359	1.287455	0.36452204	0.9637
RP11-169K16.4	1.018161	1.0157004	1.02019345	1.2861723	0.36308397	0.9637
AKR1B15	1.018157	1.0157004	1.02018532	1.2856549	0.36250345	0.9637
CTD-2297D10.1	1.018178	1.0157004	1.0202247	1.2881628	0.36531492	0.9637
LINC01290	1.018172	1.0157004	1.02021279	1.2874045	0.36446541	0.9637
RP11-80H18.4	1.018182	1.0157004	1.02023063	1.2885409	0.3657383	0.9637
STON1-GTF2A1L	1.034534	1.0319953	1.03663117	1.1448928	0.1952125	0.96371
SLC4A3	1.132368	1.1299291	1.13438171	1.0342692	0.04861172	0.96372
CDR1	1.074698	1.0771593	1.07266542	0.941758	-0.0865716	0.9638
CCDC85A	1.03288	1.0304363	1.03489808	1.1465952	0.19735617	0.96381
RP11-108M9.6	1.044939	1.047396	1.04291052	0.9053613	-0.1434345	0.96388
ATP7B	1.083323	1.0807455	1.08545145	1.0582818	0.08172379	0.96389
BAK1	1.336394	1.3391129	1.33414928	0.9853628	-0.0212731	0.96392
ZDHHC17	1.344409	1.3417863	1.34657494	1.0140106	0.02007272	0.96393
EDA	1.046009	1.0435799	1.04801517	1.1017727	0.13982665	0.96394
GIT2	1.191428	1.1889987	1.19343477	1.0234713	0.03347067	0.96399
RP11-332H18.7	1.019054	1.0214846	1.01704689	0.7934467	-0.3337948	0.96401
RP11-565A3.2	1.019037	1.0214458	1.01704689	0.7948838	-0.3311842	0.96401
PFKFB1	1.019038	1.0214483	1.01704689	0.794791	-0.3313525	0.96401
SPP2	1.019019	1.0214078	1.01704689	0.7962931	-0.3286286	0.96401
C8orf49	1.019068	1.0215155	1.01704689	0.7923069	-0.3358688	0.96401
IGSF21	1.041987	1.0395893	1.04396624	1.1105599	0.15128721	0.96419
MEI4	1.020403	1.0179515	1.02242738	1.2493293	0.32115376	0.96424
C11orf45	1.020405	1.0179659	1.02241949	1.2478939	0.31949527	0.96424
KCNQ3	1.020388	1.0179453	1.0224044	1.2484824	0.32017551	0.96424
OLAH	1.020377	1.0227629	1.01840694	0.8086389	-0.3064325	0.96424
NXF2B	1.020433	1.0228445	1.01844067	0.8072247	-0.3089577	0.96424
C11orf96	1.020399	1.022772	1.01844023	0.8097778	-0.304402	0.96424
TOLLIP-AS1	1.130055	1.1274645	1.13219464	1.0371094	0.05256804	0.96425
MTSS1	1.15879	1.1614858	1.15656452	0.9695253	-0.0446496	0.96429
RP11-626P14.2	1.021044	1.0185947	1.02306583	1.2404522	0.31086619	0.96429
ECM2	1.021049	1.018594	1.02307708	1.2411014	0.31162103	0.96429
PTPN5	1.021071	1.0186027	1.02310949	1.242263	0.31297061	0.96429
CTC-487M23.5	1.021038	1.0185928	1.02305738	1.2401257	0.31048633	0.96429
RP11-588G21.2	1.021052	1.0185975	1.02307982	1.2410208	0.31152732	0.96429
RP5-1116H23.4	1.02106	1.0186105	1.02308247	1.2402933	0.31068128	0.96429
RP11-161H23.9	1.021051	1.0185975	1.02307734	1.2408875	0.31137233	0.96429
KB-1592A4.15	1.021054	1.0185944	1.02308453	1.24148	0.31206104	0.96429
ADCYAP1R1	1.02106	1.018608	1.02308551	1.2406198	0.31106109	0.96429
RP11-324I22.2	1.021058	1.0185933	1.02309388	1.2420515	0.31272495	0.96429
GPA33	1.021045	1.0186152	1.02305091	1.2382822	0.30834011	0.96429

KCNMA1-AS3	1.019272	1.0216553	1.01730453	0.7990904	-0.3235693	0.9643
INE2	1.019313	1.0217444	1.01730453	0.7958144	-0.329496	0.9643
RP11-60H5.1	1.019283	1.0216792	1.01730453	0.7982075	-0.3251642	0.9643
DUOXA2	1.019317	1.0217541	1.01730453	0.7954591	-0.3301403	0.9643
TRIM60	1.019287	1.0216886	1.01730453	0.7978627	-0.3257876	0.9643
RP11-461L13.3	1.019271	1.0216518	1.01730453	0.7992176	-0.3233398	0.9643
CTD-2369P2.8	1.019292	1.0216982	1.01730453	0.7975094	-0.3264265	0.9643
ZNF257	1.021391	1.0238128	1.0193919	0.8143486	-0.2962815	0.96431
RP11-381N20.1	1.0214	1.023831	1.0193919	0.8137273	-0.2973827	0.96431
CTD-2047H16.5	1.021392	1.0238136	1.0193919	0.8143212	-0.2963302	0.96431
DCLK3	1.021369	1.0237638	1.0193919	0.8160283	-0.2933089	0.96431
TRAF6	1.112853	1.1150805	1.11101412	0.964665	-0.0519001	0.96436
UBE2QL1	1.027997	1.0255646	1.03000618	1.1737413	0.23111441	0.96437
CD101	1.027981	1.0255351	1.03000153	1.1749145	0.23255576	0.96437
ABCG4	1.028014	1.0255402	1.03005648	1.1768284	0.23490395	0.96437
DBH-AS1	1.022327	1.0247753	1.02030583	0.819601	-0.2870063	0.96446
AC005477.1	1.022277	1.0246525	1.02031518	0.8240604	-0.279178	0.96446
RP11-325L12.7	1.019329	1.0217202	1.01735489	0.7990212	-0.3236942	0.96449
KCNS1	1.019347	1.0217587	1.01735489	0.7976059	-0.3262521	0.96449
PTF1A	1.019334	1.0217314	1.01735489	0.7986084	-0.3244399	0.96449
NEUROG2	1.019344	1.0217534	1.01735489	0.7978028	-0.3258959	0.96449
RP11-429E11.3	1.01932	1.0217006	1.01735489	0.7997436	-0.3223906	0.96449
SPG11	1.346317	1.3435122	1.34863334	1.0149081	0.0213491	0.96456
CNTNAP5	1.027927	1.0255264	1.02990934	1.1717001	0.22860333	0.96457
RASD2	1.02791	1.0254416	1.02994811	1.177132	0.23527606	0.96457
TAPT1-AS1	1.020128	1.0176976	1.02213508	1.2507395	0.32278133	0.96463
CTC-575N7.1	1.027623	1.0300065	1.02565449	0.8549634	-0.2260654	0.96475
RP11-262H14.3	1.027567	1.029967	1.02558582	0.8537996	-0.2280305	0.96475
TMEM74B	1.065262	1.0676254	1.06331079	0.936198	-0.0951144	0.9648
ZNF385B	1.028179	1.0257718	1.03016737	1.170557	0.22719517	0.96482
ZNF157	1.028265	1.0258532	1.0302567	1.1703275	0.22691233	0.96482
FAM72A	1.027624	1.0252125	1.02961476	1.1746063	0.23217723	0.96485
CCDC113	1.158666	1.1560469	1.16082807	1.0306393	0.04353951	0.96489
AC021188.4	1.029024	1.0265632	1.03105645	1.1691549	0.2254661	0.96489
RAB26	1.028974	1.0265571	1.03096988	1.166164	0.22177064	0.96489
MFSD5	1.136898	1.1344514	1.13891809	1.0332215	0.04714952	0.96489
DAB2IP	1.246112	1.2437453	1.24806581	1.0177256	0.0253486	0.9649
NPIPB11	1.020568	1.0181815	1.02253924	1.2396779	0.30996534	0.96491
CTB-78F1.1	1.020556	1.0181241	1.02256382	1.2449643	0.31610434	0.96491
TNNI3K	1.020568	1.0181538	1.02256099	1.242768	0.31355696	0.96491
C4orf19	1.020538	1.018143	1.02251666	1.2410689	0.31158315	0.96491
RNF31	1.02321	1.0207568	1.02523654	1.2158188	0.28192824	0.96496
DCAF4	1.081066	1.0786123	1.08309237	1.0569897	0.07996136	0.965
AC005863.1	1.01918	1.0167739	1.02116715	1.2619108	0.33560996	0.96502
SHANK3	1.019176	1.0167623	1.0211695	1.2629266	0.33677079	0.96502

PRDM12	1.019177	1.0167639	1.02117008	1.2628399	0.33667176	0.96502
TRADD	1.209696	1.2077895	1.21127118	1.0167556	0.02397297	0.96505
NEXN-AS1	1.042907	1.0404204	1.04495986	1.1123059	0.15355356	0.96512
RP11-317G6.1	1.015264	1.012846	1.01726008	1.3436117	0.42611631	0.96516
RP11-124N2.1	1.015252	1.012846	1.01723842	1.3419255	0.42430456	0.96516
RP11-307P5.1	1.015246	1.012846	1.01722754	1.341078	0.42339319	0.96516
RP11-319E12.1	1.015242	1.012846	1.017221	1.340569	0.42284543	0.96516
RP11-511B23.2	1.01526	1.012846	1.01725336	1.3430887	0.42555458	0.96516
RP11-268P4.4	1.015263	1.012846	1.01725945	1.3435622	0.42606315	0.96516
CARD16	1.015246	1.012846	1.01722742	1.3410689	0.42338333	0.96516
CTD-2376I4.1	1.015243	1.012846	1.0172226	1.3406938	0.42297973	0.96516
HTR7	1.015243	1.012846	1.01722291	1.3407183	0.42300617	0.96516
HIST1H3C	1.015245	1.012846	1.01722661	1.3410056	0.42331525	0.96516
RP11-65M17.3	1.01526	1.012846	1.01725375	1.3431189	0.42558707	0.96516
KB-1836B5.4	1.015256	1.012846	1.01724627	1.3425366	0.42496139	0.96516
RP11-575L7.4	1.01526	1.012846	1.01725407	1.3431437	0.42561368	0.96516
RFPL1	1.01525	1.012846	1.01723566	1.3417107	0.42407362	0.96516
INS	1.01525	1.012846	1.01723566	1.3417107	0.42407362	0.96516
GCM2	1.015253	1.012846	1.01724011	1.3420571	0.42444602	0.96516
P2RX7	1.015259	1.012846	1.01725232	1.3430077	0.42546763	0.96516
RP11-90E5.1	1.015246	1.012846	1.01722825	1.3411336	0.42345294	0.96516
AC243756.1	1.015255	1.012846	1.01724456	1.3424035	0.42481843	0.96516
IL7	1.015245	1.012846	1.01722583	1.3409451	0.42325015	0.96516
CTC-482H14.5	1.01525	1.012846	1.01723543	1.3416929	0.42405453	0.96516
RP11-703G6.1	1.015256	1.012846	1.01724532	1.3424622	0.42488149	0.96516
CEMP1	1.015256	1.012846	1.01724532	1.3424622	0.42488149	0.96516
PRND	1.015255	1.012846	1.01724432	1.3423844	0.42479789	0.96516
RP11-946L16.1	1.015242	1.012846	1.01722018	1.3405057	0.42277734	0.96516
ABC12-49244600F4.4	1.015262	1.012846	1.01725805	1.3434534	0.42594633	0.96516
RP11-624L4.2	1.015252	1.012846	1.01723849	1.3419309	0.42431042	0.96516
RERGL	1.015242	1.012846	1.01722071	1.3405464	0.42282113	0.96516
PUS1	1.139405	1.1417044	1.13750642	0.970375	-0.0433857	0.96516
MIR497HG	1.024775	1.0271185	1.02284052	0.842249	-0.2476813	0.96519
TRIM3	1.115952	1.1184544	1.11388577	0.9614316	-0.0567438	0.96526
RP11-474P2.6	1.033364	1.0357122	1.03142456	0.879939	-0.1845246	0.96528
NFKBIZ	1.079186	1.0766749	1.08125911	1.0597875	0.08377496	0.96532
ARHGEF4	1.203142	1.2057015	1.20102767	0.9772783	-0.0331586	0.96535
ST6GALNAC5	1.139384	1.1369476	1.14139688	1.032489	0.0461264	0.96536
TNFRSF10C	1.047935	1.0502713	1.04600521	0.915139	-0.1279372	0.96538
ZNF433	1.047902	1.0502501	1.04596265	0.9146778	-0.1286644	0.96538
CTD-3214H19.4	1.030577	1.0282167	1.03252623	1.1527315	0.20505646	0.9654
MACC1	1.055729	1.058064	1.05380135	0.9265871	-0.1100016	0.9654
DHX37	1.085915	1.0835615	1.08785881	1.0514265	0.07234799	0.96546
SLC14A2	1.02989	1.0275048	1.03185957	1.158327	0.21204255	0.96546
TYROBP	1.029881	1.027439	1.03189726	1.1624776	0.21720289	0.96546

ST8SIA4	1.089033	1.0865277	1.09110243	1.0528697	0.07432691	0.96547
FOXD2-AS1	1.031664	1.0339803	1.02975128	0.8755444	-0.1917477	0.96548
KCNJ13	1.079133	1.0815278	1.07715503	0.9463641	-0.0795327	0.96548
CAPN5	1.077349	1.0797797	1.07534169	0.9443715	-0.0825736	0.96549
SEMA4G	1.08782	1.0854137	1.08980795	1.051447	0.07237617	0.96549
CSTF2T	1.175643	1.1730884	1.1777525	1.0269466	0.0383611	0.96554
LILRB1	1.018105	1.0204856	1.01613888	0.7878149	-0.3440714	0.96555
PCDHB1	1.018087	1.0204469	1.01613888	0.789305	-0.3413452	0.96555
AC073641.2	1.018084	1.0204389	1.01613888	0.7896168	-0.3407754	0.96555
ASB12	1.018055	1.0203755	1.01613888	0.7920726	-0.3362955	0.96555
PLA2G7	1.018064	1.0203946	1.01613888	0.7913299	-0.3376489	0.96555
AF131216.5	1.018069	1.0204054	1.01613888	0.7909102	-0.3384141	0.96555
RP11-211G3.2	1.018098	1.0204714	1.01613888	0.7883629	-0.3430682	0.96555
RP11-304F15.3	1.018077	1.0204233	1.01613888	0.7902183	-0.3396769	0.96555
FBXL17	1.226573	1.2241035	1.22861327	1.0201237	0.02874409	0.96563
FAM63A	1.124009	1.1215599	1.12603192	1.0367885	0.05212165	0.96564
C1orf112	1.124832	1.1272236	1.12285664	0.9656746	-0.0503909	0.96566
SKA1	1.124752	1.1270958	1.12281566	0.9663237	-0.0494215	0.96566
CHIC1	1.115026	1.1125788	1.11704661	1.0396863	0.05614832	0.96568
KCNK15	1.041242	1.0435667	1.03932209	0.9025728	-0.1478849	0.96569
ZNF318	1.207949	1.2103159	1.2059943	0.9794519	-0.0299535	0.96573
LINC01352	1.018003	1.0156039	1.019985	1.2807693	0.3570106	0.96577
LINC01173	1.017983	1.0156039	1.01994836	1.2784214	0.35436342	0.96577
UBASH3A	1.017995	1.0156039	1.01996911	1.2797508	0.35586286	0.96577
RP11-455B3.1	1.017994	1.0156039	1.01996787	1.2796714	0.3557734	0.96577
RP11-335L23.5	1.017996	1.0156039	1.0199716	1.2799105	0.35604295	0.96577
MYRFL	1.017977	1.0156039	1.01993638	1.2776534	0.35349656	0.96577
NXF3	1.017997	1.0156039	1.01997259	1.2799739	0.35611435	0.96577
RP11-298E9.5	1.017977	1.0156039	1.01993704	1.2776957	0.35354423	0.96577
RP11-875O11.1	1.017994	1.0156039	1.01996693	1.2796114	0.35570576	0.96577
AF011889.5	1.017998	1.0156039	1.01997592	1.2801873	0.35635485	0.96577
RP11-1376P16.1	1.017986	1.0156039	1.0199535	1.2787504	0.35473468	0.96577
AC074363.1	1.017978	1.0156039	1.01993944	1.2778497	0.35371811	0.96577
RP11-1006G14.2	1.017986	1.0156039	1.01995248	1.2786855	0.35466141	0.96577
RP11-268P4.5	1.017995	1.0156039	1.01996981	1.2797959	0.35591376	0.96577
ARID3C	1.017992	1.0156039	1.01996414	1.2794328	0.35550442	0.96577
ICAM2	1.017972	1.0156039	1.01992708	1.2770572	0.3528232	0.96577
LINC00484.1	1.018003	1.0156039	1.01998383	1.2806942	0.35692599	0.96577
AC009480.3	1.017996	1.0156039	1.01997075	1.279856	0.35598154	0.96577
RP11-434D9.1	1.017978	1.0156039	1.0199385	1.2777892	0.3536499	0.96577
CYP1A1	1.017984	1.0156039	1.01994872	1.2784442	0.35438919	0.96577
FCAR	1.01798	1.0156039	1.01994149	1.2779809	0.35386633	0.96577
SPIB	1.017972	1.0156039	1.01992846	1.2771458	0.35292328	0.96577
RP1-187B23.1	1.073809	1.071506	1.07571016	1.0587942	0.08242216	0.9658
SPN	1.029627	1.0272069	1.0316249	1.162384	0.21708679	0.96585

PTPRQ	1.029687	1.0273067	1.03165352	1.1591843	0.21310995	0.96585
STEAP3	1.092682	1.0949972	1.09076944	0.9554963	-0.0656779	0.96586
SHROOM2	1.128952	1.1266565	1.13084755	1.0330896	0.04696533	0.96587
CTD-2012J19.3	1.038239	1.0405765	1.03630926	0.8948341	-0.1603078	0.96588
RP11-490B18.5	1.039495	1.0370786	1.04149045	1.1189873	0.1621937	0.96588
RAB23	1.196287	1.1937767	1.1983596	1.0236504	0.03372313	0.96589
SVOPL	1.017271	1.0148948	1.01923241	1.2912151	0.36872939	0.9659
PXDNL	1.017258	1.0148928	1.01921096	1.2899531	0.3673186	0.9659
FBP1	1.017313	1.0148892	1.01931523	1.2972643	0.37547243	0.9659
ATP10B	1.017267	1.0148973	1.01922333	1.2903863	0.36780299	0.9659
CH507-145C22.1	1.017279	1.0149078	1.01923778	1.2904483	0.36787234	0.9659
RP11-333I13.1	1.017261	1.0148892	1.01922011	1.2908758	0.36835025	0.9659
RP11-677I18.3	1.01727	1.0148843	1.01924095	1.292704	0.37039202	0.9659
PRSS45	1.017263	1.0148879	1.01922493	1.2913167	0.36884285	0.9659
IL7R	1.053556	1.0512054	1.05549725	1.0838154	0.1161191	0.96593
CAMKV	1.037755	1.0352984	1.03978385	1.1270718	0.17257946	0.96595
COBL	1.03771	1.0353615	1.03964906	1.1212489	0.16510655	0.96595
FAM120B	1.210803	1.2133465	1.20870307	0.9782354	-0.0317464	0.96596
ASMTL-AS1	1.023081	1.0207096	1.02503931	1.209067	0.27389416	0.96602
RP11-728F11.4	1.023025	1.0206524	1.02498485	1.2097787	0.27474311	0.96602
AC079117.1	1.023026	1.0206382	1.02499814	1.2112537	0.27650104	0.96602
RP11-425A6.5	1.023136	1.0207184	1.02513239	1.2130462	0.27863453	0.96602
CARD9	1.025784	1.0234224	1.02773366	1.1840636	0.24374661	0.96603
TBC1D29	1.025807	1.0233697	1.02781921	1.190399	0.25144518	0.96603
MEG8	1.016147	1.0137737	1.01810696	1.3146063	0.39463085	0.96607
IFNL1	1.016141	1.0137737	1.0180956	1.313782	0.39372589	0.96607
CTAGE1	1.016137	1.0137737	1.01808871	1.3132811	0.39317579	0.96607
RP4-668E10.4	1.016134	1.0137737	1.0180839	1.3129326	0.39279281	0.96607
CTD-3064H18.4	1.016134	1.0137737	1.01808343	1.3128979	0.39275476	0.96607
PCDHA8	1.016142	1.0137737	1.01809856	1.3139964	0.39396132	0.96607
AF067845.2	1.016142	1.0137737	1.01809856	1.3139964	0.39396132	0.96607
CKMT2	1.016137	1.0137737	1.01808902	1.3133042	0.39320117	0.96607
RP11-840I19.5	1.016137	1.0137737	1.01808805	1.3132338	0.39312378	0.96607
AC005220.3	1.016137	1.0137737	1.01808846	1.3132634	0.39315627	0.96607
CTD-2291D10.1	1.016141	1.0137737	1.0180953	1.31376	0.39370171	0.96607
XIRP2	1.016152	1.0137737	1.01811648	1.3152975	0.39538918	0.96607
LDHAL6A	1.016142	1.0137737	1.01809817	1.3139683	0.39393048	0.96607
CTA-363E6.7	1.016144	1.0137737	1.01810072	1.3141536	0.39413391	0.96607
ZIM2-AS1	1.016144	1.0137737	1.01810072	1.3141536	0.39413391	0.96607
APOBEC4	1.016137	1.0137737	1.01808925	1.313321	0.39321956	0.96607
AC009495.3	1.016134	1.0137737	1.0180836	1.3129104	0.39276842	0.96607
CTD-2525I3.5	1.016134	1.0137737	1.01808391	1.3129329	0.39279314	0.96607
RP11-21C4.1	1.016149	1.0137737	1.01810969	1.3148049	0.39484875	0.96607
LINC00494	1.016158	1.0137737	1.01812629	1.3160095	0.39616996	0.96607
LRRC26	1.023859	1.0261718	1.02194892	0.8386474	-0.2538637	0.96612

C7orf61	1.023851	1.0261638	1.02194033	0.8385764	-0.2539859	0.96612
NACAD	1.09071	1.0883918	1.09262391	1.0478791	0.06747229	0.96613
IFI30	1.028675	1.0262772	1.03065509	1.1666065	0.22231799	0.96613
RGS7BP	1.032745	1.0303862	1.03469355	1.1417549	0.19125293	0.96614
PCDH10	1.084393	1.0870872	1.0821682	0.9435165	-0.0838804	0.96615
ZNF558	1.117395	1.1197308	1.11546695	0.964388	-0.0523144	0.96616
DHRS12	1.120592	1.1229812	1.11861935	0.9645321	-0.0520988	0.96619
FAM155A	1.061089	1.0636314	1.05899023	0.9270613	-0.1092633	0.96619
TFAP4	1.109708	1.112047	1.10777592	0.9618814	-0.0560691	0.96619
LINC00638	1.048346	1.0506993	1.04640304	0.9152608	-0.1277452	0.96623
ENPP5	1.102383	1.0999934	1.1043571	1.0436398	0.06162394	0.96624
CTD-2600O9.2	1.016406	1.0187104	1.01450349	0.7751577	-0.3674382	0.96627
TECTB	1.016426	1.0187536	1.01450349	0.7733727	-0.3707643	0.96627
LINC01268	1.016422	1.018746	1.01450349	0.7736835	-0.3701845	0.96627
P2RX3	1.016407	1.0187115	1.01450349	0.7751126	-0.3675223	0.96627
AC007365.3	1.016421	1.018744	1.01450349	0.7737686	-0.3700258	0.96627
RP11-10A14.5	1.024753	1.0223973	1.02669793	1.1920132	0.25340024	0.96631
RP11-350N15.6	1.02471	1.0223525	1.02665696	1.1925702	0.25407415	0.96631
CDS2	1.480886	1.4783592	1.48297352	1.0096461	0.01384971	0.96632
UCP3	1.024953	1.0272813	1.02302977	0.8441587	-0.2444139	0.96634
SLAIN1	1.115366	1.1176825	1.11345367	0.964066	-0.0527962	0.96646
C2orf82	1.155588	1.1580439	1.15356057	0.9716325	-0.0415174	0.96647
KANK3	1.02919	1.0314957	1.02728562	0.8663294	-0.2070125	0.9665
THSD7A	1.231015	1.2286516	1.23296583	1.018868	0.02696717	0.96651
RP11-49I11.1	1.031875	1.0342249	1.02993389	0.8746232	-0.1932664	0.96655
RP11-758M4.4	1.03183	1.0342573	1.02982611	0.8706506	-0.1998342	0.96655
ANKRD6	1.138566	1.1361808	1.14053635	1.0319834	0.04541979	0.9666
RP11-17J14.2	1.018926	1.0165762	1.0208667	1.2588323	0.33208612	0.96661
RP11-879F14.2	1.01891	1.0165822	1.02083166	1.2562678	0.32914409	0.96661
BBOX1	1.018934	1.0165826	1.02087624	1.2589277	0.33219542	0.96661
RP11-114F3.4	1.018958	1.0165978	1.02090779	1.2596699	0.33304574	0.96661
RP11-646I6.6	1.018933	1.0165822	1.02087466	1.258861	0.33211898	0.96661
DPF2	1.933929	1.9367934	1.93156428	0.994418	-0.0080757	0.96661
RP11-37C7.3	1.074516	1.0768876	1.07255802	0.9436892	-0.0836164	0.96663
RASA4B	1.047115	1.044785	1.04903903	1.0949867	0.13091337	0.96663
MTF1	1.174868	1.1774005	1.17277643	0.9739344	-0.0381035	0.96664
PCDHGC3	1.036341	1.0386766	1.03441143	0.8897215	-0.1685743	0.96675
BAIAP2	1.4212	1.4240564	1.4188403	0.9876995	-0.0178559	0.9668
RP11-303E16.7	1.013667	1.0113227	1.01560258	1.3779875	0.46256275	0.96681
LINC01117	1.013667	1.0113227	1.01560258	1.3779875	0.46256275	0.96681
RP11-881M11.4	1.013654	1.0113227	1.01557897	1.3759018	0.46037748	0.96681
RP4-697K14.15	1.013649	1.0113227	1.01557081	1.3751811	0.45962165	0.96681
KLK13	1.01365	1.0113227	1.01557273	1.3753507	0.45979956	0.96681
GRM5-AS1	1.013655	1.0113227	1.0155816	1.3761338	0.4606208	0.96681
RP11-1038A11.1	1.013658	1.0113227	1.01558689	1.3766014	0.46111087	0.96681

HIST1H4A	1.013658	1.0113227	1.01558689	1.3766014	0.46111087	0.96681
AC006946.15	1.013658	1.0113227	1.01558689	1.3766014	0.46111087	0.96681
LINC00896	1.013666	1.0113227	1.01560135	1.3778785	0.46244865	0.96681
FAM71F2	1.013668	1.0113227	1.015604	1.3781121	0.46269328	0.96681
C6	1.01366	1.0113227	1.01559064	1.3769329	0.46145822	0.96681
ZAP70	1.013656	1.0113227	1.01558224	1.376191	0.46068073	0.96681
KRT72	1.013648	1.0113227	1.01556825	1.3749555	0.45938497	0.96681
RP11-5C23.1	1.013648	1.0113227	1.01556842	1.3749705	0.45940069	0.96681
CHI3L2	1.013646	1.0113227	1.0155637	1.3745537	0.45896329	0.96681
LINC01419	1.01366	1.0113227	1.01558952	1.376834	0.46135461	0.96681
RP5-940J5.6	1.013656	1.0113227	1.01558263	1.3762253	0.46071665	0.96681
RP11-1099M24.7	1.013658	1.0113227	1.01558721	1.3766293	0.46114014	0.96681
CILP	1.013653	1.0113227	1.01557713	1.3757394	0.46020719	0.96681
RP11-77E14.2	1.013657	1.0113227	1.01558399	1.3763454	0.46084261	0.96681
ACER1	1.013657	1.0113227	1.01558471	1.3764092	0.46090948	0.96681
ZPLD1	1.013654	1.0113227	1.01557899	1.3759034	0.46037917	0.96681
SYNJ2BP-COX16	1.013651	1.0113227	1.01557389	1.3754533	0.45990716	0.96681
RP11-234G16.5	1.013656	1.0113227	1.01558294	1.3762522	0.46074486	0.96681
OR51E2	1.013663	1.0113227	1.0155948	1.3772998	0.46184258	0.96681
SRPK3	1.013653	1.0113227	1.01557786	1.3758039	0.46027481	0.96681
CD3E	1.013665	1.0113227	1.0155999	1.3777501	0.46231428	0.96681
RP11-326A19.3	1.013659	1.0113227	1.01558766	1.3766696	0.46118235	0.96681
CTC-518P12.6	1.013651	1.0113227	1.01557435	1.3754939	0.45994979	0.96681
RP1-8B1.4	1.037576	1.0398234	1.03571934	0.8969427	-0.1569123	0.96684
PNO1	1.197398	1.1950021	1.19937704	1.0224351	0.0320093	0.96689
NLRP3	1.027513	1.0252081	1.02941594	1.166926	0.22271302	0.96693
RP11-173D3.1	1.027525	1.0251907	1.02945254	1.1691809	0.22549821	0.96693
ELK4	1.265357	1.2628891	1.26739464	1.0171386	0.02451622	0.96693
RP11-51J9.5	1.275436	1.2778526	1.2734405	0.9841206	-0.023093	0.96693
PEX26	1.292116	1.2945187	1.29013164	0.9851042	-0.0216518	0.967
PBLD	1.049828	1.0474328	1.05180542	1.092185	0.12721725	0.96701
RP11-403P17.3	1.03657	1.038855	1.03468333	0.8926348	-0.1638581	0.96703
RP3-466P17.1	1.0366	1.0388514	1.03474049	0.894189	-0.1613483	0.96703
NMNAT2	1.050947	1.0533184	1.04898937	0.9188078	-0.122165	0.96705
RLIM	1.296576	1.2941021	1.2986191	1.0153587	0.02198955	0.96706
RP4-594I10.3	1.029729	1.0320398	1.02782098	0.8683253	-0.2036925	0.96708
TMEM208	2.140859	2.1440098	2.13825725	0.9949716	-0.0072727	0.96713
RP11-365H22.2	1.018312	1.0205723	1.01644507	0.7993783	-0.3230497	0.96715
RP11-543D5.1	1.018348	1.0206516	1.01644507	0.7963093	-0.3285992	0.96715
TULP2	1.018366	1.0206912	1.01644507	0.7947857	-0.3313622	0.96715
RP11-728G15.1	1.018332	1.0206175	1.01644507	0.7976255	-0.3262165	0.96715
SLC17A6	1.018367	1.0206942	1.01644507	0.79467	-0.3315722	0.96715
ALDH1L1-AS2	1.018328	1.0206077	1.01644507	0.7980063	-0.325528	0.96715
RP11-1007O24.3	1.02902	1.0313073	1.0271305	0.8665862	-0.2065848	0.96717
BBS5	1.107156	1.1094813	1.10523561	0.9612201	-0.0570613	0.96717

ARRDC3-AS1	1.035287	1.0329431	1.03722292	1.129917	0.17621681	0.96721
AGER	1.058504	1.0608834	1.05653919	0.928647	-0.1067977	0.96721
RP11-629B11.5	1.039526	1.0372041	1.0414436	1.1139529	0.15568818	0.96734
WAPL	1.737249	1.7343384	1.7396531	1.0072374	0.01040378	0.96737
PAPSS2	1.122262	1.1201773	1.12398347	1.0316715	0.04498366	0.96738
ERVMER34-1	1.061804	1.0594809	1.06372331	1.0713246	0.09939567	0.9674
AC078941.1	1.01971	1.0219599	1.01785142	0.8129113	-0.2988302	0.96741
RP11-215P8.3	1.034234	1.0319252	1.03613982	1.1320154	0.17889355	0.96745
ADAM20	1.0268	1.0290656	1.0249285	0.8576648	-0.2215141	0.96757
PADI2	1.02738	1.0296311	1.02552106	0.8612917	-0.2154261	0.96761
CD37	1.027439	1.029722	1.02555276	0.8597244	-0.2180539	0.96761
XXbac-BPG181M17.6	1.02018	1.0178803	1.02207963	1.2348557	0.30434242	0.96768
AC005757.6	1.02019	1.0178884	1.02209138	1.2349534	0.30445659	0.96768
RP11-541G9.2	1.020208	1.0178853	1.02212545	1.2370711	0.30692836	0.96768
ULBP3	1.020211	1.0178732	1.02214247	1.2388656	0.30901966	0.96768
NPAS4	1.020198	1.0178803	1.02211113	1.2366193	0.30640144	0.96768
RP11-90L20.2	1.020196	1.0178871	1.02210272	1.2356794	0.30530449	0.96768
RP1-35C21.2	1.01927	1.0215073	1.01742279	0.8100878	-0.3038497	0.96768
ASB3	1.176356	1.1785955	1.17450722	0.9771087	-0.033409	0.96769
CLDN15	1.098585	1.1011289	1.09648336	0.9540628	-0.0678439	0.96772
CLEC4A	1.028141	1.0303936	1.02627997	0.8646537	-0.2098057	0.96773
SDE2	1.11091	1.1132184	1.10900456	0.9627813	-0.05472	0.96776
DHDDS	1.219969	1.2220969	1.21821139	0.9825054	-0.0254627	0.96785
RP11-567J20.2	1.022236	1.0199353	1.02413621	1.2107274	0.27587408	0.96786
ZNF559-ZNF177	1.02222	1.0199379	1.02410393	1.2089513	0.27375618	0.96786
AC092535.3	1.022241	1.0199462	1.02413669	1.2100916	0.2751163	0.96786
RP11-420N3.2	1.022229	1.0199216	1.02413375	1.2114391	0.2767219	0.96786
IL1A	1.02223	1.0199431	1.02411939	1.2094122	0.274306	0.96786
TMEM72	1.022227	1.0199332	1.02412086	1.2100864	0.27511004	0.96786
RP11-368P15.3	1.020224	1.0225167	1.01833122	0.8141157	-0.2966943	0.96787
SAP30L-AS1	1.020218	1.022503	1.01833122	0.8146123	-0.2958146	0.96787
RP11-19J5.2	1.020202	1.0224664	1.01833122	0.8159404	-0.2934643	0.96787
GTF2A1L	1.020229	1.0225277	1.01833122	0.8137181	-0.2973989	0.96787
RP13-941N14.1	1.020171	1.0223983	1.01833122	0.8184204	-0.289086	0.96787
SLC39A12	1.020139	1.0178461	1.02203193	1.2345524	0.30398809	0.96787
RP11-569D9.5	1.020141	1.0178371	1.02204316	1.2358052	0.30545136	0.96787
HNF1B	1.02014	1.0178331	1.02204463	1.2361657	0.30587219	0.96787
PKN2-AS1	1.020133	1.0178304	1.02203537	1.2358341	0.30548513	0.96787
RP1-140A9.1	1.020146	1.0178317	1.02205746	1.2369819	0.30682444	0.96787
MB	1.029147	1.0313799	1.02730217	0.8700515	-0.2008273	0.96787
WNT16	1.023149	1.0208225	1.02507062	1.2040153	0.26785375	0.96797
CCDC148	1.26885	1.2709592	1.26710775	0.9857858	-0.0206539	0.96802
TBC1D17	1.268414	1.2708069	1.26643863	0.9838694	-0.0234613	0.96802
GS1-259H13.2	1.075622	1.0778538	1.07377917	0.947663	-0.0775539	0.96805
XXbac-B476C20.9	1.02709	1.0293176	1.02525024	0.8612667	-0.215468	0.96805

HSD11B2	1.027165	1.0294432	1.02528394	0.8587369	-0.219712	0.96805
DAP	2.063506	2.066624	2.06093124	0.9946628	-0.0077205	0.96809
BCL2L2-PABPN1	1.020454	1.0181552	1.02235254	1.2311904	0.30005386	0.96812
RP11-343L5.2	1.020452	1.0181586	1.0223458	1.2305915	0.29935189	0.96812
LINC01411	1.020431	1.0181546	1.02231077	1.2289343	0.29740778	0.96812
AC006004.1	1.020463	1.0181691	1.02235745	1.2305223	0.29927082	0.96812
SYNDIG1L	1.020484	1.0181704	1.02239493	1.2324952	0.30158204	0.96812
LINC00621	1.036284	1.0339879	1.03818069	1.1233617	0.16782256	0.96812
RP5-994D16.3	1.021285	1.0189595	1.02320577	1.2239664	0.29156401	0.96813
AF131216.1	1.02126	1.0189558	1.02316296	1.2219448	0.28917916	0.96813
LINC01010	1.019883	1.0176056	1.02176324	1.2361537	0.30585813	0.96815
RP11-8L8.2	1.019904	1.0176002	1.0218063	1.2389779	0.30915045	0.96815
RP11-80H5.9	1.019936	1.0176191	1.02184909	1.2400824	0.31043598	0.96815
FAM106A	1.01988	1.0175997	1.02176226	1.236512	0.30627629	0.96815
RP11-180C16.1	1.0199	1.0175997	1.0217997	1.2386396	0.30875647	0.96815
RP11-524D16__A.3	1.019896	1.0175985	1.02179311	1.2383536	0.3084233	0.96815
RP4-781K5.2	1.019892	1.0175943	1.02178987	1.2384586	0.30854566	0.96815
RP11-356N1.2	1.019874	1.0175943	1.02175645	1.236559	0.30633106	0.96815
TMEM204	1.019898	1.0176181	1.02178036	1.2362508	0.30597149	0.96815
CIDEA	1.019912	1.0176184	1.0218065	1.2377109	0.30767436	0.96815
ZMYM3	1.464697	1.4620764	1.46686039	1.0103532	0.01485973	0.96817
FRRS1L	1.241548	1.2440883	1.23944939	0.9809949	-0.0276824	0.96819
TWSG1	1.712208	1.7093769	1.71454523	1.0072857	0.01047295	0.96821
ANKRD66	1.015423	1.0131484	1.01730071	1.3158032	0.39594373	0.96823
RP11-676J12.7	1.015449	1.0131484	1.01734822	1.3194166	0.39990015	0.96823
SERPINA3	1.015425	1.0131484	1.0173051	1.3161375	0.39631018	0.96823
RP11-103J8.2	1.015438	1.0131484	1.01732924	1.3179734	0.39832128	0.96823
SCGB3A2	1.015419	1.0131484	1.01729422	1.3153098	0.39540263	0.96823
IFNL2	1.015442	1.0131484	1.01733631	1.3185106	0.39890915	0.96823
ACTBL2	1.015411	1.0131484	1.01727903	1.3141546	0.39413501	0.96823
AC013463.2	1.015423	1.0131484	1.01730195	1.3158972	0.39604681	0.96823
GJC3	1.015441	1.0131484	1.01733347	1.3182949	0.39867319	0.96823
ITGB3	1.015411	1.0131484	1.01727996	1.3142249	0.39421215	0.96823
GPR132	1.015425	1.0131484	1.01730443	1.3160864	0.39625423	0.96823
RP11-622C24.2	1.015429	1.0131484	1.01731312	1.3167473	0.3969785	0.96823
CADM3-AS1	1.015424	1.0131484	1.01730248	1.3159379	0.39609144	0.96823
CTD-2540B15.6	1.015427	1.0131484	1.01730938	1.3164625	0.39666648	0.96823
RP11-90C1.1	1.01543	1.0131484	1.01731381	1.3167996	0.39703576	0.96823
ESM1	1.015433	1.0131484	1.01731947	1.3172299	0.39750718	0.96823
RP11-384F7.1	1.015435	1.0131484	1.01732274	1.3174787	0.39777969	0.96823
RP5-837J1.4	1.01542	1.0131484	1.01729569	1.3154211	0.39552472	0.96823
HHIPL1	1.015427	1.0131484	1.01730854	1.3163991	0.39659697	0.96823
PTPRC	1.015419	1.0131484	1.01729349	1.3152538	0.39534119	0.96823
C20orf144	1.015422	1.0131484	1.01730004	1.3157526	0.39588828	0.96823
TMPRSS11D	1.015449	1.0131484	1.01734956	1.3195184	0.40001143	0.96823

TMPRSS11E	1.015418	1.0131484	1.01729212	1.3151498	0.39522716	0.96823
RP11-84D1.2	1.015417	1.0131484	1.01729077	1.3150472	0.39511459	0.96823
ALOXE3	1.01542	1.0131484	1.01729515	1.3153806	0.39548027	0.96823
RP5-1039K5.18	1.015454	1.0131484	1.01735757	1.3201278	0.40067764	0.96823
RP4-621N11.2	1.015422	1.0131484	1.01729894	1.3156684	0.3957959	0.96823
ADARB2	1.034604	1.0322742	1.03652849	1.1318175	0.17864138	0.96824
KCTD5	1.247511	1.2451611	1.24945191	1.0175019	0.02503149	0.9683
MNS1	1.109724	1.1073969	1.11164544	1.0395595	0.05597238	0.96834
KLHL3	1.201759	1.1993355	1.20376081	1.0222005	0.03167827	0.96841
GBA	1.164423	1.1665892	1.16263338	0.9762543	-0.0346711	0.96846
CNR1	1.043809	1.0415938	1.04563789	1.0972287	0.1338642	0.96859
TMEM178B	1.063221	1.0654553	1.06137522	0.9376663	-0.0928535	0.96861
RP11-884K10.7	1.063115	1.0654517	1.06118478	0.9348088	-0.0972568	0.96861
ANGEL2	1.250084	1.2475801	1.25215124	1.0184634	0.02639418	0.96863
AC091729.8	1.018689	1.0208687	1.01688941	0.8093191	-0.3052195	0.96864
RBP5	1.0187	1.0209289	1.01685865	0.8055198	-0.3120081	0.96864
ADGRA1-AS1	1.01867	1.0208781	1.0168462	0.8068821	-0.3095702	0.96864
ANKRD55	1.018666	1.0208567	1.01685774	0.8082662	-0.3070976	0.96864
RP11-359E10.1	1.018666	1.0208669	1.01684889	0.8074472	-0.3085602	0.96864
RARA	1.508444	1.5113945	1.50600738	0.9894659	-0.0152782	0.96865
RP11-154H23.3	1.059177	1.056963	1.06100531	1.0709638	0.09890968	0.96866
SSUH2	1.022118	1.019867	1.02397646	1.2068474	0.27124324	0.96869
RP11-1250I15.3	1.022812	1.0205607	1.02467145	1.1999323	0.26295299	0.96874
SLFNL1-AS1	1.022816	1.0205464	1.02469113	1.2017231	0.26510457	0.96874
FGF10	1.022769	1.0205066	1.02463764	1.2014472	0.26477327	0.96874
AC004854.4	1.022771	1.0204904	1.024654	1.2031982	0.26687432	0.96874
C9orf147	1.02974	1.0274354	1.03164316	1.1533689	0.20585403	0.9688
CLEC18B	1.078292	1.0804893	1.07647816	0.9501658	-0.0737488	0.96881
CYHR1	2.425136	2.4288192	2.42209431	0.9952934	-0.0068062	0.96881
PARD3B	1.289651	1.2872212	1.29165791	1.0154469	0.02211479	0.96883
RP11-342K6.1	1.065561	1.0677585	1.06374654	0.9407896	-0.088056	0.96891
URGCP	1.17522	1.1727239	1.17728146	1.0263863	0.03757387	0.96892
LIMK2	1.438327	1.4361921	1.44008995	1.0089361	0.01283477	0.96894
SLC31A2	1.06596	1.0636896	1.06783401	1.0650715	0.09095033	0.96904
KCNA5	1.021867	1.0196426	1.02370366	1.2067504	0.27112727	0.96909
RP11-282O18.3	1.021876	1.0196376	1.02372507	1.2081462	0.272795	0.96909
RP1-261D10.2	1.021854	1.0196304	1.02368998	1.2068023	0.27118939	0.96909
CTB-43P18.1	1.021889	1.0196122	1.02376988	1.2119973	0.27738654	0.96909
TMC4	1.046444	1.0441734	1.04831907	1.0938493	0.129414	0.96921
RP11-121M22.1	1.030042	1.0276859	1.03198849	1.1554066	0.20840062	0.96925
C1orf105	1.029935	1.0277001	1.03178155	1.1473462	0.19830074	0.96925
CYP2C8	1.029985	1.0277612	1.03182153	1.1462573	0.19693094	0.96925
TCF4-AS2	1.015271	1.013034	1.01711816	1.3133491	0.39325045	0.96929
TERT	1.015263	1.013034	1.01710344	1.31222	0.39200958	0.96929
TXK	1.015259	1.013034	1.01709718	1.3117395	0.39148129	0.96929

SSX5	1.015264	1.013034	1.01710518	1.3123529	0.39215572	0.96929
FOXB1	1.015283	1.013034	1.01714085	1.3150897	0.39516124	0.96929
RP11-141J13.5	1.015266	1.013034	1.01710923	1.3126636	0.39249728	0.96929
DAB1-AS1	1.015276	1.013034	1.01712674	1.3140073	0.39397332	0.96929
AC108051.3	1.015273	1.013034	1.01712132	1.3135917	0.3935169	0.96929
POU6F2-AS2	1.01527	1.013034	1.01711626	1.3132031	0.39309011	0.96929
RGS9BP	1.015264	1.013034	1.0171061	1.3124235	0.39223333	0.96929
HOXA1	1.015264	1.013034	1.01710505	1.3123435	0.39214544	0.96929
WNT11	1.015264	1.013034	1.01710505	1.3123435	0.39214544	0.96929
CFAP73	1.015265	1.013034	1.01710821	1.3125857	0.39241166	0.96929
CTD-2334D19.1	1.015276	1.013034	1.01712762	1.3140752	0.39404785	0.96929
RP11-357N13.3	1.015276	1.013034	1.01712762	1.3140752	0.39404785	0.96929
RTP4	1.015272	1.013034	1.01711955	1.3134555	0.39336729	0.96929
DNMBP-AS1	1.01528	1.013034	1.01713561	1.3146882	0.39472069	0.96929
MICALCL	1.015261	1.013034	1.01709969	1.3119318	0.39169276	0.96929
MIXL1	1.015276	1.013034	1.01712833	1.3141293	0.39410724	0.96929
ACOT12	1.01527	1.013034	1.0171161	1.313191	0.39307678	0.96929
CDX1	1.015259	1.013034	1.01709555	1.3116146	0.39134387	0.96929
NMUR2	1.015282	1.013034	1.01713835	1.3148983	0.3949512	0.96929
CMKLR1	1.015257	1.013034	1.01709331	1.3114426	0.39115471	0.96929
RP11-474G23.3	1.06639	1.0641876	1.06820852	1.062643	0.08765706	0.9693
RP11-624G17.3	1.018015	1.0157693	1.01986919	1.2599886	0.33341063	0.9693
LINC00534	1.018015	1.0157693	1.01986919	1.2599886	0.33341063	0.9693
ADAM2	1.018004	1.0157693	1.01984883	1.2586975	0.33193166	0.9693
PHOSPHO1	1.018003	1.0157693	1.01984829	1.2586636	0.33189279	0.9693
KRTAP2-3	1.017996	1.0157693	1.01983507	1.2578252	0.33093141	0.9693
CTD-3234P18.6	1.018026	1.0157693	1.01989033	1.2613297	0.33494541	0.9693
RP3-380B4.1	1.018008	1.0157693	1.01985589	1.2591454	0.33244488	0.9693
TAS2R5	1.018024	1.0157693	1.01988522	1.2610054	0.3345744	0.9693
SERPINE3	1.018004	1.0157693	1.01985023	1.2587864	0.33203351	0.9693
AJ239322.1	1.017994	1.0157693	1.01983203	1.2576321	0.33070997	0.9693
AP001626.1	1.018005	1.0157693	1.01985175	1.2588829	0.33214406	0.9693
CTD-2008A1.3	1.018004	1.0157693	1.01984953	1.2587419	0.3319825	0.9693
CXorf36	1.018008	1.0157693	1.0198576	1.2592539	0.33256925	0.9693
RSPH10B2	1.018015	1.0157693	1.0198695	1.2600087	0.33343372	0.9693
RP11-173C1.1	1.018014	1.0157693	1.0198684	1.2599386	0.33335348	0.9693
CATSPERD	1.017998	1.0157693	1.01983832	1.2580311	0.3311676	0.9693
AC093620.5	1.018013	1.0157693	1.01986553	1.259757	0.33314546	0.9693
RGAG1	1.018013	1.0157693	1.01986553	1.259757	0.33314546	0.9693
CALN1	1.018004	1.0157693	1.01984968	1.2587516	0.33199363	0.9693
SERPINB2	1.018021	1.0157693	1.01988024	1.2606896	0.33421306	0.9693
CASP10	1.018002	1.0157693	1.01984573	1.2585012	0.33170662	0.9693
CTA-150C2.13	1.01801	1.0157693	1.01986031	1.2594259	0.33276629	0.9693
IL20RA	1.018001	1.0157693	1.01984357	1.2583639	0.33154914	0.9693
TMEM78	1.018016	1.0157693	1.01987134	1.2601252	0.33356711	0.9693

RP11-387H17.6	1.018018	1.0157693	1.01987479	1.2603439	0.33381742	0.9693
AC016722.3	1.018015	1.0157693	1.01986973	1.2600233	0.33345037	0.9693
HAR1B	1.018027	1.0157693	1.01989087	1.2613634	0.33498403	0.9693
AC007193.9	1.018015	1.0157693	1.01987028	1.260058	0.33349012	0.9693
RP11-758P17.3	1.018008	1.0157693	1.01985707	1.2592199	0.33253029	0.9693
LINC00403	1.018021	1.0157693	1.01988027	1.2606918	0.33421558	0.9693
LINC00863	1.079499	1.0816598	1.07771384	0.9516784	-0.071454	0.9693
TMEM150A	1.292729	1.2952921	1.29061208	0.9841513	-0.0230479	0.96933
GLCE	1.232869	1.2304633	1.23485603	1.0190605	0.02723974	0.96939
DNAJC5	1.357405	1.3553777	1.35907849	1.0104136	0.01494595	0.96939
CD58	1.200654	1.2027673	1.19890914	0.9809724	-0.0277156	0.9694
RP11-186N15.3	1.020943	1.0187283	1.02277227	1.215927	0.28205659	0.96946
ICAM4	1.020912	1.0186802	1.02275452	1.2181066	0.28464044	0.96946
ALDH1L1	1.020923	1.0186591	1.02279205	1.2214996	0.28865337	0.96946
SIT1	1.020942	1.0187114	1.02278443	1.2176759	0.28413022	0.96946
LRRC29	1.09188	1.0941857	1.08997527	0.9552964	-0.0659796	0.96952
RP11-20I23.13	1.054365	1.0521579	1.05618689	1.0772469	0.10734897	0.96955
CTD-2020K17.1	1.02307	1.0252547	1.02126648	0.8420807	-0.2479696	0.96958
RP1-59D14.5	1.01701	1.0147553	1.01887175	1.2789819	0.35499585	0.96961
FAM86C1	1.100117	1.1023586	1.09826665	0.9600235	-0.0588583	0.96964
GRAMD1C	1.053321	1.0511242	1.05513588	1.0784699	0.10898594	0.96971
ACTA2-AS1	1.022482	1.0246391	1.0207014	0.8401851	-0.2512208	0.96972
CCDC130	1.43521	1.4375497	1.43327831	0.990238	-0.0141527	0.96975
NFIL3	1.078548	1.0807308	1.07674523	0.9506309	-0.0730428	0.96976
MPP3	1.075703	1.0779695	1.07383186	0.9469324	-0.0786667	0.96977
TERF2	1.147936	1.150242	1.14603125	0.9719733	-0.0410115	0.96978
CNST	1.145901	1.1482263	1.14398074	0.9713574	-0.0419258	0.96979
DDX21	1.912514	1.9150332	1.91043371	0.9949735	-0.00727	0.96983
CEACAM19	1.047099	1.0493797	1.04521546	0.9156697	-0.1271007	0.96984
PALM3	1.032685	1.0348686	1.03088104	0.8856401	-0.1752076	0.96984
TMEM222	1.401727	1.4040532	1.39980659	0.98949	-0.0152429	0.96985
THAP11	1.437662	1.4350379	1.43982924	1.0110135	0.01580233	0.96987
RNF135	1.229608	1.2270532	1.23171824	1.0205459	0.02934104	0.96987
RP11-351I24.1	1.024974	1.0272019	1.02313496	0.8504908	-0.2336324	0.96988
ASIP	1.101919	1.1040822	1.10013296	0.9620564	-0.0558066	0.96991
DCUN1D2	1.103042	1.1007419	1.1049411	1.0416825	0.05891563	0.96994
WDR72	1.036989	1.0390653	1.03527491	0.9029722	-0.1472465	0.96996
TRIM24	1.409868	1.4073603	1.41193981	1.011242	0.01612834	0.96999
ADIPOR2	1.405457	1.4030229	1.4074675	1.0110281	0.01582308	0.97001
ACRC	1.03966	1.0418963	1.037814	0.9025611	-0.1479035	0.97004
RP11-305L7.7	1.019748	1.01752	1.02158858	1.232225	0.30126568	0.97022
TP53TG5	1.019707	1.0175018	1.02152749	1.230013	0.29867354	0.97022
CMP21-97G8.1	1.019721	1.0175239	1.02153461	1.2288725	0.29733525	0.97022
CTC-301O7.4	1.019698	1.0175063	1.02150829	1.2286006	0.29701597	0.97022
ABCG1	1.019727	1.0175314	1.02153939	1.2286174	0.29703577	0.97022

BMPR1B-AS1	1.036644	1.0387984	1.03486439	0.8986037	-0.1542431	0.97024
AC007228.9	1.042042	1.0398006	1.04389355	1.1028374	0.14122013	0.97026
CRABP2	6.627125	6.6207482	6.6323914	1.0020715	0.0029854	0.97029
LRRTM2	1.045412	1.0476002	1.04360405	0.9160478	-0.1265052	0.97029
C7orf43	1.055265	1.0532108	1.05696157	1.0704882	0.09826884	0.9703
CTD-2538C1.2	1.055213	1.0529534	1.05707835	1.0778977	0.10822025	0.9703
RP11-31I22.2	1.017283	1.0150885	1.01909615	1.2656132	0.33983656	0.97031
AJ003147.9	1.017284	1.0150885	1.01909646	1.2656342	0.33986047	0.97031
NUP210L	1.0173	1.0150885	1.0191272	1.2676717	0.34218112	0.97031
SEC62-AS1	1.017302	1.0150885	1.0191298	1.2678434	0.34237658	0.97031
RP11-949J7.8	1.017293	1.0150885	1.01911427	1.2668141	0.34120487	0.97031
RP11-403I13.7	1.017284	1.0150885	1.01909677	1.2656546	0.33988371	0.97031
C11orf16	1.017273	1.0150885	1.01907623	1.2642933	0.33833123	0.97031
RP4-736L20.3	1.017291	1.0150885	1.01910938	1.26649	0.34083574	0.97031
SMARCA5-AS1	1.017285	1.0150885	1.01909972	1.2658504	0.34010697	0.97031
RP11-10A14.4	1.017295	1.0150885	1.01911648	1.2669609	0.34137202	0.97031
FAM47E-STBD1	1.017274	1.0150885	1.01907895	1.2644739	0.33853727	0.97031
LINC01314	1.017281	1.0150885	1.01909094	1.265268	0.33944303	0.97031
RP11-385F5.5	1.017269	1.0150885	1.01907009	1.2638862	0.33786652	0.97031
RP5-1180C18.1	1.017278	1.0150885	1.01908579	1.2649271	0.33905429	0.97031
AC022182.3	1.017277	1.0150885	1.01908473	1.2648567	0.33897398	0.97031
AC079767.4	1.017285	1.0150885	1.01909961	1.2658427	0.34009812	0.97031
TCP11	1.019002	1.0168225	1.0208023	1.2365739	0.30634843	0.97034
LINC00488	1.01899	1.0167824	1.02081287	1.2401611	0.31052749	0.97034
RFX8	1.019047	1.0168409	1.02086801	1.2391286	0.30932592	0.97034
AGL	1.294188	1.291924	1.29605846	1.0141628	0.02028926	0.9704
CTD-2587H24.5	1.024717	1.0225272	1.02652553	1.1774913	0.2357164	0.97046
RP5-1024G6.7	1.022143	1.0242808	1.02037691	0.8392207	-0.2528779	0.9705
CLTCL1	1.040149	1.0379562	1.04196066	1.1055023	0.14470206	0.97051
RP11-449J10.1	1.046454	1.0486014	1.04467983	0.9193109	-0.1213753	0.97051
LVRN	1.01787	1.0156733	1.01968394	1.2558905	0.32871073	0.97053
ACKR4	1.017851	1.0156795	1.01964371	1.2528261	0.32518621	0.97053
FAM19A1	1.017852	1.015672	1.01965292	1.2540169	0.32655679	0.97053
FAM124B	1.017857	1.0156836	1.01965198	1.2530304	0.32542138	0.97053
MIR137HG	1.017883	1.0156878	1.01969574	1.2554782	0.32823699	0.97053
AHSG	1.017888	1.0156834	1.01970814	1.2566273	0.32955684	0.97053
RP11-236L14.2	1.03041	1.0281492	1.03227773	1.1466678	0.19744743	0.97055
LINC00637	1.026142	1.0282709	1.02438362	0.8624989	-0.2134055	0.97072
RP11-334J6.7	1.023241	1.0254093	1.02145065	0.8442054	-0.244334	0.97074
CTA-212A2.3	1.023203	1.0253325	1.02144424	0.8465111	-0.240399	0.97074
LAT2	1.023196	1.0253094	1.02144989	0.8475065	-0.2387037	0.97074
LINC00858	1.020446	1.0225903	1.01867488	0.8266778	-0.2746029	0.97075
RP11-365O16.6	1.020456	1.022593	1.018692	0.8273344	-0.2734575	0.97075
RP11-498C9.3	1.020443	1.0225628	1.01869262	0.8284709	-0.271477	0.97075
KLF4	1.076339	1.0740983	1.07819026	1.0552232	0.07754813	0.9708

ZNF17	1.062723	1.0605108	1.06454998	1.0667521	0.09322493	0.97082
RGS22	1.083522	1.0858313	1.08161415	0.9508673	-0.0726841	0.97096
CH507-9B2.3	1.052733	1.0549667	1.05088909	0.9258174	-0.1112003	0.97101
PRKG2	1.020649	1.0184967	1.02242567	1.2124114	0.27787929	0.97105
RP11-1212A22.4	1.020648	1.018476	1.02244221	1.2146689	0.28056311	0.97105
RP5-875H18.9	1.020635	1.0184915	1.02240577	1.2116814	0.27701041	0.97105
RP4-728D4.2	1.020686	1.0184913	1.02249862	1.2167112	0.28298673	0.97105
C19orf73	1.140994	1.1432362	1.13914255	0.9714204	-0.0418323	0.97106
APOD	1.014524	1.0123699	1.01630316	1.3179722	0.39831994	0.97117
RP11-83M16.5	1.014532	1.0123699	1.01631813	1.3191828	0.39964447	0.97117
MMP10	1.014532	1.0123699	1.01631813	1.3191828	0.39964447	0.97117
DENND6A-AS1	1.014527	1.0123699	1.01630818	1.318378	0.39876411	0.97117
CHRNA6	1.014522	1.0123699	1.01629946	1.317673	0.39799243	0.97117
AC068831.16	1.014525	1.0123699	1.01630446	1.3180773	0.39843495	0.97117
AC084809.3	1.014514	1.0123699	1.01628469	1.3164793	0.39668479	0.97117
CTC-806A22.1	1.014519	1.0123699	1.01629334	1.3171783	0.39745065	0.97117
STX19	1.014521	1.0123699	1.01629703	1.3174772	0.39777801	0.97117
SULT1B1	1.014543	1.0123699	1.01633796	1.3207855	0.4013962	0.97117
TMPRSS4	1.014525	1.0123699	1.01630523	1.3181397	0.39850323	0.97117
GS1-304P7.3	1.014525	1.0123699	1.01630523	1.3181397	0.39850323	0.97117
RP3-467K16.7	1.014529	1.0123699	1.01631113	1.3186166	0.39902518	0.97117
CACNA1C-AS1	1.014533	1.0123699	1.01631891	1.3192454	0.39971299	0.97117
RP11-727A23.10	1.01452	1.0123699	1.01629619	1.3174087	0.39770299	0.97117
CCL18	1.014528	1.0123699	1.01630963	1.3184956	0.39889279	0.97117
AC010547.9	1.014547	1.0123699	1.0163458	1.3214197	0.40208872	0.97117
SLC35G6	1.01453	1.0123699	1.01631363	1.3188185	0.39924603	0.97117
RP11-12M5.3	1.014537	1.0123699	1.01632639	1.3198501	0.40037405	0.97117
AC009060.2	1.014537	1.0123699	1.01632639	1.3198501	0.40037405	0.97117
HR	1.014514	1.0123699	1.01628523	1.3165227	0.39673235	0.97117
RP11-440G5.2	1.014518	1.0123699	1.01629134	1.3170166	0.39727348	0.97117
CDH26	1.014512	1.0123699	1.01628099	1.3161803	0.39635713	0.97117
PKD1L1	1.014523	1.0123699	1.01630028	1.3177397	0.39806545	0.97117
TIMM8A	1.283912	1.2815731	1.2858442	1.0151689	0.02171974	0.97119
MAPK11	1.08718	1.0849395	1.08902995	1.0481576	0.06785566	0.9712
RP11-116D17.1	1.015371	1.0132186	1.01714933	1.2973617	0.37558073	0.97126
AC009264.1	1.0154	1.0132287	1.01719364	1.2997199	0.37820072	0.97126
RP13-514E23.2	1.015384	1.0132168	1.01717382	1.2993884	0.37783277	0.97126
RP11-159D12.10	1.015374	1.0132213	1.01715116	1.2972341	0.37543886	0.97126
AC137932.6	1.015378	1.0132207	1.01715874	1.2978716	0.37614769	0.97126
RP11-764K9.1	1.015388	1.0132171	1.01718053	1.299871	0.37836846	0.97126
RP11-345P4.10	1.015369	1.0132213	1.01714241	1.2965725	0.37470286	0.97126
C10orf105	1.015365	1.0132175	1.0171387	1.2966667	0.37480775	0.97126
PURG	1.172981	1.1708207	1.17476579	1.0230951	0.03294029	0.97129
SAV1	1.491948	1.490034	1.49352854	1.0071312	0.01025171	0.97134
STAC2	1.029245	1.0270638	1.03104633	1.1471534	0.1980583	0.97136

RP11-996F15.5	1.029193	1.027032	1.03097724	1.1459487	0.19654247	0.97136
ZNF548	1.077314	1.0751943	1.07906392	1.0514615	0.07239597	0.97138
RP11-15E1.5	1.01351	1.0113663	1.01527977	1.3443032	0.42685861	0.97139
MID1IP1-AS1	1.013511	1.0113663	1.01528198	1.344497	0.42706655	0.97139
RP3-428L16.1	1.013514	1.0113663	1.01528681	1.3449222	0.42752268	0.97139
DAW1	1.013507	1.0113663	1.01527547	1.3439243	0.42645191	0.97139
APBB1IP	1.013505	1.0113663	1.01527029	1.343469	0.42596304	0.97139
RP11-653J6.1	1.013516	1.0113663	1.01529153	1.3453377	0.42796839	0.97139
ATP13A5	1.01352	1.0113663	1.01529822	1.3459257	0.42859876	0.97139
RP11-214N1.1	1.013509	1.0113663	1.01527895	1.3442309	0.42678099	0.97139
AC114765.1	1.013515	1.0113663	1.0152889	1.3451059	0.42771972	0.97139
AP000473.5	1.013505	1.0113663	1.01527174	1.343596	0.42609946	0.97139
ANXA13	1.013511	1.0113663	1.01528223	1.3445196	0.42709082	0.97139
S100Z	1.013504	1.0113663	1.01526988	1.3434331	0.42592448	0.97139
TNFSF15	1.013503	1.0113663	1.01526703	1.3431817	0.42565449	0.97139
PYDC1	1.013507	1.0113663	1.01527442	1.3438319	0.42635269	0.97139
MSC	1.013503	1.0113663	1.01526772	1.3432428	0.42572015	0.97139
DDX4	1.013518	1.0113663	1.01529401	1.3455553	0.42820172	0.97139
CLRN3	1.013502	1.0113663	1.01526654	1.3431386	0.42560815	0.97139
EDRF1-AS1	1.013504	1.0113663	1.01526886	1.3433434	0.4258282	0.97139
GPC6-AS1	1.013512	1.0113663	1.01528332	1.3446152	0.42719337	0.97139
RP4-585I14.3	1.013524	1.0113663	1.01530655	1.3466592	0.42938475	0.97139
TM4SF19	1.013501	1.0113663	1.01526406	1.3429209	0.42537435	0.97139
PLEK2	1.013512	1.0113663	1.01528365	1.3446438	0.42722409	0.97139
RP11-472N13.2	1.013512	1.0113663	1.01528411	1.3446843	0.42726747	0.97139
HPDL	1.013513	1.0113663	1.01528502	1.3447647	0.42735376	0.97139
TBXA2R	1.013508	1.0113663	1.01527626	1.3439937	0.42652634	0.97139
RP11-856F16.2	1.013518	1.0113663	1.01529403	1.3455572	0.42820371	0.97139
LINC01304	1.013509	1.0113663	1.01527851	1.3441923	0.42673955	0.97139
RP11-676J12.6	1.013536	1.0113663	1.01532688	1.3484478	0.43129967	0.97139
ARMC2	1.051575	1.0493804	1.05338806	1.0811594	0.11257924	0.97139
RP1-223E5.4	1.058464	1.0606138	1.05668869	0.9352436	-0.096586	0.97144
ZFYVE28	1.028521	1.0263931	1.03027884	1.1472236	0.19814657	0.97144
RBM5-AS1	1.026811	1.0246589	1.02858816	1.1593459	0.21331106	0.97149
ATG2B	1.157776	1.1555924	1.15958	1.0256288	0.03650868	0.9715
C11orf95	1.275979	1.2739539	1.2776507	1.0134943	0.01933802	0.97151
FAM13A-AS1	1.02728	1.0293719	1.02555269	0.86997	-0.2009624	0.97155
RAB40A	1.035022	1.0371172	1.03329133	0.8969252	-0.1569404	0.97159
QRICH1	1.290891	1.2932262	1.28896294	0.9854609	-0.0211294	0.97159
KIAA1161	1.034195	1.0320443	1.03597135	1.1225497	0.16677936	0.97163
SLC25A35	1.128888	1.1310204	1.12712696	0.9702839	-0.0435211	0.97168
RALB	1.402481	1.404654	1.40068568	0.9901934	-0.0142178	0.97172
BEND4	1.050916	1.0530475	1.04915521	0.9266269	-0.1099396	0.97173
ZNF622	1.28334	1.2809916	1.28527914	1.0152587	0.02184742	0.97177
FOXG1-AS1	1.020584	1.0226648	1.0188648	0.83234	-0.2647551	0.97181

RP11-640N11.2	1.020643	1.0226789	1.01896235	0.8361245	-0.2582104	0.97181
GPT	1.017948	1.0200175	1.01623936	0.8112601	-0.3017636	0.97181
SH3GL2	1.017988	1.0200796	1.01626145	0.8098503	-0.3042728	0.97181
MATN1	1.017977	1.0200546	1.0162612	0.8108447	-0.3025024	0.97181
RIBC2	1.063259	1.0653432	1.06153725	0.9417542	-0.0865776	0.97183
AC004951.6	1.126794	1.1291491	1.1248483	0.9666991	-0.0488612	0.97187
AC093375.1	1.131661	1.1337536	1.12993205	0.9714281	-0.0418209	0.97193
RP11-1008C21.1	1.014386	1.0122555	1.01614612	1.3174612	0.39776051	0.97195
CIRBP-AS1	1.014371	1.0122555	1.01611741	1.3151185	0.39519279	0.97195
RP11-264L1.4	1.014389	1.0122555	1.01614999	1.3177769	0.39810616	0.97195
ART4	1.014383	1.0122555	1.01614038	1.3169928	0.39724746	0.97195
RP11-728E14.3	1.014388	1.0122555	1.01614957	1.317743	0.39806901	0.97195
AC006273.5	1.014369	1.0122555	1.01611371	1.3148168	0.39486175	0.97195
AP000355.2	1.014372	1.0122555	1.01612037	1.3153605	0.3954583	0.97195
SLC30A3	1.014376	1.0122555	1.01612799	1.3159823	0.39614004	0.97195
C12orf80	1.014371	1.0122555	1.01611847	1.3152054	0.39528809	0.97195
NCKAP1L	1.014389	1.0122555	1.01615144	1.3178956	0.39823606	0.97195
ALDH1L1-AS1	1.014381	1.0122555	1.01613648	1.3166746	0.39689888	0.97195
KLKB1	1.014378	1.0122555	1.01613044	1.3161819	0.39635885	0.97195
FAM83E	1.014383	1.0122555	1.01614021	1.3169789	0.3972322	0.97195
KLHL10	1.014394	1.0122555	1.01615971	1.3185704	0.39897456	0.97195
RP5-1177M21.1	1.014394	1.0122555	1.01615971	1.3185704	0.39897456	0.97195
AC092580.4	1.014367	1.0122555	1.0161113	1.3146206	0.3946465	0.97195
GS1-259H13.13	1.014382	1.0122555	1.01613753	1.3167608	0.39699328	0.97195
RP11-304F15.6	1.014377	1.0122555	1.01612812	1.3159931	0.39615197	0.97195
CYP27B1	1.014377	1.0122555	1.01612812	1.3159931	0.39615197	0.97195
SERINC4	1.014367	1.0122555	1.01611076	1.3145766	0.39459816	0.97195
AC104667.3	1.01438	1.0122555	1.01613509	1.3165614	0.39677476	0.97195
RP11-34P13.8	1.01439	1.0122555	1.01615299	1.3180223	0.39837479	0.97195
MROH7	1.025647	1.0277585	1.02390358	0.8611274	-0.2157015	0.97199
NDST3	1.026466	1.0286205	1.02468639	0.8625432	-0.2133313	0.97212
LAMC3	1.026419	1.0284797	1.02471764	0.8679032	-0.204394	0.97212
RP11-245J9.4	1.021904	1.0197879	1.0236506	1.1952037	0.25725653	0.97212
RP11-363E6.3	1.021911	1.0197837	1.02366758	1.1963192	0.25860239	0.97212
SNPH	1.030131	1.0280244	1.03187042	1.1372398	0.18553647	0.97215
RP11-893F2.13	1.030193	1.0280657	1.03194899	1.1383648	0.18696301	0.97215
DEDD2	1.450503	1.4527048	1.44868393	0.991118	-0.0128713	0.97218
SLC25A42	1.313613	1.3157219	1.31187146	0.9878044	-0.0177028	0.9722
GTF3C2	1.312842	1.3147414	1.31127373	0.9889824	-0.0159832	0.9722
WFIKKN2	1.040391	1.0425736	1.03858895	0.9064045	-0.141773	0.97222
AF213884.2	1.027453	1.0295529	1.02571802	0.8702363	-0.2005209	0.97225
AC061992.2	1.027428	1.0295215	1.02569949	0.8705349	-0.200026	0.97225
ABHD15	1.095771	1.0979995	1.0939299	0.958473	-0.0611904	0.97228
ALYREF	1.095728	1.0978103	1.09400908	0.9611366	-0.0571866	0.97228
CCDC82	1.511056	1.5135374	1.50900767	0.9911793	-0.012782	0.9723

MRO	1.023975	1.0218605	1.02572157	1.176621	0.23464969	0.9723
RP11-378A13.2	1.023936	1.0218188	1.02568394	1.177145	0.23529208	0.9723
SUCLA2-AS1	1.023969	1.0218559	1.02571453	1.176547	0.23455893	0.9723
NTN1	1.023956	1.0218347	1.02570732	1.1773605	0.23555613	0.9723
AFF2	1.05835	1.0563275	1.06001987	1.0655527	0.09160199	0.97231
NEUROG1	1.021889	1.0197744	1.02363615	1.1952908	0.25736162	0.97232
HBB	1.021871	1.0197498	1.0236231	1.1961176	0.25835924	0.97232
FBLL1	1.021894	1.0197732	1.02364449	1.1957819	0.25795423	0.97232
EGR3	1.021864	1.019749	1.02361053	1.1955318	0.25765248	0.97232
UBE2O	1.249279	1.2470229	1.2511423	1.0166761	0.02386011	0.97232
CTB-55O6.10	1.014113	1.0120052	1.01585284	1.3205031	0.40108773	0.97234
AP001172.2	1.014118	1.0120052	1.01586263	1.3213182	0.4019779	0.97234
RP11-91P24.6	1.014118	1.0120052	1.01586332	1.3213754	0.4020404	0.97234
MIR7515HG	1.014118	1.0120052	1.01586332	1.3213754	0.4020404	0.97234
CTD-2550O8.5	1.01413	1.0120052	1.01588383	1.3230842	0.40390485	0.97234
C19orf38	1.014108	1.0120052	1.01584457	1.3198138	0.40033438	0.97234
CHKB-CPT1B	1.01411	1.0120052	1.0158487	1.3201583	0.40071098	0.97234
CES1	1.014115	1.0120052	1.01585813	1.3209437	0.40156895	0.97234
RP11-153K11.3	1.014106	1.0120052	1.01584087	1.3195058	0.39999769	0.97234
RP11-264B17.2	1.014117	1.0120052	1.01586045	1.3211365	0.40177952	0.97234
C16orf54	1.014127	1.0120052	1.01587912	1.3226921	0.40347729	0.97234
CTD-2358C21.5	1.014121	1.0120052	1.0158684	1.321799	0.4025028	0.97234
FAM87A	1.014112	1.0120052	1.01585137	1.3203804	0.40095368	0.97234
AC108004.3	1.014112	1.0120052	1.01585099	1.3203483	0.40091859	0.97234
CCDC122	1.208489	1.2107648	1.20661031	0.9802884	-0.0287219	0.97235
RP11-706O15.5	1.024011	1.0219026	1.02575148	1.1757283	0.23355469	0.97236
VIM	95.26241	95.373665	95.1705348	0.9978476	-0.0031086	0.97241
RP11-775C24.5	1.025367	1.0274249	1.02366742	0.8629893	-0.2125855	0.97243
CTB-133G6.2	1.025397	1.0274618	1.02369161	0.8627106	-0.2130515	0.97243
CDC42EP2	1.07404	1.0719091	1.07579889	1.0540931	0.07600227	0.97244
PRMT8	1.01788	1.0199712	1.01615299	0.8088134	-0.3061212	0.97248
RP4-633O19__A.1	1.017839	1.0198763	1.01615642	0.8128466	-0.298945	0.97248
CTD-2013N17.7	1.017834	1.0198922	1.01613375	0.8110582	-0.3021226	0.97248
MED14	1.208291	1.2060582	1.2101341	1.0197803	0.02825843	0.97256
CTA-212A2.2	1.022185	1.0200588	1.0239411	1.1935477	0.25525627	0.97256
RP11-16C1.1	1.022171	1.0200641	1.02391076	1.1917182	0.25304316	0.97256
NPIPB13	1.022197	1.020066	1.02395762	1.1939439	0.255735	0.97256
GDA	1.022217	1.0201388	1.02393366	1.1884374	0.24906593	0.97256
RP11-453A12.1	1.021627	1.0195014	1.0233822	1.1990016	0.26183359	0.9726
MRAP2	1.021608	1.0194961	1.02335216	1.197789	0.26037378	0.9726
LTB4R2	1.02165	1.0195193	1.02341	1.1993231	0.26222042	0.9726
SLC6A12	1.021657	1.0195434	1.02340194	1.1974358	0.25994831	0.9726
RP5-1186P10.2	1.014526	1.0124282	1.01625753	1.3081213	0.38749627	0.97262
RP11-15H20.7	1.014517	1.0124282	1.01624192	1.3068648	0.38610994	0.97262
AC006449.2	1.014538	1.0124282	1.01628071	1.3099861	0.38955147	0.97262

RP11-249L12.1	1.014538	1.0124282	1.01628071	1.3099861	0.38955147	0.97262
RP11-218E20.5	1.014528	1.0124282	1.01626163	1.3084513	0.3878602	0.97262
C1QL3	1.014519	1.0124282	1.01624512	1.3071224	0.38639429	0.97262
SLC15A3	1.014543	1.0124282	1.01628882	1.3106388	0.39027011	0.97262
RP11-196H14.3	1.014534	1.0124282	1.01627321	1.3093828	0.38888689	0.97262
IL1B	1.014517	1.0124282	1.01624276	1.3069328	0.38618499	0.97262
RP1-59D14.1	1.014524	1.0124282	1.01625551	1.3079587	0.38731694	0.97262
KCCAT211	1.014536	1.0124282	1.01627742	1.3097218	0.3892604	0.97262
HRH4	1.014519	1.0124282	1.01624567	1.3071671	0.38644359	0.97262
RP11-532L16.3	1.014521	1.0124282	1.01624841	1.3073871	0.38668641	0.97262
DAZL	1.014525	1.0124282	1.01625709	1.3080854	0.38745672	0.97262
SPANXD	1.014512	1.0124282	1.01623352	1.3061895	0.38536424	0.97262
FETUB	1.014533	1.0124282	1.01627034	1.3091516	0.38863219	0.97262
RP11-77H9.5	1.014525	1.0124282	1.01625752	1.3081203	0.38749526	0.97262
RP11-968A15.2	1.014516	1.0124282	1.01624075	1.3067714	0.38600677	0.97262
CYP17A1-AS1	1.014525	1.0124282	1.01625699	1.3080773	0.38744785	0.97262
LKAAEAR1	1.033649	1.0357774	1.03189059	0.8913606	-0.1659189	0.97266
CCDC182	1.017146	1.0150401	1.01888474	1.2556264	0.3284073	0.97269
RP11-624J6.2	1.017153	1.0150556	1.01888569	1.2543949	0.32699158	0.97269
ESAM	1.017135	1.0150449	1.01886095	1.253648	0.32613233	0.97269
LINC00843	1.017147	1.0150508	1.01887746	1.2542518	0.32682695	0.97269
DLEC1	1.017144	1.0150512	1.01887301	1.2539184	0.32644344	0.97269
SMPX	1.017141	1.0150641	1.01885564	1.2516928	0.32388058	0.97269
RP11-757F18.5	1.017143	1.0150448	1.01887555	1.2546266	0.3272581	0.97269
FGL2	1.017164	1.0150457	1.01891348	1.2570697	0.33006461	0.97269
DAPP1	1.017127	1.0150411	1.01884917	1.2531749	0.32558782	0.97269
LINC01270	1.01715	1.0150448	1.01888767	1.2554323	0.32818419	0.97269
RP11-162A12.4	1.017135	1.0150489	1.01885824	1.2531296	0.32553563	0.97269
CFAP100	1.017152	1.0150633	1.01887746	1.2532072	0.32562496	0.97269
AC005616.2	1.017191	1.0150429	1.0189655	1.2607619	0.33429588	0.97269
GET4	1.01715	1.0150381	1.01889419	1.2564229	0.3293221	0.97269
RP11-514P8.6	1.01716	1.0150529	1.01889917	1.2555137	0.3282778	0.97269
SLC10A4	1.01714	1.0150467	1.0188683	1.2539849	0.32651998	0.97269
RP11-693J15.5	1.053686	1.05571	1.05201505	0.9336759	-0.0990062	0.97275
CTD-2349P21.9	1.046528	1.0444673	1.04823062	1.0846312	0.11720453	0.97279
SLC16A13	1.036907	1.0348612	1.03859633	1.1071414	0.1468395	0.97279
TGFBR3	1.072673	1.0706548	1.07433957	1.0521523	0.07334361	0.97288
MYO18A	1.082966	1.0808053	1.08474998	1.048817	0.06876294	0.97297
TMEM65	1.201568	1.2039239	1.19962218	0.9789051	-0.030759	0.97301
AC144652.1	1.060947	1.0587913	1.0627263	1.0669314	0.09346745	0.97302
FBXL6	1.063671	1.0657534	1.06195114	0.9421737	-0.085935	0.97303
RP11-171I2.2	1.016955	1.0189763	1.01528588	0.8055266	-0.3119959	0.97304
RP11-723O4.9	1.016961	1.0190032	1.01527526	0.8038274	-0.3150423	0.97304
RP5-1186N24.3	1.030833	1.0328748	1.02914746	0.88662	-0.1736122	0.97309
CCDC191	1.171205	1.173124	1.16961974	0.9797589	-0.0295013	0.97313

CHKA	1.171048	1.173086	1.1693643	0.9784977	-0.0313596	0.97313
RP4-669L17.10	1.023853	1.02175	1.02558967	1.1765377	0.2345475	0.97314
C6orf223	1.024531	1.0224709	1.02623294	1.167419	0.22332251	0.97317
LILRB3	1.030291	1.0323147	1.02861918	0.8856382	-0.1752106	0.9732
PCDHA11	1.030248	1.0323356	1.02852461	0.8821421	-0.180917	0.9732
DLGAP4-AS1	1.063863	1.0658798	1.06219695	0.9440981	-0.0829914	0.97322
CTC-523E23.11	1.031453	1.0293899	1.03315754	1.1281943	0.1740156	0.97323
CIC	1.358559	1.3566613	1.36012684	1.0097167	0.01395061	0.97324
RP11-902B17.1	1.017998	1.0200587	1.01629541	0.812387	-0.299761	0.97326
ADORA2A-AS1	1.017956	1.0199674	1.01629509	0.8160861	-0.2932068	0.97326
CAPRIN2	1.428851	1.4312929	1.4268335	0.9896603	-0.0149947	0.9733
SLC22A23	1.13151	1.1293057	1.1333302	1.0311242	0.04421809	0.97332
FGF5	1.033576	1.0316008	1.03520714	1.1141203	0.15590503	0.97341
GJD2	1.033522	1.0315076	1.03518497	1.116714	0.1592597	0.97341
PODN	1.033491	1.031472	1.03515796	1.1171178	0.15978133	0.97341
ZNF252P-AS1	1.03141	1.0293398	1.03311984	1.1288366	0.17483663	0.97342
ADAR	1.937708	1.9354038	1.93961106	1.0044978	0.00647443	0.97345
AC097724.3	1.024123	1.0261536	1.02244637	0.8582519	-0.220527	0.9735
RP11-620J15.4	1.033488	1.0314142	1.03520143	1.1205572	0.16421634	0.97353
RP4-593H12.1	1.02361	1.0215476	1.02531227	1.1747143	0.23230994	0.97353
RP11-80A15.1	1.023545	1.021465	1.02526177	1.176881	0.23496844	0.97353
HIST3H2BB	1.034443	1.0323823	1.03614533	1.1162064	0.15860386	0.97353
CYP3A5	1.103807	1.1016164	1.10561643	1.0393645	0.05570165	0.97365
U2AF1	1.089414	1.0914713	1.08771533	0.9589386	-0.0604896	0.97366
LRRC37A3	1.096234	1.0941052	1.09799257	1.0413088	0.05839791	0.97367
PRR5	1.241357	1.239126	1.24319896	1.0170327	0.02436612	0.97369
ZNF134	1.379981	1.3782649	1.38139843	1.0082839	0.01190184	0.97375
RP11-613D13.5	1.016984	1.0149319	1.01867869	1.2509285	0.32299938	0.97375
ACKR1	1.016989	1.0149268	1.01869222	1.2522604	0.32453464	0.97375
AC017002.2	1.016978	1.0149371	1.01866298	1.2494416	0.32128345	0.97375
CYP51A1-AS1	1.016987	1.014937	1.0186792	1.2505349	0.32254527	0.97375
SPG20-AS1	1.016995	1.014932	1.01869882	1.2522618	0.32453616	0.97375
SRA1	1.547841	1.5502569	1.54584677	0.9919853	-0.0116093	0.97375
FZR1	1.237715	1.2396678	1.23610216	0.9851225	-0.021625	0.97375
ERICH3	1.019722	1.0176758	1.02141199	1.2113762	0.276647	0.97376
S100P	1.019726	1.01768	1.02141483	1.2112443	0.27648991	0.97376
TM6SF1	1.019739	1.0176744	1.02144365	1.2132578	0.27888612	0.97376
FDXACB1	1.052399	1.0543868	1.05075763	0.9332707	-0.0996325	0.9738
RP11-219B17.3	1.024005	1.021926	1.0257221	1.1731311	0.23036424	0.97381
STYXL1	1.330818	1.3285746	1.33267131	1.0124682	0.01787665	0.97383
RP11-962G15.1	1.026664	1.0246104	1.02835955	1.1523402	0.20456664	0.97383
RP4-555D20.4	1.026667	1.0246437	1.02833766	1.1498942	0.20150108	0.97383
CARD8-AS1	1.026646	1.0245545	1.02837402	1.1555543	0.20858501	0.97383
CTC-260E6.6	1.135018	1.1373956	1.13305462	0.9684051	-0.0463174	0.97386
AC026202.3	1.022626	1.0205972	1.0243012	1.1798314	0.23858074	0.9739

LSAMP-AS1	1.022633	1.0205888	1.02432024	1.1812336	0.24029435	0.9739
AC093382.1	1.02266	1.0206139	1.02435	1.1812412	0.24030362	0.9739
AC104654.2	1.022649	1.0205943	1.02434599	1.1821727	0.24144085	0.9739
RHCG	1.022647	1.0206049	1.02433381	1.180972	0.2399748	0.9739
CTD-2588E21.1	1.022632	1.0205711	1.02433419	1.1829287	0.24236314	0.9739
ZNF879	1.080963	1.0829782	1.0792992	0.9556631	-0.065426	0.97401
SLC25A20	1.076765	1.0787667	1.07511141	0.9535931	-0.0685542	0.97405
NDOR1	1.018685	1.0166494	1.02036682	1.2232769	0.29075098	0.97406
COL28A1	1.01871	1.0166461	1.0204139	1.2263469	0.29436712	0.97406
RP1-56K13.5	1.018696	1.0166415	1.02039179	1.2253549	0.29319966	0.97406
RP11-426L16.3	1.055075	1.0570631	1.05343354	0.9363944	-0.0948118	0.97407
LL22NC03-32F9.1	1.044567	1.0465439	1.0429346	0.9224538	-0.1164515	0.97407
TSGA10IP	1.031004	1.0330023	1.02935321	0.8894297	-0.1690474	0.97422
RERE	2.079123	2.0768493	2.08100102	1.0038554	0.00555153	0.97425
SERHL2	1.052259	1.0542143	1.05064492	0.934162	-0.0982554	0.97425
NR6A1	1.162966	1.1650967	1.16120651	0.976437	-0.0344011	0.97426
AC104653.1	1.077396	1.075323	1.07910833	1.050255	0.07073968	0.97427
CTA-14H9.5	1.077203	1.0751678	1.07888369	1.0494342	0.06961173	0.97427
MTUS2	1.023144	1.0251012	1.02152804	0.8576498	-0.2215394	0.97428
RP5-1158E12.3	1.023193	1.0252475	1.02149581	0.8514052	-0.2320822	0.97428
FBXO45	1.152308	1.1503197	1.15395082	1.0241559	0.03443532	0.97433
SLAIN2	1.465191	1.4676049	1.46319809	0.9905759	-0.0136606	0.97438
PTP4A2	2.751136	2.7544736	2.74837892	0.9965262	-0.0050204	0.97448
LZTS1	1.060395	1.0623826	1.05875353	0.9418255	-0.0864683	0.97449
ZNF850	1.042395	1.0444208	1.04072211	0.9167347	-0.1254238	0.97462
RP11-631N16.4	1.043828	1.0418324	1.04547541	1.0870847	0.12046435	0.97465
RP11-930O11.3	1.021466	1.0194147	1.02316028	1.1929244	0.25450265	0.97467
CEP295NL	1.021468	1.0194712	1.0231175	1.1872644	0.24764125	0.97467
RHD	1.021431	1.0194121	1.02309796	1.1898761	0.25081136	0.97467
LINC00907	1.021412	1.0194023	1.0230712	1.1890932	0.24986176	0.97467
RP11-977P2.1	1.021445	1.0194542	1.02308981	1.1868794	0.24717331	0.97467
ELOVL3	1.033094	1.0310132	1.03481223	1.1224964	0.16671087	0.97471
TAS1R3	1.019003	1.0169849	1.02066953	1.2169363	0.28325368	0.97477
AC008067.2	1.019012	1.0169919	1.02068008	1.2170548	0.28339409	0.97477
RP11-158I9.5	1.019014	1.0169974	1.02067923	1.2166127	0.28286992	0.97477
COL4A3	1.019018	1.016985	1.02069622	1.218501	0.28510741	0.97477
PDZRN3-AS1	1.019008	1.0169873	1.02067662	1.2171824	0.28354537	0.97477
LINC00854	1.01901	1.0169925	1.02067541	1.2167362	0.28301645	0.97477
Z83844.1	1.020729	1.0187062	1.02239994	1.1974623	0.25998021	0.97478
PLIN1	1.020488	1.0225011	1.01882609	0.836675	-0.2572607	0.97489
LACTB	1.307728	1.305427	1.30962752	1.013753	0.01970621	0.9749
CC2D1B	1.079173	1.0770003	1.08096797	1.0515286	0.07248806	0.9749
MRVI1-AS1	1.032152	1.0301777	1.03378255	1.1194543	0.16279568	0.97497
CD79A	1.019582	1.0175731	1.02124159	1.2087557	0.27352274	0.97497
INHBE	1.019577	1.0175884	1.02121908	1.2064216	0.27073411	0.97497

RP11-582J16.4	1.019586	1.017582	1.02124039	1.208077	0.27271236	0.97497
HSPA1A	1.019592	1.0175658	1.0212646	1.2105682	0.27568441	0.97497
TMSB15B	1.493351	1.4911959	1.49513075	1.0080108	0.01151109	0.97501
GABRA2	1.10545	1.1074977	1.1037594	0.9652242	-0.051064	0.97508
AP000221.1	1.024368	1.0262761	1.02279191	0.8674019	-0.2052275	0.9751
RP11-328P23.4	1.021464	1.0234231	1.01984646	0.8473037	-0.2390489	0.97514
MEI1	1.021488	1.0234768	1.01984503	0.8453028	-0.2424599	0.97514
KCNH8	1.021486	1.0234596	1.01985679	0.8464253	-0.2405454	0.97514
SPHKAP	1.0187	1.0206229	1.01711266	0.8297908	-0.2691805	0.97515
TUT1	1.15342	1.1552228	1.15193157	0.9787967	-0.0309188	0.97516
PKP2	1.18035	1.1824178	1.17864307	0.9793072	-0.0301666	0.97527
BTC	1.025744	1.0277205	1.02411196	0.8698236	-0.2012052	0.97528
GOLGA8Q	1.028353	1.0302918	1.02675184	0.8831365	-0.1792916	0.9753
RP11-315O6.1	1.042834	1.0408065	1.04450841	1.090719	0.12527952	0.97548
RASSF1-AS1	1.093371	1.0953714	1.09171973	0.9617114	-0.0563241	0.97548
LGALS9	1.022393	1.0203936	1.02404366	1.1789833	0.23754332	0.97549
SPAG9	2.1532	2.1560905	2.15081233	0.9954344	-0.0066018	0.97556
KLF9	1.102735	1.1008389	1.1043011	1.0343338	0.04870186	0.97557
IFT140	1.170979	1.1729357	1.1693623	0.9793369	-0.0301228	0.97561
CTC-420A11.2	1.01629	1.014262	1.01796469	1.2596164	0.33298444	0.97563
CIITA	1.016262	1.0142725	1.01790437	1.2544667	0.3270742	0.97563
LINC00242	1.016241	1.0142685	1.01786956	1.2523805	0.32467299	0.97563
NODAL	1.016251	1.0142712	1.01788607	1.2532994	0.32573114	0.97563
PLCB3	1.159661	1.1577011	1.16127919	1.0226889	0.03236738	0.97564
CCL25	1.017097	1.0151415	1.01871278	1.2358623	0.30551804	0.97571
ENTPD2	1.017098	1.0151334	1.01872056	1.2370358	0.30688721	0.97571
RP11-251G23.5	1.017105	1.015136	1.01873061	1.2374846	0.30741056	0.97571
LINC00976	1.017124	1.0151616	1.0187442	1.2362903	0.30601751	0.97571
KB-1043D8.6	1.017093	1.0151153	1.01872535	1.2388382	0.30898776	0.97571
RP11-755F10.1	1.017111	1.0151274	1.01874881	1.2393945	0.30963541	0.97571
CTD-2035E11.3	1.017108	1.0151258	1.01874528	1.2392948	0.30951946	0.97571
SCNN1D	1.056807	1.0588681	1.05510425	0.9360632	-0.0953221	0.97575
LINC01621	1.035233	1.0371659	1.0336363	0.9050313	-0.1439605	0.97579
WEE2-AS1	1.026236	1.028145	1.02465957	0.8761627	-0.1907292	0.97583
ATG5	1.472484	1.4700999	1.47445324	1.0092605	0.01329862	0.97584
ASCL5	1.015221	1.0132631	1.01683693	1.2694529	0.34420692	0.97585
ZNF260	1.353947	1.3518508	1.35567748	1.0108758	0.01560576	0.97585
ENGASE	1.110323	1.1121788	1.10879024	0.9697934	-0.0442506	0.97587
C5orf22	1.116049	1.1180555	1.1143928	0.9689751	-0.0454685	0.97588
RYR2	1.028499	1.0265481	1.03010946	1.134147	0.18160768	0.97593
EFHB	1.072161	1.0740827	1.0705749	0.9526504	-0.0699813	0.97604
MYBL2	1.310589	1.3124708	1.30903506	0.9890046	-0.0159509	0.97607
FUT2	1.029144	1.0271092	1.03082441	1.1370455	0.18529004	0.97609
LYG2	1.043082	1.0411	1.04471917	1.0880585	0.12175612	0.97615
KRBOX1	1.018873	1.0207762	1.01730207	0.8327825	-0.2639884	0.97621

RP11-182J1.5	1.018886	1.0207874	1.01731647	0.8330259	-0.2635667	0.97621
CDX4	1.018865	1.0207507	1.01730858	0.8341192	-0.2616746	0.97621
CAPN14	1.018873	1.0207924	1.01728758	0.8314365	-0.266322	0.97621
FLJ13224	1.018862	1.0207678	1.01728897	0.8324895	-0.264496	0.97621
RP11-507K2.2	1.018868	1.0207492	1.01731424	0.8344532	-0.2610969	0.97621
ARHGAP22	1.01624	1.0181323	1.01467703	0.809443	-0.3049985	0.97623
AP001092.4	1.016237	1.0181209	1.01468092	0.810167	-0.3037088	0.97623
AC007906.1	1.01628	1.0182122	1.01468406	0.8062753	-0.3106556	0.97623
RP4-673M15.1	1.016258	1.0181681	1.0146812	0.808075	-0.307439	0.97623
PHF20	1.611219	1.6088383	1.6131849	1.0071392	0.01026303	0.97627
ATF7IP2	1.314357	1.31218	1.31615561	1.0127348	0.01825647	0.9763
RP11-63A1.1	1.023378	1.0253004	1.02179036	0.861267	-0.2154676	0.97635
RP11-732A21.2	1.023365	1.0252818	1.02178153	0.8615497	-0.2149942	0.97635
USP2-AS1	1.0239	1.0258006	1.0223296	0.8654683	-0.2084472	0.97638
RP11-700J17.1	1.023907	1.0258265	1.02232215	0.8643107	-0.2103781	0.97638
RP5-1112D6.7	1.084111	1.0860996	1.08246911	0.9578337	-0.0621529	0.97639
U2AF1L4	1.190677	1.1887751	1.19224728	1.0183932	0.02629464	0.97639
GNE	1.069859	1.0718748	1.0681947	0.9487985	-0.0758264	0.97641
CYSLTR2	1.0161	1.0141584	1.01770416	1.2504377	0.32243314	0.97641
HEPN1	1.016096	1.0141518	1.01770136	1.2508176	0.32287143	0.97641
GGT6	1.016092	1.014159	1.01768806	1.2492426	0.32105373	0.97641
AC007879.2	1.016096	1.0141472	1.01770452	1.2514535	0.32360467	0.97641
KLHL34	1.016095	1.0141555	1.01769609	1.2501239	0.32207103	0.97641
AC092881.1	1.016109	1.014157	1.0177207	1.2517239	0.32391637	0.97641
RP4-569M23.5	1.01611	1.0141576	1.01772161	1.2517359	0.32393021	0.97641
AC009410.1	1.016093	1.0141583	1.01769138	1.2495428	0.32140029	0.97641
RP11-329J18.3	1.016104	1.0141465	1.01772099	1.2526752	0.32501244	0.97641
TPSB2	1.016102	1.0141607	1.01770453	1.2502574	0.32222509	0.97641
RP11-110I1.14	1.016107	1.0141483	1.01772464	1.2527767	0.32512924	0.97641
RP1-137D17.2	1.01583	1.0177105	1.01427749	0.8061611	-0.31086	0.9765
TEX29	1.015826	1.0176937	1.01428393	0.8072875	-0.3088455	0.9765
RP11-538D16.2	1.015862	1.0177837	1.01427455	0.8026774	-0.3171078	0.9765
CTD-2287O16.4	1.015822	1.0177014	1.01427077	0.8061942	-0.3108007	0.9765
GTPBP3	1.361621	1.3636112	1.35997708	0.9900055	-0.0144915	0.97654
LANCL3	1.023643	1.0216801	1.02526435	1.1653271	0.22073493	0.97656
RP11-656D10.3	1.047248	1.0491128	1.04570813	0.9306772	-0.1036472	0.97663
LGI4	1.052304	1.0504218	1.05385918	1.0681716	0.09514337	0.97663
RP1-122P22.4	1.025706	1.027612	1.02413236	0.873981	-0.1943262	0.97664
CYP4A22-AS1	1.025684	1.0275604	1.0241336	0.8756609	-0.1915558	0.97664
NEUROD2	1.025743	1.0237795	1.02736489	1.1507747	0.20260536	0.97674
SYN2	1.023591	1.0216361	1.02520525	1.1649651	0.22028671	0.97675
PPP1R2P3	1.023631	1.0216541	1.02526361	1.1666903	0.22242159	0.97675
SLC6A4	1.023663	1.0217475	1.02524447	1.1608001	0.2151196	0.97675
KIAA1614	1.045415	1.0472797	1.04387474	0.9279831	-0.1078296	0.97677
SCARNA2	1.015847	1.0139086	1.01744704	1.2544102	0.32700916	0.9768

CTC-431G16.2	1.01584	1.0139119	1.01743281	1.2530824	0.32548134	0.9768
CTD-2235C13.3	1.015831	1.0139016	1.01742368	1.2533582	0.32579878	0.9768
RTBDN	1.023675	1.0255888	1.02209368	0.8634111	-0.2118805	0.97682
ZNF34	1.113222	1.1113119	1.11479995	1.0313362	0.04451467	0.97686
ALG1L	1.016107	1.0179879	1.01455428	0.8091145	-0.3055842	0.97689
LINC00310	1.016084	1.0179437	1.01454819	0.8107681	-0.3026388	0.97689
RIPPLY2	1.016126	1.0180256	1.01455675	0.8075622	-0.3083548	0.97689
TEX12	1.016099	1.0179765	1.01454934	0.8093516	-0.3051616	0.97689
NWD1	1.023916	1.0219748	1.02551978	1.1613204	0.215766	0.977
CKMT1B	1.024744	1.0228108	1.02634092	1.1547555	0.20758741	0.97701
COL5A3	1.023369	1.0213931	1.02500022	1.1686123	0.22479642	0.97704
ZBTB9	1.023348	1.0214173	1.02494304	1.1646199	0.21985916	0.97704
AF127936.7	1.023335	1.0213846	1.0249455	1.1665154	0.22220531	0.97704
C1orf143	1.038031	1.0361348	1.03959623	1.0957931	0.13197544	0.97707
C6orf99	1.016253	1.0143204	1.0178498	1.2464558	0.31783167	0.97708
LINC01140	1.016249	1.0143273	1.01783524	1.2448423	0.315963	0.97708
RP11-228B15.4	1.016267	1.01433	1.01786596	1.2467508	0.3181731	0.97708
CTD-2256P15.4	1.016253	1.0143289	1.01784276	1.245229	0.31641109	0.97708
RP5-902P8.10	1.016247	1.0143239	1.0178343	1.2450684	0.31622506	0.97708
RP11-849F2.7	1.016241	1.014322	1.01782647	1.2446874	0.3157834	0.97708
LINC01013	1.016239	1.0143198	1.01782319	1.2446497	0.31573981	0.97708
DOK7	1.016246	1.0143199	1.01783635	1.2455606	0.31679522	0.97708
RP11-325L7.1	1.016248	1.014325	1.01783573	1.2450792	0.3162375	0.97708
RAMP2-AS1	1.131409	1.1333312	1.12982131	0.9736756	-0.0384869	0.97711
CBLN1	1.07768	1.07957	1.07611987	0.9566401	-0.0639518	0.97711
AADAT	1.266291	1.2682376	1.26468324	0.9867492	-0.0192447	0.97712
F2RL3	1.01889	1.0169798	1.02046726	1.2053871	0.26949652	0.97714
TRMT44	1.081815	1.0798795	1.0834137	1.0442445	0.06245949	0.97728
ZNF221	1.047111	1.0451794	1.04870537	1.0780434	0.10841523	0.97736
NBPF11	1.108339	1.1102119	1.10679318	0.9689809	-0.0454598	0.97736
LINC01116	1.142859	1.1448446	1.14121979	0.9749746	-0.0365635	0.97736
SIGLEC10	1.015228	1.0170659	1.01371107	0.8034198	-0.3157741	0.97745
CTC-527H23.3	1.015223	1.0170621	1.01370373	0.8031675	-0.3162273	0.97745
AC140912.1	1.015235	1.0170874	1.01370523	0.8020651	-0.3182088	0.97745
RP11-18I14.11	1.015231	1.0170866	1.01369784	0.8016717	-0.3189165	0.97745
LBHD1	1.015235	1.0170724	1.0137177	0.803502	-0.3156264	0.97745
RP11-439K3.3	1.015232	1.0170846	1.01370283	0.8020554	-0.3182262	0.97745
RP11-879F14.1	1.015245	1.0171141	1.01370107	0.8005703	-0.3209001	0.97745
CTC-1337H24.4	1.015233	1.0170776	1.01371029	0.8028211	-0.3168495	0.97745
CTD-2526A2.2	1.015237	1.0170876	1.01370901	0.8022795	-0.3178231	0.97745
MIR9-3HG	1.029042	1.0308889	1.02751656	0.8908247	-0.1667865	0.97747
RP1-317E23.3	1.037697	1.0394832	1.03622199	0.9174036	-0.1243716	0.97751
RP1-178F15.4	1.037675	1.0395213	1.03615007	0.9146987	-0.1286315	0.97751
CISD2	2.510603	2.5130314	2.50859837	0.9970701	-0.0042331	0.97756
RP11-150C16.1	1.025636	1.0237141	1.02722329	1.1479775	0.19909435	0.97758

AC074117.10	1.183178	1.1813226	1.18470934	1.018678	0.02669812	0.97759
RP11-62F24.2	1.028524	1.0303634	1.02700495	0.8893916	-0.1691094	0.97759
OSMR-AS1	1.028601	1.0304622	1.02706358	0.8884309	-0.1706685	0.97759
ENO1-AS1	1.041385	1.0394549	1.04297961	1.0893345	0.12344697	0.97763
RP11-295I5.3	1.041468	1.0394962	1.04309551	1.0911309	0.12582423	0.97763
RP11-571M6.18	1.017117	1.0189875	1.01557188	0.820111	-0.2861089	0.97763
HIST1H2BF	1.017096	1.0189522	1.01556309	0.821175	-0.2842383	0.97763
AC007966.1	1.017083	1.0189045	1.01557915	0.8240966	-0.2791147	0.97763
RP11-503L19.1	1.017148	1.0190297	1.01559336	0.8194222	-0.287321	0.97763
CTD-2555O16.4	1.033241	1.0313461	1.03480608	1.1103799	0.15105338	0.97764
RP11-534C12.1	1.016244	1.0180797	1.01472783	0.8146043	-0.2958287	0.97767
RP4-564F22.5	1.016231	1.0180501	1.0147286	0.8159844	-0.2933866	0.97767
GFAP	1.016242	1.0180836	1.0147208	0.8140404	-0.2968278	0.97767
FAM221B	1.016239	1.0180751	1.01472265	0.8145246	-0.2959699	0.97767
C14orf105	1.016238	1.0180704	1.01472489	0.8148612	-0.2953738	0.97767
NAPEPLD	1.373201	1.3714376	1.37465685	1.008667	0.01244989	0.97772
OXCT1	1.302597	1.3008972	1.30400059	1.0103138	0.0148034	0.97774
SCN2B	1.030205	1.0320343	1.02869431	0.8957373	-0.1588525	0.97775
AC019172.2	1.033184	1.0312669	1.03476777	1.1119674	0.15311449	0.97784
DRD5	1.022399	1.024235	1.02088258	0.8616718	-0.2147897	0.97789
ZSCAN23	1.036085	1.0342327	1.03761469	1.0987924	0.13591885	0.97794
RP11-407N17.5	1.036068	1.0342015	1.0376089	1.0996281	0.13701573	0.97794
RP11-712L6.5	1.072274	1.0703401	1.07387101	1.0501978	0.07066103	0.97795
RP11-334G22.1	1.025376	1.0234597	1.02695889	1.1491582	0.20057742	0.97797
VSIG1	1.025294	1.0233939	1.02686377	1.1483233	0.19952887	0.97797
ZNF630	1.042498	1.044305	1.04100582	0.9255355	-0.1116397	0.97803
DNAJC28	1.051375	1.0532335	1.04984013	0.9362558	-0.0950254	0.97806
RP11-128A17.1	1.051436	1.0532756	1.04991691	0.9369556	-0.0939474	0.97806
SCT	1.033468	1.0316345	1.03498278	1.1058419	0.14514519	0.97809
AFAP1L1	1.032818	1.0309677	1.03434519	1.109064	0.14934265	0.97812
AC145124.2	1.263662	1.2615973	1.2653676	1.0144127	0.02064477	0.97814
AC016738.3	1.018707	1.0168286	1.02025795	1.203779	0.26757051	0.9782
RP11-45M22.5	1.018714	1.0168413	1.02025992	1.2029871	0.26662122	0.9782
NIPAL1	1.025739	1.0239021	1.02725578	1.1403115	0.18942794	0.97825
FTCDNL1	1.025746	1.0238957	1.02727352	1.1413582	0.19075169	0.97825
SYNPO2L	1.028384	1.0265469	1.02990167	1.1263705	0.17168139	0.97826
VSIG8	1.024447	1.0225272	1.02603281	1.1556169	0.20866321	0.97832
VDR	1.019578	1.0213695	1.01809945	0.8469758	-0.2396074	0.97837
ZSCAN20	1.053273	1.0550539	1.0518022	0.9409364	-0.087831	0.97839
PLCXD2	1.056629	1.0547493	1.05818135	1.0626863	0.08771572	0.97844
ZNF761	1.113183	1.1151732	1.11153968	0.9684516	-0.0462482	0.97845
KLF15	1.020437	1.0185339	1.02200784	1.1874342	0.24784761	0.97849
DNLZ	1.020435	1.0185575	1.0219862	1.1847635	0.24459905	0.97849
ALG10B	1.163664	1.1615667	1.16539678	1.0237059	0.03380125	0.97852
C9orf139	1.019	1.0208032	1.0175106	0.8417247	-0.2485796	0.97853

LINC00645	1.019024	1.020857	1.0175106	0.839554	-0.252305	0.97853
RP11-10K17.6	1.019013	1.0208213	1.01751977	0.8414367	-0.2490734	0.97853
CTD-2256P15.1	1.019019	1.0208428	1.01751342	0.8402618	-0.2510892	0.97853
AP000769.7	1.019015	1.0208433	1.01750589	0.8398824	-0.2517408	0.97853
SLC1A2	1.019024	1.0208493	1.01751748	0.8401952	-0.2512035	0.97853
SGMS1	1.203251	1.2012829	1.20487605	1.0178511	0.0255265	0.97859
RP11-45A17.2	1.032201	1.0340783	1.03064983	0.8993939	-0.152975	0.97863
MMP24	1.035764	1.0339822	1.03723598	1.0957507	0.13191963	0.97864
AP001468.58	1.021457	1.0232834	1.01994797	0.8567457	-0.2230611	0.97868
RP11-584P21.2	1.021461	1.0233035	1.01993854	0.8556029	-0.2249868	0.97868
RP11-94B19.3	1.021452	1.0232729	1.01994901	0.8571792	-0.2223312	0.97868
MS4A2	1.021494	1.0233661	1.01994778	0.8537076	-0.2281861	0.97868
RP11-795F19.1	1.066468	1.0683343	1.0649276	0.9501459	-0.073779	0.97869
ZCCHC2	1.151646	1.1498858	1.15309942	1.0214404	0.03060501	0.97883
C1QTNF6	1.224671	1.2265698	1.22310379	0.9847022	-0.0222406	0.97884
KDM8	1.04119	1.0430008	1.03969409	0.9231019	-0.1154382	0.97901
ZNF777	1.045538	1.0436806	1.04707251	1.0776532	0.10789292	0.97904
PIGN	1.160984	1.1629479	1.15936151	0.9779906	-0.0321074	0.9791
TIAF1	1.023196	1.0213606	1.02471112	1.1568571	0.21021061	0.97911
MYLK4	1.02318	1.0213082	1.02472597	1.1603969	0.21461835	0.97911
SUSD2	1.023189	1.0213388	1.02471655	1.1582906	0.21199724	0.97911
ZNF76	1.278908	1.2770924	1.28040736	1.0119632	0.01715686	0.97915
CPA2	1.022429	1.02061	1.02393069	1.1611187	0.21551542	0.97921
PCAT29	1.02074	1.0188964	1.02226327	1.1781736	0.23655209	0.97922
MIR378D2	1.020747	1.0188874	1.02228232	1.1797461	0.23847637	0.97922
SMIM24	1.080979	1.0791266	1.0825083	1.0427384	0.0603772	0.97922
CEP112	1.262756	1.2645642	1.26126278	0.9875213	-0.0181162	0.97923
FOCAD-AS1	1.018697	1.0204616	1.01724051	0.8425801	-0.2471142	0.97929
PCDHB7	1.018698	1.0204913	1.01721742	0.840231	-0.251142	0.97929
FTSJ1	1.233437	1.235234	1.23195303	0.9860523	-0.0202639	0.97935
RP11-849F2.9	1.033831	1.0319966	1.03534651	1.1046942	0.14364701	0.97937
MT3	1.037866	1.0360723	1.03934656	1.0907703	0.12534737	0.97938
CCNE1	1.081734	1.079882	1.08326247	1.0423177	0.05979512	0.97941
SH2D6	1.021316	1.0194841	1.02282829	1.1716387	0.22852778	0.97941
LINC01616	1.021328	1.0194801	1.02285429	1.1732138	0.23046589	0.97941
HYDIN	1.08683	1.0886499	1.08532662	0.9625119	-0.0551237	0.97943
ESPNL	1.040993	1.0427864	1.03951202	0.9234726	-0.1148589	0.97945
SEPT3	1.153516	1.1518425	1.15489756	1.0201197	0.02873841	0.97947
LINC01138	1.182935	1.181064	1.18447981	1.018865	0.0269629	0.97947
AC005152.3	1.019731	1.0215094	1.01826263	0.8490537	-0.2360723	0.97955
CATSPER1	1.019746	1.0215202	1.01828042	0.849456	-0.2353889	0.97955
AC002550.5	1.019742	1.0215081	1.01828441	0.8501169	-0.2342668	0.97955
LINC00379	1.019731	1.0215012	1.01826902	0.849673	-0.2350204	0.97955
LINC01254	1.019737	1.0215039	1.01827828	0.8499965	-0.2344712	0.97955
RP5-1113E3.3	1.016991	1.0187642	1.01552647	0.827451	-0.2732541	0.97956

DEGS2	1.029911	1.0280916	1.03141282	1.1182272	0.16121332	0.97959
ZNF562	1.068586	1.0703484	1.06713023	0.9542541	-0.0675546	0.97959
STON1	1.344756	1.346511	1.34330742	0.9907547	-0.0134002	0.97965
CRY2	1.127073	1.1252566	1.12857286	1.0264761	0.03769998	0.97967
BNIPL	1.023996	1.0257489	1.02254779	0.8756806	-0.1915234	0.97968
RP3-394A18.1	1.077062	1.0788879	1.07555482	0.9577496	-0.0622796	0.97981
TTC34	1.077538	1.0793539	1.07603931	0.9582306	-0.0615553	0.97984
PAX7	1.031212	1.0329987	1.02973644	0.9011388	-0.1501788	0.97986
CFAP44	1.044582	1.0427851	1.04606597	1.0766825	0.10659284	0.97988
CPT1C	1.316453	1.3147627	1.31784814	1.0098024	0.01407298	0.97989
CTC-548K16.1	1.024153	1.0223628	1.02563084	1.1461361	0.19677832	0.97992
DPF1	1.068152	1.0663892	1.06960725	1.0484723	0.06828871	0.98004
CTD-2033A16.1	1.018035	1.0162319	1.01952342	1.2027798	0.26637256	0.98009
GLIPR1L1	1.017966	1.0161619	1.019456	1.2038156	0.2676144	0.98009
EXD1	1.017967	1.0161684	1.01945246	1.2031174	0.26677737	0.98009
RP11-435O5.4	1.018827	1.0170249	1.02031516	1.19326	0.25490847	0.98015
CTD-2083E4.6	1.018808	1.0170038	1.02029844	1.1937621	0.25551536	0.98015
ZSWIM1	1.03351	1.0351946	1.03211855	0.9125983	-0.1319482	0.98017
LINC00900	1.056124	1.0579396	1.05462485	0.9427895	-0.0849923	0.98019
FUK	1.056025	1.0578183	1.05454393	0.9433682	-0.0841071	0.98019
RP11-109D9.4	1.024519	1.0262524	1.02308741	0.8794399	-0.1853431	0.98022
HSD17B13	1.024561	1.0263112	1.02311542	0.878541	-0.1868185	0.98022
RP11-770J1.4	1.016956	1.0151608	1.01843875	1.2162085	0.28239058	0.9803
XXyac-YX155B6.7	1.016955	1.015162	1.01843657	1.2159703	0.282108	0.9803
RP11-276M12.1	1.016982	1.0151579	1.01848755	1.2196653	0.2864853	0.9803
LENEP	1.016941	1.0151679	1.01840496	1.2134151	0.27907322	0.9803
HSD17B3	1.016951	1.0151661	1.01842587	1.2149351	0.2808793	0.9803
WDR37	1.153235	1.1512672	1.15486048	1.0237545	0.03386984	0.9803
KLF16	1.115373	1.1170613	1.11397916	0.973671	-0.0384937	0.98033
ALDH3B1	1.031496	1.033216	1.03007599	0.9054677	-0.143265	0.98033
RP11-362K14.6	1.023903	1.0256876	1.02242858	0.8731301	-0.1957315	0.98034
LUCAT1	1.023866	1.0256425	1.02239832	0.8734841	-0.1951467	0.98034
PTGFR	1.023854	1.0255791	1.02242952	0.8768679	-0.1895685	0.98034
FAM117B	1.186471	1.1846347	1.18798656	1.0181539	0.02595559	0.98038
RP1-198K11.5	1.091261	1.0892956	1.0928838	1.0401835	0.05683807	0.98042
RASAL2-AS1	1.053203	1.0513904	1.05470073	1.0644153	0.09006119	0.98043
VWDE	1.037687	1.0358886	1.03917176	1.0914807	0.12628668	0.98045
RP11-850A17.1	1.040516	1.0386712	1.04203987	1.0871095	0.1204972	0.98046
NBPF9	1.210282	1.2119211	1.20892842	0.9858783	-0.0205185	0.9805
RP11-269M20.3	1.017153	1.0188885	1.01572023	0.8322662	-0.2648831	0.98062
FUT6	1.017168	1.0189182	1.01572254	0.8310785	-0.2669433	0.98062
UTF1	1.014517	1.0162216	1.01310914	0.8081287	-0.3073429	0.98065
CTD-2649C14.3	1.014516	1.0162206	1.01310914	0.8081771	-0.3072566	0.98065
RP5-1160K1.6	1.01453	1.0162516	1.01310914	0.8066372	-0.3100082	0.98065
PTGES3L-AARSD1	1.014514	1.0162153	1.01310914	0.8084422	-0.3067835	0.98065

RP11-4B16.4	1.014529	1.0162485	1.01310914	0.8067915	-0.3097322	0.98065
CTD-3193O13.14	1.014531	1.0162535	1.01310914	0.8065442	-0.3101745	0.98065
LLNLR-246C6.1	1.014529	1.0162479	1.01310914	0.80682	-0.3096813	0.98065
CTD-2561B21.7	1.014526	1.0162427	1.01310914	0.8070776	-0.3092207	0.98065
RP11-10O22.1	1.014533	1.0162563	1.01310914	0.8064035	-0.3104262	0.98065
APOA5	1.014533	1.0162563	1.01310914	0.8064035	-0.3104262	0.98065
RP11-84A19.2	1.014517	1.0162219	1.01310914	0.8081145	-0.3073683	0.98065
FAT4	1.096572	1.0983989	1.09506399	0.9661085	-0.0497429	0.98068
ZNF668	1.134355	1.1326539	1.1357602	1.0234165	0.03339345	0.9807
RP11-230C9.4	1.02162	1.0233631	1.02018144	0.8638183	-0.2112001	0.98075
DUSP13	1.021614	1.0233207	1.02020395	0.8663532	-0.2069728	0.98075
RP11-531A24.5	1.021635	1.0233779	1.02019612	0.8638991	-0.2110652	0.98075
XPO5	1.29825	1.2966163	1.29959915	1.0100563	0.01443569	0.98075
RP11-393I23.4	1.02216	1.023863	1.02075372	0.8697012	-0.2014083	0.98078
NID1	1.519611	1.5175913	1.52127825	1.0071232	0.01024021	0.98082
RUFY1	1.281596	1.2796567	1.2831975	1.0126613	0.01815177	0.98085
CLEC4C	1.017811	1.0160504	1.01926405	1.2002204	0.26329934	0.98087
SYK	1.017827	1.0160719	1.019276	1.1993576	0.26226187	0.98087
CTD-2341M24.1	1.017813	1.0160661	1.01925559	1.1985195	0.26125333	0.98087
CD8A	1.017832	1.016069	1.01928843	1.2003533	0.26345905	0.98087
RP11-69I8.3	1.014106	1.0158046	1.01270407	0.803822	-0.3150521	0.98092
RP11-259O18.5	1.014105	1.0158012	1.01270407	0.8039935	-0.3147443	0.98092
SLC7A14	1.014117	1.0158283	1.01270407	0.8026166	-0.317217	0.98092
FNDC9	1.014114	1.0158209	1.01270407	0.8029943	-0.3165383	0.98092
RP11-184A2.3	1.014107	1.0158059	1.01270407	0.8037568	-0.3151691	0.98092
SOWAHB	1.01411	1.0158136	1.01270407	0.8033653	-0.3158719	0.98092
CTA-254O6.1	1.014125	1.0158449	1.01270407	0.8017787	-0.3187241	0.98092
RP11-408O19.5	1.014116	1.0158266	1.01270407	0.802703	-0.3170617	0.98092
RP5-940J5.3	1.018455	1.0166975	1.01990653	1.1921834	0.25360623	0.98098
RP11-110I1.6	1.01846	1.0166975	1.0199151	1.1926964	0.2542269	0.98098
RP11-392P7.7	1.018456	1.0166975	1.01990782	1.1922602	0.25369912	0.98098
KB-1043D8.8	1.018461	1.0166975	1.01991789	1.1928638	0.25442929	0.98098
DCDC2B	1.018461	1.0166975	1.01991789	1.1928638	0.25442929	0.98098
RP11-17A1.3	1.018451	1.0166975	1.01989857	1.1917064	0.2530289	0.98098
NICN1	1.018462	1.0166975	1.01991911	1.1929363	0.25451702	0.98098
UBXN7-AS1	1.01846	1.0166975	1.01991488	1.1926833	0.25421106	0.98098
TMEM244	1.018451	1.0166975	1.01989965	1.1917713	0.25310741	0.98098
IGF2BP2-AS1	1.018451	1.0166975	1.0198995	1.1917619	0.25309601	0.98098
RP11-644N4.1	1.018452	1.0166975	1.01990089	1.1918451	0.25319674	0.98098
AC011738.4	1.018455	1.0166975	1.01990676	1.1921971	0.25362281	0.98098
RP11-308D13.3	1.018455	1.0166975	1.01990676	1.1921971	0.25362281	0.98098
RP11-378A12.1	1.018451	1.0166975	1.01989875	1.191717	0.25304167	0.98098
AC007126.1	1.018471	1.0166975	1.01993611	1.1939545	0.25574788	0.98098
RP11-498P14.3	1.018455	1.0166975	1.01990688	1.192204	0.25363107	0.98098
RP11-295I5.4	1.018452	1.0166975	1.01990042	1.1918173	0.25316315	0.98098

AC015849.16	1.01845	1.0166975	1.01989688	1.1916051	0.25290617	0.98098
AP000442.4	1.018449	1.0166975	1.0198958	1.1915403	0.25282778	0.98098
RP11-542B15.1	1.018456	1.0166975	1.01990754	1.1922439	0.25367939	0.98098
AC006277.2	1.018453	1.0166975	1.01990335	1.1919929	0.25337565	0.98098
F8A3	1.018459	1.0166975	1.01991282	1.1925599	0.25406179	0.98098
AF121898.3	1.018459	1.0166975	1.01991391	1.1926249	0.25414036	0.98098
PLEKHD1	1.018459	1.0166975	1.01991391	1.1926249	0.25414036	0.98098
IRX4	1.018456	1.0166975	1.01990894	1.1923275	0.2537806	0.98098
ITGB2-AS1	1.018456	1.0166975	1.01990894	1.1923275	0.2537806	0.98098
RP3-340B19.3	1.018456	1.0166975	1.01990894	1.1923275	0.2537806	0.98098
LINC01098	1.018452	1.0166975	1.0199005	1.1918221	0.25316886	0.98098
CTD-2527I21.15	1.018458	1.0166975	1.01991154	1.1924834	0.25396924	0.98098
ANKS4B	1.018458	1.0166975	1.01991154	1.1924834	0.25396924	0.98098
AL022344.7	1.018455	1.0166975	1.0199065	1.1921813	0.2536037	0.98098
C17orf77	1.018459	1.0166975	1.01991438	1.1926534	0.25417482	0.98098
PGA5	1.018456	1.0166975	1.01990727	1.1922276	0.25365966	0.98098
CTA-503F6.2	1.018465	1.0166975	1.01992544	1.1933157	0.25497576	0.98098
PDF	1.018465	1.0166975	1.01992487	1.1932812	0.25493411	0.98098
RP11-521O16.1	1.025368	1.0236039	1.02682472	1.136451	0.18453549	0.98099
RP11-775D22.2	1.025426	1.0236102	1.02692469	1.1403839	0.18951963	0.98099
RP11-679B19.2	1.023995	1.0257098	1.02257854	0.8782069	-0.1873673	0.98104
KB-1466C5.1	1.02399	1.0257403	1.02254513	0.8758675	-0.1912154	0.98104
TMEFF1	1.024016	1.0257398	1.02259171	0.877696	-0.1882068	0.98104
ERVW-1	1.024834	1.0265435	1.02342244	0.8824158	-0.1804695	0.98112
MBD3	1.385027	1.3867008	1.38364466	0.9920968	-0.0114472	0.98112
DNAJB13	1.027438	1.0256996	1.02887327	1.1234895	0.16798664	0.98117
RP11-307C12.13	1.027463	1.0256975	1.02892054	1.125421	0.17046483	0.98117
ITPKA	1.027471	1.0256991	1.0289337	1.1258652	0.1710341	0.98117
ZNF619	1.061058	1.0627577	1.05965396	0.9505436	-0.0731753	0.98117
MOB3C	1.061003	1.0627845	1.05953276	0.9482086	-0.0767236	0.98117
BOLA2	1.025295	1.0235262	1.02675541	1.1372621	0.1855648	0.98118
BACH1-AS1	1.021935	1.02366	1.02051042	0.866883	-0.2060908	0.98122
KCNC1	1.021963	1.0237033	1.02052649	0.8659751	-0.2076026	0.98122
FGD6	1.118544	1.1201669	1.11720459	0.9753486	-0.0360101	0.98123
RP11-315D16.2	1.017547	1.0157965	1.01899229	1.2023133	0.26581284	0.98126
CTB-171A8.1	1.017542	1.0158024	1.01897795	1.2009546	0.26418155	0.98126
CTB-46B19.2	1.017561	1.0158018	1.01901283	1.2032033	0.26688045	0.98126
RP1-302G2.5	1.01437	1.0160502	1.01298307	0.8089017	-0.3059636	0.98131
RP11-521B24.5	1.014386	1.0160855	1.01298307	0.8071284	-0.3091298	0.98131
AC068831.15	1.014373	1.0160552	1.01298307	0.8086522	-0.3064088	0.98131
RP4-777L9.2	1.014372	1.0160539	1.01298307	0.8087184	-0.3062906	0.98131
RP3-400B16.3	1.01438	1.016072	1.01298307	0.8078086	-0.3079146	0.98131
RP11-720L2.4	1.014376	1.0160632	1.01298307	0.8082471	-0.3071317	0.98131
RP11-452K12.4	1.014374	1.0160591	1.01298307	0.8084552	-0.3067603	0.98131
RP11-443B7.1	1.014383	1.0160774	1.01298307	0.807535	-0.3084033	0.98131

RP11-152H18.3	1.014383	1.0160787	1.01298307	0.8074687	-0.3085218	0.98131
RP11-35J1.2	1.014377	1.0160639	1.01298307	0.8082144	-0.3071901	0.98131
RP11-669C19.1	1.014366	1.0160413	1.01298307	0.8093534	-0.3051583	0.98131
LINC01060	1.014373	1.0160568	1.01298307	0.8085727	-0.3065506	0.98131
MAP3K1	1.140997	1.1427179	1.13957609	0.9779862	-0.032114	0.98138
SORCS3	1.030186	1.0319207	1.02875254	0.9007486	-0.1508036	0.98141
ALG1	1.112873	1.1109204	1.11448523	1.0321391	0.04563735	0.98143
FRY-AS1	1.025671	1.0239194	1.02711719	1.1336908	0.18102719	0.98143
RP13-49I15.6	1.025672	1.0239558	1.02708852	1.1307708	0.17730648	0.98143
FAM66D	1.026515	1.0248474	1.02789234	1.1225452	0.1667736	0.98144
WDR19	1.278883	1.2806391	1.27743343	0.9885774	-0.0165742	0.98146
LINC00284	1.025092	1.0233286	1.02654805	1.1380057	0.18650779	0.98147
HIST1H3A	1.025082	1.0233431	1.02651715	1.1359732	0.18392879	0.98147
DTX3L	1.121252	1.1195063	1.12269402	1.0266738	0.03797781	0.9815
FADS6	1.017978	1.0162348	1.01941679	1.1960004	0.25821789	0.98153
RP11-685M7.3	1.017982	1.0162524	1.0194101	1.1942892	0.25615224	0.98153
FREM1	1.017966	1.0162256	1.01940244	1.1957901	0.25796412	0.98153
RP11-240G22.5	1.017986	1.0162485	1.01942024	1.1952027	0.2572553	0.98153
RP11-370I10.12	1.017987	1.0162416	1.01942861	1.1962237	0.25848718	0.98153
VAX1	1.01797	1.016241	1.01939704	1.1943277	0.2561988	0.98153
EME2	1.090022	1.0881948	1.09153054	1.0378222	0.05355932	0.98154
RASL12	1.020634	1.0188505	1.022107	1.1727567	0.22990374	0.98158
GZF1	1.086934	1.0885593	1.08559177	0.9664909	-0.0491719	0.98163
UST-AS1	1.016591	1.0148661	1.01801481	1.2118039	0.27715621	0.98171
RP11-298A10.1	1.016599	1.0148661	1.01803031	1.2128462	0.27839662	0.98171
HOXC4	1.016597	1.0148661	1.01802575	1.2125393	0.27803145	0.98171
RP11-475C16.2	1.016596	1.0148661	1.01802543	1.2125179	0.27800607	0.98171
RP11-903H12.3	1.01659	1.0148661	1.01801389	1.2117415	0.27708201	0.98171
SSTR1	1.01659	1.0148661	1.01801389	1.2117415	0.27708201	0.98171
RP1-225E12.2	1.016596	1.0148661	1.01802481	1.2124765	0.27795678	0.98171
RP11-307O13.1	1.016589	1.0148661	1.01801226	1.2116323	0.27695189	0.98171
RP11-123B3.2	1.016589	1.0148661	1.01801226	1.2116323	0.27695189	0.98171
RP11-707G14.1	1.016588	1.0148661	1.01800965	1.2114563	0.27674231	0.98171
NLRP7	1.016599	1.0148661	1.01803065	1.212869	0.27842378	0.98171
CLDN14	1.016599	1.0148661	1.01803065	1.212869	0.27842378	0.98171
LHFPL5	1.016608	1.0148661	1.01804618	1.2139138	0.27966595	0.98171
CPO	1.016597	1.0148661	1.01802563	1.2125313	0.27802194	0.98171
RP11-149B9.2	1.016589	1.0148661	1.01801093	1.2115428	0.27684538	0.98171
AC007292.4	1.016591	1.0148661	1.0180152	1.2118297	0.27718702	0.98171
IL37	1.016588	1.0148661	1.01800949	1.2114457	0.27672974	0.98171
RIMBP3C	1.016593	1.0148661	1.01801891	1.2120795	0.2774843	0.98171
RP11-556I14.1	1.016602	1.0148661	1.01803566	1.2132058	0.27882433	0.98171
VWA5B1	1.016587	1.0148661	1.01800726	1.2112958	0.27655116	0.98171
CLCN1	1.016593	1.0148661	1.0180193	1.2121054	0.27751518	0.98171
KRTAP10-4	1.0166	1.0148661	1.01803268	1.2130058	0.27858641	0.98171

RP11-442N1.2	1.016593	1.0148661	1.01801984	1.212142	0.27755871	0.98171
RP3-395M20.9	1.016592	1.0148661	1.01801681	1.2119381	0.27731603	0.98171
NAV2-AS5	1.016597	1.0148661	1.01802707	1.2126281	0.27813718	0.98171
RP11-462G2.1	1.016597	1.0148661	1.01802707	1.2126281	0.27813718	0.98171
GJB6	1.016599	1.0148661	1.01803056	1.2128632	0.2784168	0.98171
RP11-466P24.6	1.016592	1.0148661	1.01801814	1.2120276	0.27742255	0.98171
DEFB136	1.016592	1.0148661	1.01801814	1.2120276	0.27742255	0.98171
LINC01082	1.016588	1.0148661	1.01801019	1.2114926	0.2767856	0.98171
AP001046.6	1.016588	1.0148661	1.01801019	1.2114926	0.2767856	0.98171
RP11-659P15.1	1.016587	1.0148661	1.01800742	1.2113063	0.27656372	0.98171
RP11-310E22.5	1.016591	1.0148661	1.01801558	1.2118556	0.27721784	0.98171
GLIS3-AS1	1.016591	1.0148661	1.01801558	1.2118556	0.27721784	0.98171
CLPS	1.016594	1.0148661	1.0180217	1.2122672	0.27770767	0.98171
RP3-400B16.1	1.016593	1.0148661	1.01801946	1.212116	0.27752782	0.98171
SHROOM1	1.120966	1.122765	1.1194807	0.9732469	-0.0391222	0.98179
RP11-266J6.2	1.027375	1.0290819	1.02596541	0.8928374	-0.1635306	0.98186
DCUN1D2-AS	1.036057	1.0377357	1.03467022	0.9187639	-0.1222339	0.98187
PLCXD3	1.013516	1.0151854	1.0121375	0.7992849	-0.3232183	0.98188
CTD-2521M24.6	1.013504	1.0151593	1.0121375	0.8006633	-0.3207324	0.98188
RGPD1	1.01352	1.0151933	1.0121375	0.7988706	-0.3239662	0.98188
RP11-494O16.4	1.013521	1.0151956	1.0121375	0.7987518	-0.3241808	0.98188
RP11-15K19.2	1.013512	1.0151768	1.0121375	0.799742	-0.3223935	0.98188
RP11-6N17.10	1.013519	1.0151911	1.0121375	0.7989894	-0.3237517	0.98188
RP11-259O2.1	1.013515	1.0151837	1.0121375	0.7993757	-0.3230543	0.98188
LINC01343	1.013515	1.0151837	1.0121375	0.7993757	-0.3230543	0.98188
RP11-815J21.4	1.013512	1.0151759	1.0121375	0.7997898	-0.3223071	0.98188
RP11-540A21.2	1.026753	1.0284226	1.02537387	0.8927363	-0.163694	0.98199
RP11-66H6.3	1.028023	1.026291	1.02945296	1.120268	0.1638439	0.98202
TDRD12	1.015375	1.0170261	1.01401235	0.8229921	-0.2810495	0.98204
RP11-84A19.4	1.01538	1.0170495	1.01400043	0.821166	-0.2842541	0.98204
RP13-270P17.1	1.015368	1.0170154	1.01400677	0.8231805	-0.2807193	0.98204
SLC14A2-AS1	1.015365	1.0170129	1.01400384	0.82313	-0.2808077	0.98204
RP11-1263C18.1	1.01453	1.0161939	1.01315569	0.8123854	-0.2997637	0.98209
RP11-367J11.2	1.01452	1.0161717	1.01315569	0.8134982	-0.2977889	0.98209
RP11-108K3.1	1.014526	1.0161858	1.01315569	0.8127933	-0.2990396	0.98209
RP11-133F8.2	1.014514	1.0161577	1.01315569	0.8142035	-0.2965387	0.98209
GNAT2	1.014538	1.0162122	1.01315569	0.8114697	-0.3013909	0.98209
PSORS1C2	1.014538	1.0162129	1.01315569	0.8114311	-0.3014596	0.98209
RP1-278O22.1	1.014529	1.0161913	1.01315569	0.8125179	-0.2995285	0.98209
RP11-447L10.1	1.014538	1.0162111	1.01315569	0.8115253	-0.301292	0.98209
RP11-31I22.3	1.014525	1.0161837	1.01315569	0.8128959	-0.2988576	0.98209
RP11-229E13.4	1.014525	1.0161837	1.01315569	0.8128959	-0.2988576	0.98209
RP11-17E13.2	1.014514	1.016159	1.01315569	0.8141378	-0.2966551	0.98209
LINC00538	1.014548	1.0162342	1.01315569	0.8103689	-0.3033493	0.98209
RP4-536B24.4	1.014524	1.0161815	1.01315569	0.8130089	-0.2986569	0.98209

RP11-776A13.3	1.014538	1.0162127	1.01315569	0.8114416	-0.3014408	0.98209
ARSD-AS1	1.014523	1.0161792	1.01315569	0.813122	-0.2984563	0.98209
LINC00462	1.014543	1.016223	1.01315569	0.8109294	-0.3023519	0.98209
RP11-38L15.8	1.014532	1.0161994	1.01315569	0.8121118	-0.3002498	0.98209
NR1I3	1.014532	1.0161994	1.01315569	0.8121118	-0.3002498	0.98209
AP1AR	1.143297	1.1451443	1.14177201	0.9767661	-0.033915	0.98215
ZNF295-AS1	1.029223	1.0309024	1.02783635	0.9007828	-0.1507488	0.98216
PRKCQ	1.029245	1.0309308	1.02785203	0.9004618	-0.151263	0.98216
UBFD1	1.382771	1.3845062	1.38133839	0.9917614	-0.011935	0.98217
MARCH8	1.199028	1.1973628	1.2004024	1.0154011	0.02204979	0.9822
CTD-3193K9.11	1.037028	1.0353002	1.0384542	1.0893488	0.12346602	0.98223
FAM200B	2.700789	2.7043379	2.69785745	0.9961977	-0.0054961	0.98224
SH3BGRL2	1.315049	1.31707	1.31338026	0.988363	-0.0168871	0.98225
ACSL5	1.020633	1.0222677	1.01928262	0.8659453	-0.2076522	0.98229
MAN2B2	1.119173	1.1207427	1.117876	0.9762578	-0.0346659	0.98229
PACRG	1.094331	1.0960343	1.09292445	0.967617	-0.047492	0.98234
KB-1125A3.12	1.027076	1.0253767	1.02848009	1.1222921	0.16644824	0.9824
NBAS	1.412082	1.4139371	1.41055069	0.991819	-0.0118512	0.98243
CTF1	1.035169	1.0334194	1.03661396	1.0955905	0.13170868	0.9825
ABO	1.0346	1.0329367	1.03597373	1.0922084	0.12724815	0.98253
POP7	2.091942	2.0941106	2.09015189	0.9963818	-0.0052294	0.98256
RP13-991F5.2	1.014683	1.0129919	1.01607889	1.2376068	0.30755299	0.98259
CHRM4	1.014683	1.0129919	1.01607889	1.2376068	0.30755299	0.98259
CTA-268H5.14	1.014684	1.0129919	1.01608206	1.2378509	0.30783758	0.98259
GATA6-AS1	1.014683	1.0129919	1.01607904	1.2376189	0.30756717	0.98259
RP11-616M22.11	1.014683	1.0129919	1.01607904	1.2376189	0.30756717	0.98259
GSTM5	1.014678	1.0129919	1.01607092	1.2369937	0.30683814	0.98259
KLK3	1.014677	1.0129919	1.0160678	1.2367534	0.30655781	0.98259
AC024132.1	1.014677	1.0129919	1.0160678	1.2367534	0.30655781	0.98259
RP1-224A6.9	1.014684	1.0129919	1.01608078	1.2377528	0.3077232	0.98259
RP3-406P24.3	1.014684	1.0129919	1.01608078	1.2377528	0.3077232	0.98259
CTB-30L5.1	1.014682	1.0129919	1.01607733	1.2374873	0.30741368	0.98259
RP11-154H23.5	1.014682	1.0129919	1.01607733	1.2374873	0.30741368	0.98259
FCRL2	1.014685	1.0129919	1.01608382	1.2379863	0.30799531	0.98259
RP11-244N9.4	1.014682	1.0129919	1.01607749	1.2374994	0.30742785	0.98259
IYD	1.014682	1.0129919	1.01607749	1.2374994	0.30742785	0.98259
RP5-983L19.2	1.014677	1.0129919	1.01606811	1.2367769	0.3065853	0.98259
RP11-866E20.3	1.014674	1.0129919	1.0160633	1.2364071	0.30615386	0.98259
SLC5A5	1.014681	1.0129919	1.01607509	1.2373147	0.30721244	0.98259
RP11-507K2.3	1.014689	1.0129919	1.01609133	1.2385643	0.30866875	0.98259
RP11-486L19.2	1.014677	1.0129919	1.01606861	1.2368157	0.30663059	0.98259
MAGEA8-AS1	1.014677	1.0129919	1.01606861	1.2368157	0.30663059	0.98259
WEE2	1.014677	1.0129919	1.01606948	1.2368827	0.3067087	0.98259
AC003956.1	1.014677	1.0129919	1.01606948	1.2368827	0.3067087	0.98259
ADRA2A	1.014678	1.0129919	1.01607069	1.2369755	0.30681697	0.98259

RP11-554D15.3	1.014675	1.0129919	1.01606432	1.2364856	0.30624546	0.98259
RP11-473O4.5	1.014685	1.0129919	1.01608308	1.2379294	0.307929	0.98259
AC073342.12	1.014683	1.0129919	1.01607935	1.2376426	0.30759472	0.98259
RP11-863P13.4	1.014679	1.0129919	1.01607207	1.2370818	0.30694094	0.98259
DAND5	1.014686	1.0129919	1.01608428	1.2380222	0.30803716	0.98259
RP11-758N13.1	1.014682	1.0129919	1.01607695	1.2374576	0.30737908	0.98259
RP11-6D1.3	1.014675	1.0129919	1.01606576	1.2365965	0.30637485	0.98259
RP11-240B13.2	1.014678	1.0129919	1.01607019	1.2369373	0.30677232	0.98259
AC004490.1	1.014693	1.0129919	1.01609768	1.2390533	0.30923822	0.98259
RXFP2	1.014679	1.0129919	1.01607158	1.2370441	0.30689693	0.98259
INSRR	1.014678	1.0129919	1.01607008	1.2369285	0.30676214	0.98259
CTD-2313J17.6	1.01468	1.0129919	1.01607397	1.2372284	0.30711191	0.98259
TMEM8C	1.014682	1.0129919	1.01607683	1.2374488	0.30736886	0.98259
RP11-867G2.6	1.014675	1.0129919	1.01606476	1.2365195	0.30628501	0.98259
RP11-521D12.1	1.014675	1.0129919	1.01606476	1.2365195	0.30628501	0.98259
CLDN34	1.014677	1.0129919	1.01606769	1.2367446	0.30654765	0.98259
RP11-169K17.3	1.014678	1.0129919	1.01606992	1.2369164	0.30674804	0.98259
LINC00838	1.01468	1.0129919	1.01607444	1.2372641	0.30715353	0.98259
RP11-234K24.3	1.014682	1.0129919	1.01607784	1.2375264	0.30745933	0.98259
RP11-50D16.4	1.014681	1.0129919	1.01607574	1.2373644	0.30727042	0.98259
CCR9	1.014682	1.0129919	1.0160773	1.2374846	0.30741055	0.98259
RP11-963H4.5	1.014687	1.0129919	1.01608736	1.2382589	0.30831303	0.98259
RP11-481J2.3	1.050715	1.0523379	1.04937448	0.9433784	-0.0840916	0.98262
AP001437.1	1.020445	1.0187464	1.0218472	1.1654082	0.22083533	0.98264
RP11-45A17.4	1.020465	1.0188082	1.02183248	1.1607977	0.21511658	0.98264
CTD-2031P19.5	1.020459	1.0187492	1.02187028	1.1664674	0.22214602	0.98264
ZNF737	1.066308	1.0645061	1.06779551	1.0509933	0.07175345	0.98266
C12orf49	1.543716	1.5424257	1.54478233	1.0043446	0.00625429	0.98269
LINC01549	1.026109	1.0244017	1.02751806	1.1277126	0.1733995	0.98275
AC003958.2	1.017876	1.0195201	1.01651776	0.8461946	-0.2409386	0.98278
RP11-30L15.6	1.017876	1.019527	1.01651238	0.8456156	-0.241926	0.98278
MYO1H	1.017867	1.0195061	1.01651323	0.8465698	-0.2402991	0.98278
Z83851.4	1.017862	1.0194979	1.01651084	0.8467987	-0.2399091	0.98278
KLHL25	1.06363	1.0619124	1.06504773	1.050642	0.07127121	0.98281
RP11-513M16.7	1.063657	1.0619383	1.06507641	1.0506656	0.07130359	0.98281
TMEM104	1.080765	1.0790435	1.08218696	1.0397688	0.05626274	0.9829
CHRNA9	1.022144	1.0204767	1.02352041	1.1486438	0.19993143	0.98293
SNHG22	1.022157	1.0204763	1.02354566	1.1498963	0.20150382	0.98293
MAGI1	1.541889	1.5438513	1.54026898	0.993413	-0.0095345	0.98294
LYPD5	1.017285	1.0189131	1.01593993	0.8427978	-0.2467415	0.98295
CTD-2619J13.23	1.017284	1.018911	1.01593993	0.84289	-0.2465838	0.98295
RP11-44N11.3	1.017273	1.0188872	1.01593993	0.8439561	-0.2447602	0.98295
RP3-522J7.7	1.017287	1.0189177	1.01593993	0.8425928	-0.2470925	0.98295
RP11-106M3.3	1.017283	1.0189094	1.01593993	0.842963	-0.2464587	0.98295
LINC01333	1.01729	1.0189243	1.01593993	0.8422981	-0.2475971	0.98295

VWA7	1.017278	1.0188977	1.01593993	0.8434857	-0.2455645	0.98295
RP11-337N6.1	1.017294	1.0189327	1.01593993	0.8419253	-0.2482358	0.98295
SHANK2-AS1	1.01729	1.018925	1.01593993	0.8422687	-0.2476476	0.98295
C1QTNF7	1.017271	1.0188824	1.01593993	0.8441684	-0.2443973	0.98295
AC006372.4	1.01728	1.0189029	1.01593993	0.8432541	-0.2459607	0.98295
LINC00330	1.017289	1.018922	1.01593993	0.8424036	-0.2474165	0.98295
AC009473.1	1.019025	1.0206675	1.01766826	0.8548808	-0.2262049	0.983
PRR18	1.01898	1.0205822	1.01765602	0.8578301	-0.2212362	0.983
FLNB-AS1	1.019001	1.020628	1.01765807	0.8560255	-0.2242744	0.983
AC097721.2	1.018998	1.0206317	1.0176486	0.8554099	-0.2253121	0.983
RP11-83N9.6	1.019002	1.0206129	1.01767164	0.8573088	-0.2221132	0.983
RP1-199J3.7	1.027536	1.0291845	1.02617496	0.8968802	-0.1570127	0.98301
RP11-348P10.2	1.027495	1.029095	1.02617376	0.8995951	-0.1526523	0.98301
ZDHHC11B	1.037531	1.0358603	1.03891029	1.0850535	0.11776619	0.98304
GOLGA3	1.207349	1.2056955	1.20871519	1.0146806	0.02102565	0.98308
RP11-78O7.3	1.019718	1.0213344	1.01838228	0.8616271	-0.2148645	0.98308
AC011523.2	1.019737	1.0213794	1.01838139	0.8597722	-0.2179737	0.98308
RP5-901A4.1	1.019706	1.0213143	1.01837714	0.8621984	-0.2139083	0.98308
RP11-1C8.6	1.01972	1.0213575	1.01836686	0.8599726	-0.2176374	0.98308
RCOR1	1.193653	1.1954596	1.19216081	0.9831228	-0.0245564	0.98312
CRISPLD1	1.397036	1.3991313	1.39530507	0.9904136	-0.013897	0.98312
GPAM	1.155881	1.1541858	1.15728065	1.0200724	0.02867152	0.98315
RP11-168P13.1	1.016341	1.0146773	1.0177152	1.2069781	0.27139955	0.9833
RP11-1017G21.4	1.016336	1.0146773	1.01770526	1.2063008	0.27058965	0.9833
RP11-101E5.1	1.01634	1.0146773	1.01771379	1.206882	0.27128461	0.9833
NUTM2D	1.016337	1.0146773	1.01770742	1.2064482	0.27076603	0.9833
RP5-937E21.8	1.016342	1.0146773	1.01771628	1.2070518	0.27148757	0.9833
MS4A8	1.016342	1.0146773	1.01771628	1.2070518	0.27148757	0.9833
XAF1	1.016346	1.0146773	1.01772433	1.2076004	0.2721431	0.9833
HOXC8	1.016346	1.0146773	1.01772433	1.2076004	0.2721431	0.9833
TEN1-CDK3	1.016346	1.0146773	1.01772433	1.2076004	0.2721431	0.9833
GRAPL	1.016337	1.0146773	1.01770769	1.2064667	0.2707881	0.9833
RP11-375O18.2	1.016337	1.0146773	1.01770769	1.2064667	0.2707881	0.9833
HAMP	1.016337	1.0146773	1.01770769	1.2064667	0.2707881	0.9833
RP11-567N4.3	1.016339	1.0146773	1.0177105	1.2066579	0.27101666	0.9833
RP11-24F11.5	1.016341	1.0146773	1.01771439	1.2069225	0.27133306	0.9833
AC006000.5	1.016339	1.0146773	1.01771105	1.206695	0.27106112	0.9833
WNT8B	1.01634	1.0146773	1.0177128	1.2068144	0.27120377	0.9833
LINC01592	1.016339	1.0146773	1.01771214	1.2067696	0.27115023	0.9833
ASPN	1.016335	1.0146773	1.01770484	1.2062722	0.27055549	0.9833
RP11-407G23.7	1.016345	1.0146773	1.01772169	1.2074205	0.27192825	0.9833
RP11-114H24.6	1.016341	1.0146773	1.01771493	1.2069596	0.27137739	0.9833
CCDC125	1.016337	1.0146773	1.01770677	1.2064036	0.27071265	0.9833
AC136352.2	1.016336	1.0146773	1.01770604	1.2063536	0.27065289	0.9833
LL22NC03-N95F10.1	1.016336	1.0146773	1.01770576	1.2063352	0.27063084	0.9833

KB-173C10.2	1.016336	1.0146773	1.01770576	1.2063352	0.27063084	0.9833
WFDC10A	1.01634	1.0146773	1.01771265	1.2068042	0.27119165	0.9833
RP11-599B13.3	1.016342	1.0146773	1.01771698	1.2070996	0.27154477	0.9833
NCR3	1.016342	1.0146773	1.01771698	1.2070996	0.27154477	0.9833
SLC28A1	1.016344	1.0146773	1.01772115	1.2073833	0.27188375	0.9833
RP11-863H1.1	1.016336	1.0146773	1.01770569	1.2063298	0.27062444	0.9833
HSPB7	1.016336	1.0146773	1.01770569	1.2063298	0.27062444	0.9833
AC005624.2	1.016342	1.0146773	1.01771706	1.207105	0.27155122	0.9833
RP11-61L19.2	1.016338	1.0146773	1.01770853	1.2065237	0.27085631	0.9833
RP11-360O19.4	1.016337	1.0146773	1.01770718	1.2064316	0.27074618	0.9833
RP11-1070N10.4	1.01634	1.0146773	1.01771268	1.2068066	0.27119449	0.9833
CTC-338M12.2	1.016342	1.0146773	1.01771722	1.2071158	0.27156411	0.9833
RP11-166N17.1	1.016342	1.0146773	1.01771722	1.2071158	0.27156411	0.9833
CTD-2026D20.2	1.016341	1.0146773	1.01771566	1.2070093	0.27143678	0.9833
RP11-59D5__B.2	1.016344	1.0146773	1.01772095	1.2073701	0.27186797	0.9833
HOXA7	1.016344	1.0146773	1.01772095	1.2073701	0.27186797	0.9833
RP11-73M7.6	1.016342	1.0146773	1.01771749	1.2071344	0.27158631	0.9833
ZNF525	1.109176	1.1074083	1.11063659	1.0300562	0.04272311	0.98333
MYH3	1.04033	1.0420056	1.03894581	0.9271574	-0.1091138	0.98336
ZNF358	1.431643	1.429814	1.43315289	1.0077682	0.01116386	0.98345
AC005944.2	1.024927	1.0232555	1.02630715	1.1312228	0.17788307	0.98354
CD247	1.024918	1.0232387	1.02630507	1.131951	0.1788115	0.98354
RP11-128M1.1	1.14029	1.1385588	1.14171924	1.0228097	0.03253775	0.98354
AC003090.1	1.022448	1.0208373	1.02377889	1.1411689	0.19051236	0.98367
RP11-74E22.8	1.022473	1.0208137	1.02384304	1.1455444	0.19603336	0.98367
CTD-3076O17.2	1.022474	1.0208293	1.02383235	1.1441752	0.19430797	0.98367
BSN-AS2	1.022448	1.0208098	1.0238015	1.1437651	0.19379084	0.98367
WBSCR28	1.022475	1.0208199	1.02384187	1.1451457	0.19553111	0.98367
RP11-571I18.4	1.016961	1.0185454	1.01565341	0.844061	-0.2445808	0.9837
RP5-958B11.2	1.016983	1.0186056	1.01564216	0.8407244	-0.2502951	0.9837
GSTA1	1.016971	1.0185617	1.01565685	0.8435036	-0.2455339	0.9837
RP5-956O18.3	1.016973	1.0185652	1.01565814	0.8434153	-0.2456849	0.9837
LINC00929	1.016997	1.0186235	1.01565388	0.8405431	-0.2506063	0.9837
TAPBPL	1.016965	1.0185568	1.0156509	0.8434056	-0.2457014	0.9837
RBL1	1.108698	1.1070331	1.1100724	1.028396	0.04039592	0.98374
TMEM86B	1.02999	1.0283135	1.03137478	1.1081223	0.14811706	0.98375
MGAM	1.029977	1.028287	1.03137287	1.1090916	0.14937852	0.98375
RP11-70D24.2	1.02993	1.0282523	1.03131455	1.1083879	0.14846293	0.98375
TMEM203	1.700158	1.6983901	1.70161882	1.004623	0.00665425	0.98376
PRKCE	1.05524	1.0568469	1.0539121	0.9483738	-0.0764723	0.98386
GALP	1.020901	1.0224707	1.01960387	0.8724189	-0.196907	0.98389
RP11-689P11.2	1.020988	1.0226518	1.01961358	0.8658711	-0.2077759	0.98389
CTC-338M12.6	1.018002	1.0195784	1.0166999	0.8529756	-0.2294236	0.98397
SCN1A	1.018	1.0195738	1.0166999	0.8531756	-0.2290854	0.98397
RP11-950C14.7	1.018008	1.0195913	1.0166999	0.8524154	-0.2303715	0.98397

LINC00243	1.018005	1.0195852	1.0166999	0.8526787	-0.2299259	0.98397
NFE4	1.017996	1.0195661	1.0166999	0.8535126	-0.2285156	0.98397
CD163L1	1.018007	1.0195902	1.0166999	0.8524633	-0.2302904	0.98397
RP11-164H13.1	1.018021	1.0196207	1.0166999	0.8511371	-0.2325365	0.98397
RP11-304C12.5	1.015266	1.0168429	1.01396402	0.8290764	-0.270423	0.98398
AC006076.1	1.015273	1.0168576	1.01396402	0.8283509	-0.2716861	0.98398
HLX	1.015274	1.0168601	1.01396402	0.8282263	-0.271903	0.98398
RP4-669H2.1	1.015273	1.0168572	1.01396402	0.8283697	-0.2716533	0.98398
GPAT2	1.015258	1.0168246	1.01396402	0.8299749	-0.2688605	0.98398
RP11-287D1.3	1.01526	1.0168292	1.01396402	0.8297513	-0.2692491	0.98398
RP1-144F13.3	1.015262	1.0168346	1.01396402	0.8294822	-0.2697171	0.98398
AC142119.1	1.015267	1.016844	1.01396402	0.8290203	-0.2705206	0.98398
SLCO1A2	1.090297	1.0887984	1.0915348	1.0308161	0.04378692	0.98412
HHAT	1.145572	1.1439514	1.14690951	1.0205495	0.02934617	0.98412
ZKSCAN4	1.090423	1.0919379	1.0891714	0.9699087	-0.0440792	0.98414
PTGES2-AS1	1.029459	1.0310179	1.02817126	0.9082263	-0.1388763	0.98424
MORC4	1.366092	1.3677648	1.36471034	0.9916946	-0.0120322	0.98426
LINC00957	1.046285	1.0445787	1.04769364	1.0698741	0.0974411	0.98427
BCORL1	1.12913	1.1275921	1.13039979	1.0220049	0.03140206	0.98427
CTD-2378E12.1	1.029964	1.0315554	1.02865044	0.907942	-0.139328	0.98428
ANGPT4	1.030002	1.0316274	1.0286589	0.9061414	-0.1421919	0.98428
IFIH1	1.030786	1.032399	1.0294545	0.9091171	-0.137462	0.98434
RP11-666A20.4	1.053932	1.0523182	1.05526526	1.05633	0.07906054	0.98434
AP001059.7	1.021885	1.0234821	1.02056614	0.8758233	-0.1912882	0.98436
FSCN2	1.021858	1.0234423	1.02055	0.8766201	-0.1899763	0.98436
XXbac-B444P24.8	1.017589	1.0159741	1.01892241	1.1845668	0.24435952	0.98437
CTB-191K22.6	1.017589	1.0159741	1.01892308	1.184609	0.24441089	0.98437
SIX2	1.017601	1.0159741	1.01894379	1.1859052	0.24598867	0.98437
TPSD1	1.017593	1.0159741	1.01892938	1.185003	0.24489068	0.98437
CTC-542B22.2	1.0176	1.0159741	1.01894303	1.1858573	0.24593044	0.98437
SHANK1	1.017594	1.0159741	1.01893089	1.1850973	0.24500551	0.98437
GLT6D1	1.017594	1.0159741	1.01893089	1.1850973	0.24500551	0.98437
RP11-769O8.1	1.017593	1.0159741	1.01892955	1.1850137	0.24490374	0.98437
RP11-19O2.2	1.017594	1.0159741	1.01893206	1.185171	0.24509528	0.98437
CNTF	1.017594	1.0159741	1.01893206	1.185171	0.24509528	0.98437
AC009487.4	1.017599	1.0159741	1.01893995	1.1856646	0.24569593	0.98437
MYBPC3	1.017601	1.0159741	1.01894476	1.1859661	0.24606282	0.98437
FAM3B	1.017596	1.0159741	1.0189361	1.1854238	0.24540296	0.98437
RP1-292B18.3	1.017595	1.0159741	1.01893318	1.1852408	0.24518018	0.98437
AC114877.3	1.017595	1.0159741	1.01893388	1.185285	0.24523397	0.98437
LY6G6C	1.017603	1.0159741	1.0189475	1.1861371	0.24627078	0.98437
RP11-42O4.2	1.017595	1.0159741	1.01893338	1.1852536	0.24519573	0.98437
LINC00326	1.017589	1.0159741	1.01892332	1.1846237	0.24442889	0.98437
RP11-867G23.12	1.017595	1.0159741	1.01893321	1.185243	0.24518285	0.98437
RP11-467C18.1	1.017594	1.0159741	1.01893168	1.1851468	0.24506583	0.98437

AP000487.4	1.017587	1.0159741	1.01891946	1.1843823	0.24413482	0.98437
CTC-296K1.4	1.017587	1.0159741	1.01891946	1.1843823	0.24413482	0.98437
LHX6	1.017598	1.0159741	1.01893919	1.185617	0.24563799	0.98437
RP11-482D24.3	1.017595	1.0159741	1.01893298	1.1852287	0.24516545	0.98437
AC092625.1	1.017592	1.0159741	1.01892872	1.1849616	0.24484033	0.98437
RP11-1105G2.3	1.017594	1.0159741	1.01893148	1.1851348	0.24505111	0.98437
NR1I2	1.0176	1.0159741	1.01894179	1.1857801	0.24583647	0.98437
CTA-992D9.7	1.01759	1.0159741	1.01892509	1.1847348	0.24456416	0.98437
RP11-688G15.3	1.017593	1.0159741	1.01893062	1.1850809	0.24498558	0.98437
ZGPAT	1.017593	1.0159741	1.01893062	1.1850809	0.24498558	0.98437
XXyac-YX155B6.5	1.017592	1.0159741	1.01892834	1.1849381	0.24481174	0.98437
SLC32A1	1.017592	1.0159741	1.01892834	1.1849381	0.24481174	0.98437
RBM43	1.208964	1.2071441	1.21046613	1.0160375	0.02295363	0.98445
SLC17A5	1.199842	1.1982732	1.20113729	1.0144453	0.02069106	0.98447
CTB-55O6.12	1.070739	1.0690654	1.07212125	1.0442462	0.06246194	0.98447
PRAM1	1.019703	1.0180784	1.02104553	1.1641283	0.21925007	0.98453
DNAH2	1.019633	1.0180243	1.02096203	1.1629892	0.21783768	0.98455
AC016745.3	1.019633	1.0180243	1.02096203	1.1629892	0.21783768	0.98455
OTOF	1.019644	1.0180243	1.02098136	1.1640614	0.21916721	0.98455
RP11-1399P15.1	1.019634	1.0180243	1.02096257	1.1630193	0.21787501	0.98455
LINC01618	1.01964	1.0180243	1.02097351	1.1636262	0.21862765	0.98455
TSPAN8	1.019637	1.0180243	1.02096792	1.1633159	0.21824288	0.98455
RP11-545D19.1	1.019637	1.0180243	1.02096792	1.1633159	0.21824288	0.98455
RP11-456O19.5	1.019637	1.0180243	1.02096792	1.1633159	0.21824288	0.98455
BFSP2-AS1	1.019636	1.0180243	1.02096776	1.1633071	0.218232	0.98455
RP11-34F13.2	1.019635	1.0180243	1.02096574	1.1631952	0.21809317	0.98455
SPRR2A	1.019635	1.0180243	1.02096574	1.1631952	0.21809317	0.98455
RP11-43F13.3	1.019639	1.0180243	1.0209732	1.1636086	0.21860582	0.98455
RP11-327J17.2	1.019631	1.0180243	1.02095779	1.1627537	0.21754552	0.98455
RP11-671P2.1	1.019631	1.0180243	1.02095779	1.1627537	0.21754552	0.98455
CTD-2308B18.3	1.019641	1.0180243	1.02097522	1.163721	0.21874526	0.98455
RP11-17A4.2	1.019632	1.0180243	1.0209591	1.1628263	0.21763561	0.98455
GAST	1.019629	1.0180243	1.02095502	1.1625999	0.21735475	0.98455
RP11-92C4.6	1.019632	1.0180243	1.02096018	1.1628864	0.21771015	0.98455
RP11-90C4.1	1.019632	1.0180243	1.02096018	1.1628864	0.21771015	0.98455
C8B	1.01963	1.0180243	1.02095632	1.1626724	0.21744469	0.98455
AP000350.10	1.019642	1.0180243	1.02097835	1.1638944	0.2189602	0.98455
CDH9	1.01964	1.0180243	1.02097374	1.1636389	0.21864339	0.98455
CTD-2544N14.3	1.019632	1.0180243	1.02096034	1.1628951	0.21772098	0.98455
RP1-266L20.4	1.019639	1.0180243	1.02097165	1.1635226	0.21849925	0.98455
SRGN	1.019639	1.0180243	1.02097218	1.163552	0.21853564	0.98455
RP11-432J24.2	1.019639	1.0180243	1.02097218	1.163552	0.21853564	0.98455
GNAT1	1.019643	1.0180243	1.02098068	1.1640239	0.21912071	0.98455
RP11-156K13.1	1.019631	1.0180243	1.02095849	1.1627924	0.21759357	0.98455
TRAF3IP3	1.019637	1.0180243	1.02096831	1.1633373	0.21826945	0.98455

SAMD12-AS1	1.01963	1.0180243	1.0209561	1.1626599	0.21742911	0.98455
RP3-331H24.5	1.019633	1.0180243	1.02096165	1.1629678	0.21781121	0.98455
RP11-350J20.12	1.019632	1.0180243	1.02095887	1.1628137	0.21762	0.98455
GNLY	1.019632	1.0180243	1.02095887	1.1628137	0.21762	0.98455
LURAP1L-AS1	1.019631	1.0180243	1.02095817	1.162775	0.21757194	0.98455
FGD2	1.019631	1.0180243	1.02095817	1.162775	0.21757194	0.98455
RP11-354M20.3	1.019639	1.0180243	1.02097219	1.1635529	0.21853679	0.98455
SLC3A1	1.019631	1.0180243	1.02095833	1.1627837	0.21758275	0.98455
CTD-2026G6.3	1.019631	1.0180243	1.02095833	1.1627837	0.21758275	0.98455
LINC00348	1.019631	1.0180243	1.02095833	1.1627837	0.21758275	0.98455
CTD-2525I3.3	1.019631	1.0180243	1.02095794	1.1627624	0.21755633	0.98455
ZIC4-AS1	1.019642	1.0180243	1.02097803	1.1638768	0.21893831	0.98455
RP11-14N9.2	1.019642	1.0180243	1.02097725	1.1638336	0.21888486	0.98455
QPCT	1.019642	1.0180243	1.02097725	1.1638336	0.21888486	0.98455
CTD-2118P12.1	1.019632	1.0180243	1.02095964	1.1628563	0.21767287	0.98455
MOG	1.019641	1.0180243	1.02097538	1.1637298	0.21875618	0.98455
INSL4	1.019641	1.0180243	1.02097538	1.1637298	0.21875618	0.98455
CECR6	1.03179	1.0333476	1.03050354	0.9147147	-0.1286062	0.98455
RP11-281A20.2	1.017548	1.0159362	1.01887828	1.1846126	0.24441532	0.98456
RP11-733O18.1	1.017552	1.0159362	1.01888549	1.1850647	0.24496585	0.98456
RP11-353N4.6	1.017541	1.0159362	1.01886687	1.1838968	0.24354327	0.98456
RP11-25I15.2	1.017542	1.0159362	1.01886892	1.1840255	0.24370013	0.98456
WWC2-AS1	1.017545	1.0159362	1.01887319	1.1842929	0.24402593	0.98456
PNLIPRP3	1.017545	1.0159362	1.01887264	1.1842589	0.24398452	0.98456
AL121656.5	1.017549	1.0159362	1.01888131	1.184803	0.24464722	0.98456
RP11-16E23.5	1.017542	1.0159362	1.01886764	1.1839448	0.24360184	0.98456
OSTN-AS1	1.017542	1.0159362	1.01886764	1.1839448	0.24360184	0.98456
RP11-445O3.2	1.017544	1.0159362	1.01887149	1.1841866	0.24389643	0.98456
RP1-41C23.4	1.017542	1.0159362	1.01886725	1.1839208	0.24357255	0.98456
RNF148	1.017551	1.0159362	1.01888427	1.1849884	0.2448729	0.98456
FA2H	1.017549	1.0159362	1.01888116	1.1847931	0.24463513	0.98456
ITGAM	1.017549	1.0159362	1.01888116	1.1847931	0.24463513	0.98456
RP11-342C20.2	1.017549	1.0159362	1.01888116	1.1847931	0.24463513	0.98456
RP11-86A5.1	1.017543	1.0159362	1.01887057	1.1841286	0.24382572	0.98456
ZNF366	1.01755	1.0159362	1.01888263	1.1848857	0.24474792	0.98456
CTD-2263F21.1	1.017542	1.0159362	1.01886818	1.1839787	0.24364312	0.98456
CD3D	1.017542	1.0159362	1.01886818	1.1839787	0.24364312	0.98456
TBL1XR1-AS1	1.017542	1.0159362	1.01886818	1.1839787	0.24364312	0.98456
RP11-118B22.4	1.017546	1.0159362	1.01887597	1.1844674	0.24423849	0.98456
TNFSF18	1.017546	1.0159362	1.01887597	1.1844674	0.24423849	0.98456
CFC1B	1.017546	1.0159362	1.01887597	1.1844674	0.24423849	0.98456
RP11-352D3.2	1.017545	1.0159362	1.0188728	1.1842688	0.24399655	0.98456
RP11-118G23.3	1.017551	1.0159362	1.01888372	1.1849541	0.24483123	0.98456
RP4-591N18.2	1.017554	1.0159362	1.0188891	1.1852914	0.2452418	0.98456
RP11-103H7.1	1.017541	1.0159362	1.01886633	1.1838629	0.24350202	0.98456

AC114765.2	1.017554	1.0159362	1.01889072	1.1853934	0.24536598	0.98456
SCGB1D2	1.017554	1.0159362	1.01889072	1.1853934	0.24536598	0.98456
RNF222	1.01755	1.0159362	1.01888318	1.1849199	0.24478957	0.98456
CTA-989H11.1	1.01755	1.0159362	1.01888354	1.1849429	0.24481751	0.98456
RP11-100M12.3	1.01755	1.0159362	1.01888354	1.1849429	0.24481751	0.98456
UGT2A3	1.01755	1.0159362	1.01888354	1.1849429	0.24481751	0.98456
LINC01603	1.017554	1.0159362	1.01889009	1.1853536	0.24531749	0.98456
RP11-881M11.2	1.017548	1.0159362	1.01887814	1.1846037	0.24440452	0.98456
HCN2	1.01755	1.0159362	1.0188824	1.1848714	0.24473047	0.98456
RP11-510M2.1	1.017544	1.0159362	1.01887188	1.1842107	0.24392579	0.98456
AC005339.2	1.017548	1.0159362	1.01887945	1.1846863	0.24450503	0.98456
RP11-160E2.11	1.017545	1.0159362	1.0188735	1.1843126	0.24404999	0.98456
ANKRD20A1	1.017545	1.0159362	1.0188735	1.1843126	0.24404999	0.98456
SLA	1.017547	1.0159362	1.01887681	1.1845202	0.24430282	0.98456
GDNF	1.017547	1.0159362	1.01887681	1.1845202	0.24430282	0.98456
RP11-195C7.1	1.01755	1.0159362	1.01888277	1.1848946	0.24475874	0.98456
RP11-80H5.6	1.01755	1.0159362	1.01888277	1.1848946	0.24475874	0.98456
FAM20A	1.056028	1.0544153	1.05735935	1.0541039	0.07601709	0.98458
SCO2	1.300196	1.3020655	1.29865266	0.9887018	-0.0163926	0.98459
CTC-304I17.5	1.022778	1.0243164	1.02150823	0.8845146	-0.1770421	0.98461
RP11-182J1.3	1.022762	1.0243255	1.02147161	0.8826784	-0.1800403	0.98461
PDZK1	1.022818	1.0244268	1.02149016	0.8797794	-0.1847863	0.98461
IL1RAP	1.09215	1.0906292	1.09340539	1.0306325	0.04353004	0.98463
RP11-118F19.1	1.046056	1.0444915	1.04734714	1.0641845	0.08974828	0.98466
STPG3-AS1	1.029693	1.0312359	1.02841841	0.9097983	-0.1363814	0.98472
SLC10A7	1.103184	1.1049196	1.10175026	0.969793	-0.0442512	0.98473
TSACC	1.018654	1.0170556	1.01997341	1.171073	0.22783102	0.98475
RP11-173M1.4	1.018671	1.0170835	1.01998168	1.1696469	0.22607312	0.98475
CTD-2036P10.6	1.018683	1.01708	1.02000648	1.1713408	0.22816084	0.98475
MTTP	1.022135	1.0236785	1.02086042	0.880987	-0.1828074	0.98475
CIR1	1.926656	1.9288702	1.92482785	0.9956481	-0.0062922	0.98477
MAMLD1	1.045056	1.0434346	1.04639481	1.068154	0.0951196	0.98479
DIRC2	1.111898	1.1102255	1.11327996	1.0277108	0.03943438	0.9848
CTD-2396E7.9	1.017864	1.0162589	1.01918971	1.180261	0.23910598	0.98481
AC107057.1	1.017857	1.0162589	1.0191772	1.1794913	0.23816483	0.98481
CTD-2192J16.22	1.017853	1.0162589	1.01916908	1.1789923	0.23755429	0.98481
CTC-436P18.1	1.017853	1.0162589	1.01916908	1.1789923	0.23755429	0.98481
RP1-155D22.2	1.017853	1.0162589	1.01916931	1.1790062	0.23757135	0.98481
CTD-2245E15.3	1.017853	1.0162589	1.01916931	1.1790062	0.23757135	0.98481
PLVAP	1.017853	1.0162589	1.01916931	1.1790062	0.23757135	0.98481
ADGRF5	1.017852	1.0162589	1.01916792	1.1789205	0.23746645	0.98481
RP11-506M12.1	1.017857	1.0162589	1.01917688	1.1794719	0.23814107	0.98481
C9orf84	1.017856	1.0162589	1.01917557	1.1793911	0.23804221	0.98481
NHS-AS1	1.017854	1.0162589	1.01917132	1.1791297	0.23772242	0.98481
C9orf64	1.017854	1.0162589	1.01917132	1.1791297	0.23772242	0.98481

RP11-673F18.1	1.017853	1.0162589	1.0191687	1.1789687	0.23752542	0.98481
MZB1	1.017857	1.0162589	1.01917725	1.1794943	0.23816849	0.98481
RP11-158M2.5	1.017858	1.0162589	1.01917835	1.1795625	0.23825189	0.98481
SLCO1C1	1.017863	1.0162589	1.01918759	1.1801308	0.23894673	0.98481
RP11-193H5.1	1.017855	1.0162589	1.01917355	1.1792673	0.23789077	0.98481
KB-1568E2.1	1.017858	1.0162589	1.01917797	1.1795388	0.23822286	0.98481
AF127577.12	1.017858	1.0162589	1.01917797	1.1795388	0.23822286	0.98481
HIST1H2BB	1.017859	1.0162589	1.01917951	1.1796338	0.23833901	0.98481
NOS1	1.017859	1.0162589	1.01917951	1.1796338	0.23833901	0.98481
TACR1	1.017859	1.0162589	1.01917983	1.1796532	0.2383628	0.98481
RP11-89M20.2	1.017859	1.0162589	1.01918106	1.1797289	0.23845538	0.98481
RP11-65B7.2	1.017861	1.0162589	1.01918394	1.1799063	0.23867227	0.98481
RP11-45M22.3	1.017853	1.0162589	1.01917023	1.1790631	0.23764095	0.98481
GPX2	1.017855	1.0162589	1.01917224	1.1791867	0.2377921	0.98481
SLC15A1	1.017855	1.0162589	1.01917224	1.1791867	0.2377921	0.98481
RP11-407G23.1	1.017855	1.0162589	1.01917224	1.1791867	0.2377921	0.98481
AC135178.7	1.017867	1.0162589	1.01919589	1.1806411	0.23957046	0.98481
RP5-1050D4.4	1.01786	1.0162589	1.01918224	1.1798013	0.2385439	0.98481
RP11-667K14.4	1.017857	1.0162589	1.0191765	1.1794482	0.23811207	0.98481
TMED6	1.017864	1.0162589	1.01918938	1.1802405	0.2390809	0.98481
EPGN	1.017854	1.0162589	1.01917137	1.1791327	0.23772607	0.98481
AC010524.4	1.083813	1.0853806	1.0825187	0.9664808	-0.049187	0.98481
RP11-335L23.4	1.017303	1.0157004	1.01862705	1.1864045	0.24659593	0.98484
RP11-142M10.2	1.017304	1.0157004	1.01862899	1.1865281	0.24674632	0.98484
RP11-195B3.1	1.017297	1.0157004	1.01861517	1.1856477	0.24567534	0.98484
RP11-433A10.3	1.017297	1.0157004	1.01861517	1.1856477	0.24567534	0.98484
RP11-301L8.2	1.017304	1.0157004	1.01862822	1.186479	0.24668658	0.98484
RP11-179A16.2	1.017304	1.0157004	1.0186291	1.186535	0.24675471	0.98484
AC013480.2	1.017307	1.0157004	1.01863415	1.1868567	0.24714578	0.98484
GOLGA8J	1.017307	1.0157004	1.01863415	1.1868567	0.24714578	0.98484
PRAMEF12	1.017303	1.0157004	1.01862689	1.1863944	0.24658365	0.98484
RP5-1101C3.1	1.017299	1.0157004	1.01861958	1.1859285	0.24601699	0.98484
ZCCHC16	1.017298	1.0157004	1.01861644	1.1857289	0.24577422	0.98484
RP13-188A5.1	1.017298	1.0157004	1.01861644	1.1857289	0.24577422	0.98484
RP1-111B22.3	1.017297	1.0157004	1.0186149	1.1856304	0.24565435	0.98484
GS1-204I12.4	1.017297	1.0157004	1.0186149	1.1856304	0.24565435	0.98484
CCDC105	1.0173	1.0157004	1.01862043	1.1859826	0.24608284	0.98484
RP11-275I14.4	1.017302	1.0157004	1.01862415	1.1862196	0.24637108	0.98484
AC026904.1	1.017301	1.0157004	1.01862267	1.1861256	0.24625679	0.98484
RP11-20I23.7	1.017304	1.0157004	1.01862771	1.1864463	0.24664683	0.98484
LSMEM2	1.017311	1.0157004	1.01864017	1.1872404	0.24761203	0.98484
TFDP3	1.017306	1.0157004	1.01863153	1.1866901	0.24694327	0.98484
AC020571.3	1.017298	1.0157004	1.01861717	1.1857752	0.2458305	0.98484
SLCO4A1-AS1	1.017302	1.0157004	1.0186238	1.1861972	0.24634385	0.98484
RP11-107I14.4	1.017297	1.0157004	1.01861505	1.1856399	0.2456659	0.98484

C17orf98	1.017296	1.0157004	1.01861432	1.1855937	0.24560969	0.98484
AC006116.21	1.017296	1.0157004	1.01861432	1.1855937	0.24560969	0.98484
RP11-122K13.7	1.0173	1.0157004	1.01862051	1.1859876	0.24608896	0.98484
IL12A-AS1	1.017296	1.0157004	1.01861416	1.1855837	0.2455975	0.98484
IL12RB2	1.017302	1.0157004	1.01862425	1.1862262	0.2463791	0.98484
RP11-268G12.3	1.017302	1.0157004	1.01862512	1.1862817	0.24644665	0.98484
RP11-502M1.2	1.017304	1.0157004	1.01862865	1.1865065	0.24671996	0.98484
RP11-454C18.1	1.017297	1.0157004	1.01861579	1.1856872	0.24572342	0.98484
ENDOU	1.017307	1.0157004	1.01863352	1.1868167	0.24709718	0.98484
RGPD6	1.017303	1.0157004	1.01862632	1.1863579	0.24653933	0.98484
PCDHB8	1.017296	1.0157004	1.01861278	1.1854953	0.24548997	0.98484
CTD-2035E11.4	1.017301	1.0157004	1.01862198	1.1860815	0.2462031	0.98484
NKX1-2	1.017297	1.0157004	1.01861563	1.1856771	0.24571122	0.98484
TMEM43	1.435988	1.4374894	1.43474754	0.9937328	-0.0090702	0.98492
PPIL2	1.254761	1.2530292	1.25619096	1.0124957	0.01791579	0.98496
RP11-359D14.2	1.015425	1.0169626	1.01415473	0.8344651	-0.2610764	0.98504
CH17-408M7.1	1.015431	1.0169765	1.01415473	0.8337819	-0.2622581	0.98504
ZRSR1	1.01542	1.0169518	1.01415473	0.8349987	-0.2601542	0.98504
RP1-69M21.2	1.015429	1.016973	1.01415473	0.8339568	-0.2619555	0.98504
LINC01102	1.015425	1.0169632	1.01415473	0.8344362	-0.2611264	0.98504
CTC-529L17.2	1.015433	1.0169813	1.01415473	0.8335462	-0.262666	0.98504
MACC1-AS1	1.015433	1.0169813	1.01415473	0.8335462	-0.262666	0.98504
RBP3	1.015413	1.0169364	1.01415473	0.8357599	-0.2588396	0.98504
AC107218.3	1.015432	1.0169782	1.01415473	0.8336981	-0.262403	0.98504
PRMT2	3.731283	3.7350708	3.7281557	0.9974717	-0.0036522	0.98504
CTSC	1.597388	1.5954117	1.59901958	1.0060595	0.00871567	0.9851
AC090587.5	1.019887	1.0214135	1.01862593	0.8698232	-0.201206	0.98515
ROBO4	1.019905	1.0214573	1.0186238	0.8679454	-0.2043238	0.98515
GHRHR	1.019882	1.0213998	1.01862842	0.8704946	-0.2000927	0.98515
CHRM3-AS2	1.019881	1.0213967	1.0186291	0.8706523	-0.1998313	0.98515
RP11-93G5.1	1.019887	1.0214144	1.01862524	0.8697526	-0.201323	0.98515
RP11-798K3.2	1.019871	1.0213948	1.0186122	0.8699402	-0.2010119	0.98515
C5AR2	1.019887	1.0214106	1.01862865	0.8700677	-0.2008005	0.98515
FPR1	1.020449	1.0219895	1.01917758	0.8721236	-0.1973955	0.98519
ELDR	1.020435	1.0219659	1.01917132	0.8727763	-0.1963162	0.98519
RP11-433J8.2	1.020472	1.0220449	1.01917355	0.8697514	-0.201325	0.98519
AC012074.2	1.020458	1.0220085	1.01917727	0.8713568	-0.1986646	0.98519
AC007950.2	1.02047	1.0220019	1.01920486	0.8728738	-0.196155	0.98519
ELFN2	1.020499	1.0220936	1.01918224	0.8682245	-0.20386	0.98519
PCDHA2	1.02045	1.0219913	1.01917688	0.8720205	-0.1975661	0.98519
RP3-395M20.2	1.020453	1.0220012	1.01917355	0.8714764	-0.1984665	0.98519
MMP17	1.021253	1.0227793	1.01999315	0.8776892	-0.188218	0.98532
ZDHHC1	1.227566	1.2258918	1.22894778	1.0135286	0.01938679	0.98532
HMGCR	1.380333	1.3819255	1.37901715	0.992385	-0.0110282	0.98535
TIMM29	1.258244	1.2598061	1.25695342	0.9890198	-0.0159287	0.98539

RAB11B-AS1	1.445204	1.4468453	1.44384775	0.9932918	-0.0097105	0.98539
RP11-231G3.1	1.027118	1.0255626	1.02840298	1.1111167	0.15201033	0.98542
MARCH11	1.0271	1.025526	1.02839968	1.1125803	0.15390944	0.98542
SEZ6L	1.027187	1.0255743	1.02851823	1.1151117	0.15718826	0.98542
RP11-91K8.5	1.020166	1.0185928	1.02146519	1.1544878	0.20725298	0.98542
RP13-890H12.2	1.020186	1.0185944	1.02149978	1.1562526	0.20945658	0.98542
RP11-293M10.6	1.020178	1.0185988	1.02148199	1.1550206	0.20791862	0.98542
SLFNL1	1.020187	1.0186024	1.02149524	1.1555092	0.20852871	0.98542
RP11-239A17.1	1.020195	1.0185971	1.02151521	1.1569139	0.21028154	0.98542
AC073343.13	1.020187	1.0186064	1.02149202	1.1550906	0.20800604	0.98542
MRLN	1.020172	1.0186003	1.02146962	1.1542602	0.20696851	0.98542
CCDC178	1.020194	1.0185973	1.02151223	1.1567393	0.21006378	0.98542
AC092684.1	1.020171	1.018596	1.0214722	1.1546651	0.20747442	0.98542
LINC01068	1.020195	1.018603	1.0215097	1.1562508	0.20945435	0.98542
CSGALNACT1	1.020201	1.0186333	1.02149493	1.1535756	0.20611258	0.98542
GRIN1	1.020143	1.0216555	1.01889479	0.8725164	-0.1967459	0.98544
KCNV2	1.020148	1.0216818	1.01888116	0.8708279	-0.1995404	0.98544
RP11-235E17.2	1.020144	1.0216654	1.01888839	0.8718227	-0.1978933	0.98544
MYOM3	1.022234	1.0237682	1.02096776	0.8821782	-0.1808579	0.98545
MIR3179-3	1.022229	1.0237679	1.02095871	0.8818073	-0.1814646	0.98545
CTC-510F12.7	1.022228	1.0237436	1.02097554	0.8834199	-0.1788288	0.98545
RP11-164N3.2	1.022243	1.0237671	1.02098492	0.8829398	-0.179613	0.98545
RP11-257P3.3	1.02223	1.0237692	1.0209591	0.8817739	-0.1815193	0.98545
RP11-510J16.5	1.022268	1.0237604	1.0210354	0.8853116	-0.1757428	0.98553
PLBD1-AS1	1.022387	1.0240096	1.0210471	0.8766137	-0.1899868	0.98553
RP11-25H12.1	1.029245	1.0277284	1.03049792	1.0998797	0.1373458	0.9856
AC145343.2	1.029143	1.0275674	1.03044394	1.1043476	0.14319432	0.9856
RP11-708J19.1	1.124162	1.1257555	1.12284648	0.9768674	-0.0337653	0.9856
AKAP3	1.027046	1.0254951	1.02832594	1.1110351	0.15190443	0.98561
KC6	2.1829	2.1847422	2.18137874	0.997161	-0.0041017	0.98562
PPIAL4G	1.020209	1.0217462	1.01893995	0.8709529	-0.1993334	0.98563
LINC01132	1.020183	1.0216983	1.01893148	0.8724887	-0.1967917	0.98563
GALR2	1.029172	1.0275728	1.03049241	1.105889	0.14520665	0.98565
TLL1	1.0439	1.0423278	1.04519759	1.0677992	0.09464032	0.98568
TACR3	1.019281	1.0176936	1.02059258	1.1638457	0.21889985	0.9857
CTD-2189E23.1	1.019339	1.0177167	1.02067846	1.1671761	0.22302219	0.9857
BTN3A1	1.101702	1.1000004	1.10310707	1.0310661	0.04413683	0.98571
ZNF551	1.123095	1.1213911	1.12450224	1.0256292	0.03650924	0.98573
AC002117.1	1.125726	1.1272693	1.12445228	0.9778654	-0.0322922	0.98575
PCDHA5	1.028455	1.0299941	1.0271837	0.9063019	-0.1419364	0.98579
TTC17	1.854402	1.8563591	1.85278554	0.9958271	-0.0060329	0.98587
RP11-89C3.4	1.041464	1.0398231	1.04281903	1.0752324	0.10464846	0.98589
KHDC1L	1.026808	1.0252182	1.02812165	1.1151331	0.15721586	0.98589
C1orf159	1.152923	1.1545748	1.1515587	0.980488	-0.0284281	0.98592
CSK	1.215383	1.216945	1.21409366	0.9868567	-0.0190875	0.98594

CACNA1C-AS2	1.051808	1.0502396	1.05310285	1.0569919	0.07996432	0.98595
CHODL	1.07143	1.0729761	1.07015376	0.9613255	-0.056903	0.98596
AC034243.1	1.019693	1.0181317	1.02098251	1.1572305	0.21067631	0.98598
STK31	1.019693	1.0181317	1.02098179	1.1571911	0.21062716	0.98598
LBX2-AS1	1.078226	1.0796656	1.07703651	0.966998	-0.0484153	0.98602
PHEX-AS1	1.01831	1.0167712	1.01958068	1.1675214	0.22344895	0.98615
RP5-1061H20.4	1.018307	1.0167699	1.01957723	1.1674063	0.2233067	0.98615
MYLK-AS2	1.018326	1.0167627	1.01961664	1.1702525	0.22681981	0.98615
ITGAX	1.018317	1.01676	1.01960313	1.169635	0.22605839	0.98615
RP1-178F10.1	1.018306	1.0167798	1.01956723	1.1661174	0.22171307	0.98615
RFPL2	1.01832	1.0167679	1.01960136	1.1689823	0.22525313	0.98615
FAM198A	1.018321	1.0167596	1.01960968	1.1700584	0.22658053	0.98615
CTC-297N7.1	1.018321	1.0167607	1.01960907	1.1699434	0.22643879	0.98615
XXbac-BPG249D20.9	1.018315	1.0167639	1.01959589	1.1689361	0.22519608	0.98615
CTB-127C13.1	1.018338	1.0167679	1.01963454	1.1709614	0.22769348	0.98615
TSSK4	1.018339	1.0167692	1.01963579	1.1709439	0.22767201	0.98615
ANKK1	1.018304	1.0167596	1.01957902	1.1682285	0.22432255	0.98615
MTMR10	1.08787	1.0892812	1.08670397	0.9711332	-0.0422588	0.98618
DVL1	1.145426	1.143717	1.14683713	1.0217101	0.0309859	0.98619
TXNDC11	1.164019	1.1657472	1.16259115	0.9809586	-0.0277359	0.98622
ZNF682	1.164557	1.1629026	1.16592275	1.0185394	0.02650184	0.98625
APOBEC3D	1.02565	1.0271542	1.02440729	0.898841	-0.1538621	0.98625
STPG2	1.025633	1.0270961	1.02442492	0.9014197	-0.1497291	0.98625
PARVG	1.014389	1.012846	1.0156638	1.2193488	0.28611084	0.9863
TDRD15	1.014386	1.012846	1.01565814	1.2189081	0.28558934	0.9863
ABRA	1.014392	1.012846	1.01566782	1.219662	0.2864814	0.9863
FAM86B2	1.014386	1.012846	1.01565697	1.2188177	0.28548232	0.9863
CD86	1.014388	1.012846	1.01566155	1.2191742	0.28590425	0.9863
RP11-159F24.6	1.014388	1.012846	1.01566155	1.2191742	0.28590425	0.9863
KCNH7	1.014382	1.012846	1.01564989	1.2182661	0.28482934	0.9863
RP11-256I23.1	1.014388	1.012846	1.0156612	1.2191462	0.28587113	0.9863
AC005281.1	1.014387	1.012846	1.01565886	1.2189641	0.28565566	0.9863
CTC-508F8.1	1.014381	1.012846	1.01564909	1.2182036	0.28475526	0.9863
TARID	1.014382	1.012846	1.01565015	1.2182864	0.28485333	0.9863
IDI2	1.014384	1.012846	1.01565322	1.2185254	0.28513627	0.9863
AC004947.2	1.014384	1.012846	1.01565322	1.2185254	0.28513627	0.9863
STEAP3-AS1	1.014385	1.012846	1.01565535	1.218691	0.28533237	0.9863
RP1-261G23.5	1.014385	1.012846	1.01565535	1.218691	0.28533237	0.9863
ADGRG5	1.014386	1.012846	1.01565685	1.2188082	0.28547106	0.9863
LINC01416	1.014382	1.012846	1.01565001	1.2182756	0.28484056	0.9863
H2BFS	1.014384	1.012846	1.01565488	1.2186548	0.28528958	0.9863
PDZK1IP1	1.014378	1.012846	1.01564335	1.2177574	0.28422674	0.9863
XXbac-BPG308J9.3	1.014384	1.012846	1.01565388	1.2185765	0.28519677	0.9863
RP11-227D13.4	1.014384	1.012846	1.01565461	1.2186337	0.28526455	0.9863
ENAM	1.01438	1.012846	1.01564631	1.2179877	0.28449962	0.9863

RP11-1057B8.2	1.01438	1.012846	1.01564631	1.2179877	0.28449962	0.9863
RP11-325K19.1	1.014381	1.012846	1.01564944	1.2182308	0.28478745	0.9863
RP11-22L13.1	1.014384	1.012846	1.01565341	1.2185403	0.28515401	0.9863
RP11-354K1.1	1.014379	1.012846	1.01564481	1.2178705	0.28436076	0.9863
RP13-726E6.1	1.014386	1.012846	1.01565841	1.2189293	0.28561441	0.9863
RP11-82O19.1	1.014382	1.012846	1.01565109	1.2183599	0.28494042	0.9863
SHISA8	1.014385	1.012846	1.0156558	1.2187263	0.28537419	0.9863
AC004014.3	1.014385	1.012846	1.0156558	1.2187263	0.28537419	0.9863
VGLL2	1.014385	1.012846	1.0156558	1.2187263	0.28537419	0.9863
LINC01284	1.014383	1.012846	1.01565303	1.2185104	0.28511854	0.9863
LHFPL3	1.014385	1.012846	1.01565527	1.2186848	0.28532509	0.9863
APOB	1.014382	1.012846	1.0156497	1.2182512	0.28481164	0.9863
RP11-446H18.5	1.014382	1.012846	1.01565035	1.2183022	0.28487208	0.9863
RP11-756H20.1	1.014382	1.012846	1.01565035	1.2183022	0.28487208	0.9863
RP11-11C20.3	1.014381	1.012846	1.01564932	1.2182219	0.284777	0.9863
OPN1SW	1.014382	1.012846	1.01565063	1.2183239	0.28489769	0.9863
CTC-756D1.2	1.014385	1.012846	1.015656	1.2187422	0.28539293	0.9863
LYL1	1.01438	1.012846	1.01564758	1.2180862	0.28461618	0.9863
USP51	1.09568	1.0971937	1.0944291	0.9715555	-0.0416317	0.98634
ZNF546	1.095688	1.0973734	1.09429541	0.9683895	-0.0463406	0.98634
SEL1L2	1.025049	1.0265339	1.02382279	0.8978258	-0.1554925	0.9864
DEF6	1.029668	1.0281159	1.03094903	1.1007646	0.13850595	0.98643
AC002456.2	1.029011	1.0274438	1.03030569	1.1042822	0.14310887	0.98644
PKMYT1	1.094568	1.0959727	1.09340791	0.9732754	-0.0390799	0.98645
RP11-524F11.2	1.013657	1.0151352	1.01243594	0.821658	-0.2833901	0.98646
RP11-469N6.1	1.013657	1.015136	1.01243594	0.8216119	-0.283471	0.98646
TPTE2	1.013661	1.0151454	1.01243594	0.8211042	-0.2843628	0.98646
STAU2-AS1	1.013661	1.0151454	1.01243594	0.8211042	-0.2843628	0.98646
CTD-2201E18.5	1.013666	1.0151558	1.01243594	0.8205406	-0.2853534	0.98646
TUBA3D	1.013672	1.015169	1.01243594	0.8198257	-0.2866109	0.98646
LINC01422	1.013644	1.0151069	1.01243594	0.8231941	-0.2806954	0.98646
RP11-6F2.5	1.013652	1.0151242	1.01243594	0.8222535	-0.2823448	0.98646
RP11-508N22.12	1.013663	1.0151479	1.01243594	0.8209704	-0.2845979	0.98646
RP11-191L9.4	1.013661	1.0151437	1.01243594	0.8211944	-0.2842044	0.98646
RP11-380O24.1	1.013657	1.0151347	1.01243594	0.8216834	-0.2833455	0.98646
RP11-379B8.1	1.013647	1.0151141	1.01243594	0.8228021	-0.2813825	0.98646
LRP1-AS	1.013659	1.0151405	1.01243594	0.8213674	-0.2839004	0.98646
AC093627.9	1.039526	1.0410562	1.0382625	0.9319541	-0.1016691	0.98649
NUDT12	1.249618	1.2477862	1.25113053	1.0134968	0.01934157	0.98651
CLPSL2	1.026803	1.0282996	1.0255671	0.9034453	-0.1464908	0.98651
ISL1	1.02752	1.0290107	1.02628878	0.9061752	-0.142138	0.98656
SETSIP	1.02755	1.0291177	1.02625477	0.9016785	-0.149315	0.98656
TFR2	1.093385	1.0950679	1.09199475	0.9676737	-0.0474074	0.98663
PANO1	1.073179	1.0716743	1.07442201	1.0383359	0.05427329	0.98665
MAST1	1.111944	1.1103051	1.11329757	1.0271287	0.038617	0.98665

RP11-569A11.1	1.018959	1.0204548	1.01772433	0.8665106	-0.2067107	0.9867
C10orf142	1.018928	1.0204048	1.0177087	0.8678673	-0.2044536	0.9867
CTC-513N18.7	1.018959	1.020462	1.01771722	0.8658579	-0.2077979	0.9867
CTD-2265O21.3	1.018948	1.0204326	1.01772197	0.8673367	-0.205336	0.9867
C9orf40	1.243716	1.2451055	1.24256845	0.9896491	-0.015011	0.98672
CHERP	1.313028	1.3116609	1.31415649	1.0080075	0.01150641	0.98676
POMT1	1.219682	1.2179851	1.22108422	1.0142173	0.02036684	0.98677
RP11-7F18.2	1.046811	1.0483231	1.04556304	0.9428831	-0.0848492	0.9868
RP11-327P2.5	1.028734	1.0272091	1.02999363	1.1023367	0.1405649	0.98683
PRR5-ARHGAP8	1.017114	1.0156039	1.0183608	1.1766803	0.23472235	0.98691
GCSAM	1.017117	1.0156039	1.01836644	1.1770412	0.23516485	0.98691
RP11-213H15.1	1.017116	1.0156039	1.01836381	1.1768726	0.23495821	0.98691
FAM129C	1.017124	1.0156039	1.01837979	1.177897	0.23621342	0.98691
ANKRD30BL	1.017124	1.0156039	1.01837979	1.177897	0.23621342	0.98691
LINC01625	1.017117	1.0156039	1.01836632	1.1770339	0.23515592	0.98691
AL513523.2	1.017117	1.0156039	1.01836632	1.1770339	0.23515592	0.98691
LINC00868	1.017118	1.0156039	1.01836919	1.1772175	0.23538094	0.98691
C1orf140	1.017114	1.0156039	1.01836168	1.1767367	0.23479158	0.98691
RP11-334A14.8	1.017114	1.0156039	1.01836134	1.1767149	0.23476482	0.98691
MIRLET7DHG	1.017114	1.0156039	1.01836134	1.1767149	0.23476482	0.98691
TCF24	1.017114	1.0156039	1.01836134	1.1767149	0.23476482	0.98691
LINC01350	1.017113	1.0156039	1.01835923	1.1765792	0.23459844	0.98691
RP11-315E17.1	1.017126	1.0156039	1.01838372	1.178149	0.23652197	0.98691
NPIPB7	1.017118	1.0156039	1.01836926	1.1772219	0.23538635	0.98691
RP11-429B14.4	1.017128	1.0156039	1.01838733	1.1783805	0.23680543	0.98691
RP5-1057I20.5	1.017125	1.0156039	1.01838037	1.1779343	0.23625912	0.98691
SYNE1-AS1	1.017125	1.0156039	1.01838037	1.1779343	0.23625912	0.98691
TAS2R43	1.017119	1.0156039	1.01837084	1.1773237	0.23551098	0.98691
AC114730.7	1.017119	1.0156039	1.01837084	1.1773237	0.23551098	0.98691
RP11-806K15.1	1.017119	1.0156039	1.01837084	1.1773237	0.23551098	0.98691
CYTH4	1.017119	1.0156039	1.01837084	1.1773237	0.23551098	0.98691
CTB-180A7.3	1.017119	1.0156039	1.01836976	1.1772545	0.23542623	0.98691
RP11-503E24.2	1.017122	1.0156039	1.01837636	1.1776773	0.23594429	0.98691
PDCD1LG2	1.017123	1.0156039	1.01837772	1.1777643	0.23605081	0.98691
CTC-564N23.2	1.017126	1.0156039	1.01838286	1.1780939	0.2364545	0.98691
LINC01527	1.017115	1.0156039	1.01836327	1.1768385	0.23491633	0.98691
AP001055.6	1.01712	1.0156039	1.01837282	1.1774501	0.2356659	0.98691
AC098617.1	1.017118	1.0156039	1.01836799	1.1771405	0.23528654	0.98691
TMEM171	1.017112	1.0156039	1.01835711	1.1764437	0.23443225	0.98691
RP11-255P5.2	1.017123	1.0156039	1.01837678	1.1777044	0.23597751	0.98691
RP11-665I14.1	1.017123	1.0156039	1.01837678	1.1777044	0.23597751	0.98691
HEPHL1	1.017115	1.0156039	1.01836297	1.1768189	0.23489233	0.98691
TRG-AS1	1.017121	1.0156039	1.01837395	1.1775226	0.23575471	0.98691
CTD-2270F17.1	1.017121	1.0156039	1.01837395	1.1775226	0.23575471	0.98691
ERVH48-1	1.017125	1.0156039	1.01838103	1.1779768	0.23631109	0.98691

RP11-1078H9.5	1.01712	1.0156039	1.01837208	1.1774028	0.23560798	0.98691
CACNG3	1.017121	1.0156039	1.01837426	1.1775426	0.23577925	0.98691
RTL1	1.017121	1.0156039	1.01837406	1.1775299	0.23576367	0.98691
NLRP8	1.017126	1.0156039	1.01838309	1.1781086	0.23647253	0.98691
AC147651.4	1.017115	1.0156039	1.01836308	1.1768262	0.23490124	0.98691
OBP2B	1.017115	1.0156039	1.01836308	1.1768262	0.23490124	0.98691
RP11-495K9.5	1.017117	1.0156039	1.0183669	1.1770709	0.23520127	0.98691
TRIM61	1.017117	1.0156039	1.01836569	1.1769935	0.23510631	0.98691
RP11-838N2.3	1.017113	1.0156039	1.01836006	1.1766327	0.234664	0.98691
BCL6B	1.01713	1.0156039	1.01838984	1.1785408	0.23700169	0.98691
LINC00910	1.036321	1.0348073	1.03757157	1.0794165	0.11025168	0.98693
POTEF	1.051005	1.0494268	1.05230841	1.0582997	0.08174827	0.98698
LZTS3	1.073782	1.0721856	1.07510025	1.040377	0.05710635	0.98699
RP11-242G20.1	1.016398	1.0149038	1.01763219	1.1830647	0.24252898	0.98703
RP11-507M3.1	1.01639	1.0148825	1.01763469	1.1849282	0.24479968	0.98703
SPATA45	1.016398	1.0148831	1.01764964	1.1858887	0.24596862	0.98703
LINC01241	1.016408	1.0148928	1.01765844	1.1857067	0.24574724	0.98703
SMCR2	1.016408	1.014882	1.01766826	1.1872212	0.24758882	0.98703
CTD-2619J13.27	1.016408	1.0148892	1.01766266	1.1862732	0.24643628	0.98703
CTD-2589H19.6	1.016397	1.0148856	1.01764571	1.1854205	0.24539891	0.98703
CTD-3096M3.2	1.016405	1.0148858	1.01765885	1.1862845	0.24645001	0.98703
TMPRSS7	1.016414	1.0149038	1.01766062	1.1849728	0.24485394	0.98703
TREM1	1.016401	1.014882	1.01765478	1.1863155	0.24648777	0.98703
LINC00167	1.016412	1.0149038	1.01765679	1.1847158	0.24454103	0.98703
LINC01197	1.016398	1.0148895	1.01764313	1.1849387	0.24481243	0.98703
TRIM47	1.075439	1.0740582	1.07658002	1.0340522	0.04830897	0.98704
LINC00592	1.022148	1.0206495	1.02338496	1.1324712	0.1794744	0.98708
ZNF37A	1.022165	1.0206521	1.02341409	1.1337388	0.18108832	0.98708
NFE2	1.022145	1.020647	1.02338259	1.1324928	0.17950188	0.98708
RP1-122P22.2	1.022172	1.0206592	1.02342045	1.1336575	0.18098485	0.98708
RP11-611O2.1	1.024913	1.0233785	1.02617957	1.1198127	0.16325748	0.98709
CYP1B1-AS1	1.0929	1.0943833	1.09167447	0.9712993	-0.0420122	0.98709
AC010883.5	1.060144	1.0585946	1.06142341	1.0482783	0.06802173	0.98714
RHOXF1	1.027867	1.0263248	1.02914126	1.1069883	0.14663991	0.98716
PRR35	1.039348	1.0407454	1.0381937	0.9373756	-0.0933008	0.98717
CTC-537E7.3	1.016126	1.0175617	1.01494071	0.8507543	-0.2331855	0.9872
ITIH1	1.016151	1.0176159	1.01494071	0.8481397	-0.2376263	0.9872
AL133493.2	1.016126	1.0175613	1.01494071	0.8507753	-0.23315	0.9872
CCDC13-AS1	1.016161	1.0176395	1.01494071	0.847004	-0.2395593	0.9872
TRIM74	1.016134	1.017578	1.01494071	0.8499674	-0.2345207	0.9872
RP11-33B1.3	1.016145	1.0176034	1.01494071	0.8487386	-0.2366079	0.9872
RP11-148K1.10	1.015272	1.0137737	1.01650987	1.1986544	0.26141575	0.98721
RP11-705C15.4	1.015274	1.0137737	1.01651276	1.198864	0.26166803	0.98721
CRHR2	1.015274	1.0137737	1.01651269	1.1988589	0.26166186	0.98721
LINC01337	1.015271	1.0137737	1.01650817	1.1985308	0.26126694	0.98721

RP11-383M4.6	1.015278	1.0137737	1.01652071	1.1994415	0.26236274	0.98721
RP1-302D9.1	1.015275	1.0137737	1.01651458	1.1989963	0.26182725	0.98721
RP11-508N12.2	1.015273	1.0137737	1.01651157	1.1987776	0.26156398	0.98721
AP000857.3	1.015278	1.0137737	1.01652051	1.1994268	0.2623451	0.98721
C1orf210	1.015276	1.0137737	1.01651742	1.1992021	0.26207476	0.98721
FAM83C-AS1	1.015273	1.0137737	1.01651091	1.1987299	0.26150665	0.98721
HIST1H3I	1.015273	1.0137737	1.01651091	1.1987299	0.26150665	0.98721
ABCC11	1.015275	1.0137737	1.0165156	1.1990702	0.26191607	0.98721
RP11-768G7.2	1.015274	1.0137737	1.01651315	1.1988926	0.26170241	0.98721
STK24-AS1	1.015274	1.0137737	1.01651351	1.198918	0.26173303	0.98721
DLGAP2-AS1	1.015274	1.0137737	1.01651351	1.198918	0.26173303	0.98721
AC140542.2	1.015276	1.0137737	1.01651632	1.1991225	0.261979	0.98721
RP11-205K6.1	1.015279	1.0137737	1.01652122	1.1994785	0.26240727	0.98721
LINC00216	1.015277	1.0137737	1.01651812	1.1992531	0.26213615	0.98721
FAM180B	1.01527	1.0137737	1.01650621	1.1983882	0.2610953	0.98721
AF064858.8	1.015272	1.0137737	1.01650952	1.198629	0.26138519	0.98721
PI15	1.015269	1.0137737	1.01650344	1.198187	0.26085313	0.98721
RP11-107F6.3	1.015269	1.0137737	1.01650344	1.198187	0.26085313	0.98721
RP1-228P16.5	1.015273	1.0137737	1.01651075	1.1987177	0.26149197	0.98721
RP11-385D13.1	1.015273	1.0137737	1.01651146	1.1987693	0.26155405	0.98721
ARHGAP40	1.015273	1.0137737	1.01651203	1.1988112	0.2616045	0.98721
CTD-2023N9.1	1.015269	1.0137737	1.01650398	1.1982262	0.26090032	0.98721
FAM156A	1.015271	1.0137737	1.01650798	1.1985168	0.26125009	0.98721
RP11-244F12.3	1.015269	1.0137737	1.01650436	1.198254	0.26093381	0.98721
RP11-405M12.3	1.015272	1.0137737	1.01650968	1.1986404	0.26139895	0.98721
CTD-3203P2.3	1.015274	1.0137737	1.01651323	1.1988983	0.2617093	0.98721
ATP10A	1.015278	1.0137737	1.01651944	1.1993488	0.2622513	0.98721
RP11-362A1.1	1.015277	1.0137737	1.01651793	1.1992391	0.26211927	0.98721
RP11-434H14.1	1.015271	1.0137737	1.01650663	1.1984192	0.26113266	0.98721
RP5-1056H1.2	1.015271	1.0137737	1.01650663	1.1984192	0.26113266	0.98721
RP11-417B4.2	1.015271	1.0137737	1.01650663	1.1984192	0.26113266	0.98721
AC009502.4	1.015278	1.0137737	1.01651975	1.1993711	0.26227817	0.98721
RP11-319E12.2	1.015271	1.0137737	1.01650748	1.1984807	0.26120662	0.98721
CLNK	1.015279	1.0137737	1.01652173	1.199515	0.26245115	0.98721
UBL4B	1.015279	1.0137737	1.01652173	1.199515	0.26245115	0.98721
HOXA9	1.015279	1.0137737	1.01652173	1.199515	0.26245115	0.98721
AC092415.1	1.015276	1.0137737	1.01651645	1.1991319	0.26199042	0.98721
SRD5A2	1.015278	1.0137737	1.01652032	1.1994127	0.2623282	0.98721
BLID	1.015271	1.0137737	1.01650809	1.1985251	0.26126006	0.98721
GRPEL2-AS1	1.015275	1.0137737	1.01651533	1.1990504	0.26189232	0.98721
MAMSTR	1.160321	1.1617337	1.15915526	0.9840577	-0.0231852	0.98723
CTD-2371O3.2	1.040136	1.0415377	1.03897791	0.9383739	-0.0917651	0.98724
EPM2AIP1	2.085621	2.0844995	2.08654732	1.0018882	0.00272158	0.9873
RP11-363J20.1	1.028681	1.0301512	1.02746677	0.9109687	-0.1345266	0.98739
HS3ST5	1.017284	1.0187407	1.0160807	0.8580612	-0.2208476	0.98741

CHRNA4	1.017263	1.0186948	1.0160807	0.8601709	-0.2173048	0.98741
AC015987.1	1.017284	1.0187366	1.01608382	0.8584174	-0.2202488	0.98741
CKLF-CMTM1	1.017259	1.0186956	1.01607332	0.8597386	-0.2180299	0.98741
ITFG1-AS1	1.017255	1.0186693	1.01608736	0.8617001	-0.2147422	0.98741
RP11-675F6.4	1.017274	1.0187116	1.01608642	0.8597017	-0.2180919	0.98741
CFAP57	1.025793	1.0272566	1.02458367	0.9019334	-0.1489072	0.98742
ANO3	1.023027	1.024446	1.02185543	0.8940273	-0.1616092	0.98743
RP11-385F5.4	1.023021	1.0244554	1.02183618	0.8928964	-0.1634353	0.98743
CAND2	1.055143	1.0565711	1.05396413	0.9539164	-0.0680652	0.98746
LINC01396	1.017975	1.0194025	1.01679584	0.8656538	-0.208138	0.9875
CYP4F8	1.017986	1.0194273	1.01679584	0.8645488	-0.2099806	0.9875
RP11-684B21.1	1.017986	1.0194262	1.01679584	0.8645967	-0.2099008	0.9875
RP11-248M19.1	1.017979	1.0194117	1.01679584	0.8652425	-0.2088235	0.9875
SCARA5	1.017993	1.0194426	1.01679584	0.8638681	-0.2111171	0.9875
LZTS1-AS1	1.01799	1.019435	1.01679584	0.8642079	-0.2105497	0.9875
BCL2L15	1.017995	1.0194468	1.01679584	0.8636795	-0.2114321	0.9875
RP11-497D6.3	1.017982	1.0194174	1.01679584	0.8649881	-0.2092479	0.9875
CTD-3214H19.6	1.017978	1.0194097	1.01679584	0.8653304	-0.208677	0.9875
RP11-863K10.7	1.017997	1.0194507	1.01679584	0.8635088	-0.2117172	0.9875
RP11-835E18.5	1.017997	1.0194507	1.01679584	0.8635088	-0.2117172	0.9875
USP26	1.01799	1.0194361	1.01679584	0.8641576	-0.2106337	0.9875
ANKRD20A4	1.017976	1.0194042	1.01679584	0.865579	-0.2082626	0.9875
BATF2	1.032643	1.0340679	1.03146663	0.9236441	-0.1145911	0.98752
RP11-464F9.20	1.074719	1.0732421	1.07593827	1.0368111	0.05215301	0.98757
CGGBP1	2.002511	2.0012358	2.00356365	1.002325	0.00335041	0.98767
CTD-2017F17.2	1.06217	1.0607585	1.06333633	1.0424278	0.0599474	0.98769
CD72	1.082285	1.0808238	1.08349082	1.0329978	0.04683717	0.9877
FAM181A	1.037182	1.0385706	1.03603588	0.9342836	-0.0980675	0.98771
AC109642.1	1.018063	1.0165745	1.01929294	1.1640101	0.21910355	0.98774
LRRC2	1.018063	1.0165815	1.01928579	1.1630898	0.21796253	0.98774
CTC-523E23.14	1.018058	1.0165764	1.0192816	1.1631953	0.21809337	0.98774
CTD-2292P10.4	1.018068	1.0165883	1.01929057	1.1629034	0.21773125	0.98774
RP11-69C13.1	1.018075	1.0165756	1.01931401	1.1652085	0.22058817	0.98774
OR52H1	1.018065	1.0165754	1.01929485	1.1640623	0.21916827	0.98774
RP11-234K24.6	1.018069	1.0165764	1.019301	1.1643657	0.21954425	0.98774
SCN4A	1.018075	1.0165696	1.01931881	1.1659211	0.22147019	0.98774
AC009961.3	1.037781	1.0392165	1.03659637	0.9331885	-0.0997596	0.98775
CYS1	1.037713	1.039134	1.03653985	0.9337108	-0.0989524	0.98775
AC013461.1	1.475001	1.4731937	1.47649261	1.0069715	0.01002288	0.98777
LRRC43	1.029679	1.0311654	1.0284518	0.9129299	-0.131424	0.98777
CCDC173	1.060961	1.0623679	1.05979857	0.9588042	-0.0606918	0.98782
UBAP2L	1.474139	1.4723194	1.47564186	1.0070343	0.01011287	0.98785
KIF12	1.048189	1.0467398	1.04938486	1.0565906	0.07941647	0.98786
UTP15	1.097203	1.0955688	1.09855179	1.0312127	0.04434195	0.98789
RP5-1065J22.8	1.062158	1.0636603	1.06091724	0.9569113	-0.063543	0.98792

DOLK	1.168215	1.1696531	1.16702742	0.984523	-0.0225031	0.98794
LINC01180	1.012793	1.0113227	1.01400662	1.2370349	0.30688619	0.98795
RP11-388M20.6	1.012789	1.0113227	1.01399906	1.2363674	0.30610748	0.98795
LINC01597	1.012793	1.0113227	1.01400793	1.2371508	0.3070214	0.98795
RP11-706C16.7	1.012794	1.0113227	1.01400845	1.2371969	0.30707515	0.98795
C2CD4B	1.012793	1.0113227	1.01400739	1.2371029	0.30696549	0.98795
AC009495.2	1.012794	1.0113227	1.01400956	1.2372948	0.30718922	0.98795
RP4-613A2.1	1.012794	1.0113227	1.01400877	1.2372252	0.30710811	0.98795
HOXD4	1.012795	1.0113227	1.01400994	1.2373288	0.30722894	0.98795
TBX1	1.012793	1.0113227	1.01400677	1.2370488	0.30690242	0.98795
CTD-2008P7.9	1.012793	1.0113227	1.01400677	1.2370488	0.30690242	0.98795
SPRR1B	1.012793	1.0113227	1.01400677	1.2370488	0.30690242	0.98795
CXCL11	1.012792	1.0113227	1.0140046	1.2368573	0.30667904	0.98795
Z83001.1	1.012792	1.0113227	1.0140046	1.2368573	0.30667904	0.98795
AC007381.3	1.01279	1.0113227	1.01400183	1.2366121	0.30639304	0.98795
IL36RN	1.01279	1.0113227	1.0140009	1.2365305	0.3062978	0.98795
ITGBL1	1.01279	1.0113227	1.0140009	1.2365305	0.3062978	0.98795
RP11-379F12.4	1.01279	1.0113227	1.0140009	1.2365305	0.3062978	0.98795
CTD-2555A7.3	1.012793	1.0113227	1.01400754	1.2371168	0.30698173	0.98795
AC007228.5	1.012793	1.0113227	1.01400754	1.2371168	0.30698173	0.98795
CRACR2A	1.012793	1.0113227	1.01400754	1.2371168	0.30698173	0.98795
DMP1	1.012789	1.0113227	1.01399944	1.2364012	0.30614696	0.98795
CTC-498M16.4	1.012795	1.0113227	1.01401049	1.2373768	0.30728492	0.98795
RP11-412P11.1	1.012788	1.0113227	1.0139976	1.2362382	0.3059568	0.98795
NRN1L	1.012789	1.0113227	1.01400068	1.2365105	0.30627447	0.98795
AC104076.3	1.012789	1.0113227	1.01400068	1.2365105	0.30627447	0.98795
CXCR5	1.012789	1.0113227	1.01400068	1.2365105	0.30627447	0.98795
RP11-656E20.5	1.01279	1.0113227	1.01400129	1.2365644	0.30633732	0.98795
RP11-380F14.2	1.012797	1.0113227	1.01401537	1.2378081	0.30778771	0.98795
RP1-10C16.1	1.012788	1.0113227	1.01399813	1.2362859	0.3060124	0.98795
RP11-352M15.1	1.012791	1.0113227	1.01400384	1.2367894	0.30659986	0.98795
CTB-35F21.1	1.012791	1.0113227	1.01400329	1.2367416	0.30654408	0.98795
CTC-367F4.1	1.012791	1.0113227	1.01400329	1.2367416	0.30654408	0.98795
RP5-1069C8.2	1.012791	1.0113227	1.01400329	1.2367416	0.30654408	0.98795
RP11-472G21.2	1.012795	1.0113227	1.01401142	1.237459	0.30738068	0.98795
UBE2L5P	1.012795	1.0113227	1.0140111	1.237431	0.30734813	0.98795
RP11-1060J15.9	1.012788	1.0113227	1.01399829	1.2362997	0.30602855	0.98795
E4F1	1.156597	1.1581474	1.15531657	0.9821	-0.0260581	0.98799
UNC79	1.040353	1.0417833	1.03917129	0.9374866	-0.09313	0.98801
DOCK2	1.039487	1.0408477	1.03836311	0.9391745	-0.0905349	0.98801
MDC1	1.211433	1.2128023	1.21030229	0.9882517	-0.0170495	0.98802
BRSK1	1.1375	1.1391125	1.136169	0.9788405	-0.0308542	0.98803
AC133644.2	1.025903	1.0244164	1.02713106	1.1111818	0.15209491	0.98804
FLYWCH2	1.651246	1.6498841	1.65236998	1.003825	0.00550785	0.98809
LARGE2	1.024203	1.0226997	1.02544488	1.120937	0.16470524	0.9881

AC004158.3	1.015239	1.0166389	1.01408228	0.846349	-0.2406754	0.98811
HLA-DRA	1.015249	1.0166612	1.01408228	0.8452115	-0.2426157	0.98811
CTD-2193P3.2	1.015239	1.0166389	1.01408228	0.846346	-0.2406804	0.98811
SPATA13.1	1.015242	1.0166473	1.01408228	0.8459217	-0.241404	0.98811
CHRM1	1.015244	1.0166501	1.01408228	0.8457749	-0.2416544	0.98811
RPH3A	1.015261	1.0166891	1.01408228	0.8438019	-0.2450238	0.98811
LY75	1.015241	1.0166442	1.01408228	0.8460757	-0.2411414	0.98811
RP13-895J2.3	1.015246	1.0166558	1.01408228	0.8454895	-0.2421412	0.98811
LA16c-321D4.2	1.015252	1.0166679	1.01408228	0.844875	-0.2431902	0.98811
RP11-596C23.6	1.015252	1.0166676	1.01408228	0.844889	-0.2431662	0.98811
MRGPRG-AS1	1.03068	1.032082	1.02952126	0.9201801	-0.1200118	0.98812
NTN5	1.02986	1.0312535	1.02870925	0.9185933	-0.1225018	0.98816
CYB561D1	1.069585	1.0680878	1.07082159	1.0401516	0.0567938	0.98817
CLDN2	1.034509	1.0330502	1.03571405	1.0806006	0.11183337	0.98818
C18orf8	1.074834	1.0733491	1.07606006	1.0369596	0.05235972	0.98825
RP11-309M23.1	1.024786	1.0233372	1.02598268	1.1133602	0.15492041	0.98827
RP11-549B18.1	1.019174	1.0205843	1.01800911	0.8748972	-0.1928145	0.98829
PIK3IP1-AS1	1.019168	1.0205651	1.01801389	0.8759448	-0.1910881	0.98829
RP11-473M20.5	1.019205	1.0206454	1.01801481	0.8725807	-0.1966395	0.98829
OTUD3	1.167074	1.1656558	1.16824507	1.0156306	0.02237581	0.98831
NUP188	1.147321	1.1458147	1.14856458	1.0188587	0.02695393	0.98831
SPPL2B	1.487286	1.4855229	1.48874219	1.0066307	0.00953443	0.98835
SMPD1	1.264984	1.2665818	1.26366424	0.9890557	-0.0158763	0.98838
CH507-39O4.2	1.033423	1.0320203	1.03458182	1.079998	0.11102863	0.9884
CSF1	1.088743	1.0902469	1.08750146	0.9695785	-0.0445704	0.98841
C17orf53	1.063903	1.0624171	1.06513021	1.0434682	0.06138668	0.98842
AXIN2	1.063837	1.0623371	1.06507537	1.0439263	0.06201982	0.98842
ABCA13	1.048766	1.0473949	1.04989822	1.052818	0.07425602	0.98843
ESYT3	1.023165	1.0245482	1.02202312	0.8971373	-0.1565993	0.98849
HAPLN2	1.020573	1.0219739	1.01941566	0.8835794	-0.1785682	0.9885
ELMOD1	1.020579	1.0219973	1.01940722	0.8822565	-0.18073	0.9885
RP11-486A14.2	1.027666	1.0290677	1.02650794	0.9119378	-0.1329927	0.98864
AGAP2	1.027705	1.0290888	1.02656152	0.9131189	-0.1311254	0.98864
NLRP14	1.02824	1.0296254	1.02709532	0.914597	-0.1287918	0.98867
ZBTB3	1.055632	1.0540945	1.05690162	1.0518934	0.07298855	0.9887
RP11-1263C18.2	1.020211	1.0215662	1.0190916	0.8852552	-0.1758348	0.98877
AC091878.1	1.020135	1.0215324	1.01898155	0.8815332	-0.1819131	0.98877
LRRC71	1.019315	1.0178776	1.0205018	1.1467866	0.19759698	0.98881
M1AP	1.019308	1.0178684	1.02049725	1.1471247	0.19802224	0.98881
CTD-2026J24.1	1.019325	1.0178798	1.02051881	1.1475994	0.19861915	0.98881
AC010524.2	1.019362	1.0178935	1.02057492	1.1498542	0.20145094	0.98881
GPRC6A	1.019312	1.0178723	1.02050066	1.1470651	0.19794733	0.98881
IGFL4	1.019331	1.0178721	1.02053661	1.1490847	0.2004852	0.98881
RP1-286D6.5	1.019329	1.0178805	1.02052571	1.1479368	0.19904326	0.98881
RP4-533D7.5	1.019318	1.0178875	1.02049986	1.1460414	0.19665921	0.98881

KCNJ10	1.019321	1.0178748	1.02051454	1.1476788	0.19871896	0.98881
PITPNC1	1.20414	1.205443	1.20306452	0.9884229	-0.0167997	0.98889
RP11-1029J19.2	1.02794	1.0293185	1.02680253	0.9141852	-0.1294417	0.98892
NUP43	1.153585	1.1521239	1.15479198	1.0175389	0.02508398	0.98893
RP11-290F24.6	1.030048	1.0313931	1.0289363	0.9217407	-0.1175672	0.98895
WFDC3	1.030069	1.0314868	1.0288977	0.917772	-0.1237923	0.98895
RP11-476H16.1	1.021447	1.0199686	1.02266867	1.1352181	0.18296954	0.98897
UGT8	1.021422	1.0200111	1.02258735	1.128743	0.17471707	0.98897
RP11-485G7.5	1.021416	1.0199771	1.02260356	1.1314742	0.17820375	0.98897
CAPN9	1.021358	1.0199339	1.02253466	1.1304684	0.17692061	0.98898
RP11-265N6.2	1.021371	1.0199327	1.02255886	1.1317511	0.17855665	0.98898
SLC47A2	1.02136	1.0199255	1.0225455	1.1314904	0.17822437	0.98898
RP11-77K12.10	1.021356	1.0199267	1.02253665	1.1309776	0.17757041	0.98898
RP11-432J22.2	1.021346	1.0199177	1.02252476	1.1308897	0.17745825	0.98898
RP11-129K12.1	1.021373	1.019942	1.02255387	1.1309724	0.1775637	0.98898
BPI	1.021356	1.0199164	1.0225447	1.1319649	0.17882927	0.98898
DYRK2	1.353517	1.3519686	1.35479512	1.0080305	0.01153935	0.98899
CTC-336P14.1	1.019283	1.0178455	1.02047067	1.1471034	0.19799549	0.989
RP1-167A14.2	1.019273	1.0178398	1.02045693	1.1467016	0.19749003	0.989
ZNF32-AS3	1.019265	1.0178371	1.02044456	1.1461824	0.19683669	0.989
ZFPM2	1.019274	1.0178369	1.02046105	1.1471197	0.19801593	0.989
C12orf50	1.019267	1.0178482	1.02043792	1.1450944	0.19546659	0.989
ZAR1L	1.019266	1.0178331	1.0204499	1.1467403	0.19753865	0.989
RTP1	1.0193	1.0178569	1.0204923	1.1475838	0.19859945	0.989
MMEL1	1.019281	1.0178395	1.02047189	1.147558	0.19856707	0.989
RP11-439C15.5	1.019276	1.0178347	1.02046588	1.1475289	0.19853044	0.989
PHF24	1.019267	1.0178353	1.02044867	1.1465272	0.19727062	0.989
LINC00884	1.019291	1.0178507	1.02048053	1.1473255	0.19827471	0.989
LTB4R	1.059324	1.060671	1.05821225	0.9594742	-0.0596841	0.98901
PLD6	1.022344	1.0209244	1.02351629	1.1238689	0.16847374	0.98901
PAPOLB	1.022271	1.0208421	1.02345034	1.1251421	0.17010721	0.98901
CTD-3154N5.2	1.021072	1.0224776	1.01991123	0.8858246	-0.174907	0.98902
SLC51B	1.021033	1.0223969	1.01990754	0.8888532	-0.1699828	0.98902
RP11-689C9.1	1.021044	1.0224212	1.0199065	0.887843	-0.1716236	0.98902
XXbac-BCX196D17.5	1.021089	1.0225277	1.0199005	0.8833781	-0.178897	0.98902
RP11-582E3.6	1.113662	1.1122279	1.11484646	1.0233323	0.03327465	0.98905
TOR1B	1.081942	1.0833196	1.08080397	0.9698075	-0.0442296	0.98909
HEATR5A	1.137874	1.1365368	1.138978	1.0178798	0.02556721	0.9891
DDN	1.020392	1.018967	1.02156924	1.1372008	0.18548703	0.98919
FAM189A2	1.019577	1.0181641	1.02074294	1.1419749	0.19153094	0.98925
C1QTNF1	1.019579	1.0181728	1.02074018	1.1412789	0.19065145	0.98925
TUSC5	1.019606	1.0181682	1.02079396	1.1445224	0.19474574	0.98925
RP11-276H7.2	1.019585	1.0181635	1.02075832	1.1428563	0.192644	0.98925
LINC00520	1.019584	1.0181586	1.02076055	1.1432912	0.19319292	0.98925
LINC00626	1.01958	1.0181728	1.02074152	1.1413528	0.1907448	0.98925

FITM1	1.019583	1.0181562	1.02076167	1.1435058	0.19346369	0.98925
OSR2	1.019582	1.0181637	1.02075281	1.1425437	0.1922494	0.98925
RP11-464F9.21	1.019584	1.0181552	1.02076354	1.1436673	0.1936674	0.98925
CTD-2410N18.3	1.019585	1.0181665	1.02075669	1.1425833	0.19229938	0.98925
DHRS9	1.019587	1.0181602	1.02076449	1.1434081	0.19334043	0.98925
RP3-483K16.4	1.019591	1.0181523	1.02077889	1.1446953	0.19496366	0.98925
HMX2	1.019576	1.0181712	1.02073535	1.1411135	0.19044231	0.98925
LRFN2	1.019021	1.0175955	1.02019768	1.1478912	0.19898591	0.98928
RP11-103H7.3	1.019039	1.0176092	1.02022056	1.1482933	0.19949125	0.98928
RP11-166B2.5	1.019032	1.0176043	1.02021159	1.1481081	0.19925848	0.98928
SLC10A5	1.019017	1.0175944	1.02019248	1.1476621	0.19869791	0.98928
RP11-128P10.1	1.019047	1.0176103	1.02023272	1.1489164	0.20027386	0.98928
RP11-506M13.3	1.019016	1.0175939	1.02018972	1.1475418	0.1985467	0.98928
RP11-85G18.6	1.019037	1.0176135	1.0202133	1.1475996	0.19861937	0.98928
POU5F1	1.019019	1.0175961	1.02019328	1.1475985	0.19861802	0.98928
TBX22	1.019031	1.0176065	1.02020808	1.1477653	0.19882767	0.98928
RP11-665J16.1	1.019045	1.0175943	1.02024305	1.150543	0.20231487	0.98928
CERS3	1.019019	1.0176028	1.02018866	1.1469035	0.197744	0.98928
AC079610.2	1.019046	1.0175992	1.02024058	1.1500884	0.20174478	0.98928
RP11-119D9.1	1.019028	1.017609	1.02019993	1.147135	0.19803514	0.98928
IGF2-AS	1.04827	1.0468163	1.04947108	1.0567066	0.07957481	0.98932
CHML	1.622549	1.6244352	1.62099145	0.994485	-0.0079785	0.98935
TADA2B	1.066305	1.0678745	1.06500887	0.9577805	-0.0622331	0.98935
FAM138C	1.01456	1.0131484	1.01572611	1.1960476	0.25827479	0.98937
RP11-161I2.1	1.01456	1.0131484	1.01572611	1.1960476	0.25827479	0.98937
AC009505.2	1.014557	1.0131484	1.01572042	1.1956145	0.25775224	0.98937
CTC-281F24.5	1.014564	1.0131484	1.01573283	1.1965581	0.25889046	0.98937
CEACAM8	1.014564	1.0131484	1.01573344	1.1966047	0.25894667	0.98937
RP11-573G6.8	1.014559	1.0131484	1.01572393	1.1958814	0.2580743	0.98937
IL15RA	1.014558	1.0131484	1.01572154	1.1956994	0.25785471	0.98937
SLC5A10	1.014556	1.0131484	1.01571923	1.1955236	0.25764263	0.98937
RP11-260E18.1	1.014556	1.0131484	1.01571923	1.1955236	0.25764263	0.98937
UROC1	1.014562	1.0131484	1.01572873	1.1962462	0.25851438	0.98937
RP11-622A1.2	1.014561	1.0131484	1.01572772	1.1961696	0.25842192	0.98937
LINC01392	1.014561	1.0131484	1.01572772	1.1961696	0.25842192	0.98937
NKX6-1	1.014561	1.0131484	1.01572772	1.1961696	0.25842192	0.98937
RP11-544L8__B.4	1.014565	1.0131484	1.01573555	1.1967655	0.25914048	0.98937
RP11-449J1.1	1.014555	1.0131484	1.01571692	1.1953481	0.25743083	0.98937
STYK1	1.014559	1.0131484	1.01572462	1.195934	0.25813772	0.98937
RP13-820C6.4	1.014559	1.0131484	1.01572308	1.1958169	0.25799653	0.98937
CTD-2288F12.1	1.01456	1.0131484	1.01572597	1.1960362	0.25826109	0.98937
RP11-317N8.5	1.014556	1.0131484	1.01571765	1.1954037	0.25749793	0.98937
TMEM92-AS1	1.01456	1.0131484	1.01572536	1.1959898	0.25820513	0.98937
RP11-271M24.2	1.01456	1.0131484	1.01572536	1.1959898	0.25820513	0.98937
TRPC5OS	1.014557	1.0131484	1.01572015	1.1955939	0.25772743	0.98937

EPN2-AS1	1.014557	1.0131484	1.01572015	1.1955939	0.25772743	0.98937
SIAH3	1.014557	1.0131484	1.01572015	1.1955939	0.25772743	0.98937
GABRA6	1.014563	1.0131484	1.01573096	1.1964162	0.2587194	0.98937
RP11-618K13.2	1.014565	1.0131484	1.01573481	1.1967086	0.25907191	0.98937
DNAH8	1.014566	1.0131484	1.01573583	1.1967862	0.25916544	0.98937
CIB3	1.014555	1.0131484	1.01571707	1.19536	0.25744522	0.98937
RP11-435O5.7	1.014561	1.0131484	1.0157273	1.1961376	0.25838337	0.98937
AC073316.1	1.01456	1.0131484	1.01572486	1.1959519	0.25815933	0.98937
LINC00345	1.01456	1.0131484	1.01572574	1.1960191	0.25824044	0.98937
ZC3H12D	1.01456	1.0131484	1.01572586	1.1960284	0.25825163	0.98937
RP11-715J22.4	1.014564	1.0131484	1.01573278	1.1965548	0.25888648	0.98937
SLCO2B1	1.014556	1.0131484	1.01571824	1.1954488	0.25755238	0.98937
DEFB1	1.014556	1.0131484	1.01571824	1.1954488	0.25755238	0.98937
AC005256.1	1.014556	1.0131484	1.015719	1.1955064	0.25762185	0.98937
CTC-218B8.3	1.014558	1.0131484	1.01572208	1.1957405	0.25790438	0.98937
AC009133.17	1.014558	1.0131484	1.01572208	1.1957405	0.25790438	0.98937
RP1-69D17.3	1.014557	1.0131484	1.01571957	1.1955501	0.25767463	0.98937
RP11-390K5.3	1.014572	1.0131484	1.01574794	1.1977073	0.26027543	0.98937
COL5A1-AS1	1.014558	1.0131484	1.01572142	1.1956908	0.25784431	0.98937
GOLGA6L4	1.10159	1.1000857	1.10283274	1.0274467	0.03906356	0.9894
ZNF101	1.06702	1.0683514	1.06592064	0.9644373	-0.0522406	0.98952
FEZF1	1.110203	1.1116008	1.10904957	0.9771399	-0.033363	0.98955
LGALS9C	1.018164	1.0195177	1.01704689	0.8734063	-0.1952751	0.98957
RP11-398G24.2	1.018158	1.0195038	1.01704689	0.8740277	-0.1942491	0.98957
RP11-688I9.4	1.018161	1.0195093	1.01704689	0.8737828	-0.1946534	0.98957
RP11-554E23.4	1.018171	1.0195325	1.01704689	0.8727431	-0.1963711	0.98957
AC026150.8	1.018169	1.0195276	1.01704689	0.8729652	-0.196004	0.98957
RP11-158D2.2	1.018177	1.0195455	1.01704689	0.8721664	-0.1973247	0.98957
PABPN1L	1.018173	1.0195357	1.01704689	0.8726009	-0.1966061	0.98957
RP11-686D22.5	1.018164	1.0195177	1.01704689	0.8734085	-0.1952715	0.98957
RP11-98D18.16	1.018181	1.0195539	1.01704689	0.8717906	-0.1979465	0.98957
SBK1	1.412291	1.4138511	1.41100321	0.9931186	-0.0099621	0.98958
RARRES1	1.118412	1.1169136	1.11964851	1.0233923	0.03335922	0.9896
RP11-295K2.3	1.018719	1.0200699	1.01760343	0.8771058	-0.1891771	0.9896
RP1-206D15.6	1.01873	1.0200938	1.01760343	0.8760621	-0.190895	0.9896
RP3-431P23.5	1.018715	1.0200603	1.01760343	0.8775236	-0.1884902	0.9896
RP4-657D16.3	1.018714	1.0200581	1.01760343	0.8776223	-0.1883279	0.9896
SHOX	1.018713	1.0200568	1.01760343	0.8776794	-0.188234	0.9896
CTC-490E21.14	1.018723	1.0200783	1.01760343	0.8767372	-0.1897836	0.9896
RNU11	1.018714	1.0200578	1.01760343	0.8776332	-0.1883101	0.9896
MPL	1.018727	1.0200876	1.01760343	0.8763339	-0.1904474	0.9896
TUBB1	1.018744	1.0201245	1.01760343	0.8747252	-0.1930983	0.9896
RP11-636O21.1	1.018736	1.0201072	1.01760343	0.8754783	-0.1918566	0.9896
RP5-1029F21.3	1.018758	1.0201569	1.01760343	0.8733199	-0.1954179	0.9896
DACT3-AS1	1.018758	1.0201569	1.01760343	0.8733199	-0.1954179	0.9896

RNF219-AS1	1.018714	1.0200596	1.01760343	0.8775547	-0.1884391	0.9896
ERICH6	1.01871	1.0200498	1.01760343	0.8779848	-0.1877321	0.9896
ENPP6	1.018722	1.0200768	1.01760343	0.8768023	-0.1896766	0.9896
MAGEL2	1.065205	1.0638029	1.06636375	1.0401372	0.05677391	0.98971
RP11-105C19.1	1.019563	1.0208675	1.01848606	0.8858793	-0.1748179	0.98973
RP11-415J8.5	1.019533	1.020881	1.01842069	0.8821749	-0.1808634	0.98973
AC008074.3	1.019551	1.0209091	1.01842936	0.8814049	-0.1821231	0.98973
RP11-477N3.1	1.019523	1.0208675	1.01841252	0.8823535	-0.1805713	0.98973
ZBTB24	1.183799	1.1822495	1.18507873	1.0155238	0.02222398	0.98976
PER2	1.064247	1.0655081	1.06320561	0.9648517	-0.0516208	0.98979
PDZRN3	1.274936	1.2764822	1.27365908	0.989789	-0.014807	0.98981
RP11-383I23.2	1.028867	1.0273849	1.03009008	1.0987825	0.13590579	0.98983
AC079145.4	1.021944	1.020571	1.02307727	1.1218374	0.16586358	0.98985
RP3-355L5.5	1.01843	1.0197939	1.01730453	0.8742354	-0.1939063	0.98985
CST1	1.018417	1.019764	1.01730453	0.875557	-0.191727	0.98985
AC073133.1	1.018421	1.019774	1.01730453	0.8751147	-0.192456	0.98985
RP1-166H4.2	1.018415	1.0197602	1.01730453	0.875725	-0.1914502	0.98985
CTB-191K22.5	1.018424	1.0197793	1.01730453	0.8748818	-0.1928401	0.98985
AC004257.1	1.0205	1.0218428	1.0193919	0.8877922	-0.171706	0.98986
ROPN1B	1.020497	1.0218354	1.0193919	0.8880964	-0.1712118	0.98986
AC010987.6	1.020527	1.0219013	1.0193919	0.8854221	-0.1755627	0.98986
RP11-517C16.4	1.020524	1.0218939	1.0193919	0.8857224	-0.1750736	0.98986
SYP-AS1	1.020518	1.0218823	1.0193919	0.886191	-0.1743104	0.98986
RP11-752G15.8	1.020499	1.0218395	1.0193919	0.8879264	-0.171488	0.98986
RP11-524G24.2	1.020514	1.0218732	1.0193919	0.8865598	-0.1737101	0.98986
RP11-70F11.8	1.020506	1.021856	1.0193919	0.8872573	-0.1725755	0.98986
RP11-119H12.6	1.0205	1.0218408	1.0193919	0.8878769	-0.1715685	0.98986
CXorf65	1.020519	1.0218838	1.0193919	0.8861301	-0.1744096	0.98986
ARAP1-AS1	1.020519	1.021883	1.0193919	0.8861613	-0.1743588	0.98986
RP11-126K1.9	1.020491	1.0218215	1.0193919	0.8886619	-0.1702935	0.98986
KIF4B	1.020498	1.0218376	1.0193919	0.8880071	-0.1713569	0.98986
RD3L	1.021407	1.0227464	1.02030062	0.892476	-0.1641147	0.98992
RP11-59H7.4	1.020554	1.0219238	1.01942211	0.8858905	-0.1747998	0.98995
RP11-112L6.3	1.020554	1.0218928	1.01944866	0.8883582	-0.1707866	0.98995
CFD	1.125046	1.1237435	1.12612083	1.0192122	0.02745439	0.98995
CA12	1.065337	1.0639422	1.06648903	1.0398308	0.05634881	0.98996
SRC	1.162445	1.1610244	1.16361818	1.0161079	0.02305358	0.98998
C11orf98	1.067118	1.0656753	1.06830925	1.0401051	0.05672936	0.99002
RP11-379P15.1	1.018472	1.0198252	1.01735489	0.8753943	-0.191995	0.99004
RP11-573D15.8	1.018469	1.0198185	1.01735489	0.8756928	-0.1915033	0.99004
AC116609.2	1.018452	1.0197801	1.01735489	0.8773923	-0.1887061	0.99004
RP11-109P14.10	1.018473	1.0198276	1.01735489	0.8752893	-0.1921682	0.99004
CTC-499J9.1	1.018452	1.01978	1.01735489	0.8773976	-0.1886973	0.99004
CPNE4	1.030854	1.0295555	1.03192609	1.0802069	0.11130767	0.99007
LINC00867	1.02099	1.0195939	1.02214299	1.1300964	0.17644588	0.99014

RP11-218F10.3	1.046907	1.0481932	1.04584418	0.9512583	-0.072091	0.99014
CYP2E1	1.02669	1.027999	1.02560991	0.9146721	-0.1286735	0.99019
C21orf62	1.05446	1.0558108	1.05334473	0.9558136	-0.0651988	0.99023
DNAJC22	1.060219	1.058807	1.06138438	1.0438284	0.06188459	0.99029
RAD9B	1.028583	1.0272554	1.02967966	1.0889472	0.12293401	0.99031
EFCAB13	1.021423	1.0200546	1.02255244	1.1245519	0.16935023	0.99042
LINC00620	1.014403	1.013034	1.01553422	1.1918255	0.25317303	0.99043
CTB-176F20.3	1.014403	1.013034	1.01553422	1.1918255	0.25317303	0.99043
RP11-264E20.1	1.014406	1.013034	1.01553914	1.1922025	0.25362934	0.99043
RP11-240G22.4	1.014405	1.013034	1.01553712	1.1920478	0.2534421	0.99043
LINC00578	1.014402	1.013034	1.01553228	1.1916765	0.25299263	0.99043
CTD-2545M3.2	1.014402	1.013034	1.01553113	1.1915882	0.25288579	0.99043
RP5-1028K7.2	1.014407	1.013034	1.01554104	1.1923481	0.25380544	0.99043
ART3	1.0144	1.013034	1.01552898	1.1914229	0.25268558	0.99043
C17orf107	1.014405	1.013034	1.01553755	1.1920808	0.25348198	0.99043
RP11-610P16.1	1.014401	1.013034	1.01552979	1.1914851	0.25276092	0.99043
MOGAT3	1.014409	1.013034	1.01554387	1.1925659	0.25406898	0.99043
TEX13C	1.014406	1.013034	1.01553874	1.1921723	0.25359274	0.99043
GAPDHS	1.014406	1.013034	1.01553887	1.1921817	0.25360409	0.99043
BDKRB1	1.014404	1.013034	1.01553476	1.1918665	0.25322263	0.99043
RP11-91A18.4	1.014402	1.013034	1.01553098	1.1915768	0.25287196	0.99043
LINC00923	1.014402	1.013034	1.01553209	1.1916617	0.25297479	0.99043
RP5-965G21.5	1.01441	1.013034	1.01554617	1.1927422	0.2542823	0.99043
CALHM3	1.014403	1.013034	1.01553346	1.1917665	0.25310157	0.99043
AICDA	1.01441	1.013034	1.01554664	1.1927781	0.25432565	0.99043
ZYG11A	1.014403	1.013034	1.01553338	1.1917604	0.25309426	0.99043
CTD-2297D10.2	1.014412	1.013034	1.01554916	1.1929717	0.25455981	0.99043
RP11-23P13.6	1.014401	1.013034	1.0155306	1.1915474	0.25283629	0.99043
LY6D	1.014419	1.013034	1.01556303	1.1940358	0.25584607	0.99043
KLHL41	1.014401	1.013034	1.0155299	1.1914938	0.25277145	0.99043
FCMR	1.014399	1.013034	1.01552567	1.191169	0.25237814	0.99043
CTD-2544H17.1	1.014403	1.013034	1.01553442	1.1918403	0.25319089	0.99043
RP11-145M9.5	1.01441	1.013034	1.01554601	1.19273	0.25426744	0.99043
RP11-284F21.11	1.014403	1.013034	1.01553276	1.1917128	0.25303662	0.99043
AC093159.1	1.0144	1.013034	1.01552805	1.191352	0.25259977	0.99043
RP11-429P3.3	1.014404	1.013034	1.01553554	1.1919262	0.2532949	0.99043
LL22NC03-2H8.4	1.0144	1.013034	1.01552859	1.1913935	0.25264996	0.99043
SPANXA2-OT1	1.014407	1.013034	1.01554026	1.1922887	0.25373364	0.99043
RP11-138J23.1	1.014404	1.013034	1.01553562	1.1919322	0.25330223	0.99043
DSG1-AS1	1.014404	1.013034	1.01553562	1.1919322	0.25330223	0.99043
FAM109B	1.014404	1.013034	1.01553562	1.1919322	0.25330223	0.99043
RP11-498C9.16	1.0144	1.013034	1.01552721	1.1912872	0.25252128	0.99043
LAMP5-AS1	1.014404	1.013034	1.01553577	1.1919437	0.2533161	0.99043
MGC15885	1.014402	1.013034	1.01553147	1.1916142	0.25291719	0.99043
VIPR1-AS1	1.014411	1.013034	1.0155473	1.1928288	0.25438701	0.99043

RP11-9J18.1	1.014407	1.013034	1.01554049	1.1923063	0.25375489	0.99043
PNLDC1	1.014408	1.013034	1.01554286	1.1924883	0.25397508	0.99043
RP11-617D20.2	1.017139	1.0157693	1.01827011	1.1585843	0.21236298	0.99044
CMB9-22P13.2	1.017141	1.0157693	1.0182739	1.1588247	0.21266235	0.99044
IL1RAPL2	1.017146	1.0157693	1.01828244	1.1593666	0.21333679	0.99044
RP11-117L5.4	1.017146	1.0157693	1.01828244	1.1593666	0.21333679	0.99044
MAGEE2	1.017142	1.0157693	1.01827642	1.1589844	0.21286114	0.99044
RP11-70F11.11	1.017142	1.0157693	1.01827567	1.158937	0.21280209	0.99044
TEX11	1.017142	1.0157693	1.01827567	1.158937	0.21280209	0.99044
LBP	1.01714	1.0157693	1.01827111	1.1586481	0.21244243	0.99044
RP11-552D8.1	1.017146	1.0157693	1.01828367	1.1594442	0.2134334	0.99044
RP11-404E16.1	1.017146	1.0157693	1.01828367	1.1594442	0.2134334	0.99044
VNN1	1.017139	1.0157693	1.01827045	1.1586059	0.21238995	0.99044
AC093110.3	1.017135	1.0157693	1.01826335	1.1581559	0.21182952	0.99044
GFI1	1.017135	1.0157693	1.01826335	1.1581559	0.21182952	0.99044
PRH1-PRR4	1.017147	1.0157693	1.01828381	1.1594533	0.21344466	0.99044
C1QTNF3-AMACR	1.017141	1.0157693	1.01827355	1.1588025	0.21263466	0.99044
RP11-650J17.1	1.017142	1.0157693	1.0182754	1.1589197	0.21278061	0.99044
AC105052.1	1.017143	1.0157693	1.01827789	1.159078	0.21297765	0.99044
RP11-59E19.4	1.017136	1.0157693	1.01826432	1.1582172	0.21190585	0.99044
RP11-321N4.5	1.017146	1.0157693	1.01828344	1.1594297	0.21341531	0.99044
PCDHA6	1.017139	1.0157693	1.01827053	1.1586109	0.21239618	0.99044
GATA6	1.017137	1.0157693	1.01826632	1.1583444	0.21206426	0.99044
RP1-151B14.6	1.017136	1.0157693	1.01826448	1.1582272	0.21191825	0.99044
LINC01123	1.017146	1.0157693	1.01828315	1.1594113	0.21339246	0.99044
BARHL1	1.017146	1.0157693	1.01828315	1.1594113	0.21339246	0.99044
CTD-3179P9.1	1.017143	1.0157693	1.01827797	1.159083	0.21298389	0.99044
OXCT2	1.017139	1.0157693	1.01827041	1.1586037	0.21238714	0.99044
POU2AF1	1.017139	1.0157693	1.01827041	1.1586037	0.21238714	0.99044
C7	1.017139	1.0157693	1.0182703	1.1585965	0.21237817	0.99044
IMPG1	1.017137	1.0157693	1.0182666	1.1583616	0.21208563	0.99044
RP11-886P16.6	1.01715	1.0157693	1.01829041	1.1598715	0.21396495	0.99044
ZNF705A	1.01714	1.0157693	1.01827258	1.1587414	0.21255857	0.99044
AS3MT	1.017141	1.0157693	1.01827316	1.158778	0.21260422	0.99044
RP1-221C16.8	1.017141	1.0157693	1.01827416	1.1588413	0.21268298	0.99044
RP1-45C12.1	1.017141	1.0157693	1.01827416	1.1588413	0.21268298	0.99044
RP1-269M15.3	1.017141	1.0157693	1.01827448	1.1588614	0.21270803	0.99044
CTD-2329K10.1	1.017146	1.0157693	1.01828224	1.1593537	0.21332083	0.99044
RP11-309L24.10	1.017148	1.0157693	1.01828623	1.1596069	0.21363578	0.99044
AC073657.1	1.017142	1.0157693	1.0182751	1.1589009	0.21275721	0.99044
AC138517.6	1.017138	1.0157693	1.0182687	1.1584949	0.21225173	0.99044
TSKS	1.017138	1.0157693	1.0182687	1.1584949	0.21225173	0.99044
LINC00971	1.017138	1.0157693	1.01826872	1.1584961	0.21225321	0.99044
UCHL1-AS1	1.017142	1.0157693	1.01827475	1.1588786	0.2127295	0.99044
RP11-90D11.1	1.017142	1.0157693	1.01827475	1.1588786	0.2127295	0.99044

CTD-2240J17.2	1.017143	1.0157693	1.01827773	1.1590675	0.21296455	0.99044
RP11-93I21.3	1.017147	1.0157693	1.01828454	1.1594997	0.21350243	0.99044
RP11-692C24.1	1.017147	1.0157693	1.01828454	1.1594997	0.21350243	0.99044
KLHL32	1.04716	1.0484862	1.0460642	0.9500469	-0.0739294	0.99054
SNED1	1.020042	1.0186825	1.02116387	1.1328149	0.17991221	0.99057
IL6R	1.020029	1.0186718	1.02114967	1.1327054	0.17977268	0.99057
RP11-15L13.4	1.020046	1.0186477	1.02119992	1.1368633	0.18505873	0.99057
RP11-138E2.1	1.020026	1.0186636	1.02115035	1.133241	0.1804547	0.99057
FOXF1	1.020044	1.0186671	1.02118152	1.1346955	0.18230524	0.99057
RP5-1116H23.6	1.020063	1.0186975	1.02119053	1.1333367	0.18057657	0.99057
CEP295	1.330093	1.3283658	1.33151863	1.0096015	0.01378597	0.99063
FGFR4	1.0239	1.0252224	1.02280737	0.904249	-0.145208	0.99066
SNHG10	1.315862	1.317225	1.31473586	0.9921534	-0.0113649	0.9907
RP11-283I3.2	1.016113	1.0147415	1.01724532	1.1698487	0.22632201	0.99074
AC005540.3	1.016113	1.0147483	1.01724083	1.1690027	0.22527823	0.99074
RP5-999L4.2	1.016111	1.0147503	1.01723502	1.168449	0.22459475	0.99074
LINC00636	1.016103	1.0147437	1.01722557	1.1683308	0.22444881	0.99074
RP11-553P9.2	1.016114	1.0147447	1.01724416	1.1695137	0.22590878	0.99074
SLC35E4	1.112775	1.1141128	1.11167098	0.9786018	-0.0312062	0.99075
LINC01119	1.023345	1.0246556	1.02226275	0.9029491	-0.1472835	0.99081
GGT1	1.023347	1.024661	1.02226259	0.9027462	-0.1476076	0.99081
AC004878.2	1.02333	1.0246114	1.02227135	0.904922	-0.1441347	0.99081
HCFC2	1.091471	1.0900678	1.09263042	1.0284519	0.04047427	0.99084
WWC2-AS2	1.040577	1.0420028	1.039399	0.938009	-0.0923264	0.99094
MAP3K12	1.194066	1.1929725	1.19496983	1.0103501	0.01485526	0.99098
RTN4IP1	1.074305	1.0729332	1.07543697	1.0343298	0.04869631	0.991
TMEM110	1.038306	1.0370021	1.03938235	1.0643282	0.0899431	0.99104
GMDS-AS1	1.132659	1.1313181	1.1337662	1.0186421	0.02664729	0.9911
AL022476.2	1.017195	1.0184747	1.01613888	0.8735643	-0.1950141	0.99111
RP11-348B17.1	1.017217	1.0185222	1.01613888	0.871328	-0.1987122	0.99111
RP11-385E5.5	1.017217	1.0185222	1.01613888	0.871328	-0.1987122	0.99111
ZNF177	1.017198	1.018481	1.01613888	0.8732681	-0.1955035	0.99111
RP11-556N4.1	1.017196	1.0184758	1.01613888	0.8735144	-0.1950966	0.99111
ZNF860	1.017208	1.0185034	1.01613888	0.8722131	-0.1972474	0.99111
MEIS1-AS3	1.017215	1.018517	1.01613888	0.8715686	-0.1983139	0.99111
RP11-802O23.3	1.017196	1.0184766	1.01613888	0.8734745	-0.1951625	0.99111
FOXD3-AS1	1.017231	1.0185535	1.01613888	0.8698556	-0.2011522	0.99111
CALCA	1.017193	1.0184691	1.01613888	0.8738321	-0.194572	0.99111
GJB7	1.037345	1.0386574	1.03626173	0.9380276	-0.0922978	0.99115
GRB7	1.044955	1.0462572	1.04387897	0.9485871	-0.0761479	0.99116
GID8	1.86349	1.8649006	1.862325	0.9970221	-0.0043026	0.99116
LINC00524	1.060233	1.0614528	1.05922544	0.9637547	-0.0532621	0.99121
STX17-AS1	1.059726	1.0609347	1.05872724	0.9637735	-0.0532339	0.99122
LINC01004	1.038069	1.0366493	1.03924132	1.0707241	0.09858674	0.99133
GPC5	1.018829	1.0175003	1.01992654	1.1386401	0.18731181	0.99135

MED4-AS1	1.018848	1.0174987	1.0199628	1.1408186	0.19006943	0.99135
CTD-2105E13.16	1.018856	1.0175118	1.01996591	1.1401415	0.18921283	0.99135
RP11-290H9.5	1.018835	1.0175135	1.01992715	1.1378169	0.18626847	0.99135
AC004637.1	1.018863	1.0175051	1.01998383	1.1416003	0.19105766	0.99135
RP3-382I10.7	1.018858	1.0175113	1.01997075	1.1404461	0.18959822	0.99135
TIE1	1.018863	1.0175293	1.01996414	1.1389006	0.18764179	0.99135
IZUMO2	1.01885	1.0175054	1.01996053	1.1402499	0.18935	0.99135
RP11-511B23.1	1.018841	1.0175128	1.01993758	1.1384571	0.18707993	0.99135
RP11-22C11.2	1.018847	1.0174989	1.01996052	1.1406698	0.18988118	0.99135
CTA-363E19.2	1.018841	1.0175049	1.01994477	1.1393842	0.18825429	0.99135
RASAL1	1.01884	1.0175056	1.01994132	1.1391376	0.18794202	0.99135
NAA16	1.324352	1.3231474	1.32534705	1.0068069	0.00978702	0.9914
LINC01108	1.016419	1.0150885	1.01751757	1.1609919	0.21535794	0.99145
RP11-713M15.2	1.016419	1.0150885	1.01751757	1.1609919	0.21535794	0.99145
MYH6	1.016416	1.0150885	1.01751184	1.1606118	0.21488547	0.99145
RP11-809C18.3	1.01642	1.0150885	1.01751959	1.161126	0.21552449	0.99145
CEBPE	1.016415	1.0150885	1.01751074	1.1605388	0.21479481	0.99145
LINC00244	1.016422	1.0150885	1.01752359	1.1613911	0.21585386	0.99145
DCST1	1.016416	1.0150885	1.01751145	1.1605862	0.21485368	0.99145
RP11-756G20.1	1.016413	1.0150885	1.0175067	1.1602717	0.21446264	0.99145
RP4-633I8.4	1.016412	1.0150885	1.01750589	1.1602179	0.21439577	0.99145
C8A	1.016417	1.0150885	1.01751342	1.1607169	0.21501609	0.99145
ITGB2	1.016418	1.0150885	1.01751636	1.1609115	0.21525795	0.99145
RP11-697E22.1	1.016418	1.0150885	1.01751636	1.1609115	0.21525795	0.99145
LINC00239	1.016417	1.0150885	1.01751377	1.16074	0.21504491	0.99145
RP13-57D9.3	1.016412	1.0150885	1.01750451	1.160126	0.21428152	0.99145
LINC01048	1.016424	1.0150885	1.01752731	1.1616375	0.21615989	0.99145
MUC22	1.016418	1.0150885	1.01751579	1.1608738	0.21521111	0.99145
C3orf49	1.016418	1.0150885	1.01751579	1.1608738	0.21521111	0.99145
RP11-280H21.1	1.01642	1.0150885	1.01752006	1.1611569	0.2155629	0.99145
RP11-98D18.15	1.016416	1.0150885	1.01751203	1.1606245	0.21490134	0.99145
RP3-413H6.2	1.016411	1.0150885	1.01750358	1.1600648	0.21420541	0.99145
RP11-184E9.1	1.016415	1.0150885	1.0175106	1.1605299	0.21478375	0.99145
RP11-930P14.1	1.016417	1.0150885	1.0175135	1.1607221	0.21502259	0.99145
RP11-649A16.1	1.016417	1.0150885	1.0175135	1.1607221	0.21502259	0.99145
TMPRSS15	1.016414	1.0150885	1.01750913	1.1604326	0.21466271	0.99145
C2orf71	1.016419	1.0150885	1.01751734	1.1609768	0.21533914	0.99145
SPINK8	1.016419	1.0150885	1.01751734	1.1609768	0.21533914	0.99145
RP5-867C24.5	1.016419	1.0150885	1.01751734	1.1609768	0.21533914	0.99145
ACTL8	1.016412	1.0150885	1.01750581	1.1602127	0.2143893	0.99145
CTD-2562J15.6	1.016412	1.0150885	1.01750581	1.1602127	0.2143893	0.99145
RP11-13P5.2	1.016421	1.0150885	1.01752188	1.1612777	0.21571305	0.99145
AC092431.3	1.016412	1.0150885	1.01750534	1.1601814	0.21435034	0.99145
KCNK13	1.016412	1.0150885	1.01750543	1.1601873	0.21435768	0.99145
CLYBL-AS2	1.016422	1.0150885	1.01752329	1.1613706	0.21582845	0.99145

RP11-478K15.6	1.016422	1.0150885	1.01752243	1.1613139	0.21575804	0.99145
C9orf92	1.016417	1.0150885	1.01751402	1.1607567	0.21506562	0.99145
CTD-2013N24.2	1.01642	1.0150885	1.01751916	1.1610974	0.21548905	0.99145
GAS5-AS1	1.016419	1.0150885	1.0175185	1.1610537	0.21543467	0.99145
GMNC	1.016415	1.0150885	1.01751071	1.1605375	0.21479312	0.99145
ERICD	1.01642	1.0150885	1.01752013	1.1611616	0.21556874	0.99145
AMBN	1.016424	1.0150885	1.0175271	1.1616233	0.21614226	0.99145
HCG16	1.016419	1.0150885	1.01751748	1.160986	0.21535055	0.99145
RP11-855A2.3	1.016411	1.0150885	1.01750373	1.1600747	0.21421766	0.99145
LINC01094	1.018138	1.0168332	1.0192156	1.141528	0.19096624	0.99145
RP11-287D1.2	1.01815	1.0168077	1.01925775	1.1457722	0.19632027	0.99145
CRHR1	1.023887	1.0225197	1.02501544	1.1108258	0.15163257	0.99151
LCMT1-AS1	1.023927	1.0226571	1.02497643	1.1023669	0.14060442	0.99151
BHLHE40-AS1	1.026717	1.0253611	1.02783764	1.0976529	0.13442188	0.99152
CACNA1G	1.026684	1.025347	1.02778814	1.0963082	0.13265341	0.99152
AC004221.2	1.023026	1.0243144	1.02196207	0.9032523	-0.1467991	0.99158
FSBP	1.022998	1.024243	1.02197027	0.9062524	-0.1420152	0.99158
SMPD5	1.023018	1.0242866	1.0219701	0.904619	-0.1446179	0.99158
RP11-135A1.3	1.023026	1.0243031	1.02197067	0.9040255	-0.1455646	0.99158
RP11-359E3.4	1.038339	1.0395726	1.03732078	0.9430964	-0.0845229	0.99161
PHYHIP	1.038423	1.0397099	1.03736014	0.9408277	-0.0879976	0.99161
ZXDC	1.09997	1.0986159	1.10108799	1.0250675	0.03571891	0.99161
NRBP1	1.767239	1.768553	1.76615402	0.9968786	-0.0045103	0.99163
RP11-21B23.3	1.017011	1.0156856	1.01810602	1.1543065	0.20702631	0.99165
TEX41	1.017002	1.0156682	1.01810303	1.1554025	0.20839547	0.99165
LINC00336	1.016987	1.0156712	1.01807392	1.153318	0.20579036	0.99165
RP11-347C12.11	1.016997	1.0156731	1.01809064	1.1542502	0.206956	0.99165
RP5-1103G7.10	1.016995	1.0156705	1.01808871	1.1543163	0.2070386	0.99165
AC016629.3	1.017002	1.0156754	1.01809817	1.1545586	0.20734144	0.99165
RP11-615I2.6	1.056146	1.0548308	1.05723175	1.0437892	0.06183042	0.99177
SLC1A7	1.025577	1.024286	1.02664221	1.097018	0.13358717	0.99177
UTP3	1.246678	1.2481247	1.24548387	0.9893568	-0.0154371	0.9918
SHCBP1	1.081847	1.0830547	1.0808499	0.9734535	-0.038816	0.99182
PRPH2	1.040994	1.0395997	1.04214624	1.0643079	0.08991555	0.99182
RP11-571L19.8	1.015542	1.0167986	1.01450349	0.8633746	-0.2119415	0.99182
LINC00930	1.015545	1.0168073	1.01450349	0.8629293	-0.2126858	0.99182
AC010907.2	1.015554	1.0168255	1.01450349	0.861993	-0.214252	0.99182
C1QTNF1-AS1	1.01555	1.0168165	1.01450349	0.8624565	-0.2134764	0.99182
LINC01114	1.015563	1.0168468	1.01450349	0.860904	-0.2160758	0.99182
LINC01260	1.01554	1.0167953	1.01450349	0.8635436	-0.2116591	0.99182
RP11-1223D19.4	1.015556	1.0168311	1.01450349	0.8617094	-0.2147266	0.99182
AP003025.2	1.015551	1.0168188	1.01450349	0.8623407	-0.2136701	0.99182
RP11-526A4.1	1.015553	1.0168242	1.01450349	0.8620608	-0.2141384	0.99182
AC073254.1	1.015557	1.0168335	1.01450349	0.8615869	-0.2149318	0.99182
NLRP2	1.015555	1.016828	1.01450349	0.8618672	-0.2144626	0.99182

CABP4	1.015542	1.0168002	1.01450349	0.863292	-0.2120795	0.99182
CTD-3193O13.13	1.01555	1.0168183	1.01450349	0.8623647	-0.2136299	0.99182
PCK1	1.015547	1.0168117	1.01450349	0.8627033	-0.2130636	0.99182
RP5-1142A6.2	1.024102	1.0253779	1.02304858	0.9082153	-0.1388938	0.99183
LCN2	1.021291	1.0225294	1.0202676	0.8996079	-0.1526318	0.99184
LRRC74B	1.021313	1.0225829	1.0202641	0.8973191	-0.1563069	0.99184
CTD-2349P21.12	1.030979	1.0321865	1.02998247	0.9315225	-0.1023375	0.99192
NAGLU	1.138092	1.1366119	1.13931469	1.0197845	0.02826425	0.99196
LRRC8B	1.166249	1.1675596	1.16516692	0.9857205	-0.0207495	0.99197
RP11-1348G14.8	1.035479	1.0367325	1.03444369	0.9376891	-0.0928184	0.99209
RP11-514O12.4	1.036015	1.037266	1.03498207	0.9387133	-0.0912435	0.99213
RP11-115D19.2	1.050687	1.0520313	1.04957625	0.9528161	-0.0697303	0.99216
KLK8	1.050751	1.0519947	1.04972367	0.9563209	-0.0644332	0.99216
AC093388.3	1.050652	1.0519013	1.04961956	0.9560367	-0.0648621	0.99216
FAM78A	1.019794	1.0184695	1.02088866	1.1309805	0.17757407	0.99217
RP11-546O6.4	1.019804	1.0185055	1.0208758	1.1280879	0.1738795	0.99217
SPINK6	1.019815	1.0185113	1.0208917	1.1285904	0.17452194	0.99217
RP5-958B11.1	1.019787	1.0184918	1.02085683	1.127895	0.17363277	0.99217
CATSPER3	1.01979	1.0184888	1.02086443	1.1284906	0.17439441	0.99217
LINC01012	1.050198	1.0515234	1.04910275	0.9530179	-0.0694248	0.99221
SEC16B	1.057493	1.0587055	1.05649212	0.9622972	-0.0554456	0.99226
BCL11B	1.057518	1.0588237	1.05644009	0.9594794	-0.0596762	0.99226
RP11-123O22.1	1.013656	1.0123699	1.01471842	1.1898594	0.25079111	0.99232
AP000235.3	1.013656	1.0123699	1.01471842	1.1898594	0.25079111	0.99232
BACH1-IT2	1.013656	1.0123699	1.01471842	1.1898594	0.25079111	0.99232
RP11-894P9.2	1.01366	1.0123699	1.01472489	1.1903826	0.25142535	0.99232
PDYN	1.01366	1.0123699	1.01472489	1.1903826	0.25142535	0.99232
RP11-166B2.7	1.01366	1.0123699	1.01472489	1.1903826	0.25142535	0.99232
DNAH17	1.013659	1.0123699	1.01472295	1.1902259	0.25123546	0.99232
ACSM2B	1.013659	1.0123699	1.01472295	1.1902259	0.25123546	0.99232
DPEP1	1.013658	1.0123699	1.01472157	1.1901144	0.25110024	0.99232
RP11-281O15.8	1.013663	1.0123699	1.01473037	1.1908258	0.25196238	0.99232
TOMM20L	1.013663	1.0123699	1.01473037	1.1908258	0.25196238	0.99232
CITF22-49E9.3	1.013655	1.0123699	1.01471603	1.1896667	0.25055749	0.99232
RP11-823E8.3	1.013658	1.0123699	1.01472265	1.1902019	0.25120627	0.99232
RP11-505P4.6	1.013657	1.0123699	1.01472026	1.1900087	0.25097208	0.99232
TNFAIP8L2	1.013659	1.0123699	1.01472342	1.190264	0.25128154	0.99232
CH17-125A10.2	1.013659	1.0123699	1.01472412	1.1903205	0.25135002	0.99232
RP1-232L24.3	1.013659	1.0123699	1.01472412	1.1903205	0.25135002	0.99232
C7orf65	1.013659	1.0123699	1.01472412	1.1903205	0.25135002	0.99232
THEGL	1.01366	1.0123699	1.01472543	1.1904264	0.25147846	0.99232
RP11-466I1.1	1.01366	1.0123699	1.01472543	1.1904264	0.25147846	0.99232
RP5-887A10.1	1.01366	1.0123699	1.01472543	1.1904264	0.25147846	0.99232
CTC-232P5.3	1.013662	1.0123699	1.0147286	1.1906826	0.25178883	0.99232
DCAF4L2	1.013662	1.0123699	1.01472876	1.1906953	0.25180429	0.99232

NPFFR1	1.01366	1.0123699	1.01472613	1.190483	0.25154702	0.99232
ARL14EPL	1.013657	1.0123699	1.01471972	1.189965	0.25091913	0.99232
LINC00539	1.013662	1.0123699	1.01472836	1.1906628	0.2517649	0.99232
RP1-102E24.8	1.013662	1.0123699	1.01472836	1.1906628	0.2517649	0.99232
FO538757.2	1.013657	1.0123699	1.0147208	1.1900524	0.25102505	0.99232
CSN1S1	1.013657	1.0123699	1.0147208	1.1900524	0.25102505	0.99232
LINC01247	1.013665	1.0123699	1.01473434	1.1911466	0.25235099	0.99232
CTD-2193G5.1	1.013666	1.0123699	1.01473691	1.1913544	0.25260259	0.99232
APOC2	1.013659	1.0123699	1.01472304	1.1902329	0.2512439	0.99232
AC093702.1	1.013665	1.0123699	1.01473418	1.1911338	0.25233548	0.99232
RP4-539M6.14	1.01366	1.0123699	1.01472582	1.1904575	0.25151615	0.99232
PTCHD1-AS	1.013662	1.0123699	1.01472851	1.1906756	0.25178036	0.99232
RP11-353K11.1	1.013663	1.0123699	1.01473046	1.1908328	0.25197086	0.99232
RP11-286N22.10	1.013662	1.0123699	1.01472953	1.1907577	0.25187982	0.99232
ATOH1	1.013662	1.0123699	1.01472953	1.1907577	0.25187982	0.99232
RP11-745C15.2	1.013662	1.0123699	1.01472953	1.1907577	0.25187982	0.99232
RP5-978I12.1	1.013661	1.0123699	1.01472744	1.1905891	0.2516756	0.99232
RP11-486O13.2	1.013661	1.0123699	1.01472744	1.1905891	0.2516756	0.99232
RP11-166N6.3	1.013663	1.0123699	1.01473155	1.1909208	0.25207742	0.99232
RP11-336A10.2	1.013663	1.0123699	1.01473155	1.1909208	0.25207742	0.99232
MC2R	1.013657	1.0123699	1.01471918	1.1899213	0.2508662	0.99232
AC025442.3	1.013657	1.0123699	1.01471918	1.1899213	0.2508662	0.99232
APELA	1.028421	1.0270871	1.02952328	1.0899388	0.12424714	0.99239
RP11-295K3.1	1.014527	1.0132202	1.01560618	1.1804795	0.23937303	0.99239
RP11-13P5.1	1.014513	1.0132207	1.01558024	1.1784753	0.23692153	0.99239
TMX2-CTNND1	1.014525	1.0132178	1.015604	1.1805309	0.23943579	0.99239
CTC-251D13.1	1.014506	1.0132274	1.01556163	1.1764686	0.23446287	0.99239
RP11-326N17.2	1.014516	1.0132247	1.01558325	1.178345	0.23676195	0.99239
RP11-428O18.6	1.014516	1.0132224	1.01558399	1.178601	0.23707535	0.99239
FAM138E	1.014507	1.0132202	1.01557042	1.1777744	0.23606318	0.99239
RP11-379L18.3	1.014521	1.0132292	1.01558766	1.1782778	0.23667969	0.99239
SLC34A3	1.01452	1.0132178	1.01559621	1.1799415	0.23871536	0.99239
RP11-381P6.1	1.01451	1.0132168	1.01557837	1.1786751	0.2371661	0.99239
F13A1	1.014501	1.0132144	1.0155631	1.1777357	0.23601585	0.99239
CD79B	1.014505	1.0132175	1.01556895	1.1779042	0.23622224	0.99239
RP11-881M11.1	1.014515	1.0132155	1.01558875	1.1795771	0.23826974	0.99239
LINC00845	1.014507	1.0132178	1.01557083	1.1780214	0.23636579	0.99239
RP11-585P4.5	1.014516	1.0132175	1.01558875	1.179402	0.23805555	0.99239
AHSP	1.014509	1.0132186	1.01557543	1.1782948	0.23670051	0.99239
RP11-157F20.3	1.014506	1.0132182	1.01556966	1.1778917	0.23620686	0.99239
RNU6ATAC35P	1.014521	1.0132341	1.01558399	1.1775668	0.23580886	0.99239
CTB-40H15.4	1.014517	1.0132274	1.01558286	1.1780732	0.23642924	0.99239
RP3-414A15.10	1.072598	1.0712435	1.07371651	1.0347122	0.04922949	0.99243
DSTYK	1.379064	1.3803331	1.37801552	0.9939065	-0.0088179	0.99248
SLC25A10	1.178661	1.1798688	1.17766352	0.9877394	-0.0177977	0.99249

RP4-794H19.1	1.028852	1.0301201	1.02780506	0.9231395	-0.1153794	0.99251
RP11-717F1.2	1.028861	1.0301332	1.02781028	0.9229123	-0.1157345	0.99251
GLS2	1.028872	1.0301039	1.0278546	0.9252827	-0.112034	0.99251
RP11-408A13.4	1.028833	1.0300634	1.02781765	0.9252992	-0.1120081	0.99251
U47924.32	1.012646	1.0113663	1.01370228	1.2055161	0.26965094	0.99253
RP11-173P15.3	1.012646	1.0113663	1.0137024	1.2055268	0.26966373	0.99253
MYZAP	1.012645	1.0113663	1.01370169	1.2054644	0.2695891	0.99253
RP11-33A14.1	1.012645	1.0113663	1.01370169	1.2054644	0.2695891	0.99253
AC073046.25	1.012644	1.0113663	1.01369969	1.2052883	0.26937822	0.99253
LY6K	1.012644	1.0113663	1.01369969	1.2052883	0.26937822	0.99253
C15orf32	1.012649	1.0113663	1.01370743	1.2059697	0.2701937	0.99253
RP11-757A13.1	1.012657	1.0113663	1.01372338	1.2073732	0.2718717	0.99253
RP11-745O10.4	1.012646	1.0113663	1.01370346	1.2056201	0.26977541	0.99253
CLEC1A	1.01265	1.0113663	1.01370932	1.2061361	0.27039267	0.99253
UPK3BL	1.012652	1.0113663	1.01371436	1.2065796	0.27092314	0.99253
CTD-2515C13.2	1.01265	1.0113663	1.01371029	1.2062215	0.27049491	0.99253
HLA-DQB2	1.012648	1.0113663	1.01370705	1.2059359	0.27015322	0.99253
FRMPD4	1.012647	1.0113663	1.01370547	1.2057968	0.26998675	0.99253
CTD-3126B10.1	1.012647	1.0113663	1.01370445	1.2057077	0.26988018	0.99253
KLF8	1.012651	1.0113663	1.01371262	1.2064259	0.27073935	0.99253
IL26	1.012645	1.0113663	1.01370161	1.2054575	0.26958084	0.99253
RP11-67L3.4	1.01265	1.0113663	1.01371041	1.2062316	0.27050688	0.99253
XACT	1.01265	1.0113663	1.01371086	1.2062715	0.27055464	0.99253
HOXA10	1.01265	1.0113663	1.01371086	1.2062715	0.27055464	0.99253
DPP4	1.012651	1.0113663	1.0137121	1.2063805	0.27068505	0.99253
AC009227.2	1.012651	1.0113663	1.0137121	1.2063805	0.27068505	0.99253
RP11-542G1.1	1.012647	1.0113663	1.01370425	1.2056899	0.26985886	0.99253
CTD-2589M5.4	1.012645	1.0113663	1.01370176	1.2054706	0.26959649	0.99253
RP11-135J2.3	1.012651	1.0113663	1.01371153	1.2063302	0.27062492	0.99253
LINC01538	1.012651	1.0113663	1.01371153	1.2063302	0.27062492	0.99253
RP11-445K13.2	1.012645	1.0113663	1.01370115	1.2054169	0.26953223	0.99253
RP11-864I4.4	1.012647	1.0113663	1.01370392	1.2056608	0.26982409	0.99253
RP11-6I2.3	1.012647	1.0113663	1.01370531	1.2057829	0.26997019	0.99253
AC012370.3	1.012644	1.0113663	1.01369876	1.2052071	0.26928111	0.99253
RP5-1007H16.1	1.012649	1.0113663	1.01370894	1.2061021	0.27035206	0.99253
WNT4	1.012647	1.0113663	1.01370523	1.205776	0.26996191	0.99253
AC005009.2	1.012649	1.0113663	1.01370851	1.2060644	0.270307	0.99253
KERA	1.012653	1.0113663	1.01371576	1.2067026	0.2710701	0.99253
AC003986.7	1.012653	1.0113663	1.01371576	1.2067026	0.2710701	0.99253
XXyac-YX65C7_A.3	1.012653	1.0113663	1.01371576	1.2067026	0.2710701	0.99253
RP11-46C24.3	1.012644	1.0113663	1.0136993	1.2052546	0.26933791	0.99253
RP11-681H18.2	1.012648	1.0113663	1.01370606	1.2058489	0.27004919	0.99253
NETO1	1.012648	1.0113663	1.01370606	1.2058489	0.27004919	0.99253
ZNF341-AS1	1.012648	1.0113663	1.01370593	1.2058375	0.27003548	0.99253
AF131216.6	1.012645	1.0113663	1.01370037	1.2053488	0.26945068	0.99253

RP11-295P9.12	1.012651	1.0113663	1.01371107	1.2062894	0.27057603	0.99253
DEFB135	1.012655	1.0113663	1.01371843	1.206937	0.27135035	0.99253
KLRF2	1.012653	1.0113663	1.01371575	1.2067016	0.27106899	0.99253
IL12A	1.012645	1.0113663	1.01370107	1.20541	0.26952397	0.99253
RP11-817J15.3	1.012652	1.0113663	1.01371373	1.2065241	0.27085674	0.99253
BTN1A1	1.012652	1.0113663	1.01371373	1.2065241	0.27085674	0.99253
RP11-1020A11.2	1.025887	1.0245988	1.02695059	1.0956054	0.13172822	0.99253
NIM1K	1.025936	1.0246767	1.02697662	1.0932014	0.12855915	0.99253
MBNL1-AS1	1.042975	1.0417089	1.04402139	1.0554443	0.07785039	0.99256
CLU	7.490653	7.487668	7.49311746	1.00084	0.00121132	0.99256
RGS11	1.040443	1.0391686	1.04149588	1.0594163	0.08326967	0.99262
AMDHD2	1.230718	1.2294053	1.23180161	1.0104458	0.014992	0.99268
MIRLET7BHG	1.02651	1.0252237	1.02757283	1.0931326	0.12846845	0.99269
C7orf31	1.095609	1.0970432	1.09442468	0.9730169	-0.0394632	0.9927
SLC22A9	1.017457	1.0186826	1.01644507	0.8802346	-0.1840401	0.9927
AL121578.2	1.017443	1.0186517	1.01644507	0.8816912	-0.1816546	0.9927
LINC00269	1.017473	1.0187183	1.01644507	0.8785573	-0.1867917	0.9927
RP11-91I20.3	1.017447	1.0186611	1.01644507	0.8812503	-0.1823763	0.9927
QRFP	1.017472	1.0187145	1.01644507	0.8787345	-0.1865008	0.9927
CTD-2126E3.3	1.017468	1.0187061	1.01644507	0.8791268	-0.1858568	0.9927
AP004372.1	1.017464	1.0186975	1.01644507	0.8795347	-0.1851877	0.9927
LINC01228	1.017463	1.0186961	1.01644507	0.8795969	-0.1850856	0.9927
RP11-818O24.3	1.017466	1.0187027	1.01644507	0.8792875	-0.1855932	0.9927
RP3-468B3.2	1.017446	1.018658	1.01644507	0.8813962	-0.1821375	0.9927
CTD-2017C7.1	1.017459	1.0186871	1.01644507	0.8800222	-0.1843883	0.9927
LY6G6E	1.017461	1.018692	1.01644507	0.8797915	-0.1847664	0.9927
LINC00434	1.017461	1.018692	1.01644507	0.8797915	-0.1847664	0.9927
CLEC4G	1.017443	1.0186519	1.01644507	0.8816823	-0.1816692	0.9927
C4orf51	1.017466	1.0187029	1.01644507	0.8792776	-0.1856094	0.9927
AC012314.8	1.017466	1.0187018	1.01644507	0.8793317	-0.1855206	0.9927
RP11-428G5.5	1.017457	1.0186813	1.01644507	0.8802957	-0.1839398	0.9927
CHIC2	1.331246	1.3302381	1.33207921	1.005575	0.00802074	0.99276
ZNF805	1.035052	1.0337841	1.03609851	1.0685053	0.09559402	0.9928
CTB-179K24.3	1.035105	1.0337855	1.03619478	1.0713113	0.09937775	0.9928
GRTP1	1.110566	1.1093494	1.11157089	1.0203158	0.02901581	0.99284
ZNF432	1.144867	1.1436331	1.14588594	1.015685	0.02245298	0.99289
RP4-799P18.2	1.01884	1.0200419	1.01784767	0.8905161	-0.1672864	0.99292
CTB-31O20.3	1.018826	1.0200189	1.01784021	0.8911673	-0.1662318	0.99292
CGB7	1.018821	1.0200365	1.01781696	0.8892229	-0.169383	0.99292
LA16c-OS12.2	1.018811	1.020011	1.0178205	0.8905328	-0.1672593	0.99292
PKI55	1.13372	1.1349215	1.13272857	0.9837463	-0.0236418	0.99296
CCNJL	1.071899	1.073072	1.07093065	0.9706952	-0.0429097	0.99299
GTF2H2C	1.303725	1.3049376	1.30272404	0.992741	-0.0105107	0.99302
ZNF280B	1.241155	1.2424865	1.24005564	0.9899754	-0.0145354	0.99303
RP11-798G7.6	1.025938	1.0271481	1.02493815	0.9185975	-0.1224953	0.99304

RP11-96O20.5	1.025951	1.0272093	1.02491113	0.9155379	-0.1273084	0.99304
BANCR	1.026525	1.0277448	1.02551732	0.9197142	-0.1207425	0.99307
LEAP2	1.111179	1.1123608	1.11020377	0.9808026	-0.0279653	0.99309
RP11-300E4.2	1.013515	1.0122555	1.01455428	1.1875738	0.24801721	0.99309
TTC6	1.013517	1.0122555	1.01455816	1.1878899	0.24840108	0.99309
TTLL2	1.013517	1.0122555	1.01455816	1.1878899	0.24840108	0.99309
H2BFWT	1.013515	1.0122555	1.01455576	1.1876939	0.24816302	0.99309
RP11-760H22.2	1.013509	1.0122555	1.01454488	1.1868063	0.24708454	0.99309
SLC6A13	1.013509	1.0122555	1.01454434	1.1867623	0.24703104	0.99309
RP11-693J15.6	1.013515	1.0122555	1.01455598	1.1877124	0.24818558	0.99309
AC147651.1	1.013516	1.0122555	1.01455707	1.1878011	0.2482933	0.99309
RP1-28H20.3	1.013508	1.0122555	1.01454288	1.1866432	0.24688618	0.99309
AC010761.6	1.013511	1.0122555	1.01454819	1.1870764	0.24741274	0.99309
ALK	1.013511	1.0122555	1.01454726	1.187001	0.24732113	0.99309
RP11-561O23.8	1.013516	1.0122555	1.01455675	1.1877754	0.248262	0.99309
RP11-253I19.3	1.013516	1.0122555	1.01455723	1.187814	0.24830895	0.99309
LA16c-360A4.1	1.013512	1.0122555	1.01455004	1.1872272	0.24759611	0.99309
RP11-496D24.2	1.013512	1.0122555	1.01454988	1.1872144	0.24758052	0.99309
RP11-717K11.2	1.013517	1.0122555	1.014558	1.187877	0.24838542	0.99309
RP11-460B17.3	1.013518	1.0122555	1.01456007	1.1880457	0.24859033	0.99309
ESX1	1.013515	1.0122555	1.01455521	1.1876496	0.24810919	0.99309
XXyac-YR29IB3.1	1.013516	1.0122555	1.01455644	1.1877497	0.24823086	0.99309
AC123023.1	1.01351	1.0122555	1.01454672	1.1869569	0.24726756	0.99309
FARP1-AS1	1.013512	1.0122555	1.01454911	1.1871518	0.2475044	0.99309
PGR	1.013518	1.0122555	1.01456079	1.1881048	0.24866209	0.99309
C22orf34	1.013515	1.0122555	1.01455451	1.1875924	0.24803975	0.99309
OTOG	1.013513	1.0122555	1.01455058	1.1872714	0.24764978	0.99309
IQCJ	1.013513	1.0122555	1.01455058	1.1872714	0.24764978	0.99309
AP000688.29	1.013516	1.0122555	1.01455691	1.1877882	0.24827765	0.99309
LINC00460	1.01351	1.0122555	1.01454634	1.1869256	0.24722956	0.99309
HHEX	1.01351	1.0122555	1.01454634	1.1869256	0.24722956	0.99309
RP1-125I3.2	1.013517	1.0122555	1.01455939	1.1879902	0.24852295	0.99309
OR5H15	1.013511	1.0122555	1.01454803	1.1870635	0.24739717	0.99309
CTB-76P12.1	1.013511	1.0122555	1.01454803	1.1870635	0.24739717	0.99309
RP11-6N13.1	1.013511	1.0122555	1.01454803	1.1870635	0.24739717	0.99309
RP5-947P14.1	1.013518	1.0122555	1.01456002	1.1880417	0.24858549	0.99309
LRRC19	1.013518	1.0122555	1.01456002	1.1880417	0.24858549	0.99309
RP11-260O18.1	1.013518	1.0122555	1.01456002	1.1880417	0.24858549	0.99309
RP11-497G19.1	1.013516	1.0122555	1.01455653	1.1877568	0.24823943	0.99309
AC062021.1	1.013511	1.0122555	1.01454765	1.1870323	0.24735914	0.99309
AC004953.1	1.013512	1.0122555	1.01455042	1.1872586	0.24763419	0.99309
RP11-568A7.2	1.013518	1.0122555	1.0145603	1.1880648	0.24861354	0.99309
HNF4G	1.013518	1.0122555	1.0145603	1.1880648	0.24861354	0.99309
CYP4A22	1.013515	1.0122555	1.01455467	1.1876053	0.24805538	0.99309
ZIM3	1.013515	1.0122555	1.01455467	1.1876053	0.24805538	0.99309

AC034187.2	1.013515	1.0122555	1.01455467	1.1876053	0.24805538	0.99309
AC011995.1	1.013513	1.0122555	1.01455212	1.1873968	0.24780218	0.99309
AP006285.7	1.01352	1.0122555	1.01456452	1.1884092	0.24903165	0.99309
RP11-429E11.2	1.013521	1.0122555	1.01456523	1.1884667	0.24910149	0.99309
RP11-504A18.1	1.041179	1.0423642	1.04020066	0.9489307	-0.0756253	0.99312
SPEF1	1.074304	1.073112	1.07528857	1.0297699	0.04232195	0.99313
RP11-692D12.1	1.029325	1.0279895	1.03042815	1.0871257	0.12051871	0.99317
HOXB7	1.029328	1.0281076	1.03033596	1.0792804	0.11006979	0.99317
MEF2C-AS1	1.029298	1.0280597	1.03032137	1.0806022	0.11183554	0.99317
RP11-480A16.1	1.100672	1.1019612	1.0996072	0.9769128	-0.0336984	0.99318
RP11-134K13.4	1.018438	1.0196287	1.01745407	0.8892108	-0.1694027	0.9932
ZNF528	1.018426	1.0196369	1.01742568	0.8873934	-0.1723543	0.9932
HIST2H2BF	1.018419	1.0196321	1.01741739	0.8871906	-0.172684	0.9932
PLCL1	1.018411	1.0196105	1.01742046	0.8883251	-0.1708403	0.9932
ANXA9	1.018417	1.0196437	1.01740431	0.8859996	-0.174622	0.9932
KANSL1L	1.087998	1.0891521	1.08704513	0.9763665	-0.0345053	0.9932
NLRP12	1.021052	1.0197728	1.02210797	1.1181019	0.16105171	0.99324
PMP2	1.021086	1.0198185	1.02213271	1.1167721	0.15933485	0.99324
HIST1H4F	1.021044	1.019766	1.02209981	1.1180704	0.16101109	0.99324
AC073957.15	1.021045	1.0197848	1.02208633	1.1163278	0.15876069	0.99324
DAPK1	1.303352	1.3021735	1.3043257	1.0071223	0.01023884	0.9933
LINC00997	1.059341	1.0580613	1.06039701	1.0402278	0.05689947	0.9933
RP11-746M1.1	1.026235	1.0274778	1.02520828	0.9174036	-0.1243715	0.99332
CFAP53	1.130793	1.1297687	1.13163854	1.0144092	0.02063972	0.99333
RP5-890E16.5	1.029219	1.0304636	1.02819067	0.9253894	-0.1118676	0.99335
HSPB2	1.023145	1.0218879	1.02418394	1.1049018	0.14391818	0.9934
CTD-2342J14.6	1.023115	1.0218755	1.02413888	1.1034661	0.14204235	0.9934
FRMD7	1.023138	1.0218645	1.02419054	1.1063835	0.14585157	0.99342
AC009133.21	1.023126	1.0218334	1.02419427	1.1081331	0.14813115	0.99342
RP11-394I13.1	1.023116	1.0218808	1.02413689	1.10311	0.1415766	0.99342
RP11-2H3.6	1.023119	1.0218168	1.02419427	1.1089748	0.14922653	0.99342
RP11-306I1.2	1.023123	1.0218747	1.02415429	1.1042111	0.14301597	0.99342
MYO16	1.023101	1.0218351	1.02414685	1.1058713	0.14518347	0.99342
MAP1LC3C	1.01931	1.0204963	1.01833122	0.8943664	-0.1610622	0.99343
AC092614.2	1.019323	1.0205232	1.01833122	0.893193	-0.1629561	0.99343
NAV2-AS4	1.019314	1.020505	1.01833122	0.893987	-0.1616743	0.99343
RP11-222G7.2	1.01931	1.0204949	1.01833122	0.8944278	-0.1609631	0.99343
POU4F1	1.019352	1.0205881	1.01833122	0.8903794	-0.1675079	0.99343
RP11-513N24.1	1.019315	1.0205069	1.01833122	0.8939038	-0.1618085	0.99343
CTD-2521M24.8	1.019313	1.0205009	1.01833122	0.8941649	-0.1613872	0.99343
AKR1D1	1.019323	1.0205234	1.01833122	0.8931874	-0.1629652	0.99343
AC079776.1	1.019325	1.0205286	1.01833122	0.8929601	-0.1633324	0.99343
RP11-561B11.2	1.019309	1.0204939	1.01833122	0.8944725	-0.160891	0.99343
OR7D2	1.019332	1.0205431	1.01833122	0.8923309	-0.1643492	0.99343
GPR88	1.019337	1.0205539	1.01833122	0.8918588	-0.1651127	0.99343

CTD-2616J11.11	1.019343	1.0205691	1.01833122	0.8912028	-0.1661744	0.99343
AC015849.19	1.021026	1.0197841	1.02205108	1.1145847	0.15650627	0.99343
RP11-589M4.1	1.021038	1.0197411	1.02210903	1.1199492	0.16343331	0.99343
ACSL6	1.021008	1.0197254	1.0220671	1.1187143	0.16184162	0.99343
RP11-347C12.3	1.021008	1.0197509	1.02204694	1.1162514	0.15866204	0.99343
EFCC1	1.021013	1.0197481	1.02205716	1.1169283	0.15953653	0.99343
VWA3B	1.021013	1.0197437	1.02206096	1.1173645	0.16009995	0.99343
NHEJ1	1.021004	1.0197463	1.0220418	1.1162512	0.15866174	0.99343
PLAGL2	1.257846	1.2566301	1.25884942	1.008648	0.01242279	0.99347
CTB-31O20.2	1.227968	1.2290679	1.22705987	0.9912338	-0.0127027	0.99348
C17orf99	1.013247	1.0120052	1.01427211	1.1888323	0.24954519	0.99348
SLC9C2	1.013248	1.0120052	1.01427513	1.1890835	0.24984998	0.99348
CTD-2196E14.5	1.013248	1.0120052	1.01427524	1.1890923	0.2498607	0.99348
CACNA2D3-AS1	1.01325	1.0120052	1.01427799	1.189322	0.25013941	0.99348
AC019330.1	1.013245	1.0120052	1.0142693	1.1885982	0.24926107	0.99348
CTD-2376I20.1	1.013248	1.0120052	1.01427501	1.1890733	0.24983765	0.99348
CTC-338M12.7	1.013248	1.0120052	1.01427501	1.1890733	0.24983765	0.99348
AQP2	1.013247	1.0120052	1.01427316	1.1889196	0.24965111	0.99348
LINC01322	1.013247	1.0120052	1.01427177	1.1888032	0.24950987	0.99348
RP11-773H22.4	1.013247	1.0120052	1.01427177	1.1888032	0.24950987	0.99348
RP11-159K7.2	1.013245	1.0120052	1.01426865	1.1885437	0.24919498	0.99348
RP11-646J21.4	1.01325	1.0120052	1.01427741	1.1892732	0.25008012	0.99348
CTA-992D9.8	1.013249	1.0120052	1.01427622	1.1891739	0.24995968	0.99348
RP11-25O10.2	1.013249	1.0120052	1.01427622	1.1891739	0.24995968	0.99348
FOXP3	1.013259	1.0120052	1.01429447	1.190694	0.2518027	0.99348
ALDH3B2	1.013244	1.0120052	1.0142668	1.1883899	0.24900827	0.99348
BOK-AS1	1.013244	1.0120052	1.0142668	1.1883899	0.24900827	0.99348
XIRP1	1.013262	1.0120052	1.01429958	1.1911203	0.25231918	0.99348
BIRC6-AS2	1.01325	1.0120052	1.01427753	1.1892833	0.25009247	0.99348
SHANK2-AS3	1.01325	1.0120052	1.01427753	1.1892833	0.25009247	0.99348
TAS2R13	1.013245	1.0120052	1.01426909	1.1885806	0.24923978	0.99348
RP11-173P15.5	1.01325	1.0120052	1.01427827	1.1893447	0.25016687	0.99348
RP5-1077I2.3	1.013247	1.0120052	1.01427273	1.1888839	0.24960782	0.99348
LYZ	1.013251	1.0120052	1.01428048	1.1895289	0.25039028	0.99348
AC011893.3	1.013246	1.0120052	1.01427111	1.1887486	0.24944368	0.99348
ACAN	1.013254	1.0120052	1.01428615	1.1900015	0.25096335	0.99348
SLC24A2	1.013247	1.0120052	1.01427251	1.188865	0.24958487	0.99348
PLA2G4F	1.013245	1.0120052	1.01426827	1.1885118	0.24915621	0.99348
GABRG1	1.013244	1.0120052	1.01426734	1.1884342	0.24906202	0.99348
DTHD1	1.013247	1.0120052	1.01427289	1.188897	0.24962373	0.99348
LRRC61	1.013247	1.0120052	1.01427289	1.188897	0.24962373	0.99348
LINC01354	1.013252	1.0120052	1.01428114	1.1895837	0.25045682	0.99348
JAKMIP2-AS1	1.013248	1.0120052	1.01427455	1.1890354	0.24979163	0.99348
RP11-404O13.5	1.013249	1.0120052	1.01427691	1.1892316	0.25002967	0.99348
AC107081.5	1.013249	1.0120052	1.0142755	1.1891143	0.24988742	0.99348

AC110615.1	1.013245	1.0120052	1.01426903	1.1885757	0.24923374	0.99348
GBP5	1.013248	1.0120052	1.01427381	1.1889735	0.24971654	0.99348
TSPYL6	1.013248	1.0120052	1.01427381	1.1889735	0.24971654	0.99348
IGFL3	1.013245	1.0120052	1.01426819	1.1885053	0.24914828	0.99348
AC006378.2	1.013245	1.0120052	1.01426873	1.1885503	0.24920291	0.99348
DSG1	1.013251	1.0120052	1.01428067	1.189545	0.25040979	0.99348
CCDC70	1.013246	1.0120052	1.01427142	1.1887748	0.2494754	0.99348
ALOX15B	1.013245	1.0120052	1.01426965	1.1886272	0.24929634	0.99348
C10orf91	1.013246	1.0120052	1.01427158	1.1887879	0.24949129	0.99348
IL18RAP	1.013253	1.0120052	1.01428377	1.1898035	0.2507233	0.99348
CA6	1.013253	1.0120052	1.01428377	1.1898035	0.2507233	0.99348
OVOL2	1.013252	1.0120052	1.01428094	1.1895676	0.2504373	0.99348
FGL1	1.013247	1.0120052	1.01427281	1.1888904	0.24961577	0.99348
SPRR2E	1.013247	1.0120052	1.01427281	1.1888904	0.24961577	0.99348
INAFM2	1.058357	1.0572357	1.05928253	1.0357621	0.05069266	0.99352
RP11-412D9.4	1.156496	1.1551412	1.15761438	1.0159413	0.0228171	0.99354
RP11-783K16.13	1.018682	1.0198617	1.01770706	0.8915199	-0.1656611	0.99359
MUC3A	1.018709	1.019935	1.01769602	0.8876861	-0.1718785	0.99359
RP11-715J22.3	1.035484	1.0342146	1.0365327	1.067751	0.09457523	0.99361
RP11-162A12.2	1.022117	1.020904	1.02311804	1.1059155	0.14524122	0.99362
CNTD2	1.022114	1.0208774	1.02313449	1.1081124	0.14810429	0.99362
SIN3A	1.401296	1.4027197	1.40012021	0.9935451	-0.0093426	0.99363
MATR3	1.045691	1.0444037	1.04675381	1.0529264	0.07440456	0.99364
FXYD7	1.034467	1.0356505	1.03348941	0.9393811	-0.0902175	0.99365
TRIM27	1.638775	1.6372694	1.64001745	1.0043122	0.00620775	0.99366
IL23A	1.02132	1.0201245	1.02230676	1.1084368	0.14852647	0.99368
DNASE2B	1.021325	1.0200758	1.02235597	1.1135765	0.15520067	0.99368
RP11-54O7.1	1.181898	1.1830361	1.18095902	0.9886523	-0.0164649	0.99368
SERPINA5	1.049825	1.0486427	1.05080074	1.0443661	0.06262752	0.9937
NOXO1	1.020757	1.0195274	1.02177254	1.1149739	0.15700992	0.99371
RP11-115D19.4	1.020793	1.019521	1.02184324	1.1189626	0.16216177	0.99371
LINC00346	1.020768	1.0195455	1.0217783	1.1142382	0.15605773	0.99371
RP11-318A15.2	1.036007	1.0347714	1.03702704	1.0648705	0.09067797	0.99373
HYPK	1.075322	1.0741176	1.07631569	1.0296564	0.04216297	0.99373
ITPKC	1.075143	1.0739492	1.07612884	1.0294749	0.04190862	0.99373
RHBDL1	1.060023	1.0588007	1.06103201	1.0379474	0.05373329	0.99375
TTI1	1.172882	1.1741126	1.17186503	0.9870915	-0.0187443	0.99376
CTB-73N10.1	1.013662	1.0124282	1.01468092	1.1812635	0.24033081	0.99376
DNAI2	1.013662	1.0124282	1.0146802	1.1812052	0.24025959	0.99376
DUX4	1.013659	1.0124282	1.01467599	1.1808663	0.23984563	0.99376
RP11-337L12.1	1.013661	1.0124282	1.01467954	1.1811524	0.2401951	0.99376
RP11-65D17.1	1.01366	1.0124282	1.01467676	1.1809288	0.23992195	0.99376
KCNQ1	1.01366	1.0124282	1.01467676	1.1809288	0.23992195	0.99376
RP11-3G20.2	1.013663	1.0124282	1.01468332	1.1814567	0.2405667	0.99376
RP11-517I3.1	1.01366	1.0124282	1.01467641	1.1809007	0.23988761	0.99376

RP11-872D17.4	1.013661	1.0124282	1.01467889	1.1810996	0.24013064	0.99376
RP13-895J2.2	1.013661	1.0124282	1.01467889	1.1810996	0.24013064	0.99376
SLC24A3	1.013661	1.0124282	1.01467889	1.1810996	0.24013064	0.99376
MNDA	1.013661	1.0124282	1.01467889	1.1810996	0.24013064	0.99376
RP11-410D17.2	1.013666	1.0124282	1.01468854	1.181876	0.24107871	0.99376
FOXL2	1.013666	1.0124282	1.01468854	1.181876	0.24107871	0.99376
RP3-416H24.1	1.01366	1.0124282	1.01467703	1.1809506	0.23994856	0.99376
RP11-402L1.4	1.013657	1.0124282	1.01467187	1.1805352	0.23944111	0.99376
LINC00992	1.013659	1.0124282	1.01467611	1.1808761	0.23985759	0.99376
CTD-2044J15.1	1.013659	1.0124282	1.01467611	1.1808761	0.23985759	0.99376
FOXN1	1.013659	1.0124282	1.01467611	1.1808761	0.23985759	0.99376
RP11-290L1.5	1.013661	1.0124282	1.01467862	1.1810778	0.240104	0.99376
RP11-316I3.1	1.013669	1.0124282	1.01469364	1.1822864	0.24157953	0.99376
IL33	1.013669	1.0124282	1.01469297	1.1822325	0.24151373	0.99376
CSNK1A1L	1.013663	1.0124282	1.0146826	1.1813983	0.24049539	0.99376
SLC36A2	1.013664	1.0124282	1.01468523	1.18161	0.24075391	0.99376
RP11-412H8.2	1.013658	1.0124282	1.0146738	1.1806901	0.23963038	0.99376
TMIGD3	1.013658	1.0124282	1.0146738	1.1806901	0.23963038	0.99376
GJC2	1.013659	1.0124282	1.01467564	1.1808382	0.23981131	0.99376
LINC01179	1.013658	1.0124282	1.01467399	1.1807056	0.23964923	0.99376
RP5-902P8.12	1.013658	1.0124282	1.01467399	1.1807056	0.23964923	0.99376
AIRE	1.013664	1.0124282	1.01468421	1.1815282	0.24065401	0.99376
ZFP3	1.013659	1.0124282	1.01467572	1.1808445	0.23981904	0.99376
RP11-138C9.1	1.01366	1.0124282	1.01467723	1.180966	0.23996745	0.99376
RP5-908M14.5	1.013661	1.0124282	1.0146785	1.1810687	0.24009284	0.99376
GRIN3A	1.013662	1.0124282	1.0146812	1.1812855	0.2403577	0.99376
UGT2B28	1.013663	1.0124282	1.01468202	1.1813517	0.24043857	0.99376
IMPDH1	1.290037	1.291131	1.28913292	0.9931368	-0.0099356	0.99376
CDH4	1.093987	1.0951024	1.09306626	0.9785902	-0.0312232	0.99377
C10orf53	1.016276	1.0150463	1.01729116	1.1491937	0.20062204	0.99381
HUS1B	1.016301	1.0150537	1.01733066	1.151257	0.20320996	0.99381
FGFBP1	1.016271	1.0150391	1.0172885	1.1495724	0.20109732	0.99381
AC005786.5	1.016282	1.0150499	1.01729969	1.1494857	0.20098854	0.99381
SERPINB9P1	1.016284	1.0150587	1.01729646	1.1486061	0.19988415	0.99381
ADGRG3	1.016279	1.0150464	1.01729761	1.1496151	0.20115094	0.99381
C5orf63	1.016305	1.0150556	1.01733629	1.1514833	0.20349345	0.99381
BX470102.3	1.016278	1.015051	1.01729035	1.1487829	0.20010612	0.99381
RP11-462B18.2	1.016281	1.0150441	1.01730273	1.1501335	0.2018013	0.99381
NEURL4	1.062455	1.063645	1.06147188	0.9658551	-0.0501214	0.99393
FAM134A	1.798186	1.7992225	1.7973306	0.9976328	-0.0034193	0.99405
ACSF2	1.135805	1.136936	1.13487019	0.984914	-0.0219304	0.99407
FLJ35934	1.078686	1.0798309	1.07774107	0.973822	-0.03827	0.9941
RBM25	2.80126	2.8027359	2.80004199	0.9985056	-0.0021575	0.99413
IGSF9B	1.017815	1.0190074	1.01682991	0.8854384	-0.1755361	0.99415
AQP6	1.017835	1.0189909	1.0168807	0.8888839	-0.169933	0.99415

CFAP61	1.017811	1.0189651	1.0168587	0.8889333	-0.1698529	0.99415
RP11-429J17.2	1.055673	1.05685	1.05470145	0.9622076	-0.0555799	0.99416
TEKT3	1.023015	1.0217533	1.02405702	1.1059032	0.14522515	0.99418
POU3F1	1.023008	1.0217744	1.02402591	1.1034012	0.14195742	0.99418
RP11-413H22.3	1.022981	1.0217586	1.02399109	1.1026019	0.14091194	0.99418
RNF214	1.355597	1.3545126	1.35649238	1.0055845	0.00803433	0.99421
B3GALT2	1.031161	1.0323561	1.03017426	0.9325679	-0.1007193	0.99422
SYNDIG1	1.045534	1.0467088	1.04456322	0.9540642	-0.0678418	0.99423
ZNF708	1.204476	1.2056435	1.20351214	0.9896358	-0.0150304	0.99424
KCNE2	1.030571	1.0293252	1.03159921	1.0775462	0.10774978	0.99424
GFOD1	1.030608	1.0294312	1.03158006	1.0730143	0.10166928	0.99424
RIPK3	1.023631	1.0224364	1.02461776	1.0972254	0.13385993	0.99427
SLC39A2	1.023638	1.0224033	1.02465771	1.100627	0.13832557	0.99427
CD160	1.023657	1.0224267	1.02467345	1.1001824	0.13774277	0.99427
GPRC5B	1.357104	1.3582216	1.35618086	0.994303	-0.0082425	0.99427
UNC5CL	1.048247	1.0494297	1.04727124	0.9563334	-0.0644145	0.99433
RDH16	1.019674	1.0208368	1.01871457	0.8981483	-0.1549743	0.99433
THPO	1.032789	1.0339082	1.03186404	0.939714	-0.0897063	0.99437
RP11-10N23.2	1.01885	1.0200057	1.0178956	0.894527	-0.160803	0.99437
ADARB2-AS1	1.018826	1.019966	1.01788379	0.8957101	-0.1588962	0.99437
KIAA0319	1.018813	1.019976	1.01785274	0.8937096	-0.162122	0.99437
LRRN4CL	1.018827	1.0199534	1.01789662	0.8969227	-0.1569444	0.99437
LINC00311	1.047431	1.0461998	1.04844809	1.0486637	0.06855213	0.99444
ZNF841	1.05401	1.0551513	1.0530668	0.9622048	-0.0555841	0.99446
MAPK8IP2	1.058148	1.0569984	1.05909749	1.0368268	0.05217493	0.99449
NRSN2-AS1	1.06745	1.0686377	1.06646876	0.9684004	-0.0463244	0.99456
RP11-820I16.1	1.022762	1.0215573	1.02375631	1.1020076	0.14013423	0.99458
RP11-131L23.1	1.022731	1.021528	1.02372512	1.1020569	0.14019877	0.99458
WIPF2	1.397164	1.3986787	1.39591295	0.9930627	-0.0100432	0.99458
RP11-193M21.1	1.025008	1.0261247	1.02408488	0.9219186	-0.1172887	0.99459
MRPL33	3.998752	4.0024621	3.99568896	0.9977441	-0.0032582	0.99461
WIPF3	1.055339	1.054207	1.05627403	1.0381316	0.05398929	0.99469
CNOT4	1.727337	1.7261403	1.7283258	1.0030098	0.0043357	0.99478
BCL9	1.317822	1.3189191	1.31691629	0.99372	-0.0090887	0.99479
PCDHB5	1.054068	1.0529089	1.05502494	1.0399934	0.05657439	0.99482
PCDHB15	1.054056	1.0528696	1.05503502	1.0409575	0.05791111	0.99482
OXGR1	1.023147	1.0219728	1.02411653	1.0975652	0.1343067	0.99485
HIST1H3E	1.023117	1.0219302	1.02409637	1.0987736	0.13589412	0.99485
UBXN2B	1.340637	1.3417068	1.33975382	0.9942845	-0.0082694	0.99486
CTD-3105H18.14	1.025755	1.024557	1.02674406	1.0890602	0.12308377	0.99486
RP11-281O15.4	1.01614	1.0149301	1.01713835	1.147903	0.19900069	0.99487
RAET1E	1.01613	1.014931	1.01711923	1.146555	0.1973056	0.99487
SMC1B	1.016125	1.0149319	1.01710954	1.1458368	0.19640161	0.99487
MSANTD3-TMEFF1	1.016121	1.0149293	1.01710498	1.1457322	0.19626985	0.99487
LINC00836	1.018865	1.0176782	1.01984502	1.1225712	0.1668069	0.99488

C12orf54	1.018863	1.0176701	1.01984793	1.1232508	0.16768006	0.99488
ST8SIA3	1.018866	1.0176869	1.01984031	1.1217531	0.16575519	0.99488
SOHLH1	1.018869	1.0176726	1.01985668	1.1235879	0.16811305	0.99488
HP	1.018882	1.0176722	1.01988178	1.1250321	0.16996612	0.99488
RP11-426C22.5	1.018868	1.0176797	1.01984842	1.1226654	0.16692801	0.99488
RNF123	1.107114	1.1082685	1.10616004	0.9805255	-0.028373	0.9949
ZNF587B	1.045514	1.046674	1.04455627	0.954627	-0.0669909	0.99491
C1orf101	1.021754	1.0205657	1.02273564	1.1055148	0.14471835	0.995
MS4A6E	1.021787	1.0206219	1.02274864	1.1031289	0.14160141	0.995
ACTR3C	1.021824	1.0205983	1.02283534	1.108602	0.14874151	0.995
HEATR4	1.021784	1.02067	1.02270392	1.098402	0.13540617	0.995
LEF1	1.186978	1.185889	1.18787655	1.0106922	0.01534375	0.99501
SHC3	1.0807	1.0818796	1.07972493	0.9736845	-0.0384738	0.99501
ZNF710	1.080608	1.081839	1.07959234	0.9725481	-0.0401585	0.99501
WI2-85898F10.1	1.030802	1.0319403	1.02986235	0.9349421	-0.097051	0.99502
ETFBKMT	1.069004	1.0700792	1.06811524	0.9719757	-0.0410079	0.99502
TRABD2B	1.02218	1.0233151	1.02124282	0.911118	-0.1342901	0.99507
AC005592.3	1.017839	1.0166516	1.01882018	1.1302329	0.17662009	0.99517
CH17-437K3.1	1.017824	1.0166376	1.01880393	1.1302062	0.17658595	0.99517
RP4-616B8.5	1.017825	1.0166457	1.01879815	1.1293067	0.17543739	0.99517
RP11-268G13.1	1.017833	1.016641	1.01881725	1.1307758	0.1773129	0.99517
CISTR	1.017827	1.016654	1.01879643	1.1286443	0.17459084	0.99517
ADRB1	1.140078	1.1413006	1.13906899	0.9842065	-0.022967	0.99522
RP11-285F16.1	1.021629	1.022793	1.02066742	0.9067431	-0.1412343	0.99524
IKZF3	1.021582	1.0227054	1.0206535	0.9096312	-0.1366464	0.99524
POU3F3	1.0216	1.0227012	1.02069092	0.9114443	-0.1337736	0.99524
RP5-1096D14.6	1.03184	1.0329967	1.030885	0.9360022	-0.0954161	0.99524
NXNL2	1.040108	1.0390364	1.0409923	1.0501034	0.07053135	0.99524
RP11-216B9.6	1.029018	1.0301458	1.02808685	0.9317002	-0.1020623	0.99525
PATL2	1.034786	1.0358838	1.0338788	0.9441256	-0.0829493	0.99525
SLC34A2	1.023292	1.0243889	1.02238567	0.917862	-0.1236509	0.9953
RP5-1021I20.5	1.024073	1.0251983	1.02314364	0.9184614	-0.122709	0.99537
MIR1302-10	1.106351	1.1074148	1.1054721	0.9819142	-0.0263312	0.99538
FAM131B	1.107785	1.1066795	1.10869729	1.0189146	0.02703316	0.99542
RP11-171I2.5	1.040111	1.0389834	1.04104276	1.0528279	0.07426965	0.99543
CPT1A	1.209756	1.2086358	1.21068202	1.0098078	0.01408068	0.99548
LINC00950	1.043908	1.045009	1.04299877	0.9553364	-0.0659193	0.99556
WDR90	1.198816	1.199936	1.19789051	0.9897692	-0.014836	0.9956
RP11-894P9.1	1.065345	1.0664833	1.06440576	0.9687515	-0.0458015	0.99561
SPTLC1	1.128293	1.1293482	1.12742158	0.9851056	-0.0216498	0.99568
MST1	1.275519	1.2768043	1.27445696	0.9915199	-0.0122864	0.99569
SMARCD2	1.28189	1.2807641	1.28281988	1.0073221	0.01052514	0.9957
MEG3	1.05451	1.0532556	1.05554608	1.0430088	0.06075134	0.99572
C11orf91	1.020563	1.0194149	1.0215109	1.1079592	0.14790471	0.99578
PCDHA1	1.020589	1.0194485	1.02153175	1.1071182	0.14680931	0.99578

RP11-1081M5.2	1.020611	1.0194502	1.02156925	1.1089474	0.14919089	0.99578
CD7	1.072055	1.0733605	1.07097681	0.9675076	-0.0476552	0.99579
SPDYE3	1.043514	1.0446041	1.0426144	0.9553913	-0.0658363	0.99581
DDB1	1.815857	1.8170322	1.8148866	0.9973739	-0.0037936	0.99582
DZANK1	1.122474	1.1212105	1.12351669	1.0190264	0.02719139	0.99585
KCNE1	1.019861	1.0187115	1.02080987	1.1121427	0.15334186	0.99588
ZNF556	1.019843	1.0186715	1.02080969	1.1145183	0.15642037	0.99588
SCHLAP1	1.019855	1.0187039	1.02080607	1.1123916	0.15366476	0.99588
AF196972.9	1.019886	1.0187443	1.02082848	1.1111921	0.1521082	0.99588
LHFPL4	1.019859	1.0187117	1.02080654	1.1119526	0.15309529	0.99588
ADAM8	1.019881	1.018759	1.02080792	1.109222	0.14954808	0.99588
CT55	1.018152	1.0170008	1.0191026	1.1236308	0.16816812	0.9959
CTD-3224I3.3	1.018148	1.0169937	1.01910142	1.1240313	0.16868215	0.9959
EGFLAM	1.018148	1.0169943	1.01910059	1.123944	0.16857019	0.9959
RP11-384F7.2	1.018131	1.0169894	1.0190738	1.1226862	0.1669547	0.9959
RP11-557L19.1	1.018142	1.0169932	1.01909127	1.1234674	0.1679583	0.9959
RP11-1136J12.1	1.018152	1.0169852	1.01911648	1.125481	0.17054171	0.9959
RP11-319F12.2	1.018161	1.0170134	1.01910789	1.1231085	0.16749736	0.9959
CTB-35F21.5	1.01814	1.0169857	1.0190937	1.1241049	0.16877672	0.9959
RP11-495P10.3	1.018151	1.0170014	1.01909961	1.1234119	0.16788694	0.9959
RP11-443B20.1	1.018142	1.0169842	1.01909758	1.1244351	0.16920039	0.9959
ZNF441	1.124798	1.1237053	1.12570028	1.0161268	0.02308049	0.99591
LRRC34	1.070999	1.0720683	1.0701154	0.9729021	-0.0396334	0.99592
RP11-482M8.3	1.025622	1.0244968	1.02655032	1.0838292	0.11613738	0.99593
RP11-10K16.1	1.032634	1.0315027	1.03356814	1.0655636	0.09161671	0.99593
KIAA1024	1.035962	1.0370951	1.03502672	0.9442409	-0.0827731	0.99594
LINC00887	1.031304	1.0301079	1.03229107	1.0725122	0.10099408	0.99597
RP11-462G2.2	1.036652	1.0377351	1.03575678	0.9475743	-0.0776891	0.99599
ERFE	1.021294	1.0223897	1.0203896	0.9106668	-0.1350048	0.99599
RP11-532M24.1	1.018753	1.0176201	1.0196883	1.117376	0.1601147	0.99609
RP11-286N3.2	1.018723	1.0175741	1.01967091	1.1193125	0.16261289	0.99609
LRRC66	1.018715	1.0175726	1.01965878	1.1187193	0.16184811	0.99609
CTC-325J23.2	1.018725	1.0175682	1.01967998	1.1202057	0.16376371	0.99609
ZNF878	1.018723	1.0175708	1.01967397	1.1196987	0.16311055	0.99609
RP11-386I8.6	1.018741	1.0175677	1.01971003	1.1219473	0.16600492	0.99609
RP11-531A24.3	1.080381	1.0813601	1.07957256	0.9780287	-0.0320512	0.99611
ERCC5	1.16042	1.1593163	1.16133088	1.012645	0.01812848	0.99614
CD226	1.027349	1.0262309	1.02827301	1.0778506	0.10815721	0.99618
RP4-794I6.4	1.027349	1.0262045	1.02829398	1.0797363	0.11067904	0.99618
RP11-422P24.10	1.025274	1.0264359	1.02431381	0.9197288	-0.1207196	0.99618
FAM95B1	1.025255	1.0263486	1.0243515	0.9242042	-0.1137164	0.99618
SARDH	1.04268	1.0414886	1.04366317	1.0524142	0.07370268	0.9962
WDR17	1.082653	1.0836514	1.08182764	0.9781979	-0.0318017	0.99621
RP11-126L15.4	1.022371	1.0234529	1.02147671	0.9157377	-0.1269937	0.99625
RP11-108E14.1	1.022338	1.0234255	1.0214402	0.9152523	-0.1277587	0.99625

RP3-508I15.22	1.01958	1.0206652	1.01868317	0.9040904	-0.1454611	0.99626
SLC37A2	1.026575	1.0276415	1.0256934	0.9295233	-0.105437	0.99631
C19orf57	1.06564	1.0645362	1.06655192	1.0312341	0.04437193	0.99635
EXO1	1.081862	1.082789	1.08109638	0.9795552	-0.0298012	0.99638
GABRB2	1.03503	1.0360907	1.03415344	0.9463211	-0.0795983	0.99651
MAP9	1.875749	1.8769534	1.87475507	0.9974932	-0.003621	0.99652
ZNF488	1.134075	1.1328014	1.1351271	1.0175128	0.02504687	0.99653
RP11-318A15.8	1.026278	1.0273098	1.02542546	0.9310023	-0.1031434	0.99658
RP3-406A7.7	1.02621	1.0272036	1.02538944	0.9333114	-0.0995695	0.99658
C3AR1	1.026137	1.0271934	1.02526372	0.9290371	-0.1061919	0.99658
CTD-2320G14.2	1.026229	1.027334	1.02531648	0.9261886	-0.1106222	0.99658
LMNTD1	1.021522	1.0203738	1.02246971	1.1028715	0.14126476	0.99659
PITRM1-AS1	1.021536	1.0204318	1.02244762	1.0986587	0.1357433	0.99659
CCDC141	1.021522	1.0204169	1.02243456	1.0988252	0.13596192	0.99659
RP11-462P6.1	1.021548	1.0204236	1.02247621	1.100504	0.13816437	0.99659
NALCN	1.021533	1.0204242	1.02244824	1.0990983	0.13632041	0.99659
COX6B2	1.058605	1.0597365	1.05767096	0.9654226	-0.0507675	0.9966
RNASE4	1.058562	1.0596297	1.05768079	0.9673164	-0.0479402	0.9966
RP11-457M11.5	1.058756	1.0598014	1.05789269	0.9680832	-0.0467971	0.9966
RP11-352G9.1	1.036137	1.037276	1.03519711	0.9442301	-0.0827897	0.99672
CNTNAP3	1.099211	1.1003878	1.09823905	0.9785958	-0.031215	0.99676
RP11-567C2.1	1.015385	1.014266	1.01630959	1.1432454	0.19313517	0.99676
SAMD3	1.015376	1.0142739	1.01628685	1.1410237	0.19032872	0.99676
NKG7	1.015383	1.0142685	1.01630344	1.1426198	0.1923454	0.99676
HIST1H2AK	1.015378	1.0142746	1.01628985	1.1411811	0.19052778	0.99676
ERICH6B	1.015386	1.0142685	1.01630895	1.1430058	0.19283276	0.99676
ATP4B	1.015377	1.0142732	1.01628817	1.1411751	0.19052019	0.99676
TP53AIP1	1.01538	1.0142685	1.01629866	1.1422845	0.19192199	0.99676
RP11-367H1.1	1.015386	1.0142658	1.01631145	1.1433975	0.19332705	0.99676
IKZF1	1.015393	1.0142631	1.01632699	1.1447044	0.19497507	0.99676
RP4-809F18.1	1.015376	1.0142678	1.01629094	1.1417959	0.19130475	0.99676
RP11-699A5.2	1.01538	1.0142674	1.01629889	1.1423903	0.19205562	0.99676
SPEM1	1.015385	1.0142658	1.01630879	1.1432116	0.19309246	0.99676
GDPGP1	1.063998	1.0628396	1.06495489	1.0336615	0.04776377	0.99676
ZNF425	1.063939	1.0628319	1.06485317	1.0321689	0.04567909	0.99676
RP11-686F15.2	1.034039	1.035078	1.03318079	0.9459136	-0.0802196	0.99677
RNASE1	1.071228	1.0722591	1.07037563	0.9739343	-0.0381036	0.99677
IL1RAPL1	1.036184	1.0372909	1.03526975	0.9458007	-0.0803919	0.99678
CTD-2547L16.1	1.036115	1.0371224	1.03528312	0.9504543	-0.0733108	0.99678
TRIM58	1.030115	1.0289693	1.03106173	1.0722282	0.10061193	0.9968
RP11-817G13.3	1.030146	1.0290432	1.03105599	1.069304	0.09667213	0.9968
ZNF699	1.073177	1.0719503	1.0741907	1.0311374	0.04423661	0.99681
RP11-111A22.1	1.01624	1.0151161	1.01716794	1.1357377	0.18362963	0.99682
CTD-2021J15.1	1.016223	1.0151101	1.0171421	1.1344779	0.18202851	0.99682
IRF5	1.016217	1.0151088	1.01713262	1.1339485	0.18135512	0.99682

NPIPA3	1.01624	1.0151087	1.01717444	1.1367227	0.18488041	0.99682
PIK3AP1	1.016241	1.0151336	1.017155	1.1335705	0.18087412	0.99682
PRCAT47	1.016235	1.015113	1.01716158	1.1355495	0.18339065	0.99682
RP4-660H19.1	1.02718	1.0282959	1.02625864	0.928002	-0.1078001	0.99691
FPGT-TNNI3K	1.044764	1.0436328	1.04569839	1.047341	0.06673125	0.99694
RP11-932O9.9	1.027642	1.0265054	1.02858144	1.0783241	0.10879085	0.99696
PROC	1.027644	1.0265463	1.02854979	1.0754717	0.10496959	0.99696
KRTAP5-AS1	1.014361	1.0132603	1.01526976	1.1515367	0.20356036	0.99698
ZNF32-AS2	1.014378	1.0132614	1.01529976	1.1537101	0.2062807	0.99698
TGM4	1.014371	1.0132674	1.01528201	1.1518475	0.20394973	0.99698
LINC01361	1.014366	1.0132621	1.01527771	1.151983	0.20411939	0.99698
RP11-221N13.3	1.01436	1.0132603	1.01526765	1.1513773	0.20336071	0.99698
RP5-1009E24.8	1.014361	1.0132648	1.01526654	1.1509048	0.20276846	0.99698
RP11-369E15.3	1.014367	1.0132631	1.01527846	1.1519491	0.20407693	0.99698
DENND5B-AS1	1.014374	1.0132598	1.01529433	1.1534377	0.20594002	0.99698
ANKRD62	1.014371	1.0132611	1.0152881	1.1528497	0.20520442	0.99698
CTD-2013N17.4	1.014375	1.0132686	1.01528851	1.1522365	0.2044369	0.99698
RP11-805J14.5	1.014373	1.0132623	1.01529014	1.1528989	0.20526597	0.99698
LINC01563	1.014372	1.0132705	1.01528097	1.1514973	0.20351099	0.99698
ARHGEF10L	1.107417	1.108547	1.10648445	0.9809986	-0.0276771	0.99703
CCDC71	1.262356	1.2614086	1.26313818	1.0066164	0.00951402	0.99713
CTD-2260A17.2	1.036841	1.0357451	1.03774595	1.0559761	0.07857722	0.99719
RP11-707G18.1	1.072563	1.073527	1.07176644	0.9760559	-0.0349643	0.99732
RP11-165F24.3	1.019793	1.020882	1.01889354	0.9047768	-0.1443661	0.99732
NAALADL2-AS2	1.019705	1.0207309	1.0188584	0.9096769	-0.1365739	0.99732
S100A7	1.019756	1.020839	1.01886144	0.9051052	-0.1438427	0.99732
LRRC36	1.019714	1.020738	1.01886832	0.9098446	-0.1363079	0.99732
IRX5	1.017137	1.0181854	1.01627187	0.894778	-0.1603982	0.99736
AC004878.8	1.017144	1.0182056	1.01626661	0.8934925	-0.1624725	0.99736
PANX2	1.061229	1.0601849	1.06209039	1.0316613	0.04496944	0.99739
CYP19A1	1.024252	1.0253204	1.02337022	0.9229798	-0.115629	0.99745
RP11-140M13.1	1.024265	1.0252677	1.02343614	0.9275143	-0.1085585	0.99745
AC072062.3	1.024225	1.0252818	1.02335308	0.9237107	-0.114487	0.99745
ZNF16	1.14355	1.1423621	1.14453186	1.0152415	0.02182289	0.99747
BLCAP	1.532178	1.5311529	1.53302433	1.0035234	0.00507424	0.99747
RP11-1228E12.2	1.02477	1.0258139	1.02390738	0.9261435	-0.1106924	0.99748
RP1-159A19.4	1.024771	1.0257891	1.0239308	0.927942	-0.1078935	0.99748
ZNF337	1.141283	1.1400817	1.14227484	1.0156559	0.02241173	0.99748
KIAA0408	1.039434	1.040442	1.03860243	0.9545129	-0.0671634	0.9975
RP11-381K20.2	1.039441	1.0404437	1.03861265	0.9547255	-0.0668421	0.9975
CCR6	1.025586	1.0266187	1.02473255	0.9291413	-0.1060301	0.99754
C1orf186	1.025561	1.0265564	1.02473832	0.9315392	-0.1023116	0.99754
RP11-131L12.3	1.025562	1.0265822	1.02472026	0.9299544	-0.1047681	0.99754
MYCT1	1.015231	1.0141501	1.01612406	1.1395049	0.18840714	0.99754
CPNE9	1.015246	1.014153	1.01614908	1.1410395	0.19034876	0.99754

RP4-680D5.2	1.015231	1.0141474	1.0161262	1.1398734	0.18887361	0.99754
ZNF648	1.015235	1.0141523	1.01612921	1.1396885	0.18863961	0.99754
F10-AS1	1.015251	1.0141476	1.01616187	1.1423728	0.19203352	0.99754
EQTN	1.015233	1.0141523	1.01612634	1.1394855	0.18838262	0.99754
FAM179A	1.015235	1.0141568	1.01612458	1.1390005	0.18776843	0.99754
CTD-2525I3.2	1.015227	1.0141584	1.01610984	1.1378318	0.18628729	0.99754
RP11-304L19.11	1.015238	1.014153	1.01613323	1.1399197	0.1889322	0.99754
RP11-680F20.10	1.015233	1.0141523	1.01612581	1.1394485	0.1883357	0.99754
ADRA2B	1.015233	1.0141584	1.01612125	1.1386374	0.18730845	0.99754
LINC01505	1.015233	1.0141472	1.01612897	1.1400844	0.18914067	0.99754
LINC01465	1.015228	1.0141467	1.01612164	1.1396035	0.18853196	0.99754
SSBP3-AS1	1.015236	1.0141489	1.01613402	1.1402986	0.1894116	0.99754
AC000068.10	1.015241	1.0141555	1.01613735	1.1400083	0.18904435	0.99754
UBE2Q2L	1.015247	1.0141729	1.01613339	1.1383246	0.18691206	0.99754
RP11-177J6.1	1.015243	1.0141541	1.01614163	1.140423	0.18956902	0.99754
CDH22	1.015242	1.0141648	1.01613074	1.1387882	0.18749941	0.99754
RP11-1C1.6	1.015238	1.014153	1.01613402	1.1399753	0.18900257	0.99754
CHD5	1.015225	1.0141483	1.016115	1.1390069	0.18777654	0.99754
CCDC175	1.031064	1.030025	1.03192291	1.0632115	0.08842863	0.99757
RP11-314N13.3	1.031055	1.0299546	1.03196431	1.0670916	0.09368403	0.99757
ZNF155	1.06352	1.0646929	1.0625509	0.9668894	-0.0485772	0.99758
CTD-2089N3.2	1.063494	1.0645265	1.06264112	0.9707808	-0.0427826	0.99758
STAT5A	1.038857	1.0398808	1.03801232	0.9531479	-0.069228	0.9976
HIST1H2BK	1.016678	1.0176942	1.0158388	0.8951415	-0.1598124	0.99763
LINC01143	1.016697	1.0177068	1.01586379	0.8959134	-0.1585689	0.99763
RP11-135A1.2	1.016695	1.0177262	1.0158431	0.8937682	-0.1620273	0.99763
RP11-599B13.7	1.022791	1.0216931	1.02369684	1.0923681	0.12745902	0.99766
RP11-310E22.4	1.022788	1.0216711	1.0237112	1.094138	0.12979474	0.99766
SLN	1.055978	1.0571347	1.0550222	0.9630256	-0.0543539	0.99769
ACSS2	1.193859	1.1949499	1.19295769	0.9897808	-0.014819	0.99772
HEXA-AS1	1.024488	1.0255436	1.02361644	0.9245554	-0.1131683	0.99773
RP11-505K9.4	1.024506	1.0255472	1.02364612	0.9255858	-0.1115613	0.99773
LINC01554	1.024467	1.025485	1.02362579	0.9270452	-0.1092884	0.99773
RP3-412A9.16	1.024486	1.0255349	1.02361978	0.9250002	-0.1124745	0.99773
HYAL1	1.02452	1.0256004	1.02362692	0.9229119	-0.1157352	0.99773
TMEM14B	4.112497	4.1143258	4.11098624	0.9989277	-0.0015478	0.99775
RP13-516M14.2	1.026573	1.0275447	1.02577089	0.9356038	-0.0960304	0.99776
PLLP	1.041263	1.0422688	1.04043307	0.9565707	-0.0640565	0.99781
ZCWPW2	1.03965	1.0386291	1.04049364	1.048267	0.06800621	0.99781
HIPK1-AS1	1.024864	1.0237579	1.02577654	1.0849659	0.1176497	0.99783
SMIM2-AS1	1.024832	1.023771	1.02570827	1.0814994	0.11303287	0.99783
PCDHA3	1.024938	1.0239198	1.02577806	1.0776892	0.10794117	0.99783
AC010642.1	1.02481	1.0237469	1.02568809	1.0817469	0.11336299	0.99784
TFAP2C	1.024852	1.0238089	1.02571416	1.0800219	0.11106051	0.99784
ADGRD2	1.024861	1.0237297	1.02579599	1.0870764	0.12045333	0.99784

RP4-784A16.5	1.022753	1.0216823	1.0236365	1.0901263	0.1244953	0.99785
RP11-923I11.6	1.022729	1.0216291	1.02363726	1.0928433	0.1280865	0.99785
Z69720.2	1.022734	1.0216723	1.02361023	1.0894192	0.12355915	0.99785
ZNF671	1.06	1.0588411	1.0609572	1.0359625	0.05097178	0.99787
ITGA11	1.014974	1.0139029	1.01585785	1.1406109	0.18980668	0.99793
LINC01176	1.014966	1.0139042	1.01584257	1.1394121	0.18828958	0.99793
DLG1-AS1	1.014962	1.0139072	1.01583359	1.1385175	0.1871565	0.99793
EYS	1.014969	1.0139097	1.01584285	1.1389758	0.18773705	0.99793
RP11-24N18.1	1.014967	1.013898	1.01585036	1.1404805	0.18964173	0.99793
RP11-114H23.2	1.014981	1.0138991	1.01587382	1.142077	0.1916599	0.99793
RP11-78A19.3	1.014984	1.0139042	1.01587642	1.1418463	0.19136842	0.99793
TRPC3	1.014961	1.0138991	1.01583801	1.1395004	0.18840144	0.99793
AC019186.1	1.014967	1.0138997	1.01584836	1.1401913	0.18927591	0.99793
RP11-45A12.1	1.014972	1.0139077	1.01585074	1.1397089	0.18866538	0.99793
RP11-323J4.1	1.014967	1.0139114	1.01583816	1.1385038	0.1871391	0.99793
RP11-384O8.1	1.014961	1.0139063	1.01583238	1.1385025	0.1871375	0.99793
RP13-476E20.1	1.014977	1.0139037	1.01586263	1.1408963	0.19016771	0.99793
CCDC36	1.014978	1.0139089	1.01586045	1.1403096	0.18942553	0.99793
FAM46C	1.014969	1.0138983	1.01585367	1.1406892	0.18990581	0.99793
TMPPE	1.014964	1.0139062	1.01583734	1.1388706	0.18760389	0.99793
TMEM161B	1.228277	1.2269483	1.22937471	1.0106915	0.01534263	0.99797
CLN3	1.061108	1.0621378	1.06025748	0.9697399	-0.0443303	0.998
C19orf25	1.974334	1.9734659	1.97505157	1.0016289	0.00234803	0.99801
WFDC5	1.016989	1.0179904	1.01616159	0.8983472	-0.154655	0.99802
RP11-158M2.3	1.016947	1.0179386	1.01612883	0.8991145	-0.1534233	0.99802
RP5-1139B12.2	1.016966	1.0179788	1.01613041	0.8971885	-0.156517	0.99802
ITIH4	1.016941	1.0179377	1.01611833	0.8985709	-0.1542958	0.99802
MEOX1	1.016959	1.0179529	1.01613759	0.8988871	-0.1537881	0.99802
MMP16	1.274485	1.2753593	1.27376376	0.9942055	-0.008384	0.99804
KISS1	1.023093	1.0220765	1.02393219	1.0840574	0.11644109	0.9981
MPZL3	1.023038	1.0220016	1.02389425	1.0860241	0.11905612	0.9981
RP11-334C17.6	1.023091	1.0221207	1.02389186	1.0800663	0.11111981	0.9981
KB-68A7.1	1.023062	1.0219924	1.02394473	1.0887718	0.12270158	0.9981
RP1-67K17.4	1.022518	1.0215036	1.02335492	1.0860956	0.11915114	0.99814
RP11-665G4.1	1.022462	1.021412	1.02332962	1.0895574	0.12374217	0.99814
RP1-251M9.2	1.022502	1.0214583	1.02336459	1.0888378	0.12278911	0.99814
AKNAD1	1.022492	1.0214491	1.02335405	1.0888122	0.12275515	0.99814
PVR	1.105293	1.1045387	1.10591562	1.0131714	0.0188782	0.99818
RP11-90P5.2	1.015384	1.0143357	1.01625049	1.1335647	0.1808667	0.99821
CTD-2574D22.7	1.015382	1.0143357	1.01624531	1.1332038	0.18040731	0.99821
RP11-111A21.1	1.015375	1.0143255	1.01624254	1.1338216	0.18119369	0.99821
CTD-2568A17.8	1.015384	1.0143329	1.0162512	1.1338415	0.18121901	0.99821
CTD-2037L6.2	1.015392	1.0143308	1.01626793	1.1351714	0.18291013	0.99821
LCMT1-AS2	1.015378	1.0143244	1.01624809	1.1342937	0.18179427	0.99821
CA10	1.015375	1.0143261	1.01624145	1.1336972	0.18103539	0.99821

AP001604.3	1.015387	1.0143383	1.01625385	1.1335948	0.18090507	0.99821
RP11-415I12.3	1.015375	1.0143251	1.01624215	1.133828	0.18120183	0.99821
AC015933.2	1.015394	1.0143289	1.01627391	1.135741	0.18363382	0.99821
RP11-196G11.2	1.015381	1.0143367	1.01624338	1.1329954	0.18014206	0.99821
SELPLG	1.018004	1.0169465	1.01887777	1.1139611	0.15569887	0.99825
RP11-703H8.7	1.017999	1.0169387	1.01887479	1.114301	0.15613899	0.99825
ZNF180	1.120525	1.121506	1.1197142	0.9852532	-0.0214336	0.99837
SDCBP2	1.119772	1.1187178	1.12064222	1.0162103	0.02319902	0.99838
AEBP2	1.432463	1.4318453	1.43297268	1.0026106	0.00376135	0.99839
NAXD	1.281158	1.2802412	1.28191498	1.0059728	0.00859134	0.99851
HSPBAP1	1.071321	1.0702131	1.07223594	1.02881	0.04097663	0.99851
HID1	1.126499	1.1253771	1.12742579	1.0163403	0.02338354	0.99852
TLCD1	1.129445	1.1284526	1.13026526	1.0141114	0.02021619	0.99853
LINC01535	1.016074	1.0170443	1.01527304	0.8960801	-0.1583004	0.99858
XX-FW83128A1.2	1.016101	1.0170774	1.01529553	0.8956592	-0.1589781	0.99858
LINC01460	1.024743	1.0236929	1.02561018	1.0809237	0.11226473	0.99861
RP11-312B8.1	1.024733	1.023714	1.02557375	1.0784224	0.10892233	0.99861
RP11-517A5.7	1.024743	1.0236755	1.02562516	1.0823483	0.11416485	0.99861
RAI2	1.043884	1.0449929	1.04296758	0.9549856	-0.0664491	0.99861
CTB-152G17.6	1.068523	1.0675274	1.0693459	1.0269292	0.03833675	0.99866
TEF	1.113869	1.1148161	1.11308611	0.9849325	-0.0219033	0.99867
XXbac-BPGBPG55C20.1	1.025386	1.0243601	1.02623322	1.0768934	0.1068755	0.99868
VPS53	1.402362	1.4014513	1.40311429	1.0041424	0.00596393	0.99872
FIRRE	1.313235	1.3142225	1.31241867	0.9942593	-0.0083059	0.99872
KIAA1549	1.313151	1.3142662	1.31222962	0.9935197	-0.0093796	0.99872
RP11-481J13.1	1.01796	1.0189406	1.0171506	0.905494	-0.1432231	0.99875
SAMMSON	1.017975	1.018952	1.01716754	0.9058453	-0.1426634	0.99875
RABL2B	1.685261	1.6865172	1.68422399	0.9966596	-0.0048272	0.99876
PEX5L	1.09324	1.0922502	1.09405814	1.0195979	0.02800028	0.99879
AIM1L	1.031771	1.0327066	1.03099783	0.9477556	-0.077413	0.9988
AP000223.42	1.031883	1.0329211	1.0310265	0.9424495	-0.0855127	0.9988
CTD-2587H24.10	1.017114	1.0180919	1.01630594	0.901283	-0.1499479	0.9988
MARS2	1.035196	1.0341224	1.03608296	1.0574568	0.08059871	0.99886
RP5-943J3.2	1.034405	1.0333733	1.03525621	1.0564199	0.07918333	0.99888
FOXRED1	1.196605	1.1953408	1.19764917	1.011817	0.0169484	0.99889
METTL12	1.517805	1.5169476	1.51851385	1.0030299	0.00436457	0.99891
SLC24A1	1.07599	1.0769113	1.07522905	0.9781276	-0.0319054	0.99893
DUSP16	1.173881	1.1727549	1.17481068	1.0119	0.01706672	0.99893
STYX	1.232402	1.2333033	1.23165778	0.992947	-0.0102114	0.99896
PARD3-AS1	1.051639	1.0525786	1.05086285	0.967368	-0.0478633	0.99896
CTC-340A15.2	1.023287	1.0242911	1.02245846	0.9245564	-0.1131668	0.99899
SLC8A2	1.023303	1.0243255	1.02245891	0.9232667	-0.1151806	0.99899
RP11-725P16.2	1.02325	1.0241762	1.02248469	0.9300351	-0.1046429	0.99899
SLC35D2	1.234931	1.2339104	1.23577322	1.007964	0.0114441	0.99908
RP11-307C12.12	1.061078	1.060023	1.06194924	1.032092	0.04557163	0.99912

VPS11	1.112211	1.1131382	1.11144489	0.9850336	-0.0217551	0.99922
MYBPC2	1.02485	1.0238344	1.02568946	1.0778298	0.10812932	0.99928
OLFML2B	1.043707	1.0446408	1.04293614	0.9618143	-0.0561698	0.99928
RP11-1079K10.4	1.017844	1.0168298	1.01868245	1.110081	0.150665	0.99931
CYSLTR1	1.017847	1.0168299	1.01868688	1.1103353	0.15099546	0.99931
AC006273.4	1.017867	1.0168749	1.01868632	1.1073413	0.14709994	0.99931
CXCL5	1.017854	1.016827	1.01870197	1.1114261	0.15241207	0.99931
RP11-1094H24.4	1.020571	1.0195672	1.02140049	1.0936902	0.12920416	0.99932
GPR3	1.020583	1.0195732	1.02141695	1.0941979	0.12987371	0.99932
CTD-2588C8.8	1.020605	1.0195737	1.0214559	1.0961602	0.13245867	0.99932
RP4-616B8.4	1.020597	1.0195653	1.02144928	1.0962907	0.13263047	0.99932
RP11-186B7.7	1.020612	1.0195952	1.02145223	1.0947708	0.13062883	0.99932
SLC2A11	1.420318	1.4195686	1.42093703	1.0032616	0.00469778	0.99934
TRAPPC2L	2.266853	2.2658914	2.26764628	1.0013863	0.00199864	0.9994
RP5-1102E8.3	1.023502	1.0224725	1.02435183	1.0836284	0.11587016	0.99941
RP13-1032I1.11	1.023528	1.0225381	1.02434589	1.0802123	0.11131492	0.99941
INCA1	1.044472	1.0453991	1.04370671	0.962723	-0.0548073	0.99943
SYCP3	1.02048	1.0214403	1.01968657	0.9182028	-0.1231153	0.99949
KCNF1	1.020457	1.0214027	1.01967527	0.9192883	-0.1214107	0.99949
RP4-537K23.4	1.019549	1.0185663	1.02036002	1.09661	0.13305059	0.99959
CAMK2N2	1.019555	1.0185641	1.02037421	1.0975078	0.13423121	0.99959
RP11-326C3.15	1.019558	1.0185697	1.020375	1.0972196	0.13385228	0.99959
GHET1	1.030152	1.031126	1.02934698	0.9428442	-0.0849087	0.99965
RP11-923I11.4	1.019893	1.0208609	1.01909279	0.9152426	-0.1277739	0.99967
TEX14	1.019857	1.0208025	1.01907649	0.9170284	-0.1249616	0.99967
TCF15	1.01989	1.0208696	1.01908159	0.9143228	-0.1292244	0.99967
CTB-55O6.13	1.019893	1.0208548	1.0190984	0.9157776	-0.1269309	0.99967
RP1-278C19.8	1.021615	1.0225586	1.02083608	0.9236438	-0.1145914	0.99971
RP11-110I1.11	1.021602	1.0225673	1.02080554	0.9219321	-0.1172675	0.99971
WDR62	1.069172	1.0680915	1.07006463	1.0289776	0.04121155	0.99977
PRSS57	1.022312	1.023277	1.0215155	0.9243227	-0.1135316	0.99979
SLBP	1.950062	1.9489228	1.95100233	1.0021915	0.00315823	0.99982
PDCD11	1.45146	1.4525379	1.45056961	0.9956506	-0.0062885	0.99982
SELENOT	2.366551	2.3663109	2.36674874	1.0003204	0.00046223	0.99983
ERCC6L	1.042768	1.0435934	1.04208627	0.965427	-0.050761	0.99986
PSD4	1.123622	1.1245663	1.12284188	0.9861564	-0.0201116	0.99989
SRP19	1.663225	1.6626155	1.663728	1.0016789	0.00242016	0.99991
KIAA1456	1.17517	1.1742192	1.17595608	1.0099697	0.01431206	0.99991
CCDC78	1.043924	1.0430069	1.04468053	1.0389155	0.05507826	0.99993
RP11-305O4.3	1.014676	1.0148856	1.01450349	0.9743295	-0.0375183	1
AC100830.4	1.016407	1.0168071	1.01607695	0.9565577	-0.0640761	1
RP1-90G24.6	1.016387	1.0167736	1.01606769	0.9579136	-0.0620326	1
U2AF1L5	1.069042	1.068275	1.06967563	1.0205145	0.02929665	1
RP11-757G1.6	1.016396	1.0167905	1.01606948	0.9570582	-0.0633214	1
SPESP1	1.017266	1.0168044	1.01764793	1.050195	0.07065726	1

DISP2	1.02938	1.0302116	1.02869254	0.9497207	-0.0744248	1
CTA-223H9.9	1.021572	1.0205761	1.02239448	1.0883715	0.12217107	1
SPRY4	1.061182	1.0605366	1.06171573	1.0194786	0.02783148	1
CCL27	1.038834	1.0396444	1.03816433	0.9626661	-0.0548927	1
AC069155.1	1.01382	1.0129919	1.01450349	1.1163473	0.15878588	1
RP11-574O16.1	1.014677	1.0148865	1.01450349	0.9742687	-0.0376084	1
PCDHGA7	1.016412	1.0168234	1.0160725	0.9553649	-0.0658763	1
SLC9A4	1.01382	1.0129919	1.01450349	1.1163473	0.15878588	1
MBNL3	1.032783	1.0320008	1.03342909	1.044633	0.06299617	1
LINC00305	1.019896	1.0206684	1.01925748	0.9317366	-0.1020059	1
RP5-1071N3.1	1.01382	1.0129919	1.01450349	1.1163473	0.15878588	1
IGFALS	1.017292	1.0168138	1.01768631	1.05189	0.07298389	1
TBCEL	1.107884	1.108349	1.10749971	0.9921613	-0.0113533	1
TMEM256-PLSCR3	1.029343	1.0301032	1.02871466	0.9538748	-0.0681281	1
ASAP3	1.131955	1.1312786	1.13251351	1.0094066	0.0135075	1
CTD-2091N23.1	1.017263	1.0167799	1.01766228	1.0525873	0.0739399	1
CYP2C9	1.01382	1.0129919	1.01450349	1.1163473	0.15878588	1
AC005932.1	1.043181	1.0435128	1.04290685	0.9860749	-0.0202308	1
RIMS4	1.021588	1.0206001	1.02240464	1.0876	0.12114804	1
UTAT33	1.01468	1.0148931	1.01450349	0.9738407	-0.0382423	1
RP11-643M14.1	1.022432	1.0224876	1.02238593	0.9954785	-0.0065379	1
XXbac-BPG154L12.4	1.042388	1.0417181	1.04294185	1.0293344	0.04171174	1
RP11-156E8.1	1.052588	1.0529556	1.05228392	0.9873163	-0.0184158	1
USP50	1.01554	1.0148995	1.01606919	1.0785048	0.10903256	1
ARPC4-TTLL3	1.019877	1.020639	1.01924793	0.9325981	-0.1006726	1
RP11-368I7.6	1.018123	1.01869	1.01765504	0.9446257	-0.0821853	1
CASC8	1.01727	1.0168126	1.01764695	1.0496255	0.06987467	1
RP11-72I8.1	1.014684	1.0149017	1.01450349	0.9732768	-0.0390779	1
CTD-2207P18.2	1.01382	1.0129919	1.01450349	1.1163473	0.15878588	1
RP11-529H2.1	1.014676	1.0148859	1.01450349	0.9743081	-0.03755	1
CTD-2026K11.6	1.019863	1.0206223	1.01923562	0.9327565	-0.1004276	1
AOC3	1.031088	1.0301319	1.03187837	1.0579609	0.08128633	1
KRT6A	1.01382	1.0129919	1.01450349	1.1163473	0.15878588	1
GPATCH3	1.125209	1.1256693	1.12482818	0.993307	-0.0096884	1
TRIM62	1.064797	1.0644742	1.06506305	1.0091327	0.01311585	1
HLA-DMB	1.038879	1.0397275	1.03817854	0.9610116	-0.0573743	1
P2RX5	1.018134	1.01873	1.01764122	0.9418713	-0.0863982	1
CTD-3113P16.11	1.040563	1.0396683	1.04130133	1.0411667	0.05820104	1
BOLL	1.01382	1.0129919	1.01450349	1.1163473	0.15878588	1
TMEM86A	1.039666	1.0396248	1.03969964	1.0018876	0.00272062	1
TRPM6	1.01382	1.0129919	1.01450349	1.1163473	0.15878588	1
LINGO1-AS1	1.016412	1.0168217	1.01607397	0.9555514	-0.0655947	1
RP11-121G22.3	1.014682	1.0148973	1.01450349	0.9735684	-0.0386458	1
RP11-809N8.2	1.014677	1.0148862	1.01450349	0.9742929	-0.0375725	1
LINC00964	1.01382	1.0129919	1.01450349	1.1163473	0.15878588	1

AC009299.3	1.016413	1.0168283	1.01607065	0.9549776	-0.0664611	1
RP11-774D14.1	1.015534	1.0148936	1.0160633	1.0785337	0.10907131	1
RP11-284N8.3	1.019011	1.0187184	1.01925181	1.0284973	0.04053799	1
CTD-2240J17.1	1.019869	1.0206449	1.01922848	0.9313903	-0.1025423	1
LRRD1	1.015543	1.0148964	1.01607711	1.0792645	0.11004849	1
CTD-2655K5.1	1.015543	1.0148889	1.01608269	1.0801778	0.11126885	1
LA16c-385E7.1	1.017255	1.0167974	1.01763292	1.0497415	0.07003407	1
RP11-159D12.11	1.01382	1.0129919	1.01450349	1.1163473	0.15878588	1
CBLC	1.018964	1.0186739	1.01920349	1.0283601	0.04034559	1
RP11-59C5.3	1.018145	1.0187176	1.0176718	0.9441266	-0.0829477	1
RP3-496C20.1	1.01814	1.018717	1.01766375	0.9437283	-0.0835566	1
CTD-2568P8.1	1.014678	1.0148889	1.01450349	0.9741126	-0.0378395	1
TDRG1	1.01382	1.0129919	1.01450349	1.1163473	0.15878588	1
AP000304.12	1.018152	1.0187474	1.01765998	0.9419944	-0.0862096	1
FAM209B	1.019856	1.0205958	1.0192458	0.9344536	-0.0978051	1
TPRG1L	1.108692	1.1082863	1.10902724	1.0068423	0.0098378	1
LINC00463	1.014684	1.0149034	1.01450349	0.973167	-0.0392408	1
RP11-642D21.2	1.01382	1.0129919	1.01450349	1.1163473	0.15878588	1
PFAS	1.121557	1.1214383	1.12165462	1.0017816	0.00256796	1
CTD-2527I21.7	1.015541	1.0148872	1.01608063	1.0801684	0.11125627	1
ZDHHC22	1.018142	1.0187481	1.01764136	0.9409661	-0.0877854	1
RP11-158M2.2	1.01382	1.0129919	1.01450349	1.1163473	0.15878588	1
BTNL9	1.01382	1.0129919	1.01450349	1.1163473	0.15878588	1
CXCL13	1.014675	1.0148827	1.01450349	0.9745186	-0.0372384	1
CEL	1.02159	1.0206631	1.0223552	1.0818884	0.11355169	1
SPATA9	1.019821	1.0205623	1.01920944	0.9342059	-0.0981875	1
RP11-572B2.1	1.015553	1.0148973	1.0160944	1.0803533	0.11150313	1
GRIFIN	1.01382	1.0129919	1.01450349	1.1163473	0.15878588	1
KEL	1.014682	1.0148979	1.01450349	0.9735253	-0.0387096	1
CTD-3075F15.1	1.014684	1.0149021	1.01450349	0.9732525	-0.039114	1
LRP5L	1.062217	1.0626604	1.06185066	0.9870776	-0.0187646	1
STH	1.01554	1.0148897	1.01607683	1.0797309	0.11067178	1
RP11-202D1.2	1.014675	1.014882	1.01450349	0.9745641	-0.037171	1
RP11-20G13.3	1.015535	1.0148882	1.01606948	1.0793428	0.11015313	1
RP11-572M11.1	1.014683	1.0149005	1.01450349	0.9733567	-0.0389595	1
RP11-247L20.4	1.01727	1.0168142	1.01764673	1.0495112	0.06971752	1
TMEM71	1.01382	1.0129919	1.01450349	1.1163473	0.15878588	1
SPINK2	1.016399	1.0167849	1.01608009	0.9580114	-0.0618852	1
ARL14	1.01382	1.0129919	1.01450349	1.1163473	0.15878588	1
TSGA13	1.02162	1.0206475	1.02242322	1.0860039	0.1190293	1
TMPRSS11B	1.014683	1.0148995	1.01450349	0.9734213	-0.0388638	1
TNF	1.018126	1.0187258	1.01763157	0.9415649	-0.0868676	1
RP5-968P14.2	1.018154	1.0187359	1.01767393	0.9433178	-0.0841843	1
TBX18	1.014679	1.0148924	1.01450349	0.9738836	-0.0381787	1
RP11-674P19.2	1.016417	1.0168283	1.01607703	0.9553566	-0.0658888	1

AC006116.20	1.015539	1.0148831	1.01608009	1.0804227	0.1115958	1
SLC5A4	1.018991	1.0187041	1.01922709	1.0279598	0.0397838	1
ALKBH3-AS1	1.015541	1.0148869	1.01608117	1.080225	0.11133188	1
KIF25	1.017266	1.0167902	1.01765839	1.0517068	0.07273249	1
FGD5	1.013543	1.013034	1.01396402	1.0713552	0.09943684	1
RP11-115N4.1	1.013543	1.013034	1.01396402	1.0713552	0.09943684	1
TAF8	1.249923	1.2494031	1.2503523	1.003806	0.00548045	1
IFITM10	1.025598	1.0263392	1.02498521	0.9485937	-0.0761378	1
CSF2RB	1.015267	1.0149371	1.01553976	1.0403494	0.05706817	1
RP11-9N20.3	1.017852	1.0187313	1.01712553	0.9142752	-0.1292997	1
RP11-574F21.3	1.01526	1.0149268	1.01553442	1.0407075	0.05756467	1
RP1-23E21.2	1.015261	1.014928	1.01553562	1.0407033	0.05755878	1
C5orf66	1.019606	1.0187347	1.02032541	1.0849056	0.11756946	1
CTA-221G9.12	1.01871	1.0187597	1.01866836	0.9951318	-0.0070405	1
RP11-180P8.3	1.016121	1.0168354	1.01553183	0.9225709	-0.1162683	1
SH3RF3-AS1	1.013543	1.013034	1.01396402	1.0713552	0.09943684	1
INPP5B	1.075632	1.0758629	1.07544103	0.9944392	-0.0080449	1
CST2	1.015267	1.0149316	1.01554477	1.0410633	0.05805779	1
DPH6-AS1	1.028222	1.0282749	1.02817867	0.996595	-0.0049207	1
RP11-798K3.3	1.013543	1.013034	1.01396402	1.0713552	0.09943684	1
RP11-212D19.4	1.014405	1.0149392	1.01396402	0.9347224	-0.0973901	1
RP11-335O13.7	1.014404	1.0149371	1.01396402	0.9348571	-0.0971822	1
CRYBG3	1.201176	1.2007431	1.20153431	1.0039413	0.00567491	1
GABRR2	1.013543	1.013034	1.01396402	1.0713552	0.09943684	1
TMC2	1.014408	1.0149451	1.01396402	0.9343521	-0.0979617	1
UNC13C	1.015262	1.0149347	1.01553183	1.0399843	0.05656181	1
RP11-36B15.1	1.013543	1.013034	1.01396402	1.0713552	0.09943684	1
AC005237.4	1.014401	1.0149291	1.01396402	0.9353546	-0.0964147	1
AMER3	1.01614	1.0168678	1.01553828	0.9211784	-0.1184475	1
RP1-261G23.7	1.017858	1.0187468	1.0171239	0.9134309	-0.1306326	1
AF011889.2	1.017849	1.0187375	1.01711559	0.9134387	-0.1306201	1
RNF185-AS1	1.014402	1.0149315	1.01396402	0.9352084	-0.0966403	1
HOXC9	1.013543	1.013034	1.01396402	1.0713552	0.09943684	1
CXCL17	1.023037	1.0225716	1.02342162	1.0376603	0.05333422	1
EREG	1.016118	1.0168286	1.01553137	0.9229128	-0.1157338	1
LINC01111	1.015273	1.0149422	1.01554526	1.0403585	0.05708072	1
RP11-160H22.3	1.016135	1.0168601	1.01553577	0.921449	-0.1180238	1
RP11-41O4.2	1.013543	1.013034	1.01396402	1.0713552	0.09943684	1
GPR83	1.0187	1.0187301	1.01867593	0.9971076	-0.0041789	1
RP4-647C14.2	1.014401	1.0149307	1.01396402	0.9352554	-0.0965678	1
RP11-384C4.7	1.013543	1.013034	1.01396402	1.0713552	0.09943684	1
PIK3R5	1.014402	1.0149328	1.01396402	0.9351244	-0.0967698	1
HIPK4	1.015264	1.0149312	1.01553874	1.0406929	0.05754445	1
ZMYM4-AS1	1.014403	1.0149343	1.01396402	0.9350269	-0.0969203	1
RP11-423H2.3	1.02736	1.0264679	1.0280967	1.0615394	0.08615787	1

PDE1B	1.020443	1.020662	1.02026151	0.9806196	-0.0282345	1
LIN37	1.056645	1.056864	1.05646352	0.992958	-0.0101953	1
RP3-439F8.1	1.024784	1.024553	1.02497503	1.0171888	0.02458741	1
SUCLG2-AS1	1.057541	1.0568565	1.05810554	1.021968	0.03135005	1
AC131056.3	1.014402	1.0149315	1.01396402	0.9352084	-0.0966403	1
U73166.2	1.093889	1.0932468	1.09441934	1.0125749	0.0180286	1
RP11-130L8.2	1.016114	1.0168243	1.01552658	0.9228687	-0.1158027	1
STATH	1.013543	1.013034	1.01396402	1.0713552	0.09943684	1
FCGBP	1.015261	1.0149371	1.01552906	1.0396326	0.05607385	1
RP11-6O2.2	1.014406	1.0149422	1.01396402	0.9345344	-0.0976804	1
CCR1	1.019574	1.0187397	1.0202631	1.0812939	0.11275865	1
OVOL1	1.016974	1.016826	1.01709556	1.0160185	0.02292669	1
KB-1410C5.5	1.015274	1.0149334	1.01555546	1.0416574	0.05888088	1
LMBRD2	1.120578	1.1199781	1.12107275	1.0091235	0.01310278	1
SHISA3	1.020427	1.0206248	1.02026289	0.9824542	-0.0255379	1
RP11-474G23.2	1.014402	1.0149334	1.01396402	0.9350846	-0.0968312	1
RP11-360L9.7	1.01612	1.0168257	1.01553712	0.9234141	-0.1149503	1
SCML2	1.065267	1.0644237	1.06596359	1.0239026	0.0340785	1
IL18BP	1.029089	1.0283325	1.02971293	1.0487211	0.06863109	1
AC003005.2	1.018721	1.0187515	1.01869516	0.9969935	-0.004344	1
AC098820.4	1.016122	1.0168321	1.01553573	0.9229823	-0.1156252	1
AC118754.4	1.013543	1.013034	1.01396402	1.0713552	0.09943684	1
RP11-706P11.2	1.016125	1.0168327	1.01554025	0.9232199	-0.1152537	1
SATL1	1.014402	1.0149316	1.01396402	0.9351982	-0.0966559	1
C16orf96	1.015263	1.0149292	1.01553828	1.0407971	0.05768885	1
KLHL6	1.017856	1.0187511	1.01711696	0.9128503	-0.1315497	1
RP11-334E6.10	1.015263	1.0149392	1.01553098	1.0396119	0.056045	1
RTEL1-TNFRSF6B	1.014402	1.0149323	1.01396402	0.9351569	-0.0967197	1
RP3-508I15.18	1.013543	1.013034	1.01396402	1.0713552	0.09943684	1
LA16c-390H2.4	1.029902	1.0301438	1.02970259	0.9853625	-0.0212735	1
CTB-60E11.9	1.014405	1.0149397	1.01396402	0.9346931	-0.0974353	1
RP4-650F12.2	1.016137	1.0168642	1.01553562	0.9212188	-0.1183843	1
RP11-548H3.1	1.021293	1.0206458	1.02182718	1.0572188	0.08027392	1
CTD-2014B16.3	1.015259	1.0149334	1.01552805	1.0398185	0.05633171	1
SPDYE1	1.021333	1.0207177	1.02184145	1.0542427	0.07620707	1
CHRNA10	1.021321	1.0207181	1.02181886	1.0531327	0.07468724	1
GLYATL1	1.076542	1.0759005	1.07707225	1.0154378	0.02210192	1
SLC4A11	1.01526	1.014929	1.01553257	1.0404282	0.05717734	1
AFAP1-AS1	1.01613	1.0168576	1.01552979	0.921233	-0.118362	1
RP11-932O9.7	1.023009	1.0225263	1.02340775	1.0391296	0.05537559	1
CTB-178M22.1	1.013543	1.013034	1.01396402	1.0713552	0.09943684	1
MFN1	1.230106	1.2303169	1.22993203	0.998329	-0.0024127	1
AC007292.6	1.023912	1.024482	1.02344128	0.9574893	-0.0626718	1
TRIM45	1.17332	1.1733603	1.1732864	0.9995736	-0.0006153	1
RP1-228P16.8	1.015267	1.0149371	1.01553976	1.0403494	0.05706817	1

RP11-484D2.2	1.014405	1.0149389	1.01396402	0.934743	-0.0973583	1
CTD-2561B21.5	1.015263	1.0149316	1.01553755	1.0405797	0.05738745	1
AF064860.7	1.014401	1.0149309	1.01396402	0.9352434	-0.0965862	1
LA16c-60D12.1	1.014401	1.0149293	1.01396402	0.9353433	-0.0964321	1
RP11-195F19.9	1.03768	1.0377827	1.03759494	0.9950302	-0.0071877	1
CD84	1.013543	1.013034	1.01396402	1.0713552	0.09943684	1
RP11-546K22.1	1.025609	1.0263659	1.02498366	0.9475755	-0.0776872	1
CDHR5	1.015264	1.0149268	1.01554325	1.0412993	0.05838477	1
HIST1H2AL	1.018702	1.0187223	1.01868585	0.9980554	-0.0028081	1
AF127577.8	1.014405	1.0149399	1.01396402	0.9346785	-0.0974578	1
PRDM8	1.035988	1.0359041	1.03605646	1.0042442	0.00611009	1
TNFSF14	1.013543	1.013034	1.01396402	1.0713552	0.09943684	1
RP11-159H22.2	1.013543	1.013034	1.01396402	1.0713552	0.09943684	1
SFTA3	1.019593	1.0187694	1.020274	1.0801636	0.11124988	1
MATK	1.019577	1.0187343	1.02027225	1.0820955	0.11382787	1
GFM2	1.217206	1.2169997	1.21737549	1.0017319	0.00249647	1
RP11-342M21.2	1.014403	1.0149353	1.01396402	0.9349686	-0.0970103	1
ILDR1	1.013543	1.013034	1.01396402	1.0713552	0.09943684	1
RP11-492E3.2	1.014402	1.0149325	1.01396402	0.9351441	-0.0967393	1
RP1-142L7.9	1.014401	1.0149293	1.01396402	0.9353433	-0.0964321	1
DLX4	1.024848	1.024626	1.02503142	1.0164625	0.02355699	1
FBXW12	1.022151	1.0225261	1.02184193	0.9696258	-0.0445	1
SPATA3-AS1	1.014399	1.0149257	1.01396402	0.9355707	-0.0960814	1
PRB3	1.014399	1.0149259	1.01396402	0.9355588	-0.0960997	1
RP11-122A21.2	1.014409	1.0149471	1.01396402	0.9342316	-0.0981478	1
RP11-535M15.1	1.014402	1.0149319	1.01396402	0.9351792	-0.0966852	1
ELOVL2-AS1	1.015274	1.014936	1.01555268	1.0412868	0.05836742	1
MALRD1	1.027391	1.0263914	1.02821562	1.0691214	0.09642574	1
AC128709.3	1.014401	1.0149297	1.01396402	0.9353195	-0.0964688	1
AC096669.1	1.013543	1.013034	1.01396402	1.0713552	0.09943684	1
NAV2	1.622479	1.6220748	1.62281205	1.0011851	0.0017087	1
CNNM1	1.049778	1.0492681	1.0501995	1.0189049	0.0270194	1
PCSK1	1.015259	1.0149327	1.01552759	1.0398405	0.05636229	1
RP5-858B6.3	1.050713	1.0512191	1.05029516	0.9819615	-0.0262617	1
RP5-837J1.6	1.023042	1.0225527	1.02344621	1.0396201	0.05605642	1
RP11-493L12.3	1.014402	1.0149334	1.01396402	0.9350877	-0.0968264	1
RP11-409I10.2	1.014402	1.0149319	1.01396402	0.9351792	-0.0966852	1
SLC38A11	1.013543	1.013034	1.01396402	1.0713552	0.09943684	1
RP11-360L9.8	1.014399	1.0149259	1.01396402	0.9355588	-0.0960997	1
AC002429.5	1.020447	1.0206685	1.02026464	0.9804597	-0.0284698	1
C4orf32	1.018715	1.0187473	1.01868862	0.9968708	-0.0045215	1
RP11-996F15.4	1.013506	1.0132636	1.01370539	1.0333093	0.04727212	1
RSPH10B	1.014364	1.015163	1.01370392	0.9037757	-0.1459633	1
SPIRE2	1.066969	1.0667122	1.06718027	1.0070167	0.01008756	1
AC018755.17	1.014378	1.0151933	1.01370499	0.9020409	-0.1487352	1

RP11-416N4.4	1.015237	1.0151856	1.01527861	1.0061239	0.00880805	1
GOLGA6L7P	1.016095	1.0151692	1.01685976	1.1114485	0.15244106	1
CCR4	1.01436	1.01516	1.01369923	0.9036404	-0.1461793	1
CTD-2340E1.2	1.015231	1.0151716	1.01528013	1.0071532	0.01028319	1
AC007364.1	1.011789	1.0113663	1.0121375	1.0678479	0.09470618	1
MIB1	1.879628	1.8798068	1.87948057	0.9996292	-0.0005351	1
RP5-893G23.1	1.011789	1.0113663	1.0121375	1.0678479	0.09470618	1
LARP4B	1.298022	1.2973556	1.29857166	1.0040895	0.00588785	1
RP11-367G6.3	1.011789	1.0113663	1.0121375	1.0678479	0.09470618	1
KCNG4	1.012644	1.013258	1.0121375	0.9154842	-0.1273931	1
RP11-278H7.1	1.013504	1.0132626	1.01370354	1.0332486	0.04718742	1
ZBED3-AS1	1.141887	1.1426829	1.14122939	0.9898133	-0.0147716	1
RP11-211G23.2	1.015235	1.015177	1.01528223	1.006935	0.00997057	1
AC015922.6	1.014364	1.0151609	1.01370573	0.9040183	-0.1455762	1
RP11-342K6.3	1.013504	1.0132611	1.01370422	1.0334115	0.04741491	1
HSPC324	1.012644	1.013258	1.0121375	0.9154842	-0.1273931	1
PPP2R2C	1.013504	1.0132647	1.01370228	1.0329905	0.046827	1
ANGEL1	1.107467	1.1067146	1.10808915	1.0128809	0.01846461	1
ADAM32	1.017798	1.0170527	1.01841437	1.0798529	0.11083476	1
SP9	1.012646	1.0132608	1.0121375	0.9152913	-0.1276971	1
LGI2	1.017807	1.0170493	1.01843196	1.0810978	0.11249701	1
RNF217-AS1	1.023844	1.0247293	1.02311264	0.9346264	-0.0975383	1
AC098823.3	1.011789	1.0113663	1.0121375	1.0678479	0.09470618	1
RP11-321L2.1	1.011789	1.0113663	1.0121375	1.0678479	0.09470618	1
RP11-82L20.1	1.012649	1.0132674	1.0121375	0.914837	-0.1284134	1
HIST1H2AD	1.012647	1.0132635	1.0121375	0.9151054	-0.1279902	1
RP11-436D10.3	1.012648	1.0132655	1.0121375	0.9149691	-0.1282051	1
ARNT	1.281583	1.2818108	1.28139551	0.9985265	-0.0021274	1
IFNLR1	1.041981	1.041993	1.04197164	0.9994923	-0.0007327	1
P4HA3	1.0135	1.0132587	1.01369969	1.0332632	0.04720782	1
ABCB6	1.013508	1.0132623	1.01371074	1.0338098	0.04797079	1
RP3-460G2.2	1.012649	1.0132693	1.0121375	0.9147049	-0.1286217	1
SLC7A9	1.013507	1.013261	1.01371002	1.0338619	0.04804342	1
SLC16A4	1.097956	1.0969956	1.09874899	1.018077	0.02584672	1
RP11-459O1.2	1.012646	1.0132618	1.0121375	0.9152215	-0.1278071	1
CTC-479C5.12	1.040238	1.040038	1.04040258	1.0091049	0.01307619	1
CCIN	1.011789	1.0113663	1.0121375	1.0678479	0.09470618	1
RP11-497E19.2	1.024709	1.0246817	1.0247316	1.0020203	0.00291176	1
CTD-2203K17.1	1.016956	1.0170751	1.01685718	0.9872367	-0.0185321	1
KRTDAP	1.035085	1.0342586	1.03576701	1.0440297	0.06216274	1
TH2LCRR	1.012647	1.0132638	1.0121375	0.9150857	-0.1280212	1
RP11-188P17.2	1.016102	1.0151601	1.01688049	1.1134851	0.15508224	1
RP11-109D20.2	1.012645	1.0132605	1.0121375	0.9153109	-0.1276662	1
AC007285.6	1.015229	1.0151647	1.01528129	1.0076896	0.01105125	1
ATP6V1G3	1.01782	1.0170858	1.01842684	1.0784895	0.10901218	1

RP11-420A6.2	1.029104	1.028541	1.02956893	1.0360148	0.05104462	1
GACAT3	1.016942	1.0170609	1.0168443	0.9873065	-0.0184301	1
RP1-40E16.12	1.076416	1.0760247	1.07673968	1.0094046	0.01350455	1
AC106786.1	1.01436	1.0151585	1.01370077	0.9038357	-0.1458675	1
HIST4H4	1.027311	1.0266523	1.0278558	1.0451538	0.06371521	1
GFRA3	1.022957	1.0227418	1.02313451	1.0172692	0.02470147	1
RP11-400K9.4	1.012645	1.0132605	1.0121375	0.9153109	-0.1276662	1
U91328.22	1.013503	1.0132614	1.01370214	1.0332381	0.04717269	1
MSTN	1.016984	1.0170855	1.01690033	0.989162	-0.0157213	1
AC005740.6	1.015232	1.015176	1.01527826	1.0067354	0.00968453	1
RP11-701H16.4	1.012648	1.0132661	1.0121375	0.914927	-0.1282715	1
ARHGDIG	1.031574	1.0323219	1.03095579	0.9577345	-0.0623023	1
CASS4	1.011789	1.0113663	1.0121375	1.0678479	0.09470618	1
DLGAP2	1.021243	1.0208636	1.02155643	1.0332068	0.04712907	1
AC000068.9	1.01956	1.0190157	1.02000891	1.0522296	0.07344956	1
RP11-348H3.8	1.011789	1.0113663	1.0121375	1.0678479	0.09470618	1
RP11-375A5.1	1.012645	1.0132585	1.0121375	0.9154522	-0.1274435	1
BCL2L10	1.011789	1.0113663	1.0121375	1.0678479	0.09470618	1
FAM24B	1.011789	1.0113663	1.0121375	1.0678479	0.09470618	1
APOBEC2	1.015237	1.0151886	1.01527732	1.0058409	0.0084021	1
ETV7	1.029048	1.0285129	1.02948943	1.0342484	0.0485827	1
ERVV-1	1.012645	1.0132585	1.0121375	0.9154522	-0.1274435	1
FERMT3	1.015228	1.0151653	1.01527981	1.0075485	0.01084935	1
GTSF1	1.012649	1.0132692	1.0121375	0.9147114	-0.1286115	1
CTD-2562J17.7	1.012644	1.0132583	1.0121375	0.9154616	-0.1274287	1
RP11-497G19.2	1.012648	1.0132667	1.0121375	0.9148851	-0.1283375	1
RP11-370F5.4	1.011789	1.0113663	1.0121375	1.0678479	0.09470618	1
CPEB1-AS1	1.02213	1.0228153	1.02156385	0.9451501	-0.0813846	1
RP11-144G7.2	1.012645	1.0132604	1.0121375	0.9153168	-0.127657	1
GLDN	1.018689	1.01898	1.01844884	0.9720132	-0.0409522	1
ADGRG7	1.011789	1.0113663	1.0121375	1.0678479	0.09470618	1
SZT2-AS1	1.016086	1.0151528	1.01685751	1.1125031	0.15380938	1
HIST1H3J	1.012646	1.0132622	1.0121375	0.9151951	-0.1278488	1
RP11-131L23.2	1.012649	1.0132693	1.0121375	0.9147049	-0.1286217	1
RP11-845M18.6	1.013508	1.013271	1.01370373	1.0326079	0.04629248	1
KB-1410C5.3	1.036841	1.0361784	1.03738801	1.0334349	0.04744758	1
RP11-815J21.2	1.012645	1.0132603	1.0121375	0.9153233	-0.1276466	1
HLA-DOB	1.012646	1.0132622	1.0121375	0.9151951	-0.1278488	1
ARMC12	1.032475	1.0322626	1.0326512	1.012045	0.01727351	1
PABPC5-AS1	1.013511	1.0132699	1.01370932	1.0331135	0.04699879	1
FABP9	1.012645	1.0132598	1.0121375	0.9153619	-0.1275858	1
NPHP3-ACAD11	1.011789	1.0113663	1.0121375	1.0678479	0.09470618	1
MUC1	1.083295	1.0838057	1.08287296	0.9888706	-0.0161464	1
UBALD1	1.233044	1.2321711	1.23376517	1.0068661	0.00987189	1
CYSRT1	1.0488	1.0494212	1.04828786	0.9770674	-0.03347	1

C14orf178	1.017819	1.017093	1.0184185	1.0775487	0.10775307	1
TEX38	1.012645	1.0132598	1.0121375	0.9153619	-0.1275858	1
HAND1	1.01524	1.0151747	1.01529403	1.0078614	0.01129729	1
RP11-111F5.4	1.102283	1.1026715	1.10196269	0.993096	-0.0099949	1
IRF2BP1	1.162538	1.1616568	1.16326602	1.0099545	0.01429029	1
PLA2G4D	1.012647	1.0132629	1.0121375	0.9151463	-0.1279257	1
RP11-89N17.4	1.013513	1.0132746	1.01370901	1.0327218	0.04645161	1
TMPRSS2	1.015235	1.0151851	1.01527662	1.0060297	0.00867293	1
RP11-292D4.3	1.012646	1.0132608	1.0121375	0.9152913	-0.1276971	1
RP11-431J17.1	1.012647	1.0132643	1.0121375	0.915053	-0.1280728	1
MIR29A	1.012645	1.0132588	1.0121375	0.9154267	-0.1274838	1
PRKAR2B	1.114195	1.114074	1.11429525	1.0019398	0.00279578	1
CTD-3224K15.3	1.013501	1.0132583	1.01370107	1.0333931	0.04738913	1
RP11-352D13.6	1.021283	1.0209005	1.02159842	1.0333939	0.04739027	1
CTD-2184D3.5	1.011789	1.0113663	1.0121375	1.0678479	0.09470618	1
TNFRSF17	1.014381	1.0152046	1.01370023	0.9010563	-0.1503108	1
WFDC10B	1.011789	1.0113663	1.0121375	1.0678479	0.09470618	1
SLC35F4	1.013512	1.0132705	1.01371107	1.033197	0.04711536	1
RP5-942I16.1	1.022158	1.0228665	1.02157271	0.9434205	-0.0840271	1
RP11-820I16.4	1.025567	1.0246682	1.02630944	1.066534	0.09293001	1
SCIMP	1.016081	1.0151634	1.01683812	1.110442	0.151134	1
AC016912.3	1.016257	1.0156039	1.01679584	1.0763874	0.10619743	1
RP11-362K14.7	1.017976	1.0175026	1.01836709	1.0493933	0.06955542	1
SLC35E2B	2.158298	2.1574505	2.15899781	1.0013368	0.00192735	1
GS1-594A7.3	1.016257	1.0156039	1.01679584	1.0763874	0.10619743	1
RP11-291L22.4	1.016257	1.0156039	1.01679584	1.0763874	0.10619743	1
CYP21A2	1.01886	1.0194426	1.01837932	0.9453118	-0.0811378	1
LINC01351	1.017125	1.0175244	1.01679584	0.958424	-0.0612641	1
RP11-415J8.7	1.017117	1.0175066	1.01679584	0.9594027	-0.0597916	1
RP11-593F23.1	1.016257	1.0156039	1.01679584	1.0763874	0.10619743	1
RP11-203B9.4	1.017116	1.0175028	1.01679584	0.9596081	-0.0594827	1
RP11-426A6.5	1.017114	1.0174987	1.01679584	0.9598358	-0.0591404	1
RP11-1C8.5	1.017119	1.0175098	1.01679584	0.9592255	-0.0600581	1
SLC5A11	1.017119	1.0175098	1.01679584	0.9592255	-0.0600581	1
RP11-709D24.5	1.018861	1.0194426	1.01838119	0.945408	-0.080991	1
METTL7B	1.031817	1.0308779	1.03259287	1.0555403	0.07798173	1
RP11-626G11.5	1.024896	1.0251407	1.02469454	0.9822526	-0.025834	1
RP11-480D4.1	1.017984	1.0175039	1.01838139	1.0501313	0.07056971	1
WNT7A	1.027566	1.0271252	1.02792925	1.0296427	0.04214376	1
RP11-7O11.3	1.019729	1.0194426	1.01996639	1.02694	0.03835196	1
ATP6V0A1	1.506752	1.5061917	1.50721402	1.0020197	0.00291085	1
AC008440.5	1.017114	1.0174996	1.01679584	0.9597843	-0.0592178	1
CTD-2310F14.1	1.018866	1.0194776	1.01836157	0.9427012	-0.0851275	1
RP11-50B3.2	1.017116	1.0175045	1.01679584	0.9595153	-0.0596222	1
RP11-356J5.13	1.028366	1.0290007	1.02784113	0.9600144	-0.0588721	1

RP11-863K10.2	1.020596	1.0213464	1.01997725	0.9358617	-0.0956327	1
RP11-645C24.5	1.017118	1.0175076	1.01679584	0.9593435	-0.0598807	1
CLYBL-AS1	1.017118	1.0175092	1.01679584	0.9592592	-0.0600075	1
LA16c-359F1.1	1.016257	1.0156039	1.01679584	1.0763874	0.10619743	1
LINC01502	1.019724	1.0194291	1.01996693	1.0276809	0.03939239	1
MRPL35	1.325759	1.3250989	1.32630349	1.0037052	0.0053356	1
RP11-252A24.3	1.026649	1.0271085	1.02627034	0.9690821	-0.0453092	1
RP11-173P15.9	1.017117	1.0175069	1.01679584	0.9593847	-0.0598186	1
RP11-154D17.1	1.017113	1.0174962	1.01679584	0.9599684	-0.0589412	1
EXOC3L2	1.01884	1.0194088	1.01836976	0.9464678	-0.0793746	1
HNF1A	1.046581	1.0461994	1.04689592	1.0150771	0.02158924	1
SDHAF2	1.43907	1.4397209	1.43853228	0.9972969	-0.003905	1
KCNS2	1.017117	1.0175069	1.01679584	0.9593847	-0.0598186	1
PLN	1.017118	1.0175078	1.01679584	0.9593335	-0.0598956	1
THAP10	1.127898	1.1283459	1.12752878	0.9936333	-0.0092146	1
RP5-1021I20.4	1.017983	1.0175126	1.01837178	1.0490605	0.06909784	1
RP11-399E6.1	1.017119	1.0175105	1.01679584	0.9591894	-0.0601124	1
GRP	1.031005	1.0310082	1.03100313	0.9998376	-0.0002343	1
POC1B-GALNT4	1.016257	1.0156039	1.01679584	1.0763874	0.10619743	1
AC068831.6	1.021459	1.0213571	1.02154303	1.0087056	0.01250513	1
RP11-328C8.4	1.016257	1.0156039	1.01679584	1.0763874	0.10619743	1
RP11-446N19.1	1.068251	1.0672771	1.06905573	1.0264374	0.03764558	1
RP11-83M16.6	1.026645	1.0270854	1.02628125	0.9703123	-0.0434789	1
RP11-552F3.9	1.016257	1.0156039	1.01679584	1.0763874	0.10619743	1
LINC01447	1.017114	1.0174996	1.01679584	0.9597843	-0.0592178	1
RP11-538I12.3	1.037023	1.036668	1.03731672	1.0176918	0.02530077	1
RP11-830F9.5	1.016257	1.0156039	1.01679584	1.0763874	0.10619743	1
PCDHAC2	1.022321	1.0213055	1.02315998	1.0870436	0.12040978	1
RP4-669P10.20	1.016257	1.0156039	1.01679584	1.0763874	0.10619743	1
LL09NC01-139C3.1	1.028433	1.0290126	1.02795359	0.9634988	-0.0536453	1
LINC01629	1.01798	1.0175045	1.01837353	1.0496456	0.06990229	1
VAC14-AS1	1.018839	1.0193952	1.01837907	0.9476113	-0.0776327	1
GS1-166A23.1	1.017118	1.0175088	1.01679584	0.9592789	-0.0599778	1
RP11-356C4.5	1.017117	1.0175051	1.01679584	0.959483	-0.0596709	1
AC000403.4	1.017116	1.0175038	1.01679584	0.9595542	-0.0595638	1
CTD-3037G24.5	1.016257	1.0156039	1.01679584	1.0763874	0.10619743	1
RP11-419I17.2	1.019701	1.0194002	1.01994866	1.0282728	0.04022311	1
CCDC87	1.017979	1.0175198	1.01835857	1.047873	0.06746382	1
SLC6A2	1.016257	1.0156039	1.01679584	1.0763874	0.10619743	1
RP11-262A16.1	1.01884	1.0194132	1.01836686	0.9461024	-0.0799318	1
ZNF175	1.115808	1.1150468	1.11643736	1.0120865	0.01733256	1
RP11-44N12.5	1.016257	1.0156039	1.01679584	1.0763874	0.10619743	1
CTB-129P6.11	1.017116	1.0175039	1.01679584	0.959549	-0.0595717	1
USP30-AS1	1.017117	1.0175058	1.01679584	0.9594463	-0.0597261	1
NANOS3	1.131368	1.1323375	1.13056667	0.9866187	-0.0194355	1

KLB	1.017117	1.017507	1.01679584	0.9593795	-0.0598265	1
CTD-3224K15.2	1.017117	1.0175049	1.01679584	0.9594957	-0.0596517	1
C22orf42	1.017116	1.0175042	1.01679584	0.9595333	-0.0595952	1
CST11	1.017971	1.0174996	1.01836119	1.0492345	0.06933722	1
SIGLEC11	1.017979	1.0175112	1.018365	1.0487575	0.06868112	1
AC016738.4	1.018837	1.0194069	1.01836588	0.9463572	-0.0795433	1
THRIL	1.018852	1.0194426	1.01836374	0.9445101	-0.0823619	1
LRP1B	1.074375	1.0750531	1.0738152	0.9835059	-0.0239944	1
MCPH1-AS1	1.081919	1.0824132	1.08151047	0.9890459	-0.0158906	1
RP11-80H5.2	1.024042	1.0232226	1.0247189	1.0644332	0.09008537	1
RP11-616M22.1	1.016257	1.0156039	1.01679584	1.0763874	0.10619743	1
AC099342.1	1.017114	1.0174987	1.01679584	0.9598358	-0.0591404	1
RP11-157E16.1	1.018859	1.0194542	1.01836666	0.944097	-0.0829931	1
RP11-739L10.1	1.016257	1.0156039	1.01679584	1.0763874	0.10619743	1
LINC01091	1.021437	1.0213178	1.02153487	1.010185	0.01461958	1
P4HA2-AS1	1.017121	1.0175149	1.01679584	0.9589481	-0.0604754	1
RP5-855F16.1	1.017978	1.0175042	1.01836896	1.0494041	0.0695704	1
RP11-433J8.1	1.017115	1.0175023	1.01679584	0.9596383	-0.0594373	1
RP13-516M14.10	1.017982	1.0175018	1.01837907	1.0501222	0.07055727	1
TNFSF10	1.045581	1.0461694	1.04509537	0.9767374	-0.0339573	1
RP11-143J12.3	1.016257	1.0156039	1.01679584	1.0763874	0.10619743	1
ODF3B	1.14798	1.1479267	1.14802388	1.0006567	0.00094706	1
AC011525.2	1.016257	1.0156039	1.01679584	1.0763874	0.10619743	1
MIR194-2HG	1.017981	1.0175042	1.01837406	1.0496956	0.06997107	1
QPCTL	1.032661	1.0327387	1.03259727	0.9956813	-0.006244	1
YTHDC2	1.328404	1.3288476	1.3280382	0.9975385	-0.0035555	1
RP11-546K22.3	1.037854	1.0384753	1.03734104	0.9705199	-0.0431703	1
C7orf57	1.022332	1.0213428	1.02314902	1.0846275	0.11719964	1
RP11-322E11.6	1.01885	1.0194168	1.01838186	0.9466978	-0.0790242	1
TEX40	1.018831	1.0194006	1.0183608	0.946406	-0.0794688	1
LINC01054	1.017978	1.0175042	1.01836926	1.0494211	0.06959375	1
RP11-274B21.9	1.017114	1.0174989	1.01679584	0.9598202	-0.0591639	1
ATP2A3	1.024059	1.0232659	1.02471378	1.0622334	0.08710078	1
RP11-518L10.5	1.018844	1.0194277	1.01836142	0.9451178	-0.0814339	1
RP11-399K21.14	1.0232	1.0232929	1.0231242	0.9927585	-0.0104853	1
CTC-559E9.4	1.017117	1.0175068	1.01679584	0.9593899	-0.0598108	1
RP11-554E23.2	1.017983	1.0175098	1.01837395	1.0493523	0.06949914	1
RP5-1125A11.6	1.017115	1.0175006	1.01679584	0.9597281	-0.0593023	1
SPPL2A	1.303438	1.3040519	1.3029316	0.9963153	-0.0053257	1
AC073934.6	1.017114	1.0174989	1.01679584	0.9598202	-0.0591639	1
LINC00885	1.01712	1.0175129	1.01679584	0.9590539	-0.0603162	1
TBC1D30	1.057756	1.0576007	1.05788459	1.0049283	0.00709262	1
RP11-2H8.2	1.017121	1.0175152	1.01679584	0.9589273	-0.0605066	1
RP11-114B7.6	1.016141	1.0156717	1.01652797	1.0546406	0.0767515	1
CTD-2621I17.6	1.015271	1.0156713	1.01494071	0.9533785	-0.0688791	1

CAPNS1	2.265168	2.2651682	2.26516837	1.0000001	1.70E-07	1
LINC00934	1.014413	1.0137737	1.01494071	1.0847293	0.11733506	1
LINC00911	1.014413	1.0137737	1.01494071	1.0847293	0.11733506	1
C11orf97	1.016136	1.0156804	1.01651203	1.0530353	0.07455379	1
PNCK	1.123288	1.1226654	1.12380146	1.0092616	0.01330013	1
RP1-266L20.2	1.021306	1.0214018	1.02122766	0.9918641	-0.0117856	1
CTD-2060L22.1	1.015272	1.0156721	1.01494071	0.9533329	-0.0689481	1
LINC01163	1.014413	1.0137737	1.01494071	1.0847293	0.11733506	1
SLC5A8	1.015271	1.0156705	1.01494071	0.9534293	-0.0688021	1
MYOC	1.014413	1.0137737	1.01494071	1.0847293	0.11733506	1
ANKRD7	1.014413	1.0137737	1.01494071	1.0847293	0.11733506	1
LINC01033	1.014413	1.0137737	1.01494071	1.0847293	0.11733506	1
MAFTRR	1.037686	1.038494	1.03701838	0.9616659	-0.0563923	1
RP11-410N8.3	1.026514	1.0271451	1.02599331	0.957568	-0.0625531	1
RP11-482D24.2	1.023899	1.0232855	1.02440571	1.0481097	0.06778966	1
SOX1	1.016133	1.0156716	1.01651436	1.0537786	0.07557176	1
KCNT1	1.016991	1.017578	1.01650713	0.9390799	-0.0906802	1
RP11-571I18.5	1.015273	1.0156758	1.01494071	0.953105	-0.069293	1
CCDC63	1.015272	1.0156725	1.01494071	0.9533097	-0.0689832	1
RELN	1.995063	1.9952098	1.99494227	0.9997312	-0.0003878	1
URB2	1.064469	1.0653112	1.06377391	0.9764621	-0.034364	1
LINC00605	1.040355	1.0406006	1.04015301	0.9889751	-0.0159939	1
ARSF	1.014413	1.0137737	1.01494071	1.0847293	0.11733506	1
HSPB6	1.016136	1.0156818	1.01651154	1.0529122	0.07438511	1
AC004449.6	1.015276	1.0156828	1.01494071	0.9526811	-0.0699347	1
CTC-441N14.2	1.014413	1.0137737	1.01494071	1.0847293	0.11733506	1
APOC3	1.01527	1.0156688	1.01494071	0.9535315	-0.0686475	1
ARHGEF33	1.018711	1.0194802	1.01807614	0.9279225	-0.1079238	1
RP11-576N17.5	1.016143	1.0156856	1.01652032	1.0532136	0.07479812	1
RP11-297P16.3	1.020483	1.0195453	1.02125684	1.0875699	0.12110813	1
MNX1-AS2	1.014413	1.0137737	1.01494071	1.0847293	0.11733506	1
EDC4	1.017858	1.0175825	1.01808616	1.028644	0.04074382	1
ANKRD30A	1.014413	1.0137737	1.01494071	1.0847293	0.11733506	1
MIOX	1.020463	1.0195102	1.0212494	1.0891445	0.12319542	1
CPEB3	1.036054	1.0367072	1.03551535	0.9675298	-0.0476219	1
DDTL	1.016992	1.017565	1.01651944	0.9404735	-0.0885408	1
FAM9C	1.016132	1.01567	1.01651351	1.0538274	0.07563863	1
MASTL	1.153226	1.1527613	1.15360977	1.0055544	0.00799108	1
MPP4	1.023036	1.0233336	1.02278986	0.9766955	-0.0340193	1
RP11-157I4.4	1.014413	1.0137737	1.01494071	1.0847293	0.11733506	1
BSND	1.015278	1.0156869	1.01494071	0.9524323	-0.0703116	1
KLHL33	1.017872	1.017602	1.0180945	1.0279798	0.03981189	1
RP11-404F10.2	1.017859	1.0175841	1.01808672	1.0285864	0.04066305	1
AC079779.6	1.016136	1.0156876	1.01650713	1.0522436	0.07346867	1
MEOX2	1.015277	1.0156834	1.01494071	0.9526468	-0.0699866	1

GPR17	1.014413	1.0137737	1.01494071	1.0847293	0.11733506	1
RP1-20B21.4	1.014413	1.0137737	1.01494071	1.0847293	0.11733506	1
RP11-463J10.4	1.015278	1.0156858	1.01494071	0.9525008	-0.0702077	1
GLRA1	1.015273	1.015675	1.01494071	0.9531565	-0.069215	1
PRR26	1.020449	1.0194727	1.02125524	1.0915432	0.12636928	1
TRIM72	1.016135	1.0156721	1.01651672	1.0538944	0.07573032	1
CTB-113P19.5	1.015272	1.0156721	1.01494071	0.9533329	-0.0689481	1
AC007566.10	1.017	1.0175935	1.01650968	0.938399	-0.0917266	1
RP11-154H23.4	1.016992	1.0175662	1.01651793	0.9403248	-0.0887689	1
RP11-456O19.3	1.014413	1.0137737	1.01494071	1.0847293	0.11733506	1
CCDC97	1.261358	1.2617134	1.26106538	0.9975241	-0.0035764	1
RP11-196G11.6	1.028249	1.0290197	1.02761297	0.9515246	-0.0716871	1
RP11-402J6.1	1.017001	1.0175989	1.01650719	0.9379679	-0.0923896	1
SPTBN5	1.015269	1.0156676	1.01494071	0.9536053	-0.0685358	1
MVB12B	1.229582	1.2293736	1.22975352	1.0016563	0.00238754	1
AC024592.12	1.015276	1.0156813	1.01494071	0.9527722	-0.0697968	1
RP11-359J14.2	1.016143	1.0156858	1.01651975	1.0531678	0.07473537	1
LINC00654	1.01527	1.0156694	1.01494071	0.9534974	-0.0686991	1
RP3-324O17.8	1.016131	1.0156721	1.01651075	1.0535134	0.0752086	1
PRDM10	1.090499	1.0900196	1.09089469	1.0097217	0.01395765	1
DLGAP1-AS5	1.014413	1.0137737	1.01494071	1.0847293	0.11733506	1
TSPEAR-AS1	1.044678	1.044316	1.04497787	1.0149358	0.02138845	1
FSD2	1.027376	1.0271066	1.02759826	1.018139	0.02593447	1
RP11-328J14.1	1.015272	1.0156721	1.01494071	0.9533329	-0.0689481	1
ATXN2-AS	1.014413	1.0137737	1.01494071	1.0847293	0.11733506	1
IGBP1-AS2	1.014413	1.0137737	1.01494071	1.0847293	0.11733506	1
RP11-23E10.3	1.016995	1.0175806	1.01651055	0.9391362	-0.0905937	1
RP11-486I11.2	1.025626	1.0252179	1.02596283	1.0295414	0.04200181	1
LINC00598	1.028209	1.0290207	1.02753902	0.9489436	-0.0756057	1
RP11-24D15.1	1.014413	1.0137737	1.01494071	1.0847293	0.11733506	1
PARD6G-AS1	1.019613	1.0194713	1.01972937	1.0132527	0.01899405	1
AC024560.2	1.018742	1.0195274	1.01809322	0.9265576	-0.1100474	1
AC005253.4	1.014413	1.0137737	1.01494071	1.0847293	0.11733506	1
PCDHGB7	1.022161	1.0213677	1.02281547	1.0677525	0.09457733	1
ALB	1.020429	1.0194777	1.02121502	1.0891962	0.12326387	1
FGF11	1.638837	1.6390266	1.63868089	0.9994589	-0.0007808	1
STAG3	1.048203	1.0483455	1.0480857	0.994627	-0.0077725	1
GLT8D2	1.093027	1.0938742	1.0923273	0.9835213	-0.0239718	1
RP11-314P15.2	1.014413	1.0137737	1.01494071	1.0847293	0.11733506	1
RP11-713D19.1	1.019584	1.019478	1.01967206	1.0099643	0.01430425	1
CTC-260F20.3	1.015277	1.0156838	1.01494071	0.9526204	-0.0700266	1
RP11-875O11.2	1.019606	1.0195022	1.01969239	1.0097516	0.01400045	1
KLF1	1.014413	1.0137737	1.01494071	1.0847293	0.11733506	1
TMEM156	1.015274	1.0156773	1.01494071	0.9530147	-0.0694296	1
BRSK2	1.073088	1.072862	1.07327497	1.0056682	0.00815437	1

SYPL2	1.015273	1.0156751	1.01494071	0.9531507	-0.0692238	1
OLIG1	1.015271	1.0156707	1.01494071	0.9534177	-0.0688197	1
PROB1	1.035988	1.0366669	1.03542667	0.9661768	-0.0496408	1
AC007192.6	1.014413	1.0137737	1.01494071	1.0847293	0.11733506	1
C4orf36	1.084272	1.0842275	1.08430851	1.0009619	0.00138713	1
HAVCR2	1.016144	1.0156834	1.0165252	1.053677	0.07543269	1
PRR34-AS1	1.015276	1.0156825	1.01494071	0.9526985	-0.0699083	1
RP11-13E5.2	1.017003	1.0176014	1.01650798	0.9378764	-0.0925303	1
MGAM2	1.015273	1.0156758	1.01494071	0.953105	-0.069293	1
LY86-AS1	1.017012	1.0176001	1.01652679	0.9390156	-0.090779	1
ZNF354C	1.05071	1.0501783	1.05114954	1.0193557	0.02765754	1
ZNF517	1.038628	1.0385848	1.03866328	1.0020342	0.00293168	1
AC074212.6	1.016137	1.0156847	1.01650968	1.0525983	0.07395504	1
RHBDL2	1.02395	1.0234025	1.02440207	1.0427139	0.06034332	1
RP11-169E6.1	1.017861	1.017564	1.01810602	1.0308626	0.04385206	1
SLC35G3	1.015271	1.0156705	1.01494071	0.9534293	-0.0688021	1
AKR7A3	1.024792	1.0252567	1.02440799	0.9663982	-0.0493103	1
SELL	1.019592	1.0194814	1.0196824	1.0103158	0.01480635	1
HCG20	1.017852	1.0175726	1.01808243	1.0290137	0.04126221	1
AC114730.5	1.014413	1.0137737	1.01494071	1.0847293	0.11733506	1
RP11-219B4.5	1.015273	1.0156758	1.01494071	0.953105	-0.069293	1
FREM3	1.015269	1.0156668	1.01494071	0.9536515	-0.068466	1
ACTN2	1.022159	1.0213695	1.02281124	1.0674661	0.09419025	1
SMCO2	1.015271	1.0156707	1.01494071	0.9534177	-0.0688197	1
TMEM240	1.043804	1.0443536	1.04334926	0.9773571	-0.0330423	1
TBC1D26	1.020184	1.0196973	1.02058574	1.0451067	0.06365021	1
ZNF678	1.191636	1.1911447	1.19204095	1.004689	0.00674897	1
RP11-213G2.2	1.016688	1.0158123	1.01741111	1.1011114	0.13896045	1
IZUMO1	1.012388	1.0120052	1.01270407	1.0582183	0.08163733	1
AP001172.3	1.013248	1.0139066	1.01270407	0.9135258	-0.1304826	1
SLC6A1	1.014103	1.013898	1.01427281	1.0269716	0.03839625	1
TTBK1	1.018414	1.0177287	1.01897937	1.070542	0.09834145	1
PPP2R1B	1.196724	1.1964814	1.19692446	1.0022549	0.00324953	1
SRGAP3-AS2	1.014983	1.0158422	1.01427289	0.9009432	-0.150492	1
RP11-178C3.2	1.017561	1.0177102	1.0174371	0.9845771	-0.022424	1
RP11-517P14.2	1.029598	1.0291112	1.03000076	1.0305587	0.04342669	1
SLCO4A1	1.050294	1.0500472	1.05049758	1.0089986	0.01292419	1
SMPD3	1.017543	1.0176993	1.01741303	0.9838251	-0.0235263	1
CYBB	1.016699	1.0158209	1.01742365	1.1013074	0.13921721	1
RP13-20L14.1	1.015829	1.0158003	1.01585201	1.0032752	0.00471738	1
RP4-777L9.3	1.013253	1.0139175	1.01270407	0.9128106	-0.1316126	1
AL354828.2	1.012388	1.0120052	1.01270407	1.0582183	0.08163733	1
RP6-201G10.2	1.01325	1.0139101	1.01270407	0.9132982	-0.1308421	1
RP1-153G14.4	1.021856	1.0215059	1.02214576	1.0297524	0.04229751	1
TRIM67	1.017541	1.0176953	1.01741431	0.9841192	-0.0230951	1

HS6ST1	1.212349	1.2120088	1.21263055	1.0029326	0.00422467	1
IL13RA2	1.026195	1.0254396	1.02681847	1.0541999	0.07614841	1
DUOX2	1.015845	1.0158441	1.01584596	1.0001178	0.00016997	1
TTC16	1.012388	1.0120052	1.01270407	1.0582183	0.08163733	1
TRAPPC3L	1.012388	1.0120052	1.01270407	1.0582183	0.08163733	1
RP11-170N16.3	1.013245	1.0139007	1.01270407	0.9139138	-0.12987	1
ST8SIA6	1.018394	1.0176885	1.01897714	1.0728506	0.10144914	1
TFF3	1.014974	1.0158273	1.01426873	0.9015277	-0.1495563	1
CFAP58	1.02617	1.0253188	1.02687301	1.0613873	0.08595119	1
PROSER2-AS1	1.013247	1.0139034	1.01270407	0.9137407	-0.1301433	1
DIS3L2	1.281298	1.2806456	1.28183598	1.0042416	0.00610642	1
MTRNR2L11	1.016689	1.0158126	1.01741216	1.1011555	0.13901821	1
CREBZF	1.52719	1.5264494	1.52780108	1.0025676	0.00369953	1
MYO5B	1.254338	1.2536431	1.25491135	1.0050001	0.00719571	1
LINC01135	1.015826	1.0157991	1.01584836	1.0031197	0.00449379	1
RP11-535A19.1	1.012388	1.0120052	1.01270407	1.0582183	0.08163733	1
NAPSA	1.057304	1.0579889	1.05673831	0.9784343	-0.0314531	1
MYH7	1.025324	1.0253341	1.025315	0.9992472	-0.0010865	1
OVCH2	1.013245	1.0139002	1.01270407	0.9139491	-0.1298143	1
AP000431.2	1.012388	1.0120052	1.01270407	1.0582183	0.08163733	1
SPATA25	1.012388	1.0120052	1.01270407	1.0582183	0.08163733	1
LINC00222	1.015822	1.0158047	1.01583628	1.0019952	0.00287554	1
RP11-706O15.7	1.018452	1.0177525	1.01903021	1.0719752	0.10027147	1
RP11-288L9.1	1.014106	1.0139074	1.01427019	1.0260833	0.03714787	1
RP5-1172N10.2	1.016699	1.0158428	1.01740624	1.0986825	0.13577449	1
PARP2	1.341595	1.3410956	1.34200763	1.0026739	0.00385253	1
NRG3	1.021849	1.0214769	1.02215675	1.0316537	0.04495884	1
AC002310.7	1.021093	1.0216591	1.02062561	0.9522829	-0.0705378	1
RP11-187O7.3	1.013246	1.0139012	1.01270407	0.9138845	-0.1299162	1
AP000350.5	1.019301	1.0196287	1.01902997	0.9694971	-0.0446915	1
NPIPB4	1.033101	1.0330088	1.03317755	1.0051125	0.00735693	1
CTD-3051D23.1	1.014103	1.0139008	1.01427066	1.0266045	0.03788052	1
SENP3	1.089109	1.0882783	1.08979558	1.0171879	0.02458617	1
CNGB1	1.014982	1.0158349	1.01427772	0.9016631	-0.1493396	1
RP11-88E10.4	1.012388	1.0120052	1.01270407	1.0582183	0.08163733	1
CTB-35F21.2	1.014963	1.0157998	1.01427157	0.9032767	-0.1467601	1
RP11-374A4.1	1.013251	1.0139125	1.01270407	0.9131379	-0.1310954	1
STAC3	1.047786	1.0481798	1.04746124	0.9850862	-0.0216781	1
RP11-817O13.9	1.095255	1.096169	1.09450072	0.982653	-0.025246	1
WHSC1	2.07966	2.0790303	2.08017923	1.0010648	0.0015354	1
RP3-388M5.9	1.020135	1.0196069	1.02057087	1.0491633	0.06923931	1
IL36G	1.0167	1.0157954	1.01744666	1.1045383	0.14344341	1
AC005534.8	1.01668	1.0157963	1.01740945	1.1021243	0.14028698	1
RP1-137D17.1	1.015838	1.0158015	1.01586729	1.0041624	0.00599264	1
ANKRD34B	1.018421	1.0176966	1.01901909	1.0747314	0.10397619	1

ZNF534	1.013248	1.0139076	1.01270407	0.913464	-0.1305803	1
NXPE2	1.013245	1.0138997	1.01270407	0.9139796	-0.1297661	1
RP3-339A18.6	1.013246	1.0139016	1.01270407	0.9138574	-0.1299591	1
RP11-963H4.3	1.020141	1.0195948	1.02059191	1.0508853	0.07160516	1
RP1-253P7.4	1.014111	1.0139103	1.01427741	1.0263882	0.03757644	1
ZNF300	1.257966	1.2577133	1.25817421	1.0017886	0.00257807	1
LMAN1L	1.012388	1.0120052	1.01270407	1.0582183	0.08163733	1
GRIP2	1.019272	1.019621	1.0189841	0.9675395	-0.0476075	1
RP11-389G6.5	1.012388	1.0120052	1.01270407	1.0582183	0.08163733	1
RP1L1	1.013244	1.0138984	1.01270407	0.9140699	-0.1296236	1
CTD-2616J11.2	1.019282	1.0196174	1.01900539	0.9688041	-0.0457231	1
C1orf167	1.016683	1.0158059	1.01740778	1.1013492	0.13927199	1
CTB-51A17.1	1.013246	1.0139014	1.01270407	0.9138692	-0.1299404	1
ALS2CR11	1.012388	1.0120052	1.01270407	1.0582183	0.08163733	1
RDH10	2.109125	2.1093522	2.10893786	0.9996265	-0.0005389	1
LRIT3	1.014106	1.0139094	1.01426917	1.0258682	0.03684534	1
RP11-231N3.1	1.014108	1.0139031	1.01427749	1.0269287	0.03833606	1
C20orf141	1.012388	1.0120052	1.01270407	1.0582183	0.08163733	1
LINC00518	1.012388	1.0120052	1.01270407	1.0582183	0.08163733	1
LINC01449	1.014975	1.0158273	1.01427189	0.9017272	-0.1492371	1
RP11-676J12.9	1.014104	1.0139057	1.01426811	1.0260646	0.03712158	1
RP11-463O9.9	1.012388	1.0120052	1.01270407	1.0582183	0.08163733	1
BAIAP3	1.036519	1.0367644	1.03631703	0.9878319	-0.0176625	1
SBSPON	1.061523	1.0615198	1.06152647	1.0001085	0.00015659	1
HLA-DRB5	1.013243	1.0138957	1.01270407	0.9142431	-0.1293503	1
CAGE1	1.013248	1.0139074	1.01270407	0.9134737	-0.130565	1
CH25H	1.013245	1.0138993	1.01270407	0.9140101	-0.129718	1
ZBP1	1.013245	1.0139009	1.01270407	0.913906	-0.1298823	1
ST20-MTHFS	1.013251	1.0139136	1.01270407	0.9130668	-0.1312077	1
CTD-3222D19.11	1.012388	1.0120052	1.01270407	1.0582183	0.08163733	1
TLL2	1.017565	1.0177236	1.01743358	0.983639	-0.0237991	1
DUSP2	1.04592	1.0462509	1.04564746	0.986953	-0.0189467	1
RP11-413N10.3	1.02789	1.0272032	1.02845627	1.0460617	0.06496793	1
RP4-656G21.1	1.012388	1.0120052	1.01270407	1.0582183	0.08163733	1
ADAM21	1.014101	1.0138986	1.01426865	1.0266238	0.03790758	1
DNAJC25-GNG10	1.016686	1.0157979	1.01741897	1.1026139	0.14092764	1
RP11-227D13.5	1.013244	1.0138983	1.01270407	0.9140725	-0.1296195	1
RP11-170M17.1	1.012388	1.0120052	1.01270407	1.0582183	0.08163733	1
CTB-50L17.14	1.018401	1.017698	1.01898144	1.072516	0.10099911	1
RP11-423H2.4	1.015828	1.0158176	1.01583596	1.0011604	0.00167309	1
RP11-217B7.2	1.012388	1.0120052	1.01270407	1.0582183	0.08163733	1
SYT2	1.012388	1.0120052	1.01270407	1.0582183	0.08163733	1
COLCA1	1.057233	1.0577655	1.05679285	0.9831624	-0.0244984	1
TMED7-TICAM2	1.015823	1.0157974	1.01584337	1.002907	0.00418789	1
ABCC3	1.020133	1.0196098	1.02056563	1.0487414	0.06865903	1

RP11-302F12.3	1.012388	1.0120052	1.01270407	1.0582183	0.08163733	1
VSTM1	1.013246	1.0139012	1.01270407	0.9138845	-0.1299162	1
HP09025	1.013257	1.0139276	1.01270407	0.9121497	-0.1326575	1
KLK7	1.024473	1.0253638	1.02373656	0.9358453	-0.095658	1
AC015969.3	1.012388	1.0120052	1.01270407	1.0582183	0.08163733	1
RP11-435O5.5	1.021861	1.0214865	1.0221709	1.0318544	0.04523938	1
RP11-544A12.4	1.016279	1.0157693	1.0166999	1.0590108	0.08271733	1
RP11-672A2.5	1.016279	1.0157693	1.0166999	1.0590108	0.08271733	1
RP11-316O14.1	1.028401	1.029169	1.02776633	0.9519112	-0.0711011	1
BEST3	1.046517	1.04626	1.04672863	1.0101298	0.01454073	1
SNAP91	1.053493	1.0539497	1.05311653	0.984556	-0.0224548	1
CTD-2600O9.1	1.026682	1.0272663	1.02619986	0.9608864	-0.0575623	1
GORAB	1.114775	1.1151298	1.11448292	0.9943815	-0.0081287	1
REP15	1.019729	1.0195715	1.01985882	1.0146812	0.02102657	1
OSBPL10-AS1	1.021458	1.0215022	1.02142178	0.9962595	-0.0054065	1
ARHGEF7-AS2	1.024058	1.0233928	1.02460683	1.0518962	0.07299232	1
NTN3	1.017138	1.0176675	1.0166999	0.945235	-0.0812551	1
ABCA9-AS1	1.016279	1.0157693	1.0166999	1.0590108	0.08271733	1
RP11-497G19.7	1.017996	1.0176711	1.01826432	1.0335695	0.04763541	1
ZNF503-AS2	1.139175	1.1400965	1.13841346	0.9879866	-0.0174366	1
GALNT4	1.017142	1.0176765	1.0166999	0.9447539	-0.0819895	1
RP1-310O13.7	1.020628	1.0215586	1.01985998	0.9212082	-0.1184008	1
NMT2	1.512189	1.5130534	1.51147452	0.9969226	-0.0044465	1
RP11-944L7.4	1.017141	1.017676	1.0166999	0.9447766	-0.0819549	1
RNASE7	1.016279	1.0157693	1.0166999	1.0590108	0.08271733	1
CTD-2619J13.14	1.027523	1.0272122	1.02778042	1.0208827	0.02981711	1
CTD-3131K8.3	1.017143	1.0176803	1.0166999	0.9445505	-0.0823002	1
RP11-266K4.9	1.024047	1.0233895	1.0245892	1.0512937	0.07216572	1
LINC01280	1.028522	1.0293303	1.02785405	0.9496683	-0.0745043	1
RP11-737O24.5	1.016279	1.0157693	1.0166999	1.0590108	0.08271733	1
CBLL1	1.218803	1.2184772	1.2190717	1.0027209	0.0039201	1
RP11-354P11.8	1.019755	1.0196025	1.01988027	1.0141683	0.02029714	1
IL2RG	1.017996	1.0176654	1.01826926	1.0341852	0.04849452	1
LINC01251	1.024058	1.0234022	1.02459971	1.0511717	0.07199827	1
NOX5	1.017139	1.0176713	1.0166999	0.9450295	-0.0815687	1
RP11-144G16.1	1.017141	1.0176761	1.0166999	0.9447715	-0.0819627	1
RP11-589C21.6	1.043956	1.0443682	1.04361577	0.983041	-0.0246764	1
RP11-277A4.4	1.037029	1.0368031	1.03721545	1.0112047	0.01607505	1
MUC6	1.017145	1.0176844	1.0166999	0.9443319	-0.082634	1
RP11-724N1.1	1.016279	1.0157693	1.0166999	1.0590108	0.08271733	1
CDC20B	1.018856	1.019568	1.0182687	0.9335996	-0.0991241	1
AC092839.3	1.017141	1.0176741	1.0166999	0.94488	-0.081797	1
TMOD4	1.017145	1.017685	1.0166999	0.9442966	-0.082688	1
SCGB1D4	1.019752	1.0195913	1.01988398	1.0149407	0.02139551	1
ABCC9	1.016279	1.0157693	1.0166999	1.0590108	0.08271733	1

RP4-789D17.5	1.020607	1.0215216	1.01985174	0.9224086	-0.1165222	1
GAGE10	1.017139	1.0176711	1.0166999	0.9450397	-0.0815531	1
FBXO6	1.146986	1.1477556	1.14635097	0.9904937	-0.0137804	1
CATSPER4	1.018877	1.0196103	1.01827142	0.9317243	-0.102025	1
LA16c-390E6.5	1.018874	1.0195891	1.01828377	0.9333619	-0.0994915	1
RP11-661A12.7	1.017999	1.0176662	1.01827475	1.0344496	0.04886332	1
RP11-79P5.9	1.071681	1.071239	1.07204602	1.0113287	0.01625195	1
RP11-635L1.3	1.018858	1.0195763	1.01826448	0.9329888	-0.1000683	1
CTA-384D8.33	1.017148	1.0176913	1.0166999	0.9439612	-0.0832006	1
CTB-33G10.6	1.016279	1.0157693	1.0166999	1.0590108	0.08271733	1
WNT5A-AS1	1.035346	1.0349203	1.03569845	1.022284	0.03179601	1
GVQW2	1.043946	1.044397	1.04357283	0.9814355	-0.0270346	1
SAG	1.01714	1.0176723	1.0166999	0.9449746	-0.0816526	1
PPP2R3A	1.33316	1.3333432	1.33300964	0.9989995	-0.0014442	1
RP11-220C2.1	1.017146	1.0176869	1.0166999	0.944197	-0.0828403	1
HRASLS5	1.017142	1.0176771	1.0166999	0.9447171	-0.0820457	1
RP11-268G12.1	1.020604	1.0215288	1.01984039	0.9215727	-0.1178302	1
RP11-523H24.3	1.017136	1.0176639	1.0166999	0.9454244	-0.080966	1
CD300A	1.079533	1.0791232	1.07987126	1.0094544	0.01357573	1
RP11-231I16.1	1.019763	1.0196213	1.01988034	1.0132021	0.01892203	1
KLK1	1.037913	1.0387953	1.03718384	0.9584626	-0.0612059	1
ARHGAP31-AS1	1.021479	1.0215057	1.02145695	0.9977346	-0.003272	1
RP11-104N10.1	1.043201	1.0425937	1.04370289	1.0260418	0.0370895	1
SPATA32	1.016279	1.0157693	1.0166999	1.0590108	0.08271733	1
CTD-3028N15.3	1.01714	1.0176723	1.0166999	0.9449746	-0.0816526	1
RP11-173D14.3	1.017137	1.0176662	1.0166999	0.9453046	-0.0811489	1
RP11-867G23.4	1.020607	1.0215088	1.01986174	0.9234235	-0.1149356	1
TRPV4	1.019756	1.019602	1.01988279	1.0143248	0.02051975	1
FBXW10	1.017138	1.0176688	1.0166999	0.945162	-0.0813664	1
RP11-127O4.3	1.016279	1.0157693	1.0166999	1.0590108	0.08271733	1
VWA3A	1.022342	1.021532	1.02301022	1.0686529	0.0957934	1
LINC01311	1.070835	1.0712016	1.07053308	0.9906102	-0.0136106	1
AC112715.2	1.016279	1.0157693	1.0166999	1.0590108	0.08271733	1
RP5-1142A6.9	1.016279	1.0157693	1.0166999	1.0590108	0.08271733	1
PROK2	1.017138	1.0176692	1.0166999	0.9451422	-0.0813967	1
ALOX15	1.016279	1.0157693	1.0166999	1.0590108	0.08271733	1
PLK4	1.124219	1.1243439	1.12411501	0.998159	-0.0026584	1
ZBTB20-AS4	1.02148	1.0215213	1.02144629	0.996515	-0.0050365	1
RP11-435D7.3	1.017139	1.017671	1.0166999	0.9450471	-0.0815419	1
GLP2R	1.016279	1.0157693	1.0166999	1.0590108	0.08271733	1
CGB3	1.016279	1.0157693	1.0166999	1.0590108	0.08271733	1
TTLL6	1.016279	1.0157693	1.0166999	1.0590108	0.08271733	1
LOXL3	1.228161	1.227708	1.22853427	1.0036285	0.0052254	1
RP4-694B14.8	1.024097	1.0234261	1.02465091	1.0522822	0.0735217	1
RP11-120K19.4	1.018007	1.0176728	1.01828315	1.0345364	0.04898447	1

ESRRB	1.020599	1.021478	1.01987373	0.9253041	-0.1120005	1
ODF3L2	1.02232	1.0214878	1.02300788	1.0707429	0.09861214	1
SDR42E2	1.029258	1.0291833	1.02931932	1.0046602	0.00670768	1
ABHD11-AS1	1.03276	1.0330274	1.03253859	0.9852009	-0.0215101	1
LINC01324	1.016279	1.0157693	1.0166999	1.0590108	0.08271733	1
CABP2	1.017137	1.0176654	1.0166999	0.945347	-0.0810842	1
ZNF653	1.056041	1.0558285	1.05621683	1.0069565	0.0100013	1
VAV3-AS1	1.016279	1.0157693	1.0166999	1.0590108	0.08271733	1
LINC00972	1.017142	1.0176784	1.0166999	0.9446511	-0.0821465	1
OMG	1.017139	1.0176704	1.0166999	0.9450771	-0.081496	1
CFAP47	1.042307	1.0426028	1.04206304	0.9873294	-0.0183966	1
NCR2	1.016279	1.0157693	1.0166999	1.0590108	0.08271733	1
RP11-679C8.2	1.031861	1.0310481	1.03253314	1.0478309	0.06740591	1
RP11-296K13.4	1.018003	1.0176689	1.01827801	1.0344712	0.04889354	1
LINC01451	1.020593	1.0214855	1.01985535	0.9241291	-0.1138337	1
RNASE6	1.016279	1.0157693	1.0166999	1.0590108	0.08271733	1
RP11-554A11.8	1.016279	1.0157693	1.0166999	1.0590108	0.08271733	1
CD274	1.024078	1.0234579	1.02458927	1.0482284	0.06795312	1
SFTPD	1.032683	1.0329664	1.03244882	0.9842987	-0.0228318	1
CCDC190	1.018859	1.0195703	1.01827203	0.9336597	-0.0990313	1
CTD-3037G24.3	1.016279	1.0157693	1.0166999	1.0590108	0.08271733	1
AC023469.1	1.016279	1.0157693	1.0166999	1.0590108	0.08271733	1
CCDC192	1.028371	1.0291691	1.02771252	0.9500639	-0.0739036	1
RP11-713P17.4	1.017137	1.0176662	1.0166999	0.9453046	-0.0811489	1
ADRA1B	1.017143	1.0176792	1.0166999	0.9446056	-0.082216	1
PRMT5-AS1	1.017146	1.0176872	1.0166999	0.9441821	-0.0828629	1
RP11-659E9.4	1.018004	1.0176713	1.01827789	1.0343265	0.04869163	1
FLJ12825	1.018002	1.0176713	1.01827475	1.0341469	0.04844108	1
RP11-118H15.1	1.017139	1.0176713	1.0166999	0.9450295	-0.0815687	1
ENKD1	1.185662	1.1857511	1.1855877	0.9991203	-0.0012697	1
PABPC1L2B	1.023203	1.0234229	1.02302213	0.9828883	-0.0249006	1
RPGRIP1	1.017137	1.0176654	1.0166999	0.945347	-0.0810842	1
RP11-491F9.5	1.019737	1.0195642	1.01988034	1.0161595	0.02312692	1
RP11-697E2.6	1.024918	1.0253212	1.02458593	0.9709621	-0.0425131	1
RP11-976B16.1	1.027502	1.0272106	1.02774216	1.0195331	0.02790856	1
LINC00639	1.019767	1.0196244	1.01988398	1.0132268	0.01895709	1
RP11-274H24.1	1.016279	1.0157693	1.0166999	1.0590108	0.08271733	1
RP4-583P15.15	1.017139	1.0176702	1.0166999	0.9450896	-0.081477	1
SLC22A16	1.018001	1.0176712	1.01827425	1.0341289	0.04841609	1
TLR7	1.017297	1.0175999	1.01704689	0.9685763	-0.0460624	1
AF038458.5	1.019889	1.0195302	1.0201846	1.0335094	0.04755155	1
RP11-905K4.1	1.017301	1.0176095	1.01704689	0.968053	-0.0468421	1
CTC-360G5.9	1.029415	1.0290502	1.02971587	1.0229147	0.03268577	1
C7orf77	1.017295	1.017595	1.01704689	0.9688485	-0.045657	1
IGFLR1	1.033714	1.032865	1.03441541	1.0471752	0.06650282	1

GOLGA6L1	1.017304	1.0176155	1.01704689	0.9677194	-0.0473394	1
RP11-993B23.3	1.017295	1.0175943	1.01704689	0.9688845	-0.0456033	1
CCDC126	1.235473	1.2354548	1.23548836	1.0001424	0.00020536	1
RP11-501C14.6	1.017299	1.0176052	1.01704689	0.9682878	-0.0464922	1
RP11-56G10.2	1.017306	1.0176193	1.01704689	0.9675124	-0.047648	1
CNNM2	1.101405	1.1019961	1.10091647	0.9894154	-0.0153517	1
RP11-506H21.5	1.044144	1.0443857	1.0439453	0.990078	-0.0143859	1
LINC01402	1.017303	1.0176133	1.01704689	0.9678441	-0.0471534	1
RP1-127D3.4	1.016438	1.0157004	1.01704689	1.0857602	0.11870546	1
RP4-665J23.2	1.018159	1.0176052	1.01861563	1.0573944	0.08051354	1
CTD-2192J16.11	1.017311	1.0176298	1.01704689	0.9669383	-0.0485043	1
RP4-751H13.7	1.017305	1.0176165	1.01704689	0.9676672	-0.0474171	1
RP1-309F20.4	1.018169	1.0176142	1.01862771	1.0575412	0.0807138	1
RP11-134G8.5	1.025084	1.0252742	1.0249277	0.9862888	-0.019918	1
GAS8-AS1	1.017301	1.0176092	1.01704689	0.9680658	-0.0468229	1
TRHDE	1.034567	1.0347339	1.03442997	0.9912495	-0.0126798	1
EBF2	1.019052	1.0195663	1.01862771	0.9520286	-0.0709232	1
RP11-223P11.3	1.017303	1.0176123	1.01704689	0.967898	-0.0470731	1
AC005082.12	1.016438	1.0157004	1.01704689	1.0857602	0.11870546	1
RP11-307C19.2	1.024222	1.0233326	1.02495568	1.0695648	0.09702395	1
ZP2	1.017297	1.0175992	1.01704689	0.96862	-0.0459974	1
KIAA1324	1.055345	1.0558093	1.05496171	0.9848123	-0.0220793	1
RP11-446E24.4	1.018159	1.0175995	1.01862051	1.0580144	0.08135926	1
FUT3	1.016438	1.0157004	1.01704689	1.0857602	0.11870546	1
SLC25A30-AS1	1.017294	1.0175939	1.01704689	0.9689111	-0.0455637	1
C9orf66	1.020783	1.0214884	1.0202012	0.9400959	-0.0891201	1
VWCE	1.083091	1.082569	1.08352221	1.011545	0.01656051	1
BVES-AS1	1.016438	1.0157004	1.01704689	1.0857602	0.11870546	1
AIRN	1.017303	1.0176125	1.01704689	0.9678851	-0.0470923	1
STUM	1.022489	1.0233647	1.02176646	0.9315973	-0.1022216	1
PLA2G6	1.110902	1.11147	1.11043318	0.9906988	-0.0134816	1
RP4-764O22.1	1.018163	1.0176033	1.01862593	1.0580923	0.08146543	1
TSSK3	1.030316	1.031113	1.02965769	0.9532261	-0.0691096	1
PSD2	1.046746	1.0462289	1.04717221	1.0204053	0.02914227	1
RP11-457M11.7	1.017306	1.0176193	1.01704689	0.9675124	-0.047648	1
TM4SF19-TCTEX1D2	1.019016	1.0195047	1.01861254	0.9542617	-0.0675431	1
CCDC65	1.028511	1.0290574	1.02805916	0.9656467	-0.0504327	1
AC133785.1	1.019895	1.0195143	1.02020929	1.0356133	0.0504854	1
RP11-548P2.2	1.017295	1.0175943	1.01704689	0.9688845	-0.0456033	1
RP11-181G12.4	1.016438	1.0157004	1.01704689	1.0857602	0.11870546	1
RP11-162D9.3	1.017296	1.0175984	1.01704689	0.9686636	-0.0459323	1
RP11-116O18.3	1.017303	1.0176142	1.01704689	0.9677944	-0.0472276	1
MAEL	1.064984	1.0656059	1.06447037	0.9826923	-0.0251883	1
MFSD6L	1.016438	1.0157004	1.01704689	1.0857602	0.11870546	1
RP11-426C22.8	1.016438	1.0157004	1.01704689	1.0857602	0.11870546	1

RP11-374M1.3	1.017302	1.0176105	1.01704689	0.967997	-0.0469256	1
C17orf50	1.03808	1.038588	1.03765986	0.9759477	-0.0351243	1
AC068490.2	1.019033	1.0195279	1.0186238	0.9537012	-0.0683907	1
RP11-155O18.6	1.017303	1.0176142	1.01704689	0.9677944	-0.0472276	1
RUNX2	1.055491	1.0559079	1.05514595	0.9863721	-0.0197961	1
AC084809.2	1.016438	1.0157004	1.01704689	1.0857602	0.11870546	1
C11orf84	1.132214	1.1321566	1.13226063	1.0007871	0.00113505	1
VSIG2	1.018161	1.0176048	1.01862043	1.0576891	0.08091557	1
AKAP2	1.031112	1.0309668	1.03123123	1.0085405	0.01226896	1
NMUR1	1.017296	1.0175981	1.01704689	0.968677	-0.0459125	1
OXT	1.018164	1.0176095	1.01862182	1.0574895	0.08064331	1
SLC22A11	1.01904	1.0195485	1.01862062	0.9525345	-0.0701568	1
RP5-837I24.4	1.018161	1.0175995	1.01862524	1.0582832	0.0817258	1
RP1-16A9.1	1.017297	1.0175995	1.01704689	0.9686019	-0.0460242	1
SLCO4C1	1.020765	1.0214255	1.02021998	0.9437349	-0.0835465	1
RP4-564M11.2	1.017303	1.0176123	1.01704689	0.967898	-0.0470731	1
RP5-1057J7.7	1.016438	1.0157004	1.01704689	1.0857602	0.11870546	1
CCDC69	1.062267	1.061568	1.06284452	1.020734	0.02960696	1
TEKT4	1.018159	1.0175957	1.01862415	1.0584514	0.08195498	1
NOS2	1.024247	1.0234232	1.02492809	1.0642488	0.08983547	1
LINC00299	1.017303	1.0176123	1.01704689	0.967898	-0.0470731	1
RP11-431N15.2	1.020809	1.0214993	1.02023942	0.9413998	-0.0871206	1
RP11-89C3.3	1.017303	1.0176142	1.01704689	0.9677944	-0.0472276	1
SPACA6P-AS	1.024221	1.0233376	1.02495035	1.0691052	0.09640377	1
CFP	1.019026	1.0195243	1.01861512	0.9534315	-0.0687988	1
FAM26E	1.016438	1.0157004	1.01704689	1.0857602	0.11870546	1
RP11-544D21.2	1.017301	1.0176088	1.01704689	0.9680899	-0.046787	1
RP11-12D24.10	1.017299	1.0176048	1.01704689	0.9683082	-0.0464618	1
XKR9	1.0311	1.030897	1.03126686	1.0119707	0.01716753	1
RP11-1148L6.9	1.025995	1.0253264	1.02654625	1.0481656	0.06786663	1
RP1-142L7.5	1.016438	1.0157004	1.01704689	1.0857602	0.11870546	1
AVP	1.017298	1.0176025	1.01704689	0.9684376	-0.046269	1
RP11-127L20.3	1.017297	1.0176002	1.01704689	0.9685606	-0.0460858	1
LINC01032	1.01816	1.0175983	1.01862466	1.0583234	0.08178057	1
ALDH3A1	1.022469	1.0233329	1.0217561	0.9324212	-0.1009463	1
WDFY4	1.017303	1.0176142	1.01704689	0.9677944	-0.0472276	1
TMPRSS6	1.017295	1.0175962	1.01704689	0.9687822	-0.0457557	1
TMEM72-AS1	1.020771	1.0214571	1.02020441	0.9416204	-0.0867825	1
RP11-624L4.1	1.044994	1.0443263	1.04554489	1.0274923	0.03912758	1
REC114	1.016438	1.0157004	1.01704689	1.0857602	0.11870546	1
CTD-2026K11.2	1.017301	1.017609	1.01704689	0.9680771	-0.0468062	1
CTD-2544H17.2	1.018161	1.0176005	1.01862415	1.0581596	0.08155726	1
LINC01513	1.025059	1.0252072	1.02493743	0.9892982	-0.0155226	1
NPY2R	1.017297	1.0176004	1.01704689	0.968553	-0.0460971	1
CSTF3-AS1	1.030255	1.0309429	1.02968709	0.9594144	-0.059774	1

RP1-309F20.3	1.021628	1.0214215	1.02179822	1.0175845	0.0251486	1
RP5-1159O4.2	1.027665	1.0271386	1.0280992	1.0353948	0.05018101	1
AC091814.3	1.017301	1.017608	1.01704689	0.968133	-0.0467228	1
RP11-434H6.6	1.017297	1.0175995	1.01704689	0.9686019	-0.0460242	1
RP11-9M16.2	1.016438	1.0157004	1.01704689	1.0857602	0.11870546	1
AC005753.1	1.01904	1.0195372	1.0186291	0.9535216	-0.0686625	1
RP11-27M24.1	1.019881	1.0195054	1.0201918	1.0351915	0.04989774	1
HIST1H2AM	1.029411	1.0290461	1.02971257	1.0229447	0.03272812	1
RP11-536G4.2	1.024231	1.02336	1.02494969	1.0680504	0.09497973	1
SLC35G4	1.016438	1.0157004	1.01704689	1.0857602	0.11870546	1
ANKRD61	1.017301	1.0176083	1.01704689	0.9681178	-0.0467455	1
CTD-3110H11.1	1.023353	1.023312	1.02338596	1.0031728	0.00457016	1
URB1	1.095922	1.0959092	1.09593331	1.000251	0.00036204	1
GDF9	1.024228	1.0233732	1.02493334	1.0667484	0.0932199	1
XXyac-YX65C7_A.2	1.018152	1.0175995	1.01860843	1.0573283	0.08042336	1
RP11-233G1.4	1.013663	1.0143332	1.01310914	0.9146026	-0.1287831	1
HDAC11-AS1	1.012801	1.0124282	1.01310914	1.0547941	0.07696144	1
GRM7	1.024862	1.0257145	1.02415864	0.9394935	-0.0900449	1
LINC00624	1.014529	1.0143332	1.01469051	1.0249317	0.03552776	1
RP4-635E18.6	1.030923	1.031533	1.03041962	0.9646904	-0.0518621	1
TTLL10	1.019692	1.0200209	1.0194209	0.9700303	-0.0438982	1
RP1-154K9.2	1.01452	1.0143225	1.01468352	1.0252069	0.03591504	1
GATA4	1.014516	1.0143255	1.01467399	1.0243279	0.03467761	1
CTC-435M10.3	1.013661	1.014329	1.01310914	0.9148685	-0.1283637	1
RP11-426L16.10	1.013657	1.0143205	1.01310914	0.9154111	-0.1275083	1
TMEM179	1.033549	1.033413	1.03366091	1.0074199	0.01066513	1
AC022154.7	1.017971	1.0181535	1.01782092	0.9816813	-0.0266733	1
LY6G5B	1.024877	1.0257439	1.02416162	0.9385369	-0.0915146	1
C1QTNF9B-AS1	1.065424	1.0658149	1.06510086	0.9891513	-0.0157368	1
RP13-580F15.2	1.016242	1.0162243	1.01625576	1.0019407	0.00279716	1
TAS2R19	1.016246	1.0162485	1.01624361	0.9997	-0.0004328	1
RP3-475N16.1	1.023192	1.023925	1.02258679	0.9440647	-0.0830424	1
PCAT5	1.016253	1.0162439	1.01626046	1.0010183	0.00146832	1
AGO4	1.112756	1.1133441	1.11226961	0.9905201	-0.0137418	1
KIAA0930	1.190471	1.1898691	1.19096749	1.0057851	0.00832213	1
RP11-578F21.6	1.015382	1.0162247	1.01468682	0.9052134	-0.1436701	1
CLSTN2-AS1	1.012801	1.0124282	1.01310914	1.0547941	0.07696144	1
ADAMTS16	1.052444	1.0524239	1.0524615	1.0007179	0.00103534	1
CTD-2373H9.5	1.013662	1.0143315	1.01310914	0.914711	-0.128612	1
STAR	1.048125	1.0485624	1.04776422	0.9835647	-0.0239081	1
ATP6V0A4	1.012801	1.0124282	1.01310914	1.0547941	0.07696144	1
TMEM212	1.016232	1.016222	1.01624014	1.0011195	0.00161422	1
POU2F3	1.017095	1.016229	1.01781093	1.0974786	0.1341928	1
PCDH9-AS4	1.012801	1.0124282	1.01310914	1.0547941	0.07696144	1
CPNE6	1.014521	1.0143318	1.01467703	1.0240894	0.03434172	1

AC195454.1	1.017967	1.0181197	1.01784166	0.9846532	-0.0223124	1
RP11-266K4.13	1.014521	1.0143255	1.01468202	1.0248885	0.03546695	1
ELMO1	1.049854	1.0506462	1.04919936	0.9714326	-0.0418142	1
RP1-92C4.2	1.017127	1.0162347	1.01786471	1.1004064	0.13803642	1
RP1-118J21.5	1.01452	1.0143257	1.01468104	1.0248025	0.03534594	1
RP11-130C19.3	1.013662	1.0143318	1.01310914	0.9146899	-0.1286454	1
ANKFN1	1.017111	1.0162285	1.01784018	1.0993121	0.13660104	1
GNA15	1.01366	1.0143275	1.01310914	0.9149611	-0.1282177	1
MTRNR2L6	1.012801	1.0124282	1.01310914	1.0547941	0.07696144	1
RP11-65J3.2	1.017987	1.0181733	1.01783334	0.9812927	-0.0272446	1
SALL4	1.048166	1.0486328	1.04778113	0.9824868	-0.02549	1
KRT81	1.017098	1.0162173	1.01782588	1.0991875	0.13643754	1
AC011298.2	1.016254	1.0162215	1.01628071	1.0036488	0.00525454	1
SNX1	1.348874	1.3494131	1.34842833	0.9971816	-0.0040719	1
RP11-481J2.4	1.068013	1.0677879	1.06819979	1.0060763	0.00873976	1
HRCT1	1.012801	1.0124282	1.01310914	1.0547941	0.07696144	1
ANO2	1.017978	1.0181371	1.01784617	0.9839598	-0.0233288	1
INPP5J	1.046479	1.0467415	1.04626233	0.989749	-0.0148654	1
RP11-649E7.5	1.027451	1.0277082	1.02723854	0.9830509	-0.0246619	1
MMD2	1.012801	1.0124282	1.01310914	1.0547941	0.07696144	1
SLFN14	1.012801	1.0124282	1.01310914	1.0547941	0.07696144	1
RP11-552M14.1	1.012801	1.0124282	1.01310914	1.0547941	0.07696144	1
RP11-79O8.1	1.014526	1.014329	1.01468787	1.0250459	0.03568854	1
DRICH1	1.012801	1.0124282	1.01310914	1.0547941	0.07696144	1
PWRN3	1.01366	1.014327	1.01310914	0.9149969	-0.1281613	1
CACNA1B	1.014518	1.0143267	1.01467611	1.0243852	0.03475829	1
LINC00852	1.017109	1.0162341	1.01783123	1.0983793	0.1353764	1
AP001626.2	1.017095	1.0162252	1.01781424	1.0979394	0.13479846	1
WASIR1	1.01539	1.0162434	1.01468438	0.904021	-0.1455717	1
AC022201.5	1.013659	1.0143244	1.01310914	0.9151609	-0.1279026	1
RP11-7K24.3	1.020543	1.0200291	1.02096747	1.0468491	0.06605344	1
REG1A	1.012801	1.0124282	1.01310914	1.0547941	0.07696144	1
GRM4	1.01366	1.0143267	1.01310914	0.9150117	-0.1281379	1
RP11-304M2.5	1.013658	1.0143229	1.01310914	0.915256	-0.1277527	1
RP11-371A19.2	1.02058	1.0200546	1.02101413	1.0478458	0.06742646	1
ZNF492	1.01452	1.0143257	1.01468074	1.0247819	0.03531688	1
MIR142	1.012801	1.0124282	1.01310914	1.0547941	0.07696144	1
RP5-1142A6.10	1.013662	1.0143322	1.01310914	0.9146622	-0.1286891	1
RP11-500C11.3	1.045578	1.0447568	1.04625621	1.0335021	0.04754132	1
FLT3	1.017106	1.0162415	1.01782012	1.0971994	0.13382568	1
CRKL	1.265254	1.2655563	1.26500379	0.9979194	-0.0030048	1
PF4V1	1.017103	1.0162236	1.01782902	1.0989575	0.13613563	1
ZNF324B	1.054243	1.0543886	1.05412242	0.9951065	-0.0070772	1
STT3A-AS1	1.016261	1.0162349	1.01628163	1.0028765	0.00414398	1
DTX1	1.017961	1.018132	1.01782048	0.9828182	-0.0250036	1

LINC01285	1.018836	1.0181334	1.0194156	1.0707111	0.09856923	1
CTD-2196P11.2	1.01366	1.0143263	1.01310914	0.9150414	-0.128091	1
RP11-86H7.6	1.014522	1.014327	1.01468352	1.0248856	0.03546292	1
LHX4	1.016255	1.0162668	1.01624493	0.9986572	-0.0019386	1
TCEAL6	1.015392	1.0162531	1.01468092	0.9032694	-0.1467717	1
CTD-2215E18.1	1.013663	1.0143328	1.01310914	0.9146263	-0.1287457	1
SKIV2L	1.166375	1.1668436	1.16598846	0.9948748	-0.0074131	1
RP11-6E9.5	1.012801	1.0124282	1.01310914	1.0547941	0.07696144	1
AC012358.7	1.012801	1.0124282	1.01310914	1.0547941	0.07696144	1
TPRG1-AS1	1.018836	1.0181082	1.01943755	1.0734134	0.10220582	1
GJA8	1.01538	1.0162287	1.01467881	0.9044963	-0.1448136	1
SBSN	1.01453	1.0143439	1.01468332	1.0236652	0.03374398	1
RP11-77H9.6	1.013661	1.0143285	1.01310914	0.9148982	-0.1283168	1
AP003774.6	1.018835	1.0181568	1.01939514	1.0682022	0.0951848	1
CARD14	1.021428	1.0219866	1.02096726	0.9536367	-0.0684883	1
LINC01277	1.017982	1.0181493	1.01784466	0.9832131	-0.0244239	1
UBD	1.015378	1.0162184	1.0146848	0.9054394	-0.1433099	1
AGTR1	1.015377	1.0162193	1.01468066	0.9051368	-0.1437922	1
RP11-95D17.1	1.078336	1.0791949	1.07762618	0.9801919	-0.0288638	1
ALKBH6	1.264683	1.2639195	1.26531399	1.0052836	0.00760261	1
RP11-16C18.3	1.012801	1.0124282	1.01310914	1.0547941	0.07696144	1
AF146191.4	1.019699	1.0200597	1.01940175	0.9672007	-0.0481127	1
CTD-2026K11.4	1.013659	1.0143255	1.01310914	0.9150927	-0.1280102	1
ZNF662	1.013659	1.0143242	1.01310914	0.9151758	-0.1278792	1
RP11-16L21.7	1.012801	1.0124282	1.01310914	1.0547941	0.07696144	1
DSCR8	1.019717	1.0200365	1.01945351	0.9709025	-0.0426017	1
PPP1R16B	1.02403	1.0238672	1.02416416	1.0124429	0.01784052	1
RP11-353N14.1	1.01452	1.014326	1.01468066	1.0247567	0.03528147	1
RP11-282I1.1	1.012801	1.0124282	1.01310914	1.0547941	0.07696144	1
RP11-1078H9.1	1.013662	1.0143308	1.01310914	0.9147522	-0.1285472	1
CTB-11I22.1	1.013662	1.0143308	1.01310914	0.9147522	-0.1285472	1
TTC39C-AS1	1.024857	1.0257448	1.02412447	0.9370608	-0.0937854	1
RP11-244F12.2	1.01452	1.0143285	1.01467881	1.0244465	0.03484462	1
COL19A1	1.031782	1.0314939	1.03202033	1.0167144	0.02391442	1
RP11-290F5.1	1.013664	1.0143359	1.01310914	0.9144264	-0.129061	1
AC007557.1	1.013662	1.0143316	1.01310914	0.9147022	-0.1286259	1
RP11-335K5.2	1.014516	1.0143204	1.01467703	1.0249006	0.03548402	1
SLC16A1-AS1	1.62593	1.6252581	1.62648506	1.0019624	0.00282834	1
RP11-33O4.1	1.026606	1.0257938	1.02727584	1.0574568	0.0805987	1
ZRSR2	1.350851	1.3516847	1.35016245	0.9956716	-0.0062582	1
AC092718.1	1.01366	1.0143281	1.01310914	0.9149253	-0.1282742	1
PCDHGA2	1.013658	1.0143225	1.01310914	0.9152837	-0.1277092	1
RP11-343H5.6	1.016254	1.0162516	1.01625543	1.0002361	0.00034053	1
ZACN	1.022319	1.0219557	1.02261876	1.0301992	0.04292328	1
IL27RA	1.165409	1.16476	1.16594407	1.0071866	0.01033102	1

CYP17A1	1.018455	1.0178676	1.01894061	1.0600547	0.08413876	1
RP11-576I22.2	1.01673	1.0159741	1.01735489	1.0864378	0.11960564	1
RP3-454G6.2	1.01933	1.0198145	1.01893016	0.9553689	-0.0658701	1
RP11-442N24__B.1	1.017592	1.0178798	1.01735489	0.9706439	-0.042986	1
RP11-864G5.3	1.01673	1.0159741	1.01735489	1.0864378	0.11960564	1
EDN3	1.026273	1.0255818	1.02684293	1.0492969	0.06942296	1
RP11-396F22.1	1.088498	1.0886051	1.08840865	0.9977826	-0.0032026	1
CTC-325H20.4	1.021097	1.02178	1.02053386	0.9427852	-0.084999	1
HFM1	1.062604	1.0617794	1.06328458	1.0243643	0.03472882	1
CTD-2541J13.2	1.02452	1.0236075	1.02527359	1.0705766	0.09838802	1
EXOC3L4	1.017593	1.0178807	1.01735489	0.970593	-0.0430617	1
EIF4E1B	1.017599	1.017895	1.01735489	0.9698183	-0.0442136	1
RNF224	1.01673	1.0159741	1.01735489	1.0864378	0.11960564	1
RP11-452I5.2	1.017595	1.0178853	1.01735489	0.970341	-0.0434363	1
RP11-275N1.1	1.017593	1.0178803	1.01735489	0.9706149	-0.0430291	1
ZNF726	1.018452	1.0178705	1.01893298	1.0594562	0.08332393	1
MAGEB10	1.017591	1.0178762	1.01735489	0.9708395	-0.0426953	1
RP11-667M19.2	1.01845	1.0178745	1.01892482	1.0587595	0.08237495	1
RP11-525A16.4	1.017591	1.0178762	1.01735489	0.9708395	-0.0426953	1
RP11-650P15.1	1.017588	1.0178705	1.01735489	0.9711487	-0.0422359	1
PTCD2	1.119734	1.1191197	1.12024089	1.0094125	0.0135159	1
CTC-327F10.5	1.01673	1.0159741	1.01735489	1.0864378	0.11960564	1
ZFX-AS1	1.017588	1.0178705	1.01735489	0.9711487	-0.0422359	1
FMNL2	1.452011	1.452079	1.45195559	0.9997269	-0.000394	1
LINC01031	1.01673	1.0159741	1.01735489	1.0864378	0.11960564	1
CTD-2542L18.1	1.018451	1.0178721	1.01892889	1.0591279	0.08287682	1
CASC16	1.018453	1.0178803	1.01892571	1.0584652	0.0819738	1
SDR9C7	1.01845	1.0178691	1.01892904	1.0593158	0.0831328	1
THSD4-AS1	1.01673	1.0159741	1.01735489	1.0864378	0.11960564	1
AF064858.7	1.01845	1.0178705	1.01892907	1.0592371	0.08302554	1
AP000654.4	1.01673	1.0159741	1.01735489	1.0864378	0.11960564	1
RP11-873E20.1	1.029784	1.0295159	1.03000606	1.016608	0.02376349	1
TTLL11-IT1	1.018456	1.0178814	1.01893124	1.0587102	0.08230774	1
MLH3	1.530954	1.5316763	1.53035792	0.9975203	-0.0035819	1
CELA1	1.017592	1.0178782	1.01735489	0.9707273	-0.0428621	1
TMEM229A	1.01673	1.0159741	1.01735489	1.0864378	0.11960564	1
MMS19	1.302144	1.3028769	1.30153928	0.9955835	-0.0063857	1
RP11-350J20.5	1.048813	1.0485303	1.04904622	1.0106303	0.01525536	1
CTD-2503I6.1	1.017591	1.0178776	1.01735489	0.9707613	-0.0428115	1
MMP13	1.01673	1.0159741	1.01735489	1.0864378	0.11960564	1
RP11-793H13.10	1.017589	1.0178721	1.01735489	0.9710579	-0.0423707	1
AC104135.2	1.02799	1.0274544	1.02843273	1.035634	0.05051427	1
IRAK3	1.026215	1.0254825	1.02681923	1.0524578	0.07376242	1
RP6-65G23.5	1.073838	1.0734407	1.07416527	1.009866	0.01416394	1
FLJ21408	1.028877	1.0294314	1.02841926	0.9656103	-0.050487	1

FRMD6-AS2	1.019316	1.0197826	1.01893115	0.9569612	-0.0634677	1
AC093901.1	1.026271	1.02552	1.02689128	1.0537323	0.07550844	1
AC093843.1	1.017589	1.0178723	1.01735489	0.9710512	-0.0423808	1
RP11-109J4.1	1.01673	1.0159741	1.01735489	1.0864378	0.11960564	1
SLC51A	1.023647	1.023606	1.02368135	1.0031923	0.00459825	1
SH2D5	1.02537	1.0254975	1.02526557	0.9909041	-0.0131827	1
CTC-453G23.4	1.017591	1.0178774	1.01735489	0.9707727	-0.0427945	1
LRTM2	1.017595	1.0178853	1.01735489	0.970341	-0.0434363	1
RP11-313A24.1	1.017589	1.0178728	1.01735489	0.9710212	-0.0424254	1
RP11-230C9.2	1.021938	1.0217231	1.02211545	1.0180605	0.02582329	1
RP11-13A1.1	1.017589	1.0178733	1.01735489	0.9709965	-0.042462	1
PON1	1.018461	1.0178957	1.01892842	1.0577056	0.08093807	1
AC009065.1	1.021055	1.0216967	1.02052581	0.9460323	-0.0800386	1
CTD-2033D15.3	1.017594	1.0178846	1.01735489	0.9703807	-0.0433772	1
CTB-133P21.1	1.017595	1.0178856	1.01735489	0.9703299	-0.0434528	1
RP11-297J22.1	1.037477	1.0369959	1.03787482	1.023757	0.03387327	1
RIC8B	1.180345	1.1804011	1.18029923	0.9994354	-0.0008148	1
RSPO4	1.045419	1.0449605	1.04579848	1.0186383	0.02664183	1
CNBD1	1.018454	1.0178795	1.01892889	1.0586893	0.0822793	1
AQP4-AS1	1.020213	1.0198229	1.02053583	1.0359669	0.0509779	1
RP11-175K6.1	1.01673	1.0159741	1.01735489	1.0864378	0.11960564	1
RP5-1112D6.4	1.112953	1.1136634	1.11236676	0.9885923	-0.0165525	1
KCNH6	1.020196	1.0198222	1.02050498	1.0344453	0.04885743	1
HAS3	1.023676	1.0236653	1.02368535	1.0008476	0.00122226	1
ELANE	1.017591	1.0178774	1.01735489	0.9707727	-0.0427945	1
RP13-512J5.1	1.017587	1.0178676	1.01735489	0.9713059	-0.0420024	1
MMP25	1.017591	1.0178762	1.01735489	0.9708395	-0.0426953	1
TVP23C	1.071198	1.0713018	1.07111268	0.997348	-0.0038311	1
CTD-2281M20.1	1.017593	1.0178803	1.01735489	0.9706133	-0.0430314	1
PLXNB3	1.017591	1.0178766	1.01735489	0.9708165	-0.0427295	1
PASD1	1.018451	1.0178697	1.01893168	1.0594306	0.08328906	1
RP11-394I13.2	1.021959	1.0217813	1.02210583	1.0149	0.02133757	1
AC006129.2	1.01673	1.0159741	1.01735489	1.0864378	0.11960564	1
RP11-112J3.15	1.018449	1.0178763	1.01892261	1.0585329	0.08206615	1
RP11-9N12.2	1.021062	1.0217204	1.0205184	0.9446584	-0.0821353	1
WBP1	1.073814	1.0733508	1.07419642	1.011528	0.01653625	1
RP11-379L18.1	1.017591	1.0178761	1.01735489	0.9708441	-0.0426884	1
RP11-63M22.2	1.018454	1.0178835	1.01892509	1.058244	0.08167228	1
CD6	1.017598	1.0178926	1.01735489	0.9699487	-0.0440197	1
NFKBIA	1.631679	1.6313294	1.63196851	1.0010124	0.0014598	1
PLCE1-AS1	1.01673	1.0159741	1.01735489	1.0864378	0.11960564	1
WDR49	1.042822	1.0429796	1.04269266	0.9933243	-0.0096633	1
RP11-293E1.2	1.017593	1.0178813	1.01735489	0.9705601	-0.0431105	1
RP11-160H22.5	1.01673	1.0159741	1.01735489	1.0864378	0.11960564	1
RP11-546B15.1	1.017593	1.0178804	1.01735489	0.9706085	-0.0430386	1

SLC25A31	1.017588	1.0178705	1.01735489	0.9711487	-0.0422359	1
CTC-228N24.2	1.017587	1.0178682	1.01735489	0.9712703	-0.0420552	1
DLGAP1-AS4	1.019331	1.0198185	1.01892842	0.9550901	-0.0662912	1
OFCC1	1.018467	1.0178952	1.01893995	1.0583806	0.08185852	1
AIM2	1.038314	1.0388687	1.03785669	0.9739645	-0.0380589	1
ASAP1	1.711279	1.7108642	1.71162174	1.0010656	0.00153651	1
AC010976.2	1.01673	1.0159741	1.01735489	1.0864378	0.11960564	1
PWRN4	1.017594	1.0178833	1.01735489	0.9704546	-0.0432674	1
AC006994.3	1.020212	1.0197982	1.02055444	1.0381974	0.05408084	1
CTD-3099C6.7	1.017589	1.0178732	1.01735489	0.9710017	-0.0424543	1
FMNL1	1.03667	1.0371318	1.03628909	0.9773038	-0.033121	1
TM4SF19-AS1	1.027134	1.0274821	1.02684643	0.9768683	-0.033764	1
RP11-10J21.6	1.019312	1.0197877	1.01891981	0.9561392	-0.0647074	1
RP11-292D4.1	1.01673	1.0159741	1.01735489	1.0864378	0.11960564	1
RP11-94I2.4	1.026263	1.0255505	1.02685052	1.0508825	0.07160131	1
OCLM	1.019316	1.0186077	1.01990019	1.0694596	0.09688193	1
RP11-714M23.2	1.017592	1.0166975	1.01833122	1.0978393	0.13466695	1
LINC00470	1.019321	1.0186051	1.01991309	1.0703039	0.09802043	1
RP11-66B24.1	1.018452	1.0185988	1.01833122	0.9856133	-0.0209064	1
DCDC2C	1.018456	1.0186065	1.01833122	0.9852073	-0.0215008	1
RP11-461A8.5	1.01845	1.0185928	1.01833122	0.9859322	-0.0204396	1
RP11-192H23.5	1.018452	1.0185988	1.01833122	0.9856133	-0.0209064	1
SELENOV	1.027096	1.0262156	1.02782321	1.0613241	0.08586533	1
RP11-467D6.1	1.02969	1.0300657	1.0293799	0.9771914	-0.033287	1
RP11-607P23.1	1.017592	1.0166975	1.01833122	1.0978393	0.13466695	1
KCNE1B	1.017592	1.0166975	1.01833122	1.0978393	0.13466695	1
RP11-1100L3.8	1.018453	1.018601	1.01833122	0.9854948	-0.0210798	1
RP11-588H23.3	1.019322	1.0185934	1.01992356	1.0715375	0.09968234	1
MYO1G	1.02018	1.0205115	1.01990584	0.9704722	-0.0432412	1
EMX2OS	1.03317	1.0339999	1.03248413	0.9554177	-0.0657965	1
RP11-1293J14.1	1.017592	1.0166975	1.01833122	1.0978393	0.13466695	1
SCAF4	1.400908	1.4019801	1.40002341	0.9951323	-0.0070397	1
CTC-453G23.8	1.018463	1.0186228	1.01833122	0.9843426	-0.0227676	1
CYP2C18	1.017592	1.0166975	1.01833122	1.0978393	0.13466695	1
RP11-483L5.1	1.020167	1.020496	1.0198958	0.9707139	-0.042882	1
FAM201A	1.079877	1.0797868	1.07995162	1.002066	0.00297755	1
NOX1	1.017592	1.0166975	1.01833122	1.0978393	0.13466695	1
KB-1471A8.1	1.019312	1.0186057	1.01989526	1.0693103	0.09668051	1
AP000302.58	1.017592	1.0166975	1.01833122	1.0978393	0.13466695	1
AC010884.1	1.027138	1.0263424	1.02779413	1.055109	0.0773921	1
STRN3	1.47974	1.4804361	1.47916602	0.9973563	-0.0038191	1
RP11-252I13.2	1.022834	1.0225143	1.02309725	1.0258916	0.03687823	1
AC005387.3	1.018451	1.0185955	1.01833122	0.9857889	-0.0206494	1
CLPSL1	1.017592	1.0166975	1.01833122	1.0978393	0.13466695	1
RP5-1182A14.5	1.018454	1.0186033	1.01833122	0.9853723	-0.0212591	1

LHX9	1.034897	1.0358305	1.03412599	0.9524277	-0.0703186	1
LINC01347	1.017592	1.0166975	1.01833122	1.0978393	0.13466695	1
PTGER4P2-CDK2AP2P2	1.023635	1.0243558	1.02304003	0.9459777	-0.0801219	1
KRT27	1.018451	1.0185956	1.01833122	0.9857843	-0.020656	1
EFCAB9	1.019322	1.0185901	1.01992591	1.0718546	0.10010919	1
AC034110.1	1.018454	1.0186032	1.01833122	0.9853796	-0.0212484	1
RP11-804H8.6	1.07578	1.0761046	1.07551228	0.9922164	-0.0112732	1
ZBTB7C	1.040171	1.0395988	1.0406432	1.0263738	0.03755625	1
FLJ40288	1.019319	1.0186052	1.01990867	1.070058	0.09768901	1
RP11-278A23.4	1.022798	1.0224903	1.02305139	1.0249505	0.03555424	1
AC005592.2	1.057351	1.0567518	1.0578458	1.0192772	0.02754643	1
MIR3681HG	1.027958	1.028126	1.02781939	0.9890979	-0.0158147	1
C8G	1.018449	1.018591	1.01833122	0.9860262	-0.020302	1
IL2RB	1.017592	1.0166975	1.01833122	1.0978393	0.13466695	1
YEATS2-AS1	1.018454	1.0186034	1.01833122	0.9853673	-0.0212665	1
MPP7	1.117158	1.1179945	1.1164675	0.987059	-0.0187918	1
RP4-545C24.1	1.028806	1.0281596	1.02933913	1.0418862	0.05919767	1
ADGRF1	1.018452	1.0185975	1.01833122	0.9856846	-0.0208021	1
RP11-554A11.7	1.017592	1.0166975	1.01833122	1.0978393	0.13466695	1
RP11-678G14.2	1.026231	1.0262323	1.02623008	0.9999158	-0.0001215	1
AC004895.4	1.02019	1.0205431	1.01989911	0.9686531	-0.045948	1
RP11-146F11.5	1.01846	1.0186152	1.01833122	0.9847428	-0.0221811	1
IGSF6	1.022794	1.022418	1.02310453	1.0306233	0.04351714	1
RP11-638L3.1	1.018452	1.0185977	1.01833122	0.98567	-0.0208234	1
RASSF10	1.020181	1.0205102	1.01990894	0.9706835	-0.0429271	1
ADPGK-AS1	1.019315	1.0185978	1.01990727	1.0704088	0.09816193	1
RP1-118J21.25	1.017592	1.0166975	1.01833122	1.0978393	0.13466695	1
RP5-955M13.4	1.01845	1.0185933	1.01833122	0.9859026	-0.020483	1
ITPKB-AS1	1.019322	1.0186145	1.01990676	1.0694235	0.09683329	1
RP11-552F3.10	1.01845	1.0185928	1.01833122	0.9859299	-0.020443	1
PPP5D1	1.100724	1.100825	1.10064114	0.9981764	-0.0026333	1
RP1-151F17.1	1.018451	1.0185951	1.01833122	0.985809	-0.02062	1
KLHDC7B	1.017592	1.0166975	1.01833122	1.0978393	0.13466695	1
FGF4	1.018455	1.0186051	1.01833122	0.9852801	-0.0213942	1
RP1-273N12.4	1.018449	1.018591	1.01833122	0.9860262	-0.020302	1
IL16	1.017592	1.0166975	1.01833122	1.0978393	0.13466695	1
C6orf3	1.071211	1.072097	1.07047845	0.9775503	-0.0327572	1
RP5-968D22.3	1.021939	1.0224646	1.02150457	0.9572627	-0.0630132	1
RP11-723O4.2	1.01845	1.0185933	1.01833122	0.9859026	-0.020483	1
SEMA3G	1.025418	1.0263526	1.02464577	0.9352301	-0.0966067	1
RP11-461L13.5	1.018452	1.0185993	1.01833122	0.9855881	-0.0209433	1
C8orf44-SGK3	1.023672	1.0243905	1.02307925	0.9462401	-0.0797218	1
AOX1	1.018455	1.0186057	1.01833122	0.985248	-0.0214412	1
TFB2M	1.136088	1.1350347	1.13695812	1.0142436	0.02040417	1
RP11-403A21.1	1.021035	1.0205062	1.02147259	1.0471262	0.06643529	1

TISP43	1.024567	1.0244004	1.02470454	1.0124637	0.01787013	1
RP11-178L8.3	1.017592	1.0166975	1.01833122	1.0978393	0.13466695	1
PRMT6	1.294018	1.2940366	1.29400252	0.9998841	-0.0001672	1
RORA-AS1	1.044509	1.0434957	1.04534669	1.0425565	0.06012563	1
AC142293.3	1.017592	1.0166975	1.01833122	1.0978393	0.13466695	1
ADRA1A	1.01845	1.0185928	1.01833122	0.9859299	-0.020443	1
FAM49A	1.100727	1.1010917	1.10042641	0.9934194	-0.0095252	1
BEST2	1.01846	1.0186161	1.01833122	0.9846959	-0.0222498	1
RP11-829H16.3	1.017592	1.0166975	1.01833122	1.0978393	0.13466695	1
SDS	1.018454	1.0186024	1.01833122	0.9854223	-0.0211859	1
ZMYND8	1.975529	1.9766912	1.97456939	0.9978275	-0.0031377	1
FAM182B	1.019317	1.0186112	1.01989911	1.0692012	0.09653342	1
OR13C3	1.018458	1.0186108	1.01833122	0.984977	-0.0218381	1
FAM53C	1.650742	1.6510756	1.65046572	0.9990632	-0.0013521	1
RGPD8	1.018463	1.018622	1.01833122	0.9843856	-0.0227045	1
RP11-285F7.2	1.062627	1.0625912	1.06265751	1.0010602	0.00152876	1
RP11-762I7.5	1.018451	1.0185955	1.01833122	0.9857889	-0.0206494	1
RP4-680D5.9	1.018453	1.018601	1.01833122	0.9854948	-0.0210798	1
RP3-467K16.4	1.025948	1.0265537	1.02544706	0.9583233	-0.0614156	1
AC106801.1	1.015555	1.0150885	1.01593993	1.0564324	0.07920045	1
RP11-665C16.6	1.016418	1.0169979	1.01593993	0.9377614	-0.0927072	1
SLAMF7	1.01816	1.0189516	1.01750643	0.9237463	-0.1144314	1
ANKHD1	1.142705	1.1431452	1.14234074	0.9943799	-0.008131	1
SAA2	1.016414	1.0169887	1.01593993	0.9382673	-0.091929	1
RP11-93B14.4	1.01642	1.0170014	1.01593993	0.9375658	-0.0930082	1
PRLHR	1.018167	1.0189545	1.01751614	0.9241166	-0.1138532	1
CLIC2	1.023307	1.022703	1.02380639	1.0485995	0.06846379	1
CD38	1.01727	1.0169852	1.01750543	1.0306306	0.04352736	1
RP11-266N13.2	1.023347	1.0227604	1.02383154	1.0470621	0.06634704	1
LINC01376	1.031983	1.032326	1.03169911	0.9806083	-0.0282511	1
RP11-120J1.1	1.015555	1.0150885	1.01593993	1.0564324	0.07920045	1
RP4-724E16.2	1.01641	1.0169801	1.01593993	0.9387394	-0.0912034	1
KRTAP11-1	1.019023	1.0189131	1.01911458	1.0106525	0.01528698	1
RP11-77I22.2	1.016417	1.0169938	1.01593993	0.9379834	-0.0923656	1
RP5-842K16.2	1.015555	1.0150885	1.01593993	1.0564324	0.07920045	1
KCNE3	1.025971	1.026622	1.02543424	0.9553836	-0.065848	1
RNF39	1.015555	1.0150885	1.01593993	1.0564324	0.07920045	1
RP11-29H23.4	1.015555	1.0150885	1.01593993	1.0564324	0.07920045	1
FAM157C	1.059689	1.0590994	1.06017656	1.0182264	0.02605834	1
RP11-463D19.2	1.017284	1.0169934	1.01752407	1.0312254	0.04435964	1
AC005329.7	1.016418	1.0169964	1.01593993	0.9378426	-0.0925823	1
AC241377.1	1.015555	1.0150885	1.01593993	1.0564324	0.07920045	1
TMPRSS3	1.015555	1.0150885	1.01593993	1.0564324	0.07920045	1
USP29	1.016415	1.0169894	1.01593993	0.9382263	-0.0919921	1
RP11-66D17.3	1.017273	1.0169857	1.01750993	1.0308636	0.04385345	1

RP11-21K12.3	1.017274	1.0169812	1.01751532	1.0314532	0.04467843	1
RP11-834C11.6	1.016415	1.0169904	1.01593993	0.9381706	-0.0920778	1
HIST1H3B	1.021577	1.0207821	1.02223297	1.0698144	0.09736048	1
MDH1B	1.075216	1.0742608	1.0760045	1.0234802	0.03348314	1
PCDHGB2	1.01642	1.0170009	1.01593993	0.9375922	-0.0929676	1
AC131097.4	1.016411	1.0169814	1.01593993	0.9386724	-0.0913064	1
GLRA3	1.01642	1.0170009	1.01593993	0.9375922	-0.0929676	1
PADI1	1.015555	1.0150885	1.01593993	1.0564324	0.07920045	1
ZNF350-AS1	1.016426	1.0170146	1.01593993	0.9368399	-0.0941255	1
RP11-51J9.4	1.016415	1.0169906	1.01593993	0.938163	-0.0920895	1
CCR7	1.017275	1.016987	1.01751366	1.0310006	0.04404523	1
GABRQ	1.022462	1.022712	1.02225521	0.9798885	-0.0293104	1
MCTP2	1.019856	1.0188807	1.02066194	1.0943425	0.13006433	1
FAM153B	1.016418	1.0169964	1.01593993	0.9378426	-0.0925823	1
LYST	1.116534	1.1162226	1.11679095	1.0048899	0.00703749	1
SOX21	1.017282	1.0170009	1.0175135	1.0301501	0.04285456	1
RP11-849H4.4	1.015555	1.0150885	1.01593993	1.0564324	0.07920045	1
RP11-296A18.6	1.01642	1.0170017	1.01593993	0.9375475	-0.0930362	1
ZNF674	1.03719	1.0380296	1.03649651	0.9596879	-0.0593628	1
RP11-212I21.4	1.024206	1.0246691	1.0238239	0.9657384	-0.0502957	1
CST5	1.015555	1.0150885	1.01593993	1.0564324	0.07920045	1
DUSP15	1.220432	1.2196654	1.22106444	1.0063691	0.00915955	1
MAP3K15	1.038015	1.0380065	1.03802145	1.0003926	0.00056635	1
SLC22A2	1.016412	1.0169845	1.01593993	0.9384981	-0.0915742	1
CRYAA	1.015555	1.0150885	1.01593993	1.0564324	0.07920045	1
CTB-178M22.2	1.093429	1.0933867	1.09346383	1.0008263	0.00119156	1
RP11-400N13.3	1.017273	1.016987	1.01750875	1.0307116	0.04364072	1
RP11-39E3.3	1.017282	1.0170067	1.01750913	1.0295417	0.04200231	1
CTD-2192J16.26	1.017279	1.0169894	1.01751831	1.03113	0.04422618	1
TMEM79	1.084803	1.0839702	1.08549008	1.0180997	0.02587889	1
RP11-374M1.4	1.01729	1.0169943	1.01753357	1.0317351	0.04507261	1
CTD-3193O13.1	1.016413	1.0169857	1.01593993	0.9384326	-0.0916749	1
SERPINB8	1.046732	1.0476011	1.04601513	0.9666825	-0.048886	1
AL354828.1	1.017278	1.0169873	1.01751745	1.0312096	0.0443376	1
RP11-231E6.1	1.016419	1.0169987	1.01593993	0.9377121	-0.092783	1
LINC00987	1.032009	1.0323044	1.03176458	0.9832897	-0.0243116	1
CCSER1	1.066647	1.0668455	1.06648336	0.9945828	-0.0078366	1
RAET1E-AS1	1.019	1.0188929	1.01908809	1.0103301	0.01482677	1
KB-1107E3.1	1.018168	1.0189529	1.01752013	0.9244059	-0.1134016	1
SERPINC1	1.017282	1.0169974	1.01751734	1.0305923	0.04347373	1
RP11-849I19.1	1.016417	1.0169941	1.01593993	0.9379677	-0.0923899	1
TRPM5	1.017278	1.0169859	1.01752006	1.0314456	0.04466777	1
AC025335.1	1.08039	1.0799794	1.0807288	1.00937	0.01345516	1
CTD-3088G3.8	1.015555	1.0150885	1.01593993	1.0564324	0.07920045	1
NCMAP	1.019863	1.0188795	1.02067435	1.0950702	0.13102332	1

KPNA7	1.015555	1.0150885	1.01593993	1.0564324	0.07920045	1
TNK2-AS1	1.015555	1.0150885	1.01593993	1.0564324	0.07920045	1
AGXT	1.016416	1.0169934	1.01593993	0.9380047	-0.092333	1
ADGRD1	1.027686	1.0284372	1.02706657	0.9518023	-0.0712662	1
DYSF	1.015555	1.0150885	1.01593993	1.0564324	0.07920045	1
RP4-677H15.4	1.016411	1.0169819	1.01593993	0.9386415	-0.0913539	1
CH17-140K24.5	1.017271	1.0169816	1.01751089	1.0311693	0.04428124	1
CTD-2033C11.1	1.030243	1.0303607	1.03014572	0.9929199	-0.0102507	1
DOK6	1.09507	1.0952985	1.09488053	0.9956137	-0.0063419	1
MOGAT1	1.017279	1.0169904	1.01751757	1.0310268	0.0440818	1
AP000704.5	1.048458	1.0476262	1.0491454	1.0318984	0.04530089	1
AC092667.2	1.017272	1.0169808	1.01751184	1.0312729	0.04442609	1
FRMPD1	1.016412	1.0169847	1.01593993	0.9384869	-0.0915915	1
CTNNA3	1.027685	1.0285824	1.02694444	0.9426943	-0.0851381	1
BRF1	1.176349	1.1755626	1.17699899	1.0081817	0.01175571	1
AP001065.15	1.015555	1.0150885	1.01593993	1.0564324	0.07920045	1
PDE2A	1.022502	1.022783	1.02226984	0.977478	-0.0328638	1
SCN11A	1.036395	1.0361753	1.03657706	1.0111049	0.01593267	1
CTD-2325A15.5	1.016414	1.016987	1.01593993	0.9383579	-0.0917899	1
RP11-245J9.6	1.025081	1.0246596	1.02542884	1.0311965	0.04431921	1
GABARAPL1	1.819864	1.8203197	1.81948769	0.9989858	-0.001464	1
IGLL1	1.020738	1.0208241	1.02066705	0.9924573	-0.010923	1
ARHGEF38	1.017295	1.0170041	1.01753482	1.0312125	0.04434168	1
RP5-971N18.3	1.016419	1.017	1.01593993	0.9376439	-0.092888	1
CLCA2	1.015555	1.0150885	1.01593993	1.0564324	0.07920045	1
NEURL3	1.020731	1.0208147	1.02066185	0.9926562	-0.010634	1
AC079807.2	1.029375	1.0284394	1.0301473	1.0600553	0.08413954	1
PPP1R2	1.857018	1.8580133	1.8561963	0.9978823	-0.0030584	1
RP11-301G7.1	1.017275	1.0169852	1.01751366	1.031115	0.04420522	1
AP000462.3	1.01642	1.0170019	1.01593993	0.9375399	-0.093048	1
CHADL	1.042379	1.0419389	1.04274309	1.0191748	0.0274015	1
AF186192.1	1.0251	1.0246591	1.02546371	1.0326287	0.04632153	1
MARK3	1.691109	1.6917644	1.69056693	0.9982689	-0.0024996	1
GMFG	1.037167	1.0380439	1.03644248	0.9579064	-0.0620433	1
RP11-561B11.6	1.015555	1.0150885	1.01593993	1.0564324	0.07920045	1
LINC00487	1.019864	1.0189015	1.02065911	1.0929882	0.1282778	1
AC012368.1	1.015555	1.0150885	1.01593993	1.0564324	0.07920045	1
TNN	1.019881	1.0189024	1.02068896	1.094518	0.13029565	1
CTD-2246P4.1	1.01728	1.0169996	1.01751099	1.0300816	0.04275864	1
ZP3	1.125285	1.1256902	1.12495004	0.9941115	-0.0085205	1
XPNPEP2	1.01642	1.0170014	1.01593993	0.9375641	-0.0930107	1
KANTR	1.019862	1.0188823	1.0206712	1.0947405	0.13058892	1
RAB7B	1.018142	1.0188953	1.01752006	0.9272185	-0.1090188	1
CTD-2194D22.3	1.017276	1.0169842	1.01751636	1.0313296	0.0445055	1
POMK	1.031129	1.0303559	1.03176664	1.0464719	0.06553354	1

DNAJB5-AS1	1.015555	1.0150885	1.01593993	1.0564324	0.07920045	1
C6orf222	1.016421	1.0170038	1.01593993	0.9374358	-0.0932081	1
BMP8A	1.014564	1.0150594	1.01415473	0.9399289	-0.0893764	1
RP11-473E2.2	1.014554	1.0150379	1.01415473	0.9412726	-0.0873154	1
RP3-337H4.10	1.015417	1.0150491	1.01572161	1.0446849	0.06306784	1
AC100830.5	1.016285	1.0169689	1.01572085	0.92645	-0.1102149	1
ARMC4	1.043868	1.0436286	1.04406502	1.0100039	0.01436092	1
AC005363.11	1.025861	1.0265732	1.02527348	0.9510885	-0.0723485	1
SPINK9	1.01715	1.0169398	1.01732371	1.0226633	0.03233122	1
PROM2	1.027509	1.0284194	1.02675643	0.9414836	-0.0869921	1
RP11-88E10.5	1.019728	1.0188582	1.02044691	1.0842433	0.11668847	1
FBXO40	1.0137	1.0131484	1.01415473	1.0765359	0.10639649	1
MYLK2	1.01542	1.0150451	1.01572966	1.0455013	0.06419492	1
KB-1440D3.14	1.014555	1.0150407	1.01415473	0.941092	-0.0875923	1
TBX20	1.015417	1.0150403	1.01572753	1.0456934	0.06445995	1
ACP7	1.014555	1.0150401	1.01415473	0.9411329	-0.0875296	1
AC084082.3	1.014555	1.0150407	1.01415473	0.941098	-0.0875832	1
DCAF4L1	1.018024	1.0188882	1.01731101	0.9164987	-0.1257952	1
RP11-108O10.2	1.014558	1.0150463	1.01415473	0.9407421	-0.0881288	1
CBR3	1.022281	1.0226428	1.02198282	0.9708542	-0.0426734	1
RP11-78O7.2	1.018871	1.018859	1.01888023	1.0011255	0.00162286	1
ANXA4	1.410218	1.4092261	1.41103717	1.0044256	0.00637076	1
RP5-966M1.6	1.016293	1.0169703	1.0157338	0.9271367	-0.109146	1
RP11-91P24.7	1.0137	1.0131484	1.01415473	1.0765359	0.10639649	1
RP11-309L24.4	1.0137	1.0131484	1.01415473	1.0765359	0.10639649	1
GPR62	1.018858	1.018842	1.01887151	1.0015683	0.00226082	1
LINC01136	1.021487	1.0208429	1.02201929	1.0564406	0.07921168	1
COL20A1	1.014556	1.0150411	1.01415473	0.9410682	-0.0876289	1
LINC00443	1.016272	1.016938	1.01572216	0.9282161	-0.1074673	1
RP11-560I19.1	1.017142	1.0169512	1.01729902	1.0205175	0.02930094	1
LDLRAD2	1.0137	1.0131484	1.01415473	1.0765359	0.10639649	1
CTD-2540B15.9	1.018875	1.0188494	1.01889693	1.0025213	0.00363288	1
RP3-325F22.5	1.014562	1.0150556	1.01415473	0.9401627	-0.0890176	1
RP11-214D15.2	1.014558	1.0150453	1.01415473	0.9408062	-0.0880305	1
VSTM5	1.018005	1.0188717	1.01728989	0.9161786	-0.1262993	1
RP4-777O23.1	1.014564	1.0150594	1.01415473	0.9399257	-0.0893814	1
RP11-77P6.2	1.09741	1.0970517	1.09770553	1.0067371	0.009687	1
AF165138.7	1.014555	1.015039	1.01415473	0.9412028	-0.0874225	1
RP11-805I24.1	1.016287	1.016957	1.0157338	0.9278639	-0.1080148	1
RP11-122K13.15	1.015419	1.0150489	1.01572439	1.0448856	0.06334505	1
LINCR-0001	1.015423	1.0150552	1.01572648	1.0445889	0.06293525	1
RPEL1	1.014559	1.0150492	1.01415473	0.9405609	-0.0884067	1
AIPL1	1.021452	1.0207606	1.02202298	1.0608047	0.08515902	1
RP6-24A23.6	1.0232	1.0226835	1.023626	1.0415509	0.05873337	1
RP4-564F22.7	1.0137	1.0131484	1.01415473	1.0765359	0.10639649	1

AC022007.5	1.048219	1.0475203	1.04879645	1.0268551	0.03823264	1
AC007204.2	1.0137	1.0131484	1.01415473	1.0765359	0.10639649	1
RP1-149A16.3	1.014557	1.0150441	1.01415473	0.9408822	-0.087914	1
GRK1	1.017162	1.0169408	1.01734388	1.0237919	0.03392245	1
LINC00519	1.0137	1.0131484	1.01415473	1.0765359	0.10639649	1
RP11-87C12.2	1.01801	1.0188649	1.01730486	0.9173037	-0.1245287	1
DUSP7	1.081866	1.081804	1.08191656	1.0013764	0.00198435	1
RP11-92F20.1	1.014555	1.0150395	1.01415473	0.9411692	-0.087474	1
MMP19	1.018011	1.0188597	1.01730938	0.9177965	-0.1237538	1
RP11-382A20.4	1.014561	1.015052	1.01415473	0.9403854	-0.0886759	1
RP11-61E11.2	1.018876	1.0188551	1.01889241	1.0019787	0.00285178	1
LINC01126	1.015421	1.0150532	1.01572393	1.0445591	0.06289418	1
LA16c-60D12.2	1.0223	1.0226279	1.02202958	0.9735574	-0.0386621	1
MAB21L3	1.014557	1.0150433	1.01415473	0.9409317	-0.0878381	1
C9orf170	1.0137	1.0131484	1.01415473	1.0765359	0.10639649	1
ITGA10	1.018032	1.0189182	1.01730071	0.9144991	-0.1289464	1
UCP1	1.014556	1.0150428	1.01415473	0.940964	-0.0877885	1
RP11-575F12.3	1.0137	1.0131484	1.01415473	1.0765359	0.10639649	1
C1QTNF2	1.018866	1.018867	1.01886566	0.9999306	-0.0001002	1
AC012442.6	1.014556	1.0150411	1.01415473	0.9410682	-0.0876289	1
RP5-965G21.3	1.020578	1.0207601	1.02042813	0.9840109	-0.0232538	1
NKX6-3	1.015419	1.0150461	1.0157273	1.0452743	0.06388159	1
RAX2	1.014571	1.0150759	1.01415473	0.9388984	-0.0909591	1
POLR1B	1.053334	1.0531141	1.0535153	1.0075533	0.01085616	1
RP11-323F24.3	1.02489	1.0245601	1.02516325	1.0245601	0.03500466	1
RP11-382A20.7	1.01889	1.0188877	1.01889157	1.0002044	0.00029493	1
AC011718.2	1.015426	1.0150556	1.01573225	1.0449422	0.06342308	1
FGF23	1.024893	1.0245647	1.02516435	1.0244119	0.03479592	1
HTR5A-AS1	1.0137	1.0131484	1.01415473	1.0765359	0.10639649	1
SCAMP1	1.561906	1.5625146	1.56140329	0.9980244	-0.002853	1
LINC01182	1.0137	1.0131484	1.01415473	1.0765359	0.10639649	1
RP11-123I22.1	1.0137	1.0131484	1.01415473	1.0765359	0.10639649	1
SOX30	1.0137	1.0131484	1.01415473	1.0765359	0.10639649	1
LINC01489	1.014557	1.0150452	1.01415473	0.9408122	-0.0880214	1
NCBP2-AS1	1.01456	1.0150508	1.01415473	0.9404649	-0.088554	1
WT1	1.014555	1.0150401	1.01415473	0.9411329	-0.0875296	1
AC006159.4	1.014557	1.0150441	1.01415473	0.9408822	-0.087914	1
PLSCR2	1.014554	1.0150383	1.01415473	0.9412437	-0.0873599	1
LINC00437	1.014558	1.0150458	1.01415473	0.9407771	-0.0880751	1
SLC9B1	1.053392	1.0531253	1.05361234	1.0091669	0.01316485	1
LINC00426	1.0137	1.0131484	1.01415473	1.0765359	0.10639649	1
CTD-3035K23.6	1.015418	1.0150407	1.01572973	1.0458147	0.06462722	1
PRDM13	1.018896	1.0189039	1.01888867	0.9991922	-0.0011659	1
RP11-701H24.4	1.01542	1.0150488	1.01572611	1.0450096	0.06351622	1
FAM159B	1.01542	1.0150461	1.01572873	1.0453692	0.0640126	1

KRBOX4	1.211368	1.2114081	1.21133539	0.9996559	-0.0004965	1
SP5	1.01542	1.0150503	1.01572611	1.044903	0.06336897	1
CDH12	1.039585	1.0398979	1.03932692	0.9856877	-0.0207974	1
RP5-1051H14.2	1.0137	1.0131484	1.01415473	1.0765359	0.10639649	1
RP11-182J1.13	1.0137	1.0131484	1.01415473	1.0765359	0.10639649	1
AP003419.16	1.018865	1.0188404	1.01888493	1.0023621	0.00340379	1
RP11-408H20.3	1.014559	1.0150476	1.01415473	0.9406661	-0.0882454	1
FAM90A26	1.0137	1.0131484	1.01415473	1.0765359	0.10639649	1
KLRG2	1.0137	1.0131484	1.01415473	1.0765359	0.10639649	1
RFPL3	1.015417	1.0150441	1.01572409	1.0451994	0.06377822	1
RP11-863P13.1	1.014563	1.0150571	1.01415473	0.9400699	-0.08916	1
SLC28A2	1.030108	1.0303235	1.02993093	0.9870555	-0.0187969	1
TTLL11	1.037917	1.0378349	1.03798559	1.0039829	0.00573469	1
CTB-60B18.12	1.0137	1.0131484	1.01415473	1.0765359	0.10639649	1
HGF	1.041439	1.0413525	1.0415102	1.0038136	0.00549136	1
BLZF1	1.182221	1.1823888	1.1820833	0.998325	-0.0024185	1
RP11-71E19.1	1.018068	1.018504	1.01770718	0.9569402	-0.0634993	1
RP11-93K22.6	1.020664	1.0203999	1.02088149	1.023608	0.03366331	1
RP11-274H2.3	1.022393	1.0222812	1.0224855	1.0091702	0.0131695	1
AC072062.1	1.045729	1.045223	1.0461462	1.020415	0.029156	1
TNFRSF11A	1.016333	1.016569	1.01613888	0.97404	-0.0379471	1
CYP2U1	1.177081	1.1770307	1.17712228	1.0005171	0.0007458	1
RP11-421F16.3	1.017196	1.0165759	1.01770824	1.0683114	0.09533224	1
CTD-2089N3.1	1.016337	1.0165762	1.01613888	0.9736156	-0.0385758	1
SLC4A9	1.015478	1.0146773	1.01613888	1.0995795	0.13695192	1
PRC1-AS1	1.018939	1.0184852	1.01931332	1.0448007	0.06322779	1
FGD3	1.019823	1.0204521	1.01930295	0.9438139	-0.0834257	1
RP11-398A8.5	1.016337	1.0165779	1.01613888	0.973517	-0.0387219	1
RP11-357N13.2	1.016335	1.0165726	1.01613888	0.9738292	-0.0382593	1
EEF1E1-BLOC1S5	1.016345	1.0165948	1.01613888	0.9725269	-0.0401899	1
RP11-54O7.16	1.017198	1.0165764	1.01771203	1.06851	0.09560045	1
RP11-353N14.4	1.01634	1.0165826	1.01613888	0.9732443	-0.0391261	1
AC007563.5	1.023242	1.0223236	1.02400105	1.0751425	0.10452783	1
RP11-469L4.1	1.016337	1.0165758	1.01613888	0.9736399	-0.0385398	1
AC006547.15	1.015478	1.0146773	1.01613888	1.0995795	0.13695192	1
RP11-297K8.2	1.021525	1.0223232	1.02086503	0.9346782	-0.0974583	1
PPM1B	1.525675	1.5247476	1.52644004	1.0032253	0.00464561	1
GUCA1B	1.019773	1.0203733	1.01927778	0.9462281	-0.0797401	1
RP1-136B1.1	1.017203	1.016575	1.01772169	1.0691806	0.09650554	1
LA16c-352F7.1	1.016336	1.0165754	1.01613888	0.9736672	-0.0384993	1
GJA5	1.023289	1.0223662	1.02405189	1.0753674	0.10482958	1
LINC01510	1.015478	1.0146773	1.01613888	1.0995795	0.13695192	1
CNR2	1.018072	1.0185073	1.01771195	0.9570225	-0.0633753	1
ZNF333	1.13747	1.1370688	1.13780121	1.0053434	0.0076883	1
CTD-2283N19.1	1.02241	1.0223365	1.02247052	1.0060008	0.00863144	1

RP11-736K20.6	1.031885	1.031822	1.03193763	1.0036342	0.00523358	1
HECW1	1.031897	1.0318659	1.03192179	1.0017529	0.00252675	1
ASPDH	1.017203	1.0165902	1.01770853	1.0674112	0.09411608	1
RP11-638I2.8	1.015478	1.0146773	1.01613888	1.0995795	0.13695192	1
RP11-174G6.1	1.018087	1.0185269	1.01772349	0.9566354	-0.063959	1
RP11-22A3.2	1.043177	1.0433549	1.04302993	0.9925039	-0.0108553	1
RP1-30M3.5	1.022396	1.0223209	1.0224579	1.0061371	0.00882685	1
RP11-1101H11.1	1.015478	1.0146773	1.01613888	1.0995795	0.13695192	1
CTA-126B4.7	1.016337	1.0165777	1.01613888	0.9735299	-0.0387028	1
RP11-54G14.1	1.016341	1.0165864	1.01613888	0.9730212	-0.0394569	1
AP001257.1	1.015478	1.0146773	1.01613888	1.0995795	0.13695192	1
MNX1-AS1	1.017196	1.0165844	1.01770141	1.0673519	0.09403595	1
RP4-530I15.9	1.016338	1.0165789	1.01613888	0.973459	-0.0388079	1
RP11-106M3.5	1.016343	1.0165893	1.01613888	0.9728501	-0.0397105	1
RP3-508I15.10	1.018916	1.0184697	1.01928374	1.0440723	0.06222167	1
IL3RA	1.018076	1.0185222	1.01770711	0.9559959	-0.0649237	1
INHBA-AS1	1.0172	1.0165768	1.01771493	1.0686551	0.09579638	1
NLRC3	1.018063	1.0184935	1.01770726	0.9574872	-0.0626749	1
ANXA8L1	1.045707	1.045197	1.0461289	1.0206197	0.02944542	1
RP5-1028K7.3	1.017197	1.0165772	1.01770812	1.0682202	0.09520901	1
HCN4	1.018076	1.0185196	1.01771013	0.9562887	-0.0644819	1
RP11-753C18.12	1.016338	1.0165792	1.01613888	0.9734422	-0.0388327	1
RP11-252A24.5	1.0172	1.0165745	1.01771722	1.0689414	0.09618275	1
GGN	1.038801	1.0394959	1.03822783	0.9678934	-0.04708	1
MEIG1	1.059548	1.0585177	1.06039906	1.0321501	0.04565284	1
TBC1D3D	1.015478	1.0146773	1.01613888	1.0995795	0.13695192	1
GCNT2	1.11058	1.1102336	1.11086527	1.0057302	0.0082434	1
CHRNB2	1.020654	1.0203733	1.02088501	1.0251157	0.03578674	1
LINC00613	1.015478	1.0146773	1.01613888	1.0995795	0.13695192	1
MYBBP1A	1.100234	1.1006318	1.09990532	0.9927805	-0.0104533	1
RP11-531F16.4	1.015478	1.0146773	1.01613888	1.0995795	0.13695192	1
RP11-282A11.4	1.017197	1.0165785	1.01770718	1.068082	0.09502238	1
MTRNR2L10	1.024123	1.0242178	1.02404525	0.9928756	-0.0103151	1
ARTN	1.017202	1.0165768	1.0177178	1.0688285	0.09603035	1
ITCH-AS1	1.019819	1.0204318	1.01931207	0.9451947	-0.0813166	1
RP11-557C18.4	1.022367	1.0222679	1.02244875	1.008121	0.01166884	1
AC083843.2	1.016337	1.0165768	1.01613888	0.9735796	-0.0386291	1
RP11-10C24.1	1.023226	1.0222561	1.02402726	1.0795832	0.11047438	1
RBMXL3	1.016336	1.0165756	1.01613888	0.9736536	-0.0385196	1
RP11-31K23.2	1.016341	1.0165851	1.01613888	0.9730929	-0.0393506	1
SLC22A8	1.019791	1.0203934	1.01929369	0.9460764	-0.0799713	1
AC005487.2	1.016337	1.0165759	1.01613888	0.9736343	-0.0385481	1
RP11-102M11.2	1.01982	1.02046	1.01929152	0.9428914	-0.0848365	1
TMEM249	1.017195	1.0165756	1.01770623	1.0682113	0.09519706	1
RP11-114N19.3	1.019799	1.0203917	1.01930979	0.9469449	-0.0786477	1

RP11-6O2.3	1.017205	1.0165789	1.01772236	1.0689707	0.09622228	1
MAT1A	1.024118	1.0242281	1.02402704	0.9916998	-0.0120246	1
RP11-99J16__A.2	1.015478	1.0146773	1.01613888	1.0995795	0.13695192	1
CTC-591M7.1	1.015478	1.0146773	1.01613888	1.0995795	0.13695192	1
MYO5C	1.19174	1.1924263	1.19117281	0.993486	-0.0094285	1
AIF1L	1.46224	1.4612393	1.46306566	1.0039598	0.00570147	1
RP11-540O11.1	1.015478	1.0146773	1.01613888	1.0995795	0.13695192	1
SLC8A1-AS1	1.020636	1.0203808	1.02084756	1.0229002	0.03266541	1
AC079630.4	1.017196	1.0165775	1.01770703	1.0681336	0.09509217	1
AC093642.3	1.023258	1.0223301	1.02402426	1.0758684	0.10550168	1
GFM1	1.273971	1.2744679	1.27356012	0.9966927	-0.0047794	1
CFAP45	1.027544	1.0280178	1.02715299	0.9691321	-0.0452348	1
RP4-569D19.8	1.018958	1.0184887	1.01934638	1.0463868	0.06541619	1
RASGEF1C	1.02067	1.020414	1.02088226	1.0229388	0.03271979	1
ABCA12	1.015478	1.0146773	1.01613888	1.0995795	0.13695192	1
RP11-469H8.6	1.089954	1.089301	1.0904926	1.0133432	0.01912284	1
KCNAB1-AS1	1.016333	1.016569	1.01613888	0.97404	-0.0379471	1
TMEM110-MUSTN1	1.020709	1.0204856	1.02089288	1.0198803	0.02839984	1
RP11-46O21.2	1.015478	1.0146773	1.01613888	1.0995795	0.13695192	1
RP11-427M20.1	1.016338	1.016579	1.01613888	0.9734509	-0.0388199	1
NME1-NME2	1.015478	1.0146773	1.01613888	1.0995795	0.13695192	1
ARL5C	1.016337	1.0165777	1.01613888	0.9735299	-0.0387028	1
RIBC1	1.052548	1.0527776	1.05235825	0.9920536	-0.0115101	1
UBE2F-SCLY	1.016344	1.0165919	1.01613888	0.9726983	-0.0399357	1
MAST4-AS1	1.045739	1.0452479	1.04614542	1.0198364	0.02833767	1
AC002306.1	1.016343	1.0165893	1.01613888	0.9728501	-0.0397105	1
CTB-102L5.4	1.016344	1.0165923	1.01613888	0.9726708	-0.0399765	1
PYHIN1	1.017199	1.0165769	1.01771195	1.0684691	0.09554522	1
LINC01481.1	1.024148	1.0242449	1.02406779	0.9926938	-0.0105793	1
RP11-379B18.6	1.0172	1.016577	1.01771379	1.0685741	0.09568702	1
RP4-613B23.5	1.013523	1.012846	1.01408228	1.0962353	0.13255752	1
SERPINB11	1.014381	1.0147422	1.01408228	0.9552358	-0.0660711	1
ZNF208	1.014381	1.0147424	1.01408228	0.9552235	-0.0660898	1
PCDHGA1	1.016992	1.0166753	1.01725336	1.0346647	0.04916337	1
RP11-955H22.1	1.014382	1.0147458	1.01408228	0.9550057	-0.0664187	1
TIGD7	1.105797	1.1062291	1.10544038	0.9925751	-0.0107518	1
MYL2	1.013523	1.012846	1.01408228	1.0962353	0.13255752	1
IFNL3	1.013523	1.012846	1.01408228	1.0962353	0.13255752	1
GPCPD1	1.199301	1.2001929	1.19856531	0.9918701	-0.011777	1
SPDYE5	1.01524	1.0147444	1.01565001	1.0614178	0.08599268	1
RP11-557N21.1	1.015248	1.0147553	1.01565508	1.0609807	0.08539838	1
RP11-425A23.1	1.015237	1.0147408	1.01564749	1.061509	0.08611667	1
LRMP	1.013523	1.012846	1.01408228	1.0962353	0.13255752	1
CTD-2532N20.1	1.019566	1.020487	1.01880576	0.9179364	-0.123534	1
LGALS12	1.013523	1.012846	1.01408228	1.0962353	0.13255752	1

ATP1A4	1.01524	1.014739	1.01565322	1.0620248	0.08681745	1
NPIPB3	1.013523	1.012846	1.01408228	1.0962353	0.13255752	1
AC012360.6	1.014382	1.0147451	1.01408228	0.9550482	-0.0663545	1
RP11-545G3.1	1.013523	1.012846	1.01408228	1.0962353	0.13255752	1
CTB-43E15.1	1.015239	1.014746	1.01564674	1.0610848	0.08553999	1
AC023481.1	1.020409	1.0204554	1.02037041	0.9958468	-0.0060043	1
EBF3	1.014385	1.0147517	1.01408228	0.9546208	-0.0670003	1
SEPT12	1.016104	1.0166537	1.0156497	0.9397107	-0.0897115	1
LINC00704	1.013523	1.012846	1.01408228	1.0962353	0.13255752	1
RP11-883G14.1	1.023071	1.0224831	1.02355701	1.0477646	0.06731465	1
PCDHGB5	1.014385	1.0147521	1.01408228	0.9545967	-0.0670367	1
DPPA3	1.014382	1.0147444	1.01408228	0.9550907	-0.0662904	1
RP11-192P3.4	1.014383	1.014747	1.01408228	0.9549275	-0.0665368	1
NTN4	1.024742	1.0242652	1.02513488	1.0358419	0.05080387	1
RP11-449L23.2	1.01438	1.0147401	1.01408228	0.9553747	-0.0658614	1
RFX1	1.274997	1.2740656	1.27576654	1.0062064	0.00892633	1
RP4-601P9.2	1.014387	1.0147568	1.01408228	0.9542894	-0.0675012	1
RP11-474D1.2	1.015244	1.0147521	1.01565025	1.0608849	0.0852681	1
RP3-402G11.25	1.015257	1.0147708	1.01565897	1.0601315	0.08424316	1
GNA14	1.015243	1.0147434	1.01565527	1.0618482	0.08657754	1
AC008269.2	1.017835	1.018556	1.01723889	0.9290205	-0.1062177	1
IQCC	1.097329	1.0968883	1.09769279	1.0083031	0.01192943	1
MIR670HG	1.016973	1.0166766	1.01721776	1.0324478	0.04606885	1
SLC12A5	1.192183	1.1923956	1.192008	0.9979854	-0.0029094	1
SYT3	1.027347	1.0280876	1.02673595	0.9518761	-0.0711542	1
SLA2	1.014384	1.0147491	1.01408228	0.9547877	-0.0667482	1
CGB2	1.013523	1.012846	1.01408228	1.0962353	0.13255752	1
RP11-431J24.2	1.014383	1.0147479	1.01408228	0.9548638	-0.0666332	1
GS1-24F4.2	1.023907	1.0243279	1.02355911	0.9683982	-0.0463277	1
SLC22A14	1.014381	1.0147422	1.01408228	0.9552358	-0.0660711	1
ABI3BP	1.013523	1.012846	1.01408228	1.0962353	0.13255752	1
SLC52A1	1.01524	1.014738	1.01565388	1.0621437	0.086979	1
ARSD	1.27588	1.2762187	1.27559955	0.9977584	-0.0032376	1
TMEM9B-AS1	1.039489	1.0396619	1.03934574	0.9920291	-0.0115457	1
KDR	1.05492	1.0547442	1.05506566	1.0058723	0.00844712	1
WWTR1-AS1	1.021308	1.0204945	1.0219806	1.0725112	0.10099271	1
CSAG1	1.014383	1.014747	1.01408228	0.9549275	-0.0665368	1
LRIT1	1.013523	1.012846	1.01408228	1.0962353	0.13255752	1
RP5-994D16.12	1.01438	1.01474	1.01408228	0.9553808	-0.0658521	1
RP11-556E13.1	1.015241	1.01474	1.01565461	1.0620524	0.0868549	1
GOLGA8M	1.015244	1.0147506	1.01565063	1.0610186	0.08544989	1
PLCB2	1.040278	1.0395496	1.04088019	1.0336424	0.04773717	1
RP11-797D24.4	1.016961	1.016641	1.01722566	1.0351366	0.04982118	1
RP11-370I10.10	1.0161	1.016641	1.01565291	0.9406253	-0.088308	1
DENND2D	1.013523	1.012846	1.01408228	1.0962353	0.13255752	1

RP11-406D1.2	1.014383	1.0147464	1.01408228	0.9549632	-0.0664829	1
RP11-747D18.1	1.014382	1.014746	1.01408228	0.9549906	-0.0664415	1
CTD-2616J11.14	1.016982	1.0166881	1.01722504	1.0321781	0.04569192	1
OPN4	1.014383	1.014747	1.01408228	0.9549275	-0.0665368	1
PDGFRL	1.016099	1.0166399	1.01565256	0.9406635	-0.0882494	1
BDH1	1.073073	1.073867	1.07241734	0.9803743	-0.0285954	1
RP1-12G14.7	1.016111	1.0166747	1.01564481	0.9382338	-0.0919806	1
RP11-43N5.1	1.013523	1.012846	1.01408228	1.0962353	0.13255752	1
RP11-3D4.2	1.013523	1.012846	1.01408228	1.0962353	0.13255752	1
RP1-140K8.5	1.013523	1.012846	1.01408228	1.0962353	0.13255752	1
RP3-393E18.2	1.013523	1.012846	1.01408228	1.0962353	0.13255752	1
CYP3A43	1.016098	1.0166416	1.01565001	0.9404167	-0.088628	1
ZSCAN29	1.148236	1.1483635	1.14813101	0.9984332	-0.0022622	1
AC004160.4	1.013523	1.012846	1.01408228	1.0962353	0.13255752	1
GSG2	1.027295	1.0280541	1.02666887	0.9506234	-0.0730541	1
RP11-445P19.3	1.013523	1.012846	1.01408228	1.0962353	0.13255752	1
RP11-256I23.3	1.016111	1.0166701	1.01565015	0.9388134	-0.0910897	1
RP11-479J7.2	1.015249	1.0147503	1.01566155	1.0617759	0.08647927	1
RP11-553L6.2	1.013523	1.012846	1.01408228	1.0962353	0.13255752	1
LRRIQ4	1.013523	1.012846	1.01408228	1.0962353	0.13255752	1
SLC22A4	1.022131	1.0223456	1.02195338	0.9824463	-0.0255495	1
DDX31	1.091114	1.0909763	1.09122759	1.0027619	0.00397904	1
WTH3DI	1.014379	1.014739	1.01408228	0.955441	-0.0657613	1
RP11-118E18.2	1.014382	1.0147444	1.01408228	0.9550907	-0.0662904	1
CTD-2302E22.6	1.020412	1.0204712	1.02036239	0.9946829	-0.0076914	1
MC5R	1.014385	1.0147509	1.01408228	0.9546723	-0.0669225	1
ZNF410	1.029083	1.0281673	1.02983953	1.0593678	0.08320363	1
LINC00266-1	1.014379	1.014739	1.01408228	0.95544	-0.0657628	1
RAB44	1.013523	1.012846	1.01408228	1.0962353	0.13255752	1
BMP8B	1.015238	1.014739	1.01565015	1.0618165	0.08653451	1
RP11-344B5.4	1.014381	1.0147422	1.01408228	0.9552358	-0.0660711	1
RP11-230B22.1	1.017836	1.0185645	1.01723373	0.9283167	-0.107311	1
RP11-269F20.1	1.01438	1.0147396	1.01408228	0.9554048	-0.065816	1
RP11-1348G14.5	1.015241	1.0147402	1.01565388	1.0619888	0.08676852	1
RP11-354P11.4	1.013523	1.012846	1.01408228	1.0962353	0.13255752	1
APLNR	1.025671	1.0262933	1.02515731	0.9567942	-0.0637194	1
ZXDB	1.06618	1.0662116	1.06615414	0.9991322	-0.0012525	1
MR1	1.025607	1.0261971	1.02511959	0.9588699	-0.060593	1
SMAD5-AS1	1.021265	1.0204482	1.02193871	1.0728935	0.10150684	1
C16orf89	1.013523	1.012846	1.01408228	1.0962353	0.13255752	1
LINC00504	1.029921	1.0299937	1.02986173	0.9955996	-0.0063625	1
RP4-816N1.7	1.013523	1.012846	1.01408228	1.0962353	0.13255752	1
BHMG1	1.014387	1.0147566	1.01408228	0.9543018	-0.0674825	1
LGR6	1.018683	1.0185489	1.01879341	1.0131793	0.01888949	1
IL4I1	1.017822	1.0185472	1.0172232	0.928615	-0.1068475	1

CDC14A	1.097185	1.0966835	1.09759866	1.0094654	0.01359152	1
OXNAD1	1.130078	1.1291262	1.13086412	1.0134588	0.01928744	1
CTB-22K21.2	1.016118	1.0166891	1.01564674	0.9375436	-0.0930424	1
RP11-21M24.2	1.016112	1.016664	1.01565685	0.9395597	-0.0899433	1
RP11-366L5.1	1.016099	1.0166428	1.01564944	0.9403142	-0.0887852	1
AC003088.1	1.014383	1.014747	1.01408228	0.9549275	-0.0665368	1
GNAT3	1.013523	1.012846	1.01408228	1.0962353	0.13255752	1
RP11-21L23.3	1.07831	1.0777645	1.07876033	1.0128052	0.01835674	1
RP11-624D11.2	1.029061	1.0281337	1.02982592	1.06015	0.08426843	1
RP11-444E17.6	1.015251	1.0147536	1.01566155	1.0615405	0.08615937	1
LINC00354	1.018773	1.0180243	1.0193919	1.0758773	0.10551353	1
RP11-227D13.1	1.050006	1.0505689	1.0495406	0.9796649	-0.0296397	1
XAGE3	1.019642	1.0199444	1.0193919	0.9722986	-0.0405287	1
RP11-123K3.9	1.01963	1.0199177	1.0193919	0.9735999	-0.0385991	1
RBMXL2	1.022237	1.021824	1.02257793	1.0345463	0.04899824	1
RP11-519M16.1	1.018773	1.0180243	1.0193919	1.0758773	0.10551353	1
ZNF669	1.156741	1.1560318	1.15732725	1.0083024	0.01192832	1
ANKRD20A3	1.032718	1.0335088	1.03206553	0.9569272	-0.0635189	1
RMRP	1.027484	1.0276185	1.02737304	0.9911132	-0.0128782	1
RP11-338I21.1	1.018773	1.0180243	1.0193919	1.0758773	0.10551353	1
TEX35	1.019632	1.0199233	1.0193919	0.9733263	-0.0390045	1
BAALC-AS1	1.0335	1.033351	1.03362222	1.0081332	0.01168626	1
RP11-15I11.3	1.020496	1.0199343	1.02096018	1.051463	0.07239806	1
RP1-50J22.4	1.021355	1.0218295	1.0209635	0.960328	-0.0584008	1
RP11-344F13.1	1.018773	1.0180243	1.0193919	1.0758773	0.10551353	1
ZFP92	1.018773	1.0180243	1.0193919	1.0758773	0.10551353	1
MCCC1-AS1	1.019633	1.0199251	1.0193919	0.9732395	-0.0391332	1
SFTA1P	1.023982	1.0237335	1.02418716	1.0191151	0.02731703	1
ZNF670-ZNF695	1.027496	1.0276851	1.02734013	0.98754	-0.018089	1
RP11-227H15.4	1.02225	1.0218747	1.02255917	1.0312904	0.0444506	1
RP11-456N14.4	1.018773	1.0180243	1.0193919	1.0758773	0.10551353	1
EN2	1.019639	1.0199379	1.0193919	0.9726162	-0.0400575	1
ATP5J2-PTCD1	1.021374	1.0218721	1.02096203	0.9583935	-0.06131	1
SFTPA2	1.019641	1.0199417	1.0193919	0.9724278	-0.0403369	1
PPP1R3D	1.121924	1.1213955	1.12235992	1.0079441	0.01141565	1
SYT16	1.057895	1.0584253	1.05745741	0.9834346	-0.024099	1
ANTXRL	1.023098	1.0237353	1.02257226	0.9509991	-0.0724841	1
AC009014.3	1.019632	1.0199227	1.0193919	0.9733584	-0.038957	1
NADK2-AS1	1.018773	1.0180243	1.0193919	1.0758773	0.10551353	1
RP11-465L10.10	1.051754	1.0524817	1.05115329	0.9746872	-0.0369888	1
AC006126.3	1.019634	1.0199274	1.0193919	0.9731298	-0.0392958	1
RP11-572C15.5	1.01963	1.0199184	1.0193919	0.9735679	-0.0386464	1
CTD-2589M5.5	1.019641	1.0199417	1.0193919	0.9724278	-0.0403369	1
CSMD1	1.019637	1.0199334	1.0193919	0.9728323	-0.039737	1
DDX39B-AS1	1.018773	1.0180243	1.0193919	1.0758773	0.10551353	1

RP11-15J22.8	1.021379	1.0218838	1.0209618	0.9578681	-0.0621011	1
NOS3	1.044715	1.0448215	1.04462627	0.995644	-0.0062981	1
RP11-498C9.13	1.019629	1.019916	1.0193919	0.9736865	-0.0384708	1
EHF	1.018773	1.0180243	1.0193919	1.0758773	0.10551353	1
RP11-143A12.3	1.019633	1.0199245	1.0193919	0.9732716	-0.0390857	1
KLHL6-AS1	1.020502	1.0199456	1.02096203	1.0509596	0.07170726	1
FAM3D	1.029219	1.0296001	1.02890506	0.9765186	-0.0342806	1
RP11-596D21.1	1.024853	1.025759	1.02410507	0.9357918	-0.0957405	1
KBTBD12	1.019634	1.0199274	1.0193919	0.9731298	-0.0392958	1
CTC-297N7.9	1.01963	1.0199177	1.0193919	0.9735999	-0.0385991	1
CTD-2213F21.2	1.020491	1.0199209	1.02096165	1.052244	0.07346927	1
CTD-2537I9.12	1.023973	1.0237775	1.02413378	1.0149857	0.02145942	1
APBA1	1.057798	1.0581449	1.05751153	0.9891066	-0.0158021	1
CFTR	1.018773	1.0180243	1.0193919	1.0758773	0.10551353	1
SLC43A1	1.047406	1.0467128	1.04797865	1.0270988	0.03857495	1
RP5-1039K5.17	1.018773	1.0180243	1.0193919	1.0758773	0.10551353	1
RP11-569G13.2	1.019634	1.0199279	1.0193919	0.9731028	-0.0393358	1
RP11-206L10.9	1.169774	1.1694719	1.17002311	1.0032528	0.00468511	1
AGAP9	1.114393	1.1142646	1.11449989	1.0020592	0.00296774	1
OTUD7A	1.053573	1.052566	1.05440413	1.0349678	0.04958595	1
CYP2J2	1.019636	1.0199325	1.0193919	0.972878	-0.0396693	1
RP11-309M7.1	1.019633	1.0199251	1.0193919	0.9732395	-0.0391332	1
NXNL1	1.019643	1.0199462	1.0193919	0.9722119	-0.0406573	1
RP11-452F19.3	1.11955	1.1197924	1.1193494	0.9963023	-0.0053445	1
AC012507.3	1.024849	1.0257159	1.02413402	0.9384877	-0.0915903	1
GPR50-AS1	1.021371	1.0218518	1.02097351	0.9598087	-0.0591812	1
GSN-AS1	1.020498	1.01992	1.02097593	1.05301	0.07451917	1
RP11-114O18.1	1.023985	1.0237769	1.02415752	1.0160085	0.02291242	1
RP11-758P17.2	1.018773	1.0180243	1.0193919	1.0758773	0.10551353	1
LINC00951	1.020501	1.0199258	1.02097648	1.0527312	0.07413714	1
POFUT1	1.225174	1.2247627	1.22551418	1.0033436	0.00481578	1
DHRS7C	1.021365	1.0218623	1.02095502	0.958499	-0.0611512	1
RP11-131K5.2	1.019631	1.0199211	1.0193919	0.9734358	-0.0388422	1
RAPGEF5	1.049927	1.0505044	1.0494508	0.9791377	-0.0304164	1
RP11-220I1.5	1.0231	1.0237564	1.02255838	0.9495698	-0.0746541	1
CCDC144NL	1.022249	1.0218808	1.02255261	1.0307048	0.04363113	1
CTC1	1.06914	1.0697873	1.06860511	0.9830604	-0.0246481	1
RP11-517B11.4	1.029987	1.0294526	1.03042918	1.0331562	0.04705834	1
PABPC1L2A	1.02486	1.0257252	1.0241456	0.9385957	-0.0914242	1
C17orf82	1.073472	1.073729	1.07326003	0.9936388	-0.0092067	1
ZFAND1	1.562526	1.5633825	1.56181843	0.9972238	-0.0040108	1
WBSCR27	1.053404	1.0523283	1.05429163	1.0375185	0.05313709	1
PRSS48	1.019631	1.01992	1.0193919	0.9734906	-0.0387611	1
MAS1	1.023971	1.0237413	1.02416032	1.0176485	0.02523939	1
PTPN12	1.599935	1.6011472	1.59893381	0.996318	-0.0053217	1

FAM71F1	1.019636	1.019932	1.0193919	0.9729008	-0.0396354	1
RP4-778K6.3	1.019635	1.0199296	1.0193919	0.97302	-0.0394586	1
STK32A	1.024001	1.0238158	1.02415383	1.0141938	0.02033335	1
TXNDC2	1.018773	1.0180243	1.0193919	1.0758773	0.10551353	1
RP11-767C1.1	1.018773	1.0180243	1.0193919	1.0758773	0.10551353	1
RP4-612B15.3	1.036962	1.0371857	1.03677785	0.9890318	-0.0159112	1
CTD-2349P21.11	1.0214	1.021904	1.02098398	0.9579962	-0.0619081	1
GYS1	1.258215	1.2575977	1.25872433	1.0043735	0.00629584	1
RP4-734G22.3	1.020507	1.0199401	1.02097538	1.0519175	0.07302157	1
SIGLEC14	1.019637	1.0199343	1.0193919	0.9727908	-0.0397986	1
AOAH	1.022228	1.0218217	1.02256366	1.0339993	0.04823523	1
CTC-436K13.1	1.019633	1.0199245	1.0193919	0.9732716	-0.0390857	1
CTD-3193O13.11	1.023094	1.0237591	1.02254439	0.9488751	-0.0757099	1
RP11-305F18.1	1.021383	1.0218732	1.02097858	0.9590995	-0.0602476	1
PGBD2	1.102153	1.1023947	1.10195278	0.9956846	-0.0062393	1
IGSF22	1.019629	1.0199171	1.0193919	0.9736319	-0.0385517	1
RP11-197K3.1	1.019639	1.0199379	1.0193919	0.9726162	-0.0400575	1
TBC1D3I	1.018773	1.0180243	1.0193919	1.0758773	0.10551353	1
RP11-182N22.8	1.019638	1.0199353	1.0193919	0.9727421	-0.0398707	1
RP4-760C5.3	1.019632	1.0199233	1.0193919	0.9733263	-0.0390045	1
CXorf21	1.018773	1.0180243	1.0193919	1.0758773	0.10551353	1
GSG1L	1.040412	1.0409854	1.03993779	0.9744386	-0.0373567	1
RP1-197B17.4	1.0205	1.0199251	1.02097406	1.0526446	0.07401847	1
ACOT9	1.260481	1.261464	1.25966879	0.993134	-0.0099398	1
FIBCD1	3.383011	3.3838858	3.3822878	0.9993297	-0.0009674	1
OPLAH	1.072631	1.0717904	1.07332589	1.0213892	0.03053267	1
HCLS1	1.020494	1.0199233	1.02096574	1.0523212	0.07357516	1
RP11-419I17.1	1.071554	1.0715194	1.07158264	1.0008836	0.00127422	1
PHOX2A	1.020487	1.019916	1.02095794	1.0523189	0.07357202	1
AJ239322.3	1.019634	1.0199267	1.0193919	0.9731619	-0.0392483	1
SH3D19	1.315375	1.3149564	1.31572087	1.0024273	0.00349764	1
GP6	1.017458	1.0167676	1.01802739	1.0751315	0.10451316	1
AC009299.2	1.018311	1.0186664	1.0180183	0.965281	-0.050979	1
CCDC152	1.019176	1.0186519	1.01960797	1.0512575	0.07211608	1
RP11-20I20.4	1.020041	1.0205887	1.01958921	0.9514532	-0.0717955	1
RP5-894D12.5	1.016589	1.0167639	1.01644507	0.9809833	-0.0276995	1
CITF22-1A6.3	1.022676	1.0225357	1.0227927	1.0114037	0.016359	1
SEC14L4	1.020042	1.0205695	1.01960642	0.9531791	-0.0691808	1
LIPE-AS1	1.202744	1.2022569	1.20314687	1.0044004	0.00633448	1
RP5-1050D4.5	1.023531	1.0244649	1.02275937	0.930285	-0.1042554	1
ZKSCAN8	1.171427	1.1715334	1.17133924	0.9988679	-0.0016342	1
RP11-164P12.3	1.016592	1.0167692	1.01644507	0.9806712	-0.0281585	1
SCGB1A1	1.016594	1.016775	1.01644507	0.9803311	-0.028659	1
RP11-803B1.8	1.083235	1.0838097	1.08276013	0.9874771	-0.0181808	1
GBX2	1.016597	1.0167818	1.01644507	0.9799354	-0.0292415	1

AC092295.4	1.018328	1.0187059	1.01801536	0.9630827	-0.0542685	1
RP11-136L23.2	1.019192	1.018679	1.01961623	1.0501728	0.0706267	1
VIPR1	1.016593	1.0167726	1.01644507	0.980474	-0.0284488	1
DIEXF	1.125661	1.1258182	1.12553197	0.997725	-0.0032859	1
PLD2	1.152321	1.1526866	1.15201929	0.9956293	-0.0063194	1
TSPEAR	1.016588	1.0167615	1.01644507	0.9811193	-0.0274995	1
RP4-693M11.3	1.01659	1.0167647	1.01644507	0.9809338	-0.0277723	1
IAPP	1.033864	1.0339151	1.03382118	0.9972304	-0.0040012	1
RP11-48G14.1	1.016593	1.0167712	1.01644507	0.980557	-0.0283265	1
CXCR6	1.016597	1.0167813	1.01644507	0.9799628	-0.0292011	1
RP11-122K13.12	1.016592	1.0167697	1.01644507	0.980644	-0.0281987	1
RP11-629N8.3	1.015731	1.0148661	1.01644507	1.1062118	0.14562762	1
PCDH20	1.016589	1.0167623	1.01644507	0.9810776	-0.0275609	1
RP11-871F6.3	1.017456	1.0167712	1.0180217	1.0745656	0.10375352	1
RP11-217B1.2	1.026083	1.0262937	1.02590813	0.9853372	-0.0213106	1
LINC00602	1.016599	1.0167845	1.01644507	0.9797758	-0.0294764	1
ZNF646	1.088427	1.0875889	1.08911863	1.0174644	0.02497829	1
RP11-192H23.7	1.015731	1.0148661	1.01644507	1.1062118	0.14562762	1
TGM3	1.020052	1.0206104	1.01959106	0.9505443	-0.0731742	1
AC084149.1	1.015731	1.0148661	1.01644507	1.1062118	0.14562762	1
CTD-2033D15.1	1.016597	1.0167803	1.01644507	0.9800218	-0.0291142	1
RP11-401P9.5	1.016595	1.0167757	1.01644507	0.9802938	-0.028714	1
RP11-789C17.3	1.016589	1.0167623	1.01644507	0.9810776	-0.0275609	1
MRGPRF-AS1	1.01745	1.0167712	1.01801057	1.0739019	0.10286218	1
SUN3	1.01659	1.0167654	1.01644507	0.9808955	-0.0278287	1
MLIP-AS1	1.015731	1.0148661	1.01644507	1.1062118	0.14562762	1
AMZ1	1.01659	1.0167655	1.01644507	0.9808855	-0.0278434	1
MOGAT2	1.015731	1.0148661	1.01644507	1.1062118	0.14562762	1
AP000866.1	1.026928	1.0263135	1.02743629	1.0426711	0.06028419	1
RP11-392O17.1	1.015731	1.0148661	1.01644507	1.1062118	0.14562762	1
RP11-379K17.4	1.019194	1.0187033	1.01959996	1.0479402	0.06755645	1
TECPR2	1.207501	1.2077256	1.20731526	0.9980246	-0.0028527	1
CYP26C1	1.015731	1.0148661	1.01644507	1.1062118	0.14562762	1
RHPN2	1.122219	1.1219666	1.1224281	1.0037842	0.00544913	1
RP11-62I21.1	1.016588	1.0167602	1.01644507	0.9811959	-0.0273868	1
RP11-675F6.3	1.019186	1.0186656	1.01961555	1.0508947	0.07161808	1
AC109333.10	1.015731	1.0148661	1.01644507	1.1062118	0.14562762	1
IL25	1.016599	1.0167863	1.01644507	0.979671	-0.0296308	1
KCNJ11	1.019202	1.0187033	1.01961341	1.0486591	0.06854575	1
RP3-329A5.8	1.03042	1.0301494	1.03064317	1.016378	0.02343712	1
AP002954.3	1.016589	1.0167629	1.01644507	0.9810377	-0.0276195	1
RP11-120K24.3	1.018355	1.0187598	1.01802068	0.9606004	-0.0579917	1
AC105393.1	1.015731	1.0148661	1.01644507	1.1062118	0.14562762	1
CPA3	1.015731	1.0148661	1.01644507	1.1062118	0.14562762	1
HAPLN1	1.019182	1.0186737	1.01960134	1.0496783	0.06994721	1

LINC01214	1.016587	1.0167578	1.01644507	0.9813379	-0.0271781	1
RP11-48B3.5	1.041607	1.0415295	1.04167034	1.0033921	0.00488551	1
SLC25A47	1.015731	1.0148661	1.01644507	1.1062118	0.14562762	1
AC009312.1	1.018331	1.0187033	1.01802279	0.9636146	-0.0534719	1
CFAP99	1.01659	1.0167655	1.01644507	0.9808855	-0.0278434	1
KLK11	1.019167	1.0186535	1.01959102	1.0502594	0.07074567	1
RP11-90K6.1	1.02007	1.0206367	1.01960145	0.9498349	-0.0742514	1
RP13-131K19.6	1.02093	1.0206007	1.02120246	1.0292096	0.04153678	1
RP4-597N16.4	1.026952	1.0263047	1.02748653	1.04493	0.06340635	1
SNX11	1.180914	1.1811287	1.18073731	0.9978394	-0.0031205	1
CDK2AP1	1.202317	1.2018832	1.20267445	1.0039193	0.00564331	1
RP11-362K14.5	1.021802	1.0225634	1.0211735	0.9383987	-0.0917271	1
CTD-2017F17.1	1.016591	1.0167674	1.01644507	0.9807753	-0.0280054	1
RP11-470P21.2	1.015731	1.0148661	1.01644507	1.1062118	0.14562762	1
NKPD1	1.017451	1.0167712	1.01801188	1.0739799	0.10296699	1
ZFP28	1.099709	1.0991806	1.10014518	1.009726	0.01396387	1
RP11-334J6.6	1.015731	1.0148661	1.01644507	1.1062118	0.14562762	1
AP001628.7	1.015731	1.0148661	1.01644507	1.1062118	0.14562762	1
ANKRD34C	1.018313	1.0186566	1.01802924	0.9663735	-0.0493472	1
RP11-587P21.3	1.015731	1.0148661	1.01644507	1.1062118	0.14562762	1
KRT79	1.016592	1.0167697	1.01644507	0.980644	-0.0281987	1
TBC1D26.1	1.01659	1.0167656	1.01644507	0.9808794	-0.0278524	1
CTD-3185P2.1	1.055501	1.0551251	1.05581188	1.0124587	0.01786301	1
RP11-632L2.2	1.052925	1.0530887	1.05279042	0.9943816	-0.0081285	1
ADPRHL1	1.027844	1.0282759	1.02748779	0.9721268	-0.0407836	1
RP11-332K15.1	1.018321	1.0186908	1.01801558	0.963874	-0.0530836	1
RP11-390F4.10	1.018315	1.0186786	1.01801481	0.9644602	-0.0522063	1
GTSE1-AS1	1.020914	1.0205686	1.02119899	1.030646	0.04354886	1
WDR83	1.396509	1.3971454	1.39598302	0.9970731	-0.0042287	1
RP11-428K3.1	1.018318	1.0186704	1.01802707	0.9655417	-0.0505895	1
RP11-403A3.1	1.015731	1.0148661	1.01644507	1.1062118	0.14562762	1
MIR940	1.044279	1.0435678	1.04486664	1.0298117	0.04238062	1
ASTE1	1.070251	1.0702404	1.07025926	1.0002684	0.00038724	1
SYTL4	1.021763	1.0224626	1.0211861	0.943171	-0.0844087	1
LINC00589	1.015731	1.0148661	1.01644507	1.1062118	0.14562762	1
RP11-431K24.3	1.015731	1.0148661	1.01644507	1.1062118	0.14562762	1
AC074366.3	1.015731	1.0148661	1.01644507	1.1062118	0.14562762	1
RP11-118G23.1	1.017453	1.0167607	1.01802504	1.0754353	0.10492067	1
RP11-88G17.6	1.017451	1.0167629	1.01801984	1.0749814	0.10431164	1
ALS2CR12	1.016599	1.0167844	1.01644507	0.979782	-0.0294674	1
RP11-104H15.8	1.016592	1.0167692	1.01644507	0.9806712	-0.0281585	1
CTD-2555O16.1	1.019177	1.0186732	1.01959214	1.0492106	0.06930424	1
FGF1	1.025241	1.0244202	1.02591823	1.0613427	0.08589058	1
RP1-267L14.6	1.02091	1.0205774	1.02118514	1.0295357	0.0419939	1
RP11-318C24.2	1.020045	1.0205695	1.01961263	0.9534819	-0.0687226	1

RP11-160E2.19	1.017448	1.0167616	1.0180155	1.0748062	0.10407658	1
RP11-1L9.1	1.018313	1.0186599	1.01802737	0.9660997	-0.049756	1
LINC01332	1.015731	1.0148661	1.01644507	1.1062118	0.14562762	1
RP11-116D17.5	1.016596	1.0167798	1.01644507	0.9800512	-0.0290709	1
RP4-655J12.4	1.016597	1.01678	1.01644507	0.98004	-0.0290874	1
RP11-613D13.10	1.017443	1.0167616	1.01800503	1.074182	0.10323847	1
SYBU	1.054572	1.0549181	1.05428689	0.9885064	-0.0166777	1
RP11-2E11.5	1.019192	1.0187029	1.01959515	1.0477044	0.06723171	1
GUCA2A	1.015731	1.0148661	1.01644507	1.1062118	0.14562762	1
RP11-95P2.3	1.018313	1.0186598	1.01802737	0.9661097	-0.0497411	1
OR4D10	1.016595	1.0167757	1.01644507	0.9802892	-0.0287206	1
RP11-627D16.1	1.020169	1.0198148	1.02046074	1.0325968	0.04627703	1
TMEM256-PLSCR3.1	1.017555	1.0178591	1.01730453	0.9689478	-0.0455091	1
RP11-864J10.4	1.017547	1.0178395	1.01730453	0.9700105	-0.0439277	1
PXT1	1.018412	1.0178387	1.01888549	1.058682	0.08226936	1
LINC01539	1.017547	1.0178395	1.01730453	0.9700105	-0.0439277	1
MLN	1.020151	1.0197546	1.02047818	1.0366279	0.0518982	1
RP11-89H19.2	1.016686	1.0159362	1.01730453	1.0858594	0.11883736	1
RP11-314C16.1	1.072928	1.0732282	1.07267955	0.9925075	-0.0108501	1
RP11-129M16.4	1.021851	1.0216273	1.0220366	1.018927	0.02705074	1
AF131217.1	1.040918	1.0408393	1.04098232	1.0035031	0.005045	1
FAM166B	1.045295	1.0447967	1.04570625	1.0203047	0.0290001	1
COL6A6	1.0219	1.0216939	1.02207085	1.0173748	0.02485133	1
TBC1D25	1.103953	1.1037066	1.10415682	1.0043413	0.00624957	1
RP11-254F7.3	1.016686	1.0159362	1.01730453	1.0858594	0.11883736	1
RP11-798K3.4	1.01929	1.0197907	1.01887597	0.9537808	-0.0682704	1
TRMT2B	1.071976	1.0712741	1.07255639	1.0179911	0.02572492	1
RP11-218L14.4	1.020992	1.0216495	1.02044839	0.9445182	-0.0823496	1
ABHD18	1.119828	1.1193801	1.1201981	1.0068523	0.00985207	1
RP11-262K1.1	1.016686	1.0159362	1.01730453	1.0858594	0.11883736	1
IMPG2	1.023594	1.0235978	1.0235904	0.9996881	-0.00045	1
PRR27	1.016686	1.0159362	1.01730453	1.0858594	0.11883736	1
GIPC2	1.018412	1.0178326	1.01889009	1.0593007	0.0831122	1
RP11-799M12.2	1.019286	1.0197945	1.01886633	0.9531118	-0.0692826	1
CTC-265N9.1	1.016686	1.0159362	1.01730453	1.0858594	0.11883736	1
RP11-495O11.1	1.017548	1.0178418	1.01730453	0.9698883	-0.0441096	1
FOXD1	1.018412	1.0178391	1.01888504	1.0586346	0.0822047	1
LINC01358	1.020169	1.0197784	1.0204907	1.0360155	0.05104555	1
CTD-2547L24.4	1.01755	1.0178472	1.01730453	0.9695925	-0.0445495	1
SLC13A5	1.023626	1.023612	1.0236384	1.0011173	0.00161095	1
RP11-218I7.2	1.01755	1.0178469	1.01730453	0.9696075	-0.0445272	1
RP11-242J7.1	1.019273	1.0197433	1.01888388	0.9564719	-0.0642054	1
RP11-367G18.1	1.020983	1.0216448	1.02043645	0.9441735	-0.082876	1
CD5	1.0184	1.0178297	1.01887179	1.0584462	0.0819479	1
TNS4	1.019302	1.0198009	1.01888933	0.9539633	-0.0679943	1

MROH8	1.150823	1.1515945	1.1501859	0.9907078	-0.0134685	1
LRRC8E	1.040022	1.0408056	1.0393751	0.964944	-0.0514829	1
PLA2G2C	1.017546	1.0178377	1.01730453	0.9701073	-0.0437837	1
ZSCAN5B	1.017544	1.0178331	1.01730453	0.9703617	-0.0434055	1
RTEL1	1.017553	1.0178542	1.01730453	0.9692138	-0.0451132	1
RP11-75C23.1	1.016686	1.0159362	1.01730453	1.0858594	0.11883736	1
PLA2G1B	1.016686	1.0159362	1.01730453	1.0858594	0.11883736	1
AC012456.4	1.018413	1.0178487	1.01887943	1.0577468	0.08099429	1
AC241585.1	1.016686	1.0159362	1.01730453	1.0858594	0.11883736	1
PRIMA1	1.016686	1.0159362	1.01730453	1.0858594	0.11883736	1
ZNF341	1.039114	1.0388199	1.0393566	1.0138266	0.01981089	1
ADH1C	1.016686	1.0159362	1.01730453	1.0858594	0.11883736	1
ZNF107	1.140461	1.140353	1.14054934	1.0013989	0.00201685	1
NPC1L1	1.017549	1.0178461	1.01730453	0.9696538	-0.0444583	1
RP11-700J17.2	1.030489	1.0311955	1.02990486	0.9586275	-0.0609577	1
RP11-166N6.2	1.017543	1.0178317	1.01730453	0.9704375	-0.0432928	1
CFHR1	1.025339	1.0255099	1.02519875	0.9878027	-0.0177052	1
CYP4F26P	1.017545	1.0178371	1.01730453	0.970143	-0.0437306	1
AMBP	1.017547	1.0178395	1.01730453	0.9700105	-0.0439277	1
RP11-506H20.1	1.020119	1.019729	1.02044015	1.0360438	0.05108493	1
CTB-70G10.1	1.016686	1.0159362	1.01730453	1.0858594	0.11883736	1
PTCHD1	1.020147	1.0197539	1.02047148	1.0363264	0.05147844	1
PNPLA2	1.298003	1.2969532	1.29886905	1.0064517	0.00927794	1
GDNF-AS1	1.016686	1.0159362	1.01730453	1.0858594	0.11883736	1
PGAM4	1.021022	1.0216845	1.02047564	0.9442544	-0.0827525	1
CPPED1	1.142009	1.1420286	1.1419928	0.9997482	-0.0003632	1
TNXB	1.078313	1.0792597	1.07753134	0.9781933	-0.0318086	1
LINC00458	1.021006	1.0216813	1.02044786	0.9431098	-0.0845024	1
ACOT6	1.017543	1.0178319	1.01730453	0.9704226	-0.0433149	1
HNF4A	1.016686	1.0159362	1.01730453	1.0858594	0.11883736	1
ADCY9	1.03658	1.0369683	1.03625867	0.9808047	-0.0279623	1
KCNT2	1.193575	1.1942078	1.19305327	0.9940552	-0.0086021	1
C3orf80	1.018409	1.0178371	1.01888124	1.0585386	0.08207388	1
RP1-56K13.2	1.018415	1.0178472	1.01888427	1.0581074	0.08148601	1
LPXN	1.082429	1.0829303	1.08201429	0.9889539	-0.0160248	1
AZGP1	1.020148	1.0197668	1.02046324	1.0352306	0.04995211	1
PTAFR	1.017549	1.0178461	1.01730453	0.9696538	-0.0444583	1
AC005013.5	1.017553	1.0178532	1.01730453	0.9692659	-0.0450356	1
CCDC30	1.368108	1.3675759	1.36854719	1.0026423	0.00380697	1
ACTN1-AS1	1.016686	1.0159362	1.01730453	1.0858594	0.11883736	1
CTRL	1.019266	1.0197454	1.01887057	0.9556948	-0.0653781	1
RP11-235E17.6	1.017542	1.0178291	1.01730453	0.97058	-0.043081	1
RP11-407N8.6	1.017545	1.0178353	1.01730453	0.9702397	-0.0435868	1
RP11-810M2.2	1.016686	1.0159362	1.01730453	1.0858594	0.11883736	1
RUBCNL	1.01927	1.0197352	1.01888549	0.9569434	-0.0634945	1

SHPRH	1.833256	1.8337163	1.83287538	0.9989913	-0.0014559	1
AP001469.7	1.02188	1.0216855	1.02204116	1.0164026	0.02347194	1
RP11-54O7.17	1.027059	1.0274039	1.02677489	0.9770477	-0.0334991	1
AC012360.4	1.028009	1.0275148	1.02841676	1.0327807	0.04653397	1
RP11-63L7.5	1.019269	1.0197337	1.01888582	0.9570328	-0.0633597	1
RP5-1056L3.1	1.017546	1.0178387	1.01730453	0.9700566	-0.0438591	1
RP11-583F2.2	1.018413	1.0178395	1.01888746	1.0587422	0.08235139	1
HNRNPCL2	1.016686	1.0159362	1.01730453	1.0858594	0.11883736	1
GLYAT	1.016686	1.0159362	1.01730453	1.0858594	0.11883736	1
SMIM11B	1.031392	1.0312343	1.0315223	1.0092211	0.01324226	1
FRAT2	1.050406	1.0503372	1.05046333	1.0025057	0.00361049	1
PLIN4	1.016686	1.0159362	1.01730453	1.0858594	0.11883736	1
ZBTB39	1.145582	1.145834	1.14537466	0.9968504	-0.004551	1
VAV1	1.016686	1.0159362	1.01730453	1.0858594	0.11883736	1
ANKDD1B	1.016686	1.0159362	1.01730453	1.0858594	0.11883736	1
FSTL5	1.027979	1.027413	1.02844717	1.0377246	0.05342361	1
GBP7	1.020149	1.0197319	1.02049272	1.0385598	0.0545843	1
XXbac-BPG170G13.32	1.020152	1.019774	1.02046501	1.0349447	0.04955372	1
AGBL1	1.021916	1.0216614	1.0221263	1.0214624	0.03063604	1
SLC6A14	1.016686	1.0159362	1.01730453	1.0858594	0.11883736	1
CMB9-55A18.1	1.017551	1.0178487	1.01730453	0.9695104	-0.0446718	1
PARP12	1.02361	1.0235754	1.02363819	1.0026637	0.00383787	1
RP11-254F7.4	1.018412	1.017839	1.01888504	1.0586358	0.08220633	1
RP11-111K18.2	1.021012	1.0216811	1.02045941	0.9436516	-0.0836737	1
RP11-218C14.5	1.017549	1.0178453	1.01730453	0.9696987	-0.0443915	1
RP11-17E3.1	1.018409	1.0178507	1.01887057	1.057135	0.08015963	1
RP11-20I23.8	1.024465	1.0235693	1.02520467	1.0693836	0.09677939	1
RP11-297A16.2	1.020158	1.0197744	1.02047565	1.035463	0.05027597	1
CTC-487M23.7	1.01755	1.0178482	1.01730453	0.9695369	-0.0446323	1
SRL	1.016686	1.0159362	1.01730453	1.0858594	0.11883736	1
SUV39H2	1.324873	1.3255172	1.32434112	0.996387	-0.0052219	1
MAGEA10	1.017859	1.0181676	1.01760343	0.9689471	-0.0455102	1
LINC00871	1.019594	1.0201074	1.01916969	0.9533641	-0.0689007	1
CTD-2058B24.3	1.018715	1.0181552	1.01917725	1.0562935	0.07901069	1
PLAC1	1.017861	1.0181728	1.01760343	0.9686713	-0.045921	1
ITK	1.016995	1.0162589	1.01760343	1.0826967	0.11462917	1
RP11-89G4.1	1.017861	1.0181728	1.01760343	0.9686713	-0.045921	1
TDRD10	1.018725	1.0181761	1.01917797	1.0551173	0.07740339	1
RP11-12L8.1	1.017852	1.0181535	1.01760343	0.9697013	-0.0443877	1
TBX5-AS1	1.019604	1.0201211	1.0191772	0.9530899	-0.0693157	1
LDHC	1.017852	1.018153	1.01760343	0.9697261	-0.0443508	1
RP11-463C8.7	1.017855	1.0181586	1.01760343	0.9694271	-0.0447958	1
RP11-65L3.2	1.023044	1.0239003	1.0223368	0.9345809	-0.0976086	1
LLNLR-307A6.1	1.017857	1.0181641	1.01760343	0.9691329	-0.0452336	1
KLF10	1.45028	1.4500718	1.45045184	1.0008443	0.00121757	1

RP11-547D23.1	1.016995	1.0162589	1.01760343	1.0826967	0.11462917	1
ZNF474	1.023939	1.0239869	1.02390023	0.9963872	-0.0052216	1
RP11-25G10.2	1.017856	1.018161	1.01760343	0.9692971	-0.0449892	1
KCNU1	1.017855	1.0181602	1.01760343	0.9693423	-0.0449219	1
RP3-329E20.2	1.017858	1.0181668	1.01760343	0.9689868	-0.0454511	1
RP1-292L20.3	1.023028	1.0238726	1.02233013	0.9353874	-0.0963642	1
RP11-572O6.1	1.023049	1.0239141	1.02233493	0.9339634	-0.0985621	1
SERPINA3.1	1.016995	1.0162589	1.01760343	1.0826967	0.11462917	1
RP11-499A10.3	1.017854	1.0181573	1.01760343	0.9694971	-0.0446915	1
WARS2-IT1	1.017857	1.0181641	1.01760343	0.9691329	-0.0452336	1
ACRV1	1.016995	1.0162589	1.01760343	1.0826967	0.11462917	1
RP11-794P6.6	1.018716	1.0181562	1.01917827	1.0562957	0.07901371	1
VRTN	1.017855	1.0181588	1.01760343	0.9694169	-0.0448109	1
TBPL2	1.019574	1.0200638	1.01916908	0.9554043	-0.0658167	1
DNAH12	1.017857	1.0181647	1.01760343	0.9691024	-0.045279	1
LINC00642	1.01786	1.0181707	1.01760343	0.9687814	-0.0457569	1
ERVK-28	1.017851	1.0181519	1.01760343	0.9697858	-0.0442619	1
TMC3-AS1	1.023909	1.0238976	1.02391929	1.0009072	0.00130821	1
GLIS1	1.122891	1.1238851	1.12206993	0.9853484	-0.0212942	1
EPB41L4A-AS2	1.027394	1.0277429	1.02710502	0.9770073	-0.0335587	1
ANKRD13A	1.200629	1.2000116	1.20113876	1.0056355	0.00810746	1
RP11-141O15.1	1.016995	1.0162589	1.01760343	1.0826967	0.11462917	1
RP11-46J23.1	1.043863	1.0430039	1.0445731	1.0364907	0.05170718	1
GOLGA8K	1.017856	1.0181626	1.01760343	0.9692122	-0.0451155	1
RP11-465B22.8	1.016995	1.0162589	1.01760343	1.0826967	0.11462917	1
PLPPR4	1.018739	1.0182014	1.01918262	1.0539102	0.07575194	1
RP3-523C21.2	1.016995	1.0162589	1.01760343	1.0826967	0.11462917	1
ISL2	1.019593	1.0200977	1.0191765	0.9541615	-0.0676946	1
AP000679.2	1.016995	1.0162589	1.01760343	1.0826967	0.11462917	1
LRFN5	1.068158	1.0680644	1.06823605	1.0025212	0.00363277	1
RP11-863P13.3	1.017852	1.0181535	1.01760343	0.9697013	-0.0443877	1
RP11-463J10.3	1.016995	1.0162589	1.01760343	1.0826967	0.11462917	1
RP1-293L6.1	1.017854	1.0181565	1.01760343	0.9695377	-0.0446312	1
CTD-2233C11.2	1.017858	1.018166	1.01760343	0.9690321	-0.0453836	1
AL023806.1	1.022151	1.0219617	1.0223069	1.0157174	0.02249905	1
PLG	1.017856	1.018161	1.01760343	0.9692971	-0.0449892	1
FBXL14	1.023022	1.0238639	1.02232731	0.9356106	-0.0960199	1
RP5-965G21.4	1.016995	1.0162589	1.01760343	1.0826967	0.11462917	1
HBA2	1.018723	1.0181698	1.01917967	1.0555775	0.0780325	1
ARMS2	1.017853	1.0181557	1.01760343	0.9695817	-0.0445656	1
RP11-424D14.1	1.017854	1.0181568	1.01760343	0.9695219	-0.0446546	1
TWNK	1.067284	1.0680076	1.06668619	0.9805691	-0.0283087	1
THSD1	1.035137	1.0353515	1.03495947	0.9889115	-0.0160866	1
RP11-568J23.4	1.017853	1.0181555	1.01760343	0.9695919	-0.0445504	1
LHB	1.016995	1.0162589	1.01760343	1.0826967	0.11462917	1

TPO	1.023887	1.0238583	1.02391087	1.0022022	0.00317367	1
AC016999.2	1.017855	1.0181588	1.01760343	0.9694137	-0.0448156	1
FOXR1	1.019581	1.0200603	1.01918433	0.9563339	-0.0644137	1
RP11-477D19.2	1.045513	1.0448459	1.04606409	1.0271648	0.03866773	1
SCHIP1	1.016995	1.0162589	1.01760343	1.0826967	0.11462917	1
NPHP3-AS1	1.016995	1.0162589	1.01760343	1.0826967	0.11462917	1
RS1	1.016995	1.0162589	1.01760343	1.0826967	0.11462917	1
TACR2	1.018719	1.0181651	1.01917557	1.0556247	0.07809696	1
AC016751.3	1.017854	1.0181565	1.01760343	0.9695377	-0.0446312	1
TMEM136	1.575397	1.5752687	1.57550388	1.0004089	0.00058975	1
RP11-81H14.1	1.018719	1.0181637	1.01917679	1.0557763	0.0783042	1
RP11-575I8.1	1.017853	1.0181548	1.01760343	0.9696314	-0.0444917	1
RP11-659E9.2	1.020438	1.0200834	1.02073166	1.0322761	0.04582884	1
AC007040.6	1.017857	1.0181651	1.01760343	0.969082	-0.0453094	1
SLC39A5	1.019592	1.0200987	1.0191741	0.9539956	-0.0679455	1
TRPM2	1.016995	1.0162589	1.01760343	1.0826967	0.11462917	1
RP11-524H19.2	1.016995	1.0162589	1.01760343	1.0826967	0.11462917	1
RP11-96B5.4	1.016995	1.0162589	1.01760343	1.0826967	0.11462917	1
RP11-85L21.4	1.017857	1.0181637	1.01760343	0.9691547	-0.0452012	1
GSTA3	1.016995	1.0162589	1.01760343	1.0826967	0.11462917	1
CTD-2516F10.4	1.016995	1.0162589	1.01760343	1.0826967	0.11462917	1
RP11-220I1.2	1.017857	1.0181634	1.01760343	0.969168	-0.0451813	1
ACSM6	1.018726	1.0181712	1.01918488	1.0557873	0.07831921	1
RP11-411H5.1	1.018723	1.0181533	1.01919346	1.057296	0.08037936	1
RP11-536K7.3	1.021342	1.0219967	1.02080187	0.9456827	-0.0805719	1
RP11-243A14.1	1.019571	1.0200545	1.01917132	0.9559619	-0.0649749	1
MS4A7	1.016995	1.0162589	1.01760343	1.0826967	0.11462917	1
TMEM238	1.056106	1.056722	1.05559789	0.9801819	-0.0288786	1
RP11-379F4.9	1.02477	1.0238686	1.02551422	1.0689454	0.09618823	1
RP11-260M19.1	1.017855	1.0181586	1.01760343	0.9694271	-0.0447958	1
RP3-461P17.9	1.017858	1.0181665	1.01760343	0.9690072	-0.0454207	1
AC008753.4	1.016995	1.0162589	1.01760343	1.0826967	0.11462917	1
RP11-164O23.8	1.017855	1.0181597	1.01760343	0.9693672	-0.0448849	1
CACNA1I	1.017853	1.0181562	1.01760343	0.9695569	-0.0446025	1
RP11-320M16.2	1.016995	1.0162589	1.01760343	1.0826967	0.11462917	1
RP11-2C24.7	1.023104	1.0239843	1.02237648	0.9329634	-0.1001077	1
CTD-2568A17.1	1.021328	1.0219533	1.02081174	0.9480023	-0.0770375	1
PLA2G4B	1.017866	1.0181843	1.01760343	0.9680572	-0.0468357	1
LINC00968	1.016995	1.0162589	1.01760343	1.0826967	0.11462917	1
SLC25A41	1.018717	1.0181586	1.01917797	1.0561377	0.07879794	1
RP11-320N7.2	1.017854	1.0181565	1.01760343	0.9695377	-0.0446312	1
SCRG1	1.153687	1.153875	1.15353255	0.9977747	-0.0032141	1
SPRY3	1.053489	1.0526779	1.05415946	1.0281246	0.04001513	1
CYP51A1	1.05673	1.0562931	1.05709108	1.0141761	0.02030815	1
LINC01415	1.02045	1.020075	1.0207591	1.0340778	0.04834473	1

TMIGD2	1.026473	1.0257888	1.0270384	1.048456	0.06826633	1
SNAI3-AS1	1.087055	1.0869748	1.08712082	1.0016789	0.00242011	1
SPOUT1	1.239353	1.24014	1.23870217	0.9940124	-0.0086643	1
MASP1	1.025652	1.0258428	1.02549437	0.9865175	-0.0195835	1
RP11-90L1.8	1.018722	1.0181676	1.01917967	1.0557085	0.07821157	1
RP5-823G15.5	1.016995	1.0162589	1.01760343	1.0826967	0.11462917	1
ADAMTS19-AS1	1.014523	1.0142645	1.01473598	1.0330553	0.04691748	1
U52111.14	1.0128	1.0123699	1.01315569	1.0635259	0.08885521	1
RP11-211N8.2	1.041218	1.0408913	1.04148766	1.0145831	0.02088698	1
C15orf62	1.017975	1.0180988	1.01787364	0.987561	-0.0180582	1
CD52	1.061923	1.0618203	1.06200745	1.003028	0.00436182	1
SPATA21	1.018826	1.018087	1.01943645	1.0746097	0.10381276	1
COL4A3BP	1.514523	1.5155173	1.51370099	0.9964766	-0.0050921	1
SAXO1	1.018841	1.0180889	1.01946149	1.0758775	0.1055138	1
ABCA10	1.014521	1.0142685	1.01472876	1.0322587	0.0458046	1
KLRB1	1.013662	1.0142741	1.01315569	0.9216482	-0.1177119	1
LINC01433	1.0128	1.0123699	1.01315569	1.0635259	0.08885521	1
MYOZ2	1.0128	1.0123699	1.01315569	1.0635259	0.08885521	1
RAD51AP2	1.016237	1.0161633	1.0162981	1.0083379	0.01197921	1
HAUS3	1.14642	1.1457414	1.14697961	1.0084961	0.0122055	1
AC004893.11	1.045594	1.0447524	1.04628885	1.0343322	0.04869957	1
HHIPL2	1.0128	1.0123699	1.01315569	1.0635259	0.08885521	1
BFSP2	1.016235	1.0161671	1.0162917	1.0077089	0.011079	1
CTB-111F10.1	1.01366	1.0142703	1.01315569	0.9218959	-0.1173242	1
DOK3	1.017966	1.018087	1.01786546	0.9877514	-0.0177801	1
GAL3ST1	1.016248	1.0161676	1.01631363	1.0090347	0.01297572	1
XKRX	1.015383	1.0161725	1.01473175	0.9109141	-0.1346131	1
EFCAB6-AS1	1.013658	1.014266	1.01315569	0.9221679	-0.1168987	1
RP11-29B2.6	1.02663	1.0257398	1.02736509	1.0631435	0.08833628	1
ORC1	1.058463	1.0579828	1.05885867	1.0151058	0.0216301	1
RP11-10A14.3	1.01366	1.0142707	1.01315569	0.9218658	-0.1173713	1
RP11-849F2.4	1.01366	1.0142714	1.01315569	0.9218234	-0.1174377	1
MFSD8	1.179295	1.1802927	1.1784703	0.989892	-0.014657	1
AC025165.8	1.01452	1.0142678	1.01472744	1.0322141	0.04574221	1
PRKG1-AS1	1.01366	1.0142712	1.01315569	0.9218358	-0.1174184	1
NAV2-AS1	1.013661	1.0142739	1.01315569	0.9216605	-0.1176926	1
RP11-422J15.1	1.016242	1.0161697	1.01630088	1.0081116	0.01165536	1
RP11-316M1.3	1.01624	1.0161539	1.01631113	1.0097318	0.01397217	1
ATP2B2	1.013662	1.0142752	1.01315569	0.9215756	-0.1178256	1
RP11-566J3.2	1.015386	1.0161729	1.01473705	0.9112206	-0.1341278	1
LINC00265	1.018831	1.018065	1.01946363	1.0774211	0.10758228	1
BTRC	1.201533	1.2009459	1.20201693	1.0053299	0.00766899	1
U95743.1	1.016271	1.0162012	1.01632936	1.0079105	0.01136748	1
XXbac-B33L19.4	1.013661	1.0142725	1.01315569	0.9217509	-0.1175512	1
GTF3C2-AS1	1.013655	1.0142598	1.01315569	0.9225716	-0.1162673	1

RP11-407N8.5	1.013657	1.0142649	1.01315569	0.9222402	-0.1167855	1
CACNG2	1.013662	1.0142746	1.01315569	0.9216181	-0.1177591	1
METTL7A	1.049958	1.050638	1.04939633	0.975479	-0.0358172	1
RP5-857K21.4	1.01366	1.0142712	1.01315569	0.9218358	-0.1174184	1
RP11-1M18.1	1.0128	1.0123699	1.01315569	1.0635259	0.08885521	1
RP4-784A16.2	1.0128	1.0123699	1.01315569	1.0635259	0.08885521	1
CTD-2047H16.4	1.037822	1.0372006	1.03833572	1.0305139	0.04336393	1
RP11-329N22.1	1.013658	1.0142674	1.01315569	0.9220832	-0.1170312	1
RP11-388P9.2	1.013662	1.0142754	1.01315569	0.9215633	-0.1178449	1
LPCAT1	1.401635	1.4009705	1.40218283	1.0030234	0.00435528	1
CTB-167B5.1	1.014517	1.0142622	1.0147269	1.0325809	0.04625485	1
RP11-797D24.3	1.0128	1.0123699	1.01315569	1.0635259	0.08885521	1
RP11-313P22.1	1.013666	1.0142835	1.01315569	0.9210417	-0.1186616	1
RP11-304L19.12	1.014522	1.014268	1.01473239	1.032547	0.04620742	1
STPG3	1.01366	1.0142701	1.01315569	0.9219082	-0.1173049	1
SLC6A7	1.013657	1.0142638	1.01315569	0.9223126	-0.1166724	1
AC007193.10	1.01972	1.019974	1.01950943	0.9767418	-0.0339508	1
TCN1	1.0128	1.0123699	1.01315569	1.0635259	0.08885521	1
LINC00407	1.013655	1.0142605	1.01315569	0.9225293	-0.1163333	1
RP13-379L11.2	1.013659	1.0142683	1.01315569	0.9220231	-0.1171252	1
TPSG1	1.014519	1.0142703	1.01472381	1.0317831	0.04513978	1
SATB2-AS1	1.013655	1.0142605	1.01315569	0.9225293	-0.1163333	1
TOR4A	1.062873	1.0620882	1.06352075	1.0230731	0.0329093	1
TUBB8	1.015379	1.0161722	1.01472451	0.9104826	-0.1352967	1
RP11-1103G16.1	1.014515	1.01426	1.01472543	1.0326396	0.04633679	1
ADAMTSL2	1.017962	1.0180621	1.01787949	0.9898877	-0.0146632	1
LCN15	1.019757	1.0201309	1.01944866	0.9661114	-0.0497385	1
GAN	1.385833	1.3865342	1.3852539	0.9966878	-0.0047865	1
XX-C00717C00720L.1	1.01366	1.0142703	1.01315569	0.9218959	-0.1173242	1
AC007292.7	1.0128	1.0123699	1.01315569	1.0635259	0.08885521	1
RP11-317N12.1	1.015396	1.0162057	1.0147269	0.9087492	-0.1380459	1
USH1C	1.014528	1.0142788	1.01473434	1.0319046	0.04530953	1
RP11-81A22.5	1.068058	1.0677794	1.06828845	1.0075099	0.01079395	1
LILRA2	1.015381	1.0161686	1.014731	0.9110897	-0.134335	1
RP13-465B17.5	1.017106	1.0161774	1.01787252	1.1047859	0.14376676	1
RP11-46D6.1	1.017118	1.0161994	1.01787701	1.1035632	0.14216927	1
RP11-771K4.1	1.039556	1.0390071	1.04000853	1.0256721	0.03656955	1
IDO1	1.0128	1.0123699	1.01315569	1.0635259	0.08885521	1
SLC23A3	1.016253	1.0162012	1.01629502	1.0057888	0.00832735	1
MRAP	1.01366	1.0142712	1.01315569	0.9218358	-0.1174184	1
RP11-105N14.2	1.013663	1.0142767	1.01315569	0.9214796	-0.1179759	1
LRRC38	1.016245	1.0161751	1.01630181	1.0078327	0.01125618	1
AC034228.2	1.013662	1.0142746	1.01315569	0.9216181	-0.1177591	1
RP4-635E18.9	1.013662	1.0142741	1.01315569	0.9216482	-0.1177119	1
UTS2	1.01366	1.0142712	1.01315569	0.9218358	-0.1174184	1

BSCL2	1.057609	1.0580041	1.0572831	0.987569	-0.0180465	1
ZDHHC16	1.498311	1.4982403	1.49836959	1.0002595	0.00037436	1
FAM196B	1.0128	1.0123699	1.01315569	1.0635259	0.08885521	1
RP11-1100L3.7	1.013659	1.0142685	1.01315569	0.9220108	-0.1171445	1
CTD-2201E9.1	1.01366	1.0142701	1.01315569	0.9219082	-0.1173049	1
RP11-103H7.2	1.0128	1.0123699	1.01315569	1.0635259	0.08885521	1
ADCY10	1.013658	1.0142658	1.01315569	0.9221856	-0.1168709	1
PSAPL1	1.013657	1.0142633	1.01315569	0.9223426	-0.1166254	1
RP11-555G19.1	1.0128	1.0123699	1.01315569	1.0635259	0.08885521	1
AP001891.1	1.0128	1.0123699	1.01315569	1.0635259	0.08885521	1
FYB	1.0128	1.0123699	1.01315569	1.0635259	0.08885521	1
ZNF30-AS1	1.015376	1.0161602	1.01472876	0.9114232	-0.133807	1
RP11-7F17.5	1.014523	1.0142703	1.01473175	1.0323401	0.04591828	1
RP11-600F24.7	1.035213	1.0352768	1.03516109	0.9967212	-0.004738	1
RP11-10L7.1	1.0128	1.0123699	1.01315569	1.0635259	0.08885521	1
AC113608.1	1.0128	1.0123699	1.01315569	1.0635259	0.08885521	1
CH507-42P11.7	1.013655	1.0142593	1.01315569	0.9226015	-0.1162204	1
CPEB2-AS1	1.016256	1.0162101	1.01629401	1.0051778	0.00745074	1
RAB6C	1.014516	1.0142638	1.01472412	1.0322715	0.04582245	1
KCND3	1.022283	1.021886	1.02261017	1.0330884	0.04696375	1
ZBTB45	1.165574	1.1648699	1.16615594	1.0078002	0.01120963	1
EIF2B5-AS1	1.015372	1.0161546	1.01472543	0.9115335	-0.1336325	1
RP11-479O9.4	1.019735	1.020057	1.01946924	0.9706955	-0.0429093	1
RP11-1277A3.1	1.020608	1.0199959	1.02111268	1.0558497	0.07840445	1
CTC-308K20.1	1.030944	1.0315283	1.03046232	0.9661883	-0.0496236	1
SLC26A4	1.027424	1.0276036	1.02727534	0.9881081	-0.0172592	1
CTD-2631K10.1	1.013657	1.0142651	1.01315569	0.9222279	-0.1168047	1
AWAT2	1.0128	1.0123699	1.01315569	1.0635259	0.08885521	1
GGT2	1.0128	1.0123699	1.01315569	1.0635259	0.08885521	1
RP11-271F18.1	1.013661	1.0142736	1.01315569	0.9216783	-0.1176648	1
PKHD1	1.0128	1.0123699	1.01315569	1.0635259	0.08885521	1
DSG3	1.0128	1.0123699	1.01315569	1.0635259	0.08885521	1
RBM14-RBM4	1.02315	1.0238218	1.02259435	0.9484728	-0.0763217	1
RP11-265N7.1	1.016243	1.016179	1.01629586	1.0072218	0.01038147	1
SLC7A4	1.023968	1.0237618	1.02413901	1.0158731	0.02272021	1
NCDN	1.094673	1.0943617	1.09493046	1.0060275	0.00866978	1
RP11-93K22.13	1.019676	1.0199637	1.01943869	0.9737012	-0.0384489	1
PYGM	1.092267	1.0925477	1.0920354	0.9944641	-0.0080088	1
RP4-710M16.2	1.013651	1.0132159	1.01401033	1.0601115	0.08421601	1
RP11-637O19.3	1.012791	1.0132207	1.01243594	0.9406439	-0.0882795	1
RP11-57A19.7	1.017087	1.0170354	1.0171296	1.0055291	0.00795486	1
PLA2G4E-AS1	1.012794	1.0132274	1.01243594	0.9401649	-0.0890143	1
NPR1	1.011932	1.0113227	1.01243594	1.0983165	0.13529388	1
LINC00678	1.012793	1.0132247	1.01243594	0.9403578	-0.0887183	1
C2CD2	1.19544	1.1959529	1.19501589	0.9952184	-0.0069149	1

CTD-2293H3.2	1.01279	1.0132178	1.01243594	0.9408498	-0.0879636	1
RP11-70D24.3	1.015374	1.0151327	1.01557397	1.0291569	0.0414629	1
GABRB1	1.021391	1.0208358	1.02184932	1.0486412	0.06852118	1
DENND1C	1.083452	1.0836496	1.08328886	0.9956874	-0.0062352	1
RP11-847H18.2	1.016266	1.0170921	1.01558286	0.9116966	-0.1333743	1
LINC01039	1.012791	1.0132213	1.01243594	0.9405972	-0.088351	1
C2orf80	1.015374	1.0151282	1.01557621	1.0296162	0.0421066	1
RP11-1149O23.4	1.011932	1.0113227	1.01243594	1.0983165	0.13529388	1
DNAJB7	1.015385	1.0151469	1.0155816	1.028696	0.04081674	1
RP11-527F13.1	1.012793	1.0132256	1.01243594	0.9402915	-0.08882	1
TEX22	1.062792	1.0627159	1.06285433	1.0022078	0.00318173	1
ARHGAP30	1.013654	1.0132241	1.01400886	1.0593447	0.08317208	1
CTB-129O4.1	1.074928	1.0742415	1.07549504	1.0168842	0.02415534	1
C6orf47-AS1	1.013655	1.0132287	1.01400677	1.0588151	0.0824507	1
RESP18	1.012795	1.0132294	1.01243594	0.9400246	-0.0892296	1
AMDHD1	1.0171	1.0170204	1.01716594	1.0085515	0.01228478	1
CTD-2651B20.3	1.059347	1.0589649	1.05966239	1.0118288	0.01696516	1
RP11-803P9.1	1.011932	1.0113227	1.01243594	1.0983165	0.13529388	1
RP11-65N13.8	1.012791	1.0132213	1.01243594	0.9405972	-0.088351	1
METTL24	1.017096	1.0170313	1.01714998	1.0069663	0.01001543	1
ADNP	1.96655	1.966275	1.96677774	1.0005203	0.00075048	1
KLHL1	1.012789	1.0132171	1.01243594	0.9408977	-0.0878902	1
BARX1	1.019701	1.0189259	1.02034092	1.0747653	0.10402168	1
DLEU7-AS1	1.01794	1.0170098	1.0187083	1.0998517	0.137309	1
RP11-927P21.2	1.015392	1.015136	1.01560258	1.0308239	0.0437979	1
GABRG2	1.016226	1.0170105	1.01557729	0.9157458	-0.1269809	1
RP11-98D18.17	1.014523	1.015145	1.01400845	0.9249525	-0.1125488	1
PZP	1.012789	1.0132168	1.01243594	0.9409159	-0.0878623	1
C2orf15	1.181798	1.1810466	1.18241836	1.0075766	0.01088948	1
RP11-1081M5.1	1.016244	1.0170188	1.015604	0.9168668	-0.125216	1
HIST1H2AG	1.037783	1.037987	1.0376146	0.9901972	-0.0142122	1
CTD-2008P7.1	1.011932	1.0113227	1.01243594	1.0983165	0.13529388	1
LATS2-AS1	1.030001	1.0303311	1.02972868	0.9801391	-0.0289416	1
ATP2B3	1.013651	1.0132247	1.01400329	1.0588747	0.08253193	1
LRG1	1.011932	1.0113227	1.01243594	1.0983165	0.13529388	1
RP4-545L17.11	1.018815	1.0189184	1.01873026	0.9900534	-0.0144218	1
GCKR	1.016249	1.0170541	1.01558394	0.913794	-0.130059	1
RP5-894D12.4	1.011932	1.0113227	1.01243594	1.0983165	0.13529388	1
RORC	1.013651	1.013222	1.01400553	1.0592609	0.08305799	1
POLM	1.257679	1.2571101	1.25814862	1.0040393	0.00581577	1
RP11-407N17.4	1.015371	1.0151173	1.01558078	1.0306587	0.04356661	1
RP11-70L8.4	1.013653	1.0132241	1.01400809	1.0592864	0.08309271	1
PIGB	1.101421	1.1006877	1.1020266	1.0132975	0.01905783	1
TAS2R30	1.029185	1.0285011	1.02975001	1.0438192	0.06187178	1
IRS4	1.021383	1.0208034	1.02186204	1.0508891	0.07161048	1

CTC-332L22.1	1.016242	1.0170406	1.01558177	0.9143908	-0.1291173	1
SLC25A19	1.120394	1.119794	1.12088939	1.0091444	0.01313257	1
CPZ	1.014515	1.0151215	1.01401435	0.9267819	-0.1096982	1
AMY1C	1.011932	1.0113227	1.01243594	1.0983165	0.13529388	1
EIF2B5	1.346307	1.3465412	1.34611296	0.9987643	-0.0017839	1
GAS6-AS1	1.045521	1.0456145	1.04544428	0.9962675	-0.005395	1
AC007182.6	1.011932	1.0113227	1.01243594	1.0983165	0.13529388	1
RHBG	1.011932	1.0113227	1.01243594	1.0983165	0.13529388	1
RP11-239H6.2	1.01279	1.0132178	1.01243594	0.9408498	-0.0879636	1
USP12-AS2	1.028384	1.0286236	1.02818677	0.9847397	-0.0221857	1
AC009120.4	1.013652	1.0132175	1.0140111	1.0600414	0.08412057	1
CTD-2249K22.1	1.022301	1.022756	1.02192552	0.9635035	-0.0536382	1
NCF1	1.023126	1.0227607	1.0234283	1.0293335	0.04171043	1
MFRP	1.011932	1.0113227	1.01243594	1.0983165	0.13529388	1
SERTM1	1.015371	1.0151287	1.01557027	1.0291866	0.04150459	1
RP11-21L1.1	1.014514	1.0151213	1.01401333	0.9267265	-0.1097845	1
RP11-11N5.3	1.115453	1.1162655	1.11478232	0.9872434	-0.0185223	1
RP11-196H14.2	1.012788	1.0132138	1.01243594	0.9411351	-0.0875262	1
ANO9	1.035148	1.0360611	1.03439399	0.9537694	-0.0682876	1
CTD-2619J13.9	1.012789	1.0132168	1.01243594	0.9409159	-0.0878623	1
LA16c-380H5.6	1.014505	1.0151097	1.01400499	0.9268898	-0.1095302	1
RP11-96B2.1	1.012791	1.0132207	1.01243594	0.9406439	-0.0882795	1
IL20RB	1.017089	1.0170098	1.01715369	1.0084569	0.01214944	1
RP11-277P12.10	1.012793	1.0132247	1.01243594	0.9403578	-0.0887183	1
NPM2	1.027422	1.0265681	1.02812768	1.0586995	0.08229318	1
SUCNR1	1.011932	1.0113227	1.01243594	1.0983165	0.13529388	1
CNGB3	1.014512	1.015124	1.01400639	0.9261017	-0.1107575	1
RP11-440L14.4	1.021405	1.0208321	1.02187889	1.0502478	0.07072977	1
LINC01317	1.022252	1.0227008	1.02188137	0.9639041	-0.0530385	1
ZFP57	1.011932	1.0113227	1.01243594	1.0983165	0.13529388	1
ACR	1.015396	1.0151437	1.015604	1.0303932	0.04319502	1
HIST2H2AA4	1.01279	1.0132196	1.01243594	0.9407236	-0.0881572	1
GRTP1-AS1	1.013656	1.0132247	1.01401142	1.0594889	0.08336853	1
HIGD1B	1.011932	1.0113227	1.01243594	1.0983165	0.13529388	1
RP11-119D18.1	1.012791	1.01322	1.01243594	0.9406905	-0.088208	1
EPHA10	1.02749	1.0266219	1.02820722	1.0595509	0.08345296	1
RP11-457I16.2	1.011932	1.0113227	1.01243594	1.0983165	0.13529388	1
CDH10	1.024844	1.0246649	1.02499205	1.0132648	0.01901129	1
AC004231.2	1.021405	1.0208751	1.02184279	1.046354	0.06537109	1
LINC01293	1.011932	1.0113227	1.01243594	1.0983165	0.13529388	1
PRM1	1.01279	1.0132196	1.01243594	0.9407236	-0.0881572	1
KHDC3L	1.023973	1.0246294	1.02343113	0.9513494	-0.0719528	1
AC129492.1	1.016255	1.0170525	1.01559571	0.9145702	-0.1288342	1
AC138035.2	1.011932	1.0113227	1.01243594	1.0983165	0.13529388	1
RP5-921G16.2	1.011932	1.0113227	1.01243594	1.0983165	0.13529388	1

RP11-398H6.1	1.012793	1.0132247	1.01243594	0.9403578	-0.0887183	1
RP11-542A14.2	1.011932	1.0113227	1.01243594	1.0983165	0.13529388	1
RP4-761J14.8	1.013653	1.0132344	1.01399813	1.0577116	0.08094628	1
HIST1H3F	1.015373	1.0151085	1.01559071	1.0319136	0.04532217	1
DGKD	1.225573	1.2265155	1.22479445	0.9924023	-0.0110031	1
RP11-956E11.1	1.012793	1.0132247	1.01243594	0.9403578	-0.0887183	1
RP11-755F10.3	1.014367	1.0141461	1.01454988	1.028547	0.04060768	1
TP73	1.01523	1.0160445	1.01455675	0.9072765	-0.1403857	1
RP1-101G11.3	1.013515	1.0141599	1.01298307	0.9168915	-0.1251771	1
AC007292.3	1.015232	1.0160584	1.01454873	0.9059903	-0.1424325	1
TBC1D3L	1.013515	1.0141584	1.01298307	0.916989	-0.1250236	1
RP5-1185I7.1	1.012654	1.0122555	1.01298307	1.0593688	0.08320497	1
RP11-444A22.1	1.013519	1.0141687	1.01298307	0.9163205	-0.1260759	1
RP11-111M22.5	1.024722	1.0256416	1.02396243	0.9345155	-0.0977095	1
RP11-781A6.1	1.013512	1.014153	1.01298307	0.9173401	-0.1244714	1
AC007796.1	1.012654	1.0122555	1.01298307	1.0593688	0.08320497	1
MFSD2B	1.092837	1.0922194	1.09334719	1.012229	0.01753571	1
NPPA-AS1	1.019528	1.019846	1.01926603	0.9707744	-0.042792	1
RP1-117B12.4	1.012654	1.0122555	1.01298307	1.0593688	0.08320497	1
AC007386.2	1.020426	1.0198574	1.02089473	1.0522369	0.0734595	1
CCDC39-AS1	1.013519	1.0141678	1.01298307	0.9163811	-0.1259804	1
MYL1	1.014369	1.0141505	1.01454988	1.0282221	0.04015186	1
RP11-153F10.2	1.013513	1.0141552	1.01298307	0.9171948	-0.1246999	1
PSMB9	1.0221	1.0217519	1.02238674	1.0291876	0.04150604	1
RNF103-CHMP3	1.017828	1.0179867	1.01769765	0.9839288	-0.0233741	1
WI2-1959D15.1	1.016106	1.0160893	1.01611916	1.0018578	0.00267773	1
TEC	1.031598	1.0313239	1.03182356	1.0159502	0.02282972	1
AC000032.2	1.013514	1.0141568	1.01298307	0.917092	-0.1248617	1
BORCS8-MEF2B	1.014369	1.0141483	1.01455081	1.02845	0.04047164	1
RP4-813D12.3	1.029946	1.0295115	1.03030395	1.0268515	0.03822749	1
RP11-356O9.1	1.014372	1.0141581	1.0145495	1.027645	0.03934192	1
TMEM132E	1.018669	1.0179585	1.01925626	1.0722646	0.10066094	1
PATJ	1.193113	1.1936335	1.19268231	0.9950878	-0.0071042	1
RP11-3B12.2	1.013513	1.0141547	1.01298307	0.917225	-0.1246524	1
RP11-341G23.4	1.016954	1.016067	1.01768638	1.1007909	0.1385405	1
PLCL2	1.047126	1.0465198	1.04762626	1.0237852	0.033913	1
SLC18A1	1.012654	1.0122555	1.01298307	1.0593688	0.08320497	1
NOD2	1.012654	1.0122555	1.01298307	1.0593688	0.08320497	1
RP11-257O5.4	1.012654	1.0122555	1.01298307	1.0593688	0.08320497	1
CTD-2236F14.1	1.014373	1.0141523	1.01455481	1.0284416	0.04045983	1
PDSS2	1.17051	1.1704542	1.17055585	1.0005961	0.00085969	1
RP11-455F5.4	1.013515	1.0141584	1.01298307	0.916989	-0.1250236	1
SEMA6A-AS1	1.020394	1.0198574	1.02083686	1.0493271	0.06946452	1
ADCY5	1.023014	1.0237097	1.02244028	0.9464606	-0.0793857	1
RAPSN	1.013512	1.0141516	1.01298307	0.9174251	-0.1243378	1

SPRN	1.023892	1.0237143	1.02403963	1.0137181	0.01965646	1
METTL11B	1.012654	1.0122555	1.01298307	1.0593688	0.08320497	1
CLDN19	1.068671	1.0693887	1.06807912	0.9811271	-0.0274881	1
RASGRP3	1.030714	1.0312441	1.03027555	0.9690014	-0.0454293	1
INHBB	1.016093	1.0160433	1.01613408	1.0056558	0.00813659	1
RP11-122L9.1	1.016967	1.0160458	1.01772867	1.1048818	0.1438921	1
RP11-417L19.2	1.014378	1.0141608	1.01455653	1.0279444	0.03976228	1
RP11-27K13.3	1.014377	1.0141555	1.01455985	1.0285667	0.04063542	1
BRWD3	1.375082	1.3747121	1.37538687	1.0018007	0.00259558	1
AC004069.2	1.047102	1.0465161	1.04758615	1.0230031	0.03281049	1
RP11-626K17.3	1.013517	1.0141631	1.01298307	0.9166853	-0.1255015	1
RP11-66N24.6	1.013521	1.0141729	1.01298307	0.9160476	-0.1265055	1
FFAR4	1.013512	1.014153	1.01298307	0.9173401	-0.1244714	1
BCDIN3D-AS1	1.055793	1.0561179	1.05552383	0.9894131	-0.0153551	1
C10orf128	1.016102	1.016075	1.01612442	1.0030759	0.00443075	1
LINC00670	1.014375	1.0141523	1.01455977	1.0287923	0.04095169	1
HTR3A	1.013515	1.0141588	1.01298307	0.9169588	-0.1250711	1
NKX2-5	1.01351	1.0141483	1.01298307	0.9176426	-0.1239957	1
HCN1	1.012654	1.0122555	1.01298307	1.0593688	0.08320497	1
PALM2-AKAP2	1.016956	1.0160535	1.01770142	1.1026504	0.14097546	1
CTC-345K18.2	1.013517	1.0141633	1.01298307	0.9166729	-0.125521	1
TNFRSF8	1.01437	1.0141589	1.01454434	1.0272192	0.03874408	1
CSF2RA	1.026449	1.0255871	1.02716161	1.0615336	0.08615004	1
TNFSF4	1.031621	1.0313157	1.0318727	1.0177874	0.02543626	1
CTA-339C12.1	1.012654	1.0122555	1.01298307	1.0593688	0.08320497	1
OR2B6	1.013512	1.0141521	1.01298307	0.9173949	-0.1243852	1
RP11-506F3.1	1.014372	1.0141501	1.01455458	1.0285882	0.04066549	1
RP5-1039K5.16	1.013513	1.0141539	1.01298307	0.9172798	-0.1245662	1
RP4-594A5.1	1.013515	1.0141587	1.01298307	0.9169656	-0.1250605	1
ZMYND15	1.023855	1.023693	1.02398815	1.0124564	0.01785982	1
ABCG2	1.013515	1.0141581	1.01298307	0.9170069	-0.1249956	1
RASL11A	1.013513	1.0141555	1.01298307	0.917177	-0.1247279	1
RP11-326I11.4	1.01698	1.0160493	1.01774803	1.1058429	0.1451465	1
KCNA3	1.012654	1.0122555	1.01298307	1.0593688	0.08320497	1
SLC7A3	1.017818	1.01795	1.01770963	0.9866104	-0.0194476	1
SPATA22	1.016101	1.0160591	1.0161357	1.0047674	0.00686156	1
CTD-3116E22.8	1.017827	1.0179547	1.01772063	0.9869608	-0.0189353	1
RP11-861L17.3	1.013515	1.0141584	1.01298307	0.916989	-0.1250236	1
LINC01133	1.016093	1.0160515	1.01612776	1.0047482	0.00683401	1
FOXN3-AS1	1.041994	1.0427598	1.0413608	0.967283	-0.04799	1
ARR3	1.013509	1.0141461	1.01298307	0.9177875	-0.1237679	1
HIST1H2BI	1.014372	1.0141501	1.01455598	1.0286871	0.04080425	1
LINC00551	1.019548	1.0198662	1.01928482	0.9707354	-0.0428501	1
RP11-135L13.4	1.03165	1.0313602	1.03188913	1.0168672	0.02413133	1
IRF4	1.01868	1.0179352	1.01929595	1.0758696	0.10550318	1

CTD-2528L19.6	1.03334	1.0332039	1.03345205	1.0074744	0.01074321	1
KY	1.017814	1.0179843	1.01767327	0.982703	-0.0251726	1
NPSR1	1.014371	1.0141547	1.01455042	1.0279548	0.03977678	1
KRBA1	1.115249	1.1151599	1.11532174	1.0014053	0.00202602	1
AC002398.9	1.013515	1.0141597	1.01298307	0.9169039	-0.1251576	1
CTD-2314G24.2	1.014385	1.0141729	1.01456079	1.0273669	0.03895157	1
NAT2	1.014378	1.0141637	1.01455467	1.0276021	0.03928178	1
RP11-439L18.1	1.01524	1.0160733	1.01455189	0.9053464	-0.1434582	1
NUP155	1.204887	1.2048	1.20495872	1.0007749	0.00111754	1
RP11-329B9.1	1.013512	1.014153	1.01298307	0.9173401	-0.1244714	1
LINC00445	1.012654	1.0122555	1.01298307	1.0593688	0.08320497	1
SPAG11B	1.013512	1.014153	1.01298307	0.9173401	-0.1244714	1
RP11-707M3.3	1.027369	1.0275814	1.02719441	0.9859685	-0.0203865	1
RP11-524O1.4	1.017817	1.0179541	1.01770444	0.9860962	-0.0201996	1
OR10C1	1.013512	1.0141516	1.01298307	0.9174251	-0.1243378	1
METTL21C	1.01351	1.0141483	1.01298307	0.9176426	-0.1239957	1
RP11-485G7.6	1.013509	1.0141456	1.01298307	0.9178176	-0.1237206	1
PKD2L2	1.016967	1.0160895	1.017691	1.0995384	0.13689804	1
ERC2	1.231868	1.2317691	1.23195045	1.0007825	0.00112853	1
FAT2	1.012654	1.0122555	1.01298307	1.0593688	0.08320497	1
RPRML	1.028111	1.0274343	1.02866976	1.0450324	0.06354772	1
ZNF12	1.380003	1.3801457	1.37988498	0.9993142	-0.0009897	1
SPINK4	1.012654	1.0122555	1.01298307	1.0593688	0.08320497	1
RP11-74C13.4	1.013512	1.0141528	1.01298307	0.9173525	-0.124452	1
AC007131.1	1.013512	1.0141518	1.01298307	0.9174127	-0.1243572	1
RP11-96C23.15	1.015246	1.016094	1.01454488	0.9037449	-0.1460124	1
RP11-572C21.1	1.013518	1.0141648	1.01298307	0.916571	-0.1256815	1
RP11-744I24.3	1.018679	1.0179717	1.01926265	1.0718333	0.10008054	1
NRIR	1.012654	1.0122555	1.01298307	1.0593688	0.08320497	1
RP11-900F13.3	1.015248	1.0160921	1.01455004	0.9041711	-0.1453323	1
DQX1	1.016089	1.0160406	1.01612983	1.0055605	0.00799992	1
CD300C	1.016087	1.016056	1.01611261	1.0035229	0.00507355	1

padj
6.63E-94
2.58E-82
4.77E-80
7.76E-74
6.44E-72
1.80E-71
9.32E-62
4.32E-58
3.17E-56
8.20E-56
2.53E-52
1.96E-51
2.30E-51
1.27E-49
5.54E-49
4.43E-46
7.08E-46
4.71E-44
3.98E-43
2.14E-42
4.79E-42
2.84E-40
3.12E-40
2.46E-38
5.84E-36
2.91E-35
9.59E-36
3.18E-35
4.86E-35
2.03E-34
9.91E-35
5.33E-34
1.54E-34
6.10E-34
5.63E-34
1.18E-33
1.79E-32
3.28E-32
5.68E-32
2.61E-31
1.24E-30
3.32E-31
3.24E-29

2.71E-29
2.17E-28
4.45E-28
1.03E-27
2.92E-27
2.40E-27
3.80E-26
4.04E-26
2.57E-26
4.02E-26
3.54E-26
1.12E-25
3.29E-25
4.91E-25
2.21E-24
3.28E-24
2.74E-23
8.20E-23
9.46E-23
1.28E-22
5.39E-22
2.83E-22
1.34E-22
9.31E-23
2.88E-22
1.04E-21
2.17E-21
2.32E-21
2.23E-21
1.79E-20
7.81E-21
7.85E-21
8.73E-21
1.54E-20
1.91E-20
1.91E-20
3.22E-20
5.96E-20
5.03E-20
4.23E-20
3.80E-20
4.42E-20
8.20E-20
1.12E-19
8.20E-20

1.02E-19
2.27E-19
2.51E-19
2.19E-19
2.50E-18
3.88E-19
6.27E-19
5.37E-19
2.42E-18
2.68E-18
7.69E-19
8.98E-19
1.27E-18
2.57E-18
1.07E-18
9.51E-19
3.80E-18
5.43E-18
3.50E-18
2.45E-18
3.99E-18
1.82E-17
2.64E-17
8.14E-17
4.22E-17
2.82E-17
7.43E-17
3.86E-17
4.16E-17
5.92E-17
6.94E-17
2.66E-16
2.47E-16
1.61E-16
2.13E-16
1.90E-16
2.14E-16
3.69E-16
8.11E-16
1.02E-15
4.59E-16
9.63E-16
3.84E-16
4.21E-16
9.72E-16

4.09E-16
6.91E-16
1.46E-15
5.69E-16
1.44E-15
2.90E-15
2.13E-15
2.90E-15
2.33E-15
1.45E-15
1.45E-15
2.81E-15
1.70E-15
1.70E-15
2.98E-15
2.83E-15
6.52E-15
6.34E-15
8.15E-15
8.15E-15
1.45E-14
1.95E-14
1.59E-14
1.74E-14
2.11E-14
2.12E-14
1.74E-14
1.96E-14
3.78E-14
4.10E-14
4.10E-14
4.06E-14
9.69E-14
8.93E-14
1.21E-13
9.78E-14
1.05E-13
1.59E-13
1.03E-13
1.03E-13
1.04E-13
2.22E-13
1.57E-13
4.06E-13
4.06E-13

3.53E-13
1.74E-13
3.14E-13
3.88E-13
4.57E-13
2.62E-13
3.80E-13
5.21E-13
2.92E-13
4.86E-13
2.92E-13
3.22E-13
4.57E-13
5.33E-13
4.62E-13
6.21E-13
1.24E-12
5.83E-13
5.90E-13
1.17E-12
8.75E-13
2.85E-12
1.78E-12
3.90E-12
1.01E-12
1.71E-12
1.87E-12
2.68E-12
2.42E-12
3.95E-12
4.81E-12
1.73E-12
1.74E-12
4.52E-12
1.85E-12
3.72E-12
3.31E-12
3.37E-12
6.11E-12
4.88E-12
6.92E-12
5.22E-12
1.09E-11
9.62E-12
8.63E-12

6.73E-12
4.90E-12
1.35E-11
7.71E-12
7.21E-12
1.36E-11
7.27E-12
2.22E-11
7.45E-12
1.95E-11
7.45E-12
6.85E-12
1.73E-11
1.55E-11
1.44E-11
1.65E-11
2.08E-11
1.55E-11
2.52E-11
2.73E-11
2.89E-11
3.17E-11
2.92E-11
2.93E-11
4.77E-11
3.25E-11
3.28E-11
5.36E-11
6.05E-11
4.79E-11
2.88E-11
5.42E-11
5.84E-11
8.57E-11
6.34E-11
9.22E-11
5.20E-11
1.01E-10
7.38E-11
8.62E-11
8.88E-11
8.27E-11
9.54E-11
1.38E-10
1.19E-10

1.15E-10
1.03E-10
1.49E-10
1.60E-10
1.25E-10
1.60E-10
1.16E-10
1.52E-10
2.40E-10
1.65E-10
4.39E-10
2.85E-10
1.81E-10
2.06E-10
3.07E-10
3.91E-10
2.77E-10
2.13E-10
1.70E-10
3.12E-10
3.36E-10
2.03E-10
2.87E-10
2.64E-10
3.34E-10
2.64E-10
2.14E-10
3.61E-10
4.74E-10
3.28E-10
3.98E-10
2.69E-10
5.48E-10
5.49E-10
7.07E-10
6.83E-10
6.99E-10
7.83E-10
1.21E-09
6.70E-10
1.40E-09
8.17E-10
1.52E-09
2.40E-09
8.09E-10

6.63E-10
1.33E-09
1.04E-09
7.92E-10
1.96E-09
1.41E-09
1.52E-09
1.52E-09
1.83E-09
2.34E-09
1.67E-09
2.61E-09
2.43E-09
1.31E-09
2.46E-09
2.24E-09
3.33E-09
3.07E-09
2.86E-09
2.78E-09
4.61E-09
3.64E-09
3.60E-09
3.50E-09
5.48E-09
4.29E-09
4.29E-09
5.34E-09
5.03E-09
1.08E-08
9.75E-09
4.39E-09
4.65E-09
4.93E-09
4.09E-09
7.26E-09
4.80E-09
4.35E-09
7.39E-09
1.08E-08
6.33E-09
5.99E-09
1.11E-08
5.87E-09
6.03E-09

9.28E-09
6.88E-09
6.96E-09
1.25E-08
1.13E-08
8.19E-09
1.28E-08
6.94E-09
1.26E-08
1.19E-08
1.09E-08
1.12E-08
2.67E-08
1.08E-08
1.49E-08
1.14E-08
2.77E-08
1.13E-08
2.77E-08
2.01E-08
1.56E-08
2.60E-08
2.18E-08
2.09E-08
2.01E-08
1.45E-08
2.05E-08
2.29E-08
1.79E-08
2.21E-08
1.50E-08
2.43E-08
2.81E-08
4.53E-08
3.03E-08
2.84E-08
3.80E-08
4.56E-08
7.53E-08
3.50E-08
4.71E-08
4.04E-08
3.21E-08
4.44E-08
4.20E-08

4.90E-08
5.39E-08
8.14E-08
8.30E-08
9.80E-08
6.30E-08
7.39E-08
6.20E-08
8.39E-08
1.04E-07
8.79E-08
5.96E-08
1.13E-07
7.46E-08
5.96E-08
1.61E-07
1.02E-07
8.32E-08
1.01E-07
9.56E-08
1.14E-07
1.20E-07
1.28E-07
1.02E-07
1.13E-07
8.46E-08
1.68E-07
1.26E-07
8.82E-08
2.36E-07
1.35E-07
1.12E-07
1.48E-07
1.65E-07
1.65E-07
1.95E-07
2.03E-07
1.28E-07
2.14E-07
2.24E-07
2.24E-07
2.10E-07
2.49E-07
2.15E-07
1.75E-07

2.27E-07
4.23E-07
3.55E-07
2.33E-07
2.66E-07
2.49E-07
3.03E-07
5.19E-07
2.63E-07
3.60E-07
3.51E-07
2.86E-07
2.87E-07
3.62E-07
2.99E-07
6.49E-07
4.02E-07
4.42E-07
3.64E-07
3.19E-07
7.27E-07
5.28E-07
5.14E-07
4.60E-07
7.65E-07
8.33E-07
5.55E-07
4.62E-07
3.83E-07
5.48E-07
8.06E-07
5.65E-07
8.23E-07
7.25E-07
6.64E-07
5.84E-07
6.74E-07
5.12E-07
8.72E-07
1.06E-06
8.26E-07
8.01E-07
5.48E-07
1.01E-06
7.81E-07

1.19E-06
8.62E-07
1.01E-06
7.96E-07
8.17E-07
8.84E-07
1.71E-06
1.26E-06
1.03E-06
1.03E-06
1.02E-06
1.17E-06
1.31E-06
1.74E-06
1.31E-06
1.38E-06
9.98E-07
1.29E-06
1.37E-06
1.11E-06
1.06E-06
8.83E-07
1.27E-06
2.65E-06
1.44E-06
1.66E-06
1.52E-06
1.44E-06
2.65E-06
2.65E-06
1.21E-06
9.94E-07
1.42E-06
1.48E-06
1.21E-06
1.34E-06
2.52E-06
1.59E-06
1.32E-06
2.08E-06
2.05E-06
2.25E-06
2.53E-06
2.35E-06
2.42E-06

1.84E-06
2.73E-06
3.00E-06
1.74E-06
5.03E-06
2.78E-06
3.70E-06
3.14E-06
4.91E-06
3.58E-06
3.37E-06
3.58E-06
3.53E-06
3.30E-06
4.20E-06
3.43E-06
4.06E-06
3.94E-06
3.08E-06
3.63E-06
4.13E-06
3.99E-06
3.75E-06
3.75E-06
4.50E-06
4.21E-06
3.60E-06
6.89E-06
4.31E-06
4.40E-06
4.84E-06
8.49E-06
5.69E-06
5.17E-06
5.73E-06
7.77E-06
5.18E-06
8.80E-06
5.18E-06
5.61E-06
9.38E-06
8.86E-06
6.09E-06
6.21E-06
6.52E-06

6.24E-06
7.64E-06
5.29E-06
7.16E-06
8.88E-06
1.32E-05
1.32E-05
1.18E-05
7.48E-06
6.68E-06
1.35E-05
6.81E-06
1.42E-05
8.08E-06
8.24E-06
9.10E-06
9.55E-06
9.23E-06
8.95E-06
8.00E-06
1.00E-05
1.14E-05
1.62E-05
9.72E-06
8.58E-06
1.59E-05
8.58E-06
9.85E-06
1.40E-05
8.99E-06
1.40E-05
9.15E-06
1.71E-05
1.26E-05
1.19E-05
1.05E-05
1.03E-05
2.08E-05
1.37E-05
2.25E-05
1.46E-05
1.36E-05
1.62E-05
2.46E-05
1.61E-05

2.28E-05
1.33E-05
2.28E-05
1.55E-05
1.55E-05
1.68E-05
2.60E-05
2.11E-05
2.15E-05
1.87E-05
2.94E-05
1.68E-05
1.48E-05
2.44E-05
2.15E-05
2.99E-05
2.44E-05
2.08E-05
2.22E-05
2.72E-05
2.25E-05
2.07E-05
2.03E-05
2.62E-05
2.09E-05
1.83E-05
2.30E-05
2.34E-05
2.88E-05
3.44E-05
2.52E-05
3.44E-05
2.27E-05
3.32E-05
3.52E-05
2.56E-05
4.04E-05
2.89E-05
2.92E-05
2.44E-05
2.54E-05
2.57E-05
2.52E-05
3.54E-05
3.74E-05

4.31E-05
2.63E-05
3.26E-05
2.80E-05
4.14E-05
2.79E-05
2.99E-05
2.79E-05
4.96E-05
4.15E-05
3.48E-05
2.99E-05
3.65E-05
4.09E-05
3.81E-05
6.39E-05
6.41E-05
5.27E-05
3.71E-05
4.04E-05
5.27E-05
5.25E-05
5.27E-05
6.73E-05
6.01E-05
4.16E-05
4.55E-05
4.06E-05
5.51E-05
4.20E-05
4.46E-05
7.08E-05
4.46E-05
5.38E-05
5.96E-05
6.81E-05
5.96E-05
4.78E-05
5.42E-05
7.67E-05
8.38E-05
8.38E-05
5.86E-05
5.69E-05
7.53E-05

8.07E-05
7.11E-05
9.17E-05
8.37E-05
9.44E-05
7.56E-05
6.41E-05
6.41E-05
6.23E-05
6.41E-05
7.39E-05
9.54E-05
6.43E-05
9.56E-05
7.54E-05
6.61E-05
7.64E-05
6.99E-05
0.0001
8.21E-05
9.39E-05
7.02E-05
0.00012
0.0001
0.00011
0.00011
0.00011
0.00011
0.00012
0.00011
8.24E-05
0.00011
9.47E-05
0.0001
8.67E-05
8.67E-05
0.0001
9.71E-05
0.00015
0.00012
0.00018
0.00013
0.00014
0.00018
0.00013

0.0001
0.00013
0.00016
0.00016
0.00014
0.00016
0.00011
0.00014
0.00012
0.00019
0.00017
0.00014
0.00017
0.00016
0.00012
0.00012
0.00022
0.00018
0.00017
0.00018
0.00014
0.00019
0.00017
0.00017
0.00019
0.00016
0.00019
0.0002
0.00016
0.00023
0.00021
0.00024
0.00021
0.00019
0.00016
0.00025
0.00017
0.00017
0.00024
0.00023
0.00021
0.00017
0.00019
0.00018
0.00018

0.00028
0.00026
0.0003
0.00027
0.00026
0.00027
0.00034
0.00022
0.00022
0.00027
0.00031
0.00023
0.00027
0.00038
0.00028
0.00035
0.00032
0.00028
0.00035
0.00024
0.00033
0.00031
0.00039
0.0004
0.00039
0.00027
0.00026
0.00037
0.0004
0.00038
0.00039
0.00035
0.00041
0.00027
0.0003
0.00039
0.00044
0.00039
0.00035
0.00041
0.00044
0.00033
0.00049
0.00042
0.00042

0.00034
0.00042
0.00046
0.00044
0.00041
0.00047
0.00044
0.0005
0.00044
0.00049
0.00048
0.00039
0.00043
0.00048
0.00046
0.00043
0.00051
0.00042
0.00053
0.00039
0.00054
0.00051
0.00054
0.00044
0.00044
0.00049
0.00057
0.00055
0.00057
0.00041
0.00062
0.00049
0.00057
0.0005
0.00053
0.00056
0.0005
0.00088
0.00057
0.00066
0.00052
0.00052
0.0006
0.00069
0.00047

0.00069
0.00066
0.00078
0.00065
0.00065
0.00067
0.00048
0.00067
0.00074
0.00067
0.00057
0.00067
0.0006
0.00059
0.00063
0.00063
0.00077
0.00058
0.00059
0.00062
0.00091
0.00065
0.00083
0.00067
0.00067
0.0008
0.00068
0.00068
0.00087
0.00085
0.0007
0.00092
0.00074
0.00093
0.00089
0.00072
0.00083
0.00098
0.0008
0.00091
0.00105
0.00081
0.00121
0.00103
0.00101

0.00085
0.00098
0.00108
0.00113
0.00083
0.00089
0.00166
0.00097
0.00087
0.00087
0.0011
0.00114
0.00092
0.00112
0.00089
0.00114
0.00172
0.00114
0.00116
0.00135
0.00115
0.00105
0.00135
0.00123
0.00089
0.00098
0.00108
0.00096
0.00131
0.00131
0.00186
0.00125
0.00104
0.00108
0.00131
0.00125
0.00129
0.00126
0.00114
0.00149
0.00125
0.00125
0.00129
0.00112
0.00101

0.00107
0.00139
0.00142
0.00109
0.0014
0.00139
0.00136
0.00149
0.00154
0.00138
0.00158
0.00141
0.00184
0.0017
0.00161
0.00161
0.00215
0.00173
0.00172
0.00158
0.0017
0.00178
0.00215
0.00215
0.00173
0.00215
0.00177
0.00173
0.00139
0.00133
0.00152
0.00198
0.00177
0.00168
0.00153
0.00184
0.00182
0.0017
0.0014
0.00184
0.00184
0.00188
0.00189
0.00192
0.00149

0.00201
0.00192
0.00205
0.00205
0.00192
0.00206
0.00203
0.00203
0.00192
0.00208
0.00192
0.00202
0.00198
0.00192
0.00202
0.0027
0.0027
0.00194
0.00259
0.00227
0.00201
0.00201
0.00267
0.00199
0.00376
0.00235
0.00241
0.00209
0.00199
0.00215
0.00212
0.00236
0.00201
0.00255
0.00217
0.00235
0.00235
0.00251
0.00251
0.00198
0.00288
0.00223
0.00223
0.00289
0.00243

0.0021
0.00295
0.0023
0.00245
0.00447
0.00252
0.0031
0.00303
0.00292
0.0026
0.00354
0.00354
0.00285
0.00292
0.00278
0.00278
0.00308
0.00309
0.0036
0.00264
0.00269
0.00322
0.00378
0.00275
0.00318
0.00275
0.00284
0.0032
0.00368
0.00308
0.00298
0.00368
0.00355
0.00355
0.00354
0.00302
0.00377
0.00288
0.00291
0.00593
0.00333
0.00397
0.00385
0.00393
0.00333

0.00395
0.00356
0.00399
0.00342
0.00342
0.00395
0.00363
0.00642
0.00508
0.00417
0.00369
0.00437
0.0046
0.0065
0.00377
0.0046
0.00463
0.00456
0.00542
0.00402
0.0046
0.00392
0.00395
0.00392
0.00464
0.00409
0.00467
0.00403
0.00403
0.00416
0.00429
0.00503
0.00416
0.00416
0.00429
0.00429
0.00484
0.00429
0.00517
0.00614
0.00429
0.00444
0.00511
0.00434
0.00498

0.00554
0.00423
0.00558
0.00452
0.00558
0.0066
0.00468
0.00579
0.00493
0.00576
0.00584
0.00587
0.00579
0.0058
0.00498
0.00703
0.00586
0.00637
0.0058
0.00598
0.00601
0.00519
0.00647
0.00734
0.00621
0.00536
0.0055
0.00532
0.0055
0.00749
0.00636
0.00637
0.00776
0.00706
0.00716
0.00717
0.01053
0.00603
0.00794
0.00625
0.01053
0.00724
0.00724
0.01053
0.00697

0.00736
0.0074
0.00696
0.00737
0.00623
0.00643
0.00615
0.00743
0.0084
0.00645
0.01091
0.00752
0.00732
0.00752
0.00647
0.00867
0.00754
0.00867
0.0065
0.00811
0.00665
0.00658
0.00679
0.01134
0.00767
0.00891
0.01142
0.007
0.00852
0.00832
0.00884
0.00826
0.01171
0.00858
0.00858
0.00725
0.0085
0.00846
0.0091
0.00772
0.00743
0.00757
0.00759
0.00948
0.00961

0.00909
0.00944
0.00896
0.00805
0.0081
0.00837
0.01261
0.00792
0.00807
0.00916
0.00919
0.00813
0.01261
0.01071
0.01016
0.00921
0.00815
0.00892
0.01044
0.00796
0.00796
0.0103
0.01361
0.00943
0.00977
0.01014
0.00993
0.01016
0.00823
0.01318
0.01025
0.0087
0.00865
0.01151
0.01342
0.00936
0.01107
0.01059
0.00936
0.01047
0.00916
0.01145
0.00964
0.0135
0.00964

0.01225
0.01472
0.01096
0.01005
0.01017
0.01005
0.01169
0.01334
0.01169
0.01233
0.01335
0.01312
0.01029
0.01209
0.01501
0.01158
0.01338
0.01269
0.01338
0.01094
0.01354
0.01245
0.01361
0.01309
0.013
0.01686
0.01361
0.01371
0.01358
0.01371
0.02021
0.01235
0.01341
0.01317
0.01767
0.01317
0.01767
0.01288
0.01455
0.01588
0.01475
0.01723
0.01704
0.01579
0.01819

0.01466
0.01516
0.01367
0.01612
0.02092
0.0164
0.02356
0.016
0.01669
0.01674
0.01615
0.01566
0.01443
0.01679
0.01928
0.01509
0.01594
0.01937
0.01879
0.01508
0.01551
0.01752
0.01612
0.01512
0.01995
0.02075
0.01977
0.01753
0.01928
0.02028
0.0178
0.02069
0.0267
0.02116
0.01701
0.01753
0.01992
0.01887
0.01978
0.01887
0.02288
0.0174
0.01936
0.01681
0.01746

0.02291
0.01814
0.01955
0.02052
0.02168
0.02781
0.02176
0.01789
0.01953
0.02177
0.02232
0.02232
0.01856
0.01771
0.02023
0.02195
0.02032
0.02142
0.02185
0.02338
0.02228
0.02297
0.01885
0.02236
0.02867
0.02377
0.02431
0.0203
0.0261
0.022
0.02271
0.02009
0.02488
0.02208
0.0196
0.02437
0.02077
0.02262
0.02026
0.02414
0.02356
0.02026
0.02414
0.02106
0.02524

0.02625
0.02159
0.02663
0.02425
0.03486
0.02387
0.02457
0.02463
0.02308
0.02969
0.02195
0.02599
0.02599
0.03405
0.02599
0.02453
0.02327
0.03109
0.0313
0.0236
0.0278
0.02794
0.02374
0.02801
0.0239
0.03977
0.02863
0.02539
0.02867
0.0292
0.03371
0.03773
0.02558
0.02596
0.03773
0.02721
0.02721
0.02729
0.03419
0.03021
0.03419
0.0261
0.02751
0.04225
0.03341

0.02963
0.03419
0.0261
0.0261
0.03089
0.03001
0.0266
0.03089
0.03382
0.03007
0.03164
0.03007
0.03494
0.03163
0.02702
0.03389
0.03389
0.0368
0.02786
0.03188
0.03235
0.03061
0.02808
0.03061
0.03239
0.02769
0.03288
0.03677
0.03342
0.03218
0.03583
0.02842
0.03342
0.0316
0.03039
0.02842
0.03704
0.03039
0.03176
0.03863
0.0337
0.03039
0.02846
0.03552
0.03039

0.03439
0.03552
0.02949
0.02851
0.03001
0.03242
0.04613
0.03514
0.03021
0.03527
0.03527
0.03963
0.03963
0.03527
0.03698
0.03654
0.0346
0.04031
0.03876
0.03836
0.03152
0.03836
0.03783
0.03684
0.03744
0.04121
0.04121
0.03649
0.03858
0.04456
0.05022
0.03658
0.03865
0.03648
0.04463
0.03274
0.03936
0.03597
0.03395
0.03673
0.04409
0.03793
0.03719
0.03522
0.03794

0.04303
0.03987
0.03522
0.03987
0.03987
0.03522
0.04107
0.03755
0.04066
0.0432
0.04291
0.06097
0.04029
0.04825
0.04066
0.04029
0.04335
0.0422
0.04672
0.03977
0.04374
0.03663
0.04125
0.04893
0.04029
0.04029
0.03663
0.04377
0.06281
0.05736
0.03798
0.04315
0.04564
0.04171
0.05171
0.03872
0.04126
0.04598
0.04302
0.04418
0.05907
0.05199
0.04129
0.04556
0.04624

0.04752
0.04821
0.04572
0.0423
0.04572
0.05189
0.04412
0.04839
0.04438
0.04085
0.045
0.0493
0.04106
0.0447
0.05005
0.04535
0.04128
0.0633
0.05498
0.05005
0.05027
0.04311
0.05092
0.05556
0.05124
0.05027
0.05027
0.04677
0.0585
0.05164
0.04791
0.05186
0.04919
0.04742
0.0566
0.04791
0.0566
0.0495
0.0677
0.05126
0.05206
0.0495
0.04853
0.07801
0.04903

0.04901
0.04951
0.05893
0.05893
0.0528
0.05893
0.04925
0.05078
0.0564
0.05642
0.05294
0.05305
0.05041
0.05613
0.07108
0.05936
0.05078
0.06031
0.05041
0.05078
0.05081
0.05688
0.05596
0.05084
0.05688
0.06591
0.04929
0.05688
0.05712
0.05214
0.05269
0.05952
0.05269
0.05037
0.05037
0.05295
0.07588
0.07588
0.06477
0.05378
0.05463
0.05326
0.05463
0.05908
0.0634

0.06042
0.05378
0.05378
0.07335
0.0622
0.06741
0.05628
0.07164
0.06186
0.05628
0.06445
0.06395
0.05525
0.06103
0.05393
0.06204
0.05525
0.06532
0.06208
0.07345
0.06503
0.05775
0.06972
0.06208
0.0764
0.05828
0.06667
0.07053
0.08284
0.06303
0.07069
0.06274
0.06274
0.06274
0.0659
0.06082
0.06791
0.07163
0.10264
0.05848
0.05978
0.06977
0.06971
0.10264
0.06561

0.06561
0.06138
0.06983
0.06356
0.08
0.06223
0.07156
0.05956
0.08298
0.06108
0.07017
0.08039
0.06267
0.08298
0.07105
0.0655
0.07216
0.07327
0.08298
0.08298
0.07693
0.0741
0.07235
0.06755
0.07727
0.07443
0.0727
0.07193
0.07193
0.08418
0.09471
0.07401
0.08502
0.06943
0.11209
0.07831
0.07234
0.07234
0.08551
0.09611
0.11209
0.09611
0.0797
0.07168
0.11209

0.07415
0.07415
0.06976
0.11209
0.08494
0.07051
0.08263
0.07051
0.0862
0.11489
0.07224
0.08385
0.0862
0.07742
0.07114
0.07553
0.09131
0.07553
0.08388
0.08202
0.08647
0.08131
0.08349
0.09539
0.11512
0.11512
0.08272
0.08404
0.07426
0.08404
0.09622
0.08314
0.08144
0.11592
0.07945
0.07467
0.08351
0.09697
0.08171
0.07997
0.07997
0.07997
0.07552
0.09685
0.09054

0.09775
0.09685
0.08307
0.08159
0.09077
0.09685
0.07892
0.08159
0.08769
0.11297
0.07936
0.08769
0.10549
0.09169
0.07789
0.12261
0.11297
0.09618
0.11297
0.08055
0.09915
0.08813
0.09284
0.08402
0.08621
0.09355
0.08568
0.09659
0.09547
0.09114
0.09808
0.08217
0.08568
0.10469
0.09385
0.09665
0.0816
0.09086
0.08813
0.10604
0.09643
0.08484
0.09194
0.08484
0.09806

0.09194
0.10218
0.08464
0.08464
0.09508
0.10642
0.09798
0.10311
0.1148
0.09044
0.08685
0.1148
0.12351
0.13556
0.10197
0.10335
0.10993
0.09946
0.10257
0.09397
0.13617
0.09319
0.1099
0.08837
0.12635
0.10318
0.09975
0.10103
0.10444
0.08923
0.11408
0.10401
0.10401
0.10069
0.13967
0.13967
0.10407
0.13017
0.10382
0.1105
0.10475
0.10863
0.12589
0.11257
0.09983

0.09983
0.09997
0.11589
0.10631
0.10436
0.14301
0.11012
0.10691
0.0955
0.10247
0.11012
0.0977
0.10257
0.0977
0.1098
0.11905
0.09956
0.12291
0.09638
0.09956
0.12291
0.104
0.13832
0.11015
0.09966
0.10812
0.12369
0.11877
0.11165
0.11165
0.11463
0.12392
0.11316
0.10769
0.12599
0.10041
0.1235
0.10041
0.1146
0.11577
0.11722
0.10503
0.11076
0.11599
0.15806

0.10509
0.11722
0.12605
0.11722
0.1173
0.11747
0.12629
0.11701
0.12638
0.12626
0.12346
0.13159
0.11312
0.11804
0.12346
0.11599
0.16149
0.13159
0.11362
0.12376
0.11362
0.12907
0.11966
0.12971
0.14999
0.11697
0.12591
0.11399
0.16612
0.11074
0.15849
0.15849
0.1335
0.12283
0.159
0.12652
0.12652
0.12418
0.13688
0.13443
0.11337
0.1201
0.11688
0.12418
0.12418

0.11918
0.11689
0.13487
0.16139
0.13308
0.12404
0.12624
0.13308
0.12404
0.13076
0.17445
0.1179
0.16183
0.12086
0.12404
0.12404
0.11728
0.13782
0.1321
0.14982
0.11738
0.12425
0.14389
0.16529
0.13497
0.14396
0.18064
0.13515
0.15101
0.13924
0.15101
0.15295
0.14005
0.14121
0.13924
0.13924
0.1251
0.13175
0.16962
0.15017
0.14483
0.15017
0.14839
0.13666
0.12545

0.15479
0.13397
0.18851
0.15001
0.17331
0.15354
0.14725
0.17367
0.15048
0.14463
0.14752
0.1547
0.17659
0.14805
0.13177
0.15591
0.15182
0.15534
0.15182
0.15534
0.14067
0.14829
0.14782
0.14941
0.15155
0.17994
0.19295
0.1538
0.16667
0.17994
0.14426
0.20214
0.1356
0.15884
0.15566
0.13924
0.18479
0.16667
0.20255
0.15286
0.16667
0.15901
0.16667
0.15398
0.14706

0.20282
0.18411
0.14124
0.15913
0.15913
0.15488
0.15901
0.15901
0.15512
0.14134
0.18913
0.15901
0.18159
0.16684
0.16191
0.15142
0.16944
0.16944
0.18706
0.20612
0.15197
0.18351
0.15412
0.14458
0.17276
0.161
0.16249
0.20612
0.20612
0.18706
0.18706
0.15558
0.18706
0.14634
0.20612
0.16265
0.14706
0.14634
0.17476
0.14634
0.21433
0.17261
0.17791
0.16625
0.18018

0.18286
0.18286
0.17951
0.16144
0.17368
0.19399
0.21866
0.15093
0.17368
0.16325
0.16542
0.18251
0.18089
0.21641
0.16223
0.16223
0.18266
0.17891
0.17728
0.19887
0.16749
0.17605
0.18371
0.16749
0.15599
0.18142
0.22699
0.21648
0.17648
0.2222
0.18005
0.17767
0.16671
0.15794
0.17767
0.2222
0.16235
0.16671
0.1842
0.18266
0.17147
0.17804
0.19167
0.1914
0.20838

0.19156
0.18476
0.19718
0.18549
0.16766
0.16329
0.18549
0.18565
0.18549
0.18768
0.19748
0.17641
0.18673
0.16851
0.19834
0.17641
0.16851
0.19096
0.16851
0.21393
0.1792
0.21393
0.2238
0.19979
0.17924
0.19223
0.19231
0.17924
0.20293
0.17141
0.21681
0.239
0.19318
0.239
0.17275
0.20584
0.20661
0.19751
0.19751
0.24918
0.19516
0.18652
0.21222
0.2002
0.2361

0.24447
0.25007
0.2002
0.18701
0.20302
0.2128
0.25007
0.21761
0.19853
0.20414
0.21323
0.2705
0.21323
0.18561
0.20884
0.21323
0.2705
0.20617
0.19247
0.20852
0.22523
0.2359
0.19902
0.21424
0.27146
0.21434
0.20927
0.22814
0.22327
0.27146
0.21311
0.21123
0.20141
0.2146
0.22503
0.19059
0.21123
0.22327
0.21311
0.19059
0.21194
0.21194
0.26196
0.21033
0.24101

0.22549
0.21331
0.21291
0.2318
0.23219
0.21338
0.21331
0.2578
0.27639
0.2318
0.26529
0.21394
0.21567
0.252
0.19738
0.22504
0.19738
0.281
0.27581
0.19738
0.19945
0.23822
0.27581
0.2187
0.27183
0.22144
0.23575
0.28223
0.23911
0.26499
0.26499
0.2151
0.23575
0.22258
0.22713
0.26003
0.2835
0.24046
0.23575
0.25861
0.20297
0.2335
0.24278
0.22241
0.25268

0.21844
0.22241
0.28521
0.22488
0.23405
0.2225
0.22488
0.25877
0.28521
0.27472
0.22164
0.22363
0.25877
0.24827
0.25877
0.2927
0.25877
0.23487
0.2454
0.24323
0.23487
0.26217
0.23277
0.21805
0.25315
0.2421
0.29514
0.22936
0.23303
0.22936
0.24207
0.27956
0.26349
0.23246
0.22936
0.29186
0.26047
0.29186
0.29186
0.26635
0.29186
0.22981
0.25571
0.23673
0.29186

0.27395
0.22981
0.22335
0.29353
0.27395
0.22981
0.26902
0.24835
0.30893
0.30893
0.26902
0.25904
0.27176
0.24835
0.23253
0.29718
0.30966
0.30893
0.30893
0.24448
0.2713
0.2961
0.30966
0.26263
0.23109
0.30058
0.26263
0.30966
0.24665
0.2713
0.30058
0.27855
0.2713
0.24665
0.26282
0.23766
0.27898
0.23766
0.25173
0.3063
0.31309
0.24893
0.26005
0.2357
0.25186

0.26522
0.27296
0.23679
0.28335
0.27797
0.26006
0.26006
0.26972
0.27463
0.31793
0.28164
0.25645
0.28388
0.28388
0.28388
0.28108
0.30001
0.29227
0.25686
0.27742
0.26378
0.26188
0.25181
0.32161
0.27456
0.27742
0.24963
0.27618
0.2833
0.29474
0.28246
0.25288
0.32298
0.27856
0.24647
0.32405
0.25288
0.26325
0.32616
0.29678
0.29656
0.30792
0.29226
0.32195
0.28741

0.32195
0.34271
0.28076
0.29412
0.3271
0.25583
0.32857
0.31016
0.28166
0.28416
0.32982
0.29608
0.32248
0.27444
0.27001
0.32248
0.25761
0.30197
0.28722
0.28183
0.29058
0.29058
0.29657
0.28183
0.28722
0.35126
0.28882
0.3346
0.26405
0.28199
0.29191
0.33585
0.29803
0.31672
0.33468
0.28992
0.30082
0.31672
0.2797
0.26727
0.33468
0.31672
0.28665
0.33468
0.30082

0.29515
0.29739
0.26829
0.30082
0.33422
0.35994
0.34296
0.31526
0.28725
0.33422
0.27226
0.30773
0.29774
0.28605
0.28577
0.28969
0.29868
0.28918
0.28969
0.30199
0.27312
0.36527
0.28918
0.30937
0.29182
0.27781
0.32707
0.35649
0.3345
0.3078
0.27983
0.33627
0.28462
0.29835
0.31148
0.33092
0.37752
0.3109
0.32153
0.3382
0.37752
0.3382
0.36538
0.31979
0.31791

0.28985
0.37657
0.30678
0.30711
0.32849
0.37657
0.34379
0.30678
0.28993
0.28993
0.38546
0.29576
0.37228
0.33129
0.37283
0.3212
0.29476
0.33331
0.32596
0.32763
0.32655
0.31102
0.35026
0.38879
0.32763
0.35756
0.31102
0.32738
0.35277
0.32625
0.37971
0.35924
0.35277
0.32028
0.35277
0.32524
0.39489
0.34271
0.38165
0.33558
0.30342
0.34455
0.33369
0.36514
0.34453

0.3901
0.35661
0.33369
0.38365
0.33937
0.38935
0.38935
0.36491
0.33204
0.31472
0.36491
0.38935
0.34257
0.35145
0.33755
0.38811
0.33355
0.31732
0.33755
0.33805
0.3199
0.35518
0.33719
0.34146
0.37647
0.3965
0.40022
0.32092
0.35182
0.37647
0.37647
0.42612
0.34074
0.41143
0.35882
0.35882
0.36237
0.35882
0.38842
0.42456
0.34341
0.42456
0.34341
0.38842
0.35882

0.38247
0.35882
0.35391
0.41694
0.36426
0.36426
0.36426
0.37028
0.44067
0.42064
0.41265
0.37259
0.3554
0.39513
0.41265
0.33878
0.43902
0.36679
0.41265
0.39536
0.39585
0.42285
0.4257
0.42285
0.37562
0.40519
0.39557
0.37873
0.42807
0.40588
0.37873
0.42503
0.3782
0.37873
0.39557
0.42536
0.41443
0.4298
0.39557
0.39557
0.35802
0.38409
0.35871
0.38615
0.3719

0.42767
0.38144
0.38135
0.38484
0.41125
0.38658
0.43526
0.38157
0.3572
0.38356
0.37909
0.36596
0.4121
0.46665
0.4121
0.40805
0.4121
0.40282
0.3706
0.38775
0.38836
0.44425
0.46665
0.38472
0.41292
0.43499
0.46665
0.38864
0.44499
0.36911
0.39185
0.40103
0.41499
0.40681
0.37704
0.40571
0.4488
0.40681
0.45686
0.37704
0.37213
0.46854
0.46854
0.4458
0.42222

0.37759
0.46854
0.37759
0.46854
0.41187
0.42243
0.40304
0.41216
0.41461
0.45352
0.41914
0.40738
0.41216
0.40428
0.45195
0.38183
0.42531
0.41216
0.42391
0.47162
0.42391
0.41216
0.41876
0.38413
0.44029
0.42771
0.42246
0.38418
0.47362
0.41931
0.45874
0.46454
0.42958
0.44306
0.41356
0.43037
0.43805
0.41356
0.4187
0.48823
0.41931
0.43805
0.41931
0.41356
0.43805

0.42934
0.48931
0.42037
0.48931
0.44801
0.40077
0.54001
0.48931
0.48592
0.42127
0.42961
0.41816
0.44071
0.54101
0.42807
0.45177
0.48788
0.47951
0.48788
0.44673
0.43788
0.4451
0.4259
0.44257
0.40372
0.48902
0.40956
0.44925
0.49888
0.43149
0.49888
0.46003
0.43706
0.43706
0.451
0.43836
0.45011
0.46063
0.41011
0.50082
0.55278
0.43557
0.55278
0.41011
0.44004

0.43251
0.454
0.44107
0.43251
0.43251
0.49716
0.46322
0.4669
0.55368
0.49936
0.43874
0.43944
0.46139
0.49905
0.44505
0.45918
0.43944
0.43887
0.42824
0.43887
0.55981
0.50484
0.46706
0.46706
0.46403
0.45957
0.45957
0.46318
0.44391
0.5048
0.46587
0.43456
0.47061
0.47061
0.48435
0.52327
0.4833
0.52327
0.52806
0.4617
0.44977
0.47732
0.48435
0.47061
0.53842

0.43277
0.56789
0.4617
0.44977
0.47066
0.44977
0.48713
0.56789
0.44977
0.51931
0.56789
0.5249
0.46758
0.56789
0.53142
0.48747
0.49014
0.56825
0.48768
0.54973
0.44149
0.44149
0.49233
0.48102
0.48277
0.48277
0.56977
0.50102
0.44149
0.46999
0.47681
0.48102
0.47148
0.49369
0.48297
0.55338
0.57141
0.46494
0.55338
0.48254
0.44472
0.48297
0.48353
0.50495
0.47663

0.48501
0.46866
0.55805
0.54465
0.48794
0.47679
0.48195
0.50613
0.57663
0.47679
0.57663
0.54465
0.50613
0.51271
0.48342
0.516
0.51271
0.49099
0.48124
0.48501
0.51402
0.51875
0.56381
0.50074
0.58093
0.60359
0.48432
0.4877
0.51402
0.52088
0.52088
0.52483
0.48874
0.58093
0.52117
0.60359
0.50026
0.60359
0.56381
0.56381
0.51402
0.52556
0.58118
0.51402
0.52213

0.50707
0.56381
0.50612
0.51223
0.52381
0.50196
0.60423
0.51458
0.52381
0.50612
0.5694
0.50612
0.49264
0.49624
0.50984
0.52855
0.5912
0.52381
0.56882
0.52381
0.50612
0.49876
0.60572
0.60572
0.53233
0.57091
0.52054
0.48304
0.53233
0.50097
0.57091
0.5053
0.53866
0.53106
0.59684
0.60668
0.52487
0.51781
0.53162
0.53162
0.51342
0.51655
0.52879
0.53162
0.53106

0.57265
0.52879
0.52561
0.53106
0.59821
0.53106
0.50918
0.50546
0.50805
0.52952
0.54551
0.50546
0.49286
0.61321
0.50918
0.576
0.60964
0.52889
0.58596
0.6174
0.61346
0.58596
0.49531
0.54877
0.50985
0.51354
0.49549
0.51582
0.6175
0.55137
0.51164
0.52385
0.54146
0.52385
0.54055
0.59358
0.53857
0.6281
0.53857
0.52282
0.52696
0.53857
0.54055
0.59565
0.54055

0.63017
0.54055
0.63048
0.54325
0.54055
0.52333
0.62777
0.5075
0.60049
0.56177
0.62317
0.53024
0.57494
0.55482
0.60133
0.54654
0.6357
0.62777
0.62777
0.56257
0.54654
0.54098
0.62317
0.56257
0.56617
0.62317
0.54098
0.53797
0.54964
0.60335
0.56268
0.54098
0.60879
0.67645
0.56078
0.51784
0.56078
0.54098
0.63193
0.54098
0.56304
0.62317
0.62317
0.58534
0.62317

0.56264
0.62317
0.55398
0.6126
0.63193
0.55398
0.54646
0.58586
0.54415
0.61288
0.68391
0.6126
0.65876
0.54912
0.63193
0.54761
0.5817
0.54648
0.63193
0.6246
0.54912
0.54648
0.59006
0.6126
0.6126
0.6246
0.6246
0.66457
0.56305
0.55228
0.57902
0.55261
0.69241
0.58319
0.60081
0.53607
0.58104
0.55527
0.57368
0.59499
0.62584
0.55292
0.53607
0.56145
0.56797

0.63138
0.62756
0.62272
0.58638
0.55819
0.55819
0.58039
0.64484
0.59485
0.57176
0.64484
0.64484
0.67669
0.55923
0.60664
0.59593
0.57043
0.64484
0.67669
0.57043
0.59075
0.67669
0.60939
0.59075
0.5507
0.62087
0.59593
0.60939
0.69759
0.60302
0.57594
0.59593
0.60382
0.63925
0.64983
0.57276
0.64069
0.55369
0.60939
0.57276
0.64069
0.58094
0.60939
0.65389
0.64993

0.59565
0.5794
0.59565
0.60212
0.58094
0.64069
0.59191
0.57686
0.7076
0.63961
0.58094
0.63961
0.58646
0.63961
0.73252
0.61482
0.56249
0.60759
0.64069
0.65915
0.73252
0.61411
0.61411
0.64069
0.63031
0.65915
0.73252
0.71058
0.58016
0.70069
0.64051
0.64069
0.63031
0.58751
0.64069
0.62033
0.60094
0.64051
0.61902
0.64051
0.58291
0.58291
0.71868
0.67286
0.62996

0.62996
0.60176
0.57597
0.59854
0.62495
0.62087
0.62495
0.62087
0.58537
0.61025
0.58537
0.72405
0.64982
0.71928
0.60149
0.61025
0.60972
0.63238
0.62452
0.72405
0.72144
0.61346
0.66466
0.64265
0.72405
0.72144
0.65862
0.65756
0.65756
0.65862
0.60027
0.64672
0.70034
0.64465
0.72405
0.72405
0.63846
0.60036
0.72405
0.628
0.66126
0.64565
0.66001
0.64565
0.77863

0.64625
0.66001
0.64672
0.67848
0.64672
0.73325
0.65237
0.63004
0.64672
0.73325
0.65815
0.77863
0.66556
0.65815
0.77863
0.63013
0.6379
0.77863
0.63493
0.71719
0.66626
0.65339
0.70371
0.73624
0.63461
0.63493
0.73575
0.71719
0.65602
0.65755
0.65755
0.6413
0.67163
0.73575
0.65568
0.73624
0.69399
0.65759
0.72532
0.73624
0.78738
0.65892
0.70848
0.70848
0.69503

0.66309
0.67267
0.7418
0.72002
0.65947
0.65892
0.65947
0.65892
0.664
0.6542
0.6542
0.67403
0.74343
0.72002
0.63839
0.74343
0.72002
0.72002
0.67403
0.72002
0.66596
0.67403
0.65748
0.67443
0.66596
0.63839
0.72222
0.74368
0.66006
0.67806
0.70903
0.80194
0.67806
0.63839
0.66615
0.69798
0.72185
0.69761
0.66981
0.67806
0.80194
0.66615
0.66981
0.66626
0.80194

0.66199
0.6783
0.68263
0.66199
0.66719
0.75689
0.67539
0.66749
0.67282
0.73094
0.72229
0.67282
0.67825
0.80884
0.67539
0.68953
0.6604
0.75788
0.65312
0.71053
0.6857
0.75788
0.67022
0.80884
0.67539
0.76571
0.67539
0.6857
0.78428
0.66167
0.71053
0.6857
0.69172
0.67374
0.73539
0.71053
0.67374
0.68955
0.68955
0.6857
0.73539
0.67374
0.67464
0.6795
0.66018

0.81063
0.71164
0.81063
0.73662
0.67465
0.69181
0.69722
0.69331
0.68231
0.77728
0.73539
0.68231
0.69759
0.69722
0.77602
0.68231
0.68231
0.69141
0.73539
0.69141
0.71312
0.69722
0.66898
0.71312
0.73539
0.8035
0.71312
0.69865
0.69865
0.694
0.68654
0.78234
0.73539
0.68336
0.70297
0.73539
0.68336
0.73539
0.80945
0.70639
0.76845
0.70639
0.76845
0.71245
0.75557

0.71396
0.73539
0.75847
0.68519
0.73539
0.69728
0.83774
0.73539
0.68258
0.75847
0.81253
0.73539
0.83774
0.69821
0.70372
0.69818
0.71174
0.80638
0.75847
0.70372
0.77657
0.74592
0.73745
0.69904
0.7335
0.70372
0.71162
0.75847
0.80014
0.83959
0.74592
0.71872
0.70189
0.72648
0.80014
0.71872
0.69774
0.82375
0.72087
0.83959
0.78154
0.71434
0.72087
0.83959
0.71434

0.72842
0.72842
0.82432
0.7254
0.76933
0.77982
0.78408
0.72992
0.72605
0.70609
0.78408
0.73958
0.71477
0.84957
0.71477
0.76426
0.72492
0.71244
0.85013
0.71139
0.72492
0.71725
0.71139
0.7604
0.76426
0.7361
0.74103
0.71139
0.76886
0.72492
0.7604
0.73575
0.73575
0.78238
0.73086
0.73455
0.74103
0.78238
0.84746
0.73575
0.74103
0.74103
0.7416
0.7416
0.7993

0.74728
0.71228
0.72128
0.7416
0.76301
0.76426
0.74153
0.74175
0.71228
0.73697
0.7993
0.7338
0.73604
0.7416
0.76426
0.73604
0.76325
0.7416
0.77359
0.76426
0.77359
0.76426
0.80063
0.85626
0.71523
0.75345
0.74604
0.75231
0.73548
0.75345
0.75345
0.80612
0.71972
0.71972
0.75439
0.87641
0.75011
0.76248
0.87078
0.87641
0.87641
0.80967
0.76061
0.78291
0.86597

0.7585
0.79327
0.75518
0.74876
0.80967
0.79327
0.80967
0.78458
0.74876
0.86597
0.7701
0.8243
0.80979
0.79327
0.77082
0.86597
0.86597
0.77082
0.79327
0.7532
0.77171
0.88799
0.77224
0.90539
0.79085
0.81297
0.77674
0.88799
0.77018
0.80597
0.77018
0.74515
0.87295
0.80497
0.88799
0.80597
0.76898
0.8016
0.76898
0.83181
0.83181
0.88799
0.77299
0.76898
0.83181

0.77326
0.80689
0.87722
0.77326
0.92131
0.87529
0.78309
0.79246
0.80797
0.78677
0.78677
0.78309
0.77399
0.78309
0.77419
0.79246
0.89683
0.8808
0.81227
0.75908
0.8759
0.79508
0.89715
0.92276
0.83842
0.79508
0.79564
0.81675
0.89715
0.81514
0.88253
0.78394
0.80806
0.79862
0.79997
0.80022
0.81514
0.81953
0.84529
0.88926
0.81514
0.88926
0.83116
0.80854
0.81516

0.89
0.82352
0.80501
0.80532
0.84906
0.79671
0.94929
0.80184
0.83522
0.94929
0.94568
0.83021
0.80766
0.80806
0.85325
0.78437
0.89984
0.78437
0.84064
0.89984
0.85749
0.80958
0.78442
0.88117
0.94568
0.83033
0.94568
0.81821
0.81821
0.8262
0.94568
0.81428
0.82252
0.79045
0.82812
0.84613
0.93595
0.79045
0.92165
0.81518
0.94685
0.93595
0.85721
0.81518
0.97829

0.94685
0.84613
0.83967
0.82864
0.93595
0.89823
0.84613
0.84631
0.92182
0.82864
0.85841
0.85315
0.84834
0.85841
0.84985
0.93034
0.94849
0.99529
0.96
0.96
0.84618
0.84618
0.85216
0.84739
0.93173
0.89993
0.86442
0.91829
0.90398
0.8598
0.8543
0.8598
0.86584
0.96934
0.90321
0.85657
0.90321
0.95968
0.87251
0.92338
0.86526
0.86047
0.92338
1
0.85906

0.94607
0.85906
0.96194
0.86117
0.92338
0.87147
0.9782
0.88403
0.88009
0.91888
0.94607
0.94607
1
0.96403
0.8709
1
0.87421
0.87278
1
0.86381
0.88109
0.8709
0.88257
0.87278
0.86381
0.9257
0.98225
0.98225
0.9257
0.91888
1
0.90664
0.88248
0.98809
0.88642
0.87074
0.88642
0.87532
0.87074
0.91888
0.88642
0.87603
0.86443
0.91888
0.86443

0.98712
0.87986
0.87603
0.87074
0.92868
0.93198
0.88776
0.93484
0.89426
0.93198
0.98755
0.99315
0.98518
0.96779
0.92909
0.99315
0.98755
0.88455
0.91888
0.87603
0.87603
0.8978
0.89378
0.91888
0.93031
0.87603
0.91888
0.98902
0.91888
0.87603
0.91888
0.87603
0.94437
0.87473
0.87473
0.89941
0.97733
0.90582
0.89627
0.93836
1
0.99284
0.92833
0.97733
0.94189

0.94832
0.88183
0.93836
0.91661
0.97733
0.99284
0.92833
1
1
1
0.9191
0.96425
0.99284
0.9012
0.9776
1
0.88214
0.99284
0.9191
0.88214
0.99284
1
0.9191
0.92168
0.90941
0.93601
0.92168
0.92213
0.88466
1
0.95701
0.97473
0.9232
0.92376
0.98605
0.98605
0.92615
1
0.94493
0.96114
0.9216
0.95801
0.98613
1
0.96533

0.90895
1
0.93087
0.88672
0.88672
0.96114
0.93087
0.98551
1
0.9326
0.92879
0.93439
0.94711
0.92701
0.88672
0.88672
0.93363
0.94825
1
0.93433
1
0.97261
0.94825
1
1
0.96923
0.88672
0.99287
1
0.93023
0.88672
0.94825
0.92096
0.93023
0.99072
0.94825
0.96962
0.93023
0.94367
0.96736
0.93216
0.93023
0.96736
1
0.93216

0.96736
1
0.96953
0.99072
1
0.98314
0.99072
0.96287
0.99072
0.99072
1
1
0.95273
0.98569
0.95357
1
1
0.96139
1
0.9845
1
1
0.94377
0.98014
0.97118
0.96287
1
0.90888
0.94858
1
0.96287
1
0.99267
0.96029
1
0.96287
1
0.96287
0.97238
0.9682
0.99659
1
0.96887
0.96287
1

0.96595
0.9159
0.97238
0.96887
0.96287
0.96887
0.9682
0.9159
0.96048
1
1
1
1
1
1
0.9649
0.9926
1
1
1
1
1
0.9926
1
1
1
1
1
0.98541
1
1
1
1
1
0.97075
1
0.97961
1
1
0.97611
0.9855
0.98662
1
0.97075
1

0.99818
0.97963
0.97628
0.93211
0.93211
0.99818
0.99254
0.99392
1
0.98905
1
1
1
1
1
0.99477
1
1
1
0.99672
0.99276
1
1
0.97817
1
1
0.99788
1
0.99509
0.98787
0.98787
0.99788
1
0.97817
1
1
0.99695
1
0.97817
1
0.99695
1
1
0.99509
0.99509

0.99509
1
1
1
1
1
0.997
0.99643
1
1
0.99643
1
1
1
0.97817
0.95835
1
1
1
1
1
1
0.97817
1
1
1
0.99958
0.99958
0.99918
1
1
1
0.97817
1
1
1
1
1
1
0.96035
0.99918
0.99958
1
0.96035
1

0.97817
1
0.97817
1
0.97817
1
1
1
1
1
1
1
1
1
1
1
0.9625
1
1
0.97996
1
1
1
1
1
1
1
0.98006
1
1
1
1
1
1
1
1
1
1
1
1
1
1
1
1

1
1
1
1
1
1
1
1
1
1
1
1
1
1
1
1
1
1
1
1
1
1
1
1
1
1
1
1
1
1
1
1
1
1
1
1
1
1
1
1
1
1
1
1
1

0.99962
1
1
1
1
1
1
1
1
1
1
1
1
0.99962
1
1
0.99962
1
0.99962
1
1
1
1
1
1
1
1
1
1
1
1
1
1
1
1
1
1
1
1
1
1
1
1
1
1

1
1
1
1
1
1
1
1
1
1
1
1
1
1
1
1
1
1
1
1
1
1
1
1
1
1
1
1
1
1
1
1
1
1
1
1
1
1
1
1
1
1
1
1
1

1
1
1
1
1
1
1
1
1
1
1
1
1
1
1
1
1
1
1
1
1
1
1
1
1
1
1
1
1
1
1
1
1
1
1
1
1
1
1
1
1
1
1
1
1

1
1
1
1
1
1
1
1
1
1
1
1
1
1
1
1
1
1
1
1
1
1
1
1
1
1
1
1
1
1
1
1
1
1
1
1
1
1
1
1
1
1
1
1
1

1
1
1
1
1
1
1
1
1
1
1
1
1
1
1
1
1
1
1
1
1
1
1
1
1
1
1
1
1
1
1
1
1
1
1
1
1
1
1
1
1
1
1
1
1

1
1
1
1
1
1
1
1
1
1
1
1
1
1
1
1
1
1
1
1
1
1
1
1
1
1
1
1
1
1
1
1
1
1
1
1
1
1
1
1
1
1
1
1
1

1
1
1
1
1
1
1
1
1
1
1
1
1
1
1
1
1
1
1
1
1
1
1
1
1
1
1
1
1
1
1
1
1
1
1
1
1
1
1
1
1
1
1
1
1

1
1
1
1
1
1
1
1
1
1
1
1
1
1
1
1
1
1
1
1
1
1
1
1
1
1
1
1
1
1
1
1
1
1
1
1
1
1
1
1
1
1
1
1
1

1
1
1
1
1
1
1
1
1
1
1
1
1
1
1
1
1
1
1
1
1
1
1
1
1
1
1
1
1
1
1
1
1
1
1
1
1
1
1
1
1
1
1
1
1

1
1
1
1
1
1
1
1
1
1
1
1
1
1
1
1
1
1
1
1
1
1
1
1
1
1
1
1
1
1
1
1
1
1
1
1
1
1
1
1
1
1
1
1
1

1
1
1
1
1
1
1
1
1
1
1
1
1
1
1
1
1
1
1
1
1
1
1
1
1
1
1
1
1
1
1
1
1
1
1
1
1
1
1
1
1
1
1
1
1

1
1
1
1
1
1
1
1
1
1
1
1
1
1
1
1
1
1
1
1
1
1
1
1
1
1
1
1
1
1
1
1
1
1
1
1
1
1
1
1
1
1
1
1
1

1
1
1
1
1
1
1
1
1
1
1
1
1
1
1
1
1
1
1
1
1
1
1
1
1
1
1
1
1
1
1
1
1
1
1
1
1
1
1
1
1
1
1
1
1

1
1
1
1
1
1
1
1
1
1
1
1
1
1
1
1
1
1
1
1
1
1
1
1
1
1
1
1
1
1
1
1
1
1
1
1
1
1
1
1
1
1
1
1
1

1
1
1
1
1
1
1
1
1
1
1
1
1
1
1
1
1
1
1
1
1
1
1
1
1
1
1
1
1
1
1
1
1
1
1
1
1
1
1
1
1
1
1
1
1

1
1
1
1
1
1
1
1
1
1
1
1
1
1
1
1
1
1
1
1
1
1
1
1
1
1
1
1
1
1
1
1
1
1
1
1
1
1
1
1
1
1
1
1
1

1
1
1
1
1
1
1
1
1
1
1
1
1
1
1
1
1
1
1
1
1
1
1
1
1
1
1
1
1
1
1
1
1
1
1
1
1
1
1
1
1
1
1
1
1

1
1
1
1
1
1
1
1
1
1
1
1
1
1
1
1
1
1
1
1
1
1
1
1
1
1
1
1
1
1
1
1
1
1
1
1
1
1
1
1
1
1
1
1
1

1
1
1
1
1
1
1
1
1
1
1
1
1
1
1
1
1
1
1
1
1
1
1
1
1
1
1
1
1
1
1
1
1
1
1
1
1
1
1
1
1
1
1
1
1

1
1
1
1
1
1
1
1
1
1
1
1
1
1
1
1
1
1
1
1
1
1
1
1
1
1
1
1
1
1
1
1
1
1
1
1
1
1
1
1
1
1
1
1
1

1
1
1
1
1
1
1
1
1
1
1
1
1
1
1
1
1
1
1
1
1
1
1
1
1
1
1
1
1
1
1
1
1
1
1
1
1
1
1
1
1
1
1
1
1

1
1
1
1
1
1
1
1
1
1
1
1
1
1
1
1
1
1
1
1
1
1
1
1
1
1
1
1
1
1
1
1
1
1
1
1
1
1
1
1
1
1
1
1
1

1
1
1
1
1
1
1
1
1
1
1
1
1
1
1
1
1
1
1
1
1
1
1
1
1
1
1
1
1
1
1
1
1
1
1
1
1
1
1
1
1
1
1
1
1

1
1
1
1
1
1
1
1
1
1
1
1
1
1
1
1
1
1
1
1
1
1
1
1
1
1
1
1
1
1
1
1
1
1
1
1
1
1
1
1
1
1
1
1
1

1
1
1
1
1
1
1
1
1
1
1
1
1
1
1
1
1
1
1
1
1
1
1
1
1
1
1
1
1
1
1
1
1
1
1
1
1
1
1
1
1
1
1
1
1

1
1
1
1
1
1
1
1
1
1
1
1
1
1
1
1
1
1
1
1
1
1
1
1
1
1
1
1
1
1
1
1
1
1
1
1
1
1
1
1
1
1
1
1
1

1
1
1
1
1
1
1
1
1
1
1
1
1
1
1
1
1
1
1
1
1
1
1
1
1
1
1
1
1
1
1
1
1
1
1
1
1
1
1
1
1
1
1
1
1

1
1
1
1
1
1
1
1
1
1
1
1
1
1
1
1
1
1
1
1
1
1
1
1
1
1
1
1
1
1
1
1
1
1
1
1
1
1
1
1
1
1
1
1
1

1
1
1
1
1
1
1
1
1
1
1
1
1
1
1
1
1
1
1
1
1
1
1
1
1
1
1
1
1
1
1
1
1
1
1
1
1
1
1
1
1
1
1
1
1

1
1
1
1
1
1
1
1
1
1
1
1
1
1
1
1
1
1
1
1
1
1
1
1
1
1
1
1
1
1
1
1
1
1
1
1
1
1
1
1
1
1
1
1
1

1
1
1
1
1
1
1
1
1
1
1
1
1
1
1
1
1
1
1
1
1
1
1
1
1
1
1
1
1
1
1
1
1
1
1
1
1
1
1
1
1
1
1
1
1

1
1
1
1
1
1
1
1
1
1
1
1
1
1
1
1
1
1
1
1
1
1
1
1
1
1
1
1
1
1
1
1
1
1
1
1
1
1
1
1
1
1
1
1
1

1
1
1
1
1
1
1
1
1
1
1
1
1
1
1
1
1
1
1
1
1
1
1
1
1
1
1
1
1
1
1
1
1
1
1
1
1
1
1
1
1
1
1
1
1

1
1
1
1
1
1
1
1
1
1
1
1
1
1
1
1
1
1
1
1
1
1
1
1
1
1
1
1
1
1
1
1
1
1
1
1
1
1
1
1
1
1
1
1
1

1
1
1
1
1
1
1
1
1
1
1
1
1
1
1
1
1
1
1
1
1
1
1
1
1
1
1
1
1
1
1
1
1
1
1
1
1
1
1
1
1
1
1
1
1

1
1
1
1
1
1
1
1
1
1
1
1
1
1
1
1
1
1
1
1
1
1
1
1
1
1
1
1
1
1
1
1
1
1
1
1
1
1
1
1
1
1
1
1
1

1
1
1
1
1
1
1
1
1
1
1
1
1
1
1
1
1
1
1
1
1
1
1
1
1
1
1
1
1
1
1
1
1
1
1
1
1
1
1
1
1
1
1
1
1

1
1
1
1
1
1
1
1
1
1
1
1
1
1
1
1
1
1
1
1
1
1
1
1
1
1
1
1
1
1
1
1
1
1
1
1
1
1
1
1
1
1
1
1
1

1
1
1
1
1
1
1
1
1
1
1
1
1
1
1
1
1
1
1
1
1
1
1
1
1
1
1
1
1
1
1
1
1
1
1
1
1
1
1
1
1
1
1
1
1

1
1
1
1
1
1
1
1
1
1
1
1
1
1
1
1
1
1
1
1
1
1
1
1
1
1
1
1
1
1
1
1
1
1
1
1
1
1
1
1
1
1
1
1
1

1
1
1
1
1
1
1
1
1
1
1
1
1
1
1
1
1
1
1
1
1
1
1
1
1
1
1
1
1
1
1
1
1
1
1
1
1
1
1
1
1
1
1
1
1

1
1
1
1
1
1
1
1
1
1
1
1
1
1
1
1
1
1
1
1
1
1
1
1
1
1
1
1
1
1
1
1
1
1
1
1
1
1
1
1
1
1
1
1
1

1
1
1
1
1
1
1
1
1
1
1
1
1
1
1
1
1
1
1
1
1
1
1
1
1
1
1
1
1
1
1
1
1
1
1
1
1
1
1
1
1
1
1
1
1

1
1
1
1
1
1
1
1
1
1
1
1
1
1
1
1
1
1
1
1
1
1
1
1
1
1
1
1
1
1
1
1
1
1
1
1
1
1
1
1
1
1
1
1
1

1
1
1
1
1
1
1
1
1
1
1
1
1
1
1
1
1
1
1
1
1
1
1
1
1
1
1
1
1
1
1
1
1
1
1
1
1
1
1
1
1
1
1
1
1

1
1
1
1
1
1
1
1
1
1
1
1
1
1
1
1
1
1
1
1
1
1
1
1
1
1
1
1
1
1
1
1
1
1
1
1
1
1
1
1
1
1
1
1
1

1
1
1
1
1
1
1
1
1
1
1
1
1
1
1
1
1
1
1
1
1
1
1
1
1
1
1
1
1
1
1
1
1
1
1
1
1
1
1
1
1
1
1
1
1

1
1
1
1
1
1
1
1
1
1
1
1
1
1
1
1
1
1
1
1
1
1
1
1
1
1
1
1
1
1
1
1
1
1
1
1
1
1
1
1
1
1
1
1
1

1
1
1
1
1
1
1
1
1
1
1
1
1
1
1
1
1
1
1
1
1
1
1
1
1
1
1
1
1
1
1
1
1
1
1
1
1
1
1
1
1
1
1
1
1

1
1
1
1
1
1
1
1
1
1
1
1
1
1
1
1
1
1
1
1
1
1
1
1
1
1
1
1
1
1
1
1
1
1
1
1
1
1
1
1
1
1
1
1
1

1
1
1
1
1
1
1
1
1
1
1
1
1
1
1
1
1
1
1
1
1
1
1
1
1
1
1
1
1
1
1
1
1
1
1
1
1
1
1
1
1
1
1
1
1

1
1
1
1
1
1
1
1
1
1
1
1
1
1
1
1
1
1
1
1
1
1
1
1
1
1
1
1
1
1
1
1
1
1
1
1
1
1
1
1
1
1
1
1
1

1
1
1
1
1
1
1
1
1
1
1
1
1
1
1
1
1
1
1
1
1
1
1
1
1
1
1
1
1
1
1
1
1
1
1
1
1
1
1
1
1
1
1
1
1

1
1
1
1
1
1
1
1
1
1
1
1
1
1
1
1
1
1
1
1
1
1
1
1
1
1
1
1
1
1
1
1
1
1
1
1
1
1
1
1
1
1
1
1
1

1
1
1
1
1
1
1
1
1
1
1
1
1
1
1
1
1
1
1
1
1
1
1
1
1
1
1
1
1
1
1
1
1
1
1
1
1
1
1
1
1
1
1
1
1

1
1
1
1
1
1
1
1
1
1
1
1
1
1
1
1
1
1
1
1
1
1
1
1
1
1
1
1
1
1
1
1
1
1
1
1
1
1
1
1
1
1
1
1
1

1
1
1
1
1
1
1
1
1
1
1
1
1
1
1
1
1
1
1
1
1
1
1
1
1
1
1
1
1
1
1
1
1
1
1
1
1
1
1
1
1
1
1
1
1

1
1
1
1
1
1
1
1
1
1
1
1
1
1
1
1
1
1
1
1
1
1
1
1
1
1
1
1
1
1
1
1
1
1
1
1
1
1
1
1
1
1
1
1
1

1
1
1
1
1
1
1
1
1
1
1
1
1
1
1
1
1
1
1
1
1
1
1
1
1
1
1
1
1
1
1
1
1
1
1
1
1
1
1
1
1
1
1
1
1

1
1
1
1
1
1
1
1
1
1
1
1
1
1
1
1
1
1
1
1
1
1
1
1
1
1
1
1
1
1
1
1
1
1
1
1
1
1
1
1
1
1
1
1
1

1
1
1
1
1
1
1
1
1
1
1
1
1
1
1
1
1
1
1
1
1
1
1
1
1
1
1
1
1
1
1
1
1
1
1
1
1
1
1
1
1
1
1
1
1

1
1
1
1
1
1
1
1
1
1
1
1
1
1
1
1
1
1
1
1
1
1
1
1
1
1
1
1
1
1
1
1
1
1
1
1
1
1
1
1
1
1
1
1
1

1
1
1
1
1
1
1
1
1
1
1
1
1
1
1
1
1
1
1
1
1
1
1
1
1
1
1
1
1
1
1
1
1
1
1
1
1
1
1
1
1
1
1
1
1

1
1
1
1
1
1
1
1
1
1
1
1
1
1
1
1
1
1
1
1
1
1
1
1
1
1
1
1
1
1
1
1
1
1
1
1
1
1
1
1
1
1
1
1
1

1
1
1
1
1
1
1
1
1
1
1
1
1
1
1
1
1
1
1
1
1
1
1
1
1
1
1
1
1
1
1
1
1
1
1
1
1
1
1
1
1
1
1
1
1

1
1
1
1
1
1
1
1
1
1
1
1
1
1
1
1
1
1
1
1
1
1
1
1
1
1
1
1
1
1
1
1
1
1
1
1
1
1
1
1
1
1
1
1
1

1
1
1
1
1
1
1
1
1
1
1
1
1
1
1
1
1
1
1
1
1
1
1
1
1
1
1
1
1
1
1
1
1
1
1
1
1
1
1
1
1
1
1
1
1

1
1
1
1
1
1
1
1
1
1
1
1
1
1
1
1
1
1
1
1
1
1
1
1
1
1
1
1
1
1
1
1
1
1
1
1
1
1
1
1
1
1
1
1
1

1
1
1
1
1
1
1
1
1
1
1
1
1
1
1
1
1
1
1
1
1
1
1
1
1
1
1
1
1
1
1
1
1
1
1
1
1
1
1
1
1
1
1
1
1

1
1
1
1
1
1
1
1
1
1
1
1
1
1
1
1
1
1
1
1
1
1
1
1
1
1
1
1
1
1
1
1
1
1
1
1
1
1
1
1
1
1
1
1
1

1
1
1
1
1
1
1
1
1
1
1
1
1
1
1
1
1
1
1
1
1
1
1
1
1
1
1
1
1
1
1
1
1
1
1
1
1
1
1
1
1
1
1
1
1

1
1
1
1
1
1
1
1
1
1
1
1
1
1
1
1
1
1
1
1
1
1
1
1
1
1
1
1
1
1
1
1
1
1
1
1
1
1
1
1
1
1
1
1
1

1
1
1
1
1
1
1
1
1
1
1
1
1
1
1
1
1
1
1
1
1
1
1
1
1
1
1
1
1
1
1
1
1
1
1
1
1
1
1
1
1
1
1
1
1

1
1
1
1
1
1
1
1
1
1
1
1
1
1
1
1
1
1
1
1
1
1
1
1
1
1
1
1
1
1
1
1
1
1
1
1
1
1
1
1
1
1
1
1
1

1
1
1
1
1
1
1
1
1
1
1
1
1
1
1
1
1
1
1
1
1
1
1
1
1
1
1
1
1
1
1
1
1
1
1
1
1
1
1
1
1
1
1
1
1

1
1
1
1
1
1
1
1
1
1
1
1
1
1
1
1
1
1
1
1
1
1
1
1
1
1
1
1
1
1
1
1
1
1
1
1
1
1
1
1
1
1
1
1
1

1
1
1
1
1
1
1
1
1
1
1
1
1
1
1
1
1
1
1
1
1
1
1
1
1
1
1
1
1
1
1
1
1
1
1
1
1
1
1
1
1
1
1
1
1

1
1
1
1
1
1
1
1
1
1
1
1
1
1
1
1
1
1
1
1
1
1
1
1
1
1
1
1
1
1
1
1
1
1
1
1
1
1
1
1
1
1
1
1
1

1
1
1
1
1
1
1
1
1
1
1
1
1
1
1
1
1
1
1
1
1
1
1
1
1
1
1
1
1
1
1
1
1
1
1
1
1
1
1
1
1
1
1
1
1

1
1
1
1
1
1
1
1
1
1
1
1
1
1
1
1
1
1
1
1
1
1
1
1
1
1
1
1
1
1
1
1
1
1
1
1
1
1
1
1
1
1
1
1
1

1
1
1
1
1
1
1
1
1
1
1
1
1
1
1
1
1
1
1
1
1
1
1
1
1
1
1
1
1
1
1
1
1
1
1
1
1
1
1
1
1
1
1
1
1

1
1
1
1
1
1
1
1
1
1
1
1
1
1
1
1
1
1
1
1
1
1
1
1
1
1
1
1
1
1
1
1
1
1
1
1
1
1
1
1
1
1
1
1
1

1
1
1
1
1
1
1
1
1
1
1
1
1
1
1
1
1
1
1
1
1
1
1
1
1
1
1
1
1
1
1
1
1
1
1
1
1
1
1
1
1
1
1
1
1

1
1
1
1
1
1
1
1
1
1
1
1
1
1
1
1
1
1
1
1
1
1
1
1
1
1
1
1
1
1
1
1
1
1
1
1
1
1
1
1
1
1
1
1
1

1
1
1
1
1
1
1
1
1
1
1
1
1
1
1
1
1
1
1
1
1
1
1
1
1
1
1
1
1
1
1
1
1
1
1
1
1
1
1
1
1
1
1
1
1

1
1
1
1
1
1
1
1
1
1
1
1
1
1
1
1
1
1
1
1
1
1
1
1
1
1
1
1
1
1
1
1
1
1
1
1
1
1
1
1
1
1
1
1
1

1
1
1
1
1
1
1
1
1
1
1
1
1
1
1
1
1
1
1
1
1
1
1
1
1
1
1
1
1
1
1
1
1
1
1
1
1
1
1
1
1
1
1
1
1

1
1
1
1
1
1
1
1
1
1
1
1
1
1
1
1
1
1
1
1
1
1
1
1
1
1
1
1
1
1
1
1
1
1
1
1
1
1
1
1
1
1
1
1
1

1
1
1
1
1
1
1
1
1
1
1
1
1
1
1
1
1
1
1
1
1
1
1
1
1
1
1
1
1
1
1
1
1
1
1
1
1
1
1
1
1
1
1
1
1

1
1
1
1
1
1
1
1
1
1
1
1
1
1
1
1
1
1
1
1
1
1
1
1
1
1
1
1
1
1
1
1
1
1
1
1
1
1
1
1
1
1
1
1
1

1
1
1
1
1
1
1
1
1
1
1
1
1
1
1
1
1
1
1
1
1
1
1
1
1
1
1
1
1
1
1
1
1
1
1
1
1
1
1
1
1
1
1
1
1

1
1
1
1
1
1
1
1
1
1
1
1
1
1
1
1
1
1
1
1
1
1
1
1
1
1
1
1
1
1
1
1
1
1
1
1
1
1
1
1
1
1
1
1
1

1
1
1
1
1
1
1
1
1
1
1
1
1
1
1
1
1
1
1
1
1
1
1
1
1
1
1
1
1
1
1
1
1
1
1
1
1
1
1
1
1
1
1
1
1

1
1
1
1
1
1
1
1
1
1
1
1
1
1
1
1
1
1
1
1
1
1
1
1
1
1
1
1
1
1
1
1
1
1
1
1
1
1
1
1
1
1
1
1
1

1
1
1
1
1
1
1
1
1
1
1
1
1
1
1
1
1
1
1
1
1
1
1
1
1
1
1
1
1
1
1
1
1
1
1
1
1
1
1
1
1
1
1
1
1

1
1
1
1
1
1
1
1
1
1
1
1
1
1
1
1
1
1
1
1
1
1
1
1
1
1
1
1
1
1
1
1
1
1
1
1
1
1
1
1
1
1
1
1
1

1
1
1
1
1
1
1
1
1
1
1
1
1
1
1
1
1
1
1
1
1
1
1
1
1
1
1
1
1
1
1
1
1
1
1
1
1
1
1
1
1
1
1
1
1

1
1
1
1
1
1
1
1
1
1
1
1
1
1
1
1
1
1
1
1
1
1
1
1
1
1
1
1
1
1
1
1
1
1
1
1
1
1
1
1
1
1
1
1
1

1
1
1
1
1
1
1
1
1
1
1
1
1
1
1
1
1
1
1
1
1
1
1
1
1
1
1
1
1
1
1
1
1
1
1
1
1
1
1
1
1
1
1
1
1

1
1
1
1
1
1
1
1
1
1
1
1
1
1
1
1
1
1
1
1
1
1
1
1
1
1
1
1
1
1
1
1
1
1
1
1
1
1
1
1
1
1
1
1
1

1
1
1
1
1
1
1
1
1
1
1
1
1
1
1
1
1
1
1
1
1
1
1
1
1
1
1
1
1
1
1
1
1
1
1
1
1
1
1
1
1
1
1
1
1

1
1
1
1
1
1
1
1
1
1
1
1
1
1
1
1
1
1
1
1
1
1
1
1
1
1
1
1
1
1
1
1
1
1
1
1
1
1
1
1
1
1
1
1
1

1
1
1
1
1
1
1
1
1
1
1
1
1
1
1
1
1
1
1
1
1
1
1
1
1
1
1
1
1
1
1
1
1
1
1
1
1
1
1
1
1
1
1
1
1

1
1
1
1
1
1
1
1
1
1
1
1
1
1
1
1
1
1
1
1
1
1
1
1
1
1
1
1
1
1
1
1
1
1
1
1
1
1
1
1
1
1
1
1
1

1
1
1
1
1
1
1
1
1
1
1
1
1
1
1
1
1
1
1
1
1
1
1
1
1
1
1
1
1
1
1
1
1
1
1
1
1
1
1
1
1
1
1
1
1

1
1
1
1
1
1
1
1
1
1
1
1
1
1
1
1
1
1
1
1
1
1
1
1
1
1
1
1
1
1
1
1
1
1
1
1
1
1
1
1
1
1
1
1
1

1
1
1
1
1
1
1
1
1
1
1
1
1
1
1
1
1
1
1
1
1
1
1
1
1
1
1
1
1
1
1
1
1
1
1
1
1
1
1
1
1
1
1
1
1

1
1
1
1
1
1
1
1
1
1
1
1
1
1
1
1
1
1
1
1
1
1
1
1
1
1
1
1
1
1
1
1
1
1
1
1
1
1
1
1
1
1
1
1
1

1
1
1
1
1
1
1
1
1
1
1
1
1
1
1
1
1
1
1
1
1
1
1
1
1
1
1
1
1
1
1
1
1
1
1
1
1
1
1
1
1
1
1
1
1

1
1
1
1
1
1
1
1
1
1
1
1
1
1
1
1
1
1
1
1
1
1
1
1
1
1
1
1
1
1
1
1
1
1
1
1
1
1
1
1
1
1
1
1
1

1
1
1
1
1
1
1
1
1
1
1
1
1
1
1
1
1
1
1
1
1
1
1
1
1
1
1
1
1
1
1
1
1
1
1
1
1
1
1
1
1
1
1
1
1

1
1
1
1
1
1
1
1
1
1
1
1
1
1
1
1
1
1
1
1
1
1
1
1
1
1
1
1
1
1
1
1
1
1
1
1
1
1
1
1
1
1
1
1
1

1
1
1
1
1
1
1
1
1
1
1
1
1
1
1
1
1
1
1
1
1
1
1
1
1
1
1
1
1
1
1
1
1
1
1
1
1
1
1
1
1
1
1
1
1

1
1
1
1
1
1
1
1
1
1
1
1
1
1
1
1
1
1
1
1
1
1
1
1
1
1
1
1
1
1
1
1
1
1
1
1
1
1
1
1
1
1
1
1
1

1
1
1
1
1
1
1
1
1
1
1
1
1
1
1
1
1
1
1
1
1
1
1
1
1
1
1
1
1
1
1
1
1
1
1
1
1
1
1
1
1
1
1
1
1

1
1
1
1
1
1
1
1
1
1
1
1
1
1
1
1
1
1
1
1
1
1
1
1
1
1
1
1
1
1
1
1
1
1
1
1
1
1
1
1
1
1
1
1
1

1
1
1
1
1
1
1
1
1
1
1
1
1
1
1
1
1
1
1
1
1
1
1
1
1
1
1
1
1
1
1
1
1
1
1
1
1
1
1
1
1
1
1
1
1

1
1
1
1
1
1
1
1
1
1
1
1
1
1
1
1
1
1
1
1
1
1
1
1
1
1
1
1
1
1
1
1
1
1
1
1
1
1
1
1
1
1
1
1
1

1
1
1
1
1
1
1
1
1
1
1
1
1
1
1
1
1
1
1
1
1
1
1
1
1
1
1
1
1
1
1
1
1
1
1
1
1
1
1
1
1
1
1
1
1

1
1
1
1
1
1
1
1
1
1
1
1
1
1
1
1
1
1
1
1
1
1
1
1
1
1
1
1
1
1
1
1
1
1
1
1
1
1
1
1
1
1
1
1
1

1
1
1
1
1
1
1
1
1
1
1
1
1
1
1
1
1
1
1
1
1
1
1
1
1
1
1
1
1
1
1
1
1
1
1
1
1
1
1
1
1
1
1
1
1

1
1
1
1
1
1
1
1
1
1
1
1
1
1
1
1
1
1
1
1
1
1
1
1
1
1
1
1
1
1
1
1
1
1
1
1
1
1
1
1
1
1
1
1
1

1
1
1
1
1
1
1
1
1
1
1
1
1
1
1
1
1
1
1
1
1
1
1
1
1
1
1
1
1
1
1
1
1
1
1
1
1
1
1
1
1
1
1
1
1

1
1
1
1
1
1
1
1
1
1
1
1
1
1
1
1
1
1
1
1
1
1
1
1
1
1
1
1
1
1
1
1
1
1
1
1
1
1
1
1
1
1
1
1
1

1
1
1
1
1
1
1
1
1
1
1
1
1
1
1
1
1
1
1
1
1
1
1
1
1
1
1
1
1
1
1
1
1
1
1
1
1
1
1
1
1
1
1
1
1

1
1
1
1
1
1
1
1
1
1
1
1
1
1
1
1
1
1
1
1
1
1
1
1
1
1
1
1
1
1
1
1
1
1
1
1
1
1
1
1
1
1
1
1
1

1
1
1
1
1
1
1
1
1
1
1
1
1
1
1
1
1
1
1
1
1
1
1
1
1
1
1
1
1
1
1
1
1
1
1
1
1
1
1
1
1
1
1
1
1

1
1
1
1
1
1
1
1
1
1
1
1
1
1
1
1
1
1
1
1
1
1
1
1
1
1
1
1
1
1
1
1
1
1
1
1
1
1
1
1
1
1
1
1
1

1
1
1
1
1
1
1
1
1
1
1
1
1
1
1
1
1
1
1
1
1
1
1
1
1
1
1
1
1
1
1
1
1
1
1
1
1
1
1
1
1
1
1
1
1

1
1
1
1
1
1
1
1
1
1
1
1
1
1
1
1
1
1
1
1
1
1
1
1
1
1
1
1
1
1
1
1
1
1
1
1
1
1
1
1
1
1
1
1
1

1
1
1
1
1
1
1
1
1
1
1
1
1
1
1
1
1
1
1
1
1
1
1
1
1
1
1
1
1
1
1
1
1
1
1
1
1
1
1
1
1
1
1
1
1

1
1
1
1
1
1
1
1
1
1
1
1
1
1
1
1
1
1
1
1
1
1
1
1
1
1
1
1
1
1
1
1
1
1
1
1
1
1
1
1
1
1
1
1
1

1
1
1
1
1
1
1
1
1
1
1
1
1
1
1
1
1
1
1
1
1
1
1
1
1
1
1
1
1
1
1
1
1
1
1
1
1
1
1
1
1
1
1
1
1

1
1
1
1
1
1
1
1
1
1
1
1
1
1
1
1
1
1
1
1
1
1
1
1
1
1
1
1
1
1
1
1
1
1
1
1
1
1
1
1
1
1
1
1
1

1
1
1
1
1
1
1
1
1
1
1
1
1
1
1
1
1
1
1
1
1
1
1
1
1
1
1
1
1
1
1
1
1
1
1
1
1
1
1
1
1
1
1
1
1

1
1
1
1
1
1
1
1
1
1
1
1
1
1
1
1
1
1
1
1
1
1
1
1
1
1
1
1
1
1
1
1
1
1
1
1
1
1
1
1
1
1
1
1
1

1
1
1
1
1
1
1
1
1
1
1
1
1
1
1
1
1
1
1
1
1
1
1
1
1
1
1
1
1
1
1
1
1
1
1
1
1
1
1
1
1
1
1
1
1

1
1
1
1
1
1
1
1
1
1
1
1
1
1
1
1
1
1
1
1
1
1
1
1
1
1
1
1
1
1
1
1
1
1
1
1
1
1
1
1
1
1
1
1
1

1
1
1
1
1
1
1
1
1
1
1
1
1
1
1
1
1
1
1
1
1
1
1
1
1
1
1
1
1
1
1
1
1
1
1
1
1
1
1
1
1
1
1
1
1

1
1
1
1
1
1
1
1
1
1
1
1
1
1
1
1
1
1
1
1
1
1
1
1
1
1
1
1
1
1
1
1
1
1
1
1
1
1
1
1
1
1
1
1
1

1
1
1
1
1
1
1
1
1
1
1
1
1
1
1
1
1
1
1
1
1
1
1
1
1
1
1
1
1
1
1
1
1
1
1
1
1
1
1
1
1
1
1
1
1

1
1
1
1
1
1
1
1
1
1
1
1
1
1
1
1
1
1
1
1
1
1
1
1
1
1
1
1
1
1
1
1
1
1
1
1
1
1
1
1
1
1
1
1
1

1
1
1
1
1
1
1
1
1
1
1
1
1
1
1
1
1
1
1
1
1
1
1
1
1
1
1
1
1
1
1
1
1
1
1
1
1
1
1
1
1
1
1
1
1

1
1
1
1
1
1
1
1
1
1
1
1
1
1
1
1
1
1
1
1
1
1
1
1
1
1
1
1
1
1
1
1
1
1
1
1
1
1
1
1
1
1
1
1
1

1
1
1
1
1
1
1
1
1
1
1
1
1
1
1
1
1
1
1
1
1
1
1
1
1
1
1
1
1
1
1
1
1
1
1
1
1
1
1
1
1
1
1
1
1

1
1
1
1
1
1
1
1
1
1
1
1
1
1
1
1
1
1
1
1
1
1
1
1
1
1
1
1
1
1
1
1
1
1
1
1
1
1
1
1
1
1
1
1
1

1
1
1
1
1
1
1
1
1
1
1
1
1
1
1
1
1
1
1
1
1
1
1
1
1
1
1
1
1
1
1
1
1
1
1
1
1
1
1
1
1
1
1
1
1

1
1
1
1
1
1
1
1
1
1
1
1
1
1
1
1
1
1
1
1
1
1
1
1
1
1
1
1
1
1
1
1
1
1
1
1
1
1
1
1
1
1
1
1
1

1
1
1
1
1
1
1
1
1
1
1
1
1
1
1
1
1
1
1
1
1
1
1
1
1
1
1
1
1
1
1
1
1
1
1
1
1
1
1
1
1
1
1
1
1

1
1
1
1
1
1
1
1
1
1
1
1
1
1
1
1
1
1
1
1
1
1
1
1
1
1
1
1
1
1
1
1
1
1
1
1
1
1
1
1
1
1
1
1
1

1
1
1
1
1
1
1
1
1
1
1
1
1
1
1
1
1
1
1
1
1
1
1
1
1
1
1
1
1
1
1
1
1
1
1
1
1
1
1
1
1
1
1
1
1

1
1
1
1
1
1
1
1
1
1
1
1
1
1
1
1
1
1
1
1
1
1
1
1
1
1
1
1
1
1
1
1
1
1
1
1
1
1
1
1
1
1
1
1
1

1
1
1
1
1
1
1
1
1
1
1
1
1
1
1
1
1
1
1
1
1
1
1
1
1
1
1
1
1
1
1
1
1
1
1
1
1
1
1
1
1
1
1
1
1

1
1
1
1
1
1
1
1
1
1
1
1
1
1
1
1
1
1
1
1
1
1
1
1
1
1
1
1
1
1
1
1
1
1
1
1
1
1
1
1
1
1
1
1
1

1
1
1
1
1
1
1
1
1
1
1
1
1
1
1
1
1
1
1
1
1
1
1
1
1
1
1
1
1
1
1
1
1
1
1
1
1
1
1
1
1
1
1
1
1

1
1
1
1
1
1
1
1
1
1
1
1
1
1
1
1
1
1
1
1
1
1
1
1
1
1
1
1
1
1
1
1
1
1
1
1
1
1
1
1
1
1
1
1
1

1
1
1
1
1
1
1
1
1
1
1
1
1
1
1
1
1
1
1
1
1
1
1
1
1
1
1
1
1
1
1
1
1
1
1
1
1
1
1
1
1
1
1
1
1

1
1
1
1
1
1
1
1
1
1
1
1
1
1
1
1
1
1
1
1
1
1
1
1
1
1
1
1
1
1
1
1
1
1
1
1
1
1
1
1
1
1
1
1
1

1
1
1
1
1
1
1
1
1
1
1
1
1
1
1
1
1
1
1
1
1
1
1
1
1
1
1
1
1
1
1
1
1
1
1
1
1
1
1
1
1
1
1
1
1

1
1
1
1
1
1
1
1
1
1
1
1
1
1
1
1
1
1
1
1
1
1
1
1
1
1
1
1
1
1
1
1
1
1
1
1
1
1
1
1
1
1
1
1
1

1
1
1
1
1
1
1
1
1
1
1
1
1
1
1
1
1
1
1
1
1
1
1
1
1
1
1
1
1
1
1
1
1
1
1
1
1
1
1
1
1
1
1
1
1

1
1
1
1
1
1
1
1
1
1
1
1
1
1
1
1
1
1
1
1
1
1
1
1
1
1
1
1
1
1
1
1
1
1
1
1
1
1
1
1
1
1
1
1
1

1
1
1
1
1
1
1
1
1
1
1
1
1
1
1
1
1
1
1
1
1
1
1
1
1
1
1
1
1
1
1
1
1
1
1
1
1
1
1
1
1
1
1
1
1

1
1
1
1
1
1
1
1
1
1
1
1
1
1
1
1
1
1
1
1
1
1
1
1
1
1
1
1
1
1
1
1
1
1
1
1
1
1
1
1
1
1
1
1
1

1
1
1
1
1
1
1
1
1
1
1
1
1
1
1
1
1
1
1
1
1
1
1
1
1
1
1
1
1
1
1
1
1
1
1
1
1
1
1
1
1
1
1
1
1

1
1
1
1
1
1
1
1
1
1
1
1
1
1
1
1
1
1
1
1
1
1
1
1
1
1
1
1
1
1
1
1
1
1
1
1
1
1
1
1
1
1
1
1
1

1
1
1
1
1
1
1
1
1
1
1
1
1
1
1
1
1
1
1
1
1
1
1
1
1
1
1
1
1
1
1
1
1
1
1
1
1
1
1
1
1
1
1
1
1

1
1
1
1
1
1
1
1
1
1
1
1
1
1
1
1
1
1
1
1
1
1
1
1
1
1
1
1
1
1
1
1
1
1
1
1
1
1
1
1
1
1
1
1
1

1
1
1
1
1
1
1
1
1
1
1
1
1
1
1
1
1
1
1
1
1
1
1
1
1
1
1
1
1
1
1
1
1
1
1
1
1
1
1
1
1
1
1
1
1

1
1
1
1
1
1
1
1
1
1
1
1
1
1
1
1
1
1
1
1
1
1
1
1
1
1
1
1
1
1
1
1
1
1
1
1
1
1
1
1
1
1
1
1
1

1
1
1
1
1
1
1
1
1
1
1
1
1
1
1
1
1
1
1
1
1
1
1
1
1
1
1
1
1
1
1
1
1
1
1
1
1
1
1
1
1
1
1
1
1

1
1
1
1
1
1
1
1
1
1
1
1
1
1
1
1
1
1
1
1
1
1
1
1
1
1
1
1
1
1
1
1
1
1
1
1
1
1
1
1
1
1
1
1
1

1
1
1
1
1
1
1
1
1
1
1
1
1
1
1
1
1
1
1
1
1
1
1
1
1
1
1
1
1
1
1
1
1
1
1
1
1
1
1
1
1
1
1
1
1

1
1
1
1
1
1
1
1
1
1
1
1
1
1
1
1
1
1
1
1
1
1
1
1
1
1
1
1
1
1
1
1
1
1
1
1
1
1
1
1
1
1
1
1
1

1
1
1
1
1
1
1
1
1
1
1
1
1
1
1
1
1
1
1
1
1
1
1
1
1
1
1
1
1
1
1
1
1
1
1
1
1
1
1
1
1
1
1
1
1

1
1
1
1
1
1
1
1
1
1
1
1
1
1
1
1
1
1
1
1
1
1
1
1
1
1
1
1
1
1
1
1
1
1
1
1
1
1
1
1
1
1
1
1
1

1
1
1
1
1
1
1
1
1
1
1
1
1
1
1
1
1
1
1
1
1
1
1
1
1
1
1
1
1
1
1
1
1
1
1
1
1
1
1
1
1
1
1
1
1

1
1
1
1
1
1
1
1
1
1
1
1
1
1
1
1
1
1
1
1
1
1
1
1
1
1
1
1
1
1
1
1
1
1
1
1
1
1
1
1
1
1
1
1
1

1
1
1
1
1
1
1
1
1
1
1
1
1
1
1
1
1
1
1
1
1
1
1
1
1
1
1
1
1
1
1
1
1
1
1
1
1
1
1
1
1
1
1
1
1

1
1
1
1
1
1
1
1
1
1
1
1
1
1
1
1
1
1
1
1
1
1
1
1
1
1
1
1
1
1
1
1
1
1
1
1
1
1
1
1
1
1
1
1
1

1
1
1
1
1
1
1
1
1
1
1
1
1
1
1
1
1
1
1
1
1
1
1
1
1
1
1
1
1
1
1
1
1
1
1
1
1
1
1
1
1
1
1
1
1

1
1
1
1
1
1
1
1
1
1
1
1
1
1
1
1
1
1
1
1
1
1
1
1
1
1
1
1
1
1
1
1
1
1
1
1
1
1
1
1
1
1
1
1
1

1
1
1
1
1
1
1
1
1
1
1
1
1
1
1
1
1
1
1
1
1
1
1
1
1
1
1
1
1
1
1
1
1
1
1
1
1
1
1
1
1
1
1
1
1

1
1
1
1
1
1
1
1
1
1
1
1
1
1
1
1
1
1
1
1
1
1
1
1
1
1
1
1
1
1
1
1
1
1
1
1
1
1
1
1
1
1
1
1
1

1
1
1
1
1
1
1
1
1
1
1
1
1
1
1
1
1
1
1
1
1
1
1
1
1
1
1
1
1
1
1
1
1
1
1
1
1
1
1
1
1
1
1
1
1

1
1
1
1
1
1
1
1
1
1
1
1
1
1
1
1
1
1
1
1
1
1
1
1
1
1
1
1
1
1
1
1
1
1
1
1
1
1
1
1
1
1
1
1
1

1
1
1
1
1
1
1
1
1
1
1
1
1
1
1
1
1
1
1
1
1
1
1
1
1
1
1
1
1
1
1
1
1
1
1
1
1
1
1
1
1
1
1
1
1

1
1
1
1
1
1
1
1
1
1
1
1
1
1
1
1
1
1
1
1
1
1
1
1
1
1
1
1
1
1
1
1
1
1
1
1
1
1
1
1
1
1
1
1
1

1
1
1
1
1
1
1
1
1
1
1
1
1
1
1
1
1
1
1
1
1
1
1
1
1
1
1
1
1
1
1
1
1
1
1
1
1
1
1
1
1
1
1
1
1

1
1
1
1
1
1
1
1
1
1
1
1
1
1
1
1
1
1
1
1
1
1
1
1
1
1
1
1
1
1
1
1
1
1
1
1
1
1
1
1
1
1
1
1
1

1
1
1
1
1
1
1
1
1
1
1
1
1
1
1
1
1
1
1
1
1
1
1
1
1
1
1
1
1
1
1
1
1
1
1
1
1
1
1
1
1
1
1
1
1

1
1
1
1
1
1
1
1
1
1
1
1
1
1
1
1
1
1
1
1
1
1
1
1
1
1
1
1
1
1
1
1
1
1
1
1
1
1
1
1
1
1
1
1
1

1
1
1
1
1
1
1
1
1
1
1
1
1
1
1
1
1
1
1
1
1
1
1
1
1
1
1
1
1
1
1
1
1
1
1
1
1
1
1
1
1
1
1
1
1

1
1
1
1
1
1
1
1
1
1
1
1
1
1
1
1
1
1
1
1
1
1
1
1
1
1
1
1
1
1
1
1
1
1
1
1
1
1
1
1
1
1
1
1
1

1
1
1
1
1
1
1
1
1
1
1
1
1
1
1
1
1
1
1
1
1
1
1
1
1
1
1
1
1
1
1
1
1
1
1
1
1
1
1
1
1
1
1
1
1

1
1
1
1
1
1
1
1
1
1
1
1
1
1
1
1
1
1
1
1
1
1
1
1
1
1
1
1
1
1
1
1
1
1
1
1
1
1
1
1
1
1
1
1
1

1
1
1
1
1
1
1
1
1
1
1
1
1
1
1
1
1
1
1
1
1
1
1
1
1
1
1
1
1
1
1
1
1
1
1
1
1
1
1
1
1
1
1
1
1

1
1
1
1
1
1
1
1
1
1
1
1
1
1
1
1
1
1
1
1
1
1
1
1
1
1
1
1
1
1
1
1
1
1
1
1
1
1
1
1
1
1
1
1
1

1
1
1
1
1
1
1
1
1
1
1
1
1
1
1
1
1
1
1
1
1
1
1
1
1
1
1
1
1
1
1
1
1
1
1
1
1
1
1
1
1
1
1
1
1

1
1
1
1
1
1
1
1
1
1
1
1
1
1
1
1
1
1
1
1
1
1
1
1
1
1
1
1
1
1
1
1
1
1
1
1
1
1
1
1
1
1
1
1
1

1
1
1
1
1
1
1
1
1
1
1
1
1
1
1
1
1
1
1
1
1
1
1
1
1
1
1
1
1
1
1
1
1
1
1
1
1
1
1
1
1
1
1
1
1

1
1
1
1
1
1
1
1
1
1
1
1
1
1
1
1
1
1
1
1
1
1
1
1
1
1
1
1
1
1
1
1
1
1
1
1
1
1
1
1
1
1
1
1
1

1
1
1
1
1
1
1
1
1
1
1
1
1
1
1
1
1
1
1
1
1
1
1
1
1
1
1
1
1
1
1
1
1
1
1
1
1
1
1
1
1
1
1
1
1

1
1
1
1
1
1
1
1
1
1
1
1
1
1
1
1
1
1
1
1
1
1
1
1
1
1
1
1
1
1
1
1
1
1
1
1
1
1
1
1
1
1
1
1
1

1
1
1
1
1
1
1
1
1
1
1
1
1
1
1
1
1
1
1
1
1
1
1
1
1
1
1
1
1
1
1
1
1
1
1
1
1
1
1
1
1
1
1
1
1

1
1
1
1
1
1
1
1
1
1
1
1
1
1
1
1
1
1
1
1
1
1
1
1
1
1
1
1
1
1
1
1
1
1
1
1
1
1
1
1
1
1
1
1
1

1
1
1
1
1
1
1
1
1
1
1
1
1
1
1
1
1
1
1
1
1
1
1
1
1
1
1
1
1
1
1
1
1
1
1
1
1
1
1
1
1
1
1
1
1

1
1
1
1
1
1
1
1
1
1
1
1
1
1
1
1
1
1
1
1
1
1
1
1
1
1
1
1
1
1
1
1
1
1
1
1
1
1
1
1
1
1
1
1
1

1
1
1
1
1
1
1
1
1
1
1
1
1
1
1
1
1
1
1
1
1
1
1
1
1
1
1
1
1
1
1
1
1
1
1
1
1
1
1
1
1
1
1
1
1

1
1
1
1
1
1
1
1
1
1
1
1
1
1
1
1
1
1
1
1
1
1
1
1
1
1
1
1
1
1
1
1
1
1
1
1
1
1
1
1
1
1
1
1
1

1
1
1
1
1
1
1
1
1
1
1
1
1
1
1
1
1
1
1
1
1
1
1
1
1
1
1
1
1
1
1
1
1
1
1
1
1
1
1
1
1
1
1
1
1

1
1
1
1
1
1
1
1
1
1
1
1
1
1
1
1
1
1
1
1
1
1
1
1
1
1
1
1
1
1
1
1
1
1
1
1
1
1
1
1
1
1
1
1
1

1
1
1
1
1
1
1
1
1
1
1
1
1
1
1
1
1
1
1
1
1
1
1
1
1
1
1
1
1
1
1
1
1
1
1
1
1
1
1
1
1
1
1
1
1

1
1
1
1
1
1
1
1
1
1
1
1
1
1
1
1
1
1
1
1
1
1
1
1
1
1
1
1
1
1
1
1
1
1
1
1
1
1
1
1
1
1
1
1
1

1
1
1
1
1
1
1
1
1
1
1
1
1
1
1
1
1
1
1
1
1
1
1
1
1
1
1
1
1
1
1
1
1
1
1
1
1
1
1
1
1
1
1
1
1

1
1
1
1
1
1
1
1
1
1
1
1
1
1
1
1
1
1
1
1
1
1
1
1
1
1
1
1
1
1
1
1
1
1
1
1
1
1
1
1
1
1
1
1
1

1
1
1
1
1
1
1
1
1
1
1
1
1
1
1
1
1
1
1
1
1
1
1
1
1
1
1
1
1
1
1
1
1
1
1
1
1
1
1
1
1
1
1
1
1

1
1
1
1
1
1
1
1
1
1
1
1
1
1
1
1
1
1
1
1
1
1
1
1
1
1
1
1
1
1
1
1
1
1
1
1
1
1
1
1
1
1
1
1
1

1
1
1
1
1
1
1
1
1
1
1
1
1
1
1
1
1
1
1
1
1
1
1
1
1
1
1
1
1
1
1
1
1
1
1
1
1
1
1
1
1
1
1
1
1

1
1
1
1
1
1
1
1
1
1
1
1
1
1
1
1
1
1
1
1
1
1
1
1
1
1
1
1
1
1
1
1
1
1
1
1
1
1
1
1
1
1
1
1
1

1
1
1
1
1
1
1
1
1
1
1
1
1
1
1
1
1
1
1
1
1
1
1
1
1
1
1
1
1
1
1
1
1
1
1
1
1
1
1
1
1
1
1
1
1

1
1
1
1
1
1
1
1
1
1
1
1
1
1
1
1
1
1
1
1
1
1
1
1
1
1
1
1
1
1
1
1
1
1
1
1
1
1
1
1
1
1
1
1
1

1
1
1
1
1
1
1
1
1
1
1
1
1
1
1
1
1
1
1
1
1
1
1
1
1
1
1
1
1
1
1
1
1
1
1
1
1
1
1
1
1
1
1
1
1

1
1
1
1
1
1
1
1
1
1
1
1
1
1
1
1
1
1
1
1
1
1
1
1
1
1
1
1
1
1
1
1
1
1
1
1
1
1
1
1
1
1
1
1
1

1
1
1
1
1
1
1
1
1
1
1
1
1
1
1
1
1
1
1
1
1
1
1
1
1
1
1
1
1
1
1
1
1
1
1
1
1
1
1
1
1
1
1
1
1

1
1
1
1
1
1
1
1
1
1
1
1
1
1
1
1
1
1
1
1
1
1
1
1
1
1
1
1
1
1
1
1
1
1
1
1
1
1
1
1
1
1
1
1
1

1
1
1
1
1
1
1
1
1
1
1
1
1
1
1
1
1
1
1
1
1
1
1
1
1
1
1
1
1
1
1
1
1
1
1
1
1
1
1
1
1
1
1
1
1

1
1
1
1
1
1
1
1
1
1
1
1
1
1
1
1
1
1
1
1
1
1
1
1
1
1
1
1
1
1
1
1
1
1
1
1
1
1
1
1
1
1
1
1
1

1
1
1
1
1
1
1
1
1
1
1
1
1
1
1
1
1
1
1
1
1
1
1
1
1
1
1
1
1
1
1
1
1
1
1
1
1
1
1
1
1
1
1
1
1

1
1
1
1
1
1
1
1
1
1
1
1
1
1
1
1
1
1
1
1
1
1
1
1
1
1
1
1
1
1
1
1
1
1
1
1
1
1
1
1
1
1
1
1
1

1
1
1
1
1
1
1
1
1
1
1
1
1
1
1
1
1
1
1
1
1
1
1
1
1
1
1
1
1
1
1
1
1
1
1
1
1
1
1
1
1
1
1
1
1

1
1
1
1
1
1
1
1
1
1
1
1
1
1
1
1
1
1
1
1
1
1
1
1
1
1
1
1
1
1
1
1
1
1
1
1
1
1
1
1
1
1
1
1
1

1
1
1
1
1
1
1
1
1
1
1
1
1
1
1
1
1
1
1
1
1
1
1
1
1
1
1
1
1
1
1
1
1
1
1
1
1
1
1
1
1
1
1
1
1

1
1
1
1
1
1
1
1
1
1
1
1
1
1
1
1
1
1
1
1
1
1
1
1
1
1
1
1
1
1
1
1
1
1
1
1
1
1
1
1
1
1
1
1
1

1
1
1
1
1
1
1
1
1
1
1
1
1
1
1
1
1
1
1
1
1
1
1
1
1
1
1
1
1
1
1
1
1
1
1
1
1
1
1
1
1
1
1
1
1

1
1
1
1
1
1
1
1
1
1
1
1
1
1
1
1
1
1
1
1
1
1
1
1
1
1
1
1
1
1
1
1
1
1
1
1
1
1
1
1
1
1
1
1
1

1
1
1
1
1
1
1
1
1
1
1
1
1
1
1
1
1
1
1
1
1
1
1
1
1
1
1
1
1
1
1
1
1
1
1
1
1
1
1
1
1
1
1
1
1

1
1
1
1
1
1
1
1
1
1
1
1
1
1
1
1
1
1
1
1
1
1
1
1
1
1
1
1
1
1
1
1
1
1
1
1
1
1
1
1
1
1
1
1
1

1
1
1
1
1
1
1
1
1
1
1
1
1
1
1
1
1
1
1
1
1
1
1
1
1
1
1
1
1
1
1
1
1
1
1
1
1
1
1
1
1
1
1
1
1

1
1
1
1
1
1
1
1
1
1
1
1
1
1
1
1
1
1
1
1
1
1
1
1
1
1
1
1
1
1
1
1
1
1
1
1
1
1
1
1
1
1
1
1
1

1
1
1
1
1
1
1
1
1
1
1
1
1
1
1
1
1
1
1
1
1
1
1
1
1
1
1
1
1
1
1
1
1
1
1
1
1
1
1
1
1
1
1
1
1

1
1
1
1
1
1
1
1
1
1
1
1
1
1
1
1
1
1
1
1
1
1
1
1
1
1
1
1
1
1
1
1
1
1
1
1
1
1
1
1
1
1
1
1
1

1
1
1
1
1
1
1
1
1
1
1
1
1
1
1
1
1
1
1
1
1
1
1
1
1
1
1
1
1
1
1
1
1
1
1
1
1
1
1
1
1
1
1
1
1

1
1
1
1
1
1
1
1
1
1
1
1
1
1
1
1
1
1
1
1
1
1
1
1
1
1
1
1
1
1
1
1
1
1
1
1
1
1
1
1
1
1
1
1
1

1
1
1
1
1
1
1
1
1
1
1
1
1
1
1
1
1
1
1
1
1
1
1
1
1
1
1
1
1
1
1
1
1
1
1
1
1
1
1
1
1
1
1
1
1

1
1
1
1
1
1
1
1
1
1
1
1
1
1
1
1
1
1
1
1
1
1
1
1
1
1
1
1
1
1
1
1
1
1
1
1
1
1
1
1
1
1
1
1
1

1
1
1
1
1
1
1
1
1
1
1
1
1
1
1
1
1
1
1
1
1
1
1
1
1
1
1
1
1
1
1
1
1
1
1
1
1
1
1
1
1
1
1
1
1

1
1
1
1
1
1
1
1
1
1
1
1
1
1
1
1
1
1
1
1
1
1
1
1
1
1
1
1
1
1
1
1
1
1
1
1
1
1
1
1
1
1
1
1
1

1
1
1
1
1
1
1
1
1
1
1
1
1
1
1
1
1
1
1
1
1
1
1
1
1
1
1
1
1
1
1
1
1
1
1
1
1
1
1
1
1
1
1
1
1

1
1
1
1
1
1
1
1
1
1
1
1
1
1
1
1
1
1
1
1
1
1
1
1
1
1
1
1
1
1
1
1
1
1
1
1
1
1
1
1
1
1
1
1
1

1
1
1
1
1
1
1
1
1
1
1
1
1
1
1
1
1
1
1
1
1
1
1
1
1
1
1
1
1
1
1
1
1
1
1
1
1
1
1
1
1
1
1
1
1

1
1
1
1
1
1
1
1
1
1
1
1
1
1
1
1
1
1
1
1
1
1
1
1
1
1
1
1
1
1
1
1
1
1
1
1
1
1
1
1
1
1
1
1
1

1
1
1
1
1
1
1
1
1
1
1
1
1
1
1
1
1
1
1
1
1
1
1
1
1
1
1
1
1
1
1
1
1
1
1
1
1
1
1
1
1
1
1
1
1

1
1
1
1
1
1
1
1
1
1
1
1
1
1
1
1
1
1
1
1
1
1
1
1
1
1
1
1
1
1
1
1
1
1
1
1
1
1
1
1
1
1
1
1
1

1
1
1
1
1
1
1
1
1
1
1
1
1
1
1
1
1
1
1
1
1
1
1
1
1
1
1
1
1
1
1
1
1
1
1
1
1
1
1
1
1
1
1
1
1

1
1
1
1
1
1
1
1
1
1
1
1
1
1
1
1
1
1
1
1
1
1
1
1
1
1
1
1
1
1
1
1
1
1
1
1
1
1
1
1
1
1
1
1
1

1
1
1
1
1
1
1
1
1
1
1
1
1
1
1
1
1
1
1
1
1
1
1
1
1
1
1
1
1
1
1
1
1
1
1
1
1
1
1
1
1
1
1
1
1

1
1
1
1
1
1
1
1
1
1
1
1
1
1
1
1
1
1
1
1
1
1
1
1
1
1
1
1
1
1
1
1
1
1
1
1
1
1
1
1
1
1
1
1
1

1
1
1
1
1
1
1
1
1
1
1
1
1
1
1
1
1
1
1
1
1
1
1
1
1
1
1
1
1
1
1
1
1
1
1
1
1
1
1
1
1
1
1
1
1

1
1
1
1
1
1
1
1
1
1
1
1
1
1
1
1
1
1
1
1
1
1
1
1
1
1
1
1
1
1
1
1
1
1
1
1
1
1
1
1
1
1
1
1
1

1
1
1
1
1
1
1
1
1
1
1
1
1
1
1
1
1
1
1
1
1
1
1
1
1
1
1
1
1
1
1
1
1
1
1
1
1
1
1
1
1
1
1
1
1

1
1
1
1
1
1
1
1
1
1
1
1
1
1
1
1
1
1
1
1
1
1
1
1
1
1
1
1
1
1
1
1
1
1
1
1
1
1
1
1
1
1
1
1
1

1
1
1
1
1
1
1
1
1
1
1
1
1
1
1
1
1
1
1
1
1
1
1
1
1
1
1
1
1
1
1
1
1
1
1
1
1
1
1
1
1
1
1
1
1

1
1
1
1
1
1
1
1
1
1
1
1
1
1
1
1
1
1
1
1
1
1
1
1
1
1
1
1
1
1
1
1
1
1
1
1
1
1
1
1
1
1
1
1
1

1
1
1
1
1
1
1
1
1
1
1
1
1
1
1
1
1
1
1
1
1
1
1
1
1
1
1
1
1
1
1
1
1
1
1
1
1
1
1
1
1
1
1
1
1

1
1
1
1
1
1
1
1
1
1
1
1
1
1
1
1
1
1
1
1
1
1
1
1
1
1
1
1
1
1
1
1
1
1
1
1
1
1
1
1
1
1
1
1
1

1
1
1
1
1
1
1
1
1
1
1
1
1
1
1
1
1
1
1
1
1
1
1
1
1
1
1
1
1
1
1
1
1
1
1
1
1
1
1
1
1
1
1
1
1

1
1
1
1
1
1
1
1
1
1
1
1
1
1
1
1
1
1
1
1
1
1
1
1
1
1
1
1
1
1
1
1
1
1
1
1
1
1
1
1
1
1
1
1
1

1
1
1
1
1
1
1
1
1
1
1
1
1
1
1
1
1
1
1
1
1
1
1
1
1
1
1
1
1
1
1
1
1
1
1
1
1
1
1
1
1
1
1
1
1

1
1
1
1
1
1
1
1
1
1
1
1
1
1
1
1
1
1
1
1
1
1
1
1
1
1
1
1
1
1
1
1
1
1
1
1
1
1
1
1
1
1
1
1
1

1
1
1
1
1
1
1
1
1
1
1
1
1
1
1
1
1
1
1
1
1
1
1
1
1
1
1
1
1
1
1
1
1
1
1
1
1
1
1
1
1
1
1
1
1

1
1
1
1
1
1
1
1
1
1
1
1
1
1
1
1
1
1
1
1
1
1
1
1
1
1
1
1
1
1
1
1
1
1
1
1
1
1
1
1
1
1
1
1
1

1
1
1
1
1
1
1
1
1
1
1
1
1
1
1
1
1
1
1
1
1
1
1
1
1
1
1
1
1
1
1
1
1
1
1
1
1
1
1
1
1
1
1
1
1

1
1
1
1
1
1
1
1
1
1
1
1
1
1
1
1
1
1
1
1
1
1
1
1
1
1
1
1
1
1
1
1
1
1
1
1
1
1
1
1
1
1
1
1
1

1
1
1
1
1
1
1
1
1
1
1
1
1
1
1
1
1
1
1
1
1
1
1
1
1
1
1
1
1
1
1
1
1
1
1
1
1
1
1
1
1
1
1
1
1

1
1
1
1
1
1
1
1
1
1
1
1
1
1
1
1
1
1
1
1
1
1
1
1
1
1
1
1
1
1
1
1
1
1
1
1
1
1
1
1
1
1
1
1
1

1
1
1
1
1
1
1
1
1
1
1
1
1
1
1
1
1
1
1
1
1
1
1
1
1
1
1
1
1
1
1
1
1
1
1
1
1
1
1
1
1
1
1
1
1

1
1
1
1
1
1
1
1
1
1
1
1
1
1
1
1
1
1
1
1
1
1
1
1
1
1
1
1
1
1
1
1
1
1
1
1
1
1
1
1
1
1
1
1
1

1
1
1
1
1
1
1
1
1
1
1
1
1
1
1
1
1
1
1
1
1
1
1
1
1
1
1
1
1
1
1
1
1
1
1
1
1
1
1
1
1
1
1
1
1

1
1
1
1
1
1
1
1
1
1
1
1
1
1
1
1
1
1
1
1
1
1
1
1
1
1
1
1
1
1
1
1
1
1
1
1
1
1
1
1
1
1
1
1
1

1
1
1
1
1
1
1
1
1
1
1
1
1
1
1
1
1
1
1
1
1
1
1
1
1
1
1
1
1
1
1
1
1
1
1
1
1
1
1
1
1
1
1
1
1

1
1
1
1
1
1
1
1
1
1
1
1
1
1
1
1
1
1
1
1
1
1
1
1
1
1
1
1
1
1
1
1
1
1
1
1
1
1
1
1
1
1
1
1
1

1
1
1
1
1
1
1
1
1
1
1
1
1
1
1
1
1
1
1
1
1
1
1
1
1
1
1
1
1
1
1
1
1
1
1
1
1
1
1
1
1
1
1
1
1

1
1
1
1
1
1
1
1
1
1
1
1
1
1
1
1
1
1
1
1
1
1
1
1
1
1
1
1
1
1
1
1
1
1
1
1
1
1
1
1
1
1
1
1
1

1
1
1
1
1
1
1
1
1
1
1
1
1
1
1
1
1
1
1
1
1
1
1
1
1
1
1
1
1
1
1
1
1
1
1
1
1
1
1
1
1
1
1
1
1

1
1
1
1
1
1
1
1
1
1
1
1
1
1
1
1
1
1
1
1
1
1
1
1
1
1
1
1
1
1
1
1
1
1
1
1
1
1
1
1
1
1
1
1
1

1
1
1
1
1
1
1
1
1
1
1
1
1
1
1
1
1
1
1
1
1
1
1
1
1
1
1
1
1
1
1
1
1
1
1
1
1
1
1
1
1
1
1
1
1

1
1
1
1
1
1
1
1
1
1
1
1
1
1
1
1
1
1
1
1
1
1
1
1
1
1
1
1
1
1
1
1
1
1
1
1
1
1
1
1
1
1
1
1
1

1
1
1
1
1
1
1
1
1
1
1
1
1
1
1
1
1
1
1
1
1
1
1
1
1
1
1
1
1
1
1
1
1
1
1
1
1
1
1
1
1
1
1
1
1

1
1
1
1
1
1
1
1
1
1
1
1
1
1
1
1
1
1
1
1
1
1
1
1
1
1
1
1
1
1
1
1
1
1
1
1
1
1
1
1
1
1
1
1
1

1
1
1
1
1
1
1
1
1
1
1
1
1
1
1
1
1
1
1
1
1
1
1
1
1
1
1
1
1
1
1
1
1
1
1
1
1
1
1
1
1
1
1
1
1

1
1
1
1
1
1
1
1
1
1
1
1
1
1
1
1
1
1
1
1
1
1
1
1
1
1
1
1
1
1
1
1
1
1
1
1
1
1
1
1
1
1
1
1
1

1
1
1
1
1
1
1
1
1
1
1
1
1
1
1
1
1
1
1
1
1
1
1
1
1
1
1
1
1
1
1
1
1
1
1
1
1
1
1
1
1
1
1
1
1

1
1
1
1
1
1
1
1
1
1
1
1
1
1
1
1
1
1
1
1
1
1
1
1
1
1
1
1
1
1
1
1
1
1
1
1
1
1
1
1
1
1
1
1
1

1
1
1
1
1
1
1
1
1
1
1
1
1
1
1
1
1
1
1
1
1
1
1
1
1
1
1
1
1
1
1
1
1
1
1
1
1
1
1
1
1
1
1
1
1

1
1
1
1
1
1
1
1
1
1
1
1
1
1
1
1
1
1
1
1
1
1
1
1
1
1
1
1
1
1
1
1
1
1
1
1
1
1
1
1
1
1
1
1
1

1
1
1
1
1
1
1
1
1
1
1
1
1
1
1
1
1
1
1
1
1
1
1
1
1
1
1
1
1
1
1
1
1
1
1
1
1
1
1
1
1
1
1
1
1

1
1
1
1
1
1
1
1
1
1
1
1
1
1
1
1
1
1
1
1
1
1
1
1
1
1
1
1
1
1
1
1
1
1
1
1
1
1
1
1
1
1
1
1
1

1
1
1
1
1
1
1
1
1
1
1
1
1
1
1
1
1
1
1
1
1
1
1
1
1
1
1
1
1
1
1
1
1
1
1
1
1
1
1
1
1
1
1
1
1

1
1
1
1
1
1
1
1
1
1
1
1
1
1
1
1
1
1
1
1
1
1
1
1
1
1
1
1
1
1
1
1
1
1
1
1
1
1
1
1
1
1
1
1
1

1
1
1
1
1
1
1
1
1
1
1
1
1
1
1
1
1
1
1
1
1
1
1
1
1
1
1
1
1
1
1
1
1
1
1
1
1
1
1
1
1
1
1
1
1

1
1
1
1
1
1
1
1
1
1
1
1
1
1
1
1
1
1
1
1
1
1
1
1
1
1
1
1
1
1
1
1
1
1
1
1
1
1
1
1
1
1
1
1
1

1
1
1
1
1
1
1
1
1
1
1
1
1
1
1
1
1
1
1
1
1
1
1
1
1
1
1
1
1
1
1
1
1
1
1
1
1
1
1
1
1
1
1
1
1

1
1
1
1
1
1
1
1
1
1
1
1
1
1
1
1
1
1
1
1
1
1
1
1
1
1
1
1
1
1
1
1
1
1
1
1
1
1
1
1
1
1
1
1
1

1
1
1
1
1
1
1
1
1
1
1
1
1
1
1
1
1
1
1
1
1
1
1
1
1
1
1
1
1
1
1
1
1
1
1
1
1
1
1
1
1
1
1
1
1

1
1
1
1
1
1
1
1
1
1
1
1
1
1
1
1
1
1
1
1
1
1
1
1
1
1
1
1
1
1
1
1
1
1
1
1
1
1
1
1
1
1
1
1
1

1
1
1
1
1
1
1
1
1
1
1
1
1
1
1
1
1
1
1
1
1
1
1
1
1
1
1
1
1
1
1
1
1
1
1
1
1
1
1
1
1
1
1
1
1

1
1
1
1
1
1
1
1
1
1
1
1
1
1
1
1
1
1
1
1
1
1
1
1
1
1
1
1
1
1
1
1
1
1
1
1
1
1
1
1
1
1
1
1
1

1
1
1
1
1
1
1
1
1
1
1
1
1
1
1
1
1
1
1
1
1
1
1
1
1
1
1
1
1
1
1
1
1
1
1
1
1
1
1
1
1
1
1
1
1

1
1
1
1
1
1
1
1
1
1
1
1
1
1
1
1
1
1
1
1
1
1
1
1
1
1
1
1
1
1
1
1
1
1
1
1
1
1
1
1
1
1
1
1
1

1
1
1
1
1
1
1
1
1
1
1
1
1
1
1
1
1
1
1
1
1
1
1
1
1
1
1
1
1
1
1
1
1
1
1
1
1
1
1
1
1
1
1
1
1

1
1
1
1
1
1
1
1
1
1
1
1
1
1
1
1
1
1
1
1
1
1
1
1
1
1
1
1
1
1
1
1
1
1
1
1
1
1
1
1
1
1
1
1
1

1
1
1
1
1
1
1
1
1
1
1
1
1
1
1
1
1
1
1
1
1
1
1
1
1
1
1
1
1
1
1
1
1
1
1
1
1
1
1
1
1
1
1
1
1

1
1
1
1
1
1
1
1
1
1
1
1
1
1
1
1
1
1
1
1
1
1
1
1
1
1
1
1
1
1
1
1
1
1
1
1
1
1
1
1
1
1
1
1
1

1
1
1
1
1
1
1
1
1
1
1
1
1
1
1
1
1
1
1
1
1
1
1
1
1
1
1
1
1
1
1
1
1
1
1
1
1
1
1
1
1
1
1
1
1

1
1
1
1
1
1
1
1
1
1
1
1
1
1
1
1
1
1
1
1
1
1
1
1
1
1
1
1
1
1
1
1
1
1
1
1
1
1
1
1
1
1
1
1
1

1
1
1
1
1
1
1
1
1
1
1
1
1
1
1
1
1
1
1
1
1
1
1
1
1
1
1
1
1
1
1
1
1
1
1
1
1
1
1
1
1
1
1
1
1

1
1
1
1
1
1
1
1
1
1
1
1
1
1
1
1
1
1
1
1
1
1
1
1
1
1
1
1
1
1
1
1
1
1
1
1
1
1
1
1
1
1
1
1
1

1
1
1
1
1
1
1
1
1
1
1
1
1
1
1
1
1
1
1
1
1
1
1
1
1
1
1
1
1
1
1
1
1
1
1
1
1
1
1
1
1
1
1
1
1

1
1
1
1
1
1
1
1
1
1
1
1
1
1
1
1
1
1
1
1
1
1
1
1
1
1
1
1
1
1
1
1
1
1
1
1
1
1
1
1
1
1
1
1
1

1
1
1
1
1
1
1
1
1
1
1
1
1
1
1
1
1
1
1
1
1
1
1
1
1
1
1
1
1
1
1
1
1
1
1
1
1
1
1
1
1
1
1
1
1

1
1
1
1
1
1
1
1
1
1
1
1
1
1
1
1
1
1
1
1
1
1
1
1
1
1
1
1
1
1
1
1
1
1
1
1
1
1
1
1
1
1
1
1
1

1
1
1
1
1
1
1
1
1
1
1
1
1
1
1
1
1
1
1
1
1
1
1
1
1
1
1
1
1
1
1
1
1
1
1
1
1
1
1
1
1
1
1
1
1

1
1
1
1
1
1
1
1
1
1
1
1
1
1
1
1
1
1
1
1
1
1
1
1
1
1
1
1
1
1
1
1
1
1
1
1
1
1
1
1
1
1
1
1
1

1
1
1
1
1
1
1
1
1
1
1
1
1
1
1
1
1
1
1
1
1
1
1
1
1
1
1
1
1
1
1
1
1
1
1
1
1
1
1
1
1
1
1
1
1

1
1
1
1
1
1
1
1
1
1
1
1
1
1
1
1
1
1
1
1
1
1
1
1
1
1
1
1
1
1
1
1
1
1
1
1
1
1
1
1
1
1
1
1
1

1
1
1
1
1
1
1
1
1
1
1
1
1
1
1
1
1
1
1
1
1
1
1
1
1
1
1
1
1
1
1
1
1
1
1
1
1
1
1
1
1
1
1
1
1

1
1
1
1
1
1
1
1
1
1
1
1
1
1
1
1
1
1
1
1
1
1
1
1
1
1
1
1
1
1
1
1
1
1
1
1
1
1
1
1
1
1
1
1
1

1
1
1
1
1
1
1
1
1
1
1
1
1
1
1
1
1
1
1
1
1
1
1
1
1
1
1
1
1
1
1
1
1
1
1
1
1
1
1
1
1
1
1
1
1

1
1
1
1
1
1
1
1
1
1
1
1
1
1
1
1
1
1
1
1
1
1
1
1
1
1
1
1
1
1
1
1
1
1
1
1
1
1
1
1
1
1
1
1
1

1
1
1
1
1
1
1
1
1
1
1
1
1
1
1
1
1
1
1
1
1
1
1
1
1
1
1
1
1
1
1
1
1
1
1
1
1
1
1
1
1
1
1
1
1

1
1
1
1
1
1
1
1
1
1
1
1
1
1
1
1
1
1
1
1
1
1
1
1
1
1
1
1
1
1
1
1
1
1
1
1
1
1
1
1
1
1
1
1
1

1
1
1
1
1
1
1
1
1
1
1
1
1
1
1
1
1
1
1
1
1
1
1
1
1
1
1
1
1
1
1
1
1
1
1
1
1
1
1
1
1
1
1
1
1

1
1
1
1
1
1
1
1
1
1
1
1
1
1
1
1
1
1
1
1
1
1
1
1
1
1
1
1
1
1
1
1
1
1
1
1
1
1
1
1
1
1
1
1
1

1
1
1
1
1
1
1
1
1
1
1
1
1
1
1
1
1
1
1
1
1
1
1
1
1
1
1
1
1
1
1
1
1
1
1
1
1
1
1
1
1
1
1
1
1

1
1
1
1
1
1
1
1
1
1
1
1
1
1
1
1
1
1
1
1
1
1
1
1
1
1
1
1
1
1
1
1
1
1
1
1
1
1
1
1
1
1
1
1
1

1
1
1
1
1
1
1
1
1
1
1
1
1
1
1
1
1
1
1
1
1
1
1
1
1
1
1
1
1
1
1
1
1
1
1
1
1
1
1
1
1
1
1
1
1

1
1
1
1
1
1
1
1
1
1
1
1
1
1
1
1
1
1
1
1
1
1
1
1
1
1
1
1
1
1
1
1
1
1
1
1
1
1
1
1
1
1
1
1
1

1
1
1
1
1
1
1
1
1
1
1
1
1
1
1
1
1
1
1
1
1
1
1
1
1
1
1
1
1
1
1
1
1
1
1
1
1
1
1
1
1
1
1
1
1

1
1
1
1
1
1
1
1
1
1
1
1
1
1
1
1
1
1
1
1
1
1
1
1
1
1
1
1
1
1
1
1
1
1
1
1
1
1
1
1
1
1
1
1
1

1
1
1
1
1
1
1
1
1
1
1
1
1
1
1
1
1
1
1
1
1
1
1
1
1
1
1
1
1
1
1
1
1
1
1
1
1
1
1
1
1
1
1
1
1

1
1
1
1
1
1
1
1
1
1
1
1
1
1
1
1
1
1
1
1
1
1
1
1
1
1
1
1
1
1
1
1
1
1
1
1
1
1
1
1
1
1
1
1
1

1
1
1
1
1
1
1
1
1
1
1
1
1
1
1
1
1
1
1
1
1
1
1
1
1
1
1
1
1
1
1
1
1
1
1
1
1
1
1
1
1
1
1
1
1

1
1
1
1
1
1
1
1
1
1
1
1
1
1
1
1
1
1
1
1
1
1
1
1
1
1
1
1
1
1
1
1
1
1
1
1
1
1
1
1
1
1
1
1
1

1
1
1
1
1
1
1
1
1
1
1
1
1
1
1
1
1
1
1
1
1
1
1
1
1
1
1
1
1
1
1
1
1
1
1
1
1
1
1
1
1
1
1
1
1

1
1
1
1
1
1
1
1
1
1
1
1
1
1
1
1
1
1
1
1
1
1
1
1
1
1
1
1
1
1
1
1
1
1
1
1
1
1
1
1
1
1
1
1
1

1
1
1
1
1
1
1
1
1
1
1
1
1
1
1
1
1
1
1
1
1
1
1
1
1
1
1
1
1
1
1
1
1
1
1
1
1
1
1
1
1
1
1
1
1

1
1
1
1
1
1
1
1
1
1
1
1
1
1
1
1
1
1
1
1
1
1
1
1
1
1
1
1
1
1
1
1
1
1
1
1
1
1
1
1
1
1
1
1
1

1
1
1
1
1
1
1
1
1
1
1
1
1
1
1
1
1
1
1
1
1
1
1
1
1
1
1
1
1
1
1
1
1
1
1
1
1
1
1
1
1
1
1
1
1

1
1
1
1
1
1
1
1
1
1
1
1
1
1
1
1
1
1
1
1
1
1
1
1
1
1
1
1
1
1
1
1
1
1
1
1
1
1
1
1
1
1
1
1
1

1
1
1
1
1
1
1
1
1
1
1
1
1
1
1
1
1
1
1
1
1
1
1
1
1
1
1
1
1
1
1
1
1
1
1
1
1
1
1
1
1
1
1
1
1

1
1
1
1
1
1
1
1
1
1
1
1
1
1
1
1
1
1
1
1
1
1
1
1
1
1
1
1
1
1
1
1
1
1
1
1
1
1
1
1
1
1
1
1
1

1
1
1
1
1
1
1
1
1
1
1
1
1
1
1
1
1
1
1
1
1
1
1
1
1
1
1
1
1
1
1
1
1
1
1
1
1
1
1
1
1
1
1
1
1

1
1
1
1
1
1
1
1
1
1
1
1
1
1
1
1
1
1
1
1
1
1
1
1
1
1
1
1
1
1
1
1
1
1
1
1
1
1
1
1
1
1
1
1
1

1
1
1
1
1
1
1
1
1
1
1
1
1
1
1
1
1
1
1
1
1
1
1
1
1
1
1
1
1
1
1
1
1
1
1
1
1
1
1
1
1
1
1
1
1

1
1
1
1
1
1
1
1
1
1
1
1
1
1
1
1
1
1
1
1
1
1
1
1
1
1
1
1
1
1
1
1
1
1
1
1
1
1
1
1
1
1
1
1
1

1
1
1
1
1
1
1
1
1
1
1
1
1
1
1
1
1
1
1
1
1
1
1
1
1
1
1
1
1
1
1
1
1
1
1
1
1
1
1
1
1
1
1
1
1

1
1
1
1
1
1
1
1
1
1
1
1
1
1
1
1
1
1
1
1
1
1
1
1
1
1
1
1
1
1
1
1
1
1
1
1
1
1
1
1
1
1
1
1
1

1
1
1
1
1
1
1
1
1
1
1
1
1
1
1
1
1
1
1
1
1
1
1
1
1
1
1
1
1
1
1
1
1
1
1
1
1
1
1
1
1
1
1
1
1

1
1
1
1
1
1
1
1
1
1
1
1
1
1
1
1
1
1
1
1
1
1
1
1
1
1
1
1
1
1
1
1
1
1
1
1
1
1
1
1
1
1
1
1
1

1
1
1
1
1
1
1
1
1
1
1
1
1
1
1
1
1
1
1
1
1
1
1
1
1
1
1
1
1
1
1
1
1
1
1
1
1
1
1
1
1
1
1
1
1

1
1
1
1
1
1
1
1
1
1
1
1
1
1
1
1
1
1
1
1
1
1
1
1
1
1
1
1
1
1
1
1
1
1
1
1
1
1
1
1
1
1
1
1
1

1
1
1
1
1
1
1
1
1
1
1
1
1
1
1
1
1
1
1
1
1
1
1
1
1
1
1
1
1
1
1
1
1
1
1
1
1
1
1
1
1
1
1
1
1

1
1
1
1
1
1
1
1
1
1
1
1
1
1
1
1
1
1
1
1
1
1
1
1
1
1
1
1
1
1
1
1
1
1
1
1
1
1
1
1
1
1
1
1
1

1
1
1
1
1
1
1
1
1
1
1
1
1
1
1
1
1
1
1
1
1
1
1
1
1
1
1
1
1
1
1
1
1
1
1
1
1
1
1
1
1
1
1
1
1

1
1
1
1
1
1
1
1
1
1
1
1
1
1
1
1
1
1
1
1
1
1
1
1
1
1
1
1
1
1
1
1
1
1
1
1
1
1
1
1
1
1
1
1
1

1
1
1
1
1
1
1
1
1
1
1
1
1
1
1
1
1
1
1
1
1
1
1
1
1
1
1
1
1
1
1
1
1
1
1
1
1
1
1
1
1
1
1
1
1

1
1
1
1
1
1
1
1
1
1
1
1
1
1
1
1
1
1
1
1
1
1
1
1
1
1
1
1
1
1
1
1
1
1
1
1
1
1
1
1
1
1
1
1
1

1
1
1
1
1
1
1
1
1
1
1
1
1
1
1
1
1
1
1
1
1
1
1
1
1
1
1
1
1
1
1
1
1
1
1
1
1
1
1
1
1
1
1
1
1

1
1
1
1
1
1
1
1
1
1
1
1
1
1
1
1
1
1
1
1
1
1
1
1
1
1
1
1
1
1
1
1
1
1
1
1
1
1
1
1
1
1
1
1
1

1
1
1
1
1
1
1
1
1
1
1
1
1
1
1
1
1
1
1
1
1
1
1
1
1
1
1
1
1
1
1
1
1
1
1
1
1
1
1
1
1
1
1
1
1

1
1
1
1
1
1
1
1
1
1
1
1
1
1
1
1
1
1
1
1
1
1
1
1
1
1
1
1
1
1
1
1
1
1
1
1
1
1
1
1
1
1
1
1
1

1
1
1
1
1
1
1
1
1
1
1
1
1
1
1
1
1
1
1
1
1
1
1
1
1
1
1
1
1
1
1
1
1
1
1
1
1
1
1
1
1
1
1
1
1

1
1
1
1
1
1
1
1
1
1
1
1
1
1
1
1
1
1
1
1
1
1
1
1
1
1
1
1
1
1
1
1
1
1
1
1
1
1
1
1
1
1
1
1
1

1
1
1
1
1
1
1
1
1
1
1
1
1
1
1
1
1
1
1
1
1
1
1
1
1
1
1
1
1
1
1
1
1
1
1
1
1
1
1
1
1
1
1
1
1

1
1
1
1
1
1
1
1
1
1
1
1
1
1
1
1
1
1
1
1
1
1
1
1
1
1
1
1
1
1
1
1
1
1
1
1
1
1
1
1
1
1
1
1
1

1
1
1
1
1
1
1
1
1
1
1
1
1
1
1
1
1
1
1
1
1
1
1
1
1
1
1
1
1
1
1
1
1
1
1
1
1
1
1
1
1
1
1
1
1

1
1
1
1
1
1
1
1
1
1
1
1
1
1
1
1
1
1
1
1
1
1
1
1
1
1
1
1
1
1
1
1
1
1
1
1
1
1
1
1
1
1
1
1
1

1
1
1
1
1
1
1
1
1
1
1
1
1
1
1
1
1
1
1
1
1
1
1
1
1
1
1
1
1
1
1
1
1
1
1
1
1
1
1
1
1
1
1
1
1

1
1
1
1
1
1
1
1
1
1
1
1
1
1
1
1
1
1
1
1
1
1
1
1
1
1
1
1
1
1
1
1
1
1
1
1
1
1
1
1
1
1
1
1
1

1
1
1
1
1
1
1
1
1
1
1
1
1
1
1
1
1
1
1
1
1
1
1
1
1
1
1
1
1
1
1
1
1
1
1
1
1
1
1
1
1
1
1
1
1

1
1
1
1
1
1
1
1
1
1
1
1
1
1
1
1
1
1
1
1
1
1
1
1
1
1
1
1
1
1
1
1
1
1
1
1
1
1
1
1
1
1
1
1
1

1
1
1
1
1
1
1
1
1
1
1
1
1
1
1
1
1
1
1
1
1
1
1
1
1
1
1
1
1
1
1
1
1
1
1
1
1
1
1
1
1
1
1
1
1

1
1
1
1
1
1
1
1
1
1
1
1
1
1
1
1
1
1
1
1
1
1
1
1
1
1
1
1
1
1
1
1
1
1
1
1
1
1
1
1
1
1
1
1
1

1
1
1
1
1
1
1
1
1
1
1
1
1
1
1
1
1
1
1
1
1
1
1
1
1
1
1
1
1
1
1
1
1
1
1
1
1
1
1
1
1
1
1
1
1

1
1
1
1
1
1
1
1
1
1
1
1
1
1
1
1
1
1
1
1
1
1
1
1
1
1
1
1
1
1
1
1
1
1
1
1
1
1
1
1
1
1
1
1
1

1
1
1
1
1
1
1
1
1
1
1
1
1
1
1
1
1
1
1
1
1
1
1
1
1
1
1
1
1
1
1
1
1
1
1
1
1
1
1
1
1
1
1
1
1

1
1
1
1
1
1
1
1
1
1
1
1
1
1
1
1
1
1
1
1
1
1
1
1
1
1
1
1
1
1
1
1
1
1
1
1
1
1
1
1
1
1
1
1
1

1
1
1
1
1
1
1
1
1
1
1
1
1
1
1
1
1
1
1
1
1
1
1
1
1
1
1
1
1
1
1
1
1
1
1
1
1
1
1
1
1
1
1
1
1

1
1
1
1
1
1
1
1
1
1
1
1
1
1
1
1
1
1
1
1
1
1
1
1
1
1
1
1
1
1
1
1
1
1
1
1
1
1
1
1
1
1
1
1
1

1
1
1
1
1
1
1
1
1
1
1
1
1
1
1
1
1
1
1
1
1
1
1
1
1
1
1
1
1
1
1
1
1
1
1
1
1
1
1
1
1
1
1
1
1

1
1
1
1
1
1
1
1
1
1
1
1
1
1
1
1
1
1
1
1
1
1
1
1
1
1
1
1
1
1
1
1
1
1
1
1
1
1
1
1
1
1
1
1
1

1
1
1
1
1
1
1
1
1
1
1
1
1
1
1
1
1
1
1
1
1
1
1
1
1
1
1
1
1
1
1
1
1
1
1
1
1
1
1
1
1
1
1
1
1

1
1
1
1
1
1
1
1
1
1
1
1
1
1
1
1
1
1
1
1
1
1
1
1
1
1
1
1
1
1
1
1
1
1
1
1
1
1
1
1
1
1
1
1
1

1
1
1
1
1
1
1
1
1
1
1
1
1
1
1
1
1
1
1
1
1
1
1
1
1
1
1
1
1
1
1
1
1
1
1
1
1
1
1
1
1
1
1
1
1

1
1
1
1
1
1
1
1
1
1
1
1
1
1
1
1
1
1
1
1
1
1
1
1
1
1
1
1
1
1
1
1
1
1
1
1
1
1
1
1
1
1
1
1
1

1
1
1
1
1
1
1
1
1
1
1
1
1
1
1
1
1
1
1
1
1
1
1
1
1
1
1
1
1
1
1
1
1
1
1
1
1
1
1
1
1
1
1
1
1

1
1
1
1
1
1
1
1
1
1
1
1
1
1
1
1
1
1
1
1
1
1
1
1
1
1
1
1
1
1
1
1
